# Processing and mounting phlebotomine sand flies: a consensus guideline

**DOI:** 10.1051/parasite/2026009

**Published:** 2026-04-03

**Authors:** Fano José Randrianambinintsoa, Laure Augendre, Jorian Prudhomme, Jean-Philippe Martinet, Mathieu Loyer, Nalia Mekarnia, Hocine Kerkoub, Farzana Khan Perveen, Antoine Huguenin, Emilie Kariya, Mohammad Akhoundi, Andrey José De Andrade, Eduardo Berriatua, Gioia Bongiorno, Sébastien Boyer, Vasiliki Christodoulou, Magda Clara Vieira Da Costa-Ribeiro, Lucas Alexandre Farias De Souza, Huicong Ding, Blaise Dondji, Vít Dvořák, Ozge Erisoz Kasap, Eunice Aparecida Bianchi Galati, Montserrat Gállego, Cristina Ballart, Stavroula Gouzelou, Nabil Haddad, Rezki Sabrina Masse, Asrat Hailu Mekuria, Vladimir Ivovic, Szymon Kaczmarek, Mohd Khadri Shahar, Oscar D. Kirstein, Edwin Kniha, Iva Kolářová, Lincoln Timinao, Cristian Lucanas, Ognyan Mikov, Kimsear Nov, Yusuf Özbel, Bernard Pesson, Laura Cristina Posada Lopez, Didot Budi Prasetyo, Nil Rahola, Eduardo A. Rebollar-Tellez, Bruno Leite Rodrigues, Lalita Roy, Prasanta Saini, Chizu Sanjoba, Paloma Helena Fernandes Shimabukuro, Padet Siriyasatien, Agnieszka Soszyńska, Tatiana Suleşco, Massamba Sylla, Majhalia Torno, Petr Volf, Khamsing Vongphayloth, Sinh Nam Vu, April Wardhana, Eric Yessinou, Sonia Zapata, Jean-Charles Gantier, Jérôme Depaquit

**Affiliations:** 1 Faculté de Pharmacie, Université de Reims Champagne Ardenne, UR ESCAPE-USC ANSES PETARD 51 rue Cognacq-Jay 51096 Reims Cedex France; 2 Pôle de Biologie territoriale, Laboratoire de Parasitologie-Mycologie, Centre Hospitalo-Universitaire 51092 Reims France; 3 Parasitology-Mycology Department, Avicenne Hospital, AP-HP, Bobigny, Sorbonne Paris Nord University, France; Unité des Virus Émergents (UVE: Aix-Marseille Univ, Università di Corsica, IRD 190, Inserm 1207, IRBA) 13005 Marseille France; 4 Parasitology Collection of Basic Pathology, Department of Basic Pathology, Federal University of Paraná Curitiba 19031 Brazil; 5 Department of Animal Health, University of Murcia, Campus de Espinardo 30100 Espinardo, Murcia Spain; 6 Department of Infectious Diseases, Vector-borne Diseases Unit, Istituto Superiore di Sanità 00166 Rome Italy; 7 Medical and Veterinary Entomology Unit, Institut Pasteur du Cambodge Phnom Penh 12201 Cambodia; 8 Ecology & Emergence of Arthropod-borne Pathogens Unit, Department of Global Health, Institut Pasteur, CNRS UMR2000 75015 Paris France; 9 Section Veterinary Services (1417), Laboratory for Animal Health Virology Aglantzia, Nicosia 2109 Cyprus; 10 Insects Vectors and Parasites Laboratory, Department of Basic Pathology and Postgraduate program in Microbiology, Parasitology and Pathology, Federal University of Paraná 81530-900 Curitiba Brazil; 11 Department of Biological Sciences, National University of Singapore 117558 Singapore; 12 Laboratory of the Leishmaniasis Research Project, Mokolo District Hospital, Mokolo, Cameroon; Laboratory of Cellular Immunology and Parasitology, Department of Biological Sciences, Central Washington University 98926 Ellensburg WA USA; 13 Department of Parasitology, Faculty of Science, Charles University 12800 Prague Czechia; 14 VERG Laboratories, Department of Biology, Faculty of Science, Hacettepe University Beytepe, Ankara 06800 Türkiye; 15 Faculdade de Saúde Pública da Universidade de São Paulo (FSP/USP), Pós-graduação em Saúde Pública 01246-904 São Paulo Brazil; 16 Secció de Parasitologia, Departament de Biologia, Sanitat i Medi Ambient, Facultat de Farmàcia i Ciències de l’Alimentació, Universitat de Barcelona, & Institut de Salut Global de Barcelona (ISGlobal), Centro de Investigación Biomédica en Red, Enfermedades Infecciosas (CIBERINFEC) 08028 Barcelona Spain; 17 Laboratory of Infectious Diseases and Public Health, School of Medicine, University of Cyprus, Nicosia, Cyprus & Department of Pediatrics, Archbishop Makarios III Hospital Nicosia 2115 Cyprus; 18 Faculty of Health Sciences, American University of Beirut 1107 2020 Beirut Lebanon; 19 Medical Entomology Unit, Infectious Disease Research Centre, Institute for Medical Research (IMR), National Institutes of Health (NIH), Ministry of Health Malaysia 40170 Shah Alam, Selangor Malaysia; 20 School of Medicine, Addis Ababa University 28017 – 1000 Addis Ababa Ethiopia; 21 Faculty of Mathematics, Natural Sciences and Information Technologies, University of Primorska 6000 Koper Slovenia; 22 University of Lodz, Faculty of Biology and Environmental Protection, Department of Invertebrate Zoology and Hydrobiology, Banacha 12/16 90-237 Łódź Poland; 23 Laboratory of Entomology, Ministry of Health 9134302 Jerusalem Israel; 24 Center for Pathophysiology, Infectiology and Immunology, Institute of Specific Prophylaxis and Tropical Medicine, Medical University Vienna Kinderspitalgasse 15 1090 Vienna Austria; 25 Papua New Guinea Institute of Medical Research (PNGIMR) Institute PO Box 60 Headquarter, Homate Street, 441 Goroka, Eastern Highlands Province Papua New Guinea; 26 Museum of Natural History, University of the Philippines Los Baños 4031 Laguna Philippines; 27 National Centre of Infectious and Parasitic Diseases 1504 Sofia Bulgaria; 28 Ege University, Faculty of Medicine, Department of Parasitology 35040 Bornova/Izmir Türkiye; 29 Retired, Faculté de Pharmacie, Université de Strasbourg, Strasbourg 67400 Illkirch-Graffenstaden France; 30 Program for the Study and Control of Tropical Diseases (PECET), Faculty of Medicine, University of Antioquia 050010 Medellin Colombia; 31 MIVEGEC, Univ. Montpellier, CNRS, IRD, 34394 Montpellier, France & Medical Entomology Unit, Institut Pasteur de Madagascar 101 Antananarivo Madagascar; 32 Laboratorio de Entomología Médica, Departamento de Zoología de Invertebrados, Facultad de Ciencias Biológicas, Universidad Autónoma de Nuevo León, San Nicolás de los Garza 66455 NL México; 33 Tropical and Infectious Disease Centre, BP Koirala Institute of Health Sciences Dharan 56700 Nepal; 34 ICMR-Vector Control Research Centre Puducherry 605006 India; 35 Graduate School of Agricultural and Life Sciences, The University of Tokyo Tokyo 113-8657 Japan; 36 Grupo de estudos em Leishmanioses/Coleção de Flebotomíneos (COLFLEB/Fiocruz-MG), Instituto René Rachou, Fundação Oswaldo Cruz Belo Horizonte, Minas Gerais 30190009 Brazil; 37 Center of Excellence in Vector Biology and Vector-Borne Disease, Department of Parasitology, Faculty of Medicine, Chulalongkorn University Bangkok 10330 Thailand; 38 Department of Arbovirology, Bernhard Nocht Institute for Tropical Medicine Bernhard Nocht Str. 74 20359 Hamburg Germany; 39 Laboratory Vectors & Parasites, Department of Livestock Sciences and Techniques, Sine Saloum University El Hadji Ibrahima Niasse (SSUEIN) Kaffrine Campus, C.P. 24600 Senegal; 40 Environmental Health Institute, National Environment Agency, Singapore 138667, Singapore & Department of Biological Sciences, National University of Singapore 117558 Singapore; 41 Institut Pasteur du Laos, Laboratory of Vector-Borne Diseases Samsenhai Road, Ban Kao-Gnot, Sisattanak District 3560 Vientiane Lao PDR; 42 National Institute of Hygiene and Epidemiology 1 Yec-Xanh Street, Hai Ba Trung District 100000 Hanoi Vietnam; 43 Indonesian Research Center for Veterinary Science, Indonesian Agency for Agricultural Research and Development, Ministry of Agriculture Republic Indonesia Bogor 16114 Indonesia Department of Parasitology, Faculty of Veterinary Medicine, Airlangga University Surabaya 60115 Indonesia; 44 Laboratory of Research in Applied Biology, Polytechnic School of Abomey-Calavi, University of Abomey-Calavi 01 P.O. Box 2009 00000 Cotonou Benin; 45 Instituto de Microbiología, Colegio de Ciencias Biológicas y Ambientales (COCIBA), Universidad San Francisco de Quito (USFQ) 170901 Quito Ecuador

**Keywords:** Mounting, Phlebotomine sand fly, Hoyer fluid, Marc-André solution, Chloral gum, Polyvinyl alcohol, Euparal^®^, Canada balsam, *Leishmania* isolation, Field conditions, Culture, Dissection, Molecular biology, MALDI-ToF, Type-specimens

## Abstract

This article provides a comprehensive guide for the processing and mounting of phlebotomine sand fly specimens, which is crucial for species identification and pathogen detection and isolation. It discusses a range of techniques suitable for both field and laboratory settings. The guide includes detailed instructions on sand fly collection, handling, covering, and euthanasia (recommending dry freezing or CO_2_ over chemicals) as well as conservation strategies, such as cold storage and preservation in ethanol. The quality of preparation of certain anatomical structures (genital organs, head and wings) is essential for their proper microscopic observation and is described in this work. The article also presents detailed sample processing, including the clearing process with agents such as potassium hydroxide then Marc-André solution. The mounting process compares different media, emphasizing their optical properties and preservation potential. Hoyer fluid (also known as chloral gum) is recommended for quick observation, particularly for spermathecae, due to its clarity, although it is not suitable for long-term storage. Other media discussed include polyvinyl alcohol, Euparal^®^ (for limited water tolerance), and Canada balsam (a hydrocarbon-soluble medium), with the latter two offering long-term preservation capabilities. Innovative molecular biology approaches such as DNA sequencing and MALDI-ToF, which require particular attention to sample processing, are also addressed. Furthermore, short video clips illustrating various mounting techniques as well as translations in many different languages are provided, allowing the guideline to reach the diverse needs and expectations of the global scientific community.

## Abstract (fr)

Cet article propose un guide complet pour le traitement et le montage des phlébotomes, étapes cruciales pour l’identification des espèces et la détection ou l’isolement des pathogènes. Il aborde diverses techniques adaptées au terrain comme au laboratoire. Le guide comprend des instructions détaillées sur la collecte, la manipulation, la protection et l’euthanasie des phlébotomes (avec recommandation de la congélation à sec ou du CO_2_ plutôt que de l’usage de produits chimiques), ainsi que sur les stratégies de conservation telles que la réfrigération et la conservation dans l’éthanol. La préparation des structures anatomiques spécifiques (organes génitaux, tête et ailes) pour l’observation microscopique est privilégiée dans la description des techniques de dissection. Le document présente également le traitement détaillé des échantillons, notamment la clarification à l’aide de solutions telles que la potasse puis le liquide de Marc-André. Le processus de montage compare différents milieux, en soulignant leurs propriétés optiques et leur potentiel de conservation. Le liquide de Hoyer (également appelé gomme au chloral) est recommandé pour l’observation rapide, en particulier pour les spermathèques, en raison de sa limpidité, bien qu’il ne convienne pas à la conservation à long terme. D’autres milieux de culture sont abordés, notamment l’alcool polyvinylique, l’Euparal^®^ (pour une tolérance limitée à l’eau) et le baume du Canada (un milieu soluble dans les hydrocarbures), ces deux derniers permettant une conservation à long terme. Les approches innovantes de biologie moléculaire, telles que le séquençage de l’ADN et la spectrométrie de masse MALDI-ToF, qui exigent une attention particulière lors du traitement des échantillons, sont également présentées. De plus, de courtes vidéos illustrant diverses techniques de montage et des traductions en de nombreuses langues sont disponibles, permettant ainsi de répondre aux besoins et attentes variés de la communauté scientifique internationale.

## Introduction

Phlebotomine sand flies are small dipteran insects belonging to the family Psychodidae, subfamily Phlebotominae with at least 1,063 known species [21]. They are important vectors of pathogens (*Leishmania*, arboviruses, and *Bartonella*) responsible for diseases called leishmaniasis, arbovirus infections, and bartonellosis, respectively. Their identification is primarily based on detailed microscopic examination facilitated by careful collection, appropriate storage, and careful slide mounting, requiring several specific techniques, each with its own advantages and limitations.

The identification of adult phlebotomine sand flies relies on the observation of both external (*e.g.*, antennae, palpi, male genitalia) and internal structures (*e.g.* pharynx, cibarium, and spermathecae). The dissection and isolation of the latter facilitate their observation and, consequently, accurate identification. Therefore, unlike mosquitoes or kissing bugs, phlebotomine sand flies require mounting between a slide and a cover slip prior to their identification.

Until the 1980s, microscopic observation was the only method available for sand fly identification, and it remains the most widely used approach today. The choice of process and preparation was therefore relatively straightforward and mainly based on a dichotomy: on the one hand, definitive mounting allowing for long-term preservation of the specimen, and on the other, rapid mounting for identification in a medium that does not ensure long-term preservation. The final mounting, for example, in a resin such as Canada balsam, is time-consuming, requiring complete dehydration of the samples. Moreover, the refractive index of this medium is not always optimal for easy observation of the spermathecae. Mounting in an aqueous medium (*e.g.*, Hoyer liquid), by contrast, is faster and allows better visualization of refractive spermathecae, but does not allow long-term preservation of the mountings because it tends to absorb water from the atmosphere. One option is to seal the slide with nail polish once it is completely dry. This trade-off choice persists today, affecting the choice of mounting method, depending on the intended purpose of the preparation. Since the 1980 s, sand fly identification studies have combined morphology and biochemical approaches. The first one was cuticular hydrocarbon analyses, which were quickly replaced by molecular biology techniques (*i.e.*, random amplified polymorphic DNA (RAPD), restriction fragment length polymorphism (RFLP), DNA amplification, and sequencing using the Sanger method, as well as the next-generation sequencing (NGS). Today, molecular approaches are complemented by proteomic methods such as MALDI-ToF. Moreover, molecular species identification can be combined with the detection of pathogens by PCR (*Leishmania*, *Trypanosoma*, *Bartonella*, and *Phlebovirus*) since all can be detected by both end-point and real-time PCR, requiring adaptation of the sampling and storage process to the assigned goals [3, 32]. In addition to morphological characteristics traditionally used for species discrimination, other morphological approaches can be applied (*i.e.*, wing geomorphometry).

Mostly based on the authors’ own experiences and literature data, the aim of this investigation was to provide standardized guidelines for mounting and processing adult phlebotomine sand flies to optimize morphological and molecular analyses.

The need to carry out certain analysis (*e.g.*, molecular biology or MALDI-ToF) requires retaining part of the sand fly that is not necessary for morphological identification, highlighting the need for critical protocol choice.

In this article, we focus on the methods of anesthesia and euthanasia of sand flies caught alive, their storage, and their mounting process, for rapid identification or for long-term conservation allowing subsequent studies.

## Preamble: Safety and regulatory considerations should reference the relevant Safety Data Sheets (SDS)

All chemicals presented in this guideline must be handled under strict safety conditions. The health and safety committees of the research facilities are available to provide you with information not only on the hazards of these chemicals, but also on their handling procedures and waste disposal. However, it is mandatory to follow the safety instructions regarding their use and disposal. Of note, it is the responsibility of all users to ensure compliance with good and safe laboratory practices and with the applicable legislation and regulations of their country or research institution. Moreover, some of the chemicals, or their components (*i.e.*, chloral hydrate) are regulated in some countries. A list of abbreviations used in this manuscript is provided in Table 1.

**Table 1:** List of abbreviations.

BME	Basal medium Eagle
CDC	Centers for Disease Control and Prevention
CMCP	Camphor-monochlorophenol
CMR	Carcinogenic, mutagenic, reprotoxic substance
COI	Cytochrome c oxidase subunit I gene
CytB	Cytochrome b gene
DNA	Deoxyribonucleic acid
ELISA	Enzyme-linked immunosorbent assay
EtOH	Ethanol
M199	Medium 199
MALDI-ToF MS	Matrix-assisted laser desorption/ionization time-of-flight mass spectrometry
MEM	Minimum essential media
NGS	Next-generation sequencing
NNN	Novy-MacNeal-Nicolle medium
PCR	Polymerase chain reaction
Lao PDR	Lao People’s Democratic Republic
PNOC	Prepronociceptin gene
qPCR	Quantitative PCR (real-time PCR)
RAPD	Random amplified polymorphic DNA
RFLP	Restriction fragment length polymorphism
RI	Refractive index
RNA	Ribonucleic acid
RNases	Ribonucleases
RNASS	RNA stabilization solution
RT-PCR	Reverse transcription PCR
TFA	Trifluoroacetic acid

## 1 Sand fly capture

Adult sand flies can be collected alive or dead using various methods such as CDC miniature light traps, sticky traps, and aspirators using Shannon traps, or directly from resting places in the environment (*e.g.*, animal shelters). These methods involve placing traps in suitable habitats, attracting sand flies with light or other attractants (CO_2_ or chemical lures), and collecting them for further analysis, as described in several publications [2, 3, 32, 36, 49].

Capturing live sand flies allows all downstream applications, whereas collecting dead individuals prevents the isolation of *Leishmania* or virus strains. Some capture techniques, such as sticky papers regularly result in the loss of sand fly organs (antennae, palps, wings, or legs). In addition, the castor oil coating sticky papers adheres to the sand flies and must be removed at the beginning of processing, typically using a 15-minute bath in a mixture of ethanol and diethyl ether in equal parts.

## 2 Specimen euthanasia

After collection, living sand flies must be euthanized. With some collection methods (*e.g.*, sticky papers, CDC light traps equipped with a jar containing detergent or ethanol) sand flies are dead upon collection. Molecular biology can be applied to those collected directly into ethanol and on the others if they are stored in ethanol as rapidly as possible. However, none of these killing methods allow for insect processing by MALDI-ToF. Additionally, some killing methods may cause the loss of certain morphological characters. It is therefore essential to use an appropriate standard killing agent to ensure proper identification or long-term storage as voucher specimens (*i.e.*, specimens preserved and stored for future reference or comparison). Chemicals such as ethyl acetate, ethyl ether, tetrachloroethane, and chloroform can soak into cotton wool and be placed in a recipient containing the sand flies to euthanize. These killing agents should be handled carefully, following the manufacturer’s recommendations due to their toxicity. However, we do not recommend killing sand flies using chloroform, as it is, in our experience, poorly compatible with molecular biology studies. Given the hazardous nature of all these products and their questionable suitability for molecular analyses, the use of these chemicals is generally discouraged.

The most widely used method, which preserves morphology, DNA or proteins, is dry freezing the specimens. Specimens must be frozen long enough to be fully anesthetized, but not so long that they (i) dry out, or (ii) are compromised concerning *Leishmania* viability, if the aim is to isolate them *in vitro* from the sand fly digestive tract. We therefore recommend a freezing duration of 15–20 min at −20 °C, monitoring them regularly to ensure they are only stunned without killing the *Leishmania* parasites.

If a freezer is unavailable, insects can alternatively be euthanized using CO_2_. In field conditions where CO_2_ cylinders cannot be used, specimens can be killed using small commercial CO_2_ containers used in “Soda siphons” (drink dispensers), but there may be restrictions on their transport by air. As a last resort, insects can be killed by exposure to tobacco smoke. Sand flies are captured alive in a CDC trap, collected with an aspirator, retained in the glass tube, and exposed to tobacco smoke which kills them within seconds. This method is applicable in all field conditions, even in difficult isolation conditions. However, because the glass becomes impregnated with smoke, it cannot be used for subsequent collection and handling of live sand flies without thorough cleaning. Nevertheless, the same uncleaned aspirator can still be used for euthanizing sand flies from other traps for fixation purposes. It is also necessary to check whether all specimens have been removed from the aspirator. These methods are compatible with the isolation of *Leishmania via* gut dissection.

## 3 Specimen storage before processing

There are five main methods of fixation prior to processing:

### 3.1 Freezing

This method is best performed at −20 °C or, preferably, at −80 °C. These storage methods are now more widely used than liquid nitrogen storage. In all cases, cryopreservation must be implemented as quickly as possible after stunning the specimens. Cold storage in freezers offers the advantage of fully preserving the insects themselves, as well as RNA, DNA, and proteins with full integrity throughout the storage period. Instead, liquid nitrogen can severely damage the wings, legs, palps, and antennae, often amputating them and occasionally removing key morphological characters. Dry freezer storage is less traumatic for specimens, but is not ideal for preserving their fragile organs. Importantly, at the time of thawing, wings, antennae, palps, or legs may adhere to the vials and eventually tear off due to condensation. However, preservation by freezing is not always feasible in field studies because it requires access to a freezer or a liquid nitrogen container. Freezer storage is fully compatible with pathogen detection using molecular tools without loss of sensitivity, although RNA virus detection and isolation requires freezing at −80 °C or in liquid nitrogen if long-term storage is required. However, freezing samples does not allow for isolation of *Leishmania via* gut dissection, except if sand flies are first immersed in the vapor phase and then in liquid nitrogen (for example in vials placed in a stocking), simulating the cryopreservation of *Leishmania*.

### 3.2 Storage in alcohol (ethanol or isopropyl alcohol)

**Figure 1:**
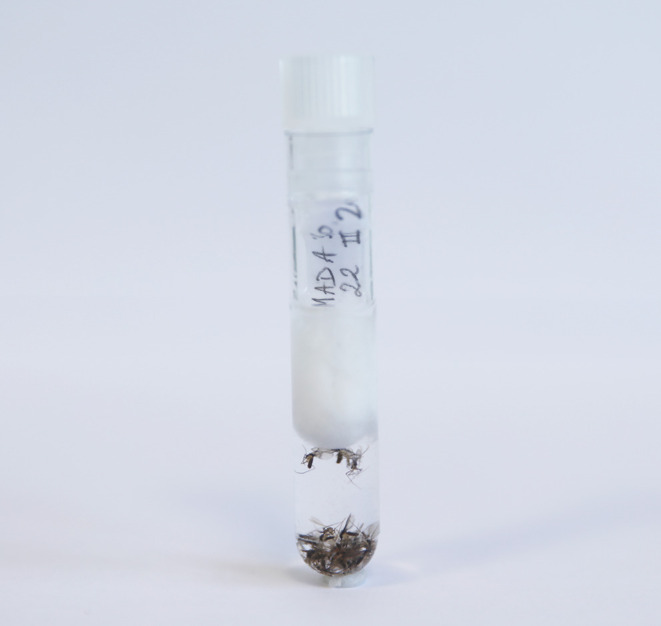
Sand flies preserved in ethanol.

This is probably the most widely used method for storing sand flies. It is easy to implement in the field, even in difficult conditions without access to a laboratory. Preservation in alcohol is particularly suitable for morphological studies, as the fragile organs (wings, legs, antennae, or palps) remain intact, if there are no air bubbles in the storage tube. Therefore, we recommend sealing the tube with a small cotton ball to remove any air bubbles and placing a label on top of the cotton plug (Fig. 1). The appropriate alcohol concentration remains a matter of debate. Generally, concentrations below 70% are not recommended [45, 66]. Higher concentrations preserve DNA more effectively and for longer periods but make the specimens more fragile and brittle for morphological studies. The use of 96% ethanol (the azeotrope mixture) ensures concentration stability over time, particularly in humid areas such as tropical countries, although 95% ethanol is often easier to obtain. Regardless of concentration, DNA is generally well preserved in ethanol (though less effectively than with freezing methods, particularly for NGS-type molecular methods). Proteins are much less stable, especially for proteomics, like MALDI-ToF applications. Sand flies preserved in alcohol for a few months can still be identified morphologically, but it is impossible to generate reference protein spectra from these specimens. Storage in alcohol or dry conditions can be improved if the sample is also frozen at −20 °C. Freezing at −20 °C mainly improves molecular preservation (*e.g.*, nucleic acids) by slowing degradation and also provides a secondary benefit for morphological preservation by reducing tissue breakdown over time, even though the effect on morphology is more limited than on molecular integrity. Storage in ethanol can also be applied for DNA and RNA virus detection when using ethanol at a concentration of at least 70% for a short storage period, less than a few months. Also, isopropyl alcohol may be readily available in some countries and preserves DNA, but does make the specimens stiff. It is not flammable like ethanol and can therefore be easily transported. If necessary, sand flies preserved in liquid nitrogen or dry-frozen can be transferred to alcohol, thereby combining the drawbacks of both methods.

### 3.3 Storage in RNA stabilization solution (RNASS)

This aqueous reagent is widely used, non-toxic, and designed to stabilize and protect RNA in fresh tissue and cell samples. It acts by rapidly penetrating the specimen and inactivating RNases (RNA-degrading enzymes), thereby preventing RNA degradation without the need for immediate freezing. Storage in RNASS is generally effective at preserving overall tissue and cellular morphology for downstream histological assessment. While RNASS is optimized for RNA stabilization rather than fixation, short- to medium-term storage typically maintains structural integrity well. RNASS allows samples to be stored at room temperature for up to 7 days, at 4 °C for several weeks, or at −20 °C/−80 °C for long-term preservation. This method is particularly valuable in fieldwork or clinical settings where cold chain infrastructure is limited. RNA extraction usually requires removing the samples from the reagent and processing them according to standard protocols.

### 3.4 Dry preservation at room temperature

This is an older method that, when applied to an *in toto* specimen (mounted whole), has the major disadvantage of poorly preserving fragile organs such as the wings, legs, antennae, and palps. However, proteomic studies using MALDI-ToF remain feasible if dehydration is carried out upon fixation with a silica gel-type desiccant. In contrast, molecular analyses targeting DNA remain difficult to perform on these samples, because DNA is often still fragmented and low in quantity, meaning analyses remain more challenging than with fresh or frozen samples, especially for nuclear genomes. However, recent techniques such as museomics could be used on samples of this type [34]. Therefore, this storage method is not recommended, unless no alternative is available. It can be combined with cold storage by placing the tubes in a freezer at −20 °C or −80 °C. The main challenge is achieving appropriate mounting of the specimens or the body parts necessary for identification. To achieve this, rehydration is essential. We recommend using a solution of Triton X-100. The duration of rehydration varies from a few hours to several days, under regular close monitoring. After complete rehydration, the specimens should be rinsed in three consecutive water baths.

### 3.5 Preservation on filter papers

The main advantage of filter papers is the long-term stability of genomic DNA within the cells of unfixed, dried whole body, or blood cells stored at room temperature. The filter paper is provided in a small card size, which makes it possible to store several hundred samples at room temperature in a volume the size of a small box. The filter paper matrix is impregnated with agents that denature infectious agents, and thus samples are no longer considered a biohazard. This allows for the storage and transport of samples without selective biohazard precautions [68].

## 4 Specimen dissection

**Figure 2:**
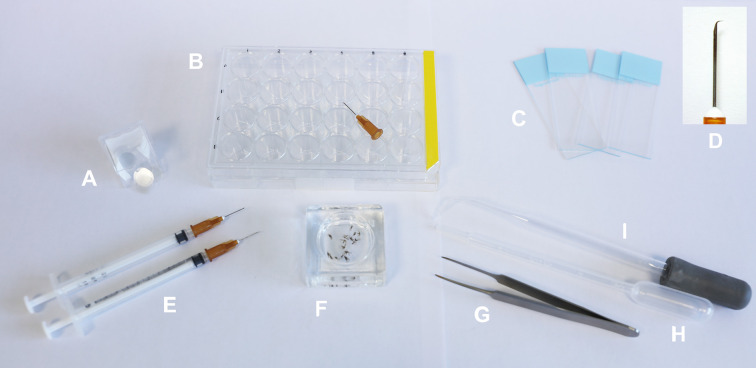
Materials required for mounting sand flies: A: round glass coverslips (10 or 12 mm diameter); B: 24-well plate and hooked needle (if using clove oil or Euparal essence to process the sand flies, do not use acrylic plates because a chemical reaction will take place and the specimens will be damaged); C: glass slides suitable for labeling; D: detail of the needle hook; E: needles attached to syringes; F: watch glass or equivalent container holding the sand flies to be mounted; G: Dumont forceps; H: plastic pipette; I: glass pipette bent by heating to facilitate transferring liquid into the wells.

**Figure 3:**
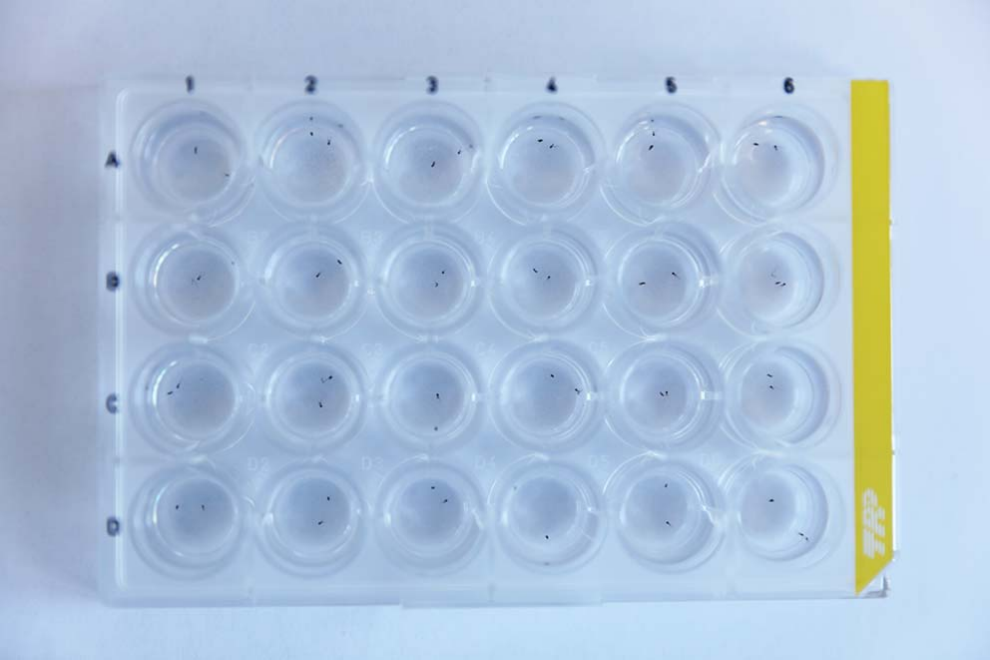
A plate with 24 wells, each containing the head and tip of the abdomen of sand flies.

Unlike many other insects, which are identified based on external characters observable on individual insects pinned *in toto*, sand flies require dissection and slide-mounting to study anatomical features for accurate identification of the species. Regardless of the chosen preparation and mounting procedure, the same dissection technique is employed (Figs. 2 and 3) (https://zenodo.org/records/18198006).

### Use of Triton X-100: non-ionic aqueous solution

Note that the mounting concerns freshly caught or adequately stored specimens. Most collectors have insect samples preserved dry (for MALDI-ToF use) or stored in alcohol for many years. Unfortunately, preservation in alcohol is not optimal over periods of years, and arthropods preserved in this way become very difficult to prepare for microscopic examination. An incident that often occurs is the degradation of plastics containing samples, followed by the evaporation of alcohol. In both cases, we have no options because the samples remain too long in alcohol or dry out. The idea therefore arose to use wetting agents that are not strong detergents. Triton X-100 is in the form of a non-ionic aqueous solution (*4-(1,1,3,3-tetramethylbutyl) phenyl-polyethylene glycol solution, or t-octylphenoxypolyethoxyethanol, polyethylene glycol tert-octylphenyl ether*), widely used as a detergent in cellular and molecular biology. It enables permeabilization of cell and nuclear membranes.

Below is a procedure using non-ionic Triton X-100 in 0.5% aqueous solution:

- Impregnate the dry sample with absolute alcohol.
- Add the necessary volume of Triton X-100 solution at 0.5% so that the entire sample is immersed.
- Allow the process to run for about 5 minutes to several days, monitoring regularly. All arthropods must become fully separated in the solution.
- Remove the Triton X-100 solution and replace with potassium hydroxide solution.

### 4.1 Head

Dissection can be performed using fine needles or entomological pins under a stereomicroscope (Figs. 2 and 3). The most commonly used needles include: 26G × 1/2″ (0.45 × 13 mm), 30G × 1/2″ (0.3 × 13 mm), or 25G × 5/8″ (0.5 × 16 mm). To prepare the specimen for identification, at a minimum, the head is separated from the body and mounted ventral side up to display the cibarium and pharynx, while the thorax and abdomen are mounted laterally following dissection. Mounting the head in a ventro-dorsal position ensures the occipital foramen is oriented upwards, so the cibarium can be observed directly. Access to these anatomical features is made easier if the head is fully separated.

### 4.2 Wings and thorax

The wings must be mounted flat. Each wing can be detached at its base and mounted independently, or one can be mounted alone, while leaving the other attached to the thorax. If geometric morphometry analysis is planned, it is essential to correctly identify and label the right and left wings before mounting. The thorax is divided into several parts, and each contains very important taxonomic information [20, 64]. Generally, it is mounted in a lateral view, to allow examination of chetotaxy and color distribution. The presence of scars of bristles in certain regions of the thorax can be used to distinguish some species of the genus *Brumptomyia*. The color distribution may be used to separate Neotropical sand flies at the genus level (*e.g.*, *Bichromomyia*), species series (*e.g.*, *Pintomyia*), or even species of the same genus (*e.g.*, *Micropygomyia*, *Nyssomyia*, *Psathyromyia*, and *Psychodopygus*) [20]. Thus, if the thorax is not used for molecular analysis, it should be mounted so as not to damage it. Importantly, it should be noted that it is not the intensity of the colors that matters, but their distribution across the thorax. Therefore, the clarification process will not eliminate the pigmentation or its pattern.

### 4.3 Genitalia

Particular care must be taken when mounting the genitalia in both males and females, as they are crucial for identifying genera, subgenera, and species. In both sexes, the genitalia are paired.

#### 4.3.1 Males

The genitalia are external and consist of paired forceps, each consisting of the gonocoxite-gonostyle articulation in its dorsal part and the epandrial lobe in its ventral part. The gonostyle bears spines and sometimes setae, which must be countable and whose insertion positions must be clearly visible. It is important to carefully observe the inner surface of the gonocoxite, which may bear a tuft of sessile setae or those carried by a lobe (= tubercle) [22]. Colleagues with less experience in dissections may perform a simple lateral mounting, without detaching the genitalia from the end of the abdomen (https://zenodo.org/records/18311158). In this case, the superposition of the two parts of the genitalia may make it difficult to count the internal setae of the gonocoxite, for example, but this avoids damaging the genitalia through a failed dissection. More experienced colleagues may attempt to open the genitalia in two, to split them. To achieve this, the beveled side of a needle (intradermal reaction needle type) must be passed through, detaching without completely cutting the genitalia to split the gonocoxite-gonostyle assemblies (https://zenodo.org/records/18311158). In this way, observation of their internal faces will be easy. This assembly also facilitates the observation of the parameres and parameral sheaths, which no longer overlap. For lateral mounting, which promotes organ superposition, the specimens must be perfectly cleared.

#### 4.3.2 Females

**Figure 4:**
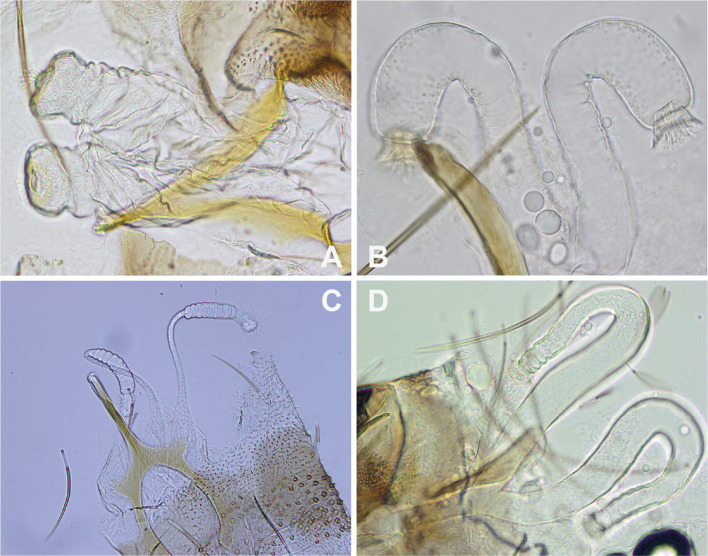
Spermathecae dissected and mounted in Marc-André fluid from fresh specimens. A: *Idiophlebotomus longiforceps* (Lao PDR); B: *Sergentomyia minuta* (France); C: *Phlebotomus ariasi* (France); D: *Sergentomyia anodontis* (Lao PDR).

The genital apparatus is internal, constituted by spermathecae. In the absence of dissection, they must be observed through the teguments and mounting the abdomen in ventral position. Regardless of the mounting medium chosen, the spermathecae itself can generally be observed correctly, especially if it is not smooth and cleared. However, observing smooth, thin-walled spermathecae can be problematic in poorly refractile media. Furthermore, observing the base of the spermathecal ducts is essential for species identification, such as in the subenus *Larroussius* [35, 37, 38], the main vectors of *Leishmania infantum* in the Old World. Without this observation, specimen identification remains impossible. To overcome these observation difficulties, the genital furca-spermathecae mounting should be removed from the abdomen (https://zenodo.org/records/18311106). Spermathecae are generally difficult to observe during dissection, but the genital furca is relatively easy to locate. Since the spermathecal ducts open into the genital furca, isolating this furca normally allows for the isolation of the spermathecae. If the spermathecae are accidentally cut during the process, they are not lost and can still be observed within the abdominal integuments (Fig. 4).

### 4.4 Midgut dissection for *Leishmania* isolation

Dissection of the digestive tract is essential for detecting and isolating *Leishmania* in female sand flies. The procedure can be performed in both field and laboratory settings, to assess vectorial competence.

It is recommended to work on freshly euthanized females. Wash the females with water or saline solution containing a mild detergent to remove excess hairs. This step helps maintain aseptic conditions for *Leishmania* isolation, while preserving the morphological features needed for identification. To find and isolate *Leishmania*, carefully remove the midgut and place it in a single drop of sterile saline solution (0.9% NaCl). After observing motile parasites under a light microscope (recommended magnification: ~200×), use an insulin syringe or micropipette to transfer them into the cultivation medium (for more details see Sect. 4.4.3).

Mount the head and genitalia directly in Marc-André fluid to clear them. Important: never allow Marc-André fluid to contact *Leishmania* – neither directly nor indirectly *via* tools or needles – as it is lethal to the parasites.

**Figure 5:**
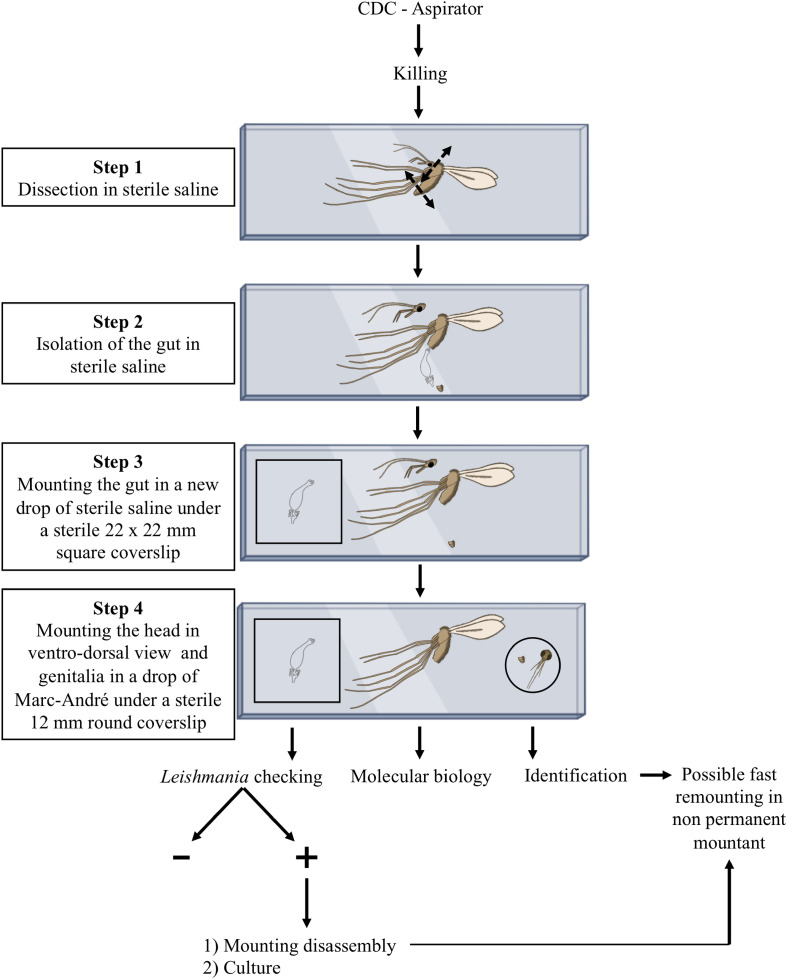
Method for *Leishmania* isolation.

Dissection of female sand flies can be performed on either one slide or two; both options have advantages and limitations (Fig. 5; https://zenodo.org/records/18311154).

#### 4.4.1 Two-slides method

The first option involves working on two separate slides: one containing sterile saline to extract the midgut, and the other to mount the head and spermathecae in Marc-André fluid. However, in field conditions, it is common for two or three people to dissect sand flies and transmit their dissections to a single researcher responsible for species identification and assessment of *Leishmania* infection in the gut. Managing two slides can cause issues with sample traceability and, in particular, make it difficult to determine with certainty which individual sand fly was infected, if a positive gut is detected (https://zenodo.org/records/18311154).

#### 4.4.2 Single-slide method

Using a single slide ensures traceability of the results. However, several precautions should be taken. To maximize sterility during this step, operators must regularly clean their hands with hydroalcoholic gel. Non-frosted slides and square coverslips (22 × 22 mm) wrapped in aluminium foil and sterilized by dry heat (using a Poupinel oven) must be used, along with sterile needles for each dissection (suggestion: 25G Ø 0.5 mm × 16 mm). The sand fly is placed in a drop of sterile saline in the middle of the slide. The head is severed while an incision is made between the 6th and 7th abdominal tergites and sternites without cutting the digestive tract (a higher cut can be made if very long spermathecae are expected). Then, the thorax must be immobilized with a needle, and the last posterior abdominal segments pulled gently with the other needle to extract the gut. If this fails, there is the possibility of blocking the end of the abdomen with a needle and pulling the digestive tract from its anterior part. If this fails again, the gut must be extracted by removing as much of the remaining tegument around it as possible. When the gut is removed, the last abdominal segments must then be separated by cutting the digestive tract. The gut is then placed in a new drop of sterile saline positioned on one side of the slide, and then gently covered with a sterile coverslip. The head and the last abdominal segments are transferred to a small drop of Marc-André liquid placed at the other end of the slide, ensuring no contact with *Leishmania*. The head is correctly oriented (occipital foramen upwards), and the spermathecae are isolated with the genital furca as indicated above and covered with a small round coverslip (Ø 12 mm, not to be confused with the sterile square coverslips). The remaining sand fly carcass and wings stay in the drop of saline in the middle of the slide (https://zenodo.org/records/18311154). In case of positivity, or for taxonomic exploration, thorax and abdomen can be preserved for molecular or proteomic studies, and the wings can be mounted in an aqueous medium. To preserve the mount, the extra volume of Marc-André liquid can be replaced with an aqueous mounting medium such as chloral gum (=Hoyer) or a polyvinyl alcohol-based medium.

Detailed videos demonstrating these procedures are available (sand fly midgut dissection: https://zenodo.org/records/18303014 and sand fly salivary glands dissection: https://zenodo.org/records/18302850), so they will not be explained here.

#### 4.4.3 *Leishmania* parasite isolation and culture from sand fly guts

Parasite isolation from the dissection of infected female sand flies is a delicate procedure requiring high skill and should initially be practiced first on parasite-free specimens. After dissection, the guts are transferred to a fresh drop of sterile saline (0.9%) or Locke’s solution for washing [4]. The dissected guts can then be processed in two ways: i) examined under a light microscope to observe the different stages of *Leishmania* promastigotes and their localization, with particular attention to the stomodeal valve, and ii) open the gut to facilitate exit of the promastigotes facilitating their mass culture [4]. Finding infective sand flies in the field is a relatively rare occurrence and therefore good practice sessions will maximize the chances of successful isolation.

If *Leishmania* parasites are observed in the gut, new sterile needles should be used and a small amount of sterile saline added around the coverslip by capillary action to release them. The gut should be carefully and quickly torn to release the parasites into the saline. Using a 100 μL micropipette or tuberculin syringe, collect the parasites and inoculate them into a properly labelled culture medium.

*In vitro* culture of *Leishmania* promastigotes: isolated parasites are initially maintained on SNB-9 blood agar slopes or in Novy, Mc Neal, Nicolle (NNN) solid medium [16] overlaid either with sterile alpha-MEM medium [16, 65] or with M199 medium, each supplemented with 10% heat-inactivated sterile fetal calf serum [FCS] (to enhance parasite growth), 1% BME vitamins, 2% sterile human urine (sterilized using a syringe filter Filtropur^®^ S 0.2 μm), 250 μg/mL amikacin (or 50 μg/mL gentamicin, or a mixture of antibiotics and amino acids (L-glutamine 200 mM-penicillin 10 000 U-streptomycin 10 mg/mL) [47]. After three days, if there is no contamination, the cultures are suspended in a properly prepared freezing medium and subsequently stored at −80 °C for 1–2 years or in liquid nitrogen at −196 °C for long-term preservation and future experimental use [7].

### 4.5 Salivary glands

Dissecting the salivary glands of sand flies is a fundamental technique for studying vector-pathogen interactions, particularly for detecting arboviruses like *Phlebovirus* (*e.g.*, Toscana virus) [44, 75]. Due to the minute size of sand flies, the procedure requires precision under a stereomicroscope, using fine forceps or microdissection needles to isolate the delicate salivary glands without causing rupture or contamination (https://zenodo.org/records/18302850) [51, 61]. Preserving gland integrity is crucial to ensure reliable downstream molecular analysis. Once extracted, the glands can be homogenized and tested *via* RT-PCR, qPCR, or immunoassays to detect viral RNA or antigens [12]. The presence of viruses in salivary glands, rather than just the gut or hemocoel, confirms that the pathogen has completed its extrinsic incubation period and is transmissible during blood feeding [71].

The dissection process is technically demanding due to the small size of sand fly salivary glands, requiring significant expertise to avoid sample degradation [1, 51]. Additionally, viral loads may be low, necessitating highly sensitive detection methods such as nested PCR or high-throughput sequencing [54]. Contamination risks further underscore the need for sterile techniques. Beyond technical hurdles, biological factors influence detection success; vector competence varies among sand fly species, and infection rates fluctuate with ecological and seasonal conditions [33, 61].

Detecting viruses in salivary glands provides critical insights into transmission risks, enabling targeted surveillance and control measures [15]. For example, identifying Toscana virus in sand flies in endemic regions has informed diagnostic protocols and public health advisories [18]. Moreover, studying virus-saliva interactions could reveal novel targets for transmission-blocking vaccines or therapeutics [15, 18].

Sand fly salivary glands can also be used as a source of antigens for measuring host antibodies against sand fly saliva using immunological methods, preferably ELISA. This method enables assessment of host exposure to sand fly bites, thus supporting evaluation of the effectiveness of vector control methods [25] and the risk of *Leishmania* transmission [40].

### 4.6 Blood meal identification

**Figure 6:**
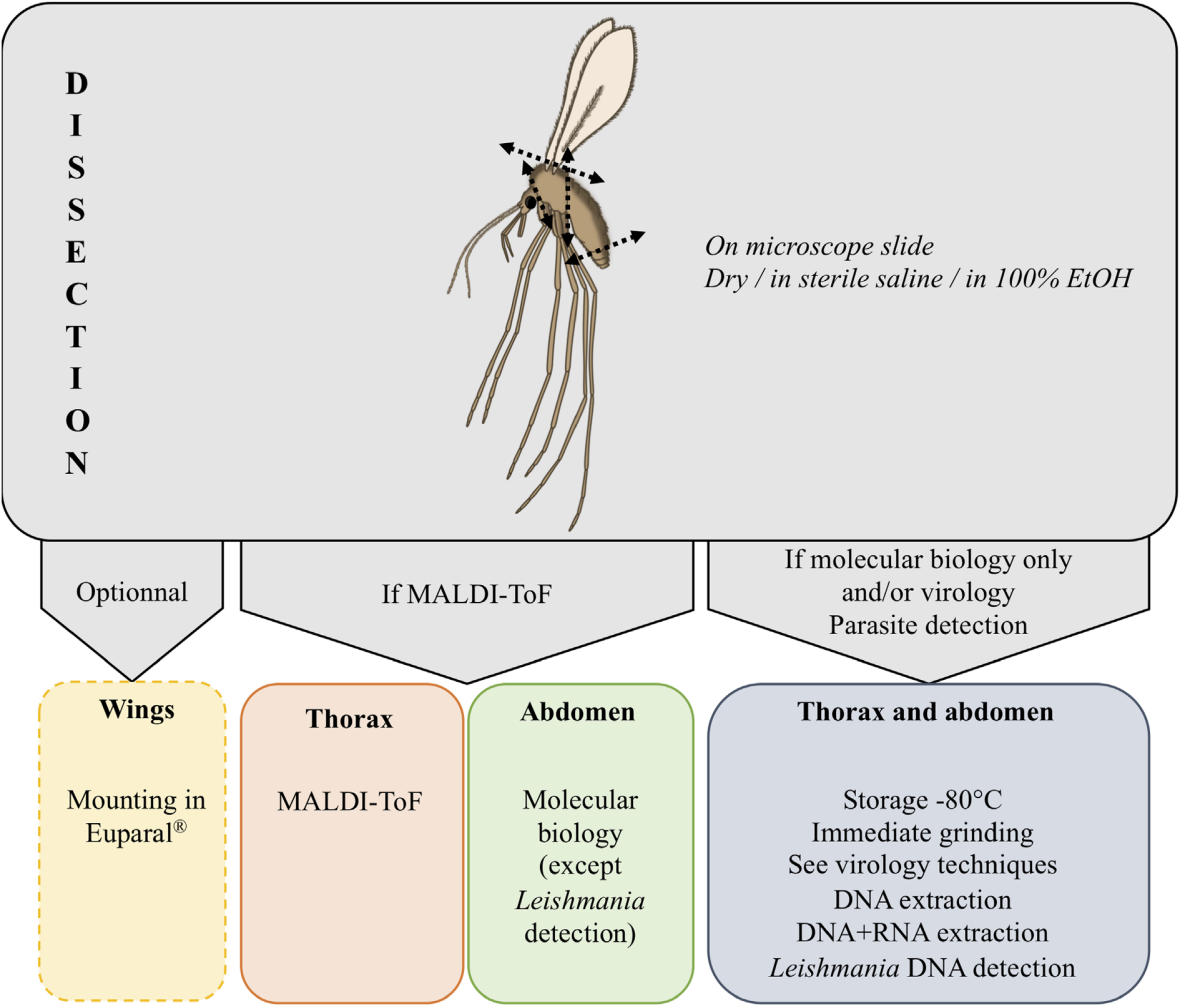
Sand fly processing for molecular biology, proteomics, and/or virology applications.

The engorged females isolated from the captures should be dissected using single-use equipment to prevent cross-contamination. Their abdomen should be examined under a stereomicroscope to assess the stage of blood meal digestion. It is recommended to select only females with a red, reddish-brown, or dark red abdomen, showing no signs of egg formation. Remove the tip of the abdomen including the spermathecae to identify the female morphologically after clearing. The main part of the abdomen (without spermathecae) should then be placed in Eppendorf^®^ tubes and stored at −20 °C until further analysis. The genetic markers commonly used for blood meal identification, such as PNOC [5, 30, 50], CytB [67], or COI [13], are well established and extensively described in the literature; therefore, they will not be further detailed in this paper (Fig. 6). Alternatively, to identify host blood, MALDI-ToF peptide mapping may be deployed [31]. It has been demonstrated experimentally that this technique enables host blood identification within a longer time frame after the blood meal uptake; therefore, it is a suitable method of choice, especially for the analysis of engorged females with visibly more advanced host blood digestion. The samples should ideally be stored at −20 °C or 4 °C, but good results may also be obtained from samples stored at room temperature for a short time. The abdomen of an engorged female should be dissected from the rest of the body shortly prior to the analysis and homogenized in distilled water. The rest of the sand fly body remains available for other molecular and morphological analyses. After the aliquot is taken from the homogenate for MALDI-ToF peptide mapping, the rest may be used for DNA isolation to confirm the host blood identification and/or screen for the presence of *Leishmania* sp. The overall time of sample preparation and analysis is very short when compared to DNA-based molecular techniques.

**Figure 7:**
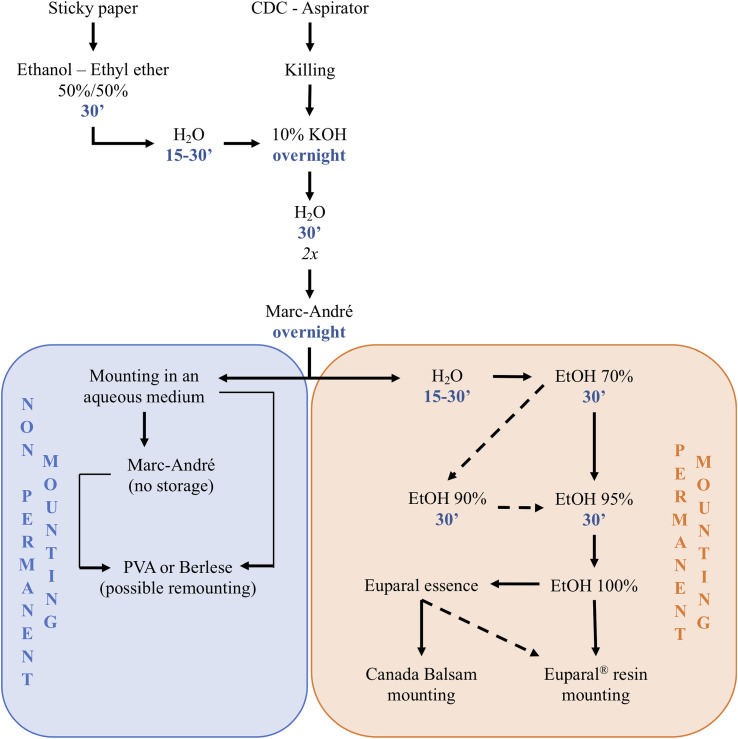
Classical method to process sand flies.

## 5 Specimen processing for morphological studies (Figs. 3, 6–8; Appendices A–D)

This section outlines the principles for preparing a sand fly specimen for mounting solely for morphological studies, followed by adaptation for applications beyond morphology. However, understanding this methodology is crucial, as it enables procedures to be adapted for specific sample types when necessary.

**Figure 8:**
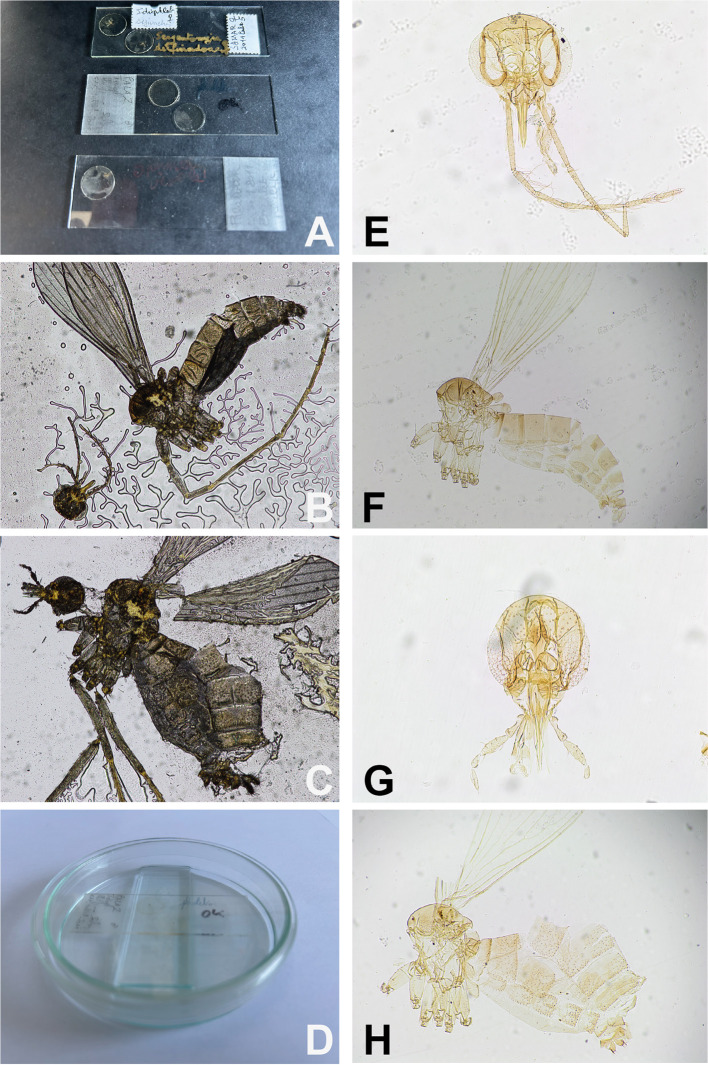
Slide remounting. A: damaged and dried slides mounted in Hoyer; B: microscopic view of a dried sand fly; C: microscopic view of another damaged sand fly; D: wet chamber containing a dried slide; E: head, and F: body of specimen B after its remounting in Euparal^®^; G: head, and H: body of damaged specimen C after its remounting in Euparal^®^.

The treatment involves successive emptying and filling steps using Pasteur pipettes equipped with flexible rubber bulbs. Round-bottomed glass containers are strongly recommended because they greatly facilitate these operations. Glass is inert to all reagents. To prevent reagent evaporation, containers should be fitted with lids and never overfilled, leading to overflow when closing or opening, and to prevent dust from falling on the samples. Chemicals required for clearing and processing are shown in Table 2.

**Table 2:** Composition of reagents used.

Potassium hydroxide 10%	Fuchsin acid 1% in distilled water
Potassium hydroxide 10 g	Acid fuchsin (as a powder) 1 g
Distilled water *qs* 100 mL	Distilled water 99 mL
	
Gum chloral mounting media (Hoyer medium)	Marc-André solution colored with acid fuchsin
Distilled water 50 mL	Marc-André solution 10mL
Chloral hydrate 200 g	Fuchsin 1% 50 μL
Gum Arabic 50 g	
Glycerol 20 mL	
	
Marc-André solution	Enecê medium
Chloral hydrate 40 g	Pure white colophony 22 g
Glacial acetic acid 30 mL	Alcohol-soluble copal gum 12 g
Distilled water 30 mL	Absolute ethanol 20 mL
	Camphor 10 g
	Turpentine essence 10 mL
	Eucalyptol 26 mL

### 5.1 Clearing

Before sand fly specimens can be prepared as permanent slide mounts, they must first be cleared by maceration using an appropriate method and clearing agent (*i.e.*, acetic acid 10% solution or Marc-André solution including chloral hydrate, which is a restricted chemical in many countries) to render them transparent. The clearing process removes body tissues, fat, secretions, and wax, making the specimen translucent, facilitating the examination of exoskeletal structures (*e.g.*, setae insertion), surface characteristics (*e.g.*, coloration) and internal features visible through the tegument (*e.g.*, spermathecae).

The two-step clearing process, which involves first using a strong base (such as potassium hydroxide), followed by a weak acid (such as acetic acid in Marc-André solution), serves distinct biochemical purposes [74]. The base breaks down soft tissues, such as proteins, fats, and muscles, through saponification and protein denaturation, leaving the chitin exoskeleton intact for structural clarity. The subsequent weak acid neutralizes any remaining alkali, preventing further degradation, and bleaches the chitin to enhance transparency [74], although washing the specimens twice in distilled water for 15 min may also be sufficient to neutralize the base. This sequential treatment combines effective tissue removal with gentle preservation, ensuring optimal specimen integrity for microscopic examination.

Two 20-minute rinses in distilled water are recommended before proceeding to the next step.

#### 5.1.1 Soft tissue lysis (Fig. 8)

Sodium hydroxide (NaOH) or potassium hydroxide (KOH) are commonly used chemical macerating agents, applied at varying concentrations and duration depending on the size and fragility of the specimens. The standard and most effective technique involves lysing soft tissues by soaking sand flies in a strong base (10% KOH or NaOH) overnight. The concentration may be increased to reduce the treatment duration (*i.e.*, KOH 20% for 6 hours) as well as heating at 37 °C.

#### 5.1.2 Clarification with or without staining

This step is followed by a lightening treatment, typically combining acetic acid and chloral hydrate (*e.g.*, Marc-André solution). After clearing, the specimens must be thoroughly rinsed in at least two successive water baths of 20 min each to remove residual chemicals.

Marc-André solution is a commonly used clearing agent for preparing sand fly specimens. Its effectiveness lies in facilitating the clearing process while minimizing significant damage to the fragile structures, such as wings and antennae.

The solution should be freshly prepared or stored in a tightly sealed container to prevent evaporation or degradation. The use of Marc-André solution is particularly advantageous when combined with brightening or staining techniques to enhance specific morphological details. Details of its composition and preparation are given in Appendix B.

For highly translucent specimens, staining may be necessary to improve visibility prior to mounting. Many stains are available, each targeting specific chemical components of the organism. It is important to select a stain that is compatible with both the specimen and the chosen mounting medium. This basic methodology can be adapted as needed, for example, by incorporating 0.1% acid fuchsin into Marc-André solution for staining. Additionally, specimens preserved in aqueous solutions and intended for resinous mounts require dehydration (see Sect. 5.2 on Dehydration), as most natural and synthetic resin mounting media are incompatible with water. New (1974) noted that some stains can deteriorate in certain mounting media [53]. For example, acid fuchsin, commonly used with Canada balsam, can also be fixed in Euparal^®^. However, acid fuchsin-stained specimens are prone to fading, particularly when remnants of clove oil, used as a final clearing fluid, remain. Specimens stored in clove oil may exhibit significant fading within a few days.

### 5.2 Dehydration

Dehydration is carried out by gradually transferring the samples through a graded ethanol solution series: 50%, 70%, 80%, 90%, or 95% and finally 100%, with each bath lasting at least 20 min. Since ethanol evaporates quickly, the container should be tightly sealed during processing. Once the specimen is completely dehydrated you can pause processing for a few days in Euparal^®^ essence, which is preferable to clove oil. Beech creosote, once widely used for this purpose, is now completely banned due to its toxicity.

The dehydration process must ensure that the fluid within the specimen is compatible with the mounting medium to prevent opacity, osmotic collapse, or distortion that could render the specimen unsuitable for taxonomic study.

### 5.3 Mounting media

#### 5.3.1 Selection and application for specimen preparation

The mounting medium should ideally have a refractive index as close as possible to that of glass, which is approximately 1.5. It must be colorless, clear, and remain perfectly transparent after drying and over time. It must be compatible with the stains used and capable of penetrating and diffusing into all tissues of the specimen. It must not dry too quickly or form a haze during mounting. It must not shrink after mounting. The selection of an appropriate mounting medium is a fundamental aspect of specimen preparation, as no single medium is ideal for all purposes. The choice should balance several key factors:

- *Optical properties*. The refractive index of the mounting medium should provide sufficient contrast and refraction of critical anatomical features used for taxonomic identification or morphological description, such as spermathecae, ascoids, Newstead sensilla, vertical cibarial teeth, and pharyngeal teeth. The visibility of these structures directly depends on the optical properties of the mounting medium.
- *Preservation*. For type specimens or materials intended for permanent collections, the medium must offer long-lasting stability and durability. In contrast, for inventory studies or epidemiological surveys, where long-term preservation is less critical, temporary or semi-permanent mounting media may be sufficient.

#### 5.3.2 Requirements for mounting media

Specialists often develop customized and complex mounting techniques tailored to specific research needs. However, these methods frequently overlook aspects such as archival quality, compatibility, standardization, or ease of handling and long-term preservation. This lack of standardization complicates the integration of donated collections and long-term curation efforts.

Scientific applications impose distinct requirements for mounting media. Taxonomists often mount whole specimens and prefer media that gently macerate inner organs to enhance the visibility of cuticular structures. The refractive index should differ sufficiently from that of the specimen and the glass slide to maximize optical clarity. Commercial mounting media are usually formulated with a refractive index close to that of glass to minimize light refraction and scattering through the slide–mounting medium–coverslip system. However, in brightfield microscopy, the natural contrast of an unstained specimen can be manipulated by deliberately choosing a mounting medium with a refractive index slightly different from that of the specimen, thereby improving its visibility against the background.

#### 5.3.3 Types of mounting media (Tables 3 and 4)

Microscopy requires the refractive index (RI) of a mounting medium to determine how light bends through the slide, medium, and specimen. When the RI is closely aligned with the coverslip glass (≈1.515), light passes uniformly, decreasing scattering and optical distortions, resulting in improved resolution and visibility of fine structures. Conversely, an RI mismatch can cause blurring, halos, or obscure unstained features. The selection of the right mounting medium is crucial to optimize contrast, clarity, and overall image quality for a given specimen due to the distinct RIs of different media.

**Table 3:** Composition of selected mounting media.

Mounting medium	Solvent	Potential pre-polymer(s) or polymer	Remarks
Hoyer = chloral gum	Glycerol, water	Compounds of gum arabic	Macerating agent: chloral hydrate
CMCP-9 (= carboxy methyl cellulose phenol)	Water (CMCP-9: 51−60%)	Fully hydrolyzed polyvinyl alcohol (CMCP-9: 0−5%)	CMC(P)-9: low viscosity: high viscosity
DMHF (dimethyl hydantoin formaldehyde)	Water	N,N′-dimethylol dimethyl hydantoin (di-methylol DMH)	
		Ether-/methylene-bridged oligomers	
		Crosslinked DMH–formaldehyde polymer network	
Canada balsam	Xylene; partly volatile components of balsam (Δ³-carene, levopimaric acid, limonene, myrcene, palustric acid, β-phellandrene, α- pinene, β-pinene)	balsam (abienol, abietic acid, isopimaric acid, sandaracopimaric acid)	Neutralization: potassium carbonate; resin from *Abies balsamea* (Linné, 1758)
Euparal^®^	Eucalyptol, paraldehyde; partly volatile components of gum sandarac (limonene, α-pinene, β-pinene)	compounds of gum sandarac (communic acid, manool, polycommunic acid, sandaracopimaric acid, 12-acetoxy-sandaracopimaric acid, sugiol, torulosic acid, torulosol, totarol)	Clearing agent: methyl salicylate; color in Euparal^®^ green: copper salt (copper abietinate); sandarac resin from *Tetraclinis articulata* (Vahl, 1791)
Enecê	Ethyl alcohol; with camphor, eucalyptol and turpentine essence	Compounds of copal gum and colophony (rosin)	

**Table 4:** Advantages and disadvantages of selected mounting media about microscope slides and unpublished observations by various persons [52].

Name	Advantages	Disadvantages
* Canada balsam	The medium is highly durable, with a lifespan exceeding 150 years.	Contains harmful components and must be handled under a hood.
	Slides can be mounted using clove oil, or phenol as mounting agents.	Requires a full, time-consuming dehydration series.
		Ethanol dehydration and transfer *via* xylene or clove oil can make some taxa brittle; alternatives (*e.g.*, isopropanol, n-butanol, Cellosolve™, 1,4-dioxane, Histoclear, terpineol) may reduce breakage.
		Specimens may blacken if xylene is replaced with phenol or if residual potassium hydroxide remains.
		High refractive indices can obscure unstained structures.
		Complete drying can take years without hot-plate drying.
		The medium yellows and darkens over time, especially when cleared with clove oil.
		Some stains weaken, and cationic dyes may fade if the medium becomes acidic – which can occur spontaneously over time.
DMHF (dimethyl hydantoin formaldehyde)	High transparency	
	Good refractive index	Possible yellowing over time
	Excellent visibility of structures	May alter some stains
	Fairly good stability of the preparations	Not suitable for formaldehyde-sensitive stains
	Compatible with many staining techniques	Air bubbles, slow drying time
	Good adhesion between slide and coverslip	Mounting medium sensitive to humidity
	Good protection of samples	Mounting is difficult to reverse
		Formaldehyde is toxic, irritant, carcinogenic
* Euparal (transparent)	Durable medium with a lifespan over 50 years.	Contains harmful components and must be handled under a hood.
	Mounting directly from 80% ethanol is possible (manufacturer recommendation).	Ethanol dehydration and transfer *via* Euparal Essence can make some taxa brittle, but using isopropanol may reduce this issue.
	Does not mask unstained structures and does not yellow or become brittle over time.	
	Has a refractive index more suitable than Canada balsam for Diptera.	
	Works well for thicker specimens due to minimal shrinkage and bubble-free drying.	
	Remains soluble in 95% ethanol, allowing re-mounting even after many years.	
Hoyer fluid	Specimens can be mounted alive or directly from water, ethanol, or formaldehyde.	Delicate plant specimens can collapse unless the medium is added gradually, which is time-consuming.
	Maceration yields excellent cuticle quality.	Cavities and crystals may form in fewer than 10 years.
	Has a favorable refractive index and can be enhanced with iodine staining for higher contrast.	Maceration can become excessive depending on chloral hydrate concentration and exposure time.
	Acetic acid in the formula can expand arthropod appendages.	Components of the medium may separate, and fine granulation can appear within months or years.
	Some specimens may remain stable for 40–60 years.	Blackening of the media has been reported.
	Water-soluble, allowing easy re-mounting.	
CMCP-9 (= carboxy methyl cellulose phenol)	Specimens can be mounted directly from media such as water, ethanol, glycerol, or formaldehyde-containing solutions, and their internal organs may be macerated when necessary to facilitate general examination or preparation.	This medium can develop crystals and darken over time, and it can sometimes macerate specimens more than intended. Unless the slide is carefully ringed, thicker samples will not do well in it because they can shrink and create gaps around the edges of the coverslip. It is not suitable for stained specimens or calcified materials, and its drying time is slower than CMC.
Eukitt™	Durable medium lasting over 30 years.	Contains harmful components and must be handled under a hood.
	Compatible with many solvents for mounting, including acetone, benzene, chloroform, dioxan, ether, isopropanol, methyl benzoate, terpineol, toluene, and xylene.	Requires a full, time-consuming dehydration series.
	Dries quickly and has a slightly acidic pH.	Not ideal for thicker specimens due to shrinkage and gas-bubble formation.
	Does not noticeably darken with age.	Coverslips may detach over time unless glass is cleaned well and sealed.
	Suitable for various stains (*e.g.*, fuchsin, hematoxylin, methyl green, methyl violet, methylene blue).	May show incomplete polymerization around collagen fibers.
	Specimens can be re-mounted after years by soaking in xylene for an extended period.	
Enecê	Highly durable medium, lasting at least 50 years.	Requires a full, time-consuming dehydration series.
	Enecê does not darken over time.	Ethanol dehydration and transfer *via* clove oil can make some specimens brittle.
	It is more malleable, allowing for the dissection of insects in the medium, as well as providing a reasonable amount of time for positioning morphological structures.	The insect continues to be clarified although very slowly; this can make it difficult to see very small structures, such as sensilla, ascoids, and simple setae.
	Low cost.	

The refractive index of the mounting medium has a significant impact on how well the fine structures can be seen when preparing sand flies for slide mounting. The delicate and lightly sclerotized features of sand flies, including cibarial armature, spermathecae, antennal segments, and wing venation, can be difficult to observe in a high refractive index mounting medium.

For sand flies, commonly used options include gum-chloral media as water-based mounting media, and Canada balsam and Enecê – Nelson Cerqueira (NC) resin as a solvent-based medium. Rawlins [60] categorized mounting media into two types: (1) permanent media: these harden over time and are suitable for long-term preservation, and (2) semi-permanent media: these do not set hard and are typically used for temporary purposes.

Mounting media may be liquid, gum-based, or resinous, soluble in water, alcohol or other solvents (*e.g.*, toluene, xylene) (Table 3). Once applied, they should be sealed from atmospheric effects using non-soluble ringing media. To clearly distinguish among the types of mounting media, the following categorization can be used:

- *Aqueous media*. These media dissolve readily in water, making them suitable for temporary or semi-permanent mounts. They are typically easy to handle, but may require sealing to prevent exposure to atmospheric moisture (*i.e.*, gum-chloral media and polyvinyl alcohol), especially in tropical humid climates.
- *Limited water-tolerant media*. These media are less affected by water, but still require protection against excessive humidity. They provide greater long-term stability compared to water-soluble options and are frequently used in semi-permanent mounts.
- *Hydrocarbon-soluble media*. These media are dissolved in organic solvents such as xylene or toluene, or essenecê (enecê solvent). They are designed for permanent mounting and offer excellent long-term stability, and resist moisture and degradation, making them ideal for archival purposes (*i.e.*, neutral Canada balsam).

In summary, water-soluble media are best for temporary mounts or cases requiring easy specimen removal; limited water-tolerant media are suited for semi-permanent mounts requiring moderate durability, and hydrocarbon-soluble media are preferred for permanent mounts intended for archival and long-term storage.

#### 5.3.4. Description of recommended mounting media (Tables 3 and 4)

##### Media for temporary observation

###### Chloral gum = Hoyer fluid/medium/solution (RI = 1.48)

Marc André fluid is the best medium for short-term (a few hours, maybe a few more if the slide is stored in a humid chamber) observation of spermathecae, including photographs (Fig. 4) or drawings. Preserving the observed spermathecae requires their remounting in an aqueous medium allowing for medium-term storage. Dehydrating them for remounting in resin is not impossible but not recommended (risk of loss). Chloral gum and Hoyer fluid are considered synonymous. This medium is commonly used for observing internal organs due to its water compatibility, simplicity, quick application, and refractive index that facilitates the examination of delicate structures such as spermathecae. However, chloral gum has significant drawbacks if not perfectly prepared or stored under controlled humidity conditions. These issues include crystallization, discoloration, and loss of viscosity. Ringing the coverslip does not resolve these problems, as the mounting media may become heavily discolored (sometimes almost black) due to interaction with the ringing medium, particularly if Euparal^®^ is used.

Hoyer medium has been considered optically the best for phlebotomine sandflies and has traditionally been used for these purposes. The medium consists of several closely related formulations, including gum arabic, glycerol, and chloral hydrate. Various formulations have been misinterpreted and misquoted [74].

Although Hoyer is a good medium for observing spermathecae in sand flies, it is not suitable for long-term preservation. It is ideal for short-term observations, including photographs, drawings, or images. Aqueous media are suitable for temporary mounts but cannot ensure long-term preservation. In contrast, resin mounting provides excellent durability often lasting for centuries, but may obscure fine details of spermathecae, since their refringence is frequently lost.

Hoyer medium degrades over the time due to dehydration (Fig. 8), resulting in the formation of small white, opaque chloral hydrate crystals. Nevertheless, specimens can be recovered from crystallized slides as the cuticle remains chemically intact, though some physical damage may occur from the growing crystals. In some cases, crystallized slides can be restored by rehydrating the mounting medium in a warm, moist environment with thymol to prevent fungal growth. Alternatively, specimens may be soaked out of the gum chloral in water, dehydrated in glacial acetic acid, and remounted in Canada balsam.

###### DMHF (dimethyl hydantoin formaldehyde) (RI = 1.48)

This water-based medium [72] performs very well optically, much like Berlese, and is as easy to use as Berlese. However, in contrast to Berlese, it does not turn black or crystalize. It works well for sand flies and other Psychodidae.

###### CMCP (camphor-mono-chlorophenol) (RI = 1.41)

This is a glycerin-based, water-soluble mounting medium used for creating transparent, permanent slides of delicate specimens, including sand flies. The advantage of this mounting medium is that specimens can be mounted directly from water or ethanol. It quickly relaxes and clears the sand fly, softening the cuticle to allow proper positioning of the specimen, which is especially useful in spreading the wings or dissecting the genitalia. Although it is reported to enable long-term storage, the exact duration of preservation remains uncertain. The major limitation of this mounting medium lies in its composition that contains phenol, a toxic and irritant substance that requires careful handling.

##### Media for permanent mounting

###### Canada balsam (RI = 1.52–1.54)

Canada balsam was first described as a suitable mounting medium for transmitted light microscopy by Andrew Pritchard in the 1830s. It remains one of the most widely used media due to its proven archival quality, with over 150 years of successful application. Unlike Hoyer fluid media, Canada balsam does not crystallize or absorb moisture. However, Canada balsam is strongly autofluorescent, which may sometimes be a disadvantage for certain microscopy techniques [60]. Using non-toxic solvents instead of xylene can reduce safety risks during preparation, but may also introduce drawbacks such as slower drying and earlier darkening of the medium.

###### Euparal^®^ (RI = 1.48)

Euparal^®^ is a widely used alternative to Canada balsam for permanent mounting, offering excellent long-term stability and a comparable refractive index. Euparal^®^ has the following characteristics: (1) dehydration requirement: before the final transfer of the mounting medium, the specimen must be dehydrated, typically transitioning from 95% to absolute ethanol, and (2) extended processing time: the final assembly in a resin, whether Canada balsam or Euparal^®^, requires dehydration, which lengthens the overall sample processing time. When dehydration with organic solvents is not feasible, samples extracted from absolute ethanol can be placed in an intermediate solution consisting of an equal mixture of Euparal ^®^ and Euparal essence, before final mounting.

###### Enecê (RI = 1.467)

Enecê is a resin-based mounting medium primarily used for small insects and is particularly popular in Brazil. Its base consists of colophony and gum copal dissolved in alcohol, camphor, essence of turpentine, and eucalyptol. Cerqueira [11] described Enecê as an alternative to Canada balsam for mounting permanent slides of larvae, exuviae of immatures, and even adult mosquitoes, and it has since been widely adopted for mounting sand flies. Enecê offers a cost effective alternative for permanent mounting, providing long-term stability and sufficient drying time, allowing dissection and accurate arrangement of morphological structures.

### 5.4 Slide preparation and drying

Proper drying of mounted slides is critical to ensure long-term stability and preservation. Slides should be dried thoroughly before considering long-term storage. For optimal results, slides mounted with permanent mounting media should be dried horizontally for 2–3 weeks, while those prepared with semi-permanent media may require only 1–2 weeks. To ensure an effective drying process, it is recommended to use an incubator set to an appropriate temperature for the mounting medium in use, avoiding excessive heat that could damage the specimens. A temperature range of 30 °C and 37 °C is recommended. This drying step is crucial to prevent slide warping, specimen deterioration, or mounting medium instability during storage.

The mounting medium used in slide preparation should always be noted on the slide label. If possible, the label should also include the specific recipe used, along with the name of the person preparing preparator and the date of preparation. Slides are initially prepared as temporary mounts and not intended for long-term preservation. However, if the status of the specimen changes, such as being designated as part of a “type” series, a more permanent mounting medium should be used to ensure the specimen’s preservation for future taxonomic study.

### 5.5 Alternative mounting techniques: card mounting

Card mounting is a technique used for several groups of insects in which specimens can either be pinned directly onto entomological cards or glued onto the surface. Given their small size and the need to observe internal organs for identification through clarification (see item 5), this method is not at all suitable for mounting sand flies.

### 5.6 Remounting damaged specimens

For rare or valuable specimens, a two-step approach is recommended according to the video accessible on: https://zenodo.org/records/18315029. 1) Rehydrate them without disassembling to allow preliminary observation. A holder for several microscope slides should be placed in a Petri dish to serve as support. The slide to be rehydrated is then placed on top, and the Petri dish is filled with a few millimeters of solvent to create a humid chamber, ensuring that the slide itself does not come into contact with the solvent (Fig. 8D). The time required for rehydration can vary from one to several days, depending on the specimen’s condition. Daily monitoring and patience are essential. Once the slide is sufficiently rehydrated, it can be removed from the humid chamber and placed in an incubator for a few hours before microscopic examination, photographing, or drawing. 2) To remount, the slide may be returned to the humid chamber for a few additional hours or overnight. Disassembly should be performed under a binocular microscope. Using fine needles, the coverslip must be carefully removed, ensuring that no sand fly elements remain attached (https://zenodo.org/records/18315029). Next, the sand fly dissected elements should be collected and rinsed with water in small wells, like those used for destructive DNA/RNA extraction (see below), before dehydration and remounting in a resin medium. When disassembling a slide, it is crucial to identify the original mounting medium to select an appropriate solvent. For aqueous mounting media, water should be used. If the mounting medium is resin-based (*e.g.*, Canada balsam or Euparal^®^), xylene should be used, under a fume hood and with appropriate personal protective equipment, including a mask.

The remounting of type or collection specimens must only be performed with consent of the curator and/or the institution owning the specimen.

## 6 Specimen identification

### 6.1 Morphology

The identification of sand flies primarily relies on the examination of their morphological characteristics, including the shape of the thorax, wings, genitalia, setae, and specific morphometric relationships among various structures. Researchers use taxonomic keys, reference collections, and original species descriptions to compare collected specimens with known taxa. Key diagnostic features, such as wing venation and head morphology in both sexes, the structure of male genitalia, and the configuration of female spermathecae, are particularly informative for species determination. Accurate identification often requires detailed microscopic examination, typically using a compound microscope to observe fine structures such as genitalia and spermathecae, or a stereomicroscope for broader morphological features.

Recent advances in imaging technology have facilitated the use of digital imaging for sand fly identification. High-resolution photographs or digital illustrations of key features can be compared with reference materials or analyzed using computer-assisted identification systems, improving both accuracy and accessibility in morphological taxonomy.

### 6.2 Wing geometry

**Figure 9:**
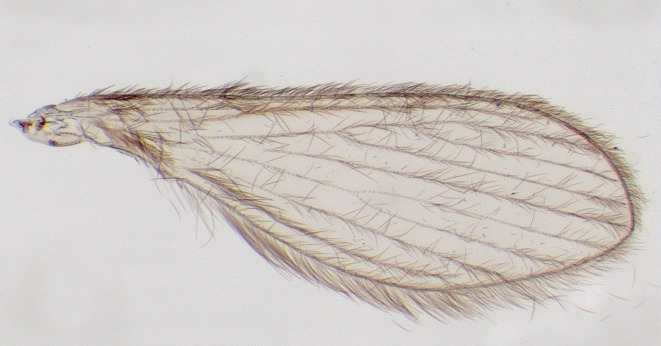
Raw wing of *Trichophoromyia ininii*.

**Figure 10:**
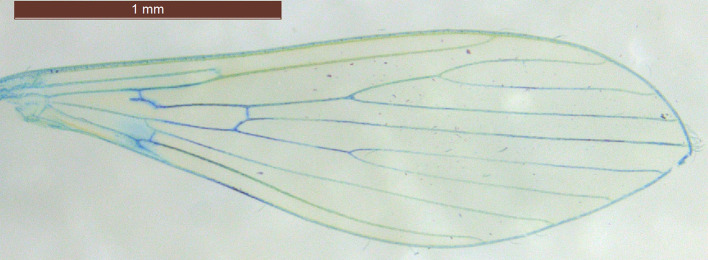
Colored wing of *Phlebotomus ariasi.*

Wing geometry is a key characteristic used in the identification and classification of different sand fly species. The wings of sand flies exhibit a unique pattern and structure, typically being long and narrow with well-developed venation (Figs. 9 and 10). The arrangement of veins forms a distinct pattern that can vary among genera and species, providing valuable diagnostic features for identification. Consequently, the study of wing geometry provides valuable insights for taxonomic purposes.

### 6.3 Wing geometric morphometrics

Researchers use various techniques, such as geometric morphometrics, to analyze and compare wing shape and size across different sand fly species or populations. Studying wing geometry provides valuable insights on behavior, habitat preferences, and flight abilities.

In the geometric morphometric approach, wings are carefully dissected, stained (if needed), and flat-mounted on slides. The prepared slides are then photographed under a stereomicroscope, digitized, and subjected to morphometric analysis. This procedure has been well described in the literature [6, 27, 42, 56, 57, 59], with a recommendation to use the right or left wing consistently for paired organs to avoid potential negative allometric effects [62].

#### Wing preparation for geometric morphometric analysis

For optimal visualization of wing veins, wings should be cleaned of scales and appropriately stained. For wing preparation, first fill small wells with the required reagents (methylene blue, ethanol, water and xylene substitute). Retrieve a wing preserved in 70% ethanol at room temperature by inverting the Eppendorf tube and emptying it over the well, then lift the wing longitudinally using a fine curved needle. Pass the wing briefly from ethanol to water and back to ethanol to remove bristles. Place the wing in methylene blue for 6 min, ensuring it floats during staining. Recover the wing carefully and immerse it in xylene substitute for 2 min (approximately one-third the time of the methylene blue). Gentle taps of the needle against the well walls can help the wing settle; xylene serves to fix the coloration. Finally, lift the wing and place it on a small drop of Euparal^®^ on a microscopic slide. Under a magnifying lens, gently unfold the wing and carefully place a coverslip. Photographs should be taken promptly before the Euparal^®^ sets, as slight adjustments of the wing position under the coverslip may be necessary to achieve optimal alignment.

### 6.4 Molecular biology techniques

In addition to morphological techniques, molecular methods are increasingly essential in entomological research, including taxonomic, population genetic, and phylogenetic studies, as well as for DNA/RNA pathogen detection, and to determine blood meal origin, vector behavior being important in the field of epidemiology [70]. DNA sequencing can be employed for species confirmation or to differentiate closely related species, providing a more accurate and reliable means of identification. Moreover, advanced molecular techniques (*i.e.*, PCR, DNA sequencing, NGS, *etc.*) and MALDI-ToF MS are gaining prominence for accurate and rapid species identification, complementing traditional morphological methods [46]. Despite these advances, morphological identification remains the reference standard for taxonomy and the basis upon which molecular data are interpreted.

#### 6.4.1 Destructive nucleic acid extraction

Nucleic acid extraction is a routine step in many biological studies, and various methods have been developed to isolate DNA from biological materials [48]. Many commercially available DNA extraction kits are designed to facilitate this process [14]. However, methods commonly used for preparing arthropod specimens for morphological identification often hinder DNA analysis, as these techniques may damage or destroy critical physical characteristics of the specimen [10]. Most DNA extraction protocols for insect tissues are destructive in nature [43], raising particular concerns for small specimens, where even limited sampling may compromise important morphological features [72]. The type and condition of the specimen play a key role in selecting an appropriate DNA isolation method [29].

The need for accurate identification of sand flies, understanding population dynamics, and minimizing non-target impacts has driven the development of molecular diagnostic tools [23]. Molecular approaches are now frequently used to complement morphological taxonomic methods for identifying sand flies. For example, the standard approach for insect barcoding involves DNA extraction, sequencing, and loss of the original specimen. Thus, there is a pressing need to explore non-destructive DNA extraction methods that preserve both the biological material and its morphological integrity.

Numerous nucleic acid extraction methods have been applied to sand flies. The quantity or quality of nucleic acids required depend on the downstream molecular analysis, as different techniques have varying sensitivity and purity requirement [9]. For example, sand fly eyes have been found to inhibit PCR amplification [69]. Beyond pathogen screening, sand fly DNA is routinely extracted for species identification purposes. Various extraction methods can be used, though yields and quality differ among techniques. Some manufacturers’ protocols have been adapted by researchers for sand flies [8], increasing the yield and/or quality of extracted nucleic acids [8, 9, 69], while other adaptations, developed for other arthropod taxa, can also be used on sand flies [58, 76]. Identification PCRs targeting small mitochondrial fragments (COI or CytB) are generally compatible with extraction methods involving high DNA fragmentation. In contrast, other long-read NGS techniques (Oxford Nanopore and PacBio) require minimal fragmentation and high-quality DNA. Spin column extractions generally yield genomic DNA fragments of up to 60 kb, whereas phenol-chloroform extraction can produce fragments up to 150 kb [77]. Table 5 summarizes different extraction techniques of sand fly DNA and indicates whether methodological adaptations have been made for these insects. Yields are not shown, as they depend on specimen size and preparation method. The modification column refers to adaptations of extraction protocols for sand flies or other small arthropods.

**Table 5:** Average cost, application, and protocol adaptation for the extraction of gDNA of phlebotomine sand flies.

Protocol	Cost	Application	Protocol adaptation for small arthropods
Spin column	2.5–3.55 US$ [39]	PCR, NGS	[9]
Phenol-chloroform	0.24 US$ [69]	PCR, NGS	[9]
HotSHOT	<0.01 US$ [69]	PCR	–
Salting out	0.12 $ [69]	PCR	–
Chelex	0.02 $4 [41]	PCR	[41, 76]

The choice of extraction method should consider several criteria, such as the number of samples, the extraction time, and the technique deployed downstream. While NGS techniques require high molecular weight genomic DNA, all the methods presented here can be used for standard PCR-based applications.

Furthermore, several studies have explored non-destructive DNA extraction methods for small terrestrial arthropods, dry-preserved museum specimens, and soft-bodied arthropods [19, 26, 28, 55, 63].

#### 6.4.2 Non-destructive nucleic acid extraction

One of the major challenges in molecular analysis of arthropods, especially sand flies, is the preservation of specimens for integration into entomological collections. Most DNA extraction protocols require maceration of the tissue, thus compromising the preservation of the original specimen. Non-destructive nucleic acid extraction methods, however, are designed to extract genetic material without physically damaging the sample, affecting its viability or altering its morphology. These methods are particularly valuable when working with precious or limited specimens, such as sand flies, where maintaining structural integrity is essential for future taxonomy, morphological or diagnostic purposes. A commonly employed technique is the non-destructive bathing method in which sand flies are immobilized, and gently immersed in a lysis buffer containing proteinase K.

The mild-vectolysis technique has been successfully applied to sand flies, particularly type specimens [24]. The technique makes use of a conventional spin column kit (in this case, a DNeasy Blood and Tissue kit, QIAGEN, Hilden, Germany) with adaptation for obtaining DNA without destroying the specimen. The modified lysis steps (volume of lysis buffer and addition of a freezing step) [17] allow for the release of nucleic acids, minimizing morphological damage [24]. Regarding sand flies, it is also possible to use a HotSHOT DNA Extraction kit (Bento Bioworks Ltd., London, United Kingdom) [73] which is rapid and inexpensive, enabling quick and low-cost processing of samples. Entomological specimens intended for morphological identification can then be rinsed. Those processed with a DNeasy Blood and Tissue kit must be cleared with Marc-André solution, whereas those processed with a HotSHOT DNA extraction kit are sufficiently clarified to be mounted in an aqueous medium, or better, in a resin after dehydration, according to the protocol detailed in this article [73]. The extracted genetic material can then be further processed for downstream analysis, such as PCR, to amplify specific genetic markers. Non-destructive nucleic acid extraction methods are crucial for studying the genetic characteristics of sand flies, including identifying potential disease-causing agents they may carry. By preserving the specimen’s integrity, researchers can obtain valuable genetic information, while retaining the sample for additional analyses or studies.

### 6.5 MALDI-ToF MS

MALDI-ToF MS (matrix-assisted laser desorption/ionization time-of-flight mass spectrometry) is a mass spectrometry-based technique designed to detect and analyze the unique protein profiles (“fingerprints”) of biological samples. MALDI-ToF is increasingly recognized as an important tool for the identification of arthropods of medical and veterinary significance. This technique has proven effective in identifying various developmental stages of sand flies, including immature forms and the blood meals of engorged females, and has been successfully applied to differentiate both male and female sand fly species under a range of storage and homogenization conditions [28, 30, 73, 74]. This method also offers high discriminatory power at the levels of subgenera, species, and populations. This technique enables researchers to achieve rapid and accurate species identification, which is essential for understanding sand fly distribution, behavior, and their role in disease transmission. By differentiating between species based on protein profiles, MALDI-ToF plays a crucial role in epidemiological studies and vector control strategies. There are two main current drawbacks of this technique that limit its routine application. First is availability of mass spectrometry equipment, which is prohibitively expensive to be readily acquired solely for the purposes of species identification of sand flies (or arthropod vectors generally). Fortunately, this limitation may be overcome by gaining machine time at mass spectrometers that have become a standard research tool in proteomic facilities and/or clinical diagnostics. Second is low representation of sand fly reference data available in open-access databases, resulting in a need to construct an in-house database with reference spectra based on unambiguously identified specimens, ideally by a combination of morphological assessment and sequencing of a suitable genetic marker (COI, cytB, or other). This limitation will hopefully soon be resolved by gradual inclusion of so far in-house sand fly reference data into the MSI Platform run by Assistance Publique-Hôpitaux de Paris, Sorbonne University, France and the BCCM/IHEM/Sciensano collection in Brussels, Belgium (https://msi.happy-dev.fr/). When MALDI-ToF protein profiling is planned to be deployed, samples should be stored preferably dry-frozen or in 70% ethanol of molecular grade and not exposed to ambient temperatures. In the absence of universal guidelines for sample preparation, users are advised to use an aqueous 60% acetonitrile/0.3% TFA solution of sinapinic acid (30 mg/mL) for the MALDI-ToF matrix preparation in order to make their protein spectra comparable with so far published sand fly data.

#### Sample Preparation for MALDI-ToF MS (Fig. 7)

Insect specimens, stored under various conditions, are first air-dried at room temperature and dissected. The head and abdomen are removed to obtain body parts containing key morphological characteristics for slide mounting and morphological analysis. The thorax can be used for MALDI-ToF and the remaining abdomen preserved for DNA extraction. For protein profiling, the thorax is manually homogenized in 1.5-mL microtubes with 10 μL of homogenization solution using disposable pestles and pellets. Two homogenization solutions are typically used: sterile distilled water and 25% formic acid.

## 7 Conclusion

In this work, we aimed to provide researchers with the most effective methods for mounting sand flies, tailored to specific research objectives, to facilitate accurate identification and pathogen detection. There is no single, universally optimal method; rather, several methods exist, each with their own advantages and limitations.

In the Appendices, we provided detailed protocols for various mounting techniques used in the preparation and identification of sand flies. These protocols, including instructional videos, offer step-by-step procedures tailored to different objectives, ensuring precise and reliable results. By offering this comprehensive resource, we aim to support researchers in selecting and applying the most appropriate mounting techniques for their specific needs.

## Data Availability

*Videos on Zenodo*: Video 1: https://zenodo.org/records/18198006. Video 2: https://zenodo.org/records/18311158. Video 3: https://zenodo.org/records/18311106. Video 4: https://zenodo.org/records/18311154. Video 5: https://zenodo.org/records/18303014. Video 6: https://zenodo.org/records/18302850. Video 7: https://zenodo.org/records/18315029.

## Supplementary material

*Supplementary File 1*: Amharic translation / ወደ አማርኛ ትርጉም *Supplementary File 2*: Arabic translation / الترجمة العربية *Supplementary File 3*: Bulgarian translation / Превод на български *Supplementary File 4*: Catalan translation / Traducció al català *Supplementary File 5*: Chinese translation / 翻译成中文 *Supplementary File 6*: Czech translation / Překlad do češtiny *Supplementary File 7*: Farsi translation / ترجمه فارسی *Supplementary File 8*: Filipino translation / Pagsasalin sa Filipino *Supplementary File 9*: Fon translation / Tintindófɔngbemɛ *Supplementary File 10*: French translation / Traduction en français *Supplementary File 11*: German translation / Deutsche Übersetzung *Supplementary File 12*: Greek translation / ελληνική μετάφραση *Supplementary File 13*: Hebrew translation / תרגום לעברית *Supplementary File 14*: Hindi translation /  हिंदी अनुवाद *Supplementary File 15*: Indonesian translation / Terjemahan Bahasa Indonesia *Supplementary File 16*: Italian translation / Traduzione in italiano *Supplementary File 17*: Japanese translation / 日本語訳 *Supplementary File 18*: Khmer translation / ការបកប្រែជាភាសាខ្មែរ *Supplementary File 19*: Lao translation / ການແປພາສາລາວ *Supplementary File 20*: Malay translation / Terjemahan dalam Bahasa Melayu *Supplementary File 21*: Malagasy translation / Dikanteny malagasy *Supplementary File 22*: Nepali translation / नेपाली अनुवाद *Supplementary File 23*: Polish translation / Tłumaczenie polskie *Supplementary File 24*: Portuguese translation / Tradução para português *Supplementary File 25*: Russian translation / Перевод на русский *Supplementary File 26*: Serbian translation / Превод на српски *Supplementary File 27*: Slovenian translation / Prevod v slovenščino *Supplementary File 28*: Spanish translation / Traducción al español *Supplementary File 29*: Thai translation / คำแปลภาษาไทย *Supplementary File 30*: Tok Pisin translation of the title, summary, and an appendix / Het tok, samari na wanpela appendix long Tok Pisin *Supplementary File 31*: Turkish translation / Türkçe çeviri *Supplementary File 32*: Urdu translation / اردو ترجمہ *Supplementary File 33*: Vietnamese translation / Bản dịch tiếng Việt *Supplementary File 34*: Wolof translation / Tekki ci lakku wolof The supplementary material of this article is available at https://www.parasite-journal.org/10.1051/parasite/2026009/olm.

## Appendix A: Biochemical theoretical foundations

The arthropods concerned are sand flies. However, the general idea can be extended to other very common arthropods for which identification can only be done on internal morphological characters. By chance, some internal organs are partially chitinised and their morphologies provide us with valuable information. This is why it is very interesting to observe food pumps, spermathecae, and their ducts. With all the reagents that we will review, it should never be forgotten that from the stage of insect fixation to assembly, we will simply apply redox reactions. The only precaution or idea that will lead us is to avoid mixing reducing reagents with oxidizing reagents.

### Ethyl alcohol; ethanol

This substance will be used in different ways. Alcohol molecules have a strong affinity for water and therefore exhibit a dehydrating effect. However, an alcohol with low concentration (*i.e.*, too rich in water) will play a role in degradation of nucleic acids (water is the enemy of nucleic acids).

When insects are placed in ethanol, it is not only to preserve them, but also to fix the tissues. In histology, we usually differentiate two important concepts: the penetration rate and the fixation rate. It is well understood that a good preservative must first quickly penetrate deep into the tissues before fixing them. For alcohol at 96%, the penetration coefficient is approximately 1.05 (in comparison, for a 0.75% aqueous solution of picric acid, the penetration coefficient is 0.45, while that of a 3% potassium dichromate solution is 1.45).

Wanting to preserve insects and other arthropods indefinitely in ethanol is a reality for entomologists. The idea is still very honorable to want to keep the field captures for subsequent studies or for future researchers. Nonetheless, this idea is not possible for a cytologist or histologist. By aiming to keep the samples in the fixative for too long, they may become practically impossible to rework. This is why samples older than 10 years are difficult or even impossible to use.

Another consideration is the ratio between the arthropod mass to be fixed and the volume of the fixator. In zoological or medical practice, it is advisable to plan for a volume 60 times greater than the volume of the pieces to be fixed. In practice, for micro-arthropods, for a given volume of specimens to be fixed, add at least 4–5 volumes of alcohol. Keep in mind that the alcohol will lose its strength as it removes all the water present in the arthropod tissues.

In conclusion:

- Ethyl alcohol is a reducing chemical agent (therefore incompatible with oxidative fixatives);
- It energetically precipitates the proteins and denatures them;
- It dissolves certain complex lipids and precipitates glycogen;
- It causes a strong contraction of the tissues and hardens them.

#### Basic potassium or sodium hydroxide solutions:

Use of these solutions in entomology has mainly focused on potassium hydroxide without a clear rationale.

Sodium hydroxide [E524] occurs in solution, either at different concentrations or with different normality. It comes in pellets, or glitter. Its major disadvantage is that it is very hygroscopic (more than KOH). When it reacts with the proteins, it dissolves them, and with lipids it transforms them into hard soaps during saponification (this is a major difference with KOH, which yields liquid soaps during saponification).

Potassium hydroxide [E525] is available as a concentrated solution, but above all, it has the advantage of being formulated in pellets of about 0.1 g., which greatly facilitates the preparation of diluted solutions when one does not specify a precision balance. For example, 1 pellet of 0.1 g in 1 mL of distilled water yields a 10% solution. The second advantage of potassium hydroxide in pellets is its lower sensitivity to carbonation (a KOH solution has high affinity for fixing CO_2_, thus creating carbonate salts).

These strong bases will be used to solubilize fatty acids by transforming them into water-soluble soaps. It should be remembered that the fixative, such as ethanol, solubilized some of the fats in the sample. However, by replacing the sample in an aqueous medium with a strong base, the fatty acids (more or less complex) will precipitate. The strong base will therefore perform a cold saponification. In some cases, when the adipose tissues are in excess, for example in females, it will be advantageous to raise the temperature to 35–40 °C to facilitate the reaction, or else to extend the contact time at room temperature.

#### Colored acid solution/colorless Marc-André solution:

Here, we will explore the advantages and inconveniences of using Marc-André solution. This solution is composed of chloral hydrate (trichloracetaldehyde monohydrate), acetic acid, and water. This solution is very oxidizing (mixture of acid and aldehyde). It will neutralize the excess potassium hydroxide that may remain in the samples, without precipitating the alkaline soaps formed during the use of potassium hydroxide. This oxidizing solution will also have an action on the secondary alcohol functions of the glucosamines that form in chitin by oxidizing them, thus softening the chitin. Another action is the dissolution of certain mineral salts present.

When the Marc-André solution is previously colored by acid fuchsine (thus in the oxidized state), it will be able to fix on the secondary alcohol functions of the structure. After the contact time of the Marc-André solution and the staining state of the samples, rinsing will be done only with ethanol. We thus begin the dehydration phase of the samples.

##### Benefits

- Neutralization of excess basic solutions
- Relaxation of chitin
- Staining of the chitin to better assess the chitinized internal structures

##### Drawbacks

Chloral hydrate is hypnotic and has been used in human medicine. It must be used under a chemical hood, and legislation on chemical risks must be complied with.

##### Dehydration solutions

Experience shows that for very small samples, it is not useful to follow the sequence of alcohol baths with increasing concentrations. If the sample is large, we will start with 80% ethanol, then 90%, 95% and finally absolute ethanol. For very small samples, use a bath with 90% ethanol followed by immersion in absolute ethanol. At this stage, we will always remember that absolute ethanol seeks to fix the water in the atmosphere.

The tradition in entomology laboratories was to finalize the dehydration of samples with a beech creosote bath. Today, this essence, widely used as a pesticide, antifungal, and wood preservative, is very strongly discouraged due to its odor (polycyclic aromatic hydrocarbons) and it is assumed to be reprotoxic, carcinogenic, a persistent organic pollutant, and ecotoxic for aquatic organisms.

A solution that we propose to prepare for mounting the samples is Euparal^®^ and Euparal essence (described in the following paragraph). A mixture of Euparal^®^ and Euparal essence is very well accepted; the samples obtained following a 90% ethanol bath.

## Appendix B: Composition of the reagents used

### Potassium hydroxide 10%

Potassium hydroxide 10 g

Distilled water q.s. 100 mL

#### Gum chloral mounting medium Hoyer medium

Distilled water 50 mL

Chloral hydrate 200 g

Gum arabic 50 g

Glycerol 20 mL

#### Marc-André solution

Chloral hydrate 40 g

Glacial acetic acid 30 mL

Distilled water 30 mL

#### Fuchsin acid 1% in distilled water

Acid fuchsin powder 1 g

Distilled water 99 mL

#### Marc-André solution colored with fuchsin

Marc-André solution 10 mL

Fuchsin 1% 50 μL

## Appendix C: Euparal^®^, Canada balsam, polyvinyl alcohol or other solutions for mounting

*Polyvinyl alcohol*: This is the ideal mounting medium when the necessary products for proper dehydration are not available. Polyvinyl alcohol is then mixed with Amman’s lactophenol. However, these assemblies exhibit the major drawbacks of either drying out or polyvinyl alcohol crystallizes because of water evaporation or blackening when phenol oxidizes. This remains a good technique for short-term mounting.

*Canada Balsam*: Its use for mounting between slide and coverslip requires dehydration of the samples to be mounted. The use of xylene or toluene is not without inconvenience.

*Enecê medium*: For mounting between slide and coverslip, such as Canada Balsam, it requires dehydration of the specimen. Enecê formulation: pure white colophony (22 g); alcohol-soluble copal gum (12 g), absolute alcohol (20 mL), camphor (10 g), turpentine essence (10 mL), and eucalyptol (26 mL). For its preparation, in a container, such as an Erlenmeyer flask, place the absolute alcohol and camphor. Then add the colophony and the copal gum. The flask is then sealed with a stopper and shaken, and only then is it heated in bain-marie at a gentle temperature so that the mixture does not boil. Once the contents are completely liquefied, the turpentine essence is added, then filtered while the mixture is still hot, and finally, the eucalyptol is added to the filtrate. When the medium becomes less fluid, it is diluted with Eenecê, having the following formula: absolute alcohol (30 mL), camphor (17 g), turpentine essence (15 mL), eucalyptol (38 mL) (Cerqueira, 1943).

*Euparal*^*®*^: This is a resin that comes from the Cypress of the Atlas *Tetraclinis articulata* (Vahl, 1791) and was studied and developed in 1906 by Gilson. Its main advantage is that it does not polymerize. The samples mounted between slides and cover-slips can easily be recovered by the action of alcohol or better still of Euparal^®^ essence. This resin, also called sandarac, accepts ethanol from 80%.

### Utilization of Triton X-100: non-ionic aqueous solution

Triton X-100 is in the form of a non-ionic aqueous solution (*4-(1,1,3,3-tetramethylbutyl) phenyl-polyethylene glycol solution, or t-octylphenoxypolyethoxyethanol, polyethylene glycol tert-octylphenyl ether*), widely used as a detergent in cellular and molecular biology. It allows for the permeabilization of cell and nuclear membranes.

Insect samples preserved in alcohol for many years are commonplace. Unfortunately, preservation in alcohol is not optimal, and the arthropods thus preserved become very difficult to prepare for microscopic examination. Plastics containing samples often degrade, followed by evaporation of alcohol. In both cases, prolonged contact with alcohol or drying of samples pose a real problem. In 2008, Jonque published a note on the rehydration of spiders with a wetting agent such as Agepon used for photographic films [26]. This led to the idea of using wetting agents that are not powerful detergents.

Below is a procedure using Triton X-100 in 0.5% aqueous solution:

- Impregnated the dry sample with absolute alcohol.
- Add the necessary volume of Triton X-100 solution at 0.5% so that the entire sample is immersed.
- Allow to stand for about 5 min or more. All the arthropods must become independent in the solution.
- The Triton X-100 solution is removed and replaced by the potassium hydroxide solution.

The technique is then followed, as described above.

## Appendix D: Euparal^®^ or Canada Balsam mounting media step by step

1. Specimens must be dehydrated (cloudy or milky appearance indicates inadequate dehydration).
2. Dehydration can be accomplished by increasing concentrations of ethyl alcohol.
3. Specimens can be transferred from 99% alcohol or absolute alcohol to a clearing agent.

Procedure:

1. Put adult sand flies in 70% ethanol.
2. Remove ethanol and replace with 10% KOH. Cover the sand flies with a glass slide.
3. Macerate until the insects become transparent.
4. Remove KOH.
5. Cover the specimen with distilled water and wait for 30–45 min.
6. Remove water and repeat washing with distilled water for 30 min (time function of numerous samples: the more samples there are to process together, the longer this time must be respected. The fewer there are, especially for those treated individually, the shorter this time can be).
7. Remove water.
8. Add Marc-André solution (potentially colored with Fuchsin acid) and wait for 24 hours (one day).
9. Remove Marc-André solution.
10. Cover the specimen with distilled water and wait for 30 to 45 min.
11. Remove water and repeat washing with distilled water for 30 min.
12. Remove water.
13. Add 70% ethanol and dissect the specimen.
  - For the head and abdomen, gently pull the head or abdomen off the thorax.
  - For the thorax, remove the wings by holding the thorax with one pair of forceps and pulling at the base of the appendages with another pair. It is possible to do a sagittal dissection, dividing the thorax into left and right sides, depending on the regions of greatest interest.
14. Dehydrate gradually through a series of aqueous ethyl alcohol solutions. 50 – 80 – 95% until absolute ethanol is reached.
15. Dehydrate the specimens by washing them twice, 10 min each, with 100% ethanol.
16. Remove ethanol and cover specimens with clove oil for 15 min at room temperature.
17. Transfer specimens from clove oil to a drop of Euparal^®^ or Canada Balsam on a clean slide.
18. Arrange as desired: The head, thorax, and abdomen of the sand fly can be dissected using fine needles or forceps under a dissecting microscope. The head must be dissected from the body in order to be mounted in a ventro-dorsal position, *i.e.*, the occipital foramen must be oriented upwards so that the cibarium can be observed directly through it.
19. The dissection is carried out in the sand fly mounting medium.
20. Leave the specimen until the surface becomes sticky.
21. Wet a clean coverslip with absolute alcohol. Drop the coverslip onto the Canada Balsam at an angle.
22. Store slides in a dry box intended for this purpose.

## Notes

**Cite this article as**: Randrianambinintsoa FJ, Augendre L, Prudhomme J, Martinet J-P, Loyer M, Mekarnia N, Kerkoub H, Perveen FK, Huguenin A, Kariya E, Akhoundi M, De Andrade AJ, Berriatua E, Bongiorno G, Boyer S, Christodoulou V, Da Costa-Ribeiro MCV, De Souza LAF, Ding H, Dondji B, Dvořák V, Erisoz Kasap O, Galati EAB, Gállego M, Ballart C, Gouzelou S, Haddad N, Masse RS, Mekuria AH, Ivovic V, Kaczmarek S, Shahar MK, Kirstein OD, Kniha E, Kolářová I, Lincoln T, Lucanas C, Mikov O, Nov K, Özbel Y, Pesson B, Posada Lopez LC, Prasetyo DB, Rahola N, Rebollar-Tellez EA, Rodrigues BL, Roy L, Saini P, Sanjoba C, Shimabukuro PH, Siriyasatien P, Soszynska A, Sulesco T, Sylla M, Torno M, Volf P, Vongphayloth K, Sinh Nam V, Wardhana A, Yessinou E, Zapata S, Gantier J-C & Depaquit J. 2026. Processing and mounting phlebotomine sand flies: a consensus guideline. Parasite **33**, 18. https://doi.org/10.1051/parasite/2026009.
