# Supplementary material for: Processing and mounting phlebotomine sand flies: a consensus guideline
Source: Parasite. 2026 Apr 3;33:18. doi: 10.1051/parasite/2026009 (PMC13047900; doi:10.1051/parasite/2026009)
Supplement: Supplementary file 1 — Amharic translation / ወደ አማርኛ ትርጉም [file parasite-33-18-s1.pdf]

Parasite 33, 18 (2026)
© F.J. Randrianambinintsoa et al., published by EDP Sciences, 2026
https://doi.org/10.1051/parasite/2026009

PARASITE
Available online at:
www.parasite-journal.org

REVIEW ARTICLE OPEN ACCESS

የፍሌቦቶሚን አሸዋ ትንኞችን ማቀነባበር እና ማንበር፡ የጋራ መግባቢያ መመሪያ

Fano José Randrianambinintsoa[1], Laure Augendre[1], Jorian Prudhomme[1], Jean-Philippe Martinet[1], Mathieu Loyer[1], Nalia Mekarnia[1], Hocine Kerkoub[1], Farzana Khan Perveen[1], Antoine Huguenin[1,2], Emilie Kariya[1,2], Mohammad Akhoundi[3], Andrey José de Andrade[4], Eduardo Berriatua[5], Gioia Bongiorno[6], Sébastien Boyer[7,8], Vasiliki Christodoulou[9], Magda Clara Vieira Da Costa-Ribeiro[10], Lucas Alexandre Farias de Souza[10], Huicong Ding[11], Blaise Dondji[12], Vít Dvořák[13], Ozge Erisoz Kasap[14], Eunice Aparecida Bianchi Galati[15], Montserrat Gállego[16], Cristina Ballart[16], Stavroula Gouzelou[17], Nabil Haddad[18], Rezki Sabrina Masse[19], Asrat Hailu Mekuria[20], Vladimir Ivovic[21], Szymon Kaczmarek[22], Mohd Khadri Shahar[19], Oscar D. Kirstein[23], Edwin Kniha[24], Iva Kolářová[13], Lincoln Timinao[25], Cristian Lucanas[26], Ognyan Mikov[27], Kimsear Nov[7], Yusuf Özbel[28], Bernard Pesson[29], Laura Cristina Posada Lopez[30], Didot Budi Prasetyo[1,7], Nil Rahola[31], Eduardo A. Rebollar-Tellez[32], Bruno Leite Rodrigues[15], Lalita Roy[33], Prasanta Saini[34], Chizu Sanjoba[35], Paloma Helena Fernandes Shimabukuro[36], Padet Siriyasatien[37], Agnieszka Soszyńska[22], Tatiana Suleşco[38], Massamba Sylla[39], Majhalia Torno[40], Petr Volf[13], Khamsing Vongphayloth[41], Vu Sinh Nam[42], April Wardhana[43], Eric Yessinou[44], Sonia Zapata[45], Jean-Charles Gantier[1], and Jérôme Depaquit[1,2,*]

[1] Faculté de Pharmacie, Université de Reims Champagne Ardenne, UR ESCAPE-USC ANSES PETARD, 51 rue Cognacq-Jay, 51096 Reims Cedex, France
[2] Pôle de Biologie territoriale, Laboratoire de Parasitologie-Mycologie, Centre Hospitalo-Universitaire, 51092 Reims, France
[3] Parasitology-Mycology Department, Avicenne Hospital, AP-HP, Bobigny, Sorbonne Paris Nord University, France; Unité des Virus Émergents (UVE: Aix-Marseille Univ, Università di Corsica, IRD 190, Inserm 1207, IRBA), 13005 Marseille, France
[4] Parasitology Collection of Basic Pathology, Department of Basic Pathology, Federal University of Paraná, Curitiba 19031, Brazil
[5] Department of Animal Health, University of Murcia, Campus de Espinardo, 30100 Espinardo, Murcia, Spain
[6] Department of Infectious Diseases, Vector-borne Diseases Unit, Istituto Superiore di Sanità, 00166 Rome, Italy
[7] Medical and Veterinary Entomology Unit, Institut Pasteur du Cambodge, Phnom Penh 12201, Cambodia
[8] Ecology & Emergence of Arthropod-borne Pathogens Unit, Department of Global Health, Institut Pasteur, CNRS UMR2000, 75015 Paris, France
[9] Section Veterinary Services (1417), Laboratory for Animal Health Virology, Aglantzia, Nicosia 2109, Cyprus
[10] Insects Vectors and Parasites Laboratory, Department of Basic Pathology and Postgraduate program in Microbiology, Parasitology and Pathology, Federal University of Paraná, 81530-900 Curitiba, Brazil
[11] Department of Biological Sciences, National University of Singapore, 117558, Singapore
[12] Laboratory of the Leishmaniasis Research Project, Mokolo District Hospital, Mokolo, Cameroon; Laboratory of Cellular Immunology and Parasitology, Department of Biological Sciences, Central Washington University, 98926 Ellensburg, WA, USA
[13] Department of Parasitology, Faculty of Science, Charles University, 12800 Prague, Czechia
[14] VERG Laboratories, Department of Biology, Faculty of Science, Hacettepe University, Beytepe, Ankara 06800, Türkiye
[15] Faculdade de Saúde Pública da Universidade de São Paulo (FSP/USP), Pós-graduação em Saúde Pública, 01246-904 São Paulo, Brazil
[16] Secció de Parasitologia, Departament de Biologia, Sanitat i Medi Ambient, Facultat de Farmàcia i Ciències de l'Alimentació, Universitat de Barcelona, & Institut de Salut Global de Barcelona (ISGlobal), Centro de Investigación Biomédica en Red, Enfermedades Infecciosas (CIBERINFEC), 08028 Barcelona, Spain
[17] Laboratory of Infectious Diseases and Public Health, School of Medicine, University of Cyprus, Nicosia, Cyprus & Department of Pediatrics, Archbishop Makarios III Hospital, Nicosia 2115, Cyprus
[18] Faculty of Health Sciences, American University of Beirut, 1107 2020 Beirut, Lebanon
[19] Medical Entomology Unit, Infectious Disease Research Centre, Institute for Medical Research (IMR), National Institutes of Health (NIH), Ministry of Health Malaysia, 40170 Shah Alam, Selangor, Malaysia
[20] School of Medicine, Addis Ababa University, 28017 - 1000 Addis Ababa, Ethiopia
[21] Faculty of Mathematics, Natural Sciences and Information Technologies, University of Primorska, 6000 Koper, Slovenia

Edited by Jean-Lou Justine
*Corresponding author: jerome.depaquit@univ-reims.fr

This is an Open Access article distributed under the terms of the Creative Commons Attribution License (https://creativecommons.org/licenses/by/4.0), which permits unrestricted use, distribution, and reproduction in any medium, provided the original work is properly cited.

[22] University of Lodz, Faculty of Biology and Environmental Protection, Department of Invertebrate Zoology and Hydrobiology, Banacha 12/16, 90-237 Łódź, Poland
[23] Laboratory of Entomology, Ministry of Health, 9134302 Jerusalem, Israel
[24] Center for Pathophysiology, Infectiology and Immunology, Institute of Specific Prophylaxis and Tropical Medicine, Medical University Vienna, Kinderspitalgasse 15, 1090 Vienna, Austria
[25] Papua New Guinea Institute of Medical Research (PNGIMR) Institute, PO Box 60, Headquarter, Homate Street, 441 Goroka, Eastern Highlands Province, Papua New Guinea
[26] Museum of Natural History, University of the Philippines Los Baños, 4031 Laguna, Philippines
[27] National Centre of Infectious and Parasitic Diseases, 1504 Sofia, Bulgaria
[28] Ege University, Faculty of Medicine, Department of Parasitology, 35040 Bornova/Izmir, Türkiye
[29] Retired, Faculté de Pharmacie, Université de Strasbourg, Strasbourg, 67400 Illkirch-Graffenstaden, France
[30] Program for the Study and Control of Tropical Diseases (PECET), Faculty of Medicine, University of Antioquia, 050010 Medellin, Colombia
[31] MIVEGEC, Univ. Montpellier, CNRS, IRD, 34394 Montpellier, France & Medical Entomology Unit, Institut Pasteur de Madagascar, 101 Antananarivo, Madagascar
[32] Laboratorio de Entomología Médica, Departamento de Zoología de Invertebrados, Facultad de Ciencias Biológicas, Universidad Autónoma de Nuevo León, San Nicolás de los Garza, 66455, NL, México
[33] Tropical and Infectious Disease Centre, BP Koirala Institute of Health Sciences, Dharan 56700, Nepal
[34] ICMR-Vector Control Research Centre, Puducherry 605006, India
[35] Graduate School of Agricultural and Life Sciences, The University of Tokyo, Tokyo 113-8657, Japan
[36] Grupo de estudos em Leishmanioses/Coleção de Flebotomíneos (COLFLEB/Fiocruz-MG), Instituto René Rachou, Fundação Oswaldo Cruz, Belo Horizonte, Minas Gerais, 30190009, Brazil
[37] Center of Excellence in Vector Biology and Vector-Borne Disease, Department of Parasitology, Faculty of Medicine, Chulalongkorn University, Bangkok 10330, Thailand
[38] Department of Arbovirology, Bernhard Nocht Institute for Tropical Medicine, Bernhard Nocht Str. 74, 20359 Hamburg, Germany [39] Laboratory Vectors & Parasites, Department of Livestock Sciences and Techniques, Sine Saloum University El Hadji Ibrahima Niasse (SSUEIN) Kaffrine Campus, C.P. 24600, Senegal.
[40] Environmental Health Institute, National Environment Agency, Singapore 138667, Singapore & Department of Biological Sciences, National University of Singapore, 117558 Singapore
[41] Institut Pasteur du Laos, Laboratory of Vector-Borne Diseases, Samsenhai Road, Ban Kao-Gnot, Sisattanak District, 3560 Vientiane, Lao PDR
[42] National Institute of Hygiene and Epidemiology, 1 Yec-Xanh Street, Hai Ba Trung District, 100000 Hanoi, Vietnam
[43] Indonesian Research Center for Veterinary Science, Indonesian Agency for Agricultural Research and Development, Ministry of Agriculture Republic Indonesia, Bogor 16114, Indonesia & Department of Parasitology, Faculty of Veterinary Medicine, Airlangga University, Surabaya 60115, Indonesia
[44] Laboratory of Research in Applied Biology, Polytechnic School of Abomey-Calavi, University of Abomey-Calavi, 01 P.O. Box 2009, 00000 Cotonou, Benin
[45] Instituto de Microbiología, Colegio de Ciencias Biológicas y Ambientales (COCIBA), Universidad San Francisco de Quito (USFQ), 170901 Quito, Ecuador

Received 1 December 2025, Accepted 29 January 2026, Published online 3 April 2026

አጭር ማጠቃለያ (Abstract)

ይህ ጽሑፍ የአሸዋ ትንኝ ናሙናዎችን ለማቀነባበር እና በስላይድ ላይ ለማንበር የሚያገለግል አጠቃላይ መመሪያ ያቀርባል፤ ይህም ለዝርያ መለያ እንዲሁም በሽታ አምጪ ተህዋስያንን ለመለየት እና ለይቶ ለማውጣት እጅግ አስፈላጊ ነው። ጽሁፉ ለመስክም ሆነ ለቤተ-ሙከራ ሁኔታዎች ተስማሚ የሆኑ የተለያዩ ዘዴዎችን ይተነትናል። ይህ መመሪያ ስለ አሸዋ ትንኞች አሰባሰብ፣ አያያዝ እና በማደንዘዝ የመግደል ሂደት (ከኬሚካሎች ይልቅ በቅዝቃዜ ማድረቅ ወይም በካርቦን ዳይ አክሳይድ (CO_2) እንዲገደሉ ተመራጭ ዘይቤ መሆኑን ያስገነዝባል) እንዲሁም የአሸዋ ትንኝ ናሙናዎችን ለረጅም ጊዜ ለማቆየት በቅዝቃዜ ወይም በኢታኖል ውስጥ ስለማቆየት ያሉ ስልቶችን ዝርዝር መመሪያዎችን ያካትታል።

የአሸዋ ትንኝ የአካል ክፍሎች (የመራቢያ አካላት፣ ጭንቅላት እና ክንፎች) የዝግጅት ጥራት ለማይክሮስኮፕ ምልከታ አስፈላጊ በመሆኑ በዚህ ሥራ ውስጥ ተገልጸዋል። ጽሁፉ የናሙና ማቀነባበሪያ ሂደቶችን በዝርዝር ይገልጻል። ለምሳሌም እንደ ፖታስየም ሃይድሮክሳይድ እና የማርክ-አንድሬ (Marc-André) ዉህዶችን በመጠቀም ናሙናዎችን የማጥራት ሂደትን (clearing process) ያካትታል። የአሸዋ ትንኝ ናሙና ማንበር ሂደቱ የተለያዩ ዘዴዎችን የሚያወዳድር ሲሆን፣ የእነሱንም ሲራዊ ባህሪያት (optical properties) እና የማቆየት አቅም (preservation pontential) ላይ ትኩረት ያደርጋል። የሆየር ፈሳሽ (Hoyer fluid - chloral gum) ወይም ክሎራል ገም ተብሎም የሚታወቀው የሴት የዘር ከረጢትን (spermathecae) በግልጽ ለማየት እና ለፈጣን ምልከታ ተመራጭ ሆኖ ሳለ ለረጅም ጊዜ ማቆያ ግን ተስማሚ አይደለም።

በተጨማሪም ሌሎች የተጠቀሱ ዉህዶች እንደ ፖሊቪኒል አልኮል (polyvinyl alcohol)፣ ዩፓራል ([Euparal®] - ውስን ውሃን የመቋቋም አቅም ላላቸው) እና ካናዳ ባልሳም ([Canada balsam] - በሃይድሮካርቦን የሚሟሟ) ሲሆኑ፣ የኋለኞቹ ሁለቱ ናሙናን ለረጅም ጊዜ የማቆየት አቅም አላቸዉ። እንዲሁም የናሙና አቀነባበር ልዩ ትኩረት የሚሹ አዳዲስ የሞለኪውላር ባዮሎጂ ዘዴዎች ለምሳሌ የዘረ መል ኮድ ቅደም ተከተል ትንተና (DNA sequencing) እና ማልዲ ቶፍ (MALDI-ToF) ተዳስሰዋል። በተጨማሪም፣ የተለያዩ የናሙና ዝግጅት ዘዴዎችን የሚያሳዩ አጫጭር የቪዲዮ ይዘቶች እና የፅሁፍ መመሪያው በ33 የተለያዩ ቋንቋዎች ተተርጉመዉ የቀረቡ ሲሆን፣ ይህም መመሪያዉ የአለም አቀፉን የሳይንስ ማህበረሰብ ፍላጎት እንዲያሟላ ያስችለዋል።

ቁልፍ ቃላት (Key words): ማንበር (Mounting)፣ ፍሌቦቶመስ የአሸዋ ትንኝ (phlebotomine sand fly)፣ የሆየር ፈሳሽ (Hoyer fluid)፣ የማርክ-አንድሬ ዉህድ (Marc-André solution)፣ ክሎራል ገም (chloral gum)፣ ፖሊቪኒል አልኮል (polyvinyl alcohol)፣ ዩፓራል (Euparal®)፣ ካናዳ ባልሳም (Canada balsam)፣ የሌሽማኒያ ንጠላ (Leishmania isolation)፣ የመስክ ሁኔታዎች (field conditions)፣ ማራባት (culture)፣ መበለት (dissection)፣ ሞለኪውላር ባዮሎጂ (molecular biology)፣ ማልዲ ቶፍ (MALDI-ToF)፣ ሙነነ-አምሳያ (Type-specimens)።

Abstract – Processing and mounting phlebotomine sand flies: a consensus guideline. This article provides a comprehensive guide for the processing and mounting of phlebotomine sand fly specimens, which is crucial for species identification and pathogen detection and isolation. It discusses a range of techniques suitable for both field and laboratory settings. The guide includes detailed instructions on sand fly collection, handling, covering, and euthanasia (recommending dry freezing or CO_2 over chemicals) as well as conservation strategies, such as cold storage and preservation in ethanol. The quality of preparation of certain anatomical structures (genital organs, head and wings) is essential for their proper microscopic observation and is described in this work. The article also presents detailed sample processing, including the clearing process with agents such as potassium hydroxide then Marc-André solution. The mounting process compares different media, emphasizing their optical properties and preservation potential. Hoyer fluid (also known as chloral gum) is recommended for quick observation, particularly for spermathecae, due to its clarity, although it is not suitable for long-term storage. Other media discussed include polyvinyl alcohol, Euparal® (for limited water tolerance), and Canada balsam (a hydrocarbon-soluble medium), with the latter two offering long-term preservation capabilities. Innovative molecular biology approaches such as DNA sequencing and MALDI-ToF, which require particular attention to sample processing, are also addressed. Furthermore, short video clips illustrating various mounting techniques as well as translations in many different languages are provided, allowing the guideline to reach the diverse needs and expectations of the global scientific community.

Key words: Mounting, Phlebotomine sand fly, Hoyer fluid, Marc-André solution, Chloral gum, Polyvinyl alcohol, Euparal®, Canada balsam, *Leishmania* isolation, Field conditions, Culture, Dissection, Molecular biology, MALDI-ToF, Type-specimens.

መግቢያ (Introduction)

ፍሌቦቶሚን አሸዋ ትንኞች (Phlebotomine sand flies) ሳይኮዲዴ (Psychodidae) በተባለው የነፍሳት ቤተሰብ እና ፍሌቦቶሚኔ (Phlebotominae) በተባለው ንዑስ ቤተሰብ ውስጥ የሚመደቡ ሆኖ ቢያንስ 1,063 የታወቁ ዝርያዎች ያሏቸው ትናንሽ ባለ ሁለት ክንፍ (dipteran) ነፍሳት ናቸው [21]። እነዚህ ትንኞች ለላይሽማኒያሲስ (leishmaniasis - ቁስለ-ደዌ)፣ ለአርቦቫይረስ ኢንፌክሽኖች እና ለባርቶኔሎሲስ (bartonellosis) በሽታዎች ተጠያቂ የሆኑ በሽታ አምጪ ተህዋስያንን (ላይሽማኒያ፣ አርቦቫይረስ እና ባርቶኔላ) የሚያስተላልፉ ተባዮች (vectors) ናቸው። የእነዚህን ትንኞች ዝርያ የመለየት ዘዴዉ በጥንቃቄ ናሙናን በመሰብሰብና በመያዝ እንዲሁም በስላይድ ላይ በማንበር፣ ከዚያም በአጉሊ መነጽር (Microscopic) ምርመራ የሚከናወን ነው። ይህ ሂደት በርካታ ቴክኒኮችን የሚጠይቅ ሲሆን፣ እያንዳንዱ ቴክኒክ የራሱ ጥቅሞች እና ገደቦች ወይም ጉድለቶች አሉት።

የአሸዋ ትንኞች መለያ ዘዴ በውጫዊ የአካል ክፍሎች (ለምሳሌ፡- አንቴና፣ ፓልፒ፣ የወንድ መራቢያ አካል) እና በውስጣዊ የአካል ክፍሎች (ለምሳሌ፡- ጉሮሮ [pharynx]፣ የምግብ መፍጫና ማስተላለፊያ ቧንቧ [cibarium] እና የሴት የዘር ከረጢት [spermathecae]) ምልከታ ላይ የተመሰረተ ነው። የውስጣዊ ክፍሎቹን መበለት እና ንጠላ ምልከታውን በማቅለል ትክክለኛ ዝርያ መለየትን ያስችላል። ስለዚህ፣ ከወባ ትንኝና ሌሎች ተባዮች ለምሳሌ ትኋን መሰል ተባይ (kissing bugs) በተለየ ሁኔታ፣ አሸዋ ትንኞችን በዝርያ ለመለየት ናሙናዎችን በስላይድ እና በሽፋን መስታወት (cover slip) መካከል ማንበር ያስፈልጋል።

እስከ 1980ዎቹ ድረስ የአጉሊ መነጽር ምልከታ አሸዋ ትንኞችን መለያ ብቸኛ ዘዴ ነበር፤ እስካሁንም ድረስ በስፋት ጥቅም ላይ እየዋለ ይገኛል። ስለዚህ የሂደቱ እና የዝግጅቱ ምርጫ በአንጻራዊ ሁኔታ ግልጽ እና በሁለት አማራጮች ላይ የተመሰረተ ነዉ። የመጀመሪያዉ ዘይቤ ናሙናው ለረጅም ጊዜ እንዲቆይ የሚያስችል ቋሚ ማንበር (definitive mounting) ሲሆን፣ ሁለተኛዉ ደግሞ ናሙናን ለረጅም ጊዜ ለማቆየት ዋስትና በማይሰጥ ፈሳሽ ውስጥ በፍጥነት ማንበር ናቸዉ። ለምሳሌ ናሙናን በቋሚነት ለማንበር እንደ ካናዳ ባልሳም (Canada balsam) ባሉ ሙጫዎች ውስጥ ማንበር፣ ናሙናው ሙሉ በሙሉ ለመድረቅ ብዙ ጊዜ የሚወስድ ነው። በተጨማሪም፣ የዚህ

ዉህድ የብርሃን ነጸብራቅ አመልካች መለኪያ (refractive index) የሴት ትንኝ የዘር ከረጢት (spermathecae) በቀላሉ ለማየት አያስችልም። በተቃራኒው በውሃማ ዉህድ (ለምሳሌ፡- በሆየር ፈሳሽ [Hoyer fluid]) ማንበር ፈጣን እና የሴት ትንኝ የዘር ከረጢት በግልጽ ለማየት ይረዳል፤ ነገር ግን ዉህዱ ውሀን ከአየር የመሳብ ባህሪ ስላለው ለረጅም ጊዜ ማቆያ ተስማሚ አይደለም። ይህን ችግር ለመቅረፍ አንደኛው አማራጭ ስላይዱ ሙሉ በሙሉ ሲደርቅ ጠርዙን በጥፍር ቀለም ማሸግ (seal) ነው። ይህ የጥቅምና ጉዳት ሚዛን (ምርጫ) እንደ ናሙናው ዝግጅት ዓላማ፣ የምንመርጠው ናሙናን የማንበር ዘዴ (Mounting method) ላይ ተጽዕኖ ያሳድራል።

ከ 1980ዎቹ ጀምሮ የአሸዋ ትንኝ መለያ ጥናቶች የአካላዊ ቅርጸት(morphology) እና የባዮ-ኬሚካል ዘዴዎችን አቀናጅተዋል። የመጀመሪያው የትንኝ ቆዳ ሽፋን የሃይድሮካርቦን ትንተና (cuticular hydrocarbon analyses) ሲሆን፣ በፍጥነትም በሞለኪውላር ባዮሎጂ ቴክኒኮች (ለምሳሌ፡- በዘፈቀደ የሚባዙ ብዙ-መልክ የዲኤንኤ ክፍሎች [Random amplified polymorphic DNA]- RAPD, በቆራጭ ኢንዛይሞች የሚፈጠር የዲኤንኤ ቁርጥራጭ ርዝመት ልዩነት [restriction fragment length polymorphism]-RFLP, ዲኤንኤ ማባዛት እና የሳንገር የዘረ መል ኮድ ቅደም-ተከተል፣ እንዲሁም የቀጣዩ ትውልድ (Next Generation) የዘረ መል ኮድ የቅደም-ተከተል ትንተና) ተተክቷል። ክቅርብ ጊዜ ወዲህም የሞለኪውላር ዘዴዎች በፕሮቲን ምርመራ ዘይቤዎች ለምሳሌ ማልዲ-ቶፍን (MALDI-ToF) በመሳሰሉ ተጠናክረዋል። ከዚህም በተጨማሪ፣ የዘረ-መል (Molecular) ዝርያ መለያ ዘዴን በሽታ አምጪ ተህዋሲያንን በፒሲአር (PCR) ከመለየት ተግባር ጋር ማቀናጀት ይቻላል። ይህም እንደ ሊሽማኒያ (Leishmania)፣ ትራይፓኖሶማ (Trypanosoma)፣ ባርቶኔላ (Bartonella) እና ፍሌቦቫይረስ (Phlebovirus) ያሉ ተህዋሲያንን በሁለቱም የፒሲአር (PCR) ዓይነቶች: ማለትም፣ ኤንድ ነይንት [End-point] እና ሪል ታይም [Real-time]) መለየት ስለሚቻል ነው። ሆኖም ይህ ሂደት ናሙና አሰባሰቡ እና አያያዙ (Storage) ከተቀመጠው ዓላማ ጋር እንዲጣጣም ማስተካከልን ይጠይቃል። ዝርያዎችን ለመለየት በልምድ ከምንጠቀምባቸው አካላዊ መዋቅሮች (Morphological characteristics) በተጨማሪ፣ ሌሎች አካላዊ ዘዴዎችንም (ለምሳሌ የክንፍ ጂኦሞርፎሜትሪ/Wing geomorphometry) ተግባራዊ ማድረግ ይቻላል።ይህ ደግሞ እንደታቀደው ግብ የናሙና አሰባሰብ እና አያያዝ ሂደትን ማስተካከል ይጠይቃል [3, 32]። ከባህላዊ የአካላዊ ቅርፀት ባህሪያት በተጨማሪ፣ ሌሎች የአካላዊ ቅርፀት ዘዴዎችም (ለምሳሌ፡- የክንፍ ጂኦ-ሞርፎሜትሪ) ጥቅም ላይ ሊውሉ ይችላሉ።

የጽሁፉ ባለቤቶችን (Authors) የግል ተሞክሮ እና በቀድሞ የምርምር ጽሑፍ መረጃዎች ላይ በመመስረት፣ የዚህ ጥናት ዓላማ አሸዋ ትንኞችን ለአካላዊ ቅርጸት እና ለሞለኪውላዊ ምርመራዎች አመቺ በሆነ መልኩ ለማንበር እና ለማዘጋጀት የሚያስችል ደረጃውን የጠበቀ መመሪያ (standardized guidelines) ማቅረብ ነው። የተወሰኑ ትንተናዎችን (ለምሳሌ፡- ሞለኪውላር ባዮሎጂ ወይም ማልዲቶፍ [MALDI-ToF]) ለማከናወን፣ ለአካላዊ ቅርፀት (Morphological) መለያ የማይፈለጉትን የአሸዋ ትንኝ አካል ክፍሎች ጠብቆ ማቆየትን ይጠይቃል። ይህም እጅግ ጥንቃቄ የተሞላበት የምርምር ቅደም ተከተል (Protocol) ምርጫ አስፈላጊ መሆኑን ያጎላል። በዚህ ጽሑፍ ውስጥ፣ በሕይወት የተያዙ የአሸዋ ትንኞችን ማደንዘዣ (Anesthesia) እና በስቃይ አልባ መግደያ (Euthanasia) ዘዴዎች፣ ክምችታቸዉን (Storage) እና ለፈጣን መለያ ወይም ለረጅም ጊዜ ማቆያ (ለቀጣይ ጥናቶች እንዲመች) የሚረዳውን የማንበር (Mounting) ሂደት ላይ ትኩረት እናደርጋለን።

1. የአሸዋ ትንኝ አሰባሰብ (Sand fly capture)

የአሸዋ ትንኞችን በተለያዩ ዘዴዎች በመጠቀም በሕይወት እያሉ ወይም ከሞቱ በኋላ መሰብሰብ ይቻላል። እነዚህም ዘዴዎች እንደ ሲዲሲ የብርሃን ወጥመዶች (CDC miniature light traps)፣ የሚያጣብቁ ወጥመዶች (sticky traps)፣ ሻኖን የሚባል ወጥመዶችን (Shannon traps) በመጠቀም፡ ወይም በቀጥታ በአካባቢው ካሉ ማረፊያ ቦታዎቻቸው (ለምሳሌ፡- ከእንስሳት መጠለያዎች) በመጣጭ መሣሪያዎች (aspirators) በመሳብ መሰብሰብን ያካትታሉ። እነዚህ ዘዴዎች ወጥመዶችን ተስማሚ በሆኑ የመኖሪያ አካባቢዎች ውስጥ ማስቀመጥን፣ ትንኞቹን በብርሃን ወይም በሌሎች ማማለያዎች (ለምሳሌ፡- ካርቦን ዳይኦክሳይድ (CO2) ወይም ኬሚካላዊ ማማለያዎች) መሳብን፣ እና ለቀጣይ ምርመራ መሰብሰብን ያካትታሉ፤ ይህም በተለያዩ የሳይንስ ጽሑፎች ላይ ተገልጿል [2, 3, 32, 36, 49]።

አሸዋ ትንኞችን በሕይወት እያሉ መሰብሰብ ሁሉንም የቀጣይ የላቦራቶሪ ምርመራዎችን ለማካሄድ ያስችላል፤ በአንጻሩ የሞቱ ትንኞችን መሰብሰብ ግን የላይሽማኒያ ጥገኛ ተህዋሲያንን ወይም የቫይረስ ዝርያዎችን ለመለየት (isolation) አስቸጋሪ ያደርገዋል። አንዳንድ የመሰብሰቢያ ዘዴዎች፣ ለምሳሌ የሚያጣብቁ ወረቀቶች (sticky papers)፣ የአሸዋ ትንኝ የአካል ክፍሎችን (አንቴና፣ ፓልፒ፣ ክንፎች ወይም እግሮች) እንዲረግፉ ወይም እንዲጠፉ ያደርጋሉ። በሚያጣብቁ ወረቀቶች ላይ የሚቀባው የጉሎ ዘይት (castor oil) ትንኞቹን ስለሚያጣብቅ፣ አስቀድሞ መወገድ አለበት፤ ይህም የሚከናወነዉ እኩል መጠን ባለው የኤታኖል እና ዳይኢትል ኢተር (diethyl ether) በእኩል መጠን በማደባለቅ ለ 15 ደቂቃ በመዘፍዘፍ ነዉ::

2. ስቃይ አልባ የናሙናዎች አገዳደል (Specimen euthanasia)

አሸዋ ትንኞች ከተሰበሰቡ በኋላ በሕይወት ካሉ መገደል አለባቸው። በአንዳንድ የአሰባሰብ ዘዴዎች (ለምሳሌ፡- አጣባቂ ወረቀቶች፣ በሳሙና ወይም በአልኮል የተሞላ ማጠራቀሚያ ያላቸው የሲዲሲ (CDC) የብርሃን ወጥመዶች) ትንኞቹ በሚያዙበት ጊዜ ወዲያዉኑ ይሞታሉ። በቀጥታ በአልኮል (Ethanol) ውስጥ ለተሰበሰቡ ወይም ከተያዙ በኋላ በአፋጣኝ አልኮል ውስጥ ለገቡ ትንኞች የዘረ-መል (Molecular biology) ምርመራ ማድረግ ይቻላል። ሆኖም፣ እነዚህ የመግደያ ዘዴዎች ናሙናውን በማልዲቶፍ (MALDI-ToF) ለመመርምር አያስችሉም። በተጨማሪም፣ አንዳንድ የመግደያ ዘዴዎች የተወሰኑ አካላዊ መለያዎችን (Morphological characters) ሊያጠፉ ይችላሉ። ስለዚህ ትክክለኛውን ዝርያ ለመለየት ወይም ናሙናዎችን ለረጅም ጊዜ እንደ ማስረጃ (Voucher specimens) ለማስቀመጥ ተገቢውን የመግደያ ዘዴ መጠቀም ወሳኝ ነው።

ኢታይል አሴቴት (Ethyl acetate)፣ ኢታይል ኢተር (Ethyl ether)፣ ቴትራክሎሮኢቴን (Tetrachloroethane) እና ክሎሮፎርም (Chloroform) የመሳሰሉ ኬሚካሎችን በጥጥ ነክሮ ትንኞቹ ባሉበት ዕቃ ውስጥ በማስቀመጥ መግደል ይቻላል። እነዚህ ኬሚካሎች መርዛማ በመሆናቸው በአምራቹ መመሪያ መሠረት በጥንቃቄ መያዝ አለባቸው። ይሁን እንጂ፣ በእኛ ተሞክሮ ክሎሮፎርምን መጠቀም ለዘረ-መል (Molecular) ጥናቶች አመቺ ስላልሆነ ትንኞችን በዚህ ኬሚካል መግደልን አንመክርም። ባጠቃላይ እነዚህ ኬሚካሎች አደገኛ በመሆናቸውና ለዘረ-መል ትንተና ያላቸው ብቃት አጠራጣሪ በመሆኑ፣ ጥቅም ላይ ባይዉሉ ይመረጣል::

አካላዊ ቅርፀትን (Morphology)፣ ዲ ኤን ኤን (DNA) ወይም ፕሮቲንን ሳይበላሽ ለመጠበቅ በብዛት ጥቅም ላይ የሚውለው ዘዴ ናሙናዎችን በደረቁ ማቀዝቀዝ (Dry freezing) ነው። ትንኞቹ ሙሉ በሙሉ እንዲደነዝዙ (Anesthetized) ለተወሰነ ጊዜ ማቀዝቀዣ ዉስጥ መቆየት አለባቸው፤ ነገር ግን እንዳይደርቁ ወይም ዓላማው የሊሽማኒያ ጥገኛ ተህዋሲያንን ከትንኟ ሆድ ውስጥ ለይቶ ማሳደግ (Culture) ከሆነ የተህዋሲያኑን ሕልውና እንዳይጎዳ ጥንቃቄ መደረግ አለበት። ስለዚህ የሊሽማኒያ ተህዋሲያን እንዳይሞቱ ክትትል በማድረግ ናሙናዉን በ -20 ድግሪ ሴንቲ ግሬድ ቅዝቃዜ ውስጥ ከ15 እስከ 20 ደቂቃዎች እንዲቆዩ ማድረግ ይመከራል::

ማቀዝቀዣ (Freezer) በማይኖርበት ጊዜ ትንኞችን በካርቦን ዳይኦክሳይድ (CO2) መግደል ይቻላል። የCO2 ሲሊንደሮችን ወደ መስክ መውሰድ በማይቻልበት ሁኔታ፣ ለመጠጥ ማከፋፈያ (Soda siphons) የሚያገለግሉ ትናንሽ የካርቦን ዳይኦክሳይድ መያዣዎችን መጠቀም ይቻላል፡፡ ነገር ግን እነዚህን በአውሮፕላን ለማጓጓዝ ገደብ ሊኖር ይችላል። እንደ መጨረሻ አማራጭ፣ ትንኞችን በሲጋራ ጭስ መግደል ይቻላል። በሲዲሲ (CDC) ወጥመድ የተያዙ ትንኞች በመምጠጫ (Aspirator) ተስበው በመስታወት ቱቦ ውስጥ እንዲቆዩ ከተደረገ በኋላ ለሲጋራ ጭስ ሲጋለጡ በሰከንዶች ውስጥ ይሞታሉ። ይህ ዘዴ በማንኛውም የመስክ ሁኔታ እና ራቅ ያሉ አካባቢዎችም ጭምር ተግባራዊ ሊሆን ይችላል። ሆኖም፣ የመስታወቱ ቱቦ በጭስ ስለሚበከል፣ በደንብ ሳይጸዳ በሕይወት ያሉ ትንኞችን ለመያዝ ዳግመኛ መጠቀም አይቻልም። ቢሆንም፣ ያልጸዳውን መምጠጫ ለሌሎች ትንኞች መግደያነት መጠቀም ይቻላል። ሁሉም ናሙናዎች ከመምጠጫው ውስጥ መውጣታቸውን ማረጋገጥ ያስፈልጋል። እነዚህ ሁሉ ዘዴዎች የትንኟን ሆድ በመበለት (Gut dissection) የሊሽማኒያ ተህዋሲያንን ለይቶ ለማውጣት ምቹ ናቸው።

መቅድም (Preface)፡-

የደህንነት እና የቁጥጥር ጉዳዮች ተገቢውን የቁስ ደህንነት መረጃ ሰነድ (Safety Data Sheets - SDS) መጥቀስ አለባቸው። በዚህ መመሪያ ውስጥ የቀረቡ ሁሉም ኬሚካሎች ጥብቅ በሆነ የደህንነት ሁኔታ ውስጥ መያዝ ይኖርባቸዋል። ስለነዚህ ኬሚካሎች አደገኛነት እንዲሁም ስለ አያያዛቸው እና ስለ አወጋገዳቸው መረጃ የሚሰጡ የምርምር ተቋማቱ የጤና እና ደህንነት ኮሚቴዎች አሉ። ኬሚካሎቹን ስለመጠቀምና ስለማስወገድ የቀረቡትን የደህንነት መመሪያዎች መከተል ግዴታ ነው። በተጨማሪም፣ መልካም እና ደህንነቱ የተጠበቀ የላቦራቶሪ አሰራርን መከተል እንዲሁም የሀገሪቱን ወይም የምርምር ተቋሙን ሕጎች እና ደንቦች ማክበር የእያንዳንዱ ተጠቃሚ ኃላፊነት መሆኑ ሊታወቅ ይገባል። ከዚህም በላይ፣ የተወሰኑ ኬሚካሎች ወይም የኬሚካሎቹ ክፍሎች (ለምሳሌ፦ ክሎራል ሃይድሬት/Chloral hydrate) በአንዳንድ ሀገራት በሕግ የተገደቡ ሊሆኑ ይችላሉ።
በዚህ ጽሑፍ ውስጥ ጥቅም ላይ የዋሉ ምህጻረ ቃላት ዝርዝር በሰንጠረዥ 1 ላይ ቀርቧል።

ሰንጠረዥ ቁጥር 1 የምህጻረ ቃላት ዝርዝር (List of abbreviations)

DNA [ዲኤንኤ]	Deoxyribonucleic acid = የዘረመል መረጃ ተሸካሚ ሞለኪዉል
RNA [አር ኤን ኤ]	Ribonucleic acid = አርኤንኤ የተባለ የዘረመል መረጃን አስተላላፊ ሞለኪዉል
BME [ቢኤምኢ]	Basal Medium Eagle = ለህዋስ ማራቢያ የሚያገለግል ፈሳሽ ዉህድ
CDC [ሲዲሲ]	Centers for Disease Control and Prevention = የበሽታ መቆጣጠሪያና መከላከያ ማዕከል
CMCP [ሲኤምሲፒ]	Camphor-monochlorophenol ካምፎር-ሞኖክሎሮፌኖል የሚባል ናሙና ማንበሪያ ኬሚካል
CMR [ሲኤምአር]	Carcinogenic, mutagenic, reprotoxic substance = ካንሰር አምጪ፣ ዘረመልን የሚለውጥ ወይም መራቢያ አካላትን የሚጎዳ ንጥረ ነገር
COI [ሲኦአይ]	Cytochrome c oxidase subunit I gene ሳይቶክሮም ሲ ኦክሲዴዝ የሚባል ለዝርያ መለያ የሚያገለግል የዘረ መል ኮድ)
CytB [ሲዋይቲ ቢ]	Cytochrome b gene = ሳይቶክሮም ቢ ጂን የሚባል ለዝርያ መለያ የሚያገለግል የዘረ መል ኮድ
ELISA [ኤላይዛ]	Enzyme-linked immunosorbent assay = ኤላይዛ የተባለ ምርመራ ዘዴ
EtOH [ኢቲኦኤች]	Ethanol = ኤታኖል የተባለ አልኮል
M199 [ኤም 199]	Medium 199 = ሚዲየም 199 ተህዋሲያን ማሳደጊያ ፈሳሽ ዉህድ
MALDI-ToF MS [ማልዲ ቶፍ ኤም ኤስ]	Matrix-assisted laser desorption/ionization time-of-flight mass spectrometry = ማልዲ-ቶፍ የተባለ በማትሪክስ የታገዘ ሌዘርን በመጠቀም የቁስ አካላትን ክብደትና ፍጥነት መለኪያ የትንተና ዘዴ
MEM [ኤም ኢ ኤም]	Minimum essential media = ሚኒመም ኤሴንሻል ሚዲያ የሚባል ህዋሳትን ለማራባት የሚያገለግል ፈሳሽ ዉህድ
NGS [ኤን ጂ ኤስ]	Next-generation sequencing = ቀጣዩ ትውልድ የዘረ መል ኮድ ቅደም-ተከተል መለያ ዘዴ
NNN [ኤን ኤን ኤን]	Novy-MacNeal-Nicolle medium = ኤን.ኤን.ኤን የተባለ ላይሽማኒያ በሽታ ህዋስን ለማሳደግ የሚጠቅም ውህድ
PCR [ፒሲአር]	Polymerase chain reaction = ፒሲአር የተባለ የዘረ መል ኮድን አባዝቶ መመርመሪያ ዘዴ)
Lao PDR[ላኦ ፒዲአር]	Lao People's Democratic Republic = የላኦስ ሕዝባዊ ዲሞክራሲያዊ ሪፐብሊክ
PNOC [ፒ ኤን ኦ ሲ]	Prepronociceptin gene = ፕሪፕሮኖሲሴፕቲን የተባለ ዘረ መል ኮድ
qPCR [ኪዉ ፒ ሲ አር]	Quantitative PCR (real-time PCR) = መጠናዊ ልኬትንና ወቅታዊ ዉጤትን የሚያሳይ የፒሲአር ምርመራ
RAPD [አር ኤ ፒ ዲ]	Random amplified polymorphic DNA = በነሲባዊ ሂደት የዘረ መል ኮድን(የዲኤንኤ)በማጉላት ዝርያን መለየት
RFLP [አር ኤፍ ኤል ፒ]	Restriction fragment length polymorphism = የዲኤንኤ ቁርጥራጮች ርዝመትን በመለካት ዝርያን መለያ ዘዴ
RI [አር አይ]	Refractive index = የብርሃን ነጸብራቅ አመልካች መለኪያ
RNases [አር ኤን ኤዝ]	Ribonucleases = የአርኤንኤ ዘረመልን አላሚ ኢንዛይሞች
RNASS [አር ኤን ኤ ኤስ ኤስ]	RNA stabilization solution = አርኤንኤን ሳይበላሽ የሚያቆይ አርጊ ዉህድ
RT-PCR [አር ቲ ፒ ሲ አር]	Reverse transcription PCR = ቅልብስ ዘረ መል ቅጅ ፒሲአር (ከአርኤንኤ ወደ ዲኤንኤ በመቀየር የሚደረግ የፒሲአር ምርመራ)
TFA [ቲ ኤፍ ኤ]	Acide trifluoroacétique = ትራይፍሎሮአሴቲክ አሲድ የተባለ ዉህድ

3. ናሙናዎችን ከማቀነባበር በፊት የማከማቸት ዘዴዎች

ናሙናዎችን ለማዘጋጀት አምስት ዋና ዋና የትክለት (Fixation) ዘዴዎች አሉ፡-

3.1. ማቀዝቀዝ (Freezing)

ይህ ዘዴ በ-20 ድግሪ ሴንቲ ግሬድ ወይም ይበልጥ ተመራጭ በሆነው በ -80ድግሪ ሴንቲ ግሬድ የሙቀት መጠን ይከናወናል። በአሁኑ ጊዜ እነዚህ ዘዴዎች ከፈሳሽ ናይትሮጅን (Liquid nitrogen) በላይ በስፋት ጥቅም ላይ ይውላሉ። በሁሉም ሁኔታዎች፣ ትንኞቹ እንዲደነዝዙ ከተደረገ በኋላ የማቀዝቀዝ ሂደቱ በአፋጣኝ መከናወን አለበት። በማቀዝቀዣ ውስጥ ማቆየት ነፍሳቱን ሙሉ በሙሉ ከመጠበቁም በላይ፣ አር ኤን ኤ (RNA)፣ ዲ ኤን ኤ (DNA) እና የፕሮቲኖች ይዘታቸው ሳይለወጥ ለረጅም ጊዜ ለማቆየት ይረዳል። በአንጻሩ፣ ፈሳሽ ናይትሮጅን የትንኞቹን ክንፎች፣ እግሮች እና አንቴናዎች ሊሰባብርና ዋና ዋና መለያ ባህሪያትን ሊያጠፋ ይችላል። ደረቅ ማቀዝቀዣ (Dry freezer) ለናሙናዎቹ ደህንነት የተሻለ ቢሆንም፣ ተፈርካሽ የሆኑ የውስጥ አካላትን ለመጠበቅ ግን ተመራጭ አይደለም። ናሙናው ከበረዶ ሲቀልጥ (Thawing) ክንፎች፣ አንቴናዎች፣ ፓልፐ የሚባለዉ የአፍ ክፍልና እግሮች ከማጠራቀሚያዉ ብልቃጥ ጋር ስለሚጣበቁ ሊቀዳደዱ ይችላሉ። ይሁን እንጂ፣ ጥናቶች በመስክ በሚከናወኑበት ጊዜ ማቀዝቀዣ ወይም ፈሳሽ ናይትሮጅን ስለማይገኝ ናሙናዎችን በጥልቅ ቅዝቃዜ ማቆየት አዳጋች ይሆናል። ናሙናን በጥልቅ ማቀዝቀዣ ውስጥ ማከማቸት ምንም ዓይነት የምርመራ ትክክለኛነት (sensitivity) ሳይቀንስ በሞለኪውላዊ ዘይቤዎች አማካኝነት በሽታ አምጪ ተህዋስያንን ለመለየት ሙሉ በሙሉ ተስማሚ ነው፡፡ የአር.ኤን.ኤ (RNA) ቫይረሶች ለመለየት እና ነጥሎ ለማውጣት (isolation) እንዲሁም ለረጅም ጊዜ ለማቆየት በ-80ድግሪ ሴንቲ ግሬድ ወይም በፈሳሽ ናይትሮጅን ውስጥ ማቀዝቀዝ ይጠይቃል፡፡ ናሙናዎችን በጥልቅ ማቀዝቀዝ የሊሽማኒያ ተህዋሲያንን ከትንኟ ሆድ ለይቶ ለማውጣት አያመችም፤ ይህን ለማድረግ የሚቻለዉ ትንኞችን መጀመሪያ በፈሳሽ ናይትሮጅን ትነት (Vapor phase) ውስጥ በማቆየት ቀጥሎም በፈሳሽ ናይትሮጅኑ ውስጥ እንዲገቡ ከተደረገ ብቻ ነው (ለምሳሌ፡- ብልቃጦቹን [Vials] በስስ ካልሲ [Stocking] ውስጥ በማስገባት)፤ ይህም ክንዋኔ የሊሽማኒያ ተህዋሲያንን በቅዝቃዜ የመጠበቅ (Cryopreservation) ሂደትን የሚመስል ተግባር ነው።

3.2. በአልኮል ውስጥ ማከማቸት (Storage in alcohol)

ይህ ምናልባትም የአሸዋ ትንኞችን ለማቆየት በብዛት ጥቅም ላይ የሚውለው ዘዴ ነው። ላቦራቶሪ በሌለባቸው ራቅ ያሉ አካባቢዎችና አስቸጋሪ የመስክ ሁኔታዎች ለመተግበር አመቺ ነው። እንዲሁም አልኮል ለአካላዊ ቅርፀት (Morphology) ጥናት በጣም ተስማሚ ነው፤ የትንኟ ክንፎች፣ እግሮች እና አንቴናዎች በማከማቻ ብልቃጥ ውስጥ የአየር አረፋ ካልተፈጠረ ሳይሰበሩ ይቆያሉ። ስለዚህ የአየር አረፋን ለማስወገድ ብልቃጡን በትንሽ ጥጥ መዝጋትና የናሙናዉን መለያ ምልክት (Label) ከጥጡ አናት ላይ ማድረግ ይመከራል (ምስል ቁጥር 1)

ናሙና ለረጅም ጊዜ በአልኮል ለማስቀመጥ የአልኮሉ ምጥነት (Concentration) የተለያዩ ግንዛቤዎች አሉ። በአጠቃላይ ከ70% በታች የሆነ አልኮል ምጥነት አይመከርም። ከፍተኛ ምጥነት ያለው አልኮል ዲ ኤን ኤን (DNA) ለረጅም ጊዜ ያቆያል፤ ነገር ግን ይህ ምጥነት ትንኞችን ተሰባባሪ (Brittle) ስለሚያደርጋቸዉ ለአካላዊ ቅርፀት ጥናቶች ተስማሚ አይደለም። የ96% ኢታኖል (Ethanol) ምጥነት ርጉዕ ቅልቅል (Azeotrope mixture) ስለሆነ በተለይ እርጥበት አዘል በሆኑ ሞቃታማ ሀገራት የአልኮሉ ምጥነት ሳይቀንስ እንዲቆይ ይረዳል። ሆኖም ግን ከ96% የአልኮል ምጥነት ይልቅ በብዛት የሚገኘዉ 95% ነዉ፡፡

የአልኮሉ ምጥነት ምንም ይሁን ምን፣ ዲ ኤን ኤ በአጠቃላይ በኢታኖል ውስጥ በጥሩ ሁኔታ ይጠበቃል (ምንም እንኳን ከማቀዝቀዝ ዘዴ ጋር ሲነፃፀር በተለይም የቀጣዩ ትውልድ የዘረ መል ኮድ ቅደም-ተከተል መለያ (NGS) ዓይነት የዘረ-መል ምርመራዎች ውጤታማነቱ ዝቅ ያለ ቢሆንም)። ፕሮቲኖች ግን በጣም ዝቅተኛ የመቆየት ባህሪ አላቸው፣ በተለይም እንደ ማልዲ-ቶፍ (MALDI-ToF) ላሉ የፕሮቲን ጥናቶች (Proteomics)። በአልኮል ውስጥ ለጥቂት ወራት የቆዩ አሸዋ ትንኞችን በቅርፃቸው መለየት ይቻላል፤ ነገር ግን ከእነዚህ ናሙናዎች የማጣቀሻ የፕሮቲን መጠነ ድግግም መረጃዎችን (Spectra) ማመንጨት አይቻልም።

ናሙናን በ-20 ዲግሪ ሴንቲ ግሬድ ውስጥ ማቀዘቀዘ በአልኮል ወይም በደረቅ ሁኔታ ውስጥ የማቆየት ሂደቱን ለማሻሻል ያስችላል። በ-20 ዲግሪ ሴንቲ ግሬድ ማቀዝቀዝ በዋነኝነት የዘረ-መል መበላሸትን በመቀነስ ሞለኪውላዊ ጥራትን (ለምሳሌ የኑክሊክ አሲዶችን) ይጠብቃል፤ እንዲሁም የሕብረ ሕዋሳትን መፈራረስ በመቀነስ ለአካል ቅርፀት ማቆያ (Morphological preservation) ተጨማሪ ጥቅም ይሰጣል። ናሙናን ቢያንስ 70% በሆነ አልኮል ምጥነት ለጥቂት ወራት ማቆየት የዲ ኤን ኤ እና አር ኤን ኤ ቫይረሶችን ለመለየት ያስችላል።

በተጨማሪም፣ አይሶፕሮፒል አልኮል (Isopropyl alcohol) በአንዳንድ አገሮች በቀላሉ ሊገኝ ይችላል፤ እሱም ዲ ኤን ኤን ይጠብቃል ነገር ግን ናሙናዎቹ እንዲጠነክሩ (Stiff) ያደርጋቸዋል። እንደ ኢታኖል በቀላሉ ተቀጣጣይ ባለመሆኑ ለማጓጓዝ ምቹ ነው። አስፈላጊ ሆኖ ሲገኝ፣ በፈሳሽ ናይትሮጅን ወይም በደረቅ ማቀዝቀዣ ውስጥ የነበሩ አሸዋ ትንኞችን ወደ አልኮል ማዛወር ይቻላል፤ ይህም የሁለቱንም ዘዴዎች ድክመቶች/ጉድለቶችን ያስወግዳል፡፡

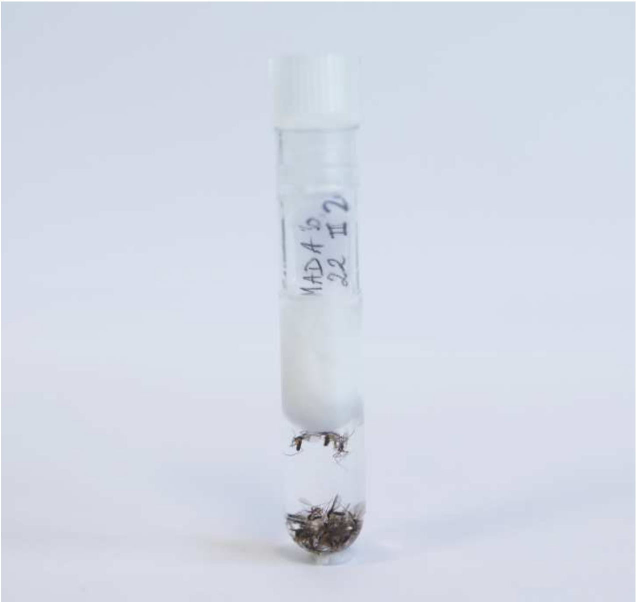

ምስል ቁጥር 1፡- በአሸዋ ትንኞች በኢታኖል (ethano) ውስጥ ተከማችተው ሲታዩ።

3.3. በአርኤንኤ ማረጋጊያ ፈሳሽ (RNASS) ውስጥ ማከማቸት

አር ኤን ኤ ኤስ ኤስ (RNASS) የተባለዉ ውሀማ ዉህድ በስፋት ጥቅም ላይ የሚውልና፣ መርዛማ ያልሆነ፣ እና በህብረ ህዋስ እና በህዋስ ናሙናዎች ውስጥ የሚገኝ አርኤንኤን (RNA) ለማረጋጋት እና ለመጠበቅ ታስቦ የተዘጋጀ ነው። ይህ ፈሳሽ በፍጥነት ወደ ናሙናው ውስጥ ዘልቆ በመግባትና አርኤንኤን የሚጎዱ ኢንዛይሞችን ሥራ በማሰናከል፣ ናሙናውን ወዲያውኑ ማቀዝቀዝ ሳያስፈልግ አርኤንኤው እንዳይበላሽ ይከላከላል። ናሙናዎችን በአር ኤን ኤ ኤስ ኤስ (RNASS) ውስጥ ማስቀመጥ የህብረ ህዋስ እና የህዋስ አካላዊ ቅርጽን በመጠበቅ ቀጣይ የስነ ህብረ ህዋሳዊ ምርመራዎች (histological assessment) ለማከናወን ይረዳል፡፡ አር ኤን ኤ ኤስ ኤስ (RNASS) ለናሙና ትክለት (Fixation) ባይጠቅምም አር ኤን ኤው እንዳይበላሽ ስለሚያደርግ፣ ለአጭር እና ለመካከለኛ ጊዜ ናሙናን ማቆየት የናሙናውን መዋቅራዊ ይዘት በጥሩ ሁኔታ እንዲጠበቅ ያግዛል፡፡

አር ኤን ኤ ኤስ ኤስ (RNASS) ናሙናዎችን በመደበኛ የሙቀት መጠን እስከ 7 ቀናት፣ በ4 ድግሪ ሴንቲ ግሬድ ቅዝቃዜ ውስጥ ለተከታታይ ሳምንታት፣ ወይም በ -20 ድግሪ ሴንቲ ግሬድ/-80 ድግሪ ሴንቲ ግሬድ በማቆየት ለረጅም ጊዜ ማከማቸት ይቻላል። ይህ ዘዴ በተለይ የቅዝቃዜ ጥበቃ ስርዓት (Cold chain) መሠረተ ልማት ውስን በሆነባቸው የመስክ ሥራዎች ወይም ክሊኒኮች ውስጥ እጅግ ጠቃሚ ነው። አር ኤን ኤን (RNA) ከናሙና ዉስጥ ለማጣራት ብዙውን ጊዜ ናሙናዎቹን ከዉህዱ ውስጥ ለይቶ ማውጣትና በመደበኛው የላቦራቶሪ ቅደም ተከተል መሠረት ማቀነባበር ይጠይቃል።

3.4. በመጠቀም ናሙናን በተመጣጣኝ ሙቀት (room temperature) ማቆየት

እርጥበት አልባ ናሙና ማቆያ ዘዴ (Dry preservation) የቆየ አሰራር ሲሆን፣ ሙሉውን ናሙና (in toto) በደረቁ ለማንበር ሲሞከር እንደ ክንፎች፣ እግሮች፣ አንቴናዎች (antennae) እና ፓልፖች (palps) ያሉ ተሰባሪ አካላትን ይጎዳል። ሆኖም፣ እንደ ሲሊካ ጄል (Silica gel) ያሉ እርጥበት መምጠጫ ንጥረ ነገሮችን በመጠቀም ፈጣን ድርቀት (Dehydration) ከተከናወነ፣ በማልዲ-ቶፍ (MALDI-ToF) ማሽን የፕሮቲን ጥናቶችን ማድረግን አያውክም።

በአንጻሩ፣ በእነዚህ ናሙናዎች ላይ የዲ ኤን ኤ (DNA) ትንተና ማድረግ አስቸጋሪ ነው፤ ምክንያቱም ዲ ኤን ኤው የተቆራረጠና (Fragmented) እና ብዛቱም ያነሰ ስለሆነ፡፡ ይህም ማለት ከአዲስ ወይም ከቀዘቀዙ ናሙናዎች ጋር ሲነጻጸር ትንተናውን ፈታኝ ያደርገዋል (በተለይም ለኑክሌር ጂኖም ምርመራ)። ቢሆንም፣ እንደ "ሚውዚዮሚክስ" (Museomics) ያሉ ዘመናዊ ዘዴዎችን በእርጥበት አልባ ማቆያ ዘዴዎች ለተከማቹ ናሙናዎች መጠቀም ይቻላል። ሌላ አማራጭ ካልጠፋ በስተቀር እርጥበት አልባ ማቆያ ማከማቻ ዘዴዎች መጠቀም አይመከርም። የእርጥበት አልባ ማቆያ ዘዴዎችን ከቅዝቃዜ ዘዴ ጋር በማቀናጀት ብልቃጦችን በ -20°C ወይም -80°C ማቀዝቀዣ ውስጥ ማስቀመጥ ይቻላል። በዚህ ዘዴ ውስጥ ዋናው ተግዳሮት ለዝርያ መለያ (Identification) የሚያስፈልጉትን የሰውነት ክፍሎች በትክክል ስላይድ ላይ ማንበር (Mounting) አለመቻል ነው። ይህንን ለማድረግ ደግሞ ናሙናውን ዳግም ማርጠብ (Rehydration) አስፈላጊ ነው። ለዚህም የትራይተን ኤክስ100 (Triton X-100) ዉህድ መጠቀም ይመከራል። የማርጠብ ሂደቱ እንደ ሁኔታው ከጥቂት ሰዓታት እስከ ጥቂት ቀናት ሊወስድ ይችላል፤ በዚህ ጊዜም ናሙናውን በቅርብ መከታተል ያስፈልጋል። ሙሉ በሙሉ ከረጠበ በኋላ፣ ናሙናዎቹ በሦስት ተከታታይ የውሃ መታጠቢያዎች (Water baths) ውስጥ መታጠብ አለባቸው።

3.5. በናሙና ማቆያ ወረቀቶች (Filter papers) ላይ ማቆየት

የናሙና ማቆያ ወረቀቶች (Filter papers) ዋና ጥቅማቸው በደረቁ የተቀመጡ ሙሉ ነፍሳትን ወይም የደም ህዋሳትን በተመጣጣኝ ሙቀት (room temperature) ሳይበላሹ በማከማቸት የዘረ-መል ዲ ኤን ኤን (Genomic DNA) ለረጅም ጊዜ በጽናት ማቆየት ማስቻላቸዉ ነው። የናሙና ማቆያ ወረቀቱ በትንሽ ካርድ መልክ የሚዘጋጅ በመሆኑ፣ በሙቶዎች የሚቆጠሩ ናሙናዎችን የአንድን ትንሽ ጠረጴዛ መሳቢያ በሚያክል ቦታ ውስጥ በመደበኛ ሙቀት ማከማቸት ይቻላል። የወረቀቱ ይዘት ተላላፊ በሽታ አምጪ ተህዋሲያንን በሚያስወግዱ (Denature) ንጥረ ነገሮች የተነከረ በመሆኑ፣ ናሙናዎቹ እንደ አደገኛ ሕይወጠንቅ (Biohazard) አይቆጠሩም። ይህ ደግሞ ናሙናዎችን ያለ ምንም ሕይወጠንቅ ጥንቃቄዎች ለማከማቸትና ለማጓጓዝ ያስችላል። [68]

4. ናሙናዎች መበለት (Specimen dissection)

ሌሎች ነፍሳት ዝርያቸው የሚለየው ሙሉ በሙሉ ተሰክተው በሚታዩ ውጫዊ ባህሪያቸው ነው፣ አሸዋ ትንኞች በአንፃሩ ትክክለኛ ዝርያቸውን ለመለየት የአካል ክፍሎች መበለት እና በጥልቅ ለማጥናት ናሙናዎችን በስላይድ ላይ ማንበር ያስፈልጋል። የተመረጠው የዝግጅት እና ናሙናን የማንበር ሂደት ምንም ይሁን ምን፣ ጥቅም ላይ የሚውለው የአካል መበለት ዘዴ ተመሳሳይ ነው (ምስል 2 እና 3 ይመልከቱ) (https://zenodo.org/records/18198006).

ትራይተን ኤክስ100 (Triton X100) አጠቃቀም፡- አዮኒክ ያልሆነ ዉሀማ ዉህድ (non-ionic aqueous solution)

በአጠቃላይ ናሙናን የማንበር ሂደት የሚመለከተዉ አዲስ የተያዙ ወይም በአግባቡ የቆዩ ናሙናዎች ነው። አብዛኞቹ የነፍሳት ናሙናዎች በደረቁ ለማልዲ-ቶፍ ጥቅም (MALDI-ToF) ወይም በአልኮል ውስጥ ለብዙ ዓመታት የተከማቹ ሊሆኑ ይችላሉ። ነገር ግን ናሙናዎችን ለረጅም ዓመታት በአልኮል ውስጥ ማቆየት ተመራጭ አይደለም፤ በዚህ መንገድ የቆዩ ነፍሳት (arthropods) በአጉሊ መነጽር ለመታየት ዝግጁ እንዲሆኑ ማድረግ በጣም አስቸጋሪ ነው። ብዙውን ጊዜ የሚያጋጥመው ችግር ናሙናዎቹ የተቀመጡበት የፕላስቲክ ዕቃ በእርጅና ምክንያት ስለሚበላሽ አልኮሉ እንዲተን ያደርጋል። በሁለቱም ሁኔታዎች ናሙናዎቹ በአልኮል ውስጥ በጣም ስለሚቆዩ ወይም ስለሚደርቁ አማራጮቻችን ጠባብ ይሆናሉ፤ ምክንያቱም ናሙናዎቹ በአልኮል ውስጥ ለረጅም ጊዜ ቆይተዋል ወይም ደርቀዋል። ስለሆነም፣ ጠንካራ ሳሙና ያልሆኑ 'ማለስለሻ ንጥረ ነገሮችን' (Wetting agents) ለምሳሌ ትራይተን ኤክስ100 (Triton X100) መጠቀም አስፈላጊ ይሆናል፡፡ ትራይተን ኤክስ100 (Triton X100) አዮኒክ ያልሆነ ዉሀማ ዉህድ (non-ionic detergent) ሲሆን፣ በሴል እና በሞለኪውላር ባዮሎጂ ዘርፍ በስፋት ጥቅም ላይ ይውላል። የህዋስ እና የህዋስ አስኳል ሽፋንን (Cell membrane) አስራጊ (permeabilization) የማድረግ ችሎታ አለው።

ከዚህ በታች 0.5% ዉሀማ ዉህድ የሆነውን ትራይተን ኤክስ100 (Triton X100) በመጠቀም የሚከናወን የአሠራር ቅደም-ተከተል ቀርቧል፡-

- የደረቀውን ናሙና በዉሀ አልባ አልኮል (absolute alcohol) ማራስ።
- ሙሉውን ናሙና ሊዘፍቅ የሚያስችል 0.5% ትራይተን ኤክስ100 (Triton X100) ዉህድ መጨምር።
- ሂደቱን በየጊዜዉ እየተከታተሉ ከ 5 ደቂቃ እስከ ብዙ ቀናት እንዲቆይ ማድረግ። ሁሉም የነፍሳቱ አካላት በዉህዱ ውስጥ ሙሉ በሙሉ መለስለስ አለባቸው።
- - ቀጥሎም የትራይተን ኤክስ100 (Triton X100) ዉህድን በማስወገድ በፖታስየም ሃይድሮክሳይድ (KOH) ዉህድ መተካት፡፡

4.1. ራስ (Head)

የአሸዋ ትንኞችን አካል መበለት የሚከናወነው ስቴሪዮ-ማይክሮስኮፕ (stereomicroscope) በመጠቀም በቀጫጭን መርፌዎች ወይም በነፍሳት መርፌዎች (entomological pins) አማካኝነት ነው (ምስል 2 እና 3)። በብዛት ጥቅም ላይ የሚውሉት መርፌዎች፡- 26G x 1/2" (0.45 × 13 ሚሜ)፣ 30G × 1/2" (0.3 × 13 ሚሜ)፣ ወይም 25G × 5/8" (0.5 × 16 ሚሜ) ናቸው። የትንኟን ዝርያ ለመለየት እንዲያመች፣ ቢያንስ ጭንቅላቱ ከሰውነቱ ተለይቶ የምግብ መፍጫና ማስተላለፊያ ቧንቧ (cibarium) እና የጉሮሮ (pharynx) ክፍሎችን ለማየት በጀርባ ማስተኛት (ventral side up) ያስፈልጋል፡፡ ደረትና ሆድ ደግሞ ከተገነጠሉ በኋላ በጎናቸው (laterally) ይቀመጣሉ። የራስ ቅሉ ቀዳዳ (occipital foramen) ወደ ላይ እንዲዞር፣ ጭንቅላቱን በፊት በኩል (ventro-dorsal) ማስተኛት ያስፈልጋል፡፡ ይህም አቀማመጥ የምግብ መፍጫና ማስተላለፊያ ቧንቧዉን (cibarium) በቀጥታ ለመመልከት ያስችላል። ጭንቅላቱ ሙሉ በሙሉ ከተገነጠል እነዚህን የሰውነት ክፍሎች በቀላሉ ለማየት ያስችላል፡፡

4.2. ክንፎች እና ደረት (Wings and thorax)

የአሸዋ ትንኞችን ክንፎች ለማንበር በቅጡ ተዘርግተው (flat) መቀመጥ አለባቸው። እያንዳንዱ ክንፍ ከሥሩ ተገንጥሎ ለብቻው ማንበር ይቻላል፣ ወይም አንደኛውን ክንፍ ብቻ ገንጥሎ በማንበር ሌላኛው ከደረት ጋር ተያይዞ እንዲቀር ማድረግ ይቻላል። የጂኦሞርፎሎጂ ልኬት (geometric morphometry) ትንተና የታቀደ ከሆነ፣ ከማንበር በፊት ቀኝ እና ግራ ክንፎችን በትክክል መለየት እና ምልክት ማድረግ አስፈላጊ ነው።

የትንኟ ደረት በበርካታ ክፍሎች የተከፈለ ሲሆን፣ እያንዳንዱ ክፍል በጣም አስፈላጊ የሆኑ የዝርያ ምደባ (Taxonomy) መረጃዎችን ይዟል [20, 64]። በአጠቃላይ የጸጉር ስሮችን (chetotaxy) እና የቀለም ስርጭትን ለመመርመር እንዲቻል ደረቱን በጎን በኩል ባለው እይታ (lateral view) ማንበር ያስፈልጋል። በአንዳንድ የደረት ክፍሎች ላይ የሚገኙ የጸጉር ጠባሳዎች (scars of bristles) የ Brumptomyia ዝርያዎችን ለመለየት ያገለግላሉ።

የቀለም ስርጭቱ የኒዮትሮፒካል አሸዋ ትንኞችን በዝርያ ደረጃ (ለምሳሌ፡- *Bichromomyia*)፣ የዝርያ ቡድኖች (ለምሳሌ፡- Pintomyia)፣ ወይም በአንድ ዝርያ ውስጥ ያሉ ንዑስ ዝርያዎችን (ለምሳሌ፡- *Micropygomyia, Nyssomyia, Psathyromyia*, እና *Psychodopygus*) ለመለየት ጥቅም ላይ ሊውል ይችላል [20]። ስለሆነም፣ ደረቱ ለሞለኪውላር ትንተና የማይውል ከሆነ፣ ሳይበላሽ በጥንቃቄ ማንበር ያስፈልጋል፡፡ አለበት።

ዋናው ነጥብ፣ የቀለሙ ድምቀት ሳይሆን ቀለሙ በደረት ላይ ያለው ስርጭት መሆኑን ልብ ማለት ያስፈልጋል። ስለዚህ ናሙናውን የማጽዳት ሂደት (clarification process) ቀለሙን ወይም የቀለም ቅርጹን አያጠፋውም።

4.3. መራቢያ አካላት (Genitalia)

የአሸዋ ትንኞችን በወገን (genera)፣ ንዑስ ወገን (subgenera) እና በዝርያ (species) ለመለየት መራቢያ አካላት ወሳኝ በመሆናቸው፣ የወንድም ሆነ የሴት መራቢያ አካላትን በጥንቃቄ ማንበር ያስፈልጋል፡፡ የመራቢያ አካላት በሁለቱም ጾታዎች ውስጥ በጥንድ በጥንድ የተቀመጡ ናቸው።

4.3.1. ወንዶች (Males)

የወንድ መራቢያ አካላት በውጭ የሚገኙ ሲሆን፣ ጥንድ የሆኑ መቆንጠጫዎችን (forceps) ይይዛሉ። እያንዳንዱ መቆንጠጫ በጀርባው ክፍል የጎኖኮክሳይት-ጎኖስታይል (gonocoxite-gonostyle) መጋጠሚያን፣ በታችኛው (ventral) ክፍሉ ደግሞ የኢፓንድሪያል ሎብን (epandrial lobe) ይይዛል። ጎኖስታይል (Gonostyle): እሾህ መሰል መዋቅሮችን (spines) እና አንዳንዴም ረዣዥም ጸጉሮችን (setae) ይይዛል። እነዚህ እሾሆች ሊቆጠሩ የሚችሉ እና የተሰኩበት ቦታ በግልጽ የሚታይ መሆን አለበት። ጎኖኮክሳይት (Gonocoxite): የውስጠኛው ገጽ በጥንቃቄ መታየት አለበት፤ ምክንያቱም በቀጥታ ከቆዳው ላይ የወጡ ወይም በትንሽ እብጠት (tubercle) ላይ ያሉ የጸጉር ስብስቦችን ሊይዝ ይችላል [22]።

በአሸዋ ትንኝ አካል ብለታ አነስተኛ ልምድ ያላቸው ባለሙያዎች፣ መራቢያ አካሉን ከሆድ ዕቃው መጨረሻ ላይ ሳይነቅሉ ቀጥታ በጎን በኩል (lateral mounting) ማንበር ይችላሉ (https://zenodo.org/records/18311158))፡፡ በዚህ ሁኔታ፣ ሁለቱ የመራቢያ አካላት አንዱ በሌላው ላይ ስለሚደረራረቡ፣ በጎኖኮክሳይት ውስጥ ያሉትን ጸጉሮች ለመቁጠር አስቸጋሪ ቢሆንም፤ ነገር ግን ይህ ዘዴ በመበለት ሂደቱ ባልመሳካቱ ምክንያት አካሉ እንዳይበላሽ ይረዳል።

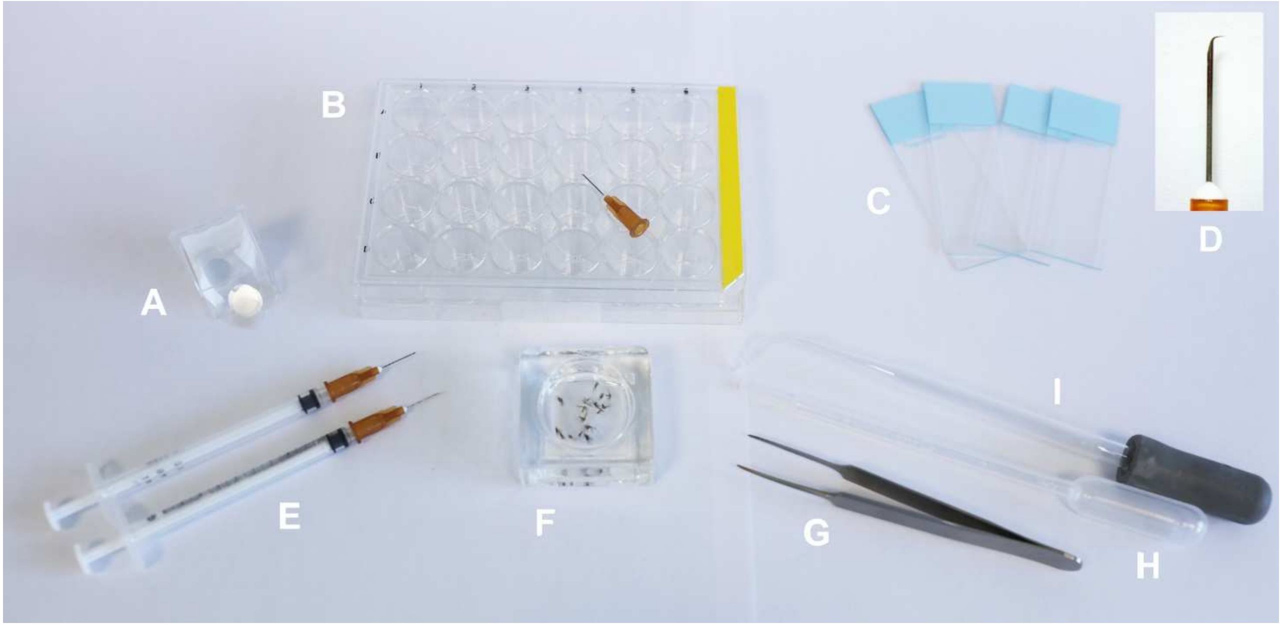

ምስል 2፡- አሸዋ ትንኞችን ለማንበር የሚያስፈልጉ ቁሳቁሶች : A፡- ክብ የሽፋን መስተዋቶች (Coverslips)፤ ከ10 እስከ 12 ሚሊ ሜትር ዲያሜትር ያላቸው።
B፡- 24 ቀዳዳዎች ያሉት ፕሌት (24-well plate) እና ጥምዝ መርፌ (hooked needle)። (ልብ ይበሉ፡- ትንኞቹን ለማቀነባበር የቅርንፉድ ዘይት (clove oil) ወይም ዩፓራል (Euparal®) የምንጠቀም ከሆነ፣ የአክሪሊክ (acrylic) ፕሌቶች አይጠቀሙ፤ ምክንያቱም ኬሚካላዊ ምላሽ ስለሚፈጠር ናሙናዎቹ ሊበላሹ ይችላሉ)።
C፡- መለያ (Label) ለመለጠፍ አመቺ የሆኑ የመስታወት ስላይዶች።
D፡- የመርፌው ጥምዝ ጫፍ ዝርዝር እይታ።
E፡- ከሲሪንጅ ጋር የተገጠሙ መርፌዎች።
F፡-አሸዋ ትንኞችን ለማስቀመጥ የሚያገለግል የእጅ ሰዓትን የሚያክል መስታወት (watch glass) ወይም ተመሳሳይ መያዣ።
G፡- የዱሞንት መቆንጠጫ (Dumont forceps)
H፡- ከላስቲክ የተሰራ ፒፔት (መምጠጫ)።
I፡- ፈሳሽን ወደ ቀዳዳዎቹ (wells) በቀላሉ ለማስተላለፍ እንዲረዳ በእሳት በማሞቅ የታጠፈ የመስታወት ፒፔት።

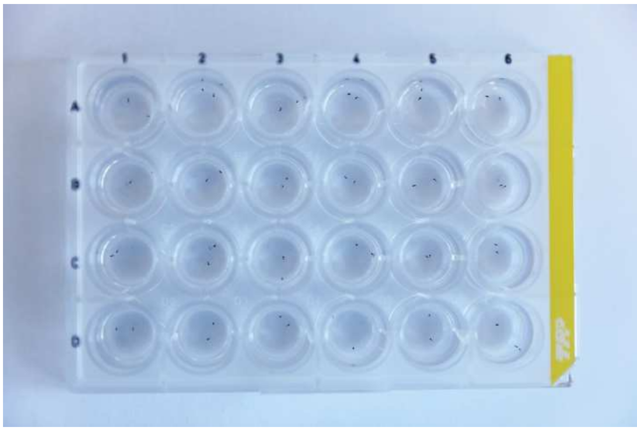

ምስል 3፡- ባለ 24 ቀዳዳ ሳህን (24-well plate). እያንዳንዱ ቀዳዳ (well) የአሸዋ ትንኝን ራስ እና የሆድ ዕቃ ጫፍ (መራቢያ አካል ያለበትን ክፍል) ይዟል። ይህ ዝግጅት ብዙ ናሙናዎችን በአንድ ጊዜ ለማቀነባበር እና ዝርያቸውን ለመለየት ይረዳል።

በሥራው ላይ ልምድ ያላቸው ባለሙያዎች ግን መራቢያ አካሉን ለሁለት ከፍሎ ለመዘርጋት ሊሞክሩ ይችላሉ። ይህንን ለማድረግ የመርፌው የተቆረጠ ሹል ጎን (እንደ ኢንትራደርማል መርፌ ዓይነት) በመጠቀም፣ የጎኖኮክሳይት-ጎኖስታይል ስብስቦችን ሙሉ በሙሉ ሳይቆርጡ በመነጠል መዘርጋት ያስፈልጋል (https://zenodo.org/records/18311158) ። በዚህ መንገድ የውስጠኛውን ገጽታ መመልከት ቀላል ይሆናል። ይህ ዓይነቱ አዘገጃጀት አንዱ በሌላው ላይ ተደራርበው የሚታዩትን ፓራሜሮች (parameres) እና የፓራሜር ሽፋኖችን (parameral sheaths) በግልጽ ለማየት ይረዳል። በጎን በኩል ለማንበር (lateral mounting) የአካል ክፍሎች መደራረብ ስለሚኖር፣ ናሙናዎቹ በደንብ የጠሩ (cleared) መሆን አለባቸው።

4.3.2. ሴቶች (Females)

የሴት መራቢያ አካላት በሰውነት ውስጥ የሚገኙ ሲሆን፣ በዋነኝነት የወንዴ ዘር ማከማቻ ከረጢትን (spermathecae) ያካትታሉ። የወንዴ ዘር ማከማቻ ከረጢትን የትንኟን አካል መበለት (dissection) ሳያስፈልግ በሰዉነት ቆዳ ዉስጥ(tegument) በቀጥታ መመልከት ይቻላል፡፡ ይህንንም ማድረግ የሚቻለዉ ሆድ ዕቃውን በፊት በኩል (ventral position) በማንበር ነዉ፡፡ የወንዴ ዘር ማከማቻ ከረጢቱ በየትኛዉም የማንበሪያ ዉህድ ቢቀመጥም

በግልፅ ይታያል፣ በተለይም የከረጢቱ አካል ሻካራና በደንብ ያልጠራ ከሆነ፡ ፡ ሆኖም ግን፣ ለስላሳና ቀጭን ግድግዳ ያላቸውን ከረጢቶችን ብርሃን በማያሳልፉ (Poorly refractile) ዉህዶች ውስጥ ማየት አስቸጋሪ ሊሆን ይችላል።
በተጨማሪም፣ በቀዳሚዉ ዓለም ክፍል (Old World) ውስጥ የላይሽማኒያ ኢንፋንተም (*Leishmania infantum*) ዋና አስተላላፊ የሆኑት እንደ *Larroussius* ያሉ ንዑስ ዝርያዎችን ለመለየት የወንዴ ዘር ማከማቻ ከረጢት ቱቦ ታችኛዉን ክፍል (base of the spermathecal ducts) መመልከት አስፈላጊ ነው [35, 37, 38]። ያለበለዚያ ዝርያን መለየት አዳጋች ነዉ፡፡ እነዚህን የምልከታ ችግሮች ለመፍታት የወንዴ ዘር ማከማቻ ከረጢት ሹካውን (genital furca-spermathecae) ከሆድ ዕቃው ለይቶ ማውጣት ያስፈልጋል። (https://zenodo.org/records/18311106)
በአጠቃላይ በትንኝ የመበለት ሂደት ውስጥ የወንዴ ዘር ማከማቻ ከረጢትን (spermathecae ለማየት አስቸጋሪ ነዉ፤ ነገር ግን የመራቢያ አካል ሹካውን (genital furca) ማግኘት በአንጻራዊ ሁኔታ ቀላል ነው። የማከማቻ ቱቦዎቹ የሚከፈቱት ወደዚህ ሹካ ውስጥ ስለሆነ፣ ሹካውን ለይቶ ማውጣት ብዙውን ጊዜ ማከማቻዎቹንም አብሮ ለማውጣት ያስችላል። በመገንጠሉ ሂደት ማከማቻዎቹ በስህተት ቢቆረጡ እንኳ በሆድ ዕቃው ቆዳ ሽፋን ውስጥ ሆነው ሊታዩ ይችላሉ (ምስል 4 ይመልከቱ)።

4.4. የላሽማኒያ ተህዋሲ ንጠላ የሚከናወን የሆድ ዕቃ (Midgut) ብለታ

በሴት አሸዋ ትንኞች ውስጥ የላሽማኒያ ጥገኛ ተህዋሲያንን ለማግኘት እና ለይቶ ለማውጣት የትንኟን የምግብ መፍጫ አካል መበለት አስፈላጊ ነው። ይህ አሠራር የትንኞቹን በሽታ የማስተላለፍ ብቃት (vectorial competence) ለመገምገም በመስክም ሆነ በላቦራቶሪ ውስጥ ሊከናወን ይችላል።

የግንጥላዉ ክንዋኔ በቅርብ በተገደሉ ሴት ትንኞች ላይ ቢደረግ ይመከራል። በቅድሚያ ትንኞቹ ላይ ያሉትን ትርፍ ጸጉሮች ለማስወገድ ለስላሳ ሳሙና ባለው ውሃ ወይም በጨው ዉህድ (saline solution) እጠባቸው። ይህ ክንዋኔ ትንኟን ለመለየት የሚያስፈልጉትን አካላዊ ባህሪያት ሳይቀይር፣ የላይሽማኒያ ተዋህሲን የመነጠል ሂደቱ በንጹህ (aseptic) ሁኔታ እንዲከናወን ይረዳል። የላይሽማኒያ ጥገኛ ተህዋሲያንን ለማግኘት እና ለመለየት፣ የሆድ ዕቃውን (midgut) በጥንቃቄ በማዉጣት ከበሽታ አምች ተዋህሲያን በፀዳ አንድ ጠብታ የጨው ዉህድ (sterile 0.9% NaCl) ውስጥ ማኖር ያስፈልጋል። ተንቀሳቃሽ የላይሽማንያ ጥገኛ ተህዋሲያንን በአጉሊ መነጽር (የሚመከር ማጉያ፦ ~200×) ከታዩ፣ የጨዉ ዉህዱን በኢንሱሊን ሲሪንጅ ወይም ሳቢ ቱቦ (micropipette) በመምጠጥ ወደ ማሳደጊያ ዉህድ (cultivation medium) መዛወር አለባቸዉ፡፡ (ለበለጠ መረጃ ምዕራፍ 4.4.3ን ይመልከቱ)።

የትንኟን ጭንቅላትና እና የመራቢያ አካል ለማጽዳት በቀጥታ ማርክ አንድሬ (Marc-André) ውስጥ ማስቀመጥ ያስፈልጋል። በዚህም ጊዜ የማርክ አንድሬ (Marc-André) ዉህድ የላይሽማኒያ ተህዋሲያን ገዳይ በመሆኑ በቀጥታም ሆነ በተዘዋዋሪ፣ በመሣሪያዎች ወይም በመርፌዎች አማካኝነት እንዳይነካካ ጥንቃቄ ማድረግ ያስፈልጋል፡፡
የሴት አሸዋ ትንኞች መበለት አንድ ወይም ሁለት ስላይዶች በመጠቀም ሊከናወን ይችላል፤ ሁለቱም አማራጮች የራሳቸው ጥቅምና ገደብ አሏቸው (ምስል 5፤ https://zenodo.org/records/18311154) ።

4.4.1. ሁለት ስላይዶች በመጠቀም ንጠላ ዘዴ (Two-slides method)

የመጀመሪያው የተዋህሲያን ንጠላ መንገድ ሁለት የተለያየ ስላይዶች በመጠቀም ሲሆን :- አንደኛው ስላይድ የምግብ መፍጫ ቱቦውን (Midgut) ለማውጣት የሚያገለግል ከበሽታ ተዋህሲያን የፀዳ ዉህድ (Sterile saline) የያዘ ሲሆን ፣ ሁለተኛው ደግሞ የትንኟን ጭንቅላት እና የዘር ማከማቻ ከረጢት (Spermathecae) በ'ማርክ-አንድሬ' (Marc-André) ዉህድ ለማንበር የሚያገለግል ነው።

ሆኖም፣ በመስክ የሥራ ሁኔታ ላይ ሁለት ወይም ሦስት ሰዎች ትንኞችን በልተዉ፣ ዝርያውን ለሚለየውና በምግብ መፍጫ ቱቦ ውስጥ የሊሽማኒያ ኢንፌክሽን መኖሩን ለሚመረምረው ሌላ ተመራማሪ ማቀበል የተለመደ ነው። በዚህ ጊዜ ሁለት ስላይዶችን በአንድ ላይ ማስተናገድ የናሙናዎችን ዱካ መከታተያ (Traceability) ሊያዘበራርቅ ይችላል፤ በተለይም በአንድ ትንኝ አንጀት ውስጥ ኢንፌክሽን ቢገኝ፣ የተበከለችው ትንኝ የትኛዋ እንደነበረች በእርግጠኝነት ለማወቅ አስቸጋሪ ያደርገዋል (https://zenodo.org/records/18311154)።

4.4.2. አንድ ስላይድን በመጠቀም ንጠላ ዘዴ (Single-slide method)

ሁለተኛዉ የተዋህሲያን ንጠላ መንገድ አንድ ስላይድ ብቻ መጠቀም ሲሆን ይህም የናሙናዎችን ዱካ ለመከታተል (traceability) ያስችላል። ሆኖም ግን፣ በርካታ ጥንቃቄዎች መወሰድ አለባቸው። ከበሽታ አምጭ ተዋህሲያን ፅዱነትን (sterility) ለማረጋገጥ፣ ባለሙያዎች እጃቸውን በየጊዜው በሃይድሮ-አልኮሆል ጄል (hydroalcoholic gel) ማጽዳት አለባቸው። ስላይዶች (non-frosted) እና ባለ አራት ማዕዘን የሽፋን መስተዋቶች (22 x 22 ሚሜ) በአሉሚኒየም ፎይል ተጠቅልለው በደረቅ ሙቀት (በፑፒኔል ምድጃ/Poupinel oven) የጸዱ መሆን አለባቸዉ ፣ እንዲሁም ለእያንዳንዱ ብለታ የሚሆኑ ንጹህ መርፌዎች (ተመራጭ : 25G Ø 0.5mm × 16mm) ጥቅም ላይ መዋል አለባቸው።

አሸዋ ትንኟ በስላይዱ መሃል ላይ ባለ ንጹህ የጨው ዉህድ (saline) ጠብታ ውስጥ ትቀመጣለች። የምግብ መፍጫ አካልን (digestive tract) ሳይቆርጡ ጭንቅላቱን መነጠል እና በ6ኛው እና በ7ኛው የሆድ ክፍሎች (abdominal tergites and sternites) መካከል ቀዳዳ መፍጠር ያስፈልጋል (በጣም ረጅም የዘር ማከማቻዎች/spermathecae ይኖራሉ ተብሎ የሚጠበቅ ከሆነ ከመገጠሚያው ከፍ ብሎ መሰንጠቅ ይቻላል)። በመቀጠል፣ ደረቱን (thorax) በአንድ መርፌ እንዳይንቀሳቀስ በማፈን፣ በሌላኛው መርፌ የሆድ ዕቃውን የመጨረሻ ክፍሎች በቀስታ በመሳብ የምግብ መፍጫ አካሉን(gut) ማውጣት ያስፈልጋል፡፡

ይህ ካልተሳካ፣ የሆድ ዕቃውን የመጨረሻ ጫፍ በመርፌ በማፈን የፊተኛዉን የምግብ መፍጫ አካል (anterior part) የመሳብ አማራጭ አለ። ይህ ካልተሳካ ደግሞ፣ በምግብ መፍጫ አካል ዙሪያ ያለውን የቀረ የሰውነት ክፍል በተቻለ መጠን በማስወገድ፣የምግብ መፍጫ አካሉን ማዉጣት ያስፈልጋል፡፡ የምግብ መፍጫ አካሉ ከወጣ በኋላ የመጨረሻዉን የሆድ ዕቃ ክፍል በመቁረጥ ነጥሎ ማዉጣት ያስፈልጋል።

በመቀጠል የምግብ መፍጫ አካሉ በስላይዱ በአንድ በኩል በተቀመጠ አዲስ ንጹህ የጨው ዉህድ ጠብታ ውስጥ ይደረጋል፣ ከዚያም በንጹህ የሽፋን መስተዋት በቀስታ ይሸፈናል። ጭንቅላቱ እና የመጨረሻዎቹ የሆድ ዕቃ ክፍሎች በስላይዱ በሌላኛው ጫፍ ላይ ባለ ትንሽ የማርክ አንድሬ (Marc-André) ዉህድ ጠብታ ውስጥ ይዘወራሉ (ይህም ከላይሽማኒያ ጋር

ምንም ዓይነት ንክኪ አለመኖሩን በማረጋገጥ ነው)። ጭንቅላቱ በትክክለኛው አቅጣጫ (የራስ ቅሉ ቀዳዳ ወደ ላይ) ይደረጋል፣ የዘር ማከማቻ ከረጢቱ (spermathecae) ከላይ እንደተጠቀሰው ከመራቢያ አካል ሹካ ጋር ሆነዉ በትንሽ ክብ የሽፋን መስተዋት [Ø 12 ሚሜ] (ማስታወሻ፣ የካሬው የሽፋን መስተዋት መጠቀም አይመከርም) ይሸፈናሉ። የቀረው የአሸዋ ትንኟ አካል እና ክንፎች በሰላይዱ መሃል ባለው የጨው ዉህድ ጠብታ ውስጥ ይቀራሉ (https://zenodo.org/records/18311154).

ጥገኛ ተህዋሲያን ከተገኙ እና የዝርያ ምደባ ጥናት ካስፈለገ፣ ደረቱ እና ሆዱ ለሞለኪውላር ወይም ለፕሮቲን ጥናቶች ሊቀመጡ ይችላሉ፣ ክንፎቹ ደግሞ በዉሀማ ዉህድ ውስጥ ማስቀመጥ ይቻላል። የተቀመጠዉን ናሙና ለረጅም ጊዜ ለማቆየት፣ ከመጠን ያለፈ የማርክ አንድሬ (Marc-André) ዉህድ በክሎራል ገም (Gum Chloral) ወይም በፖሊቪኒል (PVA) አልኮል ሊተካ ይችላል።

የሚከተሉት የቪዲዮ ቅጅዎች እነዚህን ሂደቶች በዝርዝር ስለሚያሳዩ እዚህ ላይ ዝርዝር ማብራሪያ አይሰጥባቸውም። (የአሸዋ ትንኝ የምግብ መፍጫ አካልን መበለት:- https://zenodo.org/records/18303014 እና የአሸዋ ትንኝ የምራቅ እጢ መበለት:- https://zenodo.org/records/18302850) ።

4.4.3. የላይሽማኒያ ጥገኛ ተህዋሲያንን ከአሸዋ ትንኝ ሆድ ዕቃ ንጠላ እና ማሳደግ

የላይሽማኒያ ተህዋሲ ተሸካሚ ከሆኑ ሴት አሸዋ ትንኞች ላይ ጥገኛ ተህዋሲያንን ለይቶ ማውጣት ከፍተኛ ክህሎት የሚጠይቅ ረቂቅ ተግባር ሲሆን፣ መጀመሪያ ጥገኛ ተህዋሲያን በሌለባቸው ናሙናዎች ላይ ልምምድ ሊደረግበት ይገባል። የትንኟ አካል ከተገነጣጠለ በኋላ፣ የሆድ ዕቃዎቹ (guts) እንዲታጠቡ ወደ አዲስ ከደቀቀ ህዋስ (ማይክሮብ) የጨው ዉህድ (0.9% NaCl) ወይም የሎክስ (Locke's) ዉህድ ይዛወራሉ [4]። የተገነጠሉት የሆድ ዕቃ ክፍሎች በሁለት መንገድ ሊቀነባበሩ ይችላሉ:-

ልዩ ልዩ የፕሮማስቲጎትን (promastigote) የእድገት ደረጃዎች እና መገኛ ቦታቸውን በአጉሊ መነጽር መመርመር (በተለይም ለስቶሞዲያል ቫልቭ (stomodeal valve) ልዩ ትኩረት መስጠት)።

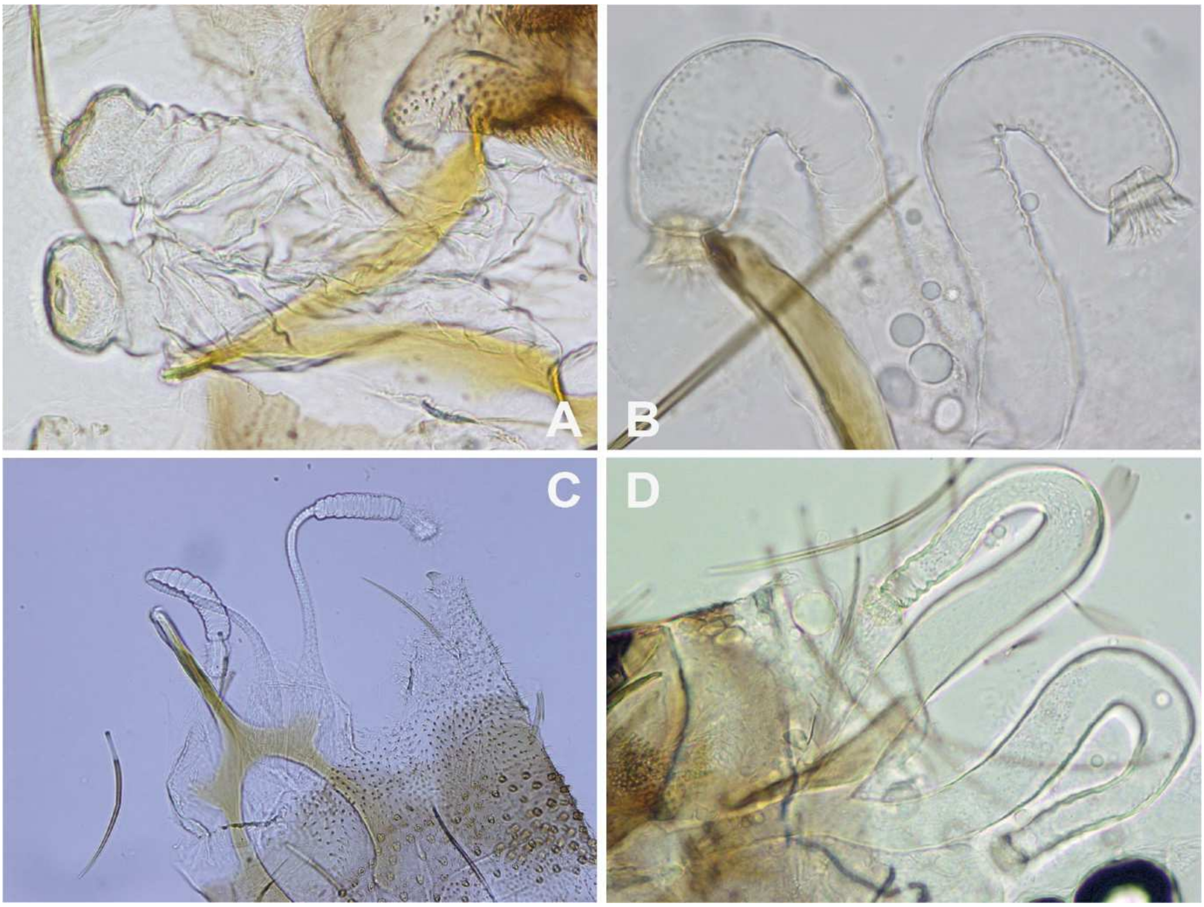

ምስል 4፡- ከተበለቱ አዳዲስ ናሙናዎች የተገኙና በ "Marc-André" ዉህድ ውስጥ የተቀመጡ የወንዴ ዘር ማከማቻ ከረጢቶች (Spermathecae).A: *Idiophlebotomus longiforceps* (RDP Laos); B: *Sergentomyia minuta* (France); C: *Phlebotomus ariasi* (France); D: *Sergentomyia anodontis* (RDP Laos).

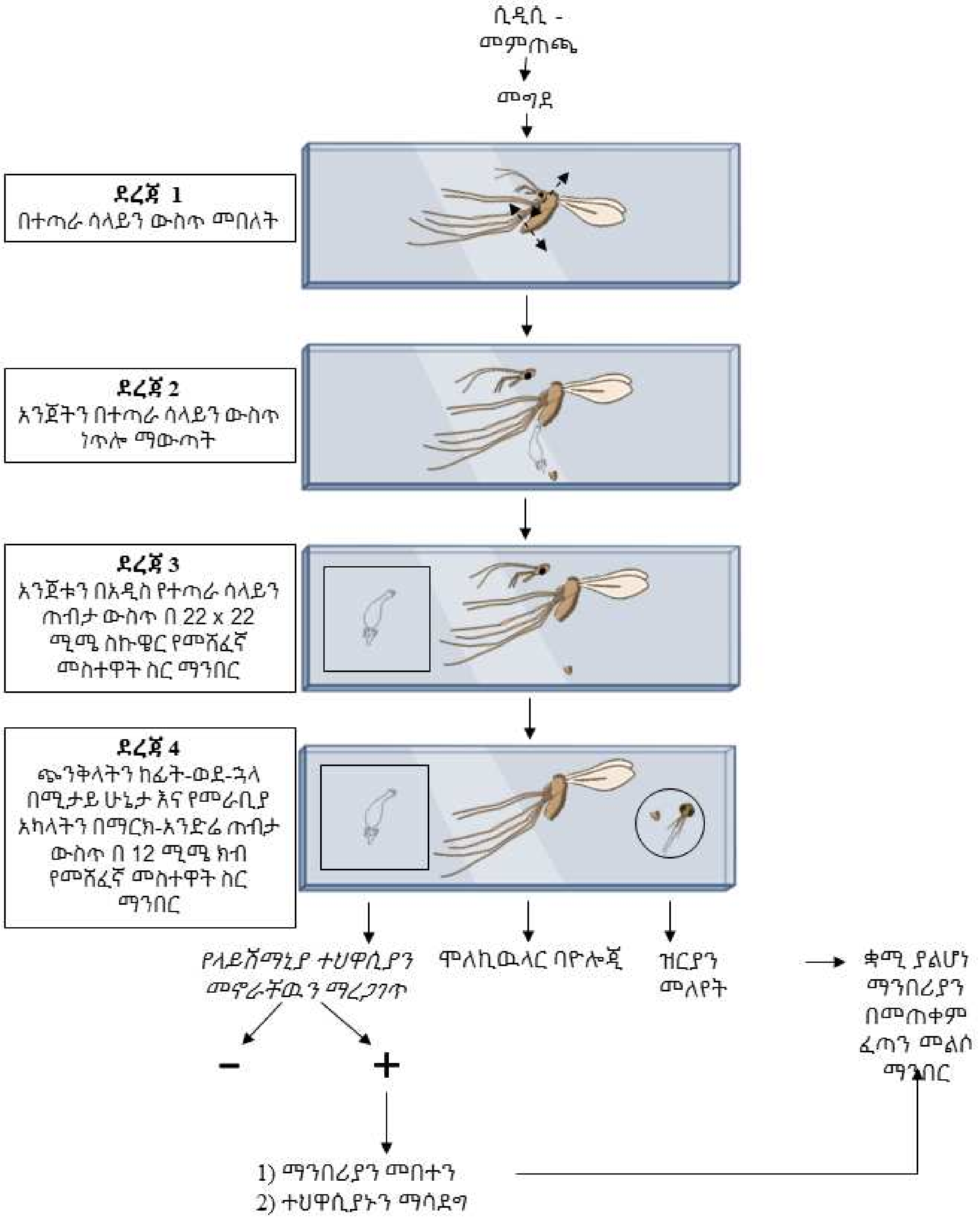

ምስል 5፡- የላሽማኒያ ተዋህሲን ንጠላ ዘዴ

ፕሮማስቲጎቶቹ በቀላሉ ወጥተው በብዛት እንዲራቡ (mass culture) ለማመቻቸት የሆድ ዕቃውን መክፈት [4]።

በመስክ ጥናት ላይ የላይሽማኒያ ተህዋሲ ተሸካሚ የሆኑ አሸዋ ትንኞችን የማግኘት ዕድል በአንጻራዊ ሁኔታ ዝቅተኛ በመሆኑ፣ አስቀድሞ ጥሩ የልምምድ ጊዜ መውሰድ ተህዋሲያኑን በተሳካ ሁኔታ የመነጠል ዕድልን ይጨምራል።

This is an Open Access article distributed under the terms of the Creative Commons Attribution License (https://creativecommons.org/licenses/by/4.0), which permits unrestricted use, distribution, and reproduction in any medium, provided the original work is properly cited.

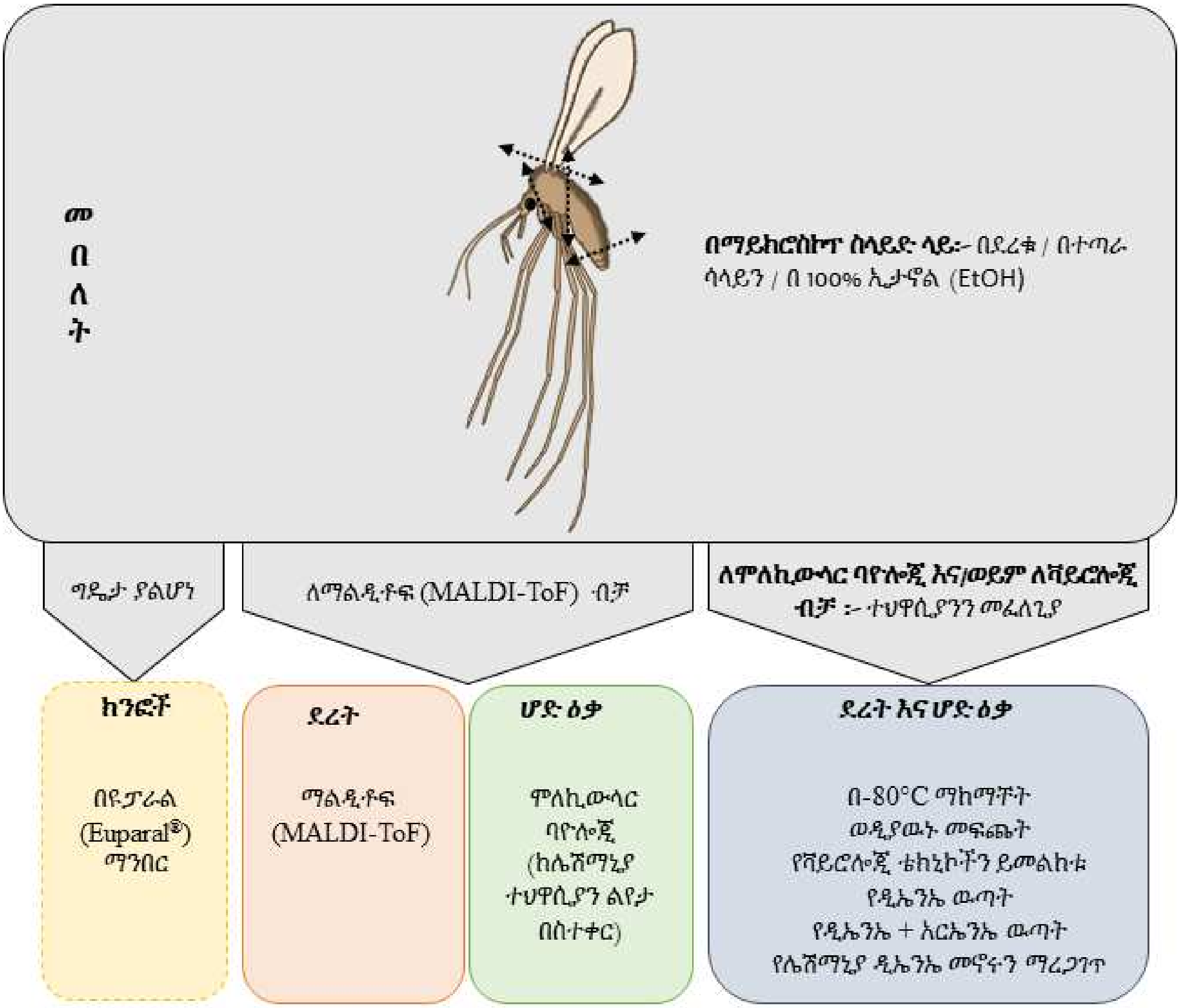

ምስል 6፡- የአሸዋ ትንኝን ለሞለኪውላር ባዮሎጂ፣ ለፕሮቲኦሚክስ እና/ወይም ለቫይሮሎጂ ምርመራዎች የማቀነባበር ሂደት ይህ ምስል አንዲትን አሸዋ ትንኝ ለተለያዩ የምርመራ አይነቶች እንዴት በክፍልፋይ መጠቀም እንደሚቻል ያሳያል።

በሆድ ዕቃ ውስጥ የላይሽማኒያ ፕገኛ ተህዋሲያን ከታዩ፣ ከደቂቀ ህዋስ (ማይክሮብ) በፀዱ መርፌዎች በመጠቀም ተህዋሲያኑ ከሆድ ዕቃዉ እንዲወጡ በማድረግ በሽፋን መስተዋቱ (coverslip) ዙሪያ የጨዉ ዉህዱ መሰስ ብሎ እንዲገባ መጨመር ያስፈልጋል። ፕገኛ ተህዋሲያኑ ወደ ጨው ዉህዱ እንዲወጡ የሆድ ዕቃው በጥንቃቄ እና በፍጥነት መቅደድ አለበት።

በ 100 µL ማይክሮ-ፒፔት ወይም በቲዩበርኩሊን (tuberculin) ሲሪንጅ በመጠቀም ፕገኛ ተህዋሲያኑን ሰብስቦ መለያ ቁጥር ወደተለጠፈለት የማሳደጊያ ዉህድ (culture medium) ውስጥ ማስገባት።

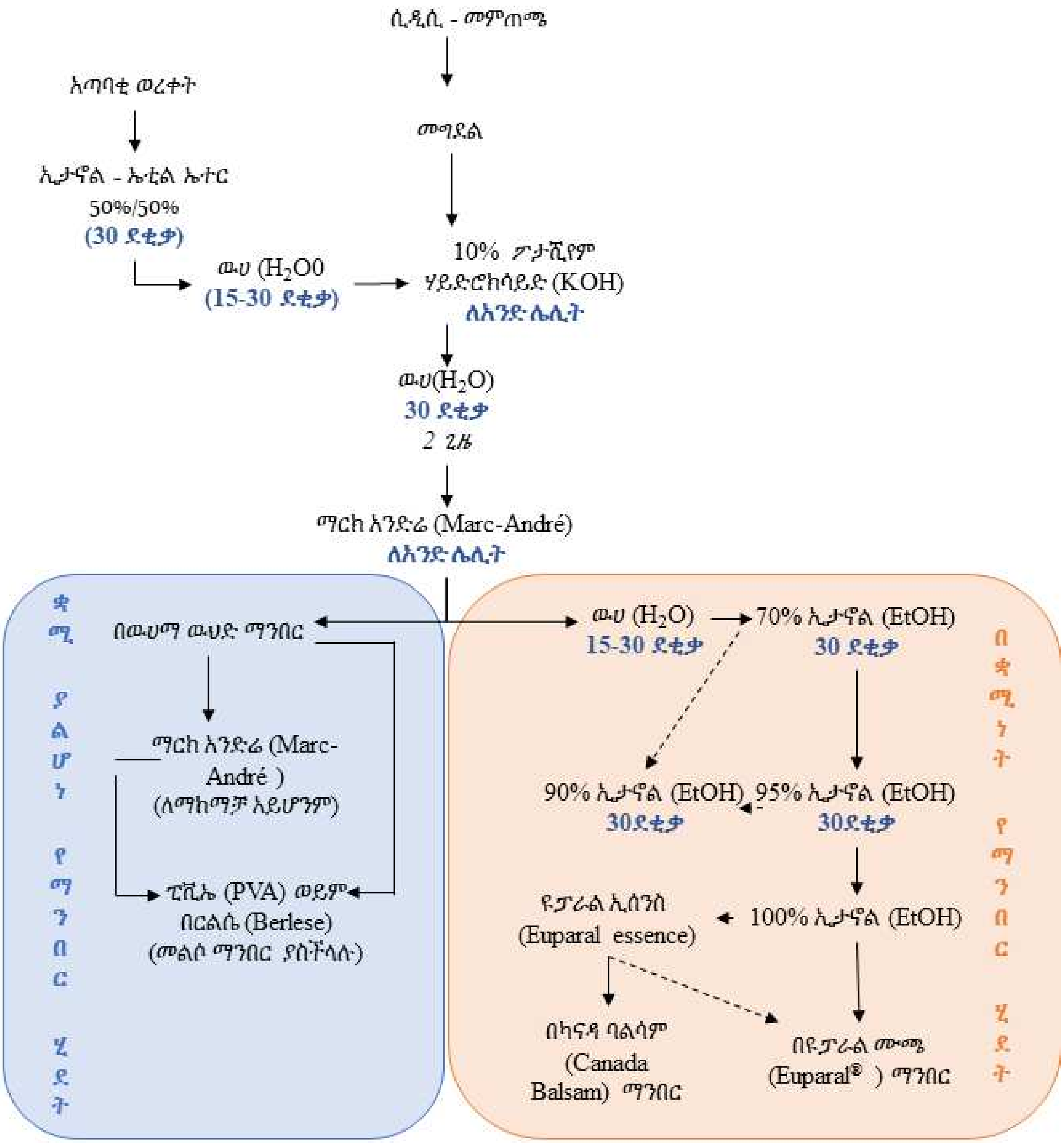

ምስል 7፡- የአሸዋ ትንኞችን ለማቀነባበር የሚከናወን መደበኛ (classical) ዘዴ

የላሽማኒያ ፐሮማስቲጎቶችን በብልቃጥ (in vitro) ማሳደግ፦ የተነጠሉት የላሽማኒያ ተህዋሲያን መጀመሪያ በኤስ ኤን ቢ9 ብለድ አጋር (SNB-9 blood agar) ወይም በኤን ኤን ኤን (NNN [Novy, Mc Neal, Nicolle]) ማሳደጊያ ዉህድ ዉስጥ ይቀመጣሉ[16]። እነዚህ የማሳደጊያ ዉህዶች በደቂቅ ተውሳክ (microbe) ባልተበከለ አልፋ ኤም ኢ ኤም (alpha-MEM) ዉህድ [16, 65] ወይም በኤም199 (M199) ዉህድ ተጨምሮባቸዉ መሆን አለበት። እያንዳንዱ ዉህድ ከሚከተሉት ጋር መቀላቀል አለበት፦

ሠንጠረዥ 2፡- ጥቅም ላይ የሚዉሉ የኬሚካል ውህዶች (Reagents) ይዘት

**የውህዱ ስም እና ይዘት**	**የውህዱ ስም እና ይዘት**
**ፖታስየም ሃይድሮክሳይድ 10% (Potassium hydroxide 10%)**<br>- ፖታስየም ሃይድሮክሳይድ 10 ግራም<br>- የተጣራ ውሃ እስከ 100 ሚሊ ሊትር (qs 100 mL)	**ፉክሲን አሲድ 1% በተጣራ ውሃ (Fuchsin acid 1% in distilled water)**<br>- አሲድ ፉክሲን (በዱቄት መልክ) 1 ግራም<br>- የተጣራ ውሃ 99 ሚሊ ሊትር
**የገም ክሎራል ማንበሪያ ዉህድ (Hoyer medium)**<br>- የተጣራ ውሃ 50 ሚሊ ሊትር<br>- ክሎራል ሃይድሬት 200 ግራም<br>- ጋም አረቢክ 50 ግራም<br>- ግሊሰሮል 20 ሚሊ ሊትር	**በአሲድ ፉክሲን የቀለመ የማርክ-አንድሬ ዉህድ**<br>- የማርክ-አንድሬ ዉህድ 10 ሚሊ ሊትር<br>- ፉክሲን 1% (50 µL)
**የማርክ-አንድሬ ዉህድ (Marc-André solution)**<br>- ክሎራል ሃይድሬት 40 ግራም<br>- ግላሲያል አሴቲክ አሲድ 30 ሚሊ ሊትር<br>- የተጣራ ውሃ 30 ሚሊ ሊትር	**የኤኔሴ ዉህድ (Enecê medium)**<br>- ንጹህ ነጭ ኮሎፎኒ (Pure white colophony) 22 ግራም<br>- በአልኮል የሚሟሟ ኮፓል ጋም (Copal gum) 12 ግራም<br>- ፍጹም ኤታኖል (Absolute ethanol) 20 ሚሊ ሊትር<br>- ካምፎር (Camphor) 10 ግራም<br>- ተርፔንታይን (Turpentine essence) 10 ሚሊ ሊትር<br>- ዩካሊፕቶል (Eucalyptol) 26 ሚሊ ሊትር

10% በሙቀት የታከመ ንጹህ የጥጃ ሴረም (FCS) (የጥገኛ ተህዋሲያኑን እድገት ለማፋጠን)።

1% ቢ ኤም ኢ (BME) ቪታሚኖች።

2% ንጹህ የሰው ሽንት (በ 0.2 µm ሲሪንጅ ፊልተር የጸዳ)።

250 µg/mL አሚካሲን (ወይም 50 µg/mL ጀንታማይሲን፣ ወይም የፀረ ተህዋሲ እና አሚኖ አሲድ ድብልቅ

(L-glutamine 200 mM-penicillin 10 000 U-streptomycin 10 mg/mL)) [47]።

ከሦስት ቀናት በኋላ ምንም ዓይነት ብክለት (contamination) ካላጋጠመ፣ የተባዙት የላሽማኒያ ተህዋሲያን (cultures) በትክክል በተዘጋጀ ማቀዝቀዣ ዉህድ ውስጥ ተደርገው ከ1 እስከ 2 ዓመት በ -80ድግሪ ሴንቲ ግሬድ፣ ወይም ለረጅም ጊዜ ለማቆየት እና ለወደፊት ምርምር በፈሳሽ ናይትሮጅን ውስጥ በ-196 ድግሪ ሴንቲ ግሬድ ሊቀመጡ ይችላሉ [7]።

4.5. የምራቅ እጢዎች (Salivary glands)

የአሸዋ ትንኞችን የምራቅ እጢ መገንጠል በአሸዋ ትንኝ እና በበሽታ አምጪ ተህዋሲያን መካከል ያለውን ግንኙነት ለማጥናት መሠረታዊ ዘዴ ነው። በተለይም እንደ ፍሌቦቫይረስ (Phlebovirus - ለምሳሌ ቶስካና ቫይረስ) ያሉ በነፍሳት የሚተላለፉ ቫይረሶችን (arboviruses) ለመለየት ይረዳል [44, 75]። አሸዋ ትንኞች በጣም ጥቃቅን በመሆናቸው፣ ሂደቱ በስቴሪዮ-ማይክሮስኮፕ በመታገዝ፣ በጣም ቀጭን በሆነ መቆንጠጫ ወይም በመገንጠያ መርፌዎች አማካኝነት እጢዎቹ ሳይቀደዱ ወይም ሳይበከሉ በጥንቃቄ መነጠል አለባቸው (https://zenodo.org/records/18302850) [51, 61]። ለአስተማማኝ የሞለኪውላር ትንተና የእጢዎቹን ደህንነት ጠብቆ ማውጣት ወሳኝ ነው። እጢዎቹ ከወጡ በኋላ፣ ተፈጭተው (homogenized) በአር ቲ ፒሲ አር (RT-PCR)፣ ኪው ፒ ሲ አር (qPCR)፣

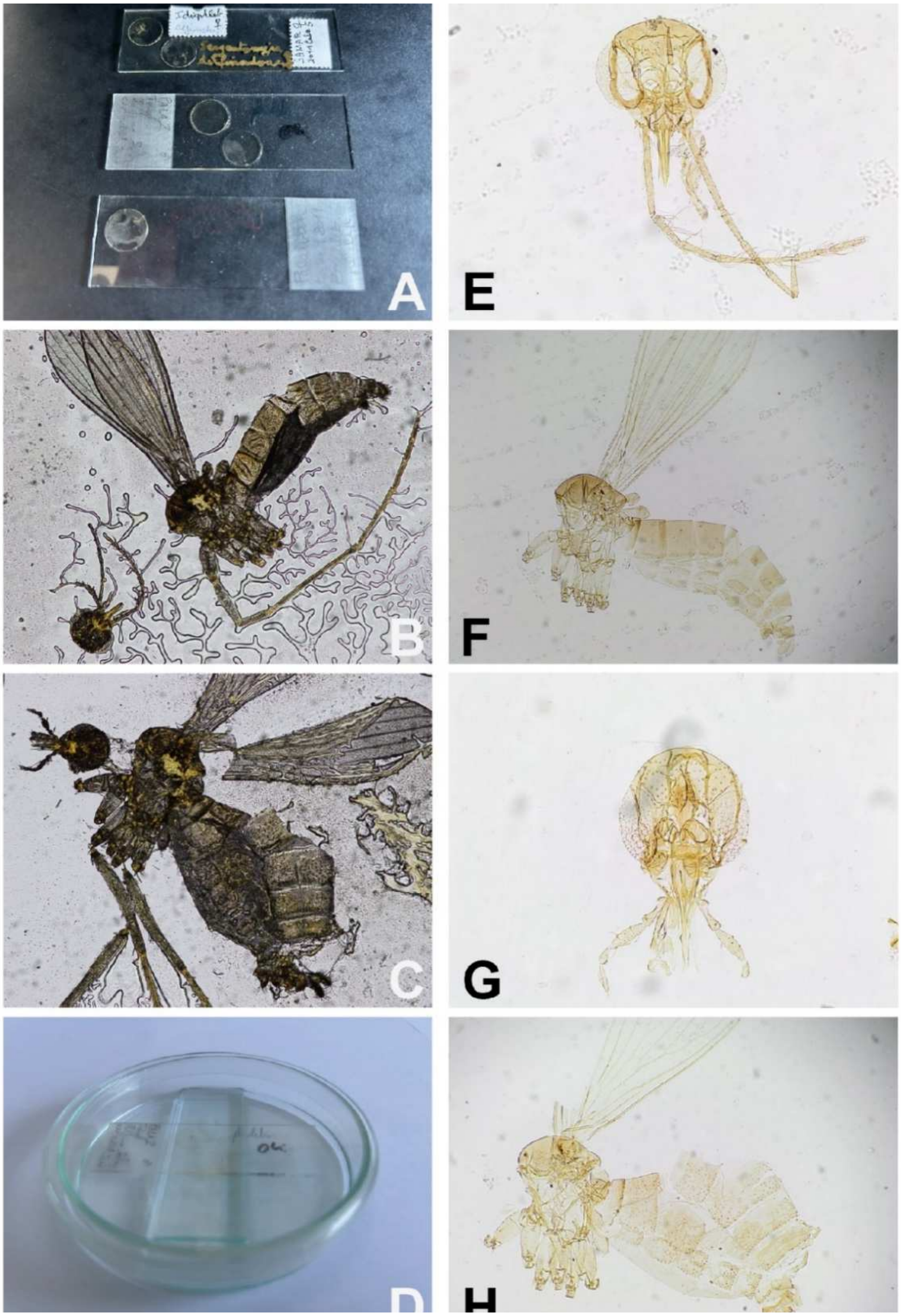

ምስል 8፡- ስላይድን በድጋሚ ማንበር። ሀ: በሆየር (Hoyer) ውህድ ተዘጋጅተው የተበላሹና የደረቁ ስላይዶች። ለ: የደረቀ የአሸዋ ትንኝ በማይክሮስኮፕ ሲታይ (በመድረቁ ምክንያት ዝርዝር ክፍሎቹ ግልጽ አይደሉም)። ሐ: ሌላኛው የተበላሸ የአሸዋ ትንኝ በማይክሮስኮፕ ሲታይ። መ: የደረቀውን ስላይድ መልሶ ለማርጠብ የሚያገለግል እርጥበት ያለው ሳጥን (Wet chamber)። ሠ እና ረ: ናሙና "ለ" በዩፓራል (Euparal®) በድጋሜ በማንበር የሚታይ ራስ (ሠ) እና አካል (ረ)፤ (እዚህ ጋር ምስሉ ምን ያህል ጥርት እንዳለ ማስተዋል ይቻላል)። ሰ እና ቀ: የተበላሸው ናሙና "ሐ" በዩፓራል (Euparal®) በድጋሜ በማንበር የሚታይ ራስ (ሰ) እና አካል (ቀ)።

ወይም በመድኄም ጠና (immunoassays) የቫይረስ አርኤንኤ (RNA) ወይም ምላሽ ቀስቃሽ ፕሮቲንን (Antigen) ለመለየት ምርመራ ማድረግ ይቻላል [12]። ቫይረሶች በሆድ ዕቃ ወይም በሰውነት ፈሳሽ (hemocoel) ውስጥ ብቻ ሳይሆን በምራቅ እጢዎች ውስጥ መገኘታቸው፣ በሽታ አምጪ ተህዋሲው ውጫዊ የመራቢያ ጊዜውን (extrinsic incubation period) ማጠናቀቁን እና ትንኟ ደም በምትመጥበት ጊዜ በሽታውን ማስተላለፍ እንደምትችል ያረጋግጣል [71]።

የአሸዋ ትንኝ የምራቅ እጢዎች በጣም ትንሽ ከመሆናቸው የተነሳ የመበለት ሂደቱ በቴክኒክ ረገድ ከፍተኛ ብቃትን የሚጠይቅ ሲሆን፣ ናሙናው እንዳይበላሽ ለማድረግ ትልቅ ልምድ ያስፈልጋል [1, 51]። በተጨማሪም፣ የቫይረሱ መጠን ዝቅተኛ ሊሆን ስለሚችል እንደ ኔስትድ ፒሲ አር (nested PCR) ወይም በፈጣንና በስፋት የሚከናወን የዲኤንኤ ቅደም ተከተል ትንተና (high-throughput sequencing) ያሉ በጣም ዉጤታማ (sensitive) የሆኑ የመለያ ዘዴዎችን መጠቀምን ይጠይቃል [54]። የመበከል (contamination) ተጋላጭነታቸዉ ደግሞ ከደቂቀ ተውሳከ ብክለት ነፃ የሚያደርጉ አጠባበቅ ዘዴዎችን መጠቀምን ይጠይቃል። ከቴክኒካዊ ችግሮች ባሻገር፣ ሥነ ህይወታዊ ሁኔታዎችም ቫይረሱን የማግኘት ስኬት ላይ ተጽዕኖ ያሳድራሉ፤ ለምሳሌ በሽታን የማስተላለፍ ብቃት (vector competence)፣ የአሸዋ ትንኝ ዝርያ እና የኢንፌክሽን መጠኑ (እንደ አካባቢያዊ እና ወቅታዊ ሁኔታዎች ይለዋወጣል) [33, 61]።

በምራቅ እጢዎች ውስጥ ቫይረሶችን መለየት ስለ በሽታው የመተላለፍ አደጋ ወሳኝ ግንዛቤን ይሰጣል፤ ይህም የታለመ የክትትል እና የቁጥጥር እርምጃዎችን ለመውሰድ ያስችላል [15]። ለምሳሌ፣ በሽታው ባለባቸው አካባቢዎች በአሸዋ ትንኞች ውስጥ የቶስካና ቫይረስ መገኘት ለምርመራ ፕሮቶኮሎች እና ለሕዝብ ጤና ምክሮች መነሻ ሆኗል [18]። ማጥናት በሽታው እንዳይተላለፍ ለሚረዱ ክትባቶች ወይም ሕክምናዎች አዳዲስ ግቦችን ለማግኘት ይረዳል [15, 18]።

የአሸዋ ትንኝ የምራቅ እጢዎች (Salivary glands)፣ አስተናጋጁ (Host - ለምሳሌ ሰው ወይም እንስሳ) ለትንኟ ፀረ ምራቅ ምላሽ (Anti-saliva antibody) ለመለካት እንደ ምላሽ ቀስቃሽ ፕሮቲን (Antigen) ምንጭነት ሊያገለግሉ ይችላሉ፤ ይህም በአብዛኛው ኤላይዛ (ELISA) በተባለ የኢሚውኖሎጂ ምርመራ ዘዴ ይከናወናል። ይህ ዘዴ አስተናጋጁ እንስሳ ወይም ሰዉ ለአሸዋ ትንኝ ንክሻ ምን ያህል እንደተጋለጠ ለመገመት ያስችላል፤ በዚህም የተባይ መቆጣጠሪያ ዘዴዎች (Vector control) ምን ያህል ውጤታማ እንደሆኑ ለመገምገም እና የሊሽማኒያ በሽታ የመተላለፍ አደጋን (Risk) ለመለየት ይረዳል።

4.6. የደም ምግብን መለየት (Blood meal identification)

ከተያዙት ትንኞች መካከል ደም መጠዉ ሆድ ዕቃቸዉ የተወጠረ (engorged) ሴቶችን አንዱ የሌላውን ናሙና እንዳይበክል (cross-contamination) የአንድ ጊዜ አገልግሎት ብቻ በሚሰጡ መሣሪያዎች መበለት ያስፈልጋል። የደም መፈጨት ደረጃውን ለመገምገም ሆድ ዕቃቸውን በስቴሪዮ-ማይክሮስኮፕ መመርመር ይገባል። ሆድ ዕቃቸው ቀይ፣ ቀይ-ቡናማ፣ ወይም ጥቁር ቀይ የሆኑ እና የእንቁላል መፈጠር ምልክት የማይታይባቸውን ሴቶች ብቻ መምረጥ ይመከራል። የትንኟን በአካላዊ ቅርፀት ለመለየት የሆድ ዕቃውን ጫፍ ከነ ዘር ማከማቻ ከረጢቱ (spermathecae) ለይቶ ማውጣት ያስፈልጋል። ቀሪው የሆድ ዕቃ ክፍል (ያለ ዘር ማከማቻዎቹ) በኢፔንዶርፍ (Eppendorf®) ቱቦዎች ውስጥ ተደርጎ ለቀጣይ ምርመራ እስከሚፈለግ በ -20ድግሪ ሴንቲ ግሬድ መቀመጥ አለበት።

የደም ምግብ ምንጭን ለመለየት በብዛት ጥቅም ላይ የሚውሉ የሥነ ዘር መለያዎች (genetic markers) ለምሳሌ ፒ ኤን ኤሲ ⍰ ሲ (PNOC) [5, 30, 50]፣ ሲ ዋይቲ CytB [67]፣ ወይም ሲ⍰ይ (COI) [13] በስፋት የታወቁ እና በተለያዩ ጽሑፎች የተገለጹ በመሆናቸው፣ በዚህ ጽሑፍ ውስጥ በዝርዝር አይብራሩም (ምስል 6)።

በአማራጭነት፣ የደም ባለቤቱን (host) ለማወቅ የማልዲ-ቶፍ (MALDI-ToF) ü ፕታይድ ዝርዝር ትንተና (peptide mapping) ዘዴን መጠቀም ይቻላል [31]። ይህ ቴክኒክ ደም ከተመጠጠ በኋላ ረዘም ላለ ጊዜ የደም ምንጭን ለመለየት እንደሚያስችል በሙከራ ተረጋግጧል፤ ስለዚህ የደም መፈጨት ሂደቱ ገፍቶ ለሚታይባቸው ሴት ትንኞች ተመራጭ ዘዴ ነው። ናሙናዎቹ በ -20ድግሪ ሴንቲ ግሬድ ወይም በ4 ድግሪ ሴንቲ ግሬድ ቢቀመጡ ይመረጣል፤ ነገር ግን ናሙናዎች በተመጣጣኝ ሙቀት ውስጥ ለአጭር ጊዜ ብቻ የቆዩ ከሆነ ጥሩ ውጤት ማግኘት ይቻላል። ደም የመጠጠችው ሴት ሆድ ዕቃ ከምርመራው ጥቂት ቀደም ብሎ ከቀሪው አካል ተለይቶ በተጣራ (distilled) ውሃ ውስጥ መፈጨት (homogenized) አለበት። ቀሪው የአሸዋ ትንኟ አካል ለሌሎች ሞለኪውላዊ እና የአካላዊ ቅርፀት ጥናት ሊውል ይችላል። ለማልዲ-ቶፍን (MALDI-ToF) የሚሆን ናሙና መጠን ከተወሰደ በኋላ፣ ቀሪውን ክፍል የዲኤንኤ (DNA) ምርመራ በማድረግ፣ የደሙ ምንጭን ለማረጋገጥ እና/ወይም የላይሽማኒያ ተህዋሲ መኖርን ለመመርመር ሊያገለግል ይችላል። ይህ የናሙና ዝግጅት እና የትንተና ዘዴ ከዲኤንኤ ምርመራዎች ጋር ሲነጻጸር ጊዜው በጣም አጭር ነው።

5. ለአካላዊ ቅርፀት ጥናቶች ናሙናዎችን ማቀነባበር (ምስል 3፣ 6፣ 7 እና 8፤ ተጨማሪዎች 1፣ 2፣ 3 እና 4)

ይህ ክፍል አንድን የአሸዋ ትንኝ ናሙና ለአካላዊ ቅርፀት ጥናት እንዲውል አድርጎ ለማዘጋጀት እና በስላይድ ላይ ለማንበር የሚያስፈልጉትን መሠረታዊ መመሪያዎች ያብራራል፤ በመቀጠልም ከአካላዊ ቅርፀት ባለፈ ለሌሎች ምርመራዎች እንዴት ማላመድ እንደሚቻል ያሳያል። ይህንን የአሠራር ዘዴ መረዳት እጅግ አስፈላጊ ነው፤ ምክንያቱም አስፈላጊ ሆኖ ሲገኝ አሠራሩን እንደ ናሙናው ዓይነት ለማስተካከል ያስችላል።

የማቀነባበሩ ሂደት ተለዋዋጭ ፈሳሽን ለመሳብና ለማፍሰስ የሚያገለግሉ የጎማ መጭመቂያ ባላቸው የፓስተር ፒፔቶች (Pasteur pipettes) አማካኝነት ፈሳሾችን በተከታታይ የማስወጣት እና የመጨመር ተግባራትን ያካትታል። ክብ ቅርጽ ያላቸው የመስታወት መያዣዎች እነዚህን ተግባራት በእጅጉ ስለሚያቀላጥፉ ተመራጭ ይሆናሉ። መስታወት ከማንኛውም ኬሚካል ጋር ምላሽ የማይሰጥ (inert) ቁስ ነው። ኬሚካሎች እንዳይተኑ ለመከላከል መያዣዎቹ ክዳን ሊኖራቸው ይገባል። እንዲሁም ክዳኑ በሚከፈት ወይም በሚዘጋበት ጊዜ ፈሳሹ እንዳይፈስ እና አቧራ በናሙናዎቹ ላይ እንዳያርፍ መያዣዎቹን ከመጠን በላይ መሙላት የለብንም። ለማጽዳት እና ለማቀነባበር የሚያስፈልጉ ኬሚካሎች በሠንጠረዥ 2 ላይ ቀርበዋል።

5.1. ማጥራት (Clearing)

የአሸዋ ትንኝ ናሙናዎች በቋሚነት በስላይድ ላይ ከማንበር በፊት፣ ተገቢውን ዘዴ እና የማጥሪያ ኬሚካል በመጠቀም (ለምሳሌ:- 10% የአሴቲክ አሲድ (acetic acid) ዉህድ ወይም በብዙ አገሮች ቁጥጥር የሚደረግበትን ክሎራል ሃይድሬት (chloral hydrate) የያዘውን የማርክ-አንድሬ (Marc Andre) ዉህድ ብርሃን አስተላላፊ እስከሚሆን ድረስ በዝግታ እንዲሟሟ መደረግ

ሠንጠረዥ 3፡- የተመረጡ ማንበሪያ ዉህዶች (Mounting Media) ይዘት

ማንበሪያ ውህድ (Mounting medium)	አሟሚ ፈሳሽ (Solvent)	መከሰት የሚችሉ ፖሊመሮች (Potential polymer(s)) ወይም ፖሊመሮች (polymer)	ተጨማሪ ማብራሪያ (Remarks)
**ሆየር (Hoyer)** = ክሎራል ገም (chloral gum)	ግሊሰሮል (glycerol)፣ ውሃ (water)	ገም አረቢክ ውህዶች (compounds of gum arabic)	ማለስለሻ ንጥረ ነገር (Macerating agent)፡- ክሎራል ሃይድሬት (chloral hydrate)
**ሲኤምሲፒ-9 (CMCP-9)** (ካርቦክሲ ሜቲል ሴሉሎስ ፌኖል (carboxy methyl cellulose phenol)	ውሃ (water) (ሲኤምሲፒ-9 (CMCP-9): 51–60%)	ሙሉ በሙሉ በዉሀ ተብላልቶ የፈራረሰ ፖሊቪኒል አልኮሆል (fully hydrolyzed polyvinyl alcohol) (ሲኤምሲፒ-9 (CMCP-9): 0–5%)	ሲኤምሲፒ-9 (CMC(P)-9)፡- ዝቅተኛ የመወፈር ባህሪ (low viscosity)፡ ከፍተኛ የመወፈር ባህሪ (high viscosity)
**ዲኤምኤችኤፍ (DMHF)** (ዲሜቲል ሃይዳንቶይን ፎርማልዲሃይድ (dimethyl hydantoin formaldehyde)	ውሃ (water)	ኤን፣ኤን-ዲሜቲሎል ዲሜቲል ሃይዳንቶይን (N,N′-dimethylol dimethyl hydantoin (di-methylol DMH))፤ በኤተር/ሜቲሊን የተሳሰሩ ኦሊጎመሮች (Ether-/methylene-bridged oligomers)፤ የተሳሰረ የዲኤምኤች-ፎርማልዲሃይድ ፖሊመር መረብ (Crosslinked DMH–formaldehyde polymer network)	
**ካናዳ ባልሳም (Canada balsam)**	ሳይሊን (xylene)፤ በከፊል ተናኝ የባልሳም ክፍሎች (partly volatile components of balsam) (ዴልታ-3-ካሬን (Δ^3-carene)፣ ሌቮፒማሪክ አሲድ (levopimaric acid)፣ ሊሞኒን (limonene)፣ ማይርሲን (myrcene)፣ ፓሉስትሪክ አሲድ (palustric acid)፣ ቤታ-ፌላንድሬን (β-phellandrene)፣ አልፋ-ፓይንን (α-pinene)፣ ቤታ-ፓይንን (β-pinene))	ባልሳም (balsam) (አቢኖል (abienol)፣ አቢቲክ አሲድ (abietic acid)፣ አይሶፒማሪክ አሲድ (isopimaric acid)፣ ሳንዳራኮፒማሪክ አሲድ (sandaracopimaric acid))	አሲድነትን ማርከስ (neutralization)፡- ፖታሲየም ካርቦኔት (potassium carbonate)፤ ከአቢየስ ባልሳሜያ (*Abies balsamea* (Linné, 1758)) የሚገኝ ሙጫ (resin)
**ዩፓራል (Euparal®)**	ዩካሊፕቶል (eucalyptol)፣ ፓራልዲሃይድ (paraldehyde)፤ በከፊል ተናኝ የገም ሳንዳራክ ክፍሎች (partly volatile components of gum sandarac) (ሊሞኒን (limonene)፣ አልፋ-ፓይንን (α-pinene)፣ ቤታ-ፓይንን (β-pinene))	የገም ሳንዳራክ ውህዶች (compounds of gum sandarac) (ኮሙኒክ አሲድ (communic acid)፣ ማኖል (manool)፣ ፖሊኮሙኒክ አሲድ (polycommunic acid)፣ ሳንዳራኮፒማሪክ አሲድ (sandaracopimaric acid)፣ 12-አሴቶክሲ-ሳንዳራኮፒማሪክ አሲድ (12-acetoxy-sandaracopimaric acid)፣ ሱጂኦል (sugiol)፣ ቶሩሎሲክ አሲድ (torulosic acid)፣ ቶሩሎሶል (torulosol)፣ ቶታሮል (totarol))	ማጥሪያ ንጥረ ነገር (clearing agent)፡- ሜቲል ሳሊሲሌት (methyl salicylate)፤ አረንጓዴማ ዩፓራል (color in Euparal® green)፡- የኮፐር ጨው (copper salt ኮፐር አቤቲኔት (copper abietinate))፤ ከቴትራክሊኒስ አርቲኩላታ (*Tetraclinis articulata* (Vahl, 1791)) የሚገኝ የሳንዳራክ ሙጫ (sandarac resin)
**ኤኔሴ (Enecê)**	ኢቲል አልኮሆል (ethyl alcohol)፤ ከካምፎር (camphor)፣ ዩካሊፕቶል (eucalyptol) እና የተርፔንቲን ቅመም (turpentine essence) ጋር	የኮፓል ገም እና ኮሎፎኒ ሙጫ ውህዶች (compounds of copal gum and colophony (rosin))	

This is an Open Access article distributed under the terms of the Creative Commons Attribution License (https://creativecommons.org/licenses/by/4.0), which permits unrestricted use, distribution, and reproduction in any medium, provided the original work is properly cited.

አለበት። የማጥራቱ ሂደት የሰውነት ህብረ ህዋሳትን፣ ስብን፣ ፈሳሾችን እና ሰም መሰል ነገሮችን ያስወግዳል፤ ይህም ናሙናው ብርሃን የሚያሳልፍ (translucent) እንዲሆን በማድረግ የውጭውን አጽም (ለምሳሌ:- የጸጉር መገጣጠሚያዎች)፣ የገጽታ ባህሪያትን (ለምሳሌ:- ቀለማትን) እና በቆዳ ውስጥ የሚታዩ የውስጥ አካላትን (ለምሳሌ:- የዘር ማከማቻ ከረጢትን) ለመመርመር ያስችላል።

ይህ በሁለት ደረጃዎች የሚከናወን የማጥራት ሂደት ሲሆን፣ በመጀመሪያ ጠንካራ ቤዝ (እንደ ፖታስየም ሃይድሮክሳይድ [KOH] ያለ)፣ በመቀጠልም ደካማ አሲድ (እንደ ማርክ-አንድሬ [Marc Andre] ውስጥ ያለ አሴቲክ አሲድ [Acetic Acid]) መጠቀምን ያካትታል፤ ይህም የተለየ ባዮኬሚካላዊ ዓላማ አለው [74]። ቤዙ እንደ ፕሮቲን፣ ስብ እና ጡንቻ ያሉ ለስላሳ ህብረ ህዋሳትን በማሟሟት (saponification) እና በፕሮቲን ፍርሰተ ቅርፅ አማካኝነት ፣ የአካል ሽፋኑን (chitin) የውጭ አጽም መዋቅራዊ ግልጽነቱን ጠብቆ እንዲቆይ ያደርጋል። ከዚያ በኋላ የሚጨመረው ደካማ አሲድ ቀሪ የሆኑ አልካሊዎችን (ቤዞችን) በማጥፋት (neutralizing) ተጨማሪ መበስበስን ይከላከላል፣ እንዲሁም አካለ ሽፋኑን በማንጻት ዕይታዊ ጥራቱን ይጨምራል[74]። እንደአማራጭም ናሙናዎቹን ለ15 ደቂቃ በተጣራ ውሃ ውስጥ ሁለት ጊዜ ማጠብ ቤዙን ለማጥፋት በቂ ሊሆን ይችላል። ይህ ተከታታይ የማጽዳት ሂደት ህብረ ህዋሳትን በማስወገድና ናሙናዎችን በጥንቃቄ በማቆየት ለአጉሊ መነፀር ምርመራ ተስማሚ ያደርጋቸዋል።

ወደ ቀጣዩ ደረጃ ከመሄድዎ በፊት ናሙናዎቹን በተጣራ ውሃ

5.1.1. ለስላሳ ህብረ ህዋሳትን ሟሟሸሽ (Soft tissue lysis) (ምስል 8)

ሶዲየም ሃይድሮክሳይድ (NaOH) ወይም ፖታስየም ሃይድሮክሳይድ (KOH) በብዛት ጥቅም ላይ የሚውሉ የህብረ ህዋስ ማለስለሻ ኬሚካሎች ናቸው። እንደ ናሙናው መጠን እና በቀላሉ የመሰበር ሁኔታ፣ በተለያየ ምጥነት (concentration) እና ለተለያየ ቆይታ ጥቅም ላይ ይውላሉ። መደበኛው እና በጣም ውጤታማው ዘዴ አሸዋ ትንኞችን በጠንካራ ቤዝ (10% ፖታስየም ሃይድሮክሳይድ [KOH] ወይም ሶዲየም ሃይድሮክሳይድ [NaOH]) ውስጥ ለአንድ ሌሊት በመዝፈቅ ለስላሳ ህብረ ህዋሳትን ማሟሟት ነው። ሂደቱ የሚወስደውን ጊዜ ለመቀነስ የኬሚካሉን መጠን መጨመር (ለምሳሌ:- 20% ፖታስየም ሃይድሮክሳይድ [KOH] ለ6 ሰዓታት) እንዲሁም እስከ 37ድግሪ ሴንቲ ግሬድ ማሞቅ ይቻላል።

5.1.2. ናሙናን በቀለም ወይም ያለ ቀለም ማጥራት (Clarification with or without staining)

ከማሟሟት ሂደት በኋላ፣ ብዙውን ጊዜ አሴቲክ አሲድ እና ክሎራል ሃይድሬትን (ለምሳሌ:- የማርክ-አንድሬ [Marc Andre] ዉህድን በማጣመር ናሙናውን የማጥራት ሥራ ይከናወናል። ከጸዳ በኋላ፣ ቀሪ ኬሚካሎችን ለማስወገድ ናሙናዎቹ ቢያንስ ለ20 ደቂቃ በሚቆዩ ሁለት ተከታታይ የውሃ መታጠቢያዎች ውስጥ በሚገባ መታጠብ አለባቸው። የማርክ-አንድሬ (Marc Andre) ዉህድ የአሸዋ ትንኝ ናሙናዎችን ለማዘጋጀት በሰፋት ጥቅም ላይ የሚውል የማጽጃ ፈሳሽ ሲሆን ጠቀሜታዉም በቀላሉ ሊሰበሩ በሚችሉ መዋቅሮች (ለምሳሌ፡ እንደ ክንፍ እና አንቴና) ላይ ከፍተኛ ጉዳት ሳያደርስ የማጽዳት ሂደቱን ማሳለጡ ነው። ዉህዱ ተዘጋጅቶ ወዲያዉኑ ጥቅም ላይ ማዋል ወይም እንዳይተን በጥብቅ በተዘጋ መያዣ ውስጥ መቀመጥ አለበት። የማርክ-አንድሬ (Marc Andre) ዉህድን መጠቀም በተለይ የተወሰኑ የአካል ክፍሎችን በግልጽ ለማየት ከሚረዱ ቀለሞች ጋር ሲቀላቀል የበለጠ ጠቃሚ ይሆናል። የውህዱ ይዘት እና አዘገጃጀት አባሪ 2 (Appendix 2) ላይ ተሰጥቷል።

ብርሃን አሳላፊ የሆኑ (translucent) ናሙናዎችን ከማንበር በፊት በደንብ እንዲታዩ ቀለም ማቅለም ሊያስፈልግ ይችላል። ለዚህም የሚያገለግሉ የተለያዩ ቀለማት የሚገኙ ሲሆን፣ እያንዳንዳቸው በነፍሳቱ ውስጥ ያሉ የተወሰኑ ኬሚካላዊ ክፍሎችን ኢላማ ያደርጋሉ። ከናሙናውም ሆነ ከተመረጠው ማንበሪያ ዉህድ ጋር ሊስማማ የሚችል ቀለም መምረጥ አስፈላጊ ነው።

ይህ መሠረታዊ ዘዴ እንደ አስፈላጊነቱ ሊስተካከል ይችላል፤ ለምሳሌ 0.1% አሲድ ፉክሲንን (acid fuchsin) በማርክ-አንድሬ (Marc Andre) ዉህድ ውስጥ በመጨመር ማቅለም ይቻላል።

በተጨማሪም፣ በዉሃማ ዉህዶች ተቀምጠው የነበሩ ናሙናዎችን እና ሙጫማ (resinous) ማንበሪያዎችን መጠቀም ከተፈለገ፣ የናሙናዎች እርጥበት ማስወገድ ሂደት (Dehydration) ያስፈልጋል (ክፍል 5.2 ይመልከቱ)፤ ምክንያቱም አብዛኞቹ ተፈጥሯዊ እና ሰው ሰራሽ ሙጫዎች ከውሃ ጋር አይስማሙም። ኒው [New] የተባለ (1974) ፀሀፊ እንደገለጸው፣ አንዳንድ ማቅለሚያዎች በተወሰኑ ማንበሪያ ዉህዶች ጋር ሲቀላቀሉ ሊበላሹ ይችላሉ [53]። ለምሳሌ በካናዳ ባልሳም (Canada balsam) ውስጥ ጥቅም ላይ የሚውለው አሲድ ፉክሲን (acid fuchsin) ዩፓራል (Euparal®) ውስጥም ሊቀመጥ (fixed) ይችላል። ሆኖም፣ በአሲድ ፉክሲን (acid fuchsin) የቀለሙ ናሙናዎች በቅርንፉድ ዘይት ሲጣሩ ቀለማቸው የመከሰም ዝንባሌ አለው። በአጠቃላይ በቅርንፉድ ዘይት ውስጥ የተቀመጡ ናሙናዎች በጥቂት ቀናት ውስጥ ቀለማቸው በከፍተኛ ሁኔታ ሊጠፋ ይችላል።

5.2. ዉሀን ማስወገድ (Dehydration)

ዉሀን የማስወገድ ሂደት የሚከናወነው ናሙናዎቹን በቅደም ተከተል መጠናቸው እየጨመረ በሚሄድ የኤታኖል (ethanol) ዉህዶች ውስጥ ማለትም በ50%፣ 70%፣ 80%፣ 90% ወይም 95% እና በመጨረሻም 100% ተራ በተራ እያዘዋወሩ በመዘፍዘፍ ነው ። በእያንዳንዱ መዘፍዘፊያ ዉስጥ ቢያንስ ለ20 ደቂቃ መቆየት አለበት። ኤታኖል (ethanol) በፍጥነት የሚተን በመሆኑ፣ በሂደቱ ወቅት የብልቃጡ ክዳን መጥበቅ አለበት። እርጥበቱ ሙሉ በሙሉ ከተወገደ በኋላ ናሙናዉ ለጥቂት ቀናት በዩፓራል ቅመም (Euparal® essence) ውስጥ መቆየት አለበት፤ ናሙናዉን በቅርንፉድ ዘይት (clove oil) ማስቀመጥ የሚቻል ቢሆንም ተመራጭ የሚሆነዉ Euparal® essence ነዉ። ቀደም ሲል ለዚህ ተግባር በሰፋት ጥቅም ላይ ይውል የነበረው ቢች ክሪኦሶት (Beech creosote) በመርዛማነቱ ምክንያት አሁን ሙሉ በሙሉ ታግዷል።

የእርጥበት ማስወገድ ሂደቱ (Dehydration process) በናሙናው ውስጥ ያለው ፈሳሽ ትንኟ ለማንበር ከምንጠቀምበት ዉህድ (Mounting medium) ጋር ተኳሃኝ መሆኑን ማረጋገጥ ያስፈልጋል። ይህ ካልሆነ ናሙናው ሊደፈርስ (Opacity)፣ በኦስሞቲክ ጫና ምክንያት ሊኮማተር (Osmotic collapse) ወይም ቅርጹ ሊዛባ (Distortion) ይችላል፤ ይህም የዝርያ መለያ ጥናት (Taxonomic study) ሂደቱን አዳጋች ያደርገዋል።

5.3. የማንበሪያ ዉህዶች (Mounting media)

5.3.1. ለናሙና ዝግጅት የማንበሪያ ዉህዶች መረጣና ትግበራ

የናሙና ማንበሪያ ዉህዶች የብርሃን ነጸብራቅ መለኪያዉ (refractive index) በግምት 1.5 ቢሆን ይመረጣል። ይህም ከመስታዎት የብርሃን ነጸብራቅ መለኪያ (refractive index) ጋር ተመጣጣኝ ያደርገዋል፡፡ የማንበሪያ ዉህዱ ቀለም የሌለው፣ ግልጽ እና ከደረቀ በኋላም ቢሆን ለረጅም ጊዜ ግልጽነቱ የማይለወጥ መሆን አለበት። ጥቅም ላይ ከሚዉሉት ማቅለሚያዎች ጋር መጣጣም ያለበት ሲሆን፣ ወደ ሁሉም የናሙናው ህብረ ህዋሳት ውስጥ የመግባት እና የመሰራጨት ችሎታ ሊኖረው ይገባል። በማንበር ሂደት ጊዜ ዉህዱ በፍጥነት መድረቅ ወይም መደብዘዝ የለበትም። ከማንበር ሂደቱ በኋላም መኮማተር የለበትም። ተገቢውን የማንበሪያ ዉህድ መምረጥ የናሙና ዝግጅት መሠረታዊ አካል ነው፤ ምክንያቱም ለሁሉም ዓላማ የሚሆን ብቸኛ ተስማሚ ዉህድ የለም። ምርጫው የሚከተሉትን ቁልፍ ጉዳዮች ማመጣጠን አለበት:-

This is an Open Access article distributed under the terms of the Creative Commons Attribution License (https://creativecommons.org/licenses/by/4.0), which permits unrestricted use, distribution, and reproduction in any medium, provided the original work is properly cited.

- **የሲራዊ (Optical) ባህሪያት፡-** የማንበሪያ ዉህዱ የብርሃን ነጸብራቅ መለኪያ ለዝርያ መለያ ወይም ለአካል መግለጫ የሚያገለግሉ ወሳኝ የአካል ክፍሎችን በግልጽ ለማየት የሚያስችል ንፅፅር (contrast) መስጠት አለበት። እነዚህም እንደ ዘር ማከማቻ ከረጢት (spermathecae)፣ አስኮይዶች (ascoids)፣ የኒውስተድ ሴንሲላ (Newstead sensilla)፣ የምግብ መፍጫና ማስተላለፊያ ቧንቧ ቀጥተኛ ጥርሶች (vertical cibarial teeth) እና የጉሮሮ ጥርሶች (pharyngeal teeth) የመሳሰሉት ናቸው። የእነዚህ መዋቅሮች ታይነት በቀጥታ በማንበሪያ ዉህዱ ሲራዊ ባህሪያት ላይ የተመሰረተ ነው።

- **ናሙና ማቆያ (Preservation)፡-** ለሙነነ አምሳያ ክምችት (Type specimen collections) የሚፈለጉ ናሙናዎች ከሆኑ፣ ዉህዱ ለረጅም ጊዜ የመቆየት እና የመረጋጋት ችሎታ ሊኖረው ይገባል። በተቃራኒው፣ ለፈጣን ቆጠራ ጥናቶች ወይም ለኤፒዲሚዮሎጂያዊ ቅኝቶች ለረጅም ጊዜ ማቆየት አስፈላጊ ካልሆነ፣ ጊዜያዊ ወይም በከፊል ዘላቂ በሆነ ማንበሪያ ዉህዶችን በመጠቀም ማቆየት ይቻላል፡፡

5.3.2. የማንበሪያ ዉህዶችን ለመምረጥ (Mounting media) የሚያስፈልጉ መስፈርቶች

ባለሙያዎች ብዙውን ጊዜ ለተወሰኑ የምርምር ፍላጎቶች የተዘጋጁ ውስብስብ እና የተለዩ የማንበሪያ ዘዴዎችን ያዘጋጃሉ። ሆኖም፣ እነዚህ ዘዴዎች ብዙ ጊዜ እንደ የረጅም ጊዜ ማከማቻ ጥራት (archival quality)፣ ተኳኋኝነት፣ ስልተ ምጥን (standardization) ፣የሂደቱን ምቹነትና ለረጅም ጊዜ የማቆየት ሁኔታን ግምት ዉስጥ አያስገቡም። ይህ የስልተ ምጥን መጓደል፣ የተለያዩ ናሙናዎችን በአንድ ላይ ለማቀናጀትና ለረጅም ጊዜ የሚደረገውን የክምችት እንክብካቤና አስተዳደር (Curation) ጥረት አስቸጋሪ ያደርገዋል፡፡
ሳይንሳዊ አተገባበሮች ለማንበሪያ ዉህዶች (Mounting media) የተለዩ መስፈርቶችን ያስገድዳሉ። የዝርያ መለያ ጥናት ባለሙያዎች (Taxonomists) ብዙውን ጊዜ ሙሉውን ናሙና (Specimen) በአንድ ላይ ማንበር ይመርጣሉ፤ እንዲሁም የአካል ሽፋኑን አወቃቀር (Cuticular structures) በደንብ ለማሳየት የውስጥ አካላትን ቀስ ብሎ የሚያለሰልስ (Macerate) ፈሳሽ ይመርጣሉ። የሲራዊ ጥራትን (Optical clarity) ለማሻሻል፣ የፈሳሹ የብርሃን ስብራት መጠን (Refractive index) ከናሙናው ስብራት መጠን ጋር በቂ ልዩነት ሊኖረው ይገባል። በገበያ ላይ የሚገኙ ማንበሪያ ዉህዶች፣ ብርሃን በመስታወቱና በማንበሪያ ዉህዱ ውስጥ ሲያልፍ የሚኖረውን ነፀብራቅና ሥርጭት (Refraction and scattering) ለመቀነስ ሲባል፣ የስብራት መጠናቸው ከመስታወት ጋር ተመሳሳይ እንዲሆን ተደርገው ይሠራሉ። ሆኖም ግን፣ በድምቀ ብርሃን ማይክሮስኮፕ (Brightfield microscopy) ምርመራ ወቅት፣ ያልቀለመ ናሙናን ተፈጥሯዊ ጎልቶ የመታየት ሁኔታ (Contrast) ለመቆጣጠር፣ የብርሃን ስብራት መጠኑ ከናሙናው ጥቂት ለየት ያለ ዉህድ ሆን ብሎ በመምረጥ ናሙናው ከሥር ጎልቶ እንዲታይ ማድረግ ይቻላል።

5.3.3. የናሙና ማንበሪያ ዉህድ ዓይነቶች (ሠንጠረዥ 3 እና 4)

በአጉሊ መነጽር ምርመራ ወቅት፣ ብርሃን በስላይድ፣ በማንበሪያ ዉህድ እና በናሙና ውስጥ እንዴት እንደሚታጠፍ ለመወሰን የማንበሪያ ዉህዱ የብርሃን ነጸብራቅ መለኪያ (Refractive Index - RI) ወሳኝ ነው። የዉህዱ ነጸብራቅ መለኪያዉ ከሽፋን መስተዋቱ ነፀብራቅ መለኪያ ጋር ሲቀራረብ (≈1.515)፣ ብርሃን በተመጣጣኝ ሁኔታ ያልፋል፤ ይህም የብርሃን መበታተንን እና መዛባትን በመቀነስ ጥቃቅን የናሙና አካላትን በግልጽ እንዲታዩ ያደርጋል። በአንጻሩ፣ የነጸብራቅ መለኪያዎች አለመጣጣም ምስሉ እንዲደበዝዝ (blurring) ወይም ያልቀለሙ ክፍሎች እንዳይታዩ ሊያደርግ ይችላል። ተገቢውን የእይታ ልዩነት(contrast) እና አጠቃላይ የምስል ጥራት ለማግኘት ትክክለኛውን የማንበሪያ ዉህድ መምረጥ አስፈላጊ ነው።

የአሸዋ ትንኞችን ለስላይድ ዝግጅት በሚያዘጋጁበት ጊዜ የማንበሪያ ዉህዱ ነጸብራቅ መለኪያ ጥቃቅን የናሙና አካላት በምን ያህል ጥራት እንደሚታዩ ይወስናል። ከፍተኛ የነጸብራቅ መለኪያ ያላቸዉ የማንበሪያ ዉህዶች ጥቅም ላይ ሲዉሉ እንደ የምግብ መፍጫና ማስተላለፊያ ቧንቧ ጥርሶች፣ የዘር ማከማቻ ከረጢት (spermathecae)፣ የአንቴና ክፍሎች እና የክንፍ ደም መላሾች ያሉ ስስ እና በቀላሉ የማይታዩ የአሸዋ ትንኝ ክፍሎችን ለማየት አስቸጋሪ ሊሆን ይችላል።

ለአሸዋ ትንኞች ማንበሪያ ዉህዶች በብዛት ጥቅም ላይ የሚውሉ አማራጮች እንደ በውሃ ሟሚ (Water-based) ማንበሪያ የሆነው ጋም-ክሎራል (Gum-chloral)፣ እና በሙጫ ሟሚ ዉህዶች (Resin-based) ማለትም ካናዳ ባልሳም (Canada balsam) እና ኒነሴ-ኔልሰን ሰርኬራ (Nelson Cerqueira -NC) ያካትታሉ። ራውሊንስ የተባልዉ ፀሀፊ [60] ናሙና ማንበሪያ ዉህዶችን በሁለት ይከፍላቸዋል፡- (1) ቋሚ (Permanent) ዉህዶች ሲሆኑ እነዚህ ከጊዜ ብዛት እየጠነከሩ የሚሄዱና ለረጅም ጊዜ ናሙና ማቆያ የሚመቹ ናቸው፤ (2) ከፊል-ቋሚ (Semi-permanent) ዉህዶች ደግሞ ናሙናዎች ጠንክረው የማይደርቁ ማንበሪያ ዉህዶች ፈሳሽ(Liquid)፣ ሙጫ (gum)፣ ወይም ሙጫማ (resinous) ሊሆኑ ይችላሉ፤ እንዲሁም በውሃ፣ በአልኮል ወይም በሌሎች አሟሚዎች (ለምሳሌ፡- ቶሉዊን (toluene)፣ ዛይሊን (xylene)) ሊሟሙ የሚችሉ ናቸው (ሠንጠረዥ 1)። አንዴ ጥቅም ላይ ከዋሉ በኋላ፣ በማይሟሙ የማሸጊያ ዉህዶች (ringing media) አማካኝነት ከአየር ንብረት ተጽእኖዎች ለመከላከል መታሸግ አለባቸው። የማንበሪያ ዉህድ አይነቶችን በግልጽ ለመለየት እንደሚከተለዉ መመደብ ይቻላል፡-

***ሀ. ውሃማ ዉህዶች* (Aqueous media)**: እነዚህ ዉህዶች በውሃ ውስጥ በቀላሉ የሚሟሙ በመሆናቸው ለጊዜያዊ ወይም ለከፊል-ቋሚ ማንበሪያ ያገለግላሉ። አጠቃቀማቸው ቀላል ቢሆንም፣ በተለይም እርጥበት አዘል በሆኑ ሞቃታማ አካባቢዎች፣ ለአየር እርጥበት እንዳይጋለጡ ማሸግ (sealing) ያስፈልጋል፡፡ (ለምሳሌ፡- ገም ክሎራል (gum-chloral) እና ፖሊቪኒል አልኮል (polyvinyl alcohol)።

***ለ. ውስን የውሃ መቋቋም አቅም ያላቸው ዉህዶች* (Limited water-tolerant media)**: እነዚህ ፈሳሾች በውሃ የመጎዳት ዕድላቸው አነስተኛ ቢሆንም እንኳን ከፍተኛ እርጥበት ካለው አካባቢ ማራቅ ያስፈልጋል። በውሃ ውስጥ ከሚሟሙት ዉህዶች ጋር ሲነጻጸሩ የተሻለ የረጅም ጊዜ መረጋጋት ችሎታ ያላቸዉ ሲሆን፣ በብዛት ለከፊል-ቋሚ ማንበሪያዎች ያገለግላሉ።

***ሐ. በሃይድሮካርቦን የሚሟሙ ዉህዶች* (Hydrocarbon-soluble media)**: እነዚህ ዉህዶች እንደ ዛይሊን፣ ቶሉዊን፣ ወይም ኤኔሴ (enecê solvent) ባሉ ተፈጥሯዊ አሟሚዎች ውስጥ የሚሟሙ ናቸው። እነዚህ ለቋሚ ማንበሪያ የተዘጋጁ እና እጅግ በጣም ጥሩ የረጅም ጊዜ መረጋጋት የሚሰጡ ናቸው። እርጥበትን ስለሚቋቋሙ እና ይዘታቸዉን ሳይቀይሩ የመቆየት ብቃት ስላላቸዉ ለረጅም ዘመን ማቆያ (archival) ዓላማዎች ተመራጭ ናቸው (ለምሳሌ፡- ኒውትሮል ካናዳ ባልሳም፡ ዲፒኤክስ (neutral Canada balsam፣ DPX))።

በአጠቃላይ ከላይ የተዘረዘሩት ሦስት የናሙና ማንበሪያ ዉህዶች ባህሪያትና ጥቅም እእንደሚከተለዉ መግለፅ ይቻላል፤

- በውሃ የሚሟሙ (Water-soluble) ዉህዶች ናሙናን በጊዜያዊነት ለማንበር ወይም በቀላሉ መልሶ ለማውጣት
- ውስን ውሃ የመቋቋም ችሎታ ያላቸዉ ዉህዶች (Limited water-tolerant) ናሙናን ላልተራዘመ ጊዜ ለማቆየት ከፊል-ቋሚ (Semi-permanent) ማንበርያነት።

- በሃይድሮካርቦን የሚሟሙ (Hydrocarbon-soluble) ዉህዶች ናሙናን ለታሪክ ተመዝግበው ለሚቀመጡ (Archival) እና ለረጅም ዘመን ቋሚ ማንበሪያነት።

5.3.4. ተመራጭ የሆኑ ማንበሪያ ዉህዶች መግለጫ (ሰንጠረዥ 3 እና 4)

__ለጊዜያዊ ምልከታ የሚያገለግሉ ዉህዶች__

ክሎራል ገም (Chloral gum) = የሆየር (Hoyer) ፈሳሽ/ዉህድ (RI = 1.48)
የማርክ-አንድሬ (Marc André) ፈሳሽ የዘር ማከማቻ ከረጢቶችን (spermathecae) ለጥቂት ሰዓታት (ስላይዱ እርጥበት ባለው ክፍል ውስጥ ከተቀመጠ ለተወሰኑ ተጨማሪ ሰዓታት) ለማየት፣ ፎቶግራፍ ለማንሳት (ምስል 4) ወይም ለመሳል ምርጡ ዉህድ ነው። የዘር ማከማቻ ከረጢቶችን ፣ ለመካከለኛ ጊዜ ለማቆየት በውሃማ (aqueous) ዉህድ ውስጥ በድጋሚ ማንበር ያስፈልጋል። በሙጫ (resin) ውስጥ ለማንበር ሲባል ናሙናዎቹን ማድረቅ (dehydration) የሚቻል ቢሆንም፣ ናሙናው ሊጠፋ ስለሚችል አይመከርም።
ክሎራል ገም እና የሆየር ፈሳሽ ተመሳሳይ ስያሜዎች ናቸዉ። ይህ ዉህድ ከውሃ ጋር ስለሚስማማ፣ ለቀላል እና ፈጣን ትግበራ አመቺ በመሆኑ እንዲሁም እንደ ዘር ማከማቻ ከረጢት ያሉ ስስ የአካል ክፍሎችን ለመመልከት በሚረዳው የብርሃን ስብራት አመልካች (refractive index) ምክንያት ለውስጥ አካላት ምልከታ በስፋት ጥቅም ላይ ይውላል። ሆኖም፣ ክሎራል ገም በአግባቡ ካልተዘጋጀ ወይም ተስማሚ በሆነ እርጥበት ውስጥ ካልተቀመጠ ጉልህ ድክመቶች ያስከትላል። እነዚህም ጥርኝ መፍጠር (Crystallization)፣ ቀለም መቀየር እና የመለጠጥ ባህሪን ማጣት ናቸው። የሽፋን መስተዋቱን (coverslip) ጠርዝ በሙጫ ማሰር (ringing) እነዚህን ችግሮች አይፈታቸውም፤ ምክንያቱም የማንበሪያ ዉህዱ ከማሰሪያው ሙጫ ጋር (በተለይም ዩፓራል® ጥቅም ላይ ከዋለ) በሚፈጥረው ግንኙነት ምክንያት ቀለሙ ሊለወጥ (አንዳንዴም ወደ ጥቁርነት ሊቀየር) ይችላል።
የሆየር ዉህድ ለአሸዋ ትንኞች በኦፐቲካል እይታ ረገድ ምርጡ ተደርጎ ይወሰዳል፣ በተለምዶም ለነዚህ ዓላማዎች ጥቅም ላይ ውሏል። ዉህዱ እንደ ገም አረቢክ፣ ግሊሰሮል እና ክሎራል ሃይድሬት ያሉ ተቀራራቢ ውህዶችን ይይዛል። የዉህዱ አዘገጃጀት ቀመሩ በተሳሳተ መንገድ ሲተረጎምና ሲጠቀስ ቆይቷል[74]።
ምንም እንኳን ሆየር በአሸዋ ትንኞች ውስጥ የዘር ማከማቻ ከረጢትን ለማየት ጥሩ ቢሆንም፣ ለረጅም ጊዜ ማቆያነት ግን ተስማሚ አይደለም። ለፎቶግራፍ፣ ለሥዕል ወይም ለምስል ቀረጻ ለሚደረጉ የአጭር ጊዜ ምልከታዎች ተመራጭ ነው። ውሃማ ዉህዶች ለጊዜያዊ ማንበሪያ ተስማሚ ቢሆኑም የረጅም ጊዜ ማቆያነት ተስማሚ ላይሆኑ ይችላሉ። በተቃራኒው፣ በሙጫ (resin) ማንበር ለዘመናት የሚቆይ እጅግ በጣም ጥሩ ጥንካሬ ይሰጣል፤ ነገር ግን የዘር ማከማቻ ከረጢቶች ረቂቅ አካላት የብርሃን ነጸብራቃቸው (refringence) ብዙ ጊዜ ስለሚጠፋ እይታቸዉ ሊያደበዝዝ ይችላል።
የሆየር ዉህድ በድርቀት (dehydration) ምክንያት በጊዜ ሂደት ይበላሻል (ምስል 8)፤ ይህም ትናንሽ ነጭ እና ብርሃን የማያሳልፉ የክሎራል ሃይድሬት ጥርኞች እንዲፈጠሩ ያደርጋል። ቢሆንም፣ የነፍሳቱ አካል ሽፋን (cuticle) በኬሚካል ሳይጎዳ ስለሚቆይ ናሙናዎቹን ከተበላሹ ስላይዶች ላይ መልሶ ማውጣት ይቻላል፤ ነገር ግን ጥርኞቹ እያደጉ ሲሄዱ በነፍሳቱ ላይ አካላዊ ጉዳት ሊያደርሱ ይችላሉ።

በስላይድ ላይ የጥርኝ መፈጠር (Crystalization) ችግር ሲያጋጥም ናሙናውን ለማዳን ሁለት ዋና አማራጮችን መጠቀም ይቻላል። የመጀመሪያው ዘዴ የማንበሪያ ዉህዱን ሙቀትና እርጥበት ባለው አካባቢ ውስጥ በማቆየት መልሶ ማርጠብ ሲሆን፣ በዚህ ሂደት ወቅት በእርጥበቱ ምክንያት የፈንገስ እድገት እንዳይከሰት ለመከላከል ታይሞል (Thymol) የተባል ኬሚካል መጠቀም አስፈላጊ ነው። ሁለተኛውና ይበልጥ አስተማማኝ የሆነው አማራጭ ደግሞ ናሙናውን ከደረቀው የገም ክሎራል ውህድ ውስጥ በውሃ ዘፍዝፎ በጥንቃቄ ማውጣት፣ ከዚያም በግላሲያል አሴቲክ አሲድ አማካኝነት በውስጡ ያለውን ውሃ ማስወገድና በመጨረሻም በካናዳ ባልሳም ላይ መልሶ ማንበር ነው።

ዲ ኤም ኤች ኤፍ (DMHF [dimethyl hydantoin formaldehyde]) (RI 1.48)
ይህ ዉሀማ ዉህድ [72] እንደ በርሌዝ (Berlese) ሁሉ በሲራዊ እይታ ረገድ በጣም ጥሩ እና ለመጠቀም ቀላል ነው። ሆኖም ከበርሌዝ በተለየ ወደ ጥቁርነት አይቀየርም ወይም ጥርኝ አይሰራም። ለአሸዋ ትንኞች እና ለሌሎች ሳይኮዲዳይ (Psychodidae) ዝርያዎች በሚገባ ያገለግላል።

ሲ ኤም ሲ ፒ (CMCP [camphor-mono-chlorophenol]) (RI = 1.41)
ይህ በግሊሰሪን ላይ የተመሰረተ እና በውሃ ውስጥ የሚሟሟ የማንበሪያ ውህድ ሲሆን፣ ብርሃን የሚያሳልፉና ቋሚ የሆኑ የአሸዋ ትንኝ እና ሌሎች ስስ ናሙናዎችን በስላይድ ላይ ለማንበር ያገለግላል። የዚህ ዉህድ ጥቅሙ ናሙናዎችን በቀጥታ ከውሃ ወይም ከኤታኖል ውስጥ ወስዶ ማንበር ማስቻሉ ነው። የአሸዋ ትንኙን በፍጥነት በማፍታታት ያጠራል፣ የአሸዋ ትንኟን የአካል ሽፋን በማለስለስ ናሙናው በትክክል እንዲቀመጥ ያደርጋል፤ ይህም በተለይ ክንፎችን ለመዘርጋት ወይም የመራቢያ አካላትን ለመበለት ጠቃሚ ነው። ለረጅም ጊዜ ማቆያ እንደሚረዳ ቢገለጽም፣ በትክክል ለምን ያህል ጊዜ እንደሚቆይ ግን እርግጠኛ መሆን አይቻልም። የዚህ ዉህድ ዋና ችግሩ ፌኖልን (phenol) የተባል መርዛማ እና የሚቆጠቁጥ ንጥረ ነገር የያዘ በመሆኑ ጥንቃቄ የተሞላበት አያያዝን የሚጠይቅ መሆኑ ነው።

__ለቋሚ ናሙና ማንበሪያነት የሚያገለግሉ ዉህዶች__

ካናዳ ባልሳም (Canada balsam) (RI = 1.52-1.54)

ለመጀመሪያ ጊዜ ለማይክሮስኮፕ ምርምር ተስማሚ መሆኑ ተለይቶ የታወቀው በ1830ዎቹ በአንድሪው ፕሪትቻርድ አማካኝነት ነበር። ይህ ውህድ ከአንድ መቶ አምሳ ዓመታት በላይ ለተሳካ አገልግሎት በመዋሉ እና ለረጅም ዘመን የመቆየት (Archival) ብቃቱ ስለተረጋገጠ ዛሬም ድረስ በሰፊው ጥቅም ላይ ከሚውሉ ዉህዶች መካከል አንዱ ነዉ። ከሆየር ዉህድ በተለየ፣ ካናዳ ባልሳም በጊዜ ሂደት ጥርኝ (ክሪስታል) አይሰራም ወይም እርጥበትን ወደ ውስጥ አይመጥም። ይሁን እንጂ ውህዱ ከፍተኛ የሆነ የተፈጥሮ ብርሃን የማንጸባረቅ (Autofluorescent) ባህሪ ስላለው ለአንዳንድ የማይክሮስኮፕ ምርመራ ዘዴዎች እንደ ጉድለት ሊታይ ይችላል። በተጨማሪም ዝግጅቱን በምናከናውንበት ወቅት ከዛይሊን ይልቅ መርዛማ ያልሆኑ አሟሚዎችን በመጠቀም የደህንነት ስጋትን መቀነስ ቢቻልም፣ ይህ ደግሞ የራሱ የሆኑ ጉዳቶች ሊኖሩት ይችላል (ለምሳሌ ቀስ ብሎ መድረቅ እና የውህዱ መጥቆር)።

ዩፓራል® (Euparal®) (RI = 1.48)
ዩፓራል (የብርሃን ስብራት መጠን = 1.48) ለረጅም ጊዜ ለሚቆይ ቋሚ ስላይድ ዝግጅት እንደ ካናዳ ባልሳም በሰፊው ጥቅም ላይ የሚውል ሲሆን፣ እጅግ በጣም ጥሩ የሆነ የመቆየት አቅም እና ለምርመራ ተስማሚ የሆነ የብርሃን ስብራት መጠን አለው። ዩፓራል ሁለት ዋና ዋና ባህሪያት አሉት፤

አንደኛው የውሃ ማስወገድ (Dehydration) አስፈላጊነት ሲሆን፣ ናሙናው ወደ ውህዱ ከመሸጋገሩ በፊት ብዙውን ጊዜ ከ95% እስከ 100% (Absolute) በሆነ አልኮል ውስጥ እንዲቆይ በማድረግ በውስጡ ያለውን ውሃ ማስወገድ የግድ ይላል
ሁለተኛው ባህሪው ደግሞ የሂደቱ መርዘም ነው፤ እንደ ካናዳ ባልሳም ወይም ዩፓራል ያሉ የሙጫ ውህዶችን መጠቀም የውሃ ማስወገድ ደረጃን ስለሚጠይቅ አጠቃላይ የናሙና ዝግጅት ጊዜውን ያራዝመዋል። ናሙናዎችን በተፈጥሮዊ አሟሚዎች ማድረቅ በማይቻልበት ሁኔታ ውስጥ፣ ከዉሀ ነፃ የሆነ አልኮል (Absolute ethanol) ዉስጥ የወጡ ናሙናዎችን ወደ መጨረሻው ማንበሪያ ዉህድ ከመውሰዳቸው በፊት እኩል መጠን ባለው የዩፓራል እና የዩፓራል ኤሰንስ (Euparal essence) ድብልቅ በተሰራ ዉህድ ውስጥ ማቆየት ያስፈልጋል፡፡

ኤነሴ [Enecê] (የብርሃን ስብራት መጠን = 1.467)
ኤነሴ በሙጫ ላይ የተመሰረተና በዋናነት ለጥቃቅን ነፍሳት ዝግጅት የሚያገለግል ውህድ ሲሆን፣ በተለይም በብራዚል ውስጥ ከፍተኛ ተወዳጅነት አለው። የውህዱ መሠረት በአልኮል፣ በካምፎር፣ በተርፔንታይን ኤሰንስ (Essence of turpentine) እና በዩካሊፕቶል ውስጥ የሚሟ ኮሎፎኒ እና የኮፓል ሙጫ (Gum copal) ነው። ሰርኬራ(Cerqueira)[11] የተባለ ተመራማሪ ኤነሴን ለነፍሳት ዕጭ (Larvae)፣ ለነፍሳት ውጫዊ ሽፋን (Exuviae) እና ለአዋቂ ትንኞች ቋሚ ስላይድ ዝግጅት እንደ ካናዳ ባልሳም አማራጭ አድርጎ የገለጸው ሲሆን፣ ከዚያ ጊዜ ጀምሮ ለአሽዋ ትንኞችም በሰፊው ጥቅም ላይ እየዋለ ይገኛል። ኤነሴ ለቋሚ ናሙና ዝግጅት አነስተኛ ወጪ የሚጠይቅ አማራጭ ሲሆን፣ ናሙናዎችን ርጉዕ የማድረግ እና ለረጅም ጊዜ የማቆየት ብቃት አለዉ፡፡ በተጨማሪም በቂ የመድረቂያ ጊዜ ስለሚሰጥ ናሙናዎችን በጥንቃቄ ለመበለት እና የአካል ክፍሎችን በቅደም ተከተል ለማስተካከል ያስችላል።

5.4. የስላይድ ዝግጅት እና ማድረቅ

የተዘጋጁ ስላይዶች ለረጅም ጊዜ ሳይበላሹ እንዲቆዩ ትክክለኛ የማድረቅ ሂደት መከተል አስፈላጊ ነው። ስላይዶችን ለረጅም ጊዜ ከምችት ከማስቀመጥ በፊት ሙሉ በሙሉ መድረቃቸውን ማረጋገጥ ያስፈልጋል። ለተሻለ ውጤት፣ በቋሚ የማንበሪያ ውህዶች (Permanent media) የተሰሩ ስላይዶች ከ2 እስከ 3 ሳምንታት፣ በከፊል-ቋሚ (Semi-permanent) የተሰሩት ደግሞ ከ1 እስከ 2 ሳምንታት በአግድም (Horizontally) ተቀምጠው መድረቅ አለባቸው። የማድረቅ ሂደቱ ውጤታማ እንዲሆን እንደ ውህዱ ባህሪ ተገቢው የሙቀት መጠን ያለው ኢንኩቤተር (Incubator) መጠቀም የሚመከር ሲሆን፣ ናሙናውን ሊጎዳ የሚችል ከፍተኛ ሙቀትን ማስወገድ ያስፈልጋል። ለዚህም ከ30°C እስከ 37°C ያለው የሙቀት መጠን ተመራጭ ነው። ይህ የማድረቅ ደረጃ ናሙናዉ እንዳይጣመምና እንዳይበላሽ ወይም በማከማቻ ጊዜ ውህዱ እንዳይቀጥን ለመከላከል ወሳኝ ነው።

በስላይድ ዝግጅት ወቅት ጥቅም ላይ የዋለው የማንበሪያ ውህድ ሁልጊዜ በስላይዱ መለጠፊያ (Label) ላይ መጥቀስ አለበት። ከተቻለ የተጠቀሙበትን ልዩ የውህድ ቀመር (Recipe)፣ የዝግጅቱን ቀን እና ያዘጋጀውን ሰው ስም ማካተት ይገባል። መጀመሪያ ላይ ስላይዶች ለጊዜያዊ አገልግሎት ብቻ ተዘጋጅተው ሊሆን ይችላል፤ ነገር ግን የናሙናው ሁኔታ ተቀይሮ ለምሳሌ የሙነነ አምሳያ ቅደም ተከተል (Type series) ለዝርያ ምደባ ጥናት በቋሚነት እንዲቀመጥ ከተፈለገ፣ ናሙናው ለወደፊት ምርምር እንዲቆይ ይበልጥ ቋሚ የሆኑ የማንበሪያ ውህዶችን መጠቀም ያስፈልጋል።

5.5. አማራጭ የናሙና ማንበሪያ ቴክኒኮች፡- በካርድ ላይ ማንበር (Card mounting)

በካርድ ላይ ናሙናዎች ማንበር በቀጥታ በነፍሳት መያዣ ካርዶች ላይ በሚስማር የሚሰኩበት ወይም በሙጫ የሚለጠፉበት ማንበሪያ ዘዴ ነው። ይህ ዘዴ ለበርካታ ነፍሳት ዝርያዎች ማንበሪያነት የሚያገለግል ሲሆን፣ አሸዋ ትንኞች ግን በጣም ትናንሽ በመሆናቸው እና ዝርያቸውን ለመለየት የውስጥ አካላትን በማጥራት (አባሪ 5ን ይመልከቱ) ማየት ስለሚያስፈልግ፣ ይህ ዘዴ ለአሸዋ ትንኞች በፍጹም ተስማሚ አይደለም።

5.6. የተጎዱ ናሙናዎችን መልሶ ማንበር (Remounting)

ብርቅዬ ወይም ውድ የሆኑና የተጎዱ ናሙናዎችን ለማንበር የሚከተለውን ባለ ሁለት ደረጃ ዘዴ መጠቀም ይመከራል፡-

1. **ሳይበታተኑ መልሶ ማርጠብ (Rehydration)፡-** ናሙናውን ከመበተንዎ በፊት ለመጀመሪያ ምልከታ እንዲያመች መልሶ ማርጠብ ያስፈልጋል። ለዚህም የስላይድ መያዣን በፔትሪ ዲሽ (Petri dish) ውስጥ በማስቀመጥ ስላይዱን በላዩ ላይ መደርደር። በመቀጠል በፔትሪ ዲሹ ውስጥ ጥቂት ሚሊሜትር የሚሆን አሟሚ ፈሳሽ (Solvent) መጨመር፤ በዚህ ጊዜ ስላይዱ ከፈሳሹ ጋር በቀጥታ እንዳይነካካ ጥንቃቄ ማድረግ ያስፈልጋል (ምስል 8 D)። ይህ ሂደት እርጥበት ያለው ክፍል (Humid chamber) ይፈጥራል። ናሙናው እንዲርጥብ የሚያስፈልገው ጊዜ እንደ ሁኔታው ከአንድ እስከ ብዙ ቀናት ሊፈጅ ስለሚችል ሂደቱን በየቀኑ በትዕግስት መከታተል ያስፈልጋል። ስላይዱ በቂ እርጥበት ካገኘ በኋላ፣ ከእርጥበት ክፍሉ አውጥቶ ለማይክሮስኮፕ ምርመራ፣ ለፎቶግራፍ ወይም ለስዕል እንዲያመች ለጥቂት ሰዓታት በኢንኩቤተር ውስጥ ማቆየት ይቻላል።

2. **መልሶ ማንበር (Remounting)፡-** ናሙናውን ከአሮጌው ስላይድ ላይ አንስቶ በአዲስ ለማንበር፣ ስላይዱን ለተጨማሪ ጥቂት ሰዓታት ወይም ለአንድ ሙሉ ሌሊት በእርጥበት ክፍሉ ውስጥ መልሶ ማቆየት ያስፈልጋል። አሮጌውን ስላይድ የመበተኑ ሂደት ባለ ሁለት ዓይን አጉሊ መነፀርን (Binocular microscope) በመጠቀም መከናወን አለበት። በጥቃቅን መርፌዎች በመታገዝ የሽፋን መስታወቱን (Coverslip) ማንሳት ያስፈልጋል፡ ፡ በዚህም ጊዜ የትንኟ አካላት ከመስታወቱ ጋር ተጣብቀው እንዳይቀሩ ከፍተኛ ጥንቃቄ ማድረግ ያስፈልጋል (https://zenodo.org/records/18315029)።

ሠንጠረዥ 4፦ የተመረጡ የማንበሪያ ዉህዶች ጠቀሜታዎች እና ጉድለቶች (በተለያዩ ባለሙያዎች ያልታተሙ ምልከታዎችን ጨምሮ [52])

**ስም** (Name)	**ጠቀሜታዎች** (Advantages)	**ጉድለቶች** (Disadvantages)
ካናዳ ባልሳም (Canada balsam)	የማንበሪያ ዉህዱ እጅግ ዘላቂ (durable) ስለሆነ ዕድሜውም ከ150 ዓመታት በላይ ሊቆይ ይችላል።<br>ስላይዶች በቅርንፉድ ዘይት (clove oil) ወይም በፌኖል (phenol) እንደ መጫኛ ንጥረ ነገሮች (mounting agents) በመጠቀም ሊጫኑ ይችላሉ።	ጎጂ የሆኑ ይዘቶች ስላለው ጥንቃቄ በሚፈልግ ማሽን (hood) ዉስጥ መያዝ አለበት።<br>በኤታኖል (Ethanol) ውሃን በማስወገድ ወደ ዛይሊን (xylene) ወይም ቅርንፉድ ዘይት (clove oil) ማዛወር አንዳንድ ዝርያዎችን (taxa) ተሰባባሪ (brittle) ሊያደርጋቸው ይችላል፤ አማራጮችን (ለምሳሌ፦ አይሶፕሮፓኖል (isopropanol)፣ ኤን-ቡታኖል (n-butanol)፣ ሴሎሶልቭ (Cellosolve™)፣ 1,4-ዳዮክሰን (1,4-dioxane)፣ ሂስቶክሊየር (Histoclear)፣ ተርፒንዮል (terpineol)) መጠቀም መሰባበርን ሊቀንስ ይችላል።<br>ዛይሊን (xylene) በፌኖል (phenol) ከተተካ ወይም የፖታሺየም ሃይድሮክሳይድ (potassium hydroxide) ቅሪት ካለ ናሙናዎቹ ሊጠቁሩ (blacken) ይችላሉ።<br>ያልተቀለሙ (unstained) የናሙና ቅርጾች በከፍተኛ የብርሃን ነጸብራቅ ምክንያት አካለ ቅርፃቸዉ ሊደበዝዝ ውይም ሊሸፈን ይችላል።<br>ያለ ማድረቂያ ማሽን (የማንበሪያ ዉህዱ እጅግ ዘላቂ (durable) ስለሆነ ዕድሜውም ከ150 ዓመታት በላይ ሊቆይ ይችላል።<br>ስላይዶች በቅርንፉድ ዘይት (clove oil) ወይም በፌኖል (phenol) እንደ መጫኛ ንጥረ ነገሮች (mounting agents) በመጠቀም ሊጫኑ ይችላሉ።hot-plate) ሙሉ በሙሉ ለማድረቅ ዓመታትን ሊወስድ ይችላል።<br>ማንበሪያ ዉህዱ (medium) በጊዜ ሂደት ወደ ቢጫነት ይለወጣል እንዲሁም ይጨልማል፤ በተለይ በቅርንፉድ ዘይት (clove oil) ሲጸዳ ይህ በከፍተኛ ሁኔታ ይታያል።<br>ማንበሪያ ዉህዱ በራሱ ጊዜ አሲዳማ (acidic) ሊሆን ስለሚችል፣ አንዳንድ ቀለሞች እንዲዳከሙ እና ካቲዮኒክ ቀለሞች (cationic dyes) እንዲደበዝዙ ሊያደርግ ይችላል።
DMHF (ዲሜቲል ሃይዳንቶይን ፎርማለዳይድ - dimethyl hydantoin formaldehyde)	ከፍተኛ የብርሀን አስተላላፊነት (High transparency)<br>ጥሩ የብርሃን ነጸብራቅ መለኪያ (Good refractive index)<br>የቅርጾች ታይነት እጅግ በጣም ጥሩ ነው (Excellent visibility of structures)<br><br>የናሙና ዝግጅቶች መረጋጋት ወይም ጥንካሬ በጥሩ ሁኔታ ላይ ነው (Fairly good stability of the preparations)<br>ከብዙ ማቅለሚያ ዘዴዎች (staining techniques) ጋር ይጣጣማል<br>ለናሙናዎች (samples) ተስማሚ ማቆያ ነዉ<br>በስላይድ (slide) እና በመሸፈኛ መስተዋቱ (coverslip) መካከል ጥሩ መጣበቅ (adhesion) ይፈጥራል	በጊዜ ሂደት ወደ ቢጫነት የመቀየር ዕድል አለዉ (Possible yellowing over time)<br>አንዳንድ ማቅለሚያዎችን (stains) ሊቀይር ይችላል<br>ፎርማለዳይድን ለማይቋቋሙ (formaldehyde-sensitive) ማቅለሚያዎች ተስማሚ አይደለም<br>የአየር አረፋዎች (Air bubbles) መፈጠር እና የማድረቂያ ጊዜው የዘገየ መሆን (slow drying time)<br>ማንበሪያ ዉህዱ(Mounting medium) እርጥበትን (humidity) መቋቋም አይችልም<br>አንዴ ከተነበረ በኋላ መልሶ ማውጣት ወይም ማስተካከል (reverse) አስቸጋሪ ነው<br>ፎርማለዳይድ (Formaldehyde) መርዛማ (toxic)፣ የሚቆጠቁጥ (irritant) እና ለካንሰር የሚያጋልጥ (carcinogenic) ነው
ኤውፓራል (Euparal - transparent)	ከ50 ዓመታት በላይ የሚቆይ ዘላቂ (durable) ዉህድ ነው።<br>በአምራች ተቋማት በተገለፀዉ መመሪያ መሰረት ቀጥታ ከ80% ኤታኖል ላይ ማንበር ይቻላል።<br><br>ያልተቀቡ ቅርጾችን አይሸፍንም፤ በጊዜ ሂደት አይሰባበርም (brittle) ወይም ወደ ቢጫነት አይቀየርም።<br>ለዝንቦች (Diptera) ምርመራ ከካናዳ ባልሳም የተሻለ የብርሃን ነጸብራቅ አለው።<br>ወፈር ያሉ ናሙናዎችን ለማንበር ተስማሚ ነው። ምክን ያቱም ያለመሸብሸብ (shrinkage) የአየር አረፋ ሳይፈጥር ይደርቃል።<br>በ95% ኢታኖል የሚሟሟ ሲሆን ከብዙ ዓመታት በኋላ በድጋሚ ለማንበር ያስችላል	ጎጂ ይዘቶች ስላለው ጥንቃቄ በሚፈልግ ማሽን (hood) ዉስጥ መያዝ አለበት።<br>በኤታኖል ውሃን በማስወገድ ወደ ኤውፓራል ኤሰንስ (Euparal Essence) ማዛወር አንዳንድ ዝርያዎችን ተሰባባሪ ሊያደርግ ይችላል። ሆኖም ይህን ችግር በአይሶፕሪፕሮል(isopropanol) ማስወገድ ይቻላል።

ስም (Name)	ጠቀሜታዎች (Advantages)	ጉድለቶች (Disadvantages)
ሆየር ፍሉድ<br>Hoyer fluid	ናሙናዎች (Specimens) በሕይወት እንዳሉ ወይም በቀጥታ ከውሃ (water)፣ ከኤታኖል (ethanol) ወይም ከፎርማለዳይድ (formaldehyde) ዉስጥ በማዉጣት ማንበር ይቻላል። ናሙናን ማለስለሱ (Maceration) እጅግ በጣም ጥሩ የቆዳ/ሽፋን (cuticle) ጥራት ይሰጣል። ተስማሚ የብርሃን ነጸብራቅ መለኪያ (favorable refractive index) አለው፤ እንዲሁም ለበለጠ ንጽጽር (higher contrast) በአዮዲን ማቅለም (iodine staining) ይቻላል። በውስጡ ያለው አሴቲክ አሲድ (Acetic acid) የአርትሮፖድ ብልቶችን/አካላትን (arthropod appendages) ሊያሰፋ (expand) ይችላል። አንዳንድ ናሙናዎች ለ 40–60 ዓመታት ያህል ሳይበላሹ ሊቆዩ (stable) ይችላሉ።	ማንበሪያ ዉህዱን (medium) በደረጃና በጥቂት በጥቂቱ ካልተጨመረ በስተቀር ደካማ የዕፅዋት ናሙናዎች (Delicate plant specimens) ሊጨማደዱ/ሊኮማተሩ (collapse) ይችላሉ፤ ይህ ደግሞ ጊዜ የሚወስድ (time-consuming) ሂደት ነው። ከ10 ዓመታት ባነሰ ጊዜ ውስጥ በውስጡ ክፍተቶች (Cavities) እና ጥርኞች (crystals) ሊፈጠሩ ይችላሉ። እንደ ክሎራል ሃይድሬት (chloral hydrate) መጠን እና እንደ መቆያ ጊዜው ሁኔታ ማለስለሱ (Maceration) ከተፈለገው በላይ ሊሆን (excessive) ይችላል። በማንበሪያዉ ውስጥ ያሉ ክፍሎች ሊለያዩ (separate) ይችላሉ፤ እንዲሁም በጥቂት ወራት ወይም ዓመታት ውስጥ ደቃቅ ቅንጣቶች (fine granulation) ሊታዩ ይችላሉ።
CMCP-9<br>(= ካርቦክሲ ሜቲል ሴሉሎስ ፌኖል / carboxy methyl cellulose phenol)	ናሙናዎች (Specimens) በቀጥታ ከውሃ (water)፣ ኤታኖል (ethanol)፣ ግሊሰሮል (glycerol) ወይም ፎርማለዳይድ ካላቸዉ ዉህዶች (formaldehyde-containing solutions) ዉስጥ በማዉጣት ማንበር ይቻላል፤ እንዲሁም አጠቃላይ ምርመራን ወይም ዝግጅትን ለማቀላጠፍ አስፈላጊ ሆኖ ሲገኝ የናሙናዎችን የውስጥ አካላት ማለስለስ(macerated) ይቻላል።	ይህ የማንበሪያ ዉህድ(medium) በጊዜ ሂደት ጥርኞችን (crystals) ሊያመጣና ሊጠቁር (darken) ይችላል፤ እንዲሁም አንዳንድ ጊዜ ናሙናዎችን ከተፈለገው በላይ ሊያለሰልስ (macerate) ይችላል። ስላይዱ በጥንቃቄ በጠርዙ ካልታሸገ (ringed) በስተቀር፣ ወፈር ያሉ ናሙናዎች ሊሸበሸቡ (shrink) እና በመሸፈኛ መስተዋቱ (coverslip) ጫፎች ዙሪያ ክፍተቶችን ሊፈጥሩ ስለሚችሉ በዚህ ዉህድ ውስጥ በጥሩ ሁኔታ አይቆዩም። ይህ ማንበሪያ ዉህድ ለቀለሙ ናሙናዎች (stained specimens) ወይም ለጠነከሩ ቁሶች (calcified materials) ተስማሚ አይደለም፤ እንዲሁም የማድረቂያ ጊዜው ከ CMC (ካርቦክሲ ሜቲል ሴሉሎስ / carboxy methyl cellulose) የበለጠ ዘገምተኛ (slower) ነው።
ኤውኪት (Eukitt™)	(terpineol)፣ ቶሉዊን (toluene) እና ዛይሊን (xylene) ጋር ተኳኃኝ ነዉ።<br>ዉህዱ በመጠኑ አሲዳማ የሆነና በፍጥነት የመድረቅ ባህሪ አለዉ።<br>በዕድሜ ብዛት የመጥቆር (darken) ባህሪዉ አነስተኛ ነዉ።<br>ለተለያዩ ማቅለሚያዎች (stains) ተስማሚ ነው (ለምሳሌ፦ ፉክሲን (fuchsin)፣ ሄማቶክሳይሊን (hematoxylin)፣ ሜቲል ግሪን (methyl green)፣ ሜቲል ቫዮሌት (methyl violet)፣ ሜቲሊን ብሉ (methylene blue)።<br>ናሙናዎችን (Specimens) ከዓመታት በኋላም ቢሆን በዛይሊን (xylene) ውስጥ ለረጅም ጊዜ በመዘፍዘፍ መልሶ ማንበር (re-mounted) ይቻላል።	ጎጂ የሆኑ ይዘቶች (harmful components) ስላለው የግድ ጥንቃቄ በሚፈልግ ማሽን (hood) ዉስጥ መያዝ አለበት።<br>የተሟላ እና ረጅም ጊዜ የሚወስድ የውሃ ማስወገድ ቅደም ተከተል (full, time-consuming dehydration series) ያስፈልገዋል።<br>የመሸብሸብ (shrinkage) እና የጋዝ አረፋዎችን የመፈጠር (gas-bubble formation) ባህሪ ስላለው ወፈር ላሉ ናሙናዎች (thicker specimens) ተስማሚ አይደለም።<br>መስታወቱ በደንብ ካልተጸዳ እና ካልታሸገ (sealed) በስተቀር የመሸፈኛ መስተዋቶች (Coverslips) በጊዜ ሂደት ሊላቀቁ (detach) ይችላሉ።<br>በህብረ ህዋስ ዉስጥ የሚገኝን ኮላጅን ፋይበር (collagen fibers) የማጠንከር ብቃቱ ዝቅተኛ ነዉ።
ኤነሴ (Enecê)	ቢያንስ ለ50 ዓመታት የሚቆይ እጅግ ዘላቂ ዉህድ (Highly durable medium) ነው።<br>ኤነሴ በጊዜ ሂደት የመጥቆር (darken) ባህሪ አያሳይም።<br>ይበልጥ ተጠፍጣፊ (malleable) በመሆኑ ነፍሳትን በዉህዱ ውስጥ ሆኖ ለመበለት(dissection) እንዲሁም የቅርጽ መዋቅሮችን (morphological structures) ለማስተካከል ያስችላል።	የተሟላ እና ረጅም ጊዜ የሚወስድ የውሃ ማስወገድ ቅደም ተከተል (full, time-consuming dehydration series) ያስፈልገዋል።<br>በኤታኖል (Ethanol) ውሃን በማስወገድ ወደ ቅርንፉድ ዘይት (clove oil) ማዛወር አንዳንድ ናሙናዎችን (specimens) ተሰባባሪ (brittle) ሊያደርግ ይችላል።<br>ዉህዱ የተነበሩ ነፍሳትን በዝግታ የማጥራት (clarified) ሂደቱን ስለማያቋርጥ ጥቃቅን የሆኑ

የተበለቱ የአሸዋ ትንኝ አካላት ተሰብስበው ውሃን የማስወገድ (dehydration) እና በሙጫ (resin) የማንበር ሂደት ከመከናወኑ በፊት ትናንሽ ጉድጓዶች ባሏቸው ሳህኖች(ልክ ለዲኤንኤ/አርኤንኤ (DNA/RNA) ማጣራት እንደሚያገለግሉ ሳህኖች ዓይነት) ውስጥ በውሃ መለቅለቅ አለባቸው:: ስላይዱን ከመበተን በፊት መጀመሪያ የማንበሪያ ውህድ ዓይነት ማወቅ ያስፈልጋል። ለውሃማ ውህዶች ውሃን መጠቀም ሲገባ፣ እንደ ካናዳ ባልሳም ወይም ዩፓራል ላሉ የሙጫ ውህዶች ግን ዛይሊን (Xylene) መጠቀም ያስፈልጋል። ዛይሊን መርዛማ ስለሆነ በጭስ መሳቢያ (Fume hood) ዉስጥ መጠቀምና እንደ ማስክ ያሉ የግል ጥንቃቄ መሣሪያዎችን መጠቀም ያስፈልጋል። የሙነነ አምሳያ (Type speciment) ናሙናዎችን መልሶ ማስተካከል የሚቻለው የክምችቱ ጠባቂ (Curator) ወይም የባለቤቱ ተቋም ፈቃድ ሲሰጥ ብቻ ነው።

This is an Open Access article distributed under the terms of the Creative Commons Attribution License (https://creativecommons.org/licenses/by/4.0), which permits unrestricted use, distribution, and reproduction in any medium, provided the original work is properly cited.

6. የትንኝ ዝርያዎች ልየታ (Specimen identification)

6.1. የአካል ቅርፀት ጥናት (Morphology)

የአሸዋ ትንኞችን ዝርያ ልየታ በዋነኝነት የተመሠረተው በአካላዊ ባህሪያቸው ምርመራ ላይ ነው፤ ይህም የደረታቸውን፣ የክንፋቸውን፣ የመራቢያ አካላቸውን፣ የጸጉሮቻቸውን (setae) ቅርጽ እና በተለያዩ መዋቅሮች መካከል ያለውን ልኬታዊ ዝምድና (morphometric relationships) ማየትን ያካትታል። ተመራማሪዎች የተሰበሰቡትን ናሙናዎች ከታወቁ ዝርያዎች ጋር ለማነጻጸር የዝርያ ምደባ ቁልፍ መለያዎች (keys)፣ የማጣቀሻ ስብስቦችን እና የቀደሙ የዝርያ መግለጫዎችን ይጠቀማሉ።

እንደ የክንፍ ደም መላሾች (wing venation) እና የሁለቱም ጾታዎች የራስ ቅርጽ፣ የወንዶች መራቢያ አካል መዋቅር፣ እና የሴቶች የዘር ማከማቻ ከረጢት (spermathecae) አቀማመጥ ያሉ ቁልፍ መለያዎች ለዝርያ ልየታ ውሳኔ በጣም ጠቃሚ ናቸው። ትክክለኛ ልየታ ለማግኘት ዝርዝር የአጉሊ መነጽር ምርመራ ያስፈልጋል፤ ይህም አብዛኛውን ጊዜ ጥቃቅን የአካል ክፍሎችን (እንደ መራቢያ አካላት) ለማየት ኮምፓውንድ ማይክሮስኮፕ፣ ወይም ጉልህ አካላዊ ባህሪያትን ለማየት ስቴሪዮ-ማይክሮስኮፕን መጠቀም ያስፈልጋል።

በምስል ቴክኖሎጂ (Imaging technology) በኩል የተመዘገቡት የቅርብ ጊዜ ውጤቶች በዲጂታል ምስል አማካኝነት የአሸዋ ትንኞችን የዝርያ ልየታ ጥረትን አቅልለውታል። ከፍተኛ ጥራት ያላቸው ፎቶግራፎች ወይም ቁልፍ የአካል ክፍል መለያዎችን (Key features) የሚያሳዩ ዲጂታል ስዕሎች፣ ቀድሞ ከተዘጋጁ የማጣቀሻ ሰነዶች ጋር ለማነጻጸር ወይም በኮምፒውተር የታገዙ የመለያ ዘዴዎችን (Computer-assisted identification systems) በመጠቀም ለመተንተን ያስችላሉ። ይህ አሰራር በስነ-ቅርጽ ጥናት (Morphological taxonomy) ላይ የሚታየውን ትክክለኛነት ከማሻሻሉም በላይ፣ መረጃዎቹን በቀላሉ ለማግኘት ትልቅ እድል ፈጥሯል።

6.2. የክንፍ ጂኦሜትሪ (Wing geometry)

የክንፍ ጂኦሜትሪ የተለያዩ የአሸዋ ትንኝ ዝርያዎችን ለመለየት እና ለመመደብ የሚያገለግል ቁልፍ ባህሪ ነው። የአሸዋ ትንኞች ክንፎች ልዩ ንድፍ እና መዋቅር አላቸው፤ አብዛኛውን ጊዜ ረጅም፣ ጠባብ እና በሚገባ የዳበሩ የደም መላሽ ቧንቧዎች (venation) አሏቸው (ምስል 9 እና 10)። የደም መላሾቹ አቀማመጥ እንደ ዝርያው የሚለያይ በመሆኑ ለመለያነት ጠቃሚ ግብዓት ይሆናል። በመሆኑም የክንፍ ጂኦሜትሪ ጥናት ለዝርያ ምደባ ጠቃሚ ግንዛቤዎችን ይሰጣል።

6.3. የክንፍ ጂኦሜትሪያዊ ቅርፀ ልኬት (Wing geometric morphometrics)

ተመራማሪዎች የተለያዩ የአሸዋ ትንኝ ዝርያዎችን ወይም ስብስቦችን (Populations) የክንፍ ቅርጽና መጠን ለማጥናት እና ለማነጻጸር እንደ ጂኦሜትሪያዊ ቅርፀ ልኬት (Geometric morphometrics) ያሉ ዘዴዎችን ይጠቀማሉ። የክንፍ ጂኦሜትሪን ማጥናት ስለ ትንኞች ጠባይ፣ ስለሚመርጡት የመኖሪያ አካባቢ (Habitat) እና ስለ በረራ አቅማቸው ጠቃሚ መረጃዎችን ይሰጣል።

በዚህ የጂኦሜትሪያዊ ቅርፀ ልኬት አሰራር ወቅት ክንፎቹ በጥንቃቄ ተበልተው፣ አስፈላጊ ሆኖ ሲገኝም ቀልመዉ (Stained)፣ በስላይድ ላይ ተዘረጋግተዉ ይጫናሉ። የተዘጋጁት ስላይዶች በስቴሪዮ ማይክሮስኮፕ (Stereomicroscope) አማካኝነት ፎቶግራፍ ከተነሱ በኋላ ምስላቸዉ ወደ ዲጂታል ይቀየራል፤ በመጨረሻም የጂኦሜትሪያዊ ቅርፀ ልኬት ትንተና ይደረግባቸዋል። ይህ አሠራር በሳይንሳዊ ጽሑፎች ውስጥ በሚገባ ተብራርቷል [6, 27, 42, 56, 57, 59]። የተዛባ የዕድገት ተጽዕኖን (Negative allometric effects) ለማስወገድ ሁልጊዜ የቀኝ ወይም የግራ ክንፍን በቋሚነት መጠቀም ይመከራል [62]።

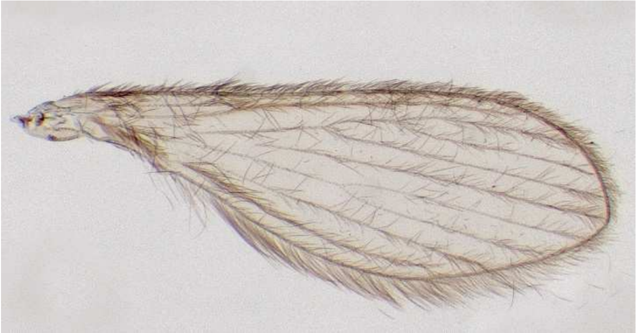

ምስል 9፦ የትሪኮፎሮማያ ኢኒኒ (Trichophoromyia ininii) ያልቀለመ ክንፍ።

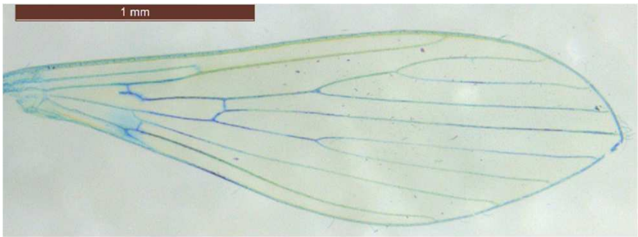

ምስል 10፦ የቀለመ የፍሌቦቶመስ አሪያሲ (Phlebotomus ariasi) ክንፍ።

ለጂኦሜትሪያዊ ቅርፀ ልኬት ትንተና የሚደረግ የክንፍ ዝግጅት (Wing preparation for geometric morphometric analysis)

የክንፍ ደም መላሾችን (wing veins) በጥሩ ሁኔታ ለማየት፣ ክንፎቹ ከቅርፊቶች (scales) መጽዳት እና በተገቢው ሁኔታ መቅለም አለባቸው። ለክንፍ ዝግጅት፣ በመጀመሪያ ትናንሽ የሳህን ጉድጓዶችን (wells) በሚያስፈልጉት ምላሽ ሰጪ ንጥረ ነገሮች (reagents) ማለትም ሜቲሊን ብሉ (methylene blue)፣ ኤታኖል (Ethanol)፣ ውሃ እና ዛይሊንን (xylene) በሚተኩ ኬሚካሎች (xylene substitute) መሞላት አለበት።

በተመጣጣኝ ሙቀት (room temperature) በ 70% ኤታኖል ውስጥ ተጠብቆ የቆየ ክንፍን፣ ከተቀመጠበት ኤፔንዶርፍ ቲዩብ (Eppendorf tube) ሙሉ በሙሉ በመገልበጥ ወደ ጉድጓዱ መጨመርና በመቀጠልም ክንፉን ረዘም ባለ ቀጭን ጠማማ መርፌ ወደ ላይ ማንሳት። በክንፉ ላይ ያሉትን ጠንካራ ፀጉሮች (bristles) ለማስወገድ ክንፉን ከአልኮል ወደ ውሃ፣ ከዚያም መልሰው ወደ አልኮል በፍጥነት ያሻግሩት። ክንፉን ለ 6 ደቂቃ ያህል በሜቲሊን ብሉ (methylene blue) ውስጥ ማቆየት፤ በዚህ ወቅት ክንፉ በቀለሙ ላይ መንሳፈፉን ማረጋገጥ ያስፈልጋል።

ክንፉን በጥንቃቄ በማውጣት ለ2 ደቂቃ ያህል (በሜቲሊን ብሉ ውስጥ የቆየበትን አንድ ሦስተኛ ያህል ጊዜ) በዛይሊን ተኪ ኬሚካል (xylene substitute) ውስጥ ይዘፍቁት። ክንፉ እንዲሰምጥ ለማድረግ መርፌውን በጉድጓዱ ግድግዳ ላይ በቀስታ መታ መታ ማድረግ ይረዳል። የዛይን ተኪ ኬሚካል የትንኟን ክንፍ ተፈሮአዊ የቀለም ይዘት እንዲጸና (fix) የማድረግ ተግባር ያከናውናል። በመጨረሻም፣ ክንፉን በማንሳት በማይክሮስኮፕ ስላይድ ላይ ባለ ትንሽ የዩፓራል (Euparal®) ጠብታ ላይ ማሳረፍ። አጉሊ መነጽር (magnifying lens) በመጠቀም፣ ክንፉን በቀስታ በመዘርጋት የሽፋን መስተዋቱን (coverslip) በጥንቃቄ ማኖር። ዩፓራሉ ደርቆ ሳይጠናከር በፊት ወዲያውኑ ፎቶግራፎች መነሳት አለባቸው። የክንፎችን ትክክለኛ አቀማመጥ (optimal alignment) ለማግኘት በሽፋን መስተዋቱ ስር የክንፉን አቀማመጥ በመጠኑ ማስተካከል ሊያስፈልግ ይችላል።

This is an Open Access article distributed under the terms of the Creative Commons Attribution License (https://creativecommons.org/licenses/by/4.0), which permits unrestricted use, distribution, and reproduction in any medium, provided the original work is properly cited.

6.4. የሞለኪውላር ባዮሎጂ ቴክኒኮች

ከአካላዊ ቅርፀት (morphological) ጥናት በተጨማሪ፣ የሞለኪውላር ዘዴዎች በሥነ ነፍሳት ምርምር ሥራ አስፈላጊ እየሆኑ መጥተዋል። እነዚህም የዝርያ ምደባ፣ የዝርያ ጄኔቲክስ እና የዝግመተ ለውጥ (phylogenetic) ጥናቶችን እንዲሁም የበሽታ አምጪ ተህዋሲያን ዲኤንኤ/አርኤንኤ (DNA/RNA) ፍለጋን እና የደም ምግብ ምንጭን ለመለየት ያገለግላሉ [70]። የዲኤንኤ ቅደም ተከተል ምርመራ (DNA sequencing) ዝርያዎችን ለማረጋገጥ ወይም ተመሳሳይ የሆኑ ዝርያዎችን ለመለየት አስተማማኝ ዘዴ ነው። በተጨማሪም፣ ፒሲአር (PCR)፣ የዲኤንኤ ቅደም ተከተል ምርመራ (DNA sequencing) እና የቀጣዩ ትውልድ የዘረ መል ኮድ ቅደም-ተከተል መለያ (NGS) የተባሉ ሞለኪዉላር ቴክኒኮች እንዲሁም ማልዲ ቶፍ ኤም ኤስ (MALDI-ToF MS) ከባህላዊው የአካላዊ ቅርፀት ጥናት ጎን ለጎን ፈጣን እና ትክክለኛ መለያ በመሆን እያገለገሉ ነው [46]። እነዚህ የቴክኖሎጂ እድገቶች ቢኖሩም፣ የአካላዊ ቅርፀት ጥናት ለዝርያ ምደባ እንደ መሠረታዊ መመዘኛ እና የሞለኪውላር መረጃዎችን ለመተርጎም እንደ መነሻ ያገለግላል።

6.4.1. አካላዊ ቅርፀት አፍራሽ የሆነ የኒውክሊክ አሲድ ዉጣት (Destructive nucleic acid extraction)

የኑክሊክ አሲድ ዉጣት (Nucleic acid extraction) በብዙ የሥነ ህይወት ጥናቶች ውስጥ የተለመደ ተግባር ሲሆን፣ ዲኤንኤን (DNA) ከናሙናዎች ዉስጥ ለይቶ ለማዉጣት የሚያስችሉ የተለያዩ ዘዴዎች አሉ። ይህንን ሂደት የሚያቀላጠፉ በገበያ ላይ የሚገኙ በርካታ የዲኤንኤ ማውጫ ኪቶች (Kits) አሉ። ሆኖም ግን፣ የአርትሮፖድ (arthropod) ናሙናዎችን ለአካላዊ ቅርፀት ጥናት (morphological identification) ለማዘጋጀት በብዛት ጥቅም ላይ የሚውሉ ዘዴዎች የናሙናውን ወሳኝ አካላዊ ባህሪያት ሊያበላሹ ወይም ሊያጠፉ ይችላሉ። አብዛኞቹ ከነፍሳት ህብረ ህዋሳት ዲኤንኤን ለማዉጣት የሚያገለግሉ ደንበ ክንዉኖች(protocols) በባህሪያቸው አፍራሽ (destructive) ናቸው፤ ስለሆነም በጥቃቅን ናሙናዎች የሚከናወኑ ሥራዎች አዳጋች ናቸዉ፣ ምክንያቱም የናሙና አወሳሰዱ ጠቃሚ የሆኑ ስነ-ቅርጻዊ መገለጫዎችን ሊያዛባ ይችላል። ተገቢውን የዲኤንኤ ዉጣት ዘዴ ለመምረጥ የናሙናው ዓይነት እና ሁኔታ ወሳኝ ሚና አላቸው[29]።
የአሸዋ ትንኞችን በትክክል የመለየት፣ የስብስብ ኩነታትን (population dynamics) የመረዳት እና በሌሎች ነፍሳት ላይ የሚደርሰውን ተጽዕኖ የመቀነስ አስፈላጊነት ለሞለኪውላር መመርመሪያ መሣሪያዎች ዕድገት መነሻ ሆኗል [23]። በአሁኑ ጊዜ የሞለኪውላር ዘዴዎች የአሸዋ ትንኞችን ለመለየት ከመደበኛ የስነ-ቅርጽ የዝርያ ምደባ ዘዴዎች ጋር እንደ ማሟያነት በሰፊው ጥቅም ላይ ይውላሉ። ለምሳሌ፣ መደበኛዉ የነፍሳት ባርኮድ (Insect barcoding) አሰራር የዲኤንኤ አመጣጥን እና ቅደም ተከተል ምርመራን (Sequencing) የሚያካትት ሲሆን፣ ይህም ብዙውን ጊዜ የዋናዉን ናሙና (Original specimen) መጥፋት ወይም መበላሸት ያስከትላል። ስለሆነም፣ ናሙናም ሆነ የናሙናዉን ስነ-ቅርጽ ይዘቱን ሳይጎዱ አፍራሽ ያልሆነ (Non-destructive) የዲኤንኤ ውጣት ዘዴዎችን መፈለግ አስፈላጊ ሆኗል።
ኑክሊክ አሲድን ከአሸዋ ትንኝ ለማውጣት በርካታ ዘዴዎች ጥቅም ላይ ይዉላሉ። የሚወጣው የኑክሊክ አሲድ መጠንና ጥራት ቀጣይ ለሆነዉ የሞለኪውላር ትንተና ውሳኝነት አለዉ። ምክንያቱም የተለያዩ ቴክኒኮች የየራሳቸው የሆነ የጥራት እና የምርመራ ክዋኔ (sensitivity) ፍላጎት ስላላቸው ነዉ[9]፣ ለምሳሌ የአሸዋ ትንኝ ዓይኖች የፒሲአር (PCR) ሂደትን የሚያስተጓጉሉ ንጥረ ነገሮች እንዳሏቸው ተደርሶበታል[69]። ከበሽታ ምርመራ ባለፈ የትንኞችን ዝርያ ለመለየት ዲኤንኤን ማውጣት የተለመደ ተግባር ቢሆንም፣ ውጤቱ እንደ አጠቃቀሙ ዘዴ ይለያያል። ተመራማሪዎች የተሻለ ጥራትና መጠን ለማግኘት ሲሉ[8፣9፣69] ፋብሪካዎች ያዘጋጇቸውን የዲኤንኤ ዉጣት መመሪያዎች ለአሸዋ ትንኝ በሚስማማ መልኩ አሻሽለው ይጠቀማሉ[8]፤ እንዲሁም ለሌሎች ነፍሳት የተዘጋጁ የዲኤንኤ (DNA) ዉጣት መመሪያዎች ለአሸዋ ትንኝ መጠቀም ይቻላል[58፣76]። አነስተኛ የሚቶኮንድሪያል ዘረ መል የሆኑትን (COI ወይም CytB) ኢላማ የሚያደርጉ መለያ የፒሲአር (PCR) ምርመራዎች፣ በአጠቃላይ የዲኤንኤ መቆራረጥን (DNA fragmentation) ከሚያስከትሉ የዉጣት ዘዴዎች ጋር ተኳሃኝ ናቸዉ። በአንፃሩ ሌሎች ረጅም-ንባብ ያላቸው የቀጣዩ ትውልድ የዘረ መል ኮድ ቅደም-ተከተል መለያ (NGS) ቴክኒኮች (Oxford Nanopore እና PacBio) በብዛት ያልተቆራረጠ እና ከፍተኛ ጥራት ያለው ዲኤንኤ (DNA) ይፈልጋሉ። በአምደ ሽክርክሪት (Spin column) የሚወጣ ዲኤንኤ እስከ 60 ኪሎ ቤዝ (kb) የሚረዝሙ ቁርጥራጮችን ሲሰጥ፣ በፊኖል-ክሎሮፎርም (Phenol-chloroform) የሚከናወን ዉጣት ግን እስከ 150 ኪሎ ቤዝ የሚደርሱ ረጅም የዲኤንኤ ቁርጥራጮችን ሊያስገኝ ይችላል[77]።
ሰንጠረዥ 5 የአሸዋ ትንኝ ዲኤንኤ ዉጣት ቴክኒኮችን የሚያጠቃልል ሲሆን፣ ለእነዚህ ነፍሳት ተብለው የተደረጉ የሥነ-ዘዴ ማስተካከያዎች (methodological adaptations) መኖራቸውን ያመለክታል። የሚገኘው የዲኤንኤ መጠን (Yields) በናሙናው መጠን እና በዝግጅት መመሪያዉ ላይ ስለሚወሰን አልተገለጸም። በሰንጠረዡ ላይ ያለዉ የማሻሻያ (modification) አምድ የሚያመለክተው ለአሸዋ ትንኞች ወይም ለሌሎች ትናንሽ አርትሮፖዶች የተደረጉ የዉጣት መመሪያ (extraction protocols) ማስተካከያዎችን ነው።
የዲኤንኤ ዉጣት ዘዴን በሚመርጡበት ጊዜ እንደ የናሙናዎች ቁጥር፣ ለዉጣት የሚፈጀዉ ጊዜ እና ከዉጣት በኋላ ጥቅም ላይ የሚውለውን ቴክኒክ (downstream technique) የመሳሰሉ በርካታ መስፈርቶችን ግምት ውስጥ ማስገባት ያስፈልጋል። ምንም እንኳን የቀጣዩ ትውልድ የዘረ መል ኮድ ቅደም-ተከተል መለያ ቴክኒኮች (NGS) ከፍተኛ ሞለኪውላዊ ክብደት ያለው ዘረ መላዊ ዲኤንኤ (Genomic DNA) ቢፈልጉም፣ እዚህ የቀረቡት የዲኤንኤ ዉጣት ዘዴዎች ለመደበኛ ፒሲአር (PCR) ተግባራት ሊውሉ ይችላሉ።
በተጨማሪም፣ በርካታ ጥናቶች ለትናንሽ የየብስ አርትሮፖዶች፣ በደረቅ ሁኔታ ለተቀመጡ የሙዚየም ናሙናዎች እና ለስላሳ አካል ላላቸው አርትሮፖዶች ናሙናውን ሳያበላሹ የዲኤንኤ ዉጣት ዘዴዎችን (non-destructive DNA extraction methods) ቃኝተዋል [19, 26, 28, 55, 63]።

6.4.2. አካላዊ ቅርፀት አፍራሽ ያልሆነ የኒውክሊክ አሲድ ዉጣት (Non-destructive nucleic acid extraction)

በአሸዋ ትንኞች ሞለኪውላዊ ጥናት ካሉት ዋና ዋና ተግዳሮቶች አንዱ፣ ናሙናዎቹ በኢንቶሞሎጂካል ስብስቦች ውስጥ እንዲካተቱ በሚያስችል መልኩ ደህንነታቸውን ጠብቆ ማቆየት አለመቻሉ ነው። አብዛኛዎቹ የዲኤንኤ (DNA) ዉጣት መመሪያዎች የትንኟን አካል ማለስለስን ስለሚጠይቁ፣ ዋና ናሙናውን ያበላሻሉ። ይሁን እንጂ፣ አፍራሽ ያልሆኑ የኒውክሊክ አሲድ ዉጣት ዘዴዎች ናሙናውን በአካል ሳይጎዱ፣ ሳይቀይዱ ወይም የአካል ቅርጹን ሳይለውጡ የዘረ መላዊ ናሙናዎችን ለማውጣት ታስበው የተዘጋጁ ናቸው። እነዚህ ዘዴዎች በተለይ እንደ አሸዋ ትንኝ ያሉ ብርቅዬ ወይም ውስን ናሙናዎች ሲያጋጥሙ፣ ለአካላዊ መለያ (Taxonomy) ወይም ለወደፊት ምርመራዎች የሰውነትን መዋቅር ጠብቆ ማቆየት አስፈላጊ በሚሆንበት ጊዜ እጅግ ጠቃሚ ናቸው። ለዚህም በብዛት ጥቅም ላይ የሚውለው ዘዴ ትንኞቹን በፕሮቲኔዝ ኬ (proteinase K) ማሟሚያ ፈሳሽ (lysis buffer) ውስጥ በጥንቃቄ የመዘፈቅ ዘዴ ነው።
በተጨማሪም መጠነኛ የህብረህዋስ መፈራረስ (Mild-vectolysis) ዘዴ የተለመደውን የዲኤን-ኢዚ ብለድ ኤንድ ቲሹ ኪት (DNeasy Blood and Tissue kit) አሰራር በመቀየርና የማቀዝቀዣ ሂደትን በመጨመር ለታወቁ ብርቅዬ ናሙናዎች ጥቅም ላይ ይውላል። በሌላ በኩል ደግሞ የሆትሾት (HotSHOT) ዘዴ ፈጣን እና ርካሽ አማራጭ በመሆን ያገለግላል።
መጠነኛ የህብረህዋስ መፈራረስ (Mild-vectolysis) የተባለው ዘዴ በአሸዋ ትንኞች፣ በተለይም በሙነነ አምሳያ (type specimen) ላይ በተሳካ ሁኔታ ተተግብሯል [24]። ይህ ቴክኒክ የተለመደ የአምደ ሽክርክሪት (spin

This is an Open Access article distributed under the terms of the Creative Commons Attribution License (https://creativecommons.org/licenses/by/4.0), which permits unrestricted use, distribution, and reproduction in any medium, provided the original work is properly cited.

ሠንጠረዥ 5፡- የአሸዋ ትንኞችን ዘረመላዊ ዲኤንኤ (gDNA) ለማውጣት የሚያገለግሉ ማሻሻያ ደንበ ክንዉኖች እንዲሁም አማካኝ ዋጋ እና አገልግሎታቸዉ

ፕሮቶኮል (Protocol)	ዋጋ (Cost)	አገልግሎት (Application)	ለጥቃቅን ነፍሳት የተመቻቸ የፕሮቶኮል ማሻሻያ ሰለመደረጉ የሚገልፅ ማጣቀሻዎች
ስፒን ኮለም (Spin column)	2.5 – 3.55 የአሜሪካ ዶላር [39]	PCR, የቀጣዩ ትውልድ የዘረ መል ኮድ ቅደም-ተከተል መለያ (NGS)	[9]
ፊኖል-ክሎሮፎርም (Phenol-chloroform)	0.24 የአሜሪካ ዶላር [69]	ፒሲአር (PCR), የቀጣዩ ትውልድ የዘረ መል ኮድ ቅደም-ተከተል መለያ (NGS)	[9]
ሆት-ሾት (HotSHOT)	<0.01 የአሜሪካ ዶላር [69]	ፒሲአር (PCR)	-
ልቲንግ አውት (Salting out)	0.12 የአሜሪካ ዶላር [69]	ፒሲአር (PCR)	-
ቼሌክስ (Chelex)	0.02 የአሜሪካ ዶላር [41]	ፒሲአር (PCR)	[41, 76]

column) ኪት ለምሳሌ፡- ዲኤን-ኢዚ ብለድ ኤንድ ቲሹ ኪት (DNeasy Blood and Tissue kit, QIAGEN, Hilden, Germany) በመጠቀም፣ ነገር ግን ናሙናውን ሳይሰባብሩ ዲኤንኤ ለማግኘት እንዲያስችል ተደርጎ የተሻሻለ ነው። የተስተካከሉ የማሟሟያ ደረጃዎች (የፈሳሹ መጠን መጨመር እና የማቀዝቀዝ ሂደት ማካተት) [17] አካላዊ ጉዳትን በትንሹ በመቀነስ ኒውክሊክ አሲዶች እንዲወጡ ያስችላሉ [24]። በተጨማሪም፣
ለአሸዋ ትንኞች ፈጣን እና ርካሽ የሆነውን የሆትሾት (HotSHOT DNA Extraction, Bento Bioworks Ltd, London, United Kingdom) ኪት መጠቀም ይቻላል [73]።
ለሥነ-ቅርጽ (morphological) መለያ የታሰቡ የኢንቶሞሎጂ ናሙናዎች ከሂደቱ በኋላ ሊታጠቡ ይችላሉ። በዲኤን-ኢዚ ብለድ ኤንድ ቲሹ ኪት (DNeasy Blood and Tissue kit) የዲኤንኤ ውጣት የተከናወኑ ናሙናዎች በማርክ-አንድሬ (Marc-André) ዉሀድ መጽዳት ያለባቸው ሲሆን፣ በሆትሾት (HotSHOT DNA extraction kit) የተከናወኑ ናሙናዎች ግን በጥራት እንዲታዩ፣ በዚህ ጽሑፍ ውስጥ በዝርዝር በቀረበው መመሪያ [73] መሠረት በውሃማ ዉሀድ ውስጥ፣ ወይም ይበልጥ በተሻለ ሁኔታ ውሃው ከተወገደ (dehydration) በኋላ በሙጫ (resin) ውስጥ ማንበር አስፈላጊ ነዉ። በመቀጠል የተጣራዉ የዘረ መል ናሙና ለተጨማሪ ትንተናዎች ሊዉል ይችላል፣ ለምሳሌ የተወሰኑ የዘረ መላዊ መለያዎችን (genetic markers) በፒሲአር (PCR) ለመመርመር።
ናሙናን ሳያጠፉ የኑክሊክ አሲድ ዉጣት ዘዴዎች የአሸዋ ትንኞችን ዘረመላዊ ባህሪያት ለማጥናት ወሳኝ ናቸዉ። ይህም ትንኞቹ ሊሸከሟቸው የሚችሉ በሽታ አምጪ ተህዋስያንን መለየትንም ያካትታል። የናሙናውን ሙሉነት (integrity) በመጠበቅ፣ ተመራማሪዎች ጠቃሚ ዘረመላዊ መረጃዎችን ማግኘት ይችላሉ፤ በተመሳሳይ ጊዜ ናሙናውን ለተጨማሪ ትንተናዎች ወይም ጥናቶች ማቆየት ያስችላቸዋል።

6.5. ማልዲ ቶፍ ኤም ኤስ (MALDI-ToF MS)

ማልዲ ቶፍ ኤም ኤስ (MALDI-ToF MS = matrix-assisted laser desorption/ionization time-of-flight mass spectrometry) ሥነ ህይወታዊ ናሙናዎችን ልዩ የፕሮቲን መገለጫ አሻራዎች (protein 'fingerprints') በመለየት ላይ የተመሠረተ የትንተና ዘዴ ነው። ይህ ዘዴ ለሕክምና እና ለእንስሳት ሕክምና ጠቀሜታ ያላቸውን ነፍሳት (arthropods) ለመለየት እንደ አስፈላጊ መሣሪያ እየታወቀ መጥቷል።

ይህ ዘዴ የአሸዋ ትንኞችን በተለያዩ የእድገት ደረጃዎች መለየት እንደሚችል ተረጋግጧል። እንዲሁም ደም የመጠጡ ሴት ትንኞች የተመገቡትን የደም ዓይነት ለመለየት ይጠቅማል። በተጨማሪም በተለያዩ የቅየጣና (homogenization) የማቆያ(storage) ሁኔታዎች ውስጥ የተቀመጡ ወንድና ሴት ናሙናዎችን በዝርያ፣ በወገን እና በመንጋ ደረጃ ለመለየት ያስችላል [28, 30, 73, 74]። ይህ ቴክኒክ ተመራማሪዎች ትንኞችን በፍጥነት እና በትክክል እንዲለዩ በማድረግ ስለ ስርጭታቸው፣ ስለ ባህሪያቸው እና በሽታን በማስተላለፍ ረገድ ስላላቸው ሚና ጠቃሚ መረጃ ይሰጣል።

ማልዲቶፍ (MALDI-ToF) የፕሮቲን መገለጫ አሻራዎች (protein profiles) ላይ በመመሥረት፣ በዝርያዎች መካከል ያለውን ልዩነት በመለየት፣ በኤፒዲሚዮሎጂያዊ ጥናቶች እና በተባይ መቆጣጠሪያ ስልቶች ውስጥ ወሳኝ ሚና ይጫወታል። ይህ ዘዴ በመደበኛነት ጥቅም ላይ እንዳይውል እንቅፋት የሆኑ ሁለት ዋና ዋና ድክመቶች አሉ።

አንደኛው፣ የመጠነ ቁስ ስፔክትሮሜትሪ (mass spectrometry equipment) ተደራሽነትን የተመለከተ ሲሆን፣ መሣሪያው እጅግ ውድ ከመሆኑ የተነሳ ለአሸዋ ትንኞች ወይም ለሌሎች ነፍሳት ዝርያ መለያ ዓላማ ብቻ መግዛት አዋጪ አለመሆኑ ነው። ይሁን እንጂ፣ በክሊኒካዊ ምርመራ ማዕከላት ወይም በፕሮቲዮሚክ የምርምር ተቋማት ውስጥ የምርመራ ጊዜ ድርሻ በማግኘት ይህንን ችግር መፍታት ይቻላል። ሁለተኛው እንቅፋት በገደብ የለሽ የመረጃ ቋቶች (Open-access databases) ውስጥ የሚገኙ የአሸዋ ትንኝ ዋቢ መረጃዎች ዉሱን መሆን ነው፤ ይህም ጉድለት ተመራማሪዎች የራሳቸውን የውስጥ መረጃ ቋት (In-house database) እንዲገነቡ ያስገድዳቸዋል። ይህንን ጉድለት ለመሙላት የስነ-ቅርጽ (Morphology) እና COI ወይም cytB ያሉ ተስማሚ የዘረ መል መለያ ዘዴዎችን መጠቀም ያስፈልጋል። ይህ ውስንነት ፓሪስ በሚገኘው ሶርቦን ዩኒቨርሲቲ (Sorbonne University) እና ብራሰልስ (Brussels) በሚገኘው BCCM/IHEM/Sciensano ናሙና ክምችት በሚያስተዳድሩት የኤም ኤስ አይ ፕላትፎርም (MSI Platform) (https://msi.happy-dev.fr/) ውስጥ እስካሁን በተለያየ የምርምር ተቋማት የሚገኙ የመረጃ ቋቶች ደረጃ በደረጃ ሲካተቱ በቅርቡ እንደሚፈታ ተስፋ ይደረጋል።

የማልዲቶፍ (MALDI-ToF) ፕሮቲን መገለጫ የአሻራ ምርመራ ለማድረግ በሚታቀድበት ጊዜ፣ ናሙናዎች በክፍል ሙቀት ሳይጋለጡ በደረቅ ሁኔታ ቀዝቅዘው (dry-frozen) ወይም ሞለኪውላዊ ጥራቱን በተጠበቀ 70% ኤታኖል ውስጥ መቀመጥ አለባቸው:: ናሙናን ለማዘጋጀት የሚረዳ ሁሉን አቀፍ መመሪያ በሌለበት ሁኔታ፣ ተመራማሪዎች ያገኟቸው የፕሮቲን መረጃዎች እስካሁን ከታተሙት የአሸዋ ትንኝ መረጃዎች ጋር ሊነጻጸሩ እንዲችሉ፣ ለማልዲቶፍ (MALDI-ToF) ማትሪክስ ዝግጅት 60% አሴቶኒትሪል/0.3% የሲናፒኒክ አሲድ ዉህድ (TFA solution of sinapinic acid) (30 mg/mL) እንዲጠቀሙ ይመከራሉ።

ለማልዲቶፍ (MALDI-ToF MS0 የሚደረግ የናሙና ዝግጅት (Sample Preparation for MALDI-ToF MS)

(ምስል 7)
በተለያዩ ሁኔታዎች ተጠብቀው የቆዩ የነፍሳት ናሙናዎች በመጀመሪያ በክፍል ሙቀት ውስጥ በአየር እንዲደርቁ ይደረጋል፤ ከዚያም ይበለታሉ (dissected)። ለስላይድ ማንበሪያ እና ለሥነ-ቅርጽ (morphological) ትንተና የሚያገለግሉ ቁልፍ መለያ ባህሪያትን የያዙ የትንኝ አካል ክፍሎችን ለማግኘት ጭንቅላት እና ሆድ ዕቃ ተለይተዉ ይወጣሉ። ደረቱ (thorax) ለማልዲቶፍ (MALDI-ToF) ምርመራ ሊያገለግል ይችላል፤ ቀሪው የሆድ ዕቃ ክፍል ደግሞ ለዲኤንኤ (DNA) ዉጣት ሊቀመጥ ይችላል። ለፕሮቲን መገለጫ ትንተና (protein profiling)፣ ደረቱን ወደ 1.5-ሚሊ ሊትር (mL) ማይክሮ-ቲዩቦች በማስተላለፍ 10 ማይክሮ ሊትር (µL) ማሟሚያ ዉህድ (homogenization solution) በመጨመር ለአንድ ጊዜ ብቻ በሚያገለግሉ መፍጫ መሳሪያዎች (pestles and pellets) አማካኝነት ይፈጫል። በተለምዶ ሁለት ዓይነት የማሟሚያ ፈሳሾች ጥቅም ላይ ይውላሉ:- እነሱም የተጣራ (sterile) ውሃ እና 25% ፎርሚክ አሲድ (formic acid) ናቸው።

7. ማጠቃለያ (Conclusion)

በዚህ ሥራ ውስጥ፣ ተመራማሪዎች የአሸዋ ትንኞችን ትክክለኛ ዝርያ ለመለየት እና በሽታ አምጪ ተህዋስያንን በቀላሉ ለመለየት እንዲችሉ፣ እንደ ምርምር ዓላማቸው የሚመረጡ ውጤታማ የናሙና ማንበር ዘዴዎችን ለማቅረብ ሞክረናል። ለሁሉም ሁኔታዎች የሚሆን አንድ ምርጥ ዘዴ የለም፤ ይልቁንም እያንዳንዳቸው የየራሳቸው ጠንካራ ጎን እና ጉድለት ያላቸው በርካታ አማራጮች አሉ።
የአሸዋ ትንኞችን ለማዘጋጀት እና ለመለየት የሚያገለግሉ የተለያዩ የማንበሪያ ቴክኒኮችን ዝርዝር መመሪያዎች በተጨማሪ መረጃዎች (Supporting data) ውስጥ አቅርበናል። እነዚህ መመሪያዎች የቪዲዮ ማሳያዎችን ጨምሮ፣ ለተለያዩ ዓላማዎች ተስማሚ የሆኑ ደረጃ በደረጃ የሚከናወኑ የአሠራር ቅደም ተከተሎችን ያሳያሉ፤ ይህም ትክክለኛ እና አስተማማኝ ውጤት ለማግኘት ይረዳል። ይህን በዝርዝር የቀረበ መመሪያ ዋና ዓላማዉ ተመራማሪዎች እንደ ፍላጎታቸው ተገቢውን የማንበሪያ ዘዴ መርጠው እንዲተገብሩ መርዳት ነው።

ምስጋና (Acknowledgements)
የዚህ ሳይንሳዊ ፅሁፍ ባለቤቶች በለንደን የተፈጥሮ ታሪክ ሙዚየም (Natural History Museum of London, UK) ለሚገኙት ሪቻርድ ሌን (Richard Lane) እና ዞዪ ጄይ አዳምስ (Zoe Jay Adams)፣ ለዚህ ጽሑፍ ጥራት መሻሻል ላደረጉት ግሩም ግምገማ ምስጋናቸውን ያቀርባሉ።

የገንዘብ ድጋፍ (Funding)
ለአጃ (AJA) የምርምር ሥራ የገንዘብ ድጋፍ ላደረጉት የብራዚል የልማት ድርጅቶች ሲኤንፒኪው (CNPq) (የመዝገብ ቁጥር:- 404395/2024-4) እና አራውካሪያ ፋውንዴሽን (Araucária Foundation) (የመዝገብ ቁጥር:- 433/2025 PDI) ምስጋናችንን እናቀርባለን።

10. የጥቅም ግጭት (Conflicts of interest)
ጄሮም ዴፓኩዊት (Jérôme Depaquit) የ "ፓራሳይት" (Parasite) ሳይንሳዊ መጽሔት ረዳት አዘጋጅ ነው፤ ሆኖም በዚህ ጽሑፍ የግምገማ ሂደት እና ውሳኔ አሰጣጥ ላይ ምንም ዓይነት ተጽዕኖ አልነበረውም። ሌሎች ደራሲዎች ምንም ዓይነት የጥቅም ግጭት እንደሌለባቸው ይገልጻሉ።

በዜኖዶ (Zenodo) ላይ የሚገኙ ቪዲዮዎች

እነዚህ ቪዲዮዎች የአሠራር ሂደቱን በዝርዝር የሚያሳዩ ናቸው:-

ቪዲዮ 1: https://zenodo.org/records/18198006
ቪዲዮ 2: https://zenodo.org/records/18311158
ቪዲዮ 3: https://zenodo.org/records/18311106
ቪዲዮ 4: https://zenodo.org/records/18311154
ቪዲዮ 5: https://zenodo.org/records/18303014
ቪዲዮ 6: https://zenodo.org/records/18302850
ቪዲዮ 7: https://zenodo.org/records/18315029

Supplementary material

The supplementary material of this article is available at https://www.parasite-journal.org/10.1051/parasite/2026009/olm

ማጣቀሻዎች (References)

1. አልካን ሲ (Alkan C)፣ አላል-ኢኽለፍ ኤቢ (Allal-Ikhlef AB)፣ አልዋሱፍ ኤስ (Alwassouf S)፣ ባክሎቲ ኤ (Baklouti A)፣ ፒዮርኮውስኪ ጂ (Piorkowski G)፣ ደ ላምባለሪ ኤክስ (de Lamballerie X)፣ ኢዝሪ ኤ (Izri A) እና ቻሬል አርኤን (Charrel RN) 2015:- በአልጄሪያ ውስጥ የቶስካና ቫይረስን ለይቶ ማውጣት፣ የጄኔቲክ ባህሪ መግለጫ እና በደም ውስጥ ያለው ስርጭት (Virus isolation, genetic characterization and seroprevalence of Toscana virus in Algeria) *ክሊኒካል ማይክሮባዮሎጂ ኤንድ ኢንፌክሽን (Clinical Microbiology and Infection)*፣ 21(11)፣ 1040 e1-9

2. አልተን ቢ (Alten B)፣ ኦዝቤል ዋይ (Ozbel Y)፣ ኤርጉናይ ኬ (Ergunay K)፣ ካሳፕ ኦኢ (Kasap OE)፣ ካል ቢ (Cull B) እና ሌሎች 2015:- በአውሮፓ ውስጥ ለፍሌቦቶሚን አሸዋ ትንኞች ናሙና አወሳሰድ ስልቶች (Sampling strategies for phlebotomine sand flies in Europe) *ቡለቲን ኦፍ ኢንቶሞሎጂካል ሪሰርች (Bulletin of Entomological Research)*፣ 105(6)፣ 664-78

3. አይሃን ኤን (Ayhan N)፣ ባክሎቲ ኤ (Baklouti A)፣ ፕሩዶም ጄ (Prudhomme J)፣ ዋልደር ጂ (Walder G)፣ አማሮ ኤፍ (Amaro F) እና ሌሎች 2017:- በአሸዋ ትንኝ በሚተላለፉ ፍሌቦቫይረሶች ላይ ለሚደረጉ ጥናቶች ተግባራዊ መመሪያዎች (Practical guidelines for studies on sandfly-borne phleboviruses)። *ቬክተር-ቦርን ኤንድ ዙኖቲክ ዲዚዝስ (Vector-Borne and Zoonotic Diseases)*፣ 17(1)፣ 73-80

4. ቤተስ ፒኤ (Bates PA) 1997:- የአሸዋ ትንኞች በሌሽማኒያ መበከል (Infection of phlebotomine sandflies with Leishmania) *ዘ ሞለኪውላር ባዮሎጂ ኦፍ ኢንሰክት ዲዚዝ ቬክተርስ (The Molecular Biology of Insect Disease Vectors)*፣ ገጽ 112-120

5. ባውም ኤም (Baum M)፣ ደ ካስትሮ ኢኤ (de Castro EA)፣ ፒንቶ ኤምሲ (Pinto MC)፣ ጎውላርት ቲኤም (Goulart TM)፣ ባውራ ደብሊው (Baura W) እና ሌሎች 2015:- በብራዚል ውስጥ የአሸዋ ትንኞች የተመገቡትን የደም ምንጭ በሞለኪውላር ዘዴ መለየት (Molecular detection of the blood meal source of sand flies in Brazil) *አክታ ትሮፒካ (Acta Tropica)*፣ 143፣ 8-12

6. ቤለን ኤ (Belen A)፣ አልተን ቢ (Alten B) እና አይተኪን ኤ (Aytekin A) 2004:- በከፍታ ልዩነት ምክንያት በ*Phlebotomus papatasi* ዝርያዎች ላይ የሚታይ የስነ-ቅርጽ እና የሞለኪውላር ባህሪያት ልዩነት (Altitudinal variation in morphometric and molecular characteristics of *Phlebotomus papatasi* populations) *ሜዲካል ኤንድ ቬተርነሪ ኢንቶሞሎጂ (Medical and Veterinary Entomology)*፣ 18(4)፣ 343-350

7. ብታቻርያ ጄ (Bhattacharya J)፣ ቻንድራ ጂ (Chandra G) እና ሃቲ ኤኬ (Hati AK) 1991:- የላሽማኒያ ዶኖቫኒ (*Leishmania Donovan)i* ፕሮማስቲጎቶችን(promastigotes) በቅዝቃዜ ለማቆየት የሚረዳ ቀላል ዘዴ (A simple method for cryopreservation of *Leishmania donovani* promastigotes) *ኢንዲያን ጆርናል ኦፍ ሜዲካል ሪሰርች (Indian Journal of Medical Research)*፣ 93፣ 245-6

8. ካሊጊዩሪ ኤልጂ (Caligiuri LG)፣ ሳንዶቫል ኤኢ (Sandoval AE)፣ ሚራንዳ ጄሲ (Miranda JC)፣ ፔሶአ ፋ (Pessoa FA)፣ ሳንቲኒ ኤምኤስ (Santini MS) እና ሌሎች 2019:- ለፒሲአር ምርመራ እንዲያመች ከነጠላ አሸዋ ትንኞች የዲኤንኤ አወጣጥ ሂደትን ማሻሻል (Optimization of DNA extraction from individual sand flies for PCR amplification) *ሜተድስ ኤንድ ፕሮቶኮልስ (Methods and Protocols)*፣ 2(2)፣ 36

9. ካሳሪል ኤኢ (Casaril AE)፣ ደ ኦሊቬራ ኤልፒ (de Oliveira LP)፣ አሎንሶ ዲፒ (Alonso DP)፣ ደ ኦሊቬራ ኢኤፍ (de Oliveira EF)፣ ጎሜስ ባሪዮስ ኤስፒ (Gomes Barrios SP) እና ሌሎች 2017:- ከአሸዋ ትንኞች ዲኤንኤ የማውጫ ዘዴን ደረጃውን የጠበቀ ማድረግ፡- ለቀጣይ ትውልድ የዝርያ ምርመራ ውሎ (Standardization of DNA extraction from sand flies: Application to genotyping by next generation sequencing) *ኤክስፐሪሜንታል ፓራሳይቶሎጂ (Experimental Parasitology)*፣ 177፣ 66-72

10. ካስታላኔሊ ኤምኤ (Castalanelli MA)፣ ሴቨርትሰን ዲኤል (Severtson DL)፣ ብሩምሌይ ሲጄ (Brumley CJ)፣ ስዚቶ ኤ (Szito A)፣ ፎቲት አርጂ (Foottit RG) እና ሌሎች 2010:- ለነፍሳት እና ለሌሎች መገጣጠሚያ ላላቸው እንስሳት ፈጣን እና አካልን የማያጠፋ የዲኤንኤ አወጣጥ ዘዴ (A rapid non-destructive DNA extraction method for insects and other arthropods)*ጆርናል ኦፍ ኤዥያ-ፓሲፊክ ኢንቶሞሎጂ (Journal of Asia-Pacific Entomology)*፣ 13(3)፣ 243-248

11. ሰርኬይራ ኤንኤል (Cerqueira NL) 1943:- ጥቃቅን ነፍሳትን በአጉሊ መነጽር መስታወት ላይ ለመሰካት የሚያገለግል አዲስ ዘዴ (Um novo meio para montagem de pequenos insetos em lâmina) *ሜሞሪያስ ዶ ኢንስቲቲውቶ ኦዝዋልዶ ክሩዝ (Memórias do Instituto Oswaldo Cruz)*፣ (39)፣ 37-41

12. ቻሬል አርኤን (Charrel RN)፣ ጋሊያን ፒ (Gallian P)፣ ናቫሮ-ማሪ ጄኤም (Navarro-Mari JM)፣ ኒኮሌቲ ኤል (Nicoletti L)፣ ፓፓ ኤ (Papa A) እና ሌሎች 2005:- በአውሮፓ ውስጥ የቶስካና ቫይረስ ድንገተኛ መከሰት (Emergence of Toscana virus in Europe) *ኢመርጂንግ ኢንፌክሸየስ ዲዚዝስ (Emerging Infectious Diseases)*፣ 11(11)፣ 1657-63

13. ቻስኮፖሉ ኤ (Chaskopoulou A)፣ ጃይንትሲስ አይኤ (Giantsis IA)፣ ደሚር ኤስ (Demir S) እና ቦን ኤምሲ (Bon MC) 2016:- በቴሳሎኒኪ ሜትሮፖሊታን አካባቢ የሚገኙ የአሸዋ ትንኞች ዝርያ ስብጥር፣ መደበኛ የእንቅስቃሴ ንድፍ እና የተመገቡት ደም ትንተና (Species composition, activity patterns and blood meal analysis of sand fly populations in Thessaloniki)*አክታ ትሮፒካ (Acta Tropica)*፣ 158፣ 170-176

14. ቼን ኤች (Chen H)፣ ራንጋሳሚ ኤም (Rangasamy M)፣ ታን ኤስዋይ (Tan SY)፣ ዋንግ ኤች (Wang H) እና ሲግፍሪድ ቢዲ (Siegfried BD) 2010:- የበቆሎ ስር ተባይ ጥንዚዛዎች ላይ ሙሉ ዲኤንኤ ለማውጣት የሚያገለግሉ አምስት ዘዴዎች ግምገማ (Evaluation of five methods for total DNA extraction from western corn rootworm beetles) *PLoS One*፣ 5(8)፣ e11963

15. ዴፓኪት ጄ (Depaquit J)፣ ግራንዳዳም ኤም (Grandadam M)፣ ፉክ ኤፍ (Fouque F)፣ አንድሪ ፒኢ (Andry PE) እና ፔይሬፊት ሲ (Peyrefitte C) 2010:- በአውሮፓ ውስጥ በአሸዋ ትንኞች የሚተላለፉ ቫይረሶች፡- ክለሳ (Arthropod-borne viruses transmitted by Phlebotomine sandflies in Europe: a review)*ዩሮሰርቬይላንስ (Eurosurveillance)*፣ 15(10)፣ 19507

16. ዳይመንድ ኤልኤስ (Diamond LS) እና ሄርማን ሲኤም (Herman CM) 1954:- በካናዳ ዝይ የአጥንት መቅኒ ውስጥ የሚታዩ ትራይፓኖሶምስ ግጥሞሽ (Incidence of Trypanosomes in the Canada Goose as revealed by bone marrow culture)*ጆርናል ኦፍ ፓራሳይቶሎጂ (Journal of Parasitology)*፣ 40(2)፣ 195-202

17. ዲንግ ኤች (Ding H)፣ ቶርኖ ኤም (Torno M)፣ ቮንግፋይሎት ኬ (Vongphayloth K)፣ ንግ ጂ (Ng G)፣ ታን ዲ (Tan D) እና ሌሎች 2025:- በግልጽ የታዩ ግን ያልተስተዋሉ፡- በሲንጋፖር የአሸዋ ትንኞች መገኘት እና ለአዲስ ሳይንሳዊ እውቀት የበቁ አራት ዝርያዎች መግለጫ (Hidden in plain sight: discovery of sand flies in Singapore and description of four species new to science) *ፓራሳይትስ ኤንድ ቬክተርስ (Parasites & Vectors)*፣ 18(1)፣ 402

18. ኤስ-ሴቴ ኤን (Es-Sette N)፣ አጃውድ ኤም (Ajaoud M)፣ ቢሻውድ ኤል (Bichaud L)፣ ሃምዲ ኤስ (Hamdi S)፣ መሉኪ ኤፍ (Mellouki F) እና ሌሎች 2014፡- *Phlebotomus sergenti* በሞሮኮ ውስጥ ለላይሽማኒያ ትሮፒካ (*Leishmania tropica)* እና ለቶስካና ቫይረስ የጋራ አስተላላፊ መሆኑ (Phlebotomus sergenti a common vector of Leishmania tropica and Toscana virus in Morocco) *ጆርናል ኦፍ ቬክተር ቦርን ዲዚዝስ (Journal of Vector Borne Diseases)*፣ 51(2)፣ 86-90

19. ፋቭሬት ሲ (Favret C) 2005፡- ለአፊድ ነፍሳት የሚያገለግል አዲስ አካልን የማያጠፋ የዲኤንኤ አወጣጥ እና ናሙናን የማጥራት ቴክኒክ (A new non-destructive DNA extraction and specimen clearing technique for aphids) *ፕሮሲዲንግስ ኦፍ ዘ ኢንቶሞሎጂካል ሶሳይቲ ኦፍ ዋሽንግተን (Proceedings of the Entomological Society of Washington)*፣ 107(2)፣ 469-470

20. ጋላቲ ኢኤቢ (Galati EAB) 2018፡- ፍሌቦቶሚኔ (Diptera, Psychodidae)፡- የአዋቂ ትንኞች ምደባ፣ ስነ-ቅርጽ እና መለያዎች (Phlebotominae: Classification, morphology and terminology of adults and identification of American taxa) በ"ብራዚሊያን ሳንድ ፍላይስ" መጽሐፍ ውስጥ፣ ገጽ 9-212

21. ጋላቲ ኢኤቢ (Galati EAB)፣ ደ አንድራዴ ኤጄ (de Andrade AJ)፣ ፐርቪን ኤፍ (Perveen F)፣ ሎየር ኤም (Loyer M)፣ ቮንግፋይሎት ኬ (Vongphayloth K) እና ሌሎች 2025፡- የዓለም የአሸዋ ትንኞች (Phlebotomine sand flies (Diptera, Psychodidae) of the world) *ፓራሳይትስ ኤንድ ቬክተርስ (Parasites & Vectors)*፣ 18(1)፣ 220

22. ጋላቲ ኢኤቢ (Galati EAB)፣ ጋልቪስ-ኦቫሎስ ኤፍ (Galvis-Ovallos F)፣ ሎየር ፒ (Lawyer P)፣ ሌገር ኤን (Leger N) እና ዴፓኪት ጄ (Depaquit J) 2017፡- ለአሸዋ ትንኞች መግለጫ የሚያገለግሉ ባህሪዎች እና ቃላቶች የተብራራ መመሪያ (An illustrated guide for characters and terminology used in descriptions of Phlebotominae)። *ፓራሳይት (Parasite)*፣ 24፣ 26

23. ጋሪፒ ቲ (Gariepy T)፣ ኩህልማን ዩ (Kuhlmann U)፣ ጊሎት ሲ (Gillott C) እና ኤርላንድሰን ኤም (Erlandson M) 2007፡- ፓራሳይቶይድስ፣ አዳኞች እና ፒሲአር፡- በመገጣጠሚያ ላላቸው እንስሳት ባዮሎጂያዊ ቁጥጥር ውስጥ የሞለኪውላር መለያዎች አጠቃቀም (Parasitoids, predators and PCR: the use of diagnostic molecular markers in biological control of Arthropods) *ጆርናል ኦፍ አፕላይድ ኢንቶሞሎጂ (Journal of Applied Entomology)*፣ 131(4)፣ 225-240

24. ጃይንትሲስ አይኤ (Giantsis IA)፣ ቻስኮፖሉ ኤ (Chaskopoulou A) እና ቦን ኤምሲ (Bon MC) 2016፡- ማይልድ-ቬክቶላይሲስ፡- ለአሸዋ ትንኞች እና ለወባ ትንኞች የሚያገለግል አካልን የማያጠፋ የዲኤንኤ አወጣጥ ዘዴ (Mild-Vectolysis: A nondestructive DNA extraction method for vouchering sand flies and mosquitoes) *ጆርናል ኦፍ ሜዲካል ኢንቶሞሎጂ (Journal of Medical Entomology)*፣ 53(3)፣ 692-695

25. ጊድዋኒ ኬ (Gidwani K)፣ ፒካዶ ኤ (Picado A)፣ ሪጃል ኤስ (Rijal S)፣ ሲንግ ኤስፒ (Singh SP)፣ ሮይ ኤል (Roy L) እና ሌሎች 2011፡- በህንድ እና ኔፓል የቪሴራል ሌሽማኒያሲስን ለመከላከል የሚያገለግሉ አጎበሮችን ለመገምገም የአሸዋ ትንኝ የደም ምርመራ ምልክቶችን መጠቀም (Serological markers of sand fly exposure to evaluate insecticidal nets against visceral leishmaniasis) *PLoS Neglected Tropical Diseases*፣ 5(9)፣ e1296

26. ጊልበርት ኤምቲፒ (Gilbert MTP)፣ ሙር ደብሊው (Moore W)፣ ሜልቺዮር ኤል (Melchior L) እና ወሮቤይ ኤም (Worobey M) 2007፡- በሙዚየም ውስጥ ከሚገኙ ደረቅ ጥንዚዛዎች ላይ ውጫዊ አካላቸውን ሳይጎዱ ዲኤንኤ የማውጣት ዘዴ (DNA extraction from dry museum beetles without conferring external morphological damage) *PLoS One*፣ 2(3)፣ e272

27. ጆርዳኒ ቢኤፍ (Giordani BF)፣ አንድራዴ ኤጄ (Andrade AJ)፣ ጋላቲ ኢኤቢ (Galati EAB) እና ጉርጌል-ጎንካልቬስ አር (Gurgel-Goncalves R) 2017፡- በሉዞማያ (*Lutzomyia*) ንዑስ ወገን ውስጥ ትንኞችን ለመለየት የክንፍ ጂኦሜትሪክ ሞርፎሜትሪክስ ያለው ሚና (The role of wing geometric morphometrics in the identification of sandflies within the subgenus *Lutzomyia*) *ሜዲካል ኤንድ ቬተርነሪ ኢንቶሞሎጂ (Medical and Veterinary Entomology)*፣ 31(4)፣ 373-380

28. ጉዝማን-ላራልዴ ኤጄ (Guzmán-Larralde AJ)፣ ሱአስቴ-ድዙል ኤፒ (Suaste-Dzul AP)፣ ጋሉ ኤ (Gallou A) እና ፔኛ-ካሪሎ ኬአይ (Peña-Carrillo KI) 2017፡-አካልን የማያጠፉ ስድስት ዘዴዎችን በመጠቀም ከጥቃቅን ነፍሳት ዲኤንኤ ማግኘት (DNA recovery from microhymenoptera using six non-destructive methodologies) *ጂኖም (Genome)*፣ 60(1)፣ 85-91

29. ሃጂባባኢ ኤም (Hajibabaei M)፣ ደዋርድ ጄአር (DeWaard JR)፣ ኢቫኖቫ ኤንቪ (Ivanova NV)፣ ራትናሲንግሃም ኤስ (Ratnasingham S) እና ሌሎች 2005፡- ከፍተኛ መጠን ያለው የዲኤንኤ ባርኮድ ለማዘጋጀት ወሳኝ የሆኑ ነጥቦች (Critical factors for assembling a high volume of DNA barcodes)። *ፊሎሶፊካል ትራንዛክሽንስ ኦፍ ዘ ሮያል ሶሳይቲ ቢ (Philosophical Transactions of the Royal Society B)*፣ 360(1462)፣ 1959-1967

30. ሃዋስ ኤን (Haouas N)፣ ፔሶን ቢ (Pesson B)፣ ቡዳቦስ አር (Boudabous R)፣ ዴዴት ጄፒ (Dedet JP) እና ሌሎች 2007፡- በትንኞች ውስጥ ያለን የደም ትንተና በመጠቀም የሌሽማኒያ በሽታ ተሸካሚ እንስሳትን ለመለየት የሚያስችል የሞለኪውላር መሣሪያ ማዘጋጀት (Development of a molecular tool for the identification of *Leishmania* reservoir hosts by blood meal analysis)። *አሜሪካን ጆርናል ኦፍ ትሮፒካል ሜዲሰን ኤንድ ሃይጂን (American Journal of Tropical Medicine and Hygiene)*፣ 77(6)፣ 1054-1059

31. ላቫክኮቫ ኬ (Hlavackova K)፣ ድቮራክ ቪ (Dvorak V)፣ ቻስኮፖሉ ኤ (Chaskopoulou A)፣ ቮልፍ ፒ (Volf P) እና ሃላዳ ፒ (Halada P) 2019፡- በነፍሳት ውስጥ የተመገቡትን የደም ዓይነት ለመለየት የሚያስችል አዲስ ማልዲቶፍ ኤም ኤ ስ (MALDI-TOF MS) ላይ የተመሠረተ ዘዴ (A novel MALDI-TOF MS-based method for blood meal identification in insect vectors) *PLoS Neglected Tropical Diseases*፣ 13(9)፣ e0007669

32. ሁመር ኤች (Huemer H)፣ ፕሩዶም ጄ (Prudhomme J)፣ አማሮ ኤፍ (Amaro F)፣ ባክሎቲ ኤ (Baklouti A) እና ሌሎች 2017፡- በአሸዋ ትንኝ በሚተላለፉ ፍሌቦቫይረሶች ላይ ለሚደረጉ ጥናቶች ተግባራዊ መመሪያ፡- ክፍል 2 (Practical guidelines for studies on sandfly-borne phleboviruses: Part II: fieldwork and virological screening)*ቬክተር-ቦርን ኤንድ ዞኖቲክ ዲዚዝስ (Vector-Borne and Zoonotic Diseases)*፣ 17(1)፣ 81-90

33. ጃንካሮቫ ኤም (Jancarova M)፣ ፖላንስካ ኤን (Polanska N)፣ ቲሰን ኤ (Thiesson A)፣ አርኖድ ኤፍ (Arnaud F) እና ሌሎች 2025፡- የተለያዩ የአሸዋ ትንኝ ዝርያዎች ለቶስካና ቫይረስ ያላቸው ተጋላጭነት (Susceptibility of diverse sand fly species to Toscana virus) *PLoS Neglected Tropical Diseases*፣ 19(5)፣ e0013031

34. ካፕ ጄዲ (Kapp JD)፣ ግሪን አርኢ (Green RE) እና ሻፒሮ ቢ (Shapiro B) 2021፡- ለመደበኛ ዲኤንኤ ምርመራ የተመቻቸ ፈጣን እና ቀልጣፋ የጂኖሚክ ላይብረሪ ዝግጅት ዘዴ (A fast and efficient single-stranded genomic library preparation method optimized for ancient DNA) *ጆርናል ኦፍ ሄሬዲቲ (Journal of Heredity)*፣ 112(3)፣ 241-249

35. ኪሊክ-ኬንድሪክ አር (Killick-Kendrick R)፣ ማሮሊ ኤም (Maroli M) እና ኪሊክ-ኬንድሪክ ኤም (Killick-Kendrick M) 1991፡- የአሸዋ ትንኞችን በላብራቶሪ ስለማላመድ የተጻፉ ጽሑፎች ዝርዝር (Bibliography of the colonization of phlebotomine sandflies) *ፓራሲቶሎጂያ (Parassitologia)*፣ 33 (suppl.)፣ 321-333

36. ሎየር ፒ (Lawyer P)፣ ኪሊክ-ኬንድሪክ ኤም (Killick-Kendrick M)፣ ሮውላንድ ቲ (Rowland T)፣ ሮውተን ኢ (Rowton E) እና ቮልፍ ፒ (Volf P) 2017፡- የአሸዋ ትንኞችን በላብራቶሪ ውስጥ ማላመድ እና በብዛት ማርባት (Laboratory colonization and mass rearing of phlebotomine sand flies (Diptera, Psychodidae)) *ፓራሳይት (Parasite)*፣ 24፣ 42

37. ሌገር ኤን (Léger N)፣ ፔሶን ቢ (Pesson B) እና ማዱሎ-ሌብሎንድ ጂ (Madulo-Leblond G) 1986፡- የግሪክ አሸዋ ትንኞች፡- 1ኛ ክፍል (Les phlébotomes de Grèce : 1ère partie) *በለቲን ዴ ላ ሶሳይቴ ዴ ፓቶሎጂ ኤግዞቲክ (Bulletin de la Société de Pathologie Exotique)*፣ 79፣ 386-397

38. ሌገር ኤን (Léger N)፣ ፔሶን ቢ (Pesson B) እና ማዱሎ-ሌብሎንድ ጂ (Madulo-Leblond G) 1986፡- የግሪክ አሸዋ ትንኞች፡- 2ኛ ክፍል (Les phlébotomes de Grèce : 2ème partie) *በለቲን ዴ ላ ሶሳይቴ ዴ ፓቶሎጂ ኤግዞቲክ (Bulletin de la Société de Pathologie Exotique)*፣ 79፣ 514-524

39. ሊዮኔል ጂኤኤፍ (Leonel JAF)፣ ቪዮቲ ጂ (Vioti G)፣ አልቬስ ኤምኤል (Alves ML)፣ ዳ ሲልቫ ዲቲ (da Silva DT) እና ሌሎች 2020፡- ከነጠላ አሸዋ ትንኞች ዲኤንኤ ማውጣት፡- የተሻለ ውጤት የሚሰጠው የትኛው ዘዴ ነው? (DNA extraction from individual Phlebotomine sand flies specimens: Which is the method with better results?) *ኤክስፔሪሜንታል ፓራሳይቶሎጂ (Experimental Parasitology)*፣ 218፣ 107981

40. ሌስቲኖቫ ቲ (Lestinova T)፣ ሮሁሶቫ አይ (Rohousova I)፣ ሲማ ኤም (Sima M)፣ ደ ኦሊቬራ ሲአይ (de Oliveira CI) እና ቮልፍ ፒ (Volf P) 2017፡- ስለ አሸዋ ትንኝ ምራቅ ግንዛቤዎች፡- በትንኞች፣ በአስተናጋጆች (HOST) እና በሌሽማኒያ መካከል ያለው የደም አመጋገብ እና የኢሚዩኖሎጂ መስተጋብር (Insights into the sand fly saliva: Blood-feeding and immune interactions) *PLoS Neglected Tropical Diseases*፣ 11(7)፣ e0005600

41. ሊንሃርድ ኤ (Lienhard A) እና ሻፈር ኤስ (Schaffer S) 2019፡- ስውር የሆነውን ማውጣት፡- ጥቃቅን ነፍሳት ውስጥ ያለውን ዲኤንኤ በጥራት ማግኘት ፈታኝ ሥራ ነው (Extracting the invisible: obtaining high quality DNA is a challenging task in small arthropods)። *PeerJ*፣ 7፣ e6753

42. ሎዛኖ-ሳርዳኔታ ዋይኤን (Lozano-Sardaneta YN)፣ ሚኬሪ-ፓቼኮ ኦኤፍ (Mikery-Pacheco OF)፣ ሁዌርታ ኤች (Huerta H) እና ሌሎች 2025፡- በሜክሲኮ ውስጥ የሌሽማኒያሲስ አስተላላፊ የሆኑ የአሸዋ ትንኝ ዝርያዎችን ለመለየት የክንፍ ጂኦሜትሪክ ሞርፎሜትሪክስ ውጤታማ ነው (Wing geometric morphometrics is effective to separate sand fly species related with leishmaniasis transmission in Mexico) *አክታ ትሮፒካ (Acta Tropica)*፣ 262፣ 107523

43. ማንደሪዮሊ ኤም (Mandrioli M)። 2008፡- የነፍሳት ስብስቦች እና የዲኤንኤ ትንተና፡- ስብስቦችን እንዴት ማስተዳደር ይቻላል? (Insect collections and DNA analyses: how to manage collections?) *ሙዚየም ማኔጅመንት ኤንድ ኩሬተርሺፕ (Museum Management and Curatorship)*፣ 23(2)፣ 193-199

44. ማሮሊ ኤም (Maroli M)፣ ፌሊቺያንጄሊ ኤምዲ (Feliciangeli MD)፣ ቢሻውድ ኤል (Bichaud L)፣ ሻሬል አርኤን (Charrel RN) እና ግራዶኒ ኤል (Gradoni L) 2013፡- አሸዋ ትንኞች እና የሌሽማኒያሲስ እንዲሁም የሌሎች የህዝብ ጤና ችግር የሆኑ በሽታዎች ስርጭት (Phlebotomine sandflies and the spreading of leishmaniases and other diseases) *ሜዲካል ኤንድ ቬተርነሪ ኢንቶሞሎጂ (Medical and Veterinary Entomology)*፣ 27(2)፣ 123-47

45. ማርኩዊና ዲ (Marquina D)፣ ቡክዘክ ኤም (Buczek M)፣ ሮንኩዊስት ኤፍ (Ronquist F) እና ሉካሲክ ፒ (Lukasik P) 2021፡- የአልኮል መጠን በነፍሳት ስነ-ቅርጽ እና በሞለኪውላር ጥበቃ ላይ ያለው ተፅዕኖ (The effect of ethanol concentration on the morphological and molecular preservation of insects) *PeerJ*፣ 9፣ e10799

46. ማቲስ ኤ (Mathis A)፣ ዴፓኪት ጄ (Depaquit J)፣ ድቮራክ ቪ (Dvorak V)፣ ቱተን ኤች (Tuten H)፣ ባኑልስ ኤኤል (Banuls AL) እና ሌሎች 2015፡- በአንድ የማልዲቶፍ ኤምኤስ (MALDI-TOF MS) ዋቢ መረጃ ቋት እና ሁለት የማስ ስፔክትሮሜትር ስርዓቶችን በመጠቀም አሸዋ ትንኞችን መለየት (Identification of phlebotomine sand flies using one MALDI-TOF MS reference database and two mass spectrometer systems) *ፓራሳይትስ ኤንድ ቬክተርስ (Parasites & Vectors)*፣ 8፣ 266

47. ሜካርኒያ ኤን (Mekarnia N)፣ ቤናላል ኬኢ (Benallal KE)፣ ሳድሎቫ ጄ (Sadlova J)፣ ቮይትኮቫ ቢ (Vojtkova B) እና ሌሎች 2024፡- የፍሌቦቶመስ ፓፓታሲ (*Phlebotomus papatasi)* በሌሽማኒያ ሜጀር (*Leishmania major Mon-25*) ተላምዶ የመኖር ብቃት፣ ተላላፊነት እና የመድኃኒት መቋቋም ባህሪ ላይ ያለው ተፅዕኖ (Effect of *Phlebotomus papatasi* on the fitness, infectivity and antimony-resistance phenotype of *Leishmania major*) *ኢንተርናሽናል ጆርናል ፎር ፓራሳይቶሎጂ - ድረግስ ኤንድ ድረግ ሪዚስታንስ (International Journal for Parasitology – Drugs and Drug resistance)*፣ 25፣ 100554

48. ሚሊጋን ቢጂ (Milligan BG) 1998፡- ሙሉ የዲኤንኤ አወጣጥ (Total DNA isolation)፤ በሆልዘል ኤአር (Hoelzel AR) አዘጋጅነት "የስብስቦች ሞለኪውላር ጄኔቲክ ትንተና፡- ተግባራዊ አቀራረብ" (Molecular Genetic Analysis of Population: A Practical Approach) በሚለው መጽሐፍ ውስጥ። ኦክስፎርድ ዩኒቨርሲቲ ፕሬስ (Oxford University Press)፡- ኦክስፎርድ

49. ሞሊና አር (Molina R)፣ ጂሜኔዝ ኤም (Jiménez M)፣ አልቫር ጄ (Alvar J)፣ ጎንዛሌዝ ኢ (González E) እና ሌሎች 2017፡- በአሸዋ ትንኝ ምርምር ውስጥ የሚተገበሩ ዘዴዎች (Methods in sand fly research) ዩኒቨርሲቲ ኦፍ አልካላ፣ ማድሪድ

50. መርፊ ደብሊውጄ (Murphy WJ)፣ ኢይዚሪክ ኢ (Eizirik E)፣ ኦብራይን ኤስጄ (O'Brien SJ)፣ ማድሰን ኦ (Madsen O) እና ሌሎች 2001፡- የቀደምት አጥቢ እንስሳት ስርጭትን በቤዢያን ፊሎጄኔቲክስ መፍታት (Resolution of the early placental mammal radiation using Bayesian phylogenetics)። *ሳይንስ (Science)*፣ 294(5550)፣ 2348-51

51 ናሲፍ-ፒሜንታ አር (Nacif-Pimenta R)፣ ፒንቶ ኤልሲ (Pinto LC)፣ ቮልፎቫ ቪ (Volfova V)፣ ቮልፍ ፒ (Volf P)፣ ፒሜንታ ፒኤፍፒ (Pimenta PFP) እና ሴኩንዲኖ ኤንኤፍሲ (Secundino NFC) 2020፡- የሌሽማኒያሲስ አስተላላፊ በሆኑ የአሸዋ ትንኞች የምራቅ እጢዎች ላይ የሚታዩ የተቀራረቡ እና የተለዩ ስነ-ቅርጻዊ ገጽታዎች፡- የአናቶሚ እና የአልትራስትራክቸር ጥናት (Conserved and distinct morphological aspects of the salivary glands of sand fly vectors of leishmaniasis: an anatomical and ultrastructural study)*ፓራሳይትስ ኤንድ ቬክተርስ (Parasites & Vectors)*፣ 13(1)፣ 441

52 ኑሃውስ ቢ (Neuhaus B)፣ ሽሚድ ቲ (Schmid T) እና ሪዴል ጄ (Riedel J) 2017፡- የአጉሊ መነጽር መስታወት (Slide) ስብስቦች አስተዳደር እና ጥናት፡- ማከማቸት፣ መረጃ ማደራጀት፣ መበላሸት፣ የእድሳት ሂደቶች እና አጠቃላይ ምክረ-ሐሳቦች (Collection management and study of microscope slides: Storage, profiling, deterioration, restoration procedures, and general recommendations) *ዙታክሳ (Zootaxa)*፣ 4322(1)፣ 1–173

53 ኒው ቲአር (New TR) 1974፡- ፕሶኮፕቴራ (Pscoptera)፤ የብሪታንያ ነፍሳትን ለመለየት የሚያገለግሉ መመሪያዎች (Handbooks for Identification of British Insects) ቅጽ 1 ለንደን፡- የለንደን ሮያል ኢንቶሞሎጂካል ሶሳይቲ (Royal Entomological Society of London) 102 ገጽ

54. ፔሬዝ-ሩይዝ ኤም (Perez-Ruiz M)፣ ኮላኦ ኤክስ (Collao X)፣ ናቫሮ-ማሪ ጄኤም (Navarro-Mari JM) እና ቴኖሪዮ ኤ (Tenorio A) 2007፡- የቶስካና

ቫይረስን ለመለየት የሚያስችል የሪል-ታይም ፒሲአር ምርመራ (Reverse transcription, real-time PCR assay for detection of Toscana virus) *ጆርናል ኦፍ ክሊኒካል ቫይሮሎጂ (Journal of Clinical Virology)*፣ 39(4)፣ 276-81

55 ፖርኮ ዲ (Porco D)፣ ሮውጀሪ አር (Rougerie R)፣ ዴሃርቬንግ ኤል (Deharveng L) እና ሄበርት ፒ (Hebert P)። 2010:- ከፍተኛ መጠን ባለው የሥራ ሂደት ውስጥ ለጥቃቅንና ለስላሳ አካል ላላቸው መገጣጠሚያ ላላቸው እንስሳት (Arthropods) አካልን የማያጠፋ የዲኤንኤ አወጣጥ እና የናሙና ጥበቃን ማጣመር:- የኮሌምቦላ (Collembola) ምሳሌ (Coupling non-destructive DNA extraction and voucher retrieval for small soft-bodied Arthropods in a high-throughput context: the example of Collembola) *ሞለኪውላር ኢኮሎጂ ሪሶርስስ (Molecular Ecology Resources)*፣ 10(6)፣ 942-945

56. ፕሩዶም ጄ (Prudhomme J)፣ ካሳን ሲ (Cassan C)፣ ሂዴ ኤም (Hide M)፣ ቶቲ ሲ (Toty C)፣ ራሆላ ኤን (Rahola N) እና ሌሎች 2016:- በፈረንሳይ ሮክደር አካባቢ የሚገኙ የፍሌቦተመስ አርያሲ - ዲፕተራ ሳይኮዲዴ ([*Phlebotomus ariasi*] , Diptera: Psychodidae) ክንፎች ስነ-ምህዳራዊ እና ስነ-ቅርጻዊ ልዩነቶች:- የጂኦሜትሪክ ሞርፎሜትሪክስ አቀራረብ (Ecology and morphological variations in wings of *Phlebotomus ariasi* (Diptera: Psychodidae) in the region of Roquedur (Gard, France): a geometric morphometrics approach)*ፓራሳይትስ ኤንድ ቬክተርስ (Parasites & Vectors)*፣ 9(1)፣ 578

57. ፕሩዶም ጄ (Prudhomme J)፣ ጉናይ ኤፍ (Gunay F)፣ ራሆላ ኤን (Rahola N)፣ ኦዋናይሚ ኤፍ (Ouanaimi F) እና ሌሎች 2012:- በሞሮኮ የአትላስ ተራሮች በሚገኙ የፍሌቦተመስ ፓፓታሲ(*Phlebotomus papatasi)* ዝርያዎች ላይ የሚታይ የክንፍ መጠንና ቅርጽ ልዩነት (Wing size and shape variation of *Phlebotomus papatasi* populations in Morocco) *ጆርናል ኦፍ ቬክተር ኢኮሎጂ (Journal of Vector Ecology)*፣ 37(1)፣ 137-47

58. ፕሩዶም ጄ (Prudhomme J)፣ ቶቲ ሲ (Toty C)፣ ካሳፕ ኦኢ (Kasap OE)፣ ራሆላ ኤን (Rahola N)፣ ቨርኝ ቢ (Vergnes B)፣ ማያ ሲ (Maia C)፣ ካምፒኖ ኤል (Campino L)፣ አንቶኒዮ ኤም (Antoniou M)፣ ጂሜኔዝ ኤም (Jimenez M)፣ ሞሊና አር (Molina R)፣ ካኔት ኤ (Cannet A)፣ አልተን ቢ (Alten B)፣ ሴሬኖ ዲ (Sereno D) እና ባኑልስ ኤኤል (Banuls AL) 2015:- በሜዲትራኒያን አካባቢ የላይሽማኒያ ኢንፋንተም (*Leishmania infantum*) አስተላላፊ በሆነው ፍሌቦተመስ አርያሲ ቶኖይር (*Phlebotomus ariasi* Tonnoir) ላይ ለሚደረጉ ዘርፈ-ብዙ የዘረመል ጥናቶች አዲስ ማይክሮሳተላይት መለያዎች (New microsatellite markers for multi-scale genetic studies on *Phlebotomus ariasi* Tonnoir, vector of *Leishmania infantum* in the Mediterranean area) *አክታ ትሮፒካ (Acta Tropica)*፣ 142፣ 79-85

59. ፕሩዶም ጄ (Prudhomme J)፣ ቬሎ ኢ (Velo E)፣ ቢኖ ኤስ (Bino S)፣ ካድሪያጅ ፒ (Kadriaj P)፣ መርሲኒ ኬ (Mersini K)፣ ጉናይ ኤፍ (Gunay F) እና አልተን ቢ (Alten B) 2019:- በአውሮፓ ለመጀመሪያ ጊዜ በተመዘገበበት የአልባኒያ ክልል ውስጥ የሚገኙ የኤደስ አልቦፒክቱስ ዲፕተራ ኩሊሲዴ (*Aedes albopictus* , Diptera, Culicidae) ከባህር ጠለል በላይ የመሬት ከፍታ በክንፍ ስነ-ቅርጽ ላይ የሚያስከትሉት ልዩነት (Altitudinal variations in wing morphology of *Aedes albopictus* (Diptera, Culicidae) in Albania, the region where it was first recorded in Europe) *ፓራሳይት (Parasite)*፣ 26፣ 55

60. ሮውሊንስ ዲጄ (Rawlins DJ) 1992:- የብርሃን አጉሊ መነጽር:- ባዮ-ቴክኒኮችን ማስተዋወቅ (Light Microscopy: An Introduction to Biotechniques) ኦክስፎርድ፡ ባዮስ ሳይንቲፊክ ፐብሊሸርስ፣ 143 ገጽ

61 ሬዲ ፒዲ (Ready PD) 2013:- የበሽታ አምጪ ተህዋስያን አስተላላፊ የሆኑት የፍሌቦቶሚን አሸዋ ትንኞች ባዮሎጂ (Biology of phlebotomine sand flies as vectors of disease agents) *አንዩዋል ሪቪው ኦፍ ኢንቶሞሎጂ (Annual Review of Entomology)*፣ 58፣ 227-50

62 ሮልፍ ኤፍጄ (Rohlf FJ) እና ስላይስ ዲ (Slice D) 1990:- የነጥቦችን (Landmarks) ትክክለኛ ተደራቢነት ለማረጋገጥ የሚያገለግሉ የፕሮክራስትስ ዘዴ ቅጥያዎች (Extensions of the Procrustes method for the optimal superimposition of landmarks) *ሲስተማቲክ ዞኦሎጂ (Systematic Zoology)*፣ 39(1)፣ 40-59

63. ሮውሊ ዲኤል (Rowley DL)፣ ኮዲንግተን ጄኤ (Coddington JA)፣ ጌትስ ኤምደብሊው (Gates MW)፣ ኖርቦም ኤኤል (Norrbom AL)፣ ኦቾአ አርኤ (Ochoa RA)፣ ቫንደንበርግ ኤንጄ (Vandenberg NJ) እና ግሪንስተን ኤምኤች (Greenstone MH) 2007:- የዲኤንኤ ባርኮድን (DNA-barcoded) ለናሙናዎች ማስረጃነት መጠቀም:- አካልን የማያጠፋ ዉጣት መመሪያ በየብስ ለሚኖሩ መገጣጠሚያ ላላቸው እንስሳት (Arthropods) ያለዉ ዉጤታማነት (Vouchering DNA-barcoded specimens: Test of a nondestructive extraction protocol for terrestrial arthropods) *ሞለኪውላር ኢኮሎጂ ኖትስ (Molecular Ecology Notes)*፣ 7(6)፣ 915-924

64. ሳቢዮ ፒቢ (Sábio PB)፣ አንድራዴ ኤጄ (Andrade AJ) እና ጋላቲ ኢኤቢ (Galati EAB) 2014:- በ"ሻኖኒ ኮምፕሌክስ" (Shannoni complex) ውስጥ የተካተቱ የአንዳንድ ዝርያዎች ታክሶኖሚያዊ ደረጃ ግምገማ እና አዲስ የሳቲሮማያ (*Psathyromyia* -Diptera: Psychodidae: Phlebotominae) ዝርያ መግለጫ (Assessment of the taxonomic status of some species included in the Shannoni complex, with the description of a new species of *Psathyromyia*) *ጆርናል ኦፍ ሜዲካል ኢንቶሞሎጂ (Journal of Medical Entomology)*፣ 51(2)፣ 331-341

65. ሳድሎቫ ጄ (Sadlova J)፣ ዮ ኤም (Yeo M)፣ ሴብሎቫ ቪ (Seblova V)፣ ሌዊስ ኤምዲ (Lewis MD)፣ ማውሪሲዮ አይ (Mauricio I)፣ ቮልፍ ፒ (Volf P) እና ማይልስ ኤምኤ (Miles MA) 2011:- በአሸዋ ትንኝ (sand fly) ውስጥ በመጀመሪያው የእድገት ደረጃ ላይ ያሉ የላይሽሜንያ ዶኖቫኒ (Leishmania donovani) ፍሎረሰንት ዲቃላዎችን በምስል ማሳየት (Visualisation of *Leishmania donovani* fluorescent hybrids during early stage development in the sand fly vector) *ፕሎስ ዋን (PLoS One)*፣ 6(5)፣ e19851

66 ሳልስ ኬ (Sales K)፣ ሚራንዳ ዲኢኦ (Miranda DEO)፣ ዳ ሲልቫ ኤፍጄ (da Silva FJ)፣ ኦትራንቶ ዲ (Otranto D)፣ ፊጌሬዶ ኤልኤ (Figueredo LA) እና ዳንታስ-ቶሬስ ኤፍ (Dantas-Torres F) 2020:- በተለያዩ የማከማቻ ጊዜያት እና የማቆያ ዘዴዎች በአሸዋ ትንኝ የዲኤንኤ (DNA) ክምችት እና ጥራት ላይ ያላቸው ተጽዕኖ ግምገማ (Evaluation of different storage times and preservation methods on phlebotomine sand fly DNA concentration and purity) *ፓራሳይትስ ኤንድ ቬክተርስ (Parasites & Vectors)*፣ 13(1)፣ 399

67. ሳልስ ኬጂ (Sales KG)፣ ኮስታ ፒኤል (Costa PL)፣ ደ ሞራይስ አርሲ (de Morais RC)፣ ኦትራንቶ ዲ (Otranto D)፣ ብራንዳኦ-ፊልሆ ኤስፒ (Brandao-Filho SP)፣ ካቫልካንቲ ኤምደፒ (Cavalcanti Mde P) እና ዳንታስ-ቶሬስ ኤፍ (Dantas-Torres F) 2015:- በአሸዋ ትንኞች ሆድ ዕቃ ውስጥ የሚገኝ የደም ምግብን በ"ሪል-ታይም ፒሲአር" (Real-time PCR) ዘዴ መለየት (Identification of phlebotomine sand fly blood meals by real-time PCR) *ፓራሳይትስ ኤንድ ቬክተርስ (Parasites & Vectors)*፣ 8፣ 230

68. ሳንት አና ኤምአር (Sant'Anna MR)፣ ጆንስ ኤንጂ (Jones NG)፣ ሂንድሌይ ጄኤ (Hindley JA)፣ ሜንዴዝ-ሶሳ ኤኤፍ (Mendes-Sousa AF)፣ ዲሎን አርጄ (Dillon RJ)፣ ካቫልካንቴ አርአር (Cavalcante RR)፣ አሌክሳንደር ቢ (Alexander B) እና ቤይትስ ፒኤ (Bates PA) 2008:- በላቦራቶሪ ውስጥ በተመገቡ እና ከመስክ በተያዙ ሉትዞማያ ሎንጂፓልፒስ (*Lutzomyia longipalpis)* አሸዋ ትንኞች ላይ የ"ኤፍቲኤ" (FTA) የመረጃ ወረቀትን በመጠቀም የደም ምግብ መለየት እና የጥገኛ ተህዋስያን ልየታ (Blood meal identification and parasite detection in laboratory-fed and field-captured *Lutzomyia longipalpis* by PCR using FTA databasing paper) *አክታ ትሮፒካ (Acta Tropica)*፣ 107(3)፣ 230-7

69. ሴኔ ኤንኤ (Senne NA)፣ ሳንቶስ ኤችኤ (Santos HA)፣ አራውጆ ቲአር (Araujo TR)፣ ፓውሊኖ ፒጂ (Paulino PG)፣ ሜንዶንሳ ኤልፒ (Mendonca LP)፣ ሞሬይራ ኤችቪኤስ (Moreira HVS)፣ ካሚሎ ቲኤ (Camilo TA) እና ዳ ኮስታ አንጄሎ አይ (da Costa Angelo I) 2022:- ደም ባልመጠጡ (Non-engorged) የፍሌቦቶሚን አሸዋ ትንኞች ላይ የተደረገ አስተማማኝ የጂኖሚክ ዲኤንኤ (gDNA) ዉጣት ዘዴዎች ንጽጽራዊ አፈጻጸም (Robust comparative performance of genomic DNA extraction methods from non-engorged phlebotomine sandflies) *ሜዲካል ኤንድ ቬተሪነሪ ኢንቶሞሎጂ (Medical and Veterinary Entomology)*፣ 36(2)፣ 203-211

70 ሾው ጄጄ (Shaw JJ) 2025፡- በአሜሪካ አሸዋ ትንኞች ውስጥ ስለሚከሰቱ የሌሽማኒያ ኢንፌክሽኖች የምርምር ፅሁፎች ዳሰሳ - ሌሽማኒያሲስን የሚያስተላልፉት ዝርያዎች የሰውን ደም ብቻ ተመጋቢ (Anthropophilic) ናቸው ወይስ የሰውንና ሌሎች እንስሳትን ደም ተመጋቢ (Anthropportunists)? (A review of *Leishmania* infections in American Phlebotomine sand flies – Are those that transmit leishmaniasis anthropophilic or anthropportunists?) *ፓራሳይት (Parasite)*፣ 32፣ 57

71. ቴሽ አርቢ (Tesh RB) እና ሞዲ ጂቢ (Modi GB) 1983፡- በፍሌቦተመስ ፓፓታሲ (*Phlebotomus papatasi)* አሸዋ ትንኞች ውስጥ የቻንዲፑራ ቫይረስ (Chandipura virus - Rhabdoviridae: Vesiculovirus) እድገት እና በእንቁላል በኩል ወደ ሚቀጥለው ትውልድ የመተላለፍ ሂደት (Growth and transovarial transmission of Chandipura virus (Rhabdoviridae: Vesiculovirus) in *Phlebotomus papatasi*) *አሜሪካን ጆርናል ኦፍ ትሮፒካል ሜዲሲን ኤንድ ሃይጂን (American Journal of Tropical Medicine and Hygiene)*፣ 32(3)፣ 621-3

72. ቶምሰን ፒኤፍ (Thomsen PF)፣ ኤሊያስ ኤስ (Elias S)፣ ጊልበርት ኤምቲፒ (Gilbert MTP)፣ ሃይሌ ጄ (Haile J)፣ ሙንች ኬ (Munch K)፣ ኩዝሚና ኤስ (Kuzmina S)፣ ፍሮዝ ዲጂ (Froese DG)፣ ሼር ኤ (Sher A)፣ ሆልዳዌይ አርኤን (Holdaway RN) እና ዊለርስሌቭ ኢ (Willerslev E) 2009፡- አካልን ሳይጎዱ ከጥንታዊ ነፍሳት የዲኤንኤ (DNA) ናሙና መውሰድ ዘዴ (Non-destructive sampling of ancient insect DNA) *ፕሎስ ዋን (PLoS One)*፣ 4(4)፣ e5048

73. ትሩኤት ጂኢ (Truett GE)፣ ሄገር ፒ (Heeger P)፣ ማይናት አርኤል (Mynatt RL)፣ ትሩኤት ኤኤ (Truett AA)፣ ዎከር ጄኤ (Walker JA) እና ዋርማን ኤምኤል (Warman ML) 2000፡- ሙቅ ሶዲየም ሃይድሮክሳይድ እና ትሪስ (HotSHOT) በመጠቀም ፒሲአር (PCR) ጥራት ያለው የአይጥ ጂኖሚክ ዲኤንኤን ማዘጋጀት (Preparation of PCR-quality mouse genomic DNA with hot sodium hydroxide and tris (HotSHOT)) *ባዮቴክኒክስ (Biotechniques)*፣ 29(1)፣ 52-54

74 አፕተን ኤምኤስ (Upton MS) 1993፡- በውሃ የሚሟሙ የ"ገም-ክሎራል" (Gum-chloral) ማንበሪያ ቅመሞች፡- የታሪክ ዳሰሳ (Aqueous gum-chloral slide mounting media: an historical review)። *ቡሌቲን ኦፍ ኢንቶሞሎጂካል ሪሰርች (Bulletin of Entomological Research)*፣ 83(2)፣ 267-274

75. ቮልፍ ፒ (Volf P) እና ሚስኮቫ ጄ (Myskova J) 2007፡- አሸዋ ትንኞች እና ሌሽማኒያ፡- የተገደቡ (Specific) ወይስ ልቅ (Permissive) አስተላላፊዎች (Sand flies and *Leishmania*: specific versus permissive vectors) *ትሬንድስ ኢን ፓራሳይቶሎጂ (Trends in Parasitology)*፣ 23(3)፣ 91-92

76. ዋንግ ኪው (Wang Q) እና ዋንግ ኤክስ (Wang X) 2012፡- ለፒሲአር (PCR) ትንተና ከአንድ የ"ኪሮኖሚድ" (Chironomid) ነፍሳት ላይ ዲኤንኤ (DNA) ለማውጣት የሚያገለግሉ ዘዴዎች ንጽጽር (Comparison of methods for DNA extraction from a single chironomid for PCR analysis) *ፓኪስታን ጆርናል ኦፍ ዞኦሎጂ (Pakistan Journal of Zoology)*፣ 44(2)

77. ዋንግ ዋይ (Wang Y)፣ ዣኦ ዋይ (Zhao Y)፣ ቦላስ ኤ (Bollas A)፣ ዋንግ ዋይ (Wang Y) እና ኦ ኬኤፍ (Au KF) 2021፡- የ"ናኖፖር" የዘረመል ቅደም ተከተል ቴክኖሎጂ (Nanopore sequencing)፣ ባዮኢንፎርማቲክስ እና ጥቅማቸዉ (Nanopore sequencing technology, bioinformatics and applications) *ኔቸር ባዮቴክኖሎጂ (Nature Biotechnology)*፣ 39(11)፣ 1348-1365

Cite this article as: Randrianambinintsoa FJ, Augendre L, Prudhomme J, Martinet J-P, Loyer M, Mekarnia N, Kerkoub H, Perveen FK, Huguenin A, Kariya E, Akhoundi M, De Andrade AJ, Berriatua E, Bongiorno G, Boyer S, Christodoulou V, Da Costa-Ribeiro MCV, De Souza LAF, Ding H, Dondji B, Dvořák V, Erisoz Kasap O, Galati EAB, Gállego M, Ballart C, Gouzelou S, Haddad N, Masse RS, Mekuria AH, Ivovic V, Kaczmarek S, Shahar MK, Kirstein OD, Kniha E, Kolářová I, Lincoln T, Lucanas C, Mikov O, Nov K, Özbel Y, Pesson B, Posada Lopez LC, Prasetyo DB, Rahola N, Rebollar-Tellez EA, Rodrigues BL, Roy L, Saini P, Sanjoba C, Shimabukuro PH, Siriyasatien P, Soszynska A, Suleşco T, Sylla M, Torno M, Volf P, Vongphayloth K, Sinh Nam V, Wardhana A, Yessinou E, Zapata S, Gantier J-C & Depaquit J. 2026. Processing and mounting phlebotomine sand flies: a consensus guideline. Parasite xx, xx. https://doi.org/10.1051/parasite/2026009.

PARASITE

An international open-access, peer-reviewed, online journal publishing high quality papers on all aspects of human and animal parasitology

Reviews, articles and short notes may be submitted. Fields include, but are not limited to: general, medical and veterinary parasitology; morphology, including ultrastructure; parasite systematics, including entomology, acarology, helminthology and protistology, and molecular analyses; molecular biology and biochemistry; immunology of parasitic diseases; host-parasite relationships; ecology and life history of parasites; epidemiology; therapeutics; new diagnostic tools.
All papers in Parasite are published in English. Manuscripts should have a broad interest and must not have been published or submitted elsewhere. No limit is imposed on the length of manuscripts.

Parasite (open-access) continues **Parasite** (print and online editions, 1994-2012) and **Annales de Parasitologie Humaine et Comparée** (1923-1993) and is the official journal of the Société Française de Parasitologie.

Editor-in-Chief: Submit your manuscript at:
[illegible]

አባሪ 1፡- የባዮኬሚካል ፅንሰ ሀሳብ መሠረቶች (Appendix 1: Biochemical theoretical foundations)

ይህ ርዕሥ የሚመለከተዉ የአሸዋ ትንኞች አርትሮፖዶች ነዉ። ሆኖም ግን፣ አጠቃላይ ሐሳቡ የዝርያ ልየታ ዘዴያቸዉ በውስጣዊ የሥነ-ቅርጽ (morphological) ባህሪያት ላይ ብቻ ለተመሰረተ ሌሎች በጣም የተለመዱ አርትሮፖዶችም ሊሠራ ይችላል። በአጋጣሚ፣ አንዳንድ የውስጥ አካላት በከፊል በቺቲን (chitin) የተገነቡ በመሆናቸው የእነሱ ቅርጽ ጠቃሚ መረጃ ይሰጠናል። ለዚህ ነው የምግብ ፓምፖችን (food pumps/cibarium)፣ የዘር ማከማቻ ከረጢቶችን (spermathecae) እና ቱቦዎቻቸውን (ducts) መመልከቱ በጣም ትኩረት የሚስብ የሚሆነው።

ቀጥለን የምንቃኛቸውን ሁሉንም ሪኤጀንቶች (reagents) በተመለከተ፣ ከነፍሳቱ ትክለት (fixation) ደረጃ ጀምሮ እስከ ማጠናቀር (assembly) ድረስ በቀላሉ የሬዶክስ አፀግብሮት (redox - oxidation-reduction reaction) እንደምንተገብር መዘንጋት የለበትም። እኛን ሊመራን የሚገባው ጥንቃቄ ወይም ሐሳብ፣ ቀላሽ ምላሽ ሰጪ ንጥረ ነገሮች (reducing reagents) ከኦክሳይድ አድራጊ (oxidizing) ሪኤጀንቶች ጋር አለመቀላቀል ነው።

ኤቲል አልኮል፤ ኢታኖል (Ethyl alcohol; ethanol)፡-

ይህ ንጥረ ነገር በተለያዩ መንገዶች ጥቅም ላይ ይውላል። የአልኮል ሞለኪውሎች ከውሃ ጋር ከፍተኛ ቁርኝት (affinity) አላቸው፤ ስለዚህም ውሃ የማስወገድ (dehydrating) ባህሪ ያሳያሉ። ሆኖም ግን፣ አነስተኛ ምጥነት ያለው አልኮል (ማለትም የውሃ መጠኑ በጣም ከፍተኛ የሆነ) የኑክሊክ አሲዶችን መበላሸት ያስከትላል (ውሃ ከኑክሊክ አሲዶች ጋር አይጣጣምም)።

ነፍሳት በኢታኖል ውስጥ ሲቀመጡ ለማቆየት ብቻ ሳይሆን፣ ህብረ ህዋሳትን ለማጠንከር (to fix the tissues) ጭምር ነው። በሥነ ህብረህዋስ (histology) ውስጥ በተለምዶ ሁለት አስፈላጊ ጽንሰ-ሐሳቦችን እንለያለን፡- እነሱም የዘልቆ መግባት መጠን (penetration rate) እና የማጠንከር መጠን (fixation rate) ናቸው። አንድ ጥሩ ማቆያ መጀመሪያ ህብረህዋሳትን ከማጠንከሩ በፊት በፍጥነት ወደ ውስጥ ዘልቆ መግባት እንዳለበት በሚገባ የታወቀ ነው። ለ 96% አልኮል፣ የዘልቆ መግባት ጠቋሚ ቁጥሩ (penetration coefficient) 1.05 ገደማ ነው (ለማነጻጸር ያህል፣ ለ 0.75% የፒክሪክ አሲድ የውሃ ውህድ የዘልቆ መግባት ጠቋሚ ቁጥሩ 0.45 ሲሆን፣ ለ 3% የፖታስየም ዲክሮሜት ዉህድ ደግሞ 1.45 ነው)።

ነፍሳትን እና ሌሎች አርትሮፖዶችን በኢታኖል ውስጥ ለዘላለም ለማቆየት መፈለግ የኢንቶሞሎጂስቶች ፅኑ ፍላጎት ነው። የመስክ ስብስቦችን ለቀጣይ ጥናቶች ወይም ለወደፊት ተመራማሪዎች ለማቆየት መፈለግ አሁንም በጣም የሚከበር ሐሳብ ነው። ቢሆንም፣ ይህ ሐሳብ ለሳይቶሎጂስት (cytologist) ወይም ለሂስቶሎጂስት (histologist) የማይቻል ነው። ናሙናዎችን በማቆያ ፈሳሽ (fixative) ውስጥ ለረጅም ጊዜ ለማቆየት በመሞከር፣ በኋላ ላይ መልሶ ለመጠቀም (to rework) በተግባር የማይቻል ሊሆኑ ይችላል። ለዚህ ነው ከ 10 ዓመት በላይ የቆዩ ናሙናዎችን ለመጠቀም አስቸጋሪ ወይም የማይቻል የሚሆነው።

ሌላው ግምት ውስጥ መግባት ያለበት ነጥብ፣ ሊጠነከር በሚፈለገው የአርትሮፖድ ክብደት (mass) እና በማቆያ ዉህዱ መጠን (volume) መካከል ያለው ጥምርታ (ratio) ነው። በሥነ-እንስሳት (zoology) ወይም በሕክምና ልምምድ፣ ሊጠነከሩ ከሚፈለጉት ቁርጥራጮች መጠን 60 እጥፍ የሚበልጥ ዉህድ ማዘጋጀት ይመከራል። በተግባር ግን፣ ለጥናንሽ አርትሮፖዶች (micro-arthropods)፣ ለተወሰነ የናሙና መጠን ቢያንስ ከ 4 እስከ 5 እጥፍ የሚበልጥ የአልኮል መጠን ይጨምሩ። አልኮሉ በአርትሮፖዱ ህብረ ህዋስ ውስጥ ያለውን ውሃ ሁሉ በሚያስወግድበት ጊዜ ጥንካሬው (strength) እየቀነሰ እንደሚሄድ መዘንጋት የለብዎትም።

ሲጠቃለል፡-

- ኤቲል አልኮል ቀላሽ ኬሚካል (reducing chemical agent) ስለሆነ ከኦክሳይድ አድራጊ ማቆያ ዉህዶች ጋር ተኳሃኝ አይደለም፤
- ፕሮቲኖችን በከፍተኛ ሁኔታ ያዘቅጣል (precipitates) እና ባህሪያቸውን ይለውጣል (denatures)፤
- የተወሰኑ ውስብስብ ቅባቶችን (complex lipids) ያሟሟል እንዲሁም ግላይኮጅንን (glycogen) ያዘቅጣል፤
- በህብረህዋሳት ላይ ከፍተኛ መኮማተርን ያስከትላል እንዲሁም ያጠነክራቸዋል።

ቤዛማ የፖታስየም ወይም የሶዲየም ሃይድሮክሳይድ ዉህዶች (Basic potassium or sodium hydroxide solutions)፡-

በኢንቶሞሎጂ (የነፍሳት ጥናት) ውስጥ የእነዚህ ዉህዶች አጠቃቀም ያለበቂ ምክንያት በዋነኝነት በፖታስየም ሃይድሮክሳይድ ላይ ያተኩራል።

- **ሶዲየም ሃይድሮክሳይድ [E524] (Sodium hydroxide):** በዉህድ መልክ በተለያየ ምጥነት ወይም የንጥረ ነገር ምጥነት ልኬት (normality) ይገኛል። በከኒኖች (pellets) ወይም በደቃቅ ዱቄት (glitter) መልክ ይመጣል። ዋናው ጉዳቱ ከፖታስየም ሃይድሮክሳይድ (KOH) በበለጠ ሁኔታ ውሃን የሚስብ (hygroscopic) መሆኑ ነው። ከፕሮቲኖች ጋር በሚገናኝበት ጊዜ ያሟሟቸዋል፤ ከቅባቶች (lipids) ጋር ደግሞ በሳፖኒፊኬሽን (saponification - የሳሙና ዝግጅት ሂደት) ወቅት ወደ ጠንካራ ሳሙና ይለውጣቸዋል። ይህም በሳፖኒፊኬሽን (Saponification) ወቅት ፈሳሽ ሳሙና ከሚሰጠው ፖታስየም ሃይድሮክሳይድ (KOH) ጋር ያለው ዋናው ልዩነት ነው።
- **ፖታስየም ሃይድሮክሳይድ [E525] (Potassium hydroxide):** በሙሉ ምጥነት (concentrated) ሊገኝ የሚችል ዉህድ ነዉ። ነገር ግን በ0.1 ግራም ገደማ በሚመዝኑ ከኒኖች (pellets) ስለሚገኝ ትክክለኛ ሚዛን በሌለበት ሁኔታ የቀጠነ ምጥነት ያላቸዉ ዉህዶችን ለማዘጋጀት ሂደቱን በጣም ያመቻቻል። ለምሳሌ 1 ከኒን (0.1 ግራም) በ 1 ሚሊ ሊትር የተጣራ ውሃ ውስጥ 10% ዉህድ ይሰጣል። የፖታስየም ሃይድሮክሳይድ ከኒኖች ሁለተኛው ጥቅማቸው ለካርቦኔሽን (carbonation) ያላቸው ዝቅተኛ ተጋላጭነት ነው። **የፖታስየም ሃይድሮክሳይድ** (KOH) ዉህድ ከአየር ላይ ካርቦን ዳይኦክሳይድን (CO_2) የመሳብ ከፍተኛ አቅም አለው፣ ይህም የካርቦኔት ጨው ይፈጥራል)።
- እነዚህ ጠንካራ ቤዞች (strong bases) የሰባ አሲዶችን (fatty acids) በውሃ ወደ ሚሟሟ ሳሙናነት ለመቀየር ያገለግላሉ። እንደ ኢታኖል ያሉ ማቆያ ዉህዶች በናሙናው ውስጥ ያሉ አንዳንድ ቅባቶችን አስቀድመው እንደሚያሟሟቸው መታወስ አለበት። ሆኖም ናሙናውን በውሃማ ዉህድ ውስጥ ባለው ጠንካራ ቤዝ በመተካት፣ የሰባ አሲዶች (በጥቂት ወይም በበዛ ሁነታ ውስብስብ የሆኑ) እንዲዘቅጡ (precipitate) ያደርጋል። ስለዚህ ጠንካራ ቤዝ ቀዝቃዛ ሳፖኒፊኬሽን (cold saponification) ያከናውናል። በአንዳንድ ሁኔታዎች፣ ለምሳሌ በሴት ነፍሳት ውስጥ የሰባ ህብረህዋሳት (adipose tissues) በብዛት በሚገኙበት ጊዜ፣ አፀግብሮትን ለማቀላጠፍ የሙቀት መጠኑን ከ35–40ድግሪ ሴንቲ ግሬድ ከፍ ማድረግ ወይም በክፍል ሙቀት ውስጥ የሚቆይበትን ጊዜ ማራዘም ጠቃሚ ይሆናል።

ባለቀለም አሲዳማ ዉህድ / ቀለም የሌለው የማርክ-አንድሬ (Marc-André) ዉህድ፡-

እዚህ ጋር የማርክ-አንድሬ ዉህድን ጠቀሜታ እና የአጠቃቀም ጉድለቶችን እንመረምራለን። ይህ ዉህድ ከክሎራል ሃይድሬት (Chloral hydrate = trichloracetaldehyde monohydrate)፣ ከአሴቲክ አሲድ እና ከውሃ የተሰራ ነው። ዉህዱ ከፍተኛ የማክሲድ (oxidizing) ባህሪ አለው (የአሲድ እና አልዲሃይድ ድብልቅ)። በናሙናዎቹ ውስጥ ሊቀር የሚችለውን ትርፍ ፖታስየም ሃይድሮክሳይድ (KOH) ያረክሳል፤ ይህንንም የሚያደርገው በ KOH አጠቃቀም ወቅት የተፈጠሩትን አልካላይን ሳሙናዎች ሳያዘቅጥ (without precipitating) ነው። ይህ ኦክሳይድ አድራጊ ዉህድ (oxidizing solution) በቺቲን ውስጥ በሚፈጠሩት ግሉኮሳሚኖች (glucosamines) የሁለተኛ ደረጃ አልኮል ተግባራት (secondary alcohol functions) ላይም ተጽዕኖ ይኖረዋል። ይህም የሚሆነዉ ዉህዱ ግሉኮሳሚኖችን ኦክሳይድ በማድረግ ቺቲኑ እንዲለሰልስ (softening) ያደርጋል። ሌላው ተግባሩ ደግሞ በውስጡ የሚገኙ የተወሰኑ ማዕድናት ጨው (mineral salts) እንዲሟሟ ማድረግ ነው።

አስቀድሞ በአሲድ ፉክሲን (acid fuchsine) የቀለመ የማርክ-አንድሬ ዉህድ (በኦክሳይድ ሁኔታ ላይ እያለ)፣ በመዋቅሩ ሁለተኛ ደረጃ ላይ የሚገኙ አካላት

መጣበቅ ይችላል። በማርክ-አንድሬ ዉህድ ውስጥ ከቆየ በኋላ እና ናሙናዎቹ የመቅለም ደረጃ ላይ ከደረሱ በኋላ፣ የማለቅለቅ ሂደቱ በኢታኖል ብቻ ይከናወናል። በዚህም የናሙናዎቹን ውሃ የማስወገድ (dehydration) ደረጃ እንጀምራለን።

ጥቅሞች፡-

- ከመጠን በላይ የሆኑ ቤዛማ (basic) ዉህዶችን ያረክሳል።
- ቺቲንን (Chitin) ማላላት (Relaxation)።
- የቺቲን (Chitin) ውስጣዊ መዋቅሮችን በተሻለ ሁኔታ ለመገምገም ቺቲኑን (Chitin) ማቅለም።

ጉድለቶች፡- ክሎራል ሃይድሬት ሰዎችን የማደንዘዝ (hypnotic) ባህሪ ያለውና በሰው ሕክምና ውስጥ ጥቅም ላይ የዋለ ነው። በኬሚካል መሳቢያ (chemical hood) ውስጥ ጥቅም ላይ መዋል አለበት፣ እንዲሁም የኬሚካል አደጋ ሕጎች መከበር አለባቸው።

ውሃን የማስወገጃ ዉህዶች (Dehydration solutions)፡-

ልምድ እንደሚያሳየው በጣም ትናንሽ ከሆኑ ናሙናዎች ዉሀን ለማስወገድ መጠናቸው እየጨመረ የሚሄድ የአልኮል መታጠቢያዎችን ቅደም ተከተል መከተል አስፈላጊ አይደለም። ናሙናው ትልቅ ከሆነ ግን በ80% ኢታኖል እንጀምራለን፣ በመቀጠልም በ 90%፣ በ 95% እና በመጨረሻም በ100% ኢታኖል (absolute) ይዘፈቃል። በጣም ትናንሽ ናሙናዎች ሲሆኑ፣ በ 90% ኢታኖል መታጠቢያ እና በመቀጠልም በንጹህ ኢታኖል ውስጥ ማዘፈቅ ይቻላል። በዚህ ደረጃ ላይ፣ ንጹህ ኢታኖል በአየር ውስጥ ያለውን እርጥበት ለመሳብ እንደሚሞክር ሁልጊዜ ማስታወስ ይገባል።

በኢንቶሞሎጂ ቤተ-ሙከራዎች ውስጥ የነበረው ልማድ የናሙናዎቹን ውሃ የማስወገድ ሂደት በቢች ክሪኦሶት (beech creosote) መታጠቢያ ማጠናቀቅ ነበር። ዛሬ ግን ይህ ንጥረ ነገር እንደ ፀረ-ተባይ፣ ፀረ-ፈንገስ እና የእንጨት ማቆያነት በስፋት የሚያገለግል ቢሆንም፣ ስሪቱ ፖሊሳይክሊክ አሮማቲክ ሀይድሮ ካርቦን (polycyclic aromatic hydrocarbons) የያዘ በመሆኑ ባለው ጠረን ምክንያት እና የመራቢያ አካላን የሚጎዳ (reprotoxic)፣ ካንሰር አምጪ፣ ቀጣይነት ያለው የኦርጋኒክ ብክለት (Persistent Organic Pollutant) እና በውሃ ውስጥ ባሉ ሕይወት ላላቸው ነገሮች ጎጂ እንደሆነ ስለሚታሰብ በጥብቅ እንዳይሠራበት ይመከራል።

ናሙናዎችን ለማንበር የሚመከረው ዉህድ ዩፓራል (Euparal®) እና የዩፓራል ኢሴንስ (Euparal essence) ነው (በሚቀጥለው አንቀጽ ተብራርቷል)። የዩፓራል እና የዩፓራል ኢሴንስ ድብልቅ በጣም ተቀባይነት ያለው ነው፤ ናሙናዎቹም ከ 90% የአልኮል መታጠቢያ በኋላ የሚገኙ ናቸው።

አባሪ 2፡- ጥቅም ላይ የሚውሉ ሪአጀንቶች ስብጥር (Appendix 2: Composition of the reagents used)

10% ፖታስየም ሃይድሮክሳይድ (Potassium hydroxide 10%)፡-

- ፖታስየም ሃይድሮክሳይድ (Potassium hydroxide) --- 10 ግራም
- የተጣራ ውሃ (Distilled water) --- በቂ መጠን እስከ 100 ሚሊ. ሊትር (mL)

የጋም ክሎራል ማንበሪያ ዉህድ - የሆየር (Hoyer) ዉህድ (Gum chloral mounting medium Hoyer medium)፡-

- የተጣራ ዉሀ ውሃ (Distilled water) --- 50 ሚሊ. ሊትር (mL)
- ክሎራል ሃይድሬት (Chloral hydrate) --- 200 ግራም
- ገም አረቢክ (Gum arabic) --- 50 ግራም
- ግሊሰሮል (Glycerol) --- 20 ሚሊ. ሊትር (mL)

የማርክ-አንድሬ ዉህድ(Marc-André solution)፡-

- ክሎራል ሃይድሬት (Chloral hydrate) --- 40 ግራም
- ግላሻል አሴቲክ አሲድ (Glacial acetic acid) --- 30 ሚሊ. ሊትር (mL)
- የተጣራ ውሃ (Distilled water) --- 30 ሚሊ. ሊትር (mL)

1% አሲድ ፉክሲን በተጣራ ውሃ (Fuchsin acid 1% in distilled water)፡-

- አሲድ ፉክሲን ዱቄት (Acid fuchsin powder) --- 1 ግራም
- የተጣራ ውሃ (Distilled water) --- 99 ሚሊ. ሊትር (mL)

በፉክሲን የቀለመ የማርክ-አንድሬ ዉህድ (Marc-André solution colored with fuchsin)፡-

- የማርክ-አንድሬ ዉህድ (Marc-André solution) --- 10 ሚሊ. ሊትር (mL)
- 1% ፉክሲን (Fuchsin 1%) --- 50 ማይክሮ ሊትር (µL)

አባሪ 3፡- ዩፓራል (Euparal®)፣ ካናዳ ባልሳም፣ ፖሊቪኒል አልኮል ወይም ሌሎች የማንበሪያ ዉህዶች (Appendix 3: Euparal®, Canada balsam, polyvinyl alcohol or other solutions for mounting)

ፖሊቪኒል አልኮል (Polyvinyl alcohol)፡- ትክክለኛ የውሃ ማስወገድ (dehydration) ሂደት ለማከናወን የሚያስፈልጉ ግብዓቶች በማይኖሩበት ጊዜ ይህ ተመራጭ የማንበሪያ ዉህድ ነው። ፖሊቪኒል አልኮል ከአማን ላክቶፌኖል (Amman's lactophenol) ጋር ይደባለቃል። ሆኖም፣ እነዚህ ስብስቦች ውሃው በመትነኑ ምክንያት የመድረቅ ወይም ፖሊቪኒል አልኮሉ ጥርኝ የመፍጠር ባህሪ ያሳያል (crystallizes)። በተጨማሪም ፌኖሉ ኦክሳይድ በሚሆንበት ጊዜ የመጥቆር ከፍተኛ ድክመቶች ይታዩባቸዋል። ይህ ዘዴ ለጥቂት ጊዜ ለሚቆይ (short-term) ማንበሪያ ጥሩ ዘዴ ነዉ።

ካናዳ ባልሳም (Canada Balsam)፡- በስላይድ እና በሽፋን መስተዋት መካከል ናሙናን ለማንበር ጥቅም ላይ ሲውል፣ ናሙናዎች ውሃቸው መወገድ (dehydration) አለበት። የዛይሊን (xylene) ወይም የቶሉዊን (toluene) አጠቃቀም የራሱ የሆኑ አስቸጋሪ ሁኔታዎች አሉት።

ኤነሴ ዉህድ (Enecê medium)፡- እንደ ካናዳ ባልሳም ሁሉ በስላይድ እና በሽፋን መስተዋት መካከል ናሙናን ለማንበር የናሙናውን ውሃ ማስወገድ ይጠይቃል። የኤነሴ ውህድ (Enecê formulation)፡- ንጹህ ነጭ ኮሎፎኒ (22 ግራም)፤ በአልኮል የሚሟሟ ኮፓል ገም (12 ግራም)፣ ንጹህ አልኮል (20 ሚሊ. ሊትር)፣ ካምፎር (10 ግራም)፣ የተርፔንታይን ኢሴንስ (10 ሚሊ. ሊትር)፣ እና ዩካሊፕቶል (26 ሚሊ. ሊትር)። ዝግጅቱ፡- እንደ ኤርለንሜየር ፍላስክ ባለ ዕቃ ውስጥ ንጹህ አልኮል እና ካምፎሩን መጨመር፤ በመቀጠል ኮሎፎኒ እና ኮፓል ገሙን መጨመር። ፍላስኩን በክዳን ዘግተው ካስከረከሩ በኋላ፣ ድብልቁ እንዳይፈላ በዝቅተኛ ሙቀት በቢን-ማሪ (bain-marie - የውሃ ትነት ሙቀት) ማሞቅ። ይዘቱ ሙሉ በሙሉ ወደ ፈሳሽነት ሲቀየር፣ የተርፔንታይን ኢሴንሱ ይጨመራል፣ ከዚያም ድብልቁ ገና ትኩስ እያለ ይጣራል (filtered)፣ በመጨረሻም ዩካሊፕቶሉ በተጣራው ፈሳሽ ላይ ይጨመራል። ዉህዱ ፈሳሽነቱ ሲቀንስ፣ በሚከተለው ቀመር በተዘጋጀ ኤነሴ ማቅጠን፡- ንጹህ አልኮል (30 ሚሊ. ሊትር)፣ ካምፎር (17 ግራም)፣ የተርፔንታይን ኢሴንስ (15 ሚሊ. ሊትር)፣ ዩካሊፕቶል (38 ሚሊ. ሊትር) (Cerqueira, 1943)።

ዩፓራል (Euparal®)፡- ይህ ከአትላስ ጥድ (*Tetraclinis articulata*) የሚገኝ ሙጫ (resin) ሲሆን በ 1906 በጊልሰን (Gilson) ጥናት ተደርጎበት የለማ ነው። ዋናው ጥቅሙ ወደ ፖሊመርነት አለመቀየሩ (does not polymerize) ነው። በስላይድ እና በሽፋን መስተዋት መካከል የተነበሩ ናሙናዎችን በአልኮል ወይም ይበልጥ በተሻለ ሁኔታ በዩፓራል ኢሴንስ (Euparal® essence) አማካኝነት በቀላሉ መልሶ ማውጣት ይቻላል። ሳንዳራክ (sandarac) ተብሎም የሚጠራው ይህ ሙጫ፣ ከ 80% ጀምሮ ያለውን ኢታኖል ይቀበላል።

ትሪቶን X100 (Triton X100) አጠቃቀም፡- አዮኒክ ያልሆነ ዉሀማ ዉህድ (non-ionic aqueous solution)፡-

ትሪቶን X100 አዮኒክ ያልሆነ ዉሃማ ዉህድ (*4-(1,1,3,3-tetramethylbutyl) phenyl-polyethylene glycol solution, or t-octylphenoxypolyethoxyethanol, polyethylene glycol tert-octylphenyl ether*) ሲሆን፣ በሴሉላር እና ሞለኪውላር ባዮሎጂ ውስጥ እንደ ማጽጃ (detergent) በስፋት ያገለግላል። ይህ የማጽጃ ዉህድ በሴል እና በኒውክሊየስ ሽፋን (membranes) አሳላፊ ቀዳዳዎችን (permeabilization) ይፈጥራል።

የነፍሳት ናሙናዎች ለብዙ ዓመታት በአልኮል ውስጥ ማቆየት የተለመደ ተግባር ነዉ። እንደ አለመታደል ሆኖ፣ በአልኮል ውስጥ ማቆየት እንከን አልባ አይደለም፣ ስለሆነም በዚህ ሁኔታ የቆዩ አርትሮፖድ ናሙናዎችን ለማይክሮስኮፕ ምርመራ ማዘጋጀት በጣም አስቸጋሪ ይሆናል። ናሙና የያዙት ፕላስቲኮች ብዙ ጊዜ ይበላሻሉ፣ ይህም አልኮሉ እንዲተን ያደርጋል። በሁለቱም ሁኔታዎች፣ ከአልኮል ጋር ለረጅም ጊዜ መገናኘት ወይም የናሙናዎቹ መድረቅ እውነተኛ ችግር ይፈጥራል። በ 2008፣ ጆንክ (Jonque) የተባለ ባለሙያ እንደየፈዉ የፎቶግራፍ ፊልሞች ለማርጠብ የሚያገለግለዉ አጌፖን (Agepon) የተባለ አርጣቢ ዉህድ (wetting agent) ሸረሪቶችን መልሶ ለማለስለስ (rehydration) እንደሚጠቀም አንድ ማስታወሻ አሳትሞ ነበር [26]። ይህ ጠንካራ ማጽጃ (detergent) ያልሆኑ አርጣቢ ውህዶችን የመጠቀም ሐሳብን አመነጨ።

ከዚህ በታች በ 0.5% የውሃ ዉህድ ውስጥ ትሪቶን X100 (Triton X100) የመጠቀም ሂደት ቀርቧል፦

- የደረቀውን ናሙና በንጹህ አልኮል (absolute alcohol) ማርጠብ።
- ናሙናው ሙሉ በሙሉ እንዲሰምጥ የሚያስፈልገውን ያህል መጠን ያለው 0.5% የትሪቶን X100 ዉህድ መጨመር።
- ለ 5 ደቂቃ ወይም ከዚያ በላይ ይተው። ሁሉም የአርትሮፖዶች ናሙናዎች በዉህዱ ውስጥ ብቻ ለብቻ መሆን አለባቸው።
- የትሪቶን X100 ዉህዱ ይወገድና በፖታስየም ሃይድሮክሳይድ (KOH) ዉህድ ይተካል።
- በመቀጠል ከላይ የተገለጸው አሰራር ይቀጥላል።

አባሪ 4፦ ዩፓራል (Euparal®) ወይም ካናዳ ባልሳም (Canada Balsam) በመጠቀም ደረጃ በደረጃ ናሙናን የማንበር ዘዴ

1. ናሙናዎች ውሃቸው በሚገባ መወገድ (dehydrated) አለበት (የደመናማ ወይም የወተት መልክ መታየት ውሃው በሚገባ አለመወገዱን ያሳያል)።
2. ውሃን የማስወገድ ሂደት የኤቲል አልኮል ምጥነትን ደረጃ በደረጃ በመጨመር ሊከናወን ይችላል።
3. ናሙናዎች ከ 99% አልኮል ወይም ከ100% አልኮል (Alcohol) ወደ ማጥሪያ ዉህድ (clearing agent) ሊዛወሩ ይችላሉ።
4.

የሥራ ቅደም ተከተል (Procedure)፦

1. የአሸዋ ዝንብዎችን በ70% ኤታኖል (Ethanol) ውስጥ ያስቀምጡ(put)።
2. ኢታኖሉን (Ethanol) በማስወገድ በ 10% ፖታሲየም ሀይድሮ ኦክሳይድ (KOH) መተካት። የአሸዋ ትንኞቹን በመስታወት ስላይድ መሸፈን።
3. ነፍሳቱ ብርሃን አሳላፊ (transparent) እስኪሆኑ ድረስ ማለስለስ (Macerate)።
4. ፖታሲየም ሀይድሮ ኦክሳይድን (KOH) ማስወገድ።
5. ናሙናውን በተጣራ ውሃ ሸፍኖ ከ 30 እስከ 45 ደቂቃ ማቆየት።
6. ውሃውን ማስወገድና በድጋሚ በተጣራ ውሃ ለ 30 ደቂቃ መዘፍዘፍ (ጊዜው እንደ ናሙናዎቹ ብዛት ይወሰናል)። ብዙ ናሙናዎች በአንድ ላይ ሲሰሩ ረጅም ጊዜ ያስፈልጋል፤ ጥቂት ሲሆኑ ወይም በተናጠል ሲሰሩ ጊዜው ሊያጥር ይችላል።
7. ውሃውን ማስወገድ።
8. የማርክ-አንድሬ (Marc-André) ዉህድ (ከተቻለ በአሲድ ፉክሲን የቀለመ) መጨመርና ለ 24 ሰዓት (አንድ ቀን) ማቆየት።
9. የማርክ-አንድሬ (Marc-André) ዉህዱን ማስወገድ።
10. ናሙናውን በተጣራ ውሃ ሸፍኖ ከ 30 እስከ 45 ደቂቃ ማቆየት።
11. ውሃውን በማስወገድ በድጋሚ በተጣራ ውሃ ዉስጥ ለ 30 ደቂቃ መዘፍዘፍ።
12. ውሃውን ማስወገድ።
13. 70% ኢታኖል (Ethanol) በመጨመር ናሙናውን መበለት፦
    - ለጭንቅላት እና ለሆድ (abdomen)፦ ጭንቅላቱን ወይም ሆዱን ከደረት (thorax) ላይ ቀስ ብሎ መሳብ።
    - ለደረት (thorax) ክፍል፦ ደረቱን በአንድ የእጅ መቆንጠጫ (forceps) በመያዝ እና የክንፎቹን መሠረት በሌላ የእጅ መቆንጠጫ በመሳብ ክንፎቹን ያስወግዱ። እንደ ፍላጎትዎ እና ይበልጥ ማየት እንደሚፈልጉት የአካል ክፍል ላይ በመመስረት፣ ደረቱን ወደ ግራ እና ቀኝ ወገን በመክፈል የጎንዮሽ መበለት (sagittal dissection) ይቻላል።
14. ደረጃ በደረጃ ምጥነቱን በሚጨምር የኤቲል አልኮል (Ethyl alcohol) ዉህዶች (50% – 80% – 95% እና በመጨረሻም 100% አልኮል[alcohol]) ውሃውን ማስወገድ።
15. ናሙናዎቹን በ 100% ኢታኖል (Ethanol) ሁለት ጊዜ፣ ለእያንዳንዱ 10 ደቂቃ በማጠብ ውሃውን ሙሉ በሙሉ ማስወገድ።
16. ኢታኖሉን (Ethanol) በማስወገድና ናሙናዎቹን በቅርንፉድ ዘይት (clove oil) በመሸፈን ለ 15 ደቂቃ በክፍል ሙቀት ማቆየት።
17. ናሙናዎቹን ከቅርንፉድ ዘይት ዉስጥ በማውጣት በንጹህ ስላይድ ላይ ወዳለ የዩፓራል (Euparal®) ወይም የካናዳ ባልሳም (Canada balsam) ጠብታ ማዛወር።
18. ናሙናን እንደሚከተለዉ ማደራጀት፦ የአሸዋ ትንኝ ጭንቅላት፣ ደረት (thorax) እና ሆድ ዕቃ (abdomen) በደቃቅ መርፌዎች ወይም በመቆንጠጫ (forceps) በመታገዝ በአጉሊ መነጽር (dissecting microscope) ስር ተለይተው ሊበለቱ ይችላሉ። ጭንቅላቱ በሆድ-ጀርባ አቅጣጫ (ventro-dorsal position) እንዲቀመጥ ከአካሉ መለየት አለበት፤ ይህም ማለት የሳይባሪየም (cibarium) ክፍል በቀጥታ እንዲታይ የጭንቅላቱ የኋላ ቀዳዳ (occipital foramen) ወደ ላይ መዞር አለበት። የመበለት ሥራው የሚከናወነው በማንበሪያ ዉህድ (mounting medium) ውስጥ ነው።
19. ናሙናዉ ተጣባቂ (sticky) እስኪሆን ድረስ ማቆየት።
20. ንጹህ የሽፋን መስተዋት (coverslip) በ100% አልኮል ማርጠብ እና የሽፋን መስተዋት በካናዳ ባልሳሙ (Canada balsam) ላይ ጋደል አድርጎ መጫን።
21. በመጨረሻም ስላይዶቹን በደረቅ ሳጥን ውስጥ ማስቀመጥ።
