# Supplementary material for: Processing and mounting phlebotomine sand flies: a consensus guideline
Source: Parasite. 2026 Apr 3;33:18. doi: 10.1051/parasite/2026009 (PMC13047900; doi:10.1051/parasite/2026009)
Supplement: Supplementary file 2 — Arabic translation / الترجمة العربية [file parasite-33-18-s2.pdf]

Parasite **33**, 18 (2026)
© F.J. Randrianambinintsoa et al., published by EDP Sciences, 2026
https://doi.org/10.1051/parasite/2026009

PARASITE
Available online at:
www.parasite-journal.org

REVIEW ARTICLE OPEN ACCESS

# تحضير ومعالجة عينات ذباب الرمل الفليبوتومي: وِفق دليل إرشادي

Fano José Randrianambinintsoa[1], Laure Augendre[1], Jorian Prudhomme[1], Jean-Philippe Martinet[1], Mathieu Loyer[1], Nalia Mekarnia[1], Hocine Kerkoub[1], Farzana Khan Perveen[1], Antoine Huguenin[1,2], Emilie Kariya[1,2], Mohammad Akhoundi[3], Andrey José de Andrade[4], Eduardo Berriatua[5], Gioia Bongiorno[6], Sébastien Boyer[7,8], Vasiliki Christodoulou[9], Magda Clara Vieira Da Costa-Ribeiro[10], Lucas Alexandre Farias de Souza[10], Huicong Ding[11], Blaise Dondji[12], Vít Dvořák[13], Ozge Erisoz Kasap[14], Eunice Aparecida Bianchi Galati[15], Montserrat Gállego[16], Cristina Ballart[16], Stavroula Gouzelou[17], Nabil Haddad[18], Rezki Sabrina Masse[19], Asrat Hailu Mekuria[20], Vladimir Ivovic[21], Szymon Kaczmarek[22], Mohd Khadri Shahar[19], Oscar D. Kirstein[23], Edwin Kniha[24], Iva Kolářová[13], Lincoln Timinao[25], Cristian Lucanas[26], Ognyan Mikov[27], Kimsear Nov[7], Yusuf Özbel[28], Bernard Pesson[29], Laura Cristina Posada Lopez[30], Didot Budi Prasetyo[1,7], Nil Rahola[31], Eduardo A. Rebollar-Tellez[32], Bruno Leite Rodrigues[15], Lalita Roy[33], Prasanta Saini[34], Chizu Sanjoba[35,36], Paloma Helena Fernandes Shimabukuro[36], Padet Siriyasatien[37], Agnieszka Soszyńska[22], Tatiana Suleşco[38], Massamba Sylla[39], Majhalia Torno[40], Petr Volf[13], Khamsing Vongphayloth[41], Vu Sinh Nam[42], April Wardhana[43], Eric Yessinou[44], Sonia Zapata[45], Jean-Charles Gantier[1], and Jérôme Depaquit[1,2,*]

[1] Faculté de Pharmacie, Université de Reims Champagne Ardenne, UR ESCAPE-USC ANSES PETARD, 51 rue Cognacq-Jay, 51096 Reims Cedex, France
[2] Pôle de Biologie territoriale, Laboratoire de Parasitologie-Mycologie, Centre Hospitalo-Universitaire, 51092 Reims, France
[3] Parasitology-Mycology Department, Avicenne Hospital, AP-HP, Bobigny, Sorbonne Paris Nord University, France; Unité des Virus Émergents (UVE: Aix-Marseille Univ, Università di Corsica, IRD 190, Inserm 1207, IRBA), 13005 Marseille, France
[4] Parasitology Collection of Basic Pathology, Department of Basic Pathology, Federal University of Paraná, Curitiba 19031, Brazil
[5] Department of Animal Health, University of Murcia, Campus de Espinardo, 30100 Espinardo, Murcia, Spain
[6] Department of Infectious Diseases, Vector-borne Diseases Unit, Istituto Superiore di Sanità, 00166 Rome, Italy
[7] Medical and Veterinary Entomology Unit, Institut Pasteur du Cambodge, Phnom Penh 12201, Cambodia
[8] Ecology & Emergence of Arthropod-borne Pathogens Unit, Department of Global Health, Institut Pasteur, CNRS UMR2000, 75015 Paris, France
[9] Section Veterinary Services (1417), Laboratory for Animal Health Virology, Aglantzia, Nicosia 2109, Cyprus
[10] Insects Vectors and Parasites Laboratory, Department of Basic Pathology and Postgraduate program in Microbiology, Parasitology and Pathology, Federal University of Paraná, 81530-900 Curitiba, Brazil
[11] Department of Biological Sciences, National University of Singapore, 117558, Singapore
[12] Laboratory of the Leishmaniasis Research Project, Mokolo District Hospital, Mokolo, Cameroon; Laboratory of Cellular Immunology and Parasitology, Department of Biological Sciences, Central Washington University, 98926 Ellensburg, WA, USA
[13] Department of Parasitology, Faculty of Science, Charles University, 12800 Prague, Czechia
[14] VERG Laboratories, Department of Biology, Faculty of Science, Hacettepe University, Beytepe, Ankara 06800, Türkiye
[15] Faculdade de Saúde Pública da Universidade de São Paulo (FSP/USP), Pós-graduação em Saúde Pública, 01246-904 São Paulo, Brazil
[16] Secció de Parasitologia, Departament de Biologia, Sanitat i Medi Ambient, Facultat de Farmàcia i Ciències de l'Alimentació, Universitat de Barcelona, & Institut de Salut Global de Barcelona (ISGlobal), Centro de Investigación Biomédica en Red, Enfermedades Infecciosas (CIBERINFEC), 08028 Barcelona, Spain
[17] Laboratory of Infectious Diseases and Public Health, School of Medicine, University of Cyprus, Nicosia, Cyprus & Department of Pediatrics, Archbishop Makarios III Hospital, Nicosia 2115, Cyprus
[18] Faculty of Health Sciences, American University of Beirut, 1107 2020 Beirut, Lebanon
[19] Medical Entomology Unit, Infectious Disease Research Centre, Institute for Medical Research (IMR), National Institutes of Health (NIH), Ministry of Health Malaysia, 40170 Shah Alam, Selangor, Malaysia
[20] School of Medicine, Addis Ababa University, 28017 - 1000 Addis Ababa, Ethiopia
[21] Faculty of Mathematics, Natural Sciences and Information Technologies, University of Primorska, 6000 Koper, Slovenia

Edited by Jean-Lou Justine
*Corresponding author: jerome.depaquit@univ-reims.fr

This is an Open Access article distributed under the terms of the Creative Commons Attribution License (https://creativecommons.org/licenses/by/4.0), which permits unrestricted use, distribution, and reproduction in any medium, provided the original work is properly cited.

[22] University of Lodz, Faculty of Biology and Environmental Protection, Department of Invertebrate Zoology and Hydrobiology, Banacha 12/16, 90-237 Łódź, Poland
[23] Laboratory of Entomology, Ministry of Health, 9134302 Jerusalem, Israel
[24] Center for Pathophysiology, Infectiology and Immunology, Institute of Specific Prophylaxis and Tropical Medicine, Medical University Vienna, Kinderspitalgasse 15, 1090 Vienna, Austria
[25] Papua New Guinea Institute of Medical Research (PNGIMR) Institute, PO Box 60, Headquarter, Homate Street, 441 Goroka, Eastern Highlands Province, Papua New Guinea
[26] Museum of Natural History, University of the Philippines Los Baños, 4031 Laguna, Philippines
[27] National Centre of Infectious and Parasitic Diseases, 1504 Sofia, Bulgaria
[28] Ege University, Faculty of Medicine, Department of Parasitology, 35040 Bornova/Izmir, Türkiye
[29] Retired, Faculté de Pharmacie, Université de Strasbourg, Strasbourg, 67400 Illkirch-Graffenstaden, France
[30] Program for the Study and Control of Tropical Diseases (PECET), Faculty of Medicine, University of Antioquia, 050010 Medellin, Colombia
[31] MIVEGEC, Univ. Montpellier, CNRS, IRD, 34394 Montpellier, France & Medical Entomology Unit, Institut Pasteur de Madagascar, 101 Antananarivo, Madagascar
[32] Laboratorio de Entomología Médica, Departamento de Zoología de Invertebrados, Facultad de Ciencias Biológicas, Universidad Autónoma de Nuevo León, San Nicolás de los Garza, 66455, NL, México
[33] Tropical and Infectious Disease Centre, BP Koirala Institute of Health Sciences, Dharan 56700, Nepal
[34] ICMR-Vector Control Research Centre, Puducherry 605006, India
[35] Graduate School of Agricultural and Life Sciences, The University of Tokyo, Tokyo 113-8657, Japan
[36] Grupo de estudos em Leishmanioses/Coleção de Flebotomíneos (COLFLEB/Fiocruz-MG), Instituto René Rachou, Fundação Oswaldo Cruz, Belo Horizonte, Minas Gerais, 30190009, Brazil
[37] Center of Excellence in Vector Biology and Vector-Borne Disease, Department of Parasitology, Faculty of Medicine, Chulalongkorn University, Bangkok 10330, Thailand
[38] Department of Arbovirology, Bernhard Nocht Institute for Tropical Medicine, Bernhard Nocht Str. 74, 20359 Hamburg, Germany [39] Laboratory Vectors & Parasites, Department of Livestock Sciences and Techniques, Sine Saloum University El Hadji Ibrahima Niasse (SSUEIN) Kaffrine Campus, C.P. 24600, Senegal.
[40] Environmental Health Institute, National Environment Agency, Singapore 138667, Singapore & Department of Biological Sciences, National University of Singapore, 117558 Singapore
[41] Institut Pasteur du Laos, Laboratory of Vector-Borne Diseases, Samsenhai Road, Ban Kao-Gnot, Sisattanak District, 3560 Vientiane, Lao PDR
[42] National Institute of Hygiene and Epidemiology, 1 Yec-Xanh Street, Hai Ba Trung District, 100000 Hanoi, Vietnam
[43] Indonesian Research Center for Veterinary Science, Indonesian Agency for Agricultural Research and Development, Ministry of Agriculture Republic Indonesia, Bogor 16114, Indonesia & Department of Parasitology, Faculty of Veterinary Medicine, Airlangga University, Surabaya 60115, Indonesia
[44] Laboratory of Research in Applied Biology, Polytechnic School of Abomey-Calavi, University of Abomey-Calavi, 01 P.O. Box 2009, 00000 Cotonou, Benin
[45] Instituto de Microbiología, Colegio de Ciencias Biológicas y Ambientales (COCIBA), Universidad San Francisco de Quito (USFQ), 170901 Quito, Ecuador

Received 1 December 2025, Accepted 29 January 2026, Published online 3 April 2026

**الملخّص –** يقدّم هذا المقال دليلاً شاملاً لمعالجة عينات ذباب الرمل الفليبوتومي وتثبيتها على الشرائح الزجاجية، وهي خطوة أساسية للتعرّف الدقيق على الأنواع، وللكشف عن العوامل الممرِضة وعزلها. ويستعرض مجموعة من التقنيات للعمل الميداني كما للمختبر. ويضمّ الدليل إرشادات مفصّلة لجمع الذباب الرملي والتعامل معه وتغطيته وإنهاء حياته بطريقة ملائمة، مع تفضيل التجميد الجاف أو استخدام ثاني أكسيد الكربون ($CO_2$) على المواد الكيميائية، إضافةً إلى طرق الحفظ مثل التخزين البارد أو الحفظ في الإيثانول .يصف المقال وسائل تحضير بعض البنى التشريحية,وخاصة الأعضاء التناسلية والرأس والأجنحة, هي العامل الحاسم لضمان ملاحظتها مجهرّياً بصورة صحيحة. كما يعرض خطوات معالجة العينات خطوةً بخطوة، بما في ذلك مرحلة التجفيف باستخدام هيدروكسيد البوتاسيوم، ثم استعمال محلول مارك–أندريه. وفيما يخص التثبيت، تقارن المقالة بين أوساط تركيب مختلفة، مع التركيز على خصائصها البصرية وقدرتها على حفظ العينة. ويوصى بسائل هوير (المعروف أيضاً بصمغ الكلورال) عندما تكون الحاجة إلى ملاحظة سريعة -ولا سيما لبنى مثل الحويصلات المنوية (spermathecae)- بفضل شفافيته العالية، لكنه لا يلائم الحفظ طويل الأمد. ومن الأوساط الأخرى المذكورة: كحول بولي فينيل، وEuparal® الذي يتحمّل الماء بقدر محدود، وبلسم كندا القابل للذوبان في المذيبات الهيدروكربونية، حيث يوفّر كلّ من Euparal® وبلسم كندا إمكانات أفضل للحفظ طويل المدى .كما تتناول المقالة تقنيات حديثة في البيولوجيا الجزيئية مثل تسلسل الحمض النووي (DNA) وتقنية MALDI-Tof، وهي مقاربات تتطلّب عناية خاصة في طريقة تجهيز العينات. وإلى جانب ذلك، أُرفقت مقاطع فيديو قصيرة تشرح طرائق تثبيت متعددة، مع ترجمات إلى عدة لغات، بما يضمن وصول هذا الدليل إلى احتياجات المجتمع العلمي العالمي وتنوّع ممارساته

**الكلمات المفتاحية :** التثبيت على شرائح زجاجية، ذباب الرمل الفليبوتومي، سائل هوير، محلول مارك–أندريه، صمغ الكلورال، كحول بولي فينيل، Euparal®، بلسم كندا، عزل الليشمانيا، ظروف ميدانية، التشريح، البيولوجيا الجزيئية، MALDI-Tof، عينات نمطية (Type-specimens).

**Abstract – Processing and mounting phlebotomine sand flies: a consensus guideline.** This article provides a comprehensive guide for the processing and mounting of phlebotomine sand fly specimens, which is crucial for species identification and pathogen detection and isolation. It discusses a range of techniques suitable for both field and laboratory settings. The guide includes detailed instructions on sand fly collection, handling, covering, and euthanasia (recommending dry freezing or $CO_2$ over chemicals) as well as conservation strategies, such as cold storage and preservation in ethanol. The quality of preparation of certain anatomical structures (genital organs, head and wings) is essential for their proper microscopic observation and is described in this work. The article also presents detailed sample processing, including the clearing process with agents such as potassium hydroxide then Marc-André solution. The mounting process compares different media, emphasizing their optical properties and preservation potential. Hoyer fluid (also known as chloral gum) is recommended for quick observation, particularly for spermathecae, due to its clarity, although it is not suitable for long-term storage. Other media discussed include polyvinyl alcohol, Euparal® (for limited water tolerance), and Canada balsam (a hydrocarbon-soluble medium), with the latter two offering long-term preservation capabilities. Innovative molecular biology approaches such as DNA sequencing and MALDI-ToF, which require particular attention to sample processing, are also addressed. Furthermore, short video clips illustrating various mounting techniques as well as translations in many different languages are provided, allowing the guideline to reach the diverse needs and expectations of the global scientific community.

**Key words:** Mounting, Phlebotomine sand fly, Hoyer fluid, Marc-André solution, Chloral gum, Polyvinyl alcohol, Euparal®, Canada balsam, *Leishmania* isolation, Field conditions, Culture, Dissection, Molecular biology, MALDI-ToF, Type-specimens.

## المقدمة

تُعدّ ذبابات الرمل الفليبوتومية حشراتٍ صغيرةً من رتبة ثنائيات الأجنحة، تنتمي إلى فصيلة *Psychodidae* وتحت-فصيلة *Phlebotominae*، ويُعرف منها ما لا يقلّ عن 1063 نوعًا [21]. تُعدّ هذه الحشرات نواقلَ ومسببات لأمراض مختلفة، من بينها الليشمانيا، البارتونيلا، وبعض الفيروسات (الأربوفيروسات) وهي المسؤولة عن أمراض مثل داء الليشمانيات، داء البارتونيلا وبعض الالتهابات الفيروسية المنقولة بالمفصليات. وتعتمد عملية التعرف على هذا الذباب أساسًا عن طريق فحصٍ مجهريٍّ دقيق، يتطلّب جمع العينات بعناية، وحفظها بطريقة مناسبة، وتحضيرها على شرائح زجاجية وفق تقنياتٍ خاصة، لكلٍّ منها مزاياها وحدودها.

يعتمد تحديد هوية ذباب الرمل البالغ على ملاحظة كلٍّ من البنى الخارجية (مثل قرون الاستشعار، ا اللوامس ، والأعضاء التناسلية الذكرية) والبنى الداخلية (مثل البلعوم، والماضغ، والحويصلات المنوية). يساهم تشريح هذه البنى الداخلية وعزلها في تسهيل ملاحظتها، وبالتالي في تحديدٍ دقيق لمختلف الأنواع. ولهذا، وعلى خلاف البعوض أو حشرة التقبيل (Triatomine)، يتطلّب ذباب الرمل الفليبوتومي تثبيته بين شريحة زجاجية مجهرية وتغطيتها قبل إجراء المُعاينة من أجل التعرف عليها.

حتى ثمانينيات القرن الماضي، كانت الملاحظة المجهرية الوسيلة الوحيدة المتاحة للتعرّف على ذباب الرمل، ولا تزال حتى اليوم الطريقة الأكثر شيوعًا. وكان اختيار طريقة التحضير آنذاك بسيطًا نسبيًا، ويقوم أساسًا على خيارين متقابلين: تثبيت نهائي يضمن الحفظ طويل المدى للعينة، أو تثبيت سريع في وسط لا يضمن ذلك. فالتثبيت النهائي، مثلًا باستخدام مواد صمغية كبلسم كندا، عمليةٌ تستغرق وقتًا طويلًا وتتطلّب تجفيفًا كاملًا للعينات، كما أنّ معامل الانكسار لهذا الوسط ليس دائمًا مثاليًا لتسهيل رؤية الحويصلات المنوية. في المقابل، يتيح التثبيت في وسطٍ مائي (مثل محلول هوير= مثبت للتحضيرات المجهرية، يتكوّن من الصمغ + الكلورال المميَّه + الغليسرين، والماء) سرعةً أكبر في التحضير، ويوفّر رؤيةً أوضح للبنى ذات الانكسار العالي كالحويصلات المنوية، إلا أنّه لا يضمن الحفظ طويل المدى، نظرًا لامتصاصه الرطوبة من الجو. ويمكن الحدّ من ذلك بإغلاق الشريحة بطبقة من طلاء الأظافر بعد جفافها التام. ولا يزال هذا التوازن بين السرعة والحفظ قائمًا حتى اليوم، مؤثّرًا في اختيار طريقة التثبيت بحسب الغاية من التحضير.

ومنذ ثمانينيات القرن الماضي، بدأت دراسات تحديد هوية ذباب الرمل تجمع بين المقاربات الشكلية والبيوكيميائية. وكانت أولى هذه المقاربات تحليل الهيدروكربونات الكيوتِكلية cuticular hydrocarbon analyses (أي الخاصة بالقشرة الخارجية للحشرة)، قبل أن تُستبدل سريعًا بتقنيات البيولوجيا الجزيئية، مثل التضخيم العشوائي للحمض النووي متعدد الأشكال (RAPD)، وتعدد أشكال أطوال القطع التقييد (RFLP)، وتضخيم الحمض النووي وتسلسله بطريقة سانغر، إضافةً إلى تقنيات الجيل الجديد من التسلسل الجيني (NGS). واليوم، تُستكمل هذه المقاربات الجزيئية بطرائق بروتيومية ، مثل مالدي - توف MALDI-Tof . كما يمكن الجمع بين تحديد الأنواع جزيئيًا والكشف عن العوامل الممرِضة بواسطة تفاعل البوليميراز المتسلسل عن طريق تضخيم الحمض النووي (PCR) مثل الليشمانيا، والتريبانوسوما، والبارتونيلا، وفيروسات الفليبوفيروس سواء باستخدام التضخيم التقليدي أو الآني، وهو ما يستلزم تكييف طرق الجمع والحفظ وفق الأهداف المحددة [3، 32]. وإلى جانب الخصائص الشكلية التقليدية المستخدمة في التمييز بين الأنواع، يمكن اعتماد مقاربات شكلية أخرى، مثل القياس الشكلي الهندسي للأجنحة .واستنادًا في الغالب إلى خبرات المؤلفين الذاتية وإلى الدراسات العلمية المنشورة، تهدف هذه الدراسة إلى تقديم إرشاداتٍ معيارية لتثبيت ومعالجة ذباب الرمل الفليبوتومي البالغ، بما يتيح تحسين التحليلات الشكلية والجزيئية على حدٍّ سواء .إنّ الحاجة إلى إجراء بعض التحاليل (كالدراسات الجزيئية أو البروتينية = / PCR MALD-Tof) تفرض الاحتفاظ بجزءٍ من الحشرة لا يُعدّ ضروريًا للتعرّف الشكلي، الأمر الذي يبرز أهمية الاختيار الدقيق للبروتوكول المناسب .وفي هذا المقال، نركّز على طرائق التخدير والقتل لذباب الرمل المصطاد حيًّا، وطرائق حفظه، وآليات تثبيته سواءً لأغراض التعرف السريع أو للحفظ طويل الأمد الذي يسمح بإجراء دراسات لاحقة.

## تمهيد: يُراعى الامتثال لمتطلبات السلامة والتنظيم وفق نشرات السلامة ذات الصلة ( Safety Data Sheets)

يؤكد هذا الدليل على ضرورة التعامل مع المواد الكيميائية وفق شروط سلامة صارمة، والالتزام بتعليمات الصحة والسلامة الخاصة باستخدامها والتخلص من نفاياتها. تتولى لجان السلامة مسؤولية تقديم المعلومات اللازمة، بينما يقع على المستخدمين واجب الالتزام بممارسات المختبر الآمنة والقوانين المعمول بها. كما يشير إلى أن بعض المواد تخضع لتنظيم قانوني في بعض الدول (مثل هيدرات الكلورال). للتوضيح، أن قائمة الاختصارات المستعملة في هذا المقال مذكورة في الجدول 1.

**الجدول 1 .** قائمة الاختصارات

| | |
|---|---|
| BME | وسط إيغل الأساسي |
| CDC | مراكز مكافحة الأمراض والوقاية منها |
| CMCP | كافور-أحادي كلوروفينول |
| CMR | مادة مسرطنة، مطفِّرة، وسامّة للتكاثر |
| COI | جين الوحدة الفرعية I لإنزيم سيتوكروم c أوكسيداز |
| CytB | جين سيتوكرومb |
| DNA | الحمض النووي الريبوزي منقوص الأكسجين |
| ELISA | اختبار المقايسة المناعية المرتبطة بالإنزيم |
| EtOH | إيثانول |
| M199 | الوسط 199 |
| MALDI-ToF MS | مطيافية الكتلة بالتأين/إزالة الامتزاز بالليزر بمساعدة المصفوفة مع محلل زمن الطيران |
| MEM | الوسط الأساسي الأدنى |
| NGS | التسلسل من الجيل التالي |
| NNN | وسط نوفي–ماكنيل–نيكول |
| PCR | تفاعل البوليميراز المتسلسل |
| Lao PDR | جمهورية لاو الديمقراطية الشعبية |
| PNOC | الجين الذي يشفر بريبرونوسيبسين (prepronociceptin) |
| qPCR | تفاعل البوليميراز المتسلسل الكمي (الزمن الحقيقي) |
| RAPD | تضخيم عشوائي للحمض النووي متعدد الأشكال |
| RFLP | تعدد أشكال أطوال قطع التقييد |
| RI | معامل الانكسار |
| RNA | الحمض النووي الريبوزي |
| RNases | إنزيمات الريبونوكلياز |
| RNASS | محلول تثبيت الحمض النووي الريبوزي |
| RT-PCR | تفاعل البوليميراز المتسلسل بالنسخ العكسي |
| TFA | حمض ثلاثي فلورو الأسيتيك |

## 1 جمع أو إصطياد ذباب الرمل

يمكن جمع ذباب الرمل البالغ حيًّا أو ميتًا باستخدام طرق مختلفة، مثل مصائد الضوء المصغّرة من نوع CDC، والمصائد اللاصقة، والشفّاطات المستخدمة مع مصائد Shannon، أو جمعها مباشرةً من أماكن استراحتها في البيئة (مثل حظائر الحيوانات). وتعتمد هذه الطرائق على وضع المصائد في المواطن المناسبة، وجذب ذباب الرمل بواسطة الضوء أو غيره من الجاذبات (مثل ثاني أكسيد الكربون أو الجاذبات الكيميائية)، ثم جمع العينات لإخضاعها للتحليل اللاحق، كما ورد في العديد من الدراسات [2، 3، 32، 36، 49].

يتيح جمع ذباب الرمل حيًّا إجراء جميع التحاليل المخبرية لاحقا، في حين أنّ جمع الأفراد الموتى يمنع عزل سلالات الليشمانيا أو الفيروسات. كما أنّ بعض تقنيات الإصطياد، مثل الأوراق اللاصقة، تؤدي في كثير من الأحيان إلى فقدان بعض الأعضاء (قرون الاستشعار، الأجنحة أو الأرجل). إضافةً إلى ذلك، يلتصق زيت الخروع المستخدم في طلاء الأوراق اللاصقة بجسم الحشرات، ويجب إزالته في بداية المعالجة، وذلك عادةً بوضع العينات في حمّام متكون من مزيج متساوٍ من الإيثانول والإيثر ثنائي الإيثيل لمدة 15 دقيقة.

## 2 إنهاء حياة العينات المصطادة

بعد الجمع، يتعين قتل ذباب الرمل الحي. إن بعض طرق الجمع (مثل الأوراق اللاصقة أو مصائد الضوء من نوع CDC المزوّدة بوعاء يحتوي على منظّف/مطهر أو إيثانول)، تكون الحشرات ميتةً بالفعل عند اصطيادها. ويمكن تطبيق تقنيات البيولوجيا الجزيئية على العينات التي جُمعت مباشرةً في الإيثانول، أو على غيرها شريطة حفظها في الإيثانول بأسرع وقت ممكن. غير أنّ أيًّا من هذه الطرائق لا يسمح بمعالجة الحشرات باستخدام تقنية MALDI-Tof. كما أنّ بعض وسائل القتل قد تؤدي إلى فقدان بعض الصفات الشكلية الخرجية المهمة. لذلك، من الضروري استخدام عامل قتل معياري مناسب يضمن تعريفًا دقيقًا أو حفظًا طويل الأمد للعينات المرجعية (أي العينات المحفوظة للرجوع إليها مستقبلًا أو للمقارنة).

يمكن استخدام مواد كيميائية مثل أسيتات الإيثيل، أو إيثر الإيثيل، أو رباعي كلورو الإيثان، أو الكلوروفورم، وذلك بتشريب قطعة قطن بها ووضعها في وعاء يحتوي على ذباب الرمل. ويجب التعامل مع هذه المواد بحذر شديد ووفق توصيات الشركة المصنّعة نظرًا لسميتها. ومع ذلك، لا نوصي باستخدام الكلوروفورم لقتل ذباب الرمل، لأنه — بحسب خبرتنا — غير متوافق جيدًا مع دراسات البيولوجيا الجزيئية. ونظرًا لخطورة هذه المواد عمومًا وللشكوك حول ملائمتها للتحليلات الجزيئية، يُنصح عادةً بتجنّب استخدامها.

وتُعدّ الطريقة الأكثر استخدامًا أيضا، التجميد الجاف للعينات وهي طريقة تحافظ على البنية الشكلية والحمض النووي أو البروتينات، ولكن يجب تجميد العينات مدةً كافية لإحداث التخدير الكامل، و أي إطالة قد تؤدي إلى: (1) جفاف الحشرات، أو (2) التأثير سلبًا في حيوية طفيلية الليشمانيا إذا كان الهدف عزلها مخبريًا من القناة الهضمية لذباب الرمل. لذلك نوصي بفترة تجميد تتراوح بين 15 و20 دقيقة عند درجة حرارة 20-°م، مع المراقبة المنتظمة للتأكد من أن الحشرات مُخدَّرة فقط دون قتل طفيليات الليشمانيا.

وفي حال عدم توفر مجمِّد، يمكن قتل الحشرات باستخدام ثاني أكسيد الكربون ($CO_2$). وفي الظروف الميدانية التي لا تتوفر فيها أسطوانات $CO_2$، يمكن استعمال عبوات $CO_2$ التجارية الصغيرة المستخدمة في أجهزة تحضير المشروبات الغازية، مع الانتباه إلى القيود المحتملة على نقلها جوًّا. وكحلٍّ أخير، يمكن قتل الحشرات بتعريضها لدخان التبغ. في هذه الحالة، يُجمع ذباب الرمل حيًّا في مصيدة CDC، ثم يُلتقط بالشفّاطة ويُحتفظ به في أنبوب زجاجي، ويُعرَّض لدخان التبغ الذي يقتله خلال ثوانٍ. وتُعدّ هذه الطريقة قابلة للتطبيق في جميع الظروف الميدانية، حتى في البيئات شديدة العزلة. غير أنّ الأنبوب الزجاجي يتشبع بالدخان، ولا يمكن استخدامه لاحقًا لجمع أو التعامل مع ذباب الرمل الحي دون تنظيفٍ دقيق. ومع ذلك، يمكن استخدام الشفّاطة نفسها غير

المنظّفة لقتل ذباب الرمل القادم من مصائد أخرى بغرض تثبيته. كما يجب التأكد من إزالة جميع العينات من الشفّاطة. وتُعدّ هذه الطرائق متوافقة مع عزل الليشمانيا عن طريق تشريح القناة الهضمية.

## 3 حفظ العينات قبل المعالجة

توجد خمس طرق رئيسية لتثبيت العينات قبل المعالجة:

### 1.3 التجميد

يُفضّل تنفيذ هذه الطريقة على درجة 20-°م، أو الأفضل على 80-°م. وتُعدّ هذه الطرائق اليوم أكثر شيوعًا من التخزين في النيتروجين السائل. وفي جميع الحالات، يجب تطبيق الحفظ بالتجميد في أسرع وقت ممكن بعد تخدير العينات. ويتميّز التخزين في المجمّد بالحفاظ الكامل على الحشرات نفسها، وكذلك على الحمض النووي (RNA & DNA) والبروتينات بكامل سلامتها طوال فترة التخزين. في المقابل، قد يؤدي التخزين في النيتروجين السائل إلى تلف شديد في الأجنحة والأرجل والملامس وقرون الاستشعار، وقد يسبب بترها أو فقدان بعض الصفات الشكلية الأساسية. التجميد الجاف أقلّ إحداثًا للضرر، لكنه ليس مثاليًا لحفظ الأعضاء الهشة. ومن المهم التنبيه إلى أنّه عند إذابة التجميد، قد تلتصق الأجنحة أو قرون الاستشعار أو الأرجل بجدران الأنابيب، وقد تتمزق بسبب التكاثف. كما أنّ التجميد ليس دائمًا ممكنًا في الدراسات الميدانية لاحتياجه إلى مجمّد أو حاوية نيتروجين سائل.

يُعدّ التخزين بالتجميد متوافقًا تمامًا مع الكشف الجزيئي عن العوامل الممرِضة دون فقدان الحساسية، غير أنّ الكشف عن فيروسات ذات الحمض النووي الريبي RNA وعزلها يتطلّب تخزينًا عند 80-°م أو في النيتروجين السائل إذا كان التخزين الطويل الأمد مطلوبًا. ومع ذلك، فإنّ تجميد العينات لا يسمح بعزل الليشمانيا عبر تشريح القناة الهضمية، إلا إذا غُمِرت ذبابات الرمل أولًا في الطور البخاري ثم في النيتروجين السائل ، لمحاكاة الحفظ بالتجميد العميق لليشمانيا.

### 2.3 الحفظ في الكحول (الإيثانول أو الكحول الأيزوبروبيلي)

تُعدّ هذه الطريقة على الأرجح الأكثر استخدامًا في حفظ ذباب الرمل، نظرًا لسهولتها وإمكانية تطبيقها في الميدان حتى في ظروف صعبة دون مختبر. وهي مناسبة بشكل خاص للدراسات الشكلية للبنية الخارجية، إذ تبقى الأعضاء الهشة (الأجنحة، الأرجل، قرون الاستشعار، أو الملامس) سليمة ما دامت أنابيب الحفظ خالية من الفقاعات الهوائية. لذلك نوصي بسدّ الأنبوب بقطعة صغيرة من القطن لإزالة أي فقاعات هواء، ووضع بطاقة تعريف فوق سدادة القطن (الشكل 1).

ولا يزال التركيز المناسب للكحول محلّ نقاش؛ فعمومًا لا يُنصح باستخدام تراكيز أقل من 70% [45، 66]. وتحافظ التراكيز الأعلى على الحمض النووي بصورة أفضل ولمدة أطول، لكنها تجعل العينات أكثر هشاشة وتكسرًا في الدراسات الشكلية للبنية الخارجية. ويضمن استخدام إيثانول بتركيز 96% (الخليط الأزيوتروبي) ثبات التركيز مع مرور الوقت، خصوصًا في المناطق الرطبة كالبلدان الاستوائية، رغم أنّ إيثانول 95% غالبًا ما يكون أسهل توفّرًا. وبغضّ النظر عن التركيز، يُحفظ الحمض النووي عادةً بصورة جيدة في الإيثانول، وإن كان أقل كفاءة مقارنةً بالتجميد، خاصةً في تطبيقات الجيل الجديد من التسلسل. أما البروتينات فهي أقل استقرارًا بكثير، لا سيما في الدراسات البروتيومية مثل MALDI-Tof ، ويمكن التعرف شكليًا على ذباب الرمل المحفوظ في الكحول لعدة أشهر، لكن لا يمكن الحصول على تسلسلات بروتينية مرجعية من هذه العينات.ويمكن تحسين الحفظ في الكحول أو حتى الحفظ الجاف إذا أُضيف التخزين بالتجميد عند 20-°م. ويُسهم التجميد عند 20-°م أساسًا في تحسين حفظ الجزيئات (كالحمضين النوويين) عبر إبطاء التحلل، كما يوفّر فائدة ثانوية للحفظ الشكلي بتقليل تدهور الأنسجة مع مرور الوقت، وإن كان تأثيره

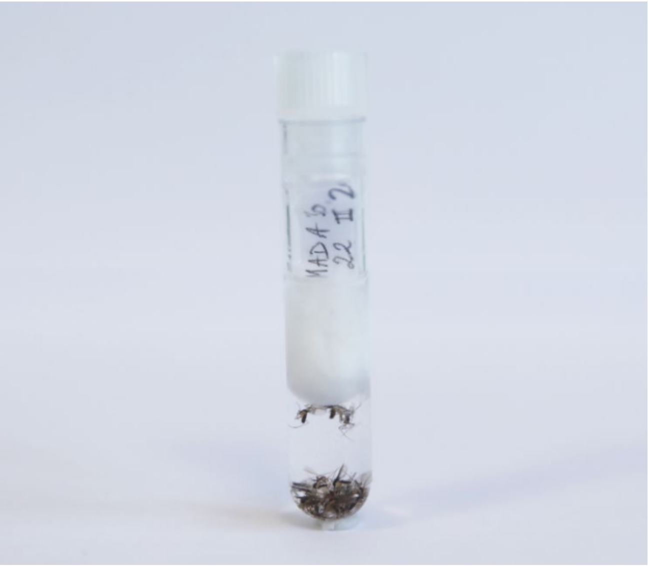


**الشكل 1.** ذباب الرمل محفوظ في الإيثانول.

على الحفظ الشكلي أقل من تأثيره على الجزيئي. كما يمكن استخدام الحفظ في الإيثانول للكشف عن الفيروسات ذات الحمض النووي الريبي و الريبي منقوص الأكسجين إذا استُخدم بتركيز لا يقل عن 70% ولفترة قصيرة لا تتجاوز بضعة أشهر. وقد يتوفر الكحول الأيزوبروبيلي بسهولة في بعض البلدان، وهو يحافظ على الحمض النووي لكنه يجعل العينات أكثر صلابة، كما أنّه غير قابل للاشتعال مثل الإيثانول، مما يسهل نقله. وإذا لزم الأمر، يمكن نقل العينات المحفوظة في النيتروجين السائل أو المجمدة جافًا لحفظها بالكحول، مما يجمع بين مساوئ الطريقتين.

### 3.3 الحفظ في محلول تثبيت الحمض النووي الريبي (RNASS)

يُستخدم هذا الكاشف المائي على نطاق واسع، وهو غير سام، ومصمَّم لتثبيت وحماية الحمض النووي الريبي في الأنسجة والخلايا الحيّة حديثة العزل. ويعمل على اختراق العينة سريعًا وتعطيل الإنزيمات المحلِّلة للحمض النووي الريبي (RNase)، مما يمنع تحلله دون الحاجة إلى تجميد العينة فوريا. ويُعدّ التخزين في هذا المحلول فعّالًا عمومًا في الحفاظ على البنية النسيجية والخلوية لأغراض التقييم النسيجي اللاحق. ورغم أنّ هدا المحلول مخصص أساسًا لتثبيت الحمض النووي الريبي (RNA) وليس للتثبيت الشكل الخارجي للحشرات، فإنّ التخزين القصير إلى المتوسط الأمد يحافظ عادةً على السلامة البنيوية بصورة جيدة. ويسمح هذا المحلول بتخزين العينات في درجة حرارة الغرفة حتى 7 أيام، أو عند 4°م لعدة أسابيع، أو عند 20-°م/80-°م للحفظ طويل الأمد. وتُعدّ هذه الطريقة ذات قيمة خاصة في العمل الميداني أو في المراكز التي تفتقر إلى بنية تبريد متكاملة. ويتطلّب استخلاص الحمض النووي الريبي (RNA) عادةً إخراج العينات من المحلول ومعالجتها وفق البروتوكولات الضرورية.

### 4.3 الحفظ الجاف في درجة حرارة الغرفة

تُعدّ هذه طريقة قديمة، ولها عيب رئيسي يتمثل في ضعف حفظ الأعضاء الهشة مثل الأجنحة والأرجل وقرون الاستشعار والملامس، خصوصًا عند حفظ العينة كاملة. ومع ذلك، تبقى دراسة البروتينات باستخدام MALDI-Tof ممكنة إذا أُجري التجفيف عند التثبيت باستخدام مادة مجفِّفة من نوع هلام السيليكا (مادة مسامية صلبة وغير سامة، تتكون من ثاني أكسيد السيليكون وتُستخدم بشكل واسع كعامل مجفف فعال لامتصاص الرطوبة وحماية المنتجات من العفن

والتلف). وعلى العكس، تبقى التحليلات الجزيئية المستهدفة للحمض النووي صعبة التنفيذ على هذه العينات، إذ يكون غالبًا مجزأً وبكميات منخفضة، مما يجعل التحليل أكثر تعقيدًا مقارنةً بالعينات الجديدة أو المجمدة. ومع ذلك، يبقى استخدام تقنيات حديثة مثل الدراسات الجينية للعينات القديمة (museomics) على هذا النوع من العينات ممكنا [34]. لكنه لا يُنصح بهذه الطريقة إلا عند غياب أي بديل. ويمكن دمجها مع التخزين البارد بوضع الأنابيب في مجمّد عند 20-°م أو 80-°م. ويتمثل التحدي الأساسي في تحقيق تثبيت مناسب للعينات أو للأجزاء الضرورية للتعريف. ولتحقيق ذلك، يُعدّ الترطيب المسبق أمرًا أساسيًا، ونوصي باستخدام محلول Triton X-100 وتختلف مدة الترطيب من بضع ساعات إلى عدة أيام، مع مراقبة منتظمة ودقيقة. وبعد اكتمال الترطيب، يجب تنظيف العينات في ثلاث حمّامات مائية متتالية.

### 5.3 الحفظ على أوراق الترشيح

تتمثل الميزة الرئيسة لأوراق الترشيح في الاستقرار طويل الأمد للحمض النووي الجينومي DNA داخل خلايا العينات غير المثبّتة والمجففة (سواء كانت أجسامًا كاملة أو خلايا دموية) المحفوظة في درجة حرارة الغرفة. وتأتي أوراق الترشيح بحجم بطاقات صغيرة، ما يسمح بتخزين مئات العينات في درجة حرارة الغرفة ضمن حجم محدود. كما أنّ مصفوفة ورق الترشيح مشبعة بعوامل تُعطِّل العوامل المُعدية، وبذلك لا تُعدّ العينات خطرًا بيولوجيًا. وهذا يتيح تخزينها ونقلها دون الحاجة إلى احتياطات بيولوجية خاصة [68].

## 4 تشريح العيّنات

على عكس العديد من الحشرات الأخرى التي يمكن التعرف عليها اعتمادًا على الصفات الخارجية الظاهرة في العينات الكاملة المثبّتة، فإن ذباب الرمل يتطلّب التشريح ووضعهِ على شرائح زجاجية لدراسة الصفات التشريحية بدقة من أجل التعرف الصحيح على مختلف الأنواع. وبغضّ النظر عن طريقة التحضير أو التجهيز المعتمدة، فإن تقنية التشريح نفسها تُستخدم دائمًا (الأشكال 2 و 3) (https://zenodo.org/records/18198006) .

### استخدام Triton X100: محلول مائي غير أيوني

يجب التنويه إلى أن عملية التركيب تخصّ العينات التي جُمعت حديثًا أو حُفظت بطريقة مناسبة. يحتفظ معظم الجامعين بعينات الحشرات إمّا محفوظة جافة لاستخدام MALDI-Tof أو محفوظة في الكحول لسنوات طويلة. ولسوء الحظ، فإن الحفظ في الكحول ليس مثاليًا على المدى الطويل، حيث تصبح المفصليات المحفوظة بهذه الطريقة صعبة التحضير للفحص المجهري. ومن الحوادث الشائعة تفكك المواد البلاستيكية الحاوية للعينات، متبوعًا بتبخّر الكحول مما يحد من وجهة استعمال هذه العينات. ومن هنا ظهرت فكرة الاعتماد على عوامل ترطيب (مركبات كيميائية غير قوية كتأثير المنظفات). ويُستخدم مركب Triton X-100 على هيئة محلول مائي غير أيوني من نوع *4-(1,1,3,3-تترامثيل بيوتيل) فينيل-بولي إيثيلين غليكول-(4-* = *(1,1,3,3tetramethylbutyl) phenyl-polyethylene glycol),*، وهو شائع الاستعمال في مجال البيولوجيا الخلوية والجزيئية كمنظف، إذ يساهم في زيادة نفاذية الأغشية الخلوية والنووية. فيما يلي بروتوكول استخدام Triton X100 بتركيز 0.5% في محلول مائي :تشبيع العينة الجافة بالكحول المطلق.

- إضافة الحجم اللازم من محلول Triton X100 بتركيز 0.5% بحيث تُغمر العينة بالكامل .
- ترك العملية من نحو 5 دقائق إلى عدة أيام مع المراقبة المنتظمة، ويجب أن تنفصل جميع المفصليات تمامًا داخل المحلول.
- إزالة محلول Triton X100 واستبداله بمحلول هيدروكسيد البوتاسيوم.

### 1.4 الرأس

يمكن إجراء التشريح باستخدام إبر دقيقة أو دبابيس حشرية تحت المجهر التشريحي (الأشكال 2 و3). ومن أكثر الإبر شيوعًا في الاستخدام:" 26G × 1/2(0.45 × 13 مم), "30G × 1/2 (0.3 × 13 مم ),"25G x 5/8 (0.5 × 16 مم) .ولتحضير العيّنة من اجل إجراء تعريف تصنيفي، يجب، كحدٍّ أدنى، فصل الرأس عن الجسم وتثبيته في وضع سفلي لإظهار الماضغ Cibarium والبلعوم Pharynx، في حين يُثبَّت الصدر والبطن جانبيًا بعد التشريح. ويضمن تثبيت الرأس في الوضع السفلي توجيه الثقبة القذالية (occipital foramen) ( فتحة تقع في مؤخرة رأس الحشرة) نحو الأعلى، مما يسمح بالملاحظة المباشرة للماضغ. كما يُسهِم الفصل الكامل للرأس في تسهيل الوصول إلى هذه البنى التشريحية.

### 2.4 الأجنحة والصدر

يجب تثبيت الأجنحة في وضعٍ مسطّح وممتد. ويمكن فصل كل جناح من قاعدته وتثبيته بشكل مستقل، أو تثبيت أحد الجناحين مع الإبقاء على الآخر متصلًا بالصدر. وعند التخطيط لإجراء تحليل القياس الشكلي الهندسي، يُعدّ من الضروري تحديد الجناحين الأيمن والأيسر بدقة ووضع تسميات واضحة قبل عملية التركيب. ينقسم الصدر إلى عدة أجزاء، يحتوي كلٌّ منها على معلومات تصنيفية مهمّة. ويُثبَّت الصدر عادةً في وضع جانبي، مما يسمح بفحص توزيع الشعيرات والالوان. ويمكن الاستعانة بندوبات الشعيرات في مناطق معيّنة من الصدر لتمييز بعض الأنواع التابعة لجنس *Brumptomyia*, كما يُستخدم نمط توزيع الألوان للفصل بين ذباب الرمل النيُوتروبي على مستوى الجنس ، أو سلسلة الأنواع، أو حتى الأنواع ضمن الجنس الواحد[20]. وتجدر الإشارة إلى أن شدّة اللون ليست العامل الحاسم في هذا السياق، بل نمط توزيعه. لذلك، فإن عملية تصفية او تفتيح العينات (Clearing) لن تزيل الصباغ أو نمطه.

### 3.4 الأعضاء التناسلية

يجب إيلاء عناية خاصة عند تشريح الأعضاء التناسلية لدى الذكور والإناث، نظرًا لأهميتها الأساسية في تحديد أنواع الذبابات وتحت الأنواع. وتتميّز الأعضاء التناسلية في كلا الجنسين بكونها مزدوجة.

### 1.3.4 الذكور

تكون الأعضاء التناسلية لدى الذكور خارجية، وتتكوّن من زوج من الملاقط، يضمّ كلٌّ منهما مركّب الغونوكوكسيت–الغونوستايل (الحريقفة-القلم التناسلي) في جزئه الظهري، والفصّ الإيبندريالي في جزئه البطني. ويحمل الغونوستايل (القلم التناسلي) أشواكًا، وأحيانًا شعيرات، يجب عدّها بدقة وتحديد مواضع ارتكازها بوضوح .كما يُعدّ فحص السطح الداخلي للغونوكوكسيت (الحريقفة) أمرًا ضروريًا، والتي قد تحمل خصلةً من أشعارٍ ثابتة (غير معنّقة)، أو أشعارًا محمولة على فصّ (أي حُديبة tubercule) [22]. وقد يكتفي غير المتمرّسين بإجراء تثبيت جانبي بسيط دون فصل الأعضاء التناسلية عن نهاية البطن (https://zenodo.org/records/18311158.)، مما يقلّل من خطر تلفها، رغم ما يترتّب على ذلك من صعوبة في عدّ بعض الشعيرات الداخلية. أمّا الأكثر خبرة، فيمكنهم فتح الأعضاء التناسلية إلى نصفين لفصل مركّبات الغونوكوكسيت–الغونوستايل، الأمر الذي يسهّل ملاحظة الأسطح الداخلية والباراميرات (رائدة جانبية) وأغلفتها بدقّة أكبر (https://zenodo.org/records/18311158).

### 2.3.4 الإناث

الجهاز التناسلي الأنثوي داخلي، ويتكوّن من الحويصلات المنوية (spermathecae). في غياب التشريح، يجب ملاحظتها عبر الأغشية الخارجية (teguments) مع تثبيت البطن في وضعية بطنية وبغضّ النظر عن وسط التثبيت المختار، يمكن عمومًا ملاحظة الحويصلات المنوية بشكل صحيح، خاصة إذا لم تكن ملساء وتمت عملية تفتيحها (clearing) جيدًا. غير أن ملاحظة الحويصلات المنوية الملساء ذات الجدران الرقيقة قد تكون إشكالية في الأوساط الضعيفة الانكسار.

علاوة على ذلك، فإن ملاحظة قاعدة قنوات الحويصلات المنوية (spermathecal ducts),أمرٌ أساسي لتحديد النوع، كما هو الحال في تحت الجنس لاروسيوس *Larroussius* [35, 37, 38] , وهو الناقل الرئيسي لطفيلي الليشمانيا الرضيعة (*Leishmania infantum*) في العالم القديم. وبدون هذه الملاحظة، يبقى تحديد العينة غير ممكن .لتجاوز صعوبات الملاحظة هذه، ينبغي فصل مركّب الشوكة التناسلية–الحويصلات المنوية (Genital Furca- Spermathecae) عن البطن .تكون الحويصلات المنوية عادةً صعبة الملاحظة أثناء التشريح غير أن الشوكة التناسلية (genital furca) سهل تحديد موضعها نسبيًا (https://zenodo.org/records/18311106). ونظرًا لأن قنوات الحويصلات المنوية تفتح في الشوكة التناسلية، فإن عزل هذه الأخيرة يسمح عادةً بعزل الحويصلات المنوية. وإذا قُطعت الحويصلات المنوية عرضًا أثناء العملية، فإنها لا تُفقد، إذ يمكن الاستمرار في ملاحظتها ضمن الغلاف البطني (الشكل 4).

### 4.4 تشريح الأمعاء الوسطى لعزل الليشمانيا

يُعد تشريح القناة الهضمية خطوة أساسية للكشف عن طفيليات الليشمانيا وعزلها من إناث ذباب الرمل. يمكن إجراء العملية في الميدان أو في المختبر بهدف تقييم الكفاءة الناقلة (vector competence).

يُعد تشريح القناة الهضمية خطوة أساسية للكشف عن طفيليات الليشمانيا وعزلها من إناث ذباب الرمل. يمكن إجراء العملية في الميدان أو في المختبر بهدف تقييم الكفاءة الناقلة (vector competence).

يُوصى بتشريح إناث حديثة القتل. تُغسل الإناث بالماء أو بمحلول ملحي يحتوي على منظف خفيف لإزالة الشعر الزائد، مما يساعد على الحفاظ على شروط عقيمة لعزل الطفيليات مع المحافظة على الصفات الشكلية اللازمة للتعريف .لعزل الليشمانيا، تُزال الأمعاء الوسطى (midgut) بعناية وتوضع في قطرة واحدة من محلول ملحي معقم (NaCl 0.9%). بعد ملاحظة الطفيليات المتحركة تحت المجهر الضوئي (تكبير تقريبي ×200)، تُنقل بواسطة حقنة أنسولين أو ماصة دقيقة إلى وسط الزرع (انظر 4.4.3).

يُثبَّت الرأس (head) والأعضاء التناسلية في سائل Marc-Andréلتوضيحها . **مهم :** يجب عدم ملامسة Marc-André لطفيليات الليشمانيا إطلاقًا لأنه يسبب بقتلها.

يمكن إجراء التشريح باستخدام شريحة واحدة أو شريحتين، ولكل طريقة مزايا وحدود (شكل 5؛ https://zenodo.org/records/18311154).

### 1.4.4 طريقة الشريحتين

يتم العمل على شريحتين منفصلتين: شريحة تحتوي على محلول ملحي معقم لاستخراج الأمعاء الوسطى (midgut), وشريحة لتثبيت الرأس (head) والحويصلات المنوية (spermathecae) في محلول Marc-André تهدف لتحديد نوع الفاصدة التي يتم تشريحها. في الظروف الميدانية حيث ما يعمل اكثر من باحث سوية، قد يسبب استخدام شريحتين ، بدلا من واحدة، أخطاء ناتجة عن صعوبة في تتبع العينات والربط بين شريحة الإمعاء الإيجابية و شريحة تحديد النوع التابعة لها (https://zenodo.org/records/18311154).

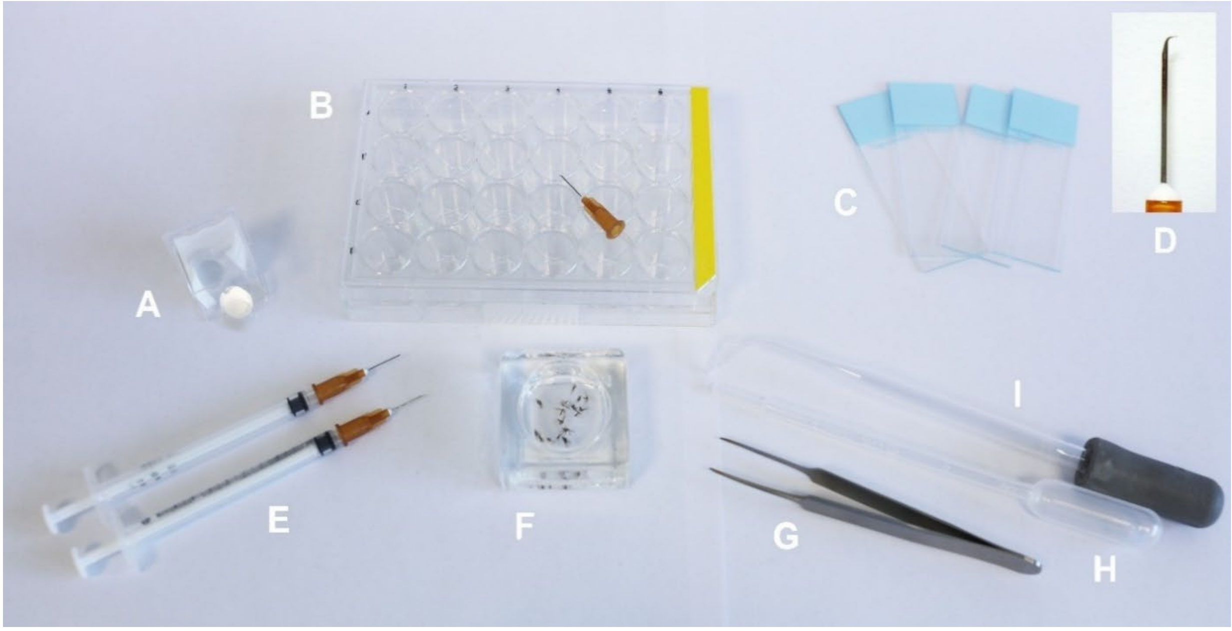


**الشكل 2.** المواد اللازمة لتركيب ذباب الرمل: A: أغطية زجاجية دائرية (قطر 10 أو 12 مم)؛ B: صفيحة ذات 24 بئرًا وإبرة معقوفة (في حال استخدام زيت القرنفل أو خلاصة ®Euparal لا تُستخدم الصفائح الأكريليكية لتجنب تفاعل كيميائي يُتلف العينات), C: شرائح زجاجية قابلة للعنونة؛D: تفاصيل رأس الإبرة المعقوفة؛ E: إبر موصولة بمحاقن؛ F: زجاج ساعة أو وعاء مشابه يحوي ذباب الرمل المراد معاينته؛ G: ملقط Dumont ؛ H: ماصة بلاستيكية؛ I: ماصة زجاجية منحنية بالتسخين لتسهيل شفط السوائل.

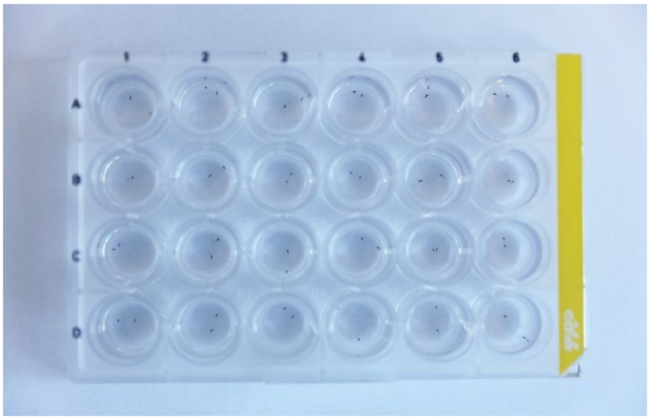


**الشكل 3.** صفيحة ذات 24 بئرًا، يحتوي كل بئر على الرأس وطرف السفلي لجسم لذباب الرمل.

### 2.4.4 طريقة الشريحة الواحدة

يضمن استخدام شريحة واحدة سهولة تتبع النتائج، لكن يجب اتخاذ احتياطات صارمة للتعقيم. يجب تنظيف اليدين بانتظام باستخدام جل كحولي مائي .تُستخدم شرائح غير معتمة الحواف وأغطية زجاجية مربعة معقمة بالحرارة الجافة باستخدام فرن بوبينيل(Poupinel oven) .يجب استخدام إبرة معقمة لكل عملية تشريح (25G Ø 0.5mm × 16mm) .توضع ذبابة الرمل في قطرة محلول ملحي معقم في منتصف الشريحة. يُفصل الرأس (head) مع إجراء شق بين القطعتين البطنيتين السادسة والسابعة 6th and 7th sternites) and sternites)دون قطع القناة الهضمية (digestive tract) يمكن رفع مستوى الشق إذا كانت الحويصلات المنوية (spermathecae) طويلة جدًا . يُثبَّت الصدر (thorax) بإبرة، ثم تُسحب القطع البطنية الخلفية بلطف لاستخراج الأمعاء (gut) . إذا فشلت الطريقة، يمكن تثبيت نهاية البطن (abdomen) وسحب القناة الهضمية (Digestive tube) من جزئها الأمامي .وإذا تعذر ذلك، تُستخرج الأمعاء (gut) بإزالة أكبر قدر ممكن من الغلاف الخارجي (tegument).بعد استخراج الأمعاء(gut) ، تُفصل القطع البطنية الأخيرة بقطع القناة الهضمية. توضع الأمعاء في قطرة جديدة من محلول ملحي معقم وتغطى بغطاء زجاجي معقم. يُنقل الرأس (head) والقطع البطنية الأخيرة إلى قطرة من Marc-André fluidفي الطرف الآخر من الشريحة، مع منع أي تماس مع الليشمانيا.يُوجَّه الرأس بحيث تكون الثقبة القذالية occipital foramen) للأعلى .تُعزل الحويصلات المنوية (spermathecae) مع الشوكة التناسلية (genital furca)وتُغطى بغطاء دائري صغير .يبقى باقي الجسم والأجنحة في قطرة المحلول الملحي في منتصف الشريحة ([https://zenodo.org/records/18311154](https://zenodo.org/records/18311154)).في حال ثبوت الإيجابية أو لأغراض تصنيفية، يمكن حفظ الصدر (thorax) والبطن (abdomen) لدراسات جزيئية أو بروتينية، كما يمكن تثبيت الأجنحة في وسط مائي .لحفظ المستحضر، يمكن استبدال فائض Marc-André fluid بوسط تثبيت مائي مثل (Chloral gum) أو وسط قائم على (polyvinyl alcohol). تم وضع مقاطع فيديو تفصيلية تشرح هذه الإجراءات : تشريح الأمعاء الوسطى لذبابة الرمل (midgut) ([https://zenodo.org/records/18303014](https://zenodo.org/records/18303014))، وتشريح الغدد اللعابية salivary glands لذبابة الرمل, لذلك لن يتم شرحها هنا . ([https://zenodo.org/records/18302850](https://zenodo.org/records/18302850)).

### 3.4.4 عزل وزراعة الليشمانيا من أمعاء ذباب الرمل

يُعدّ عزل الطفيليات من خلال تشريح إناث ذباب الرمل المصابة إجراءً دقيقًا يتطلب مهارة عالية، وينبغي في البداية التدرب عليه باستخدام عينات خالية من الطفيليات. بعد التشريح، تُنقل الأمعاء إلى قطرة جديدة من المحلول الملحي المعقم (0.9 %) أو إلى محلول لوك Locke's solution لغسلها [4]. يمكن بعد ذلك معالجة الأمعاء المُشرَّحة بطريقتين i) :فحصها تحت المجهر الضوئي لملاحظة المراحل المختلفة لطور البروميستيغوت لطفيليات الليشمانيا وتحديد تموضعها، مع إيلاء اهتمام خاص للصمام الفموي-المعدي، و ii) فتح الأمعاء لتسهيل خروج البروميستيغوتات مما يسهّل استزراعها بكميات كبيرة [4]. يُعد العثور على ذباب الرمل مُعدي في الميدان أمرًا نادرًا نسبيًا، ولذلك فإن جلسات التدريب الجيدة تزيد من فرص نجاح عملية العزل.

إذا لوحظت طفيليات الليشمانيا في الأمعاء، فيجب استخدام إبر معقمة جديدة، مع إضافة كمية صغيرة من المحلول الملحي المعقم حول الغطاء الزجاجي بواسطة الخاصية الشعرية لتحريرها. ينبغي تمزيق الأمعاء بعناية وبسرعة لتحرير الطفيليات في المحلول الملحي. باستخدام ماصة دقيقة بسعة 100 ميكرولتر أو محقنة التوبركولين tuberculine syringe، تُجمع الطفيليات وتُلقَّح في وسط زرعي موسوم بشكل صحيح.

الزراعة المخبرية لطور البروميستيغوت لليشمانيا: تُحفَظ الطفيليات المعزولة مبدئيًا على منحدرات آغار الدم SNB-9 أو في الوسط الصلب نوفي–ماك نيل–نيكول[16] (NNN) ، ويُغطّى كل منهما إما بوسط ألفا MEM-معقم [16, 65] أو بوسطM199 ، مع تدعيم كل منهما بنسبة 10% من مصل عجل جنيني معقم ومعطّل حراريًا لتعزيز نمو الطفيليات، و1% فيتامينات BME ، و2% بول بشري (مُعقَّم باستخدام مرشح حقني® Filtropur بقطر مسام 0.2 ميكرومتر)، و250 ميكروغرام/مل أميكاسين ( أو 50 ميكروغرام/مل جنتاميسين، أو مزيج من المضادات الحيوية والأحماض الأمينية : غلوتامين L 200 ملي مولار–بنسلين 10,000 وحدة–ستربتوميسين 10 ملغ/مل) [47] .بعد ثلاثة أيام، وفي حال عدم وجود تلوث، تُعلَّق المزارع في وسط تجميد مُحضَّر بشكل مناسب، ثم تُحفَظ عند -80 درجة مئوية لمدة تتراوح بين سنة إلى سنتين، أو في النيتروجين السائل عند -196 درجة مئوية للحفظ طويل الأمد والاستخدام التجريبي المستقبلي [7].

### 5.4 الغدد اللعابية

يُعدّ تشريح الغدد اللعابية لذباب الرمل تقنية أساسية لدراسة تفاعلات الناقل–الممرض، ولا سيما للكشف عن الفيروسات المنقولة بالمفصليات مثل الفليبوفيروس (Phlebovirus) (على سبيل المثال فيروس توسكانا) [44, 75]. نظرًا لصِغر حجم ذباب الرمل، يتطلب الإجراء دقة عالية تحت المجهر التشريحي، باستخدام ملاقط دقيقة أو إبر تشريح مجهرية لعزل الغدد اللعابية الرقيقة دون تمزقها, أو تلويثها [61,51] ([https://zenodo.org/records/18302850](https://zenodo.org/records/18302850)). يُعد الحفاظ على سلامة الغدد أمرًا بالغ الأهمية لضمان موثوقية التحاليل الجزيئية اللاحقة. بعد الاستخراج، يمكن تجانس الغدد وفحصها باستخدام تفاعل البوليميراز المتسلسل بالنسخ العكسي (RT-PCR) أو تفاعل البوليميراز المتسلسل الكمي (qPCR) أو الاختبارات المناعية للكشف عن الحمض النووي الريبي الفيروسي (Viral RNA) أو المستضدات (Antigens) [12]. إن وجود الفيروسات في الغدد اللعابية، وليس فقط في الأمعاء أو الجوف الدموي (الهيموسيل)، يؤكد أن العامل الممرض قد أتمّ فترة الحضانة الخارجية وأصبح قابلاً للانتقال أثناء التغذية الدموية[ 71].

تُعد عملية التشريح الغدد اللعابية لذباب الرمل متطلبة تقنيًا بسبب الحجم الصغير جدًا للغدد اللعابية، مما يستلزم خبرة كبيرة لتجنب تضرر العينة [1, 51]. إضافةً إلى ذلك، قد تكون الحمولات الفيروسية منخفضة، مما يستدعي استخدام طرائق كشف عالية الحساسية مثل تفاعل البوليميراز المتسلسل المتداخل (nested PCR)أو تقنيات التسلسل عالي الإنتاجية [54]. كما أن مخاطر التلوث تؤكد ضرورة الالتزام بتقنيات التعقيم الصارمة. إلى جانب التحديات التقنية، تؤثر العوامل البيولوجية في نجاح الكشف؛ إذ تختلف كفاءة النقل بين أنواع ذباب الرمل، كما تتباين معدلات العدوى تبعًا للظروف البيئية والموسمية [33, 61].

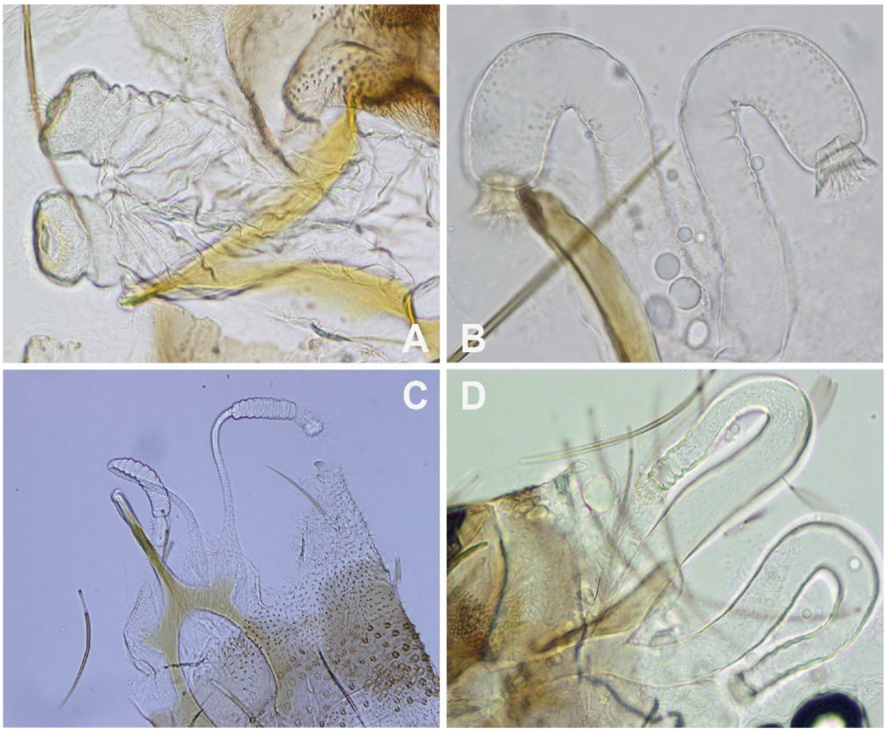


**الشكل 4.** حافظات منوية مُشرَّحة وموضوعة في سائل مارك أندريه من عينات طازجة. A: *Idiophlebotomus longiforceps* (جمهورية لاو الديمقراطية الشعبية)؛ B : *Sergentomyia minuta* (فرنسا)؛ C : *Phlebotomus ariasi* (فرنسا)؛ D : *Sergentomyia anodontis* (جمهورية لاو الديمقراطية الشعبية).

يوفّر الكشف عن الفيروسات في الغدد اللعابية معطيات حاسمة حول مخاطر الانتقال، مما يتيح توجيه برامج الترصد واتخاذ تدابير المكافحة المناسبة [15]. فعلى سبيل المثال، أسهم التعرف على فيروس توسكانا في ذباب الرمل ضمن المناطق المتوطنة في تطوير بروتوكولات التشخيص وإصدار الإرشادات الصحية العامة [18]. علاوةً على ذلك، قد تكشف دراسة التفاعلات بين الفيروس واللعاب عن أهداف جديدة لتطوير لقاحات أو علاجات تعيق الانتقال [15, 18].كما يمكن استخدام الغدد اللعابية لذباب الرمل كمصدر للمستضدات (antigens) لقياس الأجسام المضادة (antibodies) لدى العائل ضد لعاب ذباب الرمل باستخدام الطرائق المناعية، ويفضّل اختبار المقايسة المناعية المرتبطة بالإنزيم (ELISA) . تُمكّن هذه المقاربة من تقييم تعرّض العائل لدغات ذباب الرمل، مما يدعم تقدير فعالية استراتيجيات مكافحة النواقل [25] وخطر انتقال الليشمانيا [40].

## 6.4 تحديد مصدر الوجبة الدموية

يجب تشريح الإناث المحتقنة بالدم (engorged females) المعزولة من العينات الملتقطة وذلك باستخدام أدوات أحادية الاستعمال (single-use equipment)لمنع التلوث المتبادل(cross-contamination). ينبغي فحص البطن تحت مجهر تشريحي(stereomicroscope) لتقييم مرحلة هضم وجبة الدم (blood meal digestion). يُوصى باختيار الإناث فقط اللواتي يظهر بطنهن باللون الأحمر أو البني المحمر أو الأحمر الداكن، دون وجود أي علامات على تكوّن البيوض (egg formation). يتم استئصال طرف البطن بما في ذلك الحويصلات المنوية (spermathecae) من أجل التعرف المورفولوجي على الأنثى بعد عملية الإزالة والتصفية (clearing). بعد ذلك، يوضع الجزء الرئيسي من البطن من دون (spermathecae) في أنابيب Eppendorf® ويتم حفظه عند درجة حرارة - 20° م إلى حين إجراء التحاليل اللاحقة .تُعد الواسمات الجينية (genetic markers) الشائعة الاستخدام لتحديد مصدر وجبة الدم ، مثل PNOC [5, 30, 50]، و CytB[67]، و COI[13]، راسخة ومشروحة بشكل موسع في العديد من المنشورات العلمية؛ لذلك لن يتم التطرق إلى تفاصيلها في هذا الدليل (Figure 6). كبديل لذلك، يمكن استخدام تقنية MALDI- Tof (peptide mapping) لتحديد مصدر الدم العائل (host blood)[31]. وقد أُثبت تجريبياً أن هذه التقنية تمكّن من تحديد مصدر الدم ضمن إطار زمني أطول بعد امتصاص وجبة الدم ، مما يجعلها خياراً مناسباً خاصةً لتحليل الإناث المحتقنة بالدم اللواتي يُظهرن مراحل أكثر تقدماً من هضم دم العائل .يفضل حفظ العينات عند -20°م أو 4°م ، إلا أنه يمكن الحصول على نتائج جيدة أيضاً من عينات حُفظت في درجة حرارة الغرفة لفترة قصيرة. يجب فصل بطن الأنثى المحتقنة بالدم عن بقية الجسم قبل التحليل مباشرةً، ثم تجانسه (homogenization)في ماء مقطر. يبقى باقي جسم ذبابة الرمل متاحاً لإجراء تحاليل جزيئية (molecular analyses) ومورفولوجية إضافية. بعد أخذ جزء من المستخلص المتجانس (homogenate) لاستخدامه في MALDI-Tof peptide mapping، يمكن استخدام الجزء المتبقي لاستخلاص الحمض النووي (DNA isolation) لتأكيد تحديد مصدر الدم العائل و/أو للتحري عن وجود *Leishmania sp* . يُعد وقت تحضير العينات وإجراء التحليل قصيراً جداً مقارنةً بالتقنيات الجزيئية المعتمدة على الحمض النووي(DNA based).

## 5 معالجة العينات للدراسات المورفولوجية (الأشكال 3، 6، 7 و8؛ الملاحق 1، 2، 3 و4)

يستعرض هذا القسم المبادئ الأساسية لتحضير عينة من ذبابة الرمل من أجل التثبيت حصريًا للدراسات المورفولوجية، يتبع ذلك تكييف المنهجية لتطبيقات تتجاوز علم المورفولوجيا. ومع ذلك، فإن فهم هذه المنهجية يُعد أمرًا بالغ الأهمية، إذ يتيح تعديل الإجراءات لتتناسب أنواع العينات المختلفة عند الحاجة.

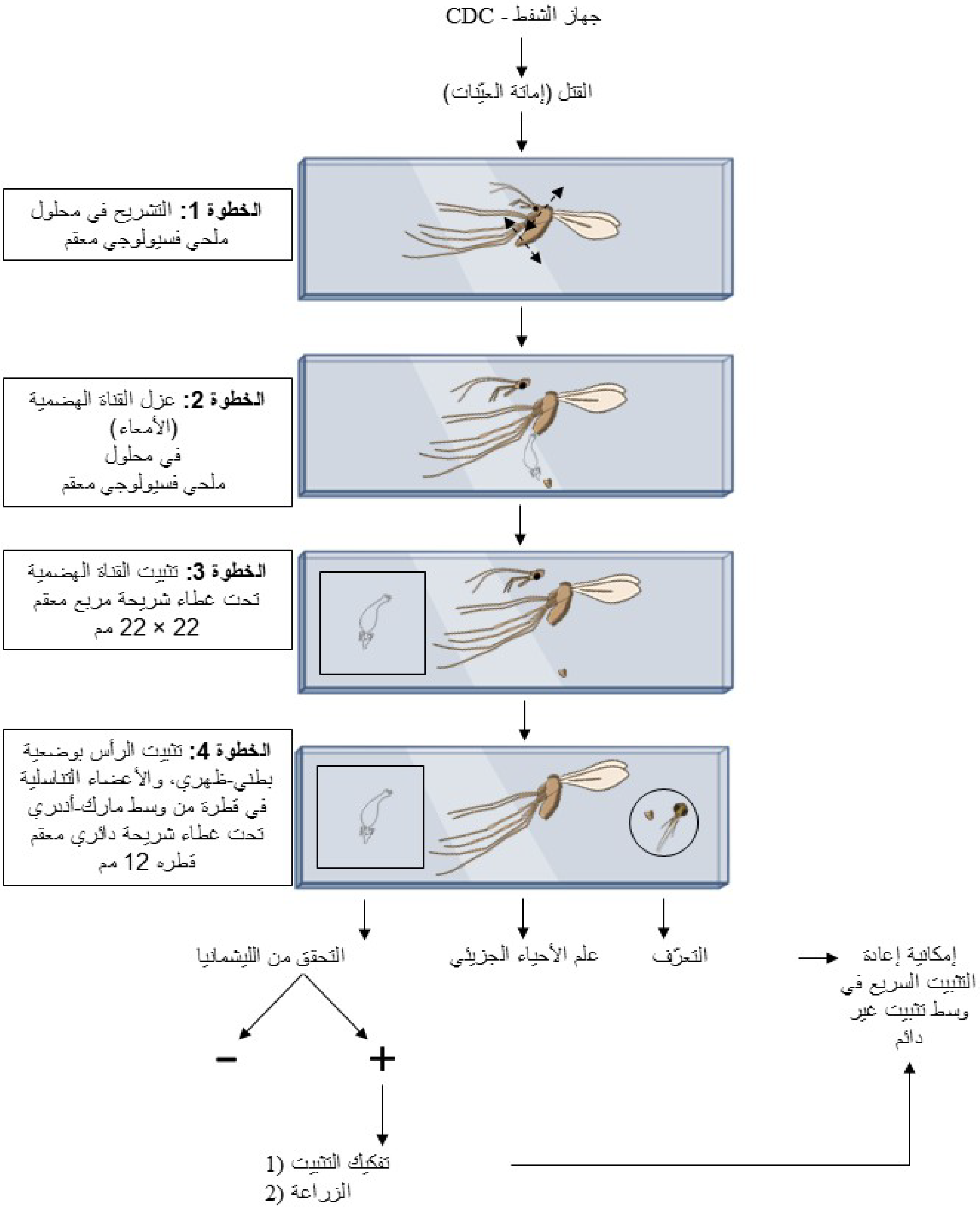


**الشكل 5.** منهجية عزل الليشمانيا.

تشمل المعالجة خطوات متعاقبة من الإفراغ والملء باستخدام ماصّات باستور المزودة بكرات مطاطية مرنة. ويوصى بشدة باستخدام أوعية زجاجية ذات قاع مستدير، نظرًا لأنها تسهّل هذه العمليات بشكل كبير. يتميز الزجاج بكونه خاملاً كيميائيًا تجاه جميع الكواشف.

ولمنع تبخر الكواشف، يجب تزويد الأوعية بأغطية وعدم ملئها بشكل مفرط، لأن ذلك قد يؤدي إلى انسكاب عند الإغلاق أو الفتح، وكذلك لمنع سقوط الغبار على العينات. المواد الكيميائية اللازمة لعمليات الإزالة والتصفية والمعالجة مبينة في الجدول 2.

### 1.5 التوضيح

قبل تحضير عينات ذباب الرمل للتثبيت الدائم على الشرائح الزجاجية، يجب إخضاعها أولًا لعملية التوضيح بواسطة الهضم الكيميائي، باستخدام طريقة مناسبة وعامل توضيح ملائم، مثل محلول حمض الأسيتيك بتركيز 10% أو محلول مارك أندريه المحتوي على كلورال هيدرات، وهو مركّب كيميائي مقيَّد الاستخدام في العديد من الدول، وذلك بهدف جعل العينات شفافة. تسهم عملية التوضيح في إزالة الأنسجة ، والدهون، والإفرازات، والمواد الشمعية، مما يجعل

العينة شبه شفافة، ويُسهّل فحص البنى الخارجية الهيكلية، مثل مواضع ارتكاز الشعيرات، والخصائص السطحية كالملونات، إضافةً إلى البنى الداخلية المرئية عبر الغلاف الخارجي، مثل المستودعات المنوية .تعتمد عملية التوضيح على مرحلتين متتاليتين، تبدأ باستخدام محلول قاعدي قوي، مثل هيدروكسيد البوتاسيوم، تليها مرحلة بحمض ضعيف، كحمض الأسيتيك ضمن محلول مارك أندريه، ولكل مرحلة دور كيميائي-حيوي مختلف. إذ يعمل المحلول القاعدي القوية على تفكيك الأنسجة الرخوة، كالبروتينات والدهون والعضلات، من خلال عمليتي التصبّن وتمسّخ البروتينات، مع الحفاظ على سلامة الهيكل الخارجي الكيتيني لضمان وضوح البنية.

بعد ذلك، يقوم الحمض الضعيف بمعادلة المواد المتبقية، مانعًا حدوث المزيد من التحلّل، كما يساهم في تبييض الكيتين وتحسين درجة الشفافية. ومع ذلك، قد يكون غسل العينات مرتين بالماء المقطّر لمدة 15 دقيقة كافيًا أيضًا لمعادلة القاعدة. ويؤدي هذا العلاج المتتابع إلى إزالة فعّالة للأنسجة مع الحفاظ اللطيف على البنية، مما يضمن سلامة العينة على نحوٍ أمثل للفحص المجهري. ويوصى بإجراء غسلتين متتاليتين لمدة 20 دقيقة في الماء المقطّر قبل الانتقال إلى المرحلة التالية.

#### 1.1.5 تحلّل الأنسجة الرخوة (الشكل 8)

يُعدّ كلٌّ من هيدروكسيد الصوديوم (NaOH) وهيدروكسيد البوتاسيوم (KOH)من العوامل الكيميائية الشائعة الاستخدام في الهضم الكيميائي باستخدام محلول قاعدي، إذ تُطبَّق بتراكيز ومدد زمنية متفاوتة تبعًا لحجم العينات ودرجة هشاشتها. وتعتمد الطريقة الأكثر فعالية على تحلّل الأنسجة الرخوة من خلال نقع ذباب الرمل في محلول قاعدي قوي، مثلKOH أو NaOH بتركيز 10%، طوال الليل. كما يمكن زيادة التركيز لتقليل مدة المعالجة، على سبيل المثال باستخدام KOH بتركيز 20% لمدة 6 ساعات. ويمكن أيضًا تسريع هذه العملية عن طريق التسخين عند درجة حرارة 37°م.

#### 2.1.5 التوضيح مع أو بدون تلوين

تعقب هذه المرحلةَ معالجةُ التفتيح، والتي غالبًا ما تجمع بين حمض الأسيتيك وكلورال هيدرات، كما في محلول مارك أندريه. وبعد الانتهاء من عملية التوضيح، يجب غسل العينات جيدًا في حمّامين مائيين متتاليين، مدة كلٍّ منهما 20 دقيقة، لإزالة بقايا المواد الكيميائية.

يُعدّ محلول مارك أندريه من عوامل التوضيح الشائعة في تحضير عينات ذباب الرمل، إذ يتميّز بفعاليته في تسهيل عملية التوضيح مع الحدّ من إلحاق أضرار كبيرة بالبنى الهشّة، مثل الأجنحة والقرون الاستشعارية.

وينبغي استعمال هذا المحلول حديثَ التحضير أو حفظه في وعاء محكم الإغلاق لمنع التبخّر أو التحلّل. وتبرز فائدته بشكل خاص عند دمجه مع تقنيات التفتيح أو التلوين لتعزيز إبراز تفاصيل مورفولوجية محددة. تُعرض تفاصيل تركيبه وتحضيره في الملحق.(2)

في حالة العينات شديدة الشفافية، قد يكون التلوين ضروريًا لتحسين الرؤية قبل التثبيت. وتتوافر العديد من الأصبغة، يستهدف كلٌّ منها مكوّنات كيميائية محددة في الكائن. ومن المهم اختيار صبغة متوافقة مع طبيعة العينة ومع وسط التركيب المستخدم. كما يمكن تكييف هذه المنهجية الأساسية حسب الحاجة، مثل إضافة الفوشين الحمضي بتركيز 0.1% إلى محلول مارك أندريه.

وتتطلّب العينات المحفوظة في محاليل مائية والمعدّة للتركيب في أوساط ذات أساس راتنجي (Resinous) (مادة لزجة طبيعية أو اصطناعية تُستعمل لتثبيت العينات وحفظها مجهريًا بعد تصلّبها) إجراء عملية تجفيف (انظر القسم 5.2)، نظرًا لعدم توافق معظم أوساط التركيب الراتنجية الطبيعية أو الاصطناعية مع الماء. وقد أشار New (1974) إلى أن بعض الأصباغ قد تفكك في أوساط تركيب معيّنة [53].

فعلى سبيل المثال، يمكن تثبيت الفوشين الحمضي المستخدم مع بلسم كندا أيضًا في وسط Euparal®، غير أنّ العينات المصبوغة بالفوشين الحمضي تكون عُرضة للبهتان، ولا سيّما عند بقاء آثار من زيت القرنفل المستخدم كسائل توضيح نهائي. كما قد تُظهر العينات المخزّنة في زيت القرنفل تلاشيًا ملحوظًا خلال بضعة أيام.

### 2.5 التجفيف

يُجرى التجفيف بنقل العينات تدريجيًا عبر سلسلة متدرجة من محاليل الإيثانول: 50%، 70%، 80%، 90% أو 95%، وأخيرًا 100%، على أن تستمر كل مرحلة مدة لا تقل عن 20 دقيقة. وبما أن الإيثانول سريع التبخر، يجب إحكام إغلاق الوعاء أثناء المعالجة. بعد اكتمال التجفيف، يمكن تعليق المعالجة مؤقتًا لعدة أيام في خلاصة Euparal®، وهي مفضلة على زيت القرنفل. أما كريوزوت الزان Beech creosote، الذي كان مستخدمًا على نطاق واسع سابقًا لهذا الغرض، فقد حُظر تمامًا حاليًا بسبب انه سام. يجب أن تضمن عملية التجفيف توافق السائل الموجود داخل العينة مع وسط التركيب، لتجنّب التعكّر أو الانهيار الأسموزي أو التشوه، وهي عوامل قد تجعل العينة غير صالحة للدراسة التصنيفية.

### 3.5 أوساط التثبيت المجهري

#### 1.3.5 اختيار وسط التثبيت المجهري وتطبيقه في تحضير العينات

يُفضَّل أن يكون لوسط التثبيت المجهري معامل انكسار قريب قدر الإمكان من معامل انكسار الزجاج، والذي يبلغ تقريبًا 1.5. كما يجب أن يكون عديم اللون، شفافًا، ويحافظ على شفافيته الكاملة بعد الجفاف وعلى المدى الطويل. وينبغي أن يكون متوافقًا مع الأصباغة المستخدمة، وقادرًا على النفاذ والانتشار داخل جميع أنسجة العينة، وألا يجف بسرعة مفرطة أو يُشكّل حويصلات او فقاعات أثناء التثبيت، وألا ينكمش بعد الجفاف. يُعد اختيار وسط التركيب المناسب عنصرًا أساسيًا في تحضير العينات، إذ لا يوجد وسط واحد مثالي لجميع الأغراض. ويجب أن يوازن الاختيار بين عدة عوامل رئيسية:

- *الخصائص البصرية* :يجب أن يوفّر معامل الانكسار تباينًا وانكسارًا كافيين لإظهار البنى التشريحية المهمّة للتعريف التصنيفي أو الوصف المورفولوجي، مثل المستودعات المنوية، والأعضاء الحسيّة الكيسية، وأعضاء نيوستيد الحسيّة، والأسنان العمودية في الماضغ (Cibarium)، وأسنان البلعوم(Pharynx). وتعتمد رؤية هذه البنى بشكل مباشر على الخصائص البصرية لوسط التثبيت المجهري.

- *الحفظ* : بالنسبة للعينات النمطية أو المواد المخصّصة للمجموعات المرجعية الدائمة، ينبغي أن يوفّر الوسط درجة عالية من الثبات والمتانة على المدى الطويل. أمّا في الدراسات الاستقصائية الوبائية، التي لا تتطلب الحفظ الطويل الأمد، فقد تكفي أوساط تثبيت مؤقتة أو شبه دائمة.

#### 2.3.5 متطلبات أوساط التثبيت المجهري

يطوّر المختصّون تقنيات تثبيت مخصّصة ومعقّدة لتلبية احتياجات بحثية محدّدة. غير أنّ هذه الطرق كثيرًا ما تُهمل جوانب مهمّة، مثل الجودة الأرشيفية، والتوافق، والتوحيد القياسي، وسهولة الاستخدام، وإمكانات الحفظ طويل الأمد، ممّا يُصعّب دمج المجموعات المتبرَّع بها وجهود الصيانة المستمرّة وتفرض التطبيقات العلمية متطلبات مختلفة على أوساط التثبيت المجهري. فغالبًا ما يقوم علماء التصنيف بتثبيت عينات كاملة، ويفضّلون أوساطًا تُحدث هضمًا كيميائيًا طفيفا للأعضاء الداخلية لتعزيز وضوح البنى الكيتينية. كما ينبغي أن يختلف معامل الانكسار بدرجة كافية عن معامل كلٍّ من العيّنة والشريحة الزجاجية لتحقيق أقصى وضوح بصري.

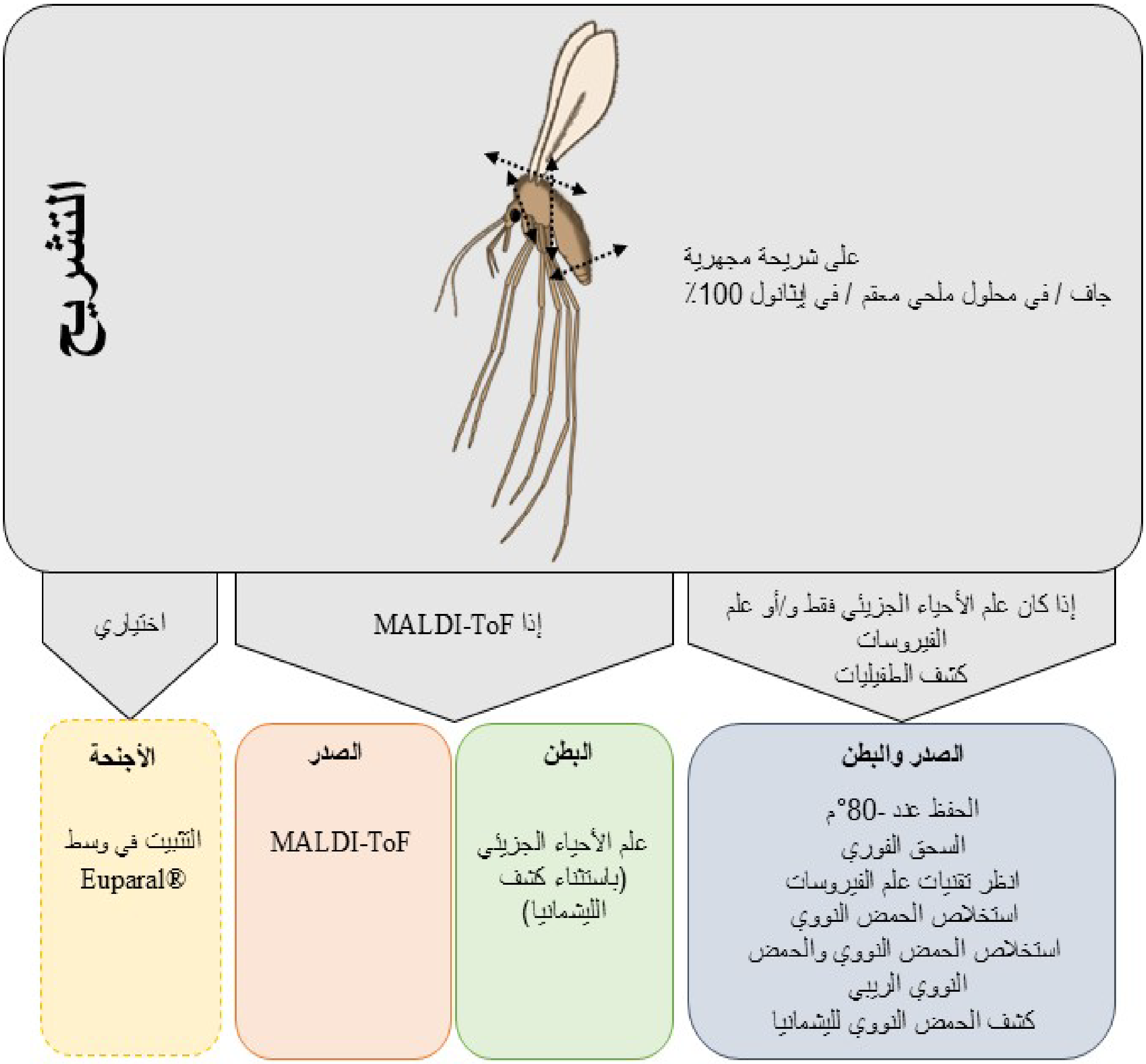


**الشكل 6.** معالجة ذبابة الرمل (Sand fly processing) لتطبيقات البيولوجيا الجزيئية (molecular biology) ، والبروتيومكس (proteomics) ، و/أو علم الفيروسات.

وتُصمَّم أوساط التثبيت التجارية عادةً بمعامل انكسار قريب من معامل الزجاج، وذلك للحدّ من انكسار الضوء وتشتّته عبر نظام الشريحة الزجاجية – وسط التثبيت–وغطائها. غير أنّه في المجهر الضوئي ذي المجال الساطع Brightfield microscopy، يمكن التحكّم في التباين الطبيعي للعينة غير المصبوغة من خلال اختيار وسط تثبيت ذي معامل انكسار يختلف قليلًا عن معامل العيّنة، ممّا يُحسّن تمييزها عن الخلفية.

### 3.3.5 أنواع أوساط التثبيت المجهري (الجداول 3 و4)

يعتمد التصوير المجهري على معامل انكسار وسط التركيب لتحديد كيفية انكسار الضوء عبر الشريحة الزجاجية والوسط والعينة. وعندما يكون معامل الانكسار قريبًا من معامل زجاج غطاء الشريحة (حوالي 1.515)، يمر الضوء بشكل متجانس، مما يقلل من التشتت والتشوهات البصرية، ويؤدي إلى تحسين الدقة ووضوح البنى الدقيقة. وعلى العكس، فإن عدم تطابق معامل الانكسار قد يسبب ضبابية أو هالات أو يحجب تفاصيل العينات غير المصبوغة. يُعد اختيار وسط التركيب المناسب أمرًا بالغ الأهمية لتحسين التباين والوضوح وجودة الصورة العامة للعينة، نظرًا لاختلاف معاملات الانكسار بين الأوساط المختلفة.

يؤثر معامل الانكسار لوسط التركيب بشكل كبير في مدى وضوح البنى الدقيقة عند تحضير ذباب الرمل للتركيب على الشرائح. فالبنى الرقيقة قليلة التصلّب، مثل البنى المتصلّبة للسيباريوم، والمستودعات المنوية، وحلقات قرون الاستشعار، أوردة الأجنحة، قد تكون صعبة الملاحظة في أوساط ذات معامل انكسار مرتفع تشمل الأوساط الشائعة الاستخدام لذباب الرمل أوساط الغراء – الكلورال المائية، وبلسم كندا، وراتنج(NC) Enecê–Nelson Cerqueira كأوساط قائمة على المذيبات. وقد صنّف Rawlins [60] أوساط التركيب إلى نوعين :(1) أوساط دائمة، تتصلّب مع مرور الوقت وتناسب الحفظ طويل الأمد؛ ( 2)أوساط شبه دائمة، لا تتصلّب تمامًا وتُستخدم عادةً لأغراض مؤقتة.

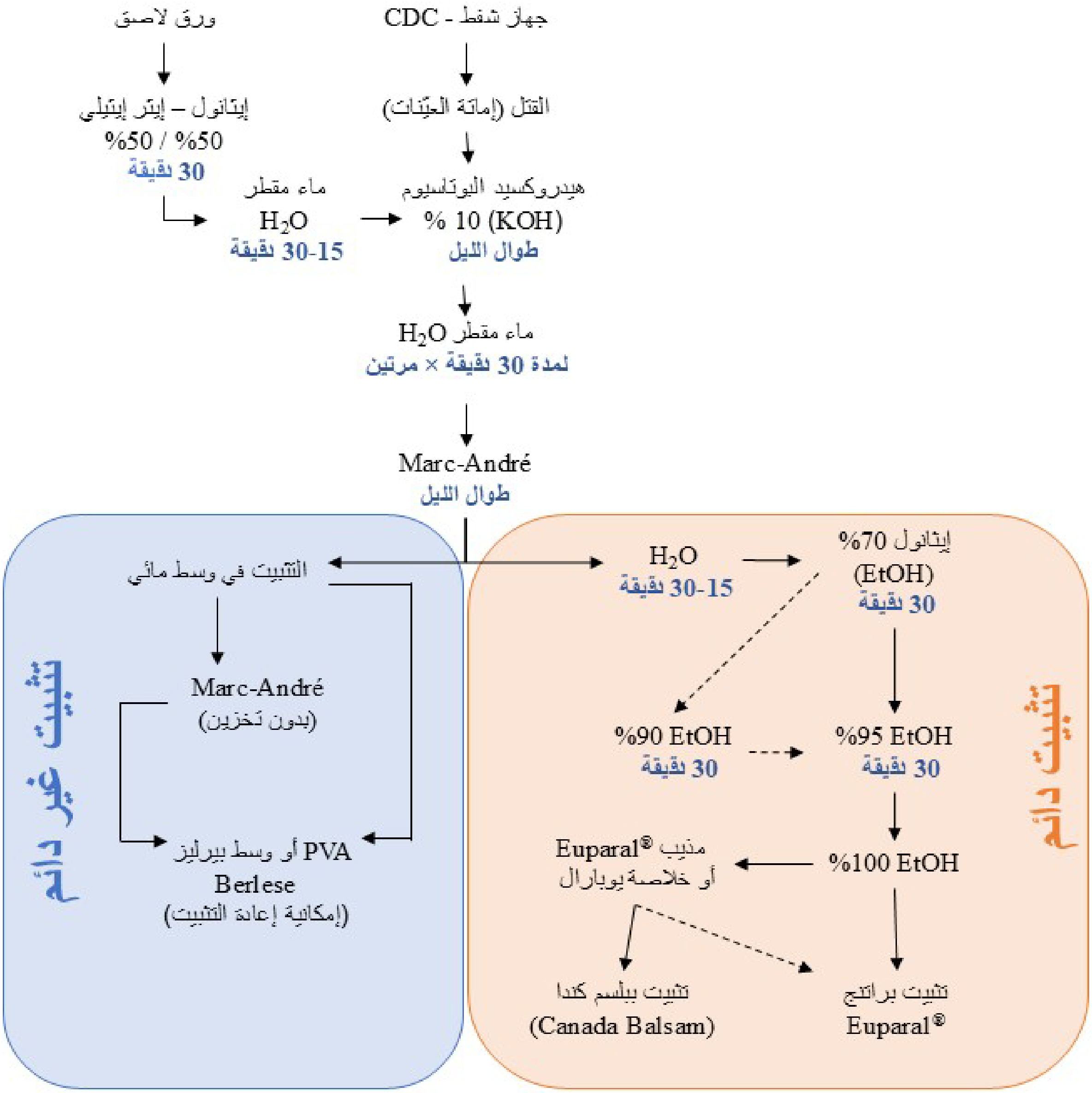


**الشكل 7.** الطريقة التقليدية لمعالجة ذباب الرمل.

يمكن أن تكون أوساط التثبيت (Mounting media) سائلة، أو صمغية، أو راتنجية (resinous)، قابلة للذوبان في الماء، الكحول، أو مذيب آخر مثل التولوين أو الزايلين (انظر الجدول 3).

بعد تطبيقها، يجب عزلها عن تأثيرات الغلاف الجوي باستخدام أوساط حاشية غير قابلة للذوبان وللتمييز بوضوح بين أنواع أوساط التثبيت المختلفة، يمكن اعتماد التصنيف الآتي:

*(أ.) الأوساط المائية:* تذوب هذه الأوساط بسهولة في الماء، مما يجعلها مناسبة للتثبيت المؤقت أو شبه الدائم. وتمتاز عادة بسهولة التعامل معها، غير أنها قد تتطلّب إحكامًا جيدًا لمنع تعرّضها للرطوبة الجوية (مثل أوساط الصمغ – الكلورال وبولي فينيل الكحول)، ولا سيّما في المناطق الاستوائية ذات الرطوبة المرتفعة.

*(ب.) الأوساط محدودة التحمّل للماء:* تكون هذه الأوساط أقل تأثّرًا بالماء، لكنها لا تزال بحاجة إلى حماية من الرطوبة المفرطة. وتوفّر ثباتًا أطول أمدًا مقارنة بالأوساط القابلة للذوبان في الماء، وتُستخدم كثيرًا في التثبيت شبه الدائم.

*(ج.) الأوساط القابلة للذوبان في الهيدروكربونات:* تذوب هذه الأوساط في مذيبات عضوية مثل الزيلين أو التولوين أو المذيبات النفطية. وهي مُصمّمة للتثبيت الدائم، وتتميّز بثبات ممتاز على المدى الطويل، ومقاومتها للرطوبة والتفكك، مما يجعلها مناسبة لأغراض الحفظ الطويل المدى (مثل بلسم كندا المتعادل).

باختصار تُعدّ الأوساط القابلة للذوبان في الماء الأنسب للتثبيت المؤقت أو في الحالات التي تتطلّب سهولة إزالة العيّنة. أمّا الأوساط محدودة التحمّل للماء، فهي مناسبة للتثبيت شبه الدائم الذي يتطلّب درجة متوسطة من المتانة. في حين تُفضّل الأوساط القابلة للذوبان في الهيدروكربونات للتثبيت الدائم المخصّص للحفظ طويل الأمد والأرشفة.

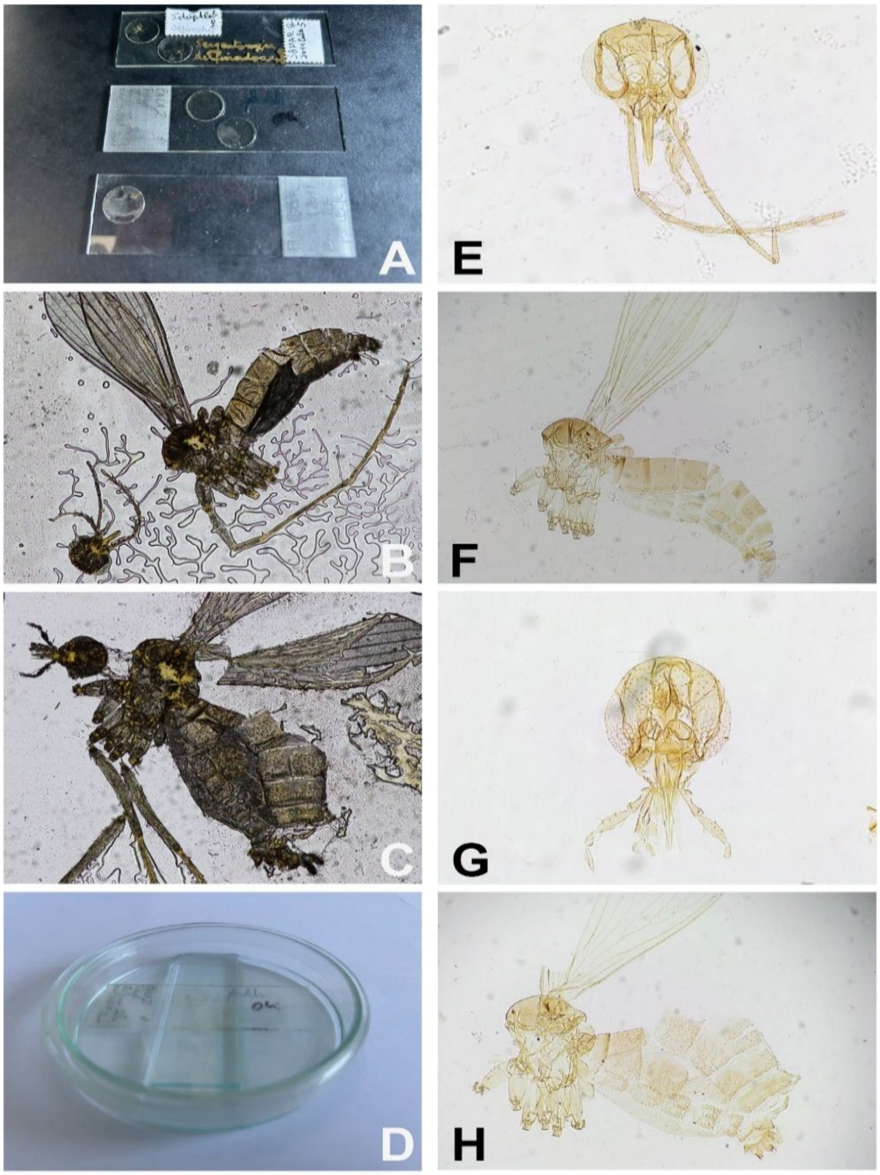


**الشكل 8.** إعادة تثبيت الشرائح (Slide remounting). أ :شرائح تالفة وجافة مُثبتة في وسط هوير(Hoyer), ب :منظر مجهري لذبابة رمل جافة, ج :منظر مجهري لذبابة رمل تالفة أخرى ,د :حجرة رطبة تحتوي على شريحة جافة ,هـ :رأس العينة ب، و :جسم العينة ب بعد إعادة تثبيتها في يوبارال (Euparal®) ,ز :رأس العينة التالفة ج، ح :جسم العينة التالفة ج بعد إعادة تثبيتها في يوبارال (Euparal®).

### 4.3.5. وصف أوساط التثبيت الموصى بها (الجدولين 3 و4)

#### *الأوساط المستخدمة للملاحظة المؤقتة*

*الصمغ الكلورال - وسط/محلول هوير (Hoyer) معامل الانكسار = 1.48).* سائل مارك أندريه (Marc André fluid) هو أفضل وسط للملاحظة قصيرة المدى (لساعات قليلة، وربما أكثر قليلًا إذا تم تخزين الشريحة في حجرة رطبة) للحويصلات المنوية(spermathecae) ، بما في ذلك التصوير الفوتوغرافي أو الرسم (الشكل 4). أما لحفظ الحويصلات المنوية بعد ملاحظتها، فيتطلب ذلك إعادة تثبيتها (remounting) في وسط مائي يسمح بالتخزين متوسط المدى. قد يكون تجفيفها لإعادة تثبيتها في راتنج (Resinous) ممكنًا لكنه غير مستحسن بسبب خطر الفقدان. يُعتبر صمغ الكلورال (Chloral gum) سائل هوير (Hoyer fluid) متكافئين تقريبًا. هذا الوسط يُستخدم عادةً لملاحظة الأعضاء الداخلية نظرًا لتوافقه مع الماء، وبساطة تطبيقه، وسرعة استخدامه، ومؤشر الانكسار (refractive index) الذي يسهل فحص الهياكل الرقيقة مثل الحويصلات المنوية. ومع ذلك، يحتوي صمغ الكلورال على مساوئ كبيرة إذا لم يُحضّر بشكل مثالي أو يُخزن في ظروف رطوبة مضبوطة، وتشمل هذه المشكلات :التبلور، تغير اللون، وفقدان اللزوجة. عزل حافة الغطاء الزجاجي (ringing) لا تحل هذه المشاكل، حيث قد يصبح الوسط مثقلًا بتغير اللون (أحيانًا أسود تقريبًا) نتيجة تفاعله مع وسط الحاشية Ringing medium ، خصوصًا عند استخدام Euparal®.

**الجدول 2.** مكونات الكواشف المستعملة

| | |
|---|---|
| فوشين حمضي 1% في ماء مقطر | هيدروكسيد البوتاسيوم 10% |
| فوشين حمضي (مسحوق) 1 غ<br>ماء مقطر 99 مل | هيدروكسيد البوتاسيوم 10 غ<br>ماء مقطر كمية كافية حتى 100 مل |
| ملوّن بالفوشين الحمضي Marc-André محلول مارك-أندري | وسط تثبيت غراء الكلورال (وسط هويرHoyer medium) |
| محلول مارك-أندري 10 مل<br>فوشين 1% 50 ميكرولتر | ماء مقطر 50 مل<br>لورال هيدرات 200 غ<br>صمغ عربي 50 غ<br>غليسرول 20 مل |
| وسط إينيسي Enecê | Marc-André محلول مارك-أندري |
| راتنج كولوفوني أبيض نقي 22 غ | كلورال هيدرات 40 غ |
| صمغ كوبال قابل للذوبان في الكحول 12 غ | حمض أسيتيك جليدي 30 مل |
| إيثانول مطلق 20 مل<br>كافور 10 غ<br>خلاصة التربنتين 10 مل<br>أوكاليبتول 26 مل | ماء مقطر 30 مل |

تم اعتبار وسط هوير (Hoyer medium) أفضل بصريًا لذباب الرمل الفلبوتوميني، وقد استُخدم تقليديًا لهذا الغرض. يتكون الوسط من عدة صيغ متقاربة تشمل الصمغ العربي، الغليسرول، وكلورال هيدرات. وقد تم سوء تفسير أو اقتباس بعض الصيغ في المنشورات العلمية [74].

على الرغم من أن هوير يعد وسطًا جيدًا لملاحظة الحويصلات المنوية في ذباب الرمل، إلا أنه غير مناسب للحفظ طويل المدى. إنه مثالي للملاحظات قصيرة المدى، بما في ذلك الصور الفوتوغرافية أو الرسوم أو الصور الرقمية. الأوساط المائية مناسبة للتركيبات المؤقتة لكنها لا تضمن الحفظ طويل المدى، بينما يوفر التثبيت بالراتنج (resin mounting) متانة ممتازة غالبًا لما قد يصل إلى قرون، لكنه قد يخفي التفاصيل الدقيقة للحويصلات المنوية لأن انعكاس الضوء فيها غالبًا ما يفقد .يتحلل وسط هوير مع مرور الوقت بسبب الجفاف (dehydration)(الشكل 8)، مما يؤدي إلى تكوّن بلورات صغيرة بيضاء معتمة من كلورال هيدرات. ومع ذلك، يمكن استعادة العينات من الشرائح المتبلورة حيث يظل الهيكل الخارجي (cuticle) سليمًا كيميائيًا، رغم أن بعض الضرر الفيزيائي قد يحدث نتيجة نمو البلورات. في بعض الحالات، يمكن إصلاح الشرائح المتبلورة عن طريق إعادة ترطيب وسط التثبيت في بيئة دافئة ورطبة مع الثيمول (thymol) لمنع نمو الفطريات. بدلاً من ذلك، يمكن نقع العينات خارج صمغ الكلورال في الماء، وتجفيفها في حمض الأسيتيك الجليدي، ثم إعادة تثبيتها في بلسم كندا.

*وسط DMHF ثنائي ميثيل هيدانتوين فورمالدهيد (معامل الانكسار= 1.48).* يُعدّ هذا الوسط المائي فعالًا بصريًا مثل وسط بيرليزي [72] ، وسهل الاستخدام، ولا يتعرض للاسوداد أو التبلور. ويُعد مناسبًا لذباب الرمل وأفراد فصيلة Psychodidae .

*وسط CMCP كافور-أحادي كلوروفينول (معامل الانكسار = 1.41).* وهو وسط تثبيت قائم على الغليسرين، قابل للذوبان في الماء، يُستخدم لإعداد شرائح زجاجية دائمة شفافة للعينات الحساسة. يمكن تثبيت العينات مباشرة من الماء أو الإيثانول. كما يعمل على تليين الكيوتكل cuticule وتوضيح العينة بسرعة، مما يساعد على ضبط الوضعية التشريحية، خاصة عند تشريح الأجنحة أو الأعضاء التناسلية .مع ذلك، يحتوي هذا الوسط على الفينول، وهو مادة سامة ومهيجة تتطلب حذرًا في التعامل.

## الأوساط المستخدمة للتثبيت الدائم

*بلسم كندا (معامل الانكسار = 1.52–1.54)* .تم وصف بلسم كندا (Canada balsam)لأول مرة كوسط تثبيت مناسب للمجهر الضوئي المنقول (transmitted light microscopy)بواسطة أندرو بريتشارد Andrew) (Pritchardفي ثلاثينيات القرن التاسع عشر. ولا يزال من أكثر الأوساط استخدامًا نظرًا لجودته المثبتة في الأرشفة، مع أكثر من 150 عامًا من التطبيق الناجح .على عكس وسائط هوير(Hoyer fluid) ، لا يتبلور بلسم كندا ولا يمتص الرطوبة .ومع ذلك، يتميز بلسم كندا بـ فلورة ذاتية قوية (strong autofluorescence)، وهو ما قد يكون أحيانًا عيبًا لبعض تقنيات المجهرية [60].استخدام مذيبات غير سامة بدل الزايلين (xylene) يمكن أن يقلل من مخاطر السلامة أثناء التحضير، لكنه قد يؤدي أيضًا إلى بعض العيوب مثل تبخر أبطأ وتغميق الوسط في وقت مبكر.

*يوبارال Euparal®* (معامل الانكسار = 1.48) .يُعتبر Euparal® بديلاً واسع الاستخدام لبلسم كندا (Canada balsam) للتثبيت الدائم، حيث يوفر ثباتًا ممتازًا طويل المدى ومؤشر انكسار مقارن مع بلسم كندا .يمتلك Euparal®الخصائص التالية :(1) الحاجة إلى التجفيف (dehydration requirement): قبل النقل النهائي لوسط التثبيت، يجب تجفيف العينة عادةً عن طريق الانتقال من كحول إيثيلي 95٪ إلى كحول إيثيلي مطلق, و (2) : extended processing timeطول زمن المعالجة: التركيب النهائي في راتنج Resinous، سواء كان بلسم كندا أوEuparal® ، يتطلب التجفيف، مما يزيد من الوقت الإجمالي لتحضير العينات .عندما لا يكون التجفيف باستخدام المذيبات العضوية ممكنًا، يمكن وضع العينات المستخرجة من كحول إيثيلي مطلق في محلول وسيط يتكون من خليط متساوي من Euparal® و Euparal essenceقبل التثبيت النهائي.

**الجدول 3.** تركيبة بعض أوساط التثبيت المختارة.

| وسط التثبيت | المذيب | البوليمر أو ما قبل البوليمر المحتمل | ملاحظات |
|---|---|---|---|
| (Hoyer fluid) هوير = صمغ الكلورال, كربوكسي ميثيل سيليلوز فينولCMCP-9 | غليسرول، ماء<br>ماء (CMCP-9 : 51–60٪) | مركبات الصمغ العربي<br>كحول بولي فينيل متحلل كليًا (CMCP-9 : 0–5٪) | عامل مُحلِّل للأنسجة:<br>كلورال هيدرات : لزوجة منخفضة أو مرتفعة |
| ثنائي ميثيل هيدانتوين فورمالدهيد DMHF | ماء | ن،ن′-ثنائي ميثيلول ثنائي ميثيل هيدانتوين؛(di-methylol DMH) قليلّات مرتبطة بجسور إيثر أو ميثيلين؛ شبكة بوليمرية متشابكة من هيدانتوين–فورمالدهيد | |
| (Canada balsam) بلسم كندا | كزيليلين؛ ومكونات متطايرة جزئيًا من البلسم (دلتا-3-كارين، حمض ليفوبيماريك، ليمونين، ميرسين، حمض بالوستريك، بيتا-فيلاندرين، ألفا-بينين، بيتا-بينين) | البلسم (أبيينول، حمض أبييتيك، حمض إيزوبيماريك، حمض سانداراكوبيماريك) | معادلة: كربونات البوتاسيوم؛ راتنج من شجرة الأبيس بلساميا *Abies balsamea* (Linné, 1758) |
| Euparal® يوبارال | يوكاليبتول، بارالدهيد؛ ومكونات متطايرة جزئيًا من صمغ السندراك (ليمونين، ألفا-بينين، بيتا-بينين) | مركبات صمغ السندراك (حمض كومونيك، مانول، حمض بوليكومونيك، حمض سانداراكوبيماريك، 12-أسيتوكسي-سانداراكوبيماريك، سوجيول، حمض تورولوزيك، تورولوزول، توتارول) | عامل إزالة: ساليسيلات الميثيل؛ اللون الأخضر: ملح نحاسي (أبييتينات النحاس)؛ راتنج السندراك من شجرة تيتراكلينيس أرتيكولاتا copper salt (copper abietinate); sandarac resin from *Tetraclinis articulata* (Vahl, 1791) |
| إينيشي Enecê | كحول إيثيلي مع كافور، يوكاليبتول وزيت التربنتين | مركبات صمغ الكوبال والكولوفونيا (الروزين)(rosin) | |

*Enecê إينيسي (معامل الانكسار = 1.467).* يُعد Enecê وسط تثبيت قائم على الراتنج يُستخدم أساسًا للحشرات الصغيرة، ويحظى بشعبية خاصة في البرازيل. يتكون أساسه من الكولوفونيا (colophony) وصمغ الكوبال مذابة في الكحول (gum copal)، مع إضافة الكافور، وزيت التربنتين، واليوكاليبتول. وصف Cerqueira Enecê [11] كبديل لبلسم كندا (Canada balsam)لتثبيت الشرائح الدائمة للبرقات، وبقايا الأطوار غير الناضجة (exuviae)، وحتى البعوض البالغ، ومنذ ذلك الحين تم اعتماده على نطاق واسع لتثبيت ذباب الرمل. يُقدّم Enecê بديلًا اقتصاديًا للتثبيت الدائم، مع توفير ثبات طويل المدى وزمن جفاف كافٍ يسمح بإجراء التشريح وترتيب الهياكل المورفولوجية بدقة.

## 4.5 تحضير الشرائح الزجاجية وتجفيفها

يُعدّ التجفيف السليم لشرائح العينات أمرًا حاسمًا لضمان ثباتها وحفظها على المدى الطويل. ويجب تجفيف الشرائح تجفيفًا كاملًا قبل التفكير في تخزينها طويل الأمد. وللحصول على أفضل النتائج، يُنصَح بتجفيف الشرائح المُحضَّرة بوسائط تثبيت دائمة في وضع أفقي لمدة تتراوح بين 2–3 أسابيع، في حين قد تتطلب الشرائح المُحضَّرة بوسائط شبه دائمة مدة أقصر تتراوح بين 1–2 أسبوع. ولضمان عملية تجفيف فعّالة، يُوصى باستخدام حاضنة مضبوطة على درجة حرارة مناسبة لنوع وسط التثبيت المستخدم، مع تجنّب الحرارة المفرطة التي قد تُلحق الضرر بالعينات. ويُوصى بنطاق حراري يتراوح بين 30 و37 درجة مئوية. وتُعدّ هذه الخطوة ضرورية لمنع تشوّه الشرائح، أو تدهور العينات، أو عدم استقرار وسط التثبيت أثناء التخزين. يجب دائمًا تدوين نوع وسط التثبيت المستخدم على ملصق الشريحة. وإذا أمكن، ينبغي أن يتضمن الملصق أيضًا الوصفة المستخدمة، واسم الشخص الذي قام بالتحضير، وتاريخ الإعداد. تُحضَّر الشرائح في البداية كتحضيرات مؤقتة، ولا يُقصد بها الحفظ طويل الأمد. غير أنه إذا تغيّرت وضع العينة، كأن تُصنَّف عينة نمطية Specimen type، فيجب استخدام وسط تثبيت أكثر ديمومة لضمان حفظ العينة لأغراض الدراسات التصنيفية المستقبلية.

## 5.5 تقنيات التثبيت البديلة: التثبيت على البطاقات

يُعدّ التثبيت على البطاقات تقنية تُستخدم في عدة مجموعات من الحشرات، حيث يمكن تثبيت العينات مباشرة على بطاقات حشرية أو لصقها على سطحها. ونظرًا لصِغَر حجم ذباب الرمل والحاجة إلى فحص الأعضاء الداخلية للتعرّف عليها عبر عملية التوضيح (انظر العنوان او العنصر 5)، فإن هذه الطريقة غير مناسبة إطلاقًا لتثبيت ذباب الرمل.

## 6.5 إعادة تثبيت العينات المتضرّرة

بالنسبة للعينات النادرة أو القيّمة، يُوصى باتباع إحدى الخطوتين، وفقًا للفيديو المتاح على1) https://zenodo.org/records/18315029. إعادة الترطيب دون التفكيك للسماح بقراءة الملاحظات الأولية. يوضع حامل لعدة شرائح مجهرية داخل طبق بتري ليعمل كدعامة، ثم توضع الشريحة المراد إعادة

**الجدول 4.** مزايا وعيوب بعض أوساط التثبيت المختارة المستخدمة في الشرائح المجهرية، استنادًا إلى ملاحظات غير منشورة لعدد من الباحثين [52].

| الاسم | المزايا | العيوب |
|---|---|---|
| بلسم كندا* (Canada balsam) | - وسط عالي المتانة، بعمرافتراضي يتجاوز 150سنة<br>- يمكن تثبيت الشرائح باستخدام زيت القرنفل أو الفينول كعوامل تثبيت | - يحتوي على مكونات ضارة ويجب التعامل معه تحت خزانة شفط.<br>- يتطلب سلسلة تجفيف كاملة تستغرق وقتًا طويلاً.<br>- قد يؤدي التجفيف بالإيثانول والنقل عبر الزايلين أو زيت القرنفل إلى جعل بعض الأصناف هشة؛ ويمكن أن تقلل البدائل على سبيل المثال(:الأيزوبروبانول، ن-بيوتانول، -1,4ديوكسان، هيستوكليير، تيربينول ,سيلوسولف™) من التكسّر<br>- قد تسودّ العينات إذا استُبدل الزايلين بالفينول أو إذا بقيت بقايا . هيدروكسيد البوتاسيوم<br>- قد تحجب معاملات الانكسار المرتفعة البُنى غير المصبوغة. قد يستغرق الجفاف التام سنوات دون استخدام صفيحة تسخين.<br>- يصفرّ الوسط ويغمق بمرور الوقت، خاصة عند التصفية بزيت.القرنفل<br>- تضعف بعض الأصباغ، وقد تتلاشى الأصباغ الكاتيونية إذا .أصبح الوسط حمضيًا ـــــوهو ما قد يحدث تلقائيًا بمرور الوقت |
| DMHF ثنائي ميثيل هيدانتوين فورمالدهيد | - يطلق الفورمالديهيد ← مخاطر صحية وسلامة واعتبارات تنظيمية.<br>- يواجه قيودًا تنظيمية وقيودًا على الملصقات البيئية-Eco) label).<br>- يحتاج إلى محفزات حمضية، مما قد يسبب تلف النسيج والتآكل.<br>- قد يسبب اصفرار الأقمشة الفاتحة.<br>- قد يقلل من نعومة النسيج.<br>- يتطلب مراقبة صارمة للعملية.<br>- غير مناسب في الحالات التي تتطلب خلوًا تامًا من الفورمالديهيد. | - يوفر مقاومة ممتازة للتجاعيد والثنيات مع أداء متين بعد الغسيل.<br>- شديد التفاعل، ويتصلب بسرعة عند درجات حرارة معتدلة.<br>- يحافظ على قوة النسيج بشكل جيد مقارنة بالراتنجات الفورمالديهيدية القديمة.<br>- قابل للذوبان في الماء وسهل التركيب مع أنظمة التشطيب النسيجي القياسية.<br>- اقتصادي ويوفر أداء صناعي متسق.<br>- متعدد الاستخدامات للقطن، والفسكوز، والكتان، والخليط منها.<br>- متوفر بدرجات منخفضة من الفورمالديهيد الحر لتلبية العديد من اللوائح |
| * يوبارال (شفاف) Euparal | - سط متين ذو عمر افتراضي يزيد عن 50 عامًا.<br>- يمكن التركيب مباشرة من محلول 80% إيثانول (وفق توصية الشركة المصنعة).<br>- لا يخفي البنى غير المصبوغة، ولا يصفرّ أو يصبح هشًا مع مرور الوقت.<br>- له معامل انكسار أكثر ملاءمة من بلسم كندا بالنسبة للحشرات ثنائية الجناح (Diptera)<br>- يعمل جيدًا مع العينات السميكة بفضل الانكماش الضئيل وتجفيفه الخالي من الفقاعات.<br>- يبقى قابلًا للذوبان في إيثانول 95%، مما يتيح إعادة التركيب حتى بعد مرور سنوات عديدة. | يحتوي على مكونات ضارة ويجب التعامل معه تحت خزانة شفط<br>قد يؤدي التجفيف بالإيثانول والنقل عبر خلاصة يوبارال<br>إلى Euparal Essence<br>جعل بعض الأصناف هشة ، لكن استخدام الأيزوبروبانول قد يقلل هذه المشكلة |
| سائل هوير (Hoyer fluid) | - يمكن تثبيت العينات حية أو .مباشرة من الماء أو الإيثانول أو الفورمالدهيد<br>- يؤدي النقع إلى .جودة ممتازة للكيوتكل<br>- له معامل انكسار مناسب ويمكن .تعزيزه بصبغة اليود لزيادة التباين<br>- يمكن لحمض الأسيتيك في التركيبة أن يمدّد زوائد مفصليات الأرجل<br>- قد تبقى بعض العينات مستقرة .لمدة 40-60سنة<br>- قابل للذوبان في الماء، مما يسمح .بإعادة التثبيت بسهولة. | - قد تنهار العينات النباتية الرقيقة ما لم يُضف الوسط تدريجيًا، وهو . إجراء يستغرق وقتًا طويلاً<br>- قد تتكوّن تجاويف وبلورات خلال أقل من 10سنوات.<br>- قد يصبح الهضم مفرطًا حسب تركيز كلورال هيدرات ومدة التعرض<br>- قد تنفصل مكونات الوسط وتظهر حبيبات دقيقة خلال أشهر أوسنوات<br>- تم الإبلاغ عن اسوداد الوسط. |

( يتواصل في الصفحة التالية)

**الجدول 4.** ( متواصل)

| الاسم | المزايا | العيوب |
|---|---|---|
| فينول كربوكسي ميثيل سليلوز CMCP | يمكن تثبيت العينات مباشرة من أوساط مثل الماء أو الإيثانول أو الغليسرول أو محاليل تحتوي على الفورمالدهيد، ويمكن هضم الأعضاء الداخلية عند الضرورة لتسهيل الفحص العام أو التحضير | - قد يُكوّن بلورات ويغمق بمرور الوقت، وقد يهضم العينات أكثر من المطلوب أحيانًا<br>- إذا لم تُحكم حواف الشريحة جيدًا، فإن العينات السميكة لا تثبت جيدًا بسبب الانكماش وتكوّن فراغات حول حواف غطاء الشريحة<br>- غير مناسب للعينات المصبوغة أو المواد المتكلسة و زمن الجفاف أبطأ منCMC |
| وسط التثبيت يوكيت Eukitt™ | - وسط متين يدوم أكثر من 30سنة<br>- متوافق مع العديد من المذيبات للتثبيت، بما في ذلك الأسيتون، البنزين، الكلوروفورم، الديوكسان، الإيثر، الأيزوبروبانول، ميثيل بنزوات، تيربينول، التولوين، والزايلين<br>- يجف بسرعة وله درجة حموضة حمضية قليلاً<br>- لا يغمق بشكل ملحوظ مع التقدم في العمر<br>- مناسب لعدة أصباغ ( مثل الفوشين، الهيماتوكسيلين، الأخضر الميثيلي، البنفسجي الميثيلي، أزرق الميثيلين).<br>- يمكن إعادة التثبيت بعد سنوات بنقع طويل في الزايلين | - يحتوي على مكونات ضارة ويجب التعامل معه تحت خزانة شفط.<br>- يتطلب سلسلة تجفيف كاملة تستغرق وقتًا طويلاً.<br>- غير مثالي للعينات السميكة بسبب الانكماش وتكوّن فقاعات غازي<br>- قد ينفصل غطاء الشريحة بمرور الوقت إذا لم يُنظف الزجاج جيدًا ويُحكم إغلاقه<br>- قد يُظهر بلمرة غير مكتملة حول ألياف الكولاجين. |
| وسط التثبيت إينيسي Enecê | - وسط عالي المتانة يدوم 50سنة .على الأقل<br>- لا يغمق بمرور الوقت.<br>- أكثر ليونة، مما يسمح بتشريح الحشرات داخل الوسط، ويوفر وقتًا مناسبًا لوضع التراكيب المورفولوجية<br>- منخفض التكلفة. | - يتطلب سلسلة تجفيف كاملة تستغرق وقتًا طويلاً.<br>- قد يؤدي التجفيف بالإيثانول والنقل عبر زيت القرنفل إلى جعل بعض العينات هشة<br>- تستمر الحشرة في التوضيح ببطء شديد، مما قد يصعّب رؤية التراكيب الصغيرة جدًا مثل الحسّاسات (sensilla) ، الأسكويدات (ascoids) والشعيرات البسيطة (simple setae). |

ترطيبها فوقه. يُملأ طبق بتري ببضعة مليمترات من المذيب لتكوين حُجرة رطبة، مع التأكد من عدم ملامسة الشريحة نفسها للمذيب) الشكل D8 .(وقد تتراوح مدة إعادة الترطيب من يوم واحد إلى عدة أيام حسب حالة العينة. وتُعدّ المراقبة اليومية والتحلّي بالصبر أمرين أساسيين. وبعد إعادة الترطيب الكافية، تُزال الشريحة من الحجرة الرطبة وتوضع في الحاضنة لبضع ساعات قبل الفحص المجهري أو التصوير أو الرسم. 2) لإعادة التثبيت، يمكن إرجاع الشريحة إلى الحجرة الرطبة لبضع ساعات إضافية أو طوال الليل. ويُجرى التفكيك تحت مجهر ثنائي العينين. وباستخدام إبر دقيقة، يجب إزالة الغطاء الزجاجي بحذر شديد، مع التأكد من عدم التصاق أي أجزاء من ذباب الرمل به . (https://zenodo.org/records/18315029)بعد ذلك، تُجمع الأجزاء المُشرَّحة من ذباب الرمل وتُشطف بالماء في عبوة صغيرة مماثلة لتلك المستخدمة في استخلاص الحمض النووي (انظر أدناه)، قبل التجفيف وإعادة التثبيت في وسط راتنجي(Resinous) ,وعند تفكيك شريحة ما، من الضروري تحديد وسط التثبيت الأصلي لاختيار المذيب المناسب؛ ففي حالة الأوساط المائية يُستخدم الماء، أما إذا كان وسط التثبيت راتنجيًا مثل بلسم كندا أو Euparal® فيُستخدم الزيلين، وذلك تحت غطاء واقي مع الشفط ومع ارتداء معدات الوقاية الشخصية المناسبة، بما في ذلك القناع .

ولا يجوز إعادة تثبيت عينات النمط أو العينات المحفوظة في المجموعات إلا بعد الحصول على موافقة القيّم و/أو المؤسسة المالكة للعينة.

## 6 تحديد هوية العينات

### 1.6 الصفات المورفولوجية

يعتمد تحديد هوية ذباب الرمل أساسًا على فحص خصائصه المورفولوجية، بما في ذلك شكل الصدر والأجنحة والأعضاء التناسلية والشعيرات، إضافةً إلى العلاقات القياسية بين مختلف البنى. ويستخدم الباحثون المفاتيح التصنيفية، والمجموعات المرجعية، والأوصاف الأصلية للأنواع لمقارنة العينات المُجمَّعة بالأنواع المعروفة. وتُعدّ السمات التشخيصية الرئيسية، مثل الأوعية الدموية للأجنحة وبنية الرأس في كلا الجنسين، وبنية الأعضاء التناسلية الذكرية، وترتيب الحويصلات المنوية لدى الإناث، ذات أهمية خاصة في تحديد الأنواع. وغالبًا ما يتطلب التحديد الدقيق فحصًا مجهريًا تفصيليًا باستخدام مجهر مركّب لملاحظة البنى الدقيقة كالأعضاء التناسلية والحويصلات المنوية، أو مجهر تشريحي (ستيريو) لفحص السمات المورفولوجية العامة.

وقد سهّلت التطورات الحديثة في تقنيات التصوير استخدام الصور الرقمية عالية الدقة في تحديد ذباب الرمل، حيث يمكن مقارنة الصور أو الرسومات الرقمية للسمات الرئيسية بالمواد المرجعية أو تحليلها باستخدام أنظمة تحديد بمساعدة الحاسوب، مما يحسّن الدقة وسهولة الوصول في التصنيف المورفولوجي.

### 2.6 هندسة الأجنحة

تُعدّ هندسة الأجنحة سمة أساسية تُستخدم في تحديد وتصنيف الأنواع المختلفة من ذباب الرمل. وتتميّز أجنحة ذباب الرمل بنمط وبنية فريدين، إذ تكون عادة طويلة وضيّقة ذات تعريق متطوّر (الأشكال 9 و10). ويُشكّل ترتيب الأوعية الدموية نمطًا مميزًا قد يختلف بين الأجناس والأنواع، مما يوفّر سمات تشخيصية قيّمة لعملية التحديد. وعليه، فإن دراسة هندسة الأجنحة تُقدّم معلومات مهمة لأغراض تصنيفية

## 3.6 القياسات الشكلية الهندسية للأجنحة

.يستخدم الباحثون تقنيات مختلفة، مثل القياسات الشكلية الهندسية، لتحليل ومقارنة شكل وحجم الأجنحة بين أنواع أو تجمعات مختلفة من ذباب الرمل.

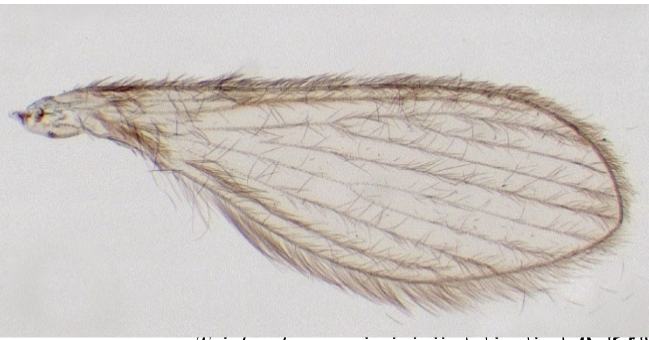

**الشكل 9.** إ جناح خام لـ *Trichophoromyia ininii*.

وتُوفّر دراسة هندسة الأجنحة رؤى قيّمة حول السلوك، والاماكن المفضلة لها، وقدرات الطيران .في هذا النهج، تُشرَّح الأجنحة بعناية، وتُصبغ عند الحاجة، وتُثبَّت مسطّحة على الشرائح. ثم تُصوَّر الشرائح المحضّرة تحت مجهر تشريحي، وتُرقم، وتُخضع للتحليل الشكلي. وقد وُصفت هذه الإجراءات بالتفصيل في السابق [6، 27، 42، 56، 57، 59]، مع التوصية باستخدام الجناح الأيمن أو الأيسر بشكل ثابت في الأعضاء المزدوجة لتجنّب التأثيرات الألومترية allometric السلبية المحتملة [62].

### تحضير الأجنحة للتحليل الشكلي الهندسي

لتحقيق أفضل وضوح للأوعية الدموية الخاصة بالأجنحة، يجب تنظيف الأجنحة من القشور وصبغها بشكل مناسب. ولتحضير الأجنحة، تُملأ أولًا انابيب صغيرة صغيرة بالكواشف اللازمة (أزرق الميثيلين، الإيثانول، الماء، وبديل الزيلين xylene). يُستخرج جناح محفوظ في إيثانول 70% في درجة حرارة الغرفة بقلب أنبوب إيبندورف وإفراغ محتواه فوق انابيب صغيرة، ثم يُرفع الجناح طوليًا باستخدام إبرة منحنية دقيقة. يُمرَّر الجناح سريعًا من الإيثانول إلى الماء ثم يعود إلى الإيثانول لإزالة الشعيرات. بعد ذلك، يُوضع الجناح في أزرق الميثيلين لمدة 6 دقائق مع التأكد من بقائه طافيًا أثناء الصبغ. ثم يُستعاد الجناح بحذر ويُغمَر في بديل الزيلين لمدة دقيقتين (حوالي ثلث مدة الصبغ). ويمكن للنقر الخفيف بالإبرة على جدران البئر أن يساعد الجناح على الاستقرار، حيث يعمل الزيلين على تثبيت اللون. أخيرًا، يُرفع الجناح ويُوضَع على قطرة صغيرة من ®Euparalعلى شريحة مجهرية. وتحت عدسة مكبّرة، يُفرد الجناح برفق ويُوضَع الغطاء الزجاجي بعناية. ويجب التقاط الصور سريعًا قبل تصلّب ®Euparal، إذ قد تكون هناك حاجة لإجراء تعديلات طفيفة على وضعية الجناح تحت الغطاء لتحقيق المحاذاة المثلى.

## 4.6 تقنيات البيولوجيا الجزيئية Molecular biology

إضافةً إلى التقنيات المورفولوجية، أصبحت الطرائق الجزيئية ذات أهمية متزايدة في أبحاث علم الحشرات، بما في ذلك الدراسات التصنيفية، والوراثة السكانية، والتطورية، فضلًا عن الكشف عن مسببات الأمراض بالحمض النووي، وتحديد مصدر الوجبة الدموية، وسلوك الناقلات، وهو أمر بالغ الأهمية في علم الوبائيات [70]. ويمكن استخدام تسلسل الحمض النووي الريبي منقوص الاوكسيجين لتأكيد هوية الأنواع أو التمييز بين الأنواع المتقاربة وراثيًا، مما يوفّر وسيلة أكثر دقة وموثوقية للتحديد. كما تكتسب التقنيات الجزيئية المتقدمة مثل تضخيم الجينات، وتسلسل الاحماض الامينية، وتقنيات NGSو، وتقنية MALDI-Tof MSأهمية متزايدة في التحديد السريع والدقيق للأنواع، مكمّلةً للطرائق المورفولوجية التقليدية [46]. وعلى الرغم من هذه التقنيات المتطورة، يظلّ التحديد المورفولوجي هو المرجع الأساسي في التصنيف والأساس الذي تُفسَّر على ضوئه البيانات الجزيئية.

### 1.4.6 الاستخلاص التدميري للأحماض النووية

يُعدّ استخلاص الأحماض النووية خطوة روتينية في العديد من الدراسات البيولوجية، وقد طُوِّرت طرائق متعددة لعزل الحمض النووي منقوص الاوكسيجين من المواد الحيوية [48]، كما تتوافر العديد من العدد التجارية (commercial kits) لتسهيل هذه العملية [14]. غير أن الطرائق الشائعة لتحضير مفصليات الأرجل لأغراض التحديد المورفولوجي غالبًا ما تُعيق التحليل الجزيئي، إذ قد تُتلف أو تُدمّر الخصائص الفيزيائية الحرجة للعينة [10]. ومعظم بروتوكولات استخلاص الحمض النووي من أنسجة الحشرات ذات طبيعة تدميرية [43]، ما يثير قلقًا خاصًا بالنسبة للعينات الصغيرة، حيث قد يؤدي حتى أخذ جزء محدود منها إلى الإضرار بسمات مورفولوجية مهمة [72]. ويلعب نوع العينة وحالتها دورًا رئيسيًا في اختيار طريقة عزل الجينات الوراثية المناسبة [29].

وقد أدّت الحاجة إلى التحديد الدقيق لذباب الرمل، وفهم ديناميات التجمعات، وتقليل التأثيرات غير المستهدفة non-target impacts، إلى تطوير أدوات تشخيص جزيئية [23]. وتُستخدم المقاربات الجزيئية اليوم على نطاق واسع لتكميل الطرائق التصنيفية المورفولوجية في تحديد
ذباب الرمل. فعلى سبيل المثال، يعتمد الترميز الشريطي barcoding للحشرات على استخلاص الحمض النووي وتسلسله، مع فقدان العينة الأصلية. ومن ثمّ، تبرز الحاجة الملحّة إلى استكشاف طرائق غير تدميرية لاستخلاصه و للمحافظة على المادة الحيوية وسلامتها المورفولوجية.

وقد طُبّقت العديد من طرائق استخلاص الأحماض النووية على ذباب الرمل. وتعتمد كمية وجودة الأحماض النووية المطلوبة على نوع التحليل الجزيئي اللاحق، إذ تختلف التقنيات في حساسيتها ومتطلبات النقاوة [9].

فعلى سبيل المثال، ثبت أن عيون ذباب الرمل قد تمنع عملية تضخيم الجينات [69]. إلى جانب فحص الميكروبات الممرضات، يُستخلص الحمض النووي لذباب الرمل كعملية روتينيّة لغرض تحديد انواعها. ويمكن استخدام طرق مختلفة للاستخلاص، مع اختلاف المردود والجودة بين التقنيات. وقد عدّل الباحثون بعض البروتوكولات الصناعية لتلائم ذباب الرمل [8]، مما حسّن مردود وجودة الأحماض النووية المستخلصة [8، 9، 69]، كما يمكن تطبيق تعديلات طُوّرت لمفصليات أرجل أخرى غير ذباب الرمل [58، 76]. تُعدّ تفاعلات تضخيم الحمض النووي التعريفية التي تستهدف مقاطع ميتوكوندرية صغيرة مثل COI أو CytBمتوافقة عمومًا مع طرائق الاستخلاص التي تُحدث تفتتًا عاليًا في الحمض النووي، في حين تتطلب تقنيات NGS ذات القراءات الطويلة Oxford Nanopore PacBio حدًا أدنى من التفتت والحمض النووي عالي الجودة. وتنتج طرائق الأعمدة الدورانية Spin columnsعادةً قطعا من الحمض النووي الجينومي تصل إلى 60 كيلوباز، بينما يمكن لاستخلاص الفينول–كلوروفورم أن يُنتج قطعا تصل إلى 150 كيلوباز [77]. ويُلخّص الجدول 5 تقنيات استخلاص الحمض النووي لذباب الرمل ويبيّن ما إذا كانت قد أُدخلت تعديلات منهجية لتناسب هذه الحشرات.ولا تُعرض المردودات من الحمض النووي نظرًا لاعتمادها على حجم العينة وطريقة تحضيرها. وتشير خانة «التعديل» إلى تكييف البروتوكولات لذباب الرمل أو لمفصليات الأرجل الصغيرة الأخرى .ويجب أن يراعي اختيار طريقة الاستخلاص عدة معايير، مثل عدد العينات، ومدة الاستخلاص، والتقنية الجزيئية اللاحقة. وبينما تتطلب تقنيات NGS DNA جينوميًا عالي الوزن الجزيئي، يمكن استخدام جميع الطرائق المعروضة هنا في تطبيقات تضخيم الجينات القياسية.وعلاوةً على ذلك، استكشفت عدة دراسات طرائق غير تدميرية لاستخلاص الحمض النووي من مفصليات الأرجل البرية الصغيرة، والعينات المتحفية المحفوظة جافّة، ومفصليات الأرجل رخوة الجسم [19، 26، 28، 55، 63].

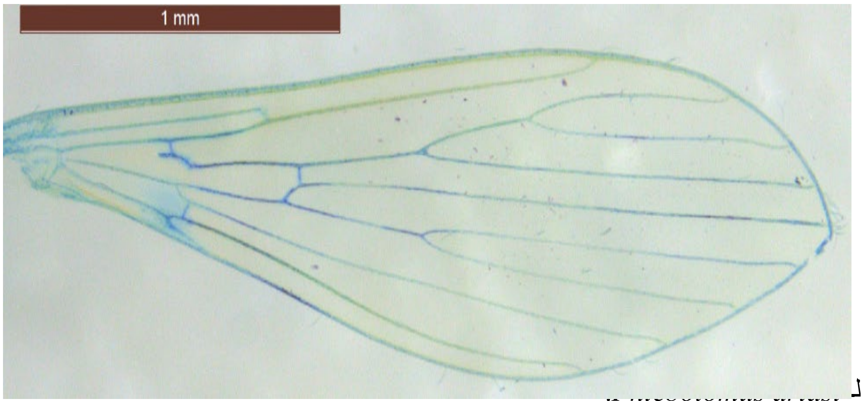


**الشكل 10.** جناح ملوّن لـ [illegible]

**الجدول 5.** متوسط التكلفة، والتطبيق، وتكييف البروتوكول لاستخلاص الحمض النووي الجيني من ذباب الرمل الفليبوتومي.

| لبروتوكول(Protocol) | التكلفة(Cost) | التطبيق(Application) | تكييف البروتوكول للحشرات المفصلية الصغيرة |
|---|---|---|---|
| سبين كولومن Spin column | 2.5 – 3.55 US$ [39] | PCR, NGS | [9] |
| فينول-كلوروفورم Phenol-chloroform | 0.24 US$ [69] | PCR, NGS | [9] |
| هوت شوت HotSHOT | <0.01 US$ [69] | PCR | – |
| سالتيغ أوت Salting out | 0.12 US$ [69] | PCR | – |
| تشيلكس Chelex | 0.02 US$ [41] | PCR | [41, 76] |

## 2.4.6 الاستخلاص غير التدميري للأحماض النووية

يُعدّ الحفاظ على العينات لإدماجها في المجموعات الحشرية أحد التحديات الرئيسية في التحليل الجزيئي لمفصليات الأرجل، ولا سيما ذباب الرمل. إذ تتطلب معظم بروتوكولات استخلاص الحمض النووي سحق الأنسجة، مما يصعب عملية حفظ العينة الأصلية. وعلى النقيض من ذلك، صُمِّمت طرائق الاستخلاص غير التدميرية لاستخراج المادة الوراثية دون إحداث تلف مادي بالعينة أو تغيير مورفولوجيتها. وتُعدّ هذه الطرائق ذات قيمة خاصة عند التعامل مع عينات ثمينة أو محدودة، مثل ذباب الرمل، حيث يُعدّ الحفاظ على السلامة البنيوية أمرًا ضروريًا لأغراض تصنيفية أو مورفولوجية أو تشخيصية مستقبلية. ومن التقنيات الشائعة طريقة «الحمّام غير التدميري»، حيث تُثبَّت ذباب الرمل وتُغمَر بلطف في محلول تحلّل يحتوي على مدمر البروتينات = بروتيناز K. وقد طُبّقت تقنية التحلّل اللطيف (mild-vectolysis) بنجاح على ذباب الرمل، ولا سيما عينات نمطية [24]. وتعتمد هذه التقنية على طقم من الأعمدة الدورانية التقليدية DNeasy Blood and Tissue kit، من شركة QIAGEN، ألمانيا مع تكييفات تسمح بالحصول على الحمض نووي دون تدمير العينة. وتُتيح خطوات التحلّل المعدّلة (حجم محلول التحلّل وإضافة خطوة تجميد) [17] تحرير الأحماض النووية مع تقليل الضرر المورفولوجي [24]. وبالنسبة لذباب الرمل، يمكن أيضًا استخدام طقم HotSHOT لاستخلاص الحمض النووي (Bento Bioworks Ltd، لندن، المملكة المتحدة[73] (، وهو سريع ومنخفض التكلفة، ما يسمح بمعالجة العينات بسرعة وبتكلفة محدودة. ويمكن بعد ذلك شطف العينات الحشرية المخصّصة للتحديد المورفولوجي. وتُصفّى العينات المعالجة بطقم DNeasy باستخدام محلول مارك-أندريه، في حين تكون العينات المعالجة بطقم HotSHOT مُصفّاة بما يكفي لتثبيتها في وسط مائي أو، ويفضّل، في وسط راتنجي بعد التجفيف، وفقًا للبروتوكول المفصّل في هذا المقال [73].

ويمكن بعد ذلك إخضاع المادة الوراثية المستخلصة للتحليلات اللاحقة، مثل تضخيم واسمات جينية محددة. وتُعدّ طرائق الاستخلاص غير التدميرية ضرورية لدراسة الخصائص الوراثية لذباب الرمل، بما في ذلك تحديد العوامل الممرِضة المحتملة التي قد يحملها. ومن خلال الحفاظ على سلامة العينة، يمكن للباحثين الحصول على معلومات جينية قيّمة مع الاحتفاظ بالعينة لإجراء تحليلات أو دراسات إضافية.

## 5.6 تقنية MALDI-Tof MS

تُعدّ تقنية MALDI-Tof MS (تقنية تحليل المواد بالليزر وقياس وقت وصول الجزيئات) تقنية قائمة على مطيافية الكتلة Mass Spectrometry، صُمِّمت لاكتشاف وتحليل البصمات البروتينية الفريدة للعينات الحيوية. وقد أصبحت MALDI-Tof معترفًا بها بشكل متزايد كأداة مهمة لتحديد مفصليات الأرجل ذات الأهمية الطبية والبيطرية. وقد أثبتت هذه التقنية فعاليتها في تحديد المراحل التطورية المختلفة لذباب الرمل، بما في ذلك الأطوار غير البالغة والوجبات الدموية لدى الإناث، كما استُخدمت بنجاح للتمييز بين الأنواع الذكرية والأنثوية تحت ظروف تخزين وتجهيز مختلفة [28، 30، 73، 74]. وتوفّر هذه الطريقة قدرة تمييز عالية على مستوى تحت الأجناس والأنواع والتجمعات. وتُمكّن الباحثين من تحقيق تحديد سريع ودقيق للأنواع، وهو أمر أساسي لفهم توزّع ذباب الرمل وسلوكه ودوره في نقل الأمراض. ومن خلال التمييز بين الأنواع استنادًا إلى البصمات البروتينية، تؤدي MALDI-Tof دورًا محوريًا في الدراسات الوبائية واستراتيجيات مكافحة النواقل. غير أن لهذه التقنية عيبين رئيسيين يحدّان من تطبيقها الروتيني. أولهما توافر أجهزة قياس الكتلة الجزيئية ، إذ تُعدّ مرتفعة التكلفة ولا يمكن اقتناؤها بسهولة لغرض تحديد أنواع ذباب الرمل وحده (أو نواقل المفصليات عمومًا). ولحسن الحظ، يمكن تجاوز هذا القيد بالحصول على وقت تشغيل على أجهزة قياس الكتلة المتاحة في مرافق

البروتيوميات أو التشخيصات السريرية. أما العيب الثاني فهو ضعف تمثيل بيانات ذباب الرمل المرجعية في قواعد البيانات مفتوحة الوصول، مما يستلزم إنشاء قاعدة بيانات داخلية تضم أطيافًا مرجعية مستندة إلى عينات محددة الهوية بشكل لا لبس فيه، ويفضَّل أن يكون ذلك بمزيج من التقييم المورفولوجي وتسلسل واسم جيني مناسب مثل COI أو cytB أو غيره .(ومن المؤمّل أن يُعالَج هذا القيد قريبًا عبر الإدراج التدريجي لبيانات ذباب الرمل المرجعية الداخلية في منصة MSI التابعة لمستشفيات باريس العامة – جامعة السوربون (فرنسا)، ومجموعة BCCM/IHEM/Sciensano في بروكسل (بلجيكا). وعند التخطيط لاستخدام توصيف البروتينات بتقنية MALDI-Tof، يُفضَّل تخزين العينات مجمّدة جافة أو في إيثانول 70% بدرجة نقاوة جزيئية، مع عدم تعريضها لدرجات الحرارة المحيطة. وفي غياب إرشادات موحّدة لتحضير العينات، يُنصَح باستخدام محلول مائي من الأسيتونتريل 60% / TFA 0.3% مع حمض السينابينيك (30 ملغ/مل) لتحضير مصفوفة MALDI-Tof، بهدف جعل الأطياف البروتينية قابلة للمقارنة مع البيانات المنشورة سابقًا لذباب الرمل.

### تحضير العينات لتقنية MALDI-Tof MS (الشكل 7)

تُجفَّف العينات الحشرية، المحفوظة تحت ظروف خاصة، هوائيًا في درجة حرارة الغرفة ثم تُشرَّح. ويُزال الرأس والبطن للحصول على أجزاء الجسم التي تحتوي على السمات المورفولوجية الأساسية لتثبيتها على الشرائح الزجاجية وإجراء التحليل المورفولوجي. ويمكن استخدام الصدر لتقنية MALDI-Tof، بينما يُحفَظ ما تبقّى من البطن لاستخلاص الحمض النووي. وللتوصيف البروتيني، يُجانس الصدر يدويًا في أنابيب دقيقة سعة 1.5 مل باستخدام مدقّات وحبيبات أحادية الاستعمال مع 10 ميكرولتر من محلول التجانس. ويُستخدم عادةً محلولان للتجانس: ماء مقطّر معقّم وحمض الفورميك بتركيز 25%.

## 7 الخاتمة

هدفنا في هذا العمل هو تزويد الباحثين بأكثر الطرائق فعالية لتثبيت, تشريح و تصنيف ذباب الرمل، بما يتلائم مع أهداف البحث المختلفة، وذلك لتسهيل التحديد الدقيق والكشف عن الممرضات. ولا توجد طريقة واحدة مثلى عالميًا؛ بل تتوافر عدة طرائق، لكل منها مميزات وسلبيات. وفي الملحقات المرفوقة، قدّمنا بروتوكولات مفصّلة لمختلف تقنيات التثبيت المستخدمة في تحضير وتحديد ذباب الرمل. وتوفّر هذه البروتوكولات، بما في ذلك مقاطع الفيديو التعليمية، إجراءات خطوة بخطوة مكيّفة لأهداف متعددة، بما يضمن نتائج دقيقة وموثوقة. ومن خلال إتاحة هذا المورد الشامل، نهدف إلى دعم الباحثين في اختيار وتطبيق تقنيات التثبيت الأنسب لاحتياجاتهم الخاصة.


## الشكر والتقدير

يشكر المؤلفون ريتشارد لين (Richard Lane) وزوي جاي آدامز (Zoe Jay Adams) من المتحف الطبيعي في لندن، المملكة المتحدة على مراجعتهم الممتازة التي ساهمت في رفع جودة هذا المخطوط.

## التمويل

نشكر وكالات التنمية البرازيلية CNPq (رقم الحالة: 2024/404395-4) ومؤسسة أراوكاريا (Araucária) رقم الحالة: 2025/433 (PDI على تمويل أبحاث AJA.


## تضارب المصالح

يُعد جيروم ديباكوي (Jérôme Depaquit) محررًا مشاركًا في مجلة Parasite؛ ولم يكن له أي تأثير على عملية المراجعة أو اتخاذ القرار بشأن هذا المخطوط. يُصرح باقي المؤلفين بعدم وجود أي تضارب مصالح.

## بيان إتاحة البيانات

*الفيديوهات على Zenodo*
*الفيديو 1*: https://zenodo.org/records/18198006
*الفيديو2*: https://zenodo.org/records/18311158
*الفيديو 3*: https://zenodo.org/records/18311106
*الفيديو 4*: https://zenodo.org/records/18311154
*الفيديو 5*: https://zenodo.org/records/18303014
*الفيديو 6*: https://zenodo.org/records/18302850
*الفيديو 7*: https://zenodo.org/records/18315029

## المواد الإضافية

المواد الإضافية لهذه المقالة متوفرة عبر الإنترنت على الرابط التالي : https://www.parasite-journal.org/10.1051/parasite/2026009/olm

## المراجع


1. Alkan C, Allal-Ikhlef AB, Alwassouf S, Baklouti A, Piorkowski G, de Lamballerie X, Izri A, Charrel RN. 2015. Virus isolation, genetic characterization and seroprevalence of Toscana virus in Algeria. Clinical Microbiology and Infection, 21(11), 1040 e1-9.
2. Alten B, Ozbel Y, Ergunay K, Kasap OE, Cull B, Antoniou M, Velo E, Prudhomme J, Molina R, Banuls AL, Schaffner F, Hendrickx G, Van Bortel W, Medlock JM. 2015. Sampling strategies for phlebotomine sand flies (Diptera: Psychodidae) in Europe. Bulletin of Entomological Research 105(6), 664–678.
3. Ayhan N, Baklouti A, Prudhomme J, Walder G, Amaro F, Alten B, Moutailler S, Ergunay K, Charrel RN, Huemer H. 2017. Practical guidelines for studies on sandfly-borne phleboviruses: Part I: Important points to consider *ante* field work. Vector-Borne and Zoonotic Diseases 17(1), 73–80.
4. Bates PA. 1997. Infection of phlebotomine sandflies with *Leishmania*, in The Molecular Biology of Insect Disease Vectors: A Methods Manual. Springer. p. 112–120.5.
5. Baum M, de Castro EA, Pinto MC, Goulart TM, Baura W, Klisiowicz Ddo R, Vieira da Costa-Ribeiro MC. 2015. Molecular detection of the blood meal source of sand flies (Diptera: Psychodidae) in a transmission area of American cutaneous leishmaniasis, Parana State, Brazil. Acta Tropica, 143, 8–12.
6. Belen A, Alten B, Aytekin A. 2004. Altitudinal variation in morphometric and molecular characteristics of *Phlebotomus papatasi* populations. Medical and Veterinary Entomology, 18(4), 343–350.
7. Bhattacharya J, Chandra G, Hati AK. 1991. A simple method for cryopreservation of *Leishmania donovani* promastigotes, Indian Journal of Medical Research, 93, 245–246.
8. Caligiuri LG, Sandoval AE, Miranda JC, Pessoa FA, Santini MS, Salomón OD, Secundino NF, McCarthy CB. 2019. Optimization of DNA extraction from individual sand flies for PCR amplification. Methods and Protocols, 2(2), 36.
9. Casaril AE, de Oliveira LP, Alonso DP, de Oliveira EF, Gomes Barrios SP, de Oliveira Moura Infran J, Fernandes WS, Oshiro ET, Ferreira AMT, Ribolla PEM, de Oliveira AG. 2017. Standardization of DNA extraction from sand flies: Application to

P, Sundar S, Boelaert M, Rogers ME. 2011. Serological markers of sand fly exposure to evaluate insecticidal nets against visceral leishmaniasis in India and Nepal: a cluster-randomized trial. PLoS Neglected Tropical Diseases, 5(9), e1296.

26. Gilbert MTP, Moore W, Melchior L, Worobey M. 2007. DNA extraction from dry museum beetles without conferring external morphological damage. PLoS One, 2(3), e272.

27. Giordani BF, Andrade AJ, Galati EAB, Gurgel-Goncalves R. 2017. The role of wing geometric morphometrics in the identification of sandflies within the subgenus *Lutzomyia.* Medical and Veterinary Entomology, 31(4), 373–380.

28. Guzmán-Larralde AJ, Suaste-Dzul AP, Gallou A, Peña-Carrillo KI. 2017. DNA recovery from microhymenoptera using six non-destructive methodologies with considerations for subsequent preparation of museum slides. Genome, 60(1), 85–91.

29. Hajibabaei M, DeWaard JR, Ivanova NV, Ratnasingham S, Dooh RT, Kirk SL, Mackie PM, Hebert PD. 2005. Critical factors for assembling a high volume of DNA barcodes. Philosophical Transactions of the Royal Society B: Biological Sciences, 360(1462), 1959–1967.

30. Haouas N, Pesson B, Boudabous R, Dedet JP, Babba H, Ravel C. 2007. Development of a molecular tool for the identification of *Leishmania* reservoir hosts by blood meal analysis in the insect vectors. American Journal of Tropical Medicine and Hygiene, 77(6), 1054–1059.

31. Hlavackova K, Dvorak V, Chaskopoulou A, Volf P, Halada P. 2019. A novel MALDI-TOF MS-based method for blood meal identification in insect vectors: A proof of concept study on phlebotomine sand flies. PLoS Neglected Tropical Diseases, 13 (9), e0007669.

32. Huemer H, Prudhomme J, Amaro F, Baklouti A, Walder G, Alten B, Moutailler S, Ergunay K, Charrel RN, Ayhan N. 2017. Practical guidelines for studies on sandfly-borne phleboviruses: Part II: Important points to consider for fieldwork and subsequent virological screening. Vector-Borne and Zoonotic Diseases, 17(1), 81–90.

33. Jancarova M, Polanska N, Thiesson A, Arnaud F, Stejskalova M, Rehbergerova M, Kohl A, Viginier B, Volf P, Ratinier M. 2025. Susceptibility of diverse sand fly species to Toscana virus. PLoS Neglected Tropical Diseases, 19(5), e0013031.

34. Kapp JD, Green RE, Shapiro B. 2021. A fast and efficient single-stranded genomic library preparation method optimized for ancient DNA. Journal of Heredity, 112(3), 241–249.

35. Killick-Kendrick R, Maroli M, Killick-Kendrick M. 1991. Bibliography of the colonization of phlebotomine sandflies. Parassitologia, 33(suppl.), 321–333.

36. Lawyer P, Killick-Kendrick M, Rowland T, Rowton E, Volf P. 2017. Laboratory colonization and mass rearing of phlebotomine sand flies (Diptera, Psychodidae). Parasite, 24, 42.

37. Léger N, Pesson B, Madulo-Leblond G. 1986. Les phlébotomes de Grèce : 1ère partie. Bulletin de la Société de Pathologie Exotique, 79, 386–397.

38. Léger N, Pesson B, Madulo-Leblond G. 1986. Les phlébotomes de Grèce : 2ème partie. Bulletin de la Société de Pathologie Exotique, 79, 514–524.

39. Leonel JAF, Vioti G, Alves ML, da Silva DT, Meneghesso PA, Benassi JC, Spada JCP, Galvis-Ovallos F, Soares RM, Oliveira T. 2020. DNA extraction from individual Phlebotomine sand flies (Diptera: Psychodidae: Phlebotominae) specimens: Which is the method with better results? Experimental Parasitology, 218, 107981.

genotyping by next generation sequencing. Experimental Parasitology, 177, 66–72.

10. Castalanelli MA, Severtson DL, Brumley CJ, Szito A, Foottit RG, Grimm M, Munyard K, Groth DM. 2010. A rapid non-destructive DNA extraction method for insects and other arthropods. Journal of Asia-Pacific Entomology, 13(3), 243–248.

11. Cerqueira NL. 1943. Um novo meio para montagem de pequenos insetos em lâmina. Memórias do Instituto Oswaldo Cruz, (39), 37–41.

12. Charrel RN, Gallian P, Navarro-Mari JM, Nicoletti L, Papa A, Sanchez-Seco MP, Tenorio A, de Lamballerie X. 2005. Emergence of Toscana virus in Europe. Emerging Infectious Diseases, 11(11), 1657–1663.

13. Chaskopoulou A, Giantsis IA, Demir S, Bon MC. 2016. Species composition, activity patterns and blood meal analysis of sand fly populations (Diptera: Psychodidae) in the metropolitan region of Thessaloniki, an endemic focus of canine leishmaniasis. Acta Tropica, 158, 170–176.

14. Chen H, Rangasamy M, Tan SY, Wang H, Siegfried BD. 2010. Evaluation of five methods for total DNA extraction from western corn rootworm beetles. PLoS One, 5(8), e11963.

15. Depaquit J, Grandadam M, Fouque F, Andry PE, Peyrefitte C. 2010. Arthropod-borne viruses transmitted by Phlebotomine sandflies in Europe: a review. Eurosurveillance, 15(10), 19507.

16. Diamond LS, Herman CM. 1954. Incidence of Trypanosomes in the Canada Goose as revealed by bone marrow culture. Journal of Parasitology, 40(2), 195–202.

17. Ding H, Torno M, Vongphayloth K, Ng G, Tan D, Sng W, Ho K, Randrianambinintsoa FJ, Depaquit J, Tan CH. 2025. Hidden in plain sight: discovery of sand flies in Singapore and description of four species new to science. Parasites & Vectors, 18(1), 402.

18. Es-Sette N, Ajaoud M, Bichaud L, Hamdi S, Mellouki F, Charrel RN, Lemrani M. 2014. *Phlebotomus sergenti* a common vector of *Leishmania tropica* and Toscana virus in Morocco. Journal of Vector Borne Diseases, 51(2), 86–90.

19. Favret C. 2005. A new non-destructive DNA extraction and specimen clearing technique for aphids (Hemiptera). Proceedings of the Entomological Society of Washington, 107(2), 469–470.

20. Galati EAB. 2018. Phlebotominae (Diptera, Psychodidae): Classification, morphology and terminology of adults and identification of American taxa, in Brazilian Sand Flies: Biology, Taxonomy, Medical Importance and Control, Rangel EF, Shaw JJ, Editors. Cham: Springer International Publishing. pp. 9–212.

21. Galati EAB, de Andrade AJ, Perveen F, Loyer M, Vongphayloth K, Randrianambinintsoa FJ, Prudhomme J, Rahola N, Akhoundi M, Shimabukuro PHF, Depaquit J. 2025. Phle-botomine sand flies (Diptera, Psychodidae) of the world. Parasites & Vectors, 18(1), 220.

22. Galati EAB, Galvis-Ovallos F, Lawyer P, Leger N, Depaquit J. 2017. An illustrated guide for characters and terminology used in descriptions of Phlebotominae (Diptera, Psychodidae). Par-asite, 24, 26.

23. Gariepy T, Kuhlmann U, Gillott C, Erlandson M. 2007. Parasitoids, predators and PCR: the use of diagnostic molecular markers in biological control of Arthropods. Journal of Applied Entomology, 131(4), 225–240.

24. Giantsis IA, Chaskopoulou A, Bon MC. 2016. Mild-Vectolysis: A nondestructive DNA extraction method for vouchering sand flies and mosquitoes. Journal of Medical Entomology, 53(3), 692–695.

25. Gidwani K, Picado A, Rijal S, Singh SP, Roy L, Volfova V, Andersen EW, Uranw S, Ostyn B, Sudarshan M, Chakravarty J, Volf

bodied Arthropods in a high-throughput context: the example of Collembola. Molecular Ecology Resources, 10(6), 942–945.

56. Prudhomme J, Cassan C, Hide M, Toty C, Rahola N, Vergnes B, Dujardin JP, Alten B, Sereno D, Banuls AL. 2016. Ecology and morphological variations in wings of *Phlebotomus ariasi* (Diptera: Psychodidae) in the region of Roquedur (Gard, France): a geometric morphometrics approach. Parasites & Vectors, 9(1), 578.
57. Prudhomme J, Gunay F, Rahola N, Ouanaimi F, Guernaoui S, Boumezzough A, Banuls AL, Sereno D, Alten B. 2012. Wing size and shape variation of *Phlebotomus papatasi* (Diptera: Psychodidae) populations from the south and north slopes of the Atlas Mountains in Morocco. Journal of Vector Ecology, 37(1), 137–147.
58. Prudhomme J, Toty C, Kasap OE, Rahola N, Vergnes B, Maia C, Campino L, Antoniou M, Jimenez M, Molina R, Cannet A, Alten B, Sereno D, Banuls AL. 2015. New microsatellite markers for multi-scale genetic studies on *Phlebotomus ariasi* Tonnoir, vector of *Leishmania infantum* in the Mediterranean area. Acta Tropica, 142, 79–85.
59. Prudhomme J, Velo E, Bino S, Kadriaj P, Mersini K, Gunay F, Alten B. 2019. Altitudinal variations in wing morphology of *Aedes albopictus* (Diptera, Culicidae) in Albania, the region where it was first recorded in Europe. Parasite, 26, 55.
60. Rawlins DJ. 1992. Light Microscopy: An Introduction to Biotechniques. Oxford: Bios Scientific publishers. 143 pp.
61. Ready PD. 2013. Biology of phlebotomine sand flies as vectors of disease agents. Annual Review of Entomology, 58, 227–250.
62. Rohlf FJ, Slice D. 1990. Extensions of the Procrustes method for the optimal superimposition of landmarks. Systematic Zoology, 39(1), 40–59.
63. Rowley DL, Coddington JA, Gates MW, Norrbom AL, Ochoa RA, Vandenberg NJ, Greenstone MH. 2007. Vouchering DNA-barcoded specimens: Test of a nondestructive extraction proto-col for terrestrial arthropods. Molecular Ecology Notes, 7(6), 915–924.
64. Sábio PB, Andrade AJ, Galati EAB. 2014. Assessment of the taxonomic status of some species included in the *Shannoni* complex, with the description of a new species of *Psathyromyia* (Diptera: Psychodidae: Phlebotominae). Journal of Medical Entomology, 51(2), 331–341.
65. Sadlova J, Yeo M, Seblova V, Lewis MD, Mauricio I, Volf P, Miles MA. 2011. Visualisation of *Leishmania donovani* fluo-rescent hybrids during early stage development in the sand fly vector. PLoS One, 6(5), e19851.
66. Sales K, Miranda DEO, da Silva FJ, Otranto D, Figueredo LA, Dantas-Torres F. 2020. Evaluation of different storage times and preservation methods on phlebotomine sand fly DNA concen-tration and purity. Parasites & Vectors, 13(1), 399
67. Sales KG, Costa PL, de Morais RC, Otranto D, Brandao-Filho SP, Cavalcanti Mde P, Dantas-Torres F. 2015. Identification of phlebotomine sand fly blood meals by real-time PCR. Parasites& Vectors, 8, 230.
68. Sant'Anna MR, Jones NG, Hindley JA, Mendes-Sousa AF, Dillon RJ, Cavalcante RR, Alexander B, Bates PA. 2008. Blood meal identification and parasite detection in laboratory-fed and field-captured *Lutzomyia longipalpis* by PCR using FTA databasing paper. Acta Tropica, 107(3), 230–237.
69. Senne NA, Santos HA, Araujo TR, Paulino PG, Mendonca LP, Moreira HVS, Camilo TA, da Costa Angelo I. 2022. Robust comparative performance of genomic DNA extraction methods from non-engorged phlebotomine sandflies. Medical and Veterinary Entomology, 36(2), 203–211.
40. Lestinova T, Rohousova I, Sima M, de Oliveira CI, Volf P. 2017. Insights into the sand fly saliva: Blood-feeding and immune interactions between sand flies, hosts, and *Leishmania.* PLoS Neglected Tropical Diseases, 11(7), e0005600.
41. Lienhard A, Schaffer S. 2019. Extracting the invisible: obtaining high quality DNA is a challenging task in small arthropods. PeerJ, 7, e6753.
42. Lozano-Sardaneta YN, Mikery-Pacheco OF, Huerta H, Rojas-Soriano JE, Contreras-Ramos A. 2025. Wing geometric morphometrics is effective to separate sand fly species (Diptera, Psychodidae, Phlebotominae) related with leishmaniasis transmission in Mexico. Acta Tropica, 262, 107523.
43. Mandrioli M. 2008. Insect collections and DNA analyses: how to manage collections? Museum Management and Curatorship, 23(2), 193–199.
44. Maroli M, Feliciangeli MD, Bichaud L, Charrel RN, Gradoni L. 2013. Phlebotomine sandflies and the spreading of leishmaniases and other diseases of public health concern. Medical and Veterinary Entomology, 27(2), 123–147.
45. Marquina D, Buczek M, Ronquist F, Lukasik P. 2021. The effect of ethanol concentration on the morphological and molecular preservation of insects for biodiversity studies. PeerJ, 9, e10799.
46. Mathis A, Depaquit J, Dvorak V, Tuten H, Banuls AL, Halada P, Zapata S, Lehrter V, Hlavackova K, Prudhomme J, Volf P, Sereno D, Kaufmann C, Pfluger V, Schaffner F. 2015. Identification of phlebotomine sand flies using one MALDI-TOF MS reference database and two mass pectrometer systems., Parasites & Vectors, 8, 266.
47. Mekarnia N, Benallal KE, Sadlova J, Vojtkova B, Mauras A, Imbert N, Longhitano M, Harrat Z, Volf P, Loiseau PM, Cojean S. 2024. Effect of *Phlebotomus papatasi* on the fitness, infectivity and antimony-resistance phenotype of antimony-resistant *Leishmania* major Mon-25. International Journal for Parasitology – Drugs and Drug Resistance, 25, 100554.
48. Milligan BG. 1998. Total DNA isolation, in Molecular Genetic Analysis of Population: A Practical Approach, Hoelzel AR, Editor. Oxford: Oxford University Press.
49. Molina R, Jiménez M, Alvar J, González E, Hernández-Taberna S, Ines MM. 2017. Methods in sand fly research. Madrid: Servicio de publicaciones Universidad de Alcalá de Henares, Madrid.
50. Murphy WJ, Eizirik E, O'Brien SJ, Madsen O, Scally M, Douady CJ, Teeling E, Ryder OA, Stanhope MJ, de Jong WW, Springer MS. 2001. Resolution of the early placental mammal radiation using Bayesian phylogenetics. Science, 294(5550), 2348–2351.
51. Nacif-Pimenta R, Pinto LC, Volfova V, Volf P, Pimenta PFP, Secundino NFC. 2020. Conserved and distinct morphological aspects of the salivary glands of sand fly vectors of leishma-niasis: an anatomical and ultrastructural study. Parasites & Vectors, 13(1), 441.
52. Neuhaus B, Schmid T, Riedel J. 2017. Collection management and study of microscope slides: Storage, profiling, deterioration, restoration procedures, and general recommendations. Zootaxa, 4322(1), 1–173.
53. New TR. 1974. Pscoptera. Handbooks for Identification of British Insects (Vol. I). London: Royal Entomological Society of London. 102 pp.
54. Perez-Ruiz M, Collao X, Navarro-Mari JM, Tenorio A. 2007. Reverse transcription, real-time PCR assay for detection of Toscana virus. Journal of Clinical Virology, 39(4), 276–281.
55. Porco D, Rougerie R, Deharveng L, Hebert P. 2010. Coupling non-destructive DNA extraction and voucher retrieval for small soft-

DNA with hot sodium hydroxide and tris (HotSHOT). Biotechniques, 29(1), 52–54.
74. Upton MS. 1993. Aqueous gum-chloral slide mounting media: an historical review. Bulletin of Entomological Research, 83(2), 267–274.
75. Volf P, Myskova J. 2007. Sand flies and Leishmania: specific versus permissive vectors. Trends in Parasitology, 23(3), 91–92.
76. Wang Q, Wang X. 2012. Comparison of methods for DNA extraction from a single chironomid for PCR analysis. Pakistan Journal of Zoology, 44(2), 421–426.
77. Wang Y, Zhao Y, Bollas A, Wang Y, Au KF. 2021. Nanopore sequencing technology, bioinformatics and applications. Nature Biotechnology, 39(11), 1348–1365.
70. Shaw JJ. 2025. A review of Leishmania infections in American Phlebotomine sand flies – Are those that transmit leishmaniasis anthropophilic or anthropportunists? Parasite, 32, 57.
71. Tesh RB, Modi GB. 1983. Growth and transovarial transmission of Chandipura virus (Rhabdoviridae: Vesiculovirus) in *Phlebotomus papatasi.* American Journal of Tropical Medicine and Hygiene, 32(3), 621–623.
72. Thomsen PF, Elias S, Gilbert MTP, Haile J, Munch K, Kuzmina S, Froese DG, Sher A, Holdaway RN, Willerslev E. 2009. Non-destructive sampling of ancient insect DNA. PLoS One, 4(4),e5048
73. Truett GE, Heeger P, Mynatt RL, Truett AA, Walker JA, Warman ML. 2000. Preparation of PCR-quality mouse genomic

**Cite this article as:** Randrianambinintsoa FJ, Augendre L, Prudhomme J, Martinet J-P, Loyer M, Mekarnia N, Kerkoub H, Perveen FK, Huguenin A, Kariya E, Akhoundi M, De Andrade AJ, Berriatua E, Bongiorno G, Boyer S, Christodoulou V, Da Costa-Ribeiro MCV, De Souza LAF, Ding H, Dondji B, Dvořák V, Erisoz Kasap O, Galati EAB, Gállego M, Ballart C, Gouzelou S, Haddad N, Masse RS, Mekuria AH, Ivovic V, Kaczmarek S, Shahar MK, Kirstein OD, Kniha E, Kolářová I, Lincoln T, Lucanas C, Mikov O, Nov K, Özbel Y, Pesson B, Posada Lopez LC, Prasetyo DB, Rahola N, Rebollar-Tellez EA, Rodrigues BL, Roy L, Saini P, Sanjoba C, Shimabukuro PH, Siriyasatien P, Soszynska A, Suleşco T, Sylla M, Torno M, Volf P, Vongphayloth K, Sinh Nam V, Wardhana A, Yessinou E, Zapata S, Gantier J-C & Depaquit J. 2026. Processing and mounting phlebotomine sand flies: a consensus guideline. Parasite xx, xx. https://doi.org/10.1051/parasite/2026009.

PARASiTE

An international open-access, peer-reviewed, online journal publishing high quality papers on all aspects of human and animal parasitology

Reviews, articles and short notes may be submitted. Fields include, but are not limited to: general, medical and veterinary parasitology; morphology, including ultrastructure; parasite systematics, including entomology, acarology, helminthology and protistology, and molecular analyses; molecular biology and biochemistry; immunology of parasitic diseases; host-parasite relationships; ecology and life history of parasites; epidemiology; therapeutics; new diagnostic tools.
All papers in Parasite are published in English. Manuscripts should have a broad interest and must not have been published or submitted elsewhere. No limit is imposed on the length of manuscripts.

**Parasite** (open-access) continues **Parasite** (print and online editions, 1994-2012) and **Annales de Parasitologie Humaine et Comparée** (1923-1993) and is the official journal of the Société Française de Parasitologie.

Editor-in-Chief:
Jean-Lou Justine, Paris

Submit your manuscript at:
https://www.editorialmanager.com/parasite

## الملحق 1: الأسس النظرية الكيميائية الحيوية

الحشرات المعنية هي ذباب الرمل. ومع ذلك، يمكن توسيع الفكرة العامة لتشمل حشرات مفصلية شائعة أخرى التي لا يمكن تحديدها إلا عبر الخصائص المورفولوجية الداخلية. بالصدفة، بعض الأعضاء الداخلية تكون جزئيًا متصلبة بالكيتين، وتوفر مورفولوجياتها معلومات قيمة. لهذا السبب من المهم جدًا ملاحظة مضخات الطعام والحويصلات المنوية وأنابيبها .مع جميع المواد الكيميائية التي سنراجعها، يجب ألا ننسى أبدًا أنه من مرحلة تثبيت الحشرات إلى التجميع، نقوم ببساطة بتطبيق تفاعلات الأكسدة والاختزال (redox reactions).الفكرة الوحيدة أو الاحتياطة التي يجب اتباعها هي تجنب خلط المواد المختزلة مع المواد المؤكسدة.

### الإيثانول (Ethyl alcohol; ethanol)

يتم استخدام هذه المادة بطرق مختلفة. لجزيئات الكحول ميل قوي للماء، وبالتالي لها تأثير تجفيف. ومع ذلك، الكحول ذو التركيز المنخفض (غني جدًا بالماء) يلعب دورًا في تحلل الأحماض النووية (الماء هو عدو الأحماض النووية) عندما توضع الحشرات في الإيثانول، ليس الهدف فقط حفظها، بل أيضًا تثبيت الأنسجة .في علم الأنسجة، نميز عادة بين مفهومين مهمين: معدل الاختراق ومعدل التثبيت .من المفهوم جيدًا أن المثبت الجيد يجب أن يخترق الأنسجة بسرعة قبل تثبيتها .بالنسبة للكحول 96%، معامل الاختراق تقريبًا 1.05 (للمقارنة: محلول حمض البيكريك المائي 0.75% → 0.45، محلول ثنائي كرومات البوتاسيوم 3% → 1.45)

رغبة الحشرات وعلماء الحشرات في الحفاظ عليها إلى أجل غير مسمى في الإيثانول هي واقع شائع. الفكرة نبيلة للحفاظ على العينات الميدانية للدراسات المستقبلية، لكنها غير ممكنة بالنسبة للسيتولوجيين أو علماء الأنسجة. إذا تم الاحتفاظ بالعينات في المثبت لفترة طويلة، تصبح عمليًا غير قابلة لإعادة العمل .لهذا السبب، العينات الأقدم من 10 سنوات صعبة أو مستحيلة الاستخدام.

اعتبار آخر هو نسبة كتلة الحشرة إلى حجم المثبت .في الممارسة البيطرية أو الطبية، يُنصح بتخطيط حجم المثبت 60 ضعف حجم العينات المراد تثبيتها .في الممارسة العملية، للعضويات الصغيرة جدًا، أضف 4–5 أضعاف حجم العينة من الإيثانول .يجب تذكر أن الكحول يفقد قوته أثناء إزالة الماء من الأنسجة.

الخلاصة:

- الإيثانول عامل مختزل (غير متوافق مع المثبتات المؤكسدة).
- يترسب البروتينات ويغير بنيتها.
- يذيب بعض الدهون المعقدة ويرسب الجليكوجين.
- يسبب انكماش الأنسجة وتصلبها.

### محاليل هيدروكسيد البوتاسيوم أو الصوديوم الأساسية:

استخدام هذه المحاليل في علم الحشرات ركز أساسًا على KOH بدون مبرر واضح.

NaOH [E524] يوجد في محلول، بتراكيز مختلفة أو بتنوع في النورمالية، على شكل حبيبات أو رقائق لامعة. أهم عيوبه :شديدة الامتصاص للرطوبة (أكثر من KOH). عند تفاعله مع البروتينات يذيبها، ومع الدهون يحولها إلى صابون صلب أثناء التصبن.

KOH [E525] متاح كمحلول مركز، لكنه مميز بوجوده على شكل حبيبات 0.1 جم : يسهل تحضير محاليل مخففة بدون ميزان دقيق. مثال: 1 حبة 0.1 جم في 1 mL ماء مقطر , محلول 10%. الميزة الثانية: ( أقل حساسية للكربنة ) يمتص أقل$CO_2$ .

تُستخدم هذه القواعد القوية لإذابة الأحماض الدهنية وتحويلها إلى صابون قابل للذوبان بالماء .يجب تذكر أن الإيثانول قد ذوب بعض الدهون مسبقًا .عند وضع العينة في وسط مائي مع قاعدة قوية، ستترسب الأحماض الدهنية → يحدث تصبن بارد .أحيانًا، إذا كانت الدهون زائدة، مثلًا في الإناث، يفضل رفع الحرارة 35–40 °م لتسهيل التفاعل أو تمديد زمن التلامس في درجة حرارة الغرفة.

### محلول Marc-André الملون/عديم اللون:

هنا، سنستعرض مزايا وعيوب استخدام محلول Marc-André. يتكون هذا المحلول من كلورال هيدرات ، حمض الأسيتيك، والماء. هذا المحلول قوي التأكسد، أي مزيج من حمض وألدهيد. يقوم بتعادل KOH الزائد الذي قد يبقى في العينات، دون أن يترسب الصابون القلوي الناتج عن استخدام KOH.يعمل هذا المحلول المؤكسد أيضًا على وظائف الكحول الثانوي في الجلوكوزامينات المكوّنة للكيتين عن طريق أكسدتها، مما يؤدي إلى تليين الكيتين، كما يذيب بعض الأملاح المعدنية الموجودة في العينة .عندما يُلوَّن محلول

Marc-André مسبقًا بحمض فوشسين، أي في الحالة المؤكسدة، فإنه يصبح قادرًا على التثبيت على وظائف الكحول الثانوي في البنية. بعد فترة التلامس مع المحلول وحالة تلوين العينات، يتم الشطف باستخدام الإيثانول فقط، وبهذه الطريقة نبدأ مرحلة تجفيف العينات.

***الفوائد:***

- تشمل تحييد المحاليل القاعدية الزائدة،
- تليين الكيتين،
- تلوين الكيتين لتسهيل تقييم الهياكل الداخلية المتصلبة بالكيتين.

***العيوب:***

كلورال هيدرات مادة منومة وقد استخدمت في الطب البشري. يجب استخدامها تحت غطاء كيميائي، ويجب الالتزام بالتشريعات الخاصة بالمخاطر الكيميائية.

***محاليل التجفيف:***

تُظهر التجربة أنه بالنسبة للعينات الصغيرة جدًا، لا فائدة من اتباع تسلسل أحواض الإيثانول بتركيزات متزايدة. إذا كانت العينة كبيرة، نبدأ بـ 80% إيثانول، ثم 90%، 95%، وأخيرًا الإيثانول المطلق. بالنسبة للعينات الصغيرة جدًا، يُستخدم حمام 90% إيثانول يليه الغمر في الإيثانول المطلق. في هذه المرحلة، يجب دائمًا تذكر أن الإيثانول المطلق يسعى لتثبيت الماء الموجود في الجو .

التقليد في مختبرات علم الحشرات كان إنهاء تجفيف العينات باستخدام حمام beech creosoteالكريوزوت .اليوم، يُمنع استخدام هذه المادة بشدة نظرًا لرائحتها (هيدروكربونات عطرية متعددة الحلقات)، ويُفترض أنها سامة للخصوبة، مسرطنة، ملوّث عضوي مستمر، وسمية بيئية للكائنات المائية.

الحل المقترح لتحضير العينات للتثبيت هو ®Euparalو Euparal essence(موضح في الفقرة التالية). يُقبل خليط ®Euparal و Euparal essenceبشكل جيد جدًا؛ ويتم استخدامه للعينات بعد حمام إيثانول 90%.

## الملحق 2: تركيبة المواد الكيميائية

**هيدروكسيد البوتاسيوم 10%**

هيدروكسيد البوتاسيوم 10 جم

ماء مقطر حتى 100 مل

**وسط التثبيت (Hoyer medium (Gum chloral**

ماء مقطر 50 مل

كلورال هيدرات 200 جم

صمغ عربي 50 جم
غليسرول 20 مل

**محلول Marc-André**
كلورال هيدرات 40 جم
حمض الأسيتيك الجليدي 30 مل
ماء مقطر 30 مل

**حمض فوشسين 1% في الماء المقطر**
مسحوق حمض الفوشسين 1 جم
ماء مقطر 99 مل

محلول **Marc-André** ملون بالفوشسين
محلول **Marc-André 10** مل
فوشسين 1% 50 ميكرولتر

## الملحق 3: أوساط التثبيت Euparal®, Canada balsam, Polyvinyl alcohol

بوليفينيل الكحول: هذا هو الوسط المثالي للتثبيت عندما لا تتوفر المنتجات اللازمة للتجفيف الصحيح. يُخلط بوليفينيل الكحول مع lactophenol Amman.ومع ذلك، تظهر لهذه التركيبات عيوب رئيسية، مثل الجفاف أو تبلور بوليفينيل الكحول بسبب تبخر الماء أو السواد عند أكسدة الفينول. تظل هذه التقنية جيدة للتثبيت قصير المدى.

كندا بلسم Canada Balsam: استخدامه للتثبيت بين الشريحة والغطاء الزجاجي يتطلب تجفيف العينات أولاً. استخدام زيلين أو تولين ليس خالٍ من العيوب.

وسط إينيسي Enecê: للتثبيت بين الشريحة والغطاء الزجاجي، كما في Canada Balsam، يتطلب تجفيف العينة. تركيب Enecê: كولوفونيا بيضاء نقية (22 جم)؛ صمغ كوبال قابل للذوبان في الكحول (12 جم)؛ إيثانول مطلق (20 مل)؛ كافور (10 جم)؛ زيت تيربينتين (10 مل)؛ ويوكاليبتول (26 مل). لتحضيره، في وعاء مثل قارورة Erlenmeyer، ضع الإيثانول المطلق والكافور، ثم أضف الكولوفونيا وصمغ الكوبال. تغلق القارورة بسدادة ويُهز، ثم يُسخن في bain-marie بدرجة حرارة خفيفة دون غليان. بعد أن يذوب المزيج بالكامل، يُضاف زيت التيربينتين، ثم يُصفى بينما المزيج لا يزال ساخنًا، وأخيرًا يُضاف اليوكاليبتول. عندما يصبح الوسط أقل سيولة، يُخفف بـ Enecê بالتركيبة التالية: إيثانول مطلق (30 مل)، كافور (17 جم)، زيت تيربينتين (15 مل)، يوكاليبتول (38 مل)(Cerqueira, 1943).

يوبارال Euparal®: هذه راتنج مستخلص من شجرة السرو الأطلسية Tetraclinis articulata (Vahl, 1791)، ودُرست وطُورت في 1906 بواسطة Gilson. ميزتها الرئيسية أنها لا تتصلب بالبلمرة. يمكن استعادة العينات المثبتة بين الشرائح والغطاء الزجاجي بسهولة باستخدام الكحول أو أفضل باستخدام Euparal® essence. يُسمى هذا الراتنج أيضًا sandarac، ويقبل الإيثانول من 80%.

**استخدام محلول مائي غير أيوني: X100 Triton :**
X100 Triton على شكل محلول مائي غير أيوني (4-(1,1,3,3-tetramethylbutyl)phenyl-polyethylene glycol، أو t-octylphenoxypolyethoxyethanol يُستخدم على نطاق واسع كمُنظف في علم الأحياء الخلوي والجزيئي. يسمح بزيادة نفاذية أغشية الخلية والنواة.
لتقسيم الصدر إلى جانبي اليسار واليمين حسب المناطق الأكثر أهمية.

العينات الحشرية المحفوظة في الكحول لسنوات عديدة شائعة، لكن الحفظ في الكحول ليس مثاليًا، وتجعل هذه الطريقة التحضير للفحص المجهري صعبًا. غالبًا ما تتحلل العبوات البلاستيكية المحتوية على العينات، ويتبخر الكحول، وفي كلتا الحالتين يشكل التعرض الطويل للكحول أو جفاف العينات مشكلة كبيرة. في 2008، نشر Jonque ملاحظة حول إعادة ترطيب العناكب باستخدام عوامل ترطيب مثل Agepon المستخدمة في الأفلام الفوتوغرافية، مما أدى إلى فكرة استخدام عوامل ترطيب غير قوية كمُنظفات.

الإجراء باستخدام محلول Triton X100 بنسبة 0.5% مائي:

- يُشبع العينة الجافة بالكحول المطلق،
- ثم يُضاف الحجم اللازم من محلول 0.5% Triton X100 حتى تغمر العينة بالـكامل.
- يُترك لمدة 5 دقائق أو أكثر حتى تصبح جميع المفصليات مستقلة في المحلول .
- بعد ذلك يُزال محلول Triton X100 ويُستبدل بمحلول هيدروكسيد البوتاسيوم،

ويُتبع باقي التقنية كما هو موصوف أعلاه.

## الملحق 4: خطوات تثبيت العينات باستخدام Euparal® أو Canada Balsam

1. يجب تجفيف العينات (المظهر الغائم أو الحليبي يدل على تجفيف غير كافٍ).
2. يمكن إجراء التجفيف عن طريق زيادة تركيز الإيثانول تدريجيًا.
3. يمكن نقل العينات من إيثانول 99% أو الإيثانول المطلق إلى عامل تنظيف.

الإجراء التفصيلي:

1. تشريح الذباب الرملي البالغ في إيثانول 70%.
2. إزالة الإيثانول واستبداله بـ KOH 10% قم بتغطية الذباب بشريحة زجاجية.
3. سحق العينات حتى تصبح شفافة.
4. إزالة KOH.
5. تغطية العينة بالماء المقطر والانتظار من 30 إلى 45 دقيقة.
6. إزالة الماء وإعادة الغسل بالماء المقطر لمدة 30 دقيقة (مدة الغسل تعتمد على عدد العينات؛ كلما زاد العدد، زادت مدة الغسل؛ والعكس صحيح للعينات الفردية).
7. إزالة الماء.
8. إضافة محلول Marc-André (قد يكون ملونًا بحمض الفوشسين) والانتظار 24 ساعة.
9. إزالة محلول Marc-André.
10. تغطية العينة بالماء المقطر والانتظار من 30 إلى 45 دقيقة.
11. إزالة الماء وإعادة الغسل بالماء المقطر لمدة 30 دقيقة.
12. إزالة الماء.
13. إضافة إيثانول 70% وتشريح العينة.
 a. للرأس والبطن، اسحب الرأس أو البطن برفق من الصدر.
 b. للصدر، أزل الأجنحة عن طريق إمساك الصدر بملاقط وسحب قاعدة الزوائد بملاقط أخرى. يمكن إجراء تشريح سهمي Sagittal
14. تجفيف العينات تدريجيًا عبر سلسلة من محاليل الإيثانول المائي: 50% – 80% – 95% حتى الوصول إلى الإيثانول المطلق.
15. تجفيف العينات بغسلها مرتين، 10 دقائق لكل غسلة، بالإيثانول 100%.

16. إزالة الإيثانول وتغطية العينات بزيت القرنفل لمدة 15 دقيقة في درجة حرارة الغرفة.
17. نقل العينات من زيت القرنفل إلى نقطة من ®Euparal أو Canada Balsamعلى شريحة نظيفة.
18. ترتيب العينات حسب الرغبة: يمكن تشريح الرأس والصدر والبطن باستخدام إبر دقيقة أو ملاقط تحت مجهر تشريح. يجب فصل الرأس عن الجسم لتثبيته في الوضع البطني الظهري، أي يجب توجيه الثقبة القذالية (occipital foramen)للأعلى لتمكين رؤية cibarium مباشرة من خلالها.

يُجرى التشريح داخل وسط تثبيت الذباب الرملي.

19. ترك العينة حتى تصبح السطح لزجًا.
20. ترطيب غطاء شريحة نظيف بالإيثانول المطلق، ووضع الغطاء على Canada Balsam على زاوية.
21. تخزين الشرائح في صندوق جاف مخصص لهذا الغرض.
