# Supplementary material for: Processing and mounting phlebotomine sand flies: a consensus guideline
Source: Parasite. 2026 Apr 3;33:18. doi: 10.1051/parasite/2026009 (PMC13047900; doi:10.1051/parasite/2026009)
Supplement: Supplementary file 3 — Bulgarian translation / Превод на български [file parasite-33-18-s3.pdf]

Parasite **33**, 18 (2026)
© F.J. Randrianambinintsoa et al., published by EDP Sciences, 2026
https://doi.org/10.1051/parasite/2026009

PARASITE
Available online at:
www.parasite-journal.org

REVIEW ARTICLE OPEN ACCESS

Обработка и монтиране на флеботомни папатацийни мушици: консенсусно ръководство

Fano José Randrianambinintsoa[1], Laure Augendre[1], Jorian Prudhomme[1], Jean-Philippe Martinet[1], Mathieu Loyer[1], Nalia Mekarnia[1], Hocine Kerkoub[1], Farzana Khan Perveen[1], Antoine Huguenin[1,2], Emilie Kariya[1,2], Mohammad Akhoundi[3], Andrey José de Andrade[4], Eduardo Berriatua[5], Gioia Bongiorno[6], Sébastien Boyer[7,8], Vasiliki Christodoulou[9], Magda Clara Vieira Da Costa-Ribeiro[10], Lucas Alexandre Farias de Souza[10], Huicong Ding[11], Blaise Dondji[12], Vít Dvořák[13], Ozge Erisoz Kasap[14], Eunice Aparecida Bianchi Galati[15], Montserrat Gállego[16], Cristina Ballart[16], Stavroula Gouzelou[17], Nabil Haddad[18], Rezki Sabrina Masse[19], Asrat Hailu Mekuria[20], Vladimir Ivovic[21], Szymon Kaczmarek[22], Mohd Khadri Shahar[19], Oscar D. Kirstein[23], Edwin Kniha[24], Iva Kolářová[13], Lincoln Timinao[25], Cristian Lucanas[26], Ognyan Mikov[27], Kimsear Nov[7], Yusuf Özbel[28], Bernard Pesson[29], Laura Cristina Posada Lopez[30], Didot Budi Prasetyo[1,7], Nil Rahola[31], Eduardo A. Rebollar-Tellez[32], Bruno Leite Rodrigues[15], Lalita Roy[33], Prasanta Saini[34], Chizu Sanjoba[35], Paloma Helena Fernandes Shimabukuro[36], Padet Siriyasatien[37], Agnieszka Soszyńska[22], Tatiana Suleşco[38], Massamba Sylla[39], Majhalia Torno[40], Petr Volf[13], Khamsing Vongphayloth[41], Vu Sinh Nam[42], April Wardhana[43], Eric Yessinou[44], Sonia Zapata[45], Jean-Charles Gantier[1], and Jérôme Depaquit[1,2,*]

[1] Faculté de Pharmacie, Université de Reims Champagne Ardenne, UR ESCAPE-USC ANSES PETARD, 51 rue Cognacq-Jay, 51096 Reims Cedex, France
[2] Pôle de Biologie territoriale, Laboratoire de Parasitologie-Mycologie, Centre Hospitalo-Universitaire, 51092 Reims, France
[3] Parasitology-Mycology Department, Avicenne Hospital, AP-HP, Bobigny, Sorbonne Paris Nord University, France; Unité des Virus Émergents (UVE: Aix-Marseille Univ, Università di Corsica, IRD 190, Inserm 1207, IRBA), 13005 Marseille, France
[4] Parasitology Collection of Basic Pathology, Department of Basic Pathology, Federal University of Paraná, Curitiba 19031, Brazil
[5] Department of Animal Health, University of Murcia, Campus de Espinardo, 30100 Espinardo, Murcia, Spain
[6] Department of Infectious Diseases, Vector-borne Diseases Unit, Istituto Superiore di Sanità, 00166 Rome, Italy
[7] Medical and Veterinary Entomology Unit, Institut Pasteur du Cambodge, Phnom Penh 12201, Cambodia
[8] Ecology & Emergence of Arthropod-borne Pathogens Unit, Department of Global Health, Institut Pasteur, CNRS UMR2000, 75015 Paris, France
[9] Section Veterinary Services (1417), Laboratory for Animal Health Virology, Aglantzia, Nicosia 2109, Cyprus
[10] Insects Vectors and Parasites Laboratory, Department of Basic Pathology and Postgraduate program in Microbiology, Parasitology and Pathology, Federal University of Paraná, 81530-900 Curitiba, Brazil
[11] Department of Biological Sciences, National University of Singapore, 117558, Singapore
[12] Laboratory of the Leishmaniasis Research Project, Mokolo District Hospital, Mokolo, Cameroon; Laboratory of Cellular Immunology and Parasitology, Department of Biological Sciences, Central Washington University, 98926 Ellensburg, WA, USA
[13] Department of Parasitology, Faculty of Science, Charles University, 12800 Prague, Czechia
[14] VERG Laboratories, Department of Biology, Faculty of Science, Hacettepe University, Beytepe, Ankara 06800, Türkiye
[15] Faculdade de Saúde Pública da Universidade de São Paulo (FSP/USP), Pós-graduação em Saúde Pública, 01246-904 São Paulo, Brazil
[16] Secció de Parasitologia, Departament de Biologia, Sanitat i Medi Ambient, Facultat de Farmàcia i Ciències de l'Alimentació, Universitat de Barcelona, & Institut de Salut Global de Barcelona (ISGlobal), Centro de Investigación Biomédica en Red, Enfermedades Infecciosas (CIBERINFEC), 08028 Barcelona, Spain
[17] Laboratory of Infectious Diseases and Public Health, School of Medicine, University of Cyprus, Nicosia, Cyprus & Department of Pediatrics, Archbishop Makarios III Hospital, Nicosia 2115, Cyprus
[18] Faculty of Health Sciences, American University of Beirut, 1107 2020 Beirut, Lebanon
[19] Medical Entomology Unit, Infectious Disease Research Centre, Institute for Medical Research (IMR), National Institutes of Health (NIH), Ministry of Health Malaysia, 40170 Shah Alam, Selangor, Malaysia

Edited by Jean-Lou Justine
*Corresponding author: jerome.depaquit@univ-reims.fr

This is an Open Access article distributed under the terms of the Creative Commons Attribution License (https://creativecommons.org/licenses/by/4.0), which permits unrestricted use, distribution, and reproduction in any medium, provided the original work is properly cited.

[20] School of Medicine, Addis Ababa University, 28017 - 1000 Addis Ababa, Ethiopia
[21] Faculty of Mathematics, Natural Sciences and Information Technologies, University of Primorska, 6000 Koper, Slovenia
[22] University of Lodz, Faculty of Biology and Environmental Protection, Department of Invertebrate Zoology and Hydrobiology, Banacha 12/16, 90-237 Łódź, Poland
[23] Laboratory of Entomology, Ministry of Health, 9134302 Jerusalem, Israel
[24] Center for Pathophysiology, Infectiology and Immunology, Institute of Specific Prophylaxis and Tropical Medicine, Medical University Vienna, Kinderspitalgasse 15, 1090 Vienna, Austria
[25] Papua New Guinea Institute of Medical Research (PNGIMR) Institute, PO Box 60, Headquarter, Homate Street, 441 Goroka, Eastern Highlands Province, Papua New Guinea
[26] Museum of Natural History, University of the Philippines Los Baños, 4031 Laguna, Philippines
[27] National Centre of Infectious and Parasitic Diseases, 1504 Sofia, Bulgaria
[28] Ege University, Faculty of Medicine, Department of Parasitology, 35040 Bornova/Izmir, Türkiye
[29] Retired, Faculté de Pharmacie, Université de Strasbourg, Strasbourg, 67400 Illkirch-Graffenstaden, France
[30] Program for the Study and Control of Tropical Diseases (PECET), Faculty of Medicine, University of Antioquia, 050010 Medellin, Colombia
[31] MIVEGEC, Univ. Montpellier, CNRS, IRD, 34394 Montpellier, France & Medical Entomology Unit, Institut Pasteur de Madagascar, 101 Antananarivo, Madagascar
[32] Laboratorio de Entomología Médica, Departamento de Zoología de Invertebrados, Facultad de Ciencias Biológicas, Universidad Autónoma de Nuevo León, San Nicolás de los Garza, 66455, NL, México
[33] Tropical and Infectious Disease Centre, BP Koirala Institute of Health Sciences, Dharan 56700, Nepal
[34] ICMR-Vector Control Research Centre, Puducherry 605006, India
[35] Graduate School of Agricultural and Life Sciences, The University of Tokyo, Tokyo 113-8657, Japan
[36] Grupo de estudos em Leishmanioses/Coleção de Flebotomíneos (COLFLEB/Fiocruz-MG), Instituto René Rachou, Fundação Oswaldo Cruz, Belo Horizonte, Minas Gerais, 30190009, Brazil
[37] Center of Excellence in Vector Biology and Vector-Borne Disease, Department of Parasitology, Faculty of Medicine, Chulalongkorn University, Bangkok 10330, Thailand
[38] Department of Arbovirology, Bernhard Nocht Institute for Tropical Medicine, Bernhard Nocht Str. 74, 20359 Hamburg, Germany [39] Laboratory Vectors & Parasites, Department of Livestock Sciences and Techniques, Sine Saloum University El Hadji Ibrahima Niasse (SSUEIN) Kaffrine Campus, C.P. 24600, Senegal.
[40] Environmental Health Institute, National Environment Agency, Singapore 138667, Singapore & Department of Biological Sciences, National University of Singapore, 117558 Singapore
[41] Institut Pasteur du Laos, Laboratory of Vector-Borne Diseases, Samsenhai Road, Ban Kao-Gnot, Sisattanak District, 3560 Vientiane, Lao PDR
[42] National Institute of Hygiene and Epidemiology, 1 Yec-Xanh Street, Hai Ba Trung District, 100000 Hanoi, Vietnam
[43] Indonesian Research Center for Veterinary Science, Indonesian Agency for Agricultural Research and Development, Ministry of Agriculture Republic Indonesia, Bogor 16114, Indonesia & Department of Parasitology, Faculty of Veterinary Medicine, Airlangga University, Surabaya 60115, Indonesia
[44] Laboratory of Research in Applied Biology, Polytechnic School of Abomey-Calavi, University of Abomey-Calavi, 01 P.O. Box 2009, 00000 Cotonou, Benin
[45] Instituto de Microbiología, Colegio de Ciencias Biológicas y Ambientales (COCIBA), Universidad San Francisco de Quito (USFQ), 170901 Quito, Ecuador

Received 1 December 2025, Accepted 29 January 2026, Published online 3 April 2026

Резюме – Тази статия предоставя изчерпателно ръководство за обработката и монтирането на екземпляри от флеботомни папатацийни мушици, което е от съществено значение за определяне на видовете и откриване и изолиране на патогени. В нея се обсъждат редица техники, подходящи както за полеви, така и за лабораторни условия. Ръководството включва подробни инструкции за събиране, обработка, покриване и евтаназия на папатацийните мушици (препоръчва се сухо замразяване или CO2 вместо химикали), както и стратегии за съхранение, като съхранение в хладилник и консервиране в етанол. Качеството на препариране на определени анатомични структури (полови органи, глава и крила) е от съществено значение за правилното им микроскопско наблюдение и е описано в този труд. Статията представя и подробна обработка на пробите, включително процеса на просветляване с агенти като калиев хидроксид и след това разтвор на Марк-Андре. Процесът на монтиране сравнява различни среди, като се подчертават техните оптични свойства и потенциал за съхранение. Течността на Хойер (известна още като хлорална смола) се препоръчва за бързо наблюдение, особено за сперматеки, поради своята прозрачност, въпреки че не е подходяща за дългосрочно съхранение. Другите обсъждани среди включват поливинилов алкохол, Euparal® (за ограничена толерантност към вода) и канадски балсам (среда, разтворима във въглеводороди), като последните две предлагат възможности за

дългосрочно съхранение. Разгледани са и иновативни подходи в молекулярната биология, като секвениране на ДНК и MALDI-ToF, които изискват специално внимание при обработката на пробите. Освен това са предоставени кратки видеоклипове, илюстриращи различни техники за монтиране, както и преводи на 33 различни езика, което позволява ръководството да отговори на разнообразните нужди и очаквания на световната научна общност.

Ключови думи: Монтиране, флеботом, папатацийна мушица, течност на Хойер, разтвор на Марк-Андре, хлорална смола, поливинилов алкохол, Euparal®, канадски балсам, изолиране на *Leishmania*, полеви условия, култура, дисекция, молекулярна биология, MALDI-ToF, типови екземпляри

Abstract – Processing and mounting phlebotomine sand flies: a consensus guideline. This article provides a comprehensive guide for the processing and mounting of phlebotomine sand fly specimens, which is crucial for species identification and pathogen detection and isolation. It discusses a range of techniques suitable for both field and laboratory settings. The guide includes detailed instructions on sand fly collection, handling, covering, and euthanasia (recommending dry freezing or CO_2 over chemicals) as well as conservation strategies, such as cold storage and preservation in ethanol. The quality of preparation of certain anatomical structures (genital organs, head and wings) is essential for their proper microscopic observation and is described in this work. The article also presents detailed sample processing, including the clearing process with agents such as potassium hydroxide then Marc-André solution. The mounting process compares different media, emphasizing their optical properties and preservation potential. Hoyer fluid (also known as chloral gum) is recommended for quick observation, particularly for spermathecae, due to its clarity, although it is not suitable for long-term storage. Other media discussed include polyvinyl alcohol, Euparal® (for limited water tolerance), and Canada balsam (a hydrocarbon-soluble medium), with the latter two offering long-term preservation capabilities. Innovative molecular biology approaches such as DNA sequencing and MALDI-ToF, which require particular attention to sample processing, are also addressed. Furthermore, short video clips illustrating various mounting techniques as well as translations in many different languages are provided, allowing the guideline to reach the diverse needs and expectations of the global scientific community.

Key words: Mounting, Phlebotomine sand fly, Hoyer fluid, Marc-André solution, Chloral gum, Polyvinyl alcohol, Euparal®, Canada balsam, *Leishmania* isolation, Field conditions, Culture, Dissection, Molecular biology, MALDI-ToF, Type-specimens.

Въведение

Флеботомните папатацийни мушици са малки двукрили насекоми, принадлежащи към семейство Psychodidae, подсемейство Phlebotominae, с най-малко 1063 известни вида [21]. Те са важни преносители на патогени (Leishmania, арбовируси и Bartonella), отговорни за заболявания, наречени съответно лайшманиоза, арбовирусни инфекции и бартонелоза. Видовото им определяне се основава предимно на подробно микроскопско изследване, улеснено от внимателно събиране, подходящо съхранение и внимателно монтиране на предметни стъкла, което изисква няколко специфични техники, всяка от които има свои предимства и ограничения.

Видовото определяне на възрастните флеботоми се основава на наблюдение както на външни (например антени, палпи, мъжки полови органи), така и на вътрешни структури (например фаринкс, цибариум и сперматека). Дисекцията и изолирането на последните улесняват тяхното наблюдение, а в последствие и точното им идентифициране. Следователно, за разлика от комарите или дървениците, папатацийните мушици изискват монтиране между предметно и покривно стъкло преди видовото им определяне.

До 80-те години на миналия век микроскопското наблюдение беше единственият метод за идентифициране на флеботомите и остава най-широко използваният подход и днес. Изборът на процес и подготовка беше относително прост и се основаваше главно на избор от две възможности: от една страна – окончателно монтиране, позволяващо дългосрочно съхранение на пробата, и от друга – бързо поставяне за определяне в среда, която не осигурява дългосрочно съхранение. Окончателното монтиране, например в смола от сорта на канадския балсам, отнема много време и изисква пълно обезводняване на пробите. Освен това, коефициентът на пречупване на тази среда не винаги е оптимален за лесно наблюдение на сперматеките. Монтирането в среда на водна основа (например течност на Хойер), от друга страна, е по-бързо и позволява по-добра визуализация на рефрактивните сперматеки, но не позволява дългосрочно съхранение на монтираните образци, тъй като има тенденция да абсорбира вода от атмосферата. Една възможност е препаратът да се запечата с лак за нокти, след като изсъхне напълно. Този компромисен избор продължава и днес, като влияе върху избора на метод на монтиране в зависимост от предназначението на препарата. От 80-те години насам проучванията

върху видовото определяне на флеботоми комбинират морфологични и биохимични подходи. Пръв беше анализът на кутикулните въглеводороди, който бързо беше заменен от молекулярно-биологични техники (например случайно амплифицирана полиморфна ДНК (RAPD), полиморфизъм на дължината на рестрикционните фрагменти (RFLP), амплификация на ДНК и секвениране по метода на Сангер, както и секвениране от ново поколение (NGS). Днес молекулярните подходи се допълват от протеомни методи като MALDI-ToF. Освен това молекулярната идентификация на видовете може да се комбинира с откриването на патогени чрез PCR (*Leishmania*, *Trypanosoma*, *Bartonella* и *Phlebovirus*), тъй като всички те могат да бъдат открити както чрез конвенционален, така и чрез PCR в реално време, което изисква адаптиране на процеса на вземане на проби и съхранение към зададените цели [3, 32]. В допълнение към морфологичните характеристики, традиционно използвани за разграничаване на видовете, могат да се приложат и други морфологични подходи (например, геометрична морфометрия на крилата).

Основано предимно на собствения опит на авторите и данни от литературата, целта на това проучване бе да се предоставят стандартизирани насоки за монтиране и обработка на възрастни флеботоми, за да се оптимизират морфологичните и молекулярните анализи.

Необходимостта от извършване на определени анализи (например молекулярно-биологични или MALDI-ToF) изисква запазване на част от папатацийната мушица, която не е необходима за морфологична идентификация, което подчертава необходимостта от критичен избор на протокол.

В тази статия се фокусираме върху методите за анестезия и евтаназия на уловените живи флеботоми, тяхното съхранение и процеса на монтиране за бърза идентификация или за дългосрочно съхранение, позволяващо последващи проучвания.

Преамбюл: Съображенията за безопасност и нормативните изисквания трябва да се позовават на съответните информационни листове за безопасност (ИЛБ).

С всички химикали, представени в настоящото ръководство, трябва да се борави при строги условия за безопасност. Комисиите по здраве и безопасност на изследователските структури са на разположение, за да ви предоставят информация не само за опасностите, свързани с тези химикали, но и за процедурите за работа с тях и за изхвърлянето на отпадъците. Въпреки това е задължително да се спазват инструкциите за безопасност относно тяхното използване и изхвърляне. Важно е да се отбележи, че всички потребители носят отговорност за спазването на добрите и безопасни лабораторни практики, както и на приложимите закони и регламенти на тяхната страна или изследователска институция. Освен това някои от химикалите или техните компоненти (например хлорал хидрат) са регулирани в някои страни. Списъкът със съкращенията, използвани в настоящия документ, е представен в таблица 1.

Таблица 1: Списък със съкращения.

**BME**	Базова среда Игъл
**CDC**	Център за контрол и превенция на заболяванията
**CMCP**	Камфор-монохлорфенол
**CMR**	Канцерогенно, мутагенно, репродуктивно токсично вещество
**COI**	Ген за субединица I на цитохром C оксидаза
**CytB**	Ген на цитохром b
**ДНК**	Дезоксирибонуклеинова киселина
**ELISA**	Ензимно-свързан имуносорбентен тест
**EtOH**	Етанол
**M199**	Среда 199
**MALDI-ToF MS**	Матрично-асистирана лазерна десорбция/йонизация, времева масова спектрометрия
**MEM**	Минимална необходима среда
**NGS**	Секвениране от ново поколение
**NNN**	Среда на Нови-МакНийл-Никол
**PCR**	Полимеразна верижна реакция
**Лаоска НДР**	Лаоска народна демократична република
**PNOC**	Препроноцицептинов ген
**qPCR**	Количествен PCR (PCR в реално време)
**RAPD**	Случайно амплифицирана полиморфна ДНК
**RFLP**	Полиморфизъм на дължината на рестрикционните фрагменти
**КП**	Коефициент на пречупване
**РНК**	Рибонуклеинова киселина
**РНКази**	Рибонуклеази
**RNASS**	Разтвор за стабилизиране на РНК
**RT-PCR**	PCR с обратна транскрипция
**TFA**	Трифлуороцетна киселина

1. Улавяне на папатацийни мушици

Възрастните папатацийни мушици могат да бъдат събирани живи или мъртви, използвайки различни методи, като миниатюрни светлинни капани CDC, лепливи капани и аспиратори, използващи капани Шанън, или директно от местата за укритие в околната среда (например обори за животни). Тези методи включват поставяне на капани в подходящи местообитания, привличане на флеботомите със светлина или други атрактанти (CO_2 или химически примамки) и събирането им за по-нататъшен анализ, както е описано в няколко публикации [2, 3, 32, 36, 49].

Улавянето на живи флеботоми позволява всички последващи приложения, докато събирането на мъртви екземпляри пречи на изолирането на *Leishmania* или вирусни щамове. Някои техники за улавяне, като лепливи хартии, често водят до загуба на органи на флеботомите (антени, палпи, крила или крака). Освен това, покритието от рициново масло на лепливите хартии се залепва за мушиците и трябва да бъде отстранено в началото на обработката, обикновено чрез 15-минутна баня в смес от етанол и диетилов етер в равни части.

2. Евтаназия на екземплярите

След събирането, живите флеботоми трябва да бъдат евтаназирани. При някои методи на събиране (например лепливи хартии, светлинни капани CDC, оборудвани с буркан, съдържащ препарат или етанол) флеботомите са мъртви при събирането. Молекулярната биология може да се приложи към тези, събрани направо в етанол и към останалите, ако се съхраняват в етанол възможно най-бързо. Никой от тези методи на умъртвяване обаче не позволява обработка на насекомите чрез MALDI-ToF. Освен това някои методи за умъртвяване могат да доведат до загуба на определени морфологични характеристики. Ето защо е от съществено значение да се използва подходящ стандартен агент за умъртвяване, за да се гарантира правилната идентификация или дългосрочно съхранение като ваучерни проби (т.е. проби, съхранени за бъдеща справка или сравнение). Химикали като етилацетат, диетилов етер, тетрахлоретан и хлороформ могат да се напоят в памук и да се поставят в съд, съдържащ флеботомите за евтаназиране. Тези средства за умъртвяване трябва да се използват внимателно, като се следват препоръките на производителя, поради токсичността им. Въпреки това, ние не препоръчваме умъртвяването на флеботоми с хлороформ, тъй като според нашия опит той е слабо съвместим с молекулярно-биологичните изследвания. Предвид опасния характер на всички тези продукти и тяхната съмнителна пригодност за молекулярни анализи, използването на тези химикали като цяло не се препоръчва.

Най-широко използваният метод, който запазва морфологията, ДНК или протеините, е сухото замразяване на пробите. Пробите трябва да бъдат замразени достатъчно дълго, за да бъдат напълно анестезирани, но не толкова дълго, че (i) да изсъхнат или (ii) да бъдат компрометирани по отношение на жизнеспособността *на Leishmania*, ако целта е да бъдат изолирани *in vitro* от храносмилателния тракт на флеботомите. **Ето защо препоръчваме замразяване с продължителност от 15 до 20 минути при -20°C, като се наблюдават редовно, за да се гарантира, че са само зашеметени, без да се убиват паразитите *Leishmania*.**

Ако няма фризер, насекомите могат да бъдат евтаназирани с CO_2. В полеви условия, където не могат да се използват бутилки с CO_2, пробите могат да бъдат умъртвени с малки търговски балончета с CO_2, използвани в сифоните за газирана вода (диспенсери за напитки), но може да има ограничения за транспортирането им по въздух. Като последна мярка, насекомите могат да бъдат умъртвени чрез излагане на тютюнев дим. Флеботомите се улавят живи в капан CDC, събират се с аспиратор, задържат се в стъклена тръба и се излагат на тютюнев дим, който ги убива за секунди. Този метод е приложим във всички полеви условия, дори и в тежки и изолирани условия. Тъй като стъклото се импрегнира с дим, то не може да се използва за последващо събиране и обработка на живи флеботоми без цялостно почистване. Въпреки това, същият непочистен аспиратор може да се използва за евтаназиране на флеботоми от други капани с цел фиксиране. Необходимо е също да се провери дали всички екземпляри са били извадени от аспиратора. Тези методи са съвместими с изолирането на *Leishmania* чрез дисекция на червото.

3. Съхранение на пробите преди обработка

Има 5 основни метода за фиксиране преди обработка:

3.1. Замразяване

Този метод се прилага най-добре при -20°C или, за предпочитане, при -80°C. Тези методи на съхранение се използват по-широко от съхранението в течен азот. При всички случаи криоконсервацията трябва да се извърши възможно най-бързо след зашеметяването на екземплярите. Съхранението в хладилници предлага предимството на пълно запазване на самите насекоми, както и на РНК, ДНК и протеини в пълна цялост през целия период на съхранение. Вместо това, течният азот може да увреди сериозно крилата, краката, палпите и антените, често ампутирайки ги и понякога премахвайки ключови морфологични белези. Съхранението в сух фризер е по-малко травматично за екземплярите, но не е идеално за запазване на техните

крехки органи. Важно е да се отбележи, че по време на размразяването крилата, антените, палпите или краката могат да залепнат за епруветките и в крайна сметка да се откъснат поради кондензация. Съхранението чрез замразяване обаче не винаги е възможно при полеви проучвания, тъй като изисква достъп до фризер или контейнер с течен азот. Съхранението във фризер е напълно съвместимо с откриването на патогени с помощта на молекулярни инструменти без загуба на чувствителност, въпреки че откриването и изолирането на РНК вируси изисква замразяване при -80 °C или в течен азот, ако е необходимо дългосрочно съхранение. Замразяването на пробите обаче не позволява изолирането на *Leishmania* чрез дисекция на червото, освен ако флеботомите първо не бъдат потопени в парната фаза и след това в течния азот (например във флакони, поставени в чорап), симулирайки криоконсервацията на *Leishmania*.

3.2. Съхранение в алкохол (етанол или изопропилов алкохол)

Това е вероятно най-широко използваният метод за съхранение на флеботоми. Той е лесен за прилагане на място, дори в трудни условия без достъп до лаборатория. Съхранението в алкохол е особено подходящо за морфологични изследвания, тъй като крехките органи (крила, крака, антени или палпи) остават непокътнати, ако в епруветката за съхранение няма въздушни мехурчета. Ето защо препоръчваме епруветката да се запечата с малък памучен тампон, за да се отстранят въздушните мехурчета, и да се постави етикет върху памучния тампон (Фигура 1). Подходящата концентрация на алкохол остава предмет на дискусия. Като цяло не се препоръчват концентрации под 70% [45, 66]. По-високите концентрации съхраняват ДНК по-ефективно и за по-дълги периоди, но правят пробите по-крехки и чупливи за морфологични изследвания. Използването на 96% етанол (азеотропна смес) осигурява стабилност на концентрацията във времето, особено във влажни райони като тропическите страни, въпреки че 95% етанол често е по-достъпен за купуване. Независимо от концентрацията, ДНК обикновено се съхранява добре в етанол (макар и по-малко ефективно, отколкото при методите на замразяване, особено за молекулярни методи от типа NGS). Протеините са много по-малко стабилни, особено при протеомни анализи, като например приложението на MALDI-ToF. Флеботомите, съхранявани в алкохол в продължение на няколко месеца, все още могат да бъдат идентифицирани морфологично, но от тези проби е невъзможно да се генерират референтни протеинови спектри. Съхранението в алкохол или сухи условия може да бъде подобрено, ако пробата също бъде замразена при -20°C. Замразяването при -20°C подобрява основно молекулното съхранение (например на нуклеинови киселини) чрез забавяне на разграждането, а също така осигурява вторична полза за морфологичното съхранение чрез намаляване на разграждането на тъканите с течение на времето, въпреки че ефектът върху морфологията е по-ограничен, отколкото върху молекулната цялост. Съхранението в етанол може да се приложи и за откриване на ДНК и РНК вируси, когато се използва етанол с концентрация от най-малко 70% за кратък период на съхранение, по-къс от няколко месеца. Освен това, изопропиловият алкохол може да бъде лесно достъпен в някои страни и съхранява ДНК, но прави пробите твърди. Той не е запалим като етанола и затова може лесно да се транспортира. При необходимост, флеботомите, съхранявани в течен азот или замразени на сухо, могат да бъдат прехвърлени в алкохол, като по този начин се комбинират недостатъците на двата метода.

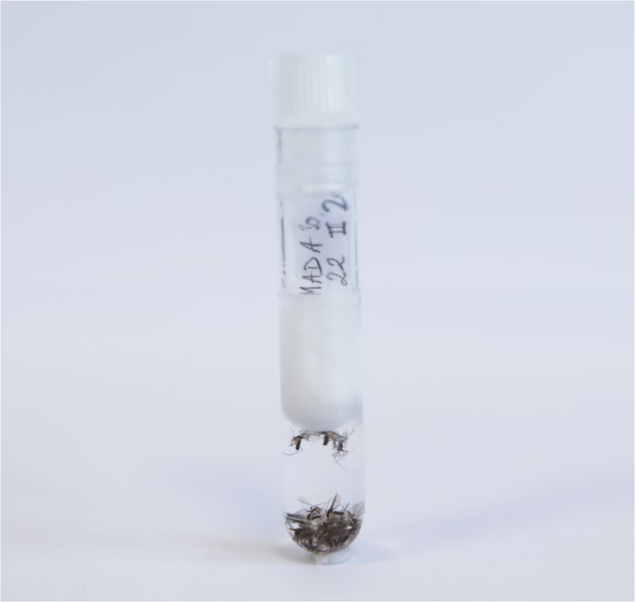

Фигура 1: Флеботоми, съхранявани в етанол.

3.3. Съхранение в разтвор за стабилизиране на РНК (RNASS)

Този воден реагент е широко използван, нетоксичен и предназначен за стабилизиране и защита на РНК в свежи тъканни и клетъчни проби. Той действа, като бързо прониква в пробата и деактивира РНКазите (ензими, разграждащи РНК), като по този начин предотвратява разграждането на РНК, без да е необходимо незабавно замразяване. Съхранението в RNASS обикновено е ефективно за запазване на цялостната морфология на тъканите и клетките за последваща хистологична оценка. Макар че RNASS е оптимизиран за стабилизиране на РНК, а не за фиксиране, краткосрочното до средносрочно съхранение обикновено поддържа добре структурната

цялост. RNASS позволява пробите да се съхраняват при стайна температура до 7 дни, при 4°C в продължение на няколко седмици или при -20°C/-80°C за дългосрочно съхранение. Този метод е особено ценен при работа на терен или в клинични условия , където инфраструктурата за хладилна верига е ограничена. Екстракцията на РНК обикновено изисква изваждане на пробите от реагента и обработката им съгласно стандартните протоколи.

3.4. Сухо съхранение при стайна температура

Това е по-стар метод, който, приложен върху *in toto* екземпляр (монтиран цял), има основния недостатък, че не съхранява добре крехките органи като крила, крака, антени и палпи. Въпреки това, протеомните изследвания с MALDI-ToF остават възможни, ако обезводняването се извършва след фиксиране със сушител от типа на силикагел. Противоположно на това, молекулярните анализи, насочени към ДНК, остават трудни за изпълнение върху тези проби, защото ДНК често е все още фрагментирана и в малко количество, което означава, че анализите остават по-трудни, отколкото при свежи или замразени проби, особено за ядрени геноми. Въпреки това, по-нови техники като музеомика могат да се използват върху проби от този тип [34]. Следователно, този метод на съхранение не се препоръчва, освен ако няма друга алтернатива. Той може да се комбинира със съхранение на студено, като епруветките се поставят във фризер при -20°C или -80°C. Основното предизвикателство е да се постигне подходящо монтиране на пробите или частите от тялото, необходими за идентифициране. За да се постигне това, рехидратацията е от съществено значение. Препоръчваме да се използва разтвор на Triton X-100. Продължителността на рехидратацията варира от няколко часа до няколко дни, при редовно внимателно наблюдение. След пълна рехидратация пробите трябва да се изплакнат в три последователни водни бани.

3.5. Консервиране върху филтърна хартия

Основното предимство на филтърната хартия е дългосрочната стабилност на геномната ДНК в клетките на нефиксирани, изсушени цели тела или кръвни клетки, съхранявани при стайна температура. Филтърната хартия се предлага в размер на малки листове, което позволява съхранението на няколкостотин проби при стайна температура в обем с размерите на малка папка. Матрицата на филтърната хартия е импрегнирана с агенти, които денатурират инфекциозните агенти, като по този начин пробите вече не се считат за биологична опасност. Това позволява съхранението и транспортирането на пробите без специални предпазни мерки срещу биологична опасност [68].

4. Дисекция на пробите

За разлика от много други насекоми, които се определят видово въз основа на външни белези, наблюдавани върху отделни насекоми, набодени *in toto*, флеботомите изискват дисекция и монтиране на предметно стъкло, за да се изучат анатомичните характеристики за точно определяне на вида. Независимо от избраната процедура за подготовка и монтиране, се използва една и съща техника за дисекция (Фигури 2 и 3) (https://zenodo.org/records/18198006).

Използване на Triton X100: нейонен воден разтвор

Имайте предвид, че монтирането се отнася за прясно уловени или подходящо съхранени екземпляри. Повечето учени съхраняват пробите от насекоми в сухо състояние (за използване с MALDI-ToF) или в алкохол в продължение на много години. За съжаление, съхранението в алкохол не е оптимално за продължителни периоди от време и членестоногите, съхранявани по този начин, стават много трудни за подготовка за микроскопско изследване. Често срещан инцидент е разграждането на пластмасовите съдове, съдържащи пробите, последвано от изпаряване на алкохола. И в двата случая нямаме избор, защото пробите остават прекалено дълго в алкохол или изсъхват. Затова възниква идеята да се използват овлажняващи агенти, които не са силни детергенти. Triton X100 е под формата на нейонен воден разтвор (*4-(1,1,3,3-тетраметилбутил) фенил-полиетилен гликол разтвор или т-октилфеноксиполитетоксиетанол, полиетилен гликол трет-октилфенил етер*), широко използван като детергент в клетъчната и молекулярната биология. Той позволява проникване през клетъчните и ядрените мембрани.

По-долу е описана процедура, при която се използва нейонен Triton X100 в 0,5% воден разтвор:

- Напойте сухата проба с абсолютен алкохол.
- Добавете необходимото количество 0,5% разтвор на Triton X100, така че цялата проба да бъде потопена.
- Оставете процеса да протича от около 5 минути до няколко дни, като го наблюдавате редовно. Всички членестоноги трябва да се отделят напълно в разтвора.
- Отстранете разтвора Triton X100 и го заместете с разтвор на калиев хидроксид.

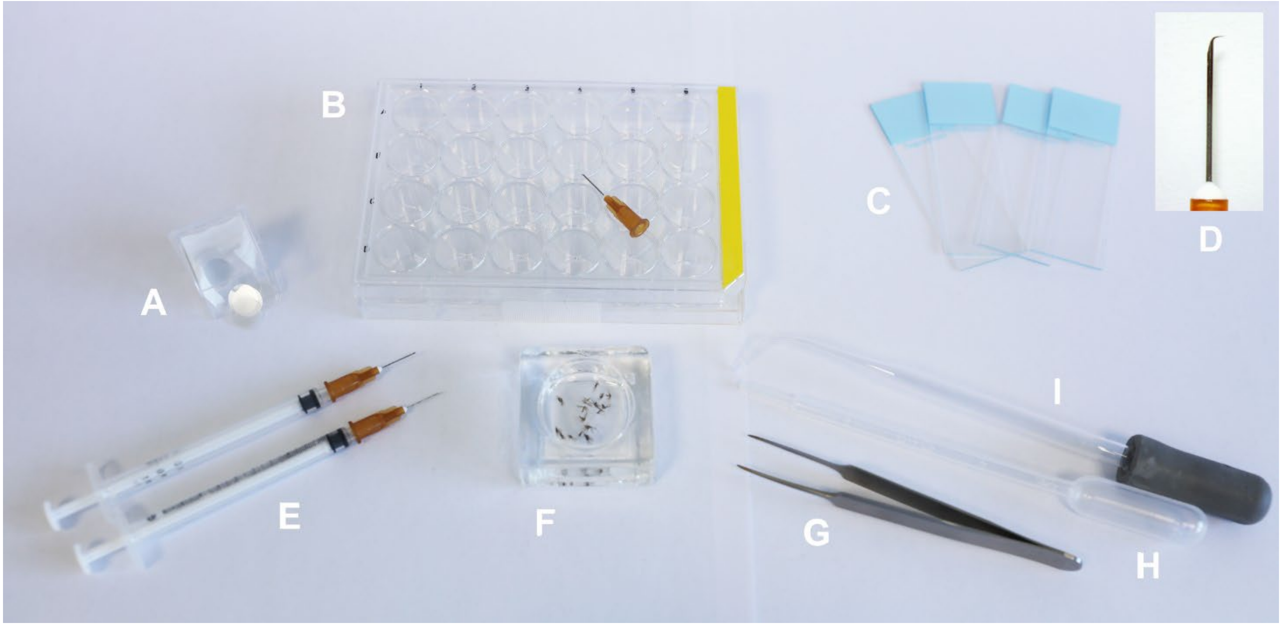

Фигура 2: Необходими материали за монтиране на флеботоми: A: кръгли покривни стъкла (с диаметър 10 или 12 мм); B: 24-ямкова плака и игла с кука (ако използвате карамфилово масло или Euparal® есенция за обработка на флеботомите, не използвайте акрилни плаки, тъй като ще протече химична реакция и пробите ще бъдат повредени); C: предметни стъкла, подходящи за етикетиране; D: детайл от куката на иглата; E: игли, прикрепени към спринцовки; F: часовниково стъкло или подобен съд, в който се съхраняват флеботомите за монтиране; G: пинсети Дюмонт; H: пластмасова пипета; I: стъклена пипета, извита чрез нагряване, за да се улесни прехвърлянето на течност в ямките.

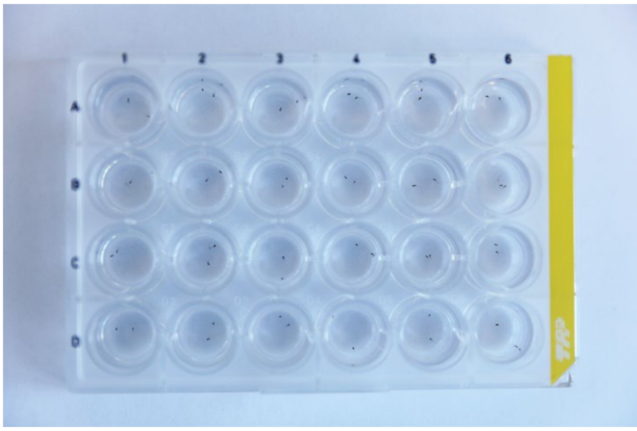

Фигура 3: Плака с 24 ямки, всяка от които съдържа главата и върха на корема на флеботомите.

4.1. Глава

Дисекцията може да се извърши с помощта на фини игли или ентомологични игли под стереомикроскоп (Фигури 2 и 3). Най-често използваните игли са: 26G x 1/2" (0,45 × 13 мм), 30G × 1/2" (0,3 × 13 мм) или 25G × 5/8" (0,5 × 16 мм). За да се подготви екземплярът за определяне, като минимум главата се отделя от тялото и се монтира с коремната страна нагоре, за да се покажат цибариумът и фаринксът, докато гърдите и коремчето се монтират странично след дисекцията. Монтирането на главата във вентро-дорзална позиция гарантира, че тилният отвор е ориентиран нагоре, така че цибариумът да може да се наблюдава директно. Достъпът до тези анатомични характеристики се улеснява, ако главата е напълно отделена.

4.2. Крила и гърди

Крилата трябва да бъдат монтирани плоско. Всяко крило може да бъде отделено в основата си и монтирано самостоятелно, или едното може да бъде монтирано самостоятелно, докато другото остава прикрепено към гърдите. Ако се планира геометричен морфометричен анализ, е от съществено значение да се идентифицират и маркират правилно дясното и лявото крило преди монтирането. Гърдите са разделени на няколко части, като всяка от тях съдържа много важна таксономична информация [20, 64]. Обикновено се монтират в страничен изглед, за да се позволи изследване на хетотаксията и разпределението на цветовете. Наличието на белези от четинки в определени области на гърдите може да се използва за разграничаване на някои видове от род *Brumptomyia*. Разпределението на цветовете може да се използва за разделяне на неотропическите флеботоми на ниво род (напр. *Bichromomyia*), видова серия (напр. *Pintomyia*) или дори видове от един и същ род (напр. *Micropygomyia*,

Nyssomyia, *Psathyromyia* и *Psychodopygus*) [20]. Следователно, ако гърдите не се използват за молекулярен анализ, те трябва да се монтират така, че да не се повредят. Важно е да се отбележи, че не интензивността на цветовете е от значение, а тяхното разпределение по гърдите. Следователно процесът на просветляване няма да елиминира пигментацията или шарката ѝ.

4.3. Полови органи

Особено внимание трябва да се обърне при монтирането на половите органи както при мъжките, така и при женските екземпляри, тъй като те са от решаващо значение за идентифицирането на рода, подрода и вида. И при двата пола гениталиите са чифтни.

4.3.1. Мъжки

Половите органи са външни и се състоят от двойка щипки, всяка от които се състои от съчленението гонококсит-гоностил в гръбната си част и епандриален лоб в коремната си част. Гоностилът има шипове и понякога четинки, които трябва да могат да се преброят и чиито места на прикрепване трябва да бъдат ясно видими. Важно е внимателно да се наблюдава вътрешната повърхност на гонококсита, която може да има кичур от уседнали четинки или такива, носени от лоб (= туберкул) [22]. Колегите с по-малко опит в дисекциите могат да извършат просто странично монтиране, без да отделят гениталиите от края на корема (https://zenodo.org/records/18311158). В този случай, наслагването на двете части на гениталиите може да затрудни преброяването на вътрешните четинки на гонококсита, но това избягва увреждането на гениталиите чрез неуспешна дисекция. По-опитните колеги могат да опитат да отворят гениталиите на две, за да ги разделят. За да се постигне това, трябва да се прокара скосената страна на игла (от типа на игла за подкожна реакция), отделяйки без напълно разрязване гениталиите, за да се разделят съчлененията на гонококсита и гоностила (https://zenodo.org/records/18311158). По този начин наблюдението на вътрешните им повърхности ще бъде лесно. Това препариране улеснява и наблюдението на парамерите и парамерните обвивки, които вече не се припокриват. За странично монтиране, което предполага наслагването на органите, екземплярите трябва да бъдат напълно просветлени.

4.3.2. Женски

Половият апарат е вътрешен и се състои от сперматеки. При липса на дисекция, те трябва да се наблюдават през телесните покривки и монтиране на корема във вентрална позиция. Независимо от избраната среда за включване, самите сперматеки обикновено могат да се наблюдават правилно, особено ако не са гладки и просветлени. Въпреки това, наблюдението на гладки, тънкостенни сперматеки може да бъде проблематично в слабо пречупващи среди. Освен това, наблюдението на основата на протоците на сперматеките е от съществено значение за определянето на видовете, например при подрод *Larroussius* [35, 37, 38], основните преносители на *Leishmania infantum* в Стария свят. Без това наблюдение определянето на екземплярите остава невъзможно. За да се преодолеят тези трудности при наблюдението, препаратът на гениталната фурка и сперматеките трябва да бъде отстранен от корема. (https://zenodo.org/records/18311106). Сперматеките обикновено са трудни за наблюдение по време на дисекцията, но гениталната фурка е относително лесна за локализиране. Тъй като протоците на сперматеките се отварят в гениталната фурка, изолирането на тази фурка обикновено позволява изолирането на сперматеките. Ако сперматеките бъдат случайно прерязани по време на процедурата, те не се губят и все още могат да бъдат наблюдавани в коремните обвивки. (Фигура 4).

4.4. Дисекция на средното черво за изолиране на *Leishmania*

Дисекцията на храносмилателния тракт е от съществено значение за откриването и изолирането на *Leishmania* при женските флеботоми. Процедурата може да се извърши както на място, така и в лабораторни условия, за да се оцени векторната компетентност.

Препоръчва се да се работи с прясно умъртвени женски екземпляри. Измийте женските екземпляри с вода или физиологичен разтвор, съдържащ мек детергент, за да премахнете излишните космици. Тази стъпка помага за поддържане на асептични условия за изолиране на *Leishmania* , като същевременно запазва морфологичните характеристики, необходими за определянето. За да намерите и изолирате *Leishmania*, внимателно отстранете средното черво и го поставете в капка стерилен физиологичен разтвор (0,9% NaCl). След като наблюдавате подвижните паразити под светлинен микроскоп (препоръчително увеличение: ~200×), използвайте инсулинова спринцовка или микропипета, за да ги прехвърлите в култивационната среда (за повече подробности вижте глава 4.4.3).

Поставете главата и гениталиите директно в течността на Марк-Андре, за да ги просветлите. Важно: никога не позволявайте течността на Марк-Андре да влезе в контакт с *Leishmania* – нито пряко, нито непряко чрез инструменти или игли – тъй като тя е смъртоносна за паразитите.

Дисекцията на женските флеботоми може да се извърши върху едно или два предметни стъкла; като двата варианта имат предимства и ограничения (Фигура 5; https://zenodo.org/records/18311154).

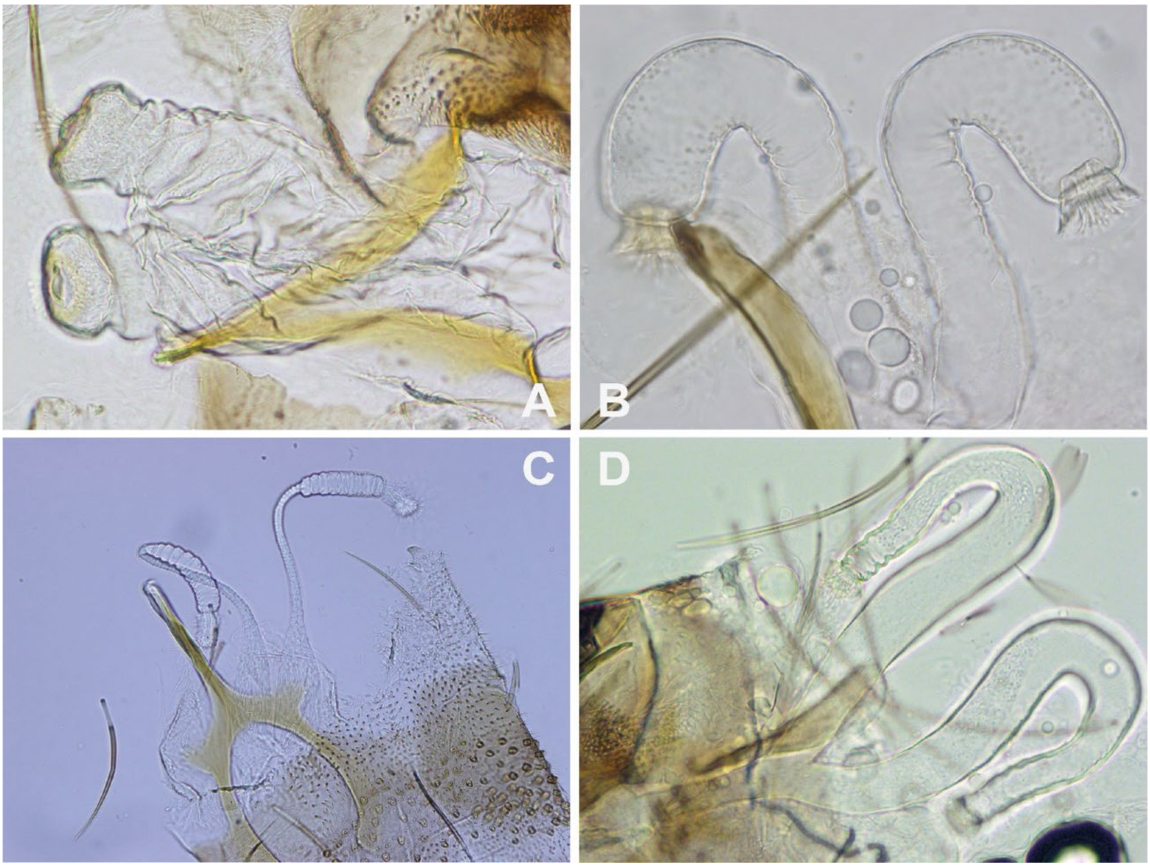

Фигура 4: Сперматеки, дисецирани и монтирани в течност на Марк-Андре от пресни екземпляри. A: *Idiophlebotomus longiforceps* (Лаоска НДР); B: *Sergentomyia minuta* (Франция); C: *Phlebotomus ariasi* (Франция); D: *Sergentomyia anodontis* (Лаоска НДР).

4.4.1. Метод с две предметни стъкла

Първият вариант включва работа с две отделни предметни стъкла: едно, съдържащо стерилен физиологичен разтвор за извличане на средното черво, и друго за поставяне на главата и сперматеките в течността на Марк-Андре. В полеви условия обаче е обичайно двама или трима души да дисецират флеботомите и да предадат дисекциите си на един изследовател, отговорен за определянето на видовете и оценката на инфекцията с *Leishmania* в червото. Работата с две предметни стъкла може да доведе до проблеми с проследяемостта на пробите и, по-специално, да затрудни определянето със сигурност кой флеботом е бил заразен, ако се открие положително черво (https://zenodo.org/records/18311154).

4.4.2. Метод с едно предметно стъкло

Използването на едно предметно стъкло гарантира проследимостта на резултатите. Въпреки това трябва да се вземат няколко предпазни мерки. За да се постигне максимална стерилност по време на тази стъпка, операторите трябва редовно да почистват ръцете си с водно-алкохолен гел. Трябва да се използват нематирани предметни стъкла и квадратни покривни стъкла (22 × 22 mm), увити в алуминиево фолио и стерилизирани чрез суха топлина (с помощта на сух стерилизатор тип Пупинел), както и стерилни игли за всяка дисекция. (препоръчително 25G Ø 0,5 мм × 16 мм). Папатацийната мушица се поставя в капка стерилен физиологичен разтвор в средата на покривно стъкло. Главата се отделя, докато се прави разрез между$6^{тия}$ и $7^{мия}$ коремни тергити и стернити, без да се прерязва храносмилателният тракт (може да се направи по-висок разрез, ако се очакват много дълги сперматеки). След това гърдите трябва да се фиксират с игла, а последните задни коремни сегменти да се изтеглят внимателно с другата игла, за да се извади червото. Ако това не успее, има възможност да се блокира краят на корема с игла и да се изтегли храносмилателният тракт от предната му част. Ако и това не успее, червото трябва да се извади, като се отстрани колкото се може повече от останалата телесна

покривка около него. След като червото е извадено, последните коремни сегменти трябва да бъдат отделени чрез разрязване на храносмилателния тракт. След това червото се поставя в нова капка стерилен физиологичен разтвор, разположена от едната страна на предметното стъкло, и се покрива внимателно със стерилно покривно стъкло. Главата и последните коремни сегменти се прехвърлят в малка капка течност на Марк-Андре, поставена в другия край на предметното стъкло, като се гарантира, че няма контакт с *Leishmania*. Главата се ориентира правилно (тилният отвор нагоре) и сперматеките се изолират с гениталната фурка, както е посочено по-горе, и се покриват с малко кръгло покривно стъкло (Ø 12 мм, да не се бърка със стерилните квадратни покривни стъкла). Остатъците от мушицата и крилата остават в капката физиологичен разтвор в средата на предметното стъкло (https://zenodo.org/records/18311154). В случай на положителна реакция или за таксономично изследване, гърдите и коремчето могат да бъдат запазени за молекулярни или протеомни изследвания, а крилата могат да бъдат монтирани във водна среда. За да се запази препаратът, излишният обем от течността на Марк-Андре може да бъде заменен с водна среда за монтиране, като хлорална смола (= течност на Хойер) или среда на базата на поливинилов алкохол.

Налични са подробни видеоклипове, демонстриращи тези процедури (дисекция на средното черво на папатацийна мушица: https://zenodo.org/records/18303014 и дисекция на слюнчените жлези на папатацийна мушица: https://zenodo.org/records/18302850), така че те няма да бъдат обяснявани тук.

4.4.3. Изолиране и култивиране на паразита *Leishmania* от червото на папатацийните мушици

Изолирането на паразити от дисекцията на заразени женски флеботоми е деликатна процедура, изискваща високи умения, и първоначално трябва да се практикува върху проби, които не съдържат паразити. След дисекцията червото се прехвърля в прясна капка стерилен физиологичен разтвор (0,9 %) или разтвор на Лок за измиване [4]. Дисецираното черво може да бъде обработено по два начина: i) да бъде изследвано под светлинен микроскоп, за да се наблюдават различните стадии на промастиготите *на Leishmania* и тяхното локализиране, с особено внимание към стомодеалната клапа, и ii) да се отвори червото, за да се улесни излизането на промастиготите, което улеснява тяхното масово култивиране [4]. Намирането на заразени флеботоми в полеви условия е сравнително рядко явление и затова добрите практически сесии ще увеличат шансовете за успешна изолация.

Ако в червото се наблюдават паразити *Leishmania*, трябва да се използват нови стерилни игли и да се добави малко количество стерилен физиологичен разтвор около покривното стъкло чрез капилярен ефект, за да се освободят. Червото трябва да се разкъса внимателно и бързо, за да се освободят паразитите във физиологичния разтвор. Съберете паразитите с помощта на 100 мкл микропипета или туберкулинова спринцовка и ги инокулирайте в подходящо етикетирана културелна среда.

Ин витро култура на промастиготи на *Leishmania*: изолираните паразити първоначално се поддържат на SNB-9 наклонен кръвен агар или в твърда среда Novy-McNeal-Nicolle (NNN) [16], покрити със стерилна среда alpha-MEM [16, 65] или със среда M199, всяка от които е с 10% термично инактивиран стерилен фетален телешки серум (FCS) (за стимулиране на растежа на паразитите), 1% BME витамини, 2% стерилна човешка урина (стерилизирана с помощта на спринцовков филтър Filtropur® S 0,2 мкм), 250 мкг/мл амикацин (или 50 мкг/мл гентамицин, или смес от антибиотици и аминокиселини (L-глутамин 200 мМ-пеницилин 10 000 U-стрептомицин 10 мг/мл) [47]. След три дни, ако няма замърсяване, културите се суспендират в подходящо подготвена среда за замразяване и след това се съхраняват при -80°C в продължение на 1-2 години или в течен азот при -196°C за дългосрочно съхранение и бъдеща експериментална употреба [7].

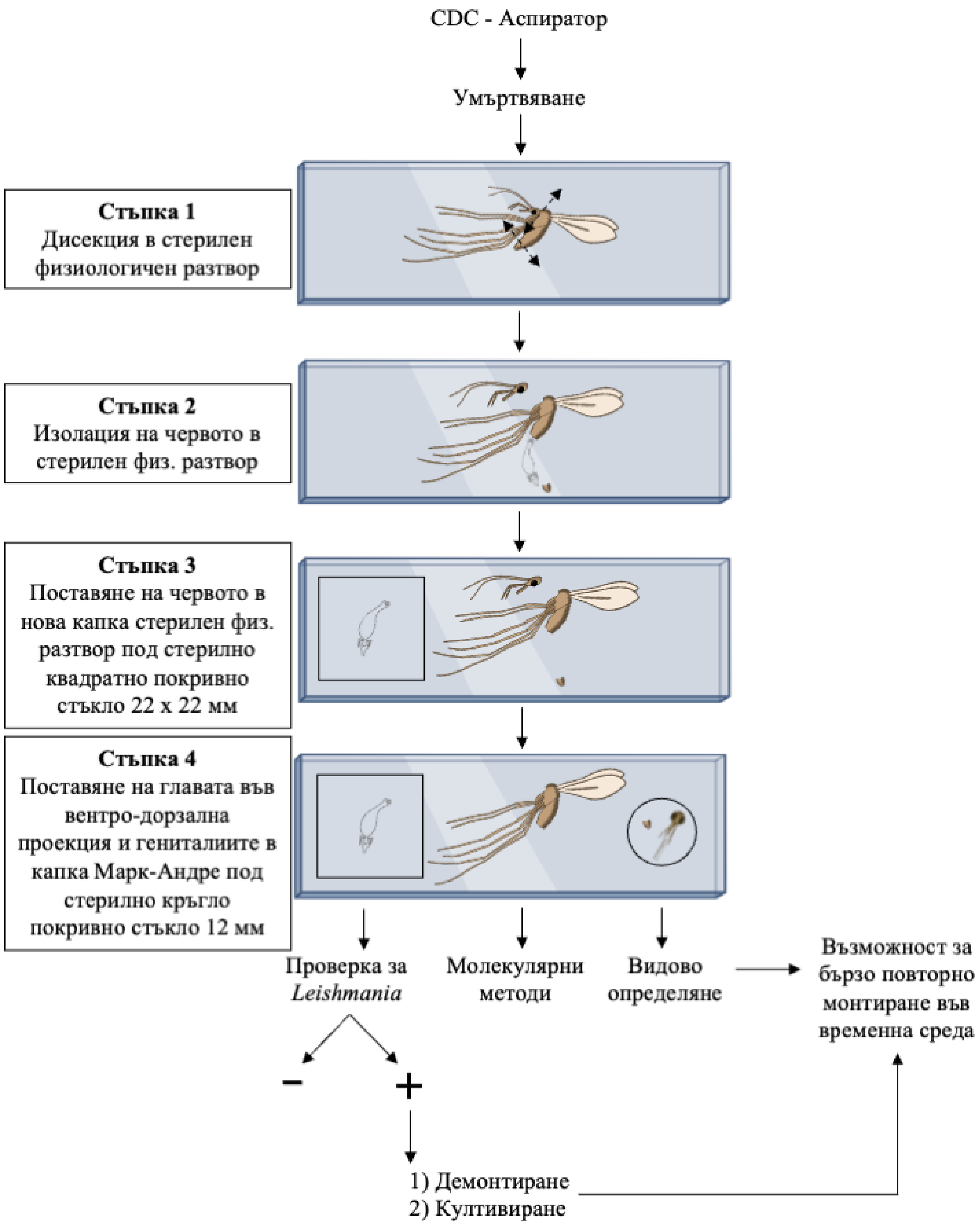

Фигура 5: Метод за изолиране *на Leishmania*

4.5. Слюнчени жлези

Дисекцията на слюнчените жлези на флеботомите е основна техника за изучаване на взаимодействията между вектори и патогени, особено за откриване на арбовируси като *Phlebovirus* (например вируса Тоскана) [44, 75]. Поради миниатюрния размер на флеботомите, процедурата изисква прецизност под стереомикроскоп, като се използват фини пинсети или игли за микродисекция, за да се изолират деликатните слюнчени жлези, без да се причинява разкъсване или замърсяване (https://zenodo.org/records/18302850) [51, 61]. Запазването на целостта на жлезите е от решаващо значение за осигуряване на надежден молекулярен анализ нататък по веригата. След извличането им, жлезите могат да бъдат хомогенизирани и тествани чрез RT-PCR, qPCR или имунологично за откриване на вирусна РНК или антигени [12]. Наличието на вируси в слюнчените жлези, а не само в червата или хемоцела, потвърждава, че патогенът е завършил своя външен инкубационен период и е преносим по време на кръвосмукане [71].

Процесът на дисекция е технически труден поради малкия размер на слюнчените жлези на флеботомите,

This is an Open Access article distributed under the terms of the Creative Commons Attribution License (https://creativecommons.org/licenses/by/4.0), which permits unrestricted use, distribution, and reproduction in any medium, provided the original work is properly cited.

което изисква значителен опит, за да се избегне разграждането на пробите [1, 51]. Освен това вирусният товар може да е нисък, което налага използването на високочувствителни методи за откриване, като nested PCR или секвениране с висока производителност [54]. Рискът от замърсяване допълнително подчертава необходимостта от стерилни техники. Освен техническите пречки, биологичните фактори също влияят върху успеха на откриването; векторната компетентност варира между видовете флеботоми, а степента на заразяване се променя в зависимост от екологичните и сезонните условия [33, 61].

Откриването на вируси в слюнчените жлези предоставя важни данни за рисковете от предаване, което позволява целенасочени мерки за наблюдение и контрол [15]. Например, идентифицирането на вируса Тоскана във флеботомите в ендемични региони е послужило за изготвянето на диагностични протоколи и препоръки за обществено здраве [18]. Освен това, изучаването на взаимодействията между вируса и слюнката може да разкрие нови цели за ваксини или терапевтични средства, блокиращи предаването [15, 18].

Слюнчените жлези на флеботомите могат да се използват и като източник на антигени за измерване на антителата на гостоприемника срещу слюнката на папатацийните мушици с помощта на имунологични методи, за предпочитане ELISA. Този метод позволява оценка на излагането на гостоприемника на ухапвания от флеботоми, като по този начин подпомага оценката на ефективността на методите за контрол на преносителите [25] и риска от предаване *на Leishmania* [40].

4.6. Идентифициране на поетата кръв

Насмуканите с кръв женски екземпляри, изолирани от уловите, трябва да бъдат дисецирани с помощта на еднократно оборудване, за да се предотврати кръстосано замърсяване. Коремчето им трябва да бъде изследвано под стереомикроскоп, за да се оцени стадият на смилане на поетата кръв. Препоръчва се да се подбират само женски екземпляри с червено, червеникаво-кафяво или тъмночервено коремче, без признаци на образуване на яйца. Отстранете върха на коремчето, включително сперматеките, за да определите женския екземпляр морфологично след просветляване. След това основната част на коремчето (без сперматеките) трябва да се постави в епруветка Eppendorf® и да се съхранява при -20°C до по-нататъшен анализ. Генетичните маркери, които обикновено се използват за идентифициране на поетата кръв, като PNOC [5, 30, 50], CytB [67] или COI [13], са добре установени и подробно описани в литературата; поради което няма да бъдат подробно разгледани в настоящата статия (Фигура 6). Алтернативно, за идентифициране на кръвта на гостоприемника може да се използва MALDI-ToF пептидно картиране [31]. Експериментално е доказано, че тази техника позволява идентифициране на кръвта на гостоприемника в по-дълъг период от време след кръвосмукането; следователно тя е подходящ метод, особено за анализ на насмукани женски екземпляри с видимо по-напреднало разграждане на кръвта на гостоприемника. В идеалния случай пробите трябва да се съхраняват при -20°C или 4°C, но добри резултати могат да се получат и от проби, съхранявани при стайна температура за кратко време. Коремчето на насмуканата с кръв женска трябва да се отдели от останалата част на тялото малко преди анализа и да се хомогенизира в дестилирана вода. Останалата част от тялото на насекомото остава на разположение за други молекулярни и морфологични анализи. След като се вземе аликвотна проба от хомогената за MALDI-ToF пептидно картиране, останалата част може да се използва за изолиране на ДНК, за да се потвърди идентификацията на кръвта на гостоприемника и/или да се провери за наличието на *Leishmania* sp. Общото време за подготовка и анализ на пробите е много кратко в сравнение с молекулярните техники, основани на ДНК.

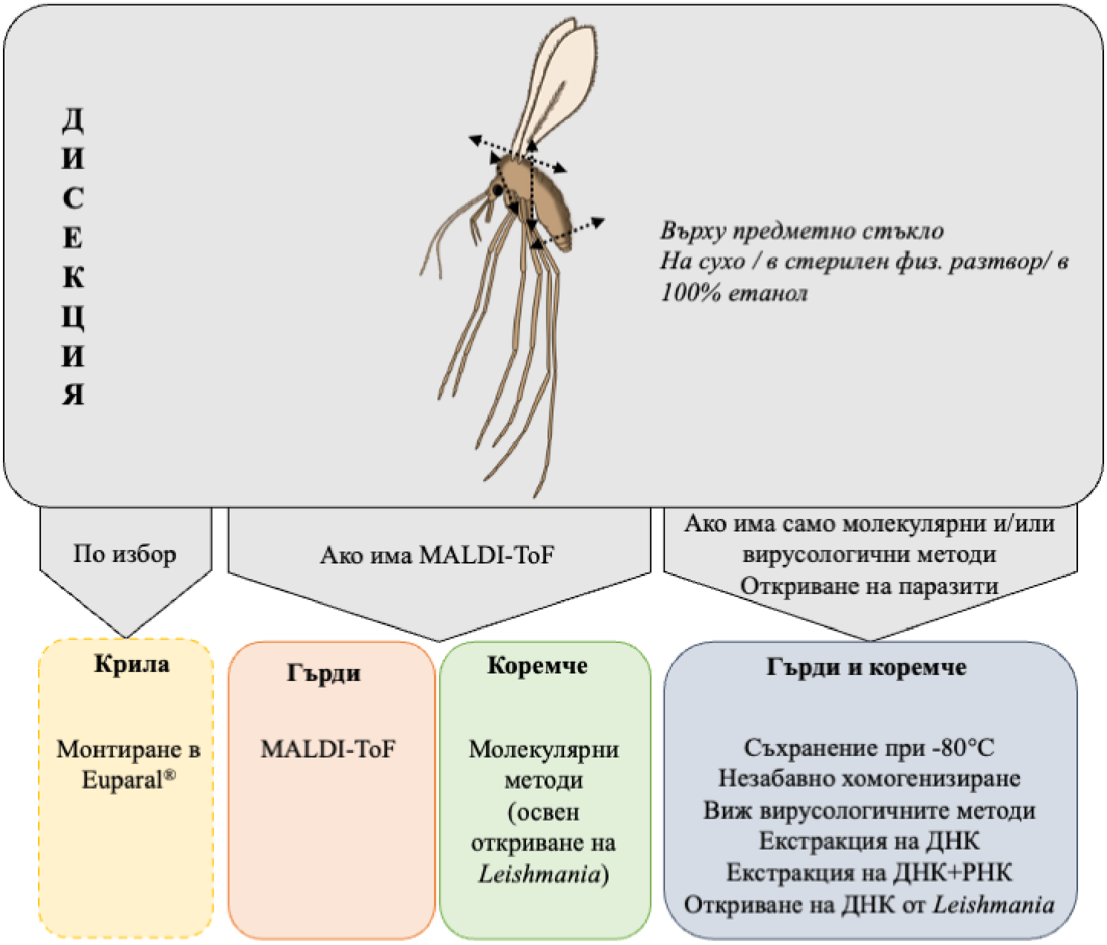

Фигура 6: Обработка на флеботоми за приложения в молекулярната биология, протеомиката и/или вирусологията.

5. Обработка на проби за морфологични изследвания (Фигури 3, 6, 7 и 8; Приложения 1, 2, 3 и 4)

В тази секция са описани принципите за подготовка на екземпляр флеботом за монтиране единствено за морфологични изследвания, последвано от адаптиране за приложения извън морфологията. Разбирането на тази методология е от решаващо значение, тъй като позволява процедурите да бъдат адаптирани за конкретни видове проби, когато е необходимо.

Обработката включва последователни стъпки на изпразване и пълнене с помощта на пастьорови пипети, оборудвани с гъвкави гумени балончета. Силно се препоръчват стъклени съдове с кръгло дъно, тъй като значително улесняват тези операции. Стъклото е инертно към всички реагенти. За да се предотврати изпаряването на реагентите, съдовете трябва да бъдат снабдени с капаци и никога да не се препълват, което води до преливане при затваряне или отваряне, както и да се предотврати попадането на прах върху пробите. Химикалите, необходими за просветляване и обработка, са изброени в Таблица 2.

This is an Open Access article distributed under the terms of the Creative Commons Attribution License (https://creativecommons.org/licenses/by/4.0), which permits unrestricted use, distribution, and reproduction in any medium, provided the original work is properly cited.

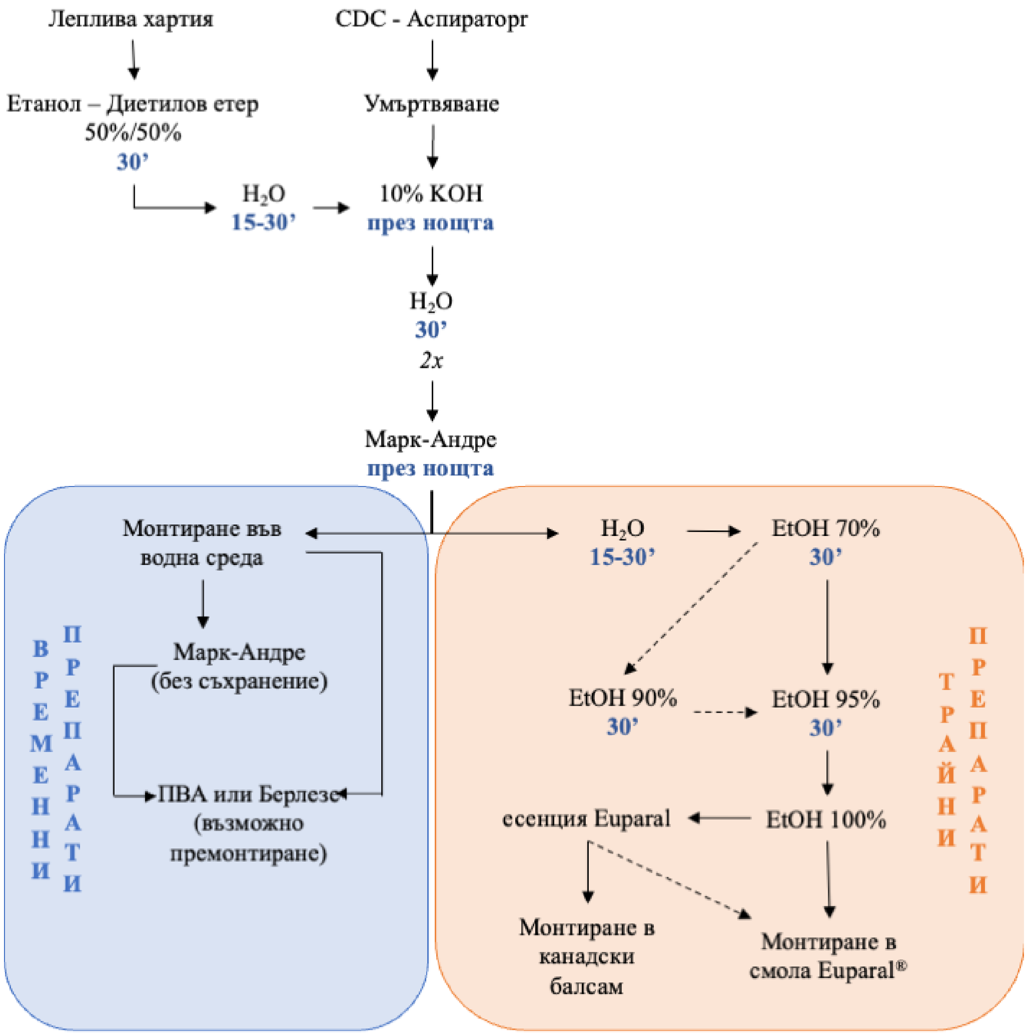

Фигура 7: Класически метод за обработка на флеботоми.

Таблица 2: Състав на използваните реагенти.

**Калиев хидроксид 10%**	**Кисел фуксин 1% в дестилирана вода**
Калиев хидроксид 10 г	Кисел фуксин (под формата на прах) 1 г
Дестилирана вода *qs* 100 мл	Дестилирана вода 99 мл
**Хлорална смола (среда на Хойер)**	**Разтвор на Марк-Андре, оцветен с кисел фуксин**
Дестилирана вода 50 мл	Разтвор на Марк-Андре 10 мл
Хлорал хидрат 200 г	Фуксин 1% 50 мкл
Гума арабика 50 г	
Глицерин 20 мл	
**Разтвор на Марк-Андре**	**Среда Enecê**
Хлорал хидрат 40 г	Чист бял колофон 22 г
Ледена оцетна киселина 30 мл	Копалова смола, разтворима в алкохол 12 г
Дестилирана вода 30 мл	Абсолютен етанол 20 мл
	Камфор 10 г
	Терпентинова есенция 10 мл
	Евкалиптол 26 мл

This is an Open Access article distributed under the terms of the Creative Commons Attribution License (https://creativecommons.org/licenses/by/4.0), which permits unrestricted use, distribution, and reproduction in any medium, provided the original work is properly cited.

5.1. Просветляване

Преди да бъдат подготвени като постоянни препарати, екземплярите от флеботоми трябва първо да бъдат просветлени чрез мацерация, като се използва подходящ метод и просветляващ агент (например 10% разтвор на оцетна киселина или разтвор на Марк-Андре, включващ хлорал хидрат, който е химикал с ограничено използване в много страни), за да станат прозрачни. Процесът на просветляване премахва телесните тъкани, мазнините, секретите и восъка, като прави екземпляра прозрачен, което улеснява изследването на екзоскелетните структури (например местата на закрепване на четинки), характеристиките на повърхността (например оцветяване) и вътрешните характеристики, видими през тегумента (например сперматеки).

Двуетапният процес на просветляване, който включва първо използване на силна основа (като калиев хидроксид), последвано от слаба киселина (като оцетна киселина в разтвор на Марк-Андре), служи за различни биохимични цели [74]. Основата разгражда меките тъкани, като протеини, мазнини и мускули, чрез осапуняване и денатурация на протеините, оставяйки хитиновия екзоскелет непокътнат за структурна яснота. Следващата слаба киселина неутрализира останалата основа, предотвратявайки по-нататъшно разграждане, и избелва хитина, за да подобри прозрачността [74], въпреки че измиването на пробите два пъти в дестилирана вода за 15 минути също може да бъде достатъчно да неутрализира основата. Това последователно третиране комбинира ефективно отстраняване на тъкани с деликатно съхранение, осигурявайки оптимална цялост на пробите за микроскопско изследване. Препоръчват се две 20-минутни изплаквания в дестилирана вода, преди да се премине към следващата стъпка.

5.1.1. Разграждане на меките тъкани (Фигура 8)

Натриевият хидроксид (NaOH) или калиевият хидроксид (KOH) са често използвани химически мацериращи агенти, които се прилагат в различни концентрации и за различно време в зависимост от размера и крехкостта на екземплярите. Стандартната и най-ефективна техника включва разграждане на меките тъкани чрез накисване на флеботомите в силна основа (10% KOH или NaOH) през нощта. Концентрацията може да се увеличи, за да се намали продължителността на третирането (т.е. KOH 20% за 6 часа), както и да се загрее до 37°C.

5.1.2. Просветляване с или без оцветяване

Тази стъпка се последва от просветляване, обикновено чрез комбинация от оцетна киселина и хлорал хидрат (напр. разтвор на Марк-Андре). След просветляването пробите трябва да се изплакнат обилно в поне две последователни водни бани по 20 минути, за да се отстранят остатъците от химикали.

Разтворът на Марк-Андре е често използван просветляващ агент за подготовка на проби от флеботоми. Неговата ефективност се състои в улесняване на процеса на просветляване, като същевременно се минимизира значителното увреждане на крехките структури, като крила и антени.

Разтворът трябва да бъде прясно приготвен или съхраняван в плътно затворен съд, за да се предотврати изпаряване или разграждане. Използването на разтвор на Марк-Андре е особено полезно, когато се комбинира с техники за просветляване или оцветяване, за да се подчертаят специфични морфологични детайли. Подробности за състава и приготвянето му са дадени в Приложение 2.

За силно прозрачни екземпляри може да е необходимо оцветяване, за да се подобри видимостта преди монтиране. Налични са много оцветители, всеки от които е насочен към конкретни химични компоненти на организма. Важно е да се избере оцветител, който е съвместим както с екземпляра, така и с избраната среда за включване. Тази основна методология може да се адаптира според нуждите, например чрез добавяне на 0,1% кисел фуксин в разтвора на Марк-Андре за оцветяване. Освен това, пробите, консервирани във водни разтвори и предназначени за смолисти среди за включване, изискват обезводняване (виж раздел 5.2. Обезводняване), тъй като повечето естествени и синтетични смолисти микроскопски среди са несъвместими с вода. New (1974) отбелязва, че някои оцветители могат да се разградят в определени среди за включване [53]. Например, киселият фуксин, който се използва често с канадски балсам, може да се фиксира и в Euparal®. Оцветените с кисел фуксин проби обаче са податливи на избледняване, особено когато има останки от карамфилово масло, използвано като последна просветляваща течност. Пробите, съхранявани в карамфилово масло, могат да избледнеят значително в рамките на няколко дни.

This is an Open Access article distributed under the terms of the Creative Commons Attribution License (https://creativecommons.org/licenses/by/4.0), which permits unrestricted use, distribution, and reproduction in any medium, provided the original work is properly cited.

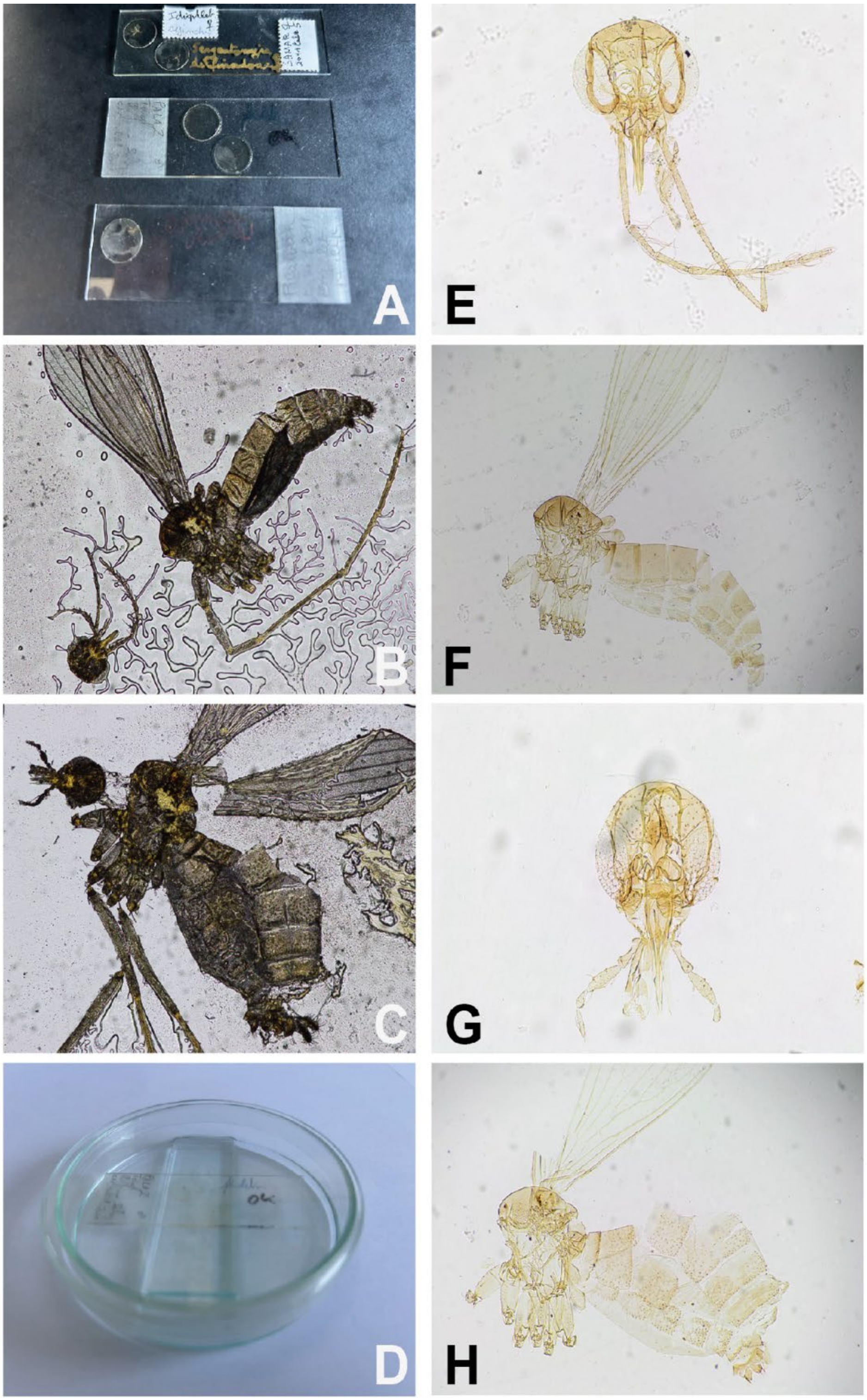

Фигура 8: Повторно монтиране на микроскопски препарат. A: повредени и изсушени препарати, монтирани в среда на Хойер; B: микроскопски изглед на изсъхнала папатацийна мушица; C: микроскопски изглед на друга повредена папатацийна мушица; D: влажна камера с изсъхнал микроскопски препарат; E: глава и F: тяло на образец B след повторно монтиране в Euparal® ; G: глава и H: тяло на повреден образец C след повторно монтиране в Euparal®.

5.2. Обезводняване

Обезводняването се извършва чрез постепенно прехвърляне на пробите през серия от разтвори на етанол с различна концентрация: 50%, 70%, 80%, 90% или 95% и накрая 100%, като всяка баня трае най-малко 20 минути. Тъй като етанолът се изпарява бързо, съдът трябва да бъде плътно затворен по време на обработката. След като пробата е напълно обезводнена, можете да прекъснете обработката за няколко дни в Euparal® есенция, която е за предпочитане пред карамфилово масло. Буковият креозот, който някога се използваше широко за тази цел, сега е напълно забранен поради токсичността си.

Процесът на обезводняване трябва да гарантира, че течността в пробата е съвместима със средата за включване, за да се предотврати потъмняване, осмотичен колапс или деформация, които биха могли да направят пробата неподходяща за таксономично изследване.

5.3. Микроскопски среди

5.3.1. Избор и приложение за препариране на образци

В идеалния случай средата за включване трябва да има коефициент на пречупване, колкото се може по-близък до този на стъклото, който е приблизително 1,5. Тя трябва да е безцветна, бистра и да остава напълно прозрачна след изсъхване и с течение на времето. Трябва да е съвместима с използваните оцветители и да може да прониква и да се разпространява във всички тъкани на образеца. Не трябва да изсъхва прекалено бързо или да помътнява по време на монтирането. Не трябва да се свива след монтирането. Изборът на подходяща среда за включване е основен аспект от подготовката на пробата, тъй като няма една среда, която да е идеална за всички цели. Изборът трябва да балансира няколко ключови фактора:

- **Оптични свойства**. Коефициентът на пречупване на микроскопската среда трябва да осигурява достатъчен контраст и пречупване за критични анатомични характеристики, използвани за таксономично определяне или морфологично описание, като сперматеки, аскоиди, сензили на Нюстед, вертикални цибариални зъби и фарингеални зъби. Видимостта на тези структури зависи пряко от оптичните свойства на микроскопската среда.

- **Съхранение**. За типови екземпляри или материали, предназначени за постоянни колекции, средата трябва да осигурява дълготрайна стабилност и издръжливост. За разлика от това, за инвентаризационни проучвания или епидемиологични изследвания, при които дългосрочното съхранение е по-малко важно, временни или полутрайни микроскопски среди могат да бъдат достатъчни.

5.3.2. Изисквания към микроскопските среди

Специалистите често разработват персонализирани и сложни техники за монтиране, съобразени с конкретните нужди на изследванията. Тези методи обаче често пренебрегват аспекти като архивно качество, съвместимост, стандартизация или лекота на работа и дългосрочно съхранение. Липсата на стандартизация затруднява интегрирането на дарени колекции и дългосрочните усилия за съхранение.

Научните приложения налагат специфични изисквания към микроскопските среди. Таксономите често монтират цели екземпляри и предпочитат средства, които нежно мацерират вътрешните органи, за да подобрят видимостта на кутикулните структури. Коефициентът на пречупване трябва да се различава достатъчно от този на екземпляра и стъклото на препарата, за да се постигне максимална оптична яснота. Търговските микроскопски среди обикновено се формулират с коефициент на пречупване, близък до този на стъклото, за да се сведе до минимум пречупването и разсейването на светлината през системата предметно стъкло – микроскопска среда – покривно стъкло. При микроскопията в светло поле обаче, естественият контраст на неоцветен образец може да се манипулира чрез целенасочен избор на микроскопска среда с коефициент на пречупване, леко различаващ се от този на образеца, като по този начин се подобрява неговата видимост на фона.

5.3.3. Видове микроскопски среди (Таблици 3 и 4)

Микроскопията изисква познаване на коефициента на пречупване (КП) на микроскопската среда, за да се определи как светлината се пречупва при преминаването си през покривното стъкло, средата и пробата. Когато КП е близък до този на покривното стъкло (≈1,515), светлината преминава равномерно, намалявайки разсейването и оптичните изкривявания, което води до по-добра разделителна способност и видимост на фините структури. Обратно, несъответствие в КП може да причини замъгляване, ореоли или неясни неоцветени структури. Изборът на подходяща микроскопска среда е от решаващо значение за оптимизиране на контраста, яснотата и цялостното качество на изображението за даден образец, поради различните КП на различните среди.

Коефициентът на пречупване на микроскопската среда има значително влияние върху това колко добре се виждат фините структури при подготовката на флеботоми за монтиране на предметно стъкло. Деликатните и леко склеротизирани белези на флеботомите, включително цибариалната арматура, сперматеките, антенните сегменти и жилкуването на крилата, могат да бъдат трудни за наблюдение в микроскопска среда с висок коефициент на пречупване.

За флеботомите често използваните опции включват хлорална смола като среда за монтиране на водна

основа и канадски балсам и смола Enecê - Nelson Cerqueira (NC) като среда на основа органичен разтворител. Rawlins [60] класифицира микроскопските среди в два типа: (1) трайни среди: втвърдяват се с времето и са подходящи за дългосрочно съхранение, и (2) полутрайни среди: не се втвърдяват и обикновено се използват за временни цели.

Микроскопските среди могат да бъдат течни, на основа смола или гума, разтворими във вода, алкохол или други разтворители (напр. толуол, ксилол) (Таблица 3). След нанасянето им, те трябва да бъдат изолирани от атмосферните влияния с помощта на неразтворими средства за запечатване. За да се направи ясно разграничение между типовете микроскопски среди, може да се използва следната категоризация:

а. Водни среди. Тези среди се разтварят лесно във вода, което ги прави подходящи за временни или полутрайни препарати. Обикновено са лесни за употреба, но може да се наложи да бъдат запечатани, за да се предотврати излагането им на атмосферна влага (т.е. средства на базата на хлорална смола и поливинилов алкохол), особено в тропичен влажен климат.

б. Среди с ограничена водна поносимост. Тези среди се влияят по-малко от вода, но все пак изискват защита от прекомерна влажност. Те осигуряват по-голяма дългосрочна стабилност в сравнение с водоразтворимите варианти и често се използват за полутрайни препарати.

в. Среди, разтворими във въглеводороди. Тези среди се разтварят в органични разтворители като ксилол или толуол, или essenecê (Enecê разтворител). Те са предназначени за трайни препарати и предлагат отлична дългосрочна стабилност, устойчиви са на влага и разграждане, което ги прави идеални за архивни цели (например неутрален канадски балсам, DPX).

В обобщение, водоразтворимите среди са най-подходящи за временни препарати или случаи, изискващи лесно отстраняване на пробата; средите с ограничена водоустойчивост са подходящи за полутрайни препарати, изискващи умерена трайност, а разтворимите във въглеводороди среди се предпочитат за трайни препарати, предназначени за архивиране и дългосрочно съхранение.

Таблица 3: Състав на избрани монтажни средства.

**Монтажно средство**	**Разтворител**	**Потенциални преполимери или полимери**	**Забележки**
Хойер = хлорална смола	глицерин, вода	съединения на гума арабика	Мацериращ агент: хлорал хидрат
CMCP-9 (= карбоксиметил целулоза фенол)	вода (CMCP-9: 51−60%)	напълно хидролизиран поливинилов алкохол (CMCP-9: 0−5%)	CMC(P)-9: нисък вискозитет: висок вискозитет
DMHF (диметил хидантоин формалдехид)	вода	N,N′-диметилол диметил хидантоин (ди-метилол DMH)<br>Етер-/метилен-свързани олигомери<br>Кръстосана DMH–формалдехидна полимерна мрежа	
Канадски балсам	ксилол; частично летливи компоненти на балсама (Δ^3-карен, левопимарова киселина, лимонен, мирцен, палустринова киселина, β-феландрен, α-пинен, β-пинен)	балсам (абиенол, абиетинова киселина, изопимарова киселина, сандаракопимарова киселина)	неутрализация: калиев карбонат;<br>смола от *Abies balsamea* (Linné, 1758)
Euparal®	евкалиптол, паралдехид; частично летливи компоненти на смолата сандарак (лимонен, α-пинен, β-пинен)	съединения на смола сандарак (комуникова киселина, маноол, поликомуникова киселина, сандаракопимарова киселина, 12-ацетокси-сандаракопимарова киселина, сугиол, торулозова киселина, торулозол, тотарол)	просветляващ агент: метил салицилат; оцветител в Euparal® зелен: медна сол (меден абиетинат); смола сандарак от *Tetraclinis articulata* (Vahl, 1791)
Enecê	етилов алкохол; с камфор, евкалиптол и терпентинова есенция	Съединения на копалова смола и колофон (росин))	

Таблица 4: Предимства и недостатъци на избрани среди за микроскопски препарати и непубликувани наблюдения на различни лица [52].

Име	Предимства	Недостатъци
* Канадски балсам	Средата е много издръжлива, с живот над 150 години.<br>Препаратите могат да се монтират с помощта на карамфилово масло или фенол като монтажни агенти.	Съдържа вредни компоненти и с него трябва да се борави в аспирационна камера.<br>Изисква пълна, времеемка обезводняваща серия.<br>Обезводняването с етанол и прехвърлянето чрез ксилол или карамфилово масло може да направи някои таксони крехки; алтернативите (например изопропанол, н-бутанол, Cellosolve™, 1,4-диоксан, Histoclear, терпинеол) могат да намалят счупването.<br>Пробите могат да почернеят, ако ксилолът бъде заменен с фенол или ако останат остатъци от калиев хидроксид.<br>Високите коефициенти на пречупване могат да скрият неоцветените структури.<br>Пълното изсушаване може да отнеме години без сушене на нагряваща плоча.<br>Средата пожълтява и потъмнява с времето, особено когато е просветлена с карамфилово масло.<br>Някои бои отслабват, а катионните багрила могат да избледнеят, ако средата стане кисела, което може да се случи спонтанно с течение на времето.
DMHF (диметил хидантоин формалдехид)	Висока прозрачност<br>Добър коефициент на пречупване<br>Отлична видимост на структурите<br>Сравнително добра стабилност на препаратите<br>Съвместим с много техники за оцветяване<br>Добра защита на пробите<br>Добра адхезия между предметното и покривното стъкло	Възможно пожълтяване с течение на времето<br>Може да промени някои бои<br>Не е подходящ за оцветяване, чувствително към формалдехид<br>Въздушни мехурчета, бавно изсъхване<br>Микроскопската среда е чувствителна към влажност<br>Монтирането е почти необратимо<br>Формалдехидът е токсичен, дразнещ, канцерогенен
* Euparal (прозрачен)	Устойчива среда с живот над 50 години.<br>Възможно е монтиране директно от 80% етанол (препоръка на производителя).<br>Не маскира неоцветени структури и не пожълтява или не става крехък с течение на времето.<br>Има по-подходящ коефициент на пречупване от канадския балсам за Diptera.<br>Подходящ за по-дебели образци, благодарение на минимално свиване и изсъхване без мехурчета.<br>Остава разтворим в 95% етанол, което позволява повторно монтиране дори след много години.	Съдържа вредни компоненти и с него трябва да се борави в аспирационна камера.<br>Обезводняването с етанол и прехвърлянето чрез Euparal есенция може да направи някои таксони крехки, но използването на изопропанол може да намали този проблем.
Течност на Хойер	Пробите могат да се монтират живи или направо от вода, етанол или формалдехид.<br>Мацерацията дава отлично качество на кутикулата.<br>Има благоприятен коефициент на пречупване и може да бъде подобрен с оцветяване с йод за по-висок контраст.<br>Оцетната киселина във формулата може да	Деликатните растителни образци могат да се разпаднат, освен ако средата не се добавя постепенно, което отнема много време.<br>В рамките на по-малко от 10 години могат да се образуват кухини и кристали.<br>Мацерацията може да стане прекомерна в зависимост от концентрацията на хлорал хидрат и времето на експозиция.

This is an Open Access article distributed under the terms of the Creative Commons Attribution License (https://creativecommons.org/licenses/by/4.0), which permits unrestricted use, distribution, and reproduction in any medium, provided the original work is properly cited.

	разшири придатъците на членестоногите.<br>Някои проби могат да останат стабилни в продължение на 40–60 години.<br>Водоразтворима, което позволява лесно повторно монтиране.	Компонентите на средата могат да се разделят и в рамките на месеци или години може да се появи фина гранулация.<br>Съобщава се за почерняване на средата.
CMCP-9 (= карбоксиме тил целулоза фенол)	Пробите могат да се монтират направо от среди като вода, етанол, глицерол или разтвори, съдържащи формалдехид, а вътрешните им органи могат да бъдат мацерирани, когато е необходимо, за да се улесни общото изследване или препариране.	Тази среда може да образува кристали и да потъмнява с течение на времето, а понякога може да мацерира пробите повече от необходимото. Освен ако покривно стъкло не е внимателно кантирано, по-дебелите проби не се представят добре в нея, защото могат да се свият и да създадат празнини около краищата на покривното стъкло. Не е подходяща за оцветени проби или калцифицирани материали, а времето за изсъхване е по-бавно от това на CMC.
Eukitt™	Устойчива среда с трайност над 30 години.<br>Съвместима с много разтворители за монтиране, включително ацетон, бензен, хлороформ, диоксан, етер, изопропанол, метил бензоат, терпинеол, толуол и ксилол.<br>Изсъхва бързо и има леко кисела pH стойност.<br>Не потъмнява забележимо с времето.<br>Подходяща за различни оцветители (например фуксин, хематоксилин, метил зелено, метил виолетово, метиленово синьо).<br>Пробите могат да бъдат монтирани отново след години, като се накиснат в ксилол за по-дълъг период от време.	Съдържа вредни компоненти и с нея трябва да се борави в аспирационна камера.<br>Изисква пълна, времеемка обезводняваща серия.<br>Не е идеален за по-дебели проби поради свиване и образуване на мехурчета.<br>Покривните стъкла могат да се отлепят с времето, освен ако стъклото не е добре почистено и запечатано.<br>Може да се наблюдава непълна полимеризация около колагеновите влакна.
Enecê	Изключително издръжлива среда, с дълготрайност от поне 50 години.<br>Enecê не потъмнява с течение на времето.<br>По-пластична е, което позволява дисекция на насекоми в средата, както и предоставя достатъчно време за позициониране на морфологичните структури.<br>Ниска цена.	Изисква пълна, времеемка обезводняваща серия.<br>Обезводняването с етанол и прехвърлянето чрез карамфилово масло може да направи някои екземпляри крехки.<br>Насекомото продължава да се просветлява, макар и много бавно; което може да затрудни виждането на много малки структури, като сенсили, аскоиди и прости четинки.

5.3.4. Описание на препоръчителните микроскопски среди (Таблици 3 и 4)

<u>*Среди за временно наблюдение*</u>

Хлорална смола = течност/среда/разтвор на Хойер (КП = 1,48)

Течността на Марк-Андре е най-добрата среда за краткосрочно (няколко часа, може би малко повече, ако препаратът се съхранява във влажна камера) наблюдение на сперматеките, включително фотографиране (Фигура 4) или рисуване. За да се съхранят наблюдаваните сперматеки, е необходимо да се монтират отново във водна среда, която позволява средносрочно съхранение. Обезводняването им за повторно монтиране в смола не е невъзможно, но не се препоръчва (риск от загуба). Хлоралната смола и течността на Хойер се считат за синоними. Тази среда се използва често за наблюдение на вътрешни органи поради своята съвместимост с вода, простота, бързо нанасяне и коефициент на пречупване, който улеснява изследването на деликатни структури като сперматеките. Хлоралната смола обаче има значителни недостатъци, ако не е перфектно подготвена или съхранявана при контролирани условия на влажност. Тези проблеми включват кристализация, обезцветяване и загуба на вискозитет. Кантирането на покривното стъкло не решава тези проблеми, тъй като микроскопската среда може да се обезцвети силно (понякога почти почернява) поради взаимодействието с кантиращата среда, особено ако се използва Euparal®.

Средата на Хойер се счита за най-добрата от оптична гледна точка за флеботомни мушици и традиционно се използва за тези цели. Средата се състои от няколко сродни формули, включващи гума арабика, глицерол и хлорал хидрат. Различните формули са били погрешно интерпретирани и цитирани [74].

Въпреки че Хойер е добра среда за наблюдение на сперматеките при флеботомите, тя не е подходяща за дългосрочно съхранение. Тя е идеална за краткосрочни наблюдения, включително фотографии, рисунки или изображения. Водните среди са подходящи за временни препарати, но не могат да гарантират дългосрочно съхранение. За разлика от това, монтирането в смола осигурява отлична трайност, често продължаваща векове, но може да скрие фините детайли на сперматеките, тъй като тяхната рефракция често се губи.

Средата на Хойер се разгражда с времето поради обезводняване (Фигура 8), което води до образуването на малки бели, непрозрачни кристали от хлорал хидрат. Въпреки това, пробите могат да бъдат възстановени от кристализиралите препарати, тъй като кутикулата остава химически непокътната, макар че може да настъпи известно физическо увреждане от растящите кристали. В някои случаи кристализиралите препарати могат да бъдат възстановени чрез рехидратиране на микроскопската среда в топла, влажна камера с тимол, за да се предотвврати растежът на гъбички. Алтернативно, пробите могат да бъдат разкиснати от хлоралната смола във вода, обезводнени в ледена оцетна киселина и монтирани отново в канадски балсам.

DMHF (диметилхидантоин формалдехид) (КП 1,48)

Тази среда на водна основа [72] има много добри оптични характеристики, подобно на средата на Берлезе, и е също толкова лесна за употреба. Въпреки това, за разлика от средата на Берлезе, тя не почернява и не кристализира. Подходяща е за флеботоми и други Psychodidae.

CMCP (камфор-монохлорфенол) (КП = 1,41)

Това е водоразтворими среда за монтиране на основата на глицерин, която се използва за създаване на прозрачни, трайни препарати на деликатни екземпляри, включително флеботоми. Предимството на тази микроскопска среда е, че екземплярите могат да се монтират директно от вода или етанол. Тя бързо отпуска и просветлява насекомото, омекотявайки кутикулата, за да позволи правилното разполагане на екземпляра, което е особено полезно при разпъване на крилата или дисекция на гениталиите. Въпреки че се съобщава, че позволява дългосрочно съхранение, точната продължителност остава неясна. Основното ограничение на тази микроскопска среда се състои в състава ѝ, който съдържа фенол – токсично и дразнещо вещество, което изисква внимателно боравене.

<u>*Среди за трайно монтиране*</u>

Канадски балсам (КП = 1,52-1,54)

Канадският балсам е описан за първи път като подходяща среда за включване за микроскопия с преминаваща светлина от Андрю Причард през 1830-те години. Той остава един от най-широко използваните материали благодарение на доказаното си архивно качество, с над 150 години успешно приложение. За разлика от течността на Хойер, канадският балсам не кристализира и не абсорбира влага. Той обаче е силно автофлуоресцентен, което понякога може да бъде недостатък за определени микроскопски техники [60]. Използването на нетоксични разтворители вместо ксилол може да намали рисковете за безопасността по време на подготовката, но може да доведе и до някои недостатъци, като по-бавно изсъхване и по-ранно потъмняване на средата.

Euparal® (КП = 1,48)

Euparal® е широко използвана алтернатива на канадския балсам за трайно монтиране, предлагаща отлична дългосрочна стабилност и сравним коефициент на пречупване. Euparal® има следните характеристики: (1) изискване за обезводняване: преди окончателното прехвърляне в микроскопската среда, пробата трябва да бъде обезводнявана, обикновено преминавайки от 95% към абсолютен етанол, и (2) удължено време за обработка: окончателното сглобяване в смола, независимо дали е канадски балсам или Euparal® , изисква обезводняване, което удължава общото време за обработка на пробата. Когато обезводняването с органични разтворители не е възможно, пробите, извлечени от абсолютен етанол, могат да бъдат поставени в междинен разтвор, състоящ се от равна смес от Euparal® и Euparal есенция, преди окончателното монтиране.

Enecê (КП = 1,467)

Enecê е смола, използвана предимно за малки насекоми и особено популярна в Бразилия. В основата ѝ стоят колофон и копал, разтворени в алкохол, камфор, терпентинова есенция и евкалиптол. Cerqueira [11] описва Enecê като алтернатива на канадския балсам за монтиране на постоянни препарати от ларви, съблекла на ларви и дори възрастни комари, и оттогава се използва широко за монтиране на флеботоми. Enecê предлага икономична алтернатива за постоянно монтиране, осигуряваща дългосрочна стабилност и достатъчно време за изсъхване, което позволява дисекция и точно подреждане на морфологичните структури.

5.4. Изготвяне и сушене на препарати

Правилното изсушаване на микроскопските препарати е от критично значение за осигуряване на дългосрочна стабилност и съхранение. Препаратите трябва да се изсушат напълно, преди да се обмисли дългосрочното им съхранение. За оптимални резултати препаратите, монтирани в трайни микроскопски среди, трябва да се сушат хоризонтално в продължение на 2–3 седмици, докато тези, подготвени в полутрайни среди, може да се наложи да се сушат само 1–2 седмици. За да се гарантира ефективен процес на сушене, се препоръчва да се използва инкубатор, настроен на

подходяща за използваната среда температура, като се избягва прекомерна топлина, която може да повреди пробите. Препоръчва се температурен диапазон от 30°C до 37°C. Тази стъпка на сушене е от решаващо значение за предотвратяване на деформация на препарата, влошаване на качеството на образците или нестабилност на микроскопската среда по време на съхранение.

Микроскопската среда, използвана при подготовката на препарата, трябва винаги да се отбелязва на етикета на стъклото. Ако е възможно, етикетът трябва да включва и конкретната използвана рецепта, заедно с името на лицето, което е подготвило препаратите, и датата на изготвяне. Стъклата първоначално се подготвят като временни препарати и не са предназначени за дългосрочно съхранение. Ако обаче състоянието на пробата се промени, например ако бъде определена като част от типова серия, трябва да се използва по-трайна микроскопска среда, за да се гарантира съхранението на образеца за бъдещи таксономични изследвания.

5.5. Алтернативни техники за монтиране: монтиране върху картончета

Монтирането върху картончета е техника, използвана за няколко групи насекоми, при която образците могат да бъдат забодени направо върху ентомологични картончета или залепени върху повърхността им. Предвид малкия им размер и необходимостта от наблюдение на вътрешните органи за видово определяне след просветляване (вж. точка 5), този метод изобщо не е подходящ за монтиране на флеботоми.

5.6. Повторно монтиране на повредени екземпляри

За редки или ценни екземпляри се препоръчва двуетапен подход, съгласно видеото, достъпно на: https://zenodo.org/records/18315029. 1) Рехидратирайте ги, без да ги разглобявате, за да можете да ги наблюдавате предварително. В петриева паничка трябва да се постави държач за няколко микроскопски препарата, който да служи като опора. Стъклото, което трябва да се рехидратира, се поставя отгоре, а петриевата паничка се напълва с няколко милиметра разтворител, за да се създаде влажна камера, като се гарантира, че самото стъкло не влиза в контакт с разтворителя (Фигура 8 D). Времето, необходимо за рехидратиране, може да варира от един до няколко дни, в зависимост от състоянието на екземпляра. Необходими са ежедневно наблюдение и търпение. След като предметното стъкло е достатъчно рехидратирано, то може да бъде извадено от влажната камера и поставено в инкубатор за няколко часа преди микроскопско изследване, фотографиране или рисуване. 2) За да се монтира отново, предметното стъкло може да бъде върнато във влажната камера за още няколко часа или за цяла нощ. Разглобяването трябва да се извърши под стереомикроскоп. С помощта на фини игли покривното стъкло трябва да се отстрани внимателно, като се гарантира, че няма останали елементи от насекомото (https://zenodo.org/records/18315029). След това дисецираните елементи от мушицата трябва да се съберат и изплакнат с вода в малки ямки, като тези, използвани за деструктивна екстракция на ДНК/РНК (виж по-долу), преди обезводняване и повторно монтиране в смола. При разглобяване на предметното стъкло е от решаващо значение да се идентифицира оригиналната среда за включване, за да се избере подходящ разтворител. За водни среди за включване трябва да се използва вода. Ако микроскопската среда е на смолна основа (напр. канадски балсам или Euparal®), трябва да се използва ксилол, под аспирационна камера и с подходящо лично предпазно оборудване, включително маска.

Повторното монтиране на типови или колекционни екземпляри трябва да се извършва само със съгласието на куратора и/или институцията, която притежава екземпляра.

6. Видово определяне на образците

6.1. Морфология

Видовото определяне на флеботомите се основава предимно на изследване на техните морфологични белези, включително формата на гърдите, крилата, гениталиите, четинките и специфичните морфометрични взаимоотношения между различните структури. Изследователите използват таксономични ключове, референтни колекции и оригинални описания на видовете, за да сравнят събраните екземпляри с известни таксони. Ключовите диагностични характеристики, като структурата на жилките на крилата и морфологията на главата при двата пола, структурата на мъжките генитали и конфигурацията на женските сперматеки, са особено информативни за определянето на вида. Точната идентификация често изисква подробно микроскопско изследване, обикновено с помощта на светлинен микроскоп, за наблюдение на фини структури като генитали и сперматеки, или стереомикроскоп за по-общи морфологични характеристики.

Последните постижения в областта на технологиите за образна диагностика улесниха използването на дигитални изображения за видово определяне на флеботоми. Снимки с висока разделителна способност или цифрови илюстрации на ключови характеристики могат да бъдат сравнени с референтни материали или анализирани с помощта на компютърни системи за

идентификация, което подобрява както точността, така и достъпността в морфологичната таксономия.

6.2. Геометрия на крилата

Геометрията на крилата е ключова характеристика, използвана при идентифицирането и класифицирането на различни видове флеботоми. Крилата на флеботомите имат уникална шарка и структура, като обикновено са дълги и тесни с добре развито жилкуване (Фигури 9 и 10). Разположението на жилките образува отличителна шарка, която може да варира между родовете и видовете, като предоставя ценни диагностични характеристики за видово определяне. Поради това изучаването на геометрията на крилата предоставя ценна информация за таксономични цели.

6.3. Геометрична морфометрия на крилото

Изследователите използват различни техники, като геометрична морфометрия, за да анализират и сравняват формата и размера на крилата при различни видове или популации флеботоми. Изследването на геометрията на крилата предоставя ценна информация за поведението, предпочитанията за местообитание и способността за полет.

При геометричния морфометричен подход крилата се отрязват внимателно, оцветяват се (ако е необходимо) и се монтират плоско върху предметното стъкло. Изготвените препарати се фотографират под стереомикроскоп, дигитализират се и се подлагат на морфометричен анализ. Тази процедура е добре описана в литературата [6, 27, 42, 56, 57, 59], с препоръка да се използва предимно или дясното, или лявото крило за чифтни органи, за да се избегнат потенциални отрицателни алометрични ефекти [62].

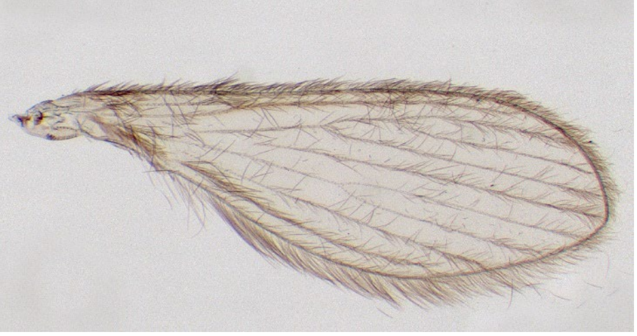

Фигура 9: Необработено крило на *Trichophoromyia ininii*.

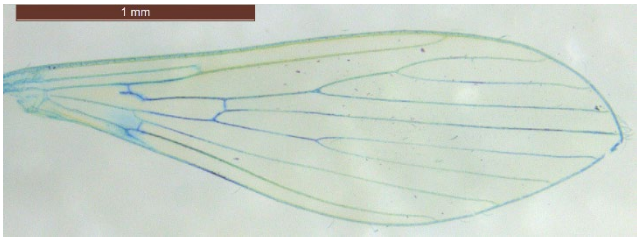

Фигура 10: Оцветено крило на *Phlebotomus ariasi.*

Подготовка на крилото за геометричен морфометричен анализ

За оптимална визуализация на крилните жилки, крилата трябва да бъдат почистени от люспи и подходящо оцветени. За подготовка на крилото, първо напълнете малки ямки с необходимите реагенти (метиленово синьо, етанол, вода и заместител на ксилол). Извадете крилото, съхранявано в 70% етанол при стайна температура, като обърнете епруветката Eppendorf и я изпразните над ямката, след което повдигнете крилото надлъжно с помощта на фина извита игла. Прекарайте крилото за кратко от етанол във вода и обратно в етанол, за да премахнете власинките. Поставете крилото в метиленово синьо за 6 минути, като се уверите, че плава по време на оцветяването. Извадете крилото внимателно и го потопете в заместител на ксилол за 2 минути (приблизително една трета от времето на метиленовото синьо). Леко потупване на стените на ямката с иглата може да помогне на крилото да се утаи; ксилолът служи за фиксиране на оцветяването. Накрая извадете крилото и го поставете върху малка капка Euparal® върху предметно стъкло. Под увеличителна лупа полека разгънете крилото и внимателно поставете покривно стъкло. Снимките трябва да се направят незабавно, преди Euparal® да се втвърди, тъй като може да се наложи леко коригиране на позицията на крилото под покривното стъкло, за да се постигне оптимално подравняване.

6.4. Молекулярно-биологични техники

В допълнение към морфологичните техники, молекулярните методи стават все по-важни в ентомологичните изследвания, включително таксономични, популационно-генетични и филогенетични проучвания, както и за откриване на ДНК/РНК на патогени и за определяне на произхода на поетата кръв, като поведението на векторите е важно в областта на епидемиологията [70]. Секвенирането на ДНК може да се използва за потвърждаване на видовете или за разграничаване на близкородствени видове, като предоставя по-точно и надеждно средство за видово определяне. Освен това, усъвършенстваните молекулярни техники (т.е. PCR, ДНК секвениране, NGS и др.) и MALDI-ToF MS придобиват все по-голямо

значение за точното и бързо определяне на видовете, допълвайки традиционните морфологични методи [46]. Въпреки тези постижения, морфологичната идентификация остава референтният стандарт за таксономията и основата, върху която се интерпретират молекулярните данни.

6.4.1. Деструктивно изолиране на нуклеинови киселини

Изолирането на нуклеинова киселина е рутинна стъпка в много биологични изследвания, като са разработени различни методи за изолиране на ДНК от биологични материали [48]. Много от предлаганите на пазара набори за изолиране на ДНК са предназначени да улеснят този процес [14]. Методите, които обикновено се използват за подготовка на екземпляри от членестоноги за морфологична идентификация, обаче често затрудняват анализа на ДНК, тъй като тези техники могат да повредят или унищожат важни физически характеристики на екземпляра [10]. Повечето протоколи за екстракция на ДНК от тъкани на насекоми са деструктивни по своята същност [43], което поражда особени опасения при дребните екземпляри, където дори ограниченото вземане на проби може да компрометира важни морфологични характеристики [72]. Видът и състоянието на екземпляра играят ключова роля при избора на подходящ метод за изолиране на ДНК [29].

Необходимостта от точно видово определяне на флеботомите, разбиране на динамиката на популациите и минимизиране на нежеланите въздействия е довела до разработването на молекулярни диагностични инструменти [23]. Молекулярните подходи се използват често в допълнение към морфологичните таксономични методи за идентифициране на флеботоми. Например, стандартният подход за баркодиране на насекоми включва извличане на ДНК, секвениране и загуба на оригиналния образец. Поради това съществува наложителна необходимост от проучване на недеструктивни методи за извличане на ДНК, които запазват както биологичния материал, така и неговата морфологична цялост.

Към флеботомите са били приложени многобройни методи за изолиране на нуклеинови киселини. Количеството или качеството на необходимите нуклеинови киселини зависи от последващия молекулярен анализ, тъй като различните техники имат различни изисквания за чувствителност и чистота [9]. Установено е, например, че очите на флеботомите инхибират PCR амплификацията [69]. Освен за скрининг на патогени, ДНК от флеботоми се изолира рутинно за целите на видовото определяне. Могат да се използват различни методи за изолиране, въпреки че добивът и качеството се различават между техниките. Протоколите на някои производители са адаптирани от изследователи за флеботоми [8], което увеличава добива и/или качеството на изолираните нуклеинови киселини [8, 9, 69], докато други адаптации, разработени за други таксони членестоноги, могат да се използват и за флеботоми [58, 76]. Идентификационните PCR, насочени към малки митохондриални фрагменти (COI или CytB), обикновено са съвместими с методи за изолиране, включващи висока фрагментация на ДНК. За разлика от тях, други техники за дълго четене при NGS (Oxford Nanopore и PacBio) изискват минимална фрагментация и висококачествена ДНК. Екстракциите с колонки обикновено дават геномни ДНК фрагменти до 60 kb, докато екстракцията с фенол-хлороформ може да произведе фрагменти до 150 kb [77]. Таблица 5 обобщава различните техники за изолиране на ДНК от флеботоми и посочва дали са направени методологични адаптации за тези насекоми. Добивът не е показан, тъй като зависи от размера на пробата и метода на подготовка. Колоната „Модификация" се отнася до адаптации на протоколите за екстракция за флеботоми или други дребни членестоноги.

При избора на метод за изолиране трябва да се вземат предвид няколко критерия, като броя на пробите, времето за екстракция и техниката, използвана в последващите етапи. Докато техниките NGS изискват геномна ДНК с високо молекулно тегло, всички методи, представени тук, могат да се използват за стандартни приложения, основани на PCR.

Освен това, няколко проучвания изследват недеструктивни методи за екстракция на ДНК за дребни сухоземни членестоноги, сухи музейни екземпляри и членестоноги с меко тяло [19, 26, 28, 55, 63].

Таблица 5: Средна цена, приложение и адаптиране на протокола за извличане на гДНК от флеботоми

Протокол	Цена	Приложение	Адаптация на протокола за дребни членестоноги
Центрофужни колонки	2,5 – 3,55 $ [39]	PCR, NGS	[9]
Фенол-хлороформ	0,24 $ [69]	PCR, NGS	[9]
HotSHOT	<0,01 $ [69]	PCR	-
Изсолване	0,12 $3 [69]	PCR	-
Chelex	0,02 $4 [41]	PCR	[41, 76]

6.4.2. Недеструктивно изолиране на нуклеинови киселини

Едно от основните предизвикателства при молекулярния анализ на членестоноги, особено на

флеботоми, е съхранението на екземплярите за включване в ентомологични колекции. Повечето протоколи за изолиране на ДНК изискват мацерация на тъканта, което компрометира съхранението на оригиналния екземпляр. Недеструктивните методи за извличане на нуклеинова киселина обаче са предназначени да извличат генетичен материал, без да увреждат физически пробата, да засягат жизнеспособността ѝ или да променят морфологията ѝ. Тези методи са особено ценни при работа с ценни или ограничени на брой екземпляри, като флеботомите, при които запазването на структурната цялост е от съществено значение за бъдещи таксономични, морфологични или диагностични цели. Често използвана техника е недеструктивният метод на баня, при който флеботомите се обездвижват и внимателно се потапят в лизисен буфер, съдържащ протеиназа К.

Техниката на меката вектолиза е била успешно прилагана при флеботоми, особено при типови проби [24]. Техниката използва конвенционален набор за центрофугиране (в този случай набор DNeasy Blood and Tissue, QIAGEN, Хилден, Германия) с адаптация за получаване на ДНК без унищожаване на пробата. Модифицираните етапи на лизис (обем на лизисния буфер и добавяне на етап на замразяване) [17] позволяват освобождаването на нуклеинови киселини, като се минимизират морфологичните увреждания [24]. По отношение на флеботомите е възможно да се използва и набор HotSHOT DNA Extraction (Bento Bioworks Ltd, Лондон, Великобритания) [73], който позволява бърза и евтина обработка на пробите. Ентомологичните образци, предназначени за морфологична идентификация, могат след това да бъдат изплакнати. Тези, които са обработени с набора DNeasy Blood and Tissue, трябва да бъдат просветлени с разтвор на Марк-Андре, докато обработените с набора HotSHOT DNA Extraction, са достатъчно просветлени, за да бъдат монтирани във водна среда или, за предпочитане, в смола след обезводняване, съгласно протокола, описан в тази статия [73]. Извлеченият генетичен материал може да бъде допълнително обработен за последващ анализ, като PCR, за да се амплифицират специфични генетични маркери. Недеструктивните методи за екстракция на нуклеинови киселини са от решаващо значение за изучаването на генетичните характеристики на флеботомите, включително идентифицирането на потенциални причинители на заболявания, които те могат да пренасят. Чрез запазване на целостта на пробата изследователите могат да получат ценна генетична информация, като същевременно запазват пробата за допълнителни анализи или изследвания.

6.5. MALDI-ToF MS

MALDI-ToF MS (матрично-асистирана лазерна десорбция/йонизация, времева масова спектрометрия) е техника, основана на масова спектрометрия, предназначена за откриване и анализ на уникалните протеинови профили („отпечатъци“) на биологични проби. MALDI-ToF все повече се признава като важно средство за видово определяне на членестоноги с медицинско и ветеринарно значение. Тази техника се е доказала като ефективна при определянето на различни стадии на развитие на флеботомите, включително предимагиналните форми и кръвта в насмуканите женски, и е била успешно прилагана за разграничаване на мъжки и женски видове флеботоми при различни условия на съхранение и хомогенизация [28, 30, 73, 74]. Този метод предлага и висока разделителна способност на ниво подрод, вид и популация. Тази техника позволява на изследователите да постигнат бърза и точна идентификация на видовете, което е от съществено значение за разбирането на разпространението, поведението и ролята на флеботомите в предаването на болести. Чрез разграничаване на видовете въз основа на протеинови профили, MALDI-ToF играе решаваща роля в епидемиологичните проучвания и стратегиите за контрол на преносителите. В момента има два основни недостатъка на тази техника, които ограничават рутинното ѝ приложение. Първият е достъпността на оборудване за масова спектрометрия, което е прекалено скъпо, за да бъде закупено единствено за целите на идентифицирането на видовете флеботоми (или по-общо на членестоноги преносители). За щастие, това ограничение може да бъде преодоляно чрез използване на машинно време на масспектрометри, които са станали стандартен изследователски инструмент в протеомни съоръжения и/или клинична диагностика. Второто е ниската представителност на референтните данни за флеботоми, налични в бази данни с отворен достъп, което води до необходимостта от създаване на вътрешна база данни с референтни спектри, базирани на недвусмислено идентифицирани екземпляри, в идеалния случай чрез комбинация от морфологична оценка и секвениране на подходящ генетичен маркер (COI, cytB или друг). Надяваме се, че това ограничение скоро ще бъде преодоляно чрез постепенното включване на досегашните вътрешни референтни бази данни за флеботоми в платформата MSI, управлявана от Assistance Publique-Hôpitaux de Paris, Университета Сорбона, Франция, и колекцията BCCM/IHEM/Sciensano в Брюксел, Белгия (https://msi.happy-dev.fr/). Когато се планира използване на MALDI-ToF протеиново профилиране, пробите трябва да се съхраняват за предпочитане в сух замразен вид или в 70% етанол от молекулярен клас и да не се излагат на околна температура. При липса на универсални указания за подготовка на пробите, на потребителите се препоръчва да използват воден 60 % ацетонитрил/0,3 % TFA разтвор на синапинова киселина (30 мг/мл) за подготовка на MALDI-ToF

матрицата, за да направят протеиновите си спектри съпоставими с публикуваните досега данни за флеботомите.

Подготовка на проби за MALDI-ToF MS (Фигура 7)

Насекомите, съхранявани при различни условия, първо се изсушават на въздух при стайна температура и се дисецират. Главата и коремчето се отстраняват, за да се отделят частите на тялото, съдържащи ключовите морфологични характеристики, за монтиране на покривно стъкло и морфологичен анализ. Гърдите може да се използват за MALDI-ToF, а останалата част от коремчето се запазва за извличане на ДНК. За профилиране на протеини гърдите се хомогенизират ръчно в 1,5-милилитрови микроепруветки с 10 мкл хомогенизиращ разтвор, като се използват еднократни пелетни пестици. Обикновено се използват два хомогенизиращи разтвора: стерилна дестилирана вода и 25% мравчена киселина.

7. Заключение

В този труд се стремим да предоставим на изследователите най-ефективните методи за монтиране на флеботоми, съобразени с конкретни изследователски цели, за да улесним точното видово определяне и откриването на патогени. Няма един универсален оптимален метод, а съществуват няколко, всеки от които има свои предимства и ограничения.

В допълнителните материали предоставяме подробни протоколи за различни техники за монтиране, използвани при препарирането и видовото определяне на флеботоми. Тези протоколи, включително обучителни видеоклипове, представят стъпка по стъпка процедурите, съобразени с различните цели, които гарантират прецизни и надеждни резултати. Чрез предоставянето на този изчерпателен ресурс целим да подкрепим изследователите при избора и прилагането на най-подходящите техники за монтиране за техните конкретни нужди.

Благодарности

Авторите благодарят на Ричард Лейн и Зоуи Джей Адамс от Природонаучния музей в Лондон, Великобритания, за отличната им рецензия, която повиши качеството на тази статия.

Финансиране

Изразяваме благодарност на бразилските агенции за развитие CNPq (номер на делото: 404395/2024-4) и Фондация Араукария (номер на делото: 433/2025 PDI) за финансирането на изследванията на AJA.

Конфликт на интереси

Жером Дьопаки е асоцииран редактор на Parasite; той не е оказал влияние върху процеса на рецензиране и вземането на решения по този ръкопис. Останалите автори декларират, че нямат конфликт на интереси.

Data availability statement

Видеоклипове в Zenodo.

Видео 1: https://zenodo.org/records/18198006

Видео 2: https://zenodo.org/records/18311158

Видео 3: https://zenodo.org/records/18311106

Видео 4: https://zenodo.org/records/18311154

Видео 5: https://zenodo.org/records/18303014

Видео 6: https://zenodo.org/records/18302850

Видео 7: https://zenodo.org/records/18315029

Допълнителни материали

Допълнителните материали към тази статия са достъпни на адрес https://www.parasite-journal.org/10.1051/parasite/2026009/olm

Литература

1. Alkan C, Allal-Ikhlef AB, Alwassouf S, Baklouti A, Piorkowski G, de Lamballerie X, Izri A, Charrel RN. 2015. Virus isolation, genetic characterization and seroprevalence of Toscana virus in Algeria. Clinical Microbiology and Infection, 21(11), 1040 e1-9.
2. Alten B, Ozbel Y, Ergunay K, Kasap OE, Cull B, Antoniou M, Velo E, Prudhomme J, Molina R, Banuls AL, Schaffner F, Hendrickx G, Van Bortel W, Medlock JM. 2015. Sampling strategies for phlebotomine sand flies (Diptera: Psychodidae) in Europe. Bulletin of Entomological Research 105(6), 664–678.
3. Ayhan N, Baklouti A, Prudhomme J, Walder G, Amaro F, Alten B, Moutailler S, Ergunay K, Charrel RN, Huemer H. 2017. Practical guidelines for studies on sandfly-borne phleboviruses: Part I: Important points to consider *ante* field work. Vector-Borne and Zoonotic Diseases 17(1), 73–80.
4. Bates PA. 1997. Infection of phlebotomine sandflies with *Leishmania*, in The Molecular Biology of Insect Disease Vectors: A Methods Manual. Springer. p. 112–120.5.
5. Baum M, de Castro EA, Pinto MC, Goulart TM, Baura W, Klisiowicz Ddo R, Vieira da Costa-Ribeiro MC. 2015. Molecular detection of the blood meal source of sand flies (Diptera: Psychodidae) in a transmission area of American cutaneous leishmaniasis, Parana State, Brazil. Acta Tropica, 143, 8–12.
6. Belen A, Alten B, Aytekin A. 2004. Altitudinal variation in morphometric and molecular characteristics of *Phlebotomus papatasi* populations. Medical and Veterinary Entomology, 18(4), 343–350.
7. Bhattacharya J, Chandra G, Hati AK. 1991. A simple method for cryopreservation of *Leishmania donovani* promastigotes, Indian Journal of Medical Research, 93, 245–246.
8. Caligiuri LG, Sandoval AE, Miranda JC, Pessoa FA, Santini MS, Salomón OD, Secundino NF, McCarthy CB. 2019. Optimization of DNA extraction from individual sand flies for PCR amplification. Methods and Protocols, 2(2), 36.
9. Casaril AE, de Oliveira LP, Alonso DP, de Oliveira EF, Gomes Barrios SP, de Oliveira Moura Infran J, Fernandes WS, Oshiro ET, Ferreira AMT, Ribolla PEM, de Oliveira AG. 2017. Standardization of DNA extraction from sand flies: Application to

genotyping by next generation sequencing. Experimental Parasitology, 177, 66–72.
10. Castalanelli MA, Severtson DL, Brumley CJ, Szito A, Foottit RG, Grimm M, Munyard K, Groth DM. 2010. A rapid non-destructive DNA extraction method for insects and other arthropods. Journal of Asia-Pacific Entomology, 13(3), 243–248.
11. Cerqueira NL. 1943. Um novo meio para montagem de pequenos insetos em lâmina. Memórias do Instituto Oswaldo Cruz, (39), 37–41.
12. Charrel RN, Gallian P, Navarro-Mari JM, Nicoletti L, Papa A, Sanchez-Seco MP, Tenorio A, de Lamballerie X. 2005. Emergence of Toscana virus in Europe. Emerging Infectious Diseases, 11(11), 1657–1663.
13. Chaskopoulou A, Giantsis IA, Demir S, Bon MC. 2016. Species composition, activity patterns and blood meal analysis of sand fly populations (Diptera: Psychodidae) in the metropolitan region of Thessaloniki, an endemic focus of canine leishmaniasis. Acta Tropica, 158, 170–176.
14. Chen H, Rangasamy M, Tan SY, Wang H, Siegfried BD. 2010. Evaluation of five methods for total DNA extraction from western corn rootworm beetles. PLoS One, 5(8), e11963.
15. Depaquit J, Grandadam M, Fouque F, Andry PE, Peyrefitte C. 2010. Arthropod-borne viruses transmitted by Phlebotomine sandflies in Europe: a review. Eurosurveillance, 15(10), 19507.
16. Diamond LS, Herman CM. 1954. Incidence of Trypanosomes in the Canada Goose as revealed by bone marrow culture. Journal of Parasitology, 40(2), 195–202.
17. Ding H, Torno M, Vongphayloth K, Ng G, Tan D, Sng W, Ho K, Randrianambinintsoa FJ, Depaquit J, Tan CH. 2025. Hidden in plain sight: discovery of sand flies in Singapore and description of four species new to science. Parasites & Vectors, 18(1), 402.
18. Es-Sette N, Ajaoud M, Bichaud L, Hamdi S, Mellouki F, Charrel RN, Lemrani M. 2014. *Phlebotomus sergenti* a common vector of *Leishmania tropica* and Toscana virus in Morocco. Journal of Vector Borne Diseases, 51(2), 86–90.
19. Favret C. 2005. A new non-destructive DNA extraction and specimen clearing technique for aphids (Hemiptera). Proceedings of the Entomological Society of Washington, 107(2), 469–470.
20. Galati EAB. 2018. Phlebotominae (Diptera, Psychodidae): Classification, morphology and terminology of adults and identification of American taxa, in Brazilian Sand Flies: Biology, Taxonomy, Medical Importance and Control, Rangel EF, Shaw JJ, Editors. Cham: Springer International Publishing. pp. 9–212.
21. Galati EAB, de Andrade AJ, Perveen F, Loyer M, Vongphayloth K, Randrianambinintsoa FJ, Prudhomme J, Rahola N, Akhoundi M, Shimabukuro PHF, Depaquit J. 2025. Phle-botomine sand flies (Diptera, Psychodidae) of the world. Parasites & Vectors, 18(1), 220.
22. Galati EAB, Galvis-Ovallos F, Lawyer P, Leger N, Depaquit J. 2017. An illustrated guide for characters and terminology used in descriptions of Phlebotominae (Diptera, Psychodidae). Par-asite, 24, 26.
23. Gariepy T, Kuhlmann U, Gillott C, Erlandson M. 2007. Parasitoids, predators and PCR: the use of diagnostic molecular markers in biological control of Arthropods. Journal of Applied Entomology, 131(4), 225–240.
24. Giantsis IA, Chaskopoulou A, Bon MC. 2016. Mild-Vectolysis: A nondestructive DNA extraction method for vouchering sand flies and mosquitoes. Journal of Medical Entomology, 53(3), 692–695.
25. Gidwani K, Picado A, Rijal S, Singh SP, Roy L, Volfova V, Andersen EW, Uranw S, Ostyn B, Sudarshan M, Chakravarty J, Volf P, Sundar S, Boelaert M, Rogers ME. 2011. Serological markers of sand fly exposure to evaluate insecticidal nets against visceral leishmaniasis in India and Nepal: a cluster-randomized trial. PLoS Neglected Tropical Diseases, 5(9), e1296.
26. Gilbert MTP, Moore W, Melchior L, Worobey M. 2007. DNA extraction from dry museum beetles without conferring external morphological damage. PLoS One, 2(3), e272.
27. Giordani BF, Andrade AJ, Galati EAB, Gurgel-Goncalves R. 2017. The role of wing geometric morphometrics in the identification of sandflies within the subgenus *Lutzomyia.* Medical and Veterinary Entomology, 31(4), 373–380.
28. Guzmán-Larralde AJ, Suaste-Dzul AP, Gallou A, Peña-Carrillo KI. 2017. DNA recovery from microhymenoptera using six non-destructive methodologies with considerations for subsequent preparation of museum slides. Genome, 60(1), 85–91.
29. Hajibabaei M, DeWaard JR, Ivanova NV, Ratnasingham S, Dooh RT, Kirk SL, Mackie PM, Hebert PD. 2005. Critical factors for assembling a high volume of DNA barcodes. Philosophical Transactions of the Royal Society B: Biological Sciences, 360(1462), 1959–1967.
30. Haouas N, Pesson B, Boudabous R, Dedet JP, Babba H, Ravel C. 2007. Development of a molecular tool for the identification of *Leishmania* reservoir hosts by blood meal analysis in the insect vectors. American Journal of Tropical Medicine and Hygiene, 77(6), 1054–1059.
31. Hlavackova K, Dvorak V, Chaskopoulou A, Volf P, Halada P. 2019. A novel MALDI-TOF MS-based method for blood meal identification in insect vectors: A proof of concept study on phlebotomine sand flies. PLoS Neglected Tropical Diseases, 13 (9), e0007669.
32. Huemer H, Prudhomme J, Amaro F, Baklouti A, Walder G, Alten B, Moutailler S, Ergunay K, Charrel RN, Ayhan N. 2017. Practical guidelines for studies on sandfly-borne phleboviruses: Part II: Important points to consider for fieldwork and subsequent virological screening. Vector-Borne and Zoonotic Diseases, 17(1), 81–90.
33. Jancarova M, Polanska N, Thiesson A, Arnaud F, Stejskalova M, Rehbergerova M, Kohl A, Viginier B, Volf P, Ratinier M. 2025. Susceptibility of diverse sand fly species to Toscana virus. PLoS Neglected Tropical Diseases, 19(5), e0013031.
34. Kapp JD, Green RE, Shapiro B. 2021. A fast and efficient single-stranded genomic library preparation method optimized for ancient DNA. Journal of Heredity, 112(3), 241–249.
35. Killick-Kendrick R, Maroli M, Killick-Kendrick M. 1991. Bibliography of the colonization of phlebotomine sandflies. Parassitologia, 33(suppl.), 321–333.
36. Lawyer P, Killick-Kendrick M, Rowland T, Rowton E, Volf P. 2017. Laboratory colonization and mass rearing of phlebotomine sand flies (Diptera, Psychodidae). Parasite, 24, 42.
37. Léger N, Pesson B, Madulo-Leblond G. 1986. Les phlébotomes de Grèce : 1ère partie. Bulletin de la Société de Pathologie Exotique, 79, 386–397.
38. Léger N, Pesson B, Madulo-Leblond G. 1986. Les phlébotomes de Grèce : 2ème partie. Bulletin de la Société de Pathologie Exotique, 79, 514–524.
39. Leonel JAF, Vioti G, Alves ML, da Silva DT, Meneghesso PA, Benassi JC, Spada JCP, Galvis-Ovallos F, Soares RM, Oliveira T. 2020. DNA extraction from individual Phlebotomine sand flies (Diptera: Psychodidae: Phlebotominae) specimens: Which is the method with better results? Experimental Parasitology, 218, 107981.

40. Lestinova T, Rohousova I, Sima M, de Oliveira CI, Volf P. 2017. Insights into the sand fly saliva: Blood-feeding and immune interactions between sand flies, hosts, and *Leishmania.* PLoS Neglected Tropical Diseases, 11(7), e0005600.
41. Lienhard A, Schaffer S. 2019. Extracting the invisible: obtaining high quality DNA is a challenging task in small arthropods. PeerJ, 7, e6753.
42. Lozano-Sardaneta YN, Mikery-Pacheco OF, Huerta H, Rojas-Soriano JE, Contreras-Ramos A. 2025. Wing geometric morphometrics is effective to separate sand fly species (Diptera, Psychodidae, Phlebotominae) related with leishmaniasis transmission in Mexico. Acta Tropica, 262, 107523.
43. Mandrioli M. 2008. Insect collections and DNA analyses: how to manage collections? Museum Management and Curatorship, 23(2), 193–199.
44. Maroli M, Feliciangeli MD, Bichaud L, Charrel RN, Gradoni L. 2013. Phlebotomine sandflies and the spreading of leishmaniases and other diseases of public health concern. Medical and Veterinary Entomology, 27(2), 123–147.
45. Marquina D, Buczek M, Ronquist F, Lukasik P. 2021. The effect of ethanol concentration on the morphological and molecular preservation of insects for biodiversity studies. PeerJ, 9, e10799.
46. Mathis A, Depaquit J, Dvorak V, Tuten H, Banuls AL, Halada P, Zapata S, Lehrter V, Hlavackova K, Prudhomme J, Volf P, Sereno D, Kaufmann C, Pfluger V, Schaffner F. 2015. Identification of phlebotomine sand flies using one MALDI-TOF MS reference database and two mass pectrometer systems., Parasites & Vectors, 8, 266.
47. Mekarnia N, Benallal KE, Sadlova J, Vojtkova B, Mauras A, Imbert N, Longhitano M, Harrat Z, Volf P, Loiseau PM, Cojean S. 2024. Effect of *Phlebotomus papatasi* on the fitness, infectivity and antimony-resistance phenotype of antimony-resistant *Leishmania* major Mon-25. International Journal for Parasitology – Drugs and Drug Resistance, 25, 100554.
48. Milligan BG. 1998. Total DNA isolation, in Molecular Genetic Analysis of Population: A Practical Approach, Hoelzel AR, Editor. Oxford: Oxford University Press.
49. Molina R, Jiménez M, Alvar J, González E, Hernández-Taberna S, Ines MM. 2017. Methods in sand fly research. Madrid: Servicio de publicaciones Universidad de Alcalá de Henares, Madrid.
50. Murphy WJ, Eizirik E, O'Brien SJ, Madsen O, Scally M, Douady CJ, Teeling E, Ryder OA, Stanhope MJ, de Jong WW, Springer MS. 2001. Resolution of the early placental mammal radiation using Bayesian phylogenetics. Science, 294(5550), 2348–2351.
51. Nacif-Pimenta R, Pinto LC, Volfova V, Volf P, Pimenta PFP, Secundino NFC. 2020. Conserved and distinct morphological aspects of the salivary glands of sand fly vectors of leishma-niasis: an anatomical and ultrastructural study. Parasites & Vectors, 13(1), 441.
52. Neuhaus B, Schmid T, Riedel J. 2017. Collection management and study of microscope slides: Storage, profiling, deterioration, restoration procedures, and general recommendations. Zootaxa, 4322(1), 1–173.
53. New TR. 1974. Pscoptera. Handbooks for Identification of British Insects (Vol. I). London: Royal Entomological Society of London. 102 pp.
54. Perez-Ruiz M, Collao X, Navarro-Mari JM, Tenorio A. 2007. Reverse transcription, real-time PCR assay for detection of Toscana virus. Journal of Clinical Virology, 39(4), 276–281.
55. Porco D, Rougerie R, Deharveng L, Hebert P. 2010. Coupling non-destructive DNA extraction and voucher retrieval for small soft-bodied Arthropods in a high-throughput context: the example of Collembola. Molecular Ecology Resources, 10(6), 942–945.
56. Prudhomme J, Cassan C, Hide M, Toty C, Rahola N, Vergnes B, Dujardin JP, Alten B, Sereno D, Banuls AL. 2016. Ecology and morphological variations in wings of *Phlebotomus ariasi* (Diptera: Psychodidae) in the region of Roquedur (Gard, France): a geometric morphometrics approach. Parasites & Vectors, 9(1), 578.
57. Prudhomme J, Gunay F, Rahola N, Ouanaimi F, Guernaoui S, Boumezzough A, Banuls AL, Sereno D, Alten B. 2012. Wing size and shape variation of *Phlebotomus papatasi* (Diptera: Psychodidae) populations from the south and north slopes of the Atlas Mountains in Morocco. Journal of Vector Ecology, 37(1), 137–147.
58. Prudhomme J, Toty C, Kasap OE, Rahola N, Vergnes B, Maia C, Campino L, Antoniou M, Jimenez M, Molina R, Cannet A, Alten B, Sereno D, Banuls AL. 2015. New microsatellite markers for multi-scale genetic studies on *Phlebotomus ariasi* Tonnoir, vector of *Leishmania infantum* in the Mediterranean area. Acta Tropica, 142, 79–85.
59. Prudhomme J, Velo E, Bino S, Kadriaj P, Mersini K, Gunay F, Alten B. 2019. Altitudinal variations in wing morphology of *Aedes albopictus* (Diptera, Culicidae) in Albania, the region where it was first recorded in Europe. Parasite, 26, 55.
60. Rawlins DJ. 1992. Light Microscopy: An Introduction to Biotechniques. Oxford: Bios Scientific publishers. 143 pp.
61. Ready PD. 2013. Biology of phlebotomine sand flies as vectors of disease agents. Annual Review of Entomology, 58, 227–250.
62. Rohlf FJ, Slice D. 1990. Extensions of the Procrustes method for the optimal superimposition of landmarks. Systematic Zoology, 39(1), 40–59.
63. Rowley DL, Coddington JA, Gates MW, Norrbom AL, Ochoa RA, Vandenberg NJ, Greenstone MH. 2007. Vouchering DNA-barcoded specimens: Test of a nondestructive extraction proto-col for terrestrial arthropods. Molecular Ecology Notes, 7(6), 915–924.
64. Sábio PB, Andrade AJ, Galati EAB. 2014. Assessment of the taxonomic status of some species included in the *Shannoni* complex, with the description of a new species of *Psathyromyia* (Diptera: Psychodidae: Phlebotominae). Journal of Medical Entomology, 51(2), 331–341.
65. Sadlova J, Yeo M, Seblova V, Lewis MD, Mauricio I, Volf P, Miles MA. 2011. Visualisation of *Leishmania donovani* fluo-rescent hybrids during early stage development in the sand fly vector. PLoS One, 6(5), e19851.
66. Sales K, Miranda DEO, da Silva FJ, Otranto D, Figueredo LA, Dantas-Torres F. 2020. Evaluation of different storage times and preservation methods on phlebotomine sand fly DNA concen-tration and purity. Parasites & Vectors, 13(1), 399
67. Sales KG, Costa PL, de Morais RC, Otranto D, Brandao-Filho SP, Cavalcanti Mde P, Dantas-Torres F. 2015. Identification of phlebotomine sand fly blood meals by real-time PCR. Parasites& Vectors, 8, 230.
68. Sant'Anna MR, Jones NG, Hindley JA, Mendes-Sousa AF, Dillon RJ, Cavalcante RR, Alexander B, Bates PA. 2008. Blood meal identification and parasite detection in laboratory-fed and field-captured *Lutzomyia longipalpis* by PCR using FTA databasing paper. Acta Tropica, 107(3), 230–237.
69. Senne NA, Santos HA, Araujo TR, Paulino PG, Mendonca LP, Moreira HVS, Camilo TA, da Costa Angelo I. 2022. Robust

comparative performance of genomic DNA extraction methods from non-engorged phlebotomine sandflies. Medical and Veterinary Entomology, 36(2), 203–211.
70. Shaw JJ. 2025. A review of Leishmania infections in American Phlebotomine sand flies – Are those that transmit leishmaniasis anthropophilic or anthropportunists? Parasite, 32, 57.
71. Tesh RB, Modi GB. 1983. Growth and transovarial transmission of Chandipura virus (Rhabdoviridae: Vesiculovirus) in *Phlebotomus papatasi.* American Journal of Tropical Medicine and Hygiene, 32(3), 621–623.
72. Thomsen PF, Elias S, Gilbert MTP, Haile J, Munch K, Kuzmina S, Froese DG, Sher A, Holdaway RN, Willerslev E. 2009. Non-destructive sampling of ancient insect DNA. PLoS One, 4(4),e5048
73. Truett GE, Heeger P, Mynatt RL, Truett AA, Walker JA, Warman ML. 2000. Preparation of PCR-quality mouse genomic DNA with hot sodium hydroxide and tris (HotSHOT). Biotechniques, 29(1), 52–54.
74. Upton MS. 1993. Aqueous gum-chloral slide mounting media: an historical review. Bulletin of Entomological Research, 83(2), 267–274.
75. Volf P, Myskova J. 2007. Sand flies and Leishmania: specific versus permissive vectors. Trends in Parasitology, 23(3), 91–92.
76. Wang Q, Wang X. 2012. Comparison of methods for DNA extraction from a single chironomid for PCR analysis. Pakistan Journal of Zoology, 44(2), 421–426.
77. Wang Y, Zhao Y, Bollas A, Wang Y, Au KF. 2021. Nanopore sequencing technology, bioinformatics and applications. Nature Biotechnology, 39(11), 1348–1365.

Cite this article as: Randrianambinintsoa FJ, Augendre L, Prudhomme J, Martinet J-P, Loyer M, Mekarnia N, Kerkoub H, Perveen FK, Huguenin A, Kariya E, Akhoundi M, De Andrade AJ, Berriatua E, Bongiorno G, Boyer S, Christodoulou V, Da Costa-Ribeiro MCV, De Souza LAF, Ding H, Dondji B, Dvořák V, Erisoz Kasap O, Galati EAB, Gállego M, Ballart C, Gouzelou S, Haddad N, Masse RS, Mekuria AH, Ivovic V, Kaczmarek S, Shahar MK, Kirstein OD, Kniha E, Kolářová I, Lincoln T, Lucanas C, Mikov O, Nov K, Özbel Y, Pesson B, Posada Lopez LC, Prasetyo DB, Rahola N, Rebollar-Tellez EA, Rodrigues BL, Roy L, Saini P, Sanjoba C, Shimabukuro PH, Siriyasatien P, Soszynska A, Suleşco T, Sylla M, Torno M, Volf P, Vongphayloth K, Sinh Nam V, Wardhana A, Yessinou E, Zapata S, Gantier J-C & Depaquit J. 2026. Processing and mounting phlebotomine sand flies: a consensus guideline. Parasite xx, xx. https://doi.org/10.1051/parasite/2026009.

PARASITE

An international open-access, peer-reviewed, online journal publishing high quality papers on all aspects of human and animal parasitology

Reviews, articles and short notes may be submitted. Fields include, but are not limited to: general, medical and veterinary parasitology; morphology, including ultrastructure; parasite systematics, including entomology, acarology, helminthology and protistology, and molecular analyses; molecular biology and biochemistry; immunology of parasitic diseases; host-parasite relationships; ecology and life history of parasites; epidemiology; therapeutics; new diagnostic tools.
All papers in Parasite are published in English. Manuscripts should have a broad interest and must not have been published or submitted elsewhere. No limit is imposed on the length of manuscripts.

Parasite (open-access) continues **Parasite** (print and online editions, 1994-2012) and **Annales de Parasitologie Humaine et Comparée** (1923-1993) and is the official journal of the Société Française de Parasitologie.

Editor-in-Chief:
Jean-Lou Justine, Paris

Submit your manuscript at:
https://www.editorialmanager.com/parasite

Приложение 1: Биохимични теоретични основи.

Целевите членестоноги са флеботоми. Общата идея обаче може да бъде разширена и към други много разпространени членестоноги, чието видово определяне може да се извършва само въз основа на вътрешни морфологични характеристики. По случайност някои вътрешни органи са частично хитинизирани и тяхната морфология ни предоставя ценна информация. Ето защо е много интересно да се наблюдават глътъчните помпи, сперматеките и техните канали. При всички реагенти, които ще разгледаме, не трябва да се забравя, че от етапа на фиксиране на насекомото до сглобяването му ще прилагаме просто окислително-редукционни реакции. Единствената предпазна мярка или идея, която ще ни води, е да избягваме смесването на редуциращи с окисляващи реагенти.

Етилов алкохол; етанол:

Това вещество се използва по различни начини. Алкохолните молекули имат силен афинитет към водата и затова проявяват обезводняващ ефект. Въпреки това, алкохол с ниска концентрация (т.е. прекалено богат на вода) ще играе роля в разграждането на нуклеиновите киселини (водата е враг на нуклеиновите киселини).

Когато насекомите се поставят в етанол, това не е само за да се съхранят, но и за да се фиксират тъканите. В хистологията обикновено се разграничават две важни понятия: степен на проникване и степен на фиксиране. Добре известно е, че един добър консервант трябва първо да проникне бързо дълбоко в тъканите, преди да ги фиксира. За алкохол с концентрация 96% коефициентът на проникване е приблизително 1,05 (за сравнение, за 0,75% воден разтвор на пикринова киселина коефициентът на проникване е 0,45, а за 3% разтвор на калиев бихромат е 1,45).

Желанието да се съхраняват насекоми и други членестоноги за неопределено време в етанол е реалност за ентомолозите. Идеята да се съхраняват уловените в полето екземпляри за последващи проучвания или за бъдещи изследователи е много похвална. Въпреки това, тази идея не е възможна за цитолозите или хистолозите. Ако се стремим да съхраняваме пробите във фиксатора прекалено дълго, те могат да станат практически невъзможни за обработка. Ето защо пробите, по-стари от 10 години, са трудни или дори невъзможни за използване.

Друго съображение е съотношението между масата на фиксираните членестоноги и обема на фиксатора. В зоологичната или медицинската практика е препоръчително да се планира обем, 60 пъти по-голям от обема на фиксираните части. На практика, за дребните членестоноги, за даден обем на фиксираните екземпляри добавете поне 4–5 обема алкохол. Имайте предвид, че алкохолът ще загуби силата си, тъй като отстранява цялата вода, присъстваща в тъканите на членестоногите.

В заключение:

- Етиловият алкохол е редуциращ химичен агент (поради което е несъвместим с окислителни фиксатори);
- Той енергично утаява протеините и ги денатурира;
- Разтваря някои сложни липиди и преципитира гликогена;
- Причинява силно свиване на тъканите и ги втвърдява.

Основни разтвори на калиев или натриев хидроксид:

Използването на тези разтвори в ентомологията се фокусира главно върху калиевия хидроксид, без ясна обосновка.

Натриевият хидроксид [E524] се среща в разтвори с различни концентрации или с различна нормалност. Предлага се под формата на пелети или гранули. Основният му недостатък е, че е много хигроскопичен (повече от KOH). Когато реагира с протеините, той ги разтваря, а липидите превръща в твърди сапуни по време на осапуняването (това е основната разлика с KOH, който дава течни сапуни по време на осапуняването).

Калиевият хидроксид [E525] се предлага като концентриран разтвор, но преди всичко има предимството, че се предлага под формата на гранули от около 0,1 г, което значително улеснява приготвянето на разредени разтвори, когато не се изисква прецизно съотношение. Например, 1 гранула от 0,1 г в 1 мл дестилирана вода дава 10% разтвор. Второто предимство на калиевия хидроксид на гранули е по-ниската му чувствителност към карбонизация (разтворът на KOH има висока афинитет към фиксиране на CO_2, като по този начин създава карбонатни соли).

Тези силни основи се използват за разтваряне на мастните киселини, като ги превръщат във водоразтворими сапуни. Трябва да се има предвид, че фиксаторът, като например етанол, разтваря част от мазнините в пробата. Въпреки това, при прехвърляне на пробата във водна среда със силна основа мастните киселини (с различна степен на сложност) ще се утаят. Силната основа ще извърши следователно студено осапуняване. В някои случаи, когато мастните тъкани са в излишък, например при женските, ще бъде предимство да се повиши температурата до 35–40 °C, за да се улесни реакцията, или да се удължи времето на контакт при стайна температура.

Оцветен киселинен разтвор/безцветен разтвор на Марк-Андре:

Тук ще разгледаме предимствата и недостатъците на използването на разтвор на Марк-Андре. Този разтвор

се състои от хлорал хидрат (трихлорацеталдехид монохидрат), оцетна киселина и вода. Този разтвор е силно окислителен (смес от киселина и алдехид). Той неутрализира излишния калиев хидроксид, който може да остане в пробите, без да преципитира алкалните сапуни, образувани при използването на калиев хидроксид. Този окислителен разтвор също така въздейства върху вторичните алкохолни функции на глюкозамините, които се образуват в хитина, като ги окислява и по този начин омекотява хитина. Друго действие е разтварянето на някои присъстващи минерални соли.

Когато разтворът на Марк-Андре е предварително оцветен с кисел фуксин (т.е. в окислено състояние), той ще може да се фиксира върху вторичните алкохолни функции на структурата. След времето за контакт на разтвора на Марк-Андре и оцветяването на пробите, изплакването се извършва само с етанол. По този начин започваме фазата на обезводняване на пробите.

Предимства:

- Неутрализиране на излишните основни разтвори
- Омекотяване на хитина
- Оцветяване на хитина за по-добра оценка на хитинизираните вътрешни структури

Недостатъци:

Хлорал хидратът е хипнотично средство и се използва в хуманната медицина. Трябва да се използва в аспирационна камера и да се спазват законовите изисквания относно химическите рискове.

Обезводняващи разтвори:

Опитът показва, че за много дребни проби не е полезно да се следва последователността от алкохолни бани с нарастваща концентрация. Ако пробата е голяма, започваме с 80% етанол, след това 90%, 95% и накрая абсолютен етанол. За много дребни проби използвайте баня с 90% етанол, последвана от потапяне в абсолютен етанол. На този етап винаги трябва да помним, че абсолютният етанол се стреми да улови вода от атмосферата.

Традицията в ентомологичните лаборатории беше да се завърши обезводняването на пробите в баня с буков креозот. Днес тази есенция, широко използвана като пестицид, противогъбично средство и консервант за дърво, се препоръчва да не се използва поради миризмата си (полициклични ароматни въглеводороди) и се счита, че е репродуктивно токсична, канцерогенна, устойчиво органично замърсяващо вещество и екотоксична за водните организми.

Разтворът, който предлагаме да се приготви за монтиране на пробите, е Euparal® и Euparal есенция (описана в следващия абзац). Сместа от Euparal® и Euparal есенция е много добре приета от пробите след баня с 90% етанол.

Приложение 2: Състав на използваните реагенти.

Калиев хидроксид 10 %

Калиев хидроксид 10 г

Дестилирана вода q.s. 100 мл

Хлорална смола/Среда на Хойер

Дестилирана вода 50 мл

Хлорал хидрат 200 г

Гума арабика 50 г

Глицерин 20 мл

Разтвор на Марк-Андре

Хлорал хидрат 40 г

Ледена оцетна киселина 30 мл

Дестилирана вода 30 мл

Кисел фуксин 1% в дестилирана вода

Кисел фуксин на прах 1 г

Дестилирана вода 99 мл

Разтвор на Марк-Андре, оцветен с фуксин

Разтвор на Марк-Андре 10 мл

Фуксин 1% 50 мкл

Приложение 3: Euparal® , канадски балсам, поливинилов алкохол или други разтвори за монтиране

Поливинилов алкохол: Това е идеалната среда за включване, когато необходимите продукти за правилно обезводняване не са налични. Поливиниловият алкохол се смесва с лактофенол на Аман. Тези съединения обаче имат сериозни недостатъци, като изсъхване или кристализация на поливиниловия алкохол поради изпаряване на водата или почерняване при окисляване на фенола. Това остава добра техника за краткосрочен монтаж.

Канадски балсам: Използването му за монтиране между предметно и покривно стъкло изисква обезводняване на пробите, които ще се монтират. Използването на ксилол или толуол не е без неудобства.

Среда Enecê: За монтиране между предметно и покривно стъкло, подобно на канадския балсам, се изисква обезводняване на пробата. Формула на Enecê: чист бял колофон (22 г); разтворима в алкохол копалова смола (12 г), абсолютен алкохол (20 мл), камфор (10 г), терпентинова есенция (10 мл) и евкалиптол (26 мл). За приготвянето му абсолютният алкохол и камфорът се поставят в съд, като например колба на Ерленмайер. След това се добавят колофонът и копаловата смола. Колбата се затваря с тапа и се разклаща, и едва тогава се нагрява във водна баня на ниска температура, така че сместа да не заври. След като съдържанието се втечни напълно, се добавя терпентиновата есенция, след което се филтрира, докато сместа е още гореща, и накрая към филтрата се добавя евкалиптол. Когато средата стане по-малко течна, се разрежда с Eenecê със следната формула: абсолютен алкохол (30 мл), камфор (17 г), терпентинова есенция (15 мл), евкалиптол (38 мл) (Cerqueira, 1943).

Euparal®: Това е смола, която се получава от кипариса на Атлас *Tetraclinis articulata* (Vahl, 1791) и е проучена и разработена през 1906 г. от Gilson. Основното ѝ предимство е, че не полимеризира. Пробите, монтирани между предметно и покривно стъкло, могат лесно да бъдат възстановени чрез действието на алкохол или, още по-добре, на Euparal® есенция. Тази смола, наричана още сандарак, приема етанол от 80%.

Използване на Triton X100: нейонен воден разтвор:

Triton X100 е под формата на нейонен воден разтвор (*4-(1,1,3,3-тетраметилбутил) фенил-полиетилен гликол разтвор, или т-октилфеноксиполитетоксиетанол, полиетилен гликол трет-октилфенил етер*), широко използван като детергент в клетъчната и молекулярната биология. Той позволява проникването на клетъчните и ядрените мембрани.

Пробите от насекоми, съхранявани в алкохол в продължение на много години, са често срещани. За съжаление, съхранението в алкохол не е оптимално и така съхраняваните членестоноги стават много трудни за подготовка за микроскопско изследване. Пластмасовите съдове, съдържащи пробите, често се разграждат, последвано от изпаряване на алкохола. И в двата случая продължителният контакт с алкохол или изсушаването на пробите представляват реален проблем. През 2008 г. Jonque публикува бележка за рехидратацията на паяци с овлажняващ агент като Agepon, използван за фотографски филми [26]. Това довежда до идеята за използване на овлажняващи агенти, които не са силни детергенти.

По-долу е описана процедура, при която се използва Triton X100 в 0,5% воден разтвор:

- Напойте сухата проба с абсолютен алкохол.
- Добавете необходимото количество 0,5% разтвор на Triton X100, така че цялата проба да бъде потопена.
- Оставете да престои около 5 минути или повече. Всички членестоноги трябва да се отделят напълно в разтвора.
- Разтворът Triton X100 се отстранява и се замества с разтвор на калиев хидроксид.

След това се следва техниката, както е описано по-горе.

Приложение 4: Монтиране в среда Euparal® или канадски балсам стъпка по стъпка

1. Пробите трябва да бъдат обезводнени (мътен или млечен вид показва недостатъчна обезводняване).

2. Обезводняването може да се постигне чрез увеличаване на концентрацията на етилов алкохол.

3. Пробите могат да се прехвърлят от 99% алкохол или абсолютен алкохол в просветляващ агент.

Процедура:

1. Поставете възрастните флеботоми в 70% етанол.

2. Отстранете етанола и го заместете с 10% KOH. Покрийте флеботомите със стъкло.

3. Мацерирайте, докато насекомите станат прозрачни.

4. Отстранете KOH.

5. Покрийте пробата с дестилирана вода и изчакайте 30 до 45 минути.

6. Отстранете водата и повторете измиването с дестилирана вода за 30 минути (времето зависи от броя на пробите: колкото повече проби се обработват заедно, толкова по-дълго трябва да се спазва това време. Колкото по-малко са пробите, особено тези, които се обработват поотделно, толкова по-кратко може да бъде това време).

7. Отстранете водата.

8. Добавете разтвор на Марк-Андре (потенциално оцветен с кисел фуксин) и изчакайте 24 часа (един ден).

9. Отстранете разтвора на Марк-Андре.

10. Покрийте пробата с дестилирана вода и изчакайте 30 до 45 минути.

11. Отстранете водата и повторете измиването с дестилирана вода за 30 минути.

12. Отстранете водата

13. Добавете 70% етанол и дисецирайте екземпляра.

а. За главата и коремчето, внимателно издърпайте главата или коремчето от гърдите.

б. За гърдите, отстранете крилата, като задържите гърдите с една пинсета и издърпате основата на придатъците с друга пинсета. Възможно е да се направи сагитална дисекция, разделяйки гърдите на лява и дясна половина, в зависимост от областите, които представляват най-голям интерес.

14. Обезводнете постепенно чрез серия от водни разтвори на етилов алкохол. 50 – 80 – 95%, докато се достигне абсолютен етанол.

15. Обезводнете пробите, като ги промиете два пъти, по 10 минути всеки път, със 100% етанол.

16. Отстранете етанола и покрийте пробите с карамфилово масло за 15 минути при стайна температура.

17. Прехвърлете пробите от карамфиловото масло в капка Euparal® или канадски балсам върху чисто предметно стъкло.

18. Подредете по желание: Главата, гърдите и коремчето на флеботома могат да бъдат дисецирани с фини игли или пинсети под дисекционен микроскоп. Главата трябва да бъде дисецирана от тялото, за да бъде монтирана във вентро-дорзална позиция, т.е. тилният отвор трябва да бъде ориентиран нагоре, така че цибариумът да може да се наблюдава директно през него.

Разрязването се извършва в средата за монтиране на насекомото.

19. Оставете пробата, докато повърхността стане лепкава.

20. Намокрете чисто покривно стъкло с абсолютен алкохол. Поставете покривното стъкло под ъгъл върху канадския балсам.

21. Съхранявайте микроскопските препарати в суха кутия, предназначена за тази цел.
