# Supplementary material for: Processing and mounting phlebotomine sand flies: a consensus guideline
Source: Parasite. 2026 Apr 3;33:18. doi: 10.1051/parasite/2026009 (PMC13047900; doi:10.1051/parasite/2026009)
Supplement: Supplementary file 4 — Catalan translation / Traducció al català [file parasite-33-18-s4.pdf]

Parasite **33**, 18 (2026)
© F.J. Randrianambinintsoa et al., published by EDP Sciences, 2026
https://doi.org/10.1051/parasite/2026009

PARASITE

Available online at:
www.parasite-journal.org

REVIEW ARTICLE OPEN ACCESS

Processament i muntatge de flebòtoms: una guia consensuada

Fano José Randrianambinintsoa[1], Laure Augendre[1], Jorian Prudhomme[1], Jean-Philippe Martinet[1], Mathieu Loyer[1], Nalia Mekarnia[1], Hocine Kerkoub[1], Farzana Khan Perveen[1], Antoine Huguenin[1,2], Emilie Kariya[1,2], Mohammad Akhoundi[3], Andrey José de Andrade[4], Eduardo Berriatua[5], Gioia Bongiorno[6], Sébastien Boyer[7,8], Vasiliki Christodoulou[9], Magda Clara Vieira Da Costa-Ribeiro[10], Lucas Alexandre Farias de Souza[10], Huicong Ding[11], Blaise Dondji[12], Vít Dvořák[13], Ozge Erisoz Kasap[14], Eunice Aparecida Bianchi Galati[15], Montserrat Gállego[16], Cristina Ballart[16], Stavroula Gouzelou[17], Nabil Haddad[18], Rezki Sabrina Masse[19], Asrat Hailu Mekuria[20], Vladimir Ivovic[21], Szymon Kaczmarek[22], Mohd Khadri Shahar[19], Oscar D. Kirstein[23], Edwin Kniha[24], Iva Kolářová[13], Lincoln Timinao[25], Cristian Lucanas[26], Ognyan Mikov[27], Kimsear Nov[7], Yusuf Özbel[28], Bernard Pesson[29], Laura Cristina Posada Lopez[30], Didot Budi Prasetyo[1,7], Nil Rahola[31], Eduardo A. Rebollar-Tellez[32], Bruno Leite Rodrigues[15], Lalita Roy[33], Prasanta Saini[34], Chizu Sanjoba[35], Paloma Helena Fernandes Shimabukuro[36], Padet Siriyasatien[37], Agnieszka Soszyńska[22], Tatiana Suleşco[38], Massamba Sylla[39], Majhalia Torno[40], Petr Volf[13], Khamsing Vongphayloth[41], Vu Sinh Nam[42], April Wardhana[43], Eric Yessinou[44], Sonia Zapata[45], Jean-Charles Gantier[1], and Jérôme Depaquit[1,2,*]

[1] Faculté de Pharmacie, Université de Reims Champagne Ardenne, UR ESCAPE-USC ANSES PETARD, 51 rue Cognacq-Jay, 51096 Reims Cedex, France
[2] Pôle de Biologie territoriale, Laboratoire de Parasitologie-Mycologie, Centre Hospitalo-Universitaire, 51092 Reims, France
[3] Parasitology-Mycology Department, Avicenne Hospital, AP-HP, Bobigny, Sorbonne Paris Nord University, France; Unité des Virus Émergents (UVE: Aix-Marseille Univ, Università di Corsica, IRD 190, Inserm 1207, IRBA), 13005 Marseille, France
[4] Parasitology Collection of Basic Pathology, Department of Basic Pathology, Federal University of Paraná, Curitiba 19031, Brazil
[5] Department of Animal Health, University of Murcia, Campus de Espinardo, 30100 Espinardo, Murcia, Spain
[6] Department of Infectious Diseases, Vector-borne Diseases Unit, Istituto Superiore di Sanità, 00166 Rome, Italy
[7] Medical and Veterinary Entomology Unit, Institut Pasteur du Cambodge, Phnom Penh 12201, Cambodia
[8] Ecology & Emergence of Arthropod-borne Pathogens Unit, Department of Global Health, Institut Pasteur, CNRS UMR2000, 75015 Paris, France
[9] Section Veterinary Services (1417), Laboratory for Animal Health Virology, Aglantzia, Nicosia 2109, Cyprus
[10] Insects Vectors and Parasites Laboratory, Department of Basic Pathology and Postgraduate program in Microbiology, Parasitology and Pathology, Federal University of Paraná, 81530-900 Curitiba, Brazil
[11] Department of Biological Sciences, National University of Singapore, 117558, Singapore
[12] Laboratory of the Leishmaniasis Research Project, Mokolo District Hospital, Mokolo, Cameroon; Laboratory of Cellular Immunology and Parasitology, Department of Biological Sciences, Central Washington University, 98926 Ellensburg, WA, USA
[13] Department of Parasitology, Faculty of Science, Charles University, 12800 Prague, Czechia
[14] VERG Laboratories, Department of Biology, Faculty of Science, Hacettepe University, Beytepe, Ankara 06800, Türkiye
[15] Faculdade de Saúde Pública da Universidade de São Paulo (FSP/USP), Pós-graduação em Saúde Pública, 01246-904 São Paulo, Brazil
[16] Secció de Parasitologia, Departament de Biologia, Sanitat i Medi Ambient, Facultat de Farmàcia i Ciències de l'Alimentació, Universitat de Barcelona, & Institut de Salut Global de Barcelona (ISGlobal), Centro de Investigación Biomédica en Red, Enfermedades Infecciosas (CIBERINFEC), 08028 Barcelona, Spain
[17] Laboratory of Infectious Diseases and Public Health, School of Medicine, University of Cyprus, Nicosia, Cyprus & Department of Pediatrics, Archbishop Makarios III Hospital, Nicosia 2115, Cyprus
[18] Faculty of Health Sciences, American University of Beirut, 1107 2020 Beirut, Lebanon
[19] Medical Entomology Unit, Infectious Disease Research Centre, Institute for Medical Research (IMR), National Institutes of Health (NIH), Ministry of Health Malaysia, 40170 Shah Alam, Selangor, Malaysia
[20] School of Medicine, Addis Ababa University, 28017 - 1000 Addis Ababa, Ethiopia
[21] Faculty of Mathematics, Natural Sciences and Information Technologies, University of Primorska, 6000 Koper, Slovenia

Edited by Jean-Lou Justine
*Corresponding author: jerome.depaquit@univ-reims.fr

This is an Open Access article distributed under the terms of the Creative Commons Attribution License https://creativecommons.org/licenses/by/4.0
which permits unrestricted use, distribution, and reproduction in any medium, provided the original work is properly cited.

[22] University of Lodz, Faculty of Biology and Environmental Protection, Department of Invertebrate Zoology and Hydrobiology, Banacha 12/16, 90-237 Łódź, Poland
[23] Laboratory of Entomology, Ministry of Health, 9134302 Jerusalem, Israel
[24] Center for Pathophysiology, Infectiology and Immunology, Institute of Specific Prophylaxis and Tropical Medicine, Medical University Vienna, Kinderspitalgasse 15, 1090 Vienna, Austria
[25] Papua New Guinea Institute of Medical Research (PNGIMR) Institute, PO Box 60, Headquarter, Homate Street, 441 Goroka, Eastern Highlands Province, Papua New Guinea
[26] Museum of Natural History, University of the Philippines Los Baños, 4031 Laguna, Philippines
[27] National Centre of Infectious and Parasitic Diseases, 1504 Sofia, Bulgaria
[28] Ege University, Faculty of Medicine, Department of Parasitology, 35040 Bornova/Izmir, Türkiye
[29] Retired, Faculté de Pharmacie, Université de Strasbourg, Strasbourg, 67400 Illkirch-Graffenstaden, France
[30] Program for the Study and Control of Tropical Diseases (PECET), Faculty of Medicine, University of Antioquia, 050010 Medellin, Colombia
[31] MIVEGEC, Univ. Montpellier, CNRS, IRD, 34394 Montpellier, France & Medical Entomology Unit, Institut Pasteur de Madagascar, 101 Antananarivo, Madagascar
[32] Laboratorio de Entomología Médica, Departamento de Zoología de Invertebrados, Facultad de Ciencias Biológicas, Universidad Autónoma de Nuevo León, San Nicolás de los Garza, 66455, NL, México
[33] Tropical and Infectious Disease Centre, BP Koirala Institute of Health Sciences, Dharan 56700, Nepal
[34] ICMR-Vector Control Research Centre, Puducherry 605006, India
[35] Graduate School of Agricultural and Life Sciences, The University of Tokyo, Tokyo 113-8657, Japan
[36] Grupo de estudos em Leishmanioses/Coleção de Flebotomíneos (COLFLEB/Fiocruz-MG), Instituto René Rachou, Fundação Oswaldo Cruz, Belo Horizonte, Minas Gerais, 30190009, Brazil
[37] Center of Excellence in Vector Biology and Vector-Borne Disease, Department of Parasitology, Faculty of Medicine, Chulalongkorn University, Bangkok 10330, Thailand
[38] Department of Arbovirology, Bernhard Nocht Institute for Tropical Medicine, Bernhard Nocht Str. 74, 20359 Hamburg, Germany [39] Laboratory Vectors & Parasites, Department of Livestock Sciences and Techniques, Sine Saloum University El Hadji Ibrahima Niasse (SSUEIN) Kaffrine Campus, C.P. 24600, Senegal.
[40] Environmental Health Institute, National Environment Agency, Singapore 138667, Singapore & Department of Biological Sciences, National University of Singapore, 117558 Singapore
[41] Institut Pasteur du Laos, Laboratory of Vector-Borne Diseases, Samsenhai Road, Ban Kao-Gnot, Sisattanak District, 3560 Vientiane, Lao PDR
[42] National Institute of Hygiene and Epidemiology, 1 Yec-Xanh Street, Hai Ba Trung District, 100000 Hanoi, Vietnam
[43] Indonesian Research Center for Veterinary Science, Indonesian Agency for Agricultural Research and Development, Ministry of Agriculture Republic Indonesia, Bogor 16114, Indonesia & Department of Parasitology, Faculty of Veterinary Medicine, Airlangga University, Surabaya 60115, Indonesia
[44] Laboratory of Research in Applied Biology, Polytechnic School of Abomey-Calavi, University of Abomey-Calavi, 01 P.O. Box 2009, 00000 Cotonou, Benin
[45] Instituto de Microbiología, Colegio de Ciencias Biológicas y Ambientales (COCIBA), Universidad San Francisco de Quito (USFQ), 170901 Quito, Ecuador

Received 1 December 2025, Accepted 29 January 2026, Published online 3 April 2026

Resum – Aquest article proporciona una guia completa per al processament i muntatge de mostres de flebòtoms, fet que és crucial per a la identificació d'espècies i la detecció i aïllament de patògens. Es discuteix sobre una sèrie de tècniques adequades tant per a entorns de camp com de laboratori. La guia inclou instruccions detallades sobre la recollida, manipulació, cobertura i eutanàsia de flebòtoms (recomanant congelació en sec o CO_2 en lloc de productes químics), així com estratègies de conservació, com ara l'emmagatzematge en fred i la conservació en etanol. La qualitat de la preparació de certes estructures anatòmiques (òrgans genitals, cap i ales) és essencial per a la seva correcta observació microscòpica i es descriu en aquest treball. L'article també presenta un processament detallat de mostres, inclòs el procés de clarificació amb agents com l'hidròxid de potassi i després la solució de Marc-André. El procés de muntatge compara diferents medis, emfatitzant les seves propietats òptiques i el seu potencial de conservació. El líquid de Hoyer (també conegut com a goma cloral) es recomana per a una observació ràpida, especialment per a les espermateques, a causa de la seva claredat, tot i que no és adequat per a l'emmagatzematge a llarg termini. Altres medis que s'han tractat inclouen l'alcohol polivinílic, l'Euparal® (per a una tolerància limitada a l'aigua) i el bàlsam del Canadà (un medi soluble en hidrocarburs), i els dos últims ofereixen capacitats de conservació a llarg termini. També s'aborden enfocaments innovadors de biologia molecular com la seqüenciació d'ADN i el MALDI-ToF, que requereixen una atenció especial al processament de mostres. A més, es proporcionen videoclips curts que il·lustren diverses tècniques de muntatge, així com traduccions en 33 idiomes diferents, fet que permet que la guia assoleixi les diverses necessitats i expectatives de la comunitat científica mundial.

Paraules clau: Muntatge, flebòtom, líquid de Hoyer, solució de Marc-André, goma de cloral, alcohol polivinílic, Euparal®, bàlsam del Canadà, aïllament de *Leishmania*, condicions de camp, cultiu, dissecció, biologia molecular, MALDI-ToF, espècimens tipus.

Abstract – Processing and mounting phlebotomine sand flies: a consensus guideline. This article provides a comprehensive guide for the processing and mounting of phlebotomine sand fly specimens, which is crucial for species identification and pathogen detection and isolation. It discusses a range of techniques suitable for both field and laboratory settings. The guide includes detailed instructions on sand fly collection, handling, covering, and euthanasia (recommending dry freezing or CO_2 over chemicals) as well as conservation strategies, such as cold storage and preservation in ethanol. The quality of preparation of certain anatomical structures (genital organs, head and wings) is essential for their proper microscopic observation and is described in this work. The article also presents detailed sample processing, including the clearing process with agents such as potassium hydroxide then Marc-André solution. The mounting process compares different media, emphasizing their optical properties and preservation potential. Hoyer fluid (also known as chloral gum) is recommended for quick observation, particularly for spermathecae, due to its clarity, although it is not suitable for long-term storage. Other media discussed include polyvinyl alcohol, Euparal® (for limited water tolerance), and Canada balsam (a hydrocarbon-soluble medium), with the latter two offering long-term preservation capabilities. Innovative molecular biology approaches such as DNA sequencing and MALDI-ToF, which require particular attention to sample processing, are also addressed. Furthermore, short video clips illustrating various mounting techniques as well as translations in many different languages are provided, allowing the guideline to reach the diverse needs and expectations of the global scientific community.

Key words: Mounting, Phlebotomine sand fly, Hoyer fluid, Marc-André solution, Chloral gum, Polyvinyl alcohol, Euparal®, Canada balsam, *Leishmania* isolation, Field conditions, Culture, Dissection, Molecular biology, MALDI-ToF, Type-specimens.

Introducció

Els flebòtoms són petits insectes dípters que pertanyen a la família Psychodidae, subfamília Phlebotominae, amb almenys 1.063 espècies conegudes [21]. Són vectors importants de patògens (*Leishmania*, arbovirus i *Bartonella*) responsables de malalties com les leishmaniosis, infeccions per arbovirus i bartonel·losi, respectivament. La seva identificació es basa principalment en un examen microscòpic detallat facilitat per una recollida acurada, un emmagatzematge adequat i un muntatge acurat en portaobjectes, que requereix diverses tècniques específiques, cadascuna amb els seus propis avantatges i limitacions.

La identificació dels flebòtoms adults es basa en l'observació tant d'estructures externes (per exemple, antenes, palps, genitalia masculina) com internes (per exemple, faringe, cibarium o cibari i espermateques). La dissecció i l'aïllament d'aquestes últimes faciliten la seva observació i, en conseqüència, la seva identificació precisa. Per tant, a diferència dels mosquits o les xinxes besadores, els flebòtoms requereixen el muntatge entre un portaobjectes i un cobreobjectes abans de la seva identificació.

Fins a la dècada del 1980, l'observació microscòpica era l'únic mètode disponible per a la identificació de flebòtoms, i continua sent l'enfocament més utilitzat avui dia. L'elecció del procés i la preparació era, per tant, relativament senzilla i es basava principalment en una dicotomia: d'una banda, el muntatge definitiu que permetia la preservació a llarg termini de la mostra, i de l'altra, un muntatge ràpid per a la identificació en un medi que no assegurava la preservació a llarg termini. El muntatge final, per exemple, en una resina com el bàlsam del Canadà, requereix molt de temps i una deshidratació completa de les mostres. A més, l'índex de refracció (IR) d'aquest medi no sempre és òptim per a una fàcil observació de les espermateques. El muntatge en un medi aquós (per exemple, líquid de Hoyer), en canvi, és més ràpid i permet una millor visualització de les espermateques refractives, però no permet la preservació a llarg termini dels muntatges perquè tendeix a absorbir aigua de l'atmosfera. Una opció és segellar el portaobjectes amb esmalt d'ungles un cop estigui completament sec. Aquest compromís persisteix avui dia, afectant l'elecció del mètode de muntatge, depenent de la finalitat prevista de la preparació. Des de la dècada del 1980, els estudis d'identificació dels flebòtoms han combinat enfocaments morfològics i bioquímics. El primer van ser les anàlisis d'hidrocarburs cuticulars, que van ser ràpidament substituïdes per tècniques de biologia molecular (és a dir, ADN polimòrfic amplificat aleatori (RAPD), polimorfisme de longitud de fragments de restricció (RFLP), amplificació d'ADN i seqüenciació mitjançant el mètode Sanger, així com la seqüenciació de nova generació (NGS)). Avui dia, els enfocaments moleculars es complementen amb mètodes proteòmics com ara el MALDI-ToF. A més, la identificació d'espècies moleculars es pot combinar amb la detecció de patògens per PCR (*Leishmania*, *Trypanosoma*, *Bartonella* i *Phlebovirus*), ja que tots es poden detectar tant per PCR de punt final com en temps real, cosa que requereix l'adaptació del procés de mostreig i emmagatzematge als objectius assignats [3, 32]. A més de les característiques morfològiques tradicionalment utilitzades per a la

discriminació d'espècies, es poden aplicar altres enfocaments morfològics (és a dir, geomorfometria de l'ala). Basant-se principalment en les pròpies experiències dels autors i dades bibliogràfiques, l'objectiu d'aquesta investigació era proporcionar pautes estandarditzades per al muntatge i processament de flebòtoms adults per optimitzar les anàlisis morfològiques i moleculars.

La necessitat de dur a terme certes anàlisis (per exemple, biologia molecular o MALDI-ToF) requereix conservar una part dels flebòtoms que no és necessària per a la identificació morfològica, fet que destaca la necessitat d'una elecció correcta del protocol.

En aquest article, ens centrem en els mètodes d'anestèsia i eutanàsia dels flebòtoms capturats vius, el seu emmagatzematge i el seu procés de muntatge, per a una identificació ràpida o per a la conservació a llarg termini que permeti estudis posteriors.

Preàmbul: Les consideracions de seguretat i reglamentàries han de fer referència a les Fitxes de Dades de Seguretat (FDS) pertinents.

Tots els productes químics presentats en aquesta guia s'han de manipular en condicions de seguretat estrictes. Els comitès de salut i seguretat de les instal·lacions de recerca estan disponibles per proporcionar-vos informació no només sobre els perills d'aquests productes químics, sinó també sobre els seus procediments de manipulació i eliminació de residus. Tanmateix, és obligatori seguir les instruccions de seguretat pel que fa al seu ús i eliminació. Cal destacar que és responsabilitat de tots els usuaris garantir el compliment de les bones i segures pràctiques de laboratori i de la legislació i les regulacions aplicables del seu país o institució de recerca. A més, alguns dels productes químics o els seus components (és a dir, l'hidrat de cloral) estan regulats en alguns països. A la Taula 1 es proporciona una llista de les abreviatures utilitzades en aquest manuscrit.

1. Captura de flebòtoms

Els flebòtoms adults es poden recollir vius o morts mitjançant diversos mètodes com ara trampes de llum miniatura tipus CDC, trampes adhesives i aspiradors amb trampes Shannon, o directament als seus llocs de repòs al medi ambient (per exemple, refugis d'animals). Aquests mètodes impliquen col·locar trampes en hàbitats adequats, atraure els flebòtoms amb llum o altres atraients (CO_2 o esquers químics) i recollir-los per a una anàlisi posterior, tal com es descriu en diverses publicacions [2, 3, 32, 36, 49].

La captura de flebòtoms vius permet totes les aplicacions posteriors, mentre que la recollida d'individus morts impedeix l'aïllament de soques de *Leishmania* o virus. Algunes tècniques de captura, com ara els papers adhesius, solen provocar la pèrdua d'òrgans dels flebòtoms (antenes, palps, ales o potes). A més, l'oli de ricí dels papers adhesius recobreix als flebòtoms i s'ha d'eliminar al començament del processament, normalment mitjançant un bany de 15 minuts en una barreja d'etanol i èter dietílic a parts iguals.

Taula 1. Llista d'abreviatures.

**ADN**	Àcid desoxiribonucleic
**ARN**	Àcid ribonucleic
**BME**	Medi Basal Eagle
**CDC**	Centre per al Control i la Prevenció de Malalties
**CMCP**	Monoclorofenol càmforat
**CMR**	Substància carcinogènica, mutagènica i reprotòxica
**COI**	Gen de la subunitat I del citocrom c oxidasa
**CytB**	Gen del citocrom b
**ELISA**	Assaig immunoabsorbent lligat a enzims
**EtOH**	Etanol
**IR**	Índex de refracció
**M199**	Medi 199
**MALDI-ToF MS**	Espectrometria de masses amb desorció/ionització làser assistida per matriu i temps de vol
**MEM**	Medi essencial mínim
**NGS**	Seqüenciació de nova generació
**NNN**	Medi Novy-MacNeal-Nicolle
**PCR**	Reacció en cadena de la polimerasa
**Lao PDR**	República Democràtica Popular de Laos
**PNOC**	Gen de la prepronociceptina
**qPCR**	PCR quantitativa (PCR en temps real)
**RAPD**	ADN polimòrfic amplificat aleatòriament
**RFLP**	Polimorfisme de longitud de fragments de restricció
**RNases**	Ribonucleases
**RNASS**	Solució d'Estabilització de l'ARN
**RT-PCR**	PCR amb transcripció inversa
**TFA**	Àcid trifluoroacètic

2. Eutanàsia de mostres

Després de la recollida, els flebòtoms vius s'han d'eutanasiar. Amb alguns mètodes de captura (per exemple, papers adhesius, trampes de llum tipus CDC equipades amb un pot que conté detergent o etanol), els flebòtoms moren en el moment de la recollida. La tecnologia de biologia molecular es pot aplicar als espècimens recollitss directament en etanol i als altres si aquets s'emmagatzemen en etanol el més ràpidament possible. Tanmateix, cap d'aquests mètodes d'eutanàsia permet el processament d'insectes mitjançant MALDI-ToF. A més, alguns mètodes d'eutanàsia poden causar la pèrdua de certs caràcters morfològics. Per tant, és essencial utilitzar un agent

d'eutanàsia estàndard adequat per garantir una identificació correcta o un emmagatzematge a llarg termini com a mostres de referència (és a dir, mostres conservades i emmagatzemades per a futures consultes o comparacions). Els productes químics com l'acetat d'etil, l'èter etílic, el tetracloroetà i el cloroform poden penetrar en el cotó fluix i col·locar-se en un recipient que contingui els flebòtoms per a l'eutanàsia. Aquests agents d'eutanàsia s'han de manipular amb cura, seguint les recomanacions del fabricant a causa de la seva toxicitat. Tanmateix, no recomanem matar els flebòtoms amb cloroform, ja que, segons la nostra experiència, és poc compatible amb els estudis de biologia molecular. Atesa la naturalesa perillosa de tots aquests productes i la seva qüestionable idoneïtat per a les anàlisis moleculars, generalment es desaconsella l'ús d'aquests productes químics.

El mètode més utilitzat, que preserva la morfologia, l'ADN o les proteïnes, és la congelació en sec de les mostres. Les mostres s'han de congelar el temps suficient per poder-les anestesiar completament, però no tant que (i) s'assequin o (ii) es vegin compromeses pel que fa a la viabilitat de la *Leishmania*, si l'objectiu és aïllar-les in vitro del tracte digestiu dels flebòtoms. **Per tant, recomanem una durada de congelació de 15 a 20 minuts a -20 °C, controlant-les regularment per assegurar-se que només s'atordeixen sense matar les leishmànies paràsites.**

Si no es disposa de congelador, els insectes poden ser sacrificats alternativament amb CO_2. En condicions de camp on no es poden utilitzar cilindres de CO_2, les mostres es poden matar utilitzant petits contenidors comercials de CO_2 utilitzats en "sifons de soda" (dispensadors de begudes), però hi pot haver restriccions en el seu transport per via aèria. Com a últim recurs, els insectes poden morir per exposició al fum del tabac. Els flebòtoms es capturen vius en una trampa CDC, es recullen amb un aspirador, es retenen al tub de vidre i s'exposen al fum del tabac, que els mata en qüestió de segons. Aquest mètode és aplicable en totes les condicions de camp, fins i tot en condicions d'aïllament difícils. Tanmateix, com que el vidre s'impregna de fum, no es pot utilitzar per a la recollida i manipulació posteriors de flebòtoms vius sense una neteja a fons. No obstant això, el mateix aspirador sense netejar encara es pot utilitzar per sacrificar flebòtoms d'altres trampes amb finalitats de fixació. També cal comprovar si s'han tret totes les mostres de l'aspirador. Aquests mètodes són compatibles amb l'aïllament de *Leishmania* mitjançant la dissecció intestinal.

3. Emmagatzematge de mostres abans del processament

Hi ha cinc mètodes principals de fixació abans del processament:

3.1. Congelació

Aquest mètode es realitza idealment a -20 °C o, preferiblement, a -80 °C. Aquests mètodes d'emmagatzematge ara s'utilitzen més àmpliament que l'emmagatzematge amb nitrogen líquid. En tots els casos, la criopreservació s'ha d'implementar el més aviat possible després d'atordir els espècimens. L'emmagatzematge en fred en congeladors ofereix l'avantatge de preservar completament els propis insectes, així com l'ARN, l'ADN i les proteïnes amb plena integritat durant tot el període d'emmagatzematge. En canvi, el nitrogen líquid pot danyar greument les ales, les potes, els palps i les antenes, sovint amputant-los i ocasionalment eliminant caràcters morfològics clau. L'emmagatzematge en sec al congelador és menys traumàtic per a les mostres, però no és ideal per preservar els seus òrgans fràgils. És important destacar que, en el moment de la descongelació, les ales, les antenes, els palps o les potes es poden adherir als vials i finalment esquinçar a causa de la condensació. Tanmateix, la conservació mitjançant congelació no sempre és factible en estudis de camp, ja que requereix accés a un congelador o a un recipient de nitrogen líquid. L'emmagatzematge per congelació és totalment compatible amb la detecció de patògens mitjançant eines moleculars sense pèrdua de sensibilitat, tot i que la detecció i l'aïllament de virus d'ARN requereix la congelació a -80 °C o en nitrogen líquid si es necessari un emmagatzematge a llarg termini. Tanmateix, la congelació de mostres no permet l'aïllament de *Leishmania* mitjançant la dissecció intestinal, excepte si els flebòtoms s'immergeixen primer en la fase de vapor i després en nitrogen líquid (per exemple, en vials col·locats en una mitja), simulant la criopreservació de *Leishmania*.

3.2. Emmagatzematge en alcohol (etanol o alcohol isopropílic)

Aquest és probablement el mètode més utilitzat per emmagatzemar els flebòtoms. És fàcil d'implementar al camp, fins i tot en condicions difícils sense accés a un laboratori. La conservació en alcohol és particularment adequada per a estudis morfològics, ja que els òrgans fràgils (ales, potes, antenes o palps) romanen intactes si no hi ha bombolles d'aire al tub d'emmagatzematge. Per tant, recomanem segellar el tub amb una petita bola de cotó per eliminar les bombolles d'aire i col·locar una etiqueta a la part superior del tap de cotó (Figura 1). La concentració d'alcohol adequada continua sent un tema de debat. Generalment, no es recomanen concentracions inferiors al 70% [45, 66]. Concentracions més altes preserven l'ADN de manera més eficaç i durant períodes més llargs, però fan que les mostres siguin més fràgils i trencadisses per a estudis morfològics. L'ús d'etanol al 96% (la barreja azeòtropa) garanteix l'estabilitat de la concentració al llarg del temps, especialment en zones humides com els països tropicals, tot i que l'etanol al 95% sovint és més fàcil d'obtenir. Independentment de la concentració, l'ADN generalment es conserva bé en etanol (encara que menys eficaçment que amb els mètodes de congelació, especialment per als mètodes moleculars de tipus NGS). Les proteïnes són molt menys estables, especialment per a la proteòmica, com les aplicacions MALDI-ToF. Els flebòtoms conservats en

alcohol durant uns mesos encara es poden identificar morfològicament, però és impossible generar espectres de proteïnes de referència a partir d'aquestes mostres. L'emmagatzematge en alcohol o en condicions seques es pot millorar si la mostra també es congela a -20 °C. La congelació a -20 °C millora principalment la preservació molecular (per exemple, àcids nucleics) en alentir la degradació i també proporciona un benefici secundari per a la preservació morfològica en reduir la degradació dels teixits al llarg del temps, tot i que l'efecte sobre la morfologia és més limitat que sobre la integritat molecular. L'emmagatzematge en etanol també es pot aplicar per a la detecció de virus d'ADN i ARN quan s'utilitza etanol a una concentració d'almenys el 70% durant un període d'emmagatzematge curt, menys d'uns quants mesos. A més, l'alcohol isopropílic pot estar fàcilment disponible en alguns països i conserva l'ADN, però fa que les mostres siguin rígides. No és inflamable com l'etanol i, per tant, es pot transportar fàcilment. Si cal, els flebòtoms conservats en nitrogen líquid o congelats en sec es poden transferir a alcohol, combinant així els inconvenients d'ambdós mètodes.

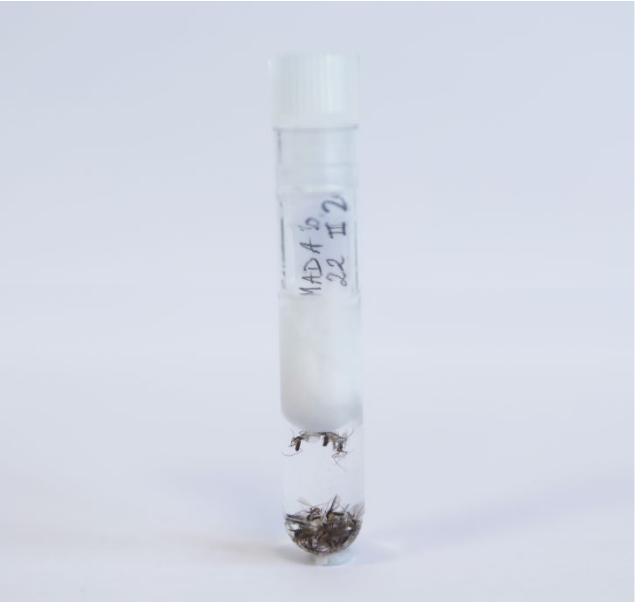

Figura 1: Flebòtoms conservats en etanol.

3.3. Emmagatzematge en una solució d'estabilització de l'ARN (RNASS)

Aquest reactiu aquós s'utilitza àmpliament, no és tòxic i està dissenyat per estabilitzar i protegir l'ARN en mostres de teixit i cèl·lules fresques. Actua penetrant ràpidament a la mostra i inactivant les RNases (enzims que degraden l'ARN), evitant així la degradació de l'ARN sense necessitat de congelació immediata. L'emmagatzematge en RNASS és generalment eficaç per preservar la morfologia general del teixit i la cel·lular per a l'avaluació histològica posterior. Tot i que la RNASS està optimitzat per a l'estabilització de l'ARN en lloc de la fixació, l'emmagatzematge a curt i mitjà termini normalment manté bé la integritat estructural. La RNASS permet emmagatzemar mostres a temperatura ambient fins a 7 dies, a 4 °C durant diverses setmanes o a -20 °C/-80 °C per a la conservació a llarg termini. Aquest mètode és particularment valuós en treballs de camp o entorns clínics on la infraestructura de la cadena de fred és limitada. L'extracció d'ARN normalment requereix treure les mostres del reactiu i processar-les segons els protocols estàndard.

3.4. Conservació en sec a temperatura ambient

Aquest és un mètode més antic que, quan s'aplica a una mostra *in toto* (muntada sencera), té el principal desavantatge de conservar malament òrgans fràgils com les ales, les potes, les antenes i els palps. Tanmateix, els estudis proteòmics mitjançant MALDI-ToF continuen sent factibles si es duu a terme deshidratació després de la fixació amb un dessecant tipus gel de sílice. En canvi, les anàlisis moleculars dirigides a l'ADN continuen sent difícils de realitzar en aquestes mostres, perquè l'ADN sovint encara està fragmentat i en baixa quantitat, cosa que significa que les anàlisis continuen sent més difícils que amb mostres fresques o congelades, especialment per a genomes nuclears. Tanmateix, tècniques recents com la museòmica es podrien utilitzar en mostres d'aquest tipus [34]. Per tant, no es recomana aquest mètode d'emmagatzematge, tret que no hi hagi cap alternativa disponible. Es pot combinar amb l'emmagatzematge en fred col·locant els tubs en un congelador a -20 °C o -80 °C. El principal repte és aconseguir un muntatge adequat de les mostres o de les parts del cos necessàries per a la identificació. Per aconseguir-ho, la rehidratació és essencial. Recomanem utilitzar una solució de Triton X-100. La durada de la rehidratació varia des d'unes poques hores fins a diversos dies, sota un control regular i acurat. Després d'una rehidratació completa, les mostres s'han d'esbandir en tres banys d'aigua consecutius.

3.5. Conservació en papers de filtre

El principal avantatge dels papers de filtre és l'estabilitat a llarg termini de l'ADN genòmic dins de les cèl·lules de sang sencera no fixada i assecada o de cèl·lules sanguínies emmagatzemades a temperatura ambient. El paper de filtre es proporciona en una mida de targeta petita, cosa que permet emmagatzemar diversos centenars de mostres a temperatura ambient en un volum equivalent al d'un petit calaix d'escriptori. La matriu del paper de filtre està impregnada amb agents que desnaturalitzen els agents infecciosos i, per tant, les mostres ja no es consideren un risc biològic. Això permet l'emmagatzematge i el transport de mostres sense necessitat de precaucions selectives de risc biològic [68].

4. Dissecció de mostres

A diferència de molts altres insectes, que s'identifiquen a partir de caràcters externs observables en individus fixats en la seva totalitat, els flebòtoms requereixen dissecció i muntatge en portaobjectes per estudiar les característiques anatòmiques per a una identificació precisa de l'espècie. Independentment del procediment de preparació i muntatge escollit, s'utilitza la mateixa tècnica de dissecció (Figures 2 i 3) (https://zenodo.org/records/18198006).

Ús de Triton X100: solució aquosa no iònica

Cal tenir en compte que el muntatge es refereix a mostres acabades de capturar o emmagatzemades adequadament. La majoria dels col·leccionistes tenen mostres d'insectes conservades en sec (per a ús MALDI-ToF) o emmagatzemades en alcohol durant molts anys. Malauradament, la conservació en alcohol no és òptima durant períodes d'anys, i els artròpodes conservats d'aquesta manera es tornen molt difícils de preparar per a l'examen microscòpic. Un incident que es produeix sovint és la degradació dels plàstics que contenen mostres, seguida de l'evaporació de l'alcohol. En ambdós casos, no tenim opcions perquè les mostres romanen massa temps en alcohol o s'assequen. Per tant, va sorgir la idea d'utilitzar agents humectants que no siguin detergents forts. El Triton X100 es presenta en forma de solució aquosa no iònica (solució de 4-(1,1,3,3-tetrametilbutil)fenol–polietilenglicol, o t-octilfenoxipolietoxietanol, èter de polietilenglicol tert-octilfenil, àmpliament utilitzada com a detergent en biologia cel·lular i molecular. Permet la permeabilització de les membranes cel·lulars i nuclears.

A continuació es mostra un procediment que utilitza el Triton X100 no iònic en una solució aquosa al 0,5%:

- Impregneu la mostra seca amb alcohol absolut.
- Afegiu el volum necessari de solució de Triton X100 al 0,5% de manera que tota la mostra quedi submergida.
- Deixeu que el procés funcioni durant uns 5 minuts o diversos dies, controlant-lo regularment. Tots els artròpodes s'han de separar completament en la solució.
- Traieu la solució de Triton X100 i substituïu-la per una solució d'hidròxid de potassi.

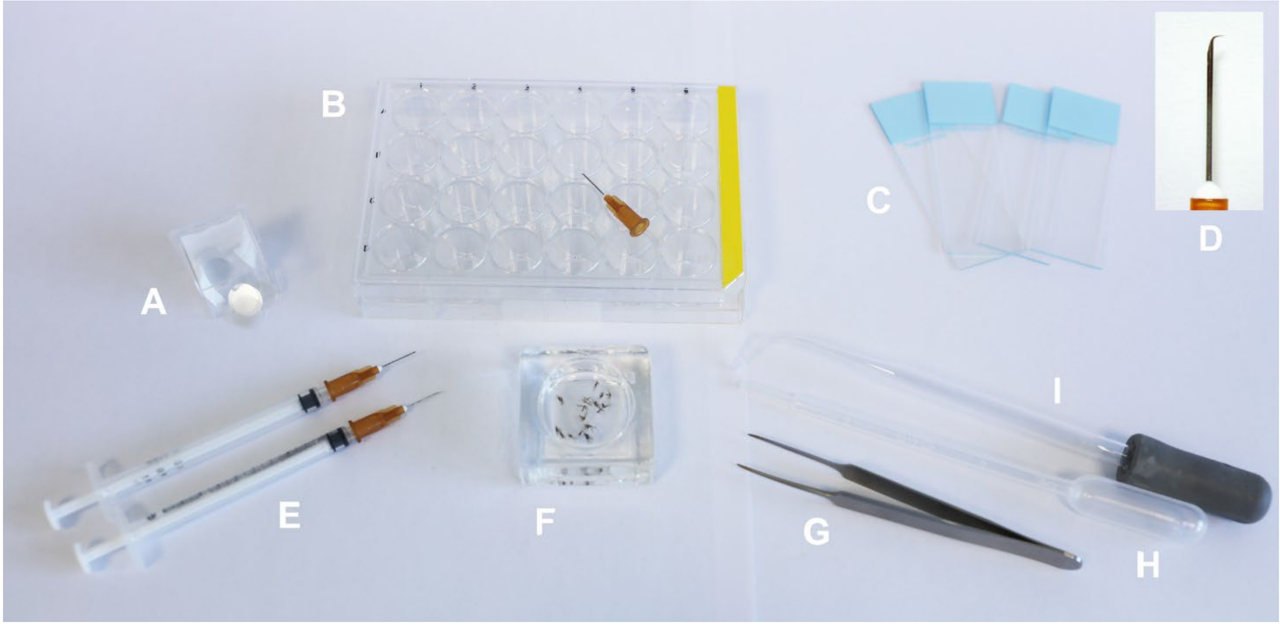

Figura 2: Materials necessaris per al muntatge de flebòtoms: A: cobreobjectes rodons de vidre (10 o 12 mm de diàmetre); B: placa de 24 pous i agulla amb ganxo (si utilitzeu oli de clau o essència Euparal® per processar els flebòtoms, no utilitzeu plaques acríliques perquè es produirà una reacció química i les mostres es faran malbé); C: portaobjectes de vidre adequats per a l'etiquetatge; D: detall del ganxo de l'agulla; E: agulles enganxades a xeringues; F: vidre de rellotge o recipient equivalent que conté els flebòtoms que s'han de muntar; G: pinces Dumont; H: pipeta de plàstic; I: pipeta de vidre doblegada per escalfament per facilitar la transferència de líquid als pous.

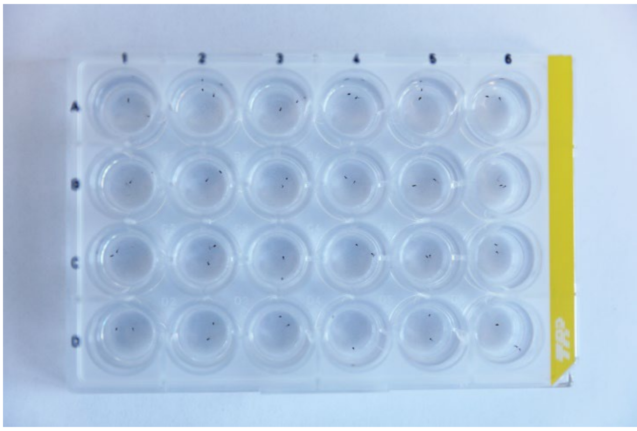

Figura 3: Una placa amb 24 pous, cadascun dels quals conté el cap i la l'extrem terminal de l'abdomen dels flebòtoms.

4.1. Cap

La dissecció es pot realitzar amb agulles fines o agulles entomològiques sota un estereomicroscopi (figures 2 i 3). Les agulles més utilitzades inclouen: 26G x 1/2" (0,45 × 13 mm), 30G × 1/2" (0,3 × 13 mm) o 25G × 5/8" (0,5 × 16 mm). Per preparar la mostra per a la identificació, com a mínim, el cap se separa del cos i es munta ventralment cap amunt per mostrar el cibari i la faringe, mentre que el tòrax i l'abdomen es munten lateralment després de la dissecció. Muntar el cap en posició ventrodorsal garanteix que el foramen occipital estigui orientat cap amunt, de manera que el cibari es pot observar directament. L'accés a aquestes característiques anatòmiques es facilita si el cap està completament separat.

4.2. Ales i tòrax

Les ales s'han de muntar planes. Cada ala es pot separar per la base i muntar-se de manera independent, o es pot muntar una sola, deixant l'altra unida al tòrax. Si es preveu una anàlisi de morfometria geomètrica, és essencial identificar i etiquetar correctament les ales dreta i esquerra abans de muntar-les. El tòrax es divideix en diverses parts, i cadascuna conté informació taxonòmica molt important [20, 64]. Generalment, es munta en vista lateral, per permetre l'examen de la quetotàxia i la distribució del color. La presència de cicatrius d'inserció de setes en certes regions del tòrax es pot utilitzar per distingir algunes espècies del gènere *Brumptomyia*. La distribució del color es pot utilitzar per separar els flebòtoms neotropicals a nivell de gènere (per exemple, *Bichromomyia*), sèries d'espècies (per exemple, *Pintomyia*) o fins i tot espècies del mateix gènere (per exemple, *Micropygomyia*, *Nyssomyia*, *Psathyromyia* i *Psychodopygus*) [20]. Per tant, si el tòrax no s'utilitza per a anàlisis moleculars, s'ha de muntar de manera que no es faci malbé. És important destacar que no és la intensitat dels colors el que importa, sinó la seva distribució pel tòrax. Per tant, el procés de clarificació no eliminarà la pigmentació ni el seu patró.

4.3. Genitalia

Cal tenir especial cura a l'hora de muntar la genitàlia, tant en mascles com en femelles, ja que són crucials per identificar gèneres, subgèneres i espècies. En ambdós sexes, les estructures genitals estan aparellades.

4.3.1. Mascles

Le genitalia és externa i consta de pinces parelles, cadascuna de les quals consta de l'articulació gonocoxita-estil a la seva part dorsal i el lòbul epandrial a la seva part ventral. L'estil porta espines i de vegades setes, que han de ser comptables i les posicions d'inserció de les quals han de ser clarament visibles. És important observar acuradament la superfície interna de la gonocoxita, que pot tenir un feix de setes sèssils o inserides en un lòbul (= tubercle) [22]. Els col·legues amb menys experiència en disseccions poden realitzar un muntatge lateral simple, sense separar la genitàlia de l'extrem de l'abdomen (https://zenodo.org/records/18311158). En aquest cas, la superposició de les dues parts de la genitàlia pot dificultar el recompte de les setes internes de la gonocoxita, per exemple, però això evita danyar la genitàlia a través d'una dissecció fallida. Els col·legues més experimentats poden intentar obrir els genitals en dos, per partir-los. Per aconseguir-ho, cal passar el costat bisellat d'una agulla (tipus agulla de reacció intradèrmica), separant-la sense tallar completament la genitàlia per dividir els conjunts gonocoxita-estil (https://zenodo.org/records/18311158). D'aquesta manera, l'observació de les seves cares internes serà fàcil. Aquest muntatge també facilita l'observació dels paràmers i les beines paramerals, que ja no se superposen. Per al muntatge lateral, que afavoreix la superposició d'òrgans, les mostres han d'estar perfectament clarificades.

4.3.2. Femelles

L'aparell genital és intern, constituït per espermateques. En absència de dissecció, s'han d'observar a través dels teguments i muntant l'abdomen en posició ventral. Independentment del medi de muntatge escollit, les espermateques en si mateixes generalment es poden observar correctament, sobretot si no són llises i clares. Tanmateix, observar espermateques llises i de parets primes pot ser problemàtic en medis poc refractius. A més, observar la base dels conductes espermatecals és essencial per a la identificació d'espècies, com en el subgènere *Larroussius* [35, 37, 38], els principals vectors de *Leishmania infantum* al Vell Món. Sense aquesta observació, la identificació de l'espècimen continua sent impossible. Per superar aquestes dificultats d'observació, el muntatge furca genital-espermateca s'ha de treure de l'abdomen (https://zenodo.org/records/18311106). Les espermateques generalment són difícils d'observar durant la dissecció, però la furca genital és relativament fàcil de localitzar. Com que els conductes espermatecals s'obren a la furca genital, l'aïllament d'aquesta furca normalment permet l'aïllament de les espermateques. Si les espermateques es tallen accidentalment durant el procés, no es perden i encara es poden observar dins dels teguments abdominals (Figura 4).

This is an Open Access article distributed under the terms of the Creative Commons Attribution License https://creativecommons.org/licenses/by/4.0 which permits unrestricted use, distribution, and reproduction in any medium, provided the original work is properly cited.

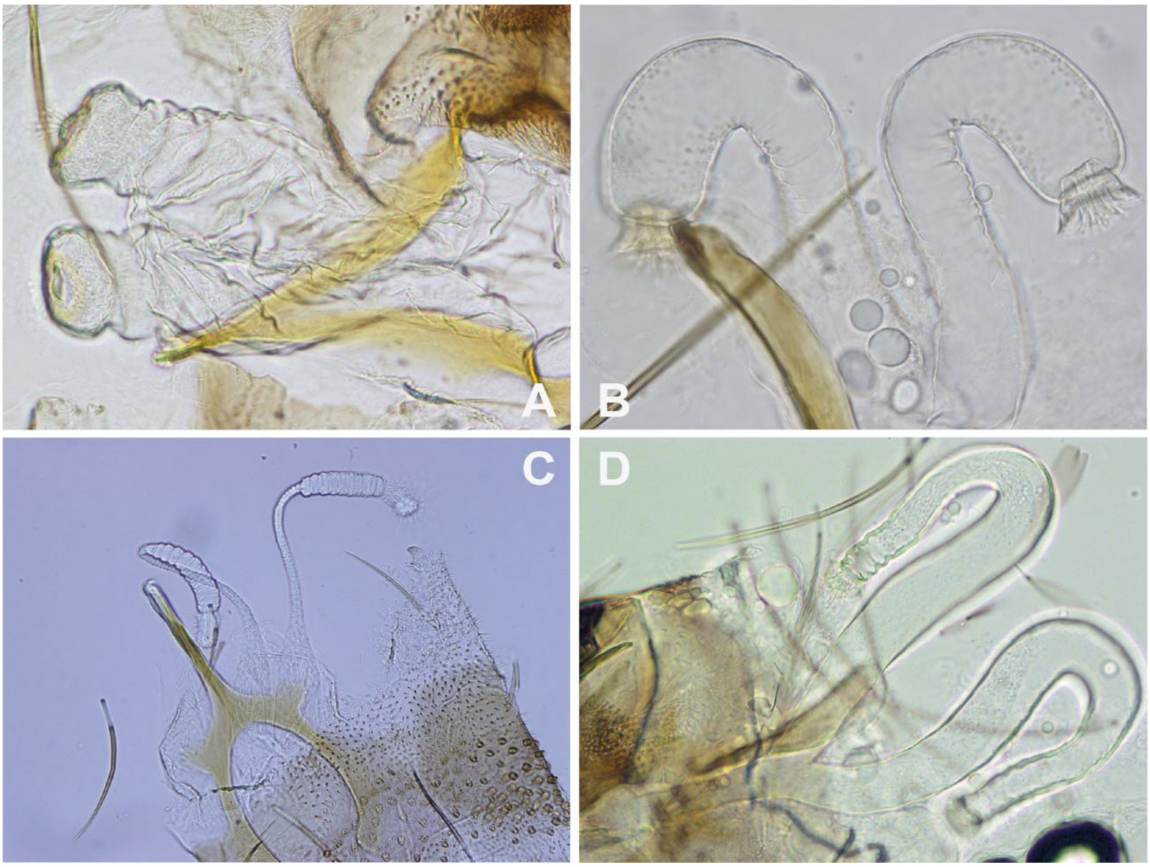

Figura 4: Espermateques disseccionades i muntades en líquid de Marc-André a partir de mostres fresques. A: *Idiophlebotomus longiforceps* (Lao PDR); B: *Sergentomyia minuta* (França); C: *Phlebotomus ariasi* (França); D: *Sergentomyia anodontis* (Lao PDR).

4.4. Dissecció de l'intestí mitjà per a l'aïllament de *Leishmania*

La dissecció del tracte digestiu és essencial per detectar i aïllar *Leishmania* en les femelles de flebòtoms El procediment es pot realitzar tant al camp com al laboratori per avaluar la competència vectorial.

Es recomana treballar amb femelles recentment sacrificades. Rentar les femelles amb aigua o una solució salina que contingui un detergent suau per eliminar l'excés de pèls. Aquest pas ajuda a mantenir les condicions asèptiques per a l'aïllament de *Leishmania*, alhora que preserva les característiques morfològiques necessàries per a la identificació. Per trobar i aïllar *Leishmania*, retireu amb cura l'intestí mitjà i col·loqueu-lo en una sola gota de solució salina estèril (0,9% de NaCl). Després d'observar els paràsits mòbils sota un microscopi òptic (augment recomanat: ~200×), utilitzeu una xeringa d'insulina o una micropipeta per transferir-los al medi de cultiu (per a més detalls, vegeu el capítol 4.4.3).

Munteu el cap i la genitàlia directament en líquid de Marc-André per clarificar-los. Important: no permeteu mai que el líquid de Marc-André entri en contacte amb la *Leishmania*, ni directament ni indirectament mitjançant eines o agulles, ja que és letal per als paràsits.

La dissecció dels flebòtoms femella es pot realitzar en un o dos portaobjectes; ambdues opcions tenen avantatges i limitacions (Figura 5; https://zenodo.org/records/18311154).

This is an Open Access article distributed under the terms of the Creative Commons Attribution License https://creativecommons.org/licenses/by/4.0 which permits unrestricted use, distribution, and reproduction in any medium, provided the original work is properly cited.

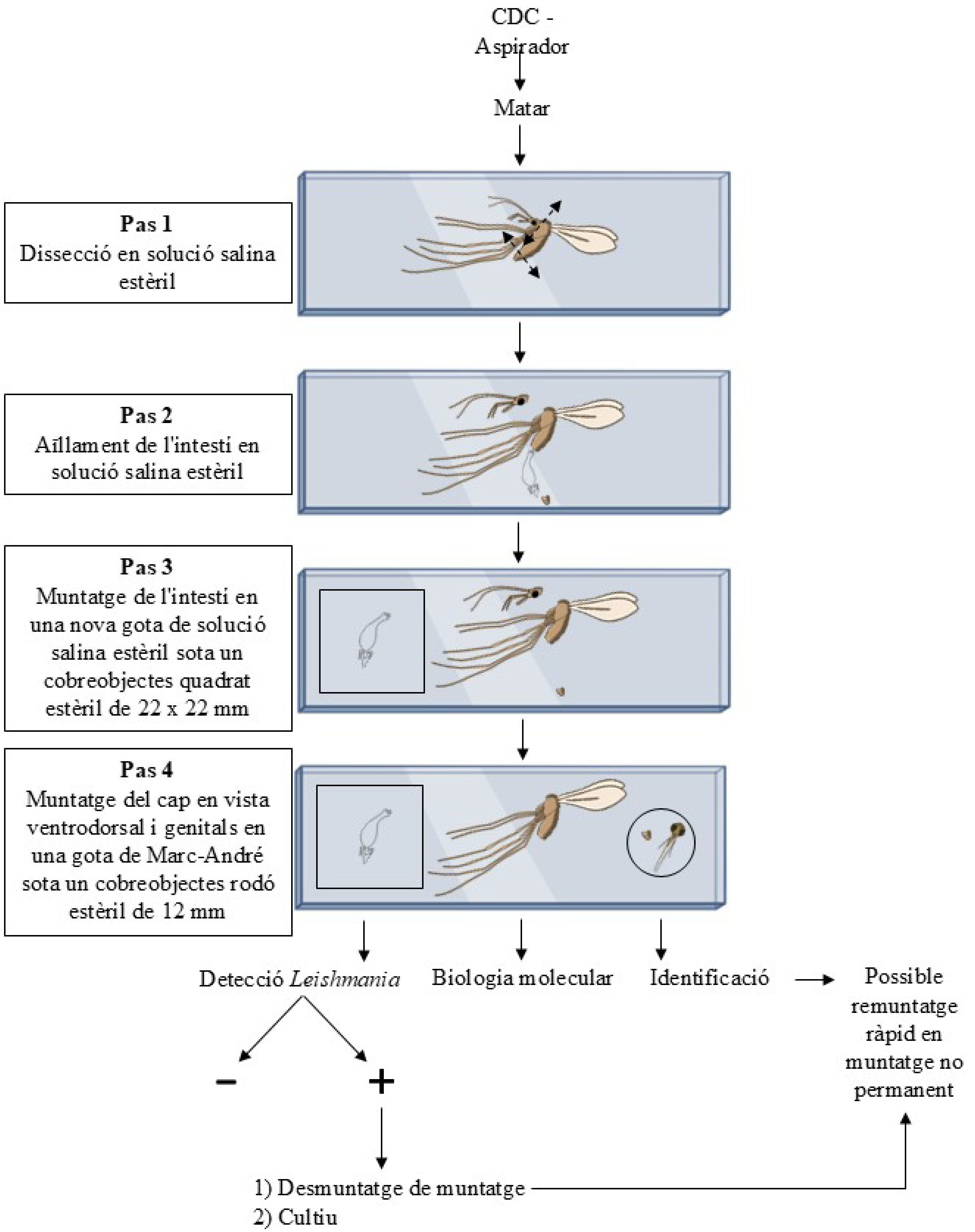

Figura 5: Mètode per a l'aïllament de la *Leishmania*

This is an Open Access article distributed under the terms of the Creative Commons Attribution License https://creativecommons.org/licenses/by/4.0 which permits unrestricted use, distribution, and reproduction in any medium, provided the original work is properly cited.

4.4.1. Mètode de dos portaobjectes

La primera opció consisteix a treballar en dos portaobjectes separats: un que conté solució salina estèril per extreure l'intestí mitjà i l'altre per muntar el cap i les espermateques en líquid de Marc-André. Tanmateix, en condicions de camp, és habitual que dues o tres persones disseccionin els flebòtoms i transmetin les seves disseccions a un sol investigador responsable de la identificació de les espècies i l'avaluació de la infecció per *Leishmania* a l'intestí. La gestió de dos portaobjectes pot causar problemes amb la traçabilitat de la mostra i, en particular, dificultar la determinació amb certesa de quin flebòtom individual es va infectar, si es detecta un intestí positiu (https://zenodo.org/records/18311154).

4.4.2. Mètode d'un sol portaobjectes

L'ús d'un sol portaobjectes garanteix la traçabilitat dels resultats. Tanmateix, cal prendre diverses precaucions. Per maximitzar l'esterilitat durant aquest pas, els operadors s'han de netejar regularment les mans amb gel hidroalcohòlic. S'han d'utilitzar portaobjectes no glaçats o esmerilats i cobreobjectes quadrats (22 x 22 mm) embolicats en paper d'alumini i esterilitzats per calor seca (mitjançant un forn de Poupinel o estufa de calor seca), juntament amb agulles estèrils per a cada dissecció (suggeriment: 25G Ø 0,5 mm × 16 mm). El flebòtom es col·loca en una gota de solució salina estèril al mig del portaobjectes. Es talla el cap mentre es fa una incisió entre els terguits i esternits abdominals 6è i 7è sense tallar el tracte digestiu (es pot fer un tall més alt si s'esperen espermateques molt llargues). A continuació, s'ha d'immobilitzar el tòrax amb una agulla i s'han de tirar suaument els últims segments abdominals posteriors amb l'altra agulla per extreure l'intestí. Si això falla, hi ha la possibilitat de bloquejar l'extrem de l'abdomen amb una agulla i estirar el tracte digestiu des de la seva part anterior. Si això falla de nou, s'ha d'extreure l'intestí eliminant tant com sigui possible el tegument restant que l'envolta. Quan s'extreu l'intestí, s'han de separar els últims segments abdominals tallant el tracte digestiu. A continuació, es col·loca l'intestí en una nova gota de solució salina estèril col·locada a un costat del portaobjectes i després es cobreix suaument amb un cobreobjectes estèril. El cap i els últims segments abdominals es transfereixen a una petita gota de líquid Marc-André col·locada a l'altre extrem del portaobjectes, assegurant-se que no hi hagi contacte amb *Leishmania*. El cap s'orienta correctament (foramen occipital cap amunt) i les espermateques s'aïllen amb la furca genital tal com s'ha indicat anteriorment i es cobreixen amb un petit cobreobjectes rodó (Ø 12 mm, no s'ha de confondre amb els cobreobjectes quadrats estèrils). La carcassa i les ales restants del flebòtom romanen a la gota de solució salina al mig del portaobjectes (https://zenodo.org/records/18311154). En cas de positivitat, o per a l'estudi taxonòmic, es poden preservar el tòrax i l'abdomen per a estudis moleculars o proteòmics, i les ales es poden muntar en un medi aquós. Per preservar el muntatge, el volum addicional de líquid Marc-André es pot substituir per un medi de muntatge aquós com ara la goma de cloral (=Hoyer) o un medi a base d'alcohol polivinílic.

Hi ha vídeos detallats que demostren aquests procediments (dissecció de l'intestí mitjà del flebòtom: https://zenodo.org/records/18303014 i dissecció de les glàndules salivals del flebòtom: https://zenodo.org/records/18302850), per la qual cosa no s'explicaran aquí.

4.4.3. Aïllament i cultiu del paràsit *Leishmania* a partir d'intestins de flebòtoms

L'aïllament del paràsit a partir de la dissecció de flebòtoms femelles infectades és un procediment delicat que requereix molta habilitat i s'ha de practicar inicialment amb mostres lliures de paràsits. Després de la dissecció, els intestins es transfereixen a una gota fresca de solució salina estèril (0,9%) o solució de Locke per rentar-los [4]. Els intestins disseccionats es poden processar de dues maneres: i) examinar-los sota un microscopi òptic per observar les diferents etapes dels promastigots de *Leishmania* i la seva localització, amb especial atenció a la vàlvula estomodeal, i ii) obrir l'intestí per facilitar la sortida dels promastigots, facilitant el seu cultiu massiu [4]. Trobar flebòtoms infecciosos al camp és un fet relativament rar i, per tant, les bones sessions de pràctica maximitzaran les possibilitats d'un aïllament amb èxit.

Si s'observen paràsits de *Leishmania* a l'intestí, s'han d'utilitzar noves agulles estèrils i afegir una petita quantitat de solució salina estèril al voltant del cobreobjectes per capil·laritat per alliberar-los. L'intestí s'ha d'esquinçar amb cura i rapidesa per alliberar els paràsits a la solució salina. Amb una micropipeta de 100 µL o una xeringa de tuberculina, recolliu els paràsits i inoculeu-los en un medi de cultiu degudament etiquetat. Cultiu *in vitro* de promastigots de *Leishmania*: els paràsits aïllats es mantenen inicialment en la pendent d'agar sang SNB-9 o en medi sòlid Novy, Mc Neal, Nicolle (NNN) [16] enriquits amb medi alfa-MEM estèril [16, 65] o amb medi M199, cadascun suplementat amb un 10% de sèrum boví fetal estèril inactivat per calor [SBF] (per millorar el creixement del paràsit), un 1% de vitamines Medi Basal Eagle (BME), un 2% d'orina humana estèril (esterilitzada amb un filtre de xeringa Filtropur® S 0,2 µm), 250 µg/mL d'amikacina (o 50 µg/mL de gentamicina, o una barreja d'antibiòtics i aminoàcids (L-glutamina 200 mM-penicil·lina 10.000 U-estreptomicina 10 mg/mL) [47]. Després de tres dies, si no hi ha contaminació, els cultius es suspenen en un medi de congelació preparat adequadament i posteriorment s'emmagatzemen a -80 °C durant 1 o 2 anys o en nitrogen líquid a -196 °C per a la conservació a llarg termini i el futur ús experimental [7].

4.5. Glàndules salivals

La dissecció de les glàndules salivals dels flebòtoms és una tècnica fonamental per estudiar les interaccions vector-patogen, en particular per detectar arbovirus com el flebovirus (per exemple, el virus Toscana) [44, 75]. A causa de la mida minúscula dels flebòtoms, el procediment requereix precisió sota un estereomicroscopi, utilitzant pinces fines o agulles de microdissecció per aïllar les delicades glàndules salivals sense causar ruptura ni contaminació (https://zenodo.org/records/18302850) [51, 61]. Preservar la integritat de la glàndula és crucial per garantir una anàlisi molecular posterior fiable. Un cop extretes, les glàndules es poden homogeneïtzar i provar mitjançant RT-PCR, qPCR o immunoassajos per detectar ARN o antígens virals [12]. La presència de virus a les glàndules salivals, en lloc de només a l'intestí o l'hemocel, confirma que el patogen ha completat el seu període d'incubació extrínseca i és transmissible durant l'alimentació sanguínia [71]. El procés de dissecció és tècnicament exigent a causa de la petita mida de les glàndules salivals dels flebòtoms, cosa que requereix una experiència significativa per evitar la degradació de la mostra [1, 51]. A més, les càrregues virals poden ser baixes, cosa que requereix mètodes de detecció altament sensibles com la PCR imbricada o la seqüenciació d'alt rendiment [54]. Els riscos de contaminació subratllen encara més la necessitat de tècniques estèrils. Més enllà dels obstacles tècnics, els factors biològics influeixen en l'èxit de la detecció; la competència del vector varia entre les espècies de flebòtoms i les taxes d'infecció fluctuen amb les condicions ecològiques i estacionals [33, 61].

La detecció de virus a les glàndules salivals proporciona informació crítica sobre els riscos de transmissió, permetent mesures de vigilància i control específiques [15]. Per exemple, la identificació del virus Toscana en flebòtoms a les regions endèmiques contribueix a elaborar protocols de diagnòstic informats i avisos de salut pública [18]. A més, l'estudi de les interaccions virus-saliva podria revelar noves dianes per a vacunes o teràpies que bloquegen la transmissió [15, 18].

Les glàndules salivals dels flebòtoms també es poden utilitzar com a font d'antígens per mesurar els anticossos de l'hoste contra la saliva dels flebòtoms mitjançant mètodes immunològics, preferiblement ELISA. Aquest mètode permet avaluar l'exposició de l'hoste a les picades dels flebòtoms, cosa que dóna suport a l'avaluació de l'eficàcia dels mètodes de control de vectors [25] i el risc de transmissió de *Leishmania* [40].

4.6. Identificació de la ingesta de sang

Les femelles alimentades aïllades de les captures s'han de disseccionar amb equips d'un sol ús per evitar la contaminació creuada. El seu abdomen s'ha d'examinar amb un estereomicroscopi per avaluar l'etapa de digestió de la ingesta de sang. Es recomana seleccionar només femelles amb un abdomen vermell, marró vermellós o vermell fosc, que no mostri signes de formació d'ous. Extrèieu l'extrem final de l'abdomen, incloses les espermateques, per identificar morfològicament la femella després de la clarificació. La part principal de l'abdomen (sense espermateques) s'ha de col·locar en tubs Eppendorf® i emmagatzemar-la a -20 °C fins a una anàlisi posterior. Els marcadors genètics que s'utilitzen habitualment per a la identificació de la ingesta de sang, com ara PNOC [5, 30, 50], CytB [67] o COI [13], estan ben establerts i descrits àmpliament a la literatura; per tant, no es detallaran més en aquest article (Figura 6). Alternativament, per identificar la sang de l'hoste, es pot utilitzar el mapatge de pèptids MALDI-ToF [31]. S'ha demostrat experimentalment que aquesta tècnica permet la identificació de la sang de l'hoste en un període de temps més llarg després de la ingesta de sang; per tant, és un mètode d'elecció adequat, especialment per a l'anàlisi de femelles ingurgitades amb una digestió de la sang de l'hoste visiblement més avançada. Idealment, les mostres s'haurien d'emmagatzemar a -20 °C o 4 °C, però també es poden obtenir bons resultats de mostres emmagatzemades a temperatura ambient durant un curt període de temps. L'abdomen d'una femella ingurgitada s'ha de disseccionar de la resta del cos poc abans de l'anàlisi i homogeneïtzar-lo en aigua destil·lada. La resta del cos del flebòtom queda disponible per a altres anàlisis moleculars i morfològiques. Després de prendre l'alíquota de l'homogenat per al mapatge de pèptids MALDI-ToF, la resta es pot utilitzar per a l'aïllament d'ADN per confirmar la identificació de la sang de l'hoste i/o detectar la presència de *Leishmania* sp. El temps total de preparació i anàlisi de mostres és molt curt en comparació amb les tècniques moleculars basades en ADN.

This is an Open Access article distributed under the terms of the Creative Commons Attribution License https://creativecommons.org/licenses/by/4.0
which permits unrestricted use, distribution, and reproduction in any medium, provided the original work is properly cited.

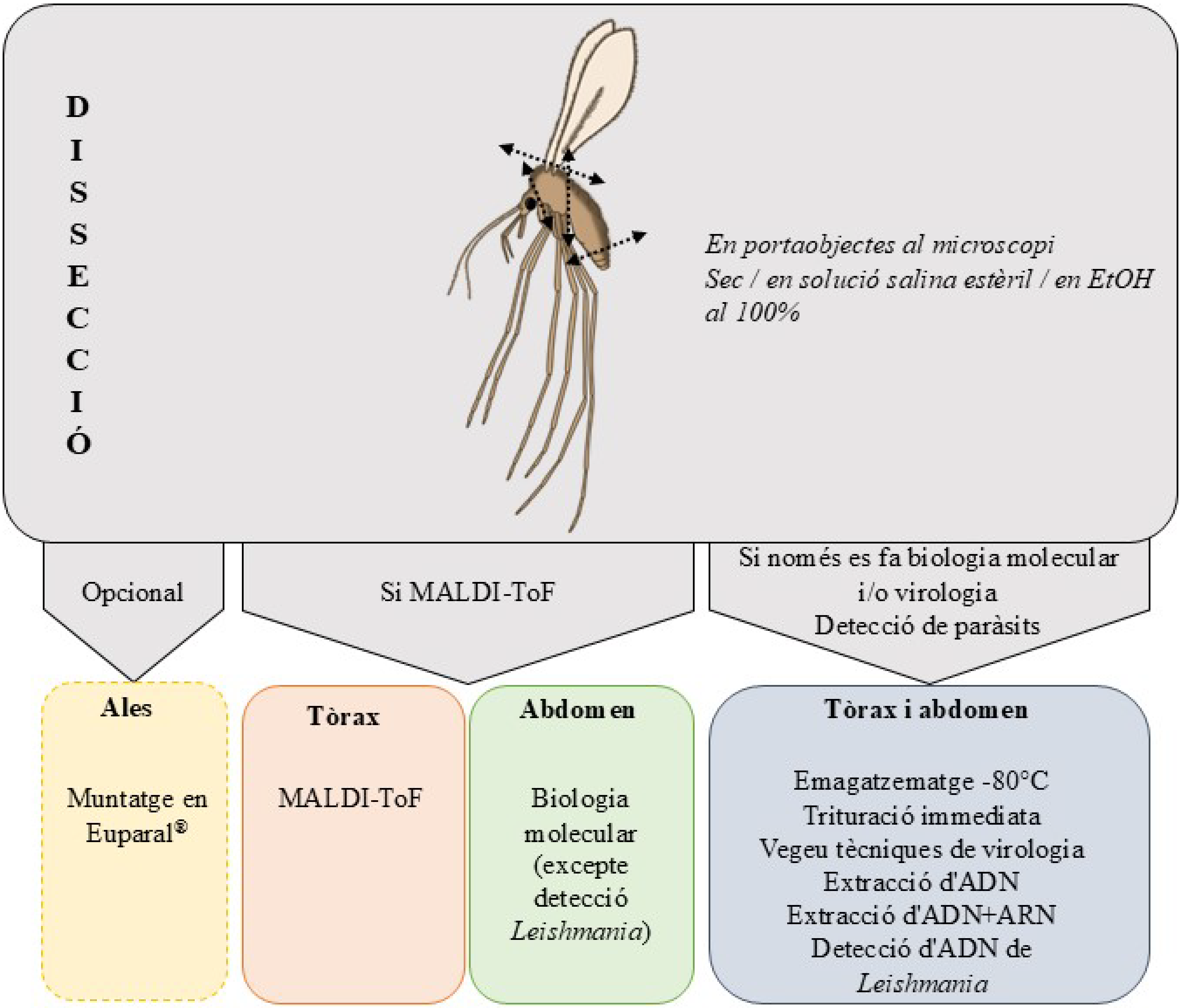

Figura 6: Processament de flebòtoms per a aplicacions de biologia molecular, proteòmica i/o virologia.

5. Processament de mostres per a estudis morfològics (Figures 3, 6, 7 i 8; Apèndixs 1, 2, 3 i 4)

Aquesta secció descriu els principis per a la preparació d'una mostra de flebòtom per al muntatge únicament per a estudis morfològics, seguit de l'adaptació per a aplicacions més enllà de la morfologia. Tanmateix, comprendre aquesta metodologia és crucial, ja que permet adaptar els procediments per a tipus de mostra específics quan sigui necessari.

El tractament implica passos successius de buidatge i ompliment mitjançant pipetes Pasteur equipades amb tetines de goma flexibles. Es recomanen fermament els recipients de vidre de fons rodó perquè faciliten enormement aquestes operacions. El vidre és inert a tots els reactius. Per evitar l'evaporació dels reactius, els recipients han de tenir tapes i mai s'han d'omplir massa, cosa que provocarà un desbordament en tancar o obrir, i per evitar que caigui pols sobre les mostres. Els productes químics necessaris per a la neteja i el processament es mostren a la Taula 2.

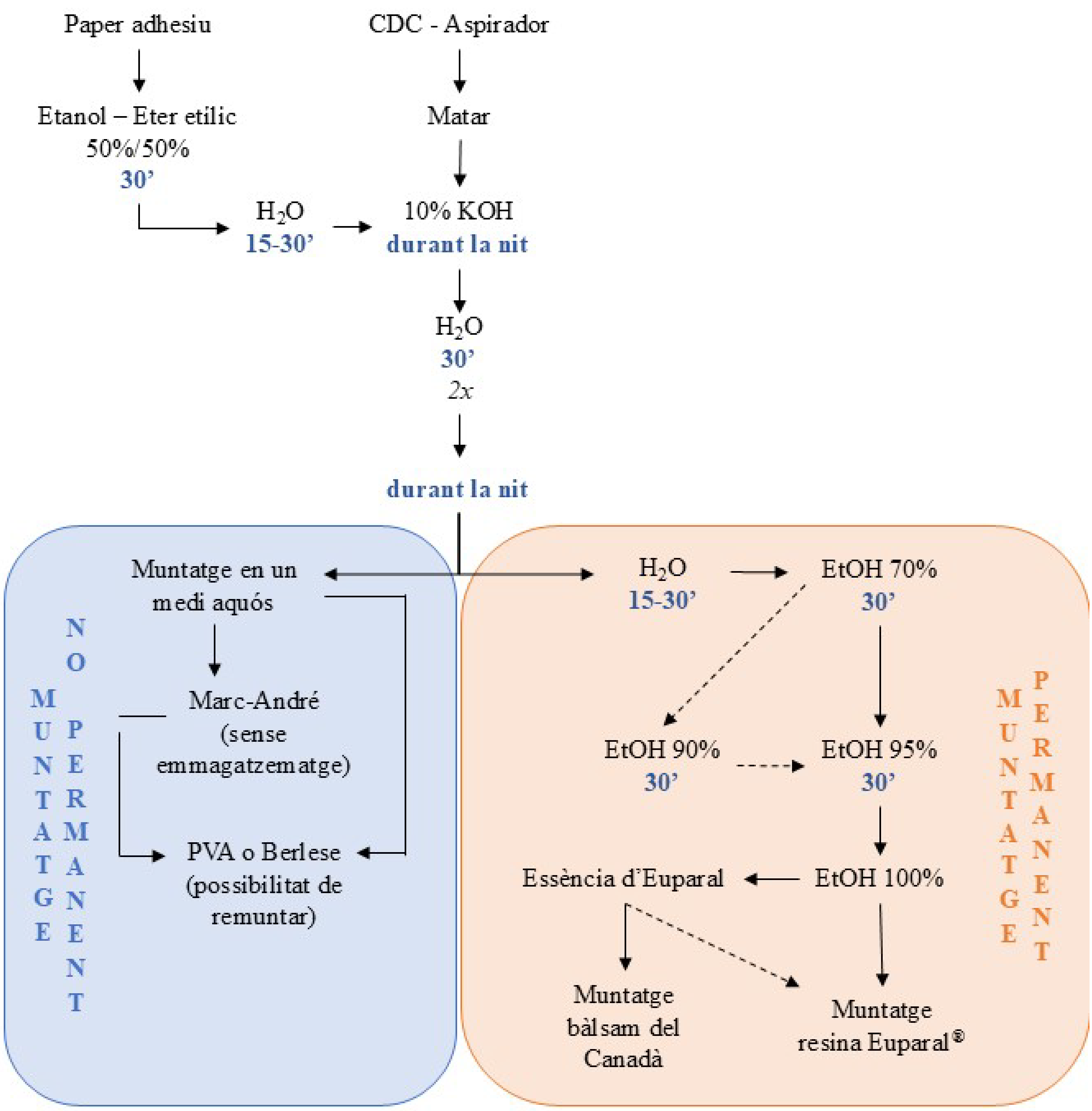

Figura 7: Mètode clàssic per processar els flebòtoms.

Taula 2: Composició dels reactius utilitzats.

**Hidròxid de potassi 10%**	**Fucsina àcida 1% en aigua destil·lada**
Hidròxid de potassi 10 g	Fucsina àcida (en pols) 1 g
Aigua destil·lada *qs* 100 mL	Aigua destil·lada 99 mL
**Medi de muntatge de goma de cloral (medi Hoyer)**	**Solució Marc-André acolorida amb fucsina àcida**
Aigua destil·lada 50 mL	Solució Marc-André 1 0mL
Hidrat de cloral 200 g	Fucsina 1% 50 µL
Goma aràbiga 50 g	
Glicerol 20 mL	
	**Medi Enecê**
**Solució de Marc-André**	Colofònia blanca pura 22 g
Hidrat de cloral 40 g	Goma de Copal soluble en alcohol 12 g
Àcid acètic glacial 30 mL	Etanol absolut 20 mL
Aigua destil·lada 30 mL	Càmfora 10 g
	Essència de trementina 10 mL
	Eucaliptol 26 mL

5.1. Clarificació

Abans que es puguin preparar mostres de flebòtoms per preparacions permanents amb portaobjectes, primer s'han de clarificar mitjançant maceració utilitzant un mètode i un agent clarificador adequats (és a dir, una solució d'àcid acètic al 10% o una solució de Marc-André que inclou hidrat de cloral, que és un producte químic restringit en molts països) per fer-les transparents. El procés de clarificació elimina els teixits corporals, el greix, les secrecions i la cera, fent que la mostra sigui translúcida i facilitant l'examen de les estructures exoesquelètiques (per exemple, la inserció de setes), les característiques de la superfície (per exemple, la coloració) i les característiques internes visibles a través del tegument (per exemple, les espermateques).

El procés de clarificació en dos passos, que implica primer utilitzar una base forta (com ara l'hidròxid de potassi), seguit d'un àcid feble (com ara l'àcid acètic en la solució de Marc-André), té finalitats bioquímiques diferents [74]. La base descompon els teixits tous, com ara les proteïnes, els greixos i els músculs, mitjançant la saponificació i la desnaturalització de proteïnes, deixant l'exoesquelet de quitina intacte per a la claredat estructural. L'àcid feble posterior neutralitza qualsevol àlcali restant, evitant una major degradació, i blanqueja la quitina per millorar la transparència [74], tot i que rentar les mostres dues vegades amb aigua destil·lada durant 15 minuts també pot ser suficient per neutralitzar la base. Aquest tractament seqüencial combina l'eliminació eficaç del teixit amb una preservació suau, garantint una integritat òptima de la mostra per a l'examen microscòpic.

Es recomanen dos rentats de 20 minuts amb aigua destil·lada abans de procedir al pas següent.

5.1.1. Lisi de teixits tous (Figura 8)

L'hidròxid de sodi (NaOH) o l'hidròxid de potassi (KOH) són agents maceradors químics d'ús comú, que s'apliquen a concentracions i durades variables segons la mida i la fragilitat de les mostres. La tècnica estàndard i més eficaç consisteix a lisar els teixits tous submergint els flebòtoms en una base forta (10% de KOH o NaOH) durant la nit. Es pot augmentar la concentració per reduir la durada del tractament (és a dir, KOH al 20% durant 6 hores), així com escalfar-los a 37 °C.

5.1.2. Clarificació amb o sense tinció

Aquest pas va seguit d'un tractament d'aclariment, que normalment combina àcid acètic i hidrat de cloral (per exemple, solució de Marc-André). Després de la clarificació, les mostres s'han d'esbandir a fons en almenys dos banys d'aigua successius de 20 minuts cadascun per eliminar els productes químics residuals.

La solució de Marc-André és un agent clarificador que s'utilitza habitualment per preparar mostres de flebòtoms. La seva eficàcia rau a facilitar el procés de clarificació alhora que minimitza els danys significatius a les estructures fràgils, com ara les ales i les antenes.

La solució s'ha de preparar al moment o emmagatzemar en un recipient hermèticament tancat per evitar l'evaporació o la degradació. L'ús de la solució de Marc-André és particularment avantatjós quan es combina amb tècniques d'aclariment o tinció per millorar detalls morfològics específics. Els detalls de la seva composició i preparació es donen a l'Apèndix 2.

Per a mostres altament translúcides, pot ser necessari tenyir per millorar la visibilitat abans del muntatge. Hi ha moltes tincions disponibles, cadascuna dirigida a components químics específics de l'organisme. És important seleccionar una tinció que sigui compatible tant amb la mostra com amb el medi de muntatge escollit.

Aquesta metodologia bàsica es pot adaptar segons calgui, per exemple, incorporant fucsina àcida al 0,1% a la solució de Marc-André per a la tinció. A més, les mostres conservades en solucions aquoses i destinades a muntatges resinosos requereixen deshidratació (vegeu la secció 5.2 sobre deshidratació), ja que la majoria dels medis de muntatge de resines naturals i sintètiques són incompatibles amb l'aigua. New (1974) va observar que algunes tincions es poden deteriorar en certs medis de muntatge [53]. Per exemple, la fucsina àcida, que s'utilitza habitualment amb el bàlsam del Canadà, també es pot fixar en Euparal®. Tanmateix, les mostres tenyides amb fucsina àcida són propenses a la decoloració, sobretot quan queden restes d'oli de clau, utilitzat com a fluid clarificador final. Les mostres emmagatzemades en oli de clau poden presentar una decoloració significativa en pocs dies.

5.2. Deshidratació

La deshidratació es duu a terme transferint gradualment les mostres a través d'una sèrie de solucions graduades d'etanol: 50%, 70%, 80%, 90% o 95% i finalment 100%, amb cada bany d'almenys 20 minuts. Com que l'etanol s'evapora ràpidament, el recipient s'ha de segellar hermèticament durant el processament. Un cop la mostra estigui completament deshidratada, podeu aturar el processament durant uns dies en essència Euparal®, que és preferible a l'oli de clau. La creosota de faig, que abans s'utilitzava àmpliament per a aquest propòsit, ara està completament prohibida a causa de la seva toxicitat.

El procés de deshidratació ha de garantir que el fluid dins de la mostra sigui compatible amb el medi de muntatge per evitar l'opacitat, el col·lapse osmòtic o la distorsió que podrien fer que la mostra no sigui adequada per a l'estudi taxonòmic.

5.3. Medis de muntatge

5.3.1. Selecció i aplicació per a la preparació de mostres

Idealment, el medi de muntatge hauria de tenir un IR el més proper possible al del vidre, que és aproximadament d'1,5. Ha de ser incolor, clar i romandre perfectament transparent després de l'assecat i amb el pas del temps. Ha de ser compatible amb les tincions utilitzades i capaç de penetrar i difondre's en tots els teixits de la mostra. No s'ha d'assecar massa ràpidament ni formar una terbolesa durant el muntatge. No s'ha de retraure després del muntatge. La selecció d'un medi de muntatge adequat és un aspecte fonamental de la preparació de mostres, ja que cap medi és ideal per a tots els propòsits. L'elecció ha d'equilibrar diversos factors clau:

- Propietats òptiques. L'IR del medi de muntatge ha de proporcionar un contrast i una refracció suficients de les característiques anatòmiques crítiques utilitzades per a la identificació taxonòmica o la descripció morfològica, com ara les espermateques, els ascoïdes, les sensílies de Newstead, les dents cibarials verticals i les dents faríngies. La visibilitat d'aquestes estructures depèn directament de les propietats òptiques del medi de muntatge.

- Preservació. Per a espècimens tipus o materials destinats a col·leccions permanents, el medi ha d'oferir estabilitat i durabilitat a llarg termini. En canvi, per a estudis d'inventari o enquestes epidemiològiques, on la conservació a llarg termini és menys crítica, poden ser suficients els medis de muntatge temporals o semipermanents.

5.3.2. Requisits per als medis de muntatge

Els especialistes sovint desenvolupen tècniques de muntatge personalitzades i complexes adaptades a les necessitats de recerca específiques. Tanmateix, aquests mètodes sovint passen per alt aspectes com la qualitat arxivística, la compatibilitat, l'estandardització o la facilitat de maneig i la preservació a llarg termini. Aquesta manca d'estandardització complica la integració de col·leccions cedides i els esforços de conservació a llarg termini.

Les aplicacions científiques imposen requisits diferents per als medis de muntatge. Els taxonomistes sovint munten exemplars sencers i prefereixen medis que maceren suaument els òrgans interns per millorar la visibilitat de les estructures cuticulars. L'IR ha de diferir prou del de l'espècimen i del portaobjectes de vidre per maximitzar la claredat òptica. Els medis de muntatge comercials solen formular-se amb un IR proper al del vidre per minimitzar la refracció i la dispersió de la llum a través del sistema portaobjectes-medi de muntatge-cobreobjectes. Tanmateix, en la microscòpia de camp clar, el contrast natural d'una mostra sense tenyir es pot manipular escollint deliberadament un medi de muntatge amb un IR lleugerament diferent del de la mostra, millorant així la seva visibilitat sobre el fons.

5.3.3. Tipus de medis de muntatge (Taules 3 i 4)

La microscòpia requereix l'IR d'un medi de muntatge per determinar com es desvia la llum a través del portaobjectes, el medi i la mostra. Quan l'IR està estretament alineat amb el vidre del cobreobjectes (≈1,515), la llum passa uniformement, disminuint la dispersió i les distorsions òptiques, cosa que resulta en una millor resolució i visibilitat de les estructures fines. Per contra, una discrepància d'IR pot causar desenfocament, halos o característiques no tenyides obscures. La selecció del medi de muntatge adequat és crucial per optimitzar el contrast, la claredat i la qualitat general de la imatge per a una mostra determinada a causa dels diferents IR dels diferents medis.

L'IR del medi de muntatge té un impacte significatiu en la capacitat de veure les estructures fines en preparar els flebòtoms per al muntatge del portaobjectes. Les característiques delicades i lleugerament esclerotitzades dels flebòtoms, incloent-hi l'armadura cibarial, les espermateques, els segments antenals i la venació de les ales, poden ser difícils d'observar en un medi de muntatge d'alt IR.

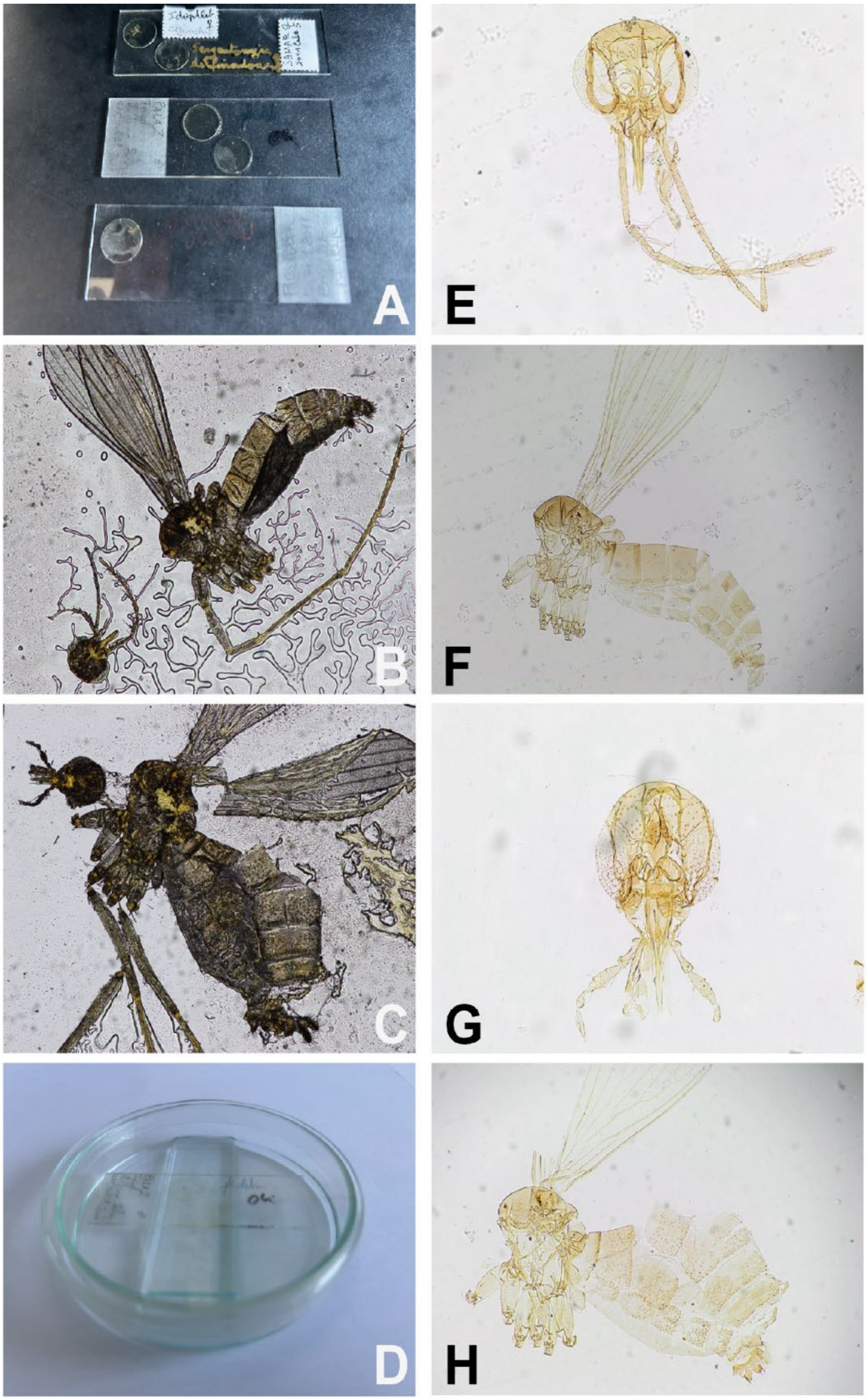

Figura 8: Remuntatge de portaobjectes. A: portaobjectes danyats i secs muntats en Hoyer; B: vista microscòpica d'un flebòtom sec; C: vista microscòpica d'un altre flebòtom danyat; D: cambra humida que conté un portaobjectes sec; E: cap i F: cos de la mostra B després del seu remuntatge en Euparal®; G: cap i H: cos de la mostra C danyat després del seu remuntatge en Euparal®.

Per als flebòtoms, les opcions més utilitzades inclouen medis de goma-cloral com a medis de muntatge a base d'aigua, i bàlsam del Canadà i resina Enecê-Nelson Cerqueira (NC) com a medi a base de dissolvent. Rawlins [60] va classificar els medis de muntatge en dos tipus: (1) medis permanents: aquests s'endureixen amb el temps i són adequats per a la conservació a llarg termini, i (2) medis semipermanents: aquests no s'endureixen i normalment s'utilitzen amb finalitats temporals

Els medis de muntatge poden ser líquids, a base de goma o resinosos, solubles en aigua, alcohol o altres dissolvents (per exemple, toluè, xilè) (Taula 3). Un cop aplicats, s'han de segellar dels efectes atmosfèrics mitjançant medis de resonància no solubles. Per distingir clarament entre els tipus de medis de muntatge, es pot utilitzar la següent categorització:

a. Medis aquosos. Aquests medis es dissolen fàcilment en aigua, cosa que els fa adequats per a muntatges temporals o semipermanents. Normalment són fàcils de manipular, però poden requerir segellat per evitar l'exposició a la humitat atmosfèrica (és a dir, medis de goma-cloral i alcohol polivinílic), especialment en climes tropicals humits.

b. Medis amb tolerància limitada a l'aigua. Aquests medis es veuen menys afectats per l'aigua, però encara requereixen protecció contra la humitat excessiva. Proporcionen una major estabilitat a llarg termini en comparació amb les opcions solubles en aigua i s'utilitzen freqüentment en muntatges semipermanents.

c. Medis solubles en hidrocarburs. Aquests medis es dissolen en dissolvents orgànics com el xilè o el toluè, o eseneçè (dissolvent enecè). Estan dissenyats per a muntatges permanents i ofereixen una excel·lent estabilitat a llarg termini, i resisteixen la humitat i la degradació, cosa que els fa ideals per a fins arxivístics (és a dir, bàlsam neutre del Canadà, DPX).

En resum, els medis solubles en aigua són els millors per a muntatges temporals o caixes que requereixen una fàcil extracció de mostres; els medis amb tolerància limitada a l'aigua són adequats per a muntatges semipermanents que requereixen una durabilitat moderada, i els medis solubles en hidrocarburs són preferibles per a muntatges permanents destinats a l'emmagatzematge arxivístic i a llarg termini.

Taula 3: Composició dels medis de muntatge seleccionats.

Medi de muntatge	Solvent	Possibles prepolímers o polímers	Observacions
Hoyer = goma de chloral	glicerol, aigua	compostos de goma aràbiga	Agent macerant: hidrat de cloral
CMCP-9 (=carboximetilcel·lulosa fenol)	aigua (CMCP-9: 51−60%)	alcohol polivinílic completament hidrolitzat (CMCP-9: 0−5%)	CMC(P)-9: baixa viscositat: alta viscositat
DMHF (dimetilhidantoïna formaldehid)	aigua	N,N′-dimetilol dimetil hidantoïna (di-metilol DMH)<br>Oligòmers amb pont d'èter/metilè<br>Xarxa de polímers DMH-formaldehid reticulats	
Bàlsam del Canada	xilè; components parcialment volàtils del bàlsam (Δ^3-carene, àcid levopimàric, limonè, mircè, àcid palústric, β-felandrè, α-pinè, β-pinè)	bàlsam (abienol, àcid abiètic, àcid isopimàric, àcid sandaracopimàric)	neutralització: carbonat de potassi; resina d'*Abies balsamea* (Linné, 1758)
Euparal®	eucaliptol, paraldehid; components parcialment volàtils de la goma sandaraca (limonè, α-pinè, β-pinè)	compostos de goma sandaraca (àcid comuní, manool, àcid policòmònic, àcid sandaracopimàric, àcid 12-acetoxi-sandaracopimàric, sugiol, àcid torulòsic, torulosol, totarol)	agent aclaridor: salicilat de metil; colorant en verd Euparal®: sal de coure (abietinat de coure); resina de sandaraca de *Tetraclinis articulata* (Vahl, 1791)
Enecê	alcohol etílic; amb càmfora, eucaliptol i essència de trementina	Compostos de goma copal i colofònia (resina)	

This is an Open Access article distributed under the terms of the Creative Commons Attribution License https://creativecommons.org/licenses/by/4.0 which permits unrestricted use, distribution, and reproduction in any medium, provided the original work is properly cited.

Taula 4: Avantatges i desavantatges de diversos medis de muntatge sobre portaobjectes de microscopi i observacions no publicades de diverses persones [52].

Nom	Avantatges	Desavantatges
* Bàlsam del Canadà	El medi és molt resistent, amb una vida útil superior als 150 anys.<br>Els portaobjectes es poden muntar utilitzant oli de clau o fenol com a agents de muntatge.	Conté components nocius i s'ha de manipular sota una campana.<br>Requereix una sèrie de deshidratació completa i que requereix molt de temps.<br>La deshidratació i la transferència d'etanol a través de xilè o oli de clau poden fer que alguns tàxons siguin fràgils; les alternatives (per exemple, isopropanol, n-butanol, Cellosolve™, 1,4-dioxà, Histoclear, terpineol) poden reduir la ruptura.<br>Les mostres es poden ennegrir si el xilè es substitueix per fenol o si queda hidròxid de potassi residual.<br>Els IR alts poden enfosquir les estructures no tenyides.<br>L'assecat complet pot trigar anys sense assecat amb placa calenta.<br>El medi es torna groguenc i enfosquit amb el temps, especialment quan es neteja amb oli de clau.<br>Algunes taques s'afebleixen i els colorants catiònics poden esvair-se si el medi es torna àcid, cosa que pot ocórrer espontàniament amb el temps.
DMHF (dimetilhidantoïna formaldehid)	Alta transparència<br>Bon IR<br>Excel·lent visibilitat de les estructures<br>Bastant bona estabilitat de les preparacions<br>Compatible amb moltes tècniques de tinció<br>Bona protecció de les mostres<br>Bona adherència entre el portaobjectes i el cobreobjectes	Possible engroguiment amb el temps<br>Pot alterar algunes tincions<br>No apte per a tincions sensibles al formaldehid<br>Formació freqüent de bombolles d'aire, temps d'assecat lent<br>Medi de muntatge sensible a la humitat<br>El muntatge és difícil de revertir<br>El formaldehid és tòxic, irritant i cancerigen
* Euparal (transparent)	Medi durador amb una vida útil de més de 50 anys.<br>És possible el muntatge directament des d'etanol al 80% (recomanació del fabricant).<br>No emmascara les estructures no tenyides i no es torna groc ni trencadís amb el temps.<br>Té un IR més adequat que el bàlsam del Canadà per a dípters.<br>Funciona bé per a mostres més gruixudes a causa de la mínima contracció i l'assecat sense bombolles.<br>Roman soluble en etanol al 95%, cosa que permet el muntatge fins i tot després de molts anys.	Conté components nocius i s'ha de manipular sota una campana extractora.<br>La deshidratació i la transferència d'etanol mitjançant Euparal Essence poden fer que alguns tàxons siguin fràgils, però l'ús d'isopropanol pot reduir aquest problema.
Medi de Hoyer	Els exemplars es poden muntar vius o directament a partir d'aigua, etanol o formaldehid.<br>La maceració proporciona una excel·lent qualitat de la cutícula.<br>Té un IR favorable i es pot millorar amb tinció de iode per a un contrast més alt.<br>L'àcid acètic de la fórmula pot expandir els apèndixs dels artròpodes.<br>Algunes mostres poden romandre estables durant 40-60 anys.<br>Solubles en aigua, cosa que permet un muntatge fàcil..	Els exemplars de plantes delicats poden col·lapsar-se si el medi no s'afegeix gradualment, cosa que requereix molt de temps.<br>Es poden formar cavitats i cristalls en menys de 10 anys.<br>La maceració pot arribar a ser excessiva depenent de la concentració d'hidrat de cloral i el temps d'exposició.<br>Els components del medi es poden separar i pot aparèixer una granulació fina en qüestió de mesos o anys.<br>S'ha informat d'ennegriment del medi.
CMCP-9	Els exemplars es poden muntar directament a partir	Aquest medi pot desenvolupar cristalls i enfosquir-se

(=Carboximetilcel·lulosa fenolada))	de medis com ara aigua, etanol, glicerol o solucions que contenen formaldehid, i els seus òrgans interns es poden macerar quan sigui necessari per facilitar l'examen general o la preparació.	amb el temps, i de vegades pot macerar les mostres més del previst. A menys que el portaobjectes estigui segellat amb cura, les mostres més gruixudes no s'hi conservaran bé perquè es poden encongir i crear espais al voltant de les vores del cobreobjectes. No és adequat per a mostres tenyides o materials calcificats, i el seu temps d'assecat és més lent que el CMC.
Eukitt™	Medi durador de més de 30 anys. Compatible amb molts dissolvents per al muntatge, com ara acetona, benzè, cloroform, dioxà, èter, isopropanol, benzoat de metil, terpineol, toluè i xilè. S'asseca ràpidament i té un pH lleugerament àcid. No s'enfosqueix notablement amb el temps. Apte per a diverses tincions (per exemple, fucsina, hematoxilina, verd de metil, violeta de metil, blau de metilè). Les mostres es poden tornar a muntar després d'anys submergint-les en xilè durant un període prolongat.	Conté components nocius i s'ha de manipular sota una campana. Requereix una sèrie de deshidratació completa i que requereix molt de temps. No és ideal per a mostres més gruixudes a causa de la contracció i la formació de bombolles de gas. Els cobreobjectes es poden separar amb el temps, tret que el vidre es netegi bé i es segelli. Pot mostrar una polimerització incompleta al voltant de les fibres de col·lagen.
Enecè	Medi molt resistent, amb una durada mínima de 50 anys. L'enecè no s'enfosqueix amb el temps. És més mal·leable, cosa que permet la dissecció d'insectes en el medi, a més de proporcionar un temps raonable per posicionar les estructures morfològiques. Baix cost.	Requereix una sèrie de deshidratació completa i que requereix molt de temps. La deshidratació d'etanol i la transferència a través d'oli de clau poden fer que alguns exemplars siguin fràgils. L'insecte continua clarificant-se, tot i que molt lentament; això pot dificultar la visualització d'estructures molt petites, com ara sensílies, ascoïdes i setes simples.

5.3.4. Descripció dels suports de muntatge recomanats (taules 3 i 4)

Medis per a observació temporal

Goma cloral = fluid/medi/solució de Hoyer (IR = 1,48)

El medi de Marc André és el millor medi per a l'observació a curt termini (unes poques hores, potser unes quantes més si el portaobjectes s'emmagatzema en una cambra humida) d'espermateques, incloent-hi fotografies (Figura 4) o dibuixos. La preservació de les espermateques observades requereix el seu muntatge en un medi aquós que permeti l'emmagatzematge a mitjà termini. Deshidratar-les per al seu muntatge en resina no és impossible, però no es recomana (risc de pèrdua). La goma cloral i el medi de Hoyer es consideren sinònims. Aquest medi s'utilitza habitualment per observar òrgans interns a causa de la seva compatibilitat amb l'aigua, simplicitat, aplicació ràpida i IR que facilita l'examen d'estructures delicades com les espermateques. Tanmateix, la goma cloral té inconvenients importants si no es prepara perfectament o s'emmagatzema en condicions d'humitat controlada. Aquests problemes inclouen la cristal·lització, la decoloració i la pèrdua de viscositat. Fer segellar el cobreobjectes no resol aquests problemes, ja que el medi de muntatge pot decolorar-se molt (de vegades gairebé negre) a causa de la interacció amb el medi per segellar, sobretot si s'utilitza Euparal®. El medi Hoyer s'ha considerat òpticament el millor per als flebòtoms i tradicionalment s'ha utilitzat per a aquests propòsits. El medi consta de diverses formulacions estretament relacionades, com ara goma aràbiga, glicerol i hidrat de cloral. Diverses formulacions han estat malinterpretades i citades erròniament [74].

Tot i que el Hoyer és un bon medi per observar espermateques en flebòtoms, no és adequat per a la conservació a llarg termini. És ideal per a observacions a curt termini, com ara fotografies, dibuixos o imatges. Els medis aquosos són adequats per a muntatges temporals, però no poden garantir la conservació a llarg termini. En canvi, el muntatge amb resina proporciona una excel·lent durabilitat que sovint dura segles, però pot ocultar detalls fins de les espermateques, ja que la seva refringència es perd amb freqüència.

El medi de Hoyer es degrada amb el temps a causa de la deshidratació (Figura 8), donant lloc a la formació de petits cristalls blancs i opacs d'hidrat de cloral. No obstant això, es poden recuperar mostres de portaobjectes cristal·litzats, ja que la cutícula roman químicament intacta, tot i que es poden produir alguns danys físics a causa dels cristalls en creixement. En alguns casos, els portaobjectes cristal·litzats es poden restaurar rehidratant el medi de muntatge en un ambient càlid i humit amb timol per evitar el creixement de

fongs. Alternativament, es poden treure les mostres de la goma cloral en remull en aigua, deshidratar-les en àcid acètic glacial i tornar-les a muntar en bàlsam del Canadà

DMHF (dimetilhidantoïna formaldehid) (IR 1,48)

Aquest medi a base d'aigua [72] té un rendiment òptic molt bo, de manera molt semblant al Berlese, i és tan fàcil d'utilitzar com el Berlese. Tanmateix, a diferència del Berlese, no s'ennegreix ni cristal·litza. Funciona bé per a flebòtoms i altres Psychodidae.

CMCP (Càmfora-monoclorofenol) (IR = 1,41)

Aquest és un medi de muntatge soluble en aigua a base de glicerina que s'utilitza per crear preparacions transparents i permanents de mostres delicades, inclosos els flebòtoms. L'avantatge d'aquest medi de muntatge és que les mostres es poden muntar directament des d'aigua o etanol. Relaxa i neteja ràpidament el flebòtom, suavitzant la cutícula per permetre el posicionament correcte de la mostra, cosa que és especialment útil per estendre les ales o disseccionar els genitals. Tot i que s'ha informat que permet l'emmagatzematge a llarg termini, la durada exacta de la conservació continua sent incerta. La principal limitació d'aquest medi de muntatge rau en la seva composició, que conté fenol, una substància tòxica i irritant que requereix una manipulació acurada.

Medis per a muntatge permanent

Bàlsam del Canadà (IR = 1.52-1.54)

El bàlsam del Canadà va ser descrit per primera vegada com un medi de muntatge adequat per a la microscòpia de llum transmesa per Andrew Pritchard a la dècada de 1830. Continua sent un dels medis més utilitzats a causa de la seva qualitat d'arxiu provada, amb més de 150 anys d'aplicació amb èxit. A diferència dels medis fluids de Hoyer, el bàlsam del Canadà no cristal·litza ni absorbeix humitat. Tanmateix, el bàlsam del Canadà és fortament autofluorescent, cosa que de vegades pot ser un desavantatge per a certes tècniques de microscòpia [60]. L'ús de dissolvents no tòxics en lloc de xilè pot reduir els riscos de seguretat durant la preparació, però també pot introduir inconvenients com ara un assecat més lent i un enfosquiment més primerenc del medi.

Euparal® (IR = 1.48)

L'Euparal® és una alternativa àmpliament utilitzada al bàlsam del Canadà per al muntatge permanent, que ofereix una excel·lent estabilitat a llarg termini i un IR comparable. Euparal® té les característiques següents: (1) requisit de deshidratació: abans de la transferència final del medi de muntatge, la mostra s'ha de deshidratar, normalment passant del 95% a etanol absolut, i (2) temps de processament prolongat: el muntatge final en una resina, ja sigui bàlsam del Canadà o Euparal®, requereix deshidratació, cosa que allarga el temps total de processament de la mostra. Quan la deshidratació amb dissolvents orgànics no és factible, les mostres extretes d'etanol absolut es poden col·locar en una solució intermèdia que consisteix en una barreja igual d'Euparal® i essència Euparal, abans del muntatge final.

Enecè (IR = 1.467)

L'Enecè és un medi de muntatge a base de resina que s'utilitza principalment per a insectes petits i és particularment popular al Brasil. La seva base consisteix en colofònia i goma copal dissolta en alcohol, càmfora, essència de trementina i eucaliptol. Cerqueira [11] va descriure l'Enecè com una alternativa al bàlsam del Canadà per muntar preparacions permanents de larves, exúvies d'immadurs i fins i tot mosquits adults, i des de llavors s'ha adoptat àmpliament per muntar flebòtoms. L'Enecè ofereix una alternativa rendible per al muntatge permanent, ja que proporciona estabilitat a llarg termini i un temps d'assecat suficient, permetent la dissecció i una disposició precisa de les estructures morfològiques.

5.4. Preparació i assecat de les preparacions

L'assecatge adequat dels portaobjectes muntats és fonamental per garantir l'estabilitat i la conservació a llarg termini. Els portaobjectes s'han d'assecar completament abans de considerar l'emmagatzematge a llarg termini. Per obtenir resultats òptims, els portaobjectes muntats amb medis de muntatge permanents s'han d'assecar horitzontalment durant 2-3 setmanes, mentre que els preparats amb medis semipermanents poden requerir només 1-2 setmanes. Per garantir un procés d'assecatge eficaç, es recomana utilitzar una estufa configurada a una temperatura adequada per al medi de muntatge que s'utilitzi, evitant una calor excessiva que pugui danyar les mostres. Es recomana un rang de temperatura de 30 °C i 37 °C. Aquest pas d'assecatge és crucial per evitar la deformació dels portaobjectes, el deteriorament de les mostres o la inestabilitat del medi de muntatge durant l'emmagatzematge.

El medi de muntatge utilitzat en la preparació dels portaobjectes sempre s'ha d'indicar a l'etiqueta del portaobjectes. Si és possible, l'etiqueta també ha d'incloure la recepta específica utilitzada, juntament amb el nom de la persona que el medi i la data de preparació. Els portaobjectes es preparen inicialment com a muntatges temporals i no estan destinats a la conservació a llarg termini. Tanmateix, si l'estatus de l'espècimen canvia, com ara si es designa com a part d'una sèrie "tipus", s'hauria d'utilitzar un medi de muntatge més permanent per garantir la preservació de l'espècimen per a futurs estudis taxonòmics.

5.5. Tècniques de muntatge alternatives: muntatge de targetes

El muntatge amb targetes és una tècnica utilitzada per a diversos grups d'insectes en què els exemplars es poden enganxar directament a targetes entomològiques o enganxar-los a la superfície. Atesa la seva petita mida i la

necessitat d'observar els òrgans interns per a la seva identificació mitjançant aclariments (vegeu l'ítem 5), aquest mètode no és gens adequat per al muntatge de flebòtoms.

5.6. Remuntatge de mostres danyades

Per a mostres rares o valuoses, es recomana un muntatge en dos passos segons el vídeo accessible a: https://zenodo.org/records/18315029. 1) rehidratar-les sense desmuntar-les per permetre l'observació preliminar. S'ha de col·locar un suport per a diversos portaobjectes de microscopi en una placa de Petri que serveixi de suport. A continuació, es col·loca el portaobjectes que s'ha de rehidratar a sobre i s'omple la placa de Petri amb uns quants mil·límetres de dissolvent per crear una cambra humida, garantint que el portaobjectes no entri en contacte amb el dissolvent (Figura 8 D). El temps necessari per a la rehidratació pot variar d'un a diversos dies, depenent de l'estat de la mostra. El control diari i la paciència són essencials. Un cop el portaobjectes estigui prou rehidratat, es pot treure de la cambra humida i col·locar-lo en una incubadora durant unes hores abans de l'examen microscòpic, la fotografia o el dibuix. 2) per tornar-lo a muntar, el portaobjectes es pot tornar a la cambra humida durant unes hores més o durant la nit. El desmuntatge s'ha de realitzar sota un microscopi binocular. Amb agulles fines, cal retirar amb cura el cobreobjectes, assegurant-se que no quedin elements de flebòtoms adherits (https://zenodo.org/records/18315029). A continuació, s'han de recollir els elements disseccionats de flebòtoms i esbandir-los amb aigua en pous petits, com els que s'utilitzen per a l'extracció destructiva d'ADN/ARN (vegeu més avall), abans de deshidratar-los i tornar-los a muntar en un medi de resina. Quan es desmunta un portaobjectes, és crucial identificar el medi de muntatge original per seleccionar un dissolvent adequat. Per a medis de muntatge aquosos, s'ha d'utilitzar aigua. Si el medi de muntatge és a base de resina (per exemple, bàlsam del Canadà o Euparal®), s'ha d'utilitzar xilè, sota una campana extractora i amb l'equip de protecció individual adequat, inclosa una màscara.

El muntatge d'espècimens tipus o de col·lecció només s'ha de realitzar amb el consentiment del conservador i/o de la institució propietària de la mostra.

6. Identificació dels espècimens

6.1. Morfologia

La identificació dels flebòtoms es basa principalment en l'examen de les seves característiques morfològiques, incloent-hi la forma del tòrax, les ales, els genitals, les setes i les relacions morfomètriques específiques entre diverses estructures. Els investigadors utilitzen claus taxonòmiques, col·leccions de referència i descripcions originals d'espècies per comparar els exemplars recollits amb els tàxons coneguts. Les característiques diagnòstiques clau, com la venació de les ales i la morfologia del cap en ambdós sexes, l'estructura dels genitals masculins i la configuració de les espermateques femenines, són particularment informatives per a la determinació de les espècies. La identificació precisa sovint requereix un examen microscòpic detallat, normalment utilitzant un microscopi compost per observar estructures fines com els genitals i les espermateques, o un estereomicroscopi per a característiques morfològiques més àmplies.

Els avenços recents en la tecnologia d'imatge han facilitat l'ús d'imatges digitals per a la identificació dels flebòtoms. Les fotografies d'alta resolució o les il·lustracions digitals de les característiques clau es poden comparar amb materials de referència o analitzar mitjançant sistemes d'identificació assistits per ordinador, millorant tant la precisió com l'accessibilitat en la taxonomia morfològica.

6.2. Geometria de l'ala

La geometria de les ales és una característica clau utilitzada en la identificació i classificació de diferents espècies de flebòtoms. Les ales dels flebòtoms presenten un patró i una estructura únics, normalment llargues i estretes amb una venació ben desenvolupada (figures 9 i 10). La disposició de les venes forma un patró diferent que pot variar entre gèneres i espècies, proporcionant característiques diagnòstiques valuoses per a la identificació. En conseqüència, l'estudi de la geometria de les ales proporciona informació valuosa per a finalitats taxonòmiques.

6.3. Morfometria geomètrica de les ales

Els investigadors utilitzen diverses tècniques, com ara la morfometria geomètrica, per analitzar i comparar la forma i la mida de les ales entre diferents espècies o poblacions de flebòtoms. L'estudi de la geometria de les ales proporciona informació valuosa sobre el comportament, les preferències d'hàbitat i les habilitats de vol.

En l'enfocament morfomètric geomètric, les ales es disseccionen acuradament, es tenyeixen (si cal) i es munten planes en portaobjectes. Els portaobjectes preparats es fotografien amb un estereomicroscopi, es digitalitzen i se sotmeten a una anàlisi morfomètrica. Aquest procediment ha estat ben descrit a la literatura [6, 27, 42, 56, 57, 59], amb la recomanació d'utilitzar l'ala dreta o esquerra de manera consistent per a òrgans aparellats per evitar possibles efectes al·lomètrics negatius [62].

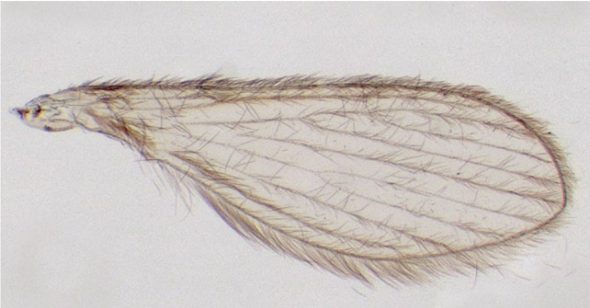

Figura 9: Ala sense tenyir de *Trichophoromyia ininii*.

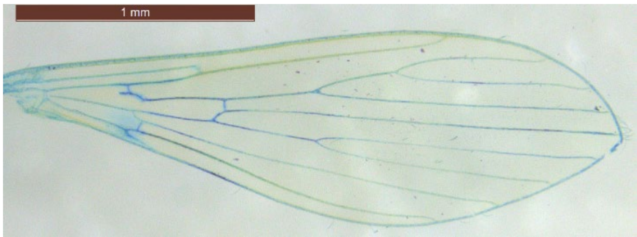

Figura 10: Ala tenyida de *Phlebotomus ariasi.*

Preparació de l'ala per a l'anàlisi geomètric morfomètric.

Per a una visualització òptima de les venes de l'ala, les ales s'han de netejar d'escates i tenyir-les adequadament. Per a la preparació de l'ala, primer ompliu els pous petits amb els reactius necessaris (blau de metilè, etanol, aigua i substitut de xilè). Recupereu una ala conservada en etanol al 70% a temperatura ambient invertint el tub Eppendorf i buidant-lo sobre el pou, després aixequeu l'ala longitudinalment amb una agulla fina corbada. Passeu l'ala breument de l'etanol a l'aigua i de tornada a l'etanol per eliminar les truges o pels rígids de l'ala. Col·loqueu l'ala en blau de metilè durant 6 minuts, assegurant-vos que suri durant la tinció. Recupereu l'ala amb cura i submergiu-la en el substitut de xilè durant 2 minuts (aproximadament un terç del temps del blau de metilè). Fent cops suaus de l'agulla contra les parets del pou poden ajudar a que l'ala s'assenti; el xilè serveix per fixar la coloració. Finalment, aixequeu l'ala i col·loqueu-la sobre una petita gota d'Euparal® en un portaobjectes microscòpics. Sota una lent d'augment, desplegueu suaument l'ala i col·loqueu amb cura un cobreobjectes. Cal fer fotografies immediatament abans que s'assequi l'Euparal®, ja que pot ser necessari ajustar lleugerament la posició de l'ala sota el cobreobjectes per aconseguir un alineament òptim.

6.4. Tècniques de biologia molecular

A més de les tècniques morfològiques, els mètodes moleculars són cada cop més essencials en la recerca entomològica, incloent-hi estudis taxonòmics, genètics de poblacions i filogenètics, així com per a la detecció de patògens d'ADN/ARN i per determinar l'origen dels àpats de sang, ja que el comportament del vector és important en el camp de l'epidemiologia [70]. La seqüenciació d'ADN es pot utilitzar per a la confirmació d'espècies o per diferenciar espècies estretament relacionades, proporcionant un mitjà d'identificació més precís i fiable. A més, les tècniques moleculars avançades (és a dir, PCR, seqüenciació d'ADN, NGS, etc.) i la MALDI-ToF MS estan guanyant protagonisme per a la identificació precisa i ràpida d'espècies, complementant els mètodes morfològics tradicionals [46]. Malgrat aquests avenços, la identificació morfològica continua sent l'estàndard de referència per a la taxonomia i la base sobre la qual s'interpreten les dades moleculars.

6.4.1. Extracció destructiva d'àcids nucleics

L'extracció d'àcids nucleics és un pas rutinari en molts estudis biològics, i s'han desenvolupat diversos mètodes per aïllar l'ADN de materials biològics [48]. Molts kits d'extracció d'ADN disponibles comercialment estan dissenyats per facilitar aquest procés [14]. Tanmateix, els mètodes que s'utilitzen habitualment per preparar mostres d'artròpodes per a la identificació morfològica sovint dificulten l'anàlisi d'ADN, ja que aquestes tècniques poden danyar o destruir característiques físiques crítiques de la mostra [10]. La majoria dels protocols d'extracció d'ADN per a teixits d'insectes són de naturalesa destructiva [43], cosa que planteja preocupacions particulars per a les mostres petites, on fins i tot un mostreig limitat pot comprometre característiques morfològiques importants [72]. El tipus i l'estat de la mostra tenen un paper clau en la selecció d'un mètode d'aïllament d'ADN adequat [29].

La necessitat d'una identificació fiable dels flebòtoms, comprendre la dinàmica de la població i minimitzar els impactes no desitjats ha impulsat el desenvolupament d'eines de diagnòstic molecular [23]. Els enfocaments moleculars ara s'utilitzen amb freqüència per complementar els mètodes taxonòmics morfològics per identificar els flebòtoms. Per exemple, l'enfocament estàndard per al codi de barres d'insectes implica l'extracció d'ADN, la seqüenciació i la pèrdua de la mostra original. Per tant, hi ha una necessitat urgent d'explorar mètodes d'extracció d'ADN no destructius que preservin tant el material biològic com la seva integritat morfològica.

S'han aplicat nombrosos mètodes d'extracció d'àcids nucleics als flebòtoms. La quantitat o la qualitat dels àcids nucleics necessaris depenen de l'anàlisi molecular posterior, ja que les diferents tècniques tenen requisits de sensibilitat i puresa variables [9]. Per exemple, s'ha descobert que els ulls dels flebòtoms inhibeixen l'amplificació per PCR [69]. Més enllà del cribratge de patògens, l'ADN dels flebòtoms s'extreu rutinàriament amb finalitats d'identificació d'espècies. Es poden utilitzar diversos mètodes d'extracció, tot i que els rendiments i la qualitat difereixen entre les tècniques. Els investigadors han adaptat alguns protocols dels fabricants per als flebòtoms [8], augmentant el rendiment i/o la qualitat dels àcids nucleics extrets [8, 9, 69], mentre que altres adaptacions, desenvolupades per a altres tàxons d'artròpodes, també es poden utilitzar en els flebòtoms [58, 76]. Les PCR d'identificació dirigides a petits fragments mitocondrials (COI o CytB) són generalment compatibles amb els mètodes d'extracció que impliquen una alta fragmentació de l'ADN. En canvi, altres tècniques de NGS de lectura llarga (Oxford Nanopore i PacBio) requereixen una fragmentació mínima i ADN d'alta qualitat. Les extraccions amb columna de centrifugació generalment produeixen fragments d'ADN genòmic de fins a 60 kb, mentre que l'extracció amb fenol-cloroform pot produir fragments de fins a 150 kb [77]. La taula 5 resumeix les diferents tècniques d'extracció d'ADN de flebòtoms i

indica si s'han fet adaptacions metodològiques per a aquests insectes. No es mostren els rendiments, ja que depenen de la mida de la mostra i del mètode de preparació. La columna de modificació fa referència a les adaptacions dels protocols d'extracció per a flebòtoms o altres artròpodes petits.

L'elecció del mètode d'extracció ha de tenir en compte diversos criteris, com ara el nombre de mostres, el temps d'extracció i la tècnica desplegada posteriorment. Si bé les tècniques de NGS requereixen ADN genòmic d'alt pes molecular, tots els mètodes presentats aquí es poden utilitzar per a aplicacions estàndard basades en PCR.

A més, diversos estudis han explorat mètodes d'extracció d'ADN no destructius per a petits artròpodes terrestres, espècimens de museu conservats en sec i artròpodes de cos tou [19, 26, 28, 55, 63].

Taula 5: Cost mitjà, aplicació i adaptació del protocol per a l'extracció de gADN de flebòtoms

Protocol	Cost	Aplicació	Protocol d'adaptació per petits artròpodes
Columna de sílice	2.5 – 3.55 US$ [39]	PCR, NGS	[9]
Fenol-cloroform	0.24 US$ [69]	PCR, NGS	[9]
HotSHOT	<0.01 US$ [69]	PCR	-
Precipitació salina	0.12 $ [69]	PCR	-
Chelex	0.02 $ [41]	PCR	[41, 76]

6.4.2. Extracció no destructiva d'àcids nucleics

Un dels principals reptes en l'anàlisi molecular d'artròpodes, especialment els flebòtoms, és la preservació d'espècimens per a la seva integració en col·leccions entomològiques. La majoria dels protocols d'extracció d'ADN requereixen la maceració del teixit, cosa que compromet la preservació de l'espècimen original. Tanmateix, els mètodes d'extracció no destructius d'àcids nucleics estan dissenyats per extreure material genètic sense danyar físicament la mostra, afectar-ne la viabilitat o alterar-ne la morfologia. Aquests mètodes són particularment valuosos quan es treballa amb espècimens preciosos o limitats, com els flebòtoms, on mantenir la integritat estructural és essencial per a futures taxonomies, morfològiques o de diagnòstic. Una tècnica habitualment emprada és el mètode de bany no destructiu en què els flebòtoms s'immobilitzen i s'immergeixen suaument en un tampó de lisi que conté proteinasa K.

La tècnica de *mild-vectolysis* s'ha aplicat amb èxit als flebòtoms, especialment als espècimens tipus [24]. La tècnica utilitza un kit estàndard de columnes de sílice (en aquest cas, un kit DNeasy Blood and Tissue, QIAGEN, Hilden, Alemanya) amb adaptació per obtenir ADN sense destruir l'espècimen. Els passos de lisi modificats (volum de tampó de lisi i addició d'un pas de congelació) [17] permeten l'alliberament d'àcids nucleics, minimitzant els danys morfològics [24]. Pel que fa als flebòtoms, també és possible utilitzar un kit d'extracció d'ADN HotSHOT (Bento Bioworks Ltd, Londres, Regne Unit) [73] que és ràpid i econòmic, permetent un processament ràpid i de baix cost de les mostres. Els espècimens entomològics destinats a la identificació morfològica es poden esbandir. Els processats amb un kit DNeasy Blood and Tissue s'han de clarificar amb una solució de Marc-André, mentre que els processats amb un kit d'extracció d'ADN HotSHOT estan prou clarificats per ser muntats en un medi aquós, o millor, en una resina després de la deshidratació, segons el protocol detallat en aquest article [73]. El material genètic extret es pot processar posteriorment per a anàlisis posteriors, com ara la PCR, per amplificar marcadors genètics específics. Els mètodes d'extracció d'àcids nucleics no destructius són crucials per estudiar les característiques genètiques dels flebòtoms, inclosa la identificació de possibles agents causants de malalties que poden portar. Preservant la integritat de la mostra, els investigadors poden obtenir informació genètica valuosa, alhora que conserven la mostra per a anàlisis o estudis addicionals.

6.5. MALDI-ToF MS

L'espectrometria de masses de temps de vol amb desorció/ionització làser assistida per matriu (MALDI-ToF MS) és una tècnica basada en l'espectrometria de masses dissenyada per detectar i analitzar els perfils proteics únics ("empremtes dactilars") de mostres biològiques. El MALDI-ToF es reconeix cada cop més com una eina important per a la identificació d'artròpodes d'importància mèdica i veterinària. Aquesta tècnica ha demostrat ser eficaç en la identificació de diverses etapes de desenvolupament dels flebòtoms, incloses les formes immadures i els àpats de sang de femelles engorjades, i s'ha aplicat amb èxit per diferenciar espècies de flebòtoms mascles i femelles en una sèrie de condicions d'emmagatzematge i homogeneïtzació [28, 30, 73, 74]. Aquest mètode també ofereix un alt poder discriminatori a nivell de subgèneres, espècies i poblacions. Aquesta tècnica permet als investigadors aconseguir una identificació ràpida i precisa de les espècies, cosa que és essencial per comprendre la distribució, el comportament i el paper dels flebòtoms en la transmissió de malalties. En diferenciar entre espècies en funció dels perfils proteics, el MALDI-ToF juga un paper crucial en els estudis epidemiològics i les estratègies de control de vectors. Hi ha dos inconvenients principals actuals d'aquesta tècnica que limiten la seva aplicació rutinària. En primer lloc, és la disponibilitat d'equips d'espectrometria de masses, que són prohibitivament cars d'adquirir fàcilment només amb la finalitat d'identificar espècies de flebòtoms (o vectors artròpodes en general). Afortunadament, aquesta limitació es pot superar accedint a espectròmetres de masses disponibles com una eina de recerca estàndard en

instal·lacions proteòmiques i/o diagnòstics clínics. En segon lloc, és la baixa representació de les dades de referència de flebòtoms disponibles en bases de dades d'accés obert, la qual cosa resulta en la necessitat de construir una base de dades interna amb espectres de referència basats en mostres identificades de manera inequívoca, idealment mitjançant una combinació d'avaluació morfològica i seqüenciació d'un marcador genètic adequat (COI, cytB o altres). Esperem que aquesta limitació es resolgui aviat mitjançant la inclusió gradual de les dades de referència de flebòtoms interns fins ara a la plataforma MSI gestionada per Assistance Publique-Hôpitaux de Paris, Universitat de la Sorbona, França i la col·lecció BCCM/IHEM/Sciensano a Brussel·les, Bèlgica (https://msi.happy-dev.fr/). Quan es planifica la implementació de perfils de proteïnes MALDI-ToF, les mostres s'han d'emmagatzemar preferiblement congelades en sec o en etanol al 70% de grau molecular i no s'han d'exposar a temperatura ambient. A falta de directrius universals per a la preparació de mostres, es recomana als usuaris que utilitzin una solució aquosa d'acetonitril al 60%/TFA al 0,3% d'àcid sinapínic (30 mg/mL) per a la preparació de la matriu MALDI-ToF per tal de fer que els seus espectres de proteïnes siguin comparables amb les dades de flebòtoms publicades fins ara.

Preparació de mostres per a MALDI-ToF MS (Figura 7)

Les mostres d'insectes, emmagatzemades en diverses condicions, primer s'assequen a l'aire a temperatura ambient i es disseccionen. Es retira el cap i l'abdomen per obtenir parts del cos que contenen característiques morfològiques clau per al muntatge de portaobjectes i l'anàlisi morfològica. El tòrax es pot utilitzar per a MALDI-ToF i l'abdomen restant es conserva per a l'extracció d'ADN. Per al perfil de proteïnes, el tòrax s'homogeneïtza manualment en microtubs d'1,5 ml amb 10 µL de solució d'homogeneïtzació mitjançant maceradors d'un sol ús (pilons). Normalment s'utilitzen dues solucions d'homogeneïtzació: aigua destil·lada estèril i àcid fòrmic al 25%.

7. Conclusió

En aquest treball, el nostre objectiu era proporcionar als investigadors els mètodes més eficaços per al muntatge de flebòtoms, adaptats a objectius de recerca específics, per facilitar la identificació precisa i la detecció de patògens. No hi ha un mètode únic i universalment òptim; més aviat, existeixen diversos mètodes, cadascun amb els seus propis avantatges i limitacions.

A les dades de suport, hem proporcionat protocols detallats per a diverses tècniques de muntatge utilitzades en la preparació i identificació de flebòtoms. Aquests protocols, inclosos vídeos instructius, ofereixen procediments pas a pas adaptats a diferents objectius, garantint resultats precisos i fiables. En oferir aquest recurs complet, pretenem ajudar els investigadors a seleccionar i aplicar les tècniques de muntatge més adequades per a les seves necessitats específiques.

Agraïments

Els autors agraeixen Richard Lane i Zoe Jay Adams del Museu d'Historia Natural de Londres, UK per la seva excel·lent revisió, que ha incrementat la qualitat d'aquest manuscrit.

Finançament

Agraïm a les agències de desenvolupament brasileres CNPq (número d'expedient: 404395/2024-4) i a la Fundació Araucária (número d'expedient: 433/2025 PDI) el finançament de la recerca d'AJA.

Conflicte d'interès

Jérôme Depaquit és editor associat de Parasite; no va tenir cap influència en el procés de revisió ni en la presa de decisions d'aquest manuscrit. Els altres autors declaren que no tenen conflictes d'interès.

Declaració de disponibilitat de dades

Vídeos a Zenodo
Video 1: https://zenodo.org/records/18198006
Video 2: https://zenodo.org/records/18311158
Video 3: https://zenodo.org/records/18311106
Video 4: https://zenodo.org/records/18311154
Video 5: https://zenodo.org/records/18303014
Video 6: https://zenodo.org/records/18302850
Video 7: https://zenodo.org/records/18315029

Materials addicionals

Més informació sobre aquest article està disponible a l'adreça següent: https://www.parasite-journal.org/10.1051/parasite/2026009/olm

Bibliografia

1. Alkan C, Allal-Ikhlef AB, Alwassouf S, Baklouti A, Piorkowski G, de Lamballerie X, Izri A, Charrel RN. 2015. Virus isolation, genetic characterization and seroprevalence of Toscana virus in Algeria. Clinical Microbiology and Infection, 21(11), 1040 e1-9.
2. Alten B, Ozbel Y, Ergunay K, Kasap OE, Cull B, Antoniou M, Velo E, Prudhomme J, Molina R, Banuls AL, Schaffner F, Hendrickx G, Van Bortel W, Medlock JM. 2015. Sampling strategies for phlebotomine sand flies (Diptera: Psychodidae) in Europe. Bulletin of Entomological Research 105(6), 664–678.
3. Ayhan N, Baklouti A, Prudhomme J, Walder G, Amaro F, Alten B, Moutailler S, Ergunay K, Charrel RN, Huemer H. 2017. Practical guidelines for studies on sandfly-borne phleboviruses: Part I: Important points to consider *ante* field work. Vector-Borne and Zoonotic Diseases 17(1), 73–80.
4. Bates PA. 1997. Infection of phlebotomine sandflies with *Leishmania*, in The Molecular Biology of Insect Disease Vectors: A Methods Manual. Springer. p. 112–120.5.

5. Baum M, de Castro EA, Pinto MC, Goulart TM, Baura W, Klisiowicz Ddo R, Vieira da Costa-Ribeiro MC. 2015. Molecular detection of the blood meal source of sand flies (Diptera: Psychodidae) in a transmission area of American cutaneous leishmaniasis, Parana State, Brazil. Acta Tropica, 143, 8–12.
6. Belen A, Alten B, Aytekin A. 2004. Altitudinal variation in morphometric and molecular characteristics of *Phlebotomus papatasi* populations. Medical and Veterinary Entomology, 18(4), 343–350.
7. Bhattacharya J, Chandra G, Hati AK. 1991. A simple method for cryopreservation of *Leishmania donovani* promastigotes, Indian Journal of Medical Research, 93, 245–246.
8. Caligiuri LG, Sandoval AE, Miranda JC, Pessoa FA, Santini MS, Salomón OD, Secundino NF, McCarthy CB. 2019. Optimization of DNA extraction from individual sand flies for PCR amplification. Methods and Protocols, 2(2), 36.
9. Casaril AE, de Oliveira LP, Alonso DP, de Oliveira EF, Gomes Barrios SP, de Oliveira Moura Infran J, Fernandes WS, Oshiro ET, Ferreira AMT, Ribolla PEM, de Oliveira AG. 2017. Standardization of DNA extraction from sand flies: Application to genotyping by next generation sequencing. Experimental Parasitology, 177, 66–72.
10. Castalanelli MA, Severtson DL, Brumley CJ, Szito A, Foottit RG, Grimm M, Munyard K, Groth DM. 2010. A rapid non-destructive DNA extraction method for insects and other arthropods. Journal of Asia-Pacific Entomology, 13(3), 243–248.
11. Cerqueira NL. 1943. Um novo meio para montagem de pequenos insetos em lâmina. Memórias do Instituto Oswaldo Cruz, (39), 37–41.
12. Charrel RN, Gallian P, Navarro-Mari JM, Nicoletti L, Papa A, Sanchez-Seco MP, Tenorio A, de Lamballerie X. 2005. Emergence of Toscana virus in Europe. Emerging Infectious Diseases, 11(11), 1657–1663.
13. Chaskopoulou A, Giantsis IA, Demir S, Bon MC. 2016. Species composition, activity patterns and blood meal analysis of sand fly populations (Diptera: Psychodidae) in the metropolitan region of Thessaloniki, an endemic focus of canine leishmaniasis. Acta Tropica, 158, 170–176.
14. Chen H, Rangasamy M, Tan SY, Wang H, Siegfried BD. 2010. Evaluation of five methods for total DNA extraction from western corn rootworm beetles. PLoS One, 5(8), e11963.
15. Depaquit J, Grandadam M, Fouque F, Andry PE, Peyrefitte C. 2010. Arthropod-borne viruses transmitted by Phlebotomine sandflies in Europe: a review. Eurosurveillance, 15(10), 19507.
16. Diamond LS, Herman CM. 1954. Incidence of Trypanosomes in the Canada Goose as revealed by bone marrow culture. Journal of Parasitology, 40(2), 195–202.
17. Ding H, Torno M, Vongphayloth K, Ng G, Tan D, Sng W, Ho K, Randrianambinintsoa FJ, Depaquit J, Tan CH. 2025. Hidden in plain sight: discovery of sand flies in Singapore and description of four species new to science. Parasites & Vectors, 18(1), 402.
18. Es-Sette N, Ajaoud M, Bichaud L, Hamdi S, Mellouki F, Charrel RN, Lemrani M. 2014. *Phlebotomus sergenti* a common vector of *Leishmania tropica* and Toscana virus in Morocco. Journal of Vector Borne Diseases, 51(2), 86–90.
19. Favret C. 2005. A new non-destructive DNA extraction and specimen clearing technique for aphids (Hemiptera). Proceedings of the Entomological Society of Washington, 107(2), 469–470.
20. Galati EAB. 2018. Phlebotominae (Diptera, Psychodidae): Classification, morphology and terminology of adults and identification of American taxa, in Brazilian Sand Flies: Biology, Taxonomy, Medical Importance and Control, Rangel EF, Shaw JJ, Editors. Cham: Springer International Publishing. pp. 9–212.
21. Galati EAB, de Andrade AJ, Perveen F, Loyer M, Vongphayloth K, Randrianambinintsoa FJ, Prudhomme J, Rahola N, Akhoundi M, Shimabukuro PHF, Depaquit J. 2025. Phlebotomine sand flies (Diptera, Psychodidae) of the world. Parasites & Vectors, 18(1), 220.
22. Galati EAB, Galvis-Ovallos F, Lawyer P, Leger N, Depaquit J. 2017. An illustrated guide for characters and terminology used in descriptions of Phlebotominae (Diptera, Psychodidae). Par-asite, 24, 26.
23. Gariepy T, Kuhlmann U, Gillott C, Erlandson M. 2007. Parasitoids, predators and PCR: the use of diagnostic molecular markers in biological control of Arthropods. Journal of Applied Entomology, 131(4), 225–240.
24. Giantsis IA, Chaskopoulou A, Bon MC. 2016. Mild-Vectolysis: A nondestructive DNA extraction method for vouchering sand flies and mosquitoes. Journal of Medical Entomology, 53(3), 692–695.
25. Gidwani K, Picado A, Rijal S, Singh SP, Roy L, Volfova V, Andersen EW, Uranw S, Ostyn B, Sudarshan M, Chakravarty J, Volf P, Sundar S, Boelaert M, Rogers ME. 2011. Serological markers of sand fly exposure to evaluate insecticidal nets against visceral leishmaniasis in India and Nepal: a cluster-randomized trial. PLoS Neglected Tropical Diseases, 5(9), e1296.
26. Gilbert MTP, Moore W, Melchior L, Worobey M. 2007. DNA extraction from dry museum beetles without conferring external morphological damage. PLoS One, 2(3), e272.
27. Giordani BF, Andrade AJ, Galati EAB, Gurgel-Goncalves R. 2017. The role of wing geometric morphometrics in the identification of sandflies within the subgenus *Lutzomyia.* Medical and Veterinary Entomology, 31(4), 373–380.
28. Guzmán-Larralde AJ, Suaste-Dzul AP, Gallou A, Peña-Carrillo KI. 2017. DNA recovery from microhymenoptera using six non-destructive methodologies with considerations for subsequent preparation of museum slides. Genome, 60(1), 85–91.
29. Hajibabaei M, DeWaard JR, Ivanova NV, Ratnasingham S, Dooh RT, Kirk SL, Mackie PM, Hebert PD. 2005. Critical factors for assembling a high volume of DNA barcodes. Philosophical Transactions of the Royal Society B: Biological Sciences, 360(1462), 1959–1967.
30. Haouas N, Pesson B, Boudabous R, Dedet JP, Babba H, Ravel C. 2007. Development of a molecular tool for the identification of *Leishmania* reservoir hosts by blood meal analysis in the insect vectors. American Journal of Tropical Medicine and Hygiene, 77(6), 1054–1059.
31. Hlavackova K, Dvorak V, Chaskopoulou A, Volf P, Halada P. 2019. A novel MALDI-TOF MS-based method for blood meal identification in insect vectors: A proof of concept study on phlebotomine sand flies. PLoS Neglected Tropical Diseases, 13 (9), e0007669.
32. Huemer H, Prudhomme J, Amaro F, Baklouti A, Walder G, Alten B, Moutailler S, Ergunay K, Charrel RN, Ayhan N. 2017. Practical guidelines for studies on sandfly-borne phleboviruses: Part II: Important points to consider for fieldwork and subsequent virological screening. Vector-Borne and Zoonotic Diseases, 17(1), 81–90.
33. Jancarova M, Polanska N, Thiesson A, Arnaud F, Stejskalova M, Rehbergerova M, Kohl A, Viginier B, Volf P, Ratinier M. 2025. Susceptibility of diverse sand fly species to Toscana virus. PLoS Neglected Tropical Diseases, 19(5), e0013031.

34. Kapp JD, Green RE, Shapiro B. 2021. A fast and efficient single-stranded genomic library preparation method optimized for ancient DNA. Journal of Heredity, 112(3), 241–249.
35. Killick-Kendrick R, Maroli M, Killick-Kendrick M. 1991. Bibliography of the colonization of phlebotomine sandflies. Parassitologia, 33(suppl.), 321–333.
36. Lawyer P, Killick-Kendrick M, Rowland T, Rowton E, Volf P. 2017. Laboratory colonization and mass rearing of phlebotomine sand flies (Diptera, Psychodidae). Parasite, 24, 42.
37. Léger N, Pesson B, Madulo-Leblond G. 1986. Les phlébotomes de Grèce : 1ère partie. Bulletin de la Société de Pathologie Exotique, 79, 386–397.
38. Léger N, Pesson B, Madulo-Leblond G. 1986. Les phlébotomes de Grèce : 2ème partie. Bulletin de la Société de Pathologie Exotique, 79, 514–524.
39. Leonel JAF, Vioti G, Alves ML, da Silva DT, Meneghesso PA, Benassi JC, Spada JCP, Galvis-Ovallos F, Soares RM, Oliveira T. 2020. DNA extraction from individual Phlebotomine sand flies (Diptera: Psychodidae: Phlebotominae) specimens: Which is the method with better results? Experimental Parasitology, 218, 107981.
40. Lestinova T, Rohousova I, Sima M, de Oliveira CI, Volf P. 2017. Insights into the sand fly saliva: Blood-feeding and immune interactions between sand flies, hosts, and *Leishmania.* PLoS Neglected Tropical Diseases, 11(7), e0005600.
41. Lienhard A, Schaffer S. 2019. Extracting the invisible: obtaining high quality DNA is a challenging task in small arthropods. PeerJ, 7, e6753.
42. Lozano-Sardaneta YN, Mikery-Pacheco OF, Huerta H, Rojas-Soriano JE, Contreras-Ramos A. 2025. Wing geometric morphometrics is effective to separate sand fly species (Diptera, Psychodidae, Phlebotominae) related with leishmaniasis transmission in Mexico. Acta Tropica, 262, 107523.
43. Mandrioli M. 2008. Insect collections and DNA analyses: how to manage collections? Museum Management and Curatorship, 23(2), 193–199.
44. Maroli M, Feliciangeli MD, Bichaud L, Charrel RN, Gradoni L. 2013. Phlebotomine sandflies and the spreading of leishmaniases and other diseases of public health concern. Medical and Veterinary Entomology, 27(2), 123–147.
45. Marquina D, Buczek M, Ronquist F, Lukasik P. 2021. The effect of ethanol concentration on the morphological and molecular preservation of insects for biodiversity studies. PeerJ, 9, e10799.
46. Mathis A, Depaquit J, Dvorak V, Tuten H, Banuls AL, Halada P, Zapata S, Lehrter V, Hlavackova K, Prudhomme J, Volf P, Sereno D, Kaufmann C, Pfluger V, Schaffner F. 2015. Identification of phlebotomine sand flies using one MALDI-TOF MS reference database and two mass pectrometer systems., Parasites & Vectors, 8, 266.
47. Mekarnia N, Benallal KE, Sadlova J, Vojtkova B, Mauras A, Imbert N, Longhitano M, Harrat Z, Volf P, Loiseau PM, Cojean S. 2024. Effect of *Phlebotomus papatasi* on the fitness, infectivity and antimony-resistance phenotype of antimony-resistant *Leishmania major* MON-25. International Journal for Parasitology – Drugs and Drug Resistance, 25, 100554.
48. Milligan BG. 1998. Total DNA isolation, in Molecular Genetic Analysis of Population: A Practical Approach, Hoelzel AR, Editor. Oxford: Oxford University Press.
49. Molina R, Jiménez M, Alvar J, González E, Hernández-Taberna S, Ines MM. 2017. Methods in sand fly research. Madrid: Servicio de publicaciones, Universidad de Alcalá de Henares, Madrid.
50. Murphy WJ, Eizirik E, O'Brien SJ, Madsen O, Scally M, Douady CJ, Teeling E, Ryder OA, Stanhope MJ, de Jong WW, Springer MS. 2001. Resolution of the early placental mammal radiation using Bayesian phylogenetics. Science, 294(5550), 2348–2351.
51. Nacif-Pimenta R, Pinto LC, Volfova V, Volf P, Pimenta PFP, Secundino NFC. 2020. Conserved and distinct morphological aspects of the salivary glands of sand fly vectors of leishmaniasis: an anatomical and ultrastructural study. Parasites & Vectors, 13(1), 441.
52. Neuhaus B, Schmid T, Riedel J. 2017. Collection management and study of microscope slides: Storage, profiling, deterioration, restoration procedures, and general recommendations. Zootaxa, 4322(1), 1–173.
53. New TR. 1974. Pscoptera. Handbooks for Identification of British Insects (Vol. I). London: Royal Entomological Society of London. 102 pp.
54. Perez-Ruiz M, Collao X, Navarro-Mari JM, Tenorio A. 2007. Reverse transcription, real-time PCR assay for detection of Toscana virus. Journal of Clinical Virology, 39(4), 276–281.
55. Porco D, Rougerie R, Deharveng L, Hebert P. 2010. Coupling non-destructive DNA extraction and voucher retrieval for small soft-bodied Arthropods in a high-throughput context: the example of Collembola. Molecular Ecology Resources, 10(6), 942–945.
56. Prudhomme J, Cassan C, Hide M, Toty C, Rahola N, Vergnes B, Dujardin JP, Alten B, Sereno D, Banuls AL. 2016. Ecology and morphological variations in wings of *Phlebotomus ariasi* (Diptera: Psychodidae) in the region of Roquedur (Gard, France): a geometric morphometrics approach. Parasites & Vectors, 9(1), 578.
57. Prudhomme J, Gunay F, Rahola N, Ouanaimi F, Guernaoui S, Boumezzough A, Banuls AL, Sereno D, Alten B. 2012. Wing size and shape variation of *Phlebotomus papatasi* (Diptera: Psychodidae) populations from the south and north slopes of the Atlas Mountains in Morocco. Journal of Vector Ecology, 37(1), 137–147.
58. Prudhomme J, Toty C, Kasap OE, Rahola N, Vergnes B, Maia C, Campino L, Antoniou M, Jimenez M, Molina R, Cannet A, Alten B, Sereno D, Banuls AL. 2015. New microsatellite markers for multi-scale genetic studies on *Phlebotomus ariasi* Tonnoir, vector of *Leishmania infantum* in the Mediterranean area. Acta Tropica, 142, 79–85.
59. Prudhomme J, Velo E, Bino S, Kadriaj P, Mersini K, Gunay F, Alten B. 2019. Altitudinal variations in wing morphology of *Aedes albopictus* (Diptera, Culicidae) in Albania, the region where it was first recorded in Europe. Parasite, 26, 55.
60. Rawlins DJ. 1992. Light Microscopy: An Introduction to Biotechniques. Oxford: Bios Scientific publishers. 143 pp.
61. Ready PD. 2013. Biology of phlebotomine sand flies as vectors of disease agents. Annual Review of Entomology, 58, 227–250.
62. Rohlf FJ, Slice D. 1990. Extensions of the Procrustes method for the optimal superimposition of landmarks. Systematic Zoology, 39(1), 40–59.
63. Rowley DL, Coddington JA, Gates MW, Norrbom AL, Ochoa RA, Vandenberg NJ, Greenstone MH. 2007. Vouchering DNA-barcoded specimens: Test of a nondestructive extraction proto-col for terrestrial arthropods. Molecular Ecology Notes, 7(6), 915–924.
64. Sábio PB, Andrade AJ, Galati EAB. 2014. Assessment of the taxonomic status of some species included in the *Shannoni* complex, with the description of a new species of *Psathyromyia*

(Diptera: Psychodidae: Phlebotominae). Journal of Medical Entomology, 51(2), 331–341.
65. Sadlova J, Yeo M, Seblova V, Lewis MD, Mauricio I, Volf P, Miles MA. 2011. Visualisation of *Leishmania donovani* fluorescent hybrids during early stage development in the sand fly vector. PLoS One, 6(5), e19851.
66. Sales K, Miranda DEO, da Silva FJ, Otranto D, Figueredo LA, Dantas-Torres F. 2020. Evaluation of different storage times and preservation methods on phlebotomine sand fly DNA concentration and purity. Parasites & Vectors, 13(1), 399
67. Sales KG, Costa PL, de Morais RC, Otranto D, Brandao-Filho SP, Cavalcanti Mde P, Dantas-Torres F. 2015. Identification of phlebotomine sand fly blood meals by real-time PCR. Parasites& Vectors, 8, 230.
68. Sant'Anna MR, Jones NG, Hindley JA, Mendes-Sousa AF, Dillon RJ, Cavalcante RR, Alexander B, Bates PA. 2008. Blood meal identification and parasite detection in laboratory-fed and field-captured *Lutzomyia longipalpis* by PCR using FTA databasing paper. Acta Tropica, 107(3), 230–237.
69. Senne NA, Santos HA, Araujo TR, Paulino PG, Mendonca LP, Moreira HVS, Camilo TA, da Costa Angelo I. 2022. Robust comparative performance of genomic DNA extraction methods from non-engorged phlebotomine sandflies. Medical and Veterinary Entomology, 36(2), 203–211.
70. Shaw JJ. 2025. A review of Leishmania infections in American Phlebotomine sand flies – Are those that transmit leishmaniasis anthropophilic or anthropportunists? Parasite, 32, 57.
71. Tesh RB, Modi GB. 1983. Growth and transovarial transmission of Chandipura virus (Rhabdoviridae: Vesiculovirus) in *Phlebotomus papatasi.* American Journal of Tropical Medicine and Hygiene, 32(3), 621–623.
72. Thomsen PF, Elias S, Gilbert MTP, Haile J, Munch K, Kuzmina S, Froese DG, Sher A, Holdaway RN, Willerslev E. 2009. Non-destructive sampling of ancient insect DNA. PLoS One, 4(4),e5048
73. Truett GE, Heeger P, Mynatt RL, Truett AA, Walker JA, Warman ML. 2000. Preparation of PCR-quality mouse genomic DNA with hot sodium hydroxide and tris (HotSHOT). Biotechniques, 29(1), 52–54.
74. Upton MS. 1993. Aqueous gum-chloral slide mounting media: an historical review. Bulletin of Entomological Research, 83(2), 267–274.
75. Volf P, Myskova J. 2007. Sand flies and *Leishmania*: specific versus permissive vectors. Trends in Parasitology, 23(3), 91–92.
76. Wang Q, Wang X. 2012. Comparison of methods for DNA extraction from a single chironomid for PCR analysis. Pakistan Journal of Zoology, 44(2), 421–426.
77. Wang Y, Zhao Y, Bollas A, Wang Y, Au KF. 2021. Nanopore sequencing technology, bioinformatics and applications. Nature Biotechnology, 39(11), 1348–1365.

Cite this article as: Randrianambinintsoa FJ, Augendre L, Prudhomme J, Martinet J-P, Loyer M, Mekarnia N, Kerkoub H, Perveen FK, Huguenin A, Kariya E, Akhoundi M, De Andrade AJ, Berriatua E, Bongiorno G, Boyer S, Christodoulou V, Da Costa-Ribeiro MCV, De Souza LAF, Ding H, Dondji B, Dvořák V, Erisoz Kasap O, Galati EAB, Gállego M, Ballart C, Gouzelou S, Haddad N, Masse RS, Mekuria AH, Ivovic V, Kaczmarek S, Shahar MK, Kirstein OD, Kniha E, Kolářová I, Lincoln T, Lucanas C, Mikov O, Nov K, Özbel Y, Pesson B, Posada Lopez LC, Prasetyo DB, Rahola N, Rebollar-Tellez EA, Rodrigues BL, Roy L, Saini P, Sanjoba C, Shimabukuro PH, Siriyasatien P, Soszynska A, Suleşco T, Sylla M, Torno M, Volf P, Vongphayloth K, Sinh Nam V, Wardhana A, Yessinou E, Zapata S, Gantier J-C & Depaquit J. 2026. Processing and mounting phlebotomine sand flies: a consensus guideline. Parasite xx, xx. https://doi.org/10.1051/parasite/2026009.

PARASITE

An international open-access, peer-reviewed, online journal publishing high quality papers on all aspects of human and animal parasitology

Reviews, articles and short notes may be submitted. Fields include, but are not limited to: general, medical and veterinary parasitology; morphology, including ultrastructure; parasite systematics, including entomology, acarology, helminthology and protistology, and molecular analyses; molecular biology and biochemistry; immunology of parasitic diseases; host-parasite relationships; ecology and life history of parasites; epidemiology; therapeutics; new diagnostic tools.
All papers in Parasite are published in English. Manuscripts should have a broad interest and must not have been published or submitted elsewhere. No limit is imposed on the length of manuscripts.

Parasite (open-access) continues **Parasite** (print and online editions, 1994-2012) and **Annales de Parasitologie Humaine et Comparée** (1923-1993) and is the official journal of the Société Française de Parasitologie.

Editor-in-Chief:
Jean-Lou Justine, Paris

Submit your manuscript at:
https://www.editorialmanager.com/parasite

Apèndix 1: Fonaments teòrics bioquímics.

Els artròpodes en qüestió són els flebòtoms. Tanmateix, la idea general es pot estendre a altres artròpodes molt comuns la identificació dels quals només es pot fer mitjançant caràcters morfològics interns. Per casualitat, alguns òrgans interns estan parcialment quitinitzats i les seves morfologies ens proporcionen informació valuosa. És per això que és molt interessant observar les bombes alimentàries, les espermateques i els seus conductes. Amb tots els reactius que revisarem, no s'ha d'oblidar mai que des de l'etapa de fixació dels insectes fins al muntatge, simplement aplicarem reaccions redox. L'única precaució o idea que ens guiarà és evitar barrejar reactius reductors amb reactius oxidants.

Alcohol etílic; etanol:

Aquesta substància s'utilitzarà de diferents maneres. Les molècules d'alcohol tenen una forta afinitat per l'aigua i, per tant, presenten un efecte deshidratant. Tanmateix, un alcohol amb baixa concentració (és a dir, massa ric en aigua) tindrà un paper en la degradació dels àcids nucleics (l'aigua és l'enemic dels àcids nucleics).

Quan es col·loquen insectes en etanol, no és només per preservar-los, sinó també per fixar els teixits. En histologia, normalment diferenciem dos conceptes importants: la taxa de penetració i la taxa de fixació. És ben sabut que un bon conservant primer ha de penetrar ràpidament i profundament als teixits abans de fixar-los. Per a l'alcohol al 96%, el coeficient de penetració és d'aproximadament 1,05 (en comparació, per a una solució aquosa d'àcid pícric al 0,75%, el coeficient de penetració és de 0,45, mentre que el d'una solució de dicromat de potassi al 3% és d'1,45).

Voler preservar insectes i altres artròpodes indefinidament en etanol és una realitat per als entomòlegs. La idea encara és molt honorable voler conservar les captures de camp per a estudis posteriors o per a futurs investigadors. No obstant això, aquesta idea no és possible per a un citòleg o històleg. Si es pretén mantenir les mostres al fixador durant massa temps, poden arribar a ser pràcticament impossibles de reelaborar. És per això que les mostres de més de 10 anys són difícils o fins i tot impossibles d'utilitzar.

Una altra consideració és la relació entre la massa d'artròpodes a fixar i el volum del fixador. En la pràctica zoològica o mèdica, és recomanable preveure un volum 60 vegades més gran que el volum de les peces a fixar. A la pràctica, per als microartròpodes, per a un volum determinat d'espècimens a fixar, afegiu almenys 4-5 volums d'alcohol. Tingueu en compte que l'alcohol perdrà la seva força a mesura que elimina tota l'aigua present als teixits dels artròpodes.

En conclusió:

- L'alcohol etílic és un agent químic reductor (per tant, incompatible amb els fixadors oxidatius);
- Precipita energèticament les proteïnes i les desnaturalitza;
- Dissol certs lípids complexos i precipita el glicogen;
- Provoca una forta contracció dels teixits i els endureix.

Solucions bàsiques d'hidròxid de potassi o sodi:

L'ús d'aquestes solucions en entomologia s'ha centrat principalment en l'hidròxid de potassi sense una justificació clara.

L'hidròxid de sodi [E524] es presenta en solució, ja sigui a diferents concentracions o amb diferent normalitat. Es presenta en grànuls o purpurina. El seu principal desavantatge és que és molt higroscòpic (més que el KOH). Quan reacciona amb les proteïnes, les dissol i amb els lípids les transforma en sabons durs durant la saponificació (aquesta és una diferència important amb el KOH, que produeix sabons líquids durant la saponificació).

L'hidròxid de potassi [E525] està disponible com a solució concentrada, però sobretot té l'avantatge de formular-se en grànuls d'uns 0,1 g, cosa que facilita enormement la preparació de solucions diluïdes quan no s'especifica una balança de precisió. Per exemple, 1 grànul de 0,1 g en 1 mL d'aigua destil·lada produeix una solució al 10%. El segon avantatge de l'hidròxid de potassi en grànuls és la seva menor sensibilitat a la carbonatació (una solució de KOH té una alta afinitat per fixar el CO_2, creant així sals de carbonat). Aquestes bases fortes s'utilitzaran per solubilitzar àcids grassos transformant-los en sabons solubles en aigua. Cal recordar que el fixador, com l'etanol, va solubilitzar alguns dels greixos de la mostra. Tanmateix, en substituir la mostra en un medi aquós per una base forta, els àcids grassos (més o menys complexos) precipitaran. La base forta, per tant, realitzarà una saponificació en fred. En alguns casos, quan els teixits adiposos són en excés, per exemple en les femelles, serà avantatjós augmentar la temperatura a 35-40 °C per facilitar la reacció, o bé allargar el temps de contacte a temperatura ambient.

Solució àcida acolorida/solució Marc-André incolora:

Aquí explorarem els avantatges i els inconvenients d'utilitzar la solució Marc-André. Aquesta solució està composta d'hidrat de cloral (Tricloracetaldehid monohidrat), àcid acètic i aigua. Aquesta solució és molt oxidant (barreja d'àcid i aldehid). Neutralitzarà l'excés d'hidròxid de potassi que pugui quedar a les mostres, sense precipitar els sabons alcalins formats durant l'ús d'hidròxid de potassi. Aquesta solució oxidant també tindrà una acció sobre les funcions d'alcohol secundari de les glucosamines que es formen a la quitina oxidant-les, suavitzant així la quitina. Una altra acció és la dissolució de certes sals minerals presents.

Quan la solució Marc-André està prèviament acolorida per fucsina àcida (per tant, en estat oxidat), podrà fixar-se sobre les funcions d'alcohol secundari de l'estructura. Després del temps de contacte de la solució Marc-André i l'estat de tinció de les mostres, l'esbandida es farà només amb etanol. Així comencem la fase de deshidratació de les mostres.

Beneficis:
- Neutralització de l'excés de solucions bàsiques
- Relaxació de la quitina
- Tinció de la quitina per avaluar millor les estructures internes quitinitzades

Inconvenients:

L'hidrat de clor és hipnòtic i s'ha utilitzat en medicina humana. S'ha d'utilitzar sota una campana química i s'ha de complir la legislació sobre riscos químics.

Solucions de deshidratació:

L'experiència demostra que per a mostres molt petites no és útil seguir la seqüència de banys d'alcohol amb concentracions creixents. Si la mostra és gran, començarem amb etanol al 80%, després amb etanol al 90%, 95% i finalment etanol absolut. Per a mostres molt petites, utilitzarem un bany amb etanol al 90% seguit d'immersió en etanol absolut. En aquesta etapa, sempre recordarem que l'etanol absolut busca fixar l'aigua a l'atmosfera.

La tradició als laboratoris d'entomologia era finalitzar la deshidratació de les mostres amb un bany de creosota de faig. Avui dia, aquesta essència, àmpliament utilitzada com a pesticida, antifúngic i conservant de la fusta, es desaconsella molt a causa de la seva olor (hidrocarburs aromàtics policíclics) i se suposa que és reprotòxica, cancerígena, contaminant orgànic persistent i ecotòxica per als organismes aquàtics.

Una solució que proposem per preparar per al muntatge de les mostres és Euparal® i essència Euparal (descrita al paràgraf següent). Una barreja d'Euparal® i essència Euparal és molt ben acceptada; mostres obtingudes després d'un bany d'etanol al 90%.

Apèndix 2: Composició dels reactius utilitzats.

Hidròxid de potassi 10%
Hidròxid de potassi 10 g
Aigua destil·lada q.s. 100 mL

Medi de muntatge de goma cloral. Medi de Hoyer
Aigua destil·lada 50 mL
Hidrat de cloral 200 g
Goma aràbiga 50 g
Glicerol 20 mL

Solució de Marc-André
Hidrat de cloral 40 g
Àcid acètic glacial 30 mL
Aigua destil·lada 30 mL

Fucsina àcida 1% en aigua destil·lada
Fucsina àcida en pols 1 g
Aigua destil·lada 99 mL

Solució de Marc-André acolorida amb fucsina
Solució de Marc-André 10 mL
Fucsina 1% 50 μL

Apèndix 3: Euparal®, bàlsam del Canadà, alcohol polivinílic o altres solucions per al muntatge.

Alcohol polivinílic: Aquest és el medi de muntatge ideal quan no es disposen dels productes necessaris per a una deshidratació adequada. L'alcohol polivinílic es barreja amb lactofenol d'Amman. Tanmateix, aquests productes presenten els principals inconvenients d'assecar-se o cristal·litzar l'alcohol polivinílic a causa de l'evaporació de l'aigua o l'ennegriment quan el fenol s'oxida. Aquesta continua sent una bona tècnica per al muntatge a curt termini.

Bàlsam del Canadà: El seu ús per al muntatge entre el portaobjectes i el cobreobjectes requereix la deshidratació de les mostres que s'han de muntar. L'ús de xilè o toluè no està exempt d'inconvenients.

Medi Enecê: Per al muntatge entre el portaobjectes i el cobreobjectes, com ara el bàlsam del Canadà, requereix la deshidratació de la mostra. Formulació Enecè: colofònia blanca pura (22 g); goma de Copal soluble en alcohol (12 g), alcohol absolut (20 mL), càmfora (10 g), essència de trementina (10 mL) i eucaliptol (26 mL). Per a la seva preparació, en un recipient, com ara un matràs Erlenmeyer, poseu-hi l'alcohol absolut i el càmfora. A continuació, afegiu-hi la colofònia i la goma Copal. A continuació, es tanca el matràs amb un tap i s'agita, i només llavors s'escalfa al bany Maria a una temperatura suau perquè la barreja no bulli. Un cop el contingut s'hagi liquat completament, s'afegeix l'essència de trementina, es filtra mentre la barreja encara està calenta i, finalment, s'afegeix l'eucaliptol al filtrat. Quan el medi es torna menys fluid, es dilueix amb Eenecè, que té la fórmula següent: alcohol absolut (30 mL), càmfora (17 g), essència de trementina (15 mL), eucaliptol (38 mL) (Cerqueira, 1943).

Euparal®: Es tracta d'una resina que prové del xiprer de l'Atles *Tetraclinis articulata* (Vahl, 1791) i va ser estudiada i desenvolupada el 1906 per Gilson. El seu principal avantatge és que no polimeritza. Les mostres muntades entre portaobjectes i cobreobjectes es poden recuperar fàcilment per l'acció de l'alcohol o, millor encara, de l'essència Euparal®. Aquesta resina, també anomenada sandaraca, accepta etanol a partir del 80%.

Utilització del Triton X100: solució aquosa no iònica:

El Triton X100 es presenta en forma de solució aquosa no iònica (solució de 4-(1,1,3,3-tetrametilbutil) fenol-polietilenglicol, t-octilfenoxipolietoxietanol, èter de polietilenglicol tert-octilfenil), àmpliament utilitzada com a detergent en biologia cel·lular i molecular. Permet la permeabilització de les membranes cel·lulars i nuclears.

Les mostres d'insectes conservades en alcohol durant molts anys són habituals. Malauradament, la conservació en alcohol no és òptima i els artròpodes així conservats es

tornen molt difícils de preparar per a l'examen microscòpic. Els plàstics que contenen mostres sovint es degraden, seguits de l'evaporació de l'alcohol. En ambdós casos, el contacte prolongat amb l'alcohol o l'assecat de les mostres representen un problema real. El 2008, Jonque va publicar una nota sobre la rehidratació d'aranyes amb un agent humectant com l'Agepon utilitzat per a pel·lícules fotogràfiques [26]. Això va conduir a la idea d'utilitzar agents humectants que no siguin detergents potents.
A continuació es mostra un procediment utilitzant Triton X100 en una solució aquosa al 0,5%:
- Impregnar la mostra seca amb alcohol absolut.
- Afegir el volum necessari de solució de Triton X100 al 0,5% de manera que tota la mostra quedi submergida.
- Deixar reposar durant uns 5 minuts o més. Tots els artròpodes s'han de separar en la solució.
- Es retira la solució de Triton X100 i es substitueix per la solució d'hidròxid de potassi.

A continuació, es segueix la tècnica descrita anteriorment.

Apèndix 4: Medi de muntatge Euparal® o Canada Balsam pas a pas

1. Les mostres s'han de deshidratar (un aspecte tèrbol o lletós indica una deshidratació inadequada).
2. La deshidratació es pot aconseguir augmentant les concentracions d'alcohol etílic.
3. Les mostres es poden transferir d'alcohol al 99% o alcohol absolut a un agent clarificador.

Procediment:

1. Plaçatz els flebòtoms adults en etanol al 70%.
2. Treure l'etanol i substituir-lo per KOH al 10%. Cobrir els flebòtoms amb un portaobjectes de vidre.
3. Macerar fins que els insectes es tornin transparents.
4. Treure el KOH.
5. Cobrir la mostra amb aigua destil·lada i esperar de 30 a 45 minuts.
6. Traieu l'aigua i repetiu el rentat amb aigua destil·lada durant 30 min (funció del temps de nombroses mostres: com més mostres hi hagi per processar juntes, més llarg s'ha de respectar aquest temps. Com menys n'hi hagi, especialment per a les tractades individualment, més curt pot ser aquest temps).
7. Traieu l'aigua.
8. Afegiu la solució de Marc-André (potencialment acolorida amb àcid fucsina) i espereu 24 hores (un dia).
9. Traieu la solució de Marc-André.
10. Cobriu la mostra amb aigua destil·lada i espereu de 30 a 45 min.
11. Traieu l'aigua i repetiu el rentat amb aigua destil·lada durant 30 min.
12. Traieu l'aigua.
13. Afegiu etanol al 70% i disseccioneu la mostra.

a. Per al cap i l'abdomen, estireu suaument el cap o l'abdomen del tòrax.

b. Per al tòrax, traieu les ales subjectant el tòrax amb un parell de pinces i estirant la base dels apèndixs amb un altre parell. És possible fer una dissecció sagital, dividint el tòrax en costats esquerre i dret, depenent de les regions de més interès.

14. Deshidrateu gradualment mitjançant una sèrie de solucions aquoses d'alcohol etílic. 50 – 80 – 95% fins que s'assoleixi etanol absolut.
15. Deshidrateu les mostres rentant-les dues vegades, 10 minuts cadascuna, amb etanol al 100%.
16. Traieu l'etanol i cobriu les mostres amb oli de clau durant 15 minuts a temperatura ambient.
17. Transferiu les mostres de l'oli de clau a una gota d'Euparal® o bàlsam del Canadà en un portaobjectes net.
18. Col·loqueu-les com vulgueu: El cap, el tòrax i l'abdomen del flebòtom es poden disseccionar amb agulles fines o pinces sota un microscopi de dissecció. El cap s'ha de disseccionar del cos per poder muntar-lo en posició ventrodorsal, és a dir, el foramen occipital ha d'estar orientat cap amunt per tal que el cibari es pugui observar directament a través d'ell.

La dissecció es duu a terme en el medi de muntatge per a flebòtoms.

19. Deixeu la mostra fins que la superfície es torni enganxosa.
20. Mulleu un cobreobjectes net amb alcohol absolut. Deixeu caure el cobreobjectes sobre el bàlsam del Canadà en angle.
21. Guardeu els portaobjectes en una caixa seca destinada a aquest propòsit.
