# Supplementary material for: Processing and mounting phlebotomine sand flies: a consensus guideline
Source: Parasite. 2026 Apr 3;33:18. doi: 10.1051/parasite/2026009 (PMC13047900; doi:10.1051/parasite/2026009)
Supplement: Supplementary file 5 — Chinese translation / 翻译成中文 [file parasite-33-18-s5.pdf]

Parasite **33**, 18 (2026)
© F.J. Randrianambinintsoa et al., published by EDP Sciences, 2026
https://doi.org/10.1051/parasite/2026009

PARASITE
Available online at:
www.parasite-journal.org

REVIEW ARTICLE OPEN ACCESS

白蛉标本处理与制片：专家共识的技术指南

Fano José Randrianambinintsoa[1], Laure Augendre[1], Jorian Prudhomme[1], Jean-Philippe Martinet[1], Mathieu Loyer[1], Nalia Mekarnia[1], Hocine Kerkoub[1], Farzana Khan Perveen[1], Antoine Huguenin[1,2], Emilie Kariya[1,2], Mohammad Akhoundi[3], Andrey José de Andrade[4], Eduardo Berriatua[5], Gioia Bongiorno[6], Sébastien Boyer[7,8], Vasiliki Christodoulou[9], Magda Clara Vieira Da Costa-Ribeiro[10], Lucas Alexandre Farias de Souza[10], Huicong Ding[11], Blaise Dondji[12], Vít Dvořák[13], Ozge Erisoz Kasap[14], Eunice Aparecida Bianchi Galati[15], Montserrat Gállego[16], Cristina Ballart[16], Stavroula Gouzelou[17], Nabil Haddad[18], Rezki Sabrina Masse[19], Asrat Hailu Mekuria[20], Vladimir Ivovic[21], Szymon Kaczmarek[22], Mohd Khadri Shahar[19], Oscar D. Kirstein[23], Edwin Kniha[24], Iva Kolářová[13], Lincoln Timinao[25], Cristian Lucanas[26], Ognyan Mikov[27], Kimsear Nov[7], Yusuf Özbel[28], Bernard Pesson[29], Laura Cristina Posada Lopez[30], Didot Budi Prasetyo[1,7], Nil Rahola[31], Eduardo A. Rebollar-Tellez[32], Bruno Leite Rodrigues[15], Lalita Roy[33], Prasanta Saini[34], Chizu Sanjoba[35], Paloma Helena Fernandes Shimabukuro[36], Padet Siriyasatien[37], Agnieszka Soszyńska[22], Tatiana Suleşco[38], Massamba Sylla[39], Majhalia Torno[40], Petr Volf[13], Khamsing Vongphayloth[41], Vu Sinh Nam[42], April Wardhana[43], Eric Yessinou[44], Sonia Zapata[45], Jean-Charles Gantier[1], and Jérôme Depaquit[1,2,*]

[1] Faculté de Pharmacie, Université de Reims Champagne Ardenne, UR ESCAPE-USC ANSES PETARD, 51 rue Cognacq-Jay, 51096 Reims Cedex, France
[2] Pôle de Biologie territoriale, Laboratoire de Parasitologie-Mycologie, Centre Hospitalo-Universitaire, 51092 Reims, France
[3] Parasitology-Mycology Department, Avicenne Hospital, AP-HP, Bobigny, Sorbonne Paris Nord University, France; Unité des Virus Émergents (UVE: Aix-Marseille Univ, Università di Corsica, IRD 190, Inserm 1207, IRBA), 13005 Marseille, France
[4] Parasitology Collection of Basic Pathology, Department of Basic Pathology, Federal University of Paraná, Curitiba 19031, Brazil
[5] Department of Animal Health, University of Murcia, Campus de Espinardo, 30100 Espinardo, Murcia, Spain
[6] Department of Infectious Diseases, Vector-borne Diseases Unit, Istituto Superiore di Sanità, 00166 Rome, Italy
[7] Medical and Veterinary Entomology Unit, Institut Pasteur du Cambodge, Phnom Penh 12201, Cambodia
[8] Ecology & Emergence of Arthropod-borne Pathogens Unit, Department of Global Health, Institut Pasteur, CNRS UMR2000, 75015 Paris, France
[9] Section Veterinary Services (1417), Laboratory for Animal Health Virology, Aglantzia, Nicosia 2109, Cyprus
[10] Insects Vectors and Parasites Laboratory, Department of Basic Pathology and Postgraduate program in Microbiology, Parasitology and Pathology, Federal University of Paraná, 81530-900 Curitiba, Brazil
[11] Department of Biological Sciences, National University of Singapore, 117558, Singapore
[12] Laboratory of the Leishmaniasis Research Project, Mokolo District Hospital, Mokolo, Cameroon; Laboratory of Cellular Immunology and Parasitology, Department of Biological Sciences, Central Washington University, 98926 Ellensburg, WA, USA
[13] Department of Parasitology, Faculty of Science, Charles University, 12800 Prague, Czechia
[14] VERG Laboratories, Department of Biology, Faculty of Science, Hacettepe University, Beytepe, Ankara 06800, Türkiye
[15] Faculdade de Saúde Pública da Universidade de São Paulo (FSP/USP), Pós-graduação em Saúde Pública, 01246-904 São Paulo, Brazil
[16] Secció de Parasitologia, Departament de Biologia, Sanitat i Medi Ambient, Facultat de Farmàcia i Ciències de l'Alimentació, Universitat de Barcelona, & Institut de Salut Global de Barcelona (ISGlobal), Centro de Investigación Biomédica en Red, Enfermedades Infecciosas (CIBERINFEC), 08028 Barcelona, Spain
[17] Laboratory of Infectious Diseases and Public Health, School of Medicine, University of Cyprus, Nicosia, Cyprus & Department of Pediatrics, Archbishop Makarios III Hospital, Nicosia 2115, Cyprus
[18] Faculty of Health Sciences, American University of Beirut, 1107 2020 Beirut, Lebanon
[19] Medical Entomology Unit, Infectious Disease Research Centre, Institute for Medical Research (IMR), National Institutes of Health (NIH), Ministry of Health Malaysia, 40170 Shah Alam, Selangor, Malaysia
[20] School of Medicine, Addis Ababa University, 28017 - 1000 Addis Ababa, Ethiopia
[21] Faculty of Mathematics, Natural Sciences and Information Technologies, University of Primorska, 6000 Koper, Slovenia
[22] University of Lodz, Faculty of Biology and Environmental Protection, Department of Invertebrate Zoology and Hydrobiology, Banacha 12/16, 90-237 Łódź, Poland
[23] Laboratory of Entomology, Ministry of Health, 9134302 Jerusalem, Israel
[24] Center for Pathophysiology, Infectiology and Immunology, Institute of Specific Prophylaxis and Tropical Medicine, Medical University

Edited by Jean-Lou Justine
*Corresponding author: jerome.depaquit@univ-reims.fr

This is an Open Access article distributed under the terms of the Creative Commons Attribution License (https://creativecommons.org/licenses/by/4.0), which permits unrestricted use, distribution, and reproduction in any medium, provided the original work is properly cited.

Vienna, Kinderspitalgasse 15, 1090 Vienna, Austria
[25] Papua New Guinea Institute of Medical Research (PNGIMR) Institute, PO Box 60, Headquarter, Homate Street, 441 Goroka, Eastern Highlands Province, Papua New Guinea
[26] Museum of Natural History, University of the Philippines Los Baños, 4031 Laguna, Philippines
[27] National Centre of Infectious and Parasitic Diseases, 1504 Sofia, Bulgaria
[28] Ege University, Faculty of Medicine, Department of Parasitology, 35040 Bornova/Izmir, Türkiye
[29] Retired, Faculté de Pharmacie, Université de Strasbourg, Strasbourg, 67400 Illkirch-Graffenstaden, France
[30] Program for the Study and Control of Tropical Diseases (PECET), Faculty of Medicine, University of Antioquia, 050010 Medellin, Colombia
[31] MIVEGEC, Univ. Montpellier, CNRS, IRD, 34394 Montpellier, France & Medical Entomology Unit, Institut Pasteur de Madagascar, 101 Antananarivo, Madagascar
[32] Laboratorio de Entomología Médica, Departamento de Zoología de Invertebrados, Facultad de Ciencias Biológicas, Universidad Autónoma de Nuevo León, San Nicolás de los Garza, 66455, NL, México
[33] Tropical and Infectious Disease Centre, BP Koirala Institute of Health Sciences, Dharan 56700, Nepal
[34] ICMR-Vector Control Research Centre, Puducherry 605006, India
[35] Graduate School of Agricultural and Life Sciences, The University of Tokyo, Tokyo 113-8657, Japan
[36] Grupo de estudos em Leishmanioses/Coleção de Flebotomíneos (COLFLEB/Fiocruz-MG), Instituto René Rachou, Fundação Oswaldo Cruz, Belo Horizonte, Minas Gerais, 30190009, Brazil
[37] Center of Excellence in Vector Biology and Vector-Borne Disease, Department of Parasitology, Faculty of Medicine, Chulalongkorn University, Bangkok 10330, Thailand
[38] Department of Arbovirology, Bernhard Nocht Institute for Tropical Medicine, Bernhard Nocht Str. 74, 20359 Hamburg, Germany [39] Laboratory Vectors & Parasites, Department of Livestock Sciences and Techniques, Sine Saloum University El Hadji Ibrahima Niasse (SSUEIN) Kaffrine Campus, C.P. 24600, Senegal.
[40] Environmental Health Institute, National Environment Agency, Singapore 138667, Singapore & Department of Biological Sciences, National University of Singapore, 117558 Singapore
[41] Institut Pasteur du Laos, Laboratory of Vector-Borne Diseases, Samsenhai Road, Ban Kao-Gnot, Sisattanak District, 3560 Vientiane, Lao PDR
[42] National Institute of Hygiene and Epidemiology, 1 Yec-Xanh Street, Hai Ba Trung District, 100000 Hanoi, Vietnam
[43] Indonesian Research Center for Veterinary Science, Indonesian Agency for Agricultural Research and Development, Ministry of Agriculture Republic Indonesia, Bogor 16114, Indonesia & Department of Parasitology, Faculty of Veterinary Medicine, Airlangga University, Surabaya 60115, Indonesia
[44] Laboratory of Research in Applied Biology, Polytechnic School of Abomey-Calavi, University of Abomey-Calavi, 01 P.O. Box 2009, 00000 Cotonou, Benin
[45] Instituto de Microbiología, Colegio de Ciencias Biológicas y Ambientales (COCIBA), Universidad San Francisco de Quito (USFQ), 170901 Quito, Ecuador

Received 1 December 2025, Accepted 29 January 2026, Published online 3 April 2026

摘要

本文为白蛉标本的处理与制片提供了一套系统而全面的技术指南，这些技术对于物种鉴定以及病原体的检测与分离至关重要。文章汇集并讨论了适用于野外条件与实验室内的多种技术方法。指南对白蛉的采集、操作处理、覆盖与安死处理（本文建议采用干冻冷冻干燥技术或二氧化碳处理，而非化学试剂处死）作了详细说明，同时介绍了多种标本保存方案，如低温保存与乙醇（ethanol）保存。文中强调，某些关键解剖结构（如生殖器、头部及翅）的制备素质会直接影响显微观察效果，并对此给出了具体技术要点。文章还详细描述标本处理流程，包括使用氢氧化钾及随后采用 Marc-André 溶液进行透明化处理。在封片方面，文中比较了多种封片介质，重点分析其光学性能与长期保存潜力。Hoyer 封片溶液（又称氯醛树胶）因透明度高，适用于快速观察，尤其受精囊等结构的检查，但不适宜长期保存。其他封片介质还包括聚乙烯醇介质（polyvinyl alcohol）、Euparal®（有限耐水性）、以及加拿大胶（Canada balsam；烃溶性封片介质），其中后两者更适合标本的长期保存。此外，文章还介绍了分子生物学新技术方法，如 DNA 测序与 MALDI-ToF 鉴定技术；这些方法对标本处理与保存条件具有较高要求。为便于白蛉标本处理与制片技术推广与统一化，指南还提供多种制片技术的简短教学视频，并附有 33 种语言译本，以满足全球科研人员的多样需求。

关键词：

制片，白蛉，Hoyer 封片溶液，Marc-André 溶液，氯醛树胶，聚乙烯醇，Euparal®，加拿大胶，利什曼原虫分离，野外条件，培养，解剖，分子生物学，MALDI-ToF，模式标本

Abstract – Abstract – This article provides a comprehensive guide for the processing and mounting of phlebotomine sand fly specimens, which is crucial for species identification and pathogen detection and isolation. It discusses a range of techniques suitable for both field and laboratory settings. The guide includes detailed instructions on sand fly collection, handling, covering, and euthanasia (recommending dry freezing or CO_2 over chemicals) as well as conservation strategies, such as cold storage and preservation in ethanol. The quality of preparation of certain anatomical structures (genital organs, head and wings) is essential for their proper microscopic observation and is described in this work. The article also presents detailed sample processing, including the clearing process with agents such as potassium hydroxide then Marc-André solution. The mounting process compares different media, emphasizing their optical properties and preservation potential. Hoyer fluid (also known as chloral gum) is recommended for quick observation, particularly for spermathecae, due to its clarity, although it is not suitable for long-term storage. Other media discussed include polyvinyl alcohol, Euparal® (for limited water tolerance), and Canada balsam (a hydrocarbon-soluble medium), with the latter two offering long-term preservation capabilities. Innovative molecular biology approaches such as DNA sequencing and MALDI-ToF, which require particular attention to sample processing, are also addressed. Furthermore, short video clips illustrating various mounting techniques as well as translations in many different languages are provided, allowing the guideline to reach the diverse needs and expectations of the global scientific community.

Key words: Mounting, Phlebotomine sand fly, Hoyer fluid, Marc-André solution, Chloral gum, Polyvinyl alcohol, Euparal®, Canada balsam, *Leishmania* isolation, Field conditions, Culture, Dissection, Molecular biology, MALDI-ToF, Type-specimens.

引言

白蛉为双翅目、蛾蚋科（Psychodidae）、白蛉亚科（Phlebotominae）的小型昆虫，目前已知物种多于 1,063 种[21]。白蛉是多种病原体的重要传播媒介，包括利什曼原虫（*Leishmania*）、虫媒病毒 arboviruses）及巴尔通体（*Bartonella*），分别可引起利什曼病、虫媒病毒感染和巴尔通体病。对白蛉的鉴定主要依赖于显微镜下的精细形态观察，而这取决于仔细采集、适当保存及标准化制片技术。所上述的流程步骤涉及多种专门技术方法，各有其优势与局限。

成虫白蛉的鉴定需要同时观察外部结构（如触角（antennae）、下颚须（palpi）、雄性生殖器）和内部结构（如咽部（pharynx）、口腔（cibarium）及受精囊（spermathecae））。对后者进行解剖与分离有助于结构清晰呈现，从而提高鉴定准确性。因此，与蚊或锥蝽不同，白蛉在鉴定前通常需要把标本置于载玻片与盖玻片之间，进行制片处理。

在 20 世纪 80 年代之前，显微形态观察是白蛉鉴定的唯一方法，至今仍为最常用技术。因此，当时的处理与制片程序相对简单，主要在两类方法之间权衡：一类为可实现长期保存的永久性封片方法，另一类为用于快速鉴定但不适于长期保存的快速制片方法。例如，采用树脂类封片溶液（如加拿大胶）进行永久封片虽可长期保存标本，但过程耗时，且需对样本进行完全脱水处理。同时，该类封片介质的折射率（refractive index）并不总是有利于受精囊结构的清晰观察。相比之下，采用水性介质（如 Hoyer 封片溶液）制片速度较快，且更有利于观察具有折光性的受精囊结构，但由于该类介质容易吸收空气中的水分，通常不适于长期保存。为了克服这一限制，科研人员可选措施之一是在制片完全干燥后以指甲油封固载玻片边缘。两类方法的利与弊至今仍然存在，制片方法的选择通常取决于标本制备的具体目的。

自 20 世纪 80 年代以来，白蛉鉴定研究逐步将形态学方法与生化方法相结合。早期应用的方法为表皮烃类分析技术，随后很快被分子生物学检测技术所取代，如随机扩增多态性 DNA（random amplified polymorphic DNA（RAPD））、限制性片段长度多态性（restriction fragment length polymorphism（RFLP））、DNA 扩增与 Sanger 测序，以及新一代测序技术（next-generation sequencing（NGS））。目前，分子方法又进一步结合蛋白质组学技术，如

MALDI-ToF。此外，基于 PCR 的分子鉴定还可同时用于病原体检测（如利什曼原虫、锥虫、巴尔通体及白蛉病毒），且均可通过终点 PCR 与实时 PCR 实现检测，因此需要根据既定研究目标对采样与保存程序进行相应调整[3, 32]。除了传统用于物种鉴定的形态特征外，科研人员还可采用其他形态学分析方法，如翅形几何形态测量（wing geomorphometry）。

本研究主要基于作者的实践经验及已发表的科学文献资料，旨在提出一套用于白蛉成虫标本处理与制片的统一化技术指南，以优化形态学与分子生物学分析。科研人员需备注，某些检测方法（如分子生物学分析或 MALDI-ToF）需保留部分不参与形态鉴定的虫体形态特征，因此在实验方案设计过程中需审慎选择处理程序。

本文重点介绍对白蛉活体样本的麻醉与安死处理方法、保存方式以及制片程序，涵盖用于快速鉴定及用于长期保存并支持后续研究的两类应用目的。

前言说明：安全与法规要求应参考相关的安全数据表（Safety Data Sheets（SDS））

本指南中涉及的所有化学试剂均须在严格的安全条件下操作。各研究机构的健康与安全委员会（health and safety committees）可提供有关相关化学品危害性、操作规范及废弃物处置流程的指导信息。同时，科研人员必须严格遵守试剂使用与处置的安全规范。所有使用者均有责任确保符合良好且安全的实验室操作规范，以及遵循所在国家或研究机构适用的法律法规。此外，部分化学品或其成分（如水合氯醛（chloral hydrate））在某些国家属于受管制物质。本文所用缩略语见表 1。

表 1： 缩略语表（**Table 1:** List of abbreviations）

缩略语	英文翻译	中文翻译
**BME**	Basal medium Eagle	基础 Eagle 培养基
**CDC**	Centers for Disease Control and Prevention	美国疾病控制与预防中心
**CMCP**	Camphor-monochlorophenol	樟脑-单氯苯酚
**CMR**	Carcinogenic, mutagenic, reprotoxic substance	致癌、致突变及生殖毒性物质
**COI**	Cytochrome c oxidase subunit I gene	细胞色素 c 氧化酶亚基 1 基因
**CytB**	Cytochrome b gene	细胞色素 b 基因
**DNA**	Deoxyribonucleic acid	脱氧核糖核酸
**ELISA**	Enzyme-linked immunosorbent assay	酶联免疫吸附试验
**EtOH**	Ethanol	乙醇
**M199**	Medium 199	199 培养基
**MALDI-ToF MS**	Matrix-assisted laser desorption/ionization time-of-flight mass spectrometry	基质辅助激光解吸/电离飞行时间质谱
**MEM**	Minimum essential media	MEM 培养基
**NGS**	Next-generation sequencing	新一代测序技术
**NNN**	Novy-MacNeal-Nicolle medium	Novy-MacNeal-Nicolle/三 N/三恩 培养基
**PCR**	Polymerase chain reaction	聚合酶链式反应
**Lao PDR**	Lao People's Democratic Republic	寮国 / 老挝人民民主共和国
**PNOC**	Prepronociceptin gene	-
**qPCR**	Quantitative PCR (real-time PCR)	实时 PCR
**RAPD**	Random amplified polymorphic DNA	如随机扩增多态性 DNA
**RFLP**	Restriction fragment length polymorphism	限制性片段长度多态性
**RI**	Refractive index	折射率
**RNA**	Ribonucleic acid	核糖核酸
**RNases**	Ribonucleases	核糖核酸酶
**RNASS**	RNA stabilization solution	RNA 稳定保存溶液
**RT-PCR**	Reverse transcription PCR	反转录 PCR
**TFA**	Trifluoroacetic acid	三氟乙酸

1. 白蛉的采集

成虫白蛉可采用多种方法进行采集，各方法可分别采集活体或已死亡的标本。常用方法包括 CDC 微型诱光灯诱捕器（CDC miniature light traps）、黏纸黏捕法（sticky traps）、或者从 Shannon 诱捕帐（Shannon traps）或直接从环境中的静息场所（如畜舍）进行吸捕采集。这些方法通常在适宜生态环境中布设诱捕器，利用光源或其他诱引物（如二氧化碳或化学诱剂）吸引白蛉，采集样本后再进一步后续分析。相关技术已在多项研究中有所报道[2, 3, 32, 36, 49]。采集活体白蛉可满足所有后续分析需求，而仅采集死亡个体则无法进行利什曼原虫或病毒株的分离。部分采集方法（如黏纸黏捕法法）在实际操作中常导致白蛉部分器官（触角、下颚须、翅或足）缺失。此外，黏纸表面的蓖麻油涂层易附着于白蛉体表面，在标本处理初期必须予以清除，通常可采用一对一乙醇（ethanol）与二乙醚（diethyl ether）混合液浸泡约 15 分钟进行处理。

2. 标本的安死处理

白蛉采集后，活体个体必须进行安死处理。某些采集方法（如黏纸黏捕法，或配备装有洗涤剂或乙醇收集瓶的 CDC 诱光灯诱捕器）在采集过程中即可使白蛉死亡。分子生物学可应用于那些直接收集于乙醇中的样本，以及若能尽快存入乙醇的其他样本。然而，上述安死方法均不适用于 MALDI-ToF 分析。此外，一些安死方法可能导致某些形态特征的缺失。因此，为确保物种鉴定的准确性以及凭证标本（即用于未来参考或比较而长期保存的标本；voucher specimens）的长期保存，选择合适且标准化的安死方法极其关键。常用的化学安死剂包括乙酸乙酯（ethyl acetate）、乙醚（ethyl ether）、四氯乙烷（tetrachloroethane）及氯仿（chloroform）。科研人员可将上述液体浸透棉棉花球后放置于装有白蛉的采集容器中进行安死处理。鉴于上述试剂均具有一定毒性，使用时须严格遵循生产厂商的安全操作建议。然而，基于作者经验，并不推荐使用氯仿对白蛉进行安死处理，因为氯仿与分子生物学研究的兼容性较差。综合考虑这些化学试剂的潜在毒害害性及其在分子分析中的局限性，本文总体上不建议采用化学试剂作为标准安死手段。

目前应用最广泛且能够较好保存白蛉形态结构、DNA 、及蛋白质的安死方法为干冻冷冻干燥技术处理。安死时间应足以使白蛉完全麻醉，但不宜过长，以避免：（i）标本发生过度干燥；或（ii）在拟从白蛉消化系统分离利什曼原虫时，影响原虫存活性。**因此，本文建议在 −20 ° C 条件下冷冻 15 - 20 分钟，并定期检查样本状态，以确保白蛉仅麻醉而不致影响利什曼原虫的活性。**

在缺乏冷冻条件的情况下，可采用二氧化碳（CO_2）进行安死处理。在野外条件下若无法使用 CO_2 钢瓶，可使用常见于苏打水抢（‘soda siphons’）中常用的小型商用 CO_2 容气瓶，但需注意其在航空运输中可能受到限制。作为最后手段，可通过烟草烟雾法对白蛉进行安死处理。具体方法为：将活体白蛉通过 CDC 诱捕器采集后，用吸捕器收集并保留于吸捕器玻璃管中，随后释放烟草烟雾将白蛉在数秒内安死。该方法适用于各种野外条件，即使在偏远或条件受限的环境中亦可实施。需要注意的是，由于玻璃器具会吸附烟雾残留，未经彻底清洗前不宜再次用于活体白蛉的采集与操作。然而，同一未经清洗的吸虫器仍可继续用于对白蛉进行安死处理并用于后续标本保存。此外，操作过程中须确认所有白蛉个体已从吸捕器中完全取出。上述安死方法均与通过解剖白蛉消化道进行利什曼原虫分离的研究目的相兼容。

3. 处理前的标本保存

在进行后续处理前，标本保存主要有四种方法：

3.1 冷冻保存

该方法通常在 −20 ° C 条件下进行，或更理想地在 −80 ° C 条件下保存。目前，这类冷冻保存方式比液氮保存方式更为常用。无论采用何种冷冻方式，均应在白蛉被麻醉后尽快实施低温保存。使用冰箱冷冻的优点在于可较完整地保存白蛉本体，同时在整个保存期间较好地维持 RNA、DNA 和蛋白质的质量。相比之下，液氮保存对白蛉形态能造成明显损伤，常见情况包括翅、足、下颚须及触角的损坏甚至断裂，有时会导致关键形态特征缺失。干冻冷冻干燥保存对标本的损伤较小，但对于保存其脆弱易损的结构仍不够理想。值得注意的是，在解冻过程中，由于冷凝过程，翅、触角、下颚须或足可能黏附于保存容器内壁，进而在取出时发生撕裂或脱落。此外，在野外研究条件

This is an Open Access article distributed under the terms of the Creative Commons Attribution License (https://creativecommons.org/licenses/by/4.0), which permits unrestricted use, distribution, and reproduction in any medium, provided the original work is properly cited.

下，冷冻保存并非总是可行，因为其需要配备冷冻设备或液氮容器。总体而言，冷冻保存与基于分子技术的病原体检测完全兼容，且不会降低检测灵敏度。但对于 RNA 病毒的检测与分离，则应在 −80 ° C 或液氮条件下长期保存样本。

然而，仅通过常规冷冻保存的标本通常不适用于通过解剖消化系统分离利什曼原虫。例外情况是：若先将白蛉置于液氮气相中预冷，随后再转入液氮中保存（例如将样本置于小瓶罐中再放入网袋内），可在一定程度上模拟利什曼原虫的低温保存条件，从而为后续分离。

3.2 酒精保存（乙醇或异丙醇；ethanol or isopropyl alcohol）

酒精保存可能是目前最常用的白蛉保存方法，且在野外条件下易于实施，即使在缺乏实验室条件的环境中也可操作。酒精保存尤其适用于形态学研究，因为在保存容器内无气泡的情况下，白蛉的脆弱器官（如翅、足、触角或下颚须）通常能够完整保存。因此，建议在保存容器内放置一小团棉花以去除气泡，并在棉塞上方放置标签（图 1）。

适宜的酒精浓度仍存在一定争议。一般不建议使用低于 70% 的酒精浓度[45, 66]。较高浓度的酒精有利于 DNA 的长期保存，但会使标本在形态学处理过程中更加脆弱和易断裂。使用 96% 乙醇（共沸混合物；azeotrope mixture）可保持浓度稳定，尤其适用于热带等高潮湿地区。不过，95% 乙醇通常更易获取。总体而言，乙醇对 DNA 的保存效果较好（但相较冷冻保存仍略逊一筹，尤其是在 NGS 等分子分析中），而对蛋白质的保存效果则明显较差，特别是在蛋白质组学研究（如 MALDI-ToF 应用）中。在酒精中保存数月的白蛉仍可进行形态学鉴定，但难以从这些标本中获得用于建立参考数据库的蛋白质组学谱图。若将酒精保存与 −20 ° C 冷冻相结合，可在一定程度上提高保存效果。−20 ° C 冷冻主要通过减缓降解代谢过程来改善生物分子的保存（如核酸），同时也可在一定程度上减少组织分解过程，从而间接有利于形态结构的保存，不过其对形态保存的改善程度不及对分子保存的影响明显。

在短期保存（通常不超过数月）且乙醇浓度不低于 70% 的条件下，酒精保存也可用于 DNA 及 RNA 病毒的检测。此外，在某些国家异丙醇更易获得，其对 DNA 保存 亦具有良好的效果，但会使标本变得较为坚硬而脆弱。备注，异丙醇不具易燃性，因此在运输方面较乙醇更为便利。如有需要，先前以液氮或干冻冷冻方式保存的白蛉也可转移至酒精中保存，但此举可能同时叠加两种保存方法的局限性。

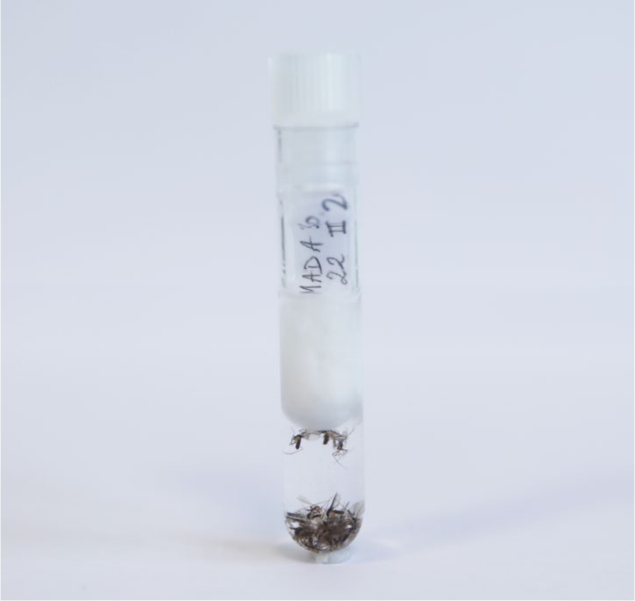

图 1：乙醇保存的白蛉标本。

3.3 RNA 稳定保存液（RNA stabilization solution; RNASS）保存

RNA 稳定保存液（RNA stabilization solution; RNASS）是一种广泛应用的水性试剂，具有无毒特点，专门用于稳定并保存新鲜组织和细胞样本中的 RNA。该试剂可迅速渗透进入标本并灭活核糖核酸酶（RNases），从而在无需立即冷冻的情况下有效防止 RNA 降解。采用 RNASS 保存通常能够较好地维持组织及细胞的整体形态结构，适用于后续组织学观察。尽管 RNASS 的主要作用在于 RNA 稳定而非组织固定保存，但在短期至中期保存过程中，标本的结构完整性通常仍能得到较好保存。使用 RNASS 时，样本可在室温条件下保存约 7 天，在 4 ° C 条件下保存数周，或在 −20 ° C / −80 ° C 条件下进行长期保存。因此，在野外调查或临床环境中，尤其是在冷链条件受限的情况下，该方法具有较高应用价值。在进行 RNA 提取时，通常需先将样本从保存液中取出，并按照常规实验流程进行后续处理。

3.4 室温干燥保存

这是一种较早期的保存方法。若将干燥保存方法用于白蛉整体标本（整虫），其主要缺点是对白蛉脆弱器官（如翅、足、触角及下颚须）的保存效果较差。然而，在使用干燥硅胶进行固定与脱水处理的情况下，仍可开展基于 MALDI-ToF 的蛋白质组学研究。相比之下，室温干燥保存的标本在分子分析方面（尤其是针对 DNA 的研究）通常较为困难，因为 DNA 往往已片段化且质量较低，因此相关分析比新鲜或冷冻保存样本更具挑战性，特别是在核基因组研究中。不过，近年来发展的一些技术，如博物馆标本基因组学(museomics)，在一定程度上可应用于此类样本[34]。因此，除非无其他可行替代方法，一般不推荐采用该保存方式。该方法也可与低温保存结合使用，即将干燥保存的标本置于 −20 °C 或 −80 °C 冷冻条件下进一步保存。此类样本在后续处理中面临的主要困难，是如何实现标本整体或用于鉴定的关键部位的有效制片。为此，需首先进行复水处理。本文推荐使用 Triton X-100 的溶液进行复水，复水时间可从数小时至数天不等，并应在过程中进行密切监督。待标本完全复水后，应依次在清水浴中进行三次冲洗处理。

3.5 滤纸保存

滤纸保存的主要优点在于，能让未固定或已经干燥处理的整虫标本和血细胞中的基因组 DNA 可在室温条件下长期且稳定保存。滤纸通常制成小型卡片形式，因此可在仅相当于一个小抽屉大小的空间内，于室温条件下存放数百份样本，便于保存与管理。该类滤纸基质通常预先浸渍可使病原体灭活的化学成分，从而使样本不再被视为生物危害物。这一特性使样本在储存和运输过程中无需采取严格的生物危害防护措施，从而提高便利性[68]。

4. 标本解剖

与许多可通过观察整虫外部形态（如：直接针插保存）进行鉴定的昆虫不同，白蛉的物种鉴定需通过解剖并进行玻片制片，以观察其解剖的内、外部形态特征，从而实现准确鉴定。无论采用何种处理与封片技术，解剖技术基本相同（图 2、图 3）(https://zenodo.org/records/18198006)。

Triton X100 的使用：非离子型水溶液

需注意，本节所述制片方法适用于新鲜采集或经适当保存的标本。许多采集者所保存的昆虫样本，往往为干燥保存（用于 MALDI-ToF 分析）或长期浸存于酒精中。然而，酒精保存多年后并非理想状态，此类节肢动物标本在解剖前常会遇到一些难题。常见问题包括：存放样本的塑料容器老化降解，以及酒精逐渐挥发。在这两种情况下，标本可能因长时间浸泡于酒精中或完全干燥而难以恢复至适宜处理状态。因此，可考虑使用湿润剂（而非强效去污剂）来改善标本状态。Triton X100 为一种非离子型水溶液（亦称 *4-(1,1,3,3-tetramethylbutyl) phenyl-polyethylene glycol solution*，或 *t-octylphenoxypolyethoxyethanol，polyethylene glycol tert-octylphenyl ether*），在细胞与分子生物学中是一种广泛使用的去污剂，可用于增加细胞膜及细胞核膜的通透性。

以下为使用 0.5% Triton X100 水溶液处理标本的推荐步骤：

- 先用无水乙醇充分浸润干燥标本。
- 加入适量 0.5% Triton X100 溶液，使整个标本完全浸没。
- 处理时间约 5 分钟至数天不等，并需定期监督，直至标本在溶液中完全分散、各个体彼此分离。
- 去除 Triton X100 溶液，并更换为氢氧化钾（potassium hydroxide）溶液进行后续处理。

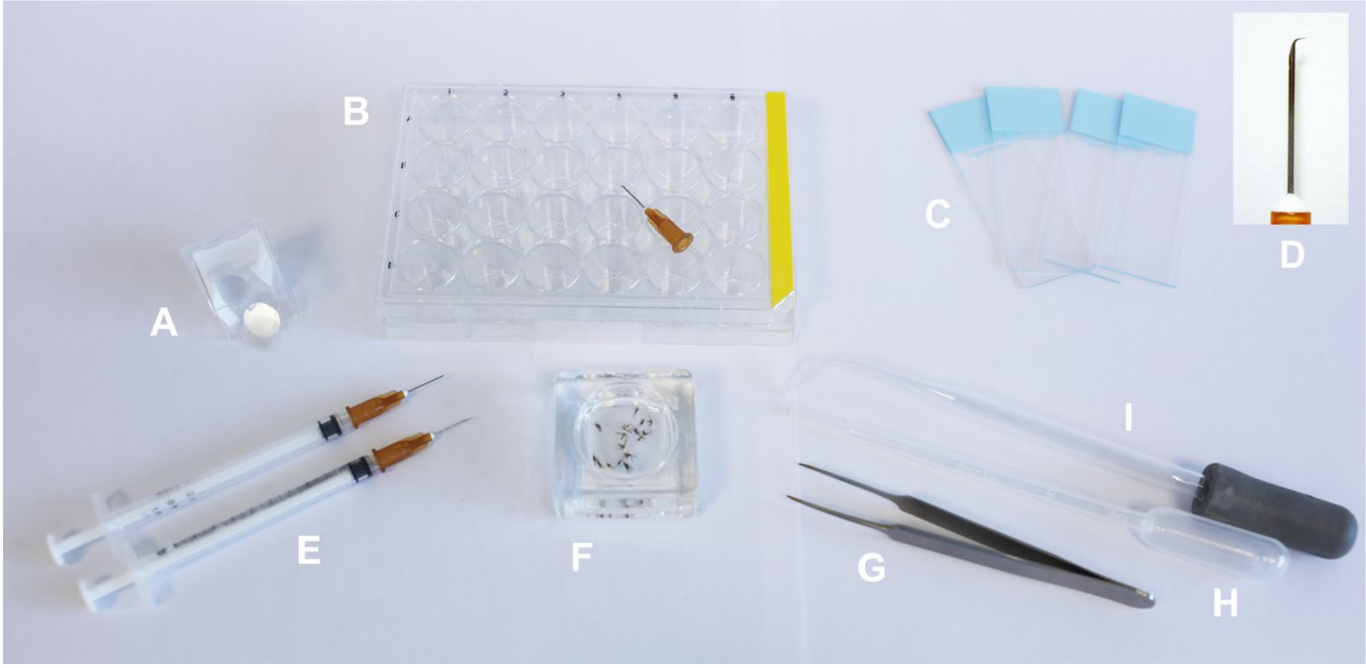

图 2：白蛉制片所需工具。A：圆形盖玻片（直径 10 或 12 mm）；B：24 孔细胞培养板及钩针（如使用丁香油（clove oil）或 Euparal® 精油处理白蛉，不应使用亚克力培养板，以免发生化学反应而损伤标本）；C：可用于标记的载玻片；D：钩针的细节；E：针头（已连接于注射器上）；F：盛放待制片白蛉的表面皿（或同类容器）；G：Dumont 镊子；H：塑料移液管/滴管（约 3-5ml），或巴斯德吸管；I：弯曲（经加热弯曲制成）的玻璃移液管，用于将液体转移至各培养板孔中。

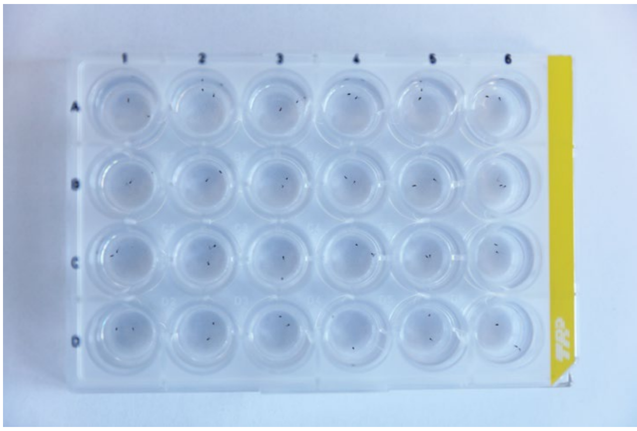

图 3： 24 孔细胞培养板示意图，每个孔中分别放置白蛉的头部及腹部末端。

4.1 头部

解剖可在体视显微镜下使用细针或昆虫针进行操作（图 2、图 3）。常用的针规格包括：26G × 1/2″（0.45 × 13 mm）、30G × 1/2″（0.3 × 13 mm）或 25G × 5/8″（0.5 × 16 mm）。

在进行物种鉴定的标本制备时，至少应将头部与虫体分离，并以腹面(ventral)朝上的方式进行制片，以便观察口腔（cibarium）和咽部（pharynx）；而胸部和腹部则在解剖后以侧面位置进行封片。将头部以腹面朝上（ventro-dorsal position）放置，可使后头大孔（occipital foramen）朝上，从而能够直接观察口腔结构。若将头部完全分离，则更有利于这些解剖结构的观察。

4.2 翅与胸部

翅应以平展状态进行封片。每侧翅可在基部将其解剖分离后分别单独封片，亦可仅取一侧翅进行制片，而将另一侧翅保留于胸部。若计划进行翅形几何形态测量（geometric morphometry）分析，则在封片前必须准确区分并标记左右翅。胸部分为多个部位，各部位均包含重要的物种鉴定信息[20, 64]。通常将胸部以侧面位置进行封片，以便观察刚毛毛序（chaetotaxy）及体色分布。在某些胸部部位存在的刚毛痕迹（bristle scars）可用于区分 *Brumptomyia* 属的部分物种。体色分布特征还可用于新热带区（Neotropical）白蛉在不同分类层级上的区分，例如属级（如 *Bichromomyia*）、

This is an Open Access article distributed under the terms of the Creative Commons Attribution License (https://creativecommons.org/licenses/by/4.0), which permits unrestricted use, distribution, and reproduction in any medium, provided the original work is properly cited.

种群系列（如 *Pintomyia*），甚至同属不同种之间的鉴别（如 *Micropygomyia*、*Nyssomyia*、*Psathyromyia* 和 *Psychodopygus*）[20]。因此，若胸部不用于分子分析，应在封片过程中尽量保持其完整，避免造成损伤。需要特别指出的是，在分类鉴定中，关键并非颜色的深浅度，而是其在胸部的分布。因此，透明化处理过程不会消除其色素或改变其分布特征。

4.3 生殖器

在对白蛉雌、雄个体进行生殖器封片时需特别谨慎，因为这些结构对于属、亚属及种级鉴定至关重要。无论雌雄，生殖器均为成对结构，在制片过程中应注意保持其完整与相对。

4.3.1 雄性生殖器

雄蛉的生殖器为外生殖器，由成对的抱器构成。每侧抱器包括背侧的上抱器基节—上抱器端节（gonocoxite–gonostyle；也称 1 和 2 节）连接结构，以及腹侧的下抱器（epandrial lobe）。上抱器端节上具有长毫结构，有时还具刚毛，这些结构应能够清晰计数，其着生位置也应明确可见。对上抱器基节内侧面的观察尤为重要，该部位有时可见一簇无柄刚毛，或附着于突起体（tubercle）上的刚毛[22]。解剖经验较少的科研人员可采用较为简单的侧位封片方法，即不需将生殖器从腹部末端完全分离（https://zenodo.org/records/18311158）。在此情况下，由于生殖器两侧结构可能发生重叠，可能会影响某些细节（如上抱器基节内侧刚毛）的计数，但可降低因解剖操作不当而损伤生殖器的风险。若采用侧位封片方式（易导致结构重叠），则必须确保标本经过充分透明化处理，以提高内部结构的可见性。对于经验较丰富的科研人员，可尝试将生殖器分开以便观察。具体操作为：使用针头斜面（如注射针）从结构间穿过，在不完全切断的情况下将其轻轻分离，从而使上抱器基节 - 上抱器端节结构分开（https://zenodo.org/records/18311158）。这样可更清楚地观察内侧面，同时也有利于观察间中附器（parameres）及阳茎结构（parameral sheaths），避免相互遮挡。

4.3.2 雌性生殖器

雌蛉的生殖器为内生殖器，主要由受精囊（spermathecae）构成。在未进行解剖的情况下，可通过腹部表皮直接观察，并将腹部以腹面朝上的方式进行封片。无论选择何种封片介质，通常均可较好地观察受精囊本体，尤其当受精囊的表面结构较为粗糙明显、且标本未经完全透明化处理。然而，对于表面光滑（不粗糙）、囊壁较薄的受精囊，在折射性较差的封片介质中观察时可能较为困难。此外，对受精囊管（spermathecal duct）基部的观察对于物种鉴定尤为关键，例如在 *Larroussius* 亚属中即是重要特征[35, 37, 38]；该类群是旧大陆利什曼原虫（*Leishmania infantum*）的主要传播媒介。若无法观察到这一结构，则难以完成准确的物种鉴定。为克服这类观察上的困难，建议将包含生殖叉（genital furca）与受精囊的结构自腹部中分离后再进行封片（https://zenodo.org/records/18311106）。在解剖过程中，受精囊通常较难直接辨认，而生殖叉相对容易定位。由于受精囊管开口于生殖叉处，通过分离生殖叉通常即可同时分离出受精囊结构。若在操作过程中不慎切断受精囊，其并不会丢失，仍可在腹部表皮内观察到（图 4）。

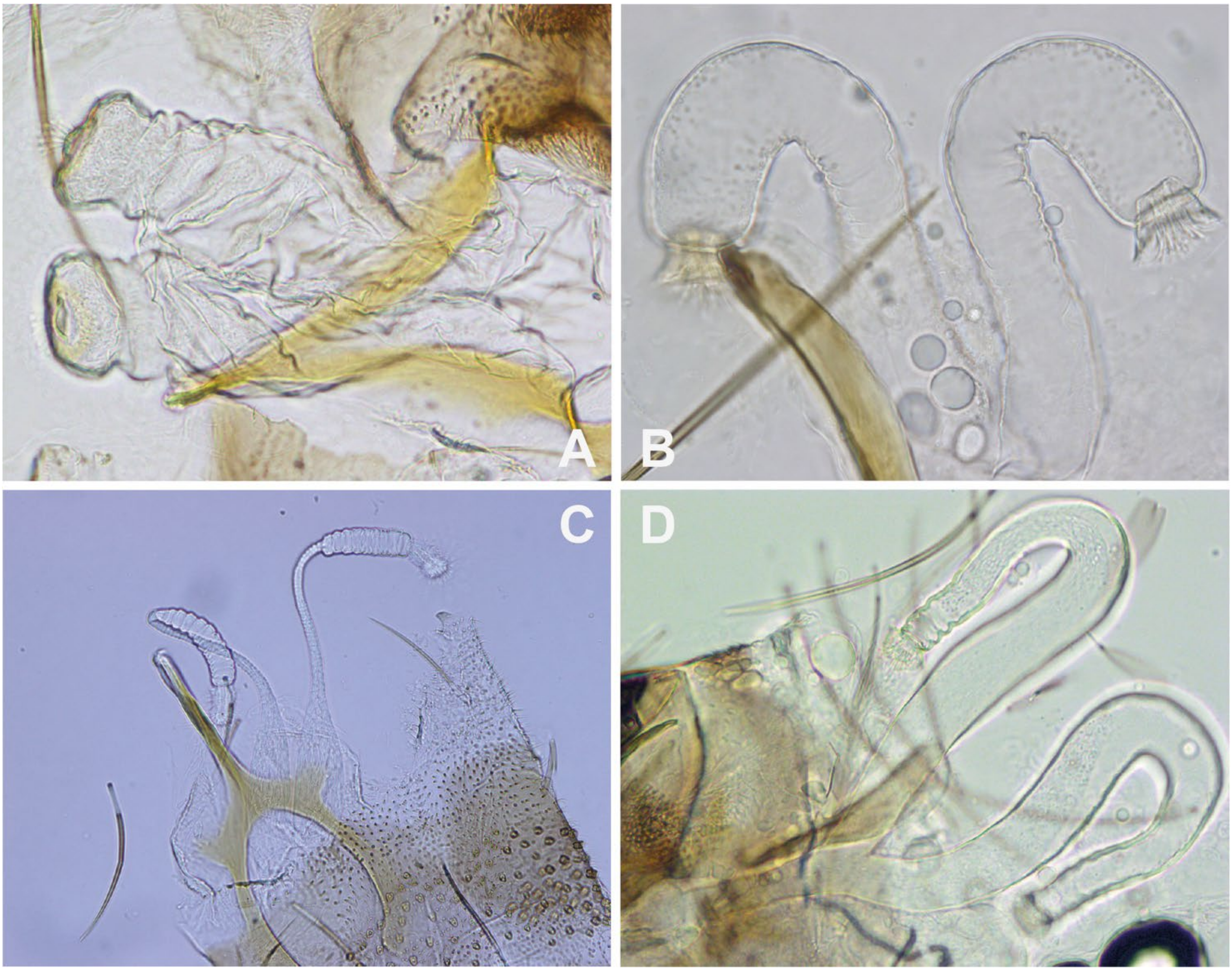

图 4： 新鲜标本解剖并以 Marc-André 溶液封片的受精囊。A：*Idiophlebotomus longiforceps*（老挝人民民主共和国）；B：*Sergentomyia minuta*（法国）；C：*Phlebotomus ariasi*（法国）；D：*Sergentomyia anodontis*（老挝人民民主共和国）。

4.4 用于利什曼原虫分离的胃（中肠）解剖

对白蛉雌虫消化道进行解剖是检测和分离利什曼原虫的关键步骤，可在野外或实验室条件下操作，以评估其传播能力。本文建议使用刚完成安死处理的雌蛉进行操作。首先用清水或含温和洗涤剂的生理盐水（saline）清洗雌蛉体表，以去除多余的体毛。该步骤有助于在分离利什曼原虫时保持无菌条件，同时尽量保留用于形态鉴定的关键结构特征。为查找并分离利什曼原虫，应小心取出中肠，并将其置于一滴无菌生理盐水（0.9% NaCl）中。在光学显微镜下观察活动的寄生虫后（推荐放大倍数约为 200×），可使用注射器或微量移液器移液管将其转移至培养基中进行培养（更多细节见第 4.4.3 节）。

头部及生殖器可直接置于 Marc-André 溶液中进行透明化处理。重要提示：必须避免 Marc-André 溶液与利什曼原虫接触—无论是直接接触，还是通过器具的间接接触——因为该溶液会导致寄生虫死亡。

雌蛉的解剖可在一张或两张载玻片上进行，两种方法各有其优缺点（图 5；https://zenodo.org/records/18311154）。

This is an Open Access article distributed under the terms of the Creative Commons Attribution License (https://creativecommons.org/licenses/by/4.0), which permits unrestricted use, distribution, and reproduction in any medium, provided the original work is properly cited.

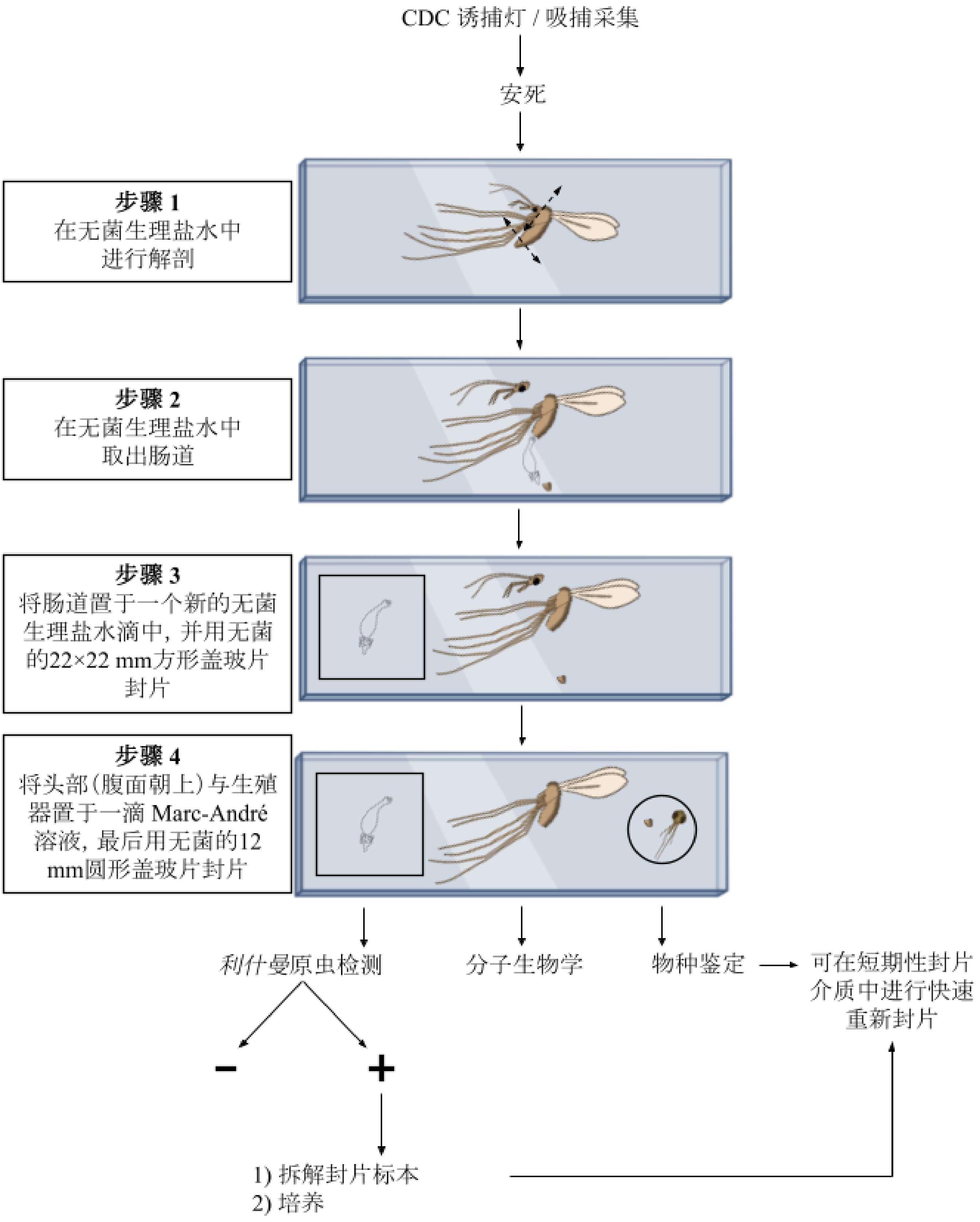

图 5：利什曼原虫分离方法示意图。

This is an Open Access article distributed under the terms of the Creative Commons Attribution License (https://creativecommons.org/licenses/by/4.0), which permits unrestricted use, distribution, and reproduction in any medium, provided the original work is properly cited.

4.4.1 双载玻片解剖法

第一种方法是使用两张独立的载玻片进行操作：一张载玻片中滴加无菌生理盐水，用于取出中肠；另一张载玻片用于将头部及受精囊置于 Marc-André 溶液中进行封片。然而，在野外条件下，通常由两至三人同时进行白蛉解剖，并将解剖所得样本交由一名研究人员统一负责物种鉴定及中肠利什曼原虫感染检测。在此情况下，同时管理两张载玻片可能会对样本的可追溯性造成困难，尤其是在发现中肠呈阳性时，可能难以准确对应回到受感染的个体（https://zenodo.org/records/18311154）。

4.4.2 单载玻片解剖法

采用单张载玻片进行操作可确保实验结果的可追溯性，但在实施过程中需注意若干关键事项。为了在操作过程中尽可能保持无菌条件，科研人员应定期使用含酒精免洗消毒液清洁双手。应使用无磨砂面的载玻片及方形盖玻片（22 × 22 mm），并以铝箔纸包裹后通过干热灭菌（Poupinel 干热灭菌箱）处理，同时每次解剖均应使用新的无菌针头（建议规格：25G，直径 0.5 mm × 16 mm）。

操作时，将白蛉置于载玻片中央的一滴无菌生理盐水中。首先切下头部，并在第 6 与第 7 腹背板（tergite）及腹板（sternite）之间切开，但应避免切断消化道（若预期受精囊较长，可在更靠前的位置切开）。随后用一根针固定胸部，另一根针轻轻牵拉腹部后端数节，将中肠缓慢抽出。若此方法未能成功，可用针固定腹部末端，从消化道前端将其牵出；若仍失败，则需尽量去除包围消化道的体壁组织（tegument），以便取出中肠。成功取出中肠后，应切断其与腹部末端节段的连接，并将中肠转移至载玻片一侧新滴加的一滴无菌生理盐水中，再轻轻覆盖无菌方形盖玻片（22 × 22 mm）。头部及腹部末端节段则转移至载玻片另一端的一小滴 Marc-André 溶液中进行处理，并确保与利什曼原虫无任何接触。头部应正确摆置（后头打孔朝上），同时按前述方法分离出包含生殖叉的受精囊结构，并覆盖小型圆形盖玻片（直径 12 mm，注意不要与用于无菌操作的方形盖玻片混淆）。其余的虫体残余部分及翅可保留在载玻片中央的生理盐水滴中（https://zenodo.org/records/18311154）。在检测结果呈阳性或需进行分类学研究时，胸部与腹部可保留用于后续分子或蛋白质组学分析，而翅则可在水性介质中进行封片。为提高封片的稳定性，可将多余的 Marc-André 溶液替换为水性封片介质，如氯醛树胶（= Hoyer 封片溶液）或基于聚乙烯醇（polyvinyl alcohol）的封片介质。

相关操作流程已有详细视频演示（白蛉中肠解剖：https://zenodo.org/records/18303014；白蛉唾液腺解剖：https://zenodo.org/records/18302850），因此本文不再对其进行详细说明。

4.4.3 白蛉胃（中肠）内利什曼原虫的分离与培养

从受感染雌蛉胃（中肠）中分离寄生虫是一项技术要求较高的操作，应先在无寄生虫的标本上进行练习以熟悉流程。解剖后，将中肠转移至一滴新的无菌生理盐水（0.9% NaCl）或 Locke 溶液中进行清洗[4]。随后，可采用两种方式进行处理：（i）在光学显微镜下观察，以识别不同发育阶段的前鞭毛体（promastigotes）及其在中肠内的分布位置，尤其应重点观察前胃瓣（stomodeal valve）区域；（ii）打开中肠以促使前鞭毛体逸出，从而便于进行规模化培养[4]。由于在野外发现受感染的白蛉个体相对少见，充分的前期操作练习有助于提高成功分离的概率。

若在中肠中观察到利什曼原虫，应更换新的无菌针头，并通过毛细作用在盖玻片边缘加入少量无菌生理盐水，使寄生虫释放出来。随后应迅速而小心地撕开中肠，使寄生虫进入盐水中。使用 100 μL 微量移液器或注射器收集寄生虫，并接种至已正确标记的培养基中。

体外（*In vitro*）培养前鞭毛体： 分离得到的寄生虫可首先在 SNB-9 血琼脂斜面（blood agar slopes）或 Novy-McNeal-Nicolle（NNN）培养基上培养[16]，并覆盖无菌 α-MEM 培养基[16, 65]或 M199 培养基。上述培养基需补充以下成分以促进寄生虫生长：10% 经热灭活的无菌胎牛血清（sterile fetal calf serum；FCS）、1% BME 维生素、2% 无菌人尿（经 Filtropur® S 0.2 μm 注射器滤膜过滤灭菌）、250 μg/mL 阿米卡星（amikacin）（或 50 μg/mL 庆大霉素（Gentamicin）），或抗生素与氨基酸混合液：L-谷氨酰胺（L-glutamine）200 mM、青霉素（Penicillin）10 000 U、

链霉素（Streptomycin）10 mg/mL）[47]。培养 3 天后，如未见生物污染，可将培养物转移至预先配制好的冷冻保存液中，并在 −80 ° C 条件下保存 1–2 年，或置于 −196 ° C 的液氮中进行长期保存，以供后续实验使用[7]。

4.5 唾液腺

对白蛉唾液腺进行解剖是研究媒介–病原相互作用（vector-pathogen interactions）的重要技术手段，尤其适用于检测虫媒病毒，如白蛉病毒属（*Phlebovirus*）（例如托斯卡纳病毒 Toscana virus）[44, 75]。由于白蛉个体极为微小，该操作需在体视显微镜下进行，并使用精细镊子或显微解剖针分离脆弱的唾液腺组织，从而避免唾液腺破裂或污染（https://zenodo.org/records/18302850）[51, 61]。保持唾液腺结构的完整性对于后续分子检测的可靠性至关重要。分离后的唾液腺可进行匀浆处理，并通过 RT-PCR、qPCR 或免疫学方法（immunoassays）检测病毒 RNA 或抗原[12]。在唾液腺中检测到病毒，而不仅是在中肠或血腔（hemocoel）中发现病毒，通常表明病原体已完成体外潜伏期，并可在吸血过程中传播[71]。

由于白蛉唾液腺体积极小，解剖过程具有较高技术难度，需要较为丰富的经验以避免样本降解[1, 51]。此外，病毒载量可能较低，因此需要采用高灵敏度的检测方法，如巢式 PCR（nested PCR）或高通量测序技术[54]。同时，样本生物污染的风险也强调了无菌操作的重要性。除技术因素外，生物因子亦会影响检测成功率，例如不同白蛉种类的媒介能力存在差异，而感染率也会随生态环境及季节变化而波动[33, 61]。

在唾液腺中检测病毒可为评估传播风险提供重要依据，从而有助于制定针对性的监测与防控措施[15]。例如，在流行区发现托斯卡纳病毒的存在，已为相关诊断流程的建立及公共卫生预警提供了依据[18]。此外，研究病毒与唾液成分之间的相互作用，还有助于发觉潜在的传播阻断疫苗或治疗靶点（therapeutics）[15, 18]。

白蛉唾液腺亦可作为抗原来源，通过免疫学方法（优选 ELISA）检测宿主对白蛉唾液的抗体反应。该方法可用于评估宿主对白蛉叮咬的暴露程度（host exposure to sand fly bites），从而支提出白蛉防控措施效果的评价[25]，并有助于评估利什曼原虫传播风险[40]。

4.6 吸血来源鉴定

从诱捕样本中分离出的吸血雌蛉应使用一次性器具进行解剖，以防止交叉污染。在体视显微镜下观察其腹部，以判断血餐的消化阶段。建议优先选择腹部呈红色、红褐色或深红色且尚未出现卵巢发育迹象的雌蛉进行分析。

为便于后续形态学鉴定，应切取腹部末端（包括受精囊）并在透明化处理后用于物种鉴定。其余主要腹部部分（不含受精囊）则应置于 Eppendorf® 离心管中，并在 −20 ° C 条件下保存以备后续分析。常用于血源鉴定的遗传标记（如 PNOC[5, 30, 50]、CytB[67] 或 COI[13]）已在文献中有充分报道，因此本文不再赘述（图 6）。此外，也可采用 MALDI-ToF 肽谱（peptide mapping）分析技术进行宿主血源鉴定[31]。研究表明，该方法可在吸血后较长时间内仍能识别宿主来源，因此尤其适用于血液已明显消化的吸血雌蛉样本。样本宜在 −20 ° C 或 4 ° C 条件下保存，但短时间室温保存亦可能获得可靠的分析结果。

在分析前，应将吸血雌蛉的腹部从其余虫体部分分离，并在蒸馏水（distilled water）中进行匀浆处理。其余虫体部分仍可用于其他分子或形态学研究。在从匀浆中取出部分样品用于 MALDI-ToF 肽谱分析后，其余部分还可用于 DNA 提取，以进一步确认宿主血源或检测是否存在利什曼原虫感染。与基于 DNA 的分子检测方法相比，该技术在样本制备及分析所需时间方面明显更短。

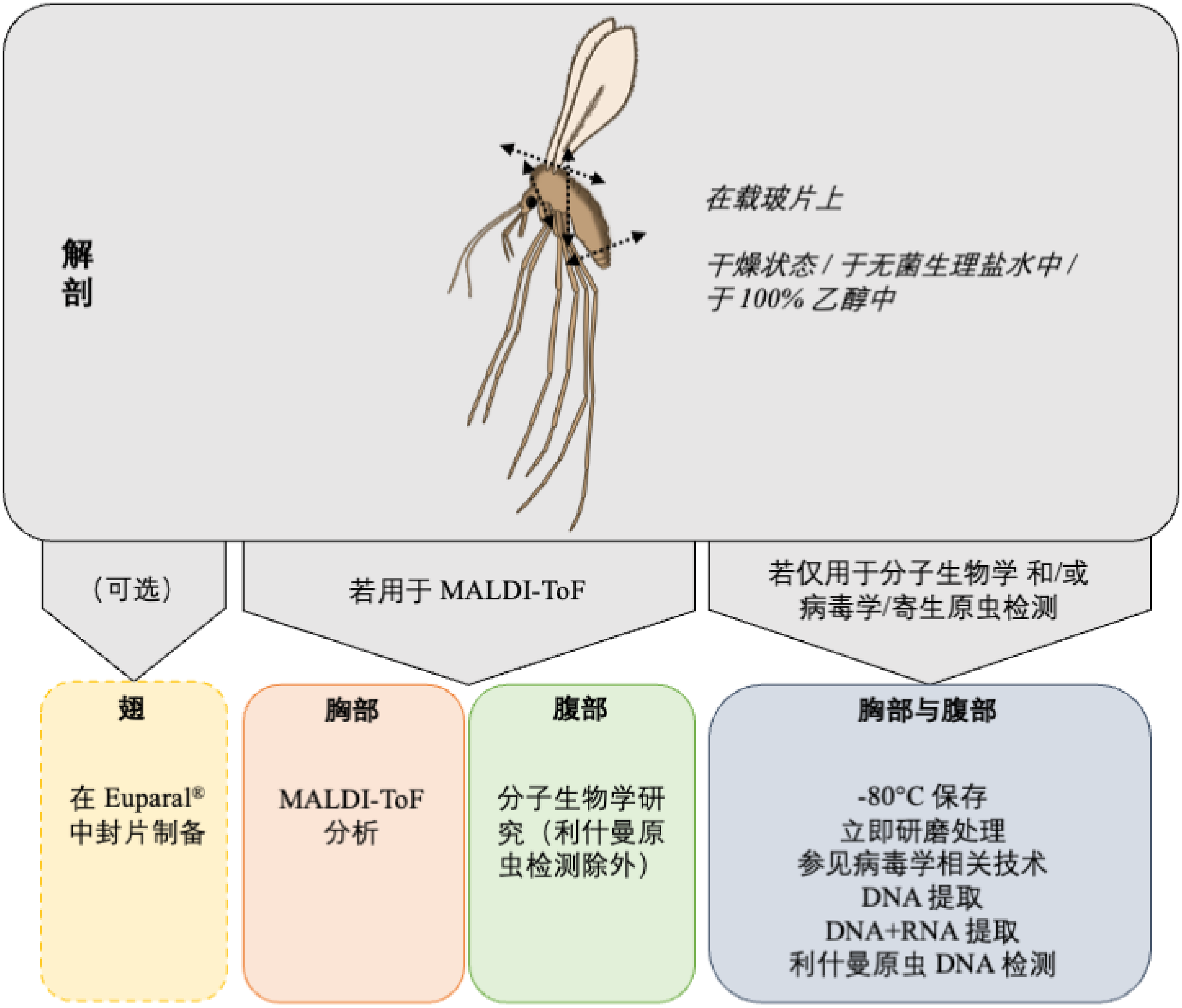

图 6： 用于分子生物学、蛋白质组学和/或病毒学研究的白蛉样本处理流程示意图。

5. 标本的形态学（morphological studies）处理流程（图 3、6、7、8；附录 1、2、3、4）

本节介绍仅用于形态学研究的白蛉标本制片前处理原则，并在此基础上说明其他用于非形态学研究的相应调整方法。然而，理解这一基础流程十分重要，因为在面对不同类型样本时，可据此对处理步骤进行灵活的调整。

该处理流程主要通过一系列连续的抽吸与注入操作完成，通常使用配有柔性橡胶吸球的巴斯德吸管进行。强烈建议使用圆底玻璃容器，因为其结构更有利于上述操作的顺利进行，同时玻璃材质对各种试剂具有良好的化学惰性。

为防止试剂挥发，容器应配备盖子，并避免装液过满，以免在开启或关闭时发生溢出；同时也可防止灰尘落入样本中。用于透明化处理及标本处理的相关试剂见表 2。

This is an Open Access article distributed under the terms of the Creative Commons Attribution License (https://creativecommons.org/licenses/by/4.0), which permits unrestricted use, distribution, and reproduction in any medium, provided the original work is properly cited.

表 2：所用试剂的配方

**氢氧化钾溶液 10%**	**酸性品红溶液（蒸馏水配制）1%**
氢氧化钾 10 g	酸性品红（粉末） 1 g
蒸馏水 100 mL	蒸馏水 99 mL
**氯醛树胶封片介质（Hoyer 封片溶液）**	**Marc-André 溶液（酸性品红染色）**
蒸馏水 50 mL	Marc-André 溶液 10mL
水合氯醛 200 g	1% 酸性品红溶液 50 µL
阿拉伯树胶 50 g	
丙三醇（又称甘油） 20 mL	
**Marc-André 溶液**	**Enecê 封片介质**
水合氯醛 40 g	纯白松香（colophony） 22 g
冰醋酸（即纯度在 99.5% 以上的无水乙酸） 30 mL	醇溶性 copal 树胶 12 g
蒸馏水 30 mL	无水乙醇 20 mL
	樟脑 10 g
	松节油 （Turpentine essence）10 mL
	桉油精（eucalyptol） 26 mL

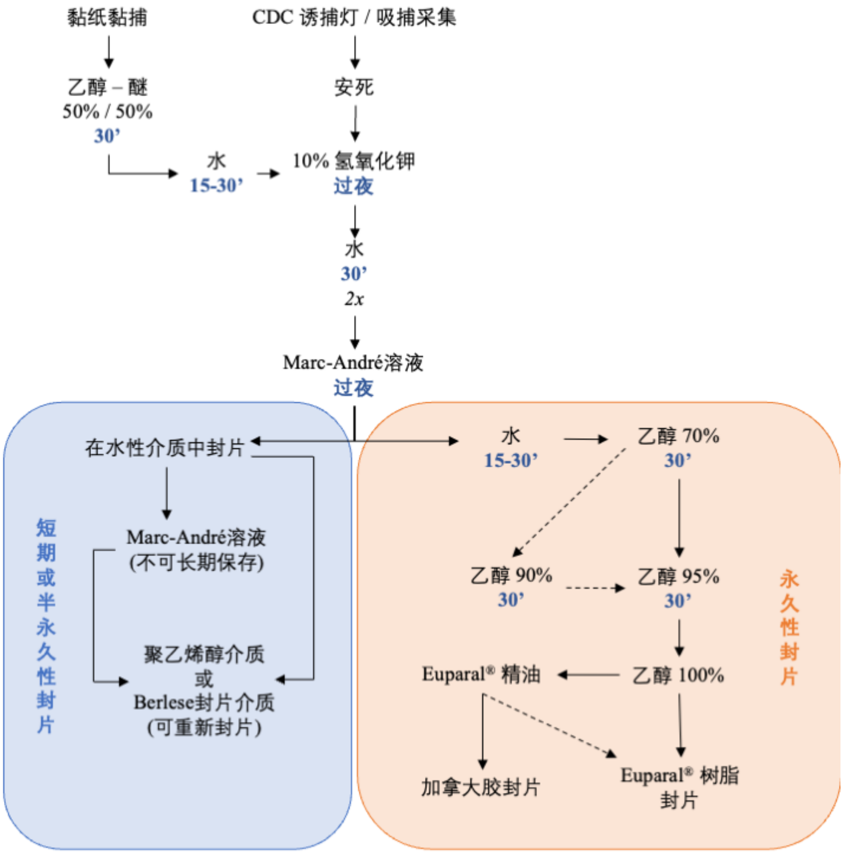

图 7： 白蛉标本处理的经典流程示意图。

5.1 透明化处理

在将白蛉标本制作为永久玻片标本之前，需先采用适当的方法和透明化试剂进行透明化处理（如 10% 醋酸溶液，或含水合氯醛的 Marc-André 溶液——后者因化学成分在许多国家属于受管制物质），以使标本透明化。透明化过程可去除体内组织、脂肪、分泌物及蜡质，使标本呈半透明状态，从而便于观察外骨骼（exoskeletal）结构（如刚毛毛序）、体表特征（如色素分布）以及透过体壁组织（tegument）可见的内部结构（如受精囊）。

该透明化流程通常分为两个步骤：首先使用强碱（如氢氧化钾），随后再使用弱酸（如含冰乙酸的 Marc-André 溶液）。两者在生化作用上各有不同功能[74]。强碱可通过皂化作用及蛋白质变性分解软组织（如蛋白质、脂肪和肌肉），同时保存几丁质外骨骼，从而使结构更加清晰便于观察。随后使用弱酸可中和残留碱性物质，防止进一步降解，并可对几丁质产生轻微漂白作用，从而提高透明度[74]。专研人员也可通过在蒸馏水中冲洗标本两次、每次 15 分钟，达到中和碱性的效果。这种依次处理的方法既能有效去除组织，又可温和地保存标本结构，从而确保其适用于显微镜观察。

在进入下一步骤前，建议先用蒸馏水冲洗两次，每次约 20 分钟。

5.1.1 软组织消解处理（图 8）

氢氧化钠（NaOH）或氢氧化钾（KOH）是常用的化学消解剂，其使用浓度及处理时间可根据标本的大小和脆弱程度进行调整。常规且较为有效的方法是将白蛉浸泡于强碱溶液中（如 10% KOH 或 NaOH）过夜，以溶解软组织。为缩短处理时间，可适当提高溶液浓度（例如使用 20% KOH 处理约 6 小时），并可在 37 ° C 条件下加温，以增强消解效果。

5.1.2 透明化与染色处理

在软组织消解之后，通常需进行进一步的透明化处理，一般采用醋酸与水合氯醛的组合（如 Marc-André 溶液）进行。透明化完成后，必须将标本在蒸馏水中彻底冲洗，至少进行两次连续水浴，每次约 20 分钟，以去除残留化学试剂。

Marc-André 溶液是制备白蛉标本时常用的透明化试剂，其优点在于既能有效提高透明度，又可在较大程度上减少对翅、触角等脆弱结构的损伤。该溶液应现配现用，或密封保存以防止挥发和变质。当与增亮或染色技术结合使用时，Marc-André 溶液尤其有助于突出特定形态结构细节。其具体配方与制备方法见附录 2。

对于透明度较高的标本，在封片前可能需要进行染色以增强结构的可见性。目前可选用多种染料，不同染料针对不同的化学成分发挥作用，因此应选择与标本性质及所用封片介质相兼容的染料。本方法可根据需要进行调整，例如可在 Marc-André 溶液中加入 0.1% 酸性品红以实现染色效果。

此外，对于保存在水性溶液中的标本，若计划使用树脂类封片介质进行封片，则需先进行脱水处理（见 5.2 脱水处理步骤），因为大多数天然或合成树脂类封片介质与水不相容。New（1974）曾指出，某些染料在特定封片介质中可能会发生变性或褪色[53]。例如，酸性品红常与加拿大胶配合使用，也可在 Euparal® 中固定。然而，使用酸性品红染色的标本容易发生褪色，尤其是在持有丁香油（用于最后透明化步骤中）残留的情况下更为明显。若标本长时间存放于丁香油中，可能在数日内出现明显褪色现象。

5.2 脱水处理

脱水过程通常通过将标本依次转移至不同浓度的乙醇溶液中逐步完成，浓度梯度为：50%、70%、80%、90% 或 95%，最后为 100% 乙醇，每一步浸泡时间至少为 20 分钟。由于乙醇挥发较快，在处理过程中应确保容器密封良好。当标本完全脱水后，若需要暂时暂停后续处理步骤，可将样本暂时置于 Euparal® 精油中保存数日。该 Euparal® 精油方法优于使用丁香油。过去曾广泛用于此处理的山毛榉杂酚油（beech creosote）由于具有毒性，目前已被全面禁用。

脱水处理的关键在于确保标本内部所含液体能够与所选封片介质相兼容，以避免出现混浊、结构塌陷或变形等现象，从而影响标本用于分类学研究的形态特征的准确观察与比对。

5.3 封片介质

This is an Open Access article distributed under the terms of the Creative Commons Attribution License (https://creativecommons.org/licenses/by/4.0), which permits unrestricted use, distribution, and reproduction in any medium, provided the original work is properly cited.

5.3.1 封片介质的选择与应用

理想的封片介质应具有尽可能接近玻璃的折射率（约为 1.5）。此外，还应满足以下基本要求：无色透明；在干燥后及长期保存过程中保持良好的透明度；与所用染料相容；能够充分渗透至标本的各组织结构；在封片过程中不应干燥过快或产生雾状混浊；封片后不应发生明显收缩。选择合适的封片介质是标本制备中的关键环节，因为并不存在一种适用于所有研究目的的理想介质。在选择时，应综合权衡以下几个主要因素：

- **光学特性：**封片介质的折射率应能够提供足够的对比度与折射效果，以清晰显示用于分类鉴定或形态描述的关键结构，如受精囊、叉形刺（ascoids）、牛氏刺（Newstead sensilla）、口腔前齿（anterior/vertical cibarial teeth）及咽甲齿（pharyngeal teeth）。这些结构的可见性在很大程度上取决于封片介质的光学性能。

- **保存性：**对于模式标本或需长期保存于标本馆的标本，所选封片介质必须具备良好的长期稳定性和耐久性。相反，对于昆虫区系调查或流行病学监测等研究，需长期保存的要求相比较低，则可使用短期性或半永久性封片介质。

5.3.2 封片介质的要求

研究人员常根据特定研究需求发展出具有针对性的复杂的制片技术。然而，这些技术往往忽视了一些关键因素，如保存质量、介质的兼容性、操作标准化程度，以及操作便利性与长期保存的稳定性。缺乏统一标准也会给受赠标本的整合与长期馆藏管理带来困难。

不同的科研应用场景，对封片介质有着各不相同的要求。分类学研究者通常对整虫标本进行封片，并倾向于选择能够温和消解内部组织的介质，以增强角质结构的可见性。此外，封片介质的折射率应与标本本身及载玻片之间存在一定差别，以提高结构的可见性。商业化封片介质通常被设计为具有接近玻璃的折射率，以减少光线在载玻片—封片介质—盖玻片系统中的折射与散射。然而，在明场显微镜观察条件下，可有意选择与标本折射率略有差异的封片介质，因而调节未染色标本的天然对比度，提高其在背景中的可见性

5.3.3 封片介质的类别（表 3、4）

在显微镜观察中，封片介质的折射率（refractive index；RI）决定了光线在载玻片、封片介质及标本之间的传播与折射情况。当封片介质的折射率与盖玻片玻璃（约 1.515）接近时，光线可较为均匀地通过。这从而减少散射和光学畸变，提高分辨率，并有助于细微结构的观察。相反，若折射率差异过大，则可能产生模糊、光晕或使未染色结构难以辨认。因此，由于不同封片介质具有不同的折射率，选择合适的介质对于优化对比度、清晰度及整体图像的质量至关重要。

在白蛉标本制片过程中，封片介质的折射率对细微结构的可见性影响尤为明显。白蛉的一些结构，如口腔前齿、受精囊、触角各节以及翅脉的排列，较为纤细况且角质化程度较低，因此在折射率较高的封片介质中往往较难清晰观察。

对于白蛉标本，常用的封片介质包括水性介质（如氯醛树胶封片介质）以及溶剂型介质（如加拿大胶和Enecê–Nelson Cerqueira（NC）树脂）。Rawlins[60]将封片介质分为两大类：（1）永久性封片介质：可随时间逐渐硬化，适用于长期保存；（2）半永久性封片介质：不会完全硬化，通常用于短期性制片。

表 3：所选封片介质的成分

封片介质	溶剂	潜在的预聚物或聚合物	备注
Hoyer（氯醛树胶）	丙三醇（glycerol），水	阿拉伯树胶相关化合物	具有组织消解作用的成分：水合氯醛（chloral hydrate）
CMCP-9（羧甲基纤维素－苯酚；carboxy methyl cellulose phenol）	水（CMCP-9：51–60%）	完全水解的聚乙烯醇（CMCP-9：0–5%）	CMCP(P)-9：低黏度型：高黏度型
DMHF（二甲基乙内酰脲甲醛；dimethyl hydantoin formaldehyde）	水	N,N′-dimethylol dimethyl hydantoin (di-methylol DMH)；<br>醚键/亚甲基桥联低聚物；Crosslinked DMH–formaldehyde polymer network	
加拿大胶	二甲苯（xylene）；树脂中部分挥发性成分（Δ³-carene、levopimaric acid、limonene、myrcene、palustric acid、β-phellandrene、α-pinene、β-pinene）	树脂成分（abienol、松香酸（abietic acid）、isopimaric acid、sandaracopimaric acid）	中和剂：碳酸钾（potassium carbonate）；*Abies balsamea*（Linné, 1758）树脂
Euparal®	桉油精（eucalyptol）、paraldehyde；以及部分挥发性的山达拉克树脂（gum sandarac）成分（limonene、α-pinene、β-pinene）	山达拉克树脂相关化合物（communic acid、manool、polycommunic acid、sandaracopimaric acid、12-acetoxy-sandaracopimaric acid、sugiol、torulosic acid、torulosol、totarol）	透明化剂：水杨酸甲酯（methyl salicylate）；Euparal® 呈绿色：因含铜盐（松香酸铜 copper abietinate）；*Tetraclinis articulata*（Vahl, 1791）树脂
Enecê	乙醇；并含樟脑、桉油精及松节油（turpentine essence）	Copal 树脂与松香（colophony）的相关成分	

封片介质可为液态、树胶型或树脂型，并可溶于水、酒精或其他溶剂（如甲苯（toulene）、二甲苯（xylene））（见表 3）。封片完成后，应使用不溶性的封边溶剂进行密封，以避免受空气中湿气等环境因素影响。为便于区分不同类别的封片介质，可参考以下方式进行分类：

a. 水溶性封片介质——该类介质（如氯醛树胶封片介质及聚乙烯醇类介质）可直接溶于水，适用于短期或半永久性封片。通常操作简便，但需进行封边处理以防止受空气湿度影响，尤其在热带高湿环境中更应注意防潮。

b. 有限耐水封片介质——该类介质对水分的敏感性较低，但仍需避免长时间接触高湿环境中。与水溶性介质相比，其长期稳定性更好，常用于半永久性封片。

c. 烃类封片介质——该类介质（如中性加拿大胶、DPX）溶于有机溶剂，如二甲苯、甲苯或 Enecê 溶剂等，主要用于永久性封片。这类介质具有优良的长期稳定性，并能有效抵抗潮湿及降解，适合用于长期保存。

总结而言，水溶性介质适用于短期性封片或需要后期取出标本的情况下使用；有限耐水介质适用于需一定耐久性的半永久性封片；而烃类封片介质则更适用于用于馆藏保存及长期保存的永久性封片标本。

表 4：所选封片介质的优缺点比较。基于多位研究者的未发表观察[52]。

封片介质	优点	缺点
*加拿大胶	介质耐久性极高，保存寿命可超过 150 年。<br>可使用丁香油或苯酚进行标本脱水，并用作制片前短期性保存标本的过渡介质。	含有有害成分，需在通风柜中操作。<br>需完整且耗时的脱水流程。<br>乙醇脱水与二甲苯或丁香油过渡可能使标本变脆弱易断裂。对此，有以下几种替代溶剂可供选择有助于降低标本破损的发生率：异丙醇（isopropanol）、n-butanol、Cellosolve™、1,4-二氧六环（1，4-dioxane）、Histoclear 或松油醇（terpineol）等。<br>若用苯酚替代二甲苯或残留氢氧化钾未完全去除，标本可能变黑。<br>折射率较高，可能降低未染色结构的可见性。<br>若不加热干燥，完全固化可能需数年。<br>若用丁香油透明化处理，可能随时间逐渐发黄、变暗。<br>某些染料会逐渐失效。
DMHF	透明度高。<br>折射率适宜。<br>结构可见性良好。<br>制片稳定性较好。<br>与多种染色方法兼容。<br>对标本保护效果好。	可能随时间发黄。<br>可能改变某些染料特质。<br>不适用于对甲醛敏感的染色。<br>易产生气泡且干燥时间较慢。<br>对湿度敏感。<br>制片后较难拆除重新封片。

	载玻片与盖玻片之间附着性强。	含甲醛，具有毒性、刺激性与致癌性。
Euparal®（透明型）	耐久性好，可保存 50 年以上。<br>可直接从 80%乙醇进行封片。<br>不掩盖未染色结构，长期不发黄、不变脆。<br>折射率比加拿大胶更适合双翅目昆虫。<br>收缩小、气泡少，适合较厚标本。<br>在 95%乙醇中仍可溶解，多年后仍可重新封片。	含有有害成分，需在通风柜中操作。<br>乙醇脱水与 Euparal® 精油过渡可能使标本变脆弱易断裂。对此，可使用异丙醇助于降低标本破损的发生率。
Hoyer 封片溶液	可直接从活体或水、乙醇、甲醛保存液中封片。<br>消解作用可产生优良的角质层观察效果。<br>折射率适中，可通过添加碘染（iodine staining）增强对比度。<br>配方中的醋酸可使节肢动物附肢伸展。<br>某些标本可稳定保存 40－60 年。<br>水溶性，便于重新封片	由于溶液注入过快易导致植物等脆弱标本塌陷，故操作较为耗时。<br>10 年内可能形成空腔（cavities）或结晶。<br>若氯醛水合物浓度过高或透明化处理时间过长，消解可能过度。<br>介质成分可能发生分离，并在数月或数年内产生细颗粒沉积。<br>介质可能逐渐变黑。
CMCP-9	可直接从水、乙醇、甘油或含甲醛溶液中封片。<br>可对内部组织产生一定消解作用，便于整体观察与制备。	可能随时间逐渐形成结晶并变暗。<br>有时对标本消解过度。<br>对于较厚标本，若封边不严，该封片介质易收缩，并在盖玻片边缘形成空隙。<br>不适用于染色标本或钙化标本。<br>干燥速度比 CMC 较慢。
Eukitt™	耐久性良好，可保存 30 年以上。<br>与多种溶剂兼容，如 acetone, benzene, chloroform, dioxan, ether, isopropanol, methyl benzoate, terpineol, toluene, and xylene。<br>干燥快，pH 略呈酸性。<br>不会随时间而明显变暗。<br>适用于多种染料，如 fuchsin, hematoxylin, methyl green, methyl violet, methylene blue。<br>可通过长期浸泡于二甲苯中重新封片。	含有有害成分，需在通风柜中操作。<br>需要完整且耗时的脱水流程。<br>对较厚标本不理想，该封片介质易收缩，并产生气泡。<br>若盖玻片未充分清洁并密封，可能随时间脱落。<br>在胶原纤维周围可能出现聚合不完全现象。
Enecê	非常耐久，可保存至少 50 年。<br>不会随时间变暗。	需要完整且耗时的脱水流程。<br>乙醇脱水与丁香油过渡可能使标本变脆弱易断裂。

	介质具可塑性，可在其中进行昆虫解剖并有足够时间调整结构位置。<br>成本较低。	标本可能持续缓慢透明化，导致极细微结构（如牛氏刺、叉形刺及细刚毛）观察困难。

5.3.4 推荐封片介质说明（表 3、4）

用于短期性观察的介质

氯醛树胶（Chloral gum） = Hoyer 封片溶液（折射率 RI = 1.48）

Marc-André 溶液是用于短期观察受精囊的最佳介质，通常可维持数小时。若将载玻片置于湿室中，甚至可延长观察时间，并适用于拍照（图 4）或绘图记录。如果需保存已观察的受精囊结构，应将其重新封片于水性介质中，以实现中期保存。不建议将其脱水后再转封于树脂类介质中（存在标本丢失的风险）。

氯醛树胶与 Hoyer 封片溶液通常视为同义名称。该类介质因其与水相容、操作简便、制片快速且折射率适中，常用于观察内部结构形态，尤其有利于显示受精囊等精细结构。然而，若配制不当或未在适宜湿度条件下保存，氯醛树胶也存在明显缺点，如结晶、变色以及黏度下降等问题。对盖玻片进行封边并不能完全避免这些问题，因为封片介质可能与封边溶液发生反应而明显变色（有时甚至接近黑色），特别是在使用 Euparal® 进行封边时更为明显。

从光学性能角度来看，Hoyer 封片溶液一直被认为是观察白蛉标本的最佳介质之一，因此被广泛使用。该介质有多种相近的配方，通常由阿拉伯树胶、甘油及水合氯醛组成，但是相关配方在文献中曾被误解或误引[74]。

尽管 Hoyer 封片溶液非常适合用于观察白蛉受精囊，但不宜用于长期保存。其优势在于短期观察，如拍照、绘图或影像记录。水性封片介质总体适用于短期性制片，但无法保证长期保存。相比之下，树脂类封片介质具有极高的耐久性，可保存数十年至数百年，但其较高的折射率可能削弱受精囊的折光性，从而使部分精细结构难以清晰观察。

Hoyer 封片溶液会随着时间而逐渐脱水而发生降解（图 8），形成细小的白色不透明水合氯醛结晶。即便如此，标本通常仍可从结晶化的载玻片中取回，因为其角质层在化学上仍保存完整，但在晶体生长过程中可能产生一定的物质损伤。在某些情况下，可通过在温暖、潮湿环境中重新对封片介质进行复水，并加入麝香草酚以防止真菌生长，从而恢复结晶化的制片。另一种方法是将载玻片浸泡于水中，再用冰醋酸脱水，随后重新封片于加拿大胶中。

DMHF（二甲基乙内酰脲甲醛；dimethyl hydantoin formaldehyde）（RI = 1.48）

这是一种水性封片介质[72]，在光学性能方面表现优良。这类介质与 Berlese 封片介质相似，且操作简便程度也与其相当。但与 Berlese 不同的是，DMHF 不会出现变黑或结晶的现象。该介质适用于白蛉及其他蛾蚋科（Psychodidae）昆虫的制片观察。

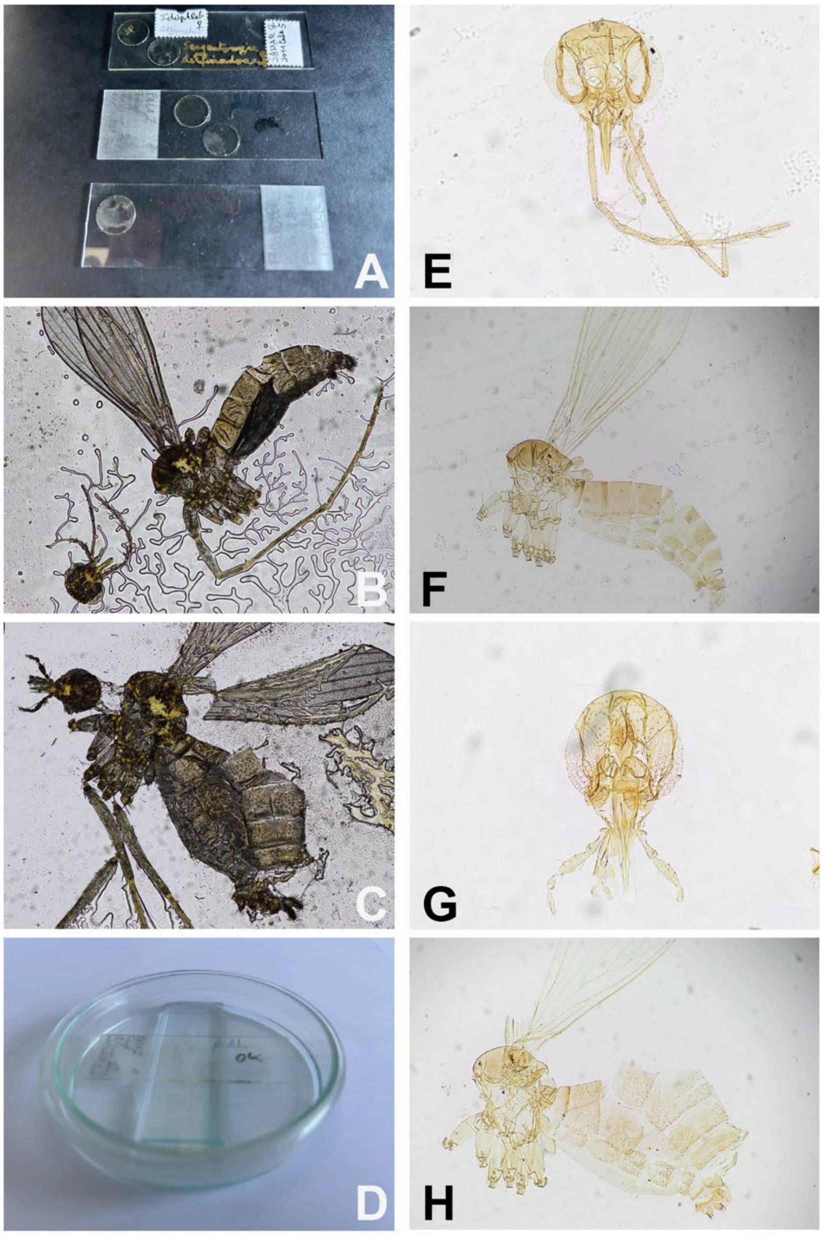

图 8：标本重新封片示意图。A：已损坏且干燥的 Hoyer 介质封片；B：干燥白蛉标本的显微图像；C：受损白蛉标本的显微图像；D：放置干燥玻片的湿室；E：标本 B 头部，于 Euparal®重新封片后；F：标本 B 胸与腹部，于 Euparal®重新封片后；G：受损标本 C 头部，于 Euparal®重新封片后；H：受损标本 C 胸与腹部，于 Euparal®重新封片后。

CMCP（樟脑－一氯苯酚；camphor-mono-chlorophenol）（RI = 1.41）

这是一种以甘油为基础的水溶性封片介质，可用于制备透明的永久性玻片标本，适用于包括白蛉在内的脆弱样本。其优点在于标本可直接从水或乙醇中转入封片。该介质可迅速使白蛉组织松弛并完成透明化处理，使角质层变软，从而便于调整标本姿态，特别适用于展开翅或进行生殖器解剖。尽管有研究指明该介质可用于长期保存标本，但其实际可保存时长仍不明确。该封片介质的主要局限在于其成分中含有苯酚，这是一种具有毒性和刺激性的物质，因此在使用过程中需谨慎操作。

This is an Open Access article distributed under the terms of the Creative Commons Attribution License (https://creativecommons.org/licenses/by/4.0), which permits unrestricted use, distribution, and reproduction in any medium, provided the original work is properly cited.

用于永久封片的介质

加拿大胶（Canada balsam）（RI = 1.52–1.54）

加拿大胶最早于 19 世纪 30 年代由 Andrew Pritchard 提出作为透射光显微镜的封片介质。因这类介质已被证实的档案级保存性能，至今仍是应用最广泛的封片介质之一，已有超过 150 年的使用历史。与 Hoyer 封片溶液不同，加拿大胶不会发生结晶，也不易吸收空气中的水分。然而，加拿大胶具有较强的自发荧光特性，这在某些显微技术中可能成为不利因素［60］。在制片过程中，使用低毒性溶剂替代二甲苯可降低操作风险，但也可能带来一些缺点，如干燥速度减慢以及封片介质较早出现变暗现象。

Euparal®（RI = 1.48）

Euparal® 是加拿大胶的常用替代介质之一，适用于永久性封片，具有良好的长期稳定性，且折射率与加拿大胶相近。其主要特点如下：（1）需进行脱水处理：在最终转入封片介质前，标本必须完成脱水，通常需经过 95% 乙醇过渡至无水乙醇；（2）处理时间较长：无论使用加拿大胶还是 Euparal® 进行树脂封片，均需先完成脱水步骤，因此整体样本处理时间会相应延长。当无法使用有机溶剂进行脱水时，可将从无水乙醇中取出的标本先转入一种过渡溶液中处理，该过渡溶液由等体积的 Euparal® 与 Euparal® essence 精油混合而成，之后再进行最终封片。

Enecê（RI = 1.467）

Enecê 是一种以树脂为基础的封片介质，主要用于小型昆虫的制片，在巴西尤为常用。其基质由松香（colophony）与 copal 树脂溶解于酒精、樟脑、松节油精及桉油精中制成。Cerqueira［11］曾将 Enecê 作为加拿大胶的替代介质，用于制作幼虫、未成熟期蜕皮以及成蚊的永久玻片标本，随后该介质也被广泛应用于白蛉标本的封片。Enecê 是一种成本较低的永久性封片替代方案，具有良好的长期稳定性，并具备较为充足的干燥时间，便于在封片过程中进行解剖操作及精确调整各形态结构的位置。

5.4 玻片制备与干燥

已封片标本的充分干燥对于确保其长期稳定性与保存质量至关重要。在考虑进行长期保存前，玻片必须彻底干燥。一般建议使用永久性封片介质制备的玻片需水平放置干燥 2–3 周，而使用半永久性封片介质制备的玻片通常干燥 1–2 周即可。为获得更理想的干燥效果，建议使用恒温培养箱，并根据所用封片介质设定适当温度，同时避免温度过高以免损伤标本。推荐的干燥温度范围为 30–37 °C。该步骤对于防止玻片变形、标本劣化或封片介质在储存期间发生不稳定现象尤为关键。

在玻片标签上应始终注明所使用的封片介质类型。如条件允许，还应记录所用配方、制片人员姓名以及制备日期。实际工作中，标本最初常仅用于短期观察制片，并未考虑长期保存。然而，当标本的身份发生变化（例如被指定为模式标本系列的一部分）时，则应改用更为永久性的封片介质，以确保其能够长期保存并供未来分类学研究使用。

5.5 其他制片方法：卡片固定法（card mounting）

卡片固定法是一种常用于多类昆虫的制片技术，可将标本直接用针固定在昆虫卡片上，或粘附于卡片表面进行保存。然而，由于白蛉个体极小，且物种鉴定需要通过透明化处理观察其内部结构（见第 5 节），因此该方法并不适用于白蛉标本的制备与保存。

5.6 受损标本的重新封片

对于稀有或珍贵标本，建议采用下述“复水-封片”两步法进行处理（参考视频：https://zenodo.org/records/18315029）。其一，在不拆解的情况下进行复水，以便进行初步观察。应于培养皿中放置一个可容纳多张载玻片的支架作为支撑。将需要复水的载玻片置于支架上方，并向培养皿内注入数毫米深的溶剂以形成湿室，同时确保载玻片本身不与溶剂接触（图 8D）。复水所需时间取决于标本的状态，可能从一天至数天不等，应每日检查并耐心等待。当载玻片充分复水后，可将其从湿室中取出，并放入恒温培养箱中放置数小时，再进行显微观察、拍照或绘图。其二，若需重新封片，可将载玻片再次放回湿室中数小时或过夜。随后在体视显微镜下进行拆片操作。使用细针小心移除盖玻片，确保没有白蛉结构粘附在盖玻片上（https://zenodo.org/records/18315029）。接着将解剖得到的各部分结构收集起来，在小型容器（例如破坏性 DNA/RNA 提取所用的小孔板）中用清水冲洗标本，然后进行脱水处理，并重新封片于树脂

类封片介质中。在拆解旧玻片时，必须首先确定原先使用的封片介质类型，以便选择合适的溶剂进行处理。对于水性封片介质，应使用水进行处理。若为树脂类封片介质（如加拿大胶或 Euparal®），则应使用二甲苯溶解，并需在通风柜内操作，同时佩戴适当的个人防护装备，包括口罩。

对于模式标本或馆藏标本的重新封片，必须事先获得馆藏负责人及/或标本所属机构的许可，方可进行操作。

6. 标本鉴定

6.1 形态学鉴定

白蛉的鉴定主要依赖其形态特征的观察，包括胸部与翅的形态、生殖器结构、刚毛的分布，以及各结构之间的特定形态测量关系。研究人员通常通过分类检索表、参考标本以及模式标本的描述，将采集到的标本与已知分类单元进行比对。在白蛉的物种鉴定中，关键特征尤为重要，例如雌雄两性的翅脉特征及头部形态、雄性生殖器的结构，以及雌性受精囊的形态等，均具有较高的分类学价值。准确鉴定通常需要借助显微镜进行精细观察：复式显微镜主要用于观察生殖器、受精囊等微细结构，而体视显微镜则适用于整体形态特征的观察。

近年来，成像技术的发展促进了数字影像在白蛉鉴定中的应用。通过获取关键形态特征的高分辨率照片或数字图像，并与参考资料进行比对，或借助计算机辅助鉴定系统进行分析，可进一步提高形态分类鉴定的准确性与可及性。

6.2 翅形几何形态

翅的几何形态是白蛉物种鉴定与分类的重要特征之一。白蛉的翅通常呈细长形，翅脉发达，并具有特征性的结构与排列模式（图 9、图 10）。翅脉的分布方式在属及物种间可存在差异，因此可作为重要的鉴别特征。因此，翅形几何形态的研究在物种鉴定与系统分类学研究中具有重要意义。

6.3 翅形几何形态测量

科研人员常采用多种方法对白蛉翅的形状与大小进行分析与比较，其中几何形态测量是一种常用技术，可用于不同物种或种群之间的比较。对翅形几何形态的研究不仅有助于分类学分析，还可为理解其行为特征、栖息地偏好及飞行能力提供重要信息。

在几何形态测量研究中，通常需先将翅小心地解剖分离，必要时进行染色处理，然后将其水平置于载玻片上，封片。制备完成的玻片在体视显微镜下拍摄图像，随后进行数字化处理，并开展形态测量分析。该方法在已发表的文献中已有较为详细的描述[6, 27, 42, 56, 57, 59] 。对于成对结构，通常建议始终使用同一侧（左翅或右翅）进行分析，以避免潜在的负向异速生长效应对研究结果产生影响［62］。

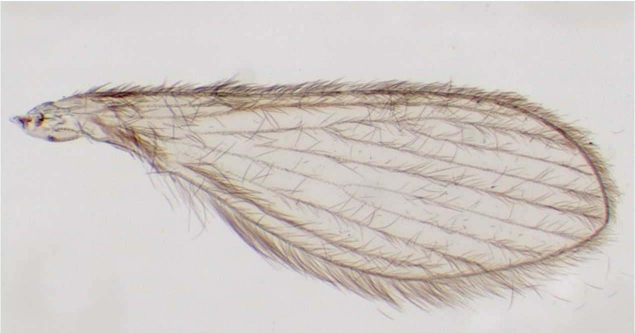

图 9： *Trichophoromyia ininii* 的翅。

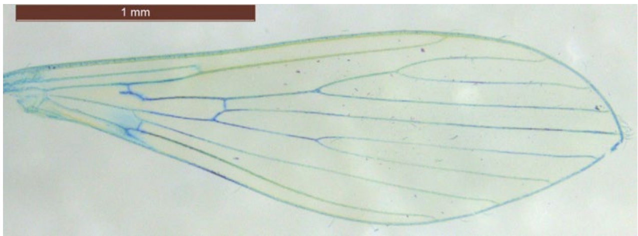

图 10： *Phlebotomus ariasi* 的翅（已染色）。

用于几何形态测量分析的翅制备

为获得清晰的翅脉显示效果，应先去除翅上的鳞片并进行适当染色。制备前，需在小型孔槽中（如细胞培养板的孔槽）分别加入所需试剂，包括亚甲蓝（methylene blue）、乙醇、水以及二甲苯替代（xylene substitute）溶剂。首先，将保存在 70% 乙醇、室温条件下的翅从 Eppendorf 管中取出，可通过将管子倒置

并将液体倒入孔槽中实现，。然后，用细钩针沿翅的纵向将其轻轻提起。将翅短暂从乙醇转入水中，再返回乙醇，以去除附着的刚毛。随后将翅置于亚甲蓝中染色 6 分钟，并确保其在染色过程中漂浮于液面。染色后，小心取出翅并置于二甲苯替代溶剂中浸泡 2 分钟（时间约为亚甲蓝染色时间的三分之一）。可轻轻用针敲击孔槽壁，使翅逐渐下沉。二甲苯替代溶剂有助于固定染色效果。最后，将翅取出并置于载玻片上一小滴 Euparal® 中。在放大镜下轻轻将翅展开，并小心覆盖盖玻片。应在 Euparal® 凝固前及时完成拍摄，因为可能需要在盖玻片下微调翅的位置以实现最佳对齐。

6.4 分子生物学技术

除了形态学方法之外，分子生物学技术在昆虫学研究中的重要性日益提升，广泛应用于分类学、群体遗传学（population genetics）及系统发生研究（phylogenetic studies），同时也用于病原体 DNA/RNA 检测、吸血来源鉴定以及媒介行为研究等流行病学相关领域[70]。DNA 测序可用于物种确认或区分形态上相近的隐存种，从而提供更为准确可靠的鉴定手段。此外，多种先进分子技术（如 PCR、DNA 测序、NGS 等）以及 MALDI-ToF 质谱分析技术在物种快速而准确的鉴定中正日益发挥重要作用，可作为传统形态学方法的重要补充[46]。尽管如此，形态学鉴定仍然是分类学研究的基础与参照标准，而分子数据的解释通常也需建立在形态学鉴定结果之上。

6.4.1 破坏性核酸提取

核酸提取是许多生物学研究中的常规步骤，目前已发展出多种从生物材料中提取 DNA 的方法[48]，并有大量商业化 DNA 提取试剂盒可用于简化这一过程[14]。然而，常用于节肢动物形态学鉴定的标本处理方法往往会对 DNA 分析造成不利影响，因为这些技术可能损伤或破坏标本的关键形态特征[10]。多数用于昆虫组织的 DNA 提取流程本质上具有破坏性[43]，这对于体型较小的标本尤为值得关注，因为这方式可能影响重要形态结构[72]。因此，在选择 DNA 提取方法时，标本的类型及其保存状态是需要重点考虑的因素[29]。

对白蛉进行准确鉴定、理解其种群动态（population dynamics）以及减少对非目标物种影响的需求，推动了分子诊断技术的发展[23]。目前，分子方法已常用于补充传统形态分类手段。例如，昆虫 DNA 条形码分析的标准流程通常包括 DNA 提取与测序，但这往往会导致原始标本的耗失。因此，开发既能获取分子数据又能尽量保留标本形态完整性的非破坏性 DNA 提取方法显得尤为重要。

除病原体检测外，白蛉 DNA 也常用于物种鉴定。目前已有多种核酸提取方法应用于白蛉研究。所需核酸的数量与质量取决于后续的分子分析类型，因为不同技术对核酸的数量与质量要求不同[9]。例如，研究发现白蛉的复眼组织可能对 PCR 扩增产生抑制效果[69]。一些商业试剂盒的操作流程已被研究人员针对白蛉进行了改进[8]，从而提高核酸提取的产量和/或质量[8, 9, 69]。此外，一些为其他节肢动物开发的改良方法同样可应用于白蛉[58, 76]。

针对小片段线粒体 DNA（如 COI 或 CytB）的鉴定 PCR 通常可兼容 DNA 高度片段化的提取方法。而某些长读长测序技术（如 Oxford Nanopore 和 PacBio）则需要完整度较高且片段较长的高质量 DNA。一般而言，基于离心柱（spin column）的提取方法可获得长度可达约 60 kb 的基因组 DNA 片段，而酚-氯仿（phenol-chloroform）提取法则可获得最长约 150 kb 的 DNA 片段[77]。表 5 总结了不同的白蛉 DNA 提取技术，并指出了这些方法是否针对白蛉 DNA 提取做出了专门调整。表中未列出核酸产量，因其取决于标本大小和制备方法。“针对小型节肢动物提取的方案改良”一栏提供了针对白蛉或其他小型节肢动物的核酸提取方案的参考文献。

在选择提取方法时，应综合考虑多种因素，如样本数量、提取所需时间以及后续所采用的技术类型。虽然 NGS 技术通常需要高分子量的基因组 DNA，但本文所述的各种方法均可用于基于 PCR 的常规分析。此外，已有多项研究探索了适用于小型陆生节肢动物、已干燥保存的博物馆标本以及软体节肢动物的非破坏性 DNA 提取方法[19, 26, 28, 55, 63]。

6.4.2 非破坏性核酸提取

在节肢动物（尤其是白蛉）的分子分析中，一个重要挑战是如何在获取 DNA 信息的同时，将标本保留并纳入昆虫标本馆收藏。多数 DNA 提取流程需要对组织进行消解处理，从而影响原始标本的保存状态。然而，

非破坏性核酸提取方法旨在提取核酸物质，而不会对样本造成物理损，并且不影响其保存价值。这类方法在处理珍贵或数量有限的标本时尤为重要，例如白蛉，因为保持其结构完整性对于未来的分类学、形态学及诊断研究至关重要。一种常用的技术是非破坏性浸泡法，即将白蛉固定后，轻柔地浸入含蛋白酶 K（proteinase K）的裂解缓冲液中。

温和裂解技术（mild-vectolysis）已成功应用于白蛉，特别是模式标本[24]。该方法基于常规离心柱试剂盒（如 DNeasy Blood and Tissue kit，QIAGEN，德国希尔登），并通过对流程进行改良，在不损坏标本的情况下提取 DNA。通过调整裂解步骤（如调整裂解缓冲液体积及增加冷冻步骤）[17]，可促进核酸释放，同时尽量减少对形态结构的损伤[24]。在白蛉研究中，还可使用 HotSHOT DNA 提取试剂盒（Bento Bioworks Ltd，英国伦敦）[73]，该方法快速且成本较低，适用于样本的高效处理。用于形态学鉴定的白蛉标本在完成核酸提取后可进行冲洗处理。接着，采用 DNeasy Blood and Tissue 试剂盒处理的标本需使用 Marc-André 溶液进行透明化；而采用 HotSHOT DNA 提取方法处理的标本通常已具有足够透明度，可直接在水性介质中封片，或按本文所述流程脱水后在树脂介质中封片[73]。提取获得的核酸物质可进一步用于后续分析，如通过 PCR 扩增特定遗传标记。

总体而言，非破坏性核酸提取方法对于研究白蛉的遗传特征具有重要意义，包括鉴定其可能携带的病原体。在保留标本完整性的同时，研究人员仍可获取关键遗传信息，并使该样本可继续用于其他分析或后续研究。

表 5：白蛉基因组 DNA（gDNA）提取方案的平均成本、应用、及小型节肢动物（如白蛉）核酸提取的方案改良

提取方案	成本	应用	针对小型节肢动物提取的方案改良
离心柱法	2.5–3.55 美元 [39]	PCR，NGS	[9]
酚－氯仿法（Phenol–chloroform）	0.24 美元 [69]	PCR，NGS	[9]
HotSHOT	<0.01 美元 [69]	PCR	-
盐析法（Salting out）	0.12 美元 [69]	PCR	-
Chelex	0.02 美元 [41]	PCR	[41, 76]

6.5 MALDI-ToF 质谱技术

MALDI-ToF MS（基质辅助激光解吸/电离飞行时间质谱）是一种基于质谱分析的技术，用于检测和分析生物样本特有的蛋白质谱图（“指纹”）。近年来，该技术逐渐成为医学与兽医学重要节肢动物鉴定中的重要工具。研究表明，该方法能够有效鉴定白蛉的不同发育阶段，包括未成熟期个体以及吸血雌虫体内的血餐来源。该方法也已在不同保存与匀浆的条件下成功区分雌雄个体及不同物种[28, 30, 73, 74]。此外，该技术在亚属、物种乃至种群水平上均具有较高的分辨能力。MALDI-ToF 能够实现快速而准确的物种鉴定，这对于理解白蛉的分布、行为及其在疾病传播中的作用具有重要意义。通过比较蛋白质谱图差异，该技术在流行病学研究及媒介控制策略制定中发挥着关键作用。

目前该技术的常规应用仍面临两项主要限制。首先是质谱设备的可获得性问题。此类仪器价格昂贵，仅为白蛉或其他节肢动物物种鉴定而专门购置往往难以实现。所幸的是，许多蛋白质组学平台或临床检测机构已将质谱仪（mass spectrometers）作为常规研究设备，研究人员可预约使用该仪器，从而部分克服这一限制。其次是公开数据库中白蛉参考数据较为匮乏。因此，研究人员通常需要基于已准确鉴定的标本建立本地参考数据库，并获取对应的参考蛋白谱图。理想情况下，这些标本应通过形态学鉴定并结合适当遗传标记（如 COI、CytB 等）的测序结果加以确认。随着更多研究团队逐步将其内部参考数据纳入由法国巴黎公立医院集团（Assistance Publique-Hôpitaux de Paris, Sorbonne University, France）运行的 MSI 平台，以及比利时布鲁塞尔（Brussels, Belgium）的 BCCM/IHEM/Sciensano

标本库，这一问题有望逐步得到改善（https://msi.happy-dev.fr/）。

当计划使用 MALDI-ToF 进行蛋白质谱分析时，样本应优先采用干冻保存，或保存在 70% 分子级乙醇中，并避免接触环境温度。由于目前尚缺乏统一的样本制备规范，建议将 30 mg 芥子酸（sinapic acid）溶于 1 mL 溶剂中（通常为 60%乙腈（acetonitrile）、40%水和 0.3% 三氟乙酸（TFA））作为 MALDI-ToF 基质制备（matrix preparation），以提高所得蛋白谱与已发表白蛉数据之间的可比性。

用于 MALDI-ToF MS 的样本制备（图 7）

在不同条件下保存的昆虫标本，首先需在室温下自然风干，然后进行解剖。将头部和腹部分离，以获取含有关键形态特征的结构，用于玻片制备及形态学分析。胸部可用于 MALDI-ToF 分析，而剩余的腹部则可保留用于 DNA 提取。在进行蛋白质谱分析时，将胸部置于 1.5 mL 微量离心管中，加入 10 μL 匀浆液，并使用一次性研磨棒（pestle）和研磨颗粒（pellets）进行手工匀浆。常用的两种匀浆液为无菌蒸馏水或 25% 甲酸溶液（formic acid）。

7. 结论

本文旨在为科研人员提供一套针对不同研究目的而优化的白蛉制片方法，以促进物种的准确鉴定及病原体检测。需要指出的是，并不存在一种普遍适用于所有情况的最佳方法，而是有多种可选方法，各自具有不同的优势与局限性。

在补充材料中，我们提供了多种用于白蛉标本制备与鉴定的详细制片操作流程。这些规范化流程（包括配套的教学视频）以分步骤形式呈现，可针对不同研究目标进行选择与应用，从而确保操作的准确性与结果的可靠性。通过整合这一套系统而全面的技术指南，我们希望能够帮助科研人员根据研究目的选择并应用最合适的制片技术。

致谢

作者感谢英国伦敦自然历史博物馆的 Richard Lane 与 Zoe Jay Adams 对本文所做的细致审阅，这些意见显著提升了论文的整体质量。

经费资助

作者感谢 Brazil development agencies CNPq（项目编号：404395/2024-4）及 Araucária Foundation（项目编号：433/2025 PDI）对 AJA 研究工作的资助。

利益冲突声明

Jérôme Depaquit 为《Parasite》期刊的 associate editor，但其未参与本稿件的审稿过程及相关决策。其他作者声明不存在任何利益冲突。

Zenodo 平台视频资料

视频 1: https://zenodo.org/records/18198006
视频 2: https://zenodo.org/records/18311158
视频 3: https://zenodo.org/records/18311106
视频 4: https://zenodo.org/records/18311154
视频 5: https://zenodo.org/records/18303014
视频 6: https://zenodo.org/records/18302850
视频 7: https://zenodo.org/records/18315029

References

1. Alkan C, Allal-Ikhlef AB, Alwassouf S, Baklouti A, Piorkowski G, de Lamballerie X, Izri A, Charrel RN. 2015. Virus isolation, genetic characterization and seroprevalence of Toscana virus in Algeria. Clinical Microbiology and Infection, 21(11), 1040 e1-9.
2. Alten B, Ozbel Y, Ergunay K, Kasap OE, Cull B, Antoniou M, Velo E, Prudhomme J, Molina R, Banuls AL, Schaffner F, Hendrickx G, Van Bortel W, Medlock JM. 2015. Sampling strategies for phlebotomine sand flies (Diptera: Psychodidae) in Europe. Bulletin of Entomological Research 105(6), 664–678.
3. Ayhan N, Baklouti A, Prudhomme J, Walder G, Amaro F, Alten B, Moutailler S, Ergunay K, Charrel RN, Huemer H. 2017. Practical guidelines for studies on sandfly-borne phleboviruses: Part I: Important points to consider *ante* field work. Vector-Borne and Zoonotic Diseases 17(1), 73–80.

4. Bates PA. 1997. Infection of phlebotomine sandflies with *Leishmania*, in The Molecular Biology of Insect Disease Vectors: A Methods Manual. Springer. p. 112–120.5.
5. Baum M, de Castro EA, Pinto MC, Goulart TM, Baura W, Klisiowicz Ddo R, Vieira da Costa-Ribeiro MC. 2015. Molecular detection of the blood meal source of sand flies (Diptera: Psychodidae) in a transmission area of American cutaneous leishmaniasis, Parana State, Brazil. Acta Tropica, 143, 8–12.
6. Belen A, Alten B, Aytekin A. 2004. Altitudinal variation in morphometric and molecular characteristics of *Phlebotomus papatasi* populations. Medical and Veterinary Entomology, 18(4), 343–350.
7. Bhattacharya J, Chandra G, Hati AK. 1991. A simple method for cryopreservation of *Leishmania donovani* promastigotes, Indian Journal of Medical Research, 93, 245–246.
8. Caligiuri LG, Sandoval AE, Miranda JC, Pessoa FA, Santini MS, Salomón OD, Secundino NF, McCarthy CB. 2019. Optimization of DNA extraction from individual sand flies for PCR amplification. Methods and Protocols, 2(2), 36.
9. Casaril AE, de Oliveira LP, Alonso DP, de Oliveira EF, Gomes Barrios SP, de Oliveira Moura Infran J, Fernandes WS, Oshiro ET, Ferreira AMT, Ribolla PEM, de Oliveira AG. 2017. Standardization of DNA extraction from sand flies: Application to genotyping by next generation sequencing. Experimental Parasitology, 177, 66–72.
10. Castalanelli MA, Severtson DL, Brumley CJ, Szito A, Foottit RG, Grimm M, Munyard K, Groth DM. 2010. A rapid non-destructive DNA extraction method for insects and other arthropods. Journal of Asia-Pacific Entomology, 13(3), 243–248.
11. Cerqueira NL. 1943. Um novo meio para montagem de pequenos insetos em lâmina. Memórias do Instituto Oswaldo Cruz, (39), 37–41.
12. Charrel RN, Gallian P, Navarro-Mari JM, Nicoletti L, Papa A, Sanchez-Seco MP, Tenorio A, de Lamballerie X. 2005. Emergence of Toscana virus in Europe. Emerging Infectious Diseases, 11(11), 1657–1663.
13. Chaskopoulou A, Giantsis IA, Demir S, Bon MC. 2016. Species composition, activity patterns and blood meal analysis of sand fly populations (Diptera: Psychodidae) in the metropolitan region of Thessaloniki, an endemic focus of canine leishmaniasis. Acta Tropica, 158, 170–176.
14. Chen H, Rangasamy M, Tan SY, Wang H, Siegfried BD. 2010. Evaluation of five methods for total DNA extraction from western corn rootworm beetles. PLoS One, 5(8), e11963.
15. Depaquit J, Grandadam M, Fouque F, Andry PE, Peyrefitte C. 2010. Arthropod-borne viruses transmitted by Phlebotomine sandflies in Europe: a review. Eurosurveillance, 15(10), 19507.
16. Diamond LS, Herman CM. 1954. Incidence of Trypanosomes in the Canada Goose as revealed by bone marrow culture. Journal of Parasitology, 40(2), 195–202.
17. Ding H, Torno M, Vongphayloth K, Ng G, Tan D, Sng W, Ho K, Randrianambinintsoa FJ, Depaquit J, Tan CH. 2025. Hidden in plain sight: discovery of sand flies in Singapore and description of four species new to science. Parasites & Vectors, 18(1), 402.
18. Es-Sette N, Ajaoud M, Bichaud L, Hamdi S, Mellouki F, Charrel RN, Lemrani M. 2014. *Phlebotomus sergenti* a common vector of *Leishmania tropica* and Toscana virus in Morocco. Journal of Vector Borne Diseases, 51(2), 86–90.
19. Favret C. 2005. A new non-destructive DNA extraction and specimen clearing technique for aphids (Hemiptera). Proceedings of the Entomological Society of Washington, 107(2), 469–470.
20. Galati EAB. 2018. Phlebotominae (Diptera, Psychodidae): Classification, morphology and terminology of adults and identification of American taxa, in Brazilian Sand Flies: Biology, Taxonomy, Medical Importance and Control, Rangel EF, Shaw JJ, Editors. Cham: Springer International Publishing. pp. 9–212.
21. Galati EAB, de Andrade AJ, Perveen F, Loyer M, Vongphayloth K, Randrianambinintsoa FJ, Prudhomme J, Rahola N, Akhoundi M, Shimabukuro PHF, Depaquit J. 2025. Phle-botomine sand flies (Diptera, Psychodidae) of the world. Parasites & Vectors, 18(1), 220.
22. Galati EAB, Galvis-Ovallos F, Lawyer P, Leger N, Depaquit J. 2017. An illustrated guide for characters and terminology used in descriptions of Phlebotominae (Diptera, Psychodidae). Par-asite, 24, 26.
23. Gariepy T, Kuhlmann U, Gillott C, Erlandson M. 2007. Parasitoids, predators and PCR: the use of diagnostic molecular markers in biological control of Arthropods. Journal of Applied Entomology, 131(4), 225–240.
24. Giantsis IA, Chaskopoulou A, Bon MC. 2016. Mild-Vectolysis: A nondestructive DNA extraction method for vouchering sand flies and mosquitoes. Journal of Medical Entomology, 53(3), 692–695.
25. Gidwani K, Picado A, Rijal S, Singh SP, Roy L, Volfova V, Andersen EW, Uranw S, Ostyn B, Sudarshan M, Chakravarty J, Volf P, Sundar S, Boelaert M, Rogers ME. 2011. Serological markers of sand fly exposure to evaluate insecticidal nets against visceral leishmaniasis in India and Nepal: a cluster-randomized trial. PLoS Neglected Tropical Diseases, 5(9), e1296.
26. Gilbert MTP, Moore W, Melchior L, Worobey M. 2007. DNA extraction from dry museum beetles without conferring external morphological damage. PLoS One, 2(3), e272.
27. Giordani BF, Andrade AJ, Galati EAB, Gurgel-Goncalves R. 2017. The role of wing geometric morphometrics in the identification of sandflies within the subgenus *Lutzomyia.* Medical and Veterinary Entomology, 31(4), 373–380.
28. Guzmán-Larralde AJ, Suaste-Dzul AP, Gallou A, Peña-Carrillo KI. 2017. DNA recovery from microhymenoptera using six non-destructive methodologies with considerations for subsequent preparation of museum slides. Genome, 60(1), 85–91.
29. Hajibabaei M, DeWaard JR, Ivanova NV, Ratnasingham S, Dooh RT, Kirk SL, Mackie PM, Hebert PD. 2005. Critical factors for assembling a high volume of DNA barcodes. Philosophical Transactions of the Royal Society B: Biological Sciences, 360(1462), 1959–1967.
30. Haouas N, Pesson B, Boudabous R, Dedet JP, Babba H, Ravel C. 2007. Development of a molecular tool for the identification of *Leishmania* reservoir hosts by blood meal analysis in the insect vectors. American Journal of Tropical Medicine and Hygiene, 77(6), 1054–1059.
31. Hlavackova K, Dvorak V, Chaskopoulou A, Volf P, Halada P. 2019. A novel MALDI-TOF MS-based method for blood meal identification in insect vectors: A proof of concept study on phlebotomine sand flies. PLoS Neglected Tropical Diseases, 13 (9), e0007669.
32. Huemer H, Prudhomme J, Amaro F, Baklouti A, Walder G, Alten B, Moutailler S, Ergunay K, Charrel RN, Ayhan N. 2017. Practical guidelines for studies on sandfly-borne phleboviruses: Part II: Important points to consider for fieldwork and subsequent virological screening. Vector-Borne and Zoonotic Diseases, 17(1), 81–90.
33. Jancarova M, Polanska N, Thiesson A, Arnaud F, Stejskalova M, Rehbergerova M, Kohl A, Viginier B, Volf P, Ratinier M. 2025.

Susceptibility of diverse sand fly species to Toscana virus. PLoS Neglected Tropical Diseases, 19(5), e0013031.

34. Kapp JD, Green RE, Shapiro B. 2021. A fast and efficient single-stranded genomic library preparation method optimized for ancient DNA. Journal of Heredity, 112(3), 241–249.
35. Killick-Kendrick R, Maroli M, Killick-Kendrick M. 1991. Bibliography of the colonization of phlebotomine sandflies. Parassitologia, 33(suppl.), 321–333.
36. Lawyer P, Killick-Kendrick M, Rowland T, Rowton E, Volf P. 2017. Laboratory colonization and mass rearing of phlebotomine sand flies (Diptera, Psychodidae). Parasite, 24, 42.
37. Léger N, Pesson B, Madulo-Leblond G. 1986. Les phlébotomes de Grèce : 1ère partie. Bulletin de la Société de Pathologie Exotique, 79, 386–397.
38. Léger N, Pesson B, Madulo-Leblond G. 1986. Les phlébotomes de Grèce : 2ème partie. Bulletin de la Société de Pathologie Exotique, 79, 514–524.
39. Leonel JAF, Vioti G, Alves ML, da Silva DT, Meneghesso PA, Benassi JC, Spada JCP, Galvis-Ovallos F, Soares RM, Oliveira T. 2020. DNA extraction from individual Phlebotomine sand flies (Diptera: Psychodidae: Phlebotominae) specimens: Which is the method with better results? Experimental Parasitology, 218, 107981.
40. Lestinova T, Rohousova I, Sima M, de Oliveira CI, Volf P. 2017. Insights into the sand fly saliva: Blood-feeding and immune interactions between sand flies, hosts, and *Leishmania.* PLoS Neglected Tropical Diseases, 11(7), e0005600.
41. Lienhard A, Schaffer S. 2019. Extracting the invisible: obtaining high quality DNA is a challenging task in small arthropods. PeerJ, 7, e6753.
42. Lozano-Sardaneta YN, Mikery-Pacheco OF, Huerta H, Rojas-Soriano JE, Contreras-Ramos A. 2025. Wing geometric morphometrics is effective to separate sand fly species (Diptera, Psychodidae, Phlebotominae) related with leishmaniasis transmission in Mexico. Acta Tropica, 262, 107523.
43. Mandrioli M. 2008. Insect collections and DNA analyses: how to manage collections? Museum Management and Curatorship, 23(2), 193–199.
44. Maroli M, Feliciangeli MD, Bichaud L, Charrel RN, Gradoni L. 2013. Phlebotomine sandflies and the spreading of leishmaniases and other diseases of public health concern. Medical and Veterinary Entomology, 27(2), 123–147.
45. Marquina D, Buczek M, Ronquist F, Lukasik P. 2021. The effect of ethanol concentration on the morphological and molecular preservation of insects for biodiversity studies. PeerJ, 9, e10799.
46. Mathis A, Depaquit J, Dvorak V, Tuten H, Banuls AL, Halada P, Zapata S, Lehrter V, Hlavackova K, Prudhomme J, Volf P, Sereno D, Kaufmann C, Pfluger V, Schaffner F. 2015. Identification of phlebotomine sand flies using one MALDI-TOF MS reference database and two mass pectrometer systems., Parasites & Vectors, 8, 266.
47. Mekarnia N, Benallal KE, Sadlova J, Vojtkova B, Mauras A, Imbert N, Longhitano M, Harrat Z, Volf P, Loiseau PM, Cojean S. 2024. Effect of *Phlebotomus papatasi* on the fitness, infectivity and antimony-resistance phenotype of antimony-resistant *Leishmania* major Mon-25. International Journal for Parasitology – Drugs and Drug Resistance, 25, 100554.
48. Milligan BG. 1998. Total DNA isolation, in Molecular Genetic Analysis of Population: A Practical Approach, Hoelzel AR, Editor. Oxford: Oxford University Press.
49. Molina R, Jiménez M, Alvar J, González E, Hernández-Taberna S, Ines MM. 2017. Methods in sand fly research. Madrid: Servicio de publicaciones Universidad de Alcalá de Henares, Madrid.
50. Murphy WJ, Eizirik E, O'Brien SJ, Madsen O, Scally M, Douady CJ, Teeling E, Ryder OA, Stanhope MJ, de Jong WW, Springer MS. 2001. Resolution of the early placental mammal radiation using Bayesian phylogenetics. Science, 294(5550), 2348–2351.
51. Nacif-Pimenta R, Pinto LC, Volfova V, Volf P, Pimenta PFP, Secundino NFC. 2020. Conserved and distinct morphological aspects of the salivary glands of sand fly vectors of leishma-niasis: an anatomical and ultrastructural study. Parasites & Vectors, 13(1), 441.
52. Neuhaus B, Schmid T, Riedel J. 2017. Collection management and study of microscope slides: Storage, profiling, deterioration, restoration procedures, and general recommendations. Zootaxa, 4322(1), 1–173.
53. New TR. 1974. Pscoptera. Handbooks for Identification of British Insects (Vol. I). London: Royal Entomological Society of London. 102 pp.
54. Perez-Ruiz M, Collao X, Navarro-Mari JM, Tenorio A. 2007. Reverse transcription, real-time PCR assay for detection of Toscana virus. Journal of Clinical Virology, 39(4), 276–281.
55. Porco D, Rougerie R, Deharveng L, Hebert P. 2010. Coupling non-destructive DNA extraction and voucher retrieval for small soft-bodied Arthropods in a high-throughput context: the example of Collembola. Molecular Ecology Resources, 10(6), 942–945.
56. Prudhomme J, Cassan C, Hide M, Toty C, Rahola N, Vergnes B, Dujardin JP, Alten B, Sereno D, Banuls AL. 2016. Ecology and morphological variations in wings of *Phlebotomus ariasi* (Diptera: Psychodidae) in the region of Roquedur (Gard, France): a geometric morphometrics approach. Parasites & Vectors, 9(1), 578.
57. Prudhomme J, Gunay F, Rahola N, Ouanaimi F, Guernaoui S, Boumezzough A, Banuls AL, Sereno D, Alten B. 2012. Wing size and shape variation of *Phlebotomus papatasi* (Diptera: Psychodidae) populations from the south and north slopes of the Atlas Mountains in Morocco. Journal of Vector Ecology, 37(1), 137–147.
58. Prudhomme J, Toty C, Kasap OE, Rahola N, Vergnes B, Maia C, Campino L, Antoniou M, Jimenez M, Molina R, Cannet A, Alten B, Sereno D, Banuls AL. 2015. New microsatellite markers for multi-scale genetic studies on *Phlebotomus ariasi* Tonnoir, vector of *Leishmania infantum* in the Mediterranean area. Acta Tropica, 142, 79–85.
59. Prudhomme J, Velo E, Bino S, Kadriaj P, Mersini K, Gunay F, Alten B. 2019. Altitudinal variations in wing morphology of *Aedes albopictus* (Diptera, Culicidae) in Albania, the region where it was first recorded in Europe. Parasite, 26, 55.
60. Rawlins DJ. 1992. Light Microscopy: An Introduction to Biotechniques. Oxford: Bios Scientific publishers. 143 pp.
61. Ready PD. 2013. Biology of phlebotomine sand flies as vectors of disease agents. Annual Review of Entomology, 58, 227–250.
62. Rohlf FJ, Slice D. 1990. Extensions of the Procrustes method for the optimal superimposition of landmarks. Systematic Zoology, 39(1), 40–59.
63. Rowley DL, Coddington JA, Gates MW, Norrbom AL, Ochoa RA, Vandenberg NJ, Greenstone MH. 2007. Vouchering DNA-barcoded specimens: Test of a nondestructive extraction proto-col for terrestrial arthropods. Molecular Ecology Notes, 7(6), 915–924.
64. Sábio PB, Andrade AJ, Galati EAB. 2014. Assessment of the taxonomic status of some species included in the *Shannoni*

complex, with the description of a new species of *Psathyromyia* (Diptera: Psychodidae: Phlebotominae). Journal of Medical Entomology, 51(2), 331–341.
65. Sadlova J, Yeo M, Seblova V, Lewis MD, Mauricio I, Volf P, Miles MA. 2011. Visualisation of *Leishmania donovani* fluorescent hybrids during early stage development in the sand fly vector. PLoS One, 6(5), e19851.
66. Sales K, Miranda DEO, da Silva FJ, Otranto D, Figueredo LA, Dantas-Torres F. 2020. Evaluation of different storage times and preservation methods on phlebotomine sand fly DNA concentration and purity. Parasites & Vectors, 13(1), 399
67. Sales KG, Costa PL, de Morais RC, Otranto D, Brandao-Filho SP, Cavalcanti Mde P, Dantas-Torres F. 2015. Identification of phlebotomine sand fly blood meals by real-time PCR. Parasites& Vectors, 8, 230.
68. Sant'Anna MR, Jones NG, Hindley JA, Mendes-Sousa AF, Dillon RJ, Cavalcante RR, Alexander B, Bates PA. 2008. Blood meal identification and parasite detection in laboratory-fed and field-captured *Lutzomyia longipalpis* by PCR using FTA databasing paper. Acta Tropica, 107(3), 230–237.
69. Senne NA, Santos HA, Araujo TR, Paulino PG, Mendonca LP, Moreira HVS, Camilo TA, da Costa Angelo I. 2022. Robust comparative performance of genomic DNA extraction methods from non-engorged phlebotomine sandflies. Medical and Veterinary Entomology, 36(2), 203–211.
70. Shaw JJ. 2025. A review of Leishmania infections in American Phlebotomine sand flies – Are those that transmit leishmaniasis anthropophilic or anthropportunists? Parasite, 32, 57.
71. Tesh RB, Modi GB. 1983. Growth and transovarial transmission of Chandipura virus (Rhabdoviridae: Vesiculovirus) in *Phlebotomus papatasi.* American Journal of Tropical Medicine and Hygiene, 32(3), 621–623.
72. Thomsen PF, Elias S, Gilbert MTP, Haile J, Munch K, Kuzmina S, Froese DG, Sher A, Holdaway RN, Willerslev E. 2009. Non-destructive sampling of ancient insect DNA. PLoS One, 4(4),e5048
73. Truett GE, Heeger P, Mynatt RL, Truett AA, Walker JA, Warman ML. 2000. Preparation of PCR-quality mouse genomic DNA with hot sodium hydroxide and tris (HotSHOT). Biotechniques, 29(1), 52–54.
74. Upton MS. 1993. Aqueous gum-chloral slide mounting media: an historical review. Bulletin of Entomological Research, 83(2), 267–274.
75. Volf P, Myskova J. 2007. Sand flies and Leishmania: specific versus permissive vectors. Trends in Parasitology, 23(3), 91–92.
76. Wang Q, Wang X. 2012. Comparison of methods for DNA extraction from a single chironomid for PCR analysis. Pakistan Journal of Zoology, 44(2), 421–426.
77. Wang Y, Zhao Y, Bollas A, Wang Y, Au KF. 2021. Nanopore sequencing technology, bioinformatics and applications. Nature Biotechnology, 39(11), 1348–1365.

Cite this article as: Randrianambinintsoa FJ, Augendre L, Prudhomme J, Martinet J-P, Loyer M, Mekarnia N, Kerkoub H, Perveen FK, Huguenin A, Kariya E, Akhoundi M, De Andrade AJ, Berriatua E, Bongiorno G, Boyer S, Christodoulou V, Da Costa-Ribeiro MCV, De Souza LAF, Ding H, Dondji B, Dvořák V, Erisoz Kasap O, Galati EAB, Gállego M, Ballart C, Gouzelou S, Haddad N, Masse RS, Mekuria AH, Ivovic V, Kaczmarek S, Shahar MK, Kirstein OD, Kniha E, Kolářová I, Lincoln T, Lucanas C, Mikov O, Nov K, Özbel Y, Pesson B, Posada Lopez LC, Prasetyo DB, Rahola N, Rebollar-Tellez EA, Rodrigues BL, Roy L, Saini P, Sanjoba C, Shimabukuro PH, Siriyasatien P, Soszynska A, Suleşco T, Sylla M, Torno M, Volf P, Vongphayloth K, Sinh Nam V, Wardhana A, Yessinou E, Zapata S, Gantier J-C & Depaquit J. 2026. Processing and mounting phlebotomine sand flies: a consensus guideline. Parasite xx, xx. https://doi.org/10.1051/parasite/2026009.

PARASITE

An international open-access, peer-reviewed, online journal publishing high quality papers on all aspects of human and animal parasitology

Reviews, articles and short notes may be submitted. Fields include, but are not limited to: general, medical and veterinary parasitology; morphology, including ultrastructure; parasite systematics, including entomology, acarology, helminthology and protistology, and molecular analyses; molecular biology and biochemistry; immunology of parasitic diseases; host-parasite relationships; ecology and life history of parasites; epidemiology; therapeutics; new diagnostic tools.
All papers in Parasite are published in English. Manuscripts should have a broad interest and must not have been published or submitted elsewhere. No limit is imposed on the length of manuscripts.

Parasite (open-access) continues **Parasite** (print and online editions, 1994-2012) and **Annales de Parasitologie Humaine et Comparée** (1923-1993) and is the official journal of the Société Française de Parasitologie.

Editor-in-Chief:
Jean-Lou Justine, Paris

Submit your manuscript at:
https://www.editorialmanager.com/parasite

附录 1：生化理论基础

本文主要讨论的节肢动物为白蛉，但其基本原理同样可适用于其他常见节肢动物，尤其是那些只能通过内部形态特征进行鉴定的类群。在这些昆虫中，一些内部器官部分发生几丁质化，其形态特征能够提供重要的分类信息。因此，观察消化系统（如口腔、咽甲）、受精囊及其受精囊管管等结构具有重要意义。在本指南所涉及的各类试剂处理中，应始终注意，从昆虫固定到最终封片的整个过程，本质上都是一系列氧化还原反应的应用。操作过程中需要遵循的基本原则是：避免将还原性试剂与氧化性试剂混合使用。

乙醇：

该试剂在标本处理过程中具有多种用途。酒精分子对水具有很强的亲和力，因此具有显著的脱水作用。然而，低浓度酒精（即含水量过高）反而可能促进核酸降解。

将昆虫置于乙醇中，不仅是为了保存标本，同时也是为了对组织进行固定。在组织学中，常区分两个重要概念：渗透速率与固定速率。理想的固定剂应当能够迅速渗透至组织深层，然后再完成固定作用。以 96% 乙醇为例，其渗透系数约为 1.05（相比之下，0.75% 苦味酸试剂（picric acid）的渗透系数为 0.45，而 3% 重铬酸钾（potassium dichromate）溶液为 1.45）。

将采集的昆虫及其他节肢动物长期保存于乙醇之中，在实践层面难以实现。诚然，将野外采集的样本留存下来，以供后续研究或留给未来的科研人员，无疑是值得尊敬的。然而，从细胞学或组织学的角度来看，这种长期保存方式并不可行。当样本在固定液中存放时间过长后，往往会变得几乎无法再次处理或利用。因此，保存时间超过 10 年的样本通常会变得难以使用，甚至完全无法再用于相关研究。

另一个需要考虑的重要因素是待固定节肢动物的体积与固定液体积之间的比例。在动物学或医学中，通常建议固定液体积应为标本体积的 60 倍。在实际操作中，对于微型节肢动物，建议每一定体积的标本至少加入 4–5 倍体积的酒精。需要注意的是，随着酒精逐渐从组织中吸收水分，其浓度会降低，从而削弱其固定与保存效果。

总结：

- 乙醇属于还原性化学试剂（因此不宜与氧化性固定剂同时使用）；
- 可强烈促使蛋白质沉淀（protein precipitation）并使其变性（denaturation）；
- 能溶解某些复杂脂类，并使糖原发生沉淀；
- 会引起组织明显收缩，并使其硬化。

氢氧化钾（potassium hydroxide）或氢氧化钠（sodium hydroxide）碱性溶液：

在昆虫学研究中，这类溶液的应用主要以氢氧化钾为主，但其使用原因往往并未被明确说明。

氢氧化钠 [E524] 通常以溶液形式存在，可配制成不同浓度或不同当量浓度，也可为颗粒状或片状。其主要缺点是极易吸湿（比 KOH 更强）。当其与蛋白质反应时，会使蛋白质溶解。与脂类反应时，则通过皂化作用将其转化为坚硬的皂类（这一点与 KOH 不同，KOH 皂化通常生成液态皂）。

氢氧化钾 [E525] 有浓缩液和颗粒状（约 0.1 克/颗粒）两种形式，后者尤其方便用于配制稀释液。例如，将一粒 0.1 g 的颗粒加入 1 mL 蒸馏水中，即可配制成约 10% KOH 溶液。氢氧化钾颗粒的另一优点是对碳酸化反应的敏感性较低（KOH 溶液对 CO_2 具有较强吸附能力，易形成碳酸盐（carbonated salts））。

这些强碱主要用于通过皂化反应将脂肪酸转化为可溶于水的皂类，从而实现脂质的溶解。需要注意的是，固定剂（如乙醇）在前期已溶解了部分脂类。然而，当样本转入含强碱的水性环境后，较为复杂的脂肪酸可能发生沉淀，此时强碱会在常温下完成皂化反应。在某些情况下，例如雌虫体内脂肪组织较多时，可适当提高温度至 35–40° C 以加快反应速度，或在室温条件下延长处理时间，以增强皂化效果。

有色酸性溶液 / 无色 Marc-André 溶液：

下文将对 Marc-André 溶液的优点与局限性进行分析。该溶液由水合氯醛（trichloracetaldehyde monohydrate）、醋酸和水组成，是一种具有较强氧化性的溶液（酸与醛的混合物）。它能够中和样本中残留的过量氢氧化钾，同时不会使在氢氧化钾处理过程中形成的碱性皂类发生沉淀。此外，这种氧化性溶液还能作用于构成几丁质的氨基葡萄糖中的仲醇基团，通过氧化作用使几丁质软化。其另一作用是溶解样本中存在的某些无机盐。

当 Marc-André 溶液预先以酸性品红进行染色（即处于氧化状态）时，它可固定于几丁质结构中的仲醇基团上。经过 Marc-André 溶液处理并完成染色后，标本仅需用乙醇进行冲洗，即可进入后续的脱水处理。

优点：

- 可中和残留的碱性溶液；
- 可使几丁质结构软化；
- 可对几丁质进行染色，从而更清晰地观察已几丁质化的内部结构。

缺点：

水合氯醛具有催眠作用，曾用于人类医学。使用时必须在通风柜内操作，并严格遵守相关化学品安全法规。

脱水溶液：

据观察，对于体型极小的样本，无需依次通过浓度递增的乙醇梯度进行脱水。若样本较大，则通常从 80% 乙醇开始，依次转入 90%、95%，最后至无水乙醇。对于极小的样本，仅需先在 90% 乙醇中处理，再转入无水乙醇即可。在此阶段需要注意，无水乙醇具有很强的吸水性，会吸收空气中的水分。

在昆虫学中，样本脱水过程常以山毛榉木焦油（beech creosote）处理作为最后一步。然而，这种物质广泛用于杀虫、防霉及木材防腐，因其强烈气味（含多环芳香烃 polycyclic aromatic hydrocarbons）以及被认为具有生殖毒性、致癌性、持久性有机污染物特性，并对水生生物具有生态毒性，如今已不推荐使用。

本文建议使用 Euparal® 与 Euparal® 精油（详见下文）作为封片前的过渡处理溶液。将样本在 90% 乙醇处理后，转入 Euparal® 与 Euparal® 精油的混合液中，通常可获得良好的处理效果，并有利于后续封片操作。

附录 2：所用试剂的配方

10% 氢氧化钾溶液

氢氧化钾 10 g

蒸馏水（q.s.）加至 100 mL

氯醛树胶封片介质（Hoyer 封片溶液）

蒸馏水 50 mL

水合氯醛 200 g

阿拉伯树胶 50 g

丙三醇（甘油）20 mL

Marc-André 溶液

水合氯醛 40 g

冰醋酸 30 mL

蒸馏水 30 mL

1% 酸性品红溶液（蒸馏水配制）

酸性品红粉末 1 g

蒸馏水 99 mL

酸性品红染色的 Marc-André 溶液

Marc-André 溶液 10 mL

1% 酸性品红溶液 50 μL

This is an Open Access article distributed under the terms of the Creative Commons Attribution License (https://creativecommons.org/licenses/by/4.0), which permits unrestricted use, distribution, and reproduction in any medium, provided the original work is properly cited.

附录 3：Euparal®、加拿大胶、聚乙烯醇及其他封片介质

聚乙烯醇（polyvinyl alcohol）：当缺乏用于充分脱水的必要试剂时，聚乙烯醇可作为替代封片介质使用。通常将聚乙烯醇与 Amman 乳酚溶液（lactophenol）混合后用于封片。然而，此类封片方式存在明显缺点。首先，随着水分蒸发，封片可能发生干燥或聚乙烯醇结晶。此外，当其中的苯酚发生氧化时，封片介质还可能出现变黑现象。因此，该方法更适用于短期性封片，而不适合长期保存。

加拿大胶（Canada balsam）：这类介质用于标本的封片。封片前必须先对标本进行充分脱水处理。其制备过程中常需使用二甲苯（xylene）或甲苯（toluene）等溶剂，但这些溶剂在操作与安全性方面均存在一定不便与风险。

Enecê 封片介质：与加拿大胶类似，使用 Enecê 在载玻片与盖玻片之间封片时，同样需要对标本进行脱水处理。Enecê 配方包括纯白松香（colophony）22 g，醇溶性 copal 树脂 12 g，无水乙醇 20 mL，樟脑（camphor）10 g，松节油精（turpentine essence）10 mL，与桉油精（eucalyptol）26 mL。

制备方法：在容器（如锥形瓶）中先加入无水乙醇与樟脑，随后加入松香与 copal 树脂。用塞子密封后摇匀，再将其置于水浴（建议 bain-marie 实验装置）中以温和温度加热，使混合物充分溶解但避免沸腾。当内容物完全液化后，加入松节油精，并在仍处于热态时进行过滤，最后向滤液中加入桉油精。当该封片介质黏度增加、流动性下降时，可使用稀释用的 Enecê 溶液进行调配，其配方如：无水乙醇 30 mL，樟脑 17 g，松节油精 15 mL，桉油精 38 mL（Cerqueira，1943）。

Euparal®：Euparal® 是一种来源于柏树 *Tetraclinis articulata*（Vahl，1791）的树脂，由 Gilson 于 1906 年进行研究并加以开发。其主要优点在于不会发生聚合反应。因此，夹在载玻片与盖玻片之间封片的样本，可通过酒精，或者更理想地通过 Euparal® 精油处理而较容易地重新取出。该树脂亦称为 sandarac，对乙醇具有较好的相容性，可接受自 80% 浓度起的乙醇处理后的标本。

Triton X100 的应用：非离子型水溶液

Triton X100 是一种非离子型水溶液（*4-(1,1,3,3-tetramethylbutyl) phenyl-polyethylene glycol solution, or t-octylphenoxypolyethoxyethanol, polyethylene glycol tert-octylphenyl ether*），在细胞生物学和分子生物学中广泛用作去污剂。它能够增加细胞膜及细胞核膜的通透性。

长期保存在酒精中的昆虫标本非常常见。然而，酒精保存并非理想的长期保存方式，往往会导致节肢动物标本逐渐难以进行标本显微观察的制备。盛放样本的塑料容器也常发生降解，随后导致酒精挥发。在这两种情况下，标本与酒精的长期接触或干燥都会带来明显问题。2008 年，Jonque 曾发表一则关于使用润湿剂（如摄影中使用的 Agepon）对蜘蛛标本进行复水的技术说明[26]，从而启发了采用非强力去污剂类润湿剂进行标本复水。

以下为使用 0.5% Triton X100 溶液的操作流程：

- 先用无水乙醇充分浸润干燥标本；
- 加入适量 0.5% Triton X100 溶液，使标本完全浸没；
- 静置约 5 分钟或更长时间，使各个节肢动物个体在溶液中充分分离；
- 移除 Triton X100 溶液，并以氢氧化钾溶液替换。

随后按前文所述流程继续进行处理。

附录 4：Euparal® 或加拿大胶封片操作流程

基本原则：

1. 标本必须充分脱水（若呈现浑浊或乳白状，说明脱水不完全）。
2. 脱水可依次通过浓度递增的乙醇梯度进行。
3. 标本可由 99% 乙醇或无水乙醇转入透明剂中进行处理。

操作步骤：

1. 将成虫白蛉放置于 70% 乙醇中。
2. 去除乙醇，加入 10% 氢氧化钾，并用载玻片覆盖白蛉标本。
3. 进行组织消解处理，直至昆虫体变为透明。
4. 去除氢氧化钾。
5. 加入蒸馏水覆盖标本，静置 30 – 45 分钟。
6. 去除水，再用蒸馏水冲洗 30 分钟。该时间取决于处理样本数量：样本越多，冲洗时间需越长；样本较少，尤其是单个处理时，可适当缩短。
7. 去除水。
8. 加入 Marc-André 溶液（可用酸性品红染色），处理 24 小时。
9. 去除 Marc-André 溶液。
10. 加入蒸馏水覆盖标本，静置 30 – 45 分钟。
11. 去除水，再用蒸馏水冲洗 30 分钟。
12. 去除水。
13. 加入 70% 乙醇并进行解剖：
    a. 对于头部与腹部：轻轻将头部或腹部从胸部分离；
    b. 对于胸部：用一把镊子固定胸部，另一把在翅基部将翅取下。也可根据研究重点进行矢状面解剖，将胸部分为左右两部分。
14. 依次通过不同浓度乙醇进行梯度脱水：50% → 80% → 95% → 无水乙醇。
15. 使用 100% 乙醇清洗标本两次，每次 10 分钟，以完成最终脱水。
16. 去除乙醇，在室温下将标本浸入丁香油中 15 分钟。
17. 将标本从丁香油转移至洁净载玻片上的一滴 Euparal® 或加拿大胶中。
18. 调整标本位置：在解剖显微镜下，用细针或镊子分离并排列头部、胸部和腹部。头部必须从身体上分离，并以腹面朝上（ventro-dorsal）方式封片，即使后头大孔朝上，以便直接观察口腔结构。解剖操作应在封片介质中进行。
19. 静置标本，直至封片介质表面变得略具黏性。
20. 用无水乙醇润湿一片洁净盖玻片，并以倾斜角度将其缓慢覆盖于 Euparal® 或加拿大胶上。
21. 将制好的玻片存放于专用的干燥保存盒中。
