# Supplementary material for: Processing and mounting phlebotomine sand flies: a consensus guideline
Source: Parasite. 2026 Apr 3;33:18. doi: 10.1051/parasite/2026009 (PMC13047900; doi:10.1051/parasite/2026009)
Supplement: Supplementary file 6 — Czech translation / Překlad do češtiny [file parasite-33-18-s6.pdf]

Parasite **33**, 18 (2026)
© F.J. Randrianambinintsoa et al., published by EDP Sciences, 2026
https://doi.org/10.1051/parasite/2026009

PARASITE
Available online at:
www.parasite-journal.org

REVIEW ARTICLE OPEN ACCESS

Zpracování a montování flebotomů: konsenzuální doporučení

Fano José Randrianambinintsoa[1], Laure Augendre[1], Jorian Prudhomme[1], Jean-Philippe Martinet[1], Mathieu Loyer[1], Nalia Mekarnia[1], Hocine Kerkoub[1], Farzana Khan Perveen[1], Antoine Huguenin[1,2], Emilie Kariya[1,2], Mohammad Akhoundi[3], Andrey José de Andrade[4], Eduardo Berriatua[5], Gioia Bongiorno[6], Sébastien Boyer[7,8], Vasiliki Christodoulou[9], Magda Clara Vieira Da Costa-Ribeiro[10], Lucas Alexandre Farias de Souza[10], Huicong Ding[11], Blaise Dondji[12], Vít Dvořák[13], Ozge Erisoz Kasap[14], Eunice Aparecida Bianchi Galati[15], Montserrat Gállego[16], Cristina Ballart[16], Stavroula Gouzelou[17], Nabil Haddad[18], Rezki Sabrina Masse[19], Asrat Hailu Mekuria[20], Vladimir Ivovic[21], Szymon Kaczmarek[22], Mohd Khadri Shahar[19], Oscar D. Kirstein[23], Edwin Kniha[24], Iva Kolářová[13], Lincoln Timinao[25], Cristian Lucanas[26], Ognyan Mikov[27], Kimsear Nov[7], Yusuf Özbel[28], Bernard Pesson[29], Laura Cristina Posada Lopez[30], Didot Budi Prasetyo[1,7], Nil Rahola[31], Eduardo A. Rebollar-Tellez[32], Bruno Leite Rodrigues[15], Lalita Roy[33], Prasanta Saini[34], Chizu Sanjoba[35], Paloma Helena Fernandes Shimabukuro[36], Padet Siriyasatien[37], Agnieszka Soszyńska[22], Tatiana Suleşco[38], Massamba Sylla[39], Majhalia Torno[40], Petr Volf[13], Khamsing Vongphayloth[41], Vu Sinh Nam[42], April Wardhana[43], Eric Yessinou[44], Sonia Zapata[45], Jean-Charles Gantier[1], and Jérôme Depaquit[1,2,*]

[1] Faculté de Pharmacie, Université de Reims Champagne Ardenne, UR ESCAPE-USC ANSES PETARD, 51 rue Cognacq-Jay, 51096 Reims Cedex, France
[2] Pôle de Biologie territoriale, Laboratoire de Parasitologie-Mycologie, Centre Hospitalo-Universitaire, 51092 Reims, France
[3] Parasitology-Mycology Department, Avicenne Hospital, AP-HP, Bobigny, Sorbonne Paris Nord University, France; Unité des Virus Émergents (UVE: Aix-Marseille Univ, Università di Corsica, IRD 190, Inserm 1207, IRBA), 13005 Marseille, France
[4] Parasitology Collection of Basic Pathology, Department of Basic Pathology, Federal University of Paraná, Curitiba 19031, Brazil
[5] Department of Animal Health, University of Murcia, Campus de Espinardo, 30100 Espinardo, Murcia, Spain
[6] Department of Infectious Diseases, Vector-borne Diseases Unit, Istituto Superiore di Sanità, 00166 Rome, Italy
[7] Medical and Veterinary Entomology Unit, Institut Pasteur du Cambodge, Phnom Penh 12201, Cambodia
[8] Ecology & Emergence of Arthropod-borne Pathogens Unit, Department of Global Health, Institut Pasteur, CNRS UMR2000, 75015 Paris, France
[9] Section Veterinary Services (1417), Laboratory for Animal Health Virology, Aglantzia, Nicosia 2109, Cyprus
[10] Insects Vectors and Parasites Laboratory, Department of Basic Pathology and Postgraduate program in Microbiology, Parasitology and Pathology, Federal University of Paraná, 81530-900 Curitiba, Brazil
[11] Department of Biological Sciences, National University of Singapore, 117558, Singapore
[12] Laboratory of the Leishmaniasis Research Project, Mokolo District Hospital, Mokolo, Cameroon; Laboratory of Cellular Immunology and Parasitology, Department of Biological Sciences, Central Washington University, 98926 Ellensburg, WA, USA
[13] Department of Parasitology, Faculty of Science, Charles University, 12800 Prague, Czechia
[14] VERG Laboratories, Department of Biology, Faculty of Science, Hacettepe University, Beytepe, Ankara 06800, Türkiye
[15] Faculdade de Saúde Pública da Universidade de São Paulo (FSP/USP), Pós-graduação em Saúde Pública, 01246-904 São Paulo, Brazil
[16] Secció de Parasitologia, Departament de Biologia, Sanitat i Medi Ambient, Facultat de Farmàcia i Ciències de l'Alimentació, Universitat de Barcelona, & Institut de Salut Global de Barcelona (ISGlobal), Centro de Investigación Biomédica en Red, Enfermedades Infecciosas (CIBERINFEC), 08028 Barcelona, Spain
[17] Laboratory of Infectious Diseases and Public Health, School of Medicine, University of Cyprus, Nicosia, Cyprus & Department of Pediatrics, Archbishop Makarios III Hospital, Nicosia 2115, Cyprus
[18] Faculty of Health Sciences, American University of Beirut, 1107 2020 Beirut, Lebanon
[19] Medical Entomology Unit, Infectious Disease Research Centre, Institute for Medical Research (IMR), National Institutes of Health (NIH), Ministry of Health Malaysia, 40170 Shah Alam, Selangor, Malaysia
[20] School of Medicine, Addis Ababa University, 28017 - 1000 Addis Ababa, Ethiopia
[21] Faculty of Mathematics, Natural Sciences and Information Technologies, University of Primorska, 6000 Koper, Slovenia
[22] University of Lodz, Faculty of Biology and Environmental Protection, Department of Invertebrate Zoology and Hydrobiology, Banacha 12/16, 90-237 Łódź, Poland
[23] Laboratory of Entomology, Ministry of Health, 9134302 Jerusalem, Israel

Edited by Jean-Lou Justine
*Corresponding author: jerome.depaquit@univ-reims.fr

This is an Open Access article distributed under the terms of the Creative Commons Attribution License (https://creativecommons.org/licenses/by/4.0), which permits unrestricted use, distribution, and reproduction in any medium, provided the original work is properly cited.

[24] Center for Pathophysiology, Infectiology and Immunology, Institute of Specific Prophylaxis and Tropical Medicine, Medical University Vienna, Kinderspitalgasse 15, 1090 Vienna, Austria
[25] Papua New Guinea Institute of Medical Research (PNGIMR) Institute, PO Box 60, Headquarter, Homate Street, 441 Goroka, Eastern Highlands Province, Papua New Guinea
[26] Museum of Natural History, University of the Philippines Los Baños, 4031 Laguna, Philippines
[27] National Centre of Infectious and Parasitic Diseases, 1504 Sofia, Bulgaria
[28] Ege University, Faculty of Medicine, Department of Parasitology, 35040 Bornova/Izmir, Türkiye
[29] Retired, Faculté de Pharmacie, Université de Strasbourg, Strasbourg, 67400 Illkirch-Graffenstaden, France
[30] Program for the Study and Control of Tropical Diseases (PECET), Faculty of Medicine, University of Antioquia, 050010 Medellin, Colombia
[31] MIVEGEC, Univ. Montpellier, CNRS, IRD, 34394 Montpellier, France & Medical Entomology Unit, Institut Pasteur de Madagascar, 101 Antananarivo, Madagascar
[32] Laboratorio de Entomología Médica, Departamento de Zoología de Invertebrados, Facultad de Ciencias Biológicas, Universidad Autónoma de Nuevo León, San Nicolás de los Garza, 66455, NL, México
[33] Tropical and Infectious Disease Centre, BP Koirala Institute of Health Sciences, Dharan 56700, Nepal
[34] ICMR-Vector Control Research Centre, Puducherry 605006, India
[35] Graduate School of Agricultural and Life Sciences, The University of Tokyo, Tokyo 113-8657, Japan
[36] Grupo de estudos em Leishmanioses/Coleção de Flebotomíneos (COLFLEB/Fiocruz-MG), Instituto René Rachou, Fundação Oswaldo Cruz, Belo Horizonte, Minas Gerais, 30190009, Brazil
[37] Center of Excellence in Vector Biology and Vector-Borne Disease, Department of Parasitology, Faculty of Medicine, Chulalongkorn University, Bangkok 10330, Thailand
[38] Department of Arbovirology, Bernhard Nocht Institute for Tropical Medicine, Bernhard Nocht Str. 74, 20359 Hamburg, Germany [39] Laboratory Vectors & Parasites, Department of Livestock Sciences and Techniques, Sine Saloum University El Hadji Ibrahima Niasse (SSUEIN) Kaffrine Campus, C.P. 24600, Senegal.
[40] Environmental Health Institute, National Environment Agency, Singapore 138667, Singapore & Department of Biological Sciences, National University of Singapore, 117558 Singapore
[41] Institut Pasteur du Laos, Laboratory of Vector-Borne Diseases, Samsenhai Road, Ban Kao-Gnot, Sisattanak District, 3560 Vientiane, Lao PDR
[42] National Institute of Hygiene and Epidemiology, 1 Yec-Xanh Street, Hai Ba Trung District, 100000 Hanoi, Vietnam
[43] Indonesian Research Center for Veterinary Science, Indonesian Agency for Agricultural Research and Development, Ministry of Agriculture Republic Indonesia, Bogor 16114, Indonesia & Department of Parasitology, Faculty of Veterinary Medicine, Airlangga University, Surabaya 60115, Indonesia
[44] Laboratory of Research in Applied Biology, Polytechnic School of Abomey-Calavi, University of Abomey-Calavi, 01 P.O. Box 2009, 00000 Cotonou, Benin
[45] Instituto de Microbiología, Colegio de Ciencias Biológicas y Ambientales (COCIBA), Universidad San Francisco de Quito (USFQ), 170901 Quito, Ecuador

Received 1 December 2025, Accepted 29 January 2026, Published online 3 April 2026

Abstrakt – Tento článek poskytuje komplexní návod pro zpracování a přípravu mikroskopických preparátů (montování) flebotomů, což je klíčové pro jejich druhové určení a pro detekci a izolaci patogenů. Pojednává o řadě technik vhodných jak pro terénní, tak pro laboratorní podmínky. Průvodce zahrnuje podrobné instrukce k odchytu flebotomů, manipulaci s nimi a jejich usmrcování (doporučuje se suché zmrazení nebo CO2 namísto chemikálií), stejně jako strategie konzervace, jako je skladování v chladu a uchovávání v ethanolu. Kvalita preparace určitých anatomických struktur (pohlavní orgány, hlava a křídla) je zásadní pro jejich správné mikroskopické pozorování. Článek také představuje detailní postup zpracování vzorků, včetně procesu projasňování pomocí činidel jako je hydroxid draselný a následně roztok Marc-André. Porovnává různé způsoby zalévání do médií s důrazem na jejich optické vlastnosti a konzervační potenciál. Hoyerovo médium je doporučováno pro rychlé pozorování, zejména spermaték, díky své čirosti, ačkoli není vhodné pro dlouhodobé skladování. Mezi další diskutovaná média patří polyvinylalkohol, Euparal® a kanadský balzám, médium rozpustné v uhlovodících, přičemž poslední dvě jmenovaná nabízejí možnost dlouhodobé konzervace. Pozornost je věnována také inovativním přístupům molekulární biologie, jako je sekvenování DNA a MALDI-ToF proteinové profilování, které vyžadují zvláštní zřetel při zpracování vzorků. Návod nabízí krátké videoklipy, ilustrující různé techniky přípravy preparátů, a také překlady do mnoha různých jazyků, což tomuto návodu umožňuje naplnit rozmanité potřeby a očekávání celosvětové vědecké komunity.

Klíčová slova: Montování, flebotomové, Hoyerovo médium, roztok Marc-André, chloral guma, polyvinylalkohol, Euparal®, kanadský balzám, izolace Leishmania, terénní podmínky, kultivace, pitva, molekulární biologie, MALDI-ToF, typové exempláře

Abstract – Processing and mounting phlebotomine sand flies: a consensus guideline. This article provides a comprehensive guide for the processing and mounting of phlebotomine sand fly specimens, which is crucial for species identification and pathogen detection and isolation. It discusses a range of techniques suitable for both field and laboratory settings. The guide includes detailed instructions on sand fly collection, handling, covering, and euthanasia (recommending dry freezing or CO_2 over chemicals) as well as conservation strategies, such as cold storage and preservation in ethanol. The quality of preparation of certain anatomical structures (genital organs, head and wings) is essential for their proper microscopic observation and is described in this work. The article also presents detailed sample processing, including the clearing process with agents such as potassium hydroxide then Marc-André solution. The mounting process compares different media, emphasizing their optical properties and preservation potential. Hoyer fluid (also known as chloral gum) is recommended for quick observation, particularly for spermathecae, due to its clarity, although it is not suitable for long-term storage. Other media discussed include polyvinyl alcohol, Euparal® (for limited water tolerance), and Canada balsam (a hydrocarbon-soluble medium), with the latter two offering long-term preservation capabilities. Innovative molecular biology approaches such as DNA sequencing and MALDI-ToF, which require particular attention to sample processing, are also addressed. Furthermore, short video clips illustrating various mounting techniques as well as translations in many different languages are provided, allowing the guideline to reach the diverse needs and expectations of the global scientific community.

Key words: Mounting, Phlebotomine sand fly, Hoyer fluid, Marc-André solution, Chloral gum, Polyvinyl alcohol, Euparal®, Canada balsam, *Leishmania* isolation, Field conditions, Culture, Dissection, Molecular biology, MALDI-ToF, Type-specimens.

Úvod

Flebotomové jsou drobný dvoukřídlý hmyz patřící do čeledi Psychodidae, podčeledi Phlebotominae, s dosud 1 063 popsanými druhy [21]. Jsou významnými přenašeči patogenů (arboviry bakterie rodu *Bartonella* a parazitičtí prvoci rodu *Leishmania*) odpovědných za onemocnění jako arbovirové infekce, bartonelóza a leishmaniózy. Jejich identifikace je primárně založena na detailním mikroskopickém posouzení morfologických znaků na preparátech, připravených po sběru, vhodném skladování a zamontování do vhodného média, což vyžaduje několik specifických technik, z nichž každá má své výhody a omezení.

Identifikace dospělých flebotomů je založena na pozorování vnějších (např. tykadla, makadla, samčí genitálie) i vnitřních struktur (např. hltan, cibarium a spermatéky). Pitva a izolace posledně jmenovaných usnadňuje jejich pozorování a následně přesnou identifikaci. Proto na rozdíl od větších zástupců hmyzu (komáři, ploštice) vyžadují flebotomové před identifikací montování mezi podložní a krycí sklíčko. Až do 80. let 20. století bylo mikroskopické pozorování jedinou dostupnou metodou pro identifikaci flebotomů a dodnes zůstává nejpoužívanějším přístupem. Volba postupu a přípravy byla tedy poměrně přímočará a založená hlavně na dichotomii: na jedné straně definitivní preparát umožňující dlouhodobé uchování vzorku a na straně druhé rychlý preparát pro identifikaci v médiu, které nezaručuje dlouhodobé uchování. Finální montování, například do pryskyřice jako je kanadský balzám, je časově náročné a vyžaduje úplnou dehydrataci vzorků. Navíc index lomu tohoto média není vždy optimální pro snadné pozorování spermaték. Montování do vodného média (např. Hoyerovo médium) je naproti tomu rychlejší a umožňuje lepší vizualizaci spermaték, ale neumožňuje dlouhodobé uchování preparátů, protože má tendenci absorbovat vodu z atmosféry. Jednou z možností je utěsnit sklíčko lakem na nehty, jakmile je zcela suché.

Tato volba kompromisu přetrvává dodnes a ovlivňuje výběr zalévací metody v závislosti na zamýšleném účelu preparátu. Od 80. let 20. století kombinují studie identifikace flebotomů morfologické a biochemické přístupy. Prvním byla analýza kutikulárních uhlovodíků, která byla rychle nahrazena technikami molekulární biologie (tj. náhodně amplifikovaná polymorfní DNA (RAPD), polymorfismus délky restrikčních fragmentů (RFLP), amplifikace DNA a sekvenování Sangerovou metodou, stejně jako sekvenování nové generace (NGS)). Dnes jsou molekulární přístupy doplňovány proteomickými metodami, jako je MALDI-ToF proteinové profilování. Navíc lze molekulární identifikaci druhů kombinovat s molekulární detekcí patogenů (*Leishmania*, *Trypanosoma*, *Bartonella*, různé fleboviry), jelikož všechny lze detekovat pomocí PCR nebo real-time PCR, což vyžaduje přizpůsobení procesu vzorkování a skladování stanoveným cílům [3, 32]. Kromě morfologických znaků, tradičně používaných pro rozlišení druhů, lze aplikovat i jiné morfologické přístupy (tj. geometrická morfometrie křídel).

Cílem této práce, založené převážně na vlastních zkušenostech autorů a údajích z literatury, bylo poskytnout standardizované pokyny pro montování a zpracování dospělých flebotomů za účelem optimalizace morfologických a molekulárních analýz. Potřeba provádět určité analýzy (např. molekulární biologie nebo MALDI-ToF proteinové profilování) vyžaduje zachování části flebotoma, která není nutná pro morfologickou identifikaci. Naše práce uvádí vhodné metody anestezie a usmrcení živě odchycených flebotomů, jejich skladování a proces

montování pro rychlou identifikaci nebo pro dlouhodobou konzervaci umožňující následné studie.

Preambule: Bezpečnostní a regulační aspekty by měly odkazovat na příslušné bezpečnostní listy (SDS)

Se všemi chemikáliemi uvedenými v tomto návodu musí být manipulováno za přísných bezpečnostních podmínek.

Tabulka 1: Seznam zkratek.

**BME**	Basal medium Eagle (Bazální médium Eagle)
**CDC**	Centers for Disease Control and Prevention (Centra pro kontrolu a prevenci nemocí)
**CMCP**	Camphor-monochlorophenol (Kafr-monochlorfenol)
**CMR**	Carcinogenic, mutagenic, reprotoxic substance (Karcinogenní, mutagenní, reprotoxická látka)
**COI**	Cytochrome c oxidase subunit I gene (Gen podjednotky I cytochrom c oxidázy)
**CytB**	Cytochrome b gene (Gen cytochromu b)
**DNA**	Deoxyribonucleic acid (Deoxyribonukleová kyselina)
**ELISA**	Enzyme-linked immunosorbent assay
**EtOH**	Ethanol
**M199**	Medium 199
**MALDI-ToF MS**	Matrix-assisted laser desorption/ionization time-of-flight mass spectrometry (Hmotnostní spektrometrie MALDI-ToF)
**MEM**	Minimum essential media (Minimální esenciální médium)
**NGS**	Next-generation sequencing (Sekvenování nové generace)
**NNN**	Novy-MacNeal-Nicolle medium
**PCR**	Polymerase chain reaction (Polymerázová řetězová reakce)
**Lao PDR**	Lao People's Democratic Republic (Laoská lidově demokratická republika)
**PNOC**	Prepronociceptin gene (Gen prepronociceptinu)
**qPCR**	Quantitative PCR (real-time PCR) (Kvantitativní PCR / real-time PCR)
**RAPD**	Random amplified polymorphic DNA (Náhodně amplifikovaná polymorfní DNA)
**RFLP**	Restriction fragment length polymorphism (Polymorfismus délky restrikčních fragmentů)
**RI**	Refractive index (Index lomu)
**RNA**	Ribonucleic acid (Ribonukleová kyselina)
**RNases**	Ribonucleases (Ribonukleázy)
**RNASS**	RNA stabilization solution (Stabilizační roztok RNA)
**RT-PCR**	Reverse transcription PCR (PCR s reverzní transkripcí)
**TFA**	Trifluoroacetic acid (Kyselina trifluoroctová)

Komise a pracovníci pro bezpečnost a ochranu zdraví ve výzkumných zařízeních jsou k dispozici pro poskytnutí informací nejen o nebezpečnosti těchto chemikálií, ale také o postupech manipulace s nimi a likvidaci odpadu. Je nutné dodržovat bezpečnostní pokyny týkající se jejich použití a likvidace. Je odpovědností všech uživatelů zajistit dodržování správné a bezpečné laboratorní praxe a platných zákonů a předpisů jejich země nebo výzkumné instituce. Navíc některé chemikálie nebo jejich složky (např. chloralhydrát) jsou v některých zemích regulovány. Seznam zkratek použitých v tomto rukopisu je uveden v Tabulce 1.

1. Odchyt flebotomů

Dospělí flebotomové mohou být sbíráni živí nebo mrtví pomocí různých metod, jako jsou CDC světelné pasti, lepové pasti a exhaustory v Shannonových pastech nebo přímo z míst odpočinku v prostředí (např. přístřešky pro zvířata). Tyto metody zahrnují umístění pastí do vhodných stanovišť, přilákání flebotomů světlem nebo jinými atraktanty (CO_2 nebo chemické návnady) a jejich sběr pro další analýzu, jak je popsáno v několika publikacích [2, 3, 32, 36, 49]. Odchyt živých flebotomů umožňuje všechny následné aplikace, zatímco sběr mrtvých jedinců znemožňuje izolaci prvoků rodu *Leishmania* nebo virů. Některé techniky odchytu, jako jsou lepové pasti, mohou vést ke ztrátě orgánů flebotomů (tykadla, makadla, křídla nebo nohy). Navíc ricinový olej pokrývající lepové papíry ulpívá na flebotomech a musí být odstraněn na začátku zpracování, obvykle pomocí patnáctiminutové lázně ve směsi ethanolu a diethyletheru ve stejném poměru.

2. Usmrcení vzorků

Po odchytu musí být živí flebotomové usmrceni. U některých metod sběru (např. lepové pasti, CDC světelné pasti vybavené nádobou obsahující detergent nebo ethanol) jsou flebotomové mrtví již při sběru. Metody molekulární biologie lze aplikovat na ty, kteří byli sbíráni přímo do ethanolu, a na ostatní, pokud jsou uloženi do ethanolu co nejrychleji. Žádná z těchto metod usmrcení však neumožňuje zpracování hmyzu pomocí MALDI-ToF proteinového profilování. Kromě toho mohou některé metody usmrcení způsobit ztrátu určitých morfologických znaků. Je proto nezbytné použít vhodné standardní usmrcovací činidlo, aby byla zajištěna správná identifikace nebo dlouhodobé uchování jako dokladové exempláře (tj. vzorky uchované a uložené pro budoucí reference nebo srovnání). Chemikálie jako ethylacetát, ethylether, tetrachloretan a chloroform lze napustit do vaty a umístit do nádoby s flebotomy k jejich usmrcení. S těmito činidly by se mělo zacházet opatrně a podle doporučení výrobce vzhledem k jejich toxicitě. Nedoporučujeme však usmrcovat flebotomy pomocí chloroformu, protože je podle našich zkušeností málo kompatibilní s metodami molekulární biologie. Vzhledem k nebezpečné povaze všech těchto produktů a jejich sporné vhodnosti pro

molekulární analýzy se používání těchto chemikálií obecně nedoporučuje.

Nejpoužívanější metodou, která zachovává morfologii, DNA i proteiny, je suché zmrazení vzorků. Vzorky musí být zmrazeny dostatečně dlouho, aby byly plně anestetizovány, ale ne tak dlouho, aby (i) vyschly, nebo (ii) byla ohrožena životaschopnost leishmanií, pokud je cílem jejich izolace in vitro z trávicího traktu flebotoma. **Doporučujeme proto dobu zmrazení 15 až 20 minut při -20 °C, s pravidelnou kontrolou, aby bylo zajištěno, že jsou pouze omráčeni, aniž by došlo k usmrcení parazitů rodu *Leishmania*.**

Pokud není k dispozici mrazák, lze hmyz alternativně usmrtit pomocí CO_2. V terénních podmínkách, kde nelze použít tlakové lahve s CO_2, lze vzorky usmrtit s pomocí malých komerčních nádobek s CO_2 používaných v sifonových lahvích, ale mohou existovat omezení pro jejich leteckou přepravu. Jako poslední možnost lze hmyz usmrtit vystavením tabákovému kouři. Flebotomové jsou odchyceni živí do CDC pasti, sebráni exhaustorem, ponecháni ve skleněné trubici a vystaveni tabákovému kouři, který je během několika sekund usmrtí. Tato metoda je použitelná i v náročných terénních podmínkách. Protože se však sklo nasytí kouřem, nelze jej použít pro následný sběr a manipulaci s živými flebotomy bez důkladného vyčištění. Nicméně tentýž nevyčištěný exhaustor lze stále použít k usmrcování flebotomů z jiných pastí pro účely fixace. Je také nutné zkontrolovat, zda byly z exhaustoru odstraněny všechny exempláře. Tyto metody jsou kompatibilní s následnou izolací leishmanií prostřednictvím pitvy střeva.

3. Skladování vzorků před zpracováním

Existují pět hlavní metody fixace před zpracováním:

3.1. Zmrazení

Tato metoda se nejlépe provádí při -20 °C nebo, pokud možno, při -80 °C. Tyto metody skladování jsou nyní používanější než skladování v kapalném dusíku. Ve všech případech musí být kryokonzervace provedena co nejrychleji po omráčení vzorků. Skladování v chladu v mrazácích nabízí výhodu plného zachování samotného hmyzu, stejně jako RNA, DNA a proteinů s plnou integritou po celou dobu skladování. Naproti tomu kapalný dusík může vážně poškodit křídla, nohy a tykadla a tím odstranit klíčové morfologické znaky. Skladování v suchém mrazu je pro vzorky méně traumatické, ale není ideální pro zachování jejich křehkých orgánů. Důležité je, že v době rozmrazování mohou křídla, tykadla nebo nohy přilnout k povrchu nádobky, a nakonec se vlivem kondenzace odtrhnout. Uchovávání zmrazením není v terénních studiích vždy proveditelné, protože vyžaduje přístup k mrazáku nebo nádobě s kapalným dusíkem. Skladování v mrazáku je plně kompatibilní s detekcí patogenů pomocí molekulárních nástrojů bez ztráty citlivosti, ačkoli detekce a izolace RNA virů vyžaduje zmrazení při -80 °C nebo v kapalném dusíku, pokud je požadováno dlouhodobé skladování. Zmrazení vzorků však neumožňuje izolaci leishmanií prostřednictvím pitvy střeva, s výjimkou případů, kdy jsou flebotomové nejprve ponořeni do plynné fáze a poté do kapalného dusíku (například v lahvičkách umístěných v punčoše), což simuluje kryokonzervaci leishmanií.

3.2. Skladování v alkoholu (ethanol nebo isopropylalkohol)

Toto je pravděpodobně nejpoužívanější metoda pro skladování flebotomů. Je snadno použitelná v terénu, i v obtížných podmínkách bez přístupu k laboratoři. Uchovávání v alkoholu je zvláště vhodné pro morfologické studie, protože křehké orgány (křídla, nohy, tykadla) zůstávají neporušené, pokud ve skladovací zkumavce nejsou vzduchové bubliny. Proto doporučujeme utěsnit zkumavku malým kouskem vaty, aby se odstranily veškeré vzduchové bubliny, a umístit štítek na horní část vatové zátky. Vhodná koncentrace alkoholu zůstává předmětem diskusí. Obecně se nedoporučují koncentrace pod 70 % [45, 66]. Vyšší koncentrace uchovávají DNA účinněji a po delší dobu, ale činí vzorky křehčími a lámavějšími pro morfologické studie. Použití 96% ethanolu (azeotropní směs) zajišťuje stabilitu koncentrace v čase, zejména ve vlhkých oblastech, jako jsou tropické země, ačkoli 95% ethanol je často snáze dostupný. Bez ohledu na koncentraci je DNA v ethanolu obecně dobře zachována (i když méně účinně než při metodách zmrazování, zejména pro molekulární metody typu NGS). Proteiny jsou mnohem méně stabilní, zejména pro proteomiku, jako jsou aplikace MALDI-ToF. Flebotomy uchovávané v alkoholu po dobu několika měsíců lze stále morfologicky identifikovat, ale je obtížné z těchto vzorků generovat kvalitní referenční proteinová spektra. Skladování v alkoholu nebo v suchých podmínkách lze zlepšit, pokud je vzorek také zmrazen na -20 °C. Zmrazení na -20 °C zlepšuje hlavně molekulární uchování (např. nukleových kyselin) zpomalením degradace a poskytuje také sekundární výhodu pro morfologické uchování snížením rozkladu tkání v čase, i když vliv na morfologii je omezenější než na molekulární integritu. Skladování v ethanolu lze také aplikovat pro detekci DNA a RNA virů při použití ethanolu v koncentraci alespoň 70 % po krátkou dobu skladování, méně než několik měsíců. Také isopropylalkohol může být v některých zemích snadno dostupný a uchovává DNA, ale způsobuje tuhost vzorků. Není hořlavý jako ethanol, a proto jej lze snadno přepravovat. V případě potřeby lze flebotomy uchované v kapalném dusíku nebo suchým zmrazením přenést do alkoholu, čímž se však kombinují nevýhody obou metod.

3.3. Skladování v RNA stabilizačním roztoku (RNASS)

Toto vodné činidlo je široce používané, netoxické a navržené tak, aby stabilizovalo a chránilo RNA v čerstvých

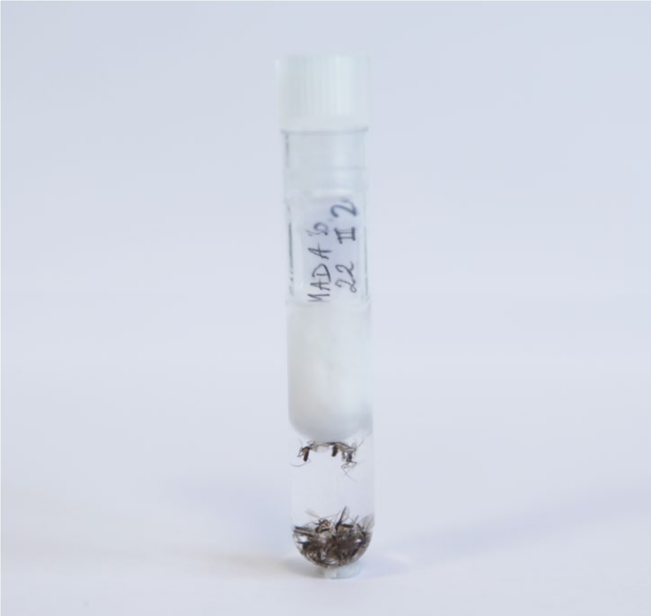

Obrázek 1. Flebotomové uchovávaní v ethanolu.

tkáních a buněčných vzorcích. Působí tak, že rychle proniká do vzorku a inaktivuje RNázy (enzymy degradující RNA), čímž zabraňuje degradaci RNA bez nutnosti okamžitého zmrazení. Skladování v RNASS je obecně účinné při zachování celkové morfologie tkání a buněk pro následné histologické hodnocení. Ačkoli je RNASS optimalizován pro stabilizaci RNA spíše než pro fixaci, krátkodobé až střednědobé skladování obvykle dobře udržuje strukturální integritu. RNASS umožňuje skladovat vzorky při pokojové teplotě po dobu až 7 dnů, při 4 °C po dobu několika týdnů nebo při -20 °C / -80 °C pro dlouhodobé uchování. Tato metoda je zvláště cenná při práci v terénu nebo v klinických podmínkách, kde je infrastruktura chladícího řetězce omezená. Extrakce RNA obvykle vyžaduje vyjmutí vzorků z činidla a jejich zpracování podle standardních protokolů.

3.4. Suché skladování při pokojové teplotě

Jedná se o klasickou metodu, která má při aplikaci na vzorek *in toto* (montovaný vcelku) hlavní nevýhodu v tom, že špatně zachovává křehké orgány, jako jsou křídla, nohy, tykadla. Proteomické studie pomocí MALDI-ToF však zůstávají proveditelné, pokud je dehydratace provedena při fixaci pomocí vysoušedla typu silikagel. Naproti tomu molekulární analýzy zaměřené na DNA se na těchto vzorcích provádějí obtížně, protože DNA je často fragmentovaná a v malém množství, což znamená, že analýzy zůstávají náročnější než u čerstvých nebo zmrazených vzorků, zejména u jaderných genomů. Nedávné techniky, jako je "museomika", by však mohly být použity na vzorcích tohoto typu [34]. Obecně se tato metoda skladování nedoporučuje, pokud není k dispozici žádná alternativa. Lze ji kombinovat se skladováním v chladu umístěním zkumavek do mrazáku při -20 °C nebo -80 °C. Hlavním problémem je úspěšné montování částí těla nezbytných pro identifikaci. K dosažení toho je nezbytná rehydratace. Doporučujeme použít roztok Triton X-100. Doba rehydratace se pohybuje od několika hodin do několika dnů za pravidelného pečlivého sledování. Po úplné rehydrataci by měly být vzorky opláchnuty ve třech po sobě jdoucích vodních lázních.

3.5. Uchovávání na filtračních papírech

Hlavní výhodou filtračních papírů je dlouhodobá stabilita genomové DNA v buňkách nefixovaného, vysušeného celého těla nebo krevních buněk skladovaných při pokojové teplotě. Filtrační papír je dodáván ve velikosti malé kartičky, což umožňuje skladovat několik set vzorků při pokojové teplotě z minimem prostoru. Matrice filtračního papíru je impregnována činidly, která denaturují infekční agens, a vzorky tak již nejsou považovány za biologické nebezpečí. To umožňuje skladování a přepravu vzorků bez nutných opatření pro biologické nebezpečí [68].

4. Pitva flebotoma

Na rozdíl od mnoha jiných druhů hmyzu, které jsou identifikovány na základě vnějších znaků pozorovatelných na jednotlivcích napíchnutých *in toto*, vyžadují flebotomové pitvu a montování na sklíčko a posouzení anatomických znaků pro přesnou identifikaci druhu. Bez ohledu na zvolený postup přípravy a montování se používá stejná pitevní technika (Obrázky 2 & 3) (https://zenodo.org/records/18198006).

Použití Tritonu X100: neiontový vodný roztok

Montování se týká čerstvě chycených nebo adekvátně uskladněných vzorků. Většina sběratelů má vzorky hmyzu uchované v suchu (pro použití MALDI-ToF) nebo uložené v alkoholu po mnoho let. Bohužel dlouhodobé uchovávání v alkoholu není optimální a členovci takto uchovaní se stávají velmi obtížnými pro přípravu k mikroskopickému určení. Často dochází k degradaci plastů obsahujících vzorky, následované odpařením alkoholu. Vzorky tak buď zůstávají příliš dlouho v alkoholu nebo vysychají. Vznikla proto myšlenka použít smáčedla, která nejsou silnými detergenty. Triton X100 je ve formě neiontového vodného roztoku široce používaný jako detergent v buněčné a molekulární biologii. Umožňuje permeabilizaci buněčných a jaderných membrán.

Níže je uveden postup s použitím neiontového Tritonu X100 v 0,5 % vodném roztoku:

- Impregnujte suchý vzorek absolutním alkoholem.
- Přidejte potřebný objem roztoku Triton X100 o koncentraci 0,5 %, aby byl celý vzorek ponořen.
- Nechte proces běžet asi 5 minut až několik dní, pravidelně kontrolujte.
- Všichni členovci se musí v roztoku od sebe plně oddělit.
- Odstraňte roztok Triton X100 a nahraďte jej roztokem hydroxidu draselného.

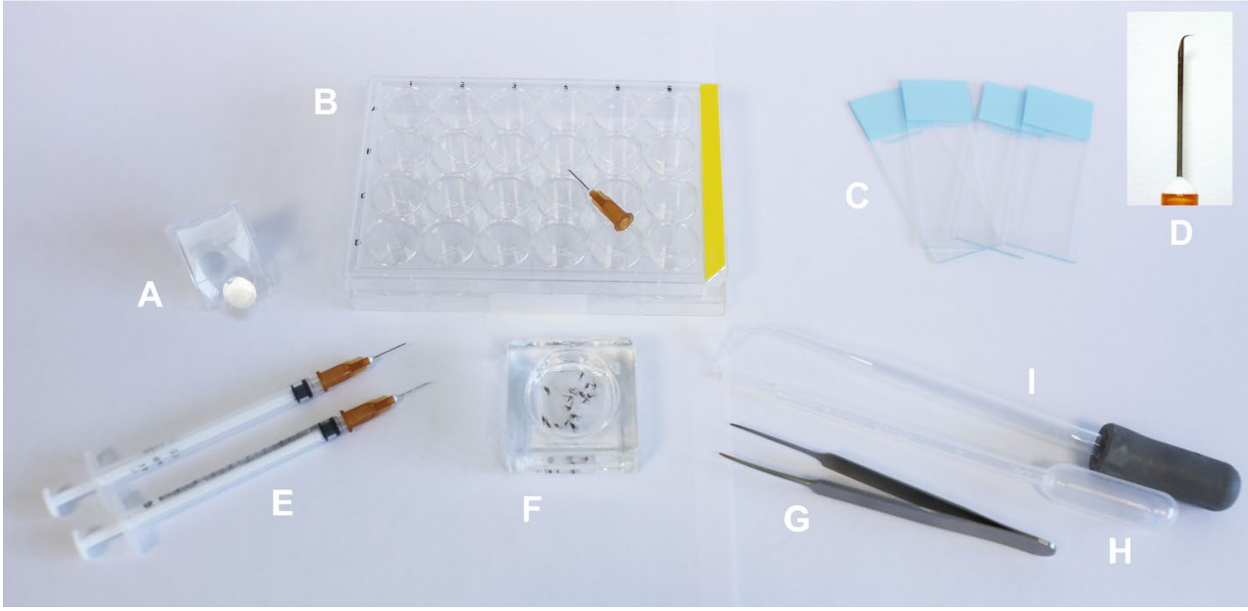

Obrázek 2. Materiály potřebné pro montování flebotomů: A: kulatá krycí sklíčka (průměr 10 nebo 12 mm); B: 24-jamková destička a zahnutá jehla (pokud používáte hřebíčkový olej nebo esenci Euparal® ke zpracování flebotomů, nepoužívejte akrylátové destičky, protože dojde k chemické reakci a vzorky budou poškozeny); C: podložní sklíčka vhodná pro popisování; D: detail zahnutí jehly; E: jehly nasazené na stříkačky; F: hodinové sklíčko nebo ekvivalentní nádoba obsahující flebotomy určené k montování; G: pinzety Dumont; H: plastová pipeta; I: skleněná pipeta ohnutá zahřátím pro usnadnění přenosu kapaliny do jamek.

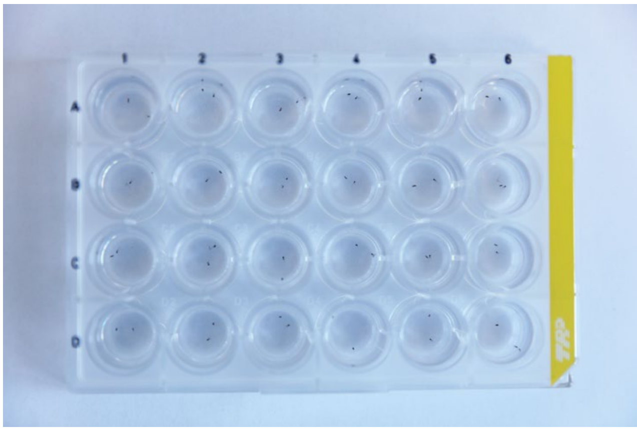

Obrázek 3. Destička s 24 jamkami, z nichž každá obsahuje hlavu a špičku zadečku flebotomů.

4.1. Hlava

Pitvu lze provádět pomocí jemných jehel nebo entomologických špendlíků pod stereolupou (Obrázky 2 & 3). Mezi nejčastěji používané jehly patří: 26G x 1/2" (0.45 × 13 mm), 30G × 1/2" (0.3 × 13 mm), nebo 25G × 5/8" (0.5 × 16 mm). K přípravě vzorku pro identifikaci se minimálně oddělí hlava od těla a montuje se ventrální stranou nahoru, aby se zobrazilo cibarium a hltan, zatímco hruď a zadeček se po pitvě montují laterálně (z boku). Montování hlavy ve ventro-dorzální poloze zajišťuje, že týlní otvor (occipital foramen) směřuje nahoru, takže cibarium lze pozorovat přímo. Přístup k těmto anatomickým znakům je snazší, pokud je hlava zcela oddělena.

4.2. Křídla a hruď

Křídla musí být montována naplocho. Každé křídlo lze oddělit u základny a montovat nezávisle, nebo lze jedno montovat samostatně, zatímco druhé se ponechá připojené k hrudníku. Pokud je plánována analýza geometrické morfometrie, je nezbytné před montováním správně identifikovat a označit pravé a levé křídlo. Hruď je rozdělena na několik částí a každá obsahuje velmi důležité taxonomické informace [20, 64]. Obecně se montuje v bočním pohledu, aby se umožnilo posouzení chaetotaxie (uspořádání štětin) a rozložení barev. Přítomnost jizev po štětinách v určitých oblastech hrudi lze použít k rozlišení některých druhů rodu *Brumptomyia*. Rozložení barev může být použito k rozdělení neotropických flebotomů na úrovni rodu (např. *Bichromomyia*), druhových sérií (např. *Pintomyia*) nebo dokonce druhů stejného rodu (např. *Micropygomyia*, *Nyssomyia*, *Psathyromyia* a *Psychodopygus*) [20]. Pokud se tedy hruď nepoužívá pro molekulární analýzu, měla by být montována tak, aby se nepoškodila. Důležité je poznamenat, že nezáleží na intenzitě barev, ale na jejich rozložení po hrudi. Proces projasňování tedy neodstraní pigmentaci ani její vzor.

4.3. Genitálie

Při montování genitálií u samců i samic je třeba dbát zvláštní opatrnosti, protože jsou klíčové pro identifikaci rodů, podrodů a druhů. U obou pohlaví jsou genitálie párové.

This is an Open Access article distributed under the terms of the Creative Commons Attribution License (https://creativecommons.org/licenses/by/4.0), which permits unrestricted use, distribution, and reproduction in any medium, provided the original work is properly cited.

4.3.1. Samci

Genitálie jsou vnější a skládají se z párových klíštěk (forceps), z nichž každá se skládá ze zakloubené dvojice gonokoxit-gonostyl v hřbetní části a epandriálního laloku ve ventrální části. Gonostyl nese trny a někdy štětiny, které musí být spočitatelné a jejichž poloha musí být jasně viditelná. Je důležité pečlivě pozorovat vnitřní povrch gonokoxitu, který může nést chomáč sesilních štětin nebo štětiny nesené lalokem (= hrbolkem) [22]. Kolegové s menšími zkušenostmi s pitvami mohou provést jednoduché laterální (boční) montování, aniž by oddělili genitálie od konce zadečku (https://zenodo.org/records/18311158). V tomto případě může superpozice (překrytí) dvou částí genitálií ztížit počítání vnitřních štětin gonokoxitu, ale zabrání se poškození genitálií nezdařenou pitvou. Zkušenější kolegové se mohou pokusit otevřít genitálie na dvě části, rozdělit je. K dosažení toho je nutné zkosenou stranou jehly oddělit bez úplného přeříznutí genitálií párovou sestavu gonokoxit-gonostyl (https://zenodo.org/records/18311158). Tímto způsobem bude pozorování jejich vnitřních stran snadné. Toto uspořádání také usnadňuje pozorování paramer a paramerálních pochev, které se již nepřekrývají. Pro laterální montování, které podporuje superpozici orgánů, musí být vzorky dokonale projasněny.

4.3.2. Samice

Genitální aparát samic je vnitřní a je tvořen spermatékami. Pokud se neprovádí pitva, musí být pozorovány přes tělní pokryv (tegument) a zadeček musí být namontován ve ventrální poloze. Bez ohledu na zvolené zalévací médium lze samotné spermatéky obecně pozorovat správně, zejména pokud nejsou hladké a jsou projasněné. Nicméně pozorování hladkých, tenkostěnných spermaték může být v málo lomivých médiích problematické. Dále je pozorování báze vývodů spermaték nezbytné pro druhovou identifikaci, například u podrodu *Larroussius* [35, 37, 38], hlavních přenašečů *Leishmania infantum* ve Starém světě. Bez tohoto pozorování zůstává jednoznačná druhová identifikace jedince nemožná. K překonání těchto obtíží při pozorování by měl být komplex genitální vidličky (furky) a spermaték vyjmut ze zadečku (https://zenodo.org/records/18311106). Spermatéky jsou během pitvy obecně obtížně pozorovatelné, ale genitální furku lze lokalizovat poměrně snadno. Vzhledem k tomu, že vývody spermaték ústí do genitální furky, izolace této furky obvykle umožní i izolaci spermaték. Pokud jsou spermatéky během procesu náhodně odděleny, nejsou ztraceny a lze je stále pozorovat uvnitř břišního integumentu (Obrázek 4).

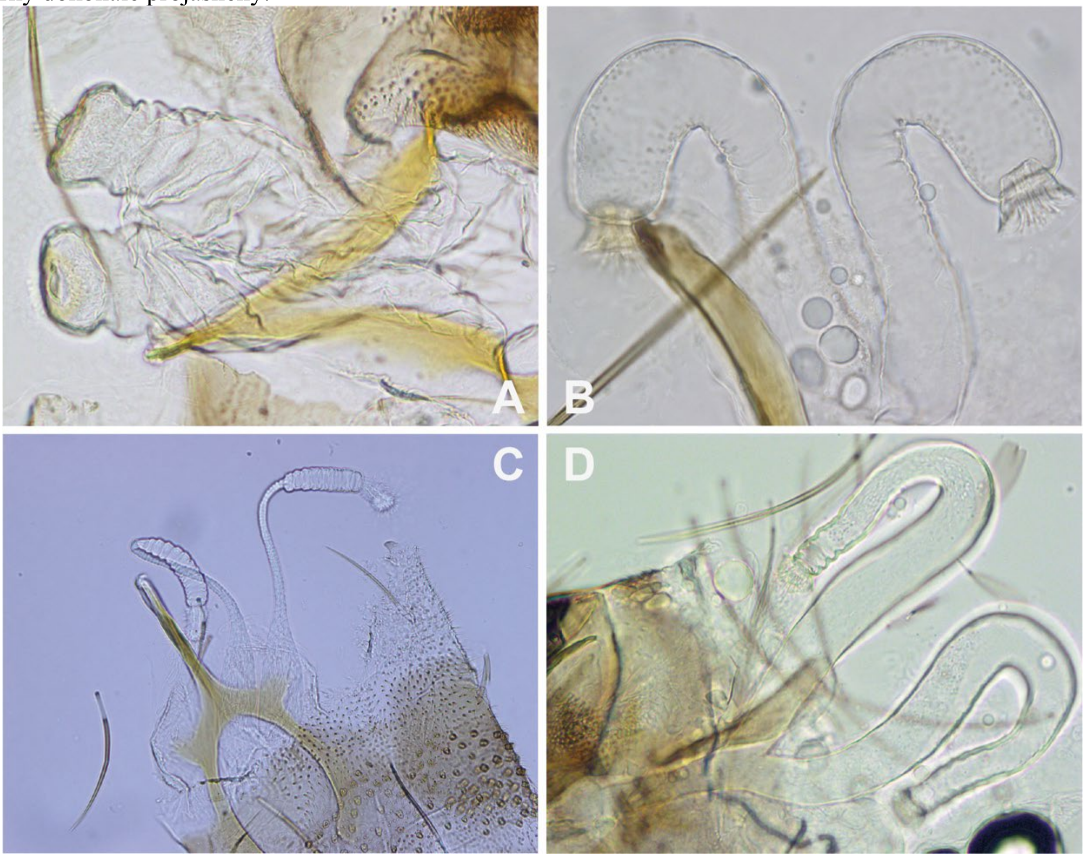

Obrázek 4. Spermatéky vypreparované a zamontované v médiu Marc-André z čerstvých jedinců. A: *Idiophlebotomus longiforceps* (Laos); B: *Sergentomyia minuta* (Francie); C: *Phlebotomus ariasi* (Francie); D: *Sergentomyia anodontis* (Laos).

This is an Open Access article distributed under the terms of the Creative Commons Attribution License (https://creativecommons.org/licenses/by/4.0), which permits unrestricted use, distribution, and reproduction in any medium, provided the original work is properly cited.

4.4. Pitva střeva pro detekci a izolaci leishmanií.

Pitva trávicího traktu je nezbytná pro detekci a izolaci leishmanií u samic flebotomů. Pitvu lze provádět jak v terénních, tak v laboratorních podmínkách pro posouzení vektorové kompetence.

Doporučuje se pracovat s čerstvě usmrcenými samicemi. Omyjte samice ve vodě nebo fyziologickém roztoku se slabým detergentem, abyste odstranili přebytečné chloupky. Tento krok pomáhá při izolaci leishmanií, a zároveň zachovává morfologické znaky potřebné pro druhovou identifikaci flebotomů. Opatrně vyjměte střevo, zejména mesenteron (střední střevo), a vložte jej do kapky sterilního fyziologického roztoku (0,9% NaCl). Po zjištění pohyblivých bičíkovců pod světelným mikroskopem (doporučené zvětšení: ~200×) je pomocí inzulinové stříkačky nebo mikropipety přeneste do kultivačního média (více podrobností viz kapitola 4.4.3).

Hlavu a genitálie montujte přímo do roztoku Marc-André pro jejich projasnění. Důležité: nikdy nedovolte, aby se roztok Marc-André dostal do kontaktu s leishmaniemi, a to ani nepřímo prostřednictvím nástrojů nebo jehel, protože roztok parazity usmrcuje.

Pitvu samic flebotomů lze provádět buď na jednom, nebo na dvou sklíčkách; obě možnosti mají své výhody a omezení (Obrázek 5; https://zenodo.org/records/18311154).

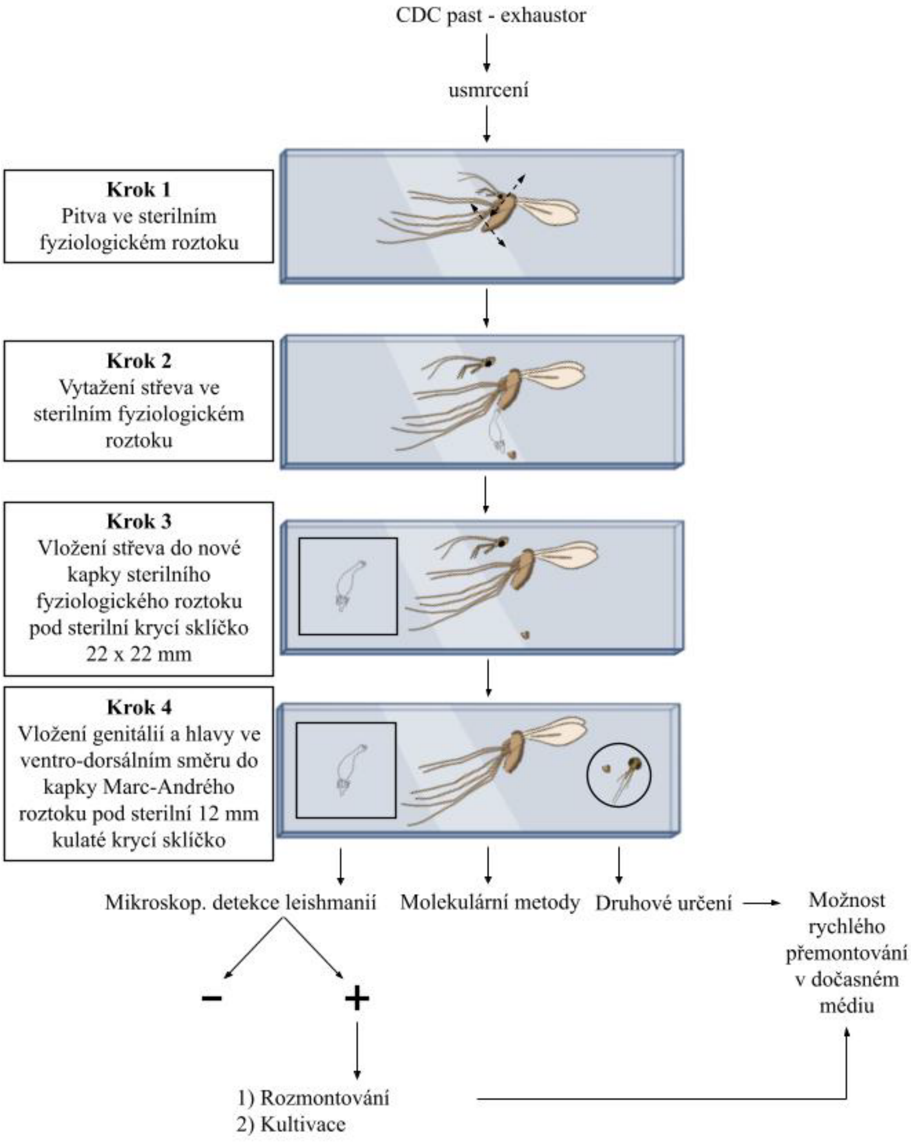

Obrázek 5. Metoda izolace leishmanií.

4.4.1. Metoda dvou sklíček

První možnost zahrnuje práci na dvou samostatných sklíčkách: jedno obsahující sterilní fyziologický roztok pro pitvu střeva a druhé pro zalití hlavy a spermaték do roztoku Marc-André. V terénních podmínkách je však běžné, že pitvají dva nebo tři lidé a předávají vypitvané flebotomy dalšímu spolupracovníkovi, odpovědnému za identifikaci druhu a posouzení leishmaniové infekce. Práce se dvěma sklíčky tak může vést k problémům s dohledatelností vzorků

This is an Open Access article distributed under the terms of the Creative Commons Attribution License (https://creativecommons.org/licenses/by/4.0), which permits unrestricted use, distribution, and reproduction in any medium, provided the original work is properly cited.

a zejména ztížit určení, který konkrétní jedinec flebotoma byl infikován v případě záchytu pozitivního střeva (https://zenodo.org/records/18311154).

4.4.2. Metoda jednoho sklíčka

Použití jediného sklíčka zajišťuje lepší dohledatelnost výsledků. Je však třeba přijmout několik opatření. Pro maximalizaci sterility je třeba si pravidelně čistit ruce dezinfekčním gelem nebo roztokem. Musí být použita hladká podložní sklíčka (bez matného okraje), zabalená v hliníkové fólii a sterilizovaná suchým teplem (pomocí horkovzdušného sterilizátoru / Poupinel), čtvercová krycí sklíčka (22 x 22 mm), spolu jehlami sterilizovanými po každé pitvě (návrh: 25G Ø 0,5 mm × 16 mm).

Flebotom se umístí do kapky sterilního fyziologického roztoku doprostřed sklíčka. Hlava se oddělí, zatímco se provede naříznutí zadečku v místě mezi 6. a 7. abdominálním tergitem a sternitem, aniž by došlo k přeříznutí trávicího traktu (lze provést naříznutí i blíže k hlavě, pokud se očekávají velmi dlouhé spermatéky). Poté musí být hrudník imobilizován jednou jehlou a poslední zadní abdominální segmenty jemně vytaženy druhou jehlou směrem dozadu. Pokud se toto nepodaří, existuje možnost zachytit konec zadečku jehlou a vytáhnout trávicí trakt dopředu za jeho přední část. Pokud i toto selže, musí být střevo vypitváno odstraněním co největšího množství zbývajícího tegumentu kolem něj. Po vypitvání střeva musí být poslední abdominální segmenty odděleny přeříznutím zadní části trávicího traktu. Střevo se poté umístí do nové kapky sterilního fyziologického roztoku umístěné po straně sklíčka a poté se jemně zakryje sterilním krycím sklíčkem. Hlava a poslední abdominální segmenty se přenesou do malé kapky kapaliny Marc-André umístěné na druhém konci sklíčka. Hlava se správně orientuje (týlní otvor směrem nahoru), spermatéky s genitální furkou se umístí zhruba do středu kapky, jak je uvedeno výše, a zakryjí se malým kulatým krycím sklíčkem (Ø 12 mm, nezaměňovat se sterilními čtvercovými krycími sklíčky). Zbývající tělo flebotoma a křídla zůstávají v kapce fyziologického roztoku uprostřed sklíčka (https://zenodo.org/records/18311154). V případě pozitivity nebo pro taxonomický průzkum lze hruď a zbytek zadečku uchovat pro molekulární nebo proteomické studie, a křídla lze namontovat do média mísitelného s vodou. Pro uchování preparátu lze roztok Marc-André nahradit zalévacím médiem mísitelným s vodou, jako je chloralhydrátové médium (= Hoyer) nebo médium na bázi polyvinylalkoholu.

Podrobněji jsou oba postupy demonstrovány na videích (pitva střeva flebotoma: https://zenodo.org/records/18303014 a pitva slinných žláz flebotoma: https://zenodo.org/records/18302850) , proto zde nebudou podrobněji vysvětlována.

4.4.3. Izolace a kultivace parazitů *Leishmania* ze střev flebotomů

Izolace parazitů z vypitvaných infikovaných samic flebotomů je delikátní procedurou, vyžadující vysokou zručnost, a je vhodné ji zpočátku trénovat na vzorcích bez parazitů. Vypitvaná střeva jsou přenesena do čerstvé kapky sterilního fyziologického roztoku (0,9 %) nebo Lockeova roztoku [4] a lze je zpracovat dvěma způsoby: i) vyšetřit celé střevo pod světelným mikroskopem na přítomnost promastigotů a jejich lokalizaci, se zvláštním zřetelem na stomodeální valvu, a ii) otevřít střevo pro uvolnění promastigotů do roztoku, což může usnadnit jejich kultivaci [4]. Nalezení infekčních flebotomů v terénu je relativně vzácným jevem, a proto kvalitní trénink v laboratoři maximalizuje šance na úspěšnou izolaci.

Pokud jsou ve střevě pozorováni bičíkovci, je třeba k další manipulaci použít nové sterilní jehly. Ke krycímu sklíčku je třeba přidat malé množství sterilního fyziologického roztoku, aby bylo možno krycí sklíčko nadzvednout. Střevo by mělo být opatrně a rychle natrženo, aby se paraziti uvolnili do fyziologického roztoku. Pomocí 100 µl mikropipety nebo tuberkulinové stříkačky pak odeberte parazity a naočkujte je do řádně označeného kultivačního média.

In vitro kultivace leishmanií: izolovaní promastigoti jsou zpočátku udržováni na šikmých krevních agarech SNB-9 nebo v pevném médiu Novy, Mc Neal, Nicolle (NNN) [16] převrstveném buď sterilním médiem alpha-MEM [16, 65] nebo médiem M199, obojí obohacené pro lepší růst leishmanií 10% tepelně inaktivovaným sterilním fetálním telecím sérem [FCS], 1% roztokem vitamínů BME, 2% sterilní lidskou močí (sterilizovanou pomocí stříkačkového filtru Filtropur® S 0,2 µm), a s přidáním antibiotik (konkrétně 250 µg/ml amikacin nebo 50 µg/ml gentamicin), nebo směsi antibiotik a aminokyselin (L-glutamin 200 mM, penicilin 10 000 U, streptomycin 10 mg/ml) [47]. Po třech dnech, pokud nedojde ke kontaminaci, se kultury mohou smíchat s řádně připraveným zamražovacím médiem a následně se skladují při -80 °C po dobu 1 až 2 let nebo v kapalném dusíku při -196 °C pro dlouhodobé uchování a budoucí experimentální použití [7].

4.5. Slinné žlázy

Pitva slinných žláz flebotomů je základní technikou pro studium interakcí vektor-patogen, zejména pro detekci arbovirů, jako jsou zástupci rodu Phlebovirus (např. virus Toscana) [44, 75]. Vzhledem k nepatrné velikosti flebotomů vyžaduje postup přesné zacházení pod stereomikroskopem za použití jemných pinzet a mikro-pitevních jehel. Slinné žlázy je třeba vypitvat, aniž by došlo k jejich prasknutí nebo kontaminaci (https://zenodo.org/records/18302850) [51, 61]. Zachování integrity žláz je klíčové i pro následné molekulární analýzy. Jakmile jsou žlázy vypitvány, mohou být homogenizovány a testovány pomocí RT-PCR, qPCR nebo imunoanalýz k detekci virové RNA, nebo na přítomnost antigenů [12]. Přítomnost virů ve slinných

žlázách, na rozdíl od střeva nebo hemocelu, potvrzuje, že patogen dokončil svůj vývoj v přenašeči a je přenosný během sání krve [71].

Proces pitvy je technicky náročný kvůli malé velikosti slinných žláz flebotomů, což vyžaduje značnou zručnost, aby se zabránilo degradaci vzorku [1, 51]. Navíc, virová nálož ve slinných žlázách může být nízká, což vyžaduje vysoce citlivé detekční metody, jako je nested PCR nebo sekvenování nové generace (high-throughput sequencing) [54]. Riziko kontaminace zvyšuje potřebu pracovat, pokud možno, sterilně. Kromě technických překážek ovlivňují úspěšnost detekce virů i biologické faktory; kompetence se liší mezi druhy flebotomů a míra infekce kolísá s ekologickými a sezónními podmínkami [33, 61].

Detekce virů ve slinných žlázách poskytuje kritické poznatky o rizicích přenosu, což umožňuje cílená kontrolní opatření [15]. Například identifikace viru Toscana u flebotomů v endemických oblastech měla vliv na diagnostické protokoly a doporučení v oblasti veřejného zdraví [18]. Studium interakcí viru a slin navíc může odhalit nové molekuly vhodné pro vývoj vakcíny nebo terapeutika blokující přenos [15, 18].

Slinné žlázy flebotomů lze také použít jako zdroj antigenů pro studium hostitelských protilátek proti slinám flebotomů pomocí imunologických metod, nejlépe ELISA. Tato metoda umožňuje posoudit míru expozice hostitele vůči pobodání flebotomy, čímž lze například ověřit účinnost metod používaných ke kontrole vektorů [25] a vyhodnotit riziko přenosu lesihmanií [40].

4.6. Identifikace krevní potravy

Nasáté samice, izolované z odchytů, by měly být pitvány pomocí jednorázového vybavení, aby se zabránilo zkřížené kontaminaci. Jejich zadeček by měl být vyšetřen pod stereomikroskopem pro posouzení stádia trávení krevní potravy. Doporučuje se vybírat pouze samice s červeným, červenohnědým nebo tmavě červeným zadečkem, nevykazující žádné známky tvorby vajíček. Odstraňte špičku zadečku včetně spermaték, abyste mohli samici po projasnění morfologicky identifikovat. Hlavní část zadečku (bez spermaték) by měla být poté umístěna do zkumavek Eppendorf® a skladována při -20 °C až do další analýzy. Genetické markery, běžně používané pro identifikaci krevní potravy, jako je PNOC [5, 30, 50], CytB [67] nebo COI [13], jsou dobře zavedeny a rozsáhle popsány v literatuře, proto nebudou v tomto článku dále podrobně rozebírány (Obrázek 6). Alternativně lze k identifikaci hostitelské krve nasadit MALDI-ToF peptidové mapování [31]. Experimentálně bylo prokázáno, že tato technika umožňuje identifikaci hostitelské krve v delším časovém rámci po nasátí krevní potravy; je tedy vhodnou metodou volby zejména pro analýzu nasátých samic s viditelně pokročilejším trávením hostitelské krve. Vzorky by měly být ideálně skladovány při -20 °C nebo 4 °C, ale dobrých výsledků lze dosáhnout i ze vzorků skladovaných krátkodobě při pokojové teplotě. Zadeček nasáté samice by měl být oddělen od zbytku těla krátce před analýzou a homogenizován v destilované vodě. Zbytek těla flebotoma zůstává k dispozici pro jiné molekulární a morfologické analýzy. Poté, co je z homogenátu odebrán alikvot pro MALDI-ToF peptidové mapování, může být zbytek použit pro izolaci DNA k potvrzení identifikace hostitelské krve a/nebo screeningu přítomnosti *Leishmania* spp. Celková doba přípravy vzorku a analýzy je velmi krátká ve srovnání s molekulárními technikami založenými na analýze DNA.

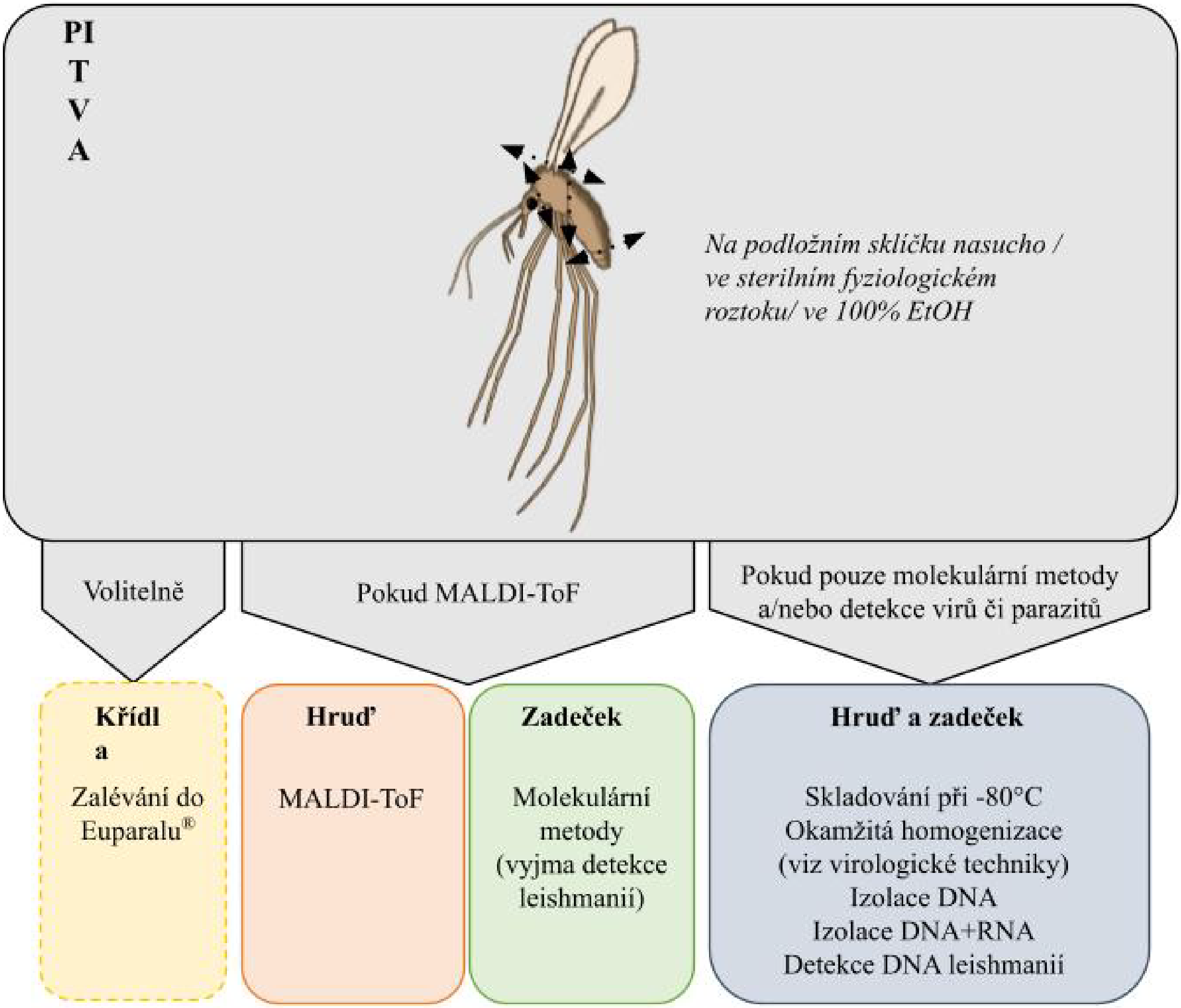

Obrázek 6. Zpracování flebotomů pro aplikace molekulární biologie, proteomiky a/nebo virologie.

5. Zpracování vzorků pro morfologické studie (Obrázky 3, 6, 7 & 8; Přílohy 1, 2, 3 & 4)

Tato část nastiňuje principy přípravy vzorku flebotoma pro montování výhradně pro morfologické studie, následované případnými úpravami pro aplikace, přesahující morfologické studie. Pochopení této metodiky je však zásadní, protože umožňuje v případě potřeby přizpůsobit postupy pro specifické typy vzorků. Zpracování zahrnuje postupné kroky vyprazdňování a plnění pomocí Pasteurových pipet, vybavených pružnými gumovými balónky. Důrazně se doporučují skleněné nádoby s kulatým dnem, protože tyto kroky značně usnadňují. Sklo je inertní vůči všem činidlům. Aby se zabránilo odpařování činidel, měly by být nádoby opatřeny víčky a nikdy by neměly být přeplňovány, což by vedlo k přetečení při zavírání nebo otevírání, a aby se zabránilo padání prachu na vzorky. Chemikálie potřebné pro projasňování a zpracování jsou uvedeny v Tabulce 2.

This is an Open Access article distributed under the terms of the Creative Commons Attribution License (https://creativecommons.org/licenses/by/4.0), which permits unrestricted use, distribution, and reproduction in any medium, provided the original work is properly cited.

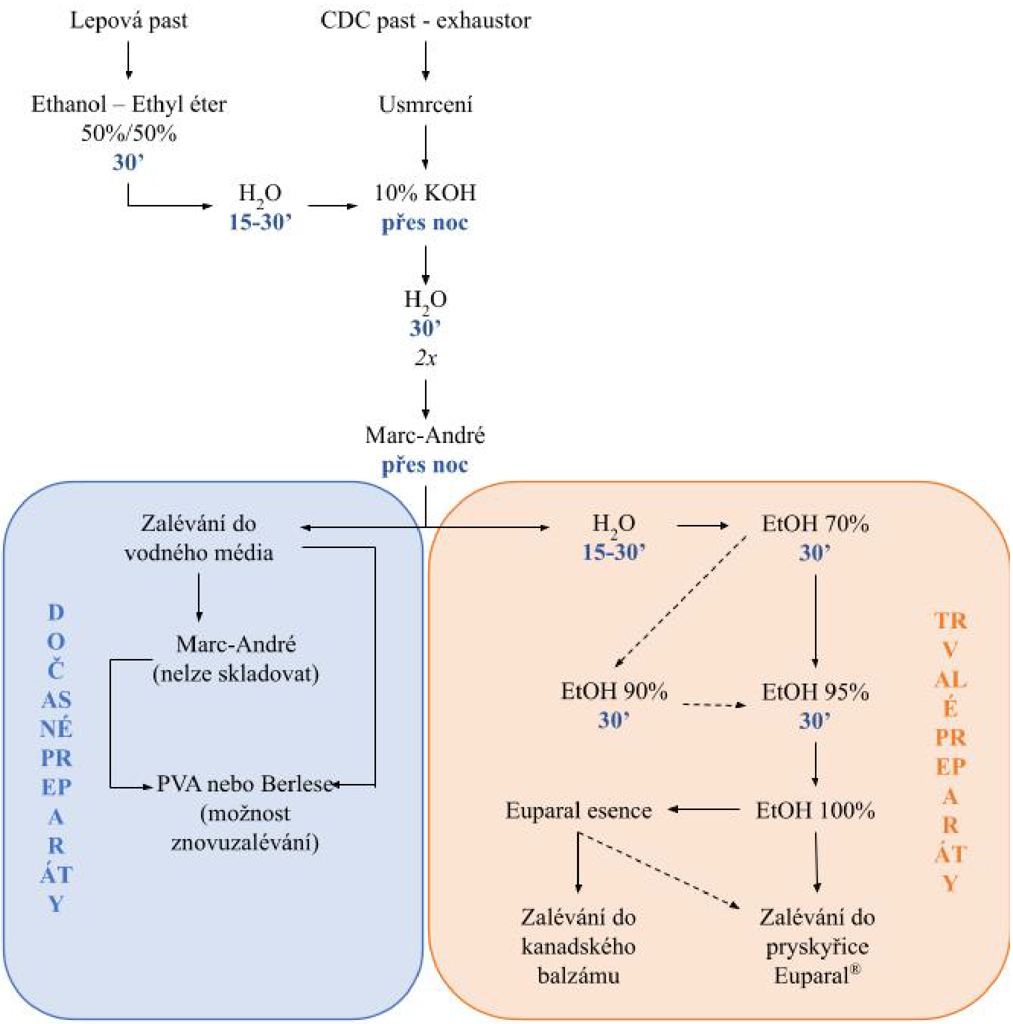

Obrázek 7. Klasická metoda zpracování flebotomů.

5.1. Projasňování

Předtím, než mohou být vzorky flebotomů připraveny jako trvalé preparáty, musí být nejprve projasněny macerací pomocí vhodné metody a projasňovacího činidla (tj. 10% roztok kyseliny octové nebo roztok Marc-André obsahující chloralhydrát, což je v mnoha zemích regulovaná chemikálie), aby se staly průsvitnými. Proces projasňování odstraňuje tělesné tkáně, tuk, sekrety a vosk, čímž se vzorek stává průsvitným, což usnadňuje vyšetření exoskeletálních struktur (např. báze štětin), povrchových charakteristik (např. zbarvení) a vnitřních znaků viditelných přes tegument (např. spermatéky).

Dvoukrokový proces projasňování, který zahrnuje nejprve použití silné zásady (jako je hydroxid draselný), následované slabou kyselinou (jako je kyselina octová v roztoku Marc-André), slouží k odlišným biochemickým účelům [74]. Zásada rozkládá měkké tkáně, jako jsou proteiny, tuky a svaly, zmýdelněním a denaturací proteinů, přičemž chitinový exoskelet ponechává nedotčený pro strukturální jasnost. Následná slabá kyselina neutralizuje veškeré zbývající alkálie, čímž zabraňuje další degradaci, a bělí chitin pro zvýšení průhlednosti [74], ačkoli dvojí promytí vzorků v destilované vodě po dobu 15 minut může také stačit k neutralizaci zásady. Toto sekvenční ošetření kombinuje účinné odstranění tkání s šetrným zachováním, což zajišťuje optimální integritu vzorku pro mikroskopické vyšetření.

Před přechodem k dalšímu kroku se doporučují dva dvacetiminutové oplachy v destilované vodě.

5.1.1. Lyze měkkých tkání (Obrázek 8)

Hydroxid sodný (NaOH) nebo hydroxid draselný (KOH) jsou běžně používaná chemická macerační činidla, aplikovaná v různých koncentracích a po různou dobu v závislosti na velikosti a křehkosti vzorků. Standardní a nejúčinnější technika zahrnuje lyzi měkkých tkání namočením flebotomů do silné zásady (10 % KOH nebo NaOH) přes noc. Koncentraci lze zvýšit pro zkrácení doby ošetření (tj. 20 % KOH po dobu 6 hodin), stejně jako zahřívání na 37 °C.

This is an Open Access article distributed under the terms of the Creative Commons Attribution License (https://creativecommons.org/licenses/by/4.0), which permits unrestricted use, distribution, and reproduction in any medium, provided the original work is properly cited.

5.1.2. Projasňování (čištění) s barvením nebo bez něj

Následujícím krokem je projasňování, obvykle využívající kombinaci kyseliny octové a chloralhydrátu (např. roztok Marc-André). Po projasnění musí být vzorky důkladně opláchnuty alespoň ve dvou po sobě jdoucích vodních lázních, každé po dobu 20 minut, aby se odstranily zbytky chemikálií.
Roztok Marc-André je běžně používané projasňovací činidlo pro přípravu vzorků flebotomů. Je účinné, a přitom nepoškozuje křehké struktury, jako jsou křídla a tykadla. Roztok by měl být čerstvě připraven nebo uchováván v těsně uzavřené nádobě, aby se zabránilo odpařování a degradaci. Použití roztoku Marc-André je obzvláště výhodné v kombinaci s barvením pro zvýraznění konkrétních morfologických detailů. Podrobnosti o jeho složení a přípravě jsou uvedeny v Příloze 2.
U velmi průsvitných vzorků může být před montáží do preparátu nutné barvení, aby se zlepšila jejich viditelnost. K dispozici je mnoho barviv, z nichž každé se váže na specifické chemické složky organismu. Je důležité zvolit takové barvivo, které je kompatibilní jak se vzorkem, tak se zvoleným zalévacím médiem. Základní metodiku lze pro účely barvení podle potřeby upravit, například přidáním 0,1% kyselého fuchsinu do roztoku Marc-André. Vzorky uchovávané ve vodných roztocích a určené pro zalévání do pryskyřice navíc vyžadují dehydrataci (viz část 5.2 Dehydratace), protože většina přírodních i syntetických pryskyřičných médií je s vodou nekompatibilní. New (1974) popisuje, že některá barviva se mohou v určitých zalévacích médiích znehodnotit [53]. Například kyselý fuchsin, běžně používaný s kanadským balzámem, lze fixovat také v médiu Euparal®. Vzorky barvené kyselým fuchsinem jsou však náchylné k blednutí, zejména pokud v nich zůstanou zbytky hřebíčkového oleje, používaného jako závěrečná projasňovací tekutina. Vzorky uchovávané v hřebíčkovém oleji mohou vykazovat výrazné vyblednutí již během několika dní.

5.2. Dehydratace

Dehydratace se provádí postupným převáděním vzorků vzestupnou řadou roztoků ethanolu: 50 %, 70 %, 80 %, 90 % nebo 95 % a nakonec 100 %, přičemž každá lázeň trvá nejméně 20 minut. Vzhledem k tomu, že se ethanol rychle odpařuje, měla by být nádoba těsně uzavřena. Jakmile je vzorek zcela dehydratován, je možné jej skladovat několik dní v médiu Euparal®, které je vhodnější než hřebíčkový olej. Dřevný kreosot, dříve pro tento účel využívaný, je nyní kvůli své toxicitě zcela zakázán.
Proces dehydratace musí zajistit, aby tekutina uvnitř vzorku byla kompatibilní se zalévacím médiem. Tím se zabrání zakalení, osmotickému kolapsu nebo deformaci, které by mohly vzorek učinit nepoužitelným pro taxonomické studium.

5.3. Zalévací média

5.3.1. Výběr a použití pro přípravu vzorků

Zalévací médium by v ideálním případě mělo mít index lomu co nejbližší indexu lomu skla, což je přibližně 1,5. Musí být téměř bezbarvé, čiré, a musí zůstat dokonale transparentní i po zaschnutí a v průběhu času. Musí být kompatibilní s použitými barvivy a schopné proniknout a difundovat do všech tkání vzorku. Nesmí zasychat příliš rychle, ani vytvářet během montáže zákal. Po montáži se nesmí smršťovat. Výběr vhodného zalévacího média je základním aspektem přípravy vzorku, protože žádné médium není ideální pro všechny účely. Volba by měla zvažovat několik klíčových faktorů:
- **Optické vlastnosti:** Index lomu zalévacího média by měl poskytovat dostatečný kontrast a lom světla u kritických anatomických znaků používaných pro taxonomickou identifikaci nebo morfologický popis. Mezi tyto znaky patří zejména spermatéky, askoidy, Newsteadova sensila, cibariální zuby a faryngeální zuby. Viditelnost těchto struktur přímo závisí na optických vlastnostech zalévacího média.
- **Konzervace:** U typových exemplářů nebo materiálů určených pro trvalé sbírky musí médium vykazovat dlouhodobou stabilitu a odolnost. Naproti tomu u inventarizačních studií nebo epidemiologických průzkumů, kde je dlouhodobé uchování méně kritické, mohou postačovat dočasná zalévací média.

5.3.2. Požadavky na zalévací média

Specialisté často vyvíjejí vlastní a složité techniky montáže, přizpůsobené konkrétním výzkumným potřebám. Tyto metody však často přehlížejí aspekty, jako je archivační kvalita, kompatibilita, standardizace nebo snadná manipulace a dlouhodobá konzervace. Tento nedostatek standardizace pak komplikuje integraci darovaných sbírek a dlouhodobou kurátorskou péči.
Vědecké aplikace kladou na zalévací média specifické požadavky. Taxonomové často montují celé exempláře a upřednostňují média, která jemně macerují vnitřní orgány, čímž se zvýrazní viditelnost kutikulárních struktur. Jejich index lomu by se měl dostatečně lišit od indexu lomu vzorku i podložního sklíčka, aby se maximalizovala optická čistota.

This is an Open Access article distributed under the terms of the Creative Commons Attribution License (https://creativecommons.org/licenses/by/4.0), which permits unrestricted use, distribution, and reproduction in any medium, provided the original work is properly cited.

Tabulka 2. Složení použitých činidel.

10% Hydroxid draselný	1% Kyselý fuchsin v destilované vodě
Hydroxid draselný 10 g	Kyselý fuchsin (práškový) 1 g
Destilovaná voda do 100 ml	Destilovaná voda 99 ml
Hoyerovo médium	Roztok Marc-André barvený kyselým fuchsinem
Destilovaná voda 50 ml	Roztok Marc-André 10ml
Chloralhydrát 200 g	1% Fuchsin 50 µl
Arabská guma 50 g	
Glycerol 20 ml	
Roztok Marc-André	Médium Enecê
Chloralhydrát 40 g	Čistá bílá kalafuna 22 g
Ledová kyselina octová 30 ml	V alkoholu rozpustná kopálová guma 12 g
Destilovaná voda 30 ml	Absolutní ethanol 20 ml
	Kafr 10 g
	Terpentýnová esence 10 ml
	Eukalyptol 26 ml

Komerční zalévací média jsou obvykle navržena s indexem lomu blízkým indexu lomu skla, aby se minimalizoval lom a rozptyl světla v systému podložní sklíčko – zalévací médium – krycí sklíčko. V mikroskopii ve světlém poli však lze s přirozeným kontrastem nebarveného vzorku manipulovat záměrným výběrem média s indexem lomu mírně odlišným od vzorku, čímž se zlepší jeho viditelnost vůči pozadí.

5.3.3. Typy zalévacích médií (Tabulky 3 & 4)

Znalost indexu lomu (RI) zalévacího media umožňuje určit, jak se světlo láme při průchodu podložním sklíčkem, médiem a vzorkem. Pokud je RI úzce sladěn s indexem krycího sklíčka (≈ 1,515), světlo prochází rovnoměrně, což snižuje rozptyl a optické zkreslení, a vede k lepšímu rozlišení a viditelnosti jemných struktur. Naopak nesoulad indexů lomu může způsobit rozmazání, haló efekty nebo zastřít nebarvené znaky. Výběr správného zalévacího média je zásadní pro optimalizaci kontrastu, jasnosti a celkové kvality obrazu daného vzorku vzhledem k rozdílným RI různých médií.
Index lomu zalévacího média má tak významný vliv na to, jak dobře jsou viditelné jemné struktury při přípravě flebotomů pro montáž na podložní sklíčko. Křehké a slabě sklerotizované znaky flebotomů, včetně cibariální armatury, spermaték, článků tykadel a žilnatiny křídel, může být v zalévacím médiu s vysokým indexem lomu obtížné pozorovat.
U flebotomů jsou běžně používána média na bázi arabské gumy a chloralhydrátu, mísitelná s vodou, a dále kanadský balzám nebo pryskyřice Enecê (NC), což jsou média rozpustná v nepolárních rozpouštědlech. Rawlins [60] rozdělil zalévací média do dvou typů: (1) trvalá média: v průběhu času tvrdnou a jsou vhodná pro dlouhodobé uchování (archivaci), a (2) dočasná média: tato média zcela neztvrdnou a obvykle se používají pro dočasné účely.
Zalévací média mohou být rozpustná ve vodě, alkoholu nebo jiných rozpouštědlech (např. toluen, xylen) (Tabulka 3). Po aplikaci by měla být izolována od okolního prostředí pomocí nerozpustných uzavíracích laků. Pro jasné rozlišení mezi typy zalévacích médií lze použít následující kategorizaci:
a. Média rozpustná ve vodě (dále vodní media). Tato média se snadno rozpouštějí ve vodě, díky čemuž jsou vhodná pro dočasné preparáty. Obvykle se s nimi snadno manipuluje, ale mohou vyžadovat uzavření, aby se zabránilo působení atmosférické vlhkosti (tj. chloralhydrátová média a polyvinylalkohol), zejména v tropickém vlhkém podnebí.

This is an Open Access article distributed under the terms of the Creative Commons Attribution License (https://creativecommons.org/licenses/by/4.0), which permits unrestricted use, distribution, and reproduction in any medium, provided the original work is properly cited.

Tabulka 3. Složení vybraných zalévacích médií.

Zalévací medium	Rozpouštědlo	Polymer	Poznámka
Hoyerovo medium	glycerol, voda	arabská guma	projasňovací složka: chloraldrát
CMCP-9 (= karboxy methyl celulose fenol)	voda (CMCP-9: 51−60%)	hydrolyzovaný polyvinyl alkohol (CMCP-9: 0−5%)	CMCP-9: méně viskózní, CMCP-10: více viskózní
DMHF (dimethyl hydantoin formaldehyd)	voda	zesíťované oligomery N,N′-dimethylol dimethylhydantoinu s methylenovými můstky.	
Kanadský balzám	xylen; částečně těkavé složky balzámu (Δ^3-karen, kyselina levopimarová a palustrová, limonen, myrcen, β-felandren, α-a β-pinen)	balzám (abienol, kyseliny abietová, isopimarová a sandarakopimarová)	pryskyřice z *Abies balsamea* (Linné, 1758), neutralizace uhličitanem draselným.
Euparal®	eukalyptol, paraldehyd; částečně těkavé složky sandarakové pryskyřice (limonen, α- a β-pinen)	složky sandarakové pryskyřice (různé organické kyseliny a alkoholy)	pryskyřice z *Tetraclinis articulata* (Vahl, 1791), projasňovacím médiem methyl salicylát.
Enecê	etylalkohol, kafr, eukalyptol a terpentýnová silice.	složky kalafuny a kopálové gumy	

b. Média s omezenou odolností vůči vodě. Tato média jsou méně mísitelná s vodou, ale stále vyžadují ochranu proti nadměrné vlhkosti. Poskytují větší dlouhodobou stabilitu ve srovnání s vodorozpustnými variantami a často se používají v dočasných preparátech.

c. Média rozpustná v uhlovodících. Tato média jsou rozpustná v organických rozpouštědlech, jako je xylen nebo toluen. Jsou navržena pro trvalou montáž a nabízejí vynikající dlouhodobou stabilitu, odolávají vlhkosti a degradaci, díky čemuž jsou ideální pro archivační účely (např. neutrální kanadský balzám, DPX).

Stručně řečeno, vodorozpustná média jsou nejlepší pro dočasné preparáty nebo případy vyžadující snadné vyjmutí vzorku; média s omezenou odolností vůči vodě jsou vhodná pro preparáty vyžadující střední trvanlivost a média rozpustná v uhlovodících jsou upřednostňována pro trvalé preparáty určené k archivaci a dlouhodobému uchovávání.

This is an Open Access article distributed under the terms of the Creative Commons Attribution License (https://creativecommons.org/licenses/by/4.0), which permits unrestricted use, distribution, and reproduction in any medium, provided the original work is properly cited.

Tabulka 4: Výhody a nevýhody vybraných zalévacích médií a nepublikované poznatky různých autorů [52].

Název	Výhody	Nevýhody
Kanadský balzám	Vysoce trvanlivé (více než 150 let), ale vyžaduje časově náročnou dehydrataci a použití toxických rozpouštědel (xylen/fenol).	Obsahuje škodlivé složky a vyžaduje manipulaci v digestoři. Dehydratační série je časově náročná a převedení přes xylen nebo hřebíčkový olej mohou způsobit křehkost; alternativy (např. isopropanol, n-butanol, Cellosolve™, 1,4-dioxan, Histoclear, terpineol) mohou snížit lámavost. Vzorky mohou zčernat, pokud je xylen nahrazen fenolem nebo pokud zůstane reziduální hydroxid draselný. Vysoké indexy lomu mohou ztížit pozorování nebarvených struktur.<br>Úplné vyschnutí může bez sušení na vyhřívané plotně trvat roky. Médium časem žloutne a tmavne, zejména pokud bylo projasněno hřebíčkovým olejem.<br>Kationtová barviva mohou vyblednout, pokud se médium stane kyselým (což může časem spontánně nastat).
DMHF (dimethyl hydantoin formaldehyd)	Vysoká transparentnost, dobrý index lomu, vynikající viditelnost struktur, poměrně dobrá stabilita preparátů.<br>Kompatibilní s mnoha barvicími technikami<br>Dobrá ochrana vzorků, dobrá adheze mezi podložním sklíčkem a krycím sklíčkem.	Možné žloutnutí v průběhu času, může měnit některé druhy barvení, nevhodné pro barvicí techniky citlivé na formaldehyd. Vzduchové bubliny, pomalá doba schnutí, citlivé na vlhkost, obtížné opětovné zalití.<br>Formaldehyd je toxický, dráždivý a karcinogenní, ve vyšších koncentracích zdravotním rizikem. Lépe pracovat v digestoři
Euparal	Odolné médium s životností přes 50 let. Je možné zalévat přímo z 80% etanolu. Časem nežloutne ani nekřehne. Má index lomu vhodnější pro Diptera než kanadský balzám. Dobře se hodí pro silnější vzorky díky minimálnímu smrštění a sušení bez tvorby bublinek. Zůstává rozpustné v 95% etanolu, což umožňuje opětovné zalití i po mnoha letech.	Obsahuje škodlivé složky a musí se s ním manipulovat v digestoři. Dehydratace etanolem a převedení přes Euparal Essence mohou u některých taxonů způsobit křehkost, ale použití isopropanolu může tento problém zmírnit.
Hoyerovo médium	Vzorky lze zalévat živé nebo přímo z vody, etanolu či formaldehydu. Macerace poskytuje vynikající kvalitu kutikuly. Má příznivý index lomu a lze jej zvýraznit jodovým barvením pro vyšší kontrast. Octová složka ve formulaci může rozšiřovat článkované končetiny členovců. Některé vzorky mohou zůstat stabilní po dobu 40–60 let. Rozpustné ve vodě, což umožňuje snadné opětovné zalití.	Může vysychat, v dutinách se mohou vytvořit krystaly již za méně než 10 let.<br>Macerace může být nadměrná v závislosti na koncentraci chloralhydrátu a době expozice. Složky média se mohou oddělovat a během měsíců či let se může objevit jemná granulace. Médium může ztmavnout až zčernat.
CMCP-9 (=karboxy methyl celulóza fenol	Vzorky lze zalévat přímo z médií, jako jsou voda, ethanol, glycerol nebo roztoky obsahující formaldehyd, a jejich vnitřní orgány mohou být podle potřeby macerovány, aby bylo usnadněno celkové pozorování nebo preparace.	Může časem vytvářet krystaly a tmavnout a někdy může vzorky macerovat více, než je zamýšleno. Pokud není preparát s tlustšími vzorky pečlivě orámován, může vysychat a vytvořit mezery kolem okrajů krycího sklíčka. Není vhodné pro barvené vzorky ani kalcifikované materiály a jeho doba schnutí je relativně dlouhá.

Tabulka 4 (pokračování)

Název	Výhody	Nevýhody
Enecê	Vysoce odolné médium s životností alespoň 50 let. Časem neztmavne. Vysychá pomalu, což umožňuje preparaci hmyzu přímo v médiu a poskytuje dostatek času pro uspořádání morfologických struktur. Levné.	Vyžaduje časově náročnou dehydratační sérii; ta a převedení přes hřebíčkový olej mohou u některých vzorků způsobit křehkost. Projasňování pokračuje, i když velmi pomalu; to může ztížit pozorování velmi malých struktur, jako jsou senzily, askoidy a štětinky.

5.3.4. Popis doporučených zalévacích médií (Tabulky 3 & 4)

Média pro dočasné pozorování

Hoyerovo médium (RI = 1,48)

Roztok Marc-André je vhodný pro krátkodobé pozorování spermaték (včetně fotografií nebo kreseb) po dobu několik hodin (možno i více, pokud je sklíčko uloženo ve vlhké komoře). Uchování spermaték však vyžaduje jejich přemontování do vodního média, umožňujícího střednědobé skladování. Jejich dehydratace pro přemontování do pryskyřic je sice možná, ale nedoporučuje se (riziko ztráty vzorku).

Hoyerovo medium a chloralhydrátové medium jsou obvykle považovány za synonyma. Obsahují arabskou gumu, glycerol a chloralhydrát a jsou jednou z variant tak zvaných Berleseho médií. Různé receptury byly v minulosti chybně interpretovány a citovány [74]. Hoyerovo médium se běžně používá k pozorování vnitřních orgánů díky své kompatibilitě s vodou, jednoduchosti, rychlé aplikaci a indexu lomu, který usnadňuje zkoumání jemných struktur, jako jsou spermatéky. Médium má však značné nevýhody, pokud není dokonale připraveno nebo skladováno v podmínkách s kontrolovanou vlhkostí. Tyto problémy zahrnují krystalizaci, změnu barvy a ztrátu viskozity. Orámování krycího sklíčka lakem tyto potíže neřeší, protože zalévací médium může v důsledku interakce s uzavíracím lakem silně změnit barvu (někdy až téměř zčernat).

Hoyerovo médium bylo považováno za opticky nejlepší pro flebotomy a tradičně se pro tyto účely používalo. Ačkoli je dobré pro pozorování spermaték u flebotomů, není vhodné pro dlouhodobou konzervaci. Je ideální pro krátkodobá pozorování, včetně pořizování fotografií, kreseb nebo snímků. Nemůže ale zajistit dlouhodobé uchování, protože v průběhu času degraduje v důsledku dehydratace (obrázek 8), což vede k tvorbě malých bílých, neprůhledných krystalů chloralhydrátu. Nicméně vzorky lze ze zkrystalizovaných sklíček zachránit, protože kutikula zůstává chemicky neporušená, i když může dojít k určitému fyzickému poškození rostoucími krystaly. V některých případech lze zkrystalizovaná sklíčka obnovit rehydratací zalévacího média v teplém a vlhkém prostředí s přídavkem thymolu, který zabraňuje růstu plísní. Alternativně lze vzorky vyplavit ve vodě, dehydratovat v ledové kyselině octové a znovu namontovat do kanadského balzámu.

DMHF (dimethylhydantoin-formaldehyd) (RI 1.48)

Toto médium na vodní bázi [72] vykazuje po optické stránce velmi dobré výsledky, podobně jako Berleseho média, a jeho použití je stejně snadné. Na rozdíl od Berleseho médií však nečerná ani nekrystalizuje. Dobře se osvědčilo u flebotomů i dalších zástupců čeledi Psychodidae.

This is an Open Access article distributed under the terms of the Creative Commons Attribution License (https://creativecommons.org/licenses/by/4.0), which permits unrestricted use, distribution, and reproduction in any medium, provided the original work is properly cited.

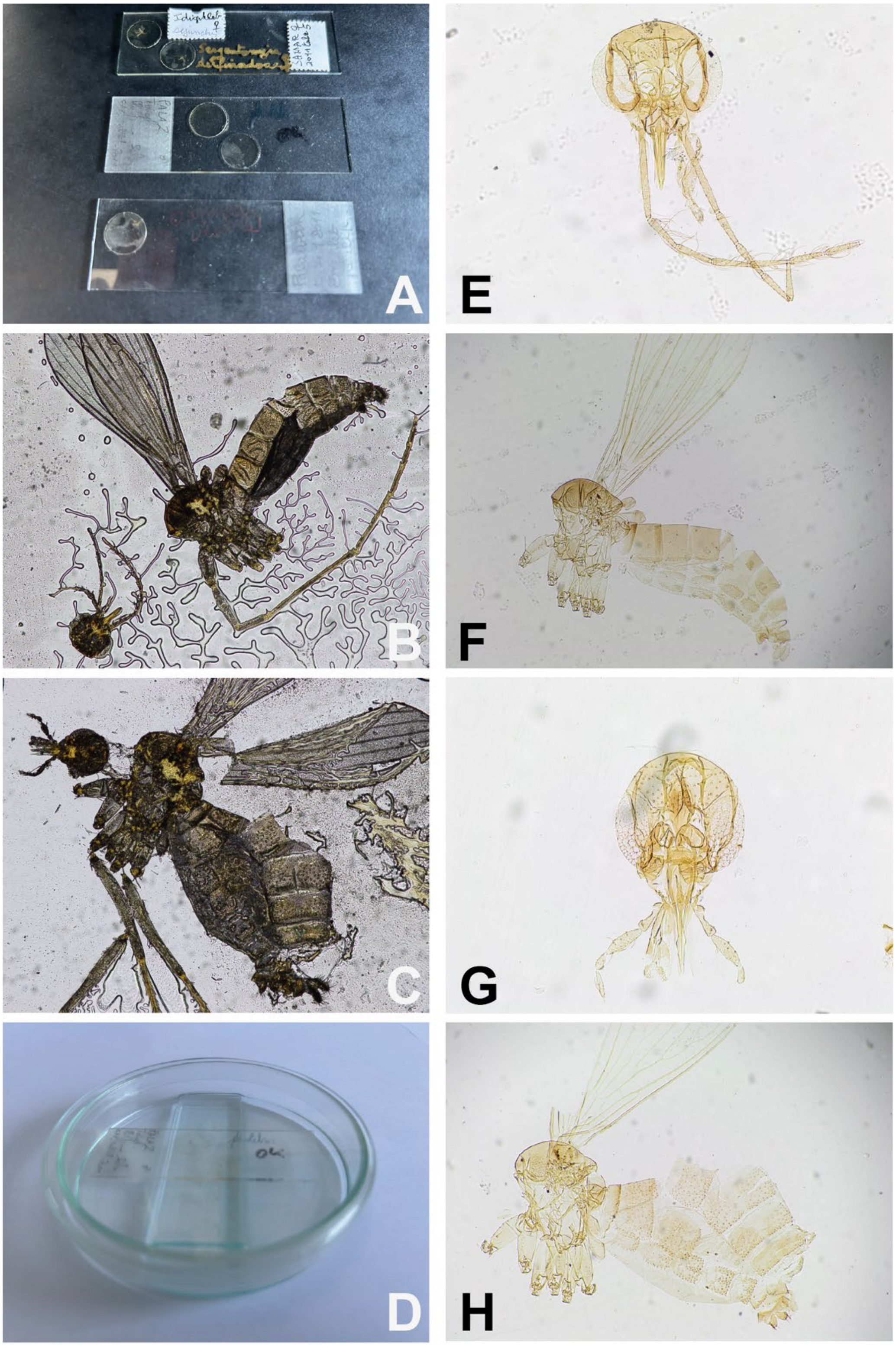

Obrázek 8: Opětovná montáž preparátu (remounting). A: poškozená a vyschlá sklíčka v Hoyerově médiu; B: mikroskopický pohled na vyschlého flebotoma; C: mikroskopický pohled na dalšího poškozeného flebotoma; D: vlhká komůrka obsahující vyschlé sklíčko; E: hlava a F: tělo exempláře B po jeho opětovném namontování do média Euparal®; G: hlava a H: tělo poškozeného exempláře C po jeho opětovném namontování do média Euparal®.

CMCP (kafr-monochlorfenol) (RI = 1.41)
Je vodorozpustným zalévacím médium na bázi glycerinu, které se používá k vytváření transparentních, trvalých preparátů jemných vzorků, včetně flebotomů. Výhodou tohoto média je, že vzorky lze montovat přímo z vody nebo ethanolu. Rychle flebotoma projasňuje, přičemž změkčuje kutikulu, což umožňuje správné polohování vzorku – to je užitečné zejména při roztahování křídel nebo pitvě genitálií. Ačkoli se uvádí, že umožňuje dlouhodobé uchování, přesná doba trvanlivosti zůstává nejistá. Hlavní omezení tohoto zalévacího média spočívá v jeho složení, které obsahuje fenol, toxickou a dráždivou látku vyžadující opatrnou manipulaci.

Média pro trvalou montáž
Kanadský balzám (RI = 1.52-1.54)
Kanadský balzám poprvé popsal jako vhodné zalévací médium pro mikroskopii v procházejícím světle Andrew Pritchard ve 30. letech 19. století. Díky své osvědčené archivační kvalitě s více než 150letou historií úspěšného používání zůstává jedním z nejrozšířenějších médií. Na rozdíl od Berleseho médií kanadský balzám nekrystalizuje ani neabsorbuje vlhkost. Kanadský balzám však vykazuje silnou autofluorescenci, což může být u některých mikroskopických technik nevýhodou [60]. Použití netoxických rozpouštědel namísto xylenu může snížit bezpečnostní rizika během přípravy, ale může také přinést nevýhody, jako je pomalejší zasychání a dřívější tmavnutí média.
Euparal® (RI = 1.48)
Euparal® je široce používanou alternativou ke kanadskému balzámu pro trvalou montáž, která nabízí vynikající dlouhodobou stabilitu a srovnatelný index lomu. Euparal® vyžaduje dehydrataci, což prodlužuje celkovou dobu zpracování vzorku: před závěrečným přenosem do zalévacího média musí být vzorek dehydratován, obvykle přechodem z 95% do absolutního ethanolu. Pokud není dehydratace organickými rozpouštědly proveditelná, lze vzorky vyjmuté z absolutního ethanolu před finální montáží umístit do přechodného roztoku sestávajícího ze směsi Euparalu® a ředidla Esence Euparal v poměru 1:1.
Enecê (RI = 1.467)
Enecê je zalévací médium na bázi pryskyřice, které se používá především pro malý hmyz a je obzvláště populární v Brazílii. Jeho základ tvoří kalafuna a kopálová guma rozpuštěné v alkoholu, kafru, terpentýnové silici a eukalyptolu. Cerqueira [11] popsal Enecê jako alternativu ke kanadskému balzámu pro montáž trvalých preparátů larev, exuvií juvenilních stadií, a dokonce i dospělců komárů; od té doby bylo toto médium široce přijato i pro montáž flebotomů. Enecê nabízí cenově výhodnou alternativu pro trvalou montáž, přičemž poskytuje dlouhodobou stabilitu a dostatečnou dobu zasychání, což umožňuje pitvu a přesné uspořádání morfologických struktur.

5.4. Příprava a sušení preparátů

Řádné vysušení sklíček se vzorky je kritické pro zajištění dlouhodobé stability a konzervace. Před uložením k dlouhodobému skladování by měla být sklíčka důkladně vysušena. Pro dosažení optimálních výsledků by se sklíčka s trvalými zalévacími médii měla sušit ve vodorovné poloze po dobu 2–3 týdnů, zatímco u preparátů s dočasnými médii mohou stačit 1–2 týdny. Pro zajištění efektivního sušení se doporučuje použít inkubátor, přičemž je třeba vyvarovat se nadměrného tepla, které by mohlo vzorky poškodit. Doporučené rozmezí teplot je 30 °C až 37 °C. Tento krok sušení je zásadní, aby se předešlo úniku zalévacího média a znehodnocení vzorku během skladování.
Použité zalévací médium by mělo být vždy uvedeno na štítku sklíčka. Pokud je to možné, štítek by měl obsahovat také použitou recepturu spolu se jménem preparátora a datem přípravy. Sklíčka mohou být zpočátku připravována jako dočasné preparáty, které nejsou určeny k dlouhodobému uchování. Pokud se však status exempláře změní, například je-li určen jako součást „typové“ série, mělo by být použito trvalejší zalévací médium, aby se zajistilo uchování vzorku pro budoucí taxonomické studie.

5.5. Alternativní techniky montáže: montáž na štítky

Montáž na štítky je technika používaná u některých skupin hmyzu, při níž mohou být exempláře buď přímo připíchnuty na entomologické štítky, nebo přilepeny na jejich povrch. Vzhledem k malé velikosti flebotomů a potřebě pozorovat vnitřní orgány pro identifikaci (viz bod 5) není tato metoda pro jejich montáž vůbec vhodná.

5.6. Opětovná montáž poškozených vzorků

U vzácných nebo cenných exemplářů se doporučuje dvoukrokový přístup podle videa dostupného na: https://zenodo.org/records/18315029. 1) Vzorek se rehydratujte, aby bylo možné provést předběžné pozorování. Do Petriho misky je umístěn držák na několik mikroskopických sklíček. Sklíčko určené k rehydrataci se položí na tuto podpěru a Petriho miska se naplní několika milimetry rozpouštědla, aby se vytvořila vlhká komůrka, přičemž je nutné zajistit, aby samotné sklíčko nepřišlo do kontaktu s rozpouštědlem (obrázek 8 D). Doba potřebná k rehydrataci se může pohybovat od jednoho do několika dnů v závislosti na stavu vzorku. Zásadní je každodenní sledování a trpělivost. Jakmile je sklíčko dostatečně rehydratováno, lze jej vyjmout z vlhké komůrky a před mikroskopickým vyšetřením, fotografováním nebo kreslením umístit na několik hodin do inkubátoru. 2) Pro opětovnou montáž lze sklíčko vrátit do vlhké komůrky na několik dalších hodin nebo přes noc. Rozebírání by mělo být prováděno pod binokulárním mikroskopem. Pomocí jemných jehel je třeba opatrně sejmout krycí sklíčko a zajistit, aby na něm nezůstaly přichyceny žádné části flebotoma (https://zenodo.org/records/18315029). Dále by měly být vypreparované části flebotoma posbírány a opláchnuty vodou v malých jamkách, podobných těm, které se používají pro destruktivní extrakci DNA/RNA (viz níže),

This is an Open Access article distributed under the terms of the Creative Commons Attribution License (https://creativecommons.org/licenses/by/4.0), which permits unrestricted use, distribution, and reproduction in any medium, provided the original work is properly cited.

a to před dehydratací a opětovnou montáží do pryskyřičného média. Při rozebírání preparátu je klíčové identifikovat původní zalévací médium, aby bylo možné zvolit vhodné rozpouštědlo. Pro vodní zalévací média by měla být použita voda. Pokud je zalévací médium na bázi pryskyřice (např. kanadský balzám nebo Euparal®), je vhodné použít xylen, a to v digestoři a s použitím vhodných osobních ochranných pomůcek, včetně masky.
Opětovná montáž typových nebo sbírkových exemplářů smí být prováděna pouze se souhlasem kurátora a/nebo instituce, která daný vzorek vlastní.

6. Druhová identifikace

6.1. Morfologie

Druhové určování flebotomů se primárně opírá o posouzení jejich morfologických znaků, včetně tvaru hrudi, křídel, genitálií, štětin a specifických morfometrických vztahů mezi různými strukturami. Výzkumníci používají taxonomické klíče, referenční sbírky a původní popisy druhů k porovnání sebraných vzorků se známými taxony. Klíčové diagnostické znaky, jako je žilnatina křídel a morfologie hlavy u obou pohlaví, struktura samčích genitálií a konfigurace samičích spermaték, jsou pro určení druhu obzvláště informativní. Přesná identifikace často vyžaduje podrobné mikroskopické pozorování drobných struktur, jako jsou genitálie a spermatéky, nebo stereomikroskopu pro širší posouzení morfologických znaků.
Nedávný pokrok v zobrazovacích technologiích usnadnil využití digitálního zobrazování k identifikaci flebotomů. Fotografie s vysokým rozlišením nebo digitální ilustrace klíčových znaků lze porovnat s referenčními materiály nebo analyzovat pomocí počítačově podporovaných identifikačních systémů, což zlepšuje jak přesnost, tak i přístupnost morfologické taxonomie.

6.2. Morfologie křídel

Morfologie křídel je důležitým znakem, používaným k druhovému určování a klasifikaci flebotomů. Jejich křídla vykazují typický vzor a strukturu, obvykle jsou dlouhá a úzká, s dobře vyvinutou žilnatinou (obrázky 9 a 10). Uspořádání žilek tvoří charakteristický vzor, který se může lišit mezi rody a druhy, což poskytuje cenné diagnostické znaky pro identifikaci. Studium morfologie křídel proto poskytuje cenné poznatky pro taxonomické účely.

6.3. Morfometrie křídel

Výzkumníci používají techniky jako je geometrická morfometrie k analýze a porovnání tvaru a velikosti křídel u různých druhů nebo populací flebotomů. Studium geometrie křídel poskytuje cenné poznatky o jejich chování, preferencích stanovišť a letových schopnostech.
Při použití geometrické morfometrie se křídla pečlivě oddělí, obarví (v případě potřeby) a umístí na podložní sklíčka. Připravená sklíčka se poté vyfotografují pod binokulární lupou, digitalizují a podrobí morfometrické analýze. Tento postup je v literatuře dobře popsán. [6, 27, 42, 56, 57, 59], s doporučením používat u párových orgánů konzistentně pravé nebo levé křídlo, aby se předešlo potenciálním negativním alometrickým účinkům [62].

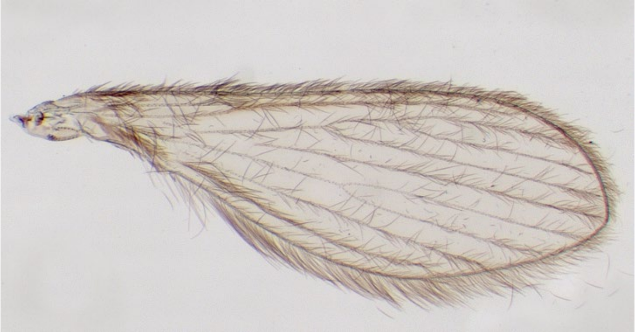

Obrázek 9: Čerstvé křídlo *Trichophoromyia ininii.*

<u>*Preparace křídla pro analýzu pomocí geometrické morfometrie*</u>
Pro optimální vizualizaci žilek na křídlech by měla být křídla očištěna od šupin a vhodně obarvena. Pro jejich preparaci nejprve naplňte malé jamky potřebnými činidly (methylenová modř, ethanol, voda a xylen). Vyjměte křídlo konzervované v 70% ethanolu při pokojové teplotě převrácením Eppendorfovy zkumavky a jejím vyprázdněním do jamky, poté křídlo podélně zvedněte pomocí jemné zahnuté jehly. Křídlo krátce protáhněte z ethanolu do vody a zpět do ethanolu, abyste odstranili chloupky. Křídlo vložte na 6 minut do methylenové modři a ujistěte se, že během barvení plave. Opatrně vyjměte křídlo a ponořte ho na 2 minuty do xylenu (přibližně na třetinu doby působení methylenové modři). Jemné poklepávání jehlou o stěny jamky může pomoci křídlu usadit se; xylen slouží k fixaci zbarvení. Nakonec křídlo zvedněte a umístěte ho na mikroskopické podložní sklíčko na malou kapku Euparalu®. Pod lupou křídlo opatrně rozložte a opatrně přiložte krycí sklíčko. Fotografie by měly být pořízeny co nejdříve před zaschnutím Euparalu®, pro dosažení optimálního zarovnání může být nutné mírně upravit polohu křídla pod krycím sklíčkem.

6.4. Techniky molekulární biologie

Kromě morfologických technik jsou molekulární metody stále důležitější v entomologickém výzkumu včetně taxonomických, populačně genetických a fylogenetických studií, stejně jako pro detekci DNA/RNA patogenů a pro určení zdrojů hostitelské krve, přičemž chování vektorů je důležité v oblasti epidemiologie [70]. Sekvenování DNA lze použít k potvrzení druhu nebo k rozlišení blízce příbuzných druhů, což poskytuje přesnější a spolehlivější způsob identifikace. Navíc pokročilé molekulární techniky (tj. PCR, sekvenování DNA, NGS atd.) a MALDI-ToF MS proteinové profilování získávají na významu pro přesnou a rychlou identifikaci druhů a doplňují tradiční morfologické

metody [46]. Navzdory tomuto pokroku zůstává morfologická identifikace referenčním standardem pro taxonomii a základem, na kterém se interpretují molekulární data.

6.4.1. Destruktivní extrakce nukleových kyselin

Extrakce nukleových kyselin je rutinním krokem v mnoha biologických studiích a byly vyvinuty různé metody pro izolaci DNA z biologických materiálů [48]. Mnoho komerčně dostupných sad pro extrakci DNA je navrženo tak, aby tento proces usnadňovalo [14]. Metody běžně používané pro přípravu vzorků členovců pro morfologickou identifikaci však často brání analýze DNA, protože tyto techniky mohou poškodit nebo zničit důležité fyzikální vlastnosti analyzovaného jedince [10]. Většina protokolů pro extrakci DNA z hmyzích tkání je destruktivní povahy [43], což vyvolává obavy zvlášť u malých vzorků, kde i omezený odběr tkáně může ohrozit důležité morfologické znaky [72]. Typ a stav vzorku hrají klíčovou roli při výběru vhodné metody izolace DNA [29].

Potřeba přesné identifikace flebotomů pro pochopení jejich populační dynamiky vedla k vývoji molekulárně diagnostických nástrojů [23]. Molekulární přístupy se nyní často používají jako doplněk morfologických taxonomických metod pro jejich druhovou identifikaci. Například standardní přístup pro DNA barcoding zahrnuje extrakci DNA, sekvenování, a přitom ztrátu původního vzorku. Existuje tedy naléhavá potřeba vyvíjet nedestruktivní metody extrakce DNA, které zachovávají jak biologický materiál, tak jeho morfologickou integritu.

U flebotomů bylo použito mnoho metod extrakce nukleových kyselin. Požadované množství nebo kvalita nukleových kyselin závisí na následné molekulární analýze, protože různé techniky mají různou citlivost a požadavky na čistotu [9]. Například bylo zjištěno, že oči fleboomů mohou inhibovat následnou PCR amplifikaci [69]. Kromě detekce DNA patogenů se DNA flebotomů běžně extrahuje pro účely identifikace druhů. Mohou být použity různé extrakční metody, ačkoli výtěžky a kvalita se u jednotlivých technik liší. Některé protokoly výrobců byly výzkumníky upraveny specificky pro flebotomy [8], čímž se zvýšil výtěžek a/nebo kvalita extrahovaných nukleových kyselin [8, 9, 69], zatímco jiné adaptace, vyvinuté původně pro jiné taxony členovců, lze použít i u flebotomů [58, 76]. Identifikační PCR cílené na malé mitochondriální fragmenty (COI nebo CytB) jsou obecně kompatibilní s extrakčními metodami zahrnujícími vysokou fragmentaci DNA. Naproti tomu jiné techniky NGS s dlouhým čtením (Oxford Nanopore a PacBio) vyžadují minimální fragmentaci a vysoce kvalitní DNA. Extrakce na stočitelných kolonkách obvykle poskytují fragmenty genomové DNA o velikosti až 60 kb, zatímco extrakce fenol-chloroformem může produkovat fragmenty až do 150 kb [77]. Tabulka 5 shrnuje různé techniky extrakce DNA a uvádí, zda byly provedeny specifické metodologické úpravy. Výtěžky nejsou uvedeny, protože závisí na velikosti vzorku a způsobu přípravy. Sloupec s modifikacemi se týká úprav extrakčních protokolů pro flebotomy nebo jiné malé členovce.

Volba extrakční metody by měla zohlednit několik kritérií, jako je počet vzorků, doba extrakce a použitá technika. Zatímco techniky NGS vyžadují genomovou DNA s vysokou molekulovou hmotností, všechny zde uvedené metody lze použít pro standardní aplikace založené na PCR. Kromě toho se několik studií zabývalo nedestruktivními metodami extrakce DNA u malých suchozemských členovců, muzejních exemplářů konzervovaných za sucha a členovců s měkkým tělem [19, 26, 28, 55, 63].

6.4.2. Nedestruktivní extrakce nukleových kyselin

Jednou z hlavních výzev v molekulární analýze členovců včetně flebotomů je konzervace vzorků pro zařazení do entomologických sbírek. Většina protokolů pro extrakci DNA vyžaduje maceraci tkáně, což ohrožuje zachování původního vzorku. Nedestruktivní metody extrakce nukleových kyselin jsou však navrženy tak, aby extrahovaly genetický materiál bez fyzického poškození vzorku, ovlivnění jeho trvanlivosti nebo změny jeho morfologie. Tyto metody jsou obzvláště cenné při práci s cennými nebo omezeně dostupnými vzorky, kde je zachování strukturální integrity nezbytné pro budoucí taxonomické, morfologické nebo diagnostické účely. Běžně používanou technikou je nedestruktivní metoda, při které jsou flebotomové znehybněni a opatrně ponořeni do lyzačního pufru obsahujícího proteinázu K.

Technika šetrné vektolýzy byla úspěšně aplikována na flebotomy, zejména na typové exempláře [24]. Tato technika využívá konvenční sadu se stočitelnými kolonkami (v tomto případě DNeasy Blood and Tissue kit, QIAGEN, Hilden, Německo) s úpravou pro získání DNA bez zničení vzorku. Modifikované lyzační kroky (objem lyzačního pufru a přidání kroku mrazení) [17] umožňují uvolnění nukleových kyselin a minimalizují morfologické poškození [24]. Je také možné použít sadu HotSHOT DNA Extraction kit (Bento Bioworks Ltd, Londýn, Spojené království) [73], která je rychlá a levná a umožňuje rychlé a nízkonákladové zpracování vzorků. Entomologické vzorky určené k morfologické identifikaci lze poté opláchnout. Vzorky zpracované sadou DNeasy Blood and Tissue kit musí být projasněny roztokem Marc-André, zatímco vzorky zpracované sadou HotSHOT DNA Extraction kit jsou vhodné pro montáž do vodného média, nebo lépe do pryskyřice po dehydrataci, dle protokolu popsaného v tomto článku [73]. Extrahovaný genetický materiál lze poté dále zpracovat pro následnou analýzu, jako je PCR, za účelem amplifikaci specifických genetických markerů. Nedestruktivní metody extrakce nukleových kyselin jsou klíčové pro studium genetických vlastností flebotomů, včetně identifikace potenciálních původců onemocnění, které mohou přenášet. Zachováním integrity vzorku mohou vědci získat cenné genetické informace a zároveň si vzorek uchovat pro další analýzy nebo studie.

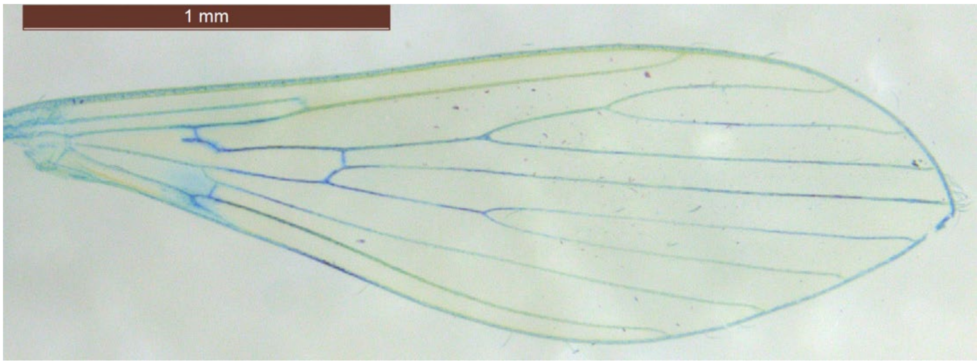

Obrázek 10. Obarvené křídlo *Phlebotomus ariasi.*

Tabulka 5. Průměrné náklady, použití a úpravy protokolů pro extrakci gDNA flebotomů

Protokol	Cena	Použití	Úpravy protokolu pro malé členovce
Stočitelné kolonky	2.5 – 3.55 US$ [39]	PCR, NGS	[9]
fenol-chloroform	0.24 US$ [69]	PCR, NGS	[9]
HotSHOT	<0.01 US$ [69]	PCR	-
Vysolení	0.12 $3 [69]	PCR	-
Chelex	0.02 $4 [41]	PCR	[41, 76]

6.5. MALDI-ToF MS

MALDI-ToF MS (matrix-assisted laser desorption/ionization time-of-flight mass spectrometry) je technika hmotnostní spektrometrie, určená k detekci a analýze jedinečných proteinových profilů („otisků prstů“) biologických vzorků. Je stále více uznávána jako užitečný nástroj pro identifikaci členovců s lékařským a veterinárním významem. Tato technika se ukázala jako účinná při identifikaci dospělců i larev flebotomů a hostitelské krve u nasátých samic a byla úspěšně použita k druhovému určení samců i samic flebotomů za různých skladovacích a homogenizačních podmínek [28, 30, 73, 74]. Umožňuje dosáhnout rychlé a přesné identifikace, což je nezbytné pro pochopení rozšíření, chování a role flebotomů v přenosu nemocí. Díky rozlišení mezi druhy na základě proteinových profilů hraje MALDI-ToF důležitou roli v epidemiologických studiích a strategiích kontroly vektorů. Rutinní použití omezují v současnosti dvě hlavní nevýhody. První je dostupnost vybavení pro hmotnostní spektrometrii, jehož snadné pořízení pouze pro účely druhové identifikace flebotomů (nebo obecně členovců) je neúnosně drahé. Naštěstí lze toto omezení překonat využitm volné kapacity hmotnostních spektrometrů, které se staly standardním výzkumným nástrojem v proteomických zařízeních a/nebo klinické diagnostice. Druhým je nízké zastoupení referenčních dostupných ve volně přístupných databázích, což vede k potřebě vytvořit interní databázi s referenčními spektry založenými na jednoznačně identifikovaných vzorcích, ideálně kombinací morfologického hodnocení a sekvenování vhodného genetického markeru (COI, cytB apod.). Toto omezení bude doufejme brzy vyřešeno postupným začleňováním dosud interních referenčních dat do platformy MSI, kterou provozuje Assistance Publique-Hôpitaux de Paris, Univerzita Sorbonna ve Francii a sbírka BCCM/IHEM/Sciensano v Bruselu v Belgii (https://msi.happy-dev.fr/). Pokud se plánuje použití proteinového profilování metodou MALDI-ToF, měly by být vzorky skladovány nejlépe zmrazené nebo v 70% ethanolu molekulární kvality a neměly by být vystaveny proměnlivým teplotám. Vzhledem k absenci univerzálních standardů pro přípravu vzorků se uživatelům doporučuje použít pro přípravu matrice MALDI-ToF vodný roztok kyseliny sinapové (30 mg/ml) o koncentraci 60% acetonitrilu a 0,3% TFA, aby byla jejich proteinová spektra srovnatelná s dosud publikovanými daty o flebotomech.

<u>*Příprava vzorku na MALDI-ToF MS*</u> (Obrázek 7)
Vzorky hmyzu skladované za různých podmínek se nejprve vysuší na vzduchu při pokojové teplotě a pitvají. Odstraní se hlava a zadeček, aby se získaly části těla obsahující klíčové morfologické charakteristiky pro montáž na sklíčka a morfologickou analýzu. Hruď lze použít pro MALDI-ToF a zbývající část zadečku se uchová pro extrakci DNA. Pro profilování proteinů se hruď ručně homogenizuje v 1,5ml mikrozkumavkách s 10 µl homogenizačního roztoku pomocí jednorázových pístků. Obvykle se používají dva

homogenizační roztoky: sterilní destilovaná voda a 25% kyselina mravenčí.

7. Shrnutí

V této práci jsme si kladli za cíl poskytnout výzkumníkům nejúčinnější metody pro zpracování flebotomů, přizpůsobené specifickým výzkumným cílům, aby se usnadnila přesná druhová identifikace a detekce patogenů. Neexistuje jediná, univerzálně optimální metoda; existuje několik metod, z nichž každá má své výhody a omezení.

V doplňujících materiálech uvádíme podrobné protokoly pro různé techniky montáže používané při přípravě a identifikaci flebotomů. Tyto protokoly, včetně instruktážních videí, nabízejí podrobné postupy přizpůsobené různým cílům a zajišťují přesné a spolehlivé výsledky. Touto komplexní prací se snažíme podpořit výzkumníky při výběru a použití nejvhodnějších technik montáže pro jejich specifické potřeby.

8. Poděkování

Autoři děkují Richardu Lane and Zoe Jay Adams z Natural History Museum of London, Velká Británie za jejich vynikající recenze, které pozvedly kvalitu tohoto rukopisu.

9. Financování

Děkujeme Brazilské rozvojové agentuře CNPq (project č.: 404395/2024-4) a Araucária Foundation (project č.: 433/2025 PDI) za financování práce AJA.

10. Střet zájmů

Jérôme Depaquit je zástupcem šéfredaktora časopisu Parasite; neměl žádný vliv na recenzní proces a rozhodování o tomto rukopisu. Ostatní autoři prohlašují, že nemají žádný střet zájmů.

11. Popisky k obrázkům

Obrázek 1: Flebotomové uchovaní v etanolu.
Obrázek 2: Materiály potřebné pro montování flebotomů: A: kulatá krycí sklíčka (průměr 10 nebo 12 mm); B: 24-jamková destička a zahnutá jehla (pokud používáte hřebíčkový olej nebo esenci Euparal® ke zpracování flebotomů, nepoužívejte akrylátové destičky, protože dojde k chemické reakci a vzorky budou poškozeny); C: podložní sklíčka vhodná pro popisování; D: detail zahnutí jehly; E: jehly nasazené na stříkačky; F: hodinové sklíčko nebo ekvivalentní nádoba obsahující flebotomy určené k montování; G: pinzety Dumont; H: plastová pipeta; I: skleněná pipeta ohnutá zahřátím pro usnadnění přenosu kapaliny do jamek.
Obrázek 3: Destička s 24 jamkami, z nichž každá obsahuje hlavu a špičku zadečku flebotomů.
Obrázek 4: Spermatéky vypreparované a zamontované v médiu Marc-André z čerstvých jedinců. A: *Idiophlebotomus longiforceps* (Laos); B: *Sergentomyia minuta* (Francie); C: *Phlebotomus ariasi* (Francie); D: *Sergentomyia anodontis* (Laos).
Obrázek 5: Metoda izolace leishmanií.
Obrázek 6: Zpracování flebotomů pro aplikace molekulární biologie, proteomiky a/nebo virologie.
Obrázek 7: Klasická metoda zpracování flebotomů.
Obrázek 8: Opětovná montáž preparátu (remounting). A: poškozená a vyschlá sklíčka v Hoyerově médiu; B: mikroskopický pohled na vyschlého flebotoma; C: mikroskopický pohled na dalšího poškozeného flebotoma; D: vlhká komůrka obsahující vyschlé sklíčko; E: hlava a F: tělo exempláře B po jeho opětovném namontování do média Euparal®; G: hlava a H: tělo poškozeného exempláře C po jeho opětovném namontování do média Euparal®.
Obrázek 9: Čerstvé křídlo *Trichophoromyia ininii*.
Obrázek 10: Obarvené křídlo *Phlebotomus ariasi.*

12. Videa na Zenodo.

Video 1: https://zenodo.org/records/18198006
Video 2: https://zenodo.org/records/18311158
Video 3: https://zenodo.org/records/18311106
Video 4: https://zenodo.org/records/18311154
Video 5: https://zenodo.org/records/18303014
Video 6: https://zenodo.org/records/18302850
Video 7: https://zenodo.org/records/18315029

This is Supplementary Material text here.

References

1. Alkan C, Allal-Ikhlef AB, Alwassouf S, Baklouti A, Piorkowski G, de Lamballerie X, Izri A, Charrel RN. 2015. Virus isolation, genetic characterization and seroprevalence of Toscana virus in Algeria. Clinical Microbiology and Infection, 21(11), 1040 e1-9.
2. Alten B, Ozbel Y, Ergunay K, Kasap OE, Cull B, Antoniou M, Velo E, Prudhomme J, Molina R, Banuls AL, Schaffner F, Hendrickx G, Van Bortel W, Medlock JM. 2015. Sampling strategies for phlebotomine sand flies (Diptera: Psychodidae) in Europe. Bulletin of Entomological Research 105(6), 664–678.
3. Ayhan N, Baklouti A, Prudhomme J, Walder G, Amaro F, Alten B, Moutailler S, Ergunay K, Charrel RN, Huemer H. 2017. Practical guidelines for studies on sandfly-borne phleboviruses: Part I: Important points to consider *ante* field work. Vector-Borne and Zoonotic Diseases 17(1), 73–80.
4. Bates PA. 1997. Infection of phlebotomine sandflies with *Leishmania*, in The Molecular Biology of Insect Disease Vectors: A Methods Manual. Springer. p. 112–120.5.

5. Baum M, de Castro EA, Pinto MC, Goulart TM, Baura W, Klisiowicz Ddo R, Vieira da Costa-Ribeiro MC. 2015. Molecular detection of the blood meal source of sand flies (Diptera: Psychodidae) in a transmission area of American cutaneous leishmaniasis, Parana State, Brazil. Acta Tropica, 143, 8–12.
6. Belen A, Alten B, Aytekin A. 2004. Altitudinal variation in morphometric and molecular characteristics of *Phlebotomus papatasi* populations. Medical and Veterinary Entomology, 18(4), 343–350.
7. Bhattacharya J, Chandra G, Hati AK. 1991. A simple method for cryopreservation of *Leishmania donovani* promastigotes, Indian Journal of Medical Research, 93, 245–246.
8. Caligiuri LG, Sandoval AE, Miranda JC, Pessoa FA, Santini MS, Salomón OD, Secundino NF, McCarthy CB. 2019. Optimization of DNA extraction from individual sand flies for PCR amplification. Methods and Protocols, 2(2), 36.
9. Casaril AE, de Oliveira LP, Alonso DP, de Oliveira EF, Gomes Barrios SP, de Oliveira Moura Infran J, Fernandes WS, Oshiro ET, Ferreira AMT, Ribolla PEM, de Oliveira AG. 2017. Standardization of DNA extraction from sand flies: Application to genotyping by next generation sequencing. Experimental Parasitology, 177, 66–72.
10. Castalanelli MA, Severtson DL, Brumley CJ, Szito A, Foottit RG, Grimm M, Munyard K, Groth DM. 2010. A rapid non-destructive DNA extraction method for insects and other arthropods. Journal of Asia-Pacific Entomology, 13(3), 243–248.
11. Cerqueira NL. 1943. Um novo meio para montagem de pequenos insetos em lâmina. Memórias do Instituto Oswaldo Cruz, (39), 37–41.
12. Charrel RN, Gallian P, Navarro-Mari JM, Nicoletti L, Papa A, Sanchez-Seco MP, Tenorio A, de Lamballerie X. 2005. Emergence of Toscana virus in Europe. Emerging Infectious Diseases, 11(11), 1657–1663.
13. Chaskopoulou A, Giantsis IA, Demir S, Bon MC. 2016. Species composition, activity patterns and blood meal analysis of sand fly populations (Diptera: Psychodidae) in the metropolitan region of Thessaloniki, an endemic focus of canine leishmaniasis. Acta Tropica, 158, 170–176.
14. Chen H, Rangasamy M, Tan SY, Wang H, Siegfried BD. 2010. Evaluation of five methods for total DNA extraction from western corn rootworm beetles. PLoS One, 5(8), e11963.
15. Depaquit J, Grandadam M, Fouque F, Andry PE, Peyrefitte C. 2010. Arthropod-borne viruses transmitted by Phlebotomine sandflies in Europe: a review. Eurosurveillance, 15(10), 19507.
16. Diamond LS, Herman CM. 1954. Incidence of Trypanosomes in the Canada Goose as revealed by bone marrow culture. Journal of Parasitology, 40(2), 195–202.
17. Ding H, Torno M, Vongphayloth K, Ng G, Tan D, Sng W, Ho K, Randrianambinintsoa FJ, Depaquit J, Tan CH. 2025. Hidden in plain sight: discovery of sand flies in Singapore and description of four species new to science. Parasites & Vectors, 18(1), 402.
18. Es-Sette N, Ajaoud M, Bichaud L, Hamdi S, Mellouki F, Charrel RN, Lemrani M. 2014. *Phlebotomus sergenti* a common vector of *Leishmania tropica* and Toscana virus in Morocco. Journal of Vector Borne Diseases, 51(2), 86–90.
19. Favret C. 2005. A new non-destructive DNA extraction and specimen clearing technique for aphids (Hemiptera). Proceedings of the Entomological Society of Washington, 107(2), 469–470.
20. Galati EAB. 2018. Phlebotominae (Diptera, Psychodidae): Classification, morphology and terminology of adults and identification of American taxa, in Brazilian Sand Flies: Biology, Taxonomy, Medical Importance and Control, Rangel EF, Shaw JJ, Editors. Cham: Springer International Publishing. pp. 9–212.
21. Galati EAB, de Andrade AJ, Perveen F, Loyer M, Vongphayloth K, Randrianambinintsoa FJ, Prudhomme J, Rahola N, Akhoundi M, Shimabukuro PHF, Depaquit J. 2025. Phle-botomine sand flies (Diptera, Psychodidae) of the world. Parasites & Vectors, 18(1), 220.
22. Galati EAB, Galvis-Ovallos F, Lawyer P, Leger N, Depaquit J. 2017. An illustrated guide for characters and terminology used in descriptions of Phlebotominae (Diptera, Psychodidae). Par-asite, 24, 26.
23. Gariepy T, Kuhlmann U, Gillott C, Erlandson M. 2007. Parasitoids, predators and PCR: the use of diagnostic molecular markers in biological control of Arthropods. Journal of Applied Entomology, 131(4), 225–240.
24. Giantsis IA, Chaskopoulou A, Bon MC. 2016. Mild-Vectolysis: A nondestructive DNA extraction method for vouchering sand flies and mosquitoes. Journal of Medical Entomology, 53(3), 692–695.
25. Gidwani K, Picado A, Rijal S, Singh SP, Roy L, Volfova V, Andersen EW, Uranw S, Ostyn B, Sudarshan M, Chakravarty J, Volf P, Sundar S, Boelaert M, Rogers ME. 2011. Serological markers of sand fly exposure to evaluate insecticidal nets against visceral leishmaniasis in India and Nepal: a cluster-randomized trial. PLoS Neglected Tropical Diseases, 5(9), e1296.
26. Gilbert MTP, Moore W, Melchior L, Worobey M. 2007. DNA extraction from dry museum beetles without conferring external morphological damage. PLoS One, 2(3), e272.
27. Giordani BF, Andrade AJ, Galati EAB, Gurgel-Goncalves R. 2017. The role of wing geometric morphometrics in the identification of sandflies within the subgenus *Lutzomyia*. Medical and Veterinary Entomology, 31(4), 373–380.
28. Guzmán-Larralde AJ, Suaste-Dzul AP, Gallou A, Peña-Carrillo KI. 2017. DNA recovery from microhymenoptera using six non-destructive methodologies with considerations for subsequent preparation of museum slides. Genome, 60(1), 85–91.
29. Hajibabaei M, DeWaard JR, Ivanova NV, Ratnasingham S, Dooh RT, Kirk SL, Mackie PM, Hebert PD. 2005. Critical factors for assembling a high volume of DNA barcodes. Philosophical Transactions of the Royal Society B: Biological Sciences, 360(1462), 1959–1967.
30. Haouas N, Pesson B, Boudabous R, Dedet JP, Babba H, Ravel C. 2007. Development of a molecular tool for the identification of *Leishmania* reservoir hosts by blood meal analysis in the insect vectors. American Journal of Tropical Medicine and Hygiene, 77(6), 1054–1059.
31. Hlavackova K, Dvorak V, Chaskopoulou A, Volf P, Halada P. 2019. A novel MALDI-TOF MS-based method for blood meal identification in insect vectors: A proof of concept study on phlebotomine sand flies. PLoS Neglected Tropical Diseases, 13 (9), e0007669.
32. Huemer H, Prudhomme J, Amaro F, Baklouti A, Walder G, Alten B, Moutailler S, Ergunay K, Charrel RN, Ayhan N. 2017. Practical guidelines for studies on sandfly-borne phleboviruses: Part II: Important points to consider for fieldwork and subsequent virological screening. Vector-Borne and Zoonotic Diseases, 17(1), 81–90.
33. Jancarova M, Polanska N, Thiesson A, Arnaud F, Stejskalova M, Rehbergerova M, Kohl A, Viginier B, Volf P, Ratinier M. 2025. Susceptibility of diverse sand fly species to Toscana virus. PLoS Neglected Tropical Diseases, 19(5), e0013031.

34. Kapp JD, Green RE, Shapiro B. 2021. A fast and efficient single-stranded genomic library preparation method optimized for ancient DNA. Journal of Heredity, 112(3), 241–249.
35. Killick-Kendrick R, Maroli M, Killick-Kendrick M. 1991. Bibliography of the colonization of phlebotomine sandflies. Parassitologia, 33(suppl.), 321–333.
36. Lawyer P, Killick-Kendrick M, Rowland T, Rowton E, Volf P. 2017. Laboratory colonization and mass rearing of phlebotomine sand flies (Diptera, Psychodidae). Parasite, 24, 42.
37. Léger N, Pesson B, Madulo-Leblond G. 1986. Les phlébotomes de Grèce : 1ère partie. Bulletin de la Société de Pathologie Exotique, 79, 386–397.
38. Léger N, Pesson B, Madulo-Leblond G. 1986. Les phlébotomes de Grèce : 2ème partie. Bulletin de la Société de Pathologie Exotique, 79, 514–524.
39. Leonel JAF, Vioti G, Alves ML, da Silva DT, Meneghesso PA, Benassi JC, Spada JCP, Galvis-Ovallos F, Soares RM, Oliveira T. 2020. DNA extraction from individual Phlebotomine sand flies (Diptera: Psychodidae: Phlebotominae) specimens: Which is the method with better results? Experimental Parasitology, 218, 107981.
40. Lestinova T, Rohousova I, Sima M, de Oliveira CI, Volf P. 2017. Insights into the sand fly saliva: Blood-feeding and immune interactions between sand flies, hosts, and *Leishmania.* PLoS Neglected Tropical Diseases, 11(7), e0005600.
41. Lienhard A, Schaffer S. 2019. Extracting the invisible: obtaining high quality DNA is a challenging task in small arthropods. PeerJ, 7, e6753.
42. Lozano-Sardaneta YN, Mikery-Pacheco OF, Huerta H, Rojas-Soriano JE, Contreras-Ramos A. 2025. Wing geometric morphometrics is effective to separate sand fly species (Diptera, Psychodidae, Phlebotominae) related with leishmaniasis transmission in Mexico. Acta Tropica, 262, 107523.
43. Mandrioli M. 2008. Insect collections and DNA analyses: how to manage collections? Museum Management and Curatorship, 23(2), 193–199.
44. Maroli M, Feliciangeli MD, Bichaud L, Charrel RN, Gradoni L. 2013. Phlebotomine sandflies and the spreading of leishmaniases and other diseases of public health concern. Medical and Veterinary Entomology, 27(2), 123–147.
45. Marquina D, Buczek M, Ronquist F, Lukasik P. 2021. The effect of ethanol concentration on the morphological and molecular preservation of insects for biodiversity studies. PeerJ, 9, e10799.
46. Mathis A, Depaquit J, Dvorak V, Tuten H, Banuls AL, Halada P, Zapata S, Lehrter V, Hlavackova K, Prudhomme J, Volf P, Sereno D, Kaufmann C, Pfluger V, Schaffner F. 2015. Identification of phlebotomine sand flies using one MALDI-TOF MS reference database and two mass pectrometer systems., Parasites & Vectors, 8, 266.
47. Mekarnia N, Benallal KE, Sadlova J, Vojtkova B, Mauras A, Imbert N, Longhitano M, Harrat Z, Volf P, Loiseau PM, Cojean S. 2024. Effect of *Phlebotomus papatasi* on the fitness, infectivity and antimony-resistance phenotype of antimony-resistant *Leishmania* major Mon-25. International Journal for Parasitology – Drugs and Drug Resistance, 25, 100554.
48. Milligan BG. 1998. Total DNA isolation, in Molecular Genetic Analysis of Population: A Practical Approach, Hoelzel AR, Editor. Oxford: Oxford University Press.
49. Molina R, Jiménez M, Alvar J, González E, Hernández-Taberna S, Ines MM. 2017. Methods in sand fly research. Madrid: Servicio de publicaciones Universidad de Alcalá de Henares, Madrid.
50. Murphy WJ, Eizirik E, O'Brien SJ, Madsen O, Scally M, Douady CJ, Teeling E, Ryder OA, Stanhope MJ, de Jong WW, Springer MS. 2001. Resolution of the early placental mammal radiation using Bayesian phylogenetics. Science, 294(5550), 2348–2351.
51. Nacif-Pimenta R, Pinto LC, Volfova V, Volf P, Pimenta PFP, Secundino NFC. 2020. Conserved and distinct morphological aspects of the salivary glands of sand fly vectors of leishma-niasis: an anatomical and ultrastructural study. Parasites & Vectors, 13(1), 441.
52. Neuhaus B, Schmid T, Riedel J. 2017. Collection management and study of microscope slides: Storage, profiling, deterioration, restoration procedures, and general recommendations. Zootaxa, 4322(1), 1–173.
53. New TR. 1974. Pscoptera. Handbooks for Identification of British Insects (Vol. I). London: Royal Entomological Society of London. 102 pp.
54. Perez-Ruiz M, Collao X, Navarro-Mari JM, Tenorio A. 2007. Reverse transcription, real-time PCR assay for detection of Toscana virus. Journal of Clinical Virology, 39(4), 276–281.
55. Porco D, Rougerie R, Deharveng L, Hebert P. 2010. Coupling non-destructive DNA extraction and voucher retrieval for small soft-bodied Arthropods in a high-throughput context: the example of Collembola. Molecular Ecology Resources, 10(6), 942–945.
56. Prudhomme J, Cassan C, Hide M, Toty C, Rahola N, Vergnes B, Dujardin JP, Alten B, Sereno D, Banuls AL. 2016. Ecology and morphological variations in wings of *Phlebotomus ariasi* (Diptera: Psychodidae) in the region of Roquedur (Gard, France): a geometric morphometrics approach. Parasites & Vectors, 9(1), 578.
57. Prudhomme J, Gunay F, Rahola N, Ouanaimi F, Guernaoui S, Boumezzough A, Banuls AL, Sereno D, Alten B. 2012. Wing size and shape variation of *Phlebotomus papatasi* (Diptera: Psychodidae) populations from the south and north slopes of the Atlas Mountains in Morocco. Journal of Vector Ecology, 37(1), 137–147.
58. Prudhomme J, Toty C, Kasap OE, Rahola N, Vergnes B, Maia C, Campino L, Antoniou M, Jimenez M, Molina R, Cannet A, Alten B, Sereno D, Banuls AL. 2015. New microsatellite markers for multi-scale genetic studies on *Phlebotomus ariasi* Tonnoir, vector of *Leishmania infantum* in the Mediterranean area. Acta Tropica, 142, 79–85.
59. Prudhomme J, Velo E, Bino S, Kadriaj P, Mersini K, Gunay F, Alten B. 2019. Altitudinal variations in wing morphology of *Aedes albopictus* (Diptera, Culicidae) in Albania, the region where it was first recorded in Europe. Parasite, 26, 55.
60. Rawlins DJ. 1992. Light Microscopy: An Introduction to Biotechniques. Oxford: Bios Scientific publishers. 143 pp.
61. Ready PD. 2013. Biology of phlebotomine sand flies as vectors of disease agents. Annual Review of Entomology, 58, 227–250.
62. Rohlf FJ, Slice D. 1990. Extensions of the Procrustes method for the optimal superimposition of landmarks. Systematic Zoology, 39(1), 40–59.
63. Rowley DL, Coddington JA, Gates MW, Norrbom AL, Ochoa RA, Vandenberg NJ, Greenstone MH. 2007. Vouchering DNA-barcoded specimens: Test of a nondestructive extraction proto-col for terrestrial arthropods. Molecular Ecology Notes, 7(6), 915–924.
64. Sábio PB, Andrade AJ, Galati EAB. 2014. Assessment of the taxonomic status of some species included in the *Shannoni* complex, with the description of a new species of *Psathyromyia*

(Diptera: Psychodidae: Phlebotominae). Journal of Medical Entomology, 51(2), 331–341.

65. Sadlova J, Yeo M, Seblova V, Lewis MD, Mauricio I, Volf P, Miles MA. 2011. Visualisation of *Leishmania donovani* fluorescent hybrids during early stage development in the sand fly vector. PLoS One, 6(5), e19851.
66. Sales K, Miranda DEO, da Silva FJ, Otranto D, Figueredo LA, Dantas-Torres F. 2020. Evaluation of different storage times and preservation methods on phlebotomine sand fly DNA concentration and purity. Parasites & Vectors, 13(1), 399
67. Sales KG, Costa PL, de Morais RC, Otranto D, Brandao-Filho SP, Cavalcanti Mde P, Dantas-Torres F. 2015. Identification of phlebotomine sand fly blood meals by real-time PCR. Parasites& Vectors, 8, 230.
68. Sant'Anna MR, Jones NG, Hindley JA, Mendes-Sousa AF, Dillon RJ, Cavalcante RR, Alexander B, Bates PA. 2008. Blood meal identification and parasite detection in laboratory-fed and field-captured *Lutzomyia longipalpis* by PCR using FTA databasing paper. Acta Tropica, 107(3), 230–237.
69. Senne NA, Santos HA, Araujo TR, Paulino PG, Mendonca LP, Moreira HVS, Camilo TA, da Costa Angelo I. 2022. Robust comparative performance of genomic DNA extraction methods from non-engorged phlebotomine sandflies. Medical and Veterinary Entomology, 36(2), 203–211.
70. Shaw JJ. 2025. A review of Leishmania infections in American Phlebotomine sand flies – Are those that transmit leishmaniasis anthropophilic or anthropportunists? Parasite, 32, 57.
71. Tesh RB, Modi GB. 1983. Growth and transovarial transmission of Chandipura virus (Rhabdoviridae: Vesiculovirus) in *Phlebotomus papatasi.* American Journal of Tropical Medicine and Hygiene, 32(3), 621–623.
72. Thomsen PF, Elias S, Gilbert MTP, Haile J, Munch K, Kuzmina S, Froese DG, Sher A, Holdaway RN, Willerslev E. 2009. Non-destructive sampling of ancient insect DNA. PLoS One, 4(4),e5048
73. Truett GE, Heeger P, Mynatt RL, Truett AA, Walker JA, Warman ML. 2000. Preparation of PCR-quality mouse genomic DNA with hot sodium hydroxide and tris (HotSHOT). Biotechniques, 29(1), 52–54.
74. Upton MS. 1993. Aqueous gum-chloral slide mounting media: an historical review. Bulletin of Entomological Research, 83(2), 267–274.
75. Volf P, Myskova J. 2007. Sand flies and Leishmania: specific versus permissive vectors. Trends in Parasitology, 23(3), 91–92.
76. Wang Q, Wang X. 2012. Comparison of methods for DNA extraction from a single chironomid for PCR analysis. Pakistan Journal of Zoology, 44(2), 421–426.
77. Wang Y, Zhao Y, Bollas A, Wang Y, Au KF. 2021. Nanopore sequencing technology, bioinformatics and applications. Nature Biotechnology, 39(11), 1348–1365.

Cite this article as: Randrianambinintsoa FJ, Augendre L, Prudhomme J, Martinet J-P, Loyer M, Mekarnia N, Kerkoub H, Perveen FK, Huguenin A, Kariya E, Akhoundi M, De Andrade AJ, Berriatua E, Bongiorno G, Boyer S, Christodoulou V, Da Costa-Ribeiro MCV, De Souza LAF, Ding H, Dondji B, Dvořák V, Erisoz Kasap O, Galati EAB, Gállego M, Ballart C, Gouzelou S, Haddad N, Masse RS, Mekuria AH, Ivovic V, Kaczmarek S, Shahar MK, Kirstein OD, Kniha E, Kolářová I, Lincoln T, Lucanas C, Mikov O, Nov K, Özbel Y, Pesson B, Posada Lopez LC, Prasetyo DB, Rahola N, Rebollar-Tellez EA, Rodrigues BL, Roy L, Saini P, Sanjoba C, Shimabukuro PH, Siriyasatien P, Soszynska A, Suleşco T, Sylla M, Torno M, Volf P, Vongphayloth K, Sinh Nam V, Wardhana A, Yessinou E, Zapata S, Gantier J-C & Depaquit J. 2026. Processing and mounting phlebotomine sand flies: a consensus guideline. Parasite xx, xx. https://doi.org/10.1051/parasite/2026009.

PARASITE

An international open-access, peer-reviewed, online journal publishing high quality papers on all aspects of human and animal parasitology

Reviews, articles and short notes may be submitted. Fields include, but are not limited to: general, medical and veterinary parasitology; morphology, including ultrastructure; parasite systematics, including entomology, acarology, helminthology and protistology, and molecular analyses; molecular biology and biochemistry; immunology of parasitic diseases; host-parasite relationships; ecology and life history of parasites; epidemiology; therapeutics; new diagnostic tools.
All papers in Parasite are published in English. Manuscripts should have a broad interest and must not have been published or submitted elsewhere. No limit is imposed on the length of manuscripts.

Parasite (open-access) continues **Parasite** (print and online editions, 1994-2012) and **Annales de Parasitologie Humaine et Comparée** (1923-1993) and is the official journal of the Société Française de Parasitologie.

Editor-in-Chief:
Jean-Lou Justine, Paris

Submit your manuscript at:
https://www.editorialmanager.com/parasite

Appendix A: Biochemické teoretické základy.

Dotyčnými členovci jsou v této práci flebotomové. Obecné principy lze však rozšířit i na další velmi běžné členovce, u kterých lze identifikaci provést pouze na základě vnitřních morfologických znaků. Shodou okolností jsou některé vnitřní orgány částečně chitinizovány a jejich morfologie nám poskytuje cenné informace. U všech používaných činidel bychom nikdy neměli zapomínat, že od fáze fixace hmyzu až po sestavení jde o redoxní reakce. Hlavní myšlenkou je vyhnout se míchání redukčních činidel s oxidačními činidly.

Etyl alkohol; etanol:
Tato látka je používána různými způsoby. Molekuly alkoholu mají silnou afinitu k vodě, a proto vykazují dehydratační účinek. Alkohol s nízkou koncentrací (tj. příliš bohatý na vodu) však bude hrát roli v degradaci nukleových kyselin (voda je nepřítelem nukleových kyselin).
Když je hmyz umístěn v etanolu, nejde jen o jeho konzervaci, ale také o fixaci tkání. V histologii obvykle rozlišujeme dva důležité pojmy: rychlost penetrace a rychlost fixace. Je dobře známo, že dobrý konzervant musí nejprve rychle proniknout hluboko do tkání, než je fixuje. Pro alkohol s koncentrací 96 % je penetrační koeficient přibližně 1,05 (pro srovnání, pro 0,75% vodný roztok kyseliny pikrové je penetrační koeficient 0,45, zatímco pro 3% roztok dichromanu draselného je to 1,45).
Snaha uchovávat hmyz a další členovce v ethanolu na dobu neurčitou je pro entomology realitou. Myšlenka uchovávat terénní vzorky pro následné studie nebo pro budoucí výzkumníky je stále velmi relevantní. Pro cytologa nebo histologa je však tato myšlenka nereálná. Pokud se vzorky uchovávají ve fixátoru příliš dlouho, může být jejich opětovné zpracování prakticky nemožné. Proto je obtížné nebo dokonce nemožné použít vzorky starší než 10 let.
Dalším faktorem, který je třeba zvážit, je poměr mezi hmotností fixovaného členovce a objemem fixátoru. V zoologické nebo lékařské praxi se doporučuje plánovat objem 60krát větší než objem fixovaných kusů. V praxi se u malých členovců pro daný objem fixovaných vzorků přidává alespoň 4–5 objemů alkoholu. Mějte na paměti, že alkohol ztratí svou sílu, protože odstraní veškerou vodu přítomnou v tkáních členovců.
Shrnuto:

- Etylalkohol je redukční chemické činidlo (proto neslučitelný s oxidačními fixativy);
- Sráží proteiny a degraduje je;
- Rozpouští určité komplexní lipidy a sráží glykogen;
- Způsobuje silnou kontrakci tkání a jejich zpevnění.

Zásadité roztoky hydroxidu draselného nebo sodného:

Aniž by k tomu byl skutečný důvod, v entomologii byl z těchto dvou roztoků používán především hydroxid draselný. Hydroxid sodný [E524] se vyskytuje v roztoku, buď v různých koncentracích, nebo s různou normalitou. Dodává se v peletách nebo šupinkách. Jeho hlavní nevýhodou je, že je velmi hygroskopický (více než KOH). Když reaguje s proteiny, rozpouští je, a u lipidů je během saponifikace přeměňuje na tuhá mýdla (to je hlavní rozdíl oproti KOH, který při saponifikaci poskytuje tekutá mýdla).
Hydroxid draselný [E525] je k dispozici jako koncentrovaný roztok, ale především má tu výhodu, že je formulován do pelet o hmotnosti přibližně 0,1 g, což značně usnadňuje přípravu zředěných roztoků, pokud není k dispozici přesná váha. Například 1 peleta o hmotnosti 0,1 g v 1 ml destilované vody vytvoří 10% roztok. Druhou výhodou hydroxidu draselného v peletách je jeho nižší citlivost ke karbonataci (roztok KOH má vysokou afinitu k vázání CO2, čímž vznikají uhličitanové soli).
Tyto silné zásady se budou používat k solubilizaci mastných kyselin jejich přeměnou na vodorozpustná mýdla. Je třeba mít na paměti, že fixační činidlo, jako je ethanol, solubilizovalo část tuků ve vzorku. Nicméně po přenesení vzorku do vodného prostředí se silnou zásadou se mastné kyseliny (více či méně komplexní) vysráží. Silná zásada proto provede saponifikaci za studena. V některých případech, kdy jsou tukové tkáně v nadbytku, například u samic, bude výhodné zvýšit teplotu na 35–40 °C pro usnadnění reakce, nebo prodloužit dobu kontaktu při pokojové teplotě.

Barevný kyselý roztok / bezbarvý Marc-Andrého roztok:
Zde prozkoumáme výhody a nevýhody používání Marc-Andrého roztoku. Tento roztok se skládá z chloralhydrátu (trichloracetaldehyd monohydrátu), kyseliny octové a vody. Je velmi oxidační (směs kyseliny a aldehydu). Bude neutralizovat přebytek hydroxidu draselného, který může ve vzorcích zůstat, aniž by došlo k vysrážení alkalických mýdel, vzniklých během použití hydroxidu draselného. Tento oxidační roztok bude mít také vliv na sekundární alkoholové funkce glukosaminů, které se tvoří v chitinu, a to jejich oxidací, čímž chitin změkčuje. Dalším účinkem je rozpuštění určitých přítomných minerálních solí.
Pokud je Marc-Andrého roztok předem obarven kyselým fuchsinem (tedy v oxidovaném stavu), bude se moci vázat na sekundární alkoholové funkce struktury. Po uplynutí doby kontaktu s Marc-Andrého roztokem a po obarvení vzorků se oplachování provádí pouze ethanolem. Tím zahájíme fázi dehydratace vzorků.

Výhody:

- Neutralizace přebytku zásaditých roztoků
- Uvolnění chitinu
- Barvení chitinu pro lepší posouzení chitinizovaných vnitřních struktur

Nevýhody:

This is an Open Access article distributed under the terms of the Creative Commons Attribution License (https://creativecommons.org/licenses/by/4.0), which permits unrestricted use, distribution, and reproduction in any medium, provided the original work is properly cited.

Chloralhydrát je hypnotikum a byl používán v humánní medicíně. Musí být používán v chemické digestoři a musí být dodržována legislativa o chemických rizicích.

Dehydratační roztoky:
Zkušenost ukazuje, že u velmi malých vzorků není nutné dodržovat sekvenci alkoholových lázní se zvyšující se koncentrací. Pokud je vzorek velký, začneme 80% ethanolem, poté 90%, 95% a nakonec absolutním ethanolem. Pro velmi malé vzorky použijte lázeň s 90% ethanolem následovanou ponořením do absolutního ethanolu. V této fázi budeme mít vždy na paměti, že absolutní ethanol má tendenci vázat vzdušnou vlhkost. Tradicí v entomologických laboratořích bylo dokončovat dehydrataci vzorků v lázni z bukového kreosotu. Dnes je tato esence, široce používaná jako pesticid, antifungicid a konzervant dřeva, velmi důrazně nedoporučována kvůli svému zápachu (polycyklické aromatické uhlovodíky) a předpokládá se, že je reprotoxická, karcinogenní, patří mezi persistentní organické polutanty a je ekotoxická pro vodní organismy.
Roztok, který navrhujeme připravit pro montování vzorků, je Euparal® a Euparal esence (popsáno v následujícím odstavci). Směs Euparalu® a Euparal esence je velmi dobře přijímána; vzorky získané po lázni v 90% ethanolu.

Příloha B: Složení použitých činidel.

10% Hydroxid draselný
Hydroxid draselný 10 g
Destilovaná voda q.s. 100 ml

Chloralhydrátové montovací médium Hoyerovo médium
Destilovaná voda 50 ml
Chloralhydrát 200 g
Arabská guma 50 g
Glycerol 20 ml

Marc-Andrého roztok
Chloralhydrát 40 g
Ledová kyselina octová ml
Destilovaná voda 30 ml

1% kyselý fuchsin v destilované vodě
Práškový kyselý fuchsin 1 g
Destilovaná voda 99 ml

Marc-Andrého roztok barvený fuchsinem
Marc-Andrého roztok 10 ml
1% Fuchsin 50 µL

Příloha C: Euparal®, kanadský balzám, polyvinylalkohol a další roztoky pro montování

Polyvinylalkohol: Jedná se o ideální montovací médium v případech, kdy nejsou k dispozici produkty nezbytné pro řádnou dehydrataci. Polyvinylalkohol se poté mísí s Ammanovým laktofenolem. Tyto preparáty však vykazují zásadní nevýhody spočívající buď ve vysychání nebo v krystalizaci polyvinylalkoholu v důsledku odpařování vody, případně v černání při oxidaci fenolu. Tato technika zůstává vhodnou metodou pro krátkodobé montování.

Kanadský balzám: Jeho použití pro montování mezi podložní a krycí sklíčko vyžaduje dehydrataci montovaných vzorků. Použití xylenu nebo toluenu není bez nevýhod.

Enecê médium: Pro montování mezi podložní a krycí sklíčko, podobně jako kanadský balzám, vyžaduje dehydrataci vzorku. Složení Enecê: čistá bílá kalafuna (22 g); v alkoholu rozpustná klovatina kopál (12 g), absolutní alkohol (20 ml), kafr (10 g), terpentýnová silice (10 ml) a eukalyptol (26 ml). Pro jeho přípravu vložte do nádoby, například do Erlenmeyerovy baňky, absolutní alkohol a kafr. Poté přidejte kalafunu a klovatinu kopál. Baňka se poté uzavře zátkou a protřepe, a teprve poté se zahřívá ve vodní lázni při mírné teplotě, aby směs nevřela. Jakmile je obsah zcela zkapalněn, přidá se terpentýnová silice, poté se směs ještě za horka přefiltruje a nakonec se k filtrátu přidá eukalyptol. Když se médium stane méně tekutým, naředí se roztokem Eenecê s následujícím složením: absolutní alkohol (30 ml), kafr (17 g), terpentýnová silice (15 ml), eukalyptol (38 ml) (Cerqueira, 1943).

Euparal®: Jedná se o pryskyřici pocházející ze sandarakovníku větvičnatého (*Tetraclinis articulate*, (Vahl, 1791), která byla studována a využita v roce 1906 Gilsonem. Její hlavní výhodou je, že nepolymeruje. Vzorky namontované mezi podložní a krycí sklíčka lze snadno získat zpět působením alkoholu, nebo ještě lépe pomocí Euparal® esence. Tato pryskyřice, nazývaná také sandarak, přijímá ethanol od 80 %

Využití Tritonu X100: neiontový vodný roztok:
Triton X100 je ve formě neiontového vodného roztoku (roztok 4-(1,1,3,3-tetramethylbutyl)fenyl-polyethylenglykolu neboli t-oktylfenoxypolyethoxyethanol, polyethylenglykol tert-oktylfenylether), široce používaný jako detergent v buněčné a molekulární biologii. Umožňuje permeabilizaci buněčných a jaderných membrán. Vzorky hmyzu uchovávané v alkoholu po mnoho let jsou běžnou záležitostí. Uchovávání v alkoholu bohužel není optimální

a členovci takto konzervovaní se stávají velmi obtížně připravitelnými pro mikroskopickou analýzu. Plasty z nádobek, použitýách k uskladnění vzorků, se v čase znehodnocují, což vede k následnému odpařování alkoholu. V obou případech představuje prodloužený kontakt s alkoholem nebo vysychání vzorků skutečný problém. V roce 2008 publikoval Jonque studii o rehydrataci pavouků pomocí smáčedla, jako je Agepon používaný pro fotografické filmy [26]. To vedlo k myšlence využít smáčedla, která nejsou silnými detergenty.
Níže je uveden postup s využitím 0,5% vodného roztoku Triton X100:

- Suchý vzorek nechte nasáknout absolutním alkoholem.
- Přidejte potřebný objem 0,5% roztoku Triton X100 tak, aby byl celý vzorek ponořen.
- Nechte odstát asi 5 minut nebo déle. Všichni členovci se musí v roztoku od sebe oddělit.
- Roztok Triton X100 se odstraní a nahradí roztokem hydroxidu draselného.

Dále se pokračuje technikou popsanou výše.

Příloha D: Podrobný postup montování do médií Euparal® nebo kanadského balzámu

1. Vzorky musí být dehydratovány (zakalený nebo mléčný vzhled indikuje nedostatečnou dehydrataci).
2. Dehydratace lze dosáhnout zvyšováním koncentrací ethylalkoholu.
3. Vzorky lze přenést z 99% alkoholu nebo absolutního alkoholu do projasňovacího činidla.

Postup:

1. Vzorky dospělé flebotomy v 70% ethanolu.
2. Odstraňte ethanol a nahraďte jej 10% KOH. Přikryjte flebotomy podložním sklíčkem.
3. Macerujte, dokud hmyz nezprůsvitní.
4. Odstraňte KOH.
5. Zalijte vzorek destilovanou vodou a počkejte 30 až 45 minut.
6. Odstraňte vodu, opakujte promývání destilovanou vodou po dobu 30 minut (doba závisí na množství vzorků: čím více vzorků se zpracovává společně, tím déle musí být tento čas dodržen. Čím méně jich je, zejména u těch zpracovávaných jednotlivě, tím kratší tento čas může být).
7. Odstraňte vodu.
8. Přidejte Marc-Andrého roztok (případně obarvený kyselým fuchsinem) a počkejte 24 hodin (jeden den).
9. Odstraňte Marc-Andrého roztok.
10. Zalijte vzorek destilovanou vodou a počkejte 30 až 45 minut.
11. Odstraňte vodu a opakujte promývání destilovanou vodou po dobu 30 minut.
12. Odstraňte vodu.
13. Přidejte 70% ethanol a proveďte pitvu vzorku.
    a. V případě hlavy a zadečku opatrně oddělte hlavu nebo zadeček od hrudi.
    b. V případě hrudi odstraňte křídla tak, že jednou pinzetou přidržíte hruď a druhou pinzetou zatáhnete za bázi přívěsků. Je možné provést sagitální pitvu, při níž se hruď rozdělí na levou a pravou stranu, v závislosti na oblastech největšího zájmu.
14. Postupně dehydratujte v řadě vodných roztoků ethylalkoholu: 50 – 80 – 95 %, dokud nedosáhnete absolutního ethanolu.
15. Vzorky dehydratujte dvojím promytím, pokaždé po dobu 10 minut, ve 100% ethanolu.
16. Odstraňte ethanol a zalijte vzorky hřebíčkovým olejem na 15 minut při pokojové teplotě.
17. Přeneste vzorky z hřebíčkového oleje do kapky Euparalu® nebo kanadského balzámu na čistém podložním sklíčku.
18. Hlavu, hruď a zadeček flebotoma lze pitvat pomocí jemných jehel nebo pinzet pod binokulární lupou. Hlava musí být od těla oddělena tak, aby mohla být namontována ve ventro-dorzální poloze, tj. týlní otvor (occipital foramen) musí směřovat nahoru, aby skrze něj bylo možné přímo pozorovat cibarium. Pitva se provádí v montovacím médiu pro flebotomy.
19. Ponechte vzorek, dokud se povrch nestane lepkavým.
20. Navlhčete čisté krycí sklíčko absolutním alkoholem. Přiložte krycí sklíčko pod úhlem na kanadský balzám.
21. Podložní sklíčka uchovávejte v suchém boxu určeném k tomuto účelu.
