# Supplementary material for: Processing and mounting phlebotomine sand flies: a consensus guideline
Source: Parasite. 2026 Apr 3;33:18. doi: 10.1051/parasite/2026009 (PMC13047900; doi:10.1051/parasite/2026009)
Supplement: Supplementary file 7 — Farsi translation / ترجمه فارسی [file parasite-33-18-s7.pdf]

Parasite **33**, 18 (2026)
© F.J. Randrianambinintsoa et al., published by EDP Sciences, 2026
https://doi.org/10.1051/parasite/2026009

PARASITE
Available online at:
www.parasite-journal.org

REVIEW ARTICLE OPEN ACCESS

پردازش و مونتاژ پشه خاکیهای فلبوتومینه: یک راهنمای توافقی

Fano José Randrianambinintsoa[1], Laure Augendre[1], Jorian Prudhomme[1], Jean-Philippe Martinet[1], Mathieu Loyer[1], Nalia Mekarnia[1], Hocine Kerkoub[1], Farzana Khan Perveen[1], Antoine Huguenin[1,2], Emilie Kariya[1,2], Mohammad Akhoundi[3], Andrey José de Andrade[4], Eduardo Berriatua[5], Gioia Bongiorno[6], Sébastien Boyer[7,8], Vasiliki Christodoulou[9], Magda Clara Vieira Da Costa-Ribeiro[10], Lucas Alexandre Farias de Souza[10], Huicong Ding[11], Blaise Dondji[12], Vít Dvořák[13], Ozge Erisoz Kasap[14], Eunice Aparecida Bianchi Galati[15], Montserrat Gállego[16], Cristina Ballart[16], Stavroula Gouzelou[17], Nabil Haddad[18], Rezki Sabrina Masse[19], Asrat Hailu Mekuria[20], Vladimir Ivovic[21], Szymon Kaczmarek[22], Mohd Khadri Shahar[19], Oscar D. Kirstein[23], Edwin Kniha[24], Iva Kolářová[13], Lincoln Timinao[25], Cristian Lucanas[26], Ognyan Mikov[27], Kimsear Nov[7], Yusuf Özbel[28], Bernard Pesson[29], Laura Cristina Posada Lopez[30], Didot Budi Prasetyo[1,7], Nil Rahola[31], Eduardo A. Rebollar-Tellez[32], Bruno Leite Rodrigues[15], Lalita Roy[33], Prasanta Saini[34], Chizu Sanjoba[35], Paloma Helena Fernandes Shimabukuro[36], Padet Siriyasatien[37], Agnieszka Soszyńska[22], Tatiana Suleşco[38], Massamba Sylla[39], Majhalia Torno[40], Petr Volf[13], Khamsing Vongphayloth[41], Vu Sinh Nam[42], April Wardhana[43], Eric Yessinou[44], Sonia Zapata[45], Jean-Charles Gantier[1], and Jérôme Depaquit[1,2,*]

[1] Faculté de Pharmacie, Université de Reims Champagne Ardenne, UR ESCAPE-USC ANSES PETARD, 51 rue Cognacq-Jay, 51096 Reims Cedex, France
[2] Pôle de Biologie territoriale, Laboratoire de Parasitologie-Mycologie, Centre Hospitalo-Universitaire, 51092 Reims, France
[3] Parasitology-Mycology Department, Avicenne Hospital, AP-HP, Bobigny, Sorbonne Paris Nord University, France; Unité des Virus Émergents (UVE: Aix-Marseille Univ, Università di Corsica, IRD 190, Inserm 1207, IRBA), 13005 Marseille, France
[4] Parasitology Collection of Basic Pathology, Department of Basic Pathology, Federal University of Paraná, Curitiba 19031, Brazil
[5] Department of Animal Health, University of Murcia, Campus de Espinardo, 30100 Espinardo, Murcia, Spain
[6] Department of Infectious Diseases, Vector-borne Diseases Unit, Istituto Superiore di Sanità, 00166 Rome, Italy
[7] Medical and Veterinary Entomology Unit, Institut Pasteur du Cambodge, Phnom Penh 12201, Cambodia
[8] Ecology & Emergence of Arthropod-borne Pathogens Unit, Department of Global Health, Institut Pasteur, CNRS UMR2000, 75015 Paris, France
[9] Section Veterinary Services (1417), Laboratory for Animal Health Virology, Aglantzia, Nicosia 2109, Cyprus
[10] Insects Vectors and Parasites Laboratory, Department of Basic Pathology and Postgraduate program in Microbiology, Parasitology and Pathology, Federal University of Paraná, 81530-900 Curitiba, Brazil
[11] Department of Biological Sciences, National University of Singapore, 117558, Singapore
[12] Laboratory of the Leishmaniasis Research Project, Mokolo District Hospital, Mokolo, Cameroon; Laboratory of Cellular Immunology and Parasitology, Department of Biological Sciences, Central Washington University, 98926 Ellensburg, WA, USA
[13] Department of Parasitology, Faculty of Science, Charles University, 12800 Prague, Czechia
[14] VERG Laboratories, Department of Biology, Faculty of Science, Hacettepe University, Beytepe, Ankara 06800, Türkiye
[15] Faculdade de Saúde Pública da Universidade de São Paulo (FSP/USP), Pós-graduação em Saúde Pública, 01246-904 São Paulo, Brazil
[16] Secció de Parasitologia, Departament de Biologia, Sanitat i Medi Ambient, Facultat de Farmàcia i Ciències de l'Alimentació, Universitat de Barcelona, & Institut de Salut Global de Barcelona (ISGlobal), Centro de Investigación Biomédica en Red, Enfermedades Infecciosas (CIBERINFEC), 08028 Barcelona, Spain
[17] Laboratory of Infectious Diseases and Public Health, School of Medicine, University of Cyprus, Nicosia, Cyprus & Department of Pediatrics, Archbishop Makarios III Hospital, Nicosia 2115, Cyprus
[18] Faculty of Health Sciences, American University of Beirut, 1107 2020 Beirut, Lebanon
[19] Medical Entomology Unit, Infectious Disease Research Centre, Institute for Medical Research (IMR), National Institutes of Health (NIH), Ministry of Health Malaysia, 40170 Shah Alam, Selangor, Malaysia
[20] School of Medicine, Addis Ababa University, 28017 - 1000 Addis Ababa, Ethiopia
[21] Faculty of Mathematics, Natural Sciences and Information Technologies, University of Primorska, 6000 Koper, Slovenia

Edited by Jean-Lou Justine
*Corresponding author: jerome.depaquit@univ-reims.fr

This is an Open Access article distributed under the terms of the Creative Commons Attribution License (https://creativecommons.org/licenses/by/4.0), which permits unrestricted use, distribution, and reproduction in any medium, provided the original work is properly cited.

[22] University of Lodz, Faculty of Biology and Environmental Protection, Department of Invertebrate Zoology and Hydrobiology, Banacha 12/16, 90-237 Łódź, Poland
[23] Laboratory of Entomology, Ministry of Health, 9134302 Jerusalem, Israel
[24] Center for Pathophysiology, Infectiology and Immunology, Institute of Specific Prophylaxis and Tropical Medicine, Medical University Vienna, Kinderspitalgasse 15, 1090 Vienna, Austria
[25] Papua New Guinea Institute of Medical Research (PNGIMR) Institute, PO Box 60, Headquarter, Homate Street, 441 Goroka, Eastern Highlands Province, Papua New Guinea
[26] Museum of Natural History, University of the Philippines Los Baños, 4031 Laguna, Philippines
[27] National Centre of Infectious and Parasitic Diseases, 1504 Sofia, Bulgaria
[28] Ege University, Faculty of Medicine, Department of Parasitology, 35040 Bornova/Izmir, Türkiye
[29] Retired, Faculté de Pharmacie, Université de Strasbourg, Strasbourg, 67400 Illkirch-Graffenstaden, France
[30] Program for the Study and Control of Tropical Diseases (PECET), Faculty of Medicine, University of Antioquia, 050010 Medellin, Colombia
[31] MIVEGEC, Univ. Montpellier, CNRS, IRD, 34394 Montpellier, France & Medical Entomology Unit, Institut Pasteur de Madagascar, 101 Antananarivo, Madagascar
[32] Laboratorio de Entomología Médica, Departamento de Zoología de Invertebrados, Facultad de Ciencias Biológicas, Universidad Autónoma de Nuevo León, San Nicolás de los Garza, 66455, NL, México
[33] Tropical and Infectious Disease Centre, BP Koirala Institute of Health Sciences, Dharan 56700, Nepal
[34] ICMR-Vector Control Research Centre, Puducherry 605006, India
[35] Graduate School of Agricultural and Life Sciences, The University of Tokyo, Tokyo 113-8657, Japan
[36] Grupo de estudos em Leishmanioses/Coleção de Flebotomíneos (COLFLEB/Fiocruz-MG), Instituto René Rachou, Fundação Oswaldo Cruz, Belo Horizonte, Minas Gerais, 30190009, Brazil
[37] Center of Excellence in Vector Biology and Vector-Borne Disease, Department of Parasitology, Faculty of Medicine, Chulalongkorn University, Bangkok 10330, Thailand
[38] Department of Arbovirology, Bernhard Nocht Institute for Tropical Medicine, Bernhard Nocht Str. 74, 20359 Hamburg, Germany [39] Laboratory Vectors & Parasites, Department of Livestock Sciences and Techniques, Sine Saloum University El Hadji Ibrahima Niasse (SSUEIN) Kaffrine Campus, C.P. 24600, Senegal.
[40] Environmental Health Institute, National Environment Agency, Singapore 138667, Singapore & Department of Biological Sciences, National University of Singapore, 117558 Singapore
[41] Institut Pasteur du Laos, Laboratory of Vector-Borne Diseases, Samsenhai Road, Ban Kao-Gnot, Sisattanak District, 3560 Vientiane, Lao PDR
[42] National Institute of Hygiene and Epidemiology, 1 Yec-Xanh Street, Hai Ba Trung District, 100000 Hanoi, Vietnam
[43] Indonesian Research Center for Veterinary Science, Indonesian Agency for Agricultural Research and Development, Ministry of Agriculture Republic Indonesia, Bogor 16114, Indonesia & Department of Parasitology, Faculty of Veterinary Medicine, Airlangga University, Surabaya 60115, Indonesia
[44] Laboratory of Research in Applied Biology, Polytechnic School of Abomey-Calavi, University of Abomey-Calavi, 01 P.O. Box 2009, 00000 Cotonou, Benin
[45] Instituto de Microbiología, Colegio de Ciencias Biológicas y Ambientales (COCIBA), Universidad San Francisco de Quito (USFQ), 170901 Quito, Ecuador

Received 1 December 2025, Accepted 29 January 2026, Published online 3 April 2026

چکیده – این مقاله یک راهنمای جامع برای پردازش و مونتاژ نمونه‌های پشه خاکی فلبوتومینه ارائه می‌دهد که برای شناسایی گونه‌ها و تشخیص و جداسازی پاتوژن‌ها بسیار مهم است. در این مقاله مجموعه‌ای از تکنیک‌ها که هم برای شرایط فیلد و هم آزمایشگاهی مناسب هستند مورد بحث قرار گرفته است. این راهنما شامل دستورالعمل‌های دقیق برای جمع‌آوری پشه‌های خاکی، جابجایی، نگهداری، بیهوش‌سازی و کشتن آن‌ها (با توصیه به انجماد خشک یا CO_2 به جای مواد شیمیایی) و همچنین روش‌های نگهداری مانند نگهداری در سرما و اتانول می‌باشد. کیفیت آماده‌سازی برخی ساختارهای آناتومیکی (اندام‌های تناسلی، سر و بال‌ها) برای مشاهده صحیح زیر میکروسکوپ بسیار مهم است که در این مطالعه توضیح داده شده است. این مقاله همچنین پردازش نمونه شامل شفاف‌سازی با استفاده از موادی مانند هیدروکسید پتاسیم و سپس محلول Marc-André را توضیح می‌دهد. در فرآیند مونتاژ، محیط‌های مختلف مونتاژ با تأکید بر ویژگی‌های نوری و قابلیت نگهداری مقایسه شده‌اند. مایع هویر(Hoyer) که به نام گام کلرال نیز شناخته می‌شود برای مشاهده سریع، به ویژه اسپرماتکاها، به دلیل شفافیت بالا توصیه می‌شود، اگرچه برای نگهداری طولانی‌مدت مناسب نیست. محیط‌های دیگر شامل پلی‌وینیل الکل، Euparal و کانادا بالزام هستند که دو مورد آخر امکان نگهداری طولانی‌مدت را فراهم می‌کنند. همچنین روش‌های نوین زیست‌شناسی مولکولی مانند تعیین توالی DNA و MALDI-ToF که نیاز به دقت خاصی در پردازش نمونه دارند، بررسی شده‌اند. علاوه بر این، ویدئوهای آموزشی کوتاه از تکنیک‌های مونتاژ و ترجمه به زبان‌های مختلف ارائه شده است تا این راهنما برای جامعه علمی جهانی قابل استفاده باشد.

کلمات کلیدی : مونتاژ روی اسلایدهای شیشه ای، پشه خاکی های فلبوتومینه، محیط هویر، محلول مارک–آندره، گام اکلرال، پلی‌وینیل الکل، ®Euparal، کانادا بالزام ، جداسازی لیشمانیا، شرایط فیلد، تشریح، آنالیز ملکولی، MALDI-Tof، نمونه های تایپ (Type-specimens).

Abstract – Processing and mounting phlebotomine sand flies: a consensus guideline. This article provides a comprehensive guide for the processing and mounting of phlebotomine sand fly specimens, which is crucial for species identification and pathogen detection and isolation. It discusses a range of techniques suitable for both field and laboratory settings. The guide includes detailed instructions on sand fly collection, handling, covering, and euthanasia (recommending dry freezing or CO_2 over chemicals) as well as conservation strategies, such as cold storage and preservation in ethanol. The quality of preparation of certain anatomical structures (genital organs, head and wings) is essential for their proper microscopic observation and is described in this work. The article also presents detailed sample processing, including the clearing process with agents such as potassium hydroxide then Marc-André solution. The mounting process compares different media, emphasizing their optical properties and preservation potential. Hoyer fluid (also known as chloral gum) is recommended for quick observation, particularly for spermathecae, due to its clarity, although it is not suitable for long-term storage. Other media discussed include polyvinyl alcohol, Euparal® (for limited water tolerance), and Canada balsam (a hydrocarbon-soluble medium), with the latter two offering long-term preservation capabilities. Innovative molecular biology approaches such as DNA sequencing and MALDI-ToF, which require particular attention to sample processing, are also addressed. Furthermore, short video clips illustrating various mounting techniques as well as translations in many different languages are provided, allowing the guideline to reach the diverse needs and expectations of the global scientific community.

Key words: Mounting, Phlebotomine sand fly, Hoyer fluid, Marc-André solution, Chloral gum, Polyvinyl alcohol, Euparal®, Canada balsam, *Leishmania* isolation, Field conditions, Culture, Dissection, Molecular biology, MALDI-ToF, Type-specimens.

مقدمه

پشه‌های خاکی فلبوتومینه حشرات دوبال کوچک متعلق به خانواده Psychodidae و زیرخانواده Phlebotominae هستند که حداقل 1063 گونه شناخته‌شده دارند [21] . این حشرات ناقل مهم پاتوژن‌هایی مانند لیشمانیا، آربوویروس‌ها و بارتونلا هستند که به ترتیب باعث بیماری‌های لیشمانیوز، عفونت‌های آربوویروسی و بارتونلوز می‌شوند. شناسایی این حشرات عمدتاً بر اساس بررسی دقیق میکروسکوپی انجام می‌شود که نیازمند جمع‌آوری صحیح، نگهداری مناسب و مونتاژ دقیق روی لام است و هر یک از این مراحل تکنیک‌ها، مزایا و محدودیت‌های خاص خود را دارند.

شناسایی پشه‌های خاکی بالغ بر اساس مشاهده ساختارهای خارجی (مانند آنتن‌ها، پالپ‌ها و دستگاه تناسلی نر) و ساختارهای داخلی (مانند فارنکس، سیباریوم و اسپرماتک) انجام می‌شود. تشریح و جدا کردن این ساختارها مشاهده آن‌ها را آسان‌تر کرده و در نتیجه شناسایی دقیق‌تر گونه را ممکن می‌سازد. بنابراین، برخلاف پشه‌ها یا ساس‌های بوسه‌زن، پشه‌های خاکی باید قبل از شناسایی بین لام و لامل مونتاژ شوند.

تا دهه ۱۹۸۰، مشاهده میکروسکوپی تنها روش موجود برای شناسایی پشه خاکیها بود و همچنان پرکاربردترین روش امروزه باقی مانده است. انتخاب فرآیند و آماده‌سازی بنابراین نسبتاً ساده بود و عمدتاً بر اساس یک دوگانگی صورت می‌گرفت: از یک سو، مونتاژ قطعی که امکان نگهداری طولانی‌مدت نمونه را فراهم می‌کرد و از سوی دیگر، مونتاژ سریع برای شناسایی و تعیین گونه در محیطی که نگهداری طولانی‌مدت را تضمین نمی‌کرد. مونتاژ نهایی، برای مثال در رزینی مانند صمغ کانادا(Canada balsam) ، وقت‌گیر است و نیازمند دهیدراسیون کامل نمونه‌هاست. علاوه بر این، ضریب شکست این محیط همیشه برای مشاهده آسان اسپرماتکها (spermathecae) ایده‌آل نیست. در مقابل، مونتاژ در محیط مایع مثلاً محلول هویر سریع‌تر است و امکان مشاهده بهتر اسپرماتکها را فراهم می‌کند، اما اجازه نگهداری طولانی‌مدت لامها را نمی‌دهد زیرا تمایل دارد آب را از هوا جذب کند. یک گزینه، مهر و موم کردن لام با لاک ناخن پس از خشک شدن کامل آن است. این انتخاب با مصالحه همچنان امروز نیز وجود دارد و بر انتخاب روش مونتاژ، بسته به هدف مورد نظر از آماده‌سازی، تأثیر می‌گذارد.

از دهه ۱۹۸۰، مطالعات شناسایی پشه خاکیها ترکیبی از رویکردهای مورفولوژیکی و بیوشیمیایی بوده است. اولین آن‌ها تحلیل‌های هیدروکربن پوستی (cuticular hydrocarbon) بود که به سرعت توسط تکنیک‌های آنالیز مولکولی جایگزین شد (RAPD، RFLP، NGS) . امروزه، رویکردهای مولکولی با روش‌های پروتئومیک مانند MALDI-ToF تکمیل می‌شوند. علاوه بر این، شناسایی مولکولی گونه‌ها می‌تواند با شناسایی پاتوژن‌ها از طریق PCR (لیشمانیا، تریپانوزوما، بارتونلا و فلبوویروس) ترکیب شود، زیرا همه این‌ها می‌توانند با روشهای PCR شناسایی شوند و نیازمند تطبیق فرآیند نمونه‌گیری و ذخیره‌سازی با اهداف مشخص شده هستند [32، 3]. علاوه بر ویژگی‌های مورفولوژیکی که به طور سنتی برای تفکیک گونه‌ها استفاده می‌شوند، دیگر رویکردهای مورفولوژیکی نیز قابل اعمال هستند (مثلاً ژئومورفومتری بال‌ها).

عمدتاً بر اساس تجربیات شخصی نویسندگان و داده‌های موجود در مقالات، هدف این مطالعه ارائه راهنمایی‌های استاندارد برای مونتاژ و پردازش پشه خاکیهای بالغ فلبوتومینه برای بهینه‌سازی تحلیل‌های مورفولوژیکی و مولکولی است. نیاز به انجام برخی تحلیل‌ها (برای مثال آنالیزهای مولکولی یا MALDI-ToF)مستلزم نگهداری بخشی از پشه خاکیها است که برای شناسایی مورفولوژیکی ضروری نیست، که بر ضرورت انتخاب دقیق پروتکل تأکید می‌کند.

در این مقاله، ما بر روش‌های بی‌حسی و کشتن پشه خاکیهای زنده، ذخیره‌سازی آن‌ها و فرآیند مونتاژ برای شناسایی سریع یا حفظ طولانی‌مدت جهت مطالعات بعدی تمرکز می‌کنیم.

پیش‌گفتار: ملاحظات ایمنی و مقرراتی باید به برگه‌های اطلاعات ایمنی (SDS) مربوطه ارجاع دهند

تمام مواد شیمیایی ارائه‌شده در این راهنما باید تحت شرایط ایمنی سختگیرانه نگهداری و استفاده شوند. کمیته‌های بهداشت و ایمنی مراکز تحقیقاتی آماده‌اند تا اطلاعاتی نه تنها درباره خطرات این مواد، بلکه درباره روش‌های کار با آن‌ها و دفع پسماندهایشان در اختیار شما قرار دهند. با این حال، پیروی از دستورالعمل‌های ایمنی مربوط به استفاده و دفع آن‌ها الزامی است. شایان ذکر است که مسئولیت همه کاربران است تا از رعایت شیوه‌های خوب و ایمن آزمایشگاهی و قوانین و مقررات قابل اجرا در کشور یا مؤسسه تحقیقاتی خود اطمینان حاصل کنند. علاوه بر این، برخی از مواد شیمیایی یا اجزای آن‌ها (مانند هیدرات کلرال) در برخی کشورها تحت نظارت هستند. فهرستی از اختصارات مورد استفاده در این مقاله در جدول 1 ارائه شده است..

جدول 1 . فهرست کلمات اختصاری

BME	محیط پایهٔ ایگل
CDC	مرکز کنترل و پیشگیری از بیماری‌ها
CMCP	کامفور–مونوکلروفنول
CMR	مادهٔ سرطان‌زا، جهش‌زا و سمی برای تولیدمثل
COI	سیتوکروم c اُکسیداز
CytB	سیتوکرومb
DNA	اسید دئوکسی‌ریبونوکلئیک
ELISA	آزمون ایمنی‌سنجی متصل به آنزیم
EtOH	اتانول
M199	محیط 199
MALDI-ToF	طیف‌سنجی جرمی زمان‌پرواز با یونش/واجذب لیزریِ
MS	کمک‌گرفته از ماتریکس
MEM	محیط کشت ضروری حداقل
NGS	توالی‌یابی نسل جدید
NNN	محیط نوفي–ماکنیل–نیکول
PCR	واکنش زنجیره‌ای پلیمراز
Lao PDR	جمهوری دموکراتیک خلق لائوس
PNOC	ژن پیش‌پرونوسیسپتین(prepronociceptin)
qPCR	واکنش زنجیره‌ای پلیمراز کمی (واکنش زنجیره‌ای پلیمراز زمان‌واقعی)
RAPD	دی‌ان‌ای پلی‌مورفیک تصادفی تکثیرشده
RFLP	پلی‌مورفیسم طول قطعات برش‌شده با آنزیم محدودکننده
RI	ضریب شکست نور
RNA	اسید ریبونوکلئیک
RNases	ریبونوکلئازها
RNASS	محلول پایدارکنندهٔRNA
RT-PCR	واکنش زنجیره‌ای پلیمراز با رونویسی معکوس
TFA	اسید تری‌فلوورواستیک

1 جمع‌آوری پشه خاکی ها

پشه‌های خاکی بالغ را می‌توان به صورت زنده یا مرده با استفاده از روش‌های مختلف جمع‌آوری کرد، مانند :تله‌های نورانی، تله چسبان یا آسپیراتور با استفاده از تله های شانون یا مستقیماً از مکان‌های استراحت آن‌ها در محیط مانند پناهگاه‌های حیوانات .این روش‌ها شامل قرار دادن تله‌ها در زیستگاه‌های مناسب، جذب پشه‌ها با نور یا جاذبه‌های دیگر مانند جاذبهای شیمیایی دی اکسید کربن و جمع‌آوری آن‌ها برای تحلیل‌های بعدی است، همان‌طور که در چندین مقاله توصیف شده است [2, 3, 32, 36, 49]

جمع‌آوری پشه‌های زنده تمام کاربردهای بعدی را امکان‌پذیر می‌کند، در حالی که جمع‌آوری نمونه های مرده مانع از جداسازی سویه‌های لایشمانیا یا ویروس می‌شود .برخی روش‌های جمع‌آوری، مانند تله های چسبان، معمولاً باعث از دست رفتن اندام‌های پشه خاکی (مثل شاخک‌ها، پالپ‌ها، بال‌ها یا پاها) می‌شوند. علاوه بر این، روکش روغن کرچک روی تله های چسبان به پشه‌ها می‌چسبد و باید در ابتدای پردازش حذف شود، که معمولاً با حمام ۱۵ دقیقه‌ای در مخلوطی از اتانول و دی‌اتیل اتر به نسبت مساوی انجام می‌گیرد.

.

2 کشتن (اتانازی) نمونه‌ها

پس از جمع‌آوری، پشه‌های خاکی زنده باید کشته شوند. در برخی روش‌های جمع‌آوری (مانند کاغذهای چسبنده، تله‌های نوری CDC مجهز به ظرف حاوی دترجنت یا اتانول) پشه‌های خاکی در زمان جمع‌آوری مرده هستند. آنالیز های مولکولی را می‌توان بر روی نمونه‌هایی که مستقیماً در اتانول جمع‌آوری شده‌اند و همچنین بر روی سایر نمونه‌ها در صورتی که در سریع‌ترین زمان ممکن در اتانول نگهداری شوند، انجام داد. با این حال، هیچ‌یک از این روش‌های کشتن، امکان آماده‌سازی حشرات برای آنالیز MALDI-ToF را فراهم نمی‌کنند. علاوه بر این، برخی روش‌های کشتن ممکن است باعث از بین رفتن برخی ویژگی‌های مورفولوژیک شوند. بنابراین استفاده از یک عامل کشتن استاندارد و مناسب برای اطمینان از شناسایی صحیح یا نگهداری طولانی‌مدت نمونه‌ها به‌عنوان نمونه‌های مرجع (voucher specimens)ضروری است (یعنی نمونه‌هایی که برای ارجاع یا مقایسه‌های آینده نگهداری و ذخیره می‌شوند).

مواد شیمیایی مانند اتیل استات، اتیل اتر، تتراکلرواتان و کلروفرم را می‌توان به پنبه آغشته کرد و در ظرف حاوی پشه‌های خاکی قرار داد تا باعث مرگ آن‌ها شود. این مواد به دلیل سمیت باید با احتیاط و مطابق توصیه‌های سازنده استفاده شوند. با این حال، استفاده از کلروفرم برای کشتن پشه‌های خاکی توصیه نمی‌شود، زیرا طبق تجربه، با مطالعات زیست‌شناسی مولکولی سازگاری کمی دارد. با توجه به خطرناک بودن این مواد و مناسب نبودن احتمالی آن‌ها برای آنالیز های مولکولی، استفاده از این مواد شیمیایی به‌طور کلی توصیه نمی‌شود.

متداول‌ترین روش که مورفولوژی، DNAیا پروتئین‌ها را حفظ می‌کند، انجماد خشک نمونه‌ها است. نمونه‌ها باید به مدت کافی منجمد شوند تا کاملاً بی‌حرکت (بیهوش) شوند، اما نه آن‌قدر طولانی که خشک شوند، یا بقای لیشمانیا در صورتی که هدف جداسازی آن‌ها به‌صورت in vitro از دستگاه گوارش پشه خاکی باشد، از بین برود .

بنابراین، انجماد به مدت ۱۵ تا ۲۰ دقیقه در دمای ۲۰- درجه سانتی‌گراد توصیه می‌شود و باید مرتباً بررسی شوند تا فقط بی‌حرکت شده باشند و انگل‌های لیشمانیا کشته نشده باشند.

اگر فریزر در دسترس نباشد، می‌توان حشرات را با گاز CO_2 کشت. در شرایط فیلد که امکان استفاده از سیلندر CO_2 وجود ندارد، می‌توان از کپسول‌های کوچک تجاری CO_2 مورد استفاده در سیفون‌های نوشابه (Soda siphons)استفاده کرد، اگرچه ممکن است حمل آن‌ها با هواپیما محدودیت داشته باشد.

به‌عنوان آخرین راه‌حل، حشرات را می‌توان با دود تنباکو کشت. در این روش، پشه‌های خاکی زنده در تله CDC به دام افتاده، با آسپیراتور جمع‌آوری شده، در لوله شیشه‌ای نگهداری می‌شوند و در معرض دود تنباکو قرار می‌گیرند که در عرض چند ثانیه آن‌ها را می‌کشد. این روش در تمامی شرایط فیلد، حتی در شرایط ایزوله و دشوار نیز قابل استفاده است. با این حال، به دلیل آغشته شدن شیشه به دود، بدون تمیز کردن کامل نمی‌توان از آن برای جمع‌آوری و نگهداری بعدی پشه‌های خاکی زنده استفاده کرد. با این وجود، همان آسپیراتور تمیزنشده را همچنان می‌توان برای کشتن پشه‌های خاکی سایر تله‌ها به‌منظور فیکس کردن نمونه‌ها استفاده کرد. همچنین لازم است بررسی شود که تمام نمونه‌ها از آسپیراتور خارج شده باشند. این روش‌ها با جداسازی لیشمانیا از طریق تشریح روده سازگار هستند.

3 نگهداری نمونه‌ها قبل از پردازش

چهار روش اصلی برای تثبیت (فیکس کردن) نمونه‌ها قبل از پردازش وجود دارد:

3.1 انجماد

این روش بهتر است در دمای ۲۰ -درجه سانتی‌گراد یا ترجیحاً ۸۰ -درجه سانتی‌گراد انجام شود. امروزه این روش‌های نگهداری بیشتر از نگهداری در نیتروژن مایع استفاده می‌شوند. در تمام موارد، انجماد باید در سریع‌ترین زمان ممکن پس از بی‌حرکت کردن نمونه‌ها انجام شود .نگهداری در فریزر این مزیت را دارد که خود حشرات و همچنین RNA، DNAو پروتئین‌ها را با حفظ کامل یکپارچگی در طول دوره نگهداری حفظ می‌کند. در مقابل، نیتروژن مایع می‌تواند به‌شدت به بال‌ها، پاها، پالپ‌ها و آنتن‌ها آسیب برساند و اغلب باعث قطع شدن آن‌ها و گاهی حذف ویژگی‌های مهم مورفولوژیک می‌شود.

نگهداری خشک در فریزر برای نمونه‌ها آسیب کمتری دارد، اما برای حفظ اندام‌های ظریف ایده‌آل نیست. نکته مهم این است که هنگام ذوب شدن، بال‌ها، آنتن‌ها، پالپ‌ها یا پاها ممکن است به دیواره ویال بچسبند و در اثر تراکم رطوبت (condensation) جدا شوند.با این حال، نگهداری با انجماد همیشه در مطالعات فیلد امکان‌پذیر نیست، زیرا نیاز به فریزر یا مخزن نیتروژن مایع دارد.

نگهداری در فریزر کاملاً با تشخیص پاتوژن‌ها با روش‌های مولکولی بدون کاهش حساسیت سازگار است، اگرچه تشخیص و جداسازی ویروس‌های RNAدر صورت نیاز به نگهداری طولانی‌مدت، به انجماد در ۸۰ -درجه سانتی‌گراد یا نیتروژن مایع نیاز دارد. با این حال، انجماد نمونه‌ها معمولاً اجازه جداسازی لیشمانیا از طریق تشریح روده را نمی‌دهد، مگر اینکه پشه‌های خاکی ابتدا در فاز بخار و سپس در نیتروژن مایع قرار داده شوند (مثلاً در ویال‌هایی داخل جوراب)، که شرایط کرایوپرزرویشن لیشمانیا را شبیه‌سازی می‌کند.

3.2 نگهداری در الکل (اتانول یا ایزوپروپیل الکل)

این روش احتمالاً رایج‌ترین روش نگهداری پشه‌های خاکی است. اجرای آن در شرایط صحرایی، حتی بدون دسترسی به آزمایشگاه، آسان است.

نگهداری در الکل به‌ویژه برای مطالعات مورفولوژیک مناسب است، زیرا اندام‌های ظریف (بال‌ها، پاها، آنتن‌ها و پالپ‌ها) در صورت نبود حباب هوا در لوله نگهداری سالم باقی می‌مانند. بنابراین توصیه می‌شود لوله با یک گلوله کوچک پنبه بسته شود تا حباب‌های هوا حذف شوند و برچسب روی قسمت حاوی پنبه قرار داده شود. (تصویر 1). غلظت مناسب الکل هنوز مورد بحث است، اما غلظت کمتر از ٪۷۰ توصیه نمی‌شود. غلظت‌های بالاتر DNA را بهتر و برای مدت طولانی‌تر حفظ می‌کنند، اما نمونه‌ها را برای مطالعات مورفولوژیک شکننده و ترد می‌کنند[45, 66]. استفاده از اتانول ٪۹۶ باعث پایداری غلظت در طول زمان، به‌ویژه در مناطق

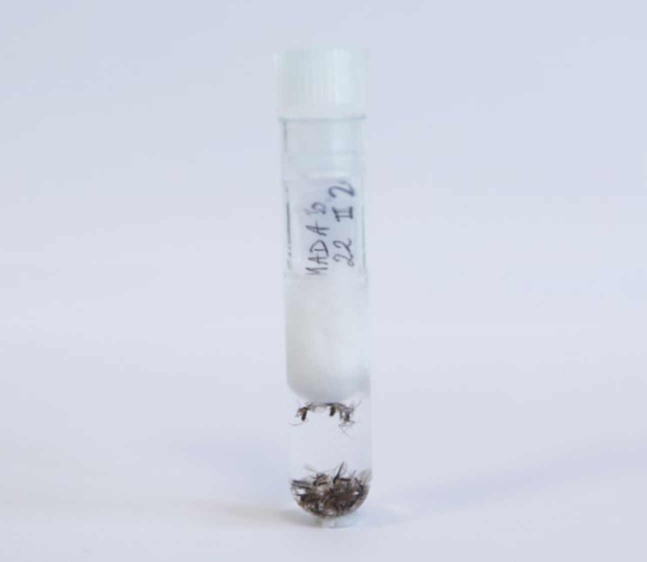

تصویر 1. نمونه های پشه خاکی نگهداری شده در اتانول.

مرطوب مانند کشورهای گرمسیری می‌شود، اگرچه اتانول ٪۹۵ معمولاً راحت‌تر در دسترس است.

صرف‌نظر از غلظت، DNA معمولاً در اتانول به‌خوبی حفظ می‌شود (اگرچه کمتر از روش انجماد، به‌ویژه برای روش‌های مولکولی مانند ان جی اس). پروتئین‌ها بسیار کمتر پایدار هستند، به‌ویژه برای مطالعات پروتئومیکس مانند MALDI-ToF. پشه‌های خاکی نگهداری‌شده در الکل پس از چند ماه هنوز از نظر مورفولوژیک قابل شناسایی هستند، اما نمی‌توان طیف‌های پروتئینی مرجع از آن‌ها تهیه کرد.

نگهداری در الکل یا به‌صورت خشک می‌تواند با انجماد در ۲۰- درجه سانتی‌گراد بهبود یابد. انجماد در این دما عمدتاً باعث حفظ بهتر مولکولی (مانند اسیدهای نوکلئیک) وکاهش سرعت تخریب می‌شود و همچنین به‌طور ثانویه به حفظ مورفولوژی کمک می‌کند، اگرچه تأثیر آن بر مورفولوژی کمتر از تأثیر آن بر یکپارچگی مولکولی است.

نگهداری در اتانول همچنین برای تشخیص DNA ونیز RNA ویروس‌ها در صورتی که از اتانول با غلظت حداقل ٪۷۰ و برای مدت کوتاه (کمتر از چند ماه) استفاده شود، مناسب است. ایزوپروپیل الکل نیز در برخی کشورها به‌راحتی در دسترس است و DNA را حفظ می‌کند، اما نمونه‌ها را سفت می‌کند. همچنین برخلاف اتانول قابل اشتعال نیست و بنابراین حمل آن آسان‌تر است.

در صورت لزوم، پشه‌های خاکی نگهداری‌شده در نیتروژن مایع یا فریزر خشک را می‌توان به الکل منتقل کرد، اگرچه این کار در واقع معایب هر دو روش را با هم ترکیب می‌کند.

3.3 نگهداری در محلول پایدارکننده RNA (RNASS)

این معرف مایع، غیرسمی و به‌طور گسترده برای پایدارسازی و حفاظت RNAدر بافت‌های تازه و نمونه‌های سلولی استفاده می‌شود. این ماده با نفوذ سریع به نمونه و غیرفعال کردن RNaseها (آنزیم‌های تخریب‌کننده RNA) از تخریبRNA بدون نیاز به انجماد فوری جلوگیری می‌کند.

نگهداری در RNASS معمولاً برای حفظ مورفولوژی کلی بافت و سلول برای بررسی‌های هیستولوژیک بعدی مؤثر است. اگرچه RNASS برای تثبیت طراحی نشده و بیشتر برای پایدارسازی RNA است، اما در نگهداری کوتاه‌مدت تا میان‌مدت، یکپارچگی ساختاری نمونه‌ها را حفظ می‌کند.

RNASSامکان نگهداری نمونه‌ها را در شرایط زیر فراهم می‌کند:

- دمای اتاق :تا ۷ روز
- ۴ درجه سانتی‌گراد :چند هفته
- ۲۰ -یا ۸۰- درجه سانتی‌گراد :نگهداری طولانی‌مدت

این روش به‌ویژه در مطالعات فیلد یا محیط‌های بالینی با محدودیت زنجیره سرد بسیار ارزشمند است. استخراج RNA معمولاً نیاز به خارج کردن نمونه از محلول و پردازش طبق پروتکل‌های استاندارد دارد.

3.4 نگهداری خشک در دمای اتاق

این روش قدیمی است و وقتی روی نمونه کامل انجام شود، اندام‌های ظریف مانند بال‌ها، پاها، آنتن‌ها و پالپ‌ها به‌خوبی حفظ نمی‌شوند .با این حال، مطالعات پروتئومیکس با MALDI-ToF در صورتی که خشک‌سازی با دسیکانت سیلیکاژل انجام شود، همچنان امکان‌پذیر است.

در مقابل، آنالیزهای مولکولی DNA روی این نمونه‌ها دشوار است، زیرا DNA معمولاً قطعه‌قطعه شده و مقدار آن کم است، بنابراین تحلیل‌ها نسبت به نمونه‌های تازه یا منجمد دشوارتر هستند، به‌ویژه برای ژنوم هسته‌ای. با این حال، تکنیک‌های جدید مانند موزئومیکس (Museomics) می‌توانند روی این نوع نمونه‌ها استفاده شوند.

بنابراین این روش توصیه نمی‌شود مگر اینکه هیچ روش دیگری در دسترس نباشد. این روش را می‌توان با نگهداری سرد ترکیب کرد، به این صورت که لوله‌ها در فریزر ۲۰ - درجه سانتی‌گراد قرار داده شوند.

برای استفاده از نمونه‌های خشک‌شده یا بخش‌های بدن برای شناسایی، آب‌دار کردن مجدد (Rehydration) ضروری است. برای این کار استفاده از محلول Triton X-100 توصیه می‌شود. مدت زمان آب‌دار کردن از چند ساعت تا چند روز متغیر است و باید مرتباً بررسی شود. پس از آب‌دار شدن کامل، نمونه‌ها باید در سه حمام آب متوالی شستشو داده شوند.

3.5 نگهداری روی کاغذ صافی

مزیت اصلی کاغذهای صافی پایداری طولانی‌مدت DNA ژنومی در سلول‌های بدن کامل خشک‌نشده یا سلول‌های خونی ذخیره‌شده در دمای اتاق است. این کاغذها به‌صورت کارت‌های کوچک هستند و می‌توان صدها نمونه را در دمای اتاق در فضایی به اندازه یک جعبه کوچک نگهداری کرد. ماتریس کاغذ صافی با موادی آغشته شده که عوامل عفونی را دناتوره می‌کنند، بنابراین نمونه‌ها دیگر خطر زیستی (Biohazard) محسوب نمی‌شوند. این موضوع امکان نگهداری و انتقال نمونه‌ها بدون نیاز به اقدامات ایمنی زیستی خاص را فراهم می‌کند[68]

.

4 تشریح نمونه ها

برخلاف بسیاری از حشرات که بر اساس ویژگی‌های ظاهری نمونه کامل (Pinned in toto) شناسایی می‌شوند، پشه‌های خاکی برای شناسایی دقیق گونه نیاز به تشریح و مونتاژ روی لام دارند تا ویژگی‌های آناتومیکی بررسی شوند. صرف‌نظر از روش آماده‌سازی و مونتاژ، روش تشریح یکسان است (تصاویر 2 و 3) (https://zenodo.org/records/18198006) .

استفاده از Triton X100: محلول مایع غیر یونی

مونتاژ معمولاً روی نمونه‌های تازه یا نمونه‌هایی که به‌خوبی نگهداری شده‌اند انجام می‌شود. بسیاری از نمونه‌ها به‌صورت خشک برای MALDI-ToF یا برای سال‌ها در الکل نگهداری شده‌اند. نگهداری طولانی در الکل مناسب نیست و بندپایان نگهداری‌شده به این روش آماده‌سازی دشواری برای بررسی میکروسکوپی دارند. گاهی پلاستیک لوله‌ها تخریب می‌شود و الکل تبخیر می‌شود؛ در این حالت نمونه‌ها خشک شده و آماده‌سازی دشوار می‌شود.
به همین دلیل استفاده از مواد مرطوب‌کننده ضعیف پیشنهاد شده است.
Triton X-100 یک محلول مایع غیر یونی است که در زیست‌شناسی سلولی و مولکولی به‌عنوان دترجنت استفاده می‌شود و باعث نفوذپذیر شدن غشای سلولی و هسته می‌شود.
روش استفاده از محلول ۰/۵٪ Triton X-100

- نمونه خشک را با الکل مطلق آغشته کنید.
- محلول Triton X-100 با غلظت ۰/۵٪ اضافه کنید تا نمونه کاملاً در محلول غوطه‌ور شود.
- اجازه دهید فرآیند از حدود ۵ دقیقه تا چند روز ادامه یابد و مرتباً بررسی شود تا بندپایان کاملاً از هم جدا شوند.
- محلول Triton X-100 را خارج کرده و با محلول هیدروکسید پتاسیم (KOH) جایگزین کنید.

4.1 سر

تشریح با سوزن‌های ظریف یا پین‌های حشره‌شناسی زیر استریومیکروسکوپ انجام می‌شود.
سوزن‌های رایج:

- 26G (0.45 × 13 mm)
- 30G (0.3 × 13 mm)
- 25G (0.5 × 16 mm)

برای شناسایی گونه، حداقل باید:

- سر از بدن جدا شود
- سر به‌صورت سطح شکمی رو به بالا مونتاژ شود تا سیباریوم (cibarium)و فارنکس دیده شوند
- قفسه سینه و شکم بعداً به‌صورت جانبی مونتاژ شوند

قرار دادن سر در موقعیت ونترودورسال باعث می‌شود سوراخ پس‌سری (occipital foramen)به سمت بالا قرار گیرد و مشاهده سیباریوم آسان شود (تصاویر 2 و 3)

4.2 بال‌ها و قفسه سینه

بال‌ها باید کاملاً صاف مونتاژ شوند. هر بال می‌تواند:

- جداگانه از قاعده جدا شود
- یا یکی جدا شود و دیگری به قفسه سینه متصل بماند

اگر مورفومتری هندسی انجام شود، باید قبل از مونتاژ بال راست و چپ مشخص و برچسب‌گذاری شوند.
قفسه سینه شامل بخش‌های مختلفی است که اطلاعات تاکسونومیک مهمی دارند [20, 64] و معمولاً به‌صورت نمای جانبی مونتاژ می‌شود تا موارد زیر بررسی شود:

- الگوی موها (Chaetotaxy)
- توزیع رنگ

در برخی گونه‌ها:

- شکل رستنگاه موها در قفسه سینه برای تشخیص گونه‌های Brumptomyia استفاده می‌شود .
- توزیع رنگ برای تشخیص جنس‌ها و گونه‌ها در پشه‌های خاکی نئوتروپیکال استفاده می‌شود [20] .

نکته مهم :شدت رنگ مهم نیست، بلکه الگوی توزیع رنگ مهم است و فرآیند شفاف‌سازی رنگدانه را از بین نمی‌برد.

4.3 اندام‌های تناسلی

اندام‌های تناسلی در نر و ماده برای شناسایی جنس، زیرجنس و گونه بسیار مهم هستند.

4.3.1 نرها

اندام تناسلی خارجی شامل:

- فورسپس‌های جفت
- Gonocoxite
- Gonostyle
- Epandrial lobe

Gonostyle دارای خارها و گاهی موهایی است که:

- باید قابل شمارش باشند
- محل اتصال آن‌ها مشخص باشد [22]

سطح داخلی Gonocoxite ممکن است:

- دسته‌ای از موها
- یا موهایی روی یک لوب (توبِرکل)

افراد کم‌تجربه می‌توانند اندام تناسلی را بدون جدا کردن از انتهای شکم به‌صورت جانبی مونتاژ کنند، اما این کار ممکن است شمارش موهای داخلی را دشوار کند. افراد باتجربه می‌توانند اندام تناسلی را باز کرده و به دو قسمت تقسیم کنند تا مشاهده ساختارهای داخلی آسان‌تر شود. (https://zenodo.org/records/18311158).

4.3.2 ماده ها

دستگاه تناسلی درونی است و از اسپرماتکها تشکیل شده است. در صورت عدم تشریح، باید آن‌ها را از طریق تگومنت‌ها و با قرار دادن شکم در وضعیت شکمی مشاهده کرد .بدون توجه به نوع مونتاژ انتخاب‌شده، اسپرمتکاها به‌طور معمول قابل مشاهده هستند، به‌ویژه اگر سطح آن‌ها ناهموار و شفاف نباشد.
با این حال، مشاهدهٔ اسپرماتکهای صاف و دیوارهٔ نازک در محیط هایی با شکست نور ضعیف می‌تواند مشکل‌ساز باشد .علاوه بر این، مشاهدهٔ پایهٔ مجاری اسپرماتک برای تعیین گونه ضروری است، مانند گونه‌های زیرگروه لاروسیوسها [35, 37, 38]، که ناقلین اصلی لیشمانیا اینفانتوم در دنیای قدیم هستند .بدون این مشاهده، تعیین گونهٔ نمونه غیرممکن است.

برای رفع این مشکلات، ساختار فوروکا-اسپرماتک از شکم جدا می‌شود (https://zenodo.org/records/18311106).اسپرماتکها در طول تشریح معمولاً دشوار به نظر می‌رسند، اما فوروکا تناسلی نسبتاً آسان قابل یافتن است .از آنجا که مجاری اسپرماتک به فوروکا تناسلی باز می‌شوند، جداسازی این فوروکا معمولاً امکان جداسازی اسپرماتکها را فراهم می‌کند .
اگر در طول این فرآیند اسپرماتکها به طور اتفاقی بریده شوند، از بین نمی‌روند و همچنان می‌توان آن‌ها را در پوشش بدنی شکم مشاهده کرد(تصویر ۴)

4.4 تشریح روده میانی برای جداسازی لیشمانیا

تشریح دستگاه گوارش برای تشخیص و جداسازی لیشمانیا در پشه‌های خاکی ماده ضروری است و در آزمایشگاه یا فیلد قابل انجام است.
مراحل:

1. کار روی ماده‌هایی که تازه کشته شده‌اند .
2. شستشو با آب یا سرم فیزیولوژی + دترجنت ملایم برای حذف موها .
3. روده میانی جدا شده و در یک قطره سرم استریل (NaCl 0.9%) قرار داده شود .
4. مشاهده انگل‌های متحرک زیر میکروسکوپ (بزرگنمایی حدود ×200) .
5. انتقال انگل‌ها به محیط کشت با سرنگ انسولین یا میکروپیپت .
6. سر و اندام تناسلی در مایع Marc-André برای شفاف‌سازی مونتاژ شوند .

نکته بسیار مهم: مایع Marc-André نباید با لیشمانیا تماس پیدا کند، چون انگل را می‌کشد (تصویر 5؛ https://zenodo.org/records/18311154).

4.4.1 روش دو لام

گزینهٔ اول شامل کار روی دو لام جداگانه است، یکی شامل نرمال سالین استریل برای استخراج رودهٔ میانی و دیگری برای مونتاژ سر و اسپرماتک در محلول Marc-André است. با این حال، در شرایط فیلد، دو یا سه نفر پشه‌های خاکی را تشریح کرده و نمونه‌ها را به یک پژوهشگر مسئول تعیین گونه و بررسی عفونت لیشمانیا در روده منتقل می کنند.
مونتاژ دو لام جداگانه می‌تواند مشکلاتی در قابلیت پیگیری نمونه‌ها ایجاد کند و به ویژه تشخیص قطعی اینکه کدام پشهٔ منفرد آلوده بوده است (https://zenodo.org/records/18311154).

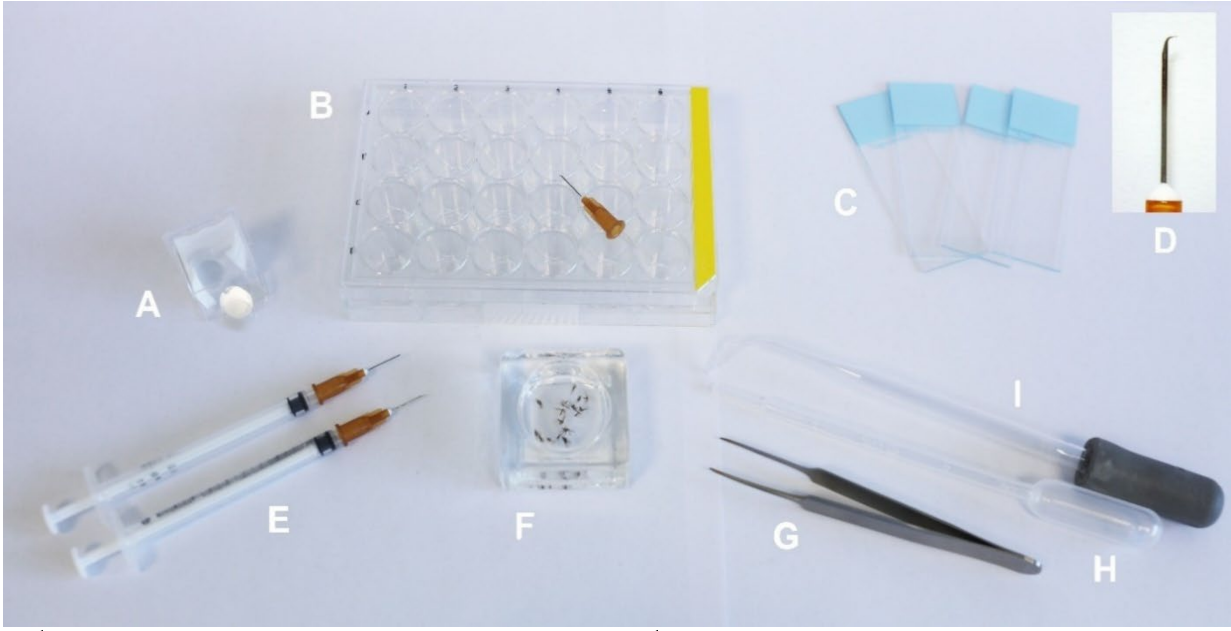

تصویر 2. مواد مورد نیاز برای مونتاژ پشه خاکیها: A: لامل های شیشه ای گرد (قطر 10 یا 12 میلیمتر)؛ B: پلیت 24 خانه ای و سوزن قلاب دار (اگر از روغن میخک یا ®Euparal برای آماده سازی پشه خاکی ها استفاده می شود، نباید از پلیت های آکریلیک استفاده کرد زیرا واکنش شیمیایی رخ داده و نمونه ها آسیب می بینند), C: لامهای شیشه ای مناسب برای برچسب گذاری؛نمای نزدیک از قلاب سوزن؛ E: سوزنهای متصل به سرنگ؛ F: شیشه ساعت یا ظرف مشابه برای نگهداری پشه خاکیهایی که قرار است مونتاژ شوند؛ G: پنس Dumont ؛ H: پیپت پلاستیکی؛ I: پیپت شیشه ای که با حرارت خم شده است تا انتقال مایع به داخل خانه های پلیت آسان تر شود.

سر جدا شده و یک برش بین ترژیت‌ها و استرنیت‌های ششم و هفتم شکم ایجاد می‌شود بدون اینکه روده بریده شود .(سپس توراکس با سوزن ثابت می‌شود و آخرین قطعات خلفی شکم با سوزن دیگر به آرامی کشیده می‌شوند تا روده استخراج شود.

اگر این روش موفق نبود، می‌توان انتهای شکم را با سوزن مسدود کرده و روده را از قسمت قدامی کشید .اگر دوباره موفق نشد، روده باید با حذف حداکثری باقی‌ماندهٔ تیگومنت اطراف آن استخراج شود .پس از خارج کردن روده، آخرین قطعات شکمی با برش روده جدا می‌شوند.

روده در یک قطرهٔ تازه نرمال سالین استریل در یک طرف لام قرار داده شده و سپس با لامل استریل به آرامی پوشانده می شود. سر و آخرین قطعات شکمی به یک قطره کوچک محلولMarc-André در طرف دیگر لام بدون تماس با لیشمانیا منتقل می‌شوند،

سر به درستی جهت‌یابی می‌شود(دهانهٔ پس‌سری رو به بالا) و اسپرماتکهای‌ها همراه با فورکا جدا شده و با یک لامل پوشانده می‌شوند، باقیماندهٔ بدن پشه و بال‌ها در قطرهٔ سالین وسط لام باقی می‌مانند .در صورت مثبت بودن یا برای کاوش تاکسونومیک، توراکس و شکم می‌توانند برای مطالعات مولکولی یا پروتئومیک حفظ شوند و بال‌ها در محیط مایع مونتاژ شوند .برای حفظ نمونه، حجم اضافی محلول Marc-Andréمی‌تواند با محیط نصب مایع مانند چوب صمغ کلرال یا محیط مبتنی بر پلی‌وینیل الکل جایگزین شود.

ویدئوهای تفصیلی این مراحل در دسترس هستند:

- تشریح رودهٔ میانی پشه خاکی : https://zenodo.org/records/18303014
- تشریح غدد بزاقی پشه خاکی : https://zenodo.org/records/18302850

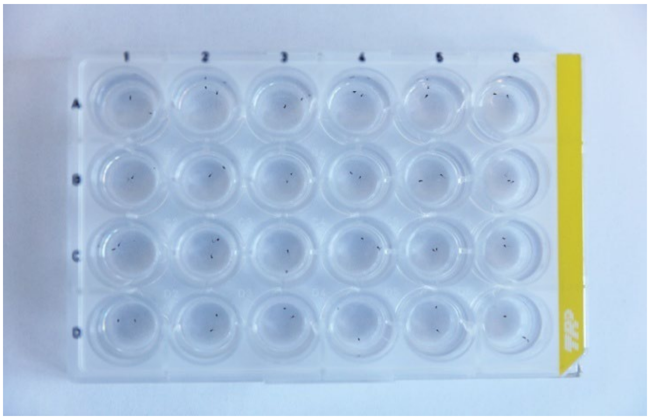

تصویر 3. یک پلیت ۲۴ خانه‌ای که در هر خانه آن، سر و انتهای شکم پشه‌های خاکی قرار داده شده است.

4.4.2 روش تک لام

استفاده از روش یک لام تضمین‌کنندهٔ قابلیت پیگیری نتایج است .با این حال، چندین احتیاط باید رعایت شود .برای حداکثر استریل بودن در این مرحله، اپراتورها باید دست‌های خود را مرتب با ژل هیدروالکلی تمیز کنند . از لام‌های بدون مات شدن و لامل‌های مربعی (22 × 22 mm) که در فویل آلومینیومی پیچیده و با حرارت خشک) کوره (Poupinel استریل شده‌اند، به همراه سوزن استریل برای هر تشریح استفاده شود) پیشنهاد: ۲۵ G Ø 0.5 mm × 16 mm).

پشهٔ خاکی در یک قطره نرمال سالین استریل در وسط لام قرار می‌گیرد .

4.4.3 جداسازی و کشت لیشمانیا

ایزولاسیون انگل از پشه‌های مادهٔ آلوده یک فرآیند حساس است که نیازمند مهارت بالا می‌باشد و توصیه می‌شود ابتدا روی نمونه‌های بدون انگل تمرین شود.

پس از تشریح، روده‌ها به قطرهٔ تازه‌ای از سالین استریل ۰.۹٪ یا محلول Lockeمنتقل می‌شوند تا شسته شوند .[4] روده‌های تشریح‌شده سپس می‌توانند به دو روش پردازش شوند:

1. بررسی زیر میکروسکوپ نوری برای مشاهدهٔ مراحل مختلف لیشمانیا پرومستیگوت‌ها و محل قرارگیری آن‌ها، با توجه ویژه به سوپتوئال ولو .(stomodeal valve)
2. باز کردن روده برای تسهیل خروج پرومستیگوت‌ها و فراهم کردن شرایط کشت انبوه آن‌ها .[4]

یافتن پشه‌های آلوده در طبیعت نسبتاً نادر است، بنابراین تمرین کافی شانس موفقیت در ایزولاسیون را افزایش می‌دهد.

اگر انگل‌های لیشمانیا در روده مشاهده شوند، باید از سوزن‌های استریل جدید استفاده شده و مقدار کمی نرمال سالین استریل دور لام اضافه شود تا از طریق اثر مویینگی (capillary action) انگل‌ها آزاد شوند .روده باید با دقت و سرعت پاره شود تا انگل‌ها وارد نرمال سالین شوند .با استفاده از مایکروپیپت ۱۰۰ میکرولیتر یا سرنگ توبرکلین، انگل‌ها جمع‌آوری شده و در محیط کشت مناسب با برچسب‌گذاری صحیح تلقیح می‌شوند.

کشت درون‌شیشه‌ای پروماستیگوت‌های لیشمانیا:

انگل‌های ایزوله‌شده ابتدا روی شیب‌های آگار خون SNB-9 یا در محیط جامد [16] Novy, Mc Neal, Nicolle (NNN) کشت داده می‌شوند و روی آن‌ها یا محیط alpha-MEM استریل [16, 65] یا محیط M199 اضافه می‌گردد که هر دو با افزودنی‌های زیر تکمیل شده‌اند:

- ۱۰٪ سرم جنین گاو استریل و غیرفعال شده حرارتی (FCS) برای افزایش رشد انگل
- ۱٪ ویتامین‌های BME
- ۲٪ ادرار انسانی استریل (استریل‌شده با فیلتر سرنگ Filtropur™ S 0.2 µm (
- ۲۵۰ µg/mL آمیکاسین یا ۵۰ µg/mL جنتامایسین یا ترکیبی از آنتی‌بیوتیک‌ها و اسیدهای آمینه [47]

پس از سه روز، در صورت عدم آلودگی، کشت‌ها در محیط فریزینگ آماده‌شده معلق می‌شوند و سپس برای نگهداری ۱–۲ سال در– ۸۰ درجه سانتی‌گراد یا در نیتروژن مایع– ۱۹۶ درجه سانتی‌گراد برای حفظ طولانی‌مدت و استفادهٔ آزمایشی بعدی ذخیره می‌شوند.[7]

4.5 غدد بزاقی

ي تشریح غدد بزاقی پشه‌های خاکی یک تکنیک اساسی برای مطالعهٔ تعاملات میزبان–پاتوژن است، به‌ویژه برای شناسایی آربوویروس‌ها مانند فلبوویروس‌ها مثلاً ویروس توسکانا .[75 ,44] به دلیل کوچکی بسیار پشه‌های خاکی، این فرآیند نیازمند دقت بالا زیر استریومیکروسکوپ است و باید از پنس‌های ظریف یا سوزن‌های میکروتشریح برای جداسازی غدد بزاقی حساس استفاده شود بدون آنکه پاره یا آلوده شوند (https://zenodo.org/records/18302850) [51, 61].حفظ یکپارچگی غدد برای تحلیل‌های مولکولی دقیق بعدی حیاتی است.

پس از استخراج، غدد می‌توانند هموژنیزه شده و با روش‌های RT-qPCR،PCR ، یا ایمونواسی‌ها برای شناسایی RNA یا آنتی‌ژن ویروسی مورد آزمایش قرار گیرند .[12] حضور ویروس‌ها در غدد بزاقی، نه تنها در روده یا هموسل، تأیید می‌کند که پاتوژن دورهٔ تکثیر خارجی خود را کامل کرده و قابلیت انتقال در هنگام تغذیه از خون را دارد.[71]

این فرآیند تشریح از نظر فنی چالش‌برانگیز است زیرا غدد بزاقی پشه‌های خاکی بسیار کوچک هستند و نیاز به تخصص بالا برای جلوگیری از تخریب نمونه دارد .[1, 51] علاوه بر این، بار ویروسی ممکن است کم باشد، بنابراین استفاده از روش‌های بسیار حساس مانند PCR تو در تو (nested PCR)یا توالی‌یابی با بازده بالا ضروری است .[54] خطرات آلودگی نیز ضرورت استفاده از تکنیک‌های استریل را بیشتر می‌کند.

عوامل بیولوژیک نیز بر موفقیت شناسایی تأثیر دارند؛ شایستگی ناقل بین گونه‌های پشه خاکی متفاوت است و نرخ ابتلا با شرایط اکولوژیکی و فصلی تغییر می‌کند.[33, 61]

شناسایی ویروس‌ها در غدد بزاقی اطلاعات حیاتی دربارهٔ ریسک انتقال ارائه می‌دهد و امکان نظارت هدفمند و اقدامات کنترلی را فراهم می‌کند .[15] برای مثال، شناسایی ویروس توسکانا در پشه‌های خاکی در مناطق بومی، به تدوین پروتکل‌های تشخیصی و توصیه‌های بهداشتی کمک کرده است .[18] همچنین، مطالعهٔ تعامل ویروس–بزاق می‌تواند اهداف جدیدی برای واکسن‌ها یا درمان‌های مسدودکنندهٔ انتقال ارائه دهد.[15, 18]

غدد بزاقی پشه‌های خاکی همچنین می‌توانند به‌عنوان منبع آنتی‌ژن برای سنجش پاسخ‌های ایمنی میزبان علیه بزاق پشه با روش‌های ایمونولوژیک، ترجیحاً ELISA ، مورد استفاده قرار گیرند .این روش امکان ارزیابی تماس میزبان با نیش پشه خاکی را فراهم می‌کند و در نتیجه ارزیابی اثربخشی کنترل ناقل [25] و ریسک انتقال لیشمانیوز [40] را پشتیبانی می‌کند.

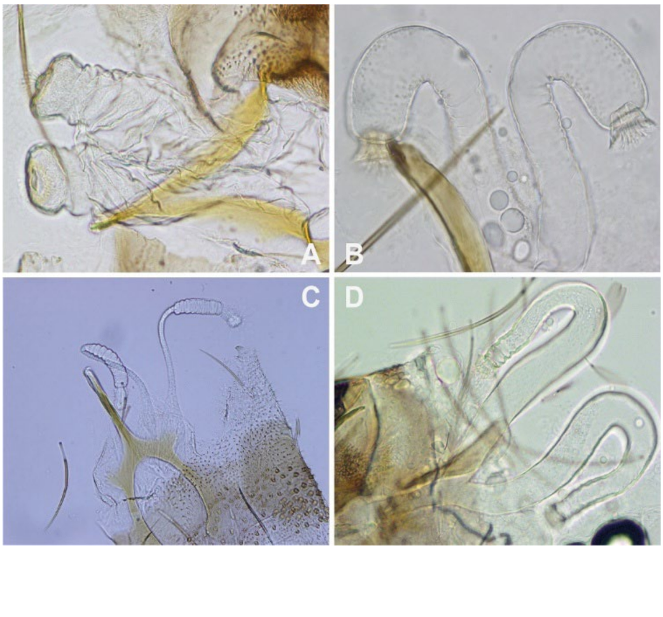

تصویر 4. اسپرماتکهایی که از نمونه‌های تازه تشریح شده و در محلول مارک–آندره (Marc-André) مونتاژ شده‌اند *Idiophlebotomus longiforceps* :A . (لائوس)؛ *Sergentomyia minuta* : B (فرانسه)؛ *Phlebotomus ariasi* : C(فرانسه)؛ *Sergentomyia anodontis* : D (لائوس).

4.6 شناسایی منبع خون

نمونه‌های مادهٔ پشه‌های پرخون باید با استفاده از تجهیزات یک‌بارمصرف تشریح شوند تا از آلودگی متقابل جلوگیری شود .شکم آن‌ها تحت استریومیکروسکوپ بررسی می‌شود تا مرحلهٔ هضم خون مشخص گردد . توصیه می‌شود تنها نمونه‌هایی با شکم قرمز، قهوه‌ای مایل به قرمز یا قرمز تیره انتخاب شوند که هیچ نشانه‌ای از تشکیل تخم نداشته باشند.

روش آماده‌سازی:

- نوک شکم شامل اسپرماتک باید برداشته شود تا نمونه ماده از نظر مورفولوژیک پس از روشن‌سازی شناسایی شود .
- بخش اصلی شکم (بدون اسپرماتک) در لوله‌های Eppendorf قرار داده شده و در دمای −۲۰ درجه سانتی‌گراد تا زمان تجزیه و تحلیل بعدی نگهداری شود .

مارکرهای ژنتیکی رایج برای شناسایی خون میزبان، مانند PNOC [5, 30, 50]، CytB [67]یا COI [13]، به‌طور گسترده در منابع علمی توصیف شده‌اند و در این مقاله توضیح مجدد داده نمی‌شوند (تصویر ۶) . به‌طور جایگزین، برای شناسایی خون میزبان، می‌توان از نقشه‌برداری پپتیدی MALDI-ToFاستفاده کرد .[31]این تکنیک نشان داده شده که حتی پس از مدت طولانی از تغذیهٔ خون، شناسایی میزبان امکان‌پذیر است و بنابراین روش مناسبی برای ماده‌های پرخون با هضم پیشرفتهٔ خون است.

شرایط نگهداری نمونه‌ها:

- ایده‌آل :دمای −۲۰ درجه سانتی‌گراد یا ۴ درجه سانتی‌گراد
- نگهداری کوتاه‌مدت در دمای محیط نیز نتایج مناسبی ارائه می‌دهد .

آماده‌سازی پیش از تحلیل:

- شکم مادهٔ پرخون باید کوتاه قبل از تحلیل از بدن جدا شود و در آب مقطر هموژنیزه شود .
- باقی‌ماندهٔ بدن پشه برای سایر تحلیل‌های مولکولی و مورفولوژیک قابل استفاده است .
- پس از برداشتن آلیکوته‌ای برای MALDI-ToF، باقی هموژنات می‌تواند برای استخراج DNAاستفاده شود تا شناسایی خون میزبان تأیید شود و/یا برای بررسی حضور Leishmania sp. استفاده گردد .

مدت زمان کلی آماده‌سازی و تحلیل نمونه‌ها در مقایسه با روش‌های مولکولی مبتنی بر DNAبسیار کوتاه است.

5 پردازش نمونه‌ها برای مطالعات مورفولوژیک (تصاویر 3، 6، 7 و8؛ پیوست های 1، 2، 3 و4)

این بخش اصول آماده‌سازی پشه‌های خاکی برای مونتاژ روی لام جهت مطالعات مورفولوژیک را توضیح می‌دهد. این روش شامل مراحل تخلیه و پر کردن متوالی با مواد شیمیایی با استفاده از پیپت پاستور است. استفاده از ظروف شیشه‌ای ته‌گرد توصیه می‌شود زیرا کار با نمونه‌ها را آسان‌تر می‌کند و شیشه با مواد شیمیایی واکنش نمی‌دهد. برای جلوگیری از تبخیر مواد، ظروف باید درب داشته باشند و بیش از حد پر نشوند.

مواد شیمیایی مورد استفاده برای شفاف‌سازی و پردازش در جدول 2 ارائه شده‌اند.

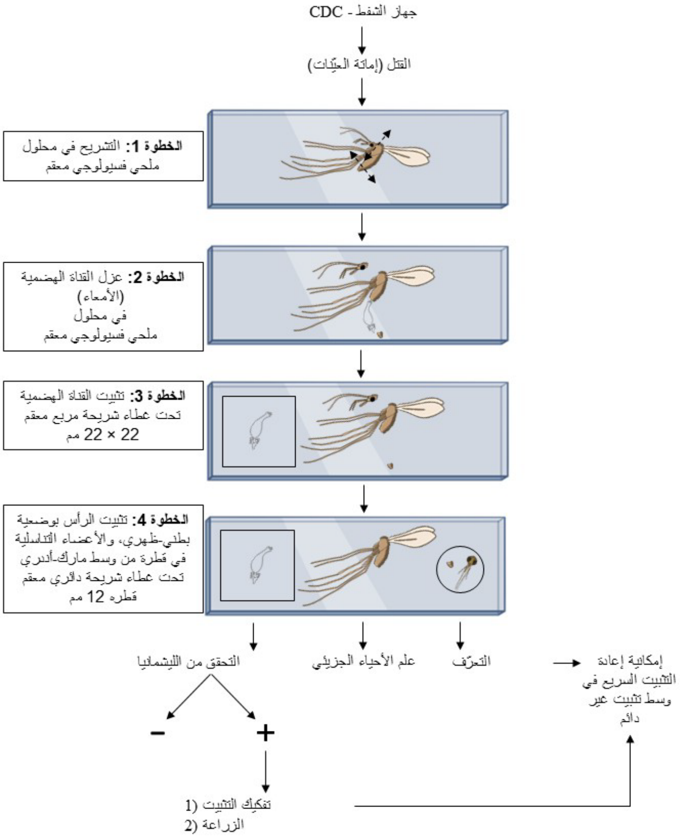

تصویر 5. روش جداسازی لیشمانیا.

5.1 شفاف‌سازی

قبل از اینکه نمونه‌های پشه خاکی بتوانند به‌صورت لام‌های دائم آماده شوند، ابتدا باید با استفاده از یک روش و عامل روشن‌کننده مناسب(مثلاً محلول ۱۰٪ اسید استیک یا محلول Marc-André شامل کلرال هیدرات که در بسیاری از کشورها یک مادهٔ محدودشده است) شفاف‌سازی شوند .فرآیند روشن‌سازی با حذف بافت‌های بدن، چربی، ترشحات و موم، نمونه را نیمه‌شفاف می‌کند و امکان بررسی ساختارهای اسکلت خارجی(مثلاً محل قرارگیری موی سطحی)، ویژگی‌های سطحی (مثلاً رنگ) و ساختارهای داخلی قابل مشاهده از طریق کوتیکول (مثل اسپرماتکها) را فراهم می‌آورد.

فرآیند روشن‌سازی دو مرحله‌ای شامل استفاده ابتدا از یک باز قوی (مثل هیدروکسید پتاسیم) و سپس یک اسید ضعیف مثل (اسید استیک) در محلول Marc-Andréاست و اهداف بیوشیمیایی متمایزی دارد:[74]

- باز قوی بافت‌های نرم مانند پروتئین‌ها، چربی‌ها و عضلات را از طریق فرایند صابونی‌شدن (saponification) و دناتوره شدن پروتئین‌ها تجزیه می‌کند، در حالی که اسکلت خارجی کیتینی را دست‌نخورده باقی می‌گذارد تا شفافیت ساختاری حفظ شود.
- اسید ضعیف باقی‌ماندهٔ باز را خنثی می‌کند تا از تخریب بیشتر جلوگیری شود و کوتیکول را سفید کرده و شفافیت آن را افزایش دهد[74] ؛ اگرچه شستشو دو بار در آب مقطر به مدت ۱۵ دقیقه نیز ممکن است برای خنثی‌سازی باز کافی باشد .

این درمان متوالی حذف مؤثر بافت‌ها را با حفظ ملایم نمونه ترکیب می‌کند و اطمینان می‌دهد که نمونه برای مشاهدهٔ میکروسکوپی بهینه و سالم باقی بماند.

قبل از ادامه به مرحله بعد، دو شستشوی ۲۰ دقیقه‌ای در آب مقطر توصیه می‌شود.

5.1.1 لیز بافت نرم (تصویر 8)

هیدروکسید سدیم (NaOH) یا هیدروکسید پتاسیم (KOH) از عوامل شیمیایی متداول برای ماسره‌سازی (تجزیه بافتی) هستند که با غلظت‌ها و مدت‌زمان‌های متفاوت، بسته به اندازه و میزان شکنندگی نمونه‌ها، به‌کار می‌روند.

روش استاندارد و مؤثرترین تکنیک شامل لیز کردن بافت‌های نرم از طریق غوطه‌ور کردن پشه‌های خاکی در یک باز قوی (KOH 10٪ یا NaOH) به مدت شبانه است .غلظت می‌تواند برای کاهش مدت زمان فرآیند افزایش یابد(مثلاً KOH ۲۰٪ برای ۶ ساعت) و همچنین می‌توان از گرمادهی در دمای ۳۷ درجه سانتی‌گراد بهره برد.

5.1.2 شفاف‌سازی با یا بدون رنگ‌آمیزی

این مرحله با فرآیند روشن‌سازی ادامه می‌یابد، که معمولاً شامل ترکیبی از اسید استیک و کلرال هیدرات (مثلاً محلول Marc-André) است .پس از روشن‌سازی، نمونه‌ها باید به‌طور کامل در حداقل دو حمام آب متوالی، هر کدام ۲۰ دقیقه، شستشو داده شوند تا مواد شیمیایی باقی‌مانده حذف شوند.

محلول Marc-André یک عامل روشن‌کنندهٔ رایج برای آماده‌سازی نمونه‌های پشهٔ خاکی است .اثربخشی آن در این است که فرآیند روشن‌سازی را تسهیل می‌کند در حالی که به ساختارهای حساس مانند بال‌ها و شاخک‌ها آسیب قابل توجهی وارد نمی‌کند .این محلول باید تازه آماده شود یا در یک ظرف کاملاً بسته نگهداری شود تا از تبخیر یا تخریب جلوگیری شود .استفاده از محلول Marc-André زمانی که با تکنیک‌های روشن‌سازی یا رنگ‌آمیزی ترکیب شود، به ویژه برای برجسته‌سازی جزئیات مورفولوژیک خاص مفید است .جزئیات ترکیب و آماده‌سازی آن در پیوست B ارائه شده است.

برای نمونه‌های بسیار شفاف، ممکن است رنگ‌آمیزی قبل از مونت ضروری باشد تا قابلیت مشاهده افزایش یابد .رنگ‌های متعددی موجود است که هر کدام ترکیبات شیمیایی خاصی از ارگانیسم را هدف قرار می‌دهند . انتخاب رنگی که با نمونه و محیط مونت انتخاب‌شده سازگار باشد، اهمیت دارد .این روش پایه می‌تواند در صورت نیاز تطبیق داده شود، مثلاً با افزودن ۰.۱٪ اسید فوکسین به محلول Marc-André برای رنگ‌آمیزی.

علاوه بر این، نمونه‌هایی که در محلول‌های آبی نگهداری شده و برای مونت رزینی در نظر گرفته شده‌اند، نیاز به دهیدراسیون دارند (به بخش ۲.۵ در مورد دهیدراسیون مراجعه کنید)، زیرا اکثر محیط‌های مونت رزینی طبیعی و مصنوعی با آب ناسازگار هستند New (1974) .ذکر کرده است که برخی رنگ‌ها می‌توانند در محیط‌های مونت خاص تخریب شوند [53]. برای مثال، اسید فوکسین که معمولاً با کانادا بالزام استفاده می‌شود، می‌تواند در Euparal نیز تثبیت شود .با این حال، نمونه‌های رنگ‌آمیزی‌شده با اسید فوکسین مستعد رنگ‌پریدگی هستند، به‌ویژه زمانی که باقی‌ماندهٔ روغن میخک که به‌عنوان محلول روشن‌کنندهٔ نهایی استفاده شده، باقی بماند .نمونه‌های ذخیره‌شده در روغن میخک ممکن است طی چند روز تغییر رنگ قابل توجهی نشان دهند.

5.2 آب‌گیری (Dehydration)

دهیدراسیون با انتقال تدریجی نمونه‌ها از طریق یک سری محلول‌های اتانول با غلظت‌های درجه‌بندی شده انجام می‌شود :۵۰٪، ۷۰٪، ۸۰٪، ۹۰٪ یا ۹۵٪ و در نهایت ۱۰۰٪، به‌طوری که هر مرحله حداقل ۲۰ دقیقه طول می‌کشد .از آنجا که اتانول به‌سرعت تبخیر می‌شود، ظرف باید در طول فرآیند کاملاً بسته باشد.

پس از دهیدراسیون کامل نمونه، می‌توان پردازش را برای چند روز در عصارهٔ Euparal متوقف کرد، که این روش ترجیح داده می‌شود نسبت به روغن میخک .کرئوزوت راش (Beech creosote) که قبلاً به‌طور گسترده برای این منظور استفاده می‌شد، اکنون به‌طور کامل به دلیل سمیتش ممنوع شده است.

فرآیند دهیدراسیون باید تضمین کند که مایع موجود در نمونه با محیط مونتاژ سازگار باشد تا از ایجاد کدری، فروپاشی اسمزی یا تغییر شکل جلوگیری شود، زیرا این مشکلات می‌توانند نمونه را برای مطالعات تاکسونومیک غیرقابل استفاده کنند.

5.3 محیط‌های مونتاژ

5.3.1 انتخاب محیط مونتاژ

محیط مونتاژ باید ایده‌آل‌ترین حالت ممکن، ضریب شکست نزدیک به شیشه داشته باشد، که تقریباً برابر ۵.۱ است .این محیط باید بی‌رنگ، شفاف و پس از خشک شدن و گذر زمان کاملاً شفاف باقی بماند .همچنین باید با رنگ‌های مورداستفاده سازگار باشد و توانایی نفوذ و پراکنش در تمام بافت‌های نمونه را داشته باشد .محیط نباید خیلی سریع خشک شود یا در هنگام مونتاژ مه‌آلود شود و نباید پس از مونت کوچک شود.

انتخاب محیط مونتاژ مناسب یک جنبهٔ اساسی در آماده‌سازی نمونه است، زیرا هیچ محیطی برای همهٔ اهداف ایده‌آل نیست .انتخاب باید بین چند عامل کلیدی تعادل برقرار کند:

- ویژگی‌های نوری :ضریب شکست محیط مونتاژ باید کنتراست و انکسار کافی برای مشاهدهٔ ویژگی‌های آناتومیکی مهم مورداستفاده در شناسایی تاکسونومیک یا توصیف مورفولوژیک، مانند اسپرماتکها، آسکوییدها، سنسیلاهای Newstead، دندان‌های سیباریال عمودی و دندان‌های حلقی، فراهم کند .قابلیت مشاهدهٔ این ساختارها مستقیماً به ویژگی‌های نوری محیط مونتاژ بستگی دارد .
- حفظ نمونه :برای نمونه‌های تیپ یا موادی که برای مجموعه‌های دائم در نظر گرفته شده‌اند، محیط باید پایداری و دوام طولانی‌مدت ارائه دهد .در مقابل، برای مطالعات موجودی یا بررسی‌های اپیدمیولوژیک که حفظ طولانی‌مدت کمتر اهمیت دارد، محیط‌های مونت موقت یا نیمه‌دائم کافی خواهند بود.

5.3.2 الزامات محیط مونتاژ

متخصصان اغلب تکنیک‌های مونتاژ سفارشی و پیچیده‌ای توسعه می‌دهند که متناسب با نیازهای خاص پژوهشی هستند .با این حال، این روش‌ها اغلب جنبه‌هایی مانند کیفیت آرشیوی، سازگاری، استانداردسازی، سهولت استفاده و نگهداری طولانی‌مدت را نادیده می‌گیرند .این کمبود استانداردسازی، یکپارچه‌سازی مجموعه‌های اهدایی و تلاش‌های بلندمدت برای نگهداری را پیچیده می‌کند.

کاربردهای علمی نیازمندی‌های خاصی برای محیط‌های مونتاژ تحمیل می‌کنند .تاکسونومیست‌ها اغلب نمونه‌های کامل را مونت می‌کنند و محیط‌هایی را ترجیح می‌دهند که اندام‌های داخلی را به آرامی نرم کنند تا وضوح ساختارهای کوتیکولی افزایش یابد .ضریب شکست محیط باید به‌قدر کافی با نمونه و لام شیشه‌ای متفاوت باشد تا شفافیت نوری حداکثر شود .محیط‌های مونتاژ تجاری معمولاً با ضریب شکستی نزدیک به شیشه فرموله می‌شوند تا انکسار و پراکندگی نور در سیستم لام–محیط مونتاژ–لامل به حداقل برسد.

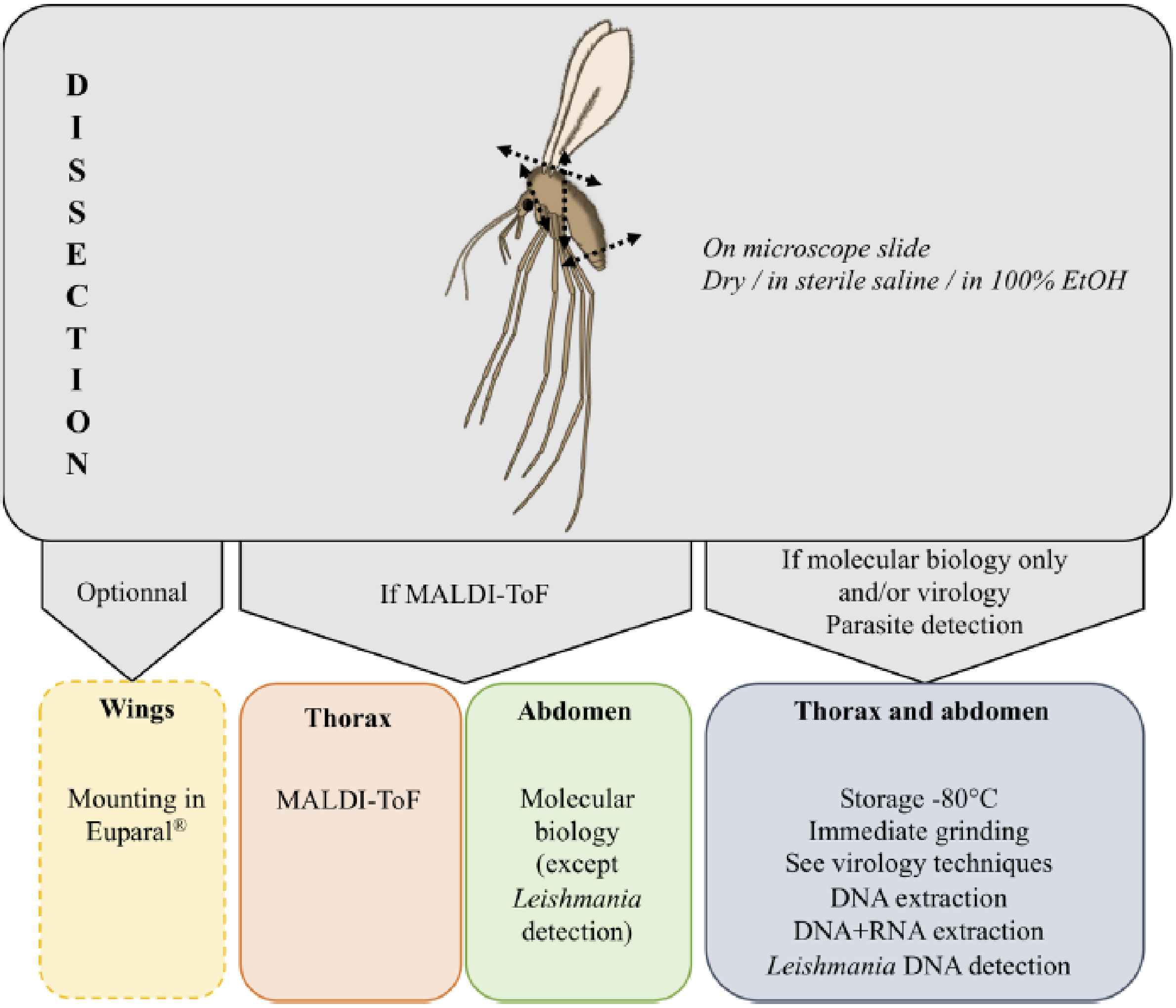

تصویر 6. آماده سازی و پردازش پشه خاکی ها (Sand fly processing) برای آنالیزهای ملکولی (molecular biology)، پروتیومکس (proteomics) ، و/یا ویروس شناسی.

با این حال، در میکروسکوپی میدان روشن (brightfield)، کنتراست طبیعی نمونهٔ بدون رنگ می‌تواند با انتخاب آگاهانهٔ محیط مونتاژ با ضریب شکست کمی متفاوت از نمونه، دستکاری شود و بدین ترتیب قابلیت مشاهدهٔ آن در برابر پس‌زمینه بهبود یابد.

5.3.3 انواع محیط‌های مونتاژ (جداول 3 و4)

در میکروسکوپی، ضریب شکست (RI) محیط مونتاژ تعیین می‌کند که نور چگونه در هنگام عبور از لام، محیط مونتاژ و نمونه شکسته می‌شود . هنگامی که RI با شیشهٔ لامل (≈ 1.515) نزدیک همراستا باشد، نور به‌طور یکنواخت عبور می‌کند، پراکندگی و اعوجاج نوری کاهش یافته و وضوح و مشاهدهٔ ساختارهای ظریف بهبود می‌یابد. برعکس، ناسازگاری RI می‌تواند باعث تاری، هاله یا پنهان شدن ویژگی‌های بدون رنگ شود.

انتخاب محیط مونتاژ مناسب برای بهینه‌سازی کنتراست، وضوح و کیفیت کلی تصویر برای هر نمونه اهمیت ویژه‌ای دارد، زیرا هر محیط RI متفاوتی دارد .ضریب شکست محیط مونتاژ تأثیر قابل توجهی بر توانایی مشاهدهٔ ساختارهای ظریف هنگام آماده‌سازی پشه‌های خاکی برای مونت روی لام دارد .ویژگی‌های ظریف و کم‌سکلریت‌شدهٔ پشه‌های خاکی، شامل آرایش سیباریال(cibarial armature) ، اسپرماتکها، قطعات آنتن و رگ‌بندی بال، ممکن است در محیطهای با ضریب شکست بالا دشوار قابل مشاهده باشند.

برای پشه‌های خاکی، گزینه‌های رایج شامل محیطهای مایع مبتنی بر کلرال گام و رزین‌های حلالی مانند کانادا بالزام و Nelson – Enecê Cerqueira (NC)هستند. راولینز [60] محیطهای مونتاژ را به دو نوع

تقسیم کرده است:

1. محیطهای دائم :این محیطها با گذر زمان سخت می‌شوند و برای نگهداری طولانی‌مدت مناسب‌اند .
2. محیطهای نیمه‌دائم :این محیطها سخت نمی‌شوند و معمولاً برای مقاصد موقت استفاده می‌شوند .

محیطهای مونتاژ می‌توانند مایع، مبتنی بر صمغ یا رزینی باشند و در آب، الکل یا سایر حلال‌ها (مانند تولوئن، زایلنحل)شوند(جدول ۱) .پس از اعمال، باید با استفاده از محیطهای حلقه‌زنی غیرقابل حل، از تأثیرات جوی محافظت شوند.

برای تمایز واضح انواع محیطهای مونتاژ، می‌توان دسته‌بندی زیر را استفاده کرد:

الف (محیطهای آبی) :این محیطها به‌راحتی در آب حل می‌شوند و برای مونت‌های موقت یا نیمه‌دائم مناسب‌اند .معمولاً استفادهٔ آسان دارند، اما ممکن است نیاز به درزگیری برای جلوگیری از تماس با رطوبت هوا باشد(مثل محیطهای gum-chloral و پلی‌وینیل الکل)، به‌ویژه در مناطق گرم و مرطوب.

ب (محیطهای با تحمل محدود نسبت به آب) :این محیطها کمتر تحت تأثیر آب قرار می‌گیرند اما هنوز نیاز به محافظت در برابر رطوبت زیاد دارند . آن‌ها پایداری طولانی‌تر نسبت به محیطهای محلول در آب ارائه می‌دهند و اغلب در مونت‌های نیمه‌دائم استفاده می‌شوند.

ج (محیطهای محلول در هیدروکربن) :این محیطها در حلال‌های آلی مانند زایلن یا تولوئن یا حلال Enecê حل می‌شوند .این محیطها برای مونت دائم طراحی شده‌اند، پایداری طولانی‌مدت عالی دارند، در برابر رطوبت و تخریب مقاوم‌اند و برای اهداف آرشیوی ایده‌آل هستند مثل(کانادا بالزام خنثی (DPX

خلاصه:

- محیطهای محلول در آب برای مونت موقت یا مواردی که نیاز به جداسازی آسان نمونه دارند، بهترین گزینه‌اند .
- محیطهای با تحمل محدود نسبت به آب برای مونت‌های نیمه‌دائم با دوام متوسط مناسب‌اند .

محیطهای محلول در هیدروکربن برای مونت‌های دائم با هدف آرشیوی و نگهداری طولانی‌مدت ترجیح داده می‌شوند .

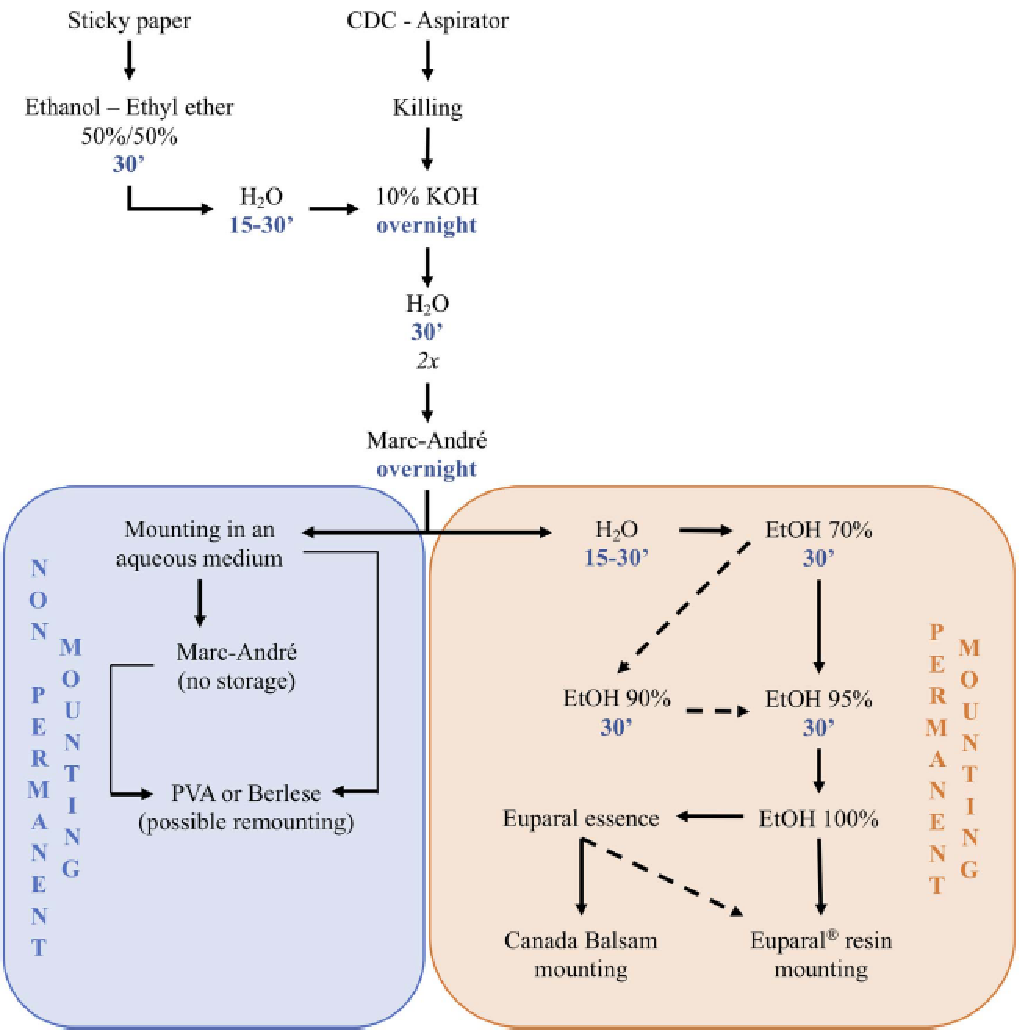

تصویر 7. روشهای کلاسیک پردازش پشه خاکیها

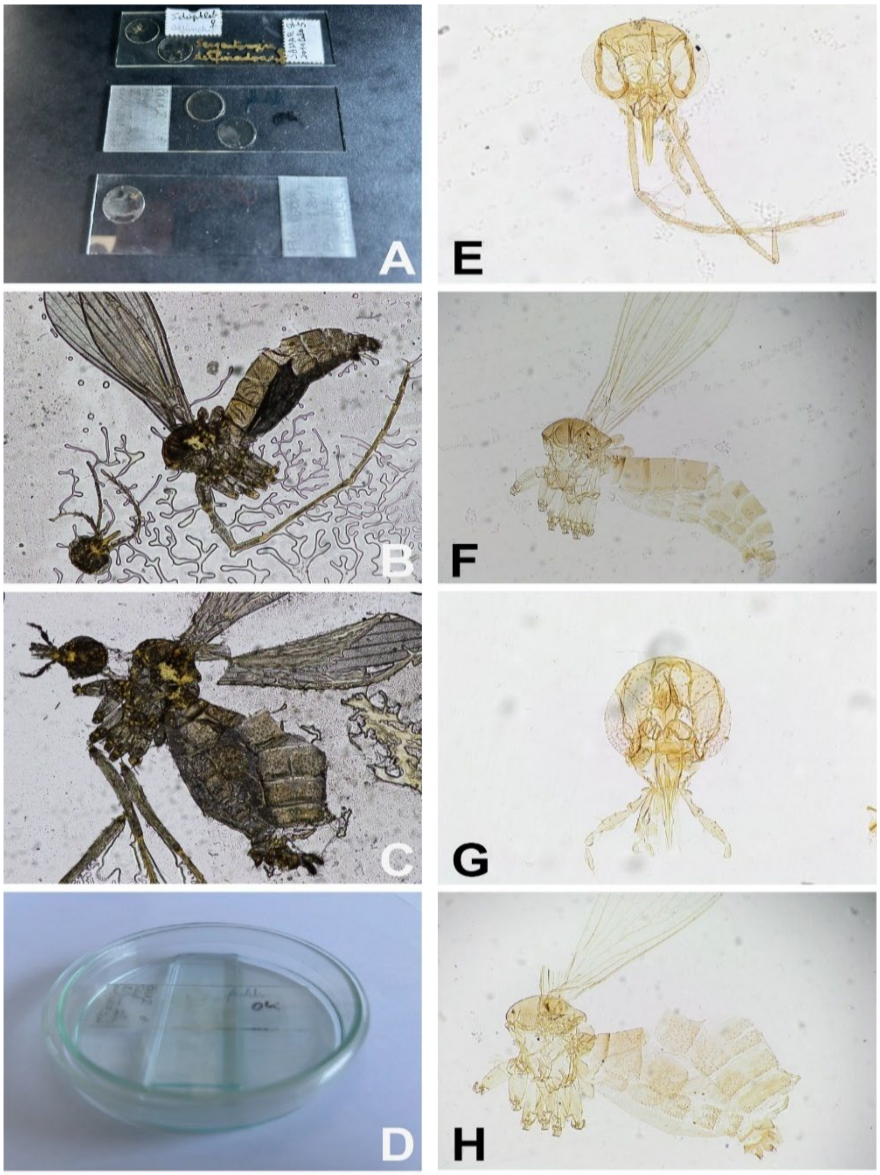

تصویر 8. مونتاژ مجدد لامها (Slide remounting). A: لام های آسیب دیده و خشک شده که در محیط Hoyer مونتاژ شده اند، B : نمای میکروسکوپی از یک پشه خاکی خشک شده، C : نمای میکروسکوپی از یک پشه خاکی آسیب دیده دیگر، D: محفظه مرطوب حاوی یک لام خشک شده, E :سر، F :بدن نمونه B پس از مونتاژ مجدد در محیط Euparal ، G: سر و H: بدن نمونه آسیب دیده C پس از مونتاژ مجدد در محیط Euparal®

5.3.4 . محیط‌های مونتاژ توصیه‌شده (جداول 3 و4)

محیط‌های موقت

کلورال‌گام/محلول هویر (Hoyer)ضریب انکسار = 1.48. محیط Marc Andréبهترین محیط برای مشاهدهٔ کوتاه‌مدت (چند ساعت)، شاید کمی بیشتر اگر لام در یک محفظهٔ مرطوب نگهداری شود) اسپرماتک است(تصویر ۴)، برای حفظ اسپرماتکهای مشاهده‌شده، باید آن‌ها را در یک محیط مایع مجدد مونتاژکرد تا نگهداری میان‌مدت ممکن شود .دهیدراسیون آن‌ها برای مونتاژ مجدد در رزین غیرممکن نیست، اما توصیه نمی‌شود (خطر از دست رفتن نمونه) . کلرال گام و محلول هویر معمولاً مترادف در نظر گرفته می‌شوند . این محیط به‌طور معمول برای مشاهدهٔ اندام‌های داخلی استفاده می‌شود، به دلیل سازگاری با آب، سادگی، کاربرد سریع، و ضریب شکست که بررسی

ساختارهای ظریف مانند اسپرماتکها را تسهیل می‌کند.
با این حال، کلرال گام معایب قابل‌توجهی دارد اگر به‌طور کامل آماده نشده باشد یا در شرایط رطوبتی کنترل‌شده نگهداری نشود .این مشکلات شامل کریستالیزه شدن، تغییر رنگ و از دست رفتن ویسکوزیته است .حلقه‌زدن (ringing)لامل این مشکلات را حل نمی‌کند، زیرا محیط مونتاژ ممکن است به شدت تغییر رنگ دهد به دلیل تعامل با محیط حلقه، به‌ویژه اگر از Euparal استفاده شود.

محیط هویر از نظر نوری برای پشه‌های خاکی فلبوتومینه بهترین در نظر گرفته شده و به‌طور سنتی برای این منظور استفاده شده است .این محیط شامل چندین فرمولاسیون نزدیک به هم است، از جمله صمغ عربی، گلیسیرول و کلرال هیدرات .برخی فرمولاسیون‌ها به اشتباه تفسیر و نقل شده‌اند.[74]

اگرچه هویر محیط خوبی برای مشاهدهٔ اسپرماتکها در پشه‌های خاکی است، برای نگهداری طولانی‌مدت مناسب نیست .این محیط برای مشاهدات کوتاه‌مدت، شامل عکسبرداری، رسم یا تصویرسازی ایده‌آل است .محیط‌های مایع برای مونتاژ موقت مناسب‌اند، اما نمی‌توانند حفظ طولانی‌مدت را تضمین کنند .در مقابل، مونتاژ رزینی دوام عالی دارد و اغلب قرن‌ها پایدار می‌ماند، اما ممکن است جزئیات ظریف اسپرماتکها را مخفی کند، زیرا شکست نور آن‌ها اغلب از بین می‌رود.

محیط هویر با گذر زمان به دلیل دهیدراسیون تخریب می‌شود(تصویر ۸) و منجر به تشکیل کریستال‌های کوچک سفید و مات کلرال هیدرات می‌گردد . با این حال، نمونه‌ها می‌توانند از لام‌های کریستالیزه بازیابی شوند زیرا کوتیکول از نظر شیمیایی سالم باقی می‌ماند، هرچند برخی آسیب‌های فیزیکی ممکن است از رشد کریستال‌ها رخ دهد .در برخی موارد، لام‌های کریستالیزه می‌توانند با آب‌دهی مجدد محیط مونتاژ در محیط مرطوب و گرم همراه با تیمول برای جلوگیری از رشد قارچ بازسازی شوند .به‌طور جایگزین، نمونه‌ها می‌توانند از کلرال گام در آب خارج شده، در اسید استیک یخچالی دهیدراته و در کانادا بالزام مجددا مونتاژ شوند.

جدول 2. ترکیب مواد شیمیایی مورد استفاده برای مونتاژ

اسید فوکسین ٪۱ در آب مقطر	هیدروکسید پتاسیوم 10%
اسید فوکسین بصورت پودر 1 گرم<br>آب مقطر 99 میلی لیتر	هیدروکسید پتاسیوم 10 گرمی<br>آب مقطر 100 میلی لیتر
محلول Marc-André رنگ آمیزی شده با اسید فوکسین	محیط مونتاژ گام کلرال (محیط هویرHoyer medium)
محلولMarc-André 10 میلی لیتر<br>اسید فوکسین 1% 50 میکرولیتر	آب مقطر 50 میلی لیتر<br>هیدرات کلرال 200 گرم<br>صمغ عربي 50 گرم<br>گلیسرول 20 میلی لیتر
محیط إينيسي Enecê	محلول Marc-André
کولوفون خالص سفید 22 گرم<br>صمغ کوبال محلول در الکل 12 گرم<br>اتانول خالص 20 میلی لیتر<br>کامفور 10 گرم<br>اسانس تربانتین 10 میلی لیتر<br>أوکالیپتول 26 میلی لیتر	هیدرات کلرال 40 گرم<br>اسید استیک یخزده 30 میلی لیتر<br>آب مقطر 30 میلی لیتر

***DMHF*ضریب انکسار= 1.48**. این محیط مبتنی بر آب [72] از نظر نوری عملکرد بسیار خوبی دارد، مشابهBerlese ، و به همان اندازه آسان برای استفاده است .با این حال، برخلافBerlese ، سیاه نمی‌شود و کریستالیزه نمی‌گردد .این محیط برای پشه‌های خاکی و دیگر خانوادهٔ Psychodidae مناسب است.

***CMCP*ضریب انکسار = 1.41** این محیط مونتاژ مبتنی بر گلیسیرین و محلول در آب است که برای ایجاد لام‌های شفاف و دائم از نمونه‌های ظریف، شامل پشه‌های خاکی استفاده می‌شود .مزیت این محیط این است که نمونه‌ها می‌توانند مستقیماً از آب یا اتانول مونتاژ شوند .این محیط به سرعت نمونه را آرام و شفاف می‌کند، کوتیکول را نرم کرده و اجازه می‌دهد نمونه به‌درستی موقعیت‌دهی شود، که به‌ویژه برای باز کردن بال‌ها یا تشریح اندام‌های تناسلی مفید است .اگرچه گزارش شده که امکان نگهداری طولانی‌مدت را فراهم می‌کند، مدت دقیق حفظ نمونه هنوز نامعلوم است .محدودیت اصلی این محیط مونتاژ، ترکیب آن است که شامل فنول، یک مادهٔ سمی و تحریک‌کننده است و نیاز به دقت در استفاده دارد.

محیط‌های دائمی

***کانادا بالزام*ضریب انکسار = 1.52–1.54** . کانادا بالزام برای اولین بار توسط اندرو پریچارد در دههٔ ۱۸۳۰ به‌عنوان یک محیط مناسب برای مونتاژ نمونه‌ها در میکروسکوپ نور عبوری توصیف شد .این ماده همچنان یکی از پرکاربردترین محیط‌هاست، زیرا کیفیت آرشیوی اثبات‌شده‌ای دارد و بیش از ۱۵۰ سال مورد استفادهٔ موفق قرار گرفته است .برخلاف محیط‌های مبتنی بر محلول هویر، کانادا بالزام کریستالیزه نمی‌شود و رطوبت را جذب نمی‌کند .با این حال، کانادا بالزام خودفلورسانس قوی دارد که گاهی ممکن است برای برخی تکنیک‌های میکروسکوپی یک عیب باشد .[60] استفاده از حلال‌های غیرسمی به جای زایلن می‌تواند خطرات ایمنی را هنگام آماده‌سازی کاهش دهد، اما ممکن است معایبی مانند خشک شدن کندتر و تیرگی زودهنگام محیط ایجاد کند.

***یوپارال Eupara®l* ضریب انکسار = 1.48**، یوپارال یک جایگزین پرکاربرد برای کانادا بالزام در مونتاژ دائم است و پایداری طولانی‌مدت و ضریب شکست مشابه ارائه می‌دهد .ویژگی‌های یوپارال شامل موارد زیر است:

1. نیاز به دهیدراسیون :قبل از انتقال نهایی محیط مونتاژ، نمونه باید دهیدراسیون شود، معمولاً با گذر از ۹۵٪ اتانول به اتانول مطلق .
2. زمان پردازش طولانی :مونتاژ نهایی در رزین، چه کانادا بالزام باشد و چه یوپارال، نیازمند دهیدراسیون است که زمان پردازش کل نمونه را افزایش می‌دهد .هنگامی که دهیدراسیون با حلال‌های آلی ممکن نباشد، نمونه‌های استخراج‌شده از اتانول مطلق می‌توانند در محلول واسطه‌ای شامل مخلوط مساوی یوپارال و عصارهٔ یوپارال قرار گیرند قبل از مونتاژ نهایی .

***Enecê*ضریب انکسار 1.467** اینسه یک محیط مونتاژ مبتنی بر رزین است که عمدتاً برای حشرات کوچک استفاده می‌شود و به‌ویژه در برزیل محبوب است .پایهٔ آن شامل کلوفونی و صمغ کوپال حل شده در الکل، کافور، عصارهٔ تربانتین و اوکالیپتول است .سرکویرا [11] اینسهٔ را به‌عنوان جایگزینی برای کانادا بالزام برای مونتاژ دائم لاروها، پوست‌اندازی مراحل نابالغ و حتی پشه‌های بالغ توصیف کرده و از آن زمان، این محیط به‌طور گسترده برای مونتاژ پشه‌های خاکی پذیرفته شده است .اینسهٔ یک گزینهٔ اقتصادی برای مونتاژ دائم ارائه می‌دهد، با پایداری طولانی‌مدت و زمان خشک شدن کافی، که امکان تشریح و چینش دقیق ساختارهای مورفولوژیک را فراهم می‌کند.

5.4 آماده‌سازی لام و خشک کردن

خشک کردن صحیح لام‌های مونت‌شده برای اطمینان از پایداری و حفظ طولانی‌مدت نمونه‌ها حیاتی است .لام‌ها باید به‌طور کامل خشک شوند قبل از آنکه برای نگهداری طولانی‌مدت در نظر گرفته شوند .برای دستیابی به نتایج بهینه، لام‌هایی که با محیط مونتاژ دائم آماده شده‌اند باید به‌صورت افقی به مدت ۲–۳ هفته خشک شوند، در حالی که لام‌هایی که با محیط نیمه‌دائم آماده شده‌اند ممکن است تنها به ۱–۲ هفته نیاز داشته باشند.
برای اطمینان از یک فرآیند خشک شدن مؤثر، توصیه می‌شود از انکوباتوری با دمای مناسب برای محیط مونتاژ استفاده شود و از گرمای بیش از حد که ممکن است به نمونه‌ها آسیب برساند، اجتناب گردد .محدودهٔ دمایی ۳۰ تا ۳۷ درجه سانتی‌گراد توصیه می‌شود .این مرحلهٔ خشک کردن برای جلوگیری از تاب خوردن لام، تخریب نمونه، یا ناپایداری محیط مونتاژ در طول نگهداری بسیار مهم است.
محیط مونتاژ استفاده‌شده در آماده‌سازی لام باید همیشه روی برچسب لام یادداشت شود .در صورت امکان، برچسب باید شامل دستورالعمل دقیق تهیه، نام فرد آماده‌کننده (preparator)و تاریخ آماده‌سازی نیز باشد .لام‌ها در ابتدا به‌عنوان مونت موقت آماده می‌شوند و برای نگهداری طولانی‌مدت در نظر گرفته نشده‌اند .با این حال، اگر وضعیت نمونه تغییر کند، مانند زمانی که به عنوان بخشی از مجموعهٔ »تیپ «تعیین شود، باید از محیط مونتاژ دائم‌تر استفاده شود تا حفظ نمونه برای مطالعات تاکسونومیک آینده تضمین گردد.

5.5 تکنیک‌های جایگزین مونتاژ :مونتاژ روی کارت

مونتاژ روی کارت یک تکنیک است که برای چندین گروه حشرات به‌کار می‌رود، که در آن نمونه‌ها یا به‌طور مستقیم روی کارت‌های حشره‌شناسی پین می‌شوند یا روی سطح چسبانده می‌شوند .با توجه به اندازهٔ کوچک آن‌ها و نیاز به مشاهدهٔ اندام‌های داخلی برای شناسایی از طریق شفاف‌سازی(به بند ۵ مراجعه کنید)، این روش اصلاً برای مونتاژ پشه‌های خاکی مناسب نیست.

5.6 مونتاژ مجدد نمونه‌های آسیب‌دیده

برای نمونه‌های نادر یا ارزشمند، یک رویکرد دو مرحله‌ای توصیه می‌شود، مطابق با ویدئوی قابل دسترس در لینک Zenodo.

1. ابتدا بدون جدا کردن اجزا، نمونه‌ها را آب‌دهی مجدد (rehydrate) کنید تا امکان مشاهدهٔ اولیه فراهم شود .یک نگهدارنده برای چند لام میکروسکوپ باید درون یک پتری‌دیش قرار داده شود تا به‌عنوان پایه عمل کند .سپس لامی که باید آب‌دهی مجدد شود روی آن قرار داده می‌شود و پتری‌دیش با چند میلی‌متر حلال پر می‌شود تا یک محفظهٔ مرطوب ایجاد شود، به‌طوری‌که خود لام با حلال تماس نداشته باشد .زمان موردنیاز برای آب‌دهی مجدد بسته به وضعیت نمونه می‌تواند از یک تا چند روز متغیر باشد .پایش روزانه و صبر بسیار مهم است .هنگامی که لام به‌طور کافی آب‌دهی مجدد شد، می‌توان آن را از محفظهٔ مرطوب خارج کرده و برای چند ساعت در انکوباتور قرار داد، سپس بررسی میکروسکوپی، عکاسی یا رسم انجام شود.
2. برای مونتاژ مجدد، لام می‌تواند برای چند ساعت دیگر یا یک شب دوباره به محفظهٔ مرطوب بازگردانده شود .جدا کردن اجزا باید زیر میکروسکوپ دوچشمی انجام شود .با استفاده از سوزن‌های ظریف، لامل باید با دقت برداشته شود و اطمینان حاصل شود که هیچ بخش از پشهٔ خاکی به لامل نچسبیده باشد . سپس اجزای تشریح‌شدهٔ پشهٔ خاکی باید جمع‌آوری شده و در چاهک‌های کوچک با آب شسته شوند، مشابه چاهک‌هایی که برای استخراج تخریبی DNA/RNAاستفاده می‌شوند، و سپس قبل از دهیدراسیون و مونتاژ مجدد در محیط رزینی آماده شوند.

هنگام باز کردن یک لام، بسیار مهم است که محیط مونتاژ اولیه شناسایی شود

تا حلال مناسب انتخاب گردد .برای محیطهای مونتاژ آبی، باید از آب استفاده شود .اگر محیط مونتاژ بر پایهٔ رزین باشد (مانند Canada balsam یا Euparal)، باید از زایلن استفاده شود، آن هم زیر هود شیمیایی و با تجهیزات حفاظت فردی مناسب، از جمله ماسک.
مونتاژ مجدد نمونه‌های تیپ یا نمونه‌های مجموعه‌ای فقط باید با رضایت مسئول مجموعه (کیوریتور/یا مؤسسهٔ مالک نمونه) انجام شود.

6 شناسایی نمونه

1.6 مرفولوژی

شناسایی پشه‌های خاکی عمدتاً بر بررسی ویژگی‌های مورفولوژیک آن‌ها متکی است، از جمله شکل قفسهٔ سینه، بال‌ها، اندام‌های تناسلی، ستاها(موها)، و روابط مورفومتریک خاص میان ساختارهای مختلف .پژوهشگران از کلیدهای رده‌بندی، مجموعه‌های مرجع، و توصیف‌های اصلی گونه‌ها برای مقایسهٔ نمونه‌های جمع‌آوری‌شده با تاکسون‌های شناخته‌شده استفاده می‌کنند . ویژگی‌های تشخیصی کلیدی، مانند رگ‌بندی بال و مورفولوژی سر در هر دو جنس، ساختار اندام تناسلی نر، و ساختار اسپرمتکا در ماده‌ها، به‌ویژه برای

جدول 3. ترکیب محیطهای مورد استفاده برای مونتاژ.

محیط مونتاژ	حلال	پلیمر یا پیش‌پلیمر(های) بالقوه	توضیحات
Hoyer fluid	گلیسرول، آب<br>آب (CMCP-9 : 51–60٪)	ترکیبات صمغ عربی<br>پلی‌وینیل الکل کاملاً هیدرولیزشده (CMCP-9 : 0–5٪)	عامل نرم‌کننده (ماسه‌کننده):<br>کلرال هیدرات: CMC(P)-9:<br>ویسکوزیتهٔ پایین؛ ویسکوزیتهٔ بالا
DMHF	آب	ان-ان′-دی‌متیلول دی‌متیل هیدانتوئین دی‌متیلول(DMH)	
Canada balsam	زایلین؛ اجزای نسبتاً فرار بالزام (دلتا-3-کارن، لووپیرامیک اسید، لیمونین، میرسین، بالوستریك اسید، بتا-فیلاندرین، ألفا-پینین، بتا-پینین)	الیگومرهای متصل‌شده با اتِر یا متیلن؛ شبکهٔ پلیمری متقاطع–DMH فرمالدهید، بالزام (ابی‌نول، اسید آبیتیک، اسید ایزوپیماریک، اسید سنداراکوپیماریک)	کربنات پتاسیم؛ رزین درخت *Abies balsamea* (Linné, 1758)
Euparal®	اوکالیپتول، بارالدهید؛ اجزای نسبتاً فرار صمغ سنداراک (لیمونین، ألفا-پینین، بیتا-پینین)	ترکیبات صمغ سنداراک (اسید کومونیک، مانول، پلی‌کومونیک اسید، اسید سنداراکوپیماریک، ۱۲-استوکسی-سنداراکوپیماریک اسید، سوگیول، اسید تورولوزیک، تورولوزول، توتارول)	عامل پاک کننده: متیل سالیسیلات رنگ شده در Eupara سبز؛ نمک مسی (أبییتینات مسی)؛ رزین سندراك درخت *Tetraclinis articulata* (Vahl, 1791)
Enecê	اتیل الکل، با کافور، اوکالیپتول و عصاره ترپنین	ترکیبات صمغ کوپال و کولوفون (رزین)	

جدول 4. مزایا و معایب محیطهای مورد استفاده برای مونتاژ لام‌های میکروسکوپی و مشاهدات منتشرنشده توسط افراد مختلف [52].

نام	مزایا	معایب
Canada balsam	- این ماده بسیار بادوام است و طول عمر آن بیش از ۱۵۰ سال است<br>- اسلایدها را می‌توان با استفاده از روغن میخک یا فنول مونتاژ کرد.	- حاوی اجزای مضر است و باید زیر هود حمل شود.<br>- نیاز به یک سری کامل و زمان‌بر آبگیری دارد .آبگیری اتانول و انتقال از طریق زایلن یا روغن میخک می‌تواند برخی از گونه‌ها را شکننده کند؛ جایگزین‌ها) مثلاً ایزوپروپانول، n-بوتانول، Cellosolve™، ۱،۴-دیوکسان، Histoclear، (terpineolممکن است آسیب را کاهش دهند.<br>- اگر زایلن با فنول جایگزین شود یا اگر هیدروکسید پتاسیم باقی بماند، نمونه‌ها ممکن است سیاه شوند.<br>- ضریب شکست بالا می‌تواند ساختارهای رنگ‌آمیزی نشده را مبهم کند.<br>- خشک کردن کامل بدون دستگاه خشک کن می‌تواند سال‌ها طول بکشد.<br>- رنگ آن به مرور زمان زرد و تیره می‌شود، به خصوص وقتی که با روغن میخک پاک شود .<br>- برخی از لکه‌ها ضعیف می‌شوند و رنگ‌های کاتیونی ممکن است در صورت اسیدی شدن محیط - که می‌تواند به مرور زمان خود به خود رخ دهد - محو شوند.
DMHF	- شفافیت بالا<br>- ضریب شکست خوب<br>- قابلیت مشاهده عالی<br>- ساختارها پایداری نسبتاً خوب<br>- آماده‌سازی‌ها سازگار با بسیاری از تکنیک‌های رنگ‌آمیزی<br>- چسبندگی خوب بین اسلاید و لامل محافظت خوب از نمونه‌ها.	- احتمال زرد شدن به مرور زمان<br>- ممکن است برخی رنگها را تغییر دهد<br>- برای رنگ های حساس به فرمالدئید مناسب نیست<br>- حباب هوا، زمان خشک شدن آهسته<br>- محیط نصب حساس به رطوبت<br>- برگرداندن مونتاژ دشوار است<br>- فرمالدئید سمی، محرک و سرطان‌زا است
Euparal	- محیط کشت بادوام با طول عمر بیش از ۵۰ سال.<br>- مونتاژ مستقیم با اتانول ۸۰٪ امکان‌پذیر است<br>- ساختارهای رنگ نشده را نمی‌پوشاند و با گذشت زمان زرد یا شکننده نمی‌شود.<br>- ضریب شکست مناسب‌تری نسبت به کانادا بالزام برای دوبالان دارد<br>- به دلیل حداقل انقباض و خشک شدن بدون حباب، برای نمونه‌های ضخیم‌تر به خوبی کار می‌کند .<br>- در اتانول ۹۵٪ محلول باقی می‌ماند و امکان مونتاژ مجدد را حتی پس از سال‌ها فراهم می‌کند..	- حاوی اجزای مضر است و باید زیر هود حمل شود<br>- آب‌زدایی اتانول و انتقال از طریق Euparal Essence می‌تواند برخی از گونه‌ها را شکننده کند، اما استفاده از ایزوپروپانول ممکن است این مشکل را کاهش دهد.
Hoyer fluid	- نمونه‌ها را می‌توان به صورت زنده یا مستقیماً از آب، اتانول یا فرمالدئید مونتاژ کرد.<br>- خیساندن منجر به بهبود کیفیت کوتیکول می شود<br>- ضریب شکست مطلوبی دارد و می‌توان آن را با رنگ‌آمیزی ید برای کنتراست بالاتر بهبود بخشید.<br>- اسید استیک موجود در فرمول می‌تواند زائده‌های بندپایان را گسترش دهد.<br>- برخی از نمونه‌ها ممکن است برای ۴۰ تا ۶۰ سال پایدار بمانند. محلول در آب، امکان مونتاژ مجدد آسان را فراهم می‌کند.	- نمونه‌های ظریف ممکن است از بین بروند، مگر اینکه محیط کشت به تدریج اضافه شود، که این کار زمان‌بر است<br>- حفره‌ها و کریستال‌ها ممکن است در کمتر از 10 سال تشکیل شوند.<br>- بسته به غلظت هیدرات کلرال و مدت زمان قرار گرفتن در معرض آن، خیساندن می‌تواند بیش از حد شود.<br>- اجزای محیط کشت ممکن است از هم جدا شوند و دانه‌بندی ریز می‌تواند ظرف ماه‌ها یا سال‌ها ظاهر شود.<br>- برخی موارد سیاه شدن محیط گزارش شده است.

(ادامه در صفحه بعد)

نام	مزایا	معایب
CMCP	نمونه‌ها را می‌توان مستقیماً از محیطهایی مانند آب، اتانول، گلیسرول یا محلول‌های حاوی فرمالدئید مانت کرد و در صورت لزوم، اندام‌های داخلی آنها را می‌توان برای تسهیل بررسی یا آماده‌سازی عمومی، خیساند.	- این محیط می‌تواند به مرور زمان کریستال تشکیل دهد و تیره شود، و گاهی اوقات می‌تواند نمونه‌ها را بیش از حد مورد نظر خیس کند .مگر اینکه اسلاید با دقت حلقه‌گذاری شود، نمونه‌های ضخیم‌تر در آن خوب عمل نمی‌کنند زیرا می‌توانند کوچک شوند و شکاف‌هایی در اطراف لبه‌های لامل ایجاد کنند .<br>- این محیط برای نمونه‌های رنگ‌آمیزی شده یا مواد کلسیفیه شده مناسب نیست و زمان خشک شدن آن کندتر از CMC است.
Eukitt™	- محیطی بادوام با ماندگاری بیش از 30 سال .<br>- سازگار با بسیاری از حلال‌ها برای نصب، از جمله استون، بنزن، کلروفرم، دی‌اکسان، اتر، ایزوپروپانول، متیل بنزوات، ترپینئول، تولوئن و زایلن.<br>- سریع خشک می‌شود و pH کمی اسیدی دارد .با گذشت زمان به طور قابل توجهی تیره نمی‌شود<br>- مناسب برای رنگ‌آمیزی‌های مختلف (مانند فوشین، هماتوکسیلین، متیل گرین، متیل ویولت، متیلن بلو) .<br>- نمونه‌ها را می‌توان پس از سال‌ها با خیساندن در زایلن برای مدت طولانی، دوباره مونتاژ کرد.	- حاوی اجزای مضر است و باید زیر هود حمل شود .<br>- نیاز به یک سری کامل و زمان‌بر آبگیری دارد .<br>- به دلیل انقباض و تشکیل حباب گاز، برای نمونه‌های ضخیم‌تر ایده‌آل نیست<br>- روکش‌ها ممکن است به مرور زمان جدا شوند، مگر اینکه شیشه به خوبی تمیز و آب‌بندی شود.
Enecê	- وسط عالي المتانة يدوم 50سنة .على الأقل<br>- لا يغمق بمرور الوقت.<br>- أكثر ليونة، مما يسمح بتشريح الحشرات داخل الوسط، ويوفر وقتًا مناسبًا لوضع التراكيب المورفولوجية<br>- منخفض التكلفة.	- نیاز به یک سری کامل و وقت‌گیر برای آبگیری دارد.<br>آب‌زدایی اتانول و انتقال از طریق روغن میخک می‌تواند برخی از نمونه‌ها را شکننده کند .<br>شفاف‌سازی حشره همچنان ادامه دارد، هرچند بسیار کند؛ این امر می‌تواند دیدن ساختارهای بسیار کوچک مانند سنسیلا، آسکوئیدها و خارهای ساده را دشوار کند.

تعیین گونه بسیار اطلاعاتی و مهم هستند .شناسایی دقیق اغلب به بررسی میکروسکوپی دقیق نیاز دارد، که معمولاً با استفاده از میکروسکوپ نوری مرکب برای مشاهدهٔ ساختارهای ظریف مانند اندام تناسلی و اسپرماتکها، یا استریومیکروسکوپ برای ویژگی‌های مورفولوژیک کلی‌تر انجام می‌شود.

پیشرفت‌های اخیر در فناوری تصویربرداری، استفاده از تصویربرداری دیجیتال را برای شناسایی پشه‌های خاکی تسهیل کرده است .عکس‌های با وضوح بالا یا تصاویر دیجیتال از ویژگی‌های کلیدی می‌توانند با مواد مرجع مقایسه شوند یا با استفاده از سیستم‌های شناسایی مبتنی بر رایانه تحلیل شوند، که این امر دقت و دسترسی‌پذیری در رده‌بندی مورفولوژیک را بهبود می‌بخشد

6.2 هندسه بال

هندسهٔ بال یک ویژگی کلیدی است که در شناسایی و طبقه‌بندی گونه‌های مختلف پشهٔ خاکی استفاده می‌شود .بال‌های پشه‌های خاکی الگو و ساختار منحصربه‌فردی نشان می‌دهند و معمولاً بلند و باریک بوده و دارای رگ‌بندی (وناسیون) به‌خوبی توسعه‌یافته هستند (اشکال ۹ و ۱۰) ، آرایش رگ‌ها یک الگوی مشخص ایجاد می‌کند که می‌تواند در میان جنس‌ها و گونه‌ها متفاوت باشد و ویژگی‌های تشخیصی ارزشمندی برای شناسایی فراهم کند .در نتیجه، مطالعهٔ هندسهٔ بال بینش‌های ارزشمندی برای اهداف رده‌بندی (تاکسونومیک) ارائه می‌دهد

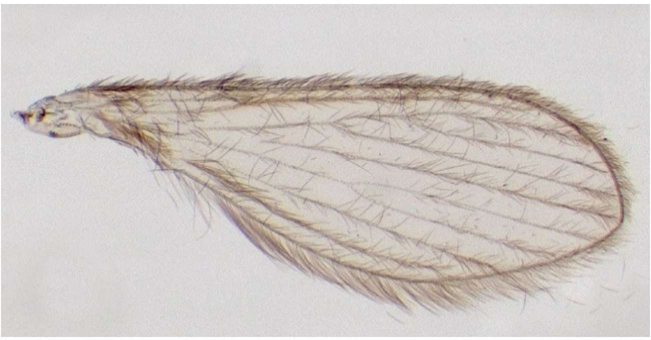

تصویر 9. تصویر بال *Trichophoromyia ininii*.

6.3 مرفومتریک هندسی بال

پژوهشگران از تکنیک‌های مختلفی مانند مورفومتریک هندسی برای تحلیل و مقایسهٔ شکل و اندازهٔ بال در میان گونه‌ها یا جمعیت‌های مختلف پشهٔ خاکی استفاده می‌کنند .مطالعهٔ هندسهٔ بال اطلاعات ارزشمندی دربارهٔ رفتار، ترجیحات زیستگاهی و توانایی‌های پروازی فراهم می‌کند.

در رویکرد مورفومتریک هندسی، بال‌ها با دقت تشریح شده، در صورت نیاز رنگ‌آمیزی شده و به‌صورت صاف روی لام‌ها مونت می‌شوند .سپس از لام‌های آماده‌شده زیر استریومیکروسکوپ عکس‌برداری می‌شود، تصاویر دیجیتالی شده و تحت آنالیز مورفومتریک قرار می‌گیرند .این روش در منابع علمی به‌خوبی توصیف شده است [27، 42، 56، 57 ، 59] و توصیه می‌شود برای اندام‌های جفت، همیشه از بال راست یا چپ به‌طور ثابت استفاده شود تا از اثرات آلومتریک منفی احتمالی جلوگیری شود.[62]

آماده‌سازی بال برای آنالیز مورفومتریک هندسی

برای مشاهدهٔ بهینهٔ رگبال‌ها(wing veins) ، بال‌ها باید از پولک‌ها تمیز شده و به‌طور مناسب رنگ‌آمیزی شوند .برای آماده‌سازی بال، ابتدا چاهک‌های کوچک را با معرف‌های موردنیاز (متیلن بلو، اتانول، آب و جایگزین زایلن) پر کنید .یک بال نگهداری‌شده در اتانول ۷۰٪ در دمای اتاق را با وارونه کردن لولهٔ اپندورف و خالی کردن آن روی چاهک خارج کنید، سپس بال را به‌صورت طولی با استفاده از یک سوزن خمیدهٔ ظریف بردارید . بال را به‌طور کوتاه از اتانول به آب و دوباره به اتانول منتقل کنید تا موهای ریز (بریستل‌ها) حذف شوند .بال را به مدت ۶ دقیقه در متیلن بلو قرار دهید و اطمینان حاصل کنید که در حین رنگ‌آمیزی روی سطح شناور بماند .بال را با دقت برداشته و به مدت ۲ دقیقه در جایگزین زایلن غوطه‌ور کنید(تقریباً یک‌سوم زمان متیلن بلو). ضربه‌های آرام سوزن به دیوارهٔ چاهک می‌تواند به نشستن بال کمک کند؛ زایلن برای تثبیت رنگ عمل می‌کند .در نهایت، بال را برداشته و روی یک قطرهٔ کوچک Euparal روی لام میکروسکوپ قرار دهید .زیر یک عدسی بزرگ‌نما، بال را به‌آرامی باز کرده و به‌دقت لامل را قرار دهید .باید بلافاصله قبل از سفت شدن Euparal از بال عکس‌برداری شود، زیرا ممکن است تنظیمات جزئی موقعیت بال زیر لامل برای دستیابی به هم‌ترازی بهینه لازم باشد.

6.4 روشهای ملکولی

علاوه بر تکنیک‌های مورفولوژیک، روش‌های مولکولی به‌طور فزاینده‌ای در پژوهش‌های حشره‌شناسی ضروری شده‌اند، از جمله در مطالعات رده‌بندی، ژنتیک جمعیت، و فیلوژنتیک، همچنین برای شناسایی پاتوژن‌های DNA/RNA و تعیین منشأ وعدهٔ خونی، که رفتار ناقل در حوزهٔ اپیدمیولوژی اهمیت دارد .[70]توالی‌یابی DNAمی‌تواند برای تأیید گونه یا تمایز گونه‌های نزدیک به هم به‌کار رود و روشی دقیق‌تر و قابل‌اعتمادتر برای شناسایی فراهم کند .علاوه بر این، تکنیک‌های مولکولی پیشرفته (مانند PCR، توالی‌یابی DNA، NGSو غیره) و MALDI-ToF MSبرای شناسایی سریع و دقیق گونه‌ها اهمیت فزاینده‌ای یافته‌اند و روش‌های سنتی مورفولوژیک را تکمیل می‌کنند .[46]با وجود این پیشرفت‌ها، شناسایی مورفولوژیک همچنان به‌عنوان استاندارد مرجع در رده‌بندی باقی مانده و مبنایی است که داده‌های مولکولی بر اساس آن تفسیر می‌شوند.

6.4.1 استخراج تخریبی DNA

استخراج اسید نوکلئیک یک مرحلهٔ معمول در بسیاری از مطالعات زیستی است، و روش‌های گوناگونی برای جداسازی DNA از مواد زیستی توسعه یافته‌اند .[48] بسیاری از کیت‌های استخراج DNA که به‌صورت تجاری در دسترس هستند برای تسهیل این فرایند طراحی شده‌اند .[14] با این حال، روش‌هایی که معمولاً برای آماده‌سازی نمونه‌های بندپایان به‌منظور شناسایی مورفولوژیک استفاده می‌شوند، اغلب مانع تحلیل DNA می‌شوند، زیرا این تکنیک‌ها ممکن است ویژگی‌های فیزیکی حیاتی نمونه را آسیب زده یا تخریب کنند .[10] بیشتر پروتکل‌های استخراج DNA از بافت‌های حشرات ماهیتی تخریبی دارند[43] ، که این موضوع به‌ویژه برای نمونه‌های کوچک نگرانی‌برانگیز است، جایی که حتی نمونه‌برداری محدود نیز ممکن است ویژگی‌های مورفولوژیک مهم را به خطر بیندازد .[72] نوع و وضعیت نمونه نقش کلیدی در انتخاب یک روش مناسب برای جداسازی DNA ایفا می‌کند.[29]

نیاز به شناسایی دقیق پشه‌های خاکی، درک پویایی جمعیت، و به حداقل رساندن اثرات بر گونه‌های غیرهدف، توسعهٔ ابزارهای تشخیصی مولکولی را هدایت کرده است .[23] رویکردهای مولکولی اکنون به‌طور مکرر برای تکمیل روش‌های رده‌بندی مورفولوژیک در شناسایی پشه‌های خاکی استفاده می‌شوند .برای مثال، رویکرد استاندارد در بارکدگذاری حشرات شامل استخراجDNA ، تعیین توالی، و از بین رفتن نمونهٔ اصلی است .بنابراین، نیاز فوری به بررسی روش‌های غیرتخریبی استخراج DNA وجود دارد که هم مادهٔ زیستی و هم یکپارچگی مورفولوژیک آن را حفظ کنند.

روش‌های متعددی برای استخراج اسید نوکلئیک از پشه‌های خاکی به‌کار گرفته شده‌اند .مقدار یا کیفیت اسیدهای نوکلئیک موردنیاز به نوع تحلیل مولکولی پایین‌دستی بستگی دارد، زیرا تکنیک‌های مختلف دارای حساسیت و الزامات خلوص متفاوتی هستند .[9] برای مثال، مشخص شده است که چشم‌های پشهٔ خاکی می‌توانند تکثیر PCR را مهار کنند .[69] فراتر از غربالگری پاتوژن، DNA پشهٔ خاکی به‌طور معمول برای اهداف شناسایی گونه استخراج می‌شود .روش‌های استخراج مختلفی قابل استفاده هستند، اگرچه بازده و کیفیت در میان تکنیک‌ها متفاوت است .برخی از پروتکل‌های شرکت‌های سازنده توسط پژوهشگران برای پشه‌های خاکی تطبیق داده شده‌اند [8]، که باعث افزایش بازده و/یا کیفیت اسیدهای نوکلئیک استخراج‌شده شده است[8 ، 9، 69] ، در حالی که سایر روش‌های اصلاح‌شده که برای دیگر رده‌های بندپایان توسعه یافته‌اند نیز می‌توانند برای پشه‌های خاکی مورد استفاده قرار گیرند. واکنش‌های PCR شناسایی که قطعات کوچک میتوکندریایی (مانند COI یا CytB) را هدف قرار می‌دهند، معمولاً با روش‌های استخراجی که باعث قطعه‌قطعه شدن زیاد DNA می‌شوند سازگار هستند .در مقابل، سایر تکنیک‌های توالی‌یابی نسل جدید با خوانش بلند (NGS) مانند Oxford Nanoporeو PacBio به حداقل میزان قطعه‌قطعه شدن و DNA با کیفیت بالا نیاز دارند .استخراج‌های مبتنی بر ستون چرخشی (spin column) معمولاً قطعات DNA ژنومی تا حدود ۶۰ کیلوباز تولید می‌کنند، در حالی که استخراج فنل-کلروفرم می‌تواند قطعاتی تا حدود ۱۵۰ کیلوباز ایجاد کند .[77] جدول ۵ تکنیک‌های مختلف استخراج DNA پشهٔ خاکی را خلاصه می‌کند و نشان می‌دهد که آیا تطبیق‌های روش‌شناختی برای این حشرات انجام شده است یا خیر .بازده‌ها نشان داده نشده‌اند، زیرا به اندازهٔ نمونه و روش آماده‌سازی بستگی دارند .ستون «اصلاح » به تطبیق پروتکل‌های استخراج برای پشه‌های خاکی یا سایر بندپایان کوچک اشاره دارد.

انتخاب روش استخراج باید چندین معیار را در نظر بگیرد، مانند تعداد نمونه‌ها، زمان استخراج، و تکنیک مورد استفاده در مراحل پایین‌دستی .در حالی که تکنیک‌های NGS به DNA ژنومی با وزن مولکولی بالا نیاز دارند، تمامی روش‌های ارائه‌شده در اینجا می‌توانند برای کاربردهای استاندارد مبتنی بر PCR استفاده شوند .علاوه بر این، چندین مطالعه روش‌های استخراج DNAغیرتخریبی را برای بندپایان کوچک خشکی‌زی، نمونه‌های موزه‌ای خشک‌شده، و بندپایان با بدن نرم بررسی کرده‌اند[19 ،26 ،28 ،55 ،63] .

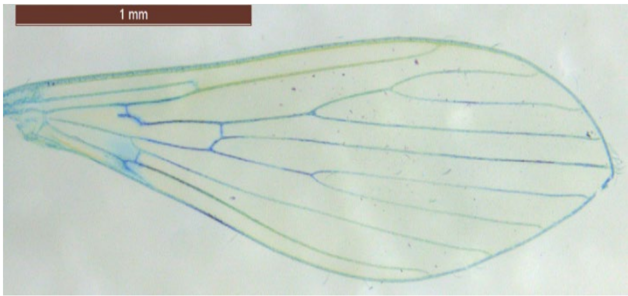

تصویر 10. بال رنگ آمیزی شده *Phlebotomus ariasi*.

جدول 5. هزینه متوسط، کاربرد و تطبیق پروتکل برای استخراج DNA ژنومی (gDNA) پشه خاکیها.

روش مورد استفاده	هزینه	کاربرد	سازگاری روش برای بندپایان کوچک
Spin column	2.5 – 3.55 US$ [39]	PCR, NGS	[9]
Phenol-chloroform	0.24 US$ [69]	PCR, NGS	[9]
HotSHOT	<0.01 US$ [69]	PCR	–
Salting out	0.12 US$ [69]	PCR	–
Chelex	0.02 US$ [41]	PCR	[41, 76]

6.4.2 استخراج غیرتخریبی

یکی از چالش‌های اصلی در آنالیزهای مولکولی بندپایان، به‌ویژه پشه خاکیها، حفظ نمونه‌ها برای ادغام در مجموعه‌های حشرشناسی است .اغلب پروتکل‌های استخراج نیازمند ماکره‌کردن (خردسازی) بافت هستند و در نتیجه، حفظ نمونه اولیه را مختل می‌کنند .با این حال، روش‌های استخراج اسیدهای نوکلئیک به‌صورت غیرتخریبی به‌گونه‌ای طراحی شده‌اند که ماده ژنتیکی را بدون آسیب فیزیکی به نمونه استخراج کنند، بدون آن‌که به قابلیت استفاده یا ویژگی‌های ریخت‌شناسی آن لطمه وارد شود.

این روش‌ها به‌ویژه در کار با نمونه‌های ارزشمند یا محدود، مانند پشه خاکیها، اهمیت دارند؛ جایی که حفظ یکپارچگی ساختاری برای مطالعات آینده در زمینه‌های رده‌بندی، مورفولوژی یا تشخیص ضروری است .یکی از تکنیک‌های رایج، روش حمام غیرتخریبی است که در آن پشه خاکی بی‌حرکت شده و به‌آرامی در بافر لیز حاوی پروتئیناز غوطه‌ور می‌شوند.

تکنیک mild-vectolysis نیز با موفقیت برای پشه خاکی ها، به‌ویژه نمونه‌های تیپ، به‌کار رفته است .این روش از یک کیت استاندارد ستون چرخشی (در اینجا، کیت DNeasy Blood and Tissue از شرکت QIAGEN، آلمان) با اصلاحاتی برای استخراج DNA بدون تخریب نمونه استفاده می‌کند .مراحل لیز اصلاح‌شده (از جمله تنظیم حجم بافر لیز و افزودن یک مرحله انجماد) امکان آزادسازی اسیدهای نوکلئیک را فراهم کرده و در عین حال آسیب ریخت‌شناسی را به حداقل می‌رساند.
در مورد پشه خاکی ها، استفاده از کیت استخراج DNA HotSHOT نیز امکان‌پذیر است که روشی سریع و کم‌هزینه بوده و پردازش نمونه‌ها را با سرعت بالا و هزینه پایین فراهم می‌کند .نمونه‌های حشره‌ای که برای شناسایی مورفولوژیک در نظر گرفته شده‌اند، می‌توانند پس از استخراج شسته شوند .نمونه‌هایی که با کیت DNeasy پردازش شده‌اند باید با محلول مارک–آندره شفاف‌سازی شوند، در حالی‌که نمونه‌های پردازش‌شده با کیت HotSHOTبه‌حد کافی شفاف هستند تا مستقیماً در محیط آبی مونتاژ شوند، یا ترجیحاً پس از دهیدراتاسیون در محیط رزینی قرار گیرند، مطابق با پروتکل ارائه‌شده در این مقاله.
ماده ژنتیکی استخراج‌شده می‌تواند برای آنالیزهای بعدی مانند PCR جهت تکثیر نشانگرهای ژنتیکی خاص مورد استفاده قرار گیرد .روش‌های استخراج غیرتخریبی اسیدهای نوکلئیک برای مطالعه ویژگی‌های ژنتیکی

پشه خاکی ها، از جمله شناسایی عوامل بیماری‌زا، بسیار حیاتی هستند . با حفظ یکپارچگی نمونه، پژوهشگران می‌توانند اطلاعات ژنتیکی ارزشمندی به‌دست آورند، در حالی که نمونه برای تحلیل‌ها یا مطالعات تکمیلی آینده نیز حفظ می‌شود

MALDI-Tof MS 6.5

MALDI-Tof MS (یک تکنیک مبتنی بر طیف‌سنجی جرمی است که برای شناسایی و تحلیل پروفایل‌های پروتئینی منحصربه‌فرد (اثر انگشت) نمونه‌های زیستی طراحی شده است .این روش به‌طور فزاینده‌ای به‌عنوان ابزاری مهم برای شناسایی بندپایان دارای اهمیت پزشکی و دامپزشکی شناخته می‌شود.

این تکنیک در شناسایی مراحل مختلف رشدی پشه خاکیها، از جمله فرم‌های نابالغ و همچنین تعیین منبع خون در ماده‌های خون‌خورده، کارآمد بوده و در تفکیک گونه‌های نر و ماده تحت شرایط مختلف نگهداری و هموژنیزاسیون نیز موفق عمل کرده است .همچنین، این روش قدرت تفکیک بالایی در سطوح زیرجنس، گونه و جمعیت دارد.

MALDI-ToFامکان شناسایی سریع و دقیق گونه‌ها را فراهم می‌کند که برای درک پراکنش، رفتار و نقش پشه خاکیها در انتقال بیماری‌ها ضروری است .با تمایز گونه‌ها بر اساس پروفایل‌های پروتئینی، این روش نقش مهمی در مطالعات اپیدمیولوژیک و راهبردهای کنترل ناقلین ایفا می‌کند.

با این حال، دو محدودیت اصلی وجود دارد که کاربرد روتین این تکنیک را محدود می‌کند .نخست، دسترسی به تجهیزات طیف‌سنجی جرمی است که هزینه بالایی دارند و تهیه آن‌ها صرفاً برای شناسایی گونه‌های پشه خاکیها معمولاً مقرون‌به‌صرفه نیست .این محدودیت می‌تواند با استفاده از امکانات موجود در مراکز پروتئومیکس یا تشخیص بالینی تا حدی برطرف شود.

دومین محدودیت، کمبود داده‌های مرجع پشه خاکی ها در پایگاه‌های داده عمومی است که منجر به نیاز به ایجاد بانک اطلاعاتی داخلی از طیف‌های مرجع، بر اساس نمونه‌هایی با شناسایی قطعی(ترجیحاً با ترکیب بررسی مورفولوژیک و تعیین توالی ژنتیکی مانند COI یا CytB) می‌شود .انتظار می‌رود این مشکل با افزودن تدریجی داده‌ها به پایگاه‌های مرجع بین‌المللی برطرف شود.

در صورتی که پروفایل‌سازی پروتئینی با MALDI-ToFمدنظر باشد، نمونه‌ها باید ترجیحاً به‌صورت منجمد خشک یا در اتانول ۷۰٪ با گرید مولکولی نگهداری شوند و در معرض دمای محیط قرار نگیرند .در نبود دستورالعمل‌های استاندارد جهانی برای آماده‌سازی نمونه، توصیه می‌شود از محلول آبی شامل ۶۰٪ استونیتریل و ۰٫۳٪ تری‌فلورواستیک اسید (TFA) حاوی اسید سیناپینیک (۳۰ میلی‌گرم بر میلی‌لیتر) برای تهیه ماتریکس MALDI-ToFاستفاده شود تا طیف‌های پروتئینی حاصل با داده‌های منتشر شده قابل مقایسه باشند.

آماده سازی نمونه برای MALDI-Tof MS (تصویر 7)

نمونه‌های حشرات که تحت شرایط مختلف نگهداری شده‌اند، ابتدا در دمای اتاق به‌صورت هواخشک خشک شده و سپس کالبدگشایی می‌شوند .سر و شکم جدا می‌شوند تا بخش‌هایی از بدن که حاوی ویژگی‌های ریخت‌شناسی کلیدی هستند برای مونتاژ روی لام و تحلیل مورفولوژیک به‌دست آید

توراکس می‌تواند برای آنالیز Maldi-Tof مورد استفاده قرار گیرد و بخش باقی‌مانده از شکم برای استخراج دی ان ای نگهداری شود .برای پروفایل‌سازی پروتئینی، توراکس به‌صورت دستی در میکروتیوب‌های ۱.۵ میلی‌لیتری حاوی ۱۰ میکرولیتر محلول هموژنیزاسیون، با استفاده از دسته‌های خردکن (پستل) یک‌بارمصرف و گلوله‌های ساینده، هموژن می‌شود. به‌طور معمول، دو نوع محلول هموژنیزاسیون مورد استفاده قرار می‌گیرد :آب مقطر استریل و اسید فرمیک ۲۵٪.

7 نتیجه گیری

در این مطالعه، هدف ما ارائه مؤثرترین روش‌های مونتاژ پشه خاکی ها به پژوهشگران، متناسب با اهداف خاص تحقیقاتی به‌منظور تسهیل شناسایی دقیق و تشخیص پاتوژن‌ها بود .هیچ روش واحد و به‌طور جهانی بهینه‌ای وجود ندارد؛ بلکه چندین روش مختلف در دسترس است که هر یک دارای مزایا و محدودیت‌های خاص خود هستند.

در پیوست‌ها، پروتکل‌های دقیق برای روش‌های مختلف مونتاژ مورد استفاده در آماده‌سازی و شناسایی پشه خاکی ها ارائه شده است .این پروتکل‌ها، همراه با ویدئوهای آموزشی، مراحل کار را به‌صورت گام‌به‌گام و متناسب با اهداف مختلف شرح می‌دهند و نتایج دقیق و قابل اعتمادی را تضمین می‌کنند . با ارائه این منبع جامع، هدف ما حمایت از پژوهشگران در انتخاب و به‌کارگیری مناسب‌ترین تکنیک‌های مونتاژ متناسب با نیازهای اختصاصی آن‌ها است.

تقدیر و تشکر

نویسندگان از ریچارد لین و زوئی جی آدامز از موزه تاریخ طبیعی لندن، انگلستان، برای بررسی عالی‌شان که کیفیت این مقاله را به میزان قابل توجهی افزایش داده است، تشکر می‌کنند.

بودجه تحقیقاتی

ما از آژانس‌های توسعه برزیلی CNPq (شماره پرونده: 2024-4/404395) و بنیاد Araucária شماره پرونده: PDI) 2025/433 برای تأمین بودجه تحقیقات AJA قدردانی می‌کنیم.

تضاد منافع

ژروم دپاکیت بعنوان سردبیر این مجله هیچ تاثیری بر روند بررسی و تصمیم‌گیری در مورد این مقاله نداشته است .سایر نویسندگان اعلام می‌کنند که هیچ تضاد منافعی ندارند.

فایلهای در دسترس

ویدیو‌ها در Zenodo

ویدیو 1: https://zenodo.org/records/18198006

ویدیو 2: https://zenodo.org/records/18311158

ویدیو 3: https://zenodo.org/records/18311106

ویدیو 4: https://zenodo.org/records/18311154

ویدیو 5: https://zenodo.org/records/18303014

ویدیو 6: https://zenodo.org/records/18302850

ویدیو 7: https://zenodo.org/records/18315029

پیوست

مطالب تکمیلی این مقاله به صورت آنلاین در لینک زیر موجود است: https://www.parasite-journal.org/10.1051/parasite/2026009/olm

منابع و رفرنس ها

1. Alkan C, Allal-Ikhlef AB, Alwassouf S, Baklouti A, Piorkowski G, de Lamballerie X, Izri A, Charrel RN. 2015. Virus isolation, genetic characterization and seroprevalence of Toscana virus in Algeria. Clinical Microbiology and Infection, 21(11), 1040 e1-9.
2. Alten B, Ozbel Y, Ergunay K, Kasap OE, Cull B, Antoniou M, Velo E, Prudhomme J, Molina R, Banuls AL, Schaffner F, Hendrickx G, Van Bortel W, Medlock JM. 2015. Sampling strategies for phlebotomine sand flies (Diptera: Psychodidae) in Europe. Bulletin of Entomological Research 105(6), 664–678.
3. Ayhan N, Baklouti A, Prudhomme J, Walder G, Amaro F, Alten B, Moutailler S, Ergunay K, Charrel RN, Huemer H. 2017. Practical guidelines for studies on sandfly-borne phleboviruses: Part I: Important points to consider *ante* field work. Vector-Borne and Zoonotic Diseases 17(1), 73–80.

identification of American taxa, in Brazilian Sand Flies: Biology, Taxonomy, Medical Importance and Control, Rangel EF, Shaw JJ, Editors. Cham: Springer International Publishing. pp. 9–212.

21. Galati EAB, de Andrade AJ, Perveen F, Loyer M, Vongphayloth K, Randrianambinintsoa FJ, Prudhomme J, Rahola N, Akhoundi M, Shimabukuro PHF, Depaquit J. 2025. Phle-botomine sand flies (Diptera, Psychodidae) of the world. Parasites & Vectors, 18(1), 220.
22. Galati EAB, Galvis-Ovallos F, Lawyer P, Leger N, Depaquit J. 2017. An illustrated guide for characters and terminology used in descriptions of Phlebotominae (Diptera, Psychodidae). Par-asite, 24, 26.
23. Gariepy T, Kuhlmann U, Gillott C, Erlandson M. 2007. Parasitoids, predators and PCR: the use of diagnostic molecular markers in biological control of Arthropods. Journal of Applied Entomology, 131(4), 225–240.
24. Giantsis IA, Chaskopoulou A, Bon MC. 2016. Mild-Vectolysis: A nondestructive DNA extraction method for vouchering sand flies and mosquitoes. Journal of Medical Entomology, 53(3), 692–695.
25. Gidwani K, Picado A, Rijal S, Singh SP, Roy L, Volfova V, Andersen EW, Uranw S, Ostyn B, Sudarshan M, Chakravarty J, Volf P, Sundar S, Boelaert M, Rogers ME. 2011. Serological markers of sand fly exposure to evaluate insecticidal nets against visceral leishmaniasis in India and Nepal: a cluster-randomized trial. PLoS Neglected Tropical Diseases, 5(9), e1296.
26. Gilbert MTP, Moore W, Melchior L, Worobey M. 2007. DNA extraction from dry museum beetles without conferring external morphological damage. PLoS One, 2(3), e272.
27. Giordani BF, Andrade AJ, Galati EAB, Gurgel-Goncalves R. 2017. The role of wing geometric morphometrics in the identification of sandflies within the subgenus *Lutzomyia.* Medical and Veterinary Entomology, 31(4), 373–380.
28. Guzmán-Larralde AJ, Suaste-Dzul AP, Gallou A, Peña-Carrillo KI. 2017. DNA recovery from microhymenoptera using six non-destructive methodologies with considerations for subsequent preparation of museum slides. Genome, 60(1), 85–91.
29. Hajibabaei M, DeWaard JR, Ivanova NV, Ratnasingham S, Dooh RT, Kirk SL, Mackie PM, Hebert PD. 2005. Critical factors for assembling a high volume of DNA barcodes. Philosophical Transactions of the Royal Society B: Biological Sciences, 360(1462), 1959–1967.
30. Haouas N, Pesson B, Boudabous R, Dedet JP, Babba H, Ravel C. 2007. Development of a molecular tool for the identification of *Leishmania* reservoir hosts by blood meal analysis in the insect vectors. American Journal of Tropical Medicine and Hygiene, 77(6), 1054–1059.
31. Hlavackova K, Dvorak V, Chaskopoulou A, Volf P, Halada P. 2019. A novel MALDI-TOF MS-based method for blood meal identification in insect vectors: A proof of concept study on phlebotomine sand flies. PLoS Neglected Tropical Diseases, 13 (9), e0007669.
32. Huemer H, Prudhomme J, Amaro F, Baklouti A, Walder G, Alten B, Moutailler S, Ergunay K, Charrel RN, Ayhan N. 2017. Practical guidelines for studies on sandfly-borne phleboviruses: Part II: Important points to consider for fieldwork and subsequent virological screening. Vector-Borne and Zoonotic Diseases, 17(1), 81–90.
33. Jancarova M, Polanska N, Thiesson A, Arnaud F, Stejskalova M, Rehbergerova M, Kohl A, Viginier B, Volf P, Ratinier M. 2025. Susceptibility of diverse sand fly species to Toscana virus. PLoS Neglected Tropical Diseases, 19(5), e0013031.

4. Bates PA. 1997. Infection of phlebotomine sandflies with *Leishmania*, in The Molecular Biology of Insect Disease Vectors: A Methods Manual. Springer. p. 112–120.5.
5. Baum M, de Castro EA, Pinto MC, Goulart TM, Baura W, Klisiowicz Ddo R, Vieira da Costa-Ribeiro MC. 2015. Molecular detection of the blood meal source of sand flies (Diptera: Psychodidae) in a transmission area of American cutaneous leishmaniasis, Parana State, Brazil. Acta Tropica, 143, 8–12.
6. Belen A, Alten B, Aytekin A. 2004. Altitudinal variation in morphometric and molecular characteristics of *Phlebotomus papatasi* populations. Medical and Veterinary Entomology, 18(4), 343–350.
7. Bhattacharya J, Chandra G, Hati AK. 1991. A simple method for cryopreservation of *Leishmania donovani* promastigotes, Indian Journal of Medical Research, 93, 245–246.
8. Caligiuri LG, Sandoval AE, Miranda JC, Pessoa FA, Santini MS, Salomón OD, Secundino NF, McCarthy CB. 2019. Optimization of DNA extraction from individual sand flies for PCR amplification. Methods and Protocols, 2(2), 36.
9. Casaril AE, de Oliveira LP, Alonso DP, de Oliveira EF, Gomes Barrios SP, de Oliveira Moura Infran J, Fernandes WS, Oshiro ET, Ferreira AMT, Ribolla PEM, de Oliveira AG. 2017. Standardization of DNA extraction from sand flies: Application to genotyping by next generation sequencing. Experimental Parasitology, 177, 66–72.
10. Castalanelli MA, Severtson DL, Brumley CJ, Szito A, Foottit RG, Grimm M, Munyard K, Groth DM. 2010. A rapid non-destructive DNA extraction method for insects and other arthropods. Journal of Asia-Pacific Entomology, 13(3), 243–248.
11. Cerqueira NL. 1943. Um novo meio para montagem de pequenos insetos em lâmina. Memórias do Instituto Oswaldo Cruz, (39), 37–41.
12. Charrel RN, Gallian P, Navarro-Mari JM, Nicoletti L, Papa A, Sanchez-Seco MP, Tenorio A, de Lamballerie X. 2005. Emergence of Toscana virus in Europe. Emerging Infectious Diseases, 11(11), 1657–1663.
13. Chaskopoulou A, Giantsis IA, Demir S, Bon MC. 2016. Species composition, activity patterns and blood meal analysis of sand fly populations (Diptera: Psychodidae) in the metropolitan region of Thessaloniki, an endemic focus of canine leishmaniasis. Acta Tropica, 158, 170–176.
14. Chen H, Rangasamy M, Tan SY, Wang H, Siegfried BD. 2010. Evaluation of five methods for total DNA extraction from western corn rootworm beetles. PLoS One, 5(8), e11963.
15. Depaquit J, Grandadam M, Fouque F, Andry PE, Peyrefitte C. 2010. Arthropod-borne viruses transmitted by Phlebotomine sandflies in Europe: a review. Eurosurveillance, 15(10), 19507.
16. Diamond LS, Herman CM. 1954. Incidence of Trypanosomes in the Canada Goose as revealed by bone marrow culture. Journal of Parasitology, 40(2), 195–202.
17. Ding H, Torno M, Vongphayloth K, Ng G, Tan D, Sng W, Ho K, Randrianambinintsoa FJ, Depaquit J, Tan CH. 2025. Hidden in plain sight: discovery of sand flies in Singapore and description of four species new to science. Parasites & Vectors, 18(1), 402.
18. Es-Sette N, Ajaoud M, Bichaud L, Hamdi S, Mellouki F, Charrel RN, Lemrani M. 2014. *Phlebotomus sergenti* a common vector of *Leishmania tropica* and Toscana virus in Morocco. Journal of Vector Borne Diseases, 51(2), 86–90.
19. Favret C. 2005. A new non-destructive DNA extraction and specimen clearing technique for aphids (Hemiptera). Proceedings of the Entomological Society of Washington, 107(2), 469–470.
20. Galati EAB. 2018. Phlebotominae (Diptera, Psychodidae): Classification, morphology and terminology of adults and

50. Murphy WJ, Eizirik E, O'Brien SJ, Madsen O, Scally M, Douady CJ, Teeling E, Ryder OA, Stanhope MJ, de Jong WW, Springer MS. 2001. Resolution of the early placental mammal radiation using Bayesian phylogenetics. Science, 294(5550), 2348–2351.
51. Nacif-Pimenta R, Pinto LC, Volfova V, Volf P, Pimenta PFP, Secundino NFC. 2020. Conserved and distinct morphological aspects of the salivary glands of sand fly vectors of leishma-niasis: an anatomical and ultrastructural study. Parasites & Vectors, 13(1), 441.
52. Neuhaus B, Schmid T, Riedel J. 2017. Collection management and study of microscope slides: Storage, profiling, deterioration, restoration procedures, and general recommendations. Zootaxa, 4322(1), 1–173.
53. New TR. 1974. Pscoptera. Handbooks for Identification of British Insects (Vol. I). London: Royal Entomological Society of London. 102 pp.
54. Perez-Ruiz M, Collao X, Navarro-Mari JM, Tenorio A. 2007. Reverse transcription, real-time PCR assay for detection of Toscana virus. Journal of Clinical Virology, 39(4), 276–281.
55. Porco D, Rougerie R, Deharveng L, Hebert P. 2010. Coupling non-destructive DNA extraction and voucher retrieval for small soft-bodied Arthropods in a high-throughput context: the example of Collembola. Molecular Ecology Resources, 10(6), 942–945.
56. Prudhomme J, Cassan C, Hide M, Toty C, Rahola N, Vergnes B, Dujardin JP, Alten B, Sereno D, Banuls AL. 2016. Ecology and morphological variations in wings of *Phlebotomus ariasi* (Diptera: Psychodidae) in the region of Roquedur (Gard, France): a geometric morphometrics approach. Parasites & Vectors, 9(1), 578.
57. Prudhomme J, Gunay F, Rahola N, Ouanaimi F, Guernaoui S, Boumezzough A, Banuls AL, Sereno D, Alten B. 2012. Wing size and shape variation of *Phlebotomus papatasi* (Diptera: Psychodidae) populations from the south and north slopes of the Atlas Mountains in Morocco. Journal of Vector Ecology, 37(1), 137–147.
58. Prudhomme J, Toty C, Kasap OE, Rahola N, Vergnes B, Maia C, Campino L, Antoniou M, Jimenez M, Molina R, Cannet A, Alten B, Sereno D, Banuls AL. 2015. New microsatellite markers for multi-scale genetic studies on *Phlebotomus ariasi* Tonnoir, vector of *Leishmania infantum* in the Mediterranean area. Acta Tropica, 142, 79–85.
59. Prudhomme J, Velo E, Bino S, Kadriaj P, Mersini K, Gunay F, Alten B. 2019. Altitudinal variations in wing morphology of *Aedes albopictus* (Diptera, Culicidae) in Albania, the region where it was first recorded in Europe. Parasite, 26, 55.
60. Rawlins DJ. 1992. Light Microscopy: An Introduction to Biotechniques. Oxford: Bios Scientific publishers. 143 pp.
61. Ready PD. 2013. Biology of phlebotomine sand flies as vectors of disease agents. Annual Review of Entomology, 58, 227–250.
62. Rohlf FJ, Slice D. 1990. Extensions of the Procrustes method for the optimal superimposition of landmarks. Systematic Zoology, 39(1), 40–59.
63. Rowley DL, Coddington JA, Gates MW, Norrbom AL, Ochoa RA, Vandenberg NJ, Greenstone MH. 2007. Vouchering DNA-barcoded specimens: Test of a nondestructive extraction proto-col for terrestrial arthropods. Molecular Ecology Notes, 7(6), 915–924.
64. Sábio PB, Andrade AJ, Galati EAB. 2014. Assessment of the taxonomic status of some species included in the *Shannoni* complex, with the description of a new species of *Psathyromyia* (Diptera: Psychodidae: Phlebotominae). Journal of Medical Entomology, 51(2), 331–341.
34. Kapp JD, Green RE, Shapiro B. 2021. A fast and efficient single-stranded genomic library preparation method optimized for ancient DNA. Journal of Heredity, 112(3), 241–249.
35. Killick-Kendrick R, Maroli M, Killick-Kendrick M. 1991. Bibliography of the colonization of phlebotomine sandflies. Parassitologia, 33(suppl.), 321–333.
36. Lawyer P, Killick-Kendrick M, Rowland T, Rowton E, Volf P. 2017. Laboratory colonization and mass rearing of phlebotomine sand flies (Diptera, Psychodidae). Parasite, 24, 42.
37. Léger N, Pesson B, Madulo-Leblond G. 1986. Les phlébotomes de Grèce : 1ère partie. Bulletin de la Société de Pathologie Exotique, 79, 386–397.
38. Léger N, Pesson B, Madulo-Leblond G. 1986. Les phlébotomes de Grèce : 2ème partie. Bulletin de la Société de Pathologie Exotique, 79, 514–524.
39. Leonel JAF, Vioti G, Alves ML, da Silva DT, Meneghesso PA, Benassi JC, Spada JCP, Galvis-Ovallos F, Soares RM, Oliveira T. 2020. DNA extraction from individual Phlebotomine sand flies (Diptera: Psychodidae: Phlebotominae) specimens: Which is the method with better results? Experimental Parasitology, 218, 107981.
40. Lestinova T, Rohousova I, Sima M, de Oliveira CI, Volf P. 2017. Insights into the sand fly saliva: Blood-feeding and immune interactions between sand flies, hosts, and *Leishmania.* PLoS Neglected Tropical Diseases, 11(7), e0005600.
41. Lienhard A, Schaffer S. 2019. Extracting the invisible: obtaining high quality DNA is a challenging task in small arthropods. PeerJ, 7, e6753.
42. Lozano-Sardaneta YN, Mikery-Pacheco OF, Huerta H, Rojas-Soriano JE, Contreras-Ramos A. 2025. Wing geometric morphometrics is effective to separate sand fly species (Diptera, Psychodidae, Phlebotominae) related with leishmaniasis transmission in Mexico. Acta Tropica, 262, 107523.
43. Mandrioli M. 2008. Insect collections and DNA analyses: how to manage collections? Museum Management and Curatorship, 23(2), 193–199.
44. Maroli M, Feliciangeli MD, Bichaud L, Charrel RN, Gradoni L. 2013. Phlebotomine sandflies and the spreading of leishmaniases and other diseases of public health concern. Medical and Veterinary Entomology, 27(2), 123–147.
45. Marquina D, Buczek M, Ronquist F, Lukasik P. 2021. The effect of ethanol concentration on the morphological and molecular preservation of insects for biodiversity studies. PeerJ, 9, e10799.
46. Mathis A, Depaquit J, Dvorak V, Tuten H, Banuls AL, Halada P, Zapata S, Lehrter V, Hlavackova K, Prudhomme J, Volf P, Sereno D, Kaufmann C, Pfluger V, Schaffner F. 2015. Identification of phlebotomine sand flies using one MALDI-TOF MS reference database and two mass pectrometer systems., Parasites & Vectors, 8, 266.
47. Mekarnia N, Benallal KE, Sadlova J, Vojtkova B, Mauras A, Imbert N, Longhitano M, Harrat Z, Volf P, Loiseau PM, Cojean S. 2024. Effect of *Phlebotomus papatasi* on the fitness, infectivity and antimony-resistance phenotype of antimony-resistant *Leishmania* major Mon-25. International Journal for Parasitology – Drugs and Drug Resistance, 25, 100554.
48. Milligan BG. 1998. Total DNA isolation, in Molecular Genetic Analysis of Population: A Practical Approach, Hoelzel AR, Editor. Oxford: Oxford University Press.
49. Molina R, Jiménez M, Alvar J, González E, Hernández-Taberna S, Ines MM. 2017. Methods in sand fly research. Madrid: Servicio de publicaciones Universidad de Alcalá de Henares, Madrid.

71. Tesh RB, Modi GB. 1983. Growth and transovarial transmission of Chandipura virus (Rhabdoviridae: Vesiculovirus) in *Phlebotomus papatasi.* American Journal of Tropical Medicine and Hygiene, 32(3), 621–623.
72. Thomsen PF, Elias S, Gilbert MTP, Haile J, Munch K, Kuzmina S, Froese DG, Sher A, Holdaway RN, Willerslev E. 2009. Non-destructive sampling of ancient insect DNA. PLoS One, 4(4),e5048
73. Truett GE, Heeger P, Mynatt RL, Truett AA, Walker JA, Warman ML. 2000. Preparation of PCR-quality mouse genomic DNA with hot sodium hydroxide and tris (HotSHOT). Biotechniques, 29(1), 52–54.
74. Upton MS. 1993. Aqueous gum-chloral slide mounting media: an historical review. Bulletin of Entomological Research, 83(2), 267–274.
75. Volf P, Myskova J. 2007. Sand flies and Leishmania: specific versus permissive vectors. Trends in Parasitology, 23(3), 91–92.
76. Wang Q, Wang X. 2012. Comparison of methods for DNA extraction from a single chironomid for PCR analysis. Pakistan Journal of Zoology, 44(2), 421–426.
77. Wang Y, Zhao Y, Bollas A, Wang Y, Au KF. 2021. Nanopore sequencing technology, bioinformatics and applications. Nature Biotechnology, 39(11), 1348–1365.
65. Sadlova J, Yeo M, Seblova V, Lewis MD, Mauricio I, Volf P, Miles MA. 2011. Visualisation of *Leishmania donovani* fluorescent hybrids during early stage development in the sand fly vector. PLoS One, 6(5), e19851.
66. Sales K, Miranda DEO, da Silva FJ, Otranto D, Figueredo LA, Dantas-Torres F. 2020. Evaluation of different storage times and preservation methods on phlebotomine sand fly DNA concentration and purity. Parasites & Vectors, 13(1), 399
67. Sales KG, Costa PL, de Morais RC, Otranto D, Brandao-Filho SP, Cavalcanti Mde P, Dantas-Torres F. 2015. Identification of phlebotomine sand fly blood meals by real-time PCR. Parasites& Vectors, 8, 230.
68. Sant'Anna MR, Jones NG, Hindley JA, Mendes-Sousa AF, Dillon RJ, Cavalcante RR, Alexander B, Bates PA. 2008. Blood meal identification and parasite detection in laboratory-fed and field-captured *Lutzomyia longipalpis* by PCR using FTA databasing paper. Acta Tropica, 107(3), 230–237.
69. Senne NA, Santos HA, Araujo TR, Paulino PG, Mendonca LP, Moreira HVS, Camilo TA, da Costa Angelo I. 2022. Robust comparative performance of genomic DNA extraction methods from non-engorged phlebotomine sandflies. Medical and Veterinary Entomology, 36(2), 203–211.
70. Shaw JJ. 2025. A review of Leishmania infections in American Phlebotomine sand flies – Are those that transmit leishmaniasis anthropophilic or anthropportunists? Parasite, 32, 57.

Cite this article as: Randrianambinintsoa FJ, Augendre L, Prudhomme J, Martinet J-P, Loyer M, Mekarnia N, Kerkoub H, Perveen FK, Huguenin A, Kariya E, Akhoundi M, De Andrade AJ, Berriatua E, Bongiorno G, Boyer S, Christodoulou V, Da Costa-Ribeiro MCV, De Souza LAF, Ding H, Dondji B, Dvořák V, Erisoz Kasap O, Galati EAB, Gállego M, Ballart C, Gouzelou S, Haddad N, Masse RS, Mekuria AH, Ivovic V, Kaczmarek S, Shahar MK, Kirstein OD, Kniha E, Kolářová I, Lincoln T, Lucanas C, Mikov O, Nov K, Özbel Y, Pesson B, Posada Lopez LC, Prasetyo DB, Rahola N, Rebollar-Tellez EA, Rodrigues BL, Roy L, Saini P, Sanjoba C, Shimabukuro PH, Siriyasatien P, Soszynska A, Suleşco T, Sylla M, Torno M, Volf P, Vongphayloth K, Sinh Nam V, Wardhana A, Yessinou E, Zapata S, Gantier J-C & Depaquit J. 2026. Processing and mounting phlebotomine sand flies: a consensus guideline. Parasite xx, xx. https://doi.org/10.1051/parasite/2026009.

PARASITE

An international open-access, peer-reviewed, online journal publishing high quality papers on all aspects of human and animal parasitology

Reviews, articles and short notes may be submitted. Fields include, but are not limited to: general, medical and veterinary parasitology; morphology, including ultrastructure; parasite systematics, including entomology, acarology, helminthology and protistology, and molecular analyses; molecular biology and biochemistry; immunology of parasitic diseases; host-parasite relationships; ecology and life history of parasites; epidemiology; therapeutics; new diagnostic tools.
All papers in Parasite are published in English. Manuscripts should have a broad interest and must not have been published or submitted elsewhere. No limit is imposed on the length of manuscripts.

Parasite (open-access) continues **Parasite** (print and online editions, 1994-2012) and **Annales de Parasitologie Humaine et Comparée** (1923-1993) and is the official journal of the Société Française de Parasitologie.

Editor-in-Chief:
Jean-Lou Justine, Paris

Submit your manuscript at:
https://www.editorialmanager.com/parasite

پیوست A : مبانی نظری بیوشیمیایی

بندپایان مورد نظر در این مطالعه پشه خاکی ها هستند. با این حال، مفهوم کلی می‌تواند به سایر بندپایان رایج نیز تعمیم یابد که شناسایی آن‌ها تنها بر اساس ویژگی‌های مورفولوژیک داخلی امکان‌پذیر است. به‌طور اتفاقی، برخی اندام‌های داخلی تا حدی کیتینه شده‌اند و مورفولوژی آن‌ها اطلاعات ارزشمندی فراهم می‌کند. به همین دلیل، مشاهده ساختارهایی مانند پمپ‌های غذایی، اسپرماتک و مجاری آن‌ها اهمیت ویژه‌ای دارد.

در تمامی مراحلی که از تثبیت حشره تا مونتاژ انجام می‌شود، اساس کار بر واکنش‌های اکسایش–کاهش (ردوکس) استوار است. نکته اساسی این است که از اختلاط مواد کاهنده با مواد اکسیدکننده اجتناب شود.

الکل اتیلیک (اتانول)

این ماده به روش‌های مختلفی مورد استفاده قرار می‌گیرد. مولکول‌های الکل تمایل بالایی به آب دارند و بنابراین خاصیت دهیدراته‌کنندگی دارند. با این حال، الکل با غلظت پایین (یعنی حاوی آب زیاد) می‌تواند باعث تخریب اسیدهای نوکلئیک شود، زیرا آب برای این مولکول‌ها مضر است.

قرار دادن حشرات در اتانول نه‌تنها برای نگهداری، بلکه برای تثبیت بافت‌ها نیز انجام می‌شود. در بافت‌شناسی، دو مفهوم مهم مطرح است: سرعت نفوذ و سرعت تثبیت. یک ماده نگهدارنده مناسب باید ابتدا به‌سرعت به عمق بافت نفوذ کرده و سپس آن را تثبیت کند. برای اتانول ۹۶٪، ضریب نفوذ حدود 1.05 است (در مقایسه، محلول آبی 0.75٪ اسید پیکریک دارای ضریب 0.45 و محلول 3٪ دی‌کرومات پتاسیم دارای ضریب 1.45 است).

نگهداری طولانی‌مدت حشرات در اتانول برای حشره‌شناسان امری رایج است، اما از دیدگاه سیتولوژی و هیستولوژی، نگهداری طولانی در فیکساتور می‌تواند باعث شود نمونه‌ها عملاً غیرقابل استفاده شوند. به همین دلیل، نمونه‌های قدیمی‌تر از ۱۰ سال اغلب به‌سختی یا اصلاً قابل استفاده نیستند.

همچنین نسبت حجم فیکساتور به حجم نمونه اهمیت دارد. در عمل، توصیه می‌شود حجم فیکساتور حدود ۶۰ برابر حجم نمونه باشد، اما برای میکروبندپایان، افزودن ۴ تا ۵ برابر حجم نمونه از الکل کافی است. باید توجه داشت که الکل با جذب آب از بافت‌ها به‌تدریج قدرت خود را از دست می‌دهد.

نتیجه‌گیری:

–اتانول یک عامل شیمیایی کاهنده است (و با مواد اکسیدکننده ناسازگار است)؛
–باعث رسوب سریع پروتئین‌ها و دناتوره‌شدن آن‌ها می‌شود؛
–برخی لیپیدهای پیچیده را حل کرده و گلیکوژن را رسوب می‌دهد؛
–موجب انقباض شدید بافت‌ها و سخت‌شدن آن‌ها می‌شود.

محلول‌های قلیایی هیدروکسید پتاسیم یا سدیم

در حشره‌شناسی، استفاده از این محلول‌ها عمدتاً به هیدروکسید پتاسیم (KOH) محدود شده است.

هیدروکسید سدیم (NaOH) به‌صورت محلول در غلظت‌های مختلف وجود دارد و به‌شدت رطوبت‌گیر است. این ماده پروتئین‌ها را حل کرده و لیپیدها را در فرآیند صابونی‌شدن به صابون‌های جامد تبدیل می‌کند.

هیدروکسید پتاسیم (KOH) معمولاً به‌صورت پلت‌های حدود ۰/۱ گرم در دسترس است که تهیه محلول‌های رقیق را آسان می‌کند (برای مثال، حل یک پلت در ۱ میلی‌لیتر آب مقطر، محلول ۱۰٪ ایجاد می‌کند). این ماده نسبت به NaOH کمتر مستعد کربناته‌شدن است.

این بازهای قوی برای حل اسیدهای چرب از طریق تبدیل آن‌ها به صابون‌های محلول در آب استفاده می‌شوند. در برخی موارد (مثلاً در نمونه‌های ماده با بافت چربی زیاد)، افزایش دما به ۳۵–۴۰ درجه سانتی‌گراد یا افزایش زمان تماس، فرآیند را تسهیل می‌کند.

محلول اسیدی رنگی / محلول بی‌رنگ مارک–آندره

محلول مارک–آندره از کلرال هیدرات، اسید استیک و آب تشکیل شده و یک عامل اکسیدکننده قوی است. این محلول:

–KOH اضافی را خنثی می‌کند بدون آن‌که صابون‌های قلیایی رسوب کنند؛
–با اکسیدکردن ساختارهای کیتینی، باعث نرم‌شدن کیتین می‌شود؛
–برخی نمک‌های معدنی را حل می‌کند.

در صورت رنگ‌آمیزی با فوکسین اسیدی، این محلول می‌تواند به ساختارهای کیتینی متصل شده و آن‌ها را برای مشاهده بهتر رنگ‌آمیزی کند. پس از این مرحله، شستشو فقط با اتانول انجام شده و فاز دهیدراتاسیون آغاز می‌شود.

مزایا:

–خنثی‌سازی بازهای اضافی
–نرم‌کردن کیتین
–افزایش وضوح ساختارهای کیتینی

معایب:

کلرال هیدرات ماده‌ای خواب‌آور است و باید تحت هود شیمیایی و با رعایت مقررات ایمنی استفاده شود.

محلول‌های دهیدراتاسیون

برای نمونه‌های بسیار کوچک، نیازی به عبور از سری کامل اتانول با غلظت‌های افزایشی نیست. در نمونه‌های بزرگ‌تر، توالی ۸۰٪، ۹۰٪، ۹۵٪ و سپس اتانول مطلق استفاده می‌شود. برای نمونه‌های کوچک، استفاده از ۹۰٪ و سپس اتانول مطلق کافی است.

باید توجه داشت که اتانول مطلق تمایل به جذب آب از محیط دارد.

در گذشته، از کرئوزوت راش برای تکمیل دهیدراتاسیون استفاده می‌شد، اما امروزه به دلیل سمیت (ترکیبات آروماتیک چندحلقه‌ای)، خاصیت سرطان‌زایی و اثرات زیست‌محیطی، استفاده از آن توصیه نمی‌شود.

به‌عنوان جایگزین، استفاده از محلول **Euparal®** و اسانس Euparal پیشنهاد می‌شود که برای مونتاژ نمونه‌ها مناسب بوده و با نمونه‌هایی که از حمام اتانول ۹۰٪ عبور کرده‌اند، سازگاری خوبی دارد.

پیوست B: ترکیب مواد و معرف‌های مورد استفاده

هیدروکسید پتاسیم ۱۰٪

هیدروکسید پتاسیم: ۱۰ گرم
آب مقطر: تا حجم ۱۰۰ میلی‌لیتر

محیط مونتاژ صمغ کلرال (محیط هویر)

آب مقطر: ۵۰ میلی‌لیتر
کلرال هیدرات: ۲۰۰ گرم
صمغ عربی: ۵۰ گرم
گلیسرول: ۲۰ میلی‌لیتر

محلول مارک–آندره

کلرال هیدرات: ۴۰ گرم
اسید استیک گلاسیال: ۳۰ میلی‌لیتر
آب مقطر: ۳۰ میلی‌لیتر

فوکسین اسیدی ۱٪ در آب مقطر

پودر فوکسین اسیدی: ۱ گرم
آب مقطر: ۹۹ میلی‌لیتر

محلول مارک–آندره رنگ‌آمیزی‌شده با فوکسین

محلول مارک–آندره: ۱۰ میلی‌لیتر
فوکسین ۱٪: ۵۰ میکرولیتر

پیوست C: Euparal® ، کانادا بالزام ، پلی‌وینیل الکل و سایر محیط‌های مونتاژ

پلی‌وینیل الکل:(PVA)

این ماده زمانی ایده‌آل است که مواد لازم برای دهیدراتاسیون کامل در دسترس نباشد. معمولاً با لاکتوفنل آمان مخلوط می‌شود. با این حال، این نوع مونتاژها دارای معایبی

مانند خشک‌شدن، تبلور PVA به‌دلیل تبخیر آب، یا سیاه‌شدن در اثر اکسیداسیون فنول هستند. بنابراین، بیشتر برای مونتاژ های کوتاه‌مدت مناسب است.

کانادا بالزام:

برای مونتاژ بین لام و لامل نیاز به دهیدراتاسیون کامل نمونه دارد. استفاده از زایلن یا تولوئن در این روش بدون مشکل نیست و می‌تواند محدودیت‌هایی ایجاد کند.

محیط:Enecê

مانند کانادا بالزام ، نیازمند دهیدراتاسیون کامل است.

ترکیب:

–کولوفونی سفید خالص: ۲۲ گرم
–صمغ کوپال محلول در الکل: ۱۲ گرم
–الکل مطلق: ۲۰ میلی‌لیتر
–کافور: ۱۰ گرم
–اسانس تربانتین: ۱۰ میلی‌لیتر
–اوکالیپتول: ۲۶ میلی‌لیتر

روش تهیه:

ابتدا الکل مطلق و کافور در ظرفی (مثلاً ارلن) قرار داده می‌شود. سپس کولوفونی و صمغ کوپال افزوده می‌شوند. ظرف بسته شده و مخلوط تکان داده می‌شود، سپس در حمام آب با دمای ملایم گرم می‌گردد (بدون جوشیدن). پس از حل کامل، اسانس تربانتین افزوده و مخلوط در حالت گرم صاف می‌شود. در نهایت اوکالیپتول به محلول افزوده می‌شود. در صورت افزایش ویسکوزیته، می‌توان آن را با محلول رقیق‌کننده Enecê (الکل مطلق، کافور، تربانتین، اوکالیپتول) تنظیم کرد.

Euparal®:

رزینی است که از درخت *Tetraclinis articulata* به‌دست می‌آید و در سال ۱۹۰۶ معرفی شد. مزیت اصلی آن عدم پلیمریزاسیون است، به‌طوری‌که نمونه‌های مونتاژشده را می‌توان با الکل یا اسانس Euparal مجدداً بازیابی کرد. این رزین (ساندارک) با اتانول ٪۸۰ نیز سازگار است.

استفاده از Triton X-100 محلول غیر یونی

Triton X-100 یک شوینده غیر یونی رایج در زیست‌شناسی سلولی و مولکولی است که برای نفوذپذیر کردن غشاهای سلولی و هسته‌ای به‌کار می‌رود.

نمونه‌های حشره‌ای که برای مدت طولانی در الکل نگهداری شده‌اند، اغلب برای بررسی میکروسکوپی دشوار می‌شوند. برای رفع این مشکل، استفاده از عوامل ترکننده توصیه شده است.

روش پیشنهادی (محلول ٪۰/۵):

–آغشته‌سازی نمونه خشک با الکل مطلق
–افزودن محلول Triton X-100 (٪۰/۵) تا غوطه‌وری کامل
–نگهداری به‌مدت حدود ۵ دقیقه
–حذف محلول Triton و جایگزینی با محلول KOH
–ادامه مراحل طبق پروتکل استاندارد

پیوست D: مونتاژ با ®Euparal یا کانادا بالزام (مرحله‌به‌مرحله)

شرایط اولیه:

۱ .نمونه‌ها باید کاملاً دهیدراته شوند (کدورت نشانه دهیدراتاسیون ناقص است).
۲ .دهیدراتاسیون با غلظت‌های افزایشی اتانول انجام می‌شود.
۳ .انتقال از الکل مطلق به عامل شفاف‌کننده امکان‌پذیر است.

مراحل:

۱ .کالبدگشایی پشه خاکی های بالغ در اتانول ٪۷۰
۲ .حذف اتانول و جایگزینی با KOH ٪۱۰) پوشاندن با لامل(
۳ .ماکرمکردن تا شفاف‌شدن نمونه
۴ .حذف KOH
۵ .شستشو با آب مقطر (۳۰–۴۵ دقیقه)
۶ .تکرار شستشو با آب مقطر (حدود ۳۰ دقیقه)
۷ .حذف آب
۸ .افزودن محلول مارک–آندره (در صورت نیاز رنگی) به‌مدت ۲۴ ساعت
۹ .حذف محلول مارک–آندره
۱۰ .شستشو مجدد با آب مقطر (۳۰–۴۵ دقیقه)
۱۱ .تکرار شستشو (۳۰ دقیقه)
۱۲ .حذف آب
۱۳ .افزودن اتانول ٪۷۰ و انجام کالبدگشایی نهایی:

الف) برای سر و شکم، جداکردن ملایم آن‌ها از توراکس
ب) برای توراکس، بال‌ها را با نگه‌داشتن توراکس توسط یک پنس و کشیدن در محل اتصال زوائد با یک پنس دیگر جدا کنید. در صورت نیاز، می‌توان برش ساژیتال انجام داد و توراکس را به دو نیمه چپ و راست تقسیم کرد، بسته به نواحی موردنظر برای مطالعه.

۱۴ .دهیدراتاسیون به‌صورت تدریجی از طریق سری محلول‌های آبی اتانول انجام شود:
٪۵۰ → ٪۸۰ → ٪۹۵ → تا رسیدن به اتانول مطلق.
۱۵ .نمونه‌ها را با دو بار شستشو، هر بار به‌مدت ۱۰ دقیقه، در اتانول ٪۱۰۰ دهیدراته کنید.
۱۶ .اتانول را حذف کرده و نمونه‌ها را به‌مدت ۱۵ دقیقه در دمای اتاق در روغن میخک قرار دهید.
۱۷ .نمونه‌ها را از روغن میخک به یک قطره از Euparal® یا کانادا بالزام روی لام تمیز منتقل کنید.
۱۸ .چیدمان نمونه:
سر، توراکس و شکم پشه خاکی می‌توانند با استفاده از سوزن‌های ظریف یا پنس، زیر میکروسکوپ استریو، کالبدگشایی شوند.
سر باید از بدن جدا شود تا در وضعیت شکمی–پشتی (ونترو-دورسال) مونتاژ گردد؛ به‌طوری‌که سوراخ پس‌سری به سمت بالا قرار گیرد تا سیباریوم مستقیماً از طریق آن قابل مشاهده باشد.
کالبدگشایی در محیط مونتاژ پشه خاکی انجام می‌شود.
۱۹ .نمونه را رها کنید تا سطح آن حالت چسبنده پیدا کند.
۲۰ .یک لامل تمیز را با اتانول مطلق مرطوب کرده و آن را با زاویه روی کانادا بالزام قرار دهید.
۲۱ .لام‌ها را در یک جعبه خشک (خشک‌کن) مخصوص نگهداری کنید.
