# Supplementary material for: Processing and mounting phlebotomine sand flies: a consensus guideline
Source: Parasite. 2026 Apr 3;33:18. doi: 10.1051/parasite/2026009 (PMC13047900; doi:10.1051/parasite/2026009)
Supplement: Supplementary file 8 — Filipino translation / Pagsasalin sa Filipino [file parasite-33-18-s8.pdf]

Parasite **33**, 18 (2026)
© F.J. Randrianambinintsoa et al., published by EDP Sciences, 2026
https://doi.org/10.1051/parasite/2026009

PARASITE
Available online at:
www.parasite-journal.org

REVIEW ARTICLE OPEN ACCESS

Pag-proseso at pag-mount ng Phlebotomine Sandflies o nik-nik: isang pinagkasunduang patnubay

Fano José Randrianambinintsoa[1], Laure Augendre[1], Jorian Prudhomme[1], Jean-Philippe Martinet[1], Mathieu Loyer[1], Nalia Mekarnia[1], Hocine Kerkoub[1], Farzana Khan Perveen[1], Antoine Huguenin[1,2], Emilie Kariya[1,2], Mohammad Akhoundi[3], Andrey José de Andrade[4], Eduardo Berriatua[5], Gioia Bongiorno[6], Sébastien Boyer[7,8], Vasiliki Christodoulou[9], Magda Clara Vieira Da Costa-Ribeiro[10], Lucas Alexandre Farias de Souza[10], Huicong Ding[11], Blaise Dondji[12], Vít Dvořák[13], Ozge Erisoz Kasap[14], Eunice Aparecida Bianchi Galati[15], Montserrat Gállego[16], Cristina Ballart[16], Stavroula Gouzelou[17], Nabil Haddad[18], Rezki Sabrina Masse[19], Asrat Hailu Mekuria[20], Vladimir Ivovic[21], Szymon Kaczmarek[22], Mohd Khadri Shahar[19], Oscar D. Kirstein[23], Edwin Kniha[24], Iva Kolářová[13], Lincoln Timinao[25], Cristian Lucanas[26], Ognyan Mikov[27], Kimsear Nov[7], Yusuf Özbel[28], Bernard Pesson[29], Laura Cristina Posada Lopez[30], Didot Budi Prasetyo[1,7], Nil Rahola[31], Eduardo A. Rebollar-Tellez[32], Bruno Leite Rodrigues[15], Lalita Roy[33], Prasanta Saini[34], Chizu Sanjoba[35], Paloma Helena Fernandes Shimabukuro[36], Padet Siriyasatien[37], Agnieszka Soszyńska[22], Tatiana Suleşco[38], Massamba Sylla[39], Majhalia Torno[40], Petr Volf[13], Khamsing Vongphayloth[41], Vu Sinh Nam[42], April Wardhana[43], Eric Yessinou[44], Sonia Zapata[45], Jean-Charles Gantier[1], and Jérôme Depaquit[1,2,*]

[1] Faculté de Pharmacie, Université de Reims Champagne Ardenne, UR ESCAPE-USC ANSES PETARD, 51 rue Cognacq-Jay, 51096 Reims Cedex, France
[2] Pôle de Biologie territoriale, Laboratoire de Parasitologie-Mycologie, Centre Hospitalo-Universitaire, 51092 Reims, France
[3] Parasitology-Mycology Department, Avicenne Hospital, AP-HP, Bobigny, Sorbonne Paris Nord University, France; Unité des Virus Émergents (UVE: Aix-Marseille Univ, Università di Corsica, IRD 190, Inserm 1207, IRBA), 13005 Marseille, France
[4] Parasitology Collection of Basic Pathology, Department of Basic Pathology, Federal University of Paraná, Curitiba 19031, Brazil
[5] Department of Animal Health, University of Murcia, Campus de Espinardo, 30100 Espinardo, Murcia, Spain
[6] Department of Infectious Diseases, Vector-borne Diseases Unit, Istituto Superiore di Sanità, 00166 Rome, Italy
[7] Medical and Veterinary Entomology Unit, Institut Pasteur du Cambodge, Phnom Penh 12201, Cambodia
[8] Ecology & Emergence of Arthropod-borne Pathogens Unit, Department of Global Health, Institut Pasteur, CNRS UMR2000, 75015 Paris, France
[9] Section Veterinary Services (1417), Laboratory for Animal Health Virology, Aglantzia, Nicosia 2109, Cyprus
[10] Insects Vectors and Parasites Laboratory, Department of Basic Pathology and Postgraduate program in Microbiology, Parasitology and Pathology, Federal University of Paraná, 81530-900 Curitiba, Brazil
[11] Department of Biological Sciences, National University of Singapore, 117558, Singapore
[12] Laboratory of the Leishmaniasis Research Project, Mokolo District Hospital, Mokolo, Cameroon; Laboratory of Cellular Immunology and Parasitology, Department of Biological Sciences, Central Washington University, 98926 Ellensburg, WA, USA
[13] Department of Parasitology, Faculty of Science, Charles University, 12800 Prague, Czechia
[14] VERG Laboratories, Department of Biology, Faculty of Science, Hacettepe University, Beytepe, Ankara 06800, Türkiye
[15] Faculdade de Saúde Pública da Universidade de São Paulo (FSP/USP), Pós-graduação em Saúde Pública, 01246-904 São Paulo, Brazil
[16] Secció de Parasitologia, Departament de Biologia, Sanitat i Medi Ambient, Facultat de Farmàcia i Ciències de l'Alimentació, Universitat de Barcelona, & Institut de Salut Global de Barcelona (ISGlobal), Centro de Investigación Biomédica en Red, Enfermedades Infecciosas (CIBERINFEC), 08028 Barcelona, Spain
[17] Laboratory of Infectious Diseases and Public Health, School of Medicine, University of Cyprus, Nicosia, Cyprus & Department of Pediatrics, Archbishop Makarios III Hospital, Nicosia 2115, Cyprus
[18] Faculty of Health Sciences, American University of Beirut, 1107 2020 Beirut, Lebanon

Edited by Jean-Lou Justine
*Corresponding author: jerome.depaquit@univ-reims.fr

This is an Open Access article distributed under the terms of the Creative Commons Attribution License (https://creativecommons.org/licenses/by/4.0), which permits unrestricted use, distribution, and reproduction in any medium, provided the original work is properly cited.

[19] Medical Entomology Unit, Infectious Disease Research Centre, Institute for Medical Research (IMR), National Institutes of Health (NIH), Ministry of Health Malaysia, 40170 Shah Alam, Selangor, Malaysia
[20] School of Medicine, Addis Ababa University, 28017 - 1000 Addis Ababa, Ethiopia
[21] Faculty of Mathematics, Natural Sciences and Information Technologies, University of Primorska, 6000 Koper, Slovenia
[22] University of Lodz, Faculty of Biology and Environmental Protection, Department of Invertebrate Zoology and Hydrobiology, Banacha 12/16, 90-237 Łódź, Poland
[23] Laboratory of Entomology, Ministry of Health, 9134302 Jerusalem, Israel
[24] Center for Pathophysiology, Infectiology and Immunology, Institute of Specific Prophylaxis and Tropical Medicine, Medical University Vienna, Kinderspitalgasse 15, 1090 Vienna, Austria
[25] Papua New Guinea Institute of Medical Research (PNGIMR) Institute, PO Box 60, Headquarter, Homate Street, 441 Goroka, Eastern Highlands Province, Papua New Guinea
[26] Museum of Natural History, University of the Philippines Los Baños, 4031 Laguna, Philippines
[27] National Centre of Infectious and Parasitic Diseases, 1504 Sofia, Bulgaria
[28] Ege University, Faculty of Medicine, Department of Parasitology, 35040 Bornova/Izmir, Türkiye
[29] Retired, Faculté de Pharmacie, Université de Strasbourg, Strasbourg, 67400 Illkirch-Graffenstaden, France
[30] Program for the Study and Control of Tropical Diseases (PECET), Faculty of Medicine, University of Antioquia, 050010 Medellin, Colombia
[31] MIVEGEC, Univ. Montpellier, CNRS, IRD, 34394 Montpellier, France & Medical Entomology Unit, Institut Pasteur de Madagascar, 101 Antananarivo, Madagascar
[32] Laboratorio de Entomología Médica, Departamento de Zoología de Invertebrados, Facultad de Ciencias Biológicas, Universidad Autónoma de Nuevo León, San Nicolás de los Garza, 66455, NL, México
[33] Tropical and Infectious Disease Centre, BP Koirala Institute of Health Sciences, Dharan 56700, Nepal
[34] ICMR-Vector Control Research Centre, Puducherry 605006, India
[35] Graduate School of Agricultural and Life Sciences, The University of Tokyo, Tokyo 113-8657, Japan
[36] Grupo de estudos em Leishmanioses/Coleção de Flebotomíneos (COLFLEB/Fiocruz-MG), Instituto René Rachou, Fundação Oswaldo Cruz, Belo Horizonte, Minas Gerais, 30190009, Brazil
[37] Center of Excellence in Vector Biology and Vector-Borne Disease, Department of Parasitology, Faculty of Medicine, Chulalongkorn University, Bangkok 10330, Thailand
[38] Department of Arbovirology, Bernhard Nocht Institute for Tropical Medicine, Bernhard Nocht Str. 74, 20359 Hamburg, Germany [39] Laboratory Vectors & Parasites, Department of Livestock Sciences and Techniques, Sine Saloum University El Hadji Ibrahima Niasse (SSUEIN) Kaffrine Campus, C.P. 24600, Senegal.
[40] Environmental Health Institute, National Environment Agency, Singapore 138667, Singapore & Department of Biological Sciences, National University of Singapore, 117558 Singapore
[41] Institut Pasteur du Laos, Laboratory of Vector-Borne Diseases, Samsenhai Road, Ban Kao-Gnot, Sisattanak District, 3560 Vientiane, Lao PDR
[42] National Institute of Hygiene and Epidemiology, 1 Yec-Xanh Street, Hai Ba Trung District, 100000 Hanoi, Vietnam
[43] Indonesian Research Center for Veterinary Science, Indonesian Agency for Agricultural Research and Development, Ministry of Agriculture Republic Indonesia, Bogor 16114, Indonesia & Department of Parasitology, Faculty of Veterinary Medicine, Airlangga University, Surabaya 60115, Indonesia
[44] Laboratory of Research in Applied Biology, Polytechnic School of Abomey-Calavi, University of Abomey-Calavi, 01 P.O. Box 2009, 00000 Cotonou, Benin
[45] Instituto de Microbiología, Colegio de Ciencias Biológicas y Ambientales (COCIBA), Universidad San Francisco de Quito (USFQ), 170901 Quito, Ecuador

Received 1 December 2025, Accepted 29 January 2026, Published online 3 April 2026

Buod – Ang papel na ito ay nagbibigay ng komprehensibong gabay sa paraan ng pagproseso at pag-mount ng mga espesimen ng phlebotomine nik-nik o nik-nik, na mahalaga para sa pagkakakilanlan ng mga sarihay (species) at sa pagtuklas at pagbubukod ng mga mikrobyong maaaaring magdulot ng sakit. Tinatalakay nito ang iba't-ibang pamamaraan na angkop sa kalagayan sa kabukiran at laboratoryo. Kasama sa gabay ang detalyadong mga tagubilin sa pagkolekta, paghawak, pagtakip at eutanasya ng mga nik-nik (inirerekomenda ang pagpapatuyo sa pamamagitan ng pagkabilad sa matinding lamig o paggamit ng CO2 kaysa sa mga kemikal) pati na rin ang mga estratehiya sa pangangalaga tulad ng pananatili sa malamig na imbakan at preserbasyon sa ethanol. Ang kalidad ng paghahanda ng ilang bahagi ng katawan (kasangkapang pangkasarian, ulo at pakpak) ay mahalaga para sa naaangkop na pagsusuri sa ilalim ng mikroskopyo na idedetalye sa gawaing ito. Ipinakita rin ng dokumento ang detalyadong pagproseso ng sample, kasama ang proseso ng paglilinaw gamit ang mga pamalibilos o reagent tulad ng potassium hydroxide na sinusundan ng Marc-André solution. Inihahambing sa proseso ng pag-mount ang iba't-ibang media, binibigyang-diin ang kanilang katangiang optikal at kakayahang mapanatili ang anking katangian ng espesimen. Inirerekomenda ang Hoyer's fluid (kilala rin bilang chloral gum) para sa mabilisang pagmamasid, lalo na para sa spermathecae, dahil sa linaw nito, bagaman hindi ito angkop para sa pang-matagalang pagtatago. Kasama sa ibang media na tinalakay ang

polyvinyl alcohol, Euparal® (para sa limitadong kakayahang tumanggap ng tubig), at Canada balsam (isang medium na nalulusaw sa hydrocarbon), kung saan ang huling dalawa ay nag-aalok ng kakayahan sa pangmatagalang preserbasyon. Tinugunan din ang mga makabagong kaparaanan sa molekular na haynayan tulad ng DNA sequencing at MALDI-TOF, na nangangailangan ng partikular na pansin sa pagproseso ng sampol. Higit pa rito, nagbigay din ng mga maikling video clips na naglalarawan sa iba't-ibang kaparaanan ng pag-mount pati na rin ang mga salin sa 33 iba't-ibang wika, na nagbibigay-daan sa pagtugon sa iba't-ibang pangangailangan at inaasahan ng pandaigdigang pamayanang pang-agham.

Mga Pangunahing Salita: Pag-mount, Phlebotomine nik-nik o nik-nik, Hoyer fluid, Marc-André solution, chloral gum, polyvinyl alcohol, Euparal®, Canada balsam, Leishmania isolation, kalagayan sa kabukiran, culture, paghihimay, molekular na haynayan, MALDI-ToF, Type-specimens.

Abstract – Processing and mounting phlebotomine sand flies: a consensus guideline. This article provides a comprehensive guide for the processing and mounting of phlebotomine sand fly specimens, which is crucial for species identification and pathogen detection and isolation. It discusses a range of techniques suitable for both field and laboratory settings. The guide includes detailed instructions on sand fly collection, handling, covering, and euthanasia (recommending dry freezing or CO_2 over chemicals) as well as conservation strategies, such as cold storage and preservation in ethanol. The quality of preparation of certain anatomical structures (genital organs, head and wings) is essential for their proper microscopic observation and is described in this work. The article also presents detailed sample processing, including the clearing process with agents such as potassium hydroxide then Marc-André solution. The mounting process compares different media, emphasizing their optical properties and preservation potential. Hoyer fluid (also known as chloral gum) is recommended for quick observation, particularly for spermathecae, due to its clarity, although it is not suitable for long-term storage. Other media discussed include polyvinyl alcohol, Euparal® (for limited water tolerance), and Canada balsam (a hydrocarbon-soluble medium), with the latter two offering long-term preservation capabilities. Innovative molecular biology approaches such as DNA sequencing and MALDI-ToF, which require particular attention to sample processing, are also addressed. Furthermore, short video clips illustrating various mounting techniques as well as translations in many different languages are provided, allowing the guideline to reach the diverse needs and expectations of the global scientific community.

Key words: Mounting, Phlebotomine sand fly, Hoyer fluid, Marc-André solution, Chloral gum, Polyvinyl alcohol, Euparal®, Canada balsam, *Leishmania* isolation, Field conditions, Culture, Dissection, Molecular biology, MALDI-ToF, Type-specimens.

Panimula

Ang mga "Phlebotomine sandflies" o nik-nik, ay mga uri ng maliliit na Diptera na kabilang sa family Psychodidae, subfamily Phlebotominae, na mayroong humigit-kumulang 1,063 na kilalang sarihay o species. Sila ay kilalang tagapagdala ng mga organismong nagdudulot ng sakit tulad ng *Leishmania*, arboviruses at *Bartonella* na responsable sa mga sakit tulad ng leishmaniasis, impeksion mula sa mga arbovirus, at bartonellosis, ayon sa pagkakabanggit. Ang pagkilanlan ng mga organismong ito ay nakabatay sa detalyadong pagsusuri sa ilalim ng mikroskopyo na napapadali sa pamamagitan ng maingat na paghuli, tamang pagsusubi at maingat na pag-mount sa microscope slides, na nangangailangan ng ilang tiyak na teknik kung saan ang bawat isa ay may sariling kahigtan at limitasyon.

Ang pagkilala ng sarihay ng mga nik-nik ay nakasalalay sa masusing pagsusuri sa iba't-ibang panlabas (hal. antenna, palp at genitalia ng lalaki) at panloob (hal. pharynx, cibarium at spermatheca) na istruktura sa katawan nito. Ang pag-daysek at paghihiwalay ng mga panloob na istruktura ay kinakailangan upang mapadali at maayos na maisagawa ang wastong pagkakilanlan. Sumakatwid, di tulad ng mga lamok at kissing bugs, nangangailangan ang mga nik-nik ng pag-mount sa pagitan ng slides at coverslips bago sila kilalanin. Hanggang sa 1980s, ang pagsilip lamang sa ilalim ng mikroskopyo ang tanging paraan upang makilala ang sarihay ng nik-nik, na siya pa rin namang pangunahing paraan na ginagamit sa panahon ngayon. Ang pagpili ng proseso at paghahanda ay naging medyo tuwiran at pangunahing nakabatay sa isang dikotomiya: sa isang banda, ang tiyak na pag-mount na nagbibigay-daan sa pangmatagalang pag-iingat ng espesimen, at sa kabilang banda, ang mabilis na pag-mount para sa pagkakakilanlan sa isang medium na hindi nagsisiguro ng pangmatagalang pag-iingat. Ang pangmatagalang pag-mount, halimbawa, sa isang resin tulad ng Canada balsam, ay nakakaubos ng oras at nangangailangan ng lubusang pag-papatuyo ng mga sampol. Bukod pa rito, ang refractive index o sukat ng pagbaluktot ng liwanag ng medium na ito ay hindi palaging pinakamainam para sa madaling pagmamasid ng mga spermathecae. Ang pag-mount sa "aqueous medium" (hal. Hoyer's liquid), sa kabilang banda, ay mas mabilis gamitin at mas malinaw kung titingnan ang spermatheca, ngunit ito ay hindi naaayon sa pangmatagalang pagdikit dahil ito ay humihigop ng tubig mula sa hangin. Isang opsyon ay ang

pagselyo ng gilid ng slide gamit ang nail polish kapag ito ay lubos nang natuyo. Ang pagtimbang ng naaangkop na gagamitin ay patuloy pa rin sa panahon ngayon, na nakakaapekto sa pagpili ng paraan ng pag-mount, depende sa layunin ng paghahanda. Simula noong 1980s, ang mga pag-aaral sa pagkakakilanlan ng nik-nik ay nakasalalay sa pinagsamang pagsusuri ng mga istrukturang pangangatawan at biokemikal na mga pamamaraan. Ang una ay ang pag-aaral ng cuticular hydrocarbon, na mabilis na napalitan ng mga teknik sa molekular na haynayan (tulad ng random amplified polymorphic DNA (RAPD), restriction fragment length polymorphism (RFLP), DNA amplification, at sequencing gamit ang Sanger method, pati na rin ang next-generation sequencing (NGS)). Sa kasalukuyan, ang mga molecular na pamamaraan ay dinaragdagan ng mga proteomic na paraan tulad ng MALDI-ToF. Bukod pa dito, ang molekular na pagkilala ng sarihay ay maaaring itambal sa pagtuklas ng mga pathogen sa pamamagitan ng PCR (*Leishmania*, *Trypanosoma*, *Bartonella*, at *Phlebovirus*) sapagkat ang lahat ng ito ay maaaring matukoy sa pamamagitan ng end-point at real-time PCR, na nangangailangan ng pagbagay ng paraan ng sampling at pagsusubi ayon sa mga nakatakdang layunin [3, 32]. Bukod sa mga karaniwang katangiang pangangatawan na ginagamit sa kinaugalian pagkakakilanlan ng sarihay, maaari ding gamitin ang iba pang pamamaraang base sa istrukturang pangangatawan (hal. wing geomorphometry).

Batay sa personal na karanasan ng mga may-akda at mga nailathalang panitikan, ang layunin ng pag-aaral na ito ay magbigay ng pamantayang alituntunin sa pag-mount at pag-proseso ng mga nik-nik, upang i-optimize ang mga pagsusuri ayon sa katangiang pangangatawan at molekular na katangian.

Sa pagsasagawa ng ilang pagsusuri (hal. molecular biology o MALDI-ToF) kinakailangan magtago ng ilang bahagi ng nik-nik na hindi mahalaga sa pagsusuri ng pangangatawan sa pagkilanlan ng sarihay, na nagbibigay-diin sa pangangailangan para sa kritikal na pagpili ng protokol. Sa artikulong ito, pinagtutuunan ang pamamaraan ng pagpapatulog at pagpatay ng mga nik-nik na nahuling buhay, ang pagsusubi at ang proseso ng pag-mount, para sa mabilisang pagkilanlan o para sa mas pangmatagalang pagtatago upang mapag-aralan pa sa mga susunod na pagkakataon.

Pambungad: Pangkaligtasan at pangregulasyong konsiderasyon ay marapat na sumanguni sa kaugnay na Safety Data Sheet (SDS).

Lahat ng mga kemikal na nabanggit sa panuntunang ito ay dapat gamitin alinsunod sa mahigpit na kondisyong pangkaligtasan. Ang mga "health and safety committees" ng mga research facilities ay nariyan upang magbigay ng mga kaalaman hindi lamang sa mga taglay na panganib ng mga kemikal na ito, kundi pati na rin sa tamang pamamaraan ng paghawak at ligtas na pagtapon ng mga nagamit na kemikal. Gayunpaman, sapilitan ang pagsunod sa mga tagubilin sa kaligtasan ukol sa paggamit at pagtatapon nito. Dapat tandaan na responsibilidad ng lahat ng gumagamit na tiyakin ang pagsunod sa mabuti at ligtas

Talahayanan 1. Listahan ng mga pagdadaglat.

**BME**	Basal medium Eagle
**CDC**	Centers for Disease Control and Prevention
**CMCP**	Camphor-monochlorophenol
**CMR**	Carcinogenic, mutagenic, reprotoxic substance
**COI**	Cytochrome c oxidase subunit I gene
**CytB**	Cytochrome b gene
**DNA**	Deoxyribonucleic acid
**ELISA**	Enzyme-linked immunosorbent assay
**EtOH**	Ethanol
**M199**	Medium 199
**MALDI-ToF MS**	Matrix-assisted laser desorption/ionization time-of-flight mass spectrometry
**MEM**	Minimum essential media
**NGS**	Next-generation sequencing
**NNN**	Novy-MacNeal-Nicolle medium
**PCR**	Polymerase chain reaction
**Lao PDR**	Lao People's Democratic Republic
**PNOC**	Prepronociceptin gene
**qPCR**	Quantitative PCR (real-time PCR)
**RAPD**	Random amplified polymorphic DNA
**RFLP**	Restriction fragment length polymorphism
**RI**	Refractive index
**RNA**	Ribonucleic acid
**RNases**	Ribonucleases
**RNASS**	RNA stabilization solution
**RT-PCR**	Reverse transcription PCR
**TFA**	Trifluoroacetic acid
**BME**	Basal medium Eagle
**CDC**	Centers for Disease Control and Prevention

na mga gawain sa laboratoryo alinsunod sa naaangkop na batas at regulasyon sa kani-kanilang bansa o research institution. Bukod pa dito, ilan sa mga kemikal o kanilang sangkap (hal., chloral hydrate) ay pinapamahalaan sa ilang mga bansa. Ang listahan ng mga pagdadaglat na ginamit sa papel na ito ay nakalahad sa Talahayanan 1.

1. Paghuli ng nik-nik

Ang magulang o adult na nik-nik ay maaring mahuli ng buhay o patay gamit sa iba't-ibang paraan tulad ng CDC miniature light traps, sticky trap, at aspirators gamit ang Shannon traps, o direkta mula sa lugar na kanilang pinagpapahingahan sa kapaligiran (hal. tirahan ng ibang hayop). Ang mga pamamaraang ito ay nagsasangkot ng paglalagay ng mga bitag sa mga naangkop na lugar, pag-akit sa mga nik-nik gamit ang ilaw o ibang pain (CO_2 o kemikal na pa-in), at pagkolekta sa kanila para sa karagdagang pagsuri, gaya ng inilarawan sa ilang mga paglilimbat [2, 3, 32, 36, 49].

Ang paghuli ng mga buhay na nik-nik ay nagbibigay-daan sa lahat ng mga karagdagang pagsusuri, samantalang ang pagkolekta ng mga patay na indibidwal ay humahadlang sa paghihiwalay ng mga strain ng *Leishmania* o ng mga virus. Ang ilang mga teknik sa paghuli, tulad ng paggamit ng malagkit na papel, ay madalas na nagre-resulta sa pagka-tanggal ng mga bahagi ng katawan ng nik-nik (antennae, palps, pakpak, o mga biyas). Dagdag pa dito, ang langis ng castor na pinahid upang maging malagkit ang papel ay dumidikit sa mga nik-nik at kailangan munang alisin sa simula ng proseso, na kadalasang tumatagal ng 15-minutong pagbabad sa pinaghalong pantay na bahagi ng ethanol at diethyl ether.

2. Pagpatay ng espesimen

Pagkatapos ng pagkolekta, ang mga buhay na nik-nik ay dapat patayin. Sa ilang pamamaraan ng pagkolekta (hal., sticky papers, CDC light traps na may kasamang lalagyan na naglalaman ng detergent o ethanol) ang mga nik-nik ay patay na nakokolekta. Ang teknik ng pagsusuri ng molekular na haynayan ay maaaring gamitin sa mga nakolekta ng direkta sa ethanol at sa iba pang sampol kung sila ay mabilis na naimbak sa ethanol. Gayunpaman, wala sa mga pamamaraang pagpatay na ito ang nagbibigay-daan sa pagpoproseso ng kulisap gamit ang MALDI-ToF. Bukod pa rito, ang ilang pamamaraan ng pagpatay ay maaaring magdulot ng pagkawala ng ilang katangiang pangangatawan. Kaya mahalaga na gumamit ng angkop na ahenteng pangpatay upang matiyak ang tamang pagkakakilanlan o pangmatagalang pag-iimbak bilang voucher espesimens (ibig sabihin, mga espesimen na napreserba at nakaimbak para sa hinaharap na sanggunian o paghahambing). Ang mga kemikal tulad ng ethyl acetate, ethyl ether, tetrachloroethane, at chloroform ay pinasisipsip sa bulak bago nilalagay sa lalagyan na naglalaman ng mga sand flies na papatayin. Ang mga ahenteng pangpatay na ito ay dapat maingat na gamitin, na sumusunod sa mga rekomendasyon ng tagagawa dahil sa kanilang taglay na panganib. Gayunpaman, hindi namin inirerekomenda ang pagpatay sa mga nik-nik gamit ang chloroform, dahil sa aming karanasan, hindi ito tugma sa mga pagsusuri ayon sa molekular na haynayan. Dahil sa mapanganib na katangian ng lahat ng mga produktong ito at sa kanilang kaduda-dudang kaangkupan para sa mga pagsusuring molekular, ang paggamit ng mga kemikal na ito ay karaniwang hindi iminumungkahi.

Ang pinaka-malawakang ginagamit na paraan, na nagpapanatili ng katangiang pangangatawan, DNA o mga protina, ay ang pagpapatuyong pagyeyelo (dry freezing) ng mga espesimen. Ang mga espesimen ay dapat napalamigan nang sapat na tagal upang maging ganap na makatulog, ngunit hindi masyadong matagal na sila ay (i) matuyuan, o (ii) makompromiso tungkol sa viability ng *Leishmania*, kung ang layunin ay ihiwalay ang mga ito in-vitro mula sa bituka ng nik-nik. **Kaya namin inirerekomenda ang tagal ng pagyeyelo mula 15 hanggang 20 minuto sa -20°C, na regular na sinusubaybayan upang matiyak na sila ay nahihilo lamang na hindi pinapatay ang mga parasitong *Leishmania*.**

Kung walang magagamit na freezer, ang mga kulisap ay maaaring patayin gamit ang CO_2. Sa mga kondisyon sa ilang (field) kung saan hindi magagamit ang mga CO_2 cylinders, ang mga espesimen ay maaaring patayin gamit ang maliliit na komersyal na CO_2 containers na ginagamit sa 'Soda siphons' (drink dispensers), ngunit maaaring may mga paghihigpit sa kanilang transportasyon sa pamamagitan ng eroplano. Bilang huling paraan, ang mga kulisap ay maaaring patayin sa pamamagitan ng paglalantad sa usok ng tabako. Ang mga nik-nik ay nahuhuli nang buhay sa CDC trap, kinokolekta gamit ang aspirator o pangsipsip, pinapanatili sa salamin na tubo (glass tube), at inilalantad sa usok ng tabako na pumapatay sa kanila sa loob ng ilang segundo. Ang paraang ito ay naaangkop sa lahat ng kondisyon sa ilang, kahit sa mga liblib na lugar. Sapagkat ang salamin ay napupuno ng usok, hindi ito magagamit para sa mga susunod na pagkolekta at ng mga buhay na nik-nik nang walang masusing paglilinis. Gayunpaman, ang hindi nalinis na aspirator ay maaari pa ring gamitin para sa pagpatay sa mga nik-nik mula sa iba pang mga bitag kung ang layunin ay para sa fixation. Kailangan din suriin kung lahat ng mga espesimen ay naalis na sa aspirator. Ang mga pamamaraang ito ay tugma sa paghihiwalay ng *Leishmania* sa pamamagitan ng daysekion ng bituka.

3. Pagiimbak ng Espesimen bago i-proseso

May lima na pangunahing pamamaraan ng fixation bago simulan ang pagpro-proseso ng nik-nik:

3.1. Freezing (pagyeyelo)

Ang pamamaraang ito ay pinakamahusay na ginagawa sa -20°C o, mas mainam sa -80°C. Ang mga pamamaraang pag-iimbak na ito ay mas malawakang ginagamit ngayon kaysa sa pag-iimbak sa liquid nitrogen. Sa lahat ng mga kaso, ang cryopreservation ay dapat ipatupad nang mabilisan sa lalong madaling panahon matapos mahilo ang mga espesimen. Ang malamig na pag-iimbak sa mga freezer ay nag-aalok ng bentahe ng ganap na pagpapanatili ng mga kulisap mismo, pati na rin ang RNA, DNA, at mga protina na may buong integridad sa buong panahon ng pag-iimbak.

Sa kabilang dako, ang liquid nitrogen ay maaaring lubhang makasira ng mga pakpak, biyas, palps, at antennae, madalas na napuputol ang mga ito at paminsan-minsan ay nadadamay na matanggal ang mga pangunahing katangian ng pangangatawan. Ang tuyong pagiimbak sa freezer ay hindi gaanong nakakasira para sa mga espesimen, ngunit hindi ito perpekto para sa pagpapanatili ng kanilang mga marupok na organo. Mahalagang tandaan, sa oras ng pagtunaw, ang mga pakpak, antennae, palps, o biyas ay maaaring dumikit sa mga vials at sa kalaunan ay mapunit dahil sa kondensasyon o hamog. Gayunpaman, ang preserbasyon sa pamamagitan ng pagyeyelo ay hindi palaging maaari sa mga pag-aaral sa ilang dahil nangangailangan ito ng access sa freezer o lagayan ng liquid nitrogen. Ang pag-iimbak sa freezer ay ganap na tugma sa pagtuklas ng pathogen gamit ang mga molekular gamit nang walang pagkawala ng pagiging sensitibo, bagaman ang pagtuklas ng RNA virus at paghihiwalay nito ay nangangailangan ng pagyeyelo sa -80°C o sa liquid nitrogen kung kailangan ang pangmatagalang pag-iimbak. Gayunpaman, ang pagyeyelo ng mga sampol ay hindi nagbibigay-daan sa paghihiwalay ng *Leishmania* sa pamamagitan ng dayseksyon ng bituka, maliban kung ang mga nik-nik ay unang ibinabad sa vapor phase at pagkatapos ay sa liquid nitrogen (halimbawa sa mga vials na inilagay sa stocking), na ginagaya ang cryopreservation ng *Leishmania*.

3.2. Pag-iimbak sa alkohol (ethanol o isopropyl na alkohol)

Ito na marahil ang pinaka-malawakang paraan na ginagamit para sa pag-iimbak ng mga nik-nik. Madali itong ipatupad sa ilang na lugar, kahit na sa mahihirap na kondisyon na walang access o kakayanang pumasok sa laboratoryo. Ang pagtatanggal sa alkohol ay naaangkop para sa pag-susuri ng mga bahagi ng katawan, dahil ang mga maseselang organo (pakpak, biyas, antennae, o palps) ay nananatiling buo, kung walang bula sa loob ng lagayang imbakan. Kaya naman inirerekomenda ang pagseselyo ng lagayan gamit ang maliit na bola ng bulak upang maalis ang anumang bula at paglalagay ng label sa ibabaw ng bulak na pamasak (Pigura 1). Ang tamang konsentrasyon ng alkohol ay nananatiling usapin ng debate. Sa pangkalahatan, ang mga konsentrasyon na mas mababa sa 70% ay hindi inirerekomenda [45, 66]. Ang mas mataas na konsentrasyon ay mas epektibong sa pag-preserba ng DNA sa mas mahabang panahon ngunit ginagawa nitong mas marupok at malutong ang mga espesimen para sa pagsusuri ng mga bahagi ng katawan. Ang paggamit ng 96% ethanol (ang azeotrope mixture) ay naniniguro ng pananatili ng konsentrasyon sa paglipas ng panahon, lalo na sa mga mahalumigmig na lugar tulad sa mga tropikal na bansa, bagaman ang 95% ethanol ay mas madaling makuha. Anuman ang konsentrasyon, ang DNA ay karaniwang mainam na na-pe-preserba sa ethanol (bagaman hindi kasing epektibo ng mga pagyeyelong pamamaraan, lalo na para sa NGS-type na molekular na pamamaraan). Ang mga protina ay mas hindi matatag, lalo na para sa proteomics, tulad ng MALDI-ToF na aplikasyon. Ang mga nik-nik na nababad sa alkohol ng ilang buwan ay maaari pa ring makilala gamit ang mga bahagi ng katawan, ngunit imposibleng makagawa ng reference protein spectra mula sa mga espesimen na ito. Ang pag-iimbak sa alkohol o tuyong mga kondisyon ay maaaring mapabuti kung ang sampol ay na-yelo din sa -20°C. Ang pagyeyelo sa -20°C ay karaniwang nagpapabuti ng molekular na preserbasyon (hal., nucleic acids) sa pamamagitan ng pagpapabagal ng pagkasira at nagbibigay din ng karagdagang benepisyo para sa preserbasyon ng pangangatawan sa pamamagitan ng pagbabawas ng pagkabulok ng kalamnan sa paglipas ng panahon, kahit na ang epekto sa pangangatawan ay mas limitado kaysa sa molekular na integridad. Ang pag-iimbak sa ethanol ay maaari ding gamitin para sa pagtuklas ng DNA at RNA virus kapag gumagamit ng ethanol sa konsentrasyon na hindi bababa sa 70% para sa maiksing panahon ng pag-iimbak, na hindi hihigit sa ilang buwan. Ang isopropyl alkohol ay maaaring madaling makuha sa ilang bansa at nag-pe-preserba ng DNA, ngunit ginagawa nitong matigas ang mga espesimen. Hindi ito madaling masunog tulad ng ethanol at nagpapadali na ito ay maibyahe. Kung kinakailangan, ang mga nik-nik na na-preserba sa liquid nitrogen o dry-frozen ay maaaring ilipat sa alkohol, sa gayon ay pinagsasama ang mga kakulangan ng mga kaparaanang nabanggit.

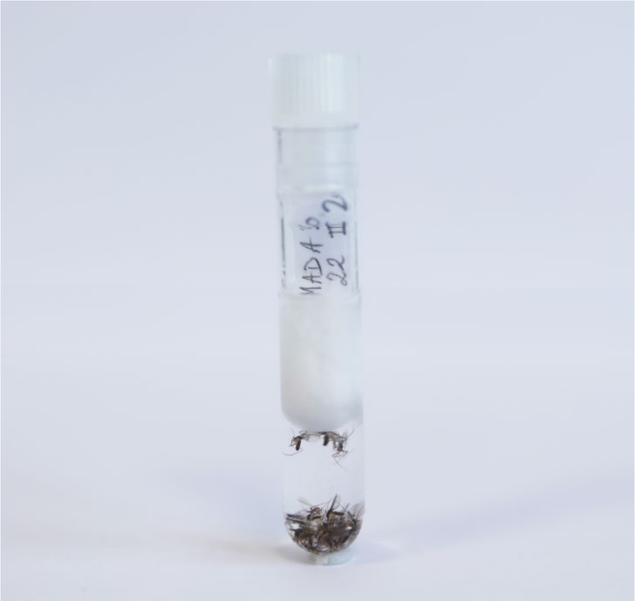

Pigura 1: Nik-nik na pinreserba sa ethanol.

3.3. Pag-iimbak sa RNA stabilization solution (RNASS)

Ang aqueous reagent na ito ay malawakang ginagamit, hindi nakakalason, at idinisenyo upang mag-stabilize at protektahan ang RNA sa mga sariwang kalamnan at mga sampol na sihay (cells). Gumagana ito sa pamamagitan ng mabilis na pagpasok sa espesimen at pag-inactivate ng mga RNases (RNA-degrading enzymes), sa gayon ay pinipigilan ang pagkasira ng RNA nang hindi na kailangang agad na i-yelo. Ang pag-iimbak sa RNASS ay karaniwang epektibo sa pag-preserba ng kabuuang kalamnan at selular na morpolohiya para sa kasunod na pagsusuri na histolohikal. Bagaman ang RNASS ay na-optimize para sa RNA stabilization sa halip na fixation, ang short- hanggang medium-term na pag-iimbak ay karaniwang napapanatili nang maayos ang istruktural na integridad. Pinapahintulutan ng RNASS na maimbak ang mga sampol sa temperaturang pang silid ng hanggang 7 araw, sa 4°C ng ilang linggo, o sa -20°C/-80°C para sa pang matagalang preserbasyon. Ang pamamaraang ito ay partikular na mahalaga sa pagtatrabaho sa ilang o sa klinikal na kapaligiran kung saan limitado ang cold chain infrastructure. Ang paghihiwalay ng RNA ay karaniwang nangangailangan ng pag-alis ng mga sampol mula sa pamalibilos at pagproseso ng mga ito ayon sa standard protokols.

3.4. Preserbasyon sa pamamagitan ng pagtutuyo sa temperaturang pang silid

Ito ay isang makalumang paraan, na kapag ginamit sa kabuuan ng espesimen (idinikit ng buo), ay nagdudulot ng malaking kawalan sa pagpapanatili ng mga maseselang bahagi tulad ng pakpak, biyas, antenna at palps. Ngunit ang mga proteonomic na pag-aaral gamit ang MALDI-ToF ay maari pa din isagawa kung lubusang natanggalan ng tubig ang katawan ng mga nik-nik noong fixation gamit ang silica gel-type na desiccant o pangtuyo. Sa kabilang banda, ang pagsusuring molekular na tinutumbok ang DNA ay nananatiling mahirap isagawa sa mga samol na ito, sapagkat ang DNA ay kadalasang putol-putol at maunti, na nangangahulugan na ang pagsusuri ay nananatiling mas mahirap kaysa sa mga bagong patay o na–yelong mga sampol, lalo na para sa mga nuclear genome. Gayunpaman, ang mga makabagong pamamaraan, tulad ng museomics, ay maaring gamitin sa mga sampol na pinatuyo sa ganitong paraan [34]. Dahil dito, ang paraan ng pag-iimbak na ito ay hindi inirerekomenda, maliban na lamang kung wala ng ibang pagpipilian. Maari din namang itambal ito sa pagiimbak sa lamig sa paraang paglalagay ng mga tubes sa freezer sa -20°C or -80°C. Ang pangunahing hamon ay ang pagkamit ng angkop na pagdikit ng mga espesimen o mga bahagi ng katawan na kinakailangan sa pagkakakilanlan. Upang makamit ito, ang muling pagbabalik ng tubig ay mahalaga. Inirerekomenda namin ang paggamit ng solusyon na Triton X-100. Ang tinatagal ng pagbabalik ng tubig ay nag-iiba mula sa ilang oras hanggang ilang araw, sa ilalim ng masusing pagsubaybay. Matapos ang lubusang pagbabalik ng tubig, ang mga espesimen ay dapat banlawan ng makatlong beses sa magkakasunod na lubluban ng tubig.

3.5. Preserbasyon sa mga filter paper

Ang pangunahing kalamangan ng pag-gamit ng filter paper ay ang matagalang katatagan ng genomic DNA sa loob ng hindi pa na-fix na selula, buong katawan na tinuyo, o selula ng dugo na nakaimbak sa temperaturang pang silid. Ang filter paper ay naihanda sa sukat ng maliliit na card, na nagbibigay-daan upang maimbak ang ilang daang sampol sa temperaturang pang silid sa lagayan na may sukat na mahalintulad sa drower ng isang maliit na mesa. Ang mga filter paper ay pinuspos ng mga kemikal na sumisira sa mga nakakahawang mikroorganismo, kaya hindi na maituturing na biohazard ang mga sampol na ito. Nagbibigay daan ito para sa pag-iimbak at pagpapadala ng mga sampol na hindi nangangailangan ng mga tiyak na pag-iingat sa panganib na biolohikal [68].

4. Pagdaysek ng mga espesimen

Di tulad ng ibang mga kulisap na ang pagkakakilanlan ay naka-basesa mga nakikitang katangiang panlabas ng mga indibidwal na kulisap nai-pin ng buo, ang mga nik-nik ay nangangailangan na ma-daysek at mai-dikit sa slides upang mapag-aralan ang mga katangiang pangangatawan para sa wastong pagkakilanlan. Kung ano man ang napiling proseso sa paghahanda at pagdikit, ang proseso ng pagda-daysek na ginagamit ay pare-parehas lamang (Pigura 2 & 3) (https://zenodo.org/records/18198006).

Paggamit ng Triton X100: non-ionic aqueous na solusyon

Tandaan ang pagdidikit o pag-mount ay ginagamit sa mga bagong huli o sa mga sapat na nakaimbak na espesimen. Karamihan ng mga kolektor ay may mga espesimen na na-preserba ng tuyo (para gamitin sa MALDI-TOF) o naka-imbak sa alkohol ng maraming taon. Sa kasamaang palad, ang preserbasyon ng espesimens sa alkohol ng matagal na panahon ay hindi mainam, at ang mga arthropods na na-preserba sa ganitong paraan ay nagiging mahirap maihanda para mikroskopikong pagsusuri. Ang kadalasang nangyayari ay ang pagkasira ng mga plastic na pinaglalagyan ng mga sampol, kasunod ang pagkatuyo ng alkohol. Sa mga pagkakataong ito, wala nang magagawa sapagkat nanatili ang sampol ng matagal sa alkohol o natuyuan na. Kaya lumitaw ang ideya na gumamit ng mga wetting agents na hindi matapang na sabon o detergent. Ang Triton X100 ay isang non-ionic aqueous na solusyon (*4-(1,1,3,3-tetramethylbutyl*) *phenyl-polyethylene glycol* na solusyon, o *t-octylphenoxypolyethoxyethanol*, *polyethylene glycol tert-octylphenyl ether*, na malawakang ginagamit bilang detergent sa selyular at molekular na haynayan. Nagbibigay daan ito sa permyabilisasyon ng mga sihay (cell) at ng nuclear membrane.

Narito ang pamamaraan sa paggamit ng non-ionic Triton X100 sa 0.5% aqueous na solusyon:

- Ibabad ang tuyong sampol sa absolute alcohol.
- Idagdag ang karampatang dami ng 0.5% Triton X100 nasolusyon hanggang sa lumubog ang kabuuan ng espesimen.
- Hayaan ang pagkababad ng espesimen sa loob ng 5 minuto hanggang ilang araw, habang sinusubaybaya ng maigi. Lahat ng arthropods ay dapat maghiwahiwalay habang nakababad sa solusyon.
- Alisin ang Triton X100 at palitan ng potassium hydroxide.

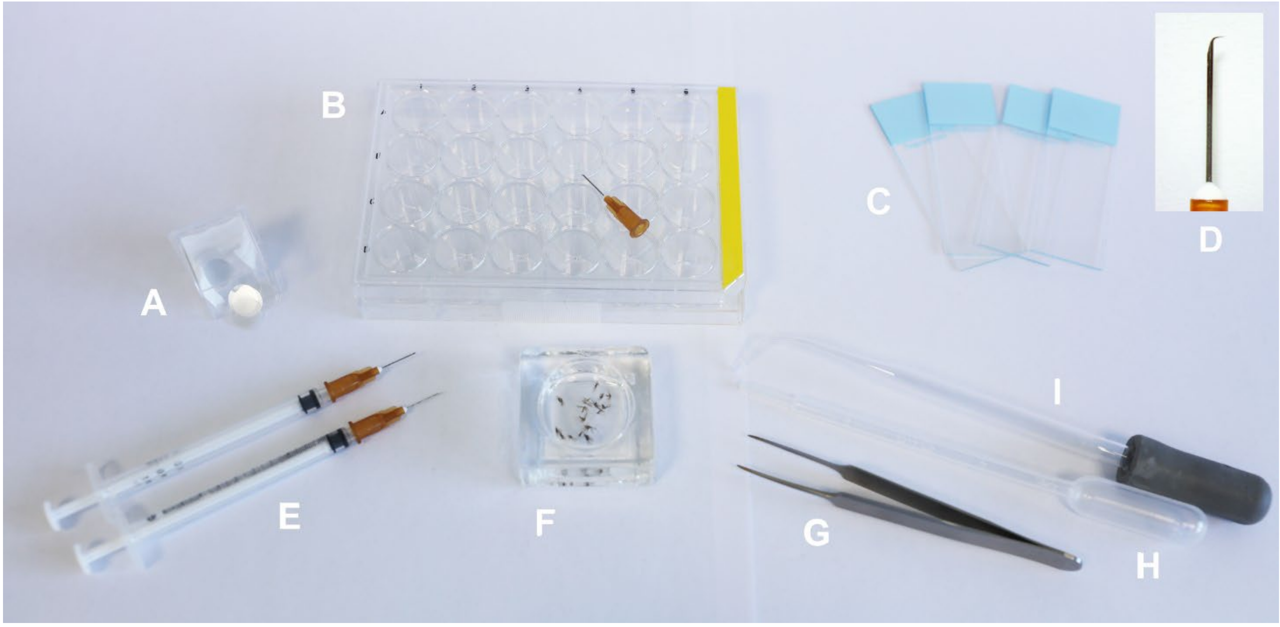

Pigura 2: Mga kinakailangang kagamitan sa pag-mount ng nik-nik: A. bilog na coverslip na gawa sa salamin (10 o 12mm ang diyametro o bantod); B. plato na may 24 na balon at karayom na may kalawit (kung langis ng clove o esensya ng Euparal® ang ginagamit sa pagproseso ng nik-nik, huwag gumamit ng acrylic na plato sapagkat ito ay magkakaroon ng kemikal na reaksyon na makakasira sa espesimen); C. slides na gawa sa salamin na maaring lagyan ng pananda; D. detalye ng karayom na may kalawit; E. mga karayom na nakakabit sa heringgilya; F. watch glass o kahalintulad na lalagyan para sa mga nik-nik na ididikit o i-mount; G. Dumont forceps o panipit; H. glass pipette na binaliko sa pamamagitan ng pag-iinit na ginagamit sa pagsasalin ng likido sa mga balon.

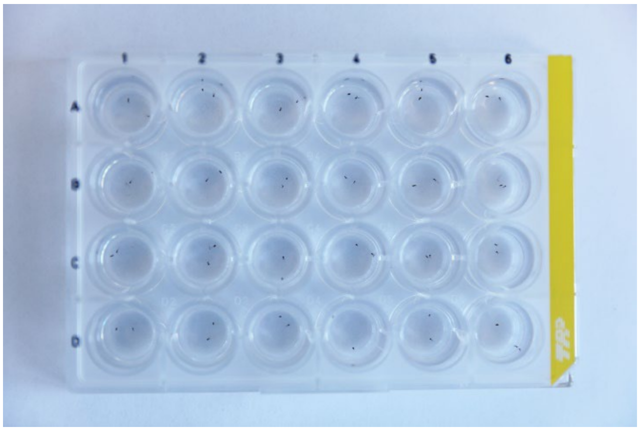

Pigura 3: Isang plato na may 24 na balon, ang bawat isa ay naglalaman ng ulo at dulo ng tiyan ng mga nik-nik.

Ang pagdaysek ay maaring gawin gamit ang maninipis na karayom o mga aspileng pang dalubkulisap sa ilalim ng stereomicropscope (Pigura 2 & 3). Kasama ang mga karayom na ito sa mga karaniwang ginagamit: 26G x 1/2" (0.45 × 13 mm), 30G × 1/2" (0.3 × 13 mm), o 25G × 5/8" (0.5 × 16 mm). Upang ihanda ang mga espesimen sa pagkakakilanlan, sa pinakamababang pangangailangan, ang ulo ay inihihiwalay sa katawan at idinidikit sa slide na nakatihaya upang mailantad ang cibarium at pharynx, habang ang dibdib (thorax) at tiyan (abdomen) ay inilalagay ng nakatagilid pagkatapos madaysek. Ang pagdidikit sa ulo ng patihaya ay tinitiyak na ang occipital foramen ay nakapaling ng pataas upang direktang makita ang cibarium. ang pag-access sa mga bahagi ng katawan na ito ay napapadali kung ang ulo ay lubusang nakahiwalay sa katawan.

4.1. Ulo

4.2. Pakpak at dibdib (thorax)

Ang mga pakpak ay dapat maidikit sa slide nang patag. Ang bawat pakpak ay maaaring ihiwalay sa katawan mula sa puno nito at idikit sa slide ng magkahiwalay, o isa lang ang tatanggalin at mag-isang ididikit sa slide habang ang kabila ay nananatiling nakakabit sa dibdib (thorax). Kung pinaplano ang pagsusuri ng geometric morphometry, mahalaga ang wastong pagkakilanlan at pagtatanda ng kaliwa at kanang pakpak bago idikit o i-mount. Ang dibdib ay nahahati sa ilang bahagi, at ang bawat isa ay naglalalaman ng napakahalagang impormasyon para sa taksonomiya [20, 64]. Karaniwang idinidikit ito ng patagilid (lateral view) upang bigyang daan ang pagsusuri ng pagkaka-ayos ng mga buhok o chaetotaxy at pagkalat ng kulay. Ang pagkakaroon ng mga peklat na pinagmulan ng mga buhok sa ilang bahagi ng dibdib ay maaaring gamitin upang makilala ang ilang sarihay ng genus *Brumptomyia*. Ang pagkaka-ayos ng kulay ay maaaring namang gamitin upang paghiwalayin ang Neotropical na nik-nik sa lebel ng genus (hal., *Bichromomyia*), hanay ng sarihay (hal., *Pintomyia*), o maging mga sarihay ng parehong genus (hal., *Micropygomyia, Nyssomyia, Psathyromyia*, at *Psychodopygus*) [20]. Kaya kung ang dibdib ay hindi gagamitin para sa molekular na pagsususri, nararapat na ito ay i-mount nang hindi ito masira. Importanteng tandaan na hindi ang tingkad ng mga kulay ang mahalaga, kundi ang kanilang pagkaka-ayos sa kabuuan ng dibdib. Samakatuwid, ang proseso ng paglilinaw ay hindi magtatanggal ng kulay o ng disenyo nito.

4.3. Henitalya o kasangkapang pangkasarian

Dapat maging napaka-ingat kapag nagma-mount ng henitalya ng lalaki at babaeng espesimen, dahil ang mga ito ay napakahalaga sa pagkakakilanlan ng mga genera, subgenera, at sarihay. Sa parehong kasarian, ang henitalya ay magkapares.

4.3.1. Kalalakihan

Ang henitalia ay panlabas at binubuo ng mga magkapares na panipit, na ang bawat isa ay binubuo ng gonocoxite-gonostyle na artikulasyon sa likod na bahagi nito at ng epandrial lobe sa harapang bahagi nito. Ang gonostyle ay may mga tinik at kung minsan ay may mga buhok (setae), na dapat mabilang at ang mga posisyon ng pagkakasingit nito ay dapat na malinaw na makita. Mahalaga na maingat na obserbahan ang loob na bahagi ng gonocoxite, na maaaring may kumpol ng buhok na direktang nakadikit (sessile setae) o mga buhok na nakadikit sa isang lobe (= tubercle) [22]. Ang mga kasamahan na may limitadong karanasan sa pag-daysek ay maaaring gumawa ng simpleng patagilid na mounting, nang hindi tinatanggal ang henitalya mula sa dulo ng tyan (https://zenodo.org/records/18311158). Sa paraang ito, ang pagkakapatong (superposition) ng dalawang bahagi ng henitalya ay maaaring magpahirap sa pagbibilang ng panloob na buhok (internal setae) ng gonocoxite. Ngunit iniiwasan nito ang pagkasira ng henitalya sa pamamagitan ng nabigong dayseksyon. Ang mga kasamahan na may mas malawak karanasan ay maaaring subukang buksan ang henitalya sa dalawa, upang hatiin ang mga ito. Upang makamit ito, ang tapyas na bahagi ng isang karayom (intradermal reaction needle type) ay dapat na ilusot sa pagitan ng henitalya, na tinatanggal nang hindi lubos na ginugupit ang henitalya upang hatiin ang mga gonocoxite-gonostyle assemblies (https://zenodo.org/records/18311158). Sa ganitong paraan, ang pagmamasid ng kanilang loob na bahagi ay magiging madali. Pinapadali din nito ang pagmamasid ng mga parameres at parameral sheaths, na hindi na magkapatong. Para sa patagilid na mounting, na nagsusulong ng pagkakapatong ng mga ng organo, ang mga espesimen ay dapat na lubusang mapalinaw.

4.3.2. Kababaihan

Ang kasangkapang pangkasarian (genital apparatus) ay panloob, na binubuo ng spermathecae. Sa kawalan ng dayseksyon, dapat silang obserbahan sa pamamagitan ng mga tegument at ang pag-mount ng tyan sa patihayang posisyon. Anuman ang mounting medium na napili, ang spermathecae ay karaniwang maaaring obserbahan ng tama, lalo na kung ito ay hindi makinis at napalinaw (cleared). Gayunpaman, ang pagmamasid sa spermathecae na makinis, na may manipis na dingding ay maaaring maging masalimuot sa mga media na mahina kakayanang magbaluktot ng liwanag (poorly refractile). Dagdag pa rito, ang pagmamasid sa puno ng mga spermathecal duct ay mahalaga para sa pagkilanlan ng mga sarihay, tulad ng sa subgenus Larroussius [35, 37, 38], ang mga pangunahing vector ng *Leishmania infantum* sa Old World. Kung hindi mapagmamasdan ito, ang pagkakilanlan ng sarihay ay nananatiling imposible. Upang malampasan ang mga hirap sa pagmamasid na ito, ang genital furca-spermathecae mounting ay dapat tanggalin mula sa tyan (https://zenodo.org/records/18311106). Ang mga spermathecae ay karaniwang mahirap obserbahan sa panahon ng dayseksyon, ngunit ang genital furca ay medyo madaling mahanap. Dahil ang mga spermathecal duct ay bumubukas sa genital furca, ang paghihiwalay ng furca na ito ay karaniwang nagbibigay-daan sa paghihiwalay ng mga spermathecae. Kung ang mga spermathecae ay aksidenteng nahiwa sa proseso, hindi sila nawawala at maaari pa ring makita sa loob ng mga abdominal integument (Pigura 4).

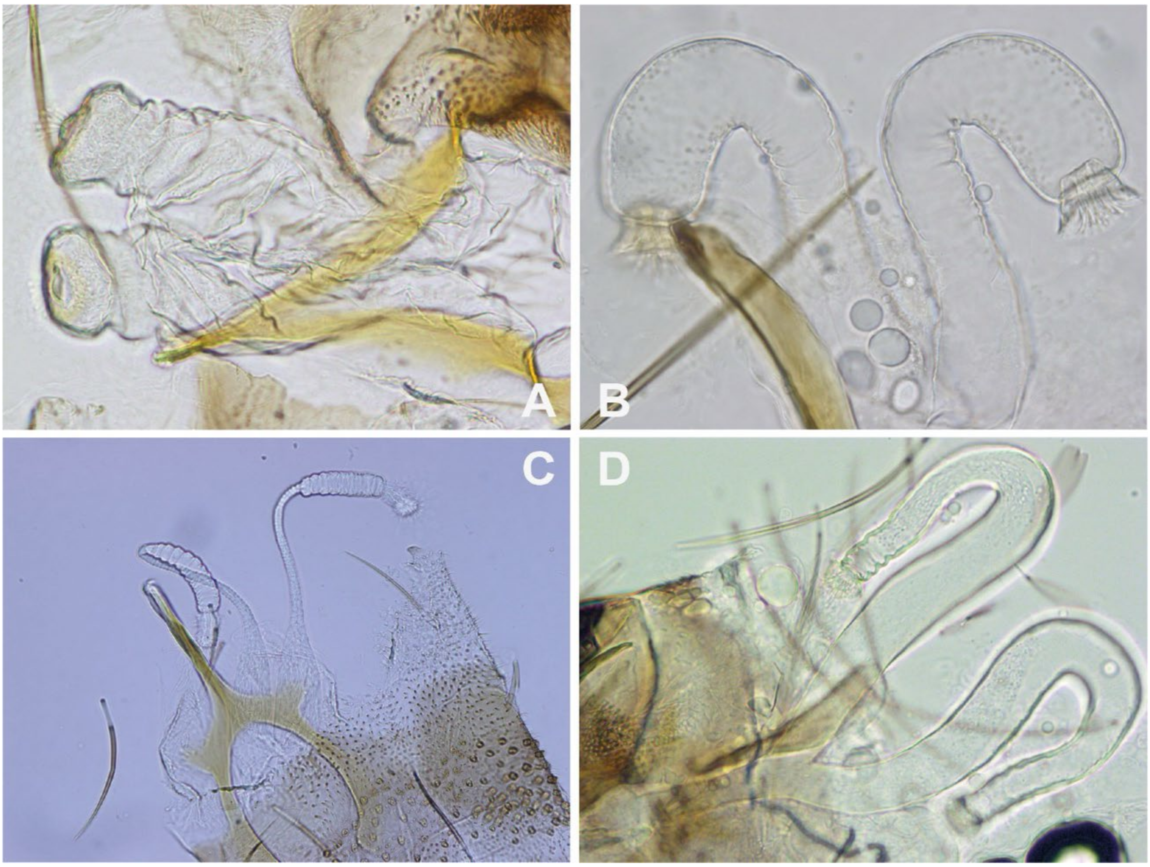

Pigura 4: Mga spermathecae na na-daysek at na-mount sa Marc-André fluid mula sa mga sariwang espesimen. A: *Idiophlebotomus longiforceps* (Lao PDR); B: *Sergentomyia minuta* (França); C: *Phlebotomus ariasi* (França); D: *Sergentomyia anodontis* (Lao PDR).

4.4. Dayseksyon ng midgut para sa aysolasyon ng *Leishmania*

Ang dayseksyon ng sistemang panunaw (digestive tract) ay mahalaga para sa pagtuklas at isolasyon ng *Leishmania* sa mga babaeng nik-nik. Ang proseso ay maaaring gawin sa labas at loob ng laboratoryo, upang masuri ang kakayanang magkalat ng sakit (vectorial competence).

Inirerekomenda na gumamit ng mga babaeng nik-nik na kakamatay pa lamang. Hugasan ang mga babaeng nik-nik gamit ang tubig o solusyong asin na may halong kaunting sabon upang matanggal ang labis na mga buhok. Ang hakbang na ito ay tumutulong na mapanatili ang aseptic na kondisyon para sa aysolasyon ng *Leishmania*, habang pine-preserba ang mga katangiang pangangatawan na kailangan para sa pagkakilanlan. Upang mahanap at ma-isolate ang *Leishmania*, maingat na tanggalin ang midgut at ilagay ito sa isang patak ng sterile na solusyong asin (0.9% NaCl). Matapos ma-obserbahan ang mga gumagalaw na parasito sa ilalim ng light microscope (inirerekomendang magnifikasyon: ~200×), gumamit ng insulin syringe o micropipette upang ilipat ang mga ito sa cultivation medium (para sa karagdagang detalye tingnan ang Kabanata 4.4.3).

I-mount ang ulo at henitalya ng direkta sa Marc-André fluid upang maging malinaw ang mga ito. Mahalaga: huwag kailanman hayaang magkaroon ng pagkakataon na mapadikit ang Marc-André fluid sa *Leishmania* – tuwiran man o hindi tuwiran sa pamamagitan ng mga kasangkapan o mga karayom – dahil nakamamatay ito sa mga parasito.

Ang dayseksyon ng mga babaeng nik-nik ay maaaring gawin sa isa o dalawang slide; ang parehong opsyon ay may mga bentahe at limitasyon (Pigura 5; https://zenodo.org/records/18311154).

This is an Open Access article distributed under the terms of the Creative Commons Attribution License (https://creativecommons.org/licenses/by/4.0), which permits unrestricted use, distribution, and reproduction in any medium, provided the original work is properly cited.

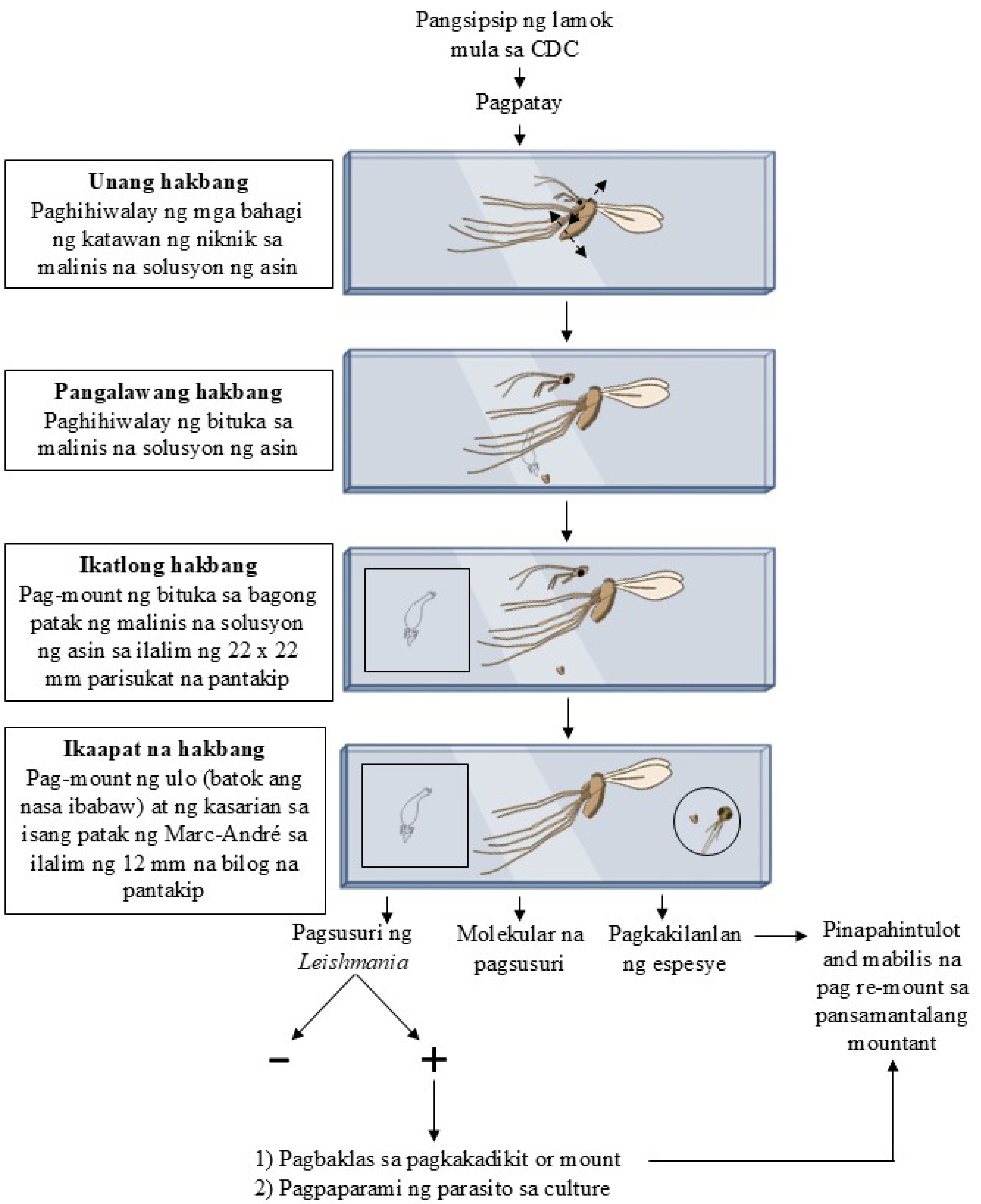

Pigura 5: Pamamaraan sa pag-isolate ng *Leishmania*

This is an Open Access article distributed under the terms of the Creative Commons Attribution License (https://creativecommons.org/licenses/by/4.0), which permits unrestricted use, distribution, and reproduction in any medium, provided the original work is properly cited.

4.4.1. Pamamaraan gamit ang dalawang slides

Ang unang paraan ang gumagamit ng dalawang magkahiwalay na slides: isa na may lamang sterile saline para sa pagkuha ng midgut, at ang isa para pagdikitan ng ulo at spermatheca sa Marc-Andre fluid. Sa kabilang banda, sa [field condition], karaniwang may dalawa o tatlong tao na nagdidisek ng nik-nik at pinapasa nila ang kanilang napagdisekan sa isang tagapag saliksik na naatasan sa pagkakakilanlan at pagtingin ng impeksyon ng Leishmania sa bituka. Ang pamamahala ng dalawang slides ay maaaring magkaroon ng problema sa pagsubaybay ng mga sampol, lalo na sa pagtukoy kung aling nik-nik ang may impeksyon o ang may bitukang nagpositibo.

4.4.2. Pamamaraan gamit ang isang slide

Ang paggamit ng isang slide ay nagsisiguro kakayahang pagsubabay ng mga resulta. Gayunpaman, dapat gumawa ng ilang pag-iingat. Upang ma-maximize ang sterility sa hakbang na ito, ang mga operator ay dapat na regular na maglinis ng kanilang mga kamay gamit ang hydroalcoholic gel. Dapat gamitin ang mga non-frosted slides at square coverslips (22 x 22 mm) na nakabalot sa aluminium foil at na-sterilize sa pamamagitan ng dry heat (gamit ang Poupinel oven), kasama ang mga sterile needles para sa bawat daysekyon (mungkahi: 25G Ø 0.5mm × 16mm). Ang nik-nik ay inilalagay sa isang patak ng sterile saline sa gitna ng slide. Ang ulo ay pinutol habang ginagawa ang hiwa sa pagitan ng ika-6 at ika-7 abdominal tergites at sternites nang hindi pinutol ang digestive tract (mas mataas na hiwa ay maaaring gawin kung inaasahan ang napakahahabang spermathecae). Pagkatapos, ang dibdib ay dapat na ma-immobilize gamit ang needle, at ang huling posterior abdominal segments ay marahan na hilahin gamit ang isa pang needle upang makuha ang gut. Kung hindi ito magtagumpay, may posibilidad na harangan ang dulo ng tyan gamit ang needle at hilahin ang digestive tract mula sa anterior part nito. Kung hindi pa rin ito magtagumpay, ang gut ay dapat na makuha sa pamamagitan ng pag-alis ng kasing dami ng natitirang tegument sa paligid nito hanggang sa maaari. Kapag naalis na ang gut, ang huling abdominal segments ay dapat na paghiwalayin sa pamamagitan ng pagputol sa digestive tract. Ang gut ay inilalagay sa bagong patak ng sterile saline na nakaposisyon sa isang gilid ng slide, at pagkatapos ay marahan na tinatakpan ng sterile coverslip. Ang ulo at ang huling abdominal segments ay inililipat sa maliit na patak ng Marc-André liquid na nakalagay sa kabilang dulo ng slide, na tinitiyak na walang contact sa Leishmania. Ang ulo ay tamang nakaorient (occipital foramen pataas), at ang spermathecae ay inihihiwalay kasama ang genital furca tulad ng nabanggit sa itaas at tinatakpan ng maliit na round coverslip (Ø 12 mm, hindi dapat malito sa mga sterile square coverslips). Ang natitirang bahagi ng katawan ng nik-nik at mga pakpak ay nananatili sa patak ng saline sa gitna ng slide (https://zenodo.org/records/18311154). Sa kaso ng positivity, o para sa taxonomic exploration, ang dibdib at tyan ay maaaring ma-preserba para sa molecular o proteomic studies, at ang mga pakpak ay maaaring i-mount sa aqueous medium. Upang ma-preserba ang mount, ang sobrang volume ng Marc-André liquid ay maaaring palitan ng aqueous mounting medium tulad ng chloral gum (=Hoyer) o polyvinyl alcohol-based medium.

Ang mga detalyadong video na nagpapakita ng mga pamamaraang ito ay available (sand fly midgut dissection: https://zenodo.org/records/18303014 at sand fly salivary glands dissection: https://zenodo.org/records/18302850), kaya hindi na ito ipapliwanag dito.

4.4.3. Aysolasyon at pagpaparami gamit ang culture ng *Leishmania* parasite mula sa bituka ng nik-nik

Ang aysolasyon ng parasito mula sa dayseksyon ng nahawaang babaeng nik-nik ay isang delikadong pamamaraan na nangangailangan ng mataas na antas ng kasanayan at dapat munang magsanay sa mga espesimen na walang parasito. Matapos ng dayseksyon, ang mga bituka ay maaari nang ilipat sa sariwang patak ng sterile saline (0.9%) o sa Locke's solution para sa mahugasan [4]. Ang mga na-daysek na bituka ay maaari nang i-proseso sa dalawang paraan: i) suriin sa ilalim ng light microscope upang makita ang iba't-ibang yugto ng *Leishmania* promastigotes at ang kanilang lokasyon, na may partikular na pansin sa stomodeal valve, at ii) buksan ang bituka upang mapadali ang paglabas ng mga promastigotes na makakatulong sa kanilang maramihang pag-culture [4]. Ang paghahanap ng nakakahawang nik-nik sa ilang ay bihirang pangyayari kaya ang magagandang pagkakataon ng pagsasanay ay makakatulong upang mapataas ang tyansa ng matagumpay na aysolasyon.

Kung nakita ang mga parasito ng *Leishmania* sa bituka, dapat gumamit ng bagong sterile na karayom at magdagdag ng kaunting sterile na saline sa paligid ng coverslip sa pamamagitan ng capillary action upang mailabas ang mga ito. Ang bituka ay dapat maingat at mabilisang mapunit upang mailabas ang mga parasito sa saline. Gamit ang 100 µL micropipette o tuberculin syringe, kolektahin ang mga parasito at ilagay sa markadong culture medium.

In vitro culture ng *Leishmania* promastigotes: ang mga na-aysoleyt na parasito ay unang pinapanatili sa SNB-9 blood agar slopes o sa Novy, Mc Neal, Nicolle (NNN) solid medium [16] na pinatungan ng sterile alpha-MEM medium [16, 65] o ng M199 medium, na ang bawat isa ay may dagdag na 10% heat-inactivated sterile fetal calf serum [FCS] (upang mapahusay ang paglaki ng mga parasito), 1% BME vitamins, 2% sterile human urine (na na-sterilize gamit ang syringe filter Filtropur® S 0.2 µm), 250 µg/mL amikacin (o 50 µg/mL gentamicin, o halo ng mga antibiotic at amino acid (L-glutamine 200 mM-penicillin 10 000 U-streptomycin 10 mg/mL) [47]. Makalipas ang tatlong araw, kung walang kontaminasyon, ang mga kultura ay isususpinde sa inihandang freezing medium na itatago sa -80°C nang 1 hanggang 2 taon o sa liquid nitrogen sa -196°C

para sa pangmatagalang pag-iingat at hinaharap na paggamit sa eksperimento [7].

4.5. Salivary glands

Ang pag-daysek ng salivary gland ng mga nik-nik ay pangunahing pamamaraan para sa pag-aaral ng interaksyon ng vector-pathogen, lalo na para sa pagdetekta ng mga arbovirus tulad ng *Phlebovirus* (hal., Toscana virus) [44, 75]. Dahil sa napakaliit na sukat ng mga nik-nik, ang pamamaraan ay nangangailangan ng presisyon sa ilalim ng stereomicroscope, gamit ang pinong panipit o microdissection na karayom upang ma-isolate ang delikadong mga salivary gland nang hindi ito mapupunit o makokontamina (https://zenodo.org/records/18302850) [51, 61]. Ang pag-iingat sa kabuuan ng gland ay mahalaga upang masiguro ang maaasahang kasunod na pagsusuring molekular. Kapag nakuha na, ang mga gland ay maaaring i-homogenize at subukan sa pamamagitan ng RT-PCR, qPCR, o immunoassay upang makita ang viral RNA o antigen [12]. Ang pagkakaroon ng mga virus sa salivary gland, hindi lamang sa bituka o hemocoel, ay nagkukumpirma na nakumpleto na ng pathogen ang extrinsic incubation period nito at maaari nang makahawa sa panahon ng blood feeding [71].

Ang proseso ng dayseksyon ay teknikal na mahirap dahil sa maliit na sukat ng salivary gland ng nik-nik, na nangangailangan ng mataas na antas ng kadalubhasaan upang maiwasan ang pagkasira ng sampol [1, 51]. Bukod pa rito, ang mga viral load ay maaaring mababa, na nangangailangan ng napakasensitibong mga paraan ng pagdetekta tulad ng nested PCR o high-throughput sequencing [54]. Ang mga panganib ng kontaminasyon ay higit pang nagbibigay-diin sa pangangailangan ng sterile na teknik. Higit sa mga teknikal na hadlang, ang mga kadahilanang pang-haynayan ay nakakaimpluwensya sa tagumpay ng pagdetekta; ang vector competence ay nag-iiba sa iba't-ibang uri ng nik-nik, at ang mga rate ng impeksyon ay nag-iiba-iba depende sa ekolohikal at panahon na mga kondisyon [33, 61].

Ang pagdetekta ng mga virus sa salivary gland ay nagbibigay ng mahalagang pananaw sa mga panganib ng transmission, na nagbibigay-daan sa targeted surveillance at mga hakbang sa kontrol [15]. Halimbawa, ang pagkilala sa Toscana virus sa mga nik-nik sa endemic na rehiyon ay naging gabay sa mga diagnostic protokol at public health advisory [18]. Higit pa rito, ang pag-aaral ng virus-saliva interaction ay maaaring magbunyag ng mga bagong target para sa transmission-blocking vaccine o therapeutics [15, 18].

Ang salivary gland ng nik-nik ay maaari ring gamitin bilang pinagkukunan ng antigen para sa pagsukat ng host antibody laban sa nik-nik saliva gamit ang mga immunological method, mas mainam ang ELISA. Ang paraang ito ay nagbibigay-daan sa pagsusuri ng exposure ng host sa mga kagat ng nik-nik, kaya sumusuporta sa pagsusuri ng bisa ng mga paraan ng vector control [25] at ang panganib ng *Leishmania* transmission [40].

4.6. Pagkakilanlan ng blood meal

Ang mga busog na mga babae na nahuli ay dapat i-daysek gamit ang minsanang-gamit na kasangkapan upang maiwasan ang cross-contamination. Ang kanilang tiyan ay dapat suriin sa ilalim ng stereomicroscope upang masuri ang yugto ng pagtunaw ng dugo. Inirerekomenda na piliin lamang ang mga babae na may pula, pula-kayumanggi, o matingkad na pulang tiyan, na walang palatandaan ng pagbubuo ng itlog. Alisin ang dulo ng tiyan kasama ang spermathecae upang makilala ang babae base sa katangiang pngangatawan pagkatapos ng paglilinaw. Ang pangunahing bahagi ng tiyan (walang spermathecae) ay dapat ilagay sa Eppendorf® tubes at itago sa -20°C hanggang sa susunod na pagsusuri. Ang mga genetic marker na karaniwang ginagamit para sa pagkilala ng blood meal, tulad ng PNOC [5, 30, 50], CytB [67], o COI [13], ay well-established at malawakang naisalarawan sa mga panitikan; samakatuwid, hindi na ito dedetalyahin pa sa papel na ito (Pigura 6). Bilang kahalili, upang makilala ang dugo ng host, maaaring gamitin ang MALDI-ToF peptide mapping [31]. Napatunayan sa eksperimental na pamamaraan na ang teknik na ito ay nagbibigay-daan sa pagkilala ng dugo ng host sa loob ng mas mahabang panahon pagkatapos na masipsip ang blood meal; samakatuwid, ito ay angkop na paraan, lalo na para sa pagsusuri ng mga busog na kababaihan na may kitang mas sulong na pag-digest ng dugo ng host. Ang mga sampol ay dapat na itago sa -20°C o 4°C, ngunit maaari ding makamit ang magandang resulta mula sa mga sampol na nakatago sa temperaturang pang-silid sa maikling panahon. Ang tiyan ng busog na babae ay dapat i-daysek mula sa iba pang bahagi ng katawan ilang sandali bago ang pagsusuri at i-homogenize sa distilled na tubig. Ang natitirang bahagi ng katawan ng nik-nik ay nananatiling handa para sa iba pang pagsusuring molekular at ng pangangatawan. Pagkatapos kunin ang aliquot mula sa homogenate para sa MALDI-ToF peptide mapping, ang tira ay maaaring gamitin para sa DNA isolation upang i-kumpirma ang pagkilanlan ng dugo ng host at/o pagtuklas sa pagkakaroon ng *Leishmania* sp. Ang kabuuang oras ng paghahanda ng sampol at pagsusuri ay napakaikli kumpara sa DNA-based na molekular na teknik.

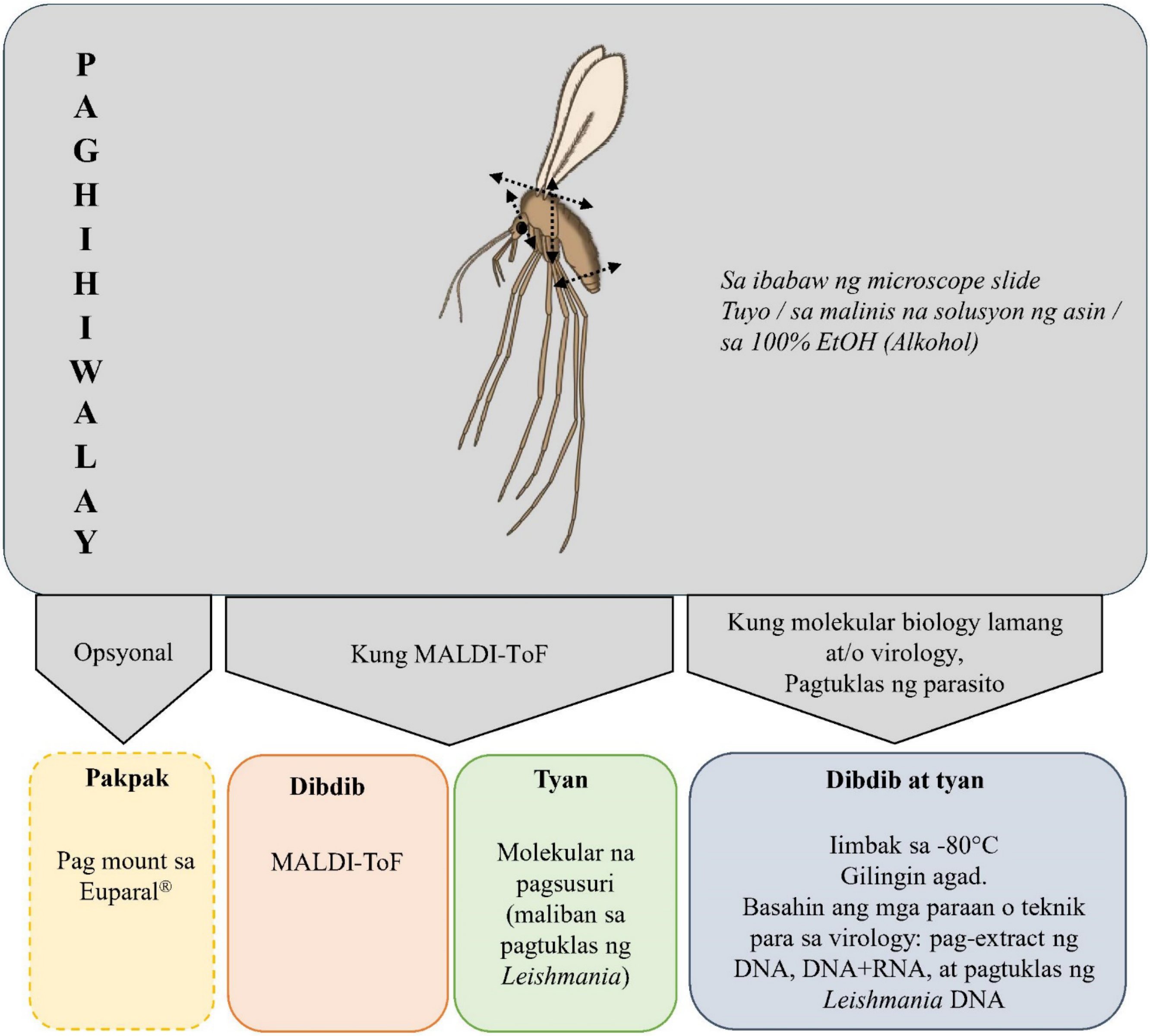

Pigura 6: Ang pagpo-proseso ng nik-nik para sa mga aplikasyong molekular na haynayan, proteomics, at/o virology.

5. Pagpoproseso ng espesimen para sa mga pag-aaral na morpolohical (Pigura 3, 6, 7 & 8; Appendices 1, 2, 3 & 4)

Ang seksyong ito ay naglalahad ng mga prinsipyo sa paghahanda ng mga nik-nik para sa pag-mount sa slides para lamang sa morpolohikal na pag-aaral, kasunod ang mga karagdagang aplikasyon bukod sa pagsusuri ng pangangatawan. Ngunit, ang pag-unawa sa mga hakbanging ito ay mahalaga sapagkat ito ang nagbibigay-daan sa mga pamamaraan na maaring gamitin sa mga partikular na uri ng sampol kung kakailanganin.

Ang prosesong ito ay gumagamit ng sunod-sunod na paglilimas at pagpupuno na hakbangin gamit ang mga Pasteur pipette na may flexible rubber bulb. Lubos na inirerekmenda ang mga lalagyang salamin na may pabilog na ilalim dahil pinapadali nito ang proseso. Ang salamin ay inert sa lahat ng kemikal. Upang maiwasan ang pag-singaw ng mga kemikal, ang mga lalalgyan ay dapat may takip at hindi dapat lubos na punuin, na magiging sanhi ng pag-apaw sa pagsasara o pagbubukas, at maiwasan ang pagkahulog ng alikabok sa mga sampol. Ang mga kemikal na kinakailangan para sa paglilinaw at pagproseso ay nakatala sa Talahayanan 2.

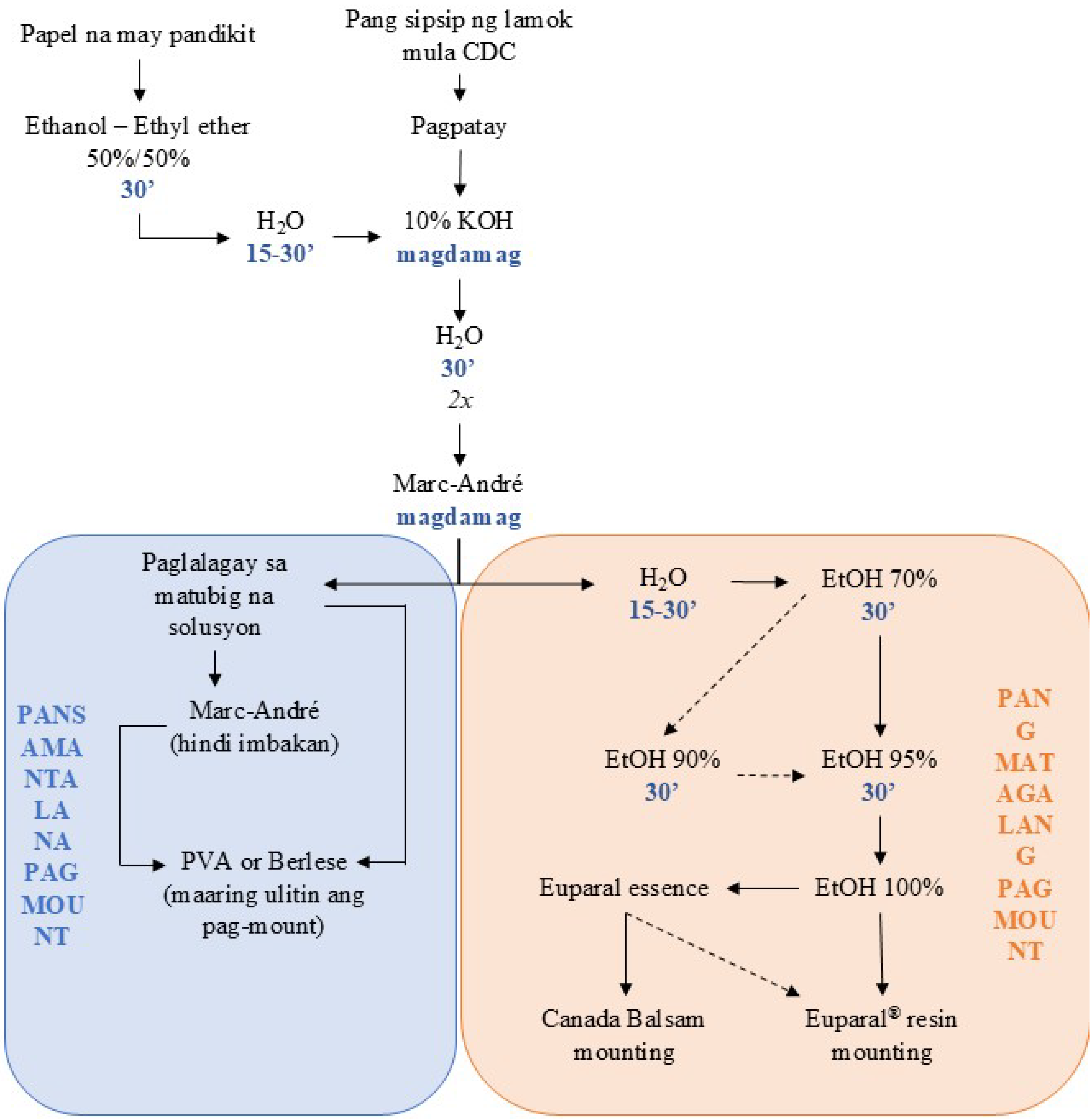

Pigura 7: Klasikong pamamaraan para sa pagproseso ng mga nik-nik.

Talahayanan 2: Komposisyon ng mga kemikal na ginamit.

**Potassium hydroxide 10%**	**Fuchsin acid 1% in distilled water**
Potassium hydroxide 10 g	Acid fuchsin (as a powder) 1 g
Distilled water *qs* 100 mL	Distilled water 99 mL
**Gum chloral mounting media (Hoyer medium)**	**Marc-André solution colored with acid fuchsin**
Distilled water 50 mL	Marc-André solution 10mL
Chloral hydrate 200 g	Fuchsin 1% 50 µL
Gum Arabic 50 g	
Glycerol 20 mL	
**Marc-André solution**	**Enecê medium**
Chloral hydrate 40 g	Pure white colophony 22 g
Glacial acetic acid 30 mL	Alcohol-soluble copal gum 12 g
Distilled water 30 mL	Absolute ethanol 20 mL
	Camphor 10 g
	Turpentine essence 10 mL
	Eucalyptol 26 mL

5.1. Paglilinaw

Bago maihanda ang mga nik-nik sa pangmatagalang pagdidikit sa slides, dapat muna silang paglinawin sa pagbababad gamit ang naaangkop na pamamaraan at mga kemikal na pangpa-linaw (hal. 10% na solusyon ng asidong asetico o Marc-Andre's solution na naglalalman ng chloral hydrate na isang pinaghihigpitan kemikal sa maraming bansa), para sila ay gawing malinaw. Ang proseso ng paglilinaw ay nagaalis ng kalamnan, taba, likidong pangangatawan, at waks, na magdudulot ng paglilinaw ng espesimen, na magpapadali ang pagsusuri ng mga istruktura sa bahagi ng exoskeleton (hal. mga pinagkabitan ng buhok), katangian ng balat (hal. kulay) at mga panloob na bahagi na makikita sa tegument (hal. spermathecae).

Ang dalawang hakbangin sa proseso ng paglilinaw, na gumagamit ng matapang na base (tulad ng Potassium hydroxide o KOH) sa umpisa, kasunod ang mahinang asido (tulad ng asidong asetico sa Marc-Andre solution), ay nagsisilbi ng natatanging biokemikal na layunin [74]. Nilulusaw ng base ang malalambot na tisyu, tulad ng protina, taba at kalamnan sa pamamagitan ng saponification at denaturation ng protina, kung saan naiiwan nitong buo ang chitin exoskeleton para sa paglilinaw ng istruktura. Ang isinunod na mahinang asido naman ang nagpapawalang-bisa sa natirang alkali, na pipigil sa pagpatuloy ng pagtunaw, at pagpapaputi ng chitin upang pagbutihin ang kalinawan[74], subalit ang pagbabanlaw ng espesimen ng dalawang beses sa distilled na tubig sa loob ng 15 minutos ay sapat na ding ipawalang-bisa ang base. Ang magkakasunod na pagbababad ay pinagsasama ang mabisang pagaalis ng tisyu at ang banayad na preserbasyon, na tinitiyak ang pinaka-mainam na kalagayan ng espesimen para masuri sa ilalim ng mikroskopyo.

Dalawang 20-minuto na pagbabanlaw sa distilled na tubig ay inirerekomenda bago magpatuloy sa susunod na hakbang.

5.1.1. Pagsira sa malambot na tisyu (Pigura 8)

Ang sodium hydroxide (NaOH) o Potassium hydroxide (KOH) ay pangkaraniwang ginagamit bilang kemikal na ahente para sa macerating, na inilalapat gamit ang iba't-ibang konsentrasyon at tagal depende sa laki at kalambutan ng mga espesimen. Ang pamantayan at pinakamabisang paraan ay kinasasangkutan ng pagsira ng malalambot na tisyu sa pagbabad ng mga nik-nik sa isang matapang na base (10% KOH o NAOH) ng magdamag. Maaring pataasin ang konsentrasyon ng base upang mapabilis ang tagal ng pagbabad (hal. 20% KOH sa loob ng 6 na oras), o maaari ding painitan hangang 37°C.

5.1.2. Paglilinaw na meron o walang pang-tina

Ang hakbang na ito ay sinusundan ng pagpapaputi, kadalasan gamit ang pinag-halong asidong asetico at chloral hydrate (hal. Mac-Andre solution). Pagkatapos palinawin, ang mga espesimen ay lubusang binabanlawan sa dalawang magkasunod na 20-minutong paglulublob sa tubig upang matanggal ang mga natitirang kemikal.

Ang Marc-Andre solution ay karaniwang ginagamit na kemikal na pangpalinaw sa paghahanda ng mga nik-nik na espesimen. Ang bisa nito ang nakapailalim sa kakayanan nitong padaliin ang proseso ng paglilinaw habang hinahadlangan ang pagkasira ng mga maseselang istruktura, tulad ng pakpak at antennae.

Ang solusyong ito ay dapat bagong gawa o naitago sa lalagyan na naisarang maigi upang maiwasan ang pag-singaw o pagkasira. Ang paggamit ng Marc-Andre solution ay nakahihigit kapag sinabayan ng paggamit ng mga

paraang nagpapakinang o nagtitina upang mapatingkad ang mga natatanging detalyeng pangangatawan. Ang mga detalye sa mga nilalaman at pagtimpla nito ay nakatala sa Appendix 2.

Para sa mga espesimen na tunay na malinaw, maaring kailanganin ang pagtitina upang mas mapagbuti ang pag-aninag ng mga ito bago i-mount. Maraming uri ng stain o tina ang maaring gamitin, ang bawat isa ay may tinutumbok na partikular na kemikal sa katawan ng organismo. Mahalaga na pumili ng stain o pang-tina na naangkop sa espesimen at sa napiling mounting medium. Ang pangkaraniwang pamamaraan na ito ay maaring i-ayon kung kinakailangan, halimbawa, pag-halo ng 0.1% acid fucshin sa Marc-Andre solution para sa pagtitina. Dagdag pa rito, ang mga espesimen na na-preserba sa solusyong aqueous na gagamitan ng mounts na gawa sa resin ay nangangailangan ng lubusang pagtatanggal ng tubig (tingnan ang Bahagi 5.2. sa Dehydration o Pagtatangal ng tubig), sapagkat ang mga natural at artipisyal na resin na ginagamit ay hindi kasundo ng tubig. Naitala ni New (1974) na may ilang mga pangtina ay kumukupas sa ilang uri ng pangdikit o mounting media [53]. Halimbawa, ang acid fuschin, na karaniwang ginagamit para sa Canada Balsam ay maari ding gamitin sa Euparal®. Ngunit ang mga espesimen na na-tina sa acid fuchsin ay madalas kumukupas, lalo na kapag may naiwang langis ng clove, na ginamit sa panghuling likidong pangpalinaw. Ang mga espesimen naimbak sa langis ng clove oil ay maaaring magpakita ng mabilisang pagkupas sa loob lamang ng ilang araw.

5.2. Dehydration o Pagtatanggal ng tubig

Ang proseso ng pagtatanggal ng tubig sa katawan ay ginagawa sa pamamagitan ng unti-unting paglipat ng mga sampol sa papataas na konsentrasyon ng alkohol: 50%, 70%, 80%, 90%, 95% at panghuli sa 100%, kung saan ang bawat pagbabad ay tumatagal ng mula 20 minuto. Sapagkat mabilis sumingaw ang ethanol, ang mga pinaglalagyan ay dapat na mahigpit na takpan sa panahon ng pagpo-proseso. Kapag lubusan nang natanggalan ng tubig ang espesimen maaring ihinto ang pag-proseso ng ilang araw sa Euparal® essence, na mas naaangkop kaysa sa langis ng clove. Ang Beech creosote, na dating pang-malawakang ginagamit para sa layuning ito, ay ipinagbawal na dahil sa ito ay nakakalason.

Sinisiguro ng proseso ng dehydration o pagtatanggal ng tubig na ang likido na nasa loob ng espesimen ay tugma sa gagamiting mounting medium, upang maiwasan ang paglabo, pagbagsak ng katawan, o pagkabaluktot na maaring gawing hindi angkop ang espesimen na magamit para sa taksonomikong pag-aaral.

5.3. Mounting media o mga Kemikal Pangdikit sa slides

5.3.1. Pagpili at aplikasyon para sa paghahanda ng espesimen

Ang mainam na mounting media ay dapat na may sukat na pangbaluktot ng liwanag o refractive index na malapit sa salamin, na mahigit kumulang na 1.5. Ito dapat ay walang kulay, malinaw at nanatiling malinaw pagkatapos nitong matuyo at sa paglipas ng panahon. Dapat din itong naaangkop sa pang-tina na ginamit at dapat may kakayahang tumagos at kumalat sa mga kalamnan ng espesimen. Hindi ito dapat mabilis matuyo o manlabo habang nagma-mount. Hindi rin ito dapat lumiit matapos i-mount. Ang pagpili ng naaangkop na mounting medium ay napakahalagang bahagi ng paghahanda ng espesimen, dahil walang iisang medium ang naangkop sa lahat ng pagkakataon. Ang pagpili ay nakasalalay sa pagbalanse ng ilang mahahalagang salik.

- **Katangiang optikal.** Ang refractive index ng mounting medium ay dapat nagbibigay ng sapat na kaibahan at repraksyon ng mga kritikal na bahagi ng katawan na ginagamit sa pagkakakilanlan taksonomikal o sa morpolohikal na pagsasalarawan, tulad ng spermatheca, ascoids, Newstead sensilla, vertical o nakatayong ngipin sa cibarium at pharyngeal na ngipin. Ang kakayahang makita ang mga istrukturang ito ng direkta ay nakasalalay sa katangiang optikal ng mounting medium.
- **Preserbasyon**. Para sa mga uri espesimen o materyales na nakalaan para sa mga pangmatagalang koleksyon, ang medium na gagamitin ay dapat na may pangmatagalang katatagan at tibay. Sa kabilang banda, para sa mga pagiimbentaryo o epidemiolohikal na pag-aral, kung saan ang pangmatagalang preserbasyon ay hindi gaanong kritikal, maaring sapat na ang pansamantala o di-permanenteng pag-mount.

5.3.2. Mga kinakailangan sa pandikit o mounting media

Ang mga mananaliksik ay madalas na nagbubuo ng pasadya at komplikadong pamamaraan ng pag-mount na iniaayon sa mga tiyak na pangangailangan ng pananaliksik. Ngunit, ang mga pamamaraan na ito ay kadalasang nakakaligtaan ang mga aspeto tulad ng kalidad pang-arkibal, pagkakatugma, istandardisasyon o kadalian ng paghawak at pangmatagalang preserbasyon. Ang kakulangan sa istandardayon ay nagpapakomplika sa pagsasama ng mga koleksyong ibinigay at mga pangmatagalang pagsisikap sa curation o pangangalaga.

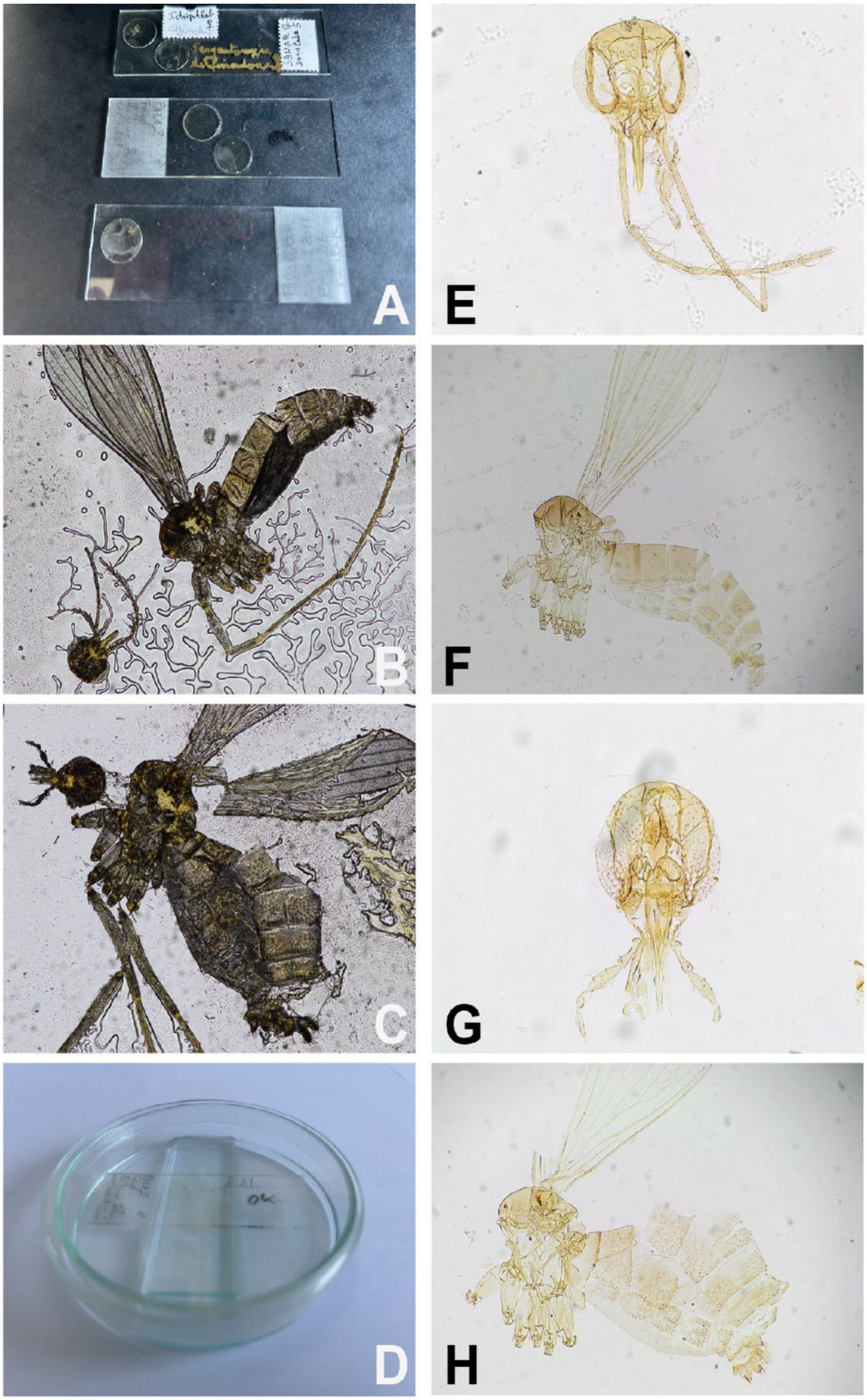

Pigura 8: Pag-remount ng slide. A: nasira at natuyong mga slide na naka-mount sa Hoyer; B: microscopic view ng natuyong nik-nik; C: microscopic view ng isa pang nasirang nik-nik; D: wet chamber na naglalaman ng natuyong slide; E: ulo, at F: katawan ng espesimen B matapos ng pag-remount nito sa Euparal®; G: ulo, at H: katawan ng nasirang espesimen C matapos ng pag-remount nito sa Euparal®.

Ang mga siyentipikong aplikasyon ay naglalapat ng tiyak na pangangailangan ng mga mounting media. Ang mga taxonomist ay kadalasang nagma-mount ng mga buong espesimen at namimili ng media na dahan-dahang tinutunaw ang lamang-loob para mapadali ang pagtingin sa mga istruktura sa balat. Ang refractive index ay dapat lubos na naiiba sa pagitan ng espesimen at sa glass slide para mapataas ang optical clarity. Ang mga nabibiling mounting media ay kadalasang tinimpla na may refractive index na halos kaparehas ng sa salamin, para mapaliit ang pagbaluktot ng ilaw at pagkalat ng liwanag sa sistema ng slide-mounting at medium-coverslip. Ngunit sa brightfield microscopy, ang natural na pagkakaiba ng espesimen na walang tina ay maaring manipulahin sa pamamagitan ng sadyang pagpili ng mounting medium na may refractive index na bahagyang naiiba sa espesimen, na nagdudulot na mapabuti ang kakahayanang makita ito laban sa kinadidikitan.

5.3.3. Mga uri ng pangdikit o mounting media (Talahayanan 3 & 4)

Ang paggamit ng microscope ay nangangailangan ng refractive index (RI) ng mounting medium para malaman kung paano lumiliko ang liwanag patagos ng slide, medium at espesimen. Kapag ang RI ay halos kaparehas ng coverslip glass (≈1.515), ang liwanag ay tumatagos nang pantay-pantay, nababawasan ang pagkakalatkalat at biswal na pagkakabaluktot na nagreresulta ng mas maayos na resolusyon at makikita ng maayos ang mga pinong istruktura. Sa kabilang banda, ang di angkop na RI ay maaring magdulot ng paglabo, mga liwanag sa palibot ng espesimen (halo) o malalabo na di nakulayang mga katangian. Ang pagpili ng tamang mounting medium ay mahalaga para mapahusay ang pag-iiba, paglilinaw at pangkalahatang kalidad ng imahen ng isang espesimen dahil sa natatanging mga RI ng iba't-ibang media.

Ang refractive index ng mounting medium ay may mahalagang epekto kung gaano kalinaw na makikita ang mga pinong istruktura kapag naghahanda ng mga nik-nik para sa pagdikit sa slide. Ang mga maseselan at di gaanong sclerotized na bahagi ng nik-nik, tulad ng cibarial armature, spermathecae, antennal segments at wing venation, ay maaring mahirap ma-obserbahan sa mounting medium na may mataas na refractive index.

Para sa mga nik-nik, karaniwang ginagamit ang gum-chloral media bilang water-based na mounting media, at Canada balsam at Enece-Nelson Cerqueira (NC) resin bilang solvent-based na pandikit. Kinategorya ni Rawlins [60] ang mga pandikit sa dalawang uri: (1) pangmatagalang pandikit: tumitigas ang mga ito sa paglipas ng panahon at naangkop sa pangmatagalang preserbasyon; at (2) semi-permanenteng pandikit: hindi lubusang tumitigas ang mga ito at kadalasan ay ginagamit sa panandaliang layunin lamang

Ang mga pandikit o mounting media ay maaring likido, gum-based o resinous, nalulusaw sa tubig, alkohol o ibang solvents o panunaw (hal. toluene, xylene) (Talahayanan 3). Kapag nilagay na, dapat itong i-selyado mula sa mga epekto ng atmospera gamit ang hindi natutunaw na ringing media. Upang malinaw na mapagiba ang iba't-ibang uri ng mga mounting media, ang sumusunod na kategorisasyon ay maaaring gamitin:

a. Aqueous media. Ang mga pandikit na ito ay madaling natutunaw sa tubig, kaya ito ay naaangkop para sa mga pansamantala o hindi permanenteng pagdikit. Ito ay kadalasang madaling gamitin, ngunit nangangailangan na iselyado upang mahadlangan na ma-apektohan ng tubig sa hangin (hal. pandikit na gum-chloral at polyvinyl alcohol), lalo na sa ma-halumigmig na klima.

b. Pandikit na bahagyang di natutunaw sa tubig. Ang mga pangdikit na ito ay hindi gaanong naapektuhan ng tubig, ngunit nangangailangan pa din ng proteksyon laban sa sobrang halumigmig. Nagbibigay ito ng mas pangmatagalang estabilidad kumpara sa mga pandikit na natutunaw agad sa tubig at mas madalas itong ginagamit para sa mga semi-permanenteng pandikit.

c. Pandikit na nalulusaw sa hydrocarbon. Ang mga pandikit na ito ay nalulusaw ng organikong solvent tulad ng xylene o toluene, o essenecê (enecê solvent). Ang mga ito ay nakadisenyo sa pangmatagalang pagdikit at nagbibigay ng pangmatagalang katatagan, at lumalaban sa kahalumigmigan at pagkasira, kaya mainam itong gamitin sa pangmatagalang pagtatago (hal. neutral Canada Balssam, DPX).

Bilang buod, and mga pandikit na natutunaw ng tubig ay pinakamainam para sa mga pansamantalang pagdikit o sa mga pagkakataon na kailangang madaling alisin ang espesimen sa; ang mga pandikit na bahagyang hindi natutunaw ng tubig ay ginagamit sa mga pagkakataon nangangailangan ng katamtamang tibay; at ang mga pandikit na natutunaw ng hydrocarbon ay mas mainam para sa mga permanenteng pagdidikit na ginagamit sa pangmatagalang pagtatago

Talahayanan 3: Mga nilalaman ng mga piling mounting media o pandikit.

Mounting medium	Solvent	Potential pre-polymer(s) or polymer	Remarks
**Hoyer** = chloral gum	glycerol, water	compounds of gum arabic	Macerating agent: chloral hydrate
**CMCP-9** (= carboxy methyl cellulose phenol)	water (CMCP-9: 51−60%)	fully hydrolyzed polyvinyl alcohol (CMCP-9: 0−5%)	CMC(P)-9: low viscosity: high viscosity
DMHF (dimethyl hydantoin formaldehyde)	water	N,N′-dimethylol dimethyl hydantoin (di-methylol DMH) Ether-/methylene-bridged oligomers Crosslinked DMH–formaldehyde polymer network	
**Canada balsam**	xylene; partly volatile components of balsam (Δ³-carene, levopimaric acid, limonene, myrcene, palustric acid, β-phellandrene, α-pinene, β-pinene)	balsam (abienol, abietic acid, isopimaric acid, sandaracopimaric acid)	neutralization: potassium carbonate; resin from *Abies balsamea* (Linné, 1758)
**Euparal®**	eucalyptol, paraldehyde; partly volatile components of gum sandarac (limonene, α-pinene, β-pinene)	compounds of gum sandarac (communic acid, manool, polycommunic acid, sandaracopimaric acid, 12-acetoxy-sandaracopimaric acid, sugiol, torulosic acid, torulosol, totarol)	clearing agent: methyl salicylate; color in Euparal® green: copper salt (copper abietinate); sandarac resin from *Tetraclinis articulata* (Vahl, 1791)
**Enecê**	ethyl alcohol; with camphor, eucalyptol and turpentine essence	Compounds of copal gum and colophony (rosin)	

Talahayanan 4: Kahigtan at kakulangan ng mga piling mounting media para sa microscope slides at mga hindi pa nailathalang obserbasyon ng iba't-ibang katauhan [52].

Pangalan ng Medium	Kahigtan	Kakulangan
* Canada balsam	Ang medium ay lubhang matibay, na tumatagal ng mahigit na 150 taon.<br>Ang slides ay maaaring i-mount gamit ang langis ng clove, o phenol bilang mounting agents.	Naglalaman ng nakapipinsalang sangkap at dapat asikasuhin sa ilalim ng hood.<br>Nangangailangan ng lubos at ubos-oras na serye ng pag-aalis ng tubig.<br>Ang pag-aalis ng tubig gamit ang ethanol at paglilipat sa pamamagitan ng xylene o langis ng clove ay maaring magpalutong ng ilang taxa; ang paggamit ng alternatibong kemikal (hal. isopropanol, n-butanol, Cellosolve™, 1,4-dioxane, Histoclear, terpineol) ay maaaring makabawas sa pagkasira.<br>Ang espesimen ay maaaring mangitim kung ang xylene ay papalitan ng phenol o kung may natitirang potassium hydroxide.<br>Ang mataas na sukat ng pagbaluktot ng liwanag o refractive indices ay maaaring panatilihing malabo ang mga istrukturang hindi na-tina.<br>Maaring tumagal ng maraming taon ang lubusang pagpapatuyo kung hindi gagamitan ng pagpapatuyo sa hot-plate.

		Ang medium ay maaaring manilaw o mangitim sa katagalan ng panahon, lalo na kung ginamitan ng langis ng clove para luminaw.<br>May ilang pang-tina na humihina, at ang mga tina na cationic ay nanglalabo kapag nagiging acidic ang medium – na maaaring mangyari nang kusa sa paglipas ng panahon.
DMHF (dimethyl hydantoin formaldehyde)	Mataas na kalidad ng kalinawan.<br>Magandang sukatan ng pagkakabaluktot ng liwanag.<br>Napakalinaw na pagpapakita ng mga istruktura.<br>May katamtamang katatagan ng mga preparasyon.<br>Katugma sa maraming teknik ng pag-titina o pag-stain.<br>Magandang proteksyon ng mga sampol.<br>Magandang pagkakadikit sa pagitan ng slide at coverslip.	Maaaring manilaw sa katagalan ng panahon.<br>Maaaring baguhin ang ilang tina.<br>Hindi naaangkop na gamitin sa tina na sensitibo sa formaldehyde.<br>Pagkakaroon ng bula, at mabagal na pagpapatuyo.<br>Ang mounting medium ay sensitibo sa kahalumigmigan o humidity.<br>Mahirap ibalik sa dati kapag nai-mount na.<br>Ang Formaldehyde ay nakakalason, nakakairita, at nagdudulot ng kanser.
* Euparal (malinaw o naaaninag)	Matibay na medium na tumatagal ng mahigit na 50 taon.<br>Maaaring direktang i-mount and espesimen mula sa 80% ethanol (ayon sa rekomendasyon ng tagagawa).<br>Hindi nagkukubli ng mga istruktura na hindi na-tina at hindi naninilaw o nagiging marupok sa katagalan ng panahon.<br>May sukatan ng pagkabaluktot ng liwanag na mas naaangkop para sa Diptera kaysa Canada Balsam.<br>Angkop na gamitin sa mas makakapal na espesimen dahil sa limitadong pagliit at hindi pamumuo ng mga bula habang pinapatuyo.<br>Nananatiling nalulusaw sa 95% ethanol, na nagbibigay daan sa pag re-mount kahit lumipas na ang maraming taon.	Naglalaman ng mga nakapipinsalang sangkap at dapat asikasuhin sa ilalim ng hood.<br>Ang pag-aalis ng tubig gamit ang ethanol at paglipat gamit ang Euparal Essence ay maaaring magpalutong ng ilang taxa, subalit ang paggamit ng isopropanol ay maaaring makabawas sa isyung ito.
Hoyer fluid	Ang mga espesimen ay maaaring i-mount ng buhay o direkta mula sa tubig, ethanol o formaldehyde.<br>Ang pagpapalambot ng laman sa pagbababad ay umaani ng magandang kaledad ng balat o kyutikel.<br>Nagtatamo ng kanais-nais na sukat ng pagbaluktot ng liwanag na maaari pang pagandahin ng pag-tina gamit ang iodine para sa mas maigting na pag-iiba ng kulay.<br>Ang paglalagay ng asidong asetiko ay nakakapagpalapad ng mga biyas ng mga arthropod.<br>Ang ilang mga espesimen ay maaaring manatiling matatag at maayos sa loob ng 40 to 60 taon.<br>Natutunaw sa tubig, na nagbibigay daan sa madaling pag re-mount.	Maseselan na halamang espesimen ay maaaring mag-collapse maliban kung dahan-dahang magdagdag ng medium na ubos-oras.<br>Ang mga lukab at kristal ay maaaring lumitaw sa paglipas ng panahon na hindi pa umabot ng 10 taon.<br>Ang pagpapalambot ng laman sa pagbababad ay maaaring mapasobra depende sa konsentrasyon ng chloral hydrate at tagal ng pagkakalantad.<br>Ang mga sangkap ng medium ay maaaring maghiwa-hiwalay, at pinong mga butil ay maaaring makita sa loob lamang ng ilang buwan o taon.<br>Pangingitim ng medium ay naiulat.
CMCP-9 (= carboxy methyl cellulose phenol)	Ang mga espesimen ay maaaring direktang i-mount mula sa pinagbabaran tulad ng tubig, ethanol, glycerol, o solusyon na naglalaman ng formaldehyde, at kung kinakailangan, ang kanilang lamang loob ay maaaring palambutin upang mapadali ang pagsusuri o paghahanda.	Ang medium na ito ay maaaring magkaroon ng mga kristal at dumilim sa katagalan ng panahon. Kung minsan, napapalambot din nito ng labis ang mga espesimen, ng hindi sinasadya. Maliban kung ang slide ay maingat na pinaikutan ng pang-selyo, ang makakapal na sampol ay hindi nagiging maayos dahil

		maaari itong umurong at magkaroon ng mga patlang sa gilid ng coverslip. Hindi ito naaangkop para sa mga na-tina na espesimen o mga calcified na materyales, at ang oras na gugugulin para sa pagpapatuyo ay mas mabagal kaysa sa CMC.
Eukitt™	Matibay na medium na tumatagal ng mahigit na 30 taon.<br>Tugma sa maraming mga pangtunaw o solvent para sa pag-mount kasama ang acetone, benzene, chloroform, dioxan, ether, isopropanol, methyl benzoate, terpineol, toluene, at xylene.<br>Mabilis matuyo at bahagyang asidik ang pH.<br>Hindi kapansin-pansin ang pagdilim sa paglipas ng panahon.<br>Angkop para sa iba't-ibang mga tina (hal. fuchsin, hematoxylin, methyl green, methyl violet, methylene blue).<br>Ang espesimen ay maaaring ulitin ang pag-mount makalipas ang maraming taon sa pagbababad sa xylene ng mahabang panahon.	Naglalaman ng nakapipinsalang sangkap at kinakailangang asikasuhin sa ilalim ng hood.<br>Nangangailangan ng lubos, at ubos oras na serye ng pag-aalis ng tubig.<br>Hindi naaangkop sa makapal na espesimen dahil sa pag-urong at pamumuo ng bula.<br>Sa katagalan ng panahon, maaaring humiwalay ang mga coverslip, maliban kung lubos na nalilinis ang salamin at na-selyado.<br>Maaaring magkaroon ng hindi lubos na polimerisasyon sa paligid ng mga hibla ng collagen.
Enecê	Lubhang matibay ang medium na ito na tumatagal ng mahigit na 50 taon.<br>Hindi nagdidilim ang Enecê sa katagalan ng panahon.<br>Ito ay mas malambot, na nagbibigay-daan sa paghihimay ng kulisap habang nasa medium, at nagbibigay ng sapat na oras para sa pagsasaayos ng mga istrukturang pangangatawan.<br>Mura.	Nangangailangan ng lubos, at ubos oras na serye ng pag-aalis ng tubig.<br>Ang pag-aalis ng tubig gamit ang ethanol at ang paglilipat gamit ang langis ng clove ay maaring magpalutong sa ilang espesimen.<br>Ang mga kulisap sa medium na ito ay patuloy na naglilinaw bagaman mabagal; maaari nitong pahirapan ang pagmamasid ng mga mumunting istruktura, tulad ng sensilla, ascoids at simpleng mga buhok.

5.3.4. Paglalarawan sa mga nirerekomendang pandikit o mounting media (Talahanayan 3 & 4)

Media para sa pansamantalang obserbasyon

Chloral gum = Hoyer fluid/medium/solution (RI=1.48)

Ang Marc André fluid ang pinakamahusay na medium para sa panandaliang (ilang oras, marahil ay higit pa kung ang slide ay nakaimbak sa humid chamber) pagmamasid ng spermathecae, kasama na ang mga pagkuha ng mga larawan (Pigura 4) o mga pag-guhit. Ang pag-preserba ng naobserbahang spermathecae ay nangangailangan ng kanilang pag-re-mount sa aqueous medium na nagbibigay-daan sa medium-term na pagsusubi. Ang lubusang pagpapatuyo sa kanila para sa pag-re-mount sa resin ay hindi imposible ngunit hindi inirerekomenda (panganib ng pagkawala). Ang chloral gum at Hoyer fluid ay itinuturing na magkatulad. Ang medium na ito ay karaniwang ginagamit para sa pagmamasid ng mga panloob na organo dahil sa pagkakatugma nito sa tubig, ka-simplehan, mabilisang aplikasyon, at refractive index na nakakatulong sa pagsusuri ng mga delikadong istruktura tulad ng spermathecae. Gayunpaman, ang chloral gum ay may malaking mga kahinaan kung hindi perpektong naihanda o nakaimbak kung saan may kontroladong mga kondisyon ng halumigmig. ang ilan sa mga isyung kakaharapin ay ang pamumuo ng kristal (crystallization), pagbabago ng kulay, at pagkawala ng pagkalapot (viscosity). Hindi nalulutas ang mga problemang ito sa pag-ring ng coverslip, dahil ang mounting media ay maaaring matingkad na makulayan (minsan halos itim) dahil sa pakikipag-ugnayan sa ringing medium, lalo na kung ang ginamit ay Euparal®.

Ang Hoyer medium ay itinuturing na pinakamahusay optically para sa phlebotomine na nik-nik at tradisyonal na ginagamit para sa mga layuning ito. Ang medium na ito ay binubuo ng ilang malapit na magkakaugnay na mga formulation, kasama ang gum arabic, glycerol, at chloral

hydrate. Iba't-ibang mga formulation ay mali ang pagkakaintindi at mali ang pagkakabanggit [74].

Bagaman ang Hoyer ay mahusay na medium para sa pagmamasid ng spermathecae sa mga nik-nik, hindi ito angkop para sa pangmatagalang preservation. Perpekto ito para sa panandaliang mga pagmamasid, kasama na ang mga larawan, pag-guhit, o mga imahen. Ang aqueous media ay angkop para sa pansamantalang mga mount ngunit hindi makakatiyak ng pangmatagalang preserbasyon Sa kabaligtaran, ang naka-mount sa resin ay nagbibigay ng napakahusay na tibay na kadalasang tumatagal ng mga siglo, ngunit maaaring magtago ng mga pinong detalye ng spermathecae, dahil ang kanilang refringence ay madalas na nawawala.

Ang Hoyer medium ay nasisira sa paglipas ng panahon dahil sa dehydration (Figure 8), na nagreresulta sa pamumuo ng maliliit na puti, opaque na kristal ng chloral hydrate. Gayunpaman, ang mga espesimen ay maaaring mabawi mula sa crystallized slides dahil ang cuticle ay nananatiling chemically intact, bagaman maaaring magkaroon ng ilang pisikal na pinsala mula sa lumalaking mga kristal. Sa ilang mga kaso, ang crystallized slides ay maaaring maibalik sa pamamagitan ng pag-rehydrate sa mounting medium sa katamtamang init, mamasa-masang kapaligiran na may thymol upang maiwasan ang fungal growth. Bilang alternatibo, ang mga espesimen ay maaaring ibabad palabas ng chloral gum sa tubig, patuyuin sa glacial acetic acid, at i-re-mount sa Canada balsam.

DMHF (dimethyl hydantoin formaldehyde) (RI 1.48)

Ang water-based medium na ito [72] ay napakahusay optically, tulad ng Berlese, at kasing daling gamitin tulad ng Berlese. Gayunpaman, sa kaibahan sa Berlese, hindi ito nangingitim o namumuo ng kristal. Mahusay ito para sa mga nik-nik at iba pang Psychodidae.

CMCP (camphor-mono-chlorophenol) (RI = 1.41)

Ito ay isang glycerin-based, natutunaw sa tubig na mounting medium na ginagamit para sa paggawa ng transparent, permanenteng mga slide ng mga delikadong espesimen, kasama na ang nik-nik. Ang bentahe ng mounting medium na ito ay ang mga espesimen ay maaaring i-mount nang direkta mula sa tubig o ethanol. Mabilis nitong pinapa-relax at pinapalinaw ang nik-nik, pinapalalambot ang cuticle upang payagan ang tamang pag-posisyon ng espesimen, na kapaki-pakinabang sa paglapat ng mga pakpak o pag-daysek ng henitalya. Bagaman naiulat na nagbibigay-daan ito sa pangmatagalang pag-iimbak, ang eksaktong tagal ng preserbasyon ay nananatiling hindi tiyak. Ang pangunahing limitasyon ng mounting medium na ito ay nasa komposisyon nito na naglalaman ng phenol, isang nakakalasong at nakakairitang sangkap na nangangailangan ng maingat na paghawak.

Media para sa permanenteng pag-mount

Canada Balsam (RI-1.52-1.54)

Ang Canada balsam ay unang inilarawan bilang angkop na mounting medium para sa transmitted light microscopy ni Andrew Pritchard noong 1830s. Nananatili ito bilang isa sa pinakamalawakang ginagamit na media dahil sa napatunayang angking archival quality, na may mahigit 150 taon ng matagumpay na aplikasyon. Hindi tulad ng Hoyer fluid media, ang Canada balsam ay hindi nag-crystallize o sumisipsip ng tubig sa hangin. Gayunpaman, ang Canada balsam ay lubhang autofluorescent, na maaaring minsan nagiging kawalan para sa ilang microscopy techniques [60]. Ang paggamit ng di nakakalasong mga panunaw sa halip na xylene ay maaaring makabawas sa mga panganib sa kaligtasan sa panahon ng paghahanda, ngunit maaari ring magdulot ng mga kahinaan tulad ng mas mabagal na pagtuyo at mas maagang pagdidilim ng medium.

Euparal® (RI = 1.48)

Ang Euparal® ay malawakang ginagamit na alternatibo sa Canada balsam para sa permanenteng mounting, na nag-aalok ng napakahusay na pangmatagalang stability at maihahambing na refractive index. Ang Euparal® ay may mga sumusunod na katangian: (1) pangangailangan ng dehydration: bago ang huling paglipat ng mounting medium, ang espesimen ay dapat tuyuin, karaniwang lumilipat mula 95% hanggang absolute ethanol, at (2) mahabang panahon ng pag-proseso: ang huling pabubuo sa resin, maging Canada balsam o Euparal®, ay nangangailangan ng dehydration o lubusang pag-aalis ng tubig, na nagpapahaba sa kabuuang oras ng pag-proseso ng sampol. Kapag ang dehydration gamit ang organic solvents ay hindi posible, ang mga sampol na nakuha mula sa absolute ethanol ay maaaring ilagay sa intermediate solution na binubuo ng pantay na halo ng Euparal® at Euparal essence, bago ang huling pag-mount.

Enecê (RI = 1.467)

Ang Enecê ay isang resin-based mounting medium na pangunahing ginagamit para sa maliliit na kulisap at partikular na sikat sa Brazil. Ang base nito ay binubuo ng colophony at gum copal na tinunaw sa alkohol, camphor, essence ng turpentine, at eucalyptol. Inilarawan ni Cerqueira [11] ang Enecê bilang alternatibo sa Canada balsam para sa pag-mount ng permanenteng mga slide ng larvae, exuviae ng mga immature, at maging adult na lamok, at mula noon ay malawakang tinanggap para sa pag-mount ng mga nik-nik. Ang Enecê ay nag-aalok ng cost effective na alternatibo para sa permanenteng pag-mount, na nagbibigay ng pangmatagalang stability at sapat na drying time, na nagpapaubaya sa dayseksyon at tumpak na pag-sasaayos ng mga istrukturang morpolohikal.

5.4. Paghahanda ng slides at pagpapatuyo

Ang tamang pagtutuyo ng mga mounted na slides ay kritkal upang masiguro ang pangmatagalang estabilidad at preserbasyon. Ang mga slide ay dapat lubusang matuyo

bago iimbak ng pangmatagalan. Para sa magandang resulta, ang mga slide na may permanenteng pandikit o mounting media ay dapat patuyuin na nakapahalang sa loob ng 2-3 linggo, samantalang ang mga ginamitan ng semi-permanenteng pandikit ay nangangailangan ng 1-2 linggo lamang. Upang masiguro ang epektibong pagpapatuyo, inirerekomenda ang paggamit ng incubator na nakatakda sa angkop na temperatura para sa ginamit na pandikit, iwasan ang paggamit ng sobrang init na maaaring makasira ng mga espesimen. Inirerekomenda ang temperatura na nasa pagitan ng 30°C at 37°C. Ang hakbang ng pagpapatuyo na ito ay mahalaga upang maiwasan ang pagbaluktot ng slide, pagkasira ng espesimen, o kawalang-tatag ng mounting media habang nakatago.

Ang mounting medium na ginamit sa paghahanda ng slide ay dapat laging nakasulat sa label ng slide. Kung kakayanin, dapat isama rin ang tiyak na pagkakatimpla ng pandikit na ginamit, kasama ang pangalan ng tao na naghanda at kung kailan ito inihanda. Ang mga slide ay unang ihinahanda bilang panandaliang mounts at hindi para sa pangmatagalang preserbasyon. Gayunpaman, kung magbabago ang katayuan ng espesimen, tulad pagkakapili bilang kasama sa mga "type" series, kinakailangan ang paggamit ng mas pangmatagalang pangdikit upang matiyak ang preserbasyon ng espesimen para sa hinaharap na pag-aaral na taksonomik.

5.5. Mga alternatibong pamamaraan ng mounting: card mounting

Ang card mounting ay isang pamamaraang ginagamit para sa ilang grupo ng mga kulisap kung saan ang mga espesimen ay maaaring direktang i-pin sa mga entomological card o idikit sa ibabaw nito. Dahil sa kanilang maliit na sukat at pangangailangan na obserbahan ang mga panloob na organo para sa pagkakakilanlan sa pamamagitan ng paglilinaw (tingnan ang item 5), ang pamamaraang ito ay hindi talaga naa-angkop para sa pag-mount ng mga nik-nik.

5.6. Pag-re-mount ng mga nasirang espesimen

Para sa mga pambihira o mahahalagang espesimen, inirerekomenda ang dalawang hakbangin na pamamaraan ayon sa video na maaaring ma-access sa: https://zenodo.org/records/18315029. 1) muling i-rehydrate ang mga ito nang hindi binubukod upang mabigyang daan ang paunang pagmamasid. Isang holder para sa ilang mga microscope slide ay dapat ilagay sa Petri dish na magsisilbing suporta. Ang slide na ire-rehydrate ay ilalagay sa ibabaw, at ang Petri dish ay pinupuno ng ilang millimetro ng solvent upang makagawa ng humid chamber, tinitiyak na ang slide mismo ay walang kontak sa solvent (Figure 8 D). Ang oras na kinakailangan para sa rehydration ay maaaring mag-iba mula sa isa hanggang ilang araw, depende sa kondisyon ng espesimen. Ang araw-araw na pagsubaybay at pasensya ay mahalaga. Kapag ang slide ay sapat na na-re-hydrate, maaari itong alisin sa humid chamber at ilagay sa incubator ng ilang oras bago ang microscopic examination, pagkuha ng larawan, o pagguhit. 2) para sa pag-remount, ang slide ay maaaring ibalik sa humid chamber ng ilang karagdagang oras o magdamag. Ang disassembly ay dapat gawin sa ilalim ng binocular microscope. Gamit ang mga pinong karayom, ang coverslip ay dapat maingat na alisin, tinitiyak na walang natitirang mga elemento ng nik-nik ang nananatiling nakakabit (https://zenodo.org/records/18315029). Kasunod, ang mga na-daysek na elemento ng nik-nik ay dapat kolektahin at banlawan ng tubig sa maliliit na balon, tulad ng mga ginagamit para sa destructive DNA/RNA extraction (tingnan sa ibaba), bago ang dehydration at pag-remount sa resin medium. Kapag dini-disassemble ang slide, mahalaga na kilalanin ang orihinal na mounting medium upang pumili ng angkop na solvent o panunaw. Para sa aqueous mounting media, dapat gamitin ang tubig. Kung ang mounting medium ay resin-based (hal., Canada balsam o Euparal®), dapat gamitin ang xylene, sa ilalim ng fume hood at may angkop na personal protective equipment, kasama na ang mask.

Ang pag-remount ng mga uri o koleksyon ng espesimen ay dapat lamang gawin na may pahintulot ng curator at/o ng institusyong nagma-may-ari ng espesimen.

6. Pagkilanlan ng mga espesimen

6.1. Morpholohiya

Ang pagkilanlan sa mga nik-nik ay panguhaning nakasalalay sa pagtingin ng mga morpholohikal na katangian, kasama ang hugis ng thorax o dibdib, pakpak, henitalya, mga buhok at mga tiyak na morpometrikang ugnayan sa iba't-ibang istruktura. Ang mga mananaliksik ay gumagamit ng taxonomic keys, reference collections, at orihinal na paglalarawan ng sarihay upang ikumpara ang mga nakolektang espesimen sa mga kilalang sarihay. Pangunahing katangiang ng pagkilanlan, tulad ng venation ng pakpak at morpolohiya ng ulo ng lalake at babae, ang istruktura ng henitalya ng lalaki, at pagkaka-ayos ng spermathecae ng babae, ay partikular na nagbibigay-kaalaman para sa pagkakakilanlan ng mga sarihay. Ang wastong pagkakakilanlan ay madalas na nangangailangan ng detalyadong pagsusuri sa mikroskopiko, kadalasan gamit ang isang compound microscope upang siyasatin ang mga maliliit na istruktura tulad ng henitalya at spermatheca o isang stereomicroscope para sa mas malawak na morpolohikal na katangian.

6.2. Geomery ng pakpak

Ang geometry ng pakpak ay isang pangunahing katangian na ginagamit sa pagkakilanlan at pag-uuri ng iba't-ibang sarihay ng nik-nik. Ang mga pakpak ng nik-nik ay may mga kakaibang disenyo at istruktura, na kadalasan ay pahaba at makitid na may malinaw na venation (Pigura 9 at 10). Ang pagkaka-ayos ng mga veins ay bumubuo ng kakaibang disenyo na nagbabago sa pagitan ng mga urihay at sarihay, kaya't maganda itong gamitin para kanilang

pagkakilanlan. Dahil dito, ang pag-aaral ng wing geometry ay nagbibigay ng mahalagang pananaw para sa layuning taksonomik.

6.3. Geometric morphometrics ng pakpak

Ginagamit ng mga mananaliksik ang iba't-ibang pamamaraan, tulad ng geometric morphometrics, upang suriin at ihambing ang hugis at laki ng pakpak sa iba't-ibangsarihay o populasyon ng nik-nik. Ang pag-aaral ng wing geometry ay nagbibigay ng mahalagang pananaw sa pag-uugali, mga likas na tirahan, at kakayahang lumipad.

Sa geometric morphometric approach, ang mga pakpak ay maingat na hinihiwa-hiwalay, tinitina (kung kinakailangan), at flat-mounted sa mga slide. Ang mga inihandang mga slide ay kinukuhanan ng larawan sa ilalim ng stereomicroscope, dini-digitize, at ginagawang paksa ng morphometric na pagsusuri. Ang pamamaraang ito ay mahusay na nailarawan sa mga panitikan [6, 27, 42, 56, 57, 59], na may rekomendasyon na gamitin nang tuloy-tuloy ang kanan o kaliwang pakpak para sa mga organong may kapares upang maiwasan ang mga potensyal na negatibong allometric effect [62].

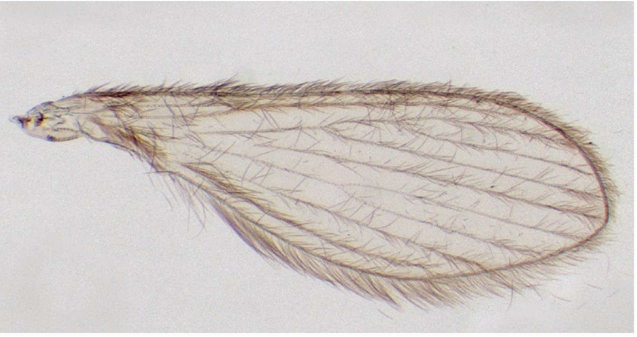

Pigura 9: Walang kulay na pakpak ng *Trichophoromyia ininii*.

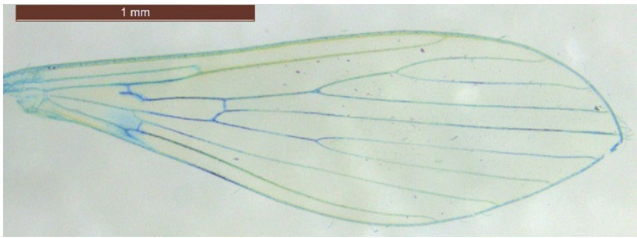

Pigura 10: Nakulayang pakpak ng *Phlebotomus ariasi.*

Paghahanda ng pakpak para sa geometric morphometric analysis

Para sa optimal na biswalisasyon sa mga vein ng mga pakpak, ang mga pakpak ay dapat alisan ng mga scale at nagamitan ng naaangkop na tina. Para sa paghahanda ng pakpak, unang punuin ang maliliit na balon ng mga kinakailangang pamalibilos (methylene blue, ethanol, tubig at xylene substitute). Kunin ang pakpak na na-preserba sa 70% ethanol sa temperaturang pang silid sa pamamagitan ng pagbaliktad ng Eppendorf tube at pagbubuhos ng laman nito sa balon, pagkatapos ay itaas ang pakpak nang pahaba gamit ang pinong karayom na nakaliko. Saglit na idaan ang pakpak mula ethanol patungo sa tubig at balik sa ethanol upang alisin ang mga bristle. Ilagay ang pakpak sa methylene blue ng 6 minuto, tiyakin na lumulutang ito habang tinitina. Maingat na kunin ang pakpak at isawsaw ito sa xylene substitute ng 2 minuto (humigit-kumulang ikatlo ng oras ng methylene blue). Ang mahinang pagtapik ng karayom sa dingding ng balon ay makakatulong sa paglubog ng pakpak; ang xylene ay nagsisilbing pang-fix ng kulay. Sa pang-wakas, itaas ang pakpak at ilagay ito sa maliit na patak ng Euparal® sa microscopic slide. Sa ilalim ng magnifying lens, dahan-dahang ilatag ang pakpak at maingat na lagyan ng coverslip. Ang mga larawan ay dapat kunan kaagad bago mag-set ang Euparal®, dahil maaaring kailanganin ang maliliit na pag-adjust sa posisyon ng pakpak sa ilalim ng coverslip upang makamit ang optimal na alignment.

6.4. Mga pamamaraan ng molekular na haynayan

Bilang karagdagan sa mga morpolohikal na teknik, ang mga molekular na pamamaraan ay lalong nagiging mahalaga sa entomological research, kasama na ang taksonomik, population genetic, at phylogenetic na pag-aaral, pati na rin ang DNA/RNA pathogen detection, at ang pagtukoy ng pinagmulan ng blood meal, kung saan ang vector behavior ay mahalaga sa larangan ng epidemiology [70]. Ang DNA sequencing ay maaaring gamitin para sa konpirmasyon ng mga sarihay o pagkilala ng mga sarihay na may malapit na ugnayan, na nagbibigay ng mas wasto at maaasahang paraan ng pagkakakilanlan. Higit pa rito, ang mga sulong na mga molecular teknik (hal., PCR, DNA sequencing, NGS, atbp.) at MALDI-ToF MS ay nagiging kilala para sa tiyak at mabilisang pagkilanlan ng mga sarihay, na sumusuporta sa mga tradisyonal na morpolohikal na pamamaraan [46]. Sa kabila ng mga pagsulong na ito, ang morpolohikal na pagkilanlan ay nananatiling pamantayan para sa taksonomi at ang batayan kung saan binibigyang-kahulugan ang molecular data.

6.4.1. Destructive nucleic acid extraction

Ang nucleic acid extraction ay isang karaniwang hakbang sa maraming pag-aaral ng haynayan, at iba't-ibang pamamaraan ang nabuo upang ihiwalay ang DNA mula sa mga biological material [48]. Maraming nabibiling DNA extraction kit ang dinisenyo upang mapadali ang prosesong ito [14]. Gayunpaman, ang mga pamamaraang karaniwang ginagamit para sa paghahanda ng mga arthropod espesimen para sa morpolohikal na pagkakilanlan ay madalas na humahadlang sa DNA analysis, dahil ang mga pamamaraang ito ay maaaring makasira o makawasak ng mga kritikal na pisikal na katangian ng espesimen [10].

Karamihan sa mga DNA extraction protokol para sa mga tisyu ng kulisap ay mapanira sa kalikasan [43], na nagdudulot ng partikular na alalahanin para sa maliliit na espesimen, kung saan maging ang limitadong sampling ay maaaring maka-kompromiso ng mga mahalagang morphological features [72]. Ang uri at kondisyon ng espesimen ay may mahalagang gampanin sa pagpili ng angkop na pamamaraan para sa DNA isolation [29].

Ang pangangailangan sa tiyak na pagkakakilanlan ng mga nik-nik, pag-unawa sa population dynamics, at pagbabawas ng mga non-target impact ay naging dahilan sa pagbuo ng mga molecular diagnostic tools [23]. Ang mga molecular approach ay madalas na ginagamit ngayon pandagdag sa mga morphological taxonomic methods para sa pagkakakilanlan ng mga nik-nik. Halimbawa, ang pamantayan para sa barcoding ng mga kulisap ay nagsasangkot ng DNA extraction, sequencing, at pagkawala ng orihinal na espesimen. Kaya, may mahigpt na pangangailangan na tuklasin ang mga non-destructive DNA extraction methods na nagpapanatili sa biological material at sa morphological integrity nito.

Maraming nucleic acid extraction method ang nailapat sa mga nik-nik. Ang dami o kalidad ng mga nucleic acid na kinakailangan ay nakadepende sa downstream molecular analysis, dahil ang iba't-ibang pamamaraan ay may iba't-ibang sensitivity at purity requirement [9]. Halimbawa, natuklasan na ang mga mata ng nik-nik ay humahadlang sa PCR amplification [69]. Higit sa pathogen screening, ang DNA ng nik-nik ay pangkaraniwang kinukuha para sa mga layunin ng pagkakilanlan ng sarihay. Iba't-ibang extraction method ang maaaring gamitin, bagaman ang mga yield at kalidad ay naiiba sa mga pamamaraan. Ang ilang protokol ng mga manufacturer ay inadapt ng mga mananaliksik para sa mga nik-nik [8], na nakadagdag sa yield at/o kalidad ng mga extracted nucleic acid [8, 9, 69], habang ang iba pang mga adaptation, na nabuo para sa iba pang arthropod taxa, ay maaari ring gamitin sa mga nik-nik [58, 76]. Ang mga PCR para sa pagkakilanlan na nakatuon sa maliliit na mitochondrial fragment (COI o CytB) ay karaniwang naaangkop sa mga extraction method na nagsasangkot ng mataas na DNA fragmentation. Sa kabaligtaran, ang iba pang long-read NGS teknik (Oxford Nanopore at PacBio) ay nangangailangan ng kaunting fragmentation at high-quality DNA. Ang mga spin column extraction ay karaniwang nagbubunga ng genomic DNA fragment na hanggang 60 kb, habang ang phenol-chloroform extraction ay maaaring makakuha ng mga fragment na hanggang 150 kb [77]. Binubuod sa Talahayanan 5 ang iba't-ibang extraction teknik ng DNA ng nik-nik at nagsasaad kung may mga adaptation ng pamamaraan na ginawa para sa mga kulisap na ito. Ang mga yield ay hindi ipinapakita, dahil nakadepende ang mga ito sa laki ng espesimen at pamamaraan ng paghahanda. Ang modification column ay tumutukoy sa mga adaptation ng extraction protokol para sa mga nik-nik o iba pang maliliit na arthropod.

Ang pagpili ng extraction method ay dapat isaalang-alang ang ilang pamantayan, tulad ng bilang ng mga sampol, ang oras ng extraction, at ang pamamaraang gagamitin sa downstream. Habang ang mga NGS teknik ay nangangailangan ng mataas na molecular weight genomic DNA, lahat ng mga pamamaraang ipinakita dito ay maaaring gamitin para sa standard PCR-based application.

Higit pa rito, ilang pag-aaral ang sumubok galugarin ang mga non-destructive DNA extraction method para sa maliliit na terrestrial arthropod, dry-preserved na espesimen sa mga museo, at arthropod na may malalambot na katawan [19, 26, 28, 55, 63].

Talahanayan 5: Average na gastos, aplikasyon, at protokol adaptation para sa extraction ng gDNA ng mga phlebotomine nik-nik

Protocol	Cost	Application	Protocol adaptation for small arthropods
Spin column	2.5 – 3.55 US$ [39]	PCR, NGS	[9]
Phenol-chloroform	0.24 US$ [69]	PCR, NGS	[9]
HotSHOT	<0.01 US$ [69]	PCR	-
Salting out	0.12 $3 [69]	PCR	-
Chelex	0.02 $4 [41]	PCR	[41, 76]

6.4.2. Non-destructive nucleic acid extraction

Isa sa mga pangunahing hamon sa molecular analysis ng mga arthropod, lalo na ng mga nik-nik, ay ang preserbasyon ng mga espesimen para sa pagsasama ng mga entomological collection. Karamihan sa mga DNA extraction protocol ay nangangailangan ng maceration ng tisyu, kaya nakokompromiso ang pagpapanatili ng orihinal na espesimen. Ang mga non-destructive nucleic acid extraction method, gayunpaman, ay dinisenyo upang makakuha ng genetic material nang hindi nakakasira sa pisikal na integridad ng sampol, na nakakaapekto sa viability nito o nagbabago sa morpology nito. Ang mga pamamaraang ito ay partikular na mahalaga kapag nagtatrabaho sa mga di pangkaraniwan o limitadong espesimen, tulad ng mga nik-nik, kung saan ang pagpapanatili ng integridad ng istruktura ay mahalaga para sa hinaharap na taxonomy, morpolohikal o diagnostic na mga layunin. Ang karaniwang ginagamit na pamamaraan ay ang non-destructive bathing method kung saan ang mga nik-nik di pinagagalaw, at dahan-dahang inilulubog sa lysis buffer na naglalaman ng proteinase K.

Ang mild-vectolysis teknik ay matagumpay na nailapat sa mga nik-nik partikular na sa mga type espesimen [24]. Ang teknik na ito ay gumagamit ng conventional spin column kit (sa kasong ito, ang DNeasy Blood and Tissue

kit, QIAGEN, Hilden, Germany) na may adaptation para sa pagkuha ng DNA nang hindi sinisira ang espesimen. Ang mga modified na hakbang para sa lysis (ang dami ng lysis buffer at pagdadagdag ng freezing step) [17] ay nagbibigay-daan sa pagpapalabas ng mga nucleic acid, na binabawasan ang pagkasira na morpolohikal [24]. Tungkol sa mga nik-nik, maaaari ring gamitin ang HotSHOT DNA Extraction kit (Bento Bioworks Ltd, London, United Kingdom) [73] na mabilis at mura, na nagpapaubaya sa mabilis at murang pagproseso ng mga sampol. Ang mga entomolohikal na espesimen na nakalaan para sa morpolohikal na pagkakakilanlan ay maaaring banlawan pagkatapos. Ang mga na-proseso gamit ang DNeasy Blood and Tissue kit ay dapat na paglinawin gamit ang Marc-André solution, habang ang mga na-proseso gamit ang HotSHOT DNA extraction kit ay sapat na napalinaw upang ma-mount sa aqueous medium, o mas mabuti, sa resin pagkatapos ng dehydration, ayon sa protokol na detalyadong nakalagay sa artikulong ito [73]. Ang nakuhang genetic material ay maaaring i-proseso pa para sa downstream analysis, tulad ng PCR, upang padamihin ang mga tiyak na genetic marker. Ang mga non-destructive nucleic acid extraction method ay mahalaga para sa pag-aaral ng mga genetic na katangian ng mga nik-nik, kasama ang pagkakakilanlan ng mga potensyal na disease-causing agent na maaari nilang dala. Sa pamamagitan ng pagpapanatili ng integridad ng espesimen, ang mga mananaliksik ay maaaring makakuha ng mahalagang genetic information, habang pinapanatili ang sampol para sa karagdagang mga analysis o pag-aaral.

6.5. MALDI-ToF MS

Ang MALDI-ToF MS (matrix-assisted laser desorption/ionization time-of-flight mass spectrometry) ay isang mass spectrometry-based na teknik na dinesenyo upang matukoy at masuri ang natatanging protein profiles (‘fingerprints’) ng mga biyolohikal na sampol. Ang MALDI-TOF ay lalong kinikilala na mahalagang kasangkapan sa pagkilanlan ng mga arthoropod na may kahalagahang pang-medikal at pang-beterinaryo. Ang pamamaraang ito ang napatunayang epektibo sa pagkilala sa iba’t-ibang anyo ng mga nik-nik, kasama ang mga di pa ganap ka-anyuan at ang mga dugo na sinipsip ng mga busog na babae, at matagumpay na nagamit sa pag-iiba ng lalaki at babaeng nik-nik sa ilalim ng iba’t-ibang kondisyon ng pag-iimbak at homogenisayson [28, 30, 73, 74]. Ang pamamaraang ito ay meron ding mataas na discriminatory power sa lebel ng sub-urihay, sarihay at sa mga populasyon. Binibigyan ng teknik na ito ng kakayahan ang mga tagapagsaliksik ng mabilis at wastong pagkakakilanlan ng nik-nik, na mahalaga upang maunawaan ang distribusyon, pag-uugali, at gampanin sa pagdadala ng sakit ng mga nik-nik. Sa pag-iiba ng mga sarihay gamit ang mga protein profile, ang MALDI-ToF ay may mahalagang gampanin sa mga pag-aaral na epidemyolohikal at mga estratehiya sa pagkontrol ng mga nagdadala ng sakit. May dalawang pangunahing kapintasan ang pamamaraan na ito na pinipigilan ang madalas na paggamit nito. Una ay ang pagkakaroon ng kagamitan pang-mass spectrometry, na marahil ang masyadong mahal upang madaling makamit para natatanging layunin sa pagkilanlan sa mga nik-nik (o sa pangkalahatan ng mga arthropod na nagdadala ng sakit). Mabuti na lang, ang limitasyong ito ay maaring malampasan sa pagkakaroon ng makinang time at mass spectrometers na pamantayang gamit sa pagsasaliksik sa pasilidad ng proteomics at/o pagsusuring klinikal. Pangalawa ay ang kakaunting datos ng mga nik-nik sa mga open-access database, na nagbubunga ng pangangailangang bumuo ng in-house database na may reference spectra na nakabatay sa nakilalang espesimen, gamit ang pinagsamang pagsusuri ng morpolohikal at pag-sequence ng angkop na genetic marker (COI, cytB o iba pa). Inaasahang ang limitasyong ito ay malapit nawang malutas sa unti-unting pagsasama ng datos ng mga in-house database sa MSI Platform na pinapatakbo ng Assistance Publique-Hopitaux de Paris, Sorbonne University, France at ng BCCM/IHEM/Sciensano koleksyon sa Brussels, Belgium (https://msi.happy-dev.fr/). Kapag ang MALDI-TOF protein profiling ay pinaplanong gamitin, ang mga sampol ay dapat na naitago na dry-frozen o sa 70% ethanol na molecular grade at hindi naihantad sa temperaturang pangkapaligiran. Sa kawalan ng pangkalahatang panuntunan para sa paghanda ng mga sampol, ang mga gagamit ng pamamaraang ito at pinapayuhan na gumamit ng aqueous 60% acetonitrile/0.3% TFA solusyon ng sinapinic acid (30 mg/mL) para sa paghahanda ng MALDI-ToF matrix upang maihahambing ang makukuhang protein spectra sa mga nakalathalang datos ng mga nik-nik.

Paghahanda ng sampol para sa MALDI-ToF MS (Pigura 7)

Ang mga espesimen na kulisap, na nakaimbak sa iba’t-ibang kalagayan, ay unang papatuyuin sa hangin sa temperaturang pang silid at ida-daysek. Ang ulo at tyan ay aalisin upang makuha ang bahagi ng katawan na may pangunahing katangiang morpolohikal para idikit sa slide at pagsusuring morpolohikal. Ang thorax o dibdib ay maaring gamitin sa MALDI-ToF at ang natirang bahagi ng tyan ay ipini-preserba para sa pagkuha ng DNA. Para sa protein profiling, ang dibdib ay mano-manong iho-homogenize sa 1.5mL microtubes na may 10 µL ng solusyon pang homogenize, gamit ang disposable na pandikdik at pellets. Dalawang solusyon pang homogenize ang karaniwang ginagamit: sterile distilled na tubig at 25% formic acid.

7. Pagtatapos

Sa lathalang ito, ninais na magbigay sa mga mananaliksik ng pinakamabisang paraan ng pag-mount ng nik-nik, nakabatay sa partikular na layunin, upang mabigyang daan ang wastong pagkakakilanlan at pagtuklas ng mga organismong nagdudulot ng sakit. Walang nag-iisang pamamaraan na maaring gamiting pangmalawakan;

sa halip, maraming mga kaparaanan ang umiiral, ang bawa't isa ay may kani-kaniyang kahigtan at limitasyon.

Sa mg sumusuportang datos, ibinigay ang detalyadong alituntunin sa iba't-ibang pag-ma-mount na ginagamit sa paghahanda at pagkakakilanlan ng mga nik-nik. Ang mga alituntuning ito, kasama ang mga intruksyonal na bidyo, ay nagbibigay ng mga susundang hakbangin na naka-akma para sa iba't-ibang layunin upang masiguro ang tiyak at mapagkakatiwalaang resulta. Sa pagkakaroon ng komprehensibong gabay, nilalayon namin na suportahan ang mga mananaliksik sa pagpili at pagsagawa ng pinakamainam na pamamaraan ng pag-mount batay sa kanilang pangangailangan.

Pasasalamat

Ang mga may akda ay nagpapasalamat kina Richard Lane at Zoey Jay Adams ng Natural History Musuem of London, UK para sa kanilang mahusay na pagsuri, na nagpataas ng kalidad ng lathalang ito.

Pondo

Kinikilala namin ang Brazilian development agencies CNPq (case number: 404395/2024-4) at ang Araucária Foundation (case number: 433/2025 PDI) na nagbigay ng pondo sa pagsasaliksik ni AJA.

Tungalian ng interes

Si Jerome Depaquit ay isang associate editor ng Parasite; hindi siya nakialam sa proseso ng pasusuri at pagdedesisyon sa lathalang ito. Ang nalalabing may akda ay naglalahad na wala silang mga tungalian ng interes.

Mga bidyo sa Zenodo

Mga bidyo sa Zenodo
Bidyo 1: https://zenodo.org/records/18198006
Bidyo 2: https://zenodo.org/records/18311158
Bidyo 3: https://zenodo.org/records/18311106
Bidyo 4: https://zenodo.org/records/18311154
Bidyo 5: https://zenodo.org/records/18303014
Bidyo 6: https://zenodo.org/records/18302850
Bidyo 7: https://zenodo.org/records/18315029

Mga karagdagang materyal

Higit pang impormasyon tungkol sa artikulong ito ay makukuha sa sumusunod na adres: https://www.parasite-journal.org/10.1051/parasite/2026009/olm

Mga Sanggunian

1. Alkan C, Allal-Ikhlef AB, Alwassouf S, Baklouti A, Piorkowski G, de Lamballerie X, Izri A, Charrel RN. 2015. Virus isolation, genetic characterization and seroprevalence of Toscana virus in Algeria. Clinical Microbiology and Infection, 21(11), 1040 e1-9.
2. Alten B, Ozbel Y, Ergunay K, Kasap OE, Cull B, Antoniou M, Velo E, Prudhomme J, Molina R, Banuls AL, Schaffner F, Hendrickx G, Van Bortel W, Medlock JM. 2015. Sampling strategies for phlebotomine sand flies (Diptera: Psychodidae) in Europe. Bulletin of Entomological Research 105(6), 664–678.
3. Ayhan N, Baklouti A, Prudhomme J, Walder G, Amaro F, Alten B, Moutailler S, Ergunay K, Charrel RN, Huemer H. 2017. Practical guidelines for studies on sandfly-borne phleboviruses: Part I: Important points to consider *ante* field work. Vector-Borne and Zoonotic Diseases 17(1), 73–80.
4. Bates PA. 1997. Infection of phlebotomine sandflies with *Leishmania*, in The Molecular Biology of Insect Disease Vectors: A Methods Manual. Springer. p. 112–120.5.
5. Baum M, de Castro EA, Pinto MC, Goulart TM, Baura W, Klisiowicz Ddo R, Vieira da Costa-Ribeiro MC. 2015. Molecular detection of the blood meal source of sand flies (Diptera: Psychodidae) in a transmission area of American cutaneous leishmaniasis, Parana State, Brazil. Acta Tropica, 143, 8–12.
6. Belen A, Alten B, Aytekin A. 2004. Altitudinal variation in morphometric and molecular characteristics of *Phlebotomus papatasi* populations. Medical and Veterinary Entomology, 18(4), 343–350.
7. Bhattacharya J, Chandra G, Hati AK. 1991. A simple method for cryopreservation of *Leishmania donovani* promastigotes, Indian Journal of Medical Research, 93, 245–246.
8. Caligiuri LG, Sandoval AE, Miranda JC, Pessoa FA, Santini MS, Salomón OD, Secundino NF, McCarthy CB. 2019. Optimization of DNA extraction from individual sand flies for PCR amplification. Methods and Protocols, 2(2), 36.
9. Casaril AE, de Oliveira LP, Alonso DP, de Oliveira EF, Gomes Barrios SP, de Oliveira Moura Infran J, Fernandes WS, Oshiro ET, Ferreira AMT, Ribolla PEM, de Oliveira AG. 2017. Standardization of DNA extraction from sand flies: Application to genotyping by next generation sequencing. Experimental Parasitology, 177, 66–72.
10. Castalanelli MA, Severtson DL, Brumley CJ, Szito A, Foottit RG, Grimm M, Munyard K, Groth DM. 2010. A rapid non-destructive DNA extraction method for insects and other arthropods. Journal of Asia-Pacific Entomology, 13(3), 243–248.
11. Cerqueira NL. 1943. Um novo meio para montagem de pequenos insetos em lâmina. Memórias do Instituto Oswaldo Cruz, (39), 37–41.
12. Charrel RN, Gallian P, Navarro-Mari JM, Nicoletti L, Papa A, Sanchez-Seco MP, Tenorio A, de Lamballerie X. 2005. Emergence of Toscana virus in Europe. Emerging Infectious Diseases, 11(11), 1657–1663.
13. Chaskopoulou A, Giantsis IA, Demir S, Bon MC. 2016. Species composition, activity patterns and blood meal analysis of sand fly populations (Diptera: Psychodidae) in the metropolitan region of Thessaloniki, an endemic focus of canine leishmaniasis. Acta Tropica, 158, 170–176.
14. Chen H, Rangasamy M, Tan SY, Wang H, Siegfried BD. 2010. Evaluation of five methods for total DNA extraction from western corn rootworm beetles. PLoS One, 5(8), e11963.
15. Depaquit J, Grandadam M, Fouque F, Andry PE, Peyrefitte C. 2010. Arthropod-borne viruses transmitted by Phlebotomine sandflies in Europe: a review. Eurosurveillance, 15(10), 19507.
16. Diamond LS, Herman CM. 1954. Incidence of Trypanosomes in the Canada Goose as revealed by bone marrow culture. Journal of Parasitology, 40(2), 195–202.
17. Ding H, Torno M, Vongphayloth K, Ng G, Tan D, Sng W, Ho K, Randrianambinintsoa FJ, Depaquit J, Tan CH. 2025. Hidden in plain sight: discovery of sand flies in Singapore and description of four species new to science. Parasites & Vectors, 18(1), 402.

18. Es-Sette N, Ajaoud M, Bichaud L, Hamdi S, Mellouki F, Charrel RN, Lemrani M. 2014. *Phlebotomus sergenti* a common vector of *Leishmania tropica* and Toscana virus in Morocco. Journal of Vector Borne Diseases, 51(2), 86–90.
19. Favret C. 2005. A new non-destructive DNA extraction and specimen clearing technique for aphids (Hemiptera). Proceedings of the Entomological Society of Washington, 107(2), 469–470.
20. Galati EAB. 2018. Phlebotominae (Diptera, Psychodidae): Classification, morphology and terminology of adults and identification of American taxa, in Brazilian Sand Flies: Biology, Taxonomy, Medical Importance and Control, Rangel EF, Shaw JJ, Editors. Cham: Springer International Publishing. pp. 9–212.
21. Galati EAB, de Andrade AJ, Perveen F, Loyer M, Vongphayloth K, Randrianambinintsoa FJ, Prudhomme J, Rahola N, Akhoundi M, Shimabukuro PHF, Depaquit J. 2025. Phle-botomine sand flies (Diptera, Psychodidae) of the world. Parasites & Vectors, 18(1), 220.
22. Galati EAB, Galvis-Ovallos F, Lawyer P, Leger N, Depaquit J. 2017. An illustrated guide for characters and terminology used in descriptions of Phlebotominae (Diptera, Psychodidae). Par-asite, 24, 26.
23. Gariepy T, Kuhlmann U, Gillott C, Erlandson M. 2007. Parasitoids, predators and PCR: the use of diagnostic molecular markers in biological control of Arthropods. Journal of Applied Entomology, 131(4), 225–240.
24. Giantsis IA, Chaskopoulou A, Bon MC. 2016. Mild-Vectolysis: A nondestructive DNA extraction method for vouchering sand flies and mosquitoes. Journal of Medical Entomology, 53(3), 692–695.
25. Gidwani K, Picado A, Rijal S, Singh SP, Roy L, Volfova V, Andersen EW, Uranw S, Ostyn B, Sudarshan M, Chakravarty J, Volf P, Sundar S, Boelaert M, Rogers ME. 2011. Serological markers of sand fly exposure to evaluate insecticidal nets against visceral leishmaniasis in India and Nepal: a cluster-randomized trial. PLoS Neglected Tropical Diseases, 5(9), e1296.
26. Gilbert MTP, Moore W, Melchior L, Worobey M. 2007. DNA extraction from dry museum beetles without conferring external morphological damage. PLoS One, 2(3), e272.
27. Giordani BF, Andrade AJ, Galati EAB, Gurgel-Goncalves R. 2017. The role of wing geometric morphometrics in the identification of sandflies within the subgenus *Lutzomyia.* Medical and Veterinary Entomology, 31(4), 373–380.
28. Guzmán-Larralde AJ, Suaste-Dzul AP, Gallou A, Peña-Carrillo KI. 2017. DNA recovery from microhymenoptera using six non-destructive methodologies with considerations for subsequent preparation of museum slides. Genome, 60(1), 85–91.
29. Hajibabaei M, DeWaard JR, Ivanova NV, Ratnasingham S, Dooh RT, Kirk SL, Mackie PM, Hebert PD. 2005. Critical factors for assembling a high volume of DNA barcodes. Philosophical Transactions of the Royal Society B: Biological Sciences, 360(1462), 1959–1967.
30. Haouas N, Pesson B, Boudabous R, Dedet JP, Babba H, Ravel C. 2007. Development of a molecular tool for the identification of *Leishmania* reservoir hosts by blood meal analysis in the insect vectors. American Journal of Tropical Medicine and Hygiene, 77(6), 1054–1059.
31. Hlavackova K, Dvorak V, Chaskopoulou A, Volf P, Halada P. 2019. A novel MALDI-TOF MS-based method for blood meal identification in insect vectors: A proof of concept study on phlebotomine sand flies. PLoS Neglected Tropical Diseases, 13 (9), e0007669.
32. Huemer H, Prudhomme J, Amaro F, Baklouti A, Walder G, Alten B, Moutailler S, Ergunay K, Charrel RN, Ayhan N. 2017. Practical guidelines for studies on sandfly-borne phleboviruses: Part II: Important points to consider for fieldwork and subsequent virological screening. Vector-Borne and Zoonotic Diseases, 17(1), 81–90.
33. Jancarova M, Polanska N, Thiesson A, Arnaud F, Stejskalova M, Rehbergerova M, Kohl A, Viginier B, Volf P, Ratinier M. 2025. Susceptibility of diverse sand fly species to Toscana virus. PLoS Neglected Tropical Diseases, 19(5), e0013031.
34. Kapp JD, Green RE, Shapiro B. 2021. A fast and efficient single-stranded genomic library preparation method optimized for ancient DNA. Journal of Heredity, 112(3), 241–249.
35. Killick-Kendrick R, Maroli M, Killick-Kendrick M. 1991. Bibliography of the colonization of phlebotomine sandflies. Parassitologia, 33(suppl.), 321–333.
36. Lawyer P, Killick-Kendrick M, Rowland T, Rowton E, Volf P. 2017. Laboratory colonization and mass rearing of phlebotomine sand flies (Diptera, Psychodidae). Parasite, 24, 42.
37. Léger N, Pesson B, Madulo-Leblond G. 1986. Les phlébotomes de Grèce : 1ère partie. Bulletin de la Société de Pathologie Exotique, 79, 386–397.
38. Léger N, Pesson B, Madulo-Leblond G. 1986. Les phlébotomes de Grèce : 2ème partie. Bulletin de la Société de Pathologie Exotique, 79, 514–524.
39. Leonel JAF, Vioti G, Alves ML, da Silva DT, Meneghesso PA, Benassi JC, Spada JCP, Galvis-Ovallos F, Soares RM, Oliveira T. 2020. DNA extraction from individual Phlebotomine sand flies (Diptera: Psychodidae: Phlebotominae) specimens: Which is the method with better results? Experimental Parasitology, 218, 107981.
40. Lestinova T, Rohousova I, Sima M, de Oliveira CI, Volf P. 2017. Insights into the sand fly saliva: Blood-feeding and immune interactions between sand flies, hosts, and *Leishmania.* PLoS Neglected Tropical Diseases, 11(7), e0005600.
41. Lienhard A, Schaffer S. 2019. Extracting the invisible: obtaining high quality DNA is a challenging task in small arthropods. PeerJ, 7, e6753.
42. Lozano-Sardaneta YN, Mikery-Pacheco OF, Huerta H, Rojas-Soriano JE, Contreras-Ramos A. 2025. Wing geometric morphometrics is effective to separate sand fly species (Diptera, Psychodidae, Phlebotominae) related with leishmaniasis transmission in Mexico. Acta Tropica, 262, 107523.
43. Mandrioli M. 2008. Insect collections and DNA analyses: how to manage collections? Museum Management and Curatorship, 23(2), 193–199.
44. Maroli M, Feliciangeli MD, Bichaud L, Charrel RN, Gradoni L. 2013. Phlebotomine sandflies and the spreading of leishmaniases and other diseases of public health concern. Medical and Veterinary Entomology, 27(2), 123–147.
45. Marquina D, Buczek M, Ronquist F, Lukasik P. 2021. The effect of ethanol concentration on the morphological and molecular preservation of insects for biodiversity studies. PeerJ, 9, e10799.
46. Mathis A, Depaquit J, Dvorak V, Tuten H, Banuls AL, Halada P, Zapata S, Lehrter V, Hlavackova K, Prudhomme J, Volf P, Sereno D, Kaufmann C, Pfluger V, Schaffner F. 2015. Identification of phlebotomine sand flies using one MALDI-TOF MS reference database and two mass pectrometer systems., Parasites & Vectors, 8, 266.
47. Mekarnia N, Benallal KE, Sadlova J, Vojtkova B, Mauras A, Imbert N, Longhitano M, Harrat Z, Volf P, Loiseau PM, Cojean S. 2024. Effect of *Phlebotomus papatasi* on the fitness,

infectivity and antimony-resistance phenotype of antimony-resistant *Leishmania* major Mon-25. International Journal for Parasitology – Drugs and Drug Resistance, 25, 100554.

48. Milligan BG. 1998. Total DNA isolation, in Molecular Genetic Analysis of Population: A Practical Approach, Hoelzel AR, Editor. Oxford: Oxford University Press.
49. Molina R, Jiménez M, Alvar J, González E, Hernández-Taberna S, Ines MM. 2017. Methods in sand fly research. Madrid: Servicio de publicaciones Universidad de Alcalá de Henares, Madrid.
50. Murphy WJ, Eizirik E, O'Brien SJ, Madsen O, Scally M, Douady CJ, Teeling E, Ryder OA, Stanhope MJ, de Jong WW, Springer MS. 2001. Resolution of the early placental mammal radiation using Bayesian phylogenetics. Science, 294(5550), 2348–2351.
51. Nacif-Pimenta R, Pinto LC, Volfova V, Volf P, Pimenta PFP, Secundino NFC. 2020. Conserved and distinct morphological aspects of the salivary glands of sand fly vectors of leishma-niasis: an anatomical and ultrastructural study. Parasites & Vectors, 13(1), 441.
52. Neuhaus B, Schmid T, Riedel J. 2017. Collection management and study of microscope slides: Storage, profiling, deterioration, restoration procedures, and general recommendations. Zootaxa, 4322(1), 1–173.
53. New TR. 1974. Pscoptera. Handbooks for Identification of British Insects (Vol. I). London: Royal Entomological Society of London. 102 pp.
54. Perez-Ruiz M, Collao X, Navarro-Mari JM, Tenorio A. 2007. Reverse transcription, real-time PCR assay for detection of Toscana virus. Journal of Clinical Virology, 39(4), 276–281.
55. Porco D, Rougerie R, Deharveng L, Hebert P. 2010. Coupling non-destructive DNA extraction and voucher retrieval for small soft-bodied Arthropods in a high-throughput context: the example of Collembola. Molecular Ecology Resources, 10(6), 942–945.
56. Prudhomme J, Cassan C, Hide M, Toty C, Rahola N, Vergnes B, Dujardin JP, Alten B, Sereno D, Banuls AL. 2016. Ecology and morphological variations in wings of *Phlebotomus ariasi* (Diptera: Psychodidae) in the region of Roquedur (Gard, France): a geometric morphometrics approach. Parasites & Vectors, 9(1), 578.
57. Prudhomme J, Gunay F, Rahola N, Ouanaimi F, Guernaoui S, Boumezzough A, Banuls AL, Sereno D, Alten B. 2012. Wing size and shape variation of *Phlebotomus papatasi* (Diptera: Psychodidae) populations from the south and north slopes of the Atlas Mountains in Morocco. Journal of Vector Ecology, 37(1), 137–147.
58. Prudhomme J, Toty C, Kasap OE, Rahola N, Vergnes B, Maia C, Campino L, Antoniou M, Jimenez M, Molina R, Cannet A, Alten B, Sereno D, Banuls AL. 2015. New microsatellite markers for multi-scale genetic studies on *Phlebotomus ariasi* Tonnoir, vector of *Leishmania infantum* in the Mediterranean area. Acta Tropica, 142, 79–85.
59. Prudhomme J, Velo E, Bino S, Kadriaj P, Mersini K, Gunay F, Alten B. 2019. Altitudinal variations in wing morphology of *Aedes albopictus* (Diptera, Culicidae) in Albania, the region where it was first recorded in Europe. Parasite, 26, 55.
60. Rawlins DJ. 1992. Light Microscopy: An Introduction to Biotechniques. Oxford: Bios Scientific publishers. 143 pp.
61. Ready PD. 2013. Biology of phlebotomine sand flies as vectors of disease agents. Annual Review of Entomology, 58, 227–250.
62. Rohlf FJ, Slice D. 1990. Extensions of the Procrustes method for the optimal superimposition of landmarks. Systematic Zoology, 39(1), 40–59.
63. Rowley DL, Coddington JA, Gates MW, Norrbom AL, Ochoa RA, Vandenberg NJ, Greenstone MH. 2007. Vouchering DNA-barcoded specimens: Test of a nondestructive extraction proto-col for terrestrial arthropods. Molecular Ecology Notes, 7(6), 915–924.
64. Sábio PB, Andrade AJ, Galati EAB. 2014. Assessment of the taxonomic status of some species included in the *Shannoni* complex, with the description of a new species of *Psathyromyia* (Diptera: Psychodidae: Phlebotominae). Journal of Medical Entomology, 51(2), 331–341.
65. Sadlova J, Yeo M, Seblova V, Lewis MD, Mauricio I, Volf P, Miles MA. 2011. Visualisation of *Leishmania donovani* fluo-rescent hybrids during early stage development in the sand fly vector. PLoS One, 6(5), e19851.
66. Sales K, Miranda DEO, da Silva FJ, Otranto D, Figueredo LA, Dantas-Torres F. 2020. Evaluation of different storage times and preservation methods on phlebotomine sand fly DNA concen-tration and purity. Parasites & Vectors, 13(1), 399
67. Sales KG, Costa PL, de Morais RC, Otranto D, Brandao-Filho SP, Cavalcanti Mde P, Dantas-Torres F. 2015. Identification of phlebotomine sand fly blood meals by real-time PCR. Parasites& Vectors, 8, 230.
68. Sant'Anna MR, Jones NG, Hindley JA, Mendes-Sousa AF, Dillon RJ, Cavalcante RR, Alexander B, Bates PA. 2008. Blood meal identification and parasite detection in laboratory-fed and field-captured *Lutzomyia longipalpis* by PCR using FTA databasing paper. Acta Tropica, 107(3), 230–237.
69. Senne NA, Santos HA, Araujo TR, Paulino PG, Mendonca LP, Moreira HVS, Camilo TA, da Costa Angelo I. 2022. Robust comparative performance of genomic DNA extraction methods from non-engorged phlebotomine sandflies. Medical and Veterinary Entomology, 36(2), 203–211.
70. Shaw JJ. 2025. A review of Leishmania infections in American Phlebotomine sand flies – Are those that transmit leishmaniasis anthropophilic or anthropportunists? Parasite, 32, 57.
71. Tesh RB, Modi GB. 1983. Growth and transovarial transmission of Chandipura virus (Rhabdoviridae: Vesiculovirus) in *Phlebotomus papatasi.* American Journal of Tropical Medicine and Hygiene, 32(3), 621–623.
72. Thomsen PF, Elias S, Gilbert MTP, Haile J, Munch K, Kuzmina S, Froese DG, Sher A, Holdaway RN, Willerslev E. 2009. Non-destructive sampling of ancient insect DNA. PLoS One, 4(4),e5048
73. Truett GE, Heeger P, Mynatt RL, Truett AA, Walker JA, Warman ML. 2000. Preparation of PCR-quality mouse genomic DNA with hot sodium hydroxide and tris (HotSHOT). Biotechniques, 29(1), 52–54.
74. Upton MS. 1993. Aqueous gum-chloral slide mounting media: an historical review. Bulletin of Entomological Research, 83(2), 267–274.
75. Volf P, Myskova J. 2007. Sand flies and Leishmania: specific versus permissive vectors. Trends in Parasitology, 23(3), 91–92.
76. Wang Q, Wang X. 2012. Comparison of methods for DNA extraction from a single chironomid for PCR analysis. Pakistan Journal of Zoology, 44(2), 421–426.
77. Wang Y, Zhao Y, Bollas A, Wang Y, Au KF. 2021. Nanopore sequencing technology, bioinformatics and applications. Nature Biotechnology, 39(11), 1348–1365.

Cite this article as: Randrianambinintsoa FJ, Augendre L, Prudhomme J, Martinet J-P, Loyer M, Mekarnia N, Kerkoub H, Perveen FK, Huguenin A, Kariya E, Akhoundi M, De Andrade AJ, Berriatua E, Bongiorno G, Boyer S, Christodoulou V, Da Costa-Ribeiro MCV, De Souza LAF, Ding H, Dondji B, Dvořák V, Erisoz Kasap O, Galati EAB, Gállego M, Ballart C, Gouzelou S, Haddad N, Masse RS, Mekuria AH, Ivovic V, Kaczmarek S, Shahar MK, Kirstein OD, Kniha E, Kolářová I, Lincoln T, Lucanas C, Mikov O, Nov K, Özbel Y, Pesson B, Posada Lopez LC, Prasetyo DB, Rahola N, Rebollar-Tellez EA, Rodrigues BL, Roy L, Saini P, Sanjoba C, Shimabukuro PH, Siriyasatien P, Soszynska A, Suleşco T, Sylla M, Torno M, Volf P, Vongphayloth K, Sinh Nam V, Wardhana A, Yessinou E, Zapata S, Gantier J-C & Depaquit J. 2026. Processing and mounting phlebotomine sand flies: a consensus guideline. Parasite xx, xx. https://doi.org/10.1051/parasite/2026009.

PARASiTE

An international open-access, peer-reviewed, online journal publishing high quality papers on all aspects of human and animal parasitology

Reviews, articles and short notes may be submitted. Fields include, but are not limited to: general, medical and veterinary parasitology; morphology, including ultrastructure; parasite systematics, including entomology, acarology, helminthology and protistology, and molecular analyses; molecular biology and biochemistry; immunology of parasitic diseases; host-parasite relationships; ecology and life history of parasites; epidemiology; therapeutics; new diagnostic tools.
All papers in Parasite are published in English. Manuscripts should have a broad interest and must not have been published or submitted elsewhere. No limit is imposed on the length of manuscripts.

Parasite (open-access) continues **Parasite** (print and online editions, 1994-2012) and **Annales de Parasitologie Humaine et Comparée** (1923-1993) and is the official journal of the Société Française de Parasitologie.

Editor-in-Chief:
Jean-Lou Justine, Paris

Submit your manuscript at:
https://www.editorialmanager.com/parasite

This is an Open Access article distributed under the terms of the Creative Commons Attribution License (https://creativecommons.org/licenses/by/4.0), which permits unrestricted use, distribution, and reproduction in any medium, provided the original work is properly cited.

Appendix 1. Pundasyon na Biokemikal na teorya.

Ang pinatutungkulang arthropod ay ang mga nik-nik. Gayunpaman, ang pangkalahatang ideya ay maaring gamitin sa iba pang mga pangkaraniwang arthropod na ang pagkilanlan ay magagawa lamang sa pamamagitan ng pagsusuri sa panloob na morpolohikal na katangian. Sa pagkakataon, ang ilang lamang loob ay maaaring partially chitinised at ang kanilang katangiang morpolohikal ay nagbibigay ng mahalagang impormasyon. Kaya interesante na obserbahan ang mga daanan ng pagkain, spermatheca at ang mga tubo nito. Sa lahat ng mga kemikal na babanggitin dito, hindi dapat makalimutan na mula sa pag-fix ng kulisap hanggang pagbubuo, aaplyan lamang ito ng mga redox reactions. Ang tanging pag-iingat o ideya na nangunguna sa atin ay ang huwag hayaang maghalo ang reducing reagents at oxidizing reagents.

Ethyl alcohol o ethanol

Ang kemikal na ito ay gagamitin na iba't-ibang paraan. Ang mga molecule ng alkohol ay may malakas na affinity sa tubig at samakatuwid ay nagpapakita ng dehydrating effect. Gayunpaman, ang isang alkohol na may mababang konsentrasyon (maraming tubig) ay makakasira ng mga nucleic acid (ang tubig ay kaaway ng mga nucleic acid).

Kapag ang mga kulisap ay inilalagay sa ethanol, hindi lamang para ma-preserba, kundi pati na rin para ma-fix ang mga tisyu. Sa histology, karaniwan pinag-iiba ang dalawang mahahalagang konsepto: ang penetration rate at ang fixation rate. Nauunawaan na ang isang mahusay na pang-preserba ay dapat na mabilis na tumagos nang malalim sa mga tisyu. Para sa 96% na alkohol, ang penetration coefficient ay humigit-kumulang 1.05 (kung ikukumpara, para sa 0.75% aqueous solution ng picric acid, ang penetration coefficient ay 0.45, habang ang 3% potassium dichromate solution ay 1.45).

Ang pagnanais na i-preserba ang mga kulisap at iba pang mga arthropod nang pang-matagalan sa ethanol ang ninanais ng mga dalubkulisap. Ang ideya ay napaka-marangal pa rin na nais na panatilihin ang mga nahuli sa kapaligiran para sa mga susunod na pag-aaral o para sa mga mananaliksik sa hinaharap. Gayunpaman, ang ideyang ito ay hindi posible para sa isang cytologist o histologist. Sa pamamagitan ng pagpuntirya na panatilihin ang mga sampol sa fixative nang masyadong matagal, maaaring maging halos imposibleng muling gamitin ang mga ito. Ito ang dahilan kung bakit ang mga sampol na mas matanda sa 10 taon ay mahirap o imposibleng gamitin.

Ang isa pang konsiderasyon ay ang ratio sa pagitan ng timbang ng arthropod na iaayos at ang dami ng fixative. Sa zoolohikal o medisina pagsusuri, ipinapayong magplano para volume na 60 beses na mas malaki kaysa sa volume ng mga piraso na ifi-fix. Para sa mga micro-arthropod, sa isang partikular na volume ng mga espesimen na iaayos, magdagdag ng hindi bababa sa 4-5 volume ng alkohol. Tandaan na ang alkohol ay humihina habang inaalis ang tubig na nasa mga tisyu ng arthropod.

Bilang konklusyon:
- Ang ethyl alcohol ay isang reducing chemical agent (Ito ay hindi angkop sa mga oxidative fixative);
- Enerhikal nitong binubuo at binabago ang mga protina;
- Tinutunaw nito ang ilang mga kumplikadong lipid at binubuo ang glycogen;
- Nagdudulot ito ng malakas na pag-urong at pinapatigas ang mga tisyu.

Solusyong basic Potassium o Sodium hydroxide

Ang paggamit ng mga solusyong ito sa dalubkulisapan ay pangunahing nakatuon sa Potassium hydroxide nang walang malinaw na katwiran.

Ang Sodium hydroxide [E524] ay isang solusyon na may iba't-ibang konsentrasyon o iba't-ibang normalidad. Ito ay maaring nasa anyo ng pellet o glitter. Ang pangunahing kakulangan nito ay ang pagiging malakas sa paghigop ng tubig sa hangin (higit pa sa KOH). Tinutunaw nito ang mga protina at pinagbubuo ang mga lipids at binabago upang maging matigas na sabon sa saponipikasyion (ito ang malaking kaibahan sa KOH na nagbubunga ng likidong sabon sa saponipikasyon).

Ang Potassium hydroxide [E525] ay mayroong purong solusyon, ngunit higit sa lahat, ito ay may kahigtan sa pagkakaroon ng pormang mga pellets na may timbang na humigit-kumulang 0.1g., na siyang nagpapadali sa paghahanda ng mas malabnaw na solusyon kung hindi nangangailangan ng balanseng timpla. Halimbawa, ang isang pellet na may 0.1g sa 1mL ng distilled water ay nagbubunga sa 10% solusyon. Ang pangalawang kalamangan nito bilang isang pellet ay mababang sensitibo sa carbonasyon (ang solusyong KOH ay may mataas na affinity sa pag-fix ng CO^2, na lumilikha ng carbonate salts).

Ito ay isang matinding base na ginagamit na pantunaw ng mga fatty acid na nagiging sabon na natutunaw sa tubig. Dapat tandaan na ang mga fixative, tulad ng alkohol, ay tumutunaw sa ilang mga taba sa mga sampol. Gayunpaman, kapag inilipat ang mga sampol sa isang aqueous medium na may matinding base, ang mga fatty acids (mas komplikado) ay mamumuo. Sa ilang pagkakataon, kapag lumalabis ang mga adipose tissue, halimbawa sa mga babae, mas mapapabilis ang reaksyon kung itataas ang temperatura ng 35-40°C o patagalin ang pagbabad sa temperaturang pang silid.

May kulay na solusyong asido/ walang kulay na solusyong Marc-Andre:

Dito, susuriin ang mga kalamangan at kakulangan ng paggamit ng solusyong Marc-Andre. Ang solusyong ito ay binubuo ng chloral hydrate (trichloracetaldehyde monohydrate), acetic acid, at tubig. Ang solusyong ito ay lubos na nag-ooxidize (pinaghalong acid at aldehyde). Pinapawalang bisa nito ang labis na Potassium hydroxide na naiwan sa sampol, nang hindi pinapahintulutan ang pamumuo ng mga sabong alkalina na nabuo habang ginagamit ang Potassium Hydroxide. Ang oxidizing na

solusyong ito ay nakakaapekto din sa mga gampaning secondaryo ng alkohol ng glucosamines na namumuo sa chitin sa pag-ooxidize nito, na nagpapalambot sa chitin. Isa pang aksyon ay ang pagtunaw din nito ng ilang mga mineral salts na naroroon.

Kapag ang solusyong Marc-Andre ay hinaluan ng acid fuchsin (kaya nasa kalagayang oxidized), magagawa nitong dumikit sa mga secondary alcohol function ng mga istruktura. Pagkatapos sa oras ng pagbabad sa Marc-Andre solution at makulayan ang mga sampol, ito ay babanlawan gamit ang alkohol lamang. At dyan ay uumpisahan na ang pagtatangal ng tubig o dehydration phase ng mga sampol.

Mga kalamangan:
- Pagpapawalang bisa ng mga labis na solusyong basic
- Pag-relax ng chitin
- Pagtitina ng chitin upang mas madaling masuri ang mga panloob na katangian

Mga kakulangan:

Ang chloral hydrate ay hypnotic at ginagamit sa medisina. Ito ay dapat gamitin sa ilalim ng isang chemical hood, at may mga batas na nangangasiwa sa panganib pang-kemikal na dapat sundin.

Solusyon sa pantanggal ng tubig (dehydration)

Ayon sa mga karanasan na para sa napakaliliit na mga sampol, hindi kapakipakinabang na striktong sundin ang pagbabad sa mga alkohol na may pataas na konsentrasyon. Kung ang sampol ay malaki, magsisimula sa 80% ethanol, tapos sa 90%, 95% at panghuli sa absolute ethanol. Para sa mga maliliit na mga sampol, ibabad sa 90% ethanol at sundan ng paglulubog sa absolute ethanol. Sa bahaging ito, dapat tandaan na ang absolute ethanol ay humihigop ng tubig sa hangin.

Ang tradisyon sa mga laboratoryo ng mga dalubkulisap ay tapusin ang dehydration ng mga sampol gamit ang beech creosote na babaran. Sa kasalukyan, ito ay malawakang ginagamit bilang pestisidyo, pangontra sa amag, at pang preserba ng kahoy, at mariing hindi na inirerekomenda dahil sa amoy nito (polycyclic aromatic hydrocarbons) at ito ay ipinapalagay na reprotoxic, carcinogenic, isang persistent organic pollutant, at nakakalason para sa mga organismo sa tubig.

Isang solusyon na iminungkahi para ihanda ang mga sampol sa pagmount ay ang Euparal® at Euparal essence (inilarawan sa kasunod na talata). Ang pinaghalong Euparal® at Euparal essence ay lubos na tinatanggap; ang mga sampol na nakuha pagkatapos ng pagbabad sa 90% ethanol

Appendix 2. Komposisyon ng mga kemikal na ginagamit.

Potassium hydroxide 10%
Potassium hydroxide 10 g
Distilled water q.s. 100 mL

Gum chloral mounting medium Hoyer medium
Distilled water 50 mL
Chloral hydrate 200 g
Gum arabic 50 g
Glycerol 20 mL

Marc-André solution
Chloral hydrate 40 g
Glacial acetic acid 30 mL
Distilled water 30 mL

Fuchsin acid 1% in distilled water
Acid fuchsin powder 1 g
Distilled water 99 mL

Marc-André solution colored with fuchsin
Marc-André solution 10 mL
Fuchsin 1% 50 µL

Appendix 3: Euparal®, Canada balsam, polyvinyl alcohol o iba pang kemikal na ginagamit sa pag-mount.

Polyvinyl alcohol: Eto ay mainam na pandikit kapag walang makuhang mga kemikal para sa wastong dehydration. Ang Polyvinyl alcohol ay hinahalo sa Amman's lactophenol. Gayunpaman, ang solusyong ito ay may mga pangunahing kakulangan alinman sa pagtutuyo o pamumuo ng kristal pagkat ang tubig sa polyvinyl alcohol sumisingaw o pangingitim kapag na oxidize ang phenol. Ito ay nananatiling isang mahusay na pamamaraan para sa panandaliang pag-mount.

Canada Balsam: Ang paggamit nito sa mounting sa pagitan ng slide at coverslip ay nangangailangan ng dehydration ng mga sampol na ididikit. Hindi nawawala ang kaakibat na abalang dala ng pag-gamit ng xylene o toluene.

Enece medium: Para sa mounting sa pagitan ng slide at coverslip, tulad ng Canada Balsam, nangangailangan ito ng dehydration ng mga espesimen. Enece formulation; purong puti na colophony (22g); copal gum na natutunaw sa alkohol (12g), absolute alcohol (20mL), camphor (10g), turpentine essence (10mL), at eucalyptol (26mL). Para sa paghahanda nito, sa isang lalagyan tulad ng Erlenmeyer flask, ilagay ang absolute alcohol at camphor. Sunod ay idagdag ang colophony at ang copal gum. Ang flask ay iseselyo gamit ang stopper at aalugin, pagkatapos lamang nito paiinitan sa baine-marie sa katamtamang init upang hindi ito kumulo. Kapag tuluyan ng nalusaw ang mga laman, idadagdag ang turpentine essence, at sasalain habang ang solusyon ay mainit pa. At panghuli ay ihahalo ang eucalyptol sa pinagsalaan. Kapag ang medium ay bahagya ng tumitigas, ito ay hahaluan ng Eenece na may sumusunod na kayarian: albsolute alcohol (30 mL), camphor (17g), turpentine essence (15 mL), eucalyptol (38mL) (Cerqueria, 1934).

Euparal®: Ito ay isang resin na nanggagaling sa Cypress ng Atlas *Tetraclinis articulata* (Vahl, 1791), na pinag-aralan at nabuo noong 1906 ni Gilson. Ang pangunahing nitong kalamangan ay hindi ito nagpo-polymerize. Ang mga sampol na nadikit sa pagitan ng slides at coverslips ay madaling makuhang muli sa pamamagitan ng alkohol o , mas mainam, ng Euparal® essence. Ang resin an ito, na tinatawag ding sandarac, ay tumatanggap ng ethanol mula sa 80%.

Paggamit ng Triton X100: solusyong non-ionic aqueous

Ang Triton X100 ay isang non-ionic aqueous na solusyon *(4-(1,1,3,3-tetramethylbutyl) phenyl-polyethylene glycol solution, or t-octylphenoxypolyethoxyethanol, polyethylene glycol tert-octylphenyl ether)*, ay malawakang ginagamit bilang detergent sa selular o haynahayang molekular. Nagbibigay daan ito sa permyabilisasyon ng mga sihay (cell) at ng mga nuclear membrane. Ang pagtatago ng maraming taon ng mga kulisap sa alkohol ay pangkaraniwam. Sa kasamaang palad, ang preserbasyon sa alkohol ay hindi mainam, at ang mga arthropods na na-preserba sa ganitong paraan ay nagiging mahirap maihanda para sa mikroskopikong pagsusuri. Ang mga plastic na naglalaman ng mga sampol ay madalas na nasisira, kasunod ang pagkatuyo ng alkohol. Sa parehas na pagkakataon, ang matagal na pagkababad sa alkohol o ang pagkatuyo ng mga sampol ay nagiging problema. Noong 2008, inilathala ni Jonque ang tala para sa rehydration ng mga gagamba gamit ang wetting agent tulad ng Agepon ginagamitin sa mga photographic films [26]. Dito pumasok ang ideya sa paggamit ng mga wetting agent na hindi matinding detergent o sabon.

Nasa ibaba ang pamamaraan sa paggamit ng Triton X100 sa 0.5% aqueous na solusyon:

- Ibabad ang tuyong sampol sa absolute alcohol.
- Idagdag ang karampatang dami ng Triton X100 na solusyon sa 0.5% hanggang sa lumubog ang kabuuan ng espesimen.
- Hayaan ang pagkababad ng espesimen sa loob ng 5 minuto o mahigit pa. Lahat ng arthropods ay dapat magkahiwahiwalay habang nakababad sa solusyon.
- Alisin ang Triton X100 at palitan ng solusyon ng potassium hydroxide.

Ang teknik na na ito ay sinusunod ayon sa pagkakalarawan sa itaas.

Appendix 4: Mga hakbang sa pag gamit ng Euparal® or Canada Balsam bilang pandikit o mounting media

1. Ang mga espesimen ay dapat na lubusang natanggalan ng tubig o na-dehydrate (ang malabo o maputing itsura ay nagpapahiwatig ng hindi sapat na dehydration).
2. Ang dehydration ay naisasagawa sa pagtataas na konsentrasyon na ethyl alcohol.
3. Ang mga espesimen ay maaring ilipat mula sa 99% alcohol o absolute alcohol papunta sa isang clearing agent.

Pamamaraan:

1. Ilagay ang magulang (adult) na nik-nik sa 70% ethanol.
2. Tanggalin ang alkohol at palitan ng 10% KOH. Takpan ang mga nik-nik ng isang glass slide.
3. Ibabad hanggang ang mga kulisap ay maging transparent o malinaw.
4. Tanggalin ang KOH.
5. Ibabad ang espesimen sa distilled na tubig at mag-intay ng 30-45 minuto.
6. Tanggalin ang tubig, at muling hugasan ng distilled na tubig sa loob ng 30 minuto (gampanin ng oras sa madaming sampol: mas maraming sampol na ipo-proseso ng magkakasama, mas mahaba ang panahon na dapat respetuhin. Sa mas maunting sampol, lalo sa mga hinahandang paisa-isa, maaring umiksi ang oras na ito).
7. Tanggalin ang tubig.
8. Ilagay ang Marc-Andre solution (maaring dagdagan ng Fuchsin acid) at maghintay ng 24 oras (1 araw).
9. Tanggalin ang Marc-Andre solution.
10. Ilubog ang espesimen sa distilled na tubig at maghintay ng 30 - 45 min.
11. Tanggalin ang tubig, at ulitin sa paghuhugas gamit ang distilled na tubig sa loob ng 30 minutos.
12. Tanggalin ang tubig.
13. Idagdag ang 70% ethanol at i-daysek ang espesimen.
    a. Para sa ulo at tyan, dahan-dahang hilahin ang ulo o tyan palayo sa dibdib.
    b. Para sa dibdib, alisin ang pakpak sa pamamagitan ng paghawak sa dibdib gamit ang panipit at hilahin mula sa puno ng mga biyas gamit ang isa pang panipit. Maari din magsagawa ng dayseksyong sagittal, na paghihiwalayin ang dibdib sa kaliwa at kanang bahagi, depende sa rehiyon na may pinaka-mataas na interes.
14. Dahan-dahang i-dehydrate o tanggalan ng tubig sa pamamagitan ng pababad sa sunod-sunod na aqueous ethyl alcohol solutions: 50 - 80 - 95% hanggang makarating sa absolute alkohol.
15. I-dehydrate ang mga espesimen sa pamamagitan ng pagbabad ng dalawang beses sa 100% ethanol sa loob ng 10 minuto.
16. Tanggalin ang ethanol at ilubog sa langis ng clove sa loob ng 15 minuto sa temperaturang pang silid.
17. Ilipat ang mga espesimen mula sa langis ng clove tungo sa isang patak ng "Euparal® o Canada Balsam sa malinis na slide.
18. I-ayos ng ayon sa kagustuhan: Ang ulo, dibdib at tyan ng nik-nik ay maaring i-daysek gamit ang pinong karayom o panipit sa ilalim ng dayseking microscope. Ang ulo ay dapat ihiwalay mula sa katawan upang maidikit ng nakatihaya (dorso-ventral position), (kung saan ang occipital foramen ay dapat na naka-psosisyon paitaas upang direktang makita ang cibarium mula sa lagusang ito.
    Ang pag-daysek ay isinasagawa sa mounting medium ng nik-nik.
19. Iwan ang espesimen hanggang maging malagkit ang ibabaw ng pandikit.
20. Basain ang isang malinis na coverslip gamit ang absolute alcohol. Ilagay ang coverslip sa ibabaw ng Canada Balsam habang naka-anggulo.
21. Itago ang mga slide sa isang tuyong kahon na nakalaan para dito.
