# Supplementary material for: Processing and mounting phlebotomine sand flies: a consensus guideline
Source: Parasite. 2026 Apr 3;33:18. doi: 10.1051/parasite/2026009 (PMC13047900; doi:10.1051/parasite/2026009)
Supplement: Supplementary file 9 — Fon translation / Tintindófɔngbemɛ [file parasite-33-18-s9.pdf]

Parasite **33**, 18 (2026)
© F.J. Randrianambinintsoa et al., published by EDP Sciences, 2026
https://doi.org/10.1051/parasite/2026009

PARASITE
Available online at:
www.parasite-journal.org

REVIEW ARTICLE

OPEN ACCESS

Azɔ̌wa dó nǔ jǐ kpódó tito ɖó nǔ jǐ flebotomu lɛ́ɛ tɔn kpó : alixlɛ́mɛwema e jí è ɖó gbè kpɔ́ dó é

Fano José Randrianambinintsoa[1], Laure Augendre[1], Jorian Prudhomme[1], Jean-Philippe Martinet[1], Mathieu Loyer[1], Nalia Mekarnia[1], Hocine Kerkoub[1], Farzana Khan Perveen[1], Antoine Huguenin[1,2], Emilie Kariya[1,2], Mohammad Akhoundi[3], Andrey José de Andrade[4], Eduardo Berriatua[5], Gioia Bongiorno[6], Sébastien Boyer[7,8], Vasiliki Christodoulou[9], Magda Clara Vieira Da Costa-Ribeiro[10], Lucas Alexandre Farias de Souza[10], Huicong Ding[11], Blaise Dondji[12], Vít Dvořák[13], Ozge Erisoz Kasap[14], Eunice Aparecida Bianchi Galati[15], Montserrat Gállego[16], Cristina Ballart[16], Stavroula Gouzelou[17], Nabil Haddad[18], Rezki Sabrina Masse[19], Asrat Hailu Mekuria[20], Vladimir Ivovic[21], Szymon Kaczmarek[22], Mohd Khadri Shahar[19], Oscar D. Kirstein[23], Edwin Kniha[24], Iva Kolářová[13], Lincoln Timinao[25], Cristian Lucanas[26], Ognyan Mikov[27], Kimsear Nov[7], Yusuf Özbel[28], Bernard Pesson[29], Laura Cristina Posada Lopez[30], Didot Budi Prasetyo[1,7], Nil Rahola[31], Eduardo A. Rebollar-Tellez[32], Bruno Leite Rodrigues[15], Lalita Roy[33], Prasanta Saini[34], Chizu Sanjoba[35], Paloma Helena Fernandes Shimabukuro[36], Padet Siriyasatien[37], Agnieszka Soszyńska[22], Tatiana Suleşco[38], Massamba Sylla[39], Majhalia Torno[40], Petr Volf[13], Khamsing Vongphayloth[41], Vu Sinh Nam[42], April Wardhana[43], Eric Yessinou[44], Sonia Zapata[45], Jean-Charles Gantier[1], and Jérôme Depaquit[1,2,*]

[1] Faculté de Pharmacie, Université de Reims Champagne Ardenne, UR ESCAPE-USC ANSES PETARD, 51 rue Cognacq-Jay, 51096 Reims Cedex, France
[2] Pôle de Biologie territoriale, Laboratoire de Parasitologie-Mycologie, Centre Hospitalo-Universitaire, 51092 Reims, France
[3] Parasitology-Mycology Department, Avicenne Hospital, AP-HP, Bobigny, Sorbonne Paris Nord University, France; Unité des Virus Émergents (UVE: Aix-Marseille Univ, Università di Corsica, IRD 190, Inserm 1207, IRBA), 13005 Marseille, France
[4] Parasitology Collection of Basic Pathology, Department of Basic Pathology, Federal University of Paraná, Curitiba 19031, Brazil
[5] Department of Animal Health, University of Murcia, Campus de Espinardo, 30100 Espinardo, Murcia, Spain
[6] Department of Infectious Diseases, Vector-borne Diseases Unit, Istituto Superiore di Sanità, 00166 Rome, Italy
[7] Medical and Veterinary Entomology Unit, Institut Pasteur du Cambodge, Phnom Penh 12201, Cambodia
[8] Ecology & Emergence of Arthropod-borne Pathogens Unit, Department of Global Health, Institut Pasteur, CNRS UMR2000, 75015 Paris, France
[9] Section Veterinary Services (1417), Laboratory for Animal Health Virology, Aglantzia, Nicosia 2109, Cyprus
[10] Insects Vectors and Parasites Laboratory, Department of Basic Pathology and Postgraduate program in Microbiology, Parasitology and Pathology, Federal University of Paraná, 81530-900 Curitiba, Brazil
[11] Department of Biological Sciences, National University of Singapore, 117558, Singapore
[12] Laboratory of the Leishmaniasis Research Project, Mokolo District Hospital, Mokolo, Cameroon; Laboratory of Cellular Immunology and Parasitology, Department of Biological Sciences, Central Washington University, 98926 Ellensburg, WA, USA
[13] Department of Parasitology, Faculty of Science, Charles University, 12800 Prague, Czechia
[14] VERG Laboratories, Department of Biology, Faculty of Science, Hacettepe University, Beytepe, Ankara 06800, Türkiye
[15] Faculdade de Saúde Pública da Universidade de São Paulo (FSP/USP), Pós-graduação em Saúde Pública, 01246-904 São Paulo, Brazil
[16] Secció de Parasitologia, Departament de Biologia, Sanitat i Medi Ambient, Facultat de Farmàcia i Ciències de l'Alimentació, Universitat de Barcelona, & Institut de Salut Global de Barcelona (ISGlobal), Centro de Investigación Biomédica en Red, Enfermedades Infecciosas (CIBERINFEC), 08028 Barcelona, Spain
[17] Laboratory of Infectious Diseases and Public Health, School of Medicine, University of Cyprus, Nicosia, Cyprus & Department of Pediatrics, Archbishop Makarios III Hospital, Nicosia 2115, Cyprus
[18] Faculty of Health Sciences, American University of Beirut, 1107 2020 Beirut, Lebanon
[19] Medical Entomology Unit, Infectious Disease Research Centre, Institute for Medical Research (IMR), National Institutes of Health (NIH), Ministry of Health Malaysia, 40170 Shah Alam, Selangor, Malaysia
[20] School of Medicine, Addis Ababa University, 28017 - 1000 Addis Ababa, Ethiopia
[21] Faculty of Mathematics, Natural Sciences and Information Technologies, University of Primorska, 6000 Koper, Slovenia

Edited by Jean-Lou Justine
*Corresponding author: jerome.depaquit@univ-reims.fr

This is an Open Access article distributed under the terms of the Creative Commons Attribution License (https://creativecommons.org/licenses/by/4.0), which permits unrestricted use, distribution, and reproduction in any medium, provided the original work is properly cited.

[22] University of Lodz, Faculty of Biology and Environmental Protection, Department of Invertebrate Zoology and Hydrobiology, Banacha 12/16, 90-237 Łódź, Poland
[23] Laboratory of Entomology, Ministry of Health, 9134302 Jerusalem, Israel
[24] Center for Pathophysiology, Infectiology and Immunology, Institute of Specific Prophylaxis and Tropical Medicine, Medical University Vienna, Kinderspitalgasse 15, 1090 Vienna, Austria
[25] Papua New Guinea Institute of Medical Research (PNGIMR) Institute, PO Box 60, Headquarter, Homate Street, 441 Goroka, Eastern Highlands Province, Papua New Guinea
[26] Museum of Natural History, University of the Philippines Los Baños, 4031 Laguna, Philippines
[27] National Centre of Infectious and Parasitic Diseases, 1504 Sofia, Bulgaria
[28] Ege University, Faculty of Medicine, Department of Parasitology, 35040 Bornova/Izmir, Türkiye
[29] Retired, Faculté de Pharmacie, Université de Strasbourg, Strasbourg, 67400 Illkirch-Graffenstaden, France
[30] Program for the Study and Control of Tropical Diseases (PECET), Faculty of Medicine, University of Antioquia, 050010 Medellin, Colombia
[31] MIVEGEC, Univ. Montpellier, CNRS, IRD, 34394 Montpellier, France & Medical Entomology Unit, Institut Pasteur de Madagascar, 101 Antananarivo, Madagascar
[32] Laboratorio de Entomología Médica, Departamento de Zoología de Invertebrados, Facultad de Ciencias Biológicas, Universidad Autónoma de Nuevo León, San Nicolás de los Garza, 66455, NL, México
[33] Tropical and Infectious Disease Centre, BP Koirala Institute of Health Sciences, Dharan 56700, Nepal
[34] ICMR-Vector Control Research Centre, Puducherry 605006, India
[35] Graduate School of Agricultural and Life Sciences, The University of Tokyo, Tokyo 113-8657, Japan
[36] Grupo de estudos em Leishmanioses/Coleção de Flebotomíneos (COLFLEB/Fiocruz-MG), Instituto René Rachou, Fundação Oswaldo Cruz, Belo Horizonte, Minas Gerais, 30190009, Brazil
[37] Center of Excellence in Vector Biology and Vector-Borne Disease, Department of Parasitology, Faculty of Medicine, Chulalongkorn University, Bangkok 10330, Thailand
[38] Department of Arbovirology, Bernhard Nocht Institute for Tropical Medicine, Bernhard Nocht Str. 74, 20359 Hamburg, Germany [39] Laboratory Vectors & Parasites, Department of Livestock Sciences and Techniques, Sine Saloum University El Hadji Ibrahima Niasse (SSUEIN) Kaffrine Campus, C.P. 24600, Senegal.
[40] Environmental Health Institute, National Environment Agency, Singapore 138667, Singapore & Department of Biological Sciences, National University of Singapore, 117558 Singapore
[41] Institut Pasteur du Laos, Laboratory of Vector-Borne Diseases, Samsenhai Road, Ban Kao-Gnot, Sisattanak District, 3560 Vientiane, Lao PDR
[42] National Institute of Hygiene and Epidemiology, 1 Yec-Xanh Street, Hai Ba Trung District, 100000 Hanoi, Vietnam
[43] Indonesian Research Center for Veterinary Science, Indonesian Agency for Agricultural Research and Development, Ministry of Agriculture Republic Indonesia, Bogor 16114, Indonesia & Department of Parasitology, Faculty of Veterinary Medicine, Airlangga University, Surabaya 60115, Indonesia
[44] Laboratory of Research in Applied Biology, Polytechnic School of Abomey-Calavi, University of Abomey-Calavi, 01 P.O. Box 2009, 00000 Cotonou, Benin
[45] Instituto de Microbiología, Colegio de Ciencias Biológicas y Ambientales (COCIBA), Universidad San Francisco de Quito (USFQ), 170901 Quito, Ecuador

Received 1 December 2025, Accepted 29 January 2026, Published online 3 April 2026

Tínmɛ - Xota élɔ na alixlémɛ ɖaxó ɖé dó lée è na w'azɔ̌ gbɔn bo na lɛ́ sɔ́ kpɔ́déwu flebotomu lɛ́ɛ tɔné ɖó nǔ jǐ gbɔn é wu, bɔ afɔɖiɖe énɛ ɖò taji tawun nú è na dó tunwun kanlin alɔkpa lɛ́ɛ bo na lɛ́ tunwun nǔ e nɔ dɔn azɔn wá lɛ́ɛ é, lobo na lɛ́ ɖè ye ɖó vo. É ɖɔ xó dó wlɛnwín tɛnmɛ tɛnmɛ e sɔgbe xá gle mɛ kpódó nǔgbéjétɛn kpó lɛ́ɛ é jí. Alixlémɛwema ɔ́ hɛn nǔkplɔ́mɛ lyɛngbe lyɛngbe lɛ́ɛ ji dó lee e nɔ cyán flebotomu lɛ́ɛ gbɔn é, lee è nɔ w'azɔ̀ dó ye ji gbɔn é, lee è na sɔ́ nǔ nú ye gbɔn é, kpo lee è na hu ye gbɔn é kpo wu (è nɔ kplɔ́n mɛ ɖɔ è ni hɛn ye dó te dó xúxú ji alǒ CO2 é ní ma zan plodwí ó) gɔ́ nú wlɛnwín e è na zán dó hɛn ye ɖ'ayi é, ɖi è ni hɛn ye ɖó fífá mɛ, bo lɛ́ hɛn ye ɖó éthanol mɛ. Nǔ sɔ́ nú wujɔnú ɖé lɛ́ɛ (avadoxwé, ta kpódó awa lɛ́ɛ kpó) ɖò taji nú è na kpɔ́n ye ganji ɖò nǔgbejenúmɛ mɛ, bɔ è tinmɛ ɖò wema elɔ mɛ. Xota ɔ́ lɛ́ xlɛ́ lee è nɔ w'azɔ̌ dó kpɔ́ndéwú lɛ́ɛ wu gbɔn é céɖécéɖé, ɖota ji ɔ́ lee é nɔ wé ye gbɔn kpódó hydroxydu potassium kpódó sin Marc-André tɔn kpán e. Wlɛnwín nǔ sɔ́ ɖó nǔ jǐ tnɔ nɔ jlɛ́ fi vovo lɛ́ɛ dó yeɖée wu, bo nɔ tɛɖě nǔwukpikpé nukúnmɛ tɔn yetɔn lɛ́ɛ kpódó nǔwukpikpé e ye ɖó bo na hɛn nǔ lɛ́ɛ ɖó te é kpo jí. Hoyer sín sìn (è nɔ lɛ́ ylɔ ɖɔ gɔmu chloralu) wɛ è nɔ kplɔ́n mɛ ɖɔ è ni yawu zan dó kpɔ́n nǔ, ɖò taji ɔ, nú spermatɛ́ku, ɖó é nɔ kɔ́n wutu, sɔ lee è na hɛn nú hwenu gegě ă. Nǔ ɖevo e jí è ɖɔ xó dó lɛ́ɛ é wɛ nyí ahan syɛnsyɛn polyvinyl tɔn, Euparal® (ɖó sin gege nɔ́ yi ji ă), kpodo balsamu Canada tɔn kpo (nɔtɛn e nɔ gba ɖo hydrocarbur mɛ lɛ́ɛ), bɔ we gudo tɔn enɛ lɛ́ɛ nɔ na nǔwukpikpé bɔ è na hɛn nǔ lɛ́ɛ ɖó te nú hwenu gegě. È lɛ́ ɖɔ xó dó wlɛnwín yɔyɔ̌ e è nɔ zán dó kplɔ́n nǔ dó nǔ e nǔɖogbɛ

lɛ́ɛ sín wujɔ nú kpó jí, ɖi ADN sín mima kpódó MALDI-ToF kpó, ee byɔ ɖɔ è ni sɔ́ ayi ɖó nǔjlɛdonǔwu lɛ́ɛ jí tawun é jí dó w'azɔ̌. Gɔ́ na ɔ́, è nɔ na yɛ klewun klewun ee xlɛ́ wlɛnwín vovo e è nɔ zán dó jla nǔ ɖó lɛ́ɛ é, bo nɔ lɛ́ lilɛ dó gbè vovo 33 mɛ lɛ́ɛ é, bɔ enɛ nɔ zɔ́n bɔ alixlɛ́mɛwema ɔ́ nɔ kpé hudo vovo e nǔnywɛ xwitixwitizɔ́watɔ́ gbè ɔ́ tɔn lɛ́ɛ ɖó nukún lɛ́ɛ é wu

Xógbe taji lɛ́ɛ : Nǔ sisɔ́ lamu lɛ́ɛ tɔn, flebotomu, sìn Hoyer tɔn, sìn Marc-André tɔn, jɛ̌ kloral tɔn, ahan polyvinyl tɔn, Euparal®, Balsamu Canada tɔn, Leishmania sɔ́ dó vo, ninɔmɛ gle mɛ tɔn lɛ́ɛ, kúnfifɔ, nǔ finfɛ́n, nǔklɔ́n dó nǔɖogbɛ lɛ́ɛ ji, MALDI-ToF, Nǔjlɛdonǔwu alɔkpa lɛ́ɛ

Abstract – Processing and mounting phlebotomine sand flies: a consensus guideline. This article provides a comprehensive guide for the processing and mounting of phlebotomine sand fly specimens, which is crucial for species identification and pathogen detection and isolation. It discusses a range of techniques suitable for both field and laboratory settings. The guide includes detailed instructions on sand fly collection, handling, covering, and euthanasia (recommending dry freezing or CO_2 over chemicals) as well as conservation strategies, such as cold storage and preservation in ethanol. The quality of preparation of certain anatomical structures (genital organs, head and wings) is essential for their proper microscopic observation and is described in this work. The article also presents detailed sample processing, including the clearing process with agents such as potassium hydroxide then Marc-André solution. The mounting process compares different media, emphasizing their optical properties and preservation potential. Hoyer fluid (also known as chloral gum) is recommended for quick observation, particularly for spermathecae, due to its clarity, although it is not suitable for long-term storage. Other media discussed include polyvinyl alcohol, Euparal® (for limited water tolerance), and Canada balsam (a hydrocarbon-soluble medium), with the latter two offering long-term preservation capabilities. Innovative molecular biology approaches such as DNA sequencing and MALDI-ToF, which require particular attention to sample processing, are also addressed. Furthermore, short video clips illustrating various mounting techniques as well as translations in many different languages are provided, allowing the guideline to reach the diverse needs and expectations of the global scientific community.

Key words: Mounting, Phlebotomine sand fly, Hoyer fluid, Marc-André solution, Chloral gum, Polyvinyl alcohol, Euparal®, Canada balsam, *Leishmania* isolation, Field conditions, Culture, Dissection, Molecular biology, MALDI-ToF, Type-specimens.

Introduction

Flebotomu lɛ́ɛ nyí jɛxlí kpɛví kpɛví e ɖò Psychodidae sín xwédo mɛ lɛ́ɛ é, xwédo kpɛví phlebotommiae tɔn e mɛ, è hɛn kanlin alɔkpa 1 063 ɖè é [21]. Ye nyí nǔ taji e nɔ hɛn azɔn wá lɛ́ɛ é (Leishmania, arbovirus, kpódó Bartonella kpó) bo nɔ dɔn azɔn e è nɔ ylɔ ɖɔ leishmaniasis, arbovirus sín azɔn lɛ́ɛ, kpódó bartonellose kpó wá lɛ́ɛ é. Nǔ taji e jí è nɔ jinjɔn bo nɔ tunwun ye é wɛ nyí ɖɔ è ni gbéjé nǔ kpɛví kpɛví lɛ́ɛ kpɔ́n céɖécéɖé, bɔ è nɔ xò ye kplé ganji, bó e nɔ́ byɔ́ ɖɔ é ní to lamu lɛ́ɛ ganji. Kɛ́nɖiɖe enɛ byɔ wlɛnwin taji ɖe lɛ́ɛ, bɔ ɖokpó ɖokpo ɖó lè tɔn lɛ́ɛ kpódó dogbó tɔn lɛ́ɛ kpó. Flebotomu mɛxó lɛ́ɛ tunwuntunwun jinjɔn wujɔnú ye tɔn wexo tɔn lɛ́ɛ ji (ɖi, zovi, bɛ, avadonú quúnnú lɛ́ɛ tɔn) kpo nǔ e ɖò xomɛ lɛ́ɛ é kpo ji (ɖi, glɔ̌, cibarium, kpódó spermatɛku kpó). Nǔ lɛ́ɛ finfɛ́n kpódó ɖiɖe ye dóvo kpó nɔ́ hɛn ye kpínkpɔ́n bɔyu ɔ, bɔ énɛ nɔ zɔ́n bɔ è nɔ tunwun ye ganji. Gbɔnvo nú zǎndukpɔ lɛ́ɛ kpó jɛxlí lɛ́ɛ kpó ɔ́ nɛ wu ɔ, phlebotomu lɛ́ɛ ɖó ná nyí sisɔ́ dó lamu kpódó lamu kpɛvi lɛ́ɛ kpó tɛntin cobɔ è na tunwùn ye. Kaka jɛ xwè 1980 lɛ́ɛ mɛ ɔ, nǔ kpɛví kpɛví lɛ́ɛ kpinkpɔn wɛ nyí wlɛnwín ɖokpo e è sixu zán dó tunwun flebotomu lɛ́ɛ é, bɔ é kpó ɖò wlɛnwín e è nɔ zán hugǎn ɖò égbé é nyí wɛ. Enɛ wu ɔ́, lee è ɖe wlɛnwín ɔ́ kpo nǔsisɔ tɔn kpó gbɔn é bɔkun tawun, bo lɛ́ jinjɔn nǔ wè jí tawun : ɖò alɔ ɖokpo mɛ ɔ́, è nǔ ɔ́ sisɔ́ dó nǔ ji nɔ zɔ́n bɔ è nɔ hɛn nǔ ɔ́ ɖó te nú hwenu gegě, bɔ ɖò alɔ ɖevo gɔ ɔ́ mɛ ɔ́, è nǔ ɔ́ sɔ́dó nǔ ji e ya e nú kɛ́nɖiɖe nɔ zɔ́n bɔ è nɔ hɛn nǔ ɔ ɖó te nú hwenu gegě ǎ. Ɖi kpɔ́ndéwú ɔ, nǔ sɔ́ dó nǔ ji gudogudo tɔn ɔ́ ɖó balsamu Canada tɔn é nɔ ɖu hwenu, bo nɔ byɔ ɖɔ è ni ɖè sìn sín nǔ e è kpɔ́n lɛ́ɛ é mɛ bǐ mlɛ́mlɛ́. Gɔ́ na ɔ́, nǔ e è nɔ mɔ ɖò nǔ enɛ mɛ é nɔ nyɔ́ hugǎn hwebǐnu bonu è na dó sixu kpɔ́n spermatɛ́ku lɛ́ɛ bɔ é na bɔwǔ ǎ. É ɖò mɔ̌ có, enyi è sɔ́ nǔ dó nǔ e mɛ sin ɖè é ɖé mɛ (kpɔ́ndéwú ɔ́, sin Hoyer tɔn) ɔ́, é nɔ yawu bo nɔ zɔ́n bɔ è nɔ mɔ spermatéku e nɔ kɔ́n lɛ́ɛ é ganji, amɔ̌, é nɔ zɔ́n bɔ è nɔ hɛn nǔ e è sɔ́ ɖó nǔ jǐ lɛ́ɛ é ɖó te nú hwenu gegě ǎ, ɖó é nɔ jló na yí sìn sín jɔhɔn mɛ. Nǔ e è sixu wà é ɖokpó wɛ nyí ɖɔ è ni sɔ́ lamu dó sú ɖiɖe ɔ́ na, enyi é ko xú bǐ mlɛ́mlɛ́ ɔ́ nɛ. Nǔ enɛ e è sɔ́ ɖ'ayǐ bo na dó ɖyɔ nǔ é kpó ɖò te kaka jɛ égbé, bo nɔ wà nǔ dó zinzin dó wlɛnwín e è na sɔ́ ɖó nǔ jǐ é wu. Sín xwè 1980 lɛ́ɛ mɛ ɔ́, nǔkplɔ́nmɛ dó tunwùn Flebotomu lɛ́ɛ nyí xò nǔ e è nɔ ylɔ ɖɔ morphologii kpódó biochimii kpó é kplé. Nukɔntɔn ɔ wɛ nyí ɖɔ è ni gbéjé akán gláglá sín xúxú lɛ́ɛ kpɔ́n, bɔ è yawu ɖyɔ ye kpódó wlɛnwín nǔɖogbɛ lɛ́ɛ tɔn lɛ́ɛ kpó (é wɛ nyí ɖɔ, hlɔ̌nhlɔ́n nína ma xwédó nǔɖe ADN wumɛ-tɛnmɛtɛnmɛnɔ tɔn (RAPD), wumɛ-tɛnmɛtɛnmɛ ga tɔn nú nǔ e fɛ́n dó vo e lɛ́ɛ (RFLP), ADN ɖiɖe ɖ'ayǐ, kpodo ɖiɖe ɖ'ayǐ

gbɔn Sanger sín wlɛnwín ɔ́ gblamɛ, gɔ́ nú ɖiɖe ɖ'ayĭ e bɔ d'ewu é (sequecing-NGS). Égbé ɔ́, è nɔ sɔ́ gɔ́ nú wlɛnwín kéɖiɖe nǔvínúví lɛ́ɛ tɔn gbɔn wlɛnwín proteomiku lɛ́ɛ ɖi MALDI-ToF gblamɛ. É ɖò mɔ̌ có, è sixu bló bɔ è na mɔ nǔ jɛ nǔ e nɔ dɔn azɔn wá lɛ́ɛ é wu gbɔn PCR (Leishmania, Trypanosoma, Bartonella, kpo Phlebovirus kpo) gblamɛ, ɖó è sixu mɔ ye bǐ gbɔn PCR e nɔ wá vivɔnu é kpódó ee nɔ nyí hwenu jɔ hwenu é kpó gblamɛ, bo nɔ byɔ ɖɔ è ni jla nǔ e è sɔ́ ɖ'ayĭ lɛ́ɛ é ɖó [,3]. Gɔ́ nú nu agbaza tɔn e è nɔ zán ɖò aca mɛ bo nɔ ɖè kanlin lɛ́ɛ ɖó vo é ɔ́, è sixu lɛ́ zán wlɛnwín agbaza tɔn ɖevo lɛ́ɛ (é wɛ nyí ɖɔ, wing geomorphométrie). É ɖò mɔ̌ có, è ɖó na hɛn nǔ e mɛ e wlan wema ɔ́ lɛ́ɛ é mɔ kpɔ́n dó ayi mɛ, bɔ nǔ e gbé nya wɛ è ɖè é wɛ nyí ɖɔ è ni na alixlɛ́mɛ e sɔgbe lɛ́ɛ é dó lee è na sɔ́ flebotomu mɛxomɔ e nɔ nyí phlebotomu lɛ́ɛ é ɖó bo na lɛ́ wà nǔ xá ye gbɔn é wu, bo na dó sixu gbéjé nǔ lɛ́ɛ kpɔ́n ganji.

Hudo e è ɖó bo na gbéjé nǔ ɖé lɛ́ɛ kpɔ́n é (kpɔ́ndéwú ɔ́, nǔnywɛ xwitixwiti sín nǔ lɛ́ɛ alǒ MALDI-ToF) byɔ ɖɔ è ni hɛn flebotomu ɔ́ sín akpáxwé ɖé ɖ'ayĭ, bɔ é ma ɖò dandan nú è na tuùn lee é cí é ǎ, bo nɔ tɛɖɛ̌ hudo e è ɖó bo na sɔ́ tuto ɖé lɛ́ɛ é jí.

Ðò xota elɔ mɛ ɔ́, mǐ sɔ́ ayi ɖó wlɛnwín e è nɔ zán dó dó anesthesie kpódó nuǔfinfɛ́n kpó nú flebotomu e è wlí ɖò gbɛ̀ lɛ́ɛ é jí, lee è nɔ hɛn ye ɖó é kpódó lee è nɔ sɔ́ ye ɖó nǔ jǐ gbɔn é kpó jí, bo na dó yawu tunwùn ye alǒ hɛn ye ɖó te nú hwenu gegě bɔ enɛ na zɔ́n bɔ è na kplɔ́n nǔ ɖò nukɔnmɛ.

Nǔjɛnukɔn tɔn: Nǔ e è na gbéjé kpɔ́n dó ayijayǐ kpo sɛ́n lɛ́ɛ kpo wu lɛ́ɛ é ɖó na sɔgbe xá wema e kúnkplá ayijayǐ lɛ́ɛ é (SDS).

È ɖó na zán plodwi e ɖo alixlɛ́mɛwena elɔ mɛ lɛ́ɛ é bǐ ɖò ninɔmɛ ayijayǐ tɔn syɛnsyɛn lɛ́ɛ glɔ́. Wěɖegbɛ́ lanmɛ na nɔ ganji kpódó ayijayǐ kpó tɔn e ɖò dobanúnǔtɛn lɛ́ɛ é ɖò gbesisɔmɛ bo na na we nǔ e kúnkplá awovinú e nǔ enɛ lɛ́ɛ nɔ dɔn wá lɛ́ɛ é kɛɖɛ ǎ, loɔ́, ye na lɛ́ na we lee è na kpé nukún dó ye wu gbɔn é kpódó lee è na bɛ́ nǔ kwijikwiji lɛ́ɛ gbɔn é kpó. Amɔ̌, é ɖò dandan ɖɔ è ni xwedó wlɛnxi e kúnkplá lee è na zán ye gbɔn é kpódó lee è na bɛ́ ye nyì gbě gbɔn é kpó é. É ɖò taji nú wemaɔ́zantɔ́ ɖokpó ɖokpó ɖɔ è ni xwédó walɔ ɖagbe nǔgbeje kpɔ́n tɔn lɛ́ɛ ɖɔ lɛ́ɛ bǐ bo lɛ́ xwedó sɛ́n kpódó tuto e ɖò tò yetɔn alǒ tutoblonunu dobanúnǔ tɔn mɛ lɛ́ɛ é kpo. Ðevo ɔ́, è nɔ kpé nukún dó nǔ e nɔ hɛn nǔ glé dó mɛ wu lɛ́ɛ (plodwi) é ɖé lɛ́ɛ, alǒ nǔ e ɖò ye mɛ lɛ́ɛ é wu (é wɛ nyí ɖɔ, chloral hydrate) ɖò tò ɖé lɛ́ɛ mɛ. È sɔ́ xókwín e sɛ sin xwe na lɛ́ɛ ɖò alɔnuwema elɔ mɛ lɛ́ɛ é sín wěma ɖé ɖó Tablo 1 mɛ.

Tablo 1. Xókwín e sɛ sin xwe na lɛ́ɛ.

BME	Hɔnsuhɔnsu basal kpɛvi
CDC	Tɛn e mɛ è nɔ kpé nukún dó azɔn lɛ́ɛ wu ɖè é kpódó ee nɔ sɔ́nǔ ɖ'ayi nú azɔn lɛ́ɛ é kpó
CMCP	Kanfɔ́lu monoklorofenɔlu
CMR	Nǔ e nɔ dɔn kansɛ́ɛzɔn wá é, nǔ e nɔ ɖyɔ nǔ é, nǔ e nɔ vɔ́ nǔ glé dó mɛ wu é
COI	Wɛnhɛnnú akpáxwé kpɛvi nukɔn tɔn protéine c tɔn e nɔ gba e
CytB	Wɛnhɛnnú protéine b tɔn
DNA	Nǔ e nɔ hɛn nǔ gble ɖo wu nú mɛ déoxyribonucléiques tɔn
ELISA	Nǔ e nɔ ɖí xwi xá azɔn é tɛ́nkpɔ́n e cá kàn xá enzyme é
EtOH	Etanɔlu
M199	199 ɖò tɛntin
MALDI-ToF MS	Laser e nɔ hɛn nǔ glé dó mɛ wu é/ionization hwenu e è nɔ j'ayĭ é sín spectrométrie
MEM	Xójlawema taji kpɛví bǐ ɔ́
NGS	Jijimɛ yɔyɔ e bɔ d'ewu é sín tutomɛ
NNN	Novi-MacNeal-Nicolle sín tɛnmɛ
PCR	Nǔwiwa polymérase tɔn
Lao PDR	Togun to wɛ nɔ sɔ́ gan to wɛ nɔ ɖu gan tɔn kpàa Lao tɔn
PNOC	Wɛnhɛnnú peptide taglomɛ tɔn
qPCR	PCR ɖaxó (PCR hwenu jɔ hwenu)
RAPD	ADN e ɖó alɔkpa gègě é e è jlaɖó ɖò jlě jí é
RFLP	E ɖó alɔkpa gègě é gaga fɛ́nnú ɖé tɔn
RI	Xókwín kpɛví kpɛví lɛ sín wěma
RNA	Asidu ribonucléiques
RNases	Ribɔnukleaz
RNASS	Nǔ e nɔ zɔ́n bɔ ARN nɔ nɔte é
RT-PCR	PCR e nɔ wlan nǔ dó wema jí é
TFA	Asidu trifluoroacétiques

1. Flebotomu lɛ́ɛ wlíwlí

È sixu wli flebotomu mɛxomɔ lɛ́ɛ kplé gbɛɖe gbɛɖe alǒ kúkú tɔn gbɔn wlɛnwín vovo lɛ́ɛ gblamɛ ɖi CDC sín mɔ e nɔ hɛn weziza lɛ́ɛ é, mɔ e nɔ tɛ́ nǔ lɛ́ɛ é, kpódó nǔgbɔnnu lɛ́ɛ e nɔ zán mɔ Shannon tɔn lɛ́ɛ, alǒ gbɔjɛtɛn ye t ɔn e ɖò ayikúngban ɔ́ jí lɛ́ɛ é mɛ (kpɔ́ndéwú ɔ́, kanlin lɛ́ɛ sín kpó mɛ). Wlɛnwín enɛ lɛ́ɛ wɛ nyí ɖɔ è ni sɔ́ mɔ lɛ́ɛ ɖó fí e jɛxa lɛ́ɛ é, bo dɔn flebotomu lɛ́ɛ kpódó weziza alǒ nǔ ɖevo e nɔ dɔn mɛ lɛ́ɛ é kpó (CO_2 alǒ nǔ e nɔ dɔn mɛ lɛ́ɛ é), lobo xò ye kplé bo na dó gbéjé ye kpɔ́n d'eji, lee è tinmɛ gbɔn ɖò wema gègě mɛ é [2, 3, 32, 36, 49].

Enyi è wlíwlí flebotomu gbɛɛɖe lɛ́ɛ nɔ zɔ́n bɔ è nɔ zán nǔ e ɖò dò lɛ́ɛ é bǐ, loɔ́ e kú lɛ́ɛ é kplékplé nɔ zɔ́n bɔ è nɔ sixú ɖè Leishmania alǒ azɔnkwín sín atínkwín lɛ́ɛ ɖó vo ǎ. Wlɛnwín e è nɔ zán dó wlí nǔ lɛ́ɛ é ɖé lɛ́ɛ, ɖi wema e è nɔ tɛ́ dó nǔ wu lɛ́ɛ é nɔ zɔ́n bɔ è nɔ hɛn nǔ wujɔnú ɖe lɛ́ɛ bú (zovi, bɛ̌, awa, alǒ afɔ) hwɛhwɛ. Gɔ́ na ɔ́, wema e è sɔ́ ami dó bló na lɛ́ɛ é nɔ tɛ́ dó flebotomu lɛ́ɛ wu, bɔ è ɖó na ɖè ye sîn ɖò azɔ̌ ɔ́ sín bǐbɛ̌mɛ, bɔ hwɛhwɛ ɔ́, è nɔ zán cɛju 15 sín dó klɔ́ ɖò éthanol kpódó éther diéthyl kpó e è xò kplé é mɛ ɖò akpáxwé ɖokpó ɔ́ lɛ́ɛ mɛ.

2. é nɔ zɔ́n bɔ kpɔ́ndéwú lɛ nɔ kú

Enyi è xò flebotomu e ɖò gbè lɛ́ɛ é kplé gudo ɔ́, è ɖó na hu ye. Kpódó wlɛnwín nǔxixa tɔn ɖé lɛ́ɛ kpó (kpɔ́ndéwú ɔ́, wema e nɔ tɛ́ lɛ́ɛ é, CDC sín mɔ wezizanɔ e mɛ è sɔ́ gannu e mɛ nǔsísɔ́ sín nǔ alǒ éthanol ɖè é ɖé dó é) flebotomu lɛ́ɛ nɔ kú azɔn ɖokpó hwenu e è xò ye kplé é. È sixu zán nǔnywɛ xwitixwiti sín nǔ lɛ́ɛ dó mɛ ɖěɖee è xò kplé tlɔlɔ dó éthanol mɛ lɛ́ɛ é jí, bo lɛ́ zán ye dó mɛ ɖevo lɛ́ɛ jí, enyi è hɛn ye dó éthanol mɛ b'ɛ na yá lee é nyɔ́ bló gbɔn é ɔ́ nɛ. Amɔ̌, wlɛnwín mɛhuhu tɔn enɛ lɛ́ɛ ɖě nɔ na gbè bɔ è nɔ zán MALDI-ToF dó wà nǔ dó nǔvínúví lɛ́ɛ wu ǎ. Gɔ́ na ɔ́, wlɛnwín mɛhuhu tɔn ɖé lɛ́ɛ sixu zinzin dó bɔ ninɔmɛ agbaza tɔn ɖé lɛ́ɛ ji. Enɛ wu ɔ, é ɖò taji ɖɔ è ni zán nǔ e nɔ hu mɛ é ɖé bo na dó tunwun í ganji alǒ e hɛn ɖó fí ɖé nú hwenu gegě ɖi kpɔ́ndéwú taji tɔn lɛ́ɛ ɖɔhun (é wɛ nyí ɖɔ, kpɔ́ndéwú e è hɛn ɖ'ayǐ bo na dó sixu kpɔ́n alǒ jlɛ́ ye dó yeɖée wu ɖò sɔgudo lɛ́ɛ é). Nǔ e nɔ hɛn nǔ gblé dó mɛ wu lɛ́ɛ (plodwi) é ɖi éthyl acétate, éther éthyl, tétrachloroéthane kpódó chloroforme kpó sixu byɔ avɔkanfún mɛ, bo na sɔ́ dó nǔ e mɛ è nɔ yí flebotomu lɛ́ɛ ɖè é ɖé mɛ, bo na dó hu ye. È ɖó na kpé nukún dó nǔ e nɔ hu mɛ lɛ́ɛ é wu ganji, bo na xwedó aliɖenúmɛ e mɛ e bló ye é ɖó aɖǐ e ye nɔ hɛn wu. Amɔ̌, mǐ nɔ kplɔ́n mɛ ɖɔ è ni zán klorofɔmu dó hu flebotomu lɛ́ɛ ǎ, ɖó ɖò nǔ e mǐ mɔ kpɔ́n lɛ́ɛ é mɛ ɔ, é nɔ sɔgbe xá nǔkplɔnmɛ nǔɖogbɛ lɛ́ɛ tɔn e nɔ nyí molekwilu é ganji ǎ. Ðó nǔ enɛ lɛ́ɛ bǐ nɔ hɛn nǔ gblé dó mɛ wu, bɔ è nɔ lɛ́ xò nǔ kpɔ́n dó lee ye sɔgbe gbɔn bo na gbéjé nǔ e ɖò mɔ ɔ́ mɛ lɛ́ɛ é kpɔ́n é wu ɔ́, è nɔ gbɛ́ ɖɔ è ni ma zán nǔ enɛ lɛ́ɛ ɔ́. Wlɛnwín e è nɔ zán hugǎn, bo nɔ hɛn ɖiɖe, ADN alǒ proteine lɛ́ɛ ɖó te é wɛ nyí ɖɔ è nɔ hɛn nǔ lɛ́ɛ ɖó te xúxú tɔn. È ɖó na hɛn nǔ e è kpɔ́n lɛ́ɛ é ɖó fifa nú hwenu gegě cobɔ è na bló bɔ ye na kú atli bǐ mlɛ́mlɛ́ ǎ, amɔ̌, é ɖó na nyí mɔ̌ kaka bɔ ye na (i) xú, alǒ (ii) na hɛn nǔ gblé dó lee *Leishmania* sixu nɔ gbè gbɔn é wu ǎ, enyi linlin ɔ́ wɛ nyí ɖɔ è ni ɖè ye ɖó vo gbeɖe sín adɔgbo flebotomu tɔn lɛ́ɛ mɛ ɔ́ nɛ́. Enɛ wu ɔ́, mǐ byɔ ɖɔ è ni bló bɔ ye na nɔ nǔfanú mɛ nú cɛju 15 jɛ 20 ɖò -20°C, bo na nɔ kpɔ́n ye hwɛhwɛ bo na dó tunwun ɖɔ ye kú tó kpowun, bo ma ka na hu nǔvínúví *Leishmania* tɔn lɛ́ɛ ǎ.

Enyi è ma ɖó nǔfanú ɖé ǎ ɔ́, è sixu hu nǔvínúví lɛ́ɛ gbɔn CO_2 gblamɛ. Ðò ninɔmɛ gle tɔn e mɛ è ma sixu zán CO_2 sín kɔ́fu lɛ́ɛ ɖè ǎ lɛ́ɛ é mɛ ɔ́, è hɛn ɔ́ e na hu kpɔ́ndéwú lɛ́ɛ gbɔn CO_2 sín kɔ́fu ajɔ̌wiwa tɔn kpɛví kpɛví e è nɔ zán ɖò 'Soda siphons' (ahan syɛnsyɛn lɛ́ɛ) mɛ é gblamɛ, amɔ̌, è sixu ɖó gbɛ́ hinhɛn ye gbɔn jɔhɔnmɛ. Nǔ gudogudo tɔn e è sixu wà é wɛ nyí ɖɔ è sixu hu nǔvínúví lɛ́ɛ gbɔn azɔ glaglá mɛ ninɔ gblamɛ. È nɔ wlí flebotomu lɛ́ɛ gbɛɖe gbɛɖe ɖò mɔ CDC tɔn ɖé mɛ, bo nɔ xò ye kplé kpódó nǔgbɔnnu kpó, bo nɔ hɛn ye dó kɔ́fu ɔ́ mɛ, lobo nɔ sɔ́ ye ɖó azɔ glaglá e nɔ hu ye ɖò cɛju klewun ɖé mɛ é mɛ. Wlɛnwín enɛ nyɔ́ zán ɖò ninɔmɛ gle tɔn lɛ́ɛ bǐ mɛ, é na bo tlɛ nyí ɖò ninɔmɛ e vɛwǔ bɔ è na ɖè mɛɖé ɖó vo lɛ́ɛ é mɛ ɔ́ nɛ. Amɔ̌, ɖó azɔ glaglá nɔ gɔ́ kɔ́fu ɔ́ mɛ wutu ɔ́, è sixu zán dó xò flebotomu e ɖò gbè lɛ́ɛ é kplé bo lɛ́ kpé nukún dó ye wu ɖò nukɔnmɛ ǎ, e nyi è ma klɔ́ ye ganji ǎ ɔ́ nɛ. É ɖò mɔ̌ có, è sixu kpó ɖò nǔgbɔnnu ɖokpo ɔ́ e è ma ko slá wǔ na ǎ é zán wɛ bo na dó hu flebotomu e ɖò mɔ ɖevo lɛ́ɛ mɛ lɛ́ɛ é, bo na dó hɛn nǔ lɛ́ɛ ɖó te. É lɛ́ ɖò dandan ɖɔ è ni kpɔ́n ɖɔ è ko ɖè nǔ e è kpɔ́n lɛ́ɛ é bǐ sín nǔgbɔnnu ɔ́ mɛ à jí. Wlɛnwín enɛ lɛ́ɛ sɔgbe xá lee è nɔ ɖè Leishmania ɖó vo gbɔn xomɛ ɖiɖe tɔ́n gblamɛ é.

3. Hinhɛn kpɔ́ndéwú lɛ́ɛ ɖó te cobɔ wà azɔ̌ ɖó ye ji

Wlɛnwín taji ɛnɛ wɛ è nɔ zán dó hɛn nǔ lɛ́ɛ ɖó te cobɔ è nɔ wà dó ye ji :

3.1. Hinhɛn dó fífámɛ

Wlɛnwín enɛ nyɔ́ hugǎn bɔ è na zán ɖò -20°C abǐ e jló mɛ ɔ́ ɖò -80°C. È nɔ zán wlɛnwín nǔhinhéntɛn tɔn enɛ lɛ́ɛ dìn hú lee è nɔ hɛn nǔ dó azote sin mɛn gbɔn. Ðò ninɔmɛ lɛ́ɛ bǐ mɛ ɔ́, è ɖó na yawu bló nǔ e è nɔ ylɔ ɖɔ cryopreservation é hwenu e è ko blo bɔ kpɔ́ndéwu lɛ́ɛ ko kú atli e. Nǔ hɛn dó nǔfánúmɛ ɔ́ nɔ wa ɖagbe bo nɔ hɛn nǔvínúví lɛ́ɛ glebu, gɔ́ nú ARN, ADN kpódó proteine lɛ́ɛ kpó ɖó mimɛ̌ jí bǐ mlɛ́mlɛ́ ɖò hwenu e è na hɛn nǔ ɔ́ ɖó é bǐ mɛ é. Loɔ́, azotu sìn sixu hɛn awa lɛ́ɛ, afɔ lɛ́ɛ, alɔví lɛ́ɛ kpo hǔnví lɛ́ɛ kpo gblé, bɔ hwɛhwɛ ɔ́, é nɔ sɛn ye, bo nɔ lɛ́ ɖè nǔ taji e ɖò agbaza ɔ́ wu lɛ́ɛ é sîn hweɖelenu. Nǔ e è nɔ hɛn ɖó nǔfanu xúxú mɛ é nɔ dóya nú nǔvínúví lɛ́ɛ sɔmɔ̌ ǎ, amɔ̌, é nyɔ́ bo nú e na dó hɛn agbaza yetɔn sín wǔjɔnú e ma syɛn sɔmɔ̌ ǎ lɛ́ɛ é ɖó te ǎ. Nǔ taji ɔ́ wɛ nyí ɖɔ hwenu e è na ɖò xúxú wɛ é ɔ́, awa lɛ́ɛ, zovi lɛ́ɛ, alɔví lɛ́, alǒ afɔ lɛ́ɛ sixu tɛ́ dó gannu lɛ́ɛ wu bo é wá fɛ́n. Amɔ̌, è sixu hɛn nǔ lɛ́ɛ ɖó fífá mɛ hwebǐnu ɖò gle lɛ́ɛ mɛ ǎ,

ɖó é nɔ́ byɔ ɖɔ è ni ɖó nuǔfánú ɖé alǒ nǔ e mɛ è nɔ bɛ́ azotu sín sìn dó é ɖé. Nǔ hɛn ɖó fífámɛ ɔ́ sɔgbe bǐ mlɛ́mlɛ́ xá nǔ e nɔ dɔn azɔn wá lɛ́ɛ é ɖiɖe gbɔn wlɛnwi nǔvíɖogbɛ tɔn gblamɛ, é ɖò mɔ̌ có, è nɔ hɛn nǔ e nɔ hɛn nǔ glé dó mɛ wu lɛ́ɛ é ɖó kpɔ́ bo nɔ lɛ́ ɖè ye ɖó vo, é ɖò mɔ̌ có, nuǔɖiɖe kpódó nǔɖe dó vovo kpó azɔnkwi ARN lɛ́ɛ tɔn nɔ byɔ́ nǔ hɛn dó fífá ji ɖò -80°C alǒ ɖò azotu sinnɔ mɛ, nú hɛn dó te nú hwenu gegě. Mɔ̌ ɖokpó ɔ́, kpɔ́ndéwú lɛ́ɛ dó fífámɛ é nɔ zɔ́n bɔ è nɔ ɖè Leishmania ɖó vo gbɔn xomɛnǔ ɖiɖe gblamɛ ǎ, afi nú è nylɔ flebotomu lɛ́ɛ dó azizɔnǔ ɔ́ mɛ jɛ nukɔn, bo wá nylɔ́ ye dó azotu sìnnɔ ɔ mɛ (ɖò gannu e è sɔ́ dó kɔ́fu mɛ lɛ́ɛ é mɛ), bo nɔ jlɛ́ lee è nɔ hɛn Leishmania ɖó te do jɔhɔnǔ mɛ gbɔn é.

3.2. Nǔ hɛn ɖ'ayi dó ahan mɛ (éthanol alǒ ahan isopropyl)

E wɛ nyí wlɛnwín e è nɔ zán hugǎn bo nɔ hɛn flebotmu lɛ́ɛ ɖ'ayi e. É bɔwǔ bɔ è na zán ɖò gle mɛ, é na bo tlɛ nyí ɖò ninɔmɛ vɛwǔ lɛ́ɛ mɛ bɔ è ma ɖó nǔgbéjéten ɖé ǎ ɔ́ nɛ. Nǔ hɛn dó ahan mɛ nyɔ́ tawun nú nǔkplɔnmɛ dó agbaza sín wǔjɔnú lɛ́ɛ (awa lɛ́ɛ, afɔ lɛ́ɛ, zovi lɛ́ɛ, alǒ alɔví lɛ́ɛ) nɔ kpó ɖò mɔ̌, enyi jɔhɔn e nɔ tɔ́n sín nǔ ɔ́ mɛ é ɖě ma ɖò nǔ e è nɔ hɛn ɖ'ayǐ é mɛ ǎ ɔ́ nɛ. Enɛ wu ɔ́, e byɔ ɖɔ è ni sɔ́ avɔkanfún kpɛví ɖé dó sú tuto ɔ́ na, bo na dó ɖè jɔhɔn e ɖò jǐ tɔn é bǐ sîn, lobo na lɛ́ sɔ́ wuntun ɖé ɖó avɔkanfún ɔ́ jí (ɖiɖe 1gɔ́ ɔ́). Ahan syɛnsyɛn ɔ́ kpó ɖò nǔ dindɔn nyí wɛ. Ðò kpaa mɛ ɔ́, è nɔ kplɔ́n mɛ ɖɔ è ni ma hɛn nǔ e ɖò 70% mɛ ó [45, 66]. E syɛn lɛ́ɛ é nɔ hɛn ADN ɖó te ganji bo nɔ lɛ́ nɔ ayǐ nú hwenu gegě, amɔ̌, é nɔ zɔ́n bɔ nǔ e è kpɔ́n lɛ́ɛ é nɔ fɛ́n bo nɔ lɛ́ zɔ́n nú nǔkplɔ́nkplɔ́n dó lee ye cí é wu. Éthanol 96% (azeotrope mixture) zinzan nɔ zɔ́n bɔ è nɔ hɛn nǔ lɛ́ɛ ɖó te ɖò hwenu gege, ɖò taji ɔ́, ɖò fí e jɔhɔn nɔ ja ɖè lɛ́ɛ é ɖi tò e mɛ jɔhɔn nɔ ja ɖè lɛ́ɛ é, é ɖò mɔ̌ có, é nɔ bɔkun hwɛhwɛ bɔ è na mɔ éthanol 95% ǎ. É na bo nyí nǔ ɖebǔ wɛ é na bo nyí ɔ, ADN nɔ hɛn ganji ɖò éthanol mɛ (é ɖò mɔ̌ có, é nɔ w'azɔ̌ ganji hú wlɛnwín nufífá tɔn lɛ́ɛ, ɖò taji ɔ́, nú wlɛnwín molekwilu NGS-type tɔn lɛ́ɛ). Plotéinu lɛ́ɛ nɔ syɛn sɔmɔ̌ ǎ, ɖò taji ɔ́, nú ploteomiques, ɖi MALDI-ToF sín nǔwiwa lɛ́ɛ ɖɔhun. È sixu kpó ɖò flebotomu e è hɛn ɖó ahan mɛ nú sun yɔywɛ ɖé lɛ́ɛ é tunwùn wɛ ɖò lee ye cí é linu, amɔ̌, è sixu bló bɔ è na mɔ nǔ jɛ nǔ e è nɔ ylɔ ɖɔ ploteinu é wu ɖò nǔ enɛ lɛ́ɛ mɛ ǎ. È sixu hɛn nǔ e è kpɔ́n é ɖó ahan alǒ fí e xú ɖè é ganji, enyi è lɛ́ hɛn nǔ e è kpɔ́n é ɖó -20°C jí ɔ́ nɛ. Fífá ɖò -20 °C nɔ zɔ́n bɔ è nɔ hɛn nǔ lɛ́ɛ ɖó te (kpɔ́ndéwú ɔ́, acide nucléique) bo nɔ hɛn nǔ glé é ɖiɖekpo gblamɛ, bo nɔ lɛ́ na lè devo ɖé mɛ e nɔ hɛn nǔ lɛ́ɛ ɖó te é gbɔn nǔ e nɔ hɛn nǔ glé dó mɛ wu lɛ́ɛ é ɖiɖekpo gblamɛ ɖò hwenu e ɖò yiyi wɛ é mɛ, é na bo tlɛ nyí ɖɔ nǔ e é nɔ wà dó nǔ lɛ́ɛ wu é hwe hú nǔ e é nɔ wà dó nǔ lɛ́ɛ wu é. È sixu lɛ́ zán nǔ e è hɛn ɖó éthanol mɛ é dó tunwùn azɔnkwín ADN kpo ARN kpo tɔn hwenu e è ɖò éthanol zán wɛ ɖò 70% mɔ̌ mɛ nú hwenu, bɔ é ma nɔ bló sun yɔywɛ ɖé ǎ é. Gɔ́ na ɔ́, ahan isopropyl sixu bɔkun bɔ è na mɔ ɖò tò ɖé lɛ́ɛ mɛ, bo na lɛ́ hɛn ADN ɖó te, amɔ̌, é nɔ zɔ́n bɔ nǔ e è kpɔ́n lɛ́ɛ é nɔ syɛn. É nɔ fyɔ́ éthanol ɖɔhun ǎ, enɛ wu ɔ́, è sixu hɛn yì fí ɖevo bɔ é na bɔwǔ. Enyi é byɔ mɔ̌ ɔ́, è sixu sɔ́ flebotomu e è hɛn ɖó azotu sín mɛ alǒ e xú lɛ́ɛ é dó ahan mɛ, bɔ mɔ̌ mɛ ɔ́, è na xò nǔ nyanya e ɖò wlɛnwín wè lɛ́ɛ mɛ é kplé.

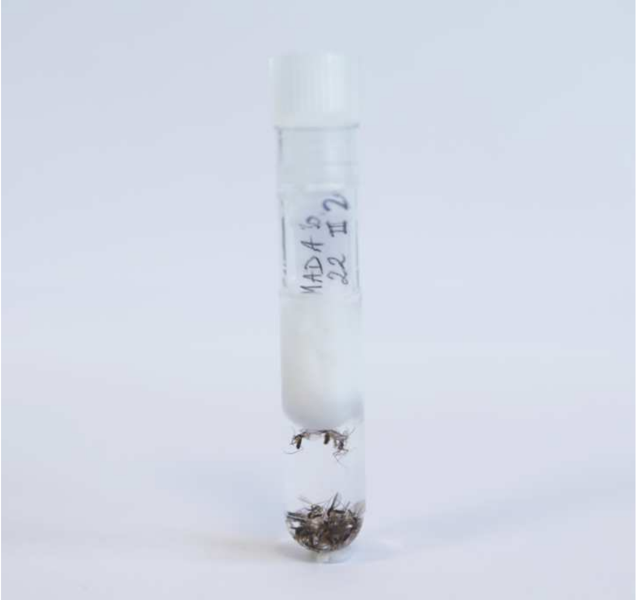

Ðiɖe 1gɔ́ ɔ́. flebotomu e è hɛn ɖó éthanol mɛ lɛ́ɛ é.

3.3. Nǔhɛn ɖò te ɖó ARN sín sìn mɛ é (RNASS)

È nɔ enɛ e nɔ dó nǔ ji é ɖò fí gègě, é nɔ hɛn nǔ glé dó mɛ wu ǎ, bɔ è bló b'ɛ nɔ hɛn ARN ɖó te bo nɔ lɛ́ cyɔn alɔ wujɔnu yɔ̌yɔ lɛ́ɛ. É nɔ yawu byɔ́ kpɔ́déwu lɛ́ɛ mɛ gbɔn acɛ hu nu RNases (enzymu e nɔ hɛn ARN glé lɛ́ɛ é), bɔ mɔ̌ mɛ ɔ, é nɔ bló bɔ ARN nɔ glé ǎ, bɔ è sɔ́ nɔ ɖó hudo hinhɛbɛ́ dó fífá mɛ ǎ. Nǔ e è nɔ hɛn ɖó RNASS mɛ é nɔ w'azɔ̌ ganji dó hɛn wujɔnú lɛ́ɛblée ye ɖe gbɔn e, bo na dó gbéjé lée ye ɖe gbɔn ɖ'ayi e kpɔ́n. Hwenu e è bló bɔ RNASS nyɔ́ hugǎn nú ARN sín hlɔnhlɔn hú ɖɔ è ni hɛn ɖó te é ɔ́, nǔ e è nɔ hɛn ɖó hwenu klewun ɖé jɛ hwenu klewun ɖé mɛ é nɔ hɛn bǔninɔ e ɖò tutoblonunu ɔ́ mɛ é ɖó te ganji. RNASS nɔ zɔ́n bɔ è nɔ hɛn nǔ e è kpɔ́n lɛ́ɛ é ɖó xɔmɛ sín fifà jí kaka nú azǎn 7, ɖò 4°C jí nú aklunɔzán gblamɛ gegě, alǒ ɖò -20°C/-80°C jí bo na dó sixu hɛn ye ɖó te nú hwenu gegě. Wlɛnwín enɛ xɔ akwɛ tawun ɖò azɔ̌ e è nɔ wà ɖò gle mɛ lɛ́ɛ é alǒ ɖò dotóoxwé lɛ́ɛ mɛ fí e è fífá ma ɖè ǎ é. Hwɛhwɛ ɔ́, cobonu è na ɖè ARN tɔ́n ɔ, é nɔ byɔ ɖɔ è ni ɖè nǔ e è kpɔ́n lɛ́ɛ é sín nǔ e è nɔ zán dó gbéjé nǔ kpɔ́n é mɛ, bo wà nǔ xá ye sɔgbe xá tuto e è sɔ́ ɖ'ayǐ lɛ́ɛ é.

3.4. Nǔhɛn ɖò te ɖò jɔhɔn nu yaya dó xúxú ji.

Wlɛnwín xóxó ɖé wɛ nyí enɛ, bɔ enyi è zán dó nǔ e è sɔ́ ɖó nǔ jǐ é ɖé jí ɔ́, é ɖó nǔ nyanya ɖaxó ɖo wujɔnú e ma syɛn sɔmɔ̌ ǎ lɛ́ɛ hinhɛn dó te mɛ, afɔ lɛ́ɛ, hǔn lɛ́ɛ kpo alɔví lɛ́ɛ kpo. É ɖò mɔ̌ có, nǔkplɔ́nmɛ proteominu tɔn e è zán MALDI-ToF dó bló lɛ́ɛ é kpó ɖò nǔ wà wɛ, enyi è bló bɔ è ɖè sìn sín agbaza mɛ hwenu e è sɔ́ nǔ e nɔ xú nǔ é ɖé ɖó te

é ɔ́ nɛ. Ðò vogbingbɔn mɛ ɔ́, nǔ gbéjé kpɔ́n molekwilu e nɔ sɔ́ ayi ɖó ADN jí lɛ́ɛ é kpó ɖò vɛwǔ wɛ bɔ è na wà ɖò kpɔ́ndéwú enɛ lɛ́ɛ jí, ɖó hwɛhwɛ ɔ́, ADN ɔ́ kpó ɖò finfén wɛ bo lɛ́ɛ ɖò ɖiɖekpo wɛ, enɛ wɛ nyí ɖɔ nǔ gbéjé kpɔ́n kpó ɖò wǔvɛ wɛ hú kpɔ́ndéwú yɔyɔ̌ lɛ́ɛ alǒ ee è hɛn dó fífá ji lɛ́ɛ é, ɖò taji ɔ́, nú genomes nucléaire lɛ́ɛ. É ɖò mɔ̌ có, è sixu zán wlɛnwín yɔyɔ̌ lɛ́ɛ ɖi museomics dó kpɔ́ndéwú alɔkpa enɛ jí [34]. Enɛ wu ɔ́, è nɔ kplɔ́n mɛ ɖɔ è ni zán wlɛnwín nǔhéntɛn tɔn enɛ ǎ, afi nú è ma mɔ wlɛnwín ɖevo ǎ ɔ́ nɛ. È sixu xò kplé xá nǔ e è nɔ hɛn ɖó fífá mɛ é gbɔn tuto lɛ́ɛ ɖiɖó dó fífá ɖé mɛ ɖò -20°C alǒ -80°C jí gblamɛ. Tagba taji ɔ́ wɛ nyí ɖɔ è ni kpéwú bo sɔ́ nǔ e è kpɔ́n lɛ́ɛ é alǒ agbaza sín wǔjɔnú e ɖò dandan bɔ è na tunwun ye lɛ́ɛ é ɖó tɛn e jɛxa é mɛ. Bo na dó kpé enɛ wu ɔ́, é ɖò taji ɖɔ è ni vɔ́ sìn na ye. E byɔ́ ɖɔ e ni zan Triton X-100 sin. Ganxixo e è nɔ vɔ́ sìn na mɛ é nɔ gbɔn vo sín ganxixo klewun ɖé yi jí jɛ azǎn gegě jí, bɔ è nɔ ɖò nukún kpé dó wǔ tɔn wɛ ganji hwɛhwɛ. Enyi è vɔ́ sìn ná nǔ e è kpɔ́n lɛ́ɛ é bǐ mlémlɛ́ gudo ɔ́, è ɖó na klɔ́ ye ɖò sin vovo atɔn mɛ.

3.5. Nǔhɛn ɖò te ɖò wema e jí è nɔ xò nǔ dó é jí é

Nǔ taji e ɖò wema e jí è nɔ xò nǔ dó é jí tɔn lɛ́ɛ mɛ é wɛ nyí ɖɔ ADN génomique ɔ́ nɔ nɔ ayǐ nú hwenu gegě ɖò agbaza ɔ́ bǐ sín kpɔ́ndewu e ma ko syɛn ǎ, (agbazam blebu abǐ hun) e è hɛn ɖó fifà jí lɛ́ɛ é mɛ. Wema e jí è nɔ xò nǔ dó é bo ɖó ninɔmɛ kati kpɛví tɔn, bɔ enɛ nɔ zɔ́n bɔ è nɔ hɛn nǔjlɛdonǔwu gege ɖó gnavisín fifà mɛ. È nɔ sɔ́ nǔ e nɔ hɛn nǔ gblé lɛ́ɛ é dó wema fitlu tɔn ɔ́ mɛ, bɔ mɔ̌ mɛ ɔ́, è sɔ́ nɔ kpɔ́n kpɔ́ndéwú lɛ́ɛ dó mɔ nǔ e nɔ hɛn nǔ gblé dó mɛ wu é ɖé ǎ. Enɛ nɔ zɔ́n bɔ è nɔ hɛn nǔ e è kpɔ́n lɛ́ɛ é bo nɔ lɛ́ hɛn yì fí ɖevo lɛ́ɛ, bo nɔ lɛ́ hɛn nǔ e è kpɔ́n lɛ́ɛ é ɖó mimɛ̌ jí ǎ [68].

4. Ðiɖe wujɔnú Kpɔ́dewu ɔ́ tɔn lɛ́ɛ

Gbɔn vo nú nǔvínúví gègě ɖevo lɛ́ɛ è nɔ tunwùn gbɔn nǔ e ɖò agbaza ye tɔn tu bɔ è sixu mɔ ɖò nǔvínúví ɖokpo ɖokpo e è tɛ́ dó tuto jí lɛ́ɛ é gblamɛ ɔ́, flebotomu lɛ́ɛ nɔ byɔ ɖɔ è ni ɖe wujɔnú ye tɔn lɛ́ɛ ye bo lɛ́ sɔ́ ye ɖó nǔ jǐ bo na dó kplɔ́n nǔ dó agbaza yetɔn wu, bo na dó tunwun nǔvínúví alɔkpa ye nyí e ganji. Wlɛ́nwín ɖebǔ é sɔ́ bo na dó sɔnǔ na abǐ bo lɛ́ ye sɔ́ ɖó lamu jǐ é ɔ́, è nɔ zán wlɛnwín wunjɔnú ɖiɖe tɔn ɖokpó ɔ́ (ɖiɖe 2 & 3) (https://zenodo.org/records/18198006).

Triton X100 zinzan: sìn e ma nyí ioniki ǎ é

Ðǒ ayi wu ɖɔ nǔ ɖɔ́ sɔ́ dó nǔ ji ɔ́ kúnkplá kpɔ́dewu è wlí yɔyɔ̌ lɛ́ɛ é alǒ e è hɛn ɖ'ayǐ ganji lɛ́ɛ é. Mɛ e nɔ xò nǔ kplé lɛ́ɛ é gègě nɔ hɛn nǔvínúví lɛ́ɛ sín kpɔ́ndéwú lɛ́ɛ ɖó te bɔ ye nɔ xú (bo na dó zán MALDI-ToF) alǒ nɔ hɛn ye ɖó ahan mɛ nú xwè mɔkpan. É blawu ɖɔ è kún sixú hɛn nǔ dó ahan mɛ ganji nú xwè gěge ó, bɔ nǔ e è hɛn ɖ'ayǐ gbɔn mɔ̌ lɛ́ɛ nɔ vɛwǔ tawun bɔ è na sɔnǔ nú ye bonu è na gbéjé ye kpɔ́n ɖò nǔgbejekpɔ́n sín lɛ́ɛ mɛ. Nǔ e nɔ jɛ hwɛhwɛ é wɛ nyí ɖɔ alǎ e mɛ è sɔ́ kpɔ́ndéwú lɛ́ɛ dó é nɔ gblé, bɔ enɛ gudo ɔ́, ahan ɔ́ nɔ xú. Ðò ninɔmɛ wè lɛ́ɛ bǐ mɛ ɔ́, mǐ ɖó nǔ ɖebǔ bo na wà ǎ, ɖó kpɔ́ndéwú lɛ́ɛ nɔ́ nɔ ahan mɛ nú hwenu gegě alǒ nɔ́ xú. Enɛ wu ɔ́, linlin ɔ́ wá tɔ́n ɖɔ è ni zán nǔ e nɔ́ hɛn nǔ xú lɛ́ɛ é, bɔ ye ma nyí nǔ e nɔ hɛn nǔ syɛnsyɛn lɛ́ɛ é ǎ. Triton X100 dí sin e ma nyí ioniki ǎ é ɖé (4-(1,1,3,3-tetraméthylbutyl) fenil-poliéthylène glycol linfín, alǒ t-oktilfenoksipolyetoxyéthanol, poliéthylène glikol tert-oktilfenyle birgent éther mɛ ɔ́, è nɔ zán ɖò fí gègě. É nɔ zɔ́n bɔ è nɔ bló bɔ nǔ e nɔ do klɔ́ nuǔ na lɛ́ɛ é kpó nɔ gbɔn mɛ.

Ðò dò ɔ́, è nɔ zán Triton X100 e ma nyí ioniki ǎ é ɖò sìn 0,5% mɛ:

- Mi kɔn ahan akú dó kpɔ́ndéwú xúxú ɔ́ ji.

- Mi kɔn é dó Triton X100 sín sìn mɛ ɖò 0,5% e jɛxá e de jí bonu kpɔ́ndéwú ɔ́ bǐ na sixú fá,.

- Mi jo nu ɔ́ do bónú e nɔ ayi nu ceju 5 mɔ̌ nú azan gege, bo na nɔ cɔ́ ɛ hwɛhwɛ. Afɔ lɛ́ɛ bǐ ɖó na jɛvovo ɖò sìn ɔ́ mɛ.

- Mi ɖe Triton X100 sin sin ɔ́ sin bo sɔ́ sin hydroxyde potassiumu tɔn do ɖyɔ na.

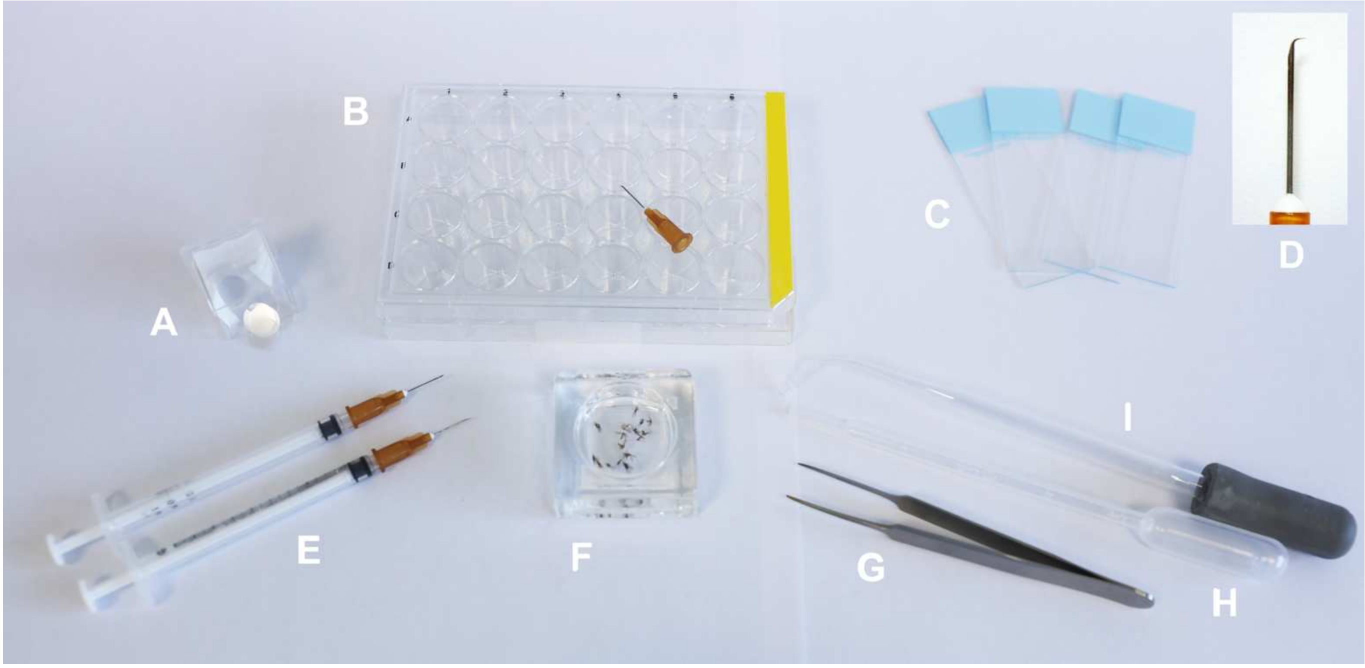

Ɖiɖe 2gɔ́ ɔ́. Nǔ e è na zán dó sɔ́ flebotomu lɛ́ɛ ɖó nǔ jí lɛ́ɛ é: A: lamu e sɔ́ go e sin toboo dó bló ná e (10 alǒ 12 mm ɖò gblǒ mɛ); B: 24- pláki kpódó yɛnvi e xá ɖo nu e kpó (enyi a zán amì atínkɛn gba ɖota tɔn alǒ sensin Euparal® tɔn dó w'azɔ̀ dó flebotomu lɛ́ɛ ji ɔ́, ma zán plaki acrylique tɔn lɛ́ɛ ó ɖó nǔ e nɔ hɛn nǔ gblé dó mɛ wu é ɖé na jɛ bɔ nǔ e è kpɔ́n lɛ́ɛ é na gblé); C: lamu go tɔn e jɛxa bɔ è na wlan nǔ dó jí lɛ́ɛ é; D: nǔtínmɛ dó yɛnvi ɔ́ sín nu ji. E: yɛ̌nvi e sɔ́ dó yɛ̌dónú nú e; F: kɔ́fu e mɛ è nɔ kpɔ́n gan ɖè é alǒ gannú é hɛn flebotomu e è na sɔ́ ɖó nǔ jí lɛ́ɛ é; G: Dumont sín nǔhɛn nú; H: pipɛti ala tɔn; I: pipɛti go tɔn e flɔ́ zo do na dó dun sin do zɛn lɛ́ɛ mɛ.

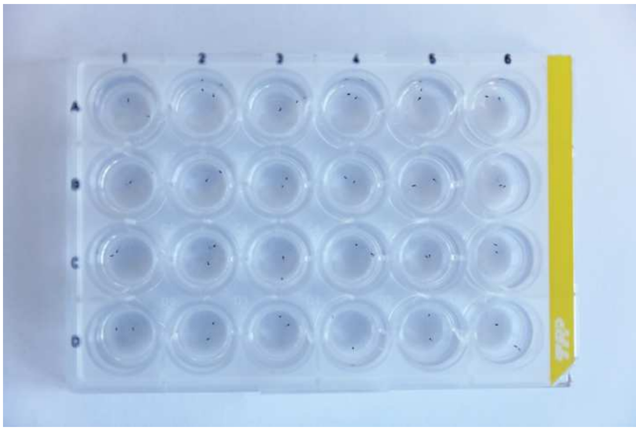

Ɖiɖe 3gɔ́ ɔ́. Plaki e mɛ dotɔ 24 ɖè é ɖé, bɔ flebotomu lɛ́ɛ sín ta kpódó adɔgo yetɔn sín nuvínu kpó ɖò ɖokpo ɖokpo mɛ.

4.1. Ta

È hɛn ɔ́ e na ɖe wujɔnú lɛ́ɛ kpódó yɛ̌nvi e wíní tawu kpó ɖò stereomicroscopu glɔ́ (ɖiɖe 2 & 3). Yɛ̌nví hugǎn lɛ́ɛ é ɖíe: 26G x 1/2" (0,45 × 13 mm), 30G × 1/2" (0,3 × 13 mm), alǒ 25G × 5/8" (0,5 × 16 mm). Nú e ná sɔ́nǔ nú kpɔ́ndéwu ɔ́ e ɖo ná ɖe ta ɔ́ sín adɔgo ɔ́ wu hwɛ bó ná sɔ́ dó nǔ ji agbɔnnusúxwedo kpanta aga cóbó síxú kpɔ́n cibarumu kpódó pharynx kpó, bɔ akɔ́n kpódó adɔgo kpó nɔ ɖò akpáxwé lɛ́ɛ jí ɖò ɖiɖe lɛ́ɛ gudo. Enyi è sɔ́ ta ɔ́ ɖó tɛn e mɛ è nɔ bló ventro-dorsal ɖè é ɔ́, é nɔ zɔ́n bɔ è nɔ mɔ ɖɔ occipital foramen ɔ́ ɖò jǐ, enɛ wu ɔ́, è sixu kpɔ́n cibariumu ɔ́ tlɔlɔ. Enyi è sɔ́ ta ɔ́ dó vo ɔ́, é nɔ bɔwǔ bɔ è na mɔ nǔ jɛ wujɔnu agbaza tɔn lɛ́ɛ wu.

4.2. Awa lɛ́ɛ kpódó akɔ́n kpó

Awa lɛ́ɛ ɖó na ɖò tité. È sixu ɖè awa ɖokpó ɖokpó sín dò tɔn nu bo sɔ́ ɖó nǔ jí éɖokponɔ, alǒ è sixu sɔ́ ɖokpó ɖó nǔ jí , bo jó wegɔ ɔ́ dó akɔ́n wu. Enyi è ɖò tuto bló wɛ bo na gbéjé nǔ e ɖò nǔ jǐ lɛ́ɛ é kpɔ́n ɔ́, é ɖò taji ɖɔ è ni tunwun awa ɖisíxwé tɔn kpódó amyɔxwé tɔn kpó ganji bo lɛ́ ɖó wuntun ye jí cobo sɔ́ nǔ jǐ. È má akɔ́nnú ɔ́ ɖó akpáxwé gègě jí, bɔ nǔ wɛn e kúnkplá nǔ e è nɔ ylɔ ɖɔ taxonomii é ɖò ɖokpo ɖokpo mɛ [20, 64]. Ɖò kpaa mɛ ɔ́, è nɔ sɔ́ ɛ dó aja ji ɖo nǔ ji, bo na dó sixu gbéjé chetotaxie kpódó lee è nɔ má sinmɛ lɛ́ɛ gbɔn é kpó kpɔ́n.

Wuntun xá tɔn ɖo adɔgoɔ́ sín akpáxwé ɖe lɛ́ɛn ɔ́ zɔn bɔ e nɔ tunwun jɛxlí alɔkpa ɖe lɛ́ɛ ɖi *Brumptomyia* lɛ́ɛ. È sixu zán sinmɛ e è má é dó klán flebotomu Neotropicalu tɔn lɛ́ɛ ɖó vo ɖò hɛ̌nnu lɛ́ɛ sín tínmɛ (kpɔ́ndéwú ɔ́, Bichromomyia), hɛ̌nnu alɔkpa lɛ́ɛ (kpɔ́ndéwú ɔ́, *Pintomyia*), alǒ hɛ̌nnu ɖokpo ɔ́ tɔn lɛ́ɛ (kpɔ́ndéwú ɔ́, *Micropygomyia*, *Nyssomyia*, Psathyromyia, kpódó Psychodogupy kpó]. Enɛ wu ɔ́, enyi è ma zán akɔ́nnú ɔ́ dó gbéjé nǔ kpɔ́n ǎ ɔ́, è ɖó na sɔ́ ɖó nǔ jǐ kpódó yɛyi kpó bo ma hɛn gblé ó. Nǔ taji ɔ́ wɛ nyí ɖɔ è ni tunwun ɖɔ é kún nyí lee sinmɛ lɛ́ɛ syɛn sɔ́ é wɛ ɖò taji ó, loɔ́,

This is an Open Access article distributed under the terms of the Creative Commons Attribution License (https://creativecommons.org/licenses/by/4.0), which permits unrestricted use, distribution, and reproduction in any medium, provided the original work is properly cited.

lee ye gbakpé gbɔn ɖò akɔ́n mɛ é wɛ ɖò taji. Enɛ wu ɔ́, lee è na bló bɔ nǔ lɛ́ɛ na ɖò wɛn gbɔn é na ɖè nǔ e ɖò jǐ tɔn é alǒ lee é cí é sîn ǎ.

4.3. Avadonú lɛ́ɛ

È ɖó na cɔ́ mɛɖée tawun hwenu e è ɖò avadonú asú lɛ́ɛ kpódó así lɛ́ɛ kpó tɔn sɔ́ ɖó nǔ jǐ wɛ é, ɖó ye ɖò taji tawun bo na dó tunwùn hɛ̌nnu lɛ́ɛ, hɛ̌nnu kpɛví lɛ́ɛ kpo kanlin alɔkpa lɛ́ɛ kpo. Ðò asú lɛ́ɛ kpódó asi lɛ́ɛ kpó mɛ ɔ́, avadonú lɛ́ɛ nɔ ɖò kpɔ́.

4.3.1. Asú lɛ́ɛ

Avadonú lɛ́ɛ wexo bɔ ɖokpó ɖokpó nɔ ɖó goflémɛ gonocoxite-gonostyle ɖò nɛngbe, bɔ epandrial ɖò akɔ́n tɔn mɛ. Gonostyle ɔ́ nɔ ɖó xú kpódó kpó hweɖelɛnu, bɔ è ɖó na xà ye, bɔ fí e è nɔ sɔ́ ye dó lɛ́ɛ é ɖó na ɖò wěxo ganji. É ɖò taji ɖɔ è ni kpɔ́n gonocoxite ɔ́ sín xomɛ ganji, ɖó é sixu hɛn xú e nɔ nɔ ayǐ é ɖé alǒ ɖěɖee lobu (= tubercule) ɖé nɔ hɛn lɛ́ɛ é [22]. Azɔ̌gbɛ́ e ma ko ɖó jɔdómɛwu gege ɖo wujɔnú ɖiɖe mɛ ǎ lɛ́ɛ sixu bló nǔ e è nɔ sɔ́ do aja ji é mɔ̌ kpowu ǎ, ma ɖe avadonú lɛ́ɛ sín adɔgo ɔ́ mɛ (https://zenodo.org/records/18311158). Ðò ninɔmɛ enɛ mɛ ɔ́, akpáxwé avadonú asi lɛ́ɛ tɔn wè lɛ́ɛ e è sɔ́ ɖó kpɔ́ é sixu zɔ́n bɔ é vɛwǔ bɔ è na xà hǔn e ɖò gonocoxitu ɔ́ mɛ lɛ́ɛ é, amɔ̌, enɛ nɔ zɔ́n bɔ è nɔ nyì alɔ nú nǔ e è nɔ ylɔ ɖɔ gonocoxitu é sín nǔ gblégblé gbɔn wujɔnúɖiɖe e è ma kpéwú bo ɖè ǎ é gblamɛ. Azɔ̌gbɛ́ e ɖó jɔdómɛwu gege lɛ́ɛ é sixu tɛnkpɔn bo na zɛ avadonú lɛ́ɛ ɖó wè, bo na má ye. Bo na dó kpé enɛ wu ɔ́, è ɖó na sɔ́ akpáxwé yěnvi tɔn ɖokpó de mɛ (nǔ e nɔ hɛn nǔ gblé dó mɛ wu ɖò agbaza mɛ é sín alɔkpa), bo ma na ɖe avadonú ma sɛ́n ye bǐ mlémlɛ́, bo na dó klán kplékplé gonocositu-gonostyle tɔn lɛ́ɛ (https://zenodo.org/records/18311158). Mɔ̌ mɛ ɔ́, nukúnmɛ yetɔn e ɖò xomɛ lɛ́ɛ é kpinkpɔn na bɔwǔ. Kplékplé enɛ nɔ lɛ́ zɔ́n bɔ è nɔ kpɔ́n paramɛ́ɛ lɛ́ɛ akpáxwé lɛ́ɛ ɖe jɛ ɖe gudó. Nú è na sɔ́ nǔ lɛ́ɛ ɖó akpáxwé, ee nɔ zɔ́n bɔ è nɔ sɔ́ agbaza sín wǔjɔnú lɛ́ɛ ɖó kpɔ́ é ɔ́, è ɖó na ɖè kpɔndewu e è kpɔ́n lɛ́ɛ é sîn ganji.

4.3.2. Avadonǔ asi lɛ́ɛ

Avadonú ɔ́ ɖò xomɛ, bɔ spermathecae wɛ nɔ bló. Enyi è ma na ɖe wujɔnú lɛ́ɛ ǎ ɔ́, è ɖó na kpɔ́n ye gbɔn xú lɛ́ɛ jí bo na lɛ́ sɔ́ adɔgo ɔ́ ɖó nǔ ji ɖo aja ji. Nǔ ɖebǔ e è na sɔ́ dó nǔ jǐ é ɔ́, è sixu kpɔ́n spermathecae ɔ́ ɖesu ganji ɖò kpaa mɛ, ɖò taji ɔ́, enyi é ma cí mǔ bo lɛ́ mɛ́ ǎ ɔ́ nɛ. Amɔ̌, enyi è kpɔ́n spermathecae e ɖò mǔ bo fɛ́ɖɛ́ ɔ́, é sixu nyí tagba ɖò nǔ e ma nɔ hɛn nǔ gblé ganji ǎ lɛ́ɛ é mɛ. Gɔ́ na ɔ́, è ɖó na kpɔ́n dò e mɛ spermatɛku lɛ́ɛ nɔ tɔ́n sín é ɖò taji bo na dó tunwun kanlin alɔkpa lɛ́ɛ, ɖi ɖò subenus Larroussius [35, 37, 38] mɛ, ee nyí nǔ taji e nɔ hɛn Leishmania infantum wá ɖò gbɛ̀ xóxó ɔ́ mɛ é. Enyi è ma kpɔ́n nǔ enɛ ǎ ɔ́, è sixu tunwùn spermatɛku lɛ́ɛ ǎ. Bo na dó ɖu ɖò nǔ kpínkpɔ́n sín tagba enɛ lɛ́ɛ jí ɔ́, è ɖó na ɖè fɛ́n e nɔ hɛn xú mɛtɔn ɖó te é sín adɔgo mɛ (https://zenodo.org/records/18311106). Hwɛhwɛ ɔ́, é nɔ vɛwǔ bɔ è na mɔ spermathecae lɛ́ɛ hwenu e è ɖò nǔ wujɔnú lɛ́ɛ ɖe wɛ é, amɔ̌, é bɔwǔ tawun bɔ è na mɔ fí e è nɔ mɔ avadonú furca tɔn ɖè é. Ðó spermatkuɛ lɛ́ɛ nɔ hun bo nɔ byɔ avadonú furca tɔn mɛ wutu ɔ́, furca énɛ e è ɖè ɖó vo é nɔ zɔ́n bɔ è nɔ ɖè spermathecae lɛ́ɛ ɖó vo. Enyi è gbò spermathecae lɛ́ɛ ɖò nǔwiwa ɔ́ hwenu ɖò manywɛ mɛ ɔ́, ye nɔ bú ǎ, bɔ è sixu kpó ɖò ye mɔ wɛ ɖò adɔgo ɔ́ sín xú lɛ́ɛ mɛ (Ðiɖe 4gɔ́ ɔ́).

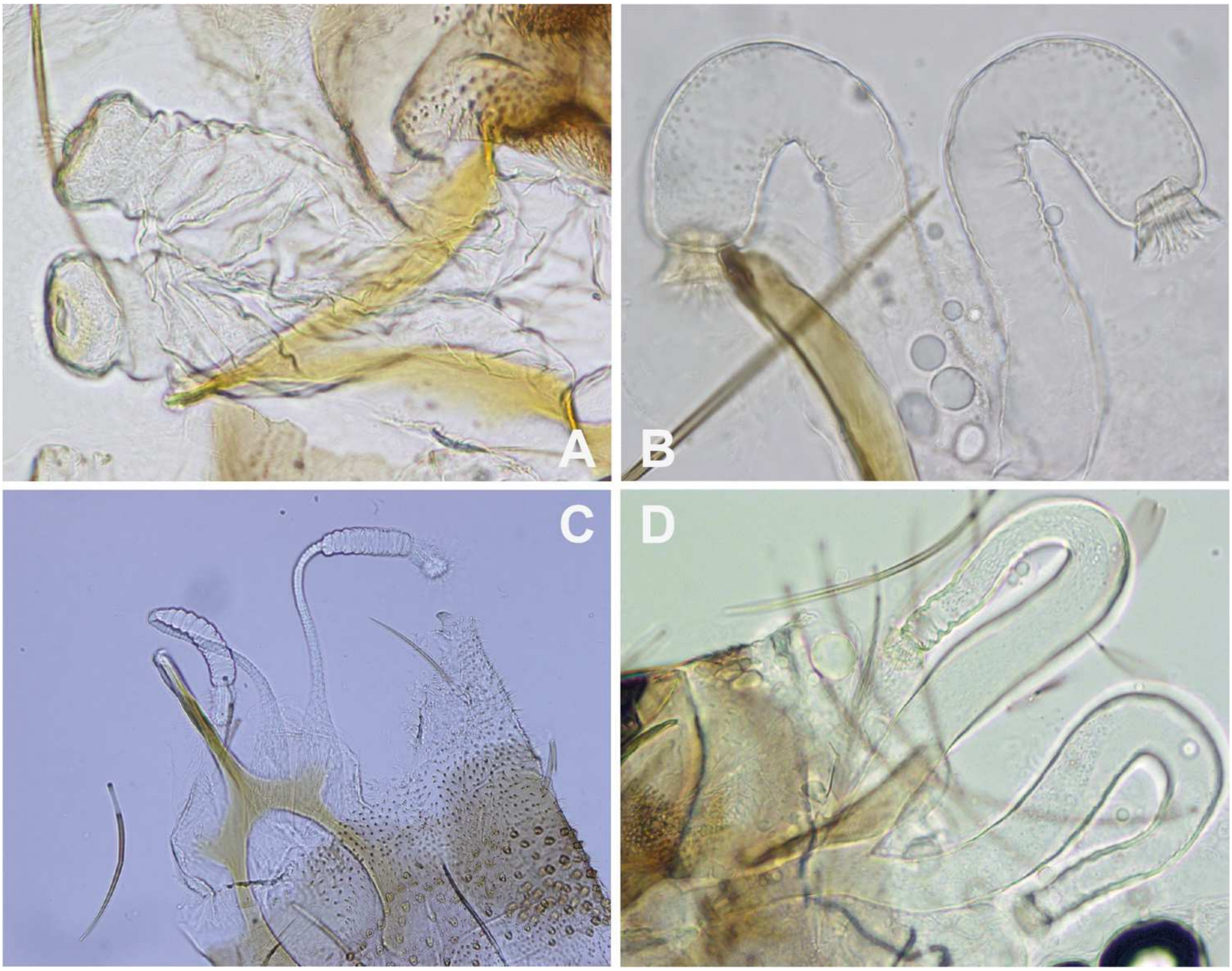

Điḍe 4gɔ́ ɔ́. È ḍe spermathecae bo sɔ́ ḍó sìn Marc-André tɔn sín nŭ yɔyɔ̌ e è kpɔ́n lɛ́ɛ é mɛ. A : *Idiophlebotomus longiforceps* (Togun to wɛ nɔ sɔ́ gan to wɛ nɔ ḍu gan tɔn kpàa Lao tɔn) ; B : *Sergentomyia minuta* (France) ; C : *Phlebotomus ariasi* (France) ; D : *Sergentomyia anodontis* (Togun to wɛ nɔ sɔ́ gan to wɛ nɔ ḍu gan tɔn kpàa Lao tɔn).

4.4. È zɛ adɔvi kpɛvi bo na dó ḍè Leishmania ḍó vo

É ḍò taji ḍɔ è ni ḍe adɔvi lɛ́ɛ bo na dó tunwùn Leishmania ḍò flebotomu asi lɛ́ɛ mɛ bo lɛ́ ḍè è ḍó vo. É ḍò mɔ̌ có, è sixu bló tuto ɔ́ ḍò gle mɛ kpo dobanúnǔtɛn kpo mɛ, bo na dó gbéjé nǔwukpikpé nǔhɛnnú tɔn kpɔ́n. È nɔ kplɔ́n mɛ ḍɔ è ni w'azɔ̌ dó asi e è hu ḍò yɔyɔ̌ lɛ́ɛ é jí. Klɔ́ asi lɛ́ɛ kpódó sin alǒ jɛ̌ e mɛ nǔ e nɔ klɔ́ nǔ é ḍé ḍè é kpó bo na dó ḍè fún e gɔ́ngɔ́n lɛ́ɛ é sîn. Afɔ enɛ nɔ d'alɔ bɔ è nɔ hɛn ninɔmɛ ḍagbe lɛ́ɛ ḍó te nú Leishmania sín, bo nɔ lɛ́ hɛn nǔ e ḍò agbaza mɛ bɔ è ḍó hudo tɔn bo na dó tunwùn ɔ́ lɛ́ɛ é ḍó te. Bo na dó mɔ Leishmania bo ḍè é ḍó vo ɔ́, è ḍó na ḍè adɔvi ɔ́ sín xomɛ ganji bo sɔ́ dó jɛ̌ e mɛ è ma nɔ mɔ nǔ jɛ nǔ mɛ ḍè ǎ é ḍokpó mɛ (0,9% NaCl). Enyi a kpɔ́n nǔvínúví e nɔ sɛ̀ tɛn lɛ́ɛ é ḍo nǔgbeje mɔ mɛ gudo ɔ́ (è kplɔ́n ḍɔ è ni bló bɔ ye na nyi ḍaxó : ~200×), zán insulinu sín jɛ̌ alǒ pipéti kpɛvi kpɛvi dó sɔ́ ye dó nǔ e mɛ è nɔ dó nǔkún ḍè é mɛ (nú a na mɔ tinmɛ ḍevo lɛ́ɛ hǔn, kpɔ́n Wemata 4.4.3). Sɔ́ ta ɔ́ kpódó avadonú ɔ́ kpó tlɔlɔ dó sìn Marc-André tɔn mɛ bo na dó ḍè ye sîn. Nu taji ɔ́ : ma lɔn gbeḍe ḍɔ sin Marc-André tɔn ni jɛ Leishmania wu ó – tlɔlɔ alǒ ma nyi tlɔlɔ gbɔn azɔwanú lɛ́ɛ alǒ nǔ lɛ́ɛ gblamɛ ǎ – ḍó é nɔ hu nǔvínúví lɛ́ɛ.

È sixu ḍe flebotomu asi lɛ́ɛ dó wè jí ; linlin wè lɛ́ɛ bǐ wɛ ḍó ḍagbe kpódó nyannya ye tɔn kpó (Điḍe 5gɔ́ ɔ́; https://zenodo.org/records/18311154).

This is an Open Access article distributed under the terms of the Creative Commons Attribution License (https://creativecommons.org/licenses/by/4.0), which permits unrestricted use, distribution, and reproduction in any medium, provided the original work is properly cited.

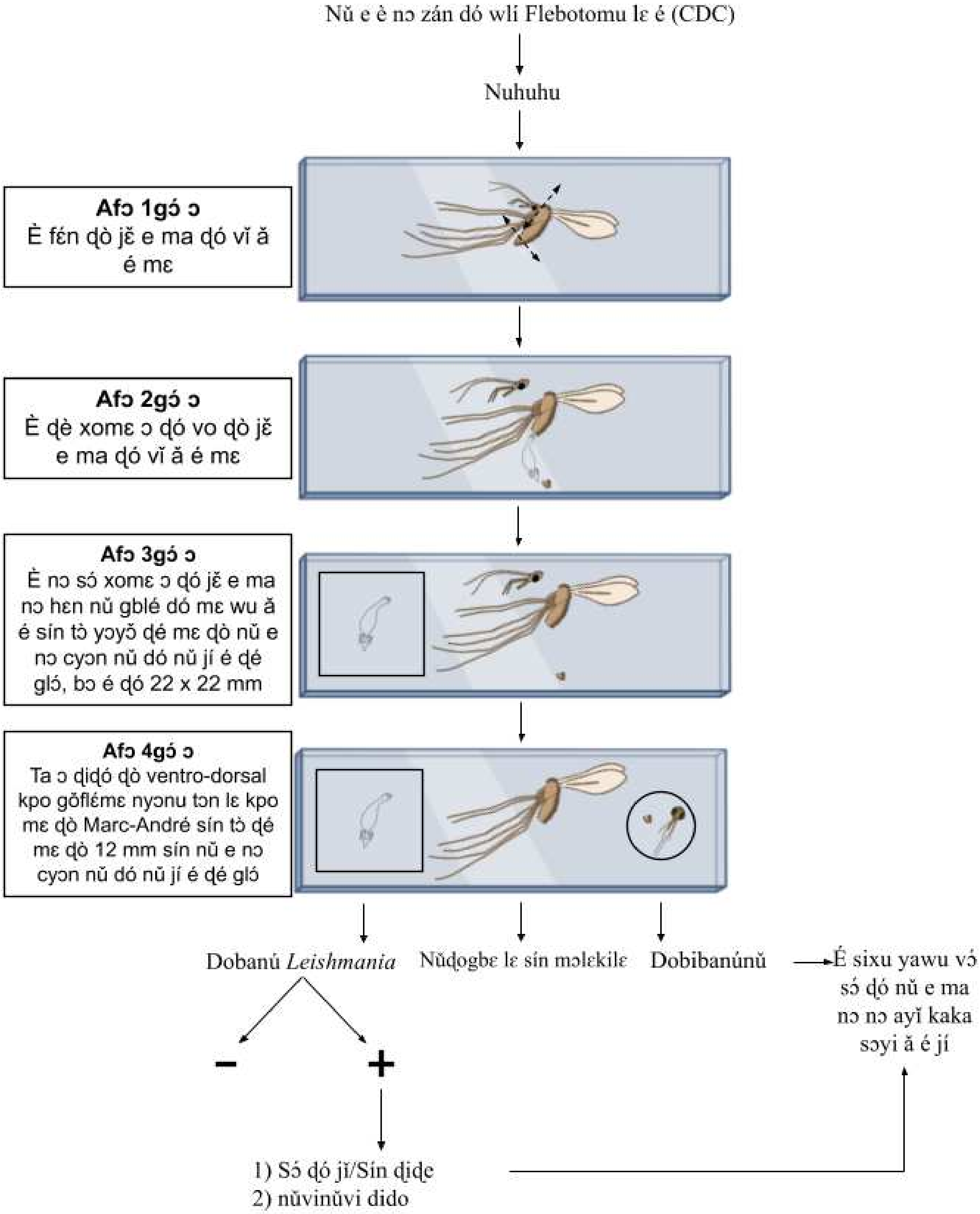

Ɖiɖe 5gɔ́ ɔ. Wlɛnwín e è nɔ zán dó ɖè *Leishmania* ɖó vo é.

This is an Open Access article distributed under the terms of the Creative Commons Attribution License (https://creativecommons.org/licenses/by/4.0), which permits unrestricted use, distribution, and reproduction in any medium, provided the original work is properly cited.

4.4.1. Wlɛnwín ɖiɖe wè tɔn

Nǔ nukɔntɔn e è sixu wà é wɛ nyí ɖɔ è ni w'azɔ̌ ɖò lamu vovo wè jí : lamu nukɔn tɔn ɔ́ seum physiologique bo ná dó ɖe adɔvi kpɛvi lɛ́ɛ, bɔ wegɔ ɔ na do sɔ́ ta ɔ́ kpó spermathecae lɛ́ɛ kpó ɖó sìn Marc-André tɔn mɛ. Amɔ̌, ɖò ninɔmɛ gle tɔn lɛ́ɛ mɛ ɔ́, é nɔ bɔwǔ bɔ mɛ wè alǒ mɛ atɔn na xò flebotomu lɛ́ɛ kplé bo na sɔ́ nǔ e ye xò lɛ́ɛ é jó nú dobanúnǔtɔ́ ɖokpo géé e nɔ kpé nukún dó nǔkún-yiya sín nǔ lɛ́ɛ wu é kpo lee è nɔ gbéjé Leishmania sín azɔn ɔ́ kpɔ́n gbɔn ɖò xomɛ é kpo. É ɖò mɔ̌ co, Lamu we zinzin na vɛwǔ bɔ è na dó tunwùn kpódó jiɖe kpó kpɔndewu ɖokpo ɖokpó e mɛ azɔn ɔ́ ɖè é, enyi è mɔ ɖɔ è hɛn xomɛ ɖagbe ɖé wá ɔ́ nɛ (https://zenodo.org/records/18311154).

4.4.2. Wlɛnwín lamu ɖokpo tɔn

Lamu ɖokpo zinzan nɔ zɔ́n bɔ è nɔ tunwùn nǔ e è mɔ lɛ́ɛ é ganji. Amɔ̌, è ɖó na cɔ́ mɛɖée ɖò nǔ gègě mɛ. Bo na dó sixu hu aɖinú nǔ lɛ́ɛ ɔ́, azɔ̌watɔ́ lɛ́ɛ ɖó na nɔ klɔ́ alɔ yetɔn lɛ́ɛ hwɛhwɛ kpódó jɛlu hydroalcooliku kpó. È ɖó na zán lamu e ma ɖó nǔ jǐ ǎ lɛ́ɛ é kpódó lamu e è sɔ́ kɔ́fu dó bló na lɛ́ɛ é kpó (22 x 22 mm) bɔ è sɔ́ aluminiumu dó blá ye na, bo sɔ́ myɔ xúxú dó bló bɔ ye na hɛn nǔ gblé dó ye wu (è na zán myɔkán Poupinel tɔn ɖé), gɔ́ nú nǔ e è ma nɔ hɛn nǔ gblé dó ye wu ǎ lɛ́ɛ é nú lamu ɖokpó ɖokpó (linlin : 25G Ø 0,5mm ×1). È nɔ sɔ́ flebotomu ɔ́ dó serumu physiologique é ɖé mɛ ɖò lamu ɔ́ tɛntin. È nɔ gbò ta ɔ́ hwenu e è ɖò fén ɖé gbò wɛ ɖò tergites kpódó sternites kpó adɔgo tɔn 6gɔ́ ɔ́ kpo 7gɔ́ ɔ́ kpo tɛntin é, bo nɔ gbò nǔ e nɔ hɛn nǔ gblé dó mɛ wu é ǎ (è sixu gbò fén ɖaxó ɖé enyi è ɖò nukún ɖó spermathecae gaga ɖé wɛ ɔ́ nɛ). Enɛ gudo ɔ́, è ɖó na sɔ́ yěnvi dó dó hɛn akɔ́n ɔ́ ɖó te, bo na dɔn adɔgo ɔ́ sín akpáxwé gudogudo tɔn e ɖò gudo lɛ́ɛ é kpó yěnvi ɖevo kpó bo na dó ɖè adɔvi ɔ́ tɔ́n. Enyi enɛ gló ɔ́, è hɛ nɔ e na sɔ́ yěnvi dó sú adɔgo ɔ́ sín vivɔnu, bo na dɔn adɔvi ɔ́ sín akpáxwé tɔn nukɔntɔn ɔ́. Enyi enɛ lɔ lɛ́ glo ɔ́, è ɖó na ɖè adɔví ɔ́ tɔ́n gbɔn nǔ e kpò ɖò xomɛ ɔ́ wu lɛ́ɛ é sísɔ́ sîn lee é nyɔ́ bló gbɔn é gblamɛ. Enyi è ɖè adɔvi ɔ́ tɔ́n ɔ́ è ɖó na ɖè adɔgo ɔ́ sín akpáxwé gudogudo tɔn lɛ́ɛ ɖó vo gbɔn serumu physiologiques gblamɛ bó ná sɔ́ akpaxwé ɖé dó lamu ji bó ná sɔ́ akpaxwe wegɔ ɔ́ dó lamu kpɛvi ji. Enɛ gudo ɔ́, è nɔ sɔ́ adɔvi ɔ́ dó jě e ma nɔ hɛn nǔ gblé dó mɛ wu ǎ é yɔyɔ̌ ɖé mɛ bɔ è nɔ sɔ́ ɖó ɖiɖe ɔ́ sín akpá ɖokpó, bo enɛ gudo ɔ́, è nɔ sɔ́ nǔ e nɔ hɛn nǔ gblé dó mɛ wu é ɖé dó cyɔn nǔ ɔ́ jí bléblé. È nɔ sɔ́ ta ɔ́ kpódó adɔgo ɔ́ sín akpáxwé gudogudo tɔn lɛ́ɛ kpo dó sìn Marc-André tɔn kpɛví ɖé mɛ bo nɔ sɔ́ ɖó ɖiɖe ɔ́ sín vivɔnu ɖevo, bo nɔ bló bɔ Leishmania nɔ xò nǔ kpɔ́n ǎ. Ta ɔ́ ɖò tɛn tɔn mɛ ganji (occipital foramen upwards), bɔ spermathecae lɛ́ɛ nɔ ɖò vo kpo avadonú furca tɔn kpo lee è xlɛ́ gbɔn ɖò aga é, bo nɔ sɔ́ nǔ kpɛví kpɛví e nɔ cyɔn nǔ dó nǔ jí é ɖé dó cyɔn ye jí (Ø 12 mm, è ma vɛdó ɖɔ lamu kpɛvi e hua ɖǐ ná wɛ ó). Nǔ e kpò ɖò wut ɔn é kpódó awa tɔn lɛ́ɛ kpó nɔ nɔ jě e ɖò kɔ́ mɛ é do lamu ji é tɛntin (https://zenodo.org/records/18311154). Nú é nyí nǔ ɖagbe, alǒ nú è na ba dò nú nǔ ɖò taxonomie mɛ ɔ́, è sixu hɛn akɔ́n kpódó adɔgo kpó ɖó te nú nǔkplɔ́nkplɔ́n molekwilu alǒ proteomic tɔn, bo sixu sɔ́ awa lɛ́ɛ ɖó nǔ e mɛ sin ɖè é ɖé mɛ. Bo na dó hɛn nǔ e e sɔ́ nǔ ɔ́ ɖó te ɔ́, è sixu ɖyɔ sin Marc-André tɔn e gɔ́ngɔ́n é kpo nǔ e nɔ hɛn sɔ́nǔ ná e ɖó sin mɛ é ɖé kpo ɖi chloral gum (=Hoyer) alǒ nǔ e è sɔ́ ahan syɛnsyɛn polyvinyl tɔn dó bló é ɖé kpó.

Yɛwunkɔnyidowu e xlɛ́ lée è nɔ wà nǔ gbɔn é céɖécéɖé lɛ́ɛ é tîn (ɖiɖe adɔvikpɛví Flebotomu tɔn: https://zenodo.org/records/18303014 kpódó atán sín gojiblanú lɛ́ɛ ɖiɖe kpó: https://zenodo.org/records/18302850 kpo), enɛ wu ɔ́, è na tinmɛ ye ɖò fí ǎ.

4.4.3. Leishmania sín nǔvínúví lɛ́ɛ ɖiɖe ɖó vo kpódó nǔkúnyiya ye tɔn kpó sín flebotomu lɛ́ɛ xomɛ mɛ

Nǔ e nɔ byɔ nǔnywɛ ɖaxó é ɖé wɛ nyí ɖɔ è ni ɖè nǔvínúví lɛ́ɛ ɖó vo ɖò flebotomu asi e è hɛn azɔn ɔ́ lɛ́ɛ é mɛ, bɔ è ɖó na bɛ́ dó nǔvínúví e mɛ nǔvínúví ma ɖè ǎ lɛ́ɛ é jí. Enyi è zɛ adɔvi ɔ́ gudo ɔ́, è nɔ sɔ́ dó jě e aɖǐ ná e mɛ (0,9 %) alǒ Locke's sin mɛ bo nɔ klɔ́ [4]. É ɖò mɔ̌ có, è sixu gbéjé adɔvi e è zɛ lɛ́ɛ é kpɔ́n ɖò ali wè nu : i) è na gbéjé ye kpɔ́n ɖò nǔgbejekpɔ́mɔ ɔ gló bo na kpɔ́n akpáxwé vovo e ɖò Leishmania promastigotes mɛ lɛ́ɛ é kpódó fí e ye ɖè é kpó, bo na ɖó ayi valve stomodeal tɔn ɔ́ wu tawun, II-) e na na zɛ adɔvi ɔ́ bo na dó bló bɔ promastigotes lɛ́ɛ na tɔ́n bo na bló bɔ è na kplɔ́n nǔ ye [4]. É ɖò mɔ̌ có, è nɔ mɔ flebotomu e nɔ hɛn azɔn wá lɛ́ɛ é ɖò gle mɛ ǎ, bɔ enɛ wu ɔ́, azɔ̌wiwa ɖagbe lɛ́ɛ na zɔ́n bɔ è na ɖó mɛɖée ɖó vo ganji. Enyi è mɔ nǔvínúví Leishmania tɔn lɛ́ɛ ɖò adɔvi ɔ́ mɛ ɔ́, è ɖó na zán yɛnví yɔyɔ̌ e hu aɖǐ lɛ́ɛ é, bo na lɛ́ sɔ́ ɖe do serumu physiologique lélɛ́ dó lamu ɔ́ bó na dó jó ye do. È ɖó na zɛ adɔvi ɔ́ ganji bo na yawu tlɛ́ bo na dó ɖè nǔvínúví lɛ́ɛ tɔ́n dó jě ɔ́ mɛ. Mi zé pipéti kpɛvi 100 µL alǒ tuberculine sín jě dó xò nǔvínúví lɛ́ɛ kplé bo dó jě dó nǔkúnyiya sín nǔ e è wlan nyikɔ tɔn ganji é ɖé mɛ.

É ɖò mɔ̌ có, è nɔ hɛn nǔvínúví e è ɖè ɖó vo lɛ́ɛ é ɖó SNB-9 sín hun agar slopes jí alǒ ɖò Novy, Mc Neal, Nicolle (NNN) sín nǔ syɛnsyɛn ɖé mɛ [16] bɔ è nɔ sɔ́ nǔ alfa-MEM e hu aɖǐ e [16, 65] alǒ nǔ e è nɔ ylɔ ɖɔ M1909 é dó gɔ́ na. nyibuví e ɖò xomɛ é sín hun e hu aɖǐ na e [FCS] (bo na dó bló bɔ nǔvínúví lɛ́ɛ na sù d'eji), vitaminu BME 1%, aɖɔ gbetɔ́ tɔn e hu aɖǐ na e 2% (è nɔ zán Filtropur® S 0,2 µm ma ɖó aɖǐ), amikacine 250 µg/mL (alǒ antibiotiques 50 µg/mL ami-glumicine, 200 mM-penicillin 10 000 U-streptomycin 10 mg/mL) [47] Azǎn atɔn gudo ɔ́, enyi nǔ kwijikwiji ɖě ma ɖò finɛ ǎ ɔ́, è nɔ sɔ́ nǔkún lɛ́ɛ ɖó fí e è sɔnǔ na ganji é ɖé mɛ, bɔ enɛ gudo ɔ́, è nɔ hɛn ye ɖó -80°C jí nú xwè 1 jɛ 2 ɖò fí e è nɔ hɛn nǔkún lɛ́ɛ ɖó é nú xwè 1 jɛ 2. è nɔ zán ɖò nǔ tén kpɔ́n mɛ [7].

4.5. Atán sín gǒjiblanú lɛ́ɛ

Zizɛ atán sín gǒjiblanú flebotomu lɛ́ɛ tɔn nyi wlɛnwin taji ɖe ɖo dobanúnǔ ɖo nǔwiwa azɔnkwín-hɛnnú tɔn, ɖo taji ɔ́, bo na do mɔ arbovirus ɖi Phlebovirus (e.g., Toscana virus) [44, 75]. Đó flebotomu lɛ́ɛ sín ga kpɛví kpɛví wutu ɔ́,

This is an Open Access article distributed under the terms of the Creative Commons Attribution License (https://creativecommons.org/licenses/by/4.0), which permits unrestricted use, distribution, and reproduction in any medium, provided the original work is properly cited.

nǔwiwa ɔ́ byɔ ɖɔ è ni bló nǔ lɛ́ɛ ganji ɖò stereomicroscope ɖé glɔ́, bo na zán yěnvi ɖagbe ɖagbe lɛ́ɛ alǒ yěnvi kpɛví kpɛví lɛ́ɛ dó ɖè atán sín gǒjiblanú lɛ́ɛ é ɖó vo, bo ma na zɔ́n bɔ ye na gbà alǒ hɛn nǔ kwiji ǎ (https://zenodo.org/records/18302850) [51, 1]. Gǒjiblanú lɛ́ɛ sín bǔninɔ hɛn ɖ'ayǐ ɖò taji tawun bo na dó sixu ɖeji dó nǔ e è nɔ gbéjé kpɔ́n ɖò dò lɛ́ɛ é wu. Enyi è ɖè ye tɔ́n ɔ́, è sixu bló bɔ gǒjiblanú lɛ́ɛ na nyí nǔ ɖokpo ɔ́ bo tɛ́n ye kpɔ́n gbɔn RT-PCR, qPCR, alǒ immunoassays gblamɛ bo na dó tunwùn ARN alǒ antigènes viral tɔn lɛ́ɛ [12]. É ɖò mɔ̌ có, enyi è mɔ ɖɔ azɔnkwín lɛ́ɛ ɖò gǒjiblanú lɛ́ɛ mɛ, é nyí ɖò adɔví alǒ hun ɔ́ kɛɖɛ mɛ ǎ ɔ́, é nɔ xlɛ́ ɖɔ azɔnkwín ɔ́ ko fó hwenu e é nɔ hɛn nǔ gblé dó mɛ wu é, bo nɔ lɛ́ hɛn mɛ gble ɖò hunɖuɖu hwenu [71]. Zizɛ atán sín gǒjiblanú flebotomu lɛ́ɛ tɔn nyi wlɛnwin taji ɖe bó hizi bó byɔ́ nǔwukpikpe ɖaxó bo na dó sixu nyì alɔ nú nǔ e è sɔ́ ɖ'ayǐ lɛ́ɛ é sín ɖiɖekpo [1, 51]. Gɔ́ na ɔ́, agban e ɖò azɔnkwín lɛ́ɛ mɛ é sixu hwe, bo na byɔ ɖɔ è ni zán wlɛnwín nǔmɔjɛnǔmɛ tɔn e nɔ hɛn nǔ gblé dó mɛ wu tawun lɛ́ɛ é ɖi PCR e è bló ɖó kpɔ́ é alǒ nǔ e è nɔ ylɔ ɖɔ high-throughput sequencing é [54]. Awě e è sixu xò mɛ lɛ́ɛ é lɛ́vɔ tɛɖɛ̌ hudo e è ɖó bo na zán wlɛnwín vɔtɔ́ lɛ́ɛ é jí. Gbɔn vo nú tagba nǔnywɛ xwitixwiti tɔn lɛ́ɛ ɔ́, nǔ e ɖò gbè ɔ́ mɛ lɛ́ɛ é wɛ nɔ wà nǔ dó lee è nɔ mɔ nǔ jɛ nǔ mɛ gbɔn é wu ; nǔwukpikpe sín nǔnywɛ nɔ gbɔn vo ɖò flebotomu alɔkpa lɛ́ɛ tentin, bɔ azɔn ɔ́ sín hlɔnhlɔn nɔ gbɔn vo sɔgbe xá ninɔmɛ e ɖò ayikúngban ɔ́ jí lɛ́ɛ é kpódó hwenu lɛ́ɛ kpó [33, 61]. Ényi è mɔ azɔnkwín lɛ́ɛ ɖò atán sín gǒjiblanú flebotomu lɛ́ɛ mɛ ɔ́, é nɔ na nukúnnúmɔjɛnǔmɛ taji dó awě e è sixu xò mɛ lɛ́ɛ é wu, bo nɔ zɔ́n bɔ è nɔ cɔ́ mɛ lɛ́ɛ bo nɔ lɛ́ kpé nukún dó ye wu [15]. Ði kpɔ́ndéwú ɔ́, è mɔ Toscana sín azɔnkwín ɖò febotomu lɛ́ɛ mɛ ɖò fí e azɔn ɔ́ gbakpé ɖè lɛ́ɛ é, bɔ enɛ zɔ́n bɔ è tunwun azɔn ɔ́ sín tuto lɛ́ɛ kpódó wěɖexámɛ e kúnkplá lanmɛ na nɔ ganji mɛ tɔn lɛ́ɛ é kpó [18]. Ðèvo ɔ́, nǔkplɔ́nkplɔ́n dó nǔ e azɔnkwín lɛ́ɛ kpódó atán lɛ́ɛ kpó nɔ wà é jí sixu ɖè nǔ yɔyɔ̌ e è na sɔ́ ɖó te lɛ́ɛ é xlɛ́ nú abɔxwí e nɔ ɖó dogbó nú azɔnkwín lɛ́ɛ é alǒ azɔngbɔnúmɛ lɛ́ɛ [15, 18]. È sixu lɛ́ zán atán sín gǒjiblanú flebotomu lɛ́ɛ dó jlɛ́ antigènes e nɔ ɖí xwi xá anticorps jonɔ tɛnkpɔn lɛ́ɛ é gbɔn wlɛnwín nǔ e nɔ ɖí xwi xá azɔn lɛ́ɛ é gblamɛ, é na nyɔ́ hugǎn ɖɔ è ni zán ELISA. Wlɛnwín enɛ nɔ zɔ́n bɔ è nɔ gbéjé lee mɛ e ɖò xwé ɔ́gbè lɛ́ɛ é nɔ ɖu nǔ gbɔn é kpɔ́n, bɔ enɛ nɔ d'alɔ bɔ è nɔ gbéjé lee wlɛnwín e è nɔ zán dó ɖu ɖò nǔ e nɔ hɛn nǔ gblé dó mɛ wu lɛ́ɛ é nɔ w'azɔ̌ gbɔn é kpɔ́n [25] kpódó lee *Leishmania* sixu hɛn mɛ gble gbɔn é kpó [40].

4.6. Kɛ́nɖiɖe hun mɛ sín nǔɖuɖu lɛ́ɛ tɔn

È ɖó na zán nǔ e è nɔ zán azɔn ɖokpo gée é dó zɛ asi lɛ́ɛ bɔ è ɖè ye ɖó vo nú nǔ e è wlí lɛ́ɛ é, bo na dó bló bɔ ye ma na hɛn nǔ kwijikwiji lɛ́ɛ wá ye jí ó. È ɖó na gbéjé adɔgo yetɔn kpɔ́n ɖò nǔ e è nɔ ylɔ ɖɔ stereomicroscope é glɔ́, bo na dó tunwùn bǎ e mɛ nǔɖuɖu e ɖò hun mɛ é nɔ ɖu ɖè é. È ɖɔ ɖɔ è ni sɔ́ asi ɖěɖee sín adɔgo vɔvɔ, vɔvɔ-akú, alǒ vɔvɔ-cécɛ́, bo ma nɔ xlɛ́ ɖɔ azìn ɖé tɔ́n ǎ lɛ́ɛ é kɛɖɛ. Sún adɔgo ɔ́ sín nuvínu e mɛ spermathecae lɛ́ɛ ɖè é bo na dó tunwùn asi ɔ ɖò ɖiɖe tɔn mɛ ɖò ɖiɖe gudo. Enɛ gudo ɔ́, è ɖó na sɔ́ adɔgo ɔ́ sín akpáxwé taji ɔ́ (e mɛ spermathecae ma ɖè ǎ é) dó Eppendorf® sín tuto lɛ́ɛ mɛ, bo na hɛn ɖò -20°C jí kaka jɛ hwenu e è na gbéjé kpɔ́n d'eji é. Nǔ e è nɔ zán dó tunwun nǔɖuɖu e ɖò hun mɛ é, ɖi PNOC [5, 30, 50], CytB [67], alǒ COI [13], è ko tunwun ganji, bɔ è tinmɛ ye tawun ɖò wema lɛ́ɛ mɛ ; enɛ wu ɔ́, è na lɛ́vɔ ɖɔ xó dó ye jí ɖò wema elɔ mɛ ǎ (ɖiɖe 6gɔ́ ɔ). Alǒ, bo na dó tunwùn hun mɛ e yí mɛ é tɔn ɔ́ è sixu zán ɖiɖe peptide MALDI-ToF tɔn [31]. È ko xlɛ́ ɖò nǔgbejekpɔ́n mɛ ɖɔ wlɛnwín enɛ nɔ zɔ́n bɔ è nɔ tunwùn hun mɛ e yí mɛ é tɔn ɖò hwenu línlín ɖé mɛ ɖò hun e è ɖu é gudo ; enɛ wu ɔ́, wlɛnwín e jɛxa é ɖé wɛ bɔ è na sɔ́, ɖò taji ɔ, nú è na gbéjé asi e è ɖu nǔ zɛ xwé wu lɛ́ɛ é kpɔ́n, bɔ è nɔ mɔ ɖɔ hun e mɛ e yí ye é ko yì nukɔn hugǎn é wɛ. É nyɔ́ wà ɔ́, è ɖó na hɛn nǔ e è kpɔ́n lɛ́ɛ é ɖó -20°C alǒ 4°C jí, amɔ̌, è sixu lɛ́ mɔ lè ɖagbe lɛ́ɛ ɖò nǔ e è hɛn ɖó adɔví sín fifà jí nú hwenu klewun ɖé lɛ́ɛ é mɛ. È ɖó na ɖè adɔgo asi e è hɛn xú é tɔn sín agbaza tɔn sín akpáxwé e kpò lɛ́ɛ é mɛ cobɔ è na gbéjé nǔ ɔ́ kpɔ́n, bo na bló bɔ é na cí nǔ ɖokpo ɔ́ ɖɔhun ɖò sìn e è hɛn xú é mɛ. Agbaza flebotomu tɔn kpò e ɖò te nú nǔ ɖevo lɛ́ɛ bo na dó gbéjé nǔ e ɖò kɔ́ mɛ lɛ́ɛ é kpo lee ye cí é kpo kpɔ́n. Enyi è ɖè alikɔtu ɔ́ sín nǔ ɖokpo ɔ́ mɛ bo na dó bló MALDI-ToF peptide mapping gudo ɔ́, è sixu zán ee kpò lɛ́ɛ é dó ɖè ADN ɖó vo bo na dó ɖexlɛ́ ɖɔ è tunwùn hun mɛ e yí mɛ é tɔn kpo/alǒ è na kpɔ́n ɖɔ Leishmania sp. Hwenu e è nɔ zán dó sɔnǔ nú kpɔ́ndéwú lɛ́ɛ bo nɔ lɛ́ gbéjé ye kpɔ́n é bǐ nɔ hwe tawun, enyi è sɔ́ jlɛ́ dó wlɛnwín nǔjlɛdonǔwu tɔn e jinjɔn ADN jí lɛ́ɛ é wu ɔ́.

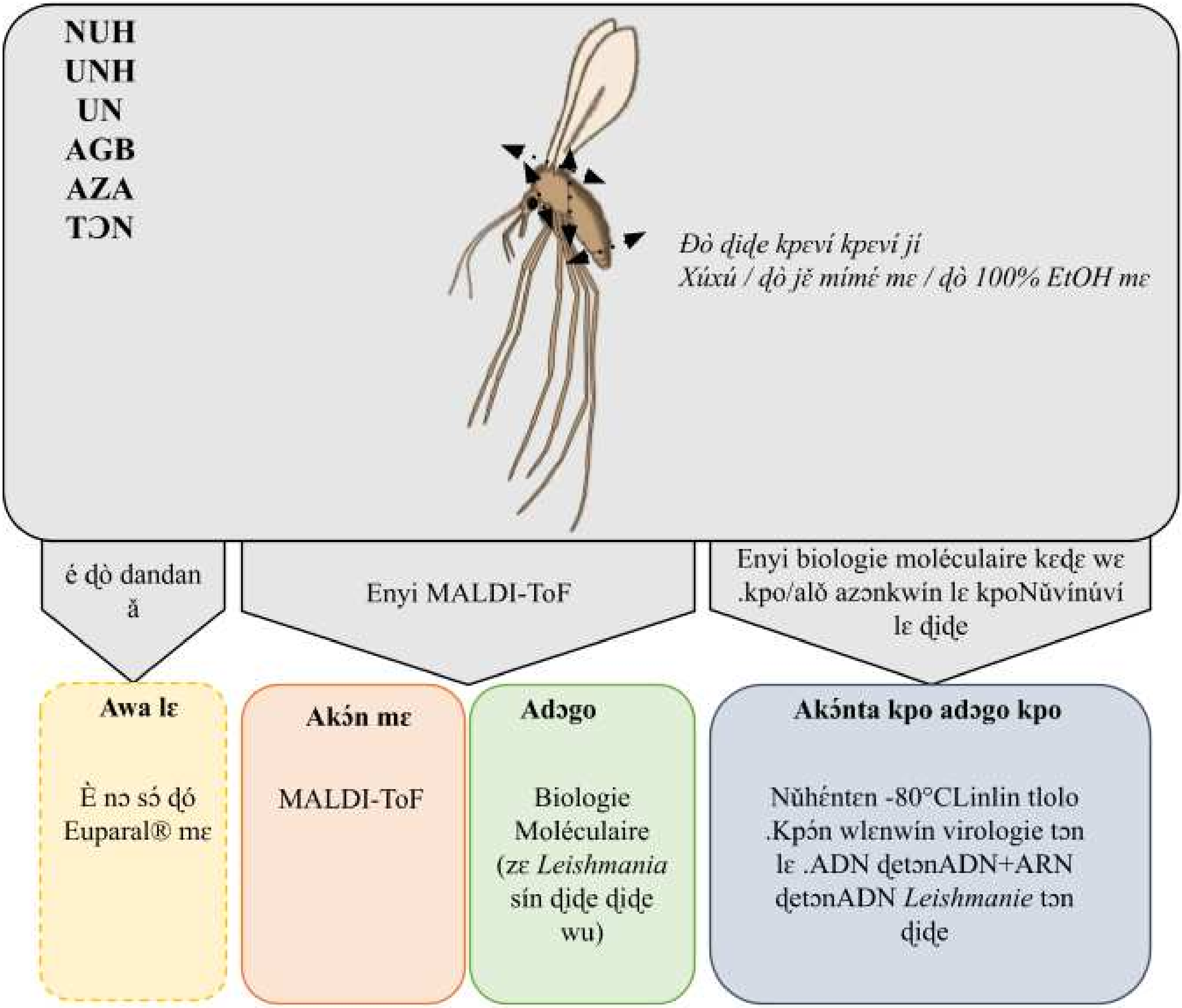

Ðiɖe 6gɔ́ ɔ́. Azɔwanu flebotomu tɔn nú biologie moléculaire, proteomique, kpodo/alǒ virusologie sín azɔ̌ lɛ́ɛ kpo.

5. Azɔ̌wiwa kpɔ́ndewú lɛ́ɛ ji nú nǔkplɔ́nmɛ dó agbaza ssín nǔ lɛ́ɛ (ɖiɖe 3, 6, 7 & 8; Akpáxwé 1, 2, 3 & 4)

Akpáxwé elɔ tinmɛ nǔgbododó e e è na zán dó sɔnǔ nú kpɔ́ndéwú flebotomu ɖé tɔn bo na sɔ́ ɖó nǔ jǐ nú nǔkplɔ́nkplɔ́n dó lee nǔ lɛ́ɛ ɖè gbɔn é kɛɖɛ wu é, bɔ enɛ gudo ɔ́, è na bló bɔ é na sɔgbe xá lee nǔ lɛ́ɛ cí é wu é. Amɔ̌, nukúnnúmɔjenǔmɛ dó wlɛnwín enɛ wu ɖò taji tawun, ɖó é nɔ zɔ́n bɔ è nɔ ɖyɔ tuto lɛ́ɛ nú kpɔ́ndéwú alɔkpa tawun tawun lɛ́ɛ hwenu e é byɔ ɖó é.

Nǔ e è nɔ wà dó gbɔ azɔn nú mɛ é wɛ nyí ɖɔ è ni ɖè nǔ e ɖò mɛ wu lɛ́ɛ é sîn bo lɛ́ gɔ́ ye mɛ, bo nɔ zán pipettes Pasteur e è sɔ́ kɔ́lu e nɔ hɛn nǔ cí mɔ̌ é dó bló na lɛ́ɛ é. È nɔ kplɔ́n mɛ tawun ɖɔ è ni zán gannu e mɛ è sɔ́ kɔ́fu dó bló bɔ dò tɔn ɖò xò é, ɖó ye nɔ zɔ́n bɔ azɔ̌ enɛ lɛ́ɛ nɔ bɔkun tawun. Go nɔ ɖí xwi xá nǔ e nɔ hɛn nǔ gblé dó mɛ wu lɛ́ɛ é bǐ ǎ. Bo na dó bló bɔ nǔ e è nɔ zán dó gbéjé nǔ kpɔ́n na lɛ́ɛ é ma wá xú ó ɔ́, è ɖó na sɔ́ nusú dó gannu lɛ́ɛ jí, bo ma na gɔ́ ye zɛ xwé wu gbeɖé ó, bɔ enyi è sú ye e alǒ hun ye ɔ, ye na gɔ́ngɔ́n, bo na lɛ́ bló bɔ kɔ́ ma na jɛ nǔ e è kpɔ́n lɛ́ɛ é jí ó. Nǔ e è nɔ zán dó ɖè nǔ lɛ́ɛ sîn bo nɔ lɛ́ wà nǔ dó ye wu lɛ́ɛ é ɖò tablo 2 mɛ.

This is an Open Access article distributed under the terms of the Creative Commons Attribution License (https://creativecommons.org/licenses/by/4.0), which permits unrestricted use, distribution, and reproduction in any medium, provided the original work is properly cited.

Tablo 2. Nǔ e è nɔ zán dó bló nǔ lɛ́ɛ é sín kplékplé.

**idrɔksidi potasiyɔmu tɔn 10%**	**Fuchsin acide 1% ɖò sin e è hu acɛ na é**
Hidrɔksidi potasiyɔmu tɔn 10 g	Asidu fuchsine (ɖò linfín mɛ) 1 g
Sin ɖiɖa qs 100 mL	Sin e è hɛn xúxú é 99 mL
**nǔsúnsúnnú chloral kpó e (Hoyer médium)**	**Marc-André sín sìn e è sɔ́ fuchsin acide dó bló na é**
Sin e è hu acɛ na é 50 mL	Marc-André sín sìn 10mL
Klolu sín sìn 200 g	Fuchsin 1% 50 µL
Nǔsúnsúnnú arabe 50 g tɔn	
Glicérol 20 mL	
**Marc-André sín xósin**	**Enecê tɛntin**
Klolu sin 40 g	Kolofoni wewé akú 22 g
Acide acétique glaciaire 30 mL	Gomme copal soluble dans l'alcool 12 g
Sin e è hu acɛ na é 30 mL	Éthanol absolu 20 mL
	Kanfɔ́ 10 g
	Tɛrpentin sin 10 mL
	Eukaliptɔlu 26 mL

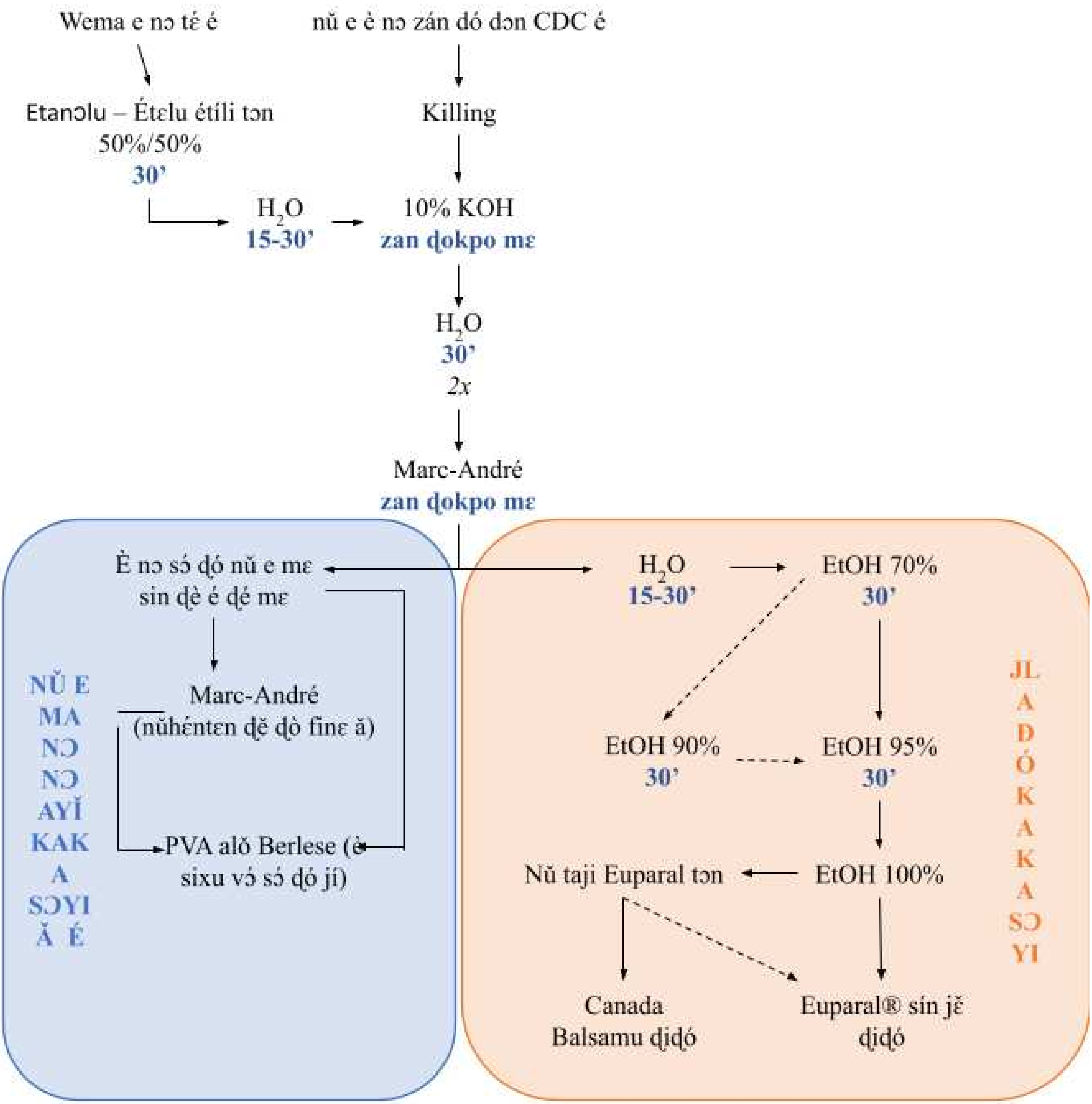

Ɖiɖe 7gɔ́ ɔ́ : Wlɛnwín hwexónu tɔn e è nɔ zán dó wà nŭ xá flebotomu lɛ́ɛ é.

5.1. Fúɖenú nŭ

Cobonu è na sɔ́nŭ nú kpɔ́ndéwú flebotomu lɛ́ɛ tɔn lɛ́ɛ dó sɔ́ ye dó nŭ ji jí kaka sɔyi ɔ́, è ɖó na ɖè ye ɖó mimě jí gbɔn wlɛnwín e sɔgbe é ɖé kpódó nŭ e nɔ slá wŭ nú ye é kpó gblamɛ (é wɛ nyí ɖo̩, acide acétique 10% sín sìn alŏ Marc-André sín sìn e mɛ chloral hydrate ɖè é, ee nyí plodwi e è ɖó dogbó na ɖò tò gègě mɛ é ɖé) bo na dó bló bɔ ye na kɔ́n. Wlɛnwí enɛ nɔ́ ɖè nŭ e ɖò agbaza mɛ lɛ́ɛ é, jŏ, nŭ e nɔ tɔ́n sín agbaza mɛ lɛ́ɛ é kpó jĕ kpó, bo nɔ zɔ́n bɔ nŭ e è kpɔ́n é nɔ kɔ́n, bo nɔ zɔ́n bɔ è nɔ gbéjé wujɔnú lɛ́ɛ kpɔ́n (kpɔ́ndéwú ɔ́, è nɔ sɔ́ fún dó mɛ), nŭ e ɖò wexo lɛ́ɛ é (kpɔ́ndéwú ɔ́, sinmɛ) kpódó nŭ e ɖò xomɛ lɛ́ɛ é kpó bɔ è nɔ mɔ gbɔn xú ɔ́ jí (kpɔ́ndéwú ɔ́, spermathecae).

Wlɛnwí ɔ́ ɖo we ji, nukɔtɔn ɔ́ nɔ zan “hydroxyde de potassium”, bo na lɛ́ zán acide e ma syɛn sɔmɔ̆ ă é ɖé (ɖi acide acétique ɖò Marc-André sín sìn mɛ), bo na dó wà nŭ

This is an Open Access article distributed under the terms of the Creative Commons Attribution License (https://creativecommons.org/licenses/by/4.0), which permits unrestricted use, distribution, and reproduction in any medium, provided the original work is properly cited.

vovo lɛ́ɛ ɖò nǔ e è nɔ ylɔ ɖɔ biochimie é mɛ [74]. Nǔ e ɖò dò é nɔ gbà agbaza e féɖé lɛ́ɛ é, ɖi proteine lɛ́ɛ, ami lɛ́ɛ kpó xú lɛ́ɛ kpó gbɔn aɖǐ ɖiɖó kpódó proteine lɛ́ɛ sín ɖiɖe kpó gblamɛ, bo nɔ jó xú e ɖò kɔ́xota é dó bɔ é nɔ ɖò wɛn. Asidu e ma ɖó hlɔnhlɔn somɔ̌ ǎ zinzin bɔ d'ewu nɔ hu acɛ nú nǔ e ɖɔ dol ɛ́ɛ bǐ, bo nɔ́ glɔ́n ali nú nǔ e ko ɖɔ ayǐ lɛ́ɛ nǔ ye ni gblé d'eji, lobo nɔ lɛ́ bló bɔ chitine ɔ́ nɔ cí wěwé bo nɔ zɔ́n bɔ nǔ lɛ́ɛ nɔ kɔ́n [74], é ɖò mɔ̌ có, enyi è klɔ́ nǔ e è kpɔ́n lɛ́ɛ é azɔn wè ɖò sìn e è hɛn xúxú é mɛ nú cɛju 15 ɔ́, é sixu lɛ́ kpé bo na hɛn nǔ e ɖò dò é gblé. Nǔ enɛ e è nɔ wà ɖò tuto jí é nɔ xò nǔ e ɖò agbaza mɛ lɛ́ɛ é sísɔ́ sîn ganji, bo nɔ lɛ́ hɛn nǔ lɛ́ɛ ɖó te kpódó wubibɔ kpó, bo nɔ zɔ́n bɔ è nɔ hɛn nǔ e è kpɔ́n lɛ́ɛ é ɖó mimɛ̌ jí ganji bo na dó gbéjé ye kpɔ́n ɖò nǔ kpɛví kpɛví lɛ́ɛ mɛ. È ɖɔ ɖɔ è ni klɔ́ sin mimɛ nú cɛju 20 cobo yì akpáxwé e bɔ d'ewu é jí.

5.1.1. Hinhɛn lanti agbaza e fá lɛ́ɛ tɔn xú (Ɖiɖe 8gɔ́ ɔ)

Hydroxyde sodium (NaOH) alǒ potassium hydroxide (KOH) wɛ nyí nǔ e è nɔ zán dó bló dó xú nǔ lɛ́ɛ é, bɔ è nɔ zán ye ɖò hlɔnhlɔn vovo mɛ, bo nɔ lɛ́ zán nú hwenu vovo sɔgbe xá ga e nǔ e è kpɔ́n lɛ́ɛ é ɖó é kpódó lee ye syɛn sɔ́ é kpó. Wlɛnwín e è sɔ́ ɖ'ayǐ bo lɛ́ nyɔ́ hugǎn é wɛ nyí ɖɔ è ni hɛn lanti e fá lɛ́ɛ é xú gbɔn flenotomu lɛ́ɛ bibɛ dó sin syɛnsyɛn ɖé mɛ (10% KOH alǒ NaOH) ɖò zǎn ɖokpo gbla mɛ. È sixu ná hlɔ̌nhlɔ́n d'eji bo na dó ɖè hwenu e è na zán dó wa azɔ̀ dó ye ji e kpò (é wɛ nyí ɖɔ, KOH 20% nú ganxixo 6) bo na lɛ́ bló bɔ é na xɔ zo ɖò 37°C jí.

5.1.2. Nǔ hɛn dó mimɛ́ kpódó sinmɛ kpó alǒ nǔ ma ɖó sinmɛ kpó

Afɔɖiɖe enɛ gudo ɔ́, è nɔ bló nǔ lɛ́ɛ nɔ mɛ́, bɔ è nɔ xò acide acétique kpódó chloral hydrate kpó kplé (kpɔ́ndéwú ɔ́, Marc-André sín sìn). Enyi è klɔ́ nǔ lɛ́ɛ gudo ɔ́, è ɖó na klɔ́ ye ganji ɖò sin e mɛ, bɔ ɖokpó ɖokpó na nɔ xɔ cɛju 20, bo na dó ɖè nǔ e kpò lɛ́ɛ é sîn. Marc-André sín sìn ɔ́, nǔ e è nɔ zán dó ɖè nǔ lɛ́ɛ ɖó mimɛ̌ jí é ɖé wɛ bo nɔ sɔnǔ nú kpɔ́ndéwú flebotomu lɛ́ɛ tɔn. Nǔ e é nɔ wà é wɛ nyí ɖɔ é nɔ zɔ́n bɔ è nɔ ɖè nǔ lɛ́ɛ sîn, bo nɔ lɛ́ ɖè nǔ e nɔ hɛn nǔ gblé dó nǔ wu lɛ́ɛ é, ɖi awa lɛ́ɛ kpódó zovi lɛ́ɛ kpó wu é kpò. È ɖó na hɛn sìn ɔ́ dó gannu e è sú ganji é ɖé mɛ, bonu é ma wá xú alǒ gblé ó. É nɔ hɛn lè wá tawun hwenu e è nɔ zán wlɛnwín Marc-André tɔn lɛ́ɛ zinzan dó bló bɔ nǔ lɛ́ɛ nɔ kɔ́n alǒ nɔ kɔ́n nǔ lɛ́ɛ é, bo na dó bló bɔ nǔ tawun tawun e ɖò agbaza mɛ lɛ́ɛ é na nyɔ́ kpɔ́n. È tinmɛ nǔ e è bló é kpódó lee è sɔnǔ na gbɔn é kpó ɖò nǔzegónúnǔ 2gɔ́ ɔ́ mɛ. Nú nǔ e nɔ kɔ́n tawun lɛ́ɛ é ɔ́, é sixu byɔ ɖɔ è ni dó nǔ dó ye jí bonu è na dó sixu mɔ nǔ ganji cobo na sɔ́ ye ɖó nǔ jǐ. Nǔ e nɔ hɛn nǔ gblé dó mɛ wu lɛ́ɛ é gègě tîn, bɔ ɖokpó ɖokpó nɔ sɔ́ ayi ɖó nǔ e ɖò nǔɖogbɛ ɔ́ mɛ lɛ́ɛ é jí. É ɖò taji ɖɔ è ni cyan nǔ e sɔgbe xá nǔ e è kpɔ́n é kpódó nǔ e è sɔ́ bo na sɔ́ ɖó nǔ jǐ é kpó é. È sixu ɖyɔ wlɛnwín taji enɛ lee é byɔ gbɔn é, ɖi kpɔ́ndéwú ɔ́, è sixu sɔ́ fuchsin acide 0,1% dó Marc-André sín sìn mɛ bo na dó hɛn nǔ dó nǔ jǐ. Ɖevo ɔ́, nǔ e è hɛn ɖ'ayǐ ɖò sìn mɛ bo sɔ́ ɖó nǔ jǐ nú nǔ e è sɔ́ jɛ̌ dó bló na lɛ́ɛ é nɔ byɔ ɖɔ è ni ɖè sìn sín ye mɛ (kpɔ́n Akpáxwé 5.2gɔ́ ɔ́ dó Nǔ E È Nɔ Sɔ́ Dó Sɔ́ D'ayǐ É Wu), ɖó nǔ e è nɔ sɔ́ jɛ̌ dó bló na lɛ́ɛ é gègě nɔ sɔgbe xá sìn ǎ. New (1974) ɖɔ ɖɔ nǔ kwijikwiji ɖé lɛ́ɛ sixu gblé ɖò nǔ e è nɔ sɔ́ ɖó nǔ jǐ lɛ́ɛ é ɖé lɛ́ɛ mɛ [53]. Ɖi kpɔ́ndéwú ɔ́, è sixu lɛ́ hɛn fuchsin acide e è nɔ zán hwɛhwɛ xá balsamu Canada tɔn é ɖó Euparal® mɛ. Amɔ̌, nǔ e è sɔ́ acide fuchsin dó bló na lɛ́ɛ é nɔ yawu xú, ɖò taji ɔ́, hwenu e ami e è nɔ zán dó ɖè nǔ lɛ́ɛ ɖó mimɛ̌ jí gudogudo tɔn ɔ́ é sín nǔ e kpò lɛ́ɛ é kpò é. Nǔ e è hɛn ɖó kɔ́fu sín amì mɛ lɛ́ɛ é sixu xlɛ́ ɖɔ emi ɖò ɖiɖekpo wɛ tawun ɖò azǎn yɔywɛ ɖé gudo.

5.2. Sinxwíxwí sín lanmɛ

È nɔ bló bɔ è nɔ ɖè sìn sín agbaza mɛ gbɔn nǔ e è sɔ́ ɖ'ayǐ lɛ́ɛ é ɖiɖe ɖ'ayǐ kpɛɖé kpɛɖé gbɔn jɛ̌ e è sɔ́ éthanol dó bló é ɖé gblamɛ : 50%, 70%, 80%, 90%, alǒ 95% bɔ gudo mɛ ɔ́, 100%, bɔ wǔsláslá ɖokpó ɖokpó nɔ ɖu cɛju 20 mɔ̌. Ðó éthanol nɔ yawu xú wutu ɔ́, è ɖó na sú gannu ɔ́ ganji hwenu e è ɖò azɔ̌ wà wɛ é. Enyi sin ko xú ɖò nǔ e è kpɔ́n é mɛ bǐ mlɛ́mlɛ́ ɔ́, è sixu ɖó azɔ̌ ɔ́ te nú azǎn yɔywɛ ɖé ɖò Euparal® essence mɛ, ee nyɔ́ hú amì clove tɔn é. Beech creosote e è nɔ zán ɖò fí gègě ɖ'ayǐ nú nǔ enɛ é ɔ́, è ko gbɛ́ bǐ mlɛ́mlɛ́ dìn ɖó aɖǐ e é nɔ hɛn mɛ gblé é wu.

Nǔ e è nɔ wà bo nɔ ɖè sìn sín lanmɛ é ɖó na bló bɔ sìn e ɖò kpɔ́ndewu ɔ́ mɛ é na sɔgbe xá nǔ e è sɔ́ ɖó nǔ jǐ é, bo na dó bló bɔ è ma na mɔ nǔ jɛ nǔ mɛ ǎ, è ma na hɛn nǔ gblé dó mɛ wu ó, alǒ è ma na hɛn nǔ gblé dó kpɔ́ndewu ɔ́ wu ó.

5.3. Nǔhɛnnu nǔ e è nɔ sɔ́ ɖó nǔ jǐ lɛ́ɛ é tɔn

5.3.1. Nǔcyancyan kpódó Nǔwiwa kpó nú nǔsisɔ́ nú kpɔ́ndéwú lɛ́ɛ

Nǔ e è nɔ sɔ́ ɖó nǔ jǐ é ɖó na ɖó hlɔ̌nhlɔ́n e sɛkpɔ kɔ́fu tɔn lee é nyɔ́ bló gbɔn é, é wɛ nyí 1,5 mɔ̌. É ɖó na ɖó sinmɛ ɖě ǎ, bo na nɔ kɔ́n, bo na nɔ kɔ́n bǐ mlɛ́mlɛ́ hwenu e é xú gudo é kpódó hwenu e é na wá yì é kpó. É ɖó na sɔgbe xá nǔ e è zán lɛ́ɛ é, bo na lɛ́ kpéwú bo na byɔ nǔ e è kpɔ́n é sín xú lɛ́ɛ bǐ mɛ bo na lɛ́ gbakpé. É ɖó na yawu xú dín alǒ bló ahún ɖé ɖò nǔ jǐ yiyi hwenu ǎ. É ɖó ná xú hwenu e è sɔ́ ɖó nǔ jǐ é ǎ. Nǔ taji ɖé wɛ é nyí ɖɔ è ni cyan nǔ e è na sɔ́ ɖó nǔ jǐ é ɖé, ɖó nǔ ɖokpó géé kún nyɔ́ nú nǔ lɛ́ɛ bǐ ó. Nǔ taji gègě wɛ è ɖó na sɔ́ ɖó jlɛ̌ jí :

- Ninɔmɛ wekpén tɔn lɛ́ɛ. É ɖó na hɛn nǔ taji e ɖò agbaza mɛ lɛ́ɛ é dó ayi mɛ, ɖi spermathecae, ascoïdes, Newstead sensilla, aɖu cibarial vertical, kpódó aɖu pharyngéal kpó. Nǔ e è nɔ mɔ ɖò nǔ enɛ lɛ́ɛ mɛ é nɔ sín nǔ e è nɔ sɔ́ ɖó nǔ jǐ é wu tlɔlɔ.

- Nǔ hɛn ɖ̯ayǐ. Nú è na ɖɔ xó dó kpɔ́ndéwú alɔkpa lɛ́ɛ tɔn alǒ nǔ e è sɔ́ ɖó te nú nǔ e è na xò kplé kaka sɔyi lɛ́ɛ é jí ɔ́, nǔ e è na zán é ɖó na nɔ na hlɔ̌nhlɔ́n e na nɔ ayǐ nú hwenu gegě é kpódó ee na nɔ ayǐ kaka sɔyi é kpó. É ɖò mɔ̌ có, nú nǔkplɔnmɛ nǔ lɛ́ɛ tɔn alǒ nǔkanbyɔ azɔn lɛ́ɛ tɔn, fí e è ma nɔ hɛn nǔ lɛ́ɛ ɖó te nú hwenu gegě ɖè ǎ é ɔ́, nǔ e è nɔ sɔ́ ɖó nǔ jǐ nú hwenu klewun ɖé alǒ nǔ e nɔ nɔ ayǐ nú hwenu klewun ɖé lɛ́ɛ é sixu kpé.

5.3.2. Dandanmɛnǔ e è byɔ ɖò nǔ e è nɔ sɔ́ ɖó nǔ jǐ lɛ́ɛ é sin nǔhɛnnu sí é

Hwɛhwɛ ɔ́, nǔnywɛtɔ́ lɛ́ɛ nɔ bló wlɛnwín e è sɔ́ ɖó mɛɖesunɔ jí bo lɛ́ vɛwǔ é, bɔ ye nɔ sɔgbe xá hudo dobanúnǔ tɔn tawun tawun ɔ́. Amɔ̌, wlɛnwín enɛ lɛ́ɛ nɔ wɔn nǔ elɔ lɛ́ɛ hwɛhwɛ : lee nǔ e è hɛn ɖ̯ayǐ lɛ́ɛ é nyɔ́ sɔ́ é, lee ye na sɔgbe gbɔn é, alǒ lee ye na bɔwu bɔ è na zán ye gbɔn é kpódó lee ye na hɛn nǔ lɛ́ɛ ɖó te nú hwenu gegě gbɔn é kpó. Nǔ enɛ e è ma nɔ bló ɖó jlɛ̌ jí ǎ é nɔ zɔ́n bɔ nǔ e è xò kplé bɔ è na lɛ́ɛ é kpódó gǎn e è dó bo na kpé nukún dó ye wu nú hwenu gegě é kpó nɔ vɛwǔ.

Nǔnywɛ xwitixwiti sín nǔ lɛ́ɛ nɔ byɔ nǔ vovo lɛ́ɛ ɖò nǔ e è nɔ sɔ́ ɖó nǔ jǐ lɛ́ɛ é sí. Hwɛhwɛ ɔ, mɛ e nɔ gbéjé nǔ lɛ́ɛ kpɔ́n lɛ́ɛ é nɔ sɔ́ nǔ e ɖò agbaza mɛ lɛ́ɛ é bǐ ɖó nǔ jǐ bo nɔ yí wǎn nú nǔ e nɔ xò nǔ e ɖò agbaza mɛ lɛ́ɛ é kplé bléblé lɛ́ɛ é, bo na dó sixu mɔ nǔ e ɖò agbaza mɛ lɛ́ɛ é ganji. Nǔ e nɔ hɛn nǔ cí é ɖó na gbɔn vo nú nǔ e è kpɔ́n é kpódó nǔ e è sɔ́ kɔ́fu dó bló é kpó, bo na dó sixu mɔ nǔ jɛ nǔ mɛ ganji. Hwɛhwɛ ɔ́, è nɔ bló nǔ e è nɔ sɔ́ ɖó ajɔ̌ jí lɛ́ɛ é kpódó nǔ e nɔ hɛn nǔ cí kɔ́fu ɖɔhun é ɖé kpó, bo na dó ɖè weziza e nɔ hɛn nǔ gblé dó mɛ wu é kpódó nǔ e nɔ gbakpé nǔ e è nɔ sɔ́ ɖó nǔ jǐ é kpó jí é kpò. Amɔ̌, ɖò nǔ kpɛví kpɛví e nɔ kpɔ́n nǔ lɛ́ɛ é mɛ ɔ́, è sixu ɖyɔ vogbingbɔn jɔwamɔ tɔn e ɖò nǔ e è ma ɖó nǔɖe na ǎ é mɛ é gbɔn nǔ e è na sɔ́ ɖó nǔ jǐ é ɖé xixo kpo jlǒ kpó gblamɛ, bɔ nǔ e è sɔ́ ɖó nǔ jǐ é na gbɔn vo kpɛɖé nú nǔ e è kpɔ́n é, bɔ enɛ na zɔ́n bɔ è na mɔ nǔ ganji ɖò nǔ e ɖò gudo é mɛ.

5.3.3. Nǔhɛnnu alɔkpa alɔkpa e è nɔ sɔ́ nǔ ɖó nǔ jǐ na lɛ́ɛ é (Tablo 3 & 4)

Nǔxwitixwi-kpinkpɔ́n nɔ byɔ ɖɔ è ni zán nǔ e è sɔ́ ɖó nǔ jǐ é ɖé sín ɖiɖe (RI) cobo na tunwùn lee weziza nɔ kɔ́n gbɔn ɖiɖe ɔ́, nǔ e è sɔ́ ɖó nǔ jǐ é kpó nǔ e è kpɔ́n é kpó jí gbɔn é. Enyi RI ɔ sɛkpɔ kɔ́fu e nɔ cyɔn nǔ dó nǔ jí é (≈1,515) ɔ́, weziza nɔ gbɔn mɔ̌ ɖokpó ɔ́, bo nɔ ɖè nǔ e nɔ gbakpé lɛ́ɛ é kpódó nǔ e nɔ hɛn nǔ gblé dó mɛ wu lɛ́ɛ é kpó kpò, bɔ enɛ nɔ zɔ́n bɔ è nɔ mɔ nǔ ɖagbe lɛ́ɛ ganji, bo nɔ lɛ́ mɔ ye ganji. Ɖò alɔ ɖevo mɛ ɔ́, RI e ma sɔgbe ǎ é sixu zɔ́n bɔ nǔ e ma ɖò wěxo ǎ lɛ́ɛ é na cí nǔ e ma ɖò wɛn ǎ é ɖɔhun, bɔ ye na cí nǔ e ɖo xomɛ ɖɔhun. É ɖò taji ɖɔ è ni cyan nǔ e è na sɔ́ ɖó nǔ jǐ é ɖagbe ɔ́, bo na dó bló bɔ nǔ lɛ́ɛ na gbɔn vo, bɔ nǔ lɛ́ɛ na zawě, lobo na lɛ́ nyɔ́ kpɔ́n ɖò ɖiɖe ɖé mɛ, ɖó RI vovo e ɖò nǔ vovo lɛ́ɛ mɛ é wutu.

É ɖó na hɛn nǔ e è nɔ nyi yɛ é dó ayi mɛ, bo na dó sixu mɔ nǔ ɖagbeɖagbe lɛ́ɛ ganji hwenu e è ɖò flebotomu lɛ́ɛ sɔnǔ na wɛ bo na sɔ́ ɖó nǔ jǐ é. É sixu vɛwǔ bɔ è na mɔ nǔ e ɖò tó lɛ́ɛ é, ɖi cibarial armature, spermathecae, zovi sín akpáxwé lɛ́ɛ, kpódó kan e nɔ hɛn awa lɛ́ɛ ɖó te lɛ́ɛ é kpó ɖò nǔ e è nɔ sɔ́ ɖó kɔ́mɛ bo nɔ hɛn nǔ cí mɔ̌ é ɖé mɛ.

Nú flebotomu lɛ́ɛ ɔ́, nǔ e è nɔ zán hugǎn lɛ́ɛ é wɛ nyí nǔ e è nɔ sɔ́ flebotomu dó bló na é, bo nɔ lɛ́ sɔ́ balsamu Canada tɔn kpódó Enecê - Nelson Cerqueira (NC) sín résine kpó dó bló nǔ e nɔ hɛn nǔ xú é ɖé. Rawlins [60] má nǔ e è nɔ sɔ́ ɖó nǔ jǐ lɛ́ɛ é ɖó wè : (1) nǔ e nɔ nɔ ayǐ kaka sɔyi lɛ́ɛ é : nǔ enɛ lɛ́ɛ nɔ syɛn ɖò hwenu e ɖò yiyi wɛ é, bo nɔ nyɔ́ zán bɔ è na hɛn nú hwenu gegě, kpó (2) nǔ e nɔ nɔ ayǐ kaka sɔyi akpáxwé ɖé lɛ́ɛ é kpó : nǔ enɛ lɛ́ɛ nɔ syɛn ǎ, bɔ è nɔ zán ye nú hwenu klewun ɖé.

Tablo 3gɔ́ ɔ́. Nǔ e è sɔ́ ɖó nǔ jǐ lɛ́ɛ é sín kplékplé.

Nǔ e è nɔ sɔ́ ɖó nǔ jǐ é sin tɛ	Sin	Polymères (alǒ polymères) e sixu tîn lɛ́ɛ é	Nǔ e è ɖɔ lɛ́ɛ é
Hoyer = gɔmu kloralu	glycérol, sin	nǔ e è nɔ ylɔ ɖɔ gɔmu arabe é	Nu e nɔ hɛn nu xú ɔ́: kloral hydrate
CMCP-9 (= carboxyméthylcellulose phénol)	sin (CMCP-9: 51-60%)	ahan syɛnsyɛn e è sɔ́ sìn dó bló na é (CMCP-9: 0–5%)	CMC(P)-9: hlɔnhlɔn kpɛɖe: hlɔnhlɔn ɖaxo
DMHF (diméthyl hydantoïne formaldéhyde)	sin	diméthylol diméthyl hydantoïne (diméthylol DMH) Oligomères éther-/méthylène-pont DMH-formaldehyde sín hlɔnhlɔn e è sɔ́ ɖó kpɔ́ é	

Balsam Canada tɔn	xylène; nǔ e ɖò balsamu mɛ bo nɔ hɛn nǔ gblé dó mɛ wu lɛ́ɛ é sín akpáxwé ɖé lɛ́ɛ (Δ3-carène, acide levopimarique, limonène, mircène, acide palustric, β-félandréne, α- pinène, β-pinène)	balsamu (abiɛnɔlu, asidi abiɛtiki, asidi isopimariki, asidi sandaracopimariki)	Nǔ e nɔ hɛn nǔ gblé dó mɛ wu é: potassium carbonate; jɛ̌ e è sɔ́ dó bló *Abies balsamea* (Linnée, 1758) tɔn na é
Euparal ®	eukaliptɔlu, paraldéhide; nǔ e nɔ hɛn nǔ gblé dó mɛ wu lɛ́ɛ é (limonɛn, α-pinɛn, β-pinɛn)	nǔ e è nɔ ylɔ́ ɖɔ gomme sandarac é (acidi communique, manool, acidi polycomunique, acidi sandaracopimarique, acidi 12-acétoxi-sandaracopimarique, sugiol, acidi torulosique, acidi torulosique, acidi torulosique)	nǔ e nɔ hɛn nǔ gblé dó mɛ wu é: salisila mɛtílu tɔn; sinmɛ e ɖò Euparal® amamú mɛ é: jɛ̌ ganvɔ tɔn (abietinate de cuivre); Sandarac sín atín e nɔ nyí *Tetraclinis articulata* (Vahl, 1791) é mɛ
Enɛcɛ	ahan syɛnsyɛn e nɔ nyí éthyl é; kpodo kanfɔ́ru, eukaliptɔlu kpódó terpentine kpó	Nǔ e è nɔ ylɔ́ ɖɔ copal gomme kpo colophonie kpo é (colophonie)	

Nǔ e è nɔ sɔ́ ɖó nǔ jǐ lɛ́ɛ é sixu nyí sin, nǔ e è sɔ́ jɛ̌ dó bló na é, nǔ e è sɔ́ jɛ̌ dó bló na bo e nɔ xú ɖò sìn e, ahan alǒ nǔ ɖevo e nɔ xú lɛ́ɛ é mɛ (kpɔ́ndéwú ɔ́, toluène, xylène) (Tablo 3). Enyi è sɔ́ nǔ dó ye jí gudo ɔ́, è ɖó na zán nǔ e nɔ xò nǔ kpɔ́n bo ma nɔ xú ǎ lɛ́ɛ é dó sú ye dó bɔ jɔhɔn ma na wà nǔ dó ye wu ó. Bo na dó tunwun vogbingbɔn e ɖò nǔ e è nɔ sɔ́ ɖó nǔ jǐ lɛ́ɛ é sín alɔkpa lɛ́ɛ tɛntin é céɖécéɖé ɔ́, è hɛn ɔ́ e ná zán nǔ elɔ lɛ́ɛ :

a. Fi e ɖe sin e lɛ́ɛ. Nǔ enɛ lɛ́ɛ nɔ yawu gba ɖò sìn mɛ, bɔ enɛ wu ɔ́, è sixu sɔ́ ye ɖó nǔ jǐ nú hwenu klewun ɖé alǒ nú hwenu klewun-zaan ɖé. Ye nɔ bɔwu bɔ è na hɛn, amɔ̌, é sixu byɔ ɖɔ è ni sú ye dó bonu jɔhɔn ma wá jɛ ye jí ó (é wɛ nyí ɖɔ, gum-chloral media kpódó ahan polyvinyl kpó), ɖò taji ɔ́, ɖò fí e jɔhɔn nɔ ja ɖè lɛ́ɛ é.

b. Nǔhɛnnu é e nɔ yi sín gbe kpɛɖe na lɛ́ɛ e. Sin nɔ wà nǔ dó nǔ enɛ lɛ́ɛ wu sɔmɔ̌ ǎ, amɔ̌, é kpó ɖò dandan ɖɔ è ni cyɔn alɔ ye jí bonu jɔhɔn ma nɔ gbo zɛ xwé wu ó. Ye nɔ zɔ́n bɔ è nɔ nɔte nú hwenu gegě hú ee è nɔ zán ɖò sìn mɛ lɛ́ɛ é, bɔ è nɔ lɛ́ zán ye hwɛhwɛ ɖò nǔ e nɔ nɔ ayǐ kaka sɔyi lɛ́ɛ é mɛ.

c. Nǔ e nɔ gba ɖò hydrocarbure mɛ lɛ́ɛ é. È nɔ hɛn nǔ enɛ lɛ́ɛ gba ɖò nǔ e nɔ hɛn nǔ gblé dó mɛ wu lɛ́ɛ é mɛ ɖi xylène alǒ toluène, alǒ essenecê (nǔ e nɔ hɛn nǔ gblé dó mɛ wu é). È bló ye bonu ye na nɔ tɛn yetɔn mɛ kaka sɔyi, bo nɔ lɛ́ nɔ ayǐ nú hwenu gegě, bo nɔ lɛ́ ɖí xwi xá jɔhɔn kpódó nǔ e nɔ hɛn nǔ gblé dó ye wu lɛ́ɛ é kpó, bɔ enɛ zɔ́n bɔ ye nyɔ́ tawun nú nǔ e è na hɛn ɖ'ayǐ lɛ́ɛ é (é wɛ nyí ɖɔ, balsamu Canada tɔn vɔ̌tɔ́)

Ðò klewun mɛ ɔ́, nǔ e nɔ gba ɖò sìn mɛ lɛ́ɛ é wɛ nyɔ́ hugǎn nú nǔ e è nɔ sɔ́ ɖó nǔ jǐ nú hwenu klewun ɖé lɛ́ɛ é alǒ nǔ e byɔ ɖɔ è ni ɖè kpɔ́ndewu lɛ́ɛ sîn bɔ é na bɔwǔ lɛ́ɛ é ; nǔ e nɔ dɛ ɖò sìn nu lɛ́ɛ é ɖó dogbó é nyɔ́ nú nǔ e nɔ nɔ ayǐ kaka sɔyi bo nɔ byɔ ɖɔ è ni nɔ ayǐ ɖò bǎ ɖé mɛ lɛ́ɛ é, bɔ è nɔ yí wǎn nú nǔ e nɔ hɛn hydrocarbure lɛ́ɛ é nú nǔ e nɔ nɔ ayǐ kaka sɔyi bɔ è sɔ́ ɖó te nú nǔ e è na hɛn ɖ'ayǐ lɛ́ɛ é kpódó nǔ e è na hɛn ɖ'ayǐ nú hwenu gegě lɛ́ɛ é kpó.

Tablo 4. Nǔ ɖagbe kpo nǔ nyanya kpo e ɖò nǔ e è sɔ́ ɖó nǔ jǐ lɛ́ɛ é mɛ é dó ɖiɖe kpɛví kpɛví lɛ́ɛ kpio nǔ e mɛ vovo lɛ́ɛ kpɔ́n bɔ è ma ko ɖetɔ́n ǎ lɛ́ɛ é kpó wu [52].

Nyikɔ́	Lè lɛ́ɛ	Nu nyanya lɛ́ɛ
* Balsamu Canada tɔn	Nǔ e è nɔ zán dó bló nǔ ɔ́ na é nɔ dɔ́ji tawun, bo nɔ nɔ gbɛ̀ nú xwè 150 jɛji.<br>È sixu zán ami e nɔ nyí atinkɛn gbaɖota é, alǒ phenol dó tɛ́ lamu lɛ́ɛ jí.	Nǔ e nɔ wà nǔ dó mɛ wu lɛ́ɛ é ɖò mɛ, bɔ è ɖó na hɛn ɖò kɔ́fu glɔ́.<br>É byɔ ɖɔ è ni bló tuto ɖé bo na dó ɖè sìn sín lanmɛ nú mɛ bǐ mlɛ́mlɛ́, bo na lɛ́ ɖu hwenu gègě.<br>Éthanol sín sìn ɖiɖekpo kpódó xylène alǒ atinkɛn gbaɖota sín ami kpó gblamɛ sixu zɔ́n bɔ taxa ɖé lɛ́ɛ na xú; nǔ ɖevo lɛ́ɛ (ɖi, isopropanol, n-butanol, CellosolveTM, 1,4-dioxane, Histoclear, terpineol) sixu ɖè finfɛ́n kpò.<br>Enyi è sɔ́ xylène dó ɖyɔ phenol na alǒ enyi hydroxyde potassium tɔn e kpò é kpò ɔ, nǔ e è kpɔ́n lɛ́ɛ é sixu huzu wiwi.<br>Nǔ e nɔ hɛn nǔ gblé dó mɛ wu lɛ́ɛ é sixu bló bɔ nǔ e è ma ɖó lamu ji ǎ lɛ́ɛ é na cí ablu mɛ.<br>Nǔ e è na xú bǐ mlɛ́mlɛ́ é sixu ɖu xwè mɔkpan enyi è ma xú ɖò kɔ́fu zozo mɛ ǎ ɔ́.<br>Nǔ e ɖò tɛntin é nɔ cí kɔ́lu ɖɔhun bo nɔ lɛ́ cí wiwi ɖò hwenu e ɖò yiyi wɛ é, ɖò taji ɔ, enyi è sɔ́ ami e nɔ nyí atinkɛn gbaɖota é dó súnsún na ɔ́ nɛ.<br>Nǔ e nɔ hɛn nǔ gblé dó mɛ wu lɛ́ɛ é ɖé lɛ́ɛ nɔ gló, bɔ nǔ e nɔ hɛn nǔ gblé dó mɛ wu lɛ́ɛ é sixu vɔ enyi nǔ e ɖò nǔ ɔ mɛ é huzu acide ɔ́ nɛ, bɔ enɛ sixu jɛ éɖée jí ɖò hwenu e ɖò yiyi wɛ é.
DMHF (dimɛtil hidantoin fɔrmaldehídi)	Nukúnnúmɔjɛnǔmɛ ɖaxó<br>Index de réfraction ɖagbe<br>Nǔ e è bló lɛ́ɛ é sín nukúnnúmɔjɛnǔmɛ ɖagbeɖagbe<br>Nǔsisɔ lɛ́ɛ sín syɛnsyɛn ɖagbe ɖé wɛ<br>É nɔ sɔgbe xá wlɛnwín nǔkún-yiya tɔn gègě.<br>Alɔcyɔnmɛji ɖagbe nú kpɔ́ndéwú lɛ́ɛ.<br>Nǔ e nɔ tɛ́ dó nǔ wu é kpo nǔ e nɔ cyɔn nǔ dó nǔ jí é kpo tɛntin é nyɔ́	É sixu wá huzu koklójó ɖò hwenu e ɖò yiyi wɛ é<br>É sixu ɖyɔ nǔ kwijikwiji ɖé lɛ́ɛ<br>É sɔgbe xá nǔ e nɔ hɛn formaldehyde wá lɛ́ɛ é ǎ<br>Jɔhɔn sín jɔhɔn, hwenu e é nɔ xú é nɔ syɛn děděɛ<br>Nǔ e è nɔ sɔ́ ɖó nǔ jǐ bo nɔ sè wuvɛ̌ xá jɔhɔn é<br>É vɛwǔ bɔ è na lɛkɔ nú jǐ yiyi .<br>Formaldehyde nɔ hɛn aɖi, nɔ dó xomɛsin mɛ, bo nɔ dɔn kansɛ́ɛzɔn wá
* Euparal (e nɔ kɔ́n)	Nǔ e nɔ hɛn nǔ gblé dó mɛ wu bo nɔ nɔ gbɛ̀ nú xwè 50 jɛji é.<br>É ɖò mɔ̌ có, è sixu sɔ́ éthanol 80% dó nǔ jǐ tlɔlɔ (wěɖexámɛ mɛ e bló é tɔn).<br>É nɔ cyɔn alɔ nǔ e jí è ma hɛn nǔ kwiji dó ǎ lɛ́ɛ é jí ǎ, bo nɔ lɛ́ cí kɔ́lu ɖɔhun alǒ nɔ xú ɖò hwenu e ɖò yiyi wɛ é ǎ.<br>É ɖó nǔ e nɔ hɛn nǔ cí é ɖé bɔ é sɔgbe hú balsamu Canada tɔn nú Diptera.<br>É nɔ w'azɔ̌ ganji nú kpɔ́ndéwú e gɔ́ngɔ́n hú mɔ̌ lɛ́ɛ é ɖó ɖiɖekpo kpɛví kpɛví kpódó xúxú e mɛ è ma nɔ mɔ jɔhɔn ɖè ǎ é kpó wutu.<br>É nɔ kpó ɖò 95% éthanol mɛ, bo nɔ zɔ́n bɔ è nɔ lɛ́vɔ sɔ́ ɖó nǔ jǐ ɖò xwè mɔkpan gudo.	Nǔ e nɔ wà nǔ dó mɛ wu lɛ́ɛ é ɖò mɛ, bɔ è ɖó na hɛn ɖò kɔ́fu glɔ́.<br>Ethanol sín sìn ɖiɖekpo kpodo ɖiɖe gbɔn Euparal Essence gblamɛ kpo sixu zɔ́n bɔ taxa ɖé lɛ́ɛ na xú, amɔ̌, isopropanol zinzan sixu ɖè xó enɛ kpò.
Hoyer sín sìn	È sixu sɔ́ nǔ e è kpɔ́n lɛ́ɛ é ɖó gbè alǒ tlɔlɔ sín sìn, éthanol, alǒ formaldehyde mɛ.<br>Maceration nɔ na cuticule ɖagbeɖagbe lɛ́ɛ.<br>É ɖó hlɔnhlɔn e nɔ hɛn nǔ cí é ɖagbe ɖé, bɔ è sixu lɛ́ bló bɔ é na kpɔ́n te d'eji kpo nǔ e	Atín sínsɛ́n e ma syɛn sɔmɔ̌ ǎ lɛ́ɛ é sixu gbà, afi nú è gɔ́ nǔ e nɔ hɛn nǔ gblé dó mɛ wu é kpɛɖé kpɛɖé, bɔ enɛ nɔ ɖu hwenu.<br>Xwè 10 mɔ̌ gudo ɔ́, dogbó lɛ́ɛ kpo kristalu lɛ́ɛ kpo sixu tɔ́n.

	è nɔ sɔ́ iode dó bló na é kpo bonu é na dó ɖè vogbingbɔn ɖaxó ɖé xlɛ́.<br>Acides acétiques e ɖò nŭ ɔ́ mɛ é sixu gbló ada nú xú e ɖò xú lɛ́ɛ mɛ lɛ́ɛ é.<br>Nŭ e è nɔ kpɔ́n lɛ́ɛ é ɖé lɛ́ɛ sixu nɔ tɛn yetɔn mɛ nú xwè 40–60.<br>É nɔ xú ɖò sìn mɛ, bɔ é nɔ bɔwŭ bɔ è na vɔ́ jlaɖó.	Maceration sixu huzu zɛ jlĕ wu sɔgbe xá hlɔnhlɔn e ɖò chloral hydrate mɛ é kpo hwenu e è na nɔ é kpo.<br>Nŭ e ɖò nŭ e mɛ è nɔ bló nŭ ɔ ɖè é mɛ lɛ́ɛ é sixu klán, bɔ nŭ kpɛví kpɛví lɛ́ɛ sixu tɔ́n ɖò sun alŏ xwè ɖé lɛ́ɛ vlamɛ.<br>È ko ɖɔ ɖɔ xójlajla sín nŭ lɛ́ɛ ɖò wiwi wɛ.
CMCP-9 (= karbɔksi mɛtílu sɛ́lulɔzu fɛ́nɔlu)	È sixu sɔ́ nŭ e è kpɔ́n lɛ́ɛ é tlɔlɔ sín nŭ ɖi sin, éthanol, glycérol, alŏ nŭ e mɛ formaldehyde ɖè lɛ́ɛ é jí, bo sixu lɛ́ bló bɔ nŭ e ɖò xomɛ yetɔn lɛ́ɛ é na xú hwenu e é byɔ ɖó é, bo na dó sixu gbéjé nŭ lɛ́ɛ kpɔ́n alŏ sɔnŭ nú ye.	Nŭ enɛ sixu bló bɔ kristalu lɛ́ɛ na tɔ́n bo na lɛ́ cí ablu mɛ ɖò hwenu e ɖò yiyi wɛ é, bɔ hweɖelɛnu ɔ, é sixu hɛn nŭ e è kpɔ́n lɛ́ɛ é xú hú lee è lin gbɔn é. Afi nú è bló alɔké dó lamu ɔ́ jí ganji ɔ, kpɔ́ndéwú e gɔ́ngɔ́n hú mɔ̆ lɛ́ɛ é na wà nŭ ganji ɖò mɛ ă, ɖó ye sixu xú bo na bló bɔ tɛnmɛ lɛ́ɛ na lɛlĕ dó lamu ɔ sín tó lɛ́ɛ. É sɔgbe xá nŭ e è sɔ́ kɔ́ dó bló na lɛ́ɛ é alŏ nŭ e è sɔ́ kɔ́ dó bló na lɛ́ɛ é ă, bɔ hwenu e é nɔ xú é nɔ lɛ́ hwe hú CMC tɔn.
EukitTM	Mɛdium e nɔ nɔ ayĭ nú xwè 30 jɛji é.<br>É nɔ sɔgbe xá nŭ e nɔ hɛn nŭ gblé dó mɛ wu lɛ́ɛ é gègĕ, ɖi acétone, benzène, kloroforme, dioxan, éther, isopropanol, benzoate méthylique, terpinéol, toluène kpo xylène kpo.<br>É nɔ yawu xú bo nɔ ɖó pH acide kpɛɖé.<br>É nɔ cí ablu mɛ bɔ è nɔ ɖó ayi wu hwenu e é ɖò kpikpo wɛ é ă.<br>É nyɔ́ nú nŭ tɛnmɛ tɛnmɛ (kpɔ́ndéwú ɔ́, fuchsin, hematoxyline, amamú mɛtil, violet mɛtíl, bleu mɛtil).<br>È sixu lɛ́vɔ sɔ́ nŭ e è kpɔ́n lɛ́ɛ é ɖó nŭ jĭ ɖò xwè mɔkpan gudo gbɔn xylène mɛ xixo dó nŭ jĭ nú hwenu gegĕ gblamɛ.	Nŭ e nɔ wà nŭ dó mɛ wu lɛ́ɛ é ɖò mɛ, bɔ è ɖó na hɛn ɖò lamu glɔ́.<br>É byɔ ɖɔ è ni bló tuto ɖé bo na dó ɖè sìn sín lanmɛ nú mɛ bĭ mlɛ́mlɛ́, bo na lɛ́ ɖu hwenu gègĕ.<br>É nyɔ́ nú kpɔ́ndéwú ɖaxó ɖaxó lɛ́ɛ ă ɖó ɖiɖekpo kpo gaz-bubble ɖiɖó kpo wutu.<br>Nŭ e nɔ cyɔn nŭ dó nŭ jí lɛ́ɛ é sixu tɔ́n ɖò hwenu e ɖò yiyi wɛ é, afi nú è klɔ́ lamu ɔ ganji bo sú.<br>É sixu xlɛ́ ɖɔ è kún ɖó polymérisation e ma kpé ă é lɛlĕ dó kolagen sín kàn lɛ́ɛ.
Enɛcê	Nŭ e nɔ dɔ́ji tawun é ɖé, bo nɔ nɔ ayĭ nú xwè 50 mɔ̆.<br>Enecê nɔ cí ablu mɛ ɖò hwenu e ɖò yiyi wɛ é ă.<br>É nɔ bɔkun hugăn, bo nɔ zɔ́n bɔ è nɔ fɛ́n nŭvínúví e ɖò tɛntin yetɔn lɛ́ɛ é, bo nɔ lɛ́ na hwenu e jɛxa é ɖé bɔ è na sɔ́ nŭ e ɖò agbaza mɛ lɛ́ɛ é ɖó tɛn yetɔn mɛ.<br>Akwɛ kpɛɖé.	É byɔ ɖɔ è ni bló tuto ɖé bo na dó ɖè sìn sín lanmɛ nú mɛ bĭ mlɛ́mlɛ́, bo na lɛ́ ɖu hwenu gègĕ.<br>Ethanol sín sìn ɖiɖekpo kpo ɖiɖe gbɔn amì clove tɔn gblamɛ kpo sixu zɔ́n bɔ kpɔ́ndéwú ɖé lɛ́ɛ na xú.<br>Nŭvínúví ɔ́ kpó ɖò ɖiɖexlɛ́ wɛ có, é ka ɖò ɖiɖexlɛ́ wɛ kpɛɖé kpɛɖé; enɛ sixu zɔ́n bɔ é vɛwŭ bɔ è na mɔ nŭ kpɛví kpɛví lɛ́ɛ, ɖi sensilla, ascoïdes kpo setae simple lɛ́ɛ kpo.

5.3.4 Tinmɛ e è byɔ́ nú nŭhɛnnú nŭ e sɔ́ ɖó nŭ jĭ lɛ́ɛ tɔn (Tableau 3 & 4)

<u>Nŭ e è nɔ zán dó kpɔ́n nŭ nú hwenu klewun ɖé lɛ́ɛ é</u>

Gomme chloral = Hoyer sín sìn/tɛnmɛ/linlin (RI = 1,48)

Marc André sín sìn wɛ nyí nŭ nyɔ́ hugăn bɔ è na zán dó kpɔ́n spermathecae lɛ́ɛ nú hwenu klewun ɖé (ganxixo klewun ɖé, vlafo ganxixo klewun ɖé lɛ́ɛ, enyi è hɛn ɖiɖe ɔ́ ɖó xɔ e mɛ jɔhɔn ɖè é ɖé mɛ ɔ́) ɔ́, è na lɛ́ kpɔ́n fɔtóo lɛ́ɛ (ɖiɖe 4gɔ́ ɔ́) alŏ ɖiɖe lɛ́ɛ. Bo na dó hɛn spermathecae e è mɔ lɛ́ɛ é ɖó te ɔ́, é byɔ ɖɔ è ni vɔ́ ye sɔ́ ɖó nŭ e mɛ sin ɖè é ɖé mɛ, bɔ enɛ na zɔ́n bɔ è na hɛn nú hwenu klewun ɖé. É ɖò mɔ̆ có, è nɔ kplɔ́n mɛ ɖɔ è ni ɖè sìn sín ye mɛ bo na dó vɔ́ ye blóɖó ɖò résine mɛ ă, amɔ̆, è nɔ kplɔ́n mɛ ɖɔ è ni hɛn nŭ gblé ă (risque de perte). È nɔ kpɔ́n jĕ kloral tɔn kpódó sìn Hoyer tɔn kpó dó mɔ nŭ ɖokpo ɔ́. È nɔ zán nŭ enɛ dó kpɔ́n nŭ e ɖò agbaza mɛ lɛ́ɛ é ɖó lee é nɔ sɔgbe xá sin gbɔn é, lee é bɔwu gbɔn é, lee é nɔ yawu w'azɔ̆ gbɔn é, kpódó lee é nɔ hɛn nŭ cí mɔ̆ é kpó wu, bɔ enɛ nɔ zɔ́n bɔ è nɔ gbéjé nŭ e ɖò hŭn mɛ lɛ́ɛ é kpɔ́n ganji, ɖi spermathecae. Amɔ̆, enyi è ma sɔnŭ nú jĕ chloral tɔn ganji alŏ hɛn ɖó fí e jɔhɔn nɔ gɔ́ é ă ɔ́, é ɖó nŭ nyanya ɖaxó ɖé lɛ́ɛ. Nŭ enɛ lɛ́ɛ ɖíe: kristalu, sinmɛ ɖyɔɖyɔ, kpo nŭ e nɔ hɛn nŭ cí é kpo. É ɖò mɔ̆ có, enyi è xò kɔ́fu ɔ́ sín gbè ɔ́, é nɔ ɖeɖɛ tagba enɛ lɛ́ɛ ă, ɖó nŭ e è nɔ sɔ́ ɖó kɔ́mɛ é sixu ɖyɔ sinmɛ

tawun (hweɖelɛnu ɔ́, é sixu ɖibla nyí wiwi) ɖó nǔ e è nɔ xò kɔ́fu ɔ na é wu, ɖò taji ɔ́, enyi è zán Euparal® ɔ́ nɛ. Hoyer medium wɛ è nɔ kpɔ́n dó mɔ nǔ nyɔ́ hugǎn e è nɔ zán ɖò nukúnmɛ é nú Flebotomu e nɔ nyí phlebotomine é, bɔ è ko nɔ zán nú nǔ enɛ lɛ́ɛ sín hwexónu. Nǔ e è nɔ zán dó bló nǔ e sɛkpɔ yeɖée tawun lɛ́ɛ é gègě wɛ ɖò nǔ e è nɔ zán dó bló nǔ ɔ́ na é mɛ, ɖi gɔmu arabique, glycérol kpódó chloral hydrate kpó. È ko tín xógbe vovo lɛ́ɛ mɛ nyì dò, bo lɛ́ sɔ́ xógbe e è zán lɛ́ɛ é sín xó lɛ́ɛ nyì dò [74]. Hoyer nyí nǔ ɖagbe ɖé bo na dó kpɔ́n spermathecae e ɖò flebotomu lɛ́ɛ mɛ é có, é ka sɔgbe bɔ è na hɛn ɖó te nú hwenu gegě ǎ. É nyɔ́ tawun nú nǔ e è nɔ kpɔ́n nú hwenu klewun ɖé lɛ́ɛ é, kaka jɛ fɔtóo lɛ́ɛ, nǔɖiɖó lɛ́ɛ, alǒ ɖiɖe lɛ́ɛ jí. Nǔ e mɛ sin ɖè lɛ́ɛ é nyɔ́ nú nǔ e è nɔ sɔ́ ɖó nǔ jǐ nú hwenu klewun ɖé lɛ́ɛ é, amɔ̌, ye sixu zɔ́n bɔ è na hɛn nǔ lɛ́ɛ ɖó te nú hwenu gegě ǎ. Ðò vogbingbɔn mɛ ɔ́, nǔ e è nɔ sɔ́ résine dó bló na é nɔ zɔ́n bɔ nǔ lɛ́ɛ nɔ nɔ ayǐ nú xwè kanweko mɔkpan hwɛhwɛ, amɔ̌, é sixu zɔ́n bɔ nǔ kpɛví kpɛví e ɖò spermathecae lɛ́ɛ mɛ lɛ́ɛ é na cí ablu mɛ, ɖó hwɛhwɛ ɔ́, è nɔ hɛn nǔ e ye nɔ kɔ́n lɛ́ɛ é bú. Hoyer medium nɔ gblé ɖò hwenu e ɖò yiyi wɛ é mɛ ɖó sìn e ɖò lanmɛ é wutu (ɖiɖe 8gɔ́ ɔ́), bɔ enɛ nɔ zɔ́n bɔ è nɔ ɖó kristalu chloral hydrate wewé kpɛví kpɛví e ma nɔ kɔ́n ǎ lɛ́ɛ é. É ɖò mɔ̌ có, è sixu mɔ kpɔ́ndéwú lɛ́ɛ ɖò ɖiɖe e è sɔ́ kristalu dó bló na lɛ́ɛ é mɛ, ɖó xú ɔ́ kpó ɖò nǔ e è nɔ ylɔ ɖɔ chimie é mɛ, enyi kristalu e ɖò susu wɛ lɛ́ɛ é na bo tlɛ hɛn nǔ gblé dó agbaza wu ɔ́ nɛ. Hweɖelɛnu ɔ́, è sixu vɔ́ ɖiɖe e è bló bɔ ye cí kristalu ɖɔhun lɛ́ɛ é blóɖó gbɔn nǔ e è sɔ́ ɖó nǔ jǐ é xixo dó fí e jǐ nɔ jayi ɖè é ɖé mɛ kpódó thymol kpó gblamɛ, bonu Flebotomu lɛ́ɛ ma wá sù ó. Alǒ, è sixu sɔ́ nǔ e è kpɔ́n lɛ́ɛ é dó sìn mɛ, bo sɔ́ acide acétique glacial dó bló bɔ ye na xú, lobo lɛ́vɔ sɔ́ balsamu Canada tɔn dó bló na.

DMHF (diméthyl) hydantoïne formaldéhyde) (RI 1,48)

Nǔ e è sɔ́ sin dó bló na é enɛ [72] nɔ w'azɔ̌ ganji ɖò nukúnmɛ, é cí Berlese ɖɔhun, bɔ é nɔ lɛ́ bwu bɔ è na zán Berlese ɖɔhun. Amɔ̌, é gbɔn vo nú Berlese, é nɔ huzu wiwi alǒ e nɔ huzu kristalu ǎ. E nɔ w'azɔ ganji nu flebotomu lɛ́ɛ kpódó Psychodidae ɖevo lɛ́ɛ kpo.

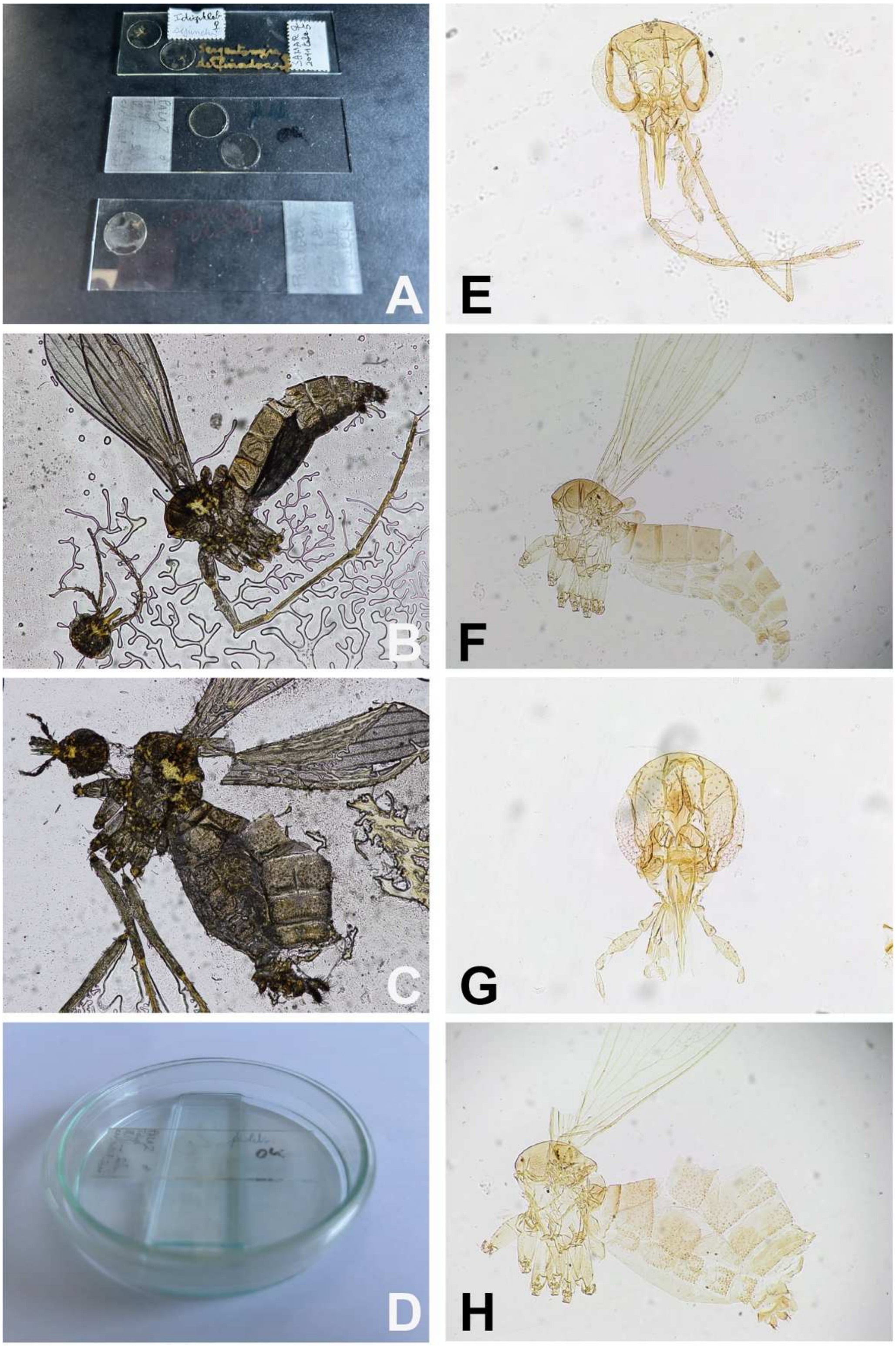

Ɖiɖe 8gɔ́ ɔ́ : È vɔ́ lamu lɛ́ɛ to. A : ɖiɖe e gblé bo xú bɔ è sɔ́ ɖó Hoyer jí lɛ́ɛ é ; B : è nɔ mɔ wujɔnú Flebotomu lɛ́ɛ tɔn ; C : ɖiɖe flebotomu ɖevo e gblé é tɔn ɖò nǔgbejekpɔ́n sin mɔ mɛ ; D : xɔ fifá e mɛ è nɔ bló ɖiɖe lamu ɖé ɖè é ; E : ta, kpó F : agbaza kpɔ́ndéwú B tɔn kpo ɖò hwenu e è vɔ́ ɛ sɔ́ ɖó Euparal® mɛ gudo é ; G : ta, kpó H : agbaza kpo nú kpɔ́ndéwú C e gblé é tɔn hwenu e è vɔ́ ɛ sɔ́ ɖó Euparal® mɛ gudo é.

This is an Open Access article distributed under the terms of the Creative Commons Attribution License (https://creativecommons.org/licenses/by/4.0), which permits unrestricted use, distribution, and reproduction in any medium, provided the original work is properly cited.

CMCP (camphre -klorofenɔlu ɖokpo) (RI = 1,41)

Nǔ enɛ nyí nǔ e è sɔ́ glycérine dó bló na, bo nɔ xú ɖò sìn mɛ é ɖé, bɔ è nɔ zán dó bló nǔ e è nɔ kpɔ́n ɖò kɔ̌ mɛ lɛ́ɛ é sín ɖiɖe e nɔ kɔ́n bo nɔ nɔ ayǐ kaka sɔyi lɛ́ɛ é, kaka jɛ flebotomu lɛ́ɛ jí. Nǔ e nyɔ́ ɖò nǔ e è nɔ sɔ́ ɖó nǔ jǐ é enɛ mɛ é wɛ nyí ɖɔ è sixu sɔ́ nǔ e è sɔ́ ɖó nǔ jǐ lɛ́ɛ é tlɔlɔ sín sìn alǒ éthanol mɛ. É nɔ yawu gbɔjɛ bo nɔ ɖè flebotomu ɔ́ sîn, bo nɔ bló bɔ flebotomu ɔ́ nɔ bɔwu bo nɔ zɔ́n bɔ è nɔ sɔ́ nǔ e è kpɔ́n é ɖó tɛn e jɛxa é mɛ, bɔ enɛ nɔ hɛn lè wá tawun ɖò awa lɛ́ɛ gbajagbaja alǒ xúxú lɛ́ɛ fénfén mɛ. È ɖɔ é nɔ zɔ́n bɔ è nɔ hɛn nǔ lɛ́ɛ ɖó te nú hwenu gegě có, hwenu nabi e è na hɛn ɖó te é ka ko ɖò wɛn ǎ. Dogbó taji e ɖò nǔ e è nɔ sɔ́ ɖó nǔ jǐ é enɛ wu é wɛ nyí ɖɔ nǔ e ɖ'emɛ lɛ́ɛ é ɖó fenol, nǔ e nɔ hɛn nǔ gblé dó mɛ wu bo nɔ lɛ́ dó xomɛsin nú mɛ é ɖé wɛ, bɔ é byɔ ɖɔ è ni kpé nukún dó wutu tɔn ganji.

<u>*Nǔ e è nɔ sɔ́ ɖó nǔ jǐ kaka sɔyi é*</u>

Balsamu Canada tɔn (RI = 1,52-1,54)

Andrew Pritchard wɛ ɖɔ xó dó balsamu Canada tɔn jí jɛ nukɔn ɖɔ é nyí nǔ e è na sɔ́ ɖó nǔ jǐ é ɖé bo na dó kpɔ́n nǔ kpɛví kpɛví e nɔ hɛn weziza wá lɛ́ɛ é ɖò xwè 1830 lɛ́ɛ mɛ. É kpó ɖò nǔjlajla sín nǔ e è nɔ zán hugǎn lɛ́ɛ é mɛ ɖó lee é nyɔ́ zán gbɔn é wu, bɔ è ko zán ɛ ganji nú xwè 150 jɛji. Balsamu Canada tɔn nɔ cí kristalu ɖɔhun alǒ nɔ yí jɔhɔn ǎ, é nɔ cí nǔ e è nɔ zán dó bló sìn na é ɖɔhun ǎ. É ɖò mɔ̌ có, balsamu Canada tɔn nɔ kɔ́n syɛnsyɛn, bɔ enɛ sixu nyí nǔ nyanya ɖé hweɖelenu nú wlɛnwín nǔ kpɛví kpɛví lɛ́ɛ kpɔ́n tɔn ɖé lɛ́ɛ [60]. Enyi è zán nǔ e ma nɔ hɛn nǔ gblé dó mɛ wu ǎ lɛ́ɛ é dó xylene tɛnmɛ ɔ́, é sixu ɖè awě e è sixu xò ɖò nǔsisɔ hwenu lɛ́ɛ é kpò, amɔ̌, é sixu lɛ́ dɔn nǔ nyanya lɛ́ɛ wá ɖi nǔ e nɔ xú kpɛɖé kpɛɖé é kpódó nǔ e mɛ nǔ ɔ́ nɔ yawu cí ablu mɛ ɖè é kpó.

Eupáli® (RI = 1,48)

Euparal® nyí nǔ e è nɔ zán tawun é ɖé bo nɔ ɖyɔ balsamu Canada tɔn bo nɔ sɔ́ ɖó nǔ jǐ kaka sɔyi, bo nɔ na hlɔ̌nhlɔ́n ɖagbe ɖé mɛ nú hwenu gegě, bo nɔ lɛ́ ɖó hlɔ̌nhlɔ́n e è sixu jlɛ́ dó nǔ wu é. Euparal® ɖó jijɔ elɔ lɛ́ɛ: (1) è ɖó na ɖè sìn sín lanmɛ: cobonu è na sɔ́ nǔ e è na sɔ́ ɖó nǔ jǐ é ɖó tɛn tɔn mɛ gudogudo tɔn ɔ́, è ɖó na ɖè sìn sín lanmɛ nú nǔ e è sɔ́ é, bɔ é nɔ nyí ɖɔ é nɔ gosin 95% jí bo nɔ wá huzu syɛ́n syɛ́n blebu, bo nɔ lɛ́ (2) hwenu e è nɔ zán dó wà nǔ na é ɖiga: kplékplé gudogudo tɔn ɔ ɖò résine ɖé mɛ, é na bo nyí Canada balsam®a, length Euparall hwenu e è nɔ w'azɔ̌ na é. Hwenu e è ma sixu bló bɔ è na ɖè sìn sín lanmɛ mɛ kpódó nǔ e nɔ hɛn nǔ gblé dó mɛ wu lɛ́ɛ é kpó ǎ é ɔ́, è sixu sɔ́ kpɔ́ndéwú e è ɖè sín éthanol absolue mɛ lɛ́ɛ é dó nǔ e nɔ hɛn nǔ gblé dó mɛ wu é ɖé mɛ, bɔ è nɔ sɔ́ Euparal ® kpódó Euparal essence kpó dó bló nǔ ɖokpo ɔ́ na, cobɔ è na sɔ́ ɖó nǔ jǐ gudogudo tɔn ɔ.

Enecê (RI = 1,467)

Enecê nyí nǔ e è nɔ sɔ́ résine dó bló bɔ è nɔ sɔ́ ɖó nǔ jǐ é ɖé, bɔ è nɔ zán jɛ nukɔn nú nǔvínúví kpɛví kpɛví lɛ́ɛ, bɔ è nɔ lɛ́ yí wǎn na tawun ɖò Brésilu. Nǔ e ɖò dò tɔn é wɛ nyí colophonie kpó gommu copal kpó e è hɛn xúxú ɖò ahan, kanfɔ́ru, terpentine sín jɛ̌ kpódó eucalyptol kpó mɛ é. Cerqueira [11] ɖɔ ɖɔ Enecê nyí nǔ ɖevo bo nɔ ɖyɔ balsamu Canada tɔn bo nɔ sɔ́ bɔkle e nɔ nɔ ayǐ kaka sɔyi lɛ́ɛ é, exuviae e ɖò bɔkle e ma ko sù ǎ lɛ́ɛ é mɛ é, kpódó bɔkle e ko sù lɛ́ɛ é kpó jí, bɔ sín hwenɛnu ɔ, è ko nɔ zán ɖò fí gègě bo nɔ sɔ́ xɛví flebotomu lɛ́ɛ tɔn ɖó nǔ jí. Enecê nɔ na wlɛnwín ɖevo e ma nɔ xɔ akwɛ sɔmɔ̌ ǎ é ɖé nú è na sɔ́ ɖó nǔ jǐ kaka sɔyi, bo nɔ zɔ́n bɔ è nɔ nɔte nú hwenu gegě, bo nɔ lɛ́ ɖó hwenu e kpé é bɔ è na xú, bo nɔ zɔ́n bɔ è nɔ fén nǔ lɛ́ɛ bo nɔ lɛ́ɛ tò nǔ e ɖò agbaza mɛ lɛ́ɛ é ganji.

5.4. Lamu lɛ́ɛ jijlaɖó kpódó xúxú kpó

É ɖò taji tawun ɖɔ è ni xúxú ɖiɖe e è sɔ́ ɖó nǔ jǐ lɛ́ɛ é ganji, bo na dó sixu nɔte hwenu e hɛn nǔ lɛ́ɛ ɖó mimɛ̌ jí. È ɖó na xú ɖiɖe lɛ́ɛ ganji cobo na lin tamɛ dó lee è na hɛn ye ɖó fí ɖé nú hwenu gegě gbɔn é jí. Nú è na mɔ lè ɖagbe hugǎn lɛ́ɛ ɔ ɔ́ è ɖó na xúxú ɖiɖe e è sɔ́ nǔ e nɔ hɛn nǔ ɖó te kaka sɔyi lɛ́ɛ é dó nǔ jǐ lɛ́ɛ é ɖò jǐxwé nú aklunɔzán gblamɛ 2–3, bɔ ɖěɖee è sɔnǔ na kpódó nǔ e nɔ hɛn nǔ ɖó te kaka sɔyi lɛ́ɛ é kpó é sixu byɔ aklunɔzán gblamɛ 1–2 kpowun. Bo na dó sixu bló bɔ nǔ lɛ́ɛ na xú ganji ɔ́, è byɔ ɖɔ è ni zán nǔ e è sɔ́ ɖó jɔhɔn e jɛxa nǔ e è na sɔ́ ɖó nǔ jǐ é jí é ɖé, bo na nyì alɔ nú zozo zɛ xwé wu e sixu hɛn nǔ e è kpɔ́n lɛ́ɛ é gblé é. È nɔ kplɔ́n mɛ ɖɔ è ni ɖó jɔhɔn 30°C kpo 37°C kpo. Afɔ enɛ e è nɔ ɖè bo nɔ xú é ɖò taji tawun bo na dó glɔ́n ali nú ɖiɖe lɛ́ɛ sín ɖiɖekpo, nǔ e è kpɔ́n lɛ́ɛ é sín ɖiɖekpo, alǒ nǔ e è sɔ́ ɖó nǔ jǐ lɛ́ɛ é sín ɖiɖekpo ɖò nǔhéntɛn hwenu.

È ɖó na wlan nǔ e è nɔ zán dó sɔnǔ nú ɖiɖe lɛ́ɛ é dó ɖiɖe lɛ́ɛ jí hwebǐnu. Enyi é nyɔ́ bló ɔ́, è ɖó na lɛ́ wlan nǔ e è zán dó ɖa nǔ ɔ́ na é dó wema ɔ́ jí, gɔ́ nú mɛ e ɖa nǔ ɔ́ é sín nyikɔ kpódó azǎn e gbè è ɖa é kpó. Ðò bǐbɛ̌mɛ ɔ́, è nɔ sɔnǔ nú ɖiɖe lɛ́ɛ bonu ye na nyí nǔ e è na sɔ́ ɖó nǔ jǐ nú hwenu klewun ɖé é, bɔ è ka sɔ́ ɖó te nú hwenu gegě ǎ. É ɖò mɔ̌ có, enyi ninɔmɛ kpɔ́dewu ɔ tɔn ɖyɔ, ɖi è sɔ́ ɛ ɖó "tinmɛ" sín tuto ɖé mɛ ɔ́, è ɖó na zán nǔ e nɔ hɛn kpɔ́dewu ɔ́ ɖó te kaka sɔyi é ɖé, bo na dó sixu hɛn kpɔ́dewu ɔ́ ɖó te nú nǔkplɔnkplɔn ɖé ɖò sɔgudo.

5.5. Wlɛnwín ɖevo lɛ́ɛ nǔsɔ́ ɖó nǔ jǐ tɔn : è nɔ sɔ́ dó kati jǐ

Kati ɖiɖó dó kpɔ́ nyí wlɛnwín ɖé bɔ è nɔ zán nú nǔvínúví gběta gègě, bɔ è sixu tɛ́ nǔvínúví lɛ́ɛ dó kati nǔvínúví lɛ́ɛ tɔn lɛ́ɛ jí tlɔlɔ alǒ tɛ́ ye dó nǔ jǐ tɔn. Ðó ye hwe tawun, bɔ è ɖó na kpɔ́n nǔ e ɖò agbaza mɛ lɛ́ɛ é bo na dó tunwùn ye gbɔn nǔ lɛ́ɛ ɖiɖexlɛ́ gblamɛ wutu ɔ́ (kpɔ́n akpáxwé 5gɔ́ ɔ́),

wlɛnwín enɛ sɔgbe ɖebǔ nú è na sɔ́ flebotomu lɛ́ɛ ɖó nǔ jǐ ǎ.

5.6. Nǔ e gblé lɛ́ɛ é vɔ́ to do nǔ jǐ

Nú nǔ e è ma nɔ mɔ ǎ lɛ́ɛ é alǒ nǔ e xɔ akwɛ lɛ́ɛ é ɔ́, è nɔ kplɔ́n mɛ ɖɔ è ni zán wlɛnwín wè sɔgbe xá yɛ e è sixu mɔ ɖò: https://zenodo.org/records/18315029 é. 1) vɔ́ sìn dó ye mɛ bo ma ɖè ye sîn ǎ, bo na dó sixu kpɔ́n ye jɛ nukɔn. È ɖó na sɔ́ nǔ e jí è na sɔ́ nǔ kpɛví kpɛví gègě dó é ɖé ɖó gannu Petri tɔn ɖé mɛ, bonu é na gɔ́ alɔ nú mɛ. Enɛ gudo ɔ́, è nɔ sɔ́ ɖiɖe e è na vɔ́ sìn na é ɖó ta, bo nɔ gɔ́ nǔ e nɔ hɛn nǔ gblé dó mɛ wu é milimɛtlu yɔywɛ ɖé dó gannu Petri tɔn mɛ, bo nɔ bló xɔ e mɛ jɔhɔn nɔ gɔ́ é ɖé, bo nɔ bló bɔ ɖiɖe ɔ́ ɖesu nɔ xò nǔ e nɔ hɛn nǔ gblé dó mɛ wu é zlɛ ǎ (ɖiɖe 8 D). Hwenu e è na zán dó vɔ́ sìn na mɛ é sixu gbɔn vo sín azǎn ɖokpo jí jɛ azǎn gègě jí, sɔgbe xá ninɔmɛ nǔ e è kpɔ́n é tɔn. Nǔ lɛ́ɛ kpinkpɔn ayihɔngbe ayihɔngbe kpódó suúluɖiɖo kpó ɖò taji. Enyi è ko gɔ́ sìn nú ɖiɖe ɔ́ ganji ɔ́, è sixu ɖè è sín xɔ e mɛ jɔhɔn ɖè é mɛ, bo sɔ́ dó nǔ e mɛ è nɔ hɛn nǔ dó é ɖé mɛ nú ganxixo klewun ɖé cobɔ è na gbéjé nǔ kpɛví kpɛví lɛ́ɛ kpɔ́n, ɖè fɔtóo, alǒ ɖè nǔ. 2) bo na dó vɔ́ jǐ ɔ́, è sixu lɛ́vɔ sɔ́ ɖiɖe ɔ́ ɖó xɔ e mɛ jɔhɔn ɖè é nú ganxixo klewun ɖé ɖevo alǒ zǎnmɛ. Nǔgbigba ɖó na nyí è nɔ wà ɖò nǔ kpɛví kpɛví e nɔ kpɔ́n nǔ lɛ́ɛ é glɔ́. È ɖó na zán lamu ɖagbeɖagbe lɛ́ɛ dó ɖè nǔ e nɔ cyɔn nǔ dó nǔ jí é sîn ganji, bo na hɛn nǔ e nɔ hɛn Flebotomu lɛ́ɛ é ɖě ɖó te ǎ (https://zenodo.org/records/18315029). Enɛ gudo ɔ́, è ɖó na xò nǔ e è fén ɖò kɔ́ mɛ lɛ́ɛ é kplé bo na klɔ́ ye kpódó sìn kpó ɖò dotɔ kpɛví kpɛví lɛ́ɛ mɛ, lee è nɔ zán dó ɖè ADN/ARN e nɔ hɛn nǔ gblé dó mɛ wu é tɔ́n gbɔn é ɖɔhun (kpɔ́n dò fí), cobɔ è na ɖè sìn sín ye mɛ bo na lɛ́ vɔ́ ye sɔ́ ɖó nǔ e mɛ è nɔ bló résine ɖè é mɛ. Hwenu e è ɖò ɖiɖe ɖé ɖè sín mɛ wɛ é ɔ́, é ɖò taji tawun ɖɔ è ni tunwun nǔ e è na sɔ́ ɖó nǔ jí jɛ nukɔn é, bo na dó sixu cyan nǔ e na hɛn nǔ gblé dó mɛ wu é ɖé. Nú è na sɔ́ nǔ e è nɔ sɔ́ ɖó sin mɛ lɛ́ɛ é dó bló nǔ na ɔ́, è ɖó na zán sìn. Enyi nǔ e è nɔ sɔ́ ɖó nǔ jǐ é nyí résine (kpɔ́ndéwú ɔ́, balsamu Canada tɔn alǒ Euparal®) ɔ́, è ɖó na zán xylène, ɖò azizɔgbá ɖé glɔ́, bo na lɛ́ zán nǔ e jɛxa lɛ́ɛ é dó cyɔn alɔ mɛɖée jí, kaka jɛ nǔ e nɔ cyɔn alɔ mɛ jí é jí.

É ɖò mɔ̌ có, mɛ e nɔ kpé nukún dó nǔ lɛ́ɛ wu é kpo/alǒ tutoblonunu e ɖó nǔ ɔ́ é kpo yí gbè nú nǔ e è xò kplé lɛ́ɛ é kɛɖɛ.

6. Kpɔ́ndéwu lɛ́ɛ tunwuntunwun

6.1. Mɔfolojíi (wujɔnǔ lɛ́ɛ)

Nǔ e nɔ zɔ́n bɔ è nɔ tunwun Flebotomu lɛ́ɛ é wɛ nyí ɖɔ è ni gbéjé lee ye cí é kpɔ́n jɛ nukɔn, kaka jɛ lee akɔ́n yetɔn cí é, awa yetɔn lɛ́ɛ, avadonú lɛ́ɛ, kpódó kancica e ɖo wujɔnú lɛ́ɛ tɛntin é kpó jí. Dobanúnǔtɔ́ lɛ́ɛ nɔ zán cávi nǔ lɛ́ɛ tunwuntunwun tɔn lɛ́ɛ, nǔ e è xò kplé lɛ́ɛ é, kpódó nǔ e è tinmɛ dó kanlin lɛ́ɛ wu dó jlɛ́ nǔ e è xò kplé lɛ́ɛ ɖe dó ɖe wu. Ninɔmɛ taji e nɔ zán dó tunwùn nǔ lɛ́ɛ é, ɖi awa lɛ́ɛ sín kan lɛ́ɛ kpódó lee ta ɔ́ cí é kpódó ɖò asú lɛ́ɛ kpódó asi lɛ́ɛ kpán mɛ, lee è bló avadonú asú lɛ́ɛ tɔn gbɔn é, kpódó lee è bló avadonú nyɔnu lɛ́ɛ tɔn gbɔn é kpó nɔ d'alɔ mɛ tawun bɔ è nɔ tunwùn kanlin alɔkpa lɛ́ɛ é. Hwɛhwɛ ɔ, è ɖó na gbéjé nǔ kpɛví kpɛví lɛ́ɛ kpɔ́n ganji cobo na tunwùn nǔ ɔ́ ganji, bɔ è nɔ zán nǔ kpɛví kpɛví e è xo klpé é dó kpɔ́n nǔ ɖagbeɖagbe lɛ́ɛ ɖi xú lɛ́ɛ kpódó spermathecae lɛ́ɛ kpó, alǒ è nɔ zán nǔgbeje kpɔ́n sín mɔ ɖé dó kpɔ́n nǔ e wíní lɛ́ɛ é.

Nukɔnyiyi e ɖò hladio sín nǔ lɛ́ɛ mɛ ɖò azǎn gudogudo tɔn elɔ lɛ́ɛ mɛ é zɔ́n bɔ è nɔ zán nǔ e è nɔ ylɔ ɖɔ dijital lɛ́ɛ é dó tunwùn flebotomu lɛ́ɛ. È sixu jlɛ́ ɖiɖe ɖaxó lɛ́ɛ é alǒ ɖiɖe e ɖò alokan jí bo xlɛ́ nǔ taji lɛ́ɛ é dó nǔ e è nɔ zán dó ba dò nú nǔ lɛ́ɛ é wu, alǒ bo gbéjé ye kpɔ́n gbɔn tuto e è nɔ zán mɔtaglomeɖo-asinanɔ dó tunwùn nǔ lɛ́ɛ na é gblamɛ, bɔ enɛ na zɔ́n bɔ è na mɔ nǔ jɛ nǔ mɛ ganji, bo na lɛ́ mɔ nǔ jɛ nǔ lɛ́ɛ wu ganji ɖò nǔ e è nɔ ylɔ ɖɔ morphonomie taxonomie é mɛ.

6.2. Awa lɛ́ɛ sín jlɛ̌

Awa lɛ́ɛ sín jlɛ̌jininɔ nyí nǔ taji ɖé bɔ è nɔ zán dó tunwùn Flebotomu alɔkpa vovo lɛ́ɛ é bo nɔ lɛ́ ɖè ye ɖó vo. Awa flebotomu lɛ́ɛ tɔn nɔ ɖè ɖiɖe kpódó tuto kpó ɖé xlɛ́, bɔ ɖò taji ɔ́, ye nɔ ɖiga bo nɔ lɛ́ hwe bo nɔ ɖó kan ganji (ɖiɖe 9gɔ́ ɔ́& 10gɔ́ ɔ́). Lee kan lɛ́ɛ ɖò tuto jí gbɔn é nɔ bló tuto vo ɖé bɔ é sixu gbɔn vo ɖò hɛ̌nnu lɛ́ɛ kpódó kanlin alɔkpa lɛ́ɛ kpo tɛntin, bo nɔ na nǔ xɔ akwɛ e è nɔ zán dó tunwun azɔn ɔ́na lɛ́ɛ é. Enɛ wu ɔ́, nǔkplɔ́nkplɔ́n dó awa lɛ́ɛ ji nɔ na nukúnnúmɔjenǔmɛ xɔ akwɛ ɖé lɛ́ɛ nú linlin e è na zán dó gbéjé nǔ lɛ́ɛ kpɔ́n é.

6.3. Awa lɛ́ɛ sín jlɛjininɔ ɖiɖe

Dobanúnǔtɔ́ lɛ́ɛ nɔ zán wlɛnwín vovo lɛ́ɛ, ɖi jlɛjininɔ sín nǔ lɛ́ɛ, bo nɔ gbéjé awa lɛ́ɛ sín ɖiɖe kpɔ́n lobo nɔ jlɛ́ ye dó Flebotomu lɛ́ɛ alǒ gběta vovo lɛ́ɛ wu. Nǔkplɔ́nkplɔ́n dó awa lɛ́ɛ sín jlɛ̌jininɔ nɔ na nukúnnúmɔjenǔmɛ xɔ akwɛ lɛ́ɛ dó walɔ, fí e ye nɔ nɔ é kpódó nǔwukpikpé e ye ɖó bo nɔ zɔn é kpó wu.

Ðò wlɛnwín jlɛjininɔ tɔn mɛ ɔ́, è nɔ fén awa lɛ́ɛ ganji, bo nɔ sɔ́ nǔ dó ye jí (enyi é byɔ mɔ̌ hǔn), lobo nɔ tɛ́ ye dó wɛn dó lamu jí. Enɛ gudo ɔ́, è nɔ ɖe fɔtóo lamu e sɔ́nǔ ná lɛ́ tɔn bó nɔ kpɔ́n ye ɖò stereomicroscope ɖé glɔ́, lobo nɔ gbéjé wujɔnú lɛ́ɛ kpɔ́n. È ko tinmɛ wlɛnwín enɛ ganji ɖò wema lɛ́ɛ mɛ [6, 27, 42, 56, 57, 59], bo lɛ́ kplɔ́n mɛ ɖɔ è ni nɔ zán awa ɖisíxwé tɔn alǒ amyɔxwé tɔn hwɛhwɛ nú agbaza sín wǔjɔnú wè lɛ́ɛ bo na dó nyì alɔ nú nǔ nyanya e sixu jɛ ɖò allométrie mɛ lɛ́ɛ é [62].

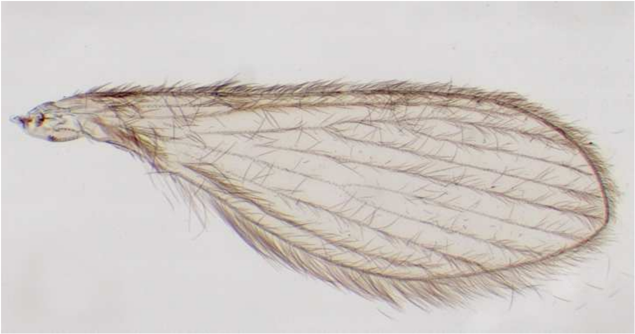

Ɖiɖe 9gɔ́ ɔ́ : *Trichophoromyia ininii* sín awa mu.

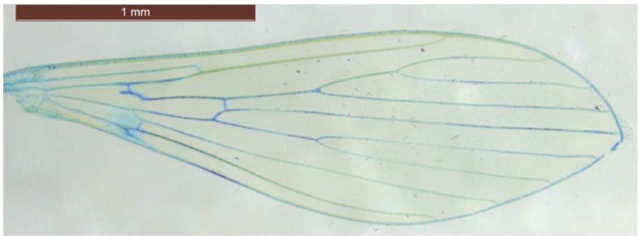

Ɖiɖe 10gɔ́ ɔ́ : *Phlebotomus ariasi* sín awa e ɖó sa sin do é.

Nǔsɔ nú Awa lɛ́ɛ nú jlɛjininɔ

Bo na dó sixu mɔ kan e ɖò awa lɛ́ɛ mɛ lɛ́ɛ é ganji ɔ́, è ɖó na klɔ́ awa lɛ́ɛ sín jě lɛ́ɛ mɛ, bo na lɛ́ sá nǔ dó ye wu lee é jɛxa gbɔn é. Nú è na sɔnǔ nú awa lɛ́ɛ ɔ́, è ɖó na gɔ́ dotɔ kpɛví kpɛví lɛ́ɛ mɛ jɛ nukɔn kpódó nǔ e è byɔ lɛ́ɛ é kpó (bleu méthylène, éthanol, sin kpódó nǔ e nɔ ɖyɔ xylène é kpó). Mi hɛn awa e è hɛn ɖ'ayǐ ɖò éthanol 70% mɛ ɖò jɔhɔn nú gbɔn Eppendorf sín tuto ɔ́ xixo bo kɔn dó dotɔ ɔ́ jí gblamɛ, enɛ gudo hǔn, mi sɔ́ awa ɔ́ j'ayǐ ɖò gaga jí gbɔn nǔ e è bló b'ɛ cí kɔ́lu ɖɔhun é ɖé zinzan gblamɛ. Sɔ́ awa ɔ́ gbɔn étanɔlu jí klewun bo sɔ́ dó sìn mɛ bo lɛ́vɔ sɔ́ dó etanɔlu jí bo ɖè jě lɛ́ɛ sîn. Sɔ́ awa ɔ́ dó blɔ mɛtilɛni tɔn mɛ nú cɛju 6, bo hɛn dó ayi mɛ ɖɔ é ɖò xixo wɛ hwenu e è ɖò nǔ dó nǔ jǐ wɛ é. Mi vɔ́ awa ɔ blóɖó ganji bo nylɔ́ ɛ dó xylene ɖyɔɖyɔ mɛ nú cɛju 2 (é ɖibla yì hwenu e è nɔ zán dó ɖyɔ xylène é sín atɔnvlɔ́ɖó mɔ̌). Enyi è xò nyǐ ɔ dó dotɔ ɔ sín dǒ lɛ́ɛ ɔ́, é sixu d'alɔ bɔ awa ɔ na j'ayǐ ; xylène nɔ d'alɔ bɔ è nɔ jla sinmɛ ɔ ɖó. Gbɔn gudo ɔ, zé awa ɔ bo sɔ́ ɖó Euparal® sín tò kpɛví ɖé jí ɖò ɖiɖe kpɛví kpɛví ɖé jí. Ðò nǔ e nɔ hɛn nǔ ɖaxó é ɖé glɔ́ ɔ, hun awa ɔ děděě bo sɔ́ nǔ e nɔ cyɔn nǔ dó nǔ jí é ɖé ɖó tɛn tɔn mɛ ganji. È ɖó na ɖè fɔtóo lɛ́ɛ tlolo cobɔ è na bló nǔ e è nɔ ylɔ ɖɔ Euparal® lɛ́ɛ é, ɖó é sixu byɔ ɖɔ è ni jla fí e awa lɛ́ɛ ɖè ɖò lamu kpɛvi kpɛvi lɛ́ɛ́ glɔ́ é kpɛɖé cobɔ è na bló bɔ ye na sɔgbe ganji.

6.4. Wlɛnwín xomɛnǔ lɛ́ɛ tɔn

Gɔ́ nú wlɛnwín wujɔnú tɔn lɛ́ɛ ɔ, wlɛnwín xomɛnǔ nǔɖogbɛ lɛ́ɛ tɔn ɖò taji d'eji wɛ ɖò dobanúnǔ dó nǔvínúví lɛ́ɛ jí mɛ, kaka jɛ taxonomie, do ba nú kunkan togun lɛ́ɛ tɔn, kpódó phylogenetique kpó jí, gɔ́ nú nǔ e nɔ zɔ́n bɔ è nɔ mɔ azɔnkwín lɛ́ɛ é ɖò ADN/RNA mɛ, bo nɔ lɛ́ tunwun fí e nǔɖuɖu hun tɔn gosin é, bɔ aaɔn hɛnnú tɔn ɖò taji ɖò azɔnkwín lɛ́ɛ kpinkpɔn mɛ [70]. È sixu zán nǔ e è nɔ ylɔ ɖɔ ADN é dó ɖexlɛ́ ɖɔ kanlin alɔkpa lɛ́ɛ tîn alǒ dó tunwun vogbingbɔn e ɖò kanlin alɔkpa e sɛkpɔ yeɖée lɛ́ɛ é mɛ é, bɔ enɛ na zɔ́n bɔ è na tunwun nǔ e ɖò kpɔ́ lɛ́ɛ é ganji bo lɛ́ ɖeji dó ye wu. Gɔ́ na ɔ, wlɛnwín xomɛnǔ lɛ́ɛ tɔn nukɔntɔn lɛ́ɛ (é wɛ nyí ɖɔ, PCR, DNA sequencing, NGS, kpódó nǔ ɖevo lɛ́ɛ kpo) kpo MALDI-ToF MS kpó ɖò tɛn nukúnɖeji mɔ wɛ bo na dó tunwun nǔkún-yiya sín nǔ lɛ́ɛ ganji bo lɛ́ yawu tunwun ye, bo na lɛ́ gɔ́ nú wlɛnwín xóxó e è nɔ zán dó tunwun nǔ lɛ́ɛ é [46]. Nukɔnyiyi enɛ lɛ́ɛ ɖò finɛ có, wujɔyúl ɛ́ɛ tunwuntunwun wɛ nyí wlɛnwi è nɔ zán dó ɖè nǔ lɛ́ɛ ɖó vo é, bo lɛ́ nyí nǔ e jí è nɔ jinjɔn bo nɔ tín nǔ e ɖò xomɛnǔ lɛ́ɛ é ji é.

6.4.1. Acides nucléiques sín ɖiɖe e nɔ hɛn nǔ gblé é.

Acide nucléique ɖiɖe sín nǔ mɛ nyí afɔɖiɖe e è nɔ ɖè hwɛhwɛ ɖò nǔkplɔnmɛ nǔɖogbɛ lɛ́ɛ tɔn gègě mɛ é, bɔ è ko ɖè wlɛnwín vovo lɛ́ɛ tɔ́n bo na dó ɖè ADN sín nǔɖogbɛ lɛ́ɛ mɛ [48]. È bló nǔ e è nɔ sà bo nɔ ɖè ADN sín mɛ lɛ́ɛ é gègě bonu ye na dó bló bɔ nǔwiwa enɛ na bɔwu [14]. É ɖò mɔ̌ có, wlɛnwín e è nɔ zán hwɛhwɛ dó sɔnǔ nú nǔ e è nɔ ylɔ ɖɔ arthropodes é bo na dó tunwun lee ye cí é nɔ ɖó dogbó nú ADN gbéjé kpɔ́n hwɛhwɛ, ɖó wlɛnwín enɛ lɛ́ɛ sixu hɛn nǔ taji e ɖò nǔ e è kpɔ́n é mɛ lɛ́ɛ é gblé [10]. ADN ɖiɖe sín xú lɛ́ɛ mɛ gègě nɔ hɛn nǔ gblé ɖò jɔwamɔ linu [43], bo nɔ fɔ́n adohu adohu taji lɛ́ɛ dó kpɔ́ndéwú kpɛví kpɛví lɛ́ɛ jí, fí e kpɔ́ndéwú kpɛɖé tlɛ sixu hɛn nǔ taji e ɖò agbaza mɛ lɛ́ɛ é gblé ɖè é [72]. Nǔ e è kpɔ́n é sín alɔkpa kpódó ninɔmɛ tɔn kpó nɔ wà nǔ taji ɖé ɖò wlɛnwín e sɔgbe bɔ è na zán dó ɖè ADN ɖó vo é ɖé xixo mɛ [29].

Hudo e ɖò flebotomu lɛ́ɛ tunwuntunwun mɛ ganji é, nukúnnúmɔjɛnǔmɛ dó lee gbɛtɔ́ lɛ́ɛ nɔ ɖyɔ gbɔn é wu, kpódó nǔ e ma nyí nǔ taji ǎ lɛ́ɛ é ɖiɖekpo kpó zɔ́n bɔ è bló azɔwanú e nɔ gbéjé nǔ kpɔ́n lɛ́ɛ é [23]. Dìn ɔ, è nɔ zán wlɛnwín xomɛnǔ tɔn lɛ́ɛ hwɛhwɛ dó gɔ́ nú wlɛnwín taxonomie morphologique tɔn lɛ́ɛ bo na dó tunwùn flebotomu lɛ́ɛ. Ði kpɔ́ndéwú ɔ́, wlɛnwín e è nɔ zán dó wlan nǔ dó nǔvínúví lɛ́ɛ jí é wɛ nyí ɖɔ è ni ɖè ADN sín mɛ, bo sɔ́ nǔ e è kpɔ́n lɛ́ɛ é ɖó tɛnmɛ tɛnmɛ, lobo lɛ́ hɛn nǔ e è kpɔ́n jɛ nukɔn é bú. Enɛ wu ɔ́, é byɔ tawun ɖɔ è ni ba wlɛnwín e ma nɔ hɛn nǔ gblé ǎ lɛ́ɛ é, bo na dó hɛn nǔ e ɖò gbè ɔ mɛ lɛ́ɛ é kpódó lee é cí é kpó ɖó te.

È ko zán wlɛnwín gègě dó ɖè acide nucléique sín kɔ́ mɛ. É ɖò mɔ̌ có, è ɖó na hɛn nǔ e è byɔ lɛ́ɛ é dó ayi mɛ, ɖó wlɛnwín vovo lɛ́ɛ ɖó nǔwukpikpé vovo bo nɔ lɛ́ ɖó hudo mimějininɔ tɔn [9]. Ɖi kpɔ́ndéwú ɔ́, è mɔ ɖɔ flebotomu sín nukún lɛ́ɛ nɔ ɖó dogbó nú PCR sín ɖiɖekpo [69]. Gɔ́ nú nǔ e nɔ dɔn azɔn wá lɛ́ɛ é kpinkpɔn gudo ɔ́, è nɔ ɖè ADN Flebotomu tɔ́n hwɛhwɛ bo na dó tunxun nǔvínúví lɛ́ɛ. È sixu zán wlɛnwín vovo lɛ́ɛ dó ɖè nǔ sín mɛ, é ɖò mɔ̌ có, nǔ e è nɔ mɔ lɛ́ɛ é kpoo lee ye nɔ nyɔ́ sɔ́ é kpó nɔ gbɔn vo ɖò wlɛnwín lɛ́ɛ tɛntin. Dobanúnǔtɔ́ lɛ́ɛ ko ɖyɔ tuto nǔbibatɔ́ ɖé lɛ́ɛ tɔn nú flebotomu lɛ́ɛ [8], bo nɔ bló bɔ nǔkún-yiya kpo/alǒ ɖagbe e è nɔ ɖè sín acide nucléique mɛ é kpo nɔ jɛji [8, 9, 69], bɔ è sixu lɛ́ zán huzuhuzu ɖevo lɛ́ɛ, bɔ è bló nú hɛ̌nnu e nɔ nyí arthropode lɛ́ɛ é ɖevo lɛ́ɛ, bo nɔ lɛ́ zán ye dó flebotomu lɛ́ɛ jí [58, 76]. PCR e nɔ sɔ́ ayi ɖó mitochondrie kpɛví kpɛví lɛ́ɛ jí lɛ́ɛ é (COI alǒ CytB) nɔ sɔgbe ɖò kpaa mɛ xá wlɛnwín e è nɔ zán dó ɖè nǔ sín mɛ lɛ́ɛ é, bɔ ye nɔ byɔ ɖɔ è ni ɖè ADN sín fɛ́nnú gègě mɛ. Ðò vogbingbɔn mɛ ɔ́, wlɛnwín NGS tɔn ɖevo e è nɔ xà nú hwenu gegě lɛ́ɛ é (Oxford Nanopore kpo PacBio kpo) nɔ byɔ ɖɔ è ni má nǔ kpɛɖé kpɛɖé, bo lɛ́ ɖó ADN ɖagbeɖagbe. É ɖò mɔ̌ có, enyi è ɖè nǔ e è nɔ ylɔ ɖɔ spin column é tɔ́n ɔ́, è nɔ mɔ ADN genomique sín akpáxwé e nɔ yì 60 kb é, bɔ è nɔ ɖè nǔ e è nɔ ylɔ ɖɔ phenol-chloroform é sín akpáxwé lɛ́ɛ mɛ kaka jɛ 150 kb jí [77]. Tablo 5gɔ́ ɔ́ xò wlɛnwín vovo e è zán dó ɖè ADN e ɖò flebotomu mɛ é tɔ́n é kplé, bo lɛ́ xlɛ́ ɖɔ è ko ɖyɔ wlɛnwín lɛ nú nǔvínúví enɛ lɛ́ɛ à jí. È nɔ xlɛ́ nǔkún-yiya lɛ́ɛ ǎ, ɖó ye nɔ sín nǔ e è kpɔ́n lɛ́ɛ é sín ɖaxó kpo lee è nɔ sɔnǔ na gbɔn é kpo wu. É ɖò mɔ̌ có, è ɖó na hɛn nǔ e è nɔ ylɔ ɖɔ “modification colonne” é dó ayi mɛ, bo na dó sixu mɔ nǔ jɛ nǔ e è nɔ ylɔ ɖɔ “protocoles d’extraction” é wu nú flebotomu lɛ́ɛ alǒ nǔvínúví kpɛví kpɛví ɖevo lɛ́ɛ.

Hwenu e è na sɔ́ wlɛnwín e è na zán dó ɖè nǔ sín mɛ é ɔ́, è ɖó na gbéjé nǔ gègě kpɔ́n, ɖi kpɔ́ndéwú nabi e è na ɖè é, hwenu e è na ɖè nǔ sín mɛ é, kpo wlɛnwín e è na zán ɖò dò é kpo. Hwenu e wlɛnwín NGS tɔn lɛ́ɛ nɔ byɔ ɖɔ è ni zán ADN génomique e ɖó hlɔ̌nhlɔ́n ɖaxó é ɔ, è sixu zán wlɛnwín e è xlɛ́ ɖò fí lɛ́ɛ é bǐ nú nǔwiwa e jinjɔn PCR jí lɛ́ɛ é.

Devo ɔ́, nǔkplɔnmɛ gègě ko ba dò nú wlɛnwín e ma nɔ hɛn nǔ gblé dó mɛ wu ǎ lɛ́ɛ é sín ADN mɛ nú nǔvínúví kpɛví kpɛví e ɖò ayikúngban jí lɛ́ɛ é, nǔ e è hɛn ɖ’ayǐ ɖò xɔgbigbázɔ́watɛn lɛ́ɛ é, kpódó nǔvínúví e ɖó agbaza bɔkun lɛ́ɛ é kpó [19, 26, 28, 55, 63].

6.4.2. Acides nucléiques sín ɖiɖe e ma nɔ hɛn nǔ gblé ǎ é

Tagba ɖaxó e ɖò nǔ e nɔ hɛn afɔɖotɔ́ lɛ́ɛ é, ɖò taji ɔ́, flebotomu lɛ́ɛ é sín nǔ lɛ́ɛ gbígbéjé kpɔ́n mɛ é ɖokpo wɛ nyí ɖɔ è ni hɛn nǔ e è kpɔ́n lɛ́ɛ é ɖó te bo na dó sixu sɔ́ dó nǔ e è xò kplé ɖò nǔvínúví lɛ́ɛ mɛ lɛ́ɛ é mɛ. Nǔ e è nɔ wà bo nɔ ɖè ADN tɔ́n lɛ́ɛ é gègě nɔ byɔ ɖɔ è ni bló bɔ nǔ e ɖò xú ɔ́ mɛ é ni xú, bɔ enɛ nɔ zɔ́n bɔ è nɔ hɛn nǔ e è sɔ́ ɖ’ayǐ é ɖó te. Amɔ̌, è bló wlɛnwín e nɔ ɖè acide nucléique e ma nɔ hɛn nǔ gblé ǎ lɛ́ɛ é tɔ́n é bonu ye na ɖè nǔ e ɖò gǔ mɛ lɛ́ɛ é tɔ́n, bo ma na hɛn nǔ e è kpɔ́n é gblé ɖò agbaza lixo ǎ, bo na wà nǔ dó lee é na nɔ gbè gbɔn é wu alǒ ɖyɔ lee é cí é ǎ. Wlɛnwín enɛ lɛ́ɛ xɔ akwɛ tawun hwenu e è ɖò azɔ̌ wà xá nǔ xɔ akwɛ alǒ nǔ e ɖó dogbó lɛ́ɛ é wɛ é, ɖi flebotomu lɛ́ɛ, fí e è na hɛn nǔ lɛ́ɛ ɖó mimɛ̌ jí ɖè é ɖò taji nú nǔ e è na gbéjé kpɔ́n ɖò sɔgudo lɛ́ɛ é, lee nǔ lɛ́ɛ cí é alǒ nǔ e è na gbéjé kpɔ́n lɛ́ɛ é. Wlɛnwín e è nɔ zán hugǎn é wɛ nyí wǔsláslá e ma nɔ hɛn nǔ gblé ǎ é, bɔ è nɔ bló bɔ flebotomu lɛ́ɛ nɔ nɔ tɛn yetɔn mɛ ǎ, bo nɔ nylɔ ye dó lysis buffer e mɛ proteinase K ɖè é mɛ bléblé.

È ko zán wlɛnwín mild-vectolyse tɔn ɔ́ ganji dó flebotomu lɛ́ɛ jí, ɖò taji ɔ́, bɔklé alɔkpa lɛ́ɛ jí [24]. Wlɛnwín ɔ́ nɔ zán nǔ e è nɔ ylɔ ɖɔ centrifuge classique (ɖò ninɔmɛ elɔ mɛ ɔ́, nǔ e è nɔ ylɔ ɖɔ DNeasy Blood and Tissue kit é, QIAGEN, Hilden, Allemagne) bɔ è nɔ bló huzuhuzu lɛ́ɛ dó mɔ ADN bo nɔ hɛn nǔ e è kpɔ́n é gblé ǎ. É ɖò mɔ̌ có, è ɖó na hɛn nǔ e è nɔ ylɔ ɖɔ lyse é ɖó mimɛ̌ jí (hlɔnhlɔn lyse tampon tɔn kpódó afɔ e nɔ hɛn jɔhɔn syɛnsyɛn é ɖé gɔ́ na kpó) [17] bo na dó sixu ɖè acide nucléique lɛ́ɛ tɔ́n, bo na dó ɖè nǔ e nɔ hɛn nǔ gblé dó agbaza wu lɛ́ɛ é kpò [24]. Ðò kɔ́mɛ sín xɛví lɛ́ɛ linu ɔ ɔ́ è sixu lɛ́ zán nǔ e nɔ ɖè ADN sín mɛ é HotSHOT (Bento Bioworks Ltd, London, Royaume-Uni) [73] e nɔ yawu bo nɔ lɛ́ xɔ akwɛ é, bo nɔ zɔ́n bɔ è nɔ yawu bló nǔ e è kpɔ́n lɛ́ɛ é bo nɔ lɛ́ xɔ akwɛ. Enɛ gudo ɔ́, è sixu klɔ́ nǔ e è sɔ́ ɖ’ayǐ nú nǔvínúví lɛ́ɛ bo na dó tunwun lee ye cí é. Mɛ e è w’azɔ̌ na kpódó DNeasy Blood and Tissue kit kpó lɛ́ɛ é ɖó na hɛn nǔ e è nɔ ylɔ ɖɔ Marc-André é sín sìn dó kɔ́n, bɔ mɛ e è w’azɔ̌ na kpo HotSHOT DNA extraction kit kpo lɛ́ɛ é ka ɖó na kɔ́n ganji bɔ è na sɔ́ ye ɖó sin mɛ, alǒ é na nyɔ́ hú mɔ̌ ɔ́, è na sɔ́ ye ɖó résine ɖé mɛ ɖò sin ɖiɖe sín lanmɛ gudo, sɔgbe xá tuto e è tinmɛ céɖécéɖé ɖò xota elɔ mɛ é [73]. Enɛ gudo ɔ́, è sixu lɛ́ zán nǔ e è ɖè sín dogbó mɛ lɛ́ɛ é dó gbéjé nǔ lɛ́ɛ kpɔ́n ɖò dò, ɖi PCR, bo na dó bló bɔ wuntun dogbó tɔn tawun tawun lɛ́ɛ na gɔ́ngɔ́n. Wlɛnwín e è nɔ zán dó ɖè acide nucléique e ma nɔ hɛn nǔ gblé ǎ é tɔ́n é ɖò taji tawun bo na dó kplɔ́n nǔ dó Flebotomu lɛ́ɛ é wu, kaka jɛ nǔ e sixu dɔn azɔn wá bɔ ye sixu hɛn lɛ́ɛ é tunwuntunwun jí. Enyi dobanúnǔtɔ́ lɛ́ɛ hɛn nǔ e è kpɔ́n é ɖó mimɛ̌ jí ɔ́, ye sixu mɔ nǔ xɔ akwɛ e kúnkplá dogbó mɛtɔn lɛ́ɛ é, bo ka na lɛ́ hɛn nǔ e è kpɔ́n é ɖó te bo na dó gbéjé kpɔ́n alǒ kplɔ́n nǔ d’eji.

Tablo 5gɔ̌ ɔ́ : Akwɛxixɔ ɖo jɛmɛ, zinzan kpódó lee è nɔ xwedo tuto nú nǔ lɛ́ɛ gbɔn é kpó bo na dó ɖè ADN flebotomu e nɔ nyí phlebotomine é tɔn tɔ́n

Sɛ́n	Akwɛ́xixɔ	Nukùnkpénùwù	Nǔjlajla nú nǔvínúví kpɛví kpɛví lɛ́ɛ
Dotín nǔ lilɛ́ lilɛ́ bó ɖe kwi dó vovo tɔn	2.5 – 3.55 US$ [39]	PCR, NGS	[9]
Fenɔlu-klorofɔmu	0.24 US$ [69]	PCR, NGS	[9]
HotSHOT	<0.01 US$ [69]	PCR	-
Jɛ ɖiɖe tɔ́n	0.12 $3 [69]	PCR	-
Chelex	0.02 $4 [41]	PCR	[41, 76]

6.5. MALDI-ToF MS

MALDI-ToF MS (wlɛnwín e nɔ zan do ɖe kén wujɔnú lɛ́ɛ e / hwenu e è nɔ zɔn é sín kénɖiɖe) nyí wlɛnwín ɖé bɔ è sɔ́ ɖó wlɛnwín e nɔ zan do ɖe kén wujɔnú lɛ́ɛ e jí, bo na dó tunwùn nǔ e ɖò proteine lɛ́ɛ mɛ é (‘alɔví’) e ɖò nǔɖogbɛ lɛ́ɛ mɛ é bo lɛ́ gbéjé ye kpɔ́n. MALDI-ToF ɔ́, è nɔ tunwùn i d’eji ɖɔ é nyí azɔwanú taji ɖé bo nɔ d’alɔ mɛ bɔ è nɔ tunwùn nǔvínúví e ɖó afɔ bo ɖò taji ɖò dotóoxwé kpódó kanlin lɛ́ɛ kpó linu lɛ́ɛ é. É ɖò mɔ̌ có, è hɛn ɔ́, è na zán wlɛnwín enɛ dó tunwun fí e flebotomu lɛ́ɛ nɔ sù ɖè é vovo lɛ́ɛ, kaka jɛ flebotomu e ma ko sù ǎ lɛ́ɛ é kpódó yinvi hun e è nɔ ɖu ɖò flebotomu asi e è gɔ́ lɛ́ɛ é mɛ é kpó jí, bɔ è ko zán ganji bo dó tunwùn vogbingbɔn e ɖò flebotomu asú lɛ́ɛ kpódó asi lɛ́ɛ kpó tɛntin é ɖò ninɔmɛ vovo e mɛ è nɔ hɛn nǔ lɛ́ɛ ɖó é kpódó ee è nɔ bló bɔ ye nɔ cí nǔ ɖokpo ɔ́ ɖɔhun lɛ́ɛ é kpó mɛ [28, 4, 30]. Wlɛnwín enɛ ɔ́ nɔ lɛ́ na hlɔ̌nhlɔ́n vogbingbɔn tɔn ɖaxó ɖò hɛ̌nnu kpɛví kpɛví lɛ́ɛ, kanlin alɔkpa lɛ́ɛ kpódó gbɛtɔ́ lɛ́ɛ kpó sín atɛji. Wlɛnwín enɛ nɔ zɔ́n bɔ dobanúnǔtɔ́ lɛ́ɛ nɔ yawu tunwùn nǔvínúví lɛ́ɛ ganji, bɔ enɛ ɖò taji bo na dó mɔ nukúnnú jɛ lee flebotomu lɛ́ɛ nɔ gbakpé gbɔn é, walɔ yetɔn lɛ́ɛ kpódó azɔ̌ e ye nɔ wà ɖò azɔn lɛ́ɛ jijla mɛ é kpó wu. MALDI-ToF nɔ wà azɔ̌ taji ɖé ɖò nǔkplɔnmɛ azɔn lɛ́ɛ tɔn kpódó wlɛnwín e è nɔ zán dó ɖu ɖò azɔn lɛ́ɛ jí lɛ́ɛ é kpó mɛ, ɖó é nɔ ɖè vogbingbɔn ɖò kanlin alɔkpa lɛ́ɛ tɛntin gbɔn proteine lɛ́ɛ jí gblamɛ. Nǔ taji wè wɛ ɖò wlɛnwín enɛ mɛ dìn bo nɔ ɖó dogbó nú lee è nɔ zán ɛ gbɔn hwɛhwɛ é. Nukɔntɔn ɔ́ wɛ nyí ɖɔ è ni ɖó nǔ e è nɔ zán dó kpɔ́n nǔ lɛ́ɛ é, bɔ ye nɔ xɔ akwɛ tawun bɔ è sixu mɔ ye bo na dó tunwun nǔ e nɔ nyí arthropodes lɛ́ɛ é keɖɛ. É nyɔ́ wà ɔ́, è sixu ɖu ɖò dogbó enɛ jí hwenu e è nɔ zán ɖò macinu jí é ɖiɖó ɖò wlɛnwín e nɔ zan do ɖe kén wujɔnú lɛ́ɛ e ko huzu azɔwanú dobanúnǔ tɔn ɖé ɖò azɔ̌xwé proteomique lɛ́ɛ kpo/alɔ̌ azɔnxwixwí lɛ́ɛ kpo mɛ é gblamɛ. Wegɔ ɔ wɛ nyí ɖɔ è nɔ xlɛ́ nǔ e è nɔ mɔ ɖò xójlawema lɛ́ɛ mɛ é kpeɖé, bɔ enɛ nɔ zɔ́n bɔ è ɖó na bló xójlawema ɖé ɖò xwégbe bo na ɖó ɖiɖe e jinjɔn nǔ e è tuùn ganji lɛ́ɛ é jí é, bɔ é na nyɔ́ hugǎn ɖɔ è ni xò nǔ kpɔ́n dó lee nǔ lɛ́ɛ ɖè gbɔn é kpo nǔ e è nɔ ylɔ ɖɔ wenhɛnnu (COI, cytB) é ɖé kpo jí. É ɖò mɔ̌ có, è ɖó na hɛn nǔ e è ɖɔ lɛ́ɛ é dó ayi mɛ, bo na dó sixu hɛn nǔ e è mɔ ɖò kɔ́ mɛ lɛ́ɛ é dó tɛn adɔkijenumɔ tɔn MSI e alɔgonǔto-dotóoxwe Paris tɔn, Wemaxɔmɛ Ɖaxó Sorbonne tɔn, France kpo nǔ e è xò kplé ɖò Bruxelles lɛ́ɛ é kpo nɔ kpé nukún dó wu é mɛ Belgique (https://msi.happy-dev.fr/). Hwenu e è ɖò tito bló wɛ bo na bló MALDI-ToF protein profiling é ɔ́, è ɖó na hɛn kpɔ́ndéwú lɛ́ɛ ɖó fí e è xúxú ɖè é alǒ ɖò éthanol 70% e ɖó hlɔ̌nhlɔ̂n molekwilu tɔn é mɛ, bo ma na sɔ́ ye ɖó jɔhɔn e ɖò fí ɔ́ é mɛ ó. É ɖò mɔ̌ có, è kplɔ́n nǔ mɛ e nɔ zán lɛ́ɛ é ɖɔ ye ni zán 60% acetonitrile/0,3% TFA sín sìn e mɛ acide sinapinique (30 mg/mL) ɖè é nú nǔsisɔ MALDI-ToF tɔn ɔ́, bo na dó sixu bló bɔ ɖiɖe proteine yetɔn lɛ́ɛ tɔn na sɔgbe xá nǔ e è ko ɖetɔ́n kaka jɛ dìn lɛ́ɛ é.

<u>*Kpɔ́ndéwú Nǔsisɔ nú MALDI-ToF MS (Ɖiɖe 7gɔ̌ ɔ)*</u>

È nɔ bɛ́ nǔvínúví e è hɛn ɖó ninɔmɛ vovo lɛ́ɛ mɛ lɛ́ɛ é dó jɔhɔnnu jɛ nukɔn bo nɔ xú ye ɖò jɔhɔnnu, bo nɔ lɛ́ fɛ́n ye. È nɔ ɖè ta kpódó adɔgo kpó sîn bo na dó mɔ agbaza sín wǔjɔnú ɖěɖee mɛ jijɔ taji e kúnkplá agbaza mɛtɔn lɛ́ɛ é ɖè lɛ́ɛ é, bo na dó sixu sɔ́ ɖiɖe lɛ́ɛ ɖó nǔ jǐ bo lɛ́ gbéjé agbaza mɛtɔn kpɔ́n. È sixu zán akɔ́n nú MALDI-ToF bo hɛn adɔgo e kpò é ɖó te bo na dó ɖè ADN tɔ́n. Nú è na bló tuto nú proteine lɛ́ɛ ɔ́, è nɔ bló bɔ akɔ́n ɔ́ nɔ cí nǔ ɖokpó ɔ́ ɖɔhun ɖò 1,5-mL ala kpɛví kpɛví lɛ́ɛ mɛ kpódó 10 µL sín nǔ e nɔ bló bɔ è nɔ bló nǔ ɖokpó ɔ́ é kpó, bo nɔ zán hwǐ kpódó lamu kpɛví kpɛví e è nɔ zán azɔn ɖokpo lɛ́ɛ é kpó. È nɔ zán nǔ e nɔ bló bɔ nǔ lɛ́ɛ nɔ cí nǔ ɖokpó ɔ́ ɖɔhun é wè : sin e è bló bɔ é ma ɖó vǐ ǎ é kpó acide formique 25% kpo.

Tasúna.

Ɖò azɔ̌ enɛ mɛ ɔ́, mǐ jló na na dobanúnǔtɔ́ lɛ́ɛ wlɛnwín e nyɔ́ hugǎn bɔ è na zán dó jla flebotomu lɛ́ɛ ɖó é, bɔ ye na sɔgbe xá nǔ e gbé nya wɛ è ɖè ɖò dobanúnǔ ɔ́ hwenu lɛ́ɛ é, bo na dó sixu tunwùn nǔ e nɔ dɔn azɔn wá lɛ́ɛ é ganji. Wlɛnwín ɖokpo géé e nyɔ́ hugǎn ɖò gbè ɔ́ bǐ mɛ é ɖě ɖè ǎ ; é nyɔ́ wà ɔ́, wlɛnwín gègě tîn, bɔ ɖokpo ɖokpo ɖó lè tɔn lɛ́ɛ kpó dogbó tɔn lɛ́ɛ kpó.
Ɖò nǔ ɖevo lɛ́ɛ mɛ ɔ́, mǐ na tuto ɖé lɛ́ɛ dó wlɛnwín vovo e è nɔ zán dó sɔnǔ nú flebotomu lɛ́ɛ bo nɔ lɛ́ tunwun ye é wu. Nǔkplɔnmɛ enɛ lɛ́ɛ, kaka jɛ yɛ nùkplɔnmɛ tɔn lɛ́ɛ jí, nɔ na tuto afɔɖiɖe ɖokpo ɖokpo tɔn e sɔgbe xá nǔ vovo e gbé nya wɛ è ɖè lɛ́ɛ é, bo nɔ zɔ́n bɔ è nɔ mɔ nǔ jɛ nǔ e è na mɔ lɛ́ɛ é wu ganji, bɔ è sixu ɖeji dó ye wu. Enyi mǐ nɔ na nǔ enɛ e góngón é ɔ́, mǐ nɔ ba bo na d’alɔ dobanúnǔtɔ́ lɛ́ɛ bɔ ye na cyan wlɛnwín nǔjlɛdonǔwu tɔn e sɔgbe hugǎn lɛ́ɛ é bo na lɛ́ zán nú hudo yetɔn tawun tawun lɛ́ɛ ɛ.

Kúdónúmɛ.

Wema wlantɔ́ lɛ́ɛ dókú nú Richard Lane kpódó Zoe Jay Adams kpó e ɖò hɔnmɛ e nɔ Kpé nukún dó tan wu É ɖò Londres, ɖò Grande-Bretagne é ɖó lee ye gbéjé wema ɔ́ kpɔ́n ganji gbɔn é wu, bɔ enɛ zɔ́n bɔ alɔnuwema elɔ nyɔ́ tawun.

Akwɛzinzan.

Mi do kú nú azɔxwe nukɔnyiyi tɔn Bresil tɔn CNPq (numɛlo : 404395/2024-4) kpódó Fondation Araucária (numɛlo : 433/2025 PDI) kpó ɖó akwɛ e ye na nú dobanunǔ AJA tɔn lɛ́ɛ wu.

Tasɔ́mɛ ɖó nǔ e ba wɛ e ɖe lɛ́ɛ wu.

Jérôme Depaquit wɛ nyí wěɖegbɛ́ wɛ kpé nukún dó wema nǔvínúví tɔn ɔ́ wú e ; é ɖó zinzín ɖebǔ dó lee è na gbéjé alɔnuwema enɛ kpɔ́n gbɔn é kpódó gbeta nǔwlanwlan tɔn ɔ́ é kpó jí ǎ. Wema wlantɔ́ ɖevo lɛ́ɛ ɖɔ emi kún ɖó nǔ ɖebǔ bo na dɔn tagba wá nú emi ó.

Xó e ɖiɖe lɛ́ɛ hɛn e

Ðiɖe 1gɔ́ ɔ́: flebotomu e è hɛn ɖó éthanol mɛ é.
Ðiɖe 2gɔ́ ɔ́: Nǔ e è na zán dó sɔ́ flebotomu lɛ́ɛ ɖó nǔ jí lɛ́ɛ é: A: lamu kɔ́fu sóbwe sóbwe nɔ (10 alǒ 12 mm ɖò glǒ mɛ); B: pláki 24 kpódó yinvi kpó (enyi è ɖò amì clove tɔn alǒ Euparal essence zán wɛ bo na dó bló flebotomu lɛ́ɛ na ɔ́, ma zán plaki acrylique tɔn lɛ́ɛ ó ɖó nǔ e nɔ nyí réaction chimique é ɖé na jɛ bɔ nǔ e è kpɔ́n lɛ́ɛ é na gblé); C: lamu kɔ́fu tɔn e jɛxa bɔ è na wlan nǔ dó jí lɛ́ɛ é; D: kɛ́nɖiɖe yinvi ɔ́ tɔn ; E: nǔ e è nɔ tɛ́ dó jě jí lɛ́ɛ é; F: ganxixo sín kɔ́fu alǒ nǔ e cí mɔ̌ é ɖé bo hɛn flebotomu e è na sɔ́ ɖó nǔ jí lɛ́ɛ é; G: Dumont sín hwǐ; H: pipɛti ala nɔ; I: pipɛti go nɔ e hozo bó hɛn sin yiyi dó nǔ mɛ bɔ wu e
Ðiɖe 3gɔ́ ɔ́: Agbàn e mɛ dotɔ 24 ɖè é ɖé, bɔ flebotomu lɛ́ɛ sín ta kpó adɔgo yetɔn sín nuvínu kpó ɖò ɖokpo ɖokpo mɛ.
Ðiɖe 4gɔ́ ɔ́: È zɛ spermathecae bo sɔ́ ɖó sìn Marc-André tɔn mɛ sín nǔ yɔyɔ̌ lɛ́ɛ mɛ. A: *Idioflébotomus longiforceps* (Lao RDP); B: *Sergentomyia minuta* (Flansé); C: *Phlebotomu ariasi* (Flansé); D: *Sergentomyia anodontis* (Lao RDP).
Ðiɖe 5gɔ́ ɔ́: Wlɛnwín e è nɔ zán dó ɖè *Leishmania* ɖó vo é.
Ðiɖe 6gɔ́ ɔ́: Azɔwanu flebotomu tɔn nú biologie moléculaire, proteomique, kpodo/alǒ virusologie sín azɔ̌ lɛ́ɛ kpo.
Ðiɖe 7gɔ́ ɔ́: Wlɛnwín hwexónu tɔn e è nɔ zán dó wà nǔ xá flebotomu lɛ́ɛ é.
Ðiɖe 8gɔ́ ɔ́: lamu lɛ́ɛ vɔ to. A: lamu e gblé bo xú bɔ è sɔ́ ɖó Hoyer jí lɛ́ɛ é; B: lamu kɔ́ xúxú ɖé tɔn ɖò nǔ kpɛví kpɛví mɛ; C: è kpɔn flebotomu ɖevo e gblé é ɖò nǔgbejekpɔ́n sin mɔ mɛ; D: xɔ e mɛ è nɔ bló lamu xúxú ɖé ɖè é; E: ta, kpódó F: agbaza kpɔ́ndéwú B tɔn kpó ɖò hwenu e è vɔ́ ɛ sɔ́ ɖó Euparal® mɛ gudo é; G: ta, kpódó H: agbaza kpó nú kpɔ́ndéwú C e gblé é tɔn hwenu e è vɔ́ ɛ sɔ́ ɖó Euparal® mɛ gudo é.
Ðiɖe 9gɔ́ ɔ́: *Trichophoromyia ininii* sín awa xúxú.
Ðiɖe 10gɔ́ ɔ́: *Flebotomus ariasi* sín awa e ɖó sinmɛ é.

12. Yɛwukɔ́nyi-dowu e ɖò Zenodo jí lɛ́ɛ é.
Yɛwukɔ́nyi-dowu 1gɔ́ ɔ́: https://zenodo.org/records/18198006.
Yɛwukɔ́nyi-dowu 2gɔ́ ɔ́: https://zenodo.org/records/18311158.
Yɛwukɔ́nyi-dowu 3gɔ́ ɔ́: https://zenodo.org/records/18311106.
Yɛwukɔ́nyi-dowu 4gɔ́ ɔ́: https://zenodo.org/records/18311154.
Yɛwukɔ́nyi-dowu 5gɔ́ ɔ́: https://zenodo.org/records/18303014.
Yɛwukɔ́nyi-dowu 6gɔ́ ɔ́: https://zenodo.org/records/18303014.
Yɛwukɔ́nyi-dowu 7gɔ́ ɔ́: https://zenodo.org/records/18315029.

Xójlawema lɛ́ɛ

1. Alkan C, Allal-Ikhlef AB, Alwassouf S, Baklouti A, Piorkowski G, de Lamballerie X, Izri A, Charrel RN. 2015. Virus isolation, genetic characterization and seroprevalence of Toscana virus in Algeria. Clinical Microbiology and Infection, 21(11), 1040 e1-9.
2. Alten B, Ozbel Y, Ergunay K, Kasap OE, Cull B, Antoniou M, Velo E, Prudhomme J, Molina R, Banuls AL, Schaffner F, Hendrickx G, Van Bortel W, Medlock JM. 2015. Sampling strategies for phlebotomine sand flies (Diptera: Psychodidae) in Europe. Bulletin of Entomological Research 105(6), 664–678.
3. Ayhan N, Baklouti A, Prudhomme J, Walder G, Amaro F, Alten B, Moutailler S, Ergunay K, Charrel RN, Huemer H. 2017. Practical guidelines for studies on sandfly-borne phleboviruses: Part I: Important points to consider *ante* field work. Vector-Borne and Zoonotic Diseases 17(1), 73–80.
4. Bates PA. 1997. Infection of phlebotomine sandflies with *Leishmania*, in The Molecular Biology of Insect Disease Vectors: A Methods Manual. Springer. p. 112–120.5.
5. Baum M, de Castro EA, Pinto MC, Goulart TM, Baura W, Klisiowicz Ddo R, Vieira da Costa-Ribeiro MC. 2015. Molecular detection of the blood meal source of sand flies (Diptera: Psychodidae) in a transmission area of American cutaneous leishmaniasis, Parana State, Brazil. Acta Tropica, 143, 8–12.
6. Belen A, Alten B, Aytekin A. 2004. Altitudinal variation in morphometric and molecular characteristics of *Phlebotomus papatasi* populations. Medical and Veterinary Entomology, 18(4), 343–350.
7. Bhattacharya J, Chandra G, Hati AK. 1991. A simple method for cryopreservation of *Leishmania donovani* promastigotes, Indian Journal of Medical Research, 93, 245–246.
8. Caligiuri LG, Sandoval AE, Miranda JC, Pessoa FA, Santini MS, Salomón OD, Secundino NF, McCarthy CB. 2019.

Optimization of DNA extraction from individual sand flies for PCR amplification. Methods and Protocols, 2(2), 36.
9. Casaril AE, de Oliveira LP, Alonso DP, de Oliveira EF, Gomes Barrios SP, de Oliveira Moura Infran J, Fernandes WS, Oshiro ET, Ferreira AMT, Ribolla PEM, de Oliveira AG. 2017. Standardization of DNA extraction from sand flies: Application to genotyping by next generation sequencing. Experimental Parasitology, 177, 66–72.
10. Castalanelli MA, Severtson DL, Brumley CJ, Szito A, Foottit RG, Grimm M, Munyard K, Groth DM. 2010. A rapid non-destructive DNA extraction method for insects and other arthropods. Journal of Asia-Pacific Entomology, 13(3), 243–248.
11. Cerqueira NL. 1943. Um novo meio para montagem de pequenos insetos em lâmina. Memórias do Instituto Oswaldo Cruz, (39), 37–41.
12. Charrel RN, Gallian P, Navarro-Mari JM, Nicoletti L, Papa A, Sanchez-Seco MP, Tenorio A, de Lamballerie X. 2005. Emergence of Toscana virus in Europe. Emerging Infectious Diseases, 11(11), 1657–1663.
13. Chaskopoulou A, Giantsis IA, Demir S, Bon MC. 2016. Species composition, activity patterns and blood meal analysis of sand fly populations (Diptera: Psychodidae) in the metropolitan region of Thessaloniki, an endemic focus of canine leishmaniasis. Acta Tropica, 158, 170–176.
14. Chen H, Rangasamy M, Tan SY, Wang H, Siegfried BD. 2010. Evaluation of five methods for total DNA extraction from western corn rootworm beetles. PLoS One, 5(8), e11963.
15. Depaquit J, Grandadam M, Fouque F, Andry PE, Peyrefitte C. 2010. Arthropod-borne viruses transmitted by Phlebotomine sandflies in Europe: a review. Eurosurveillance, 15(10), 19507.
16. Diamond LS, Herman CM. 1954. Incidence of Trypanosomes in the Canada Goose as revealed by bone marrow culture. Journal of Parasitology, 40(2), 195–202.
17. Ding H, Torno M, Vongphayloth K, Ng G, Tan D, Sng W, Ho K, Randrianambinintsoa FJ, Depaquit J, Tan CH. 2025. Hidden in plain sight: discovery of sand flies in Singapore and description of four species new to science. Parasites & Vectors, 18(1), 402.
18. Es-Sette N, Ajaoud M, Bichaud L, Hamdi S, Mellouki F, Charrel RN, Lemrani M. 2014. *Phlebotomus sergenti* a common vector of *Leishmania tropica* and Toscana virus in Morocco. Journal of Vector Borne Diseases, 51(2), 86–90.
19. Favret C. 2005. A new non-destructive DNA extraction and specimen clearing technique for aphids (Hemiptera). Proceedings of the Entomological Society of Washington, 107(2), 469–470.
20. Galati EAB. 2018. Phlebotominae (Diptera, Psychodidae): Classification, morphology and terminology of adults and identification of American taxa, in Brazilian Sand Flies: Biology, Taxonomy, Medical Importance and Control, Rangel EF, Shaw JJ, Editors. Cham: Springer International Publishing. pp. 9–212.
21. Galati EAB, de Andrade AJ, Perveen F, Loyer M, Vongphayloth K, Randrianambinintsoa FJ, Prudhomme J, Rahola N, Akhoundi M, Shimabukuro PHF, Depaquit J. 2025. Phle-botomine sand flies (Diptera, Psychodidae) of the world. Parasites & Vectors, 18(1), 220.
22. Galati EAB, Galvis-Ovallos F, Lawyer P, Leger N, Depaquit J. 2017. An illustrated guide for characters and terminology used in descriptions of Phlebotominae (Diptera, Psychodidae). Par-asite, 24, 26.
23. Gariepy T, Kuhlmann U, Gillott C, Erlandson M. 2007. Parasitoids, predators and PCR: the use of diagnostic molecular markers in biological control of Arthropods. Journal of Applied Entomology, 131(4), 225–240.
24. Giantsis IA, Chaskopoulou A, Bon MC. 2016. Mild-Vectolysis: A nondestructive DNA extraction method for vouchering sand flies and mosquitoes. Journal of Medical Entomology, 53(3), 692–695.
25. Gidwani K, Picado A, Rijal S, Singh SP, Roy L, Volfova V, Andersen EW, Uranw S, Ostyn B, Sudarshan M, Chakravarty J, Volf P, Sundar S, Boelaert M, Rogers ME. 2011. Serological markers of sand fly exposure to evaluate insecticidal nets against visceral leishmaniasis in India and Nepal: a cluster-randomized trial. PLoS Neglected Tropical Diseases, 5(9), e1296.
26. Gilbert MTP, Moore W, Melchior L, Worobey M. 2007. DNA extraction from dry museum beetles without conferring external morphological damage. PLoS One, 2(3), e272.
27. Giordani BF, Andrade AJ, Galati EAB, Gurgel-Goncalves R. 2017. The role of wing geometric morphometrics in the identification of sandflies within the subgenus *Lutzomyia*. Medical and Veterinary Entomology, 31(4), 373–380.
28. Guzmán-Larralde AJ, Suaste-Dzul AP, Gallou A, Peña-Carrillo KI. 2017. DNA recovery from microhymenoptera using six non-destructive methodologies with considerations for subsequent preparation of museum slides. Genome, 60(1), 85–91.
29. Hajibabaei M, DeWaard JR, Ivanova NV, Ratnasingham S, Dooh RT, Kirk SL, Mackie PM, Hebert PD. 2005. Critical factors for assembling a high volume of DNA barcodes. Philosophical Transactions of the Royal Society B: Biological Sciences, 360(1462), 1959–1967.
30. Haouas N, Pesson B, Boudabous R, Dedet JP, Babba H, Ravel C. 2007. Development of a molecular tool for the identification of *Leishmania* reservoir hosts by blood meal analysis in the insect vectors. American Journal of Tropical Medicine and Hygiene, 77(6), 1054–1059.
31. Hlavackova K, Dvorak V, Chaskopoulou A, Volf P, Halada P. 2019. A novel MALDI-TOF MS-based method for blood meal identification in insect vectors: A proof of concept study on phlebotomine sand flies. PLoS Neglected Tropical Diseases, 13 (9), e0007669.
32. Huemer H, Prudhomme J, Amaro F, Baklouti A, Walder G, Alten B, Moutailler S, Ergunay K, Charrel RN, Ayhan N. 2017. Practical guidelines for studies on sandfly-borne phleboviruses: Part II: Important points to consider for fieldwork and subsequent virological screening. Vector-Borne and Zoonotic Diseases, 17(1), 81–90.
33. Jancarova M, Polanska N, Thiesson A, Arnaud F, Stejskalova M, Rehbergerova M, Kohl A, Viginier B, Volf P, Ratinier M. 2025. Susceptibility of diverse sand fly species to Toscana virus. PLoS Neglected Tropical Diseases, 19(5), e0013031.
34. Kapp JD, Green RE, Shapiro B. 2021. A fast and efficient single-stranded genomic library preparation method optimized for ancient DNA. Journal of Heredity, 112(3), 241–249.
35. Killick-Kendrick R, Maroli M, Killick-Kendrick M. 1991. Bibliography of the colonization of phlebotomine sandflies. Parassitologia, 33(suppl.), 321–333.
36. Lawyer P, Killick-Kendrick M, Rowland T, Rowton E, Volf P. 2017. Laboratory colonization and mass rearing of phlebotomine sand flies (Diptera, Psychodidae). Parasite, 24, 42.
37. Léger N, Pesson B, Madulo-Leblond G. 1986. Les phlébotomes de Grèce : 1ère partie. Bulletin de la Société de Pathologie Exotique, 79, 386–397.
38. Léger N, Pesson B, Madulo-Leblond G. 1986. Les phlébotomes de Grèce : 2ème partie. Bulletin de la Société de Pathologie Exotique, 79, 514–524.

39. Leonel JAF, Vioti G, Alves ML, da Silva DT, Meneghesso PA, Benassi JC, Spada JCP, Galvis-Ovallos F, Soares RM, Oliveira T. 2020. DNA extraction from individual Phlebotomine sand flies (Diptera: Psychodidae: Phlebotominae) specimens: Which is the method with better results? Experimental Parasitology, 218, 107981.
40. Lestinova T, Rohousova I, Sima M, de Oliveira CI, Volf P. 2017. Insights into the sand fly saliva: Blood-feeding and immune interactions between sand flies, hosts, and *Leishmania.* PLoS Neglected Tropical Diseases, 11(7), e0005600.
41. Lienhard A, Schaffer S. 2019. Extracting the invisible: obtaining high quality DNA is a challenging task in small arthropods. PeerJ, 7, e6753.
42. Lozano-Sardaneta YN, Mikery-Pacheco OF, Huerta H, Rojas-Soriano JE, Contreras-Ramos A. 2025. Wing geometric morphometrics is effective to separate sand fly species (Diptera, Psychodidae, Phlebotominae) related with leishmaniasis transmission in Mexico. Acta Tropica, 262, 107523.
43. Mandrioli M. 2008. Insect collections and DNA analyses: how to manage collections? Museum Management and Curatorship, 23(2), 193–199.
44. Maroli M, Feliciangeli MD, Bichaud L, Charrel RN, Gradoni L. 2013. Phlebotomine sandflies and the spreading of leishmaniases and other diseases of public health concern. Medical and Veterinary Entomology, 27(2), 123–147.
45. Marquina D, Buczek M, Ronquist F, Lukasik P. 2021. The effect of ethanol concentration on the morphological and molecular preservation of insects for biodiversity studies. PeerJ, 9, e10799.
46. Mathis A, Depaquit J, Dvorak V, Tuten H, Banuls AL, Halada P, Zapata S, Lehrter V, Hlavackova K, Prudhomme J, Volf P, Sereno D, Kaufmann C, Pfluger V, Schaffner F. 2015. Identification of phlebotomine sand flies using one MALDI-TOF MS reference database and two mass pectrometer systems., Parasites & Vectors, 8, 266.
47. Mekarnia N, Benallal KE, Sadlova J, Vojtkova B, Mauras A, Imbert N, Longhitano M, Harrat Z, Volf P, Loiseau PM, Cojean S. 2024. Effect of *Phlebotomus papatasi* on the fitness, infectivity and antimony-resistance phenotype of antimony-resistant *Leishmania* major Mon-25. International Journal for Parasitology – Drugs and Drug Resistance, 25, 100554.
48. Milligan BG. 1998. Total DNA isolation, in Molecular Genetic Analysis of Population: A Practical Approach, Hoelzel AR, Editor. Oxford: Oxford University Press.
49. Molina R, Jiménez M, Alvar J, González E, Hernández-Taberna S, Ines MM. 2017. Methods in sand fly research. Madrid: Servicio de publicaciones Universidad de Alcalá de Henares, Madrid.
50. Murphy WJ, Eizirik E, O'Brien SJ, Madsen O, Scally M, Douady CJ, Teeling E, Ryder OA, Stanhope MJ, de Jong WW, Springer MS. 2001. Resolution of the early placental mammal radiation using Bayesian phylogenetics. Science, 294(5550), 2348–2351.
51. Nacif-Pimenta R, Pinto LC, Volfova V, Volf P, Pimenta PFP, Secundino NFC. 2020. Conserved and distinct morphological aspects of the salivary glands of sand fly vectors of leishma-niasis: an anatomical and ultrastructural study. Parasites & Vectors, 13(1), 441.
52. Neuhaus B, Schmid T, Riedel J. 2017. Collection management and study of microscope slides: Storage, profiling, deterioration, restoration procedures, and general recommendations. Zootaxa, 4322(1), 1–173.
53. New TR. 1974. Pscoptera. Handbooks for Identification of British Insects (Vol. I). London: Royal Entomological Society of London. 102 pp.
54. Perez-Ruiz M, Collao X, Navarro-Mari JM, Tenorio A. 2007. Reverse transcription, real-time PCR assay for detection of Toscana virus. Journal of Clinical Virology, 39(4), 276–281.
55. Porco D, Rougerie R, Deharveng L, Hebert P. 2010. Coupling non-destructive DNA extraction and voucher retrieval for small soft-bodied Arthropods in a high-throughput context: the example of Collembola. Molecular Ecology Resources, 10(6), 942–945.
56. Prudhomme J, Cassan C, Hide M, Toty C, Rahola N, Vergnes B, Dujardin JP, Alten B, Sereno D, Banuls AL. 2016. Ecology and morphological variations in wings of *Phlebotomus ariasi* (Diptera: Psychodidae) in the region of Roquedur (Gard, France): a geometric morphometrics approach. Parasites & Vectors, 9(1), 578.
57. Prudhomme J, Gunay F, Rahola N, Ouanaimi F, Guernaoui S, Boumezzough A, Banuls AL, Sereno D, Alten B. 2012. Wing size and shape variation of *Phlebotomus papatasi* (Diptera: Psychodidae) populations from the south and north slopes of the Atlas Mountains in Morocco. Journal of Vector Ecology, 37(1), 137–147.
58. Prudhomme J, Toty C, Kasap OE, Rahola N, Vergnes B, Maia C, Campino L, Antoniou M, Jimenez M, Molina R, Cannet A, Alten B, Sereno D, Banuls AL. 2015. New microsatellite markers for multi-scale genetic studies on *Phlebotomus ariasi* Tonnoir, vector of *Leishmania infantum* in the Mediterranean area. Acta Tropica, 142, 79–85.
59. Prudhomme J, Velo E, Bino S, Kadriaj P, Mersini K, Gunay F, Alten B. 2019. Altitudinal variations in wing morphology of *Aedes albopictus* (Diptera, Culicidae) in Albania, the region where it was first recorded in Europe. Parasite, 26, 55.
60. Rawlins DJ. 1992. Light Microscopy: An Introduction to Biotechniques. Oxford: Bios Scientific publishers. 143 pp.
61. Ready PD. 2013. Biology of phlebotomine sand flies as vectors of disease agents. Annual Review of Entomology, 58, 227–250.
62. Rohlf FJ, Slice D. 1990. Extensions of the Procrustes method for the optimal superimposition of landmarks. Systematic Zoology, 39(1), 40–59.
63. Rowley DL, Coddington JA, Gates MW, Norrbom AL, Ochoa RA, Vandenberg NJ, Greenstone MH. 2007. Vouchering DNA-barcoded specimens: Test of a nondestructive extraction proto-col for terrestrial arthropods. Molecular Ecology Notes, 7(6), 915–924.
64. Sábio PB, Andrade AJ, Galati EAB. 2014. Assessment of the taxonomic status of some species included in the *Shannoni* complex, with the description of a new species of *Psathyromyia* (Diptera: Psychodidae: Phlebotominae). Journal of Medical Entomology, 51(2), 331–341.
65. Sadlova J, Yeo M, Seblova V, Lewis MD, Mauricio I, Volf P, Miles MA. 2011. Visualisation of *Leishmania donovani* fluo-rescent hybrids during early stage development in the sand fly vector. PLoS One, 6(5), e19851.
66. Sales K, Miranda DEO, da Silva FJ, Otranto D, Figueredo LA, Dantas-Torres F. 2020. Evaluation of different storage times and preservation methods on phlebotomine sand fly DNA concen-tration and purity. Parasites & Vectors, 13(1), 399
67. Sales KG, Costa PL, de Morais RC, Otranto D, Brandao-Filho SP, Cavalcanti Mde P, Dantas-Torres F. 2015. Identification of phlebotomine sand fly blood meals by real-time PCR. Parasites& Vectors, 8, 230.

68. Sant'Anna MR, Jones NG, Hindley JA, Mendes-Sousa AF, Dillon RJ, Cavalcante RR, Alexander B, Bates PA. 2008. Blood meal identification and parasite detection in laboratory-fed and field-captured *Lutzomyia longipalpis* by PCR using FTA databasing paper. Acta Tropica, 107(3), 230–237.
69. Senne NA, Santos HA, Araujo TR, Paulino PG, Mendonca LP, Moreira HVS, Camilo TA, da Costa Angelo I. 2022. Robust comparative performance of genomic DNA extraction methods from non-engorged phlebotomine sandflies. Medical and Veterinary Entomology, 36(2), 203–211.
70. Shaw JJ. 2025. A review of Leishmania infections in American Phlebotomine sand flies – Are those that transmit leishmaniasis anthropophilic or anthropportunists? Parasite, 32, 57.
71. Tesh RB, Modi GB. 1983. Growth and transovarial transmission of Chandipura virus (Rhabdoviridae: Vesiculovirus) in *Phlebotomus papatasi.* American Journal of Tropical Medicine and Hygiene, 32(3), 621–623.
72. Thomsen PF, Elias S, Gilbert MTP, Haile J, Munch K, Kuzmina S, Froese DG, Sher A, Holdaway RN, Willerslev E. 2009. Non-destructive sampling of ancient insect DNA. PLoS One, 4(4),e5048
73. Truett GE, Heeger P, Mynatt RL, Truett AA, Walker JA, Warman ML. 2000. Preparation of PCR-quality mouse genomic DNA with hot sodium hydroxide and tris (HotSHOT). Biotechniques, 29(1), 52–54.
74. Upton MS. 1993. Aqueous gum-chloral slide mounting media: an historical review. Bulletin of Entomological Research, 83(2), 267–274.
75. Volf P, Myskova J. 2007. Sand flies and Leishmania: specific versus permissive vectors. Trends in Parasitology, 23(3), 91–92.
76. Wang Q, Wang X. 2012. Comparison of methods for DNA extraction from a single chironomid for PCR analysis. Pakistan Journal of Zoology, 44(2), 421–426.
77. Wang Y, Zhao Y, Bollas A, Wang Y, Au KF. 2021. Nanopore sequencing technology, bioinformatics and applications. Nature Biotechnology, 39(11), 1348–1365.

Cite this article as: Randrianambinintsoa FJ, Augendre L, Prudhomme J, Martinet J-P, Loyer M, Mekarnia N, Kerkoub H, Perveen FK, Huguenin A, Kariya E, Akhoundi M, De Andrade AJ, Berriatua E, Bongiorno G, Boyer S, Christodoulou V, Da Costa-Ribeiro MCV, De Souza LAF, Ding H, Dondji B, Dvořák V, Erisoz Kasap O, Galati EAB, Gállego M, Ballart C, Gouzelou S, Haddad N, Masse RS, Mekuria AH, Ivovic V, Kaczmarek S, Shahar MK, Kirstein OD, Kniha E, Kolářová I, Lincoln T, Lucanas C, Mikov O, Nov K, Özbel Y, Pesson B, Posada Lopez LC, Prasetyo DB, Rahola N, Rebollar-Tellez EA, Rodrigues BL, Roy L, Saini P, Sanjoba C, Shimabukuro PH, Siriyasatien P, Soszynska A, Suleşco T, Sylla M, Torno M, Volf P, Vongphayloth K, Sinh Nam V, Wardhana A, Yessinou E, Zapata S, Gantier J-C & Depaquit J. 2026. Processing and mounting phlebotomine sand flies: a consensus guideline. Parasite xx, xx. https://doi.org/10.1051/parasite/2026009.

PARASITE

An international open-access, peer-reviewed, online journal publishing high quality papers on all aspects of human and animal parasitology

Reviews, articles and short notes may be submitted. Fields include, but are not limited to: general, medical and veterinary parasitology; morphology, including ultrastructure; parasite systematics, including entomology, acarology, helminthology and protistology, and molecular analyses; molecular biology and biochemistry; immunology of parasitic diseases; host-parasite relationships; ecology and life history of parasites; epidemiology; therapeutics; new diagnostic tools.
All papers in Parasite are published in English. Manuscripts should have a broad interest and must not have been published or submitted elsewhere. No limit is imposed on the length of manuscripts.

Parasite (open-access) continues **Parasite** (print and online editions, 1994-2012) and **Annales de Parasitologie Humaine et Comparée** (1923-1993) and is the official journal of the Société Française de Parasitologie.

Editor-in-Chief:
Jean-Lou Justine, Paris

Submit your manuscript at:
https://www.editorialmanager.com/parasite

Appendix

Nǔzegɔnúnǔ 1gɔ́ ɔ́ : Donudónu nǔkplɔ́nmɛ e kúnkplá nǔ e nɔ nyí biochimie lɛ́ɛ é.

Nǔvínúví e xó ɖɔ wɛ è ɖè lɛ́ɛ é wɛ nyí flebotomu lɛ́ɛ. É ɖò mɔ̌ có, è sixu jla linlin ɖaxó ɔ́ ɖó nú kanlin e nɔ ɖó afɔ tawun lɛ́ɛ é ɖevo lɛ́ɛ bɔ è sixu tunwùn ye ɖò wuntun agbaza tɔn e ɖò xomɛ lɛ́ɛ é kɛɖɛ jí. Ðò nǔjlɛdonǔwu mɛ ɔ́, agbaza sín wǔjɔnú ɖé lɛ́ɛ nɔ cí xúgloxú ɖɔhun ɖò akpáxwé ɖé mɛ bɔ lee ye cí é nɔ na mǐ nǔ xɔ akwɛ ɖé lɛ́ɛ. Nǔ e wu é nɔ dɔn mɛ tawun ɖɔ è ni kpɔ́n pɔmpi nǔɖuɖu tɔn lɛ́ɛ, spermathecae lɛ́ɛ kpódó lamu kan e mɛ ye nɔ gbɔn lɛ́ɛ é kpó é nɛ. Ðó nǔ e mǐ na gbéjé kpɔ́n lɛ́ɛ é bǐ wutu ɔ́, è ɖó na wɔn gbeɖé ɖɔ sín hwenu e è na hɛn nǔvínúví lɛ́ɛ ɖó te é kaka jɛ hwenu e è na kplé ye é ɔ́, mǐ na zán nǔ e nɔ nyí redox é kpowun ǎ. Nǔ e mǐ na có mǐɖée alǒ linlin ɖokpó e na kplá mǐ é wɛ nyí ɖɔ mǐ ni ma xò nǔ e nɔ hɛn nǔ gblé dó mɛ wu lɛ́ɛ é kplé xá nǔ e nɔ hɛn nǔ gblé dó nǔ wu lɛ́ɛ é ɔ́.

Ahan syɛnsyɛn e nɔ nyí éthyl é ; etanɔlu :

È na zán nǔ enɛ ɖò ali vovo nu. Ahan syɛnsyɛn sín molekwilu lɛ́ɛ nɔ yí wǎn nú sìn tawun, enɛ wu ɔ́, ye nɔ ɖè nǔ e nɔ zɔ́n bɔ è nɔ gɔn sin mɛ é xlɛ́. Amɔ̌, ahan syɛnsyɛn e mɛ è ma nɔ mɔ nǔ gègě ɖè ǎ é (é wɛ nyí ɖɔ, sin sukpɔ́ ɖ'emɛ dín) na wà nǔ ɖé ɖò acidu nucléiques lɛ́ɛ wu (sin wɛ nyí kɛntɔ́ nú acidu nucléiques lɛ́ɛ). Nú è sɔ́ nǔvínúví lɛ́ɛ dó éthanol mɛ ɔ́, é nyí bo na dó hɛn ye ɖó te kɛɖɛ wɛ ǎ, loɔ, bo na dó jla xú lɛ́ɛ ɖó. Ðò histologie mɛ ɔ́, mǐ nɔ ɖè vogbingbɔn ɖò nǔ taji wè tɛntin hwɛhwɛ: hlɔ̌nhlɔ́n e nɔ byɔ mɛ mɛ é kpódó hlɔ̌nhlɔ́n e nɔ hɛn nǔ lidǒ é kpó. È mɔ nukúnnú jɛ wu ganji ɖɔ nǔ e nɔ hɛn nǔ ɖóte é ɖagbe ɖé ɖó na yawu byɔ xú lɛ́ɛ mɛ gɔngɔn jɛ nukɔn cobo na jla ye ɖó. Nú ahan 96% ɔ́, hlɔ̌nhlɔ́n e é nɔ byɔ mɛ é nɔ ɖibla yì 1,05 (ɖò jlɛjininɔ linu ɔ, nú è sɔ́ acide picrique sín sìn 0,75% ɔ́, hlɔ̌nhlɔ́n e é nɔ byɔ́ mɛ é nɔ nyí 0,45, bɔ 3% potassium dichromate sín hlɔ̌nhlɔ́n tɔn nɔ nyí 1,45). Jlǒ e è ɖó bo na hɛn nǔvínúví lɛ́ɛ kpódó nǔvínúví ɖevo lɛ́ɛ kpó ɖó te kaka sɔyi ɖò éthanol mɛ é nyí nǔgbo ɖé nú nǔvínúví lɛ́ɛ gbéjé kpɔ́ntɔ́ lɛ́ɛ. Linlin ɔ́ kpó ɖò yěyi ɖaxó nyí wɛ bo na jló na hɛn nǔ e è wlí ɖò gle mɛ lɛ́ɛ é ɖó te nú nǔkplɔ́nkplɔ́n e bɔ d'ewu lɛ́ɛ é alǒ nú dobanúnǔtɔ́ sɔgudo tɔn lɛ́ɛ. É ɖò mɔ̌ có, linlin enɛ sixu jɛ nú cytologiste alǒ histologiste ɖé ǎ. Enyi è jló na hɛn kpɔ́ndéwú lɛ́ɛ ɖò nǔ e è nɔ sɔ́ ɖó nǔ jí é mɛ nú hwenu gegě ɔ́, é sixu vɛwǔ bɔ è na vɔ́ ye w'azɔ̌ na. Nǔ e wu kpɔ́ndéwú e ko ɖó xwè 10 jɛji lɛ́ɛ é nɔ vɛwǔ alǒ tlɛ nɔ vɛwǔ bɔ è na zán é nɛ.

Nǔ ɖevo e è ɖó na gbéjé kpɔ́n é wɛ nyí jlɛ̌ e ɖò Flebotomu e è na hɛn ɖó te é kpódó hlɔ̌nhlɔ́n e è na sɔ́ ɖó te é kpó tɛntin é. Ðò kanlin lɛ́ɛ kpinkpɔn alǒ dotóoxwézɔ́ mɛ ɔ́, é nyɔ́ ɖɔ è ni bló tuto nú hlɔ̌nhlɔ́n e na hugǎn hlɔ̌nhlɔ́n e è na sɔ́ ɖó te é azɔn 60 é. Ðò nǔwiwa mɛ ɔ́, nú nǔvínúví kpɛví kpɛví lɛ́ɛ ɔ́, nú è na sɔ́ kpɔ́ndéwú ɖé ɖó te ɔ́, è nɔ sɔ́ ahan 4–5 gɔ́ na é hwe bǐ ɔ́. Hɛn dó ayi mɛ ɖɔ ahan syɛnsyɛn ɔ́ na gɔn hlɔ̌nhlɔ́n ɖó, ɖó é na ɖè sìn e ɖò xú e ɖò xú lɛ́ɛ mɛ é bǐ sîn.

Ðò vivɔnu ɔ́:

- Alcool éthylique ɔ́, nǔ e nɔ kɔn dó nǔ ji é wɛ (enɛ wu ɔ́, é nɔ sɔgbe xá nǔ e kɔn dó nǔ lɛ́ɛ é ǎ);
- É nɔ hɛn proteine lɛ́ɛ j'ayǐ kpódó hlɔ̌nhlɔ́n kpó bo nɔ hɛn ye dyɔ;
- É nɔ hɛn jǒ syɛnsyɛn ɖé lɛ́ɛ xú bo nɔ hɛn glycogène j'ayǐ;
- E nɔ zɔn bɔ xú lɛ́ɛ nɔ xú syɛnsyɛn bo nɔ hɛn ye syɛn.

Nǔ e è nɔ sɔ́ potassium alǒ sodium dó bló na é:

Nǔ e è nɔ zán ɖò nǔvínúví lɛ́ɛ kpinkplɔn mɛ é sɔ́ ayi ɖó hydroxyde potassium tɔn jí hugǎn, bɔ hwɛjijɔ e wu é ɖò wɛn é ɖé ɖò finɛ ǎ.

Sodium hydroxide [E524] nɔ tɔ́n ɖò sìn mɛ, é sixu nyí ɖò hlɔ̌nhlɔ́n vovo mɛ. É nɔ wá ɖò nǔywɛ, alǒ glitter mɛ. Nǔ e nyla ɖò wǔ tɔn hugǎn é wɛ nyí ɖɔ é nɔ hɛn jɔhɔn syɛnsyɛn (é nɔ hú KOH). Enyi é wà nǔ xá proteine lɛ́ɛ ɔ́, é nɔ hɛn ye xú, bɔ kpódó lipidu kpó ɔ́, é nɔ huzu ye dó aɖǐ syɛnsyɛn lɛ́ɛ mɛ ɖò aɖǐ ɖiɖó hwenu (vogbingbɔn ɖaxó ɖé wɛ nyí enɛ xá KOH, ee nɔ na aɖǐ e ɖò sìn mɛ é ɖò aɖǐ ɖiɖó hwenu é). É ɖò mɔ̌ có, è nɔ mɔ hydroxyde potassium tɔn [E525] ɖò nǔ e è nɔ ylɔ ɖɔ 0,1 g mɔ̌ é mɛ, bɔ enɛ nɔ zɔ́n bɔ è nɔ ɖe da kpò nú sin ɔ́ tawun hwenu e è ma ɖɔ jlɛ̌ e jí è na jlɛ́ nǔ dó é ǎ é. Ði kpɔ́ndéwú ɔ́, è sɔ́ 0,1 g sín kɔ́fu 1 dó sìn e è hɛn xúxú é 1 mL mɛ ɔ́, é nɔ na 10% sín kɔ́fu. Nǔ wegɔ e ɖò hydroxyde potassium tɔn mɛ ɖò akpá ɖokpó mɛ é wɛ nyí ɖɔ é nɔ hɛn nǔ gblé dó carbonate wu kpɛɖé (KOH sín sìn ɖé ɖó kancica ɖaxó bo nɔ hɛn CO2 lidǒ, bɔ mɔ̌ mɛ ɔ́, é nɔ bló jɛ̌ carbonate tɔn lɛ́ɛ). È na zán nǔ syɛnsyɛn enɛ lɛ́ɛ dó bló bɔ acide gras lɛ́ɛ na xú gbɔn ye huzuhuzu dó aɖǐ e nɔ xú ɖò sìn mɛ lɛ́ɛ é mɛ gblamɛ. È ɖó na flín ɖɔ nǔ e nɔ hɛn nǔ lidǒ é, ɖi éthanol, nɔ bló bɔ ami e ɖò kpɔ́ndéwú ɔ́ mɛ lɛ́ɛ é ɖé lɛ́ɛ nɔ xú. Amɔ̌, enyi è dyɔ kpɔ́ndéwú ɔ́ ɖò nǔ e mɛ sin ɖè é ɖé mɛ bo sɔ́ nǔ syɛnsyɛn ɖé dó dyɔ na ɔ́, acide gras (e syɛn hú mɔ̌ alǒ hwe hú mɔ̌ lɛ́ɛ é) na xú. Enɛ wu ɔ́, dodónu syɛnsyɛn ɔ́ na wà huzú aɖǐ fífá ɖé. Ðò ninɔmɛ ɖé lɛ́ɛ mɛ ɔ́, enyi jǒ lɛ́ɛ sukpɔ́ zɛ xwé wu ɔ́, ɖi kpɔ́ndéwú ɔ́, ɖò asi lɛ́ɛ mɛ ɔ́, é na nyɔ́ ɖɔ è ni sɔ́ jɔhɔn ɔ́ yì 35–40°C bo na dó bló bɔ nǔwiwa ɔ́ na bɔwu, alǒ, è na bló bɔ hwenu e è na nɔ xò nǔ kpɔ́n dó nǔ wu é na ɖi ga ɖò jɔhɔn kpàa tɔn mɛ.

Sinmɛ /Sin Marc-André tɔn e ma ɖó sinmɛ ǎ e:

Fi ɔ́, mi na ba do nǔ ɖagbe kpódó nǔ nyanya kpó e ɖɔ Marc-André sin linlin zinzan mɛ lɛ́ɛ. Klolu (tricloracetaldehyde monohydrate), acide acétique kpódó sìn kpó wɛ ɖò nǔ e è nɔ sɔ́ dó bló nǔ enɛ na é mɛ. Nǔ enɛ nɔ hɛn nǔ gblé tawun (acidu kpódó aldehyde kpó sín kpɔ́ndéwú). É na hɛn hydroxyde potassium tɔn e sixu kpò ɖò kpɔ́ndéwú lɛ́ɛ mɛ é gblé, bo na bló bɔ aɖǐ alcaline e è bló ɖò hydroxyde potassium tɔn zinzan hwenu lɛ́ɛ é na xú ǎ. Nǔ e nɔ hɛn nǔ gblé dó nǔ wu é enɛ na lɛ́ wà nǔ dó azɔ̌ e glucosamines e nɔ bló ɖò chitin mɛ lɛ́ɛ é nɔ wà ɖò ahan syɛnsyɛn mɛ é jí gbɔn ye xixo dó jǐ gblamɛ, bɔ mɔ̌ ɔ́, é na bló bɔ xúgloxú ɔ́ na bɔwu. Nǔwiwa ɖevo wɛ nyí ɖɔ è nɔ bló bɔ jɛ̌ e ɖò finɛ lɛ́ɛ é ɖé lɛ́ɛ xú. Hwenu e è ko sɔ́ acide fuchsine dó bló sinmɛ nú Marc-André sín linlin ɔ́ ɖ'ayǐ é (mɔ̌ ɔ́, é ɖò ninɔmɛ oxidé tɔn mɛ), é na kpéwú bo na tén kpɔ́n bo na hɛn azɔ̌ e è nɔ wà ɖò ahan wegɔ ɔ́ mɛ lɛ́ɛ é ɖó te. Enyi hwenu e è na xò nǔ kpɔ́n dó nǔ e è nɔ ylɔ́ ɖɔ

Marc-André é wu é kpó ninɔmɛ e mɛ è nɔ hɛn nǔ dó é kpó gudo ɔ́, éthanol kɛɖɛ wɛ è na nɔ klɔ́ nǔ ɔ́ na. Mɔ̌ ɔ́, mǐ nɔ bɛ́ sin ɖiɖekpo sín akpáxwé kpɔ́ndéwú lɛ́ɛ tɔn.

Ɖagbe e ɖe mɛ lɛ́ɛ:

- Acɛhu nú nǔ e sin taji lɛ́ɛ mɛ e zɛ xwé wu
- Xú ɔ́ sin gbɔjɛ
- Ɖɔ xó dó xú jí bo na dó gbéjé nǔ e ɖò xomɛ lɛ́ɛ é kpɔ́n ganji

Nǔ nyanya lɛ́ɛ:

Chloral hydrate nɔ hɛn mɛ blí, bɔ è ko zán ɖò azongbɔnúmɛ gbɛtɔ́ tɔn mɛ. È ɖó na zán ɖò kɔ́fu e mɛ è nɔ bló lamu lɛ́ɛ é ɖè é glɔ́, bo na lɛ́ xwedó sɛ́n ɖěɖee ɖɔ xó dó nǔ e nɔ hɛn nǔ gblé dó mɛ wu lɛ́ɛ é .

Nǔ e è nɔ wà dó ɖè sìn kpò lɛ́ɛ é:

Jɔdómɛwu lɛ́ɛ é xlɛ́ ɖɔ nú kpɔ́ndéwú kpɛví kpɛví lɛ́ɛ ɔ́, é kún nyɔ́ ɖɔ è ni xwedó lee è nɔ bló bɔ ahan syɛnsyɛn lɛ́ɛ nɔ ɖò jijɛji wɛ gbɔn é ó. Nú kpɔ́ndéwú ɖaxó ɔ́, mǐ na bɛ́ kpódó éthanol 80% kpó, enɛ gudo ɔ́, éthanol 90%, 95% bɔ gudo mɛ ɔ́, éthanol blebu. Nú kpɔ́ndéwú kpɛví kpɛví lɛ́ɛ ɔ́, è nɔ zán nǔklɔ́sin bɔ è nɔ sɔ́ éthanol 90% dó bló na, bɔ enɛ gudo ɔ́, è nɔ nylɔ dó éthanol blebu mɛ. Ɖò hwe enɛ nu ɔ́, mǐ na flín hwebǐnu ɖɔ éthanol blebu nɔ ba na hɛn sin e ɖò jɔhɔn mɛ é ɖó te. Ɖò nǔgbéjékpɔ́ntɛn e nɔ kplɔ́n nǔ dó nǔvínúví lɛ́ɛ wu lɛ́ɛ é mɛ é wɛ nyí ɖɔ è ni bló bɔ è na ɖè sìn sín nǔ e è kpɔ́n lɛ́ɛ é mɛ kpódó wǔsláslá e è nɔ ylɔ ɖɔ beech creosote é kpó. Égbé ɔ́, è nɔ gbɛ́ nǔ nú nǔ enɛ e è nɔ zán tawun dó hu nǔvínúví lɛ́ɛ, bo nɔ hu jɛ̌ lɛ́ɛ, bo nɔ lɛ́ hɛn atín lɛ́ɛ ɖó te é tawun ɖó wǎn tɔn wutu (hydrocarbures aromatiques polycycliques) bɔ è lin ɖɔ é nɔ hɛn nǔ gblé dó mɛ wu, é nɔ hɛn kansɛ́ɛzɔn wá, é nɔ hɛn nǔ gblé dó nǔ e ɖò sin mɛ lɛ́ɛ é wu kaka sɔyi, bo nɔ lɛ́ hɛn nǔ gblé dó nǔ e ɖò sin mɛ lɛ́ɛ é wu.
Nǔ e mǐ lin ɖɔ è na sɔnǔ na nú è na sɔ́ kpɔ́ndéwú lɛ́ɛ ɖó nǔ jǐ é ɖokpó wɛ nyí Euparal® kpódó Euparal essence kpó (è tinmɛ ɖò akpáxwé e bɔ d'ewu é mɛ). È nɔ yí gbè nú nǔ e è nɔ ylɔ ɖɔ Euparal® kpódó Euparal kpó é ɖé ganji; kpɔ́ndéwú e è mɔ ɖò 90% éthanol sín wǔsláslá gudo

Nǔzegɔnúnǔ 2gɔ́ ɔ: Nǔ e è sɔ́ dó bló nǔ e è nɔ zán lɛ́ɛ é na é.

Hidrɔksidi potasiyɔmu tɔn 10%
G 10 g
Sin e è hɛn xú é q.s. mL 100

Gomme chlore montage médium Hoyer médium
Sin e è hɛn xúxú é 50 mL
Klolu sin 200 g
Gomme arabe 50 g
Glicérol 20 mL

Marc-André sín xósin
Klolu sin 40 g
Acide acétique glaciaire 30 mL
Sin e è hɛn xúxú é 30 mL

Fuchsin acide 1% ɖò sin e è hɛn xúxú é mɛ
Fuchsin acide kɔ́ 1 g
Sin e è hɛn xúxú é 99 mL

Marc-André sín sìn e è sɔ́ fuchsin dó bló na é
Marc-André sín sìn 10 mL
Fuchsin 1% 50 µL

Nǔzegɔnúnǔ 3gɔ́ ɔ́: Euparal®, balsamu Canada tɔn, ahan polyvinyle tɔn alǒ nǔ ɖevo e è nɔ zán dó jla nǔ ɖó lɛ́ɛ é

Alukɔ́lu polyvinyl: Nǔ e è nɔ to ɖó nǔ jǐ é wɛ nyí nǔ e nyɔ́ hugǎn é hwenu e nǔ e sín hudo è ɖó bo na dó ɖè sìn sín lanmɛ ganji é ma ɖò finɛ ǎ é. Enɛ gudo ɔ́, è nɔ xò ahan polyvinyl tɔn dó lactophénol Amman tɔn mɛ. É ɖò mɔ̌ có, kplékplé enɛ lɛ́ɛ nɔ ɖè nǔ nyanya taji e ɖò xúxú alǒ ahan syɛnsyɛn polyvinyl tɔn e nɔ huzu kristalu é mɛ é xlɛ́, ɖó sin e nɔ xú é alǒ é nɔ cí wiwi hwenu e phenol nɔ xú é wu. Enɛ kpó ɖò wlɛnwín ɖagbe ɖé nyí wɛ nú è na sɔ́ nǔ dó nǔ jǐ nú hwenu klewun ɖé.

Balsamu Canada tɔn: È nɔ zán ɛ dó sɔ́ ɖó lamu kpo lamu kpɛvi kpo tɛntin ɔ, é nɔ byɔ ɖɔ è ni ɖè sìn sín kpɔ́ndéwú e è na sɔ́ ɖó nǔ jí lɛ́ɛ é mɛ. Xylène alǒ toluène zinzan nɔ hɛn nǔ nyannya wa.

Enecê medium: Nú è na sɔ́ ɖó lamu kpódó lamu kpɛvi kpó tɛntin, ɖi Canada Balsamu ɖɔhun ɔ́, é byɔ ɖɔ è ni ɖè sìn sín nǔ e è kpɔ́n é mɛ. Enecê sín xógbe: kolofoni wewé mímɛ́ (22 g); jɛ̌ copal e nɔ xú ɖò ahan mɛ é (12 g), ahan blebu (20 mL), kanfɔ́ (10 g), jɛ̌ e nɔ hɛn nǔ gblé dó mɛ wu é (10 mL), kpódó eucalyptol (26 mL) kpó. Bo na dó sɔnǔ na ɔ́, è nɔ sɔ́ ahan blebu kpódó kanfɔ́ kpó dó gannu ɖé mɛ, ɖi kɔ́fu Erlenmeyer tɔn ɖɔhun. Enɛ gudo hǔn, gɔ́ colophony ɔ́ kpódó jɛ̌ copal ɔ́ kpó na. Enɛ wayi ɔ́, è nɔ sɔ́ nǔ e nɔ sú nǔ dó é dó sú kɔ́fu ɔ́ na, bo nɔ húnhún, bɔ enɛ gudo jɛn è nɔ zé dó nǔklɔsin-marie tɔn mɛ ɖò zozo ɖé mɛ bonu nǔ e è xò kplé é ni ma fyɔ́ ó. Enyi nǔ e ɖ'emɛ lɛ́ɛ é huzu sìn bǐ mlɛ́mlɛ́ ɔ́, è nɔ sɔ́ jɛ̌ e nɔ nyí terpentine é dó mɛ, enɛ gudo ɔ́, è nɔ xò hwenu e nǔ e è xò kplé é kpó ɖò zo jí é, bó nɔ sɔ́ eucalyptol dó nǔ e è xò kplé é mɛ. Nú nǔ e mɛ è nɔ bló nǔ ɔ́ ɖè é ma sɔ́ nɔ xú ǎ ɔ́, è nɔ sɔ́ Eenecê dó bló na, bo nɔ ɖó linlin elɔ́: ahan blebu (30 mL), kanfɔ́ (17 g), tɛrpentin (15 mL), eucalyptol (38 mL) (Cerqueira, 1943).

Euparal®: Nǔ e è nɔ ylɔ́ ɖɔ Cyprès l'Atlas Tetraclinis articulata tɔn(Vahl, 1791) é wɛ, bɔ Gilson kplɔ́n nǔ dó wǔ tɔn bo bló ɖ'ayǐ ɖò 1906. Nǔ taji e é nɔ wà é wɛ nyí ɖɔ é nɔ bló polymérisation ǎ. É ɖò mɔ̌ có, è sixu mɔ kpɔ́ndéwú e è sɔ́ ɖó lamu lɛ́ɛ kpódó lamu e è sɔ́ dó cyɔn nǔ jí lɛ́ɛ é kpó tɛntin é bɔwu gbɔn ahan syɛnsyɛn alǒ nǔ e nyɔ́ hú mɔ̌ é gblamɛ, é wɛ nyí Euparal® essence. Resine enɛ ɔ́, e nɔ lɛ́ ylɔ́ ɖɔ sandarac ɔ́, nɔ yi etanol sin 80%.
Triton X100 sín zinzan: sìn e ma nyí ionique ǎ é:
Triton X100 ɖò ɖiɖe sin mɛ tɔn e ma nyí ionique ǎ é ɖé mɛ (4-(1,1,3,3-tetraméthylbutyl) fenil-poliéthylène glycol linfín, alǒ t-oktilfenoksipolyetoxyéthanol, poliéthylène glikol tert-oktilfenyle

birgent éther mɛ ɔ́, è nɔ zán ɖò fí gègě. É nɔ zɔ́n bɔ è nɔ bló bɔ nǔ e nɔ hɛn nǔ gblé dó mɛ wu lɛ́ɛ é kpo é kpo nɔ gbɔn mɛ.
Nǔvínúví e è hɛn dó ahan mɛ nú xwè mɔkpan lɛ́ɛ é sín kpɔ́ndéwú lɛ́ɛ nyí nǔ e è nɔ mɔ ɖò fí gègě é. É blawu ɖɔ è ni hɛn nǔ dó ahan mɛ ganji ǎ, bɔ nǔvínúví e è hɛn ɖ'ayǐ gbɔn mɔ̌ lɛ́ɛ é nɔ vɛwǔ tawun bɔ è na sɔnǔ nú ye bonu è na gbéjé ye kpɔ́n ɖò nǔgbejekpɔ́n sín mɔ lɛ́ɛ mɛ. Hwɛhwɛ ɔ́, alǎ e mɛ è sɔ́ kpɔ́ndéwú lɛ́ɛ dó é nɔ gblé, bɔ enɛ gudo ɔ́, ahan ɔ́ nɔ xú. Ðò ninɔmɛ wè lɛ́ɛ bǐ mɛ ɔ́, ahan ɖiɖó nú hwenu gegě alǒ nǔ e è kpɔ́n lɛ́ɛ é xúxú nɔ dɔn tagba ɖé wá tawun. Ðò xwè 2008 mɛ ɔ́, Jonque ɖè wema ɖé tɔ́n dó lee è nɔ vɔ́ sìn na Flebotomu lɛ́ɛ gbɔn é jí kpódó nǔ e nɔ hɛn Flebotomu lɛ́ɛ xú é ɖé kpó ɖi Agepon e è nɔ zán dó ɖè fɔtóo lɛ́ɛ é [26]. Enɛ zɔ́n bɔ è ɖó linlin ɔ́ ɖɔ è ni zán nǔ e nɔ hɛn nǔ xú lɛ́ɛ é, bɔ ye ma nyí nǔ e nɔ klɔ́ nǔ syɛnsyɛn lɛ́ɛ é ǎ.
Ðò dò ɔ́, è nɔ zán Triton X100 ɖò sìn 0,5% mɛ:
- È zé ahan blebu dó kpɔ́ndéwú xúxú ɔ́ mɛ.
- Mi zé hlɔ̌nhlɔ́n e ɖò dandan é nú Triton X100 sín sìn ɖò 0,5% jí bonu kpɔ́ndéwú ɔ́ bǐ na byɔ sin mɛ.
- Mi nɔ te nu ceju 5 mɔ alo hu mɔ. Afɔkpa lɛ́ɛ bǐ ɖó na jɛ yeɖée sí ɖò xósin ɔ́ mɛ.
- È nɔ ɖe Triton X100 sin sin ɔ́ sin mɛ bo nɔ sɔ sin e nɔ hɛn potassium hydroxyde ɔ́ wa tɛn tɔn mɛ.
Enɛ gudo ɔ, è nɔ xwedó wlɛnwín ɔ́, lee è tinmɛ gbɔn ɖò aga é.

Nǔzegɔnúnǔ 4gɔ́ ɔ́: Nǔ e è nɔ sɔ́ dó bló Euparal® alǒ Canada Balsamu na é afɔ ɖokpó ɖokpó jí

1. È ɖó na ɖè sìn sín nǔ e è kpɔ́n lɛ́ɛ é mɛ (enyi ninɔmɛ ɔ́ wlú alǒ anɔ̌sin cí mɔ̌ ɔ́, é nɔ xlɛ́ ɖɔ sìn kún ɖò ye mɛ ganji ó).
2. È sixu bló bɔ sin na gɔn ahan syɛnsyɛn e nɔ nyí éthyl alcool é jijlaɖó gblamɛ.
3. È sixu sɔ́ kpɔ́ndéwú lɛ́ɛ sín ahan 99% alǒ ahan blebu mɛ bo sɔ́ dó nǔ e nɔ hɛn nǔ gblé dó mɛ wu é ɖé mɛ.

Alixwédo dó wa azɔ̌:
1. Mi fɛ́n flebotomu mɛxó lɛ́ɛ ɖò éthanol 70% mɛ.
2. Mi ɖe ethanol sin mɛ bo sɔ KOH 10% ɖo tɛn tɔn mɛ. Sɔ́ ní dó lamu jí.
3. Mi zé dó sinmɛ kaka bɔ nǔvínúví lɛ́ɛ na kɔ́n.
4. Sún KOH sín mɛ.
5. Sɔ́ sin e è hɛn xúxú é dó súnsún nǔ e è kpɔ́n é jí bo nɔte nú cɛju 30 jɛ 45.
6. Mi ɖe sin sin mɛ
bo lɛ́ vɔ klɔ́ kpódó sin e e hu acɛ ná e kpó nu ganxixo 30 (Hwenu ɔ́ kan kpɔ́dewu nabí e wɛ e: bɔ e lɛ́ ɖe kpɔ́dewu ɖevo lɛ́ɛ bɔ e w'azɔ̌ d'e wu zɛ̌n, enyí hwenu ɔ́ ká lin ɔ́ ye sɔ́ nɔ́ sukpɔ́ ǎ, ɖo taji ɔ́ nú kpɔ́dewu e wa azɔ̌ ye tɔn vokán vokán lɛ́ɛ ɔ́, hwenu ɔ́ ɖó na nɔ kléwu)
7. Mi ɖe sin sin mɛ .
8. Mi zé Marc-André sín sìn (é sixu nyí sinmɛ Fuchsin tɔn) dó mɛ bo nɔte nú ganxixo 24 (azǎn ɖokpo).
9. Súnsún sìn Marc-André tɔn sín jǐ.
10. Sɔ́ sin e è hɛn xúxú é dó cyɔn nǔ e è kpɔ́n é jí bo nɔte nú cɛju 30 jɛ 45.
11. Mi ɖe sin bo lɛ́ vɔ klɔ́ kpódó sin e hu acɛ na e kpó nu ganxixo 30.
12. Mi ɖe sin sin .
13. Mi zé éthanol 70% dó mɛ bo fɛ́n kpɔ́ndéwú ɔ́.
A: Nú ta kpódó adɔgo kpó ɔ́, e ní yawu dɔn ta alǒ adɔgo ɔ́ sín akɔ́n jí .
B: Nú akɔ́n ɔ́, ɖè aw
a lɛ́ɛ sîn gbɔn akɔ́nnú ɔ́ hɛn kpódó sìn hwǐ ɖokpo kpó bo dɔn ɖò akɔ́nnú lɛ́ɛ sín dò kpódó hwǐ ɖevo kpó gblamɛ. È sixu fɛ́n ye cɔ́ɖwɛ cɔ́ɖwɛ, bo má akɔ́nnú ɔ́ ɖó amyɔxwé kpódó ɖisixwé kpó, sɔgbe xá lɛ́ɛ é.
14. Nɔ ɖè sìn sín ye ji kpɛɖé kpɛɖé gbɔn ahan syɛnsyɛn e nɔ nyí éthyl alcool é ɖé lɛ́ɛ gblamɛ. 50 – 80 – 95% kaka jɛ hwenu e è na mɔ éthanol blebu é.
15. Mi hɛn sin sin nu kpɔ́dewu lɛ́ɛ mɛ gbɔn ye sinsin mɛ azɔn we, min 10 ɖokpó ɖokpó, kpódó 100% ethanol kpó.
16. Mi ɖe ethanol bo cyɔn nu kpɔ́ndewu lɛ́ɛ kpódó atínkɛn gbaɖota kpó nu ganxixo 15 ɖo jɔhɔn yaya nu.
17. Sɔ́ kpɔ́ndéwú lɛ́ɛ sín amì atínkɛn gbaɖota tɔn mɛ dó Euparal® alǒ Canada Balsamu sín tɔ̀ ɖé jí ɖò lamu é ɖé jí.
18. Sɔ́ nǔ lɛ́ɛ ɖó lee jló we gbɔn é: È sixu zán nǔ kpɛví kpɛví lɛ́ɛ alǒ hwǐ kpɛví kpɛví lɛ́ɛ dó fɛ́n ta, akɔ́n kpódó adɔgo kpó nú bɔkle e nɔ nyí flebotomu é ɖò nǔgbejekpɔ́n sín mɔ kpɛ́vi lɛ́ɛ ɖé glɔ́. È ɖó na ɖè ta ɔ́ sín agbaza ɔ́ mɛ bo na dó sixu sɔ́ ɛ ɖó tɛn ventro-dorsal tɔn mɛ, enɛ wɛ nyí ɖɔ è ɖó na sɔ́ occipital foramen ɔ́ ɖó aga bonu è na dó sixu kpɔ́n cibariumu ɔ́ tlɔlɔ gbɔn mɛ.
È nɔ bló fɛ́nnú ɔ́ ɖò flebotomu e nɔ sɔ́ nǔ dó nǔ jǐ é mɛ.
19. Jǒ kpɔ́ndéwú ɔ́ dó kaka jɛ hwenu e nukún tɔn mɛ tɔn na xú é.
20. Sɔ́ ahan blebu dó kɔ́n atínkɛn gbaɖota e mɛ́ é ɖé jí. Sɔ́ nǔ e jí è nɔ cyɔn nǔ dó é dó Balsamu Canada tɔn jí ɖò zɔ̌gbe ɖé jí.
21. Sɔ́ ɖiɖe lɛ́ɛ dó gbǎví xúxú e è sɔnǔ na nú enɛ é ɖé mɛ.
