# Supplementary material for: Processing and mounting phlebotomine sand flies: a consensus guideline
Source: Parasite. 2026 Apr 3;33:18. doi: 10.1051/parasite/2026009 (PMC13047900; doi:10.1051/parasite/2026009)
Supplement: Supplementary file 10 — French translation / Traduction en français [file parasite-33-18-s10.pdf]

Parasite **33**, 18 (2026)
© F.J. Randrianambinintsoa et al., published by EDP Sciences, 2026
https://doi.org/10.1051/parasite/2026009

PARASITE
Available online at:
www.parasite-journal.org

REVIEW ARTICLE OPEN ACCESS

Traitement et montage des phlébotomes : un guide consensuel

Fano José Randrianambinintsoa[1], Laure Augendre[1], Jorian Prudhomme[1], Jean-Philippe Martinet[1], Mathieu Loyer[1], Nalia Mekarnia[1], Hocine Kerkoub[1], Farzana Khan Perveen[1], Antoine Huguenin[1,2], Emilie Kariya[1,2], Mohammad Akhoundi[3], Andrey José de Andrade[4], Eduardo Berriatua[5], Gioia Bongiorno[6], Sébastien Boyer[7,8], Vasiliki Christodoulou[9], Magda Clara Vieira Da Costa-Ribeiro[10], Lucas Alexandre Farias de Souza[10], Huicong Ding[11], Blaise Dondji[12], Vít Dvořák[13], Ozge Erisoz Kasap[14], Eunice Aparecida Bianchi Galati[15], Montserrat Gállego[16], Cristina Ballart[16], Stavroula Gouzelou[17], Nabil Haddad[18], Rezki Sabrina Masse[19], Asrat Hailu Mekuria[20], Vladimir Ivovic[21], Szymon Kaczmarek[22], Mohd Khadri Shahar[19], Oscar D. Kirstein[23], Edwin Kniha[24], Iva Kolářová[13], Lincoln Timinao[25], Cristian Lucanas[26], Ognyan Mikov[27], Kimsear Nov[7], Yusuf Özbel[28], Bernard Pesson[29], Laura Cristina Posada Lopez[30], Didot Budi Prasetyo[1,7], Nil Rahola[31], Eduardo A. Rebollar-Tellez[32], Bruno Leite Rodrigues[15], Lalita Roy[33], Prasanta Saini[34], Chizu Sanjoba[35], Paloma Helena Fernandes Shimabukuro[36], Padet Siriyasatien[37], Agnieszka Soszyńska[22], Tatiana Suleşco[38], Massamba Sylla[39], Majhalia Torno[40], Petr Volf[13], Khamsing Vongphayloth[41], Vu Sinh Nam[42], April Wardhana[43], Eric Yessinou[44], Sonia Zapata[45], Jean-Charles Gantier[1], and Jérôme Depaquit[1,2,*]

[1] Faculté de Pharmacie, Université de Reims Champagne Ardenne, UR ESCAPE-USC ANSES PETARD, 51 rue Cognacq-Jay, 51096 Reims Cedex, France
[2] Pôle de Biologie territoriale, Laboratoire de Parasitologie-Mycologie, Centre Hospitalo-Universitaire, 51092 Reims, France
[3] Parasitology-Mycology Department, Avicenne Hospital, AP-HP, Bobigny, Sorbonne Paris Nord University, France; Unité des Virus Émergents (UVE: Aix-Marseille Univ, Università di Corsica, IRD 190, Inserm 1207, IRBA), 13005 Marseille, France
[4] Parasitology Collection of Basic Pathology, Department of Basic Pathology, Federal University of Paraná, Curitiba 19031, Brazil
[5] Department of Animal Health, University of Murcia, Campus de Espinardo, 30100 Espinardo, Murcia, Spain
[6] Department of Infectious Diseases, Vector-borne Diseases Unit, Istituto Superiore di Sanità, 00166 Rome, Italy
[7] Medical and Veterinary Entomology Unit, Institut Pasteur du Cambodge, Phnom Penh 12201, Cambodia
[8] Ecology & Emergence of Arthropod-borne Pathogens Unit, Department of Global Health, Institut Pasteur, CNRS UMR2000, 75015 Paris, France
[9] Section Veterinary Services (1417), Laboratory for Animal Health Virology, Aglantzia, Nicosia 2109, Cyprus
[10] Insects Vectors and Parasites Laboratory, Department of Basic Pathology and Postgraduate program in Microbiology, Parasitology and Pathology, Federal University of Paraná, 81530-900 Curitiba, Brazil
[11] Department of Biological Sciences, National University of Singapore, 117558, Singapore
[12] Laboratory of the Leishmaniasis Research Project, Mokolo District Hospital, Mokolo, Cameroon; Laboratory of Cellular Immunology and Parasitology, Department of Biological Sciences, Central Washington University, 98926 Ellensburg, WA, USA
[13] Department of Parasitology, Faculty of Science, Charles University, 12800 Prague, Czechia
[14] VERG Laboratories, Department of Biology, Faculty of Science, Hacettepe University, Beytepe, Ankara 06800, Türkiye
[15] Faculdade de Saúde Pública da Universidade de São Paulo (FSP/USP), Pós-graduação em Saúde Pública, 01246-904 São Paulo, Brazil
[16] Secció de Parasitologia, Departament de Biologia, Sanitat i Medi Ambient, Facultat de Farmàcia i Ciències de l'Alimentació, Universitat de Barcelona, & Institut de Salut Global de Barcelona (ISGlobal), Centro de Investigación Biomédica en Red, Enfermedades Infecciosas (CIBERINFEC), 08028 Barcelona, Spain
[17] Laboratory of Infectious Diseases and Public Health, School of Medicine, University of Cyprus, Nicosia, Cyprus & Department of Pediatrics, Archbishop Makarios III Hospital, Nicosia 2115, Cyprus
[18] Faculty of Health Sciences, American University of Beirut, 1107 2020 Beirut, Lebanon
[19] Medical Entomology Unit, Infectious Disease Research Centre, Institute for Medical Research (IMR), National Institutes of Health (NIH), Ministry of Health Malaysia, 40170 Shah Alam, Selangor, Malaysia
[20] School of Medicine, Addis Ababa University, 28017 - 1000 Addis Ababa, Ethiopia

Edited by Jean-Lou Justine
*Corresponding author: jerome.depaquit@univ-reims.fr

This is an Open Access article distributed under the terms of the Creative Commons Attribution License (https://creativecommons.org/licenses/by/4.0), which permits unrestricted use, distribution, and reproduction in any medium, provided the original work is properly cited.

[21] Faculty of Mathematics, Natural Sciences and Information Technologies, University of Primorska, 6000 Koper, Slovenia
[22] University of Lodz, Faculty of Biology and Environmental Protection, Department of Invertebrate Zoology and Hydrobiology, Banacha 12/16, 90-237 Łódź, Poland
[23] Laboratory of Entomology, Ministry of Health, 9134302 Jerusalem, Israel
[24] Center for Pathophysiology, Infectiology and Immunology, Institute of Specific Prophylaxis and Tropical Medicine, Medical University Vienna, Kinderspitalgasse 15, 1090 Vienna, Austria
[25] Papua New Guinea Institute of Medical Research (PNGIMR) Institute, PO Box 60, Headquarter, Homate Street, 441 Goroka, Eastern Highlands Province, Papua New Guinea
[26] Museum of Natural History, University of the Philippines Los Baños, 4031 Laguna, Philippines
[27] National Centre of Infectious and Parasitic Diseases, 1504 Sofia, Bulgaria
[28] Ege University, Faculty of Medicine, Department of Parasitology, 35040 Bornova/Izmir, Türkiye
[29] Retired, Faculté de Pharmacie, Université de Strasbourg, Strasbourg, 67400 Illkirch-Graffenstaden, France
[30] Program for the Study and Control of Tropical Diseases (PECET), Faculty of Medicine, University of Antioquia, 050010 Medellin, Colombia
[31] MIVEGEC, Univ. Montpellier, CNRS, IRD, 34394 Montpellier, France & Medical Entomology Unit, Institut Pasteur de Madagascar, 101 Antananarivo, Madagascar
[32] Laboratorio de Entomología Médica, Departamento de Zoología de Invertebrados, Facultad de Ciencias Biológicas, Universidad Autónoma de Nuevo León, San Nicolás de los Garza, 66455, NL, México
[33] Tropical and Infectious Disease Centre, BP Koirala Institute of Health Sciences, Dharan 56700, Nepal
[34] ICMR-Vector Control Research Centre, Puducherry 605006, India
[35] Graduate School of Agricultural and Life Sciences, The University of Tokyo, Tokyo 113-8657, Japan
[36] Grupo de estudos em Leishmanioses/Coleção de Flebotomíneos (COLFLEB/Fiocruz-MG), Instituto René Rachou, Fundação Oswaldo Cruz, Belo Horizonte, Minas Gerais, 30190009, Brazil
[37] Center of Excellence in Vector Biology and Vector-Borne Disease, Department of Parasitology, Faculty of Medicine, Chulalongkorn University, Bangkok 10330, Thailand
[38] Department of Arbovirology, Bernhard Nocht Institute for Tropical Medicine, Bernhard Nocht Str. 74, 20359 Hamburg, Germany [39] Laboratory Vectors & Parasites, Department of Livestock Sciences and Techniques, Sine Saloum University El Hadji Ibrahima Niasse (SSUEIN) Kaffrine Campus, C.P. 24600, Senegal.
[40] Environmental Health Institute, National Environment Agency, Singapore 138667, Singapore & Department of Biological Sciences, National University of Singapore, 117558 Singapore
[41] Institut Pasteur du Laos, Laboratory of Vector-Borne Diseases, Samsenhai Road, Ban Kao-Gnot, Sisattanak District, 3560 Vientiane, Lao PDR
[42] National Institute of Hygiene and Epidemiology, 1 Yec-Xanh Street, Hai Ba Trung District, 100000 Hanoi, Vietnam
[43] Indonesian Research Center for Veterinary Science, Indonesian Agency for Agricultural Research and Development, Ministry of Agriculture Republic Indonesia, Bogor 16114, Indonesia & Department of Parasitology, Faculty of Veterinary Medicine, Airlangga University, Surabaya 60115, Indonesia
[44] Laboratory of Research in Applied Biology, Polytechnic School of Abomey-Calavi, University of Abomey-Calavi, 01 P.O. Box 2009, 00000 Cotonou, Benin
[45] Instituto de Microbiología, Colegio de Ciencias Biológicas y Ambientales (COCIBA), Universidad San Francisco de Quito (USFQ), 170901 Quito, Ecuador

Received 1 December 2025, Accepted 29 January 2026, Published online 3 April 2026

Résumé – Cet article propose un guide complet pour le traitement et le montage des phlébotomes, étapes cruciales pour l'identification des espèces et la détection ou l'isolement des pathogènes. Il aborde diverses techniques adaptées au terrain comme au laboratoire. Le guide comprend des instructions détaillées sur la collecte, la manipulation, la protection et l'euthanasie des phlébotomes (avec recommandation de la congélation à sec ou du CO_2 plutôt que de l'usage de produits chimiques), ainsi que sur les stratégies de conservation telles que la réfrigération et la conservation dans l'éthanol. La préparation des structures anatomiques spécifiques (organes génitaux, tête et ailes) pour l'observation microscopique est privilégiée dans la description des techniques de dissection. Le document présente également le traitement détaillé des échantillons, notamment l'éclaircissement à l'aide de solutions telles que la potasse puis le liquide de Marc-André. Le processus de montage compare différents milieux, en soulignant leurs propriétés optiques et leur potentiel de conservation. Le liquide de Hoyer (également appelé gomme au chloral) est recommandé pour l'observation rapide, en particulier pour les spermathèques, en raison de sa limpidité, bien qu'il ne convienne pas à la conservation à long terme. D'autres milieux de montage sont abordés, notamment l'alcool polyvinylique, l'Euparal® (pour une tolérance limitée à l'eau) et le baume du Canada (un milieu soluble dans les hydrocarbures), ces deux derniers permettant une conservation à long terme. Les approches innovantes de biologie moléculaire, telles que le séquençage de l'ADN et la spectrométrie de masse MALDI-ToF, qui exigent une attention particulière lors du traitement des échantillons, sont également présentées. De plus, de courtes vidéos illustrant diverses techniques de montage et des

traductions en 33 langues sont disponibles, permettant ainsi de répondre aux besoins et attentes variés de la communauté scientifique internationale.

Mots-clés : Montage, phlébotomes, liquide de Hoyer, solution de Marc-André, gomme au chloral, alcool polyvinylique, Euparal®, baume du Canada, isolement de Leishmania, conditions de terrain, culture, dissection, biologie moléculaire, MALDI-ToF, spécimens types

Abstract – Processing and mounting phlebotomine sand flies: a consensus guideline. This article provides a comprehensive guide for the processing and mounting of phlebotomine sand fly specimens, which is crucial for species identification and pathogen detection and isolation. It discusses a range of techniques suitable for both field and laboratory settings. The guide includes detailed instructions on sand fly collection, handling, covering, and euthanasia (recommending dry freezing or CO_2 over chemicals) as well as conservation strategies, such as cold storage and preservation in ethanol. The quality of preparation of certain anatomical structures (genital organs, head and wings) is essential for their proper microscopic observation and is described in this work. The article also presents detailed sample processing, including the clearing process with agents such as potassium hydroxide then Marc-André solution. The mounting process compares different media, emphasizing their optical properties and preservation potential. Hoyer fluid (also known as chloral gum) is recommended for quick observation, particularly for spermathecae, due to its clarity, although it is not suitable for long-term storage. Other media discussed include polyvinyl alcohol, Euparal® (for limited water tolerance), and Canada balsam (a hydrocarbon-soluble medium), with the latter two offering long-term preservation capabilities. Innovative molecular biology approaches such as DNA sequencing and MALDI-ToF, which require particular attention to sample processing, are also addressed. Furthermore, short video clips illustrating various mounting techniques as well as translations in many different languages are provided, allowing the guideline to reach the diverse needs and expectations of the global scientific community.

Key words: Mounting, Phlebotomine sand fly, Hoyer fluid, Marc-André solution, Chloral gum, Polyvinyl alcohol, Euparal®, Canada balsam, *Leishmania* isolation, Field conditions, Culture, Dissection, Molecular biology, MALDI-ToF, Type-specimens.

Introduction

Les phlébotomes sont des insectes diptères appartenant à la famille des Psychodidae, sous-famille des Phlebotominae, comptant au moins 1 063 espèces connues [21]. Ils constituent d'importants vecteurs d'agents pathogènes (*Leishmania*, arbovirus et *Bartonella*), responsables de pathologies telles que les leishmanioses, les arboviroses et la bartonellose. Leur identification repose principalement sur un examen microscopique détaillé, rendu possible par une collecte rigoureuse, un stockage approprié et un montage minutieux des spécimens sur lame, nécessitant plusieurs techniques spécifiques, présentant chacune ses propres avantages et limites.

L'identification des phlébotomes adultes repose sur l'observation de structures à la fois externes (e.g., antennes, palpes et génitalia mâles) et internes (e.g., pharynx, cibarium et spermathèques). La dissection et l'isolement de ces dernières facilitent leur observation et, par conséquent, une identification précise. Ainsi, contrairement aux moustiques ou aux punaises triatomes, les phlébotomes doivent être montés entre lame et lamelle avant leur identification. Jusqu'aux années 1980, l'observation microscopique constituait la seule méthode disponible pour l'identification des phlébotomes, et demeure aujourd'hui encore l'approche la plus largement utilisée. Le choix des procédés et des préparations était donc relativement simple et reposait principalement sur une dichotomie : d'une part, le montage définitif permettant la conservation à long terme des spécimens et, d'autre part, le montage rapide destiné à l'identification dans un milieu n'assurant pas une conservation prolongée. Le montage définitif, par exemple dans une résine telle que le baume du Canada, est une procédure chronophage, nécessitant une déshydratation complète des échantillons. De plus, l'indice de réfraction de ce milieu n'est pas toujours optimal pour une observation aisée des spermathèques. À l'inverse, le montage dans un milieu aqueux (e.g., liquide de Hoyer) est plus rapide et permet une meilleure visualisation des spermathèques réfringentes, mais il ne garantit pas une conservation à long terme des préparations, car ce milieu tend à absorber l'eau de l'atmosphère. Une option consiste à sceller la lame avec du vernis à ongles une fois celle-ci complètement sèche. Ce compromis demeure d'actualité et influence le choix de la méthode de montage en fonction de l'objectif poursuivi par la préparation. Depuis les années 1980, les études d'identification des phlébotomes combinent des approches morphologiques et biochimiques. La première d'entre elles reposait sur l'analyse des hydrocarbures cuticulaires, rapidement supplantée par des techniques de biologie moléculaire (i.e., amplification aléatoire d'ADN polymorphe [RAPD], polymorphisme de longueur des fragments de restriction [RFLP], amplification et séquençage de l'ADN selon la méthode de Sanger, ainsi que séquençage de nouvelle génération [NGS]). Aujourd'hui, ces approches moléculaires sont complétées par des méthodes protéomiques telles que le Matrix-Assisted Laser

Desorption-Time of Flight (MALDI-ToF). Par ailleurs, l'identification moléculaire des espèces peut être associée à la détection des agents pathogènes par PCR (Leishmania, *Trypanosoma*, *Bartonella* et Phlébovirus), tous pouvant être détectés par PCR conventionnelle ou en temps réel, ce qui nécessite une adaptation des protocoles d'échantillonnage et de conservation en fonction des objectifs assignés [3, 32]. En complément des caractères morphologiques traditionnellement utilisés pour la discrimination spécifique, d'autres approches morphologiques peuvent être appliquées (i.e., géomorphométrie alaire).

S'appuyant principalement sur l'expérience des auteurs et sur les données de la littérature, l'objectif de cette étude est de proposer des lignes directrices standardisées pour le montage et le traitement des phlébotomes adultes, afin d'optimiser les analyses morphologiques et moléculaires.

La nécessité de réaliser certaines analyses (e.g., biologie moléculaire ou MALDI-ToF) impose de conserver une partie du phlébotome non indispensable à l'identification morphologique, soulignant ainsi l'importance d'un choix éclairé des protocoles.

Dans cet article, nous nous concentrons sur les méthodes d'anesthésie et d'euthanasie des phlébotomes capturés vivants, leur conservation et leur processus de montage, que ce soit pour une identification rapide ou pour une conservation à long terme permettant des études ultérieures.

1. Capture des phlébotomes

Les phlébotomes adultes peuvent être collectés vivants ou morts à l'aide de différentes méthodes, telles que les pièges lumineux miniatures de type CDC, les pièges adhésifs, les aspirateurs utilisés avec des pièges de Shannon, ou par capture directe dans leurs lieux de repos naturels (e.g., abris pour animaux). Ces méthodes consistent à placer les pièges dans des habitats appropriés, à attirer les phlébotomes à l'aide de lumière ou d'autres attractifs (CO_2 ou attractifs chimiques), puis à les collecter pour des analyses ultérieures, comme décrit dans plusieurs publications [2, 3, 32, 36, 49]. La capture de phlébotomes vivants permet l'application de l'ensemble des techniques présentées plus loin, tandis que la collecte d'individus morts empêche l'isolement de Leishmania ou de souches virales.

Certaines techniques de capture, telles que les papiers englués, entraînent fréquemment la perte d'organes des phlébotomes (antennes, palpes, ailes ou pattes). De plus, l'huile de ricin utilisée pour enduire les papiers englués adhère aux phlébotomes et doit être éliminée dès le début du traitement, généralement par un bain de 15 minutes dans un mélange d'éthanol et d'éther diéthylique à parts égales.

2. Euthanasie des spécimens

Après la collecte, les phlébotomes vivants doivent être euthanasiés. Avec certaines méthodes de capture (e.g., papiers englués ou pièges lumineux CDC équipés d'un flacon contenant détergent ou éthanol), les phlébotomes sont déjà morts lors de la collecte. Les analyses de biologie moléculaire peuvent être appliquées aux spécimens collectés directement dans l'éthanol, ainsi qu'aux autres s'ils sont stockés dans l'éthanol le plus rapidement possible.

Toutefois, aucune de ces méthodes ne permet le traitement des insectes par MALDI-ToF. De plus, certaines méthodes d'euthanasie peuvent entraîner la perte de certains caractères morphologiques. Il est donc essentiel d'utiliser un agent d'euthanasie standard approprié afin de garantir une identification correcte ou une conservation à long terme des spécimens de référence (i.e., préservés et conservés à des fins de référence ou de comparaison ultérieures). Des substances chimiques telles que l'acétate d'éthyle, l'éther éthylique, le tétrachloroéthane et le chloroforme peuvent être imbibées sur du coton et placées dans un récipient contenant les phlébotomes afin de les euthanasier. Ces substances doivent être manipulés avec précaution, conformément aux recommandations du fabricant, en raison de leur toxicité. Nous ne recommandons pas l'utilisation du chloroforme pour l'euthanasie des phlébotomes, celui-ci étant, d'après notre expérience, peu compatible avec les études de biologie moléculaire. Compte tenu du caractère dangereux de l'ensemble de ces produits et de leur adéquation discutable aux analyses moléculaires, l'utilisation de ces substances chimiques est généralement déconseillée.

La méthode la plus largement utilisée, permettant de préserver la morphologie, l'ADN et les protéines, est la congélation à sec des spécimens. Les phlébotomes doivent être congelés suffisamment longtemps pour être complètement anesthésiés, mais pas au point de (i) se dessécher ou (ii) compromettre la viabilité de *Leishmania*, si l'objectif est leur isolement *in vitro* à partir du tube digestif du phlébotome. **Nous recommandons donc une durée de congélation de 15 à 20 minutes à -20°C, avec une surveillance régulière afin de s'assurer que les insectes sont uniquement étourdis, sans tuer les *Leishmania*.**

En l'absence de congélateur, les insectes peuvent être euthanasiés à l'aide de CO_2. En conditions de terrain, lorsque l'utilisation de bouteilles de CO_2 n'est pas possible, les spécimens peuvent être tués à l'aide de petites cartouches commerciales de CO_2 utilisées dans les siphons à soda, mais leur transport aérien peut être soumis à des restrictions. En dernier recours, les insectes peuvent être tués par exposition à la fumée de tabac. Les phlébotomes sont alors capturés vivants dans un piège CDC, récupérés à l'aide d'un aspirateur, maintenus dans le tube en verre, puis exposés à la fumée de tabac, ce qui entraîne leur mort en

quelques secondes. Cette méthode est applicable dans toutes les conditions de terrain, y compris en situation d'isolement extrême. Toutefois, le tube en verre étant imprégné de fumée, il ne peut plus être utilisé pour la capture et la manipulation ultérieures de phlébotomes vivants sans un nettoyage approfondi. Néanmoins, le même aspirateur non nettoyé peut encore être utilisé pour euthanasier des phlébotomes provenant d'autres pièges en vue de leur fixation. Il est également nécessaire de vérifier que tous les spécimens ont bien été retirés de l'aspirateur. Ces méthodes sont compatibles avec l'isolement de *Leishmania* par dissection du tube digestif.

En complément des caractères morphologiques traditionnellement utilisés pour la discrimination spécifique, d'autres approches morphologiques peuvent être appliquées (i.e., géomorphométrie alaire).

Préambule : les considérations de sécurité et de réglementation doivent se référer aux Fiches de Données de Sécurité (FDS) correspondantes.

Tous les produits chimiques présentés dans ce guide doivent être manipulés dans des conditions de sécurité strictes. Les comités d'hygiène et de sécurité des établissements de recherche sont à disposition pour fournir des informations non seulement sur les dangers associés à ces substances, mais également sur les modalités de manipulation et d'élimination des déchets. Il est toutefois impératif de respecter les consignes de sécurité relatives à leur utilisation et à leur élimination. Il convient de rappeler qu'il incombe à chaque utilisateur de veiller au respect des bonnes pratiques de laboratoire, des règles de sécurité, ainsi que de la législation et de la réglementation en vigueur dans son pays ou son institution de recherche.
Par ailleurs, certains produits chimiques ou certains de leurs composants (i.e., hydrate de chloral) sont réglementés dans certains pays. Une liste des abréviations utilisées dans ce manuscrit est fournie dans le tableau 1.

Tableau 1: Liste des abréviations.

ADN	Acide désoxyribonucléique
ARN	Acide Ribonucléique
BME	Milieu basal Eagle
CDC	Centre de contrôle et de prévention des maladies
CMCP	Monochlorophénol camphré
CMR	Substance carcinogène, mutagène, reprotoxique
COI	Sous-unité I de la Cytochrome c oxydase
CytB	Gène du cytochrome b
ELISA	Dosage immuno-enzymatique
EtOH	Éthanol
M199	Milieu 199
MALDI-ToF MS	Spectrométrie de masse à temps de vol par désorption/ionisation laser assistée par matrice
MEM	Milieu essentiel minimum
NGS	Séquençage de dernière génération
NNN	Milieu Novy-MacNeal-Nicolle
PCR	Réaction en chaîne par polymérase
RDP Laos	République démocratique populaire de Laos
PNOC	Gène de la prépronociceptine
qPCR	PCR quantitative (PCR en temps réel)
RAPD	ADN polymorphe amplifié aléatoirement
RFLP	Polymorphisme de longueur des fragments de restriction
RI	Indice de réfraction
RNases	Ribonucléases
RNASS	Solution de stabilisation des ARN
RT-PCR	Transcription inverse-PCR
TFA	Acide trifluoroacétique

3. Conservation des spécimens avant traitement

Il existe cinq principales méthodes de fixation avant le traitement :

3.1. Congélation

Cette méthode est idéalement réalisée à -20°C ou, de préférence, à -80°C. Ces modes de conservation sont aujourd'hui plus largement utilisés que le stockage dans l'azote liquide. Dans tous les cas, la cryoconservation doit être mise en œuvre le plus rapidement possible après l'étourdissement des spécimens. Le stockage à froid en congélateur présente l'avantage de préserver intégralement les insectes eux-mêmes, ainsi que l'ARN, l'ADN et les protéines, avec une intégrité optimale tout au long de la période de conservation. En revanche, l'azote liquide peut endommager sévèrement les ailes, les pattes, les palpes et les antennes, entraînant souvent leur amputation et, occasionnellement, la perte de caractères morphologiques

clés. La congélation à sec en congélateur est moins traumatisante pour les spécimens, mais elle n'est pas idéale pour la préservation de leurs organes fragiles. Il est important de noter qu'au moment de la décongélation, les ailes, antennes, palpes ou pattes peuvent adhérer aux parois des tubes et se détacher sous l'effet de la condensation. Cependant, la conservation par congélation n'est pas toujours réalisable lors des études de terrain, car elle nécessite un accès à un congélateur ou à un conteneur d'azote liquide. Le stockage en congélateur est pleinement compatible avec la détection des agents pathogènes par des outils moléculaires sans perte de sensibilité, bien que la détection et l'isolement des virus à ARN nécessitent une congélation à -80°C ou dans l'azote liquide lorsqu'une conservation à long terme est requise. En revanche, la congélation des échantillons ne permet pas l'isolement de *Leishmania* par dissection du tube digestif, sauf si les phlébotomes sont d'abord immergés dans la vapeur d'azote puis dans l'azote liquide (par exemple dans des flacons placés dans un bas), simulant ainsi la cryoconservation de *Leishmania*.

3.2. Conservation dans l'alcool (éthanol ou alcool isopropylique)

Il s'agit probablement de la méthode la plus largement utilisée pour la conservation des phlébotomes. Elle est facile à mettre en œuvre sur le terrain, même dans des conditions difficiles sans accès à un laboratoire. La conservation dans l'alcool est particulièrement adaptée aux études morphologiques, car les organes fragiles (ailes, pattes, antennes ou palpes) restent intacts en l'absence de bulles d'air dans le tube de conservation. Nous recommandons donc de sceller le tube à l'aide d'une petite boule de coton afin d'éliminer toute bulle d'air et de placer une étiquette au-dessus du bouchon de coton (Figure 1). La concentration appropriée d'alcool reste sujette à débat. En général, des concentrations inférieures à 70% ne sont pas recommandées [45, 66]. Des concentrations plus élevées assurent une meilleure conservation de l'ADN sur de longues périodes, mais rendent les spécimens plus fragiles et cassants pour les études morphologiques. L'utilisation d'éthanol à 96% (mélange azéotropique) garantit une stabilité de la concentration dans le temps, en particulier dans les régions humides telles que les pays tropicaux, bien que l'éthanol à 95 % soit souvent plus facile à obtenir. Quelle que soit la concentration, l'ADN est généralement bien conservé dans l'éthanol (bien que moins efficacement que par congélation, notamment pour les méthodes moléculaires de type NGS). En revanche, les protéines sont beaucoup moins stables, en particulier pour les applications protéomiques telles que le MALDI-ToF. Les phlébotomes conservés dans l'alcool pendant quelques mois peuvent encore être identifiés morphologiquement, mais il est impossible de générer des spectres protéiques de référence à partir de ces spécimens. La conservation dans l'alcool ou à sec peut être améliorée si l'échantillon est également congelé à -20°C. La congélation à -20°C améliore principalement la préservation moléculaire (e.g., acides nucléiques) en ralentissant la dégradation et offre également le bénéfice secondaire de la préservation morphologique en réduisant la dégradation tissulaire au fil du temps, bien que l'effet sur la morphologie soit plus limité que sur l'intégrité moléculaire. La conservation dans l'éthanol peut également être utilisée pour la détection de virus à ADN et à ARN lorsque l'éthanol est utilisé à une concentration d'au moins 70 % pour une durée de stockage courte, inférieure à quelques mois. Par ailleurs, l'alcool isopropylique peut être facilement disponible dans certains pays et permet la conservation de l'ADN, mais il rend les spécimens friables. Il n'est pas inflammable comme l'éthanol et peut donc être transporté plus facilement. Si nécessaire, les phlébotomes conservés dans l'azote liquide ou congelés à sec peuvent être transférés dans l'alcool, combinant ainsi les inconvénients des deux méthodes.

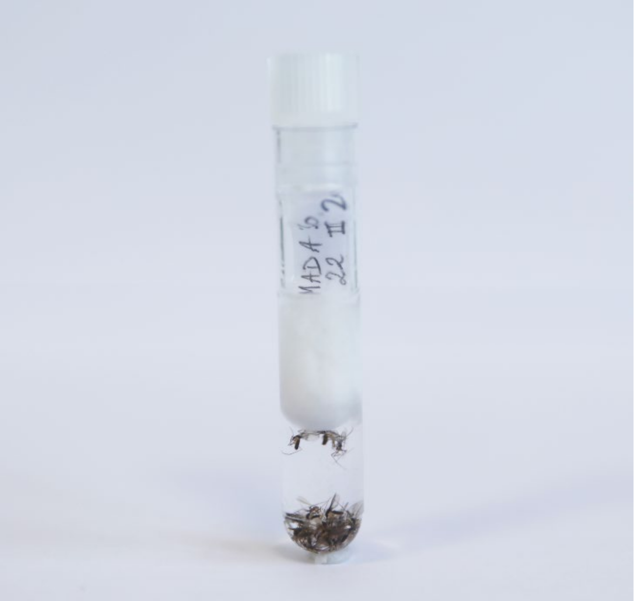

Figure 1. Phlébotomes conservés dans l'éthanol.

3.3. Conservation dans une solution de stabilisation de l'ARN (RNASS)

Ce réactif aqueux, largement utilisé et non toxique, est conçu pour stabiliser et protéger l'ARN dans les tissus frais et les échantillons cellulaires. Il agit en pénétrant rapidement dans le spécimen et en inactivant les RNases (enzymes responsables de la dégradation de l'ARN), empêchant ainsi toute dégradation de l'ARN sans nécessiter de congélation immédiate. La conservation en RNASS est généralement efficace pour préserver la morphologie globale des tissus et des cellules en vue d'analyses

histologiques ultérieures. Bien que cette solution soit optimisée pour la stabilisation de l'ARN plutôt que pour la fixation, un stockage à court ou moyen terme permet en général de maintenir correctement l'intégrité structurale. La RNASS permet le stockage des échantillons à température ambiante jusqu'à 7 jours, à 4 °C pendant plusieurs semaines, ou à –20°C/-80°C pour une conservation à long terme. Cette méthode est particulièrement précieuse lors de travaux de terrain ou en milieu clinique lorsque l'infrastructure de chaîne du froid est limitée. L'extraction de l'ARN nécessite généralement de retirer les échantillons du réactif et de les traiter selon des protocoles standard.

3.4. Conservation à sec à température ambiante

Il s'agit d'une méthode plus ancienne qui, lorsqu'elle est appliquée à des spécimens conservés in toto (montés entiers), présente l'inconvénient majeur de préserver insuffisamment les organes fragiles tels que les ailes, les pattes, les antennes et les palpes. Toutefois, des études protéomiques utilisant le MALDI-ToF restent possibles si la déshydratation est réalisée dès la fixation à l'aide d'un dessiccant de type gel de silice. En revanche, les analyses moléculaires ciblant l'ADN sont difficiles à réaliser sur ce type d'échantillons, car l'ADN est souvent fragmenté et présent en faible quantité, rendant les analyses plus complexes que sur des échantillons frais ou congelés, en particulier pour le génome nucléaire. Néanmoins, des techniques récentes telles que la muséomique peuvent être appliquées à ce type de matériel [34]. En conséquence, ce mode de conservation n'est pas recommandé, sauf en l'absence d'alternative. Il peut être combiné à un stockage à froid en plaçant les tubes dans un congélateur à -20°C ou -80°C. La principale difficulté réside dans l'obtention d'un montage correct des spécimens ou des parties du corps nécessaires à l'identification. Pour ce faire, une réhydratation est indispensable. Nous recommandons l'utilisation d'une solution de Triton X-100. La durée de réhydratation varie de quelques heures à plusieurs jours et nécessite une surveillance étroite. Après une réhydratation complète, les spécimens doivent être rincés dans trois bains d'eau consécutifs.

3.5. Conservation sur papier filtre

Le principal avantage du papier filtre réside dans la stabilité à long terme de l'ADN génomique au sein des cellules d'un corps entier non fixé et desséché, ou des cellules sanguines, conservés à température ambiante. Le papier filtre est fourni sous forme de petites cartes, permettant le stockage de plusieurs centaines d'échantillons à température ambiante dans un volume équivalent à celui d'un petit tiroir de bureau. La matrice du papier filtre est imprégnée d'agents qui dénaturent les agents infectieux ; les échantillons ne sont donc plus considérés comme un danger biologique. Cela permet le stockage et le transport des échantillons sans précautions spécifiques liées au risque biologique [68].

4. Dissection des spécimens

Contrairement à de nombreux autres insectes, qui sont identifiés sur la base de caractères externes observables sur des individus montés in toto sur épingle, les phlébotomes nécessitent une dissection et un montage sur lame afin d'étudier les caractères anatomiques indispensables à une identification spécifique adéquate. Quelle que soit la procédure de préparation et de montage choisie, la même technique de dissection est utilisée (Figs. 2 et 3) (https://zenodo.org/records/18198006).

Utilisation du Triton X-100 : solution aqueuse non ionique

Il convient de noter que le montage concerne des spécimens fraîchement capturés ou correctement conservés. De nombreux collecteurs disposent toutefois d'échantillons d'insectes conservés à sec (pour une utilisation en MALDI-ToF) ou stockés dans l'alcool depuis de nombreuses années. Malheureusement, la conservation prolongée dans l'alcool n'est pas optimale et les arthropodes ainsi conservés deviennent très difficiles à préparer pour l'examen microscopique. Un problème fréquemment rencontré est la dégradation des contenants plastiques, suivie de l'évaporation de l'alcool. Dans ces deux situations, les options de récupération sont limitées, les spécimens ayant soit séjourné trop longtemps dans l'alcool, soit complètement séché. C'est dans ce contexte qu'est apparue l'idée d'utiliser des agents mouillants n'étant pas des détergents agressifs. Le Triton X-100 se présente sous la forme d'une solution aqueuse non ionique (solution de 4-(1,1,3,3-tétraméthylbutyl)phényl-polyéthylène glycol, ou t-octylphénoxypolyéthoxyéthanol, éther de polyéthylène glycol tert-octylphénylique), largement utilisée comme détergent en biologie cellulaire et moléculaire. Il permet la perméabilisation des membranes cellulaires et nucléaires.

La procédure suivante décrit l'utilisation du Triton X-100 en solution aqueuse à 0,5 % :

- Imprégner l'échantillon sec d'alcool absolu.
- Ajouter le volume nécessaire de solution de Triton X-100 à 0,5% afin que l'échantillon soit entièrement immergé.
- Laisser agir de 5 minutes à plusieurs jours, avec une surveillance régulière. Tous les arthropodes doivent être complètement séparés dans la solution.
- Retirer la solution de Triton X100 et la remplacer par une solution d'hydroxyde de potassium.

4.1. Tête

La dissection peut être réalisée à l’aide d’aiguilles fines ou d’épingles entomologiques sous un stéréomicroscope (Figs. 2 et 3). Les aiguilles les plus couramment utilisées sont les suivantes : 26G × 1/2" (0,45 × 13 mm), 30G × 1/2" (0,3 × 13 mm) ou 25G × 5/8" (0,5 × 16 mm). Pour préparer un spécimen en vue de son identification, au minimum, la tête est séparée du corps et montée face ventrale vers le haut afin de mettre en évidence le cibarium et le pharynx, tandis que le thorax et l’abdomen sont montés latéralement après dissection. Le montage de la tête selon un axe ventro-dorsal garantit que le foramen occipital est orienté vers le haut, permettant ainsi l’observation directe du cibarium. L’accès à ces structures anatomiques est facilité lorsque la tête est entièrement séparée du reste du corps.

4.2. Ailes et thorax

Les ailes doivent être montées à plat. Chaque aile peut être détachée à sa base et montée indépendamment, ou bien une seule aile peut être montée, l’autre restant attachée au thorax. Si une analyse de morphométrie géométrique est envisagée, il est indispensable d’identifier et d’étiqueter correctement les ailes droite et gauche avant le montage. Le thorax est divisé en plusieurs parties, chacune renfermant des informations taxonomiques très importantes [20, 64]. En général, il est monté en vue latérale afin de permettre l’examen de la chétotaxie et de la distribution des couleurs. La présence d’insertions de soies dans certaines régions du thorax peut être utilisée pour distinguer certaines espèces du genre *Brumptomyia*. La distribution de la coloration peut servir à séparer les phlébotomes néotropicaux au niveau du genre (e.g., *Bichromomyia*), des séries d’espèces (e.g., *Pintomyia*), voire des espèces au sein d’un même genre (e.g., *Micropygomyia*, *Nyssomyia*, *Psathyromyia* et *Psychodopygus*) [20]. Ainsi, lorsque le thorax n’est pas destiné à des analyses moléculaires, il doit être correctement monté. ICe n’est pas l’intensité des couleurs qui importe, mais leur répartition sur le thorax. Par conséquent, l’éclaircissement n’élimine ni la pigmentation ni son motif.

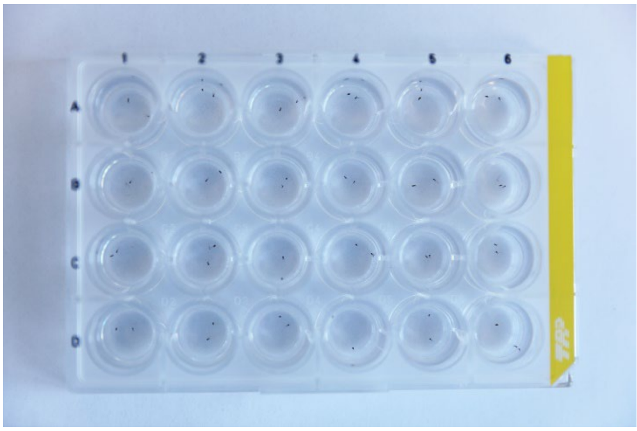

Figure 3. Plaque à 24 puits, chacun contenant tête et extrémité de l’abdomen d’un phlébotome.

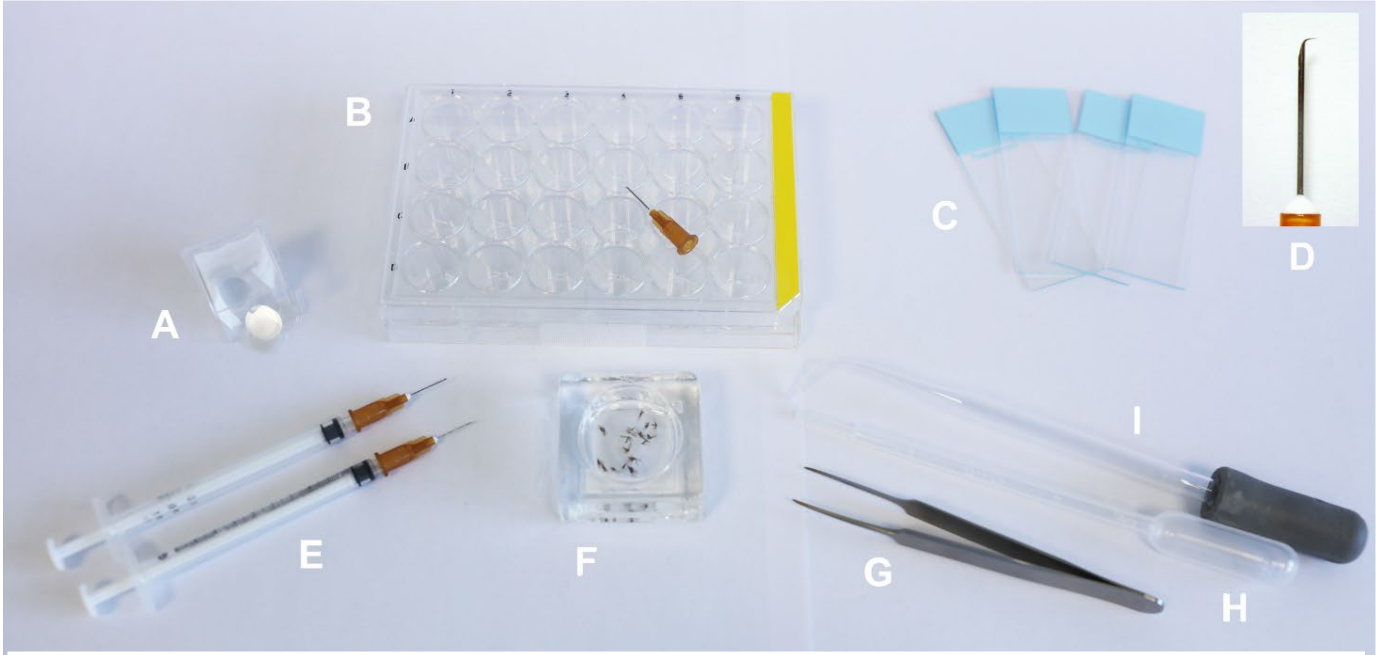

Figure 2. Matériel nécessaire au montage des phlébotomes : A: lamelles rondes en verre (diamètre 10 ou 12 mm); B: plaque à 24 puits et aiguille crochetée (si l’huile de girofle ou l’Euparal® est utilisée pour le traitement des phlébotomes, ne pas utiliser de plaques en acrylique, car une réaction chimique endommagerait les spécimens); C: lames en verre adaptées au marquage; D: détail du crochet de l’aiguille; E: aiguilles montées sur seringues; F: verre de montre ou récipient équivalent contenant les phlébotomes à monter; G: pinces Dumont; H: pipette plastique; I: pipette en verre courbée par chauffage afin de faciliter le transfert des liquides dans les puits.

This is an Open Access article distributed under the terms of the Creative Commons Attribution License (https://creativecommons.org/licenses/by/4.0), which permits unrestricted use, distribution, and reproduction in any medium, provided the original work is properly cited.

4.3. Génitalia

Un soin particulier doit être porté au montage des organes génitaux qui sont sont pairs tant chez les mâles que chez les femelles, car ils sont essentiels pour l'identification des genres, des sous-genres et des espèces.

4.3.1. Mâles

Les organes génitaux sont externes et constitués de forceps pairs, chacun comprenant, dans sa partie dorsale, l'articulation gonocoxite–gonostyle et, dans sa partie ventrale, le lobe épandrial. Le gonostyle porte des épines et parfois des soies, qui doivent être dénombrables et dont les points d'insertion doivent être clairement visibles. Il est important d'observer avec soin la face interne du gonocoxite, qui peut porter un faisceau de soies sessiles ou insérées sur un lobe (= tubercule) [22]. Les collègues moins expérimentés en dissection peuvent réaliser un simple montage latéral, sans détacher les génitalia de l'extrémité de l'abdomen (https://zenodo.org/records/18311158). Dans ce cas, la superposition des deux parties de l'appareil génital peut compliquer le décompte, par exemple, des soies internes du gonocoxite, mais cette approche permet d'éviter d'endommager les génitalia lors d'une dissection mal maîtrisée. Les collègues plus expérimentés peuvent tenter d'ouvrir l'appareil génital en deux afin de le séparer. Pour ce faire, le côté biseauté d'une aiguille (de type aiguille pour réaction intradermique) est introduit délicatement, permettant de détacher sans sectionner complètement les génitalia afin de séparer les ensembles gonocoxite–gonostyle (https://zenodo.org/records/18311158) Cette technique facilite l'observation des faces internes et permet également une meilleure visualisation des paramères et des gaines paramérales, qui ne se superposent plus. Pour un montage latéral, conduisant souvent à la superposition des organes, les spécimens doivent être parfaitement clarifiés.

4.3.2. Femelles

L'appareil génital est interne et constitué des spermathèques. En l'absence de dissection, celles-ci doivent être observées à travers les téguments en montant l'abdomen en position ventrale. Quel que soit le milieu de montage choisi, les spermathèques elles-mêmes peuvent généralement être observées correctement, en particulier lorsqu'elles ne sont ni lisses ni trop clarifiées. En revanche, l'observation de spermathèques lisses à paroi fine peut s'avérer problématique dans des milieux peu réfringents. De plus, l'observation de la base des conduits des spermathèques est indispensable à l'identification spécifique, comme chez les espèces du sous-genre *Larroussius* [35, 37, 38], principaux vecteurs de *Leishmania infantum* dans l'Ancien Monde. En l'absence de cette observation, l'identification des spécimens demeure impossible. Afin de surmonter ces difficultés, le complexe furca génitale-spermathèques doit être extrait de l'abdomen (https://zenodo.org/records/18311106). Les spermathèques sont généralement difficiles à observer lors de la dissection, mais la furca génitale est relativement facile à localiser. Étant donné que les conduits des spermathèques s'ouvrent dans la furca génitale, l'isolement de cette dernière permet normalement d'isoler également les spermathèques. Si les spermathèques sont accidentellement sectionnées au cours du processus, elles ne sont pas perdues et peuvent toujours être observées à l'intérieur des téguments abdominaux (Fig. 4).

4.4. Dissection du tube digestif pour l'isolement de *Leishmania*

La dissection du tube digestif est indispensable pour la détection et l'isolement de *Leishmania* chez les phlébotomes femelles. Cette procédure peut être réalisée aussi bien sur le terrain qu'au laboratoire afin d'évaluer la compétence vectorielle.

Il est recommandé de travailler sur des femelles fraîchement euthanasiées. Les femelles doivent être lavées à l'eau ou dans du sérum physiologique contenant un détergent doux afin d'éliminer l'excès de soies. Cette étape permet de maintenir des conditions aseptiques pour l'isolement de *Leishmania* tout en préservant les caractères morphologiques nécessaires à l'identification. Pour localiser et isoler *Leishmania*, le tube digestif moyen doit être soigneusement extrait et placé dans une goutte unique de sérum physiologique stérile (NaCl 0,9 %). Après observation de parasites mobiles sous microscope optique (grossissement recommandé : ~200×), ceux-ci sont transférés dans un milieu de culture à l'aide d'une seringue à insuline ou d'une micropipette (pour plus de détails, voir section 4.4.3).

La tête et les organes génitaux doivent être montés directement dans le liquide de Marc-André afin de les clarifier. Important : le liquide de Marc-André ne doit en aucun cas entrer en contact avec les *Leishmania*, ni directement ni indirectement par l'intermédiaire des outils ou des aiguilles, car il est létal pour les parasites.

La dissection des femelles peut être réalisée soit sur une seule lame, soit sur deux lames distinctes ; ces deux options présentent chacune des avantages et des limites (Fig. 5; https://zenodo.org/records/18311154).

4.4.1. Méthode à deux lames

La première option consiste à travailler sur deux lames distinctes : l'une contenant du sérum physiologique stérile pour l'extraction du tube digestif, et l'autre destinée au montage de la tête et des spermathèques dans le liquide de Marc-André. Toutefois, en conditions de terrain, il est fréquent que deux ou trois personnes réalisent les dissections et transmettent leurs préparations à un seul chercheur chargé de l'identification spécifique et de l'évaluation de l'infection du tube digestif par *Leishmania*. La gestion de deux lames peut alors poser des problèmes de traçabilité des échantillons et, en particulier, rendre difficile l'attribution certaine d'une infection à un individu précis lorsqu'un tube digestif positif est détecté (https://zenodo.org/records/18311154).

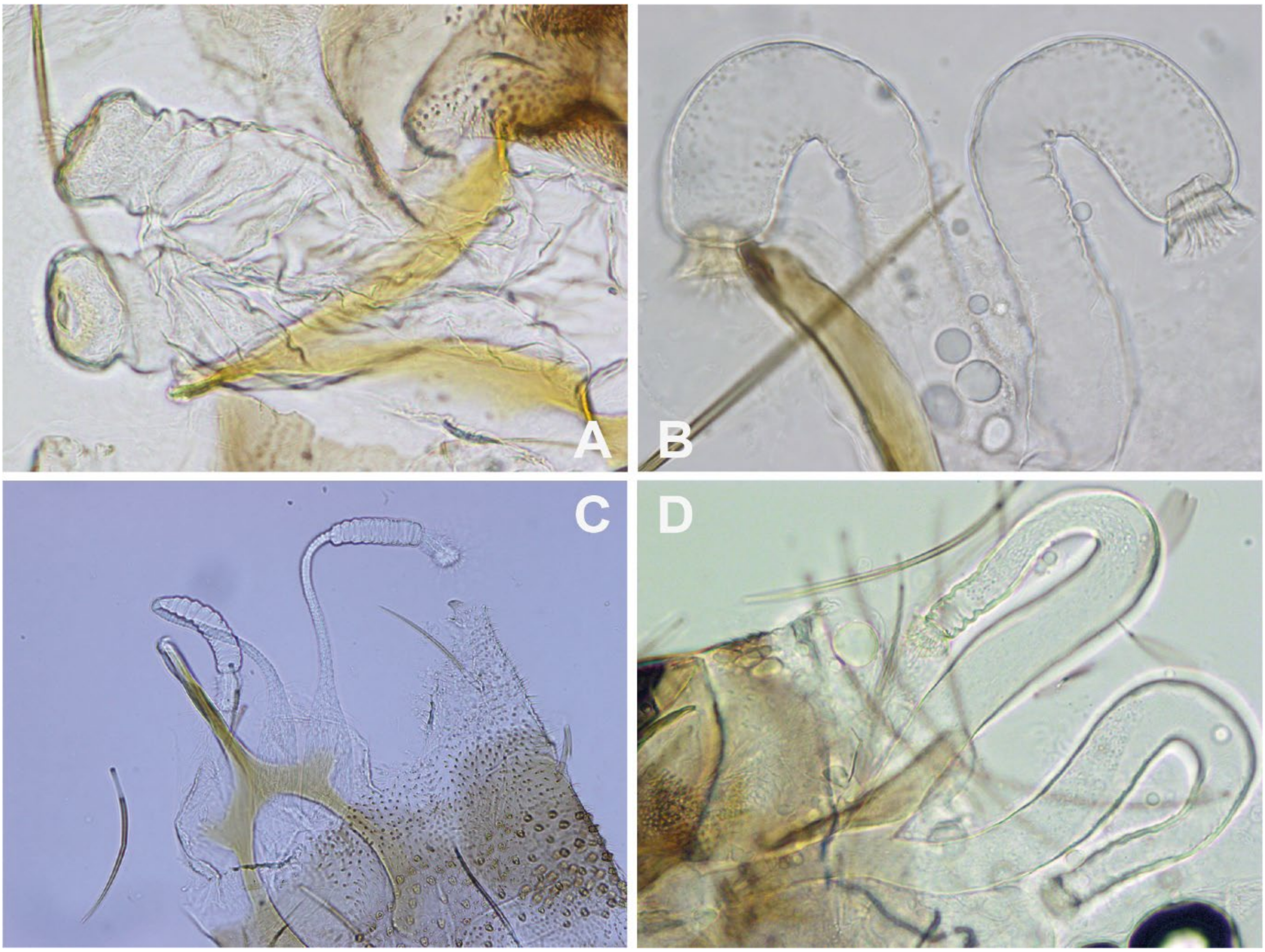

Figure 4. Spermathèques disséquées et montées dans le liquide de Marc-André à partir de spécimens frais.A: *Idiophlebotomus longiforceps* (RDP Laos); B: *Sergentomyia minuta* (France); C: *Phlebotomus ariasi* (France); D: *Sergentomyia anodontis* (RDP Laos).

4.4.2. Méthode à une seule lame

L'utilisation d'une seule lame garantit une meilleure traçabilité des résultats, mais nécessite plusieurs précautions. Afin de maximiser la stérilité au cours de cette étape, les opérateurs doivent se désinfecter régulièrement les mains avec une solution hydroalcoolique. Des lames non dépolies et des lamelles carrées (22 × 22 mm), enveloppées dans du papier aluminium et stérilisées par chaleur sèche (étuve de type Poupinel), doivent être utilisées, ainsi que des aiguilles stériles pour chaque dissection (suggestion : 25G Ø 0,5 mm × 16 mm). Le phlébotome est placé dans une goutte de sérum physiologique stérile au centre de la lame. La tête est sectionnée, tandis qu'une incision est réalisée entre les 6ème et 7ème tergites et sternites abdominaux sans sectionner le tube digestif (une incision plus antérieure peut être effectuée si des spermathèques très longues sont attendues). Le thorax est ensuite immobilisé à l'aide d'une aiguille, et les derniers segments abdominaux sont tirés délicatement avec une autre aiguille afin d'extraire le tube digestif. En cas d'échec, il est possible de bloquer l'extrémité de l'abdomen avec une aiguille et de tirer le tube digestif par sa partie antérieure. Si cette tentative échoue également, le tube digestif doit être extrait en retirant autant que possible les téguments résiduels qui l'entourent. Une fois le tube digestif extrait, les derniers segments abdominaux doivent être séparés en sectionnant le tube digestif. Le tube digestif est ensuite placé dans une nouvelle goutte de sérum physiologique stérile située sur un côté de la lame, puis délicatement recouvert d'une lamelle stérile. La tête et les derniers segments abdominaux sont transférés dans une petite goutte de liquide de Marc-André déposée à l'autre extrémité de la lame, en veillant à éviter tout contact avec les *Leishmania*. La tête est correctement orientée (foramen occipital vers le haut), et les spermathèques sont isolées avec la furca génitale comme décrit précédemment, puis recouvertes d'une petite lamelle circulaire (Ø 12 mm, à ne pas confondre avec les lamelles carrées stériles). Le reste de la carcasse du phlébotome et les ailes demeurent dans la goutte de sérum physiologique au centre de la lame (https://zenodo.org/records/18311154). En cas de résultat

This is an Open Access article distributed under the terms of the Creative Commons Attribution License (https://creativecommons.org/licenses/by/4.0), which permits unrestricted use, distribution, and reproduction in any medium, provided the original work is properly cited.

positif, ou dans le cadre d'analyses taxonomiques, le thorax et l'abdomen peuvent être conservés pour des études moléculaires ou protéomiques, et les ailes peuvent être montées dans un milieu aqueux. Afin d'assurer la conservation du montage, l'excès de liquide de Marc-André peut être remplacé par un milieu de montage aqueux tel que la gomme au chloral (= Hoyer) ou un milieu à base d'alcool polyvinylique.

Des vidéos détaillées illustrant ces procédures sont disponibles (dissection du tube digestif des phlébotomes: https://zenodo.org/records/18303014 et dissection des glandes salivaires: https://zenodo.org/records/18302850), elles ne seront donc pas détaillées ici.

4.4.3. Isolement et culture de Leishmania à partir du tube digestif des phlébotomes

L'isolement des parasites à partir de la dissection de femelles infectées est une procédure délicate nécessitant une grande technicité et devant être préalablement pratiquée sur des spécimens exempts de parasites. Après dissection, les tubes digestifs sont transférés dans une nouvelle goutte de sérum physiologique stérile (0,9 %) ou de solution de Locke pour le lavage [4]. Les tubes digestifs disséqués peuvent ensuite être traités de deux manières : i) examinés au microscope optique afin d'observer les différents stades des promastigotes de *Leishmania* et leur localisation, en portant une attention particulière à la valve stomodéale ; ii) ouverts afin de faciliter la libération des promastigotes et leur mise en culture en masse [4]. La détection de phlébotomes infectés sur le terrain étant relativement rare, des séances d'entraînement rigoureuses augmentent significativement les chances de réussite de l'isolement.

Si des *Leishmania* sont observés dans le tube digestif, de nouvelles aiguilles stériles doivent être utilisées et une petite quantité de sérum physiologique stérile ajoutée autour de la lamelle par capillarité afin de les libérer. Le tube digestif doit être délicatement et rapidement déchiré pour relâcher les parasites dans le sérum physiologique. À l'aide d'une micropipette de 100 µL ou d'une seringue à tuberculine, les parasites sont ensuite collectés et inoculés dans un milieu de culture correctement étiqueté.

Culture *in vitro* des promastigotes de *Leishmania* : les parasites isolés sont initialement maintenus sur géloses inclinées au sang SNB-9 ou dans un milieu solide de Novy, McNeal, Nicolle (NNN) [16], recouvert soit d'un milieu alpha-MEM stérile [16, 65], soit d'un milieu M199, chacun supplémenté avec 10 % de sérum de veau fœtal stérile et inactivé par la chaleur (FCS), 1 % de vitamines BME, 2 % d'urine humaine stérile (stérilisée par filtration sur seringue Filtropur® S 0,2 µm), et 250 µg/mL d'amikacine (ou 50 µg/mL de gentamicine, ou un mélange d'antibiotiques et d'acides aminés (L-glutamine 200 mM, pénicilline 10 000 U, streptomycine 10 mg/mL)) [47]. Après trois jours, en l'absence de contamination, les cultures sont mises en suspension dans un milieu de congélation approprié, puis stockées à -80°C pendant 1 à 2 ans ou dans l'azote liquide à -196°C pour une conservation à long terme et une utilisation expérimentale ultérieure [7].

4.5. Glandes salivaires

La dissection des glandes salivaires des phlébotomes est une technique fondamentale pour l'étude des interactions vecteur–pathogène, en particulier pour la détection des arbovirus tels que les Phlébovirus (e.g., virus Toscana) [44, 75]. En raison de la taille extrêmement réduite des phlébotomes, cette procédure exige une grande précision sous loupe binoculaire, à l'aide de pinces fines ou d'aiguilles de microdissection, afin d'isoler les glandes salivaires délicates sans les rompre ni les contaminer (https://zenodo.org/records/18302850) [51, 61]. La préservation de l'intégrité des glandes est essentielle pour garantir la fiabilité des analyses moléculaires ultérieures. Une fois extraites, les glandes peuvent être homogénéisées et analysées par RT-PCR, qPCR ou par des tests immunologiques afin de détecter l'ARN viral ou des antigènes [12]. La présence de virus dans les glandes salivaires, et non uniquement dans le tube digestif ou l'hémocoele, confirme que l'agent pathogène a complété sa période d'incubation extrinsèque et qu'il est transmissible lors du repas sanguin [71].

Le processus de dissection est techniquement exigeant en raison de la petite taille des glandes salivaires, et requiert une grande expérience pour éviter la dégradation des échantillons [1, 51]. De plus, les charges virales peuvent être faibles, ce qui impose l'utilisation de méthodes de détection hautement sensibles telles que la PCR nichée ou le séquençage à haut débit [54]. Les risques de contamination renforcent également la nécessité de techniques strictement aseptiques. Au-delà des contraintes techniques, des facteurs biologiques influencent le succès de la détection : la compétence vectorielle varie selon les espèces de phlébotomes, et les taux d'infection fluctuent en fonction des conditions écologiques et saisonnières [33, 61]. La détection de virus dans les glandes salivaires apporte des informations essentielles sur les risques de transmission et permet de mettre en place des mesures ciblées de surveillance et de contrôle [15]. Par exemple, l'identification du virus Toscana chez les phlébotomes dans des régions endémiques a contribué à l'élaboration de protocoles diagnostiques et de recommandations de santé publique [18]. Par ailleurs, l'étude des interactions entre les virus et la salive des phlébotomes pourrait révéler de nouvelles cibles pour le développement de vaccins ou de stratégies thérapeutiques visant à bloquer la transmission [15, 18]

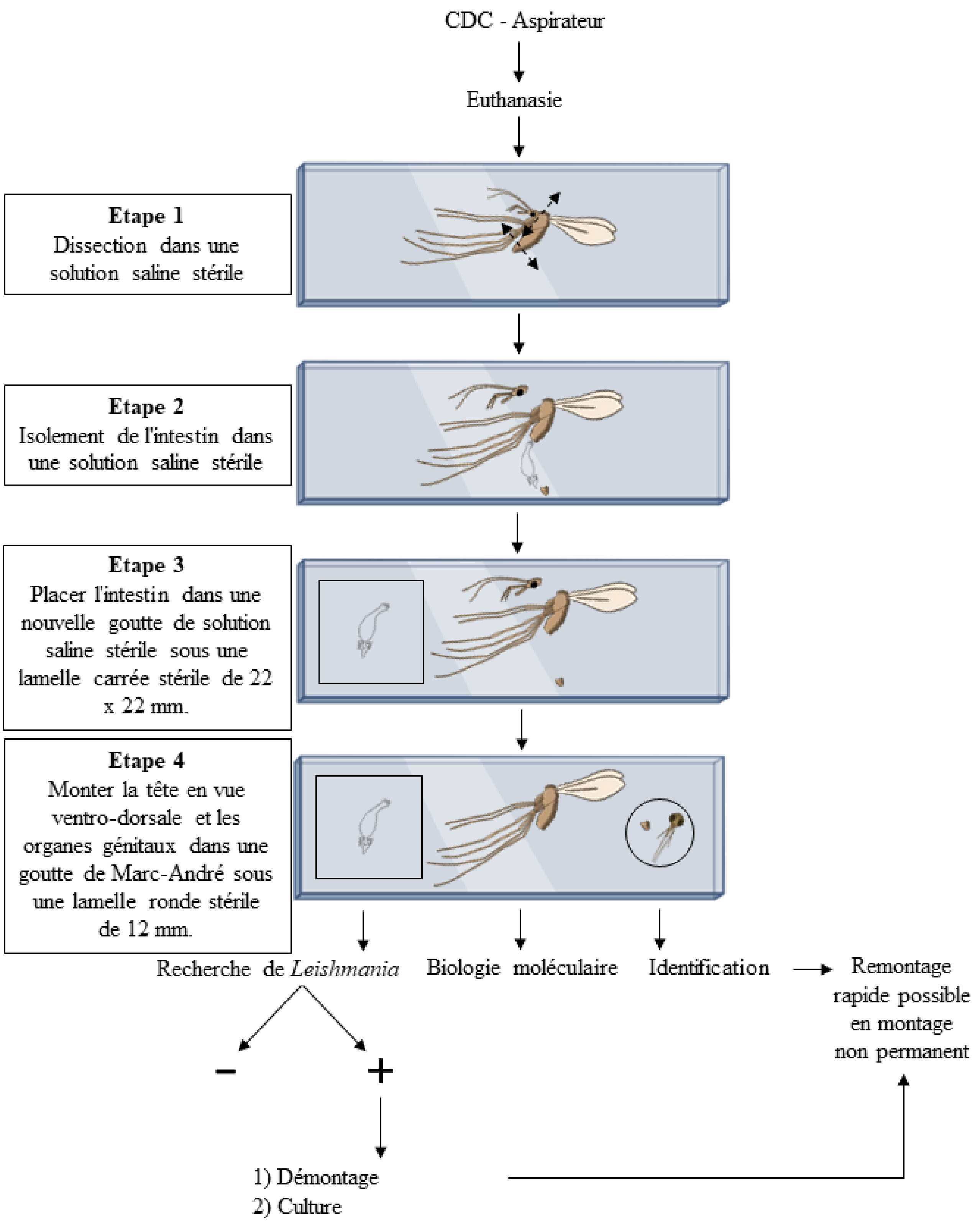

Figure 5 : Méthode d'isolement de *Leishmania*.

This is an Open Access article distributed under the terms of the Creative Commons Attribution License (https://creativecommons.org/licenses/by/4.0), which permits unrestricted use, distribution, and reproduction in any medium, provided the original work is properly cited.

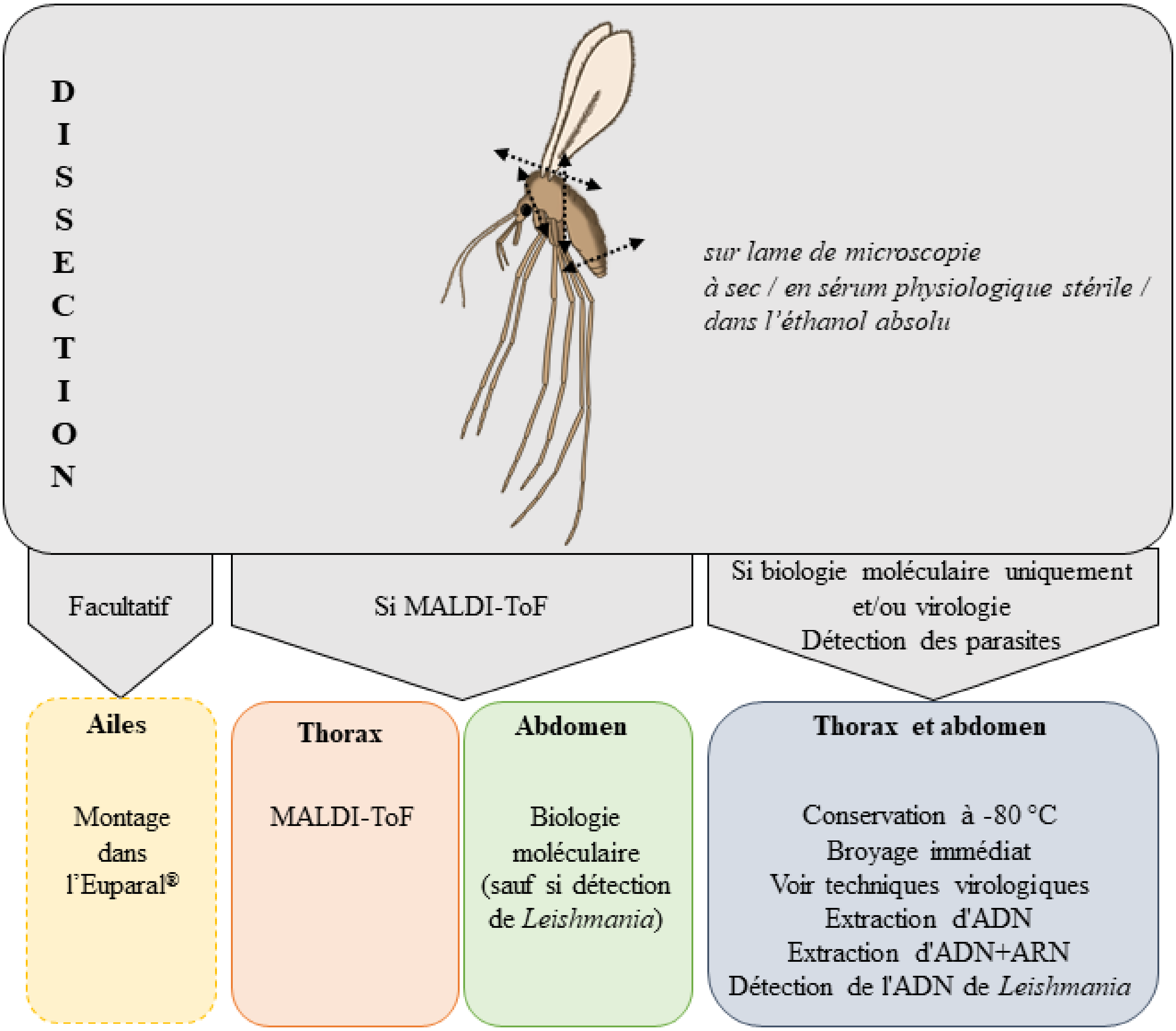

Figure 6 : Traitement des phlébotomes pour des applications en biologie moléculaire, protéomique et/ou virologique.

This is an Open Access article distributed under the terms of the Creative Commons Attribution License (https://creativecommons.org/licenses/by/4.0), which permits unrestricted use, distribution, and reproduction in any medium, provided the original work is properly cited.

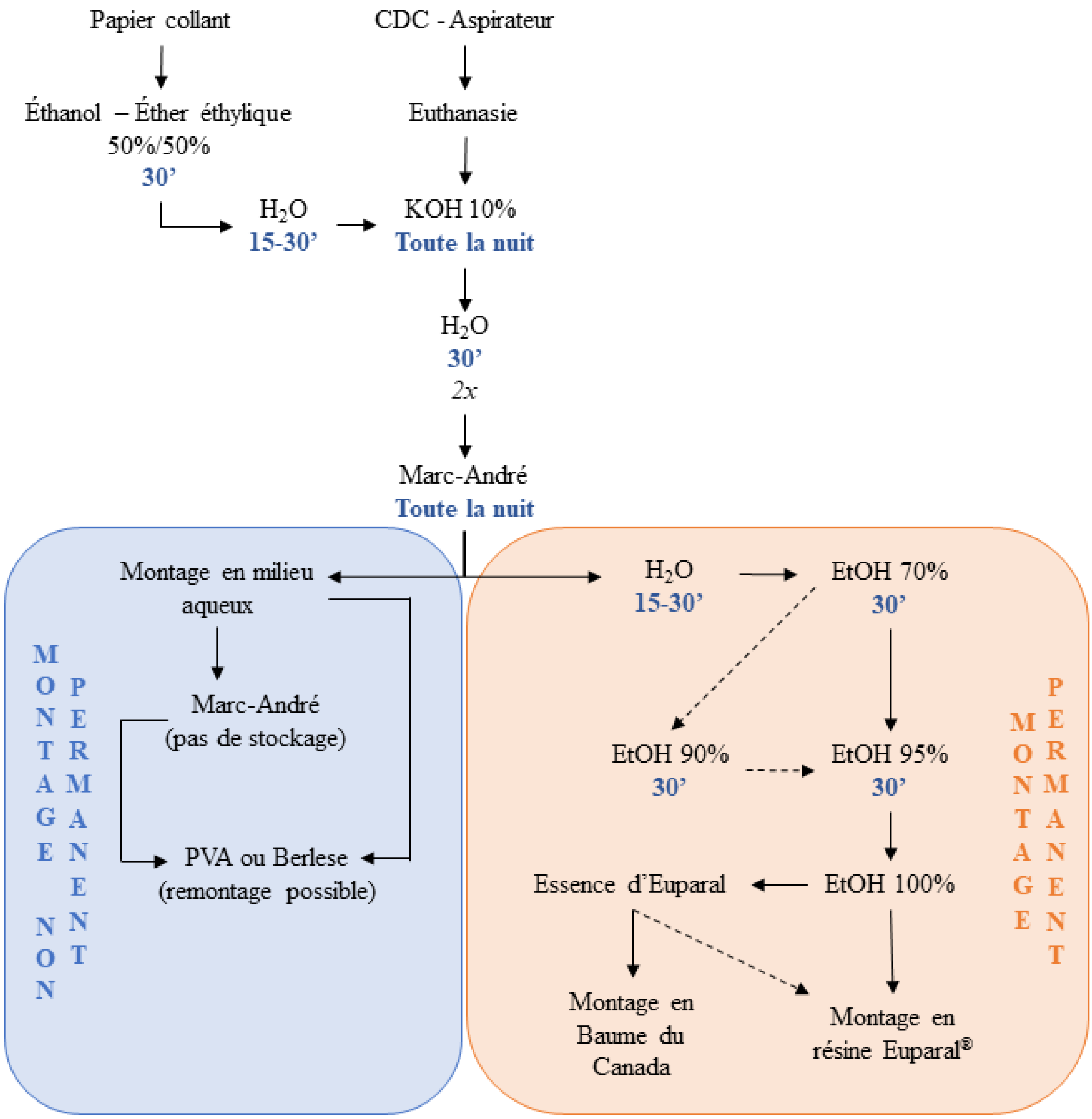

Figure 7 : Méthode classique de traitement des phlébotomes.

Les glandes salivaires des phlébotomes peuvent également être utilisées comme source d'antigènes pour mesurer, par des méthodes immunologiques, de préférence par ELISA, les anticorps de l'hôte dirigés contre la salive des phlébotomes. Cette approche permet d'évaluer l'exposition des hôtes aux piqûres de phlébotomes et de soutenir l'évaluation de l'efficacité des méthodes de lutte antivectorielle [25], ainsi que du risque de transmission de *Leishmania* [40].

4.6. Identification du repas sanguin

Les femelles gorgées isolées lors des captures doivent être disséquées à l'aide de matériel à usage unique afin de prévenir toute contamination croisée. Leur abdomen doit être examiné sous loupe binoculaire afin d'évaluer le stade de digestion du repas sanguin. Il est recommandé de ne sélectionner que des femelles présentant un abdomen rouge, rouge brunâtre ou rouge foncé, sans signe de formation d'œufs. L'extrémité de l'abdomen, incluant les

This is an Open Access article distributed under the terms of the Creative Commons Attribution License (https://creativecommons.org/licenses/by/4.0), which permits unrestricted use, distribution, and reproduction in any medium, provided the original work is properly cited.

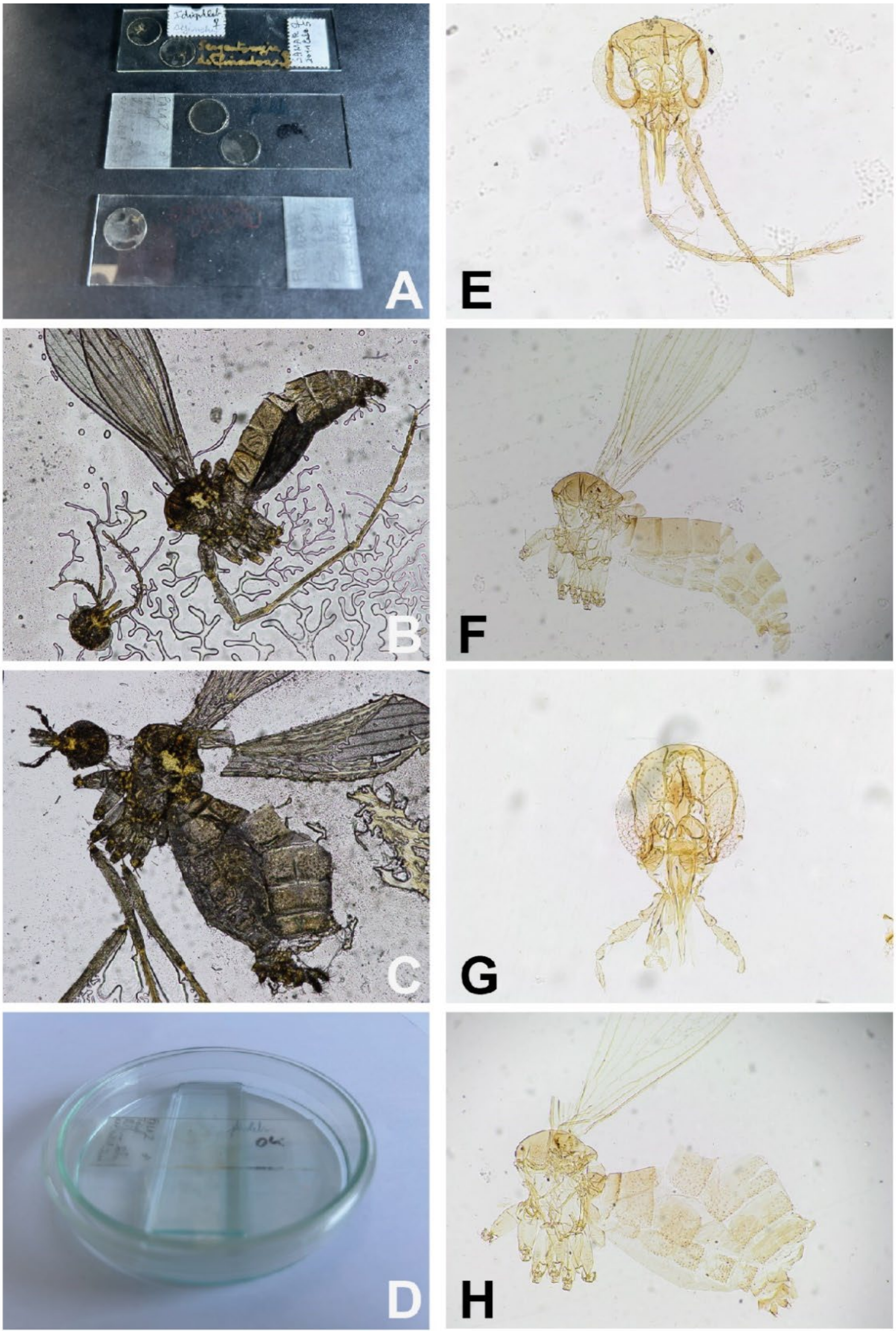

Figure 8. Remontage des lames. A : lames endommagées et desséchées montées avec du solution de Hoyer ; B : vue microscopique d'un phlébotome desséché ; C : vue microscopique d'un autre phlébotome endommagé ; D : chambre humide contenant une lame desséchée ; E : tête et F : corps du spécimen B après son remontage dans de l'Euparal® ; G : tête et H : corps du spécimen C après son remontage dans de l'Euparal®.

spermathèques, doit être retirée afin de permettre l'identification morphologique de la femelle après éclaircissement. La majeure partie de l'abdomen (sans les spermathèques) est ensuite placée dans des tubes Eppendorf® et conservée à -20°C jusqu'à l'analyse ultérieure. Les marqueurs génétiques couramment utilisés pour l'identification des repas sanguins, tels que PNOC [5, 30, 50], CytB [67] ou COI [13], sont bien établis et largement décrits dans la littérature ; ils ne seront donc pas détaillés davantage dans cet article (Figure 6). Alternativement, l'identification de l'hôte sanguin peut être réalisée par cartographie peptidique par MALDI-ToF [31]. Il a été démontré expérimentalement que cette technique permet l'identification de l'hôte sur une période plus longue après la prise du repas sanguin ; elle constitue ainsi une méthode de choix, en particulier pour l'analyse de femelles gorgées présentant un stade de digestion du sang visiblement plus avancé. Les échantillons doivent idéalement être conservés à -20°C ou -4°C, mais de bons résultats peuvent également être obtenus à partir d'échantillons conservés à température ambiante pendant une courte durée. L'abdomen d'une femelle gorgée doit être disséqué du reste du corps peu avant l'analyse, puis homogénéisé dans de l'eau distillée. Le reste du corps du phlébotome demeure disponible pour d'autres analyses morphologiques et moléculaires. Après le prélèvement d'un aliquote de l'homogénat destiné à la cartographie peptidique par MALDI-ToF, le reste de l'échantillon peut être utilisé pour l'extraction d'ADN afin de confirmer l'identification de l'hôte sanguin et/ou de rechercher la présence de *Leishmania*. Le temps global de préparation et d'analyse des échantillons est très court comparé aux techniques moléculaires basées sur l'ADN.

5. Traitement des spécimens pour les études morphologiques (Figures 3, 6, 7 et 8 ; Annexes 1, 2, 3 et 4)

Cette section présente les principes généraux de préparation d'un phlébotome en vue de son montage exclusivement destiné aux études morphologiques, puis leur adaptation à des applications allant au-delà de la morphologie. La compréhension de cette méthodologie est toutefois essentielle, car elle permet d'ajuster les procédures à des types d'échantillons spécifiques lorsque cela est nécessaire.

Le traitement repose sur une succession d'étapes de vidange et de remplissage réalisées à l'aide de pipettes Pasteur munies de poires en caoutchouc souple. L'utilisation de récipients en verre à fond arrondi est fortement recommandée, car elle facilite grandement ces manipulations. Le verre étant inerte vis-à-vis de l'ensemble des réactifs utilisés, il garantit une bonne compatibilité chimique. Afin de limiter l'évaporation des réactifs, les récipients doivent être munis de couvercles et ne jamais être trop remplis, ce qui éviterait tout débordement lors de l'ouverture ou de la fermeture, ainsi que le dépôt de poussières sur les échantillons. Les réactifs nécessaires aux étapes d'éclaircissement et de traitement sont présentés dans le Tableau 2.

Tableau 2: Composition des réactifs utilisés.

**Hydroxyde de potassium 10 %**	**Fuchsine acide 1% dans l'eau distillée**
Hydroxyde de potassium 10g	Fuchsine acide (en poudre) 1 g
Eau distillée *qsp* 100 mL	Eau distillée 99 mL
**Milieu de montage à la gomme chloral (milieu de Hoyer)**	**Solution de Marc-André colorée à la fuchsine acide**
Eau distillée 50 mL	Solution de Marc-André 10mL
Hydrate de chloral 200 g	
Gomme arabique 50 g	Fuchsine à 1 % 50 µL
Glycérol 20 mL	
**Solution de Marc-André**	**Milieu Enecê**
Hydrate de chloral 40 g	Colophane blanche pure 22 g
Acide acétique glacial 30 mL	Gomme copale soluble dans l'alcool 12 g
Eau distillée 30 mL	Éthanol absolu 20 mL
	Camphre 10 g
	Essence de térébenthine 10 mL
	Eucalyptol 26 mL

5.1. Éclaircissement

Avant que les phlébotomes puissent être préparés en montages permanents entre lame et lamelle, ils doivent être préalablement clarifiés par macération à l'aide d'une méthode et d'un agent d'éclaircissement appropriés (e.g., solution d'acide acétique à 10 % ou solution de Marc-André contenant de l'hydrate de chloral, substance réglementée dans de nombreux pays), afin de les éclaicir. Ce processus d'éclaircissement permet l'élimination des tissus corporels, des graisses, des sécrétions et des cires, rendant le spécimen translucide et facilitant l'examen des structures de l'exosquelette (e.g., insertion des soies), des caractéristiques de surface (e.g., coloration) et des structures internes visibles à travers le tégument (e.g., spermathèques).

Le processus d'éclaircissement en deux étapes, qui consiste à utiliser d'abord une base forte (telle que l'hydroxyde de potassium), suivie d'un acide faible (tel que l'acide acétique contenu dans la solution de Marc-André), répond à des objectifs biochimiques distincts [74]. La base provoque la dégradation des tissus mous, notamment des protéines, des lipides et des muscles, par saponification et dénaturation protéique, tout en laissant intact l'exosquelette chitineux, garantissant ainsi une bonne lisibilité structurale. L'acide faible neutralise ensuite les résidus alcalins, empêchant toute dégradation supplémentaire, et contribue au blanchiment de la chitine afin d'améliorer la transparence [74]. À noter que deux lavages successifs de 15 minutes dans de l'eau distillée peuvent également suffire à neutraliser la base. Ce traitement séquentiel associe une élimination efficace des tissus à une préservation douce des structures, assurant une intégrité optimale du spécimen pour l'examen microscopique.

Deux rinçages de 20 minutes chacun dans de l'eau distillée sont recommandés avant de passer à l'étape suivante.

5.1.1. Lyse des tissus mous (Figure 8)

L'hydroxyde de sodium ($NaOH$) ou l'hydroxyde de potassium (KOH) sont couramment utilisés comme agents chimiques de macération, à des concentrations et durées variables selon la taille et la fragilité des spécimens. La technique standard et la plus efficace consiste à lyser les tissus mous en immergeant les phlébotomes dans une base forte (KOH ou $NaOH$ à 10 %) pendant une nuit. La concentration peut être augmentée afin de réduire la durée du traitement (par exemple KOH à 20 % pendant 6 heures), éventuellement en combinant avec un chauffage à 37°C.

5.1.2. Éclaircissement avec ou sans coloration

Cette étape est suivie d'un traitement d'éclaircissement, généralement fondé sur l'association d'acide acétique et d'hydrate de chloral (par ex. solution de Marc-André). Après l'éclaircissement, les spécimens doivent être soigneusement rincés dans au moins deux bains successifs d'eau distillée de 20 minutes chacun, afin d'éliminer les résidus chimiques.

La solution de Marc-André est un agent d'éclaircissement largement utilisé pour la préparation des phlébotomes. Son efficacité repose sur sa capacité à clarifier les spécimens tout en limitant les dommages aux structures fragiles telles que les ailes et les antennes.

La solution doit être préparée fraîchement ou conservée dans un récipient hermétiquement fermé afin d'éviter l'évaporation ou la dégradation. L'utilisation de la solution de Marc-André est particulièrement avantageuse lorsqu'elle est associée à des techniques d'éclaircissement ou de coloration visant à améliorer la visibilité de certains caractères morphologiques. Les détails relatifs à sa composition et à sa préparation sont présentés en Annexe 2.

Pour les spécimens très translucides, une coloration peut s'avérer nécessaire afin d'améliorer la visibilité avant le montage. De nombreux colorants sont disponibles, chacun ciblant des composants chimiques spécifiques de l'organisme. Il est essentiel de choisir un colorant compatible à la fois avec le spécimen et avec le milieu de montage retenu. Cette méthodologie de base peut être adaptée, par exemple en incorporant 0,1 % de fuchsine acide à la solution de Marc-André pour obtenir une coloration. Par ailleurs, les spécimens conservés en milieu aqueux et destinés à un montage dans des milieux résineux nécessitent une déshydratation préalable (voir section 5.2), la majorité des milieux de montage résineux naturels ou synthétiques étant incompatibles avec l'eau. New (1974) a signalé que certains colorants peuvent se dégrader dans certains milieux de montage [53]. Par exemple, la fuchsine acide, couramment utilisée avec le baume du Canada, peut également être fixée dans l'Euparal®. Toutefois, les spécimens colorés à la fuchsine acide sont sujets à une décoloration progressive, en particulier lorsque des résidus d'huile de girofle, utilisée comme agent final d'éclaircissement, subsistent. Les spécimens conservés dans l'huile de girofle peuvent ainsi présenter une décoloration marquée en quelques jours.

5.2. Déshydratation

La déshydratation est réalisée par le transfert progressif des échantillons dans une série d'éthanol de concentration croissante : 50 %, 70 %, 80 %, 90 % ou 95 %, puis 100 %, chaque bain durant au minimum 20 minutes. L'éthanol s'évaporant rapidement, les récipients doivent être hermétiquement fermés pendant le traitement. Une fois le spécimen totalement déshydraté, le processus peut être interrompu pendant quelques jours en le laissant dans l'essence d'Euparal®, préférable à l'huile de girofle. La créosote de hêtre, autrefois largement utilisée à cette fin, est aujourd'hui totalement interdite en raison de sa toxicité.

Le processus de déshydratation doit garantir que le liquide présent dans le spécimen soit compatible avec le milieu de montage, afin d'éviter toute opacification, effondrement osmotique ou déformation susceptible de rendre le spécimen impropre à une étude taxonomique.

This is an Open Access article distributed under the terms of the Creative Commons Attribution License (https://creativecommons.org/licenses/by/4.0), which permits unrestricted use, distribution, and reproduction in any medium, provided the original work is properly cited.

5.3. Milieux de montage

5.3.1. Sélection et application pour la préparation des spécimens

Le milieu de montage doit idéalement posséder un indice de réfraction aussi proche que possible de celui du verre, soit environ 1,5. Il doit être incolore, limpide et rester parfaitement transparent après séchage et au cours du temps. Il doit être compatible avec les colorants utilisés et capable de pénétrer et de diffuser dans l'ensemble des tissus du spécimen. Il ne doit ni sécher trop rapidement ni former un voile lors du montage, et ne doit pas se rétracter après la mise en place. Le choix d'un milieu de montage approprié constitue un aspect fondamental de la préparation des spécimens, aucun milieu n'étant idéal pour toutes les applications. Ce choix doit résulter d'un compromis entre plusieurs facteurs clés :

- **Propriétés optiques**. L'indice de réfraction du milieu de montage doit fournir un contraste et une réfraction suffisants pour l'observation des caractères anatomiques critiques utilisés pour l'identification taxonomique ou la description morphologique, tels que les spermathèques, les ascoïdes, les sensilles de Newstead, les dents cibariales verticales et les dents pharyngiennes. La visibilité de ces structures dépend directement des propriétés optiques du milieu de montage.

- **Conservation**. Pour les spécimens types ou les matériaux destinés à des collections permanentes, le milieu doit offrir une stabilité et une durabilité à long terme. En revanche, pour les études d'inventaire ou les enquêtes épidémiologiques, pour lesquelles la conservation à long terme est moins critique, des milieux de montage temporaires ou semi-permanents peuvent être suffisants.

5.3.2. Exigences relatives aux milieux de montage

Les spécialistes développent souvent des techniques de montage personnalisées et parfois complexes, adaptées à des objectifs de recherche spécifiques. Toutefois, ces méthodes négligent fréquemment des aspects tels que la qualité archivistique, la compatibilité entre techniques, la standardisation, la facilité de manipulation ou la conservation à long terme. Cette absence de standardisation complique l'intégration de collections données et les efforts de conservation à long terme.

Les applications scientifiques imposent des exigences distinctes en matière de milieux de montage. Les taxonomistes montent souvent des spécimens entiers et privilégient des milieux capables de macérer légèrement les organes internes afin d'améliorer la visibilité des structures cuticulaires. L'indice de réfraction doit être suffisamment différent de celui du spécimen et de la lame de verre pour maximiser la clarté optique. Les milieux de montage commerciaux sont généralement formulés avec un indice de réfraction proche de celui du verre afin de minimiser la réfraction et la diffusion de la lumière à travers le système lame – milieu de montage – lamelle. Cependant, en microscopie en champ clair, le contraste naturel d'un spécimen non coloré peut être modulé en choisissant délibérément un milieu de montage dont l'indice de réfraction diffère légèrement de celui du spécimen, améliorant ainsi sa visibilité par rapport à l'arrière-plan.

5.3.3. Types de milieux de montage (Tableaux 3 et 4)

En microscopie, l'indice de réfraction (IR) d'un milieu de montage détermine la manière dont la lumière se propage à travers la lame, le milieu et le spécimen. Lorsque l'IR est proche de celui du verre de la lamelle (≈1,515), la lumière traverse l'ensemble de façon homogène, ce qui réduit la diffusion et les distorsions optiques, et améliore ainsi la résolution et la visibilité des structures fines. À l'inverse, une inadéquation des indices de réfraction peut entraîner un flou, des halos ou masquer des structures non colorées. Le choix du milieu de montage approprié est donc crucial pour optimiser le contraste, la netteté et la qualité globale de l'image d'un spécimen donné, en raison des indices de réfraction distincts des différents milieux.

L'indice de réfraction du milieu de montage a un impact majeur sur la visibilité des structures fines lors de la préparation des phlébotomes pour un montage sur lame. Les structures délicates et faiblement sclérotinisées des phlébotomes, telles que l'armature cibariale, les spermathèques, les segments antennaires et la nervation alaire, peuvent être difficiles à observer dans des milieux de montage à indice de réfraction élevé.

Chez les phlébotomes, les milieux couramment utilisés comprennent la gomme au chloral en tant que milieu aqueux, ainsi que le baume du Canada et la résine Enecê - Nelson Cerqueira (NC) en tant que milieux à base de solvants. Rawlins [60] a classé les milieux de montage en deux catégories : (1) les milieux permanents, qui durcissent avec le temps et conviennent à la conservation à long terme, et (2) les milieux semi-permanents, qui ne durcissent pas complètement et sont généralement utilisés à des fins temporaires.

This is an Open Access article distributed under the terms of the Creative Commons Attribution License (https://creativecommons.org/licenses/by/4.0), which permits unrestricted use, distribution, and reproduction in any medium, provided the original work is properly cited.

Tableau 3.: Composition de certains milieux de montage sélectionnés.

Milieu de montage	Solvant	Pré-polymère(s) ou polymère(s) potentiel(s)	Remarques
Hoyer = gomme au chloral	glycérol, eau	composés de gomme arabique	agent macérant: hydrate de chloral
CMCP-9 (= carboxyméthyl-cellulose phénol)	eau (CMCP-9: 51−60%)	alcool polyvinylique entièrement hydrolysé (CMCP-9: 0−5%)	CMCP-9: faible viscosité: forte viscosité
DMHF (diméthyl-hydantoïne-formaldéhyde)	eau	N,N′-diméthylol-diméthyl-hydantoïne (di-méthylol DMH) Oligomères pontés par éther/méthylène Réseau polymère DMH–formaldéhyde réticulé	
Baume du Canada	xylène; composants partiellement volatils du baume (Δ^3-carène, acide lévopimarique, limonène, myrcène, acide palustrique, β-phéllandrène, α-pinène, β-pinène)	baume (abiénol, acide abiétique, acide isopimarique, acide sandaracopimarique)	neutralisation : carbonate de potassium ; résine issue de *Abies balsamea* (Linné, 1758)
Euparal®	eucalyptol, paraldéhyde ; composants partiellement volatils de la gomme sandaraque (limonène, α-pinène, β-pinène)	composés de la gomme sandaraque (acide communic, manool, acide polycommunique, acide sandaracopimarique, acide 12-acétoxy-sandaracopimarique, sugiol, acide torulosique, torulosol, totarol)	agent éclaircissant : salicylate de méthyle ; colorant de l'Euparal® vert : sel de cuivre (abiétinate de cuivre) ; résine de sandaraque issue de *Tetraclinis articulata* (Vahl, 1791)
Enecê	alcool éthylique, avec camphre, eucalyptol et essence de térébenthine	composés de gomme copal et de colophane (résine)	

Tableau 4 Avantages et inconvénients de certains milieux de montage pour les préparations microscopiques, d'après des observations non publiées de plusieurs auteurs [52].

Nom	Avantages	Inconvénients
* Baume du Canada	Milieu extrêmement durable, avec une longévité dépassant 150 ans.<br>Les lames peuvent être montées après clarification au clou de girofle ou au phénol.	Contient des composants toxiques et doit être manipulé sous hotte.<br>Nécessite une déshydratation complète, longue et exigeante.<br>La déshydratation à l'éthanol et le transfert via le xylène ou le clou de girofle peuvent rendre certains taxons fragiles ; des solvants alternatifs (isopropanol, n-butanol, Cellosolve™, 1,4-dioxane, Histoclear, terpinéol) peuvent limiter la friabilité.<br>Les spécimens peuvent noircir si le xylène est remplacé par le phénol ou si des résidus de KOH subsistent.<br>L'indice de réfraction élevé peut masquer les structures non colorées.<br>Le séchage complet peut prendre plusieurs années sans plaque chauffante.<br>Jaunissement et assombrissement progressifs, surtout après clarification au clou de girofle.<br>Certaines colorations s'altèrent ; les colorants cationiques peuvent se décolorer si le milieu devient acide avec le temps.
DMHF (diméthyl-hydantoïne-formaldéhyde)	Haute transparence.<br>Bon indice de réfraction.<br>Excellente visibilité des structures.<br>Assez bonne stabilité des préparations.<br>Compatible avec de nombreuses techniques de coloration.<br>Bonne protection des échantillons.<br>Bonne adhérence entre la lame et la lamelle.	Jaunissement possible avec le temps.<br>Peut altérer certaines colorations.<br>Ne convient pas aux colorations sensibles au formaldéhyde.<br>Formation fréquente de bulles d'air, séchage lent.<br>Milieu de montage sensible à l'humidité.<br>Montage difficilement réversible.<br>Le formaldéhyde est toxique, irritant et cancérigène.
* Euparal (transparent)	Milieu durable, avec une longévité supérieure à 50 ans.<br>Montage possible directement depuis l'éthanol à 80 % (recommandation du fabricant).<br>Ne masque pas les structures non colorées et ne jaunit et ne casse pas avec le temps.<br>Indice de réfraction mieux adapté que le baume du Canada pour les Diptères.<br>Très bon comportement avec les spécimens épais (faible rétraction, peu de bulles).<br>Reste soluble dans l'éthanol à 95 %, permettant un remontage même après de nombreuses années.	Contient des composants toxiques nécessitant une manipulation sous hotte.<br>La déshydratation à l'éthanol et le transfert via l'essence d'Euparal® peuvent fragiliser certains taxons ; l'isopropanol peut limiter cet effet.

Tableau 4 (suite)

Nom	Avantages	Inconvénients
Hoyer fluid	Les spécimens peuvent être montés vivants ou directement depuis l'eau, l'éthanol ou les formaldéhydes.<br>La macération procure une excellente qualité de cuticule.<br>Indice de réfraction favorable, pouvant être renforcé par une coloration à l'iode.<br>L'acide acétique peut favoriser l'expansion des appendices des arthropodes.<br>Certains spécimens peuvent rester exploitables pendant 40–60 ans.<br>Hydrosoluble, facilitant le démontage et le remontage.	Les spécimens végétaux délicats peuvent s'effondrer si le milieu n'est pas ajouté progressivement.<br>Formation de cavités et de cristaux parfois en moins de 10 ans.<br>Risque de macération excessive selon la concentration en hydrate de chloral et la durée d'exposition.<br>Séparation des composants et apparition de granulations fines avec le temps.<br>Des cas de noircissement du milieu ont été rapportés.
CMCP-9 (= carboxyméthyl-cellulose phénol)	Montage direct possible depuis l'eau, l'éthanol, le glycérol ou des solutions contenant des formaldéhydes.<br>Les organes internes peuvent être macérés pour faciliter l'observation générale ou la préparation.	Peut cristalliser et s'assombrir avec le temps.<br>Macération parfois excessive.<br>Les échantillons épais se rétractent et créent des vides si la lame n'est pas soigneusement cerclée.<br>Inadapté aux spécimens colorés ou calcifiés.<br>Temps de séchage plus long que celui des milieux à base de CMC.
Eukitt™	Milieu durable (≥ 30 ans).<br>Compatible avec de nombreux solvants (acétone, benzène, chloroforme, dioxane, éther, isopropanol, benzoate de méthyle, terpinéol, toluène, xylène).<br>Séchage rapide, pH légèrement acide.<br>Ne s'assombrit pas sensiblement avec le temps.<br>Compatible avec de nombreuses colorations (e.g. fucshin, hématoxyline, vert de méthyle, violet de méthyle, bleu de méthylène).<br>Possibilité de remontage après plusieurs années par immersion prolongée dans le xylène.	Contient des composants toxiques nécessitant une manipulation sous hotte.<br>Exige une déshydratation complète et longue.<br>Peu adapté aux spécimens épais (rétraction, bulles de gaz).<br>Les lamelles peuvent se décoller avec le temps si le verre est mal nettoyé ou non scellé.<br>Polymérisation parfois incomplète autour des fibres riches en collagène.
Enecê	Milieu très durable (≥ 50 ans).<br>Ne s'assombrit pas avec le temps.<br>Plus malléable, permettant la dissection dans le milieu et un positionnement précis des structures durant un temps raisonnable.<br>Faible coût.	Nécessite une déshydratation complète et longue.<br>La déshydratation à l'éthanol et le transfert via le clou de girofle peuvent fragiliser certains spécimens.<br>La clarification se poursuit très lentement, ce qui peut rendre difficiles l'observation de structures très fines (sensilles, ascoïdes, soies simples).

Les milieux de montage peuvent être liquides, à base de gommes ou résineux, solubles dans l'eau, l'alcool ou d'autres solvants (par exemple le toluène ou le xylène) (Tableau 3). Après leur application, ils doivent être protégés des effets de l'atmosphère à l'aide de vernis d'étanchéité non solubles. Afin de distinguer clairement les différents

types de milieux de montage, la classification suivante peut être utilisée :

a. Milieux aqueux. Ces milieux se dissolvent facilement dans l'eau, ce qui les rend adaptés aux montages temporaires ou semi-permanents. Ils sont généralement faciles à manipuler, mais peuvent nécessiter un scellement afin d'éviter l'exposition à l'humidité atmosphérique (i.e., milieux gomme-chloralés et l'alcool polyvinylique), en particulier dans les climats tropicaux humides.

b. Milieux à tolérance limitée à l'eau. Ces milieux sont moins affectés par l'eau, mais nécessitent néanmoins une protection contre une humidité excessive. Ils offrent une meilleure stabilité à long terme que les milieux solubles dans l'eau et sont fréquemment utilisés pour des montages semi-permanents.

c. Milieux solubles dans les hydrocarbures. Ces milieux sont dissous dans des solvants organiques tels que le xylène, le toluène ou l'essenecê (solvant enecê). Ils sont conçus pour des montages permanents et offrent une excellente stabilité à long terme. Ils résistent à l'humidité et à la dégradation, ce qui les rend particulièrement adaptés à des fins archivistiques (i.e., baume du Canada neutre).

En résumé, les milieux solubles dans l'eau sont les plus adaptés aux montages temporaires ou aux situations nécessitant un retrait facile du spécimen ; les milieux à tolérance limitée à l'eau conviennent aux montages semi-permanents nécessitant une durabilité modérée ; enfin, les milieux solubles dans les hydrocarbures sont à privilégier pour les montages permanents destinés à la conservation archivistique et au stockage à long terme.

5.3.4. Description des milieux de montage recommandés (Tableaux 3 et 4)

Milieux pour observations temporaires

Gomme au chloral = liquide/milieu/solution de Hoyer (IR = 1,48). Le liquide de Marc-André constitue le meilleur milieu pour l'observation à très court terme (quelques heures, voire un peu plus si la lame est conservée dans une chambre humide) des spermathèques, notamment pour la prise de photographies (Figure 4) ou la réalisation de dessins. La conservation à plus long terme des spermathèques observées nécessite un remontage dans un milieu aqueux permettant un stockage à moyen terme. Une déshydratation suivie d'un montage en résine est techniquement possible, mais déconseillée en raison du risque élevé de perte du matériel. La gomme au chloral et le milieu de Hoyer sont considérés comme synonymes. Ce milieu est couramment utilisé pour l'observation des organes internes en raison de sa compatibilité avec l'eau, de sa simplicité de préparation, de sa mise en œuvre rapide et de son indice de réfraction favorable à l'examen de structures délicates telles que les spermathèques. Toutefois, la gomme au chloral présente des inconvénients majeurs lorsqu'elle n'est pas parfaitement préparée ou conservée sous des conditions d'humidité contrôlées, notamment la cristallisation, la décoloration et la perte de viscosité. Le cerclage de la lamelle ne permet pas de corriger ces défauts, car le milieu de montage peut subir une décoloration importante (parfois presque noire) due à des interactions avec le milieu de cerclage, en particulier lorsque l'Euparal® est utilisé.

Le milieu de Hoyer a longtemps été considéré comme offrant les meilleures propriétés optiques pour l'étude des phlébotomes et a été largement employé à cette fin. Il existe plusieurs formulations étroitement apparentées, à base de gomme arabique, de glycérol et d'hydrate de chloral, dont certaines ont été mal interprétées ou incorrectement citées dans la littérature [74].

Bien que le milieu de Hoyer soit très adapté à l'observation des spermathèques chez les phlébotomes, il ne convient pas à une conservation à long terme. Il est idéal pour des observations de courte durée, incluant la photographie, le dessin ou l'imagerie. De manière générale, les milieux aqueux conviennent aux montages temporaires mais ne garantissent pas la stabilité à long terme. À l'inverse, les milieux résineux assurent une excellente durabilité, souvent sur plusieurs siècles, mais peuvent masquer les détails fins des spermathèques en raison de la perte de leur réfringence.

Avec le temps, le milieu de Hoyer se dégrade par déshydratation (Figure 8), entraînant la formation de petits cristaux blancs et opaques d'hydrate de chloral. Les spécimens peuvent néanmoins être récupérés à partir de lames cristallisées, la cuticule restant chimiquement intacte, bien que des dommages physiques puissent survenir du fait de la croissance des cristaux. Dans certains cas, les lames cristallisées peuvent être restaurées par réhydratation du milieu de montage dans un environnement chaud et humide, avec ajout de thymol afin de prévenir le développement fongique. Alternativement, les spécimens peuvent être extraits de la gomme au chloral par immersion dans l'eau, déshydratés dans de l'acide acétique glacial, puis remontés dans du baume du Canada.

DMHF (diméthyl-hydantoïne formaldéhyde) (IR = 1.48)

Ce milieu aqueux [72] présente d'excellentes performances optiques, comparables à celles de la solution de Berlese, tout en étant aussi simple d'utilisation. Contrairement à ce dernier, le DMHF ne noircit pas et ne cristallise pas au cours du temps. Il est bien adapté au montage des phlébotomes et d'autres Psychodidae.

CMCP (camphre-mono-chlorophénol) (IR = 1.41)

C'est un milieu de montage hydrosoluble à base de glycérol, utilisé pour la réalisation de préparations transparentes et permanentes de spécimens délicats, notamment les phlébotomes. L'un de ses principaux avantages est la possibilité de monter directement les spécimens à partir de l'eau ou de l'éthanol. Il relaxe et clarifie rapidement les insectes, assouplissant la cuticule et facilitant le positionnement correct des pièces anatomiques, en particulier l'étalement des ailes ou la dissection des

génitalias. Bien que ce milieu soit décrit comme permettant une conservation à long terme, la durée réelle de cette conservation reste mal documentée. Sa principale limitation réside dans la présence de phénol, substance toxique et irritante nécessitant des précautions strictes de manipulation.

Milieux pour montage permanent

Baume du Canada (IR = 1.52-1.54) Le baume du Canada a été décrit comme milieu de montage adapté à la microscopie en lumière transmise dès les années 1830 par Andrew Pritchard. Il demeure l'un des milieux les plus utilisés en raison de ses qualités archivistiques éprouvées, avec plus de 150 ans d'utilisation réussie. Contrairement aux milieux à base de Hoyer, il ne cristallise pas et n'absorbe pas l'humidité atmosphérique. En revanche, il présente une autofluorescence marquée, pouvant constituer un inconvénient pour certaines techniques de microscopie [60]. L'utilisation de solvants non toxiques en remplacement du xylène permet de réduire les risques sanitaires, mais peut entraîner un séchage plus lent et un noircissement plus précoce du milieu.

Euparal® (IR = 1.48) ; L'Euparal® constitue une alternative largement utilisée au baume du Canada pour les montages permanents, offrant une stabilité à long terme et un indice de réfraction comparable. Son utilisation impose deux contraintes principales : (1) les spécimens doivent être déshydratés avant le montage final, généralement par un passage de l'éthanol à 95 % vers l'éthanol absolu ; (2) cette étape de déshydratation allonge la durée totale du traitement. Lorsque la déshydratation par solvants organiques n'est pas possible, les spécimens conservés dans l'éthanol absolu peuvent être transférés dans une solution intermédiaire composée d'un mélange à parts égales d'Euparal® et d'essence d'Euparal, avant le montage définitif.

Enecê (IR = 1.467). L'Enecê est un milieu de montage résineux principalement utilisé pour les petits insectes et largement répandu au Brésil. Il est composé de colophane et de gomme copal dissoutes dans de l'alcool, du camphre, de l'essence de térébenthine et de l'eucalyptol. Cerqueira [11] a décrit l'Enecê comme une alternative au baume du Canada pour le montage permanent de larves, d'exuvies de stades immatures et même d'adultes de moustiques. Il est depuis largement utilisé pour les phlébotomes. L'Enecê constitue une option économiquement avantageuse pour les montages permanents, offrant une bonne stabilité à long terme et un temps de prise suffisant pour permettre la dissection et le positionnement précis des structures morphologiques.

5.4. Préparation et séchage des lames

Un séchage adéquat des lames montées est indispensable pour garantir leur stabilité et la conservation à long terme des spécimens. Les lames doivent être complètement sèches avant toute mise en collection. Les préparations réalisées avec des milieux de montage permanents doivent être séchées à l'horizontale pendant 2 à 3 semaines, tandis que celles utilisant des milieux semi-permanents nécessitent généralement 1 à 2 semaines. Le séchage doit idéalement être effectué dans une étuve réglée à une température adaptée au milieu utilisé, en évitant toute surchauffe susceptible d'endommager les spécimens. Une plage de température comprise entre 30°C et 37°C est recommandée. Cette étape est essentielle pour prévenir le gauchissement des lames, la détérioration des spécimens ou l'instabilité du milieu de montage lors du stockage.

Le milieu de montage utilisé doit toujours être mentionné sur l'étiquette de la lame. Lorsque cela est possible, la recette exacte du milieu, le nom du préparateur et la date de préparation devraient également être indiqués. Les lames sont souvent initialement préparées comme montages temporaires ; toutefois, si le statut d'un spécimen évolue (par exemple, désignation comme élément d'une série type), un remontage dans un milieu permanent est recommandé afin d'assurer sa conservation pour les études taxonomiques futures.

5.5. Techniques alternatives de montage : montage sur carte

Le montage sur carte est une technique utilisée pour plusieurs groupes d'insectes, dans laquelle les spécimens sont soit épinglés directement sur des cartes entomologiques, soit collés à leur surface. En raison de la très petite taille des phlébotomes et de la nécessité d'observer des structures internes clarifiées pour l'identification (voir section 5), cette méthode est totalement inadaptée au montage des phlébotomes.

5.6. Remontage des spécimens endommagés

Pour les spécimens rares ou de grande valeur, une approche en deux étapes est recommandée, conformément au protocole présenté dans la vidéo disponible à l'adresse suivante : https://zenodo.org/records/18315029.

1) réhydratation sans démontage. Les lames sont d'abord réhydratées afin de permettre une observation préliminaire. Un support pouvant accueillir plusieurs lames de microscope est placé dans une boîte de Petri, et la lame à traiter est déposée au-dessus. Quelques millimètres de solvant sont ajoutés afin de créer une chambre humide, en veillant à ce que la lame n'entre pas en contact direct avec le liquide (Figure 8 D). La durée de réhydratation varie d'un à plusieurs jours selon l'état du spécimen. Une surveillance quotidienne et de la patience sont indispensables. Une fois la lame suffisamment réhydratée, elle peut être placée dans une étuve pendant quelques heures avant observation au microscope, photographie ou dessin.

2) pour le remontage, la lame peut être replacée dans la chambre humide pendant quelques heures supplémentaires ou toute une nuit. Le démontage doit être effectué sous loupe binoculaire. À l'aide d'aiguilles fines, la

lamelle est retirée avec précaution en s'assurant qu'aucun élément du phlébotome ne reste adhérent (https://zenodo.org/records/18315029). Les éléments disséqués sont ensuite récupérés et rincés à l'eau dans de petits puits, tels que ceux utilisés pour les extractions destructives d'ADN/ARN (voir ci-dessous), avant déshydratation et remontage dans un milieu résineux. Lors du démontage d'une lame, il est essentiel d'identifier le milieu de montage initial afin de choisir le solvant approprié. L'eau est utilisée pour les milieux aqueux, tandis que le xylène est nécessaire pour les milieux résineux (e.g., baume du Canada ou Euparal®). Le xylène doit être manipulé sous hotte aspirante et avec un équipement de protection individuelle adapté, incluant une protection respiratoire.

Le remontage de spécimens types ou de matériel de collection ne doit être entrepris qu'avec l'accord explicite du conservateur et/ou de l'institution détentrice du matériel.

6. Identification des spécimens

6.1. Morphologie

L'identification des phlébotomes repose principalement sur l'examen de leurs caractères morphologiques, notamment la forme du thorax, des ailes, des génitalias, des soies, ainsi que certaines relations morphométriques entre différentes structures. Les chercheurs utilisent des clés taxonomiques, des collections de référence et les descriptions originales des espèces pour comparer les spécimens collectés aux taxons connus. Les caractères diagnostiques clés, tels que la nervation alaire et la morphologie de la tête chez les deux sexes, la structure des génitalias mâles et la configuration des spermathèques chez les femelles, sont particulièrement informatifs pour la détermination spécifique. Une identification fiable nécessite souvent un examen microscopique détaillé, généralement à l'aide d'un microscope optique pour l'observation des structures fines (génitalia, spermathèques), ou d'une loupe binoculaire pour les caractères morphologiques plus généraux.

Les progrès récents en imagerie ont facilité l'utilisation de l'imagerie numérique pour l'identification des phlébotomes. Des photographies à haute résolution ou des illustrations numériques des caractères clés peuvent être comparées à des matériaux de référence ou analysées à l'aide de systèmes d'identification assistés par ordinateur, améliorant ainsi à la fois la précision et l'accessibilité de la taxonomie morphologique.

6.2. Géométrie alaire

La géométrie des ailes constitue un caractère clé pour l'identification et la classification des différentes espèces de phlébotomes. Les ailes des phlébotomes présentent une morphologie caractéristique, généralement longues et étroites, avec une nervation bien développée (Figures 9 et 10).

L'agencement des nervures forme un motif distinctif qui peut varier selon les genres et les espèces, fournissant ainsi des caractères diagnostiques précieux pour l'identification. L'étude de la géométrie alaire apporte donc des informations essentielles à des fins taxonomiques.

6.3. Morphométrie géométrique alaire

Différentes techniques, telles que la morphométrie géométrique, sont utilisées pour analyser et comparer la forme et la taille des ailes entre espèces ou populations de phlébotomes. L'étude de la géométrie alaire fournit également des informations sur le comportement, les préférences d'habitat et les capacités de vol.

Dans l'approche de morphométrie géométrique, les ailes sont soigneusement disséquées, éventuellement colorées, puis montées à plat sur lame. Les lames préparées sont ensuite photographiées sous loupe binoculaire, numérisées et soumises à une analyse morphométrique. Cette méthodologie est bien décrite dans la littérature [6, 27, 42, 56, 57, 59], avec la recommandation d'utiliser systématiquement l'aile droite ou gauche pour les organes pairs afin d'éviter d'éventuels effets allométriques négatifs [62].

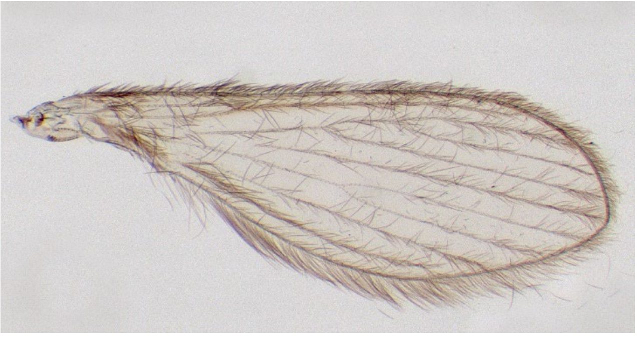

Figure 9: Aile brute de *Trichophoromyia ininii*.

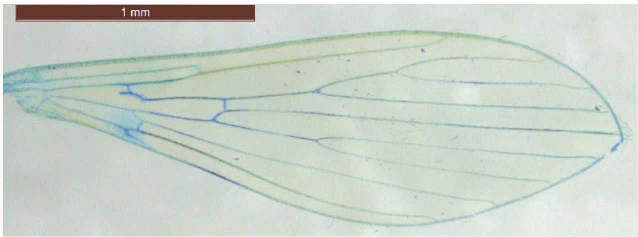

Figure 10: Aile colorée de *Phlebotomus ariasi.*

Préparation des ailes pour l'analyse morphométrique géométrique

Pour une visualisation optimale des nervures alaires, les ailes doivent être débarrassées de leurs soies et

correctement colorées. Pour la préparation, remplir d'abord de petites cupules avec les réactifs nécessaires (bleu de méthylène, éthanol, eau et substitut du xylène). Récupérer une aile conservée dans l'éthanol à 70 % à température ambiante en retournant le tube Eppendorf et en le vidant au-dessus de la cupule, puis soulever l'aile longitudinalement à l'aide d'une aiguille fine et courbe. Passer brièvement l'aile de l'éthanol à l'eau, puis de nouveau à l'éthanol afin d'éliminer les soies. Placer ensuite l'aile dans le bleu de méthylène pendant 6 minutes, en veillant à ce qu'elle reste flottante durant la coloration. Récupérer soigneusement l'aile et l'immerger dans un substitut du xylène pendant 2 minutes (environ un tiers du temps de coloration au bleu de méthylène). De légers tapotements de l'aiguille contre les parois de la cupule peuvent aider l'aile à se déposer ; le xylène sert ici à fixer la coloration. Enfin, soulever l'aile et la déposer sur une petite goutte d'Euparal® sur une lame de microscope. Sous une loupe, déplier délicatement l'aile et placer soigneusement une lamelle. Les photographies doivent être prises rapidement, avant la prise complète de l'Euparal®, car de légers ajustements de la position de l'aile sous la lamelle peuvent être nécessaires pour obtenir un alignement optimal.

6.4. Techniques de biologie moléculaire

En complément des approches morphologiques, les méthodes moléculaires sont devenues essentielles en entomologie, notamment pour les études taxonomiques, de génétique des populations et de phylogénie, ainsi que pour la détection de pathogènes à ADN/ARN et la détermination de l'origine des repas sanguins, le comportement vectoriel étant un élément clé en épidémiologie [70]. Le séquençage de l'ADN peut être utilisé pour confirmer l'identification des espèces ou différencier des espèces étroitement apparentées, offrant ainsi un moyen d'identification plus précis et plus fiable. De plus, des techniques moléculaires avancées (PCR, séquençage de l'ADN, NGS, etc.) ainsi que la spectrométrie de masse MALDI-ToF gagnent en importance pour une identification rapide et précise des espèces, en complément des méthodes morphologiques traditionnelles [46]. Malgré ces avancées, l'identification morphologique demeure la référence en taxonomie et constitue la base sur laquelle les données moléculaires sont interprétées.

6.4.1. Extraction destructive des acides nucléiques

L'extraction des acides nucléiques est une étape courante dans de nombreuses études biologiques, et diverses méthodes ont été développées pour isoler l'ADN à partir de matériaux biologiques [48]. De nombreux kits commerciaux d'extraction de l'ADN sont disponibles pour faciliter ce processus [14]. Toutefois, les méthodes classiquement utilisées pour préparer les arthropodes à l'identification morphologique entravent souvent les analyses moléculaires, car elles peuvent endommager ou détruire des caractères physiques essentiels du spécimen [10]. La plupart des protocoles d'extraction d'ADN appliqués aux tissus d'insectes sont de nature destructive [43], ce qui pose des problèmes particuliers pour les petits spécimens, chez lesquels même un prélèvement limité peut compromettre des caractères morphologiques importants [72]. Le type et l'état de conservation du spécimen jouent un rôle déterminant dans le choix d'une méthode d'isolement de l'ADN appropriée [29].

La nécessité d'une identification fiable des phlébotomes, de la compréhension des dynamiques de population et de la limitation des impacts non ciblés a favorisé le développement d'outils de diagnostic moléculaire [23]. Les approches moléculaires sont désormais fréquemment utilisées pour compléter les méthodes taxonomiques morphologiques dans l'identification des phlébotomes. Par exemple, l'approche standard du barcoding des insectes repose sur l'extraction et le séquençage de l'ADN, entraînant la perte du spécimen original. Il existe donc un besoin pressant de développer et d'explorer des méthodes d'extraction d'ADN non destructives permettant de préserver à la fois le matériel biologique et l'intégrité morphologique des spécimens.

De nombreuses méthodes d'extraction des acides nucléiques ont été appliquées aux phlébotomes. La quantité et la qualité d'ADN requises dépendent des analyses moléculaires en aval, les différentes techniques présentant des exigences variables en termes de sensibilité et de pureté [9]. Par exemple, les yeux des phlébotomes ont été identifiés comme pouvant inhiber l'amplification par PCR [69]. Au-delà du criblage des pathogènes, l'ADN des phlébotomes est couramment extrait à des fins d'identification spécifique. Différentes méthodes d'extraction peuvent être utilisées, avec des rendements et des qualités variables selon la technique. Certains protocoles proposés par les fabricants ont été adaptés par des chercheurs pour les phlébotomes [8], permettant d'augmenter le rendement et/ou la qualité des acides nucléiques extraits [8, 9, 69], tandis que d'autres adaptations, initialement développées pour d'autres groupes d'arthropodes, peuvent également être appliquées aux phlébotomes [58, 76]. Les PCR d'identification ciblant de petits fragments mitochondriaux (COI ou CytB) sont généralement compatibles avec des méthodes d'extraction entraînant une forte fragmentation de l'ADN. En revanche, certaines technologies de séquençage de longs brins (NGS), telles qu'Oxford Nanopore ou PacBio, nécessitent un ADN de haute qualité et faiblement fragmenté. Les extractions sur colonnes de silice produisent généralement des fragments d'ADN génomique allant jusqu'à 60 kb, tandis que l'extraction au phénol-chloroforme peut générer des fragments atteignant 150 kb [77]. Le Tableau 5 résume les différentes techniques d'extraction de l'ADN des phlébotomes et indique si des adaptations méthodologiques ont été mises en œuvre pour ces insectes. Les rendements ne

sont pas indiqués, car ils dépendent de la taille des spécimens et de leur mode de préparation. La colonne «modification» fait référence aux adaptations des protocoles d'extraction pour les phlébotomes ou d'autres petits arthropodes.

Le choix de la méthode d'extraction doit prendre en compte plusieurs critères, tels que le nombre d'échantillons, le temps d'extraction et les techniques utilisées en aval. Bien que les techniques de NGS nécessitent un ADN génomique de haut poids moléculaire, toutes les méthodes présentées ici sont adaptées aux applications PCR classiques.

Par ailleurs, plusieurs études ont exploré des méthodes d'extraction d'ADN non destructives appliquées à de petits arthropodes terrestres, à des spécimens de musées conservés à sec et à des arthropodes à corps mou [19, 26, 28, 55, 63].

6.4.2. Extraction non destructive des acides nucléiques

L'un des principaux défis de l'analyse moléculaire des arthropodes, et en particulier des phlébotomes, réside dans la préservation des spécimens en vue de leur intégration dans des collections entomologiques. La plupart des protocoles d'extraction de l'ADN nécessitent la macération des tissus, compromettant ainsi la conservation du spécimen original. Les méthodes d'extraction non destructives des acides nucléiques sont conçues pour extraire le matériel génétique sans endommager physiquement l'échantillon, ni altérer sa conservation ou sa morphologie. Ces méthodes sont particulièrement précieuses lorsqu'il s'agit de spécimens rares ou limités, tels que les phlébotomes, pour lesquels le maintien de l'intégrité structurale est essentiel à des fins taxonomiques, morphologiques ou diagnostiques ultérieures. Une technique couramment utilisée est la méthode de bain non destructif, dans laquelle les phlébotomes sont immobilisés puis délicatement immergés dans un tampon de lyse contenant de la protéinase K.

La technique de *mild-vectolysis* a été appliquée avec succès aux phlébotomes, notamment aux spécimens types [24]. Cette méthode repose sur l'utilisation d'un kit standard à colonnes de silice (en l'occurrence le kit DNeasy Blood and Tissue, QIAGEN, Hilden, Allemagne), avec des adaptations permettant l'obtention d'ADN sans destruction du spécimen. Les étapes de lyse modifiées (volume du tampon de lyse et ajout d'une étape de congélation) [17] permettent la libération des acides nucléiques tout en limitant les dommages morphologiques [24]. Chez les phlébotomes, il est également possible d'utiliser le kit d'extraction d'ADN HotSHOT (Bento Bioworks Ltd, Londres, Royaume-Uni) [73], qui est rapide et peu coûteux, et permet un traitement rapide et économique des échantillons. Les spécimens entomologiques destinés à l'identification morphologique peuvent ensuite être rincés. Ceux traités avec le kit DNeasy Blood and Tissue doivent être clarifiés à l'aide de la solution de Marc-André, tandis que ceux traités avec le kit HotSHOT sont suffisamment éclaircis pour être montés dans un milieu aqueux, ou de préférence dans une résine après déshydratation, selon le protocole détaillé dans cet article [73]. Le matériel génétique extrait peut ensuite être utilisé pour des analyses en aval, telles que la PCR, afin d'amplifier des marqueurs génétiques spécifiques. Les méthodes d'extraction non destructives des acides nucléiques sont essentielles pour l'étude des caractéristiques génétiques des phlébotomes, notamment pour l'identification des agents pathogènes potentiellement véhiculés. En préservant l'intégrité du spécimen, ces approches permettent d'obtenir des informations génétiques précieuses tout en conservant l'échantillon pour des analyses ou des études complémentaires.

6.5. Spectrométrie de masse MALDI-ToF

La spectrométrie de masse MALDI-ToF (Matrix-Assisted Laser Desorption/Ionization Time-of-Flight) est une technique analytique basée sur la spectrométrie de masse, conçue pour détecter et analyser les profils protéiques spécifiques (« empreintes protéiques ») des échantillons biologiques. Le MALDI-ToF est de plus en plus reconnu comme un outil majeur pour l'identification des arthropodes d'importance médicale et vétérinaire. Cette technique s'est révélée efficace pour l'identification de différents stades de développement des phlébotomes, y compris les formes immatures ainsi que les repas sanguins des femelles gorgées, et a été utilisée avec succès pour différencier les espèces mâles et femelles dans diverses conditions de conservation et d'homogénéisation [28, 30, 73, 74]. Elle offre également un fort pouvoir discriminant aux niveaux des sous-genres, des espèces et des populations. Le MALDI-ToF permet ainsi une identification rapide et précise des espèces, essentielle pour comprendre la distribution des phlébotomes, leur comportement et leur rôle dans la transmission des maladies. En distinguant les espèces sur la base de leurs profils protéiques, cette méthode joue un rôle clé dans les études épidémiologiques et les stratégies de lutte antivectorielle. Toutefois, deux limitations majeures freinent encore son application routinière. La première concerne la disponibilité des équipements de spectrométrie de masse, dont le coût est prohibitif pour une acquisition dédiée uniquement à l'identification des phlébotomes (ou des arthropodes vecteurs en général). Cette contrainte peut néanmoins être contournée par l'accès à des spectromètres de masse déjà disponibles dans des plateformes de protéomique ou des laboratoires de diagnostic clinique, où ces équipements sont devenus des outils standards. La seconde limitation est la faible représentation des données de référence concernant les phlébotomes dans les bases de données en accès libre. Cela impose la constitution de bases de données internes, fondées sur des spectres de référence obtenus à partir de spécimens identifiés sans ambiguïté, idéalement par une

Table 5: Coût moyen, applications et adaptations de protocoles pour l'extraction d'ADN génomique (ADNg) chez les phlébotomes

Protocole	Coût	Application	Adaptation du protocole pour les petits arthropodes
Colonnes de silice	2,5 – 3,55 US$ [39]	PCR, NGS	[9]
Phénol–chloroforme	0,24 US$ [69]	PCR, NGS	[9]
HotSHOT	< 0,01 US$ [69]	PCR	-
Salage	0,12 US$ [69]	PCR	-
Chelex	0,02 US$ [41]	PCR	[41, 76]

combinaison d'identification morphologique et de séquençage d'un marqueur génétique approprié (COI, cytB ou autre). Cette limitation devrait être progressivement levée grâce à l'intégration des données de référence actuellement internes dans la plateforme MSI, gérée par l'Assistance Publique–Hôpitaux de Paris et Sorbonne Université (France), ainsi que dans la collection BCCM/IHEM/Sciensano à Bruxelles (Belgique) (https://msi.happy-dev.fr/). Lorsque le profilage protéique par MALDI-ToF est envisagé, les échantillons doivent être conservés de préférence à l'état sec congelé ou dans de l'éthanol à 70 % de qualité moléculaire, et ne doivent pas être exposés à des températures ambiantes. En l'absence de protocoles universels de préparation des échantillons, il est recommandé d'utiliser une solution aqueuse composée de 60 % d'acétonitrile et de 0,3 % de TFA contenant de l'acide sinapinique (30 mg/mL) comme matrice MALDI-ToF, afin de garantir la comparabilité des spectres protéiques avec les données publiées existantes sur les phlébotomes.

Préparation des échantillons pour spectrométrie de masse MALDI-ToF MS (Figure 7)

Les spécimens d'insectes, conservés dans différentes conditions, sont d'abord séchés à l'air à température ambiante puis disséqués. La tête et l'abdomen sont retirés afin de préserver les parties contenant les caractères morphologiques essentiels pour le montage sur lame et l'analyse morphologique. Le thorax est utilisé pour l'analyse MALDI-ToF, tandis que le reste de l'abdomen peut être conservé pour l'extraction d'ADN. Pour le profilage protéique, le thorax est homogénéisé manuellement dans des microtubes de 1,5 mL avec 10 µL de solution d'homogénéisation, à l'aide de pilons jetables. Deux solutions d'homogénéisation sont couramment utilisées : de l'eau distillée stérile et de l'acide formique à 25 %.

7. Conclusion

Dans ce travail, nous avons cherché à fournir aux chercheurs les méthodes les plus efficaces pour le montage des phlébotomes, adaptées à des objectifs de recherche spécifiques, afin de faciliter une identification précise et la détection des agents pathogènes. Il n'existe pas de méthode unique et universellement optimale ; plusieurs approches sont disponibles, chacune présentant ses propres avantages et limites.

Dans les annexes, nous présentons des protocoles détaillés pour diverses techniques de montage utilisées dans la préparation et l'identification des phlébotomes. Ces protocoles, incluant des vidéos pédagogiques, proposent des procédures étape par étape adaptées à différents objectifs, garantissant des résultats précis et reproductibles. En mettant à disposition cette ressource complète, nous souhaitons aider les chercheurs à sélectionner et à appliquer les techniques de montage les plus appropriées à leurs besoins spécifiques.

Acknowledgments

Les auteurs remercient Richard Lane et Zoe Jay Adams du Natural History Museum de Londres (Royaume-Uni) pour

leur excellente relecture, qui a contribué à améliorer la qualité de ce manuscrit.

Funding

Cette étude a été financée par le projet ANRS00868-R-PR EXPECTS de l'ANRS-MIE. Nous remercions également les agences brésiliennes de développement CNPq (numéro de dossier : 404395/2024-4) et la Fondation Araucária (numéro de dossier : 433/2025 PDI) pour le financement des travaux de recherche de AJA.

Conflicts of interest

Jérôme Depaquit est éditeur associé de la revue Parasite ; il n'a exercé aucune influence sur le processus d'évaluation ni sur la prise de décision concernant ce manuscrit. Les autres auteurs déclarent ne pas avoir de conflits d'intérêts.

Data availability statement

Vidéos sur le site Zenodo:

Vidéo 1: https://zenodo.org/records/18198006

Vidéo 2: https://zenodo.org/records/18311158

Video 3: https://zenodo.org/records/18311106

Vidéo 4: https://zenodo.org/records/18311154

Vidéo 5: https://zenodo.org/records/18303014

Vidéo 6: https://zenodo.org/records/18302850

Vidéo 7: https://zenodo.org/records/18315029

Supplementary material

The supplementary material of this article is available at https://www.parasite-journal.org/10.1051/parasite/2026009/olm

References

1. Alkan C, Allal-Ikhlef AB, Alwassouf S, Baklouti A, Piorkowski G, de Lamballerie X, Izri A, Charrel RN. 2015. Virus isolation, genetic characterization and seroprevalence of Toscana virus in Algeria. Clinical Microbiology and Infection, 21(11), 1040 e1-9.
2. Alten B, Ozbel Y, Ergunay K, Kasap OE, Cull B, Antoniou M, Velo E, Prudhomme J, Molina R, Banuls AL, Schaffner F, Hendrickx G, Van Bortel W, Medlock JM. 2015. Sampling strategies for phlebotomine sand flies (Diptera: Psychodidae) in Europe. Bulletin of Entomological Research 105(6), 664–678.
3. Ayhan N, Baklouti A, Prudhomme J, Walder G, Amaro F, Alten B, Moutailler S, Ergunay K, Charrel RN, Huemer H. 2017. Practical guidelines for studies on sandfly-borne phleboviruses: Part I: Important points to consider *ante* field work. Vector-Borne and Zoonotic Diseases 17(1), 73–80.
4. Bates PA. 1997. Infection of phlebotomine sandflies with *Leishmania*, in The Molecular Biology of Insect Disease Vectors: A Methods Manual. Springer. p. 112–120.5.
5. Baum M, de Castro EA, Pinto MC, Goulart TM, Baura W, Klisiowicz Ddo R, Vieira da Costa-Ribeiro MC. 2015. Molecular detection of the blood meal source of sand flies (Diptera: Psychodidae) in a transmission area of American cutaneous leishmaniasis, Parana State, Brazil. Acta Tropica, 143, 8–12.
6. Belen A, Alten B, Aytekin A. 2004. Altitudinal variation in morphometric and molecular characteristics of *Phlebotomus papatasi* populations. Medical and Veterinary Entomology, 18(4), 343–350.
7. Bhattacharya J, Chandra G, Hati AK. 1991. A simple method for cryopreservation of *Leishmania donovani* promastigotes, Indian Journal of Medical Research, 93, 245–246.
8. Caligiuri LG, Sandoval AE, Miranda JC, Pessoa FA, Santini MS, Salomón OD, Secundino NF, McCarthy CB. 2019. Optimization of DNA extraction from individual sand flies for PCR amplification. Methods and Protocols, 2(2), 36.
9. Casaril AE, de Oliveira LP, Alonso DP, de Oliveira EF, Gomes Barrios SP, de Oliveira Moura Infran J, Fernandes WS, Oshiro ET, Ferreira AMT, Ribolla PEM, de Oliveira AG. 2017. Standardization of DNA extraction from sand flies: Application to genotyping by next generation sequencing. Experimental Parasitology, 177, 66–72.
10. Castalanelli MA, Severtson DL, Brumley CJ, Szito A, Foottit RG, Grimm M, Munyard K, Groth DM. 2010. A rapid non-destructive DNA extraction method for insects and other arthropods. Journal of Asia-Pacific Entomology, 13(3), 243–248.
11. Cerqueira NL. 1943. Um novo meio para montagem de pequenos insetos em lâmina. Memórias do Instituto Oswaldo Cruz, (39), 37–41.
12. Charrel RN, Gallian P, Navarro-Mari JM, Nicoletti L, Papa A, Sanchez-Seco MP, Tenorio A, de Lamballerie X. 2005. Emergence of Toscana virus in Europe. Emerging Infectious Diseases, 11(11), 1657–1663.
13. Chaskopoulou A, Giantsis IA, Demir S, Bon MC. 2016. Species composition, activity patterns and blood meal analysis of sand fly populations (Diptera: Psychodidae) in the metropolitan region of Thessaloniki, an endemic focus of canine leishmaniasis. Acta Tropica, 158, 170–176.
14. Chen H, Rangasamy M, Tan SY, Wang H, Siegfried BD. 2010. Evaluation of five methods for total DNA extraction from western corn rootworm beetles. PLoS One, 5(8), e11963.
15. Depaquit J, Grandadam M, Fouque F, Andry PE, Peyrefitte C. 2010. Arthropod-borne viruses transmitted by Phlebotomine sandflies in Europe: a review. Eurosurveillance, 15(10), 19507.
16. Diamond LS, Herman CM. 1954. Incidence of Trypanosomes in the Canada Goose as revealed by bone marrow culture. Journal of Parasitology, 40(2), 195–202.
17. Ding H, Torno M, Vongphayloth K, Ng G, Tan D, Sng W, Ho K, Randrianambinintsoa FJ, Depaquit J, Tan CH. 2025. Hidden in plain sight: discovery of sand flies in Singapore and description of four species new to science. Parasites & Vectors, 18(1), 402.
18. Es-Sette N, Ajaoud M, Bichaud L, Hamdi S, Mellouki F, Charrel RN, Lemrani M. 2014. *Phlebotomus sergenti* a common vector of *Leishmania tropica* and Toscana virus in Morocco. Journal of Vector Borne Diseases, 51(2), 86–90.
19. Favret C. 2005. A new non-destructive DNA extraction and specimen clearing technique for aphids (Hemiptera). Proceedings of the Entomological Society of Washington, 107(2), 469–470.
20. Galati EAB. 2018. Phlebotominae (Diptera, Psychodidae): Classification, morphology and terminology of adults and identification of American taxa, in Brazilian Sand Flies: Biology, Taxonomy, Medical Importance and Control, Rangel EF, Shaw JJ, Editors. Cham: Springer International Publishing. pp. 9–212.

21. Galati EAB, de Andrade AJ, Perveen F, Loyer M, Vongphayloth K, Randrianambinintsoa FJ, Prudhomme J, Rahola N, Akhoundi M, Shimabukuro PHF, Depaquit J. 2025. Phle-botomine sand flies (Diptera, Psychodidae) of the world. Parasites & Vectors, 18(1), 220.
22. Galati EAB, Galvis-Ovallos F, Lawyer P, Leger N, Depaquit J. 2017. An illustrated guide for characters and terminology used in descriptions of Phlebotominae (Diptera, Psychodidae). Par-asite, 24, 26.
23. Gariepy T, Kuhlmann U, Gillott C, Erlandson M. 2007. Parasitoids, predators and PCR: the use of diagnostic molecular markers in biological control of Arthropods. Journal of Applied Entomology, 131(4), 225–240.
24. Giantsis IA, Chaskopoulou A, Bon MC. 2016. Mild-Vectolysis: A nondestructive DNA extraction method for vouchering sand flies and mosquitoes. Journal of Medical Entomology, 53(3), 692–695.
25. Gidwani K, Picado A, Rijal S, Singh SP, Roy L, Volfova V, Andersen EW, Uranw S, Ostyn B, Sudarshan M, Chakravarty J, Volf P, Sundar S, Boelaert M, Rogers ME. 2011. Serological markers of sand fly exposure to evaluate insecticidal nets against visceral leishmaniasis in India and Nepal: a cluster-randomized trial. PLoS Neglected Tropical Diseases, 5(9), e1296.
26. Gilbert MTP, Moore W, Melchior L, Worobey M. 2007. DNA extraction from dry museum beetles without conferring external morphological damage. PLoS One, 2(3), e272.
27. Giordani BF, Andrade AJ, Galati EAB, Gurgel-Goncalves R. 2017. The role of wing geometric morphometrics in the identification of sandflies within the subgenus *Lutzomyia.* Medical and Veterinary Entomology, 31(4), 373–380.
28. Guzmán-Larralde AJ, Suaste-Dzul AP, Gallou A, Peña-Carrillo KI. 2017. DNA recovery from microhymenoptera using six non-destructive methodologies with considerations for subsequent preparation of museum slides. Genome, 60(1), 85–91.
29. Hajibabaei M, DeWaard JR, Ivanova NV, Ratnasingham S, Dooh RT, Kirk SL, Mackie PM, Hebert PD. 2005. Critical factors for assembling a high volume of DNA barcodes. Philosophical Transactions of the Royal Society B: Biological Sciences, 360(1462), 1959–1967.
30. Haouas N, Pesson B, Boudabous R, Dedet JP, Babba H, Ravel C. 2007. Development of a molecular tool for the identification of *Leishmania* reservoir hosts by blood meal analysis in the insect vectors. American Journal of Tropical Medicine and Hygiene, 77(6), 1054–1059.
31. Hlavackova K, Dvorak V, Chaskopoulou A, Volf P, Halada P. 2019. A novel MALDI-TOF MS-based method for blood meal identification in insect vectors: A proof of concept study on phlebotomine sand flies. PLoS Neglected Tropical Diseases, 13 (9), e0007669.
32. Huemer H, Prudhomme J, Amaro F, Baklouti A, Walder G, Alten B, Moutailler S, Ergunay K, Charrel RN, Ayhan N. 2017. Practical guidelines for studies on sandfly-borne phleboviruses: Part II: Important points to consider for fieldwork and subsequent virological screening. Vector-Borne and Zoonotic Diseases, 17(1), 81–90.
33. Jancarova M, Polanska N, Thiesson A, Arnaud F, Stejskalova M, Rehbergerova M, Kohl A, Viginier B, Volf P, Ratinier M. 2025. Susceptibility of diverse sand fly species to Toscana virus. PLoS Neglected Tropical Diseases, 19(5), e0013031.
34. Kapp JD, Green RE, Shapiro B. 2021. A fast and efficient single-stranded genomic library preparation method optimized for ancient DNA. Journal of Heredity, 112(3), 241–249.
35. Killick-Kendrick R, Maroli M, Killick-Kendrick M. 1991. Bibliography of the colonization of phlebotomine sandflies. Parassitologia, 33(suppl.), 321–333.
36. Lawyer P, Killick-Kendrick M, Rowland T, Rowton E, Volf P. 2017. Laboratory colonization and mass rearing of phlebotomine sand flies (Diptera, Psychodidae). Parasite, 24, 42.
37. Léger N, Pesson B, Madulo-Leblond G. 1986. Les phlébotomes de Grèce : 1ère partie. Bulletin de la Société de Pathologie Exotique, 79, 386–397.
38. Léger N, Pesson B, Madulo-Leblond G. 1986. Les phlébotomes de Grèce : 2ème partie. Bulletin de la Société de Pathologie Exotique, 79, 514–524.
39. Leonel JAF, Vioti G, Alves ML, da Silva DT, Meneghesso PA, Benassi JC, Spada JCP, Galvis-Ovallos F, Soares RM, Oliveira T. 2020. DNA extraction from individual Phlebotomine sand flies (Diptera: Psychodidae: Phlebotominae) specimens: Which is the method with better results? Experimental Parasitology, 218, 107981.
40. Lestinova T, Rohousova I, Sima M, de Oliveira CI, Volf P. 2017. Insights into the sand fly saliva: Blood-feeding and immune interactions between sand flies, hosts, and *Leishmania.* PLoS Neglected Tropical Diseases, 11(7), e0005600.
41. Lienhard A, Schaffer S. 2019. Extracting the invisible: obtaining high quality DNA is a challenging task in small arthropods. PeerJ, 7, e6753.
42. Lozano-Sardaneta YN, Mikery-Pacheco OF, Huerta H, Rojas-Soriano JE, Contreras-Ramos A. 2025. Wing geometric morphometrics is effective to separate sand fly species (Diptera, Psychodidae, Phlebotominae) related with leishmaniasis transmission in Mexico. Acta Tropica, 262, 107523.
43. Mandrioli M. 2008. Insect collections and DNA analyses: how to manage collections? Museum Management and Curatorship, 23(2), 193–199.
44. Maroli M, Feliciangeli MD, Bichaud L, Charrel RN, Gradoni L. 2013. Phlebotomine sandflies and the spreading of leishmaniases and other diseases of public health concern. Medical and Veterinary Entomology, 27(2), 123–147.
45. Marquina D, Buczek M, Ronquist F, Lukasik P. 2021. The effect of ethanol concentration on the morphological and molecular preservation of insects for biodiversity studies. PeerJ, 9, e10799.
46. Mathis A, Depaquit J, Dvorak V, Tuten H, Banuls AL, Halada P, Zapata S, Lehrter V, Hlavackova K, Prudhomme J, Volf P, Sereno D, Kaufmann C, Pfluger V, Schaffner F. 2015. Identification of phlebotomine sand flies using one MALDI-TOF MS reference database and two mass pectrometer systems., Parasites & Vectors, 8, 266.
47. Mekarnia N, Benallal KE, Sadlova J, Vojtkova B, Mauras A, Imbert N, Longhitano M, Harrat Z, Volf P, Loiseau PM, Cojean S. 2024. Effect of *Phlebotomus papatasi* on the fitness, infectivity and antimony-resistance phenotype of antimony-resistant *Leishmania* major Mon-25. International Journal for Parasitology – Drugs and Drug Resistance, 25, 100554.
48. Milligan BG. 1998. Total DNA isolation, in Molecular Genetic Analysis of Population: A Practical Approach, Hoelzel AR, Editor. Oxford: Oxford University Press.
49. Molina R, Jiménez M, Alvar J, González E, Hernández-Taberna S, Ines MM. 2017. Methods in sand fly research. Madrid: Servicio de publicaciones Universidad de Alcalá de Henares, Madrid.
50. Murphy WJ, Eizirik E, O'Brien SJ, Madsen O, Scally M, Douady CJ, Teeling E, Ryder OA, Stanhope MJ, de Jong WW, Springer MS. 2001. Resolution of the early placental mammal

radiation using Bayesian phylogenetics. Science, 294(5550), 2348–2351.

51. Nacif-Pimenta R, Pinto LC, Volfova V, Volf P, Pimenta PFP, Secundino NFC. 2020. Conserved and distinct morphological aspects of the salivary glands of sand fly vectors of leishma-niasis: an anatomical and ultrastructural study. Parasites & Vectors, 13(1), 441.
52. Neuhaus B, Schmid T, Riedel J. 2017. Collection management and study of microscope slides: Storage, profiling, deterioration, restoration procedures, and general recommendations. Zootaxa, 4322(1), 1–173.
53. New TR. 1974. Pscoptera. Handbooks for Identification of British Insects (Vol. I). London: Royal Entomological Society of London. 102 pp.
54. Perez-Ruiz M, Collao X, Navarro-Mari JM, Tenorio A. 2007. Reverse transcription, real-time PCR assay for detection of Toscana virus. Journal of Clinical Virology, 39(4), 276–281.
55. Porco D, Rougerie R, Deharveng L, Hebert P. 2010. Coupling non-destructive DNA extraction and voucher retrieval for small soft-bodied Arthropods in a high-throughput context: the example of Collembola. Molecular Ecology Resources, 10(6), 942–945.
56. Prudhomme J, Cassan C, Hide M, Toty C, Rahola N, Vergnes B, Dujardin JP, Alten B, Sereno D, Banuls AL. 2016. Ecology and morphological variations in wings of *Phlebotomus ariasi* (Diptera: Psychodidae) in the region of Roquedur (Gard, France): a geometric morphometrics approach. Parasites & Vectors, 9(1), 578.
57. Prudhomme J, Gunay F, Rahola N, Ouanaimi F, Guernaoui S, Boumezzough A, Banuls AL, Sereno D, Alten B. 2012. Wing size and shape variation of *Phlebotomus papatasi* (Diptera: Psychodidae) populations from the south and north slopes of the Atlas Mountains in Morocco. Journal of Vector Ecology, 37(1), 137–147.
58. Prudhomme J, Toty C, Kasap OE, Rahola N, Vergnes B, Maia C, Campino L, Antoniou M, Jimenez M, Molina R, Cannet A, Alten B, Sereno D, Banuls AL. 2015. New microsatellite markers for multi-scale genetic studies on *Phlebotomus ariasi* Tonnoir, vector of *Leishmania infantum* in the Mediterranean area. Acta Tropica, 142, 79–85.
59. Prudhomme J, Velo E, Bino S, Kadriaj P, Mersini K, Gunay F, Alten B. 2019. Altitudinal variations in wing morphology of *Aedes albopictus* (Diptera, Culicidae) in Albania, the region where it was first recorded in Europe. Parasite, 26, 55.
60. Rawlins DJ. 1992. Light Microscopy: An Introduction to Biotechniques. Oxford: Bios Scientific publishers. 143 pp.
61. Ready PD. 2013. Biology of phlebotomine sand flies as vectors of disease agents. Annual Review of Entomology, 58, 227–250.
62. Rohlf FJ, Slice D. 1990. Extensions of the Procrustes method for the optimal superimposition of landmarks. Systematic Zoology, 39(1), 40–59.
63. Rowley DL, Coddington JA, Gates MW, Norrbom AL, Ochoa RA, Vandenberg NJ, Greenstone MH. 2007. Vouchering DNA-barcoded specimens: Test of a nondestructive extraction proto-col for terrestrial arthropods. Molecular Ecology Notes, 7(6), 915–924.
64. Sábio PB, Andrade AJ, Galati EAB. 2014. Assessment of the taxonomic status of some species included in the *Shannoni* complex, with the description of a new species of *Psathyromyia* (Diptera: Psychodidae: Phlebotominae). Journal of Medical Entomology, 51(2), 331–341.
65. Sadlova J, Yeo M, Seblova V, Lewis MD, Mauricio I, Volf P, Miles MA. 2011. Visualisation of *Leishmania donovani* fluorescent hybrids during early stage development in the sand fly vector. PLoS One, 6(5), e19851.
66. Sales K, Miranda DEO, da Silva FJ, Otranto D, Figueredo LA, Dantas-Torres F. 2020. Evaluation of different storage times and preservation methods on phlebotomine sand fly DNA concentration and purity. Parasites & Vectors, 13(1), 399
67. Sales KG, Costa PL, de Morais RC, Otranto D, Brandao-Filho SP, Cavalcanti Mde P, Dantas-Torres F. 2015. Identification of phlebotomine sand fly blood meals by real-time PCR. Parasites& Vectors, 8, 230.
68. Sant'Anna MR, Jones NG, Hindley JA, Mendes-Sousa AF, Dillon RJ, Cavalcante RR, Alexander B, Bates PA. 2008. Blood meal identification and parasite detection in laboratory-fed and field-captured *Lutzomyia longipalpis* by PCR using FTA databasing paper. Acta Tropica, 107(3), 230–237.
69. Senne NA, Santos HA, Araujo TR, Paulino PG, Mendonca LP, Moreira HVS, Camilo TA, da Costa Angelo I. 2022. Robust comparative performance of genomic DNA extraction methods from non-engorged phlebotomine sandflies. Medical and Veterinary Entomology, 36(2), 203–211.
70. Shaw JJ. 2025. A review of Leishmania infections in American Phlebotomine sand flies – Are those that transmit leishmaniasis anthropophilic or anthropportunists? Parasite, 32, 57.
71. Tesh RB, Modi GB. 1983. Growth and transovarial transmission of Chandipura virus (Rhabdoviridae: Vesiculovirus) in *Phlebotomus papatasi.* American Journal of Tropical Medicine and Hygiene, 32(3), 621–623.
72. Thomsen PF, Elias S, Gilbert MTP, Haile J, Munch K, Kuzmina S, Froese DG, Sher A, Holdaway RN, Willerslev E. 2009. Non-destructive sampling of ancient insect DNA. PLoS One, 4(4),e5048
73. Truett GE, Heeger P, Mynatt RL, Truett AA, Walker JA, Warman ML. 2000. Preparation of PCR-quality mouse genomic DNA with hot sodium hydroxide and tris (HotSHOT). Biotechniques, 29(1), 52–54.
74. Upton MS. 1993. Aqueous gum-chloral slide mounting media: an historical review. Bulletin of Entomological Research, 83(2), 267–274.
75. Volf P, Myskova J. 2007. Sand flies and Leishmania: specific versus permissive vectors. Trends in Parasitology, 23(3), 91–92.
76. Wang Q, Wang X. 2012. Comparison of methods for DNA extraction from a single chironomid for PCR analysis. Pakistan Journal of Zoology, 44(2), 421–426.
77. Wang Y, Zhao Y, Bollas A, Wang Y, Au KF. 2021. Nanopore sequencing technology, bioinformatics and applications. Nature Biotechnology, 39(11), 1348–1365.

Cite this article as: Randrianambinintsoa FJ, Augendre L, Prudhomme J, Martinet J-P, Loyer M, Mekarnia N, Kerkoub H, Perveen FK, Huguenin A, Kariya E, Akhoundi M, De Andrade AJ, Berriatua E, Bongiorno G, Boyer S, Christodoulou V, Da Costa-Ribeiro MCV, De Souza LAF, Ding H, Dondji B, Dvořák V, Erisoz Kasap O, Galati EAB, Gállego M, Ballart C, Gouzelou S, Haddad N, Masse RS, Mekuria AH, Ivovic V, Kaczmarek S, Shahar MK, Kirstein OD, Kniha E, Kolářová I, Lincoln T, Lucanas C, Mikov O, Nov K, Özbel Y, Pesson B, Posada Lopez LC, Prasetyo DB, Rahola N, Rebollar-Tellez EA, Rodrigues BL, Roy L, Saini P, Sanjoba C, Shimabukuro PH, Siriyasatien P, Soszynska A, Suleşco T, Sylla M, Torno M, Volf P, Vongphayloth K, Sinh Nam V, Wardhana A, Yessinou E, Zapata S, Gantier J-C & Depaquit J. 2026. Processing and mounting phlebotomine sand flies: a consensus guideline. Parasite xx, xx. https://doi.org/10.1051/parasite/2026009.

PARASiTE

An international open-access, peer-reviewed, online journal publishing high quality papers

Reviews, articles and short notes may be submitted. Fields include, but are not limited to: general, medical and veterinary parasitology; morphology, including ultrastructure; parasite systematics, including entomology, acarology, helminthology and protistology, and molecular analyses; molecular biology and biochemistry; immunology of parasitic diseases; host-parasite relationships; ecology and life history of parasites; epidemiology; therapeutics; new diagnostic tools.
All papers in Parasite are published in English. Manuscripts should have a broad interest and must not have been published or submitted elsewhere. No limit is imposed on the length of manuscripts.

Parasite (open-access) continues **Parasite** (print and online editions, 1994-2012) and **Annales de Parasitologie Humaine et Comparée** (1923-1993) and is the official journal of the Société Française de Parasitologie.

Editor-in-Chief:
Jean-Lou Justine, Paris

Submit your manuscript at:
https://www.editorialmanager.com/parasite

Annexe 1 : Fondements théoriques biochimiques.

Les arthropodes concernés ici sont les phlébotomes. Toutefois, le principe général peut être étendu à d'autres arthropodes très courants dont l'identification repose exclusivement sur des caractères morphologiques internes. Par chance, certains organes internes sont partiellement chitinisés et leur morphologie fournit des informations précieuses. C'est pourquoi l'observation des pompes alimentaires, des spermathèques et de leurs conduits est particulièrement intéressante. Avec l'ensemble des réactifs que nous allons passer en revue, il ne faut jamais oublier que, depuis l'étape de fixation de l'insecte jusqu'au montage, nous appliquons essentiellement des réactions d'oxydoréduction. La seule précaution fondamentale consiste à éviter de mélanger des réactifs réducteurs avec des réactifs oxydants.

Alcool éthylique ; éthanol:

Cette substance est utilisée de différentes manières. Les molécules d'alcool ont une forte affinité pour l'eau et présentent donc un effet déshydratant. Toutefois, un alcool à faible concentration (i.e., trop riche en eau) jouera un rôle dans la dégradation des acides nucléiques (l'eau étant l'ennemi des acides nucléiques).

Lorsque les insectes sont placés dans l'éthanol, ce n'est pas uniquement pour les conserver, mais également pour fixer les tissus. En histologie, on distingue généralement deux notions importantes : la vitesse de pénétration et la vitesse de fixation. Il est bien établi qu'un bon fixateur doit d'abord pénétrer rapidement et profondément dans les tissus avant d'exercer son action fixatrice. Pour l'alcool à 96 %, le coefficient de pénétration est d'environ 1,05 (à titre de comparaison, une solution aqueuse d'acide picrique à 0,75 % a un coefficient de pénétration de 0,45, tandis qu'une solution de dichromate de potassium à 3 % présente un coefficient de 1,45).

La volonté de conserver indéfiniment des insectes et autres arthropodes dans l'éthanol est une réalité chez les entomologistes. L'idée de préserver les captures de terrain pour des études ultérieures ou pour de futurs chercheurs est tout à fait louable. Toutefois, cette approche n'est pas compatible avec les exigences d'un cytologiste ou d'un histologiste. En cherchant à maintenir trop longtemps les échantillons dans le fixateur, ceux-ci peuvent devenir pratiquement impossibles à retravailler. C'est pourquoi des échantillons âgés de plus de 10 ans sont difficiles, voire impossibles, à exploiter.

Un autre élément à considérer est le rapport entre la masse d'arthropodes à fixer et le volume du fixateur. En

pratique zoologique ou médicale, il est recommandé de prévoir un volume de fixateur environ 60 fois supérieur au volume des pièces à fixer. En pratique, pour les micro-arthropodes, pour un volume donné de spécimens à fixer, il convient d'ajouter au minimum 4 à 5 volumes d'alcool. Il faut garder à l'esprit que l'alcool perdra de sa concentration en absorbant l'eau contenue dans les tissus de l'arthropode. En conclusion :

- L'alcool éthylique est un agent chimique réducteur (et donc incompatible avec les fixateurs oxydants);
- Il précipite énergiquement les protéines et les dénature;
- Il dissout certains lipides complexes et précipite le glycogène;
- Il provoque une forte contraction des tissus et les durcit.

Solutions basiques d'hydroxyde de potassium ou de sodium:

L'utilisation de ces solutions en entomologie s'est principalement concentrée sur l'hydroxyde de potassium, sans justification clairement établie. L'hydroxyde de sodium [E524] se présente en solution, à différentes concentrations ou normalités, ainsi que sous forme de pastilles ou de paillettes. Son principal inconvénient est sa forte hygroscopicité (supérieure à celle du KOH). Lorsqu'il réagit avec les protéines, il les dissout, et avec les lipides, il les transforme en savons solides lors de la saponification (ce qui constitue une différence majeure avec le KOH, qui produit des savons liquides lors de cette réaction).

L'hydroxyde de potassium [E525] est disponible sous forme de solution concentrée, mais surtout sous forme de pastilles d'environ 0,1 g, ce qui facilite grandement la préparation de solutions diluées lorsqu'on ne dispose pas d'une balance de précision. Par exemple, une pastille de 0,1 g dissoute dans 1 mL d'eau distillée permet d'obtenir une solution à 10 %. Un autre avantage du KOH sous forme de pastilles est sa moindre sensibilité à la carbonatation (une solution de KOH présente une forte affinité pour le CO_2, conduisant à la formation de carbonates).

Ces bases fortes sont utilisées pour solubiliser les acides gras en les transformant en savons solubles dans l'eau. Il convient de rappeler que le fixateur, tel que l'éthanol, solubilise déjà une partie des lipides présents dans l'échantillon. Toutefois, lors du passage de l'échantillon dans un milieu aqueux contenant une base forte, les acides gras (plus ou moins complexes) précipitent. La base forte réalise ainsi une saponification à froid. Dans certains cas, lorsque les tissus adipeux sont abondants, par exemple chez les femelles, il peut être avantageux d'élever la température à 35–40 °C pour faciliter la réaction, ou d'allonger le temps de contact à température ambiante.

Solution de Marc-André colorée par la fuchsine ou non colorée:

Nous abordons ici les avantages et les inconvénients de l'utilisation de la solution de Marc-André. Cette solution est composée d'hydrate de chloral (trichloroacétaldéhyde monohydraté), d'acide acétique et d'eau. Il s'agit d'une solution fortement oxydante (mélange d'un acide et d'un aldéhyde). Elle permet de neutraliser l'excès d'hydroxyde de potassium pouvant subsister dans les échantillons, sans précipiter les savons alcalins formés lors de l'utilisation du KOH. Cette solution oxydante agit également sur les fonctions alcool secondaires des glucosamines constituant la chitine, en les oxydant, ce qui entraîne un ramollissement de la chitine. Elle permet aussi la dissolution de certains sels minéraux présents.

Lorsque la solution de Marc-André est préalablement colorée à la fuchsine acide (donc sous forme oxydée), elle peut se fixer sur les fonctions alcool secondaires des structures chitineuses. Après le temps de contact de la solution de Marc-André et selon l'état de coloration des échantillons, le rinçage est effectué uniquement à l'éthanol. On entame alors la phase de déshydratation des échantillons.

Avantages:

- Neutralisation des excès de solutions basiques
- Ramolissement de la chitine
- Coloration de la chitine permettant une meilleure appréciation des structures internes chitinisées

Inconvénients:

L'hydrate de chloral est une substance hypnotique historiquement utilisée en médecine humaine. Son emploi doit se faire sous hotte chimique et dans le respect strict de la réglementation relative aux risques chimiques.

Solutions de déshydratation:

L'expérience montre que, pour des échantillons de très petite taille, il n'est pas nécessaire de suivre une succession de bains d'alcool à concentrations croissantes. Si l'échantillon est volumineux, on commencera par de l'éthanol à 80 %, puis 90 %, 95 % et enfin de l'éthanol absolu. Pour les très petits échantillons, un bain à 90 % suivi d'une immersion dans l'éthanol absolu est suffisant. À ce stade, il faut toujours garder à l'esprit que l'éthanol absolu tend à capter l'eau présente dans l'atmosphère.

Traditionnellement, dans les laboratoires d'entomologie, la déshydratation finale des échantillons était réalisée à l'aide d'un bain de créosote de hêtre. Aujourd'hui, cette essence, autrefois largement utilisée comme pesticide, antifongique et conservateur du bois, est fortement déconseillée en raison de son odeur (liée aux hydrocarbures aromatiques polycycliques) et de sa toxicité

présumée : reprotoxique, cancérogène, polluant organique persistant et écotoxique pour les organismes aquatiques.

La solution que nous proposons pour le montage des échantillons est l'utilisation d'Euparal® et de l'essence d'Euparal (décrites dans le paragraphe suivant). Un mélange d'Euparal® et d'essence d'Euparal est très bien toléré par des échantillons ayant préalablement subi un bain d'éthanol à 90 %.

Annexe 2 : Composition des réactifs utilisés.

Hydroxyde de potassium à 10 %

Hydroxyde de potassium : 10 g
Eau distillée : q.s.p. 100 mL

Milieu de montage gomme au chloral (milieu de Hoyer)

Eau distillée : 50 mL
Hydrate de chloral : 200 g
Gomme arabique : 50 g
Glycérol : 20 mL

Solution de Marc-André

Hydrate de chloral : 40 g
Acide acétique glacial : 30 mL
Eau distillée : 30 mL

Fuchsine acide à 1 % dans l'eau distillée

Fuchsine acide (poudre) : 1 g
Eau distillée : 99 mL

Solution de Marc-André colorée à la fuchsine

Solution de Marc-André : 10 mL
Fuchsine acide à 1% 50 µL

Annexe 3 : Euparal®, baume du Canada, alcool polyvinylique et autres solutions de montage®

Alcool polyvinylique : L'alcool polyvinylique constitue un milieu de montage idéal lorsque les produits nécessaires à une déshydratation correcte ne sont pas disponibles. Dans ce cas, l'alcool polyvinylique est mélangé au lactophénol d'Amann. Toutefois, ce type de montage présente des inconvénients majeurs : soit le milieu se dessèche, soit l'alcool polyvinylique cristallise en raison de l'évaporation de l'eau, soit le montage noircit lorsque le phénol s'oxyde. Cette technique reste néanmoins adaptée pour des montages de courte durée.

Baume du Canada : L'utilisation du baume du Canada pour le montage entre lame et lamelle nécessite une déshydratation préalable des échantillons. Le recours au xylène ou au toluène pour cette étape n'est pas exempt d'inconvénients.

Milieu Enecê : À l'instar du baume du Canada, le milieu Enecê nécessite une déshydratation préalable des spécimens avant le montage entre lame et lamelle. Formulation du milieu Enecê: colophane blanche pure (22 g); gomme copal soluble dans l'alcool (12 g); alcool absolu (20 mL); camphre (10 g); essence de térébenthine (10 mL); eucalyptol : 26 mL. Préparation: Dans un récipient approprié (par exemple un erlenmeyer), introduire l'alcool absolu et le camphre, puis ajouter la colophane et la gomme copale. Le flacon est ensuite bouché et agité, puis chauffé au bain-marie à température modérée afin d'éviter toute ébullition. Lorsque le contenu est entièrement liquéfié, l'essence de térébenthine est ajoutée. Le mélange est ensuite filtré à chaud, et l'eucalyptol est ajouté au filtrat. Lorsque le milieu devient moins fluide, il est dilué avec l'essence d'Enecê, préparée selon la formule suivante : alcool absolu (30 mL), camphre (17 g), essence de térébenthine (15 mL), eucalyptol (38 mL) (Cerqueira, 1943).

Euparal : C'est une résine issue du cyprès de l'Atlas *Tetraclinis articulata* (Vahl, 1791), étudiée et développée en 1906 par Gilson. Son principal avantage est qu'elle ne polymérise pas. Les échantillons montés entre lame et lamelle peuvent être facilement récupérés par l'action de l'alcool, ou mieux encore, de l'essence d'Euparal®. Cette résine, également appelée sandaraque, accepte l'éthanol à partir de 80%.

Utilisation du Triton X100: solution aqueuse non ionique:

Le Triton X100 est une solution aqueuse non ionique (*4-(1,1,3,3-tétraméthylbutyl)phényl-polyéthylène glycol,* ou *t-octylphénoxypolyéthoxyéthanol, éther polyéthylène glycol tert-octylphénylique*), largement utilisée comme détergent en biologie cellulaire et moléculaire. Il permet la perméabilisation des membranes cellulaires et nucléaires.

Les échantillons d'insectes conservés pendant de nombreuses années dans l'alcool sont fréquents. Malheureusement, ce mode de conservation est loin d'être optimal, et les arthropodes ainsi préservés deviennent souvent très difficiles à préparer pour l'examen microscopique. Les contenants plastiques peuvent se dégrader, entraînant l'évaporation de l'alcool. Dans les deux cas, un contact prolongé avec l'alcool ou le dessèchement des échantillons constitue un problème majeur. En 2008, Jonque a publié une note concernant la réhydratation d'araignées à l'aide d'un agent mouillant tel que l'Agepon, utilisé en photographie [26]. Cette observation a conduit à l'idée d'utiliser des agents mouillants non fortement détergents.

Procédure utilisant une solution aqueuse de Triton X100 à 0,5 % :

- Imprégner l'échantillon sec avec de l'alcool absolu.
- Ajouter le volume nécessaire de solution de Triton X100 à 0,5 % afin que l'échantillon soit entièrement immergé.
- Laisser agir environ 5 minutes ou plus. Tous les arthropodes doivent devenir indépendants dans la solution.
- Retirer la solution de Triton X100 et la remplacer par la solution d'hydroxyde de potassium.

La procédure se poursuit ensuite selon le protocole décrit précédemment.

Annexe 4 : Montage pas à pas avec Euparal® ou baume du Canada

1. Les spécimens doivent être déshydratés (un aspect trouble ou laiteux indique une déshydratation insuffisante).
2. La déshydratation peut être réalisée par des concentrations croissantes d'alcool éthylique.
3. Les spécimens peuvent être transférés de l'alcool à 99 % ou de l'alcool absolu vers un agent d'éclaircissement.

Procédure :

1. Déposer les phlébotomes adultes dans de l'éthanol à 70%.
2. Retirer l'éthanol et le remplacer par une solution de KOH à 10%. Recouvrir les phlébotomes avec une lame de verre.
3. Laisser macérer jusqu'à ce que les insectes deviennent transparents.
4. Retirer la solution de KOH.
5. Recouvrir les spécimens avec de l'eau distillée et attendre 30 à 45 min.
6. Retirer l'eau et répéter le lavage à l'eau distillée pendant 30 min (le temps dépend du nombre d'échantillons traités simultanément : plus le nombre d'échantillons est élevé, plus ce temps doit être respecté ; inversement, pour un petit nombre d'échantillons, notamment traités individuellement, ce temps peut être réduit).
7. Retirer l'eau.
8. Ajouter la solution de Marc-André (éventuellement colorée à la fuchsine acide) et laisser agir pendant 24 heures (un jour).
9. Retirer la solution de Marc-André.
10. Recouvrir les spécimens avec de l'eau distillée et attendre 30 à 45 min.
11. Retirer l'eau et répéter le lavage à l'eau distillée pendant 30 min.
12. Retirer l'eau
13. Ajouter de l'éthanol à 70 % et disséquer le spécimen.

a. Pour la tête et l'abdomen, tirer délicatement la tête ou l'abdomen pour les séparer du thorax.

b. Pour le thorax, retirer les ailes en maintenant le thorax avec une paire de pinces et en tirant à la base des appendices avec une autre paire. Il est également possible de réaliser une dissection sagittale, en divisant le thorax en parties gauche et droite, selon les régions d'intérêt.

14. Déshydrater progressivement à travers une série de solutions aqueuses d'alcool éthylique : 50 – 80 – 95%, jusqu'à l'alcool absolu.
15. Finaliser la déshydratation des spécimens par deux bains successifs de 10 minutes chacun dans de l'éthanol à 100%.
16. Retirer l'éthanol et recouvrir les spécimens avec de l'huile essentielle de clou girofle pendant 15 minutes à température ambiante.
17. Transférer les spécimens de l'huile essentielle de clou girofle vers une goutte d'Euparal® ou de baume du Canada déposée sur une lame propre.
18. Disposer les pièces selon l'orientation souhaitée : la tête, le thorax et l'abdomen du phlébotome peuvent être disséqués à l'aide d'aiguilles fines ou de pinces sous loupe binoculaire. La tête doit être séparée du corps afin d'être montée en position ventro-dorsale, i.e., avec le foramen occipital orienté vers le haut, permettant l'observation directe du cibarium à travers celui-ci.

La dissection est réalisée directement dans le milieu de montage des phlébotomes.

19. Laisser le montage jusqu'à ce que la surface devienne légèrement collante.
20. Humidifier une lamelle propre avec de l'alcool absolu. Déposer la lamelle sur le baume du Canada ou l'Euparal® en l'inclinant légèrement.
21. Conserver les lames dans une boîte sèche prévue à cet effet.
