# Supplementary material for: Processing and mounting phlebotomine sand flies: a consensus guideline
Source: Parasite. 2026 Apr 3;33:18. doi: 10.1051/parasite/2026009 (PMC13047900; doi:10.1051/parasite/2026009)
Supplement: Supplementary file 11 — German translation / Deutsche Übersetzung [file parasite-33-18-s11.pdf]

Parasite **33**, 18 (2026)
© F.J. Randrianambinintsoa et al., published by EDP Sciences, 2026
https://doi.org/10.1051/parasite/2026009

PARASITE
Available online at:
www.parasite-journal.org

REVIEW ARTICLE OPEN ACCESS

Präparierung und Einbettung von Sandmücken (Phlebotominae): Eine konsensbasierte Leitlinie

Fano José Randrianambinintsoa[1], Laure Augendre[1], Jorian Prudhomme[1], Jean-Philippe Martinet[1], Mathieu Loyer[1], Nalia Mekarnia[1], Hocine Kerkoub[1], Farzana Khan Perveen[1], Antoine Huguenin[1,2], Emilie Kariya[1,2], Mohammad Akhoundi[3], Andrey José de Andrade[4], Eduardo Berriatua[5], Gioia Bongiorno[6], Sébastien Boyer[7,8], Vasiliki Christodoulou[9], Magda Clara Vieira Da Costa-Ribeiro[10], Lucas Alexandre Farias de Souza[10], Huicong Ding[11], Blaise Dondji[12], Vít Dvořák[13], Ozge Erisoz Kasap[14], Eunice Aparecida Bianchi Galati[15], Montserrat Gállego[16], Cristina Ballart[16], Stavroula Gouzelou[17], Nabil Haddad[18], Rezki Sabrina Masse[19], Asrat Hailu Mekuria[20], Vladimir Ivovic[21], Szymon Kaczmarek[22], Mohd Khadri Shahar[19], Oscar D. Kirstein[23], Edwin Kniha[24], Iva Kolářová[13], Lincoln Timinao[25], Cristian Lucanas[26], Ognyan Mikov[27], Kimsear Nov[7], Yusuf Özbel[28], Bernard Pesson[29], Laura Cristina Posada Lopez[30], Didot Budi Prasetyo[1,7], Nil Rahola[31], Eduardo A. Rebollar-Tellez[32], Bruno Leite Rodrigues[15], Lalita Roy[33], Prasanta Saini[34], Chizu Sanjoba[35], Paloma Helena Fernandes Shimabukuro[36], Padet Siriyasatien[37], Agnieszka Soszyńska[22], Tatiana Suleşco[38], Massamba Sylla[39], Majhalia Torno[40], Petr Volf[13], Khamsing Vongphayloth[41], Vu Sinh Nam[42], April Wardhana[43], Eric Yessinou[44], Sonia Zapata[45], Jean-Charles Gantier[1], and Jérôme Depaquit[1,2,*]

[1] Faculté de Pharmacie, Université de Reims Champagne Ardenne, UR ESCAPE-USC ANSES PETARD, 51 rue Cognacq-Jay, 51096 Reims Cedex, France
[2] Pôle de Biologie territoriale, Laboratoire de Parasitologie-Mycologie, Centre Hospitalo-Universitaire, 51092 Reims, France
[3] Parasitology-Mycology Department, Avicenne Hospital, AP-HP, Bobigny, Sorbonne Paris Nord University, France; Unité des Virus Émergents (UVE: Aix-Marseille Univ, Università di Corsica, IRD 190, Inserm 1207, IRBA), 13005 Marseille, France
[4] Parasitology Collection of Basic Pathology, Department of Basic Pathology, Federal University of Paraná, Curitiba 19031, Brazil
[5] Department of Animal Health, University of Murcia, Campus de Espinardo, 30100 Espinardo, Murcia, Spain
[6] Department of Infectious Diseases, Vector-borne Diseases Unit, Istituto Superiore di Sanità, 00166 Rome, Italy
[7] Medical and Veterinary Entomology Unit, Institut Pasteur du Cambodge, Phnom Penh 12201, Cambodia
[8] Ecology & Emergence of Arthropod-borne Pathogens Unit, Department of Global Health, Institut Pasteur, CNRS UMR2000, 75015 Paris, France
[9] Section Veterinary Services (1417), Laboratory for Animal Health Virology, Aglantzia, Nicosia 2109, Cyprus
[10] Insects Vectors and Parasites Laboratory, Department of Basic Pathology and Postgraduate program in Microbiology, Parasitology and Pathology, Federal University of Paraná, 81530-900 Curitiba, Brazil
[11] Department of Biological Sciences, National University of Singapore, 117558, Singapore
[12] Laboratory of the Leishmaniasis Research Project, Mokolo District Hospital, Mokolo, Cameroon; Laboratory of Cellular Immunology and Parasitology, Department of Biological Sciences, Central Washington University, 98926 Ellensburg, WA, USA
[13] Department of Parasitology, Faculty of Science, Charles University, 12800 Prague, Czechia
[14] VERG Laboratories, Department of Biology, Faculty of Science, Hacettepe University, Beytepe, Ankara 06800, Türkiye
[15] Faculdade de Saúde Pública da Universidade de São Paulo (FSP/USP), Pós-graduação em Saúde Pública, 01246-904 São Paulo, Brazil
[16] Secció de Parasitologia, Departament de Biologia, Sanitat i Medi Ambient, Facultat de Farmàcia i Ciències de l'Alimentació, Universitat de Barcelona, & Institut de Salut Global de Barcelona (ISGlobal), Centro de Investigación Biomédica en Red, Enfermedades Infecciosas (CIBERINFEC), 08028 Barcelona, Spain
[17] Laboratory of Infectious Diseases and Public Health, School of Medicine, University of Cyprus, Nicosia, Cyprus & Department of Pediatrics, Archbishop Makarios III Hospital, Nicosia 2115, Cyprus
[18] Faculty of Health Sciences, American University of Beirut, 1107 2020 Beirut, Lebanon
[19] Medical Entomology Unit, Infectious Disease Research Centre, Institute for Medical Research (IMR), National Institutes of Health (NIH), Ministry of Health Malaysia, 40170 Shah Alam, Selangor, Malaysia
[20] School of Medicine, Addis Ababa University, 28017 - 1000 Addis Ababa, Ethiopia

Edited by Jean-Lou Justine
*Corresponding author: jerome.depaquit@univ-reims.fr

This is an Open Access article distributed under the terms of the Creative Commons Attribution License (https://creativecommons.org/licenses/by/4.0), which permits unrestricted use, distribution, and reproduction in any medium, provided the original work is properly cited.

[21] Faculty of Mathematics, Natural Sciences and Information Technologies, University of Primorska, 6000 Koper, Slovenia
[22] University of Lodz, Faculty of Biology and Environmental Protection, Department of Invertebrate Zoology and Hydrobiology, Banacha 12/16, 90-237 Łódź, Poland
[23] Laboratory of Entomology, Ministry of Health, 9134302 Jerusalem, Israel
[24] Center for Pathophysiology, Infectiology and Immunology, Institute of Specific Prophylaxis and Tropical Medicine, Medical University Vienna, Kinderspitalgasse 15, 1090 Vienna, Austria
[25] Papua New Guinea Institute of Medical Research (PNGIMR) Institute, PO Box 60, Headquarter, Homate Street, 441 Goroka, Eastern Highlands Province, Papua New Guinea
[26] Museum of Natural History, University of the Philippines Los Baños, 4031 Laguna, Philippines
[27] National Centre of Infectious and Parasitic Diseases, 1504 Sofia, Bulgaria
[28] Ege University, Faculty of Medicine, Department of Parasitology, 35040 Bornova/Izmir, Türkiye
[29] Retired, Faculté de Pharmacie, Université de Strasbourg, Strasbourg, 67400 Illkirch-Graffenstaden, France
[30] Program for the Study and Control of Tropical Diseases (PECET), Faculty of Medicine, University of Antioquia, 050010 Medellin, Colombia
[31] MIVEGEC, Univ. Montpellier, CNRS, IRD, 34394 Montpellier, France & Medical Entomology Unit, Institut Pasteur de Madagascar, 101 Antananarivo, Madagascar
[32] Laboratorio de Entomología Médica, Departamento de Zoología de Invertebrados, Facultad de Ciencias Biológicas, Universidad Autónoma de Nuevo León, San Nicolás de los Garza, 66455, NL, México
[33] Tropical and Infectious Disease Centre, BP Koirala Institute of Health Sciences, Dharan 56700, Nepal
[34] ICMR-Vector Control Research Centre, Puducherry 605006, India
[35] Graduate School of Agricultural and Life Sciences, The University of Tokyo, Tokyo 113-8657, Japan
[36] Grupo de estudos em Leishmanioses/Coleção de Flebotomíneos (COLFLEB/Fiocruz-MG), Instituto René Rachou, Fundação Oswaldo Cruz, Belo Horizonte, Minas Gerais, 30190009, Brazil
[37] Center of Excellence in Vector Biology and Vector-Borne Disease, Department of Parasitology, Faculty of Medicine, Chulalongkorn University, Bangkok 10330, Thailand
[38] Department of Arbovirology, Bernhard Nocht Institute for Tropical Medicine, Bernhard Nocht Str. 74, 20359 Hamburg, Germany [39] Laboratory Vectors & Parasites, Department of Livestock Sciences and Techniques, Sine Saloum University El Hadji Ibrahima Niasse (SSUEIN) Kaffrine Campus, C.P. 24600, Senegal.
[40] Environmental Health Institute, National Environment Agency, Singapore 138667, Singapore & Department of Biological Sciences, National University of Singapore, 117558 Singapore
[41] Institut Pasteur du Laos, Laboratory of Vector-Borne Diseases, Samsenhai Road, Ban Kao-Gnot, Sisattanak District, 3560 Vientiane, Lao PDR
[42] National Institute of Hygiene and Epidemiology, 1 Yec-Xanh Street, Hai Ba Trung District, 100000 Hanoi, Vietnam
[43] Indonesian Research Center for Veterinary Science, Indonesian Agency for Agricultural Research and Development, Ministry of Agriculture Republic Indonesia, Bogor 16114, Indonesia & Department of Parasitology, Faculty of Veterinary Medicine, Airlangga University, Surabaya 60115, Indonesia
[44] Laboratory of Research in Applied Biology, Polytechnic School of Abomey-Calavi, University of Abomey-Calavi, 01 P.O. Box 2009, 00000 Cotonou, Benin
[45] Instituto de Microbiología, Colegio de Ciencias Biológicas y Ambientales (COCIBA), Universidad San Francisco de Quito (USFQ), 170901 Quito, Ecuador

Received 1 December 2025, Accepted 29 January 2026, Published online 3 April 2026

Zusammenfassung – Dieser Artikel bietet einen umfassenden Leitfaden für die Präparierung und Einbettung von Sandmücken, was für die Artidentifizierung sowie den Nachweis und die Isolierung von Pathogenen von entscheidender Bedeutung ist. Es werden eine Reihe von Techniken erörtert, die sowohl für den Einsatz im Freiland als auch im Labor geeignet sind. Der Leitfaden enthält detaillierte Anweisungen zur Sammlung, Handhabung und Tötung von Sandmücken (wobei Trockenfrieren oder CO_2 gegenüber Chemikalien empfohlen wird) sowie Konservierungsstrategien wie die Kaltlagerung und die Aufbewahrung in Ethanol.

Die Qualität der Präparierung bestimmter anatomischer Strukturen (Genitalorgane, Kopf und Flügel) ist für deren korrekte mikroskopische Untersuchung essenziell und wird in dieser Arbeit beschrieben. Der Artikel stellt zudem die detaillierte Probenverarbeitung vor, einschließlich des Aufhellungsprozesses mit Reagenzien wie Kaliumhydroxid und anschließender Marc-André-Lösung-Einbettung. Bei der Einbettung werden verschiedene Medien verglichen, wobei deren optische Eigenschaften und das Konservierungspotenzial hervorgehoben werden. Hoyer-Medium (auch bekannt als Chloralhydrat-Gummi-Medium) wird aufgrund seiner Klarheit für schnelle Beobachtungen, insbesondere von Spermatheken, empfohlen, obwohl es nicht für die Langzeitlagerung geeignet ist. Weitere diskutierte Medien umfassen Polyvinylalkohol, Euparal® (bei begrenzter Wasserverträglichkeit) und Kanadabalsam (ein kohlenwasserstofflösliches Medium), wobei die beiden letztgenannten Möglichkeiten zur Langzeitkonservierung bieten. Innovative molekularbiologische Ansätze wie DNA-Sequenzierung und MALDI-ToF, die eine besondere Aufmerksamkeit bei der

Probenaufarbeitung erfordern, werden ebenfalls behandelt. Darüber hinaus werden kurze Videoclips zur Veranschaulichung verschiedener Einbettungstechniken sowie Übersetzungen in mehreren Sprachen bereitgestellt, wodurch die Leitlinie den vielfältigen Bedürfnissen und Erwartungen der weltweiten wissenschaftlichen Gemeinschaft gerecht wird.

Schlagwörter: Präparierung, Sandmücke, Hoyer-Lösung, Marc-André-Lösung, Chloralhydrat-Gummi-Medium, Polyvinylalkohol, Euparal®, Canada-Balsam, *Leishmania*-Isolierung, Freilandbedingungen, Kultur, Präparierung, Molekularbiologie, MALDI-ToF, Typusexemplar.

Abstract – Processing and mounting phlebotomine sand flies: a consensus guideline. This article provides a comprehensive guide for the processing and mounting of phlebotomine sand fly specimens, which is crucial for species identification and pathogen detection and isolation. It discusses a range of techniques suitable for both field and laboratory settings. The guide includes detailed instructions on sand fly collection, handling, covering, and euthanasia (recommending dry freezing or CO_2 over chemicals) as well as conservation strategies, such as cold storage and preservation in ethanol. The quality of preparation of certain anatomical structures (genital organs, head and wings) is essential for their proper microscopic observation and is described in this work. The article also presents detailed sample processing, including the clearing process with agents such as potassium hydroxide then Marc-André solution. The mounting process compares different media, emphasizing their optical properties and preservation potential. Hoyer fluid (also known as chloral gum) is recommended for quick observation, particularly for spermathecae, due to its clarity, although it is not suitable for long-term storage. Other media discussed include polyvinyl alcohol, Euparal® (for limited water tolerance), and Canada balsam (a hydrocarbon-soluble medium), with the latter two offering long-term preservation capabilities. Innovative molecular biology approaches such as DNA sequencing and MALDI-ToF, which require particular attention to sample processing, are also addressed. Furthermore, short video clips illustrating various mounting techniques as well as translations in many different languages are provided, allowing the guideline to reach the diverse needs and expectations of the global scientific community.

Key words: Mounting, Phlebotomine sand fly, Hoyer fluid, Marc-André solution, Chloral gum, Polyvinyl alcohol, EuparalÒ, Canada balsam, Leishmania isolation, Field conditions, Culture, Dissection, Molecular biology, MALDI-ToF, Type-specimens.

Einleitung

Sandmücken sind kleine Insekten aus der Ordnung Diptera innerhalb der Familie Psychodidae und der Unterfamilie Phlebotominae, die mindestens 1.063 bekannte Arten umfasst [21]. Sie fungieren als bedeutende Vektoren für Pathogene (*Leishmania*, Arboviren und *Bartonella*), welche für die Erkrankungen Leishmaniose, Arbovirus-Infektionen beziehungsweise Bartonellose verantwortlich sind. Ihre Identifizierung basiert primär auf detaillierten mikroskopischen Untersuchungen, die durch eine sorgfältige Probenentnahme, angemessene Lagerung und präzise Einbettung auf Objektträgern ermöglicht werden. Dies erfordert verschiedene spezifische Techniken, die jeweils individuelle Vor- und Nachteile aufweisen.

Die Bestimmung adulter Sandmücken beruht auf der Analyse von externen (z. B. Antennen, Palpen, männliche Genitalien) als auch internen Strukturen (z. B. Pharynx, Cibarium und Spermatheken). Die Präparierung und Isolierung letzterer erleichtert deren Untersuchung und damit eine präzise Identifizierung. Im Gegensatz zu Stechmücken oder Raubwanzen müssen Sandmücken daher vor der Bestimmung zwischen einem Objektträger und einem Deckglas eingebettet werden.

Bis in die 1980er Jahre war die mikroskopische Untersuchung die einzige verfügbare Methode zur Identifizierung von Sandmücken und stellt auch heute noch den am weitesten verbreiteten Ansatz dar. Die Wahl des Verfahrens und der Präparierung war daher relativ simpel und basierte hauptsächlich auf einer Dichotomie: einerseits die dauerhafte Einbettung zur langfristigen Konservierung der Probe, andererseits die Schnelleinbettung zur Identifizierung in einem Medium, das keine Langzeitarchivierung gewährleistet. Die abschließende Einbettung, beispielsweise in einem Harz wie Kanadabalsam, ist zeitaufwendig, da sie eine vollständige Dehydrierung der Proben erfordert. Zudem ist der Brechungsindex dieses Mediums nicht immer optimal für die Visualisierung der Spermatheken. Die Einbettung in einem wässrigen Medium (z. B. Hoyer-Gemisch) ist hingegen schneller und ermöglicht eine bessere Darstellung der refraktären Spermatheken, erlaubt jedoch keine langfristige Aufbewahrung der Präparate, da diese zur Absorption von Luftfeuchtigkeit neigen. Eine Option besteht darin, den Objektträger nach vollständiger Trocknung mit Nagellack zu versiegeln. Dieser Kompromiss besteht bis heute fort und beeinflusst die Wahl der Einbettungsmethode je nach Verwendungszweck der Präparierung. Seit den 1980er Jahren kombinieren Studien zur Identifizierung von Sandmücken morphologische und biochemische Ansätze. Den Anfang machten Analysen kutikulärer Kohlenwasserstoffe, die jedoch schnell durch molekularbiologische Techniken ersetzt wurden (*d. h.*

Random Amplified Polymorphic DNA (RAPD), Restriktionsfragmentlängenpolymorphismus (RFLP), DNA-Amplifizierung und Sequenzierung nach der Sanger-Methode sowie Next-Generation Sequencing (NGS)). Heute werden molekulare Ansätze durch proteomische Methoden wie MALDI-ToF ergänzt. Darüber hinaus kann die molekulare Artidentifizierung mit dem Nachweis von Pathogenen mittels PCR (*Leishmania*, *Trypanosoma*, *Bartonella* und *Phlebovirus*) kombiniert werden, da alle mittels Endpunkt- oder Real-Time-PCR detektierbar sind. Dies erfordert eine Anpassung des Probenahme- und des Lagerungsprozesses an die jeweiligen Zielsetzungen [3, 32]. Zusätzlich zu den traditionell zur Artdifferenzierung herangezogenen morphologischen Merkmalen können weitere morphologische Ansätze angewendet werden (z. B. Geomorphometrie der Flügel).

Hauptsächlich basierend auf den Erfahrungen der Autor:innen aber auch Literaturdaten war es das Ziel dieser Untersuchung, standardisierte Leitfäden für die Einbettung und Bearbeitung adulter Sandmücken zu erstellen, um sowohl morphologische als auch molekulare Analysen zu optimieren.

Die Notwendigkeit bestimmter Analysen (z. B. Molekularbiologie oder MALDI-ToF) erfordert die Aufbewahrung von Teilen der Sandmücke, die für die morphologische Identifizierung nicht zwingend erforderlich sind, was die Bedeutung einer kritischen Protokollauswahl unterstreicht.

In diesem Artikel konzentrieren wir uns auf die Methoden der Anästhesie und Tötung lebend gefangener Sandmücken, deren Lagerung sowie den Einbettungsprozess für die Schnelldiagnostik oder die langfristige Konservierung für spätere Studien.

Präambel: Sicherheits- und regulatorische Erwägungen sollten entsprechende Sicherheitsdatenblätter (SDB) referenzieren.

Alle in dieser Richtlinie vorgestellten Chemikalien müssen unter strengen Sicherheitsvorgaben gehandhabt werden. Die Arbeitsschutzgremien der Forschungseinrichtungen stehen zur Verfügung, um Informationen über die Gefahren dieser Chemikalien sowie über deren Handhabung und Entsorgung bereitzustellen. Die Einhaltung der Sicherheitsanweisungen bezüglich Verwendung und Entsorgung ist jedoch obligatorisch. Es liegt in der Verantwortung aller Anwender, die Einhaltung guter und sicherer Laborpraktiken sowie der geltenden Gesetze und Vorschriften ihres Landes oder ihrer Forschungseinrichtung sicherzustellen. Zudem sind einige der Chemikalien oder deren Bestandteile (z. B. Chloralhydrat) in bestimmten Ländern reguliert. Eine Liste der in diesem Manuskript verwendeten Abkürzungen ist in Tabelle 1 aufgeführt.

Tabelle 1: Liste der Abkürzungen

**BI**	Brechungsindex
**BME**	Basal-Medium-Eagle
**CDC**	Centers for Disease Control and Prevention
**CMCP**	Campher-Monochlorphenol
**COI**	Cytochrom c Oxidase Untereinheit I-Gen
**CytB**	Cytochrom b-Gen
**DNA**	Desoxyribonukleinsäure
**ELISA**	Enzyme-linked immunosorbent assay
**EtOH**	Ethanol
**KMR**	Karzinogene, Mutagene, Reproduktionstoxische Stoffe
**M199**	Medium 199
**MALDI-ToF MS**	Matrix-unterstützte Laser-Desorption / Ionisation-Flugzeit-Massenspektrometrie
**MEM**	Minimum-Essential-Medium
**NGS**	Next-Generation-Sequenzierung
**NNN**	Novy-MacNeal-Nicolle-Medium
**PCR**	Polymerase-Kettenreaktion
**LVR Laos**	Demokratische Volksrepublik Laos
**PNOC**	Prepronociceptin-Gen
**qPCR**	Quantitative-PCR (real-time PCR)
**RAPD**	Random Amplified Polymorphic DNA
**RFLP**	Restriktionsfragmentlängenpoly morphismus
**RNA**	Ribonukleinsäure
**RNasen**	Ribonukleasen
**RNASL**	RNA stabilisierende Lösung
**RT-PCR**	Reverse Transkriptase-PCR
**TFA**	Trifluoressigsäure

1. Fang von Sandmücken

Adulte Sandmücken können sowohl lebend als auch tot mittels verschiedener Methoden gesammelt werden. Hierzu zählen unter anderem CDC-Miniatur-Lichtfallen, Klebefallen sowie der Einsatz von Exhaustoren in Verbindung mit Shannon-Fallen oder der Direktfang an Ruheplätzen in der Umgebung (z. B. in Tierställen). Diese Methoden beinhalten das Aufstellen von Fallen in geeigneten Habitaten, wobei Sandmücken durch Licht oder andere Lockstoffe (CO_2 oder chemische Lockstoffe) angelockt und anschließend für weiterführende Analysen gesammelt werden, wie in diversen Publikationen beschrieben [2, 3, 32, 36, 49].

Der Fang lebender Sandmücken ermöglicht alle nachfolgenden Anwendungen, wohingegen das Sammeln toter Individuen die Isolierung von *Leishmania*- oder Virusstämmen verhindert. Bestimmte Fangtechniken, wie etwa Klebefallen, führen regelmäßig zum Verlust von Organen (Antennen, Palpen, Flügel oder Beine). Zudem

haftet das Rizinusöl der Klebefallen an den Sandmücken an und muss zu Beginn der Bearbeitung entfernt werden. Dies erfolgt üblicherweise durch ein 15-minütiges Bad in einem Gemisch aus Ethanol und Diethylether zu gleichen Teilen.

2. Tötung der Individuen

Nach der Sammlung müssen lebende Sandmücken getötet werden. Bei einigen Sammelmethoden (z. B. Klebefallen oder CDC-Lichtfallen, die mit einem Gefäß mit Reinigungsmittel oder Ethanol ausgestattet sind) sind die Sandmücken bereits zum Zeitpunkt der Entnahme tot. Molekularbiologische Untersuchungen können an Proben durchgeführt werden, die direkt in Ethanol gesammelt wurden, sowie an anderen Proben, sofern diese so schnell wie möglich in Ethanol überführt werden. Keines dieser Tötungsverfahren ermöglicht jedoch eine Bearbeitung der Insekten mittels MALDI-ToF. Zudem können einige Tötungsmethoden zum Verlust bestimmter morphologischer Merkmale führen. Daher ist es essenziell, ein geeignetes Standard-Tötungsmittel zu verwenden, um eine korrekte Identifizierung oder die langfristige Aufbewahrung als Belegexemplare zu gewährleisten. Chemikalien wie Ethylacetat, Ethylether, Tetrachlorethan und Chloroform können auf Watte aufgetragen und in ein Gefäß mit den Sandmücken gegeben werden. Diese Tötungsmittel müssen aufgrund ihrer Toxizität unter strikter Beachtung der Herstellerempfehlungen gehandhabt werden. Wir raten jedoch davon ab, Sandmücken mit Chloroform zu töten, da dies nach unserer Erfahrung kaum mit molekularbiologischen Studien vereinbar ist. Angesichts der Gefährlichkeit all dieser Produkte und ihrer fragwürdigen Eignung für molekulare Analysen wird von der Verwendung dieser Chemikalien im Allgemeinen abgeraten.

Die am weitesten verbreitete Methode, welche die Morphologie, DNA und Proteine schont, ist das trockene Einfrieren der Proben. Die Exemplare müssen lange genug eingefroren werden, um vollständig anästhesiert zu sein, jedoch nicht so lange, dass sie (i) austrocknen oder (ii) die Lebensfähigkeit von *Leishmania* beeinträchtigt wird, sofern deren In-vitro-Isolierung aus dem Verdauungstrakt der Sandmücke angestrebt wird. **Wir empfehlen daher eine Gefrierdauer von 15 bis 20 Minuten bei -20 °C unter regelmäßiger Kontrolle, um sicherzustellen, dass die Tiere nur betäubt werden, ohne die *Leishmania*-Parasiten abzutöten.**

Falls kein Gefrierschrank zur Verfügung steht, können die Insekten alternativ mit CO_2 getötet werden. Unter Freilandbedingungen, in denen keine CO_2-Zylinder verwendet werden können, können die Proben mit kleinen kommerziellen CO_2-Kartuschen für „Sodasyphons“ (Getränkespender) getötet werden; hierbei sind jedoch mögliche Einschränkungen beim Lufttransport zu beachten. Als letztes Mittel können die Insekten Tabakrauch ausgesetzt werden. Dabei werden die Sandmücken lebend in einer CDC-Falle gefangen, mit einem Aspirator aufgenommen, im Glasrohr zurückgehalten und Tabakrauch ausgesetzt, der sie innerhalb von Sekunden tötet. Diese Methode ist unter allen Freilandbedingungen anwendbar, selbst unter schwierigen Isolierungsbedingungen. Da das Glas jedoch durch den Rauch imprägniert wird, kann es ohne gründliche Reinigung nicht für den nachfolgenden Fang und die Handhabung lebender Sandmücken verwendet werden. Dennoch kann derselbe ungereinigte Aspirator weiterhin zur Tötung von Sandmücken aus anderen Fallen für Fixierungszwecke verwendet werden. Zudem muss sichergestellt werden, dass alle Exemplare aus dem Aspirator entfernt wurden. Diese Methoden sind mit der Isolierung von *Leishmania* durch Darmdissektion kompatibel.

3. Probenlagerung vor der Bearbeitung

Es gibt fünf Hauptmethoden der Fixierung vor der weiteren Bearbeitung:

3.1. Einfrieren (Kryokonservierung)

Diese Methode wird idealerweise bei -20 °C oder vorzugsweise bei -80 °C durchgeführt. Diese Lagerungsverfahren sind mittlerweile weiter verbreitet als die Aufbewahrung in flüssigem Stickstoff. In jedem Fall muss die Kryokonservierung so schnell wie möglich nach der Betäubung der Proben erfolgen. Die Kaltlagerung in Gefrierschränken bietet den Vorteil, dass sowohl Insekten als auch RNA, DNA und Proteine über den gesamten Lagerungszeitraum hinweg vollständig erhalten bleiben. Im Gegensatz dazu kann flüssiger Stickstoff Flügel, Beine, Palpen und Antennen schwer beschädigen, was häufig zu Amputationen führt und gelegentlich wichtige morphologische Merkmale zerstört. Die trockene Lagerung im Gefrierschrank ist für die Proben weniger traumatisch, jedoch nicht ideal für die Erhaltung ihrer fragilen Organe. Wichtig ist, dass beim Auftauen Flügel, Antennen, Palpen oder Beine an den Gefäßwänden haften bleiben und aufgrund von Kondenswasser abreißen können.

Die Konservierung durch Einfrieren ist in Freilandstudien jedoch nicht immer durchführbar, da der Zugang zu einem Gefrierschrank oder einem Behälter mit flüssigem Stickstoff erforderlich ist. Die Lagerung im Gefrierschrank ist ohne Sensitivitätsverlust vollständig mit dem Erregernachweis mittels molekularer Techniken kompatibel, wenngleich der Nachweis und die Isolierung von RNA-Viren bei langfristiger Lagerung ein Einfrieren bei -80 °C oder in flüssigem Stickstoff erfordern. Das Einfrieren von Proben erlaubt jedoch keine Isolierung von *Leishmania* durch Darmdissektion, es sei denn, die Sandmücken werden zuerst in die Gasphase und anschließend in flüssigen Stickstoff getaucht (beispielsweise in Röhrchen, die in einem Netz platziert sind), was die Kryokonservierung von *Leishmania* simuliert.

3.2. Lagerung in Alkohol (Ethanol oder Isopropanol)

Dies ist die wahrscheinlich am weitesten verbreitete Methode zur Lagerung von Sandmücken. Sie ist im Freiland leicht umsetzbar, selbst unter schwierigen Bedingungen ohne Laborzugang. Die Konservierung in Alkohol eignet sich besonders für morphologische Studien, da die fragilen Organe (Flügel, Beine, Antennen oder Palpen) intakt bleiben, sofern sich keine Luftblasen im Lagerungsröhrchen befinden. Wir empfehlen daher, das Röhrchen mit einem kleinen Wattebausch zu verschließen, um Luftblasen zu entfernen, und ein Etikett auf dem Wattepfropfen zu platzieren (Abbildung 1). Die angemessene Alkoholkonzentration wird weiterhin kontrovers diskutiert. Im Allgemeinen werden Konzentrationen unter 70 % nicht empfohlen [45, 66]. Höhere Konzentrationen konservieren die DNA effektiver und über längere Zeiträume, machen die Proben jedoch für morphologische Studien fragiler und brüchiger. Die Verwendung von 96%igem Ethanol (das azeotrope Gemisch) gewährleistet eine zeitliche Konzentrationsstabilität, insbesondere in feuchten Regionen wie tropischen Ländern, obwohl 95%iger Ethanol oft leichter zu beschaffen ist. Unabhängig von der Konzentration bleibt die DNA in Ethanol im Allgemeinen gut erhalten (wenn auch weniger effektiv als bei Gefriermethoden, insbesondere bei molekularen Methoden vom Typ NGS). Proteine sind wesentlich weniger stabil, insbesondere für die Proteomik, wie etwa MALDI-ToF-Anwendungen. Sandmücken, die einige Monate in Alkohol konserviert wurden, können zwar noch morphologisch identifiziert werden, es ist jedoch unmöglich, aus diesen Proben Referenz-Proteinspektren zu generieren. Die Lagerung in Alkohol oder unter trockenen Bedingungen kann verbessert werden, wenn die Probe zusätzlich bei -20 °C eingefroren wird. Dies verbessert vor allem die molekulare Erhaltung (z. B. Nukleinsäuren) durch Verlangsamung des Abbaus und bietet zudem einen sekundären Vorteil für die Morphologie, indem der Gewebezerfall über die Zeit reduziert wird. Isopropanol kann in einigen Ländern leichter verfügbar sein und konserviert die DNA ebenfalls, führt jedoch zu einer Versteifung der Proben. Er ist nicht so leicht entzündlich wie Ethanol und kann daher einfacher transportiert werden. Falls erforderlich, können in flüssigem Stickstoff oder trocken eingefrorene Sandmücken in Alkohol überführt werden, wodurch jedoch die Nachteile beider Methoden kombiniert werden.

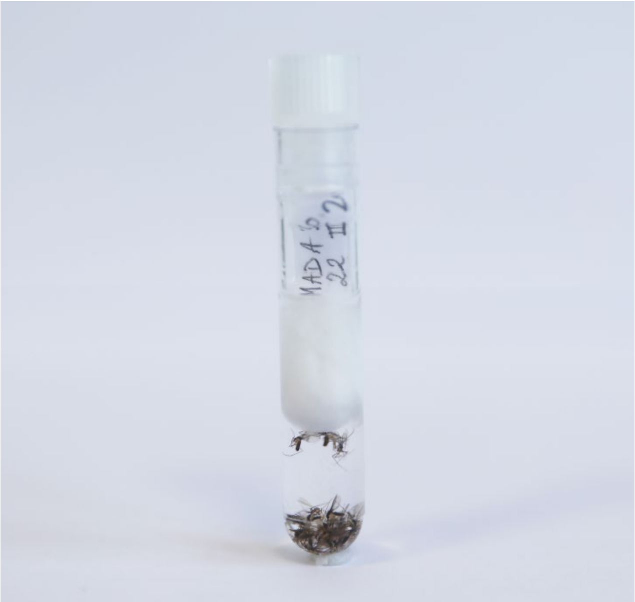

Abbildung 1: In Ethanol konservierte Sandmücken.

3.3. Lagerung in RNA-Stabilisierungslösung (RNASL)

Diese wässrige Lösung ist weit verbreitet, ungiftig und darauf ausgelegt, RNA in frischen Gewebe- und Zellproben zu stabilisieren und zu schützen. Es wirkt durch schnelles Eindringen in die Probe und Inaktivierung von RNasen (RNA-abbauende Enzyme), wodurch ein RNA-Abbau ohne sofortiges Einfrieren verhindert wird. Die Lagerung in RNASL ist im Allgemeinen effektiv für die Erhaltung der gesamten Gewebe- und Zellmorphologie für nachfolgende histologische Bewertungen. RNASL ermöglicht die Lagerung von Proben bei Raumtemperatur für bis zu 7 Tage, bei 4 °C für mehrere Wochen oder bei -20 °C/-80 °C für die Langzeitkonservierung. Diese Methode ist besonders wertvoll bei Freilandarbeiten oder im klinischen Umfeld, wo die Infrastruktur für die Kühlkette begrenzt ist.

3.4. Trockenkonservierung bei Raumtemperatur

Dies ist eine ältere Methode, die bei Anwendung auf ein *In-toto*-Exemplar (ganzpräpariert) den großen Nachteil hat, fragile Organe wie Flügel, Beine, Antennen und Palpen schlecht zu erhalten. Proteomische Studien mittels MALDI-ToF bleiben jedoch durchführbar, wenn die Dehydrierung bei der Fixierung mit einem Trockenmittel vom Typ Silica-Gel erfolgt. Im Gegensatz dazu sind molekulare DNA-Analysen bei diesen Proben schwierig durchzuführen, da die DNA oft fragmentiert und in geringer Menge vorliegt. Dennoch könnten neuere Techniken wie die „Museomics“ bei Proben dieser Art eingesetzt werden [34]. Daher wird diese Lagerungsmethode nicht empfohlen, es sei denn, es ist keine Alternative verfügbar. Sie kann mit einer Kaltlagerung kombiniert werden. Die größte Herausforderung besteht darin, eine angemessene Einbettung der Proben oder der für die Identifizierung erforderlichen Körperteile zu erreichen. Hierfür ist eine Rehydrierung unerlässlich. Wir empfehlen die Verwendung einer Triton X-100-Lösung. Die Dauer der Rehydrierung variiert von einigen Stunden bis zu mehreren Tagen unter regelmäßiger Beobachtung. Nach vollständiger

Rehydrierung sollten die Proben in drei aufeinanderfolgenden Wasserbädern gespült werden.

3.5. Konservierung auf Filterpapieren

Der Hauptvorteil von Filterpapieren ist die langfristige Stabilität genomischer DNA in den Zellen von unfixierten, getrockneten Ganzkörperpräparaten oder Blutzellen bei Raumtemperatur. Die Filterpapiermatrix ist mit Substanzen imprägniert, die Infektionserreger denaturieren, sodass die Proben nicht mehr als Biogefährdung eingestuft werden. Dies ermöglicht die Lagerung und den Transport von Proben ohne spezielle Sicherheitsvorkehrungen für biologische Gefahrenstoffe [68].

4. Präparierung der Proben (Dissektion)

Im Gegensatz zu vielen anderen Insekten, deren Identifizierung auf externen Merkmalen basiert, die an *In-toto* genadelten Individuen beobachtet werden können, erfordern Sandmücken eine Präparierung und eine Einbettung auf Objektträgern. Nur so können die anatomischen Merkmale für eine präzise Artbestimmung untersucht werden. Unabhängig vom gewählten Aufbereitungs- und Einbettungsverfahren wird stets dieselbe Präpariertechnik angewendet (Abbildungen 2 & 3) (https://zenodo.org/records/18198006).

Verwendung von Triton X-100: nichtionische wässrige Lösung

Es ist zu beachten, dass sich das Einbettungsverfahren auf frisch gefangene oder angemessen gelagerte Exemplare bezieht. Die meisten Sammler verfügen über Insektenproben, die entweder trocken (für die MALDI-ToF-Analyse) oder über viele Jahre in Alkohol gelagert wurden. Leider ist die Konservierung in Alkohol über Zeiträume von mehreren Jahren hinweg nicht optimal; derart konservierte Arthropoden lassen sich nur sehr schwer für mikroskopische Untersuchungen vorbereiten. Ein häufig auftretendes Problem ist die Zersetzung von Kunststoffgefäßen, gefolgt von der Verdunstung des Alkohols. In beiden Fällen – bei zu langer Lagerung in Alkohol oder bei Austrocknung – sind die Optionen begrenzt. Daher entstand die Idee, Netzmittel zu verwenden, die keine starken Detergenzien sind. Triton X-100 liegt in Form einer nicht-ionischen wässrigen Lösung vor (4-(1,1,3,3-Tetramethylbutyl) phenyl-polyethylenglycol-Lösung oder t-Octylphenoxypolyethoxyethanol, Polyethylenglycol-tert-octylphenylether) und wird in der Zell- und Molekularbiologie häufig als Detergens verwendet. Es ermöglicht die Permeabilisierung von Zell- und Kernmembranen.

Nachfolgend ist ein Verfahren unter Verwendung von nichtionischem Triton X-100 in 0,5%iger wässriger Lösung aufgeführt:

- Imprägnieren Sie die trockene Probe mit absolutem Alkohol.
- Geben Sie das erforderliche Volumen der 0,5%igen Triton X-100-Lösung hinzu, sodass die gesamte Probe vollständig eingetaucht ist.
- Lassen Sie den Prozess etwa 5 Minuten bis zu mehreren Tagen einwirken und kontrollieren Sie diesen regelmäßig. Alle Arthropoden müssen in der Lösung vollständig voneinander getrennt sein.
- Entfernen Sie die Triton X-100-Lösung und ersetzen Sie diese durch Kalilauge (Kaliumhydroxid-Lösung).

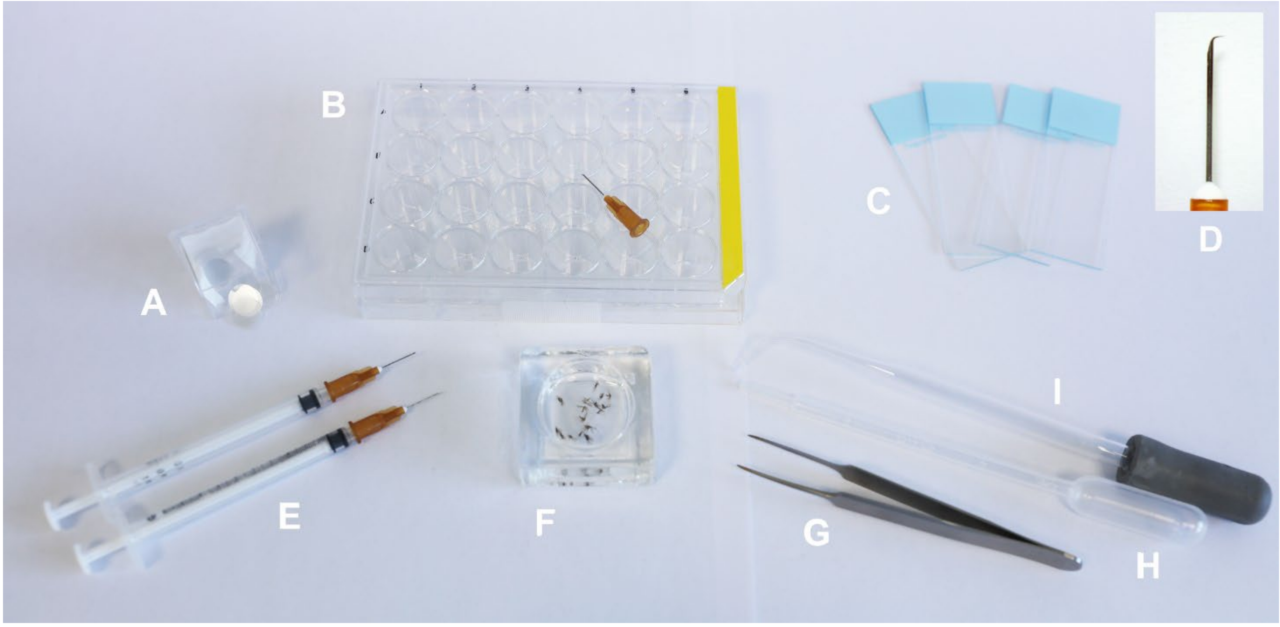

Abbildung 2: Für die Einbettung von Sandmücken benötigte Materialien: A: Runde Deckgläser (10 oder 12 mm Durchmesser); B: 24-Loch-Platte und Nadel mit Häkchen (bei Verwendung von Nelkenöl oder Euparal®-Essenz zur Bearbeitung der Sandmücken dürfen keine Acrylplatten verwendet werden, da eine chemische Reaktion stattfindet, die die Proben beschädigen würde); C: Zur Beschriftung geeignete Objektträger; D: Detailansicht der Nadel mit Häkchen; E: Auf Spritzen aufgesetzte Kanülen/Präpariernadeln; F: Uhrglas oder gleichwertiges Gefäß mit den einzubettenden Sandmücken; G: Dumont-Pinzetten; H: Kunststoffpipette; I: Durch Erhitzen gebogene Glaspipette zur Erleichterung des Flüssigkeitstransfers in die Lochplatten.

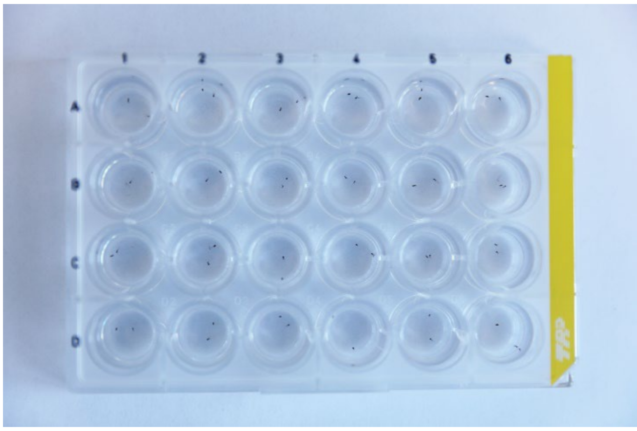

Abbildung 3: Eine 24-Loch-Platte, wobei jedes Loch den Kopf und das Abdomenende einer Sandmücke enthält.

4.1. Kopf

Die Dissektion kann unter einem Stereomikroskop mithilfe feiner Kanülen oder entomologischer Nadeln durchgeführt werden (Abbildungen 2 & 3). Zu den am häufigsten verwendeten Kanülen gehören: 26G x 1/2" (0,45 × 13 mm), 30G × 1/2" (0,3 × 13 mm) oder 25G × 5/8" (0,5 × 16 mm). Um das Exemplar für die Identifizierung vorzubereiten, wird mindestens der Kopf vom Körper getrennt und mit der Ventralseite nach oben eingebettet, um das Cibarium und den Pharynx sichtbar zu machen. Thorax und Abdomen werden hingegen nach der Dissektion lateral (seitlich) eingebettet. Die Einbettung des Kopfes in einer ventrodorsalen Position stellt sicher, dass das Hinterhauptsloch (*Foramen occipitale*) nach oben orientiert ist, sodass das Cibarium direkt beobachtet werden kann. Der Zugang zu diesen anatomischen Merkmalen wird erheblich erleichtert, wenn der Kopf vollständig abgetrennt ist.

4.2. Flügel und Thorax

Die Flügel müssen flach eingebettet werden. Jeder Flügel kann an seiner Basis abgetrennt und einzeln präpariert werden; alternativ wird nur ein Flügel abgetrennt, während der andere am Thorax verbleibt. Falls eine geometrische Morphometrie-Analyse geplant ist, ist es essenziell, den rechten und linken Flügel vor der Einbettung korrekt zu identifizieren und zu kennzeichnen. Der Thorax gliedert sich in mehrere Abschnitte, die jeweils sehr wichtige taxonomische Informationen enthalten [20, 64]. Im Allgemeinen wird er in Seitenansicht (Lateralansicht) eingebettet, um die Untersuchung der Chaetotaxie (Beborstung) und der Farbverteilung zu ermöglichen. Das Vorhandensein von Borsten-Narben in bestimmten Thoraxregionen kann zur Unterscheidung einiger Arten der Gattung *Brumptomyia* herangezogen werden. Die Verteilung der Pigmentierung dient der Differenzierung neotropischer Sandmücken auf Gattungsebene (z. B. *Bichromomyia*), Artengruppen (z. B. *Pintomyia*) oder sogar zwischen Arten derselben Gattung (z. B. *Micropygomyia*,

Nyssomyia, *Psathyromyia* und *Psychodopygus*) [20]. Sofern der Thorax nicht für molekulare Analysen verwendet wird, sollte er daher so eingebettet werden, dass er unbeschädigt bleibt. Wichtig ist hierbei, dass nicht die Intensität der Farben entscheidend ist, sondern deren Verteilung über den Thorax. Der Aufhellungsprozess zerstört weder die Pigmentierung noch deren Muster.

4.3. Genitalien

Bei der Einbettung der Genitalien ist sowohl bei Männchen als auch bei Weibchen besondere Sorgfalt geboten, da diese Merkmale entscheidend für die Identifizierung von Gattungen, Untergattungen und Arten sind. Bei beiden Geschlechtern sind die Genitalstrukturen paarig angelegt.

4.3.1. Männchen

Die Genitalien liegen extern und bestehen aus paarigen Zangen. Diese setzen sich jeweils aus der Gelenkverbindung von Gonocoxit und Gonostylus im dorsalen Teil sowie dem Epandrial-Lobus im ventralen Teil zusammen. Der Gonostylus trägt Dornen und manchmal Setae (Borsten), die zählbar sein müssen und deren Insertionspunkte klar erkennbar sein sollten. Es ist wichtig, die Innenfläche des Gonocoxits sorgfältig zu untersuchen, da diese ein Büschel aus festsitzenden Setae oder Setae auf einem Lobus (= Tuberkel) tragen kann [22]. Weniger erfahrene Kolleg:innen können eine einfache laterale Einbettung vornehmen, ohne die Genitalien vom Abdomenende abzutrennen (https://zenodo.org/records/18311158). In diesem Fall kann die Überlagerung der beiden Genitalhälften beispielsweise das Zählen der internen Setae des Gonocoxits erschweren; dies verhindert jedoch eine Beschädigung der Strukturen durch eine missglückte Dissektion. Erfahrenere Kolleg:innen können versuchen, die Genitalien zu öffnen und aufzuspalten. Hierzu muss die schräge Seite einer Nadel (Typ Intrakutannadel) so hindurchgeführt werden, dass die Gonocoxit-Gonostylus-Einheiten voneinander getrennt werden, ohne sie vollständig zu durchtrennen (https://zenodo.org/records/18311158). Auf diese Weise wird die Beobachtung ihrer Innenseiten erleichtert. Diese Anordnung begünstigt auch die Sichtbarkeit der Parameren und Parameren-Scheiden, da diese sich nicht mehr überschneiden. Für die laterale Einbettung, welche die Überlagerung von Organen begünstigt, müssen die Proben perfekt aufgehellt sein.

4.3.2. Weibchen

Der Genitalapparat ist intern und wird durch die Spermatheken gebildet. Ohne Dissektion müssen diese durch die Integumente hindurch beobachtet werden, wofür das Abdomen in ventraler Position eingebettet wird. Unabhängig vom gewählten Einbettungsmedium kann die Spermatheke selbst im Allgemeinen korrekt beobachtet werden, insbesondere wenn sie nicht glatt und gut aufgehellt ist. Die Beobachtung von glatten, dünnwandigen Spermatheken kann jedoch in Medien mit geringer Lichtbrechung problematisch sein. Darüber hinaus ist die Beobachtung der Basis der Spermathekengänge essenziell für die Artidentifizierung, wie etwa in der Untergattung *Larroussius* [35, 37, 38], den Hauptvektoren von *Leishmania infantum* in der Alten Welt. Ohne diese Beobachtung bleibt eine Identifizierung der Exemplare unmöglich. Um diese Schwierigkeiten zu überwinden, sollte die Einheit aus Furca und Spermatheken aus dem Abdomen entfernt werden. Spermatheken sind während der Dissektion im Allgemeinen schwer zu erkennen, aber die Furca ist relativ leicht zu lokalisieren. Da die Spermathekengänge in die Furca münden, ermöglicht die Isolierung der Furca normalerweise auch die Isolierung der Spermatheken. Falls die Spermatheken während des Prozesses versehentlich abgetrennt werden, gehen sie nicht verloren und können weiterhin innerhalb der abdominalen Integumente beobachtet werden (Abbildung 4).

4.4. Mitteldarmdissektion zur *Leishmania*-Isolierung

Die Dissektion des Verdauungstrakts ist für den Nachweis und die Isolierung von *Leishmania* in weiblichen Sandmücken von entscheidender Bedeutung. Das Verfahren kann sowohl unter Freilandbedingungen als auch im Labor durchgeführt werden, um die Vektorkompetenz zu bewerten.

Es wird empfohlen, frisch getötete Weibchen zu untersuchen. Waschen Sie die Weibchen mit Wasser oder einer Kochsalzlösung, die ein mildes Reinigungsmittel enthält, um überschüssige Haare (Setae) zu entfernen. Dieser Schritt trägt dazu bei, aseptische Bedingungen für die *Leishmania*-Isolierung aufrechtzuerhalten, während gleichzeitig die für die Identifizierung erforderlichen morphologischen Merkmale erhalten bleiben.

Um *Leishmania* zu finden und zu isolieren, entfernen Sie vorsichtig den Mitteldarm und platzieren Sie ihn in einem einzelnen Tropfen steriler Kochsalzlösung (0,9 % NaCl). Nach der Beobachtung beweglicher Parasiten unter einem Lichtmikroskop (empfohlene Vergrößerung: ~200×) verwenden Sie eine Insulinspritze oder eine Mikropipette, um diese in das Kultivierungsmedium zu übertragen (weitere Details siehe Kapitel 4.4.3).

Betten Sie den Kopf und die Genitalien direkt in Marc-André-Lösung ein, um sie aufzuhellen. Wichtig: Lassen Sie die Marc-André-Lösung niemals mit Leishmanien in Kontakt kommen – weder direkt noch indirekt über Instrumente oder Nadeln –, da es für die Parasiten tödlich ist.

Die Dissektion weiblicher Sandmücken kann entweder auf einem oder auf zwei Objektträgern durchgeführt werden; beide Optionen haben Vorteile und Einschränkungen (Abbildung 5, https://zenodo.org/records/18311154).

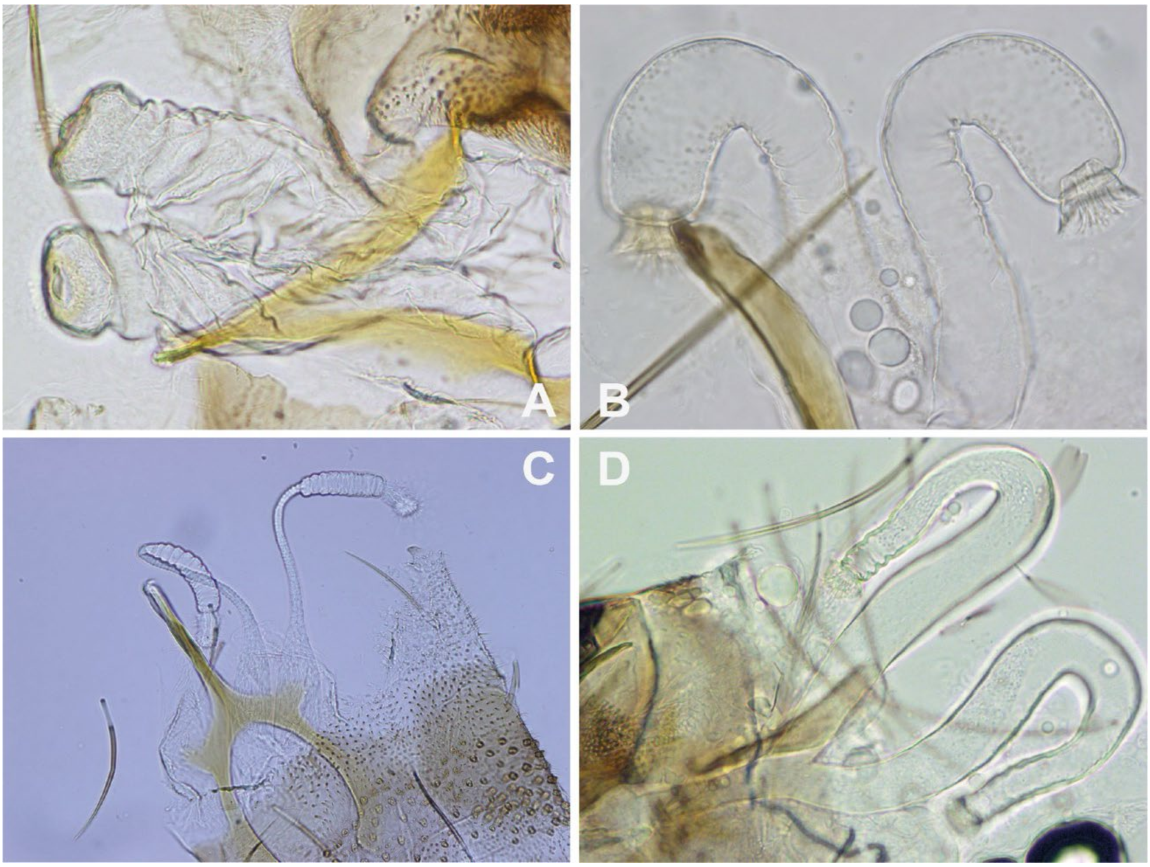

Abbildung 4: Spermatheken aus frischen Proben präpariert und in Marc-André-Lösung eingebettet. A: *Idiophlebotomus longiforceps* (Demokratische Volksrepublik Laos, LVR Laos); B: *Sergentomyia minuta* (Frankreich); C: *Phlebotomus ariasi* (Frankreich); D: *Sergentomyia anodontis* (LVR Laos).

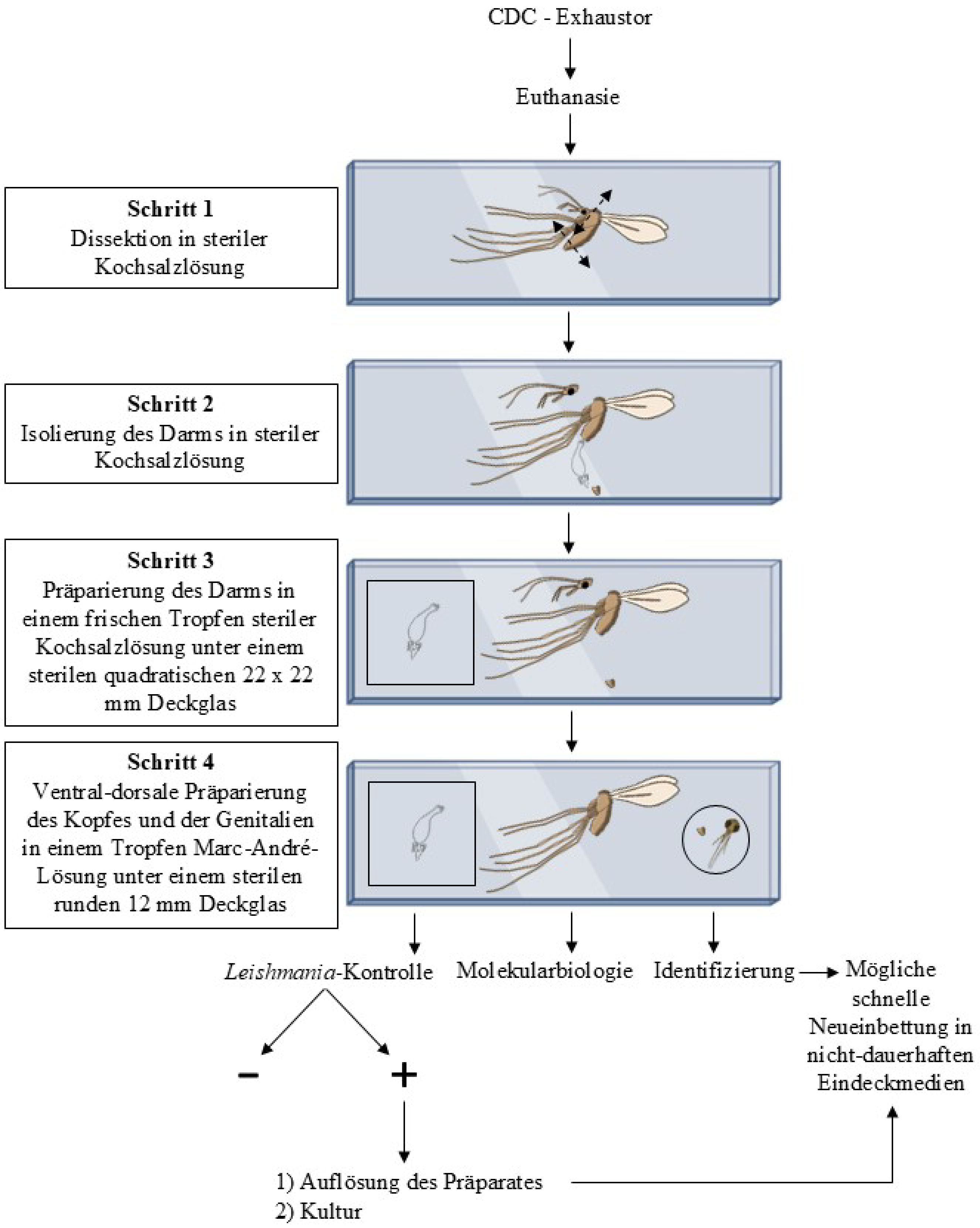

Abbildung 5: Methode zur *Leishmania*-Isolierung.

4.4.1. Zwei-Objektträger-Verfahren

Die erste Option besteht darin, auf zwei getrennten Objektträgern zu arbeiten: Einer enthält sterile Kochsalzlösung für die Extraktion des Mitteldarms, während der andere zur Einbettung von Kopf und Spermatheken in Marc-André-Lösung dient. Unter Freilandbedingungen ist es jedoch üblich, dass zwei oder drei Personen die Sandmücken sezieren und ihre Präparate an einen einzelnen Forscher weitergeben, der für die Artidentifizierung und die Bewertung der *Leishmania*-Infektion im Darm verantwortlich ist. Die Handhabung von zwei Objektträgern pro Individuum kann zu Problemen bei der Rückverfolgbarkeit der Proben führen. Insbesondere erschwert dies die zweifelsfreie Zuordnung, welche spezifische Sandmücke infiziert war, sobald ein positiver Darmbefund festgestellt wird (https://zenodo.org/records/18311154).

4.4.2. Ein-Objektträger-Methode

Die Verwendung eines einzelnen Objektträgers gewährleistet die Rückverfolgbarkeit der Ergebnisse. Hierbei müssen jedoch verschiedene Vorsichtsmaßnahmen getroffen werden. Um die Sterilität während dieses Schrittes zu maximieren, müssen die Anwender ihre Hände regelmäßig mit hydroalkoholischem Gel desinfizieren. Es sollten nicht-mattierte Objektträger und quadratische Deckgläser (22 x 22 mm) verwendet werden, die in Aluminiumfolie eingewickelt und mittels Heißluftsterilisation (z. B. in einem Poupinel-Ofen) sterilisiert wurden. Zudem sind für jede Dissektion sterile Nadeln (Empfehlung: 25G Ø 0,5 mm × 16 mm) zu verwenden. Die Sandmücke wird in einen Tropfen steriler Kochsalzlösung in der Mitte des Objektträgers gelegt. Der Kopf wird abgetrennt, während gleichzeitig ein Einschnitt zwischen dem 6. und 7. abdominalen Tergit bzw. Sternit erfolgt, ohne den Verdauungstrakt zu verletzen (ein höherer Schnitt kann vorgenommen werden, wenn besonders lange Spermatheken zu erwarten sind). Anschließend wird der Thorax mit einer Nadel fixiert, während die hintersten Abdominalsegmente mit der anderen Nadel vorsichtig weggezogen werden, um den Darm zu extrahieren. Falls dies misslingt, besteht die Möglichkeit, das Abdomenende mit einer Nadel zu fixieren und den Verdauungstrakt von seinem vorderen (anterioren) Teil aus herauszuziehen. Sollte auch dies fehlschlagen, muss der Darm extrahiert werden, indem so viel wie möglich des verbleibenden Integuments um ihn herum entfernt wird. Sobald der Darm entfernt ist, werden die letzten Abdominalsegmente durch Durchtrennen des Verdauungstrakts separiert. Der Darm wird dann in einen neuen Tropfen steriler Kochsalzlösung an einem Ende des Objektträgers platziert und vorsichtig mit einem sterilen Deckglas abgedeckt. Der Kopf und die letzten Abdominalsegmente werden in einen kleinen Tropfen Marc-André-Lösung am anderen Ende des Objektträgers übertragen, wobei jeglicher Kontakt mit den *Leishmania*-Parasiten zu vermeiden ist. Der Kopf wird korrekt ausgerichtet (Hinterhauptsloch nach oben), und die Spermatheken werden zusammen mit der Furca wie oben beschrieben isoliert und mit einem kleinen, runden Deckglas (Ø 12 mm – nicht mit den sterilen, quadratischen Deckgläsern zu verwechseln) abgedeckt. Der restliche Körper und die Flügel der Sandmücke verbleiben im Kochsalztropfen in der Mitte des Objektträgers.

Im Falle eines positiven Befundes oder für taxonomische Untersuchungen können Thorax und Abdomen für molekulare oder proteomische Studien aufbewahrt werden; die Flügel können in einem wässrigen Medium eingebettet werden. Um das Präparat dauerhaft zu erhalten, kann überschüssiges Marc-André-Lösung durch ein wässriges Einschlussmedium wie Chloral-Gummi (= Hoyer-Gemisch) oder ein Medium auf Polyvinylalkohol-Basis ersetzt werden.

Detaillierte Videos, die diese Verfahren demonstrieren, sind verfügbar (Mitteldarmdissektion: https://zenodo.org/records/18303014 und Speicheldrüsendissektion: https://zenodo.org/records/18302850) und werden daher an dieser telle nicht weiter erläutert.

4.4.3. Isolierung und Kultivierung von Leishmanien aus dem Sandmückendarm

Die Isolierung von Parasiten aus der Sektion infizierter weiblicher Sandmücken ist ein anspruchsvolles Verfahren, das hohes Geschick erfordert und zunächst an parasitenfreien Exemplaren geübt werden sollte. Nach der Sektion werden die Därme zum Waschen in einen frischen Tropfen steriler Kochsalzlösung (0,9 %) oder Ringer-Locke-Lösung überführt [4]. Die präparierten Därme können dann auf zwei Arten weiterverarbeitet werden: i) durch Untersuchung unter einem Lichtmikroskop, um die verschiedenen Stadien der *Leishmania*-Promastigoten und ihre Lokalisierung zu beobachten, mit besonderem Augenmerk auf die Stomodealklappe, und ii) durch Öffnen des Darms, um das Austreten der Promastigoten zu erleichtern, was deren Kultivierung begünstigt [4]. Das Finden infektiöser Sandmücken im Freiland ist ein relativ seltenes Ereignis; daher werden gute Übungseinheiten die Chancen auf eine erfolgreiche Isolierung maximieren.

Wenn *Leishmania*-Parasiten im Darm beobachtet werden, sollten neue sterile Nadeln verwendet und eine kleine Menge steriler Kochsalzlösung durch Kapillarwirkung um das Deckglas herum hinzugefügt werden, um sie freizusetzen. Der Darm sollte vorsichtig und schnell abgetrennt werden, um die Parasiten in die Kochsalzlösung freizusetzen. Sammeln Sie die Parasiten mit einer 100 µL-Mikropipette oder einer Tuberkulinspritze und inokulieren Sie diese in ein ordnungsgemäß beschriftetes Kulturmedium.

In-vitro-Kultur von *Leishmania*-Promastigoten: Isolierte Parasiten werden zunächst auf SNB-9-Blutagar-Schrägröhrchen oder in festem Novy-McNeal-Nicolle (NNN)-Medium gehalten [16], das entweder mit sterilem Alpha-MEM-Medium [16, 65] oder mit M199-Medium überschichtet ist. Diese sind jeweils ergänzt mit 10 % hitzeinaktiviertem, sterilem fötalem Kälberserum [FKS] zur Förderung des Parasitenwachstums, 1 % BME-Vitaminen,

This is an Open Access article distributed under the terms of the Creative Commons Attribution License (https://creativecommons.org/licenses/by/4.0), which permits unrestricted use, distribution, and reproduction in any medium, provided the original work is properly cited.

2 % sterilem Humanurin (sterilisiert mit einem Spritzenfilter Filtropur S 0,2 µm) sowie 250 µg/mL Amikacin (oder 50 µg/mL Gentamicin oder einer Mischung aus Antibiotika und Aminosäuren bestehend aus L-Glutamin 200 mM, Penicillin 10.000 U und Streptomycin 10 mg/mL) [47]. Nach drei Tagen werden die Kulturen, sofern keine Kontamination vorliegt, in einem ordnungsgemäß vorbereiteten Einfriermedium suspendiert und anschließend bei -80 °C für ein bis zwei Jahre oder in flüssigem Stickstoff bei -196 °C für die Langzeitkonservierung und künftige experimentelle Verwendung gelagert [7]

4.5. Speicheldrüsen

Die Präparierung der Speicheldrüsen von Sandmücken ist eine grundlegende Technik zur Untersuchung von Vektor-Pathogen-Interaktionen, insbesondere zum Nachweis von Arboviren wie Phleboviren (z. B. Toscana-Virus) [44, 75]. Aufgrund der geringen Größe der Sandmücken erfordert das Verfahren unter einem Stereomikroskop höchste Präzision; unter Verwendung feiner Pinzetten oder Mikrodissektionsnadeln müssen die empfindlichen Speicheldrüsen isoliert werden, ohne dass es zu Rupturen oder Kontaminationen kommt (https://zenodo.org/records/18302850) [51, 61]. Die Erhaltung der Vollständigkeit der Drüsen ist entscheidend, um zuverlässige molekularbiologische Folgeanalysen zu gewährleisten. Nach der Extraktion können die Drüsen homogenisiert und mittels RT-PCR, qPCR oder Immuno-Assays auf virale RNA oder Antigene getestet werden [12]. Der Nachweis von Viren in den Speicheldrüsen – im Gegensatz zum bloßen Vorkommen im Darm oder im Hämocoel – bestätigt, dass der Erreger seine extrinsische Inkubationszeit abgeschlossen hat und während der Blutmahlzeit übertragbar ist [71].

Der Dissektionsprozess ist aufgrund der geringen Größe der Speicheldrüsen technisch anspruchsvoll und erfordert erhebliche Expertise, um eine Zersetzung der Proben zu vermeiden [1, 51]. Zudem können die Viruslasten niedrig sein, was hochsensitive Nachweismethoden wie Nested-PCR oder Hochdurchsatzsequenzierung (HDS) erforderlich macht [54]. Kontaminationsrisiken unterstreichen zusätzlich die Notwendigkeit steriler Arbeitstechniken. Neben technischen Hürden beeinflussen biologische Faktoren den Detektionserfolg: Die Vektorkompetenz variiert zwischen den Sandmückenarten, und die Infektionsraten schwanken je nach ökologischen und saisonalen Bedingungen [33, 61].

Der Nachweis von Viren in den Speicheldrüsen liefert entscheidende Einblicke in Übertragungsrisiken und ermöglicht gezielte Überwachungs- und Kontrollmaßnahmen [15]. Beispielsweise hat die Identifizierung des Toscana-Virus in Sandmücken in Endemiegebieten zur Verbesserung von Diagnoseprotokollen und öffentlichen Gesundheitsempfehlungen beigetragen [18]. Darüber hinaus könnte die Untersuchung von Virus-Speichel-Interaktionen neue Ansatzpunkte für übertragungsblockierende Impfstoffe oder Therapeutika aufzeigen [15, 18].

Speicheldrüsen von Sandmücken können zudem als Antigenquelle zur Messung von Wirtsantikörpern gegen Sandmückenspeichel mittels immunologischer Methoden, vorzugsweise ELISA, dienen. Diese Methode ermöglicht die Bewertung der Exposition des Wirts gegenüber Sandmückenstichen und unterstützt somit die Evaluierung der Wirksamkeit von Vektorkontrollmaßnahmen [25] sowie des Risikos einer *Leishmania*-Übertragung [40].

4.6. Identifizierung der Blutmahlzeit

Vollgesogene Weibchen, die aus den Fängen isoliert wurden, sollten unter Verwendung von Einwegmaterialien seziert werden, um Kreuzkontaminationen zu vermeiden. Ihr Abdomen sollte unter einem Stereomikroskop untersucht werden, um das Stadium der Verdauung der Blutmahlzeit zu beurteilen. Es wird empfohlen, nur Weibchen mit einem roten, rötlich-braunen oder dunkelroten Abdomen auszuwählen, die keine Anzeichen von Eibildung zeigen. Die Spitze des Abdomens einschließlich der Spermatheken ist abzutrennen, um das Weibchen nach der Aufhellung morphologisch zu identifizieren. Der Hauptteil des Abdomens (ohne Spermatheken) sollte in Eppendorf®-Reaktionsgefäße überführt und bis zur weiteren Analyse bei -20 °C gelagert werden.

Die genetischen Marker, die üblicherweise zur Identifizierung der Blutmahlzeit verwendet werden – wie etwa PNOC [5, 30, 50], CytB [67] oder COI [13] – sind gut etabliert und in der Literatur ausführlich beschrieben; daher werden sie in dieser Arbeit nicht weiter im Detail erläutert (Abbildung 6). Alternativ kann zur Identifizierung des Wirtsblutes das MALDI-ToF-Peptid-Mapping eingesetzt werden [31]. Experimentell konnte nachgewiesen werden, dass diese Technik die Identifizierung des Wirtsblutes innerhalb eines längeren Zeitraums nach der Aufnahme der Blutmahlzeit ermöglicht. Daher ist sie eine geeignete Methode der Wahl, insbesondere für die Analyse von Weibchen, bei denen die Verdauung des Wirtsblutes bereits sichtlich fortgeschritten ist. Die Proben sollten idealweise bei -20 °C oder 4 °C gelagert werden, wobei jedoch auch bei kurzzeitiger Lagerung bei Raumtemperatur gute Ergebnisse erzielt werden können. Das Abdomen eines vollgesogenen Weibchens sollte kurz vor der Analyse vom Rest des Körpers getrennt und in destilliertem Wasser homogenisiert werden. Der verbleibende Körper der Sandmücke steht für weitere molekulare und morphologische Analysen zur Verfügung. Nachdem ein Aliquot aus dem Homogenisat für das MALDI-ToF-Peptid-Mapping entnommen wurde, kann der Rest für die DNA-Isolierung verwendet werden, um die Identifizierung des Wirtsblutes zu bestätigen und/oder auf das Vorhandensein von Leishmanien zu screenen. Die Gesamtzeit für

Probenvorbereitung und Analyse ist im Vergleich zu DNA-basierten molekularen Techniken sehr kurz.

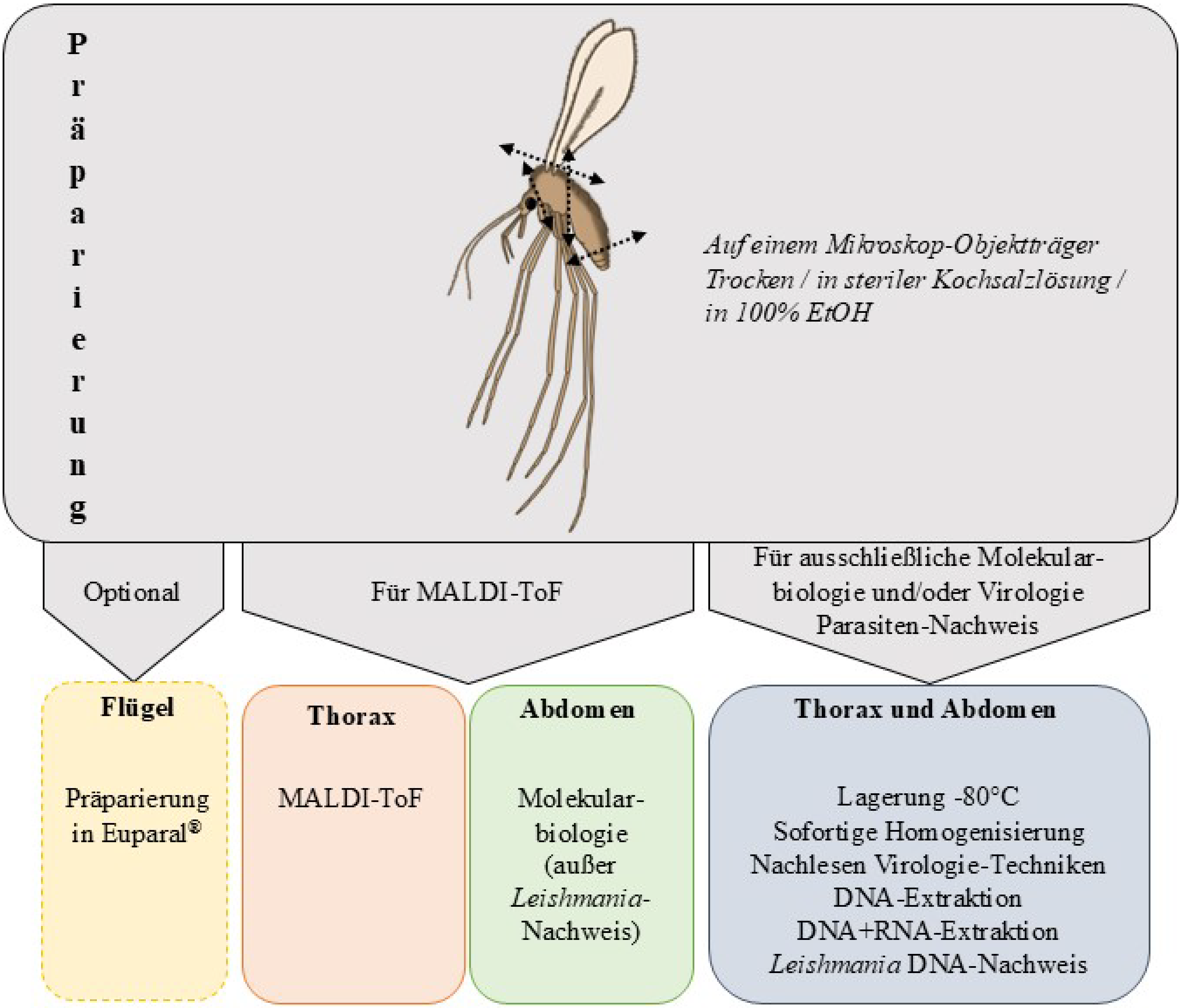

Abbildung 6: Aufbereitung von Sandmücken für Anwendungen in der Molekularbiologie, Proteomik und/oder Virologie.

5. Probenaufbereitung für morphologische Studien (Abbildungen 3, 6, 7 & 8; Anhänge 1, 2, 3 & 4)

Dieser Abschnitt beschreibt die Prinzipien zur Vorbereitung von Sandmücken-Proben für die Einbettung zu rein morphologischen Zwecken, gefolgt von Anpassungen für Anwendungen, die über die Morphologie hinausgehen. Das Verständnis dieser Methodik ist jedoch entscheidend, da sie es ermöglicht, Verfahren bei Bedarf an spezifische Probenarten anzupassen.

Die Behandlung umfasst aufeinanderfolgende Leerungs- und Füllschritte unter Verwendung von Pasteurpipetten, die mit flexiblen Gummisaugern ausgestattet sind. Die Verwendung von Glasgefäßen mit rundem Boden wird ausdrücklich empfohlen, da sie diese Vorgänge erheblich erleichtern. Glas verhält sich gegenüber allen Reagenzien inert. Um das Verdampfen von Reagenzien zu verhindern, sollten die Behälter mit Deckeln versehen und niemals überfüllt werden, um ein Überlaufen beim Schließen oder Öffnen zu vermeiden und um zu verhindern, dass Staub auf die Proben gelangt.

Die für die Aufhellung und Verarbeitung erforderlichen Chemikalien sind in Tabelle 2 aufgeführt..

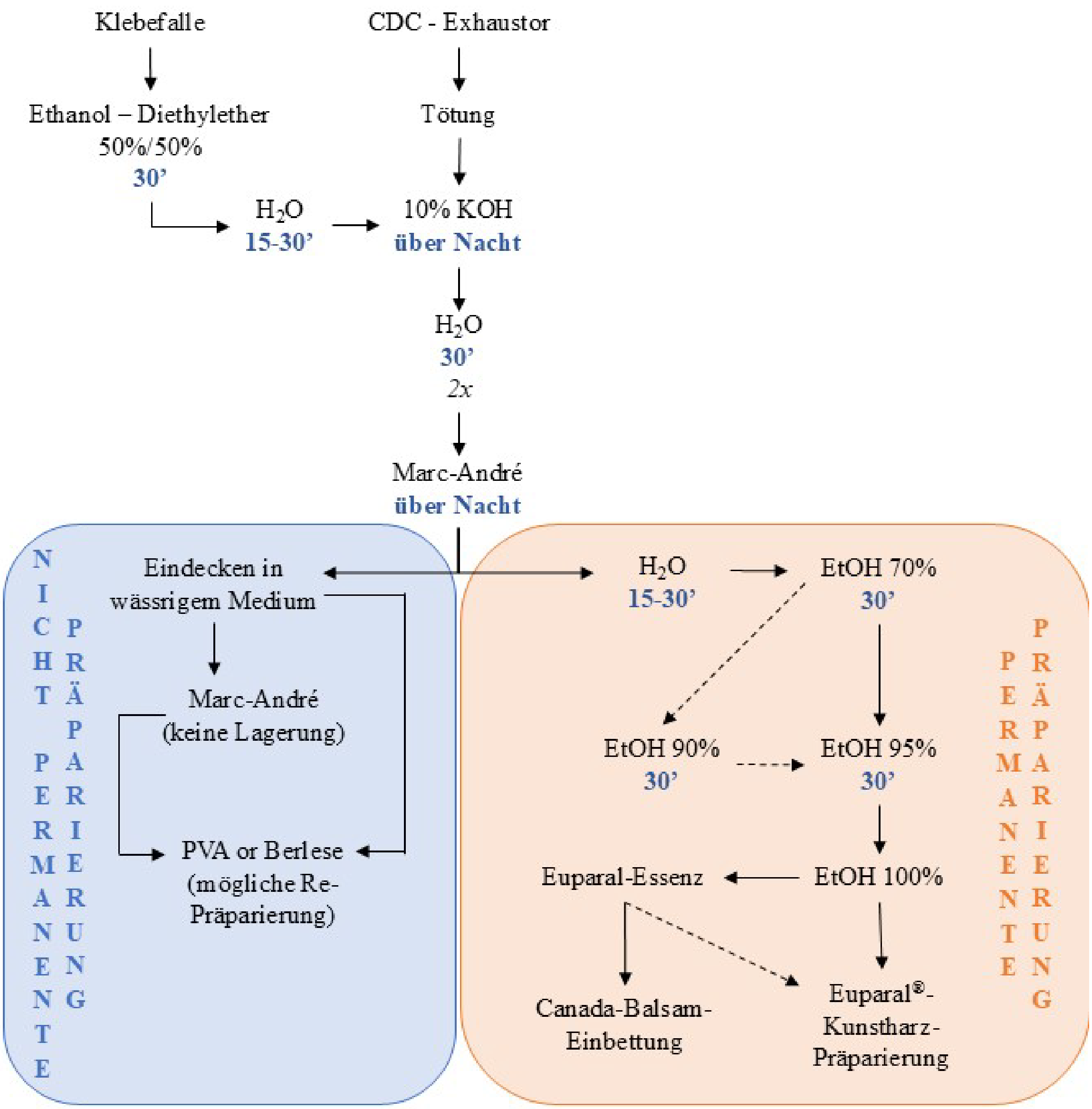

Abbildung 7: Klassisches Verfahren zur Aufbereitung von Sandmücken.

Tabelle 2: Zusammensetzung der verwendeten Reagenzien.

**Kaliumhydroxid 10%**<br>Kaliumhydroxid10 g<br>Destilliertes Wasser *ad* 100 mL	**Säurefuchsin 1% in destilliertem Wasser**<br>Säurefuchsin (als Pulver) 1 g<br>Destilliertes Wasser 99 mL
**Chloral-Gummi-Einbettungsmedium (Hoyer-Medium)**<br>Destilliertes Wasser 50 mL<br>Chloralhydrat 200 g<br>Gummi arabicum 50 g<br>Glycerin 20 mL	**Mit Säurefuchsin gefärbte Marc-André-Lösung**<br>Marc-André-Lösung10mL<br>Fuchsin 1% 50 µL
**Marc-André-Lösung**<br>Chloralhydrat 40 g<br>Eisessig (wasserfreie Essigsäure) 30 mL<br>Destilliertes Wasser 30 mL	**Enecê-Medium**<br>Reinweißes Kolophonium 22 g<br>Alkohollösliches Copal-Harz 12 g<br>Absoluter Ethanol 20 mL<br>Campher 10 g<br>Terpentinöl 10 mL<br>Eukalyptol 26 mL

This is an Open Access article distributed under the terms of the Creative Commons Attribution License (https://creativecommons.org/licenses/by/4.0), which permits unrestricted use, distribution, and reproduction in any medium, provided the original work is properly cited.

5.1. Aufhellung

Bevor Sandmücken als Dauerpräparate auf Objektträgern fixiert werden können, müssen sie zunächst durch Mazeration unter Verwendung einer geeigneten Methode und eines Aufhellungsmittels (z. B. 10%ige Essigsäurelösung oder Marc-André-Lösung mit Chloralhydrat – eine in vielen Ländern streng regulierte Chemikalie) aufgehellt werden, um sie transparent zu machen. Der Aufhellungsprozess entfernt Körpergewebe, Fett, Sekrete und Wachse. Dies macht die Probe lichtdurchlässig und erleichtert die Untersuchung von Exoskelettstrukturen (z. B. Borsteninsertionen), Oberflächenmerkmalen (z. B. Färbung) und internen Merkmalen, die durch das Integument sichtbar werden (z. B. Spermatheken).

Der zweistufige Aufhellungsprozess, bei dem zuerst eine starke Base (wie Kaliumhydroxid/Kalilauge) und anschließend eine schwache Säure (wie die Essigsäure in der Marc-André-Lösung) verwendet wird, dient unterschiedlichen biochemischen Zwecken [74]. Die Base zersetzt Weichgewebe wie Proteine, Fette und Muskeln durch Verseifung und Proteindenaturierung, wobei das Chitin-Exoskelett für die strukturelle Klarheit intakt bleibt. Die anschließende schwache Säure neutralisiert verbleibende Alkalien, verhindert weiteren Abbau und bleicht das Chitin, um die Transparenz zu erhöhen [74]. Alternativ kann ein zweimaliges Waschen der Proben in destilliertem Wasser für jeweils 15 Minuten ausreichen, um die Base zu neutralisieren. Diese sequenzielle Behandlung kombiniert eine effektive Gewebeentfernung mit einer schonenden Konservierung und gewährleistet so eine optimale Integrität der Proben für die mikroskopische Untersuchung.

Es werden zwei 20-minütige Spülgänge in destilliertem Wasser empfohlen, bevor mit dem nächsten Schritt fortgefahren wird.

5.1.1. Lyse des Weichgewebes (Abbildung 8)

Natriumhydroxid (NaOH) oder Kaliumhydroxid (KOH) sind weit verbreitete chemische Mazerationsmittel, die je nach Größe und Fragilität der Proben in unterschiedlichen Konzentrationen und für unterschiedliche Zeitspannen angewendet werden. Die standardmäßige und effektivste Technik besteht darin, das Weichgewebe zu lysieren, indem die Sandmücken über Nacht in einer starken Base (10 % KOH oder NaOH) eingeweicht werden. Die Konzentration kann erhöht werden, um die Behandlungsdauer zu verkürzen (z. B. 20 % KOH für 6 Stunden); ebenso kann der Prozess durch Erwärmen auf 37 °C beschleunigt werden.

5.1.2. Aufhellung mit oder ohne Färbung

Nach der alkalischen Lyse folgt eine aufhellende Behandlung, die typischerweise Essigsäure und Chloralhydrat kombiniert (z. B. Marc-André-Lösung). Nach diesem Schritt müssen die Proben gründlich in mindestens zwei aufeinanderfolgenden Wasserbädern von jeweils 20 Minuten gespült werden, um chemische Rückstände zu entfernen.

Die Marc-André-Lösung ist ein weit verbreitetes Mittel für Sandmücken-Präparate. Ihre Wirksamkeit beruht darauf, dass es den Aufhellungsprozess erleichtert und gleichzeitig Schäden an fragilen Strukturen wie Flügeln und Antennen minimiert.

Die Lösung sollte frisch zubereitet oder in einem fest verschlossenen Behälter gelagert werden, um Verdunstung oder Qualitätsverlust zu vermeiden. Die Verwendung ist besonders vorteilhaft, wenn sie mit Aufhellungs- oder Färbetechniken kombiniert wird, um spezifische morphologische Details hervorzuheben (Details zur Zusammensetzung siehe Anhang 2).

Bei sehr lichtdurchlässigen Exemplaren kann eine Färbung erforderlich sein, um die Sichtbarkeit vor der Einbettung zu verbessern. Es stehen viele Farbstoffe zur Verfügung, die jeweils auf spezifische chemische Komponenten des Organismus abzielen. Es ist wichtig, einen Farbstoff zu wählen, der sowohl mit der Probe als auch mit dem gewählten Einbettungsmedium kompatibel ist. Diese Basismethodik kann angepasst werden, indem beispielsweise 0,1 % Säurefuchsin in die Marc-André-Lösung integriert wird. Proben, die in wässrigen Lösungen aufbewahrt werden und für die Einbettung in Harz vorgesehen sind, müssen dehydriert werden (siehe Abschnitt 5.2 zu Dehydrierung), da die meisten natürlichen und synthetischen Harzmedien wasserunverträglich sind. New (1974) wies darauf hin, dass einige Farbstoffe in bestimmten Einbettungsmedien an Qualität verlieren können [53]. Zum Beispiel wird Säurefuchsin häufig mit Kanadabalsam verwendet, kann aber auch in Euparal® fixiert werden. Allerdings neigen mit Säurefuchsin gefärbte Präparate zum Verblassen, insbesondere wenn Reste von Nelkenöl (das als finales Klärungsmittel verwendet wird) zurückbleiben. In Nelkenöl gelagerte Proben können bereits innerhalb weniger Tage signifikant ausbleichen.

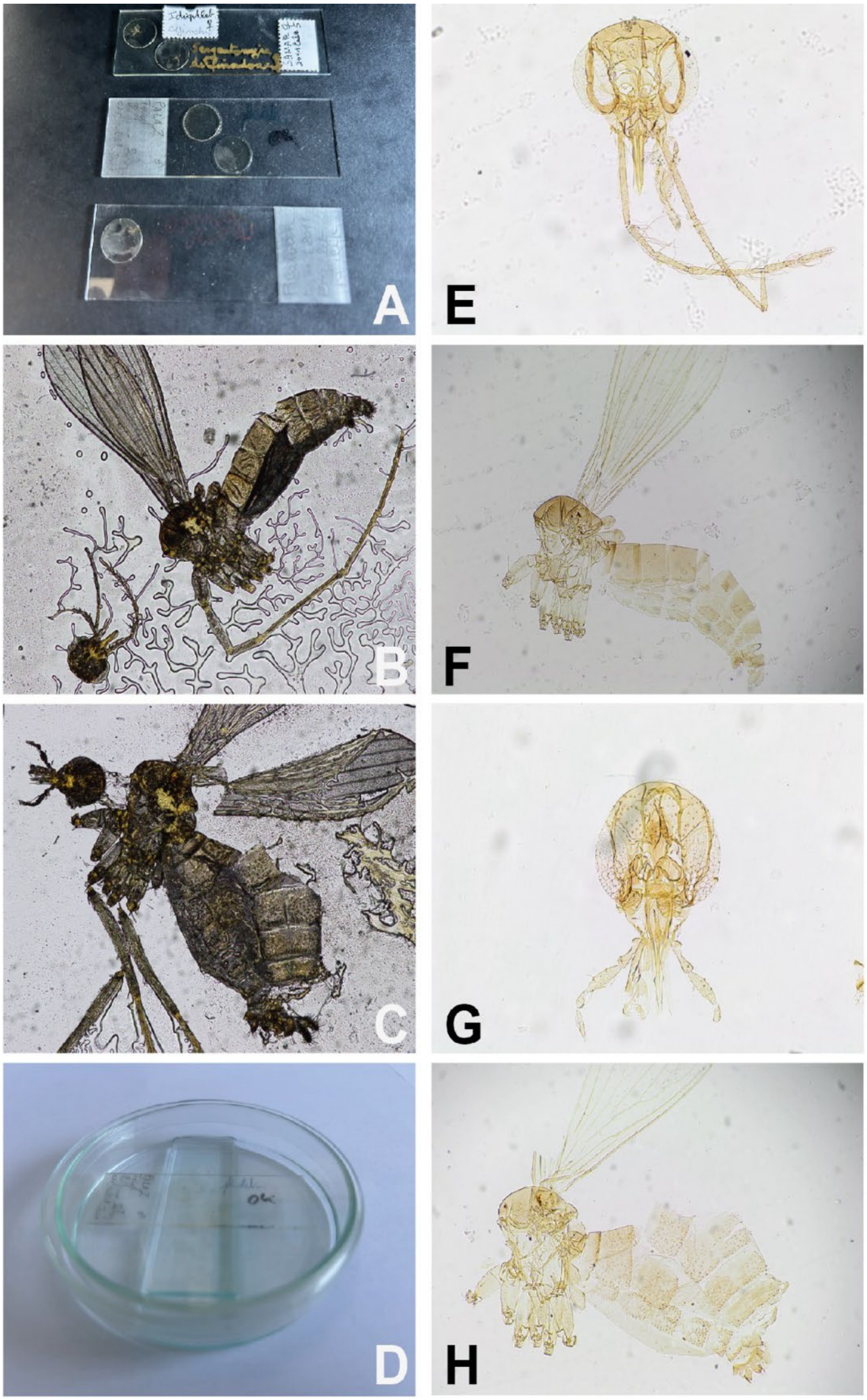

Abbildung 8: Umbettung von Objektträgern. A: Beschädigte und ausgetrocknete Objektträger, die in Hoyer eingebettet wurden; B: Mikroskopische Ansicht einer ausgetrockneten Sandmücke; C: Mikroskopische Ansicht einer weiteren beschädigten Sandmücke; D: Feuchte Kammer mit einem ausgetrockneten Objektträger; E: Kopf und F: Körper von Präparat B nach der Umbettung in Euparal®; G: Kopf und H: Körper des beschädigten Präparats.

5.2. Dehydrierung (Entwässerung)

Die Dehydrierung erfolgt durch das schrittweise Überführen der Proben durch eine aufsteigende Ethanol-Reihe: 50%, 70%, 80%, 90% (oder 95%) und schließlich 100%. Jedes Bad sollte mindestens 20 Minuten dauern. Da Ethanol schnell verdunstet, müssen die Gefäße während des gesamten Prozesses fest verschlossen bleiben. Sobald die Probe vollständig dehydriert ist, kann der Prozess für einige Tage unterbrochen werden, indem die Proben in Euparal®-Essenz gelagert werden. Dies ist gegenüber Nelkenöl vorzuziehen. Buchenholz-Kreosot, das früher für diesen Zweck weit verbreitet war, ist heute aufgrund seiner hohen Toxizität vollständig verboten.

Der Dehydrierungsprozess muss sicherstellen, dass die Flüssigkeit innerhalb der Probe mit dem späteren Einbettungsmedium kompatibel ist, um Trübung, Osmotischen Kollaps oder Verzerrungen zu vermeiden, die Proben für taxonomische Studien unbrauchbar machen.

5.3. Einbettungsmedien

5.3.1. Auswahl und Anwendung für die Probenvorbereitung

Das Einbettungsmedium sollte im Idealfall einen Brechungsindex (BI) haben, der so nah wie möglich an dem von Glas liegt (ca. 1,5). Es muss farblos und klar sein sowie nach dem Trocknen und über lange Zeiträume hinweg perfekt transparent bleiben. Zudem muss es mit den verwendeten Farbstoffen kompatibel sein und die Fähigkeit besitzen, in alle Gewebe des Exemplars einzudringen. Es darf weder zu schnell trocknen noch während des Einbettungsvorgangs eine Trübung bilden oder nach der Montage schrumpfen. Da kein einzelnes Medium für alle Zwecke ideal ist, muss die Wahl folgende Schlüsselfaktoren abwägen:

- Optische Eigenschaften. Der Brechungsindex des Mediums muss einen ausreichenden Kontrast und eine klare Lichtbrechung der kritischen anatomischen Merkmale gewährleisten, die für die taxonomische Identifizierung oder morphologische Beschreibung herangezogen werden. Dazu gehören die Spermatheken (Samentaschen), Ascoide (Sinnesorgane an den Antennen), Newstead-Sensillen, Vertikale Cibarialzähne und Pharyngealzähne. Die Sichtbarkeit dieser Strukturen hängt direkt von den optischen Eigenschaften des gewählten Mediums ab.

- Erhaltung und Dauerhaftigkeit. Für Typus-Exemplare und Material, das für permanente wissenschaftliche Sammlungen bestimmt ist, muss das Medium eine langfristige Stabilität und Haltbarkeit bieten. Gegenteilig, bei Bestandsaufnahmen oder epidemiologische Studien, für die langfristige Konservierung weniger kritisch ist, können temporäre oder semi-permanente Medien ausreichend sein, da sie oft eine schnellere Bearbeitung ermöglichen.

5.3.2. Anforderungen an Einbettungsmedien

Spezialisten entwickeln häufig maßgeschneiderte und komplexe Einbettungstechniken, die auf spezifische Forschungsbedürfnisse zugeschnitten sind. Dabei werden jedoch oft Aspekte wie die Archivqualität, Kompatibilität, Standardisierung oder die einfache Handhabung und langfristige Erhaltung vernachlässigt. Dieser Mangel an Standardisierung erschwert die Integrierung gespendeter Sammlungen in bestehende Bestände und die langfristige kuratorische Betreuung.

Wissenschaftliche Anwendungen stellen unterschiedliche Anforderungen an Einbettungsmedien. Taxonomen betten oft ganze Exemplare ein und bevorzugen Medien, die innere Organe sanft mazerieren, um die Sichtbarkeit kutikulärer Strukturen zu erhöhen. Der Brechungsindex sollte sich ausreichend von dem der Probe und des Objektträgers unterscheiden, um die optische Deutlichkeit zu maximieren. Kommerzielle Einbettungsmedien sind in der Regel mit einem Brechungsindex formuliert, der dem von Glas nahekommt, um Lichtbrechung und Streuung durch das System aus Objektträger, Medium und Deckglas zu minimieren. In der Hellfeldmikroskopie kann der natürliche Kontrast einer ungefärbten Probe jedoch manipuliert werden, indem bewusst ein Einbettungsmedium mit einem Brechungsindex gewählt wird, der sich leicht von dem der Probe unterscheidet, wodurch deren Sichtbarkeit gegenüber dem Hintergrund verbessert wird.

5.3.3. Arten von Einbettungsmedien (Tabellen 3 & 4)

In der Mikroskopie ist der Brechungsindex eines Einbettungsmediums ausschlaggebend dafür, wie das Licht durch den Objektträger, das Medium und die Probe gebrochen wird. Wenn der Brechungsindex eng auf das Glas des Deckglases abgestimmt ist (≈ 1,515), passiert das Licht das System gleichmäßig, was Streuung und optische Verzerrungen verringert. Dies führt zu einer verbesserten Auflösung und Sichtbarkeit feiner Strukturen. Im Gegensatz dazu kann eine Abweichung des Brechungsindex zu Unschärfe, Lichthöfen oder der Verdeckung unpigmentierter Merkmale führen. Die Auswahl des richtigen Einbettungsmediums ist daher entscheidend, um Kontrast, Klarheit und die allgemeine Bildqualität für eine bestimmte Probe zu optimieren

Der Brechungsindex des Mediums hat einen erheblichen Einfluss darauf, wie gut feine Strukturen bei der Vorbereitung von Sandmücken-Präparaten erkannt werden können. Die zarten und nur schwach sklerotisierten Merkmale von Sandmücken, einschließlich der Cibarialarmatur, der Spermatheken, der Antennensegmente und des Flügelgeäders, können in einem Einbettungsmedium mit hohem Brechungsindex schwer zu beobachten sein.

Für Sandmücken gehören Chloralhydrat-Gummi-Medien als wasserbasierte Einbettungsmedien sowie Kanadabalsam und Enecê - Nelson Cerqueira (NC) Harz als

lösungsmittelbasierte Medien zu den am häufigsten verwendeten Optionen. Rawlins [60] kategorisierte Einbettungsmedien in zwei Typen: (1) permanente Medien: härten mit der Zeit aus und sind für die langfristige Aufbewahrung geeignet, und (2) semi-permanente Medien: härten nicht vollständig aus und werden typischerweise für temporäre Zwecke verwendet

Einbettungsmedien können flüssig, gummibasiert oder harzig sein und sind entweder in Wasser, Alkohol oder organischen Lösungsmitteln löslich (z. B. Toluol oder Xylol) (Tabelle 3). Nach der Anwendung sollten sie durch nicht-lösliche Versiegelungsmedien vor atmosphärischen Einflüssen geschützt werden. Um die verschiedenen Arten von Einbettungsmedien klar voneinander abzugrenzen, wird die folgende Kategorisierung vorgenommen:

a. Wässrige Medien. Diese Medien lösen sich leicht in Wasser auf, was sie ideal für temporäre oder semi-permanente Präparate macht. Sie sind in der Regel einfach zu handhaben, erfordern jedoch häufig eine Versiegelung, um eine Reaktion mit der Luftfeuchtigkeit zu verhindern (z. B. Chloralhydrat-Gummi-Basis oder Polyvinylalkohol), vor allem in tropischen, feuchten Klimazonen.

b. Bedingt wasserbeständige Medien. Diese Medien werden weniger stark von Wasser beeinflusst, benötigen aber dennoch einen Schutz gegen übermäßige Feuchtigkeit. Im Vergleich zu rein wasserlöslichen Optionen bieten sie eine höhere Langzeitstabilität und finden häufig bei semi-permanenten Präparaten Verwendung.

c. Kohlenwasserstoff-lösliche Medien. Diese Medien werden in organischen Lösungsmitteln wie Xylol, Toluol oder Essenecê (Enecê-Lösungsmittel) gelöst. Sie sind speziell für die dauerhafte Einbettung konzipiert und bieten eine exzellente Langzeitstabilität. Da sie resistent gegen Feuchtigkeit und biologischen Abbau sind, stellen sie die ideale Wahl für Archivierungszwecke dar (z. B. neutraler Kanadabalsam).

Zusammenfassend lässt sich festhalten, dass wasserlösliche Medien am besten für temporäre Präparate geeignet sind, bei denen die Probe eventuell wieder entnommen werden muss. Bedingt wasserbeständige Medien bieten sich für semi-permanente Präparate mit moderater Haltbarkeit an, während kohlenwasserstoff-lösliche Medien die bevorzugte Wahl für permanente Präparate sind, die für die Archivierung und langfristige Lagerung vorgesehen sind

Tabelle 3: Zusammensetzung ausgewählter Eindeckmedien.

Eindeck-medium	Flüssigkeit	Potentielle(s) Prepolymer(e) oder Polymer	Anmerkungen
Hoyer = Chloral-hydrat	Glycerin, Wasser	Bestandteile von Gummi arabicum	Mazerationsmittel: Chloralhydrat
CMCP-9 (=Carboxymethylcellu-lose-Phenol)	Wasser (CMCP-9: 51−60%)	Polyvinylalkohol, vollständig hydrolysiert (CMCP-9: 0−5%)	CMC(P)-9: niedrige Viskosität: hohe Viskosität
DMHF (Dimethyl hydantoin-Formalde-hyd)	Wasser	N,N′-Dimethylol dimethyl hydantoin (Dimethylol DMH) Ether-/Methylen-verbrückte Oligomere Vernetztes DMH–Formaldehyd-Polymer-Netzwerk	
Canada-Balsam	Xylol; teilweise flüchtige Komponenten des Balsam (3-Caren, Levopimarsäure, Limonen, Myrcen, Palustrinsäure, β-Phellandren, α-Pinen, β-Pinen)	Balsam (Abienol, Abietinsäure, Isopimarinsäure, Sandaracopimarsäure)	Neutralisation: Kaliumcarbonat; Harz von *Abies balsamea* (Linné, 1758)
Euparal®	Eukalyptosöl, Paraldehyd; teilweise flüchtige Komponenten von Sandarak (Limonen, α-Pinen, β-Pinen)	Komponenten von Sandarak (Ameisensäure, Manool, Polycommunsäure, Sandaracopimarsäure, 12-Acetyl-Oxy-Sandaracopimarsäure, Sugiol, Torulosolsäure, Torulosol, Totarol)	Aufhellungsmedium: Methylsalicylat; Farbe in Euparal® Grün: Kupfersalz (Kupfer-Abietinate); Harz von *Tetraclinis articulata* (Vahl, 1791)
Enecê	Ethanol; mit Campher, Eukalyptusöl und Terpentinöl	Komponenten von Kauri-Harz und Kolophonium (Geigenharz)	

Tabelle 4: Vor- und Nachteile ausgewählter Einbettungsmedien für mikroskopische Präparate sowie unveröffentlichte Beobachtungen verschiedener Personen [52].

Name	Vorteile	Nachteile
* Canada-Balsam	Das Medium ist äußerst langlebig und weist eine Lebensdauer von über 150 Jahren auf.<br>Die Objektträger können unter Verwendung von Nelkenöl oder Phenol als Eindeckmedium präpariert werden.	Enthält schädliche Komponenten und muss unter einem Abzug gehandhabt werden.<br>Erfordert eine vollständige, zeitaufwendige Dehydrierungsreihe.<br>Die Ethanol-Dehydrierung und der Transfer über Xylol oder Nelkenöl können einige Taxa spröde machen; Alternativen (z. B. Isopropanol, n-Butanol, Cellosolve™, 1,4-Dioxan, Histoclear, Terpineol) können Bruchschäden reduzieren.<br>Proben können sich schwarz verfärben, wenn Xylol durch Phenol ersetzt wird oder wenn Rückstände von Kaliumhydroxid verbleiben.<br>Hohe Brechungsindizes können ungefärbte Strukturen verdecken.<br>Die vollständige Trocknung kann ohne das Trocknen auf einer Heizplatte Jahre dauern.<br>Das Medium vergilbt und dunkelt mit der Zeit nach, insbesondere wenn es mit Nelkenöl aufgehellt wurde.<br>Einige Färbungen lassen nach, und kationische Farbstoffe können verblassen, wenn das Medium sauer wird – was im Laufe der Zeit spontan geschehen kann.
DMHF (Dimethylhydantoin-Formaldehyd)	Hohe Transparenz<br>Guter Brechungsindex<br>Hervorragende Sichtbarkeit der Strukturen<br>Gute Stabilität der Präparate<br>Kompatibilität mit vielen Färbetechniken<br>Guter Schutz der Proben<br>Gute Haftung zwischen Objektträger und Deckglas	Mögliche Vergilbung im Laufe der Zeit<br>Kann einige Färbungen verändern<br>Nicht geeignet für formaldehyd-empfindliche Färbungen<br>Luftblasen, lange Trocknungszeit<br>Einbettungsmedium ist feuchtigkeitsempfindlich<br>Einbettung ist schwer rückgängig zu machen<br>Formaldehyd ist giftig, reizend, krebserregend
* Euparal (transparent)	Langlebiges Medium mit einer Lebensdauer von über 50 Jahren.<br>Einbettung direkt aus 80%igem Ethanol ist möglich (Herstellerempfehlung).<br>Verdeckt keine ungefärbten Strukturen und vergilbt oder versprödet nicht im Laufe der Zeit.<br>Besitzt einen Brechungsindex, der für Dipteren besser geeignet ist als Kanadabalsam.<br>Eignet sich gut für dickere Präparate aufgrund minimaler Schrumpfung und blasenfreier Trocknung.<br>Bleibt in 95%igem Ethanol löslich, was ein erneutes Einbetten selbst nach vielen Jahren ermöglicht.	Enthält schädliche Komponenten und muss unter einem Abzug gehandhabt werden. Die Ethanol-Dehydrierung und der Transfer über Euparal-Essenz können einige Taxa spröde machen, aber die Verwendung von Isopropanol kann dieses Problem verringern.
Hoyer-Medium	Proben können lebend oder direkt aus Wasser, Ethanol oder Formaldehyd eingebettet werden.<br>Die Mazeration liefert eine exzellente Qualität der Kutikula.<br>Besitzt einen günstigen Brechungsindex, der durch Iod-Färbung für einen höheren Kontrast weiter verbessert werden kann.<br>Die Essigsäure in der Rezeptur kann die Extremitäten von Arthropoden ausdehnen.<br>Einige Präparate können über 40–60 Jahre stabil	Empfindliche Pflanzenproben können einfallen, sofern das Medium nicht schrittweise hinzugefügt wird, was zeitaufwendig ist.<br>Hohlräume und Kristalle können sich in weniger als 10 Jahren bilden.<br>Die Mazeration kann je nach Chloralhydrat-Konzentration und Einwirkzeit übermäßig stark ausfallen.<br>Komponenten des Mediums können sich trennen, und innerhalb von Monaten oder Jahren kann eine feine

This is an Open Access article distributed under the terms of the Creative Commons Attribution License (https://creativecommons.org/licenses/by/4.0), which permits unrestricted use, distribution, and reproduction in any medium, provided the original work is properly cited.

	bleiben. Wasserlöslich, was ein einfaches erneutes Einbetten ermöglicht.	Granulation auftreten. Es wurde über eine Schwarzfärbung des Mediums berichtet.
CMCP-9 (= Carboxymethylcellulose-Phenol)	Proben können direkt aus Medien wie Wasser, Ethanol, Glycerin oder formaldehydhaltigen Lösungen eingebettet werden, und ihre inneren Organe können bei Bedarf mazeriert werden, um die allgemeine Untersuchung oder Präparation zu erleichtern.	Dieses Medium kann mit der Zeit Kristalle bilden und nachdunkeln, und es kann Proben mitunter stärker mazerieren als beabsichtigt. Sofern der Objektträger nicht sorgfältig versiegelt (umrandet) wird, eignen sich dickere Proben nicht gut für dieses Medium, da sie schrumpfen und Lücken an den Rändern des Deckglases verursachen können. Es ist weder für gefärbte Präparate noch für kalzifizierte Materialien geeignet, und seine Trocknungszeit ist langsamer als die von CMC.
Eukitt™	Langlebiges Medium mit einer Haltbarkeit von über 30 Jahren. Kompatibel mit vielen Lösungsmitteln für die Einbettung, einschließlich Aceton, Benzol, Chloroform, Dioxan, Ether, Isopropanol, Methylbenzoat, Terpineol, Toluol und Xylol. Trocknet schnell und besitzt einen leicht sauren pH-Wert. Dunkelt mit zunehmendem Alter nicht merklich nach. Geeignet für verschiedene Färbungen (z. B. Fuchsin, Hämatoxylin, Methylgrün, Methylviolett, Methylenblau). Proben können nach Jahren erneut eingebettet werden, indem sie über einen längeren Zeitraum in Xylol eingeweicht werden.	Enthält schädliche Komponenten und muss unter einem Abzug gehandhabt werden. Erfordert eine vollständige, zeitaufwendige Dehydrierungsreihe. Aufgrund von Schrumpfung und Gasblasenbildung nicht ideal für dickere Proben geeignet. Deckgläser können sich mit der Zeit lösen, sofern das Glas nicht gut gereinigt und versiegelt wurde. Kann eine unvollständige Polymerisation um Kollagenfasern herum aufweisen.
Enecê	Äußerst langlebiges Medium, das mindestens 50 Jahre hält. Enecê dunkelt im Laufe der Zeit nicht nach. Es ist formbarer, was die Dissektion von Insekten im Medium ermöglicht und zudem ausreichend Zeit für die Positionierung morphologischer Strukturen bietet. Geringe Kosten.	Erfordert eine vollständige, zeitaufwendige Dehydrierungsreihe. Die Ethanol-Dehydrierung und der Transfer über Nelkenöl können einige Proben spröde machen. Das Insekt wird weiterhin aufgehellt, wenn auch sehr langsam; dies kann es erschweren, sehr kleine Strukturen wie Sensillen, Ascoide und einfache Borsten (Setae) zu erkennen.

5.3.4. Beschreibung der empfohlenen Einbettungsmedien (Tabellen 3 & 4)

Medien für die temporäre Beobachtung

Chloral-Gummi = Hoyer-Flüssigkeit/Medium/Lösung (BI = 1,48)

Die Marc-André-Flüssigkeit ist das beste Medium für die kurzzeitige Beobachtung (einige Stunden, vielleicht etwas länger, wenn der Objektträger in einer feuchten Kammer gelagert wird) von Spermatheken, einschließlich Fotografien (Abbildung 4) oder Zeichnungen. Die Konservierung der beobachteten Spermatheken erfordert deren Umbettung in ein wässriges Medium, das eine mittelfristige Lagerung ermöglicht. Sie für die Umbettung in Harz zu dehydrieren ist nicht unmöglich, wird aber nicht empfohlen (Risiko des Verlusts). Chloral-Gummi und Hoyer-Medium werden als synonym betrachtet. Dieses Medium wird aufgrund seiner Wasserverträglichkeit, Einfachheit, schnellen Anwendung und seines Brechungsindex, der die Untersuchung empfindlicher Strukturen wie Spermatheken erleichtert, häufig für die Beobachtung innerer Organe verwendet. Chloral-Gummi weist jedoch erhebliche Nachteile auf, wenn es nicht perfekt vorbereitet oder unter kontrollierten Feuchtigkeitsbedingungen gelagert wird. Diese Probleme umfassen Kristallisation, Verfärbung und Viskositätsverlust. Das Umranden des Deckglases löst diese Probleme nicht, da sich die Einbettungsmedien aufgrund der Wechselwirkung mit dem Versiegelungsmittel stark verfärben können (manchmal fast schwarz), insbesondere wenn Euparal® verwendet wird.

Hoyer-Medium wird optisch als das beste für Sandmücken (Phlebotominae) angesehen und wurde traditionell für diese Zwecke verwendet. Das Medium besteht aus mehreren eng verwandten Formulierungen,

darunter Gummi arabicum, Glycerin und Chloralhydrat. Verschiedene Formulierungen wurden falsch interpretiert und falsch zitiert [74].

Obwohl Hoyer ein gutes Medium für die Beobachtung von Spermatheken bei Sandmücken ist, eignet es sich nicht für die langfristige Konservierung. Es ist ideal für kurzfristige Beobachtungen, einschließlich Fotografien, Zeichnungen oder Bildern. Wässrige Medien sind für temporäre Präparate geeignet, können aber keine langfristige Konservierung gewährleisten. Im Gegensatz dazu bietet die Einbettung in Harz eine hervorragende Haltbarkeit, die oft Jahrhunderte überdauert, kann jedoch feine Details der Spermatheken verdecken, da deren Lichtbrechung häufig verloren geht.

Hoyer-Medium degradiert im Laufe der Zeit durch Dehydrierung (Abbildung 8), was zur Bildung kleiner weißer, opaker Chloralhydrat-Kristalle führt. Dennoch können Proben von kristallisierten Objektträgern zurückgewonnen werden, da die Kutikula chemisch intakt bleibt, obwohl durch die wachsenden Kristalle einige physische Schäden auftreten können. In einigen Fällen können kristallisierte Objektträger wiederhergestellt werden, indem das Einbettungsmedium in einer warmen, feuchten Umgebung mit Thymol (um Pilzbefall zu verhindern) rehydriert wird. Alternativ können die Proben aus dem Chloral-Gummi in Wasser eingeweicht, in Eisessig dehydriert und erneut in Kanadabalsam eingebettet werden.

DMHF (Dimethylhydantoin-Formaldehyd) (BI = 1,48)

Dieses wasserbasierte Medium [72] erbringt optisch sehr gute Leistungen, ähnlich wie Berlese, und ist ebenso einfach in der Handhabung wie Berlese. Im Gegensatz zu Berlese verfärbt es sich jedoch nicht schwarz und kristallisiert nicht aus. Es eignet sich gut für Sandmücken und andere Psychodidae.

Abbildung 8: Umbettung von Objektträgern. A: Beschädigte und ausgetrocknete Objektträger, die in Hoyer eingebettet wurden; B: Mikroskopische Ansicht einer ausgetrockneten Sandmücke; C: Mikroskopische Ansicht einer weiteren beschädigten Sandmücke; D: Feuchte Kammer mit einem ausgetrockneten Objektträger; E: Kopf und F: Körper von Präparat B nach der Umbettung in Euparal®; G: Kopf und H: Körper des beschädigten Präparats C nach der Umbettung in Euparal®.

CMCP (Campher-Monochlorphenol) (BI = 1,41)

Dies ist ein auf Glycerin basierendes, wasserlösliches Einbettungsmedium, das zur Erstellung transparenter, permanenter Präparate von empfindlichen Proben, einschließlich Sandmücken, verwendet wird. Der Vorteil dieses Einbettungsmediums besteht darin, dass die Proben direkt aus Wasser oder Ethanol eingebettet werden können. Es entspannt und klärt die Sandmücke schnell und weicht die Kutikula auf, um eine korrekte Positionierung des Präparats zu ermöglichen, was besonders beim Ausbreiten der Flügel oder beim Dissezieren der Genitalien nützlich ist. Obwohl berichtet wird, dass es eine langfristige Lagerung ermöglicht, bleibt die genaue Dauer der Konservierung ungewiss. Die größte Einschränkung dieses Einbettungsmediums liegt in seiner Zusammensetzung, die Phenol enthält – eine giftige und reizende Substanz, die eine sorgfältige Handhabung erfordert.

Medien für permanentes Einbetten

Kanadabalsam (BI = 1,52–1,54)

Kanadabalsam wurde erstmals in den 1830er Jahren von Andrew Pritchard als geeignetes Einbettungsmedium für die Durchlichtmikroskopie beschrieben. Aufgrund seiner bewährten Archivqualität mit über 150 Jahren erfolgreicher Anwendung bleibt es eines der am weitesten verbreiteten Medien. Im Gegensatz zu Hoyer-Medien kristallisiert Kanadabalsam nicht und absorbiert keine Feuchtigkeit. Kanadabalsam ist jedoch stark autofluoreszierend, was bei bestimmten Mikroskopietechniken manchmal ein Nachteil sein kann [60]. Die Verwendung ungiftiger Lösungsmittel anstelle von Xylol kann die Sicherheitsrisiken bei der Probenherstellung verringern, kann jedoch auch Nachteile wie eine langsamere Trocknung und eine frühere Nachdunklung des Mediums mit sich bringen.

Euparal® (BI = 1,48)

Euparal® ist eine weit verbreitete Alternative zu Kanadabalsam für die dauerhafte Einbettung und bietet eine ausgezeichnete Langzeitstabilität sowie einen vergleichbaren Brechungsindex. Euparal® weist folgende Merkmale auf: (1) Erfordernis der Dehydrierung: Vor dem endgültigen Transfer in das Einbettungsmedium muss die Probe dehydriert werden, wobei typischerweise ein Übergang von 95%igem zu absolutem Ethanol erfolgt, und (2) verlängerte Bearbeitungszeit: Die endgültige Montage in einem Harz, sei es Kanadabalsam oder Euparal®, erfordert eine Dehydrierung, was die gesamte Probenbearbeitungszeit verlängert. Wenn eine Dehydrierung mit organischen Lösungsmitteln nicht möglich ist, können Proben, die aus absolutem Ethanol entnommen wurden, vor der endgültigen Einbettung in eine Zwischenlösung gelegt werden, die aus einer gleichen Mischung von Euparal® und Euparal-Essenz besteht.

Enecê (BI = 1,467)

Enecê ist ein harzbasiertes Einbettungsmedium, das primär für kleine Insekten verwendet wird und besonders in Brasilien beliebt ist. Seine Basis besteht aus Kolophonium und Copalharz, gelöst in Alkohol, Campher, Terpentinessenz und Eukalyptol. Cerqueira [11] beschrieb Enecê als Alternative zu Kanadabalsam für die Montage permanenter Präparate von Larven, Exuvien von Immaturen und sogar adulten Mücken; seither wird es in weitem Umfang für die Einbettung von Sandmücken eingesetzt. Enecê bietet eine kostengünstige Alternative für die

dauerhafte Einbettung, gewährleistet Langzeitstabilität und eine ausreichende Trocknungszeit, die eine Dissektion und präzise Anordnung morphologischer Strukturen ermöglicht.

5.4. Vorbereitung und Trocknung der Objektträger

Eine ordnungsgemäße Trocknung montierter Objektträger ist entscheidend, um die langfristige Stabilität und Erhaltung zu gewährleisten. Die Objektträger sollten gründlich getrocknet werden, bevor eine langfristige Lagerung in Betracht gezogen wird. Für optimale Ergebnisse sollten Objektträger, die mit permanenten Einbettungsmedien montiert wurden, 2–3 Wochen lang horizontal getrocknet werden, während solche, die mit semi-permanenten Medien vorbereitet wurden, nur 1–2 Wochen benötigen könnten. Um einen effektiven Trocknungsprozess zu gewährleisten, wird empfohlen, einen Inkubator zu verwenden, der auf eine für das verwendete Einbettungsmedium angemessene Temperatur eingestellt ist, wobei übermäßige Hitze, die die Proben beschädigen könnte, zu vermeiden ist. Ein Temperaturbereich zwischen 30°C und 37°C wird empfohlen. Dieser Trocknungsschritt ist von entscheidender Bedeutung, um ein Verbiegen der Objektträger, eine Verschlechterung der Probe oder eine Instabilität des Einbettungsmediums während der Lagerung zu verhindern.

Das bei der Vorbereitung des Objektträgers verwendete Einbettungsmedium sollte immer auf dem Etikett des Objektträgers vermerkt werden. Wenn möglich, sollte das Etikett auch das spezifisch verwendete Rezept enthalten, zusammen mit dem Namen des Präparators und dem Datum der Vorbereitung. Objektträger werden anfangs als temporäre Präparate erstellt und sind nicht für die langfristige Aufbewahrung gedacht. Wenn sich jedoch der Status der Probe ändert, beispielsweise wenn sie als Teil einer „Typus"-Serie bestimmt wird, sollte ein permanenteres Einbettungsmedium verwendet werden, um die Erhaltung der Probe für zukünftige taxonomische Studien zu gewährleisten.

5.5. Alternative Montagetechniken: Kartenpräparierung

Die Kartenpräparierung ist eine Technik, die für mehrere Insektengruppen verwendet wird, bei der Proben entweder direkt auf entomologische Karten genadelt oder auf die Oberfläche geklebt werden können. Angesichts ihrer geringen Größe und der Notwendigkeit, innere Organe zur Identifizierung durch Aufhellung (siehe Punkt 5) zu beobachten, ist diese Methode für die Präparierung von Sandmücken überhaupt nicht geeignet.

5.6. Umbettung beschädigter Proben

Für seltene oder wertvolle Proben wird ein zweistufiger Ansatz empfohlen, entsprechend dem Video, das unter https://zenodo.org/records/18315029 zugänglich ist. Rehydrieren Sie diese ohne Demontage, um eine vorläufige Beobachtung zu ermöglichen. Ein Halter für mehrere Objektträger sollte in eine Petrischale gestellt werden, um als Stütze zu dienen. Der zu rehydrierende Objektträger wird dann darauf platziert und die Petrischale wird mit einigen Millimetern Lösungsmittel gefüllt, um eine feuchte Kammer zu erzeugen, wobei sicherzustellen ist, dass der Objektträger selbst nicht mit dem Lösungsmittel in Kontakt kommt (Abbildung 8 D). Die für die Rehydrierung erforderliche Zeit kann je nach Zustand der Probe zwischen einem und mehreren Tagen variieren. Tägliche Überwachung und Geduld sind unerlässlich. Sobald der Objektträger ausreichend rehydriert ist, kann er aus der feuchten Kammer genommen und für einige Stunden in einen Inkubator gestellt werden, bevor er mikroskopisch untersucht, fotografiert oder gezeichnet wird. Zum Umbetten kann der Objektträger für einige zusätzliche Stunden oder über Nacht in die feuchte Kammer zurückgelegt werden. Die Demontage sollte unter einem Stereomikroskop durchgeführt werden. Mit feinen Nadeln muss das Deckglas vorsichtig entfernt werden, wobei sicherzustellen ist, dass keine Teile der Sandmücke daran hängen bleiben (https://zenodo.org/records/18315029). Als Nächstes sollten die sezierten Elemente der Sandmücke gesammelt und in kleinen Vertiefungen mit Wasser gespült werden, wie sie für die destruktive DNA/RNA-Extraktion verwendet werden (siehe unten), bevor sie dehydriert und in einem Harzmedium neu eingebettet werden. Bei der Demontage eines Objektträgers ist es entscheidend, das ursprüngliche Einbettungsmedium zu identifizieren, um ein geeignetes Lösungsmittel auszuwählen. Für wässrige Einbettungsmedien sollte Wasser verwendet werden. Wenn das Einbettungsmedium harzbasiert ist (z. B. Kanadabalsam oder Euparal®), sollte Xylol unter einem Abzug und mit angemessener persönlicher Schutzausrüstung, einschließlich einer Maske, verwendet werden.

Die Umbettung von Typus- oder Sammlungsexemplaren darf nur mit Zustimmung des Kurators und/oder der Institution erfolgen, der die Probe gehört.

6. Identifizierung der Proben

6.1. Morphologie

Die Identifizierung von Sandmücken stützt sich in erster Linie auf die Untersuchung ihrer morphologischen Merkmale, einschließlich der Form des Thorax, der Flügel, der Genitalien, der Setae (Borsten) und spezifischer morphometrischer Beziehungen zwischen verschiedenen Strukturen. Forscher nutzen Bestimmungsschlüssel, Referenzsammlungen und Originalbeschreibungen der Arten, um gesammelte Proben mit bekannten Taxa zu vergleichen. Wichtige diagnostische Merkmale wie das Flügelgeäder und die Kopfmorphologie bei beiden Geschlechtern, die Struktur der männlichen Genitalien und

die Konfiguration der weiblichen Spermatheken sind besonders aufschlussreich für die Artbestimmung. Eine genaue Identifizierung erfordert oft eine detaillierte mikroskopische Untersuchung, wobei typischerweise ein Durchlichtmikroskop zur Beobachtung feiner Strukturen wie Genitalien und Spermatheken oder ein Stereomikroskop für allgemeinere morphologische Merkmale verwendet wird.

Jüngste Fortschritte in der Bildgebungstechnologie haben die Nutzung digitaler Bildverarbeitung für die Identifizierung von Sandmücken erleichtert. Hochauflösende Fotografien oder digitale Illustrationen wichtiger Merkmale können mit Referenzmaterialien verglichen oder mithilfe computergestützter Identifizierungssysteme analysiert werden, was sowohl die Genauigkeit als auch die Zugänglichkeit in der morphologischen Taxonomie verbessert.

6.2. Flügelgeometrie

Die Flügelgeometrie ist ein Schlüsselmerkmal, das bei der Identifizierung und Klassifizierung verschiedener Sandmückenarten verwendet wird. Die Flügel von Sandmücken weisen ein einzigartiges Muster und eine besondere Struktur auf; sie sind typischerweise lang und schmal mit einem gut entwickelten Geäder (Abbildungen 9 & 10). Die Anordnung der Adern bildet ein ausgeprägtes Muster, das zwischen Gattungen und Arten variieren kann und wertvolle diagnostische Merkmale für die Identifizierung liefert. Folglich liefert die Untersuchung der Flügelgeometrie wertvolle Erkenntnisse für taxonomische Zwecke.

6.3. Geometrische Flügel-Morphometrie

Forscher nutzen verschiedene Techniken, wie die geometrische Morphometrie, um Flügelform und -größe zwischen verschiedenen Sandmückenarten oder Populationen zu analysieren und zu vergleichen. Das Studium der Flügelgeometrie liefert wertvolle Einblicke in das Verhalten, Habitatpräferenzen und Flugfähigkeiten.

Bei dem geometrisch-morphometrischen Ansatz werden die Flügel sorgfältig seziert, (bei Bedarf) gefärbt und flach auf Objektträgern montiert. Die vorbereiteten Objektträger werden dann unter einem Stereomikroskop fotografiert, digitalisiert und einer morphometrischen Analyse unterzogen. Dieses Verfahren wurde in der Literatur ausführlich beschrieben [6, 27, 42, 56, 57, 59], wobei empfohlen wird, bei paarigen Organen konsequent den rechten oder linken Flügel zu verwenden, um potenzielle negative allometrische Effekte zu vermeiden [62].

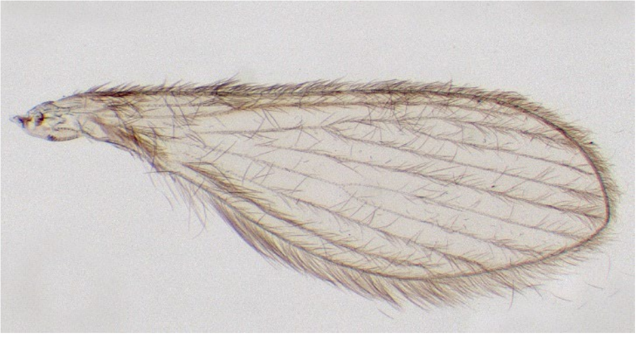

Abbildung 9: Unbearbeiteter Flügel von *Trichophoromyia ininii*.

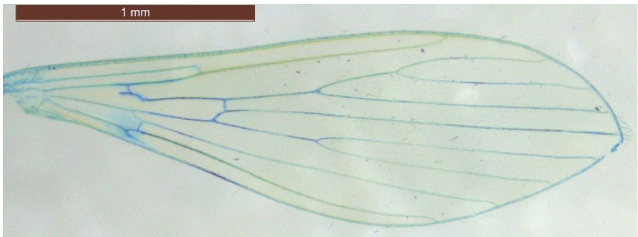

Abbildung 10: Gefärbter Flügel von *Phlebotomus ariasi.*

Flügelpräparierung für die geometrisch-morphometrische Analyse

Für eine optimale Visualisierung der Flügeladern sollten die Flügel von Schuppen befreit und angemessen gefärbt werden. Zur Vorbereitung der Flügel füllen Sie zunächst kleine Vertiefungen mit den erforderlichen Reagenzien (Methylenblau, Ethanol, Wasser und Xylol-Ersatz). Entnehmen Sie einen in 70%igem Ethanol bei Raumtemperatur konservierten Flügel, indem Sie das Eppendorf-Röhrchen umdrehen und über der Vertiefung entleeren; heben Sie den Flügel dann in Längsrichtung mit einer feinen, gebogenen Nadel an. Führen Sie den Flügel kurz von Ethanol in Wasser und zurück in Ethanol, um die Borsten zu entfernen. Legen Sie den Flügel für 6 Minuten in Methylenblau und achten Sie darauf, dass er während des Färbevorgangs schwimmt. Entnehmen Sie den Flügel vorsichtig und tauchen Sie ihn für 2 Minuten in Xylol-Ersatz (etwa ein Drittel der Zeit im Vergleich zum Methylenblau). Leichtes Klopfen der Nadel gegen die Wände der Vertiefung kann helfen, dass der Flügel absinkt; das Xylol dient dazu, die Färbung zu fixieren. Heben Sie den Flügel schließlich an und platzieren Sie ihn auf einem kleinen Tropfen Euparal® auf einem Objektträger. Entfalten Sie den Flügel unter einer Lupe vorsichtig und legen Sie behutsam ein Deckglas auf. Fotografien sollten zeitnah aufgenommen werden, bevor das Euparal® fest wird, da leichte Anpassungen der Flügelposition unter dem Deckglas notwendig sein können, um eine optimale Ausrichtung zu erreichen.

6.4. Molekularbiologische Techniken

Zusätzlich zu den morphologischen Techniken sind molekulare Methoden in der entomologischen Forschung zunehmend unverzichtbar geworden. Dazu gehören taxonomische, populationsgenetische und phylogenetische Studien sowie der Nachweis von DNA/RNA-Pathogenen und die Bestimmung der Herkunft von Blutmahlzeiten, wobei das Verhalten von Vektoren im Bereich der Epidemiologie von großer Bedeutung ist [70]. Die DNA-Sequenzierung kann zur Artbestätigung oder zur Differenzierung eng verwandter Arten eingesetzt werden und bietet ein genaueres und zuverlässigeres Mittel zur Identifizierung. Darüber hinaus gewinnen fortgeschrittene molekulare Techniken (z. B. PCR, DNA-Sequenzierung, NGS etc.) sowie MALDI-ToF MS an Bedeutung für eine präzise und schnelle Artbestimmung und ergänzen die traditionellen morphologischen Methoden [46]. Trotz dieser Fortschritte bleibt die morphologische Identifizierung der Referenzstandard für die Taxonomie und die Basis, auf der molekulare Daten interpretiert werden.

6.4.1. Destruktive Nukleinsäureextraktion

Die Extraktion von Nukleinsäuren ist ein Routineschritt in vielen biologischen Studien, und es wurden verschiedene Methoden entwickelt, um DNA aus biologischen Materialien zu isolieren [48]. Viele kommerziell erhältliche DNA-Extraktionskits sind darauf ausgelegt, diesen Prozess zu erleichtern [14]. Methoden, die üblicherweise zur Vorbereitung von Arthropoden-Proben für die morphologische Identifizierung verwendet werden, behindern jedoch oft die DNA-Analyse, da diese Techniken kritische physikalische Merkmale der Probe beschädigen oder zerstören können [10]. Die meisten DNA-Extraktionsprotokolle für Insektengewebe sind von Natur aus destruktiv [43], was insbesondere bei kleinen Proben besorgniserregend ist, da selbst eine begrenzte Probenahme wichtige morphologische Merkmale beeinträchtigen kann [72]. Die Art und der Zustand der Probe spielen eine Schlüsselrolle bei der Auswahl einer geeigneten DNA-Isolierungsmethode [29].

Die Notwendigkeit einer genauen Identifizierung von Sandmücken, das Verständnis der Populationsdynamik und und der Minimierung von Auswirkungen auf Nichtzielorganismen haben die Entwicklung molekularer Diagnosewerkzeuge vorangetrieben [23]. Molekulare Ansätze werden heute häufig eingesetzt, um morphologische taxonomische Methoden zur Identifizierung von Sandmücken zu ergänzen. Beispielsweise umfasst der Standardansatz für das Barcoding von Insekten die DNA-Extraktion, die Sequenzierung und den Verlust der ursprünglichen Probe. Daher besteht ein dringender Bedarf, zerstörungsfreie DNA-Extraktionsmethoden zu untersuchen, die sowohl das biologische Material als auch seine morphologische Integrität bewahren.

Zahlreiche Nukleinsäure-Extraktionsmethoden wurden bei Sandmücken angewendet. Die Menge oder Qualität der benötigten Nukleinsäuren hängt von der nachfolgenden molekularen Analyse ab, da verschiedene Techniken unterschiedliche Anforderungen an Empfindlichkeit und Reinheit stellen [9]. Beispielsweise wurde festgestellt, dass die Augen von Sandmücken die PCR-Amplifikation hemmen [69]. Über das Screening von Krankheitserregern hinaus wird die DNA von Sandmücken routinemäßig für Zwecke der Artenidentifizierung extrahiert. Es können verschiedene Extraktionsmethoden verwendet werden, wobei sich Ausbeute und Qualität je nach Technik unterscheiden. Einige Protokolle von Herstellern wurden von Forschern für Sandmücken angepasst [8], um die Ausbeute und/oder Qualität der extrahierten Nukleinsäuren zu erhöhen [8, 9, 69], während andere Anpassungen, die für andere Arthropodentaxa entwickelt wurden, ebenfalls für Sandmücken verwendet werden können [58, 76]. Identifizierungs-PCRs, die auf kleine mitochondriale Fragmente (COI oder CytB) abzielen, sind im Allgemeinen mit Extraktionsmethoden kompatibel, die eine hohe DNA-Fragmentierung beinhalten. Im Gegensatz dazu erfordern andere Long-Read-NGS-Techniken (Oxford Nanopore und PacBio) eine minimale Fragmentierung und qualitativ hochwertige DNA. Spin-Säulen-Extraktionen liefern im Allgemeinen genomische DNA-Fragmente von bis zu 60 kb, während die Phenol-Chloroform-Extraktion Fragmente bis zu 150 kb produzieren kann [77]. Tabelle 5 fasst verschiedene Extraktionstechniken für Sandmücken-DNA zusammen und gibt an, ob methodische Anpassungen für diese Insekten vorgenommen wurden. Die Ausbeuten werden nicht gezeigt, da sie von der Probengröße und der Vorbereitungsmethode abhängen. Die Spalte "Modifikation" bezieht sich auf Anpassungen von Extraktionsprotokollen für Sandmücken oder andere kleine Arthropoden.

Die Wahl der Extraktionsmethode sollte mehrere Kriterien berücksichtigen, wie die Anzahl der Proben, die Extraktionszeit und die nachfolgend eingesetzte Technik. Während NGS-Techniken hochmolekulare genomische DNA erfordern, können alle hier vorgestellten Methoden für Standard-PCR-basierte Anwendungen verwendet werden. Darüber hinaus haben mehrere Studien zerstörungsfreie DNA-Extraktionsmethoden für kleine terrestrische Arthropoden, trocken konservierte Museumspräparate und Arthropoden mit weichem Integument untersucht [19, 26, 28, 55, 63].

Tabelle 5: Tabelle 5: Durchschnittliche Kosten, Anwendung und Protokollanpassung für die Extraktion von genomischer (g)DNA aus Sandmücken

Protocol	Cost	Application	Protocol adaptation for small arthropods
Säulen-Extraktion	2.5 – 3.55 US$ [39]	PCR, NGS	[9]
Phenol-Chloroform	0.24 US$ [69]	PCR, NGS	[9]
HotSHOT-Extraktion	<0.01 US$ [69]	PCR	-
Salz-Präzipitation	0.12 US$ [69]	PCR	-
Chelex-Extraktion	0.02 US$ [41]	PCR	[41, 76]

6.4.2. Zerstörungsfreie Nukleinsäureextraktion

Eine der größten Herausforderungen bei der molekularen Analyse von Arthropoden, insbesondere bei Sandmücken, ist die Erhaltung der Proben für die Integration in entomologische Sammlungen. Die meisten DNA-Extraktionsprotokolle erfordern eine Mazeration des Gewebes, was die Erhaltung des ursprünglichen Exemplars beeinträchtigt. Zerstörungsfreie Nukleinsäure-Extraktionsmethoden sind jedoch darauf ausgelegt, genetisches Material zu extrahieren, ohne die Probe physisch zu beschädigen, ihre Lebensfähigkeit zu beeinträchtigen oder ihre Morphologie zu verändern. Diese Methoden sind besonders wertvoll bei der Arbeit mit kostbaren oder begrenzten Proben, wie etwa Sandmücken, bei denen die Aufrechterhaltung der strukturellen Integrität für zukünftige taxonomische, morphologische oder diagnostische Zwecke unerlässlich ist. Eine häufig angewandte Technik ist die zerstörungsfreie Bademethode, bei der die Sandmücken immobilisiert und vorsichtig in einen Lysepuffer getaucht werden, der Proteinase K enthält.

Die "Mild-Vectolyse"-Technik wurde erfolgreich bei Sandmücken angewendet, insbesondere bei Typusexemplaren [24]. Die Technik nutzt ein herkömmliches Spin-Säulen-Kit (in diesem Fall ein DNeasy Blood and Tissue Kit, QIAGEN, Hilden, Deutschland) mit einer Anpassung zur Gewinnung von DNA, ohne die Probe zu zerstören. Die modifizierten Lysis-Schritte (Volumen des Lysepuffers und Hinzufügen eines Gefrierschritts) [17] ermöglichen die Freisetzung von Nukleinsäuren bei gleichzeitiger Minimierung morphologischer Schäden [24]. Bei Sandmücken ist es auch möglich, ein HotSHOT-DNA-Extraktionskit (Bento Bioworks Ltd, London, Vereinigtes Königreich) zu verwenden [73], das schnell und kostengünstig ist und eine rasche Probenbearbeitung ermöglicht. Entomologische Proben, die für die morphologische Identifizierung bestimmt sind, können anschließend gespült werden. Proben, die mit einem DNeasy Blood and Tissue Kit bearbeitet wurden, müssen mit Marc-André-Lösung aufgehellt werden, während diejenigen, die mit einem HotSHOT-DNA-Extraktionskit bearbeitet wurden, bereits ausreichend geklärt sind, um in einem wässrigen Medium oder – besser – nach der Dehydrierung in einem Harz gemäß dem in diesem Artikel beschriebenen Protokoll eingebettet zu werden [73]. Das extrahierte genetische Material kann dann für nachfolgende Analysen wie die PCR weiterverarbeitet werden, um spezifische genetische Marker zu amplifizieren. Zerstörungsfreie Nukleinsäure-Extraktionsmethoden sind entscheidend für die Untersuchung der genetischen Eigenschaften von Sandmücken, einschließlich der Identifizierung potenzieller Krankheitserreger, die sie tragen könnten. Durch die Bewahrung der Integrität der Probe können Forscher wertvolle genetische Informationen gewinnen und gleichzeitig das Exemplar für zusätzliche Analysen oder Studien behalten.

6.5. MALDI-ToF MS

Die MALDI-ToF MS (Matrix-unterstützte Laser-Desorption/Ionisation-Flugzeit-Massenspektrometrie) ist eine auf Massenspektrometrie basierende Technik zur Erkennung und Analyse einzigartiger Proteinprofile („Fingerabdrücke“) biologischer Proben. In der Identifizierung medizinisch und veterinärmedizinisch bedeutsamer Arthropoden wird MALDI-ToF zunehmend als wichtiges Werkzeug anerkannt. Diese Technik hat sich bei der Identifizierung verschiedener Entwicklungsstadien von Sandmücken, einschließlich unreifer Formen und der Blutmahlzeiten vollgesogener Weibchen, als wirksam erwiesen. Sie wurde erfolgreich eingesetzt, um sowohl männliche als auch weibliche Sandmückenarten unter verschiedenen Lagerungs- und Homogenisierungsbedingungen zu differenzieren [28, 30, 73, 74]. Die Methode bietet zudem eine hohe Unterscheidungskraft auf der Ebene von Subgenera, Arten und Populationen. Forscher können so eine schnelle und genaue Artenbestimmung erreichen, was für das Verständnis der Verbreitung und des Verhaltens von Sandmücken sowie deren Rolle bei der Krankheitsübertragung unerlässlich ist. Durch die Differenzierung von Arten basierend auf Proteinprofilen spielt MALDI-ToF eine entscheidende Rolle in epidemiologischen Studien und Strategien zur Vektorkontrolle. Aktuell begrenzen zwei Hauptnachteile die routinemäßige Anwendung dieser Technik. Erstens ist die Verfügbarkeit von Massenspektrometern ein Problem, da diese zu teuer sind, um sie allein für die Artenbestimmung von Sandmücken oder allgemein von Arthropoden-Vektoren anzuschaffen. Glücklicherweise kann diese Einschränkung überwunden werden, indem Messzeiten an Massenspektrometern genutzt werden, die in Proteomik-Einrichtungen oder der klinischen Diagnostik zum Standard-Forschungswerkzeug geworden sind. Zweitens gibt es eine geringe Repräsentation von

Sandmücken-Referenzdaten in frei zugänglichen Datenbanken. Dies führt dazu, dass hauseigene Datenbanken mit Referenzspektren erstellt werden müssen, die auf eindeutig identifizierten Exemplaren basieren – idealerweise durch eine Kombination aus morphologischer Bewertung und Sequenzierung geeigneter genetischer Marker (COI, CytB oder andere). Es ist zu hoffen, dass diese Einschränkung bald durch die schrittweise Aufnahme bisher interner Referenzdaten in die MSI-Plattform der Assistance Publique-Hôpitaux de Paris (Sorbonne University, Frankreich) und der BCCM/IHEM/Sciensano-Sammlung in Brüssel (Belgien) behoben wird (https://msi.happy-dev.fr/). Wenn der Einsatz von MALDI-ToF-Proteinprofiling geplant ist, sollten die Proben vorzugsweise trocken-gefroren oder in 70%igem Ethanol gelagert und nicht Umgebungstemperaturen ausgesetzt werden. Mangels universeller Richtlinien für die Probenvorbereitung wird Anwendern empfohlen, für die Herstellung der MALDI-ToF-Matrix eine wässrige Lösung aus 60 % Acetonitril / 0,3 % TFA mit Sinapinsäure (30 mg/mL) zu verwenden, um ihre Proteinspektren mit den bisher veröffentlichten Daten zu Sandmücken vergleichbar zu machen.

Probenvorbereitung für MALDI-ToF MS (Abbildung 7)

Insektenproben, die unter verschiedenen Bedingungen gelagert wurden, werden zunächst bei Raumtemperatur an der Luft getrocknet und anschließend seziert. Kopf und Abdomen werden entfernt, um jene Körperteile zu erhalten, die die wichtigsten morphologischen Merkmale für die Montage auf Objektträgern und die morphologische Analyse enthalten. Der Thorax kann für MALDI-ToF verwendet werden, während das verbleibende Abdomen für die DNA-Extraktion aufbewahrt wird. Für das Protein-Profiling wird der Thorax manuell in 1,5-ml-Mikroröhrchen mit 10 µL Homogenisierungslösung unter Verwendung von Einweg-Pistillen und Pellets homogenisiert. Typischerweise werden zwei Homogenisierungslösungen verwendet: steriles destilliertes Wasser und 25%ige Ameisensäure.

7. Conclusion

In dieser Arbeit war es unser Ziel, Forschern die effektivsten Methoden für die Einbettung von Sandmücken zur Verfügung zu stellen, die auf spezifische Forschungsziele zugeschnitten sind, um eine genaue Identifizierung und den Nachweis von Krankheitserregern zu erleichtern. Es gibt keine einzelne, universell optimale Methode; vielmehr existieren mehrere Methoden, von denen jede ihre eigenen Vorteile und Einschränkungen hat.

In den ergänzenden Daten haben wir detaillierte Protokolle für verschiedene Einbettungstechniken bereitgestellt, die bei der Vorbereitung und Identifizierung von Sandmücken verwendet werden. Diese Protokolle, einschließlich Lehrvideos, bieten Schritt-für-Schritt-Verfahren, die auf unterschiedliche Zielsetzungen zugeschnitten sind und präzise sowie zuverlässige Ergebnisse gewährleisten. Durch die Bereitstellung dieser umfassenden Ressource möchten wir Forscher dabei unterstützen, die am besten geeigneten Einbettungstechniken für ihre spezifischen Bedürfnisse auszuwählen und anzuwenden.

Danksagung

Die Autorinnen danken Richard Lane und Zoe Jay Adams vom Natural History Museum in London, Großbritannien, für ihre exzellente Begutachtung, welche die Qualität dieses Manuskripts gesteigert hat.

Finanzierung

Wir danken den brasilianischen Förderagenturen CNPq (Aktenzeichen: 404395/2024-4) und der Araucária-Stiftung (Aktenzeichen: 433/2025 PDI) für die Finanzierung der Forschung von AJA.

Interessenkonflikte

Jérôme Depaquit ist Mitherausgeber von *Parasite*; er hatte keinen Einfluss auf den Begutachtungsprozess und die Entscheidungsfindung zu diesem Manuskript. Die anderen Autor:innen erklären, dass keine Interessenkonflikte bestehen.

Datenverfügbarkeits-erklärung

Vìdeos auf Zenodo
Video 1: https://zenodo.org/records/18198006
Video 2: https://zenodo.org/records/18311158
Video 3: https://zenodo.org/records/18311106
Video 4: https://zenodo.org/records/18311154
Video 5: https://zenodo.org/records/18303014
Video 6: https://zenodo.org/records/18302850
Video 7: https://zenodo.org/records/18315029

Zusätzliche Materialien

Weitere Informationen zu diesem Artikel sind unter der folgenden Adresse verfügbar: https://www.parasite-journal.org/10.1051/parasite/2026009/olm

Referenzen

1. Alkan C, Allal-Ikhlef AB, Alwassouf S, Baklouti A, Piorkowski G, de Lamballerie X, Izri A, Charrel RN. 2015. Virus isolation, genetic characterization and seroprevalence of Toscana virus in Algeria. Clinical Microbiology and Infection, 21(11), 1040 e1-9.
2. Alten B, Ozbel Y, Ergunay K, Kasap OE, Cull B, Antoniou M, Velo E, Prudhomme J, Molina R, Banuls AL, Schaffner F, Hendrickx G, Van Bortel W, Medlock JM. 2015. Sampling strategies for phlebotomine sand flies (Diptera: Psychodidae) in Europe. Bulletin of Entomological Research 105(6), 664–678.
3. Ayhan N, Baklouti A, Prudhomme J, Walder G, Amaro F, Alten B, Moutailler S, Ergunay K, Charrel RN, Huemer H. 2017. Practical guidelines for studies on sandfly-borne phleboviruses: Part I: Important points to consider *ante* field work. Vector-Borne and Zoonotic Diseases 17(1), 73–80.

4. Bates PA. 1997. Infection of phlebotomine sandflies with *Leishmania*, in The Molecular Biology of Insect Disease Vectors: A Methods Manual. Springer. p. 112–120.5.
5. Baum M, de Castro EA, Pinto MC, Goulart TM, Baura W, Klisiowicz Ddo R, Vieira da Costa-Ribeiro MC. 2015. Molecular detection of the blood meal source of sand flies (Diptera: Psychodidae) in a transmission area of American cutaneous leishmaniasis, Parana State, Brazil. Acta Tropica, 143, 8–12.
6. Belen A, Alten B, Aytekin A. 2004. Altitudinal variation in morphometric and molecular characteristics of *Phlebotomus papatasi* populations. Medical and Veterinary Entomology, 18(4), 343–350.
7. Bhattacharya J, Chandra G, Hati AK. 1991. A simple method for cryopreservation of *Leishmania donovani* promastigotes, Indian Journal of Medical Research, 93, 245–246.
8. Caligiuri LG, Sandoval AE, Miranda JC, Pessoa FA, Santini MS, Salomón OD, Secundino NF, McCarthy CB. 2019. Optimization of DNA extraction from individual sand flies for PCR amplification. Methods and Protocols, 2(2), 36.
9. Casaril AE, de Oliveira LP, Alonso DP, de Oliveira EF, Gomes Barrios SP, de Oliveira Moura Infran J, Fernandes WS, Oshiro ET, Ferreira AMT, Ribolla PEM, de Oliveira AG. 2017. Standardization of DNA extraction from sand flies: Application to genotyping by next generation sequencing. Experimental Parasitology, 177, 66–72.
10. Castalanelli MA, Severtson DL, Brumley CJ, Szito A, Foottit RG, Grimm M, Munyard K, Groth DM. 2010. A rapid non-destructive DNA extraction method for insects and other arthropods. Journal of Asia-Pacific Entomology, 13(3), 243–248.
11. Cerqueira NL. 1943. Um novo meio para montagem de pequenos insetos em lâmina. Memórias do Instituto Oswaldo Cruz, (39), 37–41.
12. Charrel RN, Gallian P, Navarro-Mari JM, Nicoletti L, Papa A, Sanchez-Seco MP, Tenorio A, de Lamballerie X. 2005. Emergence of Toscana virus in Europe. Emerging Infectious Diseases, 11(11), 1657–1663.
13. Chaskopoulou A, Giantsis IA, Demir S, Bon MC. 2016. Species composition, activity patterns and blood meal analysis of sand fly populations (Diptera: Psychodidae) in the metropolitan region of Thessaloniki, an endemic focus of canine leishmaniasis. Acta Tropica, 158, 170–176.
14. Chen H, Rangasamy M, Tan SY, Wang H, Siegfried BD. 2010. Evaluation of five methods for total DNA extraction from western corn rootworm beetles. PLoS One, 5(8), e11963.
15. Depaquit J, Grandadam M, Fouque F, Andry PE, Peyrefitte C. 2010. Arthropod-borne viruses transmitted by Phlebotomine sandflies in Europe: a review. Eurosurveillance, 15(10), 19507.
16. Diamond LS, Herman CM. 1954. Incidence of Trypanosomes in the Canada Goose as revealed by bone marrow culture. Journal of Parasitology, 40(2), 195–202.
17. Ding H, Torno M, Vongphayloth K, Ng G, Tan D, Sng W, Ho K, Randrianambinintsoa FJ, Depaquit J, Tan CH. 2025. Hidden in plain sight: discovery of sand flies in Singapore and description of four species new to science. Parasites & Vectors, 18(1), 402.
18. Es-Sette N, Ajaoud M, Bichaud L, Hamdi S, Mellouki F, Charrel RN, Lemrani M. 2014. *Phlebotomus sergenti* a common vector of *Leishmania tropica* and Toscana virus in Morocco. Journal of Vector Borne Diseases, 51(2), 86–90.
19. Favret C. 2005. A new non-destructive DNA extraction and specimen clearing technique for aphids (Hemiptera). Proceedings of the Entomological Society of Washington, 107(2), 469–470.
20. Galati EAB. 2018. Phlebotominae (Diptera, Psychodidae): Classification, morphology and terminology of adults and identification of American taxa, in Brazilian Sand Flies: Biology, Taxonomy, Medical Importance and Control, Rangel EF, Shaw JJ, Editors. Cham: Springer International Publishing. pp. 9–212.
21. Galati EAB, de Andrade AJ, Perveen F, Loyer M, Vongphayloth K, Randrianambinintsoa FJ, Prudhomme J, Rahola N, Akhoundi M, Shimabukuro PHF, Depaquit J. 2025. Phle-botomine sand flies (Diptera, Psychodidae) of the world. Parasites & Vectors, 18(1), 220.
22. Galati EAB, Galvis-Ovallos F, Lawyer P, Leger N, Depaquit J. 2017. An illustrated guide for characters and terminology used in descriptions of Phlebotominae (Diptera, Psychodidae). Par-asite, 24, 26.
23. Gariepy T, Kuhlmann U, Gillott C, Erlandson M. 2007. Parasitoids, predators and PCR: the use of diagnostic molecular markers in biological control of Arthropods. Journal of Applied Entomology, 131(4), 225–240.
24. Giantsis IA, Chaskopoulou A, Bon MC. 2016. Mild-Vectolysis: A nondestructive DNA extraction method for vouchering sand flies and mosquitoes. Journal of Medical Entomology, 53(3), 692–695.
25. Gidwani K, Picado A, Rijal S, Singh SP, Roy L, Volfova V, Andersen EW, Uranw S, Ostyn B, Sudarshan M, Chakravarty J, Volf P, Sundar S, Boelaert M, Rogers ME. 2011. Serological markers of sand fly exposure to evaluate insecticidal nets against visceral leishmaniasis in India and Nepal: a cluster-randomized trial. PLoS Neglected Tropical Diseases, 5(9), e1296.
26. Gilbert MTP, Moore W, Melchior L, Worobey M. 2007. DNA extraction from dry museum beetles without conferring external morphological damage. PLoS One, 2(3), e272.
27. Giordani BF, Andrade AJ, Galati EAB, Gurgel-Goncalves R. 2017. The role of wing geometric morphometrics in the identification of sandflies within the subgenus *Lutzomyia.* Medical and Veterinary Entomology, 31(4), 373–380.
28. Guzmán-Larralde AJ, Suaste-Dzul AP, Gallou A, Peña-Carrillo KI. 2017. DNA recovery from microhymenoptera using six non-destructive methodologies with considerations for subsequent preparation of museum slides. Genome, 60(1), 85–91.
29. Hajibabaei M, DeWaard JR, Ivanova NV, Ratnasingham S, Dooh RT, Kirk SL, Mackie PM, Hebert PD. 2005. Critical factors for assembling a high volume of DNA barcodes. Philosophical Transactions of the Royal Society B: Biological Sciences, 360(1462), 1959–1967.
30. Haouas N, Pesson B, Boudabous R, Dedet JP, Babba H, Ravel C. 2007. Development of a molecular tool for the identification of *Leishmania* reservoir hosts by blood meal analysis in the insect vectors. American Journal of Tropical Medicine and Hygiene, 77(6), 1054–1059.
31. Hlavackova K, Dvorak V, Chaskopoulou A, Volf P, Halada P. 2019. A novel MALDI-TOF MS-based method for blood meal identification in insect vectors: A proof of concept study on phlebotomine sand flies. PLoS Neglected Tropical Diseases, 13 (9), e0007669.
32. Huemer H, Prudhomme J, Amaro F, Baklouti A, Walder G, Alten B, Moutailler S, Ergunay K, Charrel RN, Ayhan N. 2017. Practical guidelines for studies on sandfly-borne phleboviruses: Part II: Important points to consider for fieldwork and subsequent virological screening. Vector-Borne and Zoonotic Diseases, 17(1), 81–90.
33. Jancarova M, Polanska N, Thiesson A, Arnaud F, Stejskalova M, Rehbergerova M, Kohl A, Viginier B, Volf P, Ratinier M. 2025.

Susceptibility of diverse sand fly species to Toscana virus. PLoS Neglected Tropical Diseases, 19(5), e0013031.
34. Kapp JD, Green RE, Shapiro B. 2021. A fast and efficient single-stranded genomic library preparation method optimized for ancient DNA. Journal of Heredity, 112(3), 241–249.
35. Killick-Kendrick R, Maroli M, Killick-Kendrick M. 1991. Bibliography of the colonization of phlebotomine sandflies. Parassitologia, 33(suppl.), 321–333.
36. Lawyer P, Killick-Kendrick M, Rowland T, Rowton E, Volf P. 2017. Laboratory colonization and mass rearing of phlebotomine sand flies (Diptera, Psychodidae). Parasite, 24, 42.
37. Léger N, Pesson B, Madulo-Leblond G. 1986. Les phlébotomes de Grèce : 1ère partie. Bulletin de la Société de Pathologie Exotique, 79, 386–397.
38. Léger N, Pesson B, Madulo-Leblond G. 1986. Les phlébotomes de Grèce : 2ème partie. Bulletin de la Société de Pathologie Exotique, 79, 514–524.
39. Leonel JAF, Vioti G, Alves ML, da Silva DT, Meneghesso PA, Benassi JC, Spada JCP, Galvis-Ovallos F, Soares RM, Oliveira T. 2020. DNA extraction from individual Phlebotomine sand flies (Diptera: Psychodidae: Phlebotominae) specimens: Which is the method with better results? Experimental Parasitology, 218, 107981.
40. Lestinova T, Rohousova I, Sima M, de Oliveira CI, Volf P. 2017. Insights into the sand fly saliva: Blood-feeding and immune interactions between sand flies, hosts, and *Leishmania.* PLoS Neglected Tropical Diseases, 11(7), e0005600.
41. Lienhard A, Schaffer S. 2019. Extracting the invisible: obtaining high quality DNA is a challenging task in small arthropods. PeerJ, 7, e6753.
42. Lozano-Sardaneta YN, Mikery-Pacheco OF, Huerta H, Rojas-Soriano JE, Contreras-Ramos A. 2025. Wing geometric morphometrics is effective to separate sand fly species (Diptera, Psychodidae, Phlebotominae) related with leishmaniasis transmission in Mexico. Acta Tropica, 262, 107523.
43. Mandrioli M. 2008. Insect collections and DNA analyses: how to manage collections? Museum Management and Curatorship, 23(2), 193–199.
44. Maroli M, Feliciangeli MD, Bichaud L, Charrel RN, Gradoni L. 2013. Phlebotomine sandflies and the spreading of leishmaniases and other diseases of public health concern. Medical and Veterinary Entomology, 27(2), 123–147.
45. Marquina D, Buczek M, Ronquist F, Lukasik P. 2021. The effect of ethanol concentration on the morphological and molecular preservation of insects for biodiversity studies. PeerJ, 9, e10799.
46. Mathis A, Depaquit J, Dvorak V, Tuten H, Banuls AL, Halada P, Zapata S, Lehrter V, Hlavackova K, Prudhomme J, Volf P, Sereno D, Kaufmann C, Pfluger V, Schaffner F. 2015. Identification of phlebotomine sand flies using one MALDI-TOF MS reference database and two mass pectrometer systems., Parasites & Vectors, 8, 266.
47. Mekarnia N, Benallal KE, Sadlova J, Vojtkova B, Mauras A, Imbert N, Longhitano M, Harrat Z, Volf P, Loiseau PM, Cojean S. 2024. Effect of *Phlebotomus papatasi* on the fitness, infectivity and antimony-resistance phenotype of antimony-resistant *Leishmania* major Mon-25. International Journal for Parasitology – Drugs and Drug Resistance, 25, 100554.
48. Milligan BG. 1998. Total DNA isolation, in Molecular Genetic Analysis of Population: A Practical Approach, Hoelzel AR, Editor. Oxford: Oxford University Press.
49. Molina R, Jiménez M, Alvar J, González E, Hernández-Taberna S, Ines MM. 2017. Methods in sand fly research. Madrid: Servicio de publicaciones Universidad de Alcalá de Henares, Madrid.
50. Murphy WJ, Eizirik E, O'Brien SJ, Madsen O, Scally M, Douady CJ, Teeling E, Ryder OA, Stanhope MJ, de Jong WW, Springer MS. 2001. Resolution of the early placental mammal radiation using Bayesian phylogenetics. Science, 294(5550), 2348–2351.
51. Nacif-Pimenta R, Pinto LC, Volfova V, Volf P, Pimenta PFP, Secundino NFC. 2020. Conserved and distinct morphological aspects of the salivary glands of sand fly vectors of leishma-niasis: an anatomical and ultrastructural study. Parasites & Vectors, 13(1), 441.
52. Neuhaus B, Schmid T, Riedel J. 2017. Collection management and study of microscope slides: Storage, profiling, deterioration, restoration procedures, and general recommendations. Zootaxa, 4322(1), 1–173.
53. New TR. 1974. Pscoptera. Handbooks for Identification of British Insects (Vol. I). London: Royal Entomological Society of London. 102 pp.
54. Perez-Ruiz M, Collao X, Navarro-Mari JM, Tenorio A. 2007. Reverse transcription, real-time PCR assay for detection of Toscana virus. Journal of Clinical Virology, 39(4), 276–281.
55. Porco D, Rougerie R, Deharveng L, Hebert P. 2010. Coupling non-destructive DNA extraction and voucher retrieval for small soft-bodied Arthropods in a high-throughput context: the example of Collembola. Molecular Ecology Resources, 10(6), 942–945.
56. Prudhomme J, Cassan C, Hide M, Toty C, Rahola N, Vergnes B, Dujardin JP, Alten B, Sereno D, Banuls AL. 2016. Ecology and morphological variations in wings of *Phlebotomus ariasi* (Diptera: Psychodidae) in the region of Roquedur (Gard, France): a geometric morphometrics approach. Parasites & Vectors, 9(1), 578.
57. Prudhomme J, Gunay F, Rahola N, Ouanaimi F, Guernaoui S, Boumezzough A, Banuls AL, Sereno D, Alten B. 2012. Wing size and shape variation of *Phlebotomus papatasi* (Diptera: Psychodidae) populations from the south and north slopes of the Atlas Mountains in Morocco. Journal of Vector Ecology, 37(1), 137–147.
58. Prudhomme J, Toty C, Kasap OE, Rahola N, Vergnes B, Maia C, Campino L, Antoniou M, Jimenez M, Molina R, Cannet A, Alten B, Sereno D, Banuls AL. 2015. New microsatellite markers for multi-scale genetic studies on *Phlebotomus ariasi* Tonnoir, vector of *Leishmania infantum* in the Mediterranean area. Acta Tropica, 142, 79–85.
59. Prudhomme J, Velo E, Bino S, Kadriaj P, Mersini K, Gunay F, Alten B. 2019. Altitudinal variations in wing morphology of *Aedes albopictus* (Diptera, Culicidae) in Albania, the region where it was first recorded in Europe. Parasite, 26, 55.
60. Rawlins DJ. 1992. Light Microscopy: An Introduction to Biotechniques. Oxford: Bios Scientific publishers. 143 pp.
61. Ready PD. 2013. Biology of phlebotomine sand flies as vectors of disease agents. Annual Review of Entomology, 58, 227–250.
62. Rohlf FJ, Slice D. 1990. Extensions of the Procrustes method for the optimal superimposition of landmarks. Systematic Zoology, 39(1), 40–59.
63. Rowley DL, Coddington JA, Gates MW, Norrbom AL, Ochoa RA, Vandenberg NJ, Greenstone MH. 2007. Vouchering DNA-barcoded specimens: Test of a nondestructive extraction proto-col for terrestrial arthropods. Molecular Ecology Notes, 7(6), 915–924.
64. Sábio PB, Andrade AJ, Galati EAB. 2014. Assessment of the taxonomic status of some species included in the *Shannoni*

complex, with the description of a new species of *Psathyromyia* (Diptera: Psychodidae: Phlebotominae). Journal of Medical Entomology, 51(2), 331–341.

65. Sadlova J, Yeo M, Seblova V, Lewis MD, Mauricio I, Volf P, Miles MA. 2011. Visualisation of *Leishmania donovani* fluorescent hybrids during early stage development in the sand fly vector. PLoS One, 6(5), e19851.
66. Sales K, Miranda DEO, da Silva FJ, Otranto D, Figueredo LA, Dantas-Torres F. 2020. Evaluation of different storage times and preservation methods on phlebotomine sand fly DNA concentration and purity. Parasites & Vectors, 13(1), 399
67. Sales KG, Costa PL, de Morais RC, Otranto D, Brandao-Filho SP, Cavalcanti Mde P, Dantas-Torres F. 2015. Identification of phlebotomine sand fly blood meals by real-time PCR. Parasites& Vectors, 8, 230.
68. Sant'Anna MR, Jones NG, Hindley JA, Mendes-Sousa AF, Dillon RJ, Cavalcante RR, Alexander B, Bates PA. 2008. Blood meal identification and parasite detection in laboratory-fed and field-captured *Lutzomyia longipalpis* by PCR using FTA databasing paper. Acta Tropica, 107(3), 230–237.
69. Senne NA, Santos HA, Araujo TR, Paulino PG, Mendonca LP, Moreira HVS, Camilo TA, da Costa Angelo I. 2022. Robust comparative performance of genomic DNA extraction methods from non-engorged phlebotomine sandflies. Medical and Veterinary Entomology, 36(2), 203–211.
70. Shaw JJ. 2025. A review of Leishmania infections in American Phlebotomine sand flies – Are those that transmit leishmaniasis anthropophilic or anthropportunists? Parasite, 32, 57.
71. Tesh RB, Modi GB. 1983. Growth and transovarial transmission of Chandipura virus (Rhabdoviridae: Vesiculovirus) in *Phlebotomus papatasi.* American Journal of Tropical Medicine and Hygiene, 32(3), 621–623.
72. Thomsen PF, Elias S, Gilbert MTP, Haile J, Munch K, Kuzmina S, Froese DG, Sher A, Holdaway RN, Willerslev E. 2009. Non-destructive sampling of ancient insect DNA. PLoS One, 4(4),e5048
73. Truett GE, Heeger P, Mynatt RL, Truett AA, Walker JA, Warman ML. 2000. Preparation of PCR-quality mouse genomic DNA with hot sodium hydroxide and tris (HotSHOT). Biotechniques, 29(1), 52–54.
74. Upton MS. 1993. Aqueous gum-chloral slide mounting media: an historical review. Bulletin of Entomological Research, 83(2), 267–274.
75. Volf P, Myskova J. 2007. Sand flies and Leishmania: specific versus permissive vectors. Trends in Parasitology, 23(3), 91–92.
76. Wang Q, Wang X. 2012. Comparison of methods for DNA extraction from a single chironomid for PCR analysis. Pakistan Journal of Zoology, 44(2), 421–426.
77. Wang Y, Zhao Y, Bollas A, Wang Y, Au KF. 2021. Nanopore sequencing technology, bioinformatics and applications. Nature Biotechnology, 39(11), 1348–1365.

Cite this article as: Randrianambinintsoa FJ, Augendre L, Prudhomme J, Martinet J-P, Loyer M, Mekarnia N, Kerkoub H, Perveen FK, Huguenin A, Kariya E, Akhoundi M, De Andrade AJ, Berriatua E, Bongiorno G, Boyer S, Christodoulou V, Da Costa-Ribeiro MCV, De Souza LAF, Ding H, Dondji B, Dvořák V, Erisoz Kasap O, Galati EAB, Gállego M, Ballart C, Gouzelou S, Haddad N, Masse RS, Mekuria AH, Ivovic V, Kaczmarek S, Shahar MK, Kirstein OD, Kniha E, Kolářová I, Lincoln T, Lucanas C, Mikov O, Nov K, Özbel Y, Pesson B, Posada Lopez LC, Prasetyo DB, Rahola N, Rebollar-Tellez EA, Rodrigues BL, Roy L, Saini P, Sanjoba C, Shimabukuro PH, Siriyasatien P, Soszynska A, Suleşco T, Sylla M, Torno M, Volf P, Vongphayloth K, Sinh Nam V, Wardhana A, Yessinou E, Zapata S, Gantier J-C & Depaquit J. 2026. Processing and mounting phlebotomine sand flies: a consensus guideline. Parasite xx, xx. https://doi.org/10.1051/parasite/2026009.

PARASITE

An international open-access, peer-reviewed, online journal publishing high quality papers on all aspects of human and animal parasitology

Reviews, articles and short notes may be submitted. Fields include, but are not limited to: general, medical and veterinary parasitology; morphology, including ultrastructure; parasite systematics, including entomology, acarology, helminthology and protistology, and molecular analyses; molecular biology and biochemistry; immunology of parasitic diseases; host-parasite relationships; ecology and life history of parasites; epidemiology; therapeutics; new diagnostic tools.
All papers in Parasite are published in English. Manuscripts should have a broad interest and must not have been published or submitted elsewhere. No limit is imposed on the length of manuscripts.

Parasite (open-access) continues **Parasite** (print and online editions, 1994-2012) and **Annales de Parasitologie Humaine et Comparée** (1923-1993) and is the official journal of the Société Française de Parasitologie.

Editor-in-Chief:
Jean-Lou Justine, Paris

Submit your manuscript at:
https://www.editorialmanager.com/parasite

Anhang 1: Biochemische theoretische Grundlagen.

Die Arthropoden der Wahl sind hier Sandmücken. Die allgemeine Idee lässt sich jedoch auf andere sehr häufige Arthropoden übertragen, deren Identifizierung ausschließlich anhand interner morphologischer Merkmale erfolgen kann. Glücklicherweise sind einige innere Organe teilweise chitinös, und morphologische Merkmale liefern wertvolle Informationen. Aus diesem Grund ist es sehr interessant, die Pharynxpumpen, Spermatheken und deren Gänge zu beobachten. Bei allen Reagenzien, die wir besprechen werden, sollte niemals vergessen werden, dass wir vom Stadium der Fixierung des Insekts bis zur Einbettung schlichtweg Redoxreaktionen anwenden. Die einzige Vorsichtsmaßnahme oder Leitidee, die uns führen wird, besteht darin, das Mischen von Reduktionsmitteln mit Oxidationsmitteln zu vermeiden.

Ethylalkohol; Ethanol:

Diese Substanz wird auf verschiedene Weise verwendet. Alkoholmoleküle haben eine starke Affinität zu Wasser und weisen daher eine dehydrierende Wirkung auf. Ein Alkohol mit niedriger Konzentration (d. h. mit zu hohem Wasseranteil) spielt jedoch eine Rolle beim Abbau von Nukleinsäuren (Wasser ist der Feind der Nukleinsäuren).

Wenn Insekten in Ethanol gelegt werden, geschieht dies nicht nur, um sie zu konservieren, sondern auch, um das Gewebe zu fixieren. In der Histologie unterscheiden wir üblicherweise zwei wichtige Konzepte: die Penetrationsrate und die Fixierungsrate. Es versteht sich von selbst, dass ein gutes Konservierungsmittel zunächst schnell tief in das Gewebe eindringen muss, bevor es dieses fixiert. Für 96%igen Alkohol beträgt der Penetrationskoeffizient etwa 1,05 (zum Vergleich: bei einer 0,75%igen wässrigen Pikrinsäurelösung liegt der Penetrationskoeffizient bei 0,45, während der einer 3%igen Kaliumdichromatlösung 1,45 beträgt).

Insekten und andere Arthropoden unbegrenzt in Ethanol aufbewahren zu wollen, ist für Entomologen Realität. Der Gedanke, Freilandfänge für spätere Studien oder für künftige Forscher aufheben zu wollen, ist immer noch sehr ehrenhaft. Dennoch ist dies für einen Zytologen oder Histologen nicht möglich. Wenn man darauf abzielt, die Proben zu lange im Fixiermittel zu belassen, können sie praktisch unmöglich nachzubearbeiten sein. Aus diesem Grund sind Proben, die älter als 10 Jahre sind, schwierig oder sogar unmöglich zu verwenden.

Ein weiterer Aspekt ist das Verhältnis zwischen der zu fixierenden Arthropodenmasse und dem Volumen des Fixiermittels. In der zoologischen oder medizinischen Praxis ist es ratsam, ein Volumen einzuplanen, das 60-mal größer ist als das Volumen der zu fixierenden Teile. In der Praxis sollte man bei Mikro-Arthropoden für ein gegebenes Volumen an zu fixierenden Exemplaren mindestens 4–5 Volumenteile Alkohol hinzufügen. Bedenken Sie, dass der Alkohol an Stärke verliert, da er das gesamte im Gewebe des Arthropoden vorhandene Wasser entzieht.

Zusammenfassend lässt sich sagen:
- Ethylalkohol ist ein reduzierendes chemisches Reagens (daher inkompatibel mit oxidativen Fixiermitteln);
- Er fällt Proteine energetisch aus und denaturiert sie;
- Er löst bestimmte komplexe Lipide und fällt Glykogen aus;
- Er verursacht eine starke Kontraktion des Gewebes und härtet es aus.

Basische Kalium- oder Natriumhydroxidlösungen

Die Verwendung dieser Lösungen in der Entomologie hat sich hauptsächlich auf Kaliumhydroxid konzentriert, ohne dass dafür eine eindeutige Begründung vorlag.

Natriumhydroxid [E524] kommt in Lösung vor, entweder in verschiedenen Konzentrationen oder mit unterschiedlicher Normalität. Es ist in Form von Pellets oder Plätzchen erhältlich. Sein Hauptnachteil besteht darin, dass es sehr hygroskopisch ist (stärker als KOH). Wenn es mit Proteinen reagiert, löst es diese auf, und Fette wandelt es während der Verseifung in harte Seifen um (dies ist ein wesentlicher Unterschied zu KOH, das bei der Verseifung flüssige Seifen liefert).

Kaliumhydroxid [E525] ist als konzentrierte Lösung erhältlich, hat aber vor allem den Vorteil, dass es in Plätzchen von etwa 0,1 g formuliert ist, was die Herstellung verdünnter Lösungen erheblich erleichtert, wenn man keine Präzisionswaage zur Verfügung hat. Zum Beispiel ergibt 1 Plätzchen von 0,1 g in 1 ml destilliertem Wasser eine 10%ige Lösung. Der zweite Vorteil von Kaliumhydroxid in Plätzchenform ist seine geringere Empfindlichkeit gegenüber Karbonisierung (eine KOH-Lösung hat eine hohe Affinität zur Bindung von CO_2, wodurch Karbonatsalze entstehen).

Diese starken Basen werden verwendet, um Fettsäuren löslich zu machen, indem sie in wasserlösliche Seifen umgewandelt werden. Es sollte bedacht werden, dass das Fixiermittel, wie etwa Ethanol, bereits einen Teil der Fette in der Probe gelöst hat. Wenn man die Probe jedoch in einem wässrigen Medium durch eine starke Base ersetzt, fallen die (mehr oder weniger komplexen) Fettsäuren aus. Die starke Base führt daher eine Kaltverseifung durch. In einigen Fällen, wenn das Fettgewebe im Übermaß vorhanden ist – zum Beispiel bei Weibchen –, ist es von Vorteil, die Temperatur auf 35–40 °C zu erhöhen, um die Reaktion zu erleichtern, oder die Kontaktzeit bei Raumtemperatur zu verlängern.

Farbstoffhaltige saure Lösung / farblose Marc-André-Lösung

Hier werden wir die Vor- und Nachteile der Verwendung der Marc-André-Lösung untersuchen. Diese Lösung setzt sich aus Chloralhydrat (Trichloracetaldehyd-Monohydrat), Essigsäure und Wasser zusammen.

Diese Lösung ist stark oxidierend (Mischung aus Säure und Aldehyd). Sie neutralisiert den Überschuss an Kaliumhydroxid, der in den Proben verbleiben kann, ohne

die alkalischen Seifen auszufällen, die während der Verwendung von Kaliumhydroxid entstanden sind. Diese oxidierende Lösung wirkt zudem auf die sekundären Alkoholfunktionen der Glucosamine, die sich im Chitin bilden, indem sie diese oxidiert und so das Chitin erweicht. Eine weitere Wirkung ist die Auflösung bestimmter vorhandener Mineralsalze.

Wenn die Marc-André-Lösung zuvor mit Säurefuchsin gefärbt wurde (sich also im oxidierten Zustand befindet), kann sie an den sekundären Alkoholfunktionen der Struktur binden. Nach der Einwirkzeit der Marc-André-Lösung und dem Erreichen des gewünschten Färbezustands der Proben erfolgt das Spülen ausschließlich mit Ethanol. Damit leiten wir die Dehydrierungsphase der Proben ein.

Vorteile:
- Neutralisation überschüssiger basischer Lösungen
- Entspannung des Chitins
- Anfärbung des Chitins zur besseren Beurteilung chitinöser innerer Strukturen

Nachteile:

Chloralhydrat wirkt hypnotisch und wurde in der Humanmedizin eingesetzt. Es muss unter einem Abzug gearbeitet werden, und die gesetzlichen Bestimmungen zu chemischen Risiken müssen strikt eingehalten werden.

Dehydrierungslösungen

Die Erfahrung zeigt, dass es bei sehr kleinen Proben nicht notwendig ist, die Abfolge von Alkoholbädern mit steigenden Konzentrationen strikt einzuhalten. Bei großen Proben beginnt man mit 80%igem Ethanol, gefolgt von 90%igem, 95%igem und schließlich absolutem Ethanol. Bei sehr kleinen Proben genügt ein Bad in 90% Ethanol mit anschließender Immersion in absolutem Ethanol. In diesem Stadium sollte man stets bedenken, dass absoluter Ethanol bestrebt ist, Wasser aus der Umgebungsatmosphäre zu binden.

In entomologischen Laboren war es Tradition, die Dehydrierung der Proben mit einem Bad in Buchenholzteerkreosot abzuschließen. Heute wird von dieser Essenz, die weit verbreitet als Pestizid, Fungizid und Holzschutzmittel eingesetzt wurde, aufgrund ihres Geruchs (polyzyklische aromatische Kohlenwasserstoffe) dringend abgeraten. Es wird davon ausgegangen, dass sie reproduktionstoxisch, krebserregend, ein persistenter organischer Schadstoff und ökotoxisch für Wasserorganismen ist.

Eine Lösung, die wir für die Einbettung der Proben vorschlagen, ist Euparal® und Euparal-Essenz (beschrieben im folgenden Abschnitt). Eine Mischung aus Euparal® und Euparal-Essenz wird sehr gut angenommen; die Proben können direkt nach einem Bad in 90% Ethanol darin überführt werden%.

Anhang 2: Zusammensetzung der verwendeten Reagenzien.

Kaliumhydroxid 10%
Kaliumhydroxid: 10 g
Destilliertes Wasser: q. s. 100 mL

Chloralhydrat-Gummi-Einbettungsmedium (Hoyer-Medium)
Destilliertes Wasser: 50 mL
Chloralhydrat: 200 g
Gummi arabicum: 50 g
Glycerin: 20 mL

Marc-André-Lösung
Chloralhydrat: 40 g
Eisessig (konzentrierte Essigsäure): 30 mL
Destilliertes Wasser: 30 mL

Säurefuchsin 1 % in destilliertem Wasser
Säurefuchsin-Pulver: 1 g
Destilliertes Wasser: 99 mL

Mit Fuchsin gefärbte Marc-André-Lösung
Marc-André-Lösung: 10 mL
Fuchsin 1%: 50 µL

Anhang 3: Euparal®, Kanadabalsam, Polyvinylalkohol oder andere Einbettungsmedien.

Polyvinylalkohol (PVA): PVA ist das ideale Einbettungsmedium, wenn die für eine ordnungsgemäße Dehydrierung erforderlichen Produkte nicht verfügbar sind. In diesem Fall wird Polyvinylalkohol mit Ammans Lactophenol gemischt. Diese Präparate weisen jedoch erhebliche Nachteile auf: Entweder trocknen sie aus, der Polyvinylalkohol kristallisiert aufgrund der Wasserverdunstung oder das Präparat schwärzt sich durch die Oxidation des Phenols. Dies bleibt eine gute Technik für die kurzfristige Einbettung.

Kanadabalsam: Die Verwendung für die Einbettung zwischen Objektträger und Deckglas erfordert eine Dehydrierung der Proben. Die Verwendung von Xylol oder Toluol als Lösungsmittel ist dabei nicht ohne Unannehmlichkeiten (Gesundheitsrisiken).

Enecê-Medium: Für die Einbettung zwischen Objektträger und Deckglas ist, wie bei Kanadabalsam, eine Dehydrierung des Präparats erforderlich. Rezeptur von Enecê: reines weißes Kolophonium (22 g), alkohollösliches Kopalharz (12 g), absoluter Alkohol (20 mL), Campher (10 g), Terpentinöl (10 mL) und Eucalyptol (26 mL). Herstellung: Geben Sie den absoluten Alkohol und den Campher in ein Gefäß (z. B. einen Erlenmeyerkolben). Fügen Sie dann das Kolophonium und das Kopalharz hinzu. Der Kolben wird mit einem Stopfen verschlossen,

geschüttelt und erst dann im Wasserbad bei milder Temperatur erhitzt, damit die Mischung nicht siedet. Sobald der Inhalt vollständig verflüssigt ist, wird das Terpentinöl hinzugefügt, die Mischung noch heiß filtriert und dem Filtrat schließlich das Eucalyptol zugesetzt. Wenn das Medium an Fließfähigkeit verliert, wird es mit „Enecê-Essenz“ verdünnt, die folgende Formel hat: absoluter Alkohol (30 mL), Campher (17 g), Terpentinöl (15 mL), Eucalyptol (38 mL) (Cerqueira, 1943).

Euparal®: Dies ist ein Harz, das von der Atlas-Zypresse *Tetraclinis articulata* (Vahl, 1791) stammt und 1906 von Gilson untersucht und entwickelt wurde. Sein Hauptvorteil besteht darin, dass es nicht polymerisiert. Zwischen Objektträger und Deckglas eingebettete Proben können leicht durch die Einwirkung von Alkohol oder – noch besser – Euparal-Essenz wiedergewonnen werden. Dieses Harz, auch Sandarak genannt, akzeptiert Ethanol ab einer Konzentration von 80 %.

Verwendung von Triton X100: Nicht-ionische wässrige Lösung:

Triton X100 liegt als nicht-ionische wässrige Lösung vor (4-(1,1,3,3-Tetramethylbutyl)phenyl-polyethylenglycol-Lösung) und wird in der Zell- und Molekularbiologie häufig als Detergens verwendet. Es ermöglicht die Permeabilisierung von Zell- und Kernmembranen.

In Alkohol konservierte Insektenproben über viele Jahre hinweg sind alltäglich. Leider ist die Konservierung in Alkohol nicht optimal, und die so konservierten Arthropoden werden sehr schwierig für die mikroskopische Untersuchung vorzubereiten. Kunststoffgefäße, die Proben enthalten, bauen sich oft ab, gefolgt von der Verdunstung des Alkohols. In beiden Fällen stellen der langjährige Kontakt mit Alkohol oder das Austrocknen der Proben ein echtes Problem dar. Im Jahr 2008 veröffentlichte Jonque eine Notiz über die Rehydrierung von Spinnen mit einem Netzmittel wie Agepon (aus der Fotografie) [26]. Dies führte zu der Idee, Netzmittel zu verwenden, die keine starken Detergenzien sind.

Verfahren unter Verwendung von Triton X100 in 0,5%iger wässriger Lösung:

- Tränken Sie die trockene Probe mit absolutem Alkohol.
- Fügen Sie das erforderliche Volumen an 0,5%iger Triton X100-Lösung hinzu, sodass die gesamte Probe eingetaucht ist.
- Lassen Sie die Probe etwa 5 Minuten oder länger stehen. Alle Arthropoden müssen in der Lösung frei beweglich sein.
- Die Triton X100-Lösung wird entfernt und durch die Kaliumhydroxidlösung ersetzt. Danach wird das oben beschriebene Standardverfahren fortgesetzt.

Anhang 4: Schritt-für-Schritt-Anleitung für die Einbettung in Euparal® oder Kanadabalsam

1. Die Proben müssen vollständig dehydriert sein (ein trübes oder milchiges Aussehen deutet auf eine unzureichende Dehydrierung hin).
2. Die Dehydrierung erfolgt durch steigende Konzentrationen von Ethylalkohol.
3. Die Proben können von 99%igem oder absolutem Alkohol in ein Klärungsmittel überführt werden.

Vorgehensweise:

1. Die ausgewachsenen Sandmücken in 70%igem Ethanol einlegen.
2. Entfernen Sie das Ethanol und ersetzen Sie es durch 10%iges KOH. Decken Sie die Sandmücken mit einem Objektträger ab.
3. Mazerieren Sie die Insekten, bis sie transparent werden.
4. Entfernen Sie das KOH.
5. Bedecken Sie die Probe mit destilliertem Wasser und warten Sie 30 bis 45 Minuten.
6. Entfernen Sie das Wasser und wiederholen Sie das Waschen mit destilliertem Wasser für 30 Minuten. (Die Zeit ist abhängig von der Probenmenge: Je mehr Proben zusammen verarbeitet werden, desto länger muss diese Zeit eingehalten werden. Bei wenigen oder einzeln behandelten Proben kann sie verkürzt werden.)
7. Entfernen Sie das Wasser.
8. Geben Sie Marc-André-Lösung hinzu (potenziell mit Säurefuchsin gefärbt) und warten Sie 24 Stunden (einen Tag).
9. Entfernen Sie die Marc-André-Lösung.
10. Bedecken Sie die Probe mit destilliertem Wasser und warten Sie 30 bis 45 Minuten.
11. Entfernen Sie das Wasser und wiederholen Sie das Waschen mit destilliertem Wasser für 30 Minuten.
12. Entfernen Sie das Wasser.
13. Geben Sie 70%igen Ethanol hinzu und sezieren Sie die Probe:
    a. Für Kopf und Abdomen: Ziehen Sie Kopf oder Abdomen vorsichtig vom Thorax ab.
    b. Für den Thorax: Entfernen Sie die Flügel, indem Sie den Thorax mit einer Pinzette fixieren und mit einer zweiten Pinzette an der Basis der Anhängsel ziehen. Je nach Interessenlage ist eine Sagittalsektion möglich, bei der der Thorax in eine linke und eine rechte Hälfte geteilt wird.
14. Dehydrieren Sie schrittweise durch eine Reihe wässriger Ethylalkohollösungen: 50% – 80% – 95% bis zum Erreichen von absolutem Ethanol.
15. Dehydrieren Sie die Proben abschließend durch zweimaliges Waschen in 100%igem Ethanol für jeweils 10 Minuten.
16. Entfernen Sie den Ethanol und bedecken Sie die Proben für 15 Minuten bei Raumtemperatur mit Nelkenöl.

17. Überführen Sie die Proben aus dem Nelkenöl in einen Tropfen Euparal® oder Kanadabalsam auf einem sauberen Objektträger.
18. Anordnung der Teile: Kopf, Thorax und Abdomen der Sandmücke können mit feinen Nadeln oder Pinzetten unter einem Seziermikroskop angeordnet werden. Der Kopf muss vom Körper getrennt werden, um ihn in ventrodorsaler Position einzubetten, d. h. das Occipitalforamen muss nach oben zeigen, damit das Cibarium direkt hindurch beobachtet werden kann. Die Präparation erfolgt direkt im Einbettungsmedium.
19. Lassen Sie die Probe ruhen, bis die Oberfläche klebrig wird.
20. Befeuchten Sie ein sauberes Deckglas mit absolutem Alkohol. Lassen Sie das Deckglas in einem Winkel auf den Kanadabalsam sinken.
21. Lagern Sie die Objektträger in einem dafür vorgesehenen Trockenkasten
