# Supplementary material for: Processing and mounting phlebotomine sand flies: a consensus guideline
Source: Parasite. 2026 Apr 3;33:18. doi: 10.1051/parasite/2026009 (PMC13047900; doi:10.1051/parasite/2026009)
Supplement: Supplementary file 12 — Greek translation / ελληνική μετάφραση [file parasite-33-18-s12.pdf]

Parasite **33**, 18 (2026)
© F.J. Randrianambinintsoa et al., published by EDP Sciences, 2026
https://doi.org/10.1051/parasite/2026009

PARASITE
Available online at:
www.parasite-journal.org

REVIEW ARTICLE OPEN ACCESS

Επεξεργασία και προετοιμασία μικροσκοπικών παρασκευασμάτων φλεβοτόμων: Οδηγός κοινής επιστημονικής αποδοχής

Fano José Randrianambinintsoa[1], Laure Augendre[1], Jorian Prudhomme[1], Jean-Philippe Martinet[1], Mathieu Loyer[1], Nalia Mekarnia[1], Hocine Kerkoub[1], Farzana Khan Perveen[1], Antoine Huguenin[1,2], Emilie Kariya[1,2], Mohammad Akhoundi[3], Andrey José de Andrade[4], Eduardo Berriatua[5], Gioia Bongiorno[6], Sébastien Boyer[7,8], Vasiliki Christodoulou[9], Magda Clara Vieira Da Costa-Ribeiro[10], Lucas Alexandre Farias de Souza[10], Huicong Ding[11], Blaise Dondji[12], Vít Dvořák[13], Ozge Erisoz Kasap[14], Eunice Aparecida Bianchi Galati[15], Montserrat Gállego[16], Cristina Ballart[16], Stavroula Gouzelou[17], Nabil Haddad[18], Rezki Sabrina Masse[19], Asrat Hailu Mekuria[20], Vladimir Ivovic[21], Szymon Kaczmarek[22], Mohd Khadri Shahar[19], Oscar D. Kirstein[23], Edwin Kniha[24], Iva Kolářová[13], Lincoln Timinao[25], Cristian Lucanas[26], Ognyan Mikov[27], Kimsear Nov[7], Yusuf Özbel[28], Bernard Pesson[29], Laura Cristina Posada Lopez[30], Didot Budi Prasetyo[1,7], Nil Rahola[31], Eduardo A. Rebollar-Tellez[32], Bruno Leite Rodrigues[15], Lalita Roy[33], Prasanta Saini[34], Chizu Sanjoba[35], Paloma Helena Fernandes Shimabukuro[36], Padet Siriyasatien[37], Agnieszka Soszyńska[22], Tatiana Suleşco[38], Massamba Sylla[39], Majhalia Torno[40], Petr Volf[13], Khamsing Vongphayloth[41], Vu Sinh Nam[42], April Wardhana[43], Eric Yessinou[44], Sonia Zapata[45], Jean-Charles Gantier[1], and Jérôme Depaquit[1,2,*]

[1] Faculté de Pharmacie, Université de Reims Champagne Ardenne, UR ESCAPE-USC ANSES PETARD, 51 rue Cognacq-Jay, 51096 Reims Cedex, France
[2] Pôle de Biologie territoriale, Laboratoire de Parasitologie-Mycologie, Centre Hospitalo-Universitaire, 51092 Reims, France
[3] Parasitology-Mycology Department, Avicenne Hospital, AP-HP, Bobigny, Sorbonne Paris Nord University, France; Unité des Virus Émergents (UVE: Aix-Marseille Univ, Università di Corsica, IRD 190, Inserm 1207, IRBA), 13005 Marseille, France
[4] Parasitology Collection of Basic Pathology, Department of Basic Pathology, Federal University of Paraná, Curitiba 19031, Brazil
[5] Department of Animal Health, University of Murcia, Campus de Espinardo, 30100 Espinardo, Murcia, Spain
[6] Department of Infectious Diseases, Vector-borne Diseases Unit, Istituto Superiore di Sanità, 00166 Rome, Italy
[7] Medical and Veterinary Entomology Unit, Institut Pasteur du Cambodge, Phnom Penh 12201, Cambodia
[8] Ecology & Emergence of Arthropod-borne Pathogens Unit, Department of Global Health, Institut Pasteur, CNRS UMR2000, 75015 Paris, France
[9] Section Veterinary Services (1417), Laboratory for Animal Health Virology, Aglantzia, Nicosia 2109, Cyprus
[10] Insects Vectors and Parasites Laboratory, Department of Basic Pathology and Postgraduate program in Microbiology, Parasitology and Pathology, Federal University of Paraná, 81530-900 Curitiba, Brazil
[11] Department of Biological Sciences, National University of Singapore, 117558, Singapore
[12] Laboratory of the Leishmaniasis Research Project, Mokolo District Hospital, Mokolo, Cameroon; Laboratory of Cellular Immunology and Parasitology, Department of Biological Sciences, Central Washington University, 98926 Ellensburg, WA, USA
[13] Department of Parasitology, Faculty of Science, Charles University, 12800 Prague, Czechia
[14] VERG Laboratories, Department of Biology, Faculty of Science, Hacettepe University, Beytepe, Ankara 06800, Türkiye
[15] Faculdade de Saúde Pública da Universidade de São Paulo (FSP/USP), Pós-graduação em Saúde Pública, 01246-904 São Paulo, Brazil
[16] Secció de Parasitologia, Departament de Biologia, Sanitat i Medi Ambient, Facultat de Farmàcia i Ciències de l'Alimentació, Universitat de Barcelona, & Institut de Salut Global de Barcelona (ISGlobal), Centro de Investigación Biomédica en Red, Enfermedades Infecciosas (CIBERINFEC), 08028 Barcelona, Spain
[17] Laboratory of Infectious Diseases and Public Health, School of Medicine, University of Cyprus, Nicosia, Cyprus & Department of Pediatrics, Archbishop Makarios III Hospital, Nicosia 2115, Cyprus
[18] Faculty of Health Sciences, American University of Beirut, 1107 2020 Beirut, Lebanon

Edited by Jean-Lou Justine
Υπεύθυνος συγγραφέας για επικοινωνία: jerome.depaquit@univ-reims.fr

This is an Open Access article distributed under the terms of the Creative Commons Attribution License (https://creativecommons.org/licenses/by/4.0), which permits unrestricted use, distribution, and reproduction in any medium, provided the original work is properly cited.

[19] Medical Entomology Unit, Infectious Disease Research Centre, Institute for Medical Research (IMR), National Institutes of Health (NIH), Ministry of Health Malaysia, 40170 Shah Alam, Selangor, Malaysia
[20] School of Medicine, Addis Ababa University, 28017 - 1000 Addis Ababa, Ethiopia
[21] Faculty of Mathematics, Natural Sciences and Information Technologies, University of Primorska, 6000 Koper, Slovenia
[22] University of Lodz, Faculty of Biology and Environmental Protection, Department of Invertebrate Zoology and Hydrobiology, Banacha 12/16, 90-237 Łódź, Poland
[23] Laboratory of Entomology, Ministry of Health, 9134302 Jerusalem, Israel
[24] Center for Pathophysiology, Infectiology and Immunology, Institute of Specific Prophylaxis and Tropical Medicine, Medical University Vienna, Kinderspitalgasse 15, 1090 Vienna, Austria
[25] Papua New Guinea Institute of Medical Research (PNGIMR) Institute, PO Box 60, Headquarter, Homate Street, 441 Goroka, Eastern Highlands Province, Papua New Guinea
[26] Museum of Natural History, University of the Philippines Los Baños, 4031 Laguna, Philippines
[27] National Centre of Infectious and Parasitic Diseases, 1504 Sofia, Bulgaria
[28] Ege University, Faculty of Medicine, Department of Parasitology, 35040 Bornova/Izmir, Türkiye
[29] Retired, Faculté de Pharmacie, Université de Strasbourg, Strasbourg, 67400 Illkirch-Graffenstaden, France
[30] Program for the Study and Control of Tropical Diseases (PECET), Faculty of Medicine, University of Antioquia, 050010 Medellin, Colombia
[31] MIVEGEC, Univ. Montpellier, CNRS, IRD, 34394 Montpellier, France & Medical Entomology Unit, Institut Pasteur de Madagascar, 101 Antananarivo, Madagascar
[32] Laboratorio de Entomología Médica, Departamento de Zoología de Invertebrados, Facultad de Ciencias Biológicas, Universidad Autónoma de Nuevo León, San Nicolás de los Garza, 66455, NL, México
[33] Tropical and Infectious Disease Centre, BP Koirala Institute of Health Sciences, Dharan 56700, Nepal
[34] ICMR-Vector Control Research Centre, Puducherry 605006, India
[35] Graduate School of Agricultural and Life Sciences, The University of Tokyo, Tokyo 113-8657, Japan
[36] Grupo de estudos em Leishmanioses/Coleção de Flebotomíneos (COLFLEB/Fiocruz-MG), Instituto René Rachou, Fundação Oswaldo Cruz, Belo Horizonte, Minas Gerais, 30190009, Brazil
[37] Center of Excellence in Vector Biology and Vector-Borne Disease, Department of Parasitology, Faculty of Medicine, Chulalongkorn University, Bangkok 10330, Thailand
[38] Department of Arbovirology, Bernhard Nocht Institute for Tropical Medicine, Bernhard Nocht Str. 74, 20359 Hamburg, Germany [39] Laboratory Vectors & Parasites, Department of Livestock Sciences and Techniques, Sine Saloum University El Hadji Ibrahima Niasse (SSUEIN) Kaffrine Campus, C.P. 24600, Senegal.
[40] Environmental Health Institute, National Environment Agency, Singapore 138667, Singapore & Department of Biological Sciences, National University of Singapore, 117558 Singapore
[41] Institut Pasteur du Laos, Laboratory of Vector-Borne Diseases, Samsenhai Road, Ban Kao-Gnot, Sisattanak District, 3560 Vientiane, Lao PDR
[42] National Institute of Hygiene and Epidemiology, 1 Yec-Xanh Street, Hai Ba Trung District, 100000 Hanoi, Vietnam
[43] Indonesian Research Center for Veterinary Science, Indonesian Agency for Agricultural Research and Development, Ministry of Agriculture Republic Indonesia, Bogor 16114, Indonesia & Department of Parasitology, Faculty of Veterinary Medicine, Airlangga University, Surabaya 60115, Indonesia
[44] Laboratory of Research in Applied Biology, Polytechnic School of Abomey-Calavi, University of Abomey-Calavi, 01 P.O. Box 2009, 00000 Cotonou, Benin
[45] Instituto de Microbiología, Colegio de Ciencias Biológicas y Ambientales (COCIBA), Universidad San Francisco de Quito (USFQ), 170901 Quito, Ecuador

Received 1 December 2025, Accepted 29 January 2026, Published online 3 April 2026

Περίληψη – Το παρόν άρθρο παρέχει έναν ολοκληρωμένο οδηγό για την επεξεργασία και προετοιμασία μικροσκοπικών παρασκευασμάτων φλεβοτόμων, διαδικασίες καθοριστικής σημασίας για την ταυτοποίηση των ειδών, καθώς και για την ανίχνευση και απομόνωση παθογόνων. Παρουσιάζεται ένα ευρύ φάσμα τεχνικών, κατάλληλων τόσο για μελέτες πεδίου όσο και για εργαστηριακές εφαρμογές. Παρέχονται αναλυτικές κατευθυντήριες οδηγίες για τη συλλογή, τον χειρισμό, τον εγκλεισμό και τη θανάτωση φλεβοτόμων (με σύσταση για χρήση βαθιάς κατάψυξης ή CO_2 έναντι χημικών μέσων), καθώς και στρατηγικές συντήρησης, όπως η φύλαξη σε χαμηλές θερμοκρασίες και η διατήρηση σε αιθανόλη. Η ποιοτική προετοιμασία των παρασκευασμάτων συγκεκριμένων ανατομικών δομών (γεννητικά όργανα, κεφαλή και πτέρυγες) είναι καθοριστική για την ορθή μικροσκοπική παρατήρηση και περιγράφεται διεξοδικά στην παρούσα εργασία. Παρουσιάζονται επίσης τα επιμέρους στάδια επεξεργασίας των δειγμάτων, συμπεριλαμβανομένης της διαδικασίας διαύγασης με παράγοντες όπως το υδροξείδιο του καλίου, ακολουθούμενη από τη χρήση του διαλύματος Marc-André. Όσον αφορά τη διαδικασία εγκλεισμού, παρουσιάζονται και συγκρίνονται ποικίλα μέσα, με έμφαση στις οπτικές τους ιδιότητες και στη δυνατότητα διατήρησης των δειγμάτων στο χρόνο. Το υγρό Hoyer (γνωστό και ως κόμμι χλωράλης) προτείνεται για ταχεία παρατήρηση, ιδίως των σπερματοθηκών, λόγω

της υψηλής διαύγειάς του, αλλά δεν ενδείκνυται για μακροχρόνια διατήρηση. Άλλα μέσα που εξετάζονται περιλαμβάνουν την πολυβινυλική αλκοόλη, το Euparal® (έχει περιορισμένη ανεκτικότητα στην υγρασία) και το βάλσαμο Καναδά (μέσο διαλυτό σε υδρογονάνθρακες), με τα δύο τελευταία να είναι κατάλληλα για μακροχρόνια διατήρηση. Παρουσιάζονται επίσης σύγχρονες προσεγγίσεις μοριακής βιολογίας, όπως η αλληλούχηση DNA και η τεχνολογία MALDI-ToF, οι οποίες απαιτούν ιδιαίτερη προσοχή και εξειδικευμένα πρωτόκολλα επεξεργασίας δειγμάτων. Συμπεριλαμβάνονται σύντομα εκπαιδευτικά βίντεο που παρουσιάζουν ποικίλες τεχνικές εγκλεισμού, καθώς και μετάφραση σε 33 γλώσσες, καθιστώντας τον οδηγό προσβάσιμο και αξιοποιήσιμο διεθνώς, καλύπτοντας τις ποικίλες ανάγκες και προσδοκίες της παγκόσμιας επιστημονικής κοινότητας.

Λέξεις-κλειδιά: Εγκλεισμός, φλεβοτόμοι, υγρό Hoyer, διάλυμα Marc-André, κόμμι χλωράλης, πολυβινυλική αλκοόλη, Euparal®, βάλσαμο Καναδά, απομόνωση *Leishmania*, συνθήκες πεδίου, καλλιέργεια, ανατομή, μοριακή βιολογία, MALDI-ToF, τυπικά δείγματα.

Abstract – Processing and mounting phlebotomine sand flies: a consensus guideline. This article provides a comprehensive guide for the processing and mounting of phlebotomine sand fly specimens, which is crucial for species identification and pathogen detection and isolation. It discusses a range of techniques suitable for both field and laboratory settings. The guide includes detailed instructions on sand fly collection, handling, covering, and euthanasia (recommending dry freezing or CO_2 over chemicals) as well as conservation strategies, such as cold storage and preservation in ethanol. The quality of preparation of certain anatomical structures (genital organs, head and wings) is essential for their proper microscopic observation and is described in this work. The article also presents detailed sample processing, including the clearing process with agents such as potassium hydroxide then Marc-André solution. The mounting process compares different media, emphasizing their optical properties and preservation potential. Hoyer fluid (also known as chloral gum) is recommended for quick observation, particularly for spermathecae, due to its clarity, although it is not suitable for long-term storage. Other media discussed include polyvinyl alcohol, Euparal® (for limited water tolerance), and Canada balsam (a hydrocarbon-soluble medium), with the latter two offering long-term preservation capabilities. Innovative molecular biology approaches such as DNA sequencing and MALDI-ToF, which require particular attention to sample processing, are also addressed. Furthermore, short video clips illustrating various mounting techniques as well as translations in many different languages are provided, allowing the guideline to reach the diverse needs and expectations of the global scientific community.

Key words: Mounting, Phlebotomine sand fly, Hoyer fluid, Marc-André solution, Chloral gum, Polyvinyl alcohol, Euparal®, Canada balsam, *Leishmania* isolation, Field conditions, Culture, Dissection, Molecular biology, MALDI-ToF, Type-specimens.

Εισαγωγή

Οι φλεβοτόμοι είναι μικρά δίπτερα έντομα που ανήκουν στην οικογένεια Psychodidae και στην υποοικογένεια Phlebotominae, με τουλάχιστον 1.063 γνωστά είδη [21]. Αποτελούν σημαντικούς φορείς παθογόνων μικροοργανισμών (*Leishmania*, αρμποϊοί και *Bartonella*) που ευθύνονται για νοσήματα όπως η λεϊσμανίαση, οι αρμποϊώσεις και η μπαρτονέλλωση. Η ταυτοποίησή τους βασίζεται κυρίως σε λεπτομερή μικροσκοπική εξέταση, η οποία προϋποθέτει προσεκτική συλλογή, κατάλληλη αποθήκευση και άρτια προετοιμασία των μικροσκοπικών παρασκευασμάτων· διαδικασίες που απαιτούν εξειδικευμένες τεχνικές, καθεμία με τα δικά της πλεονεκτήματα και περιορισμούς.

Η ταυτοποίηση των ενήλικων φλεβοτόμων βασίζεται στην παρατήρηση εξωτερικών (π.χ. κεραίες, ψηλαφίδες, αρσενικά γεννητικά όργανα) και εσωτερικών δομών (π.χ. φάρυγγας, κιβάριο, σπερματοθήκες). Η μικροανατομή και η απομόνωση των εσωτερικών οργάνων διευκολύνουν την παρατήρησή τους και κατ' επέκταση, την ακριβή ταυτοποίηση. Έτσι, σε αντίθεση με άλλα έντομα φορείς-διαβιβαστές, όπως τα κουνούπια ή οι κοριοί της υποοικογένειας Triatominae (kissing bugs), η ταυτοποίηση των φλεβοτόμων προϋποθέτει τη μικροσκοπική παρασκευή (εγκλεισμό μεταξύ αντικειμενοφόρου πλάκας και καλυπτρίδας) πριν την ταυτοποίησή τους. Έως τη δεκαετία του 1980, η μικροσκοπική παρατήρηση αποτελούσε τη μοναδική διαθέσιμη μέθοδο ταυτοποίησης φλεβοτόμων, και παραμένει έως σήμερα η πλέον διαδεδομένη και εκτενώς χρησιμοποιούμενη. Η επιλογή της μεθόδου επεξεργασίας και παρασκευής ήταν τότε απλή. Καθοριζόταν από τις εκάστοτε ανάγκες και από τον συμβιβασμό μεταξύ δύο προσεγγίσεων: του μόνιμου εγκλεισμού που επιτρέπει μακροχρόνια διατήρηση του δείγματος, ή του ταχέως εγκλεισμού που προσφέρει άμεση ταυτοποίηση αλλά περιορισμένη σταθερότητα στο χρόνο και δε διασφαλίζει μακροπρόθεσμη διατήρηση. Για

παράδειγμα, ο μόνιμος εγκλεισμός σε ρητίνη (π.χ. βάλσαμο Καναδά) είναι χρονοβόρος, καθώς προϋποθέτει την πλήρη αφυδάτωση των δειγμάτων, ενώ ο δείκτης διάθλασής του μέσου δεν είναι πάντοτε βέλτιστος για την ευκρινή παρατήρηση δομών όπως οι σπερματοθήκες. Αντιθέτως, η χρήση υδατικών μέσων εγκλεισμού (π.χ. Hoyer) πλεονεκτεί σε ταχύτητα και επιτρέπει καλύτερη οπτικοποίηση δομών με έντονες διαθλαστικές ιδιότητες όπως οι σπερματοθήκες. Ωστόσο, τα μέσα αυτά είναι υγροσκοπικά και λόγω της απορρόφησης υγρασίας από το περιβάλλον, δεν επιτρέπουν μακροχρόνια διατήρηση των δειγμάτων. Μια εναλλακτική πρακτική για την προστασία των υδατικών παρασκευασμάτων είναι η περιμετρική σφράγιση της καλυπτρίδας με βερνίκι νυχιών, αφού όμως στεγνώσουν πλήρως οι άκρες της. Η ανάγκη στάθμισης μεταξύ αυτών των παραμέτρων παραμένει επίκαιρη, καθώς ο συμβιβασμός μεταξύ ποιότητας, διάρκειας διατήρησης και ταχύτητας επεξεργασίας καθορίζει την επιλογή του κατάλληλου μέσου εγκλεισμού, ανάλογα με τον επιδιωκόμενο ερευνητικό στόχο. Από τη δεκαετία του 1980, η ταυτοποίηση φλεβοτόμων βασίζεται στον συνδυασμό μορφολογικών και βιοχημικών προσεγγίσεων. Οι πρώτες αναλύσεις επικεντρώθηκαν στην εξέταση των υδρογονανθράκων του εξωσκελετού (cuticular hydrocarbons), οι οποίες αντικαταστάθηκαν σταδιακά από τεχνικές μοριακής βιολογίας. Μεταξύ αυτών περιλαμβάνονται ο τυχαίος πολλαπλασιασμός πολυμορφικού DNA (RAPD), ο πολυμορφισμός μήκους περιοριστικών θραυσμάτων (RFLP), η αλυσιδωτή αντίδραση πολυμεράσης (PCR), η αλληλούχηση κατά Sanger, καθώς και νεότερες τεχνολογίες όπως η αλληλούχηση νέας γενιάς (NGS). Σήμερα, οι μοριακές προσεγγίσεις συμπληρώνονται από πρωτεωμικές μεθόδους (π.χ. MALDI-ToF). Επιπλέον, η μοριακή ταυτοποίηση ειδών μπορεί να συνδυαστεί με τον ταυτόχρονο προσδιορισμό παθογόνων μικροοργανισμών (π.χ. *Leishmania*, *Trypanosoma*, *Bartonella* και *Phlebovirus*), καθώς όλα είναι δυνατόν να ανιχνευθούν μέσω συμβατικής (end-point) ή ποσοτικής (qPCR) PCR, με απαραίτητη όμως προϋπόθεση την προσαρμογή των πρωτοκόλλων δειγματοληψίας και διατήρησης των δειγμάτων ανάλογα με τους ειδικούς στόχους της εκάστοτε μελέτης [3, 32]. Πέραν της κλασσικής προσέγγισης, δηλαδή της αξιοποίησης των μορφολογικών χαρακτήρων για τη διαφοροποίηση των ειδών, εφαρμόζονται πλέον και εναλλακτικές προσεγγίσεις όπως η γεωμετρική μορφομετρία των πτερύγων (wing geometric morphometrics).

Αξιοποιώντας τη συσσωρευμένη προσωπική εμπειρία των συγγραφέων σε συνδυασμό με τα διαθέσιμα βιβλιογραφικά δεδομένα, η παρούσα εργασία στοχεύει στη θέσπιση προτυποποιημένων πρωτοκόλλων και στην παροχή κατευθυντήριων οδηγιών κοινής αποδοχής για τη δημιουργία μικροσκοπικών παρασκευασμάτων και την επεξεργασία ενήλικων φλεβοτόμων, με σκοπό τη βελτιστοποίηση των μορφολογικών και μοριακών αναλύσεων.

Η ανάγκη εφαρμογής εξειδικευμένων τεχνικών (π.χ. μοριακής βιολογίας ή MALDI-ToF) συχνά επιβάλλει τη διατήρηση τμημάτων του φλεβοτόμου που δεν κρίνονται απαραίτητα για τη μορφολογική ταυτοποίηση. Η αναγκαιότητα αυτή αναδεικνύει τη σημασία της ορθής επιλογής και προσαρμογής των πρωτοκόλλων, ώστε να διασφαλίζεται η καταλληλότητα των δειγμάτων για πολλαπλές εφαρμογές.

Στο παρόν άρθρο εστιάζουμε στις μεθόδους ακινητοποίησης και θανάτωσης φλεβοτόμων που συλλέγονται ζωντανοί, στις τεχνικές αποθήκευσης και στις διαδικασίες εγκλεισμού. Οι τελευταίες εξετάζονται τόσο υπό το πρίσμα της άμεσης ή ταχείας ταυτοποίησης όσο και της μακροχρόνιας διατήρησης δειγμάτων, επιτρέποντας τη μελλοντική αξιοποίησή τους σε περαιτέρω μελέτες.

Προοίμιο: Θέματα ασφαλείας και κανονιστικές απαιτήσεις (βάσει Δελτίων Δεδομένων Ασφαλείας- ΔΔΑ)

Όλες οι χημικές ουσίες που αναφέρονται στο παρόν άρθρο πρέπει να χρησιμοποιούνται υπό αυστηρούς κανόνες ασφαλείας. Οι αρμόδιες Επιτροπές Υγείας και Ασφάλειας των ερευνητικών ιδρυμάτων παρέχουν τις απαραίτητες πληροφορίες σχετικά με την επικινδυνότητα των ουσιών, καθώς και τις ενδεδειγμένες διαδικασίες χειρισμού και απόρριψης αποβλήτων. Η συμμόρφωση με τις οδηγίες ασφαλείας κατά τον χειρισμό και την απόρριψη των υλικών είναι υποχρεωτική. Επισημαίνεται ότι η ευθύνη για την τήρηση των Αρχών Ορθής Εργαστηριακής Πρακτικής (ΟΕΠ), καθώς και της ισχύουσας νομοθεσίας και κανονισμών της χώρας ή του ιδρύματος όπου δραστηριοποιείται ο χρήστης, βαρύνει αποκλειστικά τον ίδιο. Επιπλέον, σημειώνεται ότι ορισμένες ουσίες ή επιμέρους συστατικά τους (π.χ. ένυδρη χλωράλη) ενδέχεται να υπόκεινται σε ειδικούς κανονιστικούς περιορισμούς σε ορισμένες χώρες. Ο κατάλογος των συντομογραφιών που χρησιμοποιούνται στο παρόν άρθρο παρατίθεται στον Πίνακα 1.

Πίνακας 1: Κατάλογος συντομογραφιών.

**BME**	Βασικό θρεπτικό μέσο Eagle (Basal medium Eagle)
**CDC**	Κέντρα Ελέγχου και Πρόληψης Νοσημάτων (Centers for Disease Control and Prevention)
**CMCP**	Καμφορά-μονοχλωροφαινόλη (Camphor-monochlorophenol)
**CMR**	Καρκινογόνος, μεταλλαξιογόνος και τοξική για την αναπαραγωγή ουσία (carcinogenic, mutagenic, reprotoxic substance)
**COI**	Γονίδιο υπομονάδας I της κυτοχρωμικής οξειδάσης c (Cytochrome c oxidase subunit I gene)
**CytB**	Γονίδιο κυτοχρώματος b (Cytochrome b gene)
**DNA**	Δεοξυριβονουκλεϊκό οξύ (Deoxyribonucleic acid)
**ELISA**	Ενζυμική ανοσοπροσροφητική δοκιμασία (Enzyme-linked immunosorbent assay)
**EtOH**	Αιθανόλη
**M199**	Θρεπτικό μέσο 199 (Medium 199)
**MALDI-ToF MS**	Φασματομετρία μάζας με ιονισμό/ εκρόφηση μέσω λέιζερ υποβοηθούμενου από μήτρα και αναλυτή χρόνου πτήσης (Matrix-assisted laser desorption/ionization time-of-flight mass spectrometry)
**MEM**	Ελάχιστο βασικό θρεπτικό μέσο (Minimum essential medium)
**NGS**	Αλληλούχηση νέας γενιάς (Next-generation sequencing)
**NNN**	Θρεπτικό μέσο Novy-MacNeal-Nicolle
**PCR**	Αλυσιδωτή αντίδραση πολυμεράσης (Polymerase chain reaction)
**Lao PDR**	Λαϊκή Δημοκρατία του Λάος
**PNOC**	Γονίδιο προ-προνοσιπτινίνης (Prepronociceptin gene)
**qPCR**	Ποσοτική PCR (PCR πραγματικού χρόνου)
**RAPD**	Τεχνική τυχαίου πολλαπλασιασμού πολυμορφικού DNA (Random amplified polymorphic DNA)
**RFLP**	Τεχνική πολυμορφισμού μήκους περιοριστικών θραυσμάτων (Restriction fragment length polymorphism)
**RI**	Δείκτης διάθλασης (Refractive index)
**RNA**	Ριβονουκλεϊκό οξύ (Ribonucleic acid)
**RNases**	Ριβονουκλεάσες (Ribonucleases)
**RNASS**	Διάλυμα σταθεροποίησης RNA (RNA stabilization solution)
**RT-PCR**	PCR αντίστροφης μεταγραφής (Reverse transcription PCR)
**TFA**	Τριφθοροξικό οξύ (Trifluoroacetic acid)

1. Συλλογή φλεβοτόμων

Η συλλογή ενήλικων φλεβοτόμων, ζωντανών ή νεκρών, πραγματοποιείται με ποικίλες μεθόδους, όπως με χρήση παγίδων φωτός τύπου CDC (miniature light traps), κολλητικών παγίδων (sticky traps), αναρροφητήρων σε συνδυασμό με παγίδες Shannon, ή με απευθείας συλλογή από φυσικές ή τεχνητές κρυψώνες (π.χ. φωλιές, καταλύματα ζώων όπως στάβλοι). Οι μέθοδοι αυτές περιλαμβάνουν την τοποθέτηση παγίδων σε κατάλληλα ενδιαιτήματα, την προσέλκυση φλεβοτόμων με τη χρήση φωτεινών (π.χ. φωτοπαγίδες) και χημικών (π.χ. CO_2) μέσων προσέλκυσης, την παγίδευση και συλλογή τους για περαιτέρω ανάλυση, όπως περιγράφονται εκτενώς στη βιβλιογραφία [2, 3, 32, 36, 49].

Η συλλογή ζωντανών φλεβοτόμων επιτρέπει την εφαρμογή του συνόλου των μεταγενέστερων αναλύσεων, ενώ η συλλογή νεκρών ατόμων δεν επιτρέπει την απομόνωση στελεχών Leishmania ή ιών. Ορισμένες μέθοδοι παγίδευσης και συλλογής, όπως η χρήση κολλητικών παγίδων, συχνά προκαλούν αλλοιώσεις ή απώλεια μορφολογικών χαρακτήρων (κεραίες, ψηλαφίδες, πτέρυγες ή άκρα). Επιπλέον, το καστορέλαιο με το οποίο είναι επικαλυμμένες οι κολλητικές παγίδες, προσκολλάται στα έντομα και πρέπει να απομακρύνεται στα αρχικά στάδια της επεξεργασίας, συνήθως μέσω εμβάπτισης διάρκειας 15 λεπτών σε μίγμα αιθανόλης και διαιθυλαιθέρα σε αναλογία 1:1.

2. Μέθοδοι θανάτωσης φλεβοτόμων

Μετά τη συλλογή, οι ζωντανοί φλεβοτόμοι πρέπει να υποβάλλονται σε διαδικασία θανάτωσης. Σε ορισμένες μεθόδους συλλογής (π.χ. κολλητικές επιφάνειες, παγίδες φωτός CDC με δοχείο που περιέχει απορρυπαντικό ή αιθανόλη), οι φλεβοτόμοι είναι ήδη νεκροί κατά τη συλλογή. Σε αυτά τα δείγματα, μπορούν να εφαρμοστούν τεχνικές μοριακής βιολογίας, εφόσον οι φλεβοτόμοι συλλεχθούν απευθείας σε αιθανόλη ή αποθηκευτούν σε αυτήν το συντομότερο δυνατό. Ωστόσο, οι μέθοδοι αυτές δεν είναι συμβατές με αναλύσεις MALDI-ToF και κάποιες ενδέχεται να προκαλέσουν απώλεια μορφολογικών χαρακτήρων. Για τον λόγο αυτό, είναι απαραίτητη η εφαρμογή κατάλληλης, τυποποιημένης μεθόδου θανάτωσης, ώστε να διασφαλίζεται τόσο η αξιόπιστη ταυτοποίηση όσο και η δυνατότητα μακροχρόνιας διατήρησης των δειγμάτων ως δειγμάτων αναφοράς. Χημικές ουσίες όπως οξικός αιθυλεστέρας, διαιθυλαιθέρας, τετραχλωροαιθάνιο και χλωροφόρμιο, μπορούν να χρησιμοποιηθούν εμποτίζοντας ένα βαμβάκι και τοποθετώντας το σε δοχείο που περιέχει τους συλλεγμένους

φλεβοτόμους. Οι ουσίες αυτές πρέπει να χρησιμοποιούνται με ιδιαίτερη προσοχή και σύμφωνα με τις οδηγίες του κατασκευαστή, λόγω της τοξικότητάς τους. Ωστόσο, η χρήση χλωροφορμίου δεν συνιστάται διότι δεν είναι συμβατή με εφαρμογές μοριακής βιολογίας. Συνολικά λοιπόν, λόγω της επικινδυνότητας και της περιορισμένης καταλληλότητάς τους για μοριακές αναλύσεις, η χρήση των παραπάνω χημικών κρίνεται γενικά μη ενδεδειγμένη.

Η πλέον διαδεδομένη και αποτελεσματική μέθοδος για τη διατήρηση της μορφολογικής ακεραιότητας, καθώς και της ακεραιότητας του DNA και των πρωτεϊνών είναι η ξηρή κατάψυξη των δειγμάτων. Τα δείγματα πρέπει να καταψύχονται για επαρκές χρονικό διάστημα ώστε να επιτυγχάνεται πλήρης ακινητοποίηση, χωρίς όμως να παρατείνεται σε βαθμό που (i) προκαλεί αφυδάτωση ή (ii) επηρεάζει τη βιωσιμότητα των παρασίτων Leishmania, εφόσον ο στόχος είναι η in vitro μελέτη των παρασίτων μετά την απομόνωση από το πεπτικό σύστημα των φλεβοτόμων. **Για τον λόγο αυτό, συνιστάται κατάψυξη για 15–20 λεπτά στους −20°C, με τακτικό έλεγχο, ώστε να διασφαλίζεται τόσο η ακινητοποίηση όσο και η βιωσιμότητα και η δομική ακεραιότητα των παρασίτων *Leishmania*.**

Εναλλακτικά, αν δεν υπάρχει διαθέσιμος καταψύκτης, τα έντομα μπορούν να θανατωθούν με χρήση CO_2. Σε συνθήκες πεδίου, όπου δεν είναι δυνατή η χρήση φιαλών CO_2, προτείνεται η χρήση μικρών, φορητών και εμπορικά διαθέσιμων φιαλιδίων CO_2, όπως αμπούλες για συσκευές ανθρακούχων ποτών (soda siphons), αν και ενδέχεται να υπάρχουν περιορισμοί στην αεροπορική τους μεταφορά.

Ως έσχατη λύση, τα έντομα μπορούν να θανατωθούν με έκθεση σε καπνό τσιγάρου. Οι φλεβοτόμοι συλλαμβάνονται ζωντανοί σε παγίδα CDC, συλλέγονται με τη χρήση αναρροφητήρα, μεταφέρονται σε γυάλινο σωλήνα και εκτίθενται στον καπνό, ο οποίος προκαλεί το θάνατό τους εντός ολίγων δευτερολέπτων. Η μέθοδος αυτή είναι εφαρμόσιμη στο πεδίο ακόμη και σε απομακρυσμένες περιοχές ή υπό δυσμενείς συνθήκες συλλογής και απομόνωσης των δειγμάτων. Ωστόσο, λόγω της προσρόφησης υπολειμμάτων καπνού στο γυαλί, απαιτείται σχολαστικός καθαρισμός πριν την επαναχρησιμοποίησή του για συλλογή ζωντανών εντόμων. Αντιθέτως, ο ίδιος (μη καθαρισμένος) αναρροφητήρας μπορεί να χρησιμοποιηθεί για τη θανάτωση φλεβοτόμων που συλλέχθηκαν σε άλλες παγίδες και προορίζονται για σκοπούς μονιμοποίησης. Σε κάθε περίπτωση, είναι απαραίτητο να ελέγχεται η πλήρης απομάκρυνση όλων των δειγμάτων από τον αναρροφητήρα. Οι παραπάνω μέθοδοι είναι συμβατές με την απομόνωση *Leishmania* μέσω της διατομής του εντέρου.

3. Διατήρηση και αποθήκευση δειγμάτων πριν από την επεξεργασία

Υπάρχουν πέντε κύριες μέθοδοι καθήλωσης (χημική ή φυσική σταθεροποίηση ιστών) πριν από την επεξεργασία:

3.1. Κατάψυξη

Η μέθοδος αυτή εφαρμόζεται ιδανικά στους −20°C ή κατά προτίμηση, στους −80°C. Οι μέθοδοι αποθήκευσης σε καταψύκτη υπερέχουν πλέον σε χρήση έναντι της φύλαξης σε υγρό άζωτο. Σε κάθε περίπτωση, η κρυοσυντήρηση θα πρέπει να πραγματοποιείται το συντομότερο δυνατό μετά την αναισθητοποίηση και ακινητοποίηση των δειγμάτων. Η αποθήκευση σε βαθιά κατάψυξη προσφέρει το πλεονέκτημα της πλήρους διατήρησης των εντόμων, καθώς και των νουκλεϊκών οξέων (RNA, DNA) και πρωτεϊνών, διατηρώντας την ακεραιότητά τους καθ' όλη τη διάρκεια της αποθήκευσης. Αντίθετα, το υγρό άζωτο ενδέχεται να προκαλέσει σοβαρές βλάβες σε εύθραυστες μορφολογικές δομές (πτέρυγες, άκρα, ψηλαφίδες και κεραίες), οδηγώντας συχνά σε αποκοπή ή απώλεια κρίσιμων μορφολογικών χαρακτήρων. Η ξηρή κατάψυξη είναι λιγότερο επιβαρυντική για τα δείγματα, ωστόσο δεν είναι ιδανική για τη διατήρηση ιδιαίτερα εύθραυστων οργάνων. Επιπλέον, κατά την απόψυξη, οι υδρατμοί που σχηματίζονται λόγω συμπύκνωσης ενδέχεται να προκαλέσουν την προσκόλληση των ευαίσθητων εξαρτημάτων του εντόμου στα τοιχώματα των φιαλιδίων, με κίνδυνο την καταστροφή τους. Η διατήρηση μέσω κατάψυξης δεν είναι πάντοτε εφικτή σε μελέτες πεδίου, καθώς απαιτεί πρόσβαση σε καταψύκτη ή δοχείο υγρού αζώτου. Παρόλα αυτά, είναι πλήρως συμβατή με την μοριακή ανίχνευση παθογόνων, χωρίς απώλεια ευαισθησίας. Ειδικά για την ανίχνευση και απομόνωση ιών RNA η αποθήκευση στους −80°C ή σε υγρό άζωτο κρίνεται απαραίτητη, ιδίως όταν πρόκειται για μακροχρόνια διατήρηση. Τέλος, η κατάψυξη των δειγμάτων δεν επιτρέπει την απομόνωση παρασίτων *Leishmania* μέσω ανατομής του εντέρου, εκτός εάν ακολουθηθεί ελεγχόμενη διαδικασία εμβάπτισης (αρχικά στη φάση ατμών και στη συνέχεια σε υγρό άζωτο, π.χ. σε φιαλίδια τοποθετημένα μέσα σε κάλτσα), προκειμένου να προσομοιωθεί το πρωτόκολλο κρυοσυντήρησης παρασίτων *Leishmania*.

3.2. Διατήρηση σε αλκοόλη (αιθανόλη ή ισοπροπυλική αλκοόλη)

Αποτελεί πιθανώς την πλέον διαδεδομένη μέθοδο αποθήκευσης φλεβοτόμων, καθώς εφαρμόζεται εύκολα στο πεδίο, ακόμη και υπό αντίξοες συνθήκες χωρίς πρόσβαση σε εργαστηριακές υποδομές. Η διατήρηση σε αλκοόλη ενδείκνυται για μορφολογικές μελέτες, καθώς οι εύθραυστες ανατομικές δομές (πτέρυγες, άκρα, κεραίες ή ψηλαφίδες) παραμένουν ακέραιες, εφόσον δεν εγκλωβιστούν φυσαλίδες αέρα στο σωληνάριο αποθήκευσης. Για τον λόγο αυτό, συνιστάται η σφράγιση του σωληναρίου με μικρό βαμβακερό πώμα για την απομάκρυνση του αέρα και η τοποθέτηση της ετικέτας επάνω στο βαμβάκι (Σχήμα 1). Η κατάλληλη συγκέντρωση αλκοόλης παραμένει αντικείμενο συζήτησης, ωστόσο συγκεντρώσεις κάτω του 70% δεν συνιστώνται [45, 66]. Υψηλότερες συγκεντρώσεις διασφαλίζουν αποτελεσματικότερα τη σταθερότητα του DNA σε βάθος χρόνου αλλά καθιστούν τα δείγματα πιο εύθραυστα και δύσκαμπτα, δυσχεραίνοντας τη μορφολογική ανάλυση. Η χρήση αιθανόλης 96% (αζεοτροπικό μίγμα) υπερέχει στη διασφάλιση της μακροχρόνιας σταθερότητας των δειγμάτων, ιδιαίτερα σε τροπικές χώρες και περιοχές με υψηλά επίπεδα υγρασίας, παρόλο που η αιθανόλη 95% αποτελεί συχνά την ευρύτερα διαθέσιμη επιλογή. Ανεξαρτήτως συγκέντρωσης, το DNA διατηρείται ικανοποιητικά σε αιθανόλη (αν και λιγότερο αποτελεσματικά σε σύγκριση με την κατάψυξη, ιδίως για μοριακές τεχνικές, όπως NGS). Αντίθετα, οι πρωτεΐνες παρουσιάζουν χαμηλή σταθερότητα, περιορίζοντας εφαρμογές πρωτεομικής, όπως MALDI-ToF. Δείγματα φλεβοτόμων που διατηρούνται σε αλκοόλη για αρκετούς μήνες παραμένουν κατάλληλα για μορφολογική ταυτοποίηση, αλλά δεν επιτρέπουν την παραγωγή αξιόπιστων πρωτεϊνικών φασμάτων αναφοράς. Η διατήρηση ενός δείγματος σε αλκοόλη ή σε ξηρή μορφή μπορεί να βελτιωθεί με παράλληλη κατάψυξή του στους −20°C. Η πρακτική αυτή συμβάλλει στη διαφύλαξη των νουκλεϊκών οξέων, επιβραδύνοντας την αποικοδόμησή τους και δευτερευόντως περιορίζει την ιστολογική αλλοίωση, ενισχύοντας τη μορφολογική σταθερότητα του δείγματος. Κύριο όφελος της μεθόδου παραμένει, ωστόσο, η διασφάλιση της μοριακής ακεραιότητας. Η αποθήκευση σε αιθανόλη επιτρέπει επίσης τη μεταγενέστερη ανίχνευση ιικού DNA και RNA, υπό την προϋπόθεση ότι η συγκέντρωση αιθανόλης είναι ≥70% και η διάρκεια φύλαξης δεν υπερβαίνει τους λίγους μήνες. Επιπλέον, η ισοπροπυλική αλκοόλη (ισοπροπανόλη), η οποία είναι ευρύτερα διαθέσιμη σε ορισμένες χώρες, διατηρεί το DNA, αλλά καθιστά τα δείγματα ακόμη πιο δύσκαμπτα. Καθώς δεν εμπίπτει στους ίδιους περιορισμούς διακίνησης με την αιθανόλη, η μεταφορά της είναι ευκολότερη. Εφόσον απαιτείται, δείγματα φλεβοτόμων που έχουν διατηρηθεί σε υγρό άζωτο ή έχουν καταψυχθεί σε ξηρή μορφή μπορούν να μεταφερθούν σε αλκοόλη. Ωστόσο, αυτό συνεπάγεται τον συνδυασμό των μειονεκτημάτων και των δύο μεθόδων.

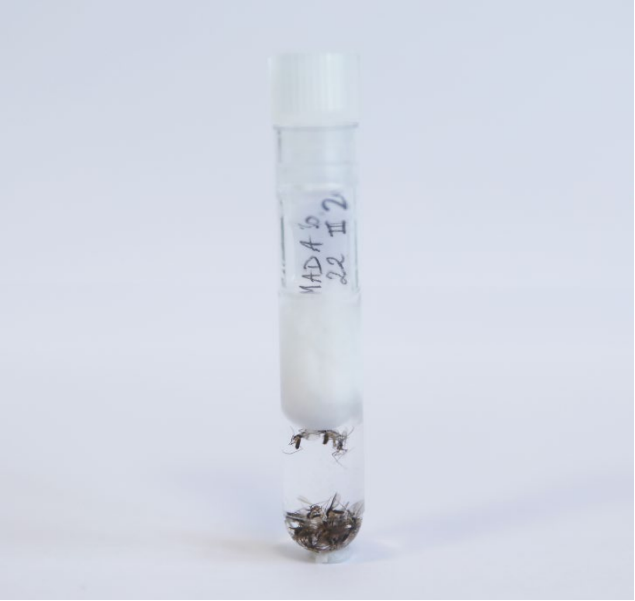

Σχήμα 1: Δείγματα φλεβοτόμων διατηρημένα σε αιθανόλη.

3.3. Αποθήκευση σε διάλυμα σταθεροποίησης RNA (RNASS)

Το συγκεκριμένο ειδικό διάλυμα χρησιμοποιείται εκτενώς, είναι μη τοξικό και σχεδιασμένο για τη σταθεροποίηση και προστασία του RNA σε φρέσκα, μη επεξεργασμένα δείγματα ιστών και κυττάρων. Η δράση του βασίζεται στην ταχεία διείσδυση στο δείγμα και την αδρανοποίηση των RNασών (ενζύμων που αποδομούν το RNA), αποτρέποντας έτσι την αποικοδόμηση του γενετικού υλικού χωρίς την ανάγκη άμεσης κατάψυξης. Η αποθήκευση σε RNASS είναι γενικά αποτελεσματική για τη διατήρηση της συνολικής μορφολογίας ιστών και κυττάρων, επιτρέποντας μεταγενέστερη ιστολογική αξιολόγηση. Παρόλο που το RNASS είναι βελτιστοποιημένο για τη σταθεροποίηση RNA και όχι για ιστολογική μονιμοποίηση, η βραχυπρόθεσμη έως μεσοπρόθεσμη φύλαξη διασφαλίζει συνήθως σε ικανοποιητικό βαθμό τη δομική ακεραιότητα των δειγμάτων. Το RNASS επιτρέπει την αποθήκευση δειγμάτων σε θερμοκρασία περιβάλλοντος έως 7 ημέρες, στους 4°C για αρκετές εβδομάδες, στους −20°C ή −80°C για μακροχρόνια διατήρηση. Η μέθοδος αυτή είναι ιδιαίτερα χρήσιμη σε μελέτες πεδίου ή σε κλινικά περιβάλλοντα όπου οι υποδομές για διατήρηση ψυκτικής αλυσίδας είναι περιορισμένες. Σχετικά με την εκχύλιση RNA, η διαδικασία προϋποθέτει την απομάκρυνση των δειγμάτων από το μέσο συντήρησης και την επεξεργασία τους σύμφωνα με τυποποιημένα εργαστηριακά πρωτόκολλα.

3.4. Ξηρή διατήρηση σε θερμοκρασία περιβάλλοντος

Πρόκειται για παλαιότερη μέθοδο, η οποία όταν εφαρμόζεται σε ακέραια δείγματα (in toto) παρουσιάζει το σημαντικό μειονέκτημα της ανεπαρκούς διατήρησης των εύθραυστων μορφολογικών δομών, όπως πτέρυγες, άκρα, κεραίες και ψηλαφίδες. Ωστόσο, οι πρωτεωμικές αναλύσεις με MALDI-ToF παραμένουν εφικτές, εφόσον το δείγμα αφυδατωθεί άμεσα κατά τη συλλογή με τη χρήση ξηραντικού μέσου όπως silica gel. Αντιθέτως, μοριακές αναλύσεις DNA είναι δύσκολο να εφαρμοστούν σε τέτοια δείγματα, καθώς το γενετικό υλικό συχνά είναι χαμηλής συγκέντρωσης και έντονα κατακερματισμένο. Αυτό καθιστά την ανάλυση – ιδιαίτερα όσον αφορά το πυρηνικό γονιδίωμα – πολύ πιο δυσχερή σε σύγκριση με φρέσκα ή κρυοσυντηρημένα δείγματα. Παρόλα αυτά, αναδυόμενες προσεγγίσεις όπως η museomics επιτρέπουν πλέον την αξιοποίηση τέτοιου τύπου δειγμάτων [34]. Συνεπώς, αυτή η μέθοδος διατήρησης δεν συνιστάται, εκτός εάν δεν υπάρχει εναλλακτική επιλογή. Στην περίπτωση αυτή, μπορεί να συνδυαστεί με αποθήκευση σε χαμηλές θερμοκρασίες, τοποθετώντας τα σωληνάρια στους −20°C ή −80°C. Η κύρια πρόκληση έγκειται στην κατάλληλη προετοιμασία (εγκλεισμό) των δειγμάτων ή των επιμέρους ανατομικών τμημάτων που απαιτούνται για ταυτοποίηση. Για τον σκοπό αυτό, είναι απαραίτητη η επανυδάτωση, κατά προτίμηση με χρήση διαλύματος Triton X-100. Η διάρκεια της επανυδάτωσης κυμαίνεται από μερικές ώρες έως αρκετές ημέρες, υπό τακτή και προσεκτική παρακολούθηση. Μετά την πλήρη επανυδάτωση, τα δείγματα πρέπει να υποβάλλονται σε τρεις διαδοχικές εκπλύσεις με νερό.

3.5. Διατήρηση σε διηθητικό χαρτί

Το κύριο πλεονέκτημα των διηθητικών χαρτιών έγκειται στη μακροχρόνια σταθερότητα του γονιδιωματικού DNA σε μη μονιμοποιημένα, ξηρά δείγματα ολόκληρων εντόμων ή αιμοκυττάρων, τα οποία διατηρούνται σε θερμοκρασία περιβάλλοντος. Το διηθητικό χαρτί διατίθεται σε μορφή μικρών ειδικών καρτών, γεγονός που επιτρέπει την αποθήκευση εκατοντάδων δειγμάτων σε θερμοκρασία δωματίου, καταλαμβάνοντας όγκο αντίστοιχο με εκείνον ενός μικρού συρταριού εργαστηρίου. Το υπόστρωμα του διηθητικού χαρτιού είναι εμποτισμένο με παράγοντες λύσης που αδρανοποιούν μολυσματικούς παράγοντες, με αποτέλεσμα τα δείγματα να μην κατατάσσονται πλέον ως επικίνδυνα βιολογικά υλικά. Αυτό καθιστά δυνατή την αποθήκευση και μεταφορά των δειγμάτων χωρίς ειδικές προφυλάξεις βιοασφάλειας [68].

4. Ανατομή δειγμάτων

Σε αντίθεση με πολλά άλλα έντομα, τα οποία ταυτοποιούνται βάσει εξωτερικών μορφολογικών χαρακτηριστικών σε ακέραια, ξηρά δείγματα στερεωμένα με καρφίδες, οι φλεβοτόμοι απαιτούν ανατομή και μονιμοποίηση σε αντικειμενοφόρο πλάκα. Αυτό κρίνεται απαραίτητο για τη μελέτη των εσωτερικών ανατομικών χαρακτηριστικών που επιτρέπουν την ακριβή ταυτοποίηση του είδους. Ανεξαρτήτως της μεθόδου προετοιμασίας που επιλέγεται, εφαρμόζεται η ίδια τεχνική ανατομής (Σχήματα 2 & 3) (https://zenodo.org/records/18198006).

Χρήση Triton X100: μη ιοντικό υδατικό διάλυμα

Επισημαίνεται ότι η διαδικασία εγκλεισμού αφορά νωπά ή κατάλληλα συντηρημένα δείγματα. Οι περισσότερες συλλογές περιλαμβάνουν δείγματα εντόμων, τα οποία είτε έχουν διατηρηθεί σε ξηρή μορφή (π.χ. για ανάλυση MALDI-ToF) είτε φυλάσσονται σε αλκοόλη για διάστημα ετών. Ωστόσο, η διατήρηση σε αλκοόλη δεν αποτελεί βέλτιστη μέθοδο για μακροχρόνια αποθήκευση, καθώς τα αρθρόποδα που συντηρούνται με αυτόν τον τρόπο καθίστανται ιδιαίτερα δύσκαμπτα, δυσχεραίνοντας την προετοιμασία τους για μικροσκοπική εξέταση. Συχνά παρατηρείται αλλοίωση των πλαστικών δοχείων που περιέχουν τα δείγματα, καθώς και εξάτμιση της αλκοόλης. Ως αποτέλεσμα, τα δείγματα καθίστανται ακατάλληλα για επεξεργασία, είτε λόγω της παρατεταμένης παραμονής τους σε αλκοόλη, είτε λόγω της πλήρους αφυδάτωσής τους. Για τον λόγο αυτό προτάθηκε η χρήση διαβρεκτικών παραγόντων (wetting agents) που δεν έχουν ισχυρή απορρυπαντική δράση. Το Triton X100 είναι ένα μη ιοντικό υδατικό διάλυμα [4-(1,1,3,3-tetramethylbutyl) phenyl-polyethylene glycol solution, γνωστό και ως t-octylphenoxypolyethoxyethanol, polyethylene glycol tert-octylphenyl ether], το οποίο χρησιμοποιείται εκτενώς ως τασιενεργός, απορρυπαντική ουσία στην κυτταρική και μοριακή βιολογία. Η δράση του ενισχύει τη διαπερατότητα των κυτταρικών και πυρηνικών μεμβρανών.

Ακολουθεί πρωτόκολλο χρήσης μη ιοντικού Triton X100 σε υδατικό διάλυμα συγκέντρωσης 0,5%:

- Εμποτισμός του ξηρού δείγματος σε απόλυτη αιθανόλη.
- Προσθήκη επαρκούς όγκου διαλύματος Triton X-100 (0,5%) μέχρι την πλήρη κάλυψη του δείγματος.
- Επώαση για χρονικό διάστημα από 5 λεπτά έως μερικά εικοσιτετράωρα, υπό τακτική παρακολούθηση. Όλα τα αρθρόποδα πρέπει να διαχωριστούν πλήρως μέσα στο διάλυμα.
- Απομάκρυνση του διαλύματος Triton X-100 και αντικατάστασή του με διάλυμα υδροξειδίου του καλίου.

4.1. Κεφαλή

Η ανατομή πραγματοποιείται μέσω στερεοσκοπίου χρησιμοποιώντας λεπτές βελόνες ή εντομολογικές καρφίδες (Σχήματα 2 & 3). Οι πιο συχνά χρησιμοποιούμενες βελόνες είναι: 26G × 1/2" (0,45 × 13 mm), 30G × 1/2" (0,3 × 13 mm) ή 25G × 5/8" (0,5 × 16 mm). Για την προετοιμασία του δείγματος προς ταυτοποίηση, απαιτείται τουλάχιστον ο διαχωρισμός της

κεφαλής από το σώμα και η τοποθέτησή της με την κοιλιακή πλευρά προς τα επάνω, ώστε να είναι εμφανές το κιβάριο (cibarium, στοματική κοιλότητα) και ο φάρυγγας. Αντίθετα, ο θώρακας και η κοιλία τοποθετούνται σε πλάγια θέση μετά την ανατομή. Η τοποθέτηση της κεφαλής σε κοιλιοραχιαία (ventro-dorsal) θέση διασφαλίζει ότι το ινιακό τρήμα (occipital foramen) είναι προσανατολισμένο προς τα πάνω, επιτρέποντας την απευθείας παρατήρηση του κιβαρίου. Η πρόσβαση σε αυτά τα ανατομικά χαρακτηριστικά διευκολύνεται σημαντικά εάν η κεφαλή έχει αποκοπεί πλήρως.

4.2. Πτέρυγες και θώρακας

Οι πτέρυγες πρέπει να τοποθετούνται σε επίπεδη διάταξη. Κάθε πτέρυγα αποσπάται από τη βάση της και τοποθετείται ανεξάρτητα ή η μία πτέρυγα παραμείνει προσαρτημένη στον θώρακα και η άλλη πτέρυγα τοποθετείται μεμονωμένα. Για αναλύσεις γεωμετρικής μορφομετρίας (geometric morphometrics), είναι σημαντικό να αναγνωρίζεται ορθά ο προσανατολισμός των πτερύγων και να σημειώνεται σωστά η δεξιά και η αριστερή πτέρυγα πριν από τον εγκλεισμό. Ο θώρακας χωρίζεται σε επιμέρους τμήματα, καθένα από τα οποία παρέχει πληροφορίες μεγάλης ταξινομικής αξίας [20, 64]. Κατά κανόνα, τοποθετείται σε πλάγια όψη (lateral view), ώστε να καταστεί δυνατή η εξέταση της χαιτοταξίας (chetotaxy, διάταξη χαιτών ή τριχών) και των χρωματικών μοτίβων (παρουσία και κατανομή χρωματικών ζωνών, κηλίδων ή σκιάσεων). Η παρουσία ουλών από τριχίδια σε συγκεκριμένες περιοχές του θώρακα αποτελεί κριτήριο για τη διάκριση ορισμένων ειδών του γένους Brumptomyia. Η κατανομή του χρώματος μπορεί να χρησιμοποιηθεί για τον διαχωρισμό φλεβοτόμων του Νέου Κόσμου σε επίπεδο γένους (π.χ., *Bichromomyia*), σειράς ειδών (π.χ., *Pintomyia*) ή ακόμη και ειδών του ίδιου γένους (π.χ., *Micropygomyia, Nyssomyia, Psathyromyia, Psychodopygus*) [20]. Συνεπώς, εάν ο θώρακας δεν προορίζεται για μοριακή ανάλυση, θα πρέπει να τοποθετείται με τρόπο ώστε να μην υποστεί φθορά. Είναι σημαντικό να σημειωθεί ότι δεν έχει σημασία η ένταση του χρώματος, αλλά η κατανομή του στον θώρακα. Επομένως, η διαδικασία διαύγασης δεν απομακρύνει τη χρωστική ούτε αλλοιώνει το πρότυπο κατανομής της

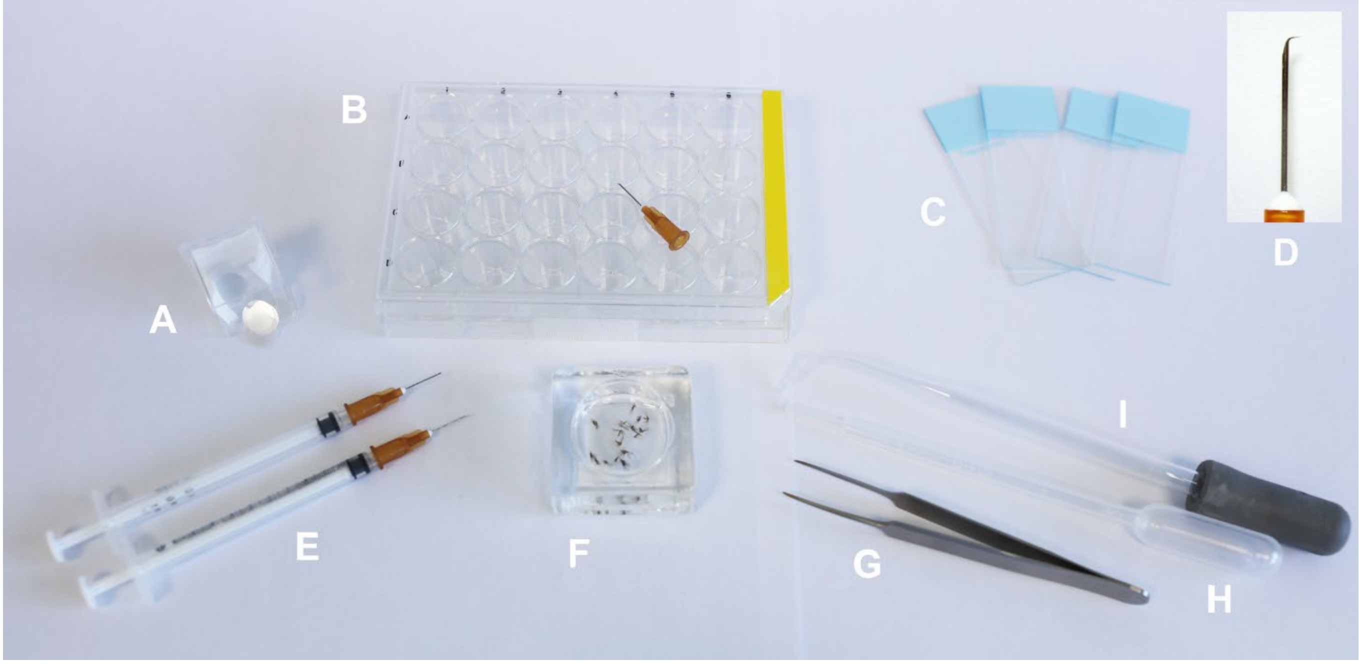

Σχήμα 2: Απαιτούμενος εξοπλισμός και υλικά για την προετοιμασία και δημιουργία παρασκευασμάτων φλεβοτόμων. A: Στρόγγυλες γυάλινες καλυπτρίδες (διαμέτρου 10 ή 12 mm). B: Πλάκα 24 φρεατίων και βελόνα με άγκιστρο (σε περίπτωση χρήσης γαρυφαλέλαιου ή Euparal® essence για την επεξεργασία των σκνιπών, αποφεύγεται η χρήση ακρυλικών πλακών, καθώς η χημική αντίδραση καταστρέφει τα δείγματα). C: Γυάλινες αντικειμενοφόροι πλάκες με επιφάνεια εύκολης σήμανσης. D: Λεπτομέρεια του αγκίστρου της βελόνας. E: Βελόνες προσαρμοσμένες σε σύριγγες. F: Ύαλος ωρολογίου (watch glass) ή ανάλογο γυάλινο δοχείο ή ισοδύναμο για την τοποθέτηση των φλεβοτόμων προς επεξεργασία. G: Λαβίδες τύπου Dumont. H: Πλαστική πιπέτα. I: Γυάλινη πιπέτα, κεκαμμένη μέσω θέρμανσης, για τη διευκόλυνση της μεταφοράς υγρού στα φρεάτια.

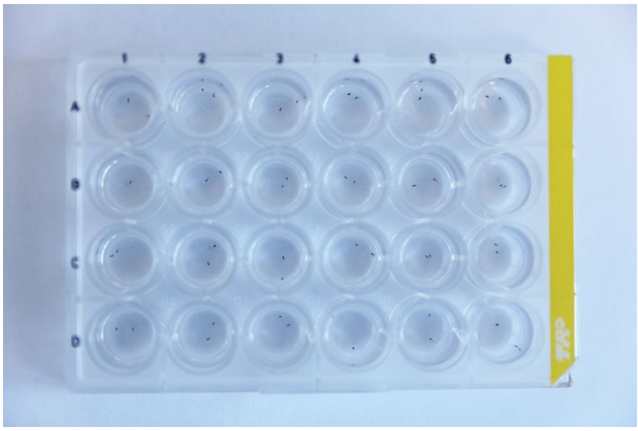

Σχήμα 3: Πλάκα 24 φρεατίων, όπου σε κάθε φρεάτιο περιέχεται η κεφαλή και το άκρο της κοιλίας φλεβοτόμων.

4.3. Γεννητικά όργανα

Ιδιαίτερη προσοχή απαιτείται κατά τον εγκλεισμό των γεννητικών οργάνων τόσο στα αρσενικά όσο και στα θηλυκά άτομα, καθώς αποτελούν κρίσιμα μορφολογικά γνωρίσματα για την ταυτοποίηση σε επίπεδο γένους, υπογένους και είδους. Και στα δύο φύλα, τα γεννητικά όργανα είναι σε ζεύγη αποτελούμενα από δύο συμμετρικά, ξεχωριστά μέρη.

4.3.1. Αρσενικά

Τα γεννητικά όργανα είναι εξωτερικά και αποτελούνται από ζεύγος λαβίδων (forceps), καθεμία από τις οποίες περιλαμβάνει, το σύμπλεγμα γονοκοξίτη–γονοστυλίου (gonocoxite-gonostyle) στο ραχιαίο τμήμα, και τον επανδριακό λοβό (epandrial lobe) στο κοιλιακό τμήμα (κάτω). Το γονοστύλιο φέρει άκανθες (spines) και σε ορισμένες περιπτώσεις, αισθητήρια τριχίδια (ή χαίτες γεννητικών οργάνων, setae), οι οποίες θα πρέπει να μπορούν να καταμετρηθούν και τα σημεία έκφυσή τους να είναι σαφώς ορατά. Είναι σημαντικό να παρατηρείται προσεκτικά η εσωτερική επιφάνεια του γονοκοξίτη, η οποία μπορεί να φέρει θύσανο εδραίων τριχών (sessile setae tuft) ή τριχών που εδράζονται σε λοβό (= φύμα, tubercle) [22]. Ερευνητές με περιορισμένη εμπειρία στην ανατομή μπορούν να εφαρμόσουν απλό πλάγιο εγκλεισμό, χωρίς να αποσπάσουν τα γεννητικά όργανα από το άκρο της κοιλίας (https://zenodo.org/records/18311158). Στην περίπτωση αυτή, η επικάλυψη των δύο τμημάτων των γεννητικών οργάνων ενδέχεται να δυσχεράνει την παρατήρηση ορισμένων μορφολογικών χαρακτήρων (π.χ. την καταμέτρηση των εσωτερικών τριχιδίων του γονοκοξίτη), ωστόσο αποφεύγεται ο κίνδυνος φθοράς των γεννητικών οργάνων λόγω ανεπιτυχούς ανατομής. Πιο έμπειροι συνάδελφοι μπορούν να επιχειρήσουν τη διάνοιξη, τον μερικό διαχωρισμό των γεννητικών οργάνων σε δύο μέρη. Για τον σκοπό αυτό, η λοξή πλευρά βελόνας (τύπου ενδοδερμικής έγχυσης) εισάγεται προσεκτικά, ώστε να επιτευχθεί διαχωρισμός, χωρίς πλήρη αποκοπή, των συμπλεγμάτων γονοκοξίτη–γονοστυλίου (https://zenodo.org/records/18311158). Η τεχνική αυτή διευκολύνει την παρατήρηση των εσωτερικών επιφανειών τους. Η διάταξη αυτή επιτρέπει επίσης σαφέστερη παρατήρηση των παραμερών (parameres) και των ελύτρων των παραμερών (parameral sheaths), καθώς δεν επικαλύπτονται πλέον. Στην περίπτωση πλάγιου εγκλεισμού, όπου ευνοείται η επικάλυψη των οργάνων, τα δείγματα πρέπει να είναι πλήρως διαυγασμένα.

4.3.2. Θηλυκά

Το γεννητικό σύστημα θηλυκών φλεβοτόμων είναι εσωτερικό και αποτελείται από τις σπερματοθήκες. Ελλείψει ανατομής, οι σπερματοθήκες πρέπει να παρατηρούνται διαμέσου των περιβλημάτων (teguments), με εγκλεισμό της κοιλίας, τοποθετημένη σε κοιλιακή διάταξη. Ανεξαρτήτως του μέσου εγκλεισμού που χρησιμοποιείται, οι σπερματοθήκες είναι συνήθως δυνατόν να παρατηρηθούν ικανοποιητικά, ιδίως όταν παρουσιάζουν επιφανειακή μορφολογία (μη λείες) και έχει προηγηθεί επαρκής διαύγαση. Αντιθέτως, η παρατήρηση λείων σπερματοθηκών με λεπτά τοιχώματα ενδέχεται να είναι προβληματική σε μέσα εγκλεισμού με χαμηλό δείκτη διάθλασης. Επιπλέον, η παρατήρηση της βάσης των πόρων των σπερματοθηκών είναι απαραίτητη για την ταυτοποίηση των ειδών, όπως στην περίπτωση του υπογένους Larroussius [35, 37, 38], το οποίο περιλαμβάνει τους κύριους φορείς-διαβιβαστές του είδους Leishmania infantum στον Παλαιό Κόσμο. Η απουσία αυτής της παρατήρησης, καθιστά την ταυτοποίηση των δειγμάτων αδύνατη. Για την αντιμετώπιση αυτών των δυσκολιών παρατήρησης, συνιστάται η απόσπαση του συμπλέγματος γεννητικής διχάλας (furca) και σπερματοθηκών από την κοιλία (https://zenodo.org/records/18311106). Οι σπερματοθήκες είναι, κατά κανόνα, δυσδιάκριτες κατά την ανατομή, ενώ η γεννητική φούρκα εντοπίζεται σχετικά εύκολα. Δεδομένου ότι οι πόροι των σπερματοθηκών εκβάλλουν στη γεννητική φούρκα, η απομόνωσή της οδηγεί συνήθως και στην ταυτόχρονη απομόνωση των σπερματοθηκών. Σε περίπτωση που οι σπερματοθήκες αποκοπούν κατά λάθος κατά τη διαδικασία ανατομής, αυτές δεν χάνονται καθώς παραμένουν εντός των κοιλιακών περιβλημάτων και μπορούν να εντοπιστούν και να παρατηρηθούν (Σχήμα 4).

4.4. Ανατομή μέσου εντέρου για απομόνωση *Leishmania*

Η ανατομή του πεπτικού σωλήνα είναι απαραίτητη για την ανίχνευση και απομόνωση παρασίτων Leishmania από θηλυκές σκνίπες. Η διαδικασία μπορεί να πραγματοποιηθεί τόσο στο πεδίο όσο και στο εργαστήριο, προκειμένου να αξιολογηθεί η ικανότητα μετάδοσης από το έντομο.

Συνιστάται η χρήση πρόσφατα θανατωμένων θηλυκών ατόμων. Τα θηλυκά άτομα υποβάλλονται σε ήπιο καθαρισμό με νερό ή διάλυμα φυσιολογικού ορού που περιέχει ήπιο απορρυπαντικό, για την απομάκρυνση των περιττών τριχιδίων. Αυτό το βήμα διασφαλίζει τη διατήρηση ασηπτικών συνθηκών για την απομόνωση του

This is an Open Access article distributed under the terms of the Creative Commons Attribution License (https://creativecommons.org/licenses/by/4.0), which permits unrestricted use, distribution, and reproduction in any medium, provided the original work is properly cited.

Leishmania, ενώ παράλληλα διαφυλάσσει τα μορφολογικά χαρακτηριστικά που απαιτούνται για την ταυτοποίηση.

Για την εύρεση και απομόνωση του Leishmania, το μέσο έντερο αφαιρείται προσεκτικά και τοποθετείται σε σταγόνα αποστειρωμένου φυσιολογικού ορού (0,9% NaCl). Μετά την παρατήρηση ζωντανών προμαστιγωτών Leishmania σε οπτικό μικροσκόπιο (συνιστώμενη μεγέθυνση: ~200×), τα παράσιτα συλλέγονται με σύριγγα ινσουλίνης ή μικροπιπέτα και μεταφέρονται σε θρεπτικό υλικό κυτταροκαλλιέργειας (για περισσότερες λεπτομέρειες, βλ. Κεφάλαιο 4.4.3).

Οι κεφαλές και τα γεννητικά όργανα τοποθετούνται απευθείας σε υγρό Marc-André για σκοπούς διαύγασης. Σημαντικό: Το υγρό δεν πρέπει να έρθει ποτέ σε επαφή με τα παράσιτα Leishmania– ούτε άμεσα ούτε έμμεσα μέσω εργαλείων ή βελόνας – καθώς είναι θανατηφόρο για τα παράσιτα.

Η ανατομή των θηλυκών σκνιπών μπορεί να πραγματοποιηθεί είτε σε μία αντικειμενοφόρο πλάκα είτε σε δύο, με κάθε επιλογή να παρουσιάζει συγκεκριμένα πλεονεκτήματα και περιορισμούς (Σχήμα 5; https://zenodo.org/records/18311154).

4.4.1. Μέθοδος με δύο αντικειμενοφόρες πλάκες

Η πρώτη προσέγγιση περιλαμβάνει τη χρήση δύο ξεχωριστών αντικειμενοφόρων πλακών, όπου η μία περιέχει αποστειρωμένο φυσιολογικό ορό για την απομόνωση του μέσου εντέρου, και η άλλη προορίζεται για τον εγκλεισμό της κεφαλής και των σπερματοθηκών σε διάλυμα Marc-André. Ωστόσο, σε συνθήκες πεδίου είναι σύνηθες δύο ή τρία άτομα να αναλαμβάνουν την ανατομή των σκνιπών και έπειτα να παραδίδουν τα δείγματα σε άλλο ερευνητή για την ταυτοποίηση των ειδών και την αξιολόγηση τυχόν μόλυνσης με παράσιτα Leishmania. Έτσι, η διαχείριση δύο πλακών ενδέχεται να προκαλέσει προβλήματα ιχνηλασιμότητας των δειγμάτων και, ειδικότερα σε περιπτώσεις ανίχνευσης μολυσμένου εντέρου, να καταστήσει δύσκολο τον ακριβή συσχετισμό του με το αντίστοιχο άτομο σκνίπας από το οποίο προήλθε (https://zenodo.org/records/18311154).

4.4.2. Μέθοδος με χρήση μίας αντικειμενοφόρου πλάκας

Η χρήση μίας μόνο αντικειμενοφόρου πλάκας διασφαλίζει την πλήρη ιχνηλασιμότητα των αποτελεσμάτων. Ωστόσο, απαιτούνται ορισμένες αυστηρές προφυλάξεις για τη μέγιστη διατήρηση ασηψίας. Οι χειριστές πρέπει να απολυμαίνουν τακτικά τα χέρια τους με αντισηπτικό διάλυμα. Επίσης, πρέπει να χρησιμοποιούνται απλές, τελείως διάφανες γυάλινες αντικειμενοφόρες πλάκες (non-frosted) και τετράγωνες καλυπτρίδες, τυλιγμένες σε αλουμινόχαρτο και αποστειρωμένες σε κλίβανο ξηράς αποστείρωσης (κλίβανος Poupinel), καθώς και αποστειρωμένες βελόνες για κάθε ανατομή (προτείνεται βελόνα 25G Ø 0,5 mm × 16 mm). Η σκνίπα τοποθετείται σε σταγόνα αποστειρωμένου φυσιολογικού ορού στο κέντρο της πλάκας. Το κεφάλι αποκόπτεται ενώ πραγματοποιείται τομή μεταξύ του 6ου και 7ου κοιλιακού τεργίτη (ραχιαίες πλάκες, abdominal tergites) και στερνίτη (κοιλιακές πλάκες, sternites) χωρίς να διαρρηχθεί ο πεπτικός σωλήνας (η τομή μπορεί να γίνει υψηλότερα, για παράδειγμα προς τον 4ο ή 5ο κοιλιακό τεργίτη, σε περίπτωση που αναμένονται μακριές σπερματοθήκες). Στη συνέχεια, ο θώρακας σταθεροποιείται με βελόνα, και τα τελευταία οπίσθια κοιλιακά τμήματα τραβιούνται προσεκτικά με δεύτερη βελόνα για την εξαγωγή του εντέρου. Αν αυτό αποτύχει, υπάρχει η δυνατότητα να ακινητοποιηθεί το άκρο της κοιλίας με μία βελόνα και να τραβηχτεί ο πεπτικός σωλήνας από το πρόσθιο τμήμα του. Αν αποτύχει ξανά, το έντερο εξάγεται αφαιρώντας γύρω από αυτό, όσο το δυνατόν περισσότερο από το εναπομείναν περίβλημα. Όταν αφαιρεθεί το έντερο, τα τελευταία κοιλιακά τμήματα αποκόπτονται, ενώ το έντερο τοποθετείται σε νέα σταγόνα αποστειρωμένου φυσιολογικού ορού, στο ένα άκρο της πλάκας, και καλύπτεται απαλά και προσεκτικά με αποστειρωμένη καλυπτρίδα. Η κεφαλή και τα τελευταία κοιλιακά τμήματα μεταφέρονται σε μικρή σταγόνα διαλύματος Marc-André στο άλλο άκρο της πλάκας, διασφαλίζοντας την πλήρη αποφυγή επαφής με παράσιτα *Leishmania*. Η κεφαλή τοποθετείται σωστά (με το ινιακό τρήμα προς τα επάνω), και οι σπερματοθήκες απομονώνονται μαζί με τη γεννητική διχάλα και καλύπτονται με μικρή στρογγυλή καλυπτρίδα (διαμέτρου Ø 12 mm, να μη συγχέεται με τις αποστειρωμένες τετράγωνες καλυπτρίδες. Το υπόλοιπο σώμα της σκνίπας, συμπεριλαμβανομένων των πτερύγων, παραμένει στη σταγόνα φυσιολογικού ορού στο κέντρο της αντικειμενοφόρου πλάκας. Σε περίπτωση ανίχνευσης παρασίτων *Leishmania*, ή στο πλαίσιο ταξινομικών αναλύσεων, ο θώρακας και η κοιλία μπορούν να διατηρηθούν για περαιτέρω μοριακές ή πρωτεϊνικές αναλύσεις, ενώ οι πτέρυγες μπορούν να τοποθετηθούν σε υδατικό μέσο εγκλεισμού. Για τη διατήρηση του παρασκευάσματος, η περίσσεια του διαλύματος Marc-André μπορεί να αντικατασταθεί από κατάλληλο υδατικό μέσο εγκλεισμού, όπως κόμμι χλωράλης (= Hoyer) ή μέσο βασισμένο σε πολυβινυλική αλκοόλη.

Λεπτομερή εκπαιδευτικά βίντεο που παρουσιάζουν αυτές τις διαδικασίες είναι διαθέσιμα (ανατομή μέσου εντέρου σνίπας: https://zenodo.org/records/18303014 και ανατομή σιελογόνων αδένων: https://zenodo.org/records/18302850), ως εκ τούτου δεν περιγράφονται περαιτέρω στην παρούσα ενότητα.

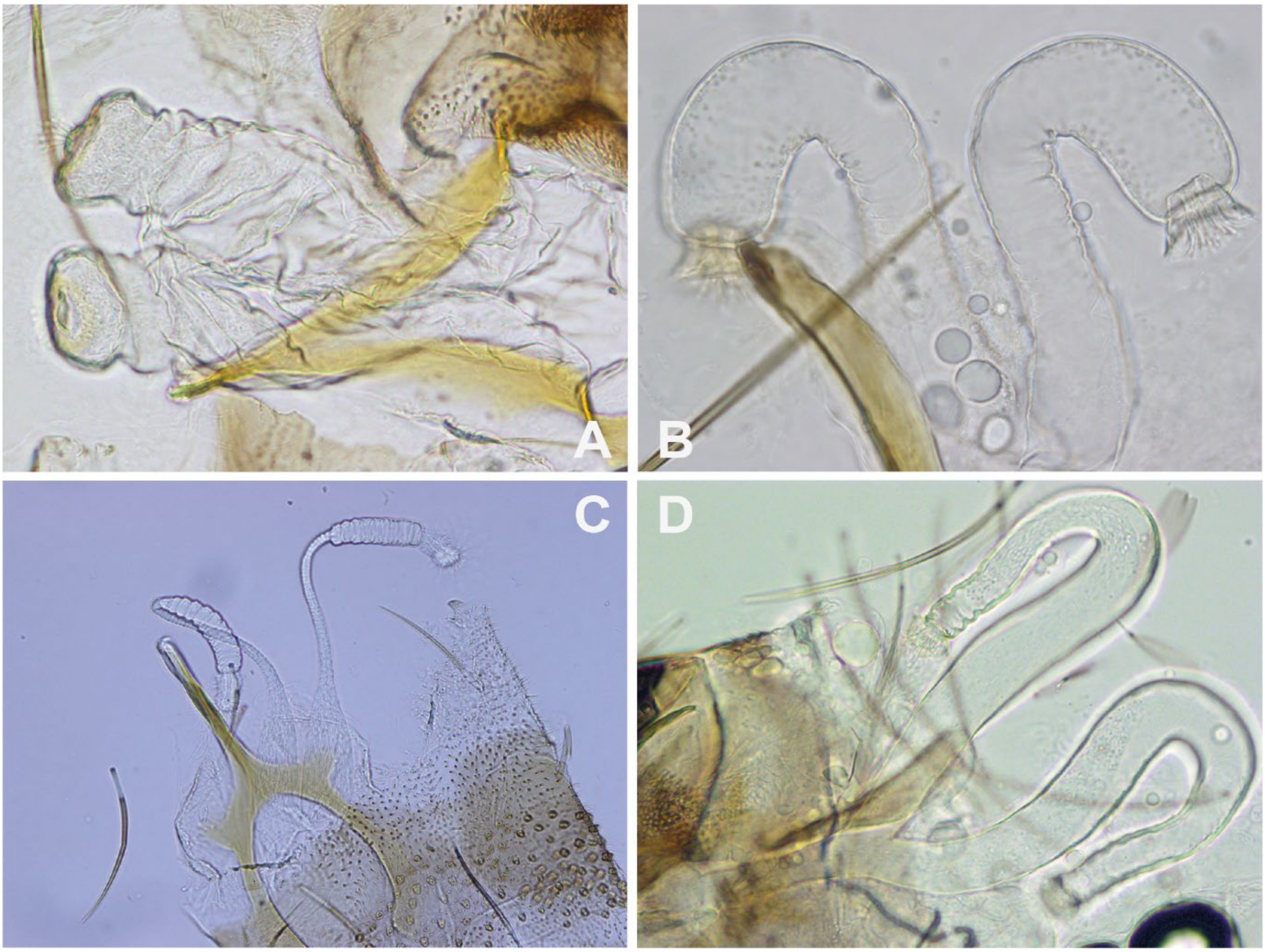

Σχήμα 4: Σπερματοθήκες απομονωμένες με ανατομή από φρέσκα δείγματα σκνιπών και εγκλεισμένες σε διάλυμα Marc-André. A: *Idiophlebotomus longiforceps* (Λαϊκή Δημοκρατία του Λάος). B: *Sergentomyia minuta* (Γαλλία). C: *Phlebotomus ariasi* (Γαλλία). D: *Sergentomyia anodontis* (Λαϊκή Δημοκρατία του Λάος).

4.4.3. Απομόνωση και καλλιέργεια παρασίτων Leishmania από το έντερο φλεβοτόμων

Η απομόνωση παρασίτων μέσω ανατομής μολυσμένων θηλυκών φλεβοτόμων αποτελεί λεπτή και απαιτητική τεχνική, η οποία προϋποθέτει υψηλή δεξιοτεχνία και θα πρέπει αρχικά να εξασκείται σε μη μολυσμένα δείγματα. Μετά την μικροανατομή, ο εντερικός σωλήνας απομονώνεται και μεταφέρεται για έκπλυση σε νέα σταγόνα αποστειρωμένου φυσιολογικού ορού (0,9%) ή διαλύματος Locke [4]. Τα ανατομημένα έντερα μπορούν στη συνέχεια να υποβληθούν σε δύο τύπους επεξεργασίας: i) μικροσκοπική εξέταση (οπτική μικροσκοπία) για την παρατήρηση των διαφορετικών σταδίων των προμαστιγωτών μορφών Leishmania καθώς και για τον ακριβή εντοπισμό τους, με ιδιαίτερη έμφαση στη συσσώρευσή τους στη στομοδεακή βαλβίδα (stomodeal valve), και ii) διάνοιξη του εντέρου για τη διευκόλυνση της απομόνωσης των προμαστιγωτών και, κατ' επέκταση, της μαζικής καλλιέργειά τους [4]. Η ανεύρεση μολυσμένων φλεβοτόμων στο πεδίο είναι σχετικά σπάνια, συνεπώς η επαρκής τεχνική κατάρτιση και εξάσκηση αυξάνει σημαντικά τις πιθανότητες επιτυχούς απομόνωσης.

Σε περίπτωση εντοπισμού παρασίτων Leishmania στο έντερο, χρησιμοποιούνται νέες αποστειρωμένες μικροβελόνες και προστίθεται μικρή ποσότητα αποστειρωμένου φυσιολογικού ορού περιμετρικά της καλυπτρίδας, ο οποίος εισχωρεί μέσω τριχοειδούς φαινομένου, προκαλώντας την έκλουση και απελευθέρωσή των παρασίτων. Το έντερο θα πρέπει να διανοιχθεί προσεκτικά και άμεσα, ώστε να απελευθερωθούν τα παράσιτα στο διάλυμα. Με τη χρήση μικροπιπέτας 100 μL ή σύριγγας ινσουλίνης/φυματίνης, τα παράσιτα συλλέγονται και ενοφθαλμίζονται σε θρεπτικό μέσο καλλιέργειας που φέρει πλήρη σήμανση.

This is an Open Access article distributed under the terms of the Creative Commons Attribution License (https://creativecommons.org/licenses/by/4.0), which permits unrestricted use, distribution, and reproduction in any medium, provided the original work is properly cited.

Πίνακας 2: Σύσταση των αντιδραστηρίων.

**Υδροξείδιο του καλίου 10%**<br>Υδροξείδιο του καλίου 10 g<br>Απεσταγμένο νερό q.s. έως 100ml	**Όξινη φουξίνη 1% σε απεσταγμένο νερό**<br>Όξινη φουξίνη (σκόνη) 1 g<br>Απεσταγμένο νερό 99 mL
**Μέσο εγκλεισμού Chloral gum (Hoyer)**<br>Απεσταγμένο νερό 50 mL<br>Ένυδρη χλωράλη 200 g<br>Αραβικό κόμμι 50 g<br>Γλυκερόλη 20 mL	**Διάλυμα Marc-André με όξινη φουξίνη**<br>Διάλυμα Marc-André 10mL<br>Όξινη φουξίνη 1% 50 μL
**Διάλυμα Marc-André**<br>Ένυδρη χλωράλη 40 g<br>Παγόμορφο οξικό οξύ 30 mL<br>Απεσταγμένο νερό 30 mL	**Μέσο Enecê**<br>Καθαρό λευκό κολοφώνιο 22 g<br>Κόμμι κοπάλ (διαλυτό σε αλκοόλη) 12 g<br>Απόλυτη αιθανόλη 20 mL<br>Καμφορά 10 g

In vitro καλλιέργεια προμαστιγωτών μορφών *Leishmania*: Τα απομονωμένα παράσιτα διατηρούνται αρχικά σε επικλινή θρεπτικά υποστρώματα αίματος SNB-9 ή σε στερεό θρεπτικό μέσο Novy–McNeal–Nicolle (NNN) [16], τα οποία καλύπτονται με υγρή φάση αποστειρωμένου μέσο α-MEM [16, 65] ή M199. Και τα δύο μέσα εμπλουτίζονται με 10% θερμικά αδρανοποιημένο εμβρυϊκό ορό μόσχου (FCS) (για ενίσχυση της ανάπτυξης των παρασίτων), 1% βιταμίνες BME, 2% αποστειρωμένα ανθρώπινα ούρα (διηθημένα μέσω φίλτρου σύριγγας Filtropur® S 0,2 μm), 250 μg/mL αμικασίνη (εναλλακτικά 50 μg/mL γενταμικίνη ή μείγμα αντιβιοτικών/ αμινοξέων που περιέχει L-γλουταμίνη 200 mM, πενικιλίνη 10.000 U, στρεπτομυκίνη 10 mg/mL) [47]. Μετά από τρεις ημέρες, εφόσον δεν παρατηρηθεί επιμόλυνση, οι καλλιέργειες μεταφέρονται σε κατάλληλο μέσο κρυοσυντήρησης και στη συνέχεια φυλάσσονται στους −80°C για 1–2 έτη ή σε υγρό άζωτο (−196°C) για μακροχρόνια διατήρηση και μελλοντική πειραματική χρήση [7].

4.5. Σιελογόνοι αδένες

Η ανατομή και απομόνωση των σιελογόνων αδένων των φλεβοτόμων αποτελεί θεμελιώδη τεχνική για τη μελέτη των αλληλεπιδράσεων φορέα-διαβιβαστή και παθογόνου, ιδιαίτερα για την ανίχνευση αρμποϊών, όπως οι Phlebovirus (π.χ. ιός Toscana) [44, 75]. Λόγω του εξαιρετικά μικρού μεγέθους των φλεβοτόμων, η διαδικασία απαιτεί υψηλή ακρίβεια υπό στερεοσκόπιο, με χρήση λεπτών λαβίδων, ακίδων ή βελόνων μικροανατομής, ώστε οι ευαίσθητοι σιελογόνοι αδένες να απομονωθούν ακέραιοι, χωρίς ρήξη ή επιμόλυνση (https://zenodo.org/records/18302850) [51, 61]. Η διατήρηση της ακεραιότητας των αδένων είναι καθοριστικής σημασίας για τη διασφάλιση αξιόπιστων μοριακών αναλύσεων σε μεταγενέστερο στάδιο. Μετά την απομόνωση, οι αδένες ομογενοποιούνται και αναλύονται μέσω RT-PCR, qPCR ή ανοσολογικών δοκιμών, για την ανίχνευση ιικού RNA ή αντιγόνων [12]. Η παρουσία ιών στους σιελογόνους αδένες -και όχι περιοριστικά στο έντερο ή το αιμοκοίλωμα- επιβεβαιώνει ότι το παθογόνο έχει ολοκληρώσει την εξωγενή περίοδο επώασης (extrinsic incubation period, ο χρόνος μεταξύ λήψης μολυσμένου αίματος μέχρι το έντομο να καταστεί μεταδοτικό) και είναι ικανό να διαβιβαστεί κατά τη διάρκεια της αιμομύζησης [71].Η ανατομή των σιελογόνων αδένων είναι τεχνικά απαιτητική λόγω του μικρού μεγέθους των φλεβοτόμων και προϋποθέτει σημαντική εργαστηριακή εμπειρία για την αποφυγή αλλοίωσης των δειγμάτων [1, 51]. Επιπλέον, τα ιικά φορτία ενδέχεται να είναι χαμηλά, καθιστώντας αναγκαία τη χρήση μεθόδων υψηλής ευαισθησίας, όπως η nested PCR ή η αλληλούχηση υψηλής απόδοσης (high-throughput sequencing) [54]. Οι κίνδυνοι επιμόλυνσης υπογραμμίζουν την ανάγκη εφαρμογής αυστηρών άσηπτων τεχνικών. Πέραν των τεχνικών δυσκολιών, η επιτυχία ανίχνευσης επηρεάζεται και από βιολογικούς παράγοντες, η ικανότητα μετάδοσης ποικίλλει μεταξύ των ειδών φλεβοτόμων, ενώ τα ποσοστά μόλυνσης μεταβάλλονται ανάλογα με τις οικολογικές και εποχικές συνθήκες [33, 61].

Η ανίχνευση ιών στους σιελογόνους αδένες παρέχει κρίσιμα δεδομένα για την εκτίμηση του κινδύνου διαβίβασης-μετάδοσης, επιτρέποντας στοχευμένη επιδημιολογική επιτήρηση και την εφαρμογή μέτρων ελέγχου [15]. Ενδεικτικά, η ανίχνευση του ιού Toscana σε φλεβοτόμους σε ενδημικές περιοχές έχει συμβάλει καθοριστικά στη διαμόρφωση διαγνωστικών πρωτοκόλλων και κατευθυντήριων οδηγιών δημόσιας υγείας [18]. Επιπλέον, η διερεύνηση των αλληλεπιδράσεων ιών-σιέλου ενδέχεται

This is an Open Access article distributed under the terms of the Creative Commons Attribution License (https://creativecommons.org/licenses/by/4.0), which permits unrestricted use, distribution, and reproduction in any medium, provided the original work is properly cited.

να αναδείξει νέους στόχους για την ανάπτυξη εμβολίων ή θεραπευτικών μέσων που παρεμποδίζουν τη διασπορά του παθογόνου [15, 18

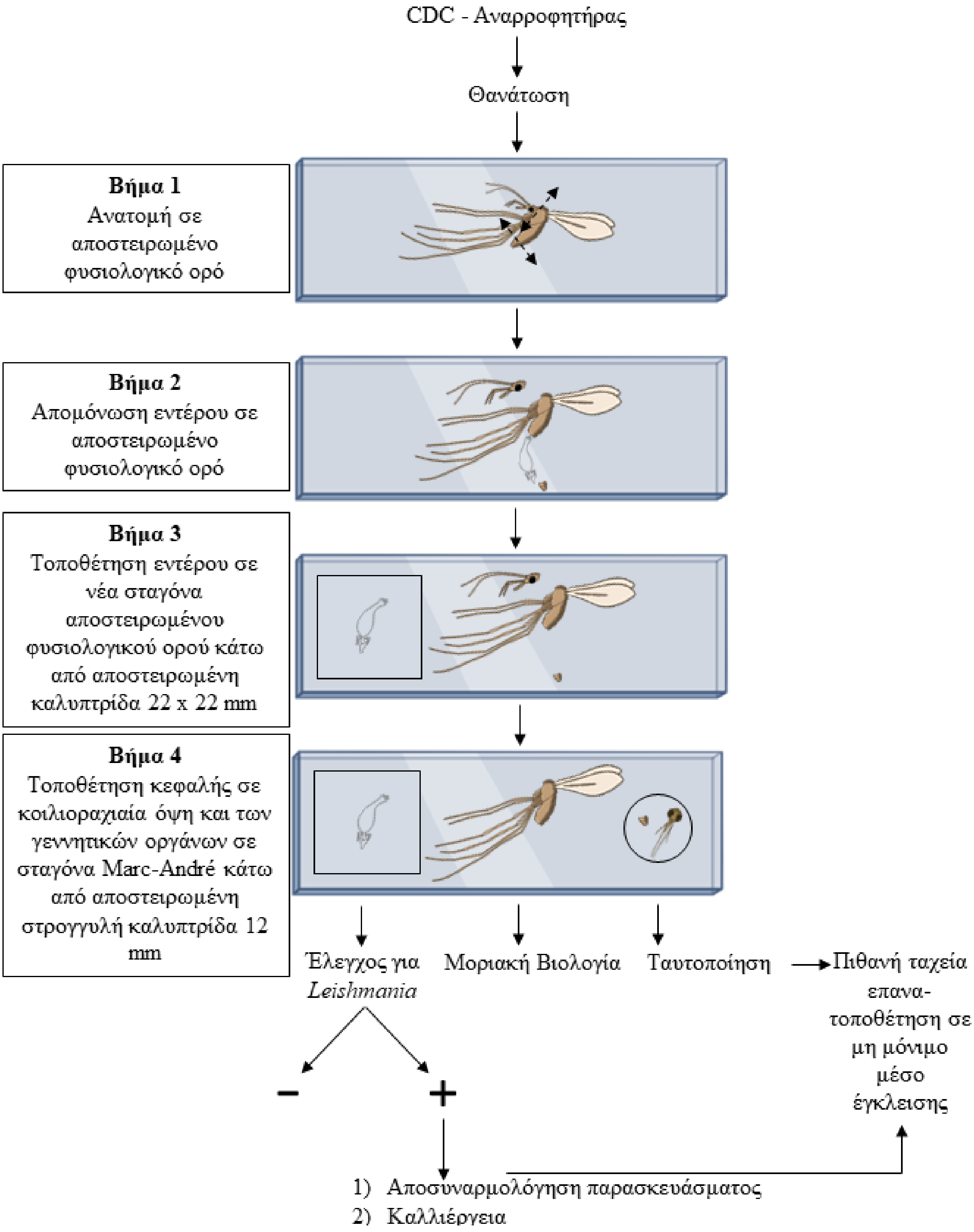

Σχήμα 5: Μέθοδος απομόνωσης παρασίτων *Leishmania*.

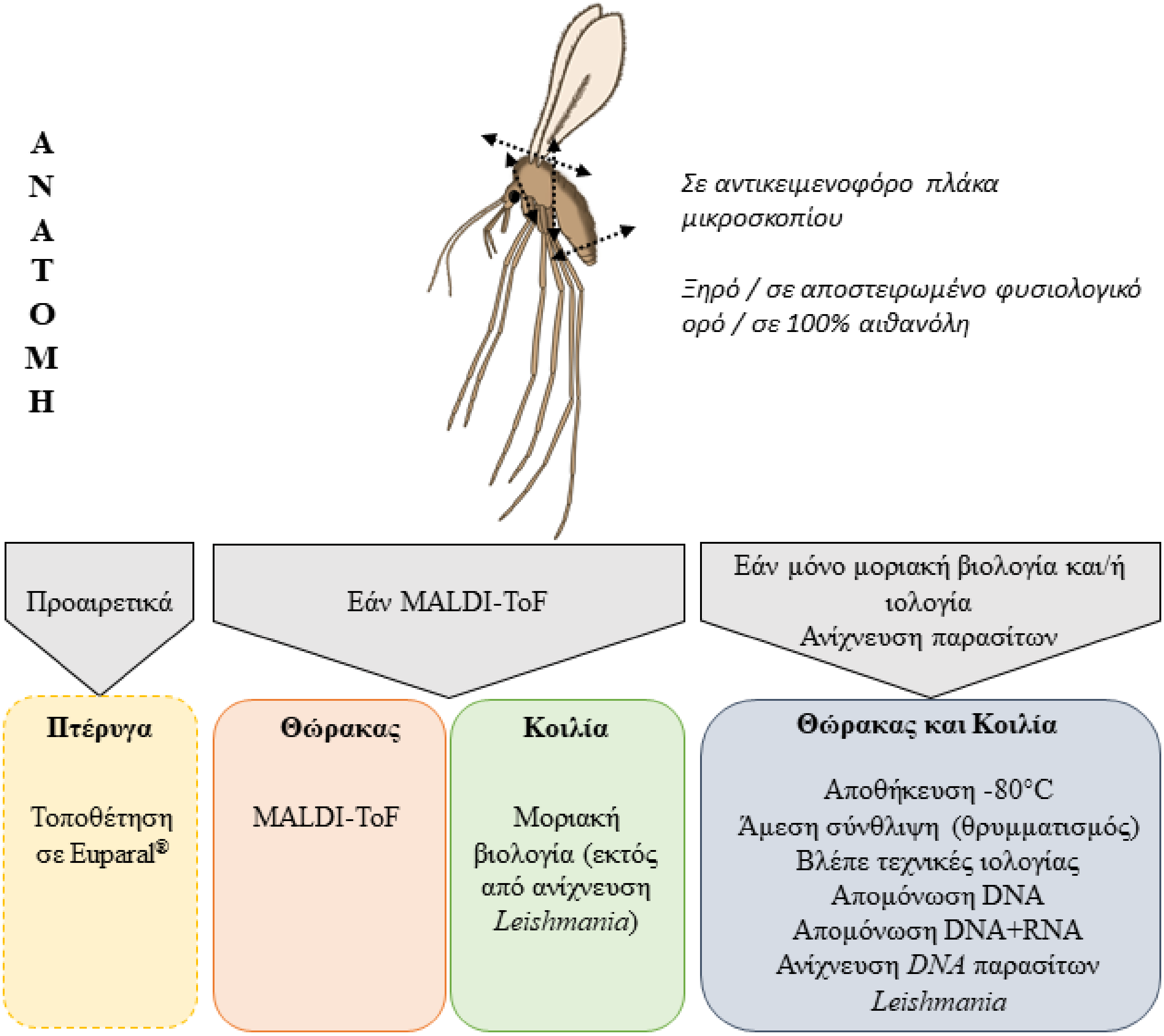

Σχήμα 6: Επεξεργασία φλεβοτόμων για εφαρμογές μοριακής βιολογίας, πρωτεομικής και/ή ιολογίας.

This is an Open Access article distributed under the terms of the Creative Commons Attribution License (https://creativecommons.org/licenses/by/4.0), which permits unrestricted use, distribution, and reproduction in any medium, provided the original work is properly cited.

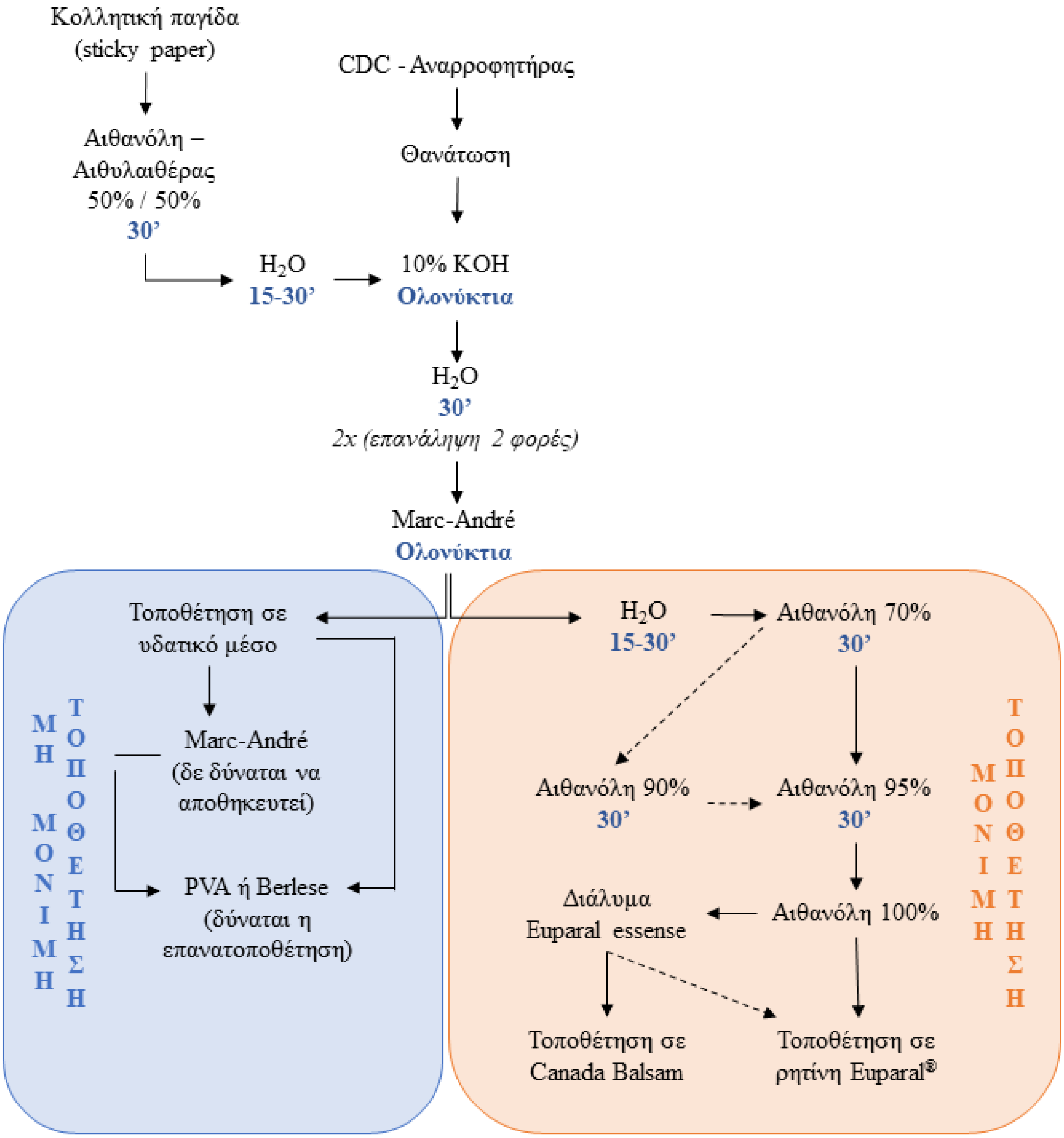

Σχήμα 7: Κλασσική μέθοδος επεξεργασίας φλεβοτόμων.

Παράλληλα, οι σιελογόνοι αδένες των φλεβοτόμων αποτελούν πηγή αντιγόνων για τον προσδιορισμό ειδικών αντισωμάτων του ξενιστή έναντι της σιέλου των φλεβοτόμων, μέσω ανοσολογικών μεθόδων (κατά προτίμηση ELISA). Η προσέγγιση αυτή επιτρέπει την ποσοτική εκτίμηση της έκθεσης των ξενιστών σε νύγματα φλεβοτόμων, συμβάλλοντας στην αξιολόγηση της αποτελεσματικότητας των μέτρων ελέγχου και καταπολέμησης των φορέων-διαβιβαστών [25], καθώς και στην εκτίμηση του κινδύνου μετάδοσης παρασίτων *Leishmania* [40]..

4.6. Προσδιορισμός του γεύματος αίματος

Τα θηλυκά που έχουν τραφεί με αίμα και έχουν απομονώνονται κατά την συλλογή θα πρέπει να ανατέμνονται με εξοπλισμό μίας χρήσης, ώστε να αποφεύγεται η διασταυρούμενη επιμόλυνση. Η κοιλία τους εξετάζεται υπό στερεοσκόπιο για την εκτίμηση του σταδίου πέψης του αίματος. Συνιστάται η επιλογή αποκλειστικά θηλυκών με ερυθρή, ερυθροκάστανη ή βαθυκόκκινη κοιλία, χωρίς ενδείξεις σχηματισμού ωαρίων. Το οπίσθιο άκρο της κοιλίας, συμπεριλαμβανομένων των σπερματοθηκών, θα πρέπει να αφαιρείται για να είναι δυνατή η μορφολογική ταυτοποίηση του θηλυκού μετά τη

This is an Open Access article distributed under the terms of the Creative Commons Attribution License (https://creativecommons.org/licenses/by/4.0), which permits unrestricted use, distribution, and reproduction in any medium, provided the original work is properly cited.

διαύγαση. Στη συνέχεια, το κύριο τμήμα της κοιλίας (χωρίς τις σπερματοθήκες) τοποθετείται σε σωληνάρια Eppendorf® και φυλάσσεται στους −20°C μέχρι την περαιτέρω επεξεργασία. Οι γενετικοί δείκτες που χρησιμοποιούνται συνήθως για τον προσδιορισμό του γεύματος αίματος, όπως PNOC [5, 30, 50], CytB [67] ή COI [13], είναι καθιερωμένοι και περιγράφονται εκτενώς στη βιβλιογραφία, επομένως δεν θα αναλυθούν περαιτέρω στην παρούσα εργασία (Σχήμα 6). Εναλλακτικά, για την ταυτοποίηση του ξενιστή από τον οποίο τράφηκε η σκνίπα, μπορεί να εφαρμοστεί πεπτιδική χαρτογράφηση με φασματομετρία μάζας που βασίζεται στην ταυτοποίηση πρωτεϊνών (MALDI-ToF peptide mapping) [31]. Έχει αποδειχθεί πειραματικά ότι η τεχνική αυτή επιτρέπει την ταυτοποίηση του ξενιστή σε διευρυμένο χρονικό παράθυρο μετά τη λήψη του γεύματος αίματος. Συνεπώς, αποτελεί την μέθοδο εκλογής, ιδίως για την ανάλυση θηλυκών που έχουν τραφεί, με εμφανώς προχωρημένο στάδιο πέψης του αίματος. Τα δείγματα θα πρέπει ιδανικά να φυλάσσονται στους −20°C ή στους 4°C, αν και ικανοποιητικά αποτελέσματα μπορούν επίσης να προκύψουν από δείγματα που διατηρήθηκαν σε θερμοκρασία περιβάλλοντος για σύντομο χρονικό διάστημα. Η κοιλία του θηλυκού ανατέμνεται από το υπόλοιπο σώμα λίγο πριν από την ανάλυση και ομογενοποιείται σε απεσταγμένο νερό. Το υπόλοιπο σώμα παραμένει διαθέσιμο για περαιτέρω μοριακές και μορφολογικές αναλύσεις. Μετά τη λήψη κλάσματος από το ομογενοποίημα για την χαρτογράφηση MALDI-ToF, το υπόλοιπο υλικό μπορεί να χρησιμοποιηθεί για εκχύλιση DNA, προκειμένου να επιβεβαιωθεί η ταυτοποίηση του ξενιστή ή/και να ελεγχθεί η παρουσία παρασίτων Leishmania. Ο συνολικός χρόνος προετοιμασίας και ανάλυσης των δειγμάτων είναι σημαντικά μικρότερος σε σύγκριση με τις μοριακές τεχνικές που βασίζονται στην ανάλυση DNA.

5. Προετοιμασία δειγμάτων για μορφολογική ανάλυση (Σχήματα 3, 6, 7 & 8 - Παραρτήματα 1, 2, 3 & 4)

Η ενότητα αυτή περιγράφει τις βασικές αρχές προετοιμασίας δειγμάτων φλεβοτόμων για εγκλεισμό και μονιμοποίηση, εστιάζοντας αποκλειστικά για μορφολογικές μελέτες, καθώς και τις απαραίτητες προσαρμογές της μεθοδολογίας για εφαρμογές πέραν της μορφολογικής ανάλυσης. Η κατανόηση της συγκεκριμένης μεθοδολογίας είναι καθοριστική, καθώς επιτρέπει την τροποποίηση των διαδικασιών για ειδικούς τύπους δειγμάτων, όταν αυτό κρίνεται απαραίτητο.

Η επεξεργασία περιλαμβάνει διαδοχικά στάδια αφαίρεσης και προσθήκης υγρών, τα οποία πραγματοποιούνται με πιπέτες Pasteur εφοδιασμένες με ελαστική θηλή αναρρόφησης (εξάρτημα poire). Συνιστάται ιδιαίτερα η χρήση γυάλινων δοχείων με κοίλο πυθμένα, καθώς διευκολύνουν σημαντικά τον χειρισμό των δειγμάτων. Το γυαλί επιλέγεται ως χημικά αδρανές υλικό έναντι όλων των χρησιμοποιούμενων αντιδραστηρίων. Για την αποφυγή εξάτμισης των αντιδραστηρίων, τα δοχεία θα πρέπει να φέρουν πώματα, ενώ συστήνεται η αποφυγή υπερπλήρωσή τους για πρόληψη τυχόν υπερχείλισης κατά τον χειρισμό τους ή εναπόθεσης σκόνης στα δείγματα. Τα χημικά αντιδραστήρια που απαιτούνται για τη διαύγαση και επεξεργασία των δειγμάτων παρουσιάζονται στον Πίνακα 2.

5.1. Διαύγαση

Πριν τα δείγματα φλεβοτόμων προετοιμαστούν ως μόνιμα μικροσκοπικά παρασκευάσματα, πρέπει πρώτα να υποβληθούν σε διαδικασία διαύγασης, η οποία περιλαμβάνει την αποδόμηση των μαλακών ιστών με χρήση κατάλληλων μεθόδων και διαυγαστικών παραγόντων (π.χ. διάλυμα οξικού οξέος 10% ή διάλυμα Marc-André που περιέχει ένυδρη χλωράλη, η χρήση της οποίας είναι περιορισμένη σε πολλές χώρες), ώστε τα δείγματα να καταστούν διαφανή. Η διαδικασία της διαύγασης απομακρύνει μαλακούς ιστούς, λιπίδια, εκκρίσεις και κηρώδεις ουσίες, καθιστώντας το δείγμα ημιδιαφανές και διευκολύνοντας την παρατήρηση εξωσκελετικών δομών (π.χ. σημεία έκφυσης των τριχών, setae insertion), επιφανειακών χαρακτηριστικών (π.χ. χρωματισμός) καθώς και εσωτερικών μορφολογικών στοιχείων που είναι ορατά μέσω του εξωτερικού περιβλήματος (tegument), όπως οι σπερματοθήκες (spermathecae).

Η διαδικασία διαύγασης πραγματοποιείται σε δύο στάδια, αρχικά με ισχυρή βάση (όπως υδροξείδιο του καλίου) και στη συνέχεια με ασθενές οξύ (όπως οξικό οξύ στο διάλυμα Marc-André), εξυπηρετώντας διαφορετικούς βιοχημικούς σκοπούς [74]. Η βάση διασπά τους μαλακούς ιστούς (πρωτεΐνες, λίπη, μυϊκό ιστό) μέσω σαπωνοποίησης και αποδιάταξης των πρωτεϊνών, αφήνοντας άθικτο τον χιτινώδη εξωσκελετό και επιτρέποντας καθαρή απεικόνιση των δομών. Το ασθενές οξύ εξουδετερώνει τα υπολείμματα της αλκαλικής βάσης, αποτρέποντας περαιτέρω αποδόμηση, και ταυτόχρονα αποχρωματίζει τη χιτίνη και ενισχύει τη διαφάνεια του δείγματος [74]. Ωστόσο, δύο διαδοχικές εκπλύσεις των δειγμάτων σε απεσταγμένο νερό, διάρκειας 15 λεπτών η καθεμία, αρκούν για την εξουδετέρωση της βάσης. Η διαδοχική αυτή επεξεργασία αφαιρεί αποτελεσματικά τους ιστούς, διατηρώντας παράλληλα τη μορφολογική ακεραιότητα και διασφαλίζοντας τη βέλτιστη κατάσταση των δειγμάτων για μικροσκοπική εξέταση.

Πριν από το επόμενο στάδιο, προτείνονται δύο επιπλέον εκπλύσεις σε απεσταγμένο νερό, διάρκειας 20 λεπτών η καθεμία.

5.1.1. Λύση μαλακών ιστών (Σχήμα 8)

Το υδροξείδιο του νατρίου (NaOH) και το υδροξείδιο του καλίου (KOH) χρησιμοποιούνται συχνά ως χημικοί παράγοντες για την αποδόμηση και λύση των μαλακών ιστών, εφαρμόζοντας διαφορετικές συγκεντρώσεις και χρόνους επώασης ανάλογα με το μέγεθος και την ευθραυστότητα των δειγμάτων. Η τυπική και πιο αποτελεσματική τεχνική περιλαμβάνει εμβάπτιση των φλεβοτόμων σε διάλυμα ισχυρής βάσης (10% KOH ή NaOH) και επώαση για περίπου 12-16 ώρες. Η αύξηση της συγκέντρωσης μπορεί να μειώσει τη διάρκεια της επεξεργασίας (π.χ. 20% KOH για 6 ώρες), ενώ η ήπια θέρμανση στους 37°C μπορεί να επιταχύνει περαιτέρω τη διαδικασία.

5.1.2. Διαύγαση με ή χωρίς χρώση

Στο στάδιο αυτό ακολουθεί η διαδικασία διαύγασης, η οποία συνήθως πραγματοποιείται με χρήση διαυγαστικού διαλύματος που περιέχει οξικό οξύ και ένυδρη χλωράλη (π.χ. διάλυμα Marc-André). Μετά τη διαύγαση, τα δείγματα πρέπει να εκπλυθούν σχολαστικά σε τουλάχιστον δύο διαδοχικά λουτρά νερού, διάρκειας 20 λεπτών το καθένα, ώστε να απομακρυνθούν πλήρως τα υπολείμματα των χημικών ουσιών.

Το διάλυμα Marc-André αποτελεί έναν ευρέως χρησιμοποιούμενο διαυγαστικό παράγοντα για την προετοιμασία δειγμάτων φλεβοτόμων. Η αποτελεσματικότητά του έγκειται στο ότι διευκολύνει τη διαδικασία διαύγασης, περιορίζοντας ταυτόχρονα την πρόκληση βλαβών στις εύθραυστες δομές, όπως οι πτέρυγες και οι κεραίες.

Το διάλυμα συνιστάται να παρασκευάζεται αμέσως πριν από τη χρήση ή να φυλάσσεται σε ερμητικά κλειστό δοχείο, προς αποφυγή εξάτμισης ή χημικής αποικοδόμησης. Η χρήση του διαλύματος Marc-André είναι ιδιαίτερα πλεονεκτική όταν συνδυάζεται με τεχνικές διαύγασης ή χρώσης που αναδεικνύουν συγκεκριμένα μορφολογικά χαρακτηριστικά. Λεπτομέρειες σχετικά με τη σύσταση και την παρασκευή του παρατίθενται στο Παράρτημα 2.

Σε ιδιαίτερα διαφανή δείγματα, ενδέχεται να απαιτείται χρώση για τη βελτίωση της ορατότητας πριν από τον εγκλεισμό. Υπάρχει πληθώρα διαθέσιμων χρωστικών, καθεμία από τις οποίες στοχεύει σε συγκεκριμένα χημικά συστατικά του οργανισμού. Επομένως, είναι σημαντικό να επιλέγεται χρωστική συμβατή τόσο με το δείγμα όσο και με το επιλεγμένο μέσο εγκλεισμού. Η βασική αυτή μεθοδολογία μπορεί να προσαρμοστεί ανάλογα με τις ανάγκες, για παράδειγμα με την προσθήκη 0,1% όξινης φουξίνης στο διάλυμα Marc-André για σκοπούς χρώσης. Επιπλέον, δείγματα που έχουν διατηρηθεί σε υδατικά διαλύματα και προορίζονται για εγκλεισμό σε ρητινώδη μέσα πρέπει να υποβληθούν σε αφυδάτωση (βλ. Ενότητα 5.2 «Αφυδάτωση»), δεδομένου ότι τα περισσότερα φυσικά και συνθετικά ρητινώδη μέσα εγκλεισμού δεν είναι συμβατά με το νερό. Ο New (1974) επισήμανε ότι ορισμένες χρωστικές ενδέχεται να υποστούν αλλοίωση σε συγκεκριμένα μέσα εγκλεισμού [53]. Για παράδειγμα, η όξινη φουξίνη, που χρησιμοποιείται συχνά με το βάλσαμο του Καναδά (Canada balsam), μπορεί επίσης να σταθεροποιηθεί σε Euparal®. Ωστόσο, δείγματα που έχουν χρωματιστεί με όξινη φουξίνη είναι επιρρεπή σε αποχρωματισμό, ιδιαίτερα όταν παραμένουν υπολείμματα γαρυφαλέλαιου, το οποίο χρησιμοποιείται ως τελικό διαυγαστικό υγρό. Δείγματα που αποθηκεύονται σε γαρυφαλέλαιο μπορεί να εμφανίσουν σημαντική εξασθένηση της χρώσης εντός ολίγων ημερών.

5.2. Αφυδάτωση

Η αφυδάτωση πραγματοποιείται σταδιακά με διαδοχικές εμβαπτίσεις σε διαλύματα αιθανόλης αυξανόμενης συγκέντρωσης: 50%, 70%, 80%, 90% ή 95% και τέλος 100%, με κάθε εμβάπτιση να διαρκεί τουλάχιστον 20 λεπτά. Δεδομένου ότι η αιθανόλη εξατμίζεται γρήγορα, το δοχείο πρέπει να παραμείνει ερμητικά κλειστό κατά τη διάρκεια της διαδικασίας. Όταν το δείγμα αφυδατωθεί πλήρως, η διαδικασία προετοιμασίας μπορεί να διακοπεί προσωρινά. Σε αυτή την περίπτωση, προτείνεται το δείγμα να παραμείνει για λίγες ημέρες σε Euparal® essence, το οποίο είναι προτιμότερο από το γαρυφαλέλαιο. Το κρεόζωτο οξιάς, που παλαιότερα χρησιμοποιούνταν ευρέως για τον σκοπό αυτό, απαγορεύεται πλέον λόγω της υψηλής τοξικότητάς του.

Η διαδικασία αφυδάτωσης πρέπει να διασφαλίζει ότι το υγρό που παραμένει εντός του δείγματος είναι συμβατό με το μέσο εγκλεισμού. Αυτό είναι απαραίτητο για την αποφυγή θόλωσης, ωσμωτικής βλάβης ή συρρίκνωσης των ιστών ή παραμόρφωση των δομών - αλλοιώσεων που θα μπορούσαν να καταστήσουν το δείγμα ακατάλληλο για ταξινομική μελέτη.

This is an Open Access article distributed under the terms of the Creative Commons Attribution License (https://creativecommons.org/licenses/by/4.0), which permits unrestricted use, distribution, and reproduction in any medium, provided the original work is properly cited.

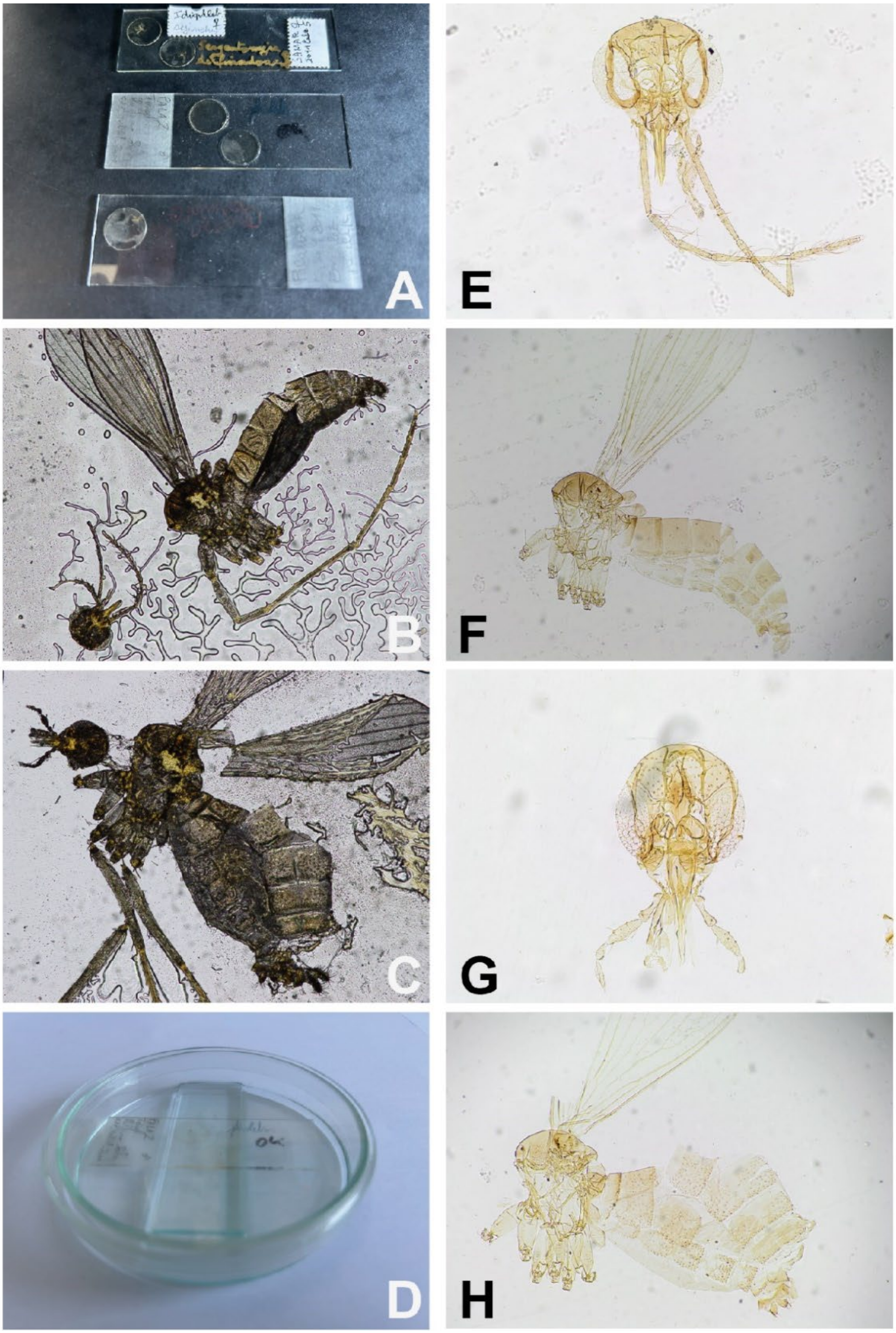

Σχήμα 8: Επανεγκλεισμός σε αντικειμενοφόρο πλάκα. A: Δείγματα με εκτεταμένες φθορές σε αντικειμενοφόρους πλάκες με μέσο Hoyer. B: Μικροσκοπική απεικόνιση δείγματος σκνίπας με μορφολογικές αλλοιώσεις. C: Μικροσκοπική απεικόνιση έτερου δείγματος σκνίπας με απώλεια χαρακτήρων. D: Ξηρό δείγμα σκνίπας σε αντικειμενοφόρο πλάκα, εντός υγρού θαλάμου (wet chamber) για επανυδάτωση. E: Κεφαλή και F: Θώρακας και κοιλία του δείγματος σκνίπας της εικόνας B, κατόπιν επανεγκλεισμού σε μέσο Euparal®. G: Κεφαλή και H: Θώρακας και κοιλία του δείγματος σκνίπας της εικόνας C, κατόπιν επανεγκλεισμού σε μέσο Euparal®.

This is an Open Access article distributed under the terms of the Creative Commons Attribution License (https://creativecommons.org/licenses/by/4.0), which permits unrestricted use, distribution, and reproduction in any medium, provided the original work is properly cited.

5.3. Μέσα εγκλεισμού

5.3.1. Επιλογή και εφαρμογή για την προετοιμασία δειγμάτων

Το μέσο εγκλεισμού θα πρέπει ιδανικά να διαθέτει δείκτη διάθλασης παραπλήσιο με εκείνον του γυαλιού, ο οποίος είναι περίπου 1,5. Πρέπει να είναι άχρωμο, διαυγές, και να παραμένει πλήρως διαφανές μετά την ξήρανση και με την πάροδο του χρόνου. Επίσης, πρέπει να είναι συμβατό με τις χρησιμοποιούμενες χρωστικές και ικανό να διεισδύει και να διαχέεται ομοιόμορφα σε όλους τους ιστούς του δείγματος. Δεν θα πρέπει να ξηραίνεται υπερβολικά γρήγορα, να προκαλεί θόλωση ή να συρρικνώνεται μετά τον εγκλεισμό. Η επιλογή του κατάλληλου μέσου εγκλεισμού αποτελεί καθοριστικό στάδιο στη διαδικασία προετοιμασίας δειγμάτων, δεδομένου ότι δεν υφίσταται ένα καθολικά ιδανικό μέσο για όλες τις εφαρμογές. Συνεπώς, η επιλογή του θα πρέπει να βασίζεται στην εξισορρόπηση των εξής βασικών παραγόντων:

- **Οπτικές ιδιότητες**. Ο δείκτης διάθλασης του μέσου εγκλεισμού πρέπει να εξασφαλίζει επαρκή αντίθεση και διάθλαση για την ανάδειξη των βασικών ανατομικών χαρακτηριστικών που χρησιμοποιούνται για ταξινομική ταυτοποίηση ή μορφολογική περιγραφή. Τέτοια χαρακτηριστικά περιλαμβάνουν τις σπερματοθήκες, τα ασκοειδή (αισθητήρια όργανα κεραίων), τα αισθητήρια όργανα Newstead (Newstead sensilla), τα κατακόρυφα δόντια του κιβαρίου ή τροφικού θαλάμου (vertical cibarial teeth) και τα φαρυγγικά δόντια (pharyngeal teeth). Η ορατότητα αυτών των δομών εξαρτάται άμεσα από τις οπτικές ιδιότητες του μέσου εγκλεισμού.
- **Διατήρηση**. Για δείγματα αναφοράς ή υλικό που προορίζεται για μόνιμες συλλογές, το μέσο πρέπει να προσφέρει μακροχρόνια σταθερότητα και ανθεκτικότητα. Αντίθετα, σε μελέτες καταγραφής ή επιδημιολογικές έρευνες, όπου η μακροβιότητα του δείγματος δεν αποτελεί πρωταρχικό στόχο, η χρήση προσωρινών ή ημιμόνιμων μέσων ενδέχεται να είναι επαρκής.

5.3.2. Απαιτήσεις για τα μέσα εγκλεισμού ή μονιμοποίησης

Οι ειδικοί συχνά αναπτύσσουν εξειδικευμένες και σύνθετες τεχνικές εγκλεισμού, σχεδιασμένες και προσαρμοσμένες σε συγκεκριμένες ερευνητικές ανάγκες ή εξατομικευμένες για το δείγμα που μελετούν. Ωστόσο, αυτές οι μέθοδοι συχνά παραβλέπουν σημαντικούς παράγοντες όπως η αρχειακή ποιότητα, συμβατότητα, τυποποίηση ή δεν λαμβάνουν υπόψη πρακτικά ζητήματα όπως την ευκολία χειρισμού και τη μακροχρόνια διατήρηση. Η απουσία μίας κοινής τυποποιημένης μεθόδου δυσχεραίνει την ενσωμάτωση συλλογών όταν αυτές δωρίζονται σε μουσεία ή ιδρύματα, καθώς και τις προσπάθειες για μακροχρόνια συντήρηση και διαχείριση των συλλογών.
Οι διαφορετικές επιστημονικές εφαρμογές ή χρήσεις ενός παρασκευάσματος καθορίζουν τις απαιτήσεις που πρέπει να πληροί ένα μέσο εγκλεισμού, θέτοντας συγκεκριμένες προδιαγραφές. Οι ταξινομιστές συχνά προβαίνουν στον εγκλεισμό (mounting) ολόκληρων δειγμάτων και προτιμούν μέσα που προκαλούν ήπια διαβροχή των εσωτερικών ιστών, ώστε να βελτιώνεται η ορατότητα των δομών του υμενιδίου (ή εξωσκελετικών δομών, cuticular structures). Ο δείκτης διάθλασης του μέσου πρέπει να διαφέρει επαρκώς από εκείνον του δείγματος και του γυαλιού της αντικειμενοφόρου πλάκας, ώστε να μεγιστοποιείται η οπτική ευκρίνεια. Τα εμπορικά μέσα εγκλεισμού συνήθως έχουν δείκτη διάθλασης κοντά σε αυτόν του γυαλιού, ώστε να ελαχιστοποιείται η διάθλαση και η σκέδαση του φωτός στο σύστημα «αντικειμενοφόρος πλάκα – μέσο – καλυπτρίδα». Ωστόσο, στη μικροσκοπία φωτεινού πεδίου, η φυσική αντίθεση ενός μη χρωσμένου δείγματος μπορεί να ενισχυθεί επιλέγοντας σκόπιμα ένα μέσο με δείκτη διάθλασης ελαφρώς διαφορετικό από αυτόν του δείγματος, βελτιώνοντας έτσι την ορατότητά του σε σχέση με το φόντο.

5.3.3. Τύποι μέσων εγκλεισμού (Πίνακες 3 & 4)

Στη μικροσκοπία, η επιλογή μέσου εγκλεισμού εξαρτάται σε μεγάλο βαθμό από τον δείκτη διάθλασης (RI), ο οποίος καθορίζει πώς διαθλάται το φως μέσω της αντικειμενοφόρου πλάκας, του μέσου και του δείγματος. Όταν ο δείκτης διάθλασης πλησιάζει αυτόν του γυαλιού της καλυπτρίδας (≈1,515), το φως διέρχεται ομοιόμορφα, μειώνοντας τη σκέδαση και τις οπτικές παραμορφώσεις, βελτιώνοντας την ανάλυση και την ευκρίνεια των λεπτών δομών. Αντίθετα, τυχόν αποκλίσεις ή ασυμφωνία μεταξύ RI του μέσου εγκλεισμού και του γυαλιού ή του δείγματος, μπορεί να προκαλέσει θόλωση, εμφάνιση «αλώ» ή απόκρυψη των μη χρωματισμένων δομών. Η επιλογή του κατάλληλου μέσου εγκλεισμού είναι καθοριστική γιατί οι διαφορές στους δείκτες διάθλασης επηρεάζουν άμεσα την αντίθεση, την καθαρότητα και τη συνολική ποιότητα της μικροσκοπικής εικόνας.
Ο δείκτης διάθλασης του μέσου επηρεάζει καθοριστικά την ορατότητα λεπτών ανατομικών δομών κατά την προετοιμασία φλεβοτόμων. Δομές όπως ο κιβαριακός οπλισμός (cibarial armature), οι σπερματοθήκες, οι αρθρώσεις των κεραιών και η νεύρωση των πτερύγων ενδέχεται να είναι δυσδιάκριτες όταν χρησιμοποιούνται μέσα με υψηλό δείκτη διάθλασης.
Για τους φλεβοτόμους, συνήθως χρησιμοποιούνται υδατοδιαλυτά μέσα εγκλεισμού τύπου κόμμι χλωράλης και μέσα διαλυτά σε οργανικούς διαλύτες (ρητίνες) όπως το βάλσαμο του Καναδά ή το Enecê–Nelson Cerqueira (NC). Ο Rawlins [60] κατηγοριοποίησε τα μέσα εγκλεισμού σε δύο τύπους: (1) Μόνιμα μέσα: Σκληραίνουν με την πάροδο του χρόνου και είναι κατάλληλα για μακροχρόνια διατήρηση των παρασκευασμάτων, και (2) Ημιμόνιμα μέσα: Δεν στερεοποιούνται πλήρως και χρησιμοποιούνται κυρίως για προσωρινή ή βραχυπρόθεσμη διατήρηση παρασκευασμάτων.

This is an Open Access article distributed under the terms of the Creative Commons Attribution License (https://creativecommons.org/licenses/by/4.0), which permits unrestricted use, distribution, and reproduction in any medium, provided the original work is properly cited.

Πίνακας 3.: Χημική σύσταση επιλεγμένων μέσων εγκλεισμού για παρασκευάσματα μικροσκοπίας.

Μέσο εγκλεισμού	Διαλύτης	Πιθανά προπολυμερή ή πολυμερή	Παρατηρήσεις
Hoyer = Κόμμι χλωράλης	Γλυκερόλη, νερό	Ενώσεις αραβικού κόμμεος (arabic gum)	Παράγοντας διαβροχής: ένυδρη χλωράλη
CMCP-9 (= carboxy methyl cellulose phenol)	Νερό (CMCP-9: 51−60%)	Πλήρως υδρολυμένη πολυβινυλική αλκοόλη (CMCP-9: 0−5%)	CMC(P)-9: χαμηλό έως υψηλό ιξώδες
DMHF (dimethyl hydantoin formaldehyde)	Νερό	N,N′-dimethylol dimethyl hydantoin (di-methylol DMH), ολιγομερή συνδεδεμένα με αιθέρα ή μεθυλένιο, διασταυρωμένο δίκτυο πολυμερούς DMH–φορμαλδεΰδης	
Βάλσαμο του Καναδά (Canada balsam)	Ξυλόλιο, μερικώς πτητικά συστατικά του βάλσαμου (Δ^3-carene, levopimaric acid, limonene, myrcene, palustric acid, β-phellandrene, α- pinene, β-pinene)	Βάλσαμο (abienol, abietic acid, isopimaric acid, sandaracopimaric acid)	Εξουδετέρωση: ανθρακικό κάλιο, ρητίνη από *Abies balsamea* (Linné, 1758)
Euparal®	Ευκαλυπτόλη, παραλδεΰδη, μερικώς πτητικά συστατικά ρητίνης sandarac (limonene, α-pinene, β-pinene)	Ενώσεις ρητίνης sandarac (communic acid, manool, polycommunic acid, sandaracopimaric acid, 12-acetoxy-sandaracopimaric acid, sugiol, torulosic acid, torulosol, totarol)	Μέσο διαύγασης: σαλικυλικό μεθύλιο (methyl salicylate). Χρωστική στο Euparal® Green: άλας χαλκού (copper abietinate), ρητίνη sandarac από *Tetraclinis articulata* (Vahl, 1791)
Enecê	Αιθυλική αλκοόλη, με καμφορά, ευκαλυπτόλη και τερεβινθέλαιο	Ενώσεις κόμμεος κοπάλ (copal gum) και κολοφωνίου (rosin)	

Πίνακας 4: Πλεονεκτήματα, μειονεκτήματα και αδημοσίευτες παρατηρήσεις ερευνητών για επιλεγμένα μέσα εγκλεισμού [52].

Ονομασία μέσου	Πλεονεκτήματα	Μειονεκτήματα
* Βάλσαμο Καναδά (Canada balsam)	Το μέσο εμφανίζει υψηλή ανθεκτικότητα, με διάρκεια ζωής που υπερβαίνει τα 150 έτη.<br><br>Οι πλάκες μικροσκοπίας μπορούν να παρασκευαστούν χρησιμοποιώντας γαρυφαλέλαιο ή φαινόλη ως μέσα διαύγασης πριν τον τελικό εγκλεισμό.	Περιέχει επιβλαβή συστατικά και πρέπει να χρησιμοποιείται εντός απαγωγού χημικών.<br><br>Απαιτεί πλήρη αφυδάτωση μέσω διαδοχικών και χρονοβόρων σταδίων.<br><br>Η αφυδάτωση με αιθανόλη και η μεταφορά με χρήση οργανικού μέσο (π.χ. ξυλένιο ή γαρυφαλέλαιο) μπορεί να αυξήσει την ευθραυστότητα ορισμένων ταξινομικών μονάδων (taxa). Η χρήση εναλλακτικών διαλυτών (π.χ. ισοπροπανόλη, n-βουτανόλη, Cellosolve™, 1,4-διοξάνιο, Histoclear, τερπινεόλη) μειώνει τον κίνδυνο θραύσης των δειγμάτων.<br><br>Τα δείγματα ενδέχεται να σκουρύνουν εάν το ξυλένιο αντικατασταθεί απόδ. φαινόλη ή αν παραμείνουν υπολείμματα υδροξειδίου του καλίου.<br><br>Ο υψηλός δείκτης διάθλασης του μέσου μπορεί να εμποδίσει την απεικόνιση μη χρωματισμένων δομών.<br><br>Χωρίς τη χρήση θερμαινόμενης πλάκας, η πλήρης ξήρανση των δειγμάτων ενδέχεται να διαρκέσει χρόνια.<br><br>Το μέσο τείνει να κιτρινίζει και να σκουραίνει με την πάροδο του χρόνου, ιδιαίτερα όταν η διαύγαση πραγματοποιείται με γαρυφαλέλαιο.<br><br>Ορισμένες χρωστικές ενδέχεται να εξασθενίσουν και οι κατιονικές βαφές μπορεί να αποχρωματιστούν, εάν το μέσο αποκτήσει όξινο pH, όπως συμβαίνει φυσικά με την πάροδο του χρόνου.
DMHF (dimethyl hydantoin formaldehyde)	Υψηλή διαφάνεια.<br><br>Κατάλληλος δείκτης διάθλασης.<br><br>Εξαιρετική ευκρίνεια των μορφολογικών δομών.<br><br>Σχετικά σταθερά παρασκευάσματα.<br><br>Συμβατό με ποικίλες τεχνικές χρώσης.<br><br>Αποτελεσματική προστασία των δειγμάτων.<br><br>Σταθερή πρόσφυση μεταξύ αντικειμενοφόρου πλάκας και καλυπτρίδας.	Κίνδυνος κιτρινίσματος με την πάροδο του χρόνου.<br><br>Κίνδυνος αλλοίωσης ορισμένων χρωστικών, πιθανή μεταβολή χρωματικής έντασης.<br><br>Ακατάλληλο για χρωστικές ευαίσθητες στη φορμαλδεΰδη.<br><br>Σχηματισμός φυσαλίδων και σχετικά αργός χρόνος ξήρανσης.<br><br>Το μέσο είναι ευαίσθητο στην υγρασία.<br><br>Η αφαίρεση ή αντικατάσταση του μέσου εγκλεισμού είναι ιδιαίτερα δύσκολη.<br><br>Η φορμαλδεΰδη είναι τοξική, ερεθιστική και καρκινογόνος ουσία.
* Euparal (διαφανές)	Ανθεκτικό μέσο με διάρκεια ζωής άνω των 50 ετών.<br><br>Επιτρέπει την απευθείας μεταφορά δειγμάτων από αιθανόλη 80% (σύμφωνα με τις οδηγίες του κατασκευαστή).<br><br>Δεν κιτρινίζει, παραμένει διαφανές και διατηρεί μακροχρόνια τη σταθερότητά του (μη εύθρυπτο), επιτρέποντας καθαρή παρατήρηση ακόμη και των μη χρωματισμένων δομών.<br><br>Για τα Δίπτερα, παρουσιάζει καταλληλότερο δείκτη διάθλασης σε σύγκριση με το βάλσαμο του Καναδά.<br><br>Κατάλληλο για παχύτερα δείγματα, καθώς δεν προκαλεί συρρίκνωσή τους ούτε δημιουργεί φυσαλίδες κατά την ξήρανση (στέγνωμα).<br><br>Παραμένει διαλυτό σε αιθανόλη 95%, επιτρέποντας εκ νέου επεξεργασία και εγκλεισμό κόμη και μετά από πολλά χρόνια.	Περιέχει επιβλαβή συστατικά και πρέπει να χρησιμοποιείται εντός απαγωγού χημικών.<br><br>Η αφυδάτωση με αιθανόλη και διαύγαση μέσω Euparal Essence ενδέχεται να καταστήσει ορισμένες ταξινομικές μονάδες εύθραυστες. Ωστόσο, η χρήση ισοπροπανόλης μπορεί να περιορίσει το πρόβλημα.

Πίνακας 4 (συνέχεια)

Ονομασία μέσου	Πλεονεκτήματα	Μειονεκτήματα
Υγρό Hoyer	Τα δείγματα μπορούν να τοποθετηθούν άμεσα στο μέσο είτε ζωντανά είτε απευθείας από το μέσο συντήρησης (νερό, αιθανόλη ή φορμαλδεΰδη).<br><br>Η διαβροχή (διαφοροποίηση, maceration) προσφέρει άριστη διατήρηση και απεικόνιση του εξωσκελετού.<br><br>Διαθέτει κατάλληλο δείκτη διάθλασης που βελτιώνει την ορατότητα των δομών του δείγματος. Η οπτική αντίθεση μπορεί να ενισχυθεί περαιτέρω με χρώση ιωδίου.<br><br>Το οξικό οξύ που περιέχει το μέσο διαστέλλει τα εξαρτήματα των αρθροπόδων, διευκολύνοντας την μορφολογική εξέταση.<br><br>Ορισμένα παρασκευάσματα παραμένουν σταθερά και διατηρούνται για 40–60 έτη.<br><br>Υδατοδιαλυτό μέσο που επιτρέπει εύκολη αφαίρεση και εκ νέου εγκλεισμό του δείγματος.	Οι ευαίσθητοι ιστοί μπορεί να διαλυθούν εάν δεν προστεθεί σταδιακά το μέσο, διαδικασία που είναι χρονοβόρα.<br><br>Πιθανόν να σχηματιστούν κενοτόπια (κοιλότητες) και κρύσταλλοι στο παρασκεύασμα, σε διάστημα μικρότερο των 10 ετών.<br><br>Ενδέχεται να προκληθεί υπερβολική διαβροχή (διάλυση ιστών) ανάλογα με τη συγκέντρωση της χλωράλης και τον χρόνο έκθεσης.<br><br>Τα συστατικά του μέσου μπορεί να διαχωριστούν, εμφανίζοντας λεπτή κοκκίωση μετά από μήνες ή χρόνια.<br><br>Υπάρχουν αναφορές για σκουρόχρωμη αλλοίωση ή μαύρισμα του μέσου με την πάροδο του χρόνου.
CMCP-9<br><br>(= carboxy methyl cellulose phenol)	Επιτρέπει απευθείας τοποθέτηση των δειγμάτων από το υλικό συντήρησής τους (νερό, αιθανόλη, γλυκερόλη ή διαλύματα φορμαλδεΰδης).<br><br>Παρέχει τη δυνατότητα διαβροχής των εσωτερικών οργάνων όταν απαιτείται να μαλακώσουν ή διαφανοποιηθούν, διευκολύνοντας την παρατήρηση ή την προετοιμασία.	Το μέσο μπορεί να κρυσταλλώσει ή να σκουρύνει με την πάροδο του χρόνου, καθώς και να προκαλέσει εντονότερη διαβροχή των δειγμάτων από την επιθυμητή. Εάν η αντικειμενοφόρος πλάκα δεν σφραγιστεί επαρκώς, τα παχύτερα δείγματα πιθανόν να συρρικνωθούν και να δημιουργηθούν κενοτόπια γύρω από την καλυπτρίδα. Είναι ακατάλληλο για δείγματα στα οποία έχει εφαρμοστεί χρωστική και για δείγματα που περιέχουν ασβέστιο ή άλατα ασβεστίου. Ο χρόνος ξήρανσης (στεγνώματος) είναι μεγαλύτερος συγκριτικά με το καθαρό CMC.
Eukitt™	Ανθεκτικό μέσο με διάρκεια ζωής άνω των 30 ετών.<br><br>Συμβατό με πολλούς διαλύτες (π.χ. ακετόνη, βενζόλιο, χλωροφόρμιο, διοξάνιο, αιθέρα, ισοπροπανόλη, βενζοϊκό μεθυλεστέρα, τερπινεόλη, τολουόλιο και ξυλένιο).<br><br>Στεγνώνει γρήγορα και παρουσιάζει ελαφρώς όξινο pH.<br><br>Δεν εμφανίζει αισθητή σκουρό-χρωση με την πάροδο του χρόνου.<br><br>Συμβατό με διάφορες χρωστικές (π.χ. φουξίνη, αιματοξυλίνη, methyl green, methyl violet, methylene blue).<br><br>Τα δείγματα μπορούν να υποβληθούν σε εκ νέου εγκλεισμό (remounting) ακόμη και μετά από χρόνια, έπειτα από παρατεταμένη εμβάπτιση σε ξυλένιο.	Περιέχει επιβλαβή συστατικά και πρέπει να χρησιμοποιείται εντός απαγωγού χημικών.<br><br>Απαιτεί πλήρη αφυδάτωση μέσω διαδοχικών και χρονοβόρων σταδίων.<br><br>Δεν θεωρείται ιδανικό για παχύτερα δείγματα, καθώς προκαλεί συρρίκνωση και σχηματισμό φυσαλίδων κατά το στέγνωμα.<br><br>Οι καλυπτρίδες ενδέχεται να αποκολληθούν με την πάροδο του χρόνου, εάν η επιφάνεια του γυαλιού δεν έχει καθαριστεί σωστά και σφραγιστεί κατάλληλα.<br><br>Μπορεί να εμφανιστεί ατελής πολυμερισμός του μέσου γύρω από τις ίνες κολλαγόνου, με κίνδυνο το παρασκεύασμα να παραμείνει ημίρευστο ή ασταθές.
Enecê	Ιδιαίτερα ανθεκτικό μέσο με διάρκεια ζωής τουλάχιστον 50 ετών.<br><br>Δεν παρουσιάζει σκουρόχρωση με την πάροδο του χρόνου.<br><br>Είναι ιδιαίτερα εύπλαστο, επιτρέποντας τη μικροτόμηση των εντόμων εντός του μέσου και παρέχοντας επαρκή χρόνο για τη διάταξη και ορθή τοποθέτηση των μορφολογικών δομών.	Απαιτεί πλήρη αφυδάτωση μέσω διαδοχικών και χρονοβόρων σταδίων.<br><br>Η αφυδάτωση με αιθανόλη και η μεταφορά με χρήση γαρυφαλέλαιου, ενδέχεται να καταστήσει εύθραυστα ορισμένα δείγματα.<br><br>Το έντομο συνεχίζει να διαφανοποιείται, έστω με πολύ αργό ρυθμό, γεγονός που ενδέχεται να δυσχεράνει την παρατήρηση πολύ λεπτών δομών, όπως τα αισθητήρια όργανα ή τριχίδια (sensilla), ασκοειδή αισθητήρια (ascoids) και τις απλές χαίτες ή σέτες (simple setae).

This is an Open Access article distributed under the terms of the Creative Commons Attribution License (https://creativecommons.org/licenses/by/4.0), which permits unrestricted use, distribution, and reproduction in any medium, provided the original work is properly cited.

Τα μέσα εγκλεισμού μπορεί να είναι υγρά, να προέρχονται από φυσικές ρητίνες ή κόμμεα (gum), ή να είναι συνθετικά ρητινώδη, και να είναι διαλυτά σε νερό, αλκοόλη ή άλλους οργανικούς διαλύτες (π.χ. τολουόλιο, ξυλόλιο) (Πίνακας 1). Μόλις το μέσο εγκλεισμού καλύψει το δείγμα, πρέπει να σφραγίζεται με αδιάβροχα προστατευτικά υλικά ώστε να προφυλάσσεται από οξυγόνο, σκόνη, ατμοσφαιρική υγρασία και να αποφεύγεται η εξάτμιση. Για να γίνει σαφής η διάκριση μεταξύ των διαφόρων τύπων μέσων εγκλεισμού, ακολουθεί η εξής κατηγοριοποίηση:

α. Υδατοδιαλυτά μέσα. Διαλύονται εύκολα στο νερό και είναι κατάλληλα για προσωρινά ή ημιμόνιμα παρασκευάσματα. Συνήθως είναι εύχρηστα, αλλά απαιτούν σφράγιση για προστασία από την ατμοσφαιρική υγρασία (π.χ. όταν χρησιμοποιείται μέσο κόμμι χλωράλης ή πολυβινυλική αλκοόλη), ιδιαίτερα σε συνθήκες όπως τα υγρά περιβάλλοντα ή τροπικά κλίματα.

β. Μέσα με περιορισμένη αντοχή στο νερό. Δεν επηρεάζονται ή αλλοιώνονται εύκολα από μικρές ποσότητες νερού, αλλά χρειάζονται προστασία από την υπερβολική υγρασία. Παρέχουν μεγαλύτερη μακροχρόνια σταθερότητα σε σύγκριση με τα πλήρως υδατοδιαλυτά μέσα και χρησιμοποιούνται κυρίως σε ημιμόνιμα παρασκευάσματα.

γ. Μέσα διαλυτά σε οργανικούς διαλύτες (υδρογονάνθρακες). Διαλύονται σε οργανικούς διαλύτες όπως ξυλόλιο, τολουόλιο ή στο Essenecê (που λειτουργεί ως διαλύτης Enecê). Προορίζονται για μόνιμα παρασκευάσματα, προσφέρουν εξαιρετική σταθερότητα, αντοχή στην υγρασία και στην αποδόμηση, καθώς δεν διασπώνται ή σκουραίνουν με την πάροδο του χρόνου, καθιστώντας τα ιδανικά για μακροχρόνια αρχειοθέτηση (π.χ. ουδέτερο Canada balsam, σύγχρονο συνθετικό).

Συνοψίζοντας, τα υδατοδιαλυτά μέσα είναι ιδανικά για προσωρινά παρασκευάσματα ή όταν απαιτείται εύκολη αφαίρεση του δείγματος. Τα μέσα με περιορισμένη αντοχή στο νερό προτιμώνται για ημιμόνιμα παρασκευάσματα που χρειάζονται μέτρια σταθερότητα και αντοχή, ενώ τα μέσα που διαλύονται σε οργανικούς διαλύτες χρησιμοποιούνται για μόνιμα παρασκευάσματα, ειδικά για μακροχρόνια αποθήκευση και αρχειοθέτηση.

5.3.4. Περιγραφή των προτεινόμενων μέσων εγκλεισμού (Πίνακες 3 & 4)

Μέσα για προσωρινή παρατήρηση

Κόμμι χλωράλης (Chloral gum) = Υγρό/ μέσο/ διάλυμα Hoyer (RI = 1,48). Το υγρό Marc André αποτελεί το καταλληλότερο μέσο για βραχυπρόθεσμη (για λίγες ώρες, με δυνατότητα παράτασης εφόσον η αντικειμενοφόρος πλάκα διατηρείται σε υγρό θάλαμο) παρατήρηση των σπερματοθηκών, συμπεριλαμβανομένης της φωτογράφησης (Σχήμα 4) ή της σχεδίασής τους. Για τη διατήρηση των σπερματοθηκών απαιτείται επανατοποθέτηση και εκ νέου εγκλεισμός (remounting) σε υδατικό μέσο κατάλληλο για μεσοπρόθεσμη αποθήκευση. Η αφυδάτωσή τους με σκοπό τον επανεγκλεισμό σε ρητινώδες μέσο είναι εφικτή, ωστόσο δε συνιστάται (λόγω αυξημένου κίνδυνου απώλειας του δείγματος). Το κόμμι χλωράλης και υγρό Hoyer θεωρούνται συνώνυμα και χρησιμοποιούνται ευρέως για την παρατήρηση των εσωτερικών οργάνων λόγω της υδατικής τους συμβατότητας (απευθείας χρήση σε δείγματα εντός υδατικού διαλύματος ή νερού), της απλότητας και της ταχύτητας εφαρμογής τους. Επίσης, τα μέσα αυτά διαθέτουν κατάλληλο δείκτη διάθλασης (παρόμοιο με αυτόν του δείγματος) που διευκολύνει την εξέταση λεπτών μορφολογικών δομών, όπως οι σπερματοθήκες. Ωστόσο, το κόμμι χλωράλης παρουσιάζει σημαντικά μειονεκτήματα όταν δεν παρασκευάζεται σωστά ή δεν αποθηκεύεται υπό ελεγχόμενες συνθήκες υγρασίας. Τα προβλήματα αυτά περιλαμβάνουν κρυστάλλωση, αποχρωματισμό και μείωση του ιξώδους. Η περιμετρική σφράγιση της καλυπτρίδας (ringing) δεν επαρκεί για την αντιμετώπιση αυτών των προβλημάτων, καθώς το μέσο εγκλεισμού μπορεί να σκουρύνει έντονα (ενίοτε να μαυρίσει) λόγω αλληλεπίδρασης με το υλικό σφράγισης, ιδίως όταν χρησιμοποιείται Euparal®.

Το μέσο Hoyer, από πλευράς οπτικών ιδιοτήτων και ποιότητας της οπτικής απεικόνισης, θεωρείται το πλέον κατάλληλο για φλεβοτόμους και χρησιμοποιείται παραδοσιακά για τον συγκεκριμένο σκοπό. Στη βιβλιογραφία περιγράφονται διάφορες παραλλαγές της βασικής σύνθεσης, η οποία περιλαμβάνει αραβικό κόμμι, γλυκερόλη και ένυδρη χλωράλη. Ορισμένες συνθέσεις έχουν παρερμηνευτεί ή αποδοθεί ανακριβώς [74], με αποτέλεσμα να εμφανίζονται αποκλίσεις στις αναλογίες συστατικών.

Παρότι το μέσο Hoyer είναι κατάλληλο για την μικροσκοπική παρατήρηση των σπερματοθηκών φλεβοτόμων, δεν ενδείκνυται για μακροχρόνια διατήρηση των παρασκευασμάτων. Είναι ιδανικό για βραχυπρόθεσμη παρατήρηση, επιτρέποντας μεταξύ άλλων την φωτογραφική καταγραφή ή τη σχεδίαση των δειγμάτων. Τα υδατικά μέσα, αν και εξυπηρετούν για προσωρινά παρασκευάσματα, δεν διασφαλίζουν μακροχρόνια σταθερότητα και διατήρηση. Αντιθέτως, τα ρητινώδη μέσα προσφέρουν υψηλή ανθεκτικότητα, η οποία μπορεί να εκτείνεται ακόμη και σε διάστημα αιώνων. Ωστόσο, τα μέσα αυτά ενδέχεται να μειώσουν τη διακριτότητα των λεπτών μορφολογικών δομών των σπερματοθηκών, καθώς συχνά προκαλούν απώλεια της διαθλαστικής τους αντίθεσης.

Το μέσο Hoyer υφίσταται σταδιακή αποδόμηση λόγω εξάτμισης ή αφυδάτωσης (Σχήμα 8), γεγονός που οδηγεί

This is an Open Access article distributed under the terms of the Creative Commons Attribution License (https://creativecommons.org/licenses/by/4.0), which permits unrestricted use, distribution, and reproduction in any medium, provided the original work is properly cited.

στον σχηματισμό μικρών, αδιαφανών κρυστάλλων ένυδρης χλωράλης. Παρά ταύτα, η ανάκτηση των δειγμάτων από κρυσταλλωμένες αντικειμενοφόρες πλάκες είναι εφικτή, καθώς το υμενίδιό τους (cuticle) παραμένει χημικά άθικτο. Ωστόσο, η ανάπτυξη κρυστάλλων μπορεί να προκαλέσει μηχανικές βλάβες. Σε ορισμένες περιπτώσεις, οι κρυσταλλωμένες πλάκες μπορούν να αποκατασταθούν με επανυδάτωση του μέσου σε θερμό και υγρό περιβάλλον, με προσθήκη θυμόλης για την αναστολή ανάπτυξης μυκήτων. Εναλλακτικά, το κόμμι χλωράλης μπορεί να απομακρυνθεί από τα δείγματα με εμβάπτιση σε νερό· στη συνέχεια τα δείγματα αφυδατώνονται σε παγόμορφο οξικό οξύ (glacial acetic acid) και υποβάλλονται σε επανεγκλεισμό σε βάλσαμο του Καναδά.

DMHF (dimethyl hydantoin formaldehyde) (RI 1,48). Αυτό το υδατοδιαλυτό μέσο [72] παρουσιάζει εξαιρετική οπτική απόδοση, συγκρίσιμη με εκείνη του μέσου Berlese, και είναι εξίσου εύχρηστο. Σε αντίθεση με το Berlese, το DMHF δεν σκουραίνει ούτε κρυσταλλώνεται με την πάροδο του χρόνου, χάρη στη σταθερή χημικού του σύσταση. Το DMHF έχει αποδειχτεί ιδιαίτερα κατάλληλο για φλεβοτόμους και άλλα είδη της οικογένειας Psychodidae.

CMCP (camphor-mono-chlorophenol) (RI = 1,41). Πρόκειται για γλυκερινούχο, υδατοδιαλυτό μέσο εγκλεισμού, το οποίο χρησιμοποιείται για την παρασκευή διάφανων, μόνιμων παρασκευασμάτων ευαίσθητων δειγμάτων, όπως οι φλεβοτόμοι. Πλεονέκτημά του αποτελεί η δυνατότητα άμεσου εγκλεισμού των δειγμάτων από νερό ή αιθανόλη, χωρίς την ανάγκη προηγούμενης αφυδάτωσης ή άλλης προετοιμασίας. Το CMCP χαλαρώνει και διαυγάζει γρήγορα το έντομο, μαλακώνοντας το υμενίδιο (εξωσκελετό) και διευκολύνοντας τη σωστή τοποθέτηση του δείγματος, γεγονός ιδιαίτερα χρήσιμο για την έκπτυξη των πτερύγων ή την ανατομή των γεννητικών οργάνων. Παρότι αναφέρεται ότι επιτρέπει μακροχρόνια αποθήκευση, η ακριβής διάρκεια διατήρησης παραμένει ασαφής. Ο κύριος περιορισμός του μέσου CMCP έγκειται στη σύστασή του, καθώς περιέχει φαινόλη, ουσία τοξική και ερεθιστική που απαιτεί προσεκτικό χειρισμό.

Μέσα μόνιμου εγκλεισμού

Βάλσαμο του Καναδά (RI = 1,52-1,54) Το βάλσαμο του Καναδά (Canada balsam, κεχριμπαρέλαιο) περιγράφεται για πρώτη φορά ως κατάλληλο μέσο για παρατήρηση με οπτικό μικροσκόπιο από τον Andrew Pritchard τη δεκαετία του 1830. Παραμένει ιστορικά ένα από τα πλέον διαδομένα μέσα λόγω της αποδεδειγμένης ποιότητας του ως σταθερό και ανθεκτικό μέσο για τη μακροχρόνια φύλαξη μικροσκοπικών παρασκευασμάτων, με περισσότερα από 150 χρόνια επιτυχούς εφαρμογής. Σε αντίθεση με μέσα τύπου Hoyer, το βάλσαμο του Καναδά δεν κρυσταλλώνει ούτε απορροφά υγρασία. Ωστόσο, παρουσιάζει ισχυρό αυτοφθορισμό, γεγονός που αποτελεί σημαντικό μειονέκτημα σε ορισμένες μικροσκοπικές τεχνικές, όπως σε εφαρμογές φθορισμού [60]. Η χρήση μη τοξικών διαλυτών αντί ξυλενίου μπορεί να μειώσει τους κινδύνους για την εργαστηριακή ασφάλεια κατά την προετοιμασία, αλλά μπορεί να εισαγάγει μειονεκτήματα, όπως βραδύτερο χρόνο στεγνώματος, συρρίκνωση κατά την ξήρανση και πρόωρη σκουρόχρωση του μέσου (μεταβολή χρώματος σε σκούρο καφέ).

Euparal® (RI = 1,48). Το Euparal® αποτελεί μία ευρέως χρησιμοποιούμενη εναλλακτική του Canada balsam για μόνιμο εγκλεισμό, προσφέροντας εξαιρετική μακροχρόνια σταθερότητα και παρόμοιο δείκτη διάθλασης (RI). Το Euparal® παρουσιάζει τα ακόλουθα χαρακτηριστικά: (1) Απαιτεί ελεγχόμενη αφυδάτωση (ξήρανση). Πριν από τη μεταφορά του δείγματος στο τελικό μέσο εγκλεισμού, είναι απαραίτητη η αφυδάτωσή του, συνήθως με σταδιακή αύξηση της συγκέντρωσης αιθανόλης από 95% σε απόλυτη αιθανόλη, (2) Μεγαλύτερος χρόνος επεξεργασίας. Η τελική παρασκευή σε ρητίνη, είτε με Canada balsam είτε με Euparal®, απαιτεί ξήρανση, γεγονός που παρατείνει συνολικά τον χρόνο επεξεργασίας του δείγματος. Όταν η αφυδάτωση με οργανικούς διαλύτες δεν είναι εφικτή, τα δείγματα που έχουν εκπλυθεί σε απόλυτη αιθανόλη μπορούν να τοποθετηθούν σε ενδιάμεσο διάλυμα προσαρμογής (ίσα μέρη Euparal® και Euparal essence) πριν από τον τελικό εγκλεισμό.

Enecê (RI = 1,467). Το Enecê είναι ένα ρητινώδες μέσο, το οποίο χρησιμοποιείται κυρίως για τον μόνιμο εγκλεισμό μικρών εντόμων, και είναι ιδιαίτερα δημοφιλές στη Βραζιλία. Η σύνθεσή του περιλαμβάνει κολοφώνιο και ρητίνη copal (ή κόμμι κοπάλ) διαλυμένα σε αλκοόλη, καμφορά, τερεβινθέλαιο και ευκαλυπτόλη. Σύμφωνα με τους Cerqueira και συνεργάτες [11] το Enecê αποτελεί εναλλακτική λύση έναντι του Canada balsam για μόνιμα παρασκευάσματα προνυμφών, εκδυμάτων (exuviae) προνυμφικών και νυμφικών σταδίων, καθώς και ενήλικων ατόμων κουνουπιών. Έκτοτε, χρησιμοποιείται ευρέως για μόνιμα μικροσκοπικά παρασκευάσματα σκνιπών. Το Enecê προσφέρει μια οικονομική επιλογή για μόνιμο εγκλεισμό, διατηρεί τα έντομα σταθερά για μεγάλο χρονικό διάστημα και παρέχει επαρκή χρόνο ξήρανσης. Ο αργό αυτός ρυθμός ξήρανσης επιτρέπει την ανατομή, αποσυναρμολόγηση και την ακριβή διάταξη των μορφολογικών τους δομών με ακρίβεια, προτού το μέσο σταθεροποιηθεί πλήρως.

5.4. Προετοιμασία και ξήρανση αντικειμενοφόρων πλακών

Η σωστή και επαρκής ξήρανση (στέγνωμα) των παρασκευασμένων πλακών είναι κρίσιμη για τη διασφάλιση της μακροχρόνιας σταθερότητας και

διατήρησης των δειγμάτων. Οι πλάκες πρέπει να ξηραίνονται σχολαστικά πριν από οποιαδήποτε μακροχρόνια αποθήκευση. Για βέλτιστα αποτελέσματα, οι πλάκες με μόνιμο μέσο εγκλεισμού πρέπει να παραμένουν σε οριζόντια θέση για 2–3 εβδομάδες, ενώ αυτές με ημιμόνιμο μέσο απαιτούν μόνο 1–2 εβδομάδες. Για να επιτευχθεί ομοιόμορφη και αποτελεσματική ξήρανση, συνιστάται η χρήση επωαστικού θαλάμου (incubator) με δυνατότητα ρύθμισης της θερμοκρασίας, ώστε να διατηρείται η κατάλληλη θερμοκρασία για το εκάστοτε μέσο και να αποφεύγεται η υπερβολική θέρμανση που θα μπορούσε να αλλοιώσει ή καταστρέψει τα δείγματα. Το προτεινόμενο εύρος θερμοκρασίας είναι 30-37°C. Αυτή η διαδικασία είναι απαραίτητη για την αποφυγή μετακίνησης της καλυπτρίδας, παραμόρφωσης της πλάκας, φθοράς των δειγμάτων ή απώλειας της σταθερότητας του μέσου εγκλεισμού κατά την αποθήκευση.

Το μέσο εγκλεισμού που χρησιμοποιείται κατά την προετοιμασία της πλάκας πρέπει πάντα να αναγράφεται στην ετικέτα. Επιπλέον, όπου είναι δυνατό, η ετικέτα πρέπει να περιλαμβάνει λεπτομερή περιγραφή του μέσου, το όνομα του παρασκευαστή και την ημερομηνία προετοιμασίας. Αρχικά, οι πλάκες προετοιμάζονται για προσωρινή τοποθέτηση (temporary mount) των δειγμάτων και δεν προορίζονται για μακροχρόνια διατήρηση. Ωστόσο, αν αλλάξει η υπόσταση ενός δείγματος - π.χ. αν οριστεί ως δείγμα αναφοράς ή ενταχθεί σε μια σημαντική συλλογή - θα πρέπει να χρησιμοποιηθεί μόνιμο μέσο εγκλεισμού, ώστε να διασφαλιστεί η ακεραιότητά του για μελλοντικές μελέτες ταξινόμησης.

5.5. Εναλλακτικές τεχνικές τοποθέτησης: Τοποθέτηση σε κάρτα

Η τοποθέτηση εντόμων σε κάρτα (card mounting) είναι μια ειδική τεχνική που χρησιμοποιείται για διάφορες ομάδες εντόμων, κατά την οποία τα δείγματα είτε καρφώνονται απευθείας πάνω σε ειδικές εντομολογικές κάρτες, είτε επικολλούνται στην επιφάνειά τους. Λόγω του μικρού μεγέθους των σκνιπών και της ανάγκης για παρατήρηση των εσωτερικών τους οργάνων κατόπιν διαύγασης (βλ. παράγραφο 5) για σκοπούς ταυτοποίησης, η μέθοδος αυτή κρίνεται ακατάλληλη για τον εγκλεισμό σκνιπών.

5.6. Επανεγκλεισμός κατεστραμμένων δειγμάτων

Για σπάνια ή πολύτιμα δείγματα, συνιστάται η διαδικασία δύο σταδίων, σύμφωνα με το αναρτημένο βίντεο στη διεύθυνση: https://zenodo.org/records/18315029.

1) Επανυδάτωση (rehydration) διατηρώντας την αρχική τοποθέτηση για να διεξαχθεί προκαταρκτική παρατήρηση. Μια βάση, η οποία μπορεί να συγκρατήσει αρκετές αντικειμενοφόρες πλάκες, τοποθετείται ως στήριγμα εντός τριβλίου Petri, δημιουργώντας ένα προστατευτικό πλαίσιο ώστε η αντικειμενοφόρος πλάκα που θα ενυδατωθεί να στέκεται υπερυψωμένη χωρίς να έρχεται σε άμεση επαφή με το υγρό (Σχήμα 8 D). Ο χρόνος επανυδάτωσης κυμαίνεται από μία έως αρκετές ημέρες, ανάλογα με την κατάσταση του δείγματος. Η καθημερινή παρακολούθηση και η υπομονή είναι απαραίτητες. Μόλις η πλάκα επανυδατωθεί επαρκώς, μπορεί να μεταφερθεί σε επωαστικό θάλαμο για μερικές ώρες πριν από τη μικροσκοπική εξέταση ή τη φωτογράφιση.

2) Επανεγκλεισμός (remounting): Η αντικειμενοφόρος πλάκα μεταφέρεται σε περιβάλλον με υψηλή υγρασία (wet chamber) για μερικές επιπλέον ώρες ή καθ' όλη τη διάρκεια της νύχτας. Η αποσυναρμολόγηση (disassembly) της πλάκας πρέπει να πραγματοποιείται ενόσω το δείγμα εξετάζεται υπό μικροσκόπιο. Η καλυπτρίδα αφαιρείται προσεκτικά με τη βοήθεια λεπτών βελόνων, διασφαλίζοντας ότι δεν παραμένουν προσκολλημένα τμήματα της σκνίπας (https://zenodo.org/records/18315029). Στη συνέχεια, τα αποκομμένα και διαχωρισμένα μέρη συλλέγονται προσεκτικά και εκπλένονται με νερό σε μικρά φρεάτια, όπως αυτά που χρησιμοποιούνται κατά τη διαδικασία εκχύλισης DNA/RNA (βλ. παρακάτω), ώστε να αφαιρεθούν τυχόν υπολείμματα. Ακολουθεί αφυδάτωση των δειγμάτων και μόνιμος επανεγκλεισμός τους σε μέσο ρητίνης. Κατά την αποσυναρμολόγηση της παρασκευασμένης πλάκας, η γνώση του αρχικού μέσου εγκλεισμού είναι καθοριστική για την επιλογή του κατάλληλου διαλύτη. Η ορθή επιλογή διασφαλίζει την αφαίρεση του μέσου χωρίς να προκληθούν δομικές αλλοιώσεις ή καταστροφή των διαγνωστικών μορφολογικών χαρακτήρων. Στην περίπτωση υδατικών μέσων εγκλεισμού (aqueous mounting media), η απομάκρυνση του δείγματος επιτυγχάνεται με τη χρήση απεσταγμένου νερού. Αντιθέτως, για ρητινώδη μέσα (π.χ., Canada balsam ή Euparal®), απαιτείται η χρήση ξυλολίου για να μαλακώσει και να απελευθερωθεί το δείγμα. Για λόγους ασφαλείας, η διαδικασία αυτή εκτελείται υποχρεωτικά εντός απαγωγού χημικών (fume hood), με χρήση του προβλεπόμενου ατομικού εξοπλισμού προστασίας, συμπεριλαμβανομένης της προστατευτικής μάσκας.

Ο επανεγκλεισμός δειγμάτων τύπου (type specimens), βάσει των οποίων έγινε η αρχική περιγραφή ενός είδους ή άλλων πολύτιμων μουσειακών δειγμάτων ή συλλογών (collection specimens), επιτρέπεται αποκλειστικά κατόπιν έγγραφης άδειας ή συναίνεσης του επιμελητή της συλλογής ή του αρμόδιου ιδρύματος..

6. Ταυτοποίηση δειγμάτων

6.1. Μορφολογία

Η ταυτοποίηση των φλεβοτόμων βασίζεται κυρίως στη μελέτη των μορφολογικών χαρακτηριστικών τους, όπως του σχήματος του θώρακα, των πτερύγων, των γεννητικών οργάνων, των τριχιδίων ή χαιτών (setae) και των συγκεκριμένων μορφομετρικών σχέσεων μεταξύ διαφόρων δομών. Οι ερευνητές χρησιμοποιούν κλειδιά ταξινόμησης, συλλογές αναφοράς και πρωτότυπες επιστημονικές περιγραφές ειδών για να συγκρίνουν τα συλλεγμένα δείγματα με γνωστά γένη ή ήδη περιγραφόμενα είδη. Σημαντικά χαρακτηριστικά, όπως η νεύρωση των πτερύγων (wing venation), η μορφολογία της κεφαλής και στα δύο φύλα, η δομή των αρσενικών γεννητικών οργάνων και η διάταξη των σπερματοθηκών των θηλυκών, παρέχουν ιδιαίτερα πολύτιμες πληροφορίες για την ταυτοποίηση των ειδών. Η ακριβής ταυτοποίηση συχνά απαιτεί λεπτομερή μικροσκοπική εξέταση, συνήθως με χρήση οπτικού μικροσκοπίου για την εξέταση λεπτομερών δομών όπως γεννητικά όργανα και σπερματοθήκες, ή στερεοσκοπίου για την παρατήρηση ευρύτερων μορφολογικών χαρακτηριστικών.

Οι πρόσφατες εξελίξεις στην απεικονιστική τεχνολογία έχουν διευκολύνει τη χρήση ψηφιακής τεκμηρίωσης για την ταυτοποίηση των φλεβοτόμων σκνιπών. Φωτογραφίες υψηλής ανάλυσης ή ψηφιακές απεικονίσεις των βασικών χαρακτηριστικών μπορούν να συγκριθούν με υλικό αναφοράς ή να αναλυθούν με τη χρήση υπολογιστικά υποβοηθούμενων συστημάτων ταυτοποίησης, βελτιώνοντας τόσο την ακρίβεια όσο και την προσβασιμότητα σε μορφολογικά δεδομένα ταξινόμησης.

6.2. Γεωμετρία πτερύγων

Η γεωμετρία των πτερύγων αποτελεί βασικό χαρακτηριστικό για την ταυτοποίηση και ταξινόμηση των διαφόρων ειδών φλεβοτόμων. Οι πτέρυγές τους εμφανίζουν μοναδικό μοτίβο και χαρακτηριστική δομή, συνήθως παρουσιάζονται επιμήκεις και σχετικά στενές με καλά σχηματισμένο δίκτυο νεύρων (Σχήματα 9 & 10). Η διάταξη των νεύρων (vein arrangement/ pattern) δημιουργεί ένα διακριτό μοτίβο, το οποίο μπορεί να διαφέρει ανά γένος και είδος, παρέχοντας σημαντικά μορφομετρικά χαρακτηριστικά που διευκολύνουν την ταυτοποίηση των ειδών. Συνεπώς, η μελέτη της γεωμετρίας των πτερύγων προσφέρει σημαντικές πληροφορίες για σκοπούς ταξινόμησης.

6.3. Γεωμετρική μορφομετρία πτερύγων

Οι ερευνητές χρησιμοποιούν διάφορες τεχνικές, όπως τη γεωμετρική μορφομετρία, για την ανάλυση και σύγκριση του σχήματος και του μεγέθους των πτερύγων μεταξύ διαφορετικών ειδών ή πληθυσμών φλεβοτόμων. Η μελέτη της γεωμετρίας των πτερύγων παρέχει πολύτιμες πληροφορίες σχετικά με τη συμπεριφορά, τις προτιμήσεις ενδιαιτήματος και την πτητική ικανότητα των σκνιπών.

Κατά τη γεωμετρική μορφομετρική προσέγγιση, οι πτέρυγες αποσπώνται προσεκτικά από το σώμα, υποβάλλονται σε χρώση (όπου απαιτείται) και τοποθετούνται επίπεδα σε αντικειμενοφόρο πλάκα. Τα παρασκευάσματα φωτογραφίζονται στη συνέχεια υπό στερεοσκόπιο, ψηφιοποιούνται και υποβάλλονται σε μορφομετρική ανάλυση. Η διαδικασία περιγράφεται εκτενώς στη βιβλιογραφία [6, 27, 42, 56, 57, 59], με σύσταση να χρησιμοποιείται πάντοτε η ίδια πτέρυγα (δεξιά ή αριστερή) στα ζεύγη οργάνων, ώστε να μην επηρεάζονται τα αποτελέσματα από τυχόν αλλομετρικές διαφορές (συσχέτιση μεγέθους-σχήματος) [62].

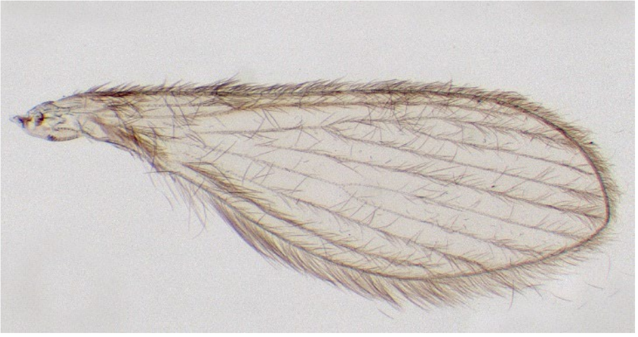

Σχήμα 9: Μορφολογία πτέρυγας φυσικού δείγματος του είδους *Trichophoromyia ininii*.

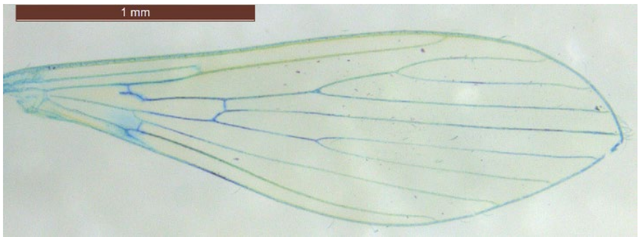

Σχήμα 10: Πτέρυγα δείγματος του είδους *Phlebotomus ariasi* κατόπιν χρώσης.

Προετοιμασία πτερύγων για γεωμετρική μορφομετρική ανάλυση

Για τη βέλτιστη απεικόνιση των νεύρων, οι πτέρυγες καθαρίζονται από λεπιδοειδείς δομές και χρωματίζονται κατάλληλα. Για την προετοιμασία των πτερύγων, αρχικά γεμίζονται μικρά φρεάτια με τα απαιτούμενα αντιδραστήρια (μπλε του μεθυλενίου, αιθανόλη, νερό και υποκατάστατο ξυλολίου). Στη συνέχεια, αφαιρείται μία πτέρυγα, που ήταν διατηρημένη σε αιθανόλη 70% σε θερμοκρασία δωματίου, με αναστροφή του μικροσωλήνα τύπου Eppendorf και με έκχυση του περιεχομένου στο φρεάτιο. Έπειτα, η πτέρυγα ανασηκώνεται κατά μήκος χρησιμοποιώντας μια λεπτή καμπυλωτή βελόνα και μεταφέρεται διαδοχικά από αιθανόλη σε νερό και εκ νέου σε αιθανόλη, για την απομάκρυνση των χαιτών.

Ακολούθως, τοποθετείται σε μπλε του μεθυλενίου (methylene blue) για 6 λεπτά, διασφαλίζοντας ότι επιπλέει κατά τη διάρκεια της χρώσης. Κατόπιν, η πτέρυγα αφαιρείται με προσοχή και εμβαπτίζεται σε υποκατάστατο ξυλολίου για 2 λεπτά (περίπου στο 1/3 του χρόνου χρώσης). Με ήπιες, προσεκτικές κινήσεις λεπτής βελόνας προς τα τοιχώματα του φρεατίου, διευκολύνεται η ομοιόμορφη βύθιση, χρώση και σταθεροποίηση της πτέρυγας. Το υποκατάστατο ξυλολίου χρησιμοποιείται στη συνέχεια για τη μονιμοποίηση της χρώσης, σταθεροποιώντας το, διασφαλίζοντας ότι δεν θα αλλοιωθεί με την πάροδο του χρόνου). Τέλος, η πτέρυγα τοποθετείται σε μικρή σταγόνα μέσου Euparal® επί αντικειμενοφόρου πλάκας. Με τη βοήθεια μεγεθυντικού φακού, η πτέρυγα ξεδιπλώνεται προσεκτικά, εκτείνεται και καλύπτεται με την καλυπτρίδα. Οι φωτογραφίες θα πρέπει να λαμβάνονται άμεσα πριν στερεοποιηθεί το Euparal®, καθώς ενδέχεται να χρειαστεί ελαφρά μετατόπιση της πτέρυγας κάτω από την καλυπτρίδα για βέλτιστη ευθυγράμμιση.

6.4. Τεχνικές μοριακής βιολογίας

Πέρα από τις μορφολογικές τεχνικές, οι μοριακές μέθοδοι διαδραματίζουν ολοένα σημαντικότερο ρόλο στην εντομολογική έρευνα, περιλαμβάνοντας μελέτες ταξινόμησης, πληθυσμιακής γενετικής και φυλογένεσης, καθώς και εφαρμογές για την ανίχνευση DNA/RNA παθογόνων παραγόντων και τον προσδιορισμό της προέλευσης του γεύματος αίματος. Το τελευταίο συνδέεται με τη συμπεριφορά των εντόμων φορέων-διαβιβαστών, η οποία αποτελεί κρίσιμο παράγοντα στον τομέα της επιδημιολογίας [70]. Η αλληλούχηση DNA μπορεί να χρησιμοποιηθεί για την επιβεβαίωση ενός είδους ή για τη διάκριση στενά συγγενικών ειδών, παρέχοντας έναν πιο ακριβή και αξιόπιστο τρόπο ταυτοποίησης. Επιπλέον, προηγμένες μοριακές τεχνικές (όπως PCR, αλληλούχηση DNA, τεχνολογίες NGS) καθώς και η τεχνική MALDI-ToF MS αποκτούν ολοένα μεγαλύτερη σημασία για την ταχεία και ακριβή ταυτοποίηση ειδών, συμπληρώνοντας τις παραδοσιακές μορφολογικές μεθόδους [46]. Παρά τις εξελίξεις αυτές, η μορφολογική ταυτοποίηση παραμένει το χρυσό πρότυπο, η μέθοδος αναφοράς για το πεδίο της ταξινομικής και αποτελεί τη βάση για την ερμηνεία των μοριακών δεδομένων.

6.4.1. Λυτική μέθοδος απομόνωσης νουκλεϊκών οξέων

Η εκχύλιση νουκλεϊκών οξέων αποτελεί κρίσιμο βήμα σε πολλές βιολογικές μελέτες και έχουν αναπτυχθεί ποικίλες μέθοδοι για την απομόνωση DNA από διαφορετικά βιολογικά υλικά [48]. Πολλά εμπορικά διαθέσιμα κιτ εκχύλισης DNA έχουν σχεδιαστεί ώστε να διευκολύνουν τη διαδικασία και να εξασφαλίζουν σταθερή ποιότητα γενετικού υλικού [14]. Ωστόσο, οι μέθοδοι που εφαρμόζονται συνήθως για την προετοιμασία δειγμάτων αρθροπόδων με σκοπό τη μορφολογική ταυτοποίηση ενδέχεται να δυσχεραίνουν τη μετέπειτα ανάλυση DNA είτε προκαλώντας μερική αλλοίωση είτε ολική απώλεια σημαντικών μορφολογικών χαρακτηριστικών του δείγματος [10]. Η διαδικασία που ακολουθείται στα περισσότερα πρωτόκολλα εκχύλισης DNA από ιστούς εντόμων έχει εγγενώς καταστρεπτικό χαρακτήρα [43], γεγονός που προκαλεί ιδιαίτερη ανησυχία σε περιπτώσεις μικρού μεγέθους δειγμάτων, όπου ακόμη και η λήψη περιορισμένης ποσότητας ιστού ενδέχεται να αλλοιώσει κρίσιμα μορφολογικά στοιχεία [72]. Ο τύπος και η κατάσταση του δείγματος διαδραματίζουν καθοριστικό ρόλο στην επιλογή της κατάλληλης μεθόδου απομόνωσης DNA [29].

Η ανάγκη για ακριβή ταυτοποίηση των φλεβοτόμων, για κατανόηση της δυναμικής των πληθυσμών τους και για ελαχιστοποίηση των ανεπιθύμητων επιπτώσεων σε μη στοχευόμενους οργανισμούς ή είδη, έχει οδηγήσει στην ανάπτυξη μοριακών διαγνωστικών εργαλείων [23]. Πλέον, οι μοριακές προσεγγίσεις συμπληρώνουν συστηματικά τις παραδοσιακές μεθόδους ταξινόμησης που βασίζονται σε μορφολογικά κριτήρια. Για παράδειγμα, η τυπική προσέγγιση ταυτοποίησης ειδών εντόμων μέσω αλληλούχησης DNA (DNA barcoding) περιλαμβάνει εκχύλιση, αλληλούχηση και συχνά απώλεια του αρχικού δείγματος. Συνεπώς, αναδεικνύεται η ανάγκη ανάπτυξης λιγότερο επεμβατικών ή μη καταστροφικών (μη λυτικών) μεθόδων εκχύλισης DNA, οι οποίες θα διατηρούν τόσο το βιολογικό υλικό όσο και τη μορφολογική ακεραιότητα του δείγματος.

Διάφορες μέθοδοι εκχύλισης νουκλεϊκών οξέων έχουν εφαρμοστεί σε δείγματα φλεβοτόμων. Η απαιτούμενη ποσότητα ή ποιότητα νουκλεϊκών οξέων εξαρτάται από τη μοριακή ανάλυση που θα ακολουθήσει, καθώς διαφορετικές τεχνικές παρουσιάζουν διαφορετικές απαιτήσεις ως προς την ευαισθησία και την καθαρότητα [9]. Για παράδειγμα, έχει αναφερθεί ότι οι οφθαλμοί των φλεβοτόμων μπορεί να αναστείλουν την τεχνική PCR [69]. Πέραν του ελέγχου για την ανίχνευση παθογόνων, το DNA των σκνιπών εκχυλίζεται συστηματικά για σκοπούς ταυτοποίησης ειδών. Διατίθενται ποικίλες τεχνικές εκχύλισης, με την απόδοση και την ποιότητα να διαφοροποιούνται σημαντικά μεταξύ πρωτοκόλλων. Ορισμένα πρωτόκολλα εμπορικών κιτ έχουν προσαρμοστεί ειδικά για εφαρμογή σε δείγματα φλεβοτόμων [8], βελτιώνοντας την απόδοση και/ή την ποιότητα του εκχυλισμένου DNA [8, 9, 69], ενώ τροποποιήσεις που αναπτύχθηκαν για άλλα αρθρόποδα μπορούν να εφαρμοστούν εξίσου σε δείγματα φλεβοτόμων [58, 76]. Μοριακές τεχνικές ταυτοποίησης, όπως η PCR που στοχεύει σε μικρά μιτοχονδριακά τμήματα (π.χ. COI, CytB) θεωρούνται συμβατές με μεθόδους εκχύλισης που προκαλούν σημαντική θραύση του DNA. Αντίθετα, τεχνολογίες αλληλούχησης μεγάλου μήκους DNA (NGS, Oxford Nanopore και PacBio) απαιτούν DNA υψηλού μοριακού βάρους και ποιότητας (ελάχιστη θραύση). Οι εκχυλίσεις με τη χρήση φυγοκεντιζόμενων στηλών (spin

column) παράγουν συνήθως θραύσματα DNA έως 60 kb, ενώ αντίστοιχα η εκχύλιση με χρήση φαινόλης-χλωροφορμίου μπορεί να αποδώσει θραύσματα έως 150 kb [77]. Στον Πίνακα 5 συνοψίζονται οι τεχνικές εκχύλισης DNA από δείγματα φλεβοτόμων και υποδεικνύονται σχετικές ειδικές τροποποιήσεις τους. Οι αποδόσεις δεν παρατίθενται διότι εξαρτώνται από το μέγεθος και τη μέθοδο προετοιμασίας του δείγματος. Η στήλη "Προσαρμογή" αφορά σε ειδικές τροποποιήσεις πρωτοκόλλων για σκνίπες ή άλλα μικρά αρθρόποδα.

Η επιλογή της κατάλληλης μεθόδου εκχύλισης πρέπει να λαμβάνει υπόψη διάφορα κριτήρια, όπως τον αριθμό δειγμάτων, τον απαιτούμενο χρόνο και την τεχνική που θα εφαρμοστεί σε επόμενα στάδια. Ενώ οι τεχνικές NGS απαιτούν gDNA υψηλού μοριακού βάρους, όλες οι αναφερόμενες μέθοδοι είναι κατάλληλες για συμβατικές εφαρμογές βασισμένες στη μέθοδο PCR.

Τέλος, αρκετές μελέτες έχουν εξετάσει ήπιες ή λιγότερο παρεμβατικές προσεγγίσεις εκχύλισης DNA για μικρά αρθρόποδα ξηράς, για δείγματα συλλογών διατηρημένα σε ξηρή μορφή και για αρθρόποδα μαλακού σώματος [19, 26, 28, 55, 63].

6.4.2. Μη λυτική μέθοδος απομόνωσης νουκλεϊκών οξέων

Μία από τις μεγαλύτερες προκλήσεις στη μοριακή ανάλυση των αρθροπόδων, ιδιαίτερα των φλεβοτόμων, είναι η διατήρηση των δειγμάτων για ένταξή τους σε εντομολογικές συλλογές. Τα περισσότερα πρωτόκολλα εκχύλισης DNA απαιτούν τη διάσπαση του ιστού, γεγονός που θέτει σε κίνδυνο τη διατήρηση του αρχικού δείγματος. Οι μη καταστρεπτικές μέθοδοι εκχύλισης νουκλεϊκών οξέων, ωστόσο, σχεδιάζονται για να εξάγουν γενετικό υλικό χωρίς να προκαλούν φυσική βλάβη στο δείγμα και χωρίς να επηρεάζουν τη δομική και μορφολογική ακεραιότητά του. Αυτές οι μέθοδοι είναι ιδιαίτερα σημαντικές κατά τη διαχείριση πολύτιμων, σπάνιων ή περιορισμένων δειγμάτων, όπως οι σκνίπες, όπου η διατήρηση της ακεραιότητας του εξωσκελετού είναι απαραίτητη για μελλοντική ταξινόμηση, μορφολογική ανάλυση ή για διαγνωστικούς σκοπούς. Μία διαδεδομένη προσέγγιση, μη καταστρεπτική, είναι η μέθοδος εμβάπτισης (immersion-based extraction), στην οποία οι σκνίπες ακινητοποιούνται και βυθίζονται προσεκτικά σε διάλυμα λύσης (lysis buffer) που περιέχει πρωτεϊνάση Κ.

Η τεχνική ήπιας λύσης (vectolysis) έχει εφαρμοστεί επιτυχώς σε σκνίπες, ιδιαίτερα σε δείγματα τύπου (δείγματα που χρησιμοποιούνται για την αρχική περιγραφή ενός είδους) [24]. Η τεχνική χρησιμοποιεί συμβατικά κιτ με στήλες φυγοκέντρησης (spin columns), όπως το DNeasy Blood and Tissue kit (QIAGEN, Hilden, Γερμανία) με κατάλληλες τροποποιήσεις ώστε να επιτυγχάνεται εκχύλιση DNA χωρίς να καταστρέφεται το δείγμα. Συγκεκριμένα, οι προσαρμογή στο στάδιο της λύσης κυττάρων (όγκος διαλύματος λύσης και προσθήκη ενός βήματος κατάψυξης) [17] επιτρέπουν την απελευθέρωση των νουκλεϊκών οξέων, ελαχιστοποιώντας τις μορφολογικές φθορές [24]. Όσον αφορά τις σκνίπες, είναι επίσης δυνατή η χρήση του HotSHOT DNA Extraction kit (Bento Bioworks Ltd, Λονδίνο, Ηνωμένο Βασίλειο) [73], το οποίο είναι γρήγορο και οικονομικό, επιτρέποντας την ταχεία και χαμηλού κόστους επεξεργασία των δειγμάτων. Μετά την εκχύλιση, τα δείγματα εκπλένονται και υποβάλλονται σε περαιτέρω επεξεργασία για μορφολογική ταυτοποίηση. Τα δείγματα που επεξεργάζονται με το DNeasy Blood and Tissue kit υποβάλλονται υποχρεωτικά σε διαύγαση με διάλυμα Marc-André, ενώ εκείνα που επεξεργάζονται με το HotSHOT DNA Extraction kit είναι ήδη επαρκώς διαυγή ώστε να τοποθετηθούν σε υδατικό μέσο ή, ακόμα καλύτερα, σε ρητίνη μετά από αφυδάτωση, σύμφωνα με το πρωτόκολλο που περιγράφεται στο παρόν άρθρο [73]. Το εκχυλισμένο γενετικό υλικό μπορεί στη συνέχεια να υποβληθεί σε επεξεργασία για περαιτέρω αναλύσεις, όπως PCR για την ενίσχυση συγκεκριμένων γενετικών δεικτών. Η εφαρμογή μη καταστρεπτικών μεθόδων εκχύλισης νουκλεϊκών οξέων είναι ιδιαίτερα σημαντική για τη μελέτη των γενετικών χαρακτηριστικών των φλεβοτόμων, καθώς και για την ανίχνευση και ταυτοποίηση παθογόνων που πιθανώς μεταφέρουν. Διατηρώντας τη δομική ακεραιότητα ενός δείγματος, οι ερευνητές μπορούν να αποκτήσουν πολύτιμες γενετικές πληροφορίες, να συνδυάσουν δεδομένα μοριακής και μορφολογικής ανάλυσης για το ίδιο άτομο, ενισχύοντας την αξιοπιστία της ταυτοποίησης και διατηρώντας παράλληλα το υλικό για μελλοντική αξιοποίηση.

6.5. Φασματομετρία μάζας MALDI-ToF (MALDI-ToF MS)

Η MALDI-ToF MS (Matrix-Assisted Laser Desorption/Ionization Time-of-Flight Mass Spectrometry- Φασματομετρία μάζας με εκρόφηση/ ιονισμό μέσω λέιζερ υποβοηθούμενου από μήτρα και αναλυτή χρόνου πτήσης) αποτελεί τεχνική φασματομετρίας μάζας σχεδιασμένη για την ανίχνευση και ανάλυση των μοναδικών πρωτεϊνικών προφίλ («πρωτεϊνικών αποτυπωμάτων») βιολογικών δειγμάτων. Η MALDI-ToF αναγνωρίζεται ολοένα και περισσότερο ως αξιόπιστο εργαλείο για την ταυτοποίηση αρθροπόδων ιατρικής και κτηνιατρικής σημασίας. Η τεχνική έχει αποδειχθεί αποτελεσματική στην αναγνώριση φλεβοτόμων σε διάφορα στάδια ανάπτυξης, συμπεριλαμβανομένων των ανώριμων σταδίων (πριν φτάσουν σε ενήλικη μορφή) και στην ανάλυση του γεύματος αίματος που έχει καταναλωθεί από θηλυκά άτομα φλεβοτόμων. Επίσης, εφαρμόζεται με επιτυχία για τη διαφοροποίηση τόσο αρσενικών όσο και θηλυκών φλεβοτόμων διαφόρων ειδών, ακόμη κι όταν τα δείγματα έχουν υποβληθεί σε διαφορετικές συνθήκες αποθήκευσης και πρωτόκολλα ομογενοποίησης [28, 30, 73, 74]. Η MALDI-ToF MS παρουσιάζει επίσης υψηλή διακριτική ικανότητα σε επίπεδο υπογένους, είδους και πληθυσμού επιτρέποντας ταχεία και ακριβή ταυτοποίηση, στοιχείο

απαραίτητο για την κατανόηση της κατανομής, της συμπεριφοράς και του ρόλου των φλεβοτόμων στη μετάδοση νοσημάτων. Η δυνατότητα διαφοροποίησης των ειδών βάσει των πρωτεϊνικών τους προφίλ την καθιστά ιδιαίτερα χρήσιμη σε επιδημιολογικές μελέτες και στρατηγικές ελέγχου εντόμων-διαβιβαστών. Ώστόσο, δύο βασικοί περιορισμοί εμποδίζουν επί του παρόντος την εφαρμογή της ως τεχνική ρουτίνας, μέρος της εργαστηριακής πρακτικής ή καθημερινό εργαλείο. Ο πρώτος περιορισμός αφορά το υψηλό κόστος και τη διαθεσιμότητα του εξοπλισμού φασματομετρίας μάζας, ιδίως όταν προορίζεται αποκλειστικά για την ταυτοποίηση φλεβοτόμων (ή αρθροπόδων-διαβιβαστών γενικότερα). Ωστόσο, ο περιορισμός αυτός μπορεί να μετριαστεί μέσω συνεργασίας με ερευνητικές μονάδες πρωτεομικής ανάλυσης και/ ή κλινικά διαγνωστικά εργαστήρια, όπου οι φασματογράφοι μάζας αποτελούν ήδη βασικό εξοπλισμό. Το δεύτερο μειονέκτημα είναι η περιορισμένη διαθεσιμότητα φασμάτων αναφοράς σε βάσεις δεδομένων ανοικτής πρόσβασης, γεγονός που καθιστά αναγκαία τη δημιουργία εσωτερικής (in-house) βάσης δεδομένων με φάσματα αναφοράς από σαφώς ταυτοποιημένα δείγματα, ιδανικά μέσω συνδυασμού μορφολογικής εξέτασης και αλληλούχησης κατάλληλων γενετικών δεικτών (όπως COI, cytB). Ο περιορισμός αυτός αναμένεται να αμβλυνθεί σταδιακά με την ενσωμάτωση υφιστάμενων, in-house δεδομένων αναφοράς στην πλατφόρμα MSI (Assistance Publique-Hôpitaux de Paris, Sorbonne University, Γαλλία) και στη συλλογή BCCM/IHEM/Sciensano (Βρυξέλλες, Βέλγιο) (https://msi.happy-dev.fr/). Για βέλτιστα αποτελέσματα, τα δείγματα που προορίζονται για ανάλυση MALDI-ToF θα πρέπει να αποθηκεύονται σε συνθήκες ξηρής κατάψυξης ή σε αιθανόλη 70% υψηλής μοριακής καθαρότητας, αποφεύγοντας την παρατεταμένη έκθεσή τους σε θερμοκρασία δωματίου. Δεδομένης της απουσίας διεθνώς τυποποιημένων πρωτοκόλλων και κατευθυντήριων οδηγιών για την προετοιμασία δειγμάτων, συνιστάται η χρήση υδατικού διαλύματος 60% ακετονιτριλίου/ 0.3% TFA (τριφθοροξικό οξύ) με σιναπινικό οξύ (30 mg/mL) για την προετοιμασία της μήτρας MALDI-ToF (MALDI-ToF matrix), ώστε τα παραγόμενα πρωτεϊνικά φάσματα να είναι συγκρίσιμα με τα ήδη διαθέσιμα δεδομένα για φλεβοτόμους.

Προετοιμασία δειγμάτων για MALDI-ToF MS (Σχήμα 7)

Τα δείγματα εντόμων, ανεξαρτήτως συνθηκών αποθήκευσης, αφήνονται αρχικά να ξηρανθούν σε θερμοκρασία δωματίου και στη συνέχεια διαχωρίζονται. Η κεφαλή και η κοιλία αφαιρούνται, ώστε να ληφθούν τα μέρη που φέρουν βασικά μορφολογικά χαρακτηριστικά, και διατηρούνται για εγκλεισμό σε αντικειμενοφόρο πλάκα (slide mounting) και μορφολογική ανάλυση. Ο θώρακας χρησιμοποιείται για ανάλυση MALDI-ToF, ενώ το υπόλοιπο τμήμα της κοιλίας διατηρείται για εξαγωγή DNA. Για την πρωτεϊνική ανάλυση, ο θώρακας ομογενοποιείται χειροκίνητα σε μικροσωληνάριο 1,5 mL με προσθήκη 10 μL διαλύματος ομογενοποίησης, χρησιμοποιώντας αναλώσιμα γουδιά και σφαιρίδια ομογενοποίησης. Συνήθως χρησιμοποιούνται δύο διαλύματα ομογενοποίησης: αποστειρωμένο απεσταγμένο νερό και μυρμηκικό οξύ 25%.

7. Συμπεράσματα

Στην παρούσα εργασία επιδιώξαμε να παρέχουμε στους ερευνητές τις πλέον αποτελεσματικές μεθόδους εγκλεισμού (mounting) φλεβοτόμων, προσαρμοσμένες σε συγκεκριμένους ερευνητικούς στόχους, με σκοπό τη διευκόλυνση της ακριβούς ταυτοποίησης και της ανίχνευσης παθογόνων. Δεν υπάρχει μία καθολικά βέλτιστη μέθοδος· αντιθέτως, υπάρχουν διάφορες προσεγγίσεις, καθεμία από τις οποίες παρουσιάζει τα δικά της πλεονεκτήματα και περιορισμούς.

Στην ενότητα «Συμπληρωματικό Υλικό» παρέχουμε αναλυτικά πρωτόκολλα για διάφορες τεχνικές τοποθέτησης και σταθεροποίησης για την προετοιμασία και ταυτοποίηση δειγμάτων φλεβοτόμων. Τα πρωτόκολλα αυτά, συνοδευόμενα από εκπαιδευτικά βίντεο, προσφέρουν οδηγίες βήμα προς βήμα, προσαρμοσμένες σε διαφορετικούς στόχους, εξασφαλίζοντας ακριβή και αξιόπιστα αποτελέσματα. Μέσω αυτής της ολοκληρωμένης πηγής, επιδιώκουμε να υποστηρίξουμε τους ερευνητές στην επιλογή και εφαρμογή των καταλληλότερων τεχνικών ανάλογα με τις ειδικές ερευνητικές τους ανάγκες.

Ευχαριστίες

Οι συγγραφείς εκφράζουν τις ευχαριστίες τους προς τον Richard Lane και τη Zoe Jay Adams από το Μουσείο Φυσικής Ιστορίας του Λονδίνου, Ηνωμένο Βασίλειο, για την εξαιρετική τους αξιολόγηση, η οποία συνέβαλε σημαντικά στη βελτίωση της ποιότητας του παρόντος άρθρου.

Χρηματοδότηση

Η παρούσα έρευνα της AJA χρηματοδοτήθηκε από τις βραζιλιάνικες αναπτυξιακές υπηρεσίες CNPq (αριθμός έργου: 404395/2024-4) και το Ίδρυμα Araucária (αριθμός υπόθεσης: 433/2025 PDI).

Σύγκρουση συμφερόντων

Jérôme Depaquit, Συνεργαζόμενος Εκδότης του επιστημονικού περιοδικού Parasite, δεν είχε οποιαδήποτε εμπλοκή στη διαδικασία αξιολόγησης ή στη λήψη αποφάσεων σχετικά με το παρόν χειρόγραφο. Οι υπόλοιποι συγγραφείς δηλώνουν υπεύθυνα ότι δεν υφίστανται συγκρουόμενα συμφέροντα.

Διαθεσιμότητα δεδομένων

Τα εκπαιδευτικά βίντεο του άρθρου είναι διαθέσιμα μέσω της πλατφόρμας Zenodo στους ακόλουθους συνδέσμους:

Βίντεο 1: https://zenodo.org/records/18198006

Βίντεο 2: https://zenodo.org/records/18311158

Βίντεο 3: https://zenodo.org/records/18311106

Βίντεο 4: https://zenodo.org/records/18311154

Βίντεο 5: https://zenodo.org/records/18303014

Βίντεο 6: https://zenodo.org/records/18302850

Βίντεο 7: https://zenodo.org/records/18315029

Συμπληρωματικό υλικό

Το συμπληρωματικό υλικό του άρθρου είναι διαθέσιμο στη διεύθυνση https://www.parasite-journal.org/10.1051/parasite/2026009/olm

Βιβλιογραφία

1. Alkan C, Allal-Ikhlef AB, Alwassouf S, Baklouti A, Piorkowski G, de Lamballerie X, Izri A, Charrel RN. 2015. Virus isolation, genetic characterization and seroprevalence of Toscana virus in Algeria. Clinical Microbiology and Infection, 21(11), 1040 e1-9.
2. Alten B, Ozbel Y, Ergunay K, Kasap OE, Cull B, Antoniou M, Velo E, Prudhomme J, Molina R, Banuls AL, Schaffner F, Hendrickx G, Van Bortel W, Medlock JM. 2015. Sampling strategies for phlebotomine sand flies (Diptera: Psychodidae) in Europe. Bulletin of Entomological Research 105(6), 664–678.
3. Ayhan N, Baklouti A, Prudhomme J, Walder G, Amaro F, Alten B, Moutailler S, Ergunay K, Charrel RN, Huemer H. 2017. Practical guidelines for studies on sandfly-borne phleboviruses: Part I: Important points to consider *ante* field work. Vector-Borne and Zoonotic Diseases 17(1), 73–80.
4. Bates PA. 1997. Infection of phlebotomine sandflies with *Leishmania*, in The Molecular Biology of Insect Disease Vectors: A Methods Manual. Springer. p. 112–120.5.
5. Baum M, de Castro EA, Pinto MC, Goulart TM, Baura W, Klisiowicz Ddo R, Vieira da Costa-Ribeiro MC. 2015. Molecular detection of the blood meal source of sand flies (Diptera: Psychodidae) in a transmission area of American cutaneous leishmaniasis, Parana State, Brazil. Acta Tropica, 143, 8–12.
6. Belen A, Alten B, Aytekin A. 2004. Altitudinal variation in morphometric and molecular characteristics of *Phlebotomus papatasi* populations. Medical and Veterinary Entomology, 18(4), 343–350.
7. Bhattacharya J, Chandra G, Hati AK. 1991. A simple method for cryopreservation of *Leishmania donovani* promastigotes, Indian Journal of Medical Research, 93, 245–246.
8. Caligiuri LG, Sandoval AE, Miranda JC, Pessoa FA, Santini MS, Salomón OD, Secundino NF, McCarthy CB. 2019. Optimization of DNA extraction from individual sand flies for PCR amplification. Methods and Protocols, 2(2), 36.
9. Casaril AE, de Oliveira LP, Alonso DP, de Oliveira EF, Gomes Barrios SP, de Oliveira Moura Infran J, Fernandes WS, Oshiro ET, Ferreira AMT, Ribolla PEM, de Oliveira AG. 2017. Standardization of DNA extraction from sand flies: Application to genotyping by next generation sequencing. Experimental Parasitology, 177, 66–72.
10. Castalanelli MA, Severtson DL, Brumley CJ, Szito A, Foottit RG, Grimm M, Munyard K, Groth DM. 2010. A rapid non-destructive DNA extraction method for insects and other arthropods. Journal of Asia-Pacific Entomology, 13(3), 243–248.
11. Cerqueira NL. 1943. Um novo meio para montagem de pequenos insetos em lâmina. Memórias do Instituto Oswaldo Cruz, (39), 37–41.
12. Charrel RN, Gallian P, Navarro-Mari JM, Nicoletti L, Papa A, Sanchez-Seco MP, Tenorio A, de Lamballerie X. 2005. Emergence of Toscana virus in Europe. Emerging Infectious Diseases, 11(11), 1657–1663.
13. Chaskopoulou A, Giantsis IA, Demir S, Bon MC. 2016. Species composition, activity patterns and blood meal analysis of sand fly populations (Diptera: Psychodidae) in the metropolitan region of Thessaloniki, an endemic focus of canine leishmaniasis. Acta Tropica, 158, 170–176.
14. Chen H, Rangasamy M, Tan SY, Wang H, Siegfried BD. 2010. Evaluation of five methods for total DNA extraction from western corn rootworm beetles. PLoS One, 5(8), e11963.
15. Depaquit J, Grandadam M, Fouque F, Andry PE, Peyrefitte C. 2010. Arthropod-borne viruses transmitted by Phlebotomine sandflies in Europe: a review. Eurosurveillance, 15(10), 19507.
16. Diamond LS, Herman CM. 1954. Incidence of Trypanosomes in the Canada Goose as revealed by bone marrow culture. Journal of Parasitology, 40(2), 195–202.
17. Ding H, Torno M, Vongphayloth K, Ng G, Tan D, Sng W, Ho K, Randrianambinintsoa FJ, Depaquit J, Tan CH. 2025. Hidden in plain sight: discovery of sand flies in Singapore and description of four species new to science. Parasites & Vectors, 18(1), 402.
18. Es-Sette N, Ajaoud M, Bichaud L, Hamdi S, Mellouki F, Charrel RN, Lemrani M. 2014. *Phlebotomus sergenti* a common vector of *Leishmania tropica* and Toscana virus in Morocco. Journal of Vector Borne Diseases, 51(2), 86–90.
19. Favret C. 2005. A new non-destructive DNA extraction and specimen clearing technique for aphids (Hemiptera). Proceedings of the Entomological Society of Washington, 107(2), 469–470.
20. Galati EAB. 2018. Phlebotominae (Diptera, Psychodidae): Classification, morphology and terminology of adults and identification of American taxa, in Brazilian Sand Flies: Biology, Taxonomy, Medical Importance and Control, Rangel EF, Shaw JJ, Editors. Cham: Springer International Publishing. pp. 9–212.
21. Galati EAB, de Andrade AJ, Perveen F, Loyer M, Vongphayloth K, Randrianambinintsoa FJ, Prudhomme J, Rahola N, Akhoundi M, Shimabukuro PHF, Depaquit J. 2025. Phle-botomine sand flies (Diptera, Psychodidae) of the world. Parasites & Vectors, 18(1), 220.
22. Galati EAB, Galvis-Ovallos F, Lawyer P, Leger N, Depaquit J. 2017. An illustrated guide for characters and terminology used in descriptions of Phlebotominae (Diptera, Psychodidae). Par-asite, 24, 26.
23. Gariepy T, Kuhlmann U, Gillott C, Erlandson M. 2007. Parasitoids, predators and PCR: the use of diagnostic molecular markers in biological control of Arthropods. Journal of Applied Entomology, 131(4), 225–240.
24. Giantsis IA, Chaskopoulou A, Bon MC. 2016. Mild-Vectolysis: A nondestructive DNA extraction method for vouchering sand flies and mosquitoes. Journal of Medical Entomology, 53(3), 692–695.
25. Gidwani K, Picado A, Rijal S, Singh SP, Roy L, Volfova V, Andersen EW, Uranw S, Ostyn B, Sudarshan M, Chakravarty J, Volf P, Sundar S, Boelaert M, Rogers ME. 2011. Serological markers of sand fly exposure to evaluate insecticidal nets against visceral leishmaniasis in India and Nepal: a cluster-randomized trial. PLoS Neglected Tropical Diseases, 5(9), e1296.

26. Gilbert MTP, Moore W, Melchior L, Worobey M. 2007. DNA extraction from dry museum beetles without conferring external morphological damage. PLoS One, 2(3), e272.
27. Giordani BF, Andrade AJ, Galati EAB, Gurgel-Goncalves R. 2017. The role of wing geometric morphometrics in the identification of sandflies within the subgenus *Lutzomyia*. Medical and Veterinary Entomology, 31(4), 373–380.
28. Guzmán-Larralde AJ, Suaste-Dzul AP, Gallou A, Peña-Carrillo KI. 2017. DNA recovery from microhymenoptera using six non-destructive methodologies with considerations for subsequent preparation of museum slides. Genome, 60(1), 85–91.
29. Hajibabaei M, DeWaard JR, Ivanova NV, Ratnasingham S, Dooh RT, Kirk SL, Mackie PM, Hebert PD. 2005. Critical factors for assembling a high volume of DNA barcodes. Philosophical Transactions of the Royal Society B: Biological Sciences, 360(1462), 1959–1967.
30. Haouas N, Pesson B, Boudabous R, Dedet JP, Babba H, Ravel C. 2007. Development of a molecular tool for the identification of *Leishmania* reservoir hosts by blood meal analysis in the insect vectors. American Journal of Tropical Medicine and Hygiene, 77(6), 1054–1059.
31. Hlavackova K, Dvorak V, Chaskopoulou A, Volf P, Halada P. 2019. A novel MALDI-TOF MS-based method for blood meal identification in insect vectors: A proof of concept study on phlebotomine sand flies. PLoS Neglected Tropical Diseases, 13 (9), e0007669.
32. Huemer H, Prudhomme J, Amaro F, Baklouti A, Walder G, Alten B, Moutailler S, Ergunay K, Charrel RN, Ayhan N. 2017. Practical guidelines for studies on sandfly-borne phleboviruses: Part II: Important points to consider for fieldwork and subsequent virological screening. Vector-Borne and Zoonotic Diseases, 17(1), 81–90.
33. Jancarova M, Polanska N, Thiesson A, Arnaud F, Stejskalova M, Rehbergerova M, Kohl A, Viginier B, Volf P, Ratinier M. 2025. Susceptibility of diverse sand fly species to Toscana virus. PLoS Neglected Tropical Diseases, 19(5), e0013031.
34. Kapp JD, Green RE, Shapiro B. 2021. A fast and efficient single-stranded genomic library preparation method optimized for ancient DNA. Journal of Heredity, 112(3), 241–249.
35. Killick-Kendrick R, Maroli M, Killick-Kendrick M. 1991. Bibliography of the colonization of phlebotomine sandflies. Parassitologia, 33(suppl.), 321–333.
36. Lawyer P, Killick-Kendrick M, Rowland T, Rowton E, Volf P. 2017. Laboratory colonization and mass rearing of phlebotomine sand flies (Diptera, Psychodidae). Parasite, 24, 42.
37. Léger N, Pesson B, Madulo-Leblond G. 1986. Les phlébotomes de Grèce : 1ère partie. Bulletin de la Société de Pathologie Exotique, 79, 386–397.
38. Léger N, Pesson B, Madulo-Leblond G. 1986. Les phlébotomes de Grèce : 2ème partie. Bulletin de la Société de Pathologie Exotique, 79, 514–524.
39. Leonel JAF, Vioti G, Alves ML, da Silva DT, Meneghesso PA, Benassi JC, Spada JCP, Galvis-Ovallos F, Soares RM, Oliveira T. 2020. DNA extraction from individual Phlebotomine sand flies (Diptera: Psychodidae: Phlebotominae) specimens: Which is the method with better results? Experimental Parasitology, 218, 107981.
40. Lestinova T, Rohousova I, Sima M, de Oliveira CI, Volf P. 2017. Insights into the sand fly saliva: Blood-feeding and immune interactions between sand flies, hosts, and *Leishmania.* PLoS Neglected Tropical Diseases, 11(7), e0005600.
41. Lienhard A, Schaffer S. 2019. Extracting the invisible: obtaining high quality DNA is a challenging task in small arthropods. PeerJ, 7, e6753.
42. Lozano-Sardaneta YN, Mikery-Pacheco OF, Huerta H, Rojas-Soriano JE, Contreras-Ramos A. 2025. Wing geometric morphometrics is effective to separate sand fly species (Diptera, Psychodidae, Phlebotominae) related with leishmaniasis transmission in Mexico. Acta Tropica, 262, 107523.
43. Mandrioli M. 2008. Insect collections and DNA analyses: how to manage collections? Museum Management and Curatorship, 23(2), 193–199.
44. Maroli M, Feliciangeli MD, Bichaud L, Charrel RN, Gradoni L. 2013. Phlebotomine sandflies and the spreading of leishmaniases and other diseases of public health concern. Medical and Veterinary Entomology, 27(2), 123–147.
45. Marquina D, Buczek M, Ronquist F, Lukasik P. 2021. The effect of ethanol concentration on the morphological and molecular preservation of insects for biodiversity studies. PeerJ, 9, e10799.
46. Mathis A, Depaquit J, Dvorak V, Tuten H, Banuls AL, Halada P, Zapata S, Lehrter V, Hlavackova K, Prudhomme J, Volf P, Sereno D, Kaufmann C, Pfluger V, Schaffner F. 2015. Identification of phlebotomine sand flies using one MALDI-TOF MS reference database and two mass pectrometer systems., Parasites & Vectors, 8, 266.
47. Mekarnia N, Benallal KE, Sadlova J, Vojtkova B, Mauras A, Imbert N, Longhitano M, Harrat Z, Volf P, Loiseau PM, Cojean S. 2024. Effect of *Phlebotomus papatasi* on the fitness, infectivity and antimony-resistance phenotype of antimony-resistant *Leishmania* major Mon-25. International Journal for Parasitology – Drugs and Drug Resistance, 25, 100554.
48. Milligan BG. 1998. Total DNA isolation, in Molecular Genetic Analysis of Population: A Practical Approach, Hoelzel AR, Editor. Oxford: Oxford University Press.
49. Molina R, Jiménez M, Alvar J, González E, Hernández-Taberna S, Ines MM. 2017. Methods in sand fly research. Madrid: Servicio de publicaciones Universidad de Alcalá de Henares, Madrid.
50. Murphy WJ, Eizirik E, O'Brien SJ, Madsen O, Scally M, Douady CJ, Teeling E, Ryder OA, Stanhope MJ, de Jong WW, Springer MS. 2001. Resolution of the early placental mammal radiation using Bayesian phylogenetics. Science, 294(5550), 2348–2351.
51. Nacif-Pimenta R, Pinto LC, Volfova V, Volf P, Pimenta PFP, Secundino NFC. 2020. Conserved and distinct morphological aspects of the salivary glands of sand fly vectors of leishma-niasis: an anatomical and ultrastructural study. Parasites & Vectors, 13(1), 441.
52. Neuhaus B, Schmid T, Riedel J. 2017. Collection management and study of microscope slides: Storage, profiling, deterioration, restoration procedures, and general recommendations. Zootaxa, 4322(1), 1–173.
53. New TR. 1974. Pscoptera. Handbooks for Identification of British Insects (Vol. I). London: Royal Entomological Society of London. 102 pp.
54. Perez-Ruiz M, Collao X, Navarro-Mari JM, Tenorio A. 2007. Reverse transcription, real-time PCR assay for detection of Toscana virus. Journal of Clinical Virology, 39(4), 276–281.
55. Porco D, Rougerie R, Deharveng L, Hebert P. 2010. Coupling non-destructive DNA extraction and voucher retrieval for small soft-bodied Arthropods in a high-throughput context: the example of Collembola. Molecular Ecology Resources, 10(6), 942–945.

56. Prudhomme J, Cassan C, Hide M, Toty C, Rahola N, Vergnes B, Dujardin JP, Alten B, Sereno D, Banuls AL. 2016. Ecology and morphological variations in wings of *Phlebotomus ariasi* (Diptera: Psychodidae) in the region of Roquedur (Gard, France): a geometric morphometrics approach. Parasites & Vectors, 9(1), 578.
57. Prudhomme J, Gunay F, Rahola N, Ouanaimi F, Guernaoui S, Boumezzough A, Banuls AL, Sereno D, Alten B. 2012. Wing size and shape variation of *Phlebotomus papatasi* (Diptera: Psychodidae) populations from the south and north slopes of the Atlas Mountains in Morocco. Journal of Vector Ecology, 37(1), 137–147.
58. Prudhomme J, Toty C, Kasap OE, Rahola N, Vergnes B, Maia C, Campino L, Antoniou M, Jimenez M, Molina R, Cannet A, Alten B, Sereno D, Banuls AL. 2015. New microsatellite markers for multi-scale genetic studies on *Phlebotomus ariasi* Tonnoir, vector of *Leishmania infantum* in the Mediterranean area. Acta Tropica, 142, 79–85.
59. Prudhomme J, Velo E, Bino S, Kadriaj P, Mersini K, Gunay F, Alten B. 2019. Altitudinal variations in wing morphology of *Aedes albopictus* (Diptera, Culicidae) in Albania, the region where it was first recorded in Europe. Parasite, 26, 55.
60. Rawlins DJ. 1992. Light Microscopy: An Introduction to Biotechniques. Oxford: Bios Scientific publishers. 143 pp.
61. Ready PD. 2013. Biology of phlebotomine sand flies as vectors of disease agents. Annual Review of Entomology, 58, 227–250.
62. Rohlf FJ, Slice D. 1990. Extensions of the Procrustes method for the optimal superimposition of landmarks. Systematic Zoology, 39(1), 40–59.
63. Rowley DL, Coddington JA, Gates MW, Norrbom AL, Ochoa RA, Vandenberg NJ, Greenstone MH. 2007. Vouchering DNA-barcoded specimens: Test of a nondestructive extraction proto-col for terrestrial arthropods. Molecular Ecology Notes, 7(6), 915–924.
64. Sábio PB, Andrade AJ, Galati EAB. 2014. Assessment of the taxonomic status of some species included in the *Shannoni* complex, with the description of a new species of *Psathyromyia* (Diptera: Psychodidae: Phlebotominae). Journal of Medical Entomology, 51(2), 331–341.
65. Sadlova J, Yeo M, Seblova V, Lewis MD, Mauricio I, Volf P, Miles MA. 2011. Visualisation of *Leishmania donovani* fluorescent hybrids during early stage development in the sand fly vector. PLoS One, 6(5), e19851.
66. Sales K, Miranda DEO, da Silva FJ, Otranto D, Figueredo LA, Dantas-Torres F. 2020. Evaluation of different storage times and preservation methods on phlebotomine sand fly DNA concentration and purity. Parasites & Vectors, 13(1), 399

Cite this article as: Randrianambinintsoa FJ, Augendre L, Prudhomme J, Martinet J-P, Loyer M, Mekarnia N, Kerkoub H, Perveen FK, Huguenin A, Kariya E, Akhoundi M, De Andrade AJ, Berriatua E, Bongiorno G, Boyer S, Christodoulou V, Da Costa-Ribeiro MCV, De Souza LAF, Ding H, Dondji B, Dvořák V, Erisoz Kasap O, Galati EAB, Gállego M, Ballart C, Gouzelou S, Haddad N, Masse RS, Mekuria AH, Ivovic V, Kaczmarek S, Shahar MK, Kirstein OD, Kniha E, Kolářová I, Lincoln T, Lucanas C, Mikov O, Nov K, Özbel Y, Pesson B, Posada Lopez LC, Prasetyo DB, Rahola N, Rebollar-Tellez EA, Rodrigues BL, Roy L, Saini P, Sanjoba C, Shimabukuro PH, Siriyasatien P, Soszynska A, Suleşco T, Sylla M, Torno M, Volf P, Vongphayloth K, Sinh Nam V, Wardhana A, Yessinou E, Zapata S, Gantier J-C & Depaquit J. 2026. Processing and mounting phlebotomine sand flies: a consensus guideline. Parasite xx, xx. https://doi.org/10.1051/parasite/2026009.

67. Sales KG, Costa PL, de Morais RC, Otranto D, Brandao-Filho SP, Cavalcanti Mde P, Dantas-Torres F. 2015. Identification of phlebotomine sand fly blood meals by real-time PCR. Parasites& Vectors, 8, 230.
68. Sant'Anna MR, Jones NG, Hindley JA, Mendes-Sousa AF, Dillon RJ, Cavalcante RR, Alexander B, Bates PA. 2008. Blood meal identification and parasite detection in laboratory-fed and field-captured *Lutzomyia longipalpis* by PCR using FTA databasing paper. Acta Tropica, 107(3), 230–237.
69. Senne NA, Santos HA, Araujo TR, Paulino PG, Mendonca LP, Moreira HVS, Camilo TA, da Costa Angelo I. 2022. Robust comparative performance of genomic DNA extraction methods from non-engorged phlebotomine sandflies. Medical and Veterinary Entomology, 36(2), 203–211.
70. Shaw JJ. 2025. A review of Leishmania infections in American Phlebotomine sand flies – Are those that transmit leishmaniasis anthropophilic or anthropportunists? Parasite, 32, 57.
71. Tesh RB, Modi GB. 1983. Growth and transovarial transmission of Chandipura virus (Rhabdoviridae: Vesiculovirus) in *Phlebotomus papatasi.* American Journal of Tropical Medicine and Hygiene, 32(3), 621–623.
72. Thomsen PF, Elias S, Gilbert MTP, Haile J, Munch K, Kuzmina S, Froese DG, Sher A, Holdaway RN, Willerslev E. 2009. Non-destructive sampling of ancient insect DNA. PLoS One, 4(4),e5048
73. Truett GE, Heeger P, Mynatt RL, Truett AA, Walker JA, Warman ML. 2000. Preparation of PCR-quality mouse genomic DNA with hot sodium hydroxide and tris (HotSHOT). Biotechniques, 29(1), 52–54.
74. Upton MS. 1993. Aqueous gum-chloral slide mounting media: an historical review. Bulletin of Entomological Research, 83(2), 267–274.
75. Volf P, Myskova J. 2007. Sand flies and Leishmania: specific versus permissive vectors. Trends in Parasitology, 23(3), 91–92.
76. Wang Q, Wang X. 2012. Comparison of methods for DNA extraction from a single chironomid for PCR analysis. Pakistan Journal of Zoology, 44(2), 421–426.
77. Wang Y, Zhao Y, Bollas A, Wang Y, Au KF. 2021. Nanopore sequencing technology, bioinformatics and applications. Nature Biotechnology, 39(11), 1348–1365.

PARASiTE

An international open-access, peer-reviewed, online journal publishing high quality papers on all aspects of human and animal parasitology

Reviews, articles and short notes may be submitted. Fields include, but are not limited to: general, medical and veterinary parasitology; morphology, including ultrastructure; parasite systematics, including entomology, acarology, helminthology and protistology, and molecular analyses; molecular biology and biochemistry; immunology of parasitic diseases; host-parasite relationships; ecology and life history of parasites; epidemiology; therapeutics; new diagnostic tools.
All papers in Parasite are published in English. Manuscripts should have a broad interest and must not have been published or submitted elsewhere. No limit is imposed on the length of manuscripts.

Parasite (open-access) continues **Parasite** (print and online editions, 1994-2012) and **Annales de Parasitologie Humaine et Comparée** (1923-1993) and is the official journal of the Société Française de Parasitologie.

Editor-in-Chief:
Jean-Lou Justine, Paris

Submit your manuscript at:
https://www.editorialmanager.com/parasite

Παράρτημα 1: Θεωρητικά θεμέλια Βιοχημείας

Στην παρούσα εργασία, τα αρθρόποδα που εξετάζονται είναι οι σκνίπες. Ωστόσο, οι αρχές αυτές μπορεί να επεκταθούν σε άλλα κοινά αρθρόποδα, των οποίων η ταυτοποίηση βασίζεται αποκλειστικά σε εσωτερικά μορφολογικά χαρακτηριστικά. Ορισμένα εσωτερικά όργανα είναι μερικώς χιτινοποιημένα (σκληρυμένα με χιτίνη) και η μορφολογία τους παρέχει πολύτιμες πληροφορίες για τη μορφολογική ανάλυση. Έτσι, είναι ιδιαίτερα ενδιαφέρουσα και χρήσιμη η παρατήρηση των αντλιών τροφής, των σπερματοθηκών και των αντίστοιχων πόρων τους. Σε όλα τα αντιδραστήρια που θα εξετάσουμε, δεν πρέπει ποτέ να ξεχνάμε ότι από το στάδιο της τοποθέτησης και της στερέωσης του εντόμου μέχρι τη συναρμολόγηση και τον εγκλεισμό, ουσιαστικά εφαρμόζουμε αντιδράσεις οξειδοαναγωγής. Η βασική αρχή που οφείλουμε να ακολουθούμε είναι η αποφυγή της ανάμειξης αναγωγικών και οξειδωτικών αντιδραστηρίων.

Αιθυλική αλκοόλη (αιθανόλη) ως συντηρητικό και μονιμοποιητικό μέσο:

Η αιθανόλη παρουσιάζει ισχυρή υδρόφιλη δράση και αφυδατώνει τους ιστούς. Ωστόσο, αλκοόλες χαμηλής συγκέντρωσης, πλούσιες σε νερό, μπορεί να προκαλέσουν αποδόμηση των νουκλεϊκών οξέων (το νερό είναι εχθρός των νουκλεϊκών οξέων καθώς επιταχύνει την υδρόλυση των πολυμερών). Όταν τα έντομα τοποθετούνται σε αιθανόλη, σκοπός δεν είναι μόνο η διατήρησή αλλά και η μονιμοποίηση (fixation) ώστε να σταθεροποιηθούν οι ιστοί τους. Στην ιστολογία διακρίνουμε δύο σημαντικές παραμέτρους: τον ρυθμό διείσδυσης (penetration rate) και τον ρυθμό στερέωσης ή σταθεροποίησης (fixation rate). Είναι κατανοητό ότι ένα αποτελεσματικό συντηρητικό πρέπει να έχει την ιδιότητα να διεισδύει ταχύτατα στους ιστούς πριν τους σταθεροποιήσει. Για διάλυμα αιθανόλης 96%, ο συντελεστής διείσδυσης είναι περίπου 1,05 (συγκριτικά, ο συντελεστής διείσδυσης υδατικού διαλύματος πυκρικού οξέος 0,75% είναι 0,45 ενώ ενός διαλύματος διχρωμικού καλίου 3% είναι 1,45).

Η επ' αόριστον ή παρατεταμένη διατήρηση εντόμων και άλλων αρθροπόδων σε αιθανόλη είναι καθιερωμένη και πρακτικά εφαρμόσιμη για τους εντομολόγους, αλλά όχι για τους κυτταρολόγους ή ιστολόγους, καθώς η μακροχρόνια παραμονή σε συντηρητικό παράγοντα καθιστά τη μελλοντική επεξεργασία των ιστών δύσκολη ή πρακτικά αδύνατη. Γι' αυτό, δείγματα παλαιότερα των 10 ετών είναι συχνά δύσκολο να αξιοποιηθούν ή είναι ακόμη και ακατάλληλα για περαιτέρω ανάλυση.
Μία εξίσου σημαντική παράμετρος που πρέπει να λαμβάνεται υπόψη είναι η αναλογία της μάζας του αρθρόποδου προς τον όγκο του συντηρητικού μέσου. Στην εργαστηριακή πρακτική της Ζωολογίας και της Ιατρικής συνιστάται ο όγκος του συντηρητικού να είναι 60 φορές μεγαλύτερος από τον όγκο του δείγματος προς σταθεροποίηση, προκειμένου να διασφαλίζεται επαρκής διείσδυση και αποτελεσματική συντήρηση. Στην περίπτωση μικρών αρθρόποδων, και για συγκεκριμένο όγκο δειγμάτων, προστίθενται τουλάχιστον 4–5 όγκοι αιθανόλης ανά όγκο δείγματος. Πρέπει, ωστόσο, να λαμβάνεται υπόψη ότι η αιθανόλη σταδιακά αραιώνεται καθώς απομακρύνει το νερό από τους ιστούς του αρθρόποδου, με αποτέλεσμα να μειώνεται η αποτελεσματικότητάς της ως συντηρητικού παράγοντα.

Συμπερασματικά:

- Η αιθυλική αλκοόλη είναι αναγωγικός παράγοντας (επομένως είναι ασύμβατη και δεν πρέπει να χρησιμοποιείται με οξειδωτικά μονιμοποιητικά μέσα).
- Προκαλεί κατακρήμνιση και αποδιάταξη των πρωτεϊνών.
- Διαλύει ορισμένα σύνθετα λιπίδια και προκαλεί καθίζηση γλυκογόνου.
- Προκαλεί έντονη συστολή και σκλήρυνση των ιστών.

Βασικά διαλύματα υδροξειδίου του καλίου ή του νατρίου:

Η χρήση ισχυρών βάσεων στην εντομολογία επικεντρώνεται κυρίως στο υδροξείδιο καλίου (KOH), συχνά χωρίς σαφή αιτιολόγηση. Το υδροξείδιο του νατρίου [NaOH, E524] διατίθεται σε διαλύματα διαφορετικών συγκεντρώσεων ή κανονικότητας, σε μορφή σφαιριδίων (pellets) ή σκόνης (glitter). Το κύριο μειονέκτημά του είναι ότι είναι ιδιαίτερα υγροσκοπικό (περισσότερο από το KOH) και κατά την αντίδρασή του με πρωτεΐνες προκαλεί διάλυσή τους, ενώ με λιπίδια σχηματίζει σκληρούς σάπωνες μέσω σαπωνοποίησης. Αυτή είναι μία βασική διαφορά από το KOH, το οποίο παράγει υγρούς σάπωνες.

Το υδροξείδιο του καλίου (E525) διατίθεται σε συμπυκνωμένο διάλυμα αλλά κυρίως σε σφαιρίδια περίπου 0,1 g, διευκολύνοντας την παρασκευή υδατικών διαλυμάτων χωρίς ζυγαριά ακριβείας. Για παράδειγμα, ένα σφαιρίδιο 0,1 g διαλυμένο σε 1 mL απεσταγμένου νερού δίνει διάλυμα περιεκτικότητας 10%. Το δεύτερο πλεονέκτημα του KOH σε μορφή σφαιριδίων είναι η χαμηλότερη ευαισθησία του στην ενανθράκωση, δηλαδή στη δέσμευση CO_2 και τον σχηματισμό ανθρακικών αλάτων.

Αυτές οι ισχυρές βάσεις χρησιμοποιούνται για τη μετατροπή λιπαρών οξέων σε υδατοδιαλυτούς σάπωνες. Σημειώνεται ότι η αιθανόλη που χρησιμοποιείται ως σταθεροποιητικό ή μονιμοποιητικό μέσο, διαλύει ήδη μέρος των λιπών, αλλά κατά την επιστροφή των δειγμάτων σε υδατικό μέσο ισχυρής βάσης, τα λιπαρά οξέα (απλά ή σύνθετα) σαπωνοποιούνται. Σε δείγματα με υψηλή περιεκτικότητα σε λιπώδη ιστό, για παράδειγμα σε θηλυκά άτομα, η θερμοκρασία μπορεί να αυξηθεί στους 35–40°C ή να παραταθεί ο χρόνος επαφής σε θερμοκρασία δωματίου για την επιτάχυνση της αντίδρασης.

Διάλυμα Marc-André (Άχρωμο ή χρωματισμένο με όξινη φουξίνη)

Στην παρούσα ενότητα θα αναλυθούν τα πλεονεκτήματα και τα μειονεκτήματα της χρήσης του διαλύματος Marc-André. Το διάλυμα Marc-André αποτελεί μείγμα ένυδρης χλωράλης (chloral hydrate, μονοϋδρική τριχλωροακεταλδεΰδη), οξικού οξέος και νερού. Είναι ισχυρό οξειδωτικό διάλυμα λόγω του συνδυασμού αλδεΰδης και οξέος. Εξουδετερώνει τυχόν περίσσεια KOH που παραμένει στα δείγματα, χωρίς να προκαλεί καθίζηση των αλάτων των λιπαρών οξέων (σαπώνων). Επίσης, οξειδώνει τις δευτερογενείς αλκοόλες των γλυκοζαμινών που σχηματίζονται στη χιτίνη, μαλακώνοντας τον εξωσκελετό και διαλύοντας ορισμένα ανόργανα άλατα των δειγμάτων.

Η προσθήκη όξινης φουξίνης σταθεροποιεί τις δευτερογενείς αλκοόλες και χρωματίζει τη χιτίνη, διευκολύνοντας την παρατήρηση των εσωτερικών χιτινοποιημένων δομών. Μετά την επώαση σε διάλυμα Marc-André και τον χρωματισμό, τα δείγματα εκπλένονται με αιθανόλη, ξεκινώντας έτσι τη φάση αφυδάτωσης.

Πλεονεκτήματα:

- Εξουδετερώνει την περίσσεια βάσης
- Χαλαρώνει τη χιτίνη
- Χρωματίζει τη χιτίνη για καλύτερη παρατήρηση των εσωτερικών δομών

Μειονεκτήματα:

- Η χλωράλη είναι τοξική ουσία με υπνωτική δράση που χρησιμοποιείται στην Ιατρική και πρέπει να χρησιμοποιείται αποκλειστικά εντός απαγωγού και πάντα σύμφωνα με τη νομοθεσία περί επικίνδυνων χημικών ουσιών.

Διαλύματα αφυδάτωσης:

Εμπειρικά διαφαίνεται ότι η εμβάπτιση σε διαδοχικά διαλύματα αλκοόλης με αυξανόμενη συγκέντρωση δεν είναι απαραίτητη για πολύ μικρά δείγματα. Για μεγαλύτερα δείγματα, η σειρά ξεκινά με εμβάπτιση σε διάλυμα αιθανόλης 80%, ακολουθούμενη από 90%, 95% και τελικά σε απόλυτη αιθανόλη. Για πολύ μικρά δείγματα, αρκεί εμβάπτιση σε αιθανόλη 90% και στη συνέχεια σε απόλυτη αιθανόλη. Σημειώνεται ότι σε αυτό το στάδιο, η απόλυτη αιθανόλη δεσμεύει τους υδρατμούς από την ατμόσφαιρα.

Παραδοσιακά στα εργαστήρια εντομολογίας, η αφυδάτωση δειγμάτων ολοκληρωνόταν σε κρεόζωτο οξιάς (beech creosote), μία ουσία που χρησιμοποιείται ευρέως ως φυτοφάρμακο, αντιμυκητιασικό και συντηρητικό ξύλου. Σήμερα η χρήση του αποφεύγεται λόγω οσμής και υψηλής τοξικότητας (πολυκυκλικοί αρωματικοί υδρογονάνθρακες), θεωρείται καρκινογόνος ουσία, τοξική για την αναπαραγωγή, προκαλεί επίμονη οργανική ρύπανση και είναι οικοτοξική για υδρόβιους οργανισμούς.

Ως εναλλακτικό μέσο για τον εγκλεισμό δειγμάτων (mounting) προτείνουμε να παρασκευάζεται διάλυμα Euparal® και Euparal essence (περιγράφεται στην επόμενη παράγραφο) μετά από εμβάπτιση των δειγμάτων σε αιθανόλη 90%. Το μείγμα είναι καλά ανεκτό και ιδανικό για μόνιμο εγκλεισμό και μικροσκόπηση.

Παράρτημα 2: Σύνθεση αντιδραστηρίων.

Διάλυμα KOH 10%
Υδροξείδιο του καλίου (KOH) 10 g
Απιονισμένο νερό q.s. 100 mL

Μέσο Hoyer (κόμμι χλωράλης)
Νερό 50 mL
Ένυδρη χλωράλη 200 g
Αραβικό κόμμι 50 g
Γλυκερόλη 20 mL

Διάλυμα Marc-André (βασικό)
Ένυδρη χλωράλη 40 g
Παγόμορφο οξικό οξύ 30 mL
Νερό 30 mL

Διάλυμα όξινης φουξίνης 1%
Σκόνη όξινης φουξίνης 1 g
Νερό 99 mL

Marc-André χρωματισμένο με φουξίνη
Διάλυμα Marc-André 10 mL
Διάλυμα όξινης φουξίνης 1% 50 μL

Παράρτημα 3: Μέσα εγκλεισμού Euparal®, Canada balsam και πολυβινυλική αλκοόλη

Πολυβινυλική αλκοόλη: Αποτελεί ιδανικό μέσο εγκλεισμού όταν τα τυπικά μέσα αφυδάτωσης δεν είναι διαθέσιμα. Σε αυτή την περίπτωση, αναμειγνύεται με γαλακτοφαινόλη του Amman (Amman's lactophenol). Ωστόσο, παρουσιάζει τα εξής κύρια μειονεκτήματα: συρρίκνωση ή κρυστάλλωση λόγω εξάτμισης του νερού καθώς και σκλήρυνση ή μαύρισμα (οξείδωση) του παρασκευάσματος όταν η φαινόλη οξειδώνεται. Παρά τα μειονεκτήματα, η τεχνική παραμένει κατάλληλη για βραχυπρόθεσμο εγκλεισμό δειγμάτων.

Βάλσαμο του Καναδά (Canada Balsam): Η χρήση του Βαλσάμου του Καναδά ως μέσου εγκλεισμού των δειγμάτων μεταξύ αντικειμενοφόρου πλάκας και καλυπτρίδας προϋποθέτει την πλήρη αφυδάτωση των δειγμάτων. Η χρήση ξυλολίου ή τολουολίου παρουσιάζει μειονεκτήματα και απαιτεί προσοχή κατά την εφαρμογή.

Μέσο Enecê: Όπως και το Βάλσαμο του Καναδά, η χρήση του μέσου Enecê προϋποθέτει πλήρη αφυδάτωση των δειγμάτων. Σύνθεση: 22 g καθαρό λευκό κολοφώνιο (pure white colophony), 12 g ρητίνη copal (alcohol-soluble copal gum), 20 mL απόλυτη αιθανόλη, 10 g καμφορά, 10 mL τερεβινθέλαιο (turpentine essence), 26 mL ευκαλυπτόλη. Διαδικασία παρασκευής: Σε κωνική φιάλη τύπου Erlenmeyer προστίθενται η απόλυτη αιθανόλη και η καμφορά. Στη συνέχεια προστίθενται το κολοφώνιο και η ρητίνη copal. Η φιάλη σφραγίζεται με πώμα, ανακινείται και θερμαίνεται σε υδατόλουτρο σε χαμηλή θερμοκρασία, ώστε το μείγμα να μη φτάσει σε βρασμό. Μόλις τα συστατικά διαλυθούν πλήρως, προστίθεται το τερεβινθέλαιο. Το μείγμα φιλτράρεται ενώ είναι ακόμη θερμό και στο τέλος, προστίθεται στο διήθημα η ευκαλυπτόλη. Όταν το μέσο αποκτήσει μεγαλύτερο ιξώδες (γίνει λιγότερο ρευστό), αραιώνεται με διάλυμα αραίωσης Enecê σύμφωνα με τη φόρμουλα: 30 mL απόλυτη αιθανόλη, 17 g καμφορά, 15 mL τερεβινθέλαιο, 38 mL ευκαλυπτόλη (Cerqueira, 1943).

Euparal®: Πρόκειται για ρητίνη προερχόμενη από το κυπαρίσσι του Άτλαντα, *Tetraclinis articulata* (Vahl, 1791), η οποία μελετήθηκε και αναπτύχθηκε από τον Gilson το 1906. Το κύριο πλεονέκτημά της είναι οτι δεν πολυμερίζεται, εξασφαλίζοντας μακροχρόνια σταθερότητα στα παρασκευάσματα. Τα δείγματα που έχουν εγκλειστεί μεταξύ πλάκας και καλυπτρίδας μπορούν εύκολα να αφαιρεθούν με αιθανόλη ή, ακόμη καλύτερα, με Euparal® essence. Αυτή η ρητίνη, γνωστή και ως sandarac, μπορεί να αναμιχθεί ή να διαλυθεί σε αιθανόλη συγκέντρωσης ≥80%, διασφαλίζοντας την ορθή τοποθέτηση και σταθεροποίηση του δείγματος.

Χρήση Triton X-100: Πρόκειται για μη ιοντικό επιφανειοδραστικό παράγοντα [4-(1,1,3,3-τετραμεθυλοβουτυλο) φαινυλο-πολυαιθυλενογλυκόλη], ο οποίος χρησιμοποιείται ευρέως ως απορρυπαντικό στην κυτταρική και μοριακή βιολογία. Αυξάνει τη διαπερατότητα των κυτταρικών και πυρηνικών μεμβρανών.

Είναι σύνηθες τα δείγματα εντόμων να διατηρούνται σε αλκοόλη για πολλά χρόνια. Δυστυχώς, η συντήρηση σε αλκοόλη δεν αποτελεί ιδανική μέθοδο για μορφολογική ανάλυση και για αρθρόποδα που διατηρούνται με αυτόν τον τρόπο, καθώς η προετοιμασία τους για μικροσκοπική εξέταση είναι δύσκολη. Τα πλαστικά δοχεία στα οποία φυλάσσονται τα δείγματα, ενδέχεται να παρουσιάσουν φθορές ή απώλεια στεγανότητας, με αποτέλεσμα την εξάτμιση της αλκοόλης και την ξήρανση των δειγμάτων. Τόσο η παρατεταμένη παραμονή σε αλκοόλη όσο και η ξήρανση αποτελούν σοβαρό πρόβλημα στην προετοιμασία των παρασκευασμάτων. Ο Jonque σε σχετική ανακοίνωση (2008), αναφέρθηκε στην επανυδάτωση ξηρών δειγμάτων αραχνών με χρήση διαβρεκτικού παράγοντα, όπως το Agepon που χρησιμοποιείται στα φωτογραφικά φιλμ [26]. Η παρατήρηση αυτή οδήγησε στη διερεύνηση της χρήσης διαβρεκτικών παραγόντων που δεν παρουσιάζουν ισχυρή απορρυπαντική δράση.

Ακολουθεί η διαδικασία επανυδάτωσης δειγμάτων με Triton X-100 σε υδατικό διάλυμα 0,5%:

- Εμβαπτίστε πλήρως το δείγμα σε απόλυτη αιθανόλη.
- Προσθέστε επαρκή όγκο διαλύματος Triton X-100 0,5%, ώστε το δείγμα να καλύπτεται πλήρως.
- Αφήστε το διάλυμα να δράσει για τουλάχιστον 5 λεπτά. Τα αρθρόποδα και τα ανατομικά τους μέρη πρέπει να διαχωρίζονται πλήρως εντός του διαλύματος.
- Απομακρύνετε το διάλυμα Triton X-100 και αντικαταστήστε με διάλυμα υδροξειδίου του καλίου (KOH).

Η διαδικασία συνεχίζεται σύμφωνα με το πρωτόκολλο που περιγράφεται παραπάνω.

Παράρτημα 4: Διαδικασία βήμα προς βήμα για τον εγκλεισμό δειγμάτων με Euparal® ή Canada Balsam

1. Απαιτείται η πλήρης αφυδάτωση των δειγμάτων (θολή ή γαλακτώδης όψη υποδηλώνει ατελή αφυδάτωση).
2. Η αφυδάτωση πραγματοποιείται με διαδοχικά αυξανόμενες συγκεντρώσεις αιθανόλης (αιθυλικής αλκοόλης).
3. Τα δείγματα μεταφέρονται από αλκοόλη 99% ή απόλυτη αιθανόλη σε κατάλληλο παράγοντα διαύγασης.

Διαδικασία:

1. Τοποθετήστε τις ενήλικες σκνίπες σε αιθανόλη 70%.
2. Αφαιρέστε την αιθανόλη και προσθέστε διάλυμα KOH 10%. Τοποθετήστε και καλύψτε τα δείγματα σε αντικειμενοφόρο πλάκα.
3. Αφήστε τα έντομα εντός του διαλύματος μέχρι οι ιστοί να μαλακώσουν και να καταστούν επαρκώς διαυγείς (διαφανείς), ώστε να φαίνονται οι εσωτερικές τους δομές.
4. Αφαιρέστε το διάλυμα KOH.
5. Καλύψτε με απιονισμένο νερό για 30–45 λεπτά.
6. Αφαιρέστε το νερό και επαναλάβετε την πλύση με απεσταγμένο νερό για 30 λεπτά. (ο χρόνος παραμονής προσαρμόζεται ανάλογα με τον αριθμό των δειγμάτων· όσο περισσότερα τα δείγματα που συνυπάρχουν προς επεξεργασία, τόσο αυξάνεται ο χρόνος. Για λιγότερα ή μεμονωμένα δείγματα, ο χρόνος μειώνεται αναλογικά.
7. Αφαιρέστε το νερό.
8. Προσθέστε διάλυμα Marc-André (ενδεχομένως χρωματισμένο με όξινη φουξίνη) και αφήστε να δράσει για 24 ώρες.
9. Αφαιρέστε το διάλυμα Marc-André.
10. Καλύψτε το δείγμα με απεσταγμένο νερό και αφήστε το για 30–45 λεπτά.
11. Αφαιρέστε το νερό και επαναλάβετε την πλύση με απεσταγμένο νερό για 30 λεπτά.
12. Αφαιρέστε το νερό.
13. Προσθέστε αιθανόλη 70% και με μικροτομή απομονώστε τα ανατομικά τμήματα των σκνιπών.

    A. Τραβήξτε απαλά και απομακρύνετε προσεκτικά την κεφαλή και την κοιλία από τον θώρακα.

    B. Για τον θώρακα, αφαιρέστε τις πτέρυγες κρατώντας τον θώρακα με ένα ζεύγος μικρολαβίδων και έλκοντας τη βάση των εξαρτημάτων με ένα δεύτερο ζεύγος. Ανάλογα με τις περιοχές ενδιαφέροντος, μπορεί να πραγματοποιηθεί τομή σε οβελιαίο επίπεδο, χωρίζοντας τον θώρακα σε αριστερή και δεξιά πλευρά.
14. Αφυδατώστε σταδιακά σε διαλύματα αιθανόλης 50% → 80% → 95% έως απόλυτη αιθανόλη.
15. Ολοκληρώστε την αφυδάτωση σε απόλυτη αιθανόλη με δύο διαδοχικές πλύσεις διάρκειας 10 λεπτών η καθεμία.
16. Αφαιρέστε την αιθανόλη και καλύψτε τα δείγματα με γαρυφαλέλαιο για 15 λεπτά σε θερμοκρασία δωματίου.
17. Μεταφέρετε τα δείγματα από το γαρυφαλέλαιο και τοποθετήστε τα σε καθαρή αντικειμενοφόρο πλάκα επάνω σε μια σταγόνα Euparal® ή Canada Balsam.
18. Με τη βοήθεια στερεοσκοπίου, προσανατολίστε και διατάξτε τα τμήματα του εντόμου στην επιθυμητή θέση. Η κεφαλή, ο θώρακας και η κοιλία της σκνίπας μπορούν να αποσπαστούν προσεκτικά με τη χρήση λεπτών βελόνων ή μικρολαβίδων, υπό στερεοσκοπική παρατήρηση. Η κεφαλή αποσπάται από το σώμα ώστε να τοποθετηθεί σε κοιλιοραχιαία (ventro-dorsal) θέση. Συγκεκριμένα, το ινιακό τρήμα (occipital foramen) πρέπει να προσανατολίζεται προς τα πάνω, προκειμένου να καθίσταται δυνατή η άμεση παρατήρηση του κιβαρίου (cibarium, στοματική κοιλότητα) και άλλων εσωτερικών δομών. Η παρασκευή πραγματοποιείται εντός κατάλληλου μέσου εγκλεισμού.
19. Αφήστε το μέσο να ημιπολυμεριστεί έως ότου καταστεί ελαφρώς ιξώδες (η επιφάνεια να γίνει κολλώδης).
20. Διαβρέξτε ελαφρά μία καθαρή καλυπτρίδα με απόλυτη αιθανόλη. Τοποθετήστε την καλυπτρίδα υπό γωνία πάνω στο μέσο Canada Balsam.
21. Αποθηκεύστε τις αντικειμενοφόρες πλάκες σε οριζόντια θέση εντός ειδικής θήκης για προστασία από τη σκόνη και την υγρασία.
