# Supplementary material for: Processing and mounting phlebotomine sand flies: a consensus guideline
Source: Parasite. 2026 Apr 3;33:18. doi: 10.1051/parasite/2026009 (PMC13047900; doi:10.1051/parasite/2026009)
Supplement: Supplementary file 13 — Hebrew translation / תרגום לעברית [file parasite-33-18-s13.pdf]

Parasite **33**, 18 (2026)
© F.J. Randrianambinintsoa et al., published by EDP Sciences, 2026
https://doi.org/10.1051/parasite/2026009

PARASITE
Available online at:
www.parasite-journal.org

REVIEW ARTICLE OPEN ACCESS

עיבוד והכנה לקיבוע של זבובי חול (Phlebotomine sand flies): הנחיות מקצועיות

Fano José Randrianambinintsoa[1], Laure Augendre[1], Jorian Prudhomme[1], Jean-Philippe Martinet[1], Mathieu Loyer[1], Nalia Mekarnia[1], Hocine Kerkoub[1], Farzana Khan Perveen[1], Antoine Huguenin[1,2], Emilie Kariya[1,2], Mohammad Akhoundi[3], Andrey José De Andrade[4], Eduardo Berriatua[5], Gioia Bongiorno[6], Sébastien Boyer[7,8], Vasiliki Christodoulou[9], Magda Clara Vieira Da Costa-Ribeiro[10], Lucas Alexandre Farias De Souza[10], Huicong Ding[11], Blaise Dondji[12], Vít Dvořák[13], Ozge Erisoz Kasap[14], Eunice Aparecida Bianchi Galati[15], Montserrat Gállego[16], Cristina Ballart[16], Stavroula Gouzelou[17], Nabil Haddad[18], Rezki Sabrina Masse[19], Asrat Hailu Mekuria[20], Vladimir Ivovic[21], Szymon Kaczmarek[22], Mohd Khadri Shahar[19], Oscar D. Kirstein[23], Edwin Kniha[24], Iva Kolářová[13], Timinao Lincoln[25], Cristian Lucanas[26], Ognyan Mikov[27], Kimsear Nov[7], Yusuf Özbel[28], Bernard Pesson[29], Laura Cristina Posada Lopez[30], Didot Budi Prasetyo[1,7], Nil Rahola[31], Eduardo A. Rebollar-Tellez[32], Bruno Leite Rodrigues[15], Lalita Roy[33], Prasanta Saini[34], Chizu Sanjoba[35], Paloma Helena Fernandes Shimabukuro[36], Padet Siriyasatien[37], Agnieszka Soszyńska[22], Tatiana Suleşco[38], Massamba Sylla[39], Majhalia Torno[40], Petr Volf[13], Khamsing Vongphayloth[41], Vu Sinh Nam[42], April Wardhana[43], Eric Yessinou[44], Sonia Zapata[45], Jean-Charles Gantier[1], and Jérôme Depaquit[1,2,*]

[1] Faculté de Pharmacie, Université de Reims Champagne Ardenne, UR ESCAPE-USC ANSES PETARD, 51 rue Cognacq-Jay, 51096 Reims Cedex, France
[2] Pôle de Biologie territoriale, Laboratoire de Parasitologie-Mycologie, Centre Hospitalo-Universitaire, 51092 Reims, France
[3] Parasitology-Mycology Department, Avicenne Hospital, AP-HP, Bobigny, Sorbonne Paris Nord University, France; Unité des Virus Émergents (UVE: Aix-Marseille Univ, Università di Corsica, IRD 190, Inserm 1207, IRBA), 13005 Marseille, France
[4] Parasitology Collection of Basic Pathology, Department of Basic Pathology, Federal University of Paraná, Curitiba 19031, Brazil
[5] Department of Animal Health, University of Murcia, Campus de Espinardo, 30100 Espinardo, Murcia, Spain
[6] Department of Infectious Diseases, Vector-borne Diseases Unit, Istituto Superiore di Sanità, 00166 Rome, Italy
[7] Medical and Veterinary Entomology Unit, Institut Pasteur du Cambodge, Phnom Penh 12201, Cambodia
[8] Ecology & Emergence of Arthropod-borne Pathogens Unit, Department of Global Health, Institut Pasteur, CNRS UMR2000, 75015 Paris, France
[9] Section Veterinary Services (1417), Laboratory for Animal Health Virology, Aglantzia, Nicosia 2109, Cyprus
[10] Insects Vectors and Parasites Laboratory, Department of Basic Pathology and Postgraduate program in Microbiology, Parasitology and Pathology, Federal University of Paraná, 81530-900 Curitiba, Brazil
[11] Department of Biological Sciences, National University of Singapore, 117558, Singapore
[12] Laboratory of the Leishmaniasis Research Project, Mokolo District Hospital, Mokolo, Cameroon; Laboratory of Cellular Immunology and Parasitology, Department of Biological Sciences, Central Washington University, 98926 Ellensburg, WA, USA
[13] Department of Parasitology, Faculty of Science, Charles University, 12800 Prague, Czechia
[14] VERG Laboratories, Department of Biology, Faculty of Science, Hacettepe University, Beytepe, Ankara 06800, Türkiye
[15] Faculdade de Saúde Pública da Universidade de São Paulo (FSP/USP), Pós-graduação em Saúde Pública, 01246-904 São Paulo, Brazil
[16] Secció de Parasitologia, Departament de Biologia, Sanitat i Medi Ambient, Facultat de Farmàcia i Ciències de l'Alimentació, Universitat de Barcelona, & Institut de Salut Global de Barcelona (ISGlobal), Centro de Investigación Biomédica en Red, Enfermedades Infecciosas (CIBERINFEC), 08028 Barcelona, Spain
[17] Laboratory of Infectious Diseases and Public Health, School of Medicine, University of Cyprus, Nicosia, Cyprus & Department of Pediatrics, Archbishop Makarios III Hospital, Nicosia 2115, Cyprus
[18] Faculty of Health Sciences, American University of Beirut, 1107 2020 Beirut, Lebanon
[19] Medical Entomology Unit, Infectious Disease Research Centre, Institute for Medical Research (IMR), National Institutes of Health (NIH), Ministry of Health Malaysia, 40170 Shah Alam, Selangor, Malaysia
[20] School of Medicine, Addis Ababa University, 28017 - 1000 Addis Ababa, Ethiopia
[21] Faculty of Mathematics, Natural Sciences and Information Technologies, University of Primorska, 6000 Koper, Slovenia
[22] University of Lodz, Faculty of Biology and Environmental Protection, Department of Invertebrate Zoology and Hydrobiology, Banacha 12/16, 90-237 Łódź, Poland
[23] Laboratory of Entomology, Ministry of Health, 9134302 Jerusalem, Israel

Edited by Jean-Lou Justine
*Corresponding author: jerome.depaquit@univ-reims.fr

This is an Open Access article distributed under the terms of the Creative Commons Attribution License (https://creativecommons.org/licenses/by/4.0), which permits unrestricted use, distribution, and reproduction in any medium, provided the original work is properly cited.

[24] Center for Pathophysiology, Infectiology and Immunology, Institute of Specific Prophylaxis and Tropical Medicine, Medical University Vienna, Kinderspitalgasse 15, 1090 Vienna, Austria
[25] Papua New Guinea Institute of Medical Research (PNGIMR) Institute, PO Box 60, Headquarter, Homate Street, 441 Goroka, Eastern Highlands Province, Papua New Guinea
[26] Museum of Natural History, University of the Philippines Los Baños, 4031 Laguna, Philippines
[27] National Centre of Infectious and Parasitic Diseases, 1504 Sofia, Bulgaria
[28] Ege University, Faculty of Medicine, Department of Parasitology, 35040 Bornova/Izmir, Türkiye
[29] Retired, Faculté de Pharmacie, Université de Strasbourg, Strasbourg, 67400 Illkirch-Graffenstaden, France
[30] Program for the Study and Control of Tropical Diseases (PECET), Faculty of Medicine, University of Antioquia, 050010 Medellin, Colombia
[31] MIVEGEC, Univ. Montpellier, CNRS, IRD, 34394 Montpellier, France & Medical Entomology Unit, Institut Pasteur de Madagascar, 101 Antananarivo, Madagascar
[32] Laboratorio de Entomología Médica, Departamento de Zoología de Invertebrados, Facultad de Ciencias Biológicas, Universidad Autónoma de Nuevo León, San Nicolás de los Garza, 66455, NL, México
[33] Tropical and Infectious Disease Centre, BP Koirala Institute of Health Sciences, Dharan 56700, Nepal
[34] ICMR-Vector Control Research Centre, ICMR-Vector Control Research Centre, Puducherry 605006, India
[35] Graduate School of Agricultural and Life Sciences, The University of Tokyo, Tokyo 113-8657, Japan
[36] Grupo de estudos em Leishmanioses/Coleção de Flebotomíneos (COLFLEB/Fiocruz-MG), Instituto René Rachou, Fundação Oswaldo Cruz, Belo Horizonte, Minas Gerais, 30190009, Brazil
[37] Center of Excellence in Vector Biology and Vector-Borne Disease, Department of Parasitology, Faculty of Medicine, Chulalongkorn University, Bangkok 10330, Thailand
[38] Department of Arbovirology, Bernhard Nocht Institute for Tropical Medicine, Bernhard Nocht Str. 74, 20359 Hamburg, Germany [39] Laboratory Vectors & Parasites, Department of Livestock Sciences and Techniques, Sine Saloum University El Hadji Ibrahima Niasse (SSUEIN) Kaffrine Campus, C.P. 24600, Senegal.
[40] Environmental Health Institute, National Environment Agency, Singapore 138667, Singapore & Department of Biological Sciences, National University of Singapore, 117558 Singapore
[41] Institut Pasteur du Laos, Laboratory of Vector-Borne Diseases, Samsenhai Road, Ban Kao-Gnot, Sisattanak District, 3560 Vientiane, Lao PDR
[42] National Institute of Hygiene and Epidemiology, 1 Yec-Xanh Street, Hai Ba Trung District, 100000 Hanoi, Vietnam
[43] Indonesian Research Center for Veterinary Science, Indonesian Agency for Agricultural Research and Development, Ministry of Agriculture Republic Indonesia, Bogor 16114, Indonesia & Department of Parasitology, Faculty of Veterinary Medicine, Airlangga University, Surabaya 60115, Indonesia
[44] Laboratory of Research in Applied Biology, Polytechnic School of Abomey-Calavi, University of Abomey-Calavi, 01 P.O. Box 2009, 00000 Cotonou, Benin
[45] Instituto de Microbiología, Colegio de Ciencias Biológicas y Ambientales (COCIBA), Universidad San Francisco de Quito (USFQ), 170901 Quito, Ecuador

Received 1 December 2025, Accepted 29 January 2026, Published online 3 April 2026

תקציר: מאמר זה מספק מדריך מקיף לעיבוד, ניתוח וקיבוע על גבי זכוכית נושאת של דגימות זבובי חול מתת-המשפחה Phlebotominae, תהליך החיוני לזיהוי מינים ולאיתור ובידוד מחוללי מחלות (פתוגנים). המאמר דן במגוון טכניקות המתאימות הן לתנאי שדה והן לסביבה מעבדתית. המדריך כולל הנחיות מפורטות ללכידת זבובי חול, לטיפול בהם ולהמתתם (תוך המלצה על הקפאה יבשה או שימוש ב- CO_2 במקום חומרים כימיים) וכן אסטרטגיות שימור, כגון אחסון בקירור ושימור באתנול. איכות ההכנה של מבנים אנטומיים מסוימים (איברי המין, הראש והכנפיים) חיונית לצפייה מיקרוסקופית תקינה בהם ומתוארת בעבודה זו. המאמר מציג גם תהליך עיבוד דגימות מפורט, הכולל את שלב הניקוי באמצעות חומרים כגון אשלגן הידרוקסיד ולאחר מכן תמיסת מארק-אנדרה (Marc-André). תהליך הקיבוע משווה בין מדיומי קיבוע שונים, תוך הדגשת תכונותיהם האופטיות ופוטנציאל השימור שלהם. נוזל הוייאר (Hoyer), הידוע גם כגומי כלורלי (chloral gum), מומלץ לצפייה מהירה, במיוחד בספרמטקות (spermathecae) , איברי אגירת הזרע בגוף הנקבה, בזכות שקיפותו הגבוהה, אולם הוא אינו מתאים לאחסון ארוך טווח. מדיומים נוספים הנדונים במאמר כוללים פוליוויניל אלכוהול, Euparal® (בעל סבילות מוגבלת למים), ובלזם קנדה (Canada balsam), כאשר שני האחרונים מאפשרים שימור ארוך טווח. בנוסף, נדונות גישות חדשניות בביולוגיה מולקולרית, כגון ריצוף DNA וMALDI-ToF , הדורשות תשומת לב מיוחדת לתהליך עיבוד הדגימות. בנוסף, מסופקים קטעי וידאו קצרים המדגימים טכניקות קיבוע שונות, וכן תרגומים ל-33 שפות שונות, במטרה לאפשר למסמך לענות על הצרכים והציפיות המגוונים של הקהילה המדעית הגלובלית.

מילות מפתח: קיבוע, זבוב חול פלבוטומוס, נוזל הוייאר (Hoyer), תמיסת Marc-André, גומי כלורלי (chloral gum), אלכוהול פוליוויניליִ (Polyvinyl alcohol), Canada balsam, Euparal®, בידוד טפילי *Leishmania* (*לישמניה*), תנאי שדה, תרבית, ניתוח, ביולוגיה מולקולרית, MALDI-ToF, דגימות טיפוס

Abstract – Processing and mounting phlebotomine sand flies: a consensus guideline. This article provides a comprehensive guide for the processing and mounting of phlebotomine sand fly specimens, which is crucial for species identification and pathogen detection and isolation. It discusses a range of techniques suitable for both field and laboratory settings. The guide includes detailed instructions on sand fly collection, handling, covering, and euthanasia (recommending dry freezing or CO_2 over chemicals) as well as conservation strategies, such as cold storage and

preservation in ethanol. The quality of preparation of certain anatomical structures (genital organs, head and wings) is essential for their proper microscopic observation and is described in this work. The article also presents detailed sample processing, including the clearing process with agents such as potassium hydroxide then Marc-André solution. The mounting process compares different media, emphasizing their optical properties and preservation potential. Hoyer fluid (also known as chloral gum) is recommended for quick observation, particularly for spermathecae, due to its clarity, although it is not suitable for long-term storage. Other media discussed include polyvinyl alcohol, Euparal® (for limited water tolerance), and Canada balsam (a hydrocarbon-soluble medium), with the latter two offering long-term preservation capabilities. Innovative molecular biology approaches such as DNA sequencing and MALDI-ToF, which require particular attention to sample processing, are also addressed. Furthermore, short video clips illustrating various mounting techniques as well as translations in many different languages are provided, allowing the guideline to reach the diverse needs and expectations of the global scientific community.

Key words: Mounting, Phlebotomine sand fly, Hoyer fluid, Marc-André solution, Chloral gum, Polyvinyl alcohol, Euparal®, Canada balsam, *Leishmania* isolation, Field conditions, Culture, Dissection, Molecular biology, MALDI-ToF, Type-specimens.

מבוא

זבובי החול הפלבוטומיניים הם חרקים דו-כנפיים קטנים המשתייכים למשפחת הפסיכודידיים (Psychodidae), תת-המשפחה Phlebotominae, הכוללת לפחות 1,063 מינים ידועים [21]. הם מהווים וקטורים (מעבירי גורמי מחלה) חשובים של פתוגנים כגון: טפילי *לישמניה* (*Leishmania*), ארבו-וירוסים (arboviruses) ו-*Bartonella*, הגורמים למחלות הנקראות לישמניאזיס (leishmaniasis), זיהומי ארבו-וירוסים וברטונלוזיס, בהתאמה. זיהויים מתבסס בעיקר על בדיקה מיקרוסקופית מפורטת, המתאפשרת באמצעות לכידה מוקפדת, אחסון מתאים וקיבוע זהיר על זכוכית נושאת. תהליך זה דורש שימוש במספר טכניקות ייעודיות, שלכל אחת מהן יתרונות ומגבלות.

זיהוי זבובי חול בוגרים מבוסס על תצפית במבנים חיצוניים כגון מחושים, בחנינים (palpi) ואיברי המין הזכריים (aedeagus), ובמבנים פנימיים, למשל הלוע (pharynx), הציבריום(cibarium) - חלל קדם-לועי במערכת הפה, והספרמטקות (spermathecae) - איברי אגירת הזרע בנקבה. דיסקציה ובידוד של המבנים הפנימיים מאפשרים תצפית ברורה יותר, ובכך תורמים לזיהוי מדויק. לפיכך, בשונה מיתושים או פשפשי נשיקה (Kissing bugs), זבובי חול מחייבים קיבוע בין זכוכית נושאת לזכוכית כיסוי טרם ביצוע הזיהוי.

עד שנות ה-1980 הייתה התצפית המיקרוסקופית השיטה היחידה לזיהוי זבובי חול, והיא נותרה עד היום הגישה הנפוצה ביותר. בחירת תהליך העיבוד וההכנה הייתה אפוא פשוטה יחסית והתבססה בעיקר על דיכוטומיה: מצד אחד, קיבוע סופי המאפשר שימור ארוך טווח של הדגימה; ומן הצד השני, קיבוע מהיר לצורך זיהוי במדיום שאינו מבטיח שימור ממושך. קיבוע סופי, לדוגמה בתערובות שרפיות כגון בלזם קנדה (Canada balsam) הינו תהליך ממושך הדורש ייבוש מלא של הדגימות. יתרה מזו, מקדם השבירה של המדיום אינו תמיד מיטבי לצפייה נוחה בספרמטקות. לעומת זאת, קיבוע במדיום מימי, למשל בנוזל הוייאר (Hoyer), מהיר יותר ומאפשר הדגמה טובה יותר של ספרמטקות בעלות שקיפות גבוהה, אך אינו מאפשר שימור ארוך טווח, שכן הוא נוטה לספוח מים מהאטמוספרה. אחת האפשרויות היא לאטום את הזכוכית הנושאת בלק (nail polish) לאחר ייבוש מלא. גישה פשרנית זו ממשיכה להתקיים עד היום ומשפיעה על בחירת שיטת הקיבוע בהתאם למטרת ההכנה של הפרפרט.

מאז שנות ה-1980 מחקרי הזיהוי של זבובי חול משלבים בין גישות מורפולוגיות לבין גישות ביוכימיות. הגישות הביוכימיות הראשונות כללו ניתוח של פחמימנים קוטיקולריים, אשר הוחלפו בהמשך ברובן בטכניקות של ביולוגיה מולקולרית, כגון Random Amplified Polymorphic DNA (RAPD), Restriction Fragment Length Polymorphism (RFLP), הגברת DNA וריצוף בשיטת Sanger, ובהמשך גם ריצוף מהדור הבא (NGS - Next Generation Sequencing). כיום משלימים את הגישות המולקולריות גם בשיטות פרוטאומיות, כגון MALDI-ToF. בנוסף, ניתן לשלב זיהוי מולקולרי של מינים עם גילוי פתוגנים באמצעות PCR (כגון *Leishmania*, *Trypanosoma*, *Bartonella* ו-*Phlebovirus*), כולם ניתנים לזיהוי הן ב-End Point PCR והן ב-Real Time PCR, מה שמחייב התאמה של הדגימה ותנאי האחסון למטרות המחקר או האבחון [3, 32]. מעבר למאפיינים המורפולוגיים המשמשים באופן מסורתי להבחנה בין מינים, ניתן ליישם גם גישות מורפולוגיות נוספות, כגון מורפומטריה גיאומטרית של הכנפיים (wing geomorphometry).

בהתבסס בעיקר על ניסיונם האישי של המחברים ועל נתוני הספרות, מטרת מחקר זה הייתה לספק קווים מנחים סטנדרטיים לקיבוע ולעיבוד של זבובי חול בוגרים מסוג פלבוטומוס, במטרה לייעל הן את האנליזה המורפולוגית והן את האנליזה המולקולרית. הצורך לבצע ניתוחים מסוימים (למשל בתחום הביולוגיה המולקולרית או באמצעות MALDI-ToF) מחייב שמירה על חלקים מגוף זבוב החול שאינם נדרשים לזיהוי מורפולוגי, דבר המדגיש את חשיבות הבחירה המושכלת בפרוטוקול המתאים.

הקדמה: שיקולי בטיחות ורגולציה צריכים להתבסס על גיליונות הבטיחות (Safety Data Sheets, SDS) הרלוונטיים

יש לטפל בכל הכימיקלים המוצגים במדריך זה בתנאי בטיחות מחמירים. ועדות הבטיחות והבריאות של מוסדות המחקר זמינות לספק מידע לא רק על הסיכונים הכרוכים בחומרים אלה, אלא גם על נהלי השימוש בהם ועל סילוק הפסולת באופן המתאים. חובה לפעול בהתאם להוראות הבטיחות הנוגעות לשימוש ולסילוק החומרים.

יודגש כי האחריות לוודא עמידה בכללי עבודה מעבדתית נאותה ובטוחה וכן בחקיקה ובתקנות החלות במדינת המשתמש או במוסד המחקר הרלוונטי מוטלת על כלל המשתמשים. נוסף על כך, חלק מן הכימיקלים או מרכיביהם (למשל כלורל הידרט - chloral hydrate) נתונים לרגולציה במדינות מסוימות. רשימת הקיצורים המופיעים במאמר זה מוצגת בטבלה 1.

במאמר זה אנו מתמקדים בשיטות ההרדמה וההמתה של זבובי חול שנלכדו חיים, בתנאי האחסון שלהם, ובתהליך הקיבוע שלהם על זכוכית נושאת, הן לצורך זיהוי מהיר והן לצורך שימור ארוך טווח המאפשר ביצוע מחקרים נוספים בעתיד.

טבלה 1: רשימת קיצורים וראשי תיבות

קיצור	מונח באנגלית	מונח בעברית
**BME**	Basal Medium Eagle	מדיום איגל בסיסי
**CDC**	Centers for Disease Control and Prevention	המרכזים לבקרת מחלות ומניעתן
**CMCP**	Camphor-monochlorophenol	קמפור-מונוכלורופנול
**CMR**	Carcinogenic, Mutagenic, Reprotoxic substance	חומר מסרטן, מוטגני ורעיל לפוריות ורבייה
**COI**	Cytochrome c oxidase subunit I gene	הגן לציטוכרום c אוקסידאז תת-יחידה I
**CytB**	Cytochrome b gene	הגן לציטוכרום b
**DNA**	Deoxyribonucleic acid	חומצה דאוקסיריבונוקלאית
**ELISA**	Enzyme-linked Imunosorbent Assay	בדיקת ELISA (אימונוסורבנט מקושר אנזים)
**EtOH**	Ethanol	אתנול
**M199**	Medium 199	מדיום 199
**MALDI-ToF MS**	Matrix-assisted laser desorption/ionization time-of-flight mass spectrometry	ספקטרומטריית מסה MALDI-ToF
**MEM**	Minimum Essential Medium	מדיום חיוני מינימלי
**NGS**	Next-Generation Sequencing	ריצוף מהדור הבא
**NNN**	Novy-MacNeal-Nicolle medium	מדיום נובי–מקניל–ניקול
**PCR**	Polymerase Chain Reaction	תגובת שרשרת פולימראז
**Lao PDR**	Lao People's Democratic Republic	הרפובליקה הדמוקרטית העממית של לאוס
**PNOC**	Prepronociceptin gene	הגן לפרה-פרונוציצפטין
**qPCR**	Quantitative PCR (real-time PCR	PCR כמותי - PCR בזמן אמת
**RAPD**	Random amplified polymorphic DNA	DNA פולימורפי מוגבר באקראי
**RFLP**	Restriction fragment length polymorphism	פולימורפיזם באורך מקטעי הגבלה
**RI**	Refractive index	מקדם שבירה
**RNA**	Ribonucleic acid	חומצה ריבונוקלאית
**RNases**	Ribonucleases	ריבונוקלאזות
**RNASS**	RNA stabilization solution	תמיסת ייצוב RNA
**RT-PCR**	Reverse transcription PCR	PCR לאחר שעתוק לאחור
**TFA**	Trifluoroacetic acid	חומצה טריפלואורואצטית

1. לכידת זבובי חול

ניתן ללכוד זבובי חול בוגרים, חיים או מתים, באמצעות מגוון שיטות, כגון מלכודות אור זעירות מסוג CDC, מלכודות דביקות, או שאיבה באמצעות מלכודות שאנון (Shannon traps), או באיסוף ישיר מאתרי מנוחה בסביבה (למשל מקלטי בעלי חיים). שיטות אלה כוללות הצבת מלכודות בבתי גידול מתאימים, משיכת זבובי החול באמצעות אור או חומרי משיכה אחרים כגון CO_2, או פיתיונות כימיים ואיסופם להמשך ניתוח, כפי שתואר במספר פרסומים [2, 3, 32, 36, 49]. לכידת זבובי חול בוגרים חיים מאפשרת את כלל היישומים הנדרשים בהמשך תהליך העבודה, בעוד שאיסוף פרטים מתים מונע בידוד של זני טפילי *Leishmania* או נגיפים. חלק מטכניקות הלכידה, כגון ניירות דביקים, גורמים לעיתים קרובות לאובדן איברים של זבובי החול (מחושים, כנפיים, רגליים או בחנינים [palps]). בנוסף, שכבת שמן קיק (castor oil) המצפה את הניירות הדביקים נצמדת לזבובי החול ויש להסירה בתחילת תהליך העיבוד, לרוב באמצעות השריה למשך 15 דקות בתערובת של אתנול (ethanol) ודיאתיל אתר (diethyl ether) בנפחים שווים.

2. המתת זבובי חול

לאחר הלכידה, יש להמית את זבובי החול שנלכדו חיים. בחלק משיטות האיסוף (למשל ניירות דביקים או מלכודות אור מסוג CDC המצוידות בכלי איסוף המכיל דטרגנט או אתנול), זבובי החול כבר מתים בעת האיסוף. ניתן ליישם שיטות ביולוגיה מולקולרית על דגימות שנאספו ישירות לתוך אתנול, וכן על דגימות אחרות אם אוחסנו באתנול במהירות האפשרית. עם זאת, אף אחת משיטות ההמתה הללו אינה מאפשרת עיבוד של הדגימות של זבובי חול באמצעות MALDI-ToF. בנוסף, חלק משיטות ההמתה עלולות לגרום לאובדן מאפיינים מורפולוגיים מסוימים.

לפיכך, יש להשתמש בחומרי המתה תקניים ומתאימים כדי להבטיח זיהוי תקין או אחסון ארוך טווח כדגימות ייחוס, כלומר דגימות המשומרות ומאוחסנות לצורך עיון או השוואה עתידיים. חומרים כגון אתיל אצטט (ethyl acetate), אתר אתילי (Diethyl ether), טטרכלורואתאן (tetrachloroethane) וכלורופורם (Chloroform), ניתנים להספגה בצמר גפן ולהכנסה לכלי המכיל את זבובי החול לצורך המתתם. יש לטפל בחומרים אלה בזהירות ובהתאם להנחיות היצרן בשל רעילותם. עם זאת, איננו ממליצים על המתת זבובי חול באמצעות כלורופורם, שכן מניסיוננו הוא אינו מתאים היטב למחקרי ביולוגיה מולקולרית. לאור הסיכון הכרוך בחומרים אלה והתאמתם המוגבלת לניתוחים מולקולריים, השימוש בהם בדרך כלל אינו מומלץ.

השיטה הנפוצה ביותר, המאפשרת שימור של המורפולוגיה וכן של DNA או חלבונים, היא הקפאה יבשה (ישירה) של הדגימות (ללא חומרים כימיים). יש להקפיא את הפרטים למשך זמן מספק כדי לגרום להרדמה מלאה, אך יש להימנע מהקפאה ממושכת מדי, העלולה לגרום ל-(1) ייבוש הדגימות או (2) פגיעה בחיוניות טפילי *Leishmania*, אם המטרה היא לבודדם *in vitro* ממערכת העיכול של זבוב החול. לפיכך אנו ממליצים על משך הקפאה של 15 - 20 דקות בטמפרטורה של -20°C, תוך בדיקה של הדגימות מפעם לפעם כדי לוודא שהפרטים רק מורדמים בקור, מבלי לפגוע בחיוניות טפילי *Leishmania*.

אם מקפיא אינו זמין, ניתן להמית את החרקים באמצעות CO_2. בתנאי שדה שבהם לא ניתן להשתמש במכלי CO_2, ניתן להמית את הדגימות באמצעות מכלים מסחריים קטנים המשמשים להפקת CO_2 (כגון מכלי "סיפון סודה" להכנת משקאות), אם כי ייתכן שקיימות

מגבלות על הובלתם בטיסה. כאפשרות אחרונה, ניתן להמית את החרקים באמצעות חשיפה לעשן טבק. במקרה זה, זבובי החול נלכדים חיים במלכודת CDC, נאספים באמצעות אספירטור, נשמרים בתוך צינור הזכוכית ונחשפים לעשן טבק, הגורם למותם בתוך שניות. שיטה זו ניתנת ליישום בכל תנאי שדה, גם בתנאי בידוד קשים. עם זאת, מאחר והזכוכית סופגת עשן, לא ניתן להשתמש בה לאחר מכן לאיסוף או לטיפול בזבובי חול חיים ללא ניקוי יסודי. למרות זאת, ניתן עדיין להשתמש באותו אספירטור שלא נוקה להמתת זבובי חול שנלכדו במלכודות אחרות לצורך קיבוע. כמו כן, יש לוודא שכל הדגימות הוצאו מן האספירטור. שיטות אלו תואמות גם לבידוד טפילי *Leishmania* באמצעות דיסקציה של מערכת העיכול של זבובי חול.

3. אחסון דגימות לפני עיבוד

קיימות ארבע שיטות עיקריות לשימור הדגימות לפני תחילת העיבוד:

3.1. הקפאה

שיטה זו מבוצעת באופן מיטבי בטמפרטורה של -20°C או בעדיפות בטמפרטורה של -80°C. שיטות אחסון אלו מצויות כיום בשימוש נפוץ יותר מאשר אחסון בחנקן נוזלי. בכל המקרים, יש ליישם שימור בהקפאה במהירות האפשרית לאחר לכידת הדגימות. אחסון בקור במקפיאים מציע את היתרון של שימור מלא של החרקים עצמם, וכן של DNA, RNA וחלבונים, תוך שמירה על שלמות מלאה לאורך כל תקופת האחסון. לעומת זאת, חנקן נוזלי עלול לגרום לנזק חמור לכנפיים, לרגליים, לבחנינים ולמחושים, ולעיתים קרובות להביא לקטיעתם ואף להסרת מאפיינים מורפולוגיים מרכזיים. אחסון יבש במקפיא הוא פחות טראומטי לדגימות, אך אינו אידיאלי לשימור איבריהן השבריריים. חשוב לציין כי בזמן ההפשרה, כנפיים, מחושים, בחנינים או רגליים עלולים להידבק לדפנות המבחנות ובסופו של דבר להיקרע כתוצאה מעיבוי. עם זאת, שימור באמצעות הקפאה אינו תמיד ישים במחקרי שדה, שכן הוא מחייב גישה למקפיא או למכל חנקן נוזלי. אחסון במקפיא מתאים באופן מלא לגילוי פתוגנים באמצעות שיטות מולקולריות ללא אובדן רגישות, אם כי גילוי ובידוד של נגיפי RNA דורש הקפאה מהירה ב- -80°C או בחנקן נוזלי אם נדרש אחסון ארוך טווח. עם זאת, הקפאת דגימות אינה מאפשרת בידוד של טפילי *Leishmania* באמצעות דיסקציה של המעי, למעט כאשר נעשה שימוש בחנקן נוזלי. במקרה זה, יש לחשוף תחילה את זבובי החול לשלב האדים של החנקן הנוזלי לפני הכנסתם לנוזל עצמו (למשל בתוך מבחנות המונחות בתוך שק רשת), ובכך לדמות תהליך של שימור בקור של *Leishmania*.

3.2. אחסון באלכוהול (אתנול או איזופרופיל אלכוהול)

זוהי ככל הנראה השיטה הנפוצה ביותר לאחסון זבובי חול. קל ליישמה בשדה, גם בתנאים קשים ללא גישה למעבדה. שימור באלכוהול מתאים במיוחד למחקרים מורפולוגיים, שכן האיברים השבריריים (כנפיים, רגליים, מחושים או בחנינים) נותרים שלמים, אם אין בועות אוויר במבחנות האחסון. לכן אנו ממליצים לאטום את המבחנה באמצעות פקק צמר גפן קטן להסרת בועות אוויר ולהניח מדבקה על גבי פקק הכותנה (איור 1). ריכוז האלכוהול המתאים נותר שנוי במחלוקת. באופן כללי, ריכוזים נמוכים מ-70% אינם מומלצים [45, 66]. ריכוזים גבוהים יותר משמרים DNA ביעילות רבה יותר ולמשך תקופות ארוכות יותר, אך הופכים את הדגימות לשבריריות וקשות יותר לטיפול במחקרים מורפולוגיים. שימוש באתנול 96% (התערובת האזאוטרופית) מבטיח יציבות ריכוז לאורך זמן, במיוחד באזורים לחים כגון מדינות טרופיות, אף כי אתנול 95% לרוב קל יותר להשגה. ללא קשר לריכוז, DNA נשמר בדרך כלל היטב באתנול (אם כי פחות ביעילות מאשר בהקפאה, במיוחד בשיטות מולקולריות מסוג NGS).חלבונים פחות יציבים באופן משמעותי, במיוחד ביישומי פרוטאומיקה כגון Maldi-ToF**.**

זבובי חול שנשמרו באלכוהול למשך מספר חודשים עדיין ניתנים לזיהוי מורפולוגי, אך לא ניתן להפיק מהם ספקטרום חלבוני ייחוס. ניתן לשפר אחסון באלכוהול או בתנאים יבשים אם הדגימה מוקפאת גם ב- -20°C. הקפאה ב- -20°C משפרת בעיקר את השימור המולקולרי (למשל חומצות גרעין) באמצעות האטת תהליכי פירוק, וכן מעניקה יתרון משני לשימור מורפולוגי על ידי הפחתת התפרקות רקמות לאורך זמן, אף כי השפעתה על המורפולוגיה מוגבלת יותר בהשוואה לשלמות המולקולרית. ניתן להשתמש באחסון באתנול גם לצורך גילוי נגיפי DNA ו-RNA, כאשר נעשה שימוש באתנול בריכוז של לפחות 70% לתקופת אחסון קצרה, שאינה עולה על מספר חודשים. איזופרופיל אלכוהול (isopropyl alcohol) עשוי להיות זמין בקלות במדינות מסוימות ומשמר DNA, אך גורם להקשיית הדגימות. הוא אינו דליק כמו אתנול ולכן ניתן לשינוע בקלות רבה יותר. במידת הצורך, ניתן להעביר זבובי חול שנשמרו בחנקן נוזלי או בהקפאה יבשה לאלכוהול, מה שגורם לשילוב של חסרונות שתי השיטות.

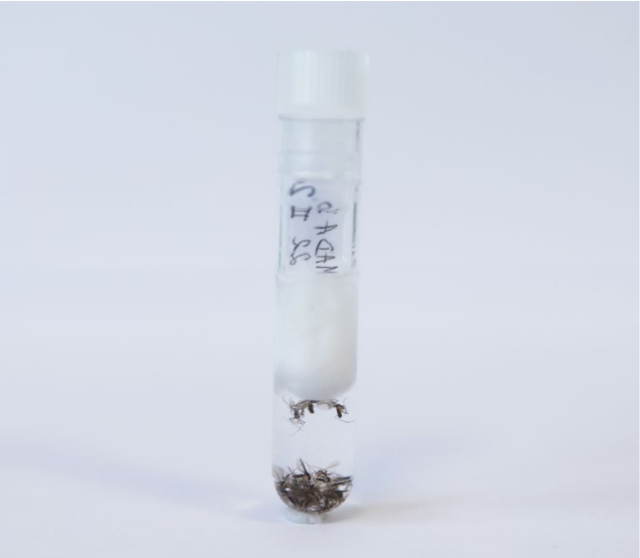

איור 1: זבובי חול שנשמרו באתנול

3.3. אחסון בתמיסת ייצוב RNA (RNASS)

ריאגנט מימי זה נמצא בשימוש נרחב, אינו רעיל, ומתוכנן לייצוב ולהגנה על RNA ברקמות ובדגימות תאים טריות. הוא פועל באמצעות חדירה מהירה לדגימה ונטרול RNases, אנזימים מפרק RNA, ובכך מונע פירוק RNA ללא צורך בהקפאה מיידית. אחסון ב-RNASS יעיל בדרך כלל בשימור המבנה הכללי של הרקמה ושל התאים לצורך הערכה היסטולוגית בהמשך. אף כי RNASS מיועד לייצוב RNA ולא לקיבוע, אחסון לטווח קצר עד בינוני שומר לרוב היטב על שלמות מבנית. RNASS מאפשר אחסון דגימות בטמפרטורת החדר עד 7 ימים, ב-4°C למשך מספר שבועות, או ב- -20°C/-80°C לשימור ארוך טווח. שיטה זו בעלת ערך מיוחד בעבודת שדה או בסביבה קלינית שבה

תשתית שרשרת קירור מוגבלת. הפקת RNA מחייבת בדרך כלל הוצאת הדגימות מהריאגנט ועיבודן בהתאם לפרוטוקולים סטנדרטיים.

3.4. שימור יבש בטמפרטורת החדר

זוהי שיטה ותיקה שבאשר מיושמת על דגימה שלמה (*in toto specimen*), חסרונה העיקרי הוא שימור לקוי של איברים שבריריים כגון כנפיים, רגליים, מחושים ובחנינים. עם זאת, מחקרי פרוטאומיקה באמצעות MALDI-ToF עדיין אפשריים אם מתבצע ייבוש בעת הקיבוע באמצעות חומר מייבש מסוג סיליקה-ג'ל. לעומת זאת, אנליזות מולקולריות המכוונות ל-DNA קשות לביצוע בדגימות אלו, משום שה-DNA לעיתים קרובות מקוטע ובכמות נמוכה, ולכן הניתוחים מאתגרים יותר בהשוואה לדגימות טריות או מוקפאות, במיוחד בכל הנוגע לגנומים גרעיניים. עם זאת, טכניקות עדכניות כגון museomics (מאפשרת בדיקה גנטית של דגימות שנשמרו לפני זמן רב) עשויות להיות ישימות בדגימות מסוג זה [34]. לפיכך, שיטת אחסון זו אינה מומלצת, אלא אם אין חלופה אחרת. ניתן לשלב אותה עם אחסון בקור באמצעות הצבת המבחנות במקפיא ב- ‎-20°C או ב- ‎-80°C. האתגר העיקרי הוא השגת קיבוע מתאים של הדגימות או של חלקי הגוף הנדרשים לצורך זיהוי. לשם כך, רהידרציה (החזרת נוזלים) היא חיונית. אנו ממליצים להשתמש בתמיסת Triton X-100. משך הרהידרציה משתנה ממספר שעות ועד מספר ימים, תוך ניטור קפדני וסדיר. לאחר רהידרציה מלאה, יש לשטוף את הדגימות בשלוש אמבטיות מים עוקבות.

3.5. שימור על גבי ניירות סינון

היתרון המרכזי של ניירות סינון הוא היציבות ארוכת הטווח של DNA גנומי בתוך תאי גוף שלמים לא מקובעים ומיובשים, או תאי דם המאוחסנים בטמפרטורת החדר. נייר הסינון מסופק בגודל כרטיס קטן, המאפשר אחסון של מאות דגימות בטמפרטורת החדר בנפח השווה לגודל מגירה קטנה. מטריצת נייר הסינון ספוגה בחומרים הגורמים לדנטורציה של גורמים זיהומיים, ולכן הדגימות אינן נחשבות עוד כסיכון ביולוגי. הדבר מאפשר אחסון ושינוע של הדגימות ללא צורך באמצעי זהירות ביולוגיים מיוחדים [68].

4. דיסקציה (ניתוח) של דגימות

בשונה מחקרים רבים אחרים, המזוהים על סמך מאפיינים חיצוניים הנצפים על פרטים בודדים המקובעים בשלמותם (*in toto*), זבובי חול מחייבים דיסקציה וקיבוע על זכוכית נושאת לצורך בחינת מאפיינים אנטומיים וזיהוי מדויק של המינים. ללא קשר לשיטת ההכנה והקיבוע שנבחרה, נעשה שימוש באותה טכניקת דיסקציה (איורים 2 ו-3). (https://zenodo.org/records/18198006)

שימוש ב- Triton X100: תמיסה מימית לא-יונית

יש לציין כי הקיבוע מתייחס לדגימות שנלכדו זה עתה או שנשמרו כראוי. לרוב האוספים קיימות דגימות חרקים שנשמרו יבשים (לשימוש ב- MALDI-ToF) או שנשמרו באלכוהול במשך שנים רבות. למרבה הצער, שימור באלכוהול אינו מיטבי לאורך שנים, ופרוקי-רגליים שנשמרו כך נעשים קשים מאוד להכנה לבדיקה מיקרוסקופית. תופעה המתרחשת לעיתים קרובות היא התפוררות הפלסטיק המכיל את הדגימות, ולאחריה התאדות האלכוהול. בשני המקרים אין פתרון יעיל, משום שהדגימות נותרות זמן רב מדי באלכוהול או מתייבשות. לפיכך עלה הרעיון להשתמש בחומרים מרטיבים שאינם דטרגנטים חזקים. Triton X-100 מצוי בצורה של תמיסה מימית לא-יונית (4-(1,1,3,3-tetramethylbutyl) phenyl-polyethylene glycol solution, or t-polyethoxyethanol, polyethylene glycol tert--octylphenoxy ether octylphenyl), ומשמש באופן נרחב כדטרגנט בביולוגיה תאית ומולקולרית. הוא מאפשר חדירות של ממברנת התא וממברנת הגרעין. להלן פרוטוקול שימוש ב- Triton X-100 לא-יוני בתמיסה מימית בריכוז 0.5%:

- להשרות את הדגימה היבשה באלכוהול מוחלט (absolute alcohol).
- להוסיף את הנפח הדרוש של תמיסת Triton X-100 בריכוז 0.5% כך שכל הדגימה תהיה שקועה.
- משך התהליך: כ-5 דקות ועד מספר ימים, תוך ניטור סדיר. כל פרוקי-הרגליים חייבים להיות מופרדים לחלוטין בתמיסה.
- להסיר את תמיסת Triton X-100 ולהחליפה בתמיסת אשלגן הידרוקסיד (potassium hydroxide).

4.1. ראש

ניתן לבצע דיסקציה באמצעות מחטים דקות או סיכות אנטומולוגיות תחת מיקרוסקופ סטריאוסקופי (איורים 2 ו-3). המחטים הנפוצות ביותר כוללות: 1/2” x G26 ‏(0.45 x 13 מ”מ), 1/2” x G30 ‏(0.3 x 13 מ”מ), או 5/8” x G25 ‏(0.5 x 16 מ”מ). לצורך הכנת הדגימה לזיהוי, לכל הפחות יש להפריד בעדינות את הראש מהגוף ולקבעו כך שהצד הוונטרלי (הגחוני) כלפי מעלה כדי להציג את הציבריום (חלל הפה הקדמי) ואת הלוע בעוד שבית החזה והבטן מקובעים לאחר הדיסקציה במבט צדי.

קיבוע הראש במנח ונטרו-דורסלי (גחוני-גבי) מבטיח כי הפתח העורפי (occipital foramen) יופנה כלפי מעלה, כך שניתן יהיה לצפות בציבריום ישירות. הגישה למבנים אנטומיים אלו קלה יותר כאשר הראש מופרד לחלוטין.

4.2. בית חזה וכנפיים

יש להניח את הכנפיים כשהן פרושות ושטוחות. ניתן לנתק כל כנף מבסיסה ולקבעה בנפרד, או לקבע אחת בלבד תוך השארת השנייה מחוברת לבית החזה. אם מתוכננת אנליזת מורפומטריה גאומטרית (geometric morphometry analysis) חיוני לזהות ולסמן כראוי את הכנף הימנית והשמאלית לפני הקיבוע. בית החזה מחולק למספר חלקים, שכל אחד מהם מכיל מידע טקסונומי חשוב מאוד [20, 64].

בדרך כלל הוא מקובע במבט צדי כדי לאפשר בחינה של דגם הזיפים (chaetotaxy) ושל התפלגות הצבע. נוכחות בסיסי הזיפים באזורים מסוימים של בית החזה יכולה לשמש להבחנה בין מינים אחדים של הסוג *Brumptomyia*. התפלגות הצבע עשויה לשמש להבדלה בין זבובי חול נאוטרופיים ברמת הסוג (לדוגמה *Bichromomyia*), סדרת מינים (לדוגמה *Pintomyia*), ואף מינים באותו סוג (לדוגמה *Psathyromyia*, *Nyssomyia*, *Micropygomyia* ו-*Psychodopygus*) [20].

לפיכך, אם בית החזה אינו משמש לאנליזה מולקולרית, יש לקבעו כך שלא ייגרם לו נזק. חשוב לציין כי אין חשיבות לעוצמת הצבעים אלא להתפלגותם על פני בית החזה. לפיכך, תהליך ההבהרה לא יבטל את הפיגמנטציה או את הדגם שלה.

4.3. איברי המין

יש להקפיד במיוחד בעת קיבוע איברי המין הן בזכרים והן בנקבות, שכן הם חיוניים לזיהוי סוגים, תת-סוגים ומינים. בשני המינים איברי המין זוגיים.

4.3.1. זכרים

איברי המין חיצוניים ומורכבים ממבנים זוגיים דמויי צבת, שכל אחד מהם כולל בחלקו הדורסלי את מפרק ה-gonocoxite–gonostyle , ובחלקה הוונטרלי את האונה האפנדריאלית (epandrial lobe).

ה-gonostyle נושא קוצים ולעיתים זיפים, שיש למנותם ואשר מיקומי החיבור שלהם חייבים להיות נראים בבירור. חשוב להתבונן בקפידה במשטח הפנימי של ה-gonocoxite, שעשוי לשאת צבר זיפים עדינים/בלתי קוציים או זיפים הנישאים על גבי גבשושית (tubercle) [22]. חוקרים בעלי פחות ניסיון בדיסקציות יכולים לבצע קיבוע צדי פשוט, ללא הפרדת איברי המין מקצה הבטן, (https://zenodo.org/records/18311158).

במקרה זה, החפיפה בין שני חלקי איברי המין עשויה להקשות על ספירת הזיפים הפנימיים של ה-gonocoxite למשל, אך שיטה זו מונעת נזק לאברי המין העלול להיגרם מדיסקציה כושלת. חוקרים מנוסים יותר יכולים לנסות לפתוח את איברי המין לשניים ולפצלם. לשם כך יש להעביר את הצד המשופע של המחט (מחט לבדיקה תוך-עורית) דרך המבנה, תוך ניתוקו מבלי לחתוך לחלוטין את איברי המין, כדי לפצל את מכלולי ה-gonocoxite–gonostyle.
(https://zenodo.org/records/18311158)

בדרך זו ניתן לצפות בקלות במשטחיהם הפנימיים. מבנה זה גם מאפשר תצפית קלה יותר על הפָּרָמֶרִים (parameres) ונדני הפרמרים (parameral sheaths), שאינם חופפים עוד אלו לאלו. בקיבוע צדי, המעודד חפיפה בין האיברים, יש להבטיח כי הדגימות הובהרו באופן מושלם.

4.3.2. נקבות

איברי המין של הנקבה הם פנימיים ומורכבים מספרמטקות. כאשר לא מבוצעת דיסקציה, יש לצפות בהן דרך מעטפות הגוף ולקבע את הבטן במנח ונטרלי (גחוני). ללא קשר למדיום הקיבוע שנבחר, ניתן בדרך כלל לצפות בספרמטקות עצמן כראוי, במיוחד אם אינן חלקות ומובהרות (הפיכת הרקמות לשקופות יותר). עם זאת, צפייה בספרמטקות חלקות ודקות-דופן עלולה להיות בעייתית במדיומים בעלי מקדם שבירה (RI) נמוך. בנוסף, צפייה בבסיס צינורות הספרמטקה חיונית לזיהוי מינים, לדוגמה בתת-הסוג *Larroussius* [35, 37, 38], הווקטורים העיקריים של *Leishmania infantum* בעולם הישן. ללא תצפית זו, זיהוי הדגימה אינו אפשרי. כדי להתגבר על קשיי תצפית אלו, ללא תצפית זו, זיהוי הדגימה אינו אפשרי. כדי להתגבר על קשיי תצפית אלו, יש להפריד מן הבטן את אברי המין כלומר הספרמטקות ואת ה-furca הגניטלית (furca genitalis) לצורך תצפית ברורה (https://zenodo.org/records/18311106).

בדרך כלל קשה לצפות בספרמטקות במהלך הדיסקציה, אך ה-furca הגניטלית קלה יחסית לאיתור. מאחר שצינורות הספרמטקה נפתחים אל ה-furca הגניטלית, בידוד ה-furca מאפשר בדרך כלל גם את בידוד הספרמטקות. אם הספרמטקות נחתכות בטעות במהלך התהליך, הן אינן אובדות וניתן עדיין לצפות בהן בתוך מעטפות הבטן (איור 4).

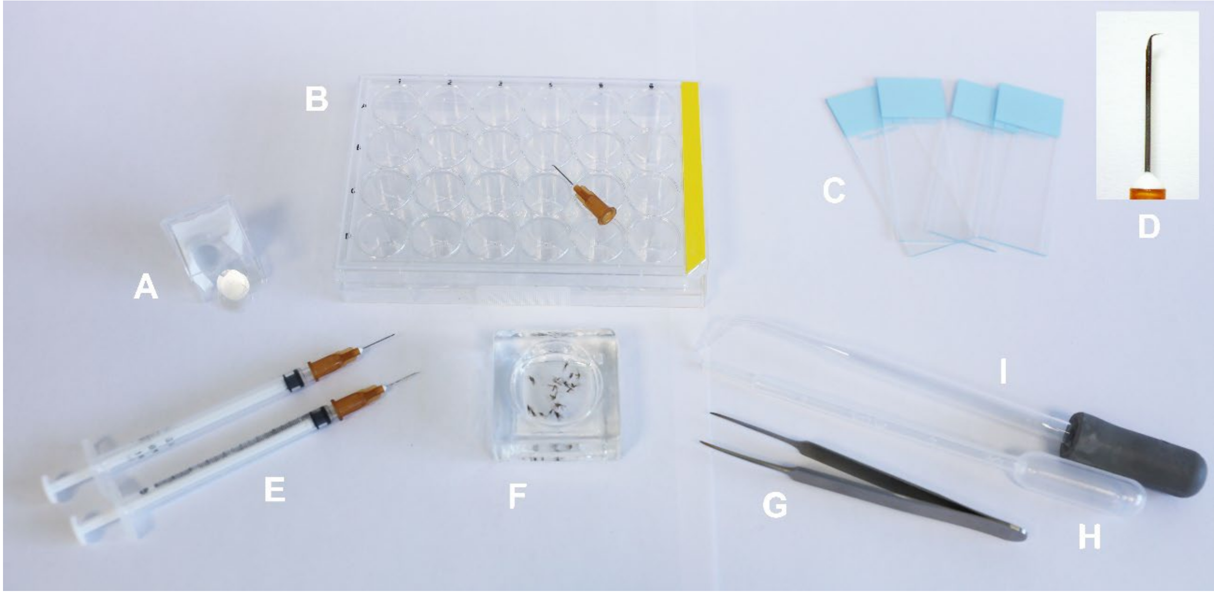

איור 2:חומרים נדרשים לקיבוע זבובי חול. **A)**- זכוכיות כיסוי עגולות מזכוכית (בקוטר 10 או 12 מ"מ), **B)**- פלטה בעלת 24 באריות ומחט מכופפת (אם נעשה שימוש בשמן ציפורן או בתמצית ®Euparal לעיבוד זבובי החול, אין להשתמש בפלטות אקריליות משום שתתרחש תגובה כימית והדגימות ייפגעו), **C)**- זכוכיות נושאות המתאימות לסימון, **D)** קרס המחט, **E)**- מחטים המחוברות למזרקים, **F)**- זכוכית שעון או מיכל מקביל המכיל את זבובי החול לקיבוע, **G)**- פינצטת Dumon, **H)**- פיפטה פלסטית, **I)**- פיפטה מזכוכית שכופפה באמצעות חימום להקלת העברת נוזלים לבאריות.

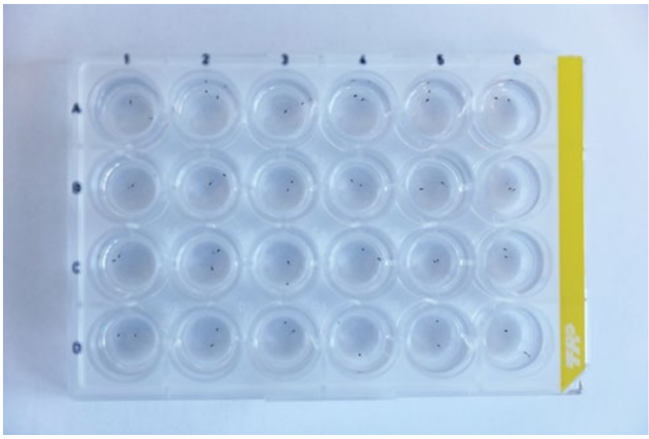

איור 3: פלטה עם 24 באריות, שכל אחת מהן מכילה את הראש ואת קצה הבטן של זבובי חול.

4.4. דיסקציה של המעי האמצעי לבידוד טפילי *Leishmania*

דיסקציה של מערכת העיכול חיונית לאיתור ולבידוד טפילי *Leishmania* בנקבות זבובי חול. ניתן לבצע את ההליך הן בתנאי שדה והן במעבדה, לצורך הערכת כשירות וקטוריאלית.

מומלץ לעבוד עם נקבות שהומתו זה עתה. יש לשטוף את הנקבות במים או בתמיסת סליין (saline) המכילה דטרגנט עדין להסרת שערות עודפות. שלב זה מסייע בשמירה על תנאים סטריליים לבידוד טפילי *Leishmania*, תוך שימור המאפיינים המורפולוגיים הדרושים לזיהוי. לצורך איתור ובידוד טפילי *Leishmania*, יש להוציא בזהירות את המעי האמצעי ולהניחו בטיפה בודדת של תמיסת סליין סטרילית (0.9% NaCl). לאחר צפייה בטפילים ניידים במיקרוסקופ אור (הגדלה מומלצת: ×200 בקירוב), יש להשתמש במזרק אינסולין או במיקרופיפטה להעברת הטפילים למצע הגידול (לפרטים נוספים ראו סעיף 4.4.3).

יש להעביר את הראש ואיברי המין ישירות לתמיסת מארק-אנדרה לצורך הבהרה. חשוב: אין לאפשר מגע של תמיסת מארק-אנדרה עם טפילי *Leishmania* (לא במישרין ולא בעקיפין באמצעות כלים או מחטים) שכן היא קטלנית לטפילים.

ניתן לבצע דיסקציה של נקבות זבובי חול על גבי זכוכית נושאת אחת או שתיים; לכל אפשרות יתרונות ומגבלות (איור 5; https://zenodo.org/records/18311154).

4.4.1. שיטת שתי זכוכיות נושאות

האפשרות הראשונה כוללת עבודה על שתי זכוכיות נושאות נפרדות: האחת מכילה סליין סטרילי להוצאת המעי האמצעי, והשנייה לקיבוע של הראש והספרמטקות בתמיסת מארק-אנדרה. עם זאת, בתנאי שדה נהוג כי שניים או שלושה חוקרים מבצעים דיסקציות ומעבירים את החלקים שניתחו לחוקר יחיד האחראי על זיהוי המין והערכת נוכחות טפילי *Leishmania* במעי. עבודה עם שתי זכוכיות נושאות עלולה ליצור בעיות במעקב אחר הדגימות, ובמיוחד להקשות על קביעה חד-משמעית איזו נקבה הייתה נגועה במקרה שמתגלה מעי חיובי (https://zenodo.org/records/18311154).

4.4.2. שיטת זכוכית נושאת אחת

שימוש בזכוכית נושאת אחת מבטיח עקיבות מלאה של התוצאות. עם זאת, יש לנקוט במספר אמצעי זהירות. לשם שמירה מרבית על סטריליות, על החוקרים לחטא את ידיהם בקביעות עם תמיסת אלכוהול לחיטוי ידיים. יש להשתמש בזכוכיות נושאות חלקות לחלוטין, ללא שוליים אטומים, ובזכוכיות כיסוי מרובעות (22×22 מ"מ) עטופות בנייר אלומיניום ומעוקרות בחום יבש (בתנור המיועד לעיקור – Poupinel oven), וכן במחטים סטריליות נפרדות לכל דיסקציה (המלצה: 25G 0.5 מ"מ × 16 מ"מ). את זבוב החול מניחים בטיפת סליין סטרילי במרכז הזכוכית הנושאת. יש לנתק את הראש ולבצע חתך בין הלוחיות הגביות והבטניות של פרקי הבטן ה-6 וה-7, מבלי לפגוע במערכת העיכול (ניתן לבצע חתך גבוה יותר אם צפויות ספרמטקות ארוכות במיוחד). לאחר מכן יש לקבע את החזה באמצעות מחט אחת, ולמשוך בעדינות את פרקי הבטן האחוריים (קצה הבטן) לאחור באמצעות המחט השנייה לשם חילוץ המעי. אם הפעולה אינה מצליחה, ניתן לקבע את קצה הבטן עם מחט ולמשוך את מערכת העיכול מחלקה הקדמי. אם גם ניסיון זה נכשל, יש להוציא את המעי באמצעות הסרה מרבית ככל הניתן של המעטפת הסובבת אותו.

לאחר בידוד המעי, יש להפריד את פרקי הבטן האחרונים באמצעות חיתוך מערכת העיכול. את המעי מניחים בטיפת סליין סטרילי חדשה בצד אחד של הזכוכית ומכסים בעדינות בזכוכית כיסוי סטרילית. את הראש ופרקי הבטן האחרונים מעבירים לטיפה קטנה של תמיסת מארק-אנדרה בקצה השני של הזכוכית, תוך הקפדה על הימנעות ממגע עם טפילי *Leishmania*. יש למקם את הראש בכיוון נכון (הנקב העורפי כלפי מעלה), לבודד את הספרמטקות יחד עם הפורקה הגניטלית (genital furca) כמתואר לעיל, ולכסות בזכוכית כיסוי עגולה קטנה (קוטר 12 מ"מ, למניעת בלבול עם זכוכיות הכיסוי המרובעות הסטריליות). יתרת גוף הזבוב והכנפיים נותרים בטיפת הסליין שבמרכז הזכוכית (https://zenodo.org/records/18311154).

במקרה של תוצאה חיובית, או לצורך מחקר טקסונומי, ניתן לשמר את החזה והבטן למחקרים מולקולריים או פרוטאומיים, ולהשקיע את הכנפיים במדיום מימי. לשימור התכשיר ניתן להחליף את עודף תמיסת מארק-אנדרה במדיום קיבוע מימי כגון נוזל הויאר או מדיום על בסיס פוליוויניל אלכוהול. קיימים סרטונים מפורטים המדגימים הליכים אלה, ולכן אינם מפורטים כאן.

ניתוח מעי זבוב חול: https://zenodo.org/records/18303014

ניתוח בלוטות רוק של זבוב חול:
https://zenodo.org/records/18302850

4.4.3. בידוד וגידול בתרבית של טפילי *Leishmania* ממעי זבובי חול

בידוד טפילים מנקבות זבובי חול נגועות הוא הליך עדין הדורש מיומנות גבוהה, ומומלץ לתרגלו תחילה על פרטים נקיים מטפילים. לאחר הדיסקציה, מועברים המעיים לטיפה טרייה של סליין סטרילי (0.9%) או לתמיסת Locke (Locke's solution) לשטיפה [4]. לאחר מכן ניתן לעבד את המעיים בשתי דרכים: (1) בדיקה במיקרוסקופ אור לצפייה בשלבים השונים של הפרומסטיגוטים (promastigotes) של טפילי Leishmania ובמיקומם, תוך תשומת לב מיוחדת לשסתום המעי הקדמי (stomodeal valve). (2) פתיחת המעי לשחרור הפרומסטיגוטים והקלה על גידולם בכמות גדולה [4]. איתור נקבות נגועות בשדה הוא נדיר יחסית, ולכן תרגול איכותי יגדיל את סיכויי הבידוד המוצלח.

אם נצפים טפילי *Leishmania* במעי, יש להשתמש במחטים סטריליות חדשות ולהוסיף מעט סליין סטרילי סביב זכוכית הכיסוי באמצעות מנגנון הנימיות, לשחרור הטפילים. יש לקרוע בזהירות ובמהירות את המעי כדי לשחרר את הטפילים לסליין. באמצעות מיקרופיפטה של 100 µL או מזרק קטן (טוברקולין), יש לאסוף את הטפילים ולזרוע אותם על מצע גידול מסומן כראוי.

גידול פרומסטיגוטים של טפילי *Leishmania* בתנאי *in vitro*: הטפילים המבודדים נשמרים תחילה על שיפועי אגר דם SNB-9 או במצע מוצק Novy, Mc Neal, Nicolle (NNN) [16]. המכוסים במדיום alpha-MEMסטרילי [16, 65], או עם מדיום M199, כל אחד בתוספת: 10% סרום עגל עוברי סטרילי (FCS) שעבר אינאקטיבציה בחום (כדי לשפר גדילה של טפילים), 1% ויטמיני BME, 2% שתן אנושי סטרילי (מסונן דרך מסנן Filtropur® S 0.2 µm), 250 µg/mL אמיקצין (amikacin), או 50 µg/mL גנטמיצין (gentamicin), או תערובת אנטיביוטיקות וחומצות אמינו (L-glutamine 200 mM-penicillin 10 000 U-streptomycin 10 mg/mL) [47]. לאחר שלושה ימים, ובהיעדר זיהום, התרביות מורחפות במדיום הקפאה מתאים ומאוחסנים ב- 80°C- למשך שנה עד שנתיים, או בחנקן נוזלי (196°C-) לשימור ארוך טווח ולשימוש ניסויי עתידי [7].

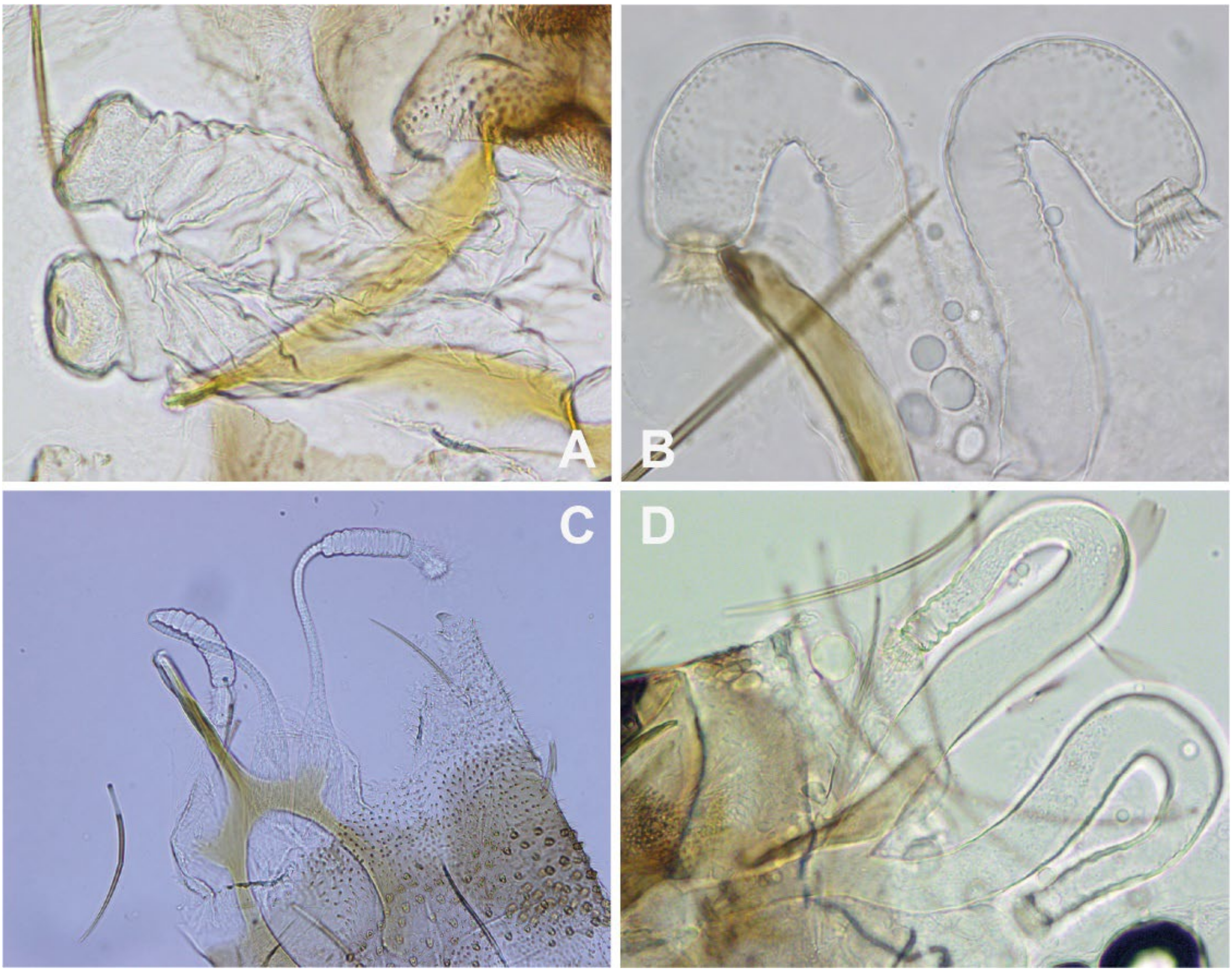

איור 4: ספרמטקות שעברו דיסקציה וקיבוע במדיום מארק-אנדרה (Marc-André) מדגימות טריות: **A-** *Idiophlebotoums longiforceps* (Lao PDR), **B-** *Sergentomyia minuta* (France), **C-** *Phlebotomus ariasi* (France), **D-** *Sergentomyia anodontis* (Lao PDR).

4.5. בלוטות רוק

דיסקציה של בלוטות הרוק בזבובי חול היא טכניקה יסודית לחקר אינטראקציות בין וקטור לפתוגן, במיוחד לאיתור ארבווירוסים כגון Phlebovirus (למשל וירוס טוסקנה - TOSV) [44, 75]. בשל גודלם הזעיר של זבובי החול, ההליך דורש דיוק תחת מיקרוסקופ סטריאוסקופי, באמצעות מלקחיים עדינים או מחטי מיקרודיסקציה, כדי לבודד את הבלוטות העדינות מבלי לגרום לקריעה או לזיהום (https://zenodo.org/records/18302850) [51, 61].

שמירה על שלמות הבלוטות חיונית להבטחת אנליזה מולקולרית אמינה בהמשך. לאחר הוצאת הבלוטות ניתן לבצע הומוגניזציה ולבדוק באמצעות qPCR ,RT-PCR או בדיקות אימונולוגיות (immunoassays) לאיתור RNA נגיפי או אנטיגנים [12]. נוכחות נגיפים בבלוטות הרוק (ולא רק במעי או בהמוצל [חלל הגוף - Hemocoel]) מאשרת כי הפתוגן

השלים את תקופת הדגירה החיצונית והוא מסוגל לעבור הלאה בעת מציצת דם [71] .

ההליך הניתוחי מורכב טכנית בגלל גודלן הקטן של בלוטות הרוק של זבובי חול ודורש מיומנות גבוהה למניעת פירוק הדגימה [1, 51]. בנוסף, עומס נגיפי עשוי להיות נמוך, ולכן נדרשות שיטות זיהוי רגישות במיוחד כגון Nested PCR או ריצוף בתפוקה גבוהה [54]. סיכוני זיהום מחייבים עבודה סטרילית מוקפדת. מעבר למכשולים טכניים, גורמים ביולוגיים כגון שונות בכשירות הווקטור בין מינים ושיעורי הדבקה המשתנים בהתאם לתנאים אקולוגיים ועונתיים, משפיעים אף הם על הצלחת הזיהוי [33, 61]. זיהוי נגיפים בבלוטות הרוק מספק תובנות קריטיות להערכת סיכוני העברה ומאפשר בתוכניות ניטור ממוקד ואמצעי בקרה. [15]. למשל, זיהוי וירוס טוסקנה בזבובי חול באזורים אנדמיים תרם לגיבוש פרוטוקולים אבחנתיים והנחיות לבריאות הציבור [18]. כמו כן, חקר אינטראקציות בין נגיף לרוק עשוי לחשוף מטרות חדשות לפיתוח חיסונים או טיפולים חוסמי-העברה [15, 18].

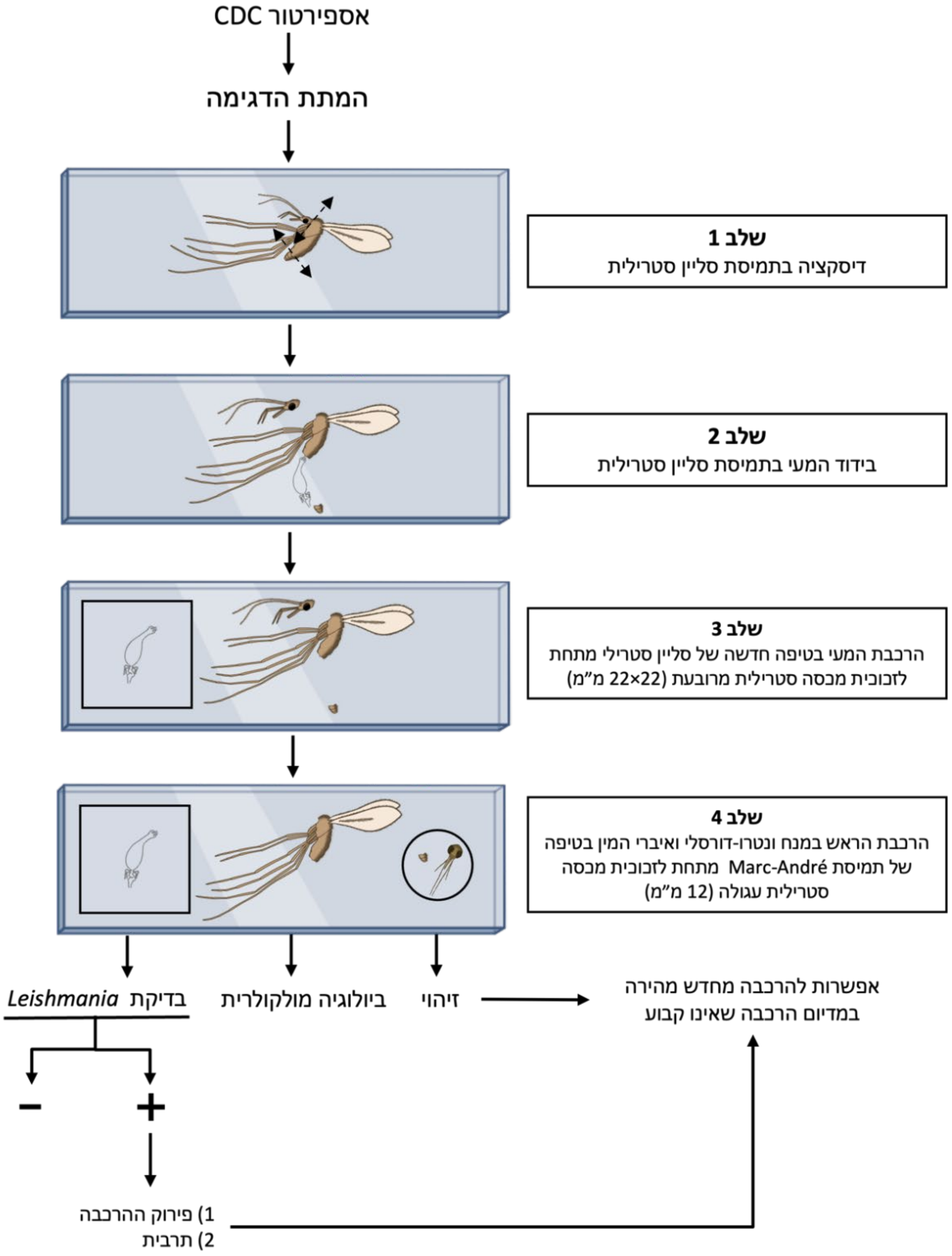

איור 5: שיטה לבידוד טפילי *Leishmania*.

ניתן להשתמש בבלוטות הרוק של זבובי חול גם כמקור לאנטיגנים למדידת נוגדני המאכסן נגד רוק זבובי חול, בשיטות אימונולוגיות ובעיקר ELISA. שיטה זו מאפשרת להעריך חשיפה של המאכסן לעקיצות זבובי חול, ולתמוך בהערכת יעילות שיטות הדברה של הוקטור [25] והסיכון להעברת טפילי *Leishmania* [40].

4.6 זיהוי מקור ארוחת דם

יש לנתח נקבות מלאות דם שנלכדו באמצעות ציוד חד-פעמי למניעת זיהום צולב. יש לבחון את הבטן שלהן תחת מיקרוסקופ סטריאוסקופי להערכת שלב עיכול ארוחת הדם. מומלץ לבחור רק נקבות עם בטן אדומה, אדומה-חומה או אדומה כהה, ללא סימני התפתחות ביצים. יש להסיר את קצה הבטן כולל הספרמטקות לצורך זיהוי מורפולוגי של הנקבה לאחר הבהרה. עיקר הבטן (ללא הספרמטקות) יועבר למבחנות Eppendorf® ויישמר ב-‎-20°C עד לאנליזות נוספות. סמנים גנטיים מקובלים לזיהוי מקור ארוחות הדם, כגון PNOC [5, 30, 50], CytB [67], או COI [13] מבוססים היטב ומתוארים בהרחבה בספרות ולכן לא יפורטו יותר במאמר זה (איור 6). לחלופין, לזיהוי דם המאכסן ניתן להשתמש באנליזת פפטידים בשיטת MALDI-ToF [31]. הוכח כי שיטה זו מאפשרת זיהוי מקור הדם גם פרק זמן ארוך יותר לאחר ארוחת הדם, ולכן היא שיטה מתאימה, במיוחד לדגימות שבהן עיכול הדם מתקדם יותר. מומלץ לאחסן דגימות ב-‎-20°C או ב-4°C , אך ניתן לקבל תוצאות טובות גם מדגימות שנשמרו זמן קצר בטמפרטורת החדר. יש לנתח את בטן נקבת הדם ולהפרידה מהגוף סמוך ככל האפשר למועד הבדיקה ולבצע הומוגניזציה במים מזוקקים. יתר גוף זבוב החול נשמר לניתוחים מולקולריים ומורפולוגיים נוספים. לאחר נטילת אליקווט (aliquot) מהבטן שעברה הומוגניזציה לצורך מיפוי פפטידים בשיטת MALDI-ToF, ניתן להשתמש ביתרת החומר לבידוד DNA לאימות זיהוי מקור הדם ו/או לסקר נוכחות טפילי *Leishmania*. משך הכנת הדגימה והאנליזה קצר משמעותית בהשוואה לשיטות מולקולריות מבוססות DNA.

5. עיבוד דגימות למחקרים מורפולוגיים (איורים 3, 6, 7 ו-8; נספחים 1, 2, 3 ו-4)

סעיף זה מתאר את העקרונות להכנת דגימת זבוב חול לקיבוע לצורך מחקרים מורפולוגיים בלבד, ולאחר מכן את התאמתם ליישומים נוספים מעבר למחקר מורפולוגי. עם זאת, הבנת מתודולוגיה זו חשובה במיוחד, שכן היא מאפשרת התאמת הפרוצדורות לסוגי דגימות ספציפיים בעת הצורך.

הטיפול כולל שלבים רצופים של ריקון ומילוי באמצעות פיפטות פסטר המצוידות בפטמות גומי גמישות. מומלץ בחום להשתמש במכלי זכוכית בעלי תחתית מעוגלת, שכן הם מקלים משמעותית על ביצוע פעולות אלו. הזכוכית היא חומר אינרטי כלפי כל הריאגנטים. כדי למנוע התאיידות של הריאגנטים, יש לצייד את המכלים במכסים ולהימנע ממילוי יתר, אשר עלול לגרום לגלישה בעת סגירה או פתיחה, וכן כדי למנוע חדירת אבק אל הדגימות. הכימיקלים הנדרשים להבהרה ולעיבוד מוצגים בטבלה 2.

5.1 הבהרה

לפני שניתן להכין את דגימות זבובי החול כמשטחים קבועים על זכוכיות נושאות, יש לבצע קודם הבהרה באמצעות ריכוך (maceration), תוך שימוש בשיטה ובחומר הבהרה מתאימים (כגון תמיסת חומצה אצטית 10% או תמיסת מארק-אנדרה הכוללת כלורל הידרט, שהוא תרכובת כימית המוגבלת לשימוש במדינות רבות), על מנת להפוך את הדגימות לשקופות. תהליך ההבהרה מסיר רקמות גוף, שומן, הפרשות ושעווה, הופך את הדגימה לשקופה למחצה ומקל על בחינת מבני השלד החיצוני (למשל נקודות החדרת זיפים), מאפיינים שעל פני השטח (צביעה) ותכונות פנימיות הנראות דרך מעטפת החרק (למשל ספרמטקות).

תהליך ההבהרה הדו-שלבי, הכולל תחילה שימוש בבסיס חזק (כגון אשלגן הידרוקסיד), ולאחר מכן בחומצה חלשה (כגון חומצה אצטית בתמיסת מארק-אנדרה), משרת מטרות ביוכימיות שונות [74]. הבסיס מפרק רקמות רכות כגון חלבונים, שומנים ושרירים, באמצעות תגובות של סבוניפיקציה ודנטורציה של חלבונים, תוך השארת השלד החיצוני הכיטיני (chitin) שלם, לשם בהירות מבנית. החומצה החלשה שמתווספת לאחר מכן מנטרלת שאריות בסיס, מונעת פירוק נוסף, ומבהירה את הכיטין כדי לשפר שקיפות [74], אף כי שטיפת הדגימות פעמיים במים מזוקקים למשך 15 דקות עשויה גם היא להספיק לנטרול הבסיס. טיפול עוקב זה משלב הסרה יעילה של רקמות עם שימור עדין, ובכך מבטיח שלמות מיטבית של הדגימה לבדיקה מיקרוסקופית. מומלץ לבצע שתי שטיפות של 20 דקות במים מזוקקים לפני המעבר לשלב הבא.

5.1.1 פירוק (ליזיס) של רקמות רכות (איור 8)

נתרן הידרוקסיד (NaOH) או אשלגן הידרוקסיד (KOH) הם חומרים נפוצים לריכוך רקמות, ומשמשים בריכוזים ובמשכי זמן שונים בהתאם לגודל הדגימות ולשבריריותן. הטכניקה הסטנדרטית והיעילה ביותר כוללת ליזיס של רקמות רכות באמצעות השריית זבובי החול בבסיס חזק (KOH או NaOH בריכוז 10%) למשך הלילה. ניתן להעלות את הריכוז כדי לקצר את משך הטיפול (למשל KOH 20% למשך 6 שעות), וכן ניתן לחמם ב-37°C.

5.1.2 הבהרה עם או ללא צביעה

שלב זה מלווה בטיפול הבהרה, בדרך כלל עם שילוב של חומצה אצטית ובכלורל הידרט (למשל תמיסת מארק-אנדרה). לאחר ההבהרה יש לשטוף היטב את הדגימות בשתי אמבטיות מים רצופות לפחות, בנות 20 דקות כל אחת, כדי להסיר שאריות כימיקלים. תמיסת מארק-אנדרה היא חומר הבהרה נפוץ להכנת דגימות זבובי חול. יעילותה נובעת מיכולתה להבהיר את הדגימות תוך צמצום נזק משמעותי למבנים שבריריים כגון כנפיים ומחושים.

יש להכין את התמיסה טרייה או לאחסן אותה במיכל אטום היטב כדי למנוע התאדות או פירוק. היתרון בשימוש בתמיסת מארק-אנדרה בולט במיוחד כאשר הוא משולב בטכניקות הבהרה או צביעה לצורך הדגשת פרטים מורפולוגיים ספציפיים. פרטי הרכב התמיסה והכנתה מופיעים בנספח 2.

לדגימות שקופות במיוחד, עשויה להידרש צביעה לשיפור הנראות לפני הקיבוע. קיימים צבעים רבים, שכל אחד מהם מכוון לרכיבים כימיים שונים של האורגניזם. חשוב לבחור צבע התואם הן לדגימה והן למדיום הקיבוע הנבחר. ניתן להתאים מתודולוגיה בסיסית זו לפי צורך, לדוגמה באמצעות הוספת פוקסין חומצי 0.1% לתמיסת מארק-אנדרה לצורך צביעה. בנוסף, דגימות שנשמרו בתמיסות מימיות ומיועדות לקיבוע במדיומים המבוססים על שרף מחייבות ייבוש (ראו סעיף 5.2 ייבוש), משום שרוב מדיומי הקיבוע השרפיים, הטבעיים והסינתטיים, אינם מתערבבים במים. New(1974), ציין כי צבעים מסוימים עלולים להיחלש במדיומי קיבוע מסוימים [53]. לדוגמה, פוקסין חומצי, המשמש לעיתים קרובות עם בלזם קנדה, ניתן גם לקיבוע ב-Euparal®.

עם זאת, דגימות שנצבעו בפוקסין חומצי נוטות לדהייה, במיוחד כאשר נותרו שאריות שמן ציפורן, המשמש כנוזל הבהרה סופי. דגימות המאוחסנות בשמן ציפורן עשויות לדהות משמעותית בתוך ימים ספורים.

5.2. ייבוש (Dehydration)

הייבוש מבוצע באמצעות העברה הדרגתית של הדגימות דרך סדרת תמיסות אתנול מדורגת: 50%, 70%, 80%, 90% או 95% ולבסוף 100%, כאשר כל אמבט נמשך לפחות 20 דקות. מאחר והאתנול מתאדה במהירות, יש לאטום היטב את המיכל במהלך העיבוד. לאחר שהדגימה עברה ייבוש הדרגתי מלא ניתן להשהות את העיבוד למספר ימים בתמצית Euparal® שהיא עדיפה על פני שמן ציפורן. החומר Beech creosote, שהיה בעבר בשימוש נרחב למטרה זו, אסור כיום לחלוטין בשל רעילותו.

תהליך הייבוש ההדרגתי חייב להבטיח שהנוזל בתוך הדגימה תואם למדיום הקיבוע, כדי למנוע עבירות, קריסה אוסמוטית או עיוות העלולים להפוך את הדגימה לבלתי מתאימה למחקר טקסונומי.

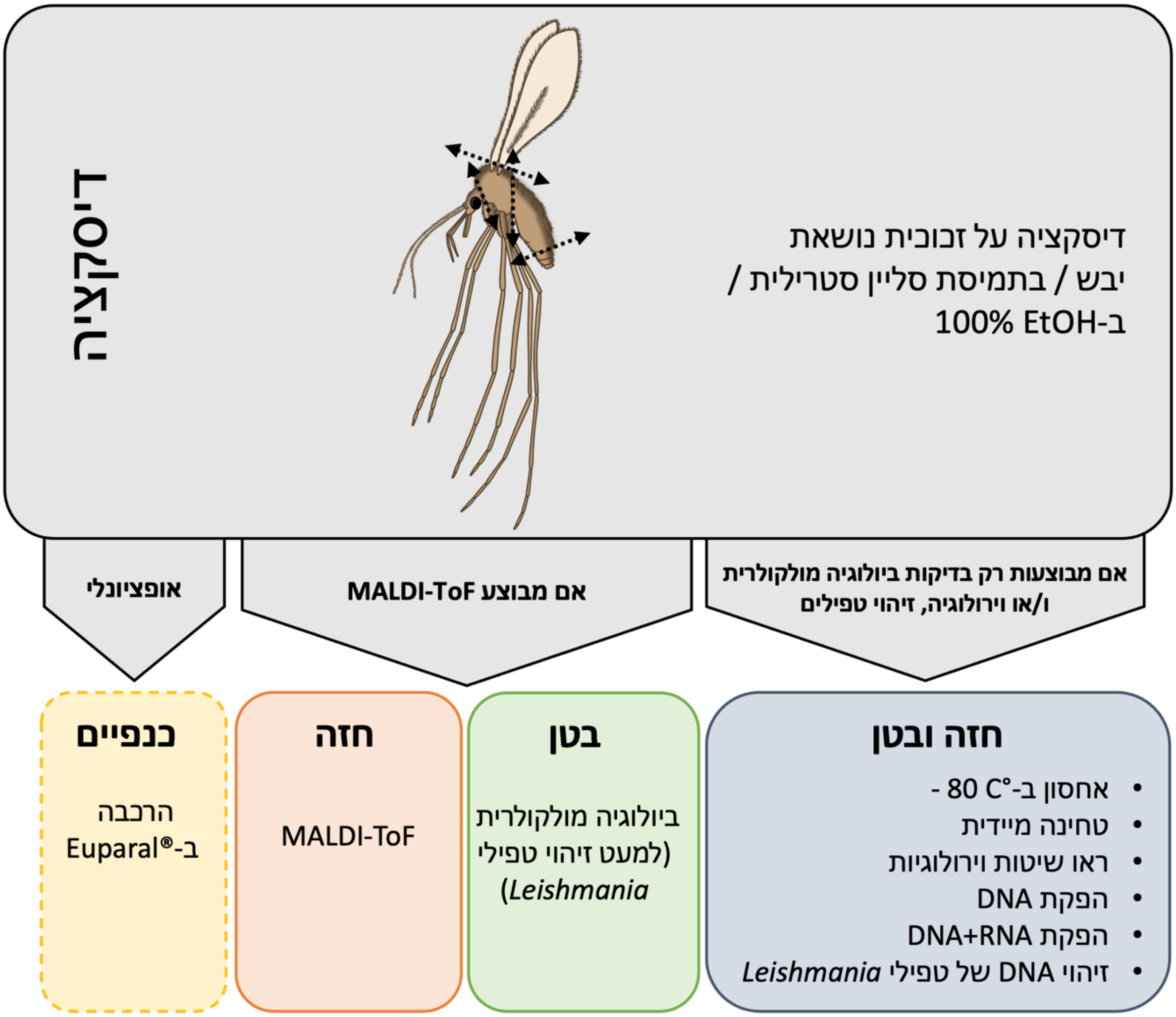

איור 6: הכנת זבוב חול ליישומי ביולוגיה מולקולרית, פרוטאומיקה ו/או וירולוגיה.

5.3. מדיומי קיבוע

5.3.1. בחירה ויישום לצורך הכנת דגימות

מדיום הקיבוע אמור, באופן אידיאלי, להיות בעל מקדם שבירה הקרוב ככל האפשר לזה של זכוכית, שהוא בעל מקדם שבירה אופטי של כ-1.5. עליו להיות חסר צבע, צלול, ולהישאר שקוף לחלוטין לאחר ייבוש ולאורך זמן. עליו להיות תואם לצבעים שבהם נעשה שימוש, ובעל יכולת לחדור ולחלחל לכל רקמות הדגימה. הוא לא אמור להתייבש מהר מדי או ליצור עבירות במהלך הקיבוע. הוא לא אמור להתכווץ לאחר הקיבוע. בחירת מדיום קיבוע מתאים היא מרכיב יסודי בהכנת דגימות, שכן אין מדיום יחיד שהוא אידיאלי לכל המטרות. הבחירה צריכה לאזן בין מספר גורמים מרכזיים:

תכונות אופטיות - מקדם השבירה של מדיום הקיבוע צריך לספק ניגודיות ושבירה מספיקות של מאפיינים אנטומיים קריטיים המשמשים לזיהוי טקסונומי או לתיאור מורפולוגי, כגון ספרמטקות, אסקואידים (ascoids), איברי חישה מסוג Newstead, השיניים האנכיות של הציבריום (vertical cibarial teeth) ושיני הלוע. נראותם של מבנים אלו תלויה באופן ישיר בתכונות האופטיות של מדיום הקיבוע.

שימור - בדגימות טיפוס (type specimens) או בחומרים המיועדים לאוספים קבועים, על המדיום לספק יציבות ועמידות לאורך זמן. לעומת זאת, במחקרי מלאי או סקרי אפידמיולוגיה, שבהם שימור ארוך טווח פחות קריטי, ייתכן שמדיומי קיבוע זמניים או חצי-קבועים יספיקו.

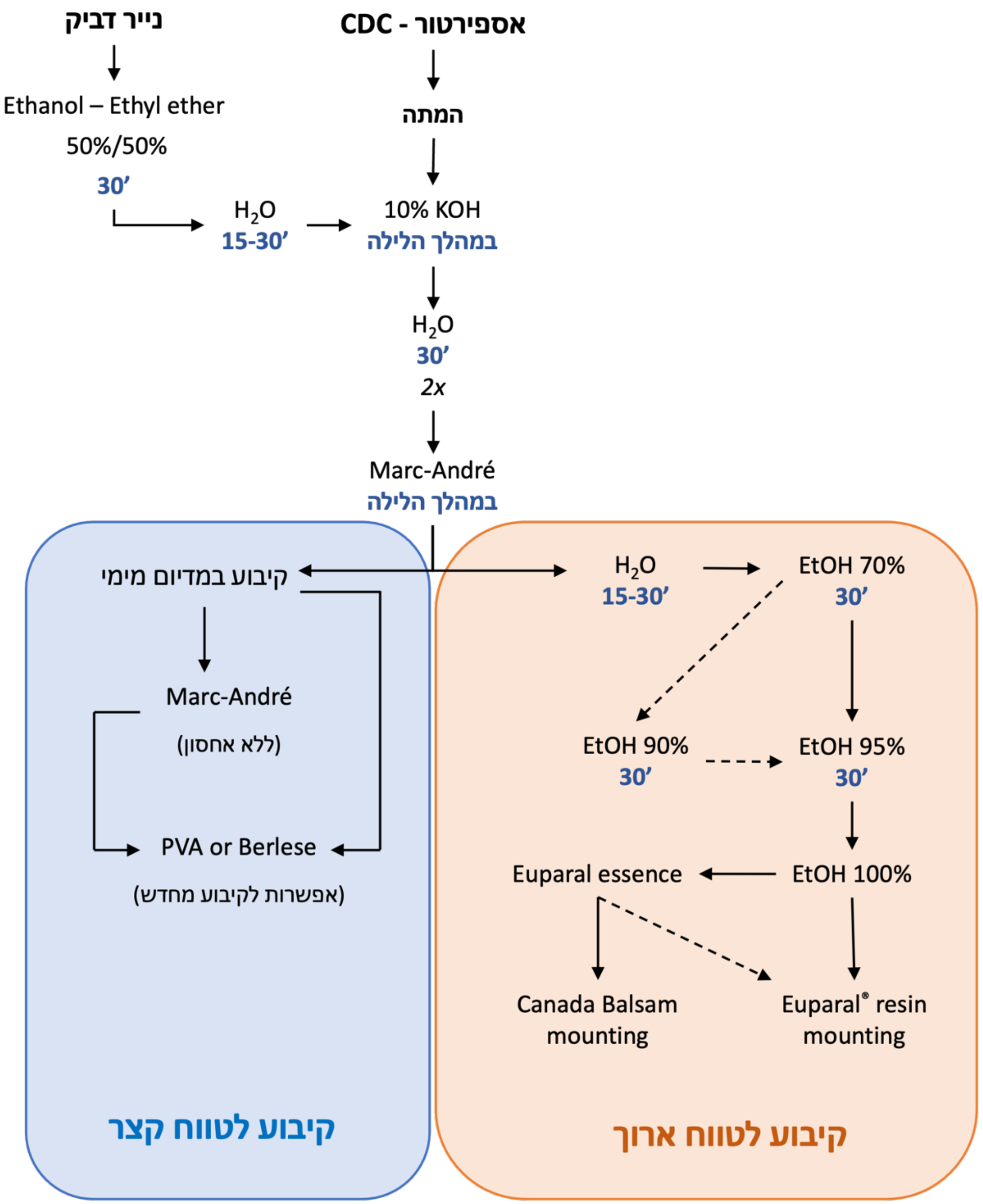

איור 7: שיטה קלאסית לעיבוד זבובי חול.

5.3.2. דרישות למדיומי קיבוע

חוקרים מנוסים מפתחים לעיתים טכניקות קיבוע מותאמות אישית ומורכבות בהתאם לצרכי מחקר ספציפיים. עם זאת, שיטות אלה מתעלמות לעיתים קרובות מהיבטים כגון איכות שימור ארכיונית, תאימות, סטנדרטיזציה, או נוחות טיפול ושימור ארוך טווח. היעדר סטנדרטיזציה זו מקשה על שילוב אוספים שנתרמו ועל מאמצי אוצרות ארוכי טווח. יישומים מדעיים מציבים דרישות שונות למדיומי קיבוע. טקסונומים מקבעים לעיתים דגימות שלמות ומעדיפים מדיומים המאפשרים ריכוך עדין של האיברים הפנימיים כדי לשפר את נראות המבנים הקוטיקולריים. מקדם השבירה צריך להיות שונה במידה מספקת מזה של הדגימה ושל הזכוכית הנושאת כדי למקסם בהירות אופטית. מדיומי קיבוע מסחריים מיוצרים בדרך כלל עם מקדם שבירה הקרוב לזה של זכוכית כדי למזער שבירת אור ופיזור דרך מערכת זכוכית נושאת–מדיום קיבוע–זכוכית כיסוי. עם זאת, במיקרוסקופיה בשדה בהיר (brightfield microscopy) ניתן לשלוט ולשפר את הניגודיות הטבעית של דגימה לא צבועה באמצעות בחירה מכוונת של מדיום קיבוע בעל מקדם שבירה השונה במעט מזה של הדגימה, וכך להגביר את יכולת ההבחנה שלה על גבי הרקע.

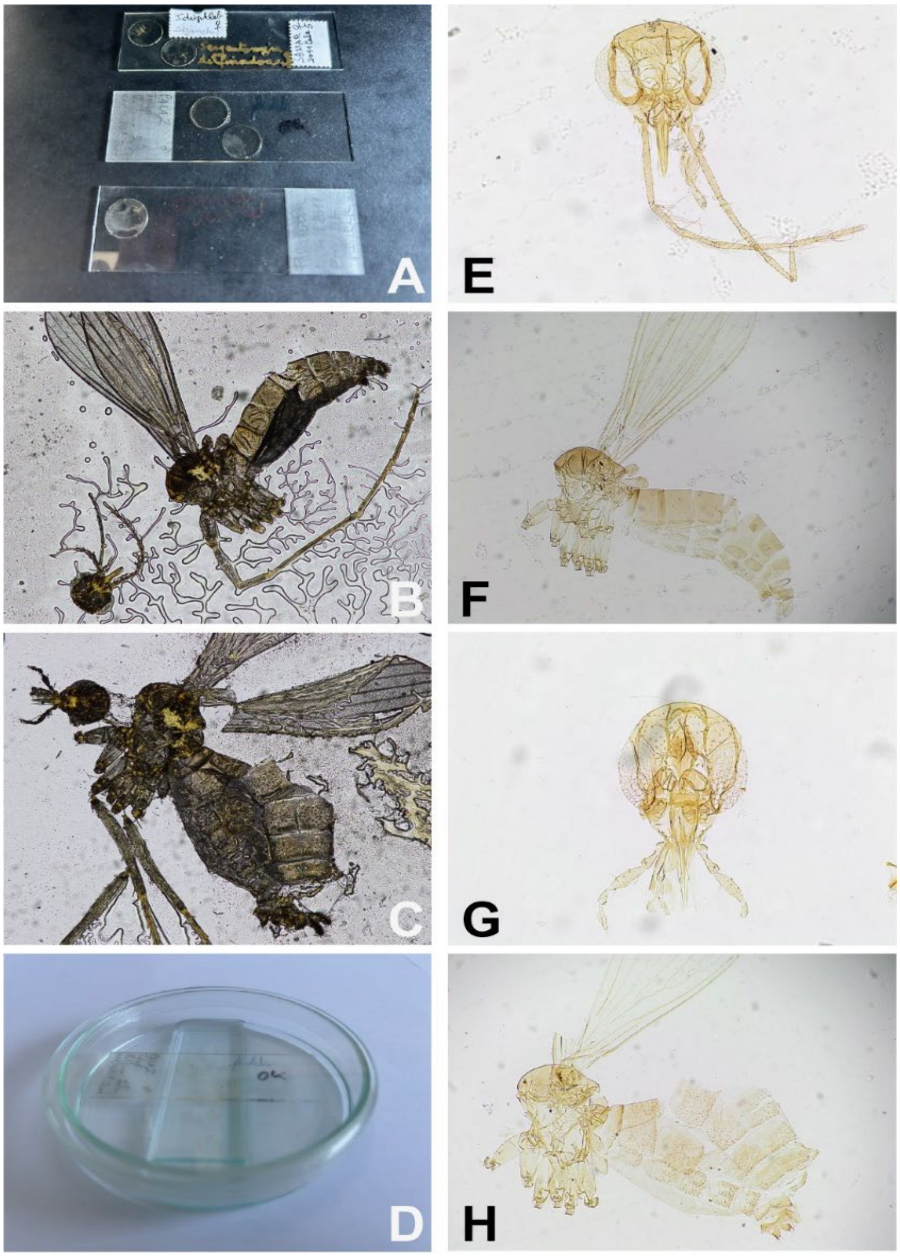

איור 8: קיבוע מחדש של סליידים: **A** - סליידים פגומים ויבשים ששוקעו במדיום הוייר; **B**- מבט מיקרוסקופי של זבוב חול שהתייבש; **C** - מבט מיקרוסקופי של זבוב חול נוסף שניזוק; **D** - תא לח המכיל סלייד יבש; **E** - הראש, ו-**F** - הגוף של דגימה B לאחר ששוקעה מחדש במדיום ®Euparal; **G** - הראש, ו-**H** - הגוף של דגימה C הפגומה לאחר ששוקעה מחדש במדיום ®Euparal.

This is an Open Access article distributed under the terms of the Creative Commons Attribution License (https://creativecommons.org/licenses/by/4.0), which permits unrestricted use, distribution, and reproduction in any medium, provided the original work is properly cited.

טבלה 2: הרכב החומרים שנעשה בהם שימוש.

ריאגנט	מונח באנגלית	רכיבים	מונח באנגלית	כמות
אשלגן הידרוקסיד 10%	Potassium hydroxide 10%	אשלגן הידרוקסיד	Potassium hydroxide	10 גרם
		מים מזוקקים	Distilled water	השלמה לנפח של 100 מ"ל
פוקסין חומצי 1% במים מזוקקים	Acid fuchsin 1% in distilled water	פוקסין חומצי (אבקה)	Acid fuchsin (powder)	1 גרם
		מים מזוקקים	Distilled water	99 מ"ל
מדיום קיבוע גאם כלורל (מדיום הויאר)	Gum chloral mounting medium (Hoyer medium)	מים מזוקקים	Distilled water	50 מ"ל
		כלורל הידרט	Chloral hydrate	200 גרם
		גומי ערבי	Gum arabic	50 גרם
		גליצרול	Glycerol	20 מ"ל
תמיסת מארק-אנדרה	Marc-André solution	כלורל הידרט	Chloral hydrate	40 גרם
		חומצה אצטית קרחונית	Glacial acetic acid	30 מ"ל
		מים מזוקקים	Distilled water	30 מ"ל
תמיסת מארק-אנדרה צבועה בפוקסין חומצי	Marc-André solution colored with acid fuchsin	תמיסת מארק-אנדרה	Marc-André solution	10 מ"ל
		פוקסין 1%	Fuchsin 1%	50 מיקרוליטר
מדיום Enecê	Enecê medium	קולופוניום לבן טהור	Pure white colophony	22 גרם
		גומי קופל מסיס באלכוהול	Alcohol-soluble copal gum	12 גרם
		אתנול מוחלט	Absolute ethanol	20 מ"ל
		קמפור	Camphor	10 גרם
		תמצית טרפנטין	Turpentine essence	10 מ"ל
		אוקליפטול	Eucalyptol	26 מ"ל

5.3.3. סוגי מדיומי קיבוע (טבלאות 3 ו-4)

מיקרוסקופיה מחייבת התייחסות למקדם השבירה (RI) של מדיום קיבוע כדי לקבוע כיצד האור נשבר דרך הזכוכית הנושאת, המדיום והדגימה. כאשר מקדם השבירה תואם בקירוב לזה של זכוכית הכיסוי (≈1.515), האור עובר באופן אחיד, פוחתים פיזור ועיוותים אופטיים ונוצר שיפור ברזולוציה ובנראות של מבנים עדינים. לעומת זאת, אי-התאמה במקדם השבירה עלולה לגרום לטשטוש, הילות, או להסתרת מאפיינים לא צבועים. בחירת מדיום הקיבוע הנכון חיונית למיטוב ניגודיות, בהירות ואיכות תמונה כללית עבור דגימה נתונה, בשל ערכי RIשונים של מדיומים שונים.

למקדם השבירה של מדיום הקיבוע השפעה משמעותית על מידת הנראות של מבנים עדינים בעת הכנת זבובי חול לקיבוע על זכוכית נושאת. מאפיינים עדינים וקשיחים קלות של זבובי חול, לרבות הציבריום (cibarial armature), ספרמטקות, פרקי מחוש ועורקי כנף, עשויים להיות קשים לצפייה במדיום קיבוע בעל מקדם שבירה גבוה. בזבובי חול, אפשרויות נפוצות כוללות מדיומי gum-chloral כמדיומי קיבוע מימיים וכן בלזם קנדה ושרף Nelson – Enecê Cerqueira (NC) כמדיום על בסיס ממס. Rawlins [60], סיווג מדיומי קיבוע לשני סוגים: (1) מדיומים קבועים: מתקשים עם הזמן ומתאימים לשימור ארוך טווח ו-(2) מדיומים חצי-קבועים: אינם מתקשים לחלוטין ומשמשים בדרך כלל למטרות זמניות.

מדיומי קיבוע יכולים להיות נוזליים, מבוססי גומי או שרפיים, מסיסים במים, באלכוהול או בממסים אחרים (למשל טולואן, קסילן (טבלה 1). לאחר שנעשה בהם שימוש, יש לאטום אותם מפני השפעות אטמוספריות באמצעות חומרי איטום שאינם מסיסים, בשולי זכוכית הכיסוי.

כדי להבחין באופן ברור בין סוגי המדיומים המשמשים לקיבוע, ניתן להשתמש בסיווג הבא:

א. מדיומים מימיים - מדיומים אלה מסיסים בקלות במים, ולכן מתאימים לקיבועים זמניים או חצי-קבועים. בדרך כלל הם נוחים לעבודה, אך עשויים לדרוש איטום כדי למנוע חשיפה ללחות אטמוספרית (לדוגמה, מדיומי Chloral-gum ומדיומים מבוססי polyvinyl alcohol) במיוחד באקלים טרופי לח.

ב. מדיומים בעלי סבילות מוגבלת למים - מדיומים אלה מושפעים פחות ממים, אך עדיין מחייבים הגנה מפני לחות עודפת. הם מספקים יציבות גבוהה יותר לאורך זמן בהשוואה לאפשרויות המסיסות במים, ומשמשים לעיתים קרובות לקיבועים חצי-קבועים.

ג. מדיומים מסיסי-פחמימנים - מדיומים אלה מומסים בממסים אורגניים כגון קסילן או טולואן, או ב-essenecê (ממס Enecê). הם מיועדים לקיבוע קבוע ומציעים יציבות מצוינת לטווח ארוך ועמידות ללחות ולפירוק, ולכן הם מתאימים במיוחד לצרכים ארכיוניים (לדוגמה בלזם קנדה ניטרלי, DPX).

לסיכום, מדיומים מסיסי-מים מתאימים במיוחד לקיבועים זמניים או למצבים שבהם נדרשת אפשרות להסרה קלה של הדגימה; מדיומים בעלי סבילות מוגבלת למים מתאימים לקיבועים חצי-קבועים הדורשים עמידות בינונית, מדיומים מסיסי-פחמימנים מתאימים לקיבועים קבועים המיועדים לשימור ארכיוני ולאחסון ארוך טווח.

This is an Open Access article distributed under the terms of the Creative Commons Attribution License (https://creativecommons.org/licenses/by/4.0), which permits unrestricted use, distribution, and reproduction in any medium, provided the original work is properly cited.

טבלה 3: הרכב מדיומי קיבוע נבחרים

מדיום קיבוע	ממס	פרה-פולימר(ים) פוטנציאלי / פולימר	הערות
מדיום הוייאר = גאם כלורל Hoyer = (chloral gum)	גליצרול, מים	תרכובות של גומי ערבי (gum arabic)	חומר לריכוך: כלורל הידרט (Chloral hydrate)
CMCP-9 (carboxy methyl cellulose phenol)	מים (CMCP-9: 51–60%)	פוליוויניל אלכוהול שעבר הידרוליזה מלאה (CMP-9: 0-5%)	CMC(P)-9: צמיגות נמוכה: צמיגות גבוהה
DMHF (Dimethyl hydantoin formaldehyde)	מים	N,N′-dimethylol dimethyl hydantoin (di-methylol DMH) Ether-/methylene-bridged oligomers רשת פולימרית מוצלבת של DMH–formaldehyde	
בלזם קנדה (Canada balsam)	קסילן (Xylene); רכיבים נדיפים חלקית של בלזם (Δ^3-carene, levopimaric acid, limonene, myrcene, palustric acid, β-phellandrene, α-pinene, β-pinene)	בלזם (abienol, abietic acid, isopimaric acid, sandaracopimaric acid)	נטרול: potassium carbonate, שרף שמקורו בעץ *Abies balsamea* (Linné, 1758)
Euparal®	Eucalyptol, paraldehyde, ובן רכיבים נדיפים חלקית של gum sandarac (limonene, α-pinene, β-pinene)	תרכובות של gum sandarac: (communic acid, manool, polycommunic acid, sandaracopimaric acid, 12-acetoxy-sandaracopimaric acid, sugiol, torulosic acid, torulosol, totarol)	חומר הבהרה: methyl salicylate; הצבע הירוק של Euparal® נובע ממלח נחושת (copper abietinate), השרף sandarac מקורו בעץ *Tetraclinis articulata* (Vahl, 1791)
Enecê	אתנול עם קמפור (camphor) ואוקליפטול (eucalyptol) ותמצית טרפנטין (turpentine essence).	תרכובות של copal gum ושל colophony (שרף)	

5.3.4. תיאור של מדיומי קיבוע מומלצים (טבלאות 3 ו-4)

מדיומים לתצפית זמנית

גאם כלורל = נוזל הוייר/מדיום/תמיסה (מקדם שבירה RI=1.48):

תמיסת מארק-אנדרה היא המדיום הטוב ביותר לתצפית קצרת טווח (מספר שעות, ואולי מעט יותר אם הסלייד נשמר בתא לח) על ספרמטקות, כולל לצילום (איור 4) או לציור. שימור הספרמטקות שנצפו מחייב קיבוע מחדש במדיום מימי המאפשר אחסון לטווח בינוני. ניתן לייבש את הדגימות לצורך קיבוע מחדש בשרף, אך זה לא מומלץ (סיכון לאובדן הדגימה). גאם כלורל ונוזל הויאר נחשבים לשמות נרדפים. מדיום זה משמש בדרך כלל לתצפית באיברים פנימיים בשל התאמתו למים, פשטות השימוש בו, יישומו המהיר ומקדם השבירה שלו, המאפשר בחינת מבנים עדינים כגון ספרמטקות. עם זאת, לגאם כלורל חסרונות משמעותיים אם לא הוכן באופן מושלם או נשמר בתנאי לחות מבוקרים. חסרונות אלה כוללים התגבשות, שינוי צבע ואובדן צמיגות. איטום שולי הכיסוי אינו פותר בעיות אלה, שכן המדיום עלול לשנות את צבעו באופן משמעותי (לעיתים כמעט שחור) עקב אינטראקציה עם חומר האיטום, במיוחד כאשר נעשה שימוש ב-Euparal®.

מדיום הואיר נחשב לטוב ביותר מבחינה אופטית עבור זבובי חול מסוג פלבוטומוס (phlebotomine sandflies), ושימש באופן מסורתי למטרות אלו. המדיום מורכב ממספר פורמולציות קרובות זו לזו, ובהן גומי ערבי (gum arabic), גליצרול וכלורל הידרט. פורמולציות שונות פורשו וצוטטו באופן שגוי [74].

למרות שהואיר מתאים לתצפית על ספרמטקות בזבובי חול, הוא אינו מתאים לשימור ארוך טווח. הוא אידיאלי לתצפיות קצרות טווח, לרבות צילום, ציור או הדמיה. מדיומים מימיים מתאימים לקיבועים זמניים אך אינם מבטיחים שימור ארוך טווח. לעומת זאת, קיבוע בשרף מספק עמידות מצוינת ולעיתים נשמר במשך מאות שנים, אך עלול לטשטש פרטים עדינים של ספרמטקות, שכן תכונות השבירה האופטיות של המדיומים אובדות לעיתים קרובות.

מדיום הויאר מתפרק עם הזמן עקב ייבוש (איור 8), דבר הגורם להיווצרות גבישי כלורל הידרט לבנים קטנים ואטומים. עם זאת, ניתן לשחזר דגימות מסליידים שעברו התגבשות, שכן הקוטיקולה נותרת שלמה מבחינה כימית, אף שעלול להיגרם נזק פיזי עקב גדילת הגבישים. במקרים מסוימים ניתן לשחזר סליידים שהתגבשו באמצעות הרטבה מחדש של מדיום הקיבוע בסביבה חמה ולחה עם תימול (thymol) למניעת גדילת פטריות. לחלופין, ניתן להוציא את הדגימות מתוך הגאם כלורל באמצעות השרייה במים, לייבש בחומצה אצטית קרחונית (glacial acetic acid), ולהרכיב מחדש בבלזם קנדה.

DMHF (dimethyl hydantoin formaldehyde) (RI= 1.48): מדיום מימי זה [72] מספק ביצועים אופטיים טובים, בדומה ל-Berlese, וקל לשימוש כמוהו. אולם בניגוד ל-Berlese, הוא אינו משחיר ואינו מתגבש. הוא מתאים לזבובי חול ולמיני Psychodidae אחרים.

CMCP (camphor-mono-chlorophenol) (RI= 1.41): זהו מדיום קיבוע מבוסס גליצרין, מסיס במים, המשמש להכנת סליידים שקופים וקבועים של דגימות עדינות, לרבות זבובי חול. יתרונו בכך שניתן לקבע דגימות ישירות ממים או מאתנול. הוא מרכך ומבהיר במהירות את זבוב החול, מרכך את הקוטיקולה ומאפשר מיקום מדויק של הדגימה, דבר המועיל במיוחד בפרישת הכנפיים או בדיסקציה של איברי המין. למרות שדווח כי הוא מאפשר אחסון ארוך טווח, משך השימור המדויק אינו ידוע. מגבלה מרכזית של מדיום זה היא נוכחות פנול בהרכבו, חומר רעיל ומגרה המחייב טיפול זהיר.

טבלה 4: יתרונות וחסרונות של מדיומי קיבוע נבחרים עבור סליידים למיקרוסקופ, ותצפיות שלא פורסמו על ידי אנשים שונים [52].

שם	יתרונות	חסרונות
**Canada balsam * (בלזם קנדה)**	– המדיום עמיד ביותר, עם אורך חיים העולה על 150 שנה. ניתן להרכיב סליידים תוך שימוש בשמן ציפורן או בפנול כחומרי קיבוע.	– מכיל רכיבים מזיקים ויש לעבוד איתו תחת מנדף.<br>– דורש סדרת דהידרציה מלאה הארוכת זמן.<br>– דהידרציה באתנול והעברה דרך קסילן או שמן ציפורן עלולה להפוך פריטים לשבירים; חלופות עשויות להפחית את השבירה (כגון- isopropanol, n-butanol, Cellosolve™, 1,4-dioxane, Histoclear, terpineol)<br>– דגימות עלולות להשחיר אם מחליפים קסילן בפנול או אם נותרו שאריות של אשלגן הידרוקסיד (KOH).<br>– מקדמי שבירה (RI) גבוהים עלולים להסתיר מבנים שאינם צבועים.<br>– ייבוש מוחלט עשוי לקחת שנים ללא שימוש בפלטת חימום.<br>– המדיום מצהיב ומתכהה לאורך זמן, במיוחד כאשר הוא עובר הבהרה עם שמן ציפורן.<br>– חלק מהצבעים נחלשים, וצבעים קטיוניים עלולים לדהות אם המדיום הופך לחומצי – דבר שעלול לקרות באופן ספונטני עם הזמן.
**DMHF (dimethyl hydantoin formaldehyde)**	– שקיפות גבוהה.<br>– מקדם שבירה מתאים.<br>– נראות מצויינת של המבנים השונים.<br>– יציבות טובה למדי של הפרפרטים.<br>– תאימות למגוון רחב של שיטות צביעה<br>– הגנה טובה על הדגימות.<br>הצמדה טובה בין הזכוכית הנושאת לזכוכית המכסה	– הצהבה אפשרית לאורך זמן.<br>– עלול לשנות חלק מהצביעות.<br>– אינו מתאים לצביעות הרגישות לפורמלדהיד<br>– היווצרות בועות אוויר וזמן ייבוש איטי<br>– מדיום ה קיבוע רגיש ללחות.<br>– הקיבוע קשה להסרה<br>פורמאלדהיד הוא חומר רעיל, מגרה ומסרטן.
**Euparal * (שקוף)**	– מדיום עמיד עם אורך חיים של מעל 50 שנה.<br>– ניתן לבצע קיבוע ישירות מאתנול 80% (המלצת היצרן).<br>– אינו מסתיר מבנים שאינם צבועים ואינו מצהיב או הופך לשביר לאורך זמן.<br>– בעל מקדם שבירה (RI) המתאים לדו-כנפיים (Diptera) יותר מאשר בלזם קנדה.<br>– עובד היטב עבור דגימות עבות הודות להתכווצות מינימלית וייבוש ללא בועות.<br>– נשאר מסיס באתנול 95%, מה שמאפשר קיבוע מחדש גם לאחר שנים רבות.	– מכיל רכיבים מזיקים ויש לטפל בו תחת מנדף.<br>– דהידרציה באתנול והעברה דרך Euparal Essence עלולה להפוך פריטים לשבירים, אך שימוש באיזופרופנול עשוי להפחית בעיה זו.

**Hoyer fluid (נוזל הויאר)**	– ניתן לשקע דגימות כשהן חיות, או ישירות ממים, אתנול או פורמאלדהיד.<br>– ריכוך כימי (Maceration) מניב איכות קוטיקולה מצוינת.<br>– בעל מדד שבירה (RI) נוח, וניתן להגבירו באמצעות צביעת יוד להשגת ניגודיות גבוהה יותר.<br>– חומצה אצטית בפורמולה יכולה לגרום להתרחבות של איברי פרוקי-רגליים.<br>– דגימות מסוימות עשויות להישאר יציבות למשך 40–60 שנה.<br>– מסיס במים, מה שמאפשר לבצע קיבוע מחדש בקלות.	– דגימות צמחים עדינות עלולות לקרוס אלא אם המדיום מתווסף בהדרגה, תהליך הדורש זמן.<br>– חללים וגבישים עלולים להיווצר בתוך פחות מ-10 שנים.<br>– עלול להתרחש ריכוך יתר, כתלות בריכוז הכלורל הידרט ובזמן החשיפה.<br>– מרכיבי המדיום עלולים להיפרד, וגירגור עדין עשוי להופיע בתוך חודשים או שנים.<br>– דווח על השחרה של המדיום.
**CMCP-9 (= carboxy methyl cellulose phenol)**	– ניתן לשקע דגימות ישירות ממדיומים כגון מים, אתנול, גליצרול או תמיסות המכילות פורמאלדהיד, וניתן לבצע ריכוך כימי של איבריהן הפנימיים בעת הצורך כדי להקל על בדיקה כללית או על הכנת הדגימה .	– מדיום זה עלול לפתח גבישים ולהתכהות עם הזמן, ולעיתים הוא עלול לבצע ריכוך כימי של הדגימות מעבר למתוכנן.<br>– דגימות עבות לא יישמרו בו היטב כיוון שהן עלולות להתכווץ וליצור רווחים סביב שולי זכוכית המכסה, אלא אם כן הסלייד נאטם בקפידה.<br>– המדיום אינו מתאים לדגימות צבועות או לחומרים מסוידים וזמן הייבוש שלו איטי יותר מזה של CMC.
**Eukitt™**	– מדיום עמיד המחזיק מעמד מעל 30 שנה.<br>– תואם לממיסים רבים לצורך קיבוע, כולל אצטון, בנזן, כלורופורם, דיוקסן, אתר, איזופרופנול, מתיל-בנזואט, טרפינאול, טולואן וקסילן.<br>– מתייבש במהירות ובעל רמת pH חומצית מעט.<br>– אינו מתכהה באופן ניכר עם הזמן.<br>– מתאים לצביעות מגוונות (כגון פוקסין, המטוקסילין, מתיל גרין, מתיל ויולט, מתילן בלו).<br>– ניתן לשקע דגימות מחדש לאחר שנים על ידי השריה ממושכת בקסילן.	– מכיל רכיבים מזיקים ויש לטפל בו תחת מנדף.<br>– דורש סדרת דהידרציה מלאה הגוזלת זמן.<br>– אינו אידיאלי לדגימות עבות בשל התכווצות והיווצרות בועות גז.<br>– זכוכיות המכסה עלולות להתנתק עם הזמן, אלא אם כן הזכוכית נוקתה היטב ונאטמה.<br>– עלול להראות פולימור לא מלא סביב סיבי קולגן.
**Enecê**	– מדיום עמיד מאוד, מחזיק מעמד לפחות 50 שנה.<br>– התכשיר אינו מתכהה עם הזמן.<br>– המדיום גמיש יותר, מה שמאפשר דיסקציה של חרקים בתוכו, וכן מספק זמן סביר למיקום של מבנים מורפולוגיים.<br>– עלות נמוכה.	– דורש סדרת דהידרציה מלאה הגוזלת זמן.<br>– דהידרציה באתנול והעברה דרך שמן ציפורן עלולות להפוך דגימות מסוימות לשבירות.<br>– הדגימה ממשיכה לעבור הבהרה, אם כי בקצב איטי מאוד; דבר זה עלול להקשות על ראיית מבנים קטנים מאוד, כגון איברי חישה (sensilla), ascoids וזיפים פשוטים.

מדיומים לקיבוע קבוע של דגימות על זכוכיות נושאות

בלזם קנדה (1.52-1.54=RI): בלזם קנדה תואר לראשונה כמדיום קיבוע מתאים למיקרוסקופיית אור על ידי Andrew Pritchard בשנות ה-1830. הוא נותר אחד המדיומים הנפוצים ביותר בזכות איכות השימור המוכחת שלו, עם למעלה מ-150 שנות שימוש מוצלח. בניגוד למדיום אויאר, בלזם קנדה אינו מתגבש ואינו סופח לחות. עם זאת, הוא אוטופלואורסצנטי (autofluorescent) באופן משמעותי, דבר שעלול להוות חסרון בטכניקות מיקרוסקופיה מסוימות [60]. שימוש בממיסים פחות רעילים במקום קסילן (xylene) עשוי להפחית סיכוני בטיחות במהלך ההכנה, אך עלול לגרום גם לחסרונות כגון ייבוש איטי יותר והכהיה מוקדמת של המדיום.

Euparal® (RI = 1.48): מהווה חלופה נפוצה לבלזם קנדה לקיבוע קבוע, ומציע יציבות מצוינת לטווח ארוך ומקדם שבירה דומה. ל-®Euparalשני מאפיינים עיקריים: (1) ייבוש הדרגתי: לפני המעבר הסופי למדיום הקיבוע יש לייבש את הדגימה, בדרך כלל במעבר מ-95% אתנול לאתנול מוחלט. בנוסף, (2) זמן עיבוד ממושך: הקיבוע הסופי בשרף, בין אם בבלזם קנדה ובין אם ב-Euparal®, מחייב ייבוש ולכן מאריך את זמן העיבוד הכולל.

כאשר ייבוש באמצעות ממסים אורגניים אינו אפשרי, ניתן להעביר דגימות שהוצאו מאתנול מוחלט לתמיסת ביניים המורכבת מתערובת שווה של ®Euparal ו- Euparal essence טרם הקיבוע הסופי.

Enecê (RI= 1.467): הוא מדיום קיבוע מבוסס שרף המשמש בעיקר לחרקים קטנים ופופולרי במיוחד בברזיל. בסיסו מורכב משרף קולופוניום (colophony) ומשרף קופל (copal gum) המומסים באלכוהול, יחד עם קמפור (comphor), תמצית טרפנטין (turpentine) ואוקליפטול. Cerqueira [11], תיאר את Enecê כחלופה לבלזם קנדה לקיבוע סליידים קבועים של זחלים, נשלים של פרטים לא בוגרים ואף של יתושים בוגרים, ומאז אומץ גם לקיבוע זבובי חול. Enecê מהווה חלופה חסכונית לקיבוע קבוע, מספק יציבות לטווח ארוך וזמן ייבוש מספק המאפשר דיסקציה וסידור מדויק של מבנים מורפולוגיים.

5.4 הכנת סלייד וייבוש

ייבוש נכון של סליידים חיוני להבטחת יציבות ושימור ארוך טווח. יש לייבש את הסליידים באופן מלא לפני אחסון ממושך. לתוצאות מיטביות, סליידים שקובעו במדיומים קבועים יש לייבש במצב אופקי במשך 2–3 שבועות, ואילו סליידים שקובעו במדיומים חצי-קבועים עשויים לדרוש 1–2 שבועות בלבד. על מנת להבטיח תהליך ייבוש יעיל, מומלץ להשתמש באינקובטור בטמפרטורה מתאימה לסוג המדיום, תוך הימנעות מחימום יתר העלול להזיק לדגימות. טווח טמפרטורות מומלץ: 30°C עד 37°C. שלב זה חיוני למניעת עיוות הסלייד, פירוק הדגימה או אי-יציבות של מדיום הקיבוע במהלך האחסון.

יש לציין תמיד על גבי תווית הסלייד את שם מדיום הקיבוע שבו נעשה שימוש. אם ניתן, יש לציין גם את המתכון המדויק, את שם מכין הפרפרט ואת תאריך ההכנה. סליידים מוכנים בתחילה בקיבועים זמניים ואינם מיועדים לשימור ארוך טווח. עם זאת, אם מעמד הדגימה משתנה, למשל אם היא מוגדרת כחלק מסדרת טיפוס, יש להשתמש במדיום קבוע יותר כדי להבטיח את שימורה של הדגימה למחקר טקסונומי עתידי.

5.5 טכניקות קיבוע חלופיות: קיבוע על כרטיס

קיבוע על גבי כרטיס הוא טכניקה הנהוגה במספר קבוצות חרקים, כאשר בטכניקה זו הדגימות מוצמדות ישירות לכרטיסים אנטומולוגיים או מודבקות על פניהם. בשל גודלם הזעיר של זבובי החול והצורך בתצפית באיברים פנימיים באמצעות הבהרה לצורך זיהוי (ראו סעיף 5), שיטה זו אינה מתאימה כלל לקיבוע זבובי חול.

5.6 קיבוע מחדש של דגימות פגומות

עבור דגימות נדירות או יקרות ערך מומלץ לנקוט בגישה דו-שלבית בהתאם לסרטון הזמין ב- https://zenodo.org/records/18315029
1- הרטבה מחדש ללא פירוק, לצורך תצפית ראשונית. יש להציב מחזיק סליידים בתוך צלחת פטרי בתמיכה. הסלייד המיועד להרטבה מונח מעל, וממלאים את צלחת הפטרי במספר מילימטרים של ממס ליצירת תא לח, תוך הקפדה שהסלייד עצמו לא יבוא במגע עם הממס (איור 8D). משך ההרטבה עשוי לנוע מיום אחד למספר ימים, בהתאם למצב הדגימה. נדרשים ניטור יומי וסבלנות. לאחר שהסלייד הורטב די הצורך, ניתן להוציאו מהתא הלח ולהכניסו לאינקובטור למספר שעות טרם בדיקה מיקרוסקופית, צילום או ציור. 2- לקיבוע מחדש, יש להחזיר את הסלייד לתא הלח למספר שעות נוספות או למשך הלילה. פירוק יש לבצע תחת מיקרוסקופ בינוקולרי. בעזרת מחטים עדינות יש להסיר בזהירות את כיסוי הזכוכית, תוך הקפדה שלא יישארו חלקי זבוב חול דבוקים. (https://zenodo.org/records/18315029)

לאחר מכן יש לאסוף את חלקי הדגימה ולשטוף במים בשקעים קטנים, כגון אלה המשמשים להפקת DNA/RNA בשיטה הרסנית (ראה להלן), לפני ייבוש הדרגתי וקיבוע מחדש במדיום על בסיס שרף. בעת פירוק סלייד יש לזהות את מדיום הקיבוע המקורי כדי לבחור ממס מתאים. במדיומים מימיים יש להשתמש במים. במדיומים שרפיים (כגון בלזם קנדה או ®Euparal) יש להשתמש בקסילן (xylene), תחת מנדף ועם ציוד מגן אישי מתאים, כולל מסכה.

קיבוע מחדש של דגימות טיפוס או דגימות מאוספים תתבצע אך ורק באישור האוצר ו/או המוסד המחזיק בדגימה.

6. זיהוי דגימות

6.1 מורפולוגיה

זיהוי זבובי חול מבוסס בראש ובראשונה על בחינת מאפייניהם המורפולוגיים, לרבות צורת החזה, הכנפיים, איברי המין, הזיפים וקשרים מורפומטריים ספציפיים בין מבנים שונים. חוקרים משתמשים במפתחות טקסונומיים, באוספי ייחוס ובתיאורי מינים מקוריים כדי להשוות דגימות שנאספו לקבוצות טקסונומיות מוכרות. מאפיינים אבחנתיים מרכזיים, כגון ורידי הכנף ומורפולוגיית הראש בשני הזוויגים, מבנה איברי המין הזכריים ותצורת הספרמטקות בנקבות, מספקים מידע חשוב במיוחד לצורך קביעת המין. זיהוי מדויק מחייב לרוב בדיקה מיקרוסקופית מפורטת, בדרך כלל באמצעות מיקרוסקופ מורכב לצפייה במבנים עדינים כגון איברי מין וספרמטקות, או באמצעות סטריאומיקרוסקופ לצורך הערכת מאפיינים מורפולוגיים כלליים יותר.

התקדמות עדכנית בטכנולוגיות הדמיה אפשרה שימוש בהדמיה דיגיטלית לצורך זיהוי זבובי חול. תצלומים ברזולוציה גבוהה או איורים דיגיטליים של מאפיינים מרכזיים ניתנים להשוואה לחומרי ייחוס או לניתוח באמצעות מערכות זיהוי ממוחשבות, ובכך משפרים הן את הדיוק והן את הנגישות בטקסונומיה מורפולוגית.

6.2 גאומטריית כנף

גאומטריית הכנף היא מאפיין מרכזי המשמש בזיהוי ובסיווג מיני זבובי חול שונים. כנפי זבובי החול מציגות דגם ומבנה ייחודיים, לרוב ארוכות וצרות עם מערך עורקים מפותח היטב (איורים 9 ו-10). סידור העורקים יוצר דגם מובחן המשתנה בין סוגים ומינים, ומהווה תכונה אבחנתית חשובה לזיהוי. לפיכך, חקר גאומטריית הכנף מספק תובנות משמעותיות לצרכים טקסונומיים.

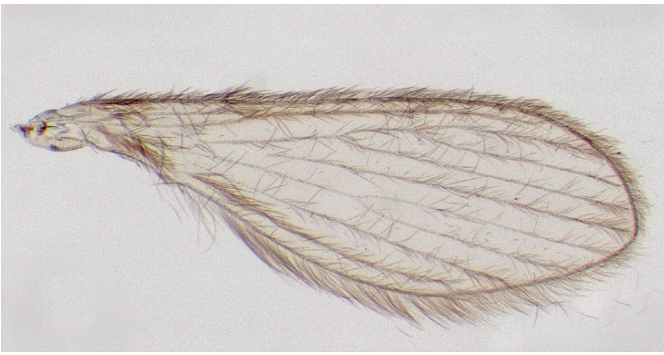

איור 9: כנף גולמית של *Trichophoromyia ininii*.

6.3. מורפומטריה גאומטרית של הכנף

חוקרים משתמשים בטכניקות שונות, כגון מורפומטריה גאומטרית, כדי לנתח ולהשוות את צורת וגודל הכנף בין מינים או אוכלוסיות שונות של זבובי חול. חקר גאומטריית הכנף מספק תובנות חשובות בנוגע להתנהגות, העדפות בית גידול ויכולות תעופה.

בגישת המורפומטריה הגאומטרית, הכנפיים מנותחות בזהירות, נצבעות (במידת הצורך), ומורכבות במצב שטוח על סליידים. לאחר מכן מצלמים את הסליידים תחת סטריאומיקרוסקופ, מבצעים דיגיטציה של התמונה, ומעבירים לניתוח מורפומטרי. פרוצדורה זו תוארה היטב בספרות [6, 27, 42, 56, 57, 59], תוך המלצה להשתמש באופן עקבי בכנף הימנית או השמאלית באיברים זוגיים, כדי למנוע השפעות אלומטריות שליליות אפשריות [62].

הכנת כנף לניתוח מורפומטריה גאומטרית

לצורך הדגמה מיטבית של עורקי הכנף, יש לנקות את הכנפיים מקשקשים ולצבוע אותן באופן מתאים. להכנת הכנף, יש למלא באריות קטנות בריאגנטים הדרושים (methylene blue, אתנול, מים ותחליף קסילן).

יש להוציא כנף שנשמרה ב-70% אתנול בטמפרטורת החדר על ידי הפיכת מבחנת אפנדורף וריקונה מעל הבארית, ולאחר מכן להרים את הכנף לאורך צירה באמצעות מחט מעוקלת עדינה.

יש להעביר את הכנף לזמן קצר מאתנול למים וחזרה לאתנול להסרת זיפים. לאחר מכן יש להניח את הכנף ב- methylene blue למשך 6 דקות, תוך הקפדה שהיא צפה במהלך הצביעה.

יש לאסוף את הכנף בזהירות ולהשרות אותה בתחליף קסילן למשך 2 דקות (כשליש מזמן הצביעה ב-methylene blue). נקישות עדינות של המחט בדפנות הבארית יסייעו לכנף להסתדר; הקסילן משמש לקיבוע הצבע.

לבסוף, יש להרים את הכנף ולהניחה על טיפה קטנה של Euparal® על גבי סלייד. תחת עדשת הגדלה יש לפרוש בעדינות את הכנף ולהניח בזהירות זכוכית כיסוי.

יש לצלם בהקדם לפני שה־ Euparal® מתקשה, שכן ייתכן ויידרשו התאמות קלות של מיקום הכנף תחת הכיסוי להשגת יישור מיטבי.

6.4. טכניקות ביולוגיה מולקולרית

בנוסף לשיטות מורפולוגיות, שיטות מולקולריות הופכות לחיוניות יותר ויותר במחקר אנטומולוגי, לרבות במחקרים טקסונומיים, גנטיקה של אוכלוסיות ופילוגנטיקה וכן לצורך זיהוי פתוגנים מבוסס RNA/DNA וקביעת מקור ארוחת הדם. מידע זה חשוב להבנת התנהגות הווקטור ולהיבטים אפידמיולוגיים של העברת מחלות [70].

ריצוף DNA מאפשר לאשר זיהוי מין או להבחין בין מינים קרובים מאוד, ומספק אמצעי מדויק ואמין יותר לזיהוי. יתרה מכך, טכניקות מולקולריות מתקדמות (כמו PCR, ריצוף DNA, NGS וכו') וכן MALDI-ToF MS צוברות חשיבות לזיהוי מהיר ומדויק של מינים, ומשלימות את השיטות המורפולוגיות המסורתיות [46]. למרות התקדמות זו, זיהוי מורפולוגי נותר תקן הייחוס בטקסונומיה ומהווה את הבסיס לפרשנות הנתונים המולקולריים.

6.4.1. הפקת חומצות גרעין בשיטה הרסנית

הפקת חומצות גרעין הוא שלב שגרתי במחקרים ביולוגיים רבים, ופותחו מגוון שיטות לבידוד DNA מחומרים ביולוגיים [48]. ערכות מסחריות רבות של הפקת DNA תוכננו להקל על תהליך זה [14]. עם זאת, שיטות הכנה המשמשות להכנת דגימות פרוקי-רגליים לזיהוי מורפולוגי לעיתים מקשות על ניתוח DNA שכן הן עלולות לפגוע או להרוס מאפיינים פיזיים קריטיים של הדגימה [10]. רוב פרוטוקולי הפקת DNA מרקמות חרקים הם הרסניים באופיים [43], דבר המעלה חשש מיוחד בדגימות קטנות, שבהן גם דיגום מצומצם עלול לפגוע במאפיינים מורפולוגיים חשובים [72]. סוג הדגימה ומצבה מהווים גורם מרכזי בבחירת שיטת בידוד DNA מתאימה [29].

הצורך בזיהוי מדויק של זבובי חול, הבנת דינמיקת אוכלוסיות וצמצום פגיעה בגורמים שאינם מטרה, הובילו לפיתוח כלי אבחון מולקולריים [23]. גישות מולקולריות משמשות כיום לעיתים קרובות כהשלמה לשיטות טקסונומיות מורפולוגיות לזיהוי זבובי חול. לדוגמה, בגישת ה-barcoding הסטנדרטית לחרקים, יש צורך בהפקת DNA וריצוף הברוכים באובדן הדגימה המקורית. לכן קיים צורך דחוף לפתח שיטות הפקת DNA לא הרסניות השומרות הן על החומר הביולוגי והן על שלמותו המורפולוגית.

שיטות רבות להפקת חומצות גרעין יושמו בזבובי חול. כמות ואיכות חומצות הגרעין הדרושות תלויות באנליזה המולקולרית המתוכננת, שכן לטכניקות שונות דרישות שונות לרגישות וטוהר [9]. לדוגמה, נמצא כי עיני זבוב החול מעכבות הגברת PCR [69]. מעבר לסקר פתוגנים, DNA של זבובי חול מופק באופן שגרתי לצורך זיהוי מינים. ניתן להשתמש בשיטות הפקה שונות, אם כי התפוקה והאיכות משתנות. פרוטוקולים מסוימים של יצרנים הותאמו במיוחד לזבובי חול [8], לשיפור התפוקה ו/או האיכות של חומצות הגרעין המופקות [8, 9, 69], וכן ניתן לעשות שימוש בהתאמות שפותחו עבור מינים אחרים של פרוקי-רגליים גם על זבובי חול [58, 76].

בדיקות PCR לזיהוי, המכוונות למקטעים מיטוכונדריאליים קצרים (CytB או COI) תואמות בדרך כלל לשיטות הפקה הכוללות פירוק משמעותי של ה-DNA למקטעים (fragmentation). לעומת זאת, טכנולוגיות NGS אחרות המבוססות על קריאה ארוכה (Oxford Nanopore ו-PacBio) דורשות פירוק מינימלי של ה-DNA ואיכות DNA גבוהה. הפקות באמצעות קולונות (spin column) מניבות בדרך כלל מקטעי DNA גנומי באורך של עד כ- 60 kb, בעוד שהפקה בשיטת phenol-chloroform יכולה להפיק מקטעים באורך של עד כ- 150 kb [77]. טבלה 5 מסכמת טכניקות הפקה שונות של DNA מזבובי חול ומציינת האם בוצעו התאמות מתודולוגיות לזבובי חול. התפוקות אינן מוצגות, שכן הן תלויות בגודל הדגימה ובשיטת ההכנה.

עמודת ההתאמה מתייחסת להתאמות של פרוטוקולי הפקה לזבובי חול או פרוקי רגליים קטנים אחרים.

בחירת שיטת ההפקה צריכה להתחשב בכמה קריטריונים, כמו מספר הדגימות, זמן ההפקה והטכניקה המולקולרית המתוכננת בהמשך. בעוד שטכניקות NGS דורשות DNA גנומי במשקל מולקולרי גבוה, כל השיטות המתוארות כאן מתאימות ליישומי PCR סטנדרטיים.

בנוסף, מספר מחקרים בחנו שיטות הפקה לא הרסניות בפרוקי-רגליים קטנים יבשתיים, בדגימות מוזאוניות בשימור יבש ובפרוקי-רגליים רכי גוף [19, 26, 28, 55, 63].

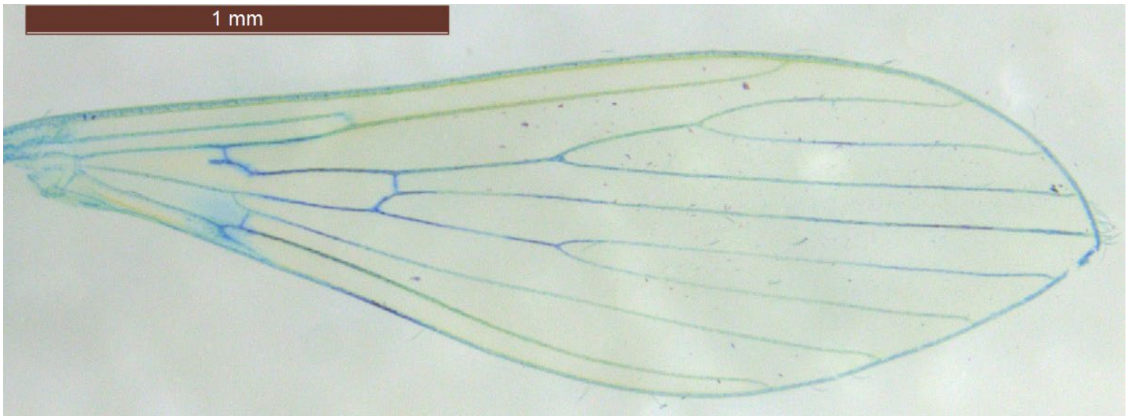

איור 10: כנף צבועה של *Phlebotomus ariasi*.

טבלה 5: עלות ממוצעת, יישום והתאמת פרוטוקול להפקת gDNA של זבובי חול מסוג פלבוטומוס (*Phlebotomus*).

פרוטוקול	עלות	יישום	התאמת פרוטוקול לפרוקי-רגליים קטנים
Spin column	2.5 – 3.55 US$ [39]	PCR, NGS	[9]
Phenol-chloroform	0.24 US$ [69]	PCR, NGS	[9]
HotSHOT	פחות מ-0.01 US$ [69]	PCR	—
Salting out	0.12 US$ [69]	PCR	—
Chelex	0.02 - 4 US$ [41]	PCR	[41, 76]

6.4.2. הפקת חומצות גרעין בשיטה לא הרסנית

אחד האתגרים המרכזיים באנליזה מולקולרית של פרוקי-רגליים, ובפרט זבובי חול, הוא שימור הדגימות לשילוב באוספים אנטומולוגיים. רוב פרוטוקולי הפקת DNA מחייבים כתישה של הרקמה ובכך פוגעים בשימור הדגימה המקורית. שיטות הפקה לא הרסניות נועדו להפיק חומר גנטי ללא פגיעה פיזית בדגימה, בהשפעה על חיוניותה או בשינוי המורפולוגיה שלה. שיטות אלו חשובות במיוחד בדגימות יקרות או מוגבלות, כמו זבובי חול, שבהן שמירה על שלמות מבנית חיונית לצרכים טקסונומיים, מורפולוגיים או דיאגנוסטיים עתידיים.

שיטה נפוצה היא אמבט ליזיס לא הרסני, שבו זבובי החול מקובעים ומושרים בעדינות בבופר ליזיס המכיל proteinase K.

טכניקת mild-vectolysis יושמה בהצלחה בזבובי חול, ובפרט בדגימות טיפוס [24]. השיטה עושה שימוש בערכת קולונות סטנדרטית (DNeasy Blood and Tissue kit, QIAGEN) עם התאמה להפקת DNA ללא הרס הדגימה. שלבי ליזיס מותאמים (נפח בופר ליזיס והוספת שלב הקפאה) [17] מאפשרים שחרור חומצות גרעין תוך מזעור נזק מורפולוגי [24].

בזבובי חול ניתן גם להשתמש בערכת הפקת HotSHOT DNA Extraction kit (Bento Bioworks Ltd) [73] שהיא מהירה וזולה. דגימות אנטומולוגיות המיועדות לזיהוי מורפולוגי יכולות לעבור לאחר מכן שטיפה. דגימות שעברו עיבוד ב-DNeasy Blood and Tissue kit דורשות הבהרה בתמיסת מארק-אנדרה ואילו דגימות שעברו עיבוד עם HotSHOT DNA מובהרות במידה מספקת לקיבוע במדיום מימי, או בעדיפות בשרף לאחר ייבוש הדרגתי, בהתאם לפרוטוקול המתואר במאמר זה [73].

החומר הגנטי המופק ניתן לעיבוד נוסף להמשך אנליזות, למשל PCR להגברת סמנים גנטיים ספציפיים. שיטות הפקת חומצות גרעין לא הרסניות חיוניות לחקר המאפיינים הגנטיים של זבובי חול ולזיהוי גורמים מחוללי מחלות פוטנציאליים שהם עשויים לשאת. בכך שנשמרת שלמות הדגימה, חוקרים יכולים להשיג מידע גנטי יקר ערך תוך שמירת הדגימה לניתוחים או מחקרים נוספים.

6.5. MALDI-ToF MS

MALDI-ToF MS (matrix-assisted laser desorption/ionization time-of-flight mass spectrometry)

היא טכניקת ספקטרומטריית מסה שמטרתהלזהות ולנתח פרופילי חלבונים ייחודיים ("טביעות אצבע") של דגימות ביולוגיות. MALDI-ToF מוכרת יותר ויותר ככלי חשוב לזיהוי פרוקי-רגליים בעלי חשיבות רפואית ווטרינרית. טכניקה זו הוכחה כיעילה בזיהוי שלבי התפתחות שונים של זבובי חול, כולל שלבים לא בוגרים וארוחות דם של נקבות דם, והודגמה כמתאימה להבחנה בין מינים של זבובי חול, זכרים ונקבות, בתנאי אחסון והומוגניזציה שונים [28, 30, 73, 74].

שיטה זו מציעה גם יכולת הבחנה גבוהה ברמת תת-סוג, מין ואוכלוסייה. הטכניקה מאפשרת זיהוי מהיר ומדויק של מינים, דבר חיוני להבנת תפוצת זבובי החול, התנהגותם ותפקידם בהעברת מחלות. באמצעות הבחנה בין מינים על בסיס פרופילי חלבון, שיטת MALDI-ToF ממלאת תפקיד מרכזי במחקרים אפידמיולוגיים ובאסטרטגיות הדברת וקטורים.

עם זאת, קיימים שני חסרונות עיקריים בשיטה זו המגבילים את יישומה השגרתי. הראשון הוא זמינות ציוד ספקטרומטריית מסה, שעלותו גבוהה מאוד ואינה מצדיקה רכישה ייעודית לזיהוי זבובי חול בלבד (או וקטורים אחרים של פרוקי־רגליים). מגבלה זו עשויה להיפתר באמצעות שימוש במכשיר במעבדות פרוטאומיקה או במערכות אבחון קליניות שבהן מכשירים אלה זמינים. החיסרון השני הוא ייצוג נמוך של נתוני ייחוס של זבובי חול במאגרי מידע פתוחים, דבר המחייב בניית

This is an Open Access article distributed under the terms of the Creative Commons Attribution License (https://creativecommons.org/licenses/by/4.0), which permits unrestricted use, distribution, and reproduction in any medium, provided the original work is properly cited.

מאגר פנימי הכולל ספקטרום ייחוס המבוסס על דגימות שזוהו באופן חד-משמעי, רצוי בשילוב זיהוי מורפולוגי וריצוף של סמן גנטי מתאים (COI, CytB או אחר). מגבלה זו צפויה להצטמצם עם שילוב הדרגתי של נתוני ייחוס במערכת MSI של Assistance Publique-Hôpitaux de Paris, Sorbonne University, צרפת, והאוסף BCCM/IHEM/Sciensano בבריסל, בלגיה (https://msi.happy-dev.fr/).

כאשר מתוכנן שימוש ב- MALDI-ToF לפרופיל חלבוני, יש לאחסן את הדגימות בהקפאה יבשה או באתנול בריכוז 70% בדרגת איכות מולקולרית, ולהימנע מחשיפה לטמפרטורת החדר. בהיעדר הנחיות אחידות להכנת דגימות, מומלץ להשתמש בתמיסה מימית המכילה 60%acetonitrile ו- 0.3% TFA של חומצה סינאפינית (sinapinic acid 30 mg/mL) להכנת מטריצה לMALDI-ToF כדי לאפשר השוואה של הספקטרום החלבוני לנתונים שכבר פורסמו בעבור זבובי חול.

הכנת דגימות ל- MALDI-ToF MS (איור 7)

דגימות חרקים, שאוחסנו בתנאים שונים, מיובשות תחילה באוויר בטמפרטורת החדר ומנותחות. הראש והבטן מוסרים לשם קבלת חלקי גוף הכוללים מאפיינים מורפולוגיים מרכזיים לצורך קיבוע על זכוכית נושאת וניתוח מורפולוגי. בית החזה יכול לשמש ל MALDI-ToF והבטן הנותרת נשמרת להפקת DNA.

לצורך פרופיל חלבוני, בית החזה עובר הומוגניזציה ידנית במבחנות מיקרו בנפח 1.5 מ"ל עם 10 µL תמיסת הומוגניזציה באמצעות עלי ומכתש חד־פעמיים. שתי תמיסות הומוגניזציה נפוצות הן מים מזוקקים סטריליים וחומצה פורמית 25%.

7. סיכום

בעבודה זו ביקשנו להציג בפני החוקרים את השיטות היעילות ביותר לקיבוע זבובי חול, בהתאם למטרות מחקר שונות, במטרה לאפשר זיהוי מדויק וגילוי פתוגנים. אין שיטה אחת מיטבית באופן אוניברסלי; קיימות מספר שיטות, שלכל אחת יתרונות ומגבלות.

בנתונים הנלווים סיפקנו פרוטוקולים מפורטים לשיטות קיבוע שונות המשמשות בהכנה ובזיהוי של זבובי חול. פרוטוקולים אלה, לרבות סרטוני הדרכה, מציגים פרוצדורות שלב-אחר-שלב המותאמות למטרות שונות ומבטיחות תוצאות מדויקות ואמינות. באמצעות משאב מקיף זה אנו מבקשים לתמוך בחוקרים בבחירה וביישום של שיטות הקיבוע המתאימות ביותר לצרכיהם.

תודות

המחברים מודים ל-Richard Lane ול-Zoe Jay Adams ממוזיאון הטבע בלונדון, בריטניה, על סקירתם המקצועית אשר שיפרה את איכות כתב היד הזה.

מימון

המחברים מודים לסוכנויות הפיתוח הברזילאיות CNPq (מספר תיק: 404395/2024-4) ול-Araucária Foundation (מספר תיק: PDI 433/2025) על מימון מחקרו של AJA.

ניגודי עניינים

Jérôme Depaquit הוא עורך משנה של *Parasite*; לא הייתה לו כל השפעה על תהליך הסקירה או קבלת ההחלטות לגבי כתב יד זה. יתר המחברים מצהירים כי אין להם ניגודי עניינים.

הצהרת זמינות נתונים

סרטונים ב-Zenodo

וידאו 1: https://zenodo.org/records/18198006
וידאו 2: https://zenodo.org/records/18311158
וידאו 3: https://zenodo.org/records/18311106
וידאו 4: https://zenodo.org/records/18311154
וידאו 5: https://zenodo.org/records/18303014
וידאו 6: https://zenodo.org/records/18302850
וידאו 7: https://zenodo.org/records/18315029

חומר משלים

החומר המשלים למאמר זה זמין ב- http://www.parasite-journal.org/10.1051/parasite/2026009/olm

מקורות

1. Alkan C, Allal-Ikhlef AB, Alwassouf S, Baklouti A, Piorkowski G, de Lamballerie X, Izri A, Charrel RN. 2015. Virus isolation, genetic characterization and seroprevalence of Toscana virus in Algeria. Clinical Microbiology and Infection, 21(11), 1040 e1-9.
2. Alten B, Ozbel Y, Ergunay K, Kasap OE, Cull B, Antoniou M, Velo E, Prudhomme J, Molina R, Banuls AL, Schaffner F, Hendrickx G, Van Bortel W, Medlock JM. 2015. Sampling strategies for phlebotomine sand flies (Diptera: Psychodidae) in Europe. Bulletin of Entomological Research 105(6), 664–678.
3. Ayhan N, Baklouti A, Prudhomme J, Walder G, Amaro F, Alten B, Moutailler S, Ergunay K, Charrel RN, Huemer H. 2017. Practical guidelines for studies on sandfly-borne phleboviruses: Part I: Important points to consider *ante* field work. Vector-Borne and Zoonotic Diseases 17(1), 73–80.
4. Bates PA. 1997. Infection of phlebotomine sandflies with *Leishmania*, in The Molecular Biology of Insect Disease Vectors: A Methods Manual. Springer. p. 112–120.5.
5. Baum M, de Castro EA, Pinto MC, Goulart TM, Baura W, Klisiowicz Ddo R, Vieira da Costa-Ribeiro MC. 2015. Molecular detection of the blood meal source of sand flies (Diptera: Psychodidae) in a transmission area of American cutaneous leishmaniasis, Parana State, Brazil. Acta Tropica, 143, 8–12.
6. Belen A, Alten B, Aytekin A. 2004. Altitudinal variation in morphometric and molecular characteristics of *Phlebotomus papatasi* populations. Medical and Veterinary Entomology, 18(4), 343–350.
7. Bhattacharya J, Chandra G, Hati AK. 1991. A simple method for cryopreservation of *Leishmania donovani* promastigotes, Indian Journal of Medical Research, 93, 245–246.
8. Caligiuri LG, Sandoval AE, Miranda JC, Pessoa FA, Santini MS, Salomón OD, Secundino NF, McCarthy CB. 2019. Optimization of DNA extraction from individual sand flies for PCR amplification. Methods and Protocols, 2(2), 36.

24. Giantsis IA, Chaskopoulou A, Bon MC. 2016. Mild-Vectolysis: A nondestructive DNA extraction method for vouchering sand flies and mosquitoes. Journal of Medical Entomology, 53(3), 692–695.
25. Gidwani K, Picado A, Rijal S, Singh SP, Roy L, Volfova V, Andersen EW, Uranw S, Ostyn B, Sudarshan M, Chakravarty J, Volf P, Sundar S, Boelaert M, Rogers ME. 2011. Serological markers of sand fly exposure to evaluate insecticidal nets against visceral leishmaniasis in India and Nepal: a cluster-randomized trial. PLoS Neglected Tropical Diseases, 5(9), e1296.
26. Gilbert MTP, Moore W, Melchior L, Worobey M. 2007. DNA extraction from dry museum beetles without conferring external morphological damage. PLoS One, 2(3), e272.
27. Giordani BF, Andrade AJ, Galati EAB, Gurgel-Goncalves R. 2017. The role of wing geometric morphometrics in the identification of sandflies within the subgenus *Lutzomyia*. Medical and Veterinary Entomology, 31(4), 373–380.
28. Guzmán-Larralde AJ, Suaste-Dzul AP, Gallou A, Peña-Carrillo KI. 2017. DNA recovery from microhymenoptera using six non-destructive methodologies with considerations for subsequent preparation of museum slides. Genome, 60(1), 85–91.
29. Hajibabaei M, DeWaard JR, Ivanova NV, Ratnasingham S, Dooh RT, Kirk SL, Mackie PM, Hebert PD. 2005. Critical factors for assembling a high volume of DNA barcodes. Philosophical Transactions of the Royal Society B: Biological Sciences, 360(1462), 1959–1967.
30. Haouas N, Pesson B, Boudabous R, Dedet JP, Babba H, Ravel C. 2007. Development of a molecular tool for the identification of *Leishmania* reservoir hosts by blood meal analysis in the insect vectors. American Journal of Tropical Medicine and Hygiene, 77(6), 1054–1059.
31. Hlavackova K, Dvorak V, Chaskopoulou A, Volf P, Halada P. 2019. A novel MALDI-TOF MS-based method for blood meal identification in insect vectors: A proof of concept study on phlebotomine sand flies. PLoS Neglected Tropical Diseases, 13 (9), e0007669.
32. Huemer H, Prudhomme J, Amaro F, Baklouti A, Walder G, Alten B, Moutailler S, Ergunay K, Charrel RN, Ayhan N. 2017. Practical guidelines for studies on sandfly-borne phleboviruses: Part II: Important points to consider for fieldwork and subsequent virological screening. Vector-Borne and Zoonotic Diseases, 17(1), 81–90.
33. Jancarova M, Polanska N, Thiesson A, Arnaud F, Stejskalova M, Rehbergerova M, Kohl A, Viginier B, Volf P, Ratinier M. 2025. Susceptibility of diverse sand fly species to Toscana virus. PLoS Neglected Tropical Diseases, 19(5), e0013031.
34. Kapp JD, Green RE, Shapiro B. 2021. A fast and efficient single-stranded genomic library preparation method optimized for ancient DNA. Journal of Heredity, 112(3), 241–249.
35. Killick-Kendrick R, Maroli M, Killick-Kendrick M. 1991. Bibliography of the colonization of phlebotomine sandflies. Parassitologia, 33(suppl.), 321–333.
36. Lawyer P, Killick-Kendrick M, Rowland T, Rowton E, Volf P. 2017. Laboratory colonization and mass rearing of phlebotomine sand flies (Diptera, Psychodidae). Parasite, 24, 42.
37. Léger N, Pesson B, Madulo-Leblond G. 1986. Les phlébotomes de Grèce : 1ère partie. Bulletin de la Société de Pathologie Exotique, 79, 386–397.
38. Léger N, Pesson B, Madulo-Leblond G. 1986. Les phlébotomes de Grèce : 2ème partie. Bulletin de la Société de Pathologie Exotique, 79, 514–524.
39. Leonel JAF, Vioti G, Alves ML, da Silva DT, Meneghesso PA, Benassi JC, Spada JCP, Galvis-Ovallos F, Soares RM, Oliveira T. 2020. DNA extraction from individual Phlebotomine sand flies
9. Casaril AE, de Oliveira LP, Alonso DP, de Oliveira EF, Gomes Barrios SP, de Oliveira Moura Infran J, Fernandes WS, Oshiro ET, Ferreira AMT, Ribolla PEM, de Oliveira AG. 2017. Standardization of DNA extraction from sand flies: Application to genotyping by next generation sequencing. Experimental Parasitology, 177, 66–72.
10. Castalanelli MA, Severtson DL, Brumley CJ, Szito A, Foottit RG, Grimm M, Munyard K, Groth DM. 2010. A rapid non-destructive DNA extraction method for insects and other arthropods. Journal of Asia-Pacific Entomology, 13(3), 243–248.
11. Cerqueira NL. 1943. Um novo meio para montagem de pequenos insetos em lâmina. Memórias do Instituto Oswaldo Cruz, (39), 37–41.
12. Charrel RN, Gallian P, Navarro-Mari JM, Nicoletti L, Papa A, Sanchez-Seco MP, Tenorio A, de Lamballerie X. 2005. Emergence of Toscana virus in Europe. Emerging Infectious Diseases, 11(11), 1657–1663.
13. Chaskopoulou A, Giantsis IA, Demir S, Bon MC. 2016. Species composition, activity patterns and blood meal analysis of sand fly populations (Diptera: Psychodidae) in the metropolitan region of Thessaloniki, an endemic focus of canine leishmaniasis. Acta Tropica, 158, 170–176.
14. Chen H, Rangasamy M, Tan SY, Wang H, Siegfried BD. 2010. Evaluation of five methods for total DNA extraction from western corn rootworm beetles. PLoS One, 5(8), e11963.
15. Depaquit J, Grandadam M, Fouque F, Andry PE, Peyrefitte C. 2010. Arthropod-borne viruses transmitted by Phlebotomine sandflies in Europe: a review. Eurosurveillance, 15(10), 19507.
16. Diamond LS, Herman CM. 1954. Incidence of Trypanosomes in the Canada Goose as revealed by bone marrow culture. Journal of Parasitology, 40(2), 195–202.
17. Ding H, Torno M, Vongphayloth K, Ng G, Tan D, Sng W, Ho K, Randrianambinintsoa FJ, Depaquit J, Tan CH. 2025. Hidden in plain sight: discovery of sand flies in Singapore and description of four species new to science. Parasites & Vectors, 18(1), 402.
18. Es-Sette N, Ajaoud M, Bichaud L, Hamdi S, Mellouki F, Charrel RN, Lemrani M. 2014. *Phlebotomus sergenti* a common vector of *Leishmania tropica* and Toscana virus in Morocco. Journal of Vector Borne Diseases, 51(2), 86–90.
19. Favret C. 2005. A new non-destructive DNA extraction and specimen clearing technique for aphids (Hemiptera). Proceedings of the Entomological Society of Washington, 107(2), 469–470.
20. Galati EAB. 2018. Phlebotominae (Diptera, Psychodidae): Classification, morphology and terminology of adults and identification of American taxa, in Brazilian Sand Flies: Biology, Taxonomy, Medical Importance and Control, Rangel EF, Shaw JJ, Editors. Cham: Springer International Publishing. pp. 9–212.
21. Galati EAB, de Andrade AJ, Perveen F, Loyer M, Vongphayloth K, Randrianambinintsoa FJ, Prudhomme J, Rahola N, Akhoundi M, Shimabukuro PHF, Depaquit J. 2025. Phle-botomine sand flies (Diptera, Psychodidae) of the world. Parasites & Vectors, 18(1), 220.
22. Galati EAB, Galvis-Ovallos F, Lawyer P, Leger N, Depaquit J. 2017. An illustrated guide for characters and terminology used in descriptions of Phlebotominae (Diptera, Psychodidae). Par-asite, 24, 26.
23. Gariepy T, Kuhlmann U, Gillott C, Erlandson M. 2007. Parasitoids, predators and PCR: the use of diagnostic molecular markers in biological control of Arthropods. Journal of Applied Entomology, 131(4), 225–240.

55. Porco D, Rougerie R, Deharveng L, Hebert P. 2010. Coupling non-destructive DNA extraction and voucher retrieval for small soft-bodied Arthropods in a high-throughput context: the example of Collembola. Molecular Ecology Resources, 10(6), 942–945.
56. Prudhomme J, Cassan C, Hide M, Toty C, Rahola N, Vergnes B, Dujardin JP, Alten B, Sereno D, Banuls AL. 2016. Ecology and morphological variations in wings of *Phlebotomus ariasi* (Diptera: Psychodidae) in the region of Roquedur (Gard, France): a geometric morphometrics approach. Parasites & Vectors, 9(1), 578.
57. Prudhomme J, Gunay F, Rahola N, Ouanaimi F, Guernaoui S, Boumezzough A, Banuls AL, Sereno D, Alten B. 2012. Wing size and shape variation of *Phlebotomus papatasi* (Diptera: Psychodidae) populations from the south and north slopes of the Atlas Mountains in Morocco. Journal of Vector Ecology, 37(1), 137–147.
58. Prudhomme J, Toty C, Kasap OE, Rahola N, Vergnes B, Maia C, Campino L, Antoniou M, Jimenez M, Molina R, Cannet A, Alten B, Sereno D, Banuls AL. 2015. New microsatellite markers for multi-scale genetic studies on *Phlebotomus ariasi* Tonnoir, vector of *Leishmania infantum* in the Mediterranean area. Acta Tropica, 142, 79–85.
59. Prudhomme J, Velo E, Bino S, Kadriaj P, Mersini K, Gunay F, Alten B. 2019. Altitudinal variations in wing morphology of *Aedes albopictus* (Diptera, Culicidae) in Albania, the region where it was first recorded in Europe. Parasite, 26, 55.
60. Rawlins DJ. 1992. Light Microscopy: An Introduction to Biotechniques. Oxford: Bios Scientific publishers. 143 pp.
61. Ready PD. 2013. Biology of phlebotomine sand flies as vectors of disease agents. Annual Review of Entomology, 58, 227–250.
62. Rohlf FJ, Slice D. 1990. Extensions of the Procrustes method for the optimal superimposition of landmarks. Systematic Zoology, 39(1), 40–59.
63. Rowley DL, Coddington JA, Gates MW, Norrbom AL, Ochoa RA, Vandenberg NJ, Greenstone MH. 2007. Vouchering DNA-barcoded specimens: Test of a nondestructive extraction proto-col for terrestrial arthropods. Molecular Ecology Notes, 7(6), 915–924.
64. Sábio PB, Andrade AJ, Galati EAB. 2014. Assessment of the taxonomic status of some species included in the *Shannoni* complex, with the description of a new species of *Psathyromyia* (Diptera: Psychodidae: Phlebotominae). Journal of Medical Entomology, 51(2), 331–341.
65. Sadlova J, Yeo M, Seblova V, Lewis MD, Mauricio I, Volf P, Miles MA. 2011. Visualisation of *Leishmania donovani* fluo-rescent hybrids during early stage development in the sand fly vector. PLoS One, 6(5), e19851.
66. Sales K, Miranda DEO, da Silva FJ, Otranto D, Figueredo LA, Dantas-Torres F. 2020. Evaluation of different storage times and preservation methods on phlebotomine sand fly DNA concentration and purity. Parasites & Vectors, 13(1), 399
67. Sales KG, Costa PL, de Morais RC, Otranto D, Brandao-Filho SP, Cavalcanti Mde P, Dantas-Torres F. 2015. Identification of phlebotomine sand fly blood meals by real-time PCR. Parasites& Vectors, 8, 230.
68. Sant'Anna MR, Jones NG, Hindley JA, Mendes-Sousa AF, Dillon RJ, Cavalcante RR, Alexander B, Bates PA. 2008. Blood meal identification and parasite detection in laboratory-fed and field-captured *Lutzomyia longipalpis* by PCR using FTA databasing paper. Acta Tropica, 107(3), 230–237.
69. Senne NA, Santos HA, Araujo TR, Paulino PG, Mendonca LP, Moreira HVS, Camilo TA, da Costa Angelo I. 2022. Robust comparative performance of genomic DNA extraction methods from non-engorged phlebotomine sandflies. Medical and Veterinary Entomology, 36(2), 203–211.
(Diptera: Psychodidae: Phlebotominae) specimens: Which is the method with better results? Experimental Parasitology, 218, 107981.
40. Lestinova T, Rohousova I, Sima M, de Oliveira CI, Volf P. 2017. Insights into the sand fly saliva: Blood-feeding and immune interactions between sand flies, hosts, and *Leishmania.* PLoS Neglected Tropical Diseases, 11(7), e0005600.
41. Lienhard A, Schaffer S. 2019. Extracting the invisible: obtaining high quality DNA is a challenging task in small arthropods. PeerJ, 7, e6753.
42. Lozano-Sardaneta YN, Mikery-Pacheco OF, Huerta H, Rojas-Soriano JE, Contreras-Ramos A. 2025. Wing geometric morphometrics is effective to separate sand fly species (Diptera, Psychodidae, Phlebotominae) related with leishmaniasis transmission in Mexico. Acta Tropica, 262, 107523.
43. Mandrioli M. 2008. Insect collections and DNA analyses: how to manage collections? Museum Management and Curatorship, 23(2), 193–199.
44. Maroli M, Feliciangeli MD, Bichaud L, Charrel RN, Gradoni L. 2013. Phlebotomine sandflies and the spreading of leishmaniases and other diseases of public health concern. Medical and Veterinary Entomology, 27(2), 123–147.
45. Marquina D, Buczek M, Ronquist F, Lukasik P. 2021. The effect of ethanol concentration on the morphological and molecular preservation of insects for biodiversity studies. PeerJ, 9, e10799.
46. Mathis A, Depaquit J, Dvorak V, Tuten H, Banuls AL, Halada P, Zapata S, Lehrter V, Hlavackova K, Prudhomme J, Volf P, Sereno D, Kaufmann C, Pfluger V, Schaffner F. 2015. Identification of phlebotomine sand flies using one MALDI-TOF MS reference database and two mass pectrometer systems., Parasites & Vectors, 8, 266.
47. Mekarnia N, Benallal KE, Sadlova J, Vojtkova B, Mauras A, Imbert N, Longhitano M, Harrat Z, Volf P, Loiseau PM, Cojean S. 2024. Effect of *Phlebotomus papatasi* on the fitness, infectivity and antimony-resistance phenotype of antimony-resistant *Leishmania* major Mon-25. International Journal for Parasitology – Drugs and Drug Resistance, 25, 100554.
48. Milligan BG. 1998. Total DNA isolation, in Molecular Genetic Analysis of Population: A Practical Approach, Hoelzel AR, Editor. Oxford: Oxford University Press.
49. Molina R, Jiménez M, Alvar J, González E, Hernández-Taberna S, Ines MM. 2017. Methods in sand fly research. Madrid: Servicio de publicaciones Universidad de Alcalá de Henares, Madrid.
50. Murphy WJ, Eizirik E, O'Brien SJ, Madsen O, Scally M, Douady CJ, Teeling E, Ryder OA, Stanhope MJ, de Jong WW, Springer MS. 2001. Resolution of the early placental mammal radiation using Bayesian phylogenetics. Science, 294(5550), 2348–2351.
51. Nacif-Pimenta R, Pinto LC, Volfova V, Volf P, Pimenta PFP, Secundino NFC. 2020. Conserved and distinct morphological aspects of the salivary glands of sand fly vectors of leishma-niasis: an anatomical and ultrastructural study. Parasites & Vectors, 13(1), 441.
52. Neuhaus B, Schmid T, Riedel J. 2017. Collection management and study of microscope slides: Storage, profiling, deterioration, restoration procedures, and general recommendations. Zootaxa, 4322(1), 1–173.
53. New TR. 1974. Pscoptera. Handbooks for Identification of British Insects (Vol. I). London: Royal Entomological Society of London. 102 pp.
54. Perez-Ruiz M, Collao X, Navarro-Mari JM, Tenorio A. 2007. Reverse transcription, real-time PCR assay for detection of Toscana virus. Journal of Clinical Virology, 39(4), 276–281.

DNA with hot sodium hydroxide and tris (HotSHOT). Biotechniques, 29(1), 52–54.
74. Upton MS. 1993. Aqueous gum-chloral slide mounting media: an historical review. Bulletin of Entomological Research, 83(2), 267–274.
75. Volf P, Myskova J. 2007. Sand flies and *Leishmania*: specific versus permissive vectors. Trends in Parasitology, 23(3), 91–92.
76. Wang Q, Wang X. 2012. Comparison of methods for DNA extraction from a single chironomid for PCR analysis. Pakistan Journal of Zoology, 44(2), 421–426.
77. Wang Y, Zhao Y, Bollas A, Wang Y, Au KF. 2021. Nanopore sequencing technology, bioinformatics and applications. Nature Biotechnology, 39(11), 1348–1365.
70. Shaw JJ. 2025. A review of *Leishmania* infections in American Phlebotomine sand flies – Are those that transmit leishmaniasis anthropophilic or anthropportunists? Parasite, 32, 57.
71. Tesh RB, Modi GB. 1983. Growth and transovarial transmission of Chandipura virus (Rhabdoviridae: Vesiculovirus) in *Phlebotomus papatasi.* American Journal of Tropical Medicine and Hygiene, 32(3), 621–623.
72. Thomsen PF, Elias S, Gilbert MTP, Haile J, Munch K, Kuzmina S, Froese DG, Sher A, Holdaway RN, Willerslev E. 2009. Non-destructive sampling of ancient insect DNA. PLoS One, 4(4),e5048
73. Truett GE, Heeger P, Mynatt RL, Truett AA, Walker JA, Warman ML. 2000. Preparation of PCR-quality mouse genomic

Cite this article as: Randrianambinintsoa FJ, Augendre L, Prudhomme J, Martinet J-P, Loyer M, Mekarnia N, Kerkoub H, Perveen FK, Huguenin A, Kariya E, Akhoundi M, De Andrade AJ, Berriatua E, Bongiorno G, Boyer S, Christodoulou V, Da Costa-Ribeiro MCV, De Souza LAF, Ding H, Dondji B, Dvořák V, Erisoz Kasap O, Galati EAB, Gállego M, Ballart C, Gouzelou S, Haddad N, Masse RS, Mekuria AH, Ivovic V, Kaczmarek S, Shahar MK, Kirstein OD, Kniha E, Kolářová I, Lincoln T, Lucanas C, Mikov O, Nov K, Özbel Y, Pesson B, Posada Lopez LC, Prasetyo DB, Rahola N, Rebollar-Tellez EA, Rodrigues BL, Roy L, Saini P, Sanjoba C, Shimabukuro PH, Siriyasatien P, Soszynska A, Suleşco T, Sylla M, Torno M, Volf P, Vongphayloth K, Sinh Nam V, Wardhana A, Yessinou E, Zapata S, Gantier J-C & Depaquit J. 2026. Processing and mounting phlebotomine sand flies: a consensus guideline. Parasite xx, xx. https://doi.org/10.1051/parasite/2026009.

PARASITE

An international open-access, peer-reviewed, online journal publishing high quality papers on all aspects of human and animal parasitology

Reviews, articles and short notes may be submitted. Fields include, but are not limited to: general, medical and veterinary parasitology; morphology, including ultrastructure; parasite systematics, including entomology, acarology, helminthology and protistology, and molecular analyses; molecular biology and biochemistry; immunology of parasitic diseases; host-parasite relationships; ecology and life history of parasites; epidemiology; therapeutics; new diagnostic tools.
All papers in Parasite are published in English. Manuscripts should have a broad interest and must not have been published or submitted elsewhere. No limit is imposed on the length of manuscripts.
Parasite (open-access) continues **Parasite** (print and online editions, 1994-2012) and **Annales de Parasitologie Humaine et Comparée** (1923-1993) and is the official journal of the Société Française de Parasitologie.

Editor-in-Chief:
Jean-Lou Justine, Paris

Submit your manuscript at:
https://www.editorialmanager.com/parasite

This article was translated by Shira Kalmus, Debora Diaz, and Oscar David Kirstein.
Medical Entomology and Parasitology Laboratory, Public Health Laboratories – Jerusalem, Public Health Services, Ministry of Health, Israel.

מאמר זה תורגם על ידי שירה קלמוס, דבורה דיאז ואוסקר דוד קירשטיין,
המעבדה לאנטומולוגיה רפואית ולפרזיטולוגיה, המעבדות לבריאות הציבור – ירושלים, שירותי בריאות הציבור, משרד הבריאות, ישראל.

נספח 1: יסודות ביוכימיים תיאורטיים

פרוקי-הרגליים הנדונים הם זבובי חול. עם זאת, ניתן להרחיב את העיקרון הכללי גם לפרוקי-רגליים נפוצים אחרים, שהזיהוי שלהם מתאפשר רק על סמך מאפיינים מורפולוגיים פנימיים. לעיתים, איברים פנימיים מסוימים הם כיטיניים באופן חלקי, והמורפולוגיה שלהם מספקת מידע בעל ערך רב. לכן, מעניין במיוחד לבחון את משאבות המזון, הספרמטקות ואת התעלות שלהן.

במהלך סקירת כלל הריאגנטים, יש לזכור כי משלב שימור החרק ועד לקיבועו על זכוכית נושאת, אנו מפעילים למעשה תהליכים כימיים של חמצון–חיזור. העיקרון המנחה היחיד הוא להימנע מערבוב ריאגנטים מחזרים עם ריאגנטים מחמצנים.

אתיל אלכוהול (Ethyl alcohol); אתנול (Ethanol)

חומר זה ישמש במספר אופנים שונים. מולקולות האלכוהול בעלות זיקה גבוהה למים ולכן מפגינות אפקט מייבש. עם זאת, אלכוהול בריכוז נמוך (כלומר עשיר מדי במים) תורם לפירוק חומצות גרעין, שכן מים הם גורם מזיק לחומצות גרעין.

כאשר חרקים מונחים באתנול, המטרה אינה רק שימורם אלא גם קיבוע הרקמות. בהיסטולוגיה נהוג להבחין בין שני מושגים חשובים: קצב חדירה וקצב קיבוע. ברור כי חומר משמר יעיל חייב תחילה לחדור במהירות לעומק הרקמות ורק לאחר מכן לקבע אותן. עבור אתנול בריכוז 96%, מקדם החדירה הוא כ-1.05 (לשם השוואה, עבור תמיסה מימית של חומצה פיקרית 0.75% מקדם החדירה הוא 0.45, ואילו עבור תמיסת אשלגן דיכרומט 3% הוא 1.45).

הרצון לשמור חרקים ופרוקי-רגליים אחרים באתנול ללא הגבלה הוא מציאות בקרב אנטומולוגים, והרצון לשמר לכידות שדה למחקרים עתידיים הוא מובן. עם זאת, גישה זו אינה מקובלת בציטולוגיה ובהיסטולוגיה, שכן השארת הדגימות בחומר המקבע לפרקי זמן ממושכים עלולה להפוך אותן לבלתי ניתנות לעיבוד חוזר. לכן דגימות בנות יותר מ-10 שנים קשות ולעיתים אף בלתי ניתנות לשימוש.

יש לשקול גם את היחס בין מסת פרוקי-הרגליים לנפח המקבע. בפרקטיקה זואולוגית או רפואית מומלץ להשתמש בנפח הגדול פי 60 מנפח הרקמות המקובעות. בפועל, עבור מיקרו-פרוקי-רגליים, יש להוסיף לפחות פי 4 - 5 נפחים של אלכוהול ביחס לנפח הדגימות. יש לזכור כי האלכוהול מאבד מחוזקו עם ספיחת המים הקיימים ברקמות.

לסיכום:

– אתיל אלכוהול הוא חומר מחזר (ולכן אינו תואם מקבעים מחמצנים).
– הוא גורם לשקיעה אנרגטית של חלבונים ולדנטורציה שלהם.
– הוא ממיס ליפידים מורכבים מסוימים ומשרה שקיעה של גליקוגן.
– הוא גורם להתכווצות חזקה של הרקמות ולהקשחתן.

תמיסות בסיסיות של אשלגן או נתרן הידרוקסיד:

השימוש בתמיסות אלו באנטומולוגיה התמקד בעיקר באשלגן הידרוקסיד (KOH) ללא היגיון ברור.

נתרן הידרוקסיד - NaOH [E524] מופיע בתמיסה, בריכוזים שונים ובנורמליות שונה. הוא מגיע בתצורה של כדוריות או פתיתים. חסרונו העיקרי הוא נטייתו החזקה לספוח מולקולות מים מהסביבה (היגרוסקופי יותר מ KOH). כשהוא מגיב עם החלבונים הוא ממיס אותם, ועם ליפידים הוא הופך אותם לסבונים קשים במהלך הסיבון – ספוניפיקציה או Saponification (זהו הבדל משמעותי מ-KOH, המניב סבונים נוזליים).

אשלגן הידרוקסיד - KOH [E525] זמין כתמיסה מרוכזת, אך יתרונו העיקרי הוא צורתו הכדורית במשקל של כ-0.1 גרם, מה שמקל מאוד על הכנת תמיסות מהולות ללא צורך במאזניים מדויקים. למשל, כדורית אחת של 0.1 גרם ב-1 מ"ל מים מזוקקים נותנת תמיסת 10%. יתרון נוסף של כדוריות KOH הוא רגישות נמוכה יותר לקרבונציה (לתמיסת KOH יש זיקה גבוהה לקיבוע פחמן דו חמצני ובכך היא יוצרת קרבונטים).

בסיסים חזקים אלו משמשים להמסת חומצות שומן על ידי הפיכתן לסבונים מסיסים במים. יש לזכור שהמקבע,כמו אתנול, המיס חלק מהשומנים בדגימה, אך החזרת הדגימה לסביבה מימית עם בסיס חזק תגרום לשקיעת חומצות השומן (מורכבות יותר או פחות). הבסיס החזק יבצע ספוניפיקציה קרה. במקרים של עודף רקמת שומן, למשל בנקבות, מומלץ להעלות את הטמפרטורה ל-35–40°C או להאריך את זמן המגע בטמפרטורת החדר כדי להקל על התגובה.

תמיסת חומצה צבועה או תמיסת מרק-אנדרה (Marc-André) ללא צבע:

נתאר את היתרונות והמגבלות בשימוש בתמיסת Marc-André.

תמיסת מרק-אנדרה מורכבת מכלורל הידרט (trichloracetaldehyde monohydrate)חומצה אצטית ומים. זוהי תמיסה מחמצנת מאוד (תערובת של חומצה ואלדהיד). היא מנטרלת את עודפי ה KOH שנותרו בדגימות מבלי להשקיע את הסבונים האלקליים שנוצרו. תמיסה זו גם מחמצנת את תפקודי האלכוהול המשניים של הגלוקוזמינים שנוצרים בכיטין, ובכך מרככת את הכיטין. פעולה נוספת שלה היא המסה של מלחים מינרליים מסוימים.

כאשר תמיסת מרק-אנדרה צבועה מראש בפוקסין חומצי (ובכך היא נמצאת במצב המחומצן), היא תוכל להיקשר לקבוצות האלכוהול המשניות במבנה. לאחר זמן החשיפה לתמיסת מרק-אנדרה ושלב הצביעה, השטיפה מתבצעת באתנול בלבד, מה שמתחיל את שלב הדהידרציה (ייבוש) של הדגימות.

יתרונות:

– ניטרול שאריות תמיסות בסיסיות
– הרפיית הכיטין
– צביעת הכיטין להדגשת מבנים כיטיניים פנימיים.

חסרונות:

– כלורל הידרט הוא חומר היפנוטי-מרדים ונעשה בו שימוש רפואי. יש להשתמש בו תחת מנדף כימי ובהתאם לחקיקה בעניין סיכונים כימיים.

תמיסות לייבוש הדגימות (דהידרציה):

הניסיון מראה שעבור דגימות קטנות מאוד, אין טעם לעקוב אחר רצף של אמבטיות אלכוהול בריכוזים עולים. אם הדגימה גדולה, נתחיל באתנול 80%, לאחר מכן 90%, 95% ולבסוף אתנול מוחלט (absolute ethanol). עבור דגימות קטנות מאוד, השתמשו באמבטיה

של אתנול 90% ולאחריה טבילה באתנול מוחלט. בשלב זה, עלינו לזכור תמיד שאתנול מוחלט "שואף" לספוח את המים הנמצאים באטמוספירה.

המסורת במעבדות אנטומולוגיה הייתה לסיים את ייבוש הדגימות באמבט של Beech creosote. כיום, השימוש בחומר זה, הנפוץ כחומר הדברה, חומר נוגד פטריות ומשמר עץ אינו מומלץ כלל בשל ריחו (פחמימנים ארומטיים רב-טבעתיים) וההנחה שהוא רעיל למערכת הרבייה, מסרטן, מהווה מזהם אורגני עמיד ורעיל לסביבה עבור אורגניזמים מימיים.

פתרון שאנו מציעים להכנת הדגימות לקיבוע הוא ®Euparal ותמצית יופרל (המתוארת בפסקה הבאה). תערובת של ®Euparal ותמצית Euparal מתאימה מאוד; הדגימות מתקבלות לאחר אמבט אתנול 90%.

נספח 2: הרכב המגיבים

אשלגן הידרוקסיד (Potassium hydroxide, KOH) 10%

– אשלגן הידרוקסיד 10 גרם
– מים מזוקקים עד להשלמה ל-100 מ"ל

מדיום קיבוע גאם כלורל (Gum chloral) - מדיום הואיר (Hoyer medium)

– מים מזוקקים 50 מ"ל
– כלורל הידרט 200 גרם
– גומי ערבי (Gum arabic) 50 גרם
– גליצרול 20 מ"ל

תמיסת מרק-אנדרה (Marc-André)

– כלורל הידרט 40 גרם
– חומצה אצטית קרחונית 30 מ"ל
– מים מזוקקים 30 מ"ל

פוקסין חומצי 1% במים מזוקקים

– אבקת פוקסין חומצי 1 גרם
– מים מזוקקים 99 מ"ל

תמיסת מרק-אנדרה (Marc-André) צבועה בפוקסין (acid fuchsin)

– תמיסת מרק-אנדרה 10 מ"ל
– פוקסין חומצי 1% 50 µL

נספח 3: ®Euparal, בלזם קנדה (Canada balsam), פוליוויניל אלכוהול ופתרונות קיבוע אחרים

פוליוויניל אלכוהול: זהו מדיום הקיבוע האידיאלי כאשר החומרים הדרושים לדהידרציה תקינה אינם זמינים. במקרה זה, פוליוויניל אלכוהול מעורבב עם Amman’s lactophenol. עם זאת, לתכשירים אלו יש חסרונות משמעותיים: הם נוטים להתייבש, הפוליוויניל אלכוהול עלול להתגבש בגלל התאיידות מים, או שהתכשיר משחיר כאשר הפנול מתחמצן. טכניקה זו נותרת טובה לקיבוע לטווח קצר בלבד.

בלזם קנדה (Canada balsam): השימוש בו לקיבוע בין זכוכית נושאת לזכוכית כיסוי מחייב דהידרציה של הדגימות. השימוש בקסילן או טולואן כממסים אינו חסין מחסרונות.

מדיום Enecê: עבור קיבוע בין זכוכית נושאת לזכוכית כיסוי, בדומה לבלזם קנדה, הוא מחייב דהידרציה של הדגימה. פורמולת ה-Enecê: שרף אורנים (colophony) לבן טהור (22 גרם); שרף קופאל (Copal gum) מסיס באלכוהול (12 גרם), אלכוהול מוחלט (20 מ"ל), קמפור (camphor, 10 גרם), תמצית טרפנטין (10 מ"ל) ואקליפטול (26 מ"ל).
אופן ההכנה: בתוך כלי, כגון בקבוק ארלנמייר, מניחים את האלכוהול המוחלט והקמפור. לאחר מכן מוסיפים את הקולופוני ואת שרף הקופאל. אוטמים את הבקבוק עם פקק ומנערים ורק אז מחממים אותו באמבט (bain-marie) בטמפרטורה עדינה כדי שהתערובת לא תרתח. ברגע שהתוכן הפך לחלוטין לנוזל, מוסיפים את תמצית הטרפנטין, מסננים בעוד התערובת חמה ולבסוף מוסיפים את האקליפטול לתסנין. כאשר המדיום נעשה צמיגי יותר, מדללים אותו באמצעות Enecê, שלו הנוסחה הבאה: אלכוהול מוחלט (30 מ"ל), קמפור (17 גרם), תמצית טרפנטין (15 מ"ל), אקליפטול (38 מ"ל) (Cerqueira, 1943).

®Euparal: זהו שרף המופק מעץ השרף המרוקאי *Tetraclinis articulata (Vahl, 1791)*, נחקר ופותח ב-1906 על ידי גילסון. יתרונו העיקרי הוא שהוא אינו עובר פולימריזציה (אינו מתקשה לצמיתות באופן בלתי הפיך). ניתן לשחזר בקלות דגימות שקובעו, באמצעות אלכוהול או טוב מכך - תמצית ®Euparal. שרף זה, הנקרא גם סנדרק (sandarac) מסיס באתנול החל מריכוז של 80%.

שימוש ב-Triton X100: תמיסה מימית לא-יונית:

Triton X100 מגיע בתצורת תמיסה מימית לא-יונית
4-(1,1,3,3-tetramethylbutyl) phenyl-polyethylene glycol solution, or t-octylphenoxypolyethoxyethanol, polyethylene glycol tert-octylphenyl ether.
הוא נמצא בשימוש נרחב כדטרגנט בביולוגיה תאית ומולקולרית.
הוא מאפשר הגברת חדירותם של קרום התא וקרום הגרעין.
דגימות חרקים שנשמרו באלכוהול במשך שנים רבות הן עניין שבשגרה. לרוע המזל, שימור באלכוהול אינו אופטימלי, פרוקי-רגליים שנשמרים כך הופכים קשים מאוד להכנה לבדיקה מיקרוסקופית. לעיתים כלי הפלסטיק המכילים את הדגימות מתבלים, מה שמוביל להתאיידות האלכוהול. בשני המקרים – מגע ממושך עם אלכוהול או התייבשות הדגימות – נוצרת בעיה ממשית. בשנת 2008 Jonque פרסם הערה על רהידרציה (השבת מים) של עכבישים באמצעות חומר הרטבה כגון Agepon המשמש לסרטי צילום [26]. הדבר הוביל לרעיון להשתמש בחומרי הרטבה שאינם דטרגנטים עוצמתיים מדי.
להלן הליך השימוש בTriton X-100 בתמיסה מימית בריכוז 0.5%:

– יש להספיג את הדגימה היבשה באלכוהול (absolute alcohol).
– הוסף את הנפח הדרוש של תמיסת Triton X-100 בריכוז 0.5%, כך שכל הדגימה תהיה טבולה לחלוטין.
– הנח לדגימה לעמוד למשך כ-5 דקות או יותר. על כל פרוקי הרגליים לנוע בחופשיות בתוך התמיסה.
– הסר את תמיסת הTriton X-100 והחלף אותה בתמיסת אשלגן הידרוקסיד (KOH).

לאחר מכן יש להמשיך בטכניקה כפי שתואר לעיל.

נספח 4: מדיום קיבוע ®Euparal או בלזם קנדה צעד אחר צעד

1. יש להעביר את הדגימות תהליך דהידרציה (מראה עבור או "חלבי" מעיד על דהידרציה לקויה).
2. ניתן לבצע את הדהידרציה באמצעות העלאת ריכוזי האתנול.
3. ניתן להעביר את הדגימות מאלכוהול 99% או מאלכוהול מוחלט אל חומר מבהיר.

תהליך:

1. בצע דיסקציה של זבובי חול בוגרים באתנול 70%.
2. החלף את האתנול ב-10% KOH. כסה את זבובי החול בזכוכית כיסוי.
3. בצע הבהרה עד שהחרקים הופכים לשקופים.
4. הסר את תמיסת ה-KOH.
5. כסה את הדגימה עם מים מזוקקים והמתן למשך 30 עד 45 דקות.
6. הסר את המים וחזור על השטיפות עם מים מזוקקים למשך 30 דקות (פונקציית הזמן עבור דגימות רבות: ככל שישנן יותר דגימות לעיבוד יחד, כך יש להקפיד על זמן ממושך יותר. ככל שישנן פחות דגימות, ובמיוחד עבור אלו המטופלות באופן אינדיבידואלי, כך זמן זה יכול להיות קצר יותר).
7. הסר את המים
8. הוסף תמיסת מרק-אנדרה (ניתן לצביעה בפוקסין) והמתן 24 שעות.
9. הסר את תמיסת מרק-אנדרה.
10. כסה את הדגימה עם מים מזוקקים והמתן למשך 30 עד 45 דקות.
11. הסר את המים וחזור על השטיפות עם מים מזוקקים למשך 30 דקות.
12. הסר את מים
13. הוסף אתנול 70% ובצע דיסקציה של הדגימה.
    א. עבור הראש והבטן, משוך בעדינות את הראש או את הבטן הרחק מהחזה.
    ב. עבור החזה, הסר את הכנפיים על ידי החזקת החזה עם פינצטה אחת ומשיכת בסיס הכנפיים עם פינצטה שנייה. ניתן לבצע חתך (דיסקציה) סגיטלית (חיתוך לאורך), המחלקת את החזה לצד ימין ושמאל, בהתאם לאזורים בעלי העניין הרב ביותר.
14. יש לבצע דהידרציה הדרגתית באמצעות סדרת תמיסות מימיות של אתנול בריכוזים עולים (50%, 80%, 95%), עד להגעה לאתנול מוחלט (100%).
15. בצע שתי שטיפות ב-100% אתנול, 10 דקות כל אחת, להשלמת הייבוש.
16. הסר את האתנול וכסה את הדגימות בשמן ציפורן (Clove oil) למשך 15 דקות בטמפרטורת החדר.
17. העבר את הדגימות משמן הציפורן אל טיפה של ®Euparal או בלזם קנדה שהונחה על זכוכית נושאת נקיה.
18. סדר את חלקי הדגימה כרצונך: ניתן לנתח את הראש, החזה והבטן של זבוב החול בעזרת מחטים דקות או פינצטות עדינות תחת מיקרוסקופ ניתוח. יש להפריד בעדינות את הראש מהגוף כדי לשקע אותו במנח גחוני-גבי (ventro-dorsal), כלומר הפתח בבסיס הראש חייב לפנות כלפי מעלה כך שניתן יהיה לצפות בציבריום ישירות דרכו.

    הדיסקציה מתבצעת בתוך מדיום הקיבוע עצמו.
19. הנח לדגימה לעמוד עד שהמשטח הופך לדביק.
20. הרטב זכוכית כיסוי נקייה באלכוהול מוחלט. הנח את זכוכית הכיסוי על מדיום הקיבוע בלזם קנדה בזווית.
21. אחסן את הסליידים בקופסה יבשה המיועדת.
