# Supplementary material for: Processing and mounting phlebotomine sand flies: a consensus guideline
Source: Parasite. 2026 Apr 3;33:18. doi: 10.1051/parasite/2026009 (PMC13047900; doi:10.1051/parasite/2026009)
Supplement: Supplementary file 14 — Hindi translation / हिंदी अनुवाद [file parasite-33-18-s14.pdf]

Parasite **33**, 18 (2026)
© F.J. Randrianambinintsoa et al., published by EDP Sciences, 2026
https://doi.org/10.1051/parasite/2026009

PARASITE
Available online at:
www.parasite-journal.org

REVIEW ARTICLE OPEN ACCESS

फ्लेबोटोमाइन बालू मक्खी का प्रसंस्करण और माउंटिंग: एक सर्वसम्मत दिशानिर्देश

Fano José Randrianambinintsoa[1], Laure Augendre[1], Jorian Prudhomme[1], Jean-Philippe Martinet[1], Mathieu Loyer[1], Nalia Mekarnia[1], Hocine Kerkoub[1], Farzana Khan Perveen[1], Antoine Huguenin[1,2], Emilie Kariya[1,2], Mohammad Akhoundi[3], Andrey José de Andrade[4], Eduardo Berriatua[5], Gioia Bongiorno[6], Sébastien Boyer[7,8], Vasiliki Christodoulou[9], Magda Clara Vieira Da Costa-Ribeiro[10], Lucas Alexandre Farias de Souza[10], Huicong Ding[11], Blaise Dondji[12], Vít Dvořák[13], Ozge Erisoz Kasap[14], Eunice Aparecida Bianchi Galati[15], Montserrat Gállego[16], Cristina Ballart[16], Stavroula Gouzelou[17], Nabil Haddad[18], Rezki Sabrina Masse[19], Asrat Hailu Mekuria[20], Vladimir Ivovic[21], Szymon Kaczmarek[22], Mohd Khadri Shahar[19], Oscar D. Kirstein[23], Edwin Kniha[24], Iva Kolářová[13], Lincoln Timinao[25], Cristian Lucanas[26], Ognyan Mikov[27], Kimsear Nov[7], Yusuf Özbel[28], Bernard Pesson[29], Laura Cristina Posada Lopez[30], Didot Budi Prasetyo[1,7], Nil Rahola[31], Eduardo A. Rebollar-Tellez[32], Bruno Leite Rodrigues[15], Lalita Roy[33], Prasanta Saini[34], Chizu Sanjoba[35], Paloma Helena Fernandes Shimabukuro[36], Padet Siriyasatien[37], Agnieszka Soszyńska[22], Tatiana Suleşco[38], Massamba Sylla[39], Majhalia Torno[40], Petr Volf[13], Khamsing Vongphayloth[41], Vu Sinh Nam[42], April Wardhana[43], Eric Yessinou[44], Sonia Zapata[45], Jean-Charles Gantier[1], and Jérôme Depaquit[1,2,*]

[1] Faculté de Pharmacie, Université de Reims Champagne Ardenne, UR ESCAPE-USC ANSES PETARD, 51 rue Cognacq-Jay, 51096 Reims Cedex, France
[2] Pôle de Biologie territoriale, Laboratoire de Parasitologie-Mycologie, Centre Hospitalo-Universitaire, 51092 Reims, France
[3] Parasitology-Mycology Department, Avicenne Hospital, AP-HP, Bobigny, Sorbonne Paris Nord University, France; Unité des Virus Émergents (UVE: Aix-Marseille Univ, Università di Corsica, IRD 190, Inserm 1207, IRBA), 13005 Marseille, France
[4] Parasitology Collection of Basic Pathology, Department of Basic Pathology, Federal University of Paraná, Curitiba 19031, Brazil
[5] Department of Animal Health, University of Murcia, Campus de Espinardo, 30100 Espinardo, Murcia, Spain
[6] Department of Infectious Diseases, Vector-borne Diseases Unit, Istituto Superiore di Sanità, 00166 Rome, Italy
[7] Medical and Veterinary Entomology Unit, Institut Pasteur du Cambodge, Phnom Penh 12201, Cambodia
[8] Ecology & Emergence of Arthropod-borne Pathogens Unit, Department of Global Health, Institut Pasteur, CNRS UMR2000, 75015 Paris, France
[9] Section Veterinary Services (1417), Laboratory for Animal Health Virology, Aglantzia, Nicosia 2109, Cyprus
[10] Insects Vectors and Parasites Laboratory, Department of Basic Pathology and Postgraduate program in Microbiology, Parasitology and Pathology, Federal University of Paraná, 81530-900 Curitiba, Brazil
[11] Department of Biological Sciences, National University of Singapore, 117558, Singapore
[12] Laboratory of the Leishmaniasis Research Project, Mokolo District Hospital, Mokolo, Cameroon; Laboratory of Cellular Immunology and Parasitology, Department of Biological Sciences, Central Washington University, 98926 Ellensburg, WA, USA
[13] Department of Parasitology, Faculty of Science, Charles University, 12800 Prague, Czechia
[14] VERG Laboratories, Department of Biology, Faculty of Science, Hacettepe University, Beytepe, Ankara 06800, Türkiye
[15] Faculdade de Saúde Pública da Universidade de São Paulo (FSP/USP), Pós-graduação em Saúde Pública, 01246-904 São Paulo, Brazil
[16] Secció de Parasitologia, Departament de Biologia, Sanitat i Medi Ambient, Facultat de Farmàcia i Ciències de l'Alimentació, Universitat de Barcelona, & Institut de Salut Global de Barcelona (ISGlobal), Centro de Investigación Biomédica en Red, Enfermedades Infecciosas (CIBERINFEC), 08028 Barcelona, Spain
[17] Laboratory of Infectious Diseases and Public Health, School of Medicine, University of Cyprus, Nicosia, Cyprus & Department of Pediatrics, Archbishop Makarios III Hospital, Nicosia 2115, Cyprus
[18] Faculty of Health Sciences, American University of Beirut, 1107 2020 Beirut, Lebanon
[19] Medical Entomology Unit, Infectious Disease Research Centre, Institute for Medical Research (IMR), National Institutes of Health (NIH), Ministry of Health Malaysia, 40170 Shah Alam, Selangor, Malaysia

Edited by Jean-Lou Justine
*: अनुरूपी लेखक: jerome.depaquit@univ-reims.fr

This is an Open Access article distributed under the terms of the Creative Commons Attribution License https://creativecommons.org/licenses/by/4.0 which permits unrestricted use, distribution, and reproduction in any medium, provided the original work is properly cited.

[20] School of Medicine, Addis Ababa University, 28017 - 1000 Addis Ababa, Ethiopia
[21] Faculty of Mathematics, Natural Sciences and Information Technologies, University of Primorska, 6000 Koper, Slovenia
[22] University of Lodz, Faculty of Biology and Environmental Protection, Department of Invertebrate Zoology and Hydrobiology, Banacha 12/16, 90-237 Łódź, Poland
[23] Laboratory of Entomology, Ministry of Health, 9134302 Jerusalem, Israel
[24] Center for Pathophysiology, Infectiology and Immunology, Institute of Specific Prophylaxis and Tropical Medicine, Medical University Vienna, Kinderspitalgasse 15, 1090 Vienna, Austria
[25] Papua New Guinea Institute of Medical Research (PNGIMR) Institute, PO Box 60, Headquarter, Homate Street, 441 Goroka, Eastern Highlands Province, Papua New Guinea
[26] Museum of Natural History, University of the Philippines Los Baños, 4031 Laguna, Philippines
[27] National Centre of Infectious and Parasitic Diseases, 1504 Sofia, Bulgaria
[28] Ege University, Faculty of Medicine, Department of Parasitology, 35040 Bornova/Izmir, Türkiye
[29] Retired, Faculté de Pharmacie, Université de Strasbourg, Strasbourg, 67400 Illkirch-Graffenstaden, France
[30] Program for the Study and Control of Tropical Diseases (PECET), Faculty of Medicine, University of Antioquia, 050010 Medellin, Colombia
[31] MIVEGEC, Univ. Montpellier, CNRS, IRD, 34394 Montpellier, France & Medical Entomology Unit, Institut Pasteur de Madagascar, 101 Antananarivo, Madagascar
[32] Laboratorio de Entomología Médica, Departamento de Zoología de Invertebrados, Facultad de Ciencias Biológicas, Universidad Autónoma de Nuevo León, San Nicolás de los Garza, 66455, NL, México
[33] Tropical and Infectious Disease Centre, BP Koirala Institute of Health Sciences, Dharan 56700, Nepal
[34] ICMR-Vector Control Research Centre, 605006, India
[35] Graduate School of Agricultural and Life Sciences, The University of Tokyo, Tokyo 113-8657, Japan
[36] Grupo de estudos em Leishmanioses/Coleção de Flebotomíneos (COLFLEB/Fiocruz-MG), Instituto René Rachou, Fundação Oswaldo Cruz, Belo Horizonte, Minas Gerais, 30190009, Brazil
[37] Center of Excellence in Vector Biology and Vector-Borne Disease, Department of Parasitology, Faculty of Medicine, Chulalongkorn University, Bangkok 10330, Thailand
[38] Department of Arbovirology, Bernhard Nocht Institute for Tropical Medicine, Bernhard Nocht Str. 74, 20359 Hamburg, Germany [39] Laboratory Vectors & Parasites, Department of Livestock Sciences and Techniques, Sine Saloum University El Hadji Ibrahima Niasse (SSUEIN) Kaffrine Campus, C.P. 24600, Senegal.
[40] Environmental Health Institute, National Environment Agency, Singapore 138667, Singapore & Department of Biological Sciences, National University of Singapore, 117558 Singapore
[41] Institut Pasteur du Laos, Laboratory of Vector-Borne Diseases, Samsenhai Road, Ban Kao-Gnot, Sisattanak District, 3560 Vientiane, Lao PDR
[42] National Institute of Hygiene and Epidemiology, 1 Yec-Xanh Street, Hai Ba Trung District, 100000 Hanoi, Vietnam
[43] Indonesian Research Center for Veterinary Science, Indonesian Agency for Agricultural Research and Development, Ministry of Agriculture Republic Indonesia, Bogor 16114, Indonesia & Department of Parasitology, Faculty of Veterinary Medicine, Airlangga University, Surabaya 60115, Indonesia
[44] Laboratory of Research in Applied Biology, Polytechnic School of Abomey-Calavi, University of Abomey-Calavi, 01 P.O. Box 2009, 00000 Cotonou, Benin
[45] Instituto de Microbiología, Colegio de Ciencias Biológicas y Ambientales (COCIBA), Universidad San Francisco de Quito (USFQ), 170901 Quito, Ecuador

Received 1 December 2025, Accepted 29 January 2026, Published online 3 April 2026

अमूर्त – यह लेख फ्लेबोटोमाइन बालू मक्खी नमूनों के प्रसंस्करण और माउंटिंग के लिए एक व्यापक मार्गदर्शिका प्रदान करता है, जो प्रजातियों की पहचान और रोगजनकों का पता लगाने और उन्हें अलग करने के लिए महत्वपूर्ण है। इसमें क्षेत्र और प्रयोगशाला दोनों स्थितियों के लिए उपयुक्त तकनीकों की एक विस्तृत श्रृंखला पर चर्चा की गई है। मार्गदर्शिका में बालू मक्खी के संग्रह, प्रबंधन, आवरण और इच्छामृत्यु (रसायनों के बजाय शुष्क फ्रीजिंग या CO2 की अनुशंसा) के साथ-साथ संरक्षण रणनीतियों, जैसे कि कोल्ड स्टोरेज और इथेनॉल में संरक्षण, पर विस्तृत निर्देश शामिल हैं। कुछ शारीरिक संरचनाओं (जननांग, सिर और पंख) की तैयारी की गुणवत्ता उनके उचित सूक्ष्मदर्शी अवलोकन के लिए आवश्यक है और इस कार्य में इसका वर्णन किया गया है। लेख में पोटेशियम हाइड्रॉक्साइड (Potassium hydroxide) और फिर मार्क-आंद्रे घोल (Marc-André solution) जैसे एजेंटों के साथ स्पष्टीकरण प्रक्रिया सहित विस्तृत नमूना प्रसंस्करण भी प्रस्तुत किया गया है। माउंटिंग प्रक्रिया में विभिन्न माध्यमों की तुलना की गई है, जिसमें उनके प्रकाशीय गुणों और संरक्षण क्षमता पर जोर दिया गया है। होयर द्रव (Hoyer fluid) (जिसे क्लोरल गम [Chloral gum] भी कहा जाता है) को इसकी स्पष्टता के कारण त्वरित अवलोकन, विशेष रूप से शुक्राणुकोशों (spermathecae) के लिए, अनुशंसित किया जाता है, हालांकि यह दीर्घकालिक भंडारण के लिए उपयुक्त नहीं है। अन्य माध्यमों में पॉलीविनाइल अल्कोहल (polyvenyl alcohol), यूपरल® (Euparol®) (सीमित जल सहनशीलता के लिए) और कनाडा बाल्सम (Canada balsam) (हाइड्रोकार्बन [Hydrocarbon]में घुलनशील माध्यम) शामिल हैं, जिनमें से अंतिम दो दीर्घकालिक संरक्षण क्षमता प्रदान करते हैं। DNA अनुक्रमण और MALDI-TOF जैसी नवीन आणविक जीव विज्ञान पद्धतियों पर भी चर्चा की गई है, जिनमें नमूना प्रसंस्करण पर विशेष ध्यान देने की

आवश्यकता होती है। इसके अलावा, विभिन्न माउंटिंग तकनीकों को दर्शाने वाले छोटे वीडियो क्लिप और 33 विभिन्न भाषाओं में अनुवाद भी उपलब्ध कराए गए हैं, जिससे यह दिशानिर्देश वैश्विक वैज्ञानिक समुदाय की विविध आवश्यकताओं और अपेक्षाओं को पूरा कर सके। बालू मक्खी

प्रमुख शब्द: माउंटिंग, फ्लेबोटोमाइन बालू मक्खी, होयर फ्लूइड, मार्क-आंद्रे सॉल्यूशन, क्लोरल गम, पॉलीविनाइल अल्कोहल, यूपरल®, कनाडा बाल्सम, लीशमैनिया अलगाव, फील्ड स्थितियां, कल्चर, विच्छेदन, आणविक जीवविज्ञान, MALDI-ToF, टाइप-नमूने

Abstract – Processing and mounting phlebotomine sand flies: a consensus guideline. This article provides a comprehensive guide for the processing and mounting of phlebotomine sand fly specimens, which is crucial for species identification and pathogen detection and isolation. It discusses a range of techniques suitable for both field and laboratory settings. The guide includes detailed instructions on sand fly collection, handling, covering, and euthanasia (recommending dry freezing or CO_2 over chemicals) as well as conservation strategies, such as cold storage and preservation in ethanol. The quality of preparation of certain anatomical structures (genital organs, head and wings) is essential for their proper microscopic observation and is described in this work. The article also presents detailed sample processing, including the clearing process with agents such as potassium hydroxide then Marc-André solution. The mounting process compares different media, emphasizing their optical properties and preservation potential. Hoyer fluid (also known as chloral gum) is recommended for quick observation, particularly for spermathecae, due to its clarity, although it is not suitable for long-term storage. Other media discussed include polyvinyl alcohol, Euparal® (for limited water tolerance), and Canada balsam (a hydrocarbon-soluble medium), with the latter two offering long-term preservation capabilities. Innovative molecular biology approaches such as DNA sequencing and MALDI-ToF, which require particular attention to sample processing, are also addressed. Furthermore, short video clips illustrating various mounting techniques as well as translations in many different languages are provided, allowing the guideline to reach the diverse needs and expectations of the global scientific community.

Key words: Mounting, Phlebotomine sand fly, Hoyer fluid, Marc-André solution, Chloral gum, Polyvinyl alcohol, Euparal®, Canada balsam, *Leishmania* isolation, Field conditions, Culture, Dissection, Molecular biology, MALDI-ToF, Type-specimens.

परिचय

फ्लेबोटोमाइन बालू मक्खी, साइकोडीडे परिवार और फ्लेबोटोमिनाई उपपरिवार से संबंधित छोटे डिप्टेरा कीट हैं, जिनकी कम से कम 1,063 ज्ञात प्रजातियाँ हैं [21]। ये लीशमैनियासिस, आर्बोवायरस संक्रमण और बार्टोनेलोसिस नामक रोगों के लिए जिम्मेदार रोगजनकों (लीशमैनिया, आर्बोवायरस और बार्टोनेला) के महत्वपूर्ण वाहक हैं। इनकी पहचान मुख्य रूप से सावधानीपूर्वक संग्रह, उचित भंडारण और स्लाइड पर सावधानीपूर्वक माउंटिंग द्वारा सुगम विस्तृत सूक्ष्मदर्शी परीक्षण पर आधारित है, जिसके लिए कई विशिष्ट तकनीकों की आवश्यकता होती है, जिनमें से प्रत्येक के अपने फायदे और सीमाएँ हैं।

वयस्क फ्लेबोटोमाइन बालू मक्खी की पहचान बाहरी (जैसे, एंटीना, पल्पी, नर जननांग) और आंतरिक संरचनाओं (जैसे, ग्रसनी, सिबेरियम और शुक्राणुकोश) दोनों के अवलोकन पर निर्भर करती है। आंतरिक संरचनाओं का विच्छेदन और पृथक्करण उनके अवलोकन और परिणामस्वरूप सटीक पहचान को आसान बनाता है। इसलिए, मच्छरों या किसिंग बग के विपरीत, फ्लेबोटोमाइन बालू मक्खी की पहचान से पहले उन्हें स्लाइड और कवर स्लिप के बीच माउंट करना आवश्यक होता है।

1980 के दशक तक, बालू मक्खी की पहचान के लिए सूक्ष्मदर्शी से अवलोकन ही एकमात्र उपलब्ध विधि थी, और आज भी यह सबसे व्यापक रूप से इस्तेमाल की जाने वाली विधि है। इसलिए प्रक्रिया और तैयारी का चुनाव अपेक्षाकृत सरल था और मुख्य रूप से दो विकल्पों पर आधारित था: एक ओर, नमूने के दीर्घकालिक संरक्षण के लिए निश्चित माउंटिंग, और दूसरी ओर, पहचान के लिए त्वरित माउंटिंग, जो दीर्घकालिक संरक्षण सुनिश्चित नहीं करती। उदाहरण के लिए, कनाडा बाल्सम जैसी रेज़िन में अंतिम माउंटिंग समय लेने वाली होती है, जिसके लिए नमूनों का पूर्ण निर्जलीकरण आवश्यक होता है। इसके अलावा, इस माध्यम का अपवर्तनांक शुक्राणुकोशिकाओं के आसान अवलोकन के लिए हमेशा अनुकूल नहीं होता। इसके विपरीत, जलीय माध्यम (जैसे, होयर लिक्विड) में माउंटिंग तेज़ होती है और अपवर्तक शुक्राणुकोशिकाओं को बेहतर ढंग से देखने की अनुमति देती है, लेकिन यह माउंटिंग के दीर्घकालिक संरक्षण की अनुमति नहीं देती क्योंकि यह वातावरण से पानी सोख लेती है। एक विकल्प यह है कि स्लाइड के पूरी तरह सूख जाने के बाद उसे नेल पॉलिश से सील कर दिया जाए। यह दुविधा आज भी बनी हुई है, जो तैयारी के उद्देश्य के आधार पर माउंटिंग विधि के चुनाव को प्रभावित करती है। 1980 के दशक से, बालू मक्खी की पहचान के अध्ययनों में आकारिकी और जैव रासायनिक दृष्टिकोणों का संयोजन किया गया है। पहला दृष्टिकोण क्यूटिकुलर हाइड्रोकार्बन विश्लेषण था, जिसे जल्द ही आणविक जीव विज्ञान तकनीकों (यानी, रैंडम एम्प्लीफाइड पॉलीमॉर्फिक DNA (RAPD)) द्वारा प्रतिस्थापित कर दिया गया।प्रतिबंध खंड लंबाई बहुरूपता (RFLP), DNA प्रवर्धन, और Sanger विधि के साथ-साथ अगली पीढ़ी के अनुक्रमण (NGS) का उपयोग करके अनुक्रमण। आज, आणविक दृष्टिकोणों को

MALDI-TOF जैसी प्रोटिओमिक विधियों द्वारा पूरक किया जाता है। इसके अलावा, आणविक प्रजाति पहचान को PCR PCR (*Leishmania*, *Trypanosoma*, *Bartonella*, और *Phlebovirus*) द्वारा रोगजनकों का पता लगाने के साथ जोड़ा जा सकता है क्योंकि इन सभी का पता एंड-पॉइंट और रियल-टाइम PCR दोनों द्वारा लगाया जा सकता है, जिसके लिए नमूनाकरण और भंडारण प्रक्रिया को निर्धारित लक्ष्यों के अनुकूल बनाने की आवश्यकता होती है [3, 32]। प्रजातियों के भेदभाव के लिए पारंपरिक रूप से उपयोग की जाने वाली रूपात्मक विशेषताओं के अलावा, अन्य रूपात्मक दृष्टिकोणों को भी लागू किया जा सकता है (जैसे, पंख भू-आकृतिमिति)।

लेखकों के स्वयं के अनुभवों और साहित्य के आंकड़ों पर आधारित इस शोध का उद्देश्य वयस्क फ्लेबोटोमाइन बालू मक्खी को माउंट करने और संसाधित करने के लिए मानकीकृत दिशानिर्देश प्रदान करना था ताकि रूपात्मक और आणविक विश्लेषण को अनुकूलित किया जा सके।

कुछ विश्लेषण (जैसे, आणविक जीवविज्ञान या MALDI-ToF) करने की आवश्यकता के लिए बालू मक्खी के उस हिस्से को बनाए रखना आवश्यक होता है जो रूपात्मक पहचान के लिए आवश्यक नहीं है, जो महत्वपूर्ण प्रोटोकॉल चयन की आवश्यकता को उजागर करता है।

इस लेख में, हम जीवित पकड़ी गई बालू मक्खियों को बेहोश करने और उन्हें इच्छामृत्यु देने की विधियों, उनके भंडारण और उनकी माउंटिंग प्रक्रिया पर ध्यान केंद्रित करते हैं, ताकि उनकी शीघ्र पहचान की जा सके या उन्हें दीर्घकालिक रूप से संरक्षित किया जा सके जिससे आगे के अध्ययन किए जा सकें।

प्रस्तावना: सुरक्षा और विनियामक संबंधी विचारों के लिए संबंधित सुरक्षा डेटा शीट (SDS) का संदर्भ लें।

इस दिशा-निर्देश में उल्लिखित सभी रसायनों का उपयोग सख्त सुरक्षा शर्तों के तहत किया जाना चाहिए। अनुसंधान संस्थानों की स्वास्थ्य एवं सुरक्षा समितियाँ आपको इन रसायनों के खतरों के साथ-साथ इनके उपयोग और अपशिष्ट निपटान की प्रक्रियाओं के बारे में भी जानकारी प्रदान करने के लिए उपलब्ध हैं। हालांकि, इनके उपयोग और निपटान संबंधी सुरक्षा निर्देशों का पालन करना अनिवार्य है। ध्यान दें कि सभी उपयोगकर्ताओं की यह जिम्मेदारी है कि वे अच्छी और सुरक्षित प्रयोगशाला पद्धतियों तथा अपने देश या अनुसंधान संस्थान के लागू कानूनों और विनियमों का अनुपालन सुनिश्चित करें। इसके अलावा, कुछ रसायन या उनके घटक (जैसे, Chloral hydrate) कुछ देशों में विनियमित हैं। इस पांडुलिपि में प्रयुक्त संक्षिप्ताक्षरों की सूची तालिका 1 में दी गई है।

1. बालू मक्खी पकड़ना

वयस्क बालू मक्खियों को जीवित या मृत अवस्था में विभिन्न विधियों जैसे CDC लघु प्रकाश जाल, चिपचिपे जाल और शैनन जाल का उपयोग करके एस्पिरेटर द्वारा एकत्र किया जा सकता है, या सीधे पर्यावरण में उनके विश्राम स्थलों (जैसे पशु आश्रय) से भी एकत्र किया जा सकता है। इन विधियों में उपयुक्त आवासों में जाल लगाना, प्रकाश या अन्य आकर्षणों (CO2 या रासायनिक प्रलोभन) से बालू मक्खियों को आकर्षित करना और आगे के विश्लेषण के लिए उन्हें एकत्र करना शामिल है, जैसा कि कई प्रकाशनों [2, 3, 32, 36, 49] में वर्णित है।

जीवित बालू मक्खियों को पकड़ने से आगे की सभी प्रक्रियाओं में आसानी होती है, जबकि मृत मक्खियों को इकट्ठा करने से लीशमैनिया या वायरस के उपभेदों को अलग करना मुश्किल हो जाता है। कुछ पकड़ने की तकनीकें, जैसे चिपचिपे कागज़, अक्सर बालू मक्खियों के अंगों (एंटेना, पल्प, पंख या पैर) के नुकसान का कारण बनती हैं। इसके अलावा, चिपचिपे कागज़ों पर लगी अरंडी के तेल की परत बालू मक्खियों से चिपक जाती है और प्रसंस्करण की शुरुआत में इसे हटाना आवश्यक होता है, जिसके लिए आमतौर पर एथेनॉल और डाइएथिल ईथर के बराबर भागों के मिश्रण में 15 मिनट के लिए डुबोया जाता है।

2. एसनमूना इच्छामृत्यु

इकट्ठा करने के बाद, जीवित बालू मक्खियों को इच्छामृत्यु देनी आवश्यक है। कुछ संग्रह विधियों (जैसे, चिपचिपे कागज़, डिटर्जेंट या इथेनॉल युक्त जार से लैस CDC लाइट ट्रैप) में रेत मक्खियाँ इकट्ठा करते ही मर जाती हैं। आणविक जीवविज्ञान उन मक्खियों पर लागू किया जा सकता है जिन्हें सीधे इथेनॉल में इकट्ठा किया जाता है और अन्य मक्खियों पर जिन्हें जितनी जल्दी हो सके इथेनॉल में संग्रहित किया जाता है। हालांकि, इनमें से कोई भी मारने की विधि MALDI-ToF द्वारा कीट प्रसंस्करण की अनुमति नहीं देती है। इसके अलावा, कुछ मारने की विधियों से कुछ रूपात्मक लक्षण नष्ट हो सकते हैं। इसलिए, उचित पहचान सुनिश्चित करने या वाउचर नमूनों के रूप में दीर्घकालिक भंडारण (अर्थात, भविष्य के संदर्भ या तुलना के लिए संरक्षित और संग्रहित नमूने) के लिए एक उपयुक्त मानक मारने वाले एजेंट का उपयोग करना आवश्यक है। एथिल एसीटेट (ethyl acetate), एथिल ईथर (ethyl ether), टेट्राक्लोरोएथेन (tetrachloroethane) और क्लोरोफॉर्म (chloroform) जैसे रसायनों को रुई में भिगोकर बालू मक्खियों वाले पात्र में इच्छामृत्यु के लिए रखा जा सकता है। इन मारने वाले एजेंटों की विषाक्तता के कारण निर्माता की सिफारिशों का पालन करते हुए इन्हें सावधानीपूर्वक संभालना चाहिए। हालांकि, हम क्लोरोफॉर्म का उपयोग करके बालू मक्खियों को मारने की सलाह नहीं देते हैं, क्योंकि हमारे अनुभव के अनुसार, यह आणविक जीवविज्ञान अध्ययनों के लिए उपयुक्त नहीं है। इन सभी उत्पादों की खतरनाक प्रकृति और आणविक विश्लेषणों के लिए इनकी संदिग्ध उपयुक्तता को देखते हुए, इन रसायनों के उपयोग को आम तौर पर हतोत्साहित किया जाता है।

तालिका 1: संकेताक्षर की सूची।

**BME**	Basal medium Eagle
**CDC**	Centers for Disease Control and Prevention
**CMCP**	Camphor-monochlorophenol
**CMR**	Carcinogenic, mutagenic, reprotoxic substance
**COI**	Cytochrome c oxidase subunit I gene
**CytB**	Cytochrome b gene
**DNA**	Deoxyribonucleic acid
**ELISA**	Enzyme-linked immunosorbent assay
**EtOH**	Ethanol
**M199**	Medium 199
**MALDI-ToF MS**	Matrix-assisted laser desorption/ionization time-of-flight mass spectrometry
**MEM**	Minimum essential media
**NGS**	Next-generation sequencing
**NNN**	Novy-MacNeal-Nicolle medium
**PCR**	Polymerase chain reaction
**Lao PDR**	Lao People's Democratic Republic
**PNOC**	Prepronociceptin gene
**qPCR**	Quantitative PCR (real-time PCR)
**RAPD**	Random amplified polymorphic DNA
**RFLP**	Restriction fragment length polymorphism
**RI**	Refractive index
**RNA**	Ribonucleic acid
**RNases**	Ribonucleases
**RNASS**	RNA stabilization solution
**RT-PCR**	Reverse transcription PCR
**TFA**	Trifluoroacetic acid
**BME**	Basal medium Eagle
**CDC**	Centers for Disease Control and Prevention

सबसे व्यापक रूप से इस्तेमाल की जाने वाली विधि, जो आकारिकी, DNA या प्रोटीन को संरक्षित करती है, नमूनों को शुष्क रूप से जमाना है। नमूनों को पूरी तरह से बेहोश करने के लिए पर्याप्त समय तक जमाना आवश्यक है, लेकिन इतना अधिक समय तक नहीं कि वे (i) सूख जाएं, या (ii) लीशमैनिया की जीवन क्षमता को प्रभावित करें, यदि उद्देश्य उन्हें रेत मक्खी के पाचन तंत्र से इन विट्रो में पृथक करना है। इसलिए हम -20°C पर 15 से 20 मिनट तक जमाने की सलाह देते हैं, और नियमित रूप से उनकी निगरानी करते रहें ताकि यह सुनिश्चित हो सके कि वे केवल बेहोश हों, लीशमैनिया परजीवियों को मारे नहीं।

यदि फ्रीजर उपलब्ध न हो, तो CO2 का उपयोग करके कीटों को इच्छामृत्यु दी जा सकती है। ऐसे क्षेत्र में जहां CO2 सिलेंडरों का उपयोग संभव न हो, नमूनों को 'सोडा साइफन' (पेय यंत्र) में उपयोग किए जाने वाले छोटे व्यावसायिक CO2 कंटेनरों का उपयोग करके मारा जा सकता है, लेकिन हवाई परिवहन पर प्रतिबंध हो सकते हैं। अंतिम उपाय के रूप में, कीटों को तंबाकू के धुएं के संपर्क में लाकर मारा जा सकता है। बालू मक्खी को CDC ट्रैप में जीवित पकड़ा जाता है, एस्पिरेटर से एकत्र किया जाता है, कांच की ट्यूब में रखा जाता है, और तंबाकू के धुएं के संपर्क में लाया जाता है जिससे वे कुछ ही सेकंड में मर जाते हैं। यह विधि सभी क्षेत्र स्थितियों में लागू होती है, यहां तक कि कठिन अलगाव स्थितियों में भी। हालांकि, कांच धुएं से भर जाने के कारण, इसे पूरी तरह से साफ किए बिना जीवित बालू मक्खी के बाद के संग्रह और हैंडलिंग के लिए उपयोग नहीं किया जा सकता है। फिर भी, उसी बिना साफ किए एस्पिरेटर का उपयोग फिक्सेशन उद्देश्यों के लिए अन्य ट्रैप से बालू मक्खी को इच्छामृत्यु देने के लिए किया जा सकता है। यह भी जांचना आवश्यक है कि एस्पिरेटर से सभी नमूने निकाल लिए गए हैं या नहीं। ये विधियां आंत विच्छेदन के माध्यम से लीशमैनिया के अलगाव के अनुकूल हैं।

3. नमूनाप्रसंस्करण से पहले भंडारण

प्रोसेसिंग से पहले फिक्सेशन की पाँच मुख्य विधियाँ हैं:

3.1. जमना

यह विधि -20°C या, अधिमानतः, -80°C पर सर्वोत्तम रूप से की जाती है। ये भंडारण विधियाँ अब तरल नाइट्रोजन भंडारण की तुलना में अधिक व्यापक रूप से उपयोग की जाती हैं। सभी मामलों में, नमूनों को बेहोश करने के बाद जितनी जल्दी हो सके क्रायोप्रिजर्वेशन लागू किया जाना चाहिए। फ्रीजर में ठंडे भंडारण का लाभ यह है कि इसमें कीटों के साथ-साथ RNA, DNA और प्रोटीन भी भंडारण अवधि के दौरान पूरी तरह से संरक्षित रहते हैं। इसके विपरीत, तरल नाइट्रोजन पंखों, पैरों, पल्प्स और एंटीना को गंभीर रूप से नुकसान पहुंचा सकता है, अक्सर उन्हें काट देता है और कभी-कभी महत्वपूर्ण रूपात्मक विशेषताओं को नष्ट कर देता है। शुष्क फ्रीजर भंडारण नमूनों के लिए कम हानिकारक है, लेकिन उनके नाजुक अंगों को संरक्षित करने के लिए आदर्श नहीं है। महत्वपूर्ण बात यह है कि पिघलने के समय, पंख, एंटीना, पल्प्स या पैर शीशियों से चिपक सकते हैं और संघनन के कारण अंततः फट सकते हैं। हालांकि, क्षेत्र अध्ययनों में फ्रीजिंग द्वारा संरक्षण हमेशा संभव नहीं होता है क्योंकि इसके लिए फ्रीजर या तरल नाइट्रोजन कंटेनर की आवश्यकता होती है। फ्रीजर में भंडारण करने से संवेदनशीलता में कोई कमी आए बिना आणविक उपकरणों का उपयोग करके रोगजनकों का पता लगाना पूरी तरह से संभव है, हालांकि RNA वायरस का पता लगाने और उसे अलग करने के लिए -80°C पर फ्रीज करना या लंबे समय तक भंडारण के लिए तरल नाइट्रोजन में फ्रीज करना आवश्यक है। हालांकि, नमूनों को फ्रीज करने से आंत के विच्छेदन द्वारा लीशमैनिया को अलग करना संभव नहीं है, सिवाय इसके कि यदि बालू मक्खियों को पहले वाष्प अवस्था में और फिर तरल नाइट्रोजन में डुबोया जाए (उदाहरण के लिए, एक मोजे में रखी शीशियों में), जो लीशमैनिया के क्रायोप्रिजर्वेशन का अनुकरण करता है।

This is an Open Access article distributed under the terms of the Creative Commons Attribution License https://creativecommons.org/licenses/by/4.0 which permits unrestricted use, distribution, and reproduction in any medium, provided the original work is properly cited.

3.2. अल्कोहल (इथेनॉल या आइसोप्रोपिल अल्कोहल) में भंडारण

बालू मक्खियों को संग्रहित करने का यह संभवतः सबसे व्यापक रूप से प्रयुक्त तरीका है। प्रयोगशाला की अनुपलब्धता के बावजूद, कठिन परिस्थितियों में भी इसे क्षेत्र में आसानी से लागू किया जा सकता है। अल्कोहल में संरक्षण विशेष रूप से आकारिकी अध्ययनों के लिए उपयुक्त है, क्योंकि यदि भंडारण ट्यूब में हवा के बुलबुले न हों तो नाजुक अंग (पंख, पैर, एंटीना या पल्प) अक्षुण्ण रहते हैं। इसलिए, हम ट्यूब को रुई के छोटे गोले से सील करने की सलाह देते हैं ताकि हवा के बुलबुले निकल जाएं और रुई के प्लग के ऊपर एक लेबल लगा दें (चित्र 1)। अल्कोहल की उपयुक्त सांद्रता अभी भी बहस का विषय है। सामान्यतः, 70% से कम सांद्रता की अनुशंसा नहीं की जाती है [45, 66]। उच्च सांद्रता DNA को अधिक प्रभावी ढंग से और लंबे समय तक संरक्षित करती है, लेकिन आकारिकी अध्ययनों के लिए नमूनों को अधिक नाजुक और भंगुर बना देती है। 96% इथेनॉल (एज़ियोट्रोप मिश्रण) का उपयोग समय के साथ सांद्रता स्थिरता सुनिश्चित करता है, विशेष रूप से उष्णकटिबंधीय देशों जैसे आर्द्र क्षेत्रों में, हालांकि 95% इथेनॉल अक्सर आसानी से प्राप्त हो जाता है। सांद्रता चाहे जो भी हो, DNA आमतौर पर इथेनॉल में अच्छी तरह संरक्षित रहता है (हालांकि फ्रीजिंग विधियों की तुलना में कम प्रभावी, विशेष रूप से एनजीएस-प्रकार की आणविक विधियों के लिए)। प्रोटीन बहुत कम स्थिर होते हैं, खासकर प्रोटिओमिक्स के लिए, जैसे कि MALDI-TOF अनुप्रयोग। कुछ महीनों तक अल्कोहल में संरक्षित बालू मक्खी की पहचान अभी भी आकारिकी के आधार पर की जा सकती है, लेकिन इन नमूनों से संदर्भ प्रोटीन स्पेक्ट्रा (reference protein spectra) उत्पन्न करना असंभव है। यदि नमूने को -20°C पर फ्रीज भी किया जाए तो अल्कोहल या शुष्क परिस्थितियों में भंडारण में सुधार किया जा सकता है। -20°C पर फ्रीज करने से मुख्य रूप से आणविक संरक्षण (जैसे, न्यूक्लिक एसिड) में सुधार होता है क्योंकि इससे क्षरण धीमा हो जाता है और समय के साथ ऊतक के टूटने को कम करके आकारिकी संरक्षण का भी लाभ मिलता है, हालांकि आकारिकी पर प्रभाव आणविक अखंडता की तुलना में सीमित होता है। इथेनॉल में भंडारण का उपयोग DNA और RNA वायरस का पता लगाने के लिए भी किया जा सकता है, जब कम से कम 70% सांद्रता वाले इथेनॉल का उपयोग कुछ महीनों से कम समय के लिए किया जाता है। इसके अलावा, आइसोप्रोपिल अल्कोहल कुछ देशों में आसानी से उपलब्ध हो सकता है और DNA को संरक्षित करता है, लेकिन इससे नमूने कठोर हो जाते हैं। यह इथेनॉल की तरह ज्वलनशील नहीं है और इसलिए इसे आसानी से परिवहन किया जा सकता है। आवश्यकता पड़ने पर, तरल नाइट्रोजन में संरक्षित या शुष्क रूप से जमाई गई बालू मक्खियों को अल्कोहल में स्थानांतरित किया जा सकता है, जिससे दोनों विधियों की कमियों का समाधान हो जाता है।

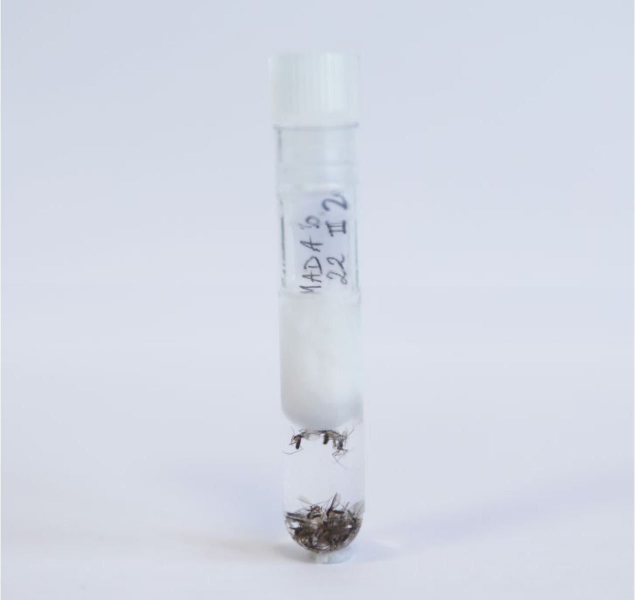

चित्र 1: बालू मक्खियों को इथेनॉल में संरक्षित किया गया।

3.3. RNA में भंडारणस्थिरीकरण समाधान (RNASS)

यह जलीय अभिकर्मक व्यापक रूप से उपयोग किया जाता है, गैर-विषाक्त है और ताजे ऊतक और कोशिका नमूनों में RNA को स्थिर और संरक्षित करने के लिए डिज़ाइन किया गया है। यह नमूने में तेजी से प्रवेश करके RNases (RNA-अपघटनकारी एंजाइम) को निष्क्रिय कर देता है, जिससे तत्काल फ्रीजिंग की आवश्यकता के बिना RNA का अपघटन रुक जाता है। RNASS में भंडारण आमतौर पर ऊतक और कोशिका की समग्र संरचना को संरक्षित करने में प्रभावी होता है, जिससे आगे के ऊतकीय विश्लेषण में मदद मिलती है। हालांकि RNASS को RNA के स्थिरीकरण के लिए अनुकूलित किया गया है, न कि उसके स्थिरीकरण के लिए, अल्प से मध्यम अवधि के भंडारण में आमतौर पर संरचनात्मक अखंडता अच्छी तरह बनी रहती है। RNASS नमूनों को कमरे के तापमान पर 7 दिनों तक, 4°C पर कई हफ्तों तक या -20°C/-80°C पर दीर्घकालिक संरक्षण के लिए संग्रहित करने की अनुमति देता है। यह विधि विशेष रूप से फील्डवर्क या नैदानिक स्थितियों में उपयोगी है जहां कोल्ड चेन अवसंरचना सीमित है। RNA निष्कर्षण के लिए आमतौर पर नमूनों को अभिकर्मक से निकालकर मानक प्रोटोकॉल के अनुसार संसाधित करना आवश्यक होता है।

3.4. कमरे के तापमान पर सूखा संरक्षण

यह एक पुरानी विधि है, जिसे संपूर्ण नमूने (पूरे रूप में माउंट किए गए) पर लागू करने पर, पंख, पैर, एंटीना और पल्प जैसे नाजुक अंगों का संरक्षण ठीक से नहीं हो पाता है। हालांकि, सिलिका जेल-प्रकार के डेसिकेंट के साथ फिक्सेशन के बाद निर्जलीकरण करने पर MALDI-ToF का उपयोग करके प्रोटिओमिक अध्ययन संभव हैं। इसके विपरीत, इन नमूनों पर DNA को लक्षित करने वाले आणविक विश्लेषण करना कठिन रहता है, क्योंकि DNA अक्सर खंडित और कम मात्रा में होता है, जिसका अर्थ है कि विश्लेषण ताजे या जमे हुए नमूनों की तुलना में

This is an Open Access article distributed under the terms of the Creative Commons Attribution License https://creativecommons.org/licenses/by/4.0
which permits unrestricted use, distribution, and reproduction in any medium, provided the original work is properly cited.

अधिक चुनौतीपूर्ण रहते हैं, विशेष रूप से नाभिकीय जीनोम के लिए। हालांकि, म्यूज़ियोमिक्स जैसी हाल की तकनीकों का उपयोग इस प्रकार के नमूनों पर किया जा सकता है [34]। इसलिए, इस भंडारण विधि की अनुशंसा नहीं की जाती है, जब तक कि कोई विकल्प उपलब्ध न हो। ट्यूबों को -20°C या -80°C पर फ्रीजर में रखकर इसे कोल्ड स्टोरेज के साथ जोड़ा जा सकता है। मुख्य चुनौती नमूनों या पहचान के लिए आवश्यक शरीर के अंगों को उचित रूप से माउंट करना है। इसे प्राप्त करने के लिए, पुनर्जलीकरण आवश्यक है। हम Triton X-100 के घोल का उपयोग करने की सलाह देते हैं। पुनर्जलीकरण की अवधि कुछ घंटों से लेकर कई दिनों तक हो सकती है, जिसके लिए नियमित रूप से गहन निगरानी आवश्यक है। पूर्ण पुनर्जलीकरण के बाद, नमूनों को लगातार तीन बार पानी से धोना चाहिए।

3.5. फ़िल्टर पेपर पर संरक्षण

फ़िल्टर पेपर का मुख्य लाभ यह है कि कमरे के तापमान पर संग्रहित अविभाजित, सूखे संपूर्ण शरीर या रक्त कोशिकाओं के भीतर जीनोमिक DNA की दीर्घकालिक स्थिरता बनी रहती है। फ़िल्टर पेपर छोटे कार्ड के आकार में उपलब्ध होता है, जिससे एक छोटे टेबल ड्राइवर के आकार के आयतन में सैकड़ों नमूनों को कमरे के तापमान पर संग्रहित करना संभव हो जाता है। फ़िल्टर पेपर मैट्रिक्स में संक्रामक एजेंटों को निष्क्रिय करने वाले पदार्थ मिलाए जाते हैं, जिससे नमूनों को अब जैव-खतरा नहीं माना जाता है। इससे चयनात्मक जैव-खतरा सावधानियों के बिना नमूनों का भंडारण और परिवहन संभव हो जाता है [68]।

4. नमूने का विच्छेदन

अन्य कई कीटों के विपरीत, जिनकी पहचान पूर्ण रूप से पिन किए गए प्रत्येक कीट पर देखे जा सकने वाले बाहरी लक्षणों के आधार पर की जाती है, बालू मक्खी की प्रजाति की सटीक पहचान के लिए विच्छेदन और स्लाइड-माउंटिंग द्वारा शारीरिक विशेषताओं का अध्ययन करना आवश्यक होता है। चुनी गई तैयारी और माउंटिंग प्रक्रिया चाहे जो भी हो, विच्छेदन की समान तकनीक का उपयोग किया जाता है (चित्र 2 और 3) (https://zenodo.org/records/18198006).

Triton X100 का उपयोग: गैर-आयनिक जलीय विलयन

ध्यान दें कि बढ़ती हुई प्रजातियाँ ताज़ा पकड़े गए या ठीक से संग्रहित नमूनों से संबंधित चिंताओं का सामना कर रही हैं। अधिकांश संग्राहक कीटों के नमूनों को सूखे रूप में (MALDI-ToF उपयोग के लिए) या कई वर्षों तक अल्कोहल में संरक्षित रखते हैं। दुर्भाग्य से, अल्कोहल में संरक्षण कई वर्षों तक इष्टतम नहीं रहता है, और इस तरह संरक्षित आर्थ्रोपोड्स को सूक्ष्मदर्शी परीक्षण के लिए तैयार करना बहुत मुश्किल हो जाता है। एक आम समस्या नमूनों वाले प्लास्टिक का क्षरण है, जिसके बाद अल्कोहल का वाष्पीकरण हो जाता है। दोनों ही मामलों में, हमारे पास कोई विकल्प नहीं होता क्योंकि नमूने बहुत लंबे समय तक अल्कोहल में रहते हैं या सूख जाते हैं। इसलिए, ऐसे वेटिंग एजेंटों का उपयोग करने का विचार आया जो प्रबल डिटर्जेंट न हों। TRITON X100 एक गैर-आयनिक जलीय विलयन (4-(1,1,3,3-tetramethylbutyl) phenyl-polyethylene glycol solution, या t-octylphenoxypolyethoxyethanol, polyethylene glycol tert-octylphenyl ether) के रूप में है, जिसका व्यापक रूप से कोशिकीय और आणविक जीव विज्ञान में डिटर्जेंट के रूप में उपयोग किया जाता है। यह कोशिका और नाभिकीय झिल्लियों के पारगम्यता को सक्षम बनाता है।

नीचे 0.5% जलीय विलयन में गैर-आयनिक TRITON X100 का उपयोग करने की प्रक्रिया दी गई है:

- सूखे नमूने को शुद्ध अल्कोहल से भिगो दें।
- 0.5% सांद्रता वाले TRITON X100 विलयन की आवश्यक मात्रा डालें ताकि पूरा नमूना उसमें डूब जाए।
- इस प्रक्रिया को लगभग 5 मिनट से लेकर कई दिनों तक चलने दें और नियमित रूप से निगरानी करते रहें। घोल में सभी आर्थ्रोपोड पूरी तरह से अलग हो जाने चाहिए।
- TRITON X100 घोल को हटा दें और उसके स्थान पर पोटेशियम हाइड्रोक्साइड घोल डालें।

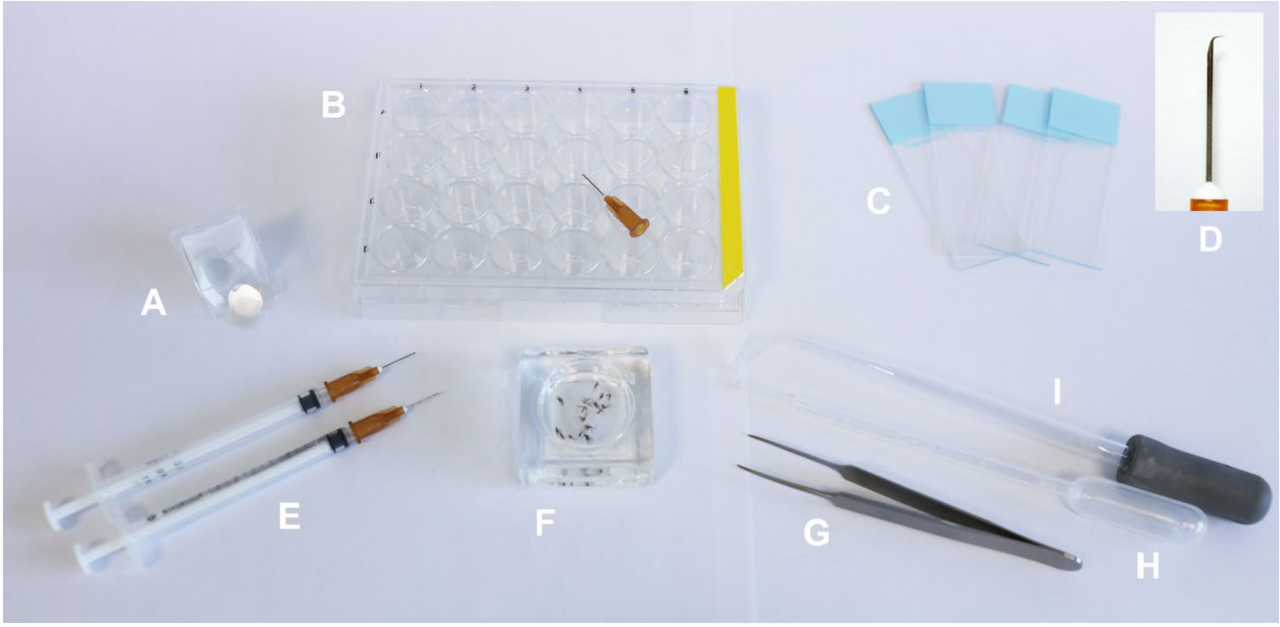

चित्र 2: बालू मक्खी को माउंट करने के लिए आवश्यक सामग्री: A: गोल कांच के कवरस्लिप (10 या 12 मिमी व्यास); B: 24-वेल प्लेट और हुक वाली सुई (यदि आप बालू मक्खी को संसाधित करने के लिए लौंग का तेल या यूपराल® एसेंस का उपयोग करते हैं, तो ऐक्रिलिक प्लेटों का उपयोग न करें क्योंकि रासायनिक प्रतिक्रिया होगी और नमूने क्षतिग्रस्त हो जाएंगे); C: कांच की स्लाइडलेबलिंग के लिए उपयुक्त; D: सुई हुक का विवरण; E: सिरिंज से जुड़ी सुइयाँ; F: माउंट किए जाने वाले बालू मक्खी को रखने के लिए वॉच ग्लास या समकक्ष कंटेनर; G: डुमोंट फोर्सेप्स; H: प्लास्टिक पिपेट; I: कुओं में तरल पदार्थ स्थानांतरित करने में सुविधा के लिए गर्म करके मोड़ा गया कांच का पिपेट।

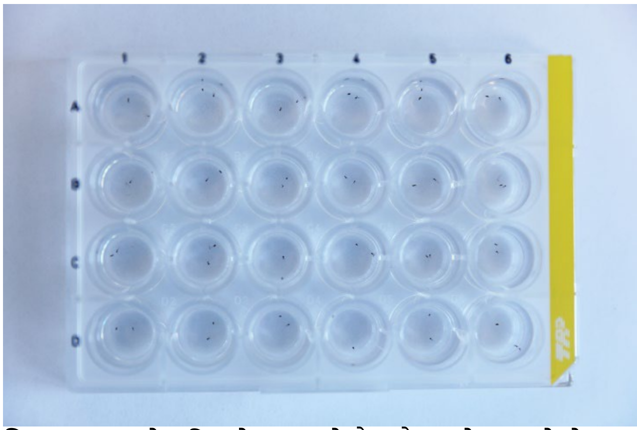

चित्र 3: एक प्लेट जिसमें 24 खांचे हैं, और प्रत्येक खांचे में बालू मक्खियों का सिर और पेट का ऊपरी भाग रखा हुआ है।

4.1. सिर

स्टीरियोमाइक्रोस्कोप के नीचे महीन सुइयों या कीटविज्ञानिक पिनों का उपयोग करके विच्छेदन किया जा सकता है (चित्र 2 और 3)। सबसे अधिक उपयोग की जाने वाली सुइयाँ हैं: 26G x 1/2" (0.45 × 13 मिमी), 30G × 1/2" (0.3 × 13 मिमी), या 25G × 5/8" (0.5 × 16 मिमी)। पहचान के लिए नमूने को तैयार करने के लिए, कम से कम, सिर को शरीर से अलग किया जाता है और सिबेरियम और ग्रसनी को प्रदर्शित करने के लिए अधर भाग ऊपर की ओर करके लगाया जाता है, जबकि वक्ष और उदर को विच्छेदन के बाद पार्श्व रूप से लगाया जाता है। सिर को अधर-पृष्ठीय स्थिति में लगाने से पश्चकपाल छिद्र ऊपर की ओर उन्मुख होता है, जिससे सिबेरियम को सीधे देखा जा सकता है। यदि सिर पूरी तरह से अलग हो तो इन शारीरिक विशेषताओं तक पहुँच आसान हो जाती है।

4.2. पंख और वक्ष

पंखों को सपाट रूप से लगाना आवश्यक है। प्रत्येक पंख को उसके आधार से अलग करके स्वतंत्र रूप से लगाया जा सकता है, या एक पंख को अकेले लगाया जा सकता है जबकि दूसरे को वक्ष से जुड़ा रहने दिया जा सकता है। यदि ज्यामितीय आकारमिति विश्लेषण की योजना है, तो लगाने से पहले दाएं और बाएं पंखों की सही पहचान और लेबलिंग करना आवश्यक है। वक्ष कई भागों में विभाजित है, और प्रत्येक भाग में महत्वपूर्ण वर्गीकरण संबंधी जानकारी होती है [20, 64]। सामान्यतः, इसे पार्श्व दृश्य में लगाया जाता है, ताकि कीटोटैक्सी और रंग वितरण की जांच की जा सके। वक्ष के कुछ क्षेत्रों में ब्रिसल्स के निशान की उपस्थिति का उपयोग Brumptomyia जीनस की कुछ प्रजातियों को अलग करने के लिए किया जा सकता है। रंग वितरण का उपयोग नियोट्रॉपिकल बालू मक्खी को जीनस स्तर (जैसे, *Bichromomyia*), प्रजाति श्रृंखला (जैसे, *Pintomyia*), या यहां तक कि एक ही जीनस की प्रजातियों (जैसे, *Micropygomyia*, *Nyssomyia*, *Psathyromyia* और

Psychodopygus) में अलग करने के लिए किया जा सकता है [20]। अतः, यदि वक्ष का उपयोग आणविक विश्लेषण के लिए नहीं किया जाता है, तो उसे क्षति से बचाने के लिए उसे सुरक्षित रूप से संजोकर रखना चाहिए। महत्वपूर्ण बात यह है कि रंगों की तीव्रता मायने नहीं रखती, बल्कि वक्ष पर उनका वितरण मायने रखता है। इसलिए, स्पष्टीकरण प्रक्रिया से रंजकता या उसका पैटर्न समाप्त नहीं होगा।

4.3. जननांग

नर और मादा दोनों में जननांगों को संजोते समय विशेष सावधानी बरतनी चाहिए, क्योंकि ये वंश, उपजनजाति और प्रजातियों की पहचान के लिए महत्वपूर्ण हैं। दोनों लिंगों में जननांग युग्मित होते हैं।

4.3.1. पुरुषों

जननांग बाह्य होते हैं और युग्मित चिमटों से बने होते हैं, जिनमें से प्रत्येक के पृष्ठीय भाग में गोनोकोक्साइट-गोनोस्टाइल जोड़ और अधर भाग में एपैंड्रियल लोब होता है। गोनोस्टाइल पर कांटे और कभी-कभी बाल (setae) होते हैं, जिनकी गणना की जा सकती है और जिनके सम्मिलन की स्थिति स्पष्ट रूप से दिखाई देनी चाहिए। गोनोकोक्साइट की आंतरिक सतह का सावधानीपूर्वक निरीक्षण करना महत्वपूर्ण है, जिस पर स्थिर बालों का गुच्छा या लोब (= ट्यूबरकल) द्वारा धारण किए गए बाल हो सकते हैं [22]। विच्छेदन में कम अनुभव वाले सहकर्मी जननांगों को उदर के सिरे से अलग किए बिना एक साधारण पार्श्व (lateral) माउंटिंग कर सकते हैं (https://zenodo.org/records/18311158)। इस स्थिति में, जननांगों के दो भागों के एक दूसरे के ऊपर होने से गोनोकोक्साइट के आंतरिक बालों की गणना करना मुश्किल हो सकता है, लेकिन इससे असफल विच्छेदन के कारण जननांगों को नुकसान होने से बचा जा सकता है। अधिक अनुभवी सहकर्मी जननांगों को दो भागों में खोलने का प्रयास कर सकते हैं। इसके लिए, सुई के नुकीले सिरे (इंट्राडर्मल रिएक्शन नीडल प्रकार) को जननांगों के बीच से गुजारना होगा, जिससे जननांग पूरी तरह से काटे बिना अलग हो जाएं और गोनोकोक्साइट-गोनोस्टाइल संयोजन अलग हो जाएं (https://zenodo.org/records/18311158)। इस तरह, उनके आंतरिक भाग का अवलोकन आसान हो जाएगा। यह संयोजन पैरामीयर और पैरामेरल शीथ के अवलोकन को भी सुगम बनाता है, जो अब एक दूसरे पर नहीं चढ़ते। पार्श्व माउंटिंग के लिए, जो अंगों के सुपरपोजिशन को बढ़ावा देता है, नमूनों को पूरी तरह से साफ किया जाना चाहिए।

4.3.2. महिलाओं

जननांग तंत्र आंतरिक होता है और शुक्राणुकोशिकाओं से बना होता है। विच्छेदन के अभाव में, इन्हें त्वचा के माध्यम से और पेट को अधर स्थिति में रखकर ही देखा जा सकता है। चुने गए माउंटिंग माध्यम के बावजूद, शुक्राणुकोशिकाओं को आमतौर पर सही ढंग से देखा जा सकता है, विशेषकर यदि वे चिकनी और स्पष्ट न हों। हालांकि, कम अपवर्तक माध्यमों में चिकनी, पतली दीवारों वाली शुक्राणुकोशिकाओं का अवलोकन करना मुश्किल हो सकता है। इसके अलावा, शुक्राणुकोशिका नलिकाओं के आधार का अवलोकन प्रजातियों की पहचान के लिए आवश्यक है, जैसे कि उपजाति Larroussius [35, 37, 38] में, जो प्राचीन विश्व में लीशमैनिया इन्फेंटम के मुख्य वाहक हैं। इस अवलोकन के बिना, नमूने की पहचान असंभव रहती है। इन अवलोकन संबंधी कठिनाइयों को दूर करने के लिए, जननांग फुर्का-शुक्राणुकोशिका माउंटिंग को पेट से हटा देना चाहिए (https://zenodo.org/records/18311106)। विच्छेदन के दौरान शुक्राणुकोशिकाओं का अवलोकन करना आमतौर पर कठिन होता है, लेकिन जननांग फुर्का को ढूंढना अपेक्षाकृत आसान होता है। क्योंकि शुक्राणु नलिकाएं जननांग के भीतरी भाग में खुलती हैं, इसलिए इस भीतरी भाग को अलग करने से सामान्यतः शुक्राणुकोशिकाओं को अलग किया जा सकता है। यदि प्रक्रिया के दौरान शुक्राणुकोशिकाएं गलती से कट भी जाएं, तो वे नष्ट नहीं होतीं और उन्हें पेट के बाहरी आवरणों के भीतर देखा जा सकता है (चित्र 4)।

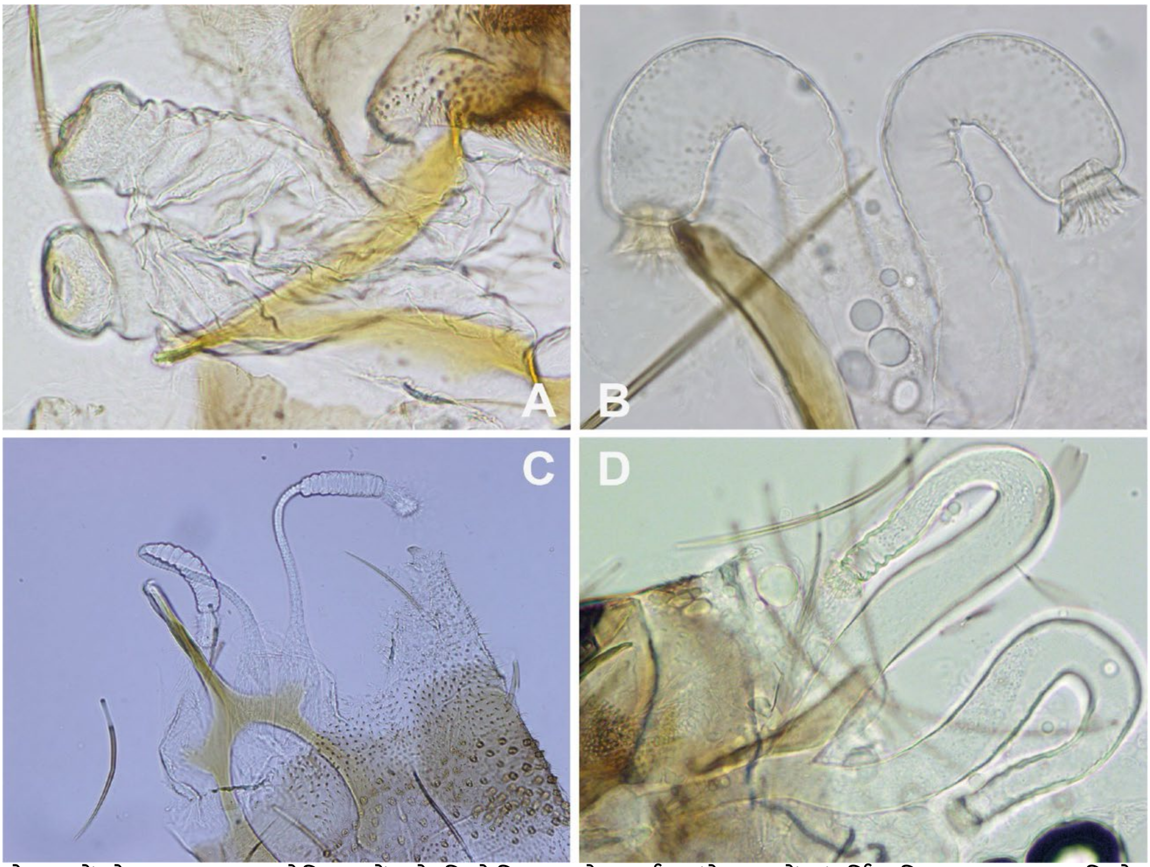

चित्र 4: ताजे नमूनों से प्राप्त शुक्राणुकोशिकाओं को विच्छेदित करके मार्क-आंद्रे द्रव में संवर्धित किया गया। A: इडियोफ्लेबोटोमस लॉन्गिफोर्सेप्स (*Idiophlebotomus longiforceps*) (लाओ पीडीआर); B: सर्जेन्टोमिया मिनुटा (*Sergentomyia minuta*) (फ्रांस); C: फ्लेबोटोमस एरियासी (*Phlebotomus ariasi*) (फ्रांस); D: सर्जेन्टोमिया एनोडोंटिस (*Sergentomyia anodontis*) (लाओ पीडीआर)।

4.4. मध्य आंत विच्छेदन के लिएलीशमैनिया एकांत

मादा बालू मक्खी में लीशमैनिया का पता लगाने और उसे अलग करने के लिए पाचन तंत्र का विच्छेदन आवश्यक है। इस प्रक्रिया को क्षेत्र और प्रयोगशाला दोनों जगहों पर किया जा सकता है, ताकि वाहक क्षमता का आकलन किया जा सके।

हाल ही में इच्छामृत्यु दी गई मादाओं पर काम करने की सलाह दी जाती है। अतिरिक्त बालों को हटाने के लिए मादाओं को पानी या हल्के डिटर्जेंट युक्त खारे घोल से धो लें। यह चरण लीशमैनिया को अलग करने के लिए रोगाणुहीन स्थितियों को बनाए रखने में मदद करता है, साथ ही पहचान के लिए आवश्यक रूपात्मक विशेषताओं को भी संरक्षित रखता है। लीशमैनिया को खोजने और अलग करने के लिए, आंत के मध्य भाग को सावधानीपूर्वक निकालें और इसे एक बूंद रोगाणु रहित खारे घोल (0.9% NaCl) में रखें। प्रकाश सूक्ष्मदर्शी (अनुशंसित आवर्धन: ~200×) के नीचे गतिशील परजीवियों का अवलोकन करने के बाद, उन्हें संवर्धन माध्यम में स्थानांतरित करने के लिए इंसुलिन सिरिंज या माइक्रोपिपेट का उपयोग करें (अधिक जानकारी के लिए अध्याय 4.4.3 देखें)।

सिर और जननांगों को मार्क-आंद्रे द्रव में सीधे डुबोकर साफ करें। महत्वपूर्ण: मार्क-आंद्रे द्रव को कभी भी लीशमैनिया के संपर्क में न आने दें – न तो सीधे और न ही औजारों या सुइयों के माध्यम से – क्योंकि यह परजीवियों के लिए घातक होता है।

मादा बालू मक्खी का विच्छेदन एक स्लाइड या दो स्लाइड पर किया जा सकता है; दोनों विकल्पों के अपने फायदे और सीमाएं हैं (चित्र 5)।(https://zenodo.org/records/18311154).

This is an Open Access article distributed under the terms of the Creative Commons Attribution License https://creativecommons.org/licenses/by/4.0 which permits unrestricted use, distribution, and reproduction in any medium, provided the original work is properly cited.

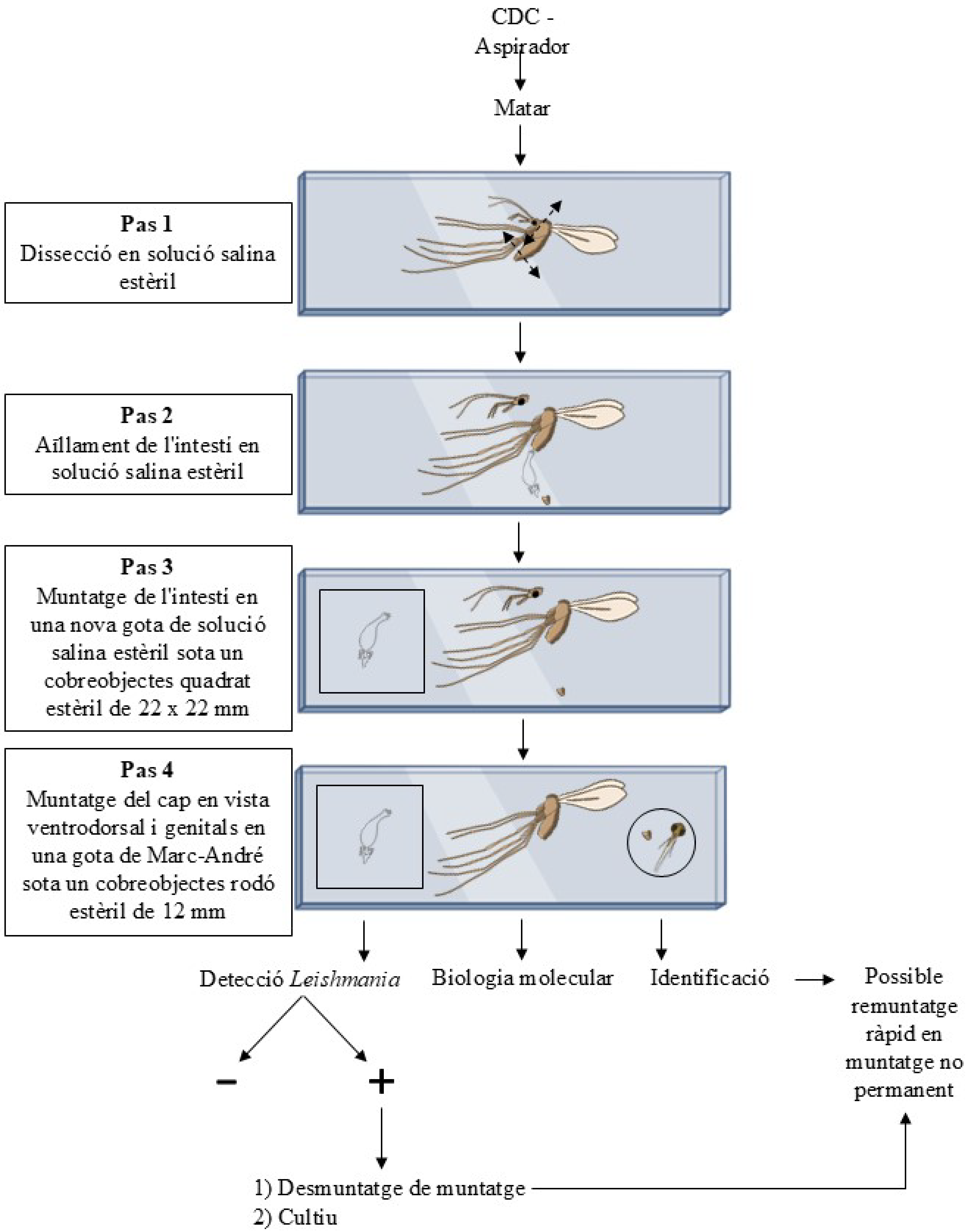

चित्र 5: लीशमैनिया को अलग करने की विधि।

This is an Open Access article distributed under the terms of the Creative Commons Attribution License https://creativecommons.org/licenses/by/4.0 which permits unrestricted use, distribution, and reproduction in any medium, provided the original work is properly cited.

4.4.1. दो-स्लाइड विधि

पहले विकल्प में दो अलग-अलग स्लाइडों पर काम करना शामिल है: एक में आंत के मध्य भाग को निकालने के लिए रोगाणु रहित खारा घोल होता है, और दूसरी में सिर और शुक्राणुकोशों को मार्क-आंद्रे द्रव में संवर्धित किया जाता है। हालांकि, क्षेत्र में, आमतौर पर दो या तीन लोग बालू मक्खियों का विच्छेदन करते हैं और अपने विच्छेदन को एक ही शोधकर्ता को सौंपते हैं जो प्रजाति की पहचान और आंत में लीशमैनिया संक्रमण के आकलन के लिए जिम्मेदार होता है। दो स्लाइडों का प्रबंधन करने से नमूने की ट्रेसबिलिटी में समस्याएँ आ सकती हैं और विशेष रूप से, यदि आंत में संक्रमण पाया जाता है, तो यह निश्चित रूप से निर्धारित करना मुश्किल हो जाता है कि कौन सी रेत मक्खी संक्रमित थी (https://zenodo.org/records/18311154).

4.4.2. एकल-स्लाइड विधि

एक ही स्लाइड का उपयोग करने से परिणामों की ट्रेसबिलिटी सुनिश्चित होती है। हालांकि, कई सावधानियां बरतनी चाहिए। इस चरण के दौरान अधिकतम रोगाणुहीनता सुनिश्चित करने के लिए, ऑपरेटरों को नियमित रूप से हाइड्रोअल्कोहलिक जेल से अपने हाथ साफ करने चाहिए। नॉन-फ्रॉस्टेड स्लाइड और वर्गाकार कवरस्लिप (22 x 22 मिमी) जिन्हें एल्युमिनियम फॉयल में लपेटकर शुष्क ऊष्मा (पौपिनेल ओवन का उपयोग करके) द्वारा रोगाणुरहित किया गया हो, का उपयोग किया जाना चाहिए, साथ ही प्रत्येक विच्छेदन के लिए रोगाणुरहित सुइयों का उपयोग किया जाना चाहिए (सुझाव: 25G Ø 0.5 मिमी × 16 मिमी)। बालू मक्खी को स्लाइड के मध्य में रोगाणुरहित खारे पानी की एक बूंद में रखा जाता है। पाचन तंत्र को काटे बिना, छठे और सातवें उदर टेरगाइट्स और स्टर्निट्स के बीच एक चीरा लगाते हुए सिर को अलग किया जाता है (यदि बहुत लंबे शुक्राणुकोशिका की उम्मीद हो तो ऊपर की ओर चीरा लगाया जा सकता है)। फिर, वक्ष को एक सुई से स्थिर किया जाना चाहिए, और आंत को निकालने के लिए अंतिम पश्च उदर खंडों को दूसरी सुई से धीरे से खींचा जाना चाहिए। यदि यह तरीका विफल हो जाता है, तो पेट के सिरे को सुई से बंद करके पाचन तंत्र को उसके अग्र भाग से खींचकर निकाला जा सकता है। यदि यह भी विफल हो जाता है, तो आंत को उसके चारों ओर बचे हुए आवरण को जितना संभव हो सके हटाकर निकालना होगा। आंत निकालने के बाद, पाचन तंत्र को काटकर पेट के अंतिम खंडों को अलग करना होगा। फिर आंत को स्लाइड के एक तरफ रखे गए रोगाणु रहित खारे पानी की एक नई बूंद में रखा जाता है, और फिर धीरे से एक रोगाणु रहित कवरस्लिप से ढक दिया जाता है। सिर और पेट के अंतिम खंडों को स्लाइड के दूसरे सिरे पर रखे गए मार्क-आंद्रे तरल की एक छोटी बूंद में स्थानांतरित किया जाता है, यह सुनिश्चित करते हुए कि उनका लीशमैनिया से कोई संपर्क न हो। सिर को सही दिशा में रखा जाता है (ऑक्सीपिटल फोरामेन ऊपर की ओर), और शुक्राणुकोषों को जननांग फुर्का के साथ ऊपर बताए अनुसार अलग किया जाता है और एक छोटी गोल कवरस्लिप (व्यास 12 मिमी, रोगाणु रहित वर्गाकार कवरस्लिप से भ्रमित न हों) से ढक दिया जाता है। बालू मक्खी का बचा हुआ शव और पंख स्लाइड के मध्य में खारे पानी की बूंद में रहते हैं (https://zenodo.org/records/18311154)। सकारात्मक परिणाम मिलने पर, या वर्गीकरण संबंधी अन्वेषण के लिए, वक्ष और उदर को आणविक या प्रोटिओमिक अध्ययन के लिए संरक्षित किया जा सकता है, और पंखों को जलीय माध्यम में माउंट किया जा सकता है। माउंट को संरक्षित करने के लिए, मार्क-आंद्रे तरल की अतिरिक्त मात्रा को क्लोरल गम (=होयर) या पॉलीविनाइल अल्कोहल-आधारित माध्यम जैसे जलीय माउंटिंग माध्यम से बदला जा सकता है।

इन प्रक्रियाओं को प्रदर्शित करने वाले विस्तृत वीडियो उपलब्ध हैं (रेत मक्खी की मध्य आंत का विच्छेदन: https://zenodo.org/records/18303014 और रेत मक्खी की लार ग्रंथियों का विच्छेदन: https://zenodo.org/records/18302850), इसलिए यहां उनकी व्याख्या नहीं की जाएगी।

4.4.3. *लीशमैनिया*बालू मक्खी की आंतों से परजीवी का पृथक्करण और संवर्धन

संक्रमित मादा बालू मक्खी के विच्छेदन से परजीवी पृथक्करण एक नाजुक प्रक्रिया है जिसके लिए उच्च कौशल की आवश्यकता होती है और प्रारंभ में इसका अभ्यास पहले परजीवी-मुक्त नमूनों पर किया जाना चाहिए। विच्छेदन के बाद, आंतों को धोने के लिए रोगाणु रहित खारे पानी (0.9%) या लॉक के घोल (Locke's solution) की एक नई बूंद में स्थानांतरित किया जाता है [4]। विच्छेदित आंतों को फिर दो तरीकों से संसाधित किया जा सकता है: i) लीशमैनिया प्रोमास्टिगोट्स के विभिन्न चरणों और उनके स्थान का निरीक्षण करने के लिए प्रकाश सूक्ष्मदर्शी के नीचे जांच करना, विशेष रूप से स्टोमोडियल वाल्व पर ध्यान देना, और ii) प्रोमास्टिगोट्स के निकास को सुगम बनाने के लिए आंत को खोलना, जिससे उनका सामूहिक संवर्धन आसान हो जाता है [4]। क्षेत्र में संक्रामक बालू मक्खी का मिलना अपेक्षाकृत दुर्लभ घटना है और इसलिए अच्छे अभ्यास सत्रों से सफल पृथक्करण की संभावना अधिकतम हो जाएगी।

यदि आंत में लीशमैनिया परजीवी दिखाई दें, तो नई रोगाणुरहित सुइयों का उपयोग करें और उन्हें मुक्त करने के लिए केशिका क्रिया द्वारा कवरस्लिप के चारों ओर थोड़ी मात्रा में रोगाणुरहित खारा घोल डालें। परजीवियों को खारे घोल में छोड़ने के लिए आंत को सावधानीपूर्वक और शीघ्रता से फाड़ें। 100 μL माइक्रोपिपेट या ट्यूबरकुलिन सिरिंज का उपयोग करके, परजीवियों को एकत्र करें और उन्हें उचित रूप से लेबल किए गए कल्चर मीडियम में डालें।

कृत्रिम परिवेशीयलीशमैनिया प्रोमास्टिगोट्स की संस्कृति: पृथक परजीवियों को प्रारंभ में SNB-9 blood agar slopes or in Novy, Mc Neal, Nicolle (NNN) ठोस माध्यम [16] में बनाए रखा जाता है, जिसके ऊपर या तो बाँझ अल्फा-MEM माध्यम [16, 65] या M199 माध्यम की परत चढ़ाई जाती है, प्रत्येक में 10% ऊष्मा-निष्क्रिय बाँझ भ्रूण बछड़ा सीरम [FCS] (परजीवी वृद्धि को बढ़ाने के लिए), 1% BME विटामिन, 2% बाँझ मानव मूत्र (फिल्ट्रोपुर® सिरिंज फिल्टर का उपयोग करके निष्फल) मिलाया जाता है। एस 0.2 μm), 250 μg/mL एमिकेसिन (या 50 μg/mL जेंटामाइसिन, या एंटीबायोटिक्स और अमीनो एसिड का मिश्रण

(एल-ग्लूटामाइन 200 mM-पेनिसिलिन 10 000 U-स्ट्रेप्टोमाइसिन 10 mg/mL) [47]। तीन दिनों के बाद, यदि कोई संदूषण नहीं है, तो संस्कृतियों को ठीक से तैयार किए गए फ्रीजिंग माध्यम में निलंबित कर दिया जाता है और बाद में 1 से 2 वर्षों के लिए -80°C पर या दीर्घकालिक संरक्षण और भविष्य के प्रयोगात्मक उपयोग के लिए -196°C पर तरल नाइट्रोजन में संग्रहीत किया जाता है [7]।

4.5. लार ग्रंथियां

बालू मक्खी की लार ग्रंथियों का विच्छेदन वेक्टर-रोगजनक अंतःक्रियाओं के अध्ययन के लिए एक मूलभूत तकनीक है, विशेष रूप से फ्लेबोवायरस (जैसे, टोस्काना वायरस) जैसे आर्बोवायरस का पता लगाने के लिए [44, 75]। बालू मक्खी के छोटे आकार के कारण, इस प्रक्रिया में स्टीरियोमाइक्रोस्कोप के नीचे सटीकता की आवश्यकता होती है, जिसमें नाजुक लार ग्रंथियों को टूटने या संदूषण के बिना अलग करने के लिए महीन चिमटी या माइक्रोडिसेक्शन सुइयों का उपयोग किया जाता है (https://zenodo.org/records/18302850) [51, 61]। विश्वसनीय आणविक विश्लेषण सुनिश्चित करने के लिए ग्रंथि की अखंडता को संरक्षित करना महत्वपूर्ण है। एक बार निकालने के बाद, ग्रंथियों को समरूप बनाया जा सकता है और वायरल RNA या एंटीजन का पता लगाने के लिए RT-PCR, qPCR या इम्यूनोएसेज़ के माध्यम से परीक्षण किया जा सकता है [12]। आंत या हीमोकोएल के बजाय लार ग्रंथियों में वायरस की उपस्थिति इस बात की पुष्टि करती है कि रोगजनक ने अपनी बाह्य ऊष्मायन अवधि पूरी कर ली है और रक्तपान के दौरान संचरणीय है [71]।

बालू मक्खी की लार ग्रंथियों के छोटे आकार के कारण विच्छेदन प्रक्रिया तकनीकी रूप से चुनौतीपूर्ण होती है, जिसके लिए नमूने के क्षरण से बचने के लिए महत्वपूर्ण विशेषज्ञता की आवश्यकता होती है [1, 51]। इसके अतिरिक्त, वायरल लोड कम हो सकता है, जिसके लिए नेस्टेड PCR या हाई-थ्रूपुट सीक्वेंसिंग जैसी अत्यधिक संवेदनशील पहचान विधियों की आवश्यकता होती है [54]। संदूषण के जोखिम रोगाणु-मुक्त तकनीकों की आवश्यकता को और भी अधिक रेखांकित करते हैं। तकनीकी बाधाओं के अलावा, जैविक कारक भी पहचान की सफलता को प्रभावित करते हैं; बालू मक्खी प्रजातियों के बीच वेक्टर क्षमता भिन्न होती है, और संक्रमण दर पारिस्थितिक और मौसमी स्थितियों के साथ घटती-बढ़ती रहती है [33, 61]।

लार ग्रंथियों में वायरस का पता लगाने से संचरण जोखिमों के बारे में महत्वपूर्ण जानकारी मिलती है, जिससे लक्षित निगरानी और नियंत्रण उपायों को सक्षम बनाया जा सकता है [15]। उदाहरण के लिए, स्थानिक क्षेत्रों में बालू मक्खियों में टोस्काना वायरस की पहचान ने नैदानिक प्रोटोकॉल और सार्वजनिक स्वास्थ्य सलाहों को सूचित किया है [18]। इसके अलावा, वायरस-लार अंतःक्रियाओं का अध्ययन संचरण-अवरोधक टीकों या उपचारों के लिए नए लक्ष्य प्रकट कर सकता है [15, 18]।

बालू मक्खी की लार ग्रंथियों का उपयोग प्रतिरक्षात्मक विधियों, अधिमानतः ELISA का उपयोग करके बालू मक्खी की लार के विरुद्ध मेजबान एंटीबॉडी को मापने के लिए प्रतिजनों के स्रोत के रूप में भी किया जा सकता है। यह विधि बालू मक्खी के काटने से मेजबान के जोखिम का आकलन करने में सक्षम बनाती है, इस प्रकार वेक्टर नियंत्रण विधियों की प्रभावशीलता [25] और लीशमैनिया संचरण के जोखिम [40] के मूल्यांकन में सहायता करती है।

4.6. रक्त भोजन की पहचान

पकड़ी गई मादाओं को अलग करने के बाद, क्रॉस-संदूषण से बचने के लिए एकल-उपयोग उपकरणों का उपयोग करके उनका विच्छेदन किया जाना चाहिए। रक्त भोजन के पाचन की अवस्था का आकलन करने के लिए उनके पेट की जांच स्टीरियोमाइक्रोस्कोप के नीचे की जानी चाहिए। यह अनुशंसा की जाती है कि केवल लाल, लाल-भूरे या गहरे लाल पेट वाली मादाओं का चयन किया जाए, जिनमें अंडे बनने के कोई लक्षण न हों। पेट के ऊपरी भाग को, जिसमें शुक्राणुकोशिकाएं भी शामिल हों, निकालकर मादा की आकारिकी पहचान की जा सकती है। पेट के मुख्य भाग (शुक्राणुकोशिकाओं के बिना) को एपेंडॉर्फ® ट्यूबों में रखा जाना चाहिए और आगे के विश्लेषण तक -20°C पर संग्रहित किया जाना चाहिए। रक्त भोजन की पहचान के लिए आमतौर पर उपयोग किए जाने वाले आनुवंशिक मार्कर, जैसे कि PNOC [5, 30, 50], CytB [67], या COI [13], अच्छी तरह से स्थापित हैं और साहित्य में व्यापक रूप से वर्णित हैं; इसलिए, इस पत्र में इनका आगे विवरण नहीं दिया जाएगा (चित्र 6)। वैकल्पिक रूप से, मेजबान रक्त की पहचान के लिए, MALDI-ToF पेप्टाइड मैपिंग का उपयोग किया जा सकता है [31]। प्रयोगों से यह सिद्ध हो चुका है कि यह तकनीक रक्तपान के बाद लंबे समय तक मेज़बान के रक्त की पहचान करने में सक्षम बनाती है; इसलिए, यह एक उपयुक्त विधि है, विशेष रूप से उन मादा मक्खियों के विश्लेषण के लिए जिनमें मेज़बान के रक्त का पाचन स्पष्ट रूप से अधिक उन्नत अवस्था में होता है। नमूनों को आदर्श रूप से -20°C या 4°C पर संग्रहित किया जाना चाहिए, लेकिन कमरे के तापमान पर थोड़े समय के लिए संग्रहित नमूनों से भी अच्छे परिणाम प्राप्त किए जा सकते हैं। विश्लेषण से ठीक पहले, रक्तपान की हुई मादा मक्खी के पेट को शरीर के शेष भाग से अलग करके आसुत जल में समरूपीकृत किया जाना चाहिए। बालू मक्खी के शरीर का शेष भाग अन्य आणविक और रूपात्मक विश्लेषणों के लिए उपलब्ध रहता है। MALDI-ToF पेप्टाइड मैपिंग के लिए समरूपीकृत मिश्रण से एक भाग निकालने के बाद, शेष भाग का उपयोग मेज़बान के रक्त की पहचान की पुष्टि करने और/या लीशमैनिया प्रजाति की उपस्थिति की जांच के लिए DNA पृथक्करण हेतु किया जा सकता है। DNA-आधारित आणविक तकनीकों की तुलना में नमूना तैयार करने और विश्लेषण का कुल समय बहुत कम है।

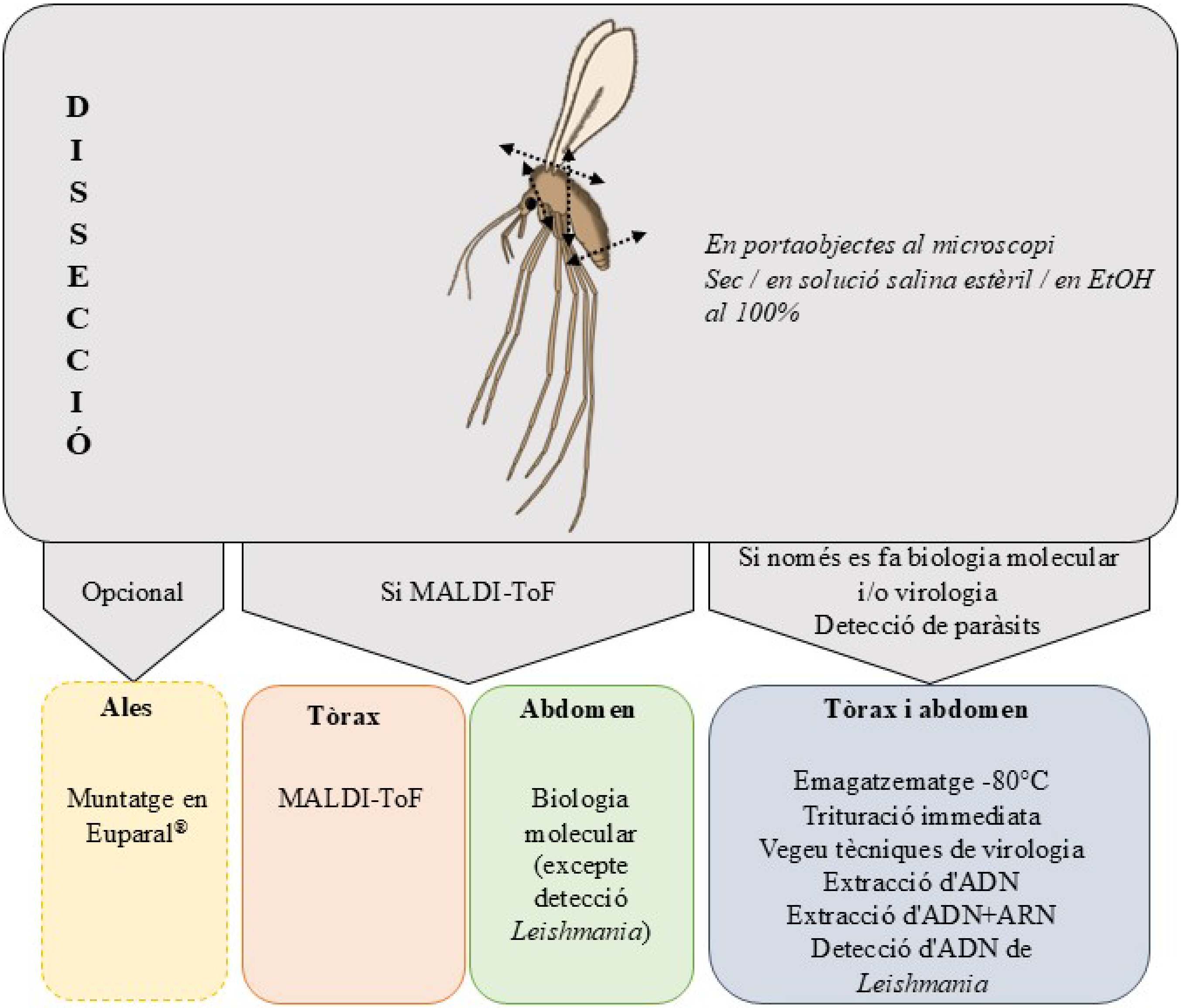

चित्र 6: आणविक जीवविज्ञान, प्रोटीओमिक्स और/या वायरोलॉजी अनुप्रयोगों के लिए बालू मक्खी का प्रसंस्करण।

5. आकारिकी अध्ययनों के लिए नमूनों का प्रसंस्करण (चित्र 3, 6, 7 और 8; परिशिष्ट 1, 2, 3 और 4)

इस खंड में आकारिकी अध्ययन के लिए बालू मक्खी के नमूने को माउंट करने हेतु तैयार करने के सिद्धांतों का वर्णन किया गया है, और इसके बाद आकारिकी से परे अन्य अनुप्रयोगों के लिए इसके अनुकूलन का भी उल्लेख है। हालांकि, इस पद्धति को समझना अत्यंत महत्वपूर्ण है, क्योंकि आवश्यकता पड़ने पर यह विशिष्ट नमूना प्रकारों के लिए प्रक्रियाओं को अनुकूलित करने में सक्षम बनाती है।

इस प्रक्रिया में लचीले रबर बल्बों से सुसज्जित पाश्चर पिपेट का उपयोग करके बार-बार खाली करने और भरने की प्रक्रिया शामिल है। गोल पेंदे वाले कांच के बर्तनों का उपयोग करने की पुरजोर सलाह दी जाती है क्योंकि इनसे ये क्रियाएं बहुत आसान हो जाती हैं। कांच सभी अभिकर्मकों के प्रति अक्रिय होता है। अभिकर्मकों के वाष्पीकरण को रोकने के लिए, बर्तनों में ढक्कन लगाना चाहिए और उन्हें कभी भी अधिक नहीं भरना चाहिए, जिससे ढक्कन खोलते या बंद करते समय वे छलक जाएं, और नमूनों पर धूल गिरने से भी रोका जा सके। शुद्धिकरण और प्रसंस्करण के लिए आवश्यक रसायनों को तालिका 2 में दर्शाया गया है।

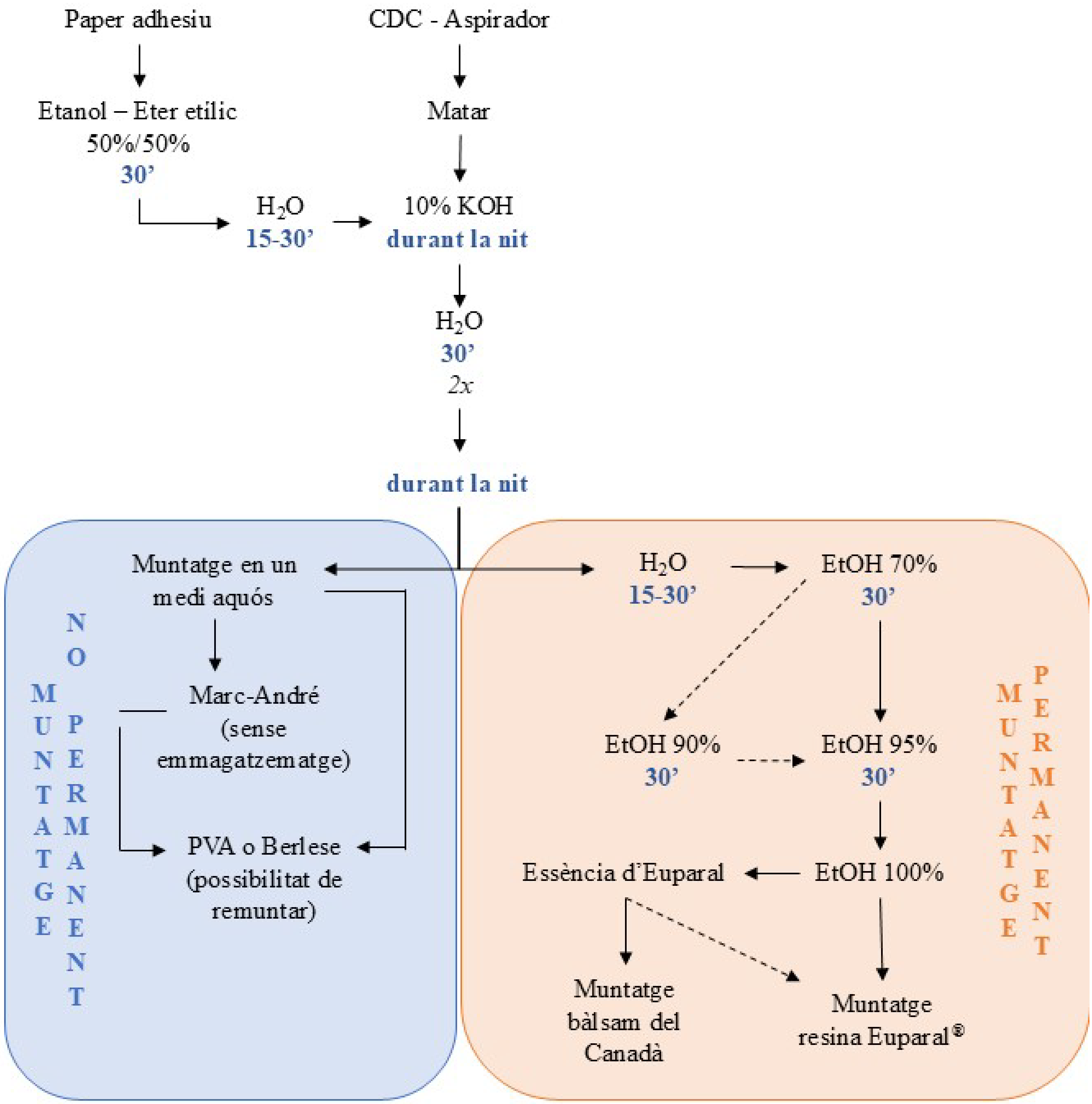

चित्र 7: बालू मक्खियों को संसाधित करने की पारंपरिक विधि।

तालिका 2:प्रयुक्त अभिकर्मकों की संरचना।

**पोटेशियम हाइड्रोक्साइड 10%**<br>पोटेशियम हाइड्रॉक्साइड 10 ग्राम<br>आसुत जल आवश्यकतानुसार 100 मिलीलीटर	**आसुत जल में 1% फुचसिन अम्ल**<br>एसिड फ्यूसिन (पाउडर के रूप में) 1 ग्राम<br>आसुत जल 99 मिलीलीटर
**गम क्लोरल माउंटिंग मीडिया (होयर मीडियम)**<br>आसुत जल 50 मिलीलीटर<br>CHLORAL HYDRATE 200 ग्राम<br>गोंद अरबी 50 ग्राम<br>ग्लिसरॉल 20 मिलीलीटर	**मार्क-आंद्रे का घोल एसिड फ्यूसिन से रंगा हुआ है**<br>मार्क-आंद्रे सॉल्यूशन 10 मिलीलीटर<br>फ्यूसिन 1% 50 μL
**मार्क-आंद्रे समाधान**<br>CHLORAL HYDRATE 40 ग्राम<br>ग्लेशियल एसिटिक एसिड 30 मिलीलीटर	**Enecê medium**<br>शुद्ध सफेद कोलोफोनी 22 ग्राम<br>अल्कोहल में घुलनशील कोपल गम 12 ग्राम<br>शुद्ध इथेनॉल 20 मिलीलीटर<br>कपूर 10 ग्राम

5.1. क्लियरिंग

बालू मक्खी के नमूनों को स्थायी स्लाइड माउंट के रूप में तैयार करने से पहले, उन्हें उपयुक्त विधि और समाशोधन एजेंट (जैसे, 10% एसिटिक एसिड घोल या CHLORAL HYDRATE युक्त मार्क-आंद्रे घोल, जो कई देशों में प्रतिबंधित रसायन है) का उपयोग करके मैसरेशन द्वारा शुद्ध किया जाना आवश्यक है ताकि वे पारदर्शी हो सकें। इस प्रक्रिया से शरीर के ऊतक, वसा, स्राव और मोम हट जाते हैं, जिससे नमूना पारभासी हो जाता है और बाह्य कंकाल संरचनाओं (जैसे, सेटे सम्मिलन), सतही विशेषताओं (जैसे, रंग) और टेगुमेंट के माध्यम से दिखाई देने वाली आंतरिक विशेषताओं (जैसे, स्पर्मेथेका) की जांच में आसानी होती है।

दो चरणों वाली शुद्धिकरण प्रक्रिया, जिसमें पहले एक प्रबल क्षार (जैसे पोटेशियम हाइड्रॉक्साइड) और फिर एक दुर्बल अम्ल (जैसे मार्क-आंद्रे विलयन में एसिटिक अम्ल) का उपयोग किया जाता है, विशिष्ट जैवरासायनिक उद्देश्यों को पूरा करती है [74]। क्षार, साबुनीकरण और प्रोटीन विकृतीकरण के माध्यम से प्रोटीन, वसा और मांसपेशियों जैसे कोमल ऊतकों को तोड़ता है, जिससे संरचनात्मक स्पष्टता के लिए काइटिन बाह्यकंकाल बरकरार रहता है। इसके बाद, दुर्बल अम्ल किसी भी शेष क्षार को निष्क्रिय कर देता है, जिससे आगे का क्षरण रुक जाता है, और काइटिन को विरंजित करके पारदर्शिता बढ़ाता है [74], हालांकि नमूनों को आसुत जल में दो बार 15 मिनट तक धोना भी क्षार को निष्क्रिय करने के लिए पर्याप्त हो सकता है। यह अनुक्रमिक उपचार प्रभावी ऊतक निष्कासन को सौम्य संरक्षण के साथ जोड़ता है, जिससे सूक्ष्मदर्शी परीक्षण के लिए नमूने की इष्टतम अखंडता सुनिश्चित होती है। अगले चरण पर आगे बढ़ने से पहले आसुत जल से दो बार 20 मिनट तक कुल्ला करने की सलाह दी जाती है।

5.1.1. नरम ऊतक का गलना (चित्र 8)

सोडियम हाइड्रॉक्साइड (NaOH) या पोटेशियम हाइड्रॉक्साइड (KOH) आमतौर पर इस्तेमाल होने वाले रासायनिक मैसरेटिंग एजेंट हैं, जिन्हें नमूनों के आकार और नाजुकता के आधार पर अलग-अलग सांद्रता और अवधि में लगाया जाता है। मानक और सबसे प्रभावी तकनीक में बालू मक्खियों को रात भर एक मजबूत क्षार (10% KOH या NaOH) में भिगोकर नरम ऊतकों को लाइस करना शामिल है। उपचार की अवधि कम करने के लिए सांद्रता को बढ़ाया जा सकता है (जैसे, 20% KOH 6 घंटे के लिए), साथ ही 37°C पर गर्म भी किया जा सकता है।

5.1.2. रंगाई के साथ या उसके बिना स्पष्टीकरण

इस चरण के बाद प्रकाश को हल्का करने की प्रक्रिया की जाती है, जिसमें आमतौर पर एसिटिक एसिड और chloral hydrate (जैसे, मार्क-आंद्रे घोल) का संयोजन किया जाता है। प्रकाश को साफ करने के बाद, अवशिष्ट रसायनों को हटाने के लिए नमूनों को कम से कम दो बार 20-20 मिनट के लिए पानी में अच्छी तरह से धोना आवश्यक है।

मार्क-आंद्रे सॉल्यूशन, बालू मक्खी के नमूनों को तैयार करने के लिए आमतौर पर इस्तेमाल किया जाने वाला एक सफाई एजेंट है। इसकी प्रभावशीलता सफाई प्रक्रिया को आसान बनाने और पंखों और एंटीना जैसी नाजुक संरचनाओं को कम से कम नुकसान पहुंचाने में निहित है।

घोल को ताज़ा तैयार किया जाना चाहिए या वाष्पीकरण या क्षरण को रोकने के लिए इसे कसकर बंद कंटेनर में संग्रहित किया जाना चाहिए। मार्क-आंद्रे घोल का उपयोग विशेष रूप से तब लाभदायक होता है जब इसे चमकाने या रंगने की तकनीकों के साथ मिलाकर विशिष्ट रूपात्मक विवरणों को निखारने के लिए

उपयोग किया जाता है। इसकी संरचना और तैयारी का विवरण परिशिष्ट 2 में दिया गया है।

अत्यधिक पारदर्शी नमूनों के लिए, माउंटिंग से पहले दृश्यता में सुधार के लिए स्टेनिंग आवश्यक हो सकती है। कई प्रकार के स्टेन उपलब्ध हैं, जिनमें से प्रत्येक जीव के विशिष्ट रासायनिक घटकों को लक्षित करता है। यह महत्वपूर्ण है कि ऐसा स्टेन चुना जाए जो नमूने और चुने गए माउंटिंग माध्यम दोनों के अनुकूल हो। इस मूल कार्यप्रणाली को आवश्यकतानुसार अनुकूलित किया जा सकता है, उदाहरण के लिए, स्टेनिंग के लिए मार्क-आंद्रे घोल में 0.1% एसिड फ्यूसिन मिलाकर। इसके अतिरिक्त, जलीय घोल में संरक्षित और रेज़िनस माउंट के लिए अभिप्रेत नमूनों को निर्जलीकरण की आवश्यकता होती है (निर्जलीकरण पर अनुभाग 5.2 देखें), क्योंकि अधिकांश प्राकृतिक और सिंथेटिक रेज़िन माउंटिंग मीडिया पानी के साथ असंगत होते हैं। न्यू (1974) ने उल्लेख किया कि कुछ स्टेन कुछ माउंटिंग मीडिया में खराब हो सकते हैं [53]। उदाहरण के लिए, कनाडा बाल्सम के साथ आमतौर पर इस्तेमाल किया जाने वाला एसिड फ्यूसिन, यूपराल® में भी स्थिर किया जा सकता है। हालांकि, एसिड फ्यूसिन से रंगे नमूने फीके पड़ने के लिए प्रवण होते हैं, विशेष रूप से जब अंतिम स्पष्टीकरण द्रव के रूप में उपयोग किए जाने वाले लौंग के तेल के अवशेष रह जाते हैं। लौंग के तेल में संग्रहित नमूनों का रंग कुछ ही दिनों में काफी फीका पड़ सकता है।

5.2. निर्जलीकरण

नमूनों को धीरे-धीरे 50%, 70%, 80%, 90%, 95% और अंत में 100% सांद्रता वाले एथेनॉल के घोल में से गुजारकर निर्जलीकरण किया जाता है, प्रत्येक घोल में कम से कम 20 मिनट का समय लगता है। चूंकि एथेनॉल जल्दी वाष्पित हो जाता है, इसलिए प्रक्रिया के दौरान बर्तन को अच्छी तरह से बंद रखना चाहिए। नमूना पूरी तरह से निर्जलित हो जाने के बाद, आप कुछ दिनों के लिए Euparal® एसेंस में प्रक्रिया रोक सकते हैं, जो लौंग के तेल से बेहतर है। बीच क्रेओसोट, जिसका उपयोग कभी इस उद्देश्य के लिए व्यापक रूप से किया जाता था, अब इसकी विषाक्तता के कारण पूरी तरह से प्रतिबंधित है।

निर्जलीकरण प्रक्रिया में यह सुनिश्चित किया जाना चाहिए कि नमूने के भीतर का तरल पदार्थ माउंटिंग माध्यम के साथ संगत हो ताकि अपारदर्शिता, परासरणीय पतन या विकृति को रोका जा सके जो नमूने को वर्गीकरण संबंधी अध्ययन के लिए अनुपयुक्त बना सकती है।

5.3. माउंटिंग मीडिया

5.3.1. नमूना तैयार करने के लिए चयन और आवेदन

आदर्श रूप से, माउंटिंग माध्यम का अपवर्तनांक कांच के अपवर्तनांक के जितना संभव हो उतना निकट होना चाहिए, जो लगभग 1.5 होता है। यह रंगहीन, स्पष्ट होना चाहिए और सूखने के बाद और समय के साथ पूरी तरह से पारदर्शी बना रहना चाहिए। यह उपयोग किए गए दागों के अनुकूल होना चाहिए और नमूने के सभी ऊतकों में प्रवेश करने और फैलने में सक्षम होना चाहिए। यह बहुत जल्दी सूखना नहीं चाहिए या माउंटिंग के दौरान धुंधलापन नहीं बनाना चाहिए। माउंटिंग के बाद यह सिकुड़ना नहीं चाहिए। उपयुक्त माउंटिंग माध्यम का चयन नमूना तैयार करने का एक मूलभूत पहलू है, क्योंकि कोई भी एक माध्यम सभी उद्देश्यों के लिए आदर्श नहीं होता है। चयन में कई प्रमुख कारकों को संतुलित करना आवश्यक है:

- प्रकाशीय गुणधर्म। माउंटिंग माध्यम का अपवर्तनांक वर्गीकरण संबंधी पहचान या आकारिकी वर्णन के लिए उपयोग किए जाने वाले महत्वपूर्ण शारीरिक संरचनाओं, जैसे कि शुक्राणुकोशिका, एस्कोइड, न्यूस्टेड संवेदी अंग, ऊर्ध्वाधर सिबेरियल दांत और ग्रसनी दांतों के पर्याप्त कंट्रास्ट और अपवर्तन को सुनिश्चित करना चाहिए। इन संरचनाओं की दृश्यता सीधे माउंटिंग माध्यम के प्रकाशीय गुणधर्मों पर निर्भर करती है।

संरक्षण। स्थायी संग्रह के लिए अभिप्रेत नमूनों या सामग्रियों के लिए, माध्यम में दीर्घकालिक स्थिरता और स्थायित्व होना आवश्यक है। इसके विपरीत, सूची अध्ययन या महामारी विज्ञान सर्वेक्षणों के लिए, जहाँ दीर्घकालिक संरक्षण उतना महत्वपूर्ण नहीं होता, अस्थायी या अर्ध-स्थायी माउंटिंग मीडिया पर्याप्त हो सकता है।

5.3.2. मीडिया को माउंट करने की आवश्यकताएँ

विशेषज्ञ अक्सर विशिष्ट शोध आवश्यकताओं के अनुरूप अनुकूलित और जटिल माउंटिंग तकनीक विकसित करते हैं। हालांकि, इन विधियों में अक्सर अभिलेखीय गुणवत्ता, अनुकूलता, मानकीकरण, या सुगम संचालन और दीर्घकालिक संरक्षण जैसे पहलुओं की अनदेखी की जाती है। मानकीकरण की यह कमी दान किए गए संग्रहों के एकीकरण और दीर्घकालिक संरक्षण प्रयासों को जटिल बना देती है।

वैज्ञानिक अनुप्रयोगों के लिए माउंटिंग मीडिया की विशिष्ट आवश्यकताएँ होती हैं। वर्गीकरण विज्ञानी अक्सर पूरे नमूनों को माउंट करते हैं और ऐसे मीडिया को प्राथमिकता देते हैं जो आंतरिक अंगों को धीरे से मैश कर दे ताकि क्यूटिकुलर संरचनाओं की दृश्यता बढ़ सके। ऑप्टिकल स्पष्टता को अधिकतम करने के लिए अपवर्तनांक नमूने और ग्लास स्लाइड के अपवर्तनांक से पर्याप्त रूप से भिन्न होना चाहिए। व्यावसायिक माउंटिंग मीडिया आमतौर पर ग्लास के अपवर्तनांक के निकट तैयार किए जाते हैं ताकि स्लाइड-माउंटिंग मीडिया-कवरस्लिप प्रणाली के माध्यम से प्रकाश के अपवर्तन और प्रकीर्णन को कम किया जा सके। हालांकि, ब्राइटफील्ड माइक्रोस्कोपी में, बिना रंगे नमूने के प्राकृतिक कंट्रास्ट को नमूने के अपवर्तनांक से थोड़ा भिन्न अपवर्तनांक वाले माउंटिंग मीडिया का चयन करके नियंत्रित किया जा सकता है, जिससे पृष्ठभूमि के सापेक्ष उसकी दृश्यता में सुधार होता है।

5.3.3. माउंटिंग मीडिया के प्रकार (तालिकाएँ)3 और 4)

सूक्ष्मदर्शी परीक्षण में, स्लाइड, माध्यम और नमूने से प्रकाश के मुड़ने का निर्धारण करने के लिए माउंटिंग माध्यम के अपवर्तनांक (RI) की आवश्यकता होती है। जब RI कवरस्लिप ग्लास (≈1.515) के साथ लगभग मेल खाता है, तो प्रकाश समान रूप से गुजरता है, जिससे प्रकीर्णन और प्रकाशीय विकृतियाँ कम हो जाती हैं, जिसके परिणामस्वरूप सूक्ष्म संरचनाओं का बेहतर रिज़ॉल्यूशन और दृश्यता प्राप्त होती है। इसके विपरीत, RI में भिन्नता के कारण धुंधलापन, प्रभामंडल या बिना रंगे हुए भाग अस्पष्ट हो सकते हैं। विभिन्न माध्यमों के अलग-अलग RI के कारण, किसी दिए गए नमूने के लिए कंट्रास्ट, स्पष्टता और समग्र छवि गुणवत्ता को अनुकूलित करने के लिए सही माउंटिंग माध्यम का चयन अत्यंत महत्वपूर्ण है।

स्लाइड माउंटिंग के लिए बालू मक्खी तैयार करते समय, माउंटिंग माध्यम का अपवर्तनांक सूक्ष्म संरचनाओं को देखने की क्षमता पर महत्वपूर्ण प्रभाव डालता है। बालू मक्खी की नाजुक और हल्की कठोर संरचनाएं, जिनमें सिबेरियल आर्मेचर, स्पर्मेथेका, एंटीना खंड और पंखों की शिराएं शामिल हैं, उच्च अपवर्तनांक वाले माउंटिंग माध्यम में देखना मुश्किल हो सकता है।

बालू मक्खियों के लिए, आमतौर पर उपयोग किए जाने वाले विकल्पों में जल-आधारित माउंटिंग मीडिया के रूप में गम-क्लोरल मीडिया और विलायक-आधारित माध्यम के रूप में कनाडा बालसम और एनेसी-नेल्सन सेर्केरा (एनसी) राल शामिल हैं। रॉलिन्स [60] ने माउंटिंग मीडिया को दो प्रकारों में वर्गीकृत किया: (1) स्थायी मीडिया: ये समय के साथ कठोर हो जाते हैं और दीर्घकालिक संरक्षण के लिए उपयुक्त होते हैं, और (2) अर्ध-स्थायी मीडिया: ये कठोर नहीं होते हैं और आमतौर पर अस्थायी उद्देश्यों के लिए उपयोग किए जाते हैं।

माउंटिंग मीडिया तरल, गोंद-आधारित या रेज़िनयुक्त हो सकता है, जो पानी, अल्कोहल या अन्य विलायकों (जैसे, टोल्यून, ज़ाइलीन) में घुलनशील होता है (तालिका 3)। एक बार लगाने के बाद, इन्हें अघुलनशील रिंगिंग मीडिया का उपयोग करके वायुमंडलीय प्रभावों से सुरक्षित किया जाना चाहिए। माउंटिंग मीडिया के प्रकारों में स्पष्ट रूप से अंतर करने के लिए, निम्नलिखित वर्गीकरण का उपयोग किया जा सकता है:

ए. जलीय माध्यम. ये माध्यम पानी में आसानी से घुल जाते हैं, जिससे ये अस्थायी या अर्ध-स्थायी माउंट के लिए उपयुक्त होते हैं। इन्हें संभालना आमतौर पर आसान होता है, लेकिन वायुमंडलीय नमी से बचाव के लिए इन्हें सील करना आवश्यक हो सकता है (जैसे कि गम-क्लोरल माध्यम और पॉलीविनाइल अल्कोहल), विशेष रूप से उष्णकटिबंधीय आर्द्र जलवायु में।

बी. सीमित जल-सहिष्णुता वाले माध्यम. ये माध्यम पानी से कम प्रभावित होते हैं, लेकिन फिर भी इन्हें अत्यधिक नमी से सुरक्षा की आवश्यकता होती है। जल-घुलनशील विकल्पों की तुलना में ये अधिक दीर्घकालिक स्थिरता प्रदान करते हैं और अक्सर अर्ध-स्थायी माउंट में उपयोग किए जाते हैं।

सी. हाइड्रोकार्बन-घुलनशील माध्यम. ये मीडिया ज़ाइलीन या टोल्यून जैसे कार्बनिक विलायकों में, या एसेनेसी (एनेसी विलायक) में घुले होते हैं। इन्हें स्थायी माउंटिंग के लिए डिज़ाइन किया गया है और ये उत्कृष्ट दीर्घकालिक स्थिरता प्रदान करते हैं, साथ ही नमी और क्षरण के प्रति प्रतिरोधी होते हैं, जिससे ये अभिलेखीय उद्देश्यों के लिए आदर्श बन जाते हैं (जैसे, तटस्थ कनाडा बाल्सम, डीपीएक्स) ।

संक्षेप में, जल में घुलनशील मीडिया अस्थायी माउंट या ऐसे केस के लिए सबसे उपयुक्त हैं जिनमें नमूने को आसानी से हटाया जा सके; सीमित जल-सहिष्णु मीडिया मध्यम स्थायित्व वाले अर्ध-स्थायी माउंट के लिए उपयुक्त हैं, और हाइड्रोकार्बन में घुलनशील मीडिया अभिलेखीय और दीर्घकालिक भंडारण के लिए बनाए गए स्थायी माउंट के लिए बेहतर हैं।

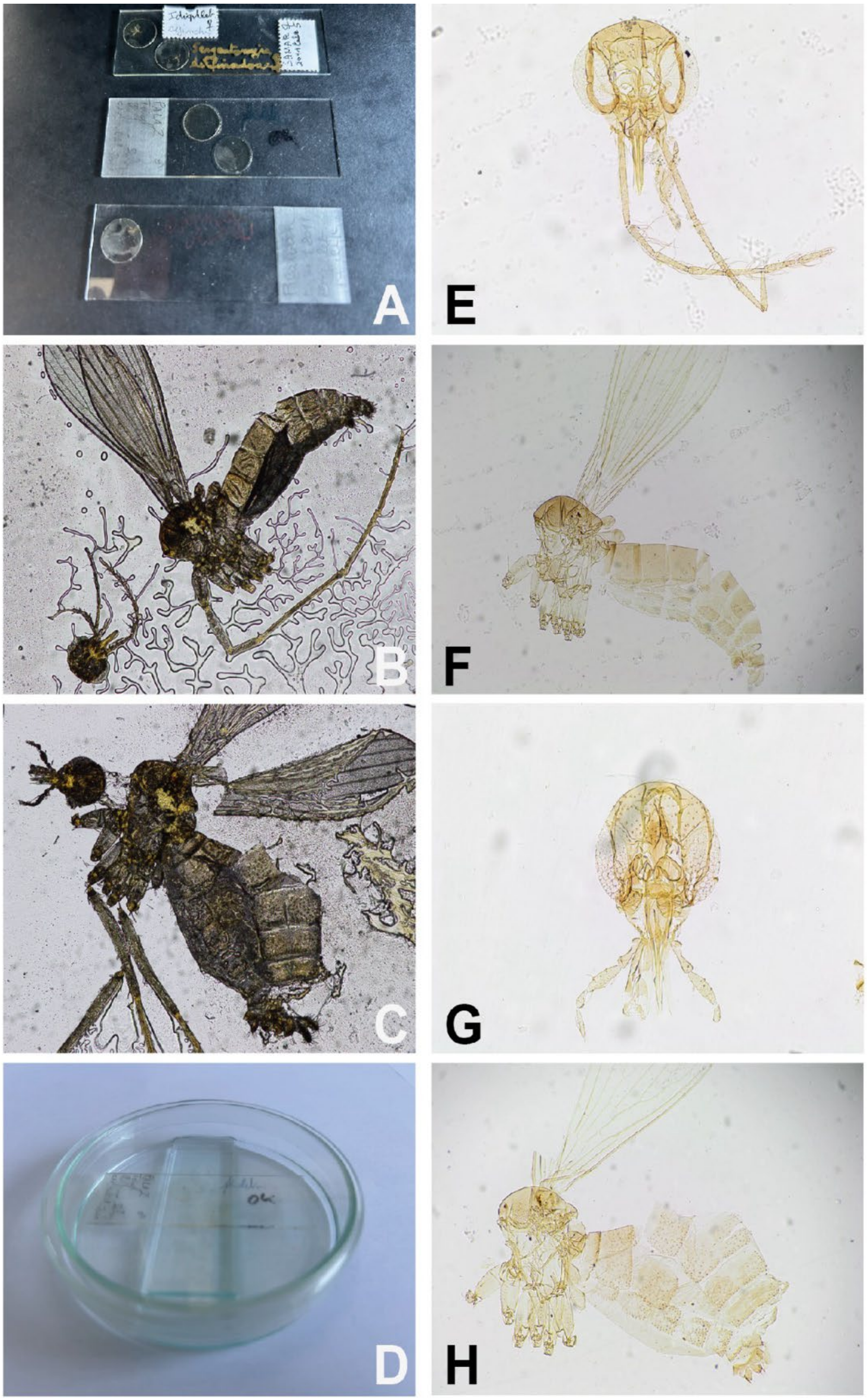

Figura 8: Remuntatge de portaobjectes. A: portaobjectes danyats i secs muntats en Hoyer; B: vista microscòpica d'un flebòtom sec; C: vista microscòpica d'un altre flebòtom danyat; D: cambra humida que conté un portaobjectes sec; E: cap i F: cos de la mostra B després del seu remuntatge en Euparal®; G: cap i H: cos de la mostra C danyat després del seu remuntatge en Euparal®.

तालिका 3: चयनित माउंटिंग मीडिया की संरचना।

माउंटिंग माध्यम	विलायक	संभावित प्री-पॉलिमर(ओं) या पॉलिमर	टिप्पणी
हॉयर = क्लोरल गम	ग्लिसरॉल, पानी	गोंद अरबी के यौगिक	मैसरेटिंग एजेंट: CHLORAL HYDRATE
सीएमसीपी-9<br><br>(= कार्बोक्सी मिथाइल सेलुलोज फिनोल)	पानी (सीएमसीपी-9: 51-60%)	पूर्णतः जल अपघटित पॉलीविनाइल अल्कोहल (सीएमसीपी-9: 0-5%)	सीएमसी(पी)-9: कम श्यानता: उच्च श्यानता
डीएमएचएफ (डाइमिथाइल हाइडेंटोइन फॉर्मेल्डिहाइड)	पानी	एन,एन'-डाइमेथिलॉल डाइमेथिल हाइडेंटोइन (डाइ-मेथिलॉल डीएमएच)<br><br>ईथर-/मेथिलीन-ब्रिज्ड ऑलिगोमर्स<br><br>क्रॉसलिंक्ड डीएमएच-फॉर्मेल्डिहाइड पॉलिमर नेटवर्क	
कनाडा बाल्सम	ज़ाइलीन; बाल्सम के आंशिक रूप से वाष्पशील घटक (Δ^3-केरेन, लेवोपिमेरिक एसिड, लिमोनेन, मिरसीन, पेलुस्ट्रिक एसिड, β-फेलैंड्रीन, α-पाइनीन, β-पीइनीन)	बाल्सम (एबीएनॉल, एबीटिक एसिड, आइसोपिमेरिक एसिड, सैंडाराकोपिमेरिक एसिड)	उदासीनीकरण: पोटेशियम कार्बोनेट;<br><br>एबिस बाल्समिया से राल (लिने, 1758)
यूपराल®	यूकेलिप्टोल, पैराल्डिहाइड; सैंडरैक गोंद के आंशिक रूप से वाष्पशील घटक (लिमोनेन, α-पाइनीन, β-पाइनीन)	सैंडारैक गम के यौगिक (कम्यूनिक एसिड, मैनूल, पॉलीकम्यूनिक एसिड, सैंडारकोपिमैरिक एसिड, 12-एसिटॉक्सी-सैंडाराकोपिमैरिक एसिड, सुगिओल, टोरुलोसिक एसिड, टोरुलोसोल, टोटारोल)	शुद्धिकरण कारक: मिथाइल सैलिसिलेट; यूपराल® ग्रीन में रंग: कॉपर लवण (कॉपर एबिएटिनेट); टेट्राक्लिनिस आर्टिकुलाटा (वाहल, 1791) से प्राप्त सैंडरैक राल
एनस	एथिल अल्कोहल; कपूर, यूकेलिप्टोल और तारपीन के अर्क के साथ	कोपल गोंद और कोलोफोनी (रोजिन) के यौगिक	
हॉयर = क्लोरल गम	ग्लिसरॉल, पानी	गोंद अरबी के यौगिक	मैसरेटिंग एजेंट: CHLORAL HYDRATE

This is an Open Access article distributed under the terms of the Creative Commons Attribution License https://creativecommons.org/licenses/by/4.0
which permits unrestricted use, distribution, and reproduction in any medium, provided the original work is properly cited.

तालिका 4: माइक्रोस्कोप स्लाइड के बारे में चयनित माउंटिंग मीडिया के फायदे और नुकसान और विभिन्न व्यक्तियों द्वारा अप्रकाशित अवलोकन [52]।

नाम	लाभ	नुकसान
कनाडा बाल्सम	यह माध्यम अत्यंत टिकाऊ है, जिसका जीवनकाल 150 वर्ष से अधिक है।	इसमें हानिकारक घटक होते हैं और इसे हुड के नीचे ही संभालना चाहिए।
	स्लाइड को माउंट करने के लिए लौंग का तेल या फिनोल का उपयोग माउंटिंग एजेंट के रूप में किया जा सकता है।	इसके लिए समय लेने वाली पूर्ण निर्जलीकरण प्रक्रिया की आवश्यकता होती है।
		इथेनॉल निर्जलीकरण और जाइलीन या लौंग के तेल के माध्यम से स्थानांतरण कुछ प्रजातियों को भंगुर बना सकता है; विकल्प (जैसे, आइसोप्रोपेनॉल, एन-ब्यूटेनॉल, सेलोसॉल्व™, 1,4-डाइऑक्सेन, हिस्टोक्लियर, टर्पिनॉल) टूटने को कम कर सकते हैं।
		यदि जाइलीन को फिनोल से बदल दिया जाए या यदि पोटेशियम हाइड्रोक्साइड का अवशिष्ट अंश शेष रह जाए तो नमूने काले पड़ सकते हैं।
		उच्च अपवर्तनांक बिना रंगी संरचनाओं को धुंधला कर सकते हैं।
		हॉट-प्लेट ड्राइंग के बिना पूरी तरह से सुखाने में वर्षों लग सकते हैं।
		समय के साथ माध्यम का रंग पीला पड़ जाता है और गहरा हो जाता है, खासकर जब इसे लौंग के तेल से साफ किया जाता है।
		यदि माध्यम अम्लीय हो जाता है - जो समय के साथ स्वतः ही हो सकता है - तो कुछ दाग कमजोर पड़ जाते हैं और धनायनिक रंग फीके पड़ सकते हैं।
डीएमएचएफ (डाइमिथाइल हाइडेंटोइन फॉर्मेल्डिहाइड)	उच्च पारदर्शिता	समय के साथ पीलापन आने की संभावना है
	अच्छा अपवर्तनांक	कुछ दागों को बदल सकता है
	संरचनाओं की उत्कृष्ट दृश्यता	फॉर्मेल्डिहाइड के प्रति संवेदनशील दागों के लिए उपयुक्त नहीं है।
	तैयारियों की स्थिरता काफी अच्छी है।	हवा के बुलबुले, सूखने में लगने वाला धीमा समय
	कई रंगाई तकनीकों के साथ संगत	माउंटिंग माध्यम नमी के प्रति संवेदनशील होता है
	नमूनों की अच्छी सुरक्षा	माउंटिंग को उलटना मुश्किल है
	स्लाइड और कवरस्लिप के बीच अच्छा आसंजन।	फॉर्मेल्डिहाइड विषैला, जलन पैदा करने वाला और कैंसरकारक होता है।

This is an Open Access article distributed under the terms of the Creative Commons Attribution License https://creativecommons.org/licenses/by/4.0
which permits unrestricted use, distribution, and reproduction in any medium, provided the original work is properly cited.

पारदर्शी (पारदर्शी)	50 वर्षों से अधिक जीवनकाल वाला टिकाऊ माध्यम।<br><br>80% इथेनॉल से सीधे माउंटिंग करना संभव है (निर्माता की अनुशंसा)।<br><br>यह बिना दाग वाली संरचनाओं को नहीं छुपाता है और समय के साथ पीला नहीं पड़ता या भंगुर नहीं होता है।<br><br>इसका अपवर्तनांक कनाडा बालसम की तुलना में डिप्टेरा के लिए अधिक उपयुक्त है।<br><br>कम सिकुड़न और बुलबुले रहित सुखाने की प्रक्रिया के कारण यह मोटे नमूनों के लिए अच्छी तरह काम करता है।<br><br>यह 95% इथेनॉल में घुलनशील रहता है, जिससे कई वर्षों बाद भी इसे दोबारा लगाया जा सकता है।	इसमें हानिकारक घटक होते हैं और इसे हुड के नीचे ही संभालना चाहिए।<br><br>इथेनॉल निर्जलीकरण और यूपरल एसेंस के माध्यम से स्थानांतरण कुछ प्रजातियों को भंगुर बना सकता है, लेकिन आइसोप्रोपेनॉल का उपयोग करने से यह समस्या कम हो सकती है।
होयर द्रव	नमूनों को जीवित अवस्था में या सीधे पानी, इथेनॉल या फॉर्मेल्डिहाइड से निकालकर माउंट किया जा सकता है।<br><br>मैसरेशन से उत्कृष्ट गुणवत्ता वाली क्यूटिकल प्राप्त होती है।<br><br>इसका अपवर्तनांक अनुकूल है और उच्च कंट्रास्ट के लिए आयोडीन स्टेनिंग से इसे बढ़ाया जा सकता है।<br><br>इस फॉर्मूले में मौजूद एसिटिक एसिड आर्थ्रोपोड के उपांगों को फैला सकता है।<br><br>कुछ नमूने 40-60 वर्षों तक स्थिर रह सकते हैं।<br><br>पानी में घुलनशील होने के कारण इसे आसानी से दोबारा लगाया जा सकता है।	यदि माध्यम को धीरे-धीरे न डाला जाए तो नाजुक पौधे मुरझा सकते हैं, जो समय लेने वाला होता है।<br><br>गुहाएं और क्रिस्टल 10 साल से भी कम समय में बन सकते हैं।<br><br>CHLORAL HYDRATE की सांद्रता और एक्सपोज़र समय के आधार पर मैसरेशन अत्यधिक हो सकता है।<br><br>माध्यम के घटक अलग हो सकते हैं, और कुछ महीनों या वर्षों के भीतर बारीक दाने दिखाई दे सकते हैं।<br><br>मीडिया को बदनाम करने की खबरें सामने आई हैं।
सीएमसीपी-9<br><br>(= कार्बोक्सी मिथाइल सेलुलोज फिनोल)	नमूनों को पानी, इथेनॉल, ग्लिसरॉल या फॉर्मेल्डिहाइड युक्त घोल जैसे माध्यमों से सीधे माउंट किया जा सकता है, और सामान्य जांच या तैयारी को सुविधाजनक बनाने के लिए आवश्यकता पड़ने पर उनके आंतरिक अंगों को मैश किया जा सकता है।	यह माध्यम समय के साथ क्रिस्टल विकसित कर सकता है और गहरा हो सकता है, और कभी-कभी यह नमूनों को अपेक्षा से अधिक गल सकता है। यदि स्लाइड को सावधानीपूर्वक घेरा न जाए, तो मोटे नमूने इसमें ठीक से नहीं टिकेंगे क्योंकि वे सिकुड़ सकते हैं और कवरस्लिप के किनारों पर अंतराल बना सकते हैं। यह रंगीन नमूनों या कैल्शियमयुक्त पदार्थों के लिए उपयुक्त नहीं है, और इसका सूखने का समय सीएमसी की तुलना में अधिक है।
यूकिट™	30 साल से अधिक समय तक चलने वाला टिकाऊ माध्यम।<br><br>यह एसीटोन, बेंजीन, क्लोरोफॉर्म, डायोक्सन, ईथर, आइसोप्रोपेनॉल, मिथाइल बेंजोएट, टर्पिनॉल, टोल्यून और जाइलीन सहित कई विलायकों के साथ संगत है।	इसमें हानिकारक घटक होते हैं और इसे हुड के नीचे ही संभालना चाहिए।<br><br>इसके लिए समय लेने वाली पूर्ण निर्जलीकरण प्रक्रिया की आवश्यकता होती है।<br><br>सिकुड़न और गैस के बुलबुले बनने के कारण यह मोटे

	यह जल्दी सूख जाता है और इसका पीएच मान थोड़ा अम्लीय होता है।<br><br>उम्र बढ़ने के साथ इसका रंग विशेष रूप से गहरा नहीं होता।<br><br>विभिन्न प्रकार के दागों के लिए उपयुक्त (जैसे, फ्यूसिन, हेमेटोक्सिलिन, मिथाइल ग्रीन, मिथाइल वायलेट, मेथिलीन ब्लू)।<br><br>नमूनों को वर्षों बाद जाइलीन में लंबे समय तक भिगोकर पुनः स्थापित किया जा सकता है।	नमूनों के लिए आदर्श नहीं है।<br><br>यदि कांच को अच्छी तरह से साफ करके सील न किया जाए तो समय के साथ कवरस्लिप निकल सकती हैं।<br><br>कोलेजन फाइबर के आसपास अपूर्ण बहुलकीकरण दिखाई दे सकता है।
एनेसे	अत्यंत टिकाऊ माध्यम, जो कम से कम 50 वर्षों तक चलता है।<br><br>समय के साथ एनेसे का रंग गहरा नहीं होता।<br><br>यह अधिक लचीला होता है, जिससे माध्यम में कीड़ों का विच्छेदन करना संभव हो जाता है, साथ ही रूपात्मक संरचनाओं को स्थिति में लाने के लिए पर्याप्त समय भी मिल जाता है।<br><br>कम लागत।	इसके लिए समय लेने वाली पूर्ण निर्जलीकरण प्रक्रिया की आवश्यकता होती है।<br><br>इथेनॉल निर्जलीकरण और लौंग के तेल के माध्यम से स्थानांतरण कुछ नमूनों को भंगुर बना सकता है।<br><br>कीट का स्वरूप धीरे-धीरे स्पष्ट होता जा रहा है; इस वजह से संवेदी अंग, एस्कोइड और साधारण सेटे जैसी बहुत छोटी संरचनाओं को देखना मुश्किल हो सकता है।

5.3.4. का विवरणअनुशंसित माउंटिंग मीडिया (तालिका 3 और 4)

अस्थायी अवलोकन के लिए मीडिया
क्लोरल गम = होयर द्रव/माध्यम/विलयन (आरआई = 1.48)

मार्क आंद्रे द्रव शुक्राणुकोशिकाओं के अल्पकालिक अवलोकन (कुछ घंटे, यदि स्लाइड को नम कक्ष में रखा जाए तो कुछ और घंटे) के लिए सर्वोत्तम माध्यम है, जिसमें फोटोग्राफ (चित्र 4) या रेखाचित्र बनाना शामिल है। देखी गई शुक्राणुकोशिकाओं को संरक्षित करने के लिए उन्हें एक जलीय माध्यम में पुनः माउंट करना आवश्यक है, जिससे उन्हें मध्यम अवधि तक संग्रहित किया जा सके। उन्हें रेज़िन में पुनः माउंट करने के लिए निर्जलित करना संभव तो है, लेकिन अनुशंसित नहीं है (नुकसान का जोखिम)। क्लोरल गम और होयर द्रव को पर्यायवाची माना जाता है। यह माध्यम आमतौर पर आंतरिक अंगों के अवलोकन के लिए उपयोग किया जाता है क्योंकि यह जल के अनुकूल है, सरल है, जल्दी लगाया जा सकता है, और इसका अपवर्तनांक शुक्राणुकोशिकाओं जैसी नाजुक संरचनाओं की जांच को आसान बनाता है। हालांकि, क्लोरल गम में महत्वपूर्ण कमियां हैं यदि इसे पूरी तरह से तैयार न किया जाए या नियंत्रित आर्द्रता की स्थिति में संग्रहित न किया जाए। इन समस्याओं में क्रिस्टलीकरण, रंग बदलना और चिपचिपाहट में कमी शामिल हैं। कवरस्लिप को रिंग करने से ये समस्याएं हल नहीं होती हैं, क्योंकि रिंगिंग माध्यम के साथ परस्पर क्रिया के कारण माउंटिंग मीडिया का रंग बहुत बदल सकता है (कभी-कभी लगभग काला हो जाता है), विशेष रूप से यदि यूपराल® का उपयोग किया जाता है।

होयर माध्यम को फ्लेबोटोमाइन सैंडफ्लाई के लिए ऑप्टिकली सबसे अच्छा माना गया है और पारंपरिक रूप से इन उद्देश्यों के लिए इसका उपयोग किया जाता रहा है। इस माध्यम में कई निकट से संबंधित फॉर्मूलेशन शामिल हैं, जिनमें गम अरेबिक, ग्लिसरॉल और chloral hydrate शामिल हैं। विभिन्न फॉर्मूलेशन की गलत व्याख्या और गलत उद्धरण किए गए हैं [74]।

हालांकि होयर माध्यम बालू मक्खियों में शुक्राणुकोशिकाओं के अवलोकन के लिए एक अच्छा माध्यम है, लेकिन यह दीर्घकालिक संरक्षण के लिए उपयुक्त नहीं है। यह अल्पकालिक अवलोकनों, जैसे फ़ोटोग्राफ़, रेखाचित्र या चित्र बनाने के लिए आदर्श है। जलीय माध्यम अस्थायी माउंट के लिए उपयुक्त होते हैं, लेकिन दीर्घकालिक संरक्षण सुनिश्चित नहीं कर सकते। इसके विपरीत, रेज़िन माउंटिंग उत्कृष्ट स्थायित्व प्रदान करती है और अक्सर सदियों तक चलती है, लेकिन शुक्राणुकोशिकाओं के सूक्ष्म विवरणों

को धुंधला कर सकती है, क्योंकि उनकी अपवर्तकता अक्सर कम हो जाती है।

समय के साथ निर्जलीकरण के कारण होयर माध्यम का क्षरण होता है (चित्र 8), जिसके परिणामस्वरूप छोटे सफेद, अपारदर्शी chloral hydrate क्रिस्टल बनते हैं। फिर भी, क्रिस्टलीकृत स्लाइड से नमूने प्राप्त किए जा सकते हैं क्योंकि क्यूटिकल रासायनिक रूप से बरकरार रहता है, हालांकि बढ़ते क्रिस्टलों से कुछ भौतिक क्षति हो सकती है। कुछ मामलों में, कवक की वृद्धि को रोकने के लिए थाइमोल के साथ गर्म, नम वातावरण में माउंटिंग माध्यम को पुनः हाइड्रेट करके क्रिस्टलीकृत स्लाइड को पुनर्स्थापित किया जा सकता है। वैकल्पिक रूप से, नमूनों को गोंद क्लोरल से पानी में भिगोकर, ग्लेशियल एसिटिक एसिड में निर्जलित करके, और कनाडा बाल्सम में पुनः माउंट किया जा सकता है।

डीएमएचएफ (डाइमिथाइल हाइडेंटोइन फॉर्मेल्डिहाइड) (आरआई 1.48)

यह जल-आधारित माध्यम [72] प्रकाशीय दृष्टि से बर्लेज़ की तरह ही बहुत अच्छा प्रदर्शन करता है और बर्लेज़ जितना ही उपयोग में आसान है। हालाँकि, बर्लेज़ के विपरीत, यह काला नहीं पड़ता और न ही क्रिस्टलीकृत होता है। यह बालू मक्खियों और अन्य साइकोडीडे के लिए अच्छा काम करता है।

चित्र 8: स्लाइड को पुनः लगाना। A: क्षतिग्रस्त और सूखी स्लाइड को होयर में लगाया गया; B: एक सूखी रेत मक्खी का सूक्ष्मदर्शी दृश्य; C: एक अन्य क्षतिग्रस्त रेत मक्खी का सूक्ष्मदर्शी दृश्य; D: एक सूखा स्लाइड युक्त गीला कक्ष; E: नमूना B का सिर, और F: शरीर, यूपारल® में पुनः लगाने के बाद; G: क्षतिग्रस्त नमूना C का सिर, और H: शरीर, यूपारल® में पुनः लगाने के बाद।

सीएमसीपी (कपूर-मोनो-क्लोरोफेनोल) (आरआई = 1.41)

यह ग्लिसरीन आधारित, पानी में घुलनशील माउंटिंग मीडियम है जिसका उपयोग बालू मक्खी सहित नाजुक नमूनों की पारदर्शी, स्थायी स्लाइड बनाने के लिए किया जाता है। इस माउंटिंग मीडियम का लाभ यह है कि नमूनों को सीधे पानी या इथेनॉल से माउंट किया जा सकता है। यह बालू मक्खी को जल्दी से शिथिल कर देता है और उसकी क्यूटिकल को नरम कर देता है, जिससे नमूने को सही स्थिति में रखना संभव हो जाता है, जो विशेष रूप से पंख फैलाने या जननांगों का विच्छेदन करने में उपयोगी होता है। हालांकि यह लंबे समय तक भंडारण को सक्षम बनाता है, लेकिन संरक्षण की सटीक अवधि अनिश्चित है। इस माउंटिंग मीडियम की प्रमुख सीमा इसकी संरचना में निहित है जिसमें फिनोल होता है, जो एक विषैला और जलन पैदा करने वाला पदार्थ है और इसके सावधानीपूर्वक उपयोग की आवश्यकता होती है।

स्थायी माउंटिंग के लिए मीडिया

कनाडा बाल्सम (आरआई = 1.52-1.54)

कनाडा बाल्सम को सर्वप्रथम 1830 के दशक में एंड्रयू प्रिचर्ड द्वारा ट्रांसमिटेड लाइट माइक्रोस्कोपी के लिए उपयुक्त माउंटिंग माध्यम के रूप में वर्णित किया गया था। 150 से अधिक वर्षों के सफल उपयोग के साथ, इसकी सिद्ध संग्रहणीय गुणवत्ता के कारण यह सबसे व्यापक रूप से उपयोग किए जाने वाले माध्यमों में से एक बना हुआ है। होयर फ्लूइड मीडिया के विपरीत, कनाडा बाल्सम क्रिस्टलीकृत नहीं होता है और न ही नमी अवशोषित करता है। हालांकि, कनाडा बाल्सम अत्यधिक स्व-प्रतिदीप्तिशील होता है, जो कभी-कभी कुछ माइक्रोस्कोपी तकनीकों के लिए एक नुकसान हो सकता है [60]। ज़ाइलीन के स्थान पर गैर-विषाक्त विलायकों का उपयोग तैयारी के दौरान सुरक्षा जोखिमों को कम कर सकता है, लेकिन इससे धीमी सुखाने की प्रक्रिया और माध्यम के जल्दी काला पड़ने जैसी कमियां भी उत्पन्न हो सकती हैं।

यूपरल®(आरआई = 1.48)

यूपराल® स्थायी माउंटिंग के लिए कनाडा बालसम का एक व्यापक रूप से इस्तेमाल किया जाने वाला विकल्प है, जो उत्कृष्ट दीर्घकालिक स्थिरता और तुलनीय अपवर्तनांक प्रदान करता है। यूपराल® की निम्नलिखित विशेषताएं हैं: (1) निर्जलीकरण की आवश्यकता: माउंटिंग माध्यम के अंतिम स्थानांतरण से पहले, नमूने को निर्जलित करना आवश्यक है, आमतौर पर 95% से पूर्ण इथेनॉल में परिवर्तित करके, और (2) विस्तारित प्रसंस्करण समय: रेज़िन में अंतिम संयोजन, चाहे वह कनाडा बालसम हो या यूपराल®, के लिए निर्जलीकरण की आवश्यकता होती है, जिससे समग्र नमूना प्रसंस्करण समय बढ़ जाता है। जब कार्बनिक विलायकों के साथ निर्जलीकरण संभव न हो, तो अंतिम माउंटिंग से पहले, पूर्ण इथेनॉल से निकाले गए नमूनों को यूपराल® और यूपराल एसेंस के समान मिश्रण वाले एक मध्यवर्ती घोल में रखा जा सकता है।

एनेसे (आरआई = 1.467)

एनेसी एक राल-आधारित माउंटिंग माध्यम है जिसका उपयोग मुख्य रूप से छोटे कीटों के लिए किया जाता है और यह विशेष रूप से ब्राजील में लोकप्रिय है। इसका आधार अल्कोहल, कपूर, तारपीन के अर्क और यूकेलिप्टोल में घुले हुए कोलोफोनी और गोंद कोपल से बना होता है। सेकेंरा [11] ने लार्वा, अपरिपक्व जीवों के

खोल और यहां तक कि वयस्क मच्छरों की स्थायी स्लाइड को माउंट करने के लिए कनाडा बाल्सम के विकल्प के रूप में एनेसी का वर्णन किया, और तब से इसे बालू मक्खी को माउंट करने के लिए व्यापक रूप से अपनाया गया है। एनेसी स्थायी माउंटिंग के लिए एक किफायती विकल्प प्रदान करता है, जो दीर्घकालिक स्थिरता और पर्याप्त सुखाने का समय देता है, जिससे विच्छेदन और रूपात्मक संरचनाओं का सटीक विन्यास संभव हो पाता है।

5.4. स्लाइड तैयार करना और सुखाना

माउंट की गई स्लाइडों को ठीक से सुखाना उनकी दीर्घकालिक स्थिरता और संरक्षण के लिए अत्यंत महत्वपूर्ण है। स्लाइडों को लंबे समय तक भंडारण के लिए रखने से पहले उन्हें अच्छी तरह सुखा लेना चाहिए। सर्वोत्तम परिणामों के लिए, स्थायी माउंटिंग मीडिया से माउंट की गई स्लाइडों को 2-3 सप्ताह तक क्षैतिज रूप से सुखाना चाहिए, जबकि अर्ध-स्थायी मीडिया से तैयार की गई स्लाइडों को केवल 1-2 सप्ताह की आवश्यकता हो सकती है। प्रभावी सुखाने की प्रक्रिया सुनिश्चित करने के लिए, उपयोग किए जा रहे माउंटिंग मीडिया के लिए उपयुक्त तापमान पर सेट किए गए इनक्यूबेटर का उपयोग करने की सलाह दी जाती है, जिससे नमूनों को नुकसान पहुंचा सकने वाली अत्यधिक गर्मी से बचा जा सके। 30°C और 37°C के बीच का तापमान अनुशंसित है। भंडारण के दौरान स्लाइड के मुड़ने, नमूने के खराब होने या माउंटिंग मीडिया के अस्थिर होने से बचाने के लिए यह सुखाने का चरण महत्वपूर्ण है।

स्लाइड तैयार करने में प्रयुक्त माउंटिंग मीडियम का उल्लेख स्लाइड लेबल पर अवश्य होना चाहिए। यदि संभव हो, तो लेबल पर प्रयुक्त विधि, स्लाइड तैयार करने वाले व्यक्ति का नाम और तैयारी की तिथि भी अंकित होनी चाहिए। स्लाइडें प्रारंभ में अस्थायी माउंट के रूप में तैयार की जाती हैं और इन्हें दीर्घकालिक संरक्षण के लिए नहीं बनाया जाता है। हालांकि, यदि नमूने की स्थिति में परिवर्तन होता है, जैसे कि उसे "टाइप" श्रृंखला के भाग के रूप में नामित किया जाता है, तो भविष्य के वर्गीकरण संबंधी अध्ययन के लिए नमूने के संरक्षण को सुनिश्चित करने हेतु अधिक स्थायी माउंटिंग मीडियम का उपयोग किया जाना चाहिए।

5.5. वैकल्पिक माउंटिंग तकनीकें: कार्ड माउंटिंग

कार्ड माउंटिंग एक ऐसी तकनीक है जिसका उपयोग कई कीट समूहों के लिए किया जाता है, जिसमें नमूनों को सीधे कीटविज्ञान कार्ड पर पिन किया जा सकता है या सतह पर चिपकाया जा सकता है। उनके छोटे आकार और पहचान के लिए आंतरिक अंगों का अवलोकन करने की आवश्यकता को देखते हुए (आइटम 5 देखें), यह विधि बालू मक्खी को माउंट करने के लिए बिल्कुल भी उपयुक्त नहीं है।

5.6. क्षतिग्रस्त नमूनों को पुनः स्थापित करना

दुर्लभ या मूल्यवान नमूनों के लिए, https://zenodo.org/records/18315029 पर उपलब्ध वीडियो के अनुसार दो-चरणीय दृष्टिकोण की अनुशंसा की जाती है। 1) प्रारंभिक अवलोकन के लिए उन्हें बिना खोले ही पुनः हाइड्रेट करें। कई माइक्रोस्कोप स्लाइडों के लिए एक होल्डर को पेट्री डिश में सपोर्ट के रूप में रखें। पुनः हाइड्रेट की जाने वाली स्लाइड को ऊपर रखें, और पेट्री डिश को कुछ मिलीमीटर विलायक से भरकर एक नम कक्ष बनाएं, यह सुनिश्चित करते हुए कि स्लाइड स्वयं विलायक के संपर्क में न आए (चित्र 8 D)। नमूने की स्थिति के आधार पर पुनः हाइड्रेट होने में एक से कई दिन लग सकते हैं। दैनिक निगरानी और धैर्य आवश्यक हैं। स्लाइड के पर्याप्त रूप से पुनः हाइड्रेट हो जाने पर, इसे नम कक्ष से निकालकर सूक्ष्मदर्शी से जांच, फोटोग्राफी या चित्र बनाने से पहले कुछ घंटों के लिए इनक्यूबेटर में रखा जा सकता है। 2) पुनः माउंट करने के लिए, स्लाइड को कुछ अतिरिक्त घंटों या रात भर के लिए नम कक्ष में वापस रखा जा सकता है। द्विनेत्री माइक्रोस्कोप के नीचे ही स्लाइड को खोलें। बारीक सुइयों का उपयोग करके, कवरस्लिप को सावधानीपूर्वक हटाना चाहिए, यह सुनिश्चित करते हुए कि कोई भी बालू मक्खी तत्व चिपका न रह जाए (https://zenodo.org/records/18315029)। इसके बाद, बालू मक्खी द्वारा विच्छेदित तत्वों को छोटे कुओं में इकट्ठा करके पानी से धोना चाहिए, जैसे कि DNA/RNA निष्कर्षण के लिए उपयोग किए जाने वाले कुएँ (नीचे देखें), निर्जलीकरण और रेजिन माध्यम में पुनः माउंट करने से पहले। स्लाइड को अलग करते समय, उपयुक्त विलायक का चयन करने के लिए मूल माउंटिंग माध्यम की पहचान करना महत्वपूर्ण है। जलीय माउंटिंग माध्यमों के लिए, पानी का उपयोग किया जाना चाहिए। यदि माउंटिंग माध्यम रेजिन-आधारित है (जैसे, कनाडा बाल्सम या यूपराल®), तो ज़ाइलीन का उपयोग फ्यूम हुड के नीचे और उचित व्यक्तिगत सुरक्षा उपकरण, जिसमें मास्क भी शामिल है, के साथ किया जाना चाहिए।

टाइप या संग्रह नमूनों को पुनः स्थापित करने का कार्य केवल क्यूरेटर और/या नमूने के स्वामी संस्थान की सहमति से ही किया जाना चाहिए।

6. नमूने की पहचान

6.1. आकृति विज्ञान

बालू मक्खियों की पहचान मुख्य रूप से उनके आकारिकी लक्षणों की जांच पर निर्भर करती है, जिसमें वक्ष, पंख, जननांग, बाल और विभिन्न संरचनाओं के बीच विशिष्ट आकारिकीय संबंध शामिल हैं। शोधकर्ता एकत्रित नमूनों की ज्ञात प्रजातियों से तुलना करने के लिए वर्गीकरण कुंजी, संदर्भ संग्रह और मूल प्रजाति विवरण का उपयोग करते हैं। प्रमुख नैदानिक लक्षण, जैसे कि दोनों लिंगों में पंखों की शिराओं का विन्यास और सिर की आकृति विज्ञान, नर जननांगों की संरचना और मादा शुक्राणुकोषों की संरचना, प्रजाति निर्धारण के लिए विशेष रूप से उपयोगी होते हैं। सटीक पहचान के लिए अक्सर विस्तृत सूक्ष्मदर्शी जांच की आवश्यकता होती है, आमतौर पर जननांगों और शुक्राणुकोषों जैसी सूक्ष्म संरचनाओं को देखने के लिए यौगिक सूक्ष्मदर्शी या व्यापक आकारिकी लक्षणों के लिए स्टीरियोसूक्ष्मदर्शी का उपयोग किया जाता है।

इमेजिंग तकनीक में हालिया प्रगति ने बालू मक्खी की पहचान के लिए डिजिटल इमेजिंग के उपयोग को आसान बना दिया है। प्रमुख विशेषताओं की उच्च-रिज़ॉल्यूशन वाली तस्वीरों या डिजिटल चित्रों की तुलना संदर्भ सामग्री से की जा सकती है या कंप्यूटर-सहायता प्राप्त पहचान प्रणालियों का उपयोग करके उनका विश्लेषण किया जा सकता है, जिससे आकारिकी वर्गीकरण में सटीकता और सुगमता दोनों में सुधार होता है।

6.2. पंख की ज्यामिति

पंखों की ज्यामिति विभिन्न रेत मक्खी प्रजातियों की पहचान और वर्गीकरण में एक महत्वपूर्ण विशेषता है। बालू मक्खियों के पंखों में एक विशिष्ट पैटर्न और संरचना होती है, जो आमतौर पर लंबी और संकीर्ण होती हैं और उनमें शिराओं का जाल सुविकसित होता है (चित्र 9 और 10)। शिराओं की व्यवस्था एक विशिष्ट पैटर्न बनाती है जो विभिन्न वंशों और प्रजातियों में भिन्न हो सकती है, जिससे पहचान के लिए महत्वपूर्ण नैदानिक लक्षण प्राप्त होते हैं। इसलिए, पंखों की ज्यामिति का अध्ययन वर्गीकरण के लिए बहुमूल्य जानकारी प्रदान करता है।

6.3. पंख ज्यामितीय आकारमिति

Els investigadors utilitzen diverses tècniques, com ara la mo शोधकर्ता विभिन्न बालू मक्खी प्रजातियों या आबादी में पंखों के आकार और आकृति का विश्लेषण और तुलना करने के लिए ज्यामितीय मॉर्फोमेट्रिक्स जैसी विभिन्न तकनीकों का उपयोग करते हैं। पंखों की ज्यामिति का अध्ययन व्यवहार, आवास वरीयताओं और उड़ान क्षमताओं के बारे में बहुमूल्य जानकारी प्रदान करता है।

ज्यामितीय मॉर्फोमेट्रिक दृष्टिकोण में, पंखों को सावधानीपूर्वक विच्छेदित किया जाता है, रंगा जाता है (यदि आवश्यक हो), और स्लाइड पर सपाट रूप से लगाया जाता है। तैयार स्लाइडों को फिर स्टीरियोमाइक्रोस्कोप के नीचे फोटो खींचा जाता है, डिजिटाइज़ किया जाता है, और मॉर्फोमेट्रिक विश्लेषण के लिए उपयोग किया जाता है। इस प्रक्रिया का साहित्य में अच्छी तरह से वर्णन किया गया है [6, 27, 42, 56, 57, 59], जिसमें संभावित नकारात्मक एलॉमेट्रिक प्रभावों से बचने के लिए युग्मित अंगों के लिए लगातार दाएं या बाएं पंख का उपयोग करने की सिफारिश की गई है [62]।

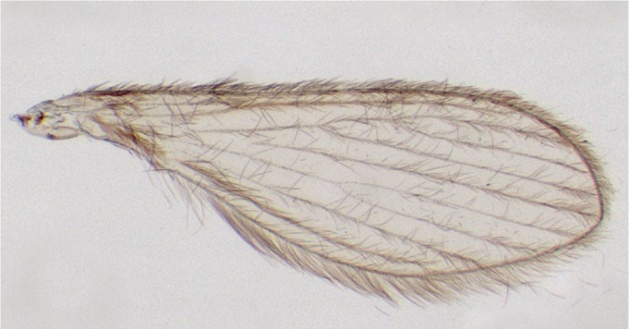

चित्र 9: *Trichophoromyia ininii* का कच्चा पंख।

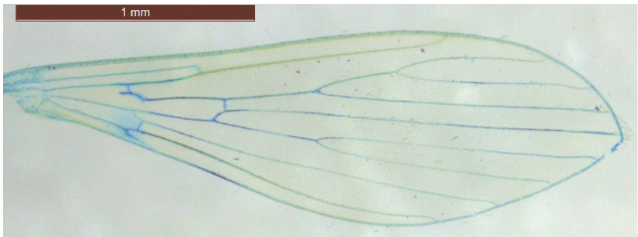

चित्र 10: *Phlebotomus ariasi* का रंगीन पंख।

ज्यामितीय आकारमितीय विश्लेषण के लिए पंख की तैयारी

पंखों की शिराओं को बेहतर ढंग से देखने के लिए, पंखों को शल्कों से साफ करके उचित रंग से रंगना चाहिए। पंख तैयार करने के लिए, सबसे पहले छोटे कुओं में आवश्यक अभिकर्मक (मेथिलीन ब्लू, इथेनॉल, पानी और ज़ाइलीन विकल्प) भरें। कमरे के तापमान पर 70% इथेनॉल में संरक्षित पंख को निकालने के लिए, एपेंडॉर्फ ट्यूब को उल्टा करके कुएँ के ऊपर खाली करें, फिर एक महीन घुमावदार सुई का उपयोग करके पंख को लंबाई में उठाएँ। पंख को इथेनॉल से पानी और फिर वापस इथेनॉल में थोड़ी देर के लिए रखें ताकि रेशे हट जाएँ। पंख को 6 मिनट के लिए मेथिलीन ब्लू में रखें, यह सुनिश्चित करते हुए कि रंगाई के दौरान यह तैरता रहे। पंख को सावधानीपूर्वक निकालें और इसे 2 मिनट के लिए ज़ाइलीन विकल्प में डुबोएँ (मेथिलीन ब्लू के समय का लगभग एक तिहाई)। कुएँ की दीवारों पर सुई से हल्के से थपथपाने से पंख को जमने में मदद मिल सकती है; ज़ाइलीन रंग को स्थिर करने का काम करता है। अंत में, पंख को उठाएँ और इसे एक सूक्ष्मदर्शी स्लाइड पर यूपराल® की एक छोटी बूंद पर रखें। आवर्धक लेंस के नीचे, पंख को धीरे से खोलें और सावधानीपूर्वक एक कवरस्लिप रखें। Euparal® के जमने से पहले ही तस्वीरें ले लेनी चाहिए, क्योंकि इष्टतम संरेखण प्राप्त करने के लिए कवरस्लिप के नीचे पंख की स्थिति में मामूली समायोजन आवश्यक हो सकता है।

6.4. आणविक जीवविज्ञान तकनीकें

आकारिकी तकनीकों के अतिरिक्त, आणविक विधियाँ कीटविज्ञान अनुसंधान में तेजी से आवश्यक होती जा रही हैं, जिनमें वर्गीकरण, जनसंख्या आनुवंशिकी और फाइलोजेनेटिक अध्ययन, साथ ही DNA/RNA रोगजनक पहचान और रक्त भोजन के स्रोत का निर्धारण शामिल है। महामारी विज्ञान के क्षेत्र में वेक्टर व्यवहार महत्वपूर्ण है [70]। प्रजातियों की पुष्टि या निकट संबंधी प्रजातियों में अंतर करने के लिए DNA अनुक्रमण का उपयोग किया जा सकता है, जो पहचान का अधिक सटीक और विश्वसनीय साधन प्रदान करता है। इसके अलावा, उन्नत आणविक तकनीकें (जैसे, PCR, DNA अनुक्रमण, एनजीएस, आदि) और MALDI-TOF एमएस सटीक और तीव्र प्रजाति पहचान के लिए प्रमुखता प्राप्त कर रही हैं, जो पारंपरिक आकारिकी विधियों की पूरक हैं [46]। इन प्रगति के बावजूद, आकारिकी पहचान वर्गीकरण के लिए संदर्भ मानक और आणविक डेटा की व्याख्या का आधार बनी हुई है।

6.4.1. विनाशकारी न्यूक्लिक एसिड निष्कर्षण

कई जैविक अध्ययनों में न्यूक्लिक एसिड निष्कर्षण एक नियमित प्रक्रिया है, और जैविक सामग्रियों से DNA को अलग करने के लिए विभिन्न विधियाँ विकसित की गई हैं [48]। कई व्यावसायिक रूप से उपलब्ध DNA निष्कर्षण किट इस प्रक्रिया को सुगम बनाने के लिए डिज़ाइन किए गए हैं [14]। हालांकि, आकारिकी पहचान के लिए आर्थ्रोपोड नमूनों को तैयार करने के लिए आमतौर पर उपयोग की जाने वाली विधियाँ अक्सर DNA विश्लेषण में बाधा डालती हैं, क्योंकि ये तकनीकें नमूने की महत्वपूर्ण भौतिक विशेषताओं को नुकसान पहुंचा सकती हैं या नष्ट कर सकती हैं [10]। कीट ऊतकों के लिए अधिकांश DNA निष्कर्षण प्रोटोकॉल प्रकृति में विनाशकारी होते हैं [43], जिससे छोटे नमूनों के लिए विशेष चिंताएँ उत्पन्न होती हैं, जहाँ सीमित नमूनाकरण भी महत्वपूर्ण आकारिकी विशेषताओं को प्रभावित कर सकता है [72]। नमूने का प्रकार और स्थिति उपयुक्त DNA पृथक्करण विधि के चयन में महत्वपूर्ण भूमिका निभाते हैं [29]।

बालू मक्खियों की सटीक पहचान, जनसंख्या गतिशीलता को समझना और गैर-लक्षित प्रभावों को कम करने की आवश्यकता ने आणविक निदान उपकरणों के विकास को बढ़ावा दिया है [23]। बालू मक्खियों की पहचान के लिए रूपात्मक वर्गीकरण विधियों के पूरक के रूप में अब आणविक दृष्टिकोणों का अक्सर उपयोग किया जाता है। उदाहरण के लिए, कीट बारकोडिंग के मानक दृष्टिकोण में DNA निष्कर्षण, अनुक्रमण और मूल नमूने का नुकसान शामिल है। इसलिए, ऐसे गैर-विनाशकारी DNA निष्कर्षण विधियों की खोज की तत्काल आवश्यकता है जो जैविक सामग्री और उसकी रूपात्मक अखंडता दोनों को संरक्षित करें।

बालू मक्खी पर न्यूक्लिक एसिड निष्कर्षण की अनेक विधियाँ लागू की गई हैं। आवश्यक न्यूक्लिक एसिड की मात्रा या गुणवत्ता आगे के आणविक विश्लेषण पर निर्भर करती है, क्योंकि विभिन्न तकनीकों की संवेदनशीलता और शुद्धता की आवश्यकताएँ भिन्न-भिन्न होती हैं [9]। उदाहरण के लिए, बालू मक्खी की आँखों में PCR प्रवर्धन को बाधित करने की क्षमता पाई गई है [69]। रोगजनक स्क्रीनिंग के अलावा, प्रजातियों की पहचान के लिए बालू मक्खी DNA का नियमित रूप से निष्कर्षण किया जाता है। विभिन्न निष्कर्षण विधियों का उपयोग किया जा सकता है, हालाँकि तकनीकों के बीच उपज और गुणवत्ता भिन्न होती है। कुछ निर्माताओं के प्रोटोकॉल को शोधकर्ताओं द्वारा बालू मक्खी के लिए अनुकूलित किया गया है [8], जिससे निष्कर्षित न्यूक्लिक एसिड की उपज और/या गुणवत्ता में वृद्धि हुई है [8, 9, 69], जबकि अन्य आर्थ्रोपोड टैक्सोन के लिए विकसित अन्य अनुकूलन का उपयोग बालू मक्खी पर भी किया जा सकता है [58, 76]। छोटे माइटोकॉन्ड्रियल खंडों (COI या CytB) को लक्षित करने वाले पहचान PCR आमतौर पर उच्च DNA विखंडन वाली निष्कर्षण विधियों के साथ संगत होते हैं। इसके विपरीत, अन्य लॉन्ग-रीड एनजीएस तकनीकें (ऑक्सफोर्ड नैनोपोर और पैकबियो) न्यूनतम विखंडन और उच्च गुणवत्ता वाले DNA की आवश्यकता होती है। स्पिन कॉलम निष्कर्षण से आमतौर पर 60 केबी तक के जीनोमिक DNA खंड प्राप्त होते हैं, जबकि फिनोल-क्लोरोफॉर्म निष्कर्षण से 150 केबी तक के खंड प्राप्त किए जा सकते हैं [77]। तालिका 5 में बालू मक्खी DNA के विभिन्न निष्कर्षण तकनीकों का सारांश दिया गया है और यह दर्शाया गया है कि क्या इन कीटों के लिए कार्यप्रणाली में कोई अनुकूलन किया गया है। उपज नहीं दर्शाई गई है, क्योंकि यह नमूने के आकार और तैयारी विधि पर निर्भर करती है। संशोधन कॉलम बालू मक्खी या अन्य छोटे आर्थ्रोपोड्स के लिए निष्कर्षण प्रोटोकॉल के अनुकूलन को संदर्भित करता है।

निष्कर्षण विधि का चयन करते समय कई मानदंडों पर विचार करना चाहिए, जैसे नमूनों की संख्या, निष्कर्षण समय और आगे उपयोग की जाने वाली तकनीक। हालांकि एनजीएस तकनीकों के लिए उच्च आणविक भार वाले जीनोमिक DNA की आवश्यकता होती है, यहाँ प्रस्तुत सभी विधियों का उपयोग मानक PCR-आधारित अनुप्रयोगों के लिए किया जा सकता है।

इसके अलावा, कई अध्ययनों ने छोटे स्थलीय आर्थ्रोपोड्स, सूखे संरक्षित संग्रहालय नमूनों और नरम शरीर वाले आर्थ्रोपोड्स के लिए गैर-विनाशकारी DNA निष्कर्षण विधियों का पता लगाया है [19, 26, 28, 55, 63]।

तालिका 5: फ्लेबोटोमाइन बालू मक्खी के जीDNA निष्कर्षण के लिए औसत लागत, अनुप्रयोग और प्रोटोकॉल अनुकूलन

शिष्टाचार	लागत	आवेदन	छोटे आर्थ्रोपोड्स के लिए प्रोटोकॉल अनुकूलन
स्पिन कॉलम	2.5 – 3.55 अमेरिकी डॉलर [39]	PCR, एनजीएस	[9]
फिनोल-क्लोरोफॉर्म	0.12 अमेरिकी डॉलर [69]	PCR, एनजीएस	[9]
हॉट शॉट	<0.01 अमेरिकी डॉलर [69]	PCR	-
नमक निकालना	0.12 $3 [69]	PCR	-
चेलेक्स	0.02 $4 [41]	PCR	[41, 76]

6.4.2. गैर-विनाशकारी न्यूक्लिक अम्ल निष्कर्षण

आर्थ्रोपोड्स, विशेषकर बालू मक्खी के आणविक विश्लेषण में एक प्रमुख चुनौती कीटविज्ञान संग्रहों में शामिल करने के लिए नमूनों का संरक्षण है। अधिकांश DNA निष्कर्षण प्रोटोकॉल में ऊतक को कुचलना आवश्यक होता है, जिससे मूल नमूने का संरक्षण प्रभावित होता है। हालांकि, गैर-विनाशकारी न्यूक्लिक एसिड निष्कर्षण विधियाँ नमूने को भौतिक रूप से नुकसान पहुँचाए बिना, उसकी व्यवहार्यता को प्रभावित किए बिना या उसकी आकृति को बदले बिना आनुवंशिक सामग्री निकालने के लिए डिज़ाइन की गई हैं। ये विधियाँ विशेष रूप से मूल्यवान होती

हैं जब बालू मक्खी जैसे कीमती या सीमित नमूनों के साथ काम किया जाता है, जहाँ संरचनात्मक अखंडता को बनाए रखना भविष्य के वर्गीकरण, आकृति विज्ञान या निदान उद्देश्यों के लिए आवश्यक है। एक सामान्य रूप से उपयोग की जाने वाली तकनीक गैर-विनाशकारी स्नान विधि है जिसमें बालू मक्खी को स्थिर किया जाता है और प्रोटीनएज़ K युक्त लाइसिस बफर में धीरे से डुबोया जाता है।

माइल्ड-वेक्टोलाइसिस तकनीक को बालू मक्खी, विशेष रूप से टाइप स्पेसिमेन [24] पर सफलतापूर्वक लागू किया गया है। यह तकनीक एक पारंपरिक स्पिन कॉलम किट (इस मामले में, एक DNeasy ब्लड एंड टिश्यू किट, QIAGEN, हिल्डेन, जर्मनी) का उपयोग करती है, जिसे नमूने को नष्ट किए बिना DNA प्राप्त करने के लिए अनुकूलित किया गया है। संशोधित लाइसिस चरण (लाइसिस बफर की मात्रा और फ्रीजिंग चरण का जोड़) [17] न्यूक्लिक एसिड को मुक्त करने की अनुमति देते हैं, जिससे आकारिकीय क्षति कम से कम होती है [24]। बालू मक्खी के संबंध में, एक हॉटशॉट DNA एक्सट्रैक्शन किट (बेंटो बायोवर्क्स लिमिटेड, लंदन, यूनाइटेड किंगडम) [73] का उपयोग करना भी संभव है, जो तीव्र और सस्ता है, जिससे नमूनों का त्वरित और कम लागत वाला प्रसंस्करण संभव हो पाता है। आकारिकीय पहचान के लिए अभिप्रेत कीटवैज्ञानिक नमूनों को फिर धोया जा सकता है। डीएनईज़ी ब्लड एंड टिश्यू किट से संसाधित नमूनों को मार्क-आंद्रे घोल से शुद्ध करना आवश्यक है, जबकि हॉटशॉट DNA निष्कर्षण किट से संसाधित नमूनों को इस लेख में विस्तृत प्रोटोकॉल [73] के अनुसार निर्जलीकरण के बाद जलीय माध्यम में, या बेहतर, रेज़िन में माउंट करने के लिए पर्याप्त रूप से शुद्ध किया जाता है। निकाले गए आनुवंशिक पदार्थ को विशिष्ट आनुवंशिक मार्करों को बढ़ाने के लिए PCR जैसे आगे के विश्लेषण के लिए संसाधित किया जा सकता है। गैर-विनाशकारी न्यूक्लिक एसिड निष्कर्षण विधियाँ बालू मक्खी की आनुवंशिक विशेषताओं का अध्ययन करने के लिए महत्वपूर्ण हैं, जिसमें उनके द्वारा ले जाए जा सकने वाले संभावित रोगजनकों की पहचान करना भी शामिल है। नमूने की अखंडता को संरक्षित करके, शोधकर्ता मूल्यवान आनुवंशिक जानकारी प्राप्त कर सकते हैं, साथ ही नमूने को अतिरिक्त विश्लेषण या अध्ययन के लिए सुरक्षित रख सकते हैं।

6.5. मालदी-टीओएफ एमएस

MALDI-ToF MS (मैट्रिक्स-असिस्टेड लेजर डिसॉर्प्शन/आयोनाइज़ेशन टाइम-ऑफ-फ्लाइट मास स्पेक्ट्रोमेट्री) एक मास स्पेक्ट्रोमेट्री-आधारित तकनीक है जिसे जैविक नमूनों के विशिष्ट प्रोटीन प्रोफाइल ('फिंगरप्रिंट') का पता लगाने और विश्लेषण करने के लिए डिज़ाइन किया गया है। MALDI-ToF को चिकित्सा और पशु चिकित्सा महत्व के आर्थ्रोपोड्स की पहचान के लिए एक महत्वपूर्ण उपकरण के रूप में तेजी से मान्यता मिल रही है। यह तकनीक बालू मक्खी के विभिन्न विकासीय चरणों की पहचान करने में प्रभावी साबित हुई है, जिसमें अपरिपक्व रूप और रक्त से भरी मादाओं का रक्त भोजन शामिल है, और भंडारण और समरूपता की विभिन्न स्थितियों में नर और मादा बालू मक्खी प्रजातियों में अंतर करने के लिए सफलतापूर्वक लागू की गई है।[28, 30, 73, 74]यह विधि उपजातियों, प्रजातियों और आबादी के स्तर पर भी उच्च विभेदक क्षमता प्रदान करती है। यह तकनीक शोधकर्ताओं को प्रजातियों की तीव्र और सटीक पहचान करने में सक्षम बनाती है, जो बालू मक्खी के वितरण, व्यवहार और रोग संचरण में उनकी भूमिका को समझने के लिए आवश्यक है। प्रोटीन प्रोफाइल के आधार पर प्रजातियों में अंतर करके, MALDI-ToF महामारी विज्ञान संबंधी अध्ययनों और वेक्टर नियंत्रण रणनीतियों में महत्वपूर्ण भूमिका निभाता है। इस तकनीक की दो मुख्य वर्तमान कमियां हैं जो इसके नियमित उपयोग को सीमित करती हैं। पहली है मास स्पेक्ट्रोमेट्री उपकरण की उपलब्धता, जो बालू मक्खी (या सामान्यतः आर्थ्रोपोड वैक्टर) की प्रजाति पहचान के लिए आसानी से प्राप्त करने के लिए अत्यधिक महंगा है। सौभाग्य से, इस सीमा को मास स्पेक्ट्रोमीटर पर मशीन समय प्राप्त करके दूर किया जा सकता है, जो प्रोटिओमिक सुविधाओं और/या नैदानिक निदान में एक मानक अनुसंधान उपकरण बन गए हैं। दूसरा कारण यह है कि ओपन-एक्सेस डेटाबेस में बालू मक्खी संदर्भ डेटा का प्रतिनिधित्व कम है, जिसके परिणामस्वरूप स्पष्ट रूप से पहचाने गए नमूनों पर आधारित संदर्भ स्पेक्ट्रा के साथ एक आंतरिक डेटाबेस बनाने की आवश्यकता है, आदर्श रूप से रूपात्मक मूल्यांकन और उपयुक्त आनुवंशिक मार्कर (COI, Cyt b, या अन्य) के अनुक्रमण के संयोजन द्वारा। उम्मीद है कि यह सीमा जल्द ही दूर हो जाएगी, जब तक कि अब तक आंतरिक रूप से एकत्र किए गए बालू मक्खी संदर्भ डेटा को असिस्टेंस पब्लिका-हॉस्पिटॉक्स डी पेरिस, सोरबोन विश्वविद्यालय, फ्रांस और ब्रुसेल्स, बेल्जियम में स्थित बीसीसीएम/आईएचईएम/साइंसनो संग्रह द्वारा संचालित एमएसआई प्लेटफॉर्म (https://msi.happy-dev.fr/) में धीरे-धीरे शामिल किया जाएगा। जब MALDI-TOF प्रोटीन प्रोफाइलिंग को लागू करने की योजना बनाई जाती है, तो नमूनों को अधिमानतः शुष्क-जमे हुए या आणविक ग्रेड के 70% इथेनॉल में संग्रहित किया जाना चाहिए और उन्हें परिवेश के तापमान के संपर्क में नहीं आना चाहिए। नमूना तैयार करने के लिए सार्वभौमिक दिशानिर्देशों के अभाव में, उपयोगकर्ताओं को सलाह दी जाती है कि वे MALDI-ToF मैट्रिक्स तैयार करने के लिए सिनापिनिक एसिड (30 मिलीग्राम/एमएल) के जलीय 60% एसीटोनिट्राइल/0.3% टीएफए घोल का उपयोग करें ताकि उनके प्रोटीन स्पेक्ट्रा को अब तक प्रकाशित बालू मक्खी डेटा के साथ तुलनीय बनाया जा सके।

MALDI-ToF MS के लिए नमूना तैयार करना (चित्र 7)

विभिन्न परिस्थितियों में संग्रहित कीट नमूनों को पहले कमरे के तापमान पर हवा में सुखाया जाता है और फिर उनका विच्छेदन किया जाता है। स्लाइड पर लगाने और आकारिकी विश्लेषण के लिए महत्वपूर्ण आकारिकी विशेषताओं वाले शरीर के भागों को प्राप्त करने के लिए सिर और उदर को अलग कर दिया जाता है। वक्ष का उपयोग MALDI-ToF के लिए किया जा सकता है और शेष उदर को DNA निष्कर्षण के लिए संरक्षित किया जाता है। प्रोटीन प्रोफाइलिंग के लिए, वक्ष को 1.5 मिलीलीटर की माइक्रो

ट्यूबों में 10 माइक्रोलीटर समरूपीकरण विलयन के साथ डिस्पोजेबल मूसल और पेलेट का उपयोग करके मैन्युअल रूप से समरूपित किया जाता है। आमतौर पर दो समरूपीकरण विलयनों का उपयोग किया जाता है: निष्फल आसुत जल और 25% फॉर्मिक अम्ल।

7. निष्कर्ष

इस शोध कार्य में हमारा उद्देश्य शोधकर्ताओं को विशिष्ट शोध उद्देश्यों के अनुरूप, बालू मक्खियों को संजोने के सबसे प्रभावी तरीके उपलब्ध कराना है, ताकि उनकी सटीक पहचान और रोगजनकों का पता लगाना आसान हो सके। कोई एक सर्वमान्य सर्वोत्तम विधि नहीं है; बल्कि कई विधियाँ मौजूद हैं, जिनमें से प्रत्येक के अपने-अपने फायदे और सीमाएँ हैं।
सहायक डेटा में, हमने बालू मक्खी की तैयारी और पहचान में उपयोग की जाने वाली विभिन्न माउंटिंग तकनीकों के लिए विस्तृत प्रोटोकॉल प्रदान किए हैं। निर्देशात्मक वीडियो सहित ये प्रोटोकॉल, विभिन्न उद्देश्यों के अनुरूप चरण-दर-चरण प्रक्रियाएं प्रस्तुत करते हैं, जिससे सटीक और विश्वसनीय परिणाम सुनिश्चित होते हैं। इस व्यापक संसाधन को उपलब्ध कराकर, हमारा उद्देश्य शोधकर्ताओं को उनकी विशिष्ट आवश्यकताओं के लिए सबसे उपयुक्त माउंटिंग तकनीकों का चयन और अनुप्रयोग करने में सहायता करना है।

स्वीकृतियाँ

लेखक इस पांडुलिपि की गुणवत्ता बढ़ाने में उत्कृष्ट समीक्षा के लिए ब्रिटेन के लंदन स्थित प्राकृतिक इतिहास संग्रहालय के Richard Lane और Zoe Jay Adams को धन्यवाद देते हैं।

अनुदान

हम एजेए के शोध को वित्त पोषित करने के लिए ब्राजील की विकास एजेंसियों सीएनपीक्यू (केस संख्या: 404395/2024-4) और अराउकारिया फाउंडेशन (केस संख्या: 433/2025 पीडीआई) को धन्यवाद देते हैं।

हितों का टकराव

Jérôme Depaquit पैरासाइट पत्रिका के सह-संपादक हैं; इस पांडुलिपि की समीक्षा प्रक्रिया और निर्णय लेने में उनका कोई योगदान नहीं था। अन्य लेखकों ने स्पष्ट किया है कि उनका कोई हित टकराव नहीं है।

1Zenodo पर वीडियो।

वीडियो 1: https://zenodo.org/records/18198006
वीडियो 2: https://zenodo.org/records/18311158
वीडियो 3: https://zenodo.org/records/18311106
वीडियो 4: https://zenodo.org/records/18311154
वीडियो 5: https://zenodo.org/records/18303014
वीडियो 6: https://zenodo.org/records/18302850
वीडियो 7: https://zenodo.org/records/18315029

Supplementary material

The supplementary material of this article is available at https://www.parasite-journal.org/10.1051/parasite/2026009/olm

Bibliografia

1. Alkan C, Allal-Ikhlef AB, Alwassouf S, Baklouti A, Piorkowski G, de Lamballerie X, Izri A, Charrel RN. 2015. Virus isolation, genetic characterization and seroprevalence of Toscana virus in Algeria. Clinical Microbiology and Infection, 21(11), 1040 e1-9.
2. Alten B, Ozbel Y, Ergunay K, Kasap OE, Cull B, Antoniou M, Velo E, Prudhomme J, Molina R, Banuls AL, Schaffner F, Hendrickx G, Van Bortel W, Medlock JM. 2015. Sampling strategies for phlebotomine sand flies (Diptera: Psychodidae) in Europe. Bulletin of Entomological Research 105(6), 664–678.
3. Ayhan N, Baklouti A, Prudhomme J, Walder G, Amaro F, Alten B, Moutailler S, Ergunay K, Charrel RN, Huemer H. 2017. Practical guidelines for studies on sandfly-borne phleboviruses: Part I: Important points to consider *ante* field work. Vector-Borne and Zoonotic Diseases 17(1), 73–80.
4. Bates PA. 1997. Infection of phlebotomine sandflies with *Leishmania*, in The Molecular Biology of Insect Disease Vectors: A Methods Manual. Springer. p. 112–120.5.
5. Baum M, de Castro EA, Pinto MC, Goulart TM, Baura W, Klisiowicz Ddo R, Vieira da Costa-Ribeiro MC. 2015. Molecular detection of the blood meal source of sand flies (Diptera: Psychodidae) in a transmission area of American cutaneous leishmaniasis, Parana State, Brazil. Acta Tropica, 143, 8–12.
6. Belen A, Alten B, Aytekin A. 2004. Altitudinal variation in morphometric and molecular characteristics of *Phlebotomus papatasi* populations. Medical and Veterinary Entomology, 18(4), 343–350.
7. Bhattacharya J, Chandra G, Hati AK. 1991. A simple method for cryopreservation of *Leishmania donovani* promastigotes, Indian Journal of Medical Research, 93, 245–246.
8. Caligiuri LG, Sandoval AE, Miranda JC, Pessoa FA, Santini MS, Salomón OD, Secundino NF, McCarthy CB. 2019. Optimization of DNA extraction from individual sand flies for PCR amplification. Methods and Protocols, 2(2), 36.
9. Casaril AE, de Oliveira LP, Alonso DP, de Oliveira EF, Gomes Barrios SP, de Oliveira Moura Infran J, Fernandes WS, Oshiro ET, Ferreira AMT, Ribolla PEM, de Oliveira AG. 2017. Standardization of DNA extraction from sand flies: Application to genotyping by next generation sequencing. Experimental Parasitology, 177, 66–72.
10. Castalanelli MA, Severtson DL, Brumley CJ, Szito A, Foottit RG, Grimm M, Munyard K, Groth DM. 2010. A rapid non-destructive DNA extraction method for insects and other arthropods. Journal of Asia-Pacific Entomology, 13(3), 243–248.
11. Cerqueira NL. 1943. Um novo meio para montagem de pequenos insetos em lâmina. Memórias do Instituto Oswaldo Cruz, (39), 37–41.
12. Charrel RN, Gallian P, Navarro-Mari JM, Nicoletti L, Papa A, Sanchez-Seco MP, Tenorio A, de Lamballerie X. 2005. Emergence of Toscana virus in Europe. Emerging Infectious Diseases, 11(11), 1657–1663.
13. Chaskopoulou A, Giantsis IA, Demir S, Bon MC. 2016. Species composition, activity patterns and blood meal analysis of sand fly populations (Diptera: Psychodidae) in the metropolitan region of Thessaloniki, an endemic focus of canine leishmaniasis. Acta Tropica, 158, 170–176.

14. Chen H, Rangasamy M, Tan SY, Wang H, Siegfried BD. 2010. Evaluation of five methods for total DNA extraction from western corn rootworm beetles. PLoS One, 5(8), e11963.
15. Depaquit J, Grandadam M, Fouque F, Andry PE, Peyrefitte C. 2010. Arthropod-borne viruses transmitted by Phlebotomine sandflies in Europe: a review. Eurosurveillance, 15(10), 19507.
16. Diamond LS, Herman CM. 1954. Incidence of Trypanosomes in the Canada Goose as revealed by bone marrow culture. Journal of Parasitology, 40(2), 195–202.
17. Ding H, Torno M, Vongphayloth K, Ng G, Tan D, Sng W, Ho K, Randrianambinintsoa FJ, Depaquit J, Tan CH. 2025. Hidden in plain sight: discovery of sand flies in Singapore and description of four species new to science. Parasites & Vectors, 18(1), 402.
18. Es-Sette N, Ajaoud M, Bichaud L, Hamdi S, Mellouki F, Charrel RN, Lemrani M. 2014. *Phlebotomus sergenti* a common vector of *Leishmania tropica* and Toscana virus in Morocco. Journal of Vector Borne Diseases, 51(2), 86–90.
19. Favret C. 2005. A new non-destructive DNA extraction and specimen clearing technique for aphids (Hemiptera). Proceedings of the Entomological Society of Washington, 107(2), 469–470.
20. Galati EAB. 2018. Phlebotominae (Diptera, Psychodidae): Classification, morphology and terminology of adults and identification of American taxa, in Brazilian Sand Flies: Biology, Taxonomy, Medical Importance and Control, Rangel EF, Shaw JJ, Editors. Cham: Springer International Publishing. pp. 9–212.
21. Galati EAB, de Andrade AJ, Perveen F, Loyer M, Vongphayloth K, Randrianambinintsoa FJ, Prudhomme J, Rahola N, Akhoundi M, Shimabukuro PHF, Depaquit J. 2025. Phlebotomine sand flies (Diptera, Psychodidae) of the world. Parasites & Vectors, 18(1), 220.
22. Galati EAB, Galvis-Ovallos F, Lawyer P, Leger N, Depaquit J. 2017. An illustrated guide for characters and terminology used in descriptions of Phlebotominae (Diptera, Psychodidae). Par-asite, 24, 26.
23. Gariepy T, Kuhlmann U, Gillott C, Erlandson M. 2007. Parasitoids, predators and PCR: the use of diagnostic molecular markers in biological control of Arthropods. Journal of Applied Entomology, 131(4), 225–240.
24. Giantsis IA, Chaskopoulou A, Bon MC. 2016. Mild-Vectolysis: A nondestructive DNA extraction method for vouchering sand flies and mosquitoes. Journal of Medical Entomology, 53(3), 692–695.
25. Gidwani K, Picado A, Rijal S, Singh SP, Roy L, Volfova V, Andersen EW, Uranw S, Ostyn B, Sudarshan M, Chakravarty J, Volf P, Sundar S, Boelaert M, Rogers ME. 2011. Serological markers of sand fly exposure to evaluate insecticidal nets against visceral leishmaniasis in India and Nepal: a cluster-randomized trial. PLoS Neglected Tropical Diseases, 5(9), e1296.
26. Gilbert MTP, Moore W, Melchior L, Worobey M. 2007. DNA extraction from dry museum beetles without conferring external morphological damage. PLoS One, 2(3), e272.
27. Giordani BF, Andrade AJ, Galati EAB, Gurgel-Goncalves R. 2017. The role of wing geometric morphometrics in the identification of sandflies within the subgenus *Lutzomyia.* Medical and Veterinary Entomology, 31(4), 373–380.
28. Guzmán-Larralde AJ, Suaste-Dzul AP, Gallou A, Peña-Carrillo KI. 2017. DNA recovery from microhymenoptera using six non-destructive methodologies with considerations for subsequent preparation of museum slides. Genome, 60(1), 85–91.
29. Hajibabaei M, DeWaard JR, Ivanova NV, Ratnasingham S, Dooh RT, Kirk SL, Mackie PM, Hebert PD. 2005. Critical factors for assembling a high volume of DNA barcodes. Philosophical Transactions of the Royal Society B: Biological Sciences, 360(1462), 1959–1967.
30. Haouas N, Pesson B, Boudabous R, Dedet JP, Babba H, Ravel C. 2007. Development of a molecular tool for the identification of *Leishmania* reservoir hosts by blood meal analysis in the insect vectors. American Journal of Tropical Medicine and Hygiene, 77(6), 1054–1059.
31. Hlavackova K, Dvorak V, Chaskopoulou A, Volf P, Halada P. 2019. A novel MALDI-TOF MS-based method for blood meal identification in insect vectors: A proof of concept study on phlebotomine sand flies. PLoS Neglected Tropical Diseases, 13 (9), e0007669.
32. Huemer H, Prudhomme J, Amaro F, Baklouti A, Walder G, Alten B, Moutailler S, Ergunay K, Charrel RN, Ayhan N. 2017. Practical guidelines for studies on sandfly-borne phleboviruses: Part II: Important points to consider for fieldwork and subsequent virological screening. Vector-Borne and Zoonotic Diseases, 17(1), 81–90.
33. Jancarova M, Polanska N, Thiesson A, Arnaud F, Stejskalova M, Rehbergerova M, Kohl A, Viginier B, Volf P, Ratinier M. 2025. Susceptibility of diverse sand fly species to Toscana virus. PLoS Neglected Tropical Diseases, 19(5), e0013031.
34. Kapp JD, Green RE, Shapiro B. 2021. A fast and efficient single-stranded genomic library preparation method optimized for ancient DNA. Journal of Heredity, 112(3), 241–249.
35. Killick-Kendrick R, Maroli M, Killick-Kendrick M. 1991. Bibliography of the colonization of phlebotomine sandflies. Parassitologia, 33(suppl.), 321–333.
36. Lawyer P, Killick-Kendrick M, Rowland T, Rowton E, Volf P. 2017. Laboratory colonization and mass rearing of phlebotomine sand flies (Diptera, Psychodidae). Parasite, 24, 42.
37. Léger N, Pesson B, Madulo-Leblond G. 1986. Les phlébotomes de Grèce : 1ère partie. Bulletin de la Société de Pathologie Exotique, 79, 386–397.
38. Léger N, Pesson B, Madulo-Leblond G. 1986. Les phlébotomes de Grèce : 2ème partie. Bulletin de la Société de Pathologie Exotique, 79, 514–524.
39. Leonel JAF, Vioti G, Alves ML, da Silva DT, Meneghesso PA, Benassi JC, Spada JCP, Galvis-Ovallos F, Soares RM, Oliveira T. 2020. DNA extraction from individual Phlebotomine sand flies (Diptera: Psychodidae: Phlebotominae) specimens: Which is the method with better results? Experimental Parasitology, 218, 107981.
40. Lestinova T, Rohousova I, Sima M, de Oliveira CI, Volf P. 2017. Insights into the sand fly saliva: Blood-feeding and immune interactions between sand flies, hosts, and *Leishmania.* PLoS Neglected Tropical Diseases, 11(7), e0005600.
41. Lienhard A, Schaffer S. 2019. Extracting the invisible: obtaining high quality DNA is a challenging task in small arthropods. PeerJ, 7, e6753.
42. Lozano-Sardaneta YN, Mikery-Pacheco OF, Huerta H, Rojas-Soriano JE, Contreras-Ramos A. 2025. Wing geometric morphometrics is effective to separate sand fly species (Diptera, Psychodidae, Phlebotominae) related with leishmaniasis transmission in Mexico. Acta Tropica, 262, 107523.
43. Mandrioli M. 2008. Insect collections and DNA analyses: how to manage collections? Museum Management and Curatorship, 23(2), 193–199.
44. Maroli M, Feliciangeli MD, Bichaud L, Charrel RN, Gradoni L. 2013. Phlebotomine sandflies and the spreading of leishmaniases and other diseases of public health concern. Medical and Veterinary Entomology, 27(2), 123–147.

45. Marquina D, Buczek M, Ronquist F, Lukasik P. 2021. The effect of ethanol concentration on the morphological and molecular preservation of insects for biodiversity studies. PeerJ, 9, e10799.
46. Mathis A, Depaquit J, Dvorak V, Tuten H, Banuls AL, Halada P, Zapata S, Lehrter V, Hlavackova K, Prudhomme J, Volf P, Sereno D, Kaufmann C, Pfluger V, Schaffner F. 2015. Identification of phlebotomine sand flies using one MALDI-TOF MS reference database and two mass pectrometer systems., Parasites & Vectors, 8, 266.
47. Mekarnia N, Benallal KE, Sadlova J, Vojtkova B, Mauras A, Imbert N, Longhitano M, Harrat Z, Volf P, Loiseau PM, Cojean S. 2024. Effect of *Phlebotomus papatasi* on the fitness, infectivity and antimony-resistance phenotype of antimony-resistant *Leishmania major* MON-25. International Journal for Parasitology – Drugs and Drug Resistance, 25, 100554.
48. Milligan BG. 1998. Total DNA isolation, in Molecular Genetic Analysis of Population: A Practical Approach, Hoelzel AR, Editor. Oxford: Oxford University Press.
49. Molina R, Jiménez M, Alvar J, González E, Hernández-Taberna S, Ines MM. 2017. Methods in sand fly research. Madrid: Servicio de publicaciones, Universidad de Alcalá de Henares, Madrid.
50. Murphy WJ, Eizirik E, O'Brien SJ, Madsen O, Scally M, Douady CJ, Teeling E, Ryder OA, Stanhope MJ, de Jong WW, Springer MS. 2001. Resolution of the early placental mammal radiation using Bayesian phylogenetics. Science, 294(5550), 2348–2351.
51. Nacif-Pimenta R, Pinto LC, Volfova V, Volf P, Pimenta PFP, Secundino NFC. 2020. Conserved and distinct morphological aspects of the salivary glands of sand fly vectors of leishmaniasis: an anatomical and ultrastructural study. Parasites & Vectors, 13(1), 441.
52. Neuhaus B, Schmid T, Riedel J. 2017. Collection management and study of microscope slides: Storage, profiling, deterioration, restoration procedures, and general recommendations. Zootaxa, 4322(1), 1–173.
53. New TR. 1974. Pscoptera. Handbooks for Identification of British Insects (Vol. I). London: Royal Entomological Society of London. 102 pp.
54. Perez-Ruiz M, Collao X, Navarro-Mari JM, Tenorio A. 2007. Reverse transcription, real-time PCR assay for detection of Toscana virus. Journal of Clinical Virology, 39(4), 276–281.
55. Porco D, Rougerie R, Deharveng L, Hebert P. 2010. Coupling non-destructive DNA extraction and voucher retrieval for small soft-bodied Arthropods in a high-throughput context: the example of Collembola. Molecular Ecology Resources, 10(6), 942–945.
56. Prudhomme J, Cassan C, Hide M, Toty C, Rahola N, Vergnes B, Dujardin JP, Alten B, Sereno D, Banuls AL. 2016. Ecology and morphological variations in wings of *Phlebotomus ariasi* (Diptera: Psychodidae) in the region of Roquedur (Gard, France): a geometric morphometrics approach. Parasites & Vectors, 9(1), 578.
57. Prudhomme J, Gunay F, Rahola N, Ouanaimi F, Guernaoui S, Boumezzough A, Banuls AL, Sereno D, Alten B. 2012. Wing size and shape variation of *Phlebotomus papatasi* (Diptera: Psychodidae) populations from the south and north slopes of the Atlas Mountains in Morocco. Journal of Vector Ecology, 37(1), 137–147.
58. Prudhomme J, Toty C, Kasap OE, Rahola N, Vergnes B, Maia C, Campino L, Antoniou M, Jimenez M, Molina R, Cannet A, Alten B, Sereno D, Banuls AL. 2015. New microsatellite markers for multi-scale genetic studies on *Phlebotomus ariasi* Tonnoir, vector of *Leishmania infantum* in the Mediterranean area. Acta Tropica, 142, 79–85.
59. Prudhomme J, Velo E, Bino S, Kadriaj P, Mersini K, Gunay F, Alten B. 2019. Altitudinal variations in wing morphology of *Aedes albopictus* (Diptera, Culicidae) in Albania, the region where it was first recorded in Europe. Parasite, 26, 55.
60. Rawlins DJ. 1992. Light Microscopy: An Introduction to Biotechniques. Oxford: Bios Scientific publishers. 143 pp.
61. Ready PD. 2013. Biology of phlebotomine sand flies as vectors of disease agents. Annual Review of Entomology, 58, 227–250.
62. Rohlf FJ, Slice D. 1990. Extensions of the Procrustes method for the optimal superimposition of landmarks. Systematic Zoology, 39(1), 40–59.
63. Rowley DL, Coddington JA, Gates MW, Norrbom AL, Ochoa RA, Vandenberg NJ, Greenstone MH. 2007. Vouchering DNA-barcoded specimens: Test of a nondestructive extraction proto-col for terrestrial arthropods. Molecular Ecology Notes, 7(6), 915–924.
64. Sábio PB, Andrade AJ, Galati EAB. 2014. Assessment of the taxonomic status of some species included in the *Shannoni* complex, with the description of a new species of *Psathyromyia* (Diptera: Psychodidae: Phlebotominae). Journal of Medical Entomology, 51(2), 331–341.
65. Sadlova J, Yeo M, Seblova V, Lewis MD, Mauricio I, Volf P, Miles MA. 2011. Visualisation of *Leishmania donovani* fluorescent hybrids during early stage development in the sand fly vector. PLoS One, 6(5), e19851.
66. Sales K, Miranda DEO, da Silva FJ, Otranto D, Figueredo LA, Dantas-Torres F. 2020. Evaluation of different storage times and preservation methods on phlebotomine sand fly DNA concentration and purity. Parasites & Vectors, 13(1), 399
67. Sales KG, Costa PL, de Morais RC, Otranto D, Brandao-Filho SP, Cavalcanti Mde P, Dantas-Torres F. 2015. Identification of phlebotomine sand fly blood meals by real-time PCR. Parasites& Vectors, 8, 230.
68. Sant'Anna MR, Jones NG, Hindley JA, Mendes-Sousa AF, Dillon RJ, Cavalcante RR, Alexander B, Bates PA. 2008. Blood meal identification and parasite detection in laboratory-fed and field-captured *Lutzomyia longipalpis* by PCR using FTA databasing paper. Acta Tropica, 107(3), 230–237.
69. Senne NA, Santos HA, Araujo TR, Paulino PG, Mendonca LP, Moreira HVS, Camilo TA, da Costa Angelo I. 2022. Robust comparative performance of genomic DNA extraction methods from non-engorged phlebotomine sandflies. Medical and Veterinary Entomology, 36(2), 203–211.
70. Shaw JJ. 2025. A review of Leishmania infections in American Phlebotomine sand flies – Are those that transmit leishmaniasis anthropophilic or anthropportunists? Parasite, 32, 57.
71. Tesh RB, Modi GB. 1983. Growth and transovarial transmission of Chandipura virus (Rhabdoviridae: Vesiculovirus) in *Phlebotomus papatasi.* American Journal of Tropical Medicine and Hygiene, 32(3), 621–623.
72. Thomsen PF, Elias S, Gilbert MTP, Haile J, Munch K, Kuzmina S, Froese DG, Sher A, Holdaway RN, Willerslev E. 2009. Non-destructive sampling of ancient insect DNA. PLoS One, 4(4),e5048
73. Truett GE, Heeger P, Mynatt RL, Truett AA, Walker JA, Warman ML. 2000. Preparation of PCR-quality mouse genomic DNA with hot sodium hydroxide and tris (HotSHOT). Biotechniques, 29(1), 52–54.

74. Upton MS. 1993. Aqueous gum-chloral slide mounting media: an historical review. Bulletin of Entomological Research, 83(2), 267–274.
75. Volf P, Myskova J. 2007. Sand flies and *Leishmania*: specific versus permissive vectors. Trends in Parasitology, 23(3), 91–92.
76. Wang Q, Wang X. 2012. Comparison of methods for DNA extraction from a single chironomid for PCR analysis. Pakistan Journal of Zoology, 44(2), 421–426.
77. Wang Y, Zhao Y, Bollas A, Wang Y, Au KF. 2021. Nanopore sequencing technology, bioinformatics and applications. Nature Biotechnology, 39(11), 1348–1365.

Cite this article as: Randrianambinintsoa FJ, Augendre L, Prudhomme J, Martinet J-P, Loyer M, Mekarnia N, Kerkoub H, Perveen FK, Huguenin A, Kariya E, Akhoundi M, De Andrade AJ, Berriatua E, Bongiorno G, Boyer S, Christodoulou V, Da Costa-Ribeiro MCV, De Souza LAF, Ding H, Dondji B, Dvořák V, Erisoz Kasap O, Galati EAB, Gállego M, Ballart C, Gouzelou S, Haddad N, Masse RS, Mekuria AH, Ivovic V, Kaczmarek S, Shahar MK, Kirstein OD, Kniha E, Kolářová I, Lincoln T, Lucanas C, Mikov O, Nov K, Özbel Y, Pesson B, Posada Lopez LC, Prasetyo DB, Rahola N, Rebollar-Tellez EA, Rodrigues BL, Roy L, Saini P, Sanjoba C, Shimabukuro PH, Siriyasatien P, Soszynska A, Suleşco T, Sylla M, Torno M, Volf P, Vongphayloth K, Sinh Nam V, Wardhana A, Yessinou E, Zapata S, Gantier J-C & Depaquit J. 2026. Processing and mounting phlebotomine sand flies: a consensus guideline. Parasite xx, xx. https://doi.org/10.1051/parasite/2026009.

PARASITE

An international open-access, peer-reviewed, online journal publishing high quality papers on all aspects of human and animal parasitology

Reviews, articles and short notes may be submitted. Fields include, but are not limited to: general, medical and veterinary parasitology; morphology, including ultrastructure; parasite systematics, including entomology, acarology, helminthology and protistology, and molecular analyses; molecular biology and biochemistry; immunology of parasitic diseases; host-parasite relationships; ecology and life history of parasites; epidemiology; therapeutics; new diagnostic tools.
All papers in Parasite are published in English. Manuscripts should have a broad interest and must not have been published or submitted elsewhere. No limit is imposed on the length of manuscripts.

Parasite (open-access) continues **Parasite** (print and online editions, 1994-2012) and **Annales de Parasitologie Humaine et Comparée** (1923-1993) and is the official journal of the Société Française de Parasitologie.

Editor-in-Chief:
Jean-Lou Justine, Paris

Submit your manuscript at:
https://www.editorialmanager.com/parasite

परिशिष्ट 1: जैव रासायनिक सैद्धांतिक आधार।

यहां जिन आर्थ्रोपोड्स की बात हो रही है, वे रेत मक्खियां हैं। हालांकि, इस सामान्य अवधारणा को अन्य बहुत आम आर्थ्रोपोड्स पर भी लागू किया जा सकता है, जिनकी पहचान केवल आंतरिक आकारिकी विशेषताओं के आधार पर ही की जा सकती है। संयोगवश, कुछ आंतरिक अंग आंशिक रूप से काइटिनयुक्त होते हैं और उनकी आकारिकी हमें बहुमूल्य जानकारी प्रदान करती है। यही कारण है कि खाद्य नलिकाओं, शुक्राणुकोशों और उनकी नलिकाओं का अवलोकन करना बहुत रोचक है। जिन सभी अभिकर्मकों की हम समीक्षा करेंगे, उनके साथ यह कभी नहीं भूलना चाहिए कि कीट के स्थिरीकरण से लेकर संयोजन तक, हम केवल रेडॉक्स अभिक्रियाओं का उपयोग करेंगे। एकमात्र सावधानी या विचार जो हमें मार्गदर्शन देगा, वह है अपचायक अभिकर्मकों को ऑक्सीकारक अभिकर्मकों के साथ मिलाने से बचना।

एथिल अल्कोहल; एथेनॉल:

इस पदार्थ का उपयोग विभिन्न तरीकों से किया जाएगा। अल्कोहल के अणुओं में पानी के प्रति प्रबल आकर्षण होता है और इसलिए वे निर्जलीकरण प्रभाव प्रदर्शित करते हैं।हालांकि, कम सांद्रता वाला अल्कोहल (यानी, पानी की मात्रा अधिक होने पर) न्यूक्लिक एसिड के अपघटन में भूमिका निभाएगा (पानी न्यूक्लिक एसिड का दुश्मन है) ।

जब कीटों को इथेनॉल में रखा जाता है, तो यह न केवल उन्हें संरक्षित करने के लिए होता है, बल्कि ऊतकों को स्थिर करने के लिए भी होता है। ऊतक विज्ञान में, हम आमतौर पर दो महत्वपूर्ण अवधारणाओं में अंतर करते हैं: प्रवेश दर और स्थिरीकरण दर। यह सर्वविदित है कि एक अच्छे परिरक्षक को ऊतकों को स्थिर करने से पहले उनमें तेजी से गहराई तक प्रवेश करना चाहिए। 96% अल्कोहल के लिए, प्रवेश गुणांक लगभग 1.05 है (तुलना में, पिक्रिक एसिड के 0.75% जलीय घोल के लिए, प्रवेश गुणांक 0.45 है, जबकि 3% पोटेशियम डाइक्रोमेट घोल के लिए यह 1.45 है)।

कीटविज्ञानियों के लिए, इथेनॉल में कीटों और अन्य आर्थ्रोपोड्स को अनिश्चित काल तक संरक्षित रखना एक वास्तविकता है। भविष्य के अध्ययनों या शोधकर्ताओं के लिए फील्ड से प्राप्त नमूनों को सुरक्षित रखना एक सराहनीय विचार है। हालांकि, कोशिकाविज्ञानी या ऊतकविज्ञानी के लिए यह संभव नहीं है। नमूनों को फिक्सेटिव में बहुत लंबे समय तक रखने से, उन पर दोबारा काम करना लगभग असंभव हो जाता है। यही कारण है कि 10 वर्ष से अधिक पुराने नमूनों का उपयोग करना कठिन या असंभव हो जाता है।

एक और महत्वपूर्ण बात यह है कि फिक्सेटर के द्रव्यमान और आयतन के बीच का अनुपात कितना होना चाहिए। प्राणी विज्ञान या चिकित्सा में, फिक्सेटर का आयतन फिक्सेटर के आयतन से 60 गुना अधिक होना उचित होता है। व्यवहार में, सूक्ष्म आर्थ्रोपोड्स के लिए, फिक्सेटर के लिए निर्धारित आयतन के लिए कम से कम 4-5 गुना अल्कोहल मिलाना चाहिए। ध्यान रखें कि अल्कोहल आर्थ्रोपोड के ऊतकों में मौजूद सारा पानी सोख लेता है, जिससे उसकी शक्ति कम हो जाती है।

निष्कर्ष के तौर पर:

- एथिल अल्कोहल एक अपचायक रासायनिक कारक है (इसलिए यह ऑक्सीकारक स्थिरीकरण पदार्थों के साथ असंगत है);
- यह ऊर्जावान तरीके से प्रोटीनों को अवक्षेपित करता है और उन्हें विकृत कर देता है;
- यह कुछ जटिल लिपिड को घोलता है और ग्लाइकोजन को अवक्षेपित करता है;
- इससे ऊतकों में तीव्र संकुचन होता है और वे सख्त हो जाते हैं।

पोटेशियम या सोडियम हाइड्रॉक्साइड के क्षारीय विलयन:

कीटविज्ञान में इन समाधानों का उपयोग मुख्य रूप से पोटेशियम हाइड्रॉक्साइड पर केंद्रित रहा है, लेकिन इसका कोई स्पष्ट तर्क नहीं है।

सोडियम हाइड्रॉक्साइड [E524] विभिन्न सांद्रताओं या अलग-अलग नॉर्मेलिटी के साथ विलयन में पाया जाता है। यह गोलियों या ग्लिटर के रूप में उपलब्ध होता है। इसकी प्रमुख कमी यह है कि यह अत्यधिक नमी सोखने वाला (KOH से भी अधिक) होता है। जब यह प्रोटीन के साथ अभिक्रिया करता है, तो उन्हें घोल देता है, और वसा के साथ अभिक्रिया करके उन्हें साबुनीकरण के दौरान कठोर साबुन में बदल देता है (यह KOH से एक प्रमुख अंतर है, जो साबुनीकरण के दौरान तरल साबुन बनाता है) ।

पोटेशियम हाइड्रॉक्साइड [E525] सांद्र विलयन के रूप में उपलब्ध है, लेकिन सबसे महत्वपूर्ण बात यह है कि यह लगभग 0.1 ग्राम की गोलियों के रूप में उपलब्ध है, जिससे सटीक मापक तराजू की आवश्यकता न होने पर भी तनु विलयन तैयार करना बहुत आसान हो जाता है। उदाहरण के लिए, 1 मिलीलीटर आसुत

जल में 0.1 ग्राम की 1 गोली डालने से 10% विलयन प्राप्त होता है। गोलियों के रूप में पोटेशियम हाइड्रॉक्साइड का दूसरा लाभ यह है कि यह कार्बोनेशन के प्रति कम संवेदनशील होता है (KOH विलयन में CO2 को स्थिर करने की उच्च क्षमता होती है, जिससे कार्बोनेट लवण बनते हैं)।

इन प्रबल क्षारों का उपयोग वसा अम्लों को जल में घुलनशील साबुन में परिवर्तित करके उन्हें घुलने योग्य बनाने के लिए किया जाएगा। यह ध्यान रखना महत्वपूर्ण है कि इथेनॉल जैसे स्थिरक पदार्थ ने नमूने में मौजूद कुछ वसा को घुलने योग्य बना दिया था। हालांकि, नमूने को एक प्रबल क्षार के साथ जलीय माध्यम में रखने पर वसा अम्ल (कमोबेश जटिल) अवक्षेपित हो जाएंगे। इस प्रकार प्रबल क्षार ठंडी साबुनीकरण प्रक्रिया को अंजाम देगा। कुछ मामलों में, जब वसा ऊतक अधिक मात्रा में होते हैं, उदाहरण के लिए महिलाओं में, तो अभिक्रिया को सुगम बनाने के लिए तापमान को 35-40 डिग्री सेल्सियस तक बढ़ाना या कमरे के तापमान पर संपर्क समय को बढ़ाना लाभकारी होगा।

रंगीन अम्लीय विलयन/रंगहीन मार्क-आंद्रे विलयन:

यहां हम मार्क-आंद्रे विलयन के लाभ और हानियों का पता लगाएंगे। यह विलयन chloral hydrate (ट्राइक्लोरोएसिटैल्डिहाइड मोनोहाइड्रेट), एसिटिक अम्ल और जल से बना होता है। यह विलयन अत्यधिक ऑक्सीकारक (अम्ल और एल्डिहाइड का मिश्रण) होता है। यह नमूनों में शेष बचे अतिरिक्त पोटेशियम हाइड्रॉक्साइड को निष्क्रिय कर देता है, बिना पोटेशियम हाइड्रॉक्साइड के उपयोग के दौरान बनने वाले क्षारीय साबुनों को अवक्षेपित किए। यह ऑक्सीकारक विलयन काइटिन में बनने वाले ग्लूकोसामाइन के द्वितीयक अल्कोहल कार्यों पर भी ऑक्सीकृत करके क्रिया करता है, जिससे काइटिन नरम हो जाता है। एक अन्य क्रिया है इसमें मौजूद कुछ खनिज लवणों का विघटन।

जब मार्क-आंद्रे विलयन को पहले से ही एसिड फ्यूसिन द्वारा रंगीन किया जाता है (अर्थात ऑक्सीकृत अवस्था में), तो यह संरचना के द्वितीयक अल्कोहल कार्यों पर स्थिर हो जाएगा। मार्क-आंद्रे विलयन और नमूनों की रंगीन अवस्था के संपर्क समय के बाद, केवल इथेनॉल से ही धोया जाएगा। इस प्रकार नमूनों के निर्जलीकरण की प्रक्रिया शुरू होती है।

फ़ायदे:

- अतिरिक्त क्षारीय विलयनों का उदासीनीकरण
- चिटिन का शिथिलन
- काइटिन की आंतरिक संरचनाओं का बेहतर आकलन करने के लिए काइटिन की रंगाई करना।
-

कमियां:

Chloral hydrate एक सम्मोहनकारी पदार्थ है और इसका उपयोग मानव चिकित्सा में किया जाता रहा है। इसका उपयोग रासायनिक आवरण के अंतर्गत ही किया जाना चाहिए, और रासायनिक जोखिमों से संबंधित कानूनों का अनुपालन अनिवार्य है।

निर्जलीकरण समाधान:

अनुभव से पता चलता है कि बहुत छोटे नमूनों के लिए, बढ़ती सांद्रता वाले अल्कोहल स्नान के क्रम का पालन करना उपयोगी नहीं है। यदि नमूना बड़ा है, तो हम 80% इथेनॉल से शुरू करेंगे, फिर 90%, 95% और अंत में शुद्ध इथेनॉल का उपयोग करेंगे। बहुत छोटे नमूनों के लिए, 90% इथेनॉल के स्नान का उपयोग करें और फिर उसे शुद्ध इथेनॉल में डुबो दें। इस चरण में, हमें हमेशा याद रखना चाहिए कि शुद्ध इथेनॉल वातावरण में मौजूद पानी को स्थिर करने का प्रयास करता है।

कीट विज्ञान प्रयोगशालाओं में नमूनों के निर्जलीकरण को बीच क्रेओसोट के घोल में डुबोकर अंतिम रूप देने की परंपरा थी। आज, कीटनाशक, कवकनाशी और लकड़ी संरक्षक के रूप में व्यापक रूप से उपयोग किए जाने वाले इस अर्क को इसकी गंध (पॉलीसाइक्लिक एरोमैटिक हाइड्रोकार्बन) के कारण सख्ती से हतोत्साहित किया जाता है और इसे प्रजनन विषैला, कैंसरकारी, एक स्थायी कार्बनिक प्रदूषक और जलीय जीवों के लिए पारिस्थितिक विषैला माना जाता है।

नमूनों को तैयार करने के लिए हम जो समाधान प्रस्तावित करते हैं, वह है यूपराल® और यूपराल एसेंस (जिसका वर्णन अगले पैराग्राफ में किया गया है) । यूपराल® और यूपराल एसेंस का मिश्रण बहुत अच्छी तरह से स्वीकार किया जाता है; 90% इथेनॉल स्नान के बाद प्राप्त नमूनों को बेहतर परिणाम मिलते हैं।

परिशिष्ट 2: प्रयुक्त अभिकर्मकों की संरचना।

पोटेशियम हाइड्रोक्साइड 10%

पोटेशियम हाइड्रॉक्साइड 10 ग्राम

आसुत जल आवश्यकतानुसार 100 मिलीलीटर

गम क्लोरल माउंटिंग मीडियम होयर मीडियम

आसुत जल 50 मिलीलीटर

CHLORAL HYDRATE 200 ग्राम

गोंद अरबी 50 ग्राम

ग्लिसरॉल 20 मिलीलीटर

मार्क-आंद्रे समाधान

CHLORAL HYDRATE 40 ग्राम

ग्लेशियल एसिटिक एसिड 30 मिलीलीटर

आसुत जल 30 मिलीलीटर

आसुत जल में 1% फुचसिन अम्ल

एसिड फ्यूसिन पाउडर 1 ग्राम

आसुत जल 99 मिलीलीटर

न घुलनेवाली तलछट-एंड्रे के घोल को फ्यूसिन से रंगा गया

मार्क-आंद्रे घोल 10 मिलीलीटर

फ्यूसिन 1% 50 µL

परिशिष्ट 3: यूपरल®कनाडा बालसम, पॉलीविनाइल अल्कोहल या अन्य माउंटिंग घोल

पॉलीविनाइल अल्कोहल: जब उचित निर्जलीकरण के लिए आवश्यक उत्पाद उपलब्ध न हों, तो यह आदर्श माउंटिंग माध्यम है। इसमें पॉलीविनाइल अल्कोहल को अम्मान के लैक्टोफेनॉल के साथ मिलाया जाता है। हालांकि, इन मिश्रणों में कुछ प्रमुख कमियां हैं, जैसे कि पानी के वाष्पीकरण के कारण पॉलीविनाइल अल्कोहल का सूख जाना या क्रिस्टलीकरण होना, या फेनॉल के ऑक्सीकरण से काला पड़ जाना। अल्पकालिक माउंटिंग के लिए यह एक अच्छी तकनीक बनी हुई है।

कनाडा बाल्सम: स्लाइड और कवरस्लिप के बीच माउंट करने के लिए इसका उपयोग करने हेतु माउंट किए जाने वाले नमूनों को निर्जलीकरण की आवश्यकता होती है। ज़ाइलीन या टोल्यून का उपयोग असुविधाजनक हो सकता है।

Enecê medium: स्लाइड और कवरस्लिप के बीच माउंट करने के लिए, जैसे कि कनाडा बाल्सम, नमूने को निर्जलीकरण की आवश्यकता होती है। एनेक फॉर्मूलेशन: शुद्ध सफेद कोलोफोनी (22 ग्राम); अल्कोहल में घुलनशील कोपल गोंद (12 ग्राम), शुद्ध अल्कोहल (20 मिलीलीटर), कपूर (10 ग्राम), तारपीन का एसेंस (10 मिलीलीटर), और यूकेलिप्टोल (26 मिलीलीटर)। इसे तैयार करने के लिए, एक बर्तन, जैसे कि एर्लेनमेयर फ्लास्क में, शुद्ध अल्कोहल और कपूर डालें। फिर कोलोफोनी और कोपल गोंद डालें। फ्लास्क को ढक्कन से बंद करके हिलाएं, और फिर इसे धीमी आंच पर बेन-मैरी में गर्म करें ताकि मिश्रण उबल न जाए। जब सामग्री पूरी तरह से तरल हो जाए, तो तारपीन का एसेंस डालें, फिर मिश्रण को गर्म रहते हुए छान लें, और अंत में, छने हुए घोल में यूकेलिप्टोल मिला दें। जब माध्यम कम तरल हो जाता है, तो इसे ईनेसी से पतला किया जाता है, जिसका सूत्र निम्नलिखित है: शुद्ध अल्कोहल (30 मिलीलीटर), कपूर (17 ग्राम), तारपीन का अर्क (15 मिलीलीटर), यूकेलिप्टोल (38 मिलीलीटर)। (सेरकेइरा, 1943)।

यूपरल®: यह एटलस टेट्राक्लिनिस आर्टिकुलाटा (वाहल, 1791) के सरू के पेड़ से प्राप्त एक राल है, जिसका अध्ययन और विकास 1906 में गिलसन द्वारा किया गया था। इसका मुख्य लाभ यह है

कि यह बहुलकित नहीं होता है। स्लाइड और कवर-स्लिप के बीच लगे नमूनों को अल्कोहल या उससे भी बेहतर यूपराल® एसेंस की क्रिया से आसानी से पुनः प्राप्त किया जा सकता है। यह राल, जिसे सैंडरैक भी कहा जाता है, 80% तक इथेनॉल को ग्रहण करता है।

TRITON X100 का उपयोग: गैर-आयनिक जलीय विलयन:

TRITON X100 एक गैर-आयनिक जलीय विलयन (4-(1,1,3,3-टेट्रामेथिलब्यूटिल) फेनिल-पॉलीइथिलीन ग्लाइकॉल विलयन, या टी-ऑक्टिलफेनोक्सीपॉलीइथॉक्सीएथेनॉल, पॉलीइथिलीन ग्लाइकॉल टर्ट-ऑक्टिलफेनिल ईथर) के रूप में है, जिसका व्यापक रूप से कोशिकीय और आणविक जीव विज्ञान में डिटर्जेंट के रूप में उपयोग किया जाता है। यह कोशिका और नाभिकीय झिल्लियों के पारगम्यता को बढ़ाता है।

कई वर्षों तक अल्कोहल में संरक्षित कीटों के नमूने आम बात हैं। दुर्भाग्य से, अल्कोहल में संरक्षण सर्वोत्तम नहीं है, और इस प्रकार संरक्षित आर्थ्रोपोड्स को सूक्ष्मदर्शी से जांच के लिए तैयार करना बहुत मुश्किल हो जाता है। नमूनों वाले प्लास्टिक अक्सर खराब हो जाते हैं, जिसके बाद अल्कोहल वाष्पित हो जाता है। दोनों ही मामलों में, अल्कोहल के साथ लंबे समय तक संपर्क या नमूनों का सूखना एक गंभीर समस्या पैदा करता है। 2008 में, जॉनक ने फोटोग्राफिक फिल्मों के लिए उपयोग किए जाने वाले एजपोन जैसे वेटिंग एजेंट के साथ मकड़ियों के पुनर्जलीकरण पर एक नोट प्रकाशित किया [26]। इससे ऐसे वेटिंग एजेंटों का उपयोग करने का विचार आया जो शक्तिशाली डिटर्जेंट नहीं हैं।

नीचे 0.5% जलीय विलयन में TRITON X100 का उपयोग करने की प्रक्रिया दी गई है:

- शुष्क नमूने को शुद्ध अल्कोहल से संतृप्त किया गया।
- 0.5% सांद्रता वाले TRITON X100 विलयन की आवश्यक मात्रा डालें ताकि पूरा नमूना उसमें डूब जाए।
- इसे लगभग 5 मिनट या उससे अधिक समय तक ऐसे ही रहने दें। सभी आर्थ्रोपोड को घोल में स्वतंत्र हो जाना चाहिए।
- TRITON X100 विलयन को हटा दिया जाता है और उसके स्थान पर पोटेशियम हाइड्रॉक्साइड विलयन डाल दिया जाता है।

इसके बाद, ऊपर वर्णित प्रक्रिया का पालन किया जाता है।

परिशिष्ट 4: यूपरल®या कनाडा बालसम माउंटिंग मीडिया को चरण दर चरण

1. नमूनों को निर्जलित किया जाना चाहिए (धुंधला या दूधिया रंग अपर्याप्त निर्जलीकरण का संकेत देता है) ।

2. एथिल अल्कोहल की सांद्रता बढ़ाकर निर्जलीकरण किया जा सकता है।

3. नमूनों को 99% अल्कोहल या शुद्ध अल्कोहल से एक समाशोधक एजेंट में स्थानांतरित किया जा सकता है।

प्रक्रिया:

1. वयस्क बालू मक्खियों को 70% इथेनॉल में विच्छेदित करें।

2. इथेनॉल को हटा दें और उसके स्थान पर 10% KOH डालें। बालू मक्खियों को कांच की स्लाइड से ढक दें।

3. कीड़ों के पारदर्शी होने तक उन्हें मसलें।

4. KOH को हटा दें।

5. नमूने को आसुत जल से ढक दें और 30 से 45 मिनट तक प्रतीक्षा करें।

6. पानी निकाल दें और आसुत जल से 30 मिनट तक दोबारा धोएं (समय नमूनों की संख्या पर निर्भर करता है: जितने अधिक नमूने एक साथ संसाधित करने होंगे, उतना ही अधिक समय लगेगा। नमूनों की संख्या जितनी कम होगी, विशेषकर जिन्हें अलग-अलग संसाधित किया जाना है, उतना ही कम समय लगेगा) ।

7. पानी निकाल दें

8. मार्क-आंद्रे घोल (संभवतः फ्यूक्सिन एसिड से रंगीन) डालें और 24 घंटे (एक दिन) प्रतीक्षा करें।

9. मार्क-आंद्रे सॉल्यूशन को हटा दें।

10. नमूने को आसुत जल से ढक दें और 30 से 45 मिनट तक प्रतीक्षा करें।

11. पानी निकाल दें और आसुत जल से 30 मिनट तक दोबारा धोएं।

12. पानी निकाल दें

13. 70% इथेनॉल मिलाएं और नमूने का विच्छेदन करें।

क. सिर और पेट के लिए, सिर या पेट को छाती से धीरे से खींचकर अलग करें।

b. वक्ष के लिए, एक चिमटी से वक्ष को पकड़ें और दूसरी चिमटी से उपांगों के आधार को खींचकर पंखों को हटा दें। रुचि के क्षेत्रों के आधार पर, वक्ष को बाएँ और दाएँ भागों में विभाजित करते हुए, सैजिटल विच्छेदन करना संभव है।

14. जलीय एथिल अल्कोहल विलयनों की एक श्रृंखला के माध्यम से धीरे-धीरे निर्जलीकरण करें। 50 – 80 – 95% तक जब तक कि पूर्ण इथेनॉल प्राप्त न हो जाए।

15. नमूनों को 100% इथेनॉल से दो बार, प्रत्येक बार 10 मिनट के लिए धोकर निर्जलित करें।

16. इथेनॉल को हटा दें और नमूनों को कमरे के तापमान पर 15 मिनट के लिए लौंग के तेल से ढक दें।

17. लौंग के तेल से नमूनों को एक साफ स्लाइड पर यूपराल® या कनाडा बालसम की एक बूंद में स्थानांतरित करें।

18. इच्छानुसार व्यवस्थित करें: बालू मक्खी के सिर, वक्ष और उदर को विच्छेदन सूक्ष्मदर्शी के नीचे महीन सुइयों या चिमटों का उपयोग करके विच्छेदित किया जा सकता है। सिर को शरीर से इस प्रकार विच्छेदित किया जाना चाहिए कि उसे अधर-पृष्ठीय स्थिति में लगाया जा सके, अर्थात् पश्चकपाल छिद्र ऊपर की ओर होना चाहिए ताकि सिबेरियम को सीधे उसके माध्यम से देखा जा सके।

यह विच्छेदन बालू मक्खी माउंटिंग मीडियम में किया जाता है।

19. नमूने को तब तक छोड़ दें जब तक कि सतह चिपचिपी न हो जाए।

20. एक साफ कवरस्लिप को शुद्ध अल्कोहल से गीला करें। कवरस्लिप को कनाडा बाल्सम पर तिरछे कोण पर रखें।

21. स्लाइड को इस उद्देश्य के लिए बनाए गए सूखे बॉक्स में रखें।
