# Supplementary material for: Processing and mounting phlebotomine sand flies: a consensus guideline
Source: Parasite. 2026 Apr 3;33:18. doi: 10.1051/parasite/2026009 (PMC13047900; doi:10.1051/parasite/2026009)
Supplement: Supplementary file 15 — Indonesian translation / Terjemahan Bahasa Indonesia [file parasite-33-18-s15.pdf]

Parasite **33**, 18 (2026)
© F.J. Randrianambinintsoa et al., published by EDP Sciences, 2026
https://doi.org/10.1051/parasite/2026009

PARASITE
Available online at:
www.parasite-journal.org

REVIEW ARTICLE OPEN ACCESS

Pedoman berbasis konsensus: Pemrosesan dan pembuatan preparat (mounting) lalat pasir (Diptera: Psychodidae)

Fano José Randrianambinintsoa[1], Laure Augendre[1], Jorian Prudhomme[1], Jean-Philippe Martinet[1], Mathieu Loyer[1], Nalia Mekarnia[1], Hocine Kerkoub[1], Farzana Khan Perveen[1], Antoine Huguenin[1,2], Emilie Kariya[1,2], Mohammad Akhoundi[3], Andrey José de Andrade[4], Eduardo Berriatua[5], Gioia Bongiorno[6], Sébastien Boyer[7,8], Vasiliki Christodoulou[9], Magda Clara Vieira Da Costa-Ribeiro[10], Lucas Alexandre Farias de Souza[10], Huicong Ding[11], Blaise Dondji[12], Vít Dvořák[13], Ozge Erisoz Kasap[14], Eunice Aparecida Bianchi Galati[15], Montserrat Gállego[16], Cristina Ballart[16], Stavroula Gouzelou[17], Nabil Haddad[18], Rezki Sabrina Masse[19], Asrat Hailu Mekuria[20], Vladimir Ivovic[21], Szymon Kaczmarek[22], Mohd Khadri Shahar[19], Oscar D. Kirstein[23], Edwin Kniha[24], Iva Kolářová[13], Lincoln Timinao[25], Cristian Lucanas[26], Ognyan Mikov[27], Kimsear Nov[7], Yusuf Özbel[28], Bernard Pesson[29], Laura Cristina Posada Lopez[30], Didot Budi Prasetyo[1,7], Nil Rahola[31], Eduardo A. Rebollar-Tellez[32], Bruno Leite Rodrigues[15], Lalita Roy[33], Prasanta Saini[34], Chizu Sanjoba[35], Paloma Helena Fernandes Shimabukuro[36], Padet Siriyasatien[37], Agnieszka Soszyńska[22], Tatiana Suleşco[38], Massamba Sylla[39], Majhalia Torno[40], Petr Volf[13], Khamsing Vongphayloth[41], Vu Sinh Nam[42], April Hari Wardhana[43], Eric Yessinou[44], Sonia Zapata[45], Jean-Charles Gantier[1], and Jérôme Depaquit[1,2]

[1] Faculté de Pharmacie, Université de Reims Champagne Ardenne, UR ESCAPE-USC ANSES PETARD, 51 rue Cognacq-Jay, 51096 Reims Cedex, France
[2] Pôle de Biologie territoriale, Laboratoire de Parasitologie-Mycologie, Centre Hospitalo-Universitaire, 51092 Reims, France
[3] Parasitology-Mycology Department, Avicenne Hospital, AP-HP, Bobigny, Sorbonne Paris Nord University, France; Unité des Virus Émergents (UVE: Aix-Marseille Univ, Università di Corsica, IRD 190, Inserm 1207, IRBA), 13005 Marseille, France
[4] Parasitology Collection of Basic Pathology, Department of Basic Pathology, Federal University of Paraná, Curitiba 19031, Brazil
[5] Department of Animal Health, University of Murcia, Campus de Espinardo, 30100 Espinardo, Murcia, Spain
[6] Department of Infectious Diseases, Vector-borne Diseases Unit, Istituto Superiore di Sanità, 00166 Rome, Italy
[7] Medical and Veterinary Entomology Unit, Institut Pasteur du Cambodge, Phnom Penh 12201, Cambodia
[8] Ecology & Emergence of Arthropod-borne Pathogens Unit, Department of Global Health, Institut Pasteur, CNRS UMR2000, 75015 Paris, France
[9] Section Veterinary Services (1417), Laboratory for Animal Health Virology, Aglantzia, Nicosia 2109, Cyprus
[10] Insects Vectors and Parasites Laboratory, Department of Basic Pathology and Postgraduate program in Microbiology, Parasitology and Pathology, Federal University of Paraná, 81530-900 Curitiba, Brazil
[11] Department of Biological Sciences, National University of Singapore, 117558, Singapore
[12] Laboratory of the Leishmaniasis Research Project, Mokolo District Hospital, Mokolo, Cameroon; Laboratory of Cellular Immunology and Parasitology, Department of Biological Sciences, Central Washington University, 98926 Ellensburg, WA, USA
[13] Department of Parasitology, Faculty of Science, Charles University, 12800 Prague, Czechia
[14] VERG Laboratories, Department of Biology, Faculty of Science, Hacettepe University, Beytepe, Ankara 06800, Türkiye
[15] Faculdade de Saúde Pública da Universidade de São Paulo (FSP/USP), Pós-graduação em Saúde Pública, 01246-904 São Paulo, Brazil
[16] Secció de Parasitologia, Departament de Biologia, Sanitat i Medi Ambient, Facultat de Farmàcia i Ciències de l'Alimentació, Universitat de Barcelona, & Institut de Salut Global de Barcelona (ISGlobal), Centro de Investigación Biomédica en Red, Enfermedades Infecciosas (CIBERINFEC), 08028 Barcelona, Spain
[17] Laboratory of Infectious Diseases and Public Health, School of Medicine, University of Cyprus, Nicosia, Cyprus & Department of Pediatrics, Archbishop Makarios III Hospital, Nicosia 2115, Cyprus
[18] Faculty of Health Sciences, American University of Beirut, 1107 2020 Beirut, Lebanon
[19] Medical Entomology Unit, Infectious Disease Research Centre, Institute for Medical Research (IMR), National Institutes of Health (NIH), Ministry of Health Malaysia, 40170 Shah Alam, Selangor, Malaysia
[20] School of Medicine, Addis Ababa University, 28017 - 1000 Addis Ababa, Ethiopia

Edited by Jean-Lou Justine
*Corresponding author: jerome.depaquit@univ-reims.fr

This is an Open Access article distributed under the terms of the Creative Commons Attribution License (https://creativecommons.org/licenses/by/4.0), which permits unrestricted use, distribution, and reproduction in any medium, provided the original work is properly cited.

[21] Faculty of Mathematics, Natural Sciences and Information Technologies, University of Primorska, 6000 Koper, Slovenia
[22] University of Lodz, Faculty of Biology and Environmental Protection, Department of Invertebrate Zoology and Hydrobiology, Banacha 12/16, 90-237 Łódź, Poland
[23] Laboratory of Entomology, Ministry of Health, 9134302 Jerusalem, Israel
[24] Center for Pathophysiology, Infectiology and Immunology, Institute of Specific Prophylaxis and Tropical Medicine, Medical University Vienna, Kinderspitalgasse 15, 1090 Vienna, Austria
[25] Papua New Guinea Institute of Medical Research (PNGIMR) Institute, PO Box 60, Headquarter, Homate Street, 441 Goroka, Eastern Highlands Province, Papua New Guinea
[26] Museum of Natural History, University of the Philippines Los Baños, 4031 Laguna, Philippines
[27] National Centre of Infectious and Parasitic Diseases, 1504 Sofia, Bulgaria
[28] Ege University, Faculty of Medicine, Department of Parasitology, 35040 Bornova/Izmir, Türkiye
[29] Retired, Faculté de Pharmacie, Université de Strasbourg, Strasbourg, 67400 Illkirch-Graffenstaden, France
[30] Program for the Study and Control of Tropical Diseases (PECET), Faculty of Medicine, University of Antioquia, 050010 Medellin, Colombia
[31] MIVEGEC, Univ. Montpellier, CNRS, IRD, 34394 Montpellier, France & Medical Entomology Unit, Institut Pasteur de Madagascar, 101 Antananarivo, Madagascar
[32] Laboratorio de Entomología Médica, Departamento de Zoología de Invertebrados, Facultad de Ciencias Biológicas, Universidad Autónoma de Nuevo León, San Nicolás de los Garza, 66455, NL, México
[33] Tropical and Infectious Disease Centre, BP Koirala Institute of Health Sciences, Dharan 56700, Nepal
[34] ICMR-Vector Control Research Centre, Puducherry 605006, India
[35] Graduate School of Agricultural and Life Sciences, The University of Tokyo, Tokyo 113-8657, Japan
[36] Grupo de estudos em Leishmanioses/Coleção de Flebotomíneos (COLFLEB/Fiocruz-MG), Instituto René Rachou, Fundação Oswaldo Cruz, Belo Horizonte, Minas Gerais, 30190009, Brazil
[37] Center of Excellence in Vector Biology and Vector-Borne Disease, Department of Parasitology, Faculty of Medicine, Chulalongkorn University, Bangkok 10330, Thailand
[38] Department of Arbovirology, Bernhard Nocht Institute for Tropical Medicine, Bernhard Nocht Str. 74, 20359 Hamburg, Germany [39] Laboratory Vectors & Parasites, Department of Livestock Sciences and Techniques, Sine Saloum University El Hadji Ibrahima Niasse (SSUEIN) Kaffrine Campus, C.P. 24600, Senegal.
[40] Environmental Health Institute, National Environment Agency, Singapore 138667, Singapore & Department of Biological Sciences, National University of Singapore, 117558 Singapore
[41] Institut Pasteur du Laos, Laboratory of Vector-Borne Diseases, Samsenhai Road, Ban Kao-Gnot, Sisattanak District, 3560 Vientiane, Lao PDR
[42] National Institute of Hygiene and Epidemiology, 1 Yec-Xanh Street, Hai Ba Trung District, 100000 Hanoi, Vietnam
[43] Indonesian Research Center for Veterinary Science, Indonesian Agency for Agricultural Research and Development, Ministry of Agriculture Republic Indonesia, Bogor 16114, Indonesia & Department of Parasitology, Faculty of Veterinary Medicine, Airlangga University, Surabaya 60115, Indonesia
[44] Laboratory of Research in Applied Biology, Polytechnic School of Abomey-Calavi, University of Abomey-Calavi, 01 P.O. Box 2009, 00000 Cotonou, Benin
[45] Instituto de Microbiología, Colegio de Ciencias Biológicas y Ambientales (COCIBA), Universidad San Francisco de Quito (USFQ), 170901 Quito, Ecuador

Received 1 December 2025, Accepted 29 January 2026, Published online 3 April 2026

Abstrak – Artikel ini menyajikan panduan komprehensif mengenai pemrosesan dan pembuatan preparat (*mounting*) spesimen lalat pasir Phlebotominae, yaitu suatu tahapan yang penting dalam melakukan identifikasi spesies, mendeteksi dan mengisolasi patogen. Berbagai teknik yang dapat diterapkan di lapangan maupun di laboratorium juga dibahas pada artikel ini secara sistematis. Pedoman ini memuat uraian rinci tentang metode koleksi, pemrosesan, pelabelan, dan eutanasia lalat pasir (dengan fokus kearah pembekuan kering atau menggunakan CO_2 dibandingkan dengan penggunaan bahan kimia lainnya), serta strategi penyimpanan spesimen seperti penyimpanan pada suhu rendah dan dalam larutan etanol. Kualitas preparasi struktur anatomi tertentu seperti organ reproduksi, kepala, dan sayap, dinilai sangat penting dalam menentukan ketepatan pengamatan secara mikroskopis dan ketepatan didalam melakukanidentifikasi spesies. Prosedur tersebut dijelaskan secara terperinci, termasuk tahapan pemrosesan spesimen dan proses penjernihan jaringan menggunakan kalium hidroksida yang dilanjutkan dengan larutan Marc-André. Proses pembuatan preparat (*mounting*) akan membandingkan penggunaan media yang berbeda berdasarkan sifat optik dan kapasitas preservasinya. Larutan Hoyer (*chloral gum*) direkomendasikan untuk observasi cepat, khususnya untuk pengamatan struktur spermateka, karena tingkat kejernihannya, meskipun kurang sesuai untuk penyimpanan jangka panjang. Media lain yang dibahas meliputi polivinil alkohol, Euparal® (dengan toleransi air yang terbatas), serta Canada balsam (media yang larut dalam hidrokarbon). Euparal® dan Canada balsam memiliki kemampuan preservasi jangka panjang. Pendekatan teknik biologi molekuler modern, seperti sekuensing DNA dan MALDI-ToF, yang memerlukan perhatian khusus pada tahap pemrosesan spesimen, juga dibahas pada artikel ini. Selain itu, video singkat yang mendemonstrasikan berbagai teknik

pembuatan preparat serta terjemahan dalam 33 bahasa akan disertakan bersama pedoman ini, sehingga dapat menjangkau beragam kebutuhan dan ekspektasi komunitas ilmiah global.

Key words: Pembuatan preparat mikroskop, lalat pasir phlebotomine, larutan Hoyer, larutan Marc-André, chloral gum, polyvinyl alcohol, Euparal®, media Canada balsam, isolasi *Leishmania*, kondisi lapangan, kultur, diseksi, biologi molekular, MALDI-ToF, Type-specimens

Abstract – Processing and mounting phlebotomine sand flies: a consensus guideline. This article provides a comprehensive guide for the processing and mounting of phlebotomine sand fly specimens, which is crucial for species identification and pathogen detection and isolation. It discusses a range of techniques suitable for both field and laboratory settings. The guide includes detailed instructions on sand fly collection, handling, covering, and euthanasia (recommending dry freezing or CO_2 over chemicals) as well as conservation strategies, such as cold storage and preservation in ethanol. The quality of preparation of certain anatomical structures (genital organs, head and wings) is essential for their proper microscopic observation and is described in this work. The article also presents detailed sample processing, including the clearing process with agents such as potassium hydroxide then Marc-André solution. The mounting process compares different media, emphasizing their optical properties and preservation potential. Hoyer fluid (also known as chloral gum) is recommended for quick observation, particularly for spermathecae, due to its clarity, although it is not suitable for long-term storage. Other media discussed include polyvinyl alcohol, Euparal® (for limited water tolerance), and Canada balsam (a hydrocarbon-soluble medium), with the latter two offering long-term preservation capabilities. Innovative molecular biology approaches such as DNA sequencing and MALDI-ToF, which require particular attention to sample processing, are also addressed. Furthermore, short video clips illustrating various mounting techniques as well as translations in many different languages are provided, allowing the guideline to reach the diverse needs and expectations of the global scientific community.

Key words: Mounting, Phlebotomine sand fly, Hoyer fluid, Marc-André solution, Chloral gum, Polyvinyl alcohol, Euparal®, Canada balsam, *Leishmania* isolation, Field conditions, Culture, Dissection, Molecular biology, MALDI-ToF, Type-specimens.

Pendahuluan

Lalat pasir Phlebotominae merupakan serangga dari ordo Diptera berukuran kecil yang termasuk dalam famili Psychodidae, subfamili Phlebotominae, dengan sedikitnya 1,063 spesies yang telah didokumentasikan [21]. Serangga ini merupakan vektor penting berbagai patogen seperti Leishmania (menyebabkan penyakit leishmaniasis), arbovirus (menyebabkan infeksi arbovirus), dan Bartonella (menyebabkan bartonellosis). Saat ini, identifikasi lalat pasir secara umum dilakukan melalui pemeriksaan mikroskopis yang difasilitasi melalui metode koleksi yang cermat, penyimpanan yang tepat, serta proses pembuatan preparat (*mounting*) yang dilakukan secara hati-hati. Prosedur tersebut memerlukan beberapa teknik khusus, dan masing-masing memiliki kelebihan dan kekurangan.

Identifikasi lalat pasir Phlebotominae dewasa mengandalkan pada pengamatan detail struktur eksternal (misalnya antena, palpus, dan genitalia jantan) serta struktur internal (misalnya faring, cibarium, dan spermateka). Preparasi dan isolasi struktur internal tersebut mempermudah pengamatan sehingga meningkatkan akurasi hasil identifikasi. Oleh karena itu, berbeda dengan nyamuk atau kepik pengisap darah (kissing bugs), lalat pasir Phlebotominae memerlukan proses *mounting* antara kaca slide dan kaca penutup (*cover glass*) sebelum proses identifikasi dilakukan.

Hingga tahun 1980-an, pengamatan mikroskopis merupakan satu-satunya metode yang tersedia untuk identifikasi lalat pasir ini, dan hingga saat ini metode tersebut masih menjadi metode yang umum digunakan. Pada saat itu, pemilihan metode pemrosesan dan preparasi relatif lebih sederhana dan terutama didasarkan pada dua pendekatan utama: yang pertama Adalah pembuatan preparat dengan media permanen yang memungkinkan preservasi spesimen jangka panjang; dan yang kedua adalah pembuatan preparat cepat untuk keperluan identifikasi menggunakan media yang tidak menjamin kualitas preservasi dalam jangka panjang. Pembuatan preparat permanen biasanya menggunakan resin seperti Canada balsam dan memerlukan waktu yang lama karena membutuhkan proses dehidrasi sampel secara menyeluruh. Selain itu, indeks bias media ini tidak selalu optimal untuk memudahkan pengamatan struktur spermateka. Sebaliknya, pembuatan preparat menggunakan media berbasis air (misalnya larutan Hoyer) memiliki proses yang lebih cepat dan memungkinkan visualisasi spermateka terlihat lebih baik karena sifat refraktifnya, namun media ini tidak memiliki kemampuan preservasi jangka panjang karena cenderung menyerap kelembapan dari atmosfer. Salah satu

alternatif solusinya adalah dengan menutup sekeliling kaca objek dengan menggunakan cat kuku (kutek) setelah preparat benar-benar kering. Kekurangan dari masing-masing media pembuatan preparat masih relevan hingga saat ini dan menjadi faktor penting dalam pemilihan metode pembuatan preparat sesuai dengan tujuan penelitian.

Sejak tahun 1980-an, identifikasi lalat pasir dilakukan dengan menggabungkan pendekatan morfologi dan biokimia. Pendekatan awal berupa analisis hidrokarbon kutikula, kemudian dengan cepat digantikan oleh teknik biologi molekuler, seperti *random amplified polymorphic* DNA (RAPD), *restriction fragment length polymorphism* (RFLP), amplifikasi DNA, dan sekuensing dengan metode Sanger, serta teknologi sekuensing generasi baru (next-generation sequencing/NGS). Saat ini, pendekatan molekuler juga dilengkapi dengan metode proteomik seperti *Matrix-Assisted Laser Desorption/Ionization-Time of Flight* (MALDI-ToF). Selain itu, identifikasi spesies melalui teknik molekuler dapat dikombinasikan dengan deteksi patogen (*Leishmania* spp., *Trypanosoma* spp., *Bartonella* spp., dan Phlebovirus), yang semuanya dapat dideteksi melalui PCR konvensional maupun *real-time* PCR. Tentu saja, hal tersebut memerlukan beberapa penyesuaian dalam prosedur pengambilan dan penyimpanan spesimen sesuai dengan tujuan analisis [3, 32]. Selain karakter morfologi yang secara tradisional digunakan untuk membedakan spesies, pendekatan morfologi lain juga dapat diterapkan, seperti geomorfometri sayap. Berdasarkan pengalaman penulis secara pribadi serta data dari beberapa literatur, tujuan dari pembuatan pedoman ini adalah untuk menerapkan standar dalam pembuatan preparat (*mounting*) dan pemrosesan spesimen lalat pasir Phlebotominae dewasa guna mengoptimalkan analisis morfologi dan bio molekuler. Analisis tertentu (misalnya biologi molekuler atau MALDI-ToF) mengharuskan bagian tubuh lalat pasir yang tidak diperlukan untuk identifikasi morfologi untuk tetap dipertahankan, sehingga menegaskan pentingnya pemilihan protokol yang tepat di tahap awal penelitian.

Dalam pedoman ini, akan dibahas metode anestesi dan eutanasia lalat pasir yang dikoleksi dalam keadaan hidup, prosedur penyimpanan, serta proses pembuatan preparat (*mounting*), baik untuk identifikasi secara cepat maupun untuk preservasi jangka panjang yang memungkinkan dilakukan studi lanjutan.

Pendahuluan : Pertimbangan Keselamatan dan Regulasi masing-masing zat kimia merujuk pada Lembar Data Keselamatan (SDS).

Semua bahan kimia yang disajikan dalam pedoman ini harus ditangani dengan kondisi keselamatan yang ketat dan sesuai aturan. Komite kesehatan dan keselamatan harus terdapat didalamfasilitas penelitian dalam rangka untuk memberikan informasi yang mencakup bahan-bahan kimia yang berbahaya dan juga prosedur penanganan dan pembuangan limbahnya. Seluruh pengguna laboratorium wajib untuk mengikuti petunjuk keselamatan terkait penggunaan dan pembuangannya. Perlu diperhatikan bahwa

Tabel 1. Daftar Singkatan.

**BME**	*Basal medium Eagle*
**CDC**	*Centers for Disease Control and Prevention*
**CMCP**	*Camphor-monochlorophenol*
**CMR**	*Carcinogenic, mutagenic, reprotoxic substance*
**COI**	*Cytochrome c oxidase subunit I gene*
**CytB**	*Cytochrome b gene*
**DNA**	*Deoxyribonucleic acid*
**ELISA**	*Enzyme-linked immunosorbent assay*
**EtOH**	*Ethanol*
**M199**	*Medium 199*
**MALDI-ToF MS**	*Matrix-assisted laser desorption/ionization time-of-flight mass spectrometry*
**MEM**	*Minimum essential media*
**NGS**	*Next-generation sequencing*
**NNN**	*Novy-MacNeal-Nicolle medium*
**PCR**	*Polymerase chain reaction*
**Lao PDR**	*Lao People's Democratic Republic*
**PNOC**	*Prepronociceptin gene*
**qPCR**	*Quantitative PCR (real-time PCR)*
**RAPD**	*Random amplified polymorphic DNA*
**RFLP**	*Restriction fragment length polymorphism*
**BME**	*Basal medium Eagle*
**CDC**	*Centers for Disease Control and Prevention*
**CMCP**	*Camphor-monochlorophenol*
**CMR**	*Carcinogenic, mutagenic, reprotoxic substance*
**COI**	*Cytochrome c oxidase subunit I gene*
**CytB**	*Cytochrome b gene*
**DNA**	*Deoxyribonucleic acid*
**ELISA**	*Enzyme-linked immunosorbent assay*

setiap pengguna laboratorium memiliki tanggung jawab untuk memastikan kepatuhan terhadap praktik laboratorium yang baik dan aman serta terhadap peraturan perundang-undangan yang berlaku di negara atau institusitempat penelitian dilakukan. Selain itu, beberapa bahan kimia, atau komponennya (misalnya kloral hidrat), diatur secara khusus di beberapa negara. Daftar singkatan yang digunakan dalam naskah ini disajikan pada Tabel 1.

1.Koleksi lalat pasir

Lalat pasir dewasa dapat dikoleksi dalam keadaan hidup maupun mati menggunakan berbagai metode seperti CDC *Light Trap*, *sticky trap*, dan *aspirator* yang digunakan bersama perangkap Shannon, atau secara langsung dari tempat istirahatnya di lingkungan (misalnya kandang hewan). Metode-metode ini meliputi penempatan perangkap di habitat yang sesuai, menarik lalat pasir dengan cahaya atau atraktan lain (CO_2 atau umpan kimia), serta mengoleksinya untuk analisis lebih lanjut, sebagaimana dijelaskan dalam beberapa publikasi [2, 3, 32, 36, 49].

Penangkapan lalat pasir hidup memungkinkan seluruh aplikasi lanjutan dapat dilakukan, sedangkan pengumpulan individu yang mati tidak memungkinkan isolasi strain *Leishmania* atau virus. Beberapa teknik penangkapan, seperti penggunaan kertas lengket (*sticky trap*), sering kali menyebabkan hilangnya organ lalat pasir (antena, palpus, sayap, atau kaki). Selain itu, lapisan minyak jarak (*castor oil*) pada kertas lengket akan menempel pada tubuh lalat pasir dan harus dihilangkan pada awal proses, biasanya dengan perendaman selama 15 menit dalam campuran etanol dan dietil eter dengan perbandingan yang sama.

2.Eutanasia spesimen

Setelah dikoleksi, lalat pasir hidup harus dieutanasia atau dimatikan. Pada beberapa metode koleksi (misalnya dengan *sticky trap* atau CDC *Light Trap* yang dilengkapi dengan wadah berisi deterjen atau etanol), lalat pasir yang berhasil ditangkap sudah dalam keadaan mati ketika dikoleksi. Analisis biologi molekuler dapat dilakukan pada spesimen yang langsung dikoleksi kedalam etanol, serta pada spesimen lainnya apabila segera disimpan dalam etanol.. Namun, tidak satu pun dari metode eutanasia tersebut memungkinkan pemrosesan lebih lanjut menggunakan MALDI-ToF. Selain itu, beberapa metode eutanasia dapat menyebabkan hilangnya karakter morfologi tertentu.

Oleh karena itu, penggunaan metode eutanasia standar harus sesuai untuk memastikan proses identifikasi yang tepat atau untuk penyimpanan jangka panjang sebagai spesimen voucher (yaitu spesimen yang diawetkan dan disimpan untuk referensi atau perbandingan dimasa mendatang). Bahan kimia seperti etil asetat, etil eter, tetrakloroetana, dan kloroform dapat diserapkan kekapas, kemudian ditempatkan dalam wadah berisi lalat pasir untuk proses eutanasia. Bahan kimia ini harus ditangani dengan hati-hati dan sesuai dengan rekomendasi manufaktur karena bersifat toksik.

Namun, penggunaan kloroform tidak direkomendasikan untuk mematikan lalat pasir karena bahan ini kurang sesuai untuk studi biologi molekuler. Mengingat sifatnya yang berbahaya serta ketidaksesuaiannya dalam analisis molekuler, maka penggunaan bahan kimia ini secara umum tidak dianjurkan.

Metode yang paling umum digunakan untuk eutanasia dan yang dapat mempertahankan morfologi, DNA, maupun protein, adalah pembekuan kering spesimen. Dalam proses ini, spesimen dibekukan dalam waktu yang cukup hingga benar-benar teranestesi, tetapi tidak terlalu lama yang membuat spesimen (i) mengering, atau (ii) mengganggu viabilitas *Leishmania* apabila tujuannya adalah mengisolasinya secara *in vitro* dari saluran pencernaan lalat pasir. **Oleh karena itu, kami merekomendasikan durasi pembekuan selama 15 hingga 20 menit pada suhu -20°C, dengan pemantauan secara berkala untuk memastikan spesimen hanya dalam keadaan terbius tanpa membunuh parasit *Leishmania*.**

Jika *freezer* tidak tersedia, lalat pasir dapat dimatikan menggunakan CO_2. Dalam kondisi lapangan di mana tabung CO_2 tidak dapat digunakan, spesimen dapat dimatikan menggunakan tabung kecil CO_2 komersial yang biasa digunakan pada "soda siphon" (dispenser minuman), meskipun mungkin terdapat pembatasan untuk pengangkutan melalui jalur udara. Sebagai pilihan terakhir, lalat pasir dapat dimatikan dengan paparan asap tembakau. Pada metode ini, lalat pasir yang ditangkap hidup-hidup menggunakan CDC *light trap* atau dikoleksi dengan aspirator, disimpan dalam tabung kaca, lalu dipaparkan pada asap tembakau yang akan membunuhnya dalam hitungan detik. Metode ini dapat diterapkan dalam berbagai kondisi lapangan, termasuk pada situasi di lapangan yang sulit.

Namun, karena kaca akan terimpregnasi oleh asap, maka tabung tersebut tidak dapat digunakan kembali untuk koleksi dan penanganan lalat pasir hidup tanpa dilakukan pembersihan secara menyeluruh. Meskipun demikian, aspirator yang belum dibersihkan masih dapat digunakan untuk mematikan lalat pasir dari perangkap lain untuk tujuan fiksasi. Selain itu, perlu dipastikan bahwa semua spesimen telah dikeluarkan dari aspirator. Metode-metode ini kompatibel dengan isolasi Leishmania melalui diseksi usus lalat pasir.

3.Prosedur penyimpanan spesimen sebelum pemrosesan lebih lanjut

Terdapat lima metode utama fiksasi sebelum pemrosesan:

3.1. Pembekuan

Metode ini paling baik dilakukan pada suhu -20°C atau, lebih disarankan, pada -80°C. Metode penyimpanan ini sekarang lebih banyak digunakan dibandingkan dengan penyimpanan dengan nitrogen cair. Dalam semua kasus, kriopreservasi harus dilakukan secepat mungkin setelah spesimen dibius. Penyimpanan dingin dalam freezer menawarkan keuntungan berupa pelestarian penuh serangga itu sendiri, serta mempertahankan RNA, DNA, dan protein dengan baik selama periode penyimpanan.

Sebaliknya, nitrogen cair dapat merusak sayap, kaki, antena palpus, dan secara signifikan seringkali membuat bagian-bagian tubuh tersebut patah sehingga menghilangkan karakter morfologi yang penting untuk identifikasi. Penyimpanan di *freezer* kering lebih sedikit menimbulkan kerusakan pada spesimen, tetapi tidak ideal

untuk menyimpan organ-organ yang rapuh. Penting untuk diperhatikan bahwa saat proses pencairan dari keadaan beku, bagian tubuh seperti sayap, antena, palpus, atau kaki dapat saja menempel pada tabung dan akhirnya robek atau rusak karena proses kondensasi.

Sayangnya, preservasi dengan pembekuan tidak selalu memungkinkan dalam kondisi studi lapang karena membutuhkan akses ke *freezer* atau wadah nitrogen cair. Penyimpanan dalam *freezer* ini sangat sesuai untuk tujuan mendeteksi agen patogen, terutama untuk analisis molekuler tanpa kehilangan sensitivitas, meskipun untuk mendeteksi dan mengisolasi virus RNA memerlukan pembekuan pada suhu -80°FC atau dalam nitrogen cair apabila disimpan dalam jangka panjang. Namun, pembekuan sampel ini tidak memungkinkan untuk mengisolasi *Leishmania* melalui diseksi organ usus, kecuali jika lalat pasir pertama-tama diekspos terhadap fase uap nitrogen cair terlebih dahulu sebelum dicelupkan dalam nitrogen cair. Hal ini akan menyimulasikan proses kriopreservasi pada *Leishmania*.

3.2. Penyimpanan dalam larutan alkohol (ethanol atau isopropyl alcohol)

Metode ini merupakan metode yang paling umum digunakan untuk preservasi lalat pasir. Metode ini mudah diterapkan di lapang, bahkan dalam kondisi sulit tanpa akses ke laboratorium. Preservasi dalam alkohol sangat cocok untuk studi morfologi, karena organ yang rapuh (sayap, kaki, antena, atau palpus) tetap utuh, asalkan tidak ada gelembung udara dalam tabung penyimpanan. Oleh karena itu, direkomendasikan untuk menutup tabung spesimen dengan bola kapas kecil untuk menghilangkan gelembung udara dan menempelkan label di atas penutup sumbat kapas (Gambar 1).

Konsentrasi alkohol yang tepat masih menjadi perdebatan. Secara umum, konsentrasi dibawah 70% tidak disarankan [45, 66]. Konsentrasi yang lebih tinggi lebih efektif dalam mempertahankan DNA dan sesuai untuk penyimpanan dalam jangka waktu lebih lama, tetapi akan membuat spesimen lebih rapuh untuk studi morfologi. Penggunaan etanol 96% (campuran azeotrop) ditujukan untuk memastikan kestabilan konsentrasi etanol dari waktu ke waktu, terutama di daerah lembap seperti negara tropis, meskipun etanol 95% sering lebih mudah diperoleh.

Terlepas dari konsentrasi etanol, DNA umumnya terawetkan dengan baik dalam etanol (meskipun kurang efektif dibandingkan metode pembekuan, terutama untuk metode molekuler NGS). Protein menjadi tidak stabil, terutama untuk analisis proteomik, seperti aplikasi MALDI-ToF. Lalat pasir yang diawetkan dalam alkohol selama beberapa bulan masih dapat diidentifikasi secara morfologis, tetapi tidak mungkin menghasilkan spektra protein referensi dari spesimen ini.

Penyimpanan dalam alkohol atau kondisi kering dapat ditingkatkan jika sampel juga dibekukan pada -20°C. Pembekuan pada -20°C terutama meningkatkan preservasi molekuler (misalnya asam nukleat/DNA) dengan memperlambat degradasi dan juga memberikan manfaat tambahan untuk preservasi morfologi dengan mengurangi kerusakan jaringan, meskipun efeknya pada morfologi lebih terbatas dibandingkan pada studi molekuler.

Penyimpanan dalam etanol juga dapat diterapkan untuk mendeteksi DNA dan virus RNA apabila menggunakan etanol dengan konsentrasi minimal 70% untuk periode penyimpanan singkat, kurang dari beberapa bulan. Selain itu, alkohol isopropil mungkin lebih mudah tersedia di beberapa negara dan dapat mempertahankan DNA, tetapi akan membuat spesimen menjadi kaku. Alkohol ini tidak mudah terbakar seperti etanol sehingga mudah untuk dibawa selama perjalanan. Jika diperlukan, lalat pasir yang diawetkan dalam nitrogen cair atau dibekukan kering dapat dipindahkan ke alkohol, sehingga menggabungkan kekurangan dari kedua metode tersebut.

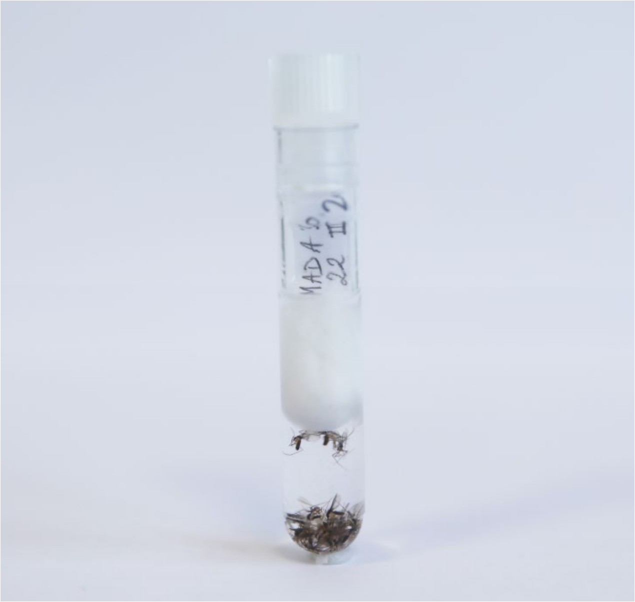

Gambar 1: Lalat pasir yang dipreservasi didalam larutan alkohol.

3.3. Penyimpanan dalam larutan penstabil RNA (RNASS)

Reagen ini umum digunakan, tidak beracun, dan dirancang untuk menstabilkan serta melindungi RNA dalam sampel jaringan dan sel. Reagen ini bekerja dengan cara menembus jaringan spesimen secara cepat dan menonaktifkan RNase (enzim yang merusak RNA), sehingga mencegah degradasi RNA tanpa perlu segera melakukan pembekuan. Penyimpanan dalam RNASS umumnya efektif untuk mempertahankan morfologi jaringan dan sel secara keseluruhan yang dapat digunakan untuk analisis histologis lebih lanjut.

Meskipun RNASS dioptimalkan untuk menjaga stabilitas RNA dibandingkan untuk fiksasi, penyimpanan

dalam jangka waktu pendek hingga menengah biasanya tetap mempertahankan integritas struktural dengan baik. RNASS memungkinkan sampel disimpan pada suhu ruang hingga 7 hari, pada 4°C selama beberapa minggu, atau pada -20°C/-80°C untuk preservasi jangka panjang. Metode ini dapat diaplikasikan terutama dalam pekerjaan di lapang atau pengaturan klinis dimana ketersediaan infrastruktur rantai dingin terbatas. Ekstraksi RNA biasanya memerlukan pengambilan sampel dari reagen dan pemrosesan sesuai dengan protokol standar.

3.4. Preservasi kering pada suhu ruang

Ini adalah metode klasik yang apabila diterapkan pada spesimen utuh (ditempatkan secara keseluruhan) memiliki kelemahan utama, yaitu kurang baik dalam preservasi organ yang rapuh seperti sayap, kaki, antena, dan palpus. Namun, studi proteomik menggunakan MALDI-ToF tetap dapat dilaksanakan jika dehidrasi dilakukan setelah fiksasi dengan pengering seperti silica gel. Sebaliknya, analisis molekuler yang menargetkan DNA relatif sulit untuk dilakukan pada sampel ini karena DNA sering kali masih terfragmentasi dan jumlahnya rendah, sehingga analisisnya lebih sulit dibandingkan dengan sampel segar atau beku, terutama untuk analisis genom inti.

Teknik terbaru seperti museomik dapat digunakan pada sampel yang diawetkan dengan metode ini [34]. Namun, metode penyimpanan ini tidak disarankan, kecuali jika tidak ada alternatif yang lain. Metode ini dapat dikombinasikan dengan penyimpanan dingin dengan menempatkan tabung didalam freezer pada -20°C atau -80°C. Tantangan utama metode ini adalah mencapai pemasangan spesimen atau bagian tubuh yang tepat untuk keperluan identifikasi.

Untuk mencapai hal ini, rehidrasi (proses pemulihan atau pengembalian cairan tubuh akibat dehidrasi) menjadi tahapan yang sangat penting. Penggunaan larutan Triton X-100 sangat direkomendasikan. Durasi rehidrasi bervariasi dari beberapa jam hingga beberapa hari, yang diikuti dengan pemantauan secara rutin dan cermat. Setelah rehidrasi selesai, spesimen harus dibilas sebanyak tiga kali secara berturut-turut dengan air.

3.5. Preservasi pada kertas saring

Keuntungan utama dari kertas saring adalah stabilitas jangka panjang DNA genomik didalam sel dari tubuh utuh yang dikeringkan tanpa fiksasi, atau sel darah yang disimpan pada suhu ruang. Kertas saring disediakan dalam ukuran kartu kecil, sehingga memungkinkan penyimpanan beberapa ratus sampel pada suhu ruang dengan luasan ruang yang tidak besar. Matriks kertas saring akan menyerap dan mendenaturasi agen infeksius, sehingga sampel tidak lagi dianggap sebagai agen hayati yang berbahaya. Hal ini memungkinkan penyimpanan dan pengangkutan sampel tanpa tindakan pencegahan biohazard khusus [68].

4.Diseksi spesimen

Berbeda dengan banyak serangga lainnya yang diidentifikasi berdasarkan karakter eksternal dan diamati pada keadaan serangga yang dipasang utuh (*pinned in toto*), lalat pasir memerlukan diseksi dan pemasangan pada preparat kaca untuk melihat fitur anatominya agar identifikasi spesies menjadi akurat. Terlepas dari prosedur persiapan dan pemasangan yang dipilih, teknik diseksi yang sama digunakan seperti pada Gambar 2 & 3 (https://zenodo.org/records/18198006).

Penggunaan Triton X100: larutan non-ionik

Sebagai catatan bahwa pembuatan preparat (*mounting*) sebaiknya menggunakan spesimen yang baru ditangkap (dalam keadaan segar) atau spesimen yang disimpan dengan baik. Sebagian besar kolektor memiliki sampel serangga yang diawetkan secara kering (untuk penggunaan MALDI-ToF) atau disimpan dalam alkohol selama bertahun-tahun. Sayangnya, preservasi dalam alkohol tidak optimal dalam jangka waktu bertahun-tahun, dan artropoda yang diawetkan dengan cara ini menjadi sangat sulit untuk pemeriksaan mikroskopis. Pada masa lampau yang sering terjadi adalah degradasi plastik yang berisi sampel, diikuti dengan penguapan alkohol. Dalam kedua kasus tersebut, tidak ada pilihan karena sampel terlalu lama berada dalam alkohol atau mengering.

Cara alternatif untuk mengatasi masalah tersebut adalah menggunakan agen basah (*wetting agents*) yang bukan deterjen kuat. Triton X100 berbentuk larutan non-ionik (larutan 4-(1,1,3,3-tetrametilbutil) fenil-polietilen glikol, atau t-octylphenoxypolyethoxyethanol, polyethylene glycol tert-octylphenyl ether), yang banyak digunakan sebagai deterjen dalam biologi seluler dan molekuler. Larutan ini memungkinkan terjadi permeabilisasi membran sel dan nukleus (inti).

Berikut prosedur penggunaan Triton X100 non-ionik dalam larutan berair 0,5%:

- Impregnasi sampel kering dengan alkohol absolut.
- Tambahkan volume larutan Triton X100 0,5% yang cukup sehingga seluruh sampel terendam.
- Biarkan proses berlangsung sekitar 5 menit hingga beberapa hari dengan pemantauan secara rutin. Semua artropoda harus terpisah sepenuhnya dalam larutan.
- Buang larutan Triton X100 dan ganti dengan larutan kalium hidroksida.

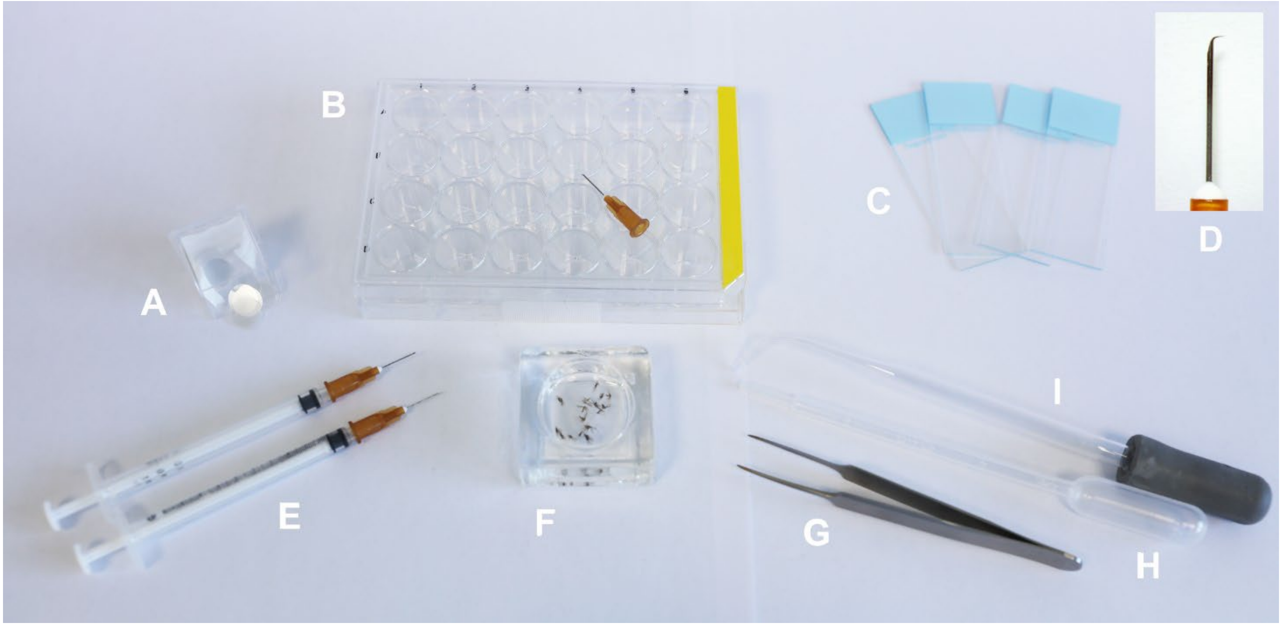

Gambar 2: Bahan yang diperlukan untuk pembuatan preparat (*mounting*) lalat pasir: A: kaca penutup bundar (diameter 10 atau 12 mm); B: pelat 24 sumur dan jarum berkait (jika menggunakan minyak cengkeh atau esens Euparal® untuk memproses lalat pasir, jangan menggunakan pelat akrilik karena akan terjadi reaksi kimia dan spesimen akan rusak); C: preparat kaca yang sesuai untuk pelabelan; D: detail kait jarum; E: jarum yang terpasang pada *syringe*; F: kaca jam atau wadah setara untuk menampung lalat pasir yang akan dibuat preparat (di *mounting*); G: pinset Dumont; H: pipet plastik; I: pipet kaca yang dibengkokkan dengan pemanasan untuk memudahkan pemindahan cairan ke sumur.

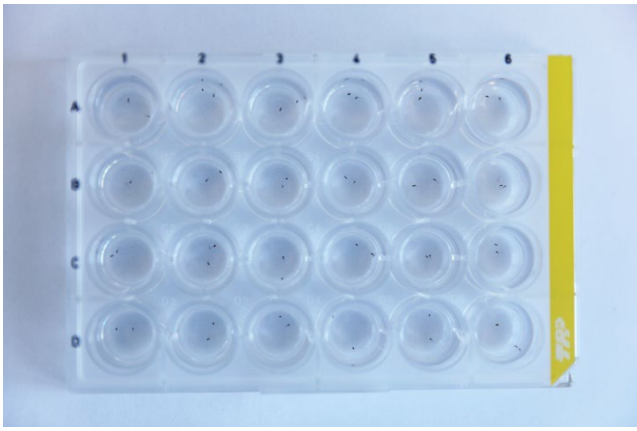

Gambar 3: Pelat dengan 24 sumur, masing-masing berisi kepala dan ujung abdomen lalat pasir.

4.1. Kepala

Diseksi atau pemotongan dapat dilakukan menggunakan jarum atau pin entomologi di bawah stereomikroskop (Gambar 2 & 3). Ukuran jarum yang paling sering digunakan adalah 26G x 1/2" (0,45 × 13 mm), 30G × 1/2" (0,3 × 13 mm), atau 25G × 5/8" (0,5 × 16 mm). Untuk mempersiapkan spesimen agar dapat diidentifikasi, minimal kepala dipisahkan dari tubuh dan dipasang dengan sisi ventral menghadap ke atas agar cibarium dan faring dapat terlihat, sedangkan toraks dan abdomen dipasang secara lateral setelah dipotong atau dibedah. Pemasangan kepala dalam posisi ventro-dorsal memastikan foramen oksipital menghadap ke atas, sehingga cibarium dapat diamati secara langsung. Akses ke bagian-bagian anatomi lalat pasir ini menjadi lebih mudah jika kepala dipisahkan sepenuhnya.

4.2. Sayap and Toraks

Sayap harus dipasang (*mounting*) dalam posisi datar. Setiap sayap dapat dilepas dari pangkalnya dan dipasang secara terpisah atau salah satu sayap dipasang sendiri sementara yang lain tetap menempel pada toraks. Apabila akan dilakukan analisis morfometri geometris maka harus dilabel dengan benar antara sayap kanan dan kiri sebelum dipasang pada kaca preparat (*mounting*). Toraks dibagi menjadi beberapa bagian, dan masing-masing mengandung informasi taksonomi yang sangat penting [20, 64]. Secara umum, toraks dipasang (*mounting*) dalam posisi lateral sehingga chetotaksi dan distribusi warna dapat diperiksa. Kehadiran bekas rambut (*bristles*) di beberapa wilayah toraks dapat digunakan untuk membedakan beberapa spesies dari genus *Brumptomyia*. Distribusi warna dapat digunakan untuk membedakan lalat pasir Neotropis pada tingkat genus (misalnya, *Bichromomyia*), seri spesies (misalnya, *Pintomyia*), atau bahkan spesies dari genus yang sama (misalnya, *Micropygomyia*, *Nyssomyia*, *Psathyromyia*, dan *Psychodopygus*) [20]. Oleh karena itu,

jika toraks tidak digunakan untuk analisis molekuler, sebaiknya dibuat preparat (mounting) sedemikian rupa agar tidak rusak. Sebagai catatan bahwa yang menjadi fokus dalam pemeriksaan ini bukanlah intensitas warna, melainkan distribusinya di seluruh toraks. Oleh karena itu, proses klarifikasi tidak akan menghilangkan pigmen atau pola warnanya.

4.3. Genitalia

Perhatian khusus harus diberikan saat memasang genitalia pada jantan maupun betina karena organ ini sangat penting untuk mengidentifikasi genus, subgenus, dan spesies. Pada kedua jenis kelamin, genitalia bersifat berpasangan.

4.3.1. Jantan

Genitalia bersifat eksternal dan terdiri dari organ forsep yang berpasangan, masing-masing terdiri dari artikulasi gonokoksit-gonostil di bagian dorsal dan lobus epandrium di bagian ventral. Gonostil memiliki duri dan kadang seta yang harus dapat dihitung dan posisi penempatannya harus terlihat dengan jelas. Pengamatan pada bagian permukaan dalam gonokoksit harus dilakukan dengan hati-hati karena mungkin terdapat jumbai seta sessile atau yang menempel pada lobus (= tuberkel) [22]. Kolega yang kurang berpengalaman dalam diseksi atau pemotongan mungkin dapat mencoba dapat melakukan pembuatan preparat (*mounting*) lateral sederhana, tanpa melepaskan genitalia dari ujung abdomen (https://zenodo.org/records/18311158). Dalam kasus ini, apabila terjadi tumpang tindih kedua bagian genitalia akan menyulitkan dalam menghitung jumlah seta internal pada gonokoksit. Namun demikian, cara ini mampu menghindari kerusakan genitalia akibat diseksi atau pemotongan yang gagal. Untuk para kolega yang lebih berpengalaman dapat mencoba membuka genitalia menjadi dua bagian. Untuk mencapai hal ini, sisi miring jarum (jenis jarum reaksi intradermal) harus dimasukkan, melepaskan tanpa memotong sepenuhnya genitalia untuk membelah rangkaian gonokoksit-gonostil (https://zenodo.org/records/18311158). Dengan cara ini, pengamatan pada permukaan internalnya menjadi lebih mudah. Rangkaian ini juga memudahkan pengamatan paramer dan selubung *parameral*, yang tidak lagi saling menumpuk. Untuk pembuatan preparat (*mounting*) lateral yang memungkinkan terjadinya tumpang tindih organ, maka spesimen harus dibersihkan dengan sempurna.

4.3.2. Betina

Alat genital betina bersifat internal, terdiri dari spermateka. Jika diseksi tidak dilakukan, maka spermateka harus diamati melalui tegumen dengan memasang abdomen dalam posisi ventral. Terlepas dari media pemasangan yang dipilih, spermateka umumnya dapat diamati dengan benar, terutama jika permukaannya tidak halus dan telah diklarifikasi. Namun, pengamatan spermateka yang halus dan dindingnya tipis dapat menjadi masalah dalam media yang kurang refraktif.

Selain itu, pengamatan pangkal saluran spermateka sangat penting untuk identifikasi spesies, misalnya pengamatan pada subgenus *Larroussius* [35, 37, 38], vektor utama *Leishmania infantum*. Tanpa pengamatan ini, identifikasi spesimen menjadi tidak mungkin. Untuk mengatasi kesulitan pengamatan ini, pemasangan furka genital-spermateka harus dilepas dari abdomen (https://zenodo.org/records/18311106).

Spermateka umumnya sulit diamati selama diseksi, tetapi furka genital relatif mudah ditemukan. Karena saluran spermateka membuka ke furka genital, isolasi furka ini biasanya memungkinkan isolasi spermateka. Jika spermateka secara tidak sengaja terpotong selama proses, spermateka tidak hilang dan masih dapat diamati di dalam tegumen abdomen (Gambar 4).

4.4. Diseksi midgut untuk isolasi parasit *Leishmania*

Diseksi saluran pencernaan sangat penting untuk mendeteksi dan mengisolasi *Leishmania* pada lalat pasir betina. Prosedur ini dapat dilakukan baik di lapang maupun di laboratorium, untuk menilai perannya sebagai kompetensi vektor. Diseksi sangat disarankan untuk dilakukan pada lalat pasir betina yang baru saja dimatikan. Spesimen lalat betina direndam dengan air atau larutan saline yang mengandung deterjen ringan untuk menghilangkan rambut atau setae berlebih. Langkah ini akan membantu menjaga kondisi tetap aseptik pada saat mengisolasi *Leishmania*, sekaligus mempertahankan fitur morfologi yang diperlukan untuk identifikasi. Untuk menemukan dan mengisolasi *Leishmania*, usus tengah dilepaskan secara hati-hati dan diletakkan dalam satu tetes larutan saline steril (0,9% NaCl). Setelah mengamati parasit yang bergerak di bawah mikroskop cahaya (perbesaran yang disarankan: ~200×), *syringe insulin* atau mikropipet digunakan untuk memindahkannya ke media kultur (untuk detail lebih lanjut lihat Bab 4.4.3). Kepala dan genitalia lalat betina langsung dibuat preparat (*mounting*) dalam larutan Marc-André agar bersih dan terlihat jelas.

Catatan : Jangan pernah membiarkan larutan Marc-André bersentuhan dengan *Leishmania* baik secara langsung maupun tidak langsung melalui alat atau jarum karena larutan ini akan mematikan parasit. Diseksi lalat pasir betina dapat dilakukan pada satu preparat kaca atau dua; kedua opsi memiliki kelebihan dan keterbatasan masing-masing (Gambar 5; https://zenodo.org/records/18311154).

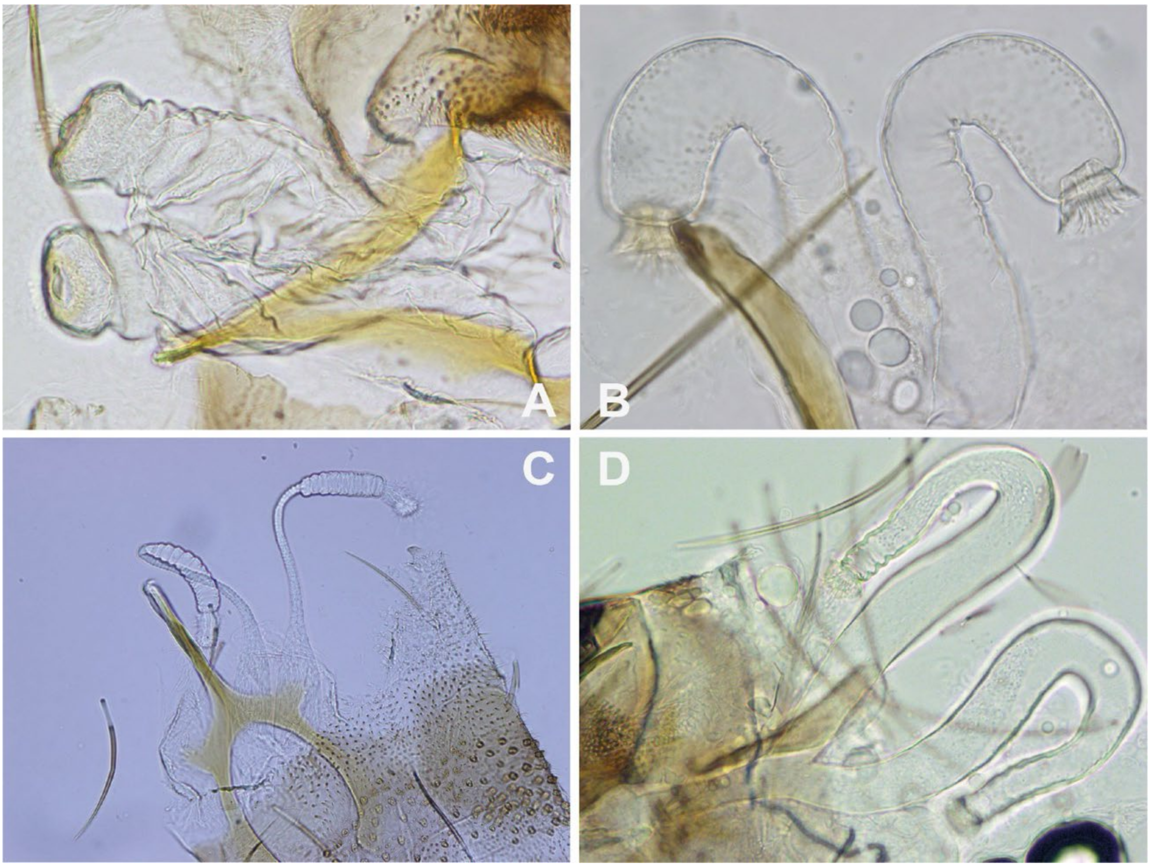

Gambar 4: Spermateka yang telah dipisahkan dari jaringan lain dan dipreservasi dalam larutan Marc-André dari spesimen segar. A: *Idiophlebotomus longiforceps* (Laos PDR); B: *Sergentomyia minuta* (Prancis); C: *Phlebotomus ariasi* (Prancis); D: *Sergentomyia anodontis* (Laos PDR)

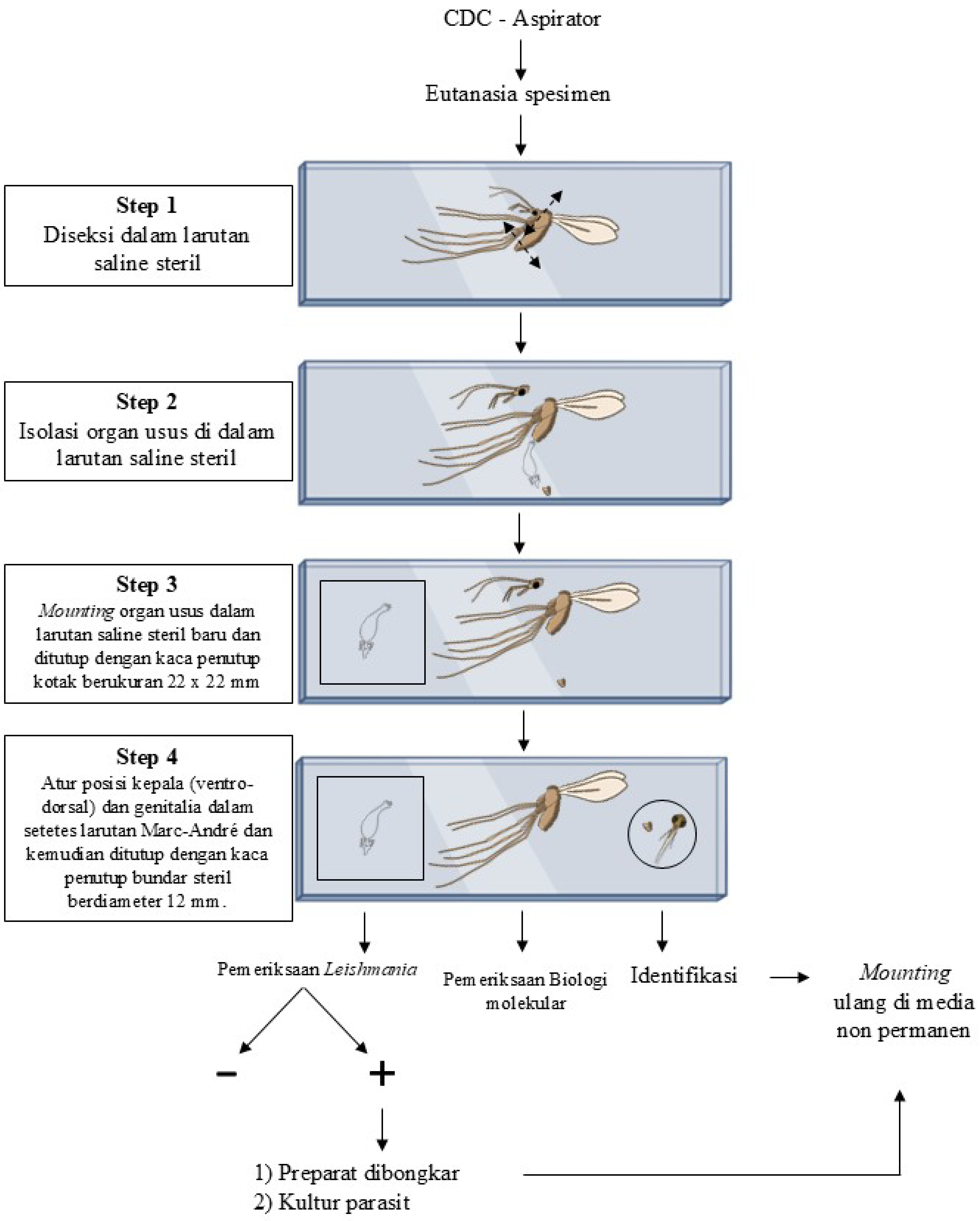

Gambar 5: Metode untuk isolasi parasit *Leishmania*

This is an Open Access article distributed under the terms of the Creative Commons Attribution License (https://creativecommons.org/licenses/by/4.0), which permits unrestricted use, distribution, and reproduction in any medium, provided the original work is properly cited.

4.4.1. Metode dua preparat kaca

Opsi pertama menggunakan dua preparat kaca yang terpisah: satu berisi saline steril untuk mengekstraksi usus tengah, dan yang lainnya untuk memasang kepala dan spermateka dalam larutan Marc-André. Namun, dalam kondisi lapangan, hal ini dilakukan oleh dua atau tiga orang untuk membedah lalat pasir dan menyerahkan hasilnya kepada seorang peneliti yang bertanggung jawab atas identifikasi spesies dan penilaian infeksi *Leishmania* di usus. Mengelola dua preparat kaca dapat menimbulkan masalah dalam melacak sampel dan khususnya menyulitkan dalam menentukan dengan pasti individu mana yang terinfeksi jika *Leishmania* ditemukan pada organ usus tengah (https://zenodo.org/records/18311154).

4.4.2. Metode satu preparat kaca

Menggunakan satu preparat kaca dapat memudahkan proses pelacakan sampel. Namun, beberapa langkah pencegahan harus dilakukan. Untuk memaksimalkan sterilisasi selama tahap ini, operator harus secara rutin membersihkan tangan dengan gel hidroalkohol. Preparat kaca yang tidak berkabut dan kaca penutup persegi (22 × 22 mm) yang dibungkus aluminium foil serta disterilkan dengan panas kering (menggunakan oven Poupinel) harus digunakan bersama dengan jarum steril untuk setiap diseksi (saran: 25G Ø 0,5 mm × 16 mm).

Lalat pasir ditempatkan dalam satu tetes larutan saline steril di tengah preparat kaca. Kepala dipisahkan, sementara sayatan dibuat di antara tergit dan sternit abdominal ke-6 dan ke-7 tanpa memotong saluran pencernaan (sayatan yang lebih tinggi dapat dibuat jika spermateka yang sangat panjang diharapkan). Kemudian, toraks harus diimobilisasi dengan jarum, dan segmen posterior abdomen terakhir ditarik perlahan dengan jarum lain untuk mengekstraksi usus. Jika cara ini gagal, ada kemungkinan menahan ujung abdomen dengan jarum dan menarik saluran pencernaan dari bagian anteriornya. Jika teknik ini juga gagal, maka usus harus diekstraksi dengan melepas sebanyak mungkin tegumen yang tersisa disekitarnya.

Setelah organ usus dikeluarkan, segmen abdomen terakhir harus dipisahkan dengan memotong saluran pencernaan. Usus kemudian ditempatkan dalam tetesan larutan saline steril baru yang diletakkan disalah satu sisi preparat kaca, lalu ditutup perlahan dengan kaca penutup steril. Kepala dan segmen abdomen terakhir dipindahkan kesatu tetes kecil larutan Marc-André yang diletakkan diujung lain preparat kaca, pastikan tidak ada kontak dengan *Leishmania*. Kepala diposisikan dengan benar (foramen oksipital menghadap ke atas), dan spermateka diisolasi bersama furka genital seperti dijelaskan sebelumnya, lalu ditutup dengan kaca penutup bundar kecil (Ø 12 mm, tidak sama dengan kaca penutup persegi steril). Lalat pasir yang tersisa dan sayap tetap berada ditetesan saline di tengah preparat kaca (https://zenodo.org/records/18311154).

Jika ditemukan hasil positif, atau untuk eksplorasi taksonomi, maka toraks dan abdomen dapat diawetkan untuk studi molekuler atau proteomik, dan sayap dapat dipasang dalam media berbasis air lainnya. Untuk menjaga hasil *mounting*, volume tambahan larutan Marc-André dapat diganti dengan media *mounting* berbasis air seperti *chloral gum* (=Hoyer) atau media lain yang berbasis poliakril alkohol.

Video-detail yang menunjukkan prosedur ini telah tersedia secara online: diseksi usus tengah lalat pasir (https://zenodo.org/records/18303014) dan diseksi kelenjar ludah lalat pasir (https://zenodo.org/records/18302850), sehingga prosedur tersebut tidak akan dijelaskan lagi pada artikel ini.

4.4.3. Isolasi dan kultur parasit Leishmania dari organ usus lalat pasir

Isolasi parasit *Leishmania* dari lalat pasir betina yang terinfeksi adalah prosedur yang sangat membutuhkan keterampilan tinggi, sehingga sebaiknya untuk awal latihan dapat dipraktikkan pada spesimen yang bebas parasit. Setelah diseksi, organ usus dipindahkan ke satu tetes larutan saline steril (0,9%) yang baru atau larutan Locke [4]. Usus yang telah diisolasi kemudian dapat diproses dengan dua cara: i) diperiksa di bawah mikroskop cahaya untuk mengamati berbagai tahap promastigot *Leishmania* dan lokasinya, dengan perhatian khusus pada katup stomodeal, dan ii) membuka usus agar promastigot mudahdikeluarkan untuk kultur massal [4]. Menemukan lalat pasir infektif di lapang merupakan kejadian yang relatif jarang, sehingga proses latihan diseksi spesimen yang baik akan memaksimalkan peluang berhasilnya mengisolasi parasit.

Jika parasit *Leishmania* terlihat di usus, maka jarum steril baru harus digunakan dan sedikit larutan saline steril ditambahkan disekitar kaca penutup yang akan masuk melalui aksi kapiler untuk melepaskan parasit. Usus harus disobek dengan hati-hati dan cepat untuk melepaskan parasit ke dalam saline. Parasit dikumpulkan dan diinokulasikan dengan menggunakan mikropipet 100 µL atau *syringe* tuberkulin ke dalam media kultur yang telah diberi label.

Kultur in vitro promastigot *Leishmania* : Parasit yang diisolasi awalnya dipelihara pada media agar darah miring SNB-9 atau pada media padat Novy, Mc Neal, Nicolle (NNN) [16] yang ditumpangi dengan media alpha-MEM steril [16, 65] atau media M199, masing-masing ditambahkan dengan 10% serum fetus sapi steril yang diinaktivasi panas [FCS] (untuk meningkatkan pertumbuhan parasit), 1% vitamin BME, 2% urin manusia steril (disterilkan menggunakan filter *syringe* Filtropur® S 0,2 µm), 250 µg/mL amikasin (atau 50 µg/mL gentamisin, atau campuran antibiotik dan asam amino (L-glutamin 200 mM-penisilin 10.000 U-streptomisin 10 mg/mL)) [47].

Setelah tiga hari, jika tidak ada kontaminasi, kultur disuspensi dalam media pembekuan yang disiapkan dengan benar dan kemudian disimpan pada -80°C selama 1 hingga 2 tahun atau dalam nitrogen cair pada -196°C untuk preservasi jangka panjang dan penggunaan eksperimen dimasa depan [7].

This is an Open Access article distributed under the terms of the Creative Commons Attribution License (https://creativecommons.org/licenses/by/4.0), which permits unrestricted use, distribution, and reproduction in any medium, provided the original work is properly cited.

4.5. Kelenjar ludah

Diseksi kelenjar ludah lalat pasir adalah teknik dasar yang digunakan untuk mempelajari interaksi vektor-patogen, terutama untuk mendeteksi arbovirus seperti Phlebovirus (misalnya, virus Toscana) [44, 75]. Karena ukuran lalat pasir yang sangat kecil, prosedur ini memerlukan ketelitian di bawah stereomikroskop, menggunakan forsep atau jarum mikrodiseksi untuk mengisolasi kelenjar ludah yang rapuh tanpa merusak atau mencemari sampel (https://zenodo.org/records/18302850) [51, 61]. Mempertahankan integritas kelenjar ludah sangat penting untuk memastikan kehandalan analisis molekuler. Setelah diekstraksi, kelenjar ludah dapat dihomogenisasi dan diuji menggunakan RT-PCR, qPCR, atau imunotest untuk mendeteksi RNA virus atau antigen [12]. Keberadaan virus di kelenjar ludah dan di usus atau hemocoel mengindikasikan bahwa patogen telah menyelesaikan periode inkubasi ekstrinsik dan dapat ditularkan selama proses pengisapan darah [71].

Proses diseksi secara teknis ini sangat menantang karena ukuran kelenjar ludah lalat pasir yang kecil, sehingga membutuhkan keahlian tinggi untuk menghindari degradasi sampel [1, 51]. Selain itu, jumlah virus secara umum rendah sehingga diperlukan metode deteksi yang sangat sensitif seperti nested PCR atau sekuensing *high throughput* [54]. Risiko kontaminasi juga dapat terjadi sehingga harus memperhatikan teknik sterilisasi. Diluar hambatan teknis, faktor biologis juga memengaruhi keberhasilan deteksi; kompetensi vektor bervariasi antar spesies lalat pasir, dan tingkat infeksi berfluktuasi sesuai kondisi ekologis dan musim [33, 61].

Mendeteksi virus di kelenjar ludah memberikan wawasan penting tentang risiko penularan, memungkinkan pengawasan dan tindakan pengendalian yang lebih terfokus [15]. Misalnya, identifikasi virus Toscana pada lalat pasir di wilayah endemik telah berkontribusi dalam penetapan protokol diagnostik dan strategi kebijakan kesehatan masyarakat [18]. Selain itu, mempelajari interaksi virus-kelenjar ludah dapat mengungkap target molekul baru untuk pengembangan vaksin atau terapi yang dapat memblokir penyebaran virus [15, 18].

Kelenjar ludah lalat pasir juga dapat digunakan sebagai sumber antigen untuk mengukur antibodi inang terhadap saliva lalat pasir dengan menggunakan metode imunologi, seperti ELISA. Metode ini memungkinkan penilaian paparan inang terhadap gigitan lalat pasir, sehingga mendukung evaluasi efektivitas metode pengendalian vektor [25] dan risiko penularan *Leishmania* [40].

4.6. Identifikasi sumber darah yang dikonsumsi lalat pasir

Lalat pasir betina yang telah menghisap darah diisolasi dari hasil tangkapan dan segera didiseksi dengan menggunakan peralatan sekali pakai untuk mencegah kontaminasi. Abdomen lalat pasir betina harus diperiksa di bawah stereomikroskop untuk menilai tahapan kandungan darah yang dikonsumsi. Untuk mengidentifikasi sumber darah disarankan untuk memilih hanya betina dengan abdomen yang berwarna merah, merah kecokelatan, atau merah gelap, tanpa tanda-tanda pembentukan telur. Ujung abdomen dan spermateka dilepaskan untuk mengidentifikasi morfologi lalat betina setelah proses pembersihan (*clearing*). Bagian utama abdomen (tanpa spermateka) kemudian ditempatkan dalam tabung Eppendorf® dan disimpan pada -20°C untuk analisis selanjutnya.

Penanda genetik yang umum digunakan untuk identifikasi darah yang dimakan, seperti gen PNOC [5, 30, 50], CytB [67], atau COI [13], sudah banyak dikenal dan dijelaskan secara luas dalam literatur; oleh karena itu, tidak akan dijelaskan lebih lanjut dalam artikel ini (Gambar 6). Alternatifnya, untuk mengidentifikasi darah inang, dapat digunakan pemetaan peptida MALDI-ToF [31]. Secara eksperimental telah ditunjukkan bahwa teknik ini mampu mengidentifikasi darah inang dalam jangka waktu lebih lama setelah pengisapan darah. Oleh karena itu, teknik ini merupakan metode yang sesuai, terutama untuk analisis lalat pasir betina yang mengandung darah inang yang telah masuk tahap lebih lanjut secara visual.

Idealnya, seluruh sampel disimpan pada suhu -20°C atau 4°C, tetapi bisa juga dari sampel yang disimpan pada suhu ruang untuk waktu singkat. Abdomen betina yang penuh dengan darah harus dipisahkan dari bagian tubuh lainnya sebelum dianalisis dan dihomogenisasi dalam air suling. Sisa tubuh lainnya dapat digunakan untuk analisis molekuler dan morfologis lainnya. Setelah aliquot diambil dari homogenat untuk pemetaan peptida MALDI-ToF, sisa sampel dapat digunakan untuk isolasi DNA guna memastikan identifikasi darah inang dan/atau memeriksa keberadaan *Leishmania*. Waktu keseluruhan persiapan sampel dan analisis sangat singkat dibandingkan dengan teknik molekuler berbasis DNA.

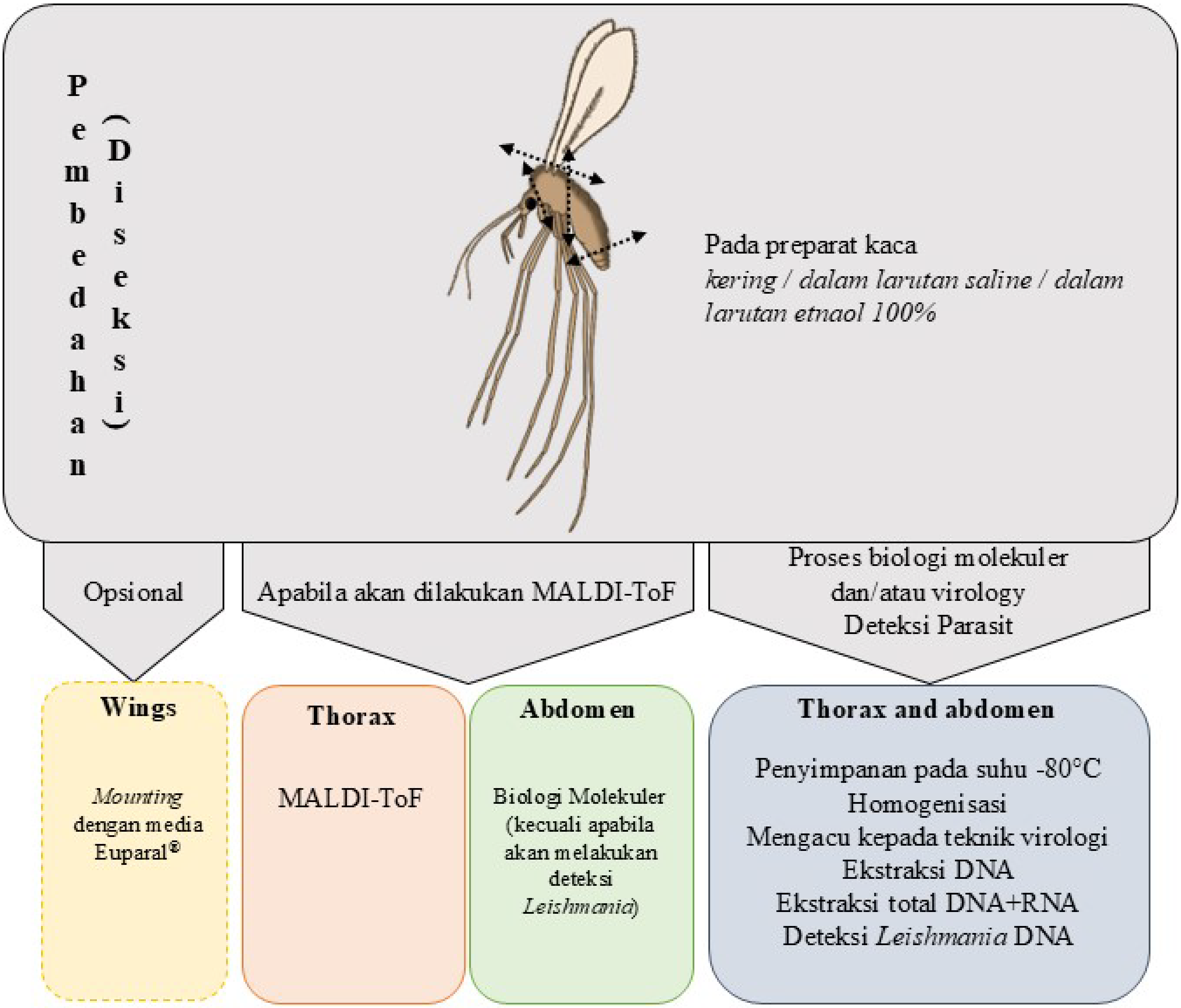

Gambar 6: Pemrosesan lalat pasir untuk aplikasi biologi molekuler, proteomik, dan/atau virologi.

5. Prosedur pemrosesan spesimen untuk kajian morfologi (Gambar 3, 6, 7 & 8; Lampiran 1, 2, 3 & 4)

Bagian ini menguraikan prinsip-prinsip penyiapan spesimen lalat pasir untuk pembuatan preparat (*mounting*) yang ditujukan khusus untuk studi morfologi, kemudian diikuti dengan adaptasi untuk aplikasi diluar kajian morfologi. Namun demikian, pemahaman terhadap metodologi ini sangat penting karena prosedurnya harus disesuaikan dengan jenis sampel tertentu apabila diperlukan. Perlakuan spesimen melibatkan tahapan pengosongan dan pengisian secara bertahap menggunakan pipet Pasteur yang dilengkapi bola karet fleksibel. Wadah kaca berbentuk dasar bulat sangat direkomendasikan karena akan memudahkan proses tersebut. Kaca sebaiknya bersifat inert terhadap seluruh reagen yang akan digunakan. Untuk mencegah penguapan reagen, wadah sebaiknya dilengkapi dengan penutup dan tidak diisi terlalu penuh, guna menghindari luapan saat penutupan atau pembukaan, serta untuk mencegah debu jatuh ke dalam sampel. Bahan kimia yang diperlukan untuk proses penjernihan (*clearing*) dan pemrosesan ditampilkan pada Tabel 2.

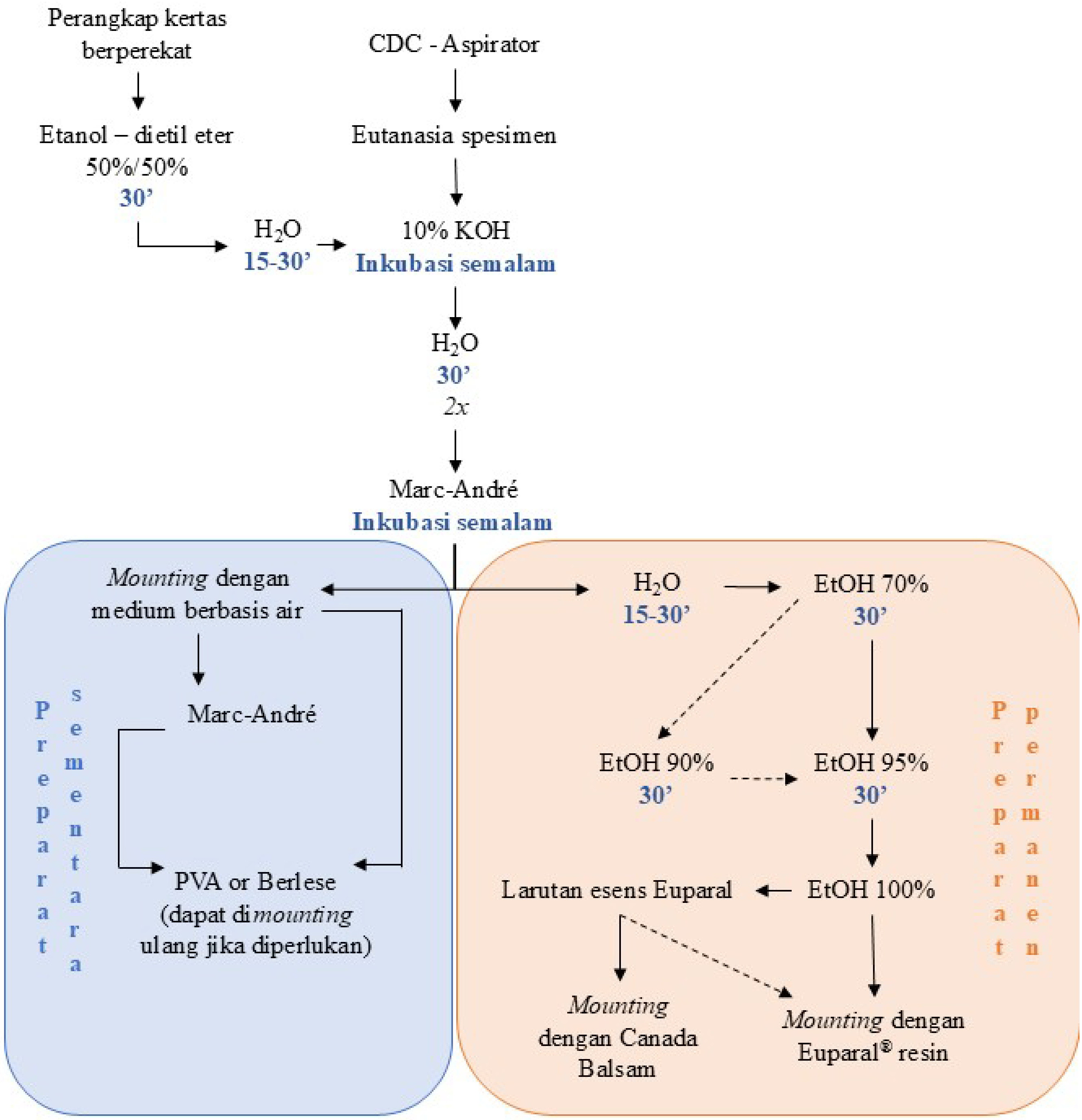

Gambar 7: Metoda klasik untuk pemrosesan spesimen lalat pasir.

Tabel 2: Komposisi reagen yang digunakan.

**Potassium hydroxide 10%**<br>Potassium hydroxide 10 g<br>Air suling *qs* 100 mL	**Fuchsin acid 1% dalam air suling**<br>Acid fuchsin (dalam bentuk serbuk) 1 g<br>Air suling 99 mL
**Gum chloral *mounting* media (Hoyer medium)**<br>Air suling 50 mL<br>Chloral hydrate 200 g<br>Gum Arabic 50 g	**Marc-André solution colored with acid fuchsin**<br>Larutan Marc-André 10mL<br>Fuchsin 1% 50 µL
**Marc-André solution**<br>Chloral hydrate 40 g<br>Asam asetat glasial 30 mL<br>Air suling 30 mL	**Enecê medium**<br>Kolofoni putih murni 22 g<br>Alcohol-soluble copal gum 12 g<br>ethanol absolut 20 mL<br>Camphor 10 g<br>Turpentine essence 10 mL<br>Eucalyptol 26 mL

5.1. Penjernihan jaringan

Sebelum spesimen lalat pasir dapat dipersiapkan sebagai preparat permanen pada kaca objek, spesimen tersebut harus terlebih dahulu dijernihkan melalui proses maserasi menggunakan metode dan agen penjernih yang sesuai (misalnya larutan asam asetat 10% atau larutan Marc-André yang mengandung kloral hidrat, yang merupakan bahan kimia terbatas di banyak negara) agar menjadi transparan. Proses penjernihan ini menghilangkan jaringan tubuh, lemak, sekresi, dan lapisan lilin, sehingga spesimen menjadi tembus cahaya dan memudahkan pengamatan struktur eksoskeleton (misalnya titik insersi seta), karakteristik permukaan (misalnya pewarnaan), serta struktur internal yang terlihat melalui tegumen (misalnya spermateka).

Proses penjernihan dua tahap, yang melibatkan penggunaan basa kuat terlebih dahulu (seperti kalium hidroksida), diikuti oleh asam lemah (seperti asam asetat dalam larutan Marc-André), memiliki tujuan biokimia yang berbeda [74]. Basa kuat akan menguraikan jaringan lunak seperti protein, lemak, dan otot melalui proses saponifikasi dan denaturasi protein, sementara eksoskeleton kitin tetap utuh sehingga struktur morfologinya tetap jelas. Selanjutnya, asam lemah berfungsi menetralkan sisa alkali, mencegah terjadinya degradasi lebih lanjut, serta memucatkan kitin untuk meningkatkan derajat transparansi [74], meskipun pencucian spesimen dua kali dalam air suling selama 15 menit juga dapat cukup untuk menetralkan basa. Perlakuan berurutan ini menggabungkan penghilangan jaringan yang efektif dengan preservasi, sehingga memastikan integritas spesimen yang optimal untuk pemeriksaan mikroskopis.

Disarankan untuk melakukan dua kali pembilasan selama 20 menit dalam air suling sebelum melanjutkan ke tahap berikutnya.

5.1.1. Lisis jaringan lunak (Gambar 8)

Natrium hidroksida (NaOH) atau kalium hidroksida (KOH) merupakan agen maserasi kimia yang umum digunakan, dengan konsentrasi dan lama perlakuan yang bervariasi tergantung pada ukuran dan kerapuhan spesimen. Teknik standar dan paling efektif melibatkan pelisisan jaringan lunak dengan merendam lalat pasir dalam basa kuat (KOH atau NaOH 10%) selama semalam. Konsentrasi dapat ditingkatkan untuk memperpendek durasi perlakuan (misalnya KOH 20% selama 6 jam), serta dapat disertai pemanasan pada suhu 37°C.

5.1.2. Penjernihan jaringan dengan atau tanpa pewarnaan

Tahap ini diikuti oleh perlakuan pencerahan (*lightening*), yang umumnya mengombinasikan asam asetat dan kloral hidrat (misalnya larutan Marc-André). Setelah proses penjernihan, spesimen harus dibilas secara menyeluruh dalam sedikitnya dua kali perendaman berturut-turut dalam air selama masing-masing 20 menit untuk menghilangkan sisa bahan kimia.

Larutan Marc-André merupakan agen penjernih yang umum digunakan dalam preparasi spesimen lalat pasir. Efektivitasnya terletak pada kemampuannya memfasilitasi proses penjernihan sekaligus meminimalkan kerusakan signifikan pada struktur yang rapuh, seperti sayap dan antena.

Larutan ini sebaiknya disiapkan dalam keadaan segar atau disimpan dalam wadah tertutup rapat untuk mencegah penguapan atau degradasi. Penggunaan larutan Marc-André sangat menguntungkan terutama bila dikombinasikan dengan teknik pencerahan tambahan atau pewarnaan untuk menonjolkan detail morfologi tertentu. Rincian komposisi dan cara pembuatannya disajikan dalam Lampiran 2.

Untuk spesimen yang sangat transparan, pewarnaan mungkin diperlukan guna meningkatkan visibilitas sebelum proses *mounting*. Tersedia berbagai jenis zat warna, masing-masing menargetkan komponen kimia tertentu dari organisme. Pemilihan zat warna yang kompatibel baik dengan spesimen maupun dengan media *mounting* yang digunakan sangat penting. Metodologi dasar ini dapat dimodifikasi sesuai dengan kebutuhan, misalnya dengan menambahkan asam fuksin 0,1% ke dalam larutan Marc-André untuk tujuan pewarnaan.

Selain itu, spesimen yang diawetkan dalam larutan berair dan akan dipasang menggunakan media resin yang memerlukan proses dehidrasi (lihat Bagian 5.2 tentang Dehidrasi), karena sebagian besar media *mounting* berbasis resin alami maupun sintetis tidak kompatibel dengan air. New (1974) mencatat bahwa beberapa zat warna dapat mengalami degradasi dalam media *mounting* tertentu [53]. Sebagai contoh, asam fuksin yang umum digunakan bersama Canada balsam juga dapat difiksasi dalam Euparal®. Namun, spesimen yang diwarnai dengan asam fuksin rentan mengalami pemudaran, terutama apabila masih terdapat sisa minyak cengkeh yang digunakan sebagai cairan penjernih akhir. Spesimen yang disimpan dalam minyak cengkeh dapat menunjukkan pemudaran yang signifikan dalam beberapa hari.

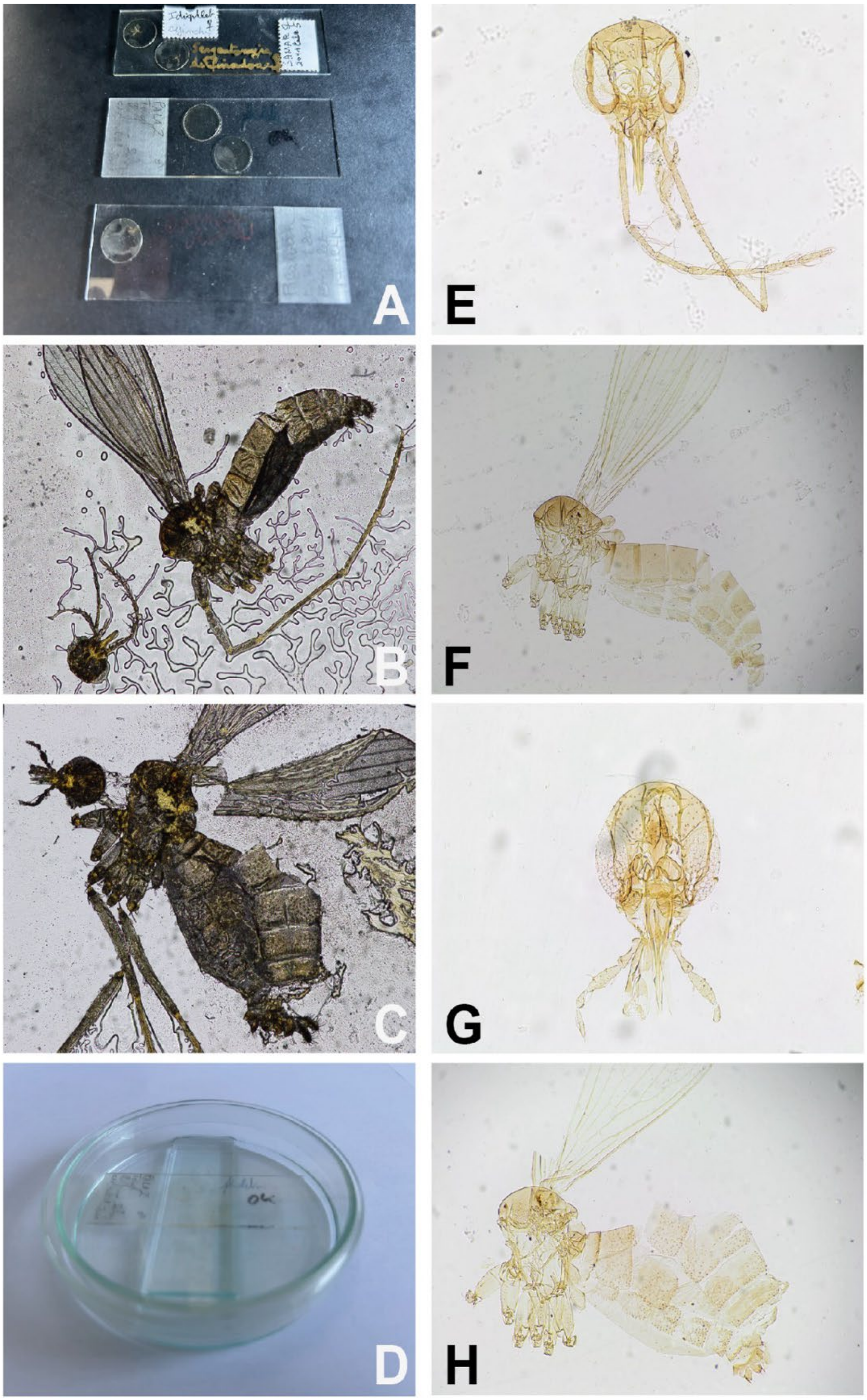

Gambar 8: Pemasangan ulang slide. A: slide yang rusak dan kering yang dipasang dengan Hoyer; B: tampilan mikroskopik lalat pasir yang kering; C: tampilan mikroskopik lalat pasir lain yang rusak; D: kamar lembap yang berisi slide kering; E: kepala, dan F: tubuh spesimen B setelah dipasang ulang dalam Euparal®; G: kepala, dan H: tubuh spesimen C yang rusak setelah dipasang ulang dalam Euparal®.

5.2. Dehidrasi spesimen

Dehidrasi dilakukan dengan memindahkan sampel secara bertahap melalui seri larutan etanol bertingkat: 50%, 70%, 80%, 90% atau 95%, dan akhirnya 100%, dengan setiap tahap perendaman berlangsung setidaknya 20 menit. Etanol mudah sekali menguap sehingga wadah harus ditutup rapat selama proses berlangsung. Setelah spesimen benar-benar terdehidrasi, proses dapat dihentikan sementara selama beberapa hari dengan merendamnya dalam esens Euparal®, yang lebih disarankan dibandingkan minyak cengkeh. Beech creosote, yang dahulu banyak digunakan untuk tujuan ini, kini dilarang karena bersifat toksik.

Proses dehidrasi harus memastikan bahwa cairan didalam spesimen sesuai dengan media *mounting* yang akan digunakan guna mencegah kekeruhan, kolaps osmotik, atau distorsi yang dapat menyebabkan spesimen tidak layak untuk studi taksonomi.

5.3. Media untuk pemasangan/pembuatan preparat (media mounting)

5.3.1. Pemilihan media dan penggunaannya dalam preparasi spesimen

Media *mounting* sebaiknya memiliki indeks bias sedekat mungkin dengan kaca, yaitu sekitar 1,5. Media tersebut harus tidak berwarna, jernih, dan tetap transparan sempurna setelah mengering maupun seiring waktu. Media ini harus kompatibel dengan zat warna yang digunakan dan mampu menembus serta menyebar ke seluruh jaringan spesimen. Media *mounting* tidak boleh mengering terlalu cepat atau membentuk kabut selama proses pemasangan, dan tidak boleh menyusut setelah preparat selesai dibuat. Pemilihan media *mounting* yang tepat merupakan aspek fundamental dalam persiapan spesimen, karena tidak ada satu media pun yang ideal untuk semua tujuan. Pilihan media harus mempertimbangkan beberapa faktor utama:

- Sifat optik. Indeks bias media *mounting* harus memberikan kontras dan pembiasan yang cukup untuk menampilkan fitur anatomi penting yang digunakan dalam identifikasi taksonomi atau deskripsi morfologi, seperti spermateka, ascoid, sensilla Newstead, gigi cibarial vertikal, dan gigi faring. Visibilitas struktur-struktur ini secara langsung bergantung pada sifat optik media *mounting*.

- Preservasi. Untuk tipe spesimen atau bahan/material yang dimaksudkan untuk koleksi permanen, media harus memberikan stabilitas dan daya tahan dalam waktu jangka panjang. Sebaliknya, untuk studi inventaris atau survei epidemiologi, di mana preservasi jangka panjang tidak terlalu penting, media *mounting* sementara atau semi-permanen mungkin sudah memadai.

5.3.2. Persyaratan untuk media *mounting*

Spesialis sering mengembangkan teknik *mounting* yang dapat disesuaikan dan teknik *mounting* yang kompleks untuk memenuhi kebutuhan penelitian tertentu. Namun, metode-metode tersebut sering mengabaikan beberapa aspek seperti kualitas arsip, kompatibilitas, standardisasi, atau kemudahan penanganan dan preservasi jangka panjang. Kurangnya standardisasi ini mempersulit integrasi dari koleksi yang akan didonasikan dan upaya-upaya kurasi dalam jangka panjang.

Aplikasi ilmiah menetapkan persyaratan khusus bagi media *mounting*. Taksonomis sering melakukan *mounting* pada spesimen utuh dan lebih menyukai media yang dapat secara lembut memaserasi organ dalam untuk meningkatkan visibilitas struktur kutikula. Indeks bias media harus berbeda cukup signifikan dari indeks bias spesimen dan kaca objek untuk memaksimalkan kejernihan optik. Media *mounting* komersial biasanya diformulasikan dengan indeks bias yang dekat dengan kaca objek untuk meminimalkan pembiasan cahaya dan penyebaran melalui sistem kaca objek–media *mounting*–kaca penutup. Namun, dalam mikroskopi *brightfield*, kontras alami spesimen yang tidak diwarnai dapat dimanipulasi dengan sengaja memilih media *mounting* yang memiliki indeks bias sedikit berbeda dari spesimen, sehingga meningkatkan visibilitasnya terhadap latar belakang.

5.3.3. Jenis-jenis media mounting (Tabel 3 & 4)

Pemeriksaan secara mikroskopi memerlukan indeks bias (RI) dari media *mounting* guna menentukan bagaimana cahaya membelok melalui kaca objek, media, dan spesimen. Ketika RI selaras dengan kaca penutup (≈1,515), cahaya melewati secara merata, mengurangi penyebaran dan distorsi optik, sehingga meningkatkan resolusi dan visibilitas struktur halus. Sebaliknya, ketidakcocokan RI dapat menyebabkan gambar buram, efek halo, atau menyamarkan fitur yang tidak diwarnai. Pemilihan media *mounting* yang tepat sangat penting untuk mengoptimalkan kontras, kejernihan, dan kualitas keseluruhan gambar untuk spesimen tertentu karena RI media yang berbeda-beda.

Indeks bias media *mounting* memiliki pengaruh signifikan terhadap seberapa jelas struktur halus dapat diamati saat menyiapkan *mounting* lalat pasir pada kaca objek. Fitur-fitur halus dan ringan tersklerotisasi pada lalat pasir, termasuk armatur cibarial, spermateka, segmen antena, dan urat sayap, dapat sulit diamati dalam media *mounting* dengan indeks bias tinggi.

Untuk lalat pasir, pilihan yang umum digunakan termasuk media *gum chloral* sebagai media *mounting* berbasis air, serta Canada balsam dan resin Enecê – Nelson Cerqueira (NC) sebagai media berbasis pelarut. Rawlins [60] mengategorikan media *mounting* menjadi dua tipe: (1) Media permanen: mengeras seiring waktu dan cocok untuk preservasi jangka panjang. (2) Media semi-permanen: tidak mengeras sepenuhnya dan biasanya digunakan untuk tujuan sementara.

Media *mounting* dapat berupa cairan, berbasis gum, atau resin, yang larut dalam air, alkohol, atau pelarut lainnya (misalnya toluena, ksilen) (Tabel 3). Setelah diaplikasikan, media tersebut harus ditutup untuk melindungi dari

pengaruh atmosfer menggunakan media pelindung yang tidak larut. Untuk membedakan secara jelas jenis-jenis media *mounting*, klasifikasi berikut dapat digunakan:

a. Media berbasis air (*aqueous media*) : Media ini mudah larut dalam air, sehingga cocok untuk *mounting* sementara atau semi-permanen. Media ini biasanya mudah ditangani, tetapi mungkin memerlukan tutup agar tidak terkena kelembapan atmosfer (misalnya media *gum-chloral* dan polivinil alkohol), terutama diiklim tropis yang lembap.

b. Media dengan toleransi air terbatas (*limited water-tolerant media*) : Media ini kurang terpengaruh oleh air, namun tetap memerlukan perlindungan terhadap kelembapan yang tinggi. Media ini mampu menjaga stabilitas jangka panjang yang lebih baik dibandingkan media larut air dan sering digunakan untuk *mounting* semi-permanen.

c. Media larut hidrokarbon (*hydrocarbon-soluble media*) : Media ini larut dalam pelarut organik seperti ksilen atau toluena, atau enecê (pelarut enecê). Media ini dirancang untuk *mounting* permanen dan memberikan stabilitas jangka panjang yang sangat baik, tahan terhadap kelembapan dan degradasi, sehingga ideal untuk tujuan arsip/penyimpanan (misalnya Canada balsam netral).

Tabel 3: Media *mounting* dan komposisinya.

**Media *mounting***	**Pelarut**	**Potensi pre-polimer atau polimer**	**Keterangan**
Hoyer = *chloral gum*	gliserol, air	Senyawa dari gum arabic	Agen maserasi: kloral hidrat
CMCP-9 (= carboxy methyl cellulose phenol)	air (CMCP-9: 51−60%)	polivinil alkohol terhidrolisis penuh (CMCP-9: 0−5%)	CMC(P)-9: viskositas rendah: viskositas tinggi
DMHF (dimethyl hydantoin formaldehyde)	air	N,N′-dimethylol dimethyl hydantoin (di-methylol DMH)<br>Ether-/methylene-bridged oligomers<br>Crosslinked DMH–formaldehyde polymer network	
Canada balsam	Ksilen; komponen balsam yang sebagian mudah menguap (Δ^3-karena, asam levopimarik, limonena, mirsena, asam palustrik, β-felandrena, α-pinena, β-pinena)	balsam (abienol, asam abietat, asam isopimarat, asam sandaracopimarat)	Netralisasi: karbonat kalium; resin dari Abies balsamea (Linné, 1758)
Euparal®	eukaliptol, paraldehida; komponen gum sandarac yang sebagian mudah menguap (limonena, α-pinena, β-pinena)	senyawa gum sandarac (asam komunik, manool, asam polikomunik, asam sandaracopimarat, 12-asetoksi-sandaracopimarat, sugiol, asam torulosik, torulosol, totarol)	Agen penjernih: metil salisilat; warna hijau pada Euparal®: garam tembaga (copper abietinate); resin sandarac dari Tetraclinis articulata (Vahl, 1791)
Enecê	alkohol etil; dengan kamper, eukaliptol, dan esens terpentin	senyawa dari gum copal dan kolofoni (rosin)	

Tabel 4: Kelebihan dan kekurangan dari berbagai media *mounting* dalam konteks kaca objek mikroskop dan pengamatan yang belum dipublikasikan oleh berbagai orang [52].

Nama media *mounting*	Kelebihan	Kekurangan
* Canada balsam	Media sangat tahan lama, umur lebih dari 150 tahun. Preparat dapat dipasang menggunakan minyak cengkeh atau fenol sebagai agen *mounting*.	Mengandung komponen berbahaya dan harus ditangani di bawah lemari asap. Memerlukan serangkaian dehidrasi lengkap yang memakan waktu. Dehidrasi dengan etanol dan transfer melalui ksilen atau minyak cengkeh dapat membuat beberapa taksa rapuh; alternatifnya menggunakan isopropanol, n-butanol, Cellosolve™, 1,4-dioxane, Histoclear, terpineol sehingga dapat mengurangi keretakan.<br><br>Spesimen menjadi hitam jika ksilen diganti dengan fenol atau jika sisa kalium hidroksida masih ada. Indeks bias tinggi dapat menyamarkan struktur yang tidak diwarnai. Pengeringan total bisa memakan waktu bertahun-tahun tanpa pemanasan. Media menjadi kekuningan dan gelap seiring waktu, terutama jika dijernihkan dengan minyak cengkeh. Beberapa zat warna melemah, dan pewarna kationik dapat memudar jika media menjadi asam, yang bisa terjadi secara alami seiring waktu.
DMHF (dimethyl hydantoin formaldehyde)	Transparansi tinggi<br>Indeks bias baik<br>Visibilitas struktur sangat baik<br>Stabilitas preparat cukup baik<br>Kompatibel dengan banyak teknik pewarnaan<br>Perlindungan spesimen baik<br>Adhesi antara kaca objek dan penutup baik	Kemungkinan menguning seiring waktu. Dapat mengubah beberapa zat warna. Tidak cocok untuk zat warna sensitif terhadap formaldehida. Membentuk gelembung udara dan waktu pengeringan lama Media *mounting* sensitif terhadap kelembapan. *Mounting* sulit dibalik. Formaldehida bersifat toksik, iritan, dan karsinogenik
* Euparal (transparent)	Media tahan lama, umur lebih dari 50 tahun. Dapat langsung digunakan pada spesimen yang berasal dari etanol 80% (rekomendasi pabrikan). Tidak menutupi struktur yang tidak diwarnai dan tidak menguning atau menjadi rapuh seiring waktu.<br>Indeks bias lebih sesuai daripada Canada balsam untuk Diptera. Cocok untuk spesimen lebih tebal karena penyusutan minimal dan pengeringan tanpa gelembung.<br>Tetap larut dalam etanol 95%, memungkinkan pemasangan ulang meskipun setelah bertahun-tahun.	Mengandung komponen berbahaya dan harus ditangani di bawah lemari asap. Dehidrasi dengan etanol dan transfer melalui Euparal Essence dapat membuat beberapa taksa rapuh, tetapi penggunaan isopropanol dapat mengurangi masalah ini.
Hoyer fluid	Spesimen dapat dipasang dalam keadaan hidup atau langsung dari air, etanol, atau formaldehida.<br>Maserasi menghasilkan kualitas kutikula yang sangat baik.<br>Indeks bias baik dan dapat ditingkatkan dengan pewarnaan iodin untuk kontras lebih tinggi.<br>Asam asetat dalam formula dapat memperluas appendage arthropoda.<br>Beberapa spesimen tetap stabil hingga 40–60 tahun.	Spesimen tumbuhan yang rapuh dapat kolaps jika media ditambahkan sekaligus; penambahan bertahap memakan waktu.<br>Ruang kosong dan kristal dapat terbentuk dalam kurang dari 10 tahun.<br>Maserasi dapat berlebihan tergantung konsentrasi kloral hidrat dan waktu paparan.<br>Komponen media dapat terpisah, dan granul halus dapat muncul dalam beberapa bulan atau tahun.

This is an Open Access article distributed under the terms of the Creative Commons Attribution License (https://creativecommons.org/licenses/by/4.0), which permits unrestricted use, distribution, and reproduction in any medium, provided the original work is properly cited.

	Larut air, memungkinkan pemasangan ulang dengan mudah.	Media dilaporkan dapat menghitam.
CMCP-9 (= carboxy methyl cellulose phenol)	Spesimen dapat dipasang langsung dari media seperti air, etanol, gliserol, atau larutan yang mengandung formaldehida, dan organ internal dapat dimaserasi jika diperlukan untuk mempermudah pemeriksaan umum atau persiapan.	Media ini dapat membentuk kristal dan menggelap seiring waktu, dan kadang memaserasi spesimen lebih dari yang diinginkan.<br>Kecuali slide dibingkai dengan hati-hati, sampel tebal akan sulit karena dapat menyusut dan menciptakan celah di sekitar penutup.<br>Tidak cocok untuk spesimen yang diwarnai atau bahan yang terkalsifikasi, dan waktu pengeringannya lebih lambat dibandingkan CMC.
Eukitt™	Media tahan lama lebih dari 30 tahun.<br>Kompatibel dengan banyak pelarut untuk *mounting*, termasuk aseton, benzena, kloroform, dioxan, eter, isopropanol, metil benzoat, terpineol, toluena, dan ksilen.<br>Mengering cepat dan pH sedikit asam.<br>Tidak menggelap secara signifikan seiring waktu.<br>Cocok untuk berbagai pewarna (misal fuksin, hematoksilin, metil hijau, metil violet, metilen biru).<br>Spesimen dapat dipasang ulang setelah bertahun-tahun dengan merendam dalam ksilen untuk waktu lama.	Mengandung komponen berbahaya dan harus ditangani di bawah lemari asap.<br>Memerlukan serangkaian dehidrasi lengkap yang memakan waktu.<br>Tidak ideal untuk spesimen tebal karena penyusutan dan terbentuknya gelembung gas.<br>Kaca penutup dapat terlepas seiring waktu jika kaca tidak dibersihkan dan disegel dengan baik. Dapat menunjukkan polimerisasi tidak sempurna di sekitar serat kolagen.
Enecê	Media sangat tahan lama, umur minimal 50 tahun.<br>Enecê tidak menggelap seiring waktu.<br>Lebih lentur, memungkinkan diseksi serangga didalam media dan memberi cukup waktu untuk memposisikan struktur morfologi.<br>Biaya rendah.	Memerlukan serangkaian dehidrasi lengkap yang memakan waktu.<br>Dehidrasi dengan etanol dan transfer melalui minyak cengkeh dapat membuat beberapa spesimen rapuh.<br>Serangga tetap mengalami pencerahan meski sangat lambat; ini dapat menyulitkan pengamatan struktur sangat kecil, seperti sensilla, ascoid, dan setae sederhana.

5.3.4. Deskripsi media mounting yang direkomendasikan (Tabel 3 & 4)

Media untuk preparat sementara (jangka pendek)

Chloral gum = Hoyer fluid/medium/solution (RI = 1.48)

Larutan Marc-André adalah media terbaik untuk pengamatan jangka pendek (beberapa jam, mungkin sedikit lebih lama jika slide disimpan dalam kamar lembap) spermateka, termasuk untuk fotografi (Gambar 4) atau gambar ilustrasi. Untuk mempertahankan spermateka yang telah diamati, perlu dilakukan pemasangan ulang dalam media berbasis air yang memungkinkan penyimpanan jangka menengah. Dehidrasi untuk pemasangan ulang dalam resin bukanlah hal yang mustahil, tetapi tidak direkomendasikan karena risiko kehilangan spesimen.

Chloral gum dan *Hoyer fluid* dianggap sinonim. Media ini umum digunakan untuk mengamati organ internal karena kompatibilitasnya dengan air, kesederhanaannya, aplikasi cepat, dan indeks biasnya yang memudahkan pemeriksaan struktur halus seperti spermateka. Namun, *chloral gum* memiliki kekurangan signifikan jika tidak disiapkan dengan sempurna atau disimpan pada kondisi kelembapan terkendali. Masalah yang terjadi antara lain kristalisasi, perubahan warna, dan hilangnya viskositas. Menyegel (ringing) penutup kaca tidak menyelesaikan masalah ini, karena media *mounting* dapat menjadi sangat berubah warna (kadang hampir hitam) akibat interaksi dengan media *sealing*/perekat, terutama jika menggunakan Euparal®.

Media Hoyer dianggap secara optik terbaik untuk lalat pasir Phlebotominae dan secara tradisional digunakan untuk tujuan ini. Media ini terdiri dari beberapa formulasi yang

saling terkait, termasuk gum arabic, gliserol, dan kloral hidrat. Berbagai formulasi ini sering disalahartikan atau salah dikutip [74].

Meskipun Hoyer merupakan media yang baik untuk mengamati spermateka pada lalat pasir, media ini tidak cocok untuk preservasi jangka panjang. Media ini ideal untuk pengamatan jangka pendek, termasuk fotografi, gambar, atau ilustrasi. Media berbasis air cocok untuk *mounting* sementara, tetapi tidak dapat menjamin preservasi dalam jangka panjang. Sebaliknya, *mounting* dengan resin memberikan daya tahan yang sangat baik, sering bertahan berabad-abad, tetapi dapat menyamarkan detail halus spermateka karena refringensi mereka sering hilang.

Media Hoyer mengalami degradasi seiring waktu akibat dehidrasi (Gambar 8), yang menghasilkan terbentuknya kristal kloral hidrat kecil berwarna putih dan buram. Meskipun demikian, spesimen masih dapat diselamatkan dari slide yang terkristalisasi karena kutikula tetap utuh secara kimia, meskipun beberapa kerusakan fisik dapat terjadi akibat pertumbuhan kristal. Dalam beberapa kasus, slide yang terkristalisasi dapat dipulihkan dengan cara menghidrasi ulang media *mounting* dalam lingkungan hangat dan lembap dengan timol untuk mencegah pertumbuhan jamur. Alternatif lain, spesimen dapat direndam keluar dari *gum chloral* dalam air, didehidrasi menggunakan asam asetat esensial, dan dipasang ulang dalam Canada balsam.

DMHF (dimethyl hydantoin formaldehyde) (RI 1.48)

Media berbasis air ini [72] memiliki performa optik yang sangat baik, mirip dengan Berlese, dan sama mudahnya digunakan seperti Berlese. Namun, berbeda dengan Berlese, media ini tidak menjadi hitam atau mengkristal. Media ini cocok untuk lalat pasir maupun anggota lain dari famili Psychodidae.

Gambar 8: Pemasangan ulang slide. A: slide yang rusak dan kering yang dipasang dengan Hoyer; B: tampilan mikroskopik lalat pasir yang kering; C: tampilan mikroskopik lalat pasir lain yang rusak; D: kamar lembap yang berisi slide kering; E: kepala, dan F: tubuh spesimen B setelah dipasang ulang dalam Euparal®; G: kepala, dan H: tubuh spesimen C yang rusak setelah dipasang ulang dalam Euparal®.

CMCP (camphor-mono-chlorophenol) (RI = 1.41)

Ini adalah media *mounting* berbasis gliserin yang larut dalam air, digunakan untuk membuat slide permanen transparan dari spesimen yang rapuh, termasuk lalat pasir. Keunggulan media *mounting* ini adalah spesimen dapat dipasang langsung dari air atau etanol. Media ini dengan cepat melonggarkan dan menjernihkan lalat pasir, melembutkan kutikula sehingga memungkinkan penempatan spesimen secara tepat, yang sangat berguna untuk melebarkan sayap atau melakukan diseksi genitalia. Meskipun dilaporkan memungkinkan penyimpanan jangka panjang, durasi pasti preservasinya masih belum pasti. Keterbatasan utama media *mounting* ini terletak pada komposisinya yang mengandung fenol, suatu zat toksik dan iritan yang memerlukan penanganan secara hati-hati.

Media untuk preparat permanen

Canada balsam (RI = 1.52-1.54)

Canada balsam pertama kali dikenalkan sebagai media *mounting* yang cocok untuk mikroskop cahaya transmisi oleh Andrew Pritchard pada tahun 1830-an. Media ini tetap menjadi salah satu media yang paling banyak digunakan karena kualitas arsipnya yang terbukti, dengan lebih dari 150 tahun penggunaan berhasil. Berbeda dengan media Hoyer fluid, Canada balsam tidak mengkristal atau menyerap kelembapan. Namun, Canada balsam memiliki autofluoresensi yang kuat, yang terkadang dapat menjadi kelemahan untuk teknik mikroskopi tertentu [60]. Menggunakan pelarut non-toksik sebagai pengganti xilena dapat mengurangi risiko keselamatan selama persiapan, tetapi juga dapat menimbulkan kekurangan seperti pengeringan lebih lambat dan penggelapan media lebih awal.

Euparal® (RI = 1.48)

Euparal® merupakan media alternatif yang banyak digunakan dibandingkan dengan Canada balsam untuk *mounting* permanen. Media ini mampu menjaga stabilitas dalam jangka panjang yang sangat baik dengan indeks bias yang sebanding. Euparal® memiliki karakteristik sebagai berikut: (1) persyaratan dehidrasi: sebelum transfer akhir media *mounting*, spesimen harus didehidrasi, biasanya melalui transisi dari etanol 95% ke etanol absolut, dan (2) waktu pemrosesan yang lebih lama: pemasangan akhir dalam resin, baik Canada balsam maupun Euparal®, memerlukan dehidrasi, yang menyebabkan keseluruhan waktu pemrosesan specimen menjadi lama. Jika proses dehidrasi menggunakan pelarut organik tidak memungkinkan, maka sampel yang diekstrak dari etanol absolut dapat ditempatkan dalam larutan antara yang terdiri dari campuran sama rata antara Euparal® dan Euparal® essence sebelum pemasangan akhir.

Enecê (RI = 1.467)

Enecê adalah media *mounting* berbasis resin yang terutama digunakan untuk serangga kecil dan sangat populer di Brasil. Basisnya terdiri dari *colophony* dan gum copal yang dilarutkan dalam alkohol, kamper, essence terpentin, dan eucalyptol. Cerqueira [11] menjelaskan Enecê sebagai alternatif Canada balsam untuk pemasangan permanen larva, eksuvia instar muda, bahkan nyamuk dewasa, dan sejak itu banyak digunakan untuk *mounting* lalat pasir. Enecê menawarkan alternatif yang ekonomis untuk *mounting* permanen, menyediakan stabilitas jangka panjang dan waktu pengeringan yang cukup,

memungkinkan diseksi dan penataan struktur morfologi secara akurat.

5.4. Persiapan dan pengeringan preparat kaca

Pengeringan slide yang dipasang dengan benar sangat penting untuk memastikan stabilitas dan preservasi jangka panjang. Slide harus dikeringkan secara menyeluruh sebelum dipertimbangkan untuk penyimpanan jangka panjang. Untuk hasil optimal, slide yang dipasang dengan media permanen sebaiknya dikeringkan secara horizontal selama 2–3 minggu, sedangkan slide yang dipersiapkan dengan media semi-permanen mungkin hanya memerlukan waktu selama 1–2 minggu.

Untuk memastikan proses pengeringan efektif, disarankan menggunakan inkubator yang diatur pada suhu yang sesuai dengan media *mounting* yang digunakan, sambil menghindari panas berlebih yang dapat merusak spesimen. Rentang suhu yang disarankan adalah 30°C hingga 37°C. Langkah pengeringan ini sangat penting untuk mencegah melengkungnya slide, kerusakan spesimen, atau ketidakstabilan media *mounting* selama penyimpanan.

Media *mounting* yang digunakan dalam persiapan slide harus selalu dicatat pada label slide. Jika memungkinkan, label juga sebaiknya mencantumkan formula spesifik yang digunakan, nama orang yang menyiapkan preparat, dan tanggal pembuatan. Slide awalnya dipersiapkan sebagai *mounting* sementara dan tidak dimaksudkan untuk preservasi jangka panjang. Namun, jika status spesimen berubah, misalnya ditetapkan sebagai bagian dari seri "type", maka media *mounting* yang lebih permanen sebaiknya digunakan untuk memastikan penyimpanan spesimen untuk studi taksonomi di masa depan.

5.5. Teknik *Mounting* Alternatif: Mounting pada Kartu

Mounting pada kartu adalah teknik yang digunakan untuk beberapa kelompok serangga dimana spesimen dapat ditusuk langsung pada kartu entomologi atau direkatkan pada permukaannya. Mengingat ukuran mereka yang kecil dan kebutuhan untuk mengamati organ internal guna identifikasi melalui proses klarifikasi (lihat poin 5), metode ini sama sekali tidak cocok untuk *mounting* lalat pasir.

5.6. Pemasangan Ulang Spesimen yang Rusak

Untuk spesimen yang langka atau bernilai, disarankan pendekatan dua langkah sesuai video yang dapat diakses di: https://zenodo.org/records/18315029. Rehidrasi tanpa membongkar dapat dilakukan untuk pengamatan awal. Sebuah penyangga untuk beberapa slide mikroskopik ditempatkan dalam cawan Petri sebagai dudukan. Slide yang akan direhidrasi diletakkan di atasnya, dan cawan Petri diisi dengan beberapa milimeter pelarut untuk menjaga kamar tetap lembap, memastikan slide itu sendiri tidak bersentuhan langsung dengan pelarut (Gambar 8 D). Waktu yang dibutuhkan untuk rehidrasi dapat bervariasi dari satu hingga beberapa hari, tergantung kondisi spesimen. Pemantauan dilakukan setiap hari. Setelah slide cukup direhidrasi, slide dapat dikeluarkan dari kamar lembap dan ditempatkan di inkubator selama beberapa jam sebelum pemeriksaan mikroskopik, pemotretan, atau pembuatan gambar.

Pemasangan ulang (re*mounting*). Slide dapat dikembalikan ke kamar lembap selama beberapa jam tambahan atau semalaman. Pembongkaran harus dilakukan di bawah mikroskop binokular. Dengan menggunakan jarum halus, *coverslip* harus dilepas dengan hati-hati, memastikan tidak ada elemen lalat pasir yang tertinggal (https://zenodo.org/records/18315029). Selanjutnya, elemen lalat pasir yang telah didiseksi dikumpulkan dan dibilas dengan air pada tempat kecil, seperti yang digunakan untuk ekstraksi DNA/RNA destruktif (lihat bagian bawah), sebelum dilakukan dehidrasi dan pemasangan ulang dalam media resin. Saat membongkar slide, sangat penting untuk mengidentifikasi media *mounting* asli yang digunakan untuk memilih pelarut yang sesuai. Jika media *mounting* berbasis air, maka harus menggunakan air kembali. Jika media *mounting* berbasis resin (misalnya, Canada balsam atau Euparal®), maka digunakan xilena, di bawah lemari asap dan dengan alat pelindung diri (APD) yang sesuai, termasuk masker. Pemasangan ulang untuk spesimen tipe atau koleksi hanya boleh dilakukan dengan izin kurator dan/atau lembaga pemilik spesimen.

6. Identifikasi spesimen

6.1. Secara morfologi

Identifikasi lalat pasir terutama bergantung pada pemeriksaan karakteristik morfologisnya, termasuk bentuk toraks, sayap, genitalia, setae, dan hubungan morfometrik spesifik antar berbagai struktur. Peneliti menggunakan kunci taksonomi, koleksi referensi, dan deskripsi spesies asli untuk membandingkan spesimen yang dikumpulkan dengan taksa yang sudah dikenal. Fitur diagnostik utama, seperti venasi sayap dan morfologi kepala pada kedua jenis kelamin, struktur genitalia jantan, dan konfigurasi spermateka pada lalat betina, sangat informatif untuk penentuan spesies. Identifikasi yang akurat sering kali memerlukan pemeriksaan mikroskopis yang detail, biasanya menggunakan mikroskop majemuk untuk mengamati struktur halus seperti genitalia dan spermateka, atau stereomikroskop untuk fitur morfologis yang lebih luas.

Kemajuan terbaru dalam teknologi pencitraan telah mempermudah penggunaan pencitraan digital untuk mengidentifikasi lalat pasir. Foto dengan resolusi tinggi atau ilustrasi digital dari fitur utama dapat dibandingkan dengan bahan referensi atau dianalisis menggunakan sistem identifikasi berbasis komputer, sehingga meningkatkan akurasi dan aksesibilitas dalam taksonomi morfologis.

6.2. Geometri sayap

Geometri sayap merupakan karakteristik kunci yang digunakan dalam identifikasi dan klasifikasi berbagai spesies lalat pasir. Sayap lalat pasir menunjukkan pola dan struktur yang unik, biasanya panjang dan sempit dengan venasi yang berkembang baik (Gambar 9 & 10). Susunan vena membentuk pola khas yang dapat berbeda antar genus dan spesies, sehingga memberikan fitur diagnostik yang berharga untuk identifikasi. Dengan demikian, studi tentang geometri sayap memberikan wawasan penting untuk tujuan taksonomi.

6.3. Morfometri geometri sayap

Para peneliti menggunakan berbagai teknik, seperti morfometri geometrik, untuk menganalisis dan membandingkan bentuk serta ukuran sayap di antara berbagai spesies atau populasi lalat pasir. Studi tentang geometri sayap memberikan wawasan penting mengenai perilaku, preferensi habitat, dan kemampuan terbang.

Dalam pendekatan morfometri geometrik, sayap dipotong dengan hati-hati, diwarnai (jika diperlukan), dan dipasang datar pada slide. Slide yang telah disiapkan kemudian difoto di bawah stereomikroskop, didigitalkan, dan dianalisis secara morfometrik. Prosedur ini telah dijelaskan dengan baik dalam literatur [6, 27, 42, 56, 57, 59], dengan rekomendasi menggunakan sayap kanan atau kiri secara konsisten pada organ berpasangan guna menghindari potensi efek alometrik negatif [62].

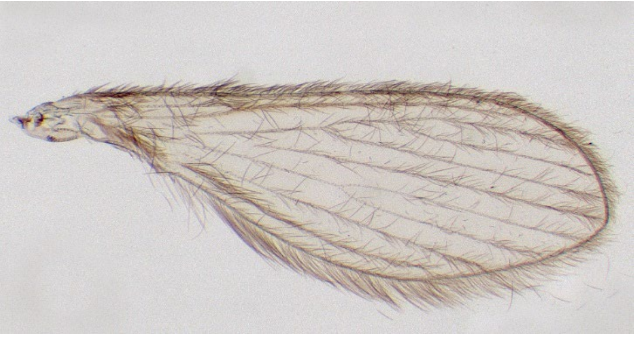

Gambar 9: Sayap *Trichophoromyia ininii* tanpa perlakuan.

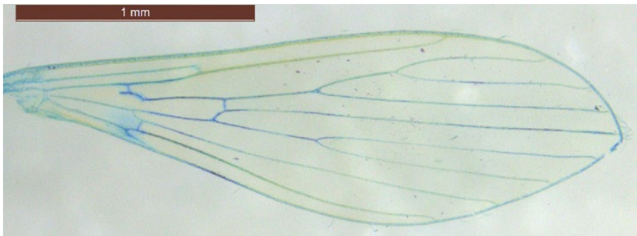

Gambar 10: Sayap *Phlebotomus ariasi* yang telah diberi perwarnaan.

Persiapan Sayap untuk Analisis Morfometri Geometrik

Untuk visualisasi vena sayap yang optimal, sayap harus dibersihkan dari sisik/rambut dan diwarnai dengan tepat. Untuk persiapan sayap, tahapan pertama adalah mengisi sumur kecil dengan reagen yang diperlukan (metilen biru, etanol, air, dan pengganti ksilen). Sayap yang disimpan dalam 70% etanol pada suhu kamar diambil dengan cara membalik tabung Eppendorf dan menuangkannya keatas sumur, lalu sayap diangkat secara longitudinal menggunakan jarum halus yang melengkung. Sayap dipindahkan secara singkat dari etanol ke air dan kembali ke etanol untuk menghilangkan bulu halus.

Sayap dimasukkan kedalam larutan metilen biru selama 6 menit, dan dipastikan sayap dalam keadaan mengapung selama pewarnaan. Sayap diambil dengan hati-hati dan direndam dalam larutan ksilen selama 2 menit (sekitar sepertiga dari waktu pewarnaan metilen biru). Ketukan lembut jarum pada dinding sumur dapat membantu sayap mengendap; ksilen berfungsi untuk menetapkan pewarnaan.

Terakhir, sayap diangkat dan diletakkan di atas setetes kecil Euparal® pada slide mikroskop. Di bawah lensa pembesar, sayap dibuka secara perlahan dan diletakkan cover slip dengan hati-hati. Pengambilan foto dapat segera dilakukan sebelum Euparal® mengeras, karena penyesuaian kecil posisi sayap di bawah cover slip mungkin diperlukan untuk mencapai penyelarasan yang optimal.

6.4. Teknik Biologi Molekular

Selain teknik morfologi, metode molekuler semakin penting dalam penelitian entomologi, termasuk studi taksonomi, genetika populasi, dan filogenetik, serta untuk deteksi DNA/RNA patogen, dan menentukan asal darah mangsa, serta perilaku vektor yang penting dalam bidang epidemiologi [70]. Sekuensing DNA dapat digunakan untuk konfirmasi spesies atau membedakan spesies yang sangat dekat, memberikan cara identifikasi yang lebih akurat dan andal. Selain itu, teknik molekuler canggih (misalnya PCR, sekuensing DNA, NGS, dll.) dan MALDI-ToF MS semakin menonjol untuk identifikasi spesies yang cepat dan tepat, melengkapi metode morfologi tradisional [46]. Meskipun ada kemajuan teknik molekuler, tetapi identifikasi morfologi tetap menjadi standar acuan dalam taksonomi dan menjadi dasar interpretasi data molekuler.

6.4.1. Ekstraksi asam nukleat secara destruktif

Ekstraksi asam nukleat adalah langkah rutin dalam banyak penelitian biologi, dan berbagai metode telah dikembangkan untuk mengisolasi DNA dari bahan biologis [48]. Banyak kit ekstraksi DNA yang tersedia secara komersial dirancang untuk mempermudah proses ini [14]. Namun, metode yang umum digunakan untuk menyiapkan spesimen arthropoda untuk identifikasi morfologis seringkali menghambat analisis DNA, karena teknik-teknik tersebut dapat merusak atau menghancurkan ciri fisik

penting dari spesimen [10]. Sebagian besar protokol ekstraksi DNA untuk jaringan serangga bersifat destruktif [43], yang menimbulkan perhatian khusus pada spesimen kecil, di mana pengambilan sampel terbatas sekalipun dapat mengurangi fitur morfologis penting [72]. Jenis dan kondisi spesimen memainkan peran kunci dalam pemilihan metode isolasi DNA yang tepat [29].

Kebutuhan untuk identifikasi yang akurat dari lalat pasir, memahami dinamika populasi, dan meminimalkan dampak pada target non-spesifik telah mendorong pengembangan alat diagnostik molekuler [23]. Pendekatan molekuler kini sering digunakan untuk melengkapi metode taksonomi morfologis dalam identifikasi lalat pasir. Misalnya, pendekatan standar untuk barcode serangga melibatkan ekstraksi DNA, pengurutan, dan hilangnya spesimen asli. Oleh karena itu, terdapat kebutuhan mendesak untuk mengeksplorasi metode ekstraksi DNA non-destruktif yang mempertahankan bahan biologis sekaligus integritas morfologisnya.

Berbagai metode ekstraksi asam nukleat telah diterapkan pada lalat pasir. Jumlah atau kualitas asam nukleat yang dibutuhkan tergantung pada analisis molekuler lanjutan, karena setiap teknik memiliki sensitivitas dan kebutuhan kemurnian yang berbeda [9]. Misalnya, mata lalat pasir ditemukan dapat menghambat amplifikasi PCR [69]. Selain untuk skrining patogen, DNA lalat pasir secara rutin diekstraksi untuk tujuan identifikasi spesies. Berbagai metode ekstraksi DNA dapat digunakan, meskipun hasil dan kualitasnya berbeda-beda diantara teknik tersebut. Beberapa protokol dari manufaktur telah diadaptasi oleh para peneliti untuk lalat pasir [8], meningkatkan hasil dan/atau kualitas asam nukleat yang diekstraksi [8, 9, 69], sementara adaptasi lain yang dikembangkan untuk taksa arthropoda lain juga dapat diterapkan pada lalat pasir [58, 76]. Identifikasi secara PCR yang menargetkan fragmen mitokondria kecil (COI atau *cyt*B) umumnya kompatibel dengan metode ekstraksi yang menghasilkan fragmentasi DNA tinggi. Sebaliknya, teknik NGS dengan long-read (Oxford Nanopore dan PacBio) memerlukan fragmentasi minimal dan DNA berkualitas tinggi. Ekstraksi menggunakan spin column umumnya menghasilkan fragmen DNA genomik hingga 60 kb, sedangkan ekstraksi fenol-kloroform dapat menghasilkan fragmen hingga 150 kb [77]. Tabel 5 merangkum berbagai teknik ekstraksi DNA lalat pasir dan menunjukkan apakah adaptasi metodologis telah dilakukan untuk serangga ini. Hasil ekstraksi tidak ditampilkan, karena tergantung pada ukuran spesimen dan metode persiapan. Kolom modifikasi merujuk pada adaptasi protokol ekstraksi untuk lalat pasir atau arthropoda kecil lainnya.

Pemilihan metode ekstraksi harus mempertimbangkan beberapa kriteria, seperti jumlah sampel, waktu ekstraksi, dan teknik yang akan diterapkan pada analisis lanjutan. Meskipun teknik NGS memerlukan DNA genomik dengan berat molekul tinggi, semua metode yang dipaparkan di sini dapat digunakan untuk aplikasi berbasis PCR standar.

Selain itu, beberapa studi telah mengeksplorasi metode ekstraksi DNA non-destruktif untuk arthropoda kecil darat, spesimen museum yang diawetkan kering, dan arthropoda bertubuh lunak [19, 26, 28, 55, 63].

Tabel 5: Rata-rata biaya, aplikasi, dan adaptasi protokol untuk ekstraksi gDNA lalat pasir

Protokol	Biaya	Aplikasi	Adaptasi protocol untuk serangga kecil
Spin column	2.5 – 3.55 US$ [39]	PCR, NGS	[9]
Phenol-chloroform	0.24 US$ [69]	PCR, NGS	[9]
HotSHOT	<0.01 US$ [69]	PCR	-
Salting out	0.12 $3 [69]	PCR	-
Chelex	0.02 $4 [41]	PCR	[41, 76]

6.4.2. Ekstraksi asam nukleat secara non-destruktif

Salah satu tantangan utama dalam analisis molekuler arthropoda, terutama lalat pasir, adalah pelestarian spesimen agar dapat diintegrasikan kedalam koleksi entomologi. Sebagian besar protokol ekstraksi DNA memerlukan pemacaran jaringan, sehingga mengorbankan pelestarian spesimen asli. Metode ekstraksi asam nukleat non-destruktif dirancang untuk mengekstraksi materi genetik tanpa merusak sampel secara fisik, memengaruhi viabilitasnya, atau mengubah morfologinya. Metode ini sangat berharga ketika bekerja dengan spesimen yang langka atau terbatas, seperti lalat pasir, dimana menjaga integritas struktural sangat penting untuk tujuan taksonomi, morfologi, atau diagnostik di masa depan.

Salah satu teknik yang umum digunakan adalah metode perendaman non-destruktif, dimana lalat pasir diimobilisasi dan direndam perlahan dalam buffer lisis yang mengandung proteinase K. Teknik mild-vectolysis telah berhasil diterapkan pada lalat pasir, terutama pada tipe-tipe spesimen [24]. Teknik ini memanfaatkan kit spin column konvensional (dalam hal ini, DNeasy Blood and Tissue kit, QIAGEN, Hilden, Jerman) dengan adaptasi untuk memperoleh DNA tanpa merusak spesimen. Tahap lisis yang dimodifikasi (volume buffer lisis dan penambahan tahap pembekuan) [17] memungkinkan pelepasan asam nukleat dengan meminimalkan kerusakan morfologis [24].

Untuk lalat pasir, juga memungkinkan menggunakan HotSHOT DNA Extraction kit (Bento Bioworks Ltd, London, Inggris) [73] yang cepat dan murah, memungkinkan pemrosesan sampel yang cepat dan hemat biaya. Spesimen entomologi yang dimaksudkan untuk identifikasi morfologis kemudian dapat dibilas. Spesimen yang diproses menggunakan DNeasy Blood and Tissue kit harus dibersihkan dengan larutan Marc-André, sedangkan yang diproses menggunakan HotSHOT DNA extraction kit

cukup jernih untuk dipasang pada media berbasis air, atau lebih baik lagi, pada resin setelah dehidrasi, sesuai protokol yang dijelaskan dalam artikel ini [73].

Materi genetik yang diekstraksi kemudian dapat diproses lebih lanjut untuk analisis lanjutan, seperti PCR yang berfungsi untuk memperbanyak penanda genetik tertentu. Metode ekstraksi asam nukleat non-destruktif sangat penting untuk mempelajari karakteristik genetik lalat pasir, termasuk mengidentifikasi agen penyebab penyakit potensial yang mungkin dibawa oleh lalat pasir. Dengan menjaga integritas spesimen, peneliti dapat memperoleh informasi genetik yang berharga, sambil mempertahankan sampel untuk analisis atau studi tambahan.

6.5. MALDI-ToF MS

MALDI-ToF MS (matrix-assisted laser desorption/ionization time-of-flight mass spectrometry) adalah teknik berbasis spektrometri massa yang dirancang untuk mendeteksi dan menganalisis profil protein unik (‘sidik jari’) dari sampel biologis. MALDI-ToF semakin diakui sebagai alat penting untuk identifikasi arthropoda yang memiliki relevansi medis dan veteriner. Teknik ini terbukti efektif dalam mengidentifikasi berbagai tahap perkembangan lalat pasir, termasuk bentuk imatur dan darah yang dikonsumsi oleh betina yang abdomennya penuh dengan darah (*engorged*), dan telah berhasil diterapkan untuk membedakan spesies lalat pasir jantan maupun betina dibawah berbagai kondisi penyimpanan dan homogenisasi [28, 30, 73, 74].

Metode ini juga menawarkan kemampuan diskriminasi yang tinggi pada tingkat subgenus, spesies, dan populasi. Teknik ini memungkinkan peneliti mencapai identifikasi spesies yang cepat dan akurat, yang penting untuk memahami distribusi lalat pasir, perilaku, serta perannya dalam penularan penyakit. Dengan membedakan spesies berdasarkan profil protein, MALDI-ToF memainkan peran penting dalam studi epidemiologi dan strategi pengendalian vektor.

Saat ini terdapat dua keterbatasan utama dari teknik MALDI-ToF yang membatasi penerapannya secara rutin. Pertama adalah ketersediaan peralatan spektrometri massa, yang biayanya sangat tinggi sehingga sulit diperoleh hanya untuk tujuan identifikasi spesies lalat pasir (atau vektor arthropoda pada umumnya). Untungnya, keterbatasan ini dapat diatasi dengan memperoleh waktu penggunaan pada spektrometer massa yang telah menjadi alat standar di fasilitas proteomik dan/atau diagnostik klinis. Kedua adalah representasi data referensi lalat pasir yang rendah dalam basis data akses terbuka, sehingga diperlukan pembuatan basis data internal dengan spektra referensi berdasarkan spesimen yang teridentifikasi secara jelas, idealnya melalui kombinasi penilaian morfologis dan sekuensing penanda genetik yang sesuai (COI, cytB, atau lainnya). Keterbatasan ini diharapkan segera teratasi melalui inklusi bertahap data referensi lalat pasir internal ke dalam MSI Platform yang dijalankan oleh Assistance Publique-Hôpitaux de Paris, Sorbonne University, Prancis dan koleksi BCCM/IHEM/Sciensano di Brussels, Belgia (https://msi.happy-dev.fr/).

Ketika profil protein MALDI-ToF direncanakan untuk digunakan, sampel sebaiknya disimpan dalam keadaan kering-dibekukan atau dalam etanol 70% kualitas molekuler, dan tidak terpapar suhu lingkungan. Karena belum ada pedoman universal untuk persiapan sampel, pengguna disarankan menggunakan larutan akueus 60% asetonitril/0,3% TFA dari asam sinapin (30 mg/mL) untuk persiapan matriks MALDI-ToF agar spektra protein mereka dapat dibandingkan dengan data lalat pasir yang telah diterbitkan.

Preparasi sample untuk\MALDI-ToF MS (Gambar 7)

Spesimen serangga, yang disimpan dalam berbagai kondisi, pertama-tama dikeringkan di udara pada suhu kamar kemudian dibedah. Kepala dan abdomen dipisahkan untuk memperoleh bagian tubuh yang mengandung karakter morfologis penting untuk pemasangan pada slide dan analisis morfologi. Thoraks dapat digunakan untuk MALDI-ToF, sedangkan abdomen yang tersisa disimpan untuk ekstraksi DNA. Untuk profil protein, toraks dihancurkan secara manual dalam tabung mikro 1,5 mL dengan 10 µL larutan homogenisasi menggunakan penghalus (pestle) dan pellet sekali pakai. Biasanya digunakan dua larutan homogenisasi: air suling steril dan asam format 25%.

7. Kesimpulan

Artikel diharapkan dapat memberikan wawasan bagi peneliti tentang metode yang paling efektif untuk pembuatan prepart (*mounting*) lalat pasir, disesuaikan dengan tujuan penelitian tertentu, guna memfasilitasi identifikasi yang akurat termasuk untuk mendeteksi agen patogen yang dibawanya. Tidak ada satu metode tunggal yang secara universal akan memberikan hasil yang optimal; sebaliknya, terdapat beberapa metode yang masing-masing memiliki keunggulan dan keterbatasannya sendiri.

Dalam data pendukung, artikel ini menyajikan protokol terperinci untuk berbagai teknik pembuatan preparat (mounting) yang digunakan dalam persiapan dan identifikasi lalat pasir. Protokol ini, termasuk video instruksional, menawarkan prosedur langkah demi langkah yang disesuaikan dengan berbagai tujuan, memastikan hasil yang tepat dan dapat diandalkan. Dengan menyediakan sumber daya yang komprehensif ini, artikel ini bertujuan untuk mendukung peneliti dalam memilih dan menerapkan teknik pembuatan preparat (*mounting*) yang paling sesuai untuk kebutuhan spesifik mereka.

Ucapan terima kasih

Para penulis mengucapkan terima kasih kepada Richard Lane dan Zoe Jay Adams dari Natural History Museum of London, UK atas tinjauan mereka yang sangat baik, yang telah meningkatkan kualitas manuskrip ini.

Sumber Dana

Kami mengucapkan terima kasih kepada lembaga pengembangan Brasil, CNPq (nomor kasus: 404395/2024-4) dan Yayasan Araucária (nomor kasus: 433/2025 PDI) atas pendanaan penelitian AJA.

Konflik Kepentingan

Jérôme Depaquit adalah editor asosiasi Parasite; ia tidak memiliki pengaruh terhadap proses peninjauan dan pengambilan keputusan terkait manuskrip ini. Penulis lainnya menyatakan bahwa mereka tidak memiliki konflik kepentingan.

Pernyataan ketersediaan data

Vìdeos di Zenodo
Video 1: https://zenodo.org/records/18198006
Video 2: https://zenodo.org/records/18311158
Video 3: https://zenodo.org/records/18311106
Video 4: https://zenodo.org/records/18311154
Video 5: https://zenodo.org/records/18303014
Video 6: https://zenodo.org/records/18302850
Video 7: https://zenodo.org/records/18315029

Materi tambahan

Informasi lebih lanjut tentang artikel ini tersedia di alamat berikut: https://www.parasite-journal.org/10.1051/parasite/2026009/olm

Daftar Pustaka

1. Alkan C, Allal-Ikhlef AB, Alwassouf S, Baklouti A, Piorkowski G, de Lamballerie X, Izri A, Charrel RN. 2015. Virus isolation, genetic characterization and seroprevalence of Toscana virus in Algeria. Clinical Microbiology and Infection, 21(11), 1040 e1-9.
2. Alten B, Ozbel Y, Ergunay K, Kasap OE, Cull B, Antoniou M, Velo E, Prudhomme J, Molina R, Banuls AL, Schaffner F, Hendrickx G, Van Bortel W, Medlock JM. 2015. Sampling strategies for phlebotomine sand flies (Diptera: Psychodidae) in Europe. Bulletin of Entomological Research 105(6), 664–678.
3. Ayhan N, Baklouti A, Prudhomme J, Walder G, Amaro F, Alten B, Moutailler S, Ergunay K, Charrel RN, Huemer H. 2017. Practical guidelines for studies on sandfly-borne phleboviruses: Part I: Important points to consider *ante* field work. Vector-Borne and Zoonotic Diseases 17(1), 73–80.
4. Bates PA. 1997. Infection of phlebotomine sandflies with *Leishmania*, in The Molecular Biology of Insect Disease Vectors: A Methods Manual. Springer. p. 112–120.5.
5. Baum M, de Castro EA, Pinto MC, Goulart TM, Baura W, Klisiowicz Ddo R, Vieira da Costa-Ribeiro MC. 2015. Molecular detection of the blood meal source of sand flies (Diptera: Psychodidae) in a transmission area of American cutaneous leishmaniasis, Parana State, Brazil. Acta Tropica, 143, 8–12.
6. Belen A, Alten B, Aytekin A. 2004. Altitudinal variation in morphometric and molecular characteristics of *Phlebotomus papatasi* populations. Medical and Veterinary Entomology, 18(4), 343–350.
7. Bhattacharya J, Chandra G, Hati AK. 1991. A simple method for cryopreservation of *Leishmania donovani* promastigotes, Indian Journal of Medical Research, 93, 245–246.
8. Caligiuri LG, Sandoval AE, Miranda JC, Pessoa FA, Santini MS, Salomón OD, Secundino NF, McCarthy CB. 2019. Optimization of DNA extraction from individual sand flies for PCR amplification. Methods and Protocols, 2(2), 36.
9. Casaril AE, de Oliveira LP, Alonso DP, de Oliveira EF, Gomes Barrios SP, de Oliveira Moura Infran J, Fernandes WS, Oshiro ET, Ferreira AMT, Ribolla PEM, de Oliveira AG. 2017. Standardization of DNA extraction from sand flies: Application to genotyping by next generation sequencing. Experimental Parasitology, 177, 66–72.
10. Castalanelli MA, Severtson DL, Brumley CJ, Szito A, Foottit RG, Grimm M, Munyard K, Groth DM. 2010. A rapid non-destructive DNA extraction method for insects and other arthropods. Journal of Asia-Pacific Entomology, 13(3), 243–248.
11. Cerqueira NL. 1943. Um novo meio para montagem de pequenos insetos em lâmina. Memórias do Instituto Oswaldo Cruz, (39), 37–41.
12. Charrel RN, Gallian P, Navarro-Mari JM, Nicoletti L, Papa A, Sanchez-Seco MP, Tenorio A, de Lamballerie X. 2005. Emergence of Toscana virus in Europe. Emerging Infectious Diseases, 11(11), 1657–1663.
13. Chaskopoulou A, Giantsis IA, Demir S, Bon MC. 2016. Species composition, activity patterns and blood meal analysis of sand fly populations (Diptera: Psychodidae) in the metropolitan region of Thessaloniki, an endemic focus of canine leishmaniasis. Acta Tropica, 158, 170–176.
14. Chen H, Rangasamy M, Tan SY, Wang H, Siegfried BD. 2010. Evaluation of five methods for total DNA extraction from western corn rootworm beetles. PLoS One, 5(8), e11963.
15. Depaquit J, Grandadam M, Fouque F, Andry PE, Peyrefitte C. 2010. Arthropod-borne viruses transmitted by Phlebotomine sandflies in Europe: a review. Eurosurveillance, 15(10), 19507.
16. Diamond LS, Herman CM. 1954. Incidence of Trypanosomes in the Canada Goose as revealed by bone marrow culture. Journal of Parasitology, 40(2), 195–202.
17. Ding H, Torno M, Vongphayloth K, Ng G, Tan D, Sng W, Ho K, Randrianambinintsoa FJ, Depaquit J, Tan CH. 2025. Hidden in plain sight: discovery of sand flies in Singapore and description of four species new to science. Parasites & Vectors, 18(1), 402.
18. Es-Sette N, Ajaoud M, Bichaud L, Hamdi S, Mellouki F, Charrel RN, Lemrani M. 2014. *Phlebotomus sergenti* a common vector of *Leishmania tropica* and Toscana virus in Morocco. Journal of Vector Borne Diseases, 51(2), 86–90.
19. Favret C. 2005. A new non-destructive DNA extraction and specimen clearing technique for aphids (Hemiptera). Proceedings of the Entomological Society of Washington, 107(2), 469–470.
20. Galati EAB. 2018. Phlebotominae (Diptera, Psychodidae): Classification, morphology and terminology of adults and identification of American taxa, in Brazilian Sand Flies: Biology, Taxonomy, Medical Importance and Control, Rangel EF, Shaw JJ, Editors. Cham: Springer International Publishing. pp. 9–212.
21. Galati EAB, de Andrade AJ, Perveen F, Loyer M, Vongphayloth K, Randrianambinintsoa FJ, Prudhomme J, Rahola N, Akhoundi M, Shimabukuro PHF, Depaquit J. 2025. Phle-botomine sand flies (Diptera, Psychodidae) of the world. Parasites & Vectors, 18(1), 220.
22. Galati EAB, Galvis-Ovallos F, Lawyer P, Leger N, Depaquit J. 2017. An illustrated guide for characters and terminology used in descriptions of Phlebotominae (Diptera, Psychodidae). Par-asite, 24, 26.

23. Gariepy T, Kuhlmann U, Gillott C, Erlandson M. 2007. Parasitoids, predators and PCR: the use of diagnostic molecular markers in biological control of Arthropods. Journal of Applied Entomology, 131(4), 225–240.
24. Giantsis IA, Chaskopoulou A, Bon MC. 2016. Mild-Vectolysis: A nondestructive DNA extraction method for vouchering sand flies and mosquitoes. Journal of Medical Entomology, 53(3), 692–695.
25. Gidwani K, Picado A, Rijal S, Singh SP, Roy L, Volfova V, Andersen EW, Uranw S, Ostyn B, Sudarshan M, Chakravarty J, Volf P, Sundar S, Boelaert M, Rogers ME. 2011. Serological markers of sand fly exposure to evaluate insecticidal nets against visceral leishmaniasis in India and Nepal: a cluster-randomized trial. PLoS Neglected Tropical Diseases, 5(9), e1296.
26. Gilbert MTP, Moore W, Melchior L, Worobey M. 2007. DNA extraction from dry museum beetles without conferring external morphological damage. PLoS One, 2(3), e272.
27. Giordani BF, Andrade AJ, Galati EAB, Gurgel-Goncalves R. 2017. The role of wing geometric morphometrics in the identification of sandflies within the subgenus *Lutzomyia.* Medical and Veterinary Entomology, 31(4), 373–380.
28. Guzmán-Larralde AJ, Suaste-Dzul AP, Gallou A, Peña-Carrillo KI. 2017. DNA recovery from microhymenoptera using six non-destructive methodologies with considerations for subsequent preparation of museum slides. Genome, 60(1), 85–91.
29. Hajibabaei M, DeWaard JR, Ivanova NV, Ratnasingham S, Dooh RT, Kirk SL, Mackie PM, Hebert PD. 2005. Critical factors for assembling a high volume of DNA barcodes. Philosophical Transactions of the Royal Society B: Biological Sciences, 360(1462), 1959–1967.
30. Haouas N, Pesson B, Boudabous R, Dedet JP, Babba H, Ravel C. 2007. Development of a molecular tool for the identification of *Leishmania* reservoir hosts by blood meal analysis in the insect vectors. American Journal of Tropical Medicine and Hygiene, 77(6), 1054–1059.
31. Hlavackova K, Dvorak V, Chaskopoulou A, Volf P, Halada P. 2019. A novel MALDI-TOF MS-based method for blood meal identification in insect vectors: A proof of concept study on phlebotomine sand flies. PLoS Neglected Tropical Diseases, 13 (9), e0007669.
32. Huemer H, Prudhomme J, Amaro F, Baklouti A, Walder G, Alten B, Moutailler S, Ergunay K, Charrel RN, Ayhan N. 2017. Practical guidelines for studies on sandfly-borne phleboviruses: Part II: Important points to consider for fieldwork and subsequent virological screening. Vector-Borne and Zoonotic Diseases, 17(1), 81–90.
33. Jancarova M, Polanska N, Thiesson A, Arnaud F, Stejskalova M, Rehbergerova M, Kohl A, Viginier B, Volf P, Ratinier M. 2025. Susceptibility of diverse sand fly species to Toscana virus. PLoS Neglected Tropical Diseases, 19(5), e0013031.
34. Kapp JD, Green RE, Shapiro B. 2021. A fast and efficient single-stranded genomic library preparation method optimized for ancient DNA. Journal of Heredity, 112(3), 241–249.
35. Killick-Kendrick R, Maroli M, Killick-Kendrick M. 1991. Bibliography of the colonization of phlebotomine sandflies. Parassitologia, 33(suppl.), 321–333.
36. Lawyer P, Killick-Kendrick M, Rowland T, Rowton E, Volf P. 2017. Laboratory colonization and mass rearing of phlebotomine sand flies (Diptera, Psychodidae). Parasite, 24, 42.
37. Léger N, Pesson B, Madulo-Leblond G. 1986. Les phlébotomes de Grèce : 1ère partie. Bulletin de la Société de Pathologie Exotique, 79, 386–397.
38. Léger N, Pesson B, Madulo-Leblond G. 1986. Les phlébotomes de Grèce : 2ème partie. Bulletin de la Société de Pathologie Exotique, 79, 514–524.
39. Leonel JAF, Vioti G, Alves ML, da Silva DT, Meneghesso PA, Benassi JC, Spada JCP, Galvis-Ovallos F, Soares RM, Oliveira T. 2020. DNA extraction from individual Phlebotomine sand flies (Diptera: Psychodidae: Phlebotominae) specimens: Which is the method with better results? Experimental Parasitology, 218, 107981.
40. Lestinova T, Rohousova I, Sima M, de Oliveira CI, Volf P. 2017. Insights into the sand fly saliva: Blood-feeding and immune interactions between sand flies, hosts, and *Leishmania.* PLoS Neglected Tropical Diseases, 11(7), e0005600.
41. Lienhard A, Schaffer S. 2019. Extracting the invisible: obtaining high quality DNA is a challenging task in small arthropods. PeerJ, 7, e6753.
42. Lozano-Sardaneta YN, Mikery-Pacheco OF, Huerta H, Rojas-Soriano JE, Contreras-Ramos A. 2025. Wing geometric morphometrics is effective to separate sand fly species (Diptera, Psychodidae, Phlebotominae) related with leishmaniasis transmission in Mexico. Acta Tropica, 262, 107523.
43. Mandrioli M. 2008. Insect collections and DNA analyses: how to manage collections? Museum Management and Curatorship, 23(2), 193–199.
44. Maroli M, Feliciangeli MD, Bichaud L, Charrel RN, Gradoni L. 2013. Phlebotomine sandflies and the spreading of leishmaniases and other diseases of public health concern. Medical and Veterinary Entomology, 27(2), 123–147.
45. Marquina D, Buczek M, Ronquist F, Lukasik P. 2021. The effect of ethanol concentration on the morphological and molecular preservation of insects for biodiversity studies. PeerJ, 9, e10799.
46. Mathis A, Depaquit J, Dvorak V, Tuten H, Banuls AL, Halada P, Zapata S, Lehrter V, Hlavackova K, Prudhomme J, Volf P, Sereno D, Kaufmann C, Pfluger V, Schaffner F. 2015. Identification of phlebotomine sand flies using one MALDI-TOF MS reference database and two mass pectrometer systems., Parasites & Vectors, 8, 266.
47. Mekarnia N, Benallal KE, Sadlova J, Vojtkova B, Mauras A, Imbert N, Longhitano M, Harrat Z, Volf P, Loiseau PM, Cojean S. 2024. Effect of *Phlebotomus papatasi* on the fitness, infectivity and antimony-resistance phenotype of antimony-resistant *Leishmania* major Mon-25. International Journal for Parasitology – Drugs and Drug Resistance, 25, 100554.
48. Milligan BG. 1998. Total DNA isolation, in Molecular Genetic Analysis of Population: A Practical Approach, Hoelzel AR, Editor. Oxford: Oxford University Press.
49. Molina R, Jiménez M, Alvar J, González E, Hernández-Taberna S, Ines MM. 2017. Methods in sand fly research. Madrid: Servicio de publicaciones Universidad de Alcalá de Henares, Madrid.
50. Murphy WJ, Eizirik E, O'Brien SJ, Madsen O, Scally M, Douady CJ, Teeling E, Ryder OA, Stanhope MJ, de Jong WW, Springer MS. 2001. Resolution of the early placental mammal radiation using Bayesian phylogenetics. Science, 294(5550), 2348–2351.
51. Nacif-Pimenta R, Pinto LC, Volfova V, Volf P, Pimenta PFP, Secundino NFC. 2020. Conserved and distinct morphological aspects of the salivary glands of sand fly vectors of leishma-niasis: an anatomical and ultrastructural study. Parasites & Vectors, 13(1), 441.
52. Neuhaus B, Schmid T, Riedel J. 2017. Collection management and study of microscope slides: Storage, profiling, deterioration,

restoration procedures, and general recommendations. Zootaxa, 4322(1), 1–173.
53. New TR. 1974. Pscoptera. Handbooks for Identification of British Insects (Vol. I). London: Royal Entomological Society of London. 102 pp.
54. Perez-Ruiz M, Collao X, Navarro-Mari JM, Tenorio A. 2007. Reverse transcription, real-time PCR assay for detection of Toscana virus. Journal of Clinical Virology, 39(4), 276–281.
55. Porco D, Rougerie R, Deharveng L, Hebert P. 2010. Coupling non-destructive DNA extraction and voucher retrieval for small soft-bodied Arthropods in a high-throughput context: the example of Collembola. Molecular Ecology Resources, 10(6), 942–945.
56. Prudhomme J, Cassan C, Hide M, Toty C, Rahola N, Vergnes B, Dujardin JP, Alten B, Sereno D, Banuls AL. 2016. Ecology and morphological variations in wings of *Phlebotomus ariasi* (Diptera: Psychodidae) in the region of Roquedur (Gard, France): a geometric morphometrics approach. Parasites & Vectors, 9(1), 578.
57. Prudhomme J, Gunay F, Rahola N, Ouanaimi F, Guernaoui S, Boumezzough A, Banuls AL, Sereno D, Alten B. 2012. Wing size and shape variation of *Phlebotomus papatasi* (Diptera: Psychodidae) populations from the south and north slopes of the Atlas Mountains in Morocco. Journal of Vector Ecology, 37(1), 137–147.
58. Prudhomme J, Toty C, Kasap OE, Rahola N, Vergnes B, Maia C, Campino L, Antoniou M, Jimenez M, Molina R, Cannet A, Alten B, Sereno D, Banuls AL. 2015. New microsatellite markers for multi-scale genetic studies on *Phlebotomus ariasi* Tonnoir, vector of *Leishmania infantum* in the Mediterranean area. Acta Tropica, 142, 79–85.
59. Prudhomme J, Velo E, Bino S, Kadriaj P, Mersini K, Gunay F, Alten B. 2019. Altitudinal variations in wing morphology of *Aedes albopictus* (Diptera, Culicidae) in Albania, the region where it was first recorded in Europe. Parasite, 26, 55.
60. Rawlins DJ. 1992. Light Microscopy: An Introduction to Biotechniques. Oxford: Bios Scientific publishers. 143 pp.
61. Ready PD. 2013. Biology of phlebotomine sand flies as vectors of disease agents. Annual Review of Entomology, 58, 227–250.
62. Rohlf FJ, Slice D. 1990. Extensions of the Procrustes method for the optimal superimposition of landmarks. Systematic Zoology, 39(1), 40–59.
63. Rowley DL, Coddington JA, Gates MW, Norrbom AL, Ochoa RA, Vandenberg NJ, Greenstone MH. 2007. Vouchering DNA-barcoded specimens: Test of a nondestructive extraction proto-col for terrestrial arthropods. Molecular Ecology Notes, 7(6), 915–924.
64. Sábio PB, Andrade AJ, Galati EAB. 2014. Assessment of the taxonomic status of some species included in the *Shannoni* complex, with the description of a new species of *Psathyromyia* (Diptera: Psychodidae: Phlebotominae). Journal of Medical Entomology, 51(2), 331–341.
65. Sadlova J, Yeo M, Seblova V, Lewis MD, Mauricio I, Volf P, Miles MA. 2011. Visualisation of *Leishmania donovani* fluorescent hybrids during early stage development in the sand fly vector. PLoS One, 6(5), e19851.
66. Sales K, Miranda DEO, da Silva FJ, Otranto D, Figueredo LA, Dantas-Torres F. 2020. Evaluation of different storage times and preservation methods on phlebotomine sand fly DNA concentration and purity. Parasites & Vectors, 13(1), 399
67. Sales KG, Costa PL, de Morais RC, Otranto D, Brandao-Filho SP, Cavalcanti Mde P, Dantas-Torres F. 2015. Identification of phlebotomine sand fly blood meals by real-time PCR. Parasites& Vectors, 8, 230.
68. Sant'Anna MR, Jones NG, Hindley JA, Mendes-Sousa AF, Dillon RJ, Cavalcante RR, Alexander B, Bates PA. 2008. Blood meal identification and parasite detection in laboratory-fed and field-captured *Lutzomyia longipalpis* by PCR using FTA databasing paper. Acta Tropica, 107(3), 230–237.
69. Senne NA, Santos HA, Araujo TR, Paulino PG, Mendonca LP, Moreira HVS, Camilo TA, da Costa Angelo I. 2022. Robust comparative performance of genomic DNA extraction methods from non-engorged phlebotomine sandflies. Medical and Veterinary Entomology, 36(2), 203–211.
70. Shaw JJ. 2025. A review of Leishmania infections in American Phlebotomine sand flies – Are those that transmit leishmaniasis anthropophilic or anthropportunists? Parasite, 32, 57.
71. Tesh RB, Modi GB. 1983. Growth and transovarial transmission of Chandipura virus (Rhabdoviridae: Vesiculovirus) in *Phlebotomus papatasi.* American Journal of Tropical Medicine and Hygiene, 32(3), 621–623.
72. Thomsen PF, Elias S, Gilbert MTP, Haile J, Munch K, Kuzmina S, Froese DG, Sher A, Holdaway RN, Willerslev E. 2009. Non-destructive sampling of ancient insect DNA. PLoS One, 4(4),e5048
73. Truett GE, Heeger P, Mynatt RL, Truett AA, Walker JA, Warman ML. 2000. Preparation of PCR-quality mouse genomic DNA with hot sodium hydroxide and tris (HotSHOT). Biotechniques, 29(1), 52–54.
74. Upton MS. 1993. Aqueous gum-chloral slide mounting media: an historical review. Bulletin of Entomological Research, 83(2), 267–274.
75. Volf P, Myskova J. 2007. Sand flies and Leishmania: specific versus permissive vectors. Trends in Parasitology, 23(3), 91–92.
76. Wang Q, Wang X. 2012. Comparison of methods for DNA extraction from a single chironomid for PCR analysis. Pakistan Journal of Zoology, 44(2), 421–426.
77. Wang Y, Zhao Y, Bollas A, Wang Y, Au KF. 2021. Nanopore sequencing technology, bioinformatics and applications. Nature Biotechnology, 39(11), 1348–1365.

Cite this article as: Randrianambinintsoa FJ, Augendre L, Prudhomme J, Martinet J-P, Loyer M, Mekarnia N, Kerkoub H, Perveen FK, Huguenin A, Kariya E, Akhoundi M, De Andrade AJ, Berriatua E, Bongiorno G, Boyer S, Christodoulou V, Da Costa-Ribeiro MCV, De Souza LAF, Ding H, Dondji B, Dvořák V, Erisoz Kasap O, Galati EAB, Gállego M, Ballart C, Gouzelou S, Haddad N, Masse RS, Mekuria AH, Ivovic V, Kaczmarek S, Shahar MK, Kirstein OD, Kniha E, Kolářová I, Lincoln T, Lucanas C, Mikov O, Nov K, Özbel Y, Pesson B, Posada Lopez LC, Prasetyo DB, Rahola N, Rebollar-Tellez EA, Rodrigues BL, Roy L, Saini P, Sanjoba C, Shimabukuro PH, Siriyasatien P, Soszynska A, Suleşco T, Sylla M, Torno M, Volf P, Vongphayloth K, Sinh Nam V, Wardhana A, Yessinou E, Zapata S, Gantier J-C & Depaquit J. 2026. Processing and mounting phlebotomine sand flies: a consensus guideline. Parasite xx, xx. https://doi.org/10.1051/parasite/2026009.

PARASITE

An international open-access, peer-reviewed, online journal publishing high quality papers on all aspects of human and animal parasitology

Reviews, articles and short notes may be submitted. Fields include, but are not limited to: general, medical and veterinary parasitology; morphology, including ultrastructure; parasite systematics, including entomology, acarology, helminthology and protistology, and molecular analyses; molecular biology and biochemistry; immunology of parasitic diseases; host-parasite relationships; ecology and life history of parasites; epidemiology; therapeutics; new diagnostic tools.
All papers in Parasite are published in English. Manuscripts should have a broad interest and must not have been published or submitted elsewhere. No limit is imposed on the length of manuscripts.

Parasite (open-access) continues **Parasite** (print and online editions, 1994-2012) and **Annales de Parasitologie Humaine et Comparée** (1923-1993) and is the official journal of the Société Française de Parasitologie.

Editor-in-Chief:
Jean-Lou Justine, Paris

Submit your manuscript at:
https://www.editorialmanager.com/parasite

Lampiran 1: Dasar Teoretis Biokimia.

Arthropoda yang dimaksud adalah lalat pasir. Namun, gagasan umum ini dapat diperluas ke arthropoda lain yang sangat umum, yang identifikasinya hanya dapat dilakukan berdasarkan karakter morfologi internal. Secara kebetulan, beberapa organ internal sebagian mengalami sclerotisasi dan morfologinya memberikan informasi berharga. Inilah sebabnya mengapa sangat menarik untuk mengamati pompa makanan, spermathecae, dan salurannya. Dengan semua reagen yang akan kita bahas, tidak boleh dilupakan bahwa dari tahap fiksasi serangga hingga perakitan, kita hanya menerapkan reaksi redoks. Satu-satunya tindakan pencegahan atau prinsip yang harus diikuti adalah menghindari pencampuran reagen pereduksi dengan reagen pengoksidasi.

Alkohol etil; etanol:

Zat ini akan digunakan dengan berbagai cara. Molekul alkohol memiliki afinitas tinggi terhadap air dan oleh karena itu menunjukkan efek dehidrasi. Namun, alkohol dengan konsentrasi rendah (yaitu terlalu banyak mengandung air) dapat merusak asam nukleat (air adalah musuh asam nukleat).

Saat serangga ditempatkan dalam etanol, tujuan bukan hanya untuk mengawetkannya, tetapi juga untuk men-fiksasi jaringan. Dalam histologi, biasanya kita membedakan dua konsep penting: laju penetrasi dan laju fiksasi. Diketahui bahwa pengawet yang baik harus menembus jaringan secara cepat sebelum men-fiksasinya. Untuk etanol 96%, koefisien penetrasi kira-kira 1,05 (sebagai perbandingan, untuk larutan 0,75% asam pikrat berair, koefisien penetrasi adalah 0,45, sedangkan untuk larutan 3% kalium dikromat adalah 1,45).

Keinginan untuk mengawetkan serangga dan arthropoda lainnya dalam etanol secara permanen adalah hal nyata bagi ahli entomologi. Tujuannya adalah menyimpan tangkapan lapangan untuk studi berikutnya atau bagi peneliti di masa depan. Namun, bagi ahli sitologi atau histologi, hal ini tidak memungkinkan. Jika sampel dibiarkan terlalu lama dalam fiksatif, sampel tersebut hampir tidak mungkin untuk dikerjakan ulang. Inilah sebabnya sampel yang lebih dari 10 tahun sulit atau bahkan tidak dapat digunakan.

Pertimbangan lain adalah rasio antara massa arthropoda yang akan difiksasi dan volume fiksatif. Dalam praktik zoologi atau medis, disarankan untuk menggunakan volume 60 kali lipat dari volume potongan yang akan difiksasi. Dalam praktiknya, untuk mikro-arthropoda, untuk volume tertentu sampel yang akan difiksasi, tambahkan setidaknya 4–5 volume alkohol. Ingatlah bahwa alkohol akan kehilangan kekuatannya saat menghilangkan semua air yang ada di jaringan arthropoda.

Kesimpulan:

Alkohol etil adalah agen kimia pereduksi (oleh karena itu tidak kompatibel dengan fiksatif oksidatif)

Secara energetik, ia mengendapkan protein dan mendenasikannya

Melarutkan lipid kompleks tertentu dan mengendapkan glikogen

Menyebabkan kontraksi jaringan yang kuat dan mengeraskannya

Larutan dasar kalium atau natrium hidroksida:

Penggunaan larutan ini dalam entomologi terutama fokus pada kalium hidroksida tanpa dasar yang jelas.

Natrium hidroksida [E524] hadir dalam larutan dengan berbagai konsentrasi atau normalitas. Tersedia dalam bentuk pelet atau kristal. Kekurangan utamanya adalah sangat higroskopis (lebih dari KOH). Saat bereaksi dengan protein, ia melarutkannya, dan dengan lipid mengubahnya menjadi sabun keras selama saponifikasi (ini berbeda dengan KOH, yang menghasilkan sabun cair).

Kalium hidroksida [E525] tersedia dalam bentuk larutan pekat, tetapi keunggulannya adalah tersedia dalam pelet sekitar 0,1 g, yang sangat memudahkan pembuatan larutan encer tanpa timbangan presisi. Contoh: 1 pelet 0,1 g dalam 1 mL air suling menghasilkan larutan 10%. Keunggulan kedua adalah sensitivitas KOH terhadap karbonasi lebih rendah (larutan KOH mudah mengikat CO2 membentuk garam karbonat).

This is an Open Access article distributed under the terms of the Creative Commons Attribution License (https://creativecommons.org/licenses/by/4.0), which permits unrestricted use, distribution, and reproduction in any medium, provided the original work is properly cited.

Basis kuat ini digunakan untuk melarutkan asam lemak dengan mengubahnya menjadi sabun yang larut dalam air. Fiksatif seperti etanol telah melarutkan sebagian lemak sampel. Dengan menempatkan sampel dalam medium berair dengan basa kuat, asam lemak akan mengendap, menjalankan proses saponifikasi dingin. Dalam beberapa kasus, misalnya pada jaringan adiposa berlebih pada betina, suhu dapat dinaikkan menjadi 35–40°C untuk mempercepat reaksi atau memperpanjang waktu kontak pada suhu ruang.

Larutan asam berwarna/larutan Marc-André tidak berwarna:

Larutan Marc-André terdiri dari kloral hidrat, asam asetat, dan air. Larutan ini bersifat sangat mengoksidasi (campuran asam dan aldehida). Ia menetralkan kelebihan kalium hidroksida yang mungkin tersisa, tanpa mengendapkan sabun alkali yang terbentuk sebelumnya. Larutan ini juga bekerja pada gugus alkohol sekunder glukosamin pada kitin, sehingga melunakkan kitin. Selain itu, dapat melarutkan beberapa garam mineral.

Jika larutan Marc-André sebelumnya diwarnai dengan fuksin asam (dalam kondisi teroksidasi), ia dapat menempel pada gugus alkohol sekunder struktur. Setelah waktu kontak dan pewarnaan, bilas hanya dengan etanol. Dengan demikian, fase dehidrasi sampel dimulai.

Manfaat:

Menetralkan larutan basa berlebih

Melunakkan kitin

Pewarnaan kitin untuk menilai struktur internal yang tersclerotisasi

Kekurangan:

Kloral hidrat bersifat hipnotik dan telah digunakan dalam medis manusia. Harus digunakan di bawah lemari kimia dan mematuhi peraturan risiko kimia.

Larutan dehidrasi:

Pengalaman menunjukkan, untuk sampel sangat kecil, tidak perlu mengikuti urutan mandi alkohol dengan konsentrasi meningkat. Jika sampel besar, mulai dengan etanol 80%, lalu 90%, 95%, dan akhirnya etanol absolut. Untuk sampel kecil, gunakan mandi 90% diikuti etanol absolut. Pada tahap ini, selalu diingat bahwa etanol absolut menahan air dari atmosfer.

Tradisi laboratorium entomologi adalah menyelesaikan dehidrasi dengan mandi beech creosote. Saat ini, penggunaan zat ini sangat tidak disarankan karena aromanya (hidrokarbon aromatik polisiklik) dan diduga bersifat reprotoksik, karsinogenik, polutan organik persisten, dan ekotoksik bagi organisme akuatik.

Larutan yang disarankan untuk *mounting* adalah Euparal® dan Euparal essence. Campuran Euparal® dan Euparal essence diterima dengan baik; sampel diperoleh setelah direndam dalam etanol 90%.

Lampiran 2: Komposisi reagen yang digunakan.

Kalium hidroksida 10%

Kalium hidroksida 10 g

Air suling secukupnya hingga 100 mL

Medium *mounting* Gum Chloral / Medium Hoyer

Air suling 50 mL

Kloral hidrat 200 g

Gum arabic 50 g

Gliserol 20 mL

Larutan Marc-André

Kloral hidrat 40 g

Asam asetat glasial 30 mL

Air suling 30 mL

Fuksin asam 1% dalam air suling

Bubuk fuksin asam 1 g

Air suling 99 mL

Larutan Marc-André yang diwarnai dengan fuksin

Larutan Marc-André 10 mL

Fuksin 1% 50 µL

Lampiran 3: Euparal®, Canada balsam, polyvinyl alcohol, atau larutan lain untuk mounting.

Polyvinyl alcohol: Ini adalah media *mounting* yang ideal ketika produk yang diperlukan untuk dehidrasi yang tepat tidak tersedia. Polyvinyl alcohol kemudian dicampur dengan lactophenol Amman. Namun, pemasangan ini memiliki kelemahan utama, yaitu mudah mengering atau polyvinyl alcohol mengkristal karena penguapan air, atau menghitam ketika fenol teroksidasi. Teknik ini tetap baik untuk *mounting* jangka pendek.

Canada Balsam: Penggunaannya untuk *mounting* antara slide dan cover-slip dan memerlukan dehidrasi gradual pada spesimen yang akan digunakan. Memerlukan penggunaan ksilen atau toluena yang memiliki resiko untuk kesehatan.

Media Enecê: Untuk *mounting* antara slide dan cover-slip, seperti Canada Balsam, spesimen juga harus mengalami tahap dehidrasi. Formulasi Enecê: colophony putih murni (22 g); copal gum larut alkohol (12 g), alkohol absolut (20 mL), kamper (10 g), essence terpentin (10 mL), dan eucalyptol (26 mL). Untuk persiapannya, membutuhkan sebuah wadah, misalnya labu Erlenmeyer. Masukkan alkohol absolut dan kamper ke dalam wadah dan tambahkan kemudian colophony dan copal gum. Labu ditutup dengan stopper dan digoyang, lalu dipanaskan dengan bain-marie pada suhu sedang agar campuran tidak mendidih. Setelah semua larut, tambahkan essence terpentin, kemudian saring selagi panas, dan akhirnya tambahkan eucalyptol ke filtrat. Ketika media menjadi terlalu kental, dapat diencerkan dengan Eenecê dengan formula: alkohol absolut (30 mL),

kamper (17 g), essence terpentin (15 mL), eucalyptol (38 mL) (Cerqueira, 1943).

Euparal®: Ini adalah resin yang berasal dari Cypress Atlas Tetraclinis articulata (Vahl, 1791) dan dipelajari serta dikembangkan pada tahun 1906 oleh Gilson. Keunggulan utamanya adalah tidak mengalami polimerisasi. Spesimen yang dipasang antara slide dan cover-slip dapat dengan mudah diambil kembali menggunakan alkohol atau lebih baik lagi dengan essence Euparal®. Resin ini, yang juga disebut sandarac, dapat menerima alkohol dari mulai konsentrasi 80%.

Penggunaan Triton X100: larutan non-ionik:

Triton X100 berbentuk larutan non-ionik (4-(1,1,3,3-tetramethylbutyl) phenyl-polyethylene glycol solution, atau t-octylphenoxypolyethoxyethanol, polyethylene glycol tert-octylphenyl ether), yang banyak digunakan sebagai deterjen dalam biologi seluler dan molekuler. Larutan ini memungkinkan permeabilisasi membran sel dan nukleus.

Pengawetan spesimen serangga dalam larutan alkohol selama bertahun-tahun adalah hal yang sangat umum. Sayangnya, pengawetan dalam alkohol tidak optimal, dan artropoda yang diawetkan menjadi sangat sulit dipersiapkan untuk pemeriksaan mikroskopis. Plastik yang berisi spesimen sering mengalami degradasi, diikuti penguapan alcohol yang dapat menyebabkan pengeringan spesimen. Pada 2008, Jonque mempublikasikan catatan tentang rehidrasi laba-laba dengan menggunakan agen basah seperti Agepon yang digunakan untuk film fotografi [26]. Hal ini memunculkan ide penggunaan agen basah yang bukan dari kategori deterjen kuat.

Berikut prosedur penggunaan Triton X100 dalam larutan air 0,5%:
- Impregnasi spesimen kering dengan alkohol absolut.
- Tambahkan volume larutan Triton X100 0,5% secukupnya hingga seluruh spesimen terendam.
- Diamkan selama sekitar 5 menit atau lebih. Semua artropoda harus terlepas dan bebas dalam larutan.
- Larutan Triton X100 dibuang dan diganti dengan larutan kalium hidroksida.

Teknik selanjutnya diikuti seperti yang dijelaskan sebelumnya.

Lampiran 4: Tahapan pembuatan preparat menggunakan media mounting Euparal® atau Canada Balsam

1. Spesimen harus didehidrasi (penampilan keruh pada hasil preparat menunjukkan proses dehidrasi yang tidak cukup).
2. Dehidrasi dapat dilakukan dengan meningkatkan konsentrasi alkohol secara bertahap.
3. Setelah dehidrasi, spesimen dapat dipindahkan dari alkohol 99% atau alkohol absolut ke agen pembersih jaringan.

Prosedur:

1. Tempatkan lalat pasir dewasa dalam 70% etanol.
2. Buang larutan etanol dan ganti dengan 10% KOH. Tutupi lalat pasir dengan gelas objek.
3. Inkubasi sampai jaringan serangga menjadi transparan.
4. Buang larutan KOH.
5. Tutupi seluruh bagian spesimen dengan air suling dan tunggu 30–45 menit.
6. Buang larutan air suling lama dan ulangi perendaman dengan air suling baru selama 30 menit (waktu disesuaikan dengan jumlah spesimen: semakin banyak spesimen yang diproses bersama, semakin lama waktu yang dibutuhkan. Semakin sedikit spesimen, terutama yang diproses satu per satu, semakin singkat waktu perendamannya).
7. Buang larutan air suling.
8. Tambahkan larutan Marc-André (dapat diberi warna dengan asam Fuchsin) dan inkubasi selama 24 jam (satu hari).
9. Buang larutan Marc-André.
10. Tutupi seluruh bagian spesimen dengan air suling dan tunggu 30–45 menit.
11. Buang larutan air suling lama dan ulangi pembilasan dengan air suling baru selama 30 menit.
12. Buang larutan air suling.
13. Tambahkan 70% etanol dan bedah spesimen:
    a. Untuk kepala dan abdomen, tarik perlahan kepala atau abdomen dari thorax.
    b. Untuk thoraks, lepaskan sayap dengan memegang thoraks menggunakan satu pasang pinset dan menarik pangkal sayap dengan pinset lain. Diseksi secara sagital juga dapat dilakukan, membagi thoraks menjadi sisi kiri dan kanan, tergantung pada wilayah yang paling menarik untuk diobservasi.
14. Dehidrasi secara bertahap melalui serangkaian larutan alkohol: 50% → 80% → 95% hingga mencapai etanol absolut.
15. Dehidrasi spesimen dengan membilasnya dua kali, masing-masing 10 menit, menggunakan etanol 100%.
16. Buang larutan etanol dan tutupi seluruh bagian spesimen dengan minyak cengkeh selama 15 menit pada suhu ruang.
17. Pindahkan spesimen dari minyak cengkeh ke setetes Euparal® atau Canada Balsam pada gelas objek bersih.
18. Susun sesuai keinginan: Kepala, thoraks, dan abdomen lalat pasir dapat dibedah menggunakan jarum halus atau pinset di bawah mikroskop bedah. Kepala harus dipisahkan dari tubuh agar dapat dipasang dalam posisi ventro-dorsal, yaitu foramen oksipital harus menghadap ke atas sehingga cibarium dapat diamati langsung.
19. Diseksi dilakukan dalam media *mounting* lalat pasir.
20. Biarkan spesimen dalam kondisi ini hingga permukaan media *mounting* menjadi lengket.

21. Basahi cover-slip bersih dengan alkohol absolut. Letakkan cover-slip di atas Canada Balsam atau Euparal dengan sudut miring.
22. Simpan slide dalam kotak kering yang diperuntukkan khusus.
