# Supplementary material for: Processing and mounting phlebotomine sand flies: a consensus guideline
Source: Parasite. 2026 Apr 3;33:18. doi: 10.1051/parasite/2026009 (PMC13047900; doi:10.1051/parasite/2026009)
Supplement: Supplementary file 16 — Italian translation / Traduzione in italiano [file parasite-33-18-s16.pdf]

Parasite **33**, 18 (2026)
© F.J. Randrianambinintsoa et al., published by EDP Sciences, 2026
https://doi.org/10.1051/parasite/2026009

PARASITE
Available online at:
www.parasite-journal.org

REVIEW ARTICLE OPEN ACCESS

Allestimento e montaggio dei flebotomi: linee guida condivise

Fano José Randrianambinintsoa[1], Laure Augendre[1], Jorian Prudhomme[1], Jean-Philippe Martinet[1], Mathieu Loyer[1], Nalia Mekarnia[1], Hocine Kerkoub[1], Farzana Khan Perveen[1], Antoine Huguenin[1,2], Emilie Kariya[1,2], Mohammad Akhoundi[3], Andrey José de Andrade[4], Eduardo Berriatua[5], Gioia Bongiorno[6], Sébastien Boyer[7,8], Vasiliki Christodoulou[9], Magda Clara Vieira Da Costa-Ribeiro[10], Lucas Alexandre Farias de Souza[10], Huicong Ding[11], Blaise Dondji[12], Vít Dvořák[13], Ozge Erisoz Kasap[14], Eunice Aparecida Bianchi Galati[15], Montserrat Gállego[16], Cristina Ballart[16], Stavroula Gouzelou[17], Nabil Haddad[18], Rezki Sabrina Masse[19], Asrat Hailu Mekuria[20], Vladimir Ivovic[21], Szymon Kaczmarek[22], Mohd Khadri Shahar[19], Oscar D. Kirstein[23], Edwin Kniha[24], Iva Kolářová[13], Lincoln Timinao[25], Cristian Lucanas[26], Ognyan Mikov[27], Kimsear Nov[7], Yusuf Özbel[28], Bernard Pesson[29], Laura Cristina Posada Lopez[30], Didot Budi Prasetyo[1,7], Nil Rahola[31], Eduardo A. Rebollar-Tellez[32], Bruno Leite Rodrigues[15], Lalita Roy[33], Prasanta Saini[34], Chizu Sanjoba[35], Paloma Helena Fernandes Shimabukuro[36], Padet Siriyasatien[37], Agnieszka Soszyńska[22], Tatiana Suleşco[38], Massamba Sylla[39], Majhalia Torno[40], Petr Volf[13], Khamsing Vongphayloth[41], Vu Sinh Nam[42], April Wardhana[43], Eric Yessinou[44], Sonia Zapata[45], Jean-Charles Gantier[1], and Jérôme Depaquit[1,2,*]

[1] Faculté de Pharmacie, Université de Reims Champagne Ardenne, UR ESCAPE-USC ANSES PETARD, 51 rue Cognacq-Jay, 51096 Reims Cedex, France
[2] Pôle de Biologie territoriale, Laboratoire de Parasitologie-Mycologie, Centre Hospitalo-Universitaire, 51092 Reims, France
[3] Parasitology-Mycology Department, Avicenne Hospital, AP-HP, Bobigny, Sorbonne Paris Nord University, France; Unité des Virus Émergents (UVE: Aix-Marseille Univ, Università di Corsica, IRD 190, Inserm 1207, IRBA), 13005 Marseille, France
[4] Parasitology Collection of Basic Pathology, Department of Basic Pathology, Federal University of Paraná, Curitiba 19031, Brazil
[5] Department of Animal Health, University of Murcia, Campus de Espinardo, 30100 Espinardo, Murcia, Spain
[6] Department of Infectious Diseases, Vector-borne Diseases Unit, Istituto Superiore di Sanità, 00166 Rome, Italy
[7] Medical and Veterinary Entomology Unit, Institut Pasteur du Cambodge, Phnom Penh 12201, Cambodia
[8] Ecology & Emergence of Arthropod-borne Pathogens Unit, Department of Global Health, Institut Pasteur, CNRS UMR2000, 75015 Paris, France
[9] Section Veterinary Services (1417), Laboratory for Animal Health Virology, Aglantzia, Nicosia 2109, Cyprus
[10] Insects Vectors and Parasites Laboratory, Department of Basic Pathology and Postgraduate program in Microbiology, Parasitology and Pathology, Federal University of Paraná, 81530-900 Curitiba, Brazil
[11] Department of Biological Sciences, National University of Singapore, 117558, Singapore
[12] Laboratory of the Leishmaniasis Research Project, Mokolo District Hospital, Mokolo, Cameroon; Laboratory of Cellular Immunology and Parasitology, Department of Biological Sciences, Central Washington University, 98926 Ellensburg, WA, USA
[13] Department of Parasitology, Faculty of Science, Charles University, 12800 Prague, Czechia
[14] VERG Laboratories, Department of Biology, Faculty of Science, Hacettepe University, Beytepe, Ankara 06800, Türkiye
[15] Faculdade de Saúde Pública da Universidade de São Paulo (FSP/USP), Pós-graduação em Saúde Pública, 01246-904 São Paulo, Brazil
[16] Secció de Parasitologia, Departament de Biologia, Sanitat i Medi Ambient, Facultat de Farmàcia i Ciències de l'Alimentació, Universitat de Barcelona, & Institut de Salut Global de Barcelona (ISGlobal), Centro de Investigación Biomédica en Red, Enfermedades Infecciosas (CIBERINFEC), 08028 Barcelona, Spain
[17] Laboratory of Infectious Diseases and Public Health, School of Medicine, University of Cyprus, Nicosia, Cyprus & Department of Pediatrics, Archbishop Makarios III Hospital, Nicosia 2115, Cyprus
[18] Faculty of Health Sciences, American University of Beirut, 1107 2020 Beirut, Lebanon
[19] Medical Entomology Unit, Infectious Disease Research Centre, Institute for Medical Research (IMR), National Institutes of Health (NIH), Ministry of Health Malaysia, 40170 Shah Alam, Selangor, Malaysia
[20] School of Medicine, Addis Ababa University, 28017 - 1000 Addis Ababa, Ethiopia
[21] Faculty of Mathematics, Natural Sciences and Information Technologies, University of Primorska, 6000 Koper, Slovenia

Edited by Jean-Lou Justine
*Corresponding author: jerome.depaquit@univ-reims.fr

This is an Open Access article distributed under the terms of the Creative Commons Attribution License (https://creativecommons.org/licenses/by/4.0), which permits unrestricted use, distribution, and reproduction in any medium, provided the original work is properly cited.

[22] University of Lodz, Faculty of Biology and Environmental Protection, Department of Invertebrate Zoology and Hydrobiology, Banacha 12/16, 90-237 Łódź, Poland
[23] Laboratory of Entomology, Ministry of Health, 9134302 Jerusalem, Israel
[24] Center for Pathophysiology, Infectiology and Immunology, Institute of Specific Prophylaxis and Tropical Medicine, Medical University Vienna, Kinderspitalgasse 15, 1090 Vienna, Austria
[25] Papua New Guinea Institute of Medical Research (PNGIMR) Institute, PO Box 60, Headquarter, Homate Street, 441 Goroka, Eastern Highlands Province, Papua New Guinea
[26] Museum of Natural History, University of the Philippines Los Baños, 4031 Laguna, Philippines
[27] National Centre of Infectious and Parasitic Diseases, 1504 Sofia, Bulgaria
[28] Ege University, Faculty of Medicine, Department of Parasitology, 35040 Bornova/Izmir, Türkiye
[29] Retired, Faculté de Pharmacie, Université de Strasbourg, Strasbourg, 67400 Illkirch-Graffenstaden, France
[30] Program for the Study and Control of Tropical Diseases (PECET), Faculty of Medicine, University of Antioquia, 050010 Medellin, Colombia
[31] MIVEGEC, Univ. Montpellier, CNRS, IRD, 34394 Montpellier, France & Medical Entomology Unit, Institut Pasteur de Madagascar, 101 Antananarivo, Madagascar
[32] Laboratorio de Entomología Médica, Departamento de Zoología de Invertebrados, Facultad de Ciencias Biológicas, Universidad Autónoma de Nuevo León, San Nicolás de los Garza, 66455, NL, México
[33] Tropical and Infectious Disease Centre, BP Koirala Institute of Health Sciences, Dharan 56700, Nepal
[34] ICMR-Vector Control Research Centre, Puducherry 605006, India
[35] Graduate School of Agricultural and Life Sciences, The University of Tokyo, Tokyo 113-8657, Japan
[36] Grupo de estudos em Leishmanioses/Coleção de Flebotomíneos (COLFLEB/Fiocruz-MG), Instituto René Rachou, Fundação Oswaldo Cruz, Belo Horizonte, Minas Gerais, 30190009, Brazil
[37] Center of Excellence in Vector Biology and Vector-Borne Disease, Department of Parasitology, Faculty of Medicine, Chulalongkorn University, Bangkok 10330, Thailand
[38] Department of Arbovirology, Bernhard Nocht Institute for Tropical Medicine, Bernhard Nocht Str. 74, 20359 Hamburg, Germany [39] Laboratory Vectors & Parasites, Department of Livestock Sciences and Techniques, Sine Saloum University El Hadji Ibrahima Niasse (SSUEIN) Kaffrine Campus, C.P. 24600, Senegal.
[40] Environmental Health Institute, National Environment Agency, Singapore 138667, Singapore & Department of Biological Sciences, National University of Singapore, 117558 Singapore
[41] Institut Pasteur du Laos, Laboratory of Vector-Borne Diseases, Samsenhai Road, Ban Kao-Gnot, Sisattanak District, 3560 Vientiane, Lao PDR
[42] National Institute of Hygiene and Epidemiology, 1 Yec-Xanh Street, Hai Ba Trung District, 100000 Hanoi, Vietnam
[43] Indonesian Research Center for Veterinary Science, Indonesian Agency for Agricultural Research and Development, Ministry of Agriculture Republic Indonesia, Bogor 16114, Indonesia & Department of Parasitology, Faculty of Veterinary Medicine, Airlangga University, Surabaya 60115, Indonesia
[44] Laboratory of Research in Applied Biology, Polytechnic School of Abomey-Calavi, University of Abomey-Calavi, 01 P.O. Box 2009, 00000 Cotonou, Benin
[45] Instituto de Microbiología, Colegio de Ciencias Biológicas y Ambientales (COCIBA), Universidad San Francisco de Quito (USFQ), 170901 Quito, Ecuador

Received 1 December 2025, Accepted 29 January 2026, Published online 3 April 2026

Abstract – Questo articolo fornisce una guida completa alla preparazione e al montaggio di esemplari di flebotomi, un processo fondamentale per l'identificazione delle specie e per la rilevazione e l'isolamento di patogeni. Vengono discusse diverse tecniche applicabili sia in contesti di campo sia di laboratorio. La guida include istruzioni dettagliate sulla raccolta, la manipolazione, il montaggio e l'eutanasia dei flebotomi (raccomandati il congelamento a secco o l'uso di CO_2 in alternativa ai metodi chimici), nonché sulle strategie di conservazione, come la conservazione a freddo e la preservazione in etanolo. La qualità della preparazione di specifiche strutture anatomiche (organi genitali, capo e ali) è essenziale per una corretta osservazione microscopica e viene presentata in dettaglio in questo lavoro. L'articolo descrive inoltre procedure dettagliate per il trattamento dei campioni, incluso il processo di chiarificazione mediante l'uso di agenti quali l'idrossido di potassio seguito dalla soluzione di Marc-André. Il processo di montaggio viene analizzato attraverso il confronto di diversi mezzi di inclusione, con particolare attenzione alle loro proprietà ottiche e al potenziale di conservazione. Il fluido di Hoyer (noto anche come gomma al clorato) è raccomandato per osservazioni rapide, in particolare delle spermateche, grazie alla sua elevata trasparenza, sebbene non sia adatto alla conservazione a lungo termine. Altri mezzi discussi includono l'alcol polivinilico, l'Euparal® (con tolleranza limitata all'acqua) e il balsamo del Canada (mezzo solubile in idrocarburi), questi ultimi due indicati per la conservazione dei preparati a lungo termine. Vengono inoltre affrontati approcci innovativi di biologia molecolare, quali il sequenziamento del DNA ed il MALDI-ToF, che richiedono particolare attenzione nella preparazione dei campioni. Infine, sono messi a

disposizione brevi videoclip illustrativi delle diverse tecniche di montaggio, nonché traduzioni in 33 lingue differenti, consentendo alla guida di rispondere alle diverse esigenze e aspettative della comunità scientifica globale.

Key words: Montaggio, flebotomi, liquid di Hoyer, soluzione di Marc-André, gomma cloralica, alcool polivinilico, Euparal®, balsamo del Canada, isolamento di Leishmania, condizioni di campo, colture cellulari, dissezione, biologia molecolare, MALDI-ToF, esemplari tipo

Abstract – Processing and mounting phlebotomine sand flies: a consensus guideline. This article provides a comprehensive guide for the processing and mounting of phlebotomine sand fly specimens, which is crucial for species identification and pathogen detection and isolation. It discusses a range of techniques suitable for both field and laboratory settings. The guide includes detailed instructions on sand fly collection, handling, covering, and euthanasia (recommending dry freezing or CO_2 over chemicals) as well as conservation strategies, such as cold storage and preservation in ethanol. The quality of preparation of certain anatomical structures (genital organs, head and wings) is essential for their proper microscopic observation and is described in this work. The article also presents detailed sample processing, including the clearing process with agents such as potassium hydroxide then Marc-André solution. The mounting process compares different media, emphasizing their optical properties and preservation potential. Hoyer fluid (also known as chloral gum) is recommended for quick observation, particularly for spermathecae, due to its clarity, although it is not suitable for long-term storage. Other media discussed include polyvinyl alcohol, Euparal® (for limited water tolerance), and Canada balsam (a hydrocarbon-soluble medium), with the latter two offering long-term preservation capabilities. Innovative molecular biology approaches such as DNA sequencing and MALDI-ToF, which require particular attention to sample processing, are also addressed. Furthermore, short video clips illustrating various mounting techniques as well as translations in many different languages are provided, allowing the guideline to reach the diverse needs and expectations of the global scientific community.

Key words: Mounting, Phlebotomine sand fly, Hoyer fluid, Marc-André solution, Chloral gum, Polyvinyl alcohol, Euparal®, Canada balsam, *Leishmania* isolation, Field conditions, Culture, Dissection, Molecular biology, MALDI-ToF, Type-specimens.

Introduzione

I flebotomi sono piccoli insetti ditteri appartenenti alla famiglia Psychodidae, sottofamiglia Phlebotominae, con almeno 1.063 specie conosciute [21]. Essi sono importanti vettori di patogeni (Leishmania, arbovirus e Bartonella) responsabili di malattie denominate rispettivamente leishmaniosi, infezioni da arbovirus e bartonellosi. La loro identificazione si basa principalmente su un esame microscopico dettagliato, reso possibile da una raccolta accurata, da una conservazione adeguata e da un montaggio corretto su vetrino, che richiedono diverse tecniche specifiche, ciascuna con i propri vantaggi e limiti.

L'identificazione dei flebotomi adulti si basa sull'osservazione sia delle strutture esterne (ad es. antenne, palpi, genitali maschili) sia di quelle interne (ad es. faringe, cibario e spermateche). La dissezione e l'isolamento di queste ultime ne facilitano l'osservazione e, di conseguenza, un'identificazione accurata. Pertanto, a differenza delle zanzare o delle cimici triatomine, i flebotomi richiedono il montaggio tra un vetrino-portaoggetti ed un coprioggetti prima della loro identificazione.

Fino agli anni '80, l'osservazione microscopica era l'unico metodo disponibile per l'identificazione dei flebotomi e rimane tuttora l'approccio più ampiamente utilizzato. La scelta del processo e della preparazione era quindi relativamente semplice e basata principalmente su una dicotomia: da un lato, il montaggio definitivo che consente la conservazione a lungo termine del campione e, dall'altro, il montaggio rapido per l'identificazione in un mezzo che non garantisce la conservazione a lungo termine. Il montaggio definitivo, ad esempio in una resina come il balsamo del Canada, richiede molto tempo, poiché necessita della completa disidratazione dei campioni. Inoltre, l'indice di rifrazione di questo mezzo non è sempre ottimale per una facile osservazione delle spermateche. Al contrario, il montaggio in un mezzo acquoso (ad es. liquido di Hoyer) è più rapido e consente una migliore visualizzazione delle spermateche, che sono strutture rifrangenti, ma non permette la conservazione a lungo termine dei preparati, poiché tende ad assorbire umidità dall'atmosfera. Un'opzione consiste nel sigillare il vetrino con smalto per unghie una volta che si è completamente asciugato. Questa scelta di compromesso persiste ancora oggi e influisce sulla selezione del metodo di montaggio in base allo scopo previsto della preparazione. Dagli anni '80, gli studi sull'identificazione dei flebotomi hanno combinato approcci morfologici e biochimici. Il primo approccio biochimico è stato l'analisi degli idrocarburi cuticolari, rapidamente sostituita dalle tecniche di biologia molecolare, ovvero DNA polimorfico amplificato casualmente (RAPD), polimorfismo della lunghezza dei frammenti di restrizione (RFLP), amplificazione del DNA e sequenziamento con metodo Sanger, nonché le tecnologie di sequenziamento di nuova generazione (NGS). Oggi, gli approcci molecolari sono affiancati da metodi proteomici come il MALDI-ToF. Inoltre, l'identificazione molecolare delle specie può essere

combinata con il rilevamento dei patogeni mediante PCR (Leishmania, Trypanosoma, Bartonella e Phlebovirus), poiché tutti possono essere individuati sia con PCR end-point sia real-time, richiedendo un adattamento dei processi di campionamento e conservazione in funzione degli obiettivi prefissati [3, 32]. Oltre alle caratteristiche morfologiche tradizionalmente utilizzate per la discriminazione delle specie, possono essere applicati anche altri approcci morfologici (ad es. la geomorfometria alare). Basandosi principalmente sull'esperienza diretta degli autori e sui dati della letteratura, l'obiettivo di questo studio è stato quello di fornire linee guida standardizzate per il montaggio ed il trattamento dei flebotomi adulti, al fine di ottimizzare le analisi morfologiche e molecolari. La necessità di effettuare alcune analisi (ad es. biologia molecolare o MALDI-ToF) richiede la conservazione di parti del flebotomo non indispensabili per l'identificazione morfologica, sottolineando l'importanza di una scelta critica del protocollo. In questo articolo presenteremo i metodi di anestesia ed eutanasia dei flebotomi catturati vivi, la loro conservazione e il processo di montaggio, sia per un'identificazione rapida sia per una conservazione a lungo termine che consenta studi successivi.

Premessa: le considerazioni in materia di sicurezza e regolamentazione devono fare riferimento alle Schede di Dati di Sicurezza (SDS)

Tutte le sostanze chimiche presentate in queste linee guida devono essere manipolate in condizioni di rigorosa sicurezza. I comitati per la salute e la sicurezza delle strutture di ricerca sono disponibili a fornire informazioni non solo sui pericoli associati a tali sostanze chimiche, ma anche sulle corrette procedure di manipolazione e di smaltimento dei rifiuti. Tuttavia, è obbligatorio seguire le istruzioni di sicurezza relative al loro utilizzo e smaltimento. Si sottolinea che è responsabilità di tutti gli utilizzatori garantire il rispetto delle buone e sicure pratiche di laboratorio, nonché della legislazione e dei regolamenti applicabili nel proprio Paese o istituzione di ricerca. Inoltre, alcune sostanze chimiche, o loro componenti (ad es. l'idrato di cloralio), sono regolamentate in alcuni Paesi. Un elenco delle abbreviazioni utilizzate in questo manoscritto è riportato nella Tabella 1.

Tabella 1: Lista delle abbreviazioni

**BME**	Medium di base Eagle
**CDC**	Centri per il Controllo e la Prevenzione delle Malattie
**CMCP**	Camforo-monoclorofenolo
**CMR**	Sostanza cancerogena, mutagena o tossica per la riproduzione
**COI**	Gene della subunità I della citocromo c ossidasi
**CytB**	Gene del citocromo b
**DNA**	Acido desossiribonucleico
**ELISA**	Saggio immunoenzimatico
**EtOH**	Etanolo
**M199**	Medium 199
**MALDI-ToF MS**	Spettrometria di massa a tempo di volo con desorbimento/ionizzazione laser assistito da matrice
**MEM**	Medium essenziale minimo
**NGS**	Sequenziamento di nuova generazione
**NNN**	Medium Novy-MacNeal-Nicolle
**PCR**	Reazione a catena della polimerasi
**Lao PDR**	Repubblica Democratica Popolare del Lao
**PNOC**	Gene della prepronociceptina
**qPCR**	PCR quantitative (real-time PCR)
**RAPD**	Amplificazione polimorfica casuale del DNA
**RFLP**	Polimorfismo della lunghezza dei frammenti di restrizione
**RI**	Indice di rifrazione
**RNA**	Acido ribonucleico
**RNases**	Ribonucleasi
**RNASS**	Soluzione per la stabilizzazione dell'RNA
**RT-PCR**	Reazione a catena della polimerasi con trascrizione inversa
**TFA**	Acido trifluoroacetico

1. Cattura dei flebotomi

I flebotomi adulti possono essere raccolti vivi o morti utilizzando diversi metodi, quali trappole luminose CDC in miniatura, trappole adesive e aspiratori impiegati con trappole di Shannon, oppure mediante raccolta diretta dai luoghi di riposo presenti nell'ambiente (ad es. ricoveri per animali). Questi metodi prevedono il posizionamento delle trappole in habitat idonei, l'impiego di attrattivi per la cattura dei flebotomi come la luce o altri (CO_2 o esche chimiche) e la loro raccolta per analisi successive, come descritto in numerose pubblicazioni [2, 3, 32, 36, 49]. La cattura di flebotomi vivi consente tutte le applicazioni successive, mentre la raccolta di individui morti impedisce l'isolamento di ceppi di *Leishmania* o di virus. Alcune tecniche di cattura, come l'uso di fogli adesivi, causano

regolarmente la perdita di organi dei flebotomi (antenne, palpi, ali o zampe). Inoltre, l'olio di ricino che riveste i gli di carta adesiva forma un film intorno ai flebotomi e deve essere rimosso all'inizio del processamento, solitamente tramite un bagno di 15 minuti in una miscela di etanolo ed etere etilico in parti uguali.

2. Eutanasia degli esemplari

Dopo la raccolta, i flebotomi vivi devono essere sottoposti a eutanasia. Con alcuni metodi di raccolta (ad es. fogli adesivi, trappole luminose CDC dotate di un barattolo contenente detergente o etanolo), i flebotomi sono già morti al momento della raccolta. La biologia molecolare può essere applicata a quelli raccolti direttamente in etanolo e agli altri se vengono conservati in etanolo il più rapidamente possibile. Tuttavia, nessuno di questi metodi di uccisione consente l'analisi degli insetti tramite MALDI-ToF. Inoltre, alcuni metodi di uccisione possono causare la perdita di determinati caratteri morfologici. È quindi essenziale utilizzare un agente di uccisione standard appropriato per garantire la corretta identificazione o la conservazione a lungo termine come esemplari di riferimento (ovvero esemplari conservati e depositati per riferimento o confronto futuro). Prodotti chimici come acetato di etile, etere etilico, tetracloroetano e cloroformio possono essere versati su cotone idrofilo e posti in un contenitore contenente i flebotomi per l'eutanasia. Questi agenti di uccisione devono essere maneggiati con cura, seguendo le raccomandazioni del produttore a causa della loro tossicità. Tuttavia, sconsigliamo di uccidere i flebotomi usando il cloroformio, poiché, nella nostra esperienza, è poco compatibile con gli studi di biologia molecolare. Data la natura pericolosa di tutti questi prodotti e la loro dubbia idoneità per le analisi molecolari, l'uso di questi prodotti chimici è generalmente sconsigliato. Il metodo più ampiamente utilizzato, che preserva la morfologia, il DNA o le proteine, è il congelamento a secco degli esemplari. Gli esemplari devono essere congelati per un tempo sufficiente tale da essere completamente anestetizzati, ma non così a lungo da (i) disidratarsi o (ii) compromettere la vitalità di *Leishmania*, se l'obiettivo è l'isolamento del parassita in vitro dal tratto digestivo del flebotomo. Raccomandiamo quindi una durata di congelamento da 15 a 20 minuti a -20°C, monitorando regolarmente gli esemplari per garantire che siano solo storditi garantendo la vitalità dei parassiti di *Leishmania*. Se non fosse disponibile un congelatore, gli insetti possono essere sottoposti ad eutanasia, usando la CO2. In condizioni di campo in cui non è possibile utilizzare bombole di CO2, gli esemplari possono essere uccisi utilizzando piccoli contenitori di CO2 commerciali utilizzati nei sifoni di soda (distributori di bevande), tuttavia potrebbero esserci restrizioni sul loro trasporto aereo. Come ultima risorsa, gli insetti possono essere uccisi mediante esposizione al fumo di tabacco. I flebotomi vengono catturati vivi in una trappola CDC, raccolti con un aspiratore, trattenuti nel tubo di vetro ed esposti al fumo di tabacco che li uccide in pochi secondi. Questo metodo è applicabile in tutte le condizioni di campo, anche in condizioni di isolamento difficili. Tuttavia, poiché il vetro si impregna di fumo, non può essere utilizzato per la successiva raccolta e manipolazione di flebotomi vivi senza una pulizia approfondita. Ciononostante, lo stesso aspiratore non pulito può ancora essere utilizzato per sottoporre ad eutanasia i flebotomi provenienti da altre trappole a scopo di fissazione. È anche necessario verificare che tutti gli esemplari siano stati rimossi dall'aspiratore. Questi metodi sono compatibili con l'isolamento di *Leishmania* tramite dissezione dell'intestino eseguita in tempi molto brevi.

3. Conservazione del campione prima del processamento

Esistono cinque metodi principali di fissazione prima del processamento:

3.1. Congelamento

Questo metodo viene eseguito al meglio a -20°C o, preferibilmente, a -80°C. Questi metodi di conservazione sono ora più ampiamente utilizzati rispetto allo stoccaggio in azoto liquido. In tutti i casi, la crioconservazione deve essere implementata il più rapidamente possibile dopo lo stordimento degli esemplari. La conservazione a freddo nei congelatori offre il vantaggio di preservare completamente gli insetti stessi, così come RNA, DNA e proteine con piena integrità durante tutto il periodo di conservazione. Al contrario, l'azoto liquido può danneggiare gravemente ali, zampe, palpi e antenne, spesso amputandoli e occasionalmente rimuovendo caratteri morfologici chiave. La conservazione a secco in congelatore è meno traumatica per gli esemplari, ma non è ideale per preservare i loro organi fragili. Importante: al momento dello scongelamento, ali, antenne, palpi o zampe possono aderire alle fiale ed eventualmente strapparsi a causa della condensa. Tuttavia, la conservazione tramite congelamento non è sempre fattibile negli studi sul campo perché richiede l'accesso a un congelatore o a un contenitore di azoto liquido. La conservazione in congelatore è pienamente compatibile con la rilevazione di patogeni tramite strumenti molecolari senza perdita di sensibilità, sebbene la rilevazione e l'isolamento di virus a RNA richiedano il congelamento a -80°C o in azoto liquido se è necessaria una conservazione a lungo termine. Tuttavia, il congelamento dei campioni non consente l'isolamento di *Leishmania* tramite dissezione intestinale, tranne nel caso in cui i flebotomi vengano prima immersi nella fase di vapore e poi nell'azoto liquido (ad esempio in fiale poste in una calza), simulando la crioconservazione di *Leishmania*.

3.2. Conservazione in alcol (etanolo o alcol isopropilico)

Questo è probabilmente il metodo più utilizzato per conservare i flebotomi. È facile da implementare sul campo, anche in condizioni difficili senza accesso a un laboratorio. La conservazione in alcol è particolarmente adatta per gli studi morfologici, poiché gli organi fragili (ali, zampe, antenne o palpi) rimangono intatti, a patto che non vi siano bolle d'aria nel tubo di conservazione. Pertanto, raccomandiamo di sigillare il tubo con un piccolo batuffolo di cotone per eliminare eventuali bolle d'aria e di posizionare un'etichetta sopra il tappo di cotone (Figura 1). La concentrazione alcolica appropriata rimane oggetto di dibattito.

In generale, le concentrazioni inferiori al 70% non sono raccomandate [45, 66]. Concentrazioni più elevate preservano il DNA in modo più efficace e per periodi più lunghi, ma rendono gli esemplari più fragili per gli studi morfologici. L'uso di etanolo al 96% (la miscela azeotropica) garantisce la stabilità della concentrazione nel tempo, in particolare nelle aree umide come i paesi tropicali, sebbene l'etanolo al 95% sia spesso più facile da reperire. Indipendentemente dalla concentrazione, il DNA è generalmente ben conservato in etanolo (sebbene meno efficacemente rispetto ai metodi di congelamento, in particolare per i metodi molecolari di tipo NGS). Le proteine sono molto meno stabili, specialmente per la proteomica, come le applicazioni MALDI-ToF. I flebotomi conservati in alcol per alcuni mesi possono ancora essere identificati morfologicamente, ma è impossibile generare spettri proteici di riferimento da questi esemplari.

La conservazione in alcol o in condizioni secche può essere migliorata se il campione viene anche congelato a -20°C. Il congelamento a -20°C migliora principalmente la conservazione molecolare (ad esempio, gli acidi nucleici) rallentando la degradazione e offre anche un vantaggio secondario per la conservazione morfologica riducendo la scomposizione dei tessuti nel tempo, anche se l'effetto sulla morfologia è più limitato rispetto a quello sull'integrità molecolare. La conservazione in etanolo può essere applicata anche per la rilevazione di virus a DNA e RNA quando si utilizza etanolo a una concentrazione di almeno il 70% per un breve periodo di conservazione, inferiore a pochi mesi. Inoltre, l'alcol isopropilico può essere facilmente reperibile in alcuni paesi e preserva il DNA, ma rende gli esemplari rigidi. Non è infiammabile come l'etanolo e può quindi essere trasportato facilmente. Se necessario, i flebotomi conservati in azoto liquido o congelati a secco possono essere trasferiti in alcol, sommando tuttavia in questo modo gli svantaggi di entrambi i metodi.

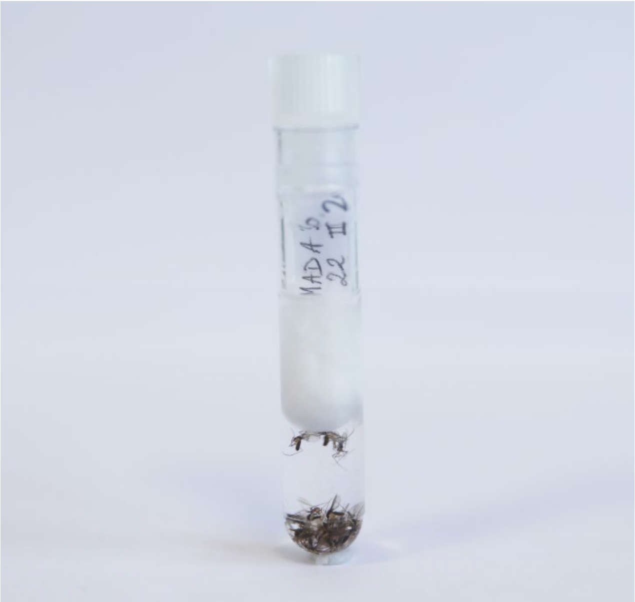

Figura 1: Flebotomi conservati in etanolo.

3.3. Conservazione in soluzione di stabilizzazione dell'RNA (RNASS)

Questo reagente acquoso è ampiamente utilizzato, non tossico e progettato per stabilizzare e proteggere l'RNA in campioni freschi di tessuti e cellule. Agisce penetrando rapidamente nel campione e inattivando le RNasi (enzimi che degradano l'RNA), prevenendo così la degradazione dell'RNA senza necessità di congelamento immediato. La conservazione in RNASS è generalmente efficace nel preservare la morfologia tissutale e cellulare complessiva per la successiva valutazione istologica. Sebbene la RNASS sia ottimizzata per la stabilizzazione dell'RNA piuttosto che per la fissazione, la conservazione a breve-medio termine mantiene solitamente bene l'integrità strutturale. La RNASS consente di conservare i campioni a temperatura ambiente fino a 7 giorni, a 4°C per diverse settimane o a -20°C/-80°C per la conservazione a lungo termine. Questo metodo è particolarmente prezioso sul campo o in contesti clinici dove le infrastrutture per la catena del freddo sono limitate. L'estrazione dell'RNA richiede solitamente la rimozione dei campioni dal reagente ed il loro trattamento secondo protocolli standard.

3.4. Conservazione a secco a temperatura ambiente

Questo è un metodo più datato che, se applicato a un esemplare in toto (montato intero), presenta il grande svantaggio di preservare scarsamente organi fragili come ali, zampe, antenne e palpi. Tuttavia, gli studi proteomici che utilizzano MALDI-ToF rimangono fattibili se la disidratazione viene effettuata previa fissazione con un disseccante di tipo gel di silice. Al contrario, le analisi molecolari mirate al DNA rimangono difficili da eseguire su questi campioni, poiché il DNA è spesso frammentato e in bassa quantità, il che rende le analisi più complesse rispetto ai campioni freschi o congelati, specialmente per i genomi nucleari. Tuttavia, tecniche recenti come la "museomica" potrebbero essere utilizzate su campioni di questo tipo [34]. Pertanto, questo metodo di conservazione non è raccomandato, a meno che non sia disponibile alcuna alternativa. Può essere combinato con la conservazione a freddo ponendo le provette in un congelatore a -20°C o -80°C. La sfida principale è ottenere un montaggio appropriato degli esemplari o delle parti del corpo necessarie per l'identificazione. Per ottenere ciò, la reidratazione è essenziale. Raccomandiamo l'uso di una soluzione di Triton X-100. La durata della reidratazione varia da poche ore a diversi giorni, sotto regolare e attento monitoraggio. Dopo la completa reidratazione, gli esemplari devono essere sciacquati in tre bagni d'acqua consecutivi.

3.5. Conservazione su carta da filtro

Il vantaggio principale delle carte da filtro è la stabilità a lungo termine del DNA genomico all'interno delle cellule di corpi interi non fissati ed essiccati, o di cellule del sangue conservate a temperatura ambiente. La carta da filtro è fornita in formato di cartoncino, il che consente di conservare diverse centinaia di campioni a temperatura ambiente in un volume pari a quello di un piccolo cassetto da tavolo. La matrice della carta da filtro è impregnata di sostanze che denaturano gli agenti infettivi, di conseguenza i campioni non sono più considerati a rischio. Questo permette la conservazione e il trasporto di campioni senza precauzioni specifiche per il rischio biologico [68].

4. Dissezione del campione

A differenza di molti altri insetti, che vengono identificati in base a caratteri esterni osservabili su esemplari singoli montati a secco (in toto), i flebotomi richiedono la dissezione e il montaggio su vetrino per studiare le caratteristiche anatomiche ai fini di un'identificazione accurata della specie. Indipendentemente dalla procedura di preparazione e montaggio scelta, viene impiegata la stessa tecnica di dissezione (Figure 2 e 3) (https://zenodo.org/records/18198006).

Uso di Triton X100: soluzione acquosa non ionica

Si noti che il montaggio riguarda esemplari appena catturati o adeguatamente conservati. La maggior parte dei collezionisti conserva i campioni di insetti a secco (per l'uso MALDI-ToF) o in alcol per molti anni. Purtroppo, la conservazione in alcol non è ottimale per periodi di anni e gli artropodi conservati in questo modo diventano molto difficili da preparare per l'esame microscopico. Un inconveniente che si verifica spesso è la degradazione delle plastiche contenenti i campioni, seguita dall'evaporazione dell'alcol. In entrambi i casi, non abbiamo alternative poiché i campioni rimangono troppo a lungo in alcol o si seccano. È nata quindi l'idea di utilizzare agenti bagnanti che non siano detergenti aggressivi. Il Triton X100 si presenta sotto forma di soluzione acquosa non ionica (soluzione di 4-(1,1,3,3-tetrametilbutil) fenil-polietilenglicole, o t-ottilfenossipolietossietanolo, polietilenglicole tert-ottilfenil etere), ampiamente utilizzato come detergente nella biologia cellulare e molecolare. Consente la permeabilizzazione delle membrane cellulari e nucleari.

Di seguito è riportata una procedura che utilizza Triton X100 non ionico in soluzione acquosa allo 0,5%:

- Impregnare il campione secco con alcol assoluto.
- Aggiungere il volume necessario di soluzione di Triton X100 allo 0,5% in modo che l'intero campione sia immerso.
- Lasciare agire il processo da circa 5 minuti a diversi giorni, monitorando regolarmente. Tutti gli artropodi devono separarsi completamente nella soluzione.
- Rimuovere la soluzione di Triton X100 e sostituirla con una soluzione di idrossido di potassio.

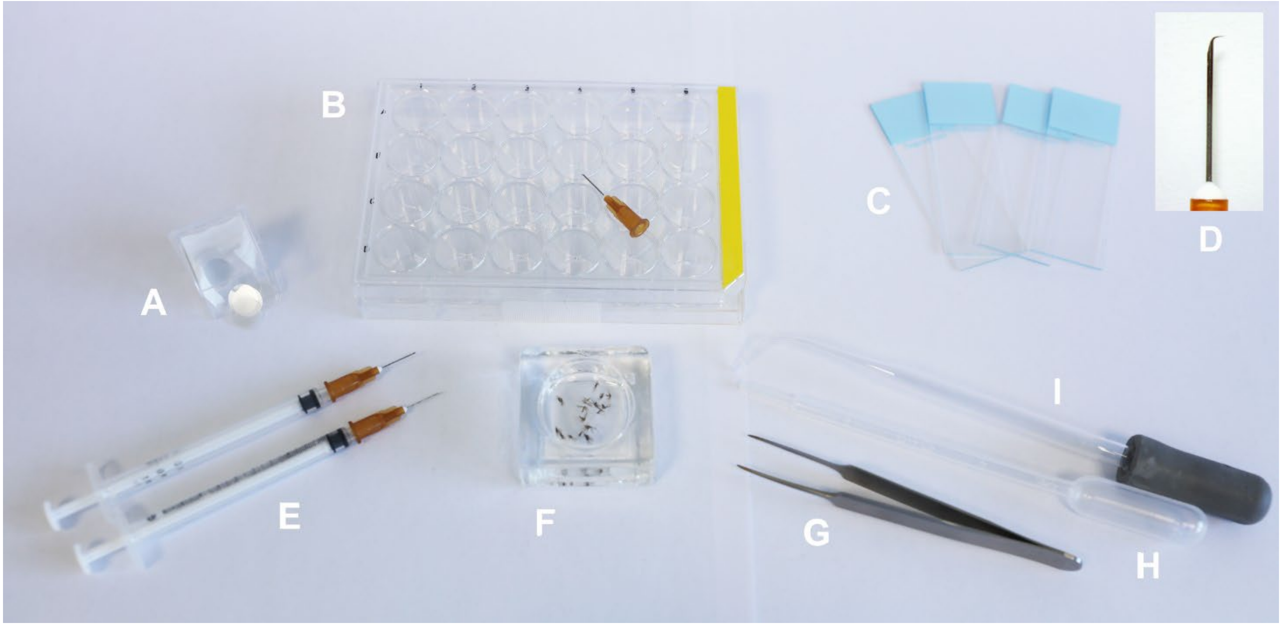

Figura 2: Materiali necessari per il montaggio dei flebotomi: A: coprioggetti in vetro rotondi (diametro 10 o 12 mm); B: piastra a 24 pozzetti e ago uncinato (se si utilizza olio di chiodi di garofano o essenza di Euparal® per trattare i flebotomi, non utilizzare piastre acriliche perché si verificherà una reazione chimica e gli esemplari verranno danneggiati); C: vetrini portaoggetti adatti per l'etichettatura; D: dettaglio dell'ago uncinato; E: aghi attaccati alle siringhe; F: vetrino ad orologio o contenitore equivalente contenente i flebotomi da montare; G: pinze Dumont; H: pipetta di plastica; I: pipetta di vetro piegata scaldandola per facilitare il trasferimento del liquido nei pozzetti.

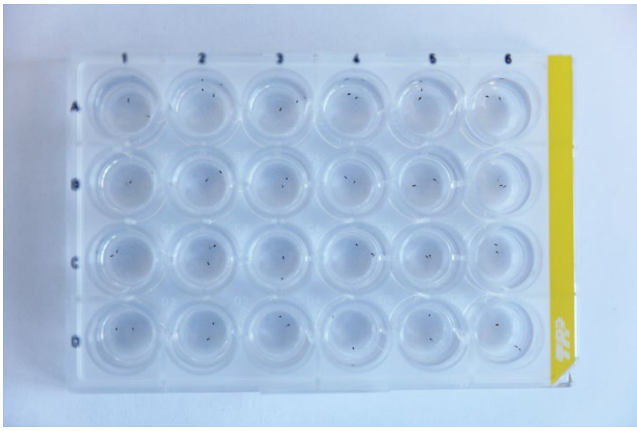

Figura 3: Una piastra con 24 pozzetti, ciascuno contenente la testa e la punta dell'addome dei flebotomi.

4.1. Capo

La dissezione può essere eseguita utilizzando aghi sottili o spilli entomologici sotto uno stereomicroscopio (Figure 2 e 3). Gli aghi più comunemente usati includono: 26G x 1/2" (0,45 × 13 mm), 30G × 1/2" (0,3 × 13 mm) o 25G × 5/8" (0,5 × 16 mm). Per preparare l'esemplare per l'identificazione, come minimo, il capo viene separato dal corpo e montato con la parte ventrale rivolta verso l'alto per mostrare il cibario ed il faringe, mentre il torace e l'addome vengono montati lateralmente a seguito della dissezione. Il montaggio del capo in posizione ventro-dorsale assicura che il forame occipitale sia orientato verso l'alto, in modo che il cibario possa essere osservato direttamente. L'accesso a queste caratteristiche anatomiche è facilitato se il capo viene completamente separato.

4.2. Ali e torace

Le ali devono essere montate in piano. Ciascuna ala può essere staccata alla base e montata indipendentemente, oppure se ne può montare una sola, lasciando l'altra attaccata al torace. Se è prevista un'analisi di morfometria geometrica, è essenziale identificare ed etichettare correttamente l'ala destra e quella sinistra prima del montaggio. Il torace è diviso in diverse parti, ciascuna delle quali contiene informazioni tassonomiche molto importanti [20, 64]. Generalmente, viene montato in vista laterale, per consentire l'esame della chetotassi e della distribuzione del colore. La presenza di cicatrici di setole in certe regioni del torace può essere utilizzata per distinguere alcune specie del genere Brumptomyia. La distribuzione del colore può essere utilizzata per separare i flebotomi neotropicali a livello di genere (es. Bichromomyia), serie di specie (es. Pintomyia) o anche specie dello stesso genere (es. Micropygomyia, Nyssomyia, Psathyromyia e Psychodopygus) [20]. Pertanto,

se il torace non viene utilizzato per l'analisi molecolare, dovrebbe essere montato in modo da non danneggiarlo. È importante notare che non è l'intensità dei colori che conta, ma la loro distribuzione sul torace. Di conseguenza, il processo di chiarificazione non eliminerà la pigmentazione né il suo pattern.

4.3. Genitali

È necessario prestare particolare attenzione quando si montano i genitali sia nei maschi che nelle femmine, poiché sono cruciali per l'identificazione di generi, sottogeneri e specie. In entrambi i sessi, i genitali sono accoppiati.

4.3.1. Maschi

I genitali sono esterni e costituiti da pinze appaiate, ognuna composta dall'articolazione gonocoxite-gonostilo nella sua parte dorsale e dal lobo epandriale nella sua parte ventrale. Il gonostilo porta spine e talvolta setole, che devono essere contabili e le cui posizioni di inserzione devono essere chiaramente visibili. È importante osservare attentamente la superficie interna del gonocoxite, che può portare un ciuffo di setole sessili o portate da un lobo (= tubercolo) [22]. I colleghi con meno esperienza nelle dissezioni possono eseguire un semplice montaggio laterale, senza staccare i genitali dall'estremità dell'addome (https://zenodo.org/records/18311158). In questo caso, la sovrapposizione delle due parti dei genitali può rendere difficile, ad esempio, il conteggio delle setole interne del gonocoxite, ma evita di danneggiare i genitali a causa di una dissezione fallita. I colleghi più esperti possono tentare di aprire i genitali in due, per scinderli. Per ottenere ciò, il lato smussato di un ago (tipo ago per reazione intradermica) deve essere passato attraverso, staccando senza tagliare completamente i genitali per scindere gli insiemi gonocoxite-gonostilo (https://zenodo.org/records/18311158). In questo modo, l'osservazione delle loro facce interne sarà facile. Questo montaggio facilita anche l'osservazione dei parameri e delle guaine paramerali, che non si sovrappongono più. Per il montaggio laterale, che favorisce la sovrapposizione degli organi, gli esemplari devono essere perfettamente chiarificati.

4.3.2. Femmine

L'apparato genitale è interno, costituito dalle spermateche. In assenza di dissezione, devono essere osservate attraverso i tegumenti montando l'addome in posizione ventrale. Indipendentemente dal mezzo di montaggio scelto, la spermateca stessa può generalmente essere osservata correttamente, specialmente se non è liscia e chiarificata. Tuttavia, l'osservazione di spermateche lisce e a pareti sottili può essere problematica in mezzi poco rifrangenti. Inoltre, l'osservazione della base dei dotti spermatecali è essenziale per l'identificazione della specie, come nel sottogenere *Larroussius* [35, 37, 38], i principali vettori di *Leishmania infantum* nel Vecchio Mondo. Senza questa osservazione, l'identificazione dell'esemplare rimane impossibile. Per superare queste difficoltà di osservazione, il montaggio della forca genitale-spermateche dovrebbe essere rimosso dall'addome (https://zenodo.org/records/18311106). Le spermateche sono generalmente difficili da osservare durante la dissezione, ma la forca genitale è relativamente facile da localizzare. Poiché i dotti spermatecali si aprono nella forca genitale, l'isolamento di questa forca consente normalmente l'isolamento delle spermateche. Se le spermateche vengono accidentalmente tagliate durante il processo, non vanno perdute e possono ancora essere osservate all'interno dei tegumenti addominali (Figura 4).

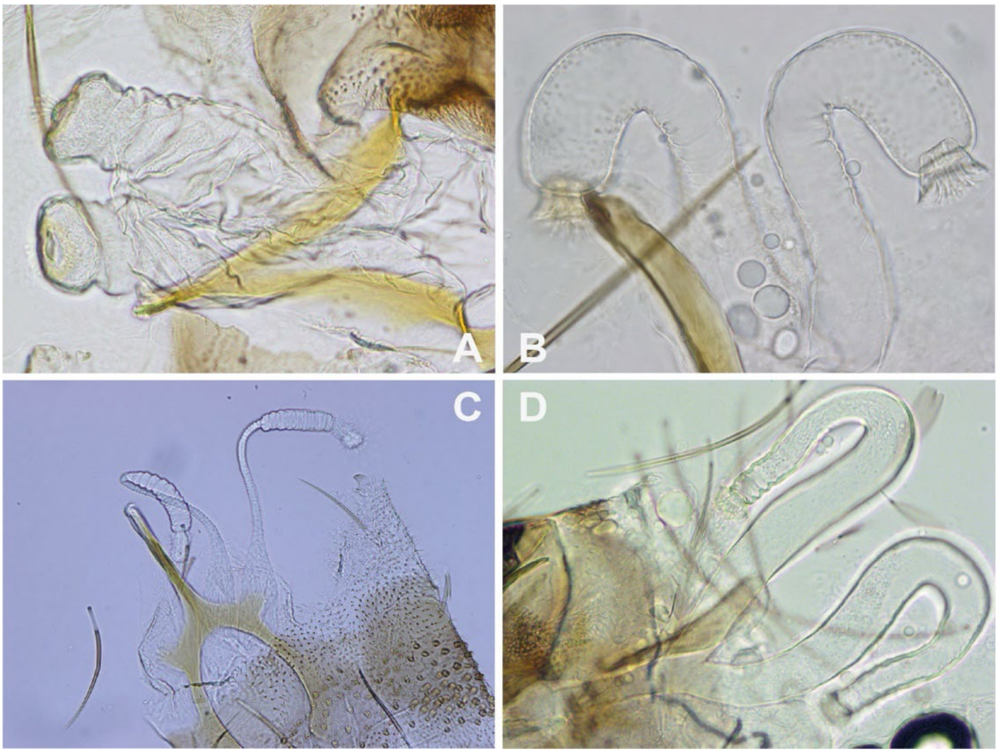

Figura 4: Spermateche disseccate e montate in liquido di Marc-André da esemplari freschi. A: *Idiophlebotomus longiforceps* (Lao PDR); B: *Sergentomyia minuta* (Francia); C: *Phlebotomus ariasi* (Francia); D: *Sergentomyia anodontis* (Lao PDR).

4.4. Dissezione dell'intestino medio per l'isolamento di *Leishmania*

La dissezione del tratto digerente è fondamentale per rilevare e isolare *Leishmania* nelle femmine di flebotomo. La procedura può essere eseguita sia sul campo che in laboratorio per valutare la competenza vettoriale.

Si raccomanda di operare su femmine appena sottoposte ad eutanasia. Lavare le femmine con acqua o una soluzione salina contenente un detergente delicato per rimuovere l'eccesso di peli. Questo passaggio aiuta a mantenere condizioni asettiche per l'isolamento di *Leishmania*, preservando al contempo le caratteristiche morfologiche necessarie per l'identificazione. Per trovare e isolare *Leishmania*, rimuovere con cura l'intestino medio e posizionarlo in una singola goccia di soluzione salina sterile (NaCl 0,9%). Dopo aver osservato i parassiti mobili al microscopio ottico (ingrandimento raccomandato: ~200×), utilizzare una siringa da insulina o una micropipetta per trasferirli nel terreno di coltura (per maggiori dettagli vedere Capitolo 4.4.3).

Montare la testa e i genitali direttamente nel liquido di Marc-André per chiarificarli. Importante: non permettere mai al liquido di Marc-André di entrare in contatto con *Leishmania* – né direttamente né indirettamente tramite strumenti o aghi – poiché è letale per i parassiti.

La dissezione delle femmine di flebotomo può essere eseguita su un solo vetrino o su due; entrambe le opzioni presentano vantaggi e limitazioni (Figura 5; https://zenodo.org/records/18311154).

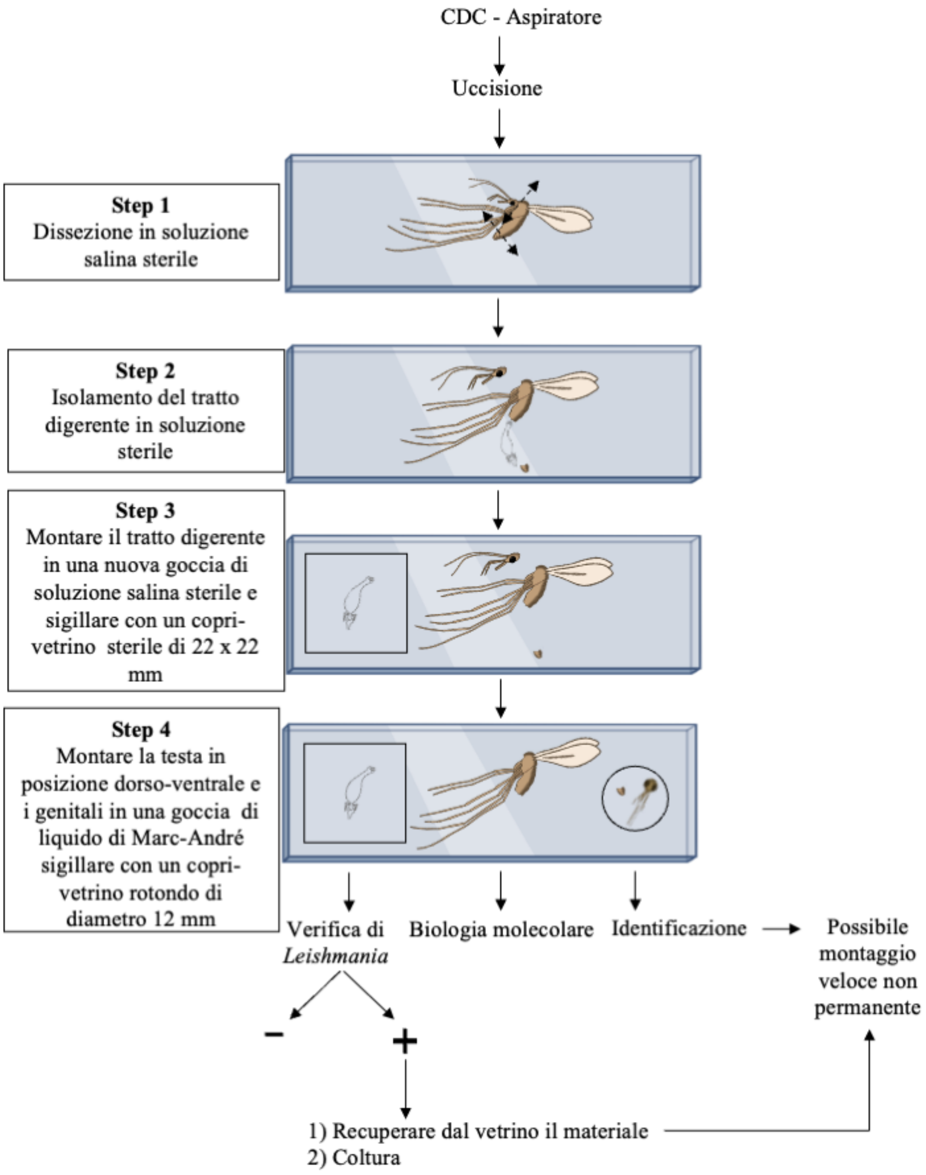

Figura 5: Metodo per l'isolamento di *Leishmania*.

4.4.1. Metodo a due vetrini

La prima opzione prevede di lavorare su due vetrini separati: uno contenente soluzione fisiologica sterile per estrarre l'intestino medio, e l'altro per montare la testa e le spermateche nel liquido di Marc-André. Tuttavia, in condizioni di campo, è comune che due o tre persone sezionino i flebotomi e trasmettano le loro dissezioni ad un unico ricercatore responsabile dell'identificazione della specie e della valutazione dell'infezione da *Leishmania* nell'intestino. La gestione di due vetrini può causare problemi di tracciabilità dei campioni e, in particolare, rendere difficile determinare con certezza quale singolo flebotomo fosse infetto, qualora venga rilevato un intestino positivo (https://zenodo.org/records/18311154).

4.4.2. Metodo a vetrino singolo

L'utilizzo di un singolo vetrino garantisce la tracciabilità dei risultati. Tuttavia, è necessario prendere diverse precauzioni. Per massimizzare la sterilità durante questa fase, gli operatori devono pulirsi regolarmente le mani con gel idroalcolico. Devono essere utilizzati vetrini non smerigliati e coprioggetti quadrati (22 x 22 mm) avvolti in fogli di alluminio e sterilizzati a calore secco (utilizzando un forno Poupinel), insieme ad aghi sterili per ogni dissezione (suggerimento: 25G Ø 0,5 mm × 16 mm). Il flebotomo viene posizionato in una goccia di soluzione fisiologica sterile al centro del vetrino. Si recide la testa mentre si effettua un'incisione tra il 6° e il 7° tergite e sternite addominale senza tagliare il tratto digerente (un taglio più alto può essere effettuato se si prevedono spermateche molto lunghe). Successivamente, il torace deve essere immobilizzato con un ago e gli ultimi segmenti addominali posteriori tirati delicatamente con l'altro ago per

This is an Open Access article distributed under the terms of the Creative Commons Attribution License (https://creativecommons.org/licenses/by/4.0), which permits unrestricted use, distribution, and reproduction in any medium, provided the original work is properly cited.

estrarre l'intestino. Se questo tentativo fallisce, c'è la possibilità di bloccare l'estremità dell'addome con un ago e tirare il tratto digerente dalla sua parte anteriore. Se fallisce ancora, l'intestino deve essere estratto rimuovendo quanto più tegumento rimanente possibile attorno ad esso. Una volta rimosso l'intestino, gli ultimi segmenti addominali devono poi essere separati tagliando il tratto digerente. L'intestino viene quindi posto in una nuova goccia di soluzione fisiologica sterile posizionata su un lato del vetrino, e poi delicatamente coperto con un coprioggetto sterile. La testa e gli ultimi segmenti addominali vengono trasferiti in una piccola goccia di liquido di Marc-André posta all'altra estremità del vetrino, assicurandosi che non entrino in contatto con la *Leishmania*. La testa viene orientata correttamente (forame occipitale verso l'alto), e le spermateche vengono isolate con la forca genitale come indicato sopra e coperte con un piccolo coprioggetto rotondo (Ø 12 mm, da non confondere con i coprioggetti quadrati sterili). La carcassa rimanente del flebotomo e le ali rimangono nella goccia di soluzione fisiologica al centro del vetrino (https://zenodo.org/records/18311154). In caso di positività, o per esplorazione tassonomica, il torace e l'addome possono essere conservati per studi molecolari o proteomici, e le ali possono essere montate in un mezzo acquoso. Per conservare il preparato, il volume extra di liquido di Marc-André può essere sostituito con un mezzo di montaggio acquoso come la gomma di cloralio (=Hoyer) o un mezzo a base di alcol polivinilico.

Video dettagliati che dimostrano queste procedure sono disponibili (dissezione dell'intestino medio del flebotomo: https://zenodo.org/records/18303014 e dissezione delle ghiandole salivari del flebotomo: https://zenodo.org/records/18302850), quindi non verranno spiegati qui.

4.4.3. Isolamento di *Leishmania* e coltura del parassita dall'intestino dei flebotomi

L'isolamento del parassita mediante la dissezione di flebotomi femmina infetti è una procedura delicata che richiede alta abilità e dovrebbe essere inizialmente praticata su esemplari privi di parassiti. Dopo la dissezione, l'intestino viene trasferito in una goccia fresca di soluzione salina sterile (0,9%) o soluzione di Locke per il lavaggio [4]. L'intestino dissezionato può quindi essere trattato in due modi: i) esaminato al microscopio ottico per osservare i diversi stadi dei promastigoti di *Leishmania* e la loro localizzazione, con particolare attenzione alla valvola stomodeale, e ii) aperto per facilitare la fuoriuscita dei promastigoti, favorendone la coltura di massa [4]. Trovare flebotomi infettivi sul campo è un evento relativamente raro, pertanto buone sessioni di pratica massimizzeranno le possibilità di un isolamento di successo.

Se vengono osservati parassiti di *Leishmania* nell'intestino, è necessario utilizzare aghi sterili nuovi e aggiungere una piccola quantità di soluzione salina sterile attorno al vetrino coprioggetto mediante capillarità per liberarli. L'intestino deve essere lacerato con cura e rapidità per rilasciare i parassiti nella soluzione salina. Utilizzando una micropipetta da 100 µL o una siringa da tubercolina, raccogliere i parassiti e inocularli in un terreno di coltura opportunamente etichettato.

Coltura in vitro dei promastigoti di *Leishmania*: i parassiti isolati vengono inizialmente mantenuti su agar sangue SNB-9 o in terreno solido Novy, McNeal, Nicolle (NNN) [16], sovrapposto con terreno sterile alpha-MEM [16, 65] o con terreno M199, ciascuno integrato con 10% di siero fetale bovino (FCS) sterile inattivato dal calore (per migliorare la crescita del parassita), 1% di vitamine BME, 2% di urina umana sterile (sterilizzata utilizzando un filtro a siringa Filtropur® S 0,2 µm), 250 µg/mL di amikacina (o 50 µg/mL di gentamicina, o una miscela di antibiotici e aminoacidi (L-glutammina 200 mM-penicillina 10 000 U-streptomicina 10 mg/mL) [47]. Dopo tre giorni, in assenza di contaminazione, le colture vengono sospese in un terreno di congelamento opportunamente preparato e successivamente conservate a -80°C per 1 o 2 anni, oppure in azoto liquido a -196°C per la conservazione a lungo termine e l'uso sperimentale futuro [7].

4.5. Ghiandole salivari

La dissezione delle ghiandole salivari dei flebotomi è una tecnica fondamentale per lo studio delle interazioni vettore-patogeno, in particolare per il rilevamento di arbovirus come i Phlebovirus (es. virus Toscana) [44, 75]. A causa delle dimensioni minute dei flebotomi, la procedura richiede precisione sotto uno stereomicroscopio, utilizzando pinze fini o aghi da microdissezione per isolare le delicate ghiandole salivari senza causarne la rottura o la contaminazione (https://zenodo.org/records/18302850) [51, 61]. Preservare l'integrità delle ghiandole è cruciale per garantire un'analisi molecolare downstream affidabile. Una volta estratte, le ghiandole possono essere omogeneizzate e testate via RT-PCR, qPCR o immunoassays per rilevare RNA virale o antigeni [12]. La presenza di virus nelle ghiandole salivari, piuttosto che solo nell'intestino o nell'emocele, conferma che il patogeno ha completato il suo periodo di incubazione estrinseca ed è trasmissibile durante l'assunzione di sangue [71].

Il processo di dissezione è tecnicamente impegnativo a causa delle piccole dimensioni delle ghiandole salivari dei flebotomi, richiedendo una notevole esperienza per evitare la degradazione del campione [1, 51]. Inoltre, la carica virale può essere bassa, rendendo necessari metodi di rilevamento altamente sensibili come la nested PCR o il sequenziamento ad alto rendimento [45]. I rischi di contaminazione sottolineano ulteriormente la necessità di tecniche sterili. Oltre agli ostacoli tecnici, fattori biologici influenzano il successo del rilevamento; la competenza vettoriale varia tra le specie di flebotomi e i tassi di infezione fluttuano con le condizioni ecologiche e stagionali [33, 61].

Il rilevamento dei virus nelle ghiandole salivari fornisce informazioni critiche sui rischi di trasmissione, consentendo l'implementazione di misure mirate di sorveglianza e controllo [15]. Ad esempio, l'identificazione del virus Toscana nei flebotomi nelle aree endemiche ha contribuito a definire i protocolli diagnostici e le raccomandazioni di sanità pubblica [18]. Inoltre, lo studio delle interazioni virus-saliva potrebbe rivelare nuovi bersagli per vaccini o terapie che bloccano la trasmissione [15, 18].

Le ghiandole salivari dei flebotomi possono anche essere utilizzate come fonte di antigeni per misurare gli anticorpi dell'ospite contro la saliva del flebotomo utilizzando metodi immunologici, preferibilmente ELISA. Questo metodo consente la valutazione dell'esposizione dell'ospite alle punture di flebotomo, supportando così la valutazione dell'efficacia dei metodi di controllo del vettore [25] e il rischio di trasmissione di *Leishmania* [40].

4.6. Identificazione del pasto di sangue

Le femmine replete isolate dalle catture devono essere dissezionate utilizzando attrezzatura monouso per prevenire la contaminazione incrociata. Il loro addome deve essere esaminato allo stereomicroscopio per valutare lo stadio di digestione del pasto di sangue. Si raccomanda di selezionare solo le femmine con addome rosso, bruno-rossastro o rosso scuro, che non mostrino segni di formazione di uova. Rimuovere la punta dell'addome, comprese le spermateche, per identificare morfologicamente la femmina dopo la diafanizzazione. La parte principale dell'addome (senza spermateche) deve poi essere posta in provette Eppendorf® e conservata a -20°C fino a successive analisi. I marcatori genetici comunemente usati per l'identificazione del pasto di sangue, come PNOC [5, 30, 50], CytB [67] o COI [13], sono ben consolidati e ampiamente descritti in letteratura; pertanto, non saranno dettagliati ulteriormente in questo articolo (Figura 6).

In alternativa, per identificare il sangue dell'ospite, può essere impiegata la mappatura peptidica MALDI-ToF [31]. È stato dimostrato sperimentalmente che questa tecnica consente l'identificazione del sangue dell'ospite entro un intervallo di tempo più lungo dopo l'assunzione del pasto di sangue; pertanto, è il metodo di scelta adatto, specialmente per l'analisi di femmine replete con una digestione del sangue dell'ospite visibilmente più avanzata. I campioni dovrebbero idealmente essere conservati a -20°C o 4°C, ma si possono ottenere buoni risultati anche da campioni conservati a temperatura ambiente per breve tempo. L'addome di una femmina repleta deve essere dissezionato dal resto del corpo poco prima dell'analisi e omogenizzato in acqua distillata. Il resto del corpo del flebotomo rimane disponibile per altre analisi molecolari e morfologiche. Dopo aver prelevato l'aliquota dall'omogenato per la mappatura peptidica MALDI-ToF, il resto può essere utilizzato per l'isolamento del DNA al fine di confermare l'identificazione del sangue dell'ospite e/o ricercare la presenza di *Leishmania spp.* Il tempo complessivo di preparazione e analisi del campione è molto breve rispetto alle tecniche molecolari basate sul DNA.

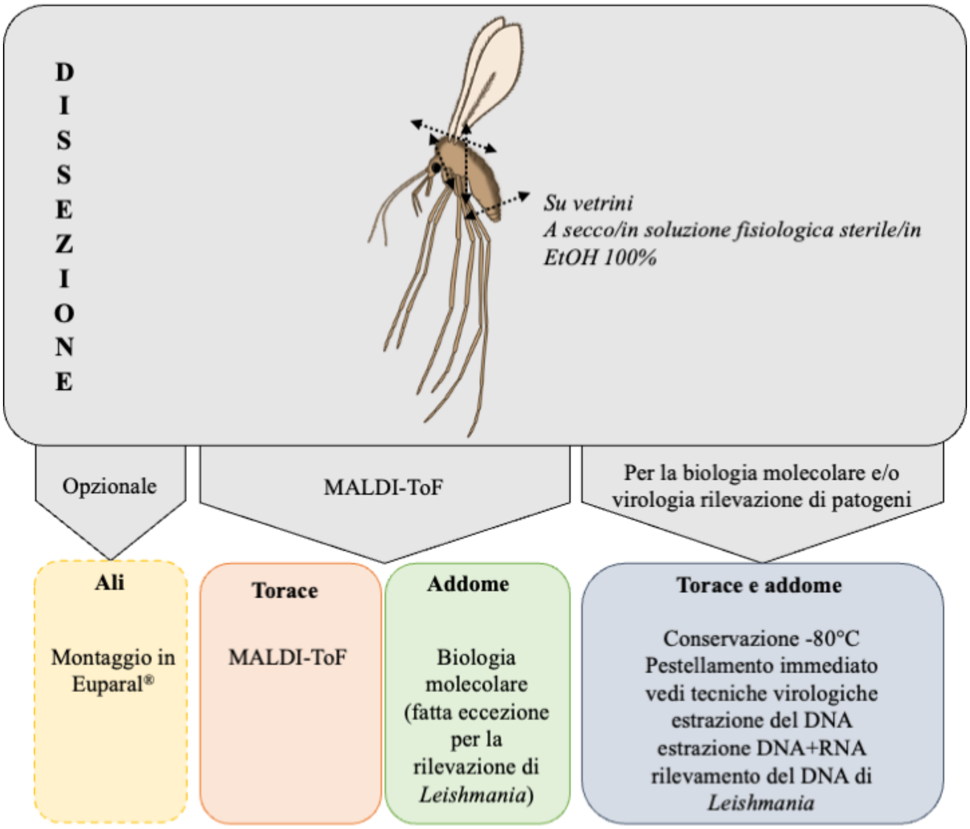

Figura 6: Processamento del flebotomo per applicazioni di biologia molecolare, proteomica e/o virologia.

5. Trattamento dei campioni per studi morfologici (Figure 3, 6, 7 e 8; Appendici 1, 2, 3 e 4)

Questa sezione delinea i principi per la preparazione di un campione di flebotomo da montare esclusivamente per studi morfologici, seguita dall'adattamento per applicazioni che vanno oltre la morfologia. Tuttavia, la comprensione di questa metodologia è cruciale, poiché consente di adattare le procedure a specifici tipi di campione quando necessario.

Il trattamento prevede fasi successive di svuotamento e riempimento utilizzando pipette Pasteur dotate di bulbi di gomma flessibili. Si raccomanda vivamente l'uso di contenitori di vetro a fondo tondo, poiché facilitano notevolmente queste operazioni. Il vetro è inerte rispetto a tutti i reagenti. Per prevenire l'evaporazione dei reagenti, i contenitori devono essere dotati di coperchi e non devono mai essere riempiti eccessivamente, per evitare traboccamenti durante la chiusura o l'apertura, e per impedire la caduta di polvere sui campioni. Le sostanze chimiche necessarie per la chiarificazione ed il trattamento sono riportate nella Tabella 2.

Tabella 2: Composizione dei reagenti utilizzati.

**Idrossido di potassio 10%**<br>Idrossido di potassio 10 g<br>Acqua distillata *qs* 100 mL	**Fucsina acida 1% in acqua distillata**<br>Fucsina acida (in polvere) 1 g<br>Acqua distillata 99 mL
**Mezzo di montaggio gomma-cloralio (mezzo di Hoyer)**<br>Acqua distillata 50 mL<br>Idrato di cloralio 200 g<br>Gomma Arabica 50 g<br>Glicerolo 20 mL	**Soluzione di Marc-André colorata con fucsina acida**<br>Soluzione Marc-André 10mL<br>Fucsina 1% 50 µL
**Soluzione di Marc-André**<br>Idrato di cloralio 40 g<br>Acido acetico glaciale 30 mL<br>Acqua distillata 30 mL	**Mezzo Enecê**<br>Colofonia bianco puro 22 g<br>Gomma copale solubile in alcool 12 g<br>Etanolo assoluto 20 mL<br>Canfora 10 g<br>Essenza di trementina 10 mL

This is an Open Access article distributed under the terms of the Creative Commons Attribution License (https://creativecommons.org/licenses/by/4.0), which permits unrestricted use, distribution, and reproduction in any medium, provided the original work is properly cited.

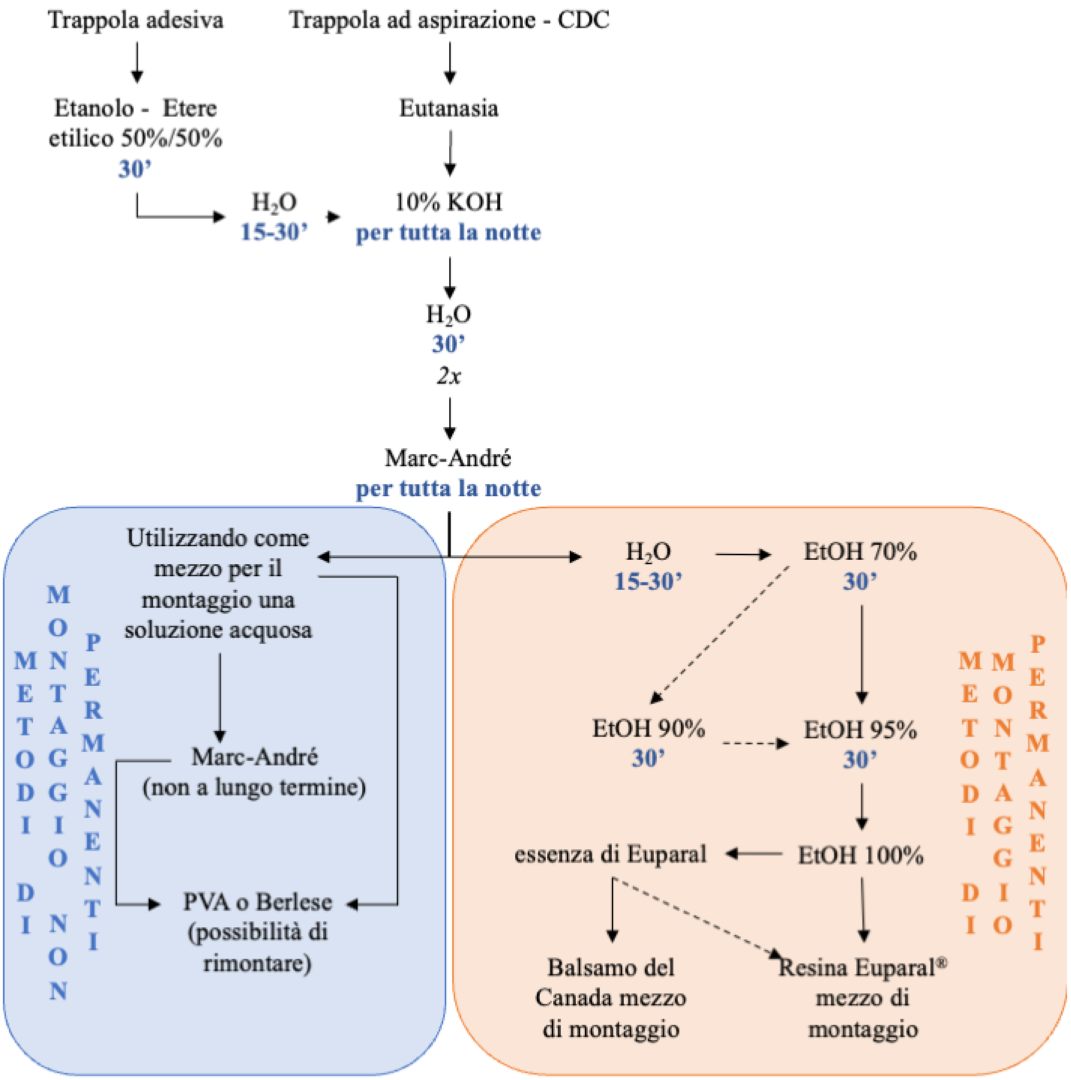

Figura 7: Metodo classico per processare i flebotomi

This is an Open Access article distributed under the terms of the Creative Commons Attribution License (https://creativecommons.org/licenses/by/4.0), which permits unrestricted use, distribution, and reproduction in any medium, provided the original work is properly cited.

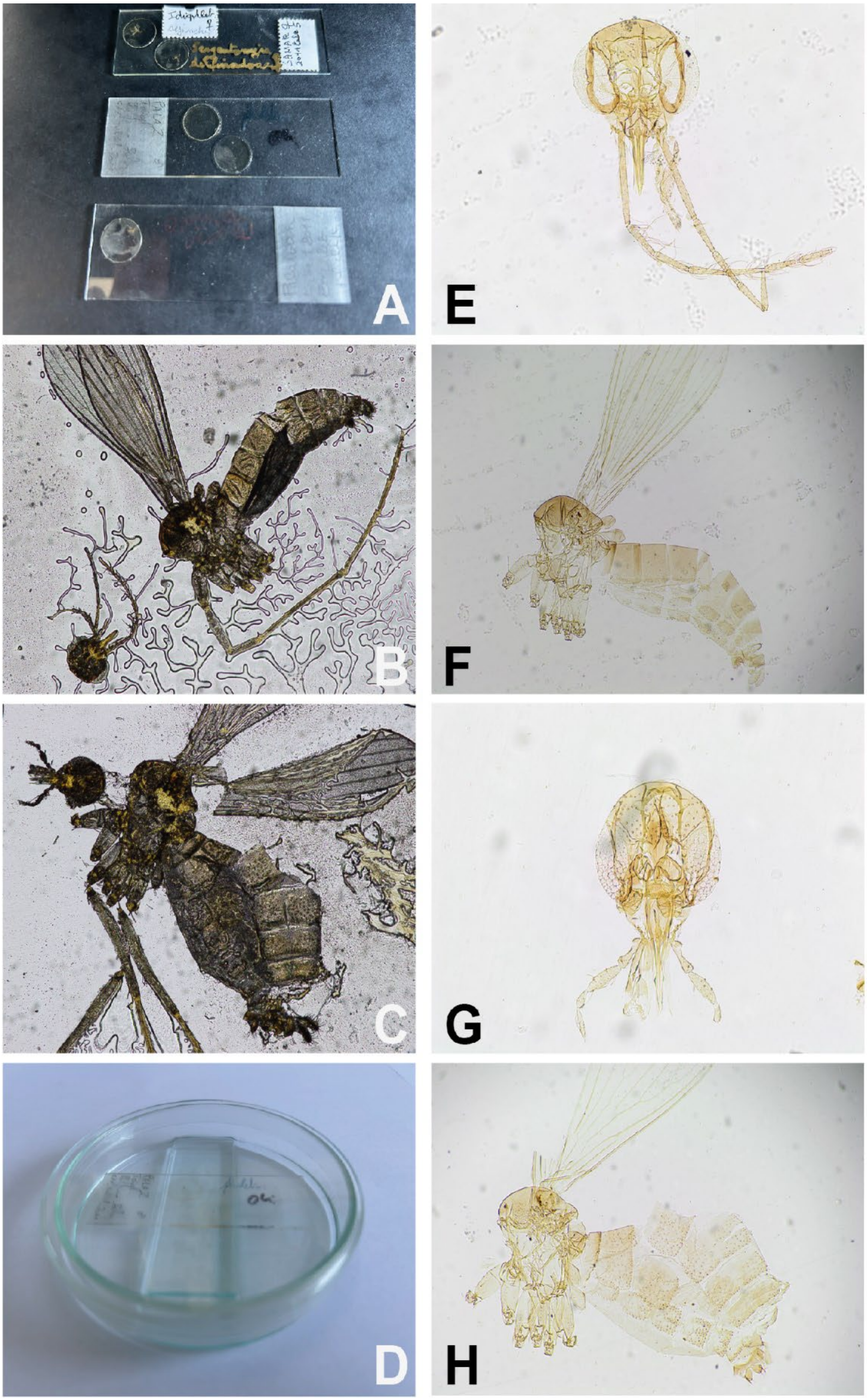

Figura 8: Rimontaggio del vetrino. A: vetrini danneggiati e secchi montati in Hoyer; B: vista microscopica di un pappatacio secco; C: vista microscopica di un altro pappatacio danneggiato; D: camera umida contenente un vetrino secco; E: testa e F: corpo del campione B dopo il rimontaggio in Euparal®; G: testa e H: corpo del campione danneggiato C dopo il rimontaggio in Euparal®.

This is an Open Access article distributed under the terms of the Creative Commons Attribution License (https://creativecommons.org/licenses/by/4.0), which permits unrestricted use, distribution, and reproduction in any medium, provided the original work is properly cited.

5.1. Chiarificazione

Prima che gli esemplari di flebotomi possano essere montati come preparati microscopici permanenti, devono essere chiarificati mediante macerazione utilizzando un metodo e un agente chiarificante appropriato (es. soluzione di acido acetico al 10% o soluzione di Marc-André contenente idrato di cloralio, che è una sostanza chimica soggetta a restrizioni in molti paesi) per renderli trasparenti. Il processo di chiarificazione rimuove i tessuti corporei, i grassi, le secrezioni e la cera, rendendo l'esemplare traslucido e facilitando l'esame delle strutture esoscheletriche (es. inserzione delle setole), delle caratteristiche superficiali (es. colorazione) e delle caratteristiche interne visibili attraverso il tegumento (es. spermateche).

Il processo di chiarificazione in due fasi, che prevede dapprima l'uso di una base forte (come l'idrossido di potassio), seguita da un acido debole (come l'acido acetico nella soluzione di Marc-André), svolge scopi biochimici distinti [74]. La base scompone i tessuti molli, come proteine, grassi e muscoli, attraverso la saponificazione e la denaturazione proteica, lasciando intatto l'esoscheletro di chitina per la chiarezza strutturale. Il successivo acido debole neutralizza eventuali residui alcalini, prevenendo un'ulteriore degradazione, e schiarisce la chitina per migliorare la trasparenza [74], tuttavia è sufficiente un duplice lavaggio degli esemplari in acqua distillata per 15 minuti pera neutralizzare la base. Questo trattamento sequenziale combina un'efficace rimozione dei tessuti con una conservazione delicata, garantendo l'integrità ottimale dell'esemplare per l'esame microscopico. Si raccomandano due risciacqui di 20 minuti in acqua distillata prima di procedere alla fase successiva.

5.1.1. Lisi dei tessuti molli (Figura 8)

L'idrossido di sodio (NaOH) o l'idrossido di potassio (KOH) sono agenti maceranti chimici comunemente usati, applicati a concentrazioni e durata variabili a seconda delle dimensioni e della fragilità degli esemplari. La tecnica standard e più efficace prevede la lisi dei tessuti molli immergendo i flebotomi in una base forte (KOH o NaOH al 10%) per una notte. La concentrazione può essere aumentata per ridurre la durata del trattamento (es. KOH al 20% per 6 ore) così come il riscaldamento a 37°C.

5.1.2. Chiarificazione con o senza colorazione

Questo passaggio è seguito da un trattamento di schiarimento, tipicamente combinando acido acetico e idrato di cloralio (es. soluzione di Marc-André). Dopo la chiarificazione, gli esemplari devono essere risciacquati accuratamente in almeno due bagni d'acqua successivi di 20 minuti ciascuno per rimuovere i residui chimici. La soluzione di Marc-André è un agente chiarificante comunemente usato per la preparazione di esemplari di flebotomi. La sua efficacia risiede nel facilitare il processo di chiarificazione riducendo al minimo danni significativi alle strutture fragili, come ali e antenne. La soluzione deve essere preparata al momento o conservata in un contenitore a chiusura ermetica per evitarne l'evaporazione o la degradazione. L'uso della soluzione di Marc-André è particolarmente vantaggioso se combinato con tecniche di schiarimento o colorazione per migliorare specifici dettagli morfologici. I dettagli sulla sua composizione e preparazione sono riportati nell'Appendice 2.

Per esemplari altamente traslucidi, la colorazione può essere necessaria per migliorare la visibilità prima del montaggio. Sono disponibili molti coloranti, ognuno dei quali mira a componenti chimici specifici dell'organismo. È importante selezionare un colorante compatibile sia con l'esemplare che con il mezzo di montaggio scelto. Questa metodologia di base può essere adattata secondo necessità, ad esempio, incorporando fucsina acida allo 0,1% nella soluzione di Marc-André per la colorazione. Inoltre, gli esemplari conservati in soluzioni acquose e destinati a montaggi in resina richiedono la disidratazione (vedere Sezione 5.2 sulla Disidratazione), poiché la maggior parte dei mezzi di montaggio resinosi naturali e sintetici è incompatibile con l'acqua. New (1974) ha notato che alcuni coloranti possono deteriorarsi in certi mezzi di montaggio [53]. Ad esempio, la fucsina acida, comunemente usata con il balsamo del Canada, può anche essere fissata in Euparal®. Tuttavia, gli esemplari colorati con fucsina acida sono inclini a sbiadire, in particolare quando rimangono residui di olio di garofano, usato come fluido chiarificante finale. Gli esemplari conservati in olio di garofano possono sbiadire notevolmente entro pochi giorni.

5.2. Disidratazione

La disidratazione viene effettuata trasferendo gradualmente i campioni attraverso una serie di soluzioni di etanolo a concentrazione crescente: 50%, 70%, 80%, 90% o 95% e infine 100%, con ogni bagno della durata di almeno 20 minuti. Poiché l'etanolo evapora rapidamente, il contenitore deve essere sigillato ermeticamente durante il processo. Una volta che il campione è completamente disidratato, è possibile mettere in pausa il processo per alcuni giorni nell'essenza di Euparal®, preferibile all'olio di garofano. Il creosoto di faggio, un tempo ampiamente utilizzato per questo scopo, è ora completamente vietato a causa della sua tossicità. Il processo di disidratazione deve garantire che il fluido all'interno del campione sia compatibile con il mezzo di montaggio, al fine di prevenire opacità, collasso osmotico o distorsioni che potrebbero rendere il campione inadatto allo studio tassonomico.

5.3. Mezzi di montaggio

5.3.1. Selezione e applicazione per la preparazione dei campioni

Il mezzo di montaggio dovrebbe idealmente avere un indice di rifrazione il più vicino possibile a quello del vetro, che è di circa 1,5. Deve essere incolore, trasparente e rimanere perfettamente limpido dopo l'asciugatura e nel tempo. Deve essere compatibile con le colorazioni utilizzate e capace di penetrare e diffondersi in tutti i tessuti del

This is an Open Access article distributed under the terms of the Creative Commons Attribution License (https://creativecommons.org/licenses/by/4.0), which permits unrestricted use, distribution, and reproduction in any medium, provided the original work is properly cited.

campione. Non deve asciugarsi troppo rapidamente né formare aloni durante il montaggio. Non deve restringersi dopo il montaggio. La selezione di un mezzo di montaggio appropriato è un aspetto fondamentale della preparazione del campione, poiché nessun singolo mezzo è ideale per tutti gli scopi. La scelta dovrebbe bilanciare diversi fattori chiave:

- **Proprietà ottiche**. L'indice di rifrazione del mezzo di montaggio dovrebbe fornire un contrasto e una rifrazione sufficienti delle caratteristiche anatomiche critiche utilizzate per l'identificazione tassonomica o la descrizione morfologica, come spermateche, ascoidi, sensilli di Newstead, dentelli verticali presenti sul cibario e denti faringei. La visibilità di queste strutture dipende direttamente dalle proprietà ottiche del mezzo di montaggio.
- **Conservazione**. Per i campioni tipo o i materiali destinati a collezioni permanenti, il mezzo deve offrire stabilità e durata nel lungo periodo. Al contrario, per studi di inventario o indagini epidemiologiche, dove la conservazione a lungo termine è meno critica, possono essere sufficienti mezzi di montaggio temporanei o semi-permanenti.

5.3.2. Requisiti per i mezzi di montaggio

Gli specialisti sviluppano spesso tecniche di montaggio personalizzate e complesse, adattate a specifiche esigenze di ricerca. Tuttavia, questi metodi spesso trascurano aspetti quali l'idoneità all'archiviazione, la compatibilità, la standardizzazione o la facilità di manipolazione e conservazione a lungo termine. Questa mancanza di standardizzazione complica l'integrazione delle collezioni donate e gli sforzi di cura a lungo termine. Le applicazioni scientifiche impongono requisiti distinti per i mezzi di montaggio. I tassonomisti spesso montano interi esemplari e preferiscono mezzi che macerano delicatamente gli organi interni per migliorare la visibilità delle strutture cuticolari. L'indice di rifrazione dovrebbe differire sufficientemente da quello del campione e del vetrino per massimizzare la chiarezza ottica. I mezzi di montaggio commerciali sono solitamente formulati con un indice di rifrazione vicino a quello del vetro per ridurre al minimo la rifrazione e la dispersione della luce attraverso il sistema vetrino-mezzo di montaggio-coprioggetto. Tuttavia, nella microscopia a campo chiaro, il contrasto naturale di un campione non colorato può essere manipolato scegliendo deliberatamente un mezzo di montaggio con un indice di rifrazione leggermente diverso da quello del campione, migliorandone così la visibilità rispetto allo sfondo.

5.3.3. Tipi di mezzi di montaggio (Tabelle 3 & 4)

La microscopia richiede che l'indice di rifrazione (IR) del mezzo di montaggio determini come la luce si piega attraverso il vetrino, il mezzo e il campione. Quando l'IR è strettamente allineato con il vetro del coprioggetto (≈1.515), la luce passa uniformemente, diminuendo la dispersione e le distorsioni ottiche, ottenendo una migliore risoluzione e visibilità delle strutture fini. Al contrario, una mancata corrispondenza dell'IR può causare sfocature, aloni o oscurare caratteristiche non colorate. La selezione del giusto mezzo di montaggio è cruciale per ottimizzare contrasto, chiarezza e qualità complessiva dell'immagine per un dato campione, a causa dei distinti IR dei diversi mezzi. L'indice di rifrazione del mezzo di montaggio ha un impatto significativo sulla visibilità delle strutture fini durante la preparazione dei flebotomi per il montaggio su vetrino. Le caratteristiche delicate e leggermente sclerotizzate dei flebotomi, tra cui l'armatura del cibario, le spermateche, i segmenti antennali e la venatura alare, possono essere difficili da osservare in un mezzo di montaggio ad alto indice di rifrazione. Per i flebotomi, le opzioni comunemente utilizzate includono mezzi gomma-cloralio come mezzi di montaggio a base acquosa, e il balsamo del Canada e la resina Enecê - Nelson Cerqueira (NC) come mezzi a base di solvente. Rawlins [60] ha classificato i mezzi di montaggio in due tipi: (1) mezzi permanenti: induriscono nel tempo e sono adatti per la conservazione a lungo termine, e (2) mezzi semi-permanenti: non induriscono completamente e sono tipicamente utilizzati per scopi temporanei.

Tabella 3: Composizione dei mezzi di montaggio selezionati.

Mezzo di montaggio	Solvente	Possibili pre-polimeri o polimeri	Note
Hoyer = gomma al cloralio	glicerolo, acqua	Composti della gomma arabica	Agente macerante: idrato di cloralio
CMCP-9 (= Carbossi-metil-cellulosa fenolo)	acqua (CMCP-9: 51−60%)	Alcol polivinilico completamente idrolizzato (CMCP-9: 0−5%)	CMC(P)-9: bassa viscosità: elevata viscosità
DMHF (Dimetilidantoin a formaldeide)	acqua	N,N′-dimetilol dimetil idantoina (dimetilol DMH)<br>Oligomeri a ponte etereo/metilenico Rete polimerica crosslinkata di DMH-formaldeide	

Balsamo del Canada	xilene; componenti parzialmente volatili del balsamo (Δ^3-carene, acid levopimarico, limonene, mircene, acido palustrico, β-fellandrene, α-pinene, β-pinene)	balsamo (abienolo, acido abietico, acido isopimarico, acido sandaracopimarico)	neutralizzazione: carbonato di potassio;<br>resina di *Abies balsamea* (Linné, 1758)
Euparal®	eucaliptolo, paraldeide; componenti parzialmente volatili della gomma sandracca (limonene, α-pinene, β-pinene)	componenti della gomma sandracca (acido communico, manoolo, acido policomunico, acido sandaracopimarico, acido 12-acetossi-sandaracopimarico, sugiolo, acido toruloso, torulosolo, totarolo)	agente schiarente: salicilato di metile; colorante in Euparal® verde: sale di rame (abietinato di rame); resina di sandaracca: da Tetraclinis articulata (Vahl, 1791)
Enecê	alcol etilico; con canfora, eucaliptolo ed essenza di trementina.	Composti di gomma copale e colofonia (rosina)	

I mezzi di montaggio possono essere liquidi, a base di gomma o resinosi, solubili in acqua, alcol o altri solventi (ad esempio, toluene, xilene) (Tabella 3). Una volta applicati, devono essere sigillati dagli effetti atmosferici utilizzando mezzi di sigillatura insolubili. Per distinguere chiaramente i tipi di mezzi di montaggio, si può utilizzare la seguente categorizzazione:

a. Mezzi acquosi. Questi mezzi si sciolgono facilmente in acqua, rendendoli adatti per montaggi temporanei o semipermanenti. Sono tipicamente facili da maneggiare, ma possono richiedere la sigillatura per prevenire l'esposizione all'umidità atmosferica (vale a dire, mezzi a base di gomma-clorale e polivinilalcool), specialmente nei climi tropicali umidi.

b. Mezzi a limitata tolleranza all'acqua. Questi mezzi sono meno influenzati dall'acqua, ma richiedono comunque protezione contro l'umidità eccessiva. Offrono una maggiore stabilità a lungo termine rispetto alle opzioni idrosolubili e sono frequentemente utilizzati in montaggi semipermanenti.

c. Mezzi solubili in idrocarburi. Questi mezzi sono sciolti in solventi organici come xilene o toluene, o essenze (solvente enecê). Sono progettati per il montaggio permanente e offrono un'eccellente stabilità a lungo termine, resistono all'umidità e al degrado, rendendoli ideali da utilizzare per l'archiviazione (vale a dire, balsamo del Canada neutro).

In sintesi, i mezzi idrosolubili sono ideali per montaggi temporanei o casi che richiedono una facile rimozione del campione; i mezzi a limitata tolleranza all'acqua sono adatti per montaggi semipermanenti che richiedono una durata moderata, mentre i mezzi solubili in idrocarburi sono preferiti per montaggi permanenti destinati all'archiviazione e alla conservazione a lungo termine.

Tabella 4: Vantaggi e svantaggi di specifici mezzi di montaggio per vetrini da microscopio e osservazioni non pubblicate da parte di varie persone [52].

nome	Vantaggi	Svantaggi
* balsamo del Canada	Il supporto è altamente durevole, con una durata superiore a 150 anni. I vetrini possono essere montati utilizzando olio di garofano o fenolo come agenti di montaggio.	Contiene componenti nocivi e deve essere manipolato sotto cappa.<br>Richiede una disidratazione completa e dispendiosa in termini di tempo.<br>La disidratazione in etanolo e il trasferimento tramite xilene o olio di garofano possono rendere alcuni taxa fragili; alternative (es. isopropanolo, n-butanolo, Cellosolve™, 1,4-diossano, Histoclear, terpineolo) possono ridurre la rottura.<br>I campioni possono annerirsi se lo xilene viene sostituito con fenolo o se residua idrossido di potassio.

		Indici di rifrazione elevati possono oscurare le strutture non colorate.<br><br>L'essiccazione completa può richiedere anni senza l'uso di una piastra riscaldante.<br>Il mezzo ingiallisce e scurisce nel tempo, specialmente quando chiarificato con olio di garofano.<br>Alcune colorazioni si indeboliscono e i coloranti cationici possono sbiadire se il mezzo diventa acido, il che può accadere spontaneamente nel tempo.
DMHF (dimetilidantoina formaldeide)	Fornisce eccellente resistenza alle pieghe e alle grinze, con prestazioni durature dopo il lavaggio.<br>Altamente reattivo, cura rapidamente a temperature moderate.<br>Buona ritenzione della resistenza del tessuto rispetto alle vecchie resine a base di formaldeide.<br>Idrosolubile e facile da formulare con i sistemi di finissaggio tessile standard.<br>Conveniente con prestazioni industriali costanti.<br>Versatile per cotone, viscosa, lino e miscele.<br>Disponibile in gradi a basso contenuto di formaldeide libera per conformarsi a molte normative.	Rilascia formaldeide → preoccupazioni per la salute, la sicurezza e la regolamentazione.<br>Affronta limitazioni normative e di etichettatura ecologica (eco-label).<br>Necessita di catalizzatori acidi, rischiando di danneggiare il tessuto e causare corrosione.<br>Può causare ingiallimento sui tessuti chiari.<br>Può ridurre la morbidezza del tessuto.<br>Richiede un rigoroso controllo del processo.<br>Non idoneo dove è richiesta l'assenza totale di formaldeide.
* Euparal (trasparente)	Mezzo durevole con una durata superiore a 50 anni.<br>Il montaggio direttamente da etanolo all'80% è possibile (raccomandazione del produttore).<br>Non maschera le strutture non colorate, non ingiallisce e non diventa fragile nel tempo.<br>Possiede un indice di rifrazione più adatto rispetto al balsamo del Canada per i Ditteri.<br>Funziona bene per esemplari più spessi grazie al minimo ritiro e all'asciugatura senza bolle.<br>Rimane solubile in etanolo al 95%, consentendo il rimontaggio anche dopo molti anni.	Contiene componenti nocivi e deve essere maneggiato sotto cappa aspirante.<br>La disidratazione con etanolo e il trasferimento tramite Essenza di Euparal possono rendere fragili alcuni taxa, ma l'uso di isopropanolo può ridurre questo problema.
Liquido di Hoyer	I campioni possono essere montati vivi o direttamente da acqua, etanolo o formaldeide.<br>La macerazione produce un'eccellente qualità della cuticola.<br>Possiede un indice di rifrazione favorevole e può essere potenziato con colorazione allo iodio per un contrasto maggiore.<br>L'acido acetico presente nella formula può espandere le appendici degli artropodi.<br>Alcuni campioni possono rimanere stabili per 40–60 anni.<br>Idrosolubile, consente una facile rimontatura.	I campioni di piante delicate possono collassare a meno che il mezzo non venga aggiunto gradualmente, il che richiede tempo.<br>Cavità e cristalli possono formarsi in meno di 10 anni.<br>La macerazione può diventare eccessiva a seconda della concentrazione di idrato di clorale e del tempo di esposizione.<br>I componenti del mezzo possono separarsi e una fine granulazione può apparire nel giro di mesi o anni.<br><br>È stato segnalato l'annerimento dei mezzi.
CMCP-9 (Carbossimetilcellulosa fenolo)	I campioni possono essere montati direttamente da terreni come acqua, etanolo, glicerolo o soluzioni contenenti formaldeide, e i loro organi interni possono essere macerati quando necessario per facilitare l'esame generale o la preparazione.	Questo mezzo può sviluppare cristalli e scurirsi nel tempo, e a volte può macerare i campioni più del previsto. A meno che il vetrino non venga sigillato attentamente, i campioni più spessi non si conservano bene perché possono restringersi e creare spazi vuoti lungo i bordi del coprioggetto.<br>Non è adatto per campioni colorati o materiali

		calcificati e il suo tempo di asciugatura è più lento rispetto al CMC.
Eukitt™	Mezzo durevole con una durata superiore a 30 anni. Compatibile con molti solventi per il montaggio, inclusi acetone, benzene, cloroformio, diossano, etere, isopropanolo, benzoato di metile, terpineolo, toluene e xilene.<br>Asciuga rapidamente e ha un pH leggermente acido.<br>Non scurisce notevolmente con il passare del tempo.<br>Adatto per varie colorazioni (es. fucsina, ematossilina, verde di metile, violetto di metile, blu di metilene).<br>I campioni possono essere rimontati dopo anni immergendoli in xilene per un periodo prolungato.	Contiene componenti nocivi e deve essere manipolato sotto cappa.<br>Richiede una serie di disidratazione completa e dispendiosa in termini di tempo.<br>Non ideale per campioni più spessi a causa del restringimento e della formazione di bolle di gas.<br>I coprioggetti possono staccarsi nel tempo a meno che il vetro non venga pulito bene e sigillato.<br>Può mostrare una polimerizzazione incompleta attorno alle fibre di collagene.
Enecê	Un mezzo altamente durevole, con una durata di almeno 50 anni. Non scurisce nel tempo. È più malleabile, consentendo la dissezione di insetti all'interno del mezzo, oltre a offrire un tempo ragionevole per il posizionamento delle strutture morfologiche. Basso costo.	Richiede una serie di disidratazione completa e dispendiosa in termini di tempo. La disidratazione in etanolo e il trasferimento tramite olio di chiodi di garofano possono rendere alcuni esemplari fragili. L'insetto continua a chiarificarsi, sebbene molto lentamente; questo può rendere difficile osservare strutture molto piccole, come sensilli, ascoidi e setole semplici.

5.3.4. Descrizione dei terreni di montaggio raccomandati (Tabelle 3 & 4)

Terreni per osservazione temporanea

Gomma all'idrato di clorale = fluido/mezzo/soluzione di Hoyer (IR = 1.48).

Il fluido di Marc André è il mezzo migliore per l'osservazione a breve termine (alcune ore, forse qualche ora in più se il vetrino è conservato in camera umida) delle spermateche, inclusa la realizzazione di fotografie (Figura 4) o disegni. La conservazione delle spermateche osservate richiede il loro rimontaggio in un mezzo acquoso che consenta una conservazione a medio termine. La disidratazione per il rimontaggio in resina non è impossibile ma sconsigliata (rischio di perdita). La gomma all'idrato di clorale e il fluido di Hoyer sono considerati sinonimi. Questo mezzo è comunemente utilizzato per l'osservazione degli organi interni grazie alla sua compatibilità con l'acqua, alla semplicità, alla rapida applicazione e all'indice di rifrazione che facilita l'esame di strutture delicate come le spermateche. Tuttavia, la gomma all'idrato di clorale presenta notevoli inconvenienti se non perfettamente preparata o conservata in condizioni di umidità controllata. Tali problemi includono cristallizzazione, scolorimento e perdita di viscosità. Sigillare il vetrino coprioggetto non risolve questi problemi, poiché i terreni di montaggio possono scurirsi notevolmente (a volte diventando quasi neri) a causa dell'interazione con il mezzo di sigillatura, in particolare se si utilizza Euparal®.

Il mezzo di Hoyer è considerato otticamente il migliore per i flebotomi ed è stato tradizionalmente utilizzato per questi scopi. Il mezzo è costituito da diverse formulazioni strettamente correlate, tra cui gomma arabica, glicerolo e idrato di clorale. Varie formulazioni sono state interpretate male e citate erroneamente [74].

Sebbene Hoyer sia un buon mezzo per osservare le spermateche nei flebotomi, non è adatto alla conservazione a lungo termine. È ideale per osservazioni a breve termine, inclusi fotografie, disegni o immagini. I mezzi acquosi sono adatti per montaggi temporanei ma non possono garantire la conservazione a lungo termine. Al contrario, il montaggio in resina offre un'eccellente durata che spesso per secoli, ma può oscurare i dettagli fini delle spermateche, poiché la loro rifrangenza viene spesso persa.

Il mezzo di Hoyer si degrada nel tempo a causa della disidratazione (Figura 8), con conseguente formazione di piccoli cristalli di idrato di clorale bianchi e opachi. Tuttavia, gli esemplari possono essere recuperati da vetrini cristallizzati poiché la cuticola rimane chimicamente intatta, sebbene possa verificarsi qualche danno fisico a causa della crescita dei cristalli. In alcuni casi, i vetrini cristallizzati possono essere ripristinati reidratando il mezzo di montaggio in un ambiente caldo e umido con timolo per prevenire la crescita fungina. In alternativa, gli esemplari possono essere estratti dalla gomma al clorale in acqua, disidratati in acido acetico glaciale e rimontati in balsamo del Canada.

DMHF (dimetil idantoina formaldeide) (IR 1.48)

Questo mezzo a base acquosa [72] funziona molto bene otticamente, proprio come il Berlese, ed è facile da usare quanto quest'ultimo. Tuttavia, a differenza del Berlese, non

diventa nero né cristallizza. Funziona bene per i flebotomi e altri Psychodidae.

Figura 8: Rimontaggio del vetrino. A: vetrini danneggiati ed essiccati montati in Hoyer; B: vista microscopica di un flebotomo essiccato; C: vista microscopica di un altro flebotomo danneggiato; D: camera umida contenente un vetrino essiccato; E: testa, e F: corpo dell'esemplare B dopo il suo rimontaggio in Euparal®; G: testa, e H: corpo dell'esemplare danneggiato C dopo il suo rimontaggio in Euparal®.

CMCP (canfora-mono-clorofenolo) (IR = 1.41) Questo è un mezzo di montaggio solubile in acqua a base di glicerina utilizzato per creare vetrini trasparenti e permanenti di esemplari delicati, inclusi i flebotomi. Il vantaggio di questo mezzo di montaggio è che gli esemplari possono essere montati direttamente dall'acqua o dall'etanolo. Ammorbidicse la cuticola e schiarisce rapidamente il flebotomo, consentendo un corretto posizionamento dell'esemplare, il che è particolarmente utile per distendere le ali o sezionare i genitali. Sebbene sia riportato che consenta la conservazione a lungo termine, l'esatta durata della conservazione rimane incerta. Il limite principale di questo mezzo di montaggio risiede nella sua composizione che contiene fenolo, una sostanza tossica e irritante che richiede una manipolazione attenta.

Terreni per montaggio permanente

Balsamo del Canada (IR = 1,52-1,54)

Il balsamo del Canada è stato descritto per la prima volta come mezzo di montaggio adatto per la microscopia a luce trasmessa da Andrew Pritchard negli anni '30 dell' Ottocento. Rimane uno dei mezzi più ampiamente utilizzati grazie alla sua comprovata affidabilità nella conservazione a lungo termine, con oltre 150 anni di applicazione di successo. A differenza dei terreni a base di liquido di Hoyer, il balsamo del Canada non cristallizza né assorbe umidità. Tuttavia, il balsamo del Canada è fortemente autofluorescente, il che a volte può rappresentare uno svantaggio per determinate tecniche di microscopia [60]. L'uso di solventi non tossici al posto dello xilene può ridurre i rischi per la sicurezza durante la preparazione, ma può anche introdurre inconvenienti quali un essiccamento più lento e un imbrunimento precoce del terreno.

Euparal® (IR = 1,48)

L'Euparal® è un'alternativa ampiamente utilizzata al balsamo del Canada per il montaggio permanente, offrendo un'eccellente stabilità a lungo termine e un indice di rifrazione paragonabile. L'Euparal® presenta le seguenti caratteristiche: (1) requisito di disidratazione: prima del trasferimento finale nel mezzo di montaggio, il campione deve essere disidratato, tipicamente passando dall'etanolo al 95% a quello assoluto, e (2) tempo di preparazione elevato: l'assemblaggio finale in resina, sia essa balsamo del Canada o Euparal®, richiede la disidratazione, il che allunga il tempo complessivo di preparazione del campione. Quando la disidratazione con solventi organici non è praticabile, i campioni estratti dall'etanolo assoluto possono essere posti in una soluzione intermedia costituita da una miscela in parti uguali di Euparal® ed essenza di Euparal, prima del montaggio finale.

Enecê (IR = 1,467)

L'Enecê è un terreno di montaggio a base di resina utilizzato principalmente per piccoli insetti ed è particolarmente popolare in Brasile. La sua base è costituita da colofonia e gomma coppale sciolte in alcol, canfora, essenza di trementina ed eucaliptolo. Cerqueira [11] ha descritto l'Enecê come un'alternativa al balsamo del Canada per il montaggio di vetrini permanenti di larve, esuvie di stadi immaturi e persino zanzare adulte, e da allora è stato ampiamente adottato per il montaggio dei flebotomi. L'Enecê offre un'alternativa economica per il montaggio permanente, garantendo stabilità a lungo termine e un tempo di essiccamento sufficiente, consentendo la dissezione e l'accurata disposizione delle strutture morfologiche.

5.4. Preparazione e asciugatura dei vetrini

L'asciugatura corretta dei vetrini montati è fondamentale per garantirne la stabilità e la conservazione a lungo termine. I vetrini devono essere asciugati accuratamente prima di prenderne in considerazione la conservazione a lungo termine. Per risultati ottimali, i vetrini montati con mezzo di montaggio permanente devono essere asciugati in orizzontale per 2-3 settimane, mentre per quelli preparati con mezzo semi-permanente possono bastare 1-2 settimane. Per garantire un processo di asciugatura efficace, si raccomanda di utilizzare un incubatore impostato ad una temperatura adeguata per il mezzo di montaggio in uso, evitando un calore eccessivo che potrebbe danneggiare i campioni. Si raccomanda un intervallo di temperatura compreso tra 30°C e 37°C. Questa fase di asciugatura è cruciale per prevenire l'imbarcamento del vetrino, il deterioramento del campione o l'instabilità del mezzo di montaggio durante la conservazione.

Il mezzo di montaggio utilizzato nella preparazione del vetrino deve essere sempre indicato sull'etichetta del vetrino stesso. Se possibile, l'etichetta dovrebbe includere anche il protocollo specifico utilizzato, insieme al nome del preparatore e alla data di preparazione. I vetrini sono inizialmente preparati come montaggi temporanei e non sono destinati alla conservazione a lungo termine. Tuttavia, se lo status del campione cambia, ad esempio se viene designato come parte di una serie "tipo", è necessario utilizzare un mezzo di montaggio permanente per garantire la conservazione del campione per futuri studi tassonomici.

5.5. Tecniche di montaggio alternative: montaggio su cartellino

Il montaggio su cartellino è una tecnica utilizzata per diversi gruppi di insetti in cui i campioni possono essere spillati direttamente su cartellini entomologici o incollati sulla superficie. Date le loro piccole dimensioni e la necessità di osservare gli organi interni per l'identificazione

attraverso la chiarificazione (vedi punto 5), questo metodo non è affatto adatto per il montaggio dei flebotomi.

5.6. Rimontaggio di campioni danneggiati

Per i campioni rari o preziosi, si raccomanda un approccio in due fasi secondo il video accessibile su: https://zenodo.org/records/18315029. 1) Reidratarli senza smontarli per consentire un'osservazione preliminare. Un supporto per diversi vetrini da microscopio deve essere posizionato in una capsula di Petri per fungere da sostegno. Il vetrino da reidratare viene quindi posizionato sopra e la capsula di Petri viene riempita con pochi millimetri di solvente per creare una camera umida, assicurandosi che il vetrino stesso non entri in contatto con il solvente (Figura 8 D). Il tempo necessario per la reidratazione può variare da uno a diversi giorni, a seconda delle condizioni del campione. Il monitoraggio quotidiano e la pazienza sono essenziali. Una volta che il vetrino è sufficientemente reidratato, può essere rimosso dalla camera umida e posto in un incubatore per poche ore prima dell'esame microscopico, della fotografia o del disegno. 2) Per rimontare, il vetrino può essere riportato nella camera umida per alcune ore aggiuntive o per tutta la notte. Lo smontaggio deve essere eseguito sotto un microscopio binoculare. Utilizzando aghi sottili, il coprioggetto deve essere rimosso con cura, assicurandosi che nessun elemento del flebotomo rimanga attaccato (https://zenodo.org/records/18315029). Successivamente, gli elementi sezionati del flebotomo devono essere raccolti e risciacquati con acqua in pozzetti piccoli, come quelli usati per l'estrazione distruttiva di DNA/RNA (vedi sotto), prima della disidratazione e del rimontaggio in un mezzo resinoso. Quando si smonta un vetrino, è fondamentale identificare il mezzo di montaggio originale per selezionare un solvente appropriato. Per i mezzi di montaggio acquosi, si deve usare acqua. Se il mezzo di montaggio è a base di resina (es. balsamo del Canada o Euparal®), si deve usare xilene, sotto cappa aspirante e con adeguati dispositivi di protezione individuale, inclusa una maschera.

Il rimontaggio di campioni tipo o di collezione deve essere eseguito solo con il consenso del curatore e/o dell'istituzione proprietaria del campione.

6. Identificazione degli esemplari

6.1. Morfologia

L'identificazione dei flebotomi si basa principalmente sull'esame delle loro caratteristiche morfologiche, tra cui la forma del torace, le ali, i genitali, le setole e specifici rapporti morfometrici tra varie strutture. I ricercatori utilizzano chiavi tassonomiche, collezioni di riferimento e descrizioni originali delle specie per confrontare gli esemplari raccolti con i taxa noti. Caratteristiche diagnostiche chiave, come la venatura alare e la morfologia del capo in entrambi i sessi, la struttura dei genitali maschili e la configurazione delle spermateche femminili, sono particolarmente informative per la determinazione della specie. L'identificazione accurata richiede spesso un esame microscopico dettagliato, tipicamente utilizzando un microscopio composto per osservare strutture fini come i genitali e le spermateche, o uno stereomicroscopio per caratteristiche morfologiche più ampie.

I recenti progressi nella tecnologia di imaging hanno facilitato l'uso dell'imaging digitale per l'identificazione dei flebotomi. Fotografie ad alta risoluzione o illustrazioni digitali di caratteristiche chiave possono essere confrontate con materiali di riferimento o analizzate utilizzando sistemi di identificazione assistiti da computer, migliorando sia l'accuratezza che l'accessibilità nella tassonomia morfologica.

6.2. Geometria alare

La geometria alare è una caratteristica chiave utilizzata nell'identificazione e nella classificazione delle diverse specie di flebotomi. Le ali dei flebotomi mostrano un motivo e una struttura unici, essendo tipicamente lunghe e strette con una venatura ben sviluppata (Figure 9 e 10). La disposizione delle vene forma un motivo distinto che può variare tra generi e specie, fornendo preziosi caratteri diagnostici per l'identificazione. Di conseguenza, lo studio della geometria alare fornisce preziose informazioni a fini tassonomici.

6.3. Morfometria geometrica alare

I ricercatori utilizzano varie tecniche, come la morfometria geometrica, per analizzare e confrontare la forma e le dimensioni delle ali tra diverse specie o popolazioni di flebotomi. Lo studio della geometria alare fornisce preziosi spunti sul comportamento, sulle preferenze di habitat e sulle capacità di volo.

Nell'approccio morfometrico geometrico, le ali vengono accuratamente disseccate, colorate (se necessario) e montate piatte su vetrini. I vetrini preparati vengono poi fotografati sotto uno stereomicroscopio, digitalizzati e sottoposti ad analisi morfometrica. Questa procedura è stata ben descritta in letteratura [6, 27, 42, 56, 57, 59], con la raccomandazione di utilizzare costantemente l'ala destra o sinistra per gli organi accoppiati, al fine di evitare potenziali effetti allometrici negativi [62].

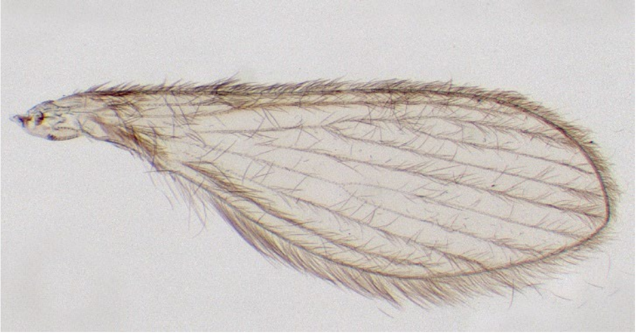

Figura 9: Ala grezza di *Trichophoromyia ininii*.

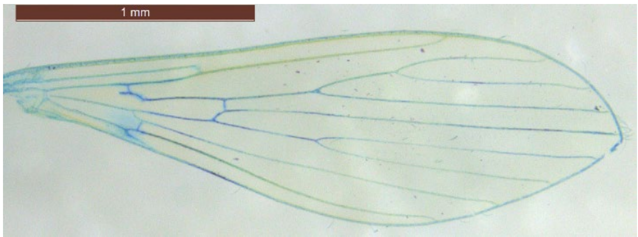

Figura 10: Ala colorata di *Phlebotomus ariasi*.

Preparazione dell'ala per l'analisi morfometrica geometrica

Per una visualizzazione ottimale delle venature alari, le ali devono essere pulite dalle scaglie e adeguatamente colorate. Per la preparazione dell'ala, innanzitutto riempire dei piccoli pozzetti con i reagenti necessari (blu di metilene, etanolo, acqua e sostituto dello xilene). Recuperare un'ala conservata in etanolo al 70% a temperatura ambiente invertendo la provetta Eppendorf e svuotandola sul pozzetto, quindi sollevare l'ala longitudinalmente utilizzando un ago sottile ricurvo. Passare l'ala brevemente dall'etanolo all'acqua e di nuovo all'etanolo per rimuovere le setole. Immergere l'ala nel blu di metilene per 6 minuti, assicurandosi che galleggi durante la colorazione. Recuperare l'ala con cura e immergerla nel sostituto dello xilene per 2 minuti (circa un terzo del tempo del blu di metilene). Piccoli colpi dell'ago contro le pareti del pozzetto possono aiutare l'ala a depositarsi; lo xilene serve a fissare la colorazione. Infine, sollevare l'ala e posizionarla su una piccola goccia di Euparal® su un vetrino microscopico. Sotto una lente d'ingrandimento, distendere delicatamente l'ala e posizionare con cura un coprioggetto. Le fotografie devono essere scattate tempestivamente prima che l'Euparal® si indurisca, poiché potrebbero essere necessari lievi aggiustamenti della posizione dell'ala sotto il coprioggetto per ottenere un allineamento ottimale.

6.4. Tecniche di biologia molecolare

Oltre alle tecniche morfologiche, i metodi molecolari sono sempre più essenziali nella ricerca entomologica, inclusi gli studi tassonomici, di genetica di popolazione e filogenetici, nonché per la rilevazione di patogeni (DNA/RNA) e per determinare l'origine del pasto di sangue, essendo il comportamento del vettore importante nel campo dell'epidemiologia [70]. Il sequenziamento del DNA può essere impiegato per la conferma della specie o per differenziare specie strettamente correlate, fornendo un mezzo di identificazione più accurato e affidabile. Inoltre, tecniche molecolari avanzate (cioè PCR, sequenziamento del DNA, NGS, ecc.) ed il MALDI-ToF MS stanno guadagnando importanza per un'identificazione rapida e accurata delle specie, completando i metodi morfologici tradizionali [46]. Nonostante questi progressi, l'identificazione morfologica rimane lo standard di riferimento per la tassonomia e la base su cui vengono interpretati i dati molecolari.

6.4.1. Estrazione distruttiva di acidi nucleici

L'estrazione di acidi nucleici è una fase di routine in molti studi biologici e sono stati sviluppati vari metodi per isolare il DNA da materiali biologici [48]. Molti kit di estrazione del DNA disponibili in commercio sono progettati per facilitare questo processo [14]. Tuttavia, i metodi comunemente usati per preparare i campioni di artropodi per l'identificazione morfologica spesso ostacolano l'analisi del DNA, poiché tali tecniche possono danneggiare o distruggere le caratteristiche fisiche critiche del campione [10]. La maggior parte dei protocolli di estrazione del DNA per i tessuti di insetti sono di natura distruttiva [43], sollevando particolari preoccupazioni per i piccoli campioni, dove anche un campionamento limitato può compromettere importanti caratteristiche morfologiche [72]. Il tipo e le condizioni del campione svolgono un ruolo chiave nella scelta di un metodo di estrazione del DNA appropriato [29].

La necessità di un'accurata identificazione dei flebotomi, di comprendere le dinamiche di popolazione e di minimizzare gli impatti non target ha guidato lo sviluppo di strumenti diagnostici molecolari [23]. Gli approcci molecolari sono ora frequentemente utilizzati per integrare i metodi tassonomici morfologici per l'identificazione dei flebotomi. Ad esempio, l'approccio standard per il barcoding degli insetti comporta l'estrazione del DNA, il sequenziamento e la perdita del campione originale. Pertanto, vi è un urgente bisogno di esplorare metodi di estrazione del DNA non distruttivi che preservino sia il materiale biologico che la sua integrità morfologica.

Numerosi metodi di estrazione di acidi nucleici sono stati applicati ai flebotomi. La quantità o la qualità degli acidi nucleici richiesti dipendono dall'analisi molecolare a valle, poiché diverse tecniche hanno vari requisiti di sensibilità e purezza [9]. Ad esempio, è stato riscontrato che gli occhi dei flebotomi inibiscono l'amplificazione PCR [69]. Oltre allo screening dei patogeni, il DNA dei flebotomi viene estratto di routine per scopi di identificazione delle specie. Possono essere utilizzati vari metodi di estrazione, sebbene le rese e la qualità differiscano tra le tecniche. Alcuni protocolli dei produttori sono stati adattati dai ricercatori per i flebotomi [8], aumentando la resa e/o la qualità degli acidi nucleici estratti [8, 9, 69], mentre altri adattamenti, sviluppati per altri taxa di artropodi, possono essere utilizzati anche sui flebotomi [58, 76]. Le PCR di identificazione che mirano a piccoli frammenti mitocondriali (COI o CytB) sono generalmente compatibili con metodi di estrazione che comportano un'elevata frammentazione del DNA. Al contrario, altre tecniche NGS a lettura lunga (Oxford Nanopore e PacBio) richiedono una frammentazione minima e DNA di alta qualità. Le estrazioni su colonna a centrifuga generalmente producono frammenti di DNA genomico fino a 60 kb, mentre l'estrazione con fenolo-cloroformio può produrre frammenti fino a 150 kb [77]. La Tabella 5 riassume le diverse tecniche

di estrazione del DNA di flebotomi e indica se sono stati apportati adattamenti metodologici per questi insetti. Le rese non sono mostrate, poiché dipendono dalle dimensioni del campione e dal metodo di preparazione. La colonna relativa alle modifiche si riferisce agli adattamenti dei protocolli di estrazione per flebotomi o altri piccoli artropodi. La scelta del metodo di estrazione dovrebbe considerare diversi criteri, come il numero di campioni, il tempo di estrazione e la tecnica utilizzata a valle. Sebbene le tecniche NGS richiedano DNA genomico ad alto peso molecolare, tutti i metodi qui presentati possono essere utilizzati per applicazioni standard basate sulla PCR. Inoltre, diversi studi hanno esplorato metodi di estrazione del DNA non distruttivi per piccoli artropodi terrestri, campioni museali conservati a secco e artropodi a corpo molle [19, 26, 28, 55, 63].

6.4.2. Estrazione non distruttiva di acidi nucleici

Una delle principali sfide nell'analisi molecolare degli artropodi, in particolare dei flebotomi, è la conservazione dei campioni per l'integrazione nelle collezioni entomologiche. La maggior parte dei protocolli di estrazione del DNA richiede la macerazione del tessuto, compromettendo così la conservazione del campione originale. I metodi di estrazione non distruttiva degli acidi nucleici, tuttavia, sono progettati per estrarre materiale genetico senza danneggiare fisicamente il campione, influenzarne la stabilità o alterarne la morfologia. Questi metodi sono particolarmente necessari quando si lavora con campioni preziosi o limitati, come i flebotomi, dove il mantenimento dell'integrità strutturale è essenziale per la tassonomia futura, per scopi morfologici o diagnostici. Una tecnica comunemente impiegata è il metodo di "bagno" non distruttivo, in cui i flebotomi vengono immobilizzati e delicatamente immersi in un tampone di lisi contenente proteinasi K. La tecnica di vectolisi lieve è stata applicata con successo ai flebotomi, in particolare ai campioni tipo [24]. La tecnica fa uso di un kit convenzionale su colonna a centrifuga (in questo caso, un kit DNeasy Blood and Tissue, QIAGEN, Hilden, Germania) con adattamento per ottenere DNA senza distruggere il campione. Le fasi di lisi modificate (volume del tampone di lisi e aggiunta di una fase di congelamento) [17] consentono il rilascio di acidi nucleici, riducendo al minimo i danni morfologici [24]. Per quanto riguarda i flebotomi, è anche possibile utilizzare un kit di estrazione del DNA HotSHOT (Bento Bioworks Ltd, Londra, Regno Unito) [73] che è rapido ed economico, consentendo un'elaborazione veloce e a basso costo dei campioni. I campioni entomologici destinati all'identificazione morfologica possono quindi essere risciacquati. Quelli trattati con un kit DNeasy Blood and Tissue devono essere chiarificati con la soluzione di Marc-André, mentre quelli trattati con un kit di estrazione del DNA HotSHOT sono sufficientemente chiarificati per essere montati in un mezzo acquoso o, meglio, in una resina dopo la disidratazione, secondo il protocollo dettagliato in questo articolo [73]. Il materiale genetico estratto può quindi essere ulteriormente elaborato per analisi a valle, come la PCR, per amplificare specifici marcatori genetici. I metodi di estrazione non distruttiva degli acidi nucleici sono cruciali per studiare le caratteristiche genetiche dei flebotomi, inclusa l'identificazione di potenziali agenti patogeni che possono trasportare. Preservando l'integrità del campione, i ricercatori possono ottenere preziose informazioni genetiche, conservando al contempo il campione per ulteriori analisi o studi.

Tabella 5: Costo medio, applicazione e adattamento del protocollo per l'estrazione del gDNA di flebotomi (Phlebotominae)

**Protocollo**	**Costo**	**Applicazione**	**Adattamento del protocollo per piccoli artropodi**
Colonna rotante	2.5 – 3.55 US$ [39]	PCR, NGS	[9]
Fenolo-cloroformio	0.24 US$ [69]	PCR, NGS	[9]
HotSHOT	<0.01 US$ [69]	PCR	-
Salting out	0.12 $3 [69]	PCR	-
Chelex	0.02 $4 [41]	PCR	[41, 76]

6.5. MALDI-ToF MS

Il MALDI-ToF MS (spettrometria di massa a desorbimento/ionizzazione laser assistita da matrice con tempo di volo) è una tecnica basata sulla spettrometria di massa progettata per rilevare e analizzare i profili proteici unici ("impronte digitali") di campioni biologici. Il MALDI-ToF è sempre più riconosciuta come uno strumento importante per l'identificazione di artropodi di interesse medico e veterinario. Questa tecnica si è dimostrata efficace nell'identificare vari stadi di sviluppo dei flebotomi, comprese le forme immature e i pasti di sangue delle femmine replete, ed è stata applicata con successo per differenziare specie di flebotomi sia maschi che femmine in una gamma di condizioni di conservazione e omogeneizzazione [28, 30, 73, 74]. Questo metodo offre inoltre un elevato potere discriminante a livello di sottogeneri, specie e popolazioni. Questa tecnica consente ai ricercatori di ottenere un'identificazione rapida e accurata delle specie, fondamentale per comprendere la distribuzione, il comportamento ed il ruolo dei flebotomi nella trasmissione delle malattie. Differenziando le specie in base ai profili proteici, il MALDI-ToF svolge un ruolo cruciale negli studi epidemiologici e nelle strategie di controllo dei vettori. Attualmente esistono due principali

inconvenienti di questa tecnica che ne limitano l'applicazione routinaria. Il primo è la disponibilità delle apparecchiature di spettrometria di massa, che sono proibitivamente costose per essere acquisite esclusivamente ai fini dell'identificazione delle specie di flebotomi (o di artropodi vettori in generale). Fortunatamente, questa limitazione può essere superata ottenendo tempo macchina su spettrometri di massa che sono diventati uno strumento di ricerca standard nelle strutture proteomiche e/o nella diagnostica clinica. Il secondo è la scarsa rappresentazione dei dati di riferimento dei flebotomi disponibili nei database ad accesso aperto, con la conseguente necessità di costruire un database interno con spettri di riferimento basati su esemplari identificati in modo inequivocabile, idealmente mediante una combinazione di valutazione morfologica e sequenziamento di un marcatore genetico adatto (COI, cytB o altro). Questa limitazione sarà auspicabilmente risolta presto dalla graduale inclusione dei dati di riferimento interni dei flebotomi nella Piattaforma MSI gestita da Assistance Publique-Hôpitaux de Paris, Sorbonne University, Francia, e dalla collezione BCCM/IHEM/Sciensano a Bruxelles, Belgio (https://msi.happy-dev.fr/). Quando si pianifica l'impiego della profilazione proteica MALDI-ToF, i campioni dovrebbero essere conservati preferibilmente congelati a secco o in etanolo al 70% di grado molecolare e non esposti a temperature ambiente. In assenza di linee guida universali per la preparazione dei campioni, si consiglia agli utenti di utilizzare una soluzione acquosa di acido sinapinico (30 mg/mL) in acetonitrile al 60%/TFA allo 0,3% per la preparazione della matrice MALDI-ToF, al fine di rendere i loro spettri proteici comparabili con i dati sui flebotomi finora pubblicati.

Preparazione del campione per MALDI-ToF MS (Figura 7)

Gli esemplari di insetti, conservati in varie condizioni, vengono prima essiccati all'aria a temperatura ambiente e dissezionati. La testa e l'addome vengono rimossi per ottenere parti del corpo contenenti caratteristiche morfologiche chiave per il montaggio su vetrino e l'analisi morfologica. Il torace può essere utilizzato per il MALDI-ToF e l'addome rimanente conservato per l'estrazione del DNA. Per la profilazione proteica, il torace viene omogeneizzato manualmente in microtubi da 1,5 mL con 10 µL di soluzione di omogeneizzazione utilizzando pestelli e pellet monouso. Tipicamente si utilizzano due soluzioni di omogeneizzazione: acqua distillata sterile e acido formico al 25%.

7. Conclusione

In questo lavoro, miriamo a fornire ai ricercatori i metodi più efficaci per il montaggio dei flebotomi, adattati a specifici obiettivi di ricerca, per facilitare un'identificazione accurata ed il rilevamento di patogeni. Non esiste un unico metodo universalmente ottimale; esistono piuttosto diversi metodi, ognuno con i propri vantaggi e limitazioni. Nei dati di supporto, abbiamo fornito protocolli dettagliati per varie tecniche di montaggio utilizzate nella preparazione e identificazione dei flebotomi. Questi protocolli, inclusi video didattici, offrono procedure passo-passo adattate a diversi obiettivi, garantendo risultati precisi e affidabili. Offrendo questa risorsa completa, miriamo a supportare i ricercatori nella selezione e nell'applicazione delle tecniche di montaggio più appropriate per le loro esigenze specifiche.

Ringraziamenti

Gli autori ringraziano Richard Lane e Zoe Jay Adams del Natural History Museum di Londra, Regno Unito, per l'eccellente revisione, che ha aumentato la qualità di questo manoscritto.

Finanziamenti

Si ringraziano le agenzie di sviluppo brasiliane CNPq (numero pratica: 404395/2024-4) e la Fondazione Araucária (numero pratica: 433/2025 PDI) per il finanziamento della ricerca di AJA.

Conflitti di interesse

Jérôme Depaquit è redattore associato di Parasite; non ha avuto alcuna influenza sul processo di revisione e sulle decisioni relative a questo manoscritto. Gli altri autori dichiarano di non avere conflitti di interesse.

Dichiarazione sulla disponibilità dei dati

Video su Zenodo

Video 1: https://zenodo.org/records/18198006

Video 2: https://zenodo.org/records/18311158

Video 3: https://zenodo.org/records/18311106

Video 4: https://zenodo.org/records/18311154

Video 5: https://zenodo.org/records/18303014

Video 6: https://zenodo.org/records/18302850

Video 7: https://zenodo.org/records/18315029

Materiali aggiuntivi

I materiali aggiuntivi relativi a questo articolo sono disponibili all'indirizzo https://www.parasite-journal.org/10.1051/parasite/2026009/olm

Referenze

1. Alkan C, Allal-Ikhlef AB, Alwassouf S, Baklouti A, Piorkowski G, de Lamballerie X, Izri A, Charrel RN. 2015. Virus isolation, genetic characterization and seroprevalence of Toscana virus in Algeria. Clinical Microbiology and Infection, 21(11), 1040 e1-9.
2. Alten B, Ozbel Y, Ergunay K, Kasap OE, Cull B, Antoniou M, Velo E, Prudhomme J, Molina R, Banuls AL, Schaffner F, Hendrickx G, Van Bortel W, Medlock JM. 2015. Sampling

strategies for phlebotomine sand flies (Diptera: Psychodidae) in Europe. Bulletin of Entomological Research 105(6), 664–678.

3. Ayhan N, Baklouti A, Prudhomme J, Walder G, Amaro F, Alten B, Moutailler S, Ergunay K, Charrel RN, Huemer H. 2017. Practical guidelines for studies on sandfly-borne phleboviruses: Part I: Important points to consider *ante* field work. Vector-Borne and Zoonotic Diseases 17(1), 73–80.
4. Bates PA. 1997. Infection of phlebotomine sandflies with *Leishmania*, in The Molecular Biology of Insect Disease Vectors: A Methods Manual. Springer. p. 112–120.5.
5. Baum M, de Castro EA, Pinto MC, Goulart TM, Baura W, Klisiowicz Ddo R, Vieira da Costa-Ribeiro MC. 2015. Molecular detection of the blood meal source of sand flies (Diptera: Psychodidae) in a transmission area of American cutaneous leishmaniasis, Parana State, Brazil. Acta Tropica, 143, 8–12.
6. Belen A, Alten B, Aytekin A. 2004. Altitudinal variation in morphometric and molecular characteristics of *Phlebotomus papatasi* populations. Medical and Veterinary Entomology, 18(4), 343–350.
7. Bhattacharya J, Chandra G, Hati AK. 1991. A simple method for cryopreservation of *Leishmania donovani* promastigotes, Indian Journal of Medical Research, 93, 245–246.
8. Caligiuri LG, Sandoval AE, Miranda JC, Pessoa FA, Santini MS, Salomón OD, Secundino NF, McCarthy CB. 2019. Optimization of DNA extraction from individual sand flies for PCR amplification. Methods and Protocols, 2(2), 36.
9. Casaril AE, de Oliveira LP, Alonso DP, de Oliveira EF, Gomes Barrios SP, de Oliveira Moura Infran J, Fernandes WS, Oshiro ET, Ferreira AMT, Ribolla PEM, de Oliveira AG. 2017. Standardization of DNA extraction from sand flies: Application to genotyping by next generation sequencing. Experimental Parasitology, 177, 66–72.
10. Castalanelli MA, Severtson DL, Brumley CJ, Szito A, Foottit RG, Grimm M, Munyard K, Groth DM. 2010. A rapid non-destructive DNA extraction method for insects and other arthropods. Journal of Asia-Pacific Entomology, 13(3), 243–248.
11. Cerqueira NL. 1943. Um novo meio para montagem de pequenos insetos em lâmina. Memórias do Instituto Oswaldo Cruz, (39), 37–41.
12. Charrel RN, Gallian P, Navarro-Mari JM, Nicoletti L, Papa A, Sanchez-Seco MP, Tenorio A, de Lamballerie X. 2005. Emergence of Toscana virus in Europe. Emerging Infectious Diseases, 11(11), 1657–1663.
13. Chaskopoulou A, Giantsis IA, Demir S, Bon MC. 2016. Species composition, activity patterns and blood meal analysis of sand fly populations (Diptera: Psychodidae) in the metropolitan region of Thessaloniki, an endemic focus of canine leishmaniasis. Acta Tropica, 158, 170–176.
14. Chen H, Rangasamy M, Tan SY, Wang H, Siegfried BD. 2010. Evaluation of five methods for total DNA extraction from western corn rootworm beetles. PLoS One, 5(8), e11963.
15. Depaquit J, Grandadam M, Fouque F, Andry PE, Peyrefitte C. 2010. Arthropod-borne viruses transmitted by Phlebotomine sandflies in Europe: a review. Eurosurveillance, 15(10), 19507.
16. Diamond LS, Herman CM. 1954. Incidence of Trypanosomes in the Canada Goose as revealed by bone marrow culture. Journal of Parasitology, 40(2), 195–202.
17. Ding H, Torno M, Vongphayloth K, Ng G, Tan D, Sng W, Ho K, Randrianambinintsoa FJ, Depaquit J, Tan CH. 2025. Hidden in plain sight: discovery of sand flies in Singapore and description of four species new to science. Parasites & Vectors, 18(1), 402.
18. Es-Sette N, Ajaoud M, Bichaud L, Hamdi S, Mellouki F, Charrel RN, Lemrani M. 2014. *Phlebotomus sergenti* a common vector of *Leishmania tropica* and Toscana virus in Morocco. Journal of Vector Borne Diseases, 51(2), 86–90.
19. Favret C. 2005. A new non-destructive DNA extraction and specimen clearing technique for aphids (Hemiptera). Proceedings of the Entomological Society of Washington, 107(2), 469–470.
20. Galati EAB. 2018. Phlebotominae (Diptera, Psychodidae): Classification, morphology and terminology of adults and identification of American taxa, in Brazilian Sand Flies: Biology, Taxonomy, Medical Importance and Control, Rangel EF, Shaw JJ, Editors. Cham: Springer International Publishing. pp. 9–212.
21. Galati EAB, de Andrade AJ, Perveen F, Loyer M, Vongphayloth K, Randrianambinintsoa FJ, Prudhomme J, Rahola N, Akhoundi M, Shimabukuro PHF, Depaquit J. 2025. Phle-botomine sand flies (Diptera, Psychodidae) of the world. Parasites & Vectors, 18(1), 220.
22. Galati EAB, Galvis-Ovallos F, Lawyer P, Leger N, Depaquit J. 2017. An illustrated guide for characters and terminology used in descriptions of Phlebotominae (Diptera, Psychodidae). Par-asite, 24, 26.
23. Gariepy T, Kuhlmann U, Gillott C, Erlandson M. 2007. Parasitoids, predators and PCR: the use of diagnostic molecular markers in biological control of Arthropods. Journal of Applied Entomology, 131(4), 225–240.
24. Giantsis IA, Chaskopoulou A, Bon MC. 2016. Mild-Vectolysis: A nondestructive DNA extraction method for vouchering sand flies and mosquitoes. Journal of Medical Entomology, 53(3), 692–695.
25. Gidwani K, Picado A, Rijal S, Singh SP, Roy L, Volfova V, Andersen EW, Uranw S, Ostyn B, Sudarshan M, Chakravarty J, Volf P, Sundar S, Boelaert M, Rogers ME. 2011. Serological markers of sand fly exposure to evaluate insecticidal nets against visceral leishmaniasis in India and Nepal: a cluster-randomized trial. PLoS Neglected Tropical Diseases, 5(9), e1296.
26. Gilbert MTP, Moore W, Melchior L, Worobey M. 2007. DNA extraction from dry museum beetles without conferring external morphological damage. PLoS One, 2(3), e272.
27. Giordani BF, Andrade AJ, Galati EAB, Gurgel-Goncalves R. 2017. The role of wing geometric morphometrics in the identification of sandflies within the subgenus *Lutzomyia*. Medical and Veterinary Entomology, 31(4), 373–380.
28. Guzmán-Larralde AJ, Suaste-Dzul AP, Gallou A, Peña-Carrillo KI. 2017. DNA recovery from microhymenoptera using six non-destructive methodologies with considerations for subsequent preparation of museum slides. Genome, 60(1), 85–91.
29. Hajibabaei M, DeWaard JR, Ivanova NV, Ratnasingham S, Dooh RT, Kirk SL, Mackie PM, Hebert PD. 2005. Critical factors for assembling a high volume of DNA barcodes. Philosophical Transactions of the Royal Society B: Biological Sciences, 360(1462), 1959–1967.
30. Haouas N, Pesson B, Boudabous R, Dedet JP, Babba H, Ravel C. 2007. Development of a molecular tool for the identification of *Leishmania* reservoir hosts by blood meal analysis in the insect vectors. American Journal of Tropical Medicine and Hygiene, 77(6), 1054–1059.
31. Hlavackova K, Dvorak V, Chaskopoulou A, Volf P, Halada P. 2019. A novel MALDI-TOF MS-based method for blood meal identification in insect vectors: A proof of concept study on phlebotomine sand flies. PLoS Neglected Tropical Diseases, 13 (9), e0007669.

32. Huemer H, Prudhomme J, Amaro F, Baklouti A, Walder G, Alten B, Moutailler S, Ergunay K, Charrel RN, Ayhan N. 2017. Practical guidelines for studies on sandfly-borne phleboviruses: Part II: Important points to consider for fieldwork and subsequent virological screening. Vector-Borne and Zoonotic Diseases, 17(1), 81–90.
33. Jancarova M, Polanska N, Thiesson A, Arnaud F, Stejskalova M, Rehbergerova M, Kohl A, Viginier B, Volf P, Ratinier M. 2025. Susceptibility of diverse sand fly species to Toscana virus. PLoS Neglected Tropical Diseases, 19(5), e0013031.
34. Kapp JD, Green RE, Shapiro B. 2021. A fast and efficient single-stranded genomic library preparation method optimized for ancient DNA. Journal of Heredity, 112(3), 241–249.
35. Killick-Kendrick R, Maroli M, Killick-Kendrick M. 1991. Bibliography of the colonization of phlebotomine sandflies. Parassitologia, 33(suppl.), 321–333.
36. Lawyer P, Killick-Kendrick M, Rowland T, Rowton E, Volf P. 2017. Laboratory colonization and mass rearing of phlebotomine sand flies (Diptera, Psychodidae). Parasite, 24, 42.
37. Léger N, Pesson B, Madulo-Leblond G. 1986. Les phlébotomes de Grèce : 1ère partie. Bulletin de la Société de Pathologie Exotique, 79, 386–397.
38. Léger N, Pesson B, Madulo-Leblond G. 1986. Les phlébotomes de Grèce : 2ème partie. Bulletin de la Société de Pathologie Exotique, 79, 514–524.
39. Leonel JAF, Vioti G, Alves ML, da Silva DT, Meneghesso PA, Benassi JC, Spada JCP, Galvis-Ovallos F, Soares RM, Oliveira T. 2020. DNA extraction from individual Phlebotomine sand flies (Diptera: Psychodidae: Phlebotominae) specimens: Which is the method with better results? Experimental Parasitology, 218, 107981.
40. Lestinova T, Rohousova I, Sima M, de Oliveira CI, Volf P. 2017. Insights into the sand fly saliva: Blood-feeding and immune interactions between sand flies, hosts, and *Leishmania.* PLoS Neglected Tropical Diseases, 11(7), e0005600.
41. Lienhard A, Schaffer S. 2019. Extracting the invisible: obtaining high quality DNA is a challenging task in small arthropods. PeerJ, 7, e6753.
42. Lozano-Sardaneta YN, Mikery-Pacheco OF, Huerta H, Rojas-Soriano JE, Contreras-Ramos A. 2025. Wing geometric morphometrics is effective to separate sand fly species (Diptera, Psychodidae, Phlebotominae) related with leishmaniasis transmission in Mexico. Acta Tropica, 262, 107523.
43. Mandrioli M. 2008. Insect collections and DNA analyses: how to manage collections? Museum Management and Curatorship, 23(2), 193–199.
44. Maroli M, Feliciangeli MD, Bichaud L, Charrel RN, Gradoni L. 2013. Phlebotomine sandflies and the spreading of leishmaniases and other diseases of public health concern. Medical and Veterinary Entomology, 27(2), 123–147.
45. Marquina D, Buczek M, Ronquist F, Lukasik P. 2021. The effect of ethanol concentration on the morphological and molecular preservation of insects for biodiversity studies. PeerJ, 9, e10799.
46. Mathis A, Depaquit J, Dvorak V, Tuten H, Banuls AL, Halada P, Zapata S, Lehrter V, Hlavackova K, Prudhomme J, Volf P, Sereno D, Kaufmann C, Pfluger V, Schaffner F. 2015. Identification of phlebotomine sand flies using one MALDI-TOF MS reference database and two mass pectrometer systems., Parasites & Vectors, 8, 266.
47. Mekarnia N, Benallal KE, Sadlova J, Vojtkova B, Mauras A, Imbert N, Longhitano M, Harrat Z, Volf P, Loiseau PM, Cojean S. 2024. Effect of *Phlebotomus papatasi* on the fitness, infectivity and antimony-resistance phenotype of antimony-resistant *Leishmania* major Mon-25. International Journal for Parasitology – Drugs and Drug Resistance, 25, 100554.
48. Milligan BG. 1998. Total DNA isolation, in Molecular Genetic Analysis of Population: A Practical Approach, Hoelzel AR, Editor. Oxford: Oxford University Press.
49. Molina R, Jiménez M, Alvar J, González E, Hernández-Taberna S, Ines MM. 2017. Methods in sand fly research. Madrid: Servicio de publicaciones Universidad de Alcalá de Henares, Madrid.
50. Murphy WJ, Eizirik E, O'Brien SJ, Madsen O, Scally M, Douady CJ, Teeling E, Ryder OA, Stanhope MJ, de Jong WW, Springer MS. 2001. Resolution of the early placental mammal radiation using Bayesian phylogenetics. Science, 294(5550), 2348–2351.
51. Nacif-Pimenta R, Pinto LC, Volfova V, Volf P, Pimenta PFP, Secundino NFC. 2020. Conserved and distinct morphological aspects of the salivary glands of sand fly vectors of leishma-niasis: an anatomical and ultrastructural study. Parasites & Vectors, 13(1), 441.
52. Neuhaus B, Schmid T, Riedel J. 2017. Collection management and study of microscope slides: Storage, profiling, deterioration, restoration procedures, and general recommendations. Zootaxa, 4322(1), 1–173.
53. New TR. 1974. Pscoptera. Handbooks for Identification of British Insects (Vol. I). London: Royal Entomological Society of London. 102 pp.
54. Perez-Ruiz M, Collao X, Navarro-Mari JM, Tenorio A. 2007. Reverse transcription, real-time PCR assay for detection of Toscana virus. Journal of Clinical Virology, 39(4), 276–281.
55. Porco D, Rougerie R, Deharveng L, Hebert P. 2010. Coupling non-destructive DNA extraction and voucher retrieval for small soft-bodied Arthropods in a high-throughput context: the example of Collembola. Molecular Ecology Resources, 10(6), 942–945.
56. Prudhomme J, Cassan C, Hide M, Toty C, Rahola N, Vergnes B, Dujardin JP, Alten B, Sereno D, Banuls AL. 2016. Ecology and morphological variations in wings of *Phlebotomus ariasi* (Diptera: Psychodidae) in the region of Roquedur (Gard, France): a geometric morphometrics approach. Parasites & Vectors, 9(1), 578.
57. Prudhomme J, Gunay F, Rahola N, Ouanaimi F, Guernaoui S, Boumezzough A, Banuls AL, Sereno D, Alten B. 2012. Wing size and shape variation of *Phlebotomus papatasi* (Diptera: Psychodidae) populations from the south and north slopes of the Atlas Mountains in Morocco. Journal of Vector Ecology, 37(1), 137–147.
58. Prudhomme J, Toty C, Kasap OE, Rahola N, Vergnes B, Maia C, Campino L, Antoniou M, Jimenez M, Molina R, Cannet A, Alten B, Sereno D, Banuls AL. 2015. New microsatellite markers for multi-scale genetic studies on *Phlebotomus ariasi* Tonnoir, vector of *Leishmania infantum* in the Mediterranean area. Acta Tropica, 142, 79–85.
59. Prudhomme J, Velo E, Bino S, Kadriaj P, Mersini K, Gunay F, Alten B. 2019. Altitudinal variations in wing morphology of *Aedes albopictus* (Diptera, Culicidae) in Albania, the region where it was first recorded in Europe. Parasite, 26, 55.
60. Rawlins DJ. 1992. Light Microscopy: An Introduction to Biotechniques. Oxford: Bios Scientific publishers. 143 pp.
61. Ready PD. 2013. Biology of phlebotomine sand flies as vectors of disease agents. Annual Review of Entomology, 58, 227–250.

62. Rohlf FJ, Slice D. 1990. Extensions of the Procrustes method for the optimal superimposition of landmarks. Systematic Zoology, 39(1), 40–59.
63. Rowley DL, Coddington JA, Gates MW, Norrbom AL, Ochoa RA, Vandenberg NJ, Greenstone MH. 2007. Vouchering DNA-barcoded specimens: Test of a nondestructive extraction proto-col for terrestrial arthropods. Molecular Ecology Notes, 7(6), 915–924.
64. Sábio PB, Andrade AJ, Galati EAB. 2014. Assessment of the taxonomic status of some species included in the *Shannoni* complex, with the description of a new species of *Psathyromyia* (Diptera: Psychodidae: Phlebotominae). Journal of Medical Entomology, 51(2), 331–341.
65. Sadlova J, Yeo M, Seblova V, Lewis MD, Mauricio I, Volf P, Miles MA. 2011. Visualisation of *Leishmania donovani* fluorescent hybrids during early stage development in the sand fly vector. PLoS One, 6(5), e19851.
66. Sales K, Miranda DEO, da Silva FJ, Otranto D, Figueredo LA, Dantas-Torres F. 2020. Evaluation of different storage times and preservation methods on phlebotomine sand fly DNA concentration and purity. Parasites & Vectors, 13(1), 399
67. Sales KG, Costa PL, de Morais RC, Otranto D, Brandao-Filho SP, Cavalcanti Mde P, Dantas-Torres F. 2015. Identification of phlebotomine sand fly blood meals by real-time PCR. Parasites& Vectors, 8, 230.
68. Sant'Anna MR, Jones NG, Hindley JA, Mendes-Sousa AF, Dillon RJ, Cavalcante RR, Alexander B, Bates PA. 2008. Blood meal identification and parasite detection in laboratory-fed and field-captured *Lutzomyia longipalpis* by PCR using FTA databasing paper. Acta Tropica, 107(3), 230–237.
69. Senne NA, Santos HA, Araujo TR, Paulino PG, Mendonca LP, Moreira HVS, Camilo TA, da Costa Angelo I. 2022. Robust comparative performance of genomic DNA extraction methods from non-engorged phlebotomine sandflies. Medical and Veterinary Entomology, 36(2), 203–211.
70. Shaw JJ. 2025. A review of Leishmania infections in American Phlebotomine sand flies – Are those that transmit leishmaniasis anthropophilic or anthropportunists? Parasite, 32, 57.
71. Tesh RB, Modi GB. 1983. Growth and transovarial transmission of Chandipura virus (Rhabdoviridae: Vesiculovirus) in *Phlebotomus papatasi.* American Journal of Tropical Medicine and Hygiene, 32(3), 621–623.
72. Thomsen PF, Elias S, Gilbert MTP, Haile J, Munch K, Kuzmina S, Froese DG, Sher A, Holdaway RN, Willerslev E. 2009. Non-destructive sampling of ancient insect DNA. PLoS One, 4(4),e5048
73. Truett GE, Heeger P, Mynatt RL, Truett AA, Walker JA, Warman ML. 2000. Preparation of PCR-quality mouse genomic DNA with hot sodium hydroxide and tris (HotSHOT). Biotechniques, 29(1), 52–54.
74. Upton MS. 1993. Aqueous gum-chloral slide mounting media: an historical review. Bulletin of Entomological Research, 83(2), 267–274.
75. Volf P, Myskova J. 2007. Sand flies and Leishmania: specific versus permissive vectors. Trends in Parasitology, 23(3), 91–92.
76. Wang Q, Wang X. 2012. Comparison of methods for DNA extraction from a single chironomid for PCR analysis. Pakistan Journal of Zoology, 44(2), 421–426.
77. Wang Y, Zhao Y, Bollas A, Wang Y, Au KF. 2021. Nanopore sequencing technology, bioinformatics and applications. Nature Biotechnology, 39(11), 1348–1365.

Cite this article as: Randrianambinintsoa FJ, Augendre L, Prudhomme J, Martinet J-P, Loyer M, Mekarnia N, Kerkoub H, Perveen FK, Huguenin A, Kariya E, Akhoundi M, De Andrade AJ, Berriatua E, Bongiorno G, Boyer S, Christodoulou V, Da Costa-Ribeiro MCV, De Souza LAF, Ding H, Dondji B, Dvořák V, Erisoz Kasap O, Galati EAB, Gállego M, Ballart C, Gouzelou S, Haddad N, Masse RS, Mekuria AH, Ivovic V, Kaczmarek S, Shahar MK, Kirstein OD, Kniha E, Kolářová I, Lincoln T, Lucanas C, Mikov O, Nov K, Özbel Y, Pesson B, Posada Lopez LC, Prasetyo DB, Rahola N, Rebollar-Tellez EA, Rodrigues BL, Roy L, Saini P, Sanjoba C, Shimabukuro PH, Siriyasatien P, Soszynska A, Suleşco T, Sylla M, Torno M, Volf P, Vongphayloth K, Sinh Nam V, Wardhana A, Yessinou E, Zapata S, Gantier J-C & Depaquit J. 2026. Processing and mounting phlebotomine sand flies: a consensus guideline. Parasite xx, xx. https://doi.org/10.1051/parasite/2026009.

PARASITE

An international open-access, peer-reviewed, online journal publishing high quality papers on all aspects of human and animal parasitology

Reviews, articles and short notes may be submitted. Fields include, but are not limited to: general, medical and veterinary parasitology; morphology, including ultrastructure; parasite systematics, including entomology, acarology, helminthology and protistology, and molecular analyses; molecular biology and biochemistry; immunology of parasitic diseases; host-parasite relationships; ecology and life history of parasites; epidemiology; therapeutics; new diagnostic tools.
All papers in Parasite are published in English. Manuscripts should have a broad interest and must not have been published or submitted elsewhere. No limit is imposed on the length of manuscripts.

Parasite (open-access) continues **Parasite** (print and online editions, 1994-2012) and **Annales de Parasitologie Humaine et Comparée** (1923-1993) and is the official journal of the Société Française de Parasitologie.

Editor-in-Chief:
Jean-Lou Justine, Paris

Submit your manuscript at:
https://www.editorialmanager.com/parasite

Appendice 1:

Fondamenti teorici biochimici. Gli artropodi in questione sono i flebotomi. Tuttavia, il concetto generale può essere esteso ad altri artropodi molto comuni per i quali l'identificazione può essere effettuata solo sulla base di caratteri morfologici interni. Fortunatamente, alcuni organi interni sono parzialmente chitinizzati e le loro morfologie ci forniscono informazioni preziose. Ecco perché è molto interessante osservare il cibario (o pompa alimentare), le spermateche e i loro dotti. Con tutti i reagenti che passeremo in rassegna, non bisogna mai dimenticare che, dalla fase di fissazione dell'insetto fino al montaggio, applicheremo semplicemente delle reazioni redox. L'unica precauzione o principio che ci guiderà è quello di evitare di mescolare reagenti riducenti con reagenti ossidanti.

Alcol etilico; etanolo

Questa sostanza verrà utilizzata in modi diversi. Le molecole di alcol hanno una forte affinità per l'acqua e quindi mostrano un effetto disidratante. Tuttavia, un alcol a bassa concentrazione (cioè troppo ricco di acqua) svolgerà un ruolo nella degradazione degli acidi nucleici (l'acqua è nemica degli acidi nucleici).

Quando gli insetti vengono posti in etanolo, non è solo per preservarli, ma anche per fissare i tessuti. In istologia, di solito differenziamo due concetti importanti: il tasso di penetrazione e il tasso di fissazione. È ben inteso che un buon conservante deve penetrare rapidamente in profondità nei tessuti prima di fissarli. Per l'alcol al 96%, il coefficiente di penetrazione è di circa 1,05 (in confronto, per una soluzione acquosa allo 0,75% di acido picrico, il coefficiente di penetrazione è 0,45, mentre quello di una soluzione di bicromato di potassio al 3% è 1,45).

La necessità di conservare insetti e altri artropodi a tempo indeterminato in etanolo è una realtà per gli entomologi. L'idea di fondo risulta onorevole voler conservare le catture sul campo per studi successivi o per futuri ricercatori. Tuttavia, questa idea non è praticabile per un citologo o un istologo. Mirando a conservare i campioni nel fissativo per troppo tempo, potrebbero diventare praticamente impossibili da rielaborare. Ecco perché i campioni con più di 10 anni sono difficili o addirittura impossibili da utilizzare.

Un'altra considerazione è il rapporto tra la massa dell'artropode da fissare e il volume del fissatore. Nella pratica zoologica o medica, è consigliabile prevedere un volume 60 volte superiore al volume dei pezzi da fissare. In pratica, per i micro-artropodi, per un dato volume di esemplari da fissare, aggiungere almeno 4-5 volumi di alcol. Tenere presente che l'alcol perderà la sua forza man mano che rimuove tutta l'acqua presente nei tessuti dell'artropode.

In conclusione:

L'alcol etilico è un agente chimico riducente (quindi incompatibile con i fissativi ossidanti); precipita energicamente le proteine e le denatura; scioglie alcuni lipidi complessi e precipita il glicogeno; causa una forte contrazione dei tessuti e li indurisce.

Soluzioni basiche di idrossido di potassio o di sodio

L'uso di queste soluzioni in entomologia si è concentrato principalmente sull'idrossido di potassio, senza una chiara giustificazione.

L'idrossido di sodio [E524] si presenta in soluzione, sia a diverse concentrazioni sia con diversa normalità. È disponibile in pellet o in scaglie. Il suo principale svantaggio è l'elevata igroscopicità (maggiore rispetto al KOH). Quando reagisce con le proteine le dissolve, mentre con i lipidi li trasforma in saponi duri durante la saponificazione (questa è una differenza importante rispetto al KOH, che produce saponi liquidi durante la saponificazione).

L'idrossido di potassio [E525] è disponibile come soluzione concentrata, ma soprattutto ha il vantaggio di essere formulato in pellet di circa 0,1 g, il che facilita notevolmente la preparazione di soluzioni diluite quando non si dispone di una bilancia di precisione. Ad esempio, 1 pellet da 0,1 g in 1 mL di acqua distillata produce una soluzione al 10%. Il secondo vantaggio dell'idrossido di potassio in pellet è la sua minore sensibilità alla carbonatazione (una soluzione di KOH ha un'elevata affinità per il fissaggio della CO_2, formando così sali carbonati).

Queste basi forti vengono utilizzate per solubilizzare gli acidi grassi trasformandoli in saponi idrosolubili. Occorre ricordare che il fissativo, come l'etanolo, solubilizza già parte dei grassi presenti nel campione. Tuttavia, riportando il campione in un mezzo acquoso con una base forte, gli acidi grassi (più o meno complessi) precipitano. La base forte realizza quindi una saponificazione a freddo. In alcuni casi, quando i tessuti adiposi sono in eccesso, ad esempio nelle femmine, può essere vantaggioso aumentare la temperatura a 35–40 °C per facilitare la reazione, oppure prolungare il tempo di contatto a temperatura ambiente.

Soluzione acida colorata / soluzione di Marc-André incolore

In questa sezione verranno esaminati i vantaggi e gli inconvenienti dell'uso della soluzione di Marc-André. Questa soluzione è composta da idrato di cloralio (tricloracetaldeide monoidrata), acido acetico e acqua. Si tratta di una soluzione fortemente ossidante (miscela di acido e aldeide). Essa neutralizza l'eccesso di idrossido di potassio eventualmente presente nei campioni, senza precipitare i saponi alcalini formati durante l'uso dell'idrossido di potassio. Questa soluzione ossidante agisce anche sulle funzioni alcoliche secondarie delle glucosamine che costituiscono la chitina, ossidandole e ammorbidendo così la chitina. Un'ulteriore azione è la dissoluzione di alcuni sali minerali presenti.

Quando la soluzione di Marc-André è precedentemente colorata con fucsina acida (quindi in stato ossidato), è in

grado di fissarsi sulle funzioni alcoliche secondarie della struttura. Dopo il tempo di contatto della soluzione di Marc-André e la fase di colorazione dei campioni, il risciacquo viene effettuato esclusivamente con etanolo. In questo modo si inizia la fase di disidratazione dei campioni.

Vantaggi:
Neutralizzazione delle soluzioni basiche in eccesso
Rilassamento della chitina
Colorazione della chitina per una migliore valutazione delle strutture interne chitinizzate

Svantaggi:
L'idrato di cloralio è un ipnotico ed è stato utilizzato in medicina umana. Deve essere impiegato sotto cappa chimica e nel rispetto della normativa sulla sicurezza e sui rischi chimici.

Soluzioni di disidratazione
L'esperienza dimostra che per campioni molto piccoli non è necessario seguire una sequenza di bagni alcolici a concentrazione crescente. Se il campione è grande, si inizierà con etanolo all'80%, poi 90%, 95% e infine etanolo assoluto. Per campioni molto piccoli, utilizzare un bagno in etanolo al 90% seguito da immersione in etanolo assoluto. In questa fase occorre sempre ricordare che l'etanolo assoluto tende ad assorbire l'acqua presente nell'atmosfera.

Tradizionalmente, nei laboratori di entomologia la disidratazione finale dei campioni veniva completata con un bagno in creosoto di faggio. Oggi questa essenza, ampiamente utilizzata come pesticida, antifungino e conservante del legno, è fortemente sconsigliata a causa del suo odore (idrocarburi policiclici aromatici) può avere effetti nocivi sulla capacità riproduttiva o sullo sviluppo embrionale, cancerogena, un inquinante organico un inquinante organico persistente e tossica per gli organismi acquatici. Una soluzione che proponiamo per il montaggio dei campioni è l'Euparal® e l'essenza di Euparal (descritti nel paragrafo seguente). Una miscela di Euparal® ed essenza di Euparal è molto ben accettata; i campioni possono essere montati dopo un bagno in etanolo al 90%.

Appendice 2: Composizione dei reagenti utilizzati

Idrossido di potassio al 10%
Idrossido di potassio 10 g
Acqua distillata q.b. a 100 mL

Mezzo di montaggio alla gomma cloralata – Mezzo di Hoyer
Acqua distillata 50 mL
Idrato di cloralio 200 g
Gomma arabica 50 g
Glicerolo 20 mL

Soluzione di Marc-André
Idrato di cloralio 40 g
Acido acetico glaciale 30 mL
Acqua distillata 30 mL

Fucsina acida all'1% in acqua distillata
Fucsina acida in polvere 1 g
Acqua distillata 99 mL

Soluzione di Marc-André colorata con fucsina
Soluzione di Marc-André 10 mL
Fucsina acida all'1% 50 µL

Appendice 3: Euparal®, balsamo del Canada, alcool polivinilico o altre soluzioni per il montaggio

Alcol polivinilico
È il mezzo di montaggio ideale quando non sono disponibili i prodotti necessari per una corretta disidratazione. L'alcol polivinilico viene quindi miscelato con il lattof enolo di Amman. Tuttavia, questi preparati presentano importanti svantaggi: tendono a seccarsi, l'alcol polivinilico può cristallizzare a causa dell'evaporazione dell'acqua oppure annerire in seguito all'ossidazione del fenolo. Resta comunque una buona tecnica per montaggi di breve durata.

Balsamo del Canada
Il suo utilizzo per il montaggio tra vetrino e coprioggetto richiede la disidratazione preventiva dei campioni. L'impiego di xilene o toluene non è privo di inconvenienti.

Mezzo Enecê
Come il balsamo del Canada, per il montaggio tra vetrino e coprioggetto richiede la disidratazione del campione.
Formulazione del mezzo Enecê:
Colofonia bianca pura 22 g
Gomma copale solubile in alcool 12 g
Alcol assoluto 20 mL
Canfora 10 g
Essenza di trementina 10 mL
Eucaliptolo 26 mL

Per la preparazione, in un contenitore (ad esempio una beuta di Erlenmeyer) si introducono l'alcol assoluto e la canfora. Si aggiungono quindi la colofonia e la gomma copale. Il recipiente viene chiuso con un tappo e agitato, quindi riscaldato a bagnomaria a temperatura moderata, evitando l'ebollizione. Una volta che il contenuto è completamente liquefatto, si aggiunge l'essenza di trementina, quindi si filtra la miscela ancora calda e infine si aggiunge l'eucaliptolo al filtrato. Quando il mezzo diventa meno fluido, viene diluito con Enecê secondo la seguente formula:
Alcol assoluto 30 mL
Canfora 17 g

Essenza di trementina 15 mL
Eucaliptolo 38 mL
(Cerqueira, 1943)

Euparal®
È una resina derivata dal cipresso dell'Atlante Tetraclinis articulata (Vahl, 1791), studiata e sviluppata nel 1906 da Gilson. Il suo principale vantaggio è che non polimerizza. I campioni montati tra vetrino e coprioggetto possono essere facilmente recuperati mediante l'azione dell'alcol o, meglio ancora, dell'essenza di Euparal®. Questa resina, detta anche sandracca, è compatibile con etanolo a partire dall'80%.

Utilizzo del Triton X100: soluzione acquosa non ionica
Il Triton X100 è una soluzione acquosa non ionica (4-(1,1,3,3-tetrametilbutil) fenil-polietilenglicole, o t-octylphenoxypolyethoxyethanol, etere polietilenglicolico del fenil-terz-ottilo), ampiamente utilizzata come detergente in biologia cellulare e molecolare. Consente la permeabilizzazione delle membrane cellulari e nucleari.

Campioni di insetti conservati in alcool per molti anni sono molto comuni. Purtroppo, la conservazione in alcool non è ottimale e gli artropodi così preservati diventano difficili da preparare per l'osservazione microscopica. I contenitori in plastica che contengono i campioni spesso si degradano, causando l'evaporazione dell'alcool. In entrambi i casi, il contatto prolungato con l'alcool o l'essiccazione dei campioni rappresentano un problema reale.

Nel 2008, Jonque ha pubblicato una nota sulla reidratazione dei ragni utilizzando un agente bagnante come l'Agepon, impiegato nella fotografia [26]. Questo ha portato all'idea di utilizzare agenti bagnanti che non siano detergenti potenti.

Di seguito è riportata una procedura che utilizza Triton X100 in soluzione acquosa allo 0,5%:

Impregnare il campione secco con alcol assoluto.

Aggiungere il volume necessario di soluzione di Triton X100 allo 0,5% in modo che il campione sia completamente immerso.

Lasciare agire per circa 5 minuti o più. Tutti gli artropodi devono separarsi e diventare indipendenti nella soluzione.

Rimuovere la soluzione di Triton X100 e sostituirla con la soluzione di idrossido di potassio.

La tecnica prosegue quindi come descritto in precedenza.

Appendice 4: Mezzi di montaggio Euparal® o balsamo del Canada – procedura passo per passo

I campioni devono essere disidratati (un aspetto torbido o lattiginoso indica una disidratazione insufficiente).

La disidratazione può essere effettuata mediante concentrazioni crescenti di alcol etilico.

I campioni possono essere trasferiti da alcol al 99% o alcol assoluto a un agente chiarificante.

Procedura:

Immergere i flebotomi adulti in etanolo al 70%.

Rimuovere l'etanolo e sostituirlo con KOH al 10%. Coprire i flebotomi con un vetrino.

Lasciare macerare fino a quando gli insetti diventano trasparenti.

Rimuovere il KOH.

Ricoprire il campione con acqua distillata e attendere 30–45 minuti.

Rimuovere l'acqua e ripetere il lavaggio con acqua distillata per 30 minuti

(tempo dipendente dal numero di campioni: maggiore è il numero di campioni trattati insieme, più lungo deve essere questo tempo; minore è il numero, soprattutto se trattati singolarmente, più breve può essere).

Rimuovere l'acqua.

Aggiungere la soluzione di Marc-André (eventualmente colorata con fucsina acida) e attendere 24 ore (un giorno).

Rimuovere la soluzione di Marc-André.

Ricoprire il campione con acqua distillata e attendere 30–45 minuti.

Rimuovere l'acqua e ripetere il lavaggio con acqua distillata per 30 minuti.

Rimuovere l'acqua.

Aggiungere etanolo al 70% e dissezionare il campione.

a. Per testa e addome, separare delicatamente la testa o l'addome dal torace.

b. Per il torace, rimuovere le ali tenendo il torace con una pinzetta e tirando alla base delle appendici con un'altra. È possibile effettuare una dissezione sagittale, dividendo il torace in metà destra e sinistra, a seconda delle regioni di maggiore interesse.

Disidratare gradualmente attraverso una serie di soluzioni acquose di etanolo: 50% – 80% – 95%, fino a raggiungere l'etanolo assoluto.

Completare la disidratazione lavando i campioni due volte, per 10 minuti ciascuna, con etanolo al 100%.

Rimuovere l'etanolo e ricoprire i campioni con olio di chiodi di garofano per 15 minuti a temperatura ambiente.

Trasferire i campioni dall'olio di chiodi di garofano a una goccia di Euparal® o balsamo del Canada su un vetrino pulito.

Disporre i campioni come desiderato: testa, torace e addome del flebotomo possono essere dissezionati utilizzando aghi fini o pinzette sotto uno stereomicroscopio.

La testa deve essere separata dal corpo per essere montata in posizione ventro-dorsale; il forame occipitale deve essere orientato verso l'alto, in modo che il cibario possa essere osservato direttamente attraverso di esso.

La dissezione viene effettuata direttamente nel mezzo di montaggio per flebotomi.

Lasciare il campione fino a quando la superficie diventa appiccicosa.

Inumidire un coprioggetto pulito con alcol assoluto. Posizionare il coprioggetto sul balsamo del Canada facendolo scendere con un'inclinazione.

Conservare i vetrini in una scatola asciutta destinata a questo scopo.
