# Supplementary material for: Processing and mounting phlebotomine sand flies: a consensus guideline
Source: Parasite. 2026 Apr 3;33:18. doi: 10.1051/parasite/2026009 (PMC13047900; doi:10.1051/parasite/2026009)
Supplement: Supplementary file 17 — Japanese translation / 日本語訳 [file parasite-33-18-s17.pdf]

Parasite **33**, 18 (2026)
© F.J. Randrianambinintsoa et al., published by EDP Sciences, 2026
https://doi.org/10.1051/parasite/2026009

PARASITE
Available online at:
www.parasite-journal.org

REVIEW ARTICLE OPEN ACCESS

サシチョウバエ研究における検体処理法と標本作製に関する指針

Fano José Randrianambinintsoa[1], Laure Augendre[1], Jorian Prudhomme[1], Jean-Philippe Martinet[1], Mathieu Loyer[1], Nalia Mekarnia[1], Hocine Kerkoub[1], Farzana Khan Perveen[1], Antoine Huguenin[1,2], Emilie Kariya[1,2], Mohammad Akhoundi[3], Andrey José de Andrade[4], Eduardo Berriatua[5], Gioia Bongiorno[6], Sébastien Boyer[7,8], Vasiliki Christodoulou[9], Magda Clara Vieira Da Costa-Ribeiro[10], Lucas Alexandre Farias de Souza[10], Huicong Ding[11], Blaise Dondji[12], Vít Dvořák[13], Ozge Erisoz Kasap[14], Eunice Aparecida Bianchi Galati[15], Montserrat Gállego[16], Cristina Ballart[16], Stavroula Gouzelou[17], Nabil Haddad[18], Rezki Sabrina Masse[19], Asrat Hailu Mekuria[20], Vladimir Ivovic[21], Szymon Kaczmarek[22], Mohd Khadri Shahar[19], Oscar D. Kirstein[23], Edwin Kniha[24], Iva Kolářová[13], Lincoln Timinao[25], Cristian Lucanas[26], Ognyan Mikov[27], Kimsear Nov[7], Yusuf Özbel[28], Bernard Pesson[29], Laura Cristina Posada Lopez[30], Didot Budi Prasetyo[1,7], Nil Rahola[31], Eduardo A. Rebollar-Tellez[32], Bruno Leite Rodrigues[15], Lalita Roy[33], Prasanta Saini[34], Chizu Sanjoba[35], Paloma Helena Fernandes Shimabukuro[36], Padet Siriyasatien[37], Agnieszka Soszyńska[22], Tatiana Suleşco[38], Massamba Sylla[39], Majhalia Torno[40], Petr Volf[13], Khamsing Vongphayloth[41], Vu Sinh Nam[42], April Wardhana[43], Eric Yessinou[44], Sonia Zapata[45], Jean-Charles Gantier[1], and Jérôme Depaquit[1,2,*]

[1] Faculté de Pharmacie, Université de Reims Champagne Ardenne, UR ESCAPE-USC ANSES PETARD, 51 rue Cognacq-Jay, 51096 Reims Cedex, France
[2] Pôle de Biologie territoriale, Laboratoire de Parasitologie-Mycologie, Centre Hospitalo-Universitaire, 51092 Reims, France
[3] Parasitology-Mycology Department, Avicenne Hospital, AP-HP, Bobigny, Sorbonne Paris Nord University, France; Unité des Virus Émergents (UVE: Aix-Marseille Univ, Università di Corsica, IRD 190, Inserm 1207, IRBA), 13005 Marseille, France
[4] Parasitology Collection of Basic Pathology, Department of Basic Pathology, Federal University of Paraná, Curitiba 19031, Brazil
[5] Department of Animal Health, University of Murcia, Campus de Espinardo, 30100 Espinardo, Murcia, Spain
[6] Department of Infectious Diseases, Vector-borne Diseases Unit, Istituto Superiore di Sanità, 00166 Rome, Italy
[7] Medical and Veterinary Entomology Unit, Institut Pasteur du Cambodge, Phnom Penh 12201, Cambodia
[8] Ecology & Emergence of Arthropod-borne Pathogens Unit, Department of Global Health, Institut Pasteur, CNRS UMR2000, 75015 Paris, France
[9] Section Veterinary Services (1417), Laboratory for Animal Health Virology, Aglantzia, Nicosia 2109, Cyprus
[10] Insects Vectors and Parasites Laboratory, Department of Basic Pathology and Postgraduate program in Microbiology, Parasitology and Pathology, Federal University of Paraná, 81530-900 Curitiba, Brazil
[11] Department of Biological Sciences, National University of Singapore, 117558, Singapore
[12] Laboratory of the Leishmaniasis Research Project, Mokolo District Hospital, Mokolo, Cameroon; Laboratory of Cellular Immunology and Parasitology, Department of Biological Sciences, Central Washington University, 98926 Ellensburg, WA, USA
[13] Department of Parasitology, Faculty of Science, Charles University, 12800 Prague, Czechia
[14] VERG Laboratories, Department of Biology, Faculty of Science, Hacettepe University, Beytepe, Ankara 06800, Türkiye
[15] Faculdade de Saúde Pública da Universidade de São Paulo (FSP/USP), Pós-graduação em Saúde Pública, 01246-904 São Paulo, Brazil
[16] Secció de Parasitologia, Departament de Biologia, Sanitat i Medi Ambient, Facultat de Farmàcia i Ciències de l'Alimentació, Universitat de Barcelona, & Institut de Salut Global de Barcelona (ISGlobal), Centro de Investigación Biomédica en Red, Enfermedades Infecciosas (CIBERINFEC), 08028 Barcelona, Spain
[17] Laboratory of Infectious Diseases and Public Health, School of Medicine, University of Cyprus, Nicosia, Cyprus & Department of Pediatrics, Archbishop Makarios III Hospital, Nicosia 2115, Cyprus
[18] Faculty of Health Sciences, American University of Beirut, 1107 2020 Beirut, Lebanon
[19] Medical Entomology Unit, Infectious Disease Research Centre, Institute for Medical Research (IMR), National Institutes of Health (NIH), Ministry of Health Malaysia, 40170 Shah Alam, Selangor, Malaysia
[20] School of Medicine, Addis Ababa University, 28017 - 1000 Addis Ababa, Ethiopia
[21] Faculty of Mathematics, Natural Sciences and Information Technologies, University of Primorska, 6000 Koper, Slovenia

Edited by Jean-Lou Justine
*Corresponding author: jerome.depaquit@univ-reims.fr

This is an Open Access article distributed under the terms of the Creative Commons Attribution License (https://creativecommons.org/licenses/by/4.0), which permits unrestricted use, distribution, and reproduction in any medium, provided the original work is properly cited.

[22] University of Lodz, Faculty of Biology and Environmental Protection, Department of Invertebrate Zoology and Hydrobiology, Banacha 12/16, 90-237 Łódź, Poland
[23] Laboratory of Entomology, Ministry of Health, 9134302 Jerusalem, Israel
[24] Center for Pathophysiology, Infectiology and Immunology, Institute of Specific Prophylaxis and Tropical Medicine, Medical University Vienna, Kinderspitalgasse 15, 1090 Vienna, Austria
[25] Papua New Guinea Institute of Medical Research (PNGIMR) Institute, PO Box 60, Headquarter, Homate Street, 441 Goroka, Eastern Highlands Province, Papua New Guinea
[26] Museum of Natural History, University of the Philippines Los Baños, 4031 Laguna, Philippines
[27] National Centre of Infectious and Parasitic Diseases, 1504 Sofia, Bulgaria
[28] Ege University, Faculty of Medicine, Department of Parasitology, 35040 Bornova/Izmir, Türkiye
[29] Retired, Faculté de Pharmacie, Université de Strasbourg, Strasbourg, 67400 Illkirch-Graffenstaden, France
[30] Program for the Study and Control of Tropical Diseases (PECET), Faculty of Medicine, University of Antioquia, 050010 Medellin, Colombia
[31] MIVEGEC, Univ. Montpellier, CNRS, IRD, 34394 Montpellier, France & Medical Entomology Unit, Institut Pasteur de Madagascar, 101 Antananarivo, Madagascar
[32] Laboratorio de Entomología Médica, Departamento de Zoología de Invertebrados, Facultad de Ciencias Biológicas, Universidad Autónoma de Nuevo León, San Nicolás de los Garza, 66455, NL, México
[33] Tropical and Infectious Disease Centre, BP Koirala Institute of Health Sciences, Dharan 56700, Nepal
[34] ICMR-Vector Control Research Centre, Puducherry 605006, India
[35] Graduate School of Agricultural and Life Sciences, The University of Tokyo, Tokyo 113-8657, Japan
[36] Grupo de estudos em Leishmanioses/Coleção de Flebotomíneos (COLFLEB/Fiocruz-MG), Instituto René Rachou, Fundação Oswaldo Cruz, Belo Horizonte, Minas Gerais, 30190009, Brazil
[37] Center of Excellence in Vector Biology and Vector-Borne Disease, Department of Parasitology, Faculty of Medicine, Chulalongkorn University, Bangkok 10330, Thailand
[38] Department of Arbovirology, Bernhard Nocht Institute for Tropical Medicine, Bernhard Nocht Str. 74, 20359 Hamburg, Germany [39] Laboratory Vectors & Parasites, Department of Livestock Sciences and Techniques, Sine Saloum University El Hadji Ibrahima Niasse (SSUEIN) Kaffrine Campus, C.P. 24600, Senegal.
[40] Environmental Health Institute, National Environment Agency, Singapore 138667, Singapore & Department of Biological Sciences, National University of Singapore, 117558 Singapore
[41] Institut Pasteur du Laos, Laboratory of Vector-Borne Diseases, Samsenhai Road, Ban Kao-Gnot, Sisattanak District, 3560 Vientiane, Lao PDR
[42] National Institute of Hygiene and Epidemiology, 1 Yec-Xanh Street, Hai Ba Trung District, 100000 Hanoi, Vietnam
[43] Indonesian Research Center for Veterinary Science, Indonesian Agency for Agricultural Research and Development, Ministry of Agriculture Republic Indonesia, Bogor 16114, Indonesia & Department of Parasitology, Faculty of Veterinary Medicine, Airlangga University, Surabaya 60115, Indonesia
[44] Laboratory of Research in Applied Biology, Polytechnic School of Abomey-Calavi, University of Abomey-Calavi, 01 P.O. Box 2009, 00000 Cotonou, Benin
[45] Instituto de Microbiología, Colegio de Ciencias Biológicas y Ambientales (COCIBA), Universidad San Francisco de Quito (USFQ), 170901 Quito, Ecuador

Received 1 December 2025, Accepted 29 January 2026, Published online 3 April 2026

要約 – 本論文は、サシチョウバエ（phlebotomine sand fly）検体の処理およびスライド標本作製について、種同定や病原体の検出・分離に不可欠となる作業手順を網羅的にまとめたガイドラインを提供するものである。本稿では、フィールドおよび実験室の両方の状況に適した多様な手法について解説する。サシチョウバエの採集、取り扱い、回収、殺処理法（化学薬品よりも乾燥冷凍または CO_2 を推奨）に加え、冷蔵保存やエタノール保存などの保存法に関する詳細な手順が含まれる。特定の解剖学的構造（生殖器、頭部、翅）の顕微鏡観察においては、標本準備の質が極めて重要であり、本稿でその方法を詳述している。また、KOH（水酸化カリウム）や Marc-André 溶液を用いた透化処理など、標本処理の詳細も示す。スライド標本作製については、複数の封入剤を比較し、その光学特性や保存性を検討している。特に Hoyer 液（chloral gum としても知られる）は透明性に優れ、メスの生殖器官（spermathecae）の迅速な観察に有用であるものの、長期保存には適さないとされる。その他の媒体として、polyvinyl alcohol、Euparal®（耐水性に限界がある）、Canada balsam（炭化水素溶解性）が取り上げられ、後者 2 つは長期保存に適している。また、DNA シークエンスや MALDI-ToF など、標本処理に特別な配慮を要する最新の分子生物学的手法についても議論している。さらに、本稿では、さまざまな封入手技を示した短い動画クリップに加えて、33

の言語への翻訳も提供しており、国際的な科学コミュニティにおける多様なニーズと期待に応える内容となっている。

キーワード: スライド標本作製、サシチョウバエ、Hoyer 液、Marc-André 溶液、chloral gum、polyvinyl alcohol、Euparal®、Canada balsam、リーシュマニア原虫分離、フィールド、培養、解剖、分子生物学、MALDI-ToF、タイプ標本

Abstract – Processing and mounting phlebotomine sand flies: a consensus guideline. This article provides a comprehensive guide for the processing and mounting of phlebotomine sand fly specimens, which is crucial for species identification and pathogen detection and isolation. It discusses a range of techniques suitable for both field and laboratory settings. The guide includes detailed instructions on sand fly collection, handling, covering, and euthanasia (recommending dry freezing or CO_2 over chemicals) as well as conservation strategies, such as cold storage and preservation in ethanol. The quality of preparation of certain anatomical structures (genital organs, head and wings) is essential for their proper microscopic observation and is described in this work. The article also presents detailed sample processing, including the clearing process with agents such as potassium hydroxide then Marc-André solution. The mounting process compares different media, emphasizing their optical properties and preservation potential. Hoyer fluid (also known as chloral gum) is recommended for quick observation, particularly for spermathecae, due to its clarity, although it is not suitable for long-term storage. Other media discussed include polyvinyl alcohol, Euparal® (for limited water tolerance), and Canada balsam (a hydrocarbon-soluble medium), with the latter two offering long-term preservation capabilities. Innovative molecular biology approaches such as DNA sequencing and MALDI-ToF, which require particular attention to sample processing, are also addressed. Furthermore, short video clips illustrating various mounting techniques as well as translations in many different languages are provided, allowing the guideline to reach the diverse needs and expectations of the global scientific community.

Key words: Mounting, Phlebotomine sand fly, Hoyer fluid, Marc-André solution, Chloral gum, Polyvinyl alcohol, Euparal®, Canada balsam, *Leishmania* isolation, Field conditions, Culture, Dissection, Molecular biology, MALDI-ToF, Type-specimens.

序文

サシチョウバエ（phlebotomine sand flies）は、ハエ目 (Diptera)・チョウバエ科(Psychodidae)サシチョウバエ亜科(Phlebotominae)に属する小型昆虫であり、これまでに少なくとも1,063種が知られている[21] 。サシチョウバエは、リーシュマニア原虫 (*Leishmania*) 、アルボウイルス(arboviruses)、バルトネラ(*Bartonella*)などの病原体を媒介し、それぞれリーシュマニア症、アルボウイルス感染症、バルトネラ症を引き起こす重要なベクターである。サシチョウバエの種同定は、主として詳細な顕微鏡観察に依存している。顕微鏡観察による種同定は、それぞれに利点と限界を有する複数の特別な技術: 慎重な採集、適切な保存、および精密なスライド標本作製によって可能となる。

サシチョウバエ成虫の同定は、外部構造(antennae、palpi、 male genitalia等)および内部構造(pharynx、cibarium、spermathecae等)の観察に基づいて行われる。解剖することで内部構造の観察が容易となり、精確な同定につながる。そのため、蚊やサシガメとは異なり、サシチョウバエでは同定前にプレパラート （スライドとカバーガラスの間）に封入する作業が必須である。

1980年代以前、サシチョウバエの同定に利用可能な手法は顕微鏡観察のみであり、現在においても最も広く使用されているアプローチである。したがって、検体処理と標本作製の選択は比較的単純であり、基本的には二分法に基づいていた。すなわち、（1）標本の長期保存を可能にする恒久的封入と、（2）長期保存性は担保されないが迅速な同定が可能な封入である。たとえば、Canada balsam などの樹脂を用いた恒久的封入では、試料の完全脱水が必須であり、時間を要する。また、この封入剤の屈折率は、メスの生殖器官の観察に必ずしも最適とはいえない。一方、Hoyer 液のような水性媒体での封入は迅速で、屈折性の強いメスの生殖器官の観察に優れるが、大気中の水分を吸収しやすいため長期保存には不向きである。完全に乾燥した後にマニキュアで封入する方法が一つの選択肢である。このトレードオフは現在も変わらず、標本作製の目的に応じて封入方法の選択に影響を与えている。

1980年代以降、サシチョウバエの同定研究は形態学と生化学的手法を組み合わせて行われるようになった。初期にはクチクラ炭化水素の分析が行われたが、すぐに分子生物学的手法に置き換えられた。すなわち、RAPD、RFLP、DNA増幅およびサンガー法によるシークエンス、さらに次世代シークエンシング（NGS）などである。現在では、これらの分子生物学的手法はMALDI-ToFによるプロテオミクスと補完的に利用されている。加えて、分子生物学的種同定は、PCRを用いた病原体検出（*Leishmania*, *Trypanosoma*, *Bartonella*, *Phlebovirus*）と組み合わせることが可能であり、エンドポイントPCR・リアルタイムPCRのいずれでも検出可能である。このため、解析の目的に応じて採集および保存方法を適応させる必要がある[3, 32]。従来の形態学的特徴に加えて、翅の形状測定など他の形態学的アプローチを適用することもできる。

本研究の目的は、著者らの経験および文献情報に基づき、サシチョウバエ成虫の形態学的および分子生物学的解析を最適化するための標準化された処理およびスライド標本作製ガイドラインを提供することである。

分子生物学やMALDI-ToFなどの特定の解析を実施するには、形態同定に必須ではないサシチョウバエの体の一部を保持する必要があり、適切なプロトコル選択が重要である。

本ガイドラインでは、生きたまま捕獲されたサシチョウバエの麻酔および安楽死の方法、保存方法、ならびに迅速な同定や長期保存・後続解析を可能とするスライド標本作製手順に焦点を当てる。

前文: 安全性および規制上の留意事項は Saftey Data Sheets を参照すること。

本ガイドラインに示すすべての化学薬品は、厳格な安全管理のもとで取り扱う必要がある。研究施設の安全衛生委員会は、それら化学薬品の危険性、適切な取り扱い手順、および廃棄方法に関する情報を提供できる。使用者はこれらの薬品の使用および廃棄に関する安全指示に従う義務がある。なお、一部の化学薬品またはその構成成分（例: chloral hydrate）は、国によって規制対象となっている場合がある。本稿で使用されている略語一覧は表1に示す。

表1：略語一覧

**BME**	Basal medium Eagle
**CDC**	Centers for Disease Control and Prevention
**CMCP**	Camphor-monochlorophenol
**CMR**	Carcinogenic, mutagenic, reprotoxic substance
**COI**	Cytochrome c oxidase subunit I gene
**CytB**	Cytochrome b gene
**DNA**	Deoxyribonucleic acid
**ELISA**	Enzyme-linked immunosorbent assay
**EtOH**	Ethanol
**M199**	Medium 199
**MALDI-ToF MS**	Matrix-assisted laser desorption/ionization time-of-flight mass spectrometry
**MEM**	Minimum essential media
**NGS**	Next-generation sequencing
**NNN**	Novy-MacNeal-Nicolle medium
**PCR**	Polymerase chain reaction
**Lao PDR**	Lao People's Democratic Republic
**PNOC**	Prepronociceptin gene
**qPCR**	Quantitative PCR (real-time PCR)
**RAPD**	Random amplified polymorphic DNA
**RFLP**	Restriction fragment length polymorphism
**RI**	Refractive index
**RNA**	Ribonucleic acid
**RNases**	Ribonucleases
**RNASS**	RNA stabilization solution
**RT-PCR**	Reverse transcription PCR
**TFA**	Trifluoroacetic acid

1. サシチョウバエの捕獲

サシチョウバエの成虫は、CDCミニチュアライトトラップ、粘着トラップ、吸虫管（シャノントラップや家畜小屋などサシチョウバエの休息場所からの直接採取）など、多様な方法を用いて生死を問わず採集することができる。これらの手法は、適切な生息環境にトラップを設置し、光やその他の誘引物質（CO_2 や化学誘引剤）を用いてサシチョウバエを誘引・捕集する。具体的な仕様方法は、複数の文献に詳述されてい

る［2, 3, 32, 36, 49］。

生きたまま捕獲した場合には、後続のすべての解析に利用可能であるが、死亡個体のみを回収した場合には リーシュマニア原虫 やウイルス株の分離は不可能となる。また、粘着トラップなど一部の捕獲手法では、触角、小顎鬚、翅、脚といった器官の欠損が頻繁に生じる。加えて、粘着トラップに塗布されているヒマシ油がサシチョウバエに付着するため、処理の開始時に除去する必要がある。通常は、エタノールとジエチルエーテルを等量混合した溶液に約15分間浸漬することで除去される。

2. 捕獲個体の殺処理

捕獲後、生きたサシチョウバエは安楽死させる必要がある。捕獲手法によっては（例：粘着トラップ、洗剤またはエタノール入り容器を備えたCDCライトトラップ）、捕獲時点で既に死個体となる場合もある。エタノールに直接投入された個体は分子生物学的解析に利用でき、他の捕獲法でも迅速にエタノール保存すれば同様の解析が可能である。しかし、これらの殺処理手法では MALDI-ToF による解析が不可能である。また、一部の殺処理法は特定の形態学的特徴を損なう可能性がある。したがって、正確な同定や将来の比較標本（voucher specimen）としての長期保存を行うには、適切な標準的殺処理法を選択することが不可欠である。

酢酸エチル、エチルエーテル、テトラクロロエタン、クロロホルムなどの化学薬品は、綿に含浸させてサシチョウバエを入れた容器に置くことで殺処理に用いることができる。これらは毒性が高いため、取り扱いは製造者の指示に従って慎重に行わなければならない。しかし、著者らの経験では、クロロホルムは分子生物学的解析との相性が悪いため推奨されない。さらに、これら化学薬品はいずれも危険性が高く、分子生物学的解析への適合性にも疑問が残るため、一般的には使用が推奨されない。

形態学的観察、DNA解析、タンパク質解析のいずれにも良好に保存できる最も広く用いられる方法は「乾燥冷凍（dry freezing）」である。標本は完全に麻酔されるまで十分に凍結させる必要があるが、（i）乾燥しすぎないこと、（ii）リーシュマニア原虫を in vitro で腸管から分離することを目的とする場合には原虫の生存性を損なわないこと、が重要である。そのため、–20°Cで15～20分間の冷凍を推奨し、サシチョウバエが気絶状態にあるだけで、原虫を殺さないよう適宜確認する必要がある。

冷凍機器が利用できない場合には、代替として CO_2 による殺処理が可能である。野外で CO_2 シリンダーが使用できない場合、小型商用 CO_2 カートリッジ（ソーダサイフォン用）を用いることも可能だが、航空機での輸送には制限がある。また、最終手段としては、タバコ煙に曝露することで殺処理する方法がある。これは、CDC ライトトラップで生きた個体を捕獲し、吸虫管で回収してガラス管内で保持し、タバコ煙に曝露することで数秒以内に殺処理するという方法である。この方法は、過酷な環境を含むあらゆる野外条件で実施可能である。ただし、ガラス器具に煙成分が付着するため、十分な洗浄を行わない限り生個体の捕獲には再利用できない。一方で、同じ未洗浄の吸虫管でも、他のトラップから得られたサシチョウバエを固定用に殺処理する目的であれば使用可能である。また、吸虫管の内部に個体が残留していないか確認する必要がある。これらの方法はいずれも、腸管解剖による リーシュマニア原虫 の分離と両立する。

3. 解析前の検体保存

解析に先立ち検体を固定する方法として、おもに以下の5種類がある。

3.1. 冷凍保存

この方法は –20°C もしくは、可能であれば –80°C で行うのが最適である。現在では液体窒素による保存よりも、これらの冷凍保存方法が広く用いられている。いずれの場合も、検体が麻酔状態になった直後、できる限り速やかに凍結保存を実施する必要がある。冷凍庫での保存は、サシチョウバエの構造だけでなく RNA、DNA、タンパク質を保存期間中良好な状態で完全に保持できるという利点がある。一方、液体窒素は翅、脚、小顎鬚、触角を著しく損傷し、断裂させ、重要な形態学的特徴を失わせることが多い。乾燥冷凍（dry freezing）は標本に対する損傷が小さいものの、これらの脆弱な器官の保存には必ずしも理想的ではない。特に注意点として、解凍時に結露により翅や触角、小顎鬚、

脚が保存容器に付着し、剥がれる恐れがある。また、冷凍保存は冷凍庫や液体窒素容器へのアクセスが必要であるため、野外研究では常に実施可能とは限らない。冷凍保存は、病原体検出を目的とした分子生物学的解析と完全に両立し、感度の低下も生じない。ただし、RNAウイルスの検出・分離には –80°C または液体窒素での長期保存が必要である。なお、冷凍標本は通常、腸管解剖によるリーシュマニア原虫の分離には適さないが、サシチョウバエをまず蒸気相、次いで液体窒素に浸漬する方法によって、リーシュマニア原虫の液体窒素保存を模倣し、分離が可能となる。

3.2. アルコール保存（エタノールまたはイソプロピルアルコール）

これはおそらく、サシチョウバエ保存に最も広く用いられている方法である。実験室にアクセスできない厳しい環境下でも野外で容易に実施可能である。アルコール保存は形態学的研究に特に適しており、翅、脚、触角、小顎鬚といった脆弱な器官が、保存チューブ内に気泡が存在しない限り良好に保たれる。そのため、気泡を除去するためにチューブ口を小さな脱脂綿で封じ、その上にラベルを配置することを推奨する（図1）。適切なアルコール濃度については議論があるが、70%未満は推奨されない［45, 66］。高濃度のアルコールはDNAをより良好かつ長期間保存する一方、形態学的研究では検体が脆くなりやすい。96%エタノール（アゼトロープ混合）は、特に湿度の高い熱帯環境において濃度が安定しやすい。なお、95%エタノールの方が入手しやすい場合が多い。濃度に関わらず、DNAは一般にエタノール中で良好に保存される（ただし、特にNGS向け分子解析では冷凍保存より劣る）。タンパク質の安定性ははるかに低く、特に MALDI-ToF などのプロテオミクス解析には適さない。数ヶ月間アルコール保存されたサシチョウバエの形態的同定は可能だが、プロテインスペクトルの取得は不可能である。アルコール（または乾燥）の状態に加えて –20°C で冷凍保存すると、DNA など分子レベルの保存性が向上し、組織分解の進行が遅れることで形態保存にも二次的効果があるが、形態への効果は分子保存ほど顕著ではない。エタノール保存は、70%以上の濃度を用い、数ヶ月以内の短期間であれば、DNAおよびRNAウイルスの検出にも利用可能である。また、イソプロピルアルコールは一部の国で入手しやすくDNA保存も可能であるが、検体を硬くする欠点がある。エタノールと異なり可燃性が低いため輸送が容易である。必要に応じて、液体窒素保存または乾燥冷凍したサシチョウバエをアルコールに移すことも可能であるが、両方法の欠点が併存するため推奨されない。

3.3. RNA 安定化溶液（RNASS）による保存

RNA 安定化溶液（RNASS）は広く使用されており、非毒性で、新鮮な組織および細胞サンプル中の RNA を安定化・保護するよう組成されている。RNASS は標本へ迅速に浸透し、RNase（RNA 分解酵素）を不活化することで、即時の凍結を必要とせず RNA の分解を防止する。RNASS での保存は、後続の組織学的解析のための組織および細胞構造の保存にも一般的に有効である。RNASS は固定液ではなく RNA 安定化を最適化した製品であるものの、短期間から中期間の保存であれば構造も良好に維持される。RNASS を使用した場合、室温（約 7 日 間）、4°C（数週間）、–20°C または –80°C（長期保存）といった温度条件で保存が可能である。このため、コールドチェーンが制限される野外などに

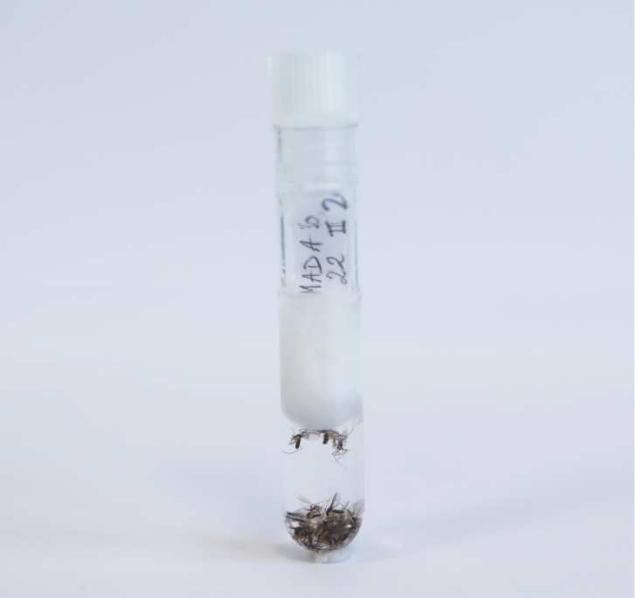

図 1：エタノール保存されたサシチョウバエ.

おいて特に有用である。RNA 抽出の際には、通常検体を RNASS から除去し、標準的なプロトコルに従って処理する。

3.4. 室温での乾燥保存

室温での乾燥保存は古くから用いられてきた手法であるが、*in toto* 標本（個体をそのまま保存）する場合、翅、脚、触角、小顎鬚といった脆弱な器官の保存が不十分になりやすいという大きな欠点がある。ただし、シリカゲルなどの乾燥剤で脱水処理を行えば、MALDI-ToF を用いたプロテオミクス解析は可能である。一方、DNA を対象とする分子生物学的解析では、DNA が断片化して量も少なくなるため、特に核ゲノム解析では新鮮検体や凍結検体と比べて明らかに不利である。それでも近年では museomics（博物館標本を対象とするゲノミクス） といった新しい技術により、このような標本を利用することも可能となっている［34］。したがって、他に選択肢がない場合を除き本保存法は推奨されない。また、この方法は –20°C または –80°C の冷凍保存と併用することも可能である。主な課題は、種同定に必要な標本や器官を適切にスライド標本が作製できる状態に戻すことである。そのためには再水和が不可欠であり、Triton X-100 溶液の使用を推奨する。再水和に要する時間は数時間から数日と幅があり、定期的な観察が必要となる。完全に再水和された後、標本は連続した3回の浸水で洗浄する。

3.5. 濾紙（フィルターペーパー）での保存

濾紙保存の主な利点は、固定されていない乾燥検体（個体全体または血液細胞）中のゲノム DNA を室温で長期間安定に保持できる点にある。濾紙は小さなカードサイズで提供され、数百の検体を小型の引き出し程度のスペースで室温保存できる。濾紙基材には感染性因子を変性させる薬剤が含浸しており、これにより検体はバイオハザードに該当しなくなる。そのため、特別なバイオハザード対策を講じることなく保存・輸送が可能となる［68］。

4. 検体の解剖

多くの昆虫では、個体全体をピン留めした標本において外部形態を観察することで同定が可能である。しかしサシチョウバエの場合、正確な種同定には、解剖およびスライド標本による内部および微細形態の観察が不可欠である。準備・封入方法がどのようなものであっても、解剖手技は基本的に同一である（図2および図3）（https://zenodo.org/records/18198006）。

Triton X-100 の使用：非イオン性水溶液

以下の手法は、新鮮検体または適切に保存された検体を前提としている。しかし、多くの収集者は、（MALDI-ToF 用に）乾燥状態で保存された検体や、長期間アルコールに保存された検体を保有している。残念ながら、アルコールによる長期保存は最適ではなく、節足動物はしばしば顕微鏡観察に適さない状態となる。よくある問題として、サンプルを入れたプラスチック容器が劣化し、アルコールが蒸発してしまうことが挙げられる。いずれの場合も、検体が長期間アルコールに浸漬され、乾燥しきってしまうため対処困難となる。そこで、浸潤剤の使用が検討された。Triton X-100 は 非イオン性水溶性界面活性剤であり、細胞生物学や分子生物学の分野で洗浄剤として広く使用されている（4-(1,1,3,3-tetramethylbutyl) phenyl–polyethylene glycol 、 t-octylphenoxypolyethoxyethanol 、 polyethylene glycol tert-octylphenyl ether）。細胞膜および核膜の透過化を可能にする。

非イオン性 Triton X-100（0.5%水溶液）を用いた処理手順は以下である。

-乾燥検体を無水エタノール（absolute alcohol）に浸す。

-検体全体が浸るように、0.5% Triton X-100 水溶液を必要量加える。

-5分～数日間浸漬し、定期的に観察する。 節足動物の体はすべて溶液中で完全に分離される必要がある。

-Triton X-100 溶液を除去し、水酸化カリウム（KOH）溶液に置き換える。

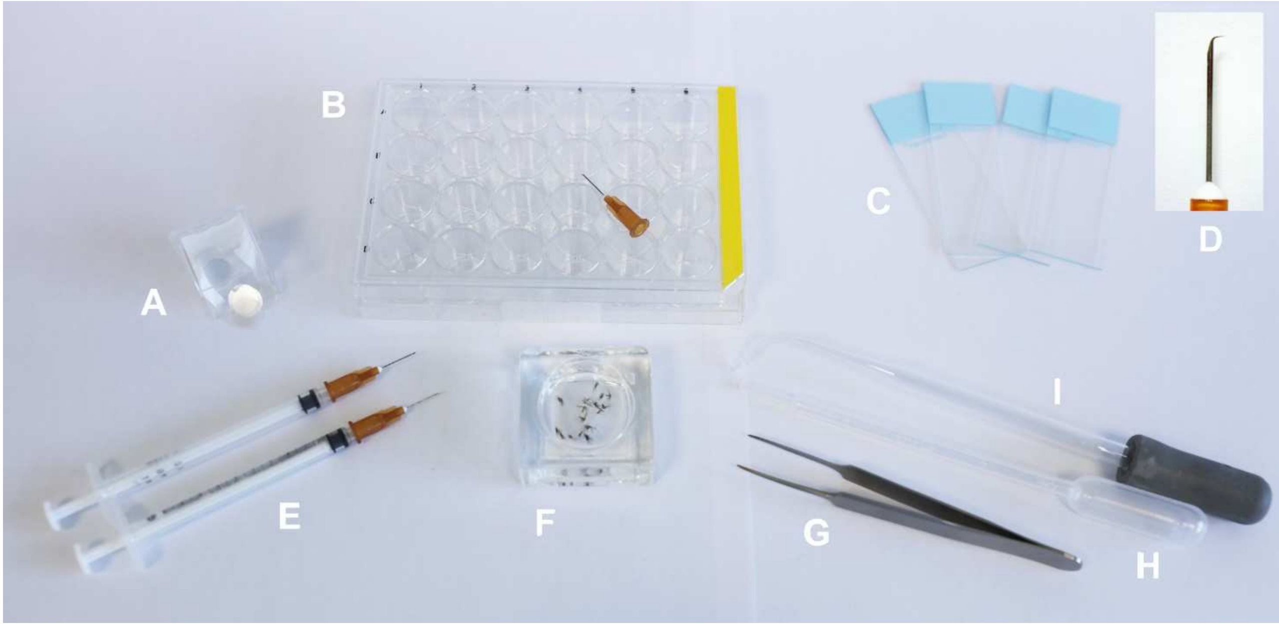

図 **2**：サシチョウバエのスライド標本作成に必要な器材**.** A：丸型カバーガラス(直径10 mmまたは12 mm); B：24ウェルプレートとフック付き針(clove oilまたは Euparal® エッセンスを用いる場合、アクリルプレートは化学反応による標本損傷のため使用不可); C：ラベリング可能なスライドグラス; D：フック針の先端; E：シリンジに装着した針; F：標本にするサシチョウバエを入れたデッシュ; G：Dumont ピンセット; H：プラスチックピペット; I：加熱して曲げたガラスピペット(ウェルへの液体移送を容易にするため).

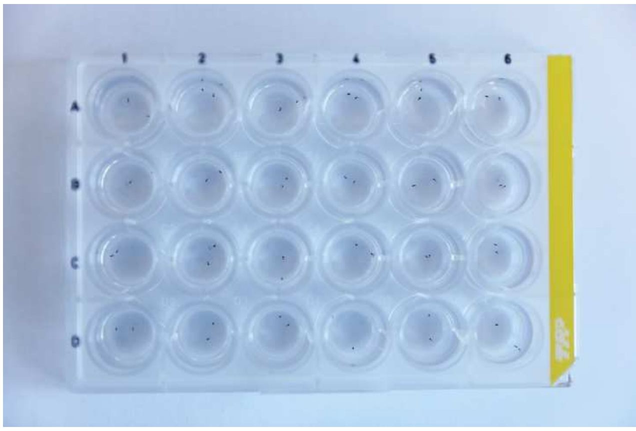

図 **3**：24ウェルプレートに配置したサシチョウバエの頭部と腹部先端.

4.1. 頭部（Head）

解剖は、実体顕微鏡下で細針または昆虫学用ピンを用いて行う（図2および図3）。一般的に使用される針には、26G × 1/2" (0.45 × 13 mm)、30G × 1/2" (0.3 × 13 mm)、25G × 5/8" (0.5 × 16 mm)などがある。同定のための最低限の準備として、頭部を胸部から切り離し、ventral side（腹側）を上にして封入し、cibarium（口器の一部）および pharynx（咽頭）を観察可能な状態にする。一方、胸部および腹部は解剖後にlateral view（側面）で封入する。頭部をventro-dorsal position（腹背方向）で封入すると、occipital foramen（後頭孔）が上向きに配置され、cibarium が直接観察できる。頭部が完全に分離されている方が、これらの形態の観察が容易である。

4.2. 翅および胸部（Wings and thorax）

翅は平らな状態で封入する必要がある。各翅は基部で切断して個別に封入してもよいし、一方の翅を胸部に付けたまま、片側だけを封入してもよい。幾何学的形態測定（geometric morphometry）を行う場合は、左右の翅を正確に識別・ラベリングしてから封入することが必須である。胸部は複数の構造からなり、非常に重要な分類学的形質を含む［20, 64］。一般に胸部

This is an Open Access article distributed under the terms of the Creative Commons Attribution License (https://creativecommons.org/licenses/by/4.0), which permits unrestricted use, distribution, and reproduction in any medium, provided the original work is properly cited.

は側面から封入し、chetaxy（刺毛配列）や色調パターンを観察する。胸部の特定領域における刺毛痕の有無は、*Brumptomyia* 属の一部の種の識別に有用である。色調パターンは、新熱帯区のサシチョウバエの分類（例： 属レベルの分類（*Bichromomyia*）、species series（*Pintomyia*等）、同一属内の種分類（*Micropygomyia*, *Nyssomyia*, *Psathyromyia*, *Psychodopygus*等））に利用される［20］。したがって、胸部を分子生物学的解析に使用しない場合は、損傷しないよう適切に封入すべきである。重要なのは 色の強さではなく、色分布パターンである点である。したがって、透化処理を行っても色素の有無や分布パターンが消失することはない。

4.3. 生殖器（Genitalia）

生殖器は雄雌ともに種・亜属・属を識別する上で最重要形態であるため、封入には特に注意を要する。雄雌とも、生殖器は対になっている。

4.3.1. 雄（Males）

雄の生殖器は外部構造であり、対の forceps（把握器）で構成される。forceps は背側の gonocoxite–gonostyleの関節部と腹側の epandrial lobe から成る。Gonostyleにはspines（棘）および必要に応じてsetae（剛毛）があり、その本数および付着位置を明瞭に観察する必要がある。Gonocoxite の内面は、無柄のsessile setae（剛毛束）または tubercle（突起）上のsessile setaeを有することがあり、詳細な観察が必要である［22］。経験の浅い研究者の場合は、腹部末端から生殖器を切り離さず、単純な側面封入でもよい（https://zenodo.org/records/18311158）。左右の生殖器が重なり、gonocoxite 内側のsetae数が数えにくい場合があるが、解剖失敗による損傷を避けられる利点がある。経験豊富な研究者の場合は、生殖器を左右に開き、分割を試みることができる。斜角を持つ針（皮内反応針型）の先端を通し、完全に切断しないように注意しながら gonocoxite–gonostyle 複合体を分離する（https://zenodo.org/records/18311158）。これにより、内部形態の観察が容易になる。また、parameres および parameral sheaths が重ならないため、これらの観察も容易になる。側面封入は構造が重なりやすいため、標本は完全に透明化されている必要がある。

4.3.2. 雌（Females）

雌の生殖器は内部構造であり、spermathecae（貯精嚢）から構成される。解剖を行わない場合は、腹部を腹側に向けて封入し、tegument（外皮）越しに観察する。選択した封入媒体にかかわらずspermathecaeが厚壁で透化されていない場合は観察可能なことが多い。しかし、薄く平滑なspermathecaeは屈折率の低い媒体では観察が困難なことがある。Spermathecal ducts の基部の観察は、特に旧 世界で *Leishmania infantum* を媒介する *Larroussius* 亜属の種識別に必須である［35, 37, 38］。Spermathecaeを確認できなければ、種同定は不可能となる。観察困難を解決するため、genital furca–spermathecae 複合体を腹部から取り出して封入する方法が推奨される（https://zenodo.org/records/18311106）。

Spermathecaeは解剖時に見つけにくい場合が多いが、genital furca は比較的容易に同定できる。Spermathecaeは genital furca に開口するため、furca を単離すれば精嚢も通常単離される。もし解剖時にspermathecaeが切断されても、腹部外皮の中に残存するため観察可能である（図4）。

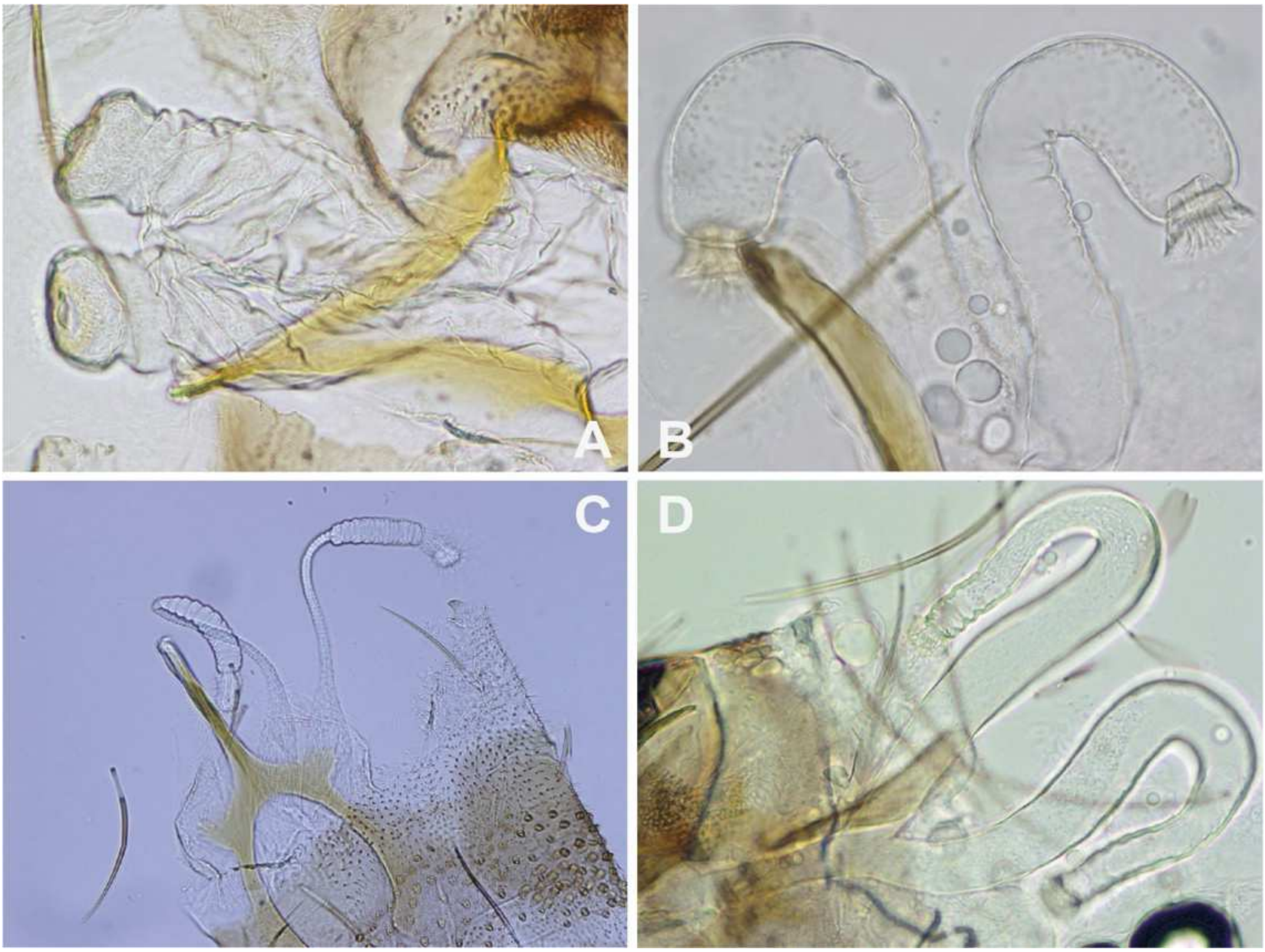

図4：新鮮検体から Marc-André 液で封入された spermathecae (貯精嚢). A：*Idiophlebotomus longiforceps* (ラオス); B：*Sergentomyia minuta* (フランス); C：*Phlebotomus ariasi* (フランス); D：*Sergentomyia anodontis* (ラオス).

4.4. リーシュマニア原虫分離のための中腸解剖

サシチョウバエの雌からリーシュマニア原虫を検出・分離するためには、消化管（特に中腸）の解剖が不可欠である。本手技は野外および実験室のどちらでも実施可能で、媒介能評価に利用される。

可能であれば、殺処理させたばかりの雌を用いることが推奨される。過剰な体毛を除去し無菌性を保つため、雌を水または軽度の界面活性剤を含む生理食塩水で洗浄する。この操作は、リーシュマニア原虫分離のための無菌条件維持と、種同定に必要な形態学的特徴の保存を両立させるために重要である。リーシュマニア原虫の検出と分離のため、中腸を慎重に取り出し、滅菌生理食塩水（0.9% NaCl）の滴中に置く。光学顕微鏡（推奨倍率：約200×）で可動性の無鞭毛型リーシュマニア原虫を確認した後、インスリンシリンジまたはマイクロピペットを用いて培地へ移す（詳細は 4.4.3 章）。頭部と生殖器は直接 Marc-André 液で封入し、透化処理を行う。

重要：Marc-André 液はリーシュマニア原虫に対して致死性であるため、直接・間接（針や器具経由）を問わず絶対に接触させてはならない。雌の解剖は、1枚のスライドで行う方法と2枚のスライドを使う方法があり、どちらにも利点と制約がある（図5; https://zenodo.org/records/18311154）。

This is an Open Access article distributed under the terms of the Creative Commons Attribution License (https://creativecommons.org/licenses/by/4.0), which permits unrestricted use, distribution, and reproduction in any medium, provided the original work is properly cited.

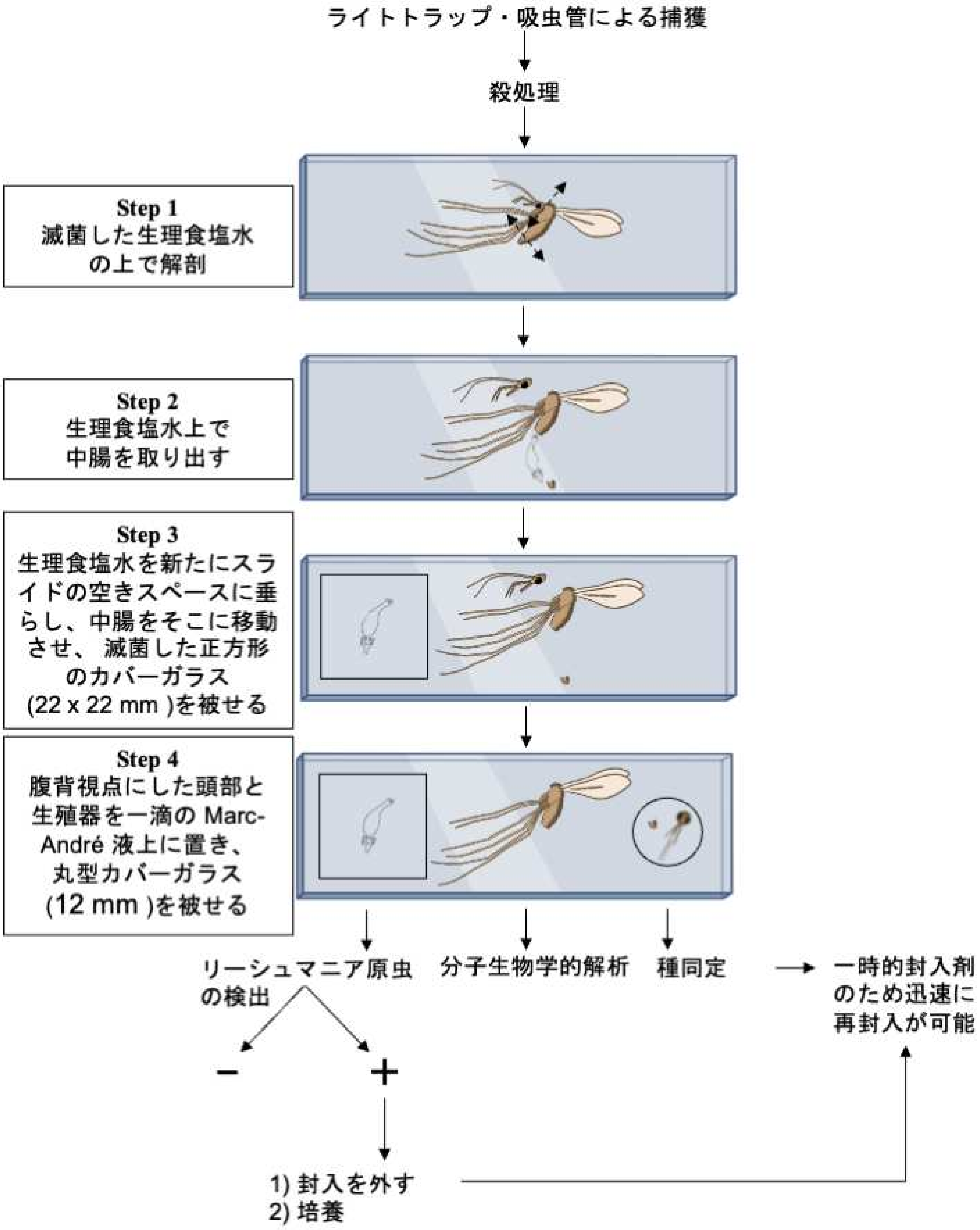

図5 ：リーシュマニア原虫分離法.

This is an Open Access article distributed under the terms of the Creative Commons Attribution License (https://creativecommons.org/licenses/by/4.0), which permits unrestricted use, distribution, and reproduction in any medium, provided the original work is properly cited.

4.4.1. 2枚スライド法（Two-slides method）

1枚目は、滅菌生理食塩水を置き、中腸を摘出するのに使用する。2枚目は、頭部およびspermathecaeを Marc-André 液で封入するために使用する。しかし、野外では複数の研究者が同時に解剖を行い、最後に1名が種同定と中腸内リーシュマニア原虫感染の有無を確認することが多い。この状況では、2枚のスライド管理が複雑化し、追跡性が損なわれる。特に、原虫陽性の中腸が見つかった際に、「どの個体が感染していたか」を確実に対応づけることが難しくなる（https://zenodo.org/records/18311154）。

4.4.2. 1枚スライド法（Single-slide method）

1枚のスライドに統合する方法では、結果の追跡性がより確保される。ただし、次のような注意点がある。

滅菌性確保：操作者は手指を定期的にアルコールジェルで消毒する。非フロストスライドとアルミホイルで包み乾熱滅菌した正方形カバーガラス（22 × 22 mm）を使用する。解剖ごとに滅菌針を用いる（例：25G, Ø 0.5 mm × 16 mm）。

手技：スライド中央に滅菌生理食塩水を垂らし、その滴の中にサシチョウバエを置く。頭部を切断、続いて第6–7腹節間で切開し、消化管を損傷しないようにする。 Spermathecaeが非常に長い種では、より前方で切開することも可能。針で胸部を固定し、もう一方の針で後腹部をゆっくり引き、中腸を抽出する。抽出が困難な場合は、 後腹部末端を針で固定し、前方から消化管を引く。それでも難しい場合、周囲の外皮をできる限り除去して中腸を取り出す。中腸が取り出せたら、消化管を切断して後腹部末端と分離する。中腸はスライド片側の新しい滅菌食塩水滴に移し、滅菌カバーガラスでやさしく覆う。頭部と後腹部末端はスライド反対側の小滴のMarc-André 液に移し、リーシュマニア原虫と接触しないよう厳重に管理する。頭部はoccipital foramen（後頭孔）が上向きになるよう配置する。Spermathecaeは genital furca とともに分離し、直径12 mm の丸カバーガラスで覆う （滅菌四角カバーガラスと混同しないよう注意）。胴体残部と翅はスライド中央の食塩水中に残す。陽性個体であった場合、または分類学的解析を行う場合には、胸部・腹部を分子生物学的またはプロテオミクス解析用に保存する。翅は水性媒体で封入が可能である。Marc-André 液の余剰分は、chloral gum（＝Hoyer）、またはポリビニルアルコール系媒体に置き換え、封入を保持できる。作業手順の詳細を示した動画が公開されている：中腸解剖：https://zenodo.org/records/18303014 ；唾液腺解剖：https://zenodo.org/records/18302850 本稿では詳細説明は割愛する。

4.4.3. サシチョウバエ中腸からの リーシュマニア原虫の分離および培養

リーシュマニア原虫感染サシチョウバエの雌からの原虫分離は高度な技術を要し、まずは非感染個体で練習することが推奨される。解剖後、中腸は新しい滅菌生理食塩水（0.9%）またはLocke 液へ移し、洗浄する［4］。その後、以下の2つプロセスがある。１）光学顕微鏡下で鞭毛型リーシュマニア原虫（プロマスチゴート）の発育段階および局在（特に stomodeal valve）を観察、２）中腸を開き、プロマスチゴートの遊出を促して大量培養へ備える［4］。野外で感染個体を得ることは稀であり、技術習熟による成功率向上が望まれる。

分離操作：中腸内にリーシュマニア原虫が確認されたら、新しい滅菌針を使用し、カバーガラス周囲に滅菌生理食塩水を毛細管現象で追加し、原虫を解放する。中腸を速やかに破り、原虫を生理食塩水中に遊離させる。100 µL マイクロピペットまたはツベルクリンシリンジを使用して原虫を回収し、ラベルされた培地へ移す。

リーシュマニア原虫の *in vitro* 培養：分離されたプロマスチゴートは、通常SNB-9 血液寒天斜面培地またはNNN（Novy–McNeal–Nicolle）固形培地のいずれかに接種され、滅菌 α-MEMまたはM199の液体培地で上層培養される。いずれも10%加熱不活化ウシ胎児血清（FCS）、1%BMEビタミン、2%滅菌ヒト尿（Filtropur® S 0.2 µmで濾過）、および適切な抗生物質を添加する。3日後、コンタミがなければ、適切に調製した凍結保存液に懸濁し–80°C で1〜2年、または液体窒素（–196°C）で長期保存が可能となり、将来の実験に利用される［7］。

4.5. 唾液腺（Salivary glands）

サシチョウバエの唾液腺解剖は、媒介昆虫と病原体の相互作用を研究するうえで極めて重要な技術であり、特に Toscana virus などの Phlebovirus 系アルボウイルスの検出 に不可欠である［44, 75］。

サシチョウバエは極めて小型であるため、この解剖は実体顕微鏡下で、精密なピンセットまたはマイクロ解剖針を用いて、脆弱な唾液腺を破損やコンタミなく単離する高い精度が求められる（https://zenodo.org/records/18302850）［51, 61］。唾液腺の完全性を保持することは、その後の分子生物学的解析の信頼性にとって必須である。抽出した唾液腺はホモジナイズ後、RT-PCR、qPCR、免疫アッセイなどによってウイルス RNA や抗原の検出が可能である［12］。重要なのは、唾液腺内でウイルスが検出されることは、病原体が媒介昆虫内で外因性潜伏期間（extrinsic incubation period）を完了し、吸血時に伝播可能な状態に達していることを示す点である［71］。

唾液腺は非常に小さく繊細であるため、解剖操作は高い熟練度を要する［1, 51］。さらに、唾液腺内のウイルス量は少ないことが多いため、nested PCR やハイスループットシーケンスのように高感度な手法が必要となる場合がある［54］。コンタミのリスクも高く、無菌的手技の徹底が求められる。また、生物学的要因として、サシチョウバエ種間での媒介能の違いや、生態条件・季節の変動がウイルス検出率に影響する［33, 61］。

唾液腺でのウイルス検出は、感染伝播リスクの評価や標的型サーベイランスおよび対策立案に重要な情報を提供する［15］。例えば、流行地のサシチョウバエから Toscana virus を検出することで、診断プロトコルや公衆衛生上の注意喚起に寄与している［18］。さらに、ウイルスと唾液成分の相互作用を研究することで、伝播阻止ワクチンや治療法の新規標的候補の発見につながる可能性がある［15, 18］。

サシチョウバエの唾液腺は、また、免疫学的手法（主に ELISA）を用いて、宿主がサシチョウバエの唾液に対する抗体を保有しているかを測定する抗原としても利用される。この方法により、宿主のサシチョウバエ咬傷への曝露評価、媒介昆虫対策の効果検証［25］、リーシュマニア原虫伝播リスクの推定［40］が可能となる。

4.6. 吸血源の同定

捕獲された吸血個体（engorged females）は、クロスコンタミネーションを避けるため必ずディスポーザブル器具を使用して解剖する必要がある。まず、腹部を実体顕微鏡下で観察し、血液消化の進行段階を評価する。選択すべきは、赤色、赤褐色、または暗赤色の腹部をもち、卵形成の徴候が見られないメスである。同定のため、まず腹部先端（spermathecaeを含む）を切除し、透化後に形態学的同定を行う。その後、spermathecae を除いた腹部主要部位を Eppendorf® チューブに入れ、–20°Cで保存して解析に用いる。吸血源同定に一般的に使用される遺伝子マーカーには、PNOC［5, 30, 50］、CytB［67］、COI［13］などがあり、既に文献で広く確立され詳細に記載されているため、本稿では詳細説明を省略する（図 6）。代替法として、MALDI-ToF によるペプチドマッピングを用いた宿主血液の同定も可能である［31］。この技術は、実験的に、吸血後より長い時間が経過した場合でも宿主血の判定が可能であることが示されており、消化が進んだ吸血個体の解析に特に有用である。試料の保存は本来 –20°C または 4°C が望ましいが、短期間であれば室温保存でも良好な結果が得られる。吸血メスの腹部は、解析の直前に体から分離し、蒸留水中でホモジナイズする。残りの体部分は、他の分子生物学的・形態学的解析に利用可能である。ホモジネートから MALDI-ToF ペプチドマッピング用の試料を採取した後、残りの試料は 宿主血の同定確認用の DNA 抽出、あるいは リーシュマニア原虫検出のためのスクリーニングに使用できる。MALDI-ToF による吸血源同定は、DNA ベースの分子的手法と比較して、試料準備から解析までの所要時間が非常に短いという利点がある。

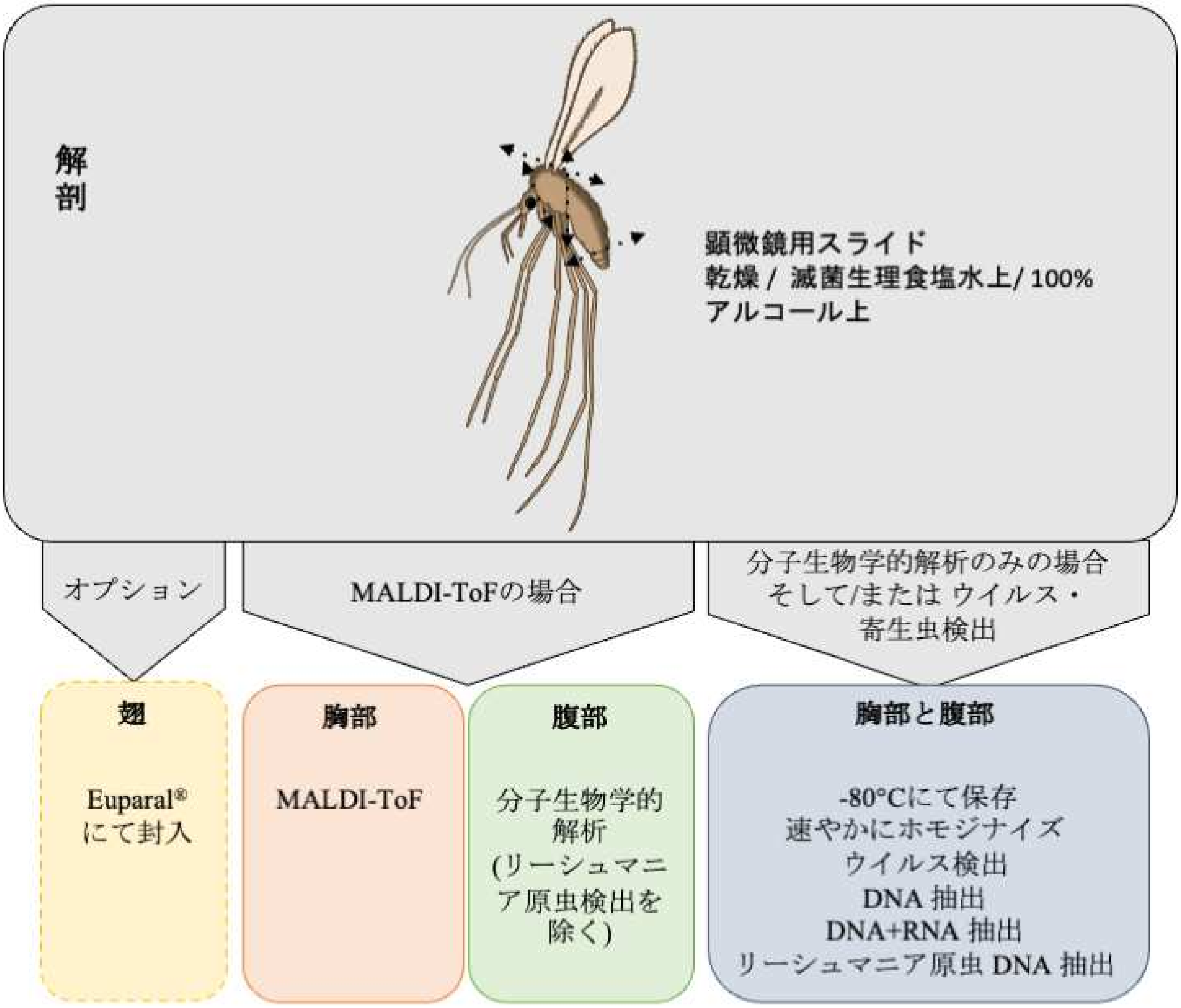

図 6： 分子生物学・プロテオミクス・ウイルス学解析のためのサシチョウバエ処理プロセス.

5. 形態学的研究のための検体処理（図 3、6、7、8；付録 1・2・3・4）

本節では、形態学的研究のみを目的としてサシチョウバエ検体を封入するための基本原理を概説し、その後、形態学以外の応用に対応するための手順の応用的調整について述べる。この一連の方法論を理解することは、必要に応じて特定のサンプルタイプに合わせて処理法を適応させるために極めて重要である。

検体処理では、パスツールピペット（柔らかいゴム球付き）を用い試薬の連続的な「排出」と「充填」操作を行う。この操作を円 滑に行うため、底が丸いガラス容器の使用が強く推奨される。ガラスは使用されるすべての試薬に対して化学的に不活性であり、反応や劣化の心配がない。試薬の蒸発を防ぐため、容器には必ず蓋を装着し、閉める・開ける際にあふれ出るほど試薬を満たさないことが重要である。また、塵埃の混入を防ぐためにも容器は常に蓋付きで扱うべきである。透化・処理に必要な試薬については 表2に示す。

This is an Open Access article distributed under the terms of the Creative Commons Attribution License (https://creativecommons.org/licenses/by/4.0), which permits unrestricted use, distribution, and reproduction in any medium, provided the original work is properly cited.

表 **2 :** 各試薬の組成

**Potassium hydroxide 10%**<br>Potassium hydroxide 10 g<br>Distilled water qs 100 mL	**Fuchsin acid 1% in distilled water**<br>Acid fuchsin (as a powder) 1 g<br>Distilled water 99 mL
**Gum chloral mounting media (Hoyer medium)**<br>Distilled water 50 mL<br>Chloral hydrate 200 g<br>Gum Arabic 50 g<br>Glycerol 20 mL	**Marc-André solution colored with acid fuchsin**<br>Marc-André solution 10mL<br>Fuchsin 1% 50 µL
**Marc-André solution**<br>Chloral hydrate 40 g<br>Glacial acetic acid 30 mL<br>Distilled water 30 mL	**Enecê medium**<br>Pure white colophony 22 g<br>Alcohol-soluble copal gum 12 g<br>Absolute ethanol 20 mL<br>Camphor 10 g<br>Turpentine essence 10 mL<br>Eucalyptol 26 mL

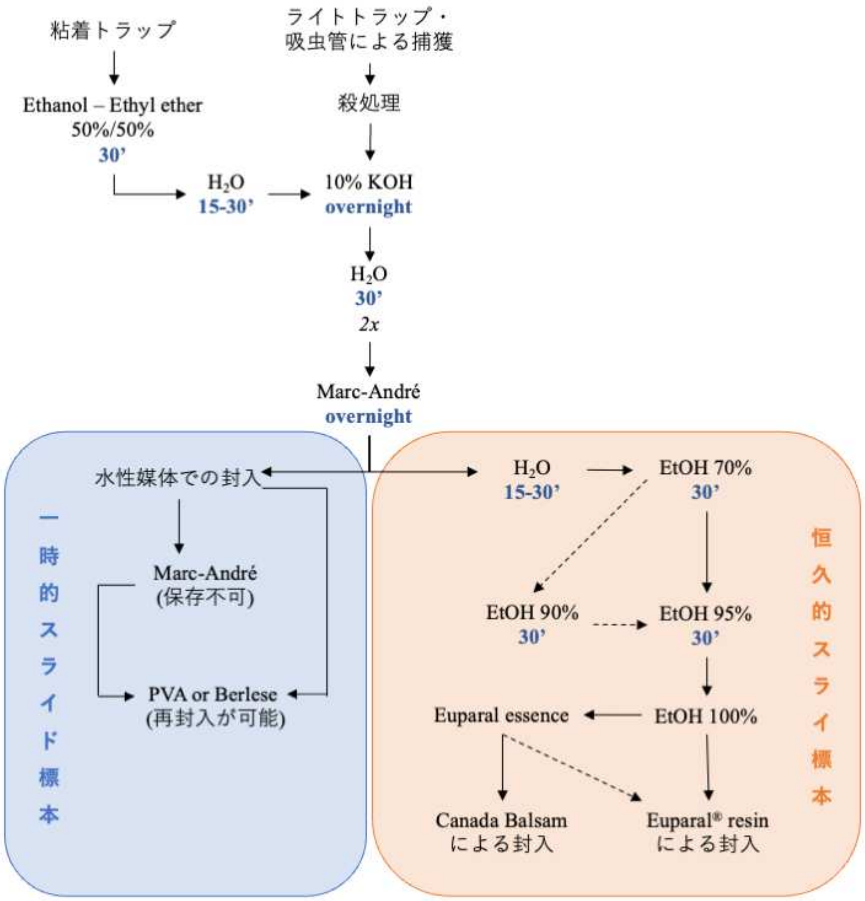

図7 : サシチョウバエ検体処理の古典的手法.

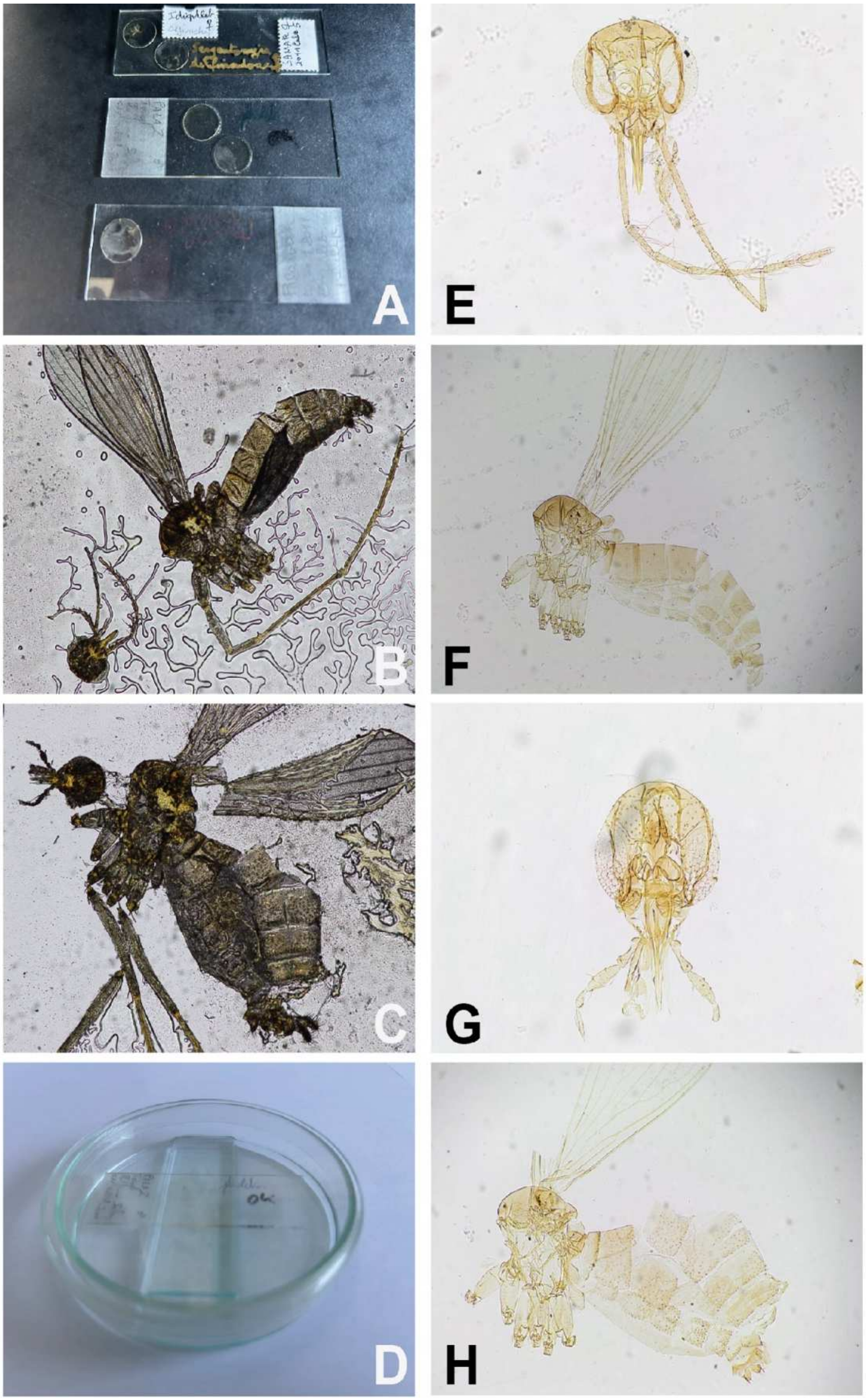

図8：スライドの再封入処理. A：Hoyer で封入され乾燥・損傷したスライド; B：乾燥した個体の顕微鏡像; C：別個体の損傷像; D：乾燥スライドを置いた湿室; E：再マウント後の標本Bの頭部; F：同標本の胴部; G：標本Cの頭部; H：同標本Cの胴部（いずれも Euparal® で再マウント）.

5.1. 透化処理

サシチョウバエ検体を恒久標本として封入する前には、まず適切な方法と透化剤（例：10%酢酸溶液、あるいは Marc-André 溶液（クロラルハイドレートを含む。多くの国で規制対象））を用いて組織をマセレーションし透明化する工程が必要である。透化処理によって、体組織、脂肪、分泌物、ワックスが除去され、標本は半透明となる。これにより外皮構造（例：刺毛の付着位置）、体表の特徴（例：色調）、外皮越しに観察可能な内部構造（例：spermathecae）の観察が容易になる。

透化は通常、最初に強塩基（例：KOH）、続いて 弱酸 （例：酢酸；Marc-André 溶液中）の 2 段階からなり、これらは異なる生化学的目的を持つ［74］。強塩基は、タンパク質、脂質、筋組織を分解するが、キチン外骨格は残り、形態構造を明瞭化する。弱酸は、残存アルカリを中和し過剰な分解を防ぎ、キチンの脱色を促進し透明性を向上（蒸留水で15分×2回の洗浄でも中和可能）させる。この連続処理により、組織除去の効率と構造保存性が両立し、顕微鏡観察に最適な標本を得ることができる。次工程へ進む前に、蒸留水で20分の洗浄を2回行うことが推奨される。

5.1.1. 軟組織の溶解（図 8）

水酸化ナトリウム（NaOH） または 水酸化カリウム（KOH） が標準的なマセレーション剤として用いられ、試料の大きさや脆弱性に応じて濃度・処理時間が調整される。一般的かつ最も効率的な方法は、10% KOH または 10% NaOH にサシチョウバエを 一晩浸漬し軟組織を溶解させる方法である。処理を短縮したい場合は、20% KOH で 6 時間浸漬、あるいは37°C の加温処理などで代替が可能である。

5.1.2. 染色の有無による明調処理

軟組織除去後、通常は酢酸とクロラルハイドレートを組み合わせた明調処理が行われ、代表的な例として Marc-André 溶液が挙げられる。透化処理の後には、残留する化学物質を確実に除去するため、標本を20分×2回以上の蒸留水洗浄が必要である。

Marc-André 溶液は、サシチョウバエ標本の作製に広く用いられる透化剤であり、処理の効率を高めつつ、翅や触角といった脆弱な構造への損傷を最小限に抑える点に大きな利点がある。Marc-André溶液は新しく調製するか、蒸発や分解を防ぐために密閉容器で保管することが望ましい。また、Marc-André溶液は、特定の形態的特徴を際立たせるための明調処理や染色法と組み合わせることで、さらに有用性を発揮する。組成および調製方法の詳細は付録2に示されている。

標本が高度に透明化されている場合には、封入前に視認性を向上させる目的で染色が必要となることがある。生物体の特定の化学成分に作用するさまざまな染色剤が存在するため、標本および選択した封入剤の双方に適合する染色剤を選ぶことが重要である。基本となる手法は目的に応じて調整することができ、例えば Marc-André 溶液に 0.1% acid fuchsinを加えて染色に用いることが可能である。

さらに、水系溶液に保存された標本で樹脂系封入剤を行う場合には、天然樹脂・合成樹脂の多くが水と不混和であるため、必ず脱水処理が必要となる（脱水については 5.2 節を参照）。New（1974）は、一部の染色剤が特定の封入剤中で劣化することを指摘しており［53］、例えば acid fuchsinは伝統的に Canada balsam と併用されるが、Euparal® による固定も可能であるものの、acid fuchsinで染色した標本は退色しやすいことが知られている。特に、最終透化段階で使用される clove oilが残留している場合に退色が顕著となり、clove oil 中で保存された標本では数日以内に強い退色が生じることがある。

5.2. 脱水

脱水は、標本を段階的にエタノールの濃度系列（50%、70%、80%、90%または 95%、そして最終的に 100%）へ移し替えて行うものであり、各工程は少なくとも20分間維持する必要がある。エタノールは揮発性が高いため、処理中は容器をしっかりと密閉しておくことが重要である。標本が完全に脱水された後は、処理を数日間中断して Euparal® エッセンスに浸しておくことが可能であり、これはclove oilよりも好ましいとされる。かつてこの目的で広く使用されていたBeech creosoteは、強い毒性のため現在では使用が全面的に禁止されている。

脱水工程では、標本内部の液体が選択した封

入剤と適合する状態となるよう注意しなければならない。液体の不適合は、標本の不透明化、浸透圧による潰れ、あるいは形態の歪みを引き起こし、分類学的研究において不適切な標本となり得るためである。

5.3. 封入剤

5.3.1. 標本作製における選択と適用

理想的な封入剤とは、ガラス（屈折率約 1.5）にできる限り近い屈折率をもち、無色透明で、乾燥後も長期にわたり完全な透明性を維持するものである。さらに、使用する染色剤と化学的に適合し、標本内部に深く浸透でき、封入作業中に急速に乾燥したり曇ったりせず、乾燥後に収縮しないことが求められる。どのような用途にも万能な封入剤は存在しないため、適切な封入剤の選択は標本作製において最も重要な判断の一つとなる。適切な封入剤を選ぶ際には、いくつかの要素を総合的に考慮する必要がある。

光学的な観点：封入剤の屈折率が分類学的同定で重要となるspermathecae、ascoids、Newstead sensilla、vertical cibarial teeth、 pharyngeal teethなどの形態を適切に捉えられることが求められる。こうした微細構造の視認性は、封入剤の光学特性に直接依存している。

保存性の観点：タイプ標本や永続的コレクションに用いる場合、長期的な安定性と耐久性を備えた封入剤である必要がある。一方で、単発的な調査や疫学調査など長期保存を重視しない目的では、一時的または半恒久的な封入剤でも十分である。

5.3.2. 封入剤に求められる条件

専門家の中には、研究目的に特化した複雑で独自 の封入技術を用いる者も少なくない。しかし、そのような方法はしばしばアーカイブ性、標準化、封入剤との互換性、扱いの容易さ、そして長期保存性といった点が十分に考慮されていないことがある。この問題は、寄贈標本の統合や長期的なコレクション管理を困難にする原因となる。

科学的用途では、封入剤には明確な要求が課される。分類学的研究では個体全体を封入することが多く、内部器官を穏やかにマセレーションして外骨格構造をより明瞭にする封入剤が好まれる。封入剤の屈折率は、標本およびガラススライドとの差が十分にあることが望ましく、これによって光学的な鮮明さが高まる。市販の封入剤の多くは、スライド—封入剤—カバーガラス系における光散乱や反射を抑えるため、ガラスと近い屈折率に調整されている。しかし、明視野顕微鏡では、標本の自 然なコントラストを強調するために、あえて標本とは異なる屈折率をもつ封入剤を選ぶことで背景との視認性を高める手法も有用である。

5.3.3. 封入剤の種類（表 3 & 4）

顕微鏡観察では、封入剤の屈折率が光の屈曲を決定するため、スライド、封入剤、標本を通過する光の挙動は媒体の屈折率によって大きく左右される。封入剤の屈折率がカバーガラスの値（約 1.515）と近い場合、光は均一に通過し散乱が抑えられ、構造の解像度と視認性が向上する。逆に大きな差があると、ぼやけ、光輪、あるいは未染色構造の不明瞭化といった問題が生じ得る。封入剤の選択は、標本の性質や目的に最適なコントラスト、鮮明度、全体的な画像品質を得るために重要である。

サシチョウバエの場合、外皮硬化が弱く非常に繊細な構造（cibarial armature、spermathecae、antennal segments、wing venation等）の観察には、封入剤の屈折率が特に強く影響する。高い屈折率の媒体では、これらの構造が観察しにくくなることがある。

一般に、サシチョウバエでよく使用される封入剤には、水性媒体としての gum-chloral 系、溶剤系媒体としての Canada balsam および Enecê – Nelson Cerqueira（NC）レジン がある。Rawlins［60］は封入剤を、時間とともに硬化して長期保存に適した「恒久的封入剤」と、硬化せず主に一時的使用を目的とした「半恒久的封入剤」の2種類に分類している。

表 **3**: 封入剤の比較

Mounting medium	Solvent	Potential pre-polymer(s) or polymer	Remarks
Hoyer = chloral gum	glycerol, water	compounds of gum arabic	Macerating agent: chloral hydrate
CMCP-9 (= carboxy methyl cellulose phenol)	Water (CMCP-9: 51–60%)	fully hydrolyzed polyvinyl alcohol (CMCP-9: 0–5%)	CMC(P)-9: low viscosity: high viscosity
DMHF (dimethyl hydantoin formaldehyde)	water	N,N′-dimethylol dimethyl hydantoin (di-methylol DMH)<br>Ether-/methylene-bridged oligomers<br>Crosslinked DMH–formaldehyde polymer network	
Canada balsam	xylene; partly volatile components of balsam (Δ³-carene, levopimaric acid, limonene, myrcene, palustric acid, β-phellandrene, α-pinene, β-pinene)	balsam (abienol, abietic acid, isopimaric acid, sandaracopimaric acid)	neutralization: potassium carbonate;<br>resin from *Abies balsamea* (Linné, 1758)
Euparal®	eucalyptol, paraldehyde; partly volatile components of gum sandarac (limonene, α-pinene, β-pinene)	compounds of gum sandarac (communic acid, manool, polycommunic acid, sandaracopimaric acid, 12-acetoxy-sandaracopimaric acid, sugiol, torulosic acid, torulosol, totarol)	clearing agent: methyl salicylate; color in Euparal® green: copper salt (copper abietinate); sandarac resin from *Tetraclinis articulata* (Vahl, 1791)
Enecê	ethyl alcohol; with camphor, eucalyptol and turpentine essence	Compounds of copal gum and colophony (rosin)	

封入剤には、水溶性、ガム系、樹脂系といった種類があり、封入剤によって水、アルコール、またはトルエンやキシレンなどの有機溶媒に可溶である場合がある（表3）。いずれの封入剤も、スライド上に適用した後は、大気中の影響を受けないように不溶性の封じ剤（ringing media）で密封する必要がある。封入剤の種類を明確に区別するために、次のような分類が用いられる。

a. 水溶性封入剤：水に容易に溶解する性質をもち、一時的または半恒久的なマウントに適している。このタイプの封入剤は一般に取り扱いが容易であるが、特に熱帯の高湿度環境では、大気中の水分の影響を受けやすいため、ガム・クロラル系媒体やポリビニルアルコール媒体などでは、湿気への曝露を防ぐためにマウント後

の封じ処理が必要となる。

b．水への耐性がある程度認められる封入剤：水溶性封入剤ほど水分による影響を受けないものの、依然として過度の湿気から保護する必要がある。こうした封入剤は、水溶性封入剤よりも長期的に安定であり、半恒久的な封入によく用いられる。

c. 炭化水素系溶媒に可溶な封入剤：キシレン、トルエン、またはessenecê（enecê solvent）などの有機溶媒に溶解するタイプで、恒久的な封入を目的として調合されている。これらの封入剤は長期安定性に優れ、湿気や劣化に強いため、アーカイブ用途に最適であり、Canada balsam やなどが典型的な例である。

総括すると、水溶性封入剤は標本の取り外しが容易で一時的な封入に最適であり、水にある程度耐性のある封入剤は適度な耐久性を求める半恒久的マウントに適している。また、炭化水素系溶媒に可溶な媒体は、長期間の保存やアーカイブを目的とする恒久的封入に最も適した選択肢である。

表 4: 顕微鏡用スライド標本作製における主要封入剤の利点と欠点（複数研究者による未発表観察を含む）[52].

名称	利点	欠点
**Canada balsam**	・この封入媒体は非常に耐久性が高く、寿命は150年以上に及ぶ。<br>・スライドは、封入剤としてクローブオイルまたはフェノールを用いて作製することができる。	・有害成分を含むため、ドラフト（排気フード）内で取り扱う必要がある。<br>・完全な脱水系列が必要であり、作業には多くの時間を要する。<br>・エタノールによる脱水と、キシレンまたはクローブオイルを介した移行は、一部の分類群を脆くすることがあり、代替溶媒（例：isopropanol, n-butanol, Cellosolve™, 1,4-dioxane, Histoclear, terpineol）を用いることで破損を軽減できる可能性がある。<br>・キシレンをフェノールに置き換えた場合、または水酸化カリウムが残留している場合、標本が黒変することがある。<br>・屈折率が高いと、未染色の構造が観察しづらくなる場合がある。<br>・ホットプレートによる乾燥を用いない場合、完全な乾燥には数年を要することがある。<br>・この封入媒体は時間の経過とともに黄変・暗色化しやすく、特にクローブオイルで透化した標本では顕著である。<br>・一部の染色剤は弱まり、陽イオン性染料は媒体が酸性化した場合に退色することがあるが、媒体の酸性化は時間の経過とともに自 然に生じ得る。

**DMHF (dimethyl hydantoin formaldehyde)**	・高い透明性。<br>・良好な屈折率。<br>・構造の視認性が非常に高い。<br>・標本の安定性が比較的良い。<br>・多くの染色法と両立する。<br>・標本の良好な保護性を提供する。<br>・スライドとカバーガラス間の接着性が高い。	・経時的に黄変する可能性がある。<br>・一部の染色を変質させる可能性がある。<br>・ホルムアルデヒドに感受性のある染色には不適切である。<br>・気泡が入りやすく、乾燥に時間を要する。<br>・湿度に影響されやすい封入媒体である。<br>・一度封入すると除去が困難である。<br>・ホルムアルデヒドは毒性・刺激性・発がん性を有する。
**Euparal (transparent)**	・耐久性に優れ、50年以上の寿命をもつ封入媒体である。<br>・製造者の推奨により、80%エタノールから直接封入することが可能である。<br>・未染色構造を覆い隠すことがなく、時間の経過によって黄変したり脆くなったりしない。<br>・ハエ目（Diptera）の標本に対しては、Canada balsam よりも適した屈折率を有する。<br>・収縮がごくわずかで、気泡のない乾燥が可能であるため、より厚みのある標本にも適している。<br>・95%エタノールに可溶な状態を保つため、長年経過した後でも再封入が可能である。	・有害成分を含むため、ドラフト（排気フード）内で取り扱う必要がある。<br>・エタノールによる脱水および Euparal®を介した移行は、一部の分類群を脆化させることがあるが、イソプロパノールを用いることでこの問題を軽減できる可能性がある。
**Hoyer fluid**	・検体は、生きた状態のまま、または水・エタノール・ホルムアルデヒドから直接封入することができる。<br>・マセレーション処理により、クチクラの状態が非常に良好に得られる。<br>・屈折率が良好であり、ヨウ素染色を併用することでコントラストをさらに高めることができる。<br>・処方中の酢酸によって、節足動物の付属肢が膨張する場合がある。<br>・標本によっては、40〜60 年にわたり安定した状態を保つことがある。<br>・水溶性であるため、再封入が容易である。	・繊細な植物標本は、媒体を徐々に添加しないと崩壊することがあり、この作業は時間を要する。<br>・10 年未満で、空隙や結晶が形成されることがある。<br>・クロラルハイドレートの濃度や曝露時間によっては、マセレーションが過度に進行する場合がある。<br>・媒体の成分が分離することがあり、数か月から数年のうちに微細な粒状物が生じることがある。<br>・媒体の黒変が報告されている。

**CMCP-9 (= carboxy methyl cellulose phenol)**	・検体は、水、エタノール、グリセロール、あるいはホルムアルデヒドを含む溶液などの媒体から直接封入することができる。<br>・必要に応じて内部器官をマセレーションし、全体的な観察や調製を容易にすることも可能である。	・この媒体は、経時的に結晶を生じたり暗色化したりすることがあり、また、意図以上に標本をマセレーションしてしまう場合がある。<br>・スライドを丁寧にリング封入しない限り、厚みのある試料は媒体中で収縮してカバーガラス周縁に隙間を生じやすく、良好な結果が得られない。<br>・染色標本や石灰化組織には不適であり、乾燥時間も CMC より遅い。
**Eukitt™**	・耐久性が高く、30年以上にわたり安定して使用できる封入媒体である。<br>・アセトン、ベンゼン、クロロホルム、ジオキサン、エーテル、イソプロパノール、安息香酸メチル、テルピネオール、トルエン、キシレンなど、多くの溶媒と併用して封入が可能である。<br>・速乾性があり、わずかに酸性の pH を示す。<br>・経年による暗色化はほとんど認められない。<br>・フクシン、ヘマトキシリン、メチルグリーン、メチルバイオレット、メチレンブルーなど、さまざまな染色剤と適合する。<br>・スライドは長期間経過後でも、キシレンに長時間浸漬することで再封入が可能である。	・有害成分を含むため、ドラフト（排気フード）内で取り扱う必要がある。<br>・完全な脱水系列を要し、その工程には時間がかかる。<br>・収縮やガス気泡の形成が生じるため、厚みのある標本には適さない。<br>・ガラス面の洗浄や封じ処理が不十分な場合、時間の経過とともにカバーガラスが剥離することがある。<br>・コラーゲン繊維の周囲で重合が不完全となる場合がある。
**Enecê**	・きわめて耐久性の高い封入媒体であり、少なくとも50年は安定して保持される。<br>・Enecê は経年による暗色化を起こさない。<br>・媒体がより可塑性をもつため、媒体中で昆虫の解剖が可能であり、形態構造を適切に配置するための十分な作業時間を確保できる。<br>・低コストである。	・完全な脱水系列が必要であり、その工程には多くの時間を要する。<br>・エタノールによる脱水およびクローブオイルを介した移行により、標本が脆くなる場合がある。<br>・昆虫は非常にゆっくりではあるものの透化が進行し続けるため、sensilla、ascoids、simple setaeなどの非常に小さな構造が観察しにくくなることがある。

5.3.4. 推奨される封入剤の解説（表 3 および表 4）

一時観察用媒体

クロラルガム（＝Hoyer fluid/medium/solution、屈折率 RI = 1.48）

Marc André 液は、spermathecaeの短時間観察（数時間、湿室に保管すればもう少し延長可能）に最適な媒体であり、写真撮影や図示（図4）にも適している。観察した spermathecaeを保存する場合には、中期的な保存が可能な水性媒体へ再マウントする必要がある。樹脂媒体への脱水再マウントも不可能ではないが、構造喪失のリスクがあるため推奨されない。クロラルガムと Hoyer fluidは同義とみなされており、水との相性の良さ、調製と使用の容易さ、迅速な適用性、そして spermathecaeのような脆弱構造の観察に適した屈折率を備えていることから、内部器官の観察によく用いられる。

しかし、クロラルガムは完全な調製や湿度管理がなされていない場合、結晶化、変色、粘性低下といった重大な欠点を示す。カバーガラスをリング剤で封入してもこれらの問題は解決せず、特に Euparal® を使用した場合にはリング剤との相互作用により媒体が著しく変色（時に黒色化）することがある。

Hoyer 媒体は古くからサシチョウバエに対して最良の光学特性を示すとされ、伝統的に使用されてきた。Hoyer 媒体はアラビアガム、グリセロール、クロラルハイドレートを含むいくつかの類似した処方から構成されるが、文献中では処方の誤認や誤引用が少なくない［74］。

サシチョウバエの spermathecae観察には非常に適しているものの、Hoyer 媒体は長期保存には不向きであり、写真撮影や描画など、短期間の観察を目的とする場合に最適である。水性媒体は本質的に一時的マウントに適するが、長期保存性は確保できない。その一方で、樹脂媒体は数世紀単位での耐久性を提供するものの、spermathecaeの微細な屈折性を失わせることがあり、観察性を損なう場合がある。

Hoyer 媒体は時間の経過とともに脱水され（図8）、白色で不透明なクロラルハイドレートの微結晶を生じて劣化する。それでも、外骨格は化学的に保持されるため、結晶化したスライドから標本を回収することは可能であるが、結晶成長により物理的損傷が生じることがある。場合によっては、温かく湿った環境下でサイモールを用いて真菌の発生を抑制しながら封入剤を再水和することで、結晶化したスライドを修復できる。また、標本をクロラルガムから水に浸して剥離させ、氷酢酸で脱水したうえで Canada balsam に再マウントする方法もある。

DMHF（dimethyl hydantoin formaldehyde、屈折率 RI = 1.48）

この水性媒体［72］は、光学的性質が Berlese 媒体に非常によく似ており、使用の容易さも同等である。しかし Berlese 媒体とは異なり、黒変や結晶化を起こさない点が大きな利点である。サシチョウバエを含む Psychodidae に対して非常に良好に機能する。

CMCP（camphor-mono-chlorophenol、屈折率 RI = 1.41）

CMCP はグリセリンを基剤とする水溶性の封入剤で、サシチョウバエを含む微細で脆弱な標本を透明な恒久プレパラートとして作製するために用いられる。この封入剤の利点は、水またはエタノールから直接マウントできる点にあり、標本を迅速に弛緩・透明化し、クチクラを軟化させることで、翅の展開や生殖器の適切な配置に特に有用である。長期保存が可能であると報告されているものの、その保存期間がどの程度かについては明確ではない。最大の欠点は、構成成分としてフェノールを含むことであり、フェノールは毒性と刺激性を有するため、取り扱いには十分な注意が必要となる。

恒久封入用媒体

Canada balsam（屈折率 RI = 1.52–1.54）

Canada balsam は、1830 年代に Andrew Pritchard によって透過光顕微鏡に適した封入剤として初めて記載され、150年以上にわたり使用されてきた実績により、現在でも最も広く用いられる恒久媒体のひとつである。Hoyer 液とは異なり、Canada balsam は結晶化せず、また湿気を吸収しない。一方で、Canada balsam は強い自家蛍光を示すため、一部の顕微鏡技術では不利となる場合がある［60］。準備の際にキシレンの代わりに無毒性溶媒を使用すると安全性は向上するが、乾燥速度の低下や媒体の早期の暗色化といった欠点が生じることがある。

Euparal®（屈折率 RI = 1.48）

Euparal® は Canada balsam に代わる恒久封入剤として広く使用されており、長期の安定性に優れ、屈折率も同程度である。Euparal® にはい

This is an Open Access article distributed under the terms of the Creative Commons Attribution License (https://creativecommons.org/licenses/by/4.0), which permits unrestricted use, distribution, and reproduction in any medium, provided the original work is properly cited.

くつかの特性がある。第一に、最終的にマウント媒体へ移す前に標本を脱水する必要があり、通常は95% エタノールから無水エタノールへと段階的に移す。第二に、樹脂マウント（Canada balsam または Euparal®）では脱水が必須となるため、全体としての処理時間が長くなる。もし有機溶媒を用いた脱水が不可能な場合には、無水エタノールから取り出した標本を Euparal® と Euparal essence を等量混合した中間溶液に移し、その後で最終封入を行う方法も利用できる。

Enecê（屈折率 RI = 1.467）

Enecê は主に小型昆虫に使用される樹脂系封入剤で、特にブラジルで広く普及している。その基材は、コロホニー（松脂）とガムコーパルをアルコール、樟脳、テレピン油、ユーカリオイルに溶解したものから構成される。Cerqueira［11］は、幼虫、脱皮殻、さらには成虫の蚊の恒久スライド標本作製において Canada balsam に代わる媒体として Enecê を紹介し、その後サシチョウバエの封入にも広く導入されてきた。Enecê は、恒久封入剤として費用対効果に優れ、十分な乾燥時間と長期安定性を提供し、解剖操作や形態構造の精密な配置を可能にする点で高く評価されている。

5.4. スライドの作製と乾燥

検体を封入したスライドを適切に乾燥させることは、長期的な安定性と標本保存を確保するうえで極めて重要である。長期保存に移行する前に、スライドは十分に乾燥させなければならない。最適な結果を得るためには、恒久封入媒体を用いたスライドは水平にして 2〜3 週間乾燥させる必要があり、半恒久媒体を用いたスライドであれば 1〜2 週間の乾燥期間で済むことが多い。乾燥工程を確実に行うためには、使用するマウント媒体に適した温度に設定したインキュベーターを利用し、過熱によって標本が損傷するのを防がねばならない。推奨される温度範囲は 30〜37°C である。この乾燥段階は、保存中のスライドの反り、標本の劣化、また封入剤の不安定化を防ぐためにも不可欠である。

スライド作製に使用した封入剤は、必ずスライドラベルに明記することが望ましい。可能であれば、その手法の詳細、標本作製者の氏名、作製日も記載すべきである。スライドは当初、一時封入として作製され、長期保存を前提としていない。しかし、標本が後にタイプ標本（type series）の構成要素として指定されるなど、標本の位置付けが変化した場合には、将来の分類学的研究に備えて、より恒久的な封入剤に切り替えることが望ましい。

5.5. 代替的封入手法：カードマウント（Card mounting）

カードマウントは、いくつかの昆虫群で用いられる方法であり、標本を昆虫カードに直接ピン留めするか、カード表面に接着して固定する手法である。しかし、サシチョウバエは体が非常に小さく、また同定に内部器官の観察が不可欠であるため（第5章参照）、この方法はサシチョウバエには全く適していない。

5.6. 破損標本の再封入

希少または価値の高い標本に対しては、https://zenodo.org/records/18315029 に示されているように、二段階のアプローチが推奨される。第一段階では、標本を分解せずに再水和して予備観察ができる状態に戻す。複数のスライドを支えるホルダーをペトリ皿内に設置し、その上に再水和すべきスライドを置き、ペトリ皿に数ミリの溶媒を加えて湿室を作る。この際、スライド自 体が溶媒に触れないように注意する（図8D）。再水和に必要な時間は標本の状態によって大きく異なり、1日から数日を要することがあるため、日々の観察と忍耐が重要である。十分に再水和されたら、スライドを湿室から取り出してインキュベーターに数時間置き、その後で顕微鏡観察、撮影、または描画を行う。

第二段階として、再封入を行う際には、必要に応じてスライドを再び湿室に戻し、数時間から一晩置く。スライドの分解は双眼実体顕微鏡下で実施し、細針を用いてカバーガラスを慎重に外し、サシチョウバエのパーツがカバーガラス側に残らないよう注意しなければならない（https://zenodo.org/records/18315029）。次に、解剖済みのパーツを集め、DNA/RNA 抽出で用いるものと同様の小さなウェル内で水洗し、その後脱水を経て樹脂媒体に再封入する。

スライドの分解に際しては、元の封入剤が何であったかを特定し、それに適合する溶媒を選

ぶことが重要である。水性媒体であれば水を、樹脂系媒体（Canada balsam や Euparal® など）であればキシレンを使用し、必ずドラフトチャンバー内で、マスクを含む適切な個人防護具を着用して作業しなければならない。

また、タイプ標本やコレクション標本の再封入を行う場合には、標本の管理者や所蔵機関から許可を得る必要がある。

6. 標本同定

6.1. 形態（Morphology）

サシチョウバエの同定は主として、その形態学的特徴の精査に基づいて行われる。具体的には、胸部、翅、生殖器、setae（剛毛）の形態、ならびに各構造間の特定の形態計測学的関係が評価対象となる。研究者は分類学的検索表、参照標本コレクション、原記載を用いて、収集標本と既知分類群とを比較する。重要な診断形質としては、雄雌に共通するwing venation（翅脈）および頭部形態、雄の生殖器の構造、雌のspermathecae（貯精嚢）の形状・配列などが挙げられ、種の決定において特に有用である。正確な同定には、しばしば詳細な顕微鏡観察が必要であり、一般に複合顕微鏡を用いて生殖器やspermathecaeといった微細構造を観察し、また立体顕微鏡によってより大きな形態特徴を概観する。

近年の撮像技術の進展により、デジタル画像を用いた同定が容易になっている。高解像度の写真や主要形質のデジタル作図は、参照資料との比較やコンピュータ支援同定システムによる解析に活用でき、形態分類の正確性とアクセス性の双方の向上に寄与している。

6.2. 翅の幾何学（Wing geometry）

翅の幾何学は、サシチョウバエ各種の同定および類別に用いられる重要な特徴である。サシチョウバエの翅は、一般に細長く、発達した翅脈を備える独特のパターンと構造を示す（図9・図10）。脈の配列は属間および種間で異なる明瞭なパターンを形成し、同定に資する有用な診断形質を提供する。このことから、翅の幾何学を精査することは、分類学的検討において重要な知見をもたらす。

6.3. 翅の幾何学的形態測定（Wing geometric morphometrics）

研究者は、幾何学的形態測定（geometric morphometrics）などの手法を用いて、種間あるいは集団間で翅の形状や大きさを解析・比較する。翅の幾何学の研究は、行動、生息環境の嗜好、飛翔能力に関する有用な示唆を与える。

幾何学的形態測定のアプローチでは、翅を慎重に解剖し、必要に応じて染色したうえで、スライド上に平坦に封入する。作製したスライドは立体顕微鏡下で撮影してデジタル化し、形態測定解析に供する。この手順は文献に詳細に記載されており［6, 27, 42, 56, 57, 59］、左右対の器官に関しては、潜在的な負のアロメトリー効果を回避するため、右翼または左翼のいずれかに統一して用いることが推奨されている［62］。

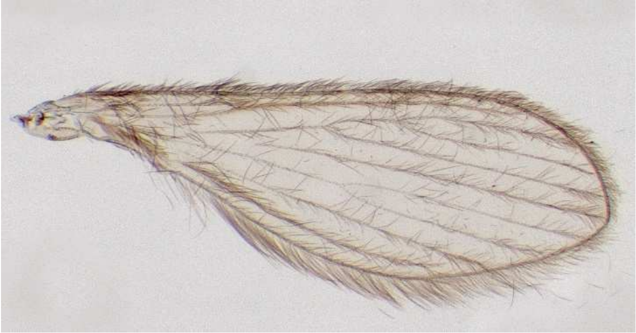

図9：*Trichophoromyia ininii* の未処理の翅.

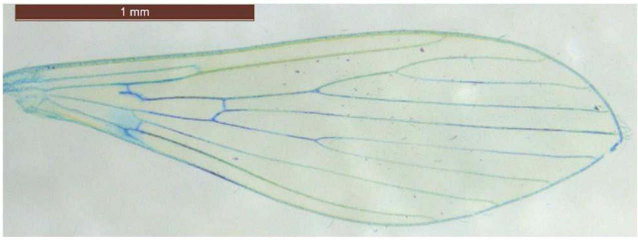

図10：*Phlebotomus ariasi* の染色済み翅.

<u>幾何学的形態測定解析のための翅の準備</u>

翅脈を最適に観察するためには、翅の鱗粉を除去し、適切に染色しておく必要がある。翅の準備にあたっては、まず小型のウェルに必要な試薬（メチレンブルー、エタノール、水、キシレン代替溶剤）を用意して満たす。70% エタノールで室温保存されている翅を、Eppendorf チューブを反転してウェルの上に内容物を落とすことで取り出し、その後、細いカーブ針を用いて翅を縦方向にすくい上げる。剛毛を除去するた

め、翅を短時間エタノールから水へ、さらに再びエタノールへと移す。

次に、翅をメチレンブルーに 6 分間浸し、その間、翅が液中に浮いた状態を保つよう注意する。その後、翅を慎重に回収し、キシレン代替溶剤に約 2 分間（メチレンブルー処理時間のおよそ 1/3）浸す。ウェルの壁に針で軽く触れて振動を加えると翅が沈みやすくなり、キシレン代替溶剤は染色の固定を助ける。

最後に、翅を拾い上げ、顕微鏡スライド上の小滴の Euparal® にそっと置く。ルーペ下で翅を丁寧に広げ、慎重にカバーガラスを載せる。Euparal® が固まり始める前に撮影を行う必要があり、最適な位置合わせを達成するため、カバーガラス下で翅の位置を微調整することが求められる。

6.4. 分子生物学的手法（Molecular biology techniques）

形態学的手法に加えて、分子生物学的手法は昆虫学研究においてますます重要性を増しており、分類学、集団遺伝学、系統学的研究に加え、DNA／RNA を用いた病原体検出、吸血源の判定、さらにはベクター行動の解析など、疫学分野における多面的な目的に利用されている［70］。DNA シークエンシングは、種の確認や近縁種間の識別に用いることができ、より高精度かつ信頼性の高い同定手段を提供する。さらに、PCR、DNA シークエンシング、次世代シークエンシング（NGS）などの高度な分子手法や MALDI-ToF MS も、迅速かつ正確な種同定のための有力な手段として普及しつつあり、伝統的な形態学的手法を補完する役割を果たしている［46］。

こうした進展にもかかわらず、形態学的同定は依然として分類学における基準（reference standard）であり、分子データの解釈は形態学的知見を基盤として行われる点に変わりはない。

6.4.1. 破壊的核酸抽出（Destructive nucleic acid extraction）

核酸抽出は多くの生物学的研究における基本的な工程であり、さまざまな生物材料から DNA を単離するための多数の方法が開発されてきた［48］。市販されている多くの DNA 抽出キットも、このプロセスを容易にすることを目的として設計されている［14］。しかし、節足動物標本を形態学的同定のために処理する際によく用いられる手法は、標本の重要な物理的特徴を損傷あるいは破壊することがあり、DNA 解析を妨げる場合がある［10］。昆虫組織の DNA 抽出法の多くは本質的に破壊的であり［43］、小型標本では、わずかなサンプリングでも重要な形態形質を失わせる危険性があるため、特に問題となる［72］。また、標本の種類や状態も、適切な DNA 抽出法を選択するうえで重要な要因となる［29］。

サシチョウバエの正確な種同定、個体群動態の理解、非標的生物への影響最小化といった目的により、分子診断ツールの開発が進んできた［23］。現在では、形態学的検索を補完する方法として、分子生物学的手法がしばしば用いられている。例えば昆虫のバーコーディングでは、DNA 抽出とシークエンスを行い、その過程で元の標本が失われることが一般的である。このため、生物材料と形態的完全性の双方を維持できる非破壊的 DNA 抽出法の開発が強く求められている。

サシチョウバエには多数の核酸抽出法が適用されてきたが、必要とされる核酸の量や品質は、その後に実施する分子解析によって異なり、各手法の要求する感度や純度もさまざまである［9］。たとえば、サシチョウバエの眼が PCR 反応を阻害することが報告されている［69］。病原体検出にとどまらず、種同定のためにサシチョウバエ DNA が日常的に抽出されているが、使用できる抽出方法は複数あり、それぞれ収量や品質に違いがある。研究者によっては、メーカーの推奨プロトコルをサシチョウバエ用に改良し、核酸の収量や品質を向上させている［8, 9, 69］。また他の節足動物群のために開発された改変法が、サシチョウバエにも応用可能であることが報告されている［58, 76］。

COI や CytB といった短いミトコンドリア断片を標的とする同定用 PCR は、DNA の断片化が大きい抽出法とも概ね適合する。一方で、Oxford Nanopore や PacBio などの長鎖リードの NGS 技術では、DNA の断片化が最小限で、かつ高品質の DNA が求められる。一般に、スピンカラム法は最大 60 kb 程度のゲノム DNA 断片を得るのに適しており、フェノール・クロロホルム

抽出法では最大 150 kb の断片が得られることがある［77］。表5には、サシチョウバエ DNA のさまざまな抽出法と、それらの手法が本種に合

わせて改変されているかどうかがまとめられている。収量は標本の大きさや処理方法に依存するため表には記載されていない。表中の「改変」は、サシチョウバエまたは他の小型節足動物に対応するための抽出プロトコルの調整を指す。

抽出法の選択にあたっては、試料数、抽出にかかる時間、その後に用いる分子解析手法など、複数の基準を考慮する必要がある。NGS は高分子量ゲノム DNA を必要とするが、ここで示したすべての方法は一般的な PCR による解析には適用可能である。さらに、小型陸生節足動物、乾燥保存された博物館標本、軟体の節足動物などを対象とした非破壊的 DNA 抽出法が複数の研究で検討されている［19, 26, 28, 55, 63］。

表5：サシチョウバエからのゲノムDNA抽出における平均コスト、用途、およびプロトコル改変の有無

Protocol	Cost	Application	Protocol adaptation for small arthropods
Spin column	2.5 – 3.55 US$ [39]	PCR, NGS	[9]
Phenol-chloroform	0.24 US$ [69]	PCR, NGS	[9]
HotSHOT	<0.01 US$ [69]	PCR	-
Salting out	0.12 US$ [69]	PCR	-
Chelex	0.02 US$ [41]	PCR	[41, 76]

6.4.2. 非破壊的核酸抽出（Non-destructive nucleic acid extraction）

節足動物、なかでもサシチョウバエの分子解析における大きな課題の一つは、将来的に昆虫学コレクションへ組み入れるために標本の保存性（形態の完全性）を確保することである。多くの DNA 抽出プロトコルは組織のマセレーションを必要とするため、元の標本の保存を犠牲にしてしまう。これに対し、非破壊的核酸抽出法は、試料の生理活性や形態を損なうことなく遺伝物質を回収するよう設計されている。この手法は、将来の分類学的検討や形態・診断用途に備えて構造的完全性の維持が不可欠な貴重または数量の限られた標本（サシチョウバエを含む）を扱う際に、とりわけ有用である。一般的な技法として、Non-destructive bathing methodが用いられており、個体を固定したのち、Proteinase K を含む溶解バッファー中に穏やかに浸漬する。

サシチョウバエ、特にタイプ標本に対しては、mild-vectolysis 技術が有効であることが示されている［24］。この方法では、通常のスピンカラムキット（例：DNeasy Blood and Tissue kit、QIAGEN, Hilden, Germany）を、標本を破壊せずに DNA を得るよう改変して用いる。具体的には、溶解バッファー量の最適化や凍結工程の追加といった溶解ステップの改変［17］によって核酸の放出を可能にし、形態的損傷を最小限に抑える［24］。サシチョウバエに関しては、HotSHOT DNA Extraction kit（Bento Bioworks Ltd, London, United Kingdom）［73］を用いることもでき、迅速かつ低コストで処理が可能である。

形態同定を予定する標本は、抽出後にリンスする。DNeasy を用いた標本は Marc-André 溶液で透化する必要がある一方、HotSHOT を用いた標本は本稿で示したプロトコル［73］に従えば、水性媒体での封入に十分な透化が得られるか、より望ましくは脱水後に樹脂媒体で封入できる。抽出したDNAは、PCR による特定遺伝マーカーの増幅などの解析に用いることができる。

このような非破壊的核酸抽出法は、サシチョウバエが保有し得る病原体の検出を含む遺伝学的特性の解明に不可欠である。標本の完全性を保持することで、研究者は価値ある遺伝情報を取得しつつ、同じ標本を追加の解析や将来の研究に供することができる。

6.5. MALDI-ToF MS（Matrix-Assisted Laser Desorption/Ionization Time of Flight Mass Spectrometry）

MALDI-ToF MSは、生物試料がもつ固有のタンパク質プロファイル（フィンガープリント）を検出・解析するために設計された質量分析技術である。近年、この手法は医療・獣医衛生上

重要な節足動物の同定において、極めて有用なツールとして認識されつつある。サシチョウバエについては、未成熟個体を含む多様な発育段階の同定や、吸血している雌の血液内容の判定に有効であることが示されており、保存条件やホモジナイズ条件が異なる状況下においても、雄雌の種判別に成功した例が報告されている［28, 30, 73, 74］。

この手法は、亜属・種・個体群といった複数の分類レベルで高い識別能力を発揮し、サシチョウバエの分布、生態行動、および病原体伝播における役割を理解するうえで不可欠な、迅速かつ高精度の種同定を可能にする。タンパク質プロファイルに基づいて種を識別できることから、疫学研究やベクター対策の立案においても重要な役割を担う。

一方で、この技術の 日常的な適用を妨げる主な制限要因が2点存在する。第一に、質量分析計の入手性の問題がある。質量分析装置は非常に高価であり、サシチョウバエ（あるいは他の節足動物ベクター）を同定する目的だけのために独自に導入することは現実的ではない。ただし、プロテオミクス施設や臨床検査機関では質量分析機器が標準的な研究装置として広く用いられているため、こうした施設における機器の共同利用によって、この問題はある程度克服可能である。

第二に、オープンアクセスのデータベースにおけるサシチョウバエの参照スペクトルが少ないことが挙げられる。このため、明確に同定された標本を基に、COI や CytB などの遺伝マーカーの配列解析および形態学的評価を組み合わせた、独自の参照データベースを構築する必要が生じる。しかし、この制限は今後改善される見込みがあり、現在、フランス・AP-HP（Assistance Publique–Hôpitaux de Paris）/ソルボンヌ大学が運営する MSI Platform や、ベルギー・ブリュッセルの BCCM/IHEM/Sciensano コレクションへの、既存のサシチョウバエ参照データの段階的な統合が予定されている（https://msi.happy-dev.fr/）。

MALDI-ToF によるタンパク質プロファイリングを予定する場合、試料は乾燥凍結または分子生物学グレードの 70% エタノールで保存し、環境温度に曝露されないようにする必要がある。試料準備に関する統一ガイドラインは現状存在しないが、既報のサシチョウバエ研究データとプロファイルの比較を可能にするため、MALDI-ToF 用マトリックスには 水系 60% acetonitrile／0.3% TFA にsinapinic acid（30 mg/mL）を溶解した溶液を使用することが推奨される。

MALDI-ToF MS のための試料調製（図7）

さまざまな条件で保存されてきた昆虫標本は、まず室温で自然乾燥させた後に解剖を行う。スライド標本および形態解析に必要な主要形態を含む体部分を得るため、頭部と腹部を切除する。胸部は MALDI-ToF に使用でき、残りの腹部は DNA 抽出用として保存する。

タンパク質プロファイリングを行う際には、胸部を 1.5 mL マイクロチューブに入れ、10 µL のホモジナイズ溶液とともにディスポーザブルのホモジナイザーペッスルおよびペレットを使用して手作業でホモジナイズする。使用されるホモジナイズ溶液には、通常、滅菌蒸留水または 25% formic acidのいずれかが用いられる。

7. 結論

本研究では、サシチョウバエの正確な同定および病原体検出を可能にするため、研究目的に応じて最適化された最も有効な標本作製法を研究者に提供することを目指した。あらゆる状況に適した単一の普遍的最適法は存在せず、それぞれの利点と限界をもつ複数の方法が存在する。

補足データでは、サシチョウバエの処理および同定に利用されるさまざまな封入技術に関する詳細なプロトコルを提示した。これらのプロトコルには、説明動画を含む段階的な手順が示されており、目的に応じて精密かつ再現性の高い結果を得ることができる。これらの総合的な情報を提供することで、研究者が自身の研究目的に最も適した標本作製技術を選択し、研究できるよう支援することを意図している。

謝辞

著者らは、ロンドン自然史博物館（Natural History Museum of London, UK）の Richard Lane 氏および Zoe Jay Adams 氏による優れた査読に謝

意を表する。両氏の助言により、本稿の質は大きく向上した。

研究資金

AJA の研究は、ブラジルの研究助成機関 CNPq（案件番号：404395/2024-4）および Araucária 財団（案件番号：433/2025 PDI）の支援を受けている。

利益相反

Jérôme Depaquit は *Parasite* 誌の副編集長であるが、本稿の査読プロセスおよび採否決定には一切関与していない。他の著者は利益相反が存在しないことを宣言する。

データ利用可能性に関する声明

Zenodo 上の動画

動画 1: https://zenodo.org/records/18198006

動画 2: https://zenodo.org/records/18311158

動画 3: https://zenodo.org/records/18311106

動画 4: https://zenodo.org/records/18311154

動画 5: https://zenodo.org/records/18303014

動画 6: https://zenodo.org/records/18302850

動画 7: https://zenodo.org/records/18315029

追加資料

この記事に関連する追加資料は、https://www.parasite-journal.org/10.1051/parasite/2026009/olm で入手できます。

引用文献

1. Alkan C, Allal-Ikhlef AB, Alwassouf S, Baklouti A, Piorkowski G, de Lamballerie X, Izri A, Charrel RN. 2015. Virus isolation, genetic characterization and seroprevalence of Toscana virus in Algeria. Clinical Microbiology and Infection, 21(11), 1040 e1-9.
2. Alten B, Ozbel Y, Ergunay K, Kasap OE, Cull B, Antoniou M, Velo E, Prudhomme J, Molina R, Banuls AL, Schaffner F, Hendrickx G, Van Bortel W, Medlock JM. 2015. Sampling strategies for phlebotomine sand flies (Diptera: Psychodidae) in Europe. Bulletin of Entomological Research 105(6), 664–678.
3. Ayhan N, Baklouti A, Prudhomme J, Walder G, Amaro F, Alten B, Moutailler S, Ergunay K, Charrel RN, Huemer H. 2017. Practical guidelines for studies on sandfly-borne phleboviruses: Part I: Important points to consider *ante* field work. Vector-Borne and Zoonotic Diseases 17(1), 73–80.
4. Bates PA. 1997. Infection of phlebotomine sandflies with *Leishmania*, in The Molecular Biology of Insect Disease Vectors: A Methods Manual. Springer. p. 112–120.5.
5. Baum M, de Castro EA, Pinto MC, Goulart TM, Baura W, Klisiowicz Ddo R, Vieira da Costa-Ribeiro MC. 2015. Molecular detection of the blood meal source of sand flies (Diptera: Psychodidae) in a transmission area of American cutaneous leishmaniasis, Parana State, Brazil. Acta Tropica, 143, 8–12.
6. Belen A, Alten B, Aytekin A. 2004. Altitudinal variation in morphometric and molecular characteristics of *Phlebotomus papatasi* populations. Medical and Veterinary Entomology, 18(4), 343–350.
7. Bhattacharya J, Chandra G, Hati AK. 1991. A simple method for cryopreservation of *Leishmania donovani* promastigotes, Indian Journal of Medical Research, 93, 245–246.
8. Caligiuri LG, Sandoval AE, Miranda JC, Pessoa FA, Santini MS, Salomón OD, Secundino NF, McCarthy CB. 2019. Optimization of DNA extraction from individual sand flies for PCR amplification. Methods and Protocols, 2(2), 36.
9. Casaril AE, de Oliveira LP, Alonso DP, de Oliveira EF, Gomes Barrios SP, de Oliveira Moura Infran J, Fernandes WS, Oshiro ET, Ferreira AMT, Ribolla PEM, de Oliveira AG. 2017. Standardization of DNA extraction from sand flies: Application to genotyping by next generation sequencing. Experimental Parasitology, 177, 66–72.
10. Castalanelli MA, Severtson DL, Brumley CJ, Szito A, Foottit RG, Grimm M, Munyard K, Groth DM. 2010. A rapid non-destructive DNA extraction method for insects and other arthropods. Journal of Asia-Pacific Entomology, 13(3), 243–248.
11. Cerqueira NL. 1943. Um novo meio para montagem de pequenos insetos em lâmina. Memórias do Instituto Oswaldo Cruz, (39), 37–41.
12. Charrel RN, Gallian P, Navarro-Mari JM, Nicoletti L, Papa A, Sanchez-Seco MP, Tenorio A, de Lamballerie X. 2005. Emergence of Toscana virus in Europe. Emerging Infectious Diseases, 11(11), 1657–1663.
13. Chaskopoulou A, Giantsis IA, Demir S, Bon MC. 2016. Species composition, activity patterns and blood meal analysis of sand fly populations (Diptera: Psychodidae) in the metropolitan region of Thessaloniki, an endemic focus of canine leishmaniasis. Acta Tropica, 158, 170–176.
14. Chen H, Rangasamy M, Tan SY, Wang H, Siegfried BD. 2010. Evaluation of five methods for total DNA extraction from western corn rootworm beetles. PLoS One, 5(8), e11963.
15. Depaquit J, Grandadam M, Fouque F, Andry PE, Peyrefitte C. 2010. Arthropod-borne viruses transmitted by Phlebotomine sandflies in Europe: a review. Eurosurveillance, 15(10), 19507.
16. Diamond LS, Herman CM. 1954. Incidence of Trypanosomes in the Canada Goose as revealed by bone marrow culture. Journal of Parasitology, 40(2), 195–202.
17. Ding H, Torno M, Vongphayloth K, Ng G, Tan D, Sng W, Ho K, Randrianambinintsoa FJ, Depaquit J, Tan CH. 2025. Hidden in plain sight: discovery of sand flies in Singapore and description of four species new to science. Parasites & Vectors, 18(1), 402.
18. Es-Sette N, Ajaoud M, Bichaud L, Hamdi S, Mellouki F, Charrel RN, Lemrani M. 2014. *Phlebotomus sergenti* a common vector of *Leishmania tropica* and Toscana virus in Morocco. Journal of Vector Borne Diseases, 51(2), 86–90.
19. Favret C. 2005. A new non-destructive DNA extraction and specimen clearing technique for aphids (Hemiptera). Proceedings of the Entomological Society of Washington, 107(2), 469–470.
20. Galati EAB. 2018. Phlebotominae (Diptera, Psychodidae): Classification, morphology and terminology of adults and identification of American taxa, in Brazilian Sand Flies: Biology, Taxonomy, Medical Importance and Control, Rangel EF, Shaw JJ, Editors. Cham: Springer International Publishing. pp. 9–212.
21. Galati EAB, de Andrade AJ, Perveen F, Loyer M, Vongphayloth K, Randrianambinintsoa FJ, Prudhomme J, Rahola N, Akhoundi M,

Shimabukuro PHF, Depaquit J. 2025. Phle-botomine sand flies (Diptera, Psychodidae) of the world. Parasites & Vectors, 18(1), 220.

22. Galati EAB, Galvis-Ovallos F, Lawyer P, Leger N, Depaquit J. 2017. An illustrated guide for characters and terminology used in descriptions of Phlebotominae (Diptera, Psychodidae). Par-asite, 24, 26.
23. Gariepy T, Kuhlmann U, Gillott C, Erlandson M. 2007. Parasitoids, predators and PCR: the use of diagnostic molecular markers in biological control of Arthropods. Journal of Applied Entomology, 131(4), 225–240.
24. Giantsis IA, Chaskopoulou A, Bon MC. 2016. Mild-Vectolysis: A nondestructive DNA extraction method for vouchering sand flies and mosquitoes. Journal of Medical Entomology, 53(3), 692–695.
25. Gidwani K, Picado A, Rijal S, Singh SP, Roy L, Volfova V, Andersen EW, Uranw S, Ostyn B, Sudarshan M, Chakravarty J, Volf P, Sundar S, Boelaert M, Rogers ME. 2011. Serological markers of sand fly exposure to evaluate insecticidal nets against visceral leishmaniasis in India and Nepal: a cluster-randomized trial. PLoS Neglected Tropical Diseases, 5(9), e1296.
26. Gilbert MTP, Moore W, Melchior L, Worobey M. 2007. DNA extraction from dry museum beetles without conferring external morphological damage. PLoS One, 2(3), e272.
27. Giordani BF, Andrade AJ, Galati EAB, Gurgel-Goncalves R. 2017. The role of wing geometric morphometrics in the identification of sandflies within the subgenus *Lutzomyia.* Medical and Veterinary Entomology, 31(4), 373–380.
28. Guzmán-Larralde AJ, Suaste-Dzul AP, Gallou A, Peña-Carrillo KI. 2017. DNA recovery from microhymenoptera using six non-destructive methodologies with considerations for subsequent preparation of museum slides. Genome, 60(1), 85–91.
29. Hajibabaei M, DeWaard JR, Ivanova NV, Ratnasingham S, Dooh RT, Kirk SL, Mackie PM, Hebert PD. 2005. Critical factors for assembling a high volume of DNA barcodes. Philosophical Transactions of the Royal Society B: Biological Sciences, 360(1462), 1959–1967.
30. Haouas N, Pesson B, Boudabous R, Dedet JP, Babba H, Ravel C. 2007. Development of a molecular tool for the identification of *Leishmania* reservoir hosts by blood meal analysis in the insect vectors. American Journal of Tropical Medicine and Hygiene, 77(6), 1054–1059.
31. Hlavackova K, Dvorak V, Chaskopoulou A, Volf P, Halada P. 2019. A novel MALDI-TOF MS-based method for blood meal identification in insect vectors: A proof of concept study on phlebotomine sand flies. PLoS Neglected Tropical Diseases, 13 (9), e0007669.
32. Huemer H, Prudhomme J, Amaro F, Baklouti A, Walder G, Alten B, Moutailler S, Ergunay K, Charrel RN, Ayhan N. 2017. Practical guidelines for studies on sandfly-borne phleboviruses: Part II: Important points to consider for fieldwork and subsequent virological screening. Vector-Borne and Zoonotic Diseases, 17(1), 81–90.
33. Jancarova M, Polanska N, Thiesson A, Arnaud F, Stejskalova M, Rehbergerova M, Kohl A, Viginier B, Volf P, Ratinier M. 2025. Susceptibility of diverse sand fly species to Toscana virus. PLoS Neglected Tropical Diseases, 19(5), e0013031.
34. Kapp JD, Green RE, Shapiro B. 2021. A fast and efficient single-stranded genomic library preparation method optimized for ancient DNA. Journal of Heredity, 112(3), 241–249.
35. Killick-Kendrick R, Maroli M, Killick-Kendrick M. 1991. Bibliography of the colonization of phlebotomine sandflies. Parassitologia, 33(suppl.), 321–333.
36. Lawyer P, Killick-Kendrick M, Rowland T, Rowton E, Volf P. 2017. Laboratory colonization and mass rearing of phlebotomine sand flies (Diptera, Psychodidae). Parasite, 24, 42.
37. Léger N, Pesson B, Madulo-Leblond G. 1986. Les phlébotomes de Grèce : 1ère partie. Bulletin de la Société de Pathologie Exotique, 79, 386–397.
38. Léger N, Pesson B, Madulo-Leblond G. 1986. Les phlébotomes de Grèce : 2ème partie. Bulletin de la Société de Pathologie Exotique, 79, 514–524.
39. Leonel JAF, Vioti G, Alves ML, da Silva DT, Meneghesso PA, Benassi JC, Spada JCP, Galvis-Ovallos F, Soares RM, Oliveira T. 2020. DNA extraction from individual Phlebotomine sand flies (Diptera: Psychodidae: Phlebotominae) specimens: Which is the method with better results? Experimental Parasitology, 218, 107981.
40. Lestinova T, Rohousova I, Sima M, de Oliveira CI, Volf P. 2017. Insights into the sand fly saliva: Blood-feeding and immune interactions between sand flies, hosts, and *Leishmania.* PLoS Neglected Tropical Diseases, 11(7), e0005600.
41. Lienhard A, Schaffer S. 2019. Extracting the invisible: obtaining high quality DNA is a challenging task in small arthropods. PeerJ, 7, e6753.
42. Lozano-Sardaneta YN, Mikery-Pacheco OF, Huerta H, Rojas-Soriano JE, Contreras-Ramos A. 2025. Wing geometric morphometrics is effective to separate sand fly species (Diptera, Psychodidae, Phlebotominae) related with leishmaniasis transmission in Mexico. Acta Tropica, 262, 107523.
43. Mandrioli M. 2008. Insect collections and DNA analyses: how to manage collections? Museum Management and Curatorship, 23(2), 193–199.
44. Maroli M, Feliciangeli MD, Bichaud L, Charrel RN, Gradoni L. 2013. Phlebotomine sandflies and the spreading of leishmaniases and other diseases of public health concern. Medical and Veterinary Entomology, 27(2), 123–147.
45. Marquina D, Buczek M, Ronquist F, Lukasik P. 2021. The effect of ethanol concentration on the morphological and molecular preservation of insects for biodiversity studies. PeerJ, 9, e10799.
46. Mathis A, Depaquit J, Dvorak V, Tuten H, Banuls AL, Halada P, Zapata S, Lehrter V, Hlavackova K, Prudhomme J, Volf P, Sereno D, Kaufmann C, Pfluger V, Schaffner F. 2015. Identification of phlebotomine sand flies using one MALDI-TOF MS reference database and two mass pectrometer systems., Parasites & Vectors, 8, 266.
47. Mekarnia N, Benallal KE, Sadlova J, Vojtkova B, Mauras A, Imbert N, Longhitano M, Harrat Z, Volf P, Loiseau PM, Cojean S. 2024. Effect of *Phlebotomus papatasi* on the fitness, infectivity and antimony-resistance phenotype of antimony-resistant *Leishmania* major Mon-25. International Journal for Parasitology – Drugs and Drug Resistance, 25, 100554.
48. Milligan BG. 1998. Total DNA isolation, in Molecular Genetic Analysis of Population: A Practical Approach, Hoelzel AR, Editor. Oxford: Oxford University Press.
49. Molina R, Jiménez M, Alvar J, González E, Hernández-Taberna S, Ines MM. 2017. Methods in sand fly research. Madrid: Servicio de publicaciones Universidad de Alcalá de Henares, Madrid.
50. Murphy WJ, Eizirik E, O'Brien SJ, Madsen O, Scally M, Douady CJ, Teeling E, Ryder OA, Stanhope MJ, de Jong WW, Springer MS. 2001. Resolution of the early placental mammal radiation using Bayesian phylogenetics. Science, 294(5550), 2348–2351.

51. Nacif-Pimenta R, Pinto LC, Volfova V, Volf P, Pimenta PFP, Secundino NFC. 2020. Conserved and distinct morphological aspects of the salivary glands of sand fly vectors of leishma-niasis: an anatomical and ultrastructural study. Parasites & Vectors, 13(1), 441.
52. Neuhaus B, Schmid T, Riedel J. 2017. Collection management and study of microscope slides: Storage, profiling, deterioration, restoration procedures, and general recommendations. Zootaxa, 4322(1), 1–173.
53. New TR. 1974. Pscoptera. Handbooks for Identification of British Insects (Vol. I). London: Royal Entomological Society of London. 102 pp.
54. Perez-Ruiz M, Collao X, Navarro-Mari JM, Tenorio A. 2007. Reverse transcription, real-time PCR assay for detection of Toscana virus. Journal of Clinical Virology, 39(4), 276–281.
55. Porco D, Rougerie R, Deharveng L, Hebert P. 2010. Coupling non-destructive DNA extraction and voucher retrieval for small soft-bodied Arthropods in a high-throughput context: the example of Collembola. Molecular Ecology Resources, 10(6), 942–945.
56. Prudhomme J, Cassan C, Hide M, Toty C, Rahola N, Vergnes B, Dujardin JP, Alten B, Sereno D, Banuls AL. 2016. Ecology and morphological variations in wings of *Phlebotomus ariasi* (Diptera: Psychodidae) in the region of Roquedur (Gard, France): a geometric morphometrics approach. Parasites & Vectors, 9(1), 578.
57. Prudhomme J, Gunay F, Rahola N, Ouanaimi F, Guernaoui S, Boumezzough A, Banuls AL, Sereno D, Alten B. 2012. Wing size and shape variation of *Phlebotomus papatasi* (Diptera: Psychodidae) populations from the south and north slopes of the Atlas Mountains in Morocco. Journal of Vector Ecology, 37(1), 137–147.
58. Prudhomme J, Toty C, Kasap OE, Rahola N, Vergnes B, Maia C, Campino L, Antoniou M, Jimenez M, Molina R, Cannet A, Alten B, Sereno D, Banuls AL. 2015. New microsatellite markers for multi-scale genetic studies on *Phlebotomus ariasi* Tonnoir, vector of *Leishmania infantum* in the Mediterranean area. Acta Tropica, 142, 79–85.
59. Prudhomme J, Velo E, Bino S, Kadriaj P, Mersini K, Gunay F, Alten B. 2019. Altitudinal variations in wing morphology of *Aedes albopictus* (Diptera, Culicidae) in Albania, the region where it was first recorded in Europe. Parasite, 26, 55.
60. Rawlins DJ. 1992. Light Microscopy: An Introduction to Biotechniques. Oxford: Bios Scientific publishers. 143 pp.
61. Ready PD. 2013. Biology of phlebotomine sand flies as vectors of disease agents. Annual Review of Entomology, 58, 227–250.
62. Rohlf FJ, Slice D. 1990. Extensions of the Procrustes method for the optimal superimposition of landmarks. Systematic Zoology, 39(1), 40–59.
63. Rowley DL, Coddington JA, Gates MW, Norrbom AL, Ochoa RA, Vandenberg NJ, Greenstone MH. 2007. Vouchering DNA-barcoded specimens: Test of a nondestructive extraction proto-col for terrestrial arthropods. Molecular Ecology Notes, 7(6), 915–924.
64. Sábio PB, Andrade AJ, Galati EAB. 2014. Assessment of the taxonomic status of some species included in the *Shannoni* complex, with the description of a new species of *Psathyromyia* (Diptera: Psychodidae: Phlebotominae). Journal of Medical Entomology, 51(2), 331–341.
65. Sadlova J, Yeo M, Seblova V, Lewis MD, Mauricio I, Volf P, Miles MA. 2011. Visualisation of *Leishmania donovani* fluorescent hybrids during early stage development in the sand fly vector. PLoS One, 6(5), e19851.
66. Sales K, Miranda DEO, da Silva FJ, Otranto D, Figueredo LA, Dantas-Torres F. 2020. Evaluation of different storage times and preservation methods on phlebotomine sand fly DNA concentration and purity. Parasites & Vectors, 13(1), 399
67. Sales KG, Costa PL, de Morais RC, Otranto D, Brandao-Filho SP, Cavalcanti Mde P, Dantas-Torres F. 2015. Identification of phlebotomine sand fly blood meals by real-time PCR. Parasites& Vectors, 8, 230.
68. Sant'Anna MR, Jones NG, Hindley JA, Mendes-Sousa AF, Dillon RJ, Cavalcante RR, Alexander B, Bates PA. 2008. Blood meal identification and parasite detection in laboratory-fed and field-captured *Lutzomyia longipalpis* by PCR using FTA databasing paper. Acta Tropica, 107(3), 230–237.
69. Senne NA, Santos HA, Araujo TR, Paulino PG, Mendonca LP, Moreira HVS, Camilo TA, da Costa Angelo I. 2022. Robust comparative performance of genomic DNA extraction methods from non-engorged phlebotomine sandflies. Medical and Veterinary Entomology, 36(2), 203–211.
70. Shaw JJ. 2025. A review of Leishmania infections in American Phlebotomine sand flies – Are those that transmit leishmaniasis anthropophilic or anthropportunists? Parasite, 32, 57.
71. Tesh RB, Modi GB. 1983. Growth and transovarial transmission of Chandipura virus (Rhabdoviridae: Vesiculovirus) in *Phlebotomus papatasi.* American Journal of Tropical Medicine and Hygiene, 32(3), 621–623.
72. Thomsen PF, Elias S, Gilbert MTP, Haile J, Munch K, Kuzmina S, Froese DG, Sher A, Holdaway RN, Willerslev E. 2009. Non-destructive sampling of ancient insect DNA. PLoS One, 4(4),e5048
73. Truett GE, Heeger P, Mynatt RL, Truett AA, Walker JA, Warman ML. 2000. Preparation of PCR-quality mouse genomic DNA with hot sodium hydroxide and tris (HotSHOT). Biotechniques, 29(1), 52–54.
74. Upton MS. 1993. Aqueous gum-chloral slide mounting media: an historical review. Bulletin of Entomological Research, 83(2), 267–274.
75. Volf P, Myskova J. 2007. Sand flies and Leishmania: specific versus permissive vectors. Trends in Parasitology, 23(3), 91–92.
76. Wang Q, Wang X. 2012. Comparison of methods for DNA extraction from a single chironomid for PCR analysis. Pakistan Journal of Zoology, 44(2), 421–426.
77. Wang Y, Zhao Y, Bollas A, Wang Y, Au KF. 2021. Nanopore sequencing technology, bioinformatics and applications. Nature Biotechnology, 39(11), 1348–1365.

Cite this article as: Randrianambinintsoa FJ, Augendre L, Prudhomme J, Martinet J-P, Loyer M, Mekarnia N, Kerkoub H, Perveen FK, Huguenin A, Kariya E, Akhoundi M, De Andrade AJ, Berriatua E, Bongiorno G, Boyer S, Christodoulou V, Da Costa-Ribeiro MCV, De Souza LAF, Ding H, Dondji B, Dvořák V, Erisoz Kasap O, Galati EAB, Gállego M, Ballart C, Gouzelou S, Haddad N, Masse RS, Mekuria AH, Ivovic V, Kaczmarek S, Shahar MK, Kirstein OD, Kniha E, Kolářová I, Lincoln T, Lucanas C, Mikov O, Nov K, Özbel Y, Pesson B, Posada Lopez LC, Prasetyo DB, Rahola N, Rebollar-Tellez EA, Rodrigues BL, Roy L, Saini P, Sanjoba C, Shimabukuro PH, Siriyasatien P, Soszynska A, Suleşco T, Sylla M, Torno M, Volf P, Vongphayloth K, Sinh Nam V, Wardhana A, Yessinou E, Zapata S, Gantier J-C & Depaquit J. 2026. Processing and mounting phlebotomine sand flies: a consensus guideline. Parasite xx, xx. https://doi.org/10.1051/parasite/2026009.

PARASITE

An international open-access, peer-reviewed, online journal publishing high quality papers on all aspects of human and animal parasitology

Reviews, articles and short notes may be submitted. Fields include, but are not limited to: general, medical and veterinary parasitology; morphology, including ultrastructure; parasite systematics, including entomology, acarology, helminthology and protistology, and molecular analyses; molecular biology and biochemistry; immunology of parasitic diseases; host-parasite relationships; ecology and life history of parasites; epidemiology; therapeutics; new diagnostic tools.
All papers in Parasite are published in English. Manuscripts should have a broad interest and must not have been published or submitted elsewhere. No limit is imposed on the length of manuscripts.

Parasite (open-access) continues **Parasite** (print and online editions, 1994-2012) and **Annales de Parasitologie Humaine et Comparée** (1923-1993) and is the official journal of the Société Française de Parasitologie.

Editor-in-Chief:
Jean-Lou Justine, Paris

Submit your manuscript at:
https://www.editorialmanager.com/parasite

This is an Open Access article distributed under the terms of the Creative Commons Attribution License (https://creativecommons.org/licenses/by/4.0), which permits unrestricted use, distribution, and reproduction in any medium, provided the original work is properly cited.

Appendix 1: 生化学的理論的基礎

対象となる節足動物はサシチョウバエである。しかし、ここに示す一般的な手法は、内部形態学的特徴によってのみ同定が可能な他の一般的な節足動物にも応用できる。いくつかの内部器官は部分的にキチン化しており、その形態は非常に重要な情報を提供してくれる。このため、food pump、spermathecae、そしてその ducts を観察することは非常に有用である。

本稿に記載する試薬について忘れてはならないのは、昆虫の固定段階からスライド標本作成まで、基本的には酸化還元反応を適用しているに過ぎないということである。唯一の注意点、または指針となる考え方は、還元剤と酸化剤を混合しないことである。

エチルアルコール（エタノール）

エチルアルコール（エタノール）はさまざまな方法で使用される。アルコール分子は水に対して強い親和性を持つため、脱水作用を示す。しかし、低濃度のアルコール（水分が多すぎるもの）は、核酸の分解を引き起こす（水は核酸の敵である）。

昆虫をエタノールに浸すのは、単に保存のためだけでなく、組織を固定するためでもある。組織学では通常、浸透速度（penetration rate）と固定速度（fixation rate） の 2 つの重要な概念を区別する。良い固定剤は、組織を固定する前に、迅速に深部まで浸透する必要がある。96% エタノールの場合、その浸透係数は約 1.05 である（比較として、0.75%水溶性ピクリン酸の浸透係数は 0.45、3%重クロム酸カリウム溶液では 1.45）。昆虫やその他の節足動物をエタノール中で長期間保存しようとするのは、昆虫学者にとっては現実的である。捕獲個体を後の研究や未来の研究者のために保存しておこうとする考えは非常に立派である。しかし、この考え方は細胞学者や組織学者には当てはまらない。長期間サンプルを固定液中に置いておくと、再処理が事実上不可能になる。そのため 10 年以上経過したサンプルは使用が困難、あるいは不可能となる。

もうひとつの重要な点は、固定すべき節足動物の量と固定液の量の比率である。動物学や医学の現場では、一般的に固定対象の体積の 60 倍の固定液量が推奨される。微小節足動物の場合、サンプル体積に対して 少なくとも 4～5 倍のアルコールを追加することが望まれる。アルコールは、節足動物組織内の水分を奪うにつれて、徐々にその濃度（強さ）を失っていくことも忘れてはならない。

結論：

エチルアルコールは 還元性化学物質（したがって酸化性固定剤とは不適合）である。
タンパク質を強力に沈殿させ、変性させる。
ある種の複合脂質を溶解し、グリコーゲンを沈殿させる。
組織を強く収縮させ、硬化させる。

塩基性の水酸化カリウム（KOH）／水酸化ナトリウム（NaOH）溶液

昆虫学における溶液の使用は、主に水酸化カリウム（KOH） に集中してきたが、明確な理由が示されているわけではない。

水酸化ナトリウム（NaOH, E524）は、濃度や規定度の異なる溶液があり、固体ではペレット状あるいはフレーク状（glitter）で販売されている。最大の欠点は、非常に吸湿性が高いこと（KOH よりも高い）である。タンパク質を溶解し、脂質と反応すると鹸化によって ハードソープに変える（これは KOH と大きく異なり、KOH は鹸化によりリキッドソープ を形成する）。

一方、水酸化カリウム（KOH, E525）は濃度の高い溶液が入手可能であるが、特に便利なのは 約 0.1 g のペレット状で販売されているもので、精密天秤を必要とせず希釈溶液を容易に調製できる点である。例として、0.1 g のペレット 1 個を 1 mL の蒸留水に溶かすと 10% 溶液が得

られる。さらに、KOH ペレットの利点として、炭酸化（CO_2 の吸収）に対する感受性が NaOH より低いことが挙げられる（KOH 溶液は CO_2 を強く固定して炭酸塩を生成しやすい）。

これらの強塩基は、脂肪酸を水溶性のソープに変えることで 脂肪酸の可溶化 に用いられる。固定剤（エタノールなど）はサンプル中の脂質を一部溶解させるが、強塩基を含む水溶液に移すと複雑な脂肪酸でも析出する。強塩基は、したがって低温（常温）での鹸化反応を引き起こす。場合によっては、特にメスのように脂肪組織が多いサンプルでは、反応を促進するために 35–40°C に加温 する方法もある。または、室温で 接触時間を延長する方法でも対応できる。

着色酸性溶液／無色 Marc-André 溶液

ここでは、Marc-André 溶液の利点と不都合について説明する。この溶液はクロラール水和物（トリクロロアセトアルデヒ ド一水和物）・酢酸・水 で構成される。Marc-André 溶液は酸とアルデヒ ドの混合物であり、強い酸化作用をもつ。そのため、サンプル中に残っている可能性のある 過剰の水酸化カリウム（KOH）を沈殿させずに中和することができる（KOH 使用時に形成されるアルカリ性ソープを沈殿させない点が重要である）。また、この酸化性溶液は、キチンを構成するグルコサミンの第二級アルコール基を酸化することでキチンを軟化させる 作用を有している。さらに、溶液中の成分がサンプル内の 一部の無機塩類を溶解 する働きもある。

Marc-André 溶液をあらかじめ 酸フクシンで着色しておく（＝酸化状態）と、構造内の第二級アルコール基に結合して染色が可能になる。Marc-André 溶液との接触時間が終わり、サンプルが染色されたら、第二級エタノールのみでリンスを行う。この段階でサンプルの脱水工程 が始まる。

利点：
過剰な塩基性溶液の中和。
キチンの軟化。
キチンを染色し、内部のキチン化構造をより明瞭に観察できる。

欠点：
クロラール水和物は催眠作用をもち、かつ医療用途で使用された化合物であるため、有害性がある。ドラフトチャンバー内で使用し、化学物質リスクに関する法令を遵守しなければならない。

脱水溶液

微少なサンプルでは、エタノール濃度を段階的に上げる伝統的な脱水系列を厳密に行う必要はないことが経験的に知られている。一方、サンプルが大きい場合は、80% エタノールからスタートし、90%、95%、無水エタノールという順に処理する。非常に小さなサンプルの場合は、90% エタノールにつけ、その後、 無水エタノール処理 で十分である。この段階で常に意識すべきなのは、無水エタノールは空気中の水分を取り込もうとするという性質である。

かつて昆虫学研究室では、脱水の最終工程にブナなどを用いたクレオソート処理を用いるのが伝統であった。しかし、この物質は殺虫剤・防カビ剤・木材防腐剤として広く使われ、強烈な臭気（多環芳香族炭化水素）、生殖毒性の懸念、発がん性、残留性有機汚染物質としての懸念、水生生物への毒性などの理由から、今日では推奨されない。

サンプルの封入（マウント）に推奨される溶液は Euparal® および Euparal essence（次の段落で説明）である。90%エタノール浴を行ったサンプルは、この Euparal とその Euparal essence の混合液と非常に相性が良い。

Appendix 2: 使用した試薬の組成

10% 水酸化カリウム溶液（Potassium hydroxide 10%）
水酸化カリウム（KOH） 10 g
蒸留水（全量 100 mL になるよう加える）

ゴム・クロラール封入剤（Hoyer medium）
蒸留水 50 mL
クロラール水和物 200 g
アラビアゴム 50 g
グリセロール 20 mL

Marc-André 溶液
クロラール水和物 40 g
氷酢酸（無水酢酸）30 mL
蒸留水 30 mL

1% 酸フクシン溶液（蒸留水溶解）
酸フクシン粉末 1 g
蒸留水 99 mL

酸フクシンで着色した Marc-André 溶液
Marc-André 溶液 10 mL
1% フクシン溶液 50 μ L

Appendix 3: Euparal®、カナダバルサム、ポリビニルアルコール等の封入剤

ポリビニルアルコール（PVA）：脱水に必要な試薬が入手できない場合に最適な封入剤である。ポリビニルアルコールを Amman のラクトフェノール と混合して使用する。ただし次のような重大な欠点がある：乾燥しやすい、水分蒸発により PVA が結晶化 する、フェノールが酸化して 黒変 する。したがって 短期的な封入 に適した技法である。

カナダバルサム（Canada balsam）：スライドとカバーガラスの間に封入する際には、サンプルの 完全な脱水が必要となる。キシレンやトルエンを使用するため、扱いに注意が必要である。

Enecê medium：カナダバルサム同様、スライドとカバーガラスの封入に使用するため、脱水操作が必須。Enecê の組成：ピュアホワイトの天然樹脂 22 g、アルコール可溶性コパル樹脂 12 g、無水アルコール（absolute alcohol）20 mL、樟脳 10 g、テレビン油（turpentine essence）10 mL、ユーカリオイル（eucalyptol）26 mL。

調製方法：無水アルコールと樟脳を三角フラスコなどのフラスコに入れる。天然樹脂とコパル樹脂を加える。栓をして振とうし、湯煎（弱火） にかけ、沸騰させないよう注意して混合物を完全に溶解させる。完全に溶けたらテレビン油を加える。温かいうちに濾過する。

最後にユーカリオイルを加える。粘度が高くなった場合は、以下の Enecê 希釈液 を加えて調整する。Enecê 希釈用液：無水アルコール 30 mL、樟脳 17 g、テレビン油 15 mL、ユーカリオイル 38 mL（Cerqueira, 1943 より）。

Euparal® ：針葉樹 *Tetraclinis articulata*（Vahl, 1791） に由来する樹脂であり、1906 年に Gilson によって研究・開発された。この樹脂の最大の利点は、重合しない ことである。そのため、スライドとカバーガラスの間に封入したサンプルは、アルコール、あるいはさらに適した Euparal® essence の作用によって容易に取り外すことができる。この樹脂（sandarac とも呼ばれる）は、80%以上のエタノールを受け入れる 性質がある。

Triton X-100 の使用：非イオン性水溶液：

Triton X-100 は、(4-(1,1,3,3-tetramethylbutyl) phenyl-polyethylene glycol solution、または t-octylphenoxypolyethoxyethanol、polyethylene glycol tert-octylphenyl ether として知られる 非イオン性界面活性剤の水溶液 であり、細胞生物学や分子生物学で 細胞膜・核膜の透過化 のために広く用いられている。

昆虫標本が長期間アルコールに保存されるのは珍しいことではない。しかし、アルコール保存は最適とは言えず、長期間保存された節足動物は、顕微鏡観察のための前処理がきわめて困難になることが多い。保存容器のプラスチックが劣化したり、アルコールが蒸発したりすることもあり、長期間のアルコール接触や乾燥は大きな問題となる。2008 年、Jonque は、写真フィルム用の湿潤剤 Agepon をクモの再水和に使用するという報告をした。この発想から、強力な洗剤ではなく、穏やかな湿潤剤を用いるというアプローチが生まれた。

0.5% Triton X-100 水溶液を用いる手順：乾燥したサンプルを 無水エタノールに浸す。サンプル全体が完全に浸るだけの 0.5% Triton X-100 溶液を加える。5 分程度（またはそれ以上）放置し、すべての節足動物が溶液中で互いに分離するまで待つ。Triton X-100 溶液を除去し、水酸化カリウム（KOH）溶液と置き換える。その後の操作は、前述の手順に従う。

Appendix 4: Euparal® またはカナダバルサムによる封入手順

１．標本は完全に脱水されていなければならない。濁りや乳白色が見られる場合は脱水不十分を示す。
２．脱水はエタノール濃度を段階的に上げることで達成できる。
３．標本は 99%アルコールまたは無水アルコールから透化剤へ移す ことができる。

手順
１．サシチョウバエ成虫を70%エタノールに入れる。
２．エタノールを除去し、10% KOH に置き換える。サシチョウバエの上にカバーガラスをかぶせる。
３．透明になるまでマセレーション（組織の軟化・溶解） を行う。
４．KOH を除去する。
５．蒸留水を注ぎ、30〜45 分待つ。
６．水を除去し、蒸留水で 30 分洗浄を繰り返す。多数の標本を同時処理する場合は時間を長く、少数または個別処理では短くしてよい）
７．水を除去する。
８．Marc-André 溶液（酸フクシンで着色してもよい）を加え、24 時間（1 日）放置する。
９．Marc-André 溶液を除去する。
１０．標本に蒸留水をかけ、30〜45 分待つ。
１１．水を除去し、再度 蒸留水で 30 分洗浄する。
１２．水を除去する。
１３．70%エタノールを加えて解剖を行う。
　a. 頭部と腹部：胸部を保持しつつ、頭または腹部を軽く引き離す。
　b. 胸部：胸部をピンセットで保持し、もう一方のピンセットで付属肢の基部を引いて翅を取り外す。必要に応じて胸部を左右に分ける矢状断（sagittal dissection）も可能。
１４．脱水系列を段階的に行う。50% → 80% → 95% → 無水エタノールへと進む。
１５．標本を 100%エタノールで 10 分× 2 回洗浄 して完全に脱水する。
１６．エタノールを除去し、クローブ油（clove oil）で標本をカバーし、室温で 15 分 。
１７．クローブ油から標本を取り出し、きれいなスライド上にEuparal® またはカナダバルサムを一滴垂らし、そこに標本を移す。
１８．配置（mounting）：サシチョウバエの頭部・胸部・腹部を、解剖顕微鏡下で細い針またはピンセットを用いて整える。頭部は、腹背位置（ventro-dorsal position） で封入する必要がある。すなわち、occipital foramenを上方に向け、cibariumが正面から観察できるようにする。この作業は封入媒質（mounting medium）中で行う。
１９．標本をそのまま置き、表面が粘着性になるまで 待つ。
２０．清潔なカバーガラスを無水アルコールで濡らし、角度をつけてカナダバルサム上に載せる。
２１．スライドを専用の乾燥箱（dry box）に保管する。
