# Supplementary material for: Processing and mounting phlebotomine sand flies: a consensus guideline
Source: Parasite. 2026 Apr 3;33:18. doi: 10.1051/parasite/2026009 (PMC13047900; doi:10.1051/parasite/2026009)
Supplement: Supplementary file 18 — Khmer translation / ការបកប្រែជាភាសាខ្មែរ [file parasite-33-18-s18.pdf]

Parasite **33**, 18 (2026)
© F.J. Randrianambinintsoa et al., published by EDP Sciences, 2026
https://doi.org/10.1051/parasite/2026009

PARASITE

Available online at:
www.parasite-journal.org

REVIEW ARTICLE

OPEN ACCESS

ទម្រង់ការណ៍ណែនាំនៃដំណើរការវិភាគ និង mounting phlebotomine sand flies

Translated by Kimsear Nov, Sovanncheypo Chao & Didot Prasetyo

Fano José Randrianambinintsoa[1], Laure Augendre[1], Jorian Prudhomme[1], Jean-Philippe Martinet[1], Mathieu Loyer[1], Nalia Mekarnia[1], Hocine Kerkoub[1], Farzana Khan Perveen[1], Antoine Huguenin[1,2], Emilie Kariya[1,2], Mohammad Akhoundi[3], Andrey José de Andrade[4], Eduardo Berriatua[5], Gioia Bongiorno[6], Sébastien Boyer[7,8], Vasiliki Christodoulou[9], Magda Clara Vieira Da Costa-Ribeiro[10], Lucas Alexandre Farias de Souza[10], Huicong Ding[11], Blaise Dondji[12], Vít Dvořák[13], Ozge Erisoz Kasap[14], Eunice Aparecida Bianchi Galati[15], Montserrat Gállego[16], Cristina Ballart[16], Stavroula Gouzelou[17], Nabil Haddad[18], Rezki Sabrina Masse[19], Asrat Hailu Mekuria[20], Vladimir Ivovic[21], Szymon Kaczmarek[22], Mohd Khadri Shahar[19], Oscar D. Kirstein[23], Edwin Kniha[24], Iva Kolářová[13], Lincoln Timinao[25], Cristian Lucanas[26], Ognyan Mikov[27], Kimsear Nov[7], Yusuf Özbel[28], Bernard Pesson[29], Laura Cristina Posada Lopez[30], Didot Budi Prasetyo[1,7], Nil Rahola[31], Eduardo A. Rebollar-Tellez[32], Bruno Leite Rodrigues[15], Lalita Roy[33], Prasanta Saini[34], Chizu Sanjoba[35], Paloma Helena Fernandes Shimabukuro[36], Padet Siriyasatien[37], Agnieszka Soszyńska[22], Tatiana Suleşco[38], Massamba Sylla[39], Majhalia Torno[40], Petr Volf[13], Khamsing Vongphayloth[41], Vu Sinh Nam[42], April Wardhana[43], Eric Yessinou[44], Sonia Zapata[45], Jean-Charles Gantier[1], and Jérôme Depaquit[1,2,*]

[1] Faculté de Pharmacie, Université de Reims Champagne Ardenne, UR ESCAPE-USC ANSES PETARD, 51 rue Cognacq-Jay, 51096 Reims Cedex, France
[2] Pôle de Biologie territoriale, Laboratoire de Parasitologie-Mycologie, Centre Hospitalo-Universitaire, 51092 Reims, France
[3] Parasitology-Mycology Department, Avicenne Hospital, AP-HP, Bobigny, Sorbonne Paris Nord University, France; Unité des Virus Émergents (UVE: Aix-Marseille Univ, Università di Corsica, IRD 190, Inserm 1207, IRBA), 13005 Marseille, France
[4] Parasitology Collection of Basic Pathology, Department of Basic Pathology, Federal University of Paraná, Curitiba 19031, Brazil
[5] Department of Animal Health, University of Murcia, Campus de Espinardo, 30100 Espinardo, Murcia, Spain
[6] Department of Infectious Diseases, Vector-borne Diseases Unit, Istituto Superiore di Sanità, 00166 Rome, Italy
[7] Medical and Veterinary Entomology Unit, Institut Pasteur du Cambodge, Phnom Penh 12201, Cambodia
[8] Ecology & Emergence of Arthropod-borne Pathogens Unit, Department of Global Health, Institut Pasteur, CNRS UMR2000, 75015 Paris, France
[9] Section Veterinary Services (1417), Laboratory for Animal Health Virology, Aglantzia, Nicosia 2109, Cyprus
[10] Insects Vectors and Parasites Laboratory, Department of Basic Pathology and Postgraduate program in Microbiology, Parasitology and Pathology, Federal University of Paraná, 81530-900 Curitiba, Brazil
[11] Department of Biological Sciences, National University of Singapore, 117558, Singapore
[12] Laboratory of the Leishmaniasis Research Project, Mokolo District Hospital, Mokolo, Cameroon; Laboratory of Cellular Immunology and Parasitology, Department of Biological Sciences, Central Washington University, 98926 Ellensburg, WA, USA
[13] Department of Parasitology, Faculty of Science, Charles University, 12800 Prague, Czechia
[14] VERG Laboratories, Department of Biology, Faculty of Science, Hacettepe University, Beytepe, Ankara 06800, Türkiye
[15] Faculdade de Saúde Pública da Universidade de São Paulo (FSP/USP), Pós-graduação em Saúde Pública, 01246-904 São Paulo, Brazil
[16] Secció de Parasitologia, Departament de Biologia, Sanitat i Medi Ambient, Facultat de Farmàcia i Ciències de l'Alimentació, Universitat de Barcelona, & Institut de Salut Global de Barcelona (ISGlobal), Centro de Investigación Biomédica en Red, Enfermedades Infecciosas (CIBERINFEC), 08028 Barcelona, Spain
[17] Laboratory of Infectious Diseases and Public Health, School of Medicine, University of Cyprus, Nicosia, Cyprus & Department of Pediatrics, Archbishop Makarios III Hospital, Nicosia 2115, Cyprus
[18] Faculty of Health Sciences, American University of Beirut, 1107 2020 Beirut, Lebanon

Edited by Jean-Lou Justine
*Corresponding author: jerome.depaquit@univ-reims.fr

This is an Open Access article distributed under the terms of the Creative Commons Attribution License (https://creativecommons.org/licenses/by/4.0), which permits unrestricted use, distribution, and reproduction in any medium, provided the original work is properly cited.

[19] Medical Entomology Unit, Infectious Disease Research Centre, Institute for Medical Research (IMR), National Institutes of Health (NIH), Ministry of Health Malaysia, 40170 Shah Alam, Selangor, Malaysia
[20] School of Medicine, Addis Ababa University, 28017 - 1000 Addis Ababa, Ethiopia
[21] Faculty of Mathematics, Natural Sciences and Information Technologies, University of Primorska, 6000 Koper, Slovenia
[22] University of Lodz, Faculty of Biology and Environmental Protection, Department of Invertebrate Zoology and Hydrobiology, Banacha 12/16, 90-237 Łódź, Poland
[23] Laboratory of Entomology, Ministry of Health, 9134302 Jerusalem, Israel
[24] Center for Pathophysiology, Infectiology and Immunology, Institute of Specific Prophylaxis and Tropical Medicine, Medical University Vienna, Kinderspitalgasse 15, 1090 Vienna, Austria
[25] Papua New Guinea Institute of Medical Research (PNGIMR) Institute, PO Box 60, Headquarter, Homate Street, 441 Goroka, Eastern Highlands Province, Papua New Guinea
[26] Museum of Natural History, University of the Philippines Los Baños, 4031 Laguna, Philippines
[27] National Centre of Infectious and Parasitic Diseases, 1504 Sofia, Bulgaria
[28] Ege University, Faculty of Medicine, Department of Parasitology, 35040 Bornova/Izmir, Türkiye
[29] Retired, Faculté de Pharmacie, Université de Strasbourg, Strasbourg, 67400 Illkirch-Graffenstaden, France
[30] Program for the Study and Control of Tropical Diseases (PECET), Faculty of Medicine, University of Antioquia, 050010 Medellin, Colombia
[31] MIVEGEC, Univ. Montpellier, CNRS, IRD, 34394 Montpellier, France & Medical Entomology Unit, Institut Pasteur de Madagascar, 101 Antananarivo, Madagascar
[32] Laboratorio de Entomología Médica, Departamento de Zoología de Invertebrados, Facultad de Ciencias Biológicas, Universidad Autónoma de Nuevo León, San Nicolás de los Garza, 66455, NL, México
[33] Tropical and Infectious Disease Centre, BP Koirala Institute of Health Sciences, Dharan 56700, Nepal
[34] ICMR-Vector Control Research Centre, Puducherry 605006, India
[35] Graduate School of Agricultural and Life Sciences, The University of Tokyo, Tokyo 113-8657, Japan
[36] Grupo de estudos em Leishmanioses/Coleção de Flebotomíneos (COLFLEB/Fiocruz-MG), Instituto René Rachou, Fundação Oswaldo Cruz, Belo Horizonte, Minas Gerais, 30190009, Brazil
[37] Center of Excellence in Vector Biology and Vector-Borne Disease, Department of Parasitology, Faculty of Medicine, Chulalongkorn University, Bangkok 10330, Thailand
[38] Department of Arbovirology, Bernhard Nocht Institute for Tropical Medicine, Bernhard Nocht Str. 74, 20359 Hamburg, Germany [39] Laboratory Vectors & Parasites, Department of Livestock Sciences and Techniques, Sine Saloum University El Hadji Ibrahima Niasse (SSUEIN) Kaffrine Campus, C.P. 24600, Senegal.
[40] Environmental Health Institute, National Environment Agency, Singapore 138667, Singapore & Department of Biological Sciences, National University of Singapore, 117558 Singapore
[41] Institut Pasteur du Laos, Laboratory of Vector-Borne Diseases, Samsenhai Road, Ban Kao-Gnot, Sisattanak District, 3560 Vientiane, Lao PDR
[42] National Institute of Hygiene and Epidemiology, 1 Yec-Xanh Street, Hai Ba Trung District, 100000 Hanoi, Vietnam
[43] Indonesian Research Center for Veterinary Science, Indonesian Agency for Agricultural Research and Development, Ministry of Agriculture Republic Indonesia, Bogor 16114, Indonesia & Department of Parasitology, Faculty of Veterinary Medicine, Airlangga University, Surabaya 60115, Indonesia
[44] Laboratory of Research in Applied Biology, Polytechnic School of Abomey-Calavi, University of Abomey-Calavi, 01 P.O. Box 2009, 00000 Cotonou, Benin
[45] Instituto de Microbiología, Colegio de Ciencias Biológicas y Ambientales (COCIBA), Universidad San Francisco de Quito (USFQ), 170901 Quito, Ecuador

Received 1 December 2025, Accepted 29 January 2026, Published online3 April 2026

មូលន័យសង្ខេប – អត្ថបទនេះផ្តល់នូវការណែនាំយ៉ាងទូលំទូលាយ សម្រាប់ដំណើរការវិភាគ និង Mounting សំណាក Phlebotomine មានសារៈសំខាន់ចំពោះការកំណត់អត្តសញ្ញាណលើប្រភេទ និងការកំណត់អត្តសញ្ញាណភ្នាក់ងារបង្ករោគ នឹងការញែកដោយឡែកនៃភ្នាក់ងារបង្ករោគ។
ទាំងនេះនឹងលើកមកពិភាក្សាអំពីបច្ចេកទេសជាច្រើនដែលសមស្របសម្រាប់ការងារទាំងនៅfield condition និងក្នុងមន្ទីរពិសោធន៍។ ទម្រង់ការណែនាំនេះរួមបញ្ចូលទាំង គោលការណែនាំយ៉ាងលម្អិតអំពីការប្រមូលសំណាក ការប្រតិបត្តិ ការទុកដាក់ និងការសម្លាប់តាមសម្រួល (ដោយណែនាំអោយប្រើវិធីបង្កកស្ងួត ឬ CO2 ជាជាងប្រើសារធាតុគីមី) នៃ Phlebotomine ព្រមទាំងយុត្តិវិធីនៃការរក្សាទុកដូចជា ការទុកដាក់នៅក្នុងសីតុណ្ហភាពត្រជាក់ និងការរក្សាទុកក្នុងអេតាណុល។ នៅក្នុងទម្រង់ការណែនាំនេះ យើងនឹងលើកយក កត្តាដែលចាំបាច់សម្រាប់ការសង្កេតដោយមីក្រូទស្សន៍ រួមមាន គុណភាពនៃការរៀបចំ រចនាសម្ព័ន្ធកាយវិភាគសាស្ត្រមួយចំនួន (សរីរាង្គបន្តពូជ, ក្បាល និងស្លាប)។ អត្ថបទនេះក៏បង្ហាញអំពីដំណើរការដ៏លម្អិតនៃការវិភាគសំណាក រួមទាំងដំណើរការសម្អាតជាមួយសារធាតុផ្សេងៗ ដូចជា ប៉ូតាស្យូមអ៊ីដ្រូស៊ីតបន្ទាប់មកសូលុយស្យុង Marc-André។ ដំណើរការវិភាគនៃការធ្វើ Mounting

នឹងលើកយកមកជជែកដោយប្រៀបធៀបរវៀងសមាសធាតុថ្នាលផ្សេងៗគ្នាដោយសង្កត់ធ្ងន់លើលក្ខណៈសម្បត្តិនៃការចាប់បាច់ពន្លឺ និងប្រសិទ្ធភាពនៃការរក្សាទុករបស់វា។ សារធាតុរាវ Hoyer Fluid (ឬហៅថាជា Chloral Gum) ត្រូវបានណែនាំសម្រាប់ការពិនិត្យរហ័ស ជាពិសេសសម្រាប់ការពិនិត្យលើថង់ផ្ទុកទឹកកាមញី (Spermathecae) ដោយសារតែភាពថ្លារបស់វាទោះបីជាវាមិនសមស្របសម្រាប់ការផ្ទុករយៈពេលវែងក៏ដោយ។ សមាសធាតុថ្នាលផ្សេងទៀតដែលត្រូវបានលើកមកពិភាក្សារួមមាន Polyvinyl Alcohol, Euparal® (ដើម្បីកំណត់កម្រិតជាតិទឹកអោយនៅតិចតួច), និង Canada Balsam (ថ្នាលដែលរលាយក្នុងអ៊ីដ្រូកាបូន) ដោយពីរប្រភេទចុងក្រោយផ្តល់នូវសមត្ថភាពរក្សាទុករយៈពេលវែង។ វិធីសាស្ត្រម៉ូលេគុលជីវសាស្ត្រដូចជា DNA Sequencing និង MALDI-ToF ត្រូវបានលើកយកមកពិភាក្សាក្នុងអត្ថបទនេះព្រោះវាវត្តបំណងសំខាន់ក្នុងដំណើរការវិភាគសំណាកផងដែរ។ លើសពីនេះទៅទៀត វីដេអូឃ្លីបខ្លីៗដែលបង្ហាញពីបច្ចេកទេសនៃការរៀបចំកញ្ចក់ឡាម ព្រមទាំងការបកប្រែជា ៣៣ ភាសាផ្សេងៗគ្នា ត្រូវបានផ្តល់អនុញ្ញាតឱ្យគោលការណ៍ណែនាំនេះឆ្លើយតបទៅនឹងតម្រូវការ និងការរំពឹងរបស់សហគមន៍វិទ្យាសាស្ត្រសកល។

ពាក្យគន្លឹះ ការតំឡើងសំណាកលើកញ្ចក់ឡាម (Mounting), phlebotomine sand fly, សារធាតុរាវ Hoyer, សូលុយស្យុង Marc-André, chloral gum, អាលកុល polyvinyl, Euparal®, Canada balsam, ការញ៉ែកបំបែក Leishmania, លក្ខ័ណនៃកន្លែងបំពេញបេសកកម្ម (Field condition), បណ្តុះ, ការកាត់បំបែក, ជីវម៉ូលេគុល, MALDI-ToF, ប្រភេទសំណាក (Type-specimens).

Abstract – Processing and mounting phlebotomine sand flies: a consensus guideline. This article provides a comprehensive guide for the processing and mounting of phlebotomine sand fly specimens, which is crucial for species identification and pathogen detection and isolation. It discusses a range of techniques suitable for both field and laboratory settings. The guide includes detailed instructions on sand fly collection, handling, covering, and euthanasia (recommending dry freezing or CO_2 over chemicals) as well as conservation strategies, such as cold storage and preservation in ethanol. The quality of preparation of certain anatomical structures (genital organs, head and wings) is essential for their proper microscopic observation and is described in this work. The article also presents detailed sample processing, including the clearing process with agents such as potassium hydroxide then Marc-André solution. The mounting process compares different media, emphasizing their optical properties and preservation potential. Hoyer fluid (also known as chloral gum) is recommended for quick observation, particularly for spermathecae, due to its clarity, although it is not suitable for long-term storage. Other media discussed include polyvinyl alcohol, Euparal® (for limited water tolerance), and Canada balsam (a hydrocarbon-soluble medium), with the latter two offering long-term preservation capabilities. Innovative molecular biology approaches such as DNA sequencing and MALDI-ToF, which require particular attention to sample processing, are also addressed. Furthermore, short video clips illustrating various mounting techniques as well as translations in many different languages are provided, allowing the guideline to reach the diverse needs and expectations of the global scientific community.

Key words: Mounting, Phlebotomine sand fly, Hoyer fluid, Marc-André solution, Chloral gum, Polyvinyl alcohol, Euparal®, Canada balsam, *Leishmania* isolation, Field conditions, Culture, Dissection, Molecular biology, MALDI-ToF, Type-specimens.

សេចក្តីផ្តើម

Phlebotomine sand flies ជាសត្វល្អិតតូចក្នុងលំដាប់ Diptera ស្ថិតក្នុងក្រុម Psychodidae អនុក្រុម Phlebotominae ដែលមានយ៉ាងហោចណាស់ 1,063 ប្រភេទដែលបានកំណត់អត្តសញ្ញាណរួចហើយ] 21]។ ពួកវាជាភ្នាក់ងារចម្លងរោគយ៉ាងសំខាន់ទៅតាមលំដាប់ ដែលរួមមាន Leishmania, arboviruses និង *Bartonella* ដែលបណ្តាលឱ្យកើតជំងឺ leishmaniasis, arboviral infections និង bartonellosis ។ ជាចម្បងនៃការកំណត់អត្តសញ្ញាណរបស់ Phlebotomine sand flies ផ្អែកលើការពិនិត្យដោយមីក្រូទស្សន៍យ៉ាងលម្អិតដែលត្រូវបានអនុវត្តក្រោមលក្ខខណ្ឌប្រមូលសំណាកយ៉ាងប្រុងប្រយ័ត្ន ការរក្សាទុកសមស្រប និងការរៀបចំកញ្ចក់ឡាម ដោយប្រើបច្ចេកទេសជំនាញជាច្រើនជំហ៊ាន ដែលដំណាក់កាលនីមួយៗមានចំណុចពិសេស និងដែនកំណត់រាងខ្លួន។

ការកំណត់អត្តសញ្ញាណ Phlebotomine sand flies ពេញវ័យផ្អែកលើការសង្កេតទាំងរចនាសម្ព័ន្ធខាងក្រៅ) ឧ . អង់តែន, palpi, ប្រដាប់បន្តពូជឈ្មោល) និងរចនាសម្ព័ន្ធខាងក្នុង) ឧ .pharynx, cibarium និង ថង់ផ្ទុកទឹកកាមញី) ។ ការបំបែកបំណែក) dissection(នឹងការញ៉ែកដាច់ដោយឡែកនៃរចនាសម្ព័ន្ធខាងក្នុងទាំង

នេះអនុញ្ញាតឱ្យមានការពិនិត្យបានច្បាស់លាស់ និងជានាការកំណត់អត្តសញ្ញាណបានត្រឹមត្រូវ។ Phlebotomine sand flies ខុសពីមូស ឬ kissing bugs ដែលវាតម្រូវឱ្យមានការ Mounting រវាងកញ្ចក់ឡាមនិងឡាម៉ែលមុននឹងធ្វើការកំណត់អត្តសញ្ញាណ។ មុនទសវត្សរ៍ 1980 ការសង្កេតមីក្រូទស្សន៍គឺជាវិធីសាស្ត្រតែមួយសម្រាប់កំណត់អត្តសញ្ញាណPhlebotomine sand flies ហើយរហូតមកដល់សព្វថ្ងៃវានៅតែជាវិធីសាស្ត្រដែលត្រូវបានប្រើប្រាស់យ៉ាងទូលំទូលាយបំផុត។ ការជ្រើសរើសដំណើរការវិភាគ និងវិធីរៀបចំសំណាកនៅពេលនោះមានលក្ខណៈសាមញ្ញ ដោយផ្អែកលើជម្រើសពីរប្រភេទ៖ 1) ការ Mounting អចិន្ត្រៃយ៍សម្រាប់ការរក្សាទុករយៈពេលវែង និង) 2) ការ Mounting រហ័សសម្រាប់ការកំណត់អត្តសញ្ញាណប៉ុន្តែវាមិនជានាការរក្សាទុករយៈពេលវែងឡើយ។ ការMounting អចិន្ត្រៃយ៍ ដូចជា ការប្រើ Canada balsam ត្រូវការពេលវេលាច្រើន ព្រោះត្រូវការដំណើរការដកទឹក ពេញលេញចេញពីសំណាកនោះ។ លើសពីនេះ សន្ទស្សន៍បាច់ពន្លឺរបស់ថ្នាលប្រភេទនេះតែងតែមិនងាយសមស្របសម្រាប់ការសង្កេតថង់ផ្ទុកទឹកកាមញីទេ។ ផ្ទុយទៅវិញ ការ Mounting ក្នុងថ្នាលរាវ) aqueous medium) ដូចជា Hoyer liquid អនុញ្ញាតឱ្យការរៀបចំបានលឿនជាង និងផ្តល់ភាពច្បាស់ល្អលើការពិនិត្យថង់ផ្ទុកទឹកកាមញី ដែលមានលក្ខណៈសម្បត្តិអុបទិកខ្ពស់ ប៉ុន្តែមិនអាចជានាការរក្សាទុករយៈពេលវែងបានទេ ព្រោះវាមានលក្ខណៈស្រូបទឹកពីបរិយាកាស។ ជម្រើសមួយផ្សេងទៀតគឺការបិទជុំវិញគែមកញ្ចក់ឡាមដោយប្រើថ្នាំបំពាក់ពណ៌ក្រចក បន្ទាប់ពីកញ្ចក់ស្ងួតទាំងស្រុង។ ជម្រើសទាំងអស់នេះមានភាពប្រកួតប្រជែង និងនៅតែមានសារៈសំខាន់ដល់សព្វថ្ងៃ ហើយការជ្រើសរើសវិធីសាស្ត្រការ Mounting អាស្រ័យលើគោលបំណងនៃការរៀបចំសំណាក។ ចាប់តាំងពីទសវត្សរ៍ 1980 ការសិក្សាកំណត់អត្តសញ្ញាណ Phlebotomine sand flies បានបញ្ចូលទាំងការវិភាគលក្ខណៈរូបសាស្ត្ររួមជាមួយវិធីសាស្ត្រគីមីជីវៈ។ ការវិភាគតាមបែប cuticular hydrocarbons គឺជាវិធីសាស្ត្រដំបូង ប៉ុន្តែត្រូវបានជំនួសយ៉ាងឆាប់រហ័សដោយបច្ចេកទេសម៉ូលេគុលជីវសាស្ត្រដូចជា random amplified polymorphic DNA (RAPD), restriction fragment length polymorphism (RFLP), DNA amplification និង DNA sequencing តាមវិធីសាស្ត្រ Sanger ក៏ដូចជា next-generation sequencing (NGS)។ បច្ចុប្បន្ន ម៉ូលេគុលជីវសាស្ត្រត្រូវបាននាវានវត្តន៍ដោយវិធីសាស្ត្រ proteomics ដូចជា MALDI-ToF។ លើសពីនេះ ការកំណត់អត្តសញ្ញាណប្រភេទតាមរយៈម៉ូលេគុលជីវសាស្ត្រអាចរួមបញ្ចូលជាមួយការរកឃើញភ្នាក់ងារបង្កជំងឺតាម PCR (ឧ. *Leishmania*, *Trypanosoma*, *Bartonella* និង *Phlebovirus*) ដោយសារតែភ្នាក់ងារទាំងនេះអាចត្រូវបានរកឃើញទាំងដោយ end-point PCR និង real-time PCR (qPCR) ដែលតម្រូវឱ្យមានការកែប្រែការប្រមូលសំណាក និងរក្សាទុកសំណាកឱ្យសមស្របទៅនឹងគោលបំណងស្រាវជ្រាវ] 3, 32] ។ ក្រៅពីការវិភាគលក្ខណៈរូបសាស្ត្រដែលបានលើកឡើងខាងលើត្រូវបានប្រើប្រាស់ជាទម្លាប់សម្រាប់ការកំណត់ប្រភេទ វិធីសាស្ត្រការវិភាគលក្ខណៈរូបសាស្ត្រផ្សេងទៀតក៏អាចអនុវត្តបានដូចជាការពិនិត្យការតម្រៀបសរសៃនៅលើស្លាប (wing-geometry) ។

ដោយផ្អែកជាចម្បងលើបទពិសោធន៍របស់អ្នកនិពន្ធ និងទិន្នន័យដែលបានមកពីអត្ថបទស្រាវជ្រាវផ្សេងៗ គោលបំណងនៃការសិក្សានេះគឺផ្តល់ទម្រង់ការណែនាំស្តង់ដារសម្រាប់ការ Mounting និងដំណើរការវិភាគសំណាក Phlebotomine sand flies ពេញវ័យដើម្បីបង្កើនប្រសិទ្ធភាពនៃការវិភាគលក្ខណៈរូបសាស្ត្រ និងជីវម៉ូលេគុល។

ការអនុវត្តវិភាគមួយចំនួន ដូចជាម៉ូលេគុលជីវសាស្ត្រ ឬ MALDI-ToF សំណាកអាចជ្រើសយកផ្នែកណាមួយនៃ Phlebotomine sand flies ដែលមិនចាំបាច់ប្រើប្រាស់ក្នុងការកំណត់អត្តសញ្ញាណបែបលក្ខណៈរូបសាស្ត្រ បង្ហាញពីតម្រូវការជ្រើសរើសវិធីសាស្ត្រក្នុងការអនុវត្តយ៉ាងយកចិត្តទុកដាក់។ ក្នុងអត្ថបទនេះ យើងកំផ្តោតលើវិធីសាស្ត្រការសណ្តំ និង ការសម្លាប់តាមសម្រួលនៃ Phlebotomine sand flies ដែលចាប់បាននៅរស់ ការរក្សាទុក និងដំណើរការ Mounting សម្រាប់ការកំណត់អត្តសញ្ញាណរហ័ស ឬសម្រាប់ការរក្សាទុករយៈពេលវែងដែលអនុញ្ញាតឱ្យអនុវត្តការសិក្សាបន្ថែមជាបន្តបន្ទាប់។

បុព្វកថា៖ ការពិចារណាសុវត្ថិភាព និងបទប្បញ្ញត្តិ (Preamble)

រាល់ការប្រើប្រាស់សារធាតុគីមីទាំងអស់ដែលបានបញ្ជាក់ក្នុងការណែនាំនេះ ហើយត្រូវអនុវត្តក្រោមលក្ខខណ្ឌសុវត្ថិភាពយ៉ាងតឹងរឹងយោងទៅលើ Safety Data Sheets (SDS) សម្រាប់ព័ត៌មានលម្អិតអំពីហានិភ័យ ការគ្រប់គ្រង និងការចោលសំណល់។ គណៈកម្មាធិការសុខភាព និងសុវត្ថិភាពរបស់មន្ទីរពិសោធន៍អាចផ្តល់ព័ត៌មានអំពីគ្រោះថ្នាក់នៃសារធាតុទាំងនេះ ក៏ដូចជាវិធីសាស្ត្រគ្រប់គ្រង និងបំបាត់សំណល់។ ទោះជាយ៉ាងណា ការអនុវត្តតាមសេចក្តីណែនាំសុវត្ថិភាព និងបទប្បញ្ញត្តិជាធរមាន គឺជាកាតព្វកិច្ចចាំបាច់។ អ្នកប្រើប្រាស់ទាំងអស់ត្រូវធានាថា

ការអនុវត្តការងារនៅមន្ទីរពិសោធន៍អនុលោមតាម Good Laboratory Practices (GLP) និងត្រូវនៅក្នុងច្បាប់ជាធរមានរបស់ប្រទេស ឬស្ថាប័ន។ សូមចំណាំថា សារធាតុគីមីខ្លះ ឬសមាសធាតុ .ឧ)Chloral hydrate ត្រូវបានគ្រប់គ្រងតាមច្បាប់នៅប្រទេសខ្លះៗ។ បញ្ជីអក្សរកាត់ដែលប្រើប្រាស់ក្នុងអត្ថបទនេះ ត្រូវបានបង្ហាញក្នុងតារាងទី 1។

តារាងទី 1៖ បញ្ជីអក្សរកាត់

**BME**	Basal medium Eagle
**CDC**	Centers for Disease Control and Prevention
**CMCP**	Camphor-monochlorophenol
**CMR**	Carcinogenic, mutagenic, reprotoxic substance
**COI**	Cytochrome c oxidase subunit I gene
**CytB**	Cytochrome b gene
**DNA**	Deoxyribonucleic acid
**ELISA**	Enzyme-linked immunosorbent assay
**EtOH**	Ethanol
**M199**	Medium 199
**MALDI-ToF MS**	Matrix-assisted laser desorption/ionization time-of-flight mass spectrometry
**MEM**	Minimum essential media
**NGS**	Next-generation sequencing
**NNN**	Novy-MacNeal-Nicolle medium
**PCR**	Polymerase chain reaction
**Lao PDR**	Lao People's Democratic Republic
**PNOC**	Prepronociceptin gene
**qPCR**	Quantitative PCR (real-time PCR)
**RAPD**	Random amplified polymorphic DNA
**RFLP**	Restriction fragment length polymorphism
**RI**	Refractive index
**RNA**	Ribonucleic acid
**RNases**	Ribonucleases
**RNASS**	RNA stabilization solution
**RT-PCR**	Reverse transcription PCR
**TFA**	Trifluoroacetic acid

1. ការចាប់ប្រមូល Phlebotomine sand flies

Phlebotomine sand flies ពេញវ័យត្រូវបានប្រមូលទាំងនៅស្ថានភាពរស់ ឬស្លាប់ដោយអាចប្រើវិធីសាស្ត្រផ្សេងគ្នាដូចជា CDC miniature light traps, អន្ទាក់ក្រដាស់ស្អិត (sticky traps), ឧបករណ៍បូម (រួមទាំង Shannon traps) ឬការប្រមូលដោយផ្ទាល់ពីមជ្ឈដ្ឋានរស់នៅ Phlebotomine sand flies) ឧ .ទ្រុងសត្វ(។ វិធីសាស្ត្រទាំងនេះពឹងផ្អែកលើការដាក់អន្ទាក់នៅក្នុងកន្លែងដែលមាន Phlebotomine sand flies រឺជម្រករបស់វាដោយការទាក់ទាញតាមរយៈពន្លឺ ឬសារធាតុទាក់ទាញផ្សេងៗ) ឧ .CO_2 ឬ chemical lures) និងការប្រមូលសំណាកសម្រាប់វិភាគបន្តដូចបានរៀបរាប់នៅក្នុងអត្ថបទស្រាវជ្រាវជាច្រើន] 2, 3, 32, 36, 49]។ ការចាប់ Phlebotomine sand flies គួរនៅស្ថានភាពរស់ដោយអនុវត្តតាមវិធីសាស្ត្រ downstream ជៀសវាងអំឡុងពេលប្រមូលសំណាកធ្វើអោយ Phlebotomine sand flies ស្លាប់បណ្តាលឱ្យប៉ះពាល់ដល់ Leishmania isolation ឬ Virus strains ។ បច្ចេកទេសប្រមូលសំណាកផ្សេងទៀតដូចជាការប្រើក្រដាស់ស្អិតអាចបណ្តាលឱ្យខូច រឺបាត់បង់សរីរាង្គ) អង់តែន, palpi, ស្លាប ឬ ជើង) ជាញឹកញាប់។ ស្រទាប់ប្រេង castor ដែលគេច្រើនប្រើបំពាក់ពណ៌លើក្រដាស់ស្អិត នឹងភ្ជាប់ជាប់ជាមួយរាងកាយ Phlebotomine sand flies ដើម្បីដកយកសំណាកបានគេត្រូវជ្រលក់វាទៅក្នុងល្បាយ អេតាណុល និង diethyl ether ក្នុងអត្រាស្មើគ្នា រយៈពេលប្រហែល 15 នាទី នេះគឺជាដំណាក់កាលដំបូងនៃដំណើរការវិភាគសំណាក។

2. ការសម្លាប់តាមសម្រួលលើសំណាក

បន្ទាប់ពីការប្រមូល Phlebotomine sand flies ដែលនៅរស់ត្រូវបានសម្លាប់តាមសម្រួល។ ក្នុងវិធីសាស្ត្រប្រមូលមួយចំនួន) ឧ .ក្រដាសស្អិត ឬ CDC light traps ដែលបំពាក់មកជាមួយកំប៉ុងមានផ្ទុកសូលុយស្យុងសាប៊ូឬអេតាណុល) គឺ Phlebotomine sand flies បានស្លាប់រួចហើយអំឡុងពេលប្រមូល។ វិធីសាស្ត្រម៉ូលេគុលជីវសាស្ត្រត្រូវបានអនុវត្តលើសំណាកដែលប្រមូលដោយផ្ទាល់ក្នុងអេតាណុល ឬលើសំណាកផ្សេងទៀត ប្រសិនបើរក្សាទុកក្នុងអេតាណុលឱ្យបានឆាប់តាមដែលអាចធ្វើទៅបាន។ ទោះជាយ៉ាងណាវិធីសាស្ត្រសម្លាប់ទាំងនេះមិនអនុញ្ញាតឱ្យដំណើរការវិភាគសំណាកដោយ MALDI-ToF ទេ។ លើសពីនេះ វិធីសាស្ត្រផ្សេងទៀតអាចបណ្តាលឱ្យបាត់បង់លក្ខណៈរូបសាស្ត្រដោយផ្នែក។ ដូច្នេះ ការជ្រើសរើសសារធាតុសម្លាប់ស្តង់ដារដែលសមស្របគឺសំខាន់ណាស់ ដើម្បីធានាការកំណត់អត្តសញ្ញាណត្រឹមត្រូវ

ឬការរក្សាទុករយៈពេលវែងជាសំណាកគំរូ ។ សារធាតុគីមីដូចជា ethyl acetate, ethyl ether, tetrachloroethane និង chloroform អោយបញ្ច្រាបលើសំឡី ហើយដាក់ក្នុងកំប៉ុងដែលមាន Phlebotomine sand flies ដើម្បីធ្វើការសម្លាប់តាមសម្រួល។ សារធាតុទាំងនេះមានភាពពុល និងត្រូវអនុវត្តតាមសេចក្តីណែនាំរបស់ក្រុមហ៊ុនផលិតយ៉ាងតឹងរឹង។ យ៉ាងណាមិញ ការមិនផ្តល់អនុសាសន៍ឱ្យប្រើ chloroform ព្រោះបទពិសោធន៍បង្ហាញថាវាមិនសមស្របសម្រាប់ការសិក្សាម៉ូលេគុលជីវិទ្យាឡើយ ។ ដោយសារភាពគ្រោះថ្នាក់ និងភាពមិនប្រាកដប្រជានៃភាពសមស្របសម្រាប់វិភាគលេគុលជីវិទ្យាការប្រើសារធាតុគីមីទាំងនេះគួរត្រូវបានកាត់បន្ថយ។

វិធីសាស្ត្រដែលប្រើប្រាស់យ៉ាងទូលំទូលាយបំផុត និងអាចរក្សាទុកសរីរវិទ្យា, DNA និងប្រូតេអ៊ីនបានល្អគឺការបង្កកស្ងួត) dry freezing)។ សំណាកត្រូវបានបង្ករយៈពេលមួយគ្រប់គ្រាន់ ដើម្បីធ្វើឱ្យ Phlebotomine sand flies សន្លប់ទាំងស្រុង ប៉ុន្តែមិនគួរយូរលើសដែលវាអាចបណ្តាលឱ្យ) i) ស្ងួត ឬ) ii) ប៉ះពាល់ដល់សមត្ថភាពរស់របស់ Leishmania ប្រសិនបើមានគោលបំណងញែក Leishmania ពីក្រពះពោះវៀន Phlebotomine sand flies ក្នុងលក្ខណ្ឌ in vitro។ ផ្តល់អនុសាសន៍គួរបង្ករយៈពេល 15–20 នាទី នៅសីតុណ្ហភាព- 20°C ដោយត្រួតពិនិត្យជាប្រចាំ ដើម្បីធានាថាសំណាកនោះបានសន្លប់ទាំងស្រុង ប៉ុន្តែប៉ារ៉ាស៊ីតក្នុងខ្លួនវាមិនត្រូវបានសម្លាប់។ ប្រសិនបើមិនមានទូទឹកកកសីតុណ្ហភាពទាប អាចជ្រើសរើសប្រើ CO_2 សម្រាប់ធ្វើការសម្លាប់តាមសម្រួលបាន។ ក្នុងលក្ខខណ្ឌនៅកន្លែងបំពេញបេសកម្ម ដែលមិនអាចប្រើធុង CO_2 ធំបាន សំណាកអាចសម្លាប់បានដោយប្រើធុង CO_2 តូចៗ Soda siphon ប៉ុន្តែវាពិបាកក្នុងការដឹកជញ្ជូនតាមយន្តហោះ។ ជាជម្រើសចុងក្រោយ អាចប្រើផ្សែងថ្នាំជក់ ដោយចាប់ Phlebotomine sand flies រស់ពី CDC trap ដោយប្រើ aspirator រួចរក្សាទុកក្នុងបំពង់ទីបកែវហើយផ្លុំផ្សែងថ្នាំជកចូល ធ្វើបែបនេះអាចសម្លាប់វាបានក្រោយប៉ុន្មានវិនាទី។ វិធីនេះអាចអនុវត្តបានក្នុងគ្រប់ស្ថានភាពនៅfield condition ប៉ុន្តែបំពង់ទីបកញ្ចក់អាចជាប់ផ្សែងថ្នាំជកពីមុនៗ យើងគួរសម្អាតវាយ៉ាងហ្មត់ចត់មុនប្រើសម្រាប់ប្រមូលសំណាករស់។ វិធីសាស្ត្រទាំងនេះអាចអនុវត្តសម្រាប់ធ្វើការញ៉ែក Leishmania តាមការបំបែកក្រពះពោះវៀន)ក្រពះពោះវៀន dissection)។

3. ការរក្សាទុកសំណាកមុនដំណើរការវិភាគ (Specimen storage before processing)

មានវិធីសាស្ត្រសំខាន់ 5 ប្រភេទសម្រាប់ការរក្សាទុកមុនដំណើរការវិភាគ៖

3.1. ការបង្កក (Freezing)

វិធីសាស្ត្រនេះអនុវត្តបានល្អបំផុតនៅសីតុណ្ហភាព- 20°C ឬល្អជាងនេះនៅ- 80°C។ បច្ចុប្បន្ន វិធីរក្សាទុកដោយទូទឹកកក ត្រូវបានប្រើប្រាស់យ៉ាងទូលំទូលាយជាងការរក្សាទុកក្នុង អាសូតរាវ។ គ្រប់ករណីទាំងអស់ ការរក្សាទុកដោយការបង្កកជាលក្ខណៈ cryopreservation ត្រូវអនុវត្តឱ្យបានឆាប់តាមដែលអាចធ្វើទៅបាន បន្ទាប់ពីបានធ្វើឱ្យសំណាកសន្លប់) stunning)។ ការរក្សាទុកក្នុងទូទឹកកកផ្តល់អត្ថប្រយោជន៍ក្នុងការរក្សាទុកសភាពដើមរបស់សត្វល្អិតទាំងមូល ព្រមទាំងរក្សាបាននូវសុពលភាព) integrity) នៃ RNA, DNA និងប្រូតេអ៊ីន នៅពេញមួយរយៈពេល។ ផ្ទុយទៅវិញ ការរក្សាទុកក្នុង អាសូតរាវ អាចបង្កការខូចខាតធ្ងន់ធ្ងរដល់ស្លាប ជើង palps និង អង់តែន ដែលជាញឹកញាប់បណ្តាលឱ្យបែករូបរាងខាងក្រៅ ឬផ្តាច់ចេញ និងអាចបាត់បង់លក្ខណៈនៃការវិភាគលក្ខណៈរូបសាស្ត្រសំខាន់ៗ។ ការរក្សាទុកដោយការបង្កកស្ងួតក្នុងទូទឹកកកមានភាពប៉ះពាល់តិចជាង នីត្រូសែនរាវ ប៉ុន្តែមិនមែនជាវិធីល្អបំផុតសម្រាប់ការរក្សាទុកសរីរាង្គដែលមានភាពផុយស្រួយនោះទេ។ ត្រូវចំនាំថានៅពេលធ្វើការលាយកំណកនៃសំណាក)thawing) ស្លាប អង់តែន palps ឬជើងអាចភ្ជាប់ជាប់នឹងជញ្ជាំងបំពង់ទីប ហើយអាចដាច់រហែកចេញដោយសារការបង្កើតសារធាតុសំណើម) condensation)។ ទោះជាយ៉ាងណា ការរក្សាទុកដោយការបង្កក មិនតែងតែអាចអនុវត្តបានក្នុងការសិក្សានៅfield condition ព្រោះត្រូវការទូទឹកកក ឬធុង អាសូតរាវ។ ការរក្សាទុកក្នុងទូទឹកកកមានភាពសមស្របពេញលេញសម្រាប់ការរកឃើញភ្នាក់ងារបង្កជម្ងឺដោយប្រើឧបករណ៍ជីវម៉ូលេគុលដោយមិនបាត់បង់ sensitivity។ ទោះបីយ៉ាងណា ការរកឃើញ និងបំបែក RNA viruses តម្រូវឱ្យរក្សាទុកនៅ -80°C ឬក្នុង អាសូតរាវ ប្រសិនបើមានគោលបំណងរក្សាទុករយៈពេលវែង។ ប៉ុន្តែ ការបង្កកសំណាកមិនអាចធ្វើ Leishmania isolation តាមរយៈdissectionក្រពះពោះវៀនទេលុះត្រាតែសំណាក Phlebotomine sand flies បានង្កកជាមុនក្នុងដំណាក់កាល vapor phase បន្ទាប់មកទើបដាក់ក្នុង អាសូតរាវ (ឧទាហរណ៍ សំណាក់ដែលដកចេញពីក្នុងស្តុក (

ដើម្បីបង្កើតលក្ខខណ្ឌស្រដៀងនឹងការរក្សាទុក Leishmania ដោយ cryopreservation។

3.2. ការរក្សាទុកសំណាកក្នុងអាល់កុល (Ethanol ឬ Isopropyl alcohol)

វិធីសាស្ត្រនេះប្រហែលជាវិធីដែលត្រូវបានប្រើប្រាស់យ៉ាងទូលំទូលាយបំផុតសម្រាប់ការរក្សាទុកPhlebotomine។ វាអាចអនុវត្តបានយ៉ាងងាយស្រួលក្នុងfield conditions ទោះបីជាស្ថិតក្នុងស្ថានភាពលំបាកដែលគ្មានលទ្ធភាពដូចក្នុងមន្ទីរពិសោធន៍ក៏ដោយ។ ការរក្សាទុកសំណាកក្នុងអាល់កុលមានភាពសមស្របជាពិសេសសម្រាប់ការសិក្សាលក្ខណៈរូបសាស្ត្រ ព្រោះសរីរាង្គដែលមានភាពផុយស្រួយដូចជាស្លាប ជើង អង់តែន ឬ palps អាចរក្សាទុកបានល្អ ប្រសិនបើមិនមានពពុះខ្យល់នៅក្នុងបំពង់ទីបផ្ទុកសំណាក។ ដូច្នេះការផ្តល់អនុសាសន៍ឱ្យបិទបំពង់ទីបដោយប្រើសំឡីកប្បាសតូចមួយ ដើម្បីបំបាត់ពពុះខ្យល់ និងដាក់ស្លាកសញ្ញានៅលើសំឡីកប្បាសនោះ) រូបទី 1) តែកម្រិតភាគរយនៃអាល់កុលនៅតែជាបញ្ហាកំពុងពិភាក្សា។ ជាទូទៅ គេមិនផ្តល់អនុសាសន៍ឱ្យប្រើកម្រិតអាល់កុលក្រោម 70% ទេ] 45, 66]។ ការប្រើកម្រិតអាល់កុលខ្ពស់ជាងនេះអាចរក្សាទុក DNA បានប្រសើរជាង និងរយៈពេលយូរជាង ប៉ុន្តែអាចធ្វើឱ្យសំណាកមានភាពផុយ និងងាយបែកបាក់ក្នុងការសិក្សាលក្ខណៈរូបសាស្ត្រ។ ការប្រើអេតាណុលកម្រិត 96% (azeotropic mixture) អាចធានាបាននូវស្ថេរភាពក្នុងរយៈពេលវែង ជាពិសេសនៅតំបន់មានសំណើមខ្ពស់ដូចជាប្រទេសនៅតំបន់ត្រូពិច ទោះបីជាអេតាណុលកម្រិត 95% មានភាពងាយស្រួលក្នុងការរកទិញក៏ដោយ។ ប្រសិនគេមិនគិតពី DNA ជាទូទៅសំណាកត្រូវបានរក្សាទុកបានល្អក្នុងអេតាណុល (ប៉ុន្តែមានប្រសិទ្ធភាពទាបជាងវិធីបង្កក ជាពិសេសសម្រាប់ការសិក្សាតាមវិធីសាស្ត្រម៉ូលេគុលជីវសាស្ត្រប្រភេទ NGS)។ ផ្ទុយទៅវិញ ប្រូតេអ៊ីនមានស្ថេរភាពទាបជាងយ៉ាងខ្លាំង ជាពិសេសសម្រាប់វិធីសាស្ត្រ proteomics ដូចជា MALDI-ToF ជាដើម។ Phlebotomine sand flies ដែលរក្សាទុកក្នុងអាល់កុលរយៈពេលពីរបីខែ នៅតែអាចកំណត់អត្តសញ្ញាណតាមការវិភាគលក្ខណៈរូបសាស្ត្របាន ប៉ុន្តែមិនអាចបង្កើតគម្រូនៃខ្សែកោងប្រូតេអ៊ីន(reference protein spectra) ពីសំណាកទាំងនេះបានទេ។ ការរក្សាទុកក្នុងអាល់កុល ឬក្នុងស្ថានភាពស្ងួតអាចធ្វើឱ្យប្រសើរឡើង ប្រសិនបើតំឡើងសីតុណ្ហភាពដល់- 20°C។ ការបង្កកនៅ- 20°C ជួយបង្កើនការរក្សាទុកសេនេទិច) ឧ .nucleic acids) ដោយបន្ថយល្បឿនការបំបែក) degradation) ហើយផ្តល់អត្ថប្រយោជន៍បន្ថែមចំពោះការរក្សាទុកលក្ខណៈរូបសាស្ត្រដោយកាត់បន្ថយការបែកបាក់ជាលិកាតាមពេលវេលា យ៉ាងណាក្តីផលប៉ះពាល់លើលក្ខណៈរូបសាស្ត្រមានដែនកម្រិតតិចជាងឥទ្ធផលលើសេនេទិច។ ការរក្សាទុកក្នុងអេតាណុលក៏អាចអនុវត្តសម្រាប់ការរកវត្តមាន DNA និង RNA វីរុសប្រសិនបើប្រើអេតាណុលក្នុងកម្រិតយ៉ាងហោចណាស់ 70% និងរក្សាទុករយៈពេលខ្លី) មិនលើសពីពីរបីខែ(។ ក្នុងប្រទេសខ្លះ isopropyl alcohol អាចរកបានងាយស្រួល និងអាចរក្សាទុក DNA បាន ប៉ុន្តែវាធ្វើឱ្យសំណាករឹង។ isopropyl alcohol មិនងាយឆេះដូច អេតាណុលទេ ដូច្នេះងាយស្រួលក្នុងការដឹកជញ្ជូន។ បើចាំបាច់ Phlebotomine sand flies ដែលបានរក្សាទុកក្នុងនីត្រូសែនរាវ ឬដោយការបង្កកស្ងួត អាចផ្ទេរទៅក្នុងអាល់កុលបាន ព្រោះវិធីនេះនឹងផ្តល់ទាំងគុណវិបត្តិនៃវិធីទាំងពីរ។

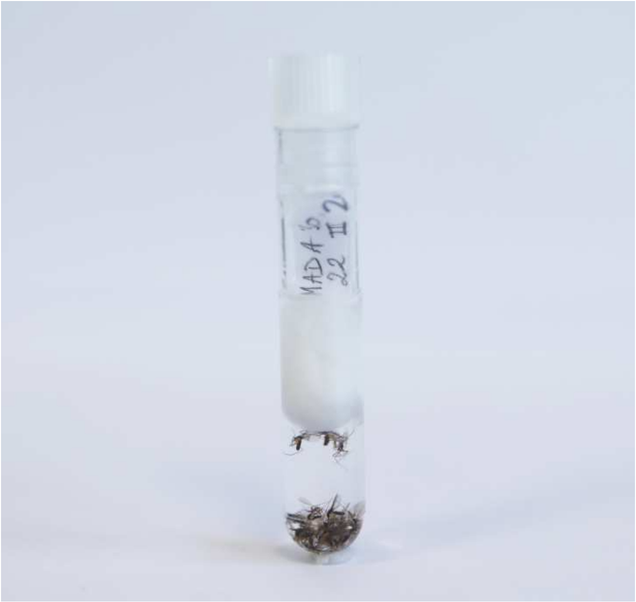

រូបទី 1៖ Phlebotomine sand fliesដែលរក្សាទុកក្នុង ethanol

3.3. ការរក្សាទុកសំណាកក្នុង RNA stabilization solution (RNASS)

RNASS ជាសារធាតុរាវដែលត្រូវបានប្រើប្រាស់យ៉ាងទូលំទូលាយ មិនមានលក្ខណៈពុល និងត្រូវបានរចនាឡើងសម្រាប់បង្កើនស្ថេរភាព និងការពារ RNA ក្នុងសំណាកជាលិកា និងកោសិកាស្រស់។ វាធ្វើសកម្មភាពដោយជ្រៀតចូលក្នុងសំណាកយ៉ាងឆាប់រហ័ស និងបង្អាក់សកម្មភាពអង់ស៊ីម RNases (RNA-degrading enzymes) ដើម្បីបង្ការពីការកម្ទេចទម្រង់ RNA ដោយមិនចាំបាច់ការបង្កកភ្លាមៗ។

ការរក្សាទុកសំណាកក្នុង RNASS ជាទូទៅមានប្រសិទ្ធភាពរក្សាទុករចនាសម្ព័នជាលិកា និងកោសិកាទាំងមូល សម្រាប់ការវិភាគប្រវត្តជាលិកានោះ។ ទោះបីជា RNASS ត្រូវបានបង្កើតឡើងសម្រាប់ស្ថេរភាព RNA ជាងការធ្វើ fixation ក៏ដោយ ការរក្សាទុករយៈពេលខ្លីទៅថ្នាលអាចរក្សាបាននូវសុពលភាពរចនាសម្ព័ន្ធយ៉ាងល្អ។ RNASS អនុញ្ញាតឱ្យរក្សាទុកសំណាកនៅសីតុណ្ហភាពបន្ទប់រហូតដល់ 7 ថ្ងៃ នៅ 4°C រយៈពេលពីរបីសប្តាហ៍ ឬនៅ- 20°C/-80°C សម្រាប់ការរក្សាទុករយៈពេលវែង។ វិធីសាស្ត្រនេះមានសារៈសំខាន់ខ្លាំងសម្រាប់ការសិក្សានៅfield condition ឬបរិបទគ្លីនិកដែលបរិយាកាសក្នុងបន្ទប់មានភាពត្រជាក់គ្រប់គ្រាន់។ ជាទូទៅ ការដកស្រង់ RNA តម្រូវឱ្យដកសំណាកចេញពី RNASS មុនពេលដំណើរការវិភាគតាមពិធីការស្តង់ដារ។

3.4. ការរក្សាទុកស្ងួតនៅសីតុណ្ហភាពបន្ទប់

នេះជាវិធីសាស្ត្រចាស់មួយដែលគេប្រើសម្រាប់រក្សាទុកលើសំណាកទាំងមូល ដែលមានគុណវិបត្តិសំខាន់គឺមិនអាចរក្សាទុកសរីរាង្គផុយស្រួយបានល្អ ដូចជា ស្លាប ជើង អង់តែន និង palps។ យ៉ាងណាក៏ដោយការសិក្សា proteomics ដោយប្រើ MALDI-ToF នៅតែអាចអនុវត្តបានប្រសិនបើការដកទឹក)dehydration) ត្រូវបានអនុវត្តនៅពេល fixation ដោយប្រើសារធាតុដកសំណើមប្រភេទ silica gel។ ផ្ទុយទៅវិញ ការវិភាគសេនេទិចដែលផ្តោតលើ DNA មានភាពលំបាកក្នុងសំណាកប្រភេទនេះ ព្រោះ DNA ជាញឹកញាប់ត្រូវបានខូច និងមានបរិមាណទាបដែលធ្វើឱ្យការវិភាគមានភាពស្មុគស្មាញជាងសំណាកថ្មី ឬសំណាកបង្កក ជាពិសេសសម្រាប់ការសិក្សា nuclear genomes។ ទោះបីជាយ៉ាងណា បច្ចេកទេសសម័យថ្មីដូចជា museomics អាចអនុវត្តលើសំណាកប្រភេទនេះបាន] 34]។ ដោយហេតុនេះ វិធីសាស្ត្ររក្សាទុកតាមរបៀបបែបនេះមិនត្រូវបានផ្តល់អនុសាសន៍ទេ លុះត្រាតែមិនមានជម្រើសផ្សេង។ វាអាចរួមបញ្ចូលជាមួយការរក្សាទុកបង្កក ដោយដាក់បំពង់ទីបក្នុងទូទឹកកក- 20°C ឬ- 80°C។ បញ្ហាសំខាន់គឺការធានាបាននូវការ Mounting អោយសមស្របទៅនឹងសំណាក ឬផ្នែករាងកាយដែលចាំបាច់សម្រាប់ការកំណត់អត្តសញ្ញាណ។ ដើម្បីសម្រេចទៅបានការធ្វើការដកជាតិទឹកឡើងវិញគឺចាំបាច់។ យើងសុំផ្តល់អនុសាស្ត្រអោយប្រើសូលូស្យុង Triton X-100។ រយៈពេលនៃការដកជាតិទឹកឡើងវិញអាចប្រព្រឹត្តទៅ២ឬ៣ម៉ោងរហូតដល់២ឬ៣ថ្ងៃដោយត្រូវត្រួតពិនិត្យជាប្រចាំ។ បន្ទាប់ពីការដកជាតិទឹកឡើងវិញទាំងស្រុងសំណាកត្រូវលាងក្នុងទឹកបិដងជាបន្តបន្ទាប់។

3.5. ការរក្សាទុកលើ Filter papers

អត្ថប្រយោជន៍សំខាន់នៃ filter papers គឺស្ថេរភាពរយៈពេលវែងនៃ genomic DNA ក្នុងកោសិកានៃសំណាក ឬក្នុងរាងកាយទាំងមូលដែលមិនបានធ្វើ fixation និងត្រូវបានស្ងួត ឬក្នុងកោសិកាឈាមដែលរក្សាទុកនៅសីតុណ្ហភាពបន្ទប់។ Filter paper មានទំហំតូចដែលអាចផ្ទុកសំណាករាប់រយនៅសីតុណ្ហភាពបន្ទប់ ក្នុងបរិមាណតូចស្មើប្រអប់តូចមួយ។ Matrix របស់ filter paper ត្រូវបានបញ្ចូលសារធាតុដែលមានសមត្ថភាពធ្វើ denaturation លើភ្នាក់ងារបង្កជំងឺបណ្តាលឱ្យំណាកនោះមិនត្រូវបានចាត់ទុកជាហានិភ័យជីវសាស្ត្រ) non-biohazardous) ហើយវាអនុញ្ញាតឱ្យរក្សាទុក និងដឹកជញ្ជូនសំណាកដោយមិនចាំបាច់អនុវត្តវិធានការពិសេសសម្រាប់សុវត្ថិភាពជីវសាស្ត្រ [68] ។

4. ការកាត់ និងបំបែកសំណាក (dissection)

ខុសពីសត្វល្អិតផ្សេងៗជាច្រើន ដែលអាចកំណត់អត្តសញ្ញាណដោយផ្អែកលើលក្ខណៈខាងក្រៅដែលមើលឃើញបាន លើសត្វល្អិតដែលរូបរាងទាំងមូលបានរៀបចំទុក (pinned in toto) sand flies ត្រូវការ dissection និង Mounting ដើម្បីសិក្សារចនាសម្ព័ន្ធរាងកាយសម្រាប់កំណត់ប្រភេទអោយបានត្រឹមត្រូវ។ ទោះវិធីសាស្ត្រនៃការរៀបចំ និងបច្ចេកទេសនៃការ Mounting ត្រូវបានជ្រើសរើសយ៉ាងដូចម្តេចក៏ដោយ បច្ចេកទេសកាត់បំបែកគឺដូចគ្នាត្រូវបានអនុវត្ត (រូបភាព ២ និង ៣) (https://zenodo.org/records/18198006) ។

ការប្រើប្រាស់ Triton X100៖ សូលុយស្យុងទឹកមិនមានអ៊ីយ៉ុង (non-ionic aqueous solution)

ចំណាំ៖ ការរៀបចំសំណាកលើកញ្ចក់ឡាមទាក់ទងនឹងសំណាកដែលទើបតែចាប់បាន ឬសំណាកដែលរក្សាទុកក្នុងអាល់កុល។ អ្នកប្រមូលសត្វល្អិតភាគច្រើនមានសំណាកជាសត្វល្អិតដែលបានរក្សាទុកស្ងួត (សម្រាប់ប្រើប្រាស់ក្នុង MALDI-ToF) ឬរក្សាទុកក្នុងអាល់កុលរយៈពេលជាច្រើនឆ្នាំ។ ជារឿងអកុសល្យ សំណាកមួយចំនួនរក្សាទុកក្នុងអាល់កុលជាច្រើនឆ្នាំដែលមិនអាចរក្សាសភាពដើមបានល្អ ធ្វើឱ្យផ្តល់ផលវិបាកក្នុងការរៀបចំសម្រាប់ពិនិត្យក្រោមមីក្រូទស្សន៍។ បញ្ហាដែលកើតឡើងញឹកញាប់គឺការបំផ្លាញដោយមានបំណែកផ្លាស្ទិចក្នុង សំណាកនោះ

ដោយសាររំហួតនៃអាល់កុល។ ក្នុងករណីទាំងពីរខាងលើនេះ គ្មានជម្រើសអ្វីផ្សេងឡើយ ពីព្រោះសំណាកត្រាំនៅក្នុងអាល់កុលរយៈពេលយូរ ឬអាល់កុលស្ងួតពេញលេញ។ ដូច្នេះហើយទើបមានការណែនាំអោយប្រើប្រាស់សារធាតុបន្ថែមសំណើម (wetting agents) ដែលមិនមែនជាប្រភេទសាប៊ូខ្លាំង។ Triton X100 មានទម្រង់ជាសូលុយស្យុង មិនមានអ៊ីយ៉ុង (4-(1,1,3,3-tetramethylbutyl) phenyl-polyethylene glycol solution ឬ t-octylphenoxypolyethoxyethanol, polyethylene glycol tert-octylphenyl ether) ដែលត្រូវបានប្រើយ៉ាងទូលំទូលាយក្នុងការសិក្សាកោសិកាសាស្ត្រនិងម៉ូលេគុលជីវវិទ្យា។

វាអនុញ្ញាតឱ្យឆ្លងស្រទាប់ស្បែកកោសិកា និងភ្នាស់ណៃយ៉ូ (cell and nuclear membranes) បានយ៉ាងងាយស្រួល។

ការប្រើប្រាស់ Triton X100 មិនមានអ៊ីយ៉ុង (កំហាប់ 0.5%)៖

– ដាក់សំណាកស្ងួតជាមួយអាល់កុលសុទ្ធ
– បន្ថែមបរិមាណ Triton X100 0.5% ដើម្បីឱ្យសំណាកទាំងមូលជ្រាមសប់ក្នុងសូលុយស្យុង
– ទុកក្នុងរយៈពេលចន្លោះប្រហែល ៥ នាទី រហូតដល់ជាច្រើនថ្ងៃ ពិនិត្យអោយទៀងទាត់រហូតដល់សំណាកទាំងអស់ត្រូវតែបែកចេញទាំងស្រុងក្នុងសូលុយស្យុង
– ដកសូលុយស្យុងTriton X100 និងជំនួសជាមួយសូលុយស្យុងប៉ូតាស្យូមហ៊ីដ្រុកស៊ីត

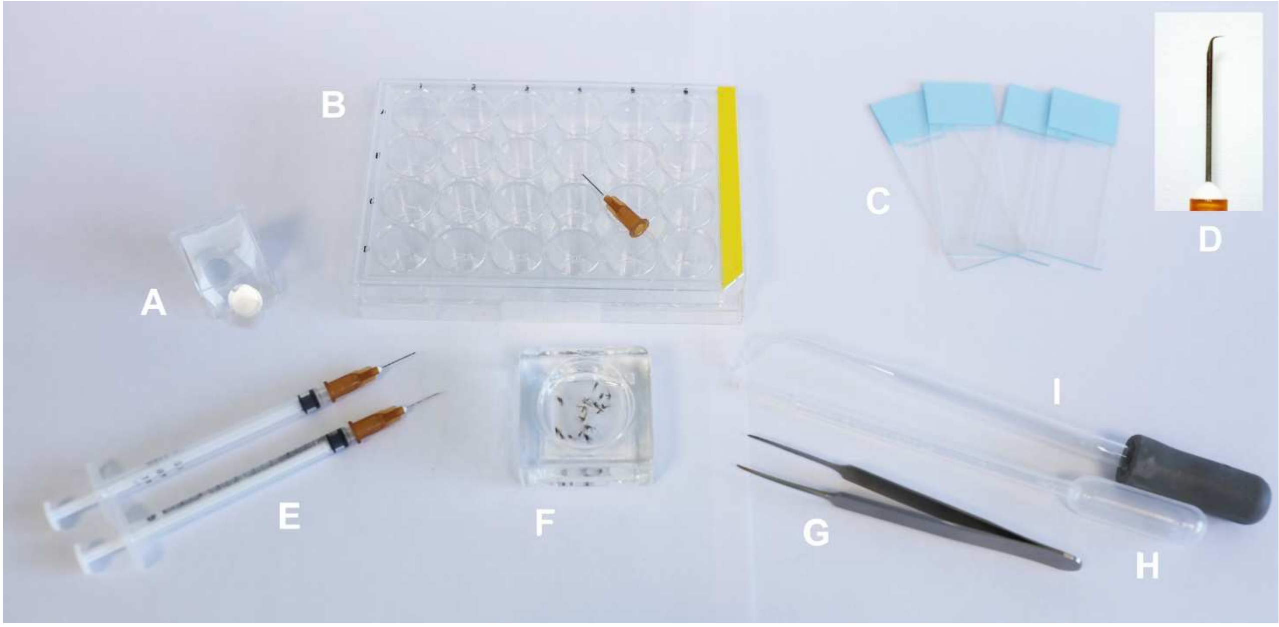

រូបភាព ២៖ សម្ភារៈសម្រាប់ដាក់ Phlebotomine លើឡាម A៖ ឡាម៉ែល ទំហំ ១០ ឬ ១២ មម B៖ មីក្រូផ្លេត ២៤រន្ធ និងម្ជុល (hooked needle) (បើអ្នកប្រើប្រាស់ clove oil ឬ Euparal® essence ដើម្បីរៀបចំPhlebotomine ហាមប្រើ acrylic plates ពីព្រោះប្រតិកម្មគីមីអាចកើតឡើង និងបំផ្លាញសំណាក) C៖ កញ្ចក់ឡាមដែលសមស្រមអាចសរសេរសញ្ញាសម្គាល់ D៖ ព័ត៌មានលម្អិតនៃម្ជុលសោត E៖ មូសភ្ជាប់ជាមួយស៊ីរ៉ាំង F៖ កញ្ចក់មើលម៉ោង ឬកំណត់ដូចម្ដេចដើម្បីកាន់Phlebotomineដែលត្រូវដាក់ស្លាយ G៖ ជំនួស Dumont forceps H៖ ពីប៉ែតប្លាស្ទិច I៖ ពីប៉ែតកញ្ចក់ដើម្បីងាយក្នុងការផ្ទេរសូលុយស្យុងទៅក្នុងរន្ធនិមយួៗ។

4.1. ក្បាល

Dissection អាចអនុវត្តដោយប្រើម្ជុលតូច ឬម្ជុលសម្រាប់ដោតសត្វល្អិត ក្រោមមីក្រូទស្សន៍ (រូបភាព ២ និង ៣(។ ម្ជុលដែលប្រើញឹកញាប់រួមមាន៖ 26G × 1/2" (0.45 × 13 មម(, 30G × 1/2" (0.3 × 13 មម (ឬ 25G × 5/8" (0.5 × 16 មម(។ ការរៀបចំសំណាកសម្រាប់ការកំណត់អត្តសញ្ញាណ យ៉ាងហោចណាស់ ត្រូវកាត់បំបែកក្បាលចេញពីរាងកាយ ហើយ Mounting ដោយដាក់សំណាកក្នុងទីតាំងផ្នែក ventral ឡើងលើដើម្បីបង្ហាញ cibarium និង pharynx ខណៈដែលផ្នែកទ្រូង និងពោះត្រូវដាក់ក្នុងទិសដៅ lateral បន្ទាប់ពី dissection។ ការដាក់ផ្នែកក្បាលក្នុងទីតាំង ventro-dorsal ដើម្បីអោយ occipital foramen ត្រូវបានតំរង់ឡើងលើដើម្បីអាចសង្កេត cibarium បានដោយផ្ទាល់។ ដំណើរការវិភាគទៅកាន់រចនាសម្ព័ន្ធកាយវិភាគទាំងនេះនឹងមានភាពងាយស្រួល ប្រសិនបើក្បាលត្រូវបានបំបែកចេញទាំងស្រុង។

4.2. ស្លាប និងទ្រូង

ស្លាបត្រូវតែដាក់ឱ្យរាបស្មើលើកញ្ចក់ឡាម។ ស្លាបនីមួយៗអាចបំបែកចេញពីគល់ ហើយដាក់ដោយឡែក ឬអាចដាក់តែមួយ ដោយទុកស្លាបមួយទៀតភ្ជាប់ជាមួយទៅនឹងទ្រូង។ ក្នុងការវិភាគ geometric morphometry គឺចាំបាច់ត្រូវកំណត់ និងដាក់ស្លាកសម្គាល់រវៀងស្លាបស្តាំ និងឆ្វេងឱ្យត្រឹមត្រូវ មុនពេល Mounting។ ផ្នែកទ្រូងត្រូវបែងចែកជាច្រើនផ្នែក ដែលផ្នែកនីមួយៗផ្ទុកព័ត៌មានសំខាន់សម្រាប់វិទ្យាសាស្ត្រនៃការកំណត់ប្រភេទ [20, 64] ។ ជាទូទៅ វាត្រូវបានដាក់ក្នុងទិសដៅ lateral ដើម្បីអនុញ្ញាតឱ្យពិនិត្យ chetotaxy និងការបែងចែកពណ៌។ វត្តមានស្នាមរោម) scars of bristles) នៅតំបន់ជាក់លាក់នៃផ្នែកទ្រូងអាចប្រើសម្រាប់បែងចែកប្រភេទខ្លះៗក្នុងពួក *Brumptomyia*។ ការបែងចែកពណ៌អាចប្រើសម្រាប់កំណត់អត្តសញ្ញាណPhlebotomine នៅតាមតំបន់ Neotropical នៅកម្រិតពួក (ឧ. *Bichromomyia*), សេរីនៃប្រភេទសត្វ (ឧ. *Pintomyia*) ឬកម្រិតប្រភេទក្នុងកម្រិតពួកដូចគ្នា) ឧ. *Micropygomyia*, *Nyssomyia*, *Psathyromyia*, និង *Psychodopygus*) [20] ។ ដូច្នេះ ប្រសិនបើផ្នែកទ្រូមិនត្រូវបានប្រើសម្រាប់វិភាគម៉ូលេគុលជីវសាស្ត្រ វាគួរត្រូវបាន Mounting ដោយប្រុងប្រយ័ត្ន ដើម្បីជៀសវាងការខូចខាត។ គួរបញ្ជាក់ថាមិនមែនអាំងតង់ស៊ីតេនៃពណ៌ដែលមានសារៈសំខាន់ទេ ប៉ុន្តែជាការបែងចែកពណ៌លើ ផ្នែកទ្រូងទាំងមូល។ ដូច្នេះ ដំណើរការនៃការធ្វើអោយសំណាកថ្លាមិនអាចបំបាត់សារធាតុពណ៌ ឬលំនាំពណ៌ឡើយ។

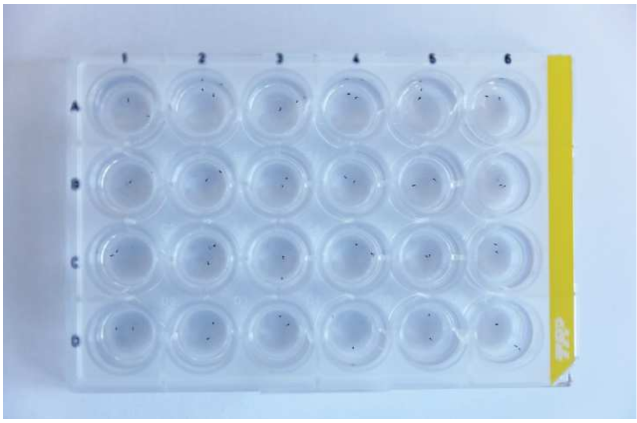

រូបភាព ៣៖ មីក្រូផ្លេត ២៤ រន្ធ ដែលរន្ធនីមួយៗផ្ទុកផ្នែកក្បាលនិងកង់ចុងក្រោយនៃផ្នែកពោះនៃ Phlebotomine

4.3. សេរីរាងបន្តពូជ (Genitalia)

ត្រូវប្រុងប្រយ័ត្នជាពិសេសនៅពេលដាក់សរីរាងបន្តរពូជទាំងភេទឈ្មោល និងភេទញី ព្រោះវាមានសារៈសំខាន់យ៉ាងខ្លាំងសម្រាប់កំណត់ ពួក, អនុពួក និង ប្រភេទ។ នៅក្នុងភេទទាំងពីរ សរីរាងបន្តរពូជមានលក្ខណៈជាគូរ (paired structures)។

4.3.1. សត្វឈ្មោល

សរីរាងបន្តរពូជឈ្មោលស្ថិតនៅផ្នែកខាងក្រៅ) external genitalia) មានសមាសភាពជាគូ forceps ដែលនីមួយមួយៗរួមមាន gonocoxite-gonostyle articulation នៅផ្នែក dorsal និង epandrial lobe នៅផ្នែក ventral។ Gonostyle មាន spines ហើយពេលខ្លះមាន setae ដែលត្រូវអាចរាប់ចំនួនបាន ហើយទីតាំងដុះរបស់វាត្រូវតែអាចមើលឃើញបានយ៉ាងច្បាស់។ វាសំខាន់ណាស់ក្នុងការសង្កេតដោយយកចិត្តទុកដាក់លើផ្ទៃខាងក្នុងរបស់ gonocoxite ដែលអាចមានរោមសរសៃដុះជាប់គ្មានគល់) sessile setae) ឬរោមដែលដុះនៅលើគល់) tubercle) [22]។

សហការីដែលមានបទពិសោធន៍តិចតួចក្នុង dissection សំណាក អាចអនុវត្ត Mounting តាមទិស lateral ដោយមិនបំបែកសរីរាងបន្តរពូជចេញពីចុងនៃផ្នែកពោះ (https://zenodo.org/records/18311158)។ ក្នុងករណីនេះការត្រួតស៊ីគ្នានៃផ្នែកទាំងពីរនៃសរីរាង្គបន្តពូជអាចធ្វើឱ្យពិបាកក្នុងការរាប់រោមសរសៃខាងក្នុងរបស់ gonocoxite ជាដើម ប៉ុន្តែវិធីនេះជៀសវាងការធ្វើឱ្យខូចខាតដល់សរីរាង្គបន្តពូជតាមរយៈ dissection បរាជ័យ។

សហការីដែលមានបទពិសោធន៍ច្រើនអាចព្យាយាមបើកសរីរាង្គបន្តពូជជាពីរដើម្បីញែកពួកវាចេញពីគ្នា។ ដើម្បីសម្រេចបាននេះ ត្រូវដាក់ចុងម្ជុលដែលបានកាត់ជារាងទ្រេត)ប្រភេទម្ជុលចាក់ក្នុងស្បែក (កាត់ចូលទៅ

This is an Open Access article distributed under the terms of the Creative Commons Attribution License (https://creativecommons.org/licenses/by/4.0), which permits unrestricted use, distribution, and reproduction in any medium, provided the original work is properly cited.

ដោយផ្តាច់ចេញ និងមិនចាំបាច់កាត់សរីរាង្គបន្តពូជទាំងស្រុងដើម្បីញែកផ្នែកផ្គុំ gonocoxite-gonostyle (https://zenodo.org/records/(18311158។ ការប្រើវិធីនេះធ្វើអោយការសង្កេតលើផ្ទៃខាងក្នុងរបស់វាយ៉ាងងាយស្រួល។ Mounting នេះក៏ជួយសម្រួលដល់ការសង្កេតមើល parameres និងស្រោម parameral sheaths ដែលលែងត្រួតស៊ីគ្នា។ សម្រាប់ Mounting ពីចំហៀង) lateral mounting) ដែលធ្វើឱ្យសរីរាង្គត្រួតស៊ីគ្នាដូចនេះសំណាកត្រូវតែធ្វើឱ្យថ្លា) perfectly cleared) ជាមុនសិន។

4.3.2. សត្វញី (Females)

ប្រព័ន្ធសរីរាងបន្តរពូជញីស្ថិតនៅខាងក្នុង) internal genital apparatus) ដែលបង្កើតឡើងដោយថង់ផ្ទុកទឹកកាមញី)spermathecae(។ ប្រសិនបើមិនមាន dissection វាត្រូវតែសង្កេតតាមរយៈ teguments ដោយដាក់ abdomen ក្នុងទីតាំង ventral។

មិនថាជ្រើសរើស mounting medium ប្រភេទណាក៏ដោយ ថង់ផ្ទុកទឹកកាមញី) spermathecae) ជាទូទៅអាចសង្កេតបានច្បាស់ប្រសិនបើវាមិនមានផ្ទៃរលោង) smooth) ហើយត្រូវបានធ្វើអោយថ្លាល្អ។ ទោះយ៉ាងណាក្តីការសង្កេតថង់ផ្ទុកទឹកកាមញី)spermathecae) ដែលមានជញ្ជាំងស្តើងនិងរលោងអាចជួបបញ្ហានៅក្នុង mediumដែលមានសមត្ថភាពចំណាំងផ្លាតពន្លឺ) refractility) ទាប។

លើសពីនេះ ការសង្កេតមើលគល់នៃបំពង់ថង់ផ្ទុកទឹកកាម) base of the spermathecal ducts) មានសារៈសំខាន់ខ្លាំងសម្រាប់កំណត់ប្រភេទដូចជាក្នុង subgenus Larroussius [35, 37, 38] ដែលជាភ្នាក់ងារចម្លងសំខាន់នៃ *Leishmania infantum* នៅតាមបណ្តាប្រទេសមួយចំនួននៃ Old World។ បើគ្មានការសង្កេតនេះ ការកំណត់អត្តសញ្ញាណសំណាកមិនអាចអនុវត្តបាន។

ដើម្បីដោះស្រាយបញ្ហានៃការសង្កេតទាំងនេះ គួរបំបែក genital furca-នៃថង់ផ្ទុកទឹកកាមញី) spermathecae) ចេញពី abdomen ពេលធ្វើmounting (https://zenodo.org/records/18311106) ។ ថង់ផ្ទុកទឹកកាមជាទូទៅពិបាកសង្កេតក្នុងអំឡុងពេលកាត់បំបែក ប៉ុន្តែ genital furca មានភាពងាយស្រួលក្នុងការរកឃើញ។ ដោយសារ spermathecal ducts លូតលាស់ចូលទៅក្នុង genital furca ការបំបែក furca នេះជាទូទៅអនុញ្ញាតឱ្យបំបែកថង់ផ្ទុកទឹកកាម)spermathecae) បានផងដែរ។

ប្រសិនបើ ថង់ផ្ទុកទឹកកាម)spermathecae) ត្រូវបានកាត់ដោយអចេតនាក្នុងអំឡុងដំណើរការវិភាគ វាមិនបាត់បង់ឡើយ ហើយនៅតែអាចសង្កេតបាននៅក្នុង abdominal integuments (រូបភាព ៤)

4.4. Dissection ក្រពះពោះវៀនកណ្តាល (Midgut dissection) សម្រាប់ញែកយក *Leishmania*

Dissection ប្រព័ន្ធរំលាយអាហារ (digestive tract) មានសារៈសំខាន់ជាអនុវត្តន៍សម្រាប់ការរកឃើញ និងញែកយក *Leishmania* នៅក្នុង Phlebotomine ភេទញី (female sand flies) ។ នីតិវិធីនេះអាចអនុវត្តបានទាំងក្នុងបរិបទfield condition (field setting) និងមន្ទីរពិសោធន៍ (laboratory setting) ដើម្បីវាយតម្លៃសមត្ថភាពចម្លងជម្ងឺ (vectorial competence) ។

ការផ្តល់អនុសាសន៍ឱ្យប្រើសត្វភេទញីដែលទើបតែបានធ្វើការសម្លាប់ថ្មីៗ។ ត្រូវលាងសត្វភេទញីដោយទឹក ឬសូលុយស្យុងសាលីន (saline solution) ដែលលាយជាមួយសាប៊ូស្រាល ដើម្បីយករោមលើសចេញ។ ជំហាននេះជួយរក្សាលក្ខខណ្ឌនៃភាពស្អាតគ្មានមេរោគ (aseptic conditions) សម្រាប់ការញែកយក *Leishmania* ខណៈពេលដែលថែរក្សា លក្ខណៈរូបសាស្ត្រ ដែលត្រូវការសម្រាប់ការកំណត់អត្តសញ្ញាណ។

ដើម្បីស្វែងរក និងញែកយក *Leishmania* ត្រូវបំបែកក្រពះពោះវៀនកណ្តាល **(dissect midgut)** ដោយប្រុងប្រយ័ត្នហើយដាក់វាទៅក្នុងចំណុចតែមួយនៃសូលុយស្យុងសាលីនកំហាប់0.9% (sterile saline solution, 0.9% NaCl)។ បន្ទាប់ពីសង្កេតឃើញប៉ារ៉ាស៊ីតដែលមានចលនា (motile parasites) ក្រោមមីក្រូទស្សន៍ (កម្រិតពង្រីកប្រហែល ~200×) ត្រូវប្រើ insulin syringe ឬ micropipette ដើម្បីផ្ទេរពួកវាទៅក្នុងថ្នាលបណ្តុះ

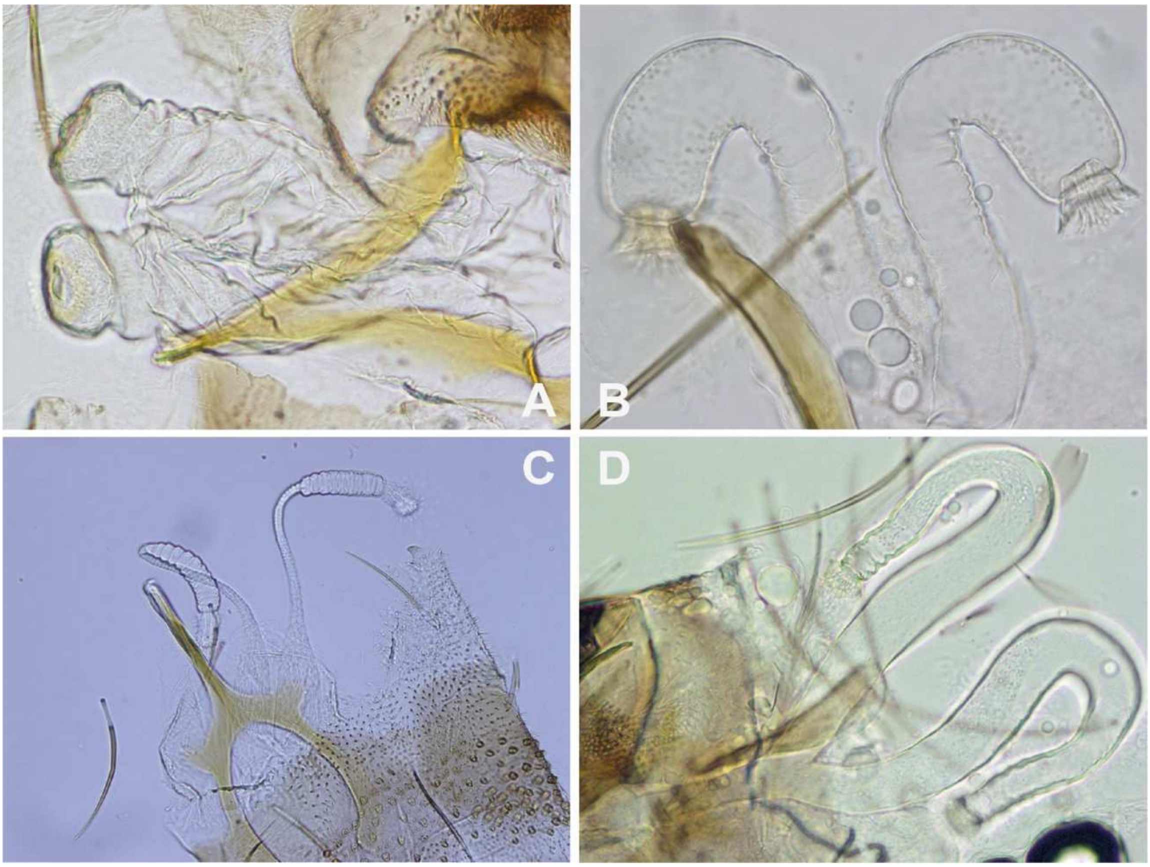

រូបភាព ៤៖ ថង់ផ្ទុកទឹកកាម(spermathecae) ដែលបានកាត់បំបែក និងការMountingក្នុង Marc-André fluid ពីសំណាកស្រស់។ A: *Idiophlebotomus longiforceps* (ឡាវ); B: *Sergentomyia minuta* (បារាំង); C: *Phlebotomus ariasi* (បារាំង); D: *Sergentomyia anodontis* (ឡាវ)

(សម្រាប់ព័ត៌មានលម្អិតបន្ថែម សូមមើលជំពូក 4.4.3)។ ត្រូវដាក់ក្បាលនិងសេរីរាងបន្ទុរពូជដោយផ្ទាល់ក្នុង Marc-André fluid ដើម្បីធ្វើអោយសំណាកថ្លា (clearing)។ **សំខាន់:** មិនត្រូវអនុញ្ញាតឱ្យ Marc-André fluid ប៉ះពាល់ជាមួយ *Leishmania* នោះឡើយទាំងដោយផ្ទាល់ ឬដោយប្រយោលតាមរយ:ឧបករណ៍ ឬម្ជុល ពីព្រោះវាអាចធ្វើអោយប៉ារ៉ាស៊ីតងាប់។ Dissection Phlebotomine ភេទញីអាចអនុវត្តលើឡាមតែមួយ ឬពីរ; ជម្រើសទាំងពីរមានអត្ថប្រយោជន៍ និងកម្រិតកំណត់ផ្ទាល់ខ្លួន (រូបភាព ៥; https://zenodo.org/records/18311154) ។

4.4.1. វិធីសាស្ត្រ Two-slides method

ជម្រើសទីមួយ គឺអនុវត្តលើឡាមពីរផ្សេងគ្នា៖ ឡាមទីមួយមានសូលុយស្យុងសាលីន (sterile saline) សម្រាប់បំបែកក្រពះពោះវៀនកណ្ដាល **(dissect midgut)** និងឡាមទីពីរសម្រាប់ដាក់ក្បាល និងថង់ផ្ទុកទឹកកាមញី (spermathecae) ក្នុង Marc-André fluid។

នោះយ៉ាងក្នុងលក្ខខណ្ឌfield conditionទាំងមានមនុស្សពីរ ឬបីនាក់ធ្វើការបំបែក Phlebotomine ហើយផ្ទេរសំណាកទៅអ្នកស្រាវជ្រាវដើម្បីទទួលខុសត្រូវលើការកំណត់ប្រភេទ និងវាយតម្លៃការឆ្លង *Leishmania* នៅក្នុងក្រពះពោះវៀនកណ្ដាល **(midgut)** ។ ការគ្រប់គ្រងឡាមទាំងពីរអាចបង្កបញ្ហាដល់ការតាមដានសំណាក (sample traceability) និងជាពិសេសធ្វើឱ្យពិបាកកំណត់ដោយភាពប្រាកដថា Phlebotomine ណាដែលមានការឆ្លងប្រសិនបើរកឃើញក្រពះពោះវៀនកណ្ដាលវិជ្ជមាន (https://zenodo.org/records/18311154) ។

4.4.2. វិធីសាស្ត្រ Single-slide method

ការប្រើស្លាយតែមួយងាយស្រួលក្នុងការតាមដានលទ្ធផលបានត្រឹមត្រូវ តែក៏ត្រូវអនុវត្តវិធានយ៉ាងប្រុងប្រយ័ត្ន។

ដើម្បីបង្កើនភាពស្អាតអាសេបទិក អ្នកអនុវត្តត្រូវសម្អាតដៃជាប្រចាំជាមួយ hydroalcoholic gel។ ត្រូវប្រើកញ្ចក់ឡាមដែរមិនមានផ្ទៃមាត់រលោង និងកញ្ចក់ឡាម៉ែលរាងការ៉េ) 22 × 22 មម(ដោយវិចខ្ចប់ជាមួយក្រដាស់អាលុយមីញ៉ូម រួចធ្វើការសម្លាប់មេរោគដោយកម្តៅស្ងួត) Poupinel oven) រួមទាំងម្ជុលផងដែរសម្រាប់ dissection នីមួយៗ) សំណើះ 25G Ø 0.5 មម × 16 មម(។

Phlebotomine ត្រូវដាក់ក្នុងដំណក់សូលុយស្យុងអំបិលគ្មានមេរោគនៅចំកណ្តាលកញ្ចក់ឡាម។ កាត់ក្បាលរបស់វា បន្ទាប់មកឆូតស្នាមវះរវាងផ្នែកកង់ខាងក្រោយ) tergites) និងផ្នែកកង់ខាងក្រោយ) sternites) ទី៦ និងទី៧ ដោយមិនត្រូវកាត់ដល់បំពង់រំលាយអាហារឡើយ)អាចកាត់ខ្ពស់ជាងនេះ ប្រសិនបើរំពឹងថាថង់ផ្ទុកទឹកកាមញី) spermathecae) វែង (។

បន្ទាប់មក ត្រូវទប់ផ្នែកទ្រូងរបស់វាជាមួយម្ជុលមួយ ហើយទាញចុងពោះខាងក្រោយថ្នមៗជាមួយម្ជុលមួយទៀត ដើម្បីទាញយកក្រពះពោះវៀនចេញ។ ប្រសិនបើមិនជោគជ័យ អាចចាក់ម្ជុលបិទចុង នៃផ្នែក ហើយទាញបំពង់រំលាយអាហារពីផ្នែកខាងមុខរបស់វា។ ប្រសិនបើបរាជ័យម្តងទៀត ត្រូវទាញយកក្រពះពោះវៀនដោយការយកចេញនូវសំណល់ស្បែកខាងក្រៅ) tegument) ជុំវិញវាឱ្យបានច្រើនតាមដែលអាចធ្វើបាន។ នៅពេលយកក្រពះពោះវៀនចេញរួច ត្រូវញែកកង់នៃផ្នែកពោះចុងក្រោយដោយកាត់បំពង់រំលាយអាហារ។ បន្ទាប់មកដាក់ក្រពះពោះវៀនក្នុងដំណក់សូលុយស្យុងសាលីនគ្មានមេរោគថ្មីមួយ ដែលដាក់នៅផ្នែកម្ខាងនៃកញ្ចក់ឡាម ហើយបិទថ្នមៗជាមួយកញ្ចក់ឡាម៉ែលគ្មានមេរោគ។ ផ្ទេរផ្នែកក្បាល និងកំណាត់កង់ផ្នែកពោះចុងក្រោយទៅកាន់ដំណក់តូចមួយនៃសូលុយស្យុង Marc-André ដែលដាក់នៅចុងម្ខាងទៀតនៃកញ្ចក់ឡាម ដោយធានាថាគ្មានប៉ះពាល់ជាមួយ Leishmania ឡើយ។ ដាក់ក្បាលឱ្យបានត្រឹមត្រូវ) occipital foramen បែរមុខឡើងលើ (ហើយញែកថង់ផ្ទុកទឹកកាម (spermathecae) ជាមួយ genital furca ដូចបានចង្អុលបង្ហាញខាងលើ ជាមួយឡាម៉ែលមូល) Ø ១២ ម.ម, កុំប្រទ្បំជាមួយឡាម៉ែលការ៉េគ្មានមេរោគ(។ គ្រោងឆ្អឹង Phlebotomine និងស្លាបដែលនៅសល់ត្រូវទុកក្នុងដំណក់សូលុយស្យុងសាលីននៅចំកណ្តាលឡាម)https://zenodo.org/records/(18311154 ។

ក្នុងករណីមានលទ្ធផលវិជ្ជមាន ឬសម្រាប់ការស្វែងយល់អំពីការសិក្សាកំណត់ប្រភេទ)taxonomic exploration) គេអាចរក្សាទុកផ្នែកទ្រូង និងពោះសម្រាប់ការសិក្សាជីវម៉ូលេគុល ឬប្រូតេអូមិក ហើយស្លាបអាចត្រូវបានគេរៀបចំក្នុង aqueous medium។ ដើម្បីធ្វើការរៀបចំស្លាបនោះអោយបានល្អ បរិមាណលើសនៃសូលុយស្យុង Marc-André អាចត្រូវបានជំនួសដោយ aqueous mounting medium ដូចជា chloral gum = Hoyer ឬប្រភេទថ្នាលដែលមានសាមាសធាតុប៉ូលីវីនីលអាល់កុល)polyvinyl alcohol-based medium)។ វីដេអូលម្អិតដែលបង្ហាញពីនីតិវិធីទាំងនេះ អាចរកបាន)ការធ្វើវះកាត់យកក្រពះពោះវៀនកណ្តាលរបស់ Phlebotomine ៖ https://zenodo.org/records/18303014 និងការធ្វើវះកាត់យកក្រពេញទឹកមាត់របស់ Phlebotomine ៖ https://zenodo.org/records/18302850 ដូច្នេះពួកវានឹងមិនត្រូវបានពន្យល់នៅទីនេះទេ។

4.4.3. ការញែកយក និងការបណ្តុះ *Leishmania* ពីក្រពះពោះវៀន Phlebotomine

ការញែកយកប៉ារ៉ាស៊ីតពីសត្វភេទញីដែលមានការឆ្លង គឺជានីតិវិធីស្មុគស្មាញនឹងទាមទារជំនាញខ្ពស់ ហើយគួរអនុវត្តសាកល្បងលើសំណាកគ្មានប៉ារ៉ាស៊ីតជាមុន។ ក្រោយ dissection ក្រពះពោះវៀនរួចត្រូវផ្ទេរទៅក្នុង sterile saline (0.9%) ឬ Locke’s solution សម្រាប់លាង] 4]។ បន្ទាប់មក អាចអនុវត្តពីរវិធី៖ i) ពិនិត្យក្រោម light microscope ដើម្បីសង្កេតដំណាក់កាលផ្សេងៗនៃ Leishmania promastigotes និងទីតាំងរបស់វា ជាពិសេសនៅ stomodeal valve ii) បើកក្រពះពោះវៀនដើម្បីឱ្យ promastigotes ចេញងាយស្រួលសម្រាប់ការបណ្តុះច្រើន] 4]។

នៅfield condition ការរកឃើញសត្វដែលមានការឆ្លងរោគគឺកម្រមាន ហេតុនេះការអនុវត្តជាប្រចាំនឹងបង្កើនឱកាសជោគជ័យ។ ប្រសិនបើសង្កេតឃើញវិជ្ជមាន Leishmania ត្រូវប្រើម្ជុលដែលសម្លាប់មេរោគរួចហើយបាញ់សូលុយស្យុងសាលីនបន្តិចជុំវិញឡាម៉ែល រួចដកយកឡាម៉ែលថ្នមៗ។ ការបំបែកក្រពះពោះវៀនត្រូវប្រុងប្រយ័ត្ន និងឆាប់រហ័ស ដើម្បីបញ្ចេញប៉ារ៉ាស៊ីតទៅក្នុង saline ។ ប្រើ micropipette 100 µL ឬ tuberculin syringe ប្រមូលប៉ារ៉ាស៊ីត ហើយ inoculate ទៅក្នុងថ្នាលបណ្តុះដែលបានដាក់ស្លាកសម្គាល់ត្រឹមត្រូវ។

ការបណ្តុះ in vitro នៃ Leishmania promastigotes ប៉ារ៉ាស៊ីតត្រូវបានញែកទៅក្នុង SNB-9 blood agar slopes ឬ Novy, Mc Neal, Nicolle (NNN) solid medium ដែលគ្របដោយ sterile alpha-MEM ឬ M199 medium បន្ថែម 10% heat-inactivated fetal calf serum (FCS), 1% BME vitamins, 2% sterile human urine (តម្រងដោយ syringe filter Filtropur® S 0.2 µm), និងប្រភេទថ្នាំផ្សះដូចជា amikacin 250 µg/mL (ឬ

This is an Open Access article distributed under the terms of the Creative Commons Attribution License (https://creativecommons.org/licenses/by/4.0), which permits unrestricted use, distribution, and reproduction in any medium, provided the original work is properly cited.

gentamicin 50 µg/mL ឬលាយ L-glutamine 200 mM–penicillin 10,000 U–streptomycin 10 mg/mL)។ បន្ទាប់ពី ៣ ថ្ងៃប្រសិនបើគ្មានការឆ្លងមេរោគពីខាងក្រៅ)contamination) ការបណ្តុះត្រូវផ្ទេរទៅក្នុងថ្នាលបង្កក ហើយរក្សាទុកនៅ- 80°C រយៈពេល ១–២ ឆ្នាំ ឬក្នុងនីត្រូសែនរាវ -196°C សម្រាប់ការរក្សាទុករយៈពេលវែង] 7]។

4.5. ក្រពេញទឹកមាត់

ការវះកាត់យកក្រពេញទឹកមាត់ Phlebotomine sand flies គឺជាបច្ចេកទេសមូលដ្ឋានសម្រាប់សិក្សាអន្តរកម្មរវាងភ្នាក់ងារចម្លងរោគនិងភ្នាក់ងារបង្ករោគ ជាពិសេសសម្រាប់ការរកឃើញវីរុសអាបូ) arboviruses) ដូចជា Phlebovirus (ឧទាហរណ៍ Toscana virus) [44, 75]។ ដោយសារតែទំហំដ៏តូចរបស់ Phlebotomine នីតិវិធីនេះទាមទារសុក្រឹតភាពខ្ពស់ក្រោមមីក្រូទស្សន៍ស្តេរ៉េអូ) stereomicroscope) ដោយប្រើដង្កៀបចាប់សត្វល្អិត ឬ ម្ជុលមានមុខតូច ដើម្បីបំបែកយកក្រពេញទឹកមាត់ដែលមានសភាពទន់ ដោយមិនបង្កឲ្យបែកបាក់ ឬ ការឆ្លងមេរោគពីខាងក្រៅ)https://zenodo.org/records/18302850) [51, 61]។ ការរក្សារូបភាពពេញលេញនៃក្រពេញមានសារៈសំខាន់ខ្លាំង ដើម្បីធានាភាពទុកចិត្តបាននៃការវិភាគម៉ូលេគុលជីវសាស្ត្រនៅដំណាក់កាលបន្ទាប់។ បន្ទាប់ពីដកចេញ ក្រពេញទឹកមាត់អាចត្រូវបានកិនបំបែក) homogenized) ហើយធ្វើតេស្តតាមរយៈ RT-PCR, qPCR ឬវិធីសេរ៉ូសាស្ត្រ)immunoassays) ដើម្បីរកវត្តមាន RNA របស់វីរុស ឬ អង់ទីហ្សែន] 12]។ ការរកឃើញវីរុសនៅក្នុងក្រពេញទឹកមាត់ មិនមែនត្រឹមតែនៅក្នុងក្រពះពោះវៀន ឬហេមូស៊ីល)hemocoel) ប៉ុណ្ណោះទេ បញ្ជាក់ថាភ្នាក់ងារបង្ករោគបានបញ្ចប់រយៈពេលបង្កកំណើតខាងក្រៅ) extrinsic incubation period) ហើយអាចចម្លងបានក្នុងអំឡុងពេលបឺតឈាម] 71]។ ដំណើរការបំបែក (dissection) មានភាពស្មុគស្មាញខាងបច្ចេកទេស ដោយសារទំហំតូចរបស់ក្រពេញទឹកមាត់ Phlebotomine ដែលទាមទារជំនាញខ្ពស់ដើម្បីជៀសវាងការខូចខាតសំណាក] 1, 51]។ បន្ថែមពីនេះ បរិមាណវីរុសអាចមានកម្រិតទាប ដូច្នេះទាមទារមាន sensitivity ខ្ពស់ ដូចជា nested PCR ឬបច្ចេកវិទ្យាកម្រិតខ្ពស់ high-throughput sequencing [54]។ ហានិភ័យនៃការឆ្លងមេរោគពីខាងក្រៅក៏បញ្ជាក់ពីតម្រូវការអនុវត្តបច្ចេកទេសសម្អាតមេរោគ) aseptic techniques) យ៉ាងតឹងរ៉ឹងផងដែរ។ លើសពីឧបសគ្គបច្ចេកទេស កត្តាជីវសាស្ត្រក៏មានឥទ្ធិពលលើភាពជោគជ័យនៃការរកឃើញផងដែរ ដោយសមត្ថភាពចម្លងរបស់ភ្នាក់ងារចម្លង)vector competence) ខុសគ្នាក្នុងចំណោមប្រភេទ Phlebotomine ហើយអត្រាឆ្លងប្រែប្រួលទៅតាមលក្ខខណ្ឌអេកូសាស្ត្រ និងរដូវកាល] 33, 61]។

ការរកឃើញវីរុសក្នុងក្រពេញទឹកមាត់ផ្តល់នូវព័ត៌មានសំខាន់សម្រាប់ការវាយតម្លៃហានិភ័យនៃការចម្លង ជួយឲ្យអនុវត្តការតាមដាន និងវិធានការគ្រប់គ្រងដោយមានគោលដៅច្បាស់លាស់]15]។ ឧទាហរណ៍ការកំណត់អត្តសញ្ញាណ Toscana virus ក្នុង Phlebotomine នៅតំបន់មានជម្ងឺរាលដាល បានជួយឲ្យកែលម្អវិធីធ្វើរោគវិនិច្ឆ័យ និងការជូនដំណឹងទៅសុខាភិបាលសាធារណៈ] 18]។ បន្ថែមទៀត ការសិក្សាអន្តរកម្មរវាងវីរុស និងទឹកមាត់អាចបង្ហាញគោលដៅថ្មីសម្រាប់វ៉ាក់សាំង ឬវិធីព្យាបាលដែលទប់ស្កាត់ការបញ្ជូនរោគ] 15, 18]។

ក្រពេញទឹកមាត់ Phlebotomine ក៏អាចប្រើជាប្រភពអង់ទីហ្សែនសម្រាប់វាស់វែងអង់ទីករប្រឆាំងនឹងទឹកមាត់ Phlebotomine ដោយប្រើវិធីសេរ៉ូសាស្ត្រ ELISA។ វិធីសាស្ត្រនេះអនុញ្ញាតឲ្យវាយតម្លៃកម្រិតការប៉ះពាល់របស់ជួលពីការខាំរបស់ Phlebotomine ដោយវាយតម្លៃប្រសិទ្ធភាពនៃវិធានការគ្រប់គ្រងភ្នាក់ងារចម្លង] 25] និងហានិភ័យនៃការចម្លង Leishmania [40]។

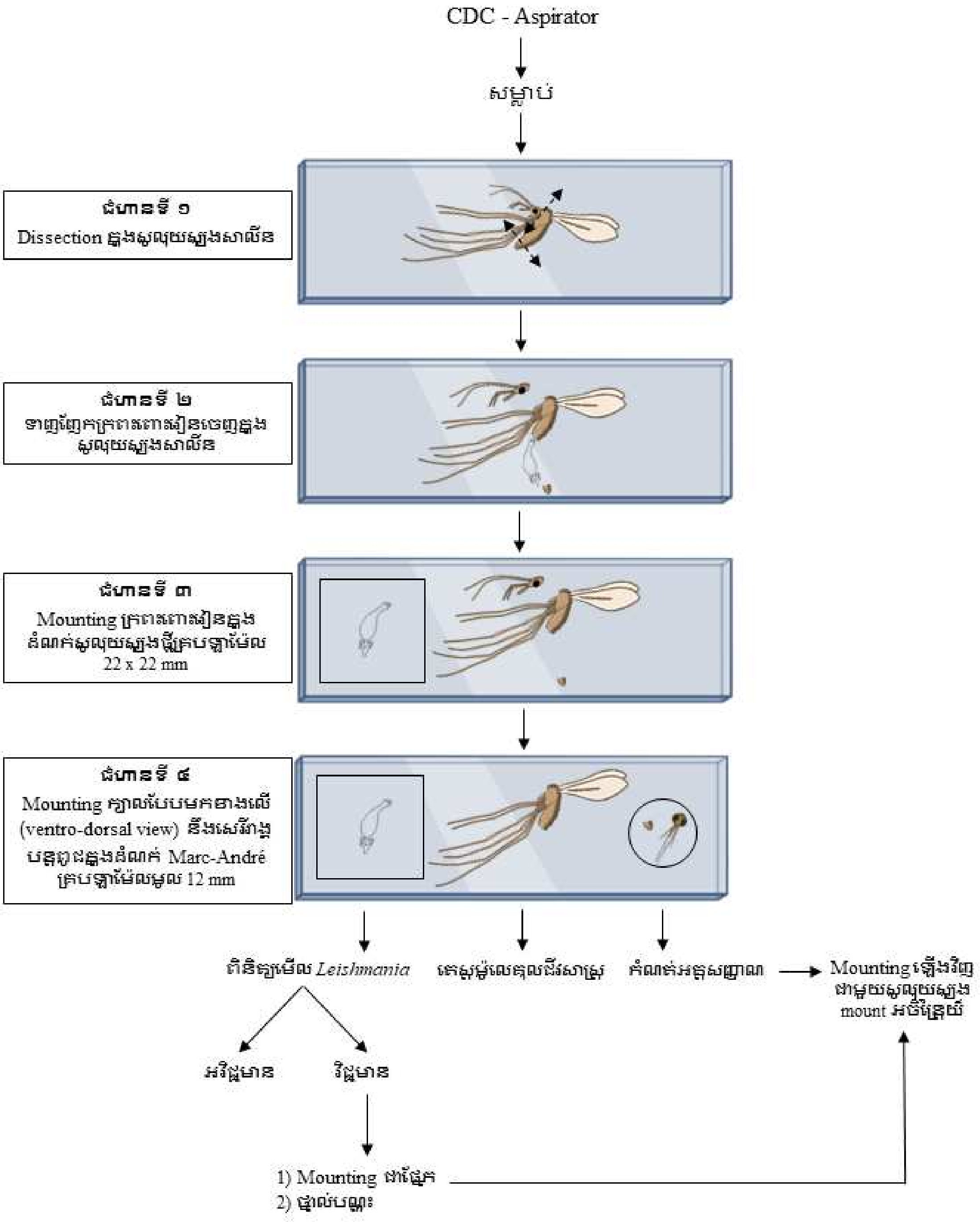

រូបភាព ៥៖ វិធីសាស្ត្រសម្រាប់ការញែកយក *Leishmania*

4.6. ការកំណត់អត្តសញ្ញាណប្រភពឈាម (Blood meal identification)

Phlebotomine ញីដែលបំពេញដោយឈាម) engorged females) ដែលបានបំបែកចេញពីសំណាកចាប់ គួរត្រូវបានវះកាត់ដោយប្រើឧបករណ៍ប្រើតែម្តង) single-use equipment) ដើម្បីការពារការឆ្លងមេរោគពីខាងក្រៅ)cross-contamination)។ ផ្នែកពោះ) abdomen) ត្រូវពិនិត្យក្រោមមីក្រូទស្សន៍ស្តេរ៉េអូ ដើម្បីវាយតម្លៃដំណាក់កាលនៃការរំលាយឈាម។ គួរជ្រើសរើសតែញីដែលមានពោះពណ៌ក្រហម ក្រហមត្នោត ឬ ក្រហមចាស់ ដោយគ្មានសញ្ញានៃការបង្កើតស៊ុត។ ត្រូវកាត់យកចុងនៃផ្នែកពោះ រួមទាំងថង់ផ្ទុកទឹកកាមញី)spermathecae) ដើម្បីកំណត់អត្តសញ្ញាណសត្វញីតាមលក្ខណៈរូបសាស្ត្រ

This is an Open Access article distributed under the terms of the Creative Commons Attribution License (https://creativecommons.org/licenses/by/4.0), which permits unrestricted use, distribution, and reproduction in any medium, provided the original work is properly cited.

បន្ទាប់ពីធ្វើការសម្អាត) clearing)។ ផ្នែកចម្បងនៃពោះ)ដោយគ្មាន ថង់ផ្ទុកទឹកកាមញី) spermathecae)) ត្រូវដាក់ក្នុងបំពង់ទីប Eppendorf® ហើយរក្សាទុកនៅសីតុណ្ហភាព- 20°C រហូតដល់ពេលធ្វើវិភាគបន្ថែម។

សញ្ញាសម្គាល់ហ្សែន) genetic markers) ដែលប្រើជាទូទៅសម្រាប់កំណត់អត្តសញ្ញាណប្រភពឈាម ដូចជា PNOC [5, 30, 50], CytB [67] ឬ COI [13] ត្រូវបានបង្កើត និងពិពណ៌នាយ៉ាងទូលំទូលាយក្នុងអត្ថបទវិទ្យាសាស្ត្រ ដូច្នេះមិនត្រូវបានពន្យល់លម្អិតបន្ថែមនៅក្នុងអត្ថបទនេះទេ) រូបទី ៦)។ ជម្រើសផ្សេងទៀតសម្រាប់កំណត់អត្តសញ្ញាណឈាមជួលអាចប្រើបច្ចេកទេស MALDI-ToF peptide mapping [31]។ ការសាកល្បងពិសោធន៍បានបង្ហាញថាបច្ចេកទេសនេះអាចកំណត់អត្តសញ្ញាណប្រភពឈាមបានក្នុងរយៈពេលយូរជាងវិធីសាស្ត្រផ្សេងបន្ទាប់ពីការបឺតឈាម ដូច្នេះវាជាជម្រើសសមស្របសម្រាប់វិភាគលើសត្វញីដែលបំពេញដោយឈាម ហើយបង្ហាញការរំលាយឈាមកម្រិតខ្ពស់ជាង។

សំណាកគួររក្សាទុកនៅ- 20°C ឬ 4°C ជាអាទិភាព ប៉ុន្តែយើងអាចទទួលបានលទ្ធផលល្អពីសំណាកដែលរក្សាទុកនៅសីតុណ្ហភាពបន្ទប់ក្នុងរយៈពេលខ្លីផងដែរ។ ពោះរបស់ញីដែលបំពេញដោយឈាម គួររះបំបែកចេញពីផ្នែកផ្សេងនៃរាងកាយមុនពេលធ្វើវិភាគ ហើយធ្វើការកិនបំបែក) homogenized) ក្នុងទឹកបិត)distilled water)។ ផ្នែករាងកាយដែលនៅសល់របស់ Phlebotomine អាចប្រើបន្តសម្រាប់វិភាគម៉ូលេគុលជីវសាស្ត្រ និងរូបសាស្ត្រផ្សេងទៀត។ បន្ទាប់ពីសម៉ាសជាគុរាវ (aliquot) ពីការកិនបំបែកសម្រាប់ធ្វើរ MALDI-ToF peptide mapping ផ្នែកដែលនៅសល់អាចប្រើសម្រាប់ចម្រាញ់ DNA ដើម្បីបញ្ជាក់ការកំណត់អត្តសញ្ញាណប្រភពឈាម និង/ឬ រកវត្តមាន Leishmania sp.។ សរុបជារួម រយៈពេលសរុបនៃការរៀបចំសំណាក និងការវិភាគគឺខ្លីជាងបើប្រៀបធៀបនឹងបច្ចេកទេសម៉ូលេគុលជីវសាស្ត្រដែលផ្អែកលើ DNA។

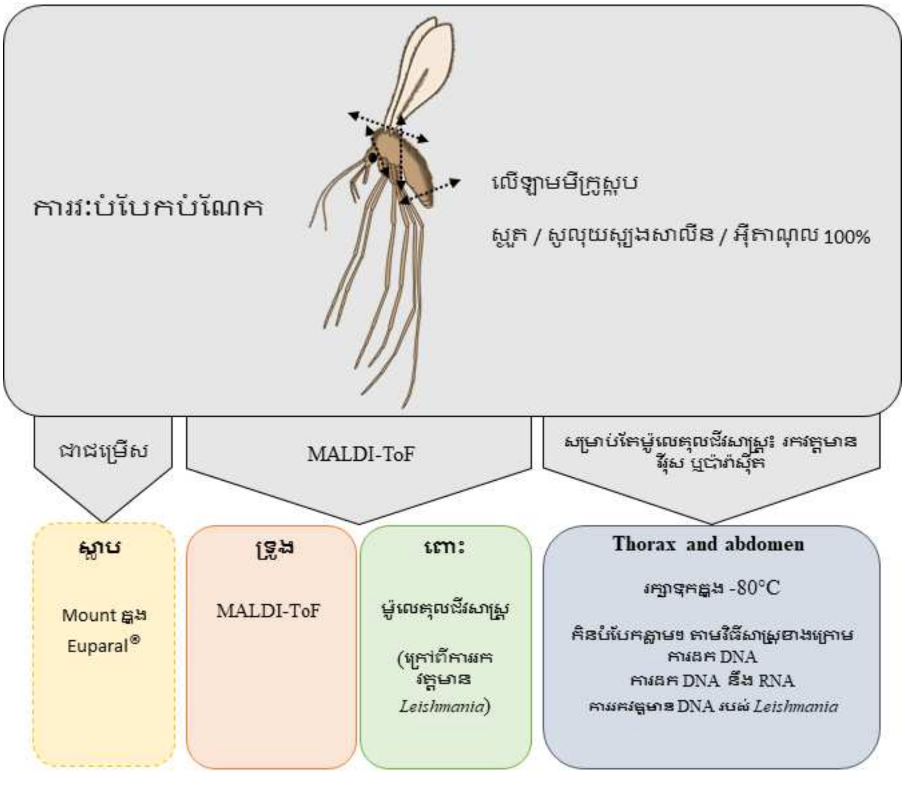

រូបទី ៦៖ ដំណើរការវិភាគPhlebotomineសម្រាប់កម្មវិធីជីវវិទ្យាmolecular biology ប្រូតេអូមិច និង/ឬ វីរុសសាស្ត្រ។

5. ការរៀបចំសំណាកសម្រាប់ការសិក្សាលក្ខណៈរូបសាស្ត្រ

ផ្នែកនេះពិពណ៌នាគោលការណ៍រៀបចំសំណាក Phlebotomine សម្រាប់ mounting ដើម្បីការសិក្សាលក្ខណៈរូបសាស្ត្រ និងការបំលែងសម្រាប់ការអនុវត្តផ្សេងៗ។ ដំណើរការវិភាគរួមមានជំហានបន្តបន្ទាប់នៃការបើតចេញ និងបំពេញសារធាតុរាវដោយប្រើ Pasteur pipettes ដែលភ្ជាប់ជាមួយ rubber bulbs។ ផ្តល់អនុសាសន៍ប្រើកញ្ចក់បាតមូល ព្រោះកញ្ចក់មិនមានប្រតិកម្មជាមួយរេសង់។ ដើម្បីជៀសវាងការរលាយរេសង់ត្រូវបិទគម្របនិងកុំបំពេញលើសកម្រិត ដើម្បីជៀសវាងការហូរ និងការធ្លាក់ចូលនៃធូលិលើសំណាក។ ទោះយ៉ាងណាក្តី ការយល់ដឹងអំពីវិធីសាស្ត្រនេះមានសារៈសំខាន់ ព្រោះវាអនុញ្ញាតកែសម្រួលនីតិវិធីឲ្យសមស្របតាមប្រភេទសំណាកជាក់លាក់នៅពេលចាំបាច់។ ការប្រតិបត្តិសំណាកពាក់ព័ន្ធនឹងជំហានបញ្ចេញ និងបំពេញសារធាតុជាបន្តបន្ទាប់ ដោយប្រើបំពង់ទីប Pasteur ដែលបំពាក់ដោយប៊ុលកៅស៊ូទន់អាចបត់បែនបាន។ គួរប្រើបំពង់ទីបកែវបាតមូល ព្រោះវាជួយឲ្យដំណើរការវិភាគទាំងនេះប្រព្រឹត្តបានងាយស្រួល។ កែវមានលក្ខណៈអិនែត (inert) ចំពោះរេសាជង់ទាំងអស់។ ដើម្បីទប់ស្កាត់ការអស់រេសាជង់ដោយការហើរចេញ ត្រូវបិទគម្របបំពង់ទីបឲ្យបានជិត ហើយមិនគួរបំពេញលើសកម្រិត ដែលអាចបណ្តាលឲ្យហូរចេញនៅពេលបិទ ឬបើក និងដើម្បីការពារធូលីធ្លាក់លើសំណាក។ សារធាតុគីមីដែលត្រូវការសម្រាប់ការសម្អាត (clearing) និងដំណើរការវិភាគសំណាក បង្ហាញក្នុងតារាងទី 2។

This is an Open Access article distributed under the terms of the Creative Commons Attribution License (https://creativecommons.org/licenses/by/4.0), which permits unrestricted use, distribution, and reproduction in any medium, provided the original work is properly cited.

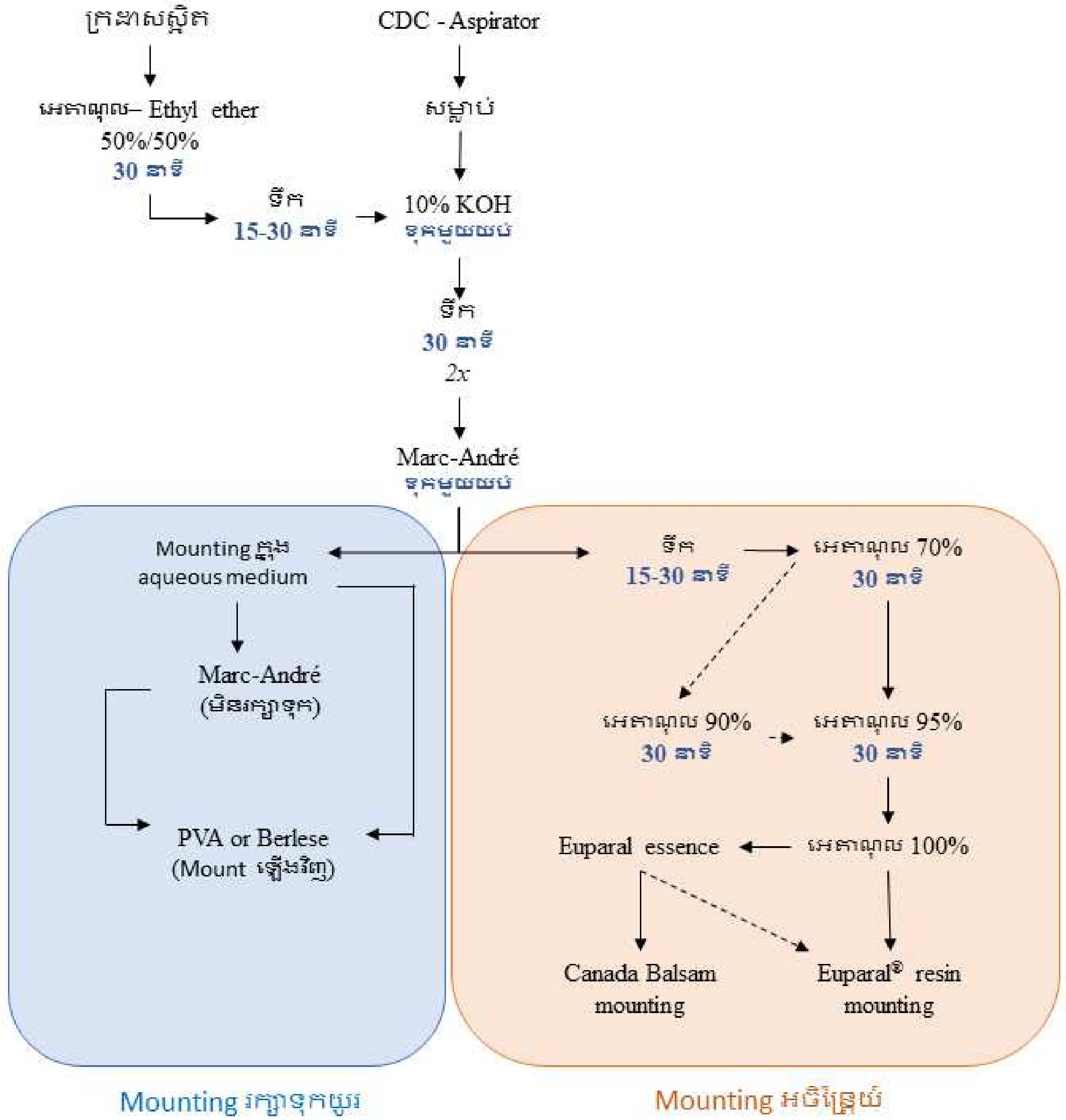

រូបទី 7៖ វិធីសាស្ត្របុរាណសម្រាប់ដំណើរការវិភាគPhlebotomine។

This is an Open Access article distributed under the terms of the Creative Commons Attribution License (https://creativecommons.org/licenses/by/4.0), which permits unrestricted use, distribution, and reproduction in any medium, provided the original work is properly cited.

តារាងទី ២៖ សមាសភាពនៃសារធាតុប្រតិកម្មដែលបានប្រើ

ឈ្មោះសារធាតុ	សមាសភាព
ប៉ូតាស្យូមអ៊ីដ្រូស៊ីត ១០%	ប៉ូតាស្យូមអ៊ីដ្រូស៊ីត ១០ ក្រាម<br>ទឹកបិត)qs) ១០០ មល.
ហ្វូចស៊ីនអាស៊ីត ១ក្នុងទឹកចម្រោះ %	ហ្វូចស៊ីនអាស៊ីត (ម្សៅក្រាម ១ (<br>ទឹកបិត៩៩ មល.
ឧបករណ៍ផ្ទុកសម្រាប់mounting ក្លរ៉ាល់ហ្គីម)Hoyer medium)	ទឹកបិត៥០ មល.<br>ក្លរ៉ាល់អ៊ីដ្រេត ២០០ ក្រាម<br>អញ្ចាញអារ៉ាប៊ីក ៥០ ក្រាម<br>គ្លីសេរីន ២០ ម.ល.
សូលុយស្យុង Marc-André ពណ៌ដោយហ្វូចស៊ីនអាស៊ីត	សូលុយស្យុង Marc-André ១០ មល.<br>ហ្វូចស៊ីន ១ ៥០ %μL
សូលុយស្យុង Marc-André	ក្លរ៉ាល់អ៊ីដ្រេត ៤០ ក្រាម<br>អាស៊ីតអាសេទិកកក ៣០ ម.ល.<br>ទឹកបិត៣០ ម.ល.
ឧបករណ៍ផ្ទុក Enecê	កូឡូហ្វានស (colophony) សុទ្ធពណ៌ស ២២ ក្រាម<br>អញ្ចាញកូប៉ាល់)copal gum) រលាយក្នុងអាល់កុល ១២ ក្រាម<br>អេតាណុលដាច់ខាត ២០ ម.ល.<br>កាហ្វួរ ១០ ក្រាម<br>អារម្មណ៍ទីពែនទីន)turpentine essence) ១០ មល.<br>អ៊ីកាលីបតូល ២៦ ម.ល.

5.1. ការធ្វើឲ្យថ្លា (Clearing)

មុនពេលរៀបចំសំណាក Phlebotomine សម្រាប់ mounting អចិន្ត្រៃយ៍ ត្រូវធ្វើឲ្យសំណាកថ្លាដោយវិធីម៉ាសីរេស្យុង (maceration) ប្រើវិធីសាស្ត្រ និងសារធាតុធ្វើឲ្យថ្លា (ឧ. សូលុយស្យុងអាស៊ីតអាសេទិក 10% ឬ សូលុយស្យុង Marc-André ដែលមាន chloral hydrate ដែលជាសារធាតុគីមីមានការគ្រប់គ្រងក្នុងប្រទេសជាច្រើន) ដើម្បីធ្វើឲ្យសំណាកក្លាយជាថ្លា។ ដំណើរការធ្វើឲ្យថ្លានេះគឺដកយកជាលិកា ខ្លាញ់ សារធាតុបញ្ចេញ និងក្រមួន បង្កើនភាពថ្លា (translucent) និងសម្រួលការពិនិត្យរចនាសម្ព័ន្ធ exoskeleton (ឧ. កន្លែងភ្ជាប់សែតា), លក្ខណៈផ្ទៃ (ឧ. ពណ៌) និងរចនាសម្ព័ន្ធខាងក្នុងដែលអាចមើលឃើញតាម tegument (ឧ. ថង់ផ្ទុកទឹកកាមញី (spermathecae))។

ដំណើរការវិភាគធ្វើឲ្យថ្លាពីរដំណាក់កាល ត្រូវប្រើសារជាតិបាស់ខ្លាំង (ឧ. potassium hydroxide) បន្ទាប់ដោយអាស៊ីតខ្សោយ (ឧ. អាស៊ីតអាសេទិក ក្នុងសូលុយស្យុង Marc-André) មានប្រតិកម្មគីមីជីវខុសៗគ្នា [74]។ សារជាតិបាស់ខ្លាំងអាចបំបែកជាលិកាទន់ (ប្រូតេអ៊ីន ខ្លាញ់ សាច់ដុំ) តាមរយៈសាប៊ូនីភីកាស្យុង (saponification) និងការបំផ្លាញរចនាសម្ព័ន្ធប្រូតេអ៊ីន ដោយរក្សាទុកស្រទាប់គីទីន (chitin) របស់ exoskeleton ឲ្យនៅស្ថិតស្ថេរ។ អាស៊ីតខ្សោយបន្ទាប់មកធ្វើអព្យាក្រឹតកម្មសារជាតិបាស់ដែលនៅសល់ និងបំបាត់ពណ៌គីទីនដើម្បីបង្កើនភាពថ្លា [74]។ ការលាងសំណាកពីរដងក្នុងទឹកបិត15 នាទីក៏អាចគ្រប់គ្រាន់សម្រាប់ធ្វើអព្យាក្រឹតកម្មផងដែរ។ ការប្រតិបត្តិជាបន្តបន្ទាប់នេះជានាការយកចេញជាលិកាប្រកបដោយប្រសិទ្ធភាព និងរក្សាភាពសុចរិតនៃសំណាកសម្រាប់ពិនិត្យមីក្រូទស្សន៍។ ផ្តល់អនុសាសន៍ឲ្យលាងពីរដងក្នុងទឹកបិត20 នាទីមុនបន្តដំណាក់កាលបន្ទាប់។

5.1.1. ការបំបែកជាលិកាទន់ (Soft tissue lysis) (រូបទី 8)

Sodium hydroxide (NaOH) ឬ potassium hydroxide (KOH) គឺជាសារធាតុម៉ាស៊ីរស្យុងដែលប្រើជាទូទៅដោយកំណត់កំហាប់ និងរយៈពេលទៅតាមទំហំ និងភាពផុយស្រួយរបស់សំណាក។ វិធីសាស្ត្រស្តង់ដារមានប្រសិទ្ធភាពខ្ពស់គឺ ជ្រលក់សំណាកក្នុងសូលុយស្យុងកំហាប់ខ្ពស់ (KOH ឬ NaOH 10%) ពេញមួយយប់។ អាចបង្កើនកំហាប់ (ឧ. KOH 20% រយៈពេល 6 ម៉ោង) ឬកម្តៅនៅ 37°C ដើម្បីកាត់បន្ថយរយៈពេលផ្ញុំ។

5.1.2. ការធ្វើឲ្យថ្លាជាមួយ ឬគ្មានការបំពាក់ពណ៌

បន្ទាប់ពីដំណាក់កាលធ្វើឲ្យថ្លា សំណាកត្រូវបានអនុវត្តន៍ការចាប់បាច់ពន្លឺបានល្អ (lightening treatment) ដែលជាការប្រមូលបញ្ចូលអាស៊ីតអាសេទិក និង chloral hydrate (ឧ. សូលុយស្យុង Marc-André)។ បន្ទាប់ពីធ្វើឲ្យថ្លា សំណាកត្រូវតែលាងឲ្យស្អាតពេញលេញក្នុង water bath យ៉ាងតិចពីរដងដោយរយៈពេល 20 នាទីក្នុងមួយដង ដើម្បីយកចេញសារធាតុគីមីសល់នៅក្នុងសំណាក។ សូលុយស្យុង Marc-André គឺជាសារធាតុធ្វើឲ្យថ្លាដែលពេញនិយមសម្រាប់រៀបចំសំណាក sand fly ។ ប្រសិទ្ធភាពរបស់វាគឺងាយស្រួលក្នុងដំណើរការវិភាគធ្វើឲ្យថ្លា នឹងកាត់បន្ថយការខូចខាតដល់ទម្រង់ទន់ជ្រាយ (fragile structures) ដូចជាស្លាប និងអង់តែន។

សូលុយស្យុងនេះគួររៀបចំថ្មី ឬទុកក្នុងដបដែលបិទជិត ដើម្បីការពារការហើរ ឬខូចខាត។ ការប្រើប្រាស់សូលុយស្យុង Marc-André មានប្រយោជន៍ច្រើនពេលបញ្ចូលជាមួយបច្ចេកទេសធ្វើអោយថ្លា (brightening) ឬបំពាក់ពណ៌ពណ៌ (staining) ដើម្បីពង្រីកព័ត៌មានរាងកាយមួយចំនួន។ ព័ត៌មានលម្អិតអំពីរចនាសម្ព័ន្ធ និងការត្រៀមផ្តល់នៅក្នុង Appendix 2។ សម្រាប់សំណាកមានភាពថ្លាខ្លាំង (highly translucent) ឬការបំពាក់ពណ៌ពណ៌អាចជារឿងចាំបាច់ ដើម្បីបង្កើនភាពមើលឃើញ មុនពេលដាក់ស្លាយ។ មានសារធាតុបំពាក់ពណ៌ពណ៌ជាច្រើនដែលផ្តោតទៅលើធាតុគីមីជាក់លាក់នៃអង្គភាព។ វាជាការសំខាន់ក្នុងការជ្រើសរើសពណ៌ដែលត្រូវគ្នាសមស្របទៅនឹងសំណាក និងថ្នាលដាក់ឡាមដែលបានជ្រើសរើស។ វិធីសាស្ត្រមូលដ្ឋាននេះអាចបត់បែនតាមតម្រូវការ ដូចជាការបញ្ចូល 0.1% acid fuchsin ទៅក្នុងសូលុយស្យុង Marc-André សម្រាប់បំពាក់ពណ៌ពណ៌។ បន្ថែមពីនេះ សំណាកដែលរក្សាក្នុងសូលុយស្យុងទឹក (aqueous solutions) និងគ្រោងទុកសម្រាប់ដាក់ឡាមរួមជាមួយទ្រទ្រង់ Resin ត្រូវការការអស់ទឹក (dehydration, មើល ផ្នែក 5.2) ព្រោះថ្នាលដាក់ស្លាយ Resin ធម្មជាតិ និងសំយោគចំរុងភាគច្រើនមិនសមស្របជាមួយទឹក។

New (1974) បានកំណត់សម្គាល់ថា សារធាតុបំពាក់ពណ៌មួយចំនួនអាចខូចខាតក្នុងថ្នាលដាក់ឡាមខ្លះៗ [53]។ ឧទាហរណ៍ acid fuchsin ដែលត្រូវបានប្រើជាទូទៅជាមួយ Canada balsam ក៏អាចត្រូវបានដាក់ក្នុង Euparal® ផងដែរ។ ទោះយ៉ាងណា សំណាកដែលបានបំពាក់ពណ៌ acid fuchsin គឺងាយបាត់ពណ៌ (fading) ជាពិសេសនៅពេលមានសំណល់ប្រេងក្លូវ (clove oil) ដែលបានប្រើជារូបមន្ត clearing ចុងក្រោយ។

5.2. ការដកជាតិទឹក (Dehydration)

ការដកជាតិទឹកត្រូវអនុវត្តដោយផ្ទេរសំណាកតាមលំដាប់ជំហាននៃសូលុយស្យុងអេតាណុលដែលមានកំហាប់បន្តបន្ទាប់៖ 50%, 70%, 80%, 90% ឬ 95% ហើយបញ្ចប់ដោយ 100% ដោយរាល់ water bath ត្រូវចំណាយពេលយ៉ាងហោចណាស់ ២០ នាទី។ ដោយសារអេតាណុលមានលក្ខណៈហួតឆាប់ ដបត្រូវបិទជិតក្នុងអំឡុងដំណើរការវិភាគ។ បន្ទាប់ពីសំណាកត្រូវបានដកជាតិទឹកពេញលេញ អាចផ្អាកដំណើរការវិភាគរយៈពេលពីរបីថ្ងៃក្នុង **Euparal® essence** ដែលត្រូវបានណែនាំឲ្យប្រើជំនួសប្រេង clove oil។ Beech creosote ដែលធ្លាប់ត្រូវបានប្រើយ៉ាងទូលំទូលាយសម្រាប់គោលបំណ

ងនេះ
បច្ចុប្បន្នត្រូវបានហាមឃាត់ទាំងស្រុងដោយសារគុណភាពពុលខ្ពស់។ ដំណើរការវិភាគដកជាតិទឹកត្រូវធានាថាសារធាតុរាវនៅក្នុងសំណាកមានភាពសមស្របគ្នាជាមួយ ថ្នាលប្រើក្នុងការរៀបចំសំណាកលើកញ្ចកឡាមដើម្បីបង្ការភាពស្រអាប់ (opacity) ការរលំដោយសម្ពាធអូស្មូស៊ីស (osmotic collapse) ឬការប្រែប្រួលរាង (distortion) ដែលអាចធ្វើឲ្យសំណាកមិនសមស្របសម្រាប់ការសិក្សាវិនិច្ឆ័យ taxonomic។

5.3. ថ្នាលប្រើក្នុងការរៀបចំសំណាកលើកញ្ចក់ឡាម (Mounting media)

5.3.1. ការជ្រើសរើស និងការអនុវត្តសម្រាប់ការរៀបចំសំណាក

ថ្នាលប្រើក្នុងការរៀបចំសំណាកលើកញ្ចក់ឡាមគួរតែមានសន្ទស្សន៍បាច់ពន្លឺ (refractive index) ជិតស្មើនឹងកញ្ចក់ដែលប្រហាក់ប្រហែល 1.5។ វាត្រូវតែគ្មានពណ៌ ស្អាតថ្លា និងរក្សាភាពថ្លាឥតខ្ចោះបន្ទាប់ពីសម្ងួតក្នុងរយៈពេលវែង។ វាត្រូវតែឆបគ្នាជាមួយសារធាតុពណ៌ (stains) ដែលបានប្រើ និងអាចជ្រៀតចូល និងចែកចាយទៅក្នុងជាលិកាទាំងអស់នៃសំណាក។
វាមិនគួរស្ងួតលឿនពេក ឬបង្កើតស្រទាប់ពពក (haze) ក្នុងអំឡុង Mountឡើយ ហើយមិនគួររួញតូចបន្ទាប់ពី Mount។

ការជ្រើសរើសថ្នាលប្រើក្នុងការរៀបចំសំណាកលើកញ្ចកឡាមសមស្របជាកត្តាមូលដ្ឋានសំខាន់ក្នុងការរៀបចំសំណាក
ព្រោះមិនមានសារធាតុតែមួយណាដែលសមស្របសម្រាប់គោលបំណងទាំងអស់ឡើយ។
ការជ្រើសរើសគួរតែធ្វើដោយធ្វើគុណភាពកត្តាសំខាន់ៗដូចខាងក្រោម៖ លក្ខណៈអុបទិក) Optical properties)៖ សន្ទស្សន៍បាច់ពន្លឺនៃថ្នាលប្រើក្នុងការរៀបចំសំណាកលើកញ្ចក់ឡាមគួរផ្តល់ភាពផ្ទុយ
និងការបាច់ពន្លឺគ្រប់គ្រាន់សម្រាប់លក្ខណៈរចនាសម្ព័ន្ធអាណាតូមីសំខាន់ៗដែលត្រូវប្រើក្នុងការកំណត់អត្តសញ្ញាណ)taxonomic identification) ឬការពិពណ៌នារូបសាស្ត្រ)morphological description) ដូចជា ថង់ផ្ទុកទឹកកាមញី) spermathecae), ascoids, Newstead sensilla, vertical cibarial teeth និង pharyngeal teeth។
ភាពអាចមើលឃើញនៃរចនាសម្ព័ន្ធទាំងនេះពឹងផ្អែកដោយផ្ទាល់លើលក្ខណៈអុបទិកនៃថ្នាលប្រើក្នុងការរៀបចំសំណាកលើកញ្ចក់ឡាម។ ការរក្សាទុក)Preservation)៖ សម្រាប់ប្រភេទសំណាក)type specimens) ឬសម្ភារៈដែលមានបំណងរក្សាទុកក្នុងសមាគមអភិរក្សអចិន្ត្រៃយ៍
ថ្នាលប្រើក្នុងការរៀបចំសំណាកលើកញ្ចក់ឡាមត្រូវផ្តល់ស្ថិរភាព និងភាពធន់យូរអង្វែង។ ផ្ទុយទៅវិញ សម្រាប់ការសិក្សាចុះបញ្ជី (inventory studies) ឬការស្ទង់មតិរោគវិទ្យា (epidemiological surveys) ដែលមិនចាំបាច់រក្សាទុករយៈពេលវែង
ថ្នាលប្រើក្នុងការរៀបចំសំណាកលើកញ្ចក់ឡាមបណ្តោះអាសន្ន ឬពាក់កណ្តាលអចិន្ត្រៃយ៍អាចគ្រប់គ្រាន់។

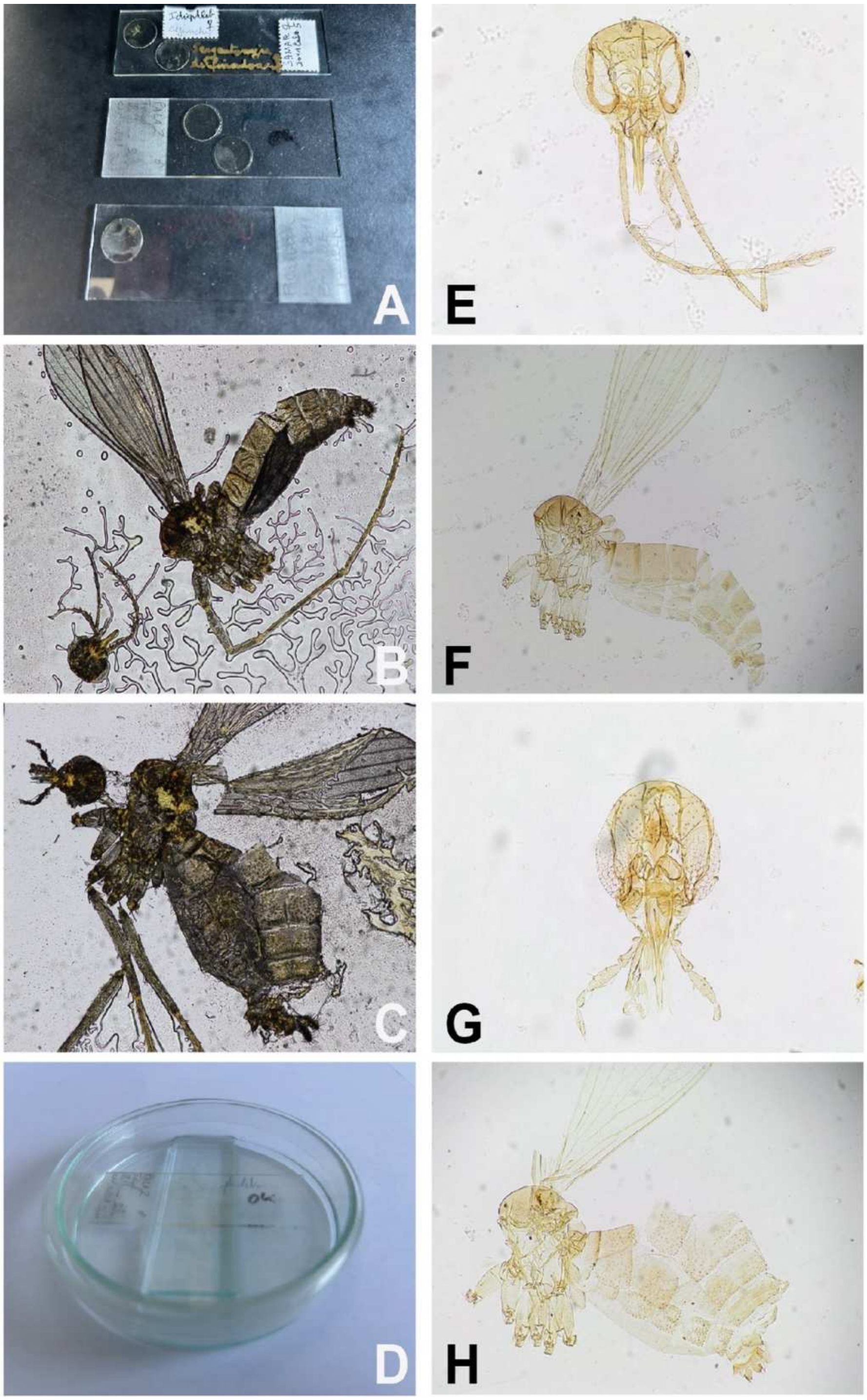

រូបភាព ៨: ការតំឡើងស្លាយឡើងវិញ៖ A: ស្លាយខូច និងស្ងួត តំឡើងក្នុង Hoyer B: មើលក្រោមកាំរស្មីម៉ៃក្រូស្កូប នៃសំខាន់ល្អិតស្ងួត C: មើលក្រោមកាំរស្មីម៉ៃក្រូស្កូប នៃសំខាន់ល្អិតខូចមួយផ្សេងទៀត D: បន្ទះសើមដែលមានស្លាយស្ងួត E: ក្បាល និង F: រាងកាយនៃសំខាន់ B បន្ទាប់ពីតំឡើងឡើងវិញក្នុង Euparal® G: ក្បាល និង H: រាងកាយនៃសំខាន់ C ខូច បន្ទាប់ពីតំឡើងឡើងវិញក្នុង Euparal®

5.3.2. តម្រូវការសម្រាប់ថ្នាលប្រើក្នុងការរៀបចំសំណាកលើកញ្ចក់ឡាម

អ្នកជំនាញជាច្រើនតែងតែអភិវឌ្ឍបច្ចេកទេស Mount ដែលបានប្តូរតាមតម្រូវការ និងមានស្នូគស្មាញ ដើម្បីឆបគ្នាជាមួយគោលបំណងស្រាវជ្រាវជាក់លាក់។ ទោះជាយ៉ាងណា វិធីសាស្ត្រទាំងនេះជាញឹកញាប់មិនបានគិតគូរពីគុណភាពអភិរក្សរយៈពេលវែង) archival quality) ភាពឆបគ្នា)compatibility) ការស្តង់ដារភាព) standardization) ឬភាពងាយស្រួលក្នុងការដោះស្រាយ និងការអភិរក្សយូរអង្វែង។ កង្វះស្តង់ដារភាពនេះបង្កការលំបាកក្នុងការបញ្ចូលសមាសធាតុបរិច្ចាគទៅក្នុងសមាគម និងការគ្រប់គ្រងអភិរក្សរយៈពេលវែង។ កម្មវិធីវិទ្យាសាស្ត្រមានតម្រូវការពិសេសខុសៗគ្នាសម្រាប់ថ្នាលប្រើក្នុងការរៀបចំសំណាកលើកញ្ចក់ឡាម។ អ្នកអាណាតូមីសាស្ត្រ) taxonomists) ជាញឹកញាប់ Mount សំណាកទាំងមូលនិងចូលចិត្តសារធាតុដែលអាចបង្កើតការរំលាយជាលិកាខាងក្នុង) gentle maceration of inner organs) ដើម្បីបង្កើនភាពច្បាស់នៃរចនាសម្ព័ន្ធ cuticular។ សន្ទស្សន៍បាចពន្លឺ) refractive index) គួរមានភាពខុសគ្នាគ្រប់គ្រាន់ពីសំណាក និងកញ្ចក់ឡាម ដើម្បីបង្កើនភាពច្បាស់អុបទិកអតិបរមា។

ថ្នាលប្រើក្នុងការរៀបចំសំណាកលើកញ្ចក់ឡាមពាណិជ្ជកម្មភាគច្រើនត្រូវបានរៀបចំឲ្យមានសន្ទស្សន៍បាចពន្លឺជិតស្មើនឹងកញ្ចក់ ដើម្បីកាត់បន្ថយការបាចពន្លឺ និងការបែកខ្ញែកពន្លឺតាមប្រព័ន្ធកញ្ចក់ឡាម–ថ្នាលប្រើក្នុងការរៀបចំសំណាកលើកញ្ចក់ឡាម–ឡាម៉ែល។ ទោះយ៉ាងណា ក្នុងមីក្រូទស្សន៍ពី brightfield ភាពផ្ទុយធម្មជាតិនៃសំណាកដែលមិនបានបំពាក់ពណ៌ពណ៌) unstained specimen) អាចត្រូវបានកែប្រែដោយជ្រើសរើសថ្នាលប្រើក្នុងការរៀបចំសំណាកលើកញ្ចក់ឡាមដែលមានសន្ទស្សន៍បាចពន្លឺខុសបន្តិចពីសំណាក ដោយវិធីនេះអាចបង្កើនភាពអាចមើលឃើញលើផ្ទៃខាងក្រោយ។

5.3.3. ប្រភេទថ្នាលប្រើក្នុងការរៀបចំសំណាកលើកញ្ចក់ឡាម (តារាង ៣ និង ៤(

មីក្រូទស្សន៍ពីត្រូវការសន្ទស្សន៍បាចពន្លឺ) Refractive Index; RI) នៃថ្នាលប្រើក្នុងការរៀបចំសំណាកលើកញ្ចក់ឡាម ដើម្បីកំណត់ពីរបៀបដែលពន្លឺបត់ឆ្លងកាត់កញ្ចក់ឡាម ថ្នាលប្រើក្នុងការរៀបចំសំណាកលើកញ្ចក់ឡាម និងសំណាក។ នៅពេល RI មានភាពស្របគ្នាជិតស្មើនឹងកញ្ចក់ឡាម៉ែល (≈1.515) ពន្លឺនឹងឆ្លងកាត់យ៉ាងស្មើភាព កាត់បន្ថយការបែកខ្ញែក និងការប្រែប្រួលអុបទិក បណ្តាលឲ្យមានភាពច្បាស់ល្អប្រសើរ និងការមើលឃើញលម្អិតរចនាសម្ព័ន្ធតូចៗបានប្រសើរ។ ផ្ទុយទៅវិញ ការមិនស្របគ្នានៃ RI អាចបង្កភាពព្រិល)blurring) រង្វង់ halo ឬបំបាំងលក្ខណៈដែលមិនបានបំពាក់ពណ៌ពណ៌។ ដូច្នេះ ការជ្រើសរើសថ្នាលប្រើក្នុងការរៀបចំសំណាកលើកញ្ចក់ឡាមសមស្របមានសារៈសំខាន់ដើម្បីបង្កើនភាពផ្ទុយ ភាពច្បាស់ និងគុណភាពរូបភាពសរុបសម្រាប់សំណាកណាមួយ ព្រោះសារធាតុនីមួយៗមាន RI ខុសគ្នា។

សន្ទស្សន៍បាចពន្លឺនៃថ្នាលប្រើក្នុងការរៀបចំសំណាកលើកញ្ចក់ឡាមមានឥទ្ធិពលយ៉ាងសំខាន់លើការមើលឃើញរចនាសម្ព័ន្ធលម្អិតនៅពេលរៀបចំ sand flies សម្រាប់ Mount លើឡាមកញ្ចក់។ លក្ខណៈស្រាល និងមានការស្លេរ៉ូទីសកម្មទាប) lightly sclerotized) របស់ sand flies រួមមាន cibarial armature, ថង់ផ្ទុកទឹកកាមញី (spermathecae), ផ្នែកអង់តែន) antennal segments) និងសរសៃស្លាប) wing venation) អាចពិបាកមើលឃើញនៅក្នុងថ្នាលប្រើក្នុងការរៀបចំសំណាកលើកញ្ចក់ឡាមដែលមាន RI ខ្ពស់។

ជម្រើសដែលត្រូវបានប្រើជាញឹកញាប់សម្រាប់ sand flies មាន gum-chloral media ជាថ្នាលប្រើក្នុងការរៀបចំសំណាកលើកញ្ចក់ឡាមមូលដ្ឋានទឹក និង Canada balsam និងជ័រ Enecê - Nelson Cerqueira (NC) resin ជាថ្នាលប្រើក្នុងការរៀបចំសំណាកលើកញ្ចក់ឡាមមូលដ្ឋានរំលាយក្នុងសូលវ៉ង់។ Rawlins [60] បានចាត់ថ្នាក់ថ្នាលប្រើក្នុងការរៀបចំសំណាកលើកញ្ចក់ឡាមជា ២ ប្រភេទ៖ (១(ថ្នាលប្រើក្នុងការរៀបចំសំណាកលើកញ្ចក់ឡាមអចិន្ត្រៃយ៍)permanent media) ដែលរឹងខ្លាំងតាមពេលវេលា និងសមស្របសម្រាប់ការអភិរក្សរយៈពេលវែង; (២ (ថ្នាលប្រើក្នុងការរៀបចំសំណាកលើកញ្ចក់ឡាមពាក់កណ្តាលអចិន្ត្រៃយ៍) semi-permanent media) ដែលមិនរឹងពេញលេញ និងត្រូវបានប្រើសម្រាប់គោលបំណងបណ្តោះអាសន្ន។

ថ្នាលប្រើក្នុងការរៀបចំសំណាកលើកញ្ចក់ឡាម)mounting media) អាចមានសភាពជារូបរាវមានមូលដ្ឋានជាកៅស៊ូ) gum-based) ឬជាជ័រ) resinous) ដែលរលាយក្នុងទឹក អាល់កុល ឬសូលវ៉ង់សរីរាង្គផ្សេងៗ) ឧ . toluene, xylene) (តារាង ៣ (។ បន្ទាប់ពីអនុវត្តលើកញ្ចក់ឡាមហើយ វាត្រូវបានបិទការប៉ះពាល់ពីបរិយាកាសដោយប្រើសារធាតុ

បិទគែម) ringing media) ដែលមិនរលាយ។ ដើម្បីបែងចែកប្រភេទថ្នាលប្រើក្នុងការរៀបចំសំណាកលើកញ្ចក់ឡាមឲ្យបានច្បាស់ អាចប្រើការចាត់ថ្នាក់ដូចខាងក្រោម៖

ក .
ថ្នាលប្រើក្នុងការរៀបចំសំណាកលើកញ្ចក់ឡាមមូលដ្ឋានទឹក) Aqueous media) សារធាតុប្រភេទនេះរលាយបានងាយក្នុងទឹក ដូច្នេះសមស្របសម្រាប់ Mount បណ្ដោះអាសន្ន ឬពាក់កណ្ដាលអចិន្ត្រៃយ៍។ ជាទូទៅ វាងាយស្រួលក្នុងការប្រើប្រាស់ ប៉ុន្តែអាចត្រូវការការបិទគែម ដើម្បីបង្ការការប៉ះពាល់ពីសំណើមបរិយាកាស) ឧ .gum-chloral media និង polyvinyl alcohol) ជាពិសេសក្នុងតំបន់អាកាសធាតុត្រូពិចដែលមានសំណើមខ្ពស់។

ខ .
ថ្នាលប្រើក្នុងការរៀបចំសំណាកលើកញ្ចក់ឡាមដែលមិនធន់ទឹក) Limited water-tolerant media) សារធាតុប្រភេទនេះត្រូវបានប៉ះពាល់ដោយទឹកតិចជាង ប៉ុន្តែត្រូវការពារពីសំណើមខ្ពស់។ វាផ្ដល់ស្ថិរភាពរយៈពេលវែងប្រសើរជាងសារធាតុរលាយក្នុងទឹក ហើយត្រូវបានប្រើញឹកញាប់សម្រាប់ម៉ោនពាក់កណ្ដាលអចិន្ត្រៃយ៍។

គ .
ថ្នាលប្រើក្នុងការរៀបចំសំណាកលើកញ្ចក់ឡាមរលាយក្នុងអ៊ីដ្រូកាបូន) Hydrocarbon-soluble media) សារធាតុប្រភេទនេះរលាយក្នុងសូលវ៉ង់សេរីរាង្គដូចជា xylene ឬ toluene ឬ essenecê (enecê solvent)។ វាត្រូវបានរចនាឡើងសម្រាប់ Mount អចិន្ត្រៃយ៍ និងផ្ដល់ស្ថិរភាពយូរអង្វែងល្អប្រសើរ ព្រមទាំងធន់នឹងសំណើម និងការប្រែប្រួលគុណភាព)degradation) ដូច្នេះសមស្របសម្រាប់គោលបំណងអភិរក្សឯកសារ)archival purposes) (ឧ .neutral Canada balsam)។

ជារួម
ថ្នាលប្រើក្នុងការរៀបចំសំណាកលើកញ្ចក់ឡាមរលាយក្នុងទឹកសមស្របបំផុតសម្រាប់ Mount បណ្ដោះអាសន្ន ឬករណីដែលត្រូវការដកសំណាកចេញបានងាយ; សារធាតុអត់ធន់ទឹកកម្រិតកំណត់សមស្របសម្រាប់ Mount ពាក់កណ្ដាលអចិន្ត្រៃយ៍ដែលត្រូវការភាពធន់ថ្នាល; ចំណែកសារធាតុរលាយក្នុងអ៊ីដ្រូកាបូនគឺជាជម្រើសអាទិភាពសម្រាប់ម៉ោនអចិន្ត្រៃយ៍ដែលមានបំណងរក្សាទុក និងរក្សាទុករយៈពេលវែង។

តារាង ៣៖ សមាសធាតុនៃថ្នាលប្រើក្នុងការរៀបចំសំណាកលើកញ្ចកឡាមដែលបានជ្រើសរើស) Composition of selected mounting media)

**ឧបករណ៍ផ្ទុកសម្រាប់mounting**	**សារធាតុរំលាយ**	**សារធាតុមុនពហុអង្គធាតុ (Pre-polymer(s)) ឬ ពហុអង្គធាតុដែលអាចមាន**	**កំណត់សម្គាល់**
ហូយើរ (Hoyer) = ក្លរ៉ាល់ហ្គ៊ម (chloral gum)	គ្លីសេរិន, ទឹក	សមាសធាតុនៃអញ្ចាញអារ៉ាប៊ីក	ភ្នាក់ងាររំលាយជាលិកា (Macerating agent)៖ ក្លរ៉ាល់អ៊ីដ្រេត
CMCP-9 (=កាបូស៊ីមេទីលសែលុយឡូសហ្វេណុល)	ទឹក (CMCP-9: ៥១–៦០%)	ប៉ូលីវីនីលអាល់កុលដែលត្រូវបានអ៊ីដ្រូលីសពេញលេញ (CMCP-9: ០–៥%)	CMC(P)-9៖ ថ្នាក់ viscosity ទាប៖ ថ្នាក់ viscosity ខ្ពស់
DMHF (ឌីមេទីលអ៊ីដង់តូអ៊ីនហ្វម៉ដេអ៊ីដ)	ទឹក	N,N′-ឌីមេទីលអុលឌីមេទីលអ៊ីដង់តូអ៊ីន (ឌី-មេទីលអុល DMH)<br>អូលីហ្គូម៉ដែលមានស្ពានអេធើរ/មេទីលីន<br>បណ្តាញពហុអង្គធាតុ DMH–ហ្វម៉ដេអ៊ីដដែលមានតំណភ្ជាប់ឆ្លងកាត់ (Crosslinked)	-
បាសាំកាណាដា (Canada balsam)	ស៊ីលីន (xylene); សមាសធាតុងាយហួតខ្លះនៃបាសាំ (Δ^3-ការិន, អាស៊ីតលេវ៉ូភីម៉ារិក, លីម៉ូណែន, មៀរស៊ីន, អាស៊ីតប៉ាលូស្ត្រិក, β-ហ្វេឡង់ឌ្រីន, α-ភីណែន, β-ភីណែន)	បាសាំ (អាប៊ីណុល, អាស៊ីតអាប៊ីអ៊ីទិក, អាស៊ីតអ៊ីសូភីម៉ារិក, អាស៊ីតសាន់ដារ៉ាកូភីម៉ារិក)	អព្យាក្រឹតកម្ម (neutralization)៖ ប៉ូតាស្យូមកាបូណាត;<br>ជ័រពីប្រភេទដើមពោធិ៍ Abies balsamea (លីនេ, ១៧៥៨)
អឺប៉ារ៉ាល់® (Euparal®)	អ៊ីកាលីបតូល, ប៉ារ៉ាល់ដេអ៊ីដ; សមាសធាតុងាយហួតខ្លះនៃអញ្ចាញសាន់ដារ៉ាក់ (លីម៉ូណែន, α-ភីណែន, β-ភីណែន)	សមាសធាតុនៃអញ្ចាញសាន់ដារ៉ាក់ (អាស៊ីតកូម្មូនិក, ម៉ាណុល, អាស៊ីតប៉ូលីកូម្មូនិក, អាស៊ីតសាន់ដារ៉ាកូភីម៉ារិក, ១២-អាសេតុកស៊ី-សាន់ដារ៉ាកូភីម៉ារិក, ស្ទ័ជីអុល, អាស៊ីតតូរូល្លូស៊ីក, តូរូឡូសុល, តូតារ៉ុល)	ភ្នាក់ងារធ្វើឱ្យថ្លា (clearing agent)៖ មេទីលសាលីស៊ីឡេត ;<br>ពណ៌បៃតងក្នុង Euparal®៖ អំបិលទង់ដែង (កូបពរអាប៊ីអ៊ីទីណេត);<br>ជ័រសាន់ដារ៉ាក់ពីប្រភេទដើម Tetraclinis articulata (វ៉ាល់, ១៧៩១)
អេណេសេ (Enecê)	អេទីលអាល់កុល; ជាមួយកាហ្វួរ, អ៊ីកាលីបតូល និងអារម្មណ៍ទីពែនទីន	សមាសធាតុនៃអញ្ចាញកូប៉ាល់ (copal gum) និងកូឡូហ្វានស (colophony = rosin)	-

តារាង ៤៖ អត្ថប្រយោជន៍និងគុណវិបត្តិនៃថ្នាលប្រើក្នុងការរៀបចំសំណាកលើកញ្ចក់ឡាមដែលបានជ្រើសរើសទាក់ទងនឹងកញ្ចក់ឡាមមីក្រូទស្សន៍និងការសង្កេតមិនបានបោះពុម្ពផ្សាយរបស់អ្នកជំនាញផ្សេងៗ [52]។

ឈ្មោះ	គុណសម្បត្តិ	គុណវិបត្តិ
**Canada balsam**	ឧបករណ៍ផ្ទុកមានភាពជាប់លាប់ខ្ពស់ អាចប្រើប្រាស់លើសពី ១៥០ ឆ្នាំ។	មានសមាសធាតុបង្កគ្រោះថ្នាក់ ត្រូវប្រុងប្រយ័ត្ន និងប្រើប្រាស់ក្រោមឧបករណ៍ស្រូបផ្សែង (hood)។
	អាចmountingស្លាយដោយប្រើប្រាស់ប្រេងក្លូវ (clove oil) ឬ ហ្វេណុល ជាភ្នាក់ងារmounting។	តម្រូវឱ្យធ្វើខ្សែសង្វាក់ខ្សោះជាតិទឹក (dehydration series) ពេញលេញ ដែលចំណាយពេលច្រើន។
		ការខ្សោះជាតិទឹកជាមួយអេតាណុល និងការផ្ទេរតាមរយៈ ស៊ីលីន ឬ ប្រេងក្លូវ អាចធ្វើឱ្យសំណាកខ្លះផុយស្រួយ។ សារធាតុជំនួស (ដូចជា អ៊ីសូប្រូផាណុល, n-ប៊ុយតាណុល, សេឡូសូល™, ១,៤-ឌីអុកសេន, ហ៊ីស្តូក្លៀ, តឺភីណេអុល) អាចកាត់បន្ថយការបាក់បែកបាន។
		សំណាកអាចប្រែជាខ្មៅ ប្រសិនបើជំនួស ស៊ីលីន ដោយ ហ្វេណុល ឬ ប្រសិនបើនៅសល់ប៉ូតាស្យូមអ៊ីដ្រូស៊ីត។
		សន្ទស្សន៍ចំណាំងបែរខ្ពស់អាចបិទបាំងរចនាសម្ព័ន្ធដែលមិនបានបំពាក់ពណ៌ពណ៌។
		ការស្ងួតពេញលេញអាចចំណាយពេលច្រើនឆ្នាំ ប្រសិនបើគ្មានការសម្ងួតលើផ្ទៃកម្តៅ (hot-plate drying)។
		ឧបករណ៍ផ្ទុកប្រែពណ៌លឿង និងងងឹតទៅតាមពេលវេលា ជាពិសេសនៅពេលធ្វើឱ្យថ្លាជាមួយប្រេងក្លូវ។
		ស្នាមប្រឡាក់ខ្លះចុះខ្សោយ ហើយពណ៌ដែលមានបន្ទុកអ៊ីយ៉ុងវិជ្ជមាន (cationic dyes) អាចរសាត់ ប្រសិនបើឧបករណ៍ផ្ទុកមានជាតិអាស៊ីត (ដែលអាចកើតឡើងដោយឯកឯងតាមពេលវេលា)។
**DMHF (ឌីមេទីលអ៊ីដង់តូអ៊ីនហ្វម័រដេអ៊ីដ)**	តម្លាភាពខ្ពស់	អាចប្រែពណ៌លឿងតាមពេលវេលា
	សន្ទស្សន៍ចំណាំងបែរល្អ	អាចប្រែប្រួលពណ៌ស្នាមប្រឡាក់ខ្លះ
	អាចមើលឃើញរចនាសម្ព័ន្ធបានយ៉ាងប្រសើរ	មិនស័ក្តិសមសម្រាប់ពណ៌ដែលងាយនឹងហ្វម័រដេអ៊ីដ
	ការត្រៀមល្អមានស្ថេរភាពគួរសម	អាចមានពពុះខ្យល់ ពេលស្ងួតយឺត
	ឆបគ្នាជាមួយបច្ចេកទេសបំពាក់ពណ៌ពណ៌ជាច្រើន	ឧបករណ៍ផ្ទុកងាយនឹងសំណើម
	ការពារសំណាកបានល្អ	ការmountingពិបាកក្នុងការធ្វើឱ្យត្រឡប់ដើមវិញ
	ភាពស្អិតជាប់ល្អរវាងស្លាយនិងគម្របកញ្ចក់	ហ្វម័រដេអ៊ីដ មានជាតិពុល, ឆាប់ខឹង, បង្កមហារីក
**Euparal**	ឧបករណ៍ផ្ទុកជាប់លាប់ អាចប្រើបានលើសពី ៥០ ឆ្នាំ។	មានសមាសធាតុបង្កគ្រោះថ្នាក់ ត្រូវប្រុងប្រយ័ត្ន និងប្រើប្រាស់ក្រោមឧបករណ៍ស្រូបផ្សែង។
	អាចmountingដោយផ្ទាល់ពីអេតាណុល ៨០% (តាមអនុសាសន៍ក្រុមហ៊ុនផលិត)។	ការខ្សោះជាតិទឹកជាមួយអេតាណុល និងការផ្ទេរតាមរយៈ អឺប៉ារ៉ាល់ អេស្សង់ (Euparal Essence) អាចធ្វើឱ្យសំណាកខ្លះផុយស្រួយ ប៉ុន្តែការប្រើ អ៊ីសូប្រូផាណុល អាចកាត់បន្ថយបញ្ហានេះបាន។
	មិនបិទបាំងរចនាសម្ព័ន្ធដែលមិនបានបំពាក់ពណ៌ពណ៌ និងមិនប្រែពណ៌លឿង ឬ ផុយតាមពេលវេលា។	

	មានសន្ទស្សន៍ចំណាំងបែរសមស្របជាង បាសាំកាណាដា សម្រាប់ពួក Diptera (សត្វរុយ)។	
	ដំណើរការល្អសម្រាប់សំណាកក្រាស់ ដោយសារការរួញតូចតិចតួចបំផុត និងការស្ងួតគ្មានពពុះ។	
	នៅតែរលាយក្នុងអេតាណុល ៩៥% ដែលអនុញ្ញាតឱ្យmountingឡើងវិញ សូម្បីតែបន្ទាប់ពីច្រើនឆ្នាំក៏ដោយ។	
	អាចmountingសំណាកដែលនៅរស់ ឬ ដោយផ្ទាល់ពីទឹក អេតាណុល ឬ ហ្វម៉ារដេអ៊ីដ។	សំណាករុក្ខជាតិដែលឆ្ងាញ់អាចបាក់បែក លើកលែងតែបន្ថែមឧបករណ៍ផ្ទុកបន្តិចម្តងៗ ដែលចំណាយពេលច្រើន។
	ការរំលាយជាលិកា (maceration) ផ្តល់នូវគុណភាពផ្ទៃខាងក្រៅ (cuticle) ល្អឥតខ្ចោះ។	ប្រហោង (cavities) និងគ្រីស្តាល់អាចបង្កើតបានក្នុងរយៈពេលតិចជាង ១០ ឆ្នាំ។
**Hoyer fluid**	មានសន្ទស្សន៍ចំណាំងបែរអំណោយផល ហើយអាចបង្កើនកម្រិតពណ៌ជាមួយការបំពាក់ពណ៌ពណ៌អ៊ីយ៉ូត។	ការរំលាយជាលិកាអាចហួសកម្រិត អាស្រ័យលើកំហាប់ក្លរ៉ាល់អ៊ីដ្រេត និងពេលវេលាប៉ះពាល់។
	អាស៊ីតអាសេទិកក្នុងរូបមន្តអាចពង្រីកសរីរាង្គនៃarthropods (arthropod appendages)។	សមាសធាតុនៃឧបករណ៍ផ្ទុកអាចបំបែកចេញពីគ្នា ហើយគ្រាប់ល្អិតៗអាចលេចឡើងក្នុងរយៈពេលប៉ុន្មានខែ ឬច្រើនឆ្នាំ។
	សំណាកខ្លះអាចនៅមានស្ថេរភាពរហូតដល់ ៤០–៦០ ឆ្នាំ។	មានរបាយការណ៍អំពីការប្រែខ្មៅនៃឧបករណ៍ផ្ទុក។
	រលាយក្នុងទឹក ងាយស្រួលក្នុងការmountingឡើងវិញ។	
**CMCP-9 (= កាបូស៊ីមេទីលសែលុយឡូសហ្វេណុល)**	អាចmountingសំណាកដោយផ្ទាល់ពីឧបករណ៍ផ្ទុកដូចជា ទឹក អេតាណុល គ្លីសេរីន ឬ សូលុយស្យុងដែលមានហ្វម៉ារដេអ៊ីដ ហើយសរីរាង្គខាងក្នុងរបស់វាអាចត្រូវបានគេរំលាយជាលិកា (macerated) នៅពេលចាំបាច់ ដើម្បីជួយសម្រួលដល់ការពិនិត្យទូទៅ ឬការត្រៀមលក្ខណៈ។	ឧបករណ៍ផ្ទុកនេះអាចបង្កើតគ្រីស្តាល់ និងងងឹតទៅតាមពេលវេលា ហើយជួនកាលអាចរំលាយជាលិកាសំណាកច្រើនជាងការចង់បាន។ លើកលែងតែស្លាយត្រូវបានព័ទ្ធជុំវិញ (ringed) ដោយប្រុងប្រយ័ត្ន សំណាកក្រាស់ៗនឹងមិនល្អជាមួយវាទេ ព្រោះវាអាចរួញ និងបង្កើតគម្លាតនៅជុំវិញគែមរបស់គម្របកញ្ចក់។ វាមិនស័ក្តិសមសម្រាប់សំណាកដែលបានបំពាក់ពណ៌ពណ៌ ឬ វត្ថុធាតុដែលមានជាតិកាល់ស្យូម ហើយពេលវេលាស្ងួតរបស់វាយឺតជាង CMC។
	ឧបករណ៍ផ្ទុកជាប់លាប់ អាចប្រើបានលើសពី ៣០ ឆ្នាំ។	មានសមាសធាតុបង្គ្រោះថ្នាក់ ត្រូវប្រុងប្រយ័ត្ន និងប្រើប្រាស់ក្រោមឧបករណ៍ស្រូបផ្សែង។
**Eukitt™**	ឆបគ្នាជាមួយសារធាតុរំលាយជាច្រើនសម្រាប់mounting រួមមាន អាសេតូន, បេនហ្ស៊ីន, ក្លរ៉ូហ្វម, ឌីអុកសាន, អេធើរ, អ៊ីសូប្រូផាណុល, មេទីលបេនហ្សូអេត, តឺភីណេអុល, តូលូអ៊ីន, និង ស៊ីលីន។	តម្រូវឱ្យធ្វើខ្សែសង្វាក់ខ្សោះជាតិទឹក ពេញលេញ ដែលចំណាយពេលច្រើន។
	ស្ងួតលឿន និងមាន pH អាស៊ីតបន្តិច។	មិនល្អសម្រាប់សំណាកក្រាស់ ដោយសារការរួញតូច និងការបង្កើតពពុះឧស្ម័ន។
	មិនងងឹតគួរឱ្យកត់សម្គាល់តាមពេលវេលា។	គម្របកញ្ចក់អាចរបូតចេញតាមពេលវេលា លើកលែងតែកញ្ចក់ត្រូវបានសម្អាតឱ្យបានល្អ និងបិទជិត។

	ស័ក្តិសមសម្រាប់ពណ៌ផ្សេងៗ (ឧទា. ហ្វូចស៊ីន, ហេម៉ាតូស៊ីលីន, មេទីលប៊ៃគង, មេទីលវីយ៉ូឡេត, មេទីលខៀវ)។	អាចបង្ហាញពីការក្លាយជាពហុអង្គធាតុ (polymerization) មិនពេញលេញនៅជុំវិញសរសៃកូឡាជែន។
	អាចmountingសំណាកឡើងវិញបន្ទាប់ពីច្រើនឆ្នាំ ដោយត្រាំក្នុង ស៊ីលីន រយៈពេលយូរ។	
	ឧបករណ៍ផ្ទុកជាប់លាប់ខ្ពស់ យ៉ាងហោចណាស់ ៥០ ឆ្នាំ។	តម្រូវឱ្យធ្វើខ្សែសង្វាក់ខ្សោះជាតិទឹក ពេញលេញ ដែលចំណាយពេលច្រើន។
	អេណេសេ មិនងងឹតទៅតាមពេលវេលាទេ។	ការខ្សោះជាតិទឹកជាមួយអេតាណុល និងការផ្ទេរតាមរយៈប្រេងក្លូវ អាចធ្វើឱ្យសំណាកខ្លះផុយស្រួយ។
**Enecê**	វាអាចបត់បែនបានច្រើនជាង ដែលអនុញ្ញាតឱ្យធ្វើវះកាត់សត្វល្អិតក្នុងឧបករណ៍ផ្ទុក ព្រមទាំងផ្តល់ពេលវេលាសមរម្យសម្រាប់កំណត់ទីតាំងរចនាសម្ព័ន្ធសរីរវិទ្យា។	សត្វល្អិតនៅតែបន្តក្លាយជាថ្លា (clarified) ទោះបីយឺតខ្លាំងក៏ដោយ ដែលអាចធ្វើឱ្យពិបាកមើលឃើញរចនាសម្ព័ន្ធតូចៗ ដូចជា សរសៃញ្ញាណ (sensilla), អាស្កូអ៊ីដ (ascoids), និង រោមសាមញ្ញ (simple setae)។
	តម្លៃទាប។	

5.3.4. សេចក្តីពិពណ៌នាថ្នាលប្រើក្នុងការរៀបចំសំណាកលើកញ្ចក់ឡាមដែលបានណែនាំ) តារាង ៣ និង ៤(

ថ្នាលប្រើក្នុងការរៀបចំសំណាកលើកញ្ចក់ឡាមសម្រាប់ការសង្កេតបណ្តោះអាសន្ន

Chloral gum = Hoyer fluid/medium/solution (RI = 1.48)

Marc André fluid គឺជាថ្នាលប្រើក្នុងការរៀបចំសំណាកលើកញ្ចក់ឡាមល្អបំផុតសម្រាប់ការសង្កេតរយៈពេលខ្លី) ពីរបីម៉ោង ហើយអាចយូរជាងនេះ ប្រសិនបើកញ្ចក់ឡាមត្រូវបានរក្សាទុកក្នុងបន្ទប់មានសំណើម (លើថង់ផ្ទុកទឹកកាមញី) spermathecae) រួមទាំងការថតរូប) រូបទី ៤ (ឬគំនូរ។ ការរក្សាទុកថង់ផ្ទុកទឹកកាមញី) spermathecae) ដែលបានសង្កេតត្រូវការ Mount ឡើងវិញក្នុងថ្នាលប្រើក្នុងការរៀបចំសំណាកលើកញ្ចក់ឡាមមូលដ្ឋានទឹក ដែលអនុញ្ញាតឲ្យរក្សាទុករយៈពេលថ្នាល។ ការដកជាតិទឹកសម្រាប់ Mount ឡើងវិញក្នុងជ័រ) resin) មិនមែនជាការមិនអាចធ្វើបានទេ ប៉ុន្តែមិនត្រូវបានណែនាំ) ហានិភ័យបាត់បង់សំណាក(។ Chloral gum និង Hoyer fluid ត្រូវបានចាត់ទុកថាជាសមនាម។ សារធាតុនេះត្រូវបានប្រើជាញ្ញីកញ្ចប់សម្រាប់សង្កេតសេរីរាង្គខាងក្នុងដោយសារតែភាពឆបគ្នាជាមួយទឹក ភាពសាមញ្ញ អនុវត្តលឿន និងសន្ទស្សន៍បាច់ពន្លឺ)refractive index) ដែលអនុគ្រោះដល់ការពិនិត្យរចនាសម្ព័ន្ធស្រាលៗ ដូចជាថង់ផ្ទុកទឹកកាមញី (spermathecae)។ ទោះជាយ៉ាងណា chloral gum មានគុណវិបត្តិសំខាន់ ប្រសិនបើមិនបានរៀបចំឲ្យបានត្រឹមត្រូវ ឬរក្សាទុកក្រោមលក្ខខណ្ឌសំណើមគ្រប់គ្រង។ បញ្ហាទាំងនេះរួមមាន ការកើតគ្រីស្តាល់) crystallization) ការប្រែពណ៌) discoloration) និងការបាត់បង់ភាពខាប់)viscosity)។ ការបិទគែម ឡាម៉េល មិនអាចដោះស្រាយបញ្ហាទាំងនេះបានទេ ព្រោះថ្នាលប្រើក្នុងការរៀបចំសំណាកលើកញ្ចក់ឡាមអាចប្រែពណ៌យ៉ាងខ្លាំង) ពេលខ្លះជិតខ្មៅ (ដោយសារប្រតិកម្មជាមួយ ringing medium ជាពិសេសនៅពេលប្រើ Euparal®។ Hoyer medium ត្រូវបានចាត់ទុកថាមានលក្ខណៈអុបទិកល្អបំផុតសម្រាប់ phlebotomine sandflies ហើយត្រូវបានប្រើតាមប្រពៃណីសម្រាប់គោលបំណងនេះ។ សារធាតុនេះមានរូបមន្តជាច្រើនដែលមានទំនាក់ទំនងជិតស្និទ្ធ រួមមាន gum arabic, glycerol និង chloral hydrate។ រូបមន្តខ្លះត្រូវបានបកស្រាយ និងដកស្រង់ខុស]74]។ទោះបីជា Hoyer ជាថ្នាលល្អសម្រាប់សង្កេតថង់ផ្ទុកទឹកកាមញី (spermathecae) ក្នុង sand flies ក៏ដោយ វាមិនសមស្របសម្រាប់ការអភិរក្សរយៈពេលវែងទេ។ វាសមស្របសម្រាប់ការសង្កេតរយៈពេលខ្លី រួមមានការថតរូប គំនូរ ឬរូបភាពផ្សេងៗ។ ថ្នាលប្រើក្នុងការរៀបចំសំណាកលើកញ្ចក់ឡាមមូលដ្ឋានទឹកសមស្របសម្រាប់ Mount បណ្តោះអាសន្ន ប៉ុន្តែមិនអាចធានាការរក្សាទុករយៈពេលវែងឡើយ។ ផ្ទុយទៅវិញ ការ Mount ដោយជ័រ) resin mounting) ផ្តល់ភាពធន់ល្អប្រសើរអាចរក្សាបានរយៈពេលជាច្រើនសតវត្សរ៍ ប៉ុន្តែអាចបំបាំងលម្អិតរចនាសម្ព័ន្ធតូចៗនៃថង់ផ្ទុកទឹកកាមញី (spermathecae) ព្រោះលក្ខណៈ refringence របស់វាជាញ្ញីកញ្ចប់ត្រូវបានបាត់បង់។ Hoyer medium

ប្រេះប្រាយតាមពេលវេលាដោយសារការបាត់បង់ទឹក)Figure 8) បណ្តាលឲ្យកើតគ្រីស្តាល់ chloral hydrate ពណ៌សស្រអាប់តូចៗ។ ទោះជាយ៉ាងណា សំណាកអាចស្តារឡើងវិញពីកញ្ចក់ឡាមដែលមានគ្រីស្តាល់ ព្រោះ cuticle នៅតែស្ថិតស្ថេរផ្នែកគីមី ទោះបីអាចមានការខូចខាតផ្នែកការវិភាគលក្ខណៈរូបសាស្ត្រខ្លះដោយសារការលូតលាស់គ្រីស្តាល់។ ក្នុងករណីខ្លះកញ្ចក់ឡាមដែលមានគ្រីស្តាល់អាចស្តារឡើងវិញដោយបន្ថែមសំណើមក្នុងបរិយាកាសក្តៅសើម និងប្រើ thymol ដើម្បីបង្ការការលូតលាស់ផ្សិត។ ជាជម្រើសផ្សេង សំណាកអាចជ្រាបចេញពី gum chloral ក្នុងទឹក បន្ទាប់មកដកជាតិទឹកក្នុង glacial acetic acid ហើយ Mount ឡើងវិញក្នុង Canada balsam។

DMHF (dimethyl hydantoin formaldehyde) (RI = 1.48)

ថ្នាលប្រើក្នុងការរៀបចំសំណាកលើកញ្ចក់ឡាមមូលដ្ឋានទឹកនេះ] 72] មានលក្ខណៈអុបទិកល្អណាស់ ស្រដៀងនឹង Berlese ហើយងាយប្រើដូច Berlese ផងដែរ។ ទោះយ៉ាងណា ខុសពី Berlese វាមិនប្រែជាពណ៌ខ្មៅ ឬបង្កើតគ្រីស្តាល់ឡើយ។ វាសមស្របសម្រាប់ sand flies និងសមាជិកផ្សេងទៀតនៃគ្រួសារ Psychodidae។

រូបភាពទី ៨៖ ការmountingស្លាយឡើងវិញ) Slide remounting) A៖ កញ្ចក់ឡាមខូចខាត និងស្ងួត ដែលបានmountingដោយថ្នាល Hoyer B៖ ទិដ្ឋភាពក្រោមមីក្រូទស្សន៍នៃ phlebotomine Phlebotomine ដែលស្ងួត C៖ ទិដ្ឋភាពក្រោមមីក្រូទស្សន៍នៃ Phlebotomine មួយផ្សេងទៀតដែលខូចខាត D៖ បន្ទប់សើម) wet chamber) ដែលផ្ទុកកញ្ចក់ឡាមស្ងួត E៖ ក្បាល និង F៖ ខ្លួនរបស់សំណាក B បន្ទាប់ពីបានmountingឡើងវិញក្នុង Euparal® G៖ ក្បាល និង H៖ ខ្លួនរបស់សំណាក C ដែលខូចខាត បន្ទាប់ពីបានmountingឡើងវិញក្នុង Euparal®

CMCP (camphor-mono-chlorophenol) (RI = 1.41)

CMCP គឺជាថ្នាលmountingមានមូលដ្ឋានលើគ្លីសេរីនដែលរលាយក្នុងទឹកត្រូវបានប្រើសម្រាប់បង្កើតកញ្ចក់ឡាមអចិន្ត្រៃយ)permanent slides) មានភាពថ្លាសម្រាប់សំណាកសេរីរាង្គមានភាពស្រាលរឹងទន់ រួមទាំង sand flies ផងដែរ។ អត្ថប្រយោជន៍នៃថ្នាលនេះគឺអាចmountingសំណាកដោយផ្ទាល់ពីទឹក ឬអេតាណុល។ វាអាចធ្វើឲ្យ Phlebotomine ទន់ និងសម្អាតបានយ៉ាងឆាប់រហ័ស ដោយបន្ថយភាពរឹងរបស់ cuticle អនុញ្ញាតឲ្យរៀបចំទីតាំងសំណាកបានត្រឹមត្រូវ ជាពិសេសមានប្រយោជន៍សម្រាប់ការពន្លាយស្លាប ឬការវះកាត់ប្រដាប់ភេទ) genitalia)។ ទោះបីមានរបាយការណ៍ថាអាចរក្សាទុកបានរយៈពេលវែងក៏ដោយ រយៈពេលពិតប្រាកដនៃការអភិរក្សនៅតែមិនប្រាកដច្បាស់។ កំណត់ហេតុចម្បងនៃថ្នាលនេះស្ថិតនៅក្នុងសមាសធាតុដែលមាន phenol ដែលជាសារធាតុពុល និងបង្កការរលាក តម្រូវឲ្យមានការប្រុងប្រយ័ត្នក្នុងការប្រើប្រាស់។

ការរៀបចំសំណាកលើកញ្ចកឡាម សម្រាប់រយៈពេលយូរ)Permanent mounting)

Canada balsam (RI = 1.52–1.54)

Canada balsam ត្រូវបានពិពណ៌នាលើកដំបូងថាជាថ្នាលmountingសមស្របសម្រាប់មីក្រូទស្សន៍ពន្លឺបញ្ជូន) transmitted light microscopy) ដោយ Andrew Pritchard នៅទសវត្សរ៍ឆ្នាំ 1830។ វានៅតែជាថ្នាលមួយដែលត្រូវបានប្រើយ៉ាងទូលំទូលាយ ដោយសារគុណភាពរក្សាទុកយូរអង្វែងដែលបានបញ្ជាក់ជាក់ស្តែងជាង 150 ឆ្នាំនៃការអនុវត្តជោគជ័យ។ ខុសពីថ្នាល Hoyer, Canada balsam មិនបង្កើតគ្រីស្តាល់និងមិនស្រូបសំណើម។ ទោះយ៉ាងណា Canada balsam មានលក្ខណៈ autofluorescent ខ្លាំង ដែលអាចក្លាយជាគុណវិបត្តិសម្រាប់បច្ចេកទេសមីក្រូទស្សន៍មួយចំនួន] 60]។ ការប្រើប្រាស់វាររំលាយមិនពុល ជំនួស xylene អាចកាត់បន្ថយហានិភ័យសុវត្ថិភាពក្នុងដំណើរការវិភាគរៀបចំ ប៉ុន្តែអាចបង្កើតគុណវិបត្តិដូចជា ពេលស្ងួតយឺត និងការប្រែពណ៌ងងឹតលឿនជាងមុន។

Euparal® (RI = 1.48)

Euparal® គឺជាជម្រើសមួយដែលត្រូវបានប្រើយ៉ាងទូលំទូលាយ ជំនួស Canada balsam សម្រាប់ការmountingថេរ ដោយផ្តល់នូវស្ថេរភាពរយៈពេលវែងល្អ និងសន្ទស្សន៍ចំណុះពន្លឺ) refractive index) ប្រហាក់ប្រហែល។ Euparal® មានលក្ខណៈសំខាន់ដូចខាងក្រោម៖

តម្រូវឲ្យដកទឹក) dehydration requirement)៖ មុនការផ្ទេរចុងក្រោយទៅថ្នាលmounting សំណាកត្រូវតែដកទឹកជាមុន ជាទូទៅផ្លាស់ប្តូរពីអេតាណុល 95% ទៅអេតាណុលបរិសុទ្ធ)absolute ethanol)។

ពេលដំណើរការវិភាគយូរ) extended processing time)៖ ការតាំងឡើងចុងក្រោយក្នុងរេស៊ីន មិនថា Canada balsam ឬ Euparal® ទេ ត្រូវការដំណើរការដកទឹក ដែលបន្ថែមរយៈពេលសរុបនៃការរៀបចំសំណាក។

នៅពេលមិនអាចដកទឹកដោយអង្គធាតុរំលាយជាមួយសេរីរាង្គ សំណាកដែលបានដកចេញពីអេតាណុលបរិសុទ្ធអាចដាក់ក្នុងសូលុយស្យុងថ្នាលមួយដែលជាល្បាយស្មើគ្នារវាង Euparal® និង Euparal essence មុន Mount ចុងក្រោយ។

Enecê (RI = 1.467)

Enecê គឺជាថ្នាលmountingមានមូលដ្ឋានលើរេស៊ីនដែលត្រូវបានប្រើជាចម្បងសម្រាប់ Sand flies ហើយមានប្រជាប្រិយភាពជាពិសេសនៅប្រទេសប្រេស៊ីល។ មូលដ្ឋានរបស់វារួមមាន colophony និង gum copal ដែលរំលាយក្នុងអាល់កុល camphor essence of turpentine និង eucalyptol។ Cerqueira [11] បានពិពណ៌នា Enecê ថាជាជម្រើសជំនួស Canada balsam សម្រាប់ការmountingកញ្ចក់ឡាមថេរនៃដំណាក់កាល larva, exuviae នៃដំណាក់កាលមិនពេញវ័យ និងសូម្បីតែសត្វមូសពេញវ័យ ហើយបន្ទាប់មកវាត្រូវបានទទួលយកយ៉ាងទូលំទូលាយសម្រាប់ការ Mount sand flies ។ Enecê ផ្តល់ជាជម្រើសមានប្រសិទ្ធភាពផ្នែកចំណាយសម្រាប់ការដាក់ Mount ថេរ ដោយផ្តល់ស្ថេរភាពរយៈពេលវែង និងពេលស្ងួតគ្រប់គ្រាន់ អនុញ្ញាតឲ្យអនុវត្តការវះបំបែក និងរៀបចំរចនាសម្ព័ន្ធរូបសាស្ត្រ) morphological structures) បានយ៉ាងត្រឹមត្រូវ។

5.4. ការរៀបចំកញ្ចក់ឡាម

និងការសម្ងួតការសម្ងួតកញ្ចក់ឡាមដែលបានmounting យ៉ាងត្រឹមត្រូវ មានសារៈសំខាន់ខ្លាំង ដើម្បីធានាស្ថេរភាព និងការអភិរក្សរយៈពេលវែង។ កញ្ចក់ឡាមត្រូវតែសម្ងួតឲ្យពេញលេញមុនពេលពិចារណាទុករក្សារយៈពេលវែង។ ដើម្បីទទួលបានលទ្ធផលល្អបំផុត ស្លាយដែលបានmountingដោយថ្នាលmountingថេរ (permanent mounting media) គួរតែសម្ងួតក្នុងទីតាំងផ្តេករយៈពេល ២–៣ សប្តាហ៍ ខណៈដែលស្លាយដែលរៀបចំដោយថ្នាលពាក់កណ្តាលថេរ (semi-permanent media) អាចត្រូវការត្រឹមតែ ១–២ សប្តាហ៍ប៉ុណ្ណោះ។

ដើម្បីធានាដំណើរការវិភាគសម្ងួតមានប្រសិទ្ធភាព គួរប្រើឧបករណ៍អាំងគុយបាទ័រ (incubator) ដែលកំណត់សីតុណ្ហភាពសមស្របទៅនឹងថ្នាល Mounting ដែលកំពុងប្រើប្រាស់ដោយជៀសវាងកម្តៅលើសកម្រិតដែលអាចបង្កការខូចខាតដល់សំណាក។ ជួរសីតុណ្ហភាពណែនាំគឺ 30°C ដល់ 37°C។ ជំហានសម្ងួតនេះមានសារៈសំខាន់ ដើម្បីបង្ការការវៀចស្លាយ (slide warping) ការខូចខាតសំណាក ឬអស្ថេរភាពនៃថ្នាលmounting ក្នុងអំឡុងពេលរក្សាទុក។

ថ្នាល Mounting ដែលប្រើក្នុងការរៀបចំកញ្ចក់ឡាម ត្រូវតែបញ្ជាក់ជានិច្ចលើស្លាកកញ្ចក់ឡាម (slide label)។ ប្រសិនបើអាចធ្វើបាន ស្លាកគួររួមបញ្ចូលរូបមន្តជាក់លាក់ដែលបានប្រើឈ្មោះអ្នករៀបចំ (preparator) និងកាលបរិច្ឆេទរៀបចំ។ ជាទូទៅ កញ្ចក់ឡាមត្រូវបានរៀបចំដំបូងជាការmountingបណ្តោះអាសន្ន (temporary mounts) មិនមានគោលបំណងសម្រាប់ការអភិរក្សរយៈពេលវែង។ ទោះយ៉ាងណា ប្រសិនបើស្ថានភាពសំណាកផ្លាស់ប្តូរ ដូចជាត្រូវបានកំណត់ជាផ្នែកមួយនៃស៊េរី "type" សម្រាប់វិទ្យាសាស្ត្រប្រភេទវិភាគ (taxonomic study) ត្រូវប្រើថ្នាលmountingថេរជាងមុន ដើម្បីធានាការអភិរក្សសម្រាប់ការសិក្សាពេលអនាគត។

5.5. បច្ចេកទេស Mount ជាជម្រើស៖ Card mounting

Card mounting គឺជាបច្ចេកទេសមួយដែលប្រើសម្រាប់ក្រុម sand flies ជាច្រើន ដោយអាចចាក់ម្ជុលភ្ជាប់សំណាកដោយផ្ទាល់លើកាតអេនតូម៉ូឡូស៊ី) entomological cards) ឬបិទភ្ជាប់លើផ្នែកកាត។ ទោះបីជាយ៉ាងណា ពីព្រោះ sand flies មានទំហំតូចខ្លាំងនិងត្រូវសង្កេតសរីរាង្គខាងក្នុងសម្រាប់ការកំណត់អត្តសញ្ញាណ តាមរយៈដំណើរការវិភាគធ្វើឲ្យថ្លា)clarification; សូមមើលចំណុច ៥(វិធីសាស្ត្រនេះមិនសមស្របឡើយសម្រាប់ការmounting sand flies ។

5.6. mountingឡើងវិញនៃសំណាកដែលខូចខាត)Remounting damaged specimens)

សម្រាប់សំណាកកម្រ ឬមានតម្លៃខ្ពស់គេណែនាំឲ្យអនុវត្តវិធីសាស្ត្រពីរដំណាក់កាល

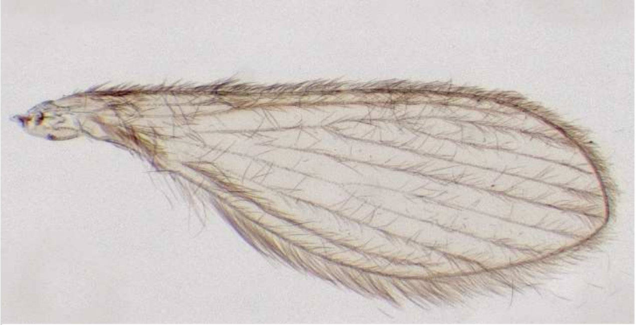

រូបភាពទី ៨៖ ស្លាបដើម) raw wing) របស់ *Trichophoromyia ininii*។

ដូចបានបង្ហាញក្នុងវីដេអូដែលអាចចូលប្រើបានតាមតំណភ្ជាប់៖ https://zenodo.org/records/18315029 ១(ការបង្កើនសំណើមឡើងវិញ) ការដកជាតិទឹកឡើងវិញ) ដោយមិនបំបែកកញ្ចក់ឡាម គួរដាក់ឧបករណ៍ទ្រាប់សម្រាប់កញ្ចក់ឡាមមីក្រូទស្សន៍ជាច្រើនក្នុងចាន Petri ដើម្បីធ្វើជាគ្រឿងទ្រាប់។ បន្ទាប់មកដាក់កញ្ចក់ឡាមដែលត្រូវបង្កើនសំណើមឡើងវិញនៅផ្នែកខាងលើ ហើយបន្ថែមអង្គធាតុរំលាយបន្តិចបន្តួច)កម្ពស់ប៉ុន្មានមីលីម៉ែត្រ (ក្នុងចាន Petri ដើម្បីបង្កើតបន្ទប់សើម) humid chamber) ដោយធានាថាកញ្ចក់ឡាមមិនប៉ះទង្គិចដោយផ្ទាល់ជាមួយ

អង្គធាតុរំលាយ) សូមមើល រូបភាពទី ៨ D)។ រយៈពេលត្រូវការសម្រាប់ការបង្កើនសំណើមឡើងវិញ អាចខុសគ្នាពីមួយថ្ងៃដល់ច្រើនថ្ងៃ អាស្រ័យលើស្ថានភាពសំណាក។ ត្រូវត្រួតពិនិត្យរៀងរាល់ថ្ងៃ និងមានការអត់ធ្មត់។ បន្ទាប់ពីកញ្ចក់ឡាមបានបង្កើនសំណើមគ្រប់គ្រាន់ អាចយកចេញពីបន្ទប់សើម ហើយដាក់ក្នុងអាំងគុយបាទ័រ រយៈពេលពីរបីម៉ោង មុនការពិនិត្យក្រោមមីក្រូទស្សន៍ ការថតរូប ឬការគូរការវិភាគលក្ខណៈរូបសាស្ត្រសាស្ត្រ។ ២ (ការmountingឡើងវិញ) Remounting) កញ្ចក់ឡាមអាចត្រូវបានដាក់ត្រឡប់ទៅបន្ទប់សើមបន្ថែមពីរបីម៉ោង ឬមួយយប់ មុនធ្វើការបំបែក។ ការបំបែកគួរអនុវត្តក្រោមមីក្រូទស្សន៍បីណូគុលារ)binocular microscope)។ ដោយប្រើម្ជុលល្អិត ត្រូវដោះគម្របឡាម៉ែលយ៉ាងប្រុងប្រយ័ត្នដើម្បីធានាថាគ្មានធាតុណាមួយនៃ Phlebotomine sand flies នៅជាប់លើគម្របកញ្ចក់។ បន្ទាប់មក ត្រូវប្រមូលសារធាតុដែលបានវះកាត់របស់ Phlebotomine sand flies រួចហើយលាងជាមួយទឹកក្នុងអាងតូចៗ) small wells) ដូចដែលប្រើសម្រាប់ការដកស្រង់ DNA/RNA ប្រភេទបំផ្លាញសំណាក) destructive extraction) មុនពេលបាត់ទឹក និងmountingឡើងវិញក្នុងថ្នាលរេស៊ីន។ នៅពេលបំបែកកញ្ចក់ឡាមមានសារៈសំខាន់ខ្លាំងក្នុងការកំណត់អត្តសញ្ញាណថ្នាល mounting ដើម្បីជ្រើសរើសរាវរំលាយសមស្រប។

– សម្រាប់ថ្នាលមានមូលដ្ឋានលើទឹក ត្រូវប្រើទឹក។
– ប្រសិនបើថ្នាលមានមូលដ្ឋានលើរេស៊ីន) ឧទាហរណ៍ Canada balsam ឬ Euparal®) ត្រូវប្រើ xylene ក្រោមទូរបង្ហូរខ្យល់ពុល) fume hood) និងត្រូវពាក់ឧបករណ៍ការពារផ្ទាល់ខ្លួនសមស្រប រួមទាំងម៉ាស់ការពារ។

ការ mounting ឡើងវិញនៃសំណាក“ type” ឬសំណាកក្នុងសារមន្ទីរត្រូវអនុវត្តតែប៉ុណ្ណោះក្រោយពេលទទួលបានការយល់ព្រមពីអ្នកថែរក្សាសំណុំសំណាក) curator) និង/ឬស្ថាប័នដែលកាន់កាប់សំណាកនោះ។

6. ការកំណត់អត្តសញ្ញាណសំណាក (Specimen identification)

6.1. រូបសាស្ត្រ(Morphology)

ការកំណត់អត្តសញ្ញាណ sand flies អាស្រ័យជាចម្បងលើការពិនិត្យលក្ខណៈរូបសាស្ត្ររួមមានរូបរាង thorax ស្លាប ប្រដាប់ភេទ) genitalia) រោម (setae) និងទំនាក់ទំនងម៉ុរហ្វូមេត្រី) morphometric relationships) រវាងរចនាសម្ព័ន្ធផ្សេងៗ។ អ្នកស្រាវជ្រាវប្រើសោរចនានុក្រមតាក់សូណូមី) taxonomic keys) សំណុំសំណាកយោង) reference collections) និងការពិពណ៌នាប្រភេទដើម ដើម្បីប្រៀបធៀបសំណាកដែលប្រមូលបានជាមួយតាក់សុងដែលបានស្គាល់រួច។ លក្ខណៈវិនិច្ឆ័យសំខាន់ៗ ដូចជាលំនាំសរសៃស្លាប) wing venation) និងម័រហ្វូឡូស៊ីក្បាល នៅទាំងភេទប្រុស និងញីរចនាសម្ព័ន្ធប្រដាប់ភេទភេទឈ្មោល និងរចនាសម្ព័ន្ធថង់ផ្ទុកទឹកកាមញី (spermathecae) ភេទញីមានសារៈសំខាន់ខ្លាំងសម្រាប់ការកំណត់ប្រភេទ។ ការកំណត់អត្តសញ្ញាណឲ្យបានត្រឹមត្រូវ តម្រូវឲ្យមានការពិនិត្យក្រោមមីក្រូទស្សន៍យ៉ាងលម្អិត ជាទូទៅប្រើមីក្រូទស្សន៍សមាស) compound microscope) សម្រាប់សង្កេតរចនាសម្ព័ន្ធល្អិតដូចជា genitalia និងថង់ផ្ទុកទឹកកាមញី (spermathecae) ឬប្រើ stereomicroscope សម្រាប់លក្ខណៈម័រហ្វូឡូស៊ីទូលំទូលាយ។ ការរីកចម្រើនបច្ចេកវិទ្យារូបភាពឌីជីថល បានជួយសម្រួលការប្រើរូបភាពគុណភាពខ្ពស់ ឬគំនូរឌីជីថលនៃលក្ខណៈសំខាន់ៗ សម្រាប់ប្រៀបធៀបជាមួយឯកសារយោង ឬវិភាគតាមប្រព័ន្ធកុំព្យូទ័រជំនួយការកំណត់អត្តសញ្ញាណ ដែលបង្កើនភាពត្រឹមត្រូវ និងភាពងាយស្រួលក្នុងតាក់សូណូមីម័រហ្វូឡូស៊ី។

6.2. ការសិក្សាពីការតំរៀបសរសៃនៅលើស្លាប) Wing geometry)

ការសិក្សាពីការតំរៀបសរសៃនៅលើស្លាប ជាលក្ខណៈសំខាន់មួយសម្រាប់ការកំណត់អត្តសញ្ញាណ និងចាត់ថ្នាក់ប្រភេទ sand flies ផ្សេងៗ។ ស្លាបរបស់ sand flies មានលំនាំនិងរចនាសម្ព័ន្ធពិសេស ជាទូទៅវែងនិងតូចចង្អៀត មានសរសៃវែននៃស្លាបអភិវឌ្ឍល្អ) រូបភាពទី ៩ និង ១០(។ ការតំរៀបសរសៃនៃស្លាបបង្កើតលំនាំពិសេស ដែលអាចខុសគ្នាតាមពួក (genus) និង ប្រភេទ (species) ដែលផ្តល់លក្ខណៈវិនិច្ឆ័យពេញលេញសម្រាប់ការកំណត់អត្តសញ្ញាណ។ ដូច្នេះការសិក្សាពីការតំរៀបសរសៃនៅលើស្លាបមានសារៈសំខាន់សម្រាប់តាក់សូណូមី។

6.3. ជីអូមេត្រីម៉ុរហ្វូមេត្រីស្លាប) Wing geometric morphometrics)

អ្នកស្រាវជ្រាវប្រើបច្ចេកទេសជាច្រើន ដូចជា geometric morphometrics ដើម្បីវិភាគ និងប្រៀបធៀបរាង និងទំហំស្លាប រវាងប្រភេទ ឬប្រជាសាស្ត្រ sand flies ផ្សេងៗ។ ការសិក្សាពីការតំរៀបសរសៃនៅលើស្លាប អាចផ្តល់ព័ត៌មានសំខាន់អំពីឥរិយាបថ ជម្រើសលំនៅដ្ឋាន និងសមត្ថភាពហោះហើរ។ ក្នុងវិធីសាស្ត្រ geometric morphometrics ស្លាបត្រូវបានវះកាត់យ៉ាងប្រុងប្រយ័ត្ន ពណ៌ស្នាម) stained) ប្រសិនបើចាំបាច់ ហើយ Mounting ផ្ទាបលើកញ្ចក់ឡាម។

បន្ទាប់មក កញ្ចក់ឡាមត្រូវថតរូបក្រោម stereomicroscope បំលែងជាទិន្នន័យឌីជីថល និងវិភាគម័រហ្វូមេត្រី។ អនុវត្តន៍នេះត្រូវបានពិពណ៌នាយ៉ាងលម្អិតក្នុងអត្ថបទយោង] 6, 27, 42, 56, 57, 59] ហើយណែនាំឲ្យប្រើស្លាបខាងស្តាំ ឬខាងឆ្វេងជាប់លាប់ សម្រាប់សរីរាង្គគូ ដើម្បីជៀសវាងផលប៉ះពាល់ allometry អវិជ្ជមាន] 62]។

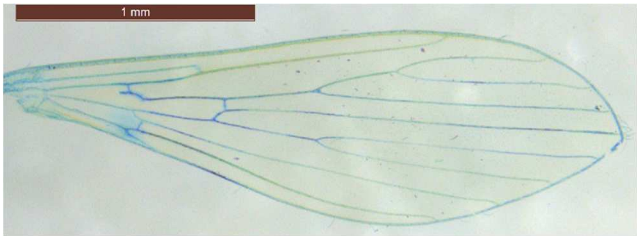

រូបភាពទី ១០៖ ស្លាបមានពណ៌ របស់ *Phlebotomus ariasi*។

ការរៀបចំស្លាបសម្រាប់ geometric morphometric analysis

ដើម្បីឲ្យមើលឃើញសរសៃនៅលើស្លាបបានច្បាស់ ស្លាបត្រូវសម្អាតស្រទាប់ស្រកា) scales(និងពណ៌ស្នាម។ ដំបូងត្រូវបំពេញអាងតូច) small wells) ដោយសមាសធាតុចាំបាច់) methylene blue អេតានុល ទឹក និង xylene substitute)។ យកស្លាបដែលរក្សាទុកក្នុងអេតាណុល 70% នៅសីតុណ្ហភាពបន្ទប់ដោយក្រឡុកបំពង់ទីប Eppendorf ហើយចាក់លើអាង បន្ទាប់មកយកស្លាបឡើងដោយម្ជុលកោងល្អិត។ ផ្លាស់ប្តូរស្លាបពីអេតាណុលទៅទឹក ហើយត្រឡប់ទៅអេតាណុលវិញ ដើម្បីយក bristles ចេញ។ ដាក់ស្លាបក្នុង methylene blue រយៈពេល ៦ នាទីដោយជានាថាវាអណ្តែត។ បន្ទាប់មកយកស្លាបដាក់ក្នុង xylene substitute រយៈពេល ២ នាទី) ប្រហែលមួយភាគបីនៃពេល methylene blue) ដើម្បីធ្វើការតំរូវពណ៌។ ចុងក្រោយដាក់ស្លាបលើបន្ទះ Euparal® តូចមួយលើកញ្ចក់ឡាម ហើយបើកស្លាបយ៉ាងប្រុងប្រយ័ត្នក្រោមកែវពង្រីកដាក់ឡាម៉ែល។ ត្រូវថតរូបឲ្យបានឆាប់មុនពេល Euparal® កក ព្រោះអាចត្រូវកែតម្រូវទីតាំងស្លាបបន្តិច។

6.4. បច្ចេកទេសម៉ូលេគុលជីវសាស្ត្រ (Molecular biology techniques)

ក្រៅពីលក្ខណៈរូបសាស្ត្រ វិធីសាស្ត្រម៉ូលេគុលជីវសាស្ត្រកាន់តែមានសារៈសំខាន់ក្នុងស្រាវជ្រាវទាក់ទងនឹង sand flies រួមមានវិទ្យាសាស្ត្រកំណត់ប្រភេទ គាក់សូល្ហមីហ្សែននៃប្រជាសាស្ត្រភីឡូហ្សេនី ការរកវត្តមាន DNA/RNA មេរោគបង្ករជម្ងឺ ការកំណត់ប្រភពឈាម និងឥរិយាបថរបស់ភ្នាក់ងារចម្លងជម្ងឺ ក្នុងវិស័យនៃវិទ្យាសាស្ត្រសិក្សាអំពីជម្ងឺឆ្លង [70]។

DNA sequencing អាចប្រើសម្រាប់បញ្ជាក់ប្រភេទ ឬញែកប្រភេទណាដែលលក្ខណៈរូបរាងស្រដៀងគ្នាខ្លាំង ដែលផ្តល់ភាពត្រឹមត្រូវខ្ពស់។ បច្ចេកទេសដូចជា PCR, DNA sequencing, NGS និង MALDI-ToF MS កំពុងកើនសារៈសំខាន់សម្រាប់ការកំណត់អត្តសញ្ញាណលឿន និងត្រឹមត្រូវ [46] ។ ទោះយ៉ាងណា ការសិក្សាលក្ខណៈរូបរាងម័រហ្វូឡូស៊ីនៅតែជាមូលដ្ឋានស្តង់ដារសម្រាប់វិទ្យាសាស្ត្រកំណត់ប្រភេទ គាក់សូល្ហមី។

6.4.1. ការដកអាស៊ីតនុយក្លេអ៊ីច (Destructive nucleic acid extraction)

ការដកអាស៊ីតនុយក្លេអ៊ីចគឺជាវិធីសាស្ត្រពេញនិយមមួយក្នុងការសិក្សាជីវវិទ្យា ហើយមានវិធីសាស្ត្រផ្សេងៗដែលបានអភិវឌ្ឍឡើងសម្រាប់ការផ្តាច់ DNA ពីសម្ភារៈជីវចម្រុះ [48]។ កញ្ចប់ដក DNA ជាច្រើនត្រូវបានរចនាឡើងដើម្បីជួយសម្រួលដំណើរការវិភាគនេះ [14]។ ទោះយ៉ាងណា វិធីសាស្ត្រដែលប្រើជាធម្មតាសម្រាប់រៀបចំសំណុំអត្ថរូបសស សម្រាប់ការកំណត់សម្ភារៈតាមការវិភាគលក្ខណៈរូបសាស្ត្រ អាចរាំងខ្ទប់ការវិភាគ DNA ព្រោះបច្ចេកទេសទាំងនេះអាចបំផ្លាញឬបំផ្លាញលក្ខណៈរាងកាយសំខាន់ៗនៃសម្ភារៈ [10]។ ប្រភេទនិងសភាពសម្ភារៈមានតួនាទីសំខាន់ក្នុងការជ្រើសរើសវិធីសាស្ត្រដក DNA ឱ្យសមស្រប [29]។

តម្រូវការសម្រាប់ការកំណត់ Sand files យ៉ាងត្រឹមត្រូវ ការយល់ដឹងអំពីសមាមាត្រពូជ និងកាត់បន្ថយហ្សែនមិនត្រូវការ ដូចនេះជំរុញឱ្យមានការអភិវឌ្ឍលើឧបករណ៍ធ្វើរោគវិនិច្ឆ័យសម្រាប់ម៉ូលេគុលជីវសាស្ត្រ [23]។ វិធីសាស្ត្រម៉ូលេគុលជីវសាស្ត្របានប្រើជាញឹកញាប់ដើម្បីបន្ថែមជាមួយវិធីសាស្ត្រទូទៅក្នុងវិទ្យាសាស្ត្រដើម្បីកំណត់ប្រភេទសត្វ (taxonomic) សម្រាប់ការកំណត់ Sand files ។ ឧទាហរណ៍វិធីស្តង់ដាសម្រាប់ insect barcoding គឺអាចរាប់បញ្ចូលការដក DNA ការលំអិតហ្សែន និងការខូចខាតសំណាកដើម។ ដូច្នេះ មានការតម្រូវយ៉ាងខ្លាំងក្នុងការស្រាវជ្រាវវិធីសាស្ត្រដក DNA ដែលមិនបំផ្លាញ (non-destructive) ដើម្បីរក្សាទុកទាំងសម្ភារៈជីវសាស្ត្រ និងភាពសំខាន់របស់រាងកាយ។

វិធីសាស្ត្រដកអាស៊ីតនុយក្លេអ៊ីចជាច្រើនត្រូវបានអនុវត្តលើ Sand files ។ បរិមាណឬគុណភាពនៃអាស៊ីតនុយក្លេអ៊ីចដែលត្រូវការអាស្រ័យលើវិភាគម៉ូលេគុលជីវសាស្ត្រនៅលំហាក្រោយ ពីព្រោះបច្ចេកទេសផ្សេងៗមានសមត្ថភាព និងតម្រូវការសុទ្ធភាពខុសគ្នា [9]។ ឧទាហរណ៍ ភ្នែក Sand files ត្រូវបានរកឃើញអាចរាំងការស្វ័យដំឡើងទ្វេ PCR

[69]។ លើសពីការត្រួតពិនិត្យភ្នាក់ងារបង្ករោគ DNA Sand files ត្រូវបានដកជាប្រចាំសម្រាប់កំណត់ប្រភេទ។ វិធីសាស្ត្រដក DNA ផ្សេងៗអាចប្រើបាន ទោះបីជាបរិមាណ និងគុណភាពខុសគ្នានៅក្នុងបច្ចេកទេសផ្សេងៗ។ ប៉ុន្តែវិធីសាស្ត្រដែលអ្នកផលិតបានបង្កើត ត្រូវបានអ្នកស្រាវជ្រាវបម្លែងសម្រាប់ Sand files [8] ដើម្បីបង្កើនបរិមាណ និង/ឬគុណភាពនៃអាស៊ីតនុយក្លេអ៊ិចដែលបានដក [8, 9, 69]។ ការបម្លែងផ្សេងទៀតដែលបានអភិវឌ្ឍសម្រាប់អត្ថរ៉ុបូសផ្សេងទៀតក៏អាចប្រើសម្រាប់ Sand files បានផងដែរ [58, 76]។ PCR សម្រាប់កំណត់ប្រភេទដោយកំណត់គោលដៅលើបំណែកហ្សែនមីតូកុងទ្រី (COI ឬ CytB) ជាទូទៅសមស្របទៅជាមួយវិធីសាស្ត្រដក DNA ដែលមានការបំបែកបំណែក DNA កម្រិតខ្ពស់។ ប៉ុន្តែ NGS បែប long-read (Oxford Nanopore និង PacBio) តម្រូវឱ្យមានកម្រិតបំបែកបំណែក DNA តិច និងគុណភាព DNA ខ្ពស់។ ការដក DNA ជាមូលដ្ឋានប្រើ spin column ជាទូទៅផ្តល់បរិមាណជារូបតំណាង DNA genomic ដល់ប្រហែល 60 kb ខណៈដែល phenol-chloroform អាចផ្តល់បានដល់ 150 kb [77]។ តារាង 5 សង្ខេបពីវិធីសាស្ត្រផ្សេងៗក្នុងការដក DNA Sand file និងបង្ហាញថាតើមានការបម្លែងវិធីសាស្ត្រសម្រាប់សត្វទាំងនេះ ឬអត្ថរ៉ុបូសតូចផ្សេងៗឬទេ។ បរិមាណមិនត្រូវបង្ហាញ ព្រោះវាអាស្រ័យលើទំហំសំណាក និងវិធីសាស្ត្ររៀបចំ។ ជួរបម្លែងសង្ខេបបញ្ជាក់ពីការបម្លែងនៃប្រព័ន្ធការដក DNA សម្រាប់ Sand file ឬអត្ថរ៉ុបូសតូចផ្សេងៗ។

ការជ្រើសរើសវិធីសាស្ត្រដក DNA គួរត្រូវគិតគូរជាមួយលក្ខខណ្ឌជាច្រើនដូចជា ចំនួនសំណាក ពេលវេលាដក និងបច្ចេកទេសដែលប្រើនៅលំហក្រោយ (downstream)។ ខណៈដែល NGS តម្រូវឲ្យមានទម្ងន់ម៉ូលេគុលរបស់ DNA genomic ក្នុងកម្រិតខ្ពស់ វិធីសាស្ត្រទាំងអស់នៅទីនេះអាចប្រើសម្រាប់កម្មវិធី PCR ធម្មតា។ លើសពីនេះក៏មានការសិក្សាច្រើនបានស្រាវជ្រាវវិធីសាស្ត្រដក DNA ដោយមិនបំផ្លាញសម្រាប់អត្ថរ៉ុបូសដីតូច សំណុំសត្វសារមន្ទីររក្សាឯកសារស្ងួត និងអត្ថរ៉ុបូសរាងខ្សែទន់ [19, 26, 28, 55, 63]។

តារាង 5: តម្លៃថ្នាល ការប្រើប្រាស់ និងការបម្លែងប្រព័ន្ធសម្រាប់ការដក gDNA របស់សត្វស្បែកខ្សាច់ phlebotomine.

ប្រូតូកូល	ថ្លៃដើម	កម្មវិធីប្រើប្រាស់	ការកែសម្រួលប្រូតូកូលសម្រាប់arthropodsតូចៗ
ជួរឈរបង្វិល)Spin column)	២ – ៥. ៥៥.៣ ដុល្លារអាមេរិក[៣៩[	PCR, NGS	[៩[
ហ្វេណុល-លរ៉ូហ្វម្◌ក (Phenol-chloroform)	០.២៤ល្លារអាមេរិក[៦៩[	PCR, NGS	[៩[
ហត់ស្ហូត់)HotSHOT)	< ០ ០១. ដុល្លារអាមេរិក[៦៩[	PCR	-
សាល់ធីងអៅត៍)Salting out)	០ ១២. ដុល្លារអាមេរិក[៦៩[	PCR	-
ឆេឡិច)Chelex)	០ ០២. ដុល្លារអាមេរិក[៤១[	PCR	[៤១, ៧៦[

6.4.2. ការដកអាស៊ីតនុយក្លេអ៊ីចមិនបំផ្លាញសំណាក (Non-destructive nucleic acid extraction)

មួយក្នុងចំណោមបញ្ហាសំខាន់ក្នុងវិភាគម៉ូលេគុលជីវនៃអត្ថរ៉ុបូស ជាពិសេស Sand file គឺការរក្សាសម្ភារៈសម្រាប់បញ្ចូលក្នុងបណ្ណាល័យសត្វវិទ្យា។ វិធីសាស្ត្រដក DNA ភាគច្រើនតម្រូវឱ្យ maceration ឬបំផ្លាញសំណាក ដូច្នេះអាចប៉ះពាល់ដល់ការរក្សាសំណាកដើម។ ទោះយ៉ាងណា វិធីសាស្ត្រដកអាស៊ីតនុយក្លេអ៊ិចដោយមិនបំផ្លាញ) non-destructive) ត្រូវបានរចនាឡើងដើម្បីដកសម្ភារៈជាតិសារព genética ដោយមិនបំផ្លាញរាងកាយសំណាក ទោះបីជាការប៉ះពាល់ដល់ជីវភាព) viability) ឬបម្លែងរាងកាយ) morphology) នោះក៏ដោយ។ វិធីសាស្ត្រទាំងនេះមានតម្លៃពិសេសពេលប្រើលើសំណាកមានតម្លៃ ឬមានកំណត់ ដូចជា Sand file ដែលការរក្សាភាពស្ថិតស្ថេរ និងរចនាសម្ព័ន្ធគឺសំខាន់សម្រាប់ការសិក្សាកំណត់ប្រភេទ)taxonomy) រាងកាយវិទ្យា) morphology) ឬគោលបំណងរោគវិនិច្ឆ័យ) diagnostic purposes) នៅអនាគត។ វិធីសាស្ត្រដែលប្រើជាញឹកញាប់មួយគឺវិធី non-destructive bathing ដែល Sand file ត្រូវបានដាក់អោយស្ងៀម ហើយត្រូវហូបក្នុង lysis buffer ដែលមាន proteinase K។

វិធីសាស្ត្រ mild-vectolysis ត្រូវបានអនុវត្តជោគជ័យលើ Sand file ជាពិសេសសម្រាប់ប្រភេទសំណាក [24]។ វិធីសាស្ត្រនេះប្រើឧបករណ៍ spin column ជាទូទៅ)ក្នុងករណីនេះ គឺ DNeasy Blood and Tissue kit, QIAGEN, Hilden, Germany) ដែលបានបម្លែងសម្រាប់ទទួល DNA ដោយមិនបំផ្លាញសំណាក។ ជំហាន lysis ដែលបានផ្លាស់ប្តូរ)បរិមាណ lysis buffer និងបន្ថែមជំហានបង្អក] 17]) អាចអនុញ្ញាតឱ្យអាស៊ីតនុយក្លេអ៊ិចត្រូវបានបញ្ចេញដើម្បីកាត់បន្ថយការខូចខាតរូបរាងកាយ] 24]។

សម្រាប់ Sand file ក៏អាចប្រើវិធីសាស្ត្រ HotSHOT DNA Extraction kit (Bento Bioworks Ltd, London, United Kingdom) [73] បានផងដែរ ដែលមានដំណើរការលឿនរហ័ស និងមានថ្លៃសមរម្យ។ សម្ភារៈអត្ថរ៉ុបូសដែលមានគោលបំណងសម្រាប់ការកំណត់រាងកាយអាចស្លក់ស្អាតក្រោយពេលដំណើរការវិភាគ។ សំណាកដែលប្រើ DNeasy Blood and Tissue kit ត្រូវបានសម្អាតដោយ Marc-André solution ខណៈដែលសំណាកដែលប្រើ HotSHOT DNA Extraction kit ត្រូវបានសម្អាតគ្រប់គ្រាន់សម្រាប់ mount ក្នុងម៉េឌៀមទឹក)aqueous medium) ឬល្អជាងនេះគឺក្នុង resin បន្ទាប់ពីការសម្ងួត) dehydration) តាមប្រព័ន្ធលំអិតក្នុងអត្ថបទនេះ] 73]។ សេនេទិចដែលបានដកអាចត្រូវបានដំណើរការវិភាគ downstream ដូចជា PCR ដើម្បីបង្កើនសញ្ញាសម្បត្តិ DNA ជាក់លាក់។ វិធីសាស្ត្រដកអាស៊ីតនុយក្លេអ៊ិចដោយមិនបំផ្លាញសំណាក មានសារៈសំខាន់ក្នុងការសិក្សាសេនេទិចរបស់ Sand file រួមទាំងការកំណត់ភ្នាក់ងារបង្ករោគដែលអាចមានក្នុងសត្វទាំងនេះផងដែរ។ ដោយរក្សាភាពស្ថិតស្ថេររបស់សំណាក អ្នកស្រាវជ្រាវអាចទទួលបានព័ត៌មានសេនេទិចដែលមានតម្លៃ ខណៈដែលរក្សាសំណាកសម្រាប់ការវិភាគបន្ថែម ឬការសិក្សា។

6.5. MALDI-ToF MS

MALDI-ToF MS (matrix-assisted laser desorption/ionization time-of-flight mass spectrometry) គឺជាបច្ចេកវិទ្យាផ្អែកលើម៉ាសស្ថិចត្រូម៉េត្រី ដែលបង្កើតឡើងសម្រាប់ស្កេននិងវិភាគប្រូតេអ៊ីន)“ស្នាមម្រាមដៃ”) របស់សំណាកជីវសាស្ត្រ។ បច្ចេកវិទ្យា MALDI-ToF កំពុងទទួលស្គាល់ថាជាឧបករណ៍សំខាន់សម្រាប់ការកំណត់ប្រភេទអត្ថរ៉ុបូសដែលមានសារៈសំខាន់ក្នុងវេជ្ជសាស្ត្រ និងវេជ្ជសាស្ត្រសត្វ។ បច្ចេកវិទ្យានេះបានបង្ហាញប្រសិទ្ធភាពក្នុងការកំណត់វគ្គអភិវឌ្ឍន៍របស់ Phlebotomine sand flies រួមទាំងទម្រង់មិនពេញវ័យ និងមូសញីដែលបំពេញដោយឈាម ហើយត្រូវបានអនុវត្តដោយជោគជ័យក្នុងការបំបែកមូស

ញី និងមូសឈ្មោលក្រោមលក្ខខ័ណ្ឌស្តុកនិងការ Homogenize [28, 30, 73, 74]។ វិធីសាស្ត្រនេះផ្តល់នូវសមត្ថភាពបែងចែកខ្ពស់នៅលើកម្រិតជាអនុពពួក) subgenera), ប្រភេទ) species), និងប្រជាជន)populations)។ បច្ចេកវិទ្យានេះអនុញ្ញាតឱ្យអ្នកស្រាវជ្រាវសម្រេចបានការកំណត់ប្រភេទយ៉ាងលឿន និងត្រឹមត្រូវដែលមានសារៈសំខាន់សម្រាប់ការយល់ដឹងអំពីការប្រមូលផ្តុំរបស់ Phlebotomine sand flies យុទ្ធសាស្ត្រផ្ទាល់ខ្លួន និងតួនាទីរបស់ពួកវានៅក្នុងការចម្លងជម្ងឺ។

ដោយភាពខុសគ្នារបស់ប្រភេទសត្វផ្អែកលើប្រូតេអ៊ីន MALDI-ToF មានតួនាទីសំខាន់ក្នុងការសិក្សាអេពីដេមីសាស្ត្រ និងយុទ្ធសាស្ត្រគ្រប់គ្រងភ្នាក់ងារចម្លងជម្ងឺ។ មានកង្វះខាតសំខាន់ពីរដែលកំណត់ការប្រើប្រាស់ប្រចាំថ្ងៃរបស់វិធីសាស្ត្រនេះ៖ ១ (គ្រឿងម៉ាសស្ពិចត្រូម៉េត្រមានតម្លៃខ្ពស់ និងពិបាកទិញសម្រាប់កំណត់ប្រភេទPhlebotomine sand flies) ឬភ្នាក់ងារចម្លង arthropod ជាទូទៅ (២ (ទិន្នន័យយោងអំពីPhlebotomine sand flies មានតិចក្នុងមូលដ្ឋានទិន្នន័យសាធារណៈដែលចាំបាច់ត្រូវបង្កើតមូលដ្ឋានទិន្នន័យផ្ទាល់ខ្លួនដោយប្រើស្តេនស្រ្តបផ្ញើ reference spectra ពីសំណាកដែលកំណត់យ៉ាងច្បាស់រួចភ្ជាប់ការវាយតម្លៃ morphological និងការតម្រៀប sequencing នៃ genetic marker សមស្រប) COI, cytB, ឬផ្សេងទៀត(។ ការកំណត់នេះអាចត្រូវបានដោះស្រាយដោយការរួមបញ្ចូលទិន្នន័យយោង Phlebotomine sand flies ផ្ទាល់ខ្លួនទៅក្នុង MSI Platform ដែលគ្រប់គ្រងដោយ Assistance Publique-Hôpitaux de Paris, Sorbonne University, France និងការប្រមូលផ្តុំ BCCM/IHEM/Sciensano នៅ Brussels, Belgium (https://msi.happy-dev.fr/)។

នៅពេលផែនការវិភាគប្រូតេអ៊ីន MALDI-ToF ត្រូវបានអនុវត្ត សំណាកគួរត្រូវបានរក្សាទុកជាបង្កកស្ងួត)dry-frozen(ឬនៅក្នុងអេតាណុល 70% នៃ molecular grade ហើយមិនត្រូវប្រើសីតុណ្ហភាពបរិយាកាស។ ប្រសិនបើគ្មានការណែនាំសាកលសម្រាប់រៀបចំសំណាក, អ្នកប្រើប្រាស់ត្រូវបានផ្តល់យោគបល់ឱ្យប្រើ aqueous solution 60% acetonitrile/0.3% TFA របស់ sinapinic acid (30 mg/mL) សម្រាប់ការរៀបចំ MALDI-ToF ដើម្បីធានាថា spectra ប្រូតេអ៊ីនអាចប្រៀបធៀបបានជាមួយទិន្នន័យដែលបានបោះពុម្ពមុននេះសម្រាប់ Phlebotomine sand flies។

ការត្រៀមសំណាកសម្រាប់ MALDI-ToF MS (រូបភាព 7) សំណាកសត្វល្អិត ដែលបានរក្សាទុកក្រោមលក្ខខ័ណ្ឌផ្សេងៗ ត្រូវបានធ្វើអោយស្ងួតក្នុងខ្យល់នៅសីតុណ្ហភាពបន្ទប់ មុននឹងធ្វើការ dissect។ ក្បាល និងពោះត្រូវបានដកចេញដើម្បីទទួលបានផ្នែករាងកាយដែលមានលក្ខណៈ morphological សំខាន់សម្រាប់ការ mount លើកញ្ចក់ឡាម និងវិភាគ morphological។ ផ្នែកទ្រូងអាចប្រើសម្រាប់ MALDI-ToF ហើយពោះដែលនៅសល់ត្រូវបានរក្សាទុកសម្រាប់ការដក DNA។ សម្រាប់ protein profiling, ផ្នែកទ្រូងត្រូវបានកិនដោយដៃក្នុង microtubes 1.5 mL ជាមួយ 10 µL នៃសារធាតុរាវសម្រាប់ homogenization ដោយប្រើ pestles និង pellets ដែលអាចបោះចោលបាន។ ជាទូទៅ មានសារធាតុរាវសម្រាប់ homogenization ពីរដែលប្រើ៖ ទឹកស្ត្រីល្អិត) sterile distilled water) និង 25% formic acid។

7. សេចក្តីសន្និដ្ឋាន

ក្នុងការស្រាវជ្រាវនេះ យើងមានគោលបំណងផ្តល់វិធីសាស្ត្រដែលមានប្រសិទ្ធភាពខ្ពស់សម្រាប់ការ mount sand flies ដែលបានប្តូរតាមគោលបំណងស្រាវជ្រាវជាក់លាក់ ដើម្បីជួយកំណត់ប្រភេទ និងសំគាល់ជម្ងឺបានត្រឹមត្រូវ។ គ្មានវិធីសាស្ត្រមួយណាដែលល្អបំផុតនោះទេ ប៉ុន្តែមានវិធីសាស្ត្រច្រើន ដែលម្នាក់ៗមានអត្ថប្រយោជន៍ និងកម្រិតកំណត់ខ្លួនឯង។

នៅក្នុងទិន្នន័យយោង យើងបានផ្តល់នូវព្រឹត្តិការណ៍លម្អិត) protocols) សម្រាប់បច្ចេកទេស mount ផ្សេងៗ ដែលប្រើសម្រាប់ការរៀបចំ និងកំណត់ប្រភេទPhlebotomine sand flies។ ព្រឹត្តិការណ៍ទាំងនេះ រួមទាំងវីដេអូបង្រៀនផងផ្តល់នូវជំហានបណ្តោះអាសន្ន) step-by-step) ដែលផ្គូផ្គងតាមគោលបំណងផ្សេងៗ ដើម្បីធានាថាលទ្ធផលមានភាពត្រឹមត្រូវ និងអាចទុកចិត្តបាន។ ដោយផ្តល់ធនធានទាំងនេះ យើងមានគោលបំណងគាំទ្រក្រុមស្រាវជ្រាវក្នុងការជ្រើសរើស និងអនុវត្តបច្ចេកទេសតំឡើងសមស្របបំផុត សម្រាប់តម្រូវការជាក់លាក់របស់ពួកគេ។

សេចក្តីអរគុណ

អ្នកនិពន្ធសូមអរគុណ Richard Lane និង Zoe Jay Adams ពីសារមន្ទីរប្រវត្តិសាស្ត្រធម្មជាតិប្រទេសអង់គ្លេស)Natural History Museum of London, UK) សម្រាប់ការត្រួតពិនិត្យយ៉ាងល្អឥតខ្ចោះ ដែលបានបង្កើនគុណភាពនៃសេចក្តីស្រាវជ្រាវនេះ។

មូលនិធិ

យើងសូមទទួលស្គាល់អាជ្ញាធរអភិវឌ្ឍន៍ប្រទេសប្រេស៊ីល CNPq (លេខករណី៖404395 /2024-4) និង Araucária Foundation (លេខករណី៖433 / 2025PDI) ដែលបានផ្តល់មូលនិធិសម្រាប់ការស្រាវជ្រាវរបស់ AJA។

ជម្លោះផលប្រយោជន៍

Jérôme Depaquit ជា associate editor នៃ Parasite មិនបានចូលរួមក្នុងដំណើរការវិភាគពិនិត្យ និងសម្រេចចិត្តលើអត្ថបទនេះទេ។ អ្នកនិពន្ធផ្សេងទៀតប្រកាសថា មិនមានជម្លោះផលប្រយោជន៍។

សេចក្តីថ្លែងការណ៍អំពីភាពអាចរកបាននៃទិន្នន័យ

វីដេអូត្រូវបានរក្សាទុកនៅក្នុង Zenodo។

វីដេអូ ១ : https://zenodo.org/records/18198006
វីដេអូ ២ : https://zenodo.org/records/18311158
វីដេអូ ៣ : https://zenodo.org/records/18311106
វីដេអូ ៤ : https://zenodo.org/records/18311154
វីដេអូ ៥ : https://zenodo.org/records/18303014
វីដេអូ ៦ : https://zenodo.org/records/18302850
វីដេអូ ៧ : https://zenodo.org/records/18315029

ឯកសារបន្ថែម

ឯកសារបន្ថែមសម្រាប់អត្ថបទនេះអាចរកបាននៅ៖ https://www.parasite-journal.org/10.1051/parasite/2026009/olm

ឯកសារយោង

1. Alkan C, Allal-Ikhlef AB, Alwassouf S, Baklouti A, Piorkowski G, de Lamballerie X, Izri A, Charrel RN. 2015. Virus isolation, genetic characterization and seroprevalence of Toscana virus in Algeria. Clinical Microbiology and Infection, 21(11), 1040 e1-9.
2. Alten B, Ozbel Y, Ergunay K, Kasap OE, Cull B, Antoniou M, Velo E, Prudhomme J, Molina R, Banuls AL, Schaffner F, Hendrickx G, Van Bortel W, Medlock JM. 2015. Sampling strategies for phlebotomine sand flies (Diptera: Psychodidae) in Europe. Bulletin of Entomological Research 105(6), 664–678.
3. Ayhan N, Baklouti A, Prudhomme J, Walder G, Amaro F, Alten B, Moutailler S, Ergunay K, Charrel RN, Huemer H. 2017. Practical guidelines for studies on sandfly-borne phleboviruses: Part I: Important points to consider *ante* field work. Vector-Borne and Zoonotic Diseases 17(1), 73–80.
4. Bates PA. 1997. Infection of phlebotomine sandflies with *Leishmania*, in The Molecular Biology of Insect Disease Vectors: A Methods Manual. Springer. p. 112–120.5.
5. Baum M, de Castro EA, Pinto MC, Goulart TM, Baura W, Klisiowicz Ddo R, Vieira da Costa-Ribeiro MC. 2015. Molecular detection of the blood meal source of sand flies (Diptera: Psychodidae) in a transmission area of American cutaneous leishmaniasis, Parana State, Brazil. Acta Tropica, 143, 8–12.
6. Belen A, Alten B, Aytekin A. 2004. Altitudinal variation in morphometric and molecular characteristics of *Phlebotomus papatasi* populations. Medical and Veterinary Entomology, 18(4), 343–350.
7. Bhattacharya J, Chandra G, Hati AK. 1991. A simple method for cryopreservation of *Leishmania donovani* promastigotes, Indian Journal of Medical Research, 93, 245–246.
8. Caligiuri LG, Sandoval AE, Miranda JC, Pessoa FA, Santini MS, Salomón OD, Secundino NF, McCarthy CB. 2019. Optimization of DNA extraction from individual sand flies for PCR amplification. Methods and Protocols, 2(2), 36.
9. Casaril AE, de Oliveira LP, Alonso DP, de Oliveira EF, Gomes Barrios SP, de Oliveira Moura Infran J, Fernandes WS, Oshiro ET, Ferreira AMT, Ribolla PEM, de Oliveira AG. 2017. Standardization of DNA extraction from sand flies: Application to genotyping by next generation sequencing. Experimental Parasitology, 177, 66–72.
10. Castalanelli MA, Severtson DL, Brumley CJ, Szito A, Foottit RG, Grimm M, Munyard K, Groth DM. 2010. A rapid non-destructive DNA extraction method for insects and other arthropods. Journal of Asia-Pacific Entomology, 13(3), 243–248.
11. Cerqueira NL. 1943. Um novo meio para montagem de pequenos insetos em lâmina. Memórias do Instituto Oswaldo Cruz, (39), 37–41.
12. Charrel RN, Gallian P, Navarro-Mari JM, Nicoletti L, Papa A, Sanchez-Seco MP, Tenorio A, de Lamballerie X. 2005. Emergence of Toscana virus in Europe. Emerging Infectious Diseases, 11(11), 1657–1663.
13. Chaskopoulou A, Giantsis IA, Demir S, Bon MC. 2016. Species composition, activity patterns and blood meal analysis of sand fly populations (Diptera: Psychodidae) in the metropolitan region of Thessaloniki, an endemic focus of canine leishmaniasis. Acta Tropica, 158, 170–176.
14. Chen H, Rangasamy M, Tan SY, Wang H, Siegfried BD. 2010. Evaluation of five methods for total DNA extraction from western corn rootworm beetles. PLoS One, 5(8), e11963.
15. Depaquit J, Grandadam M, Fouque F, Andry PE, Peyrefitte C. 2010. Arthropod-borne viruses transmitted by Phlebotomine sandflies in Europe: a review. Eurosurveillance, 15(10), 19507.
16. Diamond LS, Herman CM. 1954. Incidence of Trypanosomes in the Canada Goose as revealed by bone marrow culture. Journal of Parasitology, 40(2), 195–202.
17. Ding H, Torno M, Vongphayloth K, Ng G, Tan D, Sng W, Ho K, Randrianambinintsoa FJ, Depaquit J, Tan CH. 2025. Hidden in plain sight: discovery of sand flies in Singapore and description of four species new to science. Parasites & Vectors, 18(1), 402.
18. Es-Sette N, Ajaoud M, Bichaud L, Hamdi S, Mellouki F, Charrel RN, Lemrani M. 2014. *Phlebotomus sergenti* a common vector of *Leishmania tropica* and Toscana virus in Morocco. Journal of Vector Borne Diseases, 51(2), 86–90.
19. Favret C. 2005. A new non-destructive DNA extraction and specimen clearing technique for aphids (Hemiptera). Proceedings of the Entomological Society of Washington, 107(2), 469–470.
20. Galati EAB. 2018. Phlebotominae (Diptera, Psychodidae): Classification, morphology and terminology of adults and identification of American taxa, in Brazilian Sand Flies: Biology, Taxonomy, Medical Importance and Control, Rangel EF, Shaw JJ, Editors. Cham: Springer International Publishing. pp. 9–212.
21. Galati EAB, de Andrade AJ, Perveen F, Loyer M, Vongphayloth K, Randrianambinintsoa FJ, Prudhomme J, Rahola N, Akhoundi M, Shimabukuro PHF, Depaquit J. 2025. Phle-botomine sand flies (Diptera, Psychodidae) of the world. Parasites & Vectors, 18(1), 220.
22. Galati EAB, Galvis-Ovallos F, Lawyer P, Leger N, Depaquit J. 2017. An illustrated guide for characters and terminology used in descriptions of Phlebotominae (Diptera, Psychodidae). Par-asite, 24, 26.
23. Gariepy T, Kuhlmann U, Gillott C, Erlandson M. 2007. Parasitoids, predators and PCR: the use of diagnostic molecular markers in biological control of Arthropods. Journal of Applied Entomology, 131(4), 225–240.
24. Giantsis IA, Chaskopoulou A, Bon MC. 2016. Mild-Vectolysis: A nondestructive DNA extraction method for vouchering sand flies and mosquitoes. Journal of Medical Entomology, 53(3), 692–695.
25. Gidwani K, Picado A, Rijal S, Singh SP, Roy L, Volfova V, Andersen EW, Uranw S, Ostyn B, Sudarshan M, Chakravarty J, Volf P, Sundar S, Boelaert M, Rogers ME. 2011. Serological

This is an Open Access article distributed under the terms of the Creative Commons Attribution License (https://creativecommons.org/licenses/by/4.0), which permits unrestricted use, distribution, and reproduction in any medium, provided the original work is properly cited.

markers of sand fly exposure to evaluate insecticidal nets against visceral leishmaniasis in India and Nepal: a cluster-randomized trial. PLoS Neglected Tropical Diseases, 5(9), e1296.

26. Gilbert MTP, Moore W, Melchior L, Worobey M. 2007. DNA extraction from dry museum beetles without conferring external morphological damage. PLoS One, 2(3), e272.
27. Giordani BF, Andrade AJ, Galati EAB, Gurgel-Goncalves R. 2017. The role of wing geometric morphometrics in the identification of sandflies within the subgenus *Lutzomyia.* Medical and Veterinary Entomology, 31(4), 373–380.
28. Guzmán-Larralde AJ, Suaste-Dzul AP, Gallou A, Peña-Carrillo KI. 2017. DNA recovery from microhymenoptera using six non-destructive methodologies with considerations for subsequent preparation of museum slides. Genome, 60(1), 85–91.
29. Hajibabaei M, DeWaard JR, Ivanova NV, Ratnasingham S, Dooh RT, Kirk SL, Mackie PM, Hebert PD. 2005. Critical factors for assembling a high volume of DNA barcodes. Philosophical Transactions of the Royal Society B: Biological Sciences, 360(1462), 1959–1967.
30. Haouas N, Pesson B, Boudabous R, Dedet JP, Babba H, Ravel C. 2007. Development of a molecular tool for the identification of *Leishmania* reservoir hosts by blood meal analysis in the insect vectors. American Journal of Tropical Medicine and Hygiene, 77(6), 1054–1059.
31. Hlavackova K, Dvorak V, Chaskopoulou A, Volf P, Halada P. 2019. A novel MALDI-TOF MS-based method for blood meal identification in insect vectors: A proof of concept study on phlebotomine sand flies. PLoS Neglected Tropical Diseases, 13 (9), e0007669.
32. Huemer H, Prudhomme J, Amaro F, Baklouti A, Walder G, Alten B, Moutailler S, Ergunay K, Charrel RN, Ayhan N. 2017. Practical guidelines for studies on sandfly-borne phleboviruses: Part II: Important points to consider for fieldwork and subsequent virological screening. Vector-Borne and Zoonotic Diseases, 17(1), 81–90.
33. Jancarova M, Polanska N, Thiesson A, Arnaud F, Stejskalova M, Rehbergerova M, Kohl A, Viginier B, Volf P, Ratinier M. 2025. Susceptibility of diverse sand fly species to Toscana virus. PLoS Neglected Tropical Diseases, 19(5), e0013031.
34. Kapp JD, Green RE, Shapiro B. 2021. A fast and efficient single-stranded genomic library preparation method optimized for ancient DNA. Journal of Heredity, 112(3), 241–249.
35. Killick-Kendrick R, Maroli M, Killick-Kendrick M. 1991. Bibliography of the colonization of phlebotomine sandflies. Parassitologia, 33(suppl.), 321–333.
36. Lawyer P, Killick-Kendrick M, Rowland T, Rowton E, Volf P. 2017. Laboratory colonization and mass rearing of phlebotomine sand flies (Diptera, Psychodidae). Parasite, 24, 42.
37. Léger N, Pesson B, Madulo-Leblond G. 1986. Les phlébotomes de Grèce : 1ère partie. Bulletin de la Société de Pathologie Exotique, 79, 386–397.
38. Léger N, Pesson B, Madulo-Leblond G. 1986. Les phlébotomes de Grèce : 2ème partie. Bulletin de la Société de Pathologie Exotique, 79, 514–524.
39. Leonel JAF, Vioti G, Alves ML, da Silva DT, Meneghesso PA, Benassi JC, Spada JCP, Galvis-Ovallos F, Soares RM, Oliveira T. 2020. DNA extraction from individual Phlebotomine sand flies (Diptera: Psychodidae: Phlebotominae) specimens: Which is the method with better results? Experimental Parasitology, 218, 107981.
40. Lestinova T, Rohousova I, Sima M, de Oliveira CI, Volf P. 2017. Insights into the sand fly saliva: Blood-feeding and immune interactions between sand flies, hosts, and *Leishmania.* PLoS Neglected Tropical Diseases, 11(7), e0005600.
41. Lienhard A, Schaffer S. 2019. Extracting the invisible: obtaining high quality DNA is a challenging task in small arthropods. PeerJ, 7, e6753.
42. Lozano-Sardaneta YN, Mikery-Pacheco OF, Huerta H, Rojas-Soriano JE, Contreras-Ramos A. 2025. Wing geometric morphometrics is effective to separate sand fly species (Diptera, Psychodidae, Phlebotominae) related with leishmaniasis transmission in Mexico. Acta Tropica, 262, 107523.
43. Mandrioli M. 2008. Insect collections and DNA analyses: how to manage collections? Museum Management and Curatorship, 23(2), 193–199.
44. Maroli M, Feliciangeli MD, Bichaud L, Charrel RN, Gradoni L. 2013. Phlebotomine sandflies and the spreading of leishmaniases and other diseases of public health concern. Medical and Veterinary Entomology, 27(2), 123–147.
45. Marquina D, Buczek M, Ronquist F, Lukasik P. 2021. The effect of ethanol concentration on the morphological and molecular preservation of insects for biodiversity studies. PeerJ, 9, e10799.
46. Mathis A, Depaquit J, Dvorak V, Tuten H, Banuls AL, Halada P, Zapata S, Lehrter V, Hlavackova K, Prudhomme J, Volf P, Sereno D, Kaufmann C, Pfluger V, Schaffner F. 2015. Identification of phlebotomine sand flies using one MALDI-TOF MS reference database and two mass pectrometer systems., Parasites & Vectors, 8, 266.
47. Mekarnia N, Benallal KE, Sadlova J, Vojtkova B, Mauras A, Imbert N, Longhitano M, Harrat Z, Volf P, Loiseau PM, Cojean S. 2024. Effect of *Phlebotomus papatasi* on the fitness, infectivity and antimony-resistance phenotype of antimony-resistant *Leishmania* major Mon-25. International Journal for Parasitology – Drugs and Drug Resistance, 25, 100554.
48. Milligan BG. 1998. Total DNA isolation, in Molecular Genetic Analysis of Population: A Practical Approach, Hoelzel AR, Editor. Oxford: Oxford University Press.
49. Molina R, Jiménez M, Alvar J, González E, Hernández-Taberna S, Ines MM. 2017. Methods in sand fly research. Madrid: Servicio de publicaciones Universidad de Alcalá de Henares, Madrid.
50. Murphy WJ, Eizirik E, O'Brien SJ, Madsen O, Scally M, Douady CJ, Teeling E, Ryder OA, Stanhope MJ, de Jong WW, Springer MS. 2001. Resolution of the early placental mammal radiation using Bayesian phylogenetics. Science, 294(5550), 2348–2351.
51. Nacif-Pimenta R, Pinto LC, Volfova V, Volf P, Pimenta PFP, Secundino NFC. 2020. Conserved and distinct morphological aspects of the salivary glands of sand fly vectors of leishma-niasis: an anatomical and ultrastructural study. Parasites & Vectors, 13(1), 441.
52. Neuhaus B, Schmid T, Riedel J. 2017. Collection management and study of microscope slides: Storage, profiling, deterioration, restoration procedures, and general recommendations. Zootaxa, 4322(1), 1–173.
53. New TR. 1974. Pscoptera. Handbooks for Identification of British Insects (Vol. I). London: Royal Entomological Society of London. 102 pp.
54. Perez-Ruiz M, Collao X, Navarro-Mari JM, Tenorio A. 2007. Reverse transcription, real-time PCR assay for detection of Toscana virus. Journal of Clinical Virology, 39(4), 276–281.
55. Porco D, Rougerie R, Deharveng L, Hebert P. 2010. Coupling non-destructive DNA extraction and voucher retrieval for small

soft-bodied Arthropods in a high-throughput context: the example of Collembola. Molecular Ecology Resources, 10(6), 942–945.

56. Prudhomme J, Cassan C, Hide M, Toty C, Rahola N, Vergnes B, Dujardin JP, Alten B, Sereno D, Banuls AL. 2016. Ecology and morphological variations in wings of *Phlebotomus ariasi* (Diptera: Psychodidae) in the region of Roquedur (Gard, France): a geometric morphometrics approach. Parasites & Vectors, 9(1), 578.
57. Prudhomme J, Gunay F, Rahola N, Ouanaimi F, Guernaoui S, Boumezzough A, Banuls AL, Sereno D, Alten B. 2012. Wing size and shape variation of *Phlebotomus papatasi* (Diptera: Psychodidae) populations from the south and north slopes of the Atlas Mountains in Morocco. Journal of Vector Ecology, 37(1), 137–147.
58. Prudhomme J, Toty C, Kasap OE, Rahola N, Vergnes B, Maia C, Campino L, Antoniou M, Jimenez M, Molina R, Cannet A, Alten B, Sereno D, Banuls AL. 2015. New microsatellite markers for multi-scale genetic studies on *Phlebotomus ariasi* Tonnoir, vector of *Leishmania infantum* in the Mediterranean area. Acta Tropica, 142, 79–85.
59. Prudhomme J, Velo E, Bino S, Kadriaj P, Mersini K, Gunay F, Alten B. 2019. Altitudinal variations in wing morphology of *Aedes albopictus* (Diptera, Culicidae) in Albania, the region where it was first recorded in Europe. Parasite, 26, 55.
60. Rawlins DJ. 1992. Light Microscopy: An Introduction to Biotechniques. Oxford: Bios Scientific publishers. 143 pp.
61. Ready PD. 2013. Biology of phlebotomine sand flies as vectors of disease agents. Annual Review of Entomology, 58, 227–250.
62. Rohlf FJ, Slice D. 1990. Extensions of the Procrustes method for the optimal superimposition of landmarks. Systematic Zoology, 39(1), 40–59.
63. Rowley DL, Coddington JA, Gates MW, Norrbom AL, Ochoa RA, Vandenberg NJ, Greenstone MH. 2007. Vouchering DNA-barcoded specimens: Test of a nondestructive extraction proto-col for terrestrial arthropods. Molecular Ecology Notes, 7(6), 915–924.
64. Sábio PB, Andrade AJ, Galati EAB. 2014. Assessment of the taxonomic status of some species included in the *Shannoni* complex, with the description of a new species of *Psathyromyia* (Diptera: Psychodidae: Phlebotominae). Journal of Medical Entomology, 51(2), 331–341.
65. Sadlova J, Yeo M, Seblova V, Lewis MD, Mauricio I, Volf P, Miles MA. 2011. Visualisation of *Leishmania donovani* fluorescent hybrids during early stage development in the sand fly vector. PLoS One, 6(5), e19851.
66. Sales K, Miranda DEO, da Silva FJ, Otranto D, Figueredo LA, Dantas-Torres F. 2020. Evaluation of different storage times and preservation methods on phlebotomine sand fly DNA concentration and purity. Parasites & Vectors, 13(1), 399
67. Sales KG, Costa PL, de Morais RC, Otranto D, Brandao-Filho SP, Cavalcanti Mde P, Dantas-Torres F. 2015. Identification of phlebotomine sand fly blood meals by real-time PCR. Parasites& Vectors, 8, 230.
68. Sant'Anna MR, Jones NG, Hindley JA, Mendes-Sousa AF, Dillon RJ, Cavalcante RR, Alexander B, Bates PA. 2008. Blood meal identification and parasite detection in laboratory-fed and field-captured *Lutzomyia longipalpis* by PCR using FTA databasing paper. Acta Tropica, 107(3), 230–237.
69. Senne NA, Santos HA, Araujo TR, Paulino PG, Mendonca LP, Moreira HVS, Camilo TA, da Costa Angelo I. 2022. Robust comparative performance of genomic DNA extraction methods from non-engorged phlebotomine sandflies. Medical and Veterinary Entomology, 36(2), 203–211.
70. Shaw JJ. 2025. A review of Leishmania infections in American Phlebotomine sand flies – Are those that transmit leishmaniasis anthropophilic or anthropportunists? Parasite, 32, 57.
71. Tesh RB, Modi GB. 1983. Growth and transovarial transmission of Chandipura virus (Rhabdoviridae: Vesiculovirus) in *Phlebotomus papatasi.* American Journal of Tropical Medicine and Hygiene, 32(3), 621–623.
72. Thomsen PF, Elias S, Gilbert MTP, Haile J, Munch K, Kuzmina S, Froese DG, Sher A, Holdaway RN, Willerslev E. 2009. Non-destructive sampling of ancient insect DNA. PLoS One, 4(4),e5048
73. Truett GE, Heeger P, Mynatt RL, Truett AA, Walker JA, Warman ML. 2000. Preparation of PCR-quality mouse genomic DNA with hot sodium hydroxide and tris (HotSHOT). Biotechniques, 29(1), 52–54.
74. Upton MS. 1993. Aqueous gum-chloral slide mounting media: an historical review. Bulletin of Entomological Research, 83(2), 267–274.
75. Volf P, Myskova J. 2007. Sand flies and Leishmania: specific versus permissive vectors. Trends in Parasitology, 23(3), 91–92.
76. Wang Q, Wang X. 2012. Comparison of methods for DNA extraction from a single chironomid for PCR analysis. Pakistan Journal of Zoology, 44(2), 421–426.
77. Wang Y, Zhao Y, Bollas A, Wang Y, Au KF. 2021. Nanopore sequencing technology, bioinformatics and applications. Nature Biotechnology, 39(11), 1348–1365.

Cite this article as: Randrianambinintsoa FJ, Augendre L, Prudhomme J, Martinet J-P, Loyer M, Mekarnia N, Kerkoub H, Perveen FK, Huguenin A, Kariya E, Akhoundi M, De Andrade AJ, Berriatua E, Bongiorno G, Boyer S, Christodoulou V, Da Costa-Ribeiro MCV, De Souza LAF, Ding H, Dondji B, Dvořák V, Erisoz Kasap O, Galati EAB, Gállego M, Ballart C, Gouzelou S, Haddad N, Masse RS, Mekuria AH, Ivovic V, Kaczmarek S, Shahar MK, Kirstein OD, Kniha E, Kolářová I, Lincoln T, Lucanas C, Mikov O, Nov K, Özbel Y, Pesson B, Posada Lopez LC, Prasetyo DB, Rahola N, Rebollar-Tellez EA, Rodrigues BL, Roy L, Saini P, Sanjoba C, Shimabukuro PH, Siriyasatien P, Soszynska A, Suleşco T, Sylla M, Torno M, Volf P, Vongphayloth K, Sinh Nam V, Wardhana A, Yessinou E, Zapata S, Gantier J-C & Depaquit J. 2026. Processing and mounting phlebotomine sand flies: a consensus guideline. Parasite xx, xx. https://doi.org/10.1051/parasite/2026009.

PARASiTE

An international open-access, peer-reviewed, online journal publishing high quality papers on all aspects of human and animal parasitology

Reviews, articles and short notes may be submitted. Fields include, but are not limited to: general, medical and veterinary parasitology; morphology, including ultrastructure; parasite systematics, including entomology, acarology, helminthology and protistology, and molecular analyses; molecular biology and biochemistry; immunology of parasitic diseases; host-parasite relationships; ecology and life history of parasites; epidemiology; therapeutics; new diagnostic tools.
All papers in Parasite are published in English. Manuscripts should have a broad interest and must not have been published or submitted elsewhere. No limit is imposed on the length of manuscripts.

Parasite (open-access) continues **Parasite** (print and online editions, 1994-2012) and **Annales de Parasitologie Humaine et Comparée** (1923-1993) and is the official journal of the Société Française de Parasitologie.

Editor-in-Chief:
Jean-Lou Justine, Paris

Submit your manuscript at:
https://www.editorialmanager.com/parasite

ឧបសម្ព័ន្ធទី១: មូលដ្ឋានគ្រឹះវិទ្យាសាស្ត្រជីវគីមី (Appendix 1: Biochemical theoretical foundations)

Phlebotomine (**sand flies**) គឺជាក្រុមដែលពាក់ព័ន្ធនឹង**arthropods** ។ ទោះបីជាយ៉ាងណា គំនិតទូទៅនេះអាចពង្រីកទៅកាន់arthropodsមានជើងផ្សេងទៀតដែលមានភាពទូទៅខ្លាំង ហើយការកំណត់អត្តសញ្ញាណអាចធ្វើបានតាមលក្ខណៈរាងកាយខាងក្នុងតែប៉ុណ្ណោះ។ ដោយថៃដន្យ សរីរាងខាងក្នុងខ្លះមានសភាពជា**chitinised** និងលក្ខណៈរូបរាងរបស់វាបង្ហាញព័ត៌មានមានតម្លៃ។ នេះជាហេតុផលដែលការពិនិត្យបំពង់អាហារ (**food pumps**), **ថង់ផ្ទុកទឹកកាមញី (spermathecae)** និងបំពង់ទីបរបស់វា គឺមានចំណាប់អារម្មណ៍ខ្លាំង។ ជាមួយនឹងប្រតិករទាំងអស់ដែលយើងនឹងពិនិត្យឡើងវិញ វាមិនគួរត្រូវបានបំភ្លេចឡើយថា ចាប់ពីដំណាក់កាលនៃ insect fixationរហូតដល់ការដំឡើង (assembly) យើងនឹងអនុវត្តប្រតិកម្មអុកស៊ីតកម្ម-រីដុកស៊ីតកម្ម (redox reactions) យ៉ាងសាមញ្ញ។ ការប្រុងប្រយ័ត្ន ឬគំនិតតែមួយគត់ដែលនឹងនាំយើងគឺ ការជៀសវាងលាយសារធាតុប្រតិកម្មកាត់បន្ថយ (reducing reagents) ជាមួយសារធាតុប្រតិកម្មអុកស៊ីតកម្ម (oxidizing reagents)។

Ethyl alcohol; ethanol:
សារធាតុនេះនឹងត្រូវបានប្រើប្រាស់តាមវិធីផ្សេងៗគ្នា។ ម៉ូលេគុលអាល់កុលមានទំនាក់ទំនងខ្លាំងជាមួយទឹក ហើយដូច្នេះវាបង្ហាញពីឥទ្ធិពលខ្សោះជាតិទឹក (dehydrating effect)។ ទោះយ៉ាងណា អាល់កុលដែលមានកំហាប់ទាប (ឧ. សម្ល្បរទឹកពេក) នឹងដើរតួក្នុងការបំបែកអាស៊ីតនុយក្លេអ៊ិក (degradation of nucleic acids) (ទឹកគឺជាសត្រូវរបស់អាស៊ីតនុយក្លេអ៊ិក)។

នៅពេលដែលសត្វល្អិតត្រូវបានដាក់ក្នុងអេតាណុល វាមិនមែនគ្រាន់តែដើម្បីរក្សាទុក (preserve) វាប៉ុណ្ណោះទេ ប៉ុន្តែថែមទាំងដើម្បីជួសជុល (fix) ជាលិកាទៀតផង។ ក្នុងជាលិកាសាស្ត្រ (histology) ជាធម្មតាយើងបែងចែកគោលគំនិតសំខាន់ពីរគឺ៖

អត្រាជ្រាបចូល (penetration rate) និងfixation rate។ គេយល់យ៉ាងច្បាស់ថា សារធាតុថែរក្សាដ៏ល្អ (good preservative) ត្រូវតែជ្រាបចូលយ៉ាងលឿនទៅក្នុងជាលិកាមុនពេលfixation។ សម្រាប់អាល់កុល ៩៦% មេគុណនៃការជ្រាបចូល (penetration coefficient) គឺប្រហែល ១.០៥ (ប្រៀបធៀបទៅនឹងសូលុយស្យុងអាក្វាសនៃអាស៊ីតភីករិច (picric acid) ០.៧៥% មេគុណនៃការជ្រាបចូលគឺ ០.៤៥ រីឯសូលុយស្យុងប៉ូតាស្យូមឌីក្រូមេត (potassium dichromate) ៣% វិញ មេគុណគឺ ១.៤៥)។

ការចង់រក្សាទុកសត្វល្អិត និងarthropodsដទៃទៀតក្នុងអេតាណុលដោយគ្មានកំណត់ពេល (indefinitely) គឺជាការពិតសម្រាប់អ្នកជំនាញខាងសត្វល្អិត (entomologists)។ គំនិតដែលចង់រក្សាទុកសំណាកដែលចាប់បានពីfield(field captures) សម្រាប់ការសិក្សាជាបន្តបន្ទាប់ ឬសម្រាប់អ្នកស្រាវជ្រាវនាពេលអនាគត គឺនៅតែគួរឱ្យគោរពណាស់។ ទោះជាយ៉ាងណា គំនិតនេះមិនអាចទៅរួចទេសម្រាប់អ្នកជំនាញខាងកោសិកាវិទ្យា (cytologist) ឬជីវវិទ្យាជាលិកា (histologist)។ តាមរយៈការទុកសំណាកក្នុងសារធាតុfixative យូរពេក វាអាចក្លាយទៅជាមិនអាចធ្វើការឡើងវិញបាន (practically impossible to rework)។ នេះហើយជាមូលហេតុដែលសំណាកដែលចាស់ជាង ១០ ឆ្នាំ ពិបាក ឬស្ទើរតែមិនអាចប្រើប្រាស់បាន។

ការពិចារណាមួយទៀតគឺ សមាមាត្រ (ratio) រវាងម៉ាស់arthropodsដែលត្រូវជួសជុល និងបរិមាណនៃសារធាតុជួសជុល (fixator)។ ក្នុងការអនុវត្តផ្នែកសត្វវិទ្យា (zoological) ឬវេជ្ជសាស្ត្រ (medical) គេគួរតែរៀបចំបរិមាណធំជាង ៦០ ដងនៃបរិមាណបំណែកដែលត្រូវជួសជុល។ ក្នុងការអនុវត្តសម្រាប់មីក្រូ-arthropods (micro-arthropods) សម្រាប់បរិមាណជាក់លាក់នៃសំណាកដែលត្រូវជួសជុល ត្រូវបន្ថែមអាល់កុលយ៉ាងហោចណាស់ ៤–៥ ដងនៃបរិមាណនោះ។ ត្រូវចងចាំថា អាល់កុលនឹងបាត់បង់កម្លាំង (strength) របស់វា នៅពេលដែលវាយកទឹកទាំងអស់ដែលមាននៅក្នុងជាលិការបស់arthropodsចេញ។

សរុបសេចក្តី៖

- អេទីលអាល់កុល គឺជាភ្នាក់ងារគីមីកាត់បន្ថយ (reducing chemical agent) (ដូច្នេះវាមិនឆបគ្នាជាមួយសារធាតុជួសជុលបែបអុកស៊ីតកម្ម (oxidative fixatives));
- វាធ្វើឱ្យប្រូតេអ៊ីន precipitate យ៉ាងខ្លាំងក្លា និងធ្វើឱ្យខូចទ្រង់ទ្រាយ (denatures) ពួកវា;
- វារំលាយ (dissolves) លីពីតស្មុគស្មាញ (complex lipids) មួយចំនួន និងធ្វើឱ្យ glycogen precipitate;
- វាបណ្តាលឱ្យមានការកន្ត្រាក់ខ្លាំង (strong contraction) នៃជាលិកា និងធ្វើឱ្យពួកវារឹង (hardens)។

សូលុយស្យុងប៉ូតាស្យូម អ៊ីដ្រូស៊ីត ឬ សូដ្យូម អ៊ីដ្រូស៊ីត ៖

ការប្រើប្រាស់សូលុយស្យុងទាំងនេះក្នុងវិទ្យាសាស្ត្រនៃការសិក្សាសត្វល្អិត (Entomology) ភាគច្រើនផ្តោតលើប៉ូតាស្យូម អ៊ីដ្រូស៊ីត ដោយគ្មានបញ្ជាក់ពីមូលហេតុច្បាស់លាស់។

សូដ្យូម អ៊ីដ្រូស៊ីត [E524]: មាននៅក្នុងសូលុយស្យុងទាំងក្នុងកម្រិតកំហាប់ផ្សេងៗគ្នា ឬជាមួយនឹងសភាព (Normality) ផ្សេងគ្នា។ វាមានទម្រង់ជាគ្រាប់តូចៗ (Pellets) ឬជាផ្ទាំងភ្លឺចាំង (Glitter)។ គុណវិបត្តិចម្បងរបស់វាគឺថា

វាស្រូបយកសំណើមបានយ៉ាងខ្លាំង (Hygroscopic) (ច្រើនជាង KOH)។ នៅពេលវាប្រតិកម្មជាមួយប្រូតេអ៊ីន វារំលាយពួកវា ហើយជាមួយលីពីត (Lipids) វាបំប្លែងពួកវាទៅជាសាប៊ូរឹង (Hard Soaps) កំឡុងពេលប្រតិកម្មសាបូកម្ម (Saponification) (នេះគឺជាភាពខុសគ្នាដ៏ធំមួយពី KOH ដែលបង្កើតសាប៊ូរាវ (Liquid Soaps) កំឡុងពេលប្រតិកម្មសាបូកម្ម)។

ប៉ូតាស្យូម អ៊ីដ្រូស៊ីត [E525]:
មានលក់ជាសូលុយស្យុងកំហាប់ខ្ពស់ (Concentrated Solution) ប៉ុន្តែសំខាន់ជាងនេះទៅទៀត វាមានគុណសម្បត្តិដែលត្រូវបានផលិតជាទម្រង់គ្រាប់តូចៗ (Pellets) ប្រហែល ០.១ ក្រាម ដែលជួយសម្រួលយ៉ាងខ្លាំងដល់ការរៀបចំសូលុយស្យុងរំលាយ (Diluted Solutions) នៅពេលដែលគ្មានជញ្ជីងថ្លឹងភាពជាក់លាក់ (Precision Balance)។ ឧទាហរណ៍ គ្រាប់ ១ គ្រាប់ទម្ងន់ ០.១ ក្រាម ក្នុងទឹកបិត១ មីលីលីត្រ ផ្តល់សូលុយស្យុង ១០%។ គុណសម្បត្តិទីពីរនៃប៉ូតាស្យូម អ៊ីដ្រូស៊ីតជាគ្រាប់គឺភាពរសើបតិចជាងចំពោះការកាបូណាតនីយកម្ម (Carbonation) (សូលុយស្យុង KOH មានទំនាក់ទំនងខ្ពស់ក្នុងការចាប់យក CO_2 ដោយហេតុនោះបង្កើតអំបិលកាបូណាត)។

ការប្រើប្រាស់៖

Strong Bases
ទាំងនេះនឹងត្រូវបានប្រើដើម្បីរំលាយអាស៊ីតខ្លាញ់ (Fatty Acids) ដោយបំប្លែងពួកវាទៅជាសាប៊ូដែលរលាយក្នុងទឹក (Water-soluble Soaps)។ គួររំលឹកថា សារធាតុរក្សារូប (Fixative) ដូចជា អេតាណុល (Ethanol) បានរំលាយខ្លាញ់មួយចំនួននៅក្នុងសំណាករួចហើយ។ ទោះជាយ៉ាងណាក៏ដោយ តាមរយៈការជំនួសសំណាកនៅក្នុងឧបករណ៍ផ្ទុកអាគុយ (Aqueous Medium) ជាមួយនឹងមូលដ្ឋានរឹងមាំ អាស៊ីតខ្លាញ់ (ដែលមានលក្ខណៈស្អកស្អាញច្រើន ឬតិច) នឹងធ្លាក់ជាដីល្បាប់ (Precipitate)។ ដូច្នេះ មូលដ្ឋានរឹងមាំនឹងធ្វើសាប៊ូនីយកម្មនៅសីតុណ្ហភាពបន្ទប់ (Cold Saponification)។ ក្នុងករណីខ្លះ នៅពេលដែលជាលិកាខ្លាញ់ (Adipose Tissues) លើសឧទាហរណ៍នៅក្នុងញី វានឹងមានអត្ថប្រយោជន៍ក្នុងការបង្កើនសីតុណ្ហភាពដល់ ៣៥-៤០អង្សាសេ ដើម្បីជួយសម្រួលដល់ប្រតិកម្ម ឬក៏បង្កើនពេលវេលាប៉ះពាល់ (Contact Time) នៅសីតុណ្ហភាពបន្ទប់។

សូលុយស្យុងម៉ាក-អង់ដ្រេ (Marc-André Solution) ពណ៌/គ្មានពណ៌៖

នៅទីនេះ យើងនឹងស្វែងយល់ពីគុណសម្បត្តិ និងគុណវិបត្តិនៃការប្រើប្រាស់សូលុយស្យុងម៉ាក-អង់ដ្រេ។ សូលុយស្យុងនេះផ្សំឡើងពីក្លរ៉ាល់ អ៊ីដ្រាត (Chloral Hydrate) (ទ្រីក្លរអាសេតាល់ដេអ៊ីត ម៉ូណូអ៊ីដ្រាត - Trichloracetaldehyde Monohydrate), អាស៊ីតអាសេទិក (Acetic Acid), និងទឹក។ សូលុយស្យុងនេះមានលក្ខណៈជាអុកស៊ីតកម្មខ្លាំង (Very Oxidizing) (ល្បាយនៃអាស៊ីត និងអាល់ដេអ៊ីត)។ វានឹងបន្សាប (Neutralize) ប៉ូតាស្យូម អ៊ីដ្រូស៊ីតដែលលើសដែលអាចនៅសេសសល់ក្នុងសំណាក (Samples) ដោយមិនធ្វើឲ្យសាប៊ូអាល់កាឡាំង (Alkaline Soaps) ដែលបង្កើតឡើងអំឡុងពេលប្រើប៉ូតាស្យូម អ៊ីដ្រូស៊ីត ធ្លាក់ជាដីល្បាប់ (Precipitating) ឡើយ។

សូលុយស្យុងអុកស៊ីតកម្មនេះក៏នឹងមានសកម្មភាពលើមុខងារជាតិអាល់កុលបន្ទាប់បន្សំ (Secondary Alcohol Functions) នៃគ្លុយកូសាមីន (Glucosamines) ដែលបង្កើតជាឈីទីន (Chitin) ដោយធ្វើអុកស៊ីតកម្មពួកវា ដែលធ្វើឲ្យឈីទីនទន់ (Softening)។

សកម្មភាពមួយទៀតគឺការរំលាយ (Dissolution) អំបិលរ៉ែ (Mineral Salts) មួយចំនួនដែលមានវត្តមាន។

នៅពេលដែលសូលុយស្យុងម៉ាក-អង់ជ្រេត្រូវបានធ្វើឲ្យមានពណ៌ពីមុនដោយអាស៊ីតហ្វូស៊ីន (Acid Fuchsine) (ដូច្នេះវាស្ថិតក្នុងស្ថានភាពអុកស៊ីតកម្ម) វានឹងអាចភ្ជាប់ (Fix) ទៅលើមុខងារជាតិអាល់កុលបន្ទាប់បន្សំនៃរចនាសម្ព័ន្ធបាន។ បន្ទាប់ពីរយៈពេលប៉ះពាល់ (Contact Time) នៃសូលុយស្យុងម៉ាក-អង់ជ្រេ និងស្ថានភាពនៃការធ្វើឲ្យមានស្នាមប្រឡាក់ (Staining State) នៃសំណាករួច ការលាងសម្អាត (Rinsing) នឹងធ្វើឡើងដោយប្រើអេតាណុល (Ethanol) តែប៉ុណ្ណោះ។ ដូចនេះ យើងចាប់ផ្តើមដំណាក់កាលខ្សោះជាតិទឹក (Dehydration Phase) នៃសំណាក។ អត្ថប្រយោជន៍ (Benefits): បន្សាប (Neutralization) នូវសូលុយស្យុងមូលដ្ឋាន (Basic Solutions) ដែលលើសធ្វើឲ្យឈីទីន (Chitin) ទន់ (Relaxation) ធ្វើឲ្យឈីទីនមានស្នាមប្រឡាក់ (Staining) ដើម្បីវាយតម្លៃរចនាសម្ព័ន្ធខាងក្នុងដែលមានជាតិឈីទីន (Chitinized Internal Structures) បានកាន់តែប្រសើរគុណវិបត្តិ (Drawbacks):

ក្លរ៉ាល់ អ៊ីដ្រាត (Chloral Hydrate) គឺជាថ្នាំងងុយគេង (Hypnotic) ហើយធ្លាប់ត្រូវបានប្រើប្រាស់ក្នុងវេជ្ជសាស្ត្រមនុស្ស។ វាត្រូវតែប្រើក្រោមគំរបស្រូបផ្សែងគីមី (Chemical Hood) ហើយត្រូវតែអនុវត្តតាមច្បាប់ស្តីពីហានិភ័យគីមី (Chemical Risks)។Nសូលុយស្យុងសម្រាប់ខ្សោះជាតិទឹក (Dehydration Solutions)៖

បទពិសោធន៍បង្ហាញថា សម្រាប់សំណាកតូចៗបំផុត (Very Small Samples) វាមិនចាំបាច់ធ្វើតាមលំដាប់នៃការងូតអាល់កុល (Alcohol Baths) ដែលមានកម្រិតកំហាប់កើនឡើងជាលំដាប់នោះទេ។ ប្រសិនបើសំណាកមានទំហំធំ (Large) យើងនឹងចាប់ផ្តើមជាមួយអេតាណុល ៨០% បន្ទាប់មក ៩០%, ៩៥% និងចុងក្រោយអេតាណុលដាច់ខាត (Absolute Ethanol)។ សម្រាប់សំណាកតូចៗបំផុត សូមប្រើការងូតជាមួយអេតាណុល ៩០% បន្ទាប់មកដោយការជ្រលក់ (Immersion) ក្នុងអេតាណុលដាច់ខាត។ នៅដំណាក់កាលនេះ យើងត្រូវចងចាំជានិច្ចថា អេតាណុលដាច់ខាតស្វែងរកចាប់យក (Fix) ទឹកនៅក្នុងបរិយាកាស។

ប្រពៃណីនៅក្នុងមន្ទីរពិសោធន៍កសិករវិទ្យា (Entomology Laboratories) គឺការបញ្ចប់ការខ្សោះជាតិទឹកនៃសំណាកជាមួយនឹងការងូតក្រេអូសូត (Creosote) ពីដើមប៊ីច (Beech)។ សព្វថ្ងៃនេះ ប្រេងសំខាន់ (Essence) នេះ ដែលត្រូវបានគេប្រើប្រាស់យ៉ាងទូលំទូលាយជាថ្នាំសម្លាប់សត្វល្អិត (Pesticide), ថ្នាំប្រឆាំងផ្សិត (Antifungal), និងសារធាតុថែរក្សាឈើ (Wood Preservative) ត្រូវបានគេបង្អាក់ការប្រើប្រាស់យ៉ាងខ្លាំង ដោយសារតែក្លិនរបស់វា (អ៊ីដ្រូកាបូនក្រអូបពហុស៊ីក្លូ - Polycyclic Aromatic Hydrocarbons) ហើយវាត្រូវបានគេសន្និដ្ឋានថាជាសារធាតុពុលបន្តពូជ (Reprotoxic), បង្កមហារីក (Carcinogenic), សារធាតុឆ្លងមេរោគពីខាងក្រៅសរីរាង្គរលួយបានយូរ (Persistent Organic Pollutant), និងពុលដល់ប្រព័ន្ធអេកូឡូស៊ី (Ecotoxic) សម្រាប់សារពាង្គកាយក្នុងទឹក (Aquatic Organisms)។

សូលុយស្យុងមួយដែលយើងស្នើឡើងដើម្បីរៀបចំសម្រាប់ការmounting (Mounting) សំណាកគឺ អឺប៉ារ៉ាល់ (Euparal®) និងប្រេងសំខាន់អឺប៉ារ៉ាល់ (Euparal Essence) (បានពិពណ៌នាក្នុងកថាខណ្ឌបន្ទាប់)។ ល្បាយនៃអឺប៉ារ៉ាល់ (Euparal®) និងប្រេងសំខាន់អឺប៉ារ៉ាល់

ត្រូវបានគេទទួលយកបានយ៉ាងល្អ។
សំណាកត្រូវបានគេយកមកប្រើបន្ទាប់ពីការងូតអេតាណុល ៩០%។

ឧបសម្ព័ន្ធទី ២៖ សមាសភាពនៃសារធាតុប្រតិករ (Reagents) ដែលបានប្រើប្រាស់

ប៉ូតាស្យូម អ៊ីដ្រូស៊ីត ១០% (Potassium Hydroxide 10%)
ប៉ូតាស្យូម អ៊ីដ្រូស៊ីត ១០ ក្រាម
ទឹកបិត(Distilled Water) បន្ថែមរហូតដល់ (q.s.) ១០០ មីលីលីត្រ

Mounting Medium Hoyer Medium (Gum Chloral)
ទឹកបិត៥០ មីលីលីត្រ
Chloral Hydrate ២០០ ក្រាម
Gum Arabic ៥០ ក្រាម
Glycerol ២០ មីលីលីត្រ

សូលុយស្យុង ម៉ាក-អង់ដ្រេ (Marc-André Solution)
ក្លរ៉ាល់ អ៊ីដ្រាត ៤០ ក្រាម
អាស៊ីតអាសេទិក កក (Glacial Acetic Acid) ៣០ មីលីលីត្រ
ទឹកបិត៣០ មីលីលីត្រ

អាស៊ីតហ្វូស៊ីន (Fuchsin Acid) ១% ក្នុងទឹកចម្រោះ
ម្សៅអាស៊ីតហ្វូស៊ីន (Acid Fuchsin Powder) ១ ក្រាម
ទឹកបិត៩៩ មីលីលីត្រ

សូលុយស្យុង ម៉ាក-អង់ដ្រេ មានពណ៌ដោយហ្វូស៊ីន (Marc-André Solution Colored with Fuchsin)
សូលុយស្យុង ម៉ាក-អង់ដ្រេ ១០ មីលីលីត្រ
ហ្វូស៊ីន ១% ៥០ មីក្រូលីត្រ (μL)

ឧបសម្ព័ន្ធទី ៣៖ អឺប៉ារ៉ាល់ (Euparal®), Canada Balsam, Polyvinyl Alcohol ឬសូលុយស្យុងផ្សេងទៀតសម្រាប់ mounting

Polyvinyl Alcohol៖ នេះគឺជាMounting Medium ដ៏ល្អនៅពេលដែលផលិតផលចាំបាច់សម្រាប់ការខ្សោះជាតិទឹក (Dehydration) ត្រឹមត្រូវមិនអាចរកបាន។ Polyvinyl Alcohol ត្រូវបានលាយជាមួយ ឡាក់តូហ្វេណុល (Lactophenol) របស់អាម៉ាន់ (Amman)។
ទោះជាយ៉ាងណាក៏ដោយ ការផ្គុំ (Assemblies) ទាំងនេះបង្ហាញពីគុណវិបត្តិចម្បងគឺ រួញរឹង (Drying Out) ឬPolyvinyl Alcohol បង្កើតជាគ្រីស្តាល់ (Crystallizes) ដោយសារការហួតទឹក (Water Evaporation) ឬប្រែជាខ្មៅ (Blackening) នៅពេលហ្វេណុល (Phenol) ធ្វើអុកស៊ីតកម្ម (Oxidizes)។
នេះនៅតែជាបច្ចេកទេសដ៏ល្អសម្រាប់ការmountingរយៈពេលខ្លី (Short-term Mounting)។

Canada Balsam៖ ការប្រើប្រាស់របស់វាសម្រាប់mounting (Mounting) នៅចន្លោះស្លាយ (Slide) និងគម្របស្លាយ (ឡាម៉ែល) តម្រូវឱ្យមានការខ្សោះជាតិទឹក (Dehydration) នៃសំណាក (Samples) ដែលត្រូវmounting។ ការប្រើប្រាស់ស៊ីលីន (Xylene) ឬ តូលូអ៊ីន (Toluene) គឺមិនមែនគ្មានការលំបាក (Inconvenience) នោះទេ។

Enecê Medium៖ សម្រាប់mountingនៅចន្លោះស្លាយ និងគម្របស្លាយ ដូចជាCanada Balsamដែរ វាតម្រូវឱ្យមានការខ្សោះជាតិទឹកនៃសំណាក (Specimen)។ រូបមន្តEnecê Formulation៖ កូឡូហ្វូនីសុទ្ធពណ៌ស (Pure White Colophony) (២២ ក្រាម); Alcohol-soluble Copal

Gum (១២ ក្រាម), អាល់កុលសុទ្ធ (Absolute Alcohol) (២០ មីលីលីត្រ), កាហ្វួរ (Camphor) (១០ ក្រាម), ប្រេងសំខាន់ត្បូរប៉េនទីន (Turpentine Essence) (១០ មីលីលីត្រ), និង Eucalyptol (២៦ មីលីលីត្រ)។ សម្រាប់ការរៀបចំរបស់វា នៅក្នុងធុងមួយ ដូចជា ដបកោណអែរឡិនម៉េរ (Erlenmeyer Flask) ដាក់អាល់កុលសុទ្ធ និងកាហ្វួរ។ បន្ទាប់មកបន្ថែមកូឡូហ្វូនី និងស្ករកៅស៊ូកូប៉ាល់។ បន្ទាប់មក ដបត្រូវបានបិទដោយក្បាលឈប់ (Stopper) និងអង្រួន (Shaken) ហើយទើបតែយកទៅកម្តៅក្នុងអាងទឹកក្តៅ (Bain-marie) នៅសីតុណ្ហភាពស្រាល (Gentle Temperature) ដើម្បីកុំឱ្យល្បាយពុះ (Boil)។ នៅពេលដែលមាតិកាលាយរាវទាំងស្រុង (Completely Liquefied) រួច បន្ថែមប្រេងសំខាន់ត្បូរប៉េនទីន បន្ទាប់មកចម្រោះ (Filtered) ខណៈពេលដែលល្បាយនៅក្តៅ (Hot) ហើយចុងក្រោយបន្ថែមអឺកាលីបតូលទៅក្នុងសារធាតុរាវដែលចម្រោះរួច (Filtrate)។ នៅពេលដែលឧបករណ៍ផ្ទុក (Medium) ក្លាយទៅជារាវតិច (Less Fluid) គេរំលាយ (Diluted) វាជាមួយ អេណេក (Eenecê) ដែលមានរូបមន្តដូចខាងក្រោម៖ អាល់កុលសុទ្ធ (៣០ មីលីលីត្រ), កាហ្វួរ (១៧ ក្រាម), ប្រេងសំខាន់ត្បូរប៉េនទីន (១៥ មីលីលីត្រ), អឺកាលីបតូល (៣៨ មីលីលីត្រ) (ស៊ែរកេរ៉ា, ១៩៤៣ - Cerqueira, 1943)។

អឺប៉ារ៉ាល់ (Euparal®)៖ នេះគឺជាជ័រ (Resin) ដែលមកពីCypress of the Atlas ប្រភេទ*Tetraclinis articulata*(វ៉ាល់, ១៧៩១ - Vahl, 1791) ហើយត្រូវបានសិក្សា និងបង្កើតឡើងក្នុងឆ្នាំ ១៩០៦ ដោយGilson។ គុណសម្បត្តិចម្បងរបស់វាគឺថា វាមិនធ្វើប៉ូលីមែរនីយកម្ម (Polymerize) ទេ។ សំណាក (Samples) ដែលបានmountingនៅចន្លោះស្លាយ និងគម្របស្លាយ អាចត្រូវបានយកមកវិញ (Recovered) យ៉ាងងាយស្រួលដោយសកម្មភាពនៃជាតិអាល់កុល ឬល្អជាងនេះទៅទៀតគឺប្រេងសំខាន់អឺប៉ារ៉ាល់ (Euparal® Essence)។ ជ័រនេះ ដែលត្រូវបានគេហៅម្យ៉ាងទៀតថា សាន់ដារ៉ាក់ (Sandarac) ទទួលយកអេតាណុល (Ethanol) ចាប់ពី ៨០% ឡើងទៅ។

ការប្រើប្រាស់ទ្រីតុន X100 (Triton X100)៖ សូលុយស្យុងអាគុយ មិនមែនអ៊ីយ៉ុង (Non-ionic Aqueous Solution)
ទ្រីតុន X100 មានទម្រង់ជាAqueous Solution មិនមែនអ៊ីយ៉ុង (Non-ionic) (សូលុយស្យុង 4-(1,1,3,3-tetramethylbutyl) phenyl-polyethylene glycol, ឬ t-octylphenoxypolyethoxyethanol, polyethylene glycol tert-octylphenyl ether) ដែលត្រូវបានគេប្រើប្រាស់យ៉ាងទូលំទូលាយជាភ្នាក់ងារសម្អាត (Detergent) ក្នុងជីវវិទ្យាកោសិកា (Cellular Biology) និងជីវវិទ្យាmolecular biology (Molecular Biology)។ វាអនុញ្ញាតឱ្យមានភាពជ្រាបចូល (Permeabilization) នៃភ្នាសកោសិកា (Cell Membranes) និងភ្នាសនុយក្លេអ៊ែរ (Nuclear Membranes)។ សំណាកសត្វល្អិត (Insect Samples) ដែលរក្សាទុកក្នុងជាតិអាល់កុលអស់រយៈពេលជាច្រើនឆ្នាំ គឺជារឿងធម្មតា។ ជាអកុសល ការរក្សាទុកក្នុងអាល់កុលមិនល្អបំផុត (Optimal) ទេ ហើយarthropods (Arthropods) ដែលត្រូវបានរក្សាទុកដូច្នេះ ក្លាយទៅជាការលំបាកខ្លាំងក្នុងការរៀបចំសម្រាប់ការពិនិត្យមីក្រូទស្សន៍ (Microscopic Examination)។ ប្លាស្ទិកដែលផ្ទុកសំណាកច្រើនតែរលួយ (Degrade) បន្ទាប់មកដោយការហួតអាល់កុល (Evaporation of Alcohol)។ ក្នុងករណីទាំងពីរ ការប៉ះពាល់ជាមួយអាល់កុលយូរ (Prolonged Contact) ឬការស្ងួត (Drying) នៃសំណាកបង្កជាបញ្ហាពិតប្រាកដ។

ក្នុងឆ្នាំ ២០០៨ Jonque បានបោះពុម្ពផ្សាយកំណត់ចំណាំស្តីពីការបញ្ចូលជាតិទឹកឡើងវិញ (ការដកជាតិទឹកឡើងវិញ) នៃសត្វពីងពាង (Spiders) ជាមួយនឹងភ្នាក់ងារធ្វើឱ្យសើម (Wetting Agent) ដូចជា អាហ្គេប៉ុន (Agepon) ដែលប្រើសម្រាប់ខ្សែភាពយន្តថតរូប (Photographic Films) [២៦]។ នេះបាននាំឱ្យមានគំនិតនៃការប្រើប្រាស់ភ្នាក់ងារធ្វើឱ្យសើម (Wetting Agents) ដែលមិនមែនជាភ្នាក់ងារសម្អាតខ្លាំង (Powerful Detergents)។ ខាងក្រោមនេះគឺជានីតិវិធី (Procedure) ដោយប្រើទ្រីតុន X100 ក្នុងសូលុយស្យុងអាគុយ ០.៥%៖

- ធ្វើឱ្យសំណាកស្ងួត (Dry Sample) ឆ្អែត (Impregnated) ជាមួយអាល់កុលសុទ្ធ (Absolute Alcohol)។

- បន្ថែមបរិមាណចាំបាច់នៃសូលុយស្យុងទ្រីតុន X100 ០.៥% ដើម្បីឱ្យសំណាកទាំងមូលត្រូវបានពន្លិច (Immersed)។

- ទុកឱ្យឈរ (Allow to stand) ប្រហែល ៥ នាទី ឬច្រើនជាងនេះ។ arthropods (Arthropods) ទាំងអស់ត្រូវតែឯករាជ្យ (Become Independent) នៅក្នុងសូលុយស្យុង។

- យកសូលុយស្យុងទ្រីតុន X100 ចេញ (Removed) ហើយជំនួស (Replaced) ដោយសូលុយស្យុងប៉ូតាស្យូម អ៊ីដ្រូស៊ីត (Potassium Hydroxide Solution)។ បន្ទាប់មក អនុវត្តតាមបច្ចេកទេស (Technique) ដូចដែលបានពិពណ៌នាខាងលើ។

ឧបសម្ព័ន្ធទី ៤៖ ដំណាក់កាលនៃការប្រើប្រាស់Mounting Media អឺប៉ារ៉ាល់ (Euparal®) ឬ Canada Balsam (Canada Balsam) មួយជំហានម្តងៗ

១. សំណាក (Specimens) ត្រូវតែខ្សោះជាតិទឹក (Dehydrated) (រូបរាងពពក (Cloudy) ឬ ស្រអាប់ដូចទឹកដោះ (Milky) បង្ហាញពីការខ្សោះជាតិទឹកមិនគ្រប់គ្រាន់)។
២. ការខ្សោះជាតិទឹក (Dehydration) អាចសម្រេចបានដោយបង្កើនកម្រិតកំហាប់ (Concentrations) នៃអេទីល អាល់កុល (Ethyl Alcohol)។
៣. សំណាកអាចត្រូវបានផ្ទេរ (Transferred) ពីអាល់កុល ៩៥% ឬ អាល់កុលសុទ្ធ (Absolute Alcohol) ទៅកាន់ភ្នាក់ងារធ្វើឱ្យថ្លា (Clearing Agent)។

នីតិវិធី (Procedure)៖

១. ដាក់ Phlebotomine sand fly ក្នុងអេតាណុល ៧០%។
២. យកអេតាណុលចេញ (Remove) ហើយជំនួស (Replace) ដោយ KOH ១០%។ គ្របPhlebotomineជាមួយកញ្ចក់ឡាម(Glass Slide)។
៣. ធ្វើម៉ាសេរ៉ាត (Macerate) រហូតទាល់តែសត្វល្អិតប្រែជាថ្លា។
៤. យក KOH ចេញ។
៥. គ្របសំណាក (Specimen) ជាមួយទឹកបិត(Distilled Water) ហើយរង់ចាំ ៣០ ទៅ ៤៥ នាទី។
៦. យកទឹកចេញ ហើយធ្វើការលាងសម្អាត (Washing) ម្តងទៀតជាមួយទឹកចម្រោះរយៈពេល ៣០ នាទី (ពេលវេលាអាស្រ័យលើចំនួនសំណាក (Time Function of Numerous Samples)៖ សំណាកកាន់តែច្រើនដែលត្រូវដំណើរការវិភាគជាមួយគ្នា ពេលវេលានេះត្រូវតែគោរពឱ្យបានយូរ។ ប្រសិនបើមានតិច

ជាពិសេសសម្រាប់សំណាកដែលត្រូវបានព្យាបាលជាលក្ខណៈបុគ្គល (Treated Individually) ពេលវេលានេះអាចខ្លីជាង)
៧. យកទឹកចេញ
៨. បន្ថែមសូលុយស្យុង ម៉ាក-អង់ដ្រេ (Marc-André Solution) (ដែលអាចមានពណ៌ដោយអាស៊ីតហ្វូស៊ីន (Fuchsin Acid)) ហើយរង់ចាំ ២៤ ម៉ោង (មួយថ្ងៃ)។
៩. យកសូលុយស្យុង ម៉ាក-អង់ដ្រេ ចេញ។
១០. គ្របសំណាកជាមួយទឹកបិតហើយរង់ចាំ ៣០ ទៅ ៤៥ នាទី។
១១. យកទឹកចេញ ហើយធ្វើការលាងសម្អាតម្តងទៀតជាមួយទឹកចម្រោះរយៈពេល ៣០ នាទី។
១២. យកទឹកចេញ
១៣. បន្ថែមអេតាណុល ៧០% ហើយធ្វើវះកាត់បំបែក (Dissect) សំណាក។
ក. សម្រាប់ក្បាល (Head) និងពោះ (Abdomen) ទាញក្បាល ឬពោះចេញពីដើមទ្រូង (Thorax) ថ្នមៗ។
ខ. សម្រាប់ដើមទ្រូង (Thorax) យកស្លាប (Wings) ចេញដោយកាន់ដើមទ្រូងជាមួយកន្ទុយក្ដាម (Forceps) មួយគូ ហើយទាញនៅគល់នៃសរីរាង្គបន្ថែម (Base of the Appendages) ជាមួយកន្ទុយក្ដាមមួយគូទៀត។ វាអាចធ្វើការវះកាត់តាមបណ្ដោយពាក់កណ្ដាលខ្លួន (Sagittal Dissection) ដោយបែងចែកដើមទ្រូងជាផ្នែកខាងឆ្វេង និងស្ដាំ អាស្រ័យលើតំបន់ដែលចាប់អារម្មណ៍បំផុត (Regions of Greatest Interest)។
១៤. ខ្សោះជាតិទឹក (Dehydrate) បន្តិចម្តងៗ (Gradually) តាមរយៈស៊េរីនៃសូលុយស្យុងអេទីល អាល់កុល (Ethyl Alcohol Solutions) ក្នុងទឹក។ ៥០ – ៨០ – ៩៥% រហូតដល់អេតាណុលដាច់ខាត (Absolute Ethanol)។
១៥. ខ្សោះជាតិទឹកសំណាក (Dehydrate the Specimens) ដោយលាងសម្អាតពួកវាពីរដង ដងនីមួយៗ ១០ នាទី ជាមួយអេតាណុល ១០០%។
១៦. យកអេតាណុលចេញ ហើយគ្របសំណាកជាមួយប្រេងក្លូវ (Clove Oil) រយៈពេល ១៥ នាទី នៅសីតុណ្ហភាពបន្ទប់ (Room Temperature)។
១៧. ផ្ទេរ (Transfer) សំណាកពីប្រេងក្លូវទៅកាន់តំណក់ (Drop) នៃអឺប៉ារ៉ាល់ (Euparal®) ឬ Canada Balsam (Canada Balsam) នៅលើស្លាយស្អាត (Clean Slide)។
១៨. រៀបចំតាមការចង់បាន (Arrange as Desired)៖ ក្បាល ដើមទ្រូង និងពោះរបស់Phlebotomineអាចត្រូវបានវះកាត់បំបែក (Dissected) ដោយប្រើម្ជុលល្អ (Fine Needles) ឬ កន្ទុយក្ដាម (Forceps) ក្រោមមីក្រូទស្សន៍សម្រាប់វះកាត់ (Dissecting Microscope)។ ក្បាលត្រូវតែវះកាត់បំបែកចេញពីរាងកាយ ដើម្បីmounting (Mounted) នៅក្នុងទីតាំងពោះ-ខ្នង (Ventro-dorsal Position) ពោលគឺ ប្រហោងខាងក្រោយក្បាល (Occipital Foramen) ត្រូវតម្រង់ទៅលើ ដើម្បីឱ្យគេអាចសង្កេតឃើញស៊ីបារីយ៉ូម (Cibarium) ដោយផ្ទាល់តាមរយៈវា។ ការវះកាត់បំបែក (Dissection) ត្រូវបានអនុវត្តនៅក្នុងឧបករណ៍ផ្ទុក (Mounting Medium) របស់Phlebotomine។
១៩. ទុកសំណាក (Specimen) រហូតដល់ផ្ទៃរបស់វាក្លាយទៅជាស្អិត (Sticky)។
២០. ធ្វើឱ្យគម្របស្លាយ (ឡាម៉ែល) ស្អាត សើម (Wet) ជាមួយអាល់កុលសុទ្ធ (Absolute Alcohol)។ ទម្លាក់គម្របស្លាយទៅលើCanada Balsam (Canada Balsam) នៅមុំមួយ (At an Angle)។
២១. ទុកស្លាយ (Slides) ក្នុងប្រអប់ស្ងួត (Dry Box) ដែលមានគោលបំណងសម្រាប់ទម្រង់ការនេះ។
