# Supplementary material for: Processing and mounting phlebotomine sand flies: a consensus guideline
Source: Parasite. 2026 Apr 3;33:18. doi: 10.1051/parasite/2026009 (PMC13047900; doi:10.1051/parasite/2026009)
Supplement: Supplementary file 19 — Lao translation / ການແປພາສາລາວ [file parasite-33-18-s19.pdf]

Parasite **33**, 18 (2026)
© F.J. Randrianambinintsoa et al., published by EDP Sciences, 2026
https://doi.org/10.1051/parasite/2026009

PARASITE

Available online at:
www.parasite-journal.org

REVIEW ARTICLE OPEN ACCESS

ຂັ້ນຕອນການກະກຽມຕົວຢ່າງ ແລະ ການແຕ່ງສະໄລຕົວຢ່າງຮິ້ນຝອຍຊາຍ: ຄູ່ມືມາດຕະຖານເອກະພາບ

Fano José Randrianambinintsoa[1], Laure Augendre[1], Jorian Prudhomme[1], Jean-Philippe Martinet[1], Mathieu Loyer[1], Nalia Mekarnia[1], Hocine Kerkoub[1], Farzana Khan Perveen[1], Antoine Huguenin[1,2], Emilie Kariya[1,2], Mohammad Akhoundi[3], Andrey José de Andrade[4], Eduardo Berriatua[5], Gioia Bongiorno[6], Sébastien Boyer[7,8], Vasiliki Christodoulou[9], Magda Clara Vieira Da Costa-Ribeiro[10], Lucas Alexandre Farias de Souza[10], Huicong Ding[11], Blaise Dondji[12], Vít Dvořák[13], Ozge Erisoz Kasap[14], Eunice Aparecida Bianchi Galati[15], Montserrat Gállego[16], Cristina Ballart[16], Stavroula Gouzelou[17], Nabil Haddad[18], Rezki Sabrina Masse[19], Asrat Hailu Mekuria[20], Vladimir Ivovic[21], Szymon Kaczmarek[22], Mohd Khadri Shahar[19], Oscar D. Kirstein[23], Edwin Kniha[24], Iva Kolářová[13], Lincoln Timinao[25], Cristian Lucanas[26], Ognyan Mikov[27], Kimsear Nov[7], Yusuf Özbel[28], Bernard Pesson[29], Laura Cristina Posada Lopez[30], Didot Budi Prasetyo[1,7], Nil Rahola[31], Eduardo A. Rebollar-Tellez[32], Bruno Leite Rodrigues[15], Lalita Roy[33], Prasanta Saini[34], Chizu Sanjoba[35], Paloma Helena Fernandes Shimabukuro[36], Padet Siriyasatien[37], Agnieszka Soszyńska[22], Tatiana Suleşco[38], Massamba Sylla[39], Majhalia Torno[40], Petr Volf[13], Khamsing Vongphayloth[41], Vu Sinh Nam[42], April Wardhana[43], Eric Yessinou[44], Sonia Zapata[45], Jean-Charles Gantier[1], and Jérôme Depaquit[1,2,*]

[1] Faculté de Pharmacie, Université de Reims Champagne Ardenne, UR ESCAPE-USC ANSES PETARD, 51 rue Cognacq-Jay, 51096 Reims Cedex, France
[2] Pôle de Biologie territoriale, Laboratoire de Parasitologie-Mycologie, Centre Hospitalo-Universitaire, 51092 Reims, France
[3] Parasitology-Mycology Department, Avicenne Hospital, AP-HP, Bobigny, Sorbonne Paris Nord University, France; Unité des Virus Émergents (UVE: Aix-Marseille Univ, Università di Corsica, IRD 190, Inserm 1207, IRBA), 13005 Marseille, France
[4] Parasitology Collection of Basic Pathology, Department of Basic Pathology, Federal University of Paraná, Curitiba 19031, Brazil
[5] Department of Animal Health, University of Murcia, Campus de Espinardo, 30100 Espinardo, Murcia, Spain
[6] Department of Infectious Diseases, Vector-borne Diseases Unit, Istituto Superiore di Sanità, 00166 Rome, Italy
[7] Medical and Veterinary Entomology Unit, Institut Pasteur du Cambodge, Phnom Penh 12201, Cambodia
[8] Ecology & Emergence of Arthropod-borne Pathogens Unit, Department of Global Health, Institut Pasteur, CNRS UMR2000, 75015 Paris, France
[9] Section Veterinary Services (1417), Laboratory for Animal Health Virology, Aglantzia, Nicosia 2109, Cyprus
[10] Insects Vectors and Parasites Laboratory, Department of Basic Pathology and Postgraduate program in Microbiology, Parasitology and Pathology, Federal University of Paraná, 81530-900 Curitiba, Brazil
[11] Department of Biological Sciences, National University of Singapore, 117558, Singapore
[12] Laboratory of the Leishmaniasis Research Project, Mokolo District Hospital, Mokolo, Cameroon; Laboratory of Cellular Immunology and Parasitology, Department of Biological Sciences, Central Washington University, 98926 Ellensburg, WA, USA
[13] Department of Parasitology, Faculty of Science, Charles University, 12800 Prague, Czechia
[14] VERG Laboratories, Department of Biology, Faculty of Science, Hacettepe University, Beytepe, Ankara 06800, Türkiye
[15] Faculdade de Saúde Pública da Universidade de São Paulo (FSP/USP), Pós-graduação em Saúde Pública, 01246-904 São Paulo, Brazil
[16] Secció de Parasitologia, Departament de Biologia, Sanitat i Medi Ambient, Facultat de Farmàcia i Ciències de l'Alimentació, Universitat de Barcelona, & Institut de Salut Global de Barcelona (ISGlobal), Centro de Investigación Biomédica en Red, Enfermedades Infecciosas (CIBERINFEC), 08028 Barcelona, Spain
[17] Laboratory of Infectious Diseases and Public Health, School of Medicine, University of Cyprus, Nicosia, Cyprus & Department of Pediatrics, Archbishop Makarios III Hospital, Nicosia 2115, Cyprus
[18] Faculty of Health Sciences, American University of Beirut, 1107 2020 Beirut, Lebanon
[19] Medical Entomology Unit, Infectious Disease Research Centre, Institute for Medical Research (IMR), National Institutes of Health (NIH), Ministry of Health Malaysia, 40170 Shah Alam, Selangor, Malaysia
[20] School of Medicine, Addis Ababa University, 28017 - 1000 Addis Ababa, Ethiopia
[21] Faculty of Mathematics, Natural Sciences and Information Technologies, University of Primorska, 6000 Koper, Slovenia

Edited by Jean-Lou Justine
*Corresponding author: jerome.depaquit@univ-reims.fr

This is an Open Access article distributed under the terms of the Creative Commons Attribution License (https://creativecommons.org/licenses/by/4.0), which permits unrestricted use, distribution, and reproduction in any medium, provided the original work is properly cited.

[22] University of Lodz, Faculty of Biology and Environmental Protection, Department of Invertebrate Zoology and Hydrobiology, Banacha 12/16, 90-237 Łódź, Poland
[23] Laboratory of Entomology, Ministry of Health, 9134302 Jerusalem, Israel
[24] Center for Pathophysiology, Infectiology and Immunology, Institute of Specific Prophylaxis and Tropical Medicine, Medical University Vienna, Kinderspitalgasse 15, 1090 Vienna, Austria
[25] Papua New Guinea Institute of Medical Research (PNGIMR) Institute, PO Box 60, Headquarter, Homate Street, 441 Goroka, Eastern Highlands Province, Papua New Guinea
[26] Museum of Natural History, University of the Philippines Los Baños, 4031 Laguna, Philippines
[27] National Centre of Infectious and Parasitic Diseases, 1504 Sofia, Bulgaria
[28] Ege University, Faculty of Medicine, Department of Parasitology, 35040 Bornova/Izmir, Türkiye
[29] Retired, Faculté de Pharmacie, Université de Strasbourg, Strasbourg, 67400 Illkirch-Graffenstaden, France
[30] Program for the Study and Control of Tropical Diseases (PECET), Faculty of Medicine, University of Antioquia, 050010 Medellin, Colombia
[31] MIVEGEC, Univ. Montpellier, CNRS, IRD, 34394 Montpellier, France & Medical Entomology Unit, Institut Pasteur de Madagascar, 101 Antananarivo, Madagascar
[32] Laboratorio de Entomología Médica, Departamento de Zoología de Invertebrados, Facultad de Ciencias Biológicas, Universidad Autónoma de Nuevo León, San Nicolás de los Garza, 66455, NL, México
[33] Tropical and Infectious Disease Centre, BP Koirala Institute of Health Sciences, Dharan 56700, Nepal
[34] ICMR-Vector Control Research Centre, Puducherry 605006, India
[35] Graduate School of Agricultural and Life Sciences, The University of Tokyo, Tokyo 113-8657, Japan
[36] Grupo de estudos em Leishmanioses/Coleção de Flebotomíneos (COLFLEB/Fiocruz-MG), Instituto René Rachou, Fundação Oswaldo Cruz, Belo Horizonte, Minas Gerais, 30190009, Brazil
[37] Center of Excellence in Vector Biology and Vector-Borne Disease, Department of Parasitology, Faculty of Medicine, Chulalongkorn University, Bangkok 10330, Thailand
[38] Department of Arbovirology, Bernhard Nocht Institute for Tropical Medicine, Bernhard Nocht Str. 74, 20359 Hamburg, Germany [39] Laboratory Vectors & Parasites, Department of Livestock Sciences and Techniques, Sine Saloum University El Hadji Ibrahima Niasse (SSUEIN) Kaffrine Campus, C.P. 24600, Senegal.
[40] Environmental Health Institute, National Environment Agency, Singapore 138667, Singapore & Department of Biological Sciences, National University of Singapore, 117558 Singapore
[41] Institut Pasteur du Laos, Laboratory of Vector-Borne Diseases, Samsenhai Road, Ban Kao-Gnot, Sisattanak District, 3560 Vientiane, Lao PDR
[42] National Institute of Hygiene and Epidemiology, 1 Yec-Xanh Street, Hai Ba Trung District, 100000 Hanoi, Vietnam
[43] Indonesian Research Center for Veterinary Science, Indonesian Agency for Agricultural Research and Development, Ministry of Agriculture Republic Indonesia, Bogor 16114, Indonesia & Department of Parasitology, Faculty of Veterinary Medicine, Airlangga University, Surabaya 60115, Indonesia
[44] Laboratory of Research in Applied Biology, Polytechnic School of Abomey-Calavi, University of Abomey-Calavi, 01 P.O. Box 2009, 00000 Cotonou, Benin
[45] Instituto de Microbiología, Colegio de Ciencias Biológicas y Ambientales (COCIBA), Universidad San Francisco de Quito (USFQ), 170901 Quito, Ecuador

Received 1 December 2025, Accepted 29 January 2026, Published online 3 April 2026

ບົດຄັດຫຍໍ້ – ບົດຄວາມນີ້ນຳສະເໜີແນວທາງທີ່ຄົບຖ້ວນສຳລັບຂັ້ນຕອນການກະກຽມ ແລະ ການແຕ່ງສະໄລ ຕົວຢ່າງຮິ້ນຝອຍຊາຍ ເຊິ່ງເປັນຂັ້ນຕອນສຳຄັນສຳລັບການຈຳແນກຊະນິດ ແລະ ການກວດຫາ ຫຼື ປູກແຍກເຊື້ອພະຍາດ. ເນື້ອໃນໄດ້ກ່າວເຖິງເຕັກນິກຫຼາຍຮູບແບບ ທີ່ເໝາະສົມທັງສຳລັບການເຮັດວຽກພາກສະໜາມ ແລະ ການປະຕິບັດງານໃນຫ້ອງວິເຄາະ. ແນວທາງນີ້ປະກອບມີຄຳແນະນຳລະອຽດກ່ຽວກັບ ການເກັບຕົວຢ່າງ, ການຈັດການ, ການຂົນຍ້າຍ ແລະ ການເຮັດໃຫ້ຕາຍ (ແນະນຳໃຫ້ໃຊ້ການແຊ່ແຂງແບບແຫ້ງ ຫຼື CO_2 ແທນການໃຊ້ສານເຄມີ) ພ້ອມທັງ ຍຸດທະສາດການຮັກສາຕົວຢ່າງ ເຊັ່ນ ການເກັບໄວ້ໃນອຸນຫະພູມຕໍ່າ ຫຼື ການເກັບໃນເຫຼົ້າເອທານອນ. ຄຸນນະພາບຂອງການກະກຽມໂຄງສ້າງທາງກາຍະວິທະຍາບາງສ່ວນ (ເຊັ່ນ ອະໄວຍະວະສືບພັນ, ຫົວ ແລະ ປີກ) ມີຄວາມສຳຄັນຢ່າງຫຼວງຫຼາຍ ສຳລັບການສັງເກດດ້ວຍກ້ອງຈຸລະທັດ ແລະ ໄດ້ຖືກອະທິບາຍໄວ້ໃນບົດຄວາມນີ້. ນອກນັ້ນ ຍັງມີການອະທິບາຍລາຍລະອຽດຂັ້ນຕອນການກະກຽມແຕ່ງຕົວຢ່າງ ລວມເຖິງຂະບວນການເຮັດໃຫ້ໃສ (clearing) ໂດຍໃຊ້ສານເຊັ່ນ potassium hydroxide ແລະ ນ້ຳຢາ Marc-André. ຂັ້ນຕອນການແຕ່ງສະໄລໄດ້ປຽບທຽບໂດຍໃຊ້ນ້ຳຢາແຕ່ງຫຼາຍຊະນິດ ໂດຍເນັ້ນໃສ່ຄຸນສົມບັດທາງແສງ ແລະ ຄວາມສາມາດໃນການຮັກສາໄລຍະຍາວ. ແນະນຳໃຫ້ໃຊ້ Hoyer fluid (ຫຼື chloral gum) ສຳລັບການສັງເກດແບບຮີບດ່ວນ ໂດຍສະເພາະຖົງເກັບນ້ຳເຊື້ອເນື່ອງຈາກມີຄວາມໃສຊັດເຈນ ແຕ່ບໍ່ເໝາະສົມສຳລັບການເກັບຮັກສາໄລຍະຍາວ. ສຳລັບນ້ຳຢາແຕ່ງສະໄລອື່ນໆ ທີ່ໄດ້ກ່າວເຖິງ ປະກອບມີ polyvinyl alcohol, Euparal® (ສຳລັບຄວາມທົນທານນ້ຳທີ່ຈຳກັດ) ແລະ Canada balsam (ນ້ຳຢາທີ່ລະລາຍໃນ hydrocarbon) ໂດຍສອງຊະນິດຫຼັງນີ້ເໝາະສຳລັບການຮັກສາໄລຍະຍາວ. ບົດຄວາມຍັງໄດ້ກ່າວເຖິງແນວທາງຊີວະພັນທຸກຳສະໄໝໃໝ່ ເຊັ່ນ ການຈັດລຳດັບ DNA ແລະ MALDI-ToF ທີ່ຕ້ອງໃຫ້ຄວາມໃສ່ໃຈພິເສດໃນຂັ້ນຕອນການກະກຽມຕົວຢ່າງ. ນອກນັ້ນ ຍັງມີຄລິບວິດີໂອສັ້ນໆ ສະແດງວິທີການແຕ່ງສະໄລແບບຕ່າງໆ ພ້ອມການແປເປັນ 33 ພາສາ ເພື່ອໃຫ້ແນວທາງນີ້ສາມາດຕອບສະໜອງຄວາມຕ້ອງການຂອງຊຸມຊົນນັກວິທະຍາສາດໃນທົ່ວໂລກ.

ຄຳສັບສຳຄັນ (Key words): ການແຕ້ງສະໄລຕົວຢ້າງ, ຣິ້ນຝອຍຊາຍ, ນ້ຳຢາແຕ້ງສະໄລ Hoyer, Marc-André, Chloral gum, Polyvinyl alcohol, Euparal®, Canada balsam, ການປູກແຍກເຊື້ອ *Leishmania*, ເງື່ອນໄຂການເກັບຕົວຢ້າງໃນພາກສະໜາມ, ການປູກເຊື້ອ, ການຜ່າຕັດ, ຊີວະໂມເລກຸນ, MALDI-ToF, Type-specimens

Abstract – Processing and mounting phlebotomine sand flies: a consensus guideline. This article provides a comprehensive guide for the processing and mounting of phlebotomine sand fly specimens, which is crucial for species identification and pathogen detection and isolation. It discusses a range of techniques suitable for both field and laboratory settings. The guide includes detailed instructions on sand fly collection, handling, covering, and euthanasia (recommending dry freezing or CO_2 over chemicals) as well as conservation strategies, such as cold storage and preservation in ethanol. The quality of preparation of certain anatomical structures (genital organs, head and wings) is essential for their proper microscopic observation and is described in this work. The article also presents detailed sample processing, including the clearing process with agents such as potassium hydroxide then Marc-André solution. The mounting process compares different media, emphasizing their optical properties and preservation potential. Hoyer fluid (also known as chloral gum) is recommended for quick observation, particularly for spermathecae, due to its clarity, although it is not suitable for long-term storage. Other media discussed include polyvinyl alcohol, Euparal® (for limited water tolerance), and Canada balsam (a hydrocarbon-soluble medium), with the latter two offering long-term preservation capabilities. Innovative molecular biology approaches such as DNA sequencing and MALDI-ToF, which require particular attention to sample processing, are also addressed. Furthermore, short video clips illustrating various mounting techniques as well as translations in many different languages are provided, allowing the guideline to reach the diverse needs and expectations of the global scientific community.

Key words: Mounting, Phlebotomine sand fly, Hoyer fluid, Marc-André solution, Chloral gum, Polyvinyl alcohol, Euparal®, Canada balsam, *Leishmania* isolation, Field conditions, Culture, Dissection, Molecular biology, MALDI-ToF, Type-specimens.

ບົດນຳ

ຣິ້ນຝອຍຊາຍ (Phlebotomine sand flies) ແມ້ນແມງໄມ້ທີ່ມີສອງປີກຂະໜາດນ້ອຍ ຢູ່ໃນວົງສະກຸນ Psychodidae ແລະ ວົງຍ່ອຍ Phlebotominae ໂດຍມີຢ້າງໜ້ອຍ 1,063 ຊະນິດ ທີ່ຖືກລາຍງານແລ້ວ [21]. ພວກມັນເປັນພາຫະນະນຳເຊື້ອພະຍາດທີ່ສຳຄັນ (*Leishmania*, arboviruses ແລະ *Bartonella*) ຊຶ່ງເປັນສາເຫດຂອງພະຍາດ leishmaniasis, ການຕິດເຊື້ອ arbovirus ແລະ bartonellosis ຕາມລຳດັບ.

ການຈຳແນກຊະນິດຂອງພວກມັນອາໄສການສັງເກດລາຍລະອຽດດ້ວຍກ້ອງຈຸລະທັດເປັນຫຼັກ ໂດຍອີງໃສ້ການເກັບຕົວຢ້າງຢ້າງລະມັດລະວັງ, ການເກັບຮັກສາຢ້າງເໝາະສົມ ແລະ ການແຕ້ງສະໄລທີ່ຊຳນານ ເຊິ່ງຕ້ອງອາໄສເຕັກນິກສະເພາະຫຼາຍວິທີ ແຕ້ລະວິທີກໍມີຂໍ້ດີ ແລະ ຂໍ້ຈຳກັດທີ່ແຕກຕ່າງກັນ.

ການຈຳແນກຊະນິດ ຣິ້ນຝອຍຊາຍໂຕແກ້ ອາໄສການສັງເກດເບິ່ງໂຄງສ້າງພາຍນອກ (ເຊັ່ນ: ໜວດ (antennae), ພາກສ່ວນປາກ (palpi), ອະໄວຍະວະສືບພັນຕົວຜູ້ (male genitalia)) ແລະ ໂຄງສ້າງພາຍໃນ (ເຊັ່ນ: ທໍ່ຄໍ (pharynx), ຊີບາຣຽມ (cibarium), ແລະ ຖົງເກັບນ້ຳເຊື້ອ (spermathecae). ການຜ່າຕັດ ແລະ ການແຍກອອກຂອງໂຄງສ້າງພາຍໃນເຫຼົ່ານີ້ ຊ່ວຍໃຫ້ສາມາດສັງເກດເບິ່ງໄດ້ງ່າຍ ແລະ ນຳໄປສູ້ການຈຳແນກຊະນິດທີ່ຖືກຕ້ອງ. ດັ່ງນັ້ນ, ຕ່າງຈາກແມງໄມ້ຍຸງ ຫຼື ແມງໄມ້ຈູບ, ຣິ້ນຝອຍຊາຍ ຕ້ອງການການແຕ້ງສະໄລ ລະຫວ່າງແຜ້ນແກ້ວ ແລະ ແຜ້ນປົກ ກ່ອນທີ່ຈະດຳເນີນການຈຳແນກຊະນິດ. ຈົນເຖິງຊຸມປີ 1980, ການສັງເກດດ້ວຍກ້ອງຈຸລະທັດ (microscopic observation) ແມ້ນວິທີດຽວທີ່ມີຢູ່ ສຳລັບການຈຳແນກຊະນິດ ຣິ້ນຝອຍຊາຍ, ແລະ ມັນຍັງຄົງເປັນວິທີທີ່ນຳໃຊ້ຢ້າງກວ້າງຂວາງທີ່ສຸດໃນປັດຈຸບັນ.

ການເລືອກຂັ້ນຕອນ ແລະ ການກະກຽມຕົວຢ້າງ ຈຶ່ງຄອນຂ້າງງ່າຍກວ່າ ແລະ ອີງໃສ້ການແບ້ງເປັນສອງປະເພດຕົ້ນຕໍ: ດ້ານໜຶ່ງແມ້ນການແຕ້ງສະໄລຖາແບບວອນ (definitive mounting) ທີ່ອະນຸຍາດໃຫ້ຮັກສາຕົວຢ້າງໄດ້ໃນໄລຍະຍາວ, ແລະ ອີກດ້ານໜຶ່ງແມ້ນການແຕ້ງສະໄລແບບໄວ (rapid mounting) ສຳລັບການຈຳແນກຊະນິດ ໃນສານນ້ຳຢາທີ່ບໍ່ຮັບປະກັນການຮັກສາໄດ້ໃນໄລຍະຍາວ. ສຸດທ້າຍ, ການແຕ້ງສະໄລ ເຊັ່ນ: ໃນຢາງ (resin) ເຊັ່ນ Canada balsam, ໃຊ້ເວລາຫຼາຍ, ຕ້ອງການການດຶງນ້ຳອອກຢ້າງສົມບູນຂອງຕົວຢ້າງ (complete dehydration). ນອກຈາກນີ້, ດັດສະນີສະທ້ອນແສງ (refractive index) ຂອງສານນີ້ບໍ່ແມ້ນດີທີ່ສຸດສະເໝີໄປ ສຳລັບການສັງເກດເບິ່ງ ຖົງເກັບນ້ຳເຊື້ອ (spermathecae) ໄດ້ງ່າຍ. ໃນທາງກົງກັນຂ້າມ, ການແຕ້ງສະໄລໃນນ້ຳຢາທີ່ເປັນນ້ຳ (aqueous medium) ເຊັ່ນ Hoyer liquid ແມ້ນໄວກວ່າ ແລະ ເຮັດໃຫ້ເບິ່ງ ຖົງເກັບນ້ຳເຊື້ອ (spermathecae) ທີ່ມີດັດສະນີສະທ້ອນແສງໄດ້ດີກວ່າ, ແຕ້ບໍ່ສາມາດຮັກສາສະໄລໄດ້ໃນໄລຍະຍາວ ເພາະມັນແນວໂນ້ມທີ່ຈະດູດຊຶມນ້ຳຈາກບັນຍາກາດ. ທາງເລືອກໜຶ່ງແມ້ນການປົກແຜ້ນແກ້ວດ້ວຍນ້ຳມັນທາເລັບ (nail polish) ເມື່ອມັນແຫ້ງສົມບູນແລ້ວ. ອົງປະກອບເຫຼົ່ານີ້ມີຄວາມກ່ຽວຂ້ອງ ແລະ ມີຜົນຕໍ່ການເລືອກວິທີການກຽມຕົວຢ້າງ ໂດຍອີງຕາມຈຸດປະສົງທີ່ດຳເນີນການ. ນັບແຕ້ຊຸມປີ 1980 ເປັນຕົ້ນມາ, ການສຶກສາການຈຳແນກຊະນິດ ຣິ້ນຝອຍຊາຍ ໄດ້ປະສົມຜະສານ ວິທີການສັງເກດເບິ່ງລັກສະນະຮູບຮ່າງ (morphology) ແລະ ວິທີການຊີວະເຄມີ (biochemical approaches). ທຳອິດແມ້ນການວິເຄາະ hydrocarbon (cuticular hydrocarbon analyses), ເຊິ່ງໄດ້ຖືກປ່ຽນແທນຢ້າງໄວດ້ວຍເຕັກນິກຊີວະໂມເລກຸນ (molecular biology techniques) ເຊັ່ນ: ການເພີ້ມຈຳນວນ DNA ແບບສຸ້ມຫຼາຍຈຸດ (random amplified polymorphic DNA (RAPD)), ການຈຳກັດການວິເຄາະຄວາມຍາວເປັນທ່ອນ (restriction fragment length polymorphism (RFLP)), ການເພີ້ມຈຳນວນ DNA ແລະ ການສະແດງລຳດັບກຳມະພັນ (sequencing) ໂດຍໃຊ້ວິທີຂອງ Sanger (Sanger method), ພ້ອມທັງການສະແດງລຳດັບກຳມະພັນທັງໝົດ (next-generation sequencing (NGS)). ໃນປັດຈຸບັນ, ວິທີການຊີວະໂມເລກຸນ

ໄດ້ຖືກເສີມດ້ວຍວິທີການທາງໂປຼຕີນ proteomic ເຊັ່ນ MALDI-ToF. ນອກຈາກນີ້, ການຈຳແນກຊະນິດດ້ວຍໂມເລກຸນ ສາມາດປະສົມປະສານກັບການຊອກຫາເຊື້ອທີ່ກໍ່ໃຫ້ເກີດພະຍາດ (pathogens) ດ້ວຍ PCR (Leishmania, Trypanosoma, Bartonella, ແລະ Phlebovirus) ເນື່ອງຈາກທັງໝົດນີ້ສາມາດກວດຫາເຊື້ອໄດ້ດ້ວຍ end-point PCR ແລະ real-time PCR, ເຊິ່ງຕ້ອງການການຂັ້ນຕອນການເກັບຕົວຢ່າງທີ່ຖືກຕ້ອງ ແລະ ການເກັບຮັກສາ ໃຫ້ເໝາະສົມກັບເປົ້າໝາຍທີ່ກຳນົດໄວ້ [3, 32]. ນອກຈາກລັກສະນະຮູບຮ່າງທີ່ໃຊ້ປະຈຳໃນການແຍກຊະນິດ, ວິທີການທາງດ້ານຮູບຮ່າງອື່ນໆ ຍັງສາມາດນຳໃຊ້ໄດ້ (ເຊັ່ນ: ການວິເຄາະທາງເລຂາຄະນິດຂອງຮູບຮ່າງປີກ (wing geomorphometry)).

ອີງຕາມປະສົບການຂອງຜູ້ຂຽນເອງ ແລະ ຂໍ້ມູນຈາກເອກະສານອ້າງອີງ ຕ່າງໆ, ຈຸດປະສົງຂອງການສຶກສານີ້ແມ້ນເພື່ອສະໜອງແນວທາງການປະຕິບັດ (ຄູ່ມື) ມາດຕະຖານ (standardized guidelines) ສຳລັບການແຕ້ງສະໄລ ແລະ ການກະກຽມຕົວຢ່າງ ຮິ້ນຝອຍຊາຍໂຕແກ້ ເພື່ອເພີ່ມປະສິດທິພາບໃນການວິເຄາະລັກສະນະຮູບຮ່າງ ແລະ ການວິເຄາະທາງດ້ານຊີວະໂມເລກຸນ.

ຄວາມຕ້ອງການໃນການດຳເນີນການວິເຄາະບາງປະເພດ (ເຊັ່ນ: ການວິເຄາະທາງດ້ານຊີວະໂມເລກຸນ (molecular biology) ຫຼື MALDI-ToF) ຕ້ອງການການຮັກສາສ່ວນໜຶ່ງຂອງຮິ້ນຝອຍຊາຍທີ່ບໍ່ຈຳເປັນສຳລັບການຈຳແນກຊະນິດດ້ວຍລັກສະນະຮູບຮ່າງ (morphological identification), ເຊິ່ງເນັ້ນໃຫ້ເຫັນເຖິງຄວາມຈຳເປັນໃນການເລືອກຂັ້ນຕອນໂປຣໂຕຄອນ (protocol) ທີ່ສຳຄັນ.

ໃນບົດຄວາມນີ້, ພວກເຮົາສຸມໃສ່ວິທີການໃນການໃຫ້ຢາສະຫຼົບ (anesthesia) ແລະ ການເຮັດໃຫ້ຕາຍ (euthanasia) ຂອງ ຮິ້ນຝອຍຊາຍ ທີ່ຈັບໄດ້ຍັງມີຊີວິດ, ການເກັບຮັກສາ, ແລະ ຂັ້ນຕອນການແຕ້ງສະໄລ (mounting process), ເພື່ອການຈຳແນກຊະນິດໄວ (rapid identification) ຫຼື ເພື່ອການຮັກສາຕົວຢ່າງໃນໄລຍະຍາວ (long-term conservation) ທີ່ອະນຸຍາດໃຫ້ສາມາດດຳເນີນການສຶກສາຂັ້ນຕໍ່ໄປໄດ້.

1. ການຈັບຮິ້ນຝອຍຊາຍ

ຮິ້ນຝອຍຊາຍໂຕແກ້ ສາມາດຈັບໄດ້ທັງຍັງມີຊີວິດ ແລະ ຕາຍແລ້ວ ໂດຍໃຊ້ຫຼາຍວິທີການ ເຊັ່ນ: ກັບດັກແສງ CDC (CDC miniature light traps), ກັບດັກຕິດກາວ (sticky traps), ແລະ ເຄື່ອງດູດ (aspirators) ຮ່ວມກັບ Shannon traps, ຫຼື ຈັບໂດຍກົງຈາກບ່ອນພັກຜ່ອນໃນສະພາບແວດລ້ອມ (ເຊັ່ນ: ບ່ອນລ້ຽງສັດ). ວິທີການເຫຼົ່ານີ້ ກ່ຽວຂ້ອງກັບການວາງກັບດັກໃນທີ່ຢູ່ອາໄສທີ່ເໝາະສົມ, ດຶງດູດຮິ້ນຝອຍຊາຍ ດ້ວຍແສງ ຫຼື ສານດຶງດູດ (ສານລໍ້) ອື່ນໆ (CO_2 ຫຼື ສານລໍ້ເຄມີ), ແລະ ເກັບເອົາມາເພື່ອການວິເຄາະຕໍ່ໄປ, ດັ່ງທີ່ອະທິບາຍໄວ້ໃນເອກະສານຫຼາຍສະບັບ [2, 3, 32, 36, 49]. ການຈັບຮິ້ນຝອຍຊາຍ ທີ່ຍັງມີຊີວິດ ເຮັດໃຫ້ສາມາດດຳເນີນການການສຶກສາຕໍ່ໄດ້ທຸກຂັ້ນຕອນ ໃນຂະນະທີ່ການເກັບຕົວທີ່ຕາຍແລ້ວ ຈະບໍ່ສາມາດປູກແຍກເຊື້ອ Leishmania ຫຼື ໄວຣັສໄດ້.

ບາງວິທີການຈັບ ເຊັ່ນ: ເຈ້ຍຕິດກາວ (sticky papers) ມັກເຮັດໃຫ້ເກີດການສູນເສຍອະໄວຍະວະຂອງ ຮິ້ນຝອຍຊາຍ (ໜວດ (antennae), ພາກສ່ວນປາກ (palps), ປີກ, ຫຼື ຂາ). ນອກຈາກນີ້, ເຈ້ຍຕິດກາວທີ່ໃຊ້ນໍ້າມັນ castor oil ຈະຕິດຕົວ ຮິ້ນຝອຍຊາຍ ແລະ ຕ້ອງການການກຳຈັດອອກໃນຂັ້ນຕອນກະກຽມຕົວຢ່າງ ທຳ ອິດ, ປົກກະຕິໃຊ້ການແຊ່ໃນນໍ້າສົມປະສົມ ethanol ແລະ diethyl ether ອັດຕາສ່ວນເທົ່າກັນ ເປັນເວລາ 15 ນາທີ.

2. ການເຮັດໃຫ້ຕົວຢ້າງສະຫລົບ

ຫຼັງຈາກການເກັບຕົວຢ່າງ, ຮິ້ນຝອຍຊາຍທີ່ຍັງມີຊີວິດຕ້ອງໄດ້ເຮັດໃຫ້ຕາຍກ່ອນ. ບາງວິທີການເກັບ (ເຊັ່ນ: ເຈ້ຍຕິດ, ກັບດັກແສງ CDC ທີ່ຕິດຕັ້ງກັບກ່ອງທີ່ມີນໍ້າຢາຫຼືເຫຼົ້າເອທານອລ) ຮິ້ນຝອຍຊາຍ ຈະຕາຍທັນທີທີ່ເກັບມາໄດ້. ການວິເຄາະຊີວະໂມເລກຸນ ສາມາດນຳໃຊ້ໄດ້ ກັບຕົວຢ່າງທີ່ເກັບໃສ່ເຫຼົ້າເອທານອລໂດຍກົງ ແລະ ກັບຕົວຢ່າງທີ່ເກັບໃສ່ສານອື່ນໆ ຖ້າປ່ຽນໃສ່ເຫຼົ້າເອທານອລໃຫ້ໄວເທົ່າທີ່ໄວໄດ້ຫຼັງຈາກການເກັບຕົວຢ່າງ.

ເຖິງຢ່າງໃດກໍ່ຕາມ, ວິທີການເຮັດໃຫ້ຕາຍຂ້າງເທິງນີ້ ບໍ່ສາມາດນຳມາໃຊ້ໃນການວິເຄາະທາງດ້ານ MALDI-ToF. ນອກຈາກນີ້, ບາງວິທີການເຮັດໃຫ້ຕາຍ ອາດເຮັດໃຫ້ສູນເສຍລັກສະນະຮູບຮ່າງບາງຢ່າງ. ດັ່ງນັ້ນ, ຈຳເປັນຕ້ອງໃຊ້ສານເຮັດໃຫ້ຕາຍມາດຕະຖານທີ່ເໝາະສົມ ເພື່ອຮັບປະກັນການຈຳແນກຊະນິດທີ່ຖືກຕ້ອງ ຫຼື ການຮັກສາຕົວຢ່າງໃນໄລຍະຍາວ ເພື່ອເປັນຕົວຢ່າງອ້າງອີງ (voucher specimens) (ເຊັ່ນ: ຕົວຢ່າງທີ່ຮັກສາໄວ້ ແລະ ເກັບຮັກສາເພື່ອເປັນຕົວຢ່າງອ້າງອີງ ຫຼື ປຽບທຽບໃນອະນາຄົດ).ສານເຄມີເຊັ່ນ ethyl acetate, ethyl ether, tetrachloroethane, ແລະ chloroform ສາມາດແຊ້ໃສ່ຝ້າຍ ແລະ ວາງໃນພາຊະນະປິດທີ່ມີ ຮິ້ນຝອຍຊາຍ ເພື່ອເຮັດໃຫ້ຕາຍ. ສານເຫຼົ່ານີ້ ຕ້ອງຈັດການດ້ວຍຄວາມລະມັດລະວັງ ຕາມຄຳແນະນຳຂອງຜູ້ຜະລິດ ເນື່ອງຈາກຄວາມເປັນພິດ. ເຖິງຢ້າງໃດກໍ່ຕາມ, ພວກເຮົາບໍ່ແນະນຳໃຫ້ໃຊ້ chloroform ເພື່ອເຮັດໃຫ້ຮິ້ນຝອຍຊາຍຕາຍ ເພາະຈາກປະສົບການຂອງພວກເຮົາ ມັນບໍ່ເໝາະສົມກັບການສຶກສາຊີວະໂມເລກຸນ. ເນື່ອງຈາກຄວາມອັນຕະລາຍຂອງສານເຫຼົ່ານີ້ ແລະ ຄວາມບໍ່ແນ້ນອນໃນການນຳໃຊ້ກັບການວິເຄາະໂມເລກຸນ, ການໃຊ້ສານເຄມີເຫຼົ່ານີ້ ທົ່ວໄປແລ້ວບໍ່ໄດ້ຮັບການແນະນຳ.

ວິທີການທີ່ນຳໃຊ້ກວ້າງຂວາງທີ່ສຸດ ແລະ ຮັກສາລັກສະນະຮູບຮ່າງຂອງຕົວຢ່າງ, DNA ຫຼື ໂປຣຕີນ ໄດ້ດີ ແມ້ນການແຊ່ແຂງ (dry freezing). ຕົວຢ່າງຕ້ອງແຊ່ແຂງເປັນເວລາພຽງພໍເພື່ອໃຫ້ຕົວຢ່າງສະຫລົບຢ່າງສົມບູນ ແຕ່ບໍ່ໃຫ້ດົນເກີນໄປຈົນ (i) ຕົວຢ່າງແຂງແຫ້ງຈົນເກີນໄປ ຫຼື (ii) ສົ່ງຜົນກະທົບຕໍ່ການມີຊີວິດຂອງ ເຊື້ອ Leishmania ຖ້າຈຸດປະສົງແມ້ນການແຍກເຊື້ອໃນຫ້ອງທົດລອງ (in vitro) ຈາກລະບົບຍ່ອຍອາຫານຂອງ ຮິ້ນຝອຍຊາຍ. **ດັ່ງນັ້ນ ພວກເຮົາແນະນຳໃຫ້ແຊ້ແຂງ 15 ຫາ 20 ນາທີ ທີ່ -20°C ແລະ ຕິດຕາມຢ້າງເປັນປະຈຳ ເພື່ອຮັບປະກັນວ່າພວກມັນພຽງແຕ່ສະຫງົບພຽງພໍ ໂດຍບໍ່ເຮັດໃຫ້ *Leishmania* ຕາຍ.**

ຖ້າບໍ່ມີຕູ້ແຊ້ແຂງ, ຮິ້ນສາມາດເຮັດໃຫ້ສະຫຼົບດ້ວຍ CO_2 ໄດ້. ໃນເງື່ອນໄຂໃນພາກສະໜາມທີ່ບໍ່ສາມາດໃຊ້ກະປ໋ອງສະເພາະໃສ່ CO_2 ໄດ້, ຕົວຢ່າງສາມາດເຮັດໃຫ້ຕາຍດ້ວຍກະປ໋ອງ CO_2 ຂະໜາດນ້ອຍທີ່ໃຊ້ໃນ 'Soda siphons' (ເຄື່ອງໃສ່ເຄື່ອງດື່ມ), ແຕ່ອາດມີຂໍ້ຈຳກັດກ່ຽວກັບການຂົນສົ່ງທາງອາກາດ. ເປັນທາງເລືອກສຸດທ້າຍ, ແມງສາມາດເຮັດໃຫ້ຕາຍດ້ວຍການສຳຜັດກັບຄວັນຢາສູບ. ຮິ້ນຝອຍຊາຍທີ່ຈັບແບບມີຊີວິດໃນກັບດັກ CDC, ເກັບດ້ວຍເຄື່ອງດູດທີ່ຮັກສາໄວ້ໃນແທ່ງແກ້ວ, ແລະ

ຖ້າໄດ້ສຳຜັດກັບຄວັນຢາສູບຈະຕາຍໃນວິນາທີ. ວິທີນີ້ນຳໃຊ້ໄດ້ໃນເງື່ອນໄຂໃນພາກສະໜາມທຸກປະເພດ ແມ່ແຕ່ໃນເງື່ອນໄຂທີ່ຫ່າງໄກສອກຫຼີກ. ເຖິງຢ່າງໃດກໍ່ຕາມ, ເນື່ອງຈາກແທ່ງແກ້ວຈະມີຄວັນແທນແກ້ວ, ມັນຈະບໍ່ສາມາດນຳໃຊ້ເກັບ ແລະ ຮັກສາຮິ້ນຝອຍຊາຍທີ່ຍັງມີຊີວິດໄດ້ອີກ ຈົນກວ່າຈະເຮັດຄວາມສະອາດຢ່າງດີ. ເຖິງຢ່າງໃດກໍ່ຕາມ, ເຄື່ອງດູດດຽວກັນທີ່ບໍ່ໄດ້ເຮັດຄວາມສະອາດ ຍັງສາມາດນຳໃຊ້ເຮັດໃຫ້ຮິ້ນຝອຍຊາຍຈາກຕູ້ດັກອື່ນໆຕາຍ ເພື່ອຈຸດປະສົງການຮັກສາຕົວຢ່າງ. ນອກຈາກນີ້ ຕ້ອງກວດສອບວ່າຕົວຢ່າງທັງໝົດໄດ້ຖືກເອົາອອກຈາກເຄື່ອງດູດແລ້ວ. ວິທີການເຫຼົ່ານີ້ ສາມາດໃຊ້ໄດ້ກັບການແຍກເຊື້ອ *Leishmania* ດ້ວຍການຜ່າຕັດລະບົບຍ່ອຍອາຫານ.

ຄຳແນະນຳເພີ່ມເຕີມ: ການພິຈາລະນາເລື່ອງຄວາມປອດໄພ ແລະ ກົດລະບຽບຄວບອ້າງອີງເອກະສານໃນຂໍ້ມູນຄວາມປອດໄພທີ່ກ່ຽວຂ້ອງ (Safety Data Sheets - SDS).

ສານເຄມີທຸກຊະນິດທີ່ນຳສະເໜີໃນແນວທາງນີ້ ຕ້ອງຈັດການພາຍໃຕ້ເງື່ອນໄຂຄວາມປອດໄພຢ່າງເຂັ້ມງວດ. ຄະນະກຳມະການສຸຂະພາບ ແລະ ຄວາມປອດໄພຂອງສະຖາບັນຄົ້ນຄວ້າ ພ້ອມທີ່ຈະໃຫ້ຂໍ້ມູນແກ່ທ່ານ ບໍ່ພຽງແຕ່ກ່ຽວກັບອັນຕະລາຍຂອງສານເຄມີເຫຼົ່ານີ້ເທົ່ານັ້ນ, ແຕ່ຍັງກ່ຽວກັບຂັ້ນຕອນການຈັດການ ແລະ ການກຳຈັດສິ່ງເສດເຫຼືອອີກດ້ວຍ. ເຖິງຢ່າງໃດກໍ່ຕາມ, ການປະຕິບັດຕາມຄຳແນະນຳດ້ານຄວາມປອດໄພກ່ຽວກັບການນຳໃຊ້ ແລະ ການກຳຈັດ ແມ່ນເປັນເລື່ອງບັງຄັບ. ແນ່ນອນວ່າ, ມັນເປັນຄວາມຮັບຜິດຊອບຂອງຜູ້ໃຊ້ທຸກຄົນເພື່ອຮັບປະກັນການປະຕິບັດຕາມການປະຕິບັດການໃນຫ້ອງທົດລອງທີ່ດີ ແລະ ປອດໄພພາຍໃຕ້ກົດໝາຍ ແລະ ກົດລະບຽບທີ່ກ່ຽວຂ້ອງຂອງປະເທດ ຫຼືສະຖາບັນການຄົ້ນຄວ້າຂອງຕົນ. ນອກຈາກນັ້ນ, ສານເຄມີບາງຊະນິດ, ຫຼືອົງປະກອບຂອງພວກມັນ (ຕົວຢ່າງ chloral hydrate) ຖືກຄວບຄຸມຢູ່ໃນບາງປະເທດ. ບັນຊີລາຍຊື່ຂອງຕົວຫຍໍ້ທີ່ໃຊ້ໃນຄູ່ມືສະບັບນີ້ແມ່ນສະແດງໃຫ້ຢູ່ໃນຕາຕະລາງ 1.

ຕາຕະລາງ 1: ລາຍຊື່ຄຳຫຍໍ້ຕ່າງໆ.

ADN	Deoxyribonucleic acid
ARN	Ribonucleic acid
BME	Basal medium Eagle
CDC	Centers for Disease Control and Prevention
CMCP	Camphor-monochlorophenol
CMR	Carcinogenic, mutagenic, reprotoxic substance
COI	Cytochrome c oxidase subunit I gene
CytB	Cytochrome b gene
ELISA	Enzyme-linked immunosorbent assay
EtOH	Ethanol
M199	Medium 199
MALDI-ToF MS	Matrix-assisted laser desorption/ionization time-of-flight mass spectrometry
MEM	Minimum essential media
NGS	Next-generation sequencing
NNN	Novy-MacNeal-Nicolle medium
PCR	ການເພີ່ມຈຳນວນດ້ວຍ Polymerase chain reaction
RDP Laos	ສ ປປ ລາວ
PNOC	Prepronociceptin gene
qPCR	Quantitative PCR (real-time PCR)
RAPD	Random amplified polymorphic DNA
RFLP	Restriction fragment length polymorphism
RI	ດັດສະນີການສະທ້ອນແສງ (Refractive index)
RNases	Ribonucleases
RNASS	RNA stabilization solution
RT-PCR	Reverse transcription PCR
TFA	Trifluoroacetic acid

3. ການເກັບຮັກສາຕົວຢ່າງກ່ອນການດຳເນີນການກະກຽມຕົວຢ່າງ

ປະກອບມີຫ້າວິທີຫຼັກ ກ່ອນການດຳເນີນການກະກຽມຕົວຢ່າງ:

3.1. ການແຊ່ແຂງ

ວິທີນີ້ດີທີ່ສຸດໃນ -20°C ຫຼື ດີກວ່າໃນ -80°C. ວິທີການເກັບຮັກສານີ້ ປະຈຸບັນນຳໃຊ້ຢ່າງກວ້າງຂວາງກວ່າການເກັບໃນນ້ຳໄນໂຕຣເຈນ. ໃນທຸກກໍລະນີ, ການເກັບຮັກສາດ້ວຍຄວາມເຢັນ ຕ້ອງດຳເນີນໄວທີ່ສຸດຫຼັງຈາກສະຫຼົບຕົວຢ່າງ. ການເກັບຮັກສາໃນຕູ້ແຊ່ແຂງ ມີຂໍ້ດີຄືຮັກສາໂຕແມງໄມ້ເອງ, ພ້ອມກັບ RNA, DNA ແລະ ໂປຣຕີນ ໃຫ້ຄົງສະພາບສົມບູນຕະຫຼອດໄລຍະເກັບຮັກສາ. ສ່ວນໄນໂຕຣເຈນແຫຼວ ອາດເຮັດໃຫ້ປີກ, ຂາ, ພາກສ່ວນປາກ ແລະ ໜວດ ເສຍຫາຍຫຼາຍ, ຂາດອອກ ແລະ ບາງຄັ້ງສູນເສຍລັກສະນະທາງຮູບຮ່າງທີ່ສຳຄັນ. ການເກັບແຊ່ແຂງແຫ້ງເປັນອັນຕະລາຍຫນ້ອຍກວ່າຕໍ່ຕົວຢ່າງ, ແຕ່ບໍ່ເໝາະສົມສຳລັບການຮັກສາອະໄວຍະວະທີ່ອ່ອນ. ສຳຄັນກວ່ານັ້ນ, ປີກ, ໜວດ, ພາກສ່ວນປາກ ຫຼື ຂາ ອາດຕິດກັບຫຼອດເກັບຕົວຢ່າງ ແລະ ຖືກດຶງອອກໄດ້ເນື່ອງຈາກການເປື່ອຍຂອງນ້ຳກ້ອນ. ເຖິງຢ່າງໃດກໍ່ຕາມ, ການຮັກສາດ້ວຍການແຊ່ແຂງ ບໍ່ສະດວກສະບາຍໃນການສຶກສາໃນພາກສະໜາມ ເພາະຕ້ອງການຕູ້ແຊ່ແຂງ ຫຼື ຖັງໄນໂຕຣເຈນ. ການເກັບໃນຕູ້ແຊ່ແຂງເຂົ້າກັນໄດ້ຢ່າງສົມບູນກັບການກວດຫາພະຍາດດ້ວຍເຄື່ອງມືໂມເລກຸນ ໂດຍບໍ່ສູນເສຍຄວາມໄວໃນການກວດ, ຖ້າຕ້ອງການເກັບຮັກສາໃນໄລຍະຍາວເພື່ອ ການກວດຫາ ແລະ ປູກການແຍກໄວຣັສ RNA ຕ້ອງການແຊ່ແຂງທີ່ -80°C ຫຼື ໄນໂຕຣເຈນ. ເຖິງຢ່າງໃດກໍ່ຕາມ, ການແຊ່ແຂງຕົວຢ່າງບໍ່ອະນຸຍາດໃຫ້ແຍກ *Leishmania* ດ້ວຍການຜ່າຕັດລະບົບຍ່ອຍອາຫານ,

ຍົກເວັ້ນກໍລະນີຈຸ່ມໃນອາຍເຢັນຂອງໄນໂຕຣເຈນ ກ່ອນ ແລະ ຫຼັງຈາກນັ້ນໃສ່ນ້ຳໄນໂຕຣເຈນ (ເຊັ່ນ: ໃສ່ຫຼອດໃນຖັງ) ເພື່ອການເກັບເຊື້ອ *Leishmania* ດ້ວຍຄວາມເຢັນ.

3.2. ການເກັບຮັກສາໃນເຫຼົ້າເອທານອນ (ethanol ຫຼື isopropyl alcohol)

ອາດເປັນວິທີທີ່ນຳໃຊ້ກວ້າງຂວາງທີ່ສຸດສຳລັບການເກັບຮິ້ນຝອຍຊາຍ. ງ່າຍຕໍ່ການປະຕິບັດໃນພາກສະໜາມ ເຖິງແມ່ນວ່າໃນເງື່ອນໄຂທີ່ຍາກລຳບາກ ໂດຍບໍ່ຕ້ອງເຂົ້າຫ້ອງທົດລອງ. ການຮັກສາໃນເອທານອນ ເໝາະສົມສຳລັບການສຶກສາລັກສະນະຮູບຮ່າງ ເພາະອະໄວຍະວະອ່ອນ (ປີກ, ຂາ, ໜວດ, ຫຼື ພາກສ່ວນປາກ) ຍັງຄົງສະພາບເຕັມຖ້າບໍ່ມີຟອງອາກາດໃນຫຼອດ. ດັ່ງນັ້ນ ພວກເຮົາແນະນຳໃຫ້ປິດຫຼອດດ້ວຍກ້ອນຝ້າຍນ້ອຍເພື່ອກຳຈັດຟອງອາກາດ ແລະ ວາງປ້າຍຢູ່ເທິງຝ້າຍ (ຮູບທີ 1). ຄວາມເຂັ້ມຂຸ້ນຂອງເອທານອນທີ່ເໝາະສົມ ຍັງມີການຖົກຖຽງກັນຢູ່. ທົ່ວໄປ ຄວາມເຂັ້ມຕ່ຳກວ່າ 70% ແມ່ນບໍ່ແນະນຳ [45, 66]. ຄວາມເຂັ້ມຂຸ້ນສູງກວ່າ ຮັກສາ DNA ໄດ້ດີກວ່າ ແລະ ຍາວນານຂຶ້ນ ແຕ່ເຮັດໃຫ້ຕົວຢ່າງບອບບາງ ແລະ ແຕກຫັກງ່າຍສຳລັບການສຶກສາລັກສະນະຮູບຮ່າງ. ການໃຊ້ 96% ethanol (azeotrope mixture) ຮັບປະກັນຄວາມເຂັ້ມຂຸ້ນທີ່ສະຖຽນຕາມໄລຍະເວລາ ໂດຍສະເພາະໃນພື້ນທີ່ຊຸ່ມຊື່ນເຊັ່ນປະເທດຮ້ອນ, ແຕ່ເຫຼົ້າ 95% ethanol ມັກຫາໄດ້ງ່າຍກວ່າ. ເຖິງຢ່າງໃດກໍ່ຕາມ, DNA ຖືກຮັກສາໄດ້ດີໃນເອທານອນ (ບໍ່ດີເທົ່າກັບການແຊ່ແຂງ ໂດຍສະເພາະສຳລັບວິທີໂມເລກຸນແບບ NGS). ໂປຣຕີນ ມີຄວາມສະຖຽນໜ້ອຍກວ່າຫຼາຍ ໂດຍສະເພາະສຳລັບ proteomics ເຊັ່ນ MALDI-ToF. ຮິ້ນຝອຍຊາຍ ທີ່ເກັບໃນເອທານອນ ເປັນເວລາສອງສາມເດືອນ ຍັງສາມາດຈຳແນກຊະນິດດ້ວຍລັກສະນະຮູບຮ່າງໄດ້, ແຕ່ບໍ່ສາມາດສ້າງສະເປັກຕະຣຳຂອງໂປຣຕີນໄດ້. ການເກັບໃນເອທານອນ ຫຼື ແຫ້ງ ສາມາດເພີ່ມປະສິດພິພາບໄດ້ ຖ້າແຊ່ແຂງຕົວຢ່າງທີ່ -20°C ເພີ່ມເຕີມ. ການແຊ່ແຂງທີ່ -20°C ຊ່ວຍເພີ່ມການຮັກສາໂມເລກຸນ (ເຊັ່ນ: ກົດນິວເຄລີກ) ໂດຍຊະລໍການແຕກສະຫຼາຍຂອງໂມເລກຸນ ແລະ ຍັງມີປະໂຫຍດທີສອງຕໍ່ການຮັກສາລັກສະນະຮູບຮ່າງ ໂດຍຫຼຸດການທຳລາຍເນື້ອເຍື່ອຕາມການເວລາ ເຖິງຢ່າງໃດກໍ່ຕາມ ຜົນຕໍ່ຮູບຮ່າງມີໜ້ອຍກວ່າຜົນຕໍ່ຄວາມສະຖຽນຂອງໂມເລກຸນ. ການເກັບໃນເອທານອນ ຍັງນຳໃຊ້ໄດ້ສຳລັບການຊອກຫາ DNA ແລະ RNA virus ເມື່ອໃຊ້ເອທານອນຄວາມເຂັ້ມຢ່າງໜ້ອຍ 70% ໃນໄລຍະເກັບຮັກສາສັ້ນ ບໍ່ເກີນໜຶ່ງຫາສອງເດືອນ. ນອກຈາກນີ້, isopropyl alcohol ອາດຫາໄດ້ງ່າຍໃນບາງປະເທດ ແລະ ຮັກສາ DNA ໄດ້, ແຕ່ເຮັດໃຫ້ຕົວຢ່າງແຂງກອບ ມັນບໍ່ເປັນໄຟໄດ້ເຊັ່ນ ethanol ດັ່ງນັ້ນຈຶ່ງຂົນສົ່ງງ່າຍ. ຖ້າຈຳເປັນ, ຮິ້ນຝອຍຊາຍ ທີ່ເກັບໃນນ້ຳໄນໂຕຣເຈນ ຫຼື ແຊ່ແຂງແຫ້ງ ສາມາດຍ້າຍໄປເກັບໃນເອທານອນ ເພື່ອປະສົມຂໍ້ເສຍຂອງທັງສອງວິທີ.

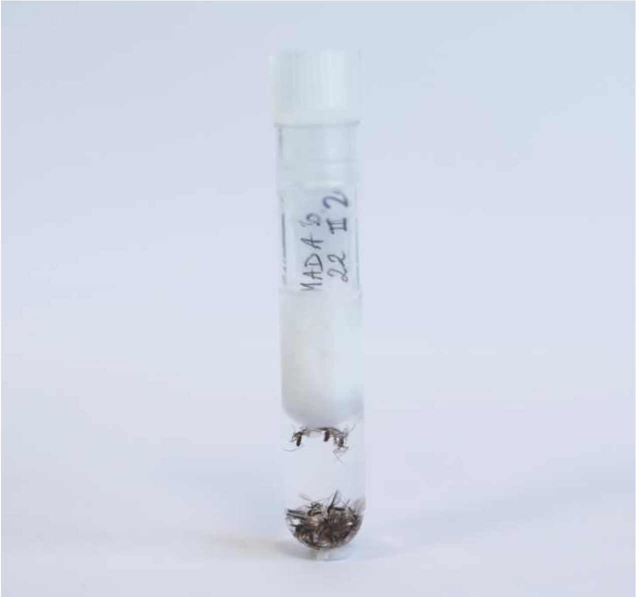

ຮູບທີ 1: ຮິ້ນຝອຍຊາຍ ທີ່ຮັກສາໄວ້ໃນເຫຼົ້າເອທານອນ.

3.3. ການເກັບຮັກສາໃນນ້ຳຢາຮັກສາສະພາບ RNA (RNASS)

ນ້ຳຢານີ້ເປັນສານນ້ຳ ທີ່ຖືກນຳໃຊ້ກວ້າງຂວາງ, ບໍ່ເປັນພິດ, ແລະ ອອກແບບມາເພື່ອຄົງສະພາບ ແລະ ປົກປ້ອງ RNA ໃນຕົວຢ່າງເນື້ອເຍື່ອ ແລະ ຕົວຢ່າງຈຸລັງ. ມັນເຮັດວຽກໂດຍການເຂົ້າໄປໃນຕົວຢ່າງຢ່າງໄວວາ ແລະ ປິດກັ້ນ RNases (ເອນໄຊທີ່ທຳລາຍ RNA), ດັ່ງນັ້ນ ຈຶ່ງປ້ອງກັນການແຕກສະຫຼາຍຂອງ RNA ໂດຍບໍ່ຕ້ອງແຊ່ແຂງທັນທີ. ການເກັບໃນ RNASS ປົກກະຕິມີປະສິດທິພາບໃນການຮັກສາຮູບຮ່າງຂອງເນື້ອເຍື່ອ ແລະ ຈຸລັງ ສຳລັບການວິເຄາະທາງການຮູບຮ່າງຕໍ່ໄປ. ເຖິງແມ່ນວ່າ RNASS ຖືກສ້າງມາສຳລັບການຄົງສະພາບຂອງໂຄງສ້າງ ແລະ ການເກັບຮັກສາໄລຍະສັ້ນເຖິງປານກາງໄດ້ດີ, RNASS ສາມາດເກັບຕົວຢ່າງໃນອຸນຫະພູມຫ້ອງໄດ້ເຖິງ 7 ວັນ, ທີ່ 4°C ເປັນເວລາຫຼາຍອາທິດ, ຫຼື ທີ່ -20°C/-80°C ເພື່ອການຮັກສາໃນໄລຍະຍາວ. ວິທີນີ້ມີຄຸນຄ່າໂດຍສະເພາະໃນການເຮັດວຽກໃນພາກສະໜາມ ຫຼື ໃນຄລີນິກ ທີ່ບໍ່ມີລະບົບເຄື່ອງເກັບຄວາມເຢັນ. ການສະກັດ RNA ປົກກະຕິແມ່ນຕ້ອງເອົາຕົວຢ່າງອອກຈາກນ້ຳຢາ ແລະ ສາມາດດຳເນີນການຕາມໂປຣໂຕຄອນມາດຕະຖານ.

3.4. ການເກັບຮັກສາແຫ້ງໃນອຸນຫະພູມຫ້ອງ

ນີ້ເປັນວິທີເກົ່າ ທີ່ນຳໃຊ້ກັບຕົວຢ່າງທັງໝົດໂຕ (mounted whole), ມີຂໍ້ເສຍຫຼັກຄືຮັກສາອະໄວຍະວະອ່ອນໄວເຊັ່ນ ປີກ, ຂາ, ໜວດ, ແລະ ພາກສ່ວນປາກ ບໍ່ດີ. ເຖິງຢ່າງໃດກໍ່ຕາມ, ການສຶກສາທາງໂປຼຕີນ ດ້ວຍ MALDI-ToF ຍັງສາມາດເຮັດໄດ້ຖ້າໃຊ້ສານປະເພດ silica gel ເຮັດໃຫ້ຕົວຢ່າງແຫ້ງ. ໃນທາງກົງກັນຂ້າມ, ການວິເຄາະທາງໂມເລກຸນທີ່ມີເປົ້າໝາຍ DNA ແມ່ນຍາກທີ່ຈະສຶກສາຕໍ່ກັບຕົວຢ່າງເຫຼົ່ານີ້, ເພາະ DNA ມັກຖືກຍ່ອຍສະຫຼາຍ ແລະ ມີປະລິມານຕ່ຳ, ເຮັດໃຫ້ການວິເຄາະຍາກກວ່າກັບຕົວຢ່າງສົດ ຫຼື ແຊ່ແຂງ, ໂດຍສະເພາະສຳລັບ ພັນທຸກຳໃນແກ່ນຈຸລັງ. ເຖິງຢ່າງໃດກໍ່ຕາມ,

ເຕັກນິກລ່າສຸດເຊັ່ນ museomics ສາມາດນໍາໃຊ້ກັບຕົວຢ່າງໃນການເກັບປະເພດນີ້ [34]. ສະນັ້ນ, ວິທີເກັບແບບນີ້ແມ່ນບໍ່ແນະນໍາ ຍົກເວັ້ນກໍລະນີບໍ່ມີທາງເລືອກອື່ນ. ສາມາດປະສົມກັບການເກັບໃນຄວາມເຢັນໂດຍຮັກສາຫຼອດຕົວຢ່າງໃນຕູ້ແຊ່ແຂງທີ່ -20°C ຫຼື -80°C, ແຕ່ບັນຫາຫຼັກກໍ່ຄືການນໍາມາແຕ່ງສະໄລຕົວຢ່າງທີ່ຖືກຕ້ອງ ຫຼື ສ່ວນຕ່າງໆຂອງຮ່າງກາຍທີ່ຈໍາເປັນສໍາລັບການຈໍາແນກຊະນິດແມ່ນມີຄວາມຫຍຸ້ງຍາກ. ເພື່ອເຮັດໄດ້, ການເຕີມນໍ້າ (rehydration) ແມ່ນມີຄວາມຈໍາເປັນ, ພວກເຮົາແນະນໍາໃຫ້ໃຊ້ນໍ້າຢາ Triton X-100ສໍາລັບການເຕີມນໍ້າ ໄລຍະເວລາໃນການເຕີມນໍ້າແມ່ນແຕກຕ່າງກັນແຕ່ສອງສາມຊົ່ວໂມງຫາເປັນຫຼາຍວັນ, ຕ້ອງໄດ້ຕິດຕາມຢ່າງໃກ້ຊິດ. ຫຼັງຈາກເຕີມນໍ້າສົມບູນ, ຕົວຢ່າງຄວນລ້າງດ້ວຍນໍ້າ 3 ເທື່ອຕິດຕໍ່ກັນ.

3.5. ການເກັບຮັກສາໃນເຈັຍກັນຕອງ

ຂໍ້ດີຫຼັກຂອງເຈັຍກັນຕອງຄືຮັກສາຄວາມສະຖຽນຂອງ DNA ໃນຈຸລັງທີ່ບໍ່ໄດ້ຮັບການຄົງສະພາບ ແລະ ເຮັດໃຫ້ແຫ້ງ ຫຼື ຈຸລັງເມັດເລືອດທີ່ເກັບໃນອຸນຫະພູມຫ້ອງ. ເຈັຍກອງມີຂະໜາດນ້ອຍ, ສາມາດເກັບຫຼາຍຮ້ອຍຕົວຢ່າງໃນອຸນຫະພູມຫ້ອງ ໃນບໍລິມາດຂະໜາດຂອງໂຕະນ້ອຍໆ. ໃນເຈັຍກອງແມ່ນຖືກຍ້ອມດ້ວຍສານທີ່ໃຊ້ອະເຊື້ອທີ່ກໍ່ໃຫ້ເກີດພະຍາດຕິດຕໍ່, ດັ່ງນັ້ນ ຕົວຢ່າງບໍ່ຖືກນັບວ່າເປັນຄວາມສ່ຽງທາງຊີວະພາບ (biohazard) ເຊິ່ງເຮັດໃຫ້ການເກັບຮັກສາ ແລະ ຂົນສົ່ງຕົວຢ່າງໂດຍບໍ່ຕ້ອງມີມາດຕະການປ້ອງກັນຄວາມສ່ຽງທາງຊີວະພາບພິເສດ [68].

4. ການຜ່າຕັດຕົວຢ່າງ

ຕ່າງຈາກແມງຫຼາຍຊະນິດທີ່ຖືກຈໍາແນກຊະນິດດ້ວຍລັກສະນະພາຍນອກທີ່ສາມາດສັງເກດເຫັນໄດ້ໃນຕົວຢ່າງແມງໄມ້ທີ່ຕິດເປັນຕົວທັງໝົດ (pinned in toto), ຮິ້ນຝອຍຊາຍ ຕ້ອງການການຜ່າຕັດ ແລະ ການແຕ່ງໃສ່ແຜ່ນແກ້ວສະໄລ ເພື່ອສຶກສາລັກສະນະທາງກາຍະວິທະຍາສໍາລັບການຈໍາແນກຊະນິດທີ່ຖືກຕ້ອງ. ເຖິງຈະເລືອກຂັ້ນຕອນກະກຽມ ແລະ ການແຕ່ງສະໄລແນວໃດກໍ່ຕາມ, ການຜ່າຕັດແມ່ນໃຊ້ເຕັກນິກດຽວກັນ (ຮູບທີ 2 ແລະ 3) (https://zenodo.org/records/18198006).

ການໃຊ້ນໍ້າ Triton X100: ນໍ້າຢານໍ້າບໍ່ມີໄອອອນ (non-ionic aqueous solution)

ເປັນທີ່ສັງເກດວ່າການແຕ່ງສະໄລແມ່ນກ່ຽວພັນກັບຕົວຢ່າງຍັງສົດທີ່ຈັບໃໝ່ ຫຼື ເກັບຮັກສາໄວ້ໄດ້ດີ. ນັກເກັບຕົວຢ່າງສ່ວນໃຫຍ່ແມ່ນມີຕົວຢ່າງທີ່ເກັບແຫ້ງ (ທີ່ສໍາລັບການໃຊ້ MALDI-ToF) ຫຼື ເກັບໃນເຫຼົ້າເອທານອນເປັນເວລາຫຼາຍປີ. ແຕ່ໜ້າເສຍດາຍທີ່ການເກັບຕົວຢ່າງໃນເຫຼົ້າເອທານອນທີ່ບໍ່ເໝາະສົມໃນໄລຍະເວລາຫຼາຍປີ, ແລະ ການຮັກສາຕົວຢ່າງແມງໄມ້ຂາຂໍອື່ນໆທີ່ເກັບແບບນີ້ ຈະກາຍເປັນຂໍ້ຫຍຸ້ງຍາກຫຼາຍຕໍ່ການກະກຽມສໍາລັບການສັງເກດດ້ວຍກ້ອງຈຸລະທັດ. ເຫດການທີ່ເກີດຂຶ້ນ ແລະ ມັກພົບເລື້ອຍໆ ແມ່ນການເສື່ອມສະພາບຄວາມຢືດຢຸ່ນຂອງຕົວຢ່າງ, ຕາມດ້ວຍການລະເຫີຍຂອງເຫຼົ້າເອທານອນ. ໃນທັງສອງກໍລະນີ, ພວກເຮົາບໍ່ມີທາງເລືອກ ເພາະຕົວຢ່າງຢູ່ໃນເຫຼົ້າເອທານອນເປັນເວລາດົນເກີນໄປ ຫຼື ແຫ້ງ. ດັ່ງນັ້ນ ຈຶ່ງເກີດແນວຄວາມຄິດໃຊ້ນໍ້າຢາເຕີມນໍ້າ (wetting agents) ທີ່ບໍ່ແມ່ນນໍ້າຢາຊັກຟອກທີ່ແຮງ. Triton X100 ເປັນນໍ້າຢານໍ້າບໍ່ມີໄອອອນ (4-(1,1,3,3-tetramethylbutyl) phenyl-polyethylene glycol solution, ຫຼື t-octylphenoxypolyethoxyethanol, polyethylene glycol tert-octylphenyl ether), ຖືກນໍາໃຊ້ກວ້າງຂວາງເປັນນໍ້າຢາຊັກຟອກໃນການສຶກສາຈຸລັງ ແລະ ຊີວະໂມເລກຸນ ມັນຊ່ວຍເຮັດໃຫ້ເຍື່ອຫຸ້ມຈຸລັງ ແລະ ເຍື່ອຫຸ້ມແກ່ນຈຸລັງສາມາດຊຶມຜ່ານໄດ້ (permeabilization).

ຂັ້ນຕອນການໃຊ້ Triton X100 ທີ່ບໍ່ມີໄອອອນໃນນໍ້າຢາ 0.5% ມີດັ່ງນີ້:

- ເອົາເຫຼົ້າເອທານອນບໍລິສຸດໃສ່ໃນຕົວຢ່າງແຫ້ງ;
- ຕື່ມປະລິມານ Triton X100 0.5% ທີ່ຈາເປັນເພື່ອໃຫ້ຕົວຢ່າງຈົມລົງຈົນໝົດ;
- ປະໄວ້ໃຫ້ມີການດໍາເນີນການປະສົມກັນໄປເປັນເວລາປະມານ 5 ນາທີ ຫາ ຫຼາຍວັນ, ຕິດຕາມຢ່າງເປັນປະຈໍາ. ຕົວຢ່າງແມງໄມ້ຂາຂໍທັງໝົດຕ້ອງແຍກອອກຈາກກັນໄດ້ຢ່າງສົມບູນໃນນໍ້າຢາ;
- ເອົານໍ້າຢາ Triton X100 ອອກ ແລະ ແທນດ້ວຍນໍ້າຢາ potassium hydroxide.

4.1. ຫົວ (Head)

ການຜ່າຕັດສາມາດດໍາເນີນການໄດ້ໂດຍໃຊ້ເຂັມນ້ອຍໆ ຫຼື ເຂັມແມງໄມ້ (entomological pins) ພາຍໃຕ້ກ້ອງສະເຕຣີໂອ (stereomicroscope) (ຮູບທີ 2 ແລະ 3). ເຂັມທີ່ນໍາໃຊ້ທົ່ວໄປປະກອບມີຂະໜາດ: 26G x 1/2" (0.45 × 13 mm), 30G × 1/2" (0.3 × 13 mm), ຫຼື 25G × 5/8" (0.5 × 16 mm). ເພື່ອກະກຽມຕົວຢ່າງສໍາລັບການຈໍາແນກຊະນິດ, ຢ່າງໜ້ອຍຕ້ອງແຍກຫົວອອກຈາກຮ່າງກາຍ ແລະ ແຕ່ງສະໄລດ້ານລຸ່ມຂຶ້ນເທິງ (ventral side up) ເພື່ອສະແດງຜົ້ງປາກ (cibarium) ແລະ ຜົ້ງຄໍ (pharynx), ໃນຂະນະທີ່ ເອິກ (thorax) ແລະ ທ້ອງ (abdomen) ແມ່ນແຕ່ງສະໄລດ້ານຂ້າງ (laterally) ຫຼັງການຜ່າຕັດ. ການແຕ່ງສະໄລຫົວໃນແບບໜ້າ-ຫຼັງ (ventro-dorsal) ແມ່ນເພື່ອຮັບປະກັນວ່າຫົວດ້ານ occipital foramen ຊີ້ຂຶ້ນເທິງ, ເຮັດໃຫ້ cibarium ສາມາດສັງເກດເບິ່ງໄດ້ໂດຍກົງ. ການເຂົ້າເຖິງລັກສະນະກາຍວິທະຍາເຫຼົ່ານີ້ ຈະງ່າຍຂຶ້ນຖ້າຫົວຖືກຕັດແຍກອອກຈາກຮ່າງກາຍຢ່າງສົມບູນ.

4.2. ປີກ ແລະ ເອິກ (thorax)

ປີກຕ້ອງແຕ້ງສະໄລໃຫ້ແຜ່ອອກແບບຮາບພຽງ ປີກແຕ່ລະຂ້າງສາມາດຕັດແຍກອອກທີ່ກົກປີກ ແລະ ແຕ້ງສະໄລແຍກກັນ, ຫຼື ແຕ້ງສະໄລປີກຂ້າງໜຶ່ງແຍກ ແລະ ອີກຂ້າງໜຶ່ງຍັງຕິດກັບເອິກ. ຖ້າມີແຜນການວິເຄາະທາງເລຂາຄະນິດ (geometric morphometry), ຈຳເປັນຕ້ອງລະບຸ ແລະ ໝາຍປ້າຍລະຫັດປີກຂວາ ແລະ ຊ້າຍໃຫ້ຖືກຕ້ອງກ່ອນແຕ້ງສະໄລ. ເອິກແບ່ງອອກເປັນຫຼາຍສ່ວນ ແລະ ແຕ່ລະສ່ວນມີຂໍ້ມູນທາງອານຸກົມວິຖານ (taxonomy)ທີ່ສາຄັນຫຼາຍ [20, 64]. ໂດຍທົ່ວໄປແຕ້ງສະໄລເອິກໃນແບບດ້ານຂ້າງ (lateral view) ເພື່ອສັງເກດເບິ່ງຂົນ chetotaxy ແລະ ການກະຈາຍສີ. ການມີຮູຂອງຂົນໃນບາງພື້ນທີ່ຂອງເອິກສາມາດໃຊ້ໃນການຈຳແນກຊະນິດຂອງບາງຊະນິດໃນ ສະກຸນ (genus) *Brumptomyia*. ການກະຈາຍສີ ອາດໃຊ້ແຍກ ຮິ້ນຝອຍຊາຍ ໃນ Neotropical ທີ່ລະດັບ ສະກຸນ (ເຊັ່ນ *Bichromomyia*), series ຊະນິດ (ເຊັ່ນ *Pintomyia*), ຫຼື ແມ້ແຕ່ຊະນິດໃນ ສະກຸນ ດຽວກັນ (ເຊັ່ນ *Micropygomyia*, *Nyssomyia*, *Psathyromyia*, ແລະ *Psychodopygus*) [20]. ດັ່ງນັ້ນ ຖ້າ ເອິກ ບໍ່ໃຊ້ສໍາລັບການວິເຄາະດ້ານໂມເລກຸນ, ຄວນແຕ້ງສະໄລບໍ່ໃຫ້ເສຍຫາຍ. ສໍາຄັນກວ່ານັ້ນ, ບໍ່ແມ່ນຄວາມເຂັ້ມຂອງສີ ແຕ່ເປັນການກະຈາຍຂອງສີຕາມເອິກ. ດັ່ງນັ້ນ ຂັ້ນຕອນການເຮັດໃຫ້ໂປ່ງໃສຈະຕ້ອງບໍ່ທຳລາຍສີ ຫຼື ຮູບແບບສີຂອງມັນ.

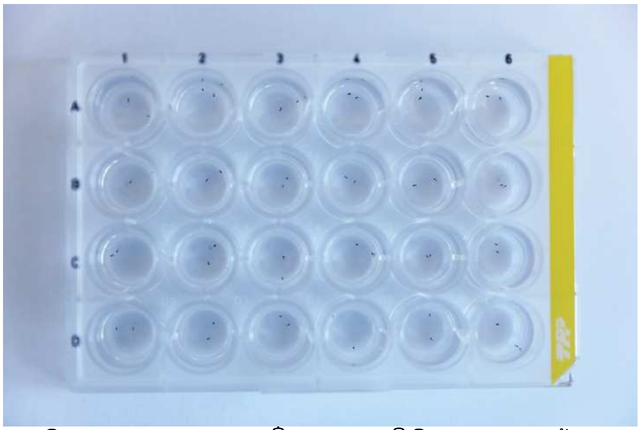

ຮູບທີ 3: ແຜ່ນ 24 ຂຸມ, ເຊິ່ງແຕ່ລະຂຸມມີຫົວ ແລະ ປາຍທ້ອງຂອງ ຮິ້ນຝອຍຊາຍ.

4.3. ອະໄວຍະວະສືບພັນ (Genitalia)

ຕ້ອງປະຕິບັດດ້ວຍຄວາມລະມັດລະວັງເປັນພິເສດເມື່ອແຕ້ງສະໄລອະໄວຍະວະສືບພັນທັງໃນຕົວຜູ້ ແລະ ຕົວແມ່, ເພາະມັນສຳຄັນຕໍ່ການຈຳແນກ ສະກຸນ (genus), ສະກຸນຍ່ອຍ (subgenus), ແລະ ຊະນິດ (species). ໃນທັງສອງເພດ ອະໄວຍະວະສືບພັນມີເປັນຄູ່.

4.3.1. ຕົວຜູ້ (Males)

ອະໄວຍະວະສືບພັນພາຍນອກ ແລະ ປະກອບດ້ວຍ forceps ຄູ່, ແຕ່ລະອັນເປັນ gonocoxite-gonostyle articulation ທີ່ດ້ານເທິງ ແລະ epandrial lobe ດ້ານລຸ່ມ. Gonostyle ມີ spines ແລະ ບາງຄັ້ງຂົນ setae, ຕ້ອງນັບໄດ້ ແລະ ເບິ່ງຕຳແໜ່ງການຕິດໄດ້ຊັດເຈນ.

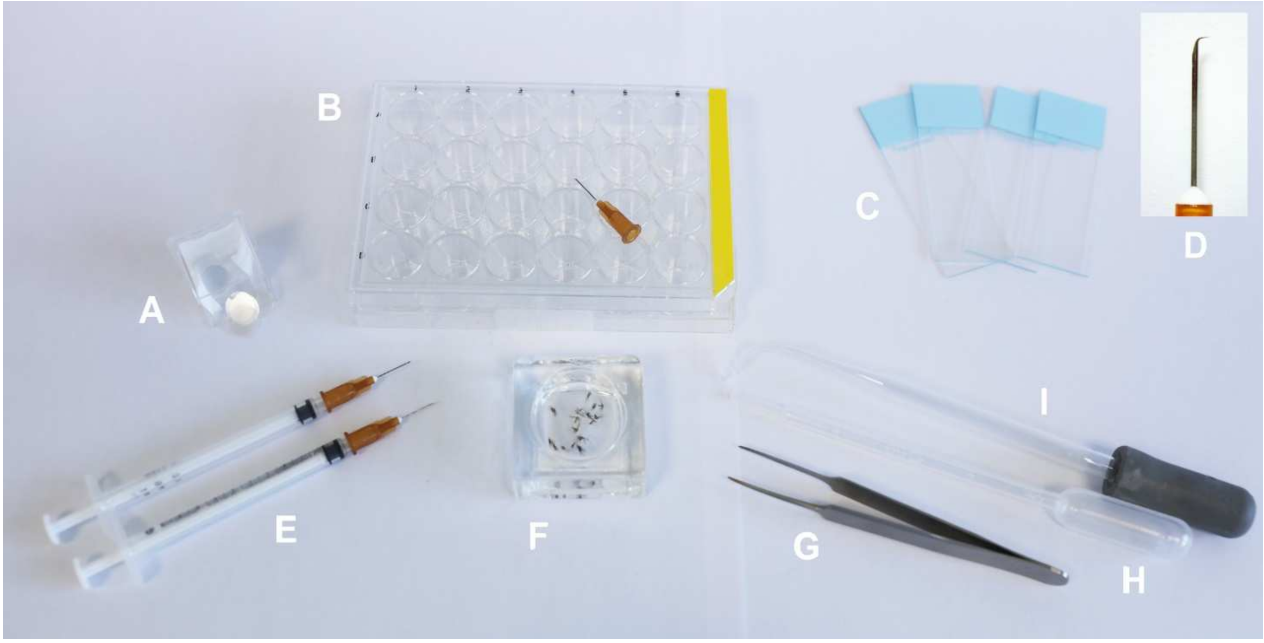

ຮູບທີ 2: ອຸປະກອນທີ່ຕ້ອງການສໍາລັບການແຕ້ງສະໄລ ຮິ້ນຝອຍຊາຍ: A: ແຜ່ນປົກແກ້ວມົນ (ເສັ້ນຜ່ານກາງ 10 ຫຼື 12 mm); B: ແຜ່ນ 24 ຂຸມ ແລະ ເຂັມມີຫົວ (ຖ້າໃຊ້ນ້ຳມັນ clove oil ຫຼື Euparal® essence ເພື່ອແຕ້ງ ຮິ້ນຝອຍຊາຍ, ຢ່າໃຊ້ແຜ່ນອະຄຣີລິກເພາະຈະເກີດປະຕິກິລິຍາທາງເຄມີ ແລະ ຕົວຢ່າງຈະເສຍຫາຍ); C: ແຜ່ນແກ້ວທີ່ເໝາະສົມສໍາລັບຕິດລະຫັດ; D: ລາຍລະອຽດຂອງເຂັມມີປາຍຂໍ; E: ເຂັມຕິດກັບດ້າມຊະລິ້ງ; F: ແກ້ວ ຫຼື ພາຊະນະທຽບເທົ່າທີ່ໃຊ້ວາງຮິ້ນຝອຍຊາຍ ທີ່ຈະແຕ້ງສະໄລ; G: ຄີບ Dumont; H: ປີແປັດດູດແບບພາດສະຕິກ; I: ປາຍແກ້ວທີ່ງໍດ້ວຍການໃຊ້ຄວາມຮ້ອນເພື່ອປ່ຽນນ້ຳຢາໃສ່ຮູງ່າຍຂຶ້ນ.

This is an Open Access article distributed under the terms of the Creative Commons Attribution License (https://creativecommons.org/licenses/by/4.0), which permits unrestricted use, distribution, and reproduction in any medium, provided the original work is properly cited.

ສໍາຄັນແມ່ນຕ້ອງສັງເກດດ້ານໃນກອງ gonocoxite, ເຊິ່ງອາດມີ tuft ຂອງ sessile setae ຫຼື ຕິດກັບ lobe (= tubercle) [22]. ຜູ້ທີ່ມີປະສົບການໜ້ອຍອາດແຕ່ງສະໄລດ້ານຂ້າງ lateral ງ່າຍໆ ໂດຍບໍ່ແຍກອະໄວຍະວະສືບພັນອອກຈາກປາຍທ້ອງ (https://zenodo.org/records/18311158). ໃນກໍລະນີນີ້, ການທັບຊ້ອນກັນຂອງສອງສ່ວນ ອາດເຮັດໃຫ້ນັບ setae ດ້ານໃນ gonocoxite ຍາກ, ແຕ່ປ້ອງກັນການເສຍຫາຍອະໄວຍະວະສືບພັນເນື່ອງຈາກຜ່າຕັດຜິດພາດ .
ຜູ້ມີປະສົບການອາດພະຍາຍາມເປີດອະໄວຍະວະສືບພັນເປັນສອງສ່ວນ ເພື່ອເຮັດໄດ້, ໃຊ້ດ້ານ bevel ຂອງເຂັມ (ປະເພດ intradermal reaction needle) ຜ່ານໄປ ໂດຍແຍກແຕ່ບໍ່ຕັດສົມບູນ ເພື່ອແຍກ gonocoxite-gonostyle (https://zenodo.org/records/18311158). ແບບນີ້ ການສັງເກດດ້ານໃນ ຈະງ່າຍຂຶ້ນ. ການແຕ່ງສະໄລນີ້ ຍັງຊ່ວຍເບິ່ງ parameres ແລະ parameral sheaths ໄດ້ງ່າຍ ເພາະບໍ່ທັບຊ້ອນກັນ.
ສໍາລັບການແຕ່ງສະໄລດ້ານຂ້າງທີ່ເຮັດໃຫ້ອະໄວຍະວະທັບຊ້ອນ, ຕົວຢ່າງຕ້ອງໄດ້ເຮັດໃຫ້ໃສຢ່າງສົມບູນ.

4.3.2. ຕົວແມ່ (Females)

ອະໄວຍະວະສືບພັນເປັນພາຍໃນ, ປະກອບດ້ວຍຖົງເກັບນໍ້າເຊື້ອ spermathecae. ຖ້າບໍ່ຜ່າຕັດ, ຕ້ອງສັງເກດຜ່ານເປືອກຫຸ້ມ (teguments) ແລະ ແຕ່ງສະໄລທ້ອງ ດ້ານລຸ່ມ (ventral position). ເຖິງຈະເລືອກ ນໍ້າຢາແຕ່ງສະໄລ (mounting medium) ແນວໃດກໍ່ຕາມ, spermathecae ປົກກະຕິສາມາດສັງເກດເບິ່ງໄດ້ດີ ໂດຍສະເພາະຖ້າບໍ່ແມ່ນ ກ້ຽງ (smooth) ແລະ ໃສ (cleared). ເຖິງຢ່າງໃດກໍ່ຕາມ, ການສັງເກດ smooth, thin-walled spermathecae ອາດຍາກໃນ medium ທີ່ refractile ບໍ່ດີ. ນອກຈາກນີ້, ການສັງເກດ ພື້ນ (base) ຂອງທໍ່ spermathecal ducts ຈໍາເປັນສໍາລັບການຈໍາແນກຊະນິດ, ເຊັ່ນໃນ ສະກຸນຍ່ອຍ (subgenus) Larroussius [35, 37, 38], ພາຫະນໍາຫຼັກຂອງ Leishmania infantum ໃນ Old World. ຖ້າບໍ່ສັງເກດໄດ້, ການຈໍາແນກຕົວຢ່າງຈະເປັນໄປບໍ່ໄດ້. ເພື່ອແກ້ໄຂຄວາມຍາກນີ້, ຄວນແຍກ genital furca-spermathecae ອອກຈາກ abdomen (https://zenodo.org/records/18311106).
Spermathecae ມັກຍາກສັງເກດໃນລະຫວ່າງຜ່າຕັດ, ແຕ່ genital furca ພົບງ່າຍກວ່າ. ເນື່ອງຈາກ spermathecal ducts ເປີດເຂົ້າ genital furca, ການແຍກ furca ນີ້ ປົກກະຕິອະນຸຍາດໃຫ້ແຍກ spermathecae ໄດ້. ຖ້າສັບສົນແລະຕັດ spermathecae ໂດຍບໍ່ຕັ້ງໃຈ, ມັນບໍ່ສູນເສຍ ແລະ ຍັງສາມາດສັງເກດໄດ້ໃນ teguments ຂອງ abdomen (ຮູບທີ 4).

4.4. ການຜ່າຕັດກະເພາະສໍາລັບການປູກແຍກເຊື້ອ Leishmania

ການຜ່າຕັດລະບົບຍ່ອຍອາຫານ ຈໍາເປັນສໍາລັບການກວດຫາ ແລະ ປູກແຍກເຊື້ອ Leishmania ໃນ ຮິ້ນຝອຍຊາຍ ຕົວແມ່. ຂັ້ນຕອນນີ້ດໍາເນີນໄດ້ທັງໃນພາກສະໜາມ ແລະ ພາຍໃນຫ້ອງທົດລອງ ເພື່ອປະເມີນຄວາມສາມາດໃນການເປັນພາຫະ vectorial competence ຂອງຮິ້ນ.

ແນະນໍາໃຫ້ເຮັດກັບຕົວແມ່ທີ່ເຮັດໃຫ້ຕາຍໃໝ່. ລ້າງຕົວແມ່ດ້ວຍນໍ້າ ຫຼື saline solution ທີ່ມີຄວາມເປັນນໍ້າຢາຂ້າເຊື້ອອ່ອນໆ ເພື່ອກໍາຈັດຂົນ. ຂັ້ນຕອນນີ້ຊ່ວຍຮັກສາຄວາມອະນາໄມສໍາລັບການແຍກ Leishmania,
ໃນຂະນະທີ່ຮັກສາລັກສະນະຮູບຮ່າງທີ່ຈໍາເປັນສໍາລັບການຈໍາແນກ.
ເພື່ອຊອກຫາ ແລະ ປູກແຍກເຊື້ອ Leishmania, ແຍກກະເພາະ midgut ອອກຢ່າງລະມັດລະວັງ ແລະ ໃສ່ໃນນໍ້າຫຍົດດຽວຂອງ sterile saline solution (0.9% NaCl). ຫຼັງຈາກສັງເກດແມ່ກາຝາກ parasites ທີ່ເຄື່ອນໄຫວດ້ວຍກ້ອງຈຸລະທັດ (magnification ປະມານ 200×), ໃຊ້ insulin syringe ຫຼື micropipette ຖ່າຍໄປໃນພູມປູກ cultivation medium (ລາຍລະອຽດເພີ່ມເຕີມເບິ່ງບົດທີ 4.4.3).

ແຕ່ງສະໄລຫົວ ແລະ ອະໄວຍະວະສືບພັນໂດຍກົງໃນນໍ້າຢາ Marc-André ເພື່ອເຮັດໃຫ້ໂປ່ງໃສ. ສໍາຄັນ: ຫ້າມໃຫ້ນໍ້າ Marc-André ສໍາຜັດກັບ Leishmania – ບໍ່ວ່າຈະໂດຍກົງ ຫຼື ຜ່ານເຄື່ອງມື ຫຼື ເຂັມ – ເພາະມັນທໍາລາຍແມ່ກາຝາກ parasites.

ການຜ່າຕັດ ຮິ້ນຝອຍຊາຍ ຕົວແມ່ ສາມາດເຮັດໃນແຜ່ນສະໄລແຜ່ນດຽວ ຫຼື ສອງແຜ່ນ; ທັງສອງວິທີມີຂໍ້ດີ ແລະ ຂໍ້ຈໍາກັດ (ຮູບທີ 5; https://zenodo.org/records/18311154).

4.4.1. ວິທີໃຊ້ແຜ່ນສະໄລສອງແຜ່ນ

ວິທີທໍາອິດແມ່ນການໃຊ້ແຜ່ນແກ້ວສອງແຜ່ນແຍກກັນ:
ແຜ່ນທີໜຶ່ງແມ່ນມີນໍ້າກັ່ນເພື່ອແຍກເຊື້ອຈາກກະເພາະລໍາໄສ້,
ແຜ່ນອີກໜຶ່ງແມ່ນເພື່ອແຕ່ງສະໄລຫົວ ແລະ ຖົງເກັບນໍ້າເຊື້ອໃນນໍ້າຢາ Marc-André. ເຖິງຢ່າງໃດກໍ່ຕາມ, ໃນເງື່ອນໄຂໃນພາກສະໜາມ, ມັກມີ 2 ຫາ 3 ຄົນ ຜ່າຕັດຮິ້ນຝອຍຊາຍ ແລະ ສົ່ງໄປໃຫ້ນັກຄົ້ນຄວ້າຄົນໜຶ່ງທີ່ຮັບຜິດຊອບການຈໍາແນກຊະນິດຮິ້ນ ແລະ ອີກຄົນນຶ່ງເພື່ອຊອກຫາເຊື້ອ Leishmania ໃນກະເພາະລໍາໄສ້.
ການປະຕິບັດງານໂດຍໃຊ້ແຜ່ນແກ້ວສອງແຜ່ນ
ອາດເຮັດໃຫ້ການຕິດຕາມຕົວຢ່າງຫຍຸ້ງຍາກ ໂດຍສະເພາະ ການນໍາຫາແຜ່ນແກ້ວຂອງຊະນິດຮິ້ນ ແລະ ແຜ່ນແກ້ວກະເພາະລໍາໄສ້ ຖ້າພົບພົບເຊື້ອ (https://zenodo.org/records/18311154).

4.4.2. ວິທີໃຊ້ແຜ່ນສະໄລແຜ່ນ ແຜ່ນດຽວ

ການໃຊ້ແຜ່ນສະໄລແຜ່ນດຽວ ແມ່ນຮັບປະກັນການຕິດຕາມຜົນ. ແຕ່ ເຖິງຢ່າງ ໃດກໍ່ຕາມ, ຕ້ອງປະຕິບັດດ້ວຍຄວາມລະມັດລະວັງເພື່ອເພີ່ມ

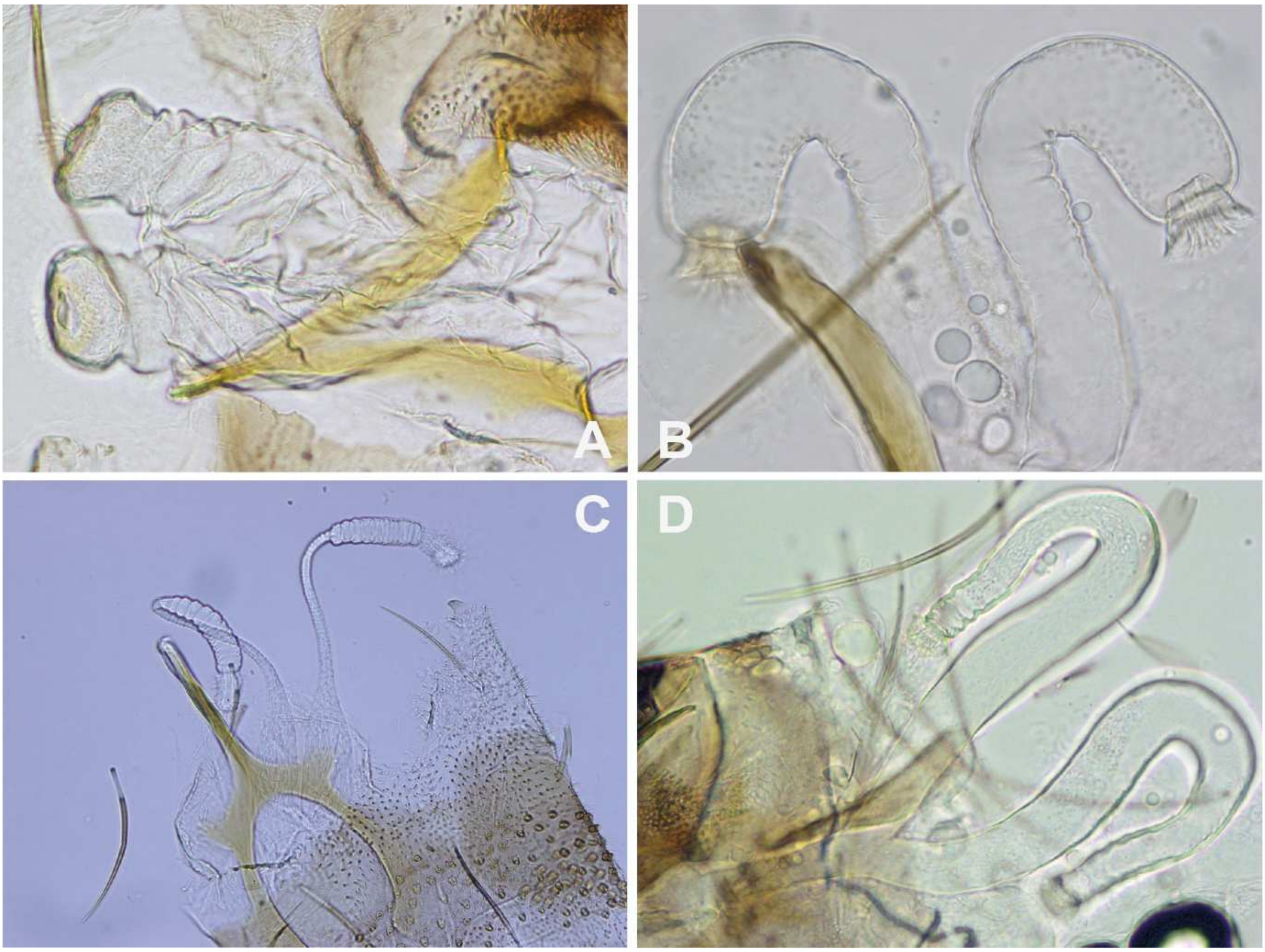

ຮູບທີ 4: Spermathecae ທີ່ຜ່າຕັດ ແລະ ແຕ່ງສະໄລໃນນ້ຳ Marc-André ຈາກຕົວຢ່າງສົດ. A: *Idiophlebotomus longiforceps* (ສ.ປ.ປ ລາວ); B: *Sergentomyia minuta* (France); C: *Phlebotomus ariasi* (France); D: *Sergentomyia anodontis* (ສ.ປ.ປ ລາວ).

ຄວາມອະເຊື້ອສູງສຸດໃນຂັ້ນຕອນນີ້, ຜູ້ດຳເນີນ ການຕ້ອງເຮັດຄວາມສະອາດມືດ້ວຍເຈວ hydroalcoholic ເປັນປະຈຳ. ຕ້ອງໃຊ້ແຜ່ນສະໄລທີ່ບໍ່ແມ່ນ frosted ແລະ ແຜ່ນປົກແບບສີ່ຫຼ່ຽມ (22 x 22 mm) ທີ່ຫໍ່ດ້ວຍແຜ່ນ aluminium foil ແລະ ເຮັດໃຫ້ sterile ດ້ວຍຄວາມຮ້ອນ (ໃຊ້ເຕົາ Poupinel), ພ້ອມກັບເຂັມ sterile ໃໝ່ສຳລັບການຜ່າຕັດແຕ່ລະຄັ້ງ (ແນະນຳ: 25G Ø 0.5mm × 16mm). ວາງ ຣິ້ນຝອຍຊາຍ ໃນນ້ຳ sterile saline ກາງແຜ່ນ. ຕັດຫົວອອກ, ຕັດລະຫວ່າງ tergites ແລະ sternites ຂອງທ້ອງ ຂໍ້ທີ 6 ແລະ 7 ໂດຍບໍ່ໃຫ້ຕັດລະບົບຍ່ອຍອາຫານ (ສາມາດຕັດສູງກວ່ານີ້ ຖ້າຄາດວ່າຖົງເກັບນ້ຳເຊື້ອຍາວຫຼາຍ). ຫຼັງຈາກນັ້ນ, ຍຶດເອິກໄວ້ດ້ວຍເຂັມໜຶ່ງ, ແລະ ດຶງສ່ວນປາຍທ້ອງ ໄປທາງດ້ານຫຼັງດ້ວຍເຂັມອີກອັນໜຶ່ງເພື່ອດຶງລຳໄສ້ອອກ. ຖ້າບໍ່ສຳເລັດ, ອາດຍຶດ ປາຍທ້ອງດ້ວຍເຂັມ ແລະ ດຶງລະບົບຍ່ອຍອາຫານຈາກສ່ວນໜ້າ. ຖ້າຍັງບໍ່ສຳເລັດ, ຕ້ອງດຶງລຳໄສ້ອອກໂດຍທຳລາຍ tegument ທີ່ເຫຼືອຢູ່ຮອບມັນໃຫ້ຫຼາຍທີ່ສຸດ. ເມື່ອດຶງ ລຳໄສ້ອອກໄດ້, ແຍກສ່ວນປາຍທ້ອງສຸດທ້າຍດ້ວຍການຕັດລະບົບຍ່ອຍ ອາຫານ. ໃສ່ລຳໄສ້ໃນນ້ຳ sterile saline ໃໝ່ ທີ່ວາງໄວ້ຂ້າງໜຶ່ງຂອງແຜ່ນ, ແລະ ປົກດ້ວຍແຜ່ນປົກ sterile ຢ່າງຄ່ອຍໆ. ຍ້າຍຫົວ ແລະ ສ່ວນປາຍທ້ອງສຸດທ້າຍໄປໃນນ້ຳຢາແຕ່ງສະໄລ Marc-André ນ້ອຍໆ ທີ່ວາງໄວ້ອີກປາຍໜຶ່ງຂອງແຜ່ນ, ຮັບປະກັນບໍ່ສຳຜັດກັບ Leishmania. ຈັດຫົວໃຫ້ຖືກຕ້ອງ (occipital foramen ຂຶ້ນເທິງ), ແລະ ແຍກຖົງເກັບນ້ຳເຊື້ອ ກັບ genital furca ຕາມທີ່ອະທິບາຍຂ້າງເທິງ ແລະ ປົກດ້ວຍແຜ່ນປົກມົນນ້ອຍ (Ø 12 mm, ຢ່າສັບສົນກັບແຜ່ນປົກສີ່ຫຼ່ຽມ sterile). ສ່ວນທີ່ເຫຼືອຂອງຮ່າງກາຍ ຣິ້ນຝອຍຊາຍ ແລະ ປົກ ຍັງຢູ່ໃນນ້ຳ saline ກາງແຜ່ນ (https://zenodo.org/records/18311154).
ໃນກໍລະນີໃຫ້ຜົນບວກ, ຫຼື ສຳລັບການສຶກສາ ການຈຳແນກຊະນິດ, ເອິກ ແລະ ທ້ອງ ສາມາດເກັບຮັກສາໄວ້ສຳລັບການວິເຄາະໂມເລກຸນ ຫຼື proteomic, ແລະ ປົກ ສາມາດແຕ່ງສະໄລໃນນ້ຳຢາທີ່ເປັນນ້ຳ. ເພື່ອຮັກສາການແຕ່ງສະໄລ, ປະລິມານເພີ່ມເຕີມຂອງນ້ຳ Marc-André ສາມາດປ່ຽນແທນດ້ວຍ ນ້ຳຢາແຕ່ງສະໄລ ທີ່ເປັນ medium ນ້ຳ ເຊັ່ນ ກົາ chloral (=Hoyer) ຫຼື medium ອີງໂພລີວີນິນ ອາລຄອຮອນ.

ວິດີໂອລະອຽດທີ່ສະແດງຂັ້ນຕອນເຫຼົ່ານີ້ມີໃຫ້ເບິ່ງ (ການຜ່າຕັດກະເພາະລຳໄສ້ຣິ້ນຝອຍຊາຍ: https://zenodo.org/records/18303014 ແລະ ການຜ່າຕັດຕ່ອມນ້ຳລາຍຣິ້ນຝອຍຊາຍ:

This is an Open Access article distributed under the terms of the Creative Commons Attribution License (https://creativecommons.org/licenses/by/4.0), which permits unrestricted use, distribution, and reproduction in any medium, provided the original work is properly cited.

https://zenodo.org/records/18302850), ດັ່ງນັ້ນ ຈຶ່ງບໍ່ອະທິບາຍຊ້ຳໃນທີ່ນີ້.

4.4.3. ການແຍກ ແລະ ປູກ *Leishmania* ຈາກກະເພາະລຳໄສ້ຂອງ ຮິ້ນຝອຍຊາຍ

ການແຍກແມ່ກາຝາກຈາກການຜ່າຕັດ ຮິ້ນຝອຍຊາຍໃນໂຕແມ່ທີ່ຕິດເຊື້ອ ເປັນຂັ້ນຕອນທີ່ລະອຽດອ່ອນ ຕ້ອງການທັກສະສູງ ແລະ ຄວນເລີ່ມຝຶກກັບຕົວຢ່າງທີ່ບໍ່ມີແມ່ກາຝາກກ່ອນ. ຫຼັງການຜ່າຕັດ, ຍ້າຍເອົາລຳໄສ້ໄປໃສ່ໃນນ້ຳເກືອທີ່ອະເຊື້ອໃໝ່ (NSS 0.9 %) ຫຼື Locke's solution ເພື່ອລ້າງ [4]. ລຳໄສ້ທີ່ຜ່າຕັດແລ້ວສາມາດດຳເນີນໄດ້ສອງວິທີ: i) ສັງເກດດ້ວຍກ້ອງຈຸລະທັດເພື່ອເບິ່ງໄລຍະຕ່າງໆຂອງ Leishmania promastigotes ແລະ ຕຳແໜ່ງບ່ອນຢູ່ຂອງພວກມັນ, ໂດຍເນັ້ນເບິ່ງວາວປິດທາງເປີດກະເພາະ stomodeal valve, ແລະ ii) ເປີດລຳໄສ້ເພື່ອໃຫ້ promastigotes ອອກງ່າຍ ສຳລັບການປູກເຊື້ອໃຫ້ໄດ້ຈຳນວນຫຼາຍ [4]. ການພົບ ຮິ້ນຝອຍຊາຍທີ່ຕິດເຊື້ອ ມີໂອກາດໜ້ອຍຫລາຍໃນພາກສະໜາມ, ດັ່ງນັ້ນ ການຝຶກຫັດທີ່ດີ ຈະເພີ່ມໂອກາດ ຄວາມສຳເລັດໃນການປູກແຍກເຊື້ອ. ຖ້າສັງເກດເຫັນ Leishmania parasites ໃນລຳໄສ້, ໃຫ້ໃຊ້ເຂັມໃໝ່ ແລະ ເພີ່ມນ້ຳເກືອທີ່ອະເຊື້ອນ້ອຍໆ ຮອບແຜ່ນປົກດ້ວຍວິທີ capillary action ເພື່ອປ່ອຍພວກມັນອອກ. ຕ້ອງລະມັດລະວັງ ແລະ ໄວໃນການຕັດລຳໄສ້ ເພື່ອປ່ອຍແມ່ກາຝາກອອກໃນນ້ຳເກືອ. ນຳໃຊ້ micropipette 100 µL ຫຼື tuberculin syringe ເພື່ອເກັບ parasites ແລະ ປູກໃນ culture medium ທີ່ໝາຍລະຫັດທີ່ຖືກຕ້ອງ.

ການປູກ Leishmania promastigotes ໃນແກ້ວທົດລອງ (in vitro): ແມ່ກາຝາກທີ່ແຍກໄດ້ ເລີ່ມເກັບຮັກສາໃນພູມປູກ SNB-9 blood agar slopes ຫຼື ໃນ Novy, McNeal, Nicolle (NNN) solid medium [16] ທີ່ປົກດ້ວຍ alpha-MEM medium sterile [16, 65] ຫຼື M199 medium, ແຕ່ລະອັນຕື່ມ 10% heat-inactivated sterile fetal calf serum [FCS] (ເພື່ອເພີ່ມການເຕີບໂຕຂອງແມ່ກາຝາກ), 1% BME vitamins, 2% sterile human urine (ເຮັດໃຫ້ອະເຊື້ອດ້ວຍການກອງດ້ວຍ syringe filter Filtropur® S 0.2 µm), 250 µg/mL amikacin (ຫຼື 50 µg/mL gentamicin, ຫຼື ສ່ວນປະສົມ antibiotics ແລະ amino acids (L-glutamine 200 mM-penicillin 10 000 U-streptomycin 10 mg/mL) [47]. ຫຼັງ 3 ວັນ, ຖ້າບໍ່ມີການປົນເປື້ອນ, ແມ່ນໃຫ້ນຳເອົາມາຍາຍໃສ່ໃນພູມປູກທີ່ແຂແຂງທີ່ກຽມຢ່າງເໝາະສົມ ແລະ ເກັບໄວ້ທີ່ -80°C ເປັນເວລາ 1-2 ປີ ຫຼື ໃນໄນໂຕຣເຈນແຫຼວ -196°C ເພື່ອການຮັກສາໃນໄລຍະຍາວ ແລະ ການໃຊ້ທົດລອງໃນອະນາຄົດ [7].

4.5. ຕ່ອມນ້ຳລາຍ (Salivary glands)

ການຜ່າຕັດ ຕ່ອມນ້ຳລາຍ ຂອງ ຮິ້ນຝອຍຊາຍ ເປັນເຕັກນິກພື້ນຖານສຳລັບການສຶກສາຄວາມສຳພັນລະຫວ່າງ vector-pathogen, ໂດຍສະເພາະການກວດຫາໄວຣັດທີ່ມີແມງໄມ້ເປັນພາຫະ(arboviruses) ເຊັ່ນ Phlebovirus (ຕົວຢ່າງ Toscana virus) [44, 75]. ເນື່ອງຈາກຮິ້ນຝອຍຊາຍມີຂະໜາດນ້ອຍ, ຂັ້ນຕອນນີ້ຕ້ອງການຄວາມແມ່ນຍຳສູງພາຍໃຕ້ກ້ອງຜ່າຕັດສະເຕຣີໂອ, ໃຊ້ ຄີບທີ່ນ້ອຍລະອຽດ ຫຼື ເຂັມຜ່າຕັດນ້ອຍໆເພື່ອແຍກ ຕ່ອມນ້ຳລາຍທີ່ອ່ອນໂດຍບໍ່ເຮັດໃຫ້ແຕກ ຫຼື ປົນເປື້ອນ (https://zenodo.org/records/18302850) [51, 61]. ການຮັກສາຄວາມສົມບູນຂອງ ຕ່ອມນ້ຳລາຍ ເປັນສິ່ງສຳຄັນຕໍ່ການວິເຄາະໂມເລກຸນຕໍ່ໄປ. ເມື່ອແຍກອອກມາໄດ້, ຕ່ອມນ້ຳລາຍ ສາມາດ ນຳມາບົດ ແລະ ກວດດ້ວຍ RT-PCR, qPCR, ຫຼື immunoassays ເພື່ອຊອກຫາ viral RNA ຫຼື antigens [12]. ການພົບ viruses ໃນ ຕ່ອມນ້ຳລາຍ (ແທນທີ່ຈະເປັນ gut ຫຼື hemocoel) ຢືນຢັນວ່າ ເຊື້ອໄດ້ຜ່ານໄລຍະການບົ່ມເຊື້ອ extrinsic incubation period ແລະ ສາມາດຕິດຕໍ່ໄດ້ໃນລະຫວ່າງການກິນເລືອດຂອງພາຫະ [71].

ການຜ່າຕັດນີ້ຍາກເນື່ອງຈາກຂະໜາດນ້ອຍຂອງ ຕ່ອມນ້ຳລາຍ ຂອງ ຮິ້ນຝອຍຊາຍ, ຕ້ອງການຄວາມຊຳນານສູງເພື່ອຫຼີກເວັ້ນການເສື່ອມສະພາບຕົວຢ່າງ [1, 51]. ນອກຈາກນີ້, viral loads ອາດຕ່ຳ, ຕ້ອງໃຊ້ວິທີຊອກຫາທີ່ມີຄວາມອ່ອນໄຫວສູງເຊັ່ນ nested PCR ຫຼື high-throughput sequencing [54]. ຄວາມສ່ຽງປົນເປື້ອນເນັ້ນໃຫ້ ເຫັນເຖິງຄວາມຈຳເປັນຂອງ sterile techniques. ນອກເໜືອຈາກຄວາມຍາກດ້ານເຕັກນິກ, ປັດໄຈທາງຊີວະພາບ ຍັງສົ່ງຜົນກະທົບຕໍ່ຄວາມສຳເລັດ; ຄວາມສາມາດເປັນ vector ແຕກຕ່າງກັນລະຫວ່າງຊະນິດ ຮິ້ນຝອຍຊາຍ, ແລະ ອັດຕາການຕິດເຊື້ອປ່ຽນແປງຕາມເງື່ອນໄຂນິເວດ ແລະ ລະດູການ [33, 61]. ການຊອກຫາ viruses ໃນ ຕ່ອມນ້ຳລາຍ ໃຫ້ຂໍ້ມູນສຳຄັນກ່ຽວກັບຄວາມສ່ຽງການຕິດຕໍ່, ອະນຸຍາດໃຫ້ມີການຕິດຕາມ ແລະ ມາດຕະການຄວບຄຸມເປົ້າໝາຍ [15]. ຕົວຢ່າງ, ການພົບ Toscana virus ໃນ ຮິ້ນຝອຍຊາຍ ໃນເຂດ endemic ໄດ້ຊ່ວຍປັບປຸງໂປຣໂຕຄອນການການກວດ ແລະ ຄຳແນະນຳສາທາລະນະສຸກ [18]. ນອກຈາກນີ້, ການສຶກສາການຕິດຕໍ່ລະຫວ່າງ virus-ການລາຍ ອາດເປີດເຜີຍເປົ້າໝາຍໃໝ່ສຳລັບ vaccines ຫຼື therapeutics ທີ່ຂັດຂວາງການຕິດຕໍ່ [15, 18].

ຕ່ອມນ້ຳລາຍ ຂອງ ຮິ້ນຝອຍຊາຍ ຍັງສາມາດໃຊ້ເປັນແຫຼ່ງ antigens ສຳລັບການວັດແທກ antibodies ຂອງ host ຕໍ່ນ້ຳລາຍ ຮິ້ນຝອຍຊາຍ ດ້ວຍວິທີ immunological, ໂດຍສະເພາະ ELISA. ວິທີນີ້ອະນຸຍາດໃຫ້ປະເມີນການສຳຜັດຂອງ host ກັບການກັດຂອງ ຮິ້ນຝອຍຊາຍ, ຊ່ວຍປະເມີນປະສິດທິພາບຂອງມາດຕະການຄວບຄຸມ vector [25] ແລະ ຄວາມສ່ຽງການຕິດຕໍ່ Leishmania [40].

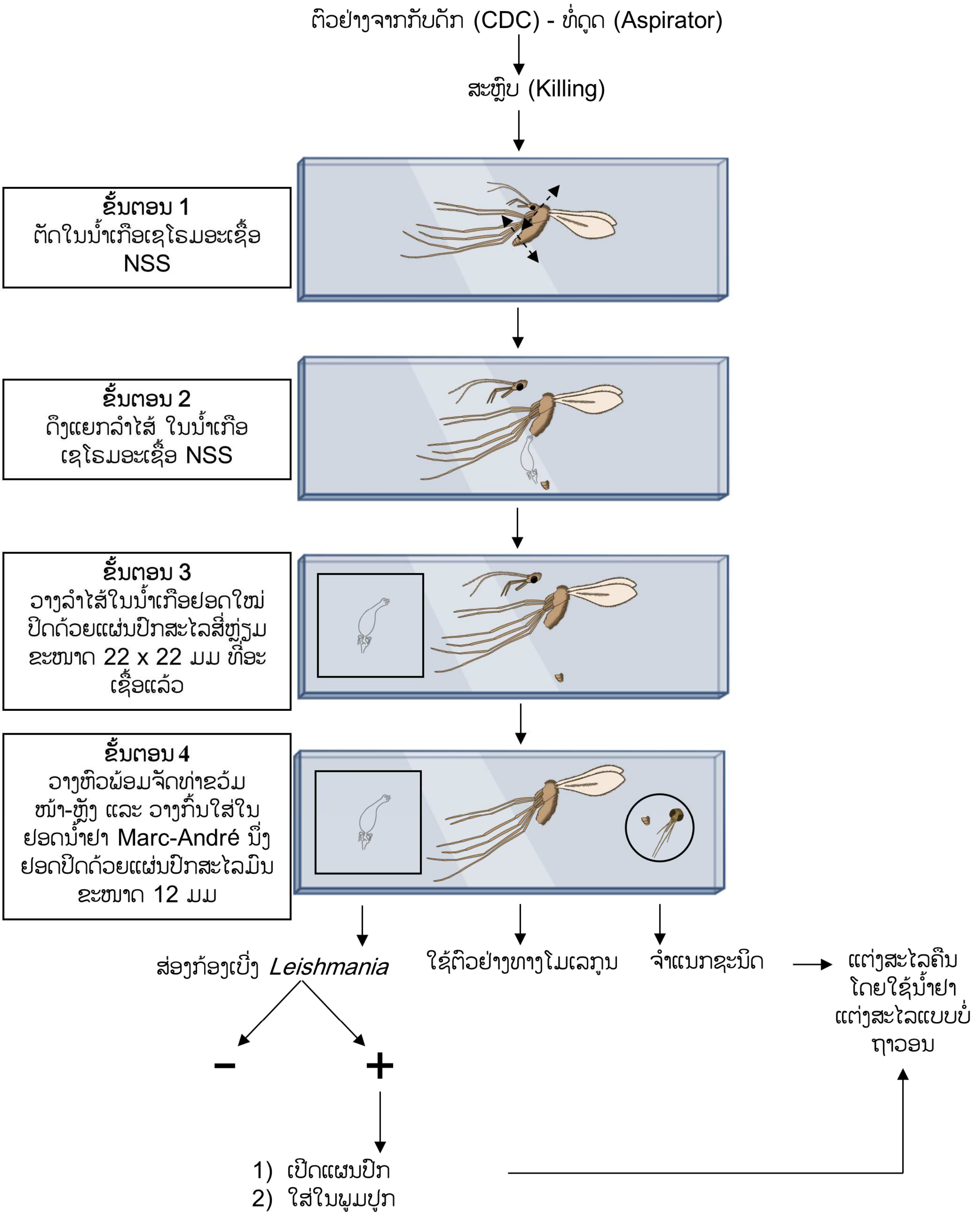

ຮູບທີ 5: ວິທີການປູກແຍກເຊື້ອ *Leishmania*.

This is an Open Access article distributed under the terms of the Creative Commons Attribution License (https://creativecommons.org/licenses/by/4.0), which permits unrestricted use, distribution, and reproduction in any medium, provided the original work is properly cited.

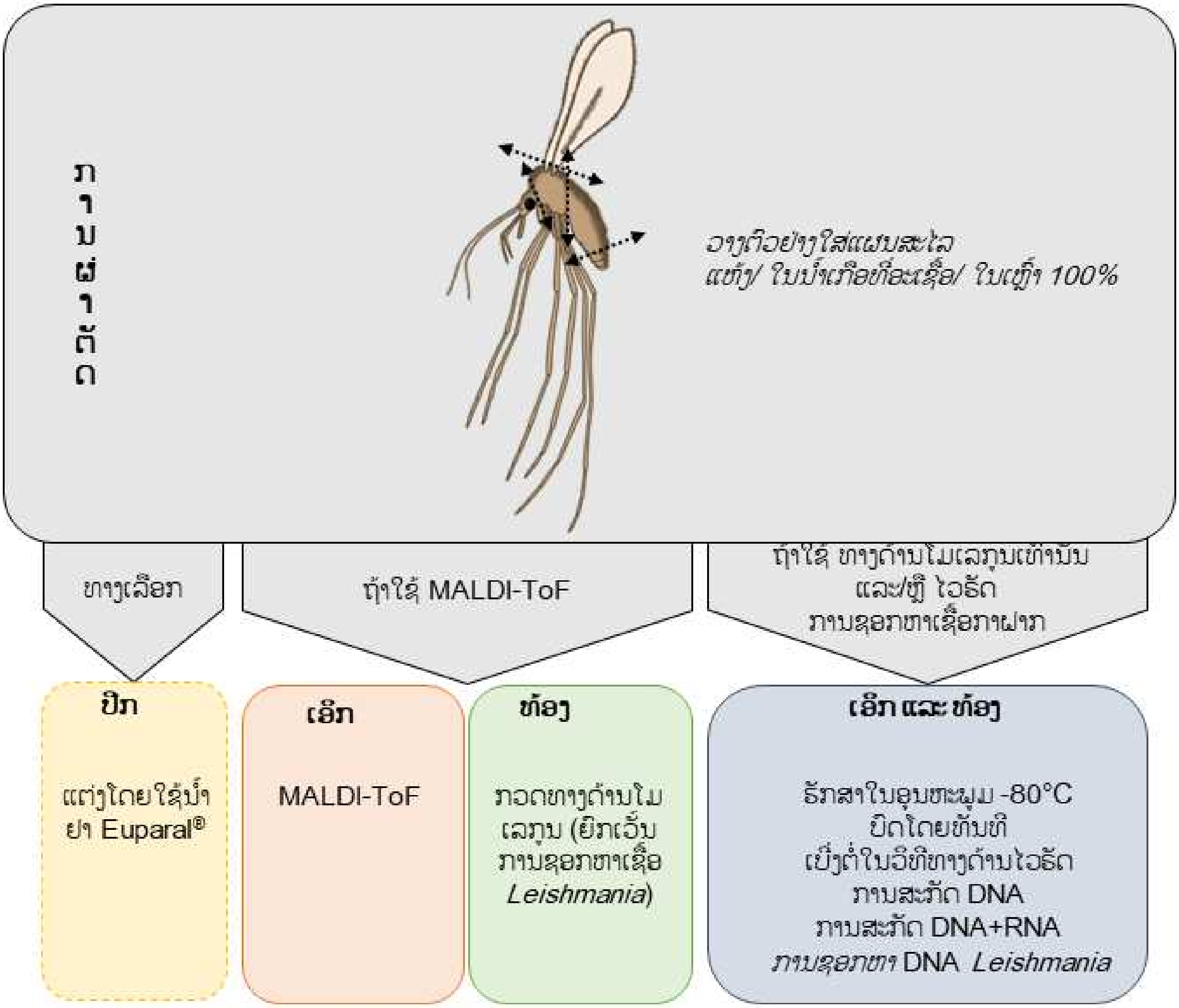

ຮູບທີ 6: ການກະກຽມຕົວຢູ່ງຮິ້ນຝອຍຊາຍ ສໍາລັບການນໍາໃຊ້ທາງຊີວະໂມເລກຸນ, proteomics, ແລະ/ຫຼື virology applications.

This is an Open Access article distributed under the terms of the Creative Commons Attribution License (https://creativecommons.org/licenses/by/4.0), which permits unrestricted use, distribution, and reproduction in any medium, provided the original work is properly cited.

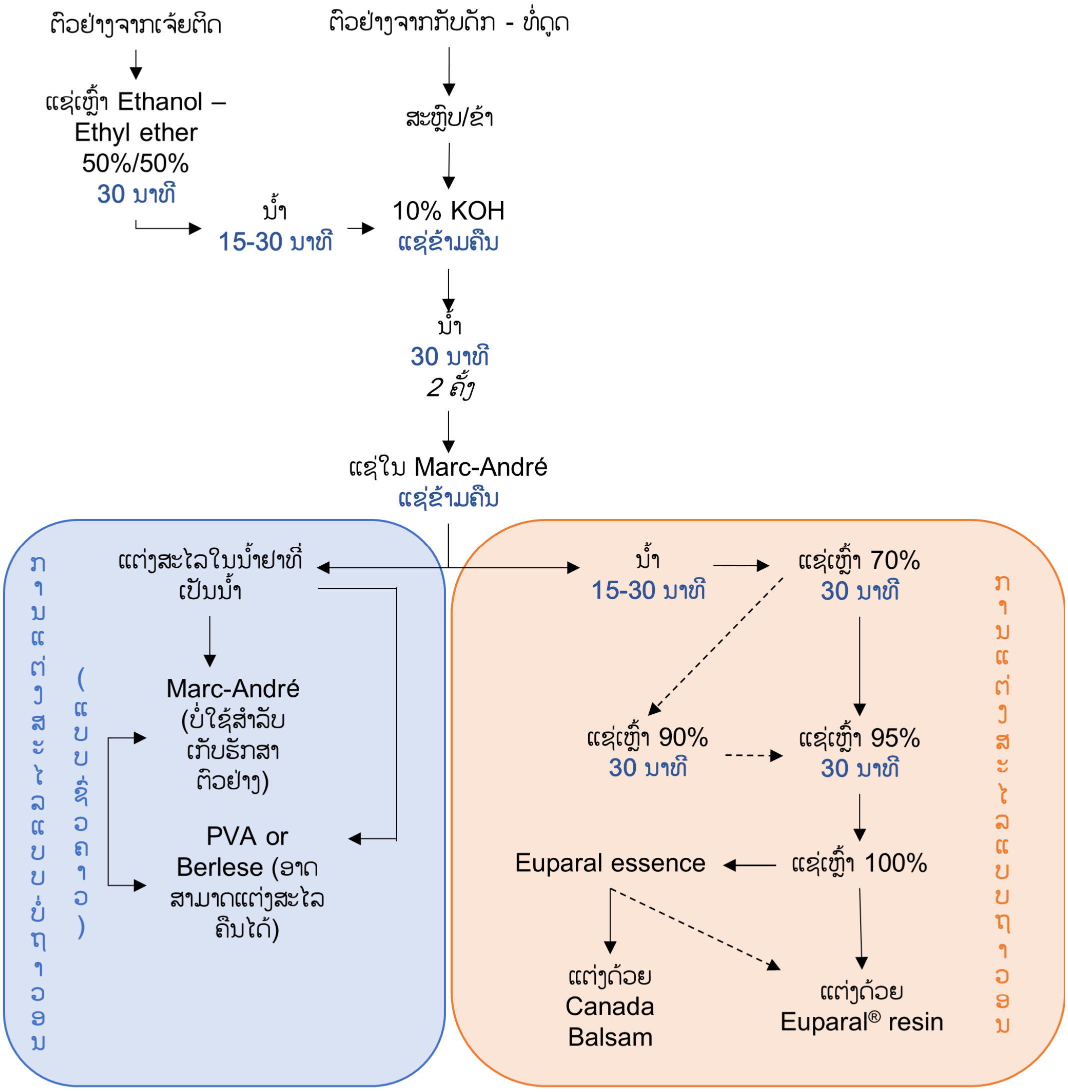

ຮູບທີ 7: ວິທີການພື້ນຖານ ສຳລັບຂັ້ນຕອນການກຽມຕົວຢ່າງຮິ້ນຝອຍຊາຍ.

4.6. ການຈຳແນກຊະນິດອາຫານຈາກເລືອດ (Blood meal identification)

ຕົວແມ່ທີ່ມີເລືອດເຕັມ (engorged females) ທີ່ແຍກອອກຈາກການຈັບ ຄວນໄດ້ຮັບການຜ່າຕັດດ້ວຍອຸປະກອນໃຊ້ຄັ້ງດຽວເພື່ອປ້ອງກັນການປົນເປື້ອນ. ທ້ອງຂອງຕົວຢ່າງຄວນໄດ້ຮັບການສັງເກດດ້ວຍກ້ອງສະເຕຣີໂອເພື່ອປະເມີນຂັ້ນຕອນການຍ່ອຍເລືອດ. ແນະນຳ ໃຫ້ເລືອກພຽງຕົວແມ່ທີ່ມີທ້ອງສີແດງ, ນ້ຳຕານອ່ອນ, ຫຼືແດງເຂັ້ມ ໂດຍບໍ່ມີສັນຍານການສ້າງໄຂ່. ແຍກປາຍທ້ອງລວມ ຖົງເກັບນ້ຳເຊື້ອ ອອກເພື່ອຈຳແນກຕົວແມ່ດ້ວຍ ຮູບຮ່າງ ຫຼັງການເຮັດໃຫ້ໃສແລ້ວ. ສ່ວນຫຼັກຂອງທ້ອງ (ບໍ່ລວມ ຖົງເກັບນ້ຳເຊື້ອ) ຄວນໃສ່ໃນຫຼອດ

This is an Open Access article distributed under the terms of the Creative Commons Attribution License (https://creativecommons.org/licenses/by/4.0), which permits unrestricted use, distribution, and reproduction in any medium, provided the original work is properly cited.

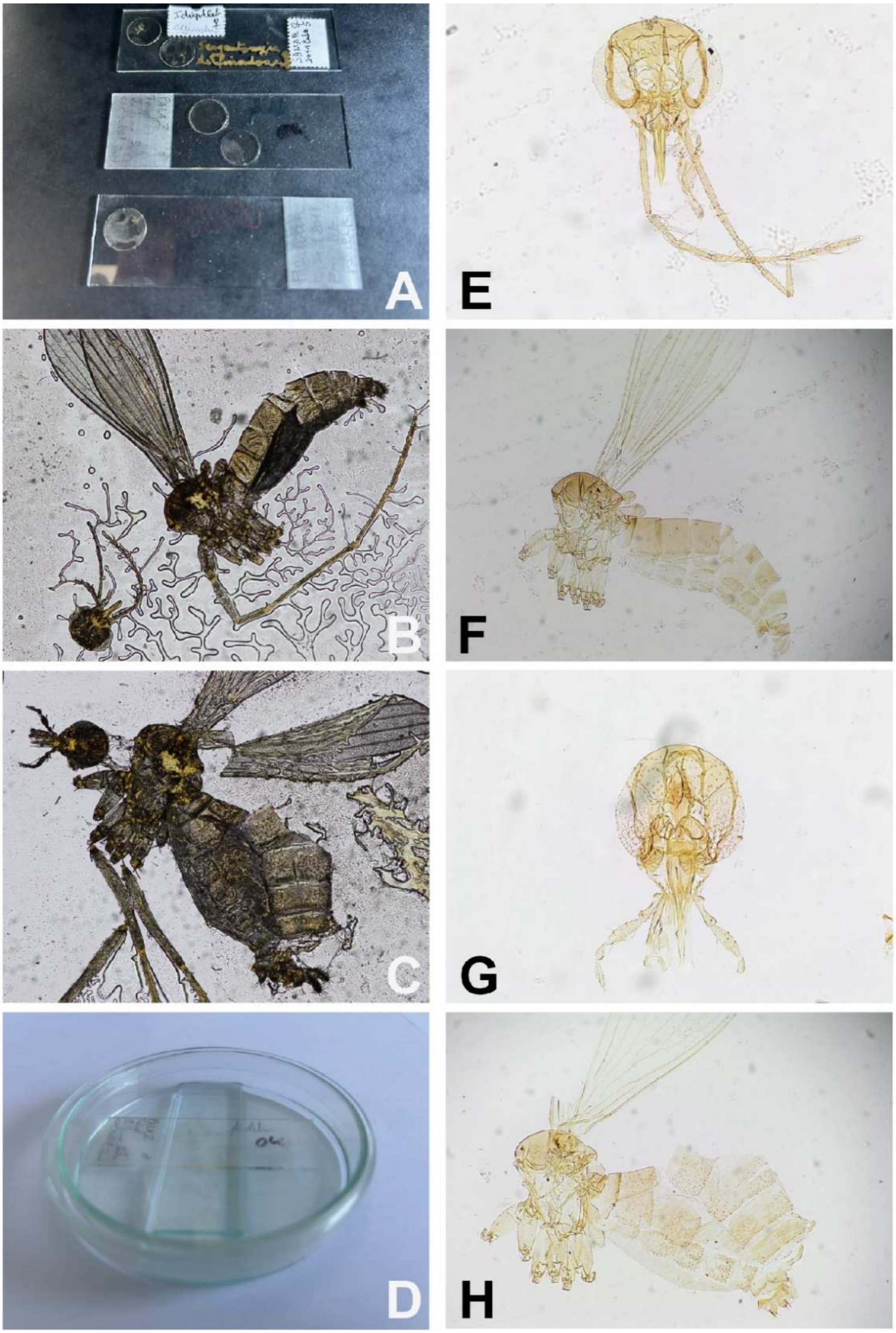

ຮູບ 8: ການແຕ່ງແຜ່ນແກ້ວສະໄລໃໝ່, A: ແຜ່ນແກ້ວສະໄລທີ່ເສຍຫາຍ ແລະ ແຫ້ງ ທີ່ແຕ່ງດ້ວຍ Hoyer, B: ພາບກ້ອງຈຸລະທັດຂອງ ຣິ້ນຝອຍຊາຍທີ່ແຫ້ງ, C: ພາບກ້ອງຈຸລະທັດຂອງ ຣິ້ນຝອຍຊາຍທີ່ເສຍຫາຍ, D: ຫ້ອງຊຸ່ມທີ່ມີແຜ່ນແກ້ວສະໄລທີ່ແຫ້ງ, E: ຫົວ ແລະ F: ລຳຕົວ ຂອງຕົວຢ່າງ B ຫຼັງຈາກແຕ່ງໃໝ່ໃນ Euparal®, G: ຫົວ ແລະ H: ລຳຕົວ ຂອງຕົວຢ່າງ C ຫຼັງຈາກແຕ່ງໃໝ່ໃນ Euparal®.

Eppendorf® ແລະ ເກັບທີ່ອຸນຫະພູມ -20°C ຈົນກວ່າຈະວິເຄາະຕໍ່ໄປ.
ຕົວຊີ້ບອກທາງກຳມະພັນທີ່ນຳໃຊ້ທົ່ວໄປສຳລັບການຈຳແນກຊະນິດອາຫານຈາກເລືອດ ເຊັ່ນ PNOC [5, 30, 50], CytB [67], ຫຼື COI [13] ໄດ້ຖືກສ້າງຕັ້ງແລະອະທິບາຍຢ່າງລະອຽດໃນເອກະສານອ້າງອີງ; ດັ່ງນັ້ນ ຈຶ່ງບໍ່ອະທິບາຍຊ້ຳໃນເອກະສານນີ້ (ຮູບທີ 6). ອີກທາງເລືອກ, ເພື່ອຈຳແນກເລືອດຂອງ host, ການແມັບ peptide ດ້ວຍ MALDI-ToF ສາມາດນຳໃຊ້ [31]. ໄດ້ພິສູດທາງການທົດລອງວ່າວິທີນີ້ອະນຸຍາດໃຫ້ຈຳແນກເລືອດຂອງ host ໄດ້ໃນໄລຍະເວລາທີ່ຍາວກວ່າຫຼັງການກິນເລືອດ; ດັ່ງນັ້ນເປັນວິທີທີ່ເໝາະສົມ,
ໂດຍສະເພາະສຳລັບການວິເຄາະຕົວແມ່ທີ່ມີເລືອດທີ່ຍ່ອຍໄປຫຼາຍຢ່າງເຫັນໄດ້ຊັດເຈນ. ຕົວຢ່າງຄວນເກັບທີ່ -20°C ຫຼື 4°C ແຕ່ຜົນດີອາດໄດ້ຈາກການເກັບໃນອຸນຫະພູມຫ້ອງໄລຍະສັ້ນ.
ທ້ອງຂອງຕົວແມ່ທີ່ມີເລືອດ ຄວນໄດ້ຮັບການຜ່າຕັດແຍກຈາກຮ່າງກາຍກ່ອນການວິເຄາະ ແລະ ບົດ ໃນນ້ຳກັ່ນ. ສ່ວນທີ່ເຫຼືອຂອງຮ່າງກາຍ ຮົ້ນຝອຍຊາຍ ຍັງພ້ອມໃຊ້ສຳລັບການວິເຄາະໂມເລກຸນ ແລະ ສຶກສາຮູບຮ່າງ ອື່ນໆ. ຫຼັງເອົາ aliquot ຈາກ homogenate ສຳລັບ MALDI-ToF peptide mapping, ສ່ວນທີ່ເຫຼືອສາມາດໃຊ້ສຳລັບການສະກັດ DNA ເພື່ອຢືນຢັນການຈຳແນກເລືອດຂອງ host ແລະ/ຫຼື ສະແກນການມີ Leishmania sp. ໂດຍລວມ, ໄລຍະເວລາກະກຽມ ແລະ ວິເຄາະຕົວຢ່າງ ສັ້ນຫຼາຍເມື່ອປຽບທຽບກັບເຕັກນິກໂມເລກຸນເຊື້ອ DNA.

ຕາຕະລາງ 2: ສ່ວນປະກອບຂອງນ້ຳຢາທີ່ນຳໃຊ້.

**Potassium hydroxide 10%**	**Fuchsin acid 1% ໃນນ້ຳກັ່ນ**
Potassium hydroxide 10 g	Acid fuchsin (as a powder) 1 g
ນ້ຳກັ່ນຕື່ມໃຫ້ໄດ້ 100 mL	ນ້ຳກັ່ນ 99 mL
**Gum chloral mounting media (Hoyer medium)**	**ສານລະລາຍ Marc-André ຍ້ອມສີດ້ວຍ acid fuchsin**
ນ້ຳກັ່ນ 50 mL	ສານລະລາຍ Marc-André 10mL
Hydrate de chloral 200 g	Fuchsine à 1 % 50 µL
**ສານລະລາຍ Marc-André**	**Enecê medium**
Hydrate de chloral 40 g	Colophane blanche pure 22 g
Acide acétique glacial 30 mL	Gomme copale soluble dans l'alcool 12 g
ນ້ຳກັ່ນ 30 mL	Éthanol absolu 20 mL
	Camphre 10 g
	Essence de térébenthine 10 mL
	Eucalyptol 26 mL

5. ຂັ້ນຕອນການກະກຽມຕົວຢ່າງສຳລັບການສຶກສາລັກສະນະຮູບຮ່າງ (ຮູບທີ 3, 6, 7 ແລະ 8; ເອກະສານຊ້ອນທ້າຍ 1, 2, 3 ແລະ 4)

ພາກສ່ວນນີ້ອະທິບາຍຫຼັກການກະກຽມຕົວຢ່າງ ຮົ້ນຝອຍຊາຍ ສຳລັບການແຕ່ງສະໄລເພື່ອການສຶກສາລັກສະນະຮູບຮ່າງເທົ່ານັ້ນ, ຕາມດ້ວຍການປັບໃຊ້ກັບການນຳໃຊ້ນອກເໜືອຈາກ ສຶກສາຮູບຮ່າງ. ເຖິງຢ່າງໃດກໍ່ຕາມ, ການເຂົ້າໃຈ methodology ນີ້ ເປັນສິ່ງສຳຄັນ ເພາະອະນຸຍາດໃຫ້ປັບຂັ້ນຕອນໃຫ້ເໝາະກັບປະເພດຕົວຢ່າງພິເສດເມື່ອຈຳເປັນ.

ການປະຕິບັດກ່ຽວຂ້ອງກັບຂັ້ນຕອນເອົາອອກ ແລະ ເຕີມເຕັມ ຕິດຕໍ່ກັນໂດຍໃຊ້ Pasteur pipettes ທີ່ຕິດຢາງອ່ອນ. ແນະນຳໃຫ້ໃຊ້ພາຊະນະແກ້ວກົມລຸ່ມ ເພາະຊ່ວຍງ່າຍຕໍ່ການດຳເນີນການເຫຼົ່ານີ້ຢ່າງຫຼາຍ. ແກ້ວບໍ່ປະຕິກິລິຍາກັບ reagents ທັງໝົດ. ເພື່ອປ້ອງກັນການລະເຫີຍ reagents, ພາຊະນະຄວນມີຝາ ແລະ ບໍ່ເຕັມເກີນໄປ ເພື່ອຫຼີກເວັ້ນການເລື່ອຍເມື່ອປິດ ຫຼື ເປີດ ແລະ ປ້ອງກັນການຕົກຂອງຝຸ່ນໃສ່ຕົວຢ່າງ. ສານເຄມີທີ່ຕ້ອງການສຳລັບການເຮັດໃຫ້ໃສ ແລະ ການປະມອນຜົນ ສະແດງໃນຕາຕະລາງ 2.

5.1. ການເຮັດໃຫ້ໃສ (Clearing)

ກ່ອນທີ່ຕົວຢ່າງ ຮິ້ນຝອຍຊາຍ ຈະສາມາດກະກຽມເປັນການແຕ່ງສະໄລຖາວອນ (permanent slide mounts), ຕ້ອງເຮັດໃຫ້ໂປ່ງໃສກ່ອນ ດ້ວຍການ maceration ໂດຍໃຊ້ວິທີ ແລະ ນ້ຳຢາເຮັດໃຫ້ໂປ່ງໃສ ທີ່ເໝາະສົມ (ເຊັ່ນ: ນ້ຳຢາ acetic acid 10% ຫຼື ນ້ຳ Marc-André ທີ່ມີ chloral hydrate ເຊິ່ງເປັນສານເຄມີທີ່ຖືກຈຳກັດໃນຫຼາຍປະເທດ) ເພື່ອເຮັດໃຫ້ຕົວຢ່າງໂປ່ງໃສ.
ຂັ້ນຕອນການເຮັດໃຫ້ໂປ່ງໃສແມ່ນການກຳຈັດເນື້ອເຍື່ອ, ໄຂມັນ, ນ້ຳຂັບຍ່ອຍ, ແລະ ໄຂຂີ້ເຜີ້ງ ເພື່ອເຮັດໃຫ້ຕົວຢ່າງໂປ່ງໃສ, ຊ່ວຍໃຫ້ສາມາດສັງເກດເບິ່ງໂຄງສ້າງ exoskeletal (ເຊັ່ນ: ຕຳແໜ່ງການຕິດຂອງຂົນ setae), ລັກສະນະພື້ນຜິວ (ເຊັ່ນ: ການກະຈາຍສີ), ແລະ ລັກສະນະພາຍໃນ ທີ່ເຫັນໄດ້ຜ່ານ tegument (ເຊັ່ນ: ຖົງເກັບນ້ຳເຊື້ອ).

ຂັ້ນຕອນການເຮັດໃຫ້ໂປ່ງໃສ 2 ຂັ້ນຕອນ ໂດຍການໃຊ້ ທາດດ່າງ (base) ແຂງ (ເຊັ່ນ potassium hydroxide) ກ່ອນ ຕາມດ້ວຍ ກົດອ່ອນ (ເຊັ່ນ acetic acid ໃນນ້ຳ Marc-André) ເຊິ່ງມີຈຸດປະສົງທາງເຄມີຊີວະທາດຕ່າງກັນ [74]. ທາດດ່າງທຳລາຍເນື້ອເຍື່ອອ່ອນ ເຊັ່ນ ໂປຣຕີນ, ໄຂມັນ, ແລະ ກ້າມເນື້ອ ຜ່ານ saponification ແລະ protein denaturation ໂດຍຮັກສາ exoskeleton chitin ໃຫ້ຄົງສະພາບເພື່ອຄວາມຊັດເຈນໂຄງສ້າງ. ກົດອ່ອນທີ່ຕາມມາເປັນການກຳຈັດ alkali ທີ່ເຫຼືອ ປ້ອງກັນການເສື່ອມສະພາບຕໍ່ໄປ ແລະ ເຮັດໃຫ້ chitin ຂາວເພື່ອເພີ່ມຄວາມໂປ່ງໃສ [74] ເຖິງຢ່າງໃດກໍ່ຕາມ ການລ້າງຕົວຢ່າງໃນ ນ້ຳກັ່ນ 2 ຄັ້ງ ເປັນເວລາ 15 ນາທີ ກໍ່ສາມາດເປັນການກຳຈັດ ທາດດ່າງໄດ້ພຽງພໍ. ການປະຕິບັດຕາມລຳດັບນີ້ ປະສົມການກຳຈັດເນື້ອເຍື່ອທີ່ມີປະສິດທິພາບກັບການຮັກສາອ່ອນໆ ຮັບປະກັນຄວາມສົມບູນຂອງຕົວຢ່າງທີ່ດີທີ່ສຸດສຳລັບການສັງເກດດ້ວຍກ້ອງຈຸລະທັດ.

ແນະນຳໃຫ້ລ້າງໃນ ນ້ຳກັ່ນ 2 ຄັ້ງ ເປັນເວລາ 20 ນາທີ ກ່ອນການດຳເນີນການຂັ້ນຕໍ່ໄປ.

5.1.1. ການຍ່ອຍສະຫຼາຍເນື້ອເຍື່ອອ່ອນ (Soft tissue lysis) (ຮູບທີ 8)

Sodium hydroxide (NaOH) ຫຼື potassium hydroxide (KOH) ເປັນນ້ຳຢາ macerating ທາງເຄມີທີ່ນຳໃຊ້ທົ່ວໄປ ໃຊ້ໃນຄວາມເຂັ້ມ ແລະ ໄລຍະເວລາຕ່າງກັນ ຂຶ້ນກັບຂະໜາດ ແລະ ຄວາມອ່ອນໄຫວຂອງຕົວຢ່າງ. ເຕັກນິກມາດຕະຖານ ແລະ ມີປະສິດທິພາບທີ່ສຸດ ກ່ຽວຂ້ອງກັບການແຕກເນື້ອເຍື່ອອ່ອນ ໂດຍແຊ່ ຮິ້ນຝອຍຊາຍ ໃນ ທາດດ່າງແຂງ (10% KOH ຫຼື NaOH) ຕະຫຼອດຄືນ. ສາມາດເພີ່ມຄວາມເຂັ້ມເພື່ອຫຼຸດໄລຍະເວລາ (ເຊັ່ນ: KOH 20% ເປັນເວລາ 6 ຊົ່ວໂມງ) ພ້ອມກັບການເຮັດຄວາມຮ້ອນ ທີ່ 37°C.

5.1.2. ການເຮັດໃຫ້ໃສ ພ້ອມດ້ວຍ ຫຼື ບໍ່ມີ ການຍ້ອມສີ

ຂັ້ນຕອນນີ້ຕາມດ້ວຍການເຮັດໃຫ້ແຈ່ງຂຶ້ນ ປົກກະຕິປະສົມ acetic acid ແລະ chloral hydrate (ເຊັ່ນ: ນ້ຳ Marc-André). ຫຼັງການເຮັດໃຫ້ໂປ່ງໃສ ຕົວຢ່າງຕ້ອງລ້າງຢ່າງລະອຽດໃນນ້ຳ ຢ່າງໜ້ອຍ 2 ຄັ້ງຕິດຕໍ່ກັນ ເປັນເວລາ 20 ນາທີ ແຕ່ລະຄັ້ງເພື່ອກາຈັດນ້ຳຢາທີ່ເຫຼືອ.

ນ້ຳຢາ Marc-André ເປັນນ້ຳຢາເຮັດໃຫ້ໂປ່ງໃສທີ່ນຳໃຊ້ທົ່ວໄປສຳລັບການກະກຽມຕົວຢ່າງ ຮິ້ນຝອຍຊາຍ.
ປະສິດທິພາບຂອງມັນຢູ່ໃນການຊ່ວຍຂັ້ນຕອນການເຮັດໃຫ້ໂປ່ງໃສ ໃນຂະນະທີ່ຫຼຸດຜ່ອນການເສຍຫາຍຢ່າງຮຸນແຮງຕໍ່ໂຄງສ້າງອ່ອນໄວ ເຊັ່ນ ປີກ ແລະ ໜວດ.

ນ້ຳຢານີ້ຄວນກຽມໃໝ່ ຫຼື ເກັບໃນພາຊະນະປິດແໜ້ນເພື່ອປ້ອງກັນການລະເຫີຍ ຫຼື ການເສື່ອມສະພາບ. ການໃຊ້ນ້ຳ Marc-André ມີຂໍ້ດີເປັນພິເສດ ເມື່ອປະສົມກັບເຕັກນິກການເຮັດໃຫ້ແຈ່ງຂຶ້ນ ຫຼື ການຍ້ອມສີ ເພື່ອເພີ່ມລາຍລະອຽດທາງດ້ານຮູບຮ່າງພິເສດ. ລາຍລະອຽດອົງປະກອບ ແລະ ການກຽມ ສະແດງໃນເອກະສານຊ້ອນທ້າຍ 2.

ສຳລັບຕົວຢ່າງທີ່ໂປ່ງໃສຫຼາຍ, ການຍ້ອມສີອາດຈຳເປັນເພື່ອເພີ່ມຄວາມສາມາດເບິ່ງເຫັນກ່ອນການແຕ່ງສະໄລ. ມີນ້ຳຍ້ອມສີຫຼາຍຊະນິດ ແຕ່ລະອັນມີເປົ້າໝາຍສະເພາະຕາມອົງປະກອບເຄມີພິເສດຂອງສິ່ງມີຊີວິດນັ້ນໆ ສຳຄັນຕ້ອງເລືອກນ້ຳທາສີທີ່ເຂົ້າກັນໄດ້ກັບຕົວຢ່າງ ແລະ ນ້ຳຢາແຕ່ງສະໄລທີ່ເລືອກ. ວິທີການພື້ນຖານນີ້ສາມາດປັບໃຊ້ໄດ້ ເຊັ່ນ: ເພີ່ມ acid fuchsin 0.1% ໃນນ້ຳ Marc-André ເພື່ອການທາສີ. ນອກຈາກນີ້, ຕົວຢ່າງທີ່ເກັບໃນນ້ຳ ແລະ ຕ້ອງການແຕ່ງສະໄລໃນ resin ຕ້ອງການ dehydration (ເບິ່ງບົດ 5.2 ກ່ຽວກັບ Dehydration) ເພາະນ້ຳຢາແຕ່ງສະໄລ resin ທຳມະຊາດ ແລະ ສັງລະສິດສ່ວນໃຫຍ່ບໍ່ເຂົ້າກັນກັບນ້ຳ. New (1974) ບັນທຶກວ່ານ້ຳທາສີບາງອັນເສື່ອມສະພາບໃນນ້ຳຢາແຕ່ງສະໄລບາງຊະນິດ [53]. ຕົວຢ່າງ acid fuchsin ທີ່ນຳໃຊ້ກັບ Canada balsam ຍັງສາມາດຄົງສະພາບໃນ Euparal®. ເຖິງຢ່າງໃດກໍ່ຕາມ ຕົວຢ່າງທີ່ທາດ້ວຍ acid fuchsin ອາດຈາງງ່າຍ ໂດຍສະເພາະເມື່ອມີ clove oil ເປັນນ້ຳຢາ cleared ສຸດທ້າຍເຫຼືອຢູ່. ຕົວຢ່າງທີ່ເກັບໃນ clove oil ອາດຈາງຢ່າງຫຼາຍໃນສອງສາມວັນ.

5.2. ການເອົານ້ຳອອກ (Dehydration)

ການເອົານ້ຳອອກ ດຳເນີນໂດຍການຖ່າຍຕົວຢ່າງຜ່ານລຳດັບນ້ຳຢາ ethanol ທີ່ເພີ່ມຄວາມເຂັ້ມທາງລະດັບ: 50%, 70%, 80%, 90%, ຫຼື 95% ແລະ ສຸດທ້າຍ 100% ແຕ່ລະອ່າງຢ່າງໜ້ອຍ 20 ນາທີ. ເນື່ອງຈາກ ethanol ລະເຫີຍໄວ ພາຊະນະຕ້ອງປິດແໜ້ນໃນລະຫວ່າງການປະມອນຜົນ.
ເມື່ອຕົວຢ່າງແຫ້ງສົມບູນແລ້ວ ສາມາດຢຸດການປະມອນຜົນເປັນເວລາສອງສາມວັນໃນ Euparal® essence ເຊິ່ງດີກວ່າ clove oil. Beech creosote ເຄີຍນຳໃຊ້ກວ້າງຂວາງໃນຈຸດນີ້ ແຕ່ປັດຈຸບັນຖືກຫ້າມຢ່າງສົມບູນເນື່ອງຈາກຄວາມເປັນພິດ.

ຂັ້ນຕອນການເອົານ້ຳອອກ ຕ້ອງຮັບປະກັນວ່ານ້ຳໃນຕົວຢ່າງເຂົ້າກັນໄດ້ກັບນ້ຳຢາແຕ່ງສະໄລ ເພື່ອປ້ອງກັນ opacity, osmotic collapse, ຫຼື distortion ທີ່ອາດເຮັດໃຫ້ຕົວຢ່າງບໍ່ເໝາະສຳລັບການສຶກສາ ການຈຳແນກຊະນິດ.

This is an Open Access article distributed under the terms of the Creative Commons Attribution License (https://creativecommons.org/licenses/by/4.0), which permits unrestricted use, distribution, and reproduction in any medium, provided the original work is properly cited.

5.3. ນ້ຳຢາແຕ້ງສະໄລ (Mounting media)

5.3.1. ການເລືອກ ແລະ ການນຳໃຊ້ສຳລັບການກະກຽມຕົວຢ່າງ

ນ້ຳຢາແຕ້ງສະໄລ ຄວນມີດັດສະນີສະທ້ອນແສງ (refractive index) ໃກ້ຄຽງກັບແກ້ວທີ່ສຸດ ຄືປະມານ 1.5. ຕ້ອງບໍ່ມີສີ, ໂປ່ງໃສ, ແລະ ຄົງຄວາມໂປ່ງໃສຢ່າງສົມບູນ ຫຼັງການແຕ້ງ ແລະ ຕາມເວລາ. ຕ້ອງເຂົ້າກັນໄດ້ກັບນ້ຳຫາສີທີ່ນຳໃຊ້ ແລະ ສາມາດເຂົ້າໄປ ແລະ ກະຈາຍໃນເນື້ອເຍື່ອທັງໝົດຂອງຕົວຢ່າງ. ຕ້ອງບໍ່ແຫ້ງໄວເກີນໄປ ຫຼື ສ້າງຄວາມມົວໃນລະຫວ່າງການແຕ້ງສະໄລ. ຕ້ອງບໍ່ຫຍໍ້ຕົວຫຼັງການແຕ້ງສະໄລ. ການເລືອກນ້ຳຢາແຕ້ງສະໄລທີ່ເໝາະສົມ ເປັນສິ່ງພື້ນຖານຂອງການກະກຽມຕົວຢ່າງ ເພາະບໍ່ມີນ້ຳຢາແຕ້ງສະໄລອັນໃດເໝາະສົມກັບທຸກຈຸດປະສົງ. ການເລືອກຄວນສົມດຸນກັບປັດໃຈສຳຄັນຫຼາຍຢ່າງ:

- ຄຸນສົມບັດດ້ານການເບິ່ງເຫັນ (Optical properties). ດັດສະນີສະທ້ອນແສງຂອງນ້ຳຢາແຕ້ງສະໄລ ຄວນໃຫ້ contrast ແລະ ການສະທ້ອນແສງພຽງພໍ ຕໍ່ລັກສະນະກາຍວິທະຍາສຳຄັນທີ່ໃຊ້ໃນການຈຳແນກຊະນິດ ຫຼື ການອະທິບາຍ ຮູບຮ່າງ ເຊັ່ນ ຖົງເກັບນ້ຳເຊື້ອ, ascoids, Newstead sensilla, vertical cibarial teeth, ແລະ pharyngeal teeth. ການເບິ່ງເຫັນຂອງໂຄງສ້າງເຫຼົ່ານີ້ ຂຶ້ນກັບຄຸນສົມບັດດ້ານການເບິ່ງເຫັນຂອງນ້ຳຢາແຕ້ງສະໄລໂດຍກົງ.

- ການຮັກສາ (Preservation). ສຳລັບຕົວຢ່າງປະເພດ (type specimens) ຫຼື ວັດສະດຸທີ່ຕ້ອງການເກັບຮັກສາໄວ້ຖາວອນ, ນ້ຳຢາແຕ້ງສະໄລຕ້ອງໃຫ້ຄວາມສະຖຽນທີ່ຍາວນານ ແລະ ຄວາມທົນທານ. ໃນທາງກົງກັນຂ້າມ ສຳລັບການສຶກສາສິນຄ້າຄົ້ນຄວ້າ ຫຼື ການສຳຫຼວດດ້ານການແພ່ກະຈາຍພະຍາດ ທີ່ການຮັກສາໄລຍະຍາວບໍ່ສຳຄັນ ນ້ຳຢາແຕ້ງສະໄລຊົ່ວຄາວ ຫຼື ກາງໆອາຍຸ ອາດພຽງພໍ.

5.3.2. ຄວາມຕ້ອງການສຳລັບນ້ຳຢາແຕ້ງສະໄລ

ນັກຊ່ຽວຊານມັກພັດທະນາເຕັກນິກການແຕ້ງສະໄລທີ່ປັບແຕ່ງ ແລະ ຊັບຊ້ອນ ຕາມຄວາມຕ້ອງການຂອງການຄົ້ນຄວ້າພິເສດ. ເຖິງຢ່າງໃດກໍ່ຕາມ ວິທີເຫຼົ່ານີ້ມັກບໍ່ໄດ້ພິຈາລະນາເລື່ອງຄຸນນະພາບການເກັບຮັກສາໄລຍະຍາວ, ການເຂົ້າກັນໄດ້, ການມາດຕະຖານ, ຄວາມງ່າຍໃນການຈັດການ ແລະ ການຮັກສາໄລຍະຍາວ. ການຂາດມາດຕະຖານນີ້ ເຮັດໃຫ້ການປະສານງານຊຸດສະສົມທີ່ໄດ້ຮັບການບໍລິຈາກ ແລະ ການຮັກສາຊຸດສະສົມໄລຍະຍາວ ຍາກຂຶ້ນ.

ການນຳໃຊ້ທາງວິທະຍາສາດ ມີຄວາມຕ້ອງການທີ່ແຕກຕ່າງກັນ. ນັກອານຸກົມວິທານມັກແຕ້ງສະໄລຕົວຢ່າງທັງໝົດ ແລະ ມັກນ້ຳຢາແຕ້ງສະໄລທີ່ເຮັດໃຫ້ເນື້ອອະໄວຍະວະພາຍໃນແຕກອ່ອນໆ ເພື່ອເພີ່ມຄວາມເບິ່ງເຫັນຂອງໂຄງສ້າງ cuticular. ດັດສະນີສະທ້ອນແສງຄວນຕ່າງກັນເທົ່າທີ່ຈະເປັນໄປໄດ້ກັບຕົວຢ່າງ ແລະ ແຜ່ນແກ້ວ ເພື່ອເພີ່ມຄວາມໂປ່ງໃສດ້ານການເບິ່ງເຫັນ. ນ້ຳຢາແຕ້ງສະໄລການຄ້າ ປົກກະຕິຖືກອອກແບບໃຫ້ມີດັດສະນີສະທ້ອນແສງໃກ້ກັບແກ້ວ ເພື່ອຫຼຸດການສະທ້ອນແລະການກະຈາຍແສງຜ່ານລະບົບແຜ່ນແກ້ວ–ນ້ຳຢາແຕ້ງສະໄລ–ແຜ່ນປົກ. ເຖິງຢ່າງໃດກໍ່ຕາມ ໃນການສັງເກດດ້ວຍ brightfield microscopy ຄວາມຕ່າງທຳມະຊາດຂອງຕົວຢ່າງທີ່ບໍ່ທາສີ ສາມາດປັບແຕ່ງໄດ້ ໂດຍການເລືອກນ້ຳຢາແຕ້ງສະໄລທີ່ມີດັດສະນີສະທ້ອນແສງຕ່າງກັນເລັກນ້ອຍກັບຕົວຢ່າງ ເພື່ອເພີ່ມຄວາມເບິ່ງເຫັນກັບພື້ນຫຼັງ.

5.3.3. ປະເພດຂອງນ້ຳຢາແຕ້ງສະໄລ (ຕາຕະລາງ 3 ແລະ 4)

ການສັງເກດດ້ວຍກ້ອງຈຸລະທັດ ຕ້ອງການດັດສະນີສະທ້ອນແສງ (RI) ຂອງນ້ຳຢາແຕ້ງສະໄລ ເພື່ອກຳນົດວ່າແສງແບນແນວໃດຜ່ານແຜ່ນແກ້ວ, ນ້ຳຢາແຕ້ງສະໄລ ແລະ ຕົວຢ່າງ. ເມື່ອ RI ໃກ້ກັບແກ້ວແຜ່ນປົກ (≈1.515) ແສງຜ່ານໄດ້ເປັນເອກະພາບ ຫຼຸດການກະຈາຍແລະການບິດເບືອນດ້ານການເບິ່ງເຫັນ ເຮັດໃຫ້ຄວາມລະອຽດ ແລະ ການເບິ່ງເຫັນໂຄງສ້າງລະອຽດດີຂຶ້ນ. ກົງກັນຂ້າມ ການບໍ່ກົງກັນຂອງ RI ອາດເຮັດໃຫ້ມົວ, ມີ halo, ຫຼື ບັງລັກສະນະທີ່ບໍ່ທາສີ. ການເລືອກນ້ຳຢາແຕ້ງສະໄລທີ່ຖືກຕ້ອງ ສຳຄັນຕໍ່ການເພີ່ມ contrast, ຄວາມໂປ່ງໃສ ແລະ ຄຸນນະພາບຮູບພາບໂດຍລວມ ເນື່ອງຈາກ RI ທີ່ແຕກຕ່າງກັນລະຫວ່າງນ້ຳຢາຕ່າງໆ.

ດັດສະນີສະທ້ອນແສງຂອງນ້ຳຢາແຕ້ງສະໄລ ມີຜົນກະທົບຢ່າງຫຼວງຫຼາຍຕໍ່ການເບິ່ງເຫັນໂຄງສ້າງລະອຽດ ເມື່ອກະກຽມ ຮິ້ນຝອຍຊາຍ ສຳລັບການແຕ້ງສະໄລ. ລັກສະນະອ່ອນໄວ ແລະ ມີ sclerotized ນ້ອຍຂອງ ຮິ້ນຝອຍຊາຍ ເຊັ່ນ cibarial armature, ຖົງເກັບນ້ຳເຊື້ອ, ສ່ວນຂອງໜວດ, ແລະ ເສັ້ນເລືອດປີກ ອາດຍາກສັງເກດໃນນ້ຳຢາແຕ້ງສະໄລທີ່ມີ RI ສູງ.

ສຳລັບ ຮິ້ນຝອຍຊາຍ ທາງເລືອກທີ່ນຳໃຊ້ທົ່ວໄປປະກອບມີ gum-chloral media ເປັນນ້ຳຢາແຕ້ງສະໄລອີງນ້ຳ ແລະ Canada balsam ກັບ Enecê - Nelson Cerqueira (NC) resin ເປັນນ້ຳຢາແຕ້ງສະໄລອີງ solvent. Rawlins [60] ແບ່ງນ້ຳຢາແຕ້ງສະໄລອອກເປັນ 2 ປະເພດ: (1) ນ້ຳຢາຖາວອນ (permanent media): ເຫຼົ່ານີ້ແຂງຕາມເວລາ ແລະ ເໝາະສຳລັບການຮັກສາໄລຍະຍາວ, ແລະ (2) ນ້ຳຢາກາງໆອາຍຸ (semi-permanent media): ບໍ່ແຂງ ແລະ ນຳໃຊ້ສຳລັບຈຸດປະສົງຊົ່ວຄາວ.

This is an Open Access article distributed under the terms of the Creative Commons Attribution License (https://creativecommons.org/licenses/by/4.0), which permits unrestricted use, distribution, and reproduction in any medium, provided the original work is properly cited.

ຕາຕະລາງ 3: ອົງປະກອບຂອງນ້ຳຢາແຕ່ງສະໄລທີ່ເລືອກມານຳໃຊ້.

ສານນ້ຳຢາແຕ່ງສະໄລ	ສານເຮັດລະລາຍ (Solvant)	Pré-polymère(s) ou polymère(s) potentiel(s)	ໝາຍເຫດ
Hoyer = chloral gum	glycérol, ນ້ຳ	ສານປະກອບຂອງຢາງອາຣາບິກ (gum Arabic)	ຕົວເຮັດໃຫ້ຊຸ່ມ: ຄລໍຣໍໄຮເດຣດ
CMCP-9 (= carboxy methyl cellulose phenol)	ນ້ຳ (CMCP-9: 51–60%)	fully hydrolyzed polyvinyl alcohol (CMCP-9: 0–5%)	CMC(P)-9: ຄວາມໜືດຕ່ຳ: ຄວາມໜືດສູງ
DMHF (dimethyl hydantoin formaldehyde)	ນ້ຳ	N,N′-dimethylol dimethyl hydantoin (di-methylol DMH)<br>Ether-/methylene-bridged oligomers<br>Crosslinked DMH–formaldehyde polymer network	
Canada balsam	xylène; ສ່ວນປະກອບທີ່ລະເຫີຍໄດ້ບາງສ່ວນ ຂອງ balsam (Δ^3-carène, acide lévopimarique, limonène, myrcène, acide palustrique, β-phéllandrène, α-pinène, β-pinène)	balsam (abienol, abietic acid, isopimaric acid, sandaracopimaric acid)	ການເຮັດໃຫ້ເປັນກາງ:: potassium carbonate;<br>resin from *Abies balsamea* (Linné, 1758)
Euparal®	eucalyptol, paraldehyde; partly volatile components of gum sandarac (limonene, α-pinene, β-pinene)	ສານປະກອບຂອງ gum sandarac (communic acid, manool, polycommunic acid, sandaracopimaric acid, 12-acetoxy-sandaracopimaric acid, sugiol, torulosic acid, torulosol, totarol)	ສານເຮັດໃຫ້ໃສ: methyl salicylate; color in Euparal® green: copper salt (copper abietinate); sandarac resin from *Tetraclinis articulata* (Vahl, 1791)
Enecê	ethyl alcohol; with camphor, eucalyptol and turpentine essence	Compounds of copal gum and colophony (rosin)	

ຊື່ (name)	ຂໍ້ດີ (Advantages)	ຂໍ້ເສຍ (Disadvantages)
* Canada balsam	ນ້ຳຢາແຕ່ງສະໄລນີ້ມີຄວາມທົນທານສູງຫຼາຍ ດ້ວຍອາຍຸການໃຊ້ງານເກີນ 150 ປີ.<br>ແຜ່ນແກ້ວສາມາດແຕ່ງສະໄລໄດ້ໂດຍໃຊ້ນ້ຳມັນ clove oil ຫຼື phenol ເປັນຕົວແຕ່ງສະໄລ.	ມີອົງປະກອບທີ່ເປັນອັນຕະລາຍ ແລະ ຕ້ອງຈັດການພາຍໃຕ້ຕູ້ດູດຄວັນ (fume hood).<br>ຕ້ອງການການເອົານ້ຳອອກ (dehydration) ຢ່າງຄົບຖ້ວນ ແລະ ໃຊ້ເວລາຫຼາຍ.<br>ການເອົານ້ຳອອກດ້ວຍ ethanol ແລະ ການຖ່າຍຜ່ານ xylene ຫຼື clove oil ອາດເຮັດໃຫ້ບາງ taxa ກາຍເປັນອ່ອນໄວ ແລະ ແຕກຫຼາຍ; ທາງເລືອກອື່ນ (ເຊັ່ນ isopropanol, n-butanol, Cellosolve™, 1,4-dioxane, Histoclear, terpineol) ອາດຊ່ວຍຫຼຸດການແຕກ.<br>ຕົວຢ່າງອາດປ່ຽນສີດຳ ຖ້າໃຊ້ phenol ແທນ xylene ຫຼື ຍັງມີ potassium hydroxide ເຫຼືອຢູ່.<br>ດັດສະນີສະທ້ອນແສງສູງ ອາດເຮັດໃຫ້ໂຄງສ້າງທີ່ບໍ່ທາສີ ບໍ່ເຫັນຊັດເຈນ.<br>ການແຫ້ງສົມບູນ ອາດໃຊ້ເວລາຫຼາຍປີ ຖ້າບໍ່ໃຊ້ hot-plate ໃນການແຫ້ງ.<br>ນ້ຳຢາແຕ່ງສະໄລນີ້ ປ່ຽນສີເຫຼືອງ ແລະ ເຂັ້ມຂຶ້ນຕາມເວລາ ໂດຍສະເພາະເມື່ອເຮັດໃຫ້ໂປ່ງໃສດ້ວຍ clove oil.<br>ນ້ຳທາສີບາງອັນ ອາດອ່ອນແລ້ງ ແລະ ນ້ຳທາສີ cationic ອາດຈາງ<br>ຖ້ານ້ຳຢາແຕ່ງສະໄລກາຍເປັນ acidic — ເຊິ່ງອາດເກີດຂຶ້ນໂດຍຕົນເອງຕາມເວລາ.
DMHF (dimethyl hydantoin formaldehyde)	ຄວາມໂປ່ງໃສສູງ<br>ດັດສະນີສະທ້ອນແສງດີ<br>ການເບິ່ງເຫັນໂຄງສ້າງເຢີ່ຍຢີ່<br>ຄວາມສະຖຽນຂອງການກະກຽມດີໃນລະດັບພໍໃຊ້<br>ເຂົ້າກັນໄດ້ກັບເຕັກນິກການທາສີຫຼາຍຢ່າງ<br>ການປົກປ້ອງຕົວຢ່າງດີ<br>ການຕິດແຜ່ນແກ້ວກັບແຜ່ນປົກດີ	ອາດປ່ຽນສີເຫຼືອງຕາມເວລາ<br>ອາດປ່ຽນແປງນ້ຳຢາຍ້ອມສີບາງອັນ<br>ບໍ່ເໝາະສຳລັບນ້ຳຢາຍ້ອມສີທີ່ອ່ອນໄຫວຕໍ່ formaldehyde<br>ມີຟອງອາກາດ, ແຫ້ງຊ້າ<br>ນ້ຳຢາແຕ່ງສະໄລອ່ອນໄຫວຕໍ່ຄວາມຊຸ່ມ<br>ການແຕ່ງສະໄລຍາກທີ່ຈະກັບຄືນ<br>Formaldehyde ເປັນພິດ, ແພ້, ກໍ່ໃຫ້ເກີດເຮັດມະເຮັງ
* Euparal (transparent)	ນ້ຳຢາແຕ່ງສະໄລທີ່ທົນທານ ດ້ວຍອາຍຸການໃຊ້ງານເກີນ 50 ປີ.<br>ສາມາດແຕ່ງສະໄລໂດຍກົງຈາກ ethanol 80% ໄດ້ (ຕາມຄຳແນະນຳຂອງຜູ້ຜະລິດ).<br>ບໍ່ບັງລັກສະນະທີ່ບໍ່ທາສີ ແລະ ບໍ່ປ່ຽນສີເຫຼືອງ ຫຼື ກາຍເປັນອ່ອນໄວຕາມເວລາ.<br>ມີດັດສະນີສະທ້ອນແສງ ເໝາະສົມກວ່າ Canada balsam ສຳລັບ Diptera.<br>ເຮັດວຽກດີກັບຕົວຢ່າງທີ່ໜາກວ່າ ເນື່ອງຈາກການຫຍໍ້ຕົວນ້ອຍ ແລະ ແຫ້ງໂດຍບໍ່ມີຟອງ.<br>[illegible] ethanol 95%	ມີອົງປະກອບທີ່ເປັນອັນຕະລາຍ ແລະ ຕ້ອງຈັດການພາຍໃຕ້ຕູ້ດູດຄວັນ (fume hood).<br>ການເອົານ້ຳອອກດ້ວຍ ethanol ແລະ ການຖ່າຍຜ່ານ Euparal Essence ອາດເຮັດໃຫ້ບາງ taxa ກາຍເປັນອ່ອນໄວ ແລະ ແຕກຫຼາຍ, ແຕ່ການໃຊ້ isopropanol ອາດຊ່ວຍຫຼຸດບັນຫານີ້ໄດ້.

ຕາຕະລາງ 4 (ຕໍ່)

ຊື່ (name)	ຂໍ້ດີ (Advantages)	ຂໍ້ເສຍ (Disadvantages)
Hoyer fluid	ຕົວຢ່າງສາມາດແຕ່ງສະໄລໄດ້ໂດຍກົງຂະນະທີ່ຍັງມີຊີວິດ ຫຼື ຈາກນ້ຳ, ເອທານອນ, ຫຼື formaldehyde ໂດຍກົງ.<br>ການ maceration ໃຫ້ຄຸນນະພາບ cuticle ທີ່ດີເລີດ.<br>ມີດັດສະນີສະທ້ອນແສງທີ່ເໝາະສົມ ແລະ ສາມາດເພີ່ມ contrast ດ້ວຍການທາສີ iodine.<br>Acetic acid ໃນສູດ ອາດເຮັດໃຫ້ອະໄວຍະວະຂອງ arthropod ຂະຫຍາຍ.<br>ບາງຕົວຢ່າງອາດຄົງສະພາບໄດ້ 40–60 ປີ.<br>ລະລາຍໃນນ້ຳ ອະນຸຍາດໃຫ້ການແຕ່ງສະໄລໃໝ່ງ່າຍ.	ຕົວຢ່າງພິດອ່ອນໄວ ອາດລົ້ມລົງ ຖ້າບໍ່ເພີ່ມນ້ຳຢາແຕ່ງສະໄລຊ້ຳ ເຊິ່ງໃຊ້ເວລາຫຼາຍ.<br>ອາດເກີດຊ່ອງວ່າງ ແລະ ເຂສຕອນໄດ້ໃນເວລາໜ້ອຍກວ່າ 10 ປີ.<br>ການ maceration ອາດເກີດຫຼາຍເກີນໄປ ຂຶ້ນກັບຄວາມເຂັ້ມຂອງ chloral hydrate ແລະ ໄລຍະເວລາສຳຜັດ.<br>ອົງປະກອບຂອງນ້ຳຢາແຕ່ງສະໄລ ອາດແຍກອອກຈາກກັນ ແລະ ເກີດເມັດລະອຽດ (fine granulation) ໄດ້ໃນພາຍໃນເດືອນ ຫຼື ປີ.<br>ມີການລາຍງານວ່ານ້ຳຢາແຕ່ງສະໄລ ປ່ຽນສີດຳ.
CMCP-9<br>(= carboxy methyl cellulose phenol)	ຕົວຢ່າງສາມາດແຕ່ງສະໄລໂດຍກົງຈາກນ້ຳຢາຕ່າງໆ ເຊັ່ນ ນ້ຳ, ເອທານອນ, glycerol, ຫຼື ນ້ຳຢາທີ່ມີ formaldehyde, ແລະ ອະໄວຍະວະພາຍໃນ ອາດໄດ້ຮັບການ macerated ເມື່ອຈຳເປັນ ເພື່ອຊ່ວຍໃນການສຳຫຼວດທົ່ວໄປ ຫຼື ການກະກຽມ.	ນ້ຳຢາແຕ່ງສະໄລນີ້ ອາດເກີດເຂສຕອນ ແລະ ປ່ຽນສີເຂັ້ມຂຶ້ນຕາມເວລາ ແລະ ບາງຄັ້ງອາດເຮັດໃຫ້ macerate ຕົວຢ່າງຫຼາຍກວ່າທີ່ຕ້ອງການ.<br>ຖ້າບໍ່ໄດ້ເຮັດການປະທັບ (ringed)<br>ແຜ່ນແກ້ວຢ່າງລະມັດລະວັງ ຕົວຢ່າງທີ່ໜາກວ່າ ຈະບໍ່ເໝາະສົມ ເພາະອາດຫຍໍ້ຕົວ ແລະ ເກີດຊ່ອງວ່າງຮອບແຜ່ນປົກ.<br>ບໍ່ເໝາະສຳລັບຕົວຢ່າງທີ່ທາສີ ຫຼື ວັດສະດຸທີ່ມີການຄິດ (calcified materials) ແລະ ໄລຍະເວລາແຫ້ງຊ້າກວ່າ CMC.
Eukitt™	ນ້ຳຢາແຕ່ງສະໄລທີ່ທົນທານ ດ້ວຍອາຍຸການໃຊ້ງານເກີນ 30 ປີ.<br>ເຂົ້າກັນໄດ້ກັບ solvent<br>ຫຼາຍຊະນິດສຳລັບການແຕ່ງສະໄລ ລວມທັງ acetone, benzene, chloroform, dioxan, ether, isopropanol, methyl benzoate, terpineol, toluene, ແລະ xylene.<br>ແຫ້ງໄວ ແລະ ມີ pH ອາດເປັນກົດເລັກນ້ອຍ.<br>ບໍ່ປ່ຽນສີເຂັ້ມຢ່າງເຫັນໄດ້ຊັດເຈນ ຕາມອາຍຸ.<br>ເໝາະສຳລັບນ້ຳທາສີຕ່າງໆ (ເຊັ່ນ fuchsin, hematoxylin, methyl green, methyl violet, methylene blue).<br>ຕົວຢ່າງສາມາດແຕ່ງສະໄລໃໝ່ໄດ້ຫຼັງຜ່ານໄປຫຼາຍປີ ໂດຍການແຊ່ໃນ xylene ເປັນເວລາດົນ.	ມີອົງປະກອບທີ່ເປັນອັນຕະລາຍ ແລະ ຕ້ອງຈັດການພາຍໃຕ້ຕູ້ດູດຄວັນ (fume hood).<br>ຕ້ອງການການເອົານ້ຳອອກ (dehydration) ຢ່າງຄົບຖ້ວນ ແລະ ໃຊ້ເວລາຫຼາຍ.<br>ບໍ່ເໝາະສຳລັບຕົວຢ່າງທີ່ໜາກວ່າ ເນື່ອງຈາກການຫຍໍ້ຕົວ ແລະ ການເກີດຟອງແກັສ.<br>ແຜ່ນປົກອາດລື່ອອອກຕາມເວລາ ຖ້າບໍ່ເຮັດຄວາມສະອາດແກ້ວໃຫ້ດີ ແລະ ປະທັບໃຫ້ແໜ້ນ.<br>ອາດສະແດງການ polymerize ບໍ່ສົມບູນ ຮອບເສັ້ນໄຍ collagen.
Enecê	ນ້ຳຢາແຕ່ງສະໄລທີ່ທົນທານສູງ ດ້ວຍອາຍຸການໃຊ້ງານຢ່າງໜ້ອຍ 50 ປີ.<br>Enecê ບໍ່ປ່ຽນສີເຂັ້ມຂຶ້ນຕາມເວລາ.<br>ມີຄວາມອ່ອນໄຫວຫຼາຍກວ່າ ອະນຸຍາດໃຫ້ດຳເນີນການຜ່າຕັດແນງໃນນ້ຳຢາແຕ່ງສະ ໄລ ແລະ<br>ໃຫ້ເວລາພຽງພໍສຳລັບການຈັດຕຳແໜ່ງໂຄງສ້າງ ຮູບຮ່າງ.<br>ລາຄາ◌ຕຕ່ຳ.	ຕ້ອງການການເອົານ້ຳອອກ (dehydration) ຢ່າງຄົບຖ້ວນ ແລະ ໃຊ້ເວລາຫຼາຍ.<br>ການເອົານ້ຳອອກດ້ວຍ ethanol ແລະ ການຖ່າຍຜ່ານ clove oil<br>ອາດເຮັດໃຫ້ບາງຕົວຢ່າງກາຍເປັນອ່ອນໄວ ແລະ ແຕກງ່າຍ.<br>ແນງຍັງຄົງເຮັດໃຫ້ໂປ່ງໃສຕໍ່ໄປ ເຖິງຈະຊ້າຫຼາຍ; ນີ້ອາດເຮັດໃຫ້ຍາກທີ່ຈະເບິ່ງໂຄງສ້າງນ້ອຍໆ ເຊັ່ນ sensilla, ascoids, ແລະ simple setae.

ນ້ຳຢາແຕ່ງສະໄລ ອາດເປັນແບບນ້ຳ (liquid), ອີງກໍາ (gum-based), ຫຼື ອີງ resin ໄດ້, ລະລາຍໄດ້ໃນນ້ຳ, ເອທານອນ ຫຼື solvent ອື່ນໆ (ເຊັ່ນ toluene, xylene) (ຕາຕະລາງ 1). ເມື່ອນຳໃຊ້ແລ້ວ ຄວນເຮັດການປະທັບ (sealed)

ດ້ວຍນ້ຳຢາປະທັບທີ່ບໍ່ລະລາຍ (non-soluble ringing media) ເພື່ອປ້ອງກັນການສຳຜັດກັບບັນຍາກາດ.
ເພື່ອແຍກປະເພດຂອງນ້ຳຢາແຕ່ງສະໄລໃຫ້ຊັດເຈນ ສາມາດໃຊ້ການແບ່ງປະເພດດັ່ງຕໍ່ໄປນີ້:

a. ນ້ຳຢາແບບນ້ຳ (Aqueous media). ນ້ຳຢາເຫຼົ່ານີ້ລະລາຍງ່າຍໃນນ້ຳ ເໝາະສຳລັບການແຕ່ງສະໄລຊົ່ວຄາວ ຫຼື ກາງໆອາຍຸ (temporary or semi-permanent mounts). ປົກກະຕິງ່າຍຕໍ່ການຈັດການ ແຕ່ອາດຕ້ອງການການປະທັບເພື່ອປ້ອງກັນການສຳຜັດກັບຄວາມຊຸ່ມຊື່ນ ໃນບັນຍາກາດ (ເຊັ່ນ gum-chloral media ແລະ polyvinyl alcohol) ໂດຍສະເພາະໃນເຂດຮ້ອນຊຸ່ມຊື່ນ.

b. ນ້ຳຢາທີ່ທົນຕໍ່ນ້ຳໄດ້ຈຳກັດ (Limited water-tolerant media). ນ້ຳຢາເຫຼົ່ານີ້ຖືກສຳຜັດກັບນ້ຳໜ້ອຍກວ່າ ແຕ່ຍັງຕ້ອງການການປ້ອງກັນຄວາມຊຸ່ມຊື່ນເກີນໄປ. ໃຫ້ຄວາມສະຖຽນທີ່ຍາວນານກວ່ານ້ຳຢາລະລາຍນ້ຳ ແລະ ນຳໃຊ້ບ່ອຍໃນການແຕ່ງສະໄລກາງໆອາຍຸ.

c. ນ້ຳຢາລະລາຍໃນ hydrocarbon (Hydrocarbon-soluble media). ນ້ຳຢາເຫຼົ່ານີ້ລະລາຍໃນ solvent ອິນຊີເຊັ່ນ xylene ຫຼື toluene, ຫຼື essenecê (enecê solvent). ອອກແບບສຳລັບການແຕ່ງສະໄລຖາວອນ ແລະ ໃຫ້ຄວາມສະຖຽນທີ່ດີເລີດໃນໄລຍະຍາວ, ຕ້ານການຊຸ່ມຊື່ນ ແລະ ການເສື່ອມສະພາບ ເໝາະສຳລັບການເກັບຮັກສາໄລຍະຍາວ (ເຊັ່ນ neutral Canada balsam, DPX).

ສະຫຼຸບ ນ້ຳຢາລະລາຍນ້ຳ ເໝາະທີ່ສຸດສຳລັບການແຕ່ງສະໄລຊົ່ວຄາວ ຫຼື ກໍລະນີທີ່ຕ້ອງການເອົາຕົວຢ່າງອອກງ່າຍ; ນ້ຳຢາທົນຕໍ່ນ້ຳໄດ້ຈຳກັດ ເໝາະສຳລັບການແຕ່ງສະໄລກາງໆອາຍຸທີ່ຕ້ອງການຄວາມທົນທານປານກາງ; ນ້ຳຢາລະລາຍໃນ hydrocarbon ເປັນທາງເລືອກທີ່ດີທີ່ສຸດສຳລັບການແຕ່ງສະໄລຖາວອນ ທີ່ຕ້ອງການເກັບຮັກສາໄລຍະຍາວ ແລະ ເປັນແຫຼ່ງອ້າງອີງ.

5.3.4. ຄຳອະທິບາຍກ່ຽວກັບນ້ຳຢາແຕ່ງສະໄລ (mounting media) (ຕາຕະລາງ 3 & 4)

<u>ນ້ຳຢາສຳລັບການສັງເກດຊົ່ວຄາວ</u>

Chloral gum = Hoyer fluid/medium/solution (RI = 1.48). Marc André fluid ເປັນສານທີ່ດີທີ່ສຸດສຳລັບການສັງເກດໃນໄລຍະສັ້ນ (ບໍ່ຫລາຍຊົ່ວໂມງ ຫຼື ອາດຈະຍາວກວ່ານັ້ນ ຖ້າ ສະໄລຖືກເກັບໃນຫ້ອງຊຸ່ມ) ສຳລັບການສັງເກດຖົງເກັບນ້ຳເຊື້ອ (spermathecae), ລວມທັງການຖ່າຍຮູບ (ຮູບ 4) ຫຼື ການແຕ້ມຮູບ. ການຮັກສາຖົງເກັບນ້ຳເຊື້ອ (spermathecae) ທີ່ໄດ້ສັງເກດແລ້ວ ຈຳເປັນຕ້ອງນຳມາແຕ່ງສະໄລໃໝ່ໃນນ້ຳຢາທີ່ເປັນນ້ຳ ເພື່ອໃຫ້ສາມາດເກັບໄດ້ໃນໄລຍະກາງ. ການດຶງນ້ຳອອກ (dehydrate) ເພື່ອແຕ່ງສະໄລໃໝ່ໃນ resin ສາມາດເຮັດໄດ້ ແຕ່ບໍ່ແນະນຳ (ມີຄວາມສ່ຽງຕໍ່ການສູນເສຍ). Chloral gum ແລະ Hoyer fluid ຖືກພິຈາລະນາວ່າເປັນຄຳສັບທີ່ມີຄວາມໝາຍຄືກັນ.
ສານນີ້ຖືກນຳໃຊ້ບໍ່ຂາດໃນການສັງເກດອະວະຍະວະພາຍໃນ ເນື່ອງຈາກມັນເຂົ້າກັນໄດ້ກັບນ້ຳ, ໃຊ້ງານງ່າຍ, ເຮັດໄດ້ໄວ ແລະ ມີດັດຊະນີຫັກເຫແສງ (refractive index) ທີ່ຊ່ວຍໃຫ້ການກວດເບິ່ງໂຄງສ້າງທີ່ບອບບາງ ເຊັ່ນ ຖົງເກັບນ້ຳເຊື້ອ (spermathecae) ເຮັດໄດ້ງ່າຍ. ແຕ່ຢ່າງໃດກໍຕາມ chloral gum ມີຂໍ້ຈຳກັດສຳຄັນ ຖ້າບໍ່ໄດ້ກຽມຫຼືເກັບຮັກສາໃນສະພາບຄວາມຊຸ່ມທີ່ຄວບຄຸມໄດ້ດີ.
ບັນຫາເຫຼົ່ານີ້ລວມມີ ການຕົກຕະກອນຄຣິສຕອນ, ການປ່ຽນສີ ແລະ ການສູນເສຍຄວາມໜືດ. ການປິດຂອບ ແຜນປົກສະໄລ coverslip (ringing) ບໍ່ສາມາດແກ້ໄຂບັນຫາເຫຼົ່ານີ້ໄດ້ ເນື່ອງຈາກ ນ້ຳຢາແຕ່ງສະໄລ ອາດຈະປ່ຽນສີເຂັ້ມຫຼາຍ (ບາງຄັ້ງເກືອບດຳ) ເນື່ອງຈາກປະຕິສຳພັນກັບສານທີ່ໃຊ້ປິດຂອບ ໂດຍສະເພາະຖ້າໃຊ້ Euparal®..

Hoyer medium ໄດ້ຖືກພິຈາລະນາວ່າເປັນນ້ຳຢາທີ່ດີທີ່ສຸດສຳລັບຮິ້ນຝອຍຊາຍ ແລະ ໄດ້ຖືກນຳໃຊ້ມາດັ່ງເດີມໃນຈຸດປະສົງນີ້.
ສານນີ້ປະກອບມີສູດຫຼາຍແບບທີ່ໃກ້ຄຽງກັນ ເຊັ່ນ gum arabic, glycerol ແລະ chloral hydrate. ສູດຫຼາຍແບບໄດ້ຖືກເຂົ້າໃຈຜິດ ຫຼື ອ້າງອີງຜິດ [74].

ເຖິງແມ່ນວ່າ Hoyer ເປັນນ້ຳຢາແຕ່ງສະໄລທີ່ດີສຳລັບການສັງເກດຖົງເກັບນ້ຳເຊື້ອ (spermathecae) ໃນຮິ້ນຝອຍຊາຍ ແຕ່ມັນບໍ່ເໝາະສຳລັບການເກັບຮັກສາໃນໄລຍະຍາວ.
ມັນເໝາະສຳລັບການສັງເກດໄລຍະສັ້ນ ເຊັ່ນ ການຖ່າຍຮູບ, ການແຕ້ມຮູບ ຫຼື ການບັນທຶກພາບ.
ນ້ຳຢາຊະນິດເປັນນ້ຳເໝາະສຳລັບການແຕ່ງສະໄລຊົ່ວຄາວ ແຕ່ບໍ່ສາມາດຮັບປະກັນການເກັບຮັກສາໄດ້ໃນໄລຍະຍາວ. ກົງກັນຂ້າມ, ການແຕ່ງສະໄລດ້ວຍ resin ໃຫ້ຄວາມຄົງທົນສູງ ແລະ ອາດຈະຢູ່ໄດ້ເຖິງຫຼາຍຮ້ອຍປີ ແຕ່ອາດຈະເຮັດໃຫ້ລາຍລະອຽດຂອງຖົງເກັບນ້ຳເຊື້ອ ເຫັນໄດ້ບໍ່ຊັດ ເນື່ອງຈາກຄຸນສົມບັດການຫັກເຫຂອງແສງມັກຈະຫາຍໄປ.

Hoyer medium ຈະເສື່ອມສະພາບໄປຕາມເວລາ ເນື່ອງຈາກການສູນເສຍນ້ຳ (dehydration) (ຮູບ 8) ສິ່ງຜົນໃຫ້ເກີດການກໍຕົວເປັນຄຣິສຕອນ chloral hydrate ສີຂາວ ແລະ ຂຸ່ນ. ແຕ່ຢ່າງໃດກໍຕາມ ຕົວຢ່າງຍັງສາມາດກູ້ຄືນໄດ້ຈາກ ສະໄລທີ່ເກີດຄຣິສຕອນ ເນື່ອງຈາກ cuticle ຍັງຄົງສະພາບທາງເຄມີ ແມ່ນແຕ່ອາດຈະມີຄວາມເສຍຫາຍທາງກາຍະພາບບາງສ່ວນຈາກການເຕີບໂຕຂອງຄຣິສຕອນ. ໃນບາງກໍລະນີ ສະໄລທີ່ເກີດຄຣິສຕອນສາມາດຟື້ນຟູໄດ້ ໂດຍການເພີ່ມຄວາມຊຸ່ມໃຫ້ນ້ຳຢາແຕ່ງສະໄລໃນສະພາບອຸ່ນ ແລະ ຊຸ່ມ ພ້ອມກັບໃຊ້ thymol ເພື່ອປ້ອງກັນເຊື້ອຣາ. ອີກທາງເລືອກໜຶ່ງ ຄື ການແຊ່ຕົວຢ່າງອອກຈາກ gum chloral ໃນນ້ຳ, ຕາມດ້ວຍການ dehydrate ໃນ glacial acetic acid ແລະ ນຳໄປ ແຕ່ງໃໝ່ໃນ Canada balsam.

DMHF (diméthyl-hydantoïne formaldéhyde) (IR = 1.48)
ນ້ຳຢາທີ່ມີນ້ຳເປັນຖານນີ້ [72] ມີປະສິດທິພາບທາງ optical ດີຫຼາຍ ຄ້າຍຄືກັບ Berlese ແລະ ໃຊ້ງານງ່າຍເທົ່າກັນ. ແຕ່ຕ່າງຈາກ Berlese, ມັນບໍ່ປ່ຽນເປັນສີດຳ ຫຼື ເກີດຄຣິສຕອນ. ມັນເໝາະສຳລັບ ຮີ້ນຝອຍຊາຍ ແລະ ແມງໃນວົງສະກຸນ Psychodidae ອື່ນໆ.

CMCP (camphre-mono-chlorophénol) (IR = 1.41)
ນີ້ແມ່ນນ້ຳຢາສຳລັບການແຕ່ງສະໄລທີ່ມີ glycerin ເປັນຖານ ແລະ ລະລາຍໃນນ້ຳ ໃຊ້ສຳລັບການແຕ່ງແຜ່ນແກ້ວສະໄລແບບຖາວອນທີ່ໃສ ສຳລັບຕົວຢ່າງທີ່ບອບບາງ ລວມທັງ ຮີ້ນຝອຍຊາຍ. ຂໍ້ດີຄື ສາມາດແຕ່ງຕົວຢ່າງໄດ້ໂດຍກົງຈາກນ້ຳ ຫຼື ethanol. ມັນຊ່ວຍໃຫ້ ຮີ້ນຝອຍຊາຍ ຄາຍຕົວ ແລະ ໃສ ຢ່າງວ່ອງໄວ ໂດຍເຮັດໃຫ້ cuticle ອ່ອນລົງ ເພື່ອໃຫ້ຈັດຕຳແໜ່ງໄດ້ຖືກຕ້ອງ ເຊັ່ນ ການກະຈາຍປີກ ຫຼື ການຜ່າອະວະຍະວະເພດ.
ເຖິງແມ່ນວ່າມີການລາຍງານວ່າສາມາດເກັບໄດ້ໄລຍະຍາວ ແຕ່ໄລຍະເວລາທີ່ແນ່ນອນຍັງບໍ່ຊັດເຈນ. ຂໍ້ຈຳກັດຫຼັກຄື ມັນມີ phenol ເຊິ່ງເປັນສານພິດ ແລະ ລະຄາຍເຄືອງ ຕ້ອງໃຊ້ດ້ວຍຄວາມລະວັງ..

<u>ນ້ຳຢາສຳລັບການ mount ແບບຖາວອນ</u>

Canada balsam (IR = 1.52-1.54)
ນ້ຳຢານີ້ໄດ້ຖືກພັນລະນາວ່າເປັນນ້ຳຢາແຕ່ງສະໄລທີ່ເໝາະສຳລັບການສິ່ງສະທ້ອນແສງໃນກ້ອງຈຸລະທັດ ໂດຍ ທ່ານ Andrew Pritchard ໃນທົດສະວັດ 1830. ມັນຍັງເປັນນ້ຳຢາທີ່ນິຍົມໃຊ້ຫຼາຍ ເນື່ອງຈາກຄຸນນະພາບໃນການເກັບຮັກສາທີ່ດີ ແລະ ມີປະຫວັດການໃຊ້ງານກວ່າ 150 ປີ. ຕ່າງຈາກ Hoyer, ມັນບໍ່ເກີດຄຣິສຕອນ ຫຼື ດູດຊຶມນ້ຳ. ແຕ່ມັນມີ autofluorescence ສູງ ເຊິ່ງອາດເປັນຂໍ້ຈຳກັດໃນບາງເຕັກນິກ microscopy [60]. ການໃຊ້ solvent ທີ່ບໍ່ມີພິດແທນ xylene ສາມາດຫຼຸດຄວາມສ່ຽງ ແຕ່ອາດເຮັດໃຫ້ແຫ້ງຊ້າ ແລະ ປ່ຽນສີໄວຂຶ້ນ.

Euparal® (IR = 1.48) ; L'Euparal® Euparal® ເປັນທາງເລືອກທີ່ນິຍົມແທນ Canada balsam ສຳລັບການແຕ່ງສະໄລແບບຖາວອນ ໂດຍມີຄວາມຄົງທົນໄລຍະຍາວທີ່ດີ ແລະ ອັດຕາການຫັກເຫແສງໃກ້ຄຽງກັນ. ມັນມີລັກສະນະສຳຄັນຄື: (1) ຕ້ອງມີການດຶງນ້ຳອອກກ່ອນ: ກ່ອນການແຕ່ງສຸດທ້າຍ ຕົວຢ່າງຕ້ອງຖືກຜ່ານເຫຼົ້າ ethanol ຈາກ 95% ໄປຫາ absolute ethanol; (2) ໃຊ້ເວລາດົນ: ການຈັດການໃນ resin ຈະເພີ່ມເວລາໃນການກະກຽມຕົວຢ່າງ. ຖ້າບໍ່ສາມາດ dehydrate ດ້ວຍ solvent ໄດ້ ສາມາດນຳຕົວຢ່າງຈາກ absolute ethanol ໄປໃສ່ນ້ຳຢາກາງ (mixture) ລະຫວ່າງ Euparal® ແລະ Euparal essence ກ່ອນການແຕ່ງສຸດທ້າຍ.

Enecê (IR = 1.467). Enecê ແມ່ນນ້ຳຢາ ແຕ່ງສະໄລ ທີ່ມີຢາງ resin ເປັນຖານ ໃຊ້ຫຼາຍສຳລັບແມງໄມ້ນ້ອຍ ແລະ ນິຍົມໃນປະເທດບຣາຊິນ. ສ່ວນປະກອບມີ colophony ແລະ gum copal ລະລາຍໃນ alcohol, camphor, essence of turpentine ແລະ eucalyptol. Cerqueira [11] ໄດ້ອະທິບາຍວ່າ Enecê ເປັນທາງເລືອກຂອງ Canada balsam ສຳລັບການແຕ່ງແຜ່ນແກ້ວສະໄລແບບຖາວອນ ຂອງໜອນແມງໄມ້ (larvae), ຄາບແມງໄມ້ (exuviae) ແລະ ແມ່ກະທັ້ງຍຸງຕົວແກ່ ແລະ ຕໍ່ມາຖືກນຳໃຊ້ຢ່າງກວ້າງຂວາງສຳລັບ ຮີ້ນຝອຍຊາຍ. Enecê ເປັນທາງເລືອກທີ່ປະຢັດ ສຳລັບການແຕ່ງສະໄລແບບຖາວອນ ໂດຍໃຫ້ຄວາມຄົງທົນໄລຍະຍາວ ແລະ ເວລາແຫ້ງທີ່ເໝາະສົມ ເພື່ອໃຫ້ສາມາດຜ່າ ແລະ ຈັດຮູບໂຄງສ້າງຮູບຮ່າງໄດ້ຢ່າງແມ່ນຍຳ.

5.4. ການກະກຽມແຜ່ນແກ້ວສະໄລ ແລະ ການອົບແຫ້ງ

ການອົບແຫ້ງແຜ່ນແກ້ວສະໄລຫຼັງຈາກການແຕ່ງສະໄລແມ່ນສິ່ງສຳຄັນຫຼາຍ ເພື່ອຮັບປະກັນຄວາມຄົງທົນ ແລະ ການເກັບຮັກສາໃນໄລຍະຍາວ.
ແຜ່ນແກ້ວສະໄລຄວນຖືກອົບໃຫ້ແຫ້ງສົມບູນ ກ່ອນຈະນຳໄປເກັບຮັກສາ. ເພື່ອໄດ້ຜົນທີ່ດີ, ແຜ່ນແກ້ວສະໄລທີ່ໃຊ້ນ້ຳຢາສຳລັບການແຕ່ງສະໄລແບບຖາວອນ ຄວນອົບແຫ້ງໃນທ່ານອນພຽງ 2–3 ອາທິດ, ສຳລັບນ້ຳຢາແບບເຄິ່ງຖາວອນ ອາດໃຊ້ເວລາ 1–2 ອາທິດ. ແນະນຳໃຫ້ໃຊ້ຕູ້ອົບ incubator ທີ່ຕັ້ງອຸນຫະພູມໃຫ້ເໝາະສົມ ກັບນ້ຳຢາທີ່ໃຊ້ ແລະ ຫຼີກລ້ຽງຄວາມຮ້ອນສູງເກີນໄປ ເພາະອາດທຳລາຍຕົວຢ່າງ. ອຸນຫະພູມທີ່ແນະນຳຄື 30–37°C. ຂັ້ນຕອນນີ້ສຳຄັນເພາະຊ່ວຍປ້ອງກັນການບິດງໍຂອງແຜ່ນແກ້ວສະໄລ, ການເສຍຫາຍຂອງຕົວຢ່າງ ຫຼື ຄວາມບໍ່ສະຖຽນຂອງນ້ຳຢາໃນໄລຍະການເກັບຮັກສາ.
ນ້ຳຢາທີ່ໃຊ້ໃນການແຕ່ງສະໄລຄວນຖືກບັນທຶກລົງໃນແຜ່ນແກ້ວສະໄລສະເໝີ. ຖ້າເປັນໄປໄດ້ ຄວນບັນທຶກສູດທີ່ໃຊ້, ຊື່ຜູ້ກະກຽມ ແລະ ວັນທີກະກຽມ. ໃນຂັ້ນຕົ້ນ ແຜ່ນແກ້ວສະໄລມັກຖືກກະກຽມເປັນແບບຊົ່ວຄາວ ແລະ ບໍ່ໄດ້ມຸ່ງເນັ້ນການເກັບຮັກສາຍາວ. ແຕ່ຖ້າສະຖານະຂອງຕົວຢ່າງປ່ຽນໄປ (ເຊັ່ນ ກາຍເປັນອ້າງອີງ "type specimen") ຄວນໃຊ້ນ້ຳຢາແບບຖາວອນເພື່ອຮັກສາສຳລັບການສຶກສາການຈຳແນກຊະນິດໃນອະນາຄົດ.

5.5. Techniques alternatives de montage : montage sur carte

Card mounting ແມ່ນວິທີທີ່ໃຊ້ກັບແມງໄມ້ຫຼາຍກຸ່ມ ໂດຍຕົວຢ່າງສາມາດຖືກປັກເຂັມໃສ່ເຈ້ຍ ຫຼື ຕິດກາວໄວ້ດ້ານເທິງເຈ້ຍ. ແຕ່ເນື່ອງຈາກ ຮີ້ນຝອຍຊາຍ ມີຂະໜາດນ້ອຍ ແລະ ຈຳເປັນຕ້ອງສັງເກດອະວະຍະວະພາຍໃນ (ເບິ່ງໃນຂໍ້ທີ 6) ວິທີນີ້ຈຶ່ງບໍ່ເໝາະສົມເລີຍກັບການຮັກສາຮີ້ນຝອຍຊາຍ.

5.6. ການແຕ່ງສະໄລໃໝ່ສຳລັບຕົວຢ່າງທີ່ເສຍຫາຍ

ສຳລັບຕົວຢ່າງທີ່ຫາຍາກ ຫຼື ມີຄ່າ ແນະນຳໃຫ້ໃຊ້ວິທີ 2 ຂັ້ນຕອນ ຕາມວິດີໂອທີ່ເຂົ້າເຖິງໄດ້ທີ່: https://zenodo.org/records/18315029.
1) ເພີ່ມຄວາມຊຸ່ມ (rehydrate) ໂດຍບໍ່ແກະແຜ່ນແກ້ວສະໄລ ເພື່ອໃຫ້ສາມາດສັງເກດເບື້ອງຕົ້ນ.
ຄວນວາງທີ່ຈັບສຳລັບແຜ່ນແກ້ວສະໄລຫຼາຍໆ ໃນ Petri dish ເພື່ອເປັນຖານຮອງ. ຈາກນັ້ນນຳແຜ່ນແກ້ວສະໄລທີ່ຈະ rehydrate ວາງໄວ້ເທິງ ແລະ ເພີ່ມ solvent ປະມານບາງມີລິແມັດໃນ Petri dish ເພື່ອສ້າງຫ້ອງຊຸ່ມ ໂດຍໃຫ້ແນ່ໃຈວ່າແຜ່ນແກ້ວສະໄລບໍ່ໄດ້ສຳຜັດກັບ solvent (ຮູບ 8 D). ເວລາທີ່ໃຊ້ໃນການ rehydrate ອາດຈະແຕກຕ່າງຈາກ 1 ຫາຫຼາຍມື້ ຂຶ້ນກັບສະພາບຂອງຕົວຢ່າງ.

This is an Open Access article distributed under the terms of the Creative Commons Attribution License (https://creativecommons.org/licenses/by/4.0), which permits unrestricted use, distribution, and reproduction in any medium, provided the original work is properly cited.

ການກວດສອບທຸກມື້ ແລະ ຄວາມອິດທົນແມ່ນສິ່ງສຳຄັນ. ເມື່ອແຜ່ນແກ້ວສະໄລມີຄວາມຊຸ່ມພຽງພໍແລ້ວ ສາມາດນຳອອກຈາກຫ້ອງຊຸ່ມ ແລະ ນຳໄປວາງໃນ incubator ສອງສາມຊົ່ວໂມງ ກ່ອນການສັງເກດດ້ວຍກ້ອງຈຸລະທັດ, ການຖ່າຍຮູບ ຫຼື ການແຕ້ມຮູບ..

2) ເພື່ອການແຕ່ງສະໄລໃໝ່ ສາມາດນຳແຜ່ນແກ້ວສະໄລກັບໄປຫ້ອງຊຸ່ມອີກສອງສາມຊົ່ວໂມງ ຫຼື ຄ້າງຄືນ. ການແກະແຜ່ນແກ້ວສະໄລຄວນເຮັດພາຍໃຕ້ກ້ອງ binocular microscope. ໃຊ້ເຂັມລະອຽດ ເພື່ອເອົາ coverslip ອອກຢ່າງລະມັດລະວັງ ໂດຍໃຫ້ແນ່ໃຈວ່າບໍ່ມີສ່ວນໃດຂອງ ຮິ້ນຝອຍຊາຍ ຕິດຢູ່ (https://zenodo.org/records/18315029). ຈາກນັ້ນ ຄວນເກັບສ່ວນທີ່ຖືກຜ່າອອກ ແລະ ລ້າງດ້ວຍນ້ຳໃນຫຼຸມນ້ອຍ ຄ້າຍກັບທີ່ໃຊ້ໃນການສະກັດ DNA/RNA ແບບທຳລາຍ (ດັ່ງລຸ່ມນີ້), ກ່ອນການ dehydrate ແລະ ການແຕ່ງສະໄລໃໝ່ໃນນ້ຳຢາປະເພດ resin. ໃນການແກະແຜ່ນແກ້ວສະໄລ ຈຳເປັນຕ້ອງຮູ້ຊະນິດນ້ຳຢາທີ່ໃຊ້ເດີມ ເພື່ອເລືອກ solvent ໃຫ້ເໝາະສົມ. ສຳລັບນ້ຳຢາປະເພດ aqueous ໃຫ້ໃຊ້ນ້ຳ. ຖ້າເປັນນ້ຳຢາປະເພດ resin (ເຊັ່ນ Canada balsam ຫຼື Euparal®) ຄວນໃຊ້ xylene ໂດຍເຮັດໃນ fume hood ແລະ ໃຊ້ອຸປະກອນປ້ອງກັນສ່ວນບຸກຄົນຢ່າງເໝາະສົມ ລວມທັງໜ້າກາກ. ການແຕ່ງສະໄລໃໝ່ຂອງ type specimen ຫຼື ຕົວຢ່າງໃນ collection ຕ້ອງໄດ້ຮັບການອະນຸຍາດຈາກ curator ຫຼື ສະຖາບັນເຈົ້າຂອງກ່ອນເທົ່ານັ້ນ.

6. ການຈຳແນກຕົວຢ່າງ

6.1. ຮູບຮ່າງ (Morphology)

ການຈຳແນກ ຮິ້ນຝອຍຊາຍ ພື້ນຖານແມ່ນອີງໃສ່ການກວດສອບລັກສະນະຮູບຮ່າງ (morphology) ເຊັ່ນ ຮູບຊົງຂອງເອິກ (thorax), ປີກ, ອະໄວຍະວະສືບພັນ (genitalia), ຂົນ (setae) ແລະ ຄວາມສຳພັນທາງ morphometric ລະຫວ່າງໂຄງສ້າງຕ່າງໆ. ນັກວິຈັຍໃຊ້ taxonomic keys, ຄັງຕົວຢ່າງອ້າງອີງ (reference collections) ແລະ ຄຳອະທິບາຍສາຍພັນດັ່ງເດີມ ເພື່ອປຽບທຽບຕົວຢ່າງທີ່ເກັບກັບ taxa ທີ່ຮູ້ຈັກແລ້ວ. ລັກສະນະສຳຄັນສຳລັບການຈຳແນກ ປະກອບມີ ເສັ້ນປີກ (wing venation) ແລະ ຮູບຮ່າງຫົວ ທັງໃນເພດຜູ້ ແລະ ເພດຍິງ, ໂຄງສ້າງ genitalia ຂອງເພດຜູ້ ແລະ ຮູບແບບ spermathecae ຂອງເພດຍິງ. ການຈຳແນກທີ່ແມ່ນຍຳ ມັກຈະຕ້ອງໃຊ້ການກວດດ້ວຍກ້ອງຈຸລະທັດ ໂດຍໃຊ້ compound microscope ເພື່ອເບິ່ງໂຄງສ້າງລະອຽດ (ເຊັ່ນ genitalia ແລະ spermathecae) ຫຼື stereomicroscope ສຳລັບການເບິ່ງຮູບຮ່າງທົ່ວໄປ.

ໃນປັດຈຸບັນ ເທັກໂນໂລຊີການຖ່າຍຮູບໄດ້ຊ່ວຍໃຫ້ການຈຳແນກມີຄວາມກ້າວໜ້າຂຶ້ນ . ຮູບຄວາມລະອຽດສູງ ຫຼື ຮູບວາດດິຈິຕອນຂອງລັກສະນະສາຄັນ ສາມາດນຳໄປປຽບທຽບກັບຂໍ້ມູນອ້າງອີງ ຫຼື ວິເຄາະດ້ວຍລະບົບຄອມພິວເຕີ ເພື່ອເພີ່ມຄວາມແມ່ນຍຳ ແລະ ຄວາມສະດວກໃນ ການຈຳແນກຊະນິດ.

6.2. ຮູບຊົງເລຂາຄະນິດຂອງປີກ (Wing geometry)

ຮູບຮ່າງປີກແມ່ນລັກສະນະສຳຄັນ ທີ່ໃຊ້ໃນການຈຳແນກ ແລະ ຈັດປະເພດຂອງ ຮິ້ນຝອຍຊາຍ ແຕ່ລະສາຍພັນ. ປີກຂອງພວກນີ້ມີຮູບແບບສະເພາະ ໂດຍທົ່ວໄປຈະຍາວ ແລະ ແຄບ ພ້ອມກັບມີເສັ້ນປີກ (venation) ທີ່ພັດທະນາດີ (ຮູບ 9 & 10).

ການຈັດຮຽງຂອງເສັ້ນປີກຈະສ້າງຮູບແບບສະເພາະ ທີ່ອາດແຕກຕ່າງກັນລະຫວ່າງ genus ແລະ species ເຮັດໃຫ້ເປັນຈຸດຊີ້ນຳສຳຄັນໃນການຈຳແນກ. ດັ່ງນັ້ນ ການສຶກສາຮູບຮ່າງປີກຈຶ່ງມີປະໂຫຍດຫຼາຍໃນດ້ານອານຸກົມວິຖານ (taxonomy).

6.3. ການວັດແທກທາງເລຂາຄະນິດຂອງປີກ (Wing geometric morphometrics)

ນັກຄົ້ນຄ້ວາໃຊ້ຫຼາຍວິທີ ເຊັ່ນ ການວັດແທກທາງເລຂາຄະນິດຂອງປີກ ເພື່ອວິເຄາະ ແລະ ປຽບທຽບຮູບຮ່າງ ແລະ ຂະໜາດຂອງປີກ ໃນ ຮິ້ນຝອຍຊາຍ ສາຍພັນ ຫຼື ປະຊາກອນທີ່ແຕກຕ່າງກັນ. ການສຶກສານີ້ຍັງຊ່ວຍໃຫ້ເຂົ້າໃຈພຶດຕິກຳ, ຄວາມມັກທີ່ຢູ່ອາໄສ (habitat preferences) ແລະ ຄວາມສາມາດໃນການບິນ.

ໃນວິທີ geometric morphometrics ປີກຈະຖືກຜ່າຕັດອອກຢ່າງລະມັດລະວັງ, ຍ້ອມສີ (ຖ້າຈຳເປັນ) ແລະ ແຕ່ງສະໄລໃຫ້ແບນແພ້ອອກໃນແຜ່ນແກ້ວສະໄລ. ຫຼັງຈາກນັ້ນ ຈະຖ່າຍຮູບດ້ວຍ stereomicroscope, ແປງເປັນຂໍ້ມູນດິຈິຕອນ ແລະ ນຳໄປວິເຄາະ morphometric. ວິທີນີ້ໄດ້ຖືກອະທິບາຍຢ່າງກວ້າງຂວາງໃນເອກະສານອ້າງອີງ [6, 27, 42, 56, 57, 59] ແລະ ແນະນຳໃຫ້ໃຊ້ປີກຂ້າງດຽວ (ຊ້າຍ ຫຼື ຂວາ) ຢ່າງສະໝ່ຳເໝີ ເພື່ອຫຼີກລ້ຽງຜົນກະທົບ allometric ທີ່ອາດເກີດຂຶ້ນ [62].

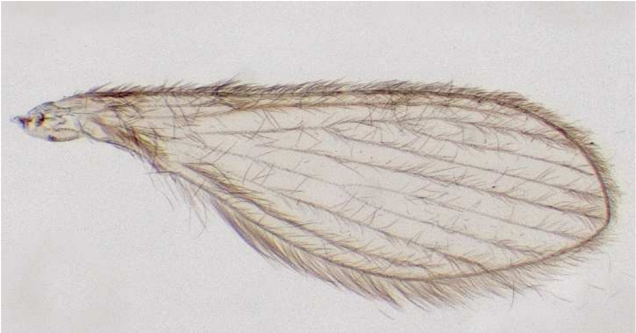

ຮູບ 9: ປີກສິດຂອງ *Trichophoromyia ininii*.

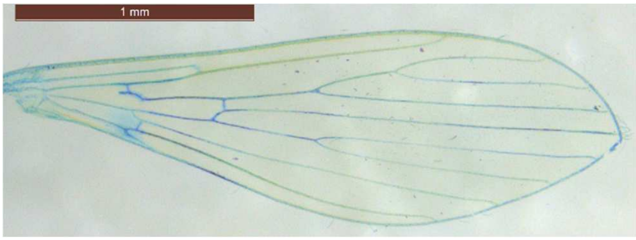

ຮູບ 10: ປີກທີ່ຍ້ອມສີຂອງ *Phlebotomus ariasi*.

ການກະກຽມປີກສຳລັບການວິເຄາະການວັດແທກທາງເລຂາຄະນິດຂອງປີກ

ເພື່ອການສັງເກດເສັ້ນປີກໃຫ້ໄດ້ດີທີ່ສຸດ, ປີກຄວນໄດ້ຮັບການເຮັດຄວາມສະອາດເອົາເກັດອອກ ແລະ ຍ້ອມສີຢ່າງເໝາະສົມ. ສຳລັບການກະກຽມປີກ, ກ່ອນອື່ນໃສ່ນ້ຳຢາທີ່ຕ້ອງການໃນຮູນ້ອຍໆ (methylene blue, ethanol, ນ້ຳ ແລະ xylene substitute). ເອົາປີກທີ່ເກັບໄວ້ໃນ ethanol 70% ໃນອຸນຫະພູມຫ້ອງ ໂດຍປຽນຫົວ Eppendorf tube ແລະ ເອົາອອກລົງໃນຮູ, ຫຼັງຈາກນັ້ນ ຍົກປີກຕາມ ລວງຍາວດ້ວຍເຂັມໂຄ້ງນ້ອຍ. ຖ່າຍປີກຜ່ານ ethanol ໄປນ້ຳ ແລະ ກັບມາ ethanol ອີກຄັ້ງເພື່ອກຳຈັດຂົນ. ໃສ່ປີກໃນ methylene blue ເປັນເວລາ 6 ນາທີ ໂດຍຮັບປະກັນວ່າມັນລອຍຢູ່ໃນລະຫວ່າງການທາສີ. ເອົາປີກອອກຢ່າງລະມັດລະວັງ ແລະ ຈຸ່ມໃນ xylene substitute ເປັນເວລາ 2 ນາທີ (ປະມານ 1/3 ຂອງເວລາ methylene blue). ການເຕັ້ນເຂັມເບົາໆກັບຝາຮູ ສາມາດຊ່ວຍໃຫ້ປີກຕັ້ງຄົງ; xylene ຊ່ວຍໃຫ້ສີຄົງຕົວ. ສຸດທ້າຍ ຍົກປີກ ແລະ ວາງໃນນ້ຳຫຍດນ້ອຍຂອງ Euparal® ໃນແຜ່ນແກ້ວຈຸລະທັດ. ພາຍໃຕ້ເລນຂະຫຍາຍ, ກະຈາຍປີກອອກຢ່າງອ່ອນໆ ແລະ ວາງແຜ່ນປົກຢ່າງລະມັດລະວັງ. ຄວນຖ່າຍຮູບທັນທີກ່ອນ Euparal® ແຂງ ເພາະອາດຕ້ອງການປັບຕຳແໜ່ງປີກເລັກນ້ອຍໃຕ້ແຜ່ນປົກເພື່ອໃຫ້ໄດ້ການຈັດຕຳແໜ່ງທີ່ດີທີ່ສຸດ.

6.4. ເຕັກນິກທາງດ້ານຊີວະໂມເລກຸນ

ນອກເໜືອຈາກເຕັກນິກທາງດ້ານຮູບຮ່າງ, ວິທີການທາງດ້ານໂມເລກຸນ ກຳລັງກາຍເປັນສິ່ງຈຳເປັນເພີ່ມຂຶ້ນໃນການຄົ້ນຄວ້າດ້ານ ແມງໄມ້ວິທະຍາລວມທັງການສຶກສາ ການຈຳແນກຊະນິດ, ພັນທຸກຳດ້ານປະຊາກອນ (population genetic), ແລະ ການສຶກສາ phylogenetic, ພ້ອມກັບການກວດຫາ DNA/RNA ຂອງເຊື້ອພະຍາດ, ແລະ ການຊອກຫາຊະນິດຂອງເລືອດທີ່ມັນກິນ, ເຊິ່ງພຶດຕິກຳຂອງພາຫະນຳເຊື້ອແມ່ນມີຄວາມສຳຄັນໃນຂະແໜງລະບາດວິທະຍາ [70]. ການລຽງລຳດັບກຳມະພັນ DNA ສາມາດນຳໃຊ້ເພື່ອຢືນຢັນຊະນິດ ຫຼື ແຍກຊະນິດທີ່ກ່ຽວຂ້ອງໃກ້ຄຽງກັນ ເພື່ອໃຫ້ຜົນການຈຳແນກທີ່ຖືກຕ້ອງ ແລະ ເຊື່ອຖືໄດ້ຫຼາຍ ກວ່າ. ຍິ່ງໄປກ່ວານັ້ນ, ເຕັກນິກໂມເລກຸນຂັ້ນສູງ (ເຊັ່ນ PCR, DNA sequencing, NGS, ແລະ ອື່ນໆ) ແລະ MALDI-ToF MS ກຳລັງໄດ້ຮັບຄວາມນິຍົມເພື່ອການຈຳແນກຊະນິດທີ່ຖືກຕ້ອງ ແລະ ໄວ ເປັນການເສີມສ້າງເຕັກນິກ ທາງດ້ານຮູບຮ່າງແບບດັ້ງເດີມ [46]. ເຖິງຢ່າງໃດກໍ່ຕາມ ການຈຳແນກດ້ວຍຮູບຮ່າງຍັງຄົງເປັນມາດຕະຖານອ້າງອີງສຳລັບການຈຳແນກຊະນິດ ແລະ ເປັນພື້ນຖານສຳລັບການອ່ານຜົນຂໍ້ມູນທາງດ້ານໂມເລກຸນ.

6.4.1. ການສະກັດ nucleic acid ແບບທຳລາຍຕົວຢ່າງ (Destructive nucleic acid extraction)

ການສະກັດສານພັນທຸກຳ nucleic acid ເປັນຂັ້ນຕອນທີ່ໃຊ້ປະຈຳໃນການສຶກສາທາງຊີວະວິທະຍາຫຼາຍໆຢ່າງ ແລະ ມີວິທີການ ຕ່າງໆໄດ້ຖືກພັດທະນາຂຶ້ນເພື່ອແຍກ DNA ອອກຈາກວັດສະດຸທາງຊີວະ [48]. ຊຸດການສະກັດ DNA ທີ່ຊື້ຂາຍທາງການຄ້າຫຼາຍໆຊຸດ ໄດ້ຖືກອອກແບບເພື່ອຊ່ວຍໃນການດຳເນີນງານໃນຂັ້ນຕອນນີ້ [14]. ເຖິງຢ່າງໃດກໍ່ຕາມ ວິທີການທີ່ນຳໃຊ້ທົ່ວໄປໃນການກະກຽມຕົວຢ່າງແມງໄມ້ຕີນຂໍ ສຳລັບການຈຳແນກດ້ວຍ ຮູບຮ່າງ ມັກຈະຂັດຂວາງການວິເຄາະ DNA ເພາະເຕັກນິກເຫຼົ່ານີ້ອາດເຮັດໃຫ້ເສຍຫາຍ ຫຼື ທຳລາຍລັກສະນະທາງກາຍພາບສຳຄັນຂອງຕົວຢ່າງ [10]. ໂປຣໂຕຄອນການສະກັດ DNA ສ່ວນໃຫຍ່ສຳລັບເນື້ອເຍື່ອແມງ ເປັນແບບທຳລາຍ [43] ເຊິ່ງເຮັດໃຫ້ເກີດຄວາມກັງວົນເປັນພິເສດກັບຕົວຢ່າງຂະໜາດນ້ອຍ ທີ່ການສຳຫຼວດແບບຈຳກັດກໍ່ອາດເຮັດໃຫ້ເສຍຫາຍລັກສະນະ ຮູບຮ່າງສຳຄັນ [72]. ປະເພດ ແລະ ສະພາບຂອງຕົວຢ່າງ ມີບົດບາດສຳຄັນໃນການເລືອກວິທີການສະກັດ DNA ທີ່ເໝາະສົມ [29].

ຄວາມຕ້ອງການການຈຳແນກຊະນິດຂອງຣິ້ນຝອຍຊາຍ ທີ່ຖືກຕ້ອງ, ການເຂົ້າໃຈພັນທຸກຳທາງດ້ານປະຊາກອນ, ແລະ ການຫຼຸດຜ່ອນຜົນກະທົບຕໍ່ເປົ້າໝາຍອື່ນ ໄດ້ຂັບເຄື່ອນການພັດທະນາເຄື່ອງມືການການກວດທາງດ້ານໂມເລກຸນ [23]. ວິທີການທາງດ້ານໂມເລກຸນ ປັດຈຸບັນນຳໃຊ້ເປັນປະຈຳເພື່ອເສີມສ້າງເຕັກນິກການຈຳແນກຊະນິດດ້ວຍຮູບຮ່າງໃນການຈຳແນກ ຣິ້ນຝອຍຊາຍ. ຕົວຢ່າງ, ວິທີມາດຕະຖານສຳລັບ insect barcoding ກ່ຽວຂ້ອງກັບການສະກັດ DNA, sequencing, ແລະ ການສູນເສຍຕົວຢ່າງຄັ້ງເດີມ. ດັ່ງນັ້ນ ມີຄວາມຕ້ອງການດ້ານການຄົ້ນຄວ້າວິທີການສະກັດ DNA ແບບບໍ່ທຳລາຍ (non-destructive) ທີ່ຮັກສາທັງວັດສະດຸທາງຊີວະ ແລະ ຄວາມສົມບູນຂອງ ຮູບຮ່າງ ໄວ້.

ມີວິທີການສະກັດ nucleic acid ຫຼາຍຢ່າງທີ່ນຳໃຊ້ກັບຣິ້ນຝອຍຊາຍ. ປະລິມານ ຫຼື ຄຸນນະພາບຂອງ nucleic acid ທີ່ຕ້ອງການ ຂຶ້ນກັບການວິເຄາະໂມເລກຸນຕໍ່ໄປ ເພາະເຕັກນິກຕ່າງໆມີຄວາມອ່ອນໄຫວ ແລະ ຄວາມບໍລິສຸດທີ່ແຕກຕ່າງກັນ [9]. ຕົວຢ່າງ, ຕາ ຣິ້ນຝອຍຊາຍໄດ້ຖືກພົບວ່າຍັບຍັ້ງການຂະຫຍາຍ PCR [69]. ນອກເໜືອຈາກການສະແກນ pathogen, DNA ຂອງ ຣິ້ນຝອຍຊາຍ ຖືກສະກັດເປັນປະຈຳເພື່ອການຈຳແນກຊະນິດ. ສາມາດນຳໃຊ້ວິທີການສະກັດຕ່າງໆໄດ້ ເຖິງວ່າຜົນໄດ້ຮັບ ແລະ ຄຸນນະພາບຈະແຕກຕ່າງກັນ. ບາງໂປຣໂຕຄອນຂອງຜູ້ຜະລິດໄດ້ຖືກປັບໃຊ້ໂດຍນັກຄົ້ນຄວ້າສຳລັບ ຣິ້ນຝອຍຊາຍ [8] ເພື່ອເພີ່ມຜົນໄດ້ຮັບ ແລະ/ຫຼື ຄຸນນະພາບຂອງ nucleic acid ທີ່ສະກັດໄດ້ [8, 9, 69] ໃນຂະນະທີ່ການປັບໃຊ້ອື່ນໆທີ່ພັດທະນາສຳລັບ ແມງໄມ້ຕີນຂໍ ອື່ນໆ ກໍ່ສາມາດນຳໃຊ້ກັບ ຣິ້ນຝອຍຊາຍ ໄດ້ [58, 76]. PCR ສຳລັບການຈຳແນກທີ່ເປົ້າໝາຍຊິ້ນ mitochondrial ນ້ອຍ (COI ຫຼື CytB) ປົກກະຕິເຂົ້າກັນໄດ້ກັບວິທີການສະກັດທີ່ເກີດການ fragment DNA

ສູງ. ກົງກັນຂ້າມ ເຕັກນິກ NGS ອ່ານຍາວອື່ນໆ (Oxford Nanopore ແລະ PacBio) ຕ້ອງການການ fragment ນ້ອຍທີ່ສຸດ ແລະ DNA ຄຸນນະພາບສູງ. ການສະກັດດ້ວຍ spin column ປົກກະຕິໃຫ້ genomic DNA fragment ສູງສຸດ 60 kb ໃນຂະນະທີ່ phenol-chloroform ສາມາດຜະລິດ fragment ສູງສຸດ 150 kb [77]. ຕາຕະລາງ 5 ສະຫຼຸບວິທີການສະກັດ DNA ຂອງ ຮິ້ນຝອຍຊາຍ ທີ່ແຕກຕ່າງກັນ ແລະ ຊີ້ບອກວ່າມີການປັບໂປຣໂຕຄອນໃດໃຫ້ເໝາະກັບແມງໄມ້ນ້ອຍເຫຼົ່ານີ້ຫຼືບໍ່. ຜົນໄດ້ຮັບບໍ່ໄດ້ສະແດງ ເພາະຂຶ້ນກັບຂະໜາດຕົວຢ່າງ ແລະ ວິທີກະກຽມ. ຖ້ງ "modification" ອ້າງເຖິງການປັບໂປຣໂຕຄອນການສະກັດສຳລັບ ຮິ້ນຝອຍຊາຍ ຫຼື ແມງໄມ້ຕີນຂໍ ນ້ອຍອື່ນໆ.

ການເລືອກວິທີການສະກັດ ຄວນພິຈາລະນາຫຼາຍເງື່ອນໄຂ ເຊັ່ນ ຈຳນວນຕົວຢ່າງ, ໄລຍະເວລາສະກັດ, ແລະ ເຕັກນິກທີ່ນຳໃຊ້ຕໍ່ໄປ. ເຖິງວ່າເຕັກນິກ NGS ຕ້ອງການ genomic DNA ທີ່ມີນ້ຳໜັກໂມເລກຸນສູງ ແຕ່ວິທີການທີ່ນຳສະເໜີທັງໝົດນີ້ ສາມາດນຳໃຊ້ກັບການນຳໃຊ້ PCR ມາດຕະຖານໄດ້.

ນອກຈາກນີ້ ຍັງໄດ້ມີການສຶກສາຢູ່າງຫຼວງຫຼາຍ ເພື່ອທົດລອງໃຊ້ວິທີການສະກັດ DNA ແບບບໍ່ທຳລາຍ ສຳລັບ ແມງໄມ້ຕີນຂໍ ຂະໜາດນ້ອຍເທິງພື້ນດິນອື່ນໆ, ຕົວຢ່າງໃນພິພິດທະພັນທີ່ເກັບແຫ້ງ, ແລະ ແມງໄມ້ຕີນຂໍ ທີ່ມີຮ່າງກາຍອ່ອນ [19, 26, 28, 55, 63].

6.4.2. ການສະກັດ nucleic acid ແບບບໍ່ທຳລາຍຕົວຢ່າງ (Non-destructive nucleic acid extraction)

ໜຶ່ງໃນບັນຫາຫຼັກໃນການວິເຄາະໂມເລກຸນຂອງ ແມງໄມ້ຕີນຂໍ, ໂດຍສະເພາະ ຮິ້ນຝອຍຊາຍ, ຄືການຮັກສາຕົວຢ່າງເພື່ອນຳໄປສົມທົບກັບຊຸດເກັບສະສົມທາງດ້ານແມງໄມ້ວິທະຍາ. ໂປຣໂຕຄອນການສະກັດ DNA ສ່ວນໃຫຍ່ຕ້ອງການ maceration ເນື້ອເຍື່ອ, ເຮັດໃຫ້ການຮັກສາຕົວຢ່າງດັ້ງເດີມຖືກທຳລາຍ. ວິທີການສະກັດ nucleic acid ແບບບໍ່ທຳລາຍ ກໍ່ຖືກອອກແບບຂຶ້ນເພື່ອສະກັດສານພັນທຸກຳ ໂດຍບໍ່ເຮັດໃຫ້ຕົວຢ່າງເສຍຫາຍທາງກາຍພາບ, ບໍ່ສົ່ງຜົນກະທົບຕໍ່ການມີຊີວິດ ຫຼື ປ່ຽນແປງຮູບຮ່າງ. ວິທີນີ້ມີຄຸນຄ່າໂດຍສະເພາະເມື່ອເຮັດກັບຕົວຢ່າງທີ່ມີຄ່າ ຫຼື ມີຈຳກັດ ເຊັ່ນ ຮິ້ນຝອຍຊາຍ ທີ່ການຮັກສາຄວາມສົມບູນຂອງໂຄງສ້າງ ຈຳເປັນຕໍ່ການການຈຳແນກຊະນິດ, ຮູບຮ່າງ ຫຼື ການການກວດຫາໃນອະນາຄົດ. ເຕັກນິກທີ່ນຳໃຊ້ທົ່ວໄປຄືການແຊ່ແບບບໍ່ທຳລາຍ (non-destructive bathing method) ໂດຍ immobilize ຮິ້ນຝອຍຊາຍ ແລະ ຈຸ່ມອ່ອນໆໃນ lysis buffer ທີ່ມີ proteinase K.

ເຕັກນິກ mild-vectolysis ໄດ້ນຳໃຊ້ສຳເລັດກັບ ຮິ້ນຝອຍຊາຍ, ໂດຍສະເພາະຕົວຢ່າງປະເພດ [24]. ເຕັກນິກນີ້ນຳໃຊ້ spin column kit ທົ່ວໄປ (ໃນກໍລະນີນີ້ຄື DNeasy Blood and Tissue kit, QIAGEN, Hilden, Germany) ດ້ວຍການປັບໃຊ້ເພື່ອສະກັດ DNA ໂດຍບໍ່ທຳລາຍຕົວຢ່າງ. ຂັ້ນຕອນ lysis ທີ່ປັບແລ້ວ (ປະລິມານ lysis buffer ແລະ ການເພີ່ມຂັ້ນຕອນ freezing) [17] ອະນຸຍາດໃຫ້ປ່ອຍ nucleic acid ອອກ ໃນຂະນະທີ່ຫຼຸດການເສຍຫາຍ ຮູບຮ່າງ [24]. ກ່ຽວກັບ ຮິ້ນຝອຍຊາຍ, ຍັງສາມາດນຳໃຊ້ HotSHOT DNA Extraction kit (Bento Bioworks Ltd, London, United Kingdom) [73] ທີ່ໄວ ແລະ ລາຄາຖືກ ເຮັດໃຫ້ການປະມວນຜົນຕົວຢ່າງໄວ ແລະ ຄ່າໃຊ້ຈ່າຍຕ່ຳ. ຕົວຢ່າງ entomological ທີ່ຕ້ອງການໃຊ້ສຳລັບການຈຳແນກ ຮູບຮ່າງ ສາມາດລ້າງຫຼັງການປະມວນຜົນ. ຕົວຢ່າງທີ່ປະມວນຜົນດ້ວຍ DNeasy Blood and Tissue kit ຕ້ອງເຮັດໃຫ້ໂປ່ງໃສດ້ວຍນ້ຳ Marc-André, ໃນຂະນະທີ່ຕົວຢ່າງທີ່ປະມວນຜົນດ້ວຍ HotSHOT DNA extraction kit ໂປ່ງໃສພຽງພໍທີ່ຈະແຕ່ງສະໄລໃນ medium ນ້ຳ, ຫຼື ດີກວ່າແມ່ນໃນ resin ຫຼັງການເອົານ້ຳອອກ ຕາມໂປຣໂຕຄອນທີ່ອະທິບາຍໃນເອກະສານນີ້ [73]. ວັດສະດຸພັນທຸກຳທີ່ສະກັດໄດ້ ສາມາດນຳໄປປະມວນຜົນຕໍ່ເພື່ອການວິເຄາະຕໍ່ໄປ ເຊັ່ນ PCR ເພື່ອຂະຫຍາຍ genetic markers ພິເສດ. ວິທີການສະກັດ nucleic acid ແບບບໍ່ທຳລາຍ ມີຄວາມສຳຄັນຕໍ່ການສຶກສາລັກສະນະພັນທຸກຳຂອງ ຮິ້ນຝອຍຊາຍ ລວມທັງການຈຳແນກເຊື້ອພະຍາດທີ່ອາດມີ. ໂດຍການຮັກສາຄວາມສົມບູນຂອງຕົວຢ່າງ, ນັກຄົ້ນຄວ້າສາມາດໄດ້ຂໍ້ມູນພັນທຸກຳທີ່ມີຄ່າ ໃນຂະນະທີ່ຮັກສາຕົວຢ່າງໄວ້ສຳລັບການວິເຄາະ ຫຼື ການສຶກສາເພີ່ມເຕີມ.

6.5. MALDI-ToF MS

MALDI-ToF MS (matrix-assisted laser desorption/ionization time-of-flight mass spectrometry) ເປັນເຕັກນິກທີ່ອີງໃສ່ mass spectrometry ອອກແບບມາເພື່ອກວດຫາ ແລະ ວິເຄາະຮູບແບບໂປຣຕີນ ທີ່ເປັນເອກະລັກຂອງຕົວຢ່າງທາງຊີວະ. MALDI-ToF ກຳລັງຖືກຍອມ ຮັບເປັນເຄື່ອງມືສຳຄັນສຳລັບການຈຳແນກ ແມງໄມ້ຕີນຂໍ ທີ່ມີຄວາມສຳຄັນທາງການແພດ ແລະ ສັດຕະວະແພດ. ເຕັກນິກນີ້ໄດ້ພິສູດປະສິດທິພາບໃນການຈຳແນກຂັ້ນຕອນການເຕີບໂຕຕ່າງໆຂອງ ຮິ້ນຝອຍຊາຍ ລວມທັງຮູບແບບ immature ແລະ blood meal ຂອງຕົວແມ່ ທີ່ມີເລືອດເຕັມ, ແລະ ໄດ້ນຳໃຊ້ສຳເລັດໃນການແຍກຊະນິດ ຮິ້ນຝອຍຊາຍ ຕົວຜູ້ ແລະ ຕົວແມ່ ພາຍໃຕ້ເງື່ອນໄຂການເກັບຮັກສາ ແລະ homogenization ທີ່ແຕກຕ່າງກັນ [28, 30, 73, 74]. ວິທີນີ້ຍັງໃຫ້ພະລັງການແຍກທີ່ສູງຢູ່ລະດັບ subgenus, ຊະນິດ, ແລະ ປະຊາກອນ. ເຕັກນິກນີ້ອະນຸຍາດໃຫ້ນັກຄົ້ນຄວ້າໄດ້ຮັບການຈຳແນກຊະນິດໄດ້ໄວ ແລະ ຖືກຕ້ອງ ເຊິ່ງຈຳເປັນສຳລັບການເຂົ້າໃຈການກະຈາຍຂອງ ຮິ້ນຝອຍຊາຍ, ພຶດຕິກຳ, ແລະ ບົດບາດໃນການຕິດຕໍ່ພະຍາດ. ດ້ວຍການແຍກຊະນິດຕາມຮູບແບບໂປຣຕີນ, MALDI-ToF ມີບົດບາດສຳຄັນໃນການສຶກສາດ້ານລະບາດວິທະຍາ ແລະ ຍຸດທະສາດການຄວບຄຸມພາຫະນຳເຊື້ອ. ປັດຈຸບັນມີຂໍ້ຈຳກັດ 2 ຢ່າງຫຼັກຂອງເຕັກນິກນີ້ທີ່ຈຳກັດການນຳໃຊ້ເປັນປະຈຳ. ປະການທຳອິດຄືຄວາມພ້ອມໃຊ້ງານຂອງເຄື່ອງ mass spectrometry ທີ່ມີລາຄາແພງຫຼາຍ ຈົນເຮັດໃຫ້ຍາກທີ່ຈະຊື້ມາໃຊ້ ເພື່ອຈຳແນກຊະນິດ ຮິ້ນຝອຍຊາຍ (ຫຼື ພາຫະແມງໄມ້ຕີນຂໍ ທົ່ວໄປ). ໂຊກດີທີ່ຂໍ້ຈຳກັດນີ້ສາມາດແກ້ໄຂໄດ້ດ້ວຍການໃຊ້ເວລາເຄື່ອງ mass spectrometer ທີ່ກາຍເປັນເຄື່ອງມືມາດຕະຖານໃນໂລກ proteomic ແລະ/ຫຼື ການການກວດຫາດ້ານຄລີນິກ. ຂໍ້ຈຳກັດທີສອງຄືການຂາດຂໍ້ມູນອ້າງອີງຂອງ ຮິ້ນຝອຍຊາຍ ໃນຖານຂໍ້ມູນເປີດ ເຮັດໃຫ້ຕ້ອງສ້າງຖານຂໍ້ມູນພາຍໃນເອງພ້ອມກັບ reference spectra ຈາກຕົວຢ່າງທີ່ຈຳແນກໄດ້ຢ່າງຊັດເຈນ ໂດຍດີທີ່ສຸດແມ່ນປະສົມການປະເມີນ ຮູບຮ່າງ ກັບ sequencing ຂອງ genetic

marker ທີ່ເໝາະສົມ (COI, cytB, ຫຼື ອື່ນໆ). ຂໍ້ຈຳກັດນີ້ຄາດວ່າຈະໄດ້ຮັບການແກ້ໄຂໃນໄວໆນີ້ ດ້ວຍການປະກອບຂໍ້ມູນອ້າງອີງຂອງ ຣິ້ນຝອຍຊາຍ ທີ່ຍັງຢູ່ພາຍໃນເຂົ້າໃນ MSI Platform ທີ່ດຳເນີນໂດຍ Assistance Publique-Hôpitaux de Paris, Sorbonne University, France ແລະ ຊຸດສະສົມ BCCM/IHEM/Sciensano ທີ່ Brussels, Belgium. ເມື່ອວາງແຜນນຳໃຊ້ MALDI-ToF protein profiling, ຕົວຢ່າງຄວນເກັບແຊ່ແຂງແຫ້ງ ຫຼື ໃນເອທານອນ 70% ຄຸນນະພາບໂມເລກຸນ ແລະ ບໍ່ສຳຜັດກັບອຸນຫະພູມຫ້ອງ. ໃນຖ້າບໍ່ມີຂໍ້ກຳນົດສາກົນສຳລັບການກະກຽມຕົວຢ່າງ, ຜູ້ໃຊ້ຄວນນຳໃຊ້ນ້ຳຢາ aqueous 60% acetonitrile/0.3% TFA ຂອງ sinapinic acid (30 mg/mL) ສຳລັບການກະກຽມ matrix MALDI-ToF ເພື່ອໃຫ້ຮູບແບບໂປຣຕີນເປັນເອກະລັກກັບຂໍ້ມູນທີ່ຖືກເຜີຍແຜ່ຂອງ ຣິ້ນຝອຍຊາຍ ທີ່ຜ່ານມາ.

7. ສະຫຼຸບ

ໃນການຄົ້ນຄວ້ານີ້, ພວກເຮົາມີຈຸດປະສົງເພື່ອສະໜອງນັກຄົ້ນຄວ້າດ້ວຍວິທີການທີ່ມີປະສິດທິພາບທີ່ສຸດສຳລັບການແຍງສະໄລ ຣິ້ນຝອຍຊາຍທີ່ປັບໃຊ້ກັບຈຸດປະສົງການຄົ້ນຄວ້າພິເສດ ເພື່ອຊ່ວຍໃຫ້ການຈຳແນກຊະນິດ ແລະ ການກວດຫາເຊື້ອພະຍາດຖືກຕ້ອງ. ບໍ່ມີວິທີດຽວທີ່ເໝາະສົມສາກົນ; ມີຫຼາຍວິທີ, ແຕ່ລະວິທີມີຂໍ້ດີ ແລະ ຂໍ້ເສຍຂອງຕົນເອງ.

ໃນຂໍ້ມູນເພີ່ມເຕີມ, ພວກເຮົາໄດ້ສະໜອງໂປຣໂຕຄອນລະອຽດສຳລັບເຕັກນິກການແຍງສະໄລຕ່າງໆທີ່ນຳໃຊ້ໃນການກະກຽມ ແລະ ການຈຳແນກຣິ້ນຝອຍຊາຍ. ໂປຣໂຕຄອນເຫຼົ່ານີ້, ລວມທັງວິດີໂອສອນ, ໃຫ້ຂັ້ນຕອນລະອຽດທີ່ປັບໃຊ້ກັບຈຸດປະສົງຕ່າງໆ ເພື່ອຮັບປະກັນຜົນທີ່ຊັດເຈນ ແລະ ເຊື່ອຖືໄດ້. ດ້ວຍການສະໜອງຊັບພະຍາກອນທີ່ຄົບຖ້ວນນີ້, ພວກເຮົາມີຈຸດປະສົງເພື່ອຊ່ວຍນັກຄົ້ນຄວ້າໃນການເລືອກ ແລະ ນຳໃຊ້ເຕັກນິກການແຍງສະໄລທີ່ເໝາະສົມທີ່ສຸດກັບຄວາມຕ້ອງການຂອງຕົນ.

ຕາຕະລາງ 5: ຄ່າໃຊ້ຈ່າຍສະເລ່ຍ, ການນຳໃຊ້, ແລະ ການປັບໂປຣໂຕຄອນ ສຳລັບການສະກັດສານພັນທຸກຳ gDNA ຂອງ ຣິ້ນຝອຍຊາຍ

ຂັ້ນຕອນວິທີການ(Protocole)	ລາຄາ	ການນຳໃຊ້	ການປັບປຸງຂັ້ນຕອນວິທີການສຳລັບແມງໄມ້ຕີນຂໍ່ນ້ອຍ
Colonnes de silice	2,5 – 3,55 US$ [39]	PCR, NGS	[9]
Phénol–chloroforme	0,24 US$ [69]	PCR, NGS	[9]
HotSHOT	< 0,01 US$ [69]	PCR	-
Salage	0,12 US$ [69]	PCR	-
Chelex	0,02 US$ [41]	PCR	[41, 76]

ການກະກຽມຕົວຢ່າງສຳລັບ MALDI-ToF MS (ຮູບທີ 7)

ຕົວຢ່າງແມງທີ່ເກັບໄວ້ໃນເງື່ອນໄຂຕ່າງໆ ກ່ອນອື່ນໃຫ້ແຫ້ງອາກາດໃນອຸນຫະພູມຫ້ອງ ແລະ ຜ່າຕັດ. ແຍກຫົວ ແລະ ທ້ອງອອກເພື່ອໄດ້ສ່ວນ ຮ່າງກາຍທີ່ມີລັກສະນະຮູບຮ່າງສຳຄັນສຳລັບການແຍງສະໄລໃນແຜ່ນ ແກ້ວ ແລະ ການວິເຄາະ ຮູບຮ່າງ. ເອິກ ສາມາດນຳໃຊ້ສຳລັບ MALDI-ToF ແລະ ທ້ອງທີ່ເຫຼືອສາມາດເກັບຮັກສາສຳລັບການສະກັດ DNA. ສຳລັບການແມັບໂປຣຕີນ, ເອິກ ຖືກບົດ ດ້ວຍມືໃນຫຼອດ microtube 1.5 mL ດ້ວຍ homogenization solution 10 µL ໂດຍໃຊ້ pestle ແລະ pellet ທີ່ໃຊ້ຄັ້ງດຽວ. ປົກກະຕິໃຊ້ homogenization solution 2 ຊະນິດ: sterile distilled water ແລະ 25% formic acid.

ຄຳຂອບໃຈ

ຜູ້ຂຽນຂອບໃຈທ່ານ Richard Lane ແລະ Zoe Jay Adams ຈາກ Natural History Museum ຂອງລອນດອນ (ສະຫະລາຊະອານາຈັກ) ສາລັບການອ່ານກວດຄືນທີ່ດີເລີດ, ເຊິ່ງຊ່ວຍປັບປຸງຄຸນນະພາບຂອງເອກະສານນີ້.

ທຶນຄົ້ນຄ້ວາ

ພວກເຮົາຂອບໃຈອົງການພັດທະນາຂອງບາຊິນ CNPq (ເລກທີໂຄງການ: 404395/2024-4) ແລະ ມູນລະນິທິ Araucária (ເລກທີໂຄງການ: 433/2025 PDI) ສໍາລັບການສະໜັບສະໜູນທາງການເງິນໃນການຄົ້ນຄວ້າຂອງ AJA.

ຂໍ້ຂັດແຍ້ງດ້ານຜົນປະໂຫຍດ (Conflicts of interest)

ທ່ານ Jérôme Depaquit ເປັນຮອງບັນນາທິການຂອງວາລະສານ Parasite; ລາວບໍ່ມີອິດທິພົນໃດໆຕໍ່ຂະບວນການປະເມີນຜົນ ຫຼື ການຕັດສິນໃຈກ່ຽວກັບການຕີພິມເອກະສານສະບັບນີ້.
ຜູ້ຂຽນຄົນອື່ນໆແຈ້ງວ່າພວກເຂົາບໍ່ມີຄວາມຂັດແຍ້ງທາງດ້ານຜົນປະໂຫຍດ.

ແຫຼ່ງຄວາມພ້ອມຂອງຂໍ້ມູນ

ວິດີໂອໃນເວັບໄຊ້ Zenodo:

ວິດີໂອ 1: https://zenodo.org/records/18198006
ວິດີໂອ 2: https://zenodo.org/records/18311158
ວິດີໂອ 3: https://zenodo.org/records/18311106
ວິດີໂອ 4: https://zenodo.org/records/18311154
ວິດີໂອ 5: https://zenodo.org/records/18303014
ວິດີໂອ 6: https://zenodo.org/records/18302850
ວິດີໂອ 7: https://zenodo.org/records/18315029

ເອກະສານເພີ່ມເຕີມ

ເອກະສານເພີ່ມເຕີມສຳລັບບົດຕີພິມສະບັບນີ້ເບິ່ງໄດ້ທີ່ https://www.parasite-journal.org/10.1051/parasite/2026009/olm

ເອກະສານອ້າງອີງ

1. Alkan C, Allal-Ikhlef AB, Alwassouf S, Baklouti A, Piorkowski G, de Lamballerie X, Izri A, Charrel RN. 2015. Virus isolation, genetic characterization and seroprevalence of Toscana virus in Algeria. Clinical Microbiology and Infection, 21(11), 1040 e1-9.
2. Alten B, Ozbel Y, Ergunay K, Kasap OE, Cull B, Antoniou M, Velo E, Prudhomme J, Molina R, Banuls AL, Schaffner F, Hendrickx G, Van Bortel W, Medlock JM. 2015. Sampling strategies for phlebotomine sand flies (Diptera: Psychodidae) in Europe. Bulletin of Entomological Research 105(6), 664–678.
3. Ayhan N, Baklouti A, Prudhomme J, Walder G, Amaro F, Alten B, Moutailler S, Ergunay K, Charrel RN, Huemer H. 2017. Practical guidelines for studies on sandfly-borne phleboviruses: Part I: Important points to consider *ante* field work. Vector-Borne and Zoonotic Diseases 17(1), 73–80.
4. Bates PA. 1997. Infection of phlebotomine sandflies with *Leishmania*, in The Molecular Biology of Insect Disease Vectors: A Methods Manual. Springer. p. 112–120.5.
5. Baum M, de Castro EA, Pinto MC, Goulart TM, Baura W, Klisiowicz Ddo R, Vieira da Costa-Ribeiro MC. 2015. Molecular detection of the blood meal source of sand flies (Diptera: Psychodidae) in a transmission area of American cutaneous leishmaniasis, Parana State, Brazil. Acta Tropica, 143, 8–12.
6. Belen A, Alten B, Aytekin A. 2004. Altitudinal variation in morphometric and molecular characteristics of *Phlebotomus papatasi* populations. Medical and Veterinary Entomology, 18(4), 343–350.
7. Bhattacharya J, Chandra G, Hati AK. 1991. A simple method for cryopreservation of *Leishmania donovani* promastigotes, Indian Journal of Medical Research, 93, 245–246.
8. Caligiuri LG, Sandoval AE, Miranda JC, Pessoa FA, Santini MS, Salomón OD, Secundino NF, McCarthy CB. 2019. Optimization of DNA extraction from individual sand flies for PCR amplification. Methods and Protocols, 2(2), 36.
9. Casaril AE, de Oliveira LP, Alonso DP, de Oliveira EF, Gomes Barrios SP, de Oliveira Moura Infran J, Fernandes WS, Oshiro ET, Ferreira AMT, Ribolla PEM, de Oliveira AG. 2017. Standardization of DNA extraction from sand flies: Application to genotyping by next generation sequencing. Experimental Parasitology, 177, 66–72.
10. Castalanelli MA, Severtson DL, Brumley CJ, Szito A, Foottit RG, Grimm M, Munyard K, Groth DM. 2010. A rapid non-destructive DNA extraction method for insects and other arthropods. Journal of Asia-Pacific Entomology, 13(3), 243–248.
11. Cerqueira NL. 1943. Um novo meio para montagem de pequenos insetos em lâmina. Memórias do Instituto Oswaldo Cruz, (39), 37–41.
12. Charrel RN, Gallian P, Navarro-Mari JM, Nicoletti L, Papa A, Sanchez-Seco MP, Tenorio A, de Lamballerie X. 2005. Emergence of Toscana virus in Europe. Emerging Infectious Diseases, 11(11), 1657–1663.
13. Chaskopoulou A, Giantsis IA, Demir S, Bon MC. 2016. Species composition, activity patterns and blood meal analysis of sand fly populations (Diptera: Psychodidae) in the metropolitan region of Thessaloniki, an endemic focus of canine leishmaniasis. Acta Tropica, 158, 170–176.
14. Chen H, Rangasamy M, Tan SY, Wang H, Siegfried BD. 2010. Evaluation of five methods for total DNA extraction from western corn rootworm beetles. PLoS One, 5(8), e11963.

15. Depaquit J, Grandadam M, Fouque F, Andry PE, Peyrefitte C. 2010. Arthropod-borne viruses transmitted by Phlebotomine sandflies in Europe: a review. Eurosurveillance, 15(10), 19507.
16. Diamond LS, Herman CM. 1954. Incidence of Trypanosomes in the Canada Goose as revealed by bone marrow culture. Journal of Parasitology, 40(2), 195–202.
17. Ding H, Torno M, Vongphayloth K, Ng G, Tan D, Sng W, Ho K, Randrianambinintsoa FJ, Depaquit J, Tan CH. 2025. Hidden in plain sight: discovery of sand flies in Singapore and description of four species new to science. Parasites & Vectors, 18(1), 402.
18. Es-Sette N, Ajaoud M, Bichaud L, Hamdi S, Mellouki F, Charrel RN, Lemrani M. 2014. *Phlebotomus sergenti* a common vector of *Leishmania tropica* and Toscana virus in Morocco. Journal of Vector Borne Diseases, 51(2), 86–90.
19. Favret C. 2005. A new non-destructive DNA extraction and specimen clearing technique for aphids (Hemiptera). Proceedings of the Entomological Society of Washington, 107(2), 469–470.
20. Galati EAB. 2018. Phlebotominae (Diptera, Psychodidae): Classification, morphology and terminology of adults and identification of American taxa, in Brazilian Sand Flies: Biology, Taxonomy, Medical Importance and Control, Rangel EF, Shaw JJ, Editors. Cham: Springer International Publishing. pp. 9–212.
21. Galati EAB, de Andrade AJ, Perveen F, Loyer M, Vongphayloth K, Randrianambinintsoa FJ, Prudhomme J, Rahola N, Akhoundi M, Shimabukuro PHF, Depaquit J. 2025. Phlebotomine sand flies (Diptera, Psychodidae) of the world. Parasites & Vectors, 18(1), 220.
22. Galati EAB, Galvis-Ovallos F, Lawyer P, Leger N, Depaquit J. 2017. An illustrated guide for characters and terminology used in descriptions of Phlebotominae (Diptera, Psychodidae). Par-asite, 24, 26.
23. Gariepy T, Kuhlmann U, Gillott C, Erlandson M. 2007. Parasitoids, predators and PCR: the use of diagnostic molecular markers in biological control of Arthropods. Journal of Applied Entomology, 131(4), 225–240.
24. Giantsis IA, Chaskopoulou A, Bon MC. 2016. Mild-Vectolysis: A nondestructive DNA extraction method for vouchering sand flies and mosquitoes. Journal of Medical Entomology, 53(3), 692–695.
25. Gidwani K, Picado A, Rijal S, Singh SP, Roy L, Volfova V, Andersen EW, Uranw S, Ostyn B, Sudarshan M, Chakravarty J, Volf P, Sundar S, Boelaert M, Rogers ME. 2011. Serological markers of sand fly exposure to evaluate insecticidal nets against visceral leishmaniasis in India and Nepal: a cluster-randomized trial. PLoS Neglected Tropical Diseases, 5(9), e1296.
26. Gilbert MTP, Moore W, Melchior L, Worobey M. 2007. DNA extraction from dry museum beetles without conferring external morphological damage. PLoS One, 2(3), e272.
27. Giordani BF, Andrade AJ, Galati EAB, Gurgel-Goncalves R. 2017. The role of wing geometric morphometrics in the identification of sandflies within the subgenus *Lutzomyia.* Medical and Veterinary Entomology, 31(4), 373–380.
28. Guzmán-Larralde AJ, Suaste-Dzul AP, Gallou A, Peña-Carrillo KI. 2017. DNA recovery from microhymenoptera using six non-destructive methodologies with considerations for subsequent preparation of museum slides. Genome, 60(1), 85–91.
29. Hajibabaei M, DeWaard JR, Ivanova NV, Ratnasingham S, Dooh RT, Kirk SL, Mackie PM, Hebert PD. 2005. Critical factors for assembling a high volume of DNA barcodes. Philosophical Transactions of the Royal Society B: Biological Sciences, 360(1462), 1959–1967.
30. Haouas N, Pesson B, Boudabous R, Dedet JP, Babba H, Ravel C. 2007. Development of a molecular tool for the identification of *Leishmania* reservoir hosts by blood meal analysis in the insect vectors. American Journal of Tropical Medicine and Hygiene, 77(6), 1054–1059.
31. Hlavackova K, Dvorak V, Chaskopoulou A, Volf P, Halada P. 2019. A novel MALDI-TOF MS-based method for blood meal identification in insect vectors: A proof of concept study on phlebotomine sand flies. PLoS Neglected Tropical Diseases, 13 (9), e0007669.
32. Huemer H, Prudhomme J, Amaro F, Baklouti A, Walder G, Alten B, Moutailler S, Ergunay K, Charrel RN, Ayhan N. 2017. Practical guidelines for studies on sandfly-borne phleboviruses: Part II: Important points to consider for fieldwork and subsequent virological screening. Vector-Borne and Zoonotic Diseases, 17(1), 81–90.
33. Jancarova M, Polanska N, Thiesson A, Arnaud F, Stejskalova M, Rehbergerova M, Kohl A, Viginier B, Volf P, Ratinier M. 2025. Susceptibility of diverse sand fly species to Toscana virus. PLoS Neglected Tropical Diseases, 19(5), e0013031.
34. Kapp JD, Green RE, Shapiro B. 2021. A fast and efficient single-stranded genomic library preparation method optimized for ancient DNA. Journal of Heredity, 112(3), 241–249.
35. Killick-Kendrick R, Maroli M, Killick-Kendrick M. 1991. Bibliography of the colonization of phlebotomine sandflies. Parassitologia, 33(suppl.), 321–333.
36. Lawyer P, Killick-Kendrick M, Rowland T, Rowton E, Volf P. 2017. Laboratory colonization and mass rearing of phlebotomine sand flies (Diptera, Psychodidae). Parasite, 24, 42.

37. Léger N, Pesson B, Madulo-Leblond G. 1986. Les phlébotomes de Grèce : 1ère partie. Bulletin de la Société de Pathologie Exotique, 79, 386–397.
38. Léger N, Pesson B, Madulo-Leblond G. 1986. Les phlébotomes de Grèce : 2ème partie. Bulletin de la Société de Pathologie Exotique, 79, 514–524.
39. Leonel JAF, Vioti G, Alves ML, da Silva DT, Meneghesso PA, Benassi JC, Spada JCP, Galvis-Ovallos F, Soares RM, Oliveira T. 2020. DNA extraction from individual Phlebotomine sand flies (Diptera: Psychodidae: Phlebotominae) specimens: Which is the method with better results? Experimental Parasitology, 218, 107981.
40. Lestinova T, Rohousova I, Sima M, de Oliveira CI, Volf P. 2017. Insights into the sand fly saliva: Blood-feeding and immune interactions between sand flies, hosts, and *Leishmania.* PLoS Neglected Tropical Diseases, 11(7), e0005600.
41. Lienhard A, Schaffer S. 2019. Extracting the invisible: obtaining high quality DNA is a challenging task in small arthropods. PeerJ, 7, e6753.
42. Lozano-Sardaneta YN, Mikery-Pacheco OF, Huerta H, Rojas-Soriano JE, Contreras-Ramos A. 2025. Wing geometric morphometrics is effective to separate sand fly species (Diptera, Psychodidae, Phlebotominae) related with leishmaniasis transmission in Mexico. Acta Tropica, 262, 107523.
43. Mandrioli M. 2008. Insect collections and DNA analyses: how to manage collections? Museum Management and Curatorship, 23(2), 193–199.
44. Maroli M, Feliciangeli MD, Bichaud L, Charrel RN, Gradoni L. 2013. Phlebotomine sandflies and the spreading of leishmaniases and other diseases of public health concern. Medical and Veterinary Entomology, 27(2), 123–147.
45. Marquina D, Buczek M, Ronquist F, Lukasik P. 2021. The effect of ethanol concentration on the morphological and molecular preservation of insects for biodiversity studies. PeerJ, 9, e10799.
46. Mathis A, Depaquit J, Dvorak V, Tuten H, Banuls AL, Halada P, Zapata S, Lehrter V, Hlavackova K, Prudhomme J, Volf P, Sereno D, Kaufmann C, Pfluger V, Schaffner F. 2015. Identification of phlebotomine sand flies using one MALDI-TOF MS reference database and two mass pectrometer systems., Parasites & Vectors, 8, 266.
47. Mekarnia N, Benallal KE, Sadlova J, Vojtkova B, Mauras A, Imbert N, Longhitano M, Harrat Z, Volf P, Loiseau PM, Cojean S. 2024. Effect of *Phlebotomus papatasi* on the fitness, infectivity and antimony-resistance phenotype of antimony-resistant *Leishmania* major Mon-25. International Journal for Parasitology – Drugs and Drug Resistance, 25, 100554.
48. Milligan BG. 1998. Total DNA isolation, in Molecular Genetic Analysis of Population: A Practical Approach, Hoelzel AR, Editor. Oxford: Oxford University Press.
49. Molina R, Jiménez M, Alvar J, González E, Hernández-Taberna S, Ines MM. 2017. Methods in sand fly research. Madrid: Servicio de publicaciones Universidad de Alcalá de Henares, Madrid.
50. Murphy WJ, Eizirik E, O'Brien SJ, Madsen O, Scally M, Douady CJ, Teeling E, Ryder OA, Stanhope MJ, de Jong WW, Springer MS. 2001. Resolution of the early placental mammal radiation using Bayesian phylogenetics. Science, 294(5550), 2348–2351.
51. Nacif-Pimenta R, Pinto LC, Volfova V, Volf P, Pimenta PFP, Secundino NFC. 2020. Conserved and distinct morphological aspects of the salivary glands of sand fly vectors of leishma-niasis: an anatomical and ultrastructural study. Parasites & Vectors, 13(1), 441.
52. Neuhaus B, Schmid T, Riedel J. 2017. Collection management and study of microscope slides: Storage, profiling, deterioration, restoration procedures, and general recommendations. Zootaxa, 4322(1), 1–173.
53. New TR. 1974. Pscoptera. Handbooks for Identification of British Insects (Vol. I). London: Royal Entomological Society of London. 102 pp.
54. Perez-Ruiz M, Collao X, Navarro-Mari JM, Tenorio A. 2007. Reverse transcription, real-time PCR assay for detection of Toscana virus. Journal of Clinical Virology, 39(4), 276–281.
55. Porco D, Rougerie R, Deharveng L, Hebert P. 2010. Coupling non-destructive DNA extraction and voucher retrieval for small soft-bodied Arthropods in a high-throughput context: the example of Collembola. Molecular Ecology Resources, 10(6), 942–945.
56. Prudhomme J, Cassan C, Hide M, Toty C, Rahola N, Vergnes B, Dujardin JP, Alten B, Sereno D, Banuls AL. 2016. Ecology and morphological variations in wings of *Phlebotomus ariasi* (Diptera: Psychodidae) in the region of Roquedur (Gard, France): a geometric morphometrics approach. Parasites & Vectors, 9(1), 578.
57. Prudhomme J, Gunay F, Rahola N, Ouanaimi F, Guernaoui S, Boumezzough A, Banuls AL, Sereno D, Alten B. 2012. Wing size and shape variation of *Phlebotomus papatasi* (Diptera: Psychodidae) populations from the south and north slopes of the Atlas Mountains in Morocco. Journal of Vector Ecology, 37(1), 137–147.
58. Prudhomme J, Toty C, Kasap OE, Rahola N, Vergnes B, Maia C, Campino L, Antoniou M, Jimenez M, Molina R, Cannet A, Alten B, Sereno D, Banuls AL. 2015. New microsatellite markers for multi-scale genetic studies on *Phlebotomus ariasi* Tonnoir, vector of *Leishmania infantum* in the Mediterranean area. Acta Tropica, 142, 79–85.

59. Prudhomme J, Velo E, Bino S, Kadriaj P, Mersini K, Gunay F, Alten B. 2019. Altitudinal variations in wing morphology of *Aedes albopictus* (Diptera, Culicidae) in Albania, the region where it was first recorded in Europe. Parasite, 26, 55.
60. Rawlins DJ. 1992. Light Microscopy: An Introduction to Biotechniques. Oxford: Bios Scientific publishers. 143 pp.
61. Ready PD. 2013. Biology of phlebotomine sand flies as vectors of disease agents. Annual Review of Entomology, 58, 227–250.
62. Rohlf FJ, Slice D. 1990. Extensions of the Procrustes method for the optimal superimposition of landmarks. Systematic Zoology, 39(1), 40–59.
63. Rowley DL, Coddington JA, Gates MW, Norrbom AL, Ochoa RA, Vandenberg NJ, Greenstone MH. 2007. Vouchering DNA-barcoded specimens: Test of a nondestructive extraction proto-col for terrestrial arthropods. Molecular Ecology Notes, 7(6), 915–924.
64. Sábio PB, Andrade AJ, Galati EAB. 2014. Assessment of the taxonomic status of some species included in the *Shannoni* complex, with the description of a new species of *Psathyromyia* (Diptera: Psychodidae: Phlebotominae). Journal of Medical Entomology, 51(2), 331–341.
65. Sadlova J, Yeo M, Seblova V, Lewis MD, Mauricio I, Volf P, Miles MA. 2011. Visualisation of *Leishmania donovani* fluo-rescent hybrids during early stage development in the sand fly vector. PLoS One, 6(5), e19851.
66. Sales K, Miranda DEO, da Silva FJ, Otranto D, Figueredo LA, Dantas-Torres F. 2020. Evaluation of different storage times and preservation methods on phlebotomine sand fly DNA concen-tration and purity. Parasites & Vectors, 13(1), 399
67. Sales KG, Costa PL, de Morais RC, Otranto D, Brandao-Filho SP, Cavalcanti Mde P, Dantas-Torres F. 2015. Identification of phlebotomine sand fly blood meals by real-time PCR. Parasites& Vectors, 8, 230.
68. Sant'Anna MR, Jones NG, Hindley JA, Mendes-Sousa AF, Dillon RJ, Cavalcante RR, Alexander B, Bates PA. 2008. Blood meal identification and parasite detection in laboratory-fed and field-captured *Lutzomyia longipalpis* by PCR using FTA databasing paper. Acta Tropica, 107(3), 230–237.
69. Senne NA, Santos HA, Araujo TR, Paulino PG, Mendonca LP, Moreira HVS, Camilo TA, da Costa Angelo I. 2022. Robust comparative performance of genomic DNA extraction methods from non-engorged phlebotomine sandflies. Medical and Veterinary Entomology, 36(2), 203–211.
70. Shaw JJ. 2025. A review of Leishmania infections in American Phlebotomine sand flies – Are those that transmit leishmaniasis anthropophilic or anthropportunists? Parasite, 32, 57.
71. Tesh RB, Modi GB. 1983. Growth and transovarial transmission of Chandipura virus (Rhabdoviridae: Vesiculovirus) in *Phlebotomus papatasi*. American Journal of Tropical Medicine and Hygiene, 32(3), 621–623.
72. Thomsen PF, Elias S, Gilbert MTP, Haile J, Munch K, Kuzmina S, Froese DG, Sher A, Holdaway RN, Willerslev E. 2009. Non-destructive sampling of ancient insect DNA. PLoS One, 4(4),e5048
73. Truett GE, Heeger P, Mynatt RL, Truett AA, Walker JA, Warman ML. 2000. Preparation of PCR-quality mouse genomic DNA with hot sodium hydroxide and tris (HotSHOT). Biotechniques, 29(1), 52–54.
74. Upton MS. 1993. Aqueous gum-chloral slide mounting media: an historical review. Bulletin of Entomological Research, 83(2), 267–274.
75. Volf P, Myskova J. 2007. Sand flies and Leishmania: specific versus permissive vectors. Trends in Parasitology, 23(3), 91–92.
76. Wang Q, Wang X. 2012. Comparison of methods for DNA extraction from a single chironomid for PCR analysis. Pakistan Journal of Zoology, 44(2), 421–426.
77. Wang Y, Zhao Y, Bollas A, Wang Y, Au KF. 2021. Nanopore sequencing technology, bioinformatics and applications. Nature Biotechnology, 39(11), 1348–1365.

Cite this article as: Randrianambinintsoa FJ, Augendre L, Prudhomme J, Martinet J-P, Loyer M, Mekarnia N, Kerkoub H, Perveen FK, Huguenin A, Kariya E, Akhoundi M, De Andrade AJ, Berriatua E, Bongiorno G, Boyer S, Christodoulou V, Da Costa-Ribeiro MCV, De Souza LAF, Ding H, Dondji B, Dvořák V, Erisoz Kasap O, Galati EAB, Gállego M, Ballart C, Gouzelou S, Haddad N, Masse RS, Mekuria AH, Ivovic V, Kaczmarek S, Shahar MK, Kirstein OD, Kniha E, Kolářová I, Lincoln T, Lucanas C, Mikov O, Nov K, Özbel Y, Pesson B, Posada Lopez LC, Prasetyo DB, Rahola N, Rebollar-Tellez EA, Rodrigues BL, Roy L, Saini P, Sanjoba C, Shimabukuro PH, Siriyasatien P, Soszynska A, Suleșco T, Sylla M, Torno M, Volf P, Vongphayloth K, Sinh Nam V, Wardhana A, Yessinou E, Zapata S, Gantier J-C & Depaquit J. 2026. Processing and mounting phlebotomine sand flies: a consensus guideline. Parasite xx, xx. https://doi.org/10.1051/parasite/2026009.

PARASITE

An international open-access, peer-reviewed, online journal publishing high quality papers on all aspects of human and animal parasitology

Reviews, articles and short notes may be submitted. Fields include, but are not limited to: general, medical and veterinary parasitology; morphology, including ultrastructure; parasite systematics, including entomology, acarology, helminthology and protistology, and molecular analyses; molecular biology and biochemistry; immunology of parasitic diseases; host-parasite relationships; ecology and life history of parasites; epidemiology; therapeutics; new diagnostic tools.
All papers in Parasite are published in English. Manuscripts should have a broad interest and must not have been published or submitted elsewhere. No limit is imposed on the length of manuscripts.

Parasite (open-access) continues **Parasite** (print and online editions, 1994-2012) and **Annales de Parasitologie Humaine et Comparée** (1923-1993) and is the official journal of the Société Française de Parasitologie.

Editor-in-Chief:
Jean-Lou Justine, Paris

Submit your manuscript at:
https://www.editorialmanager.com/parasite

ເອກະສານຊ້ອນທ້າຍ 1: ພື້ນຖານທາງເຄມີຊີວະທາດ (Biochemical theoretical foundations).

ແມງໄມ້ທີ່ກ່ຽວຂ້ອງໃນທີ່ນີ້ແມ່ນ ຮີ້ນຝອຍຊາຍ. ເຖິງຢ່າງໃດກໍ່ຕາມ, ແນວຄວາມຄິດທົ່ວໄປສາມາດຂະຫຍາຍໄປເຖິງ ແມງໄມ້ຕີນຂໍທົ່ວໄປອື່ນໆ ທີ່ການຈໍາແນກຊະນິດສາມາດດໍາເນີນໄດ້ພຽງແຕ່ດ້ວຍລັກສະນະຮູບຮ່າງພາຍໃນ. ໂດຍບັງເອີນ, ອະໄວຍະວະພາຍໃນບາງຢ່າງມີໄຄຕິໄນສ໌ບາງສ່ວນ ແລະ ຮູບຮ່າງຂອງມັນໃຫ້ຂໍ້ມູນທີ່ມີຄຸນຄ່າແກ່ພວກເຮົາ. ນີ້ເປັນເຫດຜົນທີ່ຈະຕ້ອງໄດ້ສັງເກດເບິ່ງ ປ້ໍາອາຫານ, ຖົງເກັບນ້ໍາເຊື້ອ, ແລະ ທໍ່ຂອງມັນ. ດ້ວຍ reagents ທັງໝົດທີ່ພວກເຮົາຈະກວດຄືນ, ຢ່າລືມວ່າຕັ້ງແຕ່ຂັ້ນຕອນການ fixation ຂອງແມງໄມ້ຈົນເຖິງການປະກອບຄືນ, ພວກເຮົາຈະນໍາໃຊ້ປະຕິກິລິຍາ redox ງ່າຍໆເທົ່ານັ້ນ. ຂໍ້ລະມັດລະວັງ ຫຼື ແນວຄວາມຄິດແມ່ນການຫຼີກເວັ້ນການປະສົມສານຣີດິຊ (reducing reagents) ກັບ ສານອອກຊີໄດຊ໌ (oxidizing reagents).

ເອທິລ ອາລຄອຮອລ; ເຫຼົ້າ ເອທານອນ

ສານນີ້ຈະນໍາໃຊ້ໃນຫຼາຍຮູບແບບ. ໂມເລກຸນເຫຼົ້າມີການຈັບກັບນ້ໍາສູງ ດັ່ງນັ້ນ ຈຶ່ງມີຜົນຕໍ່ການດຶງເອົານ້ໍາອອກ. ເຖິງຢ່າງໃດກໍ່ຕາມ, ເຫຼົ້າທີ່ມີຄວາມເຂັ້ມຕ່ໍາ (ຄືມີນ້ໍາຫຼາຍເກີນໄປ) ຈະມີບົດບາດຕໍ່ການເສື່ອມສະພາບສານກໍາມະພັນ nucleic acid (ນ້ໍາເປັນສັດຕູຂອງ nucleic acid).

ເມື່ອໃສ່ແມງໄມ້ໃນເຫຼົ້າເອທານອນ ບໍ່ພຽງແຕ່ເພື່ອຮັກສາແມງໄມ້ໄວ້ເທົ່ານັ້ນ, ແຕ່ຍັງເພື່ອຄົງສະພາບຂອງເນື້ອເຍື່ອ. ໃນດ້ານອະນາປັດ histology, ແມ່ນແຍກແນວຄວາມຄິດສໍາຄັນເປັນສອງແນວ: ອັດຕາການຊຶມເຂົ້າໄປ (penetration rate) ແລະ ອັດຕາການຄົງສະພາບ (fixation rate). ເປັນເລື່ອງເຂົ້າໃຈດີວ່າ ສານເກັບຮັກສາທີ່ດີຕ້ອງຊຶມເຂົ້າໄປເຖິງເນື້ອເຍື່ອຢ່າງໄວວາກ່ອນການຄົງສະພາບ. ສໍາລັບເອທານອນ 96%, ຄ່າ penetration coefficient ປະມານ 1.05 (ປຽບທຽບກັບ 0.75% aqueous solution ຂອງ picric acid ມີ 0.45, ໃນຂະນະທີ່ 3% potassium dichromate solution ມີ 1.45).

ຄວາມຕ້ອງການຮັກສາແມງໄມ້ ແລະ ແມງໄມ້ຕີນຂໍອື່ນໆໃນເອທານອນໄລຍະຍາວ ແມ່ນຊີວິດຄວາມເປັນຈິງສໍາລັບນັກຄົ້ນຄ້ວາແມງໄມ້ວິທະຍາ. ແນວຄວາມຄິດນີ້ຍັງມີຄວາມສໍາຄັນໃນການເກັບ ການຈັບ ຈາກພາກສະໜາມເພື່ອການສຶກສາຂັ້ນຕໍ່ໄປ ຫຼື ສໍາລັບນັກຄົ້ນຄວ້າໃນອະນາຄົດ. ເຖິງຢ່າງໃດກໍ່ຕາມ, ແນວຄວາມຄິດນີ້ເປັນໄປບໍ່ໄດ້ສໍາລັບ ນັກອະນາປັດ cytologist ຫຼື histologist. ເຊິ່ງການເກັບຕົວຢ່າງໃນສານຄົງສະພາບ fixative ດົນເກີນໄປ ອາດເຮັດໃຫ້ເຮັດວຽກຕໍ່ບໍ່ໄດ້. ນີ້ເປັນເຫດຜົນທີ່ຕົວຢ່າງອາຍຸເກີນ 10 ປີ ຈະນໍາໃຊ້ຍາກ ຫຼື ການນໍາໃຊ້ເປັນໄປບໍ່ໄດ້.

ອີກປັດໄຈໜຶ່ງຄືອັດຕາສ່ວນລະຫວ່າງມວນຂອງ ແມງໄມ້ຕີນຂໍ ທີ່ຕ້ອງຄົງສະພາບກັບປະລິມານສານຄົງສະພາບ (fixator). ໃນການປະຕິບັດທາງດານສັດ (zoological) ຫຼື ການແພດ, ແນະນໍາໃຫ້ຄາດຄະເນ 60 ເທົ່າຂອງປະລິມານຕົວຢ່າງ. ໃນທາງປະຕິບັດ, ສໍາລັບ ແມງໄມ້ນ້ອຍໆ, ສໍາລັບປະລິມານຕົວຢ່າງທີ່ຕ້ອງກໍານົດ, ເພີ່ມ alcohol ອີກຢ່າງໜ້ອຍ 4–5 ປະລິມານ. ຈື່ວ່າ alcohol ຈະສູນເສຍຄວາມເຂັ້ມເມື່ອດູດນ້ໍາອອກຈາກເນື້ອເຍື່ອ ແມງໄມ້ຕີນຂໍ.

ສະຫຼຸບ:

- Ethyl alcohol ເປັນ ສານຣີດິຊ (reducing reagents) (ດັ່ງນັ້ນບໍ່ເຂົ້າກັນກັບ ການຄົງສະພາບແບບອອກຊີໄດຊ໌);
- ເຮັດໃຫ້ໂປຣຕີນຕົກຕະກອນຢ່າງແຮງ ແລະ ເຮັດໃຫ້ພວກມັນເສື່ອມສະພາບ;
- ລະລາຍໄຂມັນປະສົມບາງຊະນິດ ແລະ ເຮັດໃຫ້ glycogen ຕົກຕະກອນ;
- ເຮັດໃຫ້ເກີດການຫົດຕົວຢ່າງແຮງຂອງເນື້ອເຍື່ອ ແລະ ເຮັດໃຫ້ພວກມັນແຂງຕົວ.

ສານລະລາຍດ່າງ potassium hydroxide ຫຼື sodium hydroxide:

ການນຳໃຊ້ສານລະລາຍເຫຼົ່ານີ້ໃນສາຂາແມງໄມ້ວິທະຍາ(entomology) ແມ່ນໄດ້ເນັ້ນໃສ່ potassium hydroxide ເປັນຫຼັກ ໂດຍບໍ່ມີເຫດຜົນທີ່ຊັດເຈນ. Sodium hydroxide [E524] ມີຢູ່ໃນຮູບແບບສານລະລາຍ ໃນຄວາມເຂັ້ມຂຸ້ນຫຼາຍລະດັບ ຫຼືຄ່າ normality ທີ່ແຕກຕ່າງ ແລະຍັງມີໃນຮູບແບບເມັດ ຫຼື ແຜ່ນນ້ອຍ. ຂໍ້ເສຍຫຼັກຂອງມັນຄືດູດຄວາມຊຸ່ມສູງ (ສູງກ່ວາຂອງ KOH). ເມື່ອມັນປະຕິກິລິຍາກັບໂປຣຕີນ ມັນຈະລະລາຍໂປຣຕີນ ແລະເມື່ອກັບໄຂມັນ ມັນຈະປ່ຽນເປັນສະບູແຂງໃນຂະບວນການ saponification (ນີ້ແມ່ນຄວາມແຕກຕ່າງສຳຄັນກັບ KOH ທີ່ໃຫ້ສະບູແບບເປັນທາດແຫຼວຈາກປະຕິກິລິຍານີ້).

Potassium hydroxide [E525] ມີຢູ່ໃນຮູບແບບສານລະລາຍເຂັ້ມຂຸ້ນ ແລະສ່ວນໃຫຍ່ໃນຮູບແບບເມັດປະມານ 0.1 g ເຊິ່ງຊ່ວຍໃຫ້ການກະກຽມສານລະລາຍເຈືອຈາງເຮັດໄດ້ງ່າຍ ເມື່ອບໍ່ມີຊິງສັ່ງທີ່ແມ່ນຍຳ. ຕົວຢ່າງ ເມັດ 0.1 g ລະລາຍໃນນ້ຳກັ່ນ 1 mL ຈະໄດ້ສານລະລາຍ 10%. ຂໍ້ດີອື່ນຂອງ KOH ໃນຮູບແບບເມັດຄືມັນມີຄວາມໄວຕໍ່ການກໍ່ຕົວເປັນ carbonate ຫນ້ອຍກວ່າ (ເພາະສານລະລາຍ KOH ມີຄວາມສາມາດດຶງ CO_2 ສູງ ນຳໄປສູ່ການເກີດ carbonate).

ທາດດ່າງສູງເຫຼົ່ານີ້ໃຊ້ເພື່ອລະລາຍກົດໄຂມັນ ໂດຍປ່ຽນໃຫ້ເປັນສະບູທີ່ລະລາຍໃນນ້ຳ. ຄວນນຶງວ່າສານຕຶງສະພາບ (fixateur) ເຊັ່ນ ethanol ກໍສາມາດລະລາຍໄຂມັນບາງສ່ວນໃນຕົວຢ່າງໄດ້ແລ້ວ. ແຕ່ເມື່ອນຳຕົວຢ່າງໄປຢູ່ໃນສະພາບນ້ຳທີ່ມີດ່າງສູງ ກົດໄຂມັນ (ທີ່ມີຄວາມຊັບຊ້ອນຕ່າງກັນ) ຈະຕົກຕະກອນ. ດ່າງສູງຈະເຮັດໃຫ້ເກີດ saponification ແບບເຢັນ. ໃນບາງກໍລະນີ ເມື່ອມີໄຂມັນຫຼາຍ (ເຊັ່ນ ໃນໂຕແມ່) ອາດຈະມີປະໂຫຍດທີ່ຈະເພີ່ມອຸນຫະພູມເຖິງ 35–40 °C ເພື່ອຊ່ວຍໃຫ້ປະຕິກິລິຍາເກີດໄດ້ງ່າຍຂຶ້ນ ຫຼືເພີ່ມເວລາຂຶ້ນໃນອຸນຫະພູມຫ້ອງ.

ສານລະລາຍ Marc-André **ທີ່ຍ້ອມສີດ້ວຍ** fuchsine **ຫຼື ບໍ່ຍ້ອມສີ:**

ໃນທີ່ນີ້ພວກເຮົາຈະກ່າວເຖິງຂໍ້ດີ ແລະ ຂໍ້ເສຍຂອງການນຳໃຊ້ສານລະລາຍ Marc-André. ສານນີ້ປະກອບມີ chloral hydrate (trichloroacétaldéhyde monohydraté), ກົດ acétique ແລະ ນ້ຳ. ມັນເປັນສານອອກຊີໄດຊ໌ແຮງ (ເປັນການປະສົມຂອງກົດ ແລະ aldehyde). ມັນສາມາດຊ່ວຍກຳຈັດ potassium hydroxide ທີ່ເຫຼືອຢູ່ໃນຕົວຢ່າງ ໂດຍບໍ່ເຮັດໃຫ້ສະບູດ່າງທີ່ເກີດຈາກ KOH ຕົກຕະກອນ. ນອກຈາກນັ້ນ ສານອອກຊີໄດຊ໌ນີ້ຍັງມີຜົນຕໍ່ກຸ່ມເຫຼົ້າ alcohol ຂັ້ນສອງ (secondary alcohol) ຂອງ glucosamine ໃນ chitine ໂດຍການອອກຊີໄດຊ໌ເຮັດໃຫ້ chitine ອ່ອນລົງ. ມັນຍັງສາມາດຊ່ວຍລະລາຍເກືອແຮ່ບາງຊະນິດໃນຕົວຢ່າງໄດ້.

ເມື່ອສານລະລາຍ Marc-André ຖືກຍ້ອມສີກ່ອນດ້ວຍ fuchsine acide (ຢູ່ໃນຮູບແບບທີ່ຖືກອອກຊີໄດຊ໌) ມັນສາມາດໄປຈັບກັບກຸ່ມ alcohol ຂັ້ນສອງໃນໂຄງສ້າງ chitine. ຫຼັງຈາກເວລາທີ່ໃຫ້ສານ Marc-André ສຳຜັດກັບຕົວຢ່າງ ແລະ ຂຶ້ນກັບລະດັບການຍ້ອມສີ ການລ້າງຈະເຮັດໂດຍໃຊ້ເຫຼົ້າ ethanol ເທົ່ານັ້ນ. ຫຼັງຈາກນັ້ນ ຈະເຂົ້າສູ່ຂັ້ນຕອນການດຶງນ້ຳອອກ (dehydration) ຈາກຕົວຢ່າງ.

ຂໍ້ດີ:

- ຊ່ວຍປັບສະພາບດ່າງທີ່ເຫຼືອໃຫ້ເປັນກາງ
- ເຮັດໃຫ້ chitine ອ່ອນລົງ
- ຊ່ວຍຍ້ອມສີ chitine ເຮັດໃຫ້ເຫັນໂຄງສ້າງພາຍໃນທີ່ມີ chitine ຊັດເຈນຂຶ້ນ

ຂໍ້ເສຍ:

Chloral hydrate ເປັນສານທີ່ເປັນພິດເຮັດໃຫ້ຢາກນອນ ເຄີຍຖືກນຳໃຊ້ໃນທາງການແພດໃນຄົນ. ການນຳໃຊ້ຕ້ອງເຮັດໃນຕູ້ດູດຄວັນ (hotte chimique) ແລະ ປະຕິບັດຕາມຂໍ້ກຳນົດຄວາມປອດໄພສານເຄມີຢ່າງເຄັ່ງຄັດ.

ສານລະລາຍສຳລັບການດຶງນ້ຳ ***(Dehydration):***

ຈາກປະສົບການພົບວ່າ ສຳລັບຕົວຢ່າງທີ່ມີຂະໜາດນ້ອຍຫຼາຍ ບໍ່ຈຳເປັນຕ້ອງຜ່ານຂັ້ນຕອນແຊ່ເຫຼົ້າຫຼາຍລະດັບຄວາມເຂັ້ມຂຸ້ນ. ຖ້າຕົວຢ່າງມີຂະໜາດໃຫຍ່ ຄວນເລີ່ມຈາກ ethanol 80% ຕາມດ້ວຍ 90%, 95% ແລະ ສຸດທ້າຍແມ່ນ ethanol ເຂັ້ມຂຸ້ນ (absolute ethanol). ສຳລັບຕົວຢ່າງນ້ອຍຫຼາຍ ການແຊ່ໃນ 90% ແລະ ຕໍ່ດ້ວຍ absolute ethanol ກໍພຽງພໍ. ໃນຂັ້ນຕອນນີ້ ຄວນຄຳນຶງວ່າ absolute ethanol ມີແນວໂນ້ມດຶງນ້ຳຈາກອາກາດ.

ໃນອະດີດ ຫ້ອງວິເຄາະແມງໄມ້ວິທະຍາມັກໃຊ້ créosote ໃນຂັ້ນຕອນດຶງນ້ຳສຸດທ້າຍ. ປັດຈຸບັນ ສານນີ້ບໍ່ແນະນຳໃຫ້ໃຊ້ແລ້ວ ເນື່ອງຈາກກິ່ນແຮງ (ກ່ຽວຂ້ອງກັບ polycyclic aromatic hydrocarbons) ແລະ ຄວາມເປັນພິດ: ອາດມີຜົນຕໍ່ລະບົບສືບພັນ, ກໍ່ໃຫ້ເກີດມະເຮັງ, ເປັນສານມົນລະພິດຕົກຄ້າງ ແລະ ມີຜົນຮ້າຍຕໍ່ສັດນ້ຳ.

ສານແນະນຳສຳລັບການແຕ່ງຕົວຢ່າງ ແມ່ນແນະນຳໃຫ້ໃຊ້ Euparal® ແລະ essence ຂອງ Euparal (ອະທິບາຍໃນພາກຕໍ່ໄປ). ການປະສົມ Euparal® ກັບ essence ຂອງມັນ ສາມາດໃຊ້ໄດ້ດີກັບຕົວຢ່າງທີ່ໄດ້ຜ່ານເຫຼົ້າ 90% ມາແລ້ວ.

ເອກະສານຊ້ອນທ້າຍ 2 : ສ່ວນປະກອບຂອງສານທີ່ໃຊ້.

Hydroxyde de potassium à 10 %

Hydroxyde de potassium : 10 g
ນ້ຳກັ່ນ: ເພີ່ມໃຫ້ຄົບ 100 mL

Milieu de montage gomme au chloral (milieu de Hoyer)

ນ້ຳກັ່ນ: 50 mL
Hydrate de chloral : 200 g
Gomme arabique : 50 g
Glycérol : 20 mL

ສານລະລາຍ Marc-André

Hydrate de chloral : 40 g
Acide acétique glacial : 30 mL
ນ້ຳກັ່ນ: 30 mL

Fuchsine acide à 1 % ໃນນ້ຳກັ່ນ

Fuchsine acide (poudre) : 1 g
ນ້ຳກັ່ນ: 99 mL

ສານລະລາຍ Marc-André ທີ່ຍ້ອມສີດ້ວຍ fuchsine

ສານລະລາຍ Marc-André : 10 mL
Fuchsine acide à 1% 50 µL

ເອກະສານຊ້ອນທ້າຍ 3 : Euparal®, baume du Canada, alcool polyvinylique ແລະ ສານນ້ຳຢາແຕ່ງສະໄລອື່ນໆ

ເປັນນ້ຳຢາແຕ່ງສະໄລທີ່ດີທີ່ສຸດເມື່ອບໍ່ມີຜະລິດຕະພັນທີ່ຈຳເປັນສຳລັບ dehydration ທີ່ເໝາະສົມ. Polyvinyl alcohol ຖືກປະສົມກັບ Amman's lactophenol. ເຖິງຢ່າງໃດກໍ່ຕາມ, ການປະຊຸມນີ້ມີຂໍ້ເສຍຫຼັກຄືການແຕ່ງ ຫຼື polyvinyl alcohol ກາຍເປັນເຄສຕອນເນື່ອງຈາກການລະເຫີຍນ້ຳ ຫຼື ປ່ຽນສີດຳເມື່ອ phenol oxidize. ນີ້ຍັງເປັນເຕັກນິກດີສຳລັບການແຕ່ງສະໄລຊົ່ວຄາວ.

Canada balsam: ການນຳໃຊ້ Canada balsam ສຳລັບແຕ່ງສະໄລລະຫວ່າງ ແຜ່ນແກ້ວສະໄລ ແລະ ແຜ່ນປົກສະໄລ ຈຳເປັນຕ້ອງມີການດຶງນ້ຳອອກ (dehydration) ຈາກຕົວຢ່າງກ່ອນ. ການນຳໃຊ້ xylène ຫຼື toluène ໃນຂັ້ນຕອນນີ້ ກໍມີຂໍ້ເສຍ ແລະ ຂໍ້ຈຳກັດບາງປະການ.

Enecê: ເຊັ່ນດຽວກັບສານນ້ຳຢາ Canada balsam, Enecê ຕ້ອງການໃຫ້ຕົວຢ່າງແຫ້ງໂດຍການດຶງນ້ຳອອກກ່ອນກ່ອນທີ່ຈະແຕ່ງໃສ່ແຜ່ນສະໄລ. Enecê ປະກອບດ້ວຍ: rosin ສີຂາວບໍລິສຸດ (22 ກຣາມ); copal gum ທີ່ລະລາຍໃນເຫຼົ້າ (12 ກຣາມ); ເຫຼົ້າບໍລິສຸດ (20 ມລ); camphor (10 ກຣາມ); turpentine (10 ມລ); eucalyptol: 26 ມລ. ການກະກຽມ: ໃນພາຊະນະທີ່ເໝາະສົມ (ເຊັ່ນ: ຂວດ Erlenmeyer), ປະສົມເຫຼົ້າບໍລິສຸດ ແລະ camphor, ຈາກນັ້ນຕື່ມ rosin ແລະ copal gum. ຫຼັງຈາກນັ້ນ, ປິດຝາກະຕຸກ ແລະ ສັ່ນ, ຈາກນັ້ນໃຫ້ຄວາມຮ້ອນໃນອ່າງນ້ຳທີ່ອຸນຫະພູມປານກາງເພື່ອຫຼີກເວັ້ນການຕົ້ມ. ເມື່ອເນື້ອໃນເປັນຂອງແຫຼວໜຶດແລ້ວ, turpentine ຈະຖືກຕື່ມ. ຫຼັງຈາກນັ້ນ, ສ່ວນປະສົມຈະຖືກກັ່ນຕອງໃນຂະນະທີ່ຍັງອຸ່ນຢູ່, ແລະ eucalyptol ຈະຖືກຕື່ມໃສ່ນ້ຳກອງ. ເມື່ອສານປະສົມກາຍເປັນນ້ຳໜ້ອຍລົງ, ມັນຈະຖືກເຈືອຈາງດ້ວຍນ້ຳຢາ Enecê, ເຊິ່ງກະກຽມຕາມສູດຕໍ່ໄປນີ້: ເຫຼົ້າບໍລິສຸດ (30 ມລ), ກາຟ (17 ກຣາມ), ນ້ຳຫອມນ້ຳມັນຢາງ (15 ມລ), ຢູຄາລິບຕອລ (38 ມລ) (Cerqueira, 1943).

Euparal : ນີ້ເປັນຢາງ resin ທີ່ມາຈາກ Cypress ຂອງ Atlas Tetraclinis articulata (Vahl, 1791) ແລະ ຖືກສຶກສາ ແລະ ພັດທະນາໃນ 1906 ໂດຍ ທ່ານ Gilson. ຂໍ້ດີຫຼັກຄືມັນບໍ່ polymerize. ຕົວຢ່າງທີ່ແຕ່ງສະໄລລະຫວ່າງ ແ່ນແກ້ວສະໄລ ແລະ ແຜ່ນປົກສາມາດເອົາອອກງ່າຍດ້ວຍການໃສ່ເຫຼົ້າ ຫຼື ດີກວ່ານັ້ນ ແມ່ນໃຊ້ Euparal® essence. ຢາງResin ນີ້ ເອີ້ນ sandarac ສາມາດປະສົມກັບເຫຼົ້າໄດ້ ethanol ຈາກ 80%.

ການນຳໃຊ້ Triton X100: non-ionic aqueous solution:

Triton X100 ແມ່ນສານລະລາຍນ້ຳແບບບໍ່ມີປະຈຸ (non ionique) (4-(1,1,3,3-tetramethylbutyl) phenyl-polyethylene glycol ຫຼື t-octylphenoxypolyethoxyethanol, polyethylene glycol tert-octylphenyl ether), ຖືກນຳໃຊ້ຢ່າງກວ້າງຂວາງເປັນສານຊັກຟອກໃນຊີວະວິທະຍາຂອງຈຸລັງ ແລະ ໂມເລກຸນ. ມັນຊ່ວຍໃຫ້ການຊຶມຜ່ານຂອງເຍື່ອຫຸ້ມຈຸລັງ ແລະ ເຍື່ອຫຸ້ມນິວເຄຼຍ. ຕົວຢ່າງແມງທີ່ເກັບໃນເຫຼົ້າເປັນເວລາຫຼາຍປີເປັນເລື່ອງທີ່ພົບເຫັນທົ່ວໄປ. ແຕ່ນ່າເສຍດາຍ, ວິທີການຮັກສານີ້ຍັງບໍ່ດີທີ່ສຸດ, ແລະ ແມງໄມ້ທີ່ຖືກຮັກສາໄວ້ໃນວິທີນີ້ມັກຈະເປັນເລື່ອງຍາກຫຼາຍທີ່ຈະກະກຽມສຳລັບການສັງເກດດ້ວຍກ້ອງຈຸລະທັດ. ພາຊະນະເກັບທີ່ເປັນຢາງທີ່ມີຕົວຢ່າງມັກເສື່ອມສະພາບ, ຕາມດ້ວຍການລະເຫີຍຂອງເຫຼົ້າ. ໃນທັງສອງກໍລະນີ, ການສຳຜັດກັບເຫຼົ້າເປັນເວລາດົນ ຫຼື ການແຫ້ງຂອງຕົວຢ່າງເປັນບັນຫາໃຫຍ່. ໃນປີ 2008, ທ່ານ Jonque ໄດ້ເຜີຍແຜ່ບັນທຶກກ່ຽວກັບການເຮັດໃຫ້ນ້ຳເຂົ້າຄືນໃນແມງມຸມໂດຍໃຊ້ສານເຮັດໃຫ້ຊຸ່ມເຊັ່ນ Agepon, ເພື່ອໃຊ້ໃນການຖ່າຍຮູບ [26].

ການສັງເກດການນີ້ໄດ້ນຳໄປສູ່ແນວຄວາມຄິດຂອງການໃຊ້ສານເຮັດໃຫ້ຊຸ່ມທີ່ບໍ່ແມ່ນຜົງຊັກຟອກທີ່ມີຄວາມເຂັ້ມຂຸ້ນສູງ.

.

ຂັ້ນຕອນການໃຊ້ສານລະລາຍນ້ຳ 0.5% ຂອງ Triton X100:

- ແຊ່ຕົວຢ່າງແຫ້ງດ້ວຍເຫຼົ້າບໍລິສຸດ.
- ຕື່ມສານລະລາຍ 0.5% Triton X100 ໃນປະລິມານທີ່ຕ້ອງການ ເພື່ອໃຫ້ຕົວຢ່າງຈົມຢູ່ໃນນ້ຳໝົດ.
- ປະໄວ້ປະມານ 5 ນາທີ ຫຼື ດົນກວ່ານັ້ນ. ແມງໄມ້ຕົ້ນຂໍທັງໝົດຄວນຈະເປັນອິດສະຫຼະໃນສານລະລາຍ..
- ເອົາສານລະລາຍ Triton X100 ອອກ ແລະ ປ່ຽນແທນດ້ວຍສານລະລາຍໂພແທດຊຽມໄຮດຣອກໄຊດ໌.

ຫຼັງຈາກນັ້ນ, ສືບຕໍ່ໄປຕາມຕາມຂັ້ນຕອນທີ່ອະທິບາຍຂ້າງເທິງ.

ເອກະສານຊ້ອນທ້າຍ 4 : ຂັ້ນຕອນການແຕ່ງສະໄລດ້ວຍ Euparal® ຫຼື Canada Balsam

1. ຕົວຢ່າງຕ້ອງຖືກດຶງນ້ຳອອກໃຫ້ແຫ້ງ (ລັກສະນະຂຸ່ນ ຫຼື ຄ້າຍຄືນົມຊີ້ໃຫ້ເຫັນເຖິງການດຶງນ້ຳອອກທີ່ບໍ່ພຽງພໍ).
2. ການດຶງນ້ຳອອກສາມາດເຮັດໄດ້ໂດຍການເພີ່ມຄວາມເຂັ້ມຂຸ້ນຂອງເຫຼົ້າ.
3. ຕົວຢ່າງສາມາດຍ້າຍອອກຈາກເຫຼົ້າ 99% ຫຼື ເຫຼົ້າບໍລິສຸດ (absolu) ໄປຫາສານທີ່ເຮັດໃຫ້ໃສ (agent d'éclaircissement) ໄດ້.

ຂັ້ນຕອນ (Procédure):

1. ນຳ ຮົ້ນຝອຍຊາຍ ຕົວແກ່ໃສ່ໃນພາຊະນະທີ່ມີເຫຼົ້າ ethanol 70%.
2. ດູດເອົາ ethanol ອອກ ແລະ ຕື່ມແທນດ້ວຍ KOH 10%. ປົກພາຊະນະທີ່ມີຕົວຢ່າງດ້ວຍ ແຜ່ນແກ້ວ.
3. ແຊ່ປະໄວ້ຈົນຕົວຢ່າງໃສ (transparent).
4. ດູດເອົາ KOH ອອກ.
5. ຕື່ມນ້ຳກັ່ນ ແລະ ປ່ອຍໄວ້ 30–45 ນາທີ.
6. ດູດເອົານ້ຳອອກ ແລະ ລ້າງຊ້ຳດ້ວຍນ້ຳກັ່ນອີກ 30 ນາທີ (ເວລາຂຶ້ນກັບຈານວນຕົວຢ່າງ).
7. ດູດເອົານ້ຳອອກ.
8. ເພີ່ມສານນ້ຳຢາ Marc-André (ອາດຍ້ອມດ້ວຍ fuchsine) ແລະ ປະໄວ້ 24 ຊົ່ວໂມງ.
9. ດູດເອົາສານ Marc-André ອອກ.
10. ຕື່ມນ້ຳກັ່ນ ແລະ ປະໄວ້ 30–45 ນາທີ.
11. ດູດເອົານ້ຳອອກ ແລະ ລ້າງຊ້ຳ 30 ນາທີ.
12. ດູດເອົານ້ຳອອກ.
13. ຕື່ມເຫຼົ້າ ethanol 70% ແລະ ຜ່າຕັດຕົວຢ່າງ:
    a. ຫົວ ແລະ ທ້ອງ: ດຶງອອກຢ່າງລະມັດລະວັງໃຫ້ແຍກຈາກເອິກ;
    b. ເອິກ: ຕັດປີກອອກ ໂດຍໃຊ້ເຂັມຈັບ 2 ອັນ ຫຼື ຜ່າຕັດແບບກາງ (sagittal) ຕາມຄວາມຕ້ອງການ
14. ດຶງນ້ຳອອກແບບຄ່ອຍໆ ໃນຄວາມເຂັ້ມຂຸ້ນຂອງເຫຼົ້າ ethanol ຈາກ 50 → 80 → 95% ແລະ ສຸດທ້າຍແມ່ນເຫຼົ້າເຂັ້ມຂຸ້ນ absolute ethanol.
15. ດຶງນ້ຳອອກສຸດທ້າຍ ໂດຍແຊ່ ethanol 100% 2 ຄັ້ງ (10 ນາທີ/ຄັ້ງ).
16. ດູດເອົາ ethanol ອອກ ແລະ ປົກຕົວຢ່າງດ້ວຍ clove oil ເປັນເວລາ 15 ນາທີ ໃນອຸນຫະພູມຫ້ອງ.
17. ຍ້າຍຕົວຢ່າງໄປໃສ່ຢອດ Euparal® ຫຼື baume du Canada ຢູ່ເທິງ ແຜ່ນແກ້ວສະໄລ.
18. ຈັດຕຳແໜ່ງຕາມທີ່ຕ້ອງການ:

- ແຍກຫົວ, ເອິກ, ແລະ ທ້ອງ ໂດຍໃຊ້ເຂັມ ຫຼື ຄີມຄີບ ພາຍໃຕ້ກ້ອງຜ່າຕັດແມງໄມ້
- ຫົວຄວນວາງໃນທ່າ ventro-dorsal ຄື occipital foramen ຕ້ອງຊີ້ຂຶ້ນເທິງເພື່ອໃຫ້ cibarium ສາມາດສັງເກດໄດ້ໂດຍກົງ
- ການຜ່າຕັດເຮັດໄດ້ໂດຍກົງ ໃນສານນ້ຳຢາແຕ່ງສະໄລ

19. ປ່ອຍໃຫ້ ແຫ້ງຈົນເກີດຄວາມໜຽວໜ້ອຍນຶ່ງ.
20. ຊຸບດ້ວຍເຫຼົ້າ absolute ethanol ແລະ ວາງທັບລົງໃນນ້ຳຢາ Euparal® ຫຼື baume du Canada ໂດຍຄ່ອຍວາງໃຫ້ເປັນມຸມນ້ອຍໆ.
21. ເກັບຮັກສາ ແຜ່ນແກ້ວສະໄລ ໃນກ່ອງແຫ້ງທີ່ອອກແບບມາເພື່ອຈຸດປະສົງນີ້ສະເພາະ.
