# Supplementary material for: Processing and mounting phlebotomine sand flies: a consensus guideline
Source: Parasite. 2026 Apr 3;33:18. doi: 10.1051/parasite/2026009 (PMC13047900; doi:10.1051/parasite/2026009)
Supplement: Supplementary file 20 — Malay translation / Terjemahan dalam Bahasa Melayu [file parasite-33-18-s20.pdf]

Parasite **33**, 18 (2026)
© FJ Randrianambinintsoa et al., published by EDP Sciences, 2026
https://doi.org/10.1051/parasite/2026009

PARASITE
Available online at:
www.parasite-journal.org

REVIEW ARTICLE OPEN ACCESS

Fikarakarana sy adirafitra ho an'ny mokafihitra na « phlébotomes » : torolalana iombonana

Fano José Randrianambinintsoa[1], Laure Augendre[1], Jorian Prudhomme[1], Jean-Philippe Martinet[1], Mathieu Loyer[1], Nalia Mekarnia[1], Hocine Kerkoub[1], Farzana Khan Perveen[1], Antoine Huguenin[1,2], Emilie Kariya[1,2], Mohammad Akhoundi[3], Andrey José de Andrade[4], Eduardo Berriatua[5], Gioia Bongiorno[6], Sébastien Boyer[7,8], Vasiliki Christodoulou[9], Magda Clara Vieira Da Costa-Ribeiro[10], Lucas Alexandre Farias de Souza[10], Huicong Ding[11], Blaise Dondji[12], Vít Dvořák[13], Ozge Erisoz Kasap[14], Eunice Aparecida Bianchi Galati[15], Montserrat Gállego[16], Cristina Ballart[16], Stavroula Gouzelou[17], Nabil Haddad[18], Rezki Sabrina Masse[19], Asrat Hailu Mekuria[20], Vladimir Ivovic[21], Szymon Kaczmarek[22], Mohd Khadri Shahar[19], Oscar D. Kirstein[23], Edwin Kniha[24], Iva Kolářová[13], Lincoln Timinao[25], Cristian Lucanas[26], Ognyan Mikov[27], Kimsear Nov[7], Yusuf Özbel[28], Bernard Pesson[29], Laura Cristina Posada Lopez[30], Didot Budi Prasetyo[1,7], Nil Rahola[31], Eduardo A. Rebollar-Tellez[32], Bruno Leite Rodrigues[15], Lalita Roy[33], Prasanta Saini[34], Chizu Sanjoba[35], Paloma Helena Fernandes Shimabukuro[36], Padet Siriyasatien[37], Agnieszka Soszyńska[22], Tatiana Suleşco[38], Massamba Sylla[39], Majhalia Torno[40], Petr Volf[13], Khamsing Vongphayloth[41], Vu Sinh Nam[42], April Wardhana[43], Eric Yessinou[44], Sonia Zapata[45], Jean-Charles Gantier[1], and Jérôme Depaquit[1,2,*]

[1] Faculté de Pharmacie, Université de Reims Champagne Ardenne, UR ESCAPE-USC ANSES PETARD, 51 rue Cognacq-Jay, 51096 Reims Cedex, France
[2] Pôle de Biologie territoriale, Laboratoire de Parasitologie-Mycologie, Centre Hospitalo-Universitaire, 51092 Reims, France
[3] Parasitology-Mycology Department, Avicenne Hospital, AP-HP, Bobigny, Sorbonne Paris Nord University, France; Unité des Virus Émergents (UVE: Aix-Marseille Univ, Università di Corsica, IRD 190, Inserm 1207, IRBA), 13005 Marseille, France
[4] Parasitology Collection of Basic Pathology, Department of Basic Pathology, Federal University of Paraná, Curitiba 19031, Brazil
[5] Department of Animal Health, University of Murcia, Campus de Espinardo, 30100 Espinardo, Murcia, Spain
[6] Department of Infectious Diseases, Vector-borne Diseases Unit, Istituto Superiore di Sanità, 00166 Rome, Italy
[7] Medical and Veterinary Entomology Unit, Institut Pasteur du Cambodge, Phnom Penh 12201, Cambodia
[8] Ecology & Emergence of Arthropod-borne Pathogens Unit, Department of Global Health, Institut Pasteur, CNRS UMR2000, 75015 Paris, France
[9] Section Veterinary Services (1417), Laboratory for Animal Health Virology, Aglantzia, Nicosia 2109, Cyprus
[10] Insects Vectors and Parasites Laboratory, Department of Basic Pathology and Postgraduate program in Microbiology, Parasitology and Pathology, Federal University of Paraná, 81530-900 Curitiba, Brazil
[11] Department of Biological Sciences, National University of Singapore, 117558, Singapore
[12] Laboratory of the Leishmaniasis Research Project, Mokolo District Hospital, Mokolo, Cameroon; Laboratory of Cellular Immunology and Parasitology, Department of Biological Sciences, Central Washington University, 98926 Ellensburg, WA, USA
[13] Department of Parasitology, Faculty of Science, Charles University, 12800 Prague, Czechia
[14] VERG Laboratories, Department of Biology, Faculty of Science, Hacettepe University, Beytepe, Ankara 06800, Türkiye
[15] Faculdade de Saúde Pública da Universidade de São Paulo (FSP/USP), Pós-graduação em Saúde Pública, 01246-904 São Paulo, Brazil
[16] Secció de Parasitologia, Departament de Biologia, Sanitat i Medi Ambient, Facultat de Farmàcia i Ciències de l'Alimentació, Universitat de Barcelona, & Institut de Salut Global de Barcelona (ISGlobal), Centro de Investigación Biomédica en Red, Enfermedades Infecciosas (CIBERINFEC), 08028 Barcelona, Spain
[17] Laboratory of Infectious Diseases and Public Health, School of Medicine, University of Cyprus, Nicosia, Cyprus & Department of Pediatrics, Archbishop Makarios III Hospital, Nicosia 2115, Cyprus
[18] Faculty of Health Sciences, American University of Beirut, 1107 2020 Beirut, Lebanon
[19] Medical Entomology Unit, Infectious Disease Research Centre, Institute for Medical Research (IMR), National Institutes of Health (NIH), Ministry of Health Malaysia, 40170 Shah Alam, Selangor, Malaysia
[20] School of Medicine, Addis Ababa University, 28017 - 1000 Addis Ababa, Ethiopia

Edited by Jean-Lou Justine
*Corresponding author: jerome.depaquit@univ-reims.fr

Ity dia lahatsoratra Open Access nozaraina teo ambanin'ny fepetran'ny Creative Commons Attribution License (https://creativecommons.org/licenses/by/4.0), izay mamela ny fampiasana, ny fizarana ary ny famokarana tsy misy fetra amin'ny fitaovana rehetra, raha toa ka voatanisa araka ny tokony ho izy ny asa tany am-boalohany.

[21] Faculty of Mathematics, Natural Sciences and Information Technologies, University of Primorska, 6000 Koper, Slovenia
[22] University of Lodz, Faculty of Biology and Environmental Protection, Department of Invertebrate Zoology and Hydrobiology, Banacha 12/16, 90-237 Łódź, Poland
[23] Laboratory of Entomology, Ministry of Health, 9134302 Jerusalem, Israel
[24] Center for Pathophysiology, Infectiology and Immunology, Institute of Specific Prophylaxis and Tropical Medicine, Medical University Vienna, Kinderspitalgasse 15, 1090 Vienna, Austria
[25] Papua New Guinea Institute of Medical Research (PNGIMR) Institute, PO Box 60, Headquarter, Homate Street, 441 Goroka, Eastern Highlands Province, Papua New Guinea
[26] Museum of Natural History, University of the Philippines Los Baños, 4031 Laguna, Philippines
[27] National Centre of Infectious and Parasitic Diseases, 1504 Sofia, Bulgaria
[28] Ege University, Faculty of Medicine, Department of Parasitology, 35040 Bornova/Izmir, Türkiye
[29] Retired, Faculté de Pharmacie, Université de Strasbourg, Strasbourg, 67400 Illkirch-Graffenstaden, France
[30] Program for the Study and Control of Tropical Diseases (PECET), Faculty of Medicine, University of Antioquia, 050010 Medellin, Colombia
[31] MIVEGEC, Univ. Montpellier, CNRS, IRD, 34394 Montpellier, France & Medical Entomology Unit, Institut Pasteur de Madagascar, 101 Antananarivo, Madagascar
[32] Laboratorio de Entomología Médica, Departamento de Zoología de Invertebrados, Facultad de Ciencias Biológicas, Universidad Autónoma de Nuevo León, San Nicolás de los Garza, 66455, NL, México
[33] Tropical and Infectious Disease Centre, BP Koirala Institute of Health Sciences, Dharan 56700, Nepal
[34] ICMR-Vector Control Research Centre, Puducherry 605006, India
[35] Graduate School of Agricultural and Life Sciences, The University of Tokyo, Tokyo 113-8657, Japan
[36] Grupo de estudos em Leishmanioses/Coleção de Flebotomíneos (COLFLEB/Fiocruz-MG), Instituto René Rachou, Fundação Oswaldo Cruz, Belo Horizonte, Minas Gerais, 30190009, Brazil
[37] Center of Excellence in Vector Biology and Vector-Borne Disease, Department of Parasitology, Faculty of Medicine, Chulalongkorn University, Bangkok 10330, Thailand
[38] Department of Arbovirology, Bernhard Nocht Institute for Tropical Medicine, Bernhard Nocht Str. 74, 20359 Hamburg, Germany [39] Laboratory Vectors & Parasites, Department of Livestock Sciences and Techniques, Sine Saloum University El Hadji Ibrahima Niasse (SSUEIN) Kaffrine Campus, C.P. 24600, Senegal.
[40] Environmental Health Institute, National Environment Agency, Singapore 138667, Singapore & Department of Biological Sciences, National University of Singapore, 117558 Singapore
[41] Institut Pasteur du Laos, Laboratory of Vector-Borne Diseases, Samsenhai Road, Ban Kao-Gnot, Sisattanak District, 3560 Vientiane, Lao PDR
[42] National Institute of Hygiene and Epidemiology, 1 Yec-Xanh Street, Hai Ba Trung District, 100000 Hanoi, Vietnam
[43] Indonesian Research Center for Veterinary Science, Indonesian Agency for Agricultural Research and Development, Ministry of Agriculture Republic Indonesia, Bogor 16114, Indonesia & Department of Parasitology, Faculty of Veterinary Medicine, Airlangga University, Surabaya 60115, Indonesia
[44] Laboratory of Research in Applied Biology, Polytechnic School of Abomey-Calavi, University of Abomey-Calavi, 01 P.O. Box 2009, 00000 Cotonou, Benin
[45] Instituto de Microbiología, Colegio de Ciencias Biológicas y Ambientales (COCIBA), Universidad San Francisco de Quito (USFQ), 170901 Quito, Ecuador

Received 1 December 2025, Accepted 29 January 2026, Published online 3 April 2026

Famintinana – Ity lahatsoratra ity dia manome torolalana feno momba ny fikarakarana sy fanomanana ny santionan'ny mokafihitra (« Phlébotomes »), izay tena zava-dehibe ilaina amin'ny famantarana ny karazana sy ny fitiliana na fitsirihana ireo bibikely mpanaparitaka aretina na tsimok'aretina. Miresaka teknika isan-karazany azo ampiasaina eny an-kianja na ao amin'ny laboratoara izy io. Ahitana toromarika amin'ny antsipiriany ao anatin'ny torolanana momba ny fanangonana, ny fikirakirana, ny fiarovana ary ny famonoana tsy maharary ny mokafihitra (miaraka amin'ny tolo-kevitra hampiasa fampangatsiahana maina na fampiasana ny « CO_2 » fa tsy ny fampiasana akora simika), ary koa paikady fitehirizana toy ny vata fampangatsiahana sy ny fitehirizana amin'ny alikaola toy ny « éthanol ». Asongadina ato ao amin'ity famaritana ny teknika fanomanana ireo taova mandinika sy manokana anatiny sasany (ny taovam-pananahana, ny loha ary ny elatra) izay tena ilaina tokoa ahafahana mijery azy ireo amin'ny fomba mikrôskôpika araka ny tokony ho izy, izay resahana ato anatin'ity asa ity. Manolotra torolalana amin'ny antsipiriany momba ny fikarakarana santionany ihany koa ity tahirin-kevitra ity, ao anatin'izany ny fanadiovana amin'ny alalan'ny ranon-javatra isan-karazany toy ny potasy sy ny ranoka « Marc-André ». Ny dingan'ny fametahana dia mampitaha ireo fitaovana samihafa, ka manasongadina ny toetrany ara-optika sy ny fahafahany mitahiry. Ny ranoka Hoyer (fantatra ihany koa amin'ny

anarana hoe « chloral gum ») dia atolotra ho an'ny fandinihana haingana, indrindra ho an'ny « spermathèque », noho ny fangaraharan'izy io, na dia tsy mety amin'ny fitehirizana maharitra aza. Anisan'ireo ranoka hafa resahina ny « alcool polyvinyl », ny « Euparal® » (ho an'ny fandeferana amin'ny rano voafetra), ary ny « Canada balsam » (ranoka levona amin'ny hydrocarbon), ka ireo roa farany ireo dia ahafahana mitahiry maharitra. Voaresaka ihany koa ireo fomba fiasa vaovao amin'ny biolojia molekiolera toy ny fandaharana mirindra ny « ADN » sy ny « MALDI-ToF », izay mitaky fiheverana manokana rehefa mikarakara ireo mokafihitra isankarazany. Ankoatra izany, misy horonantsary fohy mampiseho ireo karazana teknika fanamafisana isan-karazany ary koa fandikàna amin'ny fiteny 33 samy hafa, ka ahafahan'ity torolalana ity mamaly ny filàna sy ny andrasan'ny vondrom-piarahamonina siantifika maneran-tany.

Teny fanalahidy: Fampisandrahana, mokafihitra, ranok'i Hoyer, ranok'i Marc-André, « gomme au chloral », alikaola « polyvinyl », « Euparal® », « Canada balsam », fanokanana *Leishmania*, toe-javatra eny an-kianja, fiompiana, fandidiana, biôlojia môlekiolera, MALDI-ToF, santionany karazana

Abstract – Processing and mounting phlebotomine sand flies: a consensus guideline. This article provides a comprehensive guide for the processing and mounting of phlebotomine sand fly specimens, which is crucial for species identification and pathogen detection and isolation. It discusses a range of techniques suitable for both field and laboratory settings. The guide includes detailed instructions on sand fly collec-tion, handling, covering, and euthanasia (recommending dry freezing or CO_2 over chemicals) as well as conservation strategies, such as cold storage and preservation in ethanol. The quality of preparation of certain anatomical structures (genital organs, head and wings) is essential for their proper microscopic observation and is described in this work. The article also presents detailed sample processing, including the clearing process with agents such as potassium hydrox-ide then Marc-André solution. The mounting process compares different media, emphasizing their optical properties and preservation potential. Hoyer fluid (also known as chloral gum) is recommended for quick observation, particularly for spermathecae, due to its clarity, although it is not suitable for long-term storage. Other media discussed include polyvinyl alcohol, Euparal® (for limited water tolerance), and Canada balsam (a hydrocarbon-soluble medium), with the latter two offering long-term preservation capabilities. Innovative molecular biology approaches such as DNA sequencing and MALDI-ToF, which require particular attention to sample processing, are also addressed. Furthermore, short video clips illustrating various mounting techniques as well as translations in many different languages are provided, allowing the guideline to reach the diverse needs and expectations of the global scientific community.

Key words: Mounting, Phlebotomine sand fly, Hoyer fluid, Marc-André solution, Chloral gum, Polyvinyl alcohol, Euparal®, Canada balsam, *Leishmania* isolation, Field conditions, Culture, Dissection, Molecular biology, MALDI-ToF, Type-specimens.

Fampidirana

Ny mokafihitra dia bibikely manana elatra roa « diptères » ao amin'ny fianakaviana Psychodidae, zana-fianakaviana Phlebotominae, ary misy karazany 1 063 farafahakeliny fantatra [21]. Izy ireo dia mpitondra ny otrikaretina (*Leishmania*, arbovirus ary *Bartonella)* manan-danja, izay mahatonga aretina toy ny « leishmaniasis », ny aretina ateraky ny « arbovirus » ary ny « bartonellosis ». Ny famantarana azy ireo dia mifototra indrindra amin'ny fandinihana amin'ny mikraoskaopy amin'ny antsipiriany, izay azo atao noho ny fanangonana hentitra, ny fitehirizana sahaza ary ny fametahana am-pitandremana ny santionany eo ambony lamandy, izay mitaky teknika manokana maromaro, ka samy manana ny tombony sy ny fetra misy azy ireo.

Ny famantarana ny mokafihitra lehibe dia mifototra amin'ny fandinihana ny rafitra ivelany (ohatra: ny rambony, ny palps ary ny taovam-pananahan'ny lahy) sy ny rafitra anatiny (ohatra: ny tenda, ny cibarium ary ny spermathecae). Ny fandinihana anatiny sy ny fanavahana ireo taova anatiny ireo dia manamora ny fandinihana azy ireo ary, vokatr'izany, ny famantarana azy ireo amin'ny fomba marina. Noho izany, tsy toy ny lalitra na ny bibikely triatomine, dia tsy maintsy apetaka eo anelanelan'ny "slide" sy "cover slip" ny mokafihitra vao azo fantarina. Hatramin'ny taona 1980, ny fijerena amin'ny alalan'ny mikrôskôpy no hany fomba nisy hamantarana ny mokafihitra, ary mbola io no fomba be mpampiasa indrindra ankehitriny. Noho izany, somary tsotra ny fisafidianana ny fomba fiasa sy ny fanomanana ary nifototra indrindra tamin'ny fisarahana roa: etsy an-daniny, ny fametahana maharitra ho an'ny fitehirizana maharitra ny santionany, ary etsy an-kilany, ny fametahana haingana ho an'ny famantarana ao anatin'ny tontolo tsy miantoka fitehirizana maharitra. Ny fametahana maharitra, ohatra amin'ny alalan'ny résine toy ny Canada balsam, dia fomba fiasa mandany fotoana izay mitaky fanamainana tanteraka ny santionany. Ankoatra izany, ny tondron'ny fanehoan-javatra amin'ity fitaovana ity dia tsy voatery ho tsara indrindra amin'ny fijerena mora ny spermathecae. Mifanohitra

amin'izany, ny fametahana anaty ranoka (ohatra, ranoka Hoyer) dia haingana kokoa ary ahafahana mahita tsara kokoa ireo spermathecae mampiverina ny hazavana, saingy tsy miantoka ny fitahirizana maharitra ny fanomanana, satria ity ranoka ity dia mirona hisintona rano avy amin'ny rivotra manodidina. Safidy iray ny manentsina ny lamandy amin'ny lakaoly ho an'ny hoho rehefa maina tanteraka izy. Mbola manan-danja io marimaritra iraisana io ary misy fiantraikany amin'ny fisafidianana ny fomba fanamafisana arakaraka ny tanjon'ny fanomanana. Nanomboka tamin'ny taona 1980, ny fikarohana hamantarana ny mokafihitra dia nampifangaro ny fomba fiasa ara-morfolôjia sy ara-biôkimia. Ny voalohany tamin'ireo dia nifototra tamin'ny famakafakana ny hydrocarbons amin'ny kutikula, izay nosoloan'ny teknika biolojia molekiolera haingana (izany hoe, ADN polimôrika nohamafisina kisendrasendra [, restriction fragment length polymorphism [RFLP], fanamafisana ny ADN ary fandaharana ny fototarazo amin'ny fampiasana ny fomba Sanger, ary ny fandaharana fototarazo taranaka vaovao [NGS]). Ankehitriny, ampiana fomba proteômika toy ny Matrix-Assisted Laser Desorption-Time of Flight (MALDI-ToF) ireo fomba fiasa molekiolera ireo. Ankoatra izany, ny famantarana ny karazana amin'ny alalan'ny molekiola dia azo atambatra amin'ny fitiliana ireo mikraoba miteraka aretina amin'ny alalan'ny PCR (Leishmania, *Trypanosoma*, *Bartonella* ary Phlebovirus), izay samy azo fantarina avokoa amin'ny alalan'ny PCR mahazatra na mivantana, ka mitaky fanarahana ny fomba fandraisana santionany sy ny fitehirizana araka ny tanjona kasaina [3, 32]. Ankoatra ireo toetra ara-morfa fampiasa hatrizay hanavahana ireo karazana, dia azo ampiharina ihany koa ny fomba fiasa ara-morfa hafa (ohatra, ny jeomorfometria ny elatra).

Miorina indrindra amin'ny traikefan'ireo mpanoratra sy ny angon-drakitra avy amin'ny literatiora, ny tanjon'ity fikarohana ity dia ny hanolotra torolalana voarafitra tsara momba ny fametahana sy ny fikarakarana ireo mokafihitra efa lehibe mba hanatsarana ny famakafakana ara-morfa sy ara-molekiola.

Ny filàna hanaovana famakafakana sasany (ohatra, biolojia molekiolera na MALDI-ToF) dia mitaky ny fitahirizana ireo ampahany amin'ny mokafihitra izay tsy tena ilaina amin'ny famantarana ara-morfolojia, ka manasongadina ny maha-zava-dehibe ny fisafidianana protokoly voahevitra tsara.

Ato anatin'ity lahatsoratra ity, mifantoka amin'ny fomba fampatoriana sy famonoana am-pitandremana ireo mokafihitra vao tratrarina velona izahay, ny dingan'ny fitehirizana sy fametahana azy ireo, na ho an'ny famantarana haingana izany na ho an'ny fitehirizana maharitra ho an'ny fikarohana lalindalina kokoa.

1. Fisamborana ny mokafihitra

Ny moka fasika efa lehibe dia azo angonina velona na maty amin'ny fomba isan-karazany, toy ny fandrika jiro kely karazana CDC, fandrika mipetaka, mpanadio rivotra ampiasaina miaraka amin'ny fandrika Shannon, na amin'ny fisamborana mivantana ao amin'ny toerana fialan-tsasatra voajanahary (ohatra, fialofan'ny biby). Ireo fomba ireo dia ahitana ny fametrahana fandrika amin'ny toeram-ponenana mety, ny fisarihana ny mokafihitra amin'ny alalan'ny hazavana na mpamokatra hafa (CO_2 na mpamokatra simika), ary avy eo ny fanangonana azy ireo ho an'ny famakafakana fanampiny, araka ny voalaza ao amin'ny famoaham-boky maromaro [2, 3, 32, 36, 49]. Ny fakana ny mokafihitra velona dia ahafahana mampihatra ireo teknika rehetra aseho etsy ambany, raha toa kosa ny fanangonana ireo maty no manakana ny fanavahana ny karazana *Leishmania* na viriosy.

Ny teknika fakana sasany, toy ny taratasy miraikitra, dia matetika miteraka fahaverezan'ny taovan'ny mokafihitra (tenda-tsofina, rantsan-tenda, elatra na tongotra). Ankoatra izany, miraikitra amin'ny mokafihitra ny menaka castor ampiasaina hanosorana ny taratasy miraikitra ka tsy maintsy esorina amin'ny fiandohan'ny fanodinana, matetika amin'ny alalan'ny fanendrena mandritra ny 15 minitra ao anaty fangaro misy ampahany mitovy etanol sy etera di-etilika.

2. Famonoana ny santionany

Aorian'ny fanangonana, tsy maintsy vonoina ny mokafihitra mbola velona. Amin'ny fomba fanangonana sasany (ohatra, taratasy miraikitra na fandrika jiro CDC misy tavoahangy misy savony fanasan-damba na éthanol), efa maty sahady ny mokafihitra amin'ny fotoana anangonana azy. Azo atao ny fanadihadiana momba ny biolojia molekiolera amin'ireo santionany nangonina mivantana tao anaty étanol, ary koa amin'ny hafa raha toa ka tehirizina haingana araka izay azo atao ao anaty étanol izy ireo.

Na izany aza, tsy misy amin'ireo fomba ireo ahafahana manodina ny bibikely amin'ny alalan'ny MALDI-ToF. Ankoatra izany, ny fomba famonoana sasany dia mety hahatonga ny fahaverezan'ny toetra ara-batana sasany. Noho izany, tena ilaina ny fampiasana akora famonoana bibikely manara-penitra mba hiantohana ny famantarana marina na ny fitehirizana maharitra ireo santionany fototra (izany hoe, voatahiry sy voatazona ho an'ny fampiasana na fampitahana amin'ny ho avy). Azo tsentsinina amin'ny kotona ny akora simika toy ny 'ethyl acetate', 'ethyl ether', 'tetrachloroethane', ary 'chloroform' ary atao anaty fitoerana iray miaraka amin'ireo mokafihitra mba hamonoana azy ireo. Tokony hotandrovana fatratra ireo akora ireo, araka ny toromarika omen'ny mpanamboatra azy, noho ny poizin'izy ireo. Tsy manoro hevitra ny fampiasana klôroforma izahay hamonoana ny mokafihitra, satria araka ny traikefanay dia tsy mifanaraka amin'ny fandalinana biôlôjia molekiolera izany. Noho ny maha-dangerina an'ireo vokatra rehetra ireo

sy ny maha-ahiana ny fahaizany mifanaraka amin'ny famakafakana molekiolera, dia amin'ny ankapobeny dia tsy asaina mampiasa ireo akora simika ireo.

Ny fomba fanao be mpampiasa indrindra, izay mitahiry ny bika aman'endrika, ny ADN ary ny prôteinina, dia ny fampangatsiahana maina ny santionany. Tsy maintsy atao anaty vata fampangatsiahana mandritra ny fotoana ampy tsara ny mokafihitra mba ho torana tanteraka, saingy tsy tokony hahatonga azy ho (i) maina na (ii) hanimba ny fahaveloman'ny *Leishmania*, raha toa ka ny tanjona dia ny hanasaraka azy ireo *in vitro* amin'ny lalan-tsakafon'ny mokafihitra. **Noho izany dia manoro hevitra izahay ny hanondraka azy ireo mandritra ny 15 ka hatramin'ny 20 minitra amin'ny -20°C, miaraka amin'ny fanaraha-maso tsy tapaka mba hahazoana antoka fa maty fanina fotsiny ireo bibikely ary tsy maty kosa ny *Leishmania*.**

Raha tsy misy vata fampangatsiahana, azo vonoina amin'ny alalan'ny CO_2 ireo bibikely. Any an-tsaha, rehefa tsy azo ampiasaina ny fampiasana tavoahangy CO_2, dia azo vonoina amin'ny alalan'ny karatra kely fivarotana CO_2 ampiasaina amin'ny sodina fisintonana rano ireo santionany, saingy mety hisy fameperana ny fitaterana azy ireo an'habakabaka. Raha tsy misy afa-tsy izany, azo vonoina amin'ny alalan'ny setroka paraky ny bibikely. Aorian'izany dia tratra velona ao anaty fandrika CDC ny mokafihitra, alaina amin'ny alalan'ny aspiratera avy ao anaty fitoerana misy rano, tehirizina ao anaty fantsona fitaratra, ary avy eo ampidirina amin'ny setroka paraky, izay mamono azy ireo ao anatin'ny segondra vitsy. Azo ampiharina amin'ny toe-javatra rehetra eny an-tsaha ity fomba ity, anisan'izany ny toerana tena mitoka-monina. Na izany aza, satria miraikitra amin'ny setroka ny fantsona fitaratra, dia tsy azo ampiasaina intsony haka sy hikarakara ny mokafihitra velona manaraka izany raha tsy diovina tsara. Na izany aza, azo ampiasaina ihany ilay mpanadio banga tsy voadio hanomezana fahafatesana ireo mokafihitra avy amin'ny fandrika hafa mba hatao fanadihadiana. Ilaina ihany koa ny manamarina fa nesorina avokoa ny santionany rehetra tao anatin'ilay mpanadio banga. Ireo fomba ireo dia mifanaraka amin'ny fanokanana ny *Leishmania* amin'ny alalan'ny fandinihana ny lalan-tsakafo.

Ankoatra ireo toetra ara-morfa ampiasaina amin'ny fomba nentim-paharazana mba hanavahana manokana, dia azo ampiharina ihany koa ny fomba fiasa ara-morfa hafa (ohatra, ny jeomorfometria ny elatra).

Fampidirana: Ny lafiny momba ny fiarovana sy ny fitsipika dia tokony hifototra amin'ireo Taratasy Momba ny Angon-drakitra momba ny Fiarovana (SDS) mifandraika amin'izany.

Ny akora simika rehetra aseho ato amin'ity torolalana ity dia tsy maintsy karakaraina ao anatin'ny fepetra fiarovana hentitra. Ny komitin'ny fahasalamana sy ny fiarovana ao amin'ireo toeram-pikarohana dia vonona hanome fampahalalana tsy ny momba ireo loza ateraky ny akora ireo ihany, fa koa ny momba ny fomba fikarakarana sy fanariana ny fako. Na izany aza, tena ilaina ny manaraka ny toromarika momba ny fiarovana amin'ny fampiasana sy ny fanariana azy ireo. Tsara ny mitadidy fa andraikitry ny mpampiasa tsirairay ny miantoka ny fanarahana ny fomba fanao tsara ao amin'ny laboratoara, ny fitsipika momba ny fiarovana, ary ny lalàna sy ny fitsipika manan-kery ao amin'ny fireneny na ny toeram-pikarohana misy azy.
Ankoatra izany, misy akora simika sasany na ny ampahany sasany amin'izy ireo (ohatra, chloral hydrate) voafehin'ny lalàna any amin'ny firenena sasany. Omena ao amin'ny Tabilao 1 ny lisitry ny fanafohezana ampiasaina ato anatin'ity lahatsoratra ity.

Tabilao 1: Lisitry ny fanafohezan-teny.

ADN	Acide désoxyribonucléique
ARN	Acide Ribonucléique
BME	Milieu basal Eagle
CDC	Centre de contrôle et de prévention des maladies
CMCP	Monochlorophénol camphré
CMR	Substance carcinogène, mutagène, reprotoxique
COI	Sous-unité I de la Cytochrome c oxydase
CytB	Gène du cytochrome b
ELISA	Dosage immuno-enzymatique
EtOH	Éthanol
M199	Milieu 199
MALDI-ToF MS	Spectrométrie de masse à temps de vol par désorption/ionisation laser assistée par matrice
MEM	Milieu essentiel minimum
NGS	Séquençage de dernière génération
NNN	Milieu Novy-MacNeal-Nicolle
PCR	Réaction en chaîne par polymérase
RDP Laos	République démocratique populaire de Laos
PNOC	Gène de la prépronociceptine
qPCR	PCR quantitative (PCR en temps réel)
RAPD	ADN polymorphe amplifié aléatoirement
RFLP	Polymorphisme de longueur des fragments de restriction
RI	Indice de réfraction
RNases	Ribonucléases
RNASS	Solution de stabilisation des ARN
RT-PCR	Transcription inverse-PCR
TFA	Acide trifluoroacétique

3. Fitahirizana ny santionany alohan'ny fanodinana

Misy fomba dimy lehibe fampiorenana alohan'ny fanodinana:

3.1. Fampangatsiahana

Ny fomba fanao tsara indrindra dia atao amin'ny -20°C na, tsara kokoa aza, amin'ny -80°C. Ireo fomba fitehirizana ireo dia ampiasaina bebe kokoa ankehitriny noho ny fitehirizana amin'ny azota ranoka . Na ahoana na ahoana, ny fitehirizana amin'ny hatsiaka dia tsy maintsy atao haingana araka izay azo atao aorian'ny fanatoranana ny santionany. Ny fitehirizana mangatsiaka ao anaty vata fampangatsiahana dia manana tombony amin'ny fiarovana tanteraka ny bibikely, ary koa ny RNA, DNA ary ny proteinina, miaraka amin'ny fahamendrehana tsara indrindra mandritra ny fotoana fitehirizana. Etsy ankilany, mety hanimba mafy ny elatra, ny tongotra, ny palps ary ny antena ny azota ranoka, ka matetika miteraka fanapahana azy ireo ary indraindray, fahaverezan'ny toetra ara-morfolôjia manan-danja. Ny fampangatsiahana maina anaty vata fampangatsiahana dia tsy dia manimba loatra ny santionany, saingy tsy dia tsara indrindra amin'ny fitehirizana ireo taova marefo ananany. Zava-dehibe ny manamarika fa mandritra ny fanafanana dia mety hiraikitra amin'ny rindrin'ny fantsika ny elatra, ny antena, ny palps na ny tongotra ary mety hiala noho ny fiforonan'ny hamandoana. Na izany aza, tsy azo atao foana ny fitahirizana amin'ny hatsiaka be amin'ny fikarohana eny an-kianja, satria mitaky fidirana amin'ny vata fampangatsiahana na fitoeran'ny azota ranoka izany. Ny fitehirizana ao anaty vata fampangatsiahana dia mifanaraka tanteraka amin'ny fitiliana ireo otrikaretina amin'ny fampiasana fitaovana molekiolera ka tsy mihena ny fahatsapany, na dia mitaky fampangatsiahana amin'ny -80°C na anaty azota ranoka aza ny fitiliana sy ny fanokanana ireo virosy ARN rehefa ilaina ny fitehirizana maharitra. Na izany aza, ny fampangatsiahana ny santionany dia tsy ahafahana manokana ny *Leishmania* amin'ny alalan'ny fandinihana ny taovam-pandevonan-kanina, raha tsy hoe nampidirina tao anaty etona azota aloha ireo mokafihitra ary avy eo tao anaty azota ranoka (ohatra, ao anaty tavoahangy kely apetraka anaty ba kiraro), ka manahaka ny fitehirizana anaty hatsiaka ny *Leishmania*.

3.2. Fitehirizana amin'ny alikaola (etanôla na alikaola isôprôpila)

Ity angamba no fomba fampiasana be indrindra hitahirizana ny mokafihitra. Mora ampiharina eny an-toerana izy io, na dia amin'ny toe-javatra sarotra tsy misy laboratoara aza. Ny fitahirizana amin'ny alikaola dia tena mety indrindra ho an'ny fandalinana ara-morfolôjia, satria ireo taova marefo (elatra, tongotra, rambony na palps) dia mijanona ho tsy simba noho ny tsy fisian'ny bolan-drivotra ao anatin'ny fantsona fitahirizana. Noho izany dia manoro hevitra izahay ny hanentsenana ny fantsona amin'ny kotona kely iray mba hanesorana ny bolan-drivotra rehetra ary hametrahana etikety eo ambonin'ilay kotona (Sary 1). Ny fatran'ny alikaola mety dia mbola adihevitra. Amin'ny ankapobeny, tsy asaina ampiasaina ny fatra latsaky ny 70% [45, 66]. Ny fatra avo kokoa dia miantoka ny fitahirizana tsara kokoa ny ADN mandritra ny fotoana maharitra, saingy mahatonga ny santionany ho mora simba sy mora vakivaky kokoa amin'ny fandalinana ara-morfolôjia. Ny fampiasana etanol 96% (fangaro azeotropika) dia miantoka ny fahamarinan'ny fatra rehefa mandeha ny fotoana, indrindra any amin'ny faritra mando toy ny any amin'ny firenena tropikaly, na dia matetika mora kokoa aza ny mahazo etanol 95%. Na inona na inona fatra ampiasaina, amin'ny ankapobeny dia voatahiry tsara ao anaty etanol ny ADN (na dia tsy dia mahomby loatra aza izany raha oharina amin'ny fampangatsiahana, indrindra ho an'ireo fomba fiasa molekiolera karazana NGS). Mifanohitra amin'izany kosa, tsy maharitra ela loatra ny proteinina, indrindra ho an'ny fampiharana proteômika toy ny MALDI-ToF. Ny mokafihitra voatahiry anaty alikaola nandritra ny volana vitsivitsy dia mbola azo fantarina ara-morfolôjia, saingy tsy azo atao ny mamokatra spektran'ny prôteinina fakan-tahaka avy amin'ireo santionany ireo. Azo hatsaraina ny fitahirizana anaty alikaola na ny fitehirizana maina raha ampiana fampangatsiahana ny santionany amin'ny -20°C. Ny fampangatsiahana amin'ny -20°C dia manatsara indrindra ny fitahirizana ny singa molekiolera (ohatra, ny asidra nikleika) amin'ny alalan'ny fampiadana ny fahasimbana, ary manome tombony faharoa koa amin'ny fitahirizana ny bika aman'endrika amin'ny alalan'ny fampihenana ny fahasimban'ny sela rehefa mandeha ny fotoana, na dia voafetra kokoa aza ny fiantraikany amin'ny bika aman'endrika raha oharina amin'ny fahamendrehan'ny singa molekiolera. Azo ampiasaina ihany koa ny fitahirizana anaty etanol mba hamantarana ireo viriosy ADN sy ARN rehefa ampiasaina amin'ny fatra 70% farafahakeliny izy io mandritra ny fotoana fitehirizana fohy latsaky ny volana vitsivitsy. Ankoatra izany, mety ho mora hita any amin'ny firenena sasany ny alikaola izoprofilika ary ahafahana mitahiry ny ADN, saingy mahatonga ny santionany ho mora vaky izany. Tsy mora mirehitra izy io toy ny etanol ka mora entina kokoa. Raha ilaina, azo afindra ao anaty alikaola ireo karapangady voatahiry ao anaty azota ranoka na nohamainina tamin'ny fampangatsiahana, ka mitambatra ny fatiantoka avy amin'ireo fomba roa ireo.

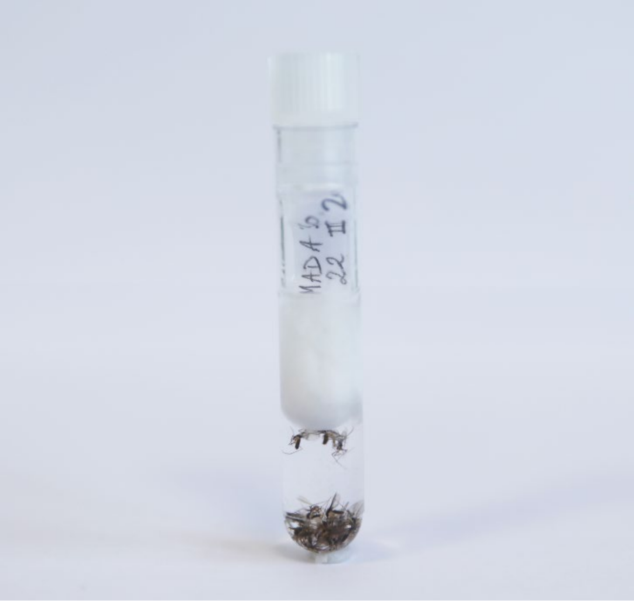

Sary 1. Mokafihitra voatahiry anaty ethanol.

3.3. Fitahirizana amin'ny ranoka mampiorina ny ARN (RNASS)

Ity akora simika rano ampiasaina matetika sy tsy misy poizina ity dia natao hanamafisana sy hiarovana ny ARN ao anatin'ny santionan-taova sy sela vao. Miasa izy io amin'ny alalan'ny fidirana haingana ao anatin'ilay santionany sy ny fanafoanana ny asan'ny RNases (enzima tompon'andraikitra amin'ny fandravana ny ARN), ka misoroka ny fandravana ny ARN nefa tsy mila atao mangatsiaka avy hatrany. Amin'ny ankapobeny dia mandaitra ny fitahirizana amin'ny RNASS amin'ny fitazonana ny bika aman'endriky ny taova sy ny sela manontolo ho an'ny famakafakana histolojika manaraka. Na dia natao manokana hampahamarina ny RNA aza ity ranon-javatra ity fa tsy hanamafy azy, ny fitahirizana mandritra ny fotoana fohy ka hatramin'ny antonony dia matetika mitazona tsara ny firafitry ny zavatra. Ny RNASS dia ahafahana mitahiry ny santionany amin'ny maripanan'ny efitrano mandritra ny hatramin'ny 7 andro, amin'ny 4°C mandritra ny herinandro maromaro, na amin'ny -20°C/-80°C ho an'ny fitahirizana maharitra. Tena sarobidy ity fomba ity amin'ny asa eny an-tsaha na any amin'ny toeram-pitsaboana izay voafetra ny fotodrafitrasa momba ny rojo mangatsiaka. Amin'ny ankapobeny, ny fakana ny RNA dia mitaky ny esorana ny santionany avy ao anatin'ilay ranoka fampiasana ary karakaraina araka ny fomba fanao mahazatra.

3.4. Fitehirizana maina amin'ny maripanan'ny efitrano

Ity dia fomba fanao efa tranainy izay, rehefa ampiharina amin'ny santionany voatahiry manontolo (napetaka manontolo), dia manana fatiantoka lehibe amin'ny tsy fahampian'ny fiarovana ireo taova marefo toy ny elatra, ny tongotra, ny rambony fofona ary ny palps. Na izany aza, mbola azo atao ny fandalinana proteômika mampiasa ny MALDI-ToF raha toa ka atao avy hatrany aorian'ny fanamafisana ny fanamainana amin'ny alalan'ny fitaovana mpanamaina karazana gel silika. Etsy ankilany, sarotra ny manao famakafakana molekiolera mikendry ny ADN amin'ity karazana santionany ity, satria matetika tapaka sady kely ny habetsahany ny ADN, ka mahatonga ny famakafakana ho sarotra kokoa noho ny amin'ny santionany vao na nangatsiaka, indrindra ho an'ny génôma niokleary. Na izany aza, azo ampiharina amin'ity karazana akora ity ny teknika vaovao toy ny museomics [34]. Noho izany, tsy atolotra ity fomba fitehirizana ity, afa-tsy raha tsy misy safidy hafa. Azo atambatra amin'ny fitehirizana anaty hatsiaka izy io, amin'ny alalan'ny fametrahana ireo fantsona ao anaty vata fampangatsiahana amin'ny -20°C na -80°C. Ny fahasahiranana lehibe dia ny fahazoana fampidirana (montage) marina an'ireo santionany na ampahany amin'ny vatana ilaina amin'ny famantarana. Mba hanaovana izany, tena ilaina ny famerenana rano (rehydration). Manoro hevitra izahay ny hampiasana ranoka Triton X-100. Miovaova manomboka amin'ny ora vitsivitsy ka hatramin'ny andro maromaro ny faharetan'ny famerenana rano ary mitaky fanaraha-maso akaiky. Aorian'ny famerenana rano tanteraka, dia tsy maintsy sasana ao anaty rano misy fandroana telo mifanesy ireo santionany.

3.5. Fitehirizana amin'ny taratasy sivana

Ny tombony lehibe indrindra amin'ny taratasy sivana dia ny faharetan'ny ADN génômika ao anatin'ny sela, na dia ao anaty vatana iray manontolo tsy voafatotra sy maina tanteraka, na ao anaty sela ra tehirizina amin'ny maripanan'ny efitrano aza. Ny taratasy sivana dia amidy amin'ny endrika karatra kely, ka ahafahana mitahiry santionany an-jatony maro amin'ny maripanan'ny efitrano ao anaty toerana mitovy habe amin'ny vatasarihana kely iray. Ny fototry ny taratasy sivana dia nasiana akora manimba ireo otrikaretina, izay midika fa tsy heverina ho loza biolojika intsony ireo santionany. Izany dia ahafahana mitahiry sy mampita ireo santionany tsy misy fepetra manokana momba ny loza biolojika [68].

3.3. Fitehirizana amin'ny ranon-javatra mampiorina ny ARN (RNASS)

Ity ranoka fampiasa matetika sy tsy misy poizina ity dia natao hanamafisana sy hiarovana ny RNA ao anatin'ny santionan-taova sy sela vao. Miasa izy io amin'ny alalan'ny fidirana haingana ao anatin'ilay santionany sy ny fanafoanana ny asan'ny RNases (enzima mandrava ny RNA), ka misoroka ny fandravana ny RNA nefa tsy mila atao mangatsiaka avy hatrany. Ny fitahirizana ao anatin'ny RNASS dia amin'ny ankapobeny mandaitra amin'ny fitehirizana ny bika aman'endriky ny taova sy ny sela manontolo ho an'ny famakafakana histolojika manaraka. Na

dia natao indrindra hampiorina ny RNA aza ity ranon-javatra ity fa tsy hanamafy azy, ny fitahirizana mandritra ny fotoana fohy ka hatramin'ny antonony dia amin'ny ankapobeny mitazona tsara ny firafitry ny zavatra. Ny RNASS dia ahafahana mitahiry ny santionany amin'ny maripanan'ny efitrano mandritra ny hatramin'ny 7 andro, amin'ny 4°C mandritra ny herinandro maromaro, na amin'ny -20°C/-80°C ho an'ny fitahirizana maharitra. Tena sarobidy ity fomba ity amin'ny asa eny an-tsaha na any amin'ny toeram-pitsaboana izay voafetra ny fotodrafitrasa momba ny rojo mangatsiaka. Amin'ny ankapobeny, ny fakana ny RNA dia mitaky ny fanesorana ny santionany avy ao anatin'ilay ranoka fampiasa sy ny fikarakarana azy araka ny paika fanao mahazatra.

3.4. Fitehirizana maina amin'ny maripanan'ny efitra

Ity dia fomba fanao efa tranainy izay, rehefa ampiharina amin'ny santionany voatahiry manontolo (napetaka manontolo), dia manana fatiantoka lehibe amin'ny tsy fahampian'ny fitahirizana ireo taova marefo toy ny elatra, ny tongotra, ny rantsan-tenda ary ny rantsan-bava. Na izany aza, mbola azo atao ny fandalinana proteômika mampiasa ny MALDI-ToF raha toa ka atao avy hatrany aorian'ny fanamafisana ny fanamainana amin'ny alalan'ny fitaovana mpanamaina karazana gel silika. Etsy ankilany, sarotra ny manao famakafakana molekiolera mikendry ny ADN amin'ity karazana santionany ity, satria matetika tapaka sady kely ny habetsahany ny ADN, ka mahatonga ny famakafakana ho sarotra kokoa noho ny amin'ny santionany vao na nangatsiaka, indrindra ho an'ny génôma nokleary ( ). Na izany aza, azo ampiharina amin'ity karazana akora ity ny teknika vaovao toy ny museomics [34]. Noho izany, tsy atolotra ity fomba fitahirizana ity, afa-tsy raha tsy misy safidy hafa. Azo atambatra amin'ny fitehirizana anaty hatsiaka izy io, amin'ny alalan'ny fametrahana ireo fantsika ao anaty vata fampangatsiahana amin'ny -20°C na -80°C. Ny fahasahiranana lehibe indrindra dia ny fahazoana fampidirana (montage) marina an'ireo santionany na ampahany amin'ny vatana ilaina amin'ny famantarana. Mba hanaovana izany, tena ilaina ny famerenana rano (rehydration). Manoro hevitra izahay ny hampiasana ranoka Triton X-100. Miovaova manomboka amin'ny ora vitsivitsy ka hatramin'ny andro maromaro ny faharetan'ny famerenana rano ary mitaky fanaraha-maso akaiky. Rehefa vita tanteraka ny famerenana rano, dia tokony hasiana rano fanasan-drano intelo misesy ireo santionany.

3.5. Fitehirizana amin'ny taratasy sivana

Ny tombony lehibe indrindra amin'ny taratasy sivana dia ny faharetan'ny ADN génômika ao anatin'ny sela, na dia ao anaty vatana iray manontolo tsy voafatotra sy maina tanteraka, na ao anaty sela ra tehirizina amin'ny maripanan'ny efitrano aza. Ny taratasy sivana dia amidy amin'ny endrika karatra kely, ka ahafahana mitahiry santionany an-jatony maro amin'ny maripanan'ny efitrano ao anaty toerana mitovy habe amin'ny vatasarihana kely iray. Ny fototra misy amin'ny taratasy sivana dia nasiana akora manimba ireo otrikaretina, izay midika fa tsy heverina ho loza biolojika intsony ireo santionany. Izany dia ahafahana mitahiry sy mampita ireo santionany tsy misy fepetra manokana momba ny loza biolojika [68].

4. Fandinihana ny santionany

Tsy toy ny bibikely maro hafa, izay fantarina mifototra amin'ny endrika ivelany hita amin'ireo voatondro iray manontolo amin'ny fanjaitra, ny mokafihitra kosa dia mitaky fandinihana anatiny sy fametahana amin'ny solaitrabe mba handinihana ireo toetra ara-batana ilaina amin'ny famantarana marina ny karazany. Na inona na inona fomba fikarakarana sy fametahana nofidiana, dia teknika fanaovana dissection iray ihany no ampiasaina (Sary 2 sy 3) (https://zenodo.org/records/18198006).

Fampiasana Triton X-100: ranoka an-drano tsy ionika

Tsara ho marihina fa ny fametahana dia natao ho an'ny santionany vao voasambotra na voatahiry tsara. Na izany aza, maro ireo mpanangona no manana santionana bibikely izay notazonina maina (ho ampiasaina amin'ny MALDI-ToF) na notehirizina anaty alikaola nandritra ny taona maro. Mampalahelo anefa fa tsy tsara ny fitehirizana maharitra anaty alikaola, ary lasa sarotra be ny fanomanana ireo bibikely misy taolana voatahiry amin'izany fomba izany mba hanaovana fizahana amin'ny mikrôskôpy. Ny olana iray matetika miseho dia ny fahasimban'ny fitoerana plastika, izay arahin'ny fiavonan'ny alikaola. Amin'ireo tranga roa ireo, voafetra ny ranoka, satria na efa ela loatra no nisy tao anaty alikaola ireo santionany na efa maina tanteraka. Ao anatin'izany tontolo izany no nipoiran'ny hevitra hampiasa akora mandofa izay tsy savony fanasan-damba mahery. Ny Triton X-100 dia tonga amin'ny endrika ranoka anaty rano tsy ionika (ranoka misy 4-(1,1,3,3-tetramethylbutyl)phenyl-polyethylene glycol, na t-octylphenoxypolyethoxyethanol, ether polietilena glikola tert-oktilfenila), izay ampiasaina matetika ho mpanadio amin'ny biolojia sela sy molekiola. Izy io dia ahafahana mandalo ny fonon'ny sela sy ny fonon'ny fototarazo.

Ny fomba fiasa manaraka ity dia mamaritra ny fampiasana ny Triton X-100 ao anaty ranoka rano 0,5%:

- Tondrahana alikaola madio tanteraka ny santionany maina.
- Arotsahy ny habetsahan'ny ranon-javatra misy Triton X-100 0.5% ilaina mba ho tototra tanteraka ilay santionany.
- Avela hiasa mandritra ny 5 minitra ka hatramin'ny andro maromaro, ary araho maso tsy tapaka. Tokony hisaraka tanteraka ao anatin'ilay ranoka ny biby mandady rehetra.

- Esory ny ranoka Triton X100 ary soloy ranoka potasiôma idrôksida.

4.1. Loha

Azo atao ny fandidiana amin'ny fampiasana fanjaitra manify na tsilo entomolojika eo ambanin'ny stereomicroscope (Sary 2 sy 3). Ny fanjaitra be mpampiasa indrindra dia: 26G × 1/2" (0.45 × 13 mm), 30G × 1/2" (0.3 × 13 mm) na 25G × 5/8" (0.5 × 16 mm). Mba hanomanana santionany ho fantarina, farafahakeliny, dia esorina amin'ny vatana ny loha ary apetaka miakatra ny lafiny aty ambany mba hampisehoana ny cibarium sy ny pharynx, raha ny tratrany sy ny kibony kosa dia apetaka mitsivalana aorian'ny fandinihana. Ny fametahana ny loha manaraka ny tsipika avy eo ambany mankany ambony dia miantoka fa mitodika miakatra ny lavaka eo ambadiky ny loha, ka ahafahana mijery mivantana ny cibarium. Mora kokoa ny miditra amin'ireo taova ireo rehefa voasaraka tanteraka amin'ny vatana sisa ny loha.

4.2. Elatra sy tratra (thorax)

Tokony hapetaka mipipy ny elatra. Azo esorina eo amin'ny fotony ny elatra tsirairay ary apetaka mitokana, na elatra iray ihany no apetaka, ary ny iray kosa avela miraikitra amin'ny tratrany. Raha kasaina atao ny famakafakana ara-morfolôjia jeometrika, dia tena ilaina ny mamantatra sy manome marika tsara ny elatra havanana sy havia alohan'ny hametahana azy. Mizara ho ampahany maromaro ny tratrany, ka ny tsirairay dia misy fampahalalana manan-danja lehibe amin'ny taksonomia [20, 64]. Amin'ny ankapobeny, apetraka amin'ny fijery an-kilany izy io mba ahafahana manadihady ny chetotaxy sy ny fitsinjaràn'ny loko. Ny fisian'ny fidiran'ny volom-bary amin'ny faritra sasany amin'ny tratrany dia azo ampiasaina hanavahana karazana sasany ao amin'ny fianakaviana *Brumptomyia*. Azo ampiasaina ny fitsinjaràn'ny loko hanavahana ireo mokafihitra Neotropikaly eo amin'ny ambaratongan'ny fianakaviana (ohatra, *Bichromomyia*), andiana karazana (ohatra, *Pintomyia*), ary na dia ireo karazana ao anatin'ny fianakaviana iray aza (ohatra, *Micropygomyia*, *Nyssomyia, Psathyromyia,* ary *Psychodopygus*) [20]. Noho izany, rehefa tsy natao hanaovana fanadihadiana molekiolera ny tratra, dia tsy maintsy apetraka amin'ny fomba izay hisorohana ny fahasimbana. Tsy maintsy asongadina fa tsy ny hamafin'ny loko no zava-dehibe, fa ny fitsinjarany eo amin'ny tratra. Vokatr'izany, ny dingana

Ny fanalefahana loko dia tsy manala ny loko na ny sorany.

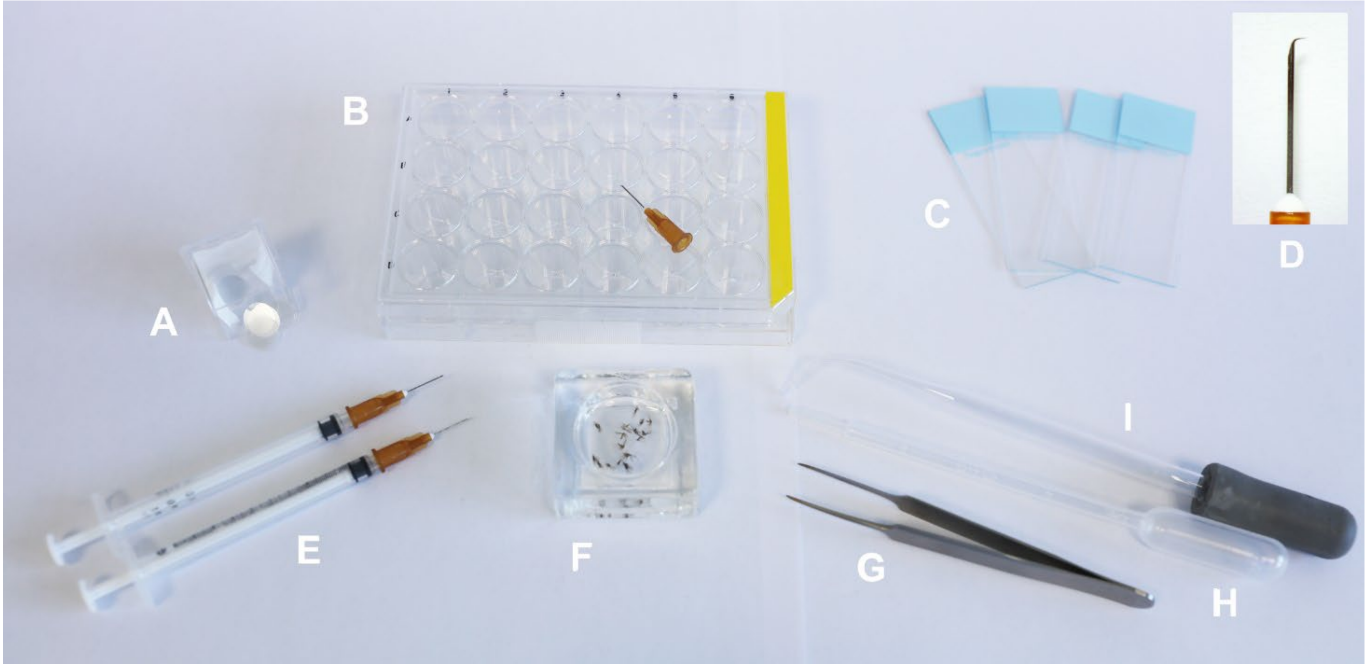

Sary 2. Fitaovana ilaina amin'ny fanangonana lalitra: A: boribory fitaratra slats (savaivony 10 na 12 mm); B: Takelaka 24 sy fanjaitra mihantona (raha misy menaka jirofo na Euparal® ampiasaina amin'ny fitsaboana ny mokafihitra, aza mampiasa takelaka akrilika, satria ny fanehoan-kevitra simika dia hanimba ny santionany); C: fitaratra mety amin'ny marika; D: antsipirian'ny farango fanjaitra; E: fanjaitra napetraka amin'ny syringes; F: fitaratra fiambenana na fitoeran-javatra mitovy amin'izany misy ny mokafihitra hapetraka; G: Dumont clamps; H: pipette plastika; I: pipette fitaratra miforitra amin'ny fanafanana mba hanamorana ny famindrana ranon-javatra ao anaty lavaka fantsakana.

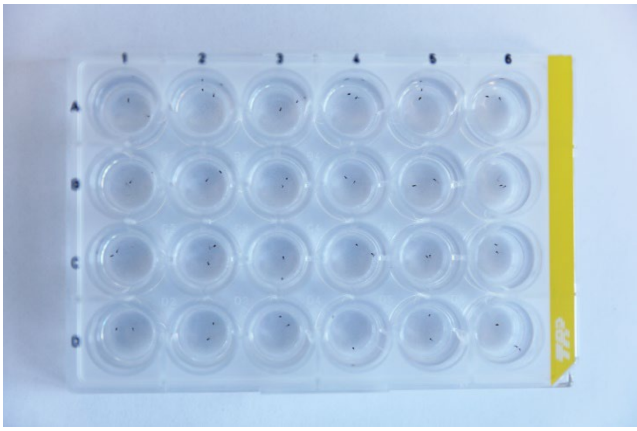

Sary 3. Takelaka misy lavaka fantsakana 24, izay samy misy ny loha sy ny faran'ny kibon'ny mokafihitra.

4.3. Ny taovam-pananahana

Tena ilaina ny fitandremana manokana amin'ny fametahana ny taovam-pananahana na amin'ny lahy na amin'ny vavy, satria tena ilaina izany amin'ny famantarana ny *genera*, *subgenera* ary ny karazany. Samy manana ny mpivady azy ny taovam-pananahana na amin'ny lahy na amin'ny vavy.

4.3.1. Mokafihitra lahy

Ny taovam-pananahana dia ivelany ary ahitana forceps mifanila roa, izay samy ahitana, ao amin'ny faritra aoriana, ny tonon-taolana gonocoxite-gonostyle ary, ao amin'ny faritra aty an-kibony, ny loba epandrialy. Ny gonostyle dia mitondra tsilo ary indraindray volombava, izay tsy maintsy azo isaina ary ny toerana ipetrahany dia tsy maintsy hita mazava tsara. Zava-dehibe ny mijery tsara ny lafiny anatiny amin'ny gonocoxite, izay mety mitondra andiana volombava tsy misy tahony na volombava napetaka amin'ny rantsana iray (= tubercle) [22]. Afaka manao fanandratana an-kilany tsotra ireo mpiara-miasa tsy dia za-draharaha amin'ny fandravana vatana, ka tsy voatery esorina amin'ny tendron'ny kibo ny taovam-pananahana (https://zenodo.org/records/18311158). Amin'izany tranga izany, ny fifanindrihan'ireo tapany roa amin'ny taovam-pananahana dia mety hanasarotra ny fanisana, ohatra, ireo seta anatiny an'ny gonocoxite, saingy ity fomba fiasa ity dia misoroka ny fahasimban'ny taovam-pananahana mandritra ny fandinihana tsy voafehy tsara. Ireo mpiara-miasa za-draharaha kokoa dia afaka manandrana manao ' ' mba hanokafana ny taovam-pananahana ho roa mba hanasarahana azy. Mba hanaovana izany, ampidirina moramora ny lafiny mitongilana amin'ny fanjaitra iray (toy ny fanjaitra fanaovana fanehoan-kevitra anaty hoditra), ka ahafahana manala ny taovam-pananahana nefa tsy tapahina tanteraka, mba hanasarahana ireo fitambarana gonocoxite-gonostyle (https://zenodo.org/records/18311158). Ity teknika ity dia manamora ny fijerena ny velaran'ny taova anatiny ary ahafahana mahita tsara kokoa ihany koa ireo parametra sy ny fonony parameraly, izay tsy mifanindry intsony. Ho an'ny fametahana amin'ny sisiny, izay matetika miteraka fifanindrihan'ny taova, dia tsy maintsy hazavaina tanteraka ny santionany.

4.3.2. Mokafihitra vavy

Anaty ny taovam-pananahana ary ahitana spermathecae. Raha tsy misy fandidiana, dia tsy maintsy jerena amin'ny alalan'ny hoditra ivelany izy ireo amin'ny alalan'ny fametahana ny kibo amin'ny lafiny ventrala. Na inona na inona fitaovana fametahana nofidiana, amin'ny ankapobeny dia azo jerena tsara ny spermathecae, indrindra fa rehefa tsy malama sady tsy voadio loatra ny atiny. Etsy ankilany, mety ho sarotra ny fijerena ireo spermathecae malama sy manify rindrina amin'ny fitaovana mampihena ny fanehoana hazavana. Ankoatra izany, tena ilaina ny fandinihana ny fototry ny lalan-drano-pananahana mba hamantarana azy amin'ny fomba manokana, toy ny amin'ireo karazana ao amin'ny zana-tsokajy *Larroussius* [35, 37, 38], izay mpitatitra lehibe ny *Leishmania infantum* any amin'ny Tany Tranainy. Raha tsy misy io fandinihana io, dia tsy azo atao ny mamantatra ireo santionany. Mba handresena ireo fahasahiranana ireo, dia tsy maintsy esorina ao amin'ny kibo ny fitambaran'ny furca genitalis sy ny spermathecae (https://zenodo.org/records/18311106). Sarotra jerena amin'ny ankapobeny ny spermathecae mandritra ny fandinihana ny biby, saingy somary mora hita kosa ny furca genitalis. Koa satria misokatra ao amin'ny genital furca ny lalan-drano mankany amin'ny spermatheca, ny fanalana mitokana an'io farany io dia matetika manala mitokana ihany koa ny spermathecae. Raha toa ka tapaka tsy nahy ny spermathecae mandritra ny dingana, dia tsy very izy ireo ary mbola azo jerena ao anatin'ny hoditry ny kibo (Sary 4).

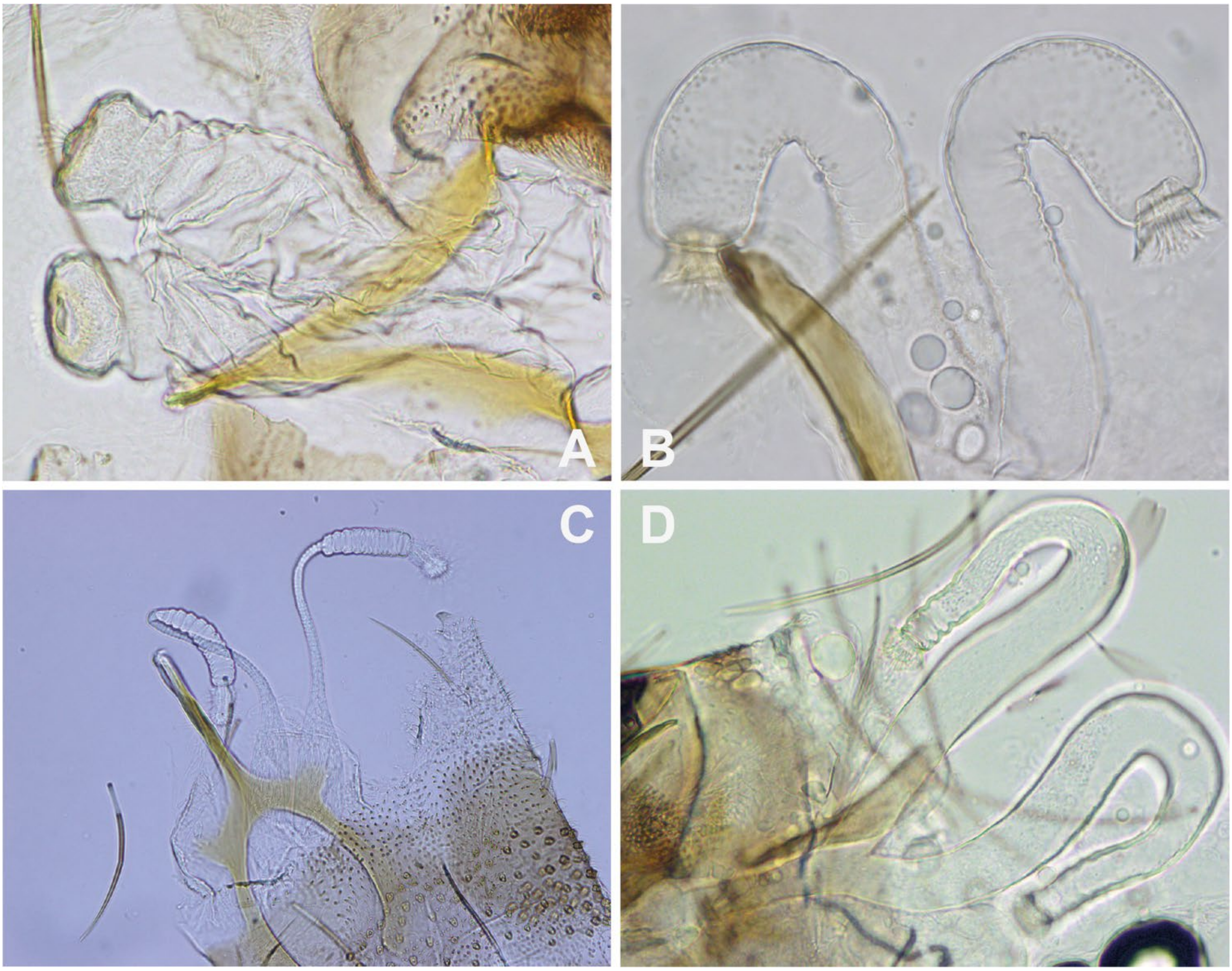

Sary 4. Spermathecae voatetika sy napetraka tao anaty ranon'i Marc-André avy amin'ny santionany vao. A: *Idiophlebotomus longiforceps* (DRP Laos); B: *Sergentomyia minuta* (Frantsa); C: *Phlebotomus ariasi* (Frantsa); D: *Sergentomyia anodontis* (DRP Laos).

4.4. Fandinihana ny taovam-pandevonan-kanina mba hanokanana ny *Leishmania*

Tena ilaina ny fandinihana ny taovam-pandevonan-kanina mba hahitana sy hanokanana ny *Leishmania* ao amin'ny mokafihitra vavy. Azo atao na eny an-toerana na ao amin'ny laboratoara ity fomba fiasa ity mba hanombanana ny fahaizan'ilay bibikely mampita ny aretina.

Soso-kevitra ny miasa amin'ireo vavy vao novonoina. Tokony hosasana amin'ny rano na ranon-tsira misy savony malefaka ny vavy mba hanalana ireo volom-biby mihoatra. Ity dingana ity dia mitazona ny fepetra tsy misy mikraoba amin'ny fanokanana ny *Leishmania* sady miaro ireo toetra ara-batana ilaina amin'ny famantarana azy. Mba hahitana sy hanokanana *ny Leishmania*, dia tsy maintsy esorina am-pitandremana ny tsinay afovoany ary apetraka ao anaty tsiranoka iray monja misy rano masira ara-batana tsy misy otrikaretina (0.9% NaCl). Aorian'ny fandinihana ireo parasy mivezivezy eo ambanin'ny mikroskaopy optika (fanalehibeazana atolotra: ~200×), dia afindràna ao anaty tontolo fambolena izy ireo amin'ny alalan'ny tsindrona insulinina na pipeta madinika (ho an'ny antsipiriany misimisy kokoa, jereo ny fizarana 4.4.3).

Tokony hapetrahana mivantana ao anaty ranon'i Marc-André ny loha sy ny taovam-pananahana mba hanazavana azy ireo. Zava-dehibe: Tsy tokony hifandray amin'ny *Leishmania* na amin'ny fomba inona na inona ny ranon'i Marc-André, na mivantana na an-kolaka amin'ny alalan'ny fitaovana na fanjaitra, satria mahafaty ireo parasy izany.

Ny fandinihana ny vavy dia azo atao na amin'ny solaitrabe iray monja na amin'ny solaitrabe roa misaraka; samy manana ny tombony sy ny fetra ireo safidy roa ireo (Sary 5; https://zenodo.org/records/18311154).

4.4.1. Fomba fanao amin'ny solaitrabe roa

Ny safidy voalohany dia ny miasa amin'ny slide roa misaraka: ny iray misy ranoka saline steril ho an'ny fakana ao amin'ny lalan-tsakafo, ary ny iray hafa ho an'ny fametrahana ny loha sy ny spermathecae ao anaty ranon'i Marc-André. Na izany aza, eny an-tsaha, mahazatra ny olona roa na telo no manao ny fanalana ary mandefa ny

Ity dia lahatsoratra Open Access nozaraina teo ambanin'ny fepetran'ny Creative Commons Attribution License (https://creativecommons.org/licenses/by/4.0), izay mamela ny fampiasana, ny fizarana ary ny famokarana tsy misy fetra amin'ny fitaovana rehetra, raha toa ka voatanisa araka ny tokony ho izy ny asa tany am-boalohany.

fanomanany amin'ny mpikaroka iray miandraikitra ny famantarana manokana sy ny fanombanana ny aretina *Leishmania* ao amin'ny lalan-tsakafo. Ny fitantanana slide roa dia mety hiteraka olana eo amin'ny fanarahana ny dian'ny santionany, ary indrindra indrindra, mety hahatonga ny fanamarinana ny fisian'ny aretina ho an'ny olona iray manokana ho sarotra rehefa misy tsinanoka voamarina fa mitondra ny aretina (https://zenodo.org/records/18311154).

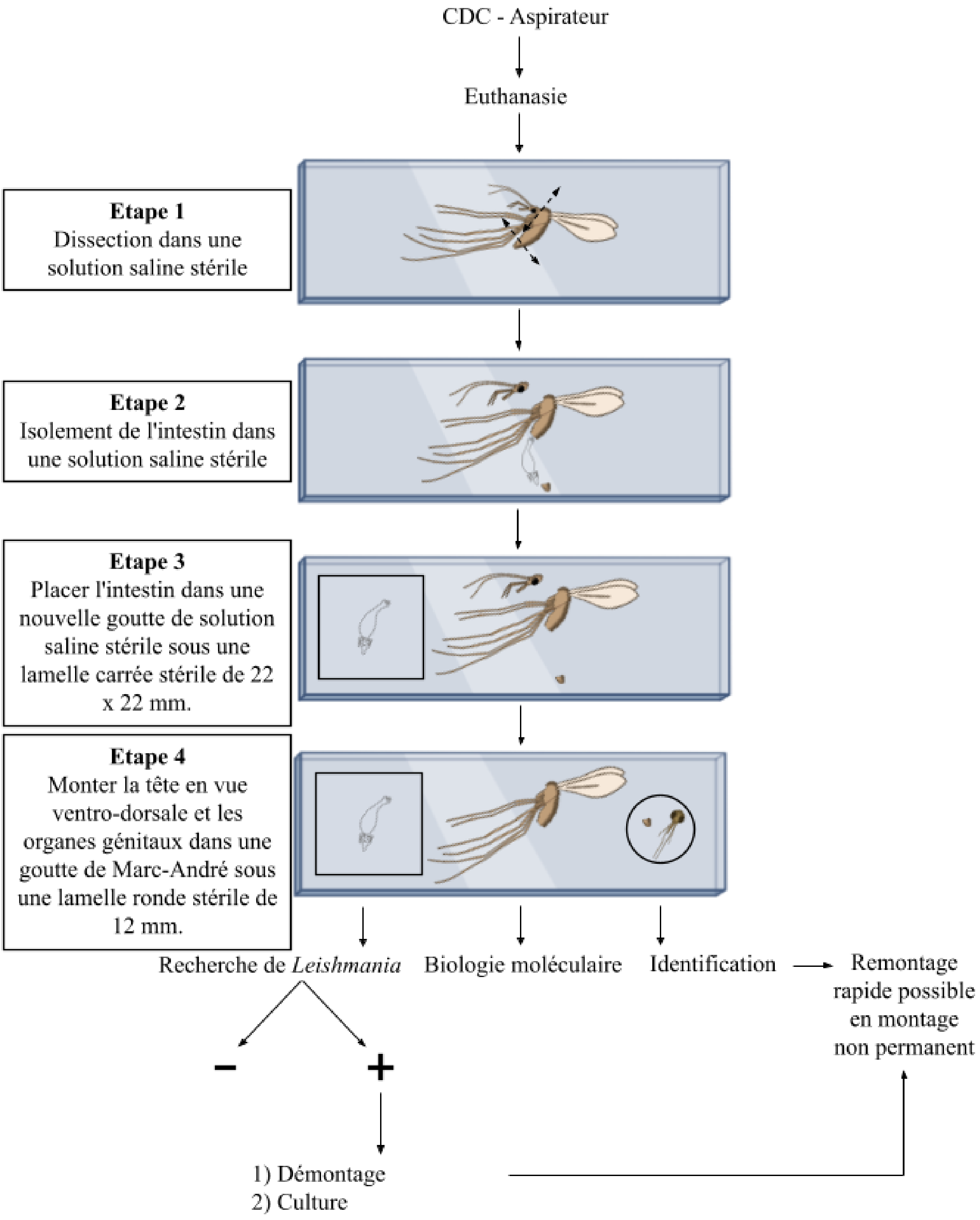

Sary 5: Fomba fanokanana *Leishmania*.

4.4.2. Fomba tokana

Ny fampiasana slide tokana dia miantoka ny fanaraha-maso tsara kokoa ny vokatra, saingy mitaky fitandremana maromaro. Mba hampitomboana ny sterility mandritra ity dingana ity, ny mpandraharaha dia tokony hanadio tsy tapaka ny tanany amin'ny fanadiovana tanana mifototra amin'ny alikaola. Ny lelany tsy misy hatsiaka sy ny tsipika toradroa (22 × 22 mm), fonosina amin'ny foil aluminium ary sterilized amin'ny hafanana maina (lafaoro karazana Poupinel), dia tokony hampiasaina, ary koa fanjaitra sterile ho an'ny fanesorana tsirairay (soso-kevitra: 25G Ø 0.5 mm × 16 mm). Ny sandfly dia apetraka ao anaty rano mitete amin'ny sira sterile eo afovoan'ny slide. Tapaka ny lohany, raha toa kosa ka misy incision eo anelanelan'ny tergites sy sternites an-kibo faha-6 sy faha-7 nefa tsy manapaka ny lalan-kanina (azo atao ny incision anterior kokoa raha toa ka andrasana ny spermathecae lava be). Ny tratra dia tsy mihetsika amin'ny fanjaitra, ary ny ampahany farany amin'ny kibo dia sintonina moramora amin'ny fanjaitra iray hafa mba hanesorana ny lalan-kanina. Raha tsy mahomby izany, dia azo atao ny manakana ny faran'ny kibo amin'ny fanjaitra ary misintona ny lalan-kanina amin'ny alàlan'ny tapany aloha. Raha tsy mahomby ihany koa io ezaka io, dia tokony hoesorina ny lalan-kanina amin'ny alàlan'ny fanesorana ny sisa tavela manodidina azy araka izay azo atao. Raha vantany vao nesorina ny lalan-kanina, dia tsy maintsy sarahina amin'ny alalan'ny fanapahana ny lalan-kanina ny ampahany farany amin'ny kibo. Ny lalan-kanina dia apetraka ao anaty rano vaovao amin'ny sira sterile eo amin'ny lafiny iray amin'ny slide, avy eo dia rakotra malefaka amin'ny fonony sterile. Ny loha sy ny kibo farany dia afindra amin'ny rano kely an'i Marc-André napetraka eo amin'ny faran'ny lelany, mitandrina mba tsy hifandray amin'i *Leishmania*. Ny loha dia mifantoka tsara (occipital foramen miakatra), ary ny spermathecae dia mitoka-monina amin'ny firaisana ara-nofo araka ny voalaza etsy ambony, avy eo rakotra lamellae boribory kely (Ø 12 mm, tsy tokony hafangaro amin'ny lamellae toradroa sterile). Ny sisa amin'ny fatin'ny fasika sy ny elatra dia mijanona ao amin'ny sira mitete eo afovoan'ny slide (https://zenodo.org/records/18311154). Raha misy valiny tsara, na ao anatin'ny fanadihadiana taxonomika, ny thorax sy ny kibo dia azo tehirizina ho an'ny fandalinana molekiola na proteomika, ary ny elatra dia azo apetraka amin'ny fitaovana aqueous. Mba hiantohana ny fitehirizana ny fametahana, ny ranon-javatra Marc-André tafahoatra dia azo soloina amin'ny fitaovana mivaingana toy ny siligaoma chloral (= Hoyer) na fitaovana mifototra amin'ny alikaola polyvinyl.

Ny horonan-tsary amin'ny antsipiriany mampiseho ireo fomba fiasa ireo dia misy (fanesorana ny lalan-kanina fasika: https://zenodo.org/records/18303014 sy ny fanesorana ny taova fihary rora: https://zenodo.org/records/18302850), noho izany dia tsy horesahina amin'ny antsipiriany eto izy ireo.

4.4.3. Ny fitokana-monina sy ny famelomana *Leishmania* amin'ny fandevonan-kanina amin'ny sandflies

Ny fitokanana ny katsentsitra amin'ny fanesorana ny vehivavy voan'ny aretina dia fomba fiasa marefo mitaky teknika be dia be ary tsy maintsy atao mialoha amin'ny santionany tsy misy katsentsitra. Aorian'ny fanesorana ny lalan-kanina dia afindra amin'ny rano vaovao amin'ny sira sterile (0.9%) na ranoka Locke ho an'ny fanasana [4]. Azo tsaboina amin'ny fomba roa ny lalan-kanina voatetika: i) dinihina amin'ny mikraoskaopy maivana mba handinihana ny dingana samihafa amin' ny promastigotes *Leishmania* sy ny toerana misy azy ireo, mifantoka manokana amin'ny valva stomodeal; ii) misokatra mba hanamorana ny famoahana ny promastigotes sy ny kolontsaina faobe [4]. Koa satria tsy fahita firy ny fahitana ireo mokafihitra voan'ny aretina any an-tsaha, ny fotoam-piofanana henjana dia mampitombo be ny vintana amin'ny fitokana-monina mahomby.
Raha hita ao amin'ny lalan-kanina ny *Leishmania*, dia tokony hampiasaina ny fanjaitra vaovao ary ampiana sira kely manodidina ny lamella amin'ny alàlan'ny hetsika capillary mba hamoahana azy ireo. Ny lalan-kanina dia tsy maintsy rovitra moramora sy haingana mba hamoahana ireo katsentsitra ao amin'ny ranoka sira. Amin'ny fampiasana micropipette 100 µL na syringe, ny katsentsitra dia angonina ary inoculated ao anaty kolontsaina voamarika tsara.

Ny kolontsaina *in vitro* an'ny *Leishmania promastigotes* : ny katsentsitra mitoka-monina dia voatazona amin'ny takelaka agar SNB-9 na amin'ny fitaovana matanjaka avy amin'ny Novy, McNeal, Nicolle (NNN) [16], rakotra ny mpanelanelana alpha-MEM sterile [16, 65] na M199, izay samy ampiana amin'ny 10% sterile, heat-inactivated fetal calf serum (SCF), 1% vitamina BME, 2% urine olombelona sterile (sterilized amin'ny filtration amin'ny 0.2 µm Filtropur® S syringe), ary 250 µg / mL amikacine (na 50 µg / mL gentamicin, na fifangaroan'ny antibiotika sy asidra amine (L-glutamine 200 mM, penicillin 10,000 U, streptomycin 10 mg / mL)) [47]. Aorian'ny telo andro, raha tsy misy ny fandotoana, ny kolontsaina dia mihantona amin'ny fitaovana mangatsiaka mety ary avy eo voatahiry amin'ny -80 ° C mandritra ny 1-2 taona na amin'ny azota ranon-javatra amin'ny -196 ° C ho an'ny fitehirizana maharitra sy ny fampiasana fanandramana manaraka [7].

4.5. Ny taova fihary rora

Ny fanesorana ny fihary rora amin'ny mokafihitra dia teknika fototra ho an'ny fandalinana ny fifandraisana vector-pathogen, indrindra ho an'ny fahitana ny arboviruses toy ny Phleboviruses (ohatra, ny virus Toscana) [44, 75]. Noho ny haben'ny fasika kely dia kely, ity fomba fiasa ity dia mitaky fametrahana mazava tsara eo ambanin'ny loupe binocular, amin'ny fampiasana forceps tsara na fanjaitra

microdissection, mba hanakanana ny fihary marefo nefa tsy manapaka na mandoto azy ireo (https://zenodo.org/records/18302850) [51, 61]. Ny fitehirizana ny tsy fivadihan'ny fihary dia tena ilaina mba hiantohana ny fahamendrehan'ny famakafakana molekiola manaraka. Raha vantany vao nalaina ny fihary dia azo homogenized sy dinihina amin'ny alàlan'ny RT-PCR, qPCR, na immunological assays mba hamantarana ny RNA virosy na ny antigen [12]. Ny fisian'ny viriosy ao amin'ny fihary rora, ary tsy ao amin'ny gastrointestinal tract na hemocoele ihany, dia manamafy fa ny pathogen dia nahavita ny vanim-potoana incubation extrinsic ary azo ampitaina amin'ny alàlan'ny sakafo ra [71].

Ny fizotran'ny fanesorana dia mitaky ara-teknika noho ny haben'ny fihary rora, ary mitaky traikefa be dia be mba hisorohana ny fahasimban'ny santionany [1, 51]. Ankoatr'izay, ny enta-mavesatra virosy dia mety ho ambany, mitaky ny fampiasana fomba fanaraha-maso tena saro-pady toy ny PCR na ny fizotran'ny fizotran'ny avo lenta [54]. Ny loza ateraky ny fandotoana koa dia manamafy ny filàna teknika aseptika hentitra. Ankoatra ny fanerena ara-teknika, ny antony biolojika dia misy fiantraikany amin'ny fahombiazan'ny fitadiavana: ny fahaiza-manaon'ny vector dia miovaova amin'ny karazana fasika, ary ny tahan'ny aretina dia miovaova arakaraka ny toe-javatra ara-tontolo iainana sy ara-potoana [33, 61].

Ny fahitana ny viriosy ao amin'ny fihary rora dia manome fampahalalana manan-danja momba ny loza ateraky ny fifindran'ny aretina ary mamela ny fanaraha-maso sy ny fanaraha-maso ny fepetra [15]. Ohatra, ny famantarana ny viriosy Toscana amin'ny mokafihitra any amin'ny faritra endemika dia nandray anjara tamin'ny famolavolana protokol diagnostika sy tolo-kevitra momba ny fahasalamam-bahoaka [18]. Ankoatr'izay, ny fandalinana ny fifandraisana misy eo amin'ny viriosy sy ny rora fasika dia mety hanambara tanjona vaovao amin'ny famolavolana vaksiny na paikady fitsaboana mikendry ny hanakanana ny fifindran'ny [15, 18].

Ny fihary rora amin'ny mokafihitra dia azo ampiasaina ihany koa ho loharanon'ny antigen mba handrefesana, amin'ny alàlan'ny fomba immunological, tsara kokoa amin'ny ELISA, ny antibody amin'ny rora sandfly. Ity fomba ity dia ahafahana manombatombana ny fipoahan'ny mpampiantrano amin'ny kaikitry ny fasika ary manohana ny fanombanana ny fahombiazan'ny fomba fanaraha-maso vector [25], ary koa ny mety hisian'ny fifindran'ny *Leishmania* [40].

4.6. Famantarana ny sakafo rà

Ny vehivavy mitoka-monina mandritra ny fisamborana dia tokony hotapahina amin'ny fampiasana fitaovana tokana mba hisorohana ny fandotoana. Ny kibony dia tokony hodinihina eo ambanin'ny loupe binocular mba hanombanana ny dingana fandevonan-kanina amin'ny sakafon'ny ra. Soso-kevitra ny hifidy mokafihitra vavy voky rà mena, mena volontsôkôlà na mena maizina tsy misy mariky ny fananganana atody. Ny faran'ny kibo, anisan'izany ny spermatheca, dia tsy maintsy esorina mba hahafahana mamantatra ny vavy aorian'ny fihenan'ny toetra. Ny ankamaroan'ny kibo (tsy misy spermatheca) dia apetraka ao anaty fantsona Eppendorf® ary voatahiry amin'ny -20 ° C mandra-pahatongan'ny famakafakana taty aoriana. Ny mari-pamantarana ara-jenetika matetika ampiasaina amin'ny famantarana ny sakafo ra, toy ny PNOC [5, 30, 50], CytB [67] na COI [13], dia miorina tsara ary voafaritra betsaka ao amin'ny literatiora; noho izany dia tsy ho antsipiriany bebe kokoa ao amin'ity lahatsoratra ity (Sary 6). Na izany aza, ny famantarana ny mpampiantrano ra dia azo tratrarina amin'ny alàlan'ny sarintany peptide amin'ny alàlan'ny MALDI-ToF [31]. Naseho tamin'ny fanandramana fa ity teknika ity dia mamela ny famantarana ny mpampiantrano mandritra ny fotoana lava kokoa aorian'ny fihinanana ra; Noho izany dia fomba iray azo isafidianana izany, indrindra ho an'ny famakafakana ny vehivavy mihodina miaraka amin'ny dingana mandroso kokoa amin'ny fandevonan-kanina. Ny santionany dia tokony hotehirizina amin'ny -20 ° C na -4 ° C, fa ny vokatra tsara dia azo avy amin'ny santionany voatahiry amin'ny hafanan'ny efitrano mandritra ny fotoana fohy. Ny kibon'ny vehivavy iray dia tokony hoesorina amin'ny sisa amin'ny vatana fotoana fohy alohan'ny fanadihadiana ary avy eo homogenized amin'ny rano voadio. Ny sisa tavela amin'ny vatan'ny fasika dia mbola azo ampiasaina amin'ny famakafakana morphological sy molekiola bebe kokoa. Aorian'ny fanesorana ny aliquot amin'ny homogenate natao ho an'ny sarintany peptide amin'ny alàlan'ny MALDI-ToF, ny sisa amin'ny santionany dia azo ampiasaina amin'ny fitrandrahana ADN mba hanamafisana ny famantarana ny mpampiantrano ra sy / na hitady ny fisian'ny *Leishmania*. Ny fotoana fanomanana sy famakafakana santionany amin'ny ankapobeny dia fohy raha oharina amin'ny teknika molekiola mifototra amin'ny ADN.

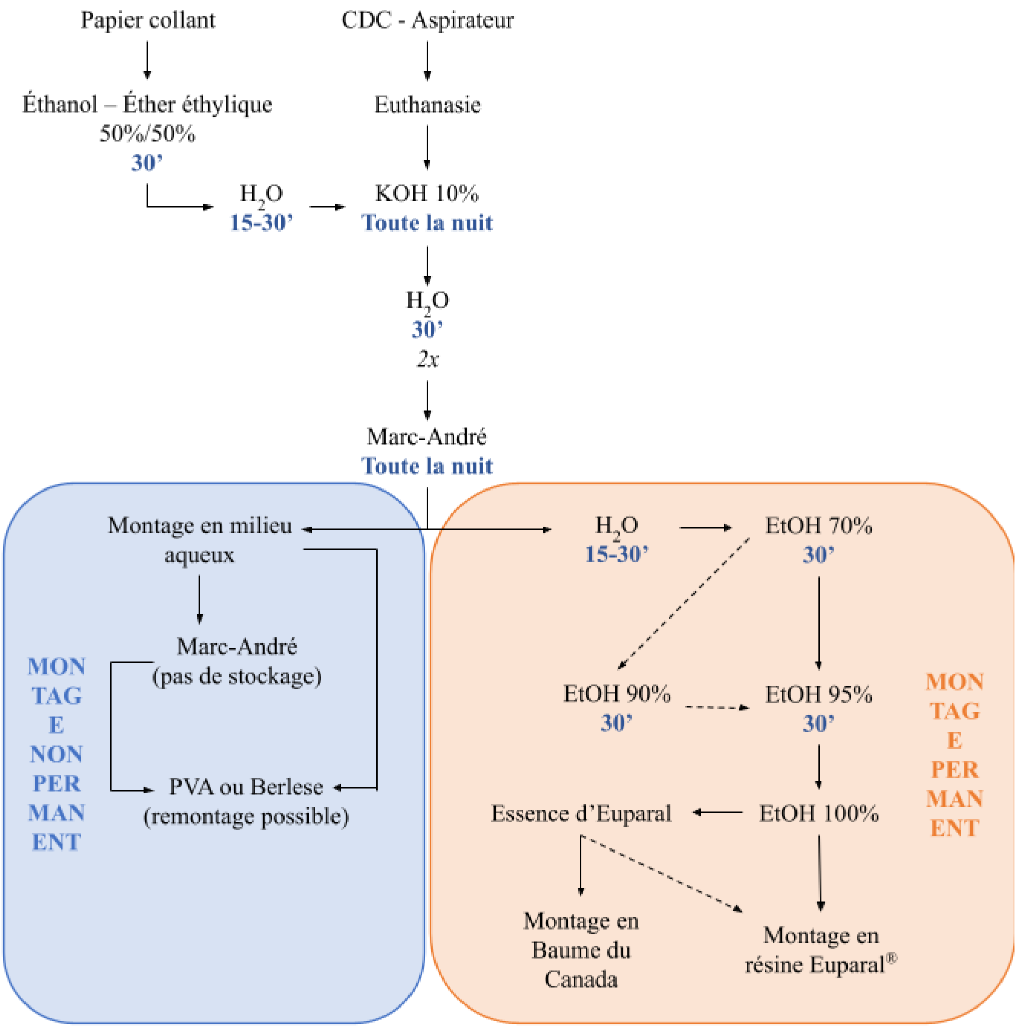

Sary 6: Fikarakarana ny mokafihitra ho an'ny fampiharana amin'ny molekiola, proteômika ary / na biolojia virolozika.

Ity dia lahatsoratra Open Access nozaraina teo ambanin'ny fepetran'ny Creative Commons Attribution License (https://creativecommons.org/licenses/by/4.0), izay mamela ny fampiasana, ny fizarana ary ny famokarana tsy misy fetra amin'ny fitaovana rehetra, raha toa ka voatanisa araka ny tokony ho izy ny asa tany am-boalohany.

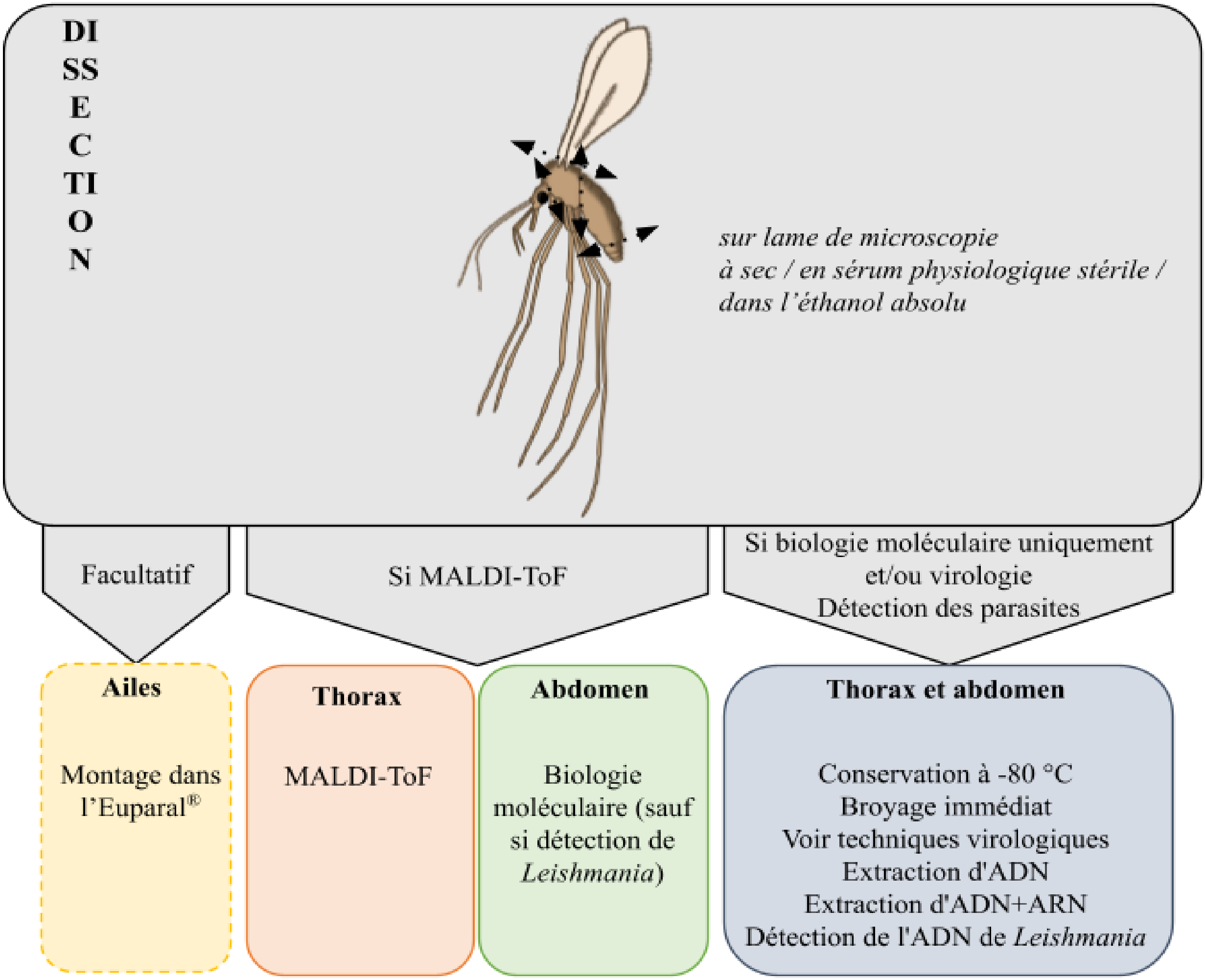

Sary 7. Fomba mahazatra amin'ny fitsaboana ny mokafihitra.

Tabilao 2: Firafitry ny famantarana simika ampiasaina.

**Potassium hydroxide 10%**<br>Potasioma Hydroxide 10g<br>Rano distillée *qsp* 100 mL	**Asidra Fuchsin 1% amin'ny rano distillation**<br>Asidra fuchsin (vovoka) 1 g<br>Rano distillée 99 mL
**Chloral Gum Mounting Media (Hoyer Medium)**<br>Rano distillée 50 mL<br>Chloral hydrate 200 g<br>Siligaoma arabo 50 g<br>Glycerol 20 mL	**Ranoka Marc-André naharo amin'ny fuchsin asidra**<br>Marc-André Solution 10mL<br>1% fuchsin 50 µL
**Ny ranoka nataon'i Marc-André**<br>Chloral hydrate 40 g<br>Asidra acetic glacial 30 mL<br>Rano distillée 30 mL	**Milieu Enecê**<br>Rosin fotsy madio 22 g<br>Siligaoma kopal mety levona amin'ny alikaola 12 g<br>Ethanol tanteraka 20 mL<br>Camphor 10 g<br>Turpentine 10 mL<br>Eucalyptol 26 mL

Ity dia lahatsoratra Open Access nozaraina teo ambanin'ny fepetran'ny Creative Commons Attribution License (https://creativecommons.org/licenses/by/4.0), izay mamela ny fampiasana, ny fizarana ary ny famokarana tsy misy fetra amin'ny fitaovana rehetra, raha toa ka voatanisa araka ny tokony ho izy ny asa tany am-boalohany.

5. Fanodinana ny santionany ho an'ny fandalinana morphological (Sary 3, 6, 7 ary 8; Annexe 1, 2, 3 ary 4)

Ity fizarana ity dia manolotra ny fitsipika ankapobeny amin'ny fanomanana ny sandfly ho an'ny fandalinana morphological manokana, ary avy eo mampifanaraka azy ireo amin'ny fampiharana mihoatra ny morphology. Na izany aza, ny fahatakarana an'io fomba fiasa io dia tena ilaina, satria mamela ny fomba fiasa hanitsy amin'ny karazana santionany manokana raha ilaina.

Ny fitsaboana dia mifototra amin'ny fifandimbiasana ny emptying sy ny famenoana dingana natao tamin'ny fampiasana Pasteur pipettes fitaovana amin'ny malefaka fingotra takamoa. Ny fampiasana kaontenera fitaratra miaraka amin'ny ambany boribory dia tena soso-kevitra, satria manamora ireo fanodinkodinana ireo. Satria ny fitaratra dia tsy misy dikany amin'ny fanajana ny reagents rehetra ampiasaina, dia miantoka ny fampifanarahana simika tsara. Mba hamerana ny fivoahan'ny reagents, ny fitoeran-javatra dia tsy maintsy omena sarony ary tsy ho feno mihitsy, izay hisorohana ny fihoaram-pefy rehefa manokatra na mikatona, ary koa ny fametrahana vovoka amin'ny santionany. Ny reagents ilaina amin'ny dingana fanamafisana sy fitsaboana dia aseho ao amin'ny Table 2.

5.1. Fanazavana

Alohan'ny hanomanana ny mokafihitra amin'ny fivoriambe maharitra, dia tsy maintsy hazavaina amin'ny alàlan'ny maceration izy ireo amin'ny alàlan'ny fomba mety sy ny fanamafisana (ohatra, ranoka asidra acetic 10% na ranoka Marc-André misy chloral hydrate, akora voafehy any amin' ny firenena maro), mba hanazavana azy ireo. Ity dingana fanamafisana ity dia ahafahana manala ny vatana, ny tavy, ny tsiambaratelo ary ny savoka, ka mahatonga ny santionany ho mangarahara ary manamora ny fandinihana ny firafitry ny exoskeleton (ohatra, ny fampidirana volo), ny endri-javatra ambonin'ny tany (ohatra, ny fandotoana), ary ny rafitra anatiny hita amin'ny alàlan'ny integument (ohatra, spermatheca).

Ny fizotran'ny fanamafisana dingana roa, izay mitaky ny fampiasana fototra matanjaka (toy ny potassium hydroxide), arahin'ny asidra malemy (toy ny asidra acetic ao amin'ny ranoka Marc-André), dia manompo tanjona biochemical miavaka [74]. Ny fototra dia miteraka ny fahasimban'ny tavy malefaka, anisan'izany ny proteinina, ny lipida ary ny hozatra, amin'ny alàlan'ny saponification sy ny denaturation proteinina, raha mamela ny exoskeleton chitinous tsy misy dikany, ka miantoka ny famakiana tsara ny rafitra. Ny asidra malemy dia manafoana ny sisa tavela amin'ny alkaline, manakana ny fahasimban'ny hafa, ary mandray anjara amin'ny fanamafisana ny chitin mba hanatsarana ny mangarahara [74]. Mariho fa ny fanasana roa misesy mandritra ny 15 minitra amin'ny rano distillée dia mety ho ampy ihany koa mba hanafoanana ny fototra. Ity fitsaboana manaraka ity dia mampifangaro ny fanesorana ny tavy mahomby amin'ny fiarovana malefaka ny rafitra, miantoka ny fahamendrehan'ny santionany tsara indrindra ho an'ny fandinihana mikroskopika.

Ny fandroana roa mandritra ny 20 minitra tsirairay amin'ny rano distillée dia atolotra alohan'ny handrosoana amin'ny dingana manaraka.

Ity dia lahatsoratra Open Access nozaraina teo ambanin'ny fepetran'ny Creative Commons Attribution License (https://creativecommons.org/licenses/by/4.0), izay mamela ny fampiasana, ny fizarana ary ny famokarana tsy misy fetra amin'ny fitaovana rehetra, raha toa ka voatanisa araka ny tokony ho izy ny asa tany am-boalohany.

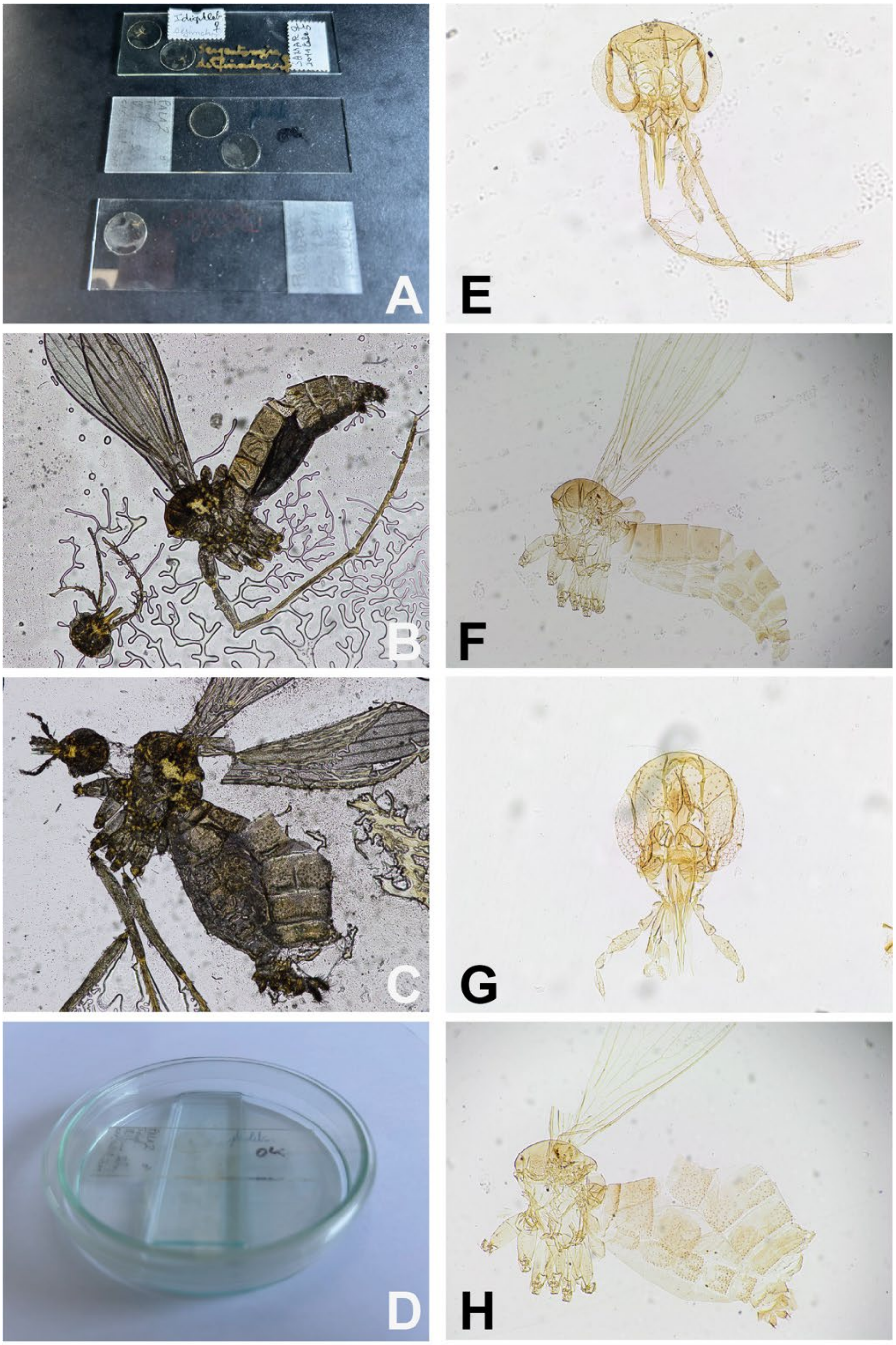

Sary 8. Famerenana ny slide. A: lelany simba sy maina napetraka tamin'ny ranoka Hoyer; B: fomba fijery mikroskopika amin'ny mokafihitra maina; C: fomba fijery mikroskopika amin'ny fasika simba iray hafa; D: efitrano mando misy lelany maina; E: loha sy F: vatan'ny santionany B taorian'ny nanamboarana azy indray tao amin'ny Euparal®; G: loha sy H: vatan'ny santionany C taorian'ny nanamboarana azy indray tao amin'ny Euparal®.

5.1.1. Lysis malefaka (Sary 8)

Sodium hydroxide (NaOH) na potassium hydroxide (KOH) dia matetika ampiasaina ho toy ny simika maceration, amin'ny fifantohana sy ny faharetan'ny isan-karazany arakaraka ny habeny sy ny fahalemen'ny santionany. Ny teknika mahazatra sy mahomby indrindra dia ny fanamafisana ny tavy malefaka amin'ny alàlan'ny fandrobana fasika amin'ny fototra matanjaka (10% KOH na NaOH) mandritra ny alina. Ny fifantohana dia azo ampitomboina mba hampihenana ny faharetan'ny fitsaboana (ohatra KOH amin'ny 20% mandritra ny ora 6),

Ity dia lahatsoratra Open Access nozaraina teo ambanin'ny fepetran'ny Creative Commons Attribution License (https://creativecommons.org/licenses/by/4.0), izay mamela ny fampiasana, ny fizarana ary ny famokarana tsy misy fetra amin'ny fitaovana rehetra, raha toa ka voatanisa araka ny tokony ho izy ny asa tany am-boalohany.

mety amin'ny alàlan'ny fampifangaroana amin'ny hafanana amin'ny 37 ° C.

5.1.2. Fanazavana miaraka amin'ny loto na tsy misy loto

Ity dingana ity dia arahin'ny fitsaboana maivana, matetika mifototra amin'ny fitambaran'ny asidra acetic sy chloral hydrate (ohatra ny ranoka Marc-André). Aorian'ny fanamafisana, ny santionany dia tokony hosasana tsara ao anaty fandroana roa misesy farafahakeliny amin'ny rano 20 minitra tsirairay, mba hanesorana ny sisa simika.

Ny ranoka nataon'i Marc-André dia mpandraharaha manify ampiasaina betsaka amin'ny fanomanana ny mokafihitra. Ny fahombiazany dia mifototra amin'ny fahafahany manazava ny santionany ary mametra ny fahasimbana amin'ny rafitra marefo toy ny elatra sy ny antena.

Ny ranoka dia tokony ho voaomana vao haingana na tehirizina ao anaty fitoeran-javatra voaisy tombo-kase mafy mba hisorohana ny etona na ny fahasimbana. Ny fampiasana ny ranoka nataon'i Marc-André dia tena mahasoa indrindra rehefa ampiarahina amin'ny teknika fanazavana na fandotoana mba hanatsarana ny fahitana ny endri-tsoratra morphological sasany. Ny antsipiriany momba ny fananganana sy ny fanomanana azy dia aseho ao amin'ny Annexe 2.

Ho an'ny santionany tena translucent, dia mety ilaina ny fandotoana mba hanatsarana ny fahitana alohan'ny hametrahana. Maro ny loko misy, ny tsirairay dia mikendry ireo singa simika manokana ao amin'ny vatana. Zava-dehibe ny mifidy loko izay mifanaraka amin'ny santionany sy ny fitaovana voafidy. Ity fomba fiasa fototra ity dia azo ampifanarahana, ohatra amin'ny fampidirana fuchsin asidra 0.1% ao amin'ny ranoka Marc-André mba hahazoana tasy. Ankoatr'izay, ny santionany voatahiry ao anaty rano ary natao ho an'ny fampitam-baovao amin'ny hazo malefaka dia mitaky ny dehydration mialoha (jereo ny fizarana 5.2), satria ny ankamaroan'ny haino aman-jery voajanahary na synthetic dia tsy mifanaraka amin'ny rano. New (1974) dia nitatitra fa ny loko sasany dia mety hihena amin'ny haino aman-jery sasany [53]. Ohatra, ny fuchsin marikivy, izay matetika ampiasaina amin'ny balsama Kanada, dia azo amboarina ao amin'ny Euparal® ihany koa. Na izany aza, ny santionany voaloto amin'ny fuchsin asidra dia mora miovaova tsikelikely, indrindra rehefa mijanona ny sisa tavela amin'ny menaka jirofino, ampiasaina ho toy ny mpandraharaha fanamafisana farany. Ny santionany voatahiry ao anaty menaka jirofo dia mety hampiseho fiovana miharihary ao anatin'ny andro vitsivitsy.

5.2. Tsy fahampian-drano

Ny tsy fahampian-drano dia tratra amin'ny alàlan'ny famindrana tsikelikely ny santionany amin'ny andiana etanol mitombo ny fifantohana: 50%, 70%, 80%, 90% na 95%, avy eo 100%, isaky ny fandroana maharitra farafahakeliny 20 minitra. Satria mihena haingana ny etanol, dia tsy maintsy asiana tombo-kase mafy ireo fitoeran-javatra mandritra ny fanodinana. Raha vantany vao maina tanteraka ny santionany, dia azo tapahina mandritra ny andro vitsivitsy ny fizotrany amin'ny alàlan'ny famelana azy ao amin'ny Euparal® essence, tsara kokoa noho ny menaka jirofino. Ny creosote Beech, izay nampiasaina betsaka tamin'ity tanjona ity, dia voarara tanteraka ankehitriny noho ny poizina.

Ny fizotran'ny dehydration dia tsy maintsy miantoka fa ny ranon-javatra ao amin'ny santionany dia mifanaraka amin'ny fitaovana fiakarana, mba hisorohana ny opacification, ny fianjeran'ny osmotika na ny deformation izay mety hahatonga ny santionany ho tsy mety amin'ny fandalinana taxonomika.

5.3. Fampidirana haino aman-jery

5.3.1. Fifantenana sy fampiharana ny fanomanana santionany

Ny fanelanelanana dia tokony hanana mari-pamantarana refractive akaiky araka izay azo atao amin'ny fitaratra, izany hoe eo amin'ny 1.5. Tsy maintsy tsy misy loko, mazava ary mijanona ho mangarahara tanteraka aorian'ny fanamainana sy rehefa mandeha ny fotoana. Tsy maintsy mifanaraka amin'ny loko ampiasaina izy io ary afaka miditra sy miparitaka manerana ny tavy amin'ny santionany. Tsy tokony ho maina haingana loatra izy io na hamorona zavona mandritra ny fivoriambe, ary tsy tokony hihena aorian'ny fametrahana. Ny fisafidianana ny fitaovana mety dia lafiny fototra amin'ny fanomanana santionany, satria tsy misy fitaovana mety tsara ho an'ny fampiharana rehetra. Ity safidy ity dia tsy maintsy vokatry ny fifanakalozana eo amin'ny lafin-javatra manan-danja maromaro:

- **Fananana optika**. Ny mari-pamantarana refractive amin'ny fitaovana dia hanome fifanoherana sy refraction ampy ho an'ny fandinihana ireo tarehin-tsoratra anatomika ilaina amin'ny famantarana ny taxonomic na ny famaritana morphological, toy ny spermatheca, ascoids, Newstead sensillae, nify cibarial mitsangana, ary nify pharyngeal. Ny fahitana ireo rafitra ireo dia miankina mivantana amin'ny fananana optika amin'ny fitaovana fiasa.
- **Fiarovana**. Ho an'ny karazana santionany na fitaovana ho an'ny fanangonana maharitra, ny tontolo iainana dia tsy maintsy manome fitoniana sy faharetana maharitra. Etsy ankilany, ho an'ny fandalinana lisitra na fanadihadiana epidemiolojika, izay tsy dia manakiana loatra ny fiarovana maharitra, dia mety ho ampy ny haino aman-jery vonjimaika na semi-maharitra.

5.3.2. Fepetra takiana amin'ny fametrahana haino aman-jery

Matetika ny manam-pahaizana manokana dia mamolavola teknika fivoriambe tsirairay ary indraindray sarotra izay mifanaraka amin'ny tanjona fikarohana manokana. Na izany aza, matetika ireo fomba ireo dia tsy

miraharaha ny lafiny toy ny kalitaon'ny tahiry, ny fifaninanana eo amin'ny teknika, ny fitsipika, ny fanamorana ny fikirakirana na ny fiarovana maharitra. Ity tsy fahampian'ny fenitra ity dia manasarotra ny fampidirana ny angon-drakitra tsirairay sy ny ezaka fiarovana maharitra.

Ny fampiharana ara-tsiansa dia mametraka fepetra miavaka amin'ny fampidirana haino aman-jery. Ny taxonomists dia matetika mametraka santionany iray manontolo ary mankasitraka ny haino aman-jery izay afaka macerate maivana ny taova anatiny mba hanatsarana ny fahitana ny rafitra cuticular. Ny mari-pamantarana refractive dia tsy maintsy tsy mitovy amin'ny an'ny santionany sy ny fitaratra mba hampitomboana ny fahazavan'ny optika. Ny haino aman-jery ara-barotra dia matetika namboarina tamin'ny index refractive akaiky ny fitaratra mba hampihenana ny fiparitahan'ny hazavana sy ny fanaparitahana ny hazavana amin'ny alàlan'ny rafitra lamel-mounting medium-lamella. Na izany aza, amin'ny mikroskopia mamirapiratra, ny fifanoherana voajanahary amin'ny santionany tsy misy pentina dia azo ovaina amin'ny alàlan'ny fisafidianana an-tsitrapo izay manana tondro refractive hafa noho ny santionany, ka manatsara ny fahitana azy mifandraika amin'ny ambadika.

5.3.3. Karazana haino aman-jery fametrahana (tabilao 3 sy 4)

Tabilao 3.: Firafitry ara-tsimika voafantina.

Fametrahana fitaovana	Solvent	Pre-polymer (s) na polymer mety	Fanamarihana
Hoyer = siligaoma chloral	glycerol, rano	Ny fitambarana arabo goma	Macerant agent: chloral hydrate
CMCP-9 (= carboxymethyl-cellulose phenol)	rano (CMCP-9: 51-60%)	alikaola polyvinyl hydrolyzed tanteraka (CMCP-9: 0-5%)	CMCP-9: Low Viscosity: High Viscosity
DMHF (dimethyl-hydantoin-formaldehyde)	Rano	N, N'-dimethylol-dimethyl-hydantoin (di-methylol DMH) Ether / methylene-bridged oligomersDMH-cross-linked formaldehyde polymer network	
Canada Balsam	xylene; ampahany miovaova singa ny balsama (Δ^3-kerene, levopimaric asidra, limonene, myrcene, palustric asidra, β-phellandrene, α-pinene, β-pinene)	Balsam (abienol, asidra abietika, asidra isopimarika, asidra sandaracopimaric)	neutralization: potassium carbonate; resin avy amin'ny *Abies balsamea* (Linnaeus, 1758)
Euparal®	eucalyptol, paraldehyde; Singa miovaova amin'ny ampahany amin'ny siligaoma sandaraque (limonene, α-pinene, β-pinene)	Sandaraque gum compounds (mampita asidra, manool, asidra polycommunication, sandaracopimaric asidra, 12-acetoxy-sandaracopimaric asidra, sugiol, torulosic asidra, torulosol, totarol)	mpandraharaha maivana: methyl salicylate; loko Euparal® maitso: sira varahina (abietinate varahina); resina sandaraque avy amin'ny *Tetraclinis articulata* (Vahl, 1791)
Enecê	alikaola ethyl, miaraka amin'ny camphor, eucalyptol ary turpentine	Fitambarana ny siligaoma copal sy rosin (resin)	

Tabilao 4 Ny tombony sy ny tsy fahampian'ny haino aman-jery voafantina ho an'ny fanomanana mikroskopika, mifototra amin'ny fandinihana tsy navoaka nataon'ny mpanoratra maro [52].

Anarana	Tombontsoa	Ny tsy fahampiana
* Balsam Kanada	Tontolo iainana tena maharitra, miaraka amin'ny androm-piainana mihoatra ny 150 taona.<br>Ny lelany dia azo apetraka aorian'ny fanazavana amin'ny jirofo na phenol.	Izy io dia misy singa misy poizina ary tokony hokarakaraina ao anaty setroka.<br>Mitaky dehydration tanteraka, lava ary mitaky dehydration.<br>Ny tsy fahampian-drano amin'ny ethanol sy ny famindrana amin'ny alàlan'ny xylene na jirofo dia mety hahatonga ny taxa sasany ho marefo; Ny solvents hafa (isopropanol, n-butanol, Cellosolve™, 1,4-dioxane, Histoclear, terpineol) dia afaka mametra ny fahasahiranana.<br>Ny santionany dia mety hivadika mainty raha toa ka soloina phenol ny xylene na raha mbola misy sisa tavela amin'ny KOH.<br>Ny mari-pamantarana avo lenta dia afaka manafina rafitra tsy misy loko.<br>Mety haharitra taona maro vao maina tanteraka raha tsy misy griddle.<br>Ny mavo sy ny maizina tsikelikely, indrindra aorian'ny fanazavana amin'ny jirofon'ny jirofony.<br>Ny loko sasany dia novaina; Ny loko cationic dia mety hiova loko raha toa ka lasa asidra ny medium rehefa mandeha ny fotoana.
DMHF (dimethyl-hydantoin-formaldehyde)	Mangarahara avo lenta.<br>Tondro refractive tsara.<br>Fahitana tsara ny rafitra.<br>Fahamarinan-toerana tsara amin'ny fanomanana.<br>Mifanaraka amin'ny teknika fandokoana maro.<br>Fiarovana santionany tsara.<br>Tsara adhesion eo amin'ny lelany sy ny slat.	Mety ho mavo rehefa mandeha ny fotoana.<br>Mety hanova ny loko sasany.<br>Tsy mety amin'ny tasy saro-pady formaldehyde.<br>Matetika ny fiforonan'ny rivotra miboiboika, maina miadana.<br>Fitaovana fiakarana mora tohina amin'ny hamandoana.<br>Sarotra ny miverina.<br>Ny formaldehyde dia misy poizina, mahasosotra ary carcinogenic.
* Euparal (mangarahara)	Tontolo iainana maharitra, miaraka amin'ny androm-piainana mihoatra ny 50 taona.<br>Azo apetraka mivantana amin'ny ethanol 80% (tolo-kevitry ny mpanamboatra).<br>Tsy hanafina ireo rafitra tsy misy loko ary tsy ho mavo na ho tapaka rehefa mandeha ny fotoana.<br>Ny mari-pamantarana refractive dia mety kokoa noho ny balsama Kanada ho an'ny Diptera.<br>Fitondran-tena tena tsara miaraka amin'ny santionany matevina (fihenan-javatra ambany, bubbles vitsivitsy).<br>Mijanona ho 95% levona amin'ny etanol, mamela ny paompy na dia efa an-taonany maro aza.	Ahitana singa misy poizina mitaky setroka hood.<br>Ny tsy fahampian-drano amin'ny ethanol sy ny famindrana amin'ny alàlan'ny lasantsy Euparal® dia mety hampihena ny taxa sasany; Ny Isopropanol dia mety hametra an'io vokatra io.

Tabilao 4 (tohiny)

Anarana	Tombontsoa	Ny tsy fahampiana
Tsiranoka Hoyer	Ny santionany dia azo apetraka velona na mivantana avy amin'ny rano, ethanol na formaldehydes.<br>Ny maceration dia manome kalitao tsara ho an'ny cuticle.<br>Ny mari-pamantarana refractive mahasoa, izay azo ampitomboina amin'ny alàlan'ny fandotoana ioda.<br>Ny asidra acetic dia afaka mampiroborobo ny fanitarana ny appendages arthropoda.<br>Ny santionany sasany dia afaka mijanona ho azo ampiasaina mandritra ny 40-60 taona.<br>Mora levona amin'ny rano, ka mora ny manala sy mamerina azy.	Ny santionan'ny zavamaniry marefo dia mety hianjera raha tsy ampiana tsikelikely ny mpanelanelana.<br>Ny fananganana lavaka sy kristaly, indraindray ao anatin'ny latsaky ny 10 taona.<br>Ny mety hisian'ny maceration be loatra arakaraka ny fifantohan'ny chloral hydrate sy ny faharetan'ny fipoahana.<br>Ny fisarahana ny singa sy ny fisehoan'ny granulations tsara rehefa mandeha ny fotoana.<br>Nisy ny tranga fanamafisana ny tontolo iainana.
CMCP-9<br>(= carboxymethyl-cellulose phenol)	Azo atao ny fametrahana mivantana avy amin'ny rano, ethanol, glycerol na formaldehyde-misy ranoka.<br>Ny taova anatiny dia azo atao macerated mba hanamorana ny fandinihana ankapobeny na ny fanomanana.	Mety hivadika sy hihamaizina rehefa mandeha ny fotoana.<br>Maceration indraindray tafahoatra.<br>Ny santionany matevina dia mihena ary mamorona banga raha tsy voafatotra tsara ny slide.<br>Tsy mety amin'ny santionany miloko na calcified.<br>Fotoana fanamainana lava kokoa noho ny haino aman-jery mifototra amin'ny CMC.
Eukitt™	Tontolo iainana maharitra (≥ 30 taona).<br>Mifanaraka amin'ny solvents maro (acetone, benzene, chloroform, dioxane, ether, isopropanol, methyl benzoate, terpineol, toluene, xylene).<br>Fanamainana haingana, pH somary asidra.<br>Tsy mihamaizina rehefa mandeha ny fotoana.<br>Mifanaraka amin'ny loko maro (ohatra fucshin, hematoxylin, methyl green, methyl violet, methylene blue).<br>Ny mety ho famerenana indray aorian'ny taona maro amin'ny alàlan'ny fanitrihana maharitra amin'ny xylene.	Ahitana singa misy poizina mitaky setroka hood.<br>Mitaky dehydration feno sy maharitra.<br>Tsy mety amin'ny santionany matevina (shrinkage, bubbles entona).<br>Ny slats dia mety hivoaka rehefa mandeha ny fotoana raha toa ka tsy voadio tsara na tsy voaisy tombo-kase ny fitaratra.<br>Ny polymerization indraindray dia tsy feno manodidina ny kofehy manankarena collagen.
Enecê	Tontolo iainana tena maharitra (≥ 50 taona).<br>Tsy ho maizina rehefa mandeha ny fotoana.<br>Mora kokoa ny miovaova, mamela ny fanesorana amin'ny toerana misy ny rafitra mandritra ny fotoana mety.<br>Vidiny ambany.	Mitaky déshydratation feno sy maharitra.<br>Ny tsy fahampian-drano amin'ny ethanol sy ny famindrana amin'ny alàlan'ny jirofo dia mety hampihena ny santionany sasany.<br>Ny fanazavana dia mitohy tsikelikely, izay mety hahatonga azy ho sarotra ny mandinika ireo rafitra tena tsara (sensilla, ascoids, volo tsotra).

Ao amin'ny mikroskopia, ny index refractive (IR) amin'ny fitaovana iray dia mamaritra ny fomba

fandehanan'ny hazavana amin'ny alàlan'ny slide, medium ary specimen. Rehefa manakaiky ny fitaratra amin'ny coverslip (≈1.515) ny IR, ny hazavana dia mandalo amin'ny homogeneously manontolo, izay mampihena ny fiparitahana sy ny fanodinkodinana optika, ary noho izany dia manatsara ny famahana sy ny fahitana ny rafitra tsara. Mifanohitra amin'izany, ny tsy fahampian'ny tondro refractive dia mety hitarika ho amin'ny fanjavozavozavo, halos, na fanafenana rafitra tsy misy loko. Ny fisafidianana ny fitaovana mety dia zava-dehibe amin'ny fanatsarana ny fifanoherana, ny maranitra ary ny kalitaon'ny sary amin'ny ankapobeny amin'ny santionany iray, noho ny tondro refractive miavaka amin'ny haino aman-jery samihafa.

Ny mari-pamantarana refractive amin'ny fitaovana mitaingina dia misy fiatraikany lehibe amin'ny fahitana ny rafitra tsara rehefa manomana fasika ho an'ny fametrahana slide. Ny rafitra marefo sy malemy amin'ny sclerotinized amin'ny mokafihitra, toy ny rafitra cibarial, spermatheca, fizarana antennal, ary ny elatra venation, dia mety ho sarotra ny mandinika amin'ny haino aman-jery avo lenta.

Ao amin'ny mokafihitra, ny haino aman-jery ampiasaina matetika dia ahitana ny siligaoma chloral ho toy ny fitaovana aqueous, ary ny balsama Kanada sy ny resina Enecê - Nelson Cerqueira (NC) ho toy ny haino aman-jery mifototra amin'ny solvent. Rawlins [60] dia nanasokajy ny haino aman-jery ho sokajy roa: (1) haino aman-jery maharitra, izay mihamafy rehefa mandeha ny fotoana ary mety amin'ny fitehirizana maharitra, ary (2) haino aman-jery semi-maharitra, izay tsy mihamafy tanteraka ary matetika ampiasaina amin'ny tanjona vonjimaika.

Ny haino aman-jery dia mety ho ranon-javatra, mifototra amin'ny siligaoma na resinous, mety levona amin'ny rano, alikaola, na solvents hafa (ohatra toluene na xylene) (Table 3). Aorian'ny fampiharana dia tsy maintsy arovana amin'ny fiantraikan'ny atmosfera izy ireo amin'ny alàlan'ny famehezana tsy mety levona. Mba hanavahana mazava tsara ireo karazana haino aman-jery samihafa, dia azo ampiasaina ireto fanasokajiana manaraka ireto:

a. Haino aman-jery aqueous. Ireo haino aman-jery ireo dia levona mora foana ao anaty rano, ka mahatonga azy ireo ho mety amin'ny fametrahana vonjimaika na semi-maharitra. Amin'ny ankapobeny dia mora ny mitantana azy ireo, saingy mety mitaky famehezana mba hisorohana ny hamandoan'ny atmosfera (izany hoe, haino aman-jery siligaoma sy alikaola polyvinyl), indrindra amin'ny toetr'andro tropikaly mando.

b. Tontolo iainana voafetra amin'ny fandeferana amin'ny rano. Ireo tontolo iainana ireo dia tsy dia voakasiky ny rano, saingy mbola mitaky fiarovana amin'ny hamandoana be loatra. Izy ireo dia manolotra fahamarinan-toerana maharitra tsara kokoa noho ny haino aman-jery mety levona amin'ny rano ary matetika ampiasaina amin'ny fametrahana semi-maharitra.

c. Haino aman-jery mety levona amin'ny hydrocarbon. Ireo haino aman-jery ireo dia levona amin'ny solvents organika toy ny xylene, toluene na essenecê (enecê solvent). Izy ireo dia natao ho an'ny tendrombohitra maharitra ary manolotra fahamarinan-toerana maharitra. Izy ireo dia mahatohitra ny hamandoana sy ny fahasimbana, ka mahatonga azy ireo ho mety indrindra amin'ny tanjona tahiry (izany hoe, balsama Kanada tsy miandany).

Raha fintinina, ny haino aman-jery mety levona amin'ny rano dia mety indrindra amin'ny fametrahana vonjimaika na toe-javatra mitaky fanesorana santionany mora; Ny tontolo iainana mahazaka rano dia mety amin'ny tendrombohitra semi-maharitra mitaky faharetana antonony; Farany, ny haino aman-jery mety levona amin'ny hydrocarbon dia tokony ho tsara kokoa amin'ny fametrahana maharitra natao ho an'ny fiarovana ny tahiry sy ny fitehirizana maharitra.

5.3.4. Famaritana ny haino aman-jery napetraka (tabilao 3 sy 4)

Tontolo iainana ho an'ny fandinihana vonjimaika

Chloral gum = ranon-javatra / medium / ranoka Hoyer (IR = 1.48). Ny ranon'i Marc-André no fitaovana tsara indrindra ho an'ny fandinihana fohy (ora vitsivitsy, na kely kokoa aza raha toa ka voatahiry ao anaty efitrano mando ny slide) amin'ny spermathecaeca, indrindra amin'ny fakana sary (Sary 4) na fanaovana sary. Ny fitehirizana maharitra ny spermathecae hita dia mitaky ny fanodinana amin'ny fitaovana aqueous mamela ny fitehirizana antonony. Ny dehydration arahin'ny resin mounting dia azo atao ara-teknika, saingy tsy atolotra noho ny loza mety hitranga amin'ny fahaverezan'ny fitaovana. Ny siligaoma chloral sy ny medium Hoyer dia heverina ho mitovy hevitra. Ity fitaovana ity dia matetika ampiasaina amin'ny fandinihana ny taova anatiny noho ny fifaninanana amin'ny rano, ny fahatsoran'ny fanomanana, ny fampiharana haingana ary ny mari-pamantarana refractive tsara amin'ny fandinihana ireo rafitra marefo toy ny spermatheca. Na izany aza, ny siligaoma chloral dia manana fatiantoka lehibe rehefa tsy voaomana tsara na voatahiry eo ambanin'ny toe-javatra mifehy ny hamandoana, anisan'izany ny crystallization, ny fiovaovan'ny loko ary ny fahaverezan'ny viscosity. Ny fametahana ny fonony dia tsy manitsy ireo lesoka ireo, satria ny fitaovana dia mety hiaina fiovana lehibe (indraindray saika mainty) noho ny fifandraisana amin'ny fitaovana fametahana, indrindra rehefa ampiasaina ny Euparal®.

Ny haino aman-jery an'i Hoyer dia noheverina hatry ny ela fa manolotra ny toetra optika tsara indrindra ho an'ny fandalinana ny mokafihitra ary nampiasaina betsaka ho an'ity tanjona ity. Misy famolavolana maromaro mifandraika akaiky, mifototra amin'ny siligaoma arabo, glycerol ary chloral hydrate, ny sasany amin'izy ireo dia diso na diso voalaza ao amin'ny literatiora [74].

Na dia mety amin'ny fandinihana spermatheca amin'ny fasika aza ny mpanelanelana an'i Hoyer, dia tsy mety amin'ny fitehirizana maharitra. Izy io dia mety tsara

amin'ny fandinihana fohy, anisan'izany ny sary, ny sary, na ny sary. Amin'ny ankapobeny, ny haino aman-jery aqueous dia mety amin'ny fametrahana vonjimaika saingy tsy manome antoka ny fahamarinan-toerana maharitra. Mifanohitra amin'izany, ny haino aman-jery resinous dia manome faharetana tsara, matetika mandritra ny taonjato maromaro, saingy afaka manafina ny antsipiriany tsara momba ny spermathecae noho ny fahaverezan'ny refringence.

Rehefa mandeha ny fotoana, ny medium an'i Hoyer dia mihena amin'ny alàlan'ny tsy fahampian-drano (Sary 8), ka miteraka fiforonan'ny kristaly kely, fotsy, manjavozavo amin'ny chloral hydrate. Na izany aza, ny santionany dia azo averina avy amin'ny kristaly kristaly, miaraka amin'ny cuticle mijanona simika, na dia mety hitranga aza ny fahasimbana ara-batana noho ny fitomboan'ny kristaly. Amin'ny toe-javatra sasany, ny slides crystallized dia azo averina amin'ny alàlan'ny famerenana ny fitaovana amin'ny tontolo mafana sy mando, miaraka amin'ny thymol mba hisorohana ny fitomboan'ny holatra. Na izany aza, ny santionany dia azo alaina avy amin'ny siligaoma chloral amin'ny alàlan'ny fanitrihana amin'ny rano, tsy misy rano amin'ny asidra acetic glacial, ary avy eo averina ao amin'ny balsama Canada.

DMHF (dimethyl-hydantoin formaldehyde) (IR = 1.48)
Ity fitaovana aqueous [72] ity dia manana fahombiazana optika tena tsara, azo ampitahaina amin'ny an'ny ranoka Berlese, nefa mora ampiasaina. Tsy toy ny farany, ny DMHF dia tsy mainty na kristaly rehefa mandeha ny fotoana. Izy io dia mety tsara amin'ny fametrahana fasika sy Psychodidae hafa.

CMCP (camphor-mono-chlorophenol) (IR = 1.41)
Izy io dia fitaovana mifototra amin'ny glycerol mety levona amin'ny rano, ampiasaina amin'ny famokarana fiomanana mangarahara sy maharitra amin'ny santionany marefo, ao anatin'izany ny mokafihitra. Ny iray amin'ireo tombony lehibe indrindra dia ny fahaizana mametraka santionany mivantana avy amin'ny rano na etanol. Izy io dia manamaivana sy manazava haingana ny bibikely, manalefaka ny cuticle ary manamora ny fametrahana marina ny faritra anatomika, indrindra ny fiparitahan'ny elatra na ny fanesorana ny firaisana ara-nofo. Na dia voalaza aza fa mamela ny fiarovana maharitra io tontolo iainana io, dia mbola tsy voarakitra tsara ny faharetan'io fiarovana io. Ny fetra lehibe indrindra dia ny fisian'ny phenol, zavatra misy poizina sy manelingelina izay mitaky fepetra hentitra amin'ny fikarakarana.

Media ho an'ny fametrahana maharitra

Canada Balsam (IR = 1.52-1.54) Canada balsam dia nofaritana ho toy ny fitaovana mety ho an'ny mikroskopia maivana nalefa tamin'ny taona 1830 nataon'i Andrew Pritchard. Izy io dia mijanona ho iray amin'ireo tontolo iainana be mpampiasa indrindra noho ny toetra voaporofo amin'ny tahiry, miaraka amin'ny fampiasana mahomby mihoatra ny 150 taona. Tsy toy ny haino aman-jery miorina amin'i Hoyer, tsy kristaly na mitroka ny hamandoana amin'ny atmosfera izy io. Etsy ankilany, manana autofluorescence voamarika izy io, izay mety ho fatiantoka ho an'ny teknika mikroskopia sasany [60]. Ny fampiasana solvents tsy misy poizina ho fanoloana ny xylene dia mampihena ny loza ara-pahasalamana, saingy mety hitarika ho amin'ny fanamainana miadana kokoa sy ny fanamainana aloha kokoa amin'ny mpanelanelana.

Euparal® (IR = 1.48); Euparal® dia safidy be mpampiasa amin'ny Balsam Kanada ho an'ny fametrahana maharitra, manolotra fitoniana maharitra sy tondro refractive azo ampitahaina. Ny fampiasana azy dia mametraka fanerena roa lehibe: (1) ny santionany dia tsy maintsy dehydrated alohan'ny fivoriambe farany, amin'ny ankapobeny amin'ny alàlan'ny fiovana avy amin'ny ethanol 95% mankany amin'ny ethanol tanteraka; (2) Ity dingana tsy fahampian-drano ity dia manitatra ny faharetan'ny fitsaboana. Rehefa tsy azo atao ny dehydration amin'ny solvents organika, ny santionany voatahiry ao amin'ny ethanol tanteraka dia azo afindra amin'ny ranoka mpanelanelana misy fifangaroan'ny solika Euparal sy Euparal®, alohan'ny fivoriambe farany.

Enecê (IR = 1,467). Enecê dia fitaovana fampitakarana resinous ampiasaina indrindra ho an'ny bibikely kely ary miparitaka be any Brezila. Izy io dia ahitana rosin sy siligaoma kopal levona amin'ny alikaola, camphor, turpentine ary eucalyptol. Cerqueira [11] dia namaritra ny Enecê ho toy ny safidy hafa amin'ny balsama Kanada ho an'ny fitomboan'ny olitra, ny exuviae tsy matotra, ary na dia ny olon-dehibe moka aza. Nanomboka teo dia nampiasaina betsaka ho an'ny mokafihitra izy io. Ny Enecê dia safidy mahasoa ara-toekarena ho an'ny mountings maharitra, manolotra fahamarinan-toerana maharitra sy fotoana fametrahana ampy mba hamela ny dissection sy ny fametrahana mazava tsara ny rafitra morphological.

5.4. Fanomanana sy fanamainana ny lelany

Ny fanamainana tsara ny slide nitaingina dia tena ilaina mba hiantohana ny fahamarinan-toerana sy ny fiarovana maharitra ny santionany. Ny takelaka dia tsy maintsy maina tanteraka alohan'ny hametrahana azy ao amin'ny fanangonana. Ny fiomanana vita amin'ny haino aman-jery maharitra dia tokony ho maina mitsivalana mandritra ny 2 ka hatramin'ny 3 herinandro, raha toa kosa ireo mampiasa haino aman-jery semi-maharitra amin'ny ankapobeny dia mitaky 1 ka hatramin'ny 2 herinandro. Ny fanamainana dia tokony hatao ao anaty lafaoro napetraka amin'ny mari-pana mifanaraka amin'ny tontolo iainana ampiasaina, misoroka ny hafanana tafahoatra izay mety hanimba ny santionany. Ny mari-pana 30 ° C ka hatramin'ny 37 ° C dia soso-kevitra. Ity dingana ity dia tena ilaina mba hisorohana ny fikorontanan'ny lelany, ny fahasimban'ny santionany, na ny tsy fandriampahalemana antonony mandritra ny fitehirizana.

Ny fitaovana ampiasaina dia tokony holazaina foana eo amin'ny mari-pamantarana ny lelany. Raha azo atao, ny fomba fahandro afovoany, ny anaran'ny mpanamboatra ary ny daty fanomanana dia tokony holazaina ihany koa. Ny lelany dia matetika nomanina voalohany ho toy ny fametrahana vonjimaika; Na izany aza, raha miova ny satan'ny santionany (ohatra, ny fanendrena ho ampahany amin'ny andian-dahatsoratra), dia atolotra ny famerenana amin'ny tontolo iainana maharitra mba hiantohana ny fiarovana azy amin'ny fandalinana taxonomic amin'ny ho avy.

5.5. Teknika fametrahana hafa: fametrahana birao

Ny fametrahana karatra dia teknika ampiasaina amin'ny vondrona bibikely maromaro, izay misy santionany na mipetaka mivantana amin'ny sarintany entomolojika na mipetaka amin'ny endriny. Noho ny haben'ny fasika kely sy ny filàna mandinika ny rafitra anatiny mazava ho an'ny famantarana (jereo ny fizarana 5), ity fomba ity dia tsy mety tanteraka amin'ny fametrahana fasika.

5.6. Famerenana ny santionany simba

Ho an'ny santionany tsy fahita firy na avo lenta, dia atolotra ny fomba fiasa roa, mifanaraka amin'ny protokol aseho ao amin'ny horonantsary azo alaina amin'ny: https://zenodo.org/records/18315029.

1) Rehydration tsy misy disassembly. Ny slide dia averina amin'ny rano voalohany mba hahafahana mandinika mialoha. Ny fijoroana izay afaka mandray mikraoskaopy maromaro dia apetraka ao anaty lovia Petri, ary ny slide hotsaboina dia apetraka eo an-tampony. Ny milimetatra vitsivitsy amin'ny solvent dia ampiana mba hamoronana efitrano lena, hahazoana antoka fa ny slide dia tsy mifandray mivantana amin'ny ranon-javatra (Sary 8 D). Ny faharetan'ny rehydration dia miovaova amin'ny iray ka hatramin'ny andro maromaro arakaraka ny toetry ny santionany. Ilaina ny fanaraha-maso isan'andro sy ny faharetana. Raha vantany vao ampy ny rano dia azo apetraka ao anaty lafaoro mandritra ny ora vitsivitsy alohan'ny hijerena azy eo ambanin'ny mikraoskaopy, sary na sary.

2) Ho an'ny famerenana indray, ny lelany dia azo averina ao amin'ny efitrano mando mandritra ny ora vitsivitsy na mandritra ny alina. Ny fanesorana dia tsy maintsy atao eo ambanin'ny fanamafisana binocular. Amin'ny fampiasana fanjaitra madinika, dia esorina tsara ny lamella, mba hahazoana antoka fa tsy misy ampahany amin'ny mokafihitra mijanona (https://zenodo.org/records/18315029). Ny singa voatetika dia averina avy eo ary voasasa amin'ny rano ao anaty lavaka kely, toy ireo ampiasaina amin'ny fitrandrahana ADN / RNA manimba (jereo etsy ambany), alohan'ny tsy fahampian-drano sy ny fanodinana amin'ny fitaovana resinous. Rehefa manala ny lelany dia zava-dehibe ny mamantatra ny fitaovana fametrahana voalohany mba hisafidianana ny solvent mety. Ny rano dia ampiasaina amin'ny haino aman-jery aqueous, raha ny xylene kosa dia ilaina amin'ny haino aman-jery malefaka (ohatra, balsama Canada na Euparal®). Ny xylene dia tokony hokarakaraina amin'ny setroka sy miaraka amin'ny fitaovana fiarovana manokana, anisan'izany ny fiarovana amin'ny taovam-pisefoana.

Ny famerenana ny karazana santionany na fitaovana fanangonana dia tsy tokony hatao afa-tsy amin'ny fanekena mazava avy amin'ny mpikarakara sy / na ny andrim-panjakana mitazona ny fitaovana.

6. Famantarana ny santionany

6.1. Endrika ivelany

Ny famantarana ny mokafihitra dia mifototra indrindra amin'ny fandinihana ny toetrany ivelany, indrindra ny endriky ny thorax, ny elatra, ny taovam-pananahana, ny volo, ary koa ny fifandraisana ny refy ivelany sasany eo amin'ny rafitra samihafa. Ny mpikaroka dia mampiasa ny fanalahidin'ny taxonomika, ny fanangonana ary ny famaritana ny karazana tany am-boalohany mba hampitahana ny santionany nangonina amin'ny taxa fantatra. Ny toetra diagnostika manan-danja, toy ny fiaviana elatra sy ny morphology amin'ny lahy sy ny vavy, ny firafitry ny firaisana ara-nofo amin'ny lahy sy ny vavy, ary ny fanamboarana spermatheca amin'ny vehivavy, dia tena mampahafantatra ny famaritana manokana. Ny famantarana azo antoka dia matetika mitaky fandinihana mikroskopika amin'ny antsipiriany, matetika mampiasa mikraoskaopy optika ho an'ny fandinihana ny rafitra tsara (firaisana ara-nofo, spermatheca), na fitaratra fanamafisana binocular ho an'ny endri-tsoratra morphological ankapobeny kokoa.

Ny fandrosoana vao haingana amin'ny sary dia nanamora ny fampiasana sary nomerika ho an'ny famantarana ny sandfly. Ny sary avo lenta na sary nomerika amin'ny endri-tsoratra manan-danja dia azo ampitahaina amin'ny fitaovana fanovozan-kevitra na dinihina amin'ny alàlan'ny rafitra famantarana amin'ny solosaina, manatsara ny fahamarinana sy ny fahazoana ny taxonomy morphological.

6.2. Jeômetry ny elatra

Ny geometry elatra dia toetra manan-danja amin'ny famantarana sy ny fanasokajiana ireo karazana fasika samihafa. Ny elatry ny mokafihitra dia manana morphology mampiavaka azy, amin'ny ankapobeny lava sy tery, miaraka amin'ny venation mivoatra tsara (Sary 9 sy 10).

Ny fandaminana ny lalan-drà dia mamorona lamina miavaka izay mety miovaova eo amin'ny karazana sy ny karazana, ka manome toetra sarobidy ho an'ny famantarana. Ny fandalinana ny geometry elatra dia manome fampahalalana manan-danja ho an'ny tanjona taxonomika.

6.3. Môrfômetry jeômetrika elatra

Ny teknika samihafa, toy ny môrfômetry jeômetrika, dia ampiasaina handinihana sy hampitahana ny endrika sy ny haben'ny elatra eo amin'ny karazana fasika na mponina. Ny fandalinana ny geometry elatra koa dia manome fampahalalana momba ny fitondran-tena, ny safidin'ny toeram-ponenana ary ny fahaiza-manidina.

Ao amin'ny fomba môrfômetry jeômetrika, ny elatra dia voavaha tsara, mety ho loko, ary avy eo napetraka amin'ny lelany. Avy eo dia alaina sary eo ambanin'ny loupe binocular, nomerika ary iharan'ny fanadihadiana morphometric. Ity fomba fiasa ity dia voafaritra tsara ao amin'ny literatiora [6, 27, 42, 56, 57, 59], miaraka amin'ny tolo-kevitra hampiasa ny elatra havanana na havia ho an'ny taova mba hisorohana ny mety ho voka-dratsin'ny allometric [62].

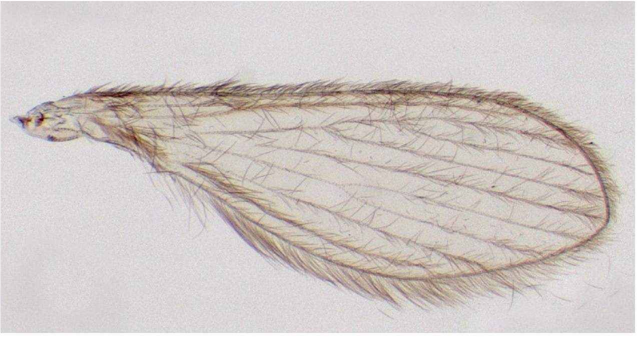

Sary 9: Elatra tsotra de *Trichophoromyia ininii.*

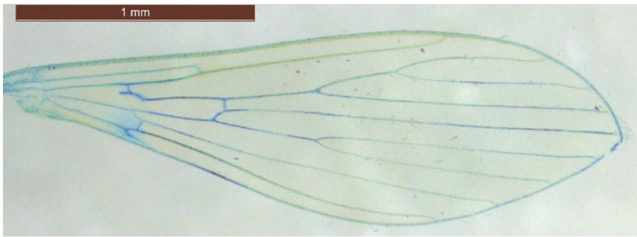

Sary 10: Elatra miloko an'i *Phlebotomus ariasi.*

Fanomanana elatra ho an'ny famakafakana morphometric geometrika

Ho an'ny fahitana tsara indrindra ny lalan-drà elatra, ny elatra dia tsy maintsy esorina amin'ny volony ary miloko tsara. Ho an'ny fiomanana, fenoy aloha ny kaopy kely amin'ny reagents ilaina (methylene blue, ethanol, rano ary xylene solon'nylena). Raiso ny elatra voatahiry ao amin'ny etanol 70% amin'ny hafanan'ny efitrano amin'ny alàlan'ny fanodinana ny fantsona Eppendorf ary manafoana azy eo ambonin'ny kaopy, avy eo manandratra ny elatra amin'ny alàlan'ny fanjaitra manify sy miolakolaka. Ampidiro vetivety ny elatra avy amin'ny etanol mankany amin'ny rano ary avy eo miverina amin'ny etanol mba hanesorana ny volo. Avy eo dia apetraho ao amin'ny methylene blue ny elatra mandritra ny 6 minitra, ary ataovy azo antoka fa mijanona ho miboiboika mandritra ny fandokoana. Angony amim-pitandremana ny elatra ary asitsoka ao anaty solon'ny xylene mandritra ny 2 minitra (eo amin'ny ampahatelon'ny fotoana fandokoana manga methylene). Ny fikapohana malefaka ny fanjaitra amin'ny rindrin'ny kaopy dia afaka manampy ny elatra hipetraka; Ampiasaina eto ny xylene mba hanamboarana ny loko. Farany, atsangano ny elatra ary apetraho eo amin'ny Euparal® kely eo amin'ny slide mikraoskaopy. Eo ambanin'ny fitaratra fanamafisana dia avelatra moramora ilay elatra ary apetraho amim-pitandremana ny takelaka. Ny sary dia tokony halaina haingana, alohan'ny handraisana tanteraka ny Euparal®, satria mety ilaina ny fanitsiana kely amin'ny toeran'ny elatra eo ambanin'ny slat mba hahatratrarana ny fampifanarahana tsara indrindra.

6.4. Teknikan'ny biolojia molekiola

Ankoatra ny fomba morphological, ny fomba molekiola dia nanjary tena ilaina amin'ny entomology, indrindra ho an'ny taxonomic, ny fototarazo ary ny phylogeny fandalinana, ary koa ny fahitana ny ADN / RNA pathogens sy ny famaritana ny niandohan'ny sakafo ra, ny fitondran-tenan'ny vector dia singa manan-danja amin'ny epidemiolojia [70]. Ny fizotran'ny ADN dia azo ampiasaina hanamafisana ny famantarana ny karazana na hanavahana ny karazana mifandray akaiky, manome fomba famantarana marina sy azo itokisana kokoa. Ankoatr'izay, ny teknika molekiola mandroso (PCR, ADN sequencing, NGS, sns) ary koa ny spectrometry MALDI-ToF dia miha-manan-danja amin'ny famantarana haingana sy marina ny karazana, ankoatry ny fomba morphological nentim-paharazana [46]. Na dia eo aza ireo fandrosoana ireo, ny famantarana morphological dia mijanona ho referansa amin'ny taxonomy ary mandrafitra ny fototry ny fandikana ny angon-drakitra molekiola.

6.4.1. Fitrandrahana asidra nokleika manimba

Ny fitrandrahana asidra nokleika dia dingana iraisana amin'ny fandalinana biolojika maro, ary fomba samihafa no novolavolaina mba hanasarahana ny ADN amin'ny fitaovana biolojika [48]. Maro ny fitaovana fitrandrahana ADN ara-barotra azo alaina mba hanamorana izany dingana izany.[14] Na izany aza, ny fomba mahazatra ampiasaina hanomanana ny arthropods ho an'ny famantarana morphological dia matetika manakana ny famakafakana molekiola, satria mety hanimba na hanimba ny toetra ara-batana manan-danja amin'ny santionany [10]. Ny ankamaroan'ny protokol fitrandrahana ADN ampiharina amin'ny tavy bibikely dia manimba ny natiora [43], izay mametraka olana manokana ho an'ny santionany kely, izay na dia ny santionany voafetra aza dia mety hanimba ny toetra morphological manan-danja [72]. Ny karazana sy ny satan'ny fiarovana ny santionany dia mitana andraikitra lehibe amin'ny fisafidianana ny fomba fitokana-monina ADN mety [29].

Ny filàna famantarana azo antoka ny mokafihitra, ny fahatakarana ny fihetsehan'ny mponina ary ny famerana ny fiantraikany tsy voafaritra dia nampiroborobo ny fampandrosoana ny fitaovana diagnostika molekiola [23]. Ny fomba fiasa molekiola dia matetika ampiasaina mba hamenoana ny fomba morphologe taxonomique amin'ny famantarana ny mokafihitra. Ohatra, ny fomba fiasa mahazatra amin'ny barcoding bibikely dia miankina amin'ny fitrandrahana ADN sy ny fizotran'ny ADN, ka miteraka fahaverezan'ny santionany tany am-boalohany. Noho izany dia ilaina maika ny mamolavola sy mitrandraka fomba fitrandrahana ADN tsy manimba izay mitahiry ny fitaovana biolojika sy ny fahamendrehan'ny morphological amin'ny santionany.

Maro ny fomba fitrandrahana asidra nokleika no ampiharina amin'ny mokafihitra. Ny habetsahana sy ny kalitaon'ny ADN takiana dia miankina amin'ny famakafakana molekiola ambany, miaraka amin'ny teknika samihafa izay manana fepetra samihafa amin'ny fahatsapana sy ny fahadiovana [9]. Ohatra, ny mason'ny mokafihitra dia fantatra fa afaka manakana ny fanamafisana ny PCR [69]. Ankoatra ny fitiliana ny pathogen, ny ADN sandfly dia matetika alaina ho an'ny tanjona manokana amin'ny famantarana. Azo ampiasaina ny fomba fitrandrahana samihafa, miaraka amin'ny vokatra sy kalitao samihafa arakaraka ny teknika. Ny protokol sasany natolotry ny mpanamboatra dia namboarin'ny mpikaroka ho an'ny moakafihitra [8], ahafahana mampitombo ny vokatra sy / na ny kalitaon'ny asidra nokleika nalaina [8, 9, 69], raha ny fampifanarahana hafa, izay novolavolaina tany am-boalohany ho an'ny vondrona arthropod hafa, dia azo ampiharina amin'ny mokafihitra ihany koa [58, 76]. Ny PCR famantarana mikendry sombintsombiny mitochondrial kely (COI na CytB) dia mifanaraka amin'ny fomba fitrandrahana izay miteraka fizarazarana ADN avo. Etsy ankilany, ny teknolojia NGS sasany, toy ny Oxford Nanopore na PacBio, dia mitaky ADN avo lenta. Ny fitrandrahana andry silika dia matetika mamokatra sombintsombin'ny ADN genomika hatramin'ny 60 kb, raha ny fitrandrahana phenol-chloroform kosa dia afaka miteraka sombintsombiny hatramin'ny 150 kb [77]. Ny tabilao 5 dia mamintina ny teknika fitrandrahana ADN samihafa avy amin'ny mokafihitra ary manondro raha toa ka nampiharina ny fampifanarahana ara-metodolojika ho an'ireo bibikely ireo. Ny vokatra dia tsy aseho satria miankina amin'ny haben'ny santionany sy ny fomba fanomanana azy ireo. Ny tsanganana "fanitsiana" dia manondro ny fampifanarahana ny protokol fitrandrahana ho an'ny mokafihitra na arthropods kely hafa.

Ny fisafidianana ny fomba fitrandrahana dia tokony handinika fepetra maromaro, toy ny isan'ny santionany, ny fotoana fitrandrahana ary ny teknika ampiasaina any ambany. Na dia mitaky ADN genomika avo lenta aza ny teknikan'ny NGS, ny fomba rehetra aseho eto dia mety amin'ny fampiharana PCR mahazatra.

Ankoatr'izay, fanadihadiana maro no nandinika ny fomba fitrandrahana ADN tsy manimba ampiharina amin'ny arthropods terestrialy kely, santionany amin'ny tranombakoka maina, ary arthropods malefaka [19, 26, 28, 55, 63].

6.4.2. Fitrandrahana asidra nokleika tsy manimba

Ny iray amin'ireo fanamby lehibe amin'ny famakafakana molekiolan'ny arthropods, ary indrindra ny mokafihitra, dia ny fitehirizana ny santionany ho an'ny fampidirana ao amin'ny fanangonana entomological. Ny ankamaroan'ny protokol fitrandrahana ADN dia mitaky ny maceration ny sela, manimba ny fiarovana ny santionany tany am-boalohany. Ny fomba fitrandrahana asidra nokleika tsy manimba dia natao hanesorana ny fitaovana ara-jenetika nefa tsy manimba ny santionany na manova ny fahavelomany na ny morphology. Ireo fomba ireo dia tena sarobidy rehefa mifandray amin'ny santionany tsy fahita firy na voafetra, toy ny mokafihitra, izay ny fitazonana ny tsy fivadihana ara-drafitra dia tena ilaina amin'ny tanjona taxonomic, morphological na diagnostika. Ny teknika mahazatra dia ny fomba fandroana tsy manimba, izay tsy mihetsika ny mokafihitra ary avy eo alentika moramora ao anaty buffer lysis misy proteinase K.

Ny teknikan'*ny mild-vectolysis* dia nampiharina tamim-pahombiazana tamin'ny mokafihitra, indrindra amin'ny karazana santionany [24]. Ity fomba ity dia mifototra amin'ny fampiasana kitapo tsanganana silika mahazatra (amin'ity tranga ity ny kitapo DNeasy Blood and Tissue, QIAGEN, Hilden, Alemana), miaraka amin'ny fampifanarahana mamela ny ADN ho azo nefa tsy manimba ny santionany. Ny dingana lysis novaina (habetsaky ny buffer lysis sy ny fampidirana dingana mangatsiaka) [17] dia mamela ny famoahana asidra nokleika raha mametra ny fahasimbana morphological [24]. Amin'ny mokafihitra, azo atao ihany koa ny mampiasa ny kitapo fitrandrahana ADN HotSHOT (Bento Bioworks Ltd, London, UK) [73], izay haingana sy tsy lafo, ary mamela ny fanodinana santionany haingana sy mahomby. Ny santionany entomological natao ho an'ny famantarana morphological dia azo sasana avy eo. Ireo izay tsaboina amin'ny kitapo DNeasy Blood and Tissue dia tokony hazavaina amin'ny alàlan'ny ranoka Marc-André, raha ireo izay tsaboina amin'ny kitapo HotSHOT kosa dia maivana ampy mba hipetraka amin'ny fitaovana misy rano, na tsara kokoa amin'ny resin aorian'ny tsy fahampian-drano, araka ny protocole amin'ny antsipiriany ato amin'ity lahatsoratra ity [73]. Ny fitaovana ara-jenetika nalaina dia azo ampiasaina amin'ny famakafakana ambany, toy ny PCR, mba hanamafisana ny marika fototarazo manokana. Ny fomba fitrandrahana asidra nokleika tsy manimba dia tena ilaina amin'ny fandalinana ny toetra ara-jenetika amin'ny mokafihitra, anisan'izany ny famantarana ireo pathogens mety ho entana. Amin'ny alàlan'ny fitehirizana ny tsy fivadihan'ny santionany, ireo fomba ireo dia manome fampahalalana sarobidy momba ny fototarazo raha mitahiry ny santionany ho an'ny fanadihadiana na fandalinana bebe kokoa.

6.5. MALDI-ToF Mass Spectrometry

Tabilao 5: Ny vidiny, ny fampiharana ary ny fampifanarahana ny protocoles ho an'ny fitrandrahana ADN genomika (gDNA) amin'nymokafihitra

Protocol	Coût	Fampiharana	Fampifanarahana ny protocole ho an'ny bibikely
Tsanganana silika	2,5 – 3,55 US$ [39]	PCR, NGS	[9]
Phenol-chloroform	0,24 US$ [69]	PCR, NGS	[9]
HotSHOT	< 0,01 US$ [69]	PCR	-
Salage	0,12 US$ [69]	PCR	-
Chelex	0,02 US$ [41]	PCR	[41, 76]

MALDI-ToF (Matrix-Assisted Laser Desorption / Ionization Time-of-Flight) dia teknika famakafakana mifototra amin'ny spectrometry faobe, natao hamantarana sy handinihana ny mombamomba ny proteinina manokana ("proteinina dian-tanana") amin'ny santionany biolojika. Ny MALDI-ToF dia ekena ho fitaovana lehibe amin'ny famantarana ny arthropods amin'ny maha-zava-dehibe ny fitsaboana sy ny veterinera. Ity teknika ity dia naseho fa mahomby amin'ny famantarana ny dingana samihafa amin'ny fivoaran'ny fasika, anisan'izany ny endrika tsy matotra ary koa ny sakafo rà amin'ny vehivavy mihodina, ary nampiasaina tamim-pahombiazana mba hanavahana ny karazana lahy sy vavy amin'ny toe-javatra samihafa amin'ny fiarovana sy ny homogenization [28, 30, 73, 74]. Izy io koa dia manome hery manavaka matanjaka eo amin'ny sehatry ny subgena, ny karazana ary ny mponina. Ny MALDI-ToF dia mamela ny famantarana haingana sy marina ny karazana, izay tena ilaina amin'ny fahatakarana ny fizarana ny mokafihitra, ny fitondran-tenany ary ny anjara asany amin'ny fifindran'ny aretina. Amin'ny alàlan'ny fanavahana ny karazana mifototra amin'ny mombamomba ny proteinina, ity fomba ity dia mitana andraikitra lehibe amin'ny fandalinana epidemiolojika sy ny paikady fanaraha-maso vector. Na izany aza, misy fetra roa lehibe mbola manakana ny fampiharana azy mahazatra. Ny voalohany dia mikasika ny fisian'ny fitaovana spectrometry faobe, ny vidiny dia voarara ho an'ny fividianana natokana ho an'ny famantarana ny mokafihitra (na arthropod vectors amin'ny ankapobeny). Na izany aza, io faneriterena io dia azo alefa amin'ny alàlan'ny fidirana amin'ny spectrometers efa misy ao amin'ny sehatra proteomics na laboratoara fitsaboana klinika, izay nanjary fitaovana mahazatra. Ny fetra faharoa dia ny tsy fahampian'ny fanehoana ny angon-drakitra fototra amin'ny mokafihitra ao amin'ny tahiry misokatra. Izany dia mitaky ny famoronana tahirin-kevitra anatiny, mifototra amin'ny spectra azo avy amin'ny santionany fantatra tsy misy fisalasalana, tsara indrindra amin'ny alàlan'ny fitambaran'ny famantarana morphological sy ny fizotran'ny mari-pamantarana ara-jenetika mety (IOC, cytB na hafa). Ity fetra ity dia tokony hoesorina tsikelikely amin'ny alàlan'ny fampidirana ny angon-drakitra amin'izao fotoana izao ao amin'ny sehatra MSI, tantanan'ny Assistance Publique-Hôpitaux de Paris sy ny Sorbonne University (France), ary koa ao amin'ny fanangonana BCCM / IHEM / Sciensano any Bruxelles (Belzika) (https://msi.happy-dev.fr/). Rehefa raisina ny profiling proteinina miaraka amin'ny MALDI-ToF, ny santionany dia tokony hotehirizina amin'ny fanjakana maina mangatsiaka na amin'ny etanol molekiola 70%, ary tsy tokony hiharihary amin'ny mari-pana manodidina. Raha tsy misy ny protokol fanomanana santionany manerantany, dia atolotra ny ranoka aqueous amin'ny 60% acetonitrile sy 0.3% TFA misy asidra sinapinic (30 mg / mL) ampiasaina ho matrix MALDI-ToF mba hiantohana ny fampitahana ny proteinina amin'ny angon-drakitra efa navoaka.

Fanomanana santionany ho an'ny MALDI-ToF MS Mass Spectrometry (Sary 7)

Ny santionan'ny bibikely, voatahiry amin'ny toe-javatra samihafa, dia maina amin'ny rivotra amin'ny hafanan'ny efitrano ary avy eo notapahina. Ny loha sy ny kibo dia esorina mba hiarovana ireo ampahany misy ny endri-

tsoratra morphological ilaina amin'ny fametrahana slide sy ny famakafakana morphological. Ny tratra dia ampiasaina amin'ny famakafakana MALDI-ToF, raha ny sisa amin'ny kibo kosa dia azo tehirizina amin'ny fitrandrahana ADN. Ho an'ny profiling proteinina, ny thorax dia homogenized amin'ny tanana amin'ny microtubes 1.5 mL miaraka amin'ny ranoka homogenization 10 µL, amin'ny fampiasana pestles azo ampiasaina. Ranoka homogenization roa no matetika ampiasaina: rano distillée sterile sy asidra formic 25%.

7. Famaranana

Amin'ity asa ity dia nitady ny hanome ny mpikaroka ny fomba mahomby indrindra amin'ny fametrahana ny mokafihitra, mifanaraka amin'ny tanjona fikarohana manokana, mba hanamorana ny famantarana marina sy ny fahitana ny pathogens. Tsy misy fomba tokana sy tsara indrindra ho an'ny rehetra; Maro ny fomba fiasa, samy manana ny tombony sy ny fetrany manokana.

Ao amin'ny appendices, ny protocoles amin'ny antsipiriany ho an'ny teknika isan-karazany ampiasaina amin'ny fanomanana sy ny famantarana ny mokafihitra dia aseho. Ireo protocoles ireo, anisan'izany ny horonan-tsary fampianarana, dia manome fomba fiasa tsikelikely ho an'ny tanjona samihafa, miantoka ny valiny marina sy azo averina. Amin'ny alàlan'ny fanaovana an'io loharano feno io dia mikendry ny hanampy ireo mpikaroka hisafidy sy hampihatra ny teknika mety indrindra ho an'ny filan'izy ireo manokana.

Fankasitrahana

Misaotra an'i Richard Lane sy Zoe Jay Adams avy ao amin'ny Natural History Museum any London, UK ireo mpanoratra noho ny fanitsiana tsara nataon'izy ireo, izay nanampy tamin'ny fanatsarana ny kalitaon'ity sora-tanana ity.

Famatsiam-bola

Misaotra ny sampan-draharahan'ny fampandrosoana Breziliana CNPq (laharan'ny raharaha: 404395/2024-4) sy ny Araucária Foundation (laharan'ny raharaha: 433/2025 PDI) izahay tamin'ny famatsiam-bola ny asa fikarohana nataon'ny AJA.

Fifandonana tombontsoa

Jérôme Depaquit dia tonian-dahatsoratra mpiara-miasa amin'ny gazety Parasite; Tsy nisy fiantraikany tamin'ny fizotran'ny fanombanana na ny fanapahan-kevitra momba ity sora-tanana ity izy. Milaza ireo mpanoratra hafa fa tsy manana fifandonana tombontsoa izy ireo.

Fanambarana momba ny fisian'ny angon-drakitra

Lahatsary ao amin'ny tranonkalan'ny Zenodo:

Lahatsary 1: https://zenodo.org/records/18198006

Lahatsary 2: https://zenodo.org/records/18311158

Lahatsary 3: https://zenodo.org/records/18311106

Lahatsary 4: https://zenodo.org/records/18311154

Lahatsary 5: https://zenodo.org/records/18303014

Lahatsary 6: https://zenodo.org/records/18302850

Lahatsary 7: https://zenodo.org/records/18315029

Fitaovana fanampiny

Azo jerena ao amin'ny https://www.parasite-journal.org/10.1051/parasite/2026009/olm ny fitaovana fanampiny amin'ity lahatsoratra ity.

Bibliôgrafia

1. Alkan C, Allal-Ikhlef AB, Alwassouf S, Baklouti A, Piorkowski G, de Lamballerie X, Izri A, Charrel RN. 2015. Virus isolation, genetic characterization and seroprevalence of Toscana virus in Algeria. Clinical Microbiology and Infection, 21(11), 1040 e1-9.
2. Alten B, Ozbel Y, Ergunay K, Kasap OE, Cull B, Antoniou M, Velo E, Prudhomme J, Molina R, Banuls AL, Schaffner F, Hendrickx G, Van Bortel W, Medlock JM. 2015. Sampling strategies for phlebotomine sand flies (Diptera: Psychodidae) in Europe. Bulletin of Entomological Research 105(6), 664–678.
3. Ayhan N, Baklouti A, Prudhomme J, Walder G, Amaro F, Alten B, Moutailler S, Ergunay K, Charrel RN, Huemer H. 2017. Practical guidelines for studies on sandfly-borne phleboviruses: Part I: Important points to consider *ante* field work. Vector-Borne and Zoonotic Diseases 17(1), 73–80.
4. Bates PA. 1997. Infection of phlebotomine sandflies with *Leishmania*, in The Molecular Biology of Insect Disease Vectors: A Methods Manual. Springer. p. 112–120.5.
5. Baum M, de Castro EA, Pinto MC, Goulart TM, Baura W, Klisiowicz Ddo R, Vieira da Costa-Ribeiro MC. 2015. Molecular detection of the blood meal source of sand flies (Diptera: Psychodidae) in a transmission area of American cutaneous leishmaniasis, Parana State, Brazil. Acta Tropica, 143, 8–12.
6. Belen A, Alten B, Aytekin A. 2004. Altitudinal variation in morphometric and molecular characteristics of *Phlebotomus papatasi* populations. Medical and Veterinary Entomology, 18(4), 343–350.
7. Bhattacharya J, Chandra G, Hati AK. 1991. A simple method for cryopreservation of *Leishmania donovani* promastigotes, Indian Journal of Medical Research, 93, 245–246.
8. Caligiuri LG, Sandoval AE, Miranda JC, Pessoa FA, Santini MS, Salomón OD, Secundino NF, McCarthy CB. 2019. Optimization of DNA extraction from individual sand flies for PCR amplification. Methods and Protocols, 2(2), 36.
9. Casaril AE, de Oliveira LP, Alonso DP, de Oliveira EF, Gomes Barrios SP, de Oliveira Moura Infran J, Fernandes WS, Oshiro ET, Ferreira AMT, Ribolla PEM, de Oliveira AG. 2017. Standardization of DNA extraction from sand flies: Application to genotyping by next generation sequencing. Experimental Parasitology, 177, 66–72.
10. Castalanelli MA, Severtson DL, Brumley CJ, Szito A, Foottit RG, Grimm M, Munyard K, Groth DM. 2010. A rapid non-destructive DNA extraction method for insects and other arthropods. Journal of Asia-Pacific Entomology, 13(3), 243–248.
11. Cerqueira NL. 1943. Um novo meio para montagem de pequenos insetos em lâmina. Memórias do Instituto Oswaldo Cruz, (39), 37–41.

12. Charrel RN, Gallian P, Navarro-Mari JM, Nicoletti L, Papa A, Sanchez-Seco MP, Tenorio A, de Lamballerie X. 2005. Emergence of Toscana virus in Europe. Emerging Infectious Diseases, 11(11), 1657–1663.
13. Chaskopoulou A, Giantsis IA, Demir S, Bon MC. 2016. Species composition, activity patterns and blood meal analysis of sand fly populations (Diptera: Psychodidae) in the metropolitan region of Thessaloniki, an endemic focus of canine leishmaniasis. Acta Tropica, 158, 170–176.
14. Chen H, Rangasamy M, Tan SY, Wang H, Siegfried BD. 2010. Evaluation of five methods for total DNA extraction from western corn rootworm beetles. PLoS One, 5(8), e11963.
15. Depaquit J, Grandadam M, Fouque F, Andry PE, Peyrefitte C. 2010. Arthropod-borne viruses transmitted by Phlebotomine sandflies in Europe: a review. Eurosurveillance, 15(10), 19507.
16. Diamond LS, Herman CM. 1954. Incidence of Trypanosomes in the Canada Goose as revealed by bone marrow culture. Journal of Parasitology, 40(2), 195–202.
17. Ding H, Torno M, Vongphayloth K, Ng G, Tan D, Sng W, Ho K, Randrianambinintsoa FJ, Depaquit J, Tan CH. 2025. Hidden in plain sight: discovery of sand flies in Singapore and description of four species new to science. Parasites & Vectors, 18(1), 402.
18. Es-Sette N, Ajaoud M, Bichaud L, Hamdi S, Mellouki F, Charrel RN, Lemrani M. 2014. *Phlebotomus sergenti* a common vector of *Leishmania tropica* and Toscana virus in Morocco. Journal of Vector Borne Diseases, 51(2), 86–90.
19. Favret C. 2005. A new non-destructive DNA extraction and specimen clearing technique for aphids (Hemiptera). Proceedings of the Entomological Society of Washington, 107(2), 469–470.
20. Galati EAB. 2018. Phlebotominae (Diptera, Psychodidae): Classification, morphology and terminology of adults and identification of American taxa, in Brazilian Sand Flies: Biology, Taxonomy, Medical Importance and Control, Rangel EF, Shaw JJ, Editors. Cham: Springer International Publishing. pp. 9–212.
21. Galati EAB, de Andrade AJ, Perveen F, Loyer M, Vongphayloth K, Randrianambinintsoa FJ, Prudhomme J, Rahola N, Akhoundi M, Shimabukuro PHF, Depaquit J. 2025. Phle-botomine sand flies (Diptera, Psychodidae) of the world. Parasites & Vectors, 18(1), 220.
22. Galati EAB, Galvis-Ovallos F, Lawyer P, Leger N, Depaquit J. 2017. An illustrated guide for characters and terminology used in descriptions of Phlebotominae (Diptera, Psychodidae). Par-asite, 24, 26.
23. Gariepy T, Kuhlmann U, Gillott C, Erlandson M. 2007. Parasitoids, predators and PCR: the use of diagnostic molecular markers in biological control of Arthropods. Journal of Applied Entomology, 131(4), 225–240.
24. Giantsis IA, Chaskopoulou A, Bon MC. 2016. Mild-Vectolysis: A nondestructive DNA extraction method for vouchering sand flies and mosquitoes. Journal of Medical Entomology, 53(3), 692–695.
25. Gidwani K, Picado A, Rijal S, Singh SP, Roy L, Volfova V, Andersen EW, Uranw S, Ostyn B, Sudarshan M, Chakravarty J, Volf P, Sundar S, Boelaert M, Rogers ME. 2011. Serological markers of sand fly exposure to evaluate insecticidal nets against visceral leishmaniasis in India and Nepal: a cluster-randomized trial. PLoS Neglected Tropical Diseases, 5(9), e1296.
26. Gilbert MTP, Moore W, Melchior L, Worobey M. 2007. DNA extraction from dry museum beetles without conferring external morphological damage. PLoS One, 2(3), e272.
27. Giordani BF, Andrade AJ, Galati EAB, Gurgel-Goncalves R. 2017. The role of wing geometric morphometrics in the identification of sandflies within the subgenus *Lutzomyia.* Medical and Veterinary Entomology, 31(4), 373–380.
28. Guzmán-Larralde AJ, Suaste-Dzul AP, Gallou A, Peña-Carrillo KI. 2017. DNA recovery from microhymenoptera using six non-destructive methodologies with considerations for subsequent preparation of museum slides. Genome, 60(1), 85–91.
29. Hajibabaei M, DeWaard JR, Ivanova NV, Ratnasingham S, Dooh RT, Kirk SL, Mackie PM, Hebert PD. 2005. Critical factors for assembling a high volume of DNA barcodes. Philosophical Transactions of the Royal Society B: Biological Sciences, 360(1462), 1959–1967.
30. Haouas N, Pesson B, Boudabous R, Dedet JP, Babba H, Ravel C. 2007. Development of a molecular tool for the identification of *Leishmania* reservoir hosts by blood meal analysis in the insect vectors. American Journal of Tropical Medicine and Hygiene, 77(6), 1054–1059.
31. Hlavackova K, Dvorak V, Chaskopoulou A, Volf P, Halada P. 2019. A novel MALDI-TOF MS-based method for blood meal identification in insect vectors: A proof of concept study on phlebotomine sand flies. PLoS Neglected Tropical Diseases, 13 (9), e0007669.
32. Huemer H, Prudhomme J, Amaro F, Baklouti A, Walder G, Alten B, Moutailler S, Ergunay K, Charrel RN, Ayhan N. 2017. Practical guidelines for studies on sandfly-borne phleboviruses: Part II: Important points to consider for fieldwork and subsequent virological screening. Vector-Borne and Zoonotic Diseases, 17(1), 81–90.
33. Jancarova M, Polanska N, Thiesson A, Arnaud F, Stejskalova M, Rehbergerova M, Kohl A, Viginier B, Volf P, Ratinier M. 2025. Susceptibility of diverse sand fly species to Toscana virus. PLoS Neglected Tropical Diseases, 19(5), e0013031.
34. Kapp JD, Green RE, Shapiro B. 2021. A fast and efficient single-stranded genomic library preparation method optimized for ancient DNA. Journal of Heredity, 112(3), 241–249.
35. Killick-Kendrick R, Maroli M, Killick-Kendrick M. 1991. Bibliography of the colonization of phlebotomine sandflies. Parassitologia, 33(suppl.), 321–333.
36. Lawyer P, Killick-Kendrick M, Rowland T, Rowton E, Volf P. 2017. Laboratory colonization and mass rearing of phlebotomine sand flies (Diptera, Psychodidae). Parasite, 24, 42.
37. Léger N, Pesson B, Madulo-Leblond G. 1986. Les phlébotomes de Grèce : 1ère partie. Bulletin de la Société de Pathologie Exotique, 79, 386–397.
38. Léger N, Pesson B, Madulo-Leblond G. 1986. Les phlébotomes de Grèce : 2ème partie. Bulletin de la Société de Pathologie Exotique, 79, 514–524.
39. Leonel JAF, Vioti G, Alves ML, da Silva DT, Meneghesso PA, Benassi JC, Spada JCP, Galvis-Ovallos F, Soares RM, Oliveira T. 2020. DNA extraction from individual Phlebotomine sand flies (Diptera: Psychodidae: Phlebotominae) specimens: Which is the method with better results? Experimental Parasitology, 218, 107981.
40. Lestinova T, Rohousova I, Sima M, de Oliveira CI, Volf P. 2017. Insights into the sand fly saliva: Blood-feeding and immune interactions between sand flies, hosts, and *Leishmania.* PLoS Neglected Tropical Diseases, 11(7), e0005600.
41. Lienhard A, Schaffer S. 2019. Extracting the invisible: obtaining high quality DNA is a challenging task in small arthropods. PeerJ, 7, e6753.
42. Lozano-Sardaneta YN, Mikery-Pacheco OF, Huerta H, Rojas-Soriano JE, Contreras-Ramos A. 2025. Wing geometric morphometrics is effective to separate sand fly species (Diptera,

Psychodidae, Phlebotominae) related with leishmaniasis transmission in Mexico. Acta Tropica, 262, 107523.

43. Mandrioli M. 2008. Insect collections and DNA analyses: how to manage collections? Museum Management and Curatorship, 23(2), 193–199.
44. Maroli M, Feliciangeli MD, Bichaud L, Charrel RN, Gradoni L. 2013. Phlebotomine sandflies and the spreading of leishmaniases and other diseases of public health concern. Medical and Veterinary Entomology, 27(2), 123–147.
45. Marquina D, Buczek M, Ronquist F, Lukasik P. 2021. The effect of ethanol concentration on the morphological and molecular preservation of insects for biodiversity studies. PeerJ, 9, e10799.
46. Mathis A, Depaquit J, Dvorak V, Tuten H, Banuls AL, Halada P, Zapata S, Lehrter V, Hlavackova K, Prudhomme J, Volf P, Sereno D, Kaufmann C, Pfluger V, Schaffner F. 2015. Identification of phlebotomine sand flies using one MALDI-TOF MS reference database and two mass pectrometer systems., Parasites & Vectors, 8, 266.
47. Mekarnia N, Benallal KE, Sadlova J, Vojtkova B, Mauras A, Imbert N, Longhitano M, Harrat Z, Volf P, Loiseau PM, Cojean S. 2024. Effect of *Phlebotomus papatasi* on the fitness, infectivity and antimony-resistance phenotype of antimony-resistant *Leishmania* major Mon-25. International Journal for Parasitology – Drugs and Drug Resistance, 25, 100554.
48. Milligan BG. 1998. Total DNA isolation, in Molecular Genetic Analysis of Population: A Practical Approach, Hoelzel AR, Editor. Oxford: Oxford University Press.
49. Molina R, Jiménez M, Alvar J, González E, Hernández-Taberna S, Ines MM. 2017. Methods in sand fly research. Madrid: Servicio de publicaciones Universidad de Alcalá de Henares, Madrid.
50. Murphy WJ, Eizirik E, O'Brien SJ, Madsen O, Scally M, Douady CJ, Teeling E, Ryder OA, Stanhope MJ, de Jong WW, Springer MS. 2001. Resolution of the early placental mammal radiation using Bayesian phylogenetics. Science, 294(5550), 2348–2351.
51. Nacif-Pimenta R, Pinto LC, Volfova V, Volf P, Pimenta PFP, Secundino NFC. 2020. Conserved and distinct morphological aspects of the salivary glands of sand fly vectors of leishma-niasis: an anatomical and ultrastructural study. Parasites & Vectors, 13(1), 441.
52. Neuhaus B, Schmid T, Riedel J. 2017. Collection management and study of microscope slides: Storage, profiling, deterioration, restoration procedures, and general recommendations. Zootaxa, 4322(1), 1–173.
53. New TR. 1974. Pscoptera. Handbooks for Identification of British Insects (Vol. I). London: Royal Entomological Society of London. 102 pp.
54. Perez-Ruiz M, Collao X, Navarro-Mari JM, Tenorio A. 2007. Reverse transcription, real-time PCR assay for detection of Toscana virus. Journal of Clinical Virology, 39(4), 276–281.
55. Porco D, Rougerie R, Deharveng L, Hebert P. 2010. Coupling non-destructive DNA extraction and voucher retrieval for small soft-bodied Arthropods in a high-throughput context: the example of Collembola. Molecular Ecology Resources, 10(6), 942–945.
56. Prudhomme J, Cassan C, Hide M, Toty C, Rahola N, Vergnes B, Dujardin JP, Alten B, Sereno D, Banuls AL. 2016. Ecology and morphological variations in wings of *Phlebotomus ariasi* (Diptera: Psychodidae) in the region of Roquedur (Gard, France): a geometric morphometrics approach. Parasites & Vectors, 9(1), 578.
57. Prudhomme J, Gunay F, Rahola N, Ouanaimi F, Guernaoui S, Boumezzough A, Banuls AL, Sereno D, Alten B. 2012. Wing size and shape variation of *Phlebotomus papatasi* (Diptera: Psychodidae) populations from the south and north slopes of the Atlas Mountains in Morocco. Journal of Vector Ecology, 37(1), 137–147.
58. Prudhomme J, Toty C, Kasap OE, Rahola N, Vergnes B, Maia C, Campino L, Antoniou M, Jimenez M, Molina R, Cannet A, Alten B, Sereno D, Banuls AL. 2015. New microsatellite markers for multi-scale genetic studies on *Phlebotomus ariasi* Tonnoir, vector of *Leishmania infantum* in the Mediterranean area. Acta Tropica, 142, 79–85.
59. Prudhomme J, Velo E, Bino S, Kadriaj P, Mersini K, Gunay F, Alten B. 2019. Altitudinal variations in wing morphology of *Aedes albopictus* (Diptera, Culicidae) in Albania, the region where it was first recorded in Europe. Parasite, 26, 55.
60. Rawlins DJ. 1992. Light Microscopy: An Introduction to Biotechniques. Oxford: Bios Scientific publishers. 143 pp.
61. Ready PD. 2013. Biology of phlebotomine sand flies as vectors of disease agents. Annual Review of Entomology, 58, 227–250.
62. Rohlf FJ, Slice D. 1990. Extensions of the Procrustes method for the optimal superimposition of landmarks. Systematic Zoology, 39(1), 40–59.
63. Rowley DL, Coddington JA, Gates MW, Norrbom AL, Ochoa RA, Vandenberg NJ, Greenstone MH. 2007. Vouchering DNA-barcoded specimens: Test of a nondestructive extraction proto-col for terrestrial arthropods. Molecular Ecology Notes, 7(6), 915–924.
64. Sábio PB, Andrade AJ, Galati EAB. 2014. Assessment of the taxonomic status of some species included in the *Shannoni* complex, with the description of a new species of *Psathyromyia* (Diptera: Psychodidae: Phlebotominae). Journal of Medical Entomology, 51(2), 331–341.
65. Sadlova J, Yeo M, Seblova V, Lewis MD, Mauricio I, Volf P, Miles MA. 2011. Visualisation of *Leishmania donovani* fluo-rescent hybrids during early stage development in the sand fly vector. PLoS One, 6(5), e19851.
66. Sales K, Miranda DEO, da Silva FJ, Otranto D, Figueredo LA, Dantas-Torres F. 2020. Evaluation of different storage times and preservation methods on phlebotomine sand fly DNA concen-tration and purity. Parasites & Vectors, 13(1), 399
67. Sales KG, Costa PL, de Morais RC, Otranto D, Brandao-Filho SP, Cavalcanti Mde P, Dantas-Torres F. 2015. Identification of phlebotomine sand fly blood meals by real-time PCR. Parasites& Vectors, 8, 230.
68. Sant'Anna MR, Jones NG, Hindley JA, Mendes-Sousa AF, Dillon RJ, Cavalcante RR, Alexander B, Bates PA. 2008. Blood meal identification and parasite detection in laboratory-fed and field-captured *Lutzomyia longipalpis* by PCR using FTA databasing paper. Acta Tropica, 107(3), 230–237.
69. Senne NA, Santos HA, Araujo TR, Paulino PG, Mendonca LP, Moreira HVS, Camilo TA, da Costa Angelo I. 2022. Robust comparative performance of genomic DNA extraction methods from non-engorged phlebotomine sandflies. Medical and Veterinary Entomology, 36(2), 203–211.
70. Shaw JJ. 2025. A review of Leishmania infections in American Phlebotomine sand flies – Are those that transmit leishmaniasis anthropophilic or anthropportunists? Parasite, 32, 57.
71. Tesh RB, Modi GB. 1983. Growth and transovarial transmission of Chandipura virus (Rhabdoviridae: Vesiculovirus) in *Phlebotomus papatasi.* American Journal of Tropical Medicine and Hygiene, 32(3), 621–623.
72. Thomsen PF, Elias S, Gilbert MTP, Haile J, Munch K, Kuzmina S, Froese DG, Sher A, Holdaway RN, Willerslev E. 2009. Non-

destructive sampling of ancient insect DNA. PLoS One, 4(4),e5048
73. Truett GE, Heeger P, Mynatt RL, Truett AA, Walker JA, Warman ML. 2000. Preparation of PCR-quality mouse genomic DNA with hot sodium hydroxide and tris (HotSHOT). Biotechniques, 29(1), 52–54.
74. Upton MS. 1993. Aqueous gum-chloral slide mounting media: an historical review. Bulletin of Entomological Research, 83(2), 267–274.
75. Volf P, Myskova J. 2007. Sand flies and Leishmania: specific versus permissive vectors. Trends in Parasitology, 23(3), 91–92.
76. Wang Q, Wang X. 2012. Comparison of methods for DNA extraction from a single chironomid for PCR analysis. Pakistan Journal of Zoology, 44(2), 421–426.
77. Wang Y, Zhao Y, Bollas A, Wang Y, Au KF. 2021. Nanopore sequencing technology, bioinformatics and applications. Nature Biotechnology, 39(11), 1348–1365.

Cite this article as: Randrianambinintsoa FJ, Augendre L, Prudhomme J, Martinet J-P, Loyer M, Mekarnia N, Kerkoub H, Perveen FK, Huguenin A, Kariya E, Akhoundi M, De Andrade AJ, Berriatua E, Bongiorno G, Boyer S, Christodoulou V, Da Costa-Ribeiro MCV, De Souza LAF, Ding H, Dondji B, Dvořák V, Erisoz Kasap O, Galati EAB, Gállego M, Ballart C, Gouzelou S, Haddad N, Masse RS, Mekuria AH, Ivovic V, Kaczmarek S, Shahar MK, Kirstein OD, Kniha E, Kolářová I, Lincoln T, Lucanas C, Mikov O, Nov K, Özbel Y, Pesson B, Posada Lopez LC, Prasetyo DB, Rahola N, Rebollar-Tellez EA, Rodrigues BL, Roy L, Saini P, Sanjoba C, Shimabukuro PH, Siriyasatien P, Soszynska A, Suleşco T, Sylla M, Torno M, Volf P, Vongphayloth K, Sinh Nam V, Wardhana A, Yessinou E, Zapata S, Gantier J-C & Depaquit J. 2026. Processing and mounting phlebotomine sand flies: a consensus guideline. Parasite xx, xx. https://doi.org/10.1051/parasite/2026009.

PARASITE

An international open-access, peer-reviewed, online journal publishing high quality papers on all aspects of human and animal parasitology

Reviews, articles and short notes may be submitted. Fields include, but are not limited to: general, medical and veterinary parasitology; morphology, including ultrastructure; parasite systematics, including entomology, acarology, helminthology and protistology, and molecular analyses; molecular biology and biochemistry; immunology of parasitic diseases; host-parasite relationships; ecology and life history of parasites; epidemiology; therapeutics; new diagnostic tools.
All papers in Parasite are published in English. Manuscripts should have a broad interest and must not have been published or submitted elsewhere. No limit is imposed on the length of manuscripts.

Parasite (open-access) continues **Parasite** (print and online editions, 1994-2012) and **Annales de Parasitologie Humaine et Comparée** (1923-1993) and is the official journal of the Société Française de Parasitologie.

Editor-in-Chief:
Jean-Lou Justine, Paris

Submit your manuscript at:
https://www.editorialmanager.com/parasite

Fanazavana fanampiny 1: Fototra teorika biosimika.

Ny arthropods voakasika eto dia mokafihitra. Na izany aza, ny fitsipika ankapobeny dia azo itatra amin'ny arthropods hafa izay mifototra amin'ny toetra morphological anatiny. Soa ihany fa ny taova anatiny sasany dia ampahany amin'ny chitinized ary ny morphology dia manome fampahalalana sarobidy. Izany no antony mahaliana indrindra ny fandinihana ny paompy sakafo, ny spermathecae ary ny fantsona. Miaraka amin'ireo reagents rehetra izay hojerentsika, dia tsy tokony hohadinointsika mihitsy fa, manomboka amin'ny dingana fanamboarana bibikely ka hatramin'ny fivoriambe, dia mampihatra ny fanehoan-kevitra redox isika. Ny hany fepetra fototra dia ny fisorohana ny fampifangaroana ny fampihenana ny reagents amin'ny oxidizing reagents.

alikaola etil; Etanol:

Ity zava-mahadomelina ity dia ampiasaina amin'ny fomba isan-karazany. Ny molekiolan'ny alikaola dia manana fifandraisana matanjaka amin'ny rano ary noho izany dia misy fiantraikany amin'ny fanalefahana rano. Na izany aza, ny alikaola amin'ny fifantohana ambany (izany hoe, manan-karena loatra amin'ny rano) dia handray anjara amin'ny fahasimban'ny asidra nokleika (ny rano no fahavalon'ny asidra nokleika).

Rehefa apetraka ao amin'ny etanol ny bibikely, dia tsy vitan'ny hoe mitahiry azy ireo fotsiny izany, fa manamboatra ny tavy ihany koa. Amin'ny ankapobeny, misy foto-kevitra roa manan-danja: ny hafainganam-pandehan'ny fidirana sy ny hafainganam-pandehan'ny fixation. Voamarina tsara fa ny fixative tsara dia tsy maintsy miditra haingana sy lalina ao amin'ny tavy alohan'ny hampiharana ny hetsika fixative. Ho an'ny alikaola 96%, ny coefficient penetration dia eo amin'ny 1.05 eo ho eo (raha ampitahaina, ny ranoka aqueous amin'ny asidra picric 0.75% dia manana coefficient penetration 0.45, raha ny ranoka potassium dichromate 3% kosa dia manana coefficient 1.45).

Ny faniriana hitazona ny bibikely sy ny arthropods hafa ao amin'ny ethanol mandritra ny fotoana tsy voafetra dia zava-misy eo amin'ny entomologists. Ny hevitra momba ny fitehirizana ny saha ho an'ny fandalinana amin'ny ho avy na ho an'ny mpikaroka ho avy dia tena mendri-piderana. Na izany aza, ity fomba ity dia tsy mifanaraka amin'ny fepetra takian'ny cytologist na histologist. Amin'ny fiezahana hitazona ny santionany ao amin'ny fixative mandritra ny fotoana ela loatra, dia mety ho lasa saika tsy azo atao ny mamerina azy ireo. Izany no antony mahatonga ny santionany mihoatra ny 10 taona ho sarotra, raha tsy tsy azo atao, ny mampiasa.

Ny singa iray hafa tokony hodinihina dia ny tahan'ny habetsaky ny arthropods hapetraka sy ny habetsaky ny fixative. Amin'ny fomba fanao ara-pitsaboana na ara-pitsaboana, dia soso-kevitra ny hanome ny habetsaky ny fixative eo amin'ny 60 heny noho ny habetsaky ny faritra tokony hapetraka. Amin'ny fampiharana, ho an'ny micro-arthropods, ho an'ny habetsaky ny santionany voafaritra, dia tokony ampiana alikaola 4 ka hatramin'ny 5 farafahakeliny. Tokony hotadidina fa ny alikaola dia ho very ny fifantohana amin'ny alàlan'ny fandraisana ny rano ao amin'ny vatan'ny arthropod.

Ho famaranana:

- Ny alikaola etil dia singa simika mampihena (ary noho izany dia tsy mifanaraka amin'ny fixatives oxidatives);
- Izy io dia mampihena ny proteinina ary manala azy ireo;
- Izy io dia manala ny lipida sarotra sasany ary mampihena ny glycogen;
- Izany dia mahatonga ny tavy hihena mafy sy hanamafy azy ireo.

Ranoka fototra amin'ny potasioma na sodium hydroxide:

Ny fampiasana ireo ranoka ireo amin'ny entomology dia nifantoka indrindra tamin'ny potassium hydroxide, tsy misy fanamarinana mazava. Sodium hydroxide [E524] dia tonga amin'ny ranoka, amin'ny fifantohana samihafa na ara-dalàna, ary koa amin'ny endriky ny pellets na flakes. Ny fatiantoka lehibe indrindra dia ny hygroscopicity avo lenta (avo kokoa noho ny an'ny KOH). Rehefa mihetsika amin'ny proteinina izy dia manala azy ireo, ary amin'ny lipida dia manova azy ireo ho savony mafy mandritra ny saponification (izay fahasamihafana lehibe amin'ny KOH, izay mamokatra savony ranon-javatra mandritra io fihetsika io).

Potassium hydroxide [E525] dia misy ho ranoka mifantoka, fa ny ankamaroany amin'ny endriky ny pellets eo amin'ny 0.1 g, izay manamora ny fanomanana ranoka dilute rehefa tsy misy ny fifandanjana marina. Ohatra, ny takelaka 0.1 g levona ao anaty rano 1 mL dia mamokatra ranoka 10%. Ny tombony iray hafa amin'ny KOH amin'ny endrika pellet dia ny fahatsapana ambany kokoa amin'ny karbaona (ny ranoka KOH dia manana fifandraisana avo lenta amin'ny CO_2, mitarika amin'ny fananganana karbaona).

Ireo fototra matanjaka ireo dia ampiasaina amin'ny famahana ny asidra matavy amin'ny alàlan'ny famadihana azy ireo ho savony mety levona amin'ny rano. Tokony hotsaroana fa ny fixative, toy ny ethanol, dia efa solubilizes ny sasany amin'ireo lipida hita ao amin'ny santionany. Na izany aza, rehefa mandalo amin'ny alàlan'ny fitaovana misy fototra matanjaka ny santionany, dia mihena ny asidra matavy (mihoatra na latsaka). Ny fototra matanjaka dia mahatratra ny saponification mangatsiaka. Amin'ny toe-javatra sasany, rehefa be dia be ny tavy matavy, ohatra amin'ny vehivavy, dia mety ho tombony ny mampiakatra ny mari-pana amin'ny 35-40 ° C mba hanamorana ny fanehoan-kevitra, na hanitatra ny fotoana fifandraisana amin'ny hafanan'ny efitrano.

Ranoka Marc-André voaloton'ny fuchsin na tsy misy loko:

Eto isika dia miresaka momba ny tombony sy ny tsy fahampian'ny fampiasana ny ranoka Marc-André. Ity ranoka ity dia ahitana chloral hydrate (trichloroacetaldehyde monohydrate), asidra acetic ary rano. Izy io dia ranoka tena oxidizing (fifangaroan'ny asidra sy aldehyde). Izy io dia manafoana ny potasioma hydroxide tafahoatra izay mety hijanona ao amin'ny santionany, nefa tsy manaparitaka ny savony alkaline niforona nandritra ny fampiasana KOH. Ity ranoka oxidative ity koa dia miasa amin'ny fiasan'ny alikaola faharoa amin'ny glucosamines izay mamorona chitin, amin'ny alàlan'ny oxidizing azy ireo, izay mitarika ho amin'ny fanalefahana ny chitin. Izy io koa dia mamela ny fanafoanana ny sira mineraly sasany.

Rehefa voaloton'ny fuchsin asidra ny ranoka Marc-André (izany hoe amin'ny endrika oxidized), dia afaka mifamatotra amin'ny fiasan'ny alikaola faharoa amin'ny rafitra chitinous. Aorian'ny fotoana fifandraisana amin'ny ranoka Marc-André ary miankina amin'ny toetry ny fandotoana ny santionany, ny fanasana dia atao amin'ny ethanol ihany. Avy eo dia manomboka ny dingana dehydration amin'ny santionany.

Tombony:

- Fanafoanana ny ranoka fototra tafahoatra
- Fanalefahana ny chitin
- Ny fandokoana chitin dia mamela ny fankasitrahana tsara kokoa ny rafitra anatiny chitinized

Lafi-ratsiny:

Chloral hydrate dia singa hypnotic ampiasaina ara-tantara amin'ny fitsaboana olombelona. Tsy maintsy ampiasaina amin'ny hood simika izy io ary amin'ny fanarahana hentitra ny fitsipika mifandraika amin'ny loza simika.

Ranoka amin'ny déshydration:

Ny traikefa dia mampiseho fa, ho an'ny santionany kely, dia tsy ilaina ny manaraka ny fandroana alikaola mifandimby amin'ny fitomboan'ny fifantohana. Raha lehibe ny santionany, dia hanomboka amin'ny ethanol 80% isika, avy eo 90%, 95% ary farany ethanol tanteraka. Ho an'ny santionany kely, ny fandroana 90% arahin'ny fanitrihana amin'ny etanol tanteraka dia ampy. Amin'izao fotoana izao, tokony hotadidina foana fa ny ethanol tanteraka dia mikendry ny hisambotra rano avy amin'ny atmosfera.

Araka ny nentim-paharazana, ao amin'ny laboratoara entomology, ny fanesorana farany ny santionany dia natao tamin'ny alalan'ny fandroana creosote beech. Ankehitriny, ity karazana ity, izay nampiasaina betsaka ho toy ny pesticide, antifungal ary wood preservative, dia tsy kivy mafy noho ny fofony (mifandray amin'ny hydrocarbons aromatika polycyclic) sy ny poizina heverina ho poizina: reprotoxic, carcinogenic, loto organika maharitra ary ecotoxic ho an'ny zavamananaina anaty rano.

Ny ranoka arosonay ho an'ny fametrahana ny santionany dia ny fampiasana ny Euparal ® sy Euparal essence (voalaza ao amin'ny andininy manaraka). Ny fifangaroan'ny lasantsy Euparal sy Euparal dia tena azo leferina amin'ny santionany izay efa nandro tamin'ny ethanol 90%.

Fanampiny 2: Firafitry ny reagents ampiasaina.

Hydroxyde de potassium à 10 %

Hydroxyde de potassium : 10 g
Eau distillée : q.s.p. 100 mL

Milieu de montage gomme au chloral (milieu de Hoyer)

Eau distillée : 50 mL
Hydrate de chloral : 200 g
Gomme arabique : 50 g
Glycérol : 20 mL

Solution de Marc-André

Hydrate de chloral : 40 g
Acide acétique glacial : 30 mL
Eau distillée : 30 mL

Fuchsine acide à 1 % dans l'eau distillée

Fuchsine acide (poudre) : 1 g
Eau distillée : 99 mL

Solution de Marc-André colorée à la fuchsine

Solution de Marc-André : 10 mL
Fuchsine acide à 1% 50 µL

Fanampiny 3: Euparal®, balsam-pahasalamana Kanada, alikaola polyvinyl ary ranoka hafa®

Alikaola polyvinyl: Ny alikaola polyvinyl dia manome fitaovana tsara indrindra rehefa tsy misy ny vokatra ilaina amin'ny dehydration mety. Amin'ity tranga ity, ny alikaola polyvinyl dia mifangaro amin'ny lactophenol's Amann. Na izany aza, ity karazana fivoriambe ity dia manana fatiantoka lehibe: na maina ny mpanelanelana, na ny alikaola polyvinyl dia kristaly noho ny fivoahan'ny rano, na ny fivoriambe dia mainty rehefa mihena ny phenol. Na izany aza, ity teknika ity dia mbola mety amin'ny fanitsiana vetivety.

Canada Balsam: Ny fampiasana ny Canada Balsam ho an'ny fivoriana eo anelanelan'ny slide sy ny coverslip dia mitaky ny dehydration mialoha ny santionany. Ny fampiasana xylene na toluene ho an'ity dingana ity dia tsy misy ny tsy fahampiana.

Enecê medium: Toy ny balsama Kanada, ny medium Enecê dia mitaky ny fanesorana mialoha ny santionany alohan'ny hametrahana eo anelanelan'ny lelany sy ny fonony. Enecê Medium Formulation: Rosin fotsy madio (22 g); siligaoma kopal mety levona amin'ny alikaola (12 g); alikaola tanteraka (20 mL); camphor (10 g); turpentine (10 mL); eucalyptol: 26 mL. Fiomanana: Ao amin'ny fitoeran-javatra mety (ohatra ny tavoahangy Erlenmeyer), ampidiro ny alikaola sy ny camphor tanteraka, avy eo ampio ny rosin sy ny siligaoma kopal. Avy eo dia voafehy ny tavoahangy ary afangaro, avy eo hafanaina ao anaty fandroana rano amin'ny hafanana antonony mba hisorohana ny mangotraka. Rehefa voafehy tanteraka ny votoatiny, dia ampiana turpentine. Avy eo dia voasivana amin'ny hafanana ny fangaro, ary ampidirina ao amin'ny filtrate ny eucalyptol. Rehefa lasa tsy dia misy rano loatra ny mpanelanelana, dia alefa amin'ny menaka Enecê, voaomana araka ireto raikipohy manaraka ireto: alikaola tanteraka (30 mL), camphor (17 g), turpentine (15 mL), eucalyptol (38 mL) (Cerqueira, 1943).

Euparal: Izy io dia resina avy amin'ny cypress an'ny Atlas *Tetraclinis articulata* (Vahl, 1791), nianatra sy novolavolain'i Gilson tamin'ny 1906. Ny tombony lehibe indrindra dia ny tsy fanaovana polymerize. Ny santionany napetraka eo anelanelan'ny slide sy ny coverslip dia azo averina mora foana amin'ny alàlan'ny asan'ny alikaola, na tsara kokoa, Euparal® essence. Ity resina ity, antsoina koa hoe sandaraque, dia manaiky etanol avy amin'ny 80%.

Fampiasana Triton X100: ranoka tsy misy rano:

Triton X100 dia ranoka aqueous tsy ionika (*4-(1,1,3,3-tetramethylbutyl) phenyl-polyethylene glycol,* na *t-octylphenoxypolyethoxyethanol, tert-octylphenyl polyethylene glycol ether*), be mpampiasa ho toy ny detergent amin'ny sela sy ny biolojia molekiola. Izy io dia mamela ny permeabilization ny sela sy ny membrane nokleary.

Ny santionan'ny bibikely voatahiry nandritra ny taona maro tao anaty alikaola dia mahazatra. Mampalahelo fa ity fomba fitehirizana ity dia lavitra ny tsara indrindra, ary ny arthropods voatahiry amin'izany fomba izany dia matetika sarotra ny miomana amin'ny fandinihana mikroskopika. Ny fitoeran-javatra plastika dia mety hihena, ka mahatonga ny alikaola ho etona. Amin'ireo tranga roa ireo, ny fifandraisana maharitra amin'ny alikaola na ny fanamainana ny santionany dia olana lehibe. Tamin'ny 2008, namoaka fanamarihana momba ny famerenana ny rano amin'ny hala i Jonque amin'ny fampiasana fitaovana mando toy ny Agepon, ampiasaina amin'ny sary [26]. Ity fandinihana ity dia nitarika ny hevitra hampiasa ireo mpandraharaha mando izay tsy dia manadio loatra.

Fomba fampiasana ranoka 0.5% an'ny Triton X100:

- Ampidiro amin'ny alikaola tanteraka ny santionany maina.
- Ampio ny habetsaky ny ranoka Triton X100 amin'ny 0.5% mba hahafahan'ny santionany milentika tanteraka.
- Avelao mandritra ny 5 minitra na mihoatra. Ny arthropods rehetra dia tsy maintsy mahaleo tena amin'ny ranoka.
- Esory ny ranoka Triton X100 ary soloy amin'ny ranoka potassium hydroxide.

Ny dingana dia mitohy araka ny protocole voalaza etsy ambony.

Fanampiny 4: Fivoriambe tsikelikely miaraka amin'ny balsama Euparal® na Canada

1. Ny santionany dia tokony ho tsy ampy rano (ny fisehoan'ny rahona na ronono dia manondro ny tsy fahampian'ny rano).
2. Ny fanalàna ny rano dia azo tratrarina amin'ny alàlan'ny fampitomboana ny fifantohana amin'ny alikaola ethyl.
3. Ny santionany dia azo afindra avy amin'ny alikaola 99% na alikaola tanteraka mankany amin'ny mpandraharaha maivana.

Fomba fiasa:

1. Ampifanaraho amin'ny ethanol 70% ny mokafihitra lehibe.
2. Esory ny etanol ary soloy ranoka KOH 10%. Sarony amin'ny slide fitaratra nymokafihitra.
3. Avelao ny macerate mandra-pahatongan'ny bibikely ho mangarahara.
4. Esory ny ranoka amin'ny KOH.
5. Sarony amin'ny rano distillée ny santionany ary miandry 30 ka hatramin'ny 45 minitra.
6. Esory ny rano ary avereno ny fanasana amin'ny rano distillée mandritra ny 30 min (ny fotoana dia miankina amin'ny isan'ny santionany nokarakaraina tamin'ny fotoana iray: ny avo kokoa ny isan'ny santionany, ny bebe kokoa amin'ity indray mitoraka ity dia tsy maintsy hajaina; mifanohitra amin'izany, ho an'ny santionany vitsivitsy, indrindra fa ny tsirairay voahodina, ity indray mitoraka ity dia azo mihena).
7. Esory ny rano.
8. Ampio ny ranoka Marc-André (mety ho voaloko amin'ny fuchsin asidra) ary avelao mandritra ny 24 ora (iray andro).
9. Esory ny ranoka nataon'i Marc-André.
10. Sarony amin'ny rano distillée ny santionany ary miandry 30 ka hatramin'ny 45 minitra.
11. Esory ny rano ary avereno ny fanasana amin'ny rano distillée mandritra ny 30 minitra.
12. Esory ny rano
13. Ampio etanol 70% ary tapaho ny santionany.
a. Ho an'ny loha sy ny kibo, esory moramora ny loha na ny kibo hiala amin'ny tratra.
b. Ho an'ny thorax, esory ny elatra amin'ny alàlan'ny fihazonana ny thorax amin'ny pliers ary misintona ny fototry ny appendages amin'ny mpivady hafa. Azo atao ihany koa ny manao dissection sagittal, mizara ny thorax ho faritra havia sy havanana, arakaraka ny faritra mahaliana.
14. Mihena tsikelikely amin'ny alàlan'ny andiana ranoka aqueous amin'ny alikaola ethyl: 50 - 80 - 95%, hatramin'ny alikaola tanteraka.
15. Famaranana ny déshydratation ny santionany amin'ny alalan'ny fandroana roa misesy ny 10 minitra tsirairay ao amin'ny 100% ethanol.
16. Esory ny etanol ary rakotra menaka manan-danja mandritra ny 15 minitra amin'ny hafanan'ny efitrano.
17. Ampidiro ny santionan'ny menaka manan-danja amin'ny menaka manan-danja amin'ny Euparal® na Canada Balsam amin'ny slide madio.
18. Alamino amin'ny fironana tadiavina ireo sombin-javatra: ny lohany, ny thorax ary ny kibon'ny fasika dia azo esorina amin'ny alàlan'ny fanjaitra tsara na forceps eo ambanin'ny loupe binocular. Ny loha dia tokony hisaraka amin'ny vatana mba hapetraka amin'ny toerana ventro-dorsal, izany hoe, miaraka amin'ny foramen occipital manatrika ny ambony, mamela ny fandinihana mivantana ny cibarium amin'ny alàlan'izany.
Ny fanesorana dia tanterahina mivantana amin'ny fitaovana fiakaran'ny fasika.
19. Avelao ny fametrahana mandra-pahatongan'ny faritra ho somary miraikitra.
20. Mandoa ny slat madio amin'ny alikaola tanteraka. Apetraho amin'ny balsama Canada na Euparal® amin'ny zoro kely ny slat.
21. Tehirizo ao anaty boaty maina natokana ho an'ity tanjona ity ny lelany.
