# Supplementary material for: Processing and mounting phlebotomine sand flies: a consensus guideline
Source: Parasite. 2026 Apr 3;33:18. doi: 10.1051/parasite/2026009 (PMC13047900; doi:10.1051/parasite/2026009)
Supplement: Supplementary file 21 — Malagasy translation / Dikanteny malagasy [file parasite-33-18-s21.pdf]

Parasite **33**, 18 (2026)
© F.J. Randrianambinintsoa et al., published by EDP Sciences, 2026
https://doi.org/10.1051/parasite/2026009

PARASITE
Available online at:
www.parasite-journal.org

REVIEW ARTICLE OPEN ACCESS

Garis panduan pemprosesan dan pelekapan lalat pasir phlebotomine

Fano José Randrianambinintsoa[1], Laure Augendre[1], Jorian Prudhomme[1], Jean-Philippe Martinet[1], Mathieu Loyer[1], Nalia Mekarnia[1], Hocine Kerkoub[1], Farzana Khan Perveen[1], Antoine Huguenin[1,2], Emilie Kariya[1,2], Mohammad Akhoundi[3], Andrey José de Andrade[4], Eduardo Berriatua[5], Gioia Bongiorno[6], Sébastien Boyer[7,8], Vasiliki Christodoulou[9], Magda Clara Vieira Da Costa-Ribeiro[10], Lucas Alexandre Farias de Souza[10], Huicong Ding[11], Blaise Dondji[12], Vít Dvořák[13], Ozge Erisoz Kasap[14], Eunice Aparecida Bianchi Galati[15], Montserrat Gállego[16], Cristina Ballart[16], Stavroula Gouzelou[17], Nabil Haddad[18], Rezki Sabrina Masse[19], Asrat Hailu Mekuria[20], Vladimir Ivovic[21], Szymon Kaczmarek[22], Mohd Khadri Shahar[19], Oscar D. Kirstein[23], Edwin Kniha[24], Iva Kolářová[13], Lincoln Timinao[25], Cristian Lucanas[26], Ognyan Mikov[27], Kimsear Nov[7], Yusuf Özbel[28], Bernard Pesson[29], Laura Cristina Posada Lopez[30], Didot Budi Prasetyo[1,7], Nil Rahola[31], Eduardo A. Rebollar-Tellez[32], Bruno Leite Rodrigues[15], Lalita Roy[33], Prasanta Saini[34], Chizu Sanjoba[35], Paloma Helena Fernandes Shimabukuro[36], Padet Siriyasatien[37], Agnieszka Soszyńska[22], Tatiana Suleşco[38], Massamba Sylla[39], Majhalia Torno[40], Petr Volf[13], Khamsing Vongphayloth[41], Vu Sinh Nam[42], April Wardhana[43], Eric Yessinou[44], Sonia Zapata[45], Jean-Charles Gantier[1], and Jérôme Depaquit[1,2,*]

[1] Faculté de Pharmacie, Université de Reims Champagne Ardenne, UR ESCAPE-USC ANSES PETARD, 51 rue Cognacq-Jay, 51096 Reims Cedex, France
[2] Pôle de Biologie territoriale, Laboratoire de Parasitologie-Mycologie, Centre Hospitalo-Universitaire, 51092 Reims, France
[3] Parasitology-Mycology Department, Avicenne Hospital, AP-HP, Bobigny, Sorbonne Paris Nord University, France; Unité des Virus Émergents (UVE: Aix-Marseille Univ, Università di Corsica, IRD 190, Inserm 1207, IRBA), 13005 Marseille, France
[4] Parasitology Collection of Basic Pathology, Department of Basic Pathology, Federal University of Paraná, Curitiba 19031, Brazil
[5] Department of Animal Health, University of Murcia, Campus de Espinardo, 30100 Espinardo, Murcia, Spain
[6] Department of Infectious Diseases, Vector-borne Diseases Unit, Istituto Superiore di Sanità, 00166 Rome, Italy
[7] Medical and Veterinary Entomology Unit, Institut Pasteur du Cambodge, Phnom Penh 12201, Cambodia
[8] Ecology & Emergence of Arthropod-borne Pathogens Unit, Department of Global Health, Institut Pasteur, CNRS UMR2000, 75015 Paris, France
[9] Section Veterinary Services (1417), Laboratory for Animal Health Virology, Aglantzia, Nicosia 2109, Cyprus
[10] Insects Vectors and Parasites Laboratory, Department of Basic Pathology and Postgraduate program in Microbiology, Parasitology and Pathology, Federal University of Paraná, 81530-900 Curitiba, Brazil
[11] Department of Biological Sciences, National University of Singapore, 117558, Singapore
[12] Laboratory of the Leishmaniasis Research Project, Mokolo District Hospital, Mokolo, Cameroon; Laboratory of Cellular Immunology and Parasitology, Department of Biological Sciences, Central Washington University, 98926 Ellensburg, WA, USA
[13] Department of Parasitology, Faculty of Science, Charles University, 12800 Prague, Czechia
[14] VERG Laboratories, Department of Biology, Faculty of Science, Hacettepe University, Beytepe, Ankara 06800, Türkiye
[15] Faculdade de Saúde Pública da Universidade de São Paulo (FSP/USP), Pós-graduação em Saúde Pública, 01246-904 São Paulo, Brazil
[16] Secció de Parasitologia, Departament de Biologia, Sanitat i Medi Ambient, Facultat de Farmàcia i Ciències de l'Alimentació, Universitat de Barcelona, & Institut de Salut Global de Barcelona (ISGlobal), Centro de Investigación Biomédica en Red, Enfermedades Infecciosas (CIBERINFEC), 08028 Barcelona, Spain
[17] Laboratory of Infectious Diseases and Public Health, School of Medicine, University of Cyprus, Nicosia, Cyprus & Department of Pediatrics, Archbishop Makarios III Hospital, Nicosia 2115, Cyprus
[18] Faculty of Health Sciences, American University of Beirut, 1107 2020 Beirut, Lebanon
[19] Medical Entomology Unit, Infectious Disease Research Centre, Institute for Medical Research (IMR), National Institutes of Health (NIH), Ministry of Health Malaysia, 40170 Shah Alam, Selangor, Malaysia
[20] School of Medicine, Addis Ababa University, 28017 - 1000 Addis Ababa, Ethiopia
[21] Faculty of Mathematics, Natural Sciences and Information Technologies, University of Primorska, 6000 Koper, Slovenia

Edited by Jean-Lou Justine
*Corresponding author: jerome.depaquit@univ-reims.fr

This is an Open Access article distributed under the terms of the Creative Commons Attribution License (https://creativecommons.org/licenses/by/4.0), which permits unrestricted use, distribution, and reproduction in any medium, provided the original work is properly cited.

[22] University of Lodz, Faculty of Biology and Environmental Protection, Department of Invertebrate Zoology and Hydrobiology, Banacha 12/16, 90-237 Łódź, Poland
[23] Laboratory of Entomology, Ministry of Health, 9134302 Jerusalem, Israel
[24] Center for Pathophysiology, Infectiology and Immunology, Institute of Specific Prophylaxis and Tropical Medicine, Medical University Vienna, Kinderspitalgasse 15, 1090 Vienna, Austria
[25] Papua New Guinea Institute of Medical Research (PNGIMR) Institute, PO Box 60, Headquarter, Homate Street, 441 Goroka, Eastern Highlands Province, Papua New Guinea
[26] Museum of Natural History, University of the Philippines Los Baños, 4031 Laguna, Philippines
[27] National Centre of Infectious and Parasitic Diseases, 1504 Sofia, Bulgaria
[28] Ege University, Faculty of Medicine, Department of Parasitology, 35040 Bornova/Izmir, Türkiye
[29] Retired, Faculté de Pharmacie, Université de Strasbourg, Strasbourg, 67400 Illkirch-Graffenstaden, France
[30] Program for the Study and Control of Tropical Diseases (PECET), Faculty of Medicine, University of Antioquia, 050010 Medellin, Colombia
[31] MIVEGEC, Univ. Montpellier, CNRS, IRD, 34394 Montpellier, France & Medical Entomology Unit, Institut Pasteur de Madagascar, 101 Antananarivo, Madagascar
[32] Laboratorio de Entomología Médica, Departamento de Zoología de Invertebrados, Facultad de Ciencias Biológicas, Universidad Autónoma de Nuevo León, San Nicolás de los Garza, 66455, NL, México
[33] Tropical and Infectious Disease Centre, BP Koirala Institute of Health Sciences, Dharan 56700, Nepal
[34] ICMR-Vector Control Research Centre, Puducherry 605006, India
[35] Graduate School of Agricultural and Life Sciences, The University of Tokyo, Tokyo 113-8657, Japan
[36] Grupo de estudos em Leishmanioses/Coleção de Flebotomíneos (COLFLEB/Fiocruz-MG), Instituto René Rachou, Fundação Oswaldo Cruz, Belo Horizonte, Minas Gerais, 30190009, Brazil
[37] Center of Excellence in Vector Biology and Vector-Borne Disease, Department of Parasitology, Faculty of Medicine, Chulalongkorn University, Bangkok 10330, Thailand
[38] Department of Arbovirology, Bernhard Nocht Institute for Tropical Medicine, Bernhard Nocht Str. 74, 20359 Hamburg, Germany [39] Laboratory Vectors & Parasites, Department of Livestock Sciences and Techniques, Sine Saloum University El Hadji Ibrahima Niasse (SSUEIN) Kaffrine Campus, C.P. 24600, Senegal.
[40] Environmental Health Institute, National Environment Agency, Singapore 138667, Singapore & Department of Biological Sciences, National University of Singapore, 117558 Singapore
[41] Institut Pasteur du Laos, Laboratory of Vector-Borne Diseases, Samsenhai Road, Ban Kao-Gnot, Sisattanak District, 3560 Vientiane, Lao PDR
[42] National Institute of Hygiene and Epidemiology, 1 Yec-Xanh Street, Hai Ba Trung District, 100000 Hanoi, Vietnam
[43] Indonesian Research Center for Veterinary Science, Indonesian Agency for Agricultural Research and Development, Ministry of Agriculture Republic Indonesia, Bogor 16114, Indonesia & Department of Parasitology, Faculty of Veterinary Medicine, Airlangga University, Surabaya 60115, Indonesia
[44] Laboratory of Research in Applied Biology, Polytechnic School of Abomey-Calavi, University of Abomey-Calavi, 01 P.O. Box 2009, 00000 Cotonou, Benin
[45] Instituto de Microbiología, Colegio de Ciencias Biológicas y Ambientales (COCIBA), Universidad San Francisco de Quito (USFQ), 170901 Quito, Ecuador

Received 1 December 2025, Accepted 29 January 2026, Published online 3 April 2026

Abstrak – Artikel ini menyediakan panduan komprehensif mengenai pemprosesan dan pelekapan spesimen lalat pasir phlebotomine, yang merupakan prosedur penting dalam pengecaman spesies serta pengesanan dan pengasingan patogen. Panduan ini membincangkan pelbagai teknik yang sesuai diaplikasikan dalam kondisi lapangan dan makmal, termasuk arahan terperinci mengenai pengumpulan, pengendalian, penutupan, dan eutanasia lalat pasir (mengesyorkan pengunaan penyejukbekuan kering atau CO_2 berbanding bahan kimia), serta strategi konservasi, seperti storan sejuk dan pengawetan dalam etanol. Kualiti penyediaan struktur anatomi tertentu (organ genital, kepala, dan sayap) adalah penting bagi memastikan pemerhatian mikroskopik yang tepat, dan perkara ini dihuraikan dengan jelas dalam artikel ini. Artikel ini juga membentangkan pemprosesan sampel secara terperinci, termasuk proses penjernihan menggunakan agen seperti kalium hidroksida yang diikuti dengan larutan Marc-André. Dalam proses pelekapan, pelbagai medium dinilai secara perbandingan dengan memberi tumpuan kepada sifat optik dan potensi pengawetan jangka panjang. Penggunaan cecair Hoyer (turut dikenali sebagai gam kloral) adalah disyorkan bagi tujuan pemerhatian pantas, khususnya bagi struktur spermateka, kerana mempunyai kejernihan yang tinggi, walaupun medium ini tidak sesuai untuk penyimpanan jangka panjang. Medium lain yang turut dibincangkan termasuk alkohol polivinil, Euparal® (untuk toleransi air yang terhad), dan balsam Kanada (medium larut hidrokarbon), dengan dua medium terakhir menawarkan keupayaan pengawetan jangka panjang. Pendekatan inovatif dalam biologi molekul, termasuk penjujukan DNA dan MALDI-ToF, turut dibincangkan dengan penekanan terhadap keperluan pengendalian teliti dalam pemprosesan

sampel. Tambahan pula, klip video pendek yang memaparkan beberapa teknik pelekapan, berserta terjemahan dalam pelbagai bahasa, turut disediakan bagi membolehkan panduan ini memenuhi keperluan dan jangkaan yang meluas komuniti saintifik di peringkat global.

Kata Kunci: Pelekapan, lalat pasir phlebotomine, cecair Hoyer, larutan Marc-André, gam kloral, alkohol polivinil, Euparal®, balsam Kanada, pengasingan *Leishmania*, kondisi lapangan, kultur, pembedahan, biologi molekul, MALDI-ToF, spesimen rujuk

Abstract – Processing and mounting phlebotomine sand flies: a consensus guideline. This article provides a comprehensive guide for the processing and mounting of phlebotomine sand fly specimens, which is crucial for species identification and pathogen detection and isolation. It discusses a range of techniques suitable for both field and laboratory settings. The guide includes detailed instructions on sand fly collection, handling, covering, and euthanasia (recommending dry freezing or CO_2 over chemicals) as well as conservation strategies, such as cold storage and preservation in ethanol. The quality of preparation of certain anatomical structures (genital organs, head and wings) is essential for their proper microscopic observation and is described in this work. The article also presents detailed sample processing, including the clearing process with agents such as potassium hydroxide then Marc-André solution. The mounting process compares different media, emphasizing their optical properties and preservation potential. Hoyer fluid (also known as chloral gum) is recommended for quick observation, particularly for spermathecae, due to its clarity, although it is not suitable for long-term storage. Other media discussed include polyvinyl alcohol, Euparal® (for limited water tolerance), and Canada balsam (a hydrocarbon-soluble medium), with the latter two offering long-term preservation capabilities. Innovative molecular biology approaches such as DNA sequencing and MALDI-ToF, which require particular attention to sample processing, are also addressed. Furthermore, short video clips illustrating various mounting techniques as well as translations in many different languages are provided, allowing the guideline to reach the diverse needs and expectations of the global scientific community.

Key words: Mounting, Phlebotomine sand fly, Hoyer fluid, Marc-André solution, Chloral gum, Polyvinyl alcohol, Euparal®, Canada balsam, *Leishmania* isolation, Field conditions, Culture, Dissection, Molecular biology, MALDI-ToF, Type-specimens.

Pengenalan

Lalat pasir phlebotomine merupakan serangga diptera kecil yang tergolong dalam famili Psychodidae, subfamili Phlebotominae, dengan sekurang-kurangnya 1,063 spesies yang diketahui [21]. Serangga ini merupakan vektor penting bagi patogen (*Leishmania*, arbovirus, dan *Bartonella*) yang bertanggungjawab terhadap penyakit yang dikenali sebagai leishmaniasis, jangkitan arbovirus, dan bartonellosis. Pengecaman spesies lalat pasir ini bergantung terutamanya pada pemeriksaan mikroskopik terperinci, yang dibantu oleh pengumpulan spesimen yang teliti, penyimpanan yang sesuai, dan pelekapan pada slaid mikroskop secara cermat, yang memerlukan beberapa teknik khusus dengan masing-masing mempunyai kelebihan dan hadnya tersendiri.

Pengecaman lalat pasir phlebotomine dewasa memerlukan kepada pemerhatian kedua-dua struktur luaran (contohnya, antena, palpi, genitalia jantan) dan struktur dalaman (contohnya, farinks, sibarium, dan spermateka). Pembedahan dan pengasingan struktur dalaman ini memudahkan pemerhatian terperinci dan seterusnya memastikan pengecaman yang tepat. Oleh sebab itu, berbeza dengan nyamuk atau pepijat cium, lalat pasir phlebotomine memerlukan pelekapan di antara slaid mikroskop dan kaca penutup sebelum pengecaman spesies dapat dilakukan. Sehingga tahun 1980-an, pemerhatian mikroskopik merupakan satu-satunya kaedah yang tersedia untuk pengecaman spesies lalat pasir, dan kaedah ini kekal sebagai pendekatan yang paling meluas digunakan sehingga hari ini. Justeru, pemilihan kaedah pemprosesan dan penyediaan spesimen adalah tidak rumit dan berasaskan dua pendekatan utama: pelekapan definitif untuk pengawetan jangka panjang spesimen; atau pelekapan pantas untuk pengecaman spesies menggunakan medium yang tidak menjamin pengawetan jangka panjang. Dalam proses pelekapan akhir, contohnya menggunakan resin seperti balsam Kanada, prosedur ini memerlukan tempoh masa yang panjang kerana spesimen perlu melalui proses penyahhidratan sepenuhnya. Selain itu, indeks biasan medium ini tidak sentiasa optimum, sekali gus menyukarkan pemerhatian mikroskopik yang jelas dan mudah terhadap struktur spermateka. Sebaliknya, pelekapan dalam medium berakua, seperti cecair Hoyer, adalah lebih cepat dan membolehkan visualisasi spermateka yang terbias dengan lebih jelas, namun medium ini tidak sesuai untuk pengawetan jangka panjang kerana cenderung menyerap kelembapan daripada atmosfera. Bagi mengatasi kekurangan tersebut, slaid mikroskop boleh dikedapkan dengan pengilat kuku setelah kering sepenuhnya. Pilihan timbal balas ini masih wujud hingga kini, dan secara langsung mempengaruhi pemilihan kaedah pelekapan mengikut keperluan dan tujuan penyediaan spesimen. Sejak tahun 1980-an, kajian pengecaman spesies lalat pasir telah menggabungkan kaedah morfologi dan pendekatan biokimia. Kaedah terawal yang digunakan ialah analisis

hidrokarbon kutikel, yang kemudiannya digantikan dengan teknik biologi molekul seperti DNA Polimorfisme Teramplifikasi Rawak (RAPD), Polimorfisme Panjang Serpihan Penyekatan (RFLP), amplifikasi DNA dan penjujukan berasaskan kaedah Sanger serta Penjujukan Generasi Baharu (NGS). Kini, pendekatan molekul tersebut turut dilengkapi dengan kaedah proteomik seperti MALDI-ToF bagi meningkatkan ketepatan pengecaman spesies lalat pasir. Tambahan pula, pengecaman spesies secara molekul boleh digabungkan dengan pengesanan patogen seperti *Leishmania*, *Trypanosoma*, *Bartonella* dan *Phlebovirus* dengan menggunakan teknik Tindak Balas Berantai Polimerase (PCR), sama ada secara konvensional (*end-point*) atau masa nyata (*real-time*), yang seterusnya memerlukan penyesuaian terhadap protokol pensampelan serta kaedah penyimpanan spesimen mengikut objektif kajian yang ditetapkan [3,32]. Selain ciri morfologi yang digunakan secara tradisional untuk tujuan diskriminasi spesies lalat pasir, pendekatan morfologi lain juga boleh digunakan, misalnya geomorfometri sayap. Oleh yang demikian, berdasarkan pengalaman penulis sendiri dan data literasi, tujuan penyiasatan ini adalah untuk menyediakan garis panduan standard bagi pemprosesan dan pelekapan lalat pasir phlebotomine dewasa, bagi mengoptimumkan analisis morfologi dan molekul. Pengekalan sebahagian struktur luaran lalat pasir, walaupun tidak digunakan dalam pengecaman morfologi, adalah perlu bagi membolehkan pelaksanaan analisis lanjutan seperti biologi molekul atau MALDI-ToF, sekali gus menekankan kepentingan pemilihan protokol yang tepat dan bersesuaian. Dalam artikel ini, kami memberi penekanan mengenai kaedah anestesia dan eutanasia lalat pasir yang ditangkap dalam keaadaan hidup, strategi penyimpanan, serta teknik pelekapan, sama ada untuk pengecaman pantas atau tujuan konservasi jangka panjang yang membolehkan analisis lanjut dilaksanakan.

Mukadimah: Semua pertimbangan keselamatan dan pematuhan peraturan hendaklah dibuat dengan merujuk kepada Lembaran Data Keselamatan (Safety Data Sheets, SDS) yang berkaitan.

Semua bahan kimia yang dinyatakan dalam garis panduan ini mesti dikendalikan di bawah syarat keselamatan yang ketat. Jawatankuasa Kesihatan dan Keselamatan di setiap fasiliti penyelidikan boleh dirujuk bagi mendapatkan maklumat bukan sahaja mengenai bahaya bahan kimia tersebut, tetapi juga berkaitan prosedur pengendalian dan pelupusan sisa. Walau bagaimanapun, pematuhan terhadap arahan keselamatan berhubung penggunaan dan pelupusannya adalah mandotori. Perlu diambil perhatian bahawa adalah menjadi tanggungjawab semua pengguna untuk memastikan pematuhan terhadap amalan makmal yang baik dan selamat serta mematuhi undang-undang dan peraturan yang berkuat kuasa di negara atau institusi penyelidikan masing-masing. Selain itu, sesetengah bahan kimia atau komponennya, seperti kloral hidrat, adalah tertakluk kepada peraturan kawal selia di beberapa negara. Senarai singkatan yang digunakan dalam manuskrip ini disediakan dalam Jadual 1.

Jadual 1: Senarai singkatan.

**BME**	Medium asas Eagle
**CDC**	Pusat Kawalan dan Pencegahan Penyakit
**CMCP**	Kamfor-monoklorofenol
**CMR**	Bahan karsinogenik, mutagenik, dan reprotoksik
**COI**	Gen subunit I sitokrom c oksidase
**CytB**	Gen sitokrom b
**DNA**	Asid deoksiribonukleik
**ELISA**	Asai imunojerapan Berpaut Enzim
**EtOH**	Etanol
**M199**	Medium 199
**MALDI-ToF MS**	Spektrometer jisim masa terbang penyaherapan/pengionan laser dibantu matriks
**MEM**	Medium penting minimum
**NGS**	Penjujukan Generasi Baharu
**NNN**	Medium Novy-MacNeal-Nicolle
**PCR**	Tindak balas berantai polimerase
**RDP Laos**	Republik Demokratik Rakyat Laos
**PNOC**	Gen prepronociceptin
**qPCR**	PCR kuantitatif (*real-time PCR*)
**RAPD**	DNA Polimorfisme Teramplifikasi Rawak
**RFLP**	Polimorfisme Panjang Serpihan Penyekatan
**RI**	Indeks biasan
**RNA**	Asid ribonukleik
**RNases**	Ribonuklease
**RNASS**	Larutan penstabilan RNA
**RT-PCR**	PCR transkripsi songsang
**TFA**	Asid trifluoroasetik

1. Penangkapan lalat pasir

Lalat pasir dewasa boleh dikumpul dalam keadaan hidup atau mati menggunakan pelbagai kaedah seperti perangkap cahaya miniatur CDC, perangkap berpelekat, aspirator menggunakan perangkap Shannon, atau secara langsung daripada tempat rehat di persekitaran (contohnya, tempat perlindungan haiwan). Kaedah-kaedah ini melibatkan pemasangan perangkap di habitat yang sesuai, penggunaan

cahaya atau bahan penarik lain (seperti CO_2 atau umpan kimia) untuk menarik lalat pasir, serta pengumpulan spesimen bagi tujuan analisis lanjut, seperti yang telah dilaporkan dalam beberapa penerbitan [2, 3, 32, 36, 49]. Penangkapan lalat pasir secara hidup membolehkan pelaksanaan pelbagai analisis lanjutan, manakala pengumpulan spesimen yang telah mati boleh menghalang pengasingan strain Leishmania atau virus. Di samping itu, beberapa teknik penangkapan, seperti penggunaan kertas berpelekat, sering mengakibatkan kehilangan bahagian tubuh lalat pasir seperti antena, palpus, sayap atau kaki. Tambahan pula, salutan minyak kastor pada kertas berpelekat akan melekat pada lalat pasir dan perlu disingkirkan pada peringkat awal pemprosesan, lazimnya melalui perendaman selama kira-kira 15 minit dalam campuran etanol dan dietil eter pada nisbah yang sama.

2. Eutanasia spesimen

Selepas proses kutipan, lalat pasir yang hidup perlu dipengsankan secara eutanasia. Beberapa kaedah kutipan (contohnya, kertas berpelekat, perangkap cahaya CDC yang dilengkapi dengan bekas berisi detergen atau etanol), mengakibatkan lalat pasir telah mati semasa proses kutipan. Biologi molekul boleh digunakan ke atas spesimen yang dikutip secara langsung ke dalam etanol dan terhadap spesimen lain jika disimpan dalam etanol secepat mungkin. Walau bagaimanapun, tiada satu pun kaedah eutanasia yang membolehkan pemprosesan serangga menggunakan MALDI-ToF. Selain itu, sesetengah kaedah eutanasia boleh menyebabkan kehilangan sebahagian ciri morfologi pada badan serangga. Oleh itu, penggunaan agen eutanasia standard yang sesuai adalah penting bagi memastikan pengecaman spesies yang tepat atau penyimpanan jangka panjang sebagai spesimen rujukan (iaitu spesimen yang dipelihara dan disimpan untuk rujukan atau perbandingan masa depan). Bahan kimia seperti etil asetat, etil eter, tetrachloroetana, dan kloroform boleh diserap ke dalam kapas dan diletakkan dalam bekas yang mengandungi lalat pasir untuk tujuan euthanasia. Agen eutanasia ini mesti dikendalikan dengan berhati-hati mengikut saranan pengilang kerana sifat toksiknya. Walau bagaimanapun, kami tidak mengesyorkan penggunaan kloroform untuk proses euthanasia lalat pasir kerana, berdasarkan pengalaman kami, bahan kimia ini kurang sesuai untuk kajian biologi molekul. Memandangkan sifat berbahaya semula jadi semua bahan kimia ini serta keraguan terhadap kesesuaian penggunaannya dalam analisis molekul, penggunaan bahan kimia tersebut secara amnya tidak digalakkan. Kaedah yang paling menyeluruh digunakan dan dapat mengekalkan morfologi spesimen, DNA, atau protein adalah kaedah pembekuan kering (*dry freezing*) spesimen. Spesimen mesti dibekukan dalam tempoh yang mencukupi untuk memastikan mereka sepenuhnya telah dipengsankan secara eutanasia, tetapi tidak terlalu lama sehingga mereka (i) menjadi kering atau (ii) mengalami kerosakan terhadap kebolehan hidup *Leishmania*, sekiranya matlamatnya adalah untuk mengasingkan parasit tersebut secara *in vitro* daripada saluran pencernaan lalat pasir. **Oleh itu, kami mengesyorkan tempoh penyejukbekuan selama 15 hingga 20 minit pada suhu -20°C, dengan pemantauan secara berkala untuk memastikan spesimen hanya dipengsankan secara eutanasia tanpa mematikan parasit *Leishmania*.** Jika penyejuk beku tidak tersedia, serangga boleh dipengsankan secara eutanasia menggunakan CO_2. Dalam keadaan lapangan di mana silinder CO_2 tidak boleh digunakan, spesimen boleh dipengsankan menggunakan bekas CO_2 komersial kecil yang digunakan dalam 'Soda siphons' (dispenser minuman), walaupun terdapat kemungkinan sekatan pengangkutannya melalui udara. Sebagai langkah terakhir, serangga juga boleh dipengsankan melalui pendedahan kepada asap rokok. Lalat pasir ditangkap dalam keadaan hidup dalam perangkap CDC, dikutip menggunakan aspirator, seterusnya disimpan dalam tiub kaca, dan didedahkan kepada asap rokok yang dapat mengakibatkan lalat pasir pengsan dalam beberapa saat. Kaedah ini sesuai digunakan dalam semua keadaan lapangan, termasuk situasi pengasingan yang sukar. Namun demikian, tiub kaca tersebut tidak sesuai digunakan untuk tujuan pengumpulan dan pengendalian lalat pasir hidup tanpa pembersihan menyeluruh, kerana residu asap terperangkap pada permukaan kaca. Walau bagaimanapun, aspirator yang sama tanpa dibersihkan masih boleh digunakan untuk proses eutanasia lalat pasir dari perangkap lain bagi tujuan fiksasi. Adalah juga penting untuk memastikan semua spesimen telah dikeluarkan daripada aspirator. Semua kaedah ini adalah sesuai untuk pengasingan *Leishmania* melalui pembedahan gut lalat pasir.

3. Penyimpanan spesimen sebelum pemprosesan

Terdapat lima kaedah utama bagi fiksasi spesimen sebelum pemprosesan:

3.1. Penyejukbekuan

Kaedah ini paling sesuai dijalankan pada suhu -20°C atau, lebih disarankan, pada suhu -80°C. Kini, kaedah penyimpanan ini lebih banyak digunakan berbanding penyimpanan dalam nitrogen cecair. Dalam semua keadaan, krioawetan perlu dilaksanakan secepat mungkin selepas proses spesimen dipengsankan. Storan sejuk dalam penyejuk beku memberi kelebihan dalam pengawetan yang menyeluruh terhadap serangga serta dapat mengekalkan keutuhan RNA, DNA, dan protein sepenuhnya sepanjang tempoh penyimpanan. Sebaliknya, nitrogen cecair boleh merosakkan sayap, kaki, palpus, dan antena, sering mengakibatkan terputus dan kadangkala kehilangan ciri morfologi utama. Penyimpanan kering dalam penyejuk beku memberi kesan yang kurang traumatik terhadap spesimen, tetapi tidak ideal untuk mengekalkan organ halus pada spesimen. Penting untuk diperhatikan bahawa semasa

proses nyahsejuk bekuan, sayap, antena, palpus, atau kaki mungkin melekat pada tiub dan akhirnya terkoyak akibat kondensasi. Walau bagaimanapun, pengawetan melalui penyejukbekuan kurang praktikal untuk dilaksanakan dalam kajian lapangan kerana memerlukan akses kepada penyejuk beku atau bekas nitrogen cecair. Penyimpanan dalam penyejuk beku adalah sepenuhnya serasi dengan pengesanan patogen menggunakan kaedah molekul tanpa mengurangkan sensitiviti, walaupun pengesanan dan pengasingan virus RNA memerlukan penyejukbekuan pada suhu -80°C atau dalam nitrogen cecair jika penyimpanan jangka panjang diperlukan. Namun, penyejukbekuan spesimen tidak membolehkan pengasingan *Leishmania* dilaksanakan melalui pembedahan gut, kecuali jika lalat pasir direndam terlebih dahulu dalam fasa wap dan kemudian dalam nitrogen cecair (contohnya dalam vail yang diletakkan dalam penstokan), bagi mensimulasikan proses krioawetan *Leishmania*.

3.2. Penyimpanan dalam alkohol (etanol atau alkohol Isopropil)

Kaedah ini merupakan antara yang paling banyak digunakan untuk penyimpanan lalat pasir. Kaedah ini juga mudah dilaksanakan di lapangan, termasuk dalam keadaan sukar tanpa akses kepada makmal. Pengawetan dalam alkohol amat sesuai untuk kajian morfologi, kerana organ halus seperti sayap, kaki, antena, atau palpus akan kekal utuh sekiranya tiub penyimpanan bebas daripada gelembung udara. Oleh itu, kami menyarankan agar tiub dikedap dengan kapas kecil bagi mengeluarkan sebarang gelembung udara dan meletakkan label di atas palam kapas (Rajah 1). Kadar kepekatan alkohol yang sesuai masih menjadi perdebatan. Secara amnya, kepekatan di bawah 70% tidak digalakkan [45, 66]. Kepekatan tinggi dapat mengekalkan keutuhan DNA dengan lebih berkesan dan untuk tempoh yang lebih lama, namun akan menjadikan spesimen lebih rapuh untuk kajian morfologi. Penggunaan etanol 96% (campuran azeotrop) dapat memastikan kestabilan kepekatan sepanjang masa, terutamanya di kawasan beriklim lembap seperti negara tropika, walaupun etanol 95% biasanya lebih mudah diperoleh. Tanpa mengira kepekatan, DNA umumnya terpelihara dengan baik dalam etanol, walaupun kurang berkesan berbanding kaedah penyejukbekuan, terutamanya untuk teknik molekul jenis NGS. Protein pula menunjukkan kestabilan yang rendah, khususnya dalam aplikasi proteomik seperti MALDI-ToF. Lalat pasir yang diawet dalam alkohol selama beberapa bulan masih boleh dikenal pasti secara morfologi, tetapi tidak boleh digunakan untuk menghasilkan spektrum protein rujukan. Penyimpanan dalam alkohol atau keadaan kering boleh dipertingkatkan dengan pembekuan sampel pada suhu -20°C. Penyejukbekuan pada suhu -20°C terutamanya akan meningkatkan pengawetan molekul (contohnya asid nukleik) dengan memperlahankan pemerosotan, serta memberikan manfaat tambahan bagi pengawetan morfologi dengan mengurangkan penguraian tisu dari semasa ke semasa, walaupun kesannya terhadap morfologi lebih terhad berbanding terhadap integriti molekul. Penyimpanan dalam etanol juga boleh digunakan untuk pengesanan DNA dan virus RNA dengan menggunakan etanol sekurang-kurangnya 70% untuk tempoh penyimpanan pendek yang kurang daripada beberapa bulan. Selain itu, alkohol isopropil mudah diperoleh di beberapa negara dan mampu mengekalkan keutuhan DNA tetapi menyebabkan spesimen menjadi kaku. Alkohol ini tidak mudah terbakar seperti etanol, sekali gus mudah untuk diangkut. Apabila diperlukan, lalat pasir yang diawet dalam nitrogen cecair atau bekuan kering boleh dipindahkan ke dalam alkohol, dengan risiko menggabungkan kelemahan kedua-dua kaedah tersebut.

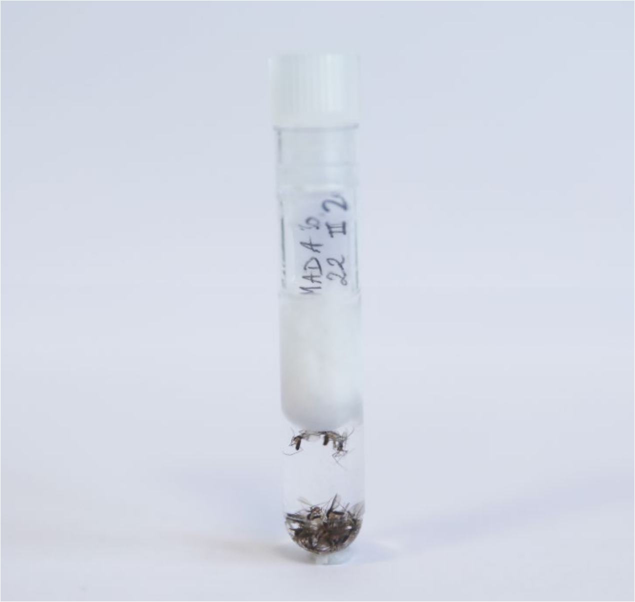

Rajah 1: Lalat pasir diawet dalam etanol.

3.3. Penyimpanan dalam larutan penstabil RNA (RNASS)

Reagen berasaskan akua ini digunakan secara meluas, bersifat tidak toksik, dan direka bentuk untuk menstabilkan serta melindungi RNA dalam sampel tisu dan sel segar. Reagen ini bertindak dengan menembusi spesimen dengan cepat dan menyahaktifkan RNase (enzim yang menguraikan RNA), sekali gus menghalang pemerosotan RNA tanpa memerlukan penyejukbekuan segera. Penyimpanan dalam RNASS secara amnya berkesan dalam mengekalkan morfologi keseluruhan tisu dan struktur sel untuk penilaian histologi seterusnya. Walaupun RNASS dioptimumkan untuk penstabilan RNA dan bukannya sebagai bahan fiksasi, penyimpanan dalam tempoh jangka pendek hingga sederhana lazimnya masih mengekalkan keutuhan struktur spesimen dengan baik. RNASS membolehkan sampel disimpan pada suhu bilik sehingga 7 hari, pada suhu 4°C selama beberapa minggu, atau pada suhu −20°C hingga

−80°C untuk penyimpanan jangka panjang. Kaedah ini amat berguna dalam kerja lapangan atau persekitaran klinikal yang mempunyai kemudahan rantaian sejuk yang terhad. Bagi tujuan pengekstrakan RNA, sampel lazimnya perlu dikeluarkan terlebih dahulu daripada reagen tersebut sebelum diproses mengikut protokol standard.

3.4. Pengawetan kering pada suhu bilik

Kekurangan utama kaedah lama ini apabila digunakan pada spesimen *in toto* (seluruhnya dilekap) ialah tidak dapat mengawet organ halus seperti sayap, kaki, antena, dan palpus dengan baik. Walau bagaimanapun, kajian proteomik menggunakan MALDI-ToF masih boleh dijalankan sekiranya penyahhidratan dilakukan semasa proses fiksasi menggunakan bahan pengering jenis gel silika. Sebaliknya, analisis molekul yang menyasarkan DNA akan sukar dilakukan pada sampel ini kerana DNA sering terserpih dan kuantitinya adalah rendah, yang akan menjadikan analisis lebih mencabar berbanding sampel segar atau beku, terutamanya bagi genom nuklear. Teknik terkini seperti museomik boleh diaplikasikan pada sampel jenis ini [34]. Oleh itu, kaedah penyimpanan ini tidak digalakkan kecuali tiada alternatif lain. Kaedah ini boleh digabungkan dengan storan sejuk dengan meletakkan tiub dalam penyejuk beku pada suhu −20°C atau −80°C. Cabaran utama kaedah ini adalah memastikan pelekapan yang sesuai bagi spesimen atau bahagian badan yang diperlukan untuk pengecaman spesies. Bagi tujuan ini, proses penghidratan semula adalah penting, dan kami mencadangkan penggunaan larutan Triton X-100. Tempoh penghidratan semula adalah berbeza, daripada beberapa jam hingga beberapa hari, dengan pemantauan rapi secara berkala. Setelah tempoh penghidratan semula lengkap, spesimen harus dibilas dalam alat mandian air sebanyak tiga kali secara berturut-turut.

3.5. Pengawetan pada kertas turas

Kelebihan utama penggunaan kertas turas ialah kestabilan jangka panjang DNA genomik dalam sel yang tidak ditetap, seluruh badan yang dikeringkan, atau sel darah yang disimpan pada suhu bilik. Kertas turas disediakan dalam saiz kad kecil, sekali gus membolehkan penyimpanan beberapa ratus sampel pada suhu bilik dalam isipadu bersamaan saiz kotak kecil. Matriks kertas turas diisitepukan dengan agen yang boleh menyahaslikan agen jangkitan, dan seterusnya menjadikan sampel pada kertas turas tidak lagi dianggap sebagai bahaya biologi. Hal ini membolehkan penyimpanan dan pengangkutan sampel tanpa memerlukan langkah keselamatan bahaya biologi yang khusus [68].

4. Pembedahan spesimen

Berbeza dengan kebanyakan serangga lain yang dikenal pasti berdasarkan ciri luaran yang dapat diperhatikan pada setiap serangga yang dipin secara *in toto*, lalat pasir memerlukan pembedahan dan pelekapan pada slaid mikroskop untuk mengkaji ciri anatomi bagi pengecaman spesies yang tepat. Tanpa mengambil kira mengenai prosedur persediaan dan pelekapan yang dipilih, teknik pembedahan yang sama akan turut digunakan (Rajah 2 & 3) (https://zenodo.org/records/18198006).

Penggunaan Triton X100: larutan berakua bukan ion

Perlu diambil perhatian bahawa proses pelekapan ini hanya melibatkan spesimen yang baru ditangkap atau disimpan dengan baik. Kebanyakan pengumpul menyimpan sampel serangga secara kering (untuk kegunaan MALDI-ToF) atau dalam alkohol untuk jangka masa bertahun-tahun. Malangnya, pengawetan dalam alkohol tidak optimum untuk tempoh yang panjang, dan artropod yang disimpan dengan cara ini menjadi sukar untuk disediakan bagi pemeriksaan mikroskopik. Kejadian yang sering berlaku ialah pereputan plastik yang mengandungi sampel, diikuti oleh penyejatan alkohol. Dalam kedua-dua kes, tiada pilihan kerana sampel terlalu lama dalam alkohol atau menjadi kering. Oleh itu, timbul idea untuk menggunakan agen pembasah yang merupakan bukan detergen kuat. Triton X-100 ialah dalam bentuk larutan berakua bukan ion (*4-(1,1,3,3-tetrametilbutil*) larutan *fenil-polietilena glikol*, atau *t-oktilfenoksipolietoksietanol*), *etilena glikol tert-oktilfenil eter*), yang banyak digunakan sebagai detergen dalam sel dan biologi molekul. Larutan ini menyebabkan peningkatan terhadap kebolehtelapan sel dan membran nukleus.

Berikut merupakan prosedur menggunakan Triton X-100 bukan ion dalam larutan berakua 0.5%:

- Sampel kering diisitepukan dengan alcohol mutlak.
- Tambahkan larutan Triton X-100 berkepekatan 0.5% pada isipadu yang mencukupi supaya keseluruhan sampel terendam sepenuhnya.
- Biarkan sampel berada dalam larutan selama 5 minit hingga beberapa hari, dengan pemantauan berkala. Semua artropod hendaklah terpisah sepenuhnya dalam larutan tersebut.
- Larutan Triton X-100 kemudian disingkirkan dan digantikan dengan larutan kalium hidroksida.

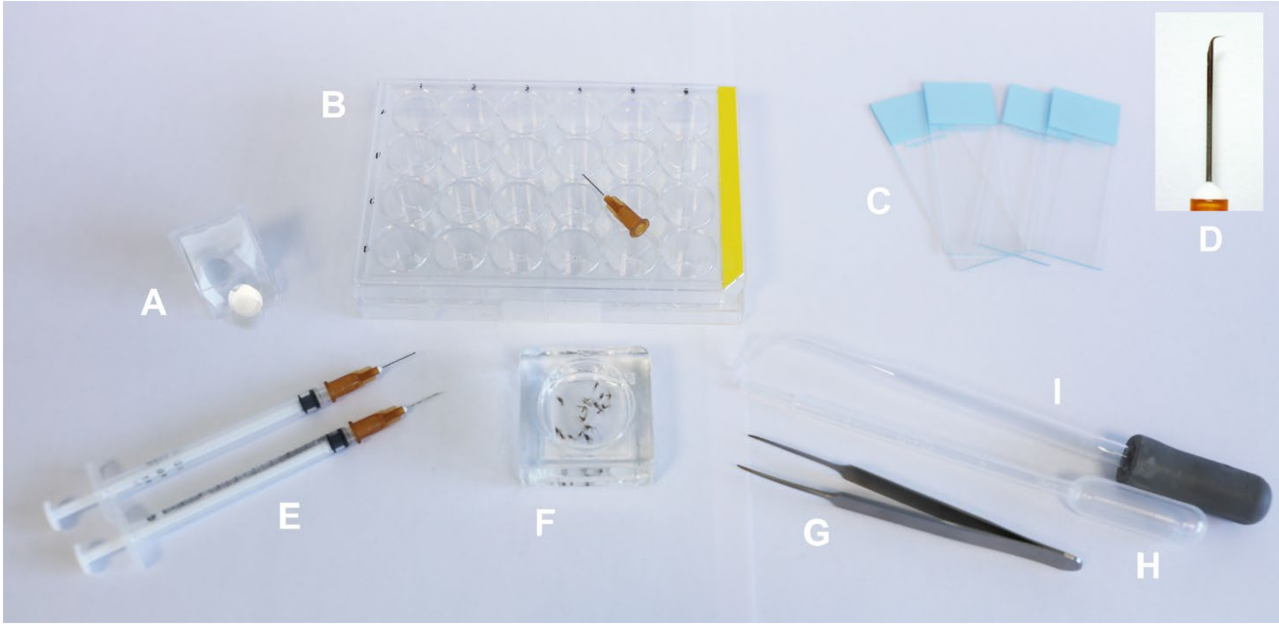

Rajah 2: Bahan-bahan yang diperlukan untuk pelekapan lalat pasir: A: Penutup kaca bulat (diameter 10mm atau 12mm); B: Plat 24-telaga dan jarum berkait (sekiranya minyak cengkih atau esen Euparal® digunakan untuk memproses lalat pasir, jangan gunakan plat akrilik kerana tindak balas kimia boleh berlaku dan spesimen akan rosak); C: Slaid kaca yang sesuai untuk pelabelan; D: perincian hujung jarum berkait; E: Jarum yang dipasang pada picagari; F: kaca tilik atau bekas setara yang dapat memegang lalat pasir untuk dilekapkan pada slaid; G: forseps Dumont; H: pipet plastik; I: pipet kaca yang dibengkokkan melalui pemanasan bagi memudahkan pemindahan cecair ke dalam telaga.

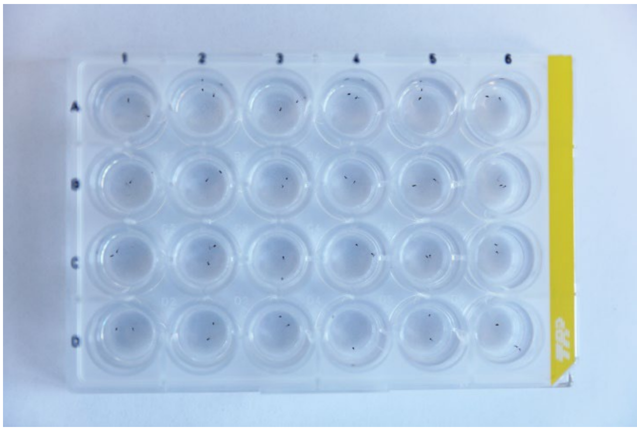

Rajah 3: Plat 24-telaga, setiap telaga mengandungi bahagian kepala dan hujung abdomen lalat pasir.

4.1. Kepala

Pembedahan boleh dilakukan menggunakan jarum halus atau pin entomologi di bawah mikroskop stereo (Rajah 2 & 3). Antara jarum yang paling lazim digunakan ialah: 26G x 1/2" (0.45 × 13 mm), 30G × 1/2" (0.3 × 13 mm), or 25G × 5/8" (0.5 × 16 mm). Bagi penyediaan spesimen untuk tujuan pengecaman, sekurang-kurangnya kepala perlu dipisahkan daripada badan dan dilekap dalam kedudukan ventral menghadap ke atas untuk memaparkan sibarium dan farinks, manakala toraks dan abdomen dilekap dalam kedudukan lateral selepas proses pembedahan. Pelekapan kepala dalam kedudukan ventro-dorsal bagi memastikan foramen oksipital berorientasikan ke atas, sekali gus membolehkan sibarium diperhatikan secara langsung. Akses kepada ciri-ciri anatomi ini menjadi lebih mudah apabila kepala dipisahkan sepenuhnya daripada badan.

4.2. Sayap dan toraks

Bahagian sayap hendaklah dilekap dalam kedudukan mendatar. Setiap bahagian sayap boleh dipisahkan pada pangkalnya dan dilekap secara berasingan, atau satu bahagian sayap sahaja dilekap manakala satu lagi dibiarkan melekat pada toraks. Jika analisis morfometri geometri adalah dirancang, sayap kanan dan kiri hendaklah dikenal pasti dan dilabel dengan tepat sebelum proses pelekapan dilakukan. Toraks terbahagi kepada beberapa bahagian, dan setiap bahagian mengandungi maklumat taksonomi yang sangat penting [20, 64]. Secara umumnya, toraks dilekap dalam kedudukan lateral bagi membolehkan pemerhatian terhadap ketotaksi dan taburan warna. Kehadiran parut seta pada kawasan tertentu toraks boleh digunakan untuk membezakan beberapa spesies dalam genus *Brumptomyia*. Taburan warna boleh digunakan untuk membezakan lalat pasir Neotropika pada peringkat genus (contohnya

Bichromomyia), siri spesies (contohnya *Pintomyia*), atau bahkan spesies dalam genus yang sama (contohnya *Micropygomyia*, *Nyssomyia*, *Psathyromyia*, dan *Psychodopygus*) [20]. Oleh itu, sekiranya toraks tidak digunakan untuk analisis molekul, toraks hendaklah dilekap dengan cara yang tidak menyebabkan kerosakan pada struktur tersebut. Perlu diperhatikan bahawa aspek yang penting bukanlah keamatan warna, tetapi taburan warna pada toraks. Justeru, proses penjernihan tidak akan menghilangkan pigmen atau corak taburan warna pada toraks.

4.3. Genitalia

Perhatian khusus perlu diberikan semasa pelekapan organ genital bagi kedua-dua jantan dan betina, kerana organ ini amat penting untuk pengecaman genus, subgenus, dan spesies lalat pasir. Kedua-dua jantina memiliki organ genital yang berpasangan.

4.3.1. Jantan

Genitalia adalah organ luaran dan terdiri daripada forceps berpasangan, masing-masing mengandungi artikulasi gonokoksit-gonostil pada bahagian dorsal dan lobus epandrium pada bahagian ventral. Gonostil mempunyai duri dan kadang-kadang seta, yang perlu boleh dikira dan kedudukan sisipannya mesti dapat dilihat dengan jelas. Penting untuk memerhatikan permukaan dalam gonokoksit, yang mungkin mempunyai sekumpulan seta sessile atau yang dibawa oleh lobus (= tuberkel) [22]. Para penyelidik yang kurang berpengalaman dalam pembedahan boleh melakukan pelekapan lateral mudah tanpa memisahkan genitalia daripada hujung abdomen (https://zenodo.org/records/18311158). Dalam kes ini, superposisi bagi kedua-dua bahagian genitalia mungkin menyukarkan pengiraan setae dalaman gonokoksit, contohnya, namun langkah ini mengelakkan kerosakan pada genitalia akibat pembedahan yang gagal. Para penyelidik yang lebih berpengalaman boleh mencuba membuka genitalia kepada dua bahagian bagi memisahkannya. Untuk melakukan ini, sisi berbucu jarum (jenis jarum reaksi intradermal) mesti dimasukkan, memisahkan tanpa memotong sepenuhnya genitalia bagi membahagikan set perhimpunan gonokoksit-gonostil (https://zenodo.org/records/18311158). Dengan cara ini, pemerhatian pada permukaan dalaman menjadi lebih mudah. Susunan ini juga memudahkan pemerhatian parameres dan selongsong parameral, yang tidak lagi bertindih. Bagi pelekapan lateral, yang menyebabkan superposisi organ, spesimen tersebut mesti dibersihkan sepenuhnya.

4.3.2. Betina

Perkakas genital adalah organ dalaman yang terdiri daripada spermateka. Jika pembedahan tidak dilakukan, spermateka mesti diperhatikan melalui tegumen dengan pelekapan abdomen dalam kedudukan ventral. Tanpa mengira medium pelekapan yang digunakan, spermateka secara amnya dapat diperhatikan dengan tepat, terutamanya jika permukaannya tidak licin dan telah dijelaskan. Namun, pemerhatian spermateka yang licin dan dindingnya nipis boleh menjadi sukar dalam medium yang kurang refraktil. Selain itu, pemerhatian pangkalan saluran spermateka adalah penting bagi pengecaman spesies, seperti pada subgenus *Larroussius* [35, 37, 38], yang merupakan vektor utama bagi *Leishmania infantum* di Dunia Lama. Tanpa pemerhatian ini, pengecaman spesimen adalah mustahil. Bagi mengatasi kesukaran pemerhatian, pelekapan furka genital-spermateka boleh dikeluarkan daripada abdomen (https://zenodo.org/records/18311106). Spermateka biasanya sukar diperhatikan semasa pembedahan, namun furka genital secara relatifnya mudah dikesan. Memandangkan saluran spermateka membuka ke dalam furka genital, pengasingan furka ini biasanya membolehkan spermateka diasingkan. Sekiranya spermateka secara tidak sengaja terpotong semasa proses tersebut, spermateka tidak hilang dan masih boleh diperhatikan dalam integumen abdomen (Rajah 4).

4.4. Pembedahan gut tengah bagi pengasingan *Leishmania*

Pembedahan saluran pencernaan adalah penting untuk mengesan dan mengasingkan *Leishmania* pada lalat pasir betina. Prosedur ini boleh dijalankan sama ada di lapangan atau makmal bagi menilai kemampuan vektor. Adalah disarankan untuk menggunakan lalat pasir betina yang baru dipengsankan secara eutanasia. Basuh lalat pasir betina dengan air atau larutan saline yang mengandungi detergen ringan bagi menyingkirkan bulu-bulu berlebihan. Langkah ini membantu mengekalkan keadaan aseptik semasa pengasingan *Leishmania*, sementara ciri morfologi yang diperlukan untuk pengecaman spesies dapat dikekalkan.

Bagi tujuan mencari dan mengasing *Leishmania*, keluarkan gut tengah dengan berhati-hati dan letakkan di atas satu titisan larutan saline steril (NaCl 0.9%). Setelah mengamati parasit yang bergerak di bawah mikroskop cahaya (pembesaran yang disyorkan: ~200×), gunakan picagari insulin atau mikropipet untuk memindahkannya ke dalam medium kultur (untuk maklumat lanjut lihat Bab 4.4.3).

Kepala dan genitalia betina dilekap secara langsung dalam larutan Marc-André untuk dijernihkan. Penting: jangan biarkan larutan Marc-André bersentuhan dengan *Leishmania* sama ada secara langsung atau tidak langsung melalui alat atau jarum, kerana larutan tersebut mampu mematikan parasit.

Pembedahan lalat pasir betina boleh dilakukan pada satu atau dua slaid; kedua-dua kaedah mempunyai kelebihan dan keterbatasan masing-masing (Rajah 5; https://zenodo.org/records/18311154).

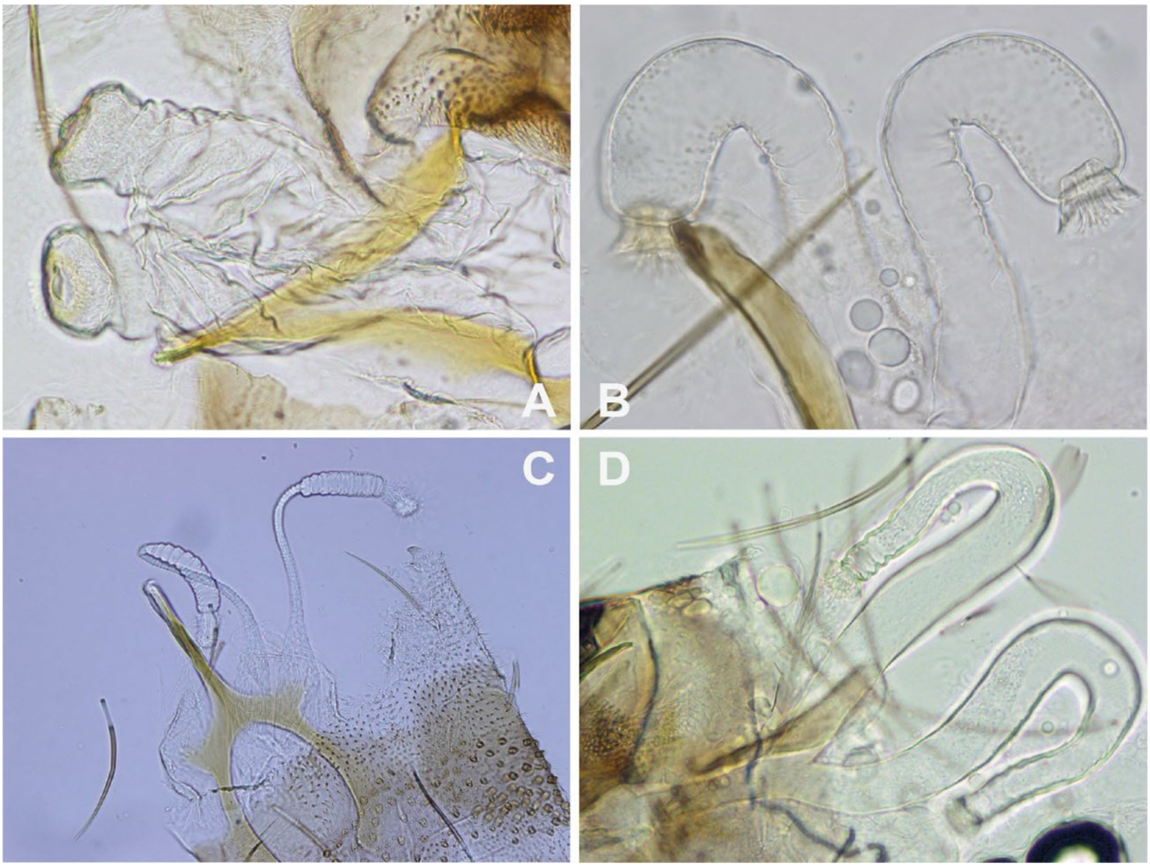

Rajah 4: Spermateka dibedah dan dilekap dalam cecair Marc-André daripada spesimen segar. A: *Idiophlebotomus longiforceps* (RDP Laos); B: *Sergentomyia minuta* (Perancis); C: *Phlebotomus ariasi* (Perancis); D: *Sergentomyia anodontis* (RDP Laos).

4.4.1. Kaedah dua slaid

Pilihan pertama melibatkan penggunaan dua slaid berasingan: satu mengandungi larutan saline steril untuk mengekstrak gut tengah, dan satu lagi untuk melekap kepala dan spermateka dalam larutan Marc-André. Namun, di lapangan, adalah perkara biasa bagi dua hingga tiga individu melakukan pembedahan lalat pasir dan menyerahkan hasilnya kepada seorang penyelidik yang bertanggungjawab untuk pengecaman spesies serta penilaian jangkitan *Leishmania* dalam gut. Pengurusan kaedah dua slaid boleh menimbulkan masalah penjejakan sampel dan, khususnya, menyukarkan pengesahan dengan tepat lalat pasir mana yang dijangkiti sekiranya gut yang positif dikesan (https://zenodo.org/records/18311154).

4.4.2. Kaedah satu slaid

Penggunaan kaedah satu slaid dapat memastikan kebolehjejakan keputusan. Namun begitu, beberapa langkah berjaga-jaga perlu diambil. Bagi memaksimumkan tahap kesterilan, operator mesti membersihkan tangan secara berkala menggunakan gel hidroalkohol. Slaid yang tidak berkabus dan kaca penutup slaid berbentuk segi empat (22 × 22 mm) yang dibalut dengan kerajang aluminium dan disterilkan menggunakan haba kering (ketuhar Poupinel) perlu digunakan, berserta jarum steril untuk setiap pembedahan (cadangan: 25G Ø 0.5 mm × 16 mm). Lalat pasir diletakkan di atas titisan larutan saline steril di tengah slaid. Kepala dipisahkan sementara pemotongan dibuat antara tergit dan sternit abdomen ke-6 dan ke-7 tanpa memotong saluran pencernaan (Jika spermateka dijangka melebihi panjang biasa, pemotongan boleh dipindahkan ke posisi lebih tinggi). Kemudian, toraks mesti dipegang pegun menggunakan jarum, manakala segmen posterior terakhir abdomen ditarik perlahan dengan jarum kedua bagi mengekstrak bahagian gut. Sekiranya kaedah ini gagal, hujung abdomen boleh disekat menggunakan jarum dan saluran pencernaan ditarik dari bahagian anterior. Jika masih gagal, gut perlu dikeluarkan dengan menyingkirkan sebahagian besar tegumen sekelilingnya. Setelah gut dikeluarkan, segmen abdomen terakhir dipisahkan dengan memotong saluran pencernaan. Gut kemudian diletakkan di titisan larutan saline steril baru di salah satu hujung slaid dan ditutup perlahan dengan penutup slaid steril.

Kepala dan segmen abdomen terakhir dipindahkan ke titisan kecil larutan Marc-André di hujung lain slaid bagi memastikan tiada sentuhan dengan *Leishmania*. Kepala diposisikan dengan betul (foramen oksipital menghala ke atas), dan spermateka diasingkan bersama furka genital seperti yang telah diterangkan, kemudian ditutup dengan penutup slaid bulat kecil (Ø 12 mm, berbeza dengan kaca penutup slaid segi empat steril). Baki bahagian badan lalat pasir dan sayap dibiarkan dalam titisan saline di tengah slaid (https://zenodo.org/records/18311154). Sekiranya ditemui positif atau bagi tujuan eksplorasi taksonomi, toraks dan abdomen boleh disimpan untuk analisis molekul atau proteomik, manakala sayap boleh dilekap dalam medium berakua. Bagi mengekalkan pelekapan, jumlah larutan Marc-André yang berlebihan boleh diganti dengan medium pelekapan berakua seperti gam kloral (=Hoyer) atau medium berasaskan alkohol polivinil.

Terdapat video terperinci yang memaparkan prosedur ini (pembedahan gut tengah lalat pasir: https://zenodo.org/records/18303014 dan pembedahan kelenjar air liur lalat pasir: https://zenodo.org/records/18302850), maka prosedur tersebut tidak akan dihuraikan di sini.

4.4.3. Pengasingan dan pengkulturan parasit *Leishmania* daripada gut lalat pasir

Pengasingan parasit daripada pembedahan lalat pasir betina yang dijangkiti merupakan prosedur halus yang memerlukan kemahiran tinggi dan disarankan untuk mula dipraktikkan pada spesimen bebas parasit terlebih dahulu. Selepas pembedahan, gut dipindahkan ke titisan baru larutan saline steril (0.9%) atau larutan Locke untuk prpses pembilasan [4]. Gut yang telah dibedah boleh diproses dengan dua cara: i) Pemeriksaan di bawah mikroskop cahaya untuk memerhatikan peringkat berbeza bagi promastigot *Leishmania* dan lokalisasinya, dengan perhatian khusus pada injap stomodeal, dan ii) membuka gut bagi memudahkan lalauan keluar promastigot untuk proses kultur secara besar-besaran [4]. Penemuan lalat pasir yang dijangkiti di lapangan adalah secara relatifnya jarang, oleh itu, sesi latihan yang baik akan meningkatkan peluang kejayaan pengasingan.

Sekiranya parasit *Leishmania* diperhatikan dalam gut, jarum steril baharu harus digunakan dan sedikit larutan saline steril ditambahkan di sekeliling kaca penutup slaid melalui tindakan kapilari bagi melepaskan parasit tersebut. Gut harus dibuka secara berhati-hati dan cepat untuk melepaskan parasit ke dalam saline. Menggunakan mikropipet 100 µL atau picagari tuberkulin, parasit dikutip dan diinokulat ke dalam medium kultur yang dilabel dengan betul.

Pengkulturan *in vitro* promastigot *Leishmania*: Promastigot *Leishmania* yang telah diasingkan pada peringkat awal dipelihara pada condong agar-agar darah SNB-9 atau dalam medium pepejal Novy, McNeal, Nicolle (NNN) [16], yang dilapisi sama ada dengan medium alfa-MEM steril [16, 65] atau medium M199. Kedua-dua medium ditambah dengan 10% serum anak lembu fetus (FCS) steril yang dinyahaktif haba (bagi meningkatkan pertumbuhan parasit), 1% vitamin BME, 2% air kencing manusia steril (disterilkan menggunakan penapis picagari Filtropur® S 0.2 µm), serta 250 µg/mL amikasin (atau 50 µg/mL gentamisin, atau campuran antibiotik dan asid amino yang mengandungi L-glutamin 200 mM, penisilin 10 000 U, dan streptomisin 10 mg/mL)[47]. Selepas tiga hari, sekiranya tiada kontaminasi, kultur dipindahkan ke medium penyejukbekuan yang disediakan dengan betul dan disimpan pada suhu −80 °C selama 1 hingga 2 tahun atau dalam nitrogen cecair pada suhu −196 °C bagi tujuan pengawetan jangka panjang serta kegunaan eksperimen pada masa hadapan [7].

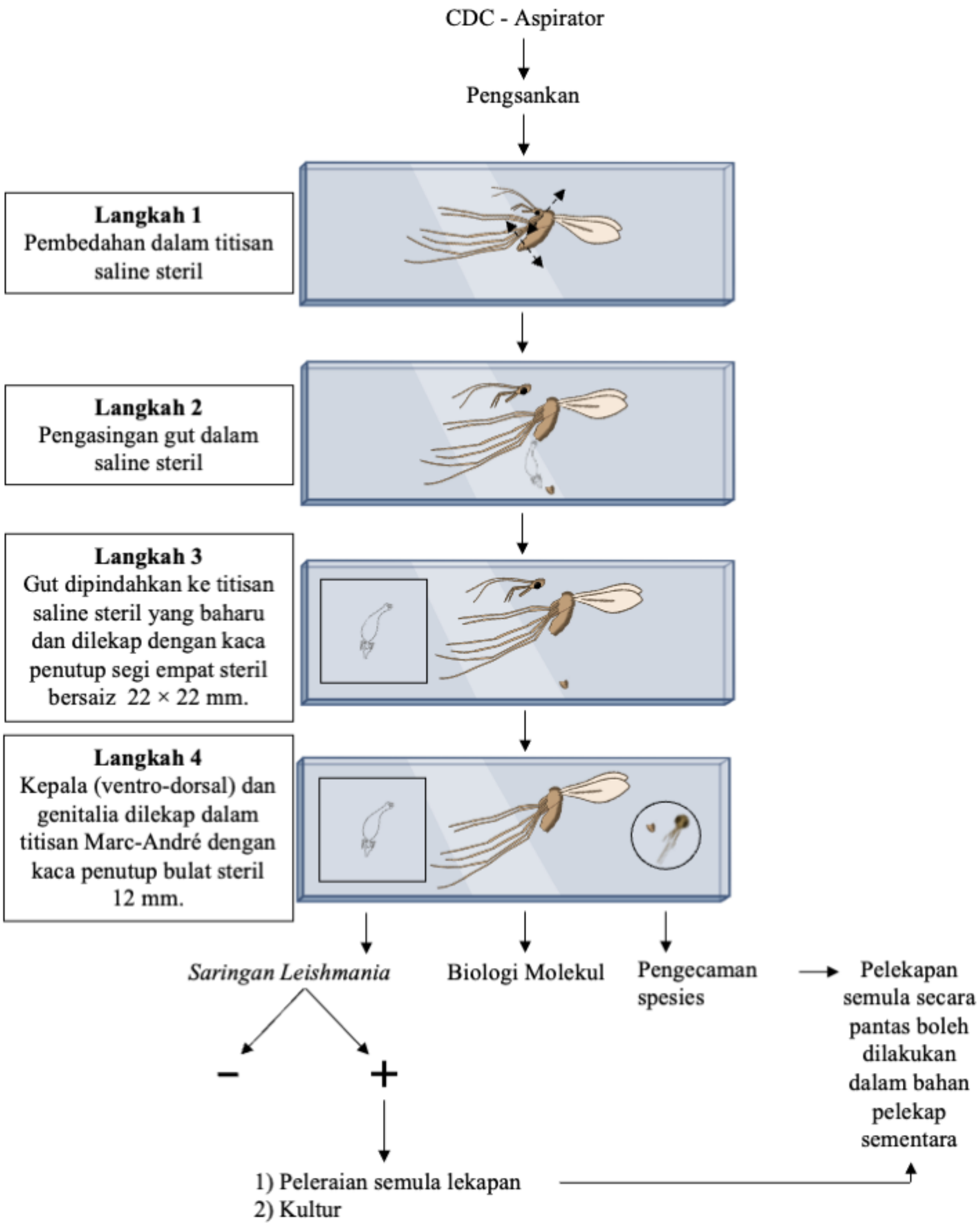

Rajah 5: Kaedah pengasingan *Leishmania*.

4.5. Kelenjar air liur

Pembedahan kelenjar air liur lalat pasir merupakan teknik asas untuk mengkaji interaksi vektor–patogen, khususnya bagi pengesanan arbovirus seperti *Phlebovirus* (contohnya virus Toscana) [44, 75]. Disebabkan saiz lalat pasir yang sangat kecil, prosedur ini memerlukan ketelitian tinggi di bawah mikroskop stereo dengan menggunakan forseps halus atau jarum mikrodiseksi untuk mengasingkan kelenjar liur yang rapuh tanpa menyebabkan pecah atau kontaminasi (https://zenodo.org/records/18302850) [51, 61]. Pengawetan keutuhan kelenjar adalah penting bagi memastikan ketepatan analisis molekul seterusnya dapat dijalankan. Selepas diekstrak, kelenjar boleh dihomogenkan dan dianalisis menggunakan RT-PCR, qPCR atau imunoasai bagi mengesan RNA virus atau antigen [12]. Kehadiran virus dalam kelenjar air liur, dan bukan hanya dalam gut atau hemosel, mengesahkan bahawa patogen telah melengkapkan tempoh inkubasi ekstrinsik dan berpotensi ditularkan semasa proses pengambilan darah [71].

Proses pembedahan ini secara teknikalnya adalah mencabar kerana saiz kelenjar liur lalat pasir yang sangat kecil dan memerlukan kepakaran yang tinggi bagi mengelakkan pemerosotan sampel [1, 51]. Selain itu, beban virus yang berkemungkinan rendah, justeru memerlukan

This is an Open Access article distributed under the terms of the Creative Commons Attribution License (https://creativecommons.org/licenses/by/4.0), which permits unrestricted use, distribution, and reproduction in any medium, provided the original work is properly cited.

kaedah pengesanan yang sangat sensitif seperti PCR bersarang (*nested PCR*) atau penjujukan berkapasiti tinggi (*high-throughput sequencing*) [54]. Risiko kontaminasi turut menekankan keperluan penggunaan teknik aseptik yang ketat.

Di samping cabaran teknikal, faktor biologi juga mempengaruhi kejayaan pengesanan; kompetensi vektor berbeza antara spesies lalat pasir, dan kadar jangkitan berubah mengikut keadaan ekologi serta musim [33, 61].

Pengesanan virus dalam kelenjar air liur lalat pasir memberikan maklumat penting mengenai risiko penularan, sekali gus membolehkan pelaksanaan pengawasan dan langkah kawalan yang lebih tertumpu [15]. Contohnya, pengesanan virus Toscana dalam lalat pasir di kawasan endemik telah mempengaruhi protokol diagnostik dan panduan kesihatan awam [18]. Selain itu, kajian interaksi virus–air liur berupaya menyingkap sasaran novel bagi pembangunan vaksin atau terapi penghalang penulara [15, 18]. Kelenjar air liur lalat pasir juga boleh digunakan sebagai sumber antigen untuk mengukur antibodi hos terhadap air liur lalat pasir menggunakan kaedah imunologi, terutamanya ELISA. Kaedah ini membolehkan penilaian pendedahan hos kepada gigitan lalat pasir, seterusnya menyokong penilaian keberkesanan langkah kawalan vektor [25] dan risiko penularan *Leishmania* [40].

4.6. Pengecaman sumber darah hos

Betina dalam keadaan kenyang yang diperoleh daripada tangkapan perlu dibedah menggunakan peralatan pakai buang bagi mengelakkan kontaminasi silang. Abdomen mereka diperiksa di bawah mikroskop stereo untuk menilai peringkat pencernaan darah. Adalah disyorkan untuk memilih hanya betina dengan abdomen berwarna merah, coklat kemerahan, atau merah gelap, tanpa tanda pembentukan telur. Potong hujung abdomen termasuk spermateka bagi membolehkan pengecaman morfologi betina selepas proses penjernihan. Bahagian utama abdomen (tanpa spermateka) kemudian dimasukkan ke dalam tiub Eppendorf® dan disimpan pada suhu −20 °C sehingga analisis selanjutnya dilakukan. Penanda genetik yang lazim digunakan untuk pengecaman sumber darah hos, seperti PNOC [5, 30, 50], CytB [67], atau COI [13], telah lama diguna pakai dan diterangkan secara meluas dalam sastera; oleh itu, penanda genetik ini tidak akan dihuraikan secara lanjut dalam kertas ini (Rajah 6). Sebagai alternatif, pemetaan peptida menggunakan MALDI-ToF boleh digunakan untuk mengenal pasti darah hos [31]. Kajian eksperimen menunjukkan bahawa teknik ini membolehkan pengecaman darah hos dalam tempoh masa yang lebih panjang selepas pengambilan darah, menjadikannya kaedah yang sesuai, terutamanya bagi analisis betina yang kenyang dengan tahap pencernaan darah hos yang lebih maju.

Sampel disyorkan disimpan pada suhu −20 °C atau 4 °C, walaupun keputusan yang baik juga boleh diperoleh daripada sampel yang disimpan pada suhu bilik untuk tempoh singkat. Abdomen betina yang kenyang perlu dibedah daripada badan sebelum analisis dan dihomogenkan dalam air suling. Bahagian badan lalat pasir yang selebihnya masih boleh digunakan untuk analisis molekul dan morfologi lain. Selepas alikuot diambil daripada homogenat untuk pemetaan peptida MALDI-ToF, baki homogenat boleh digunakan untuk pengasingan DNA bagi mengesahkan pengecaman darah hos dan/atau untuk saringan kehadiran *Leishmania* sp. Keseluruhan masa persediaan dan analisis sampel adalah sangat singkat jika dibandingkan dengan teknik molekul berasaskan DNA.

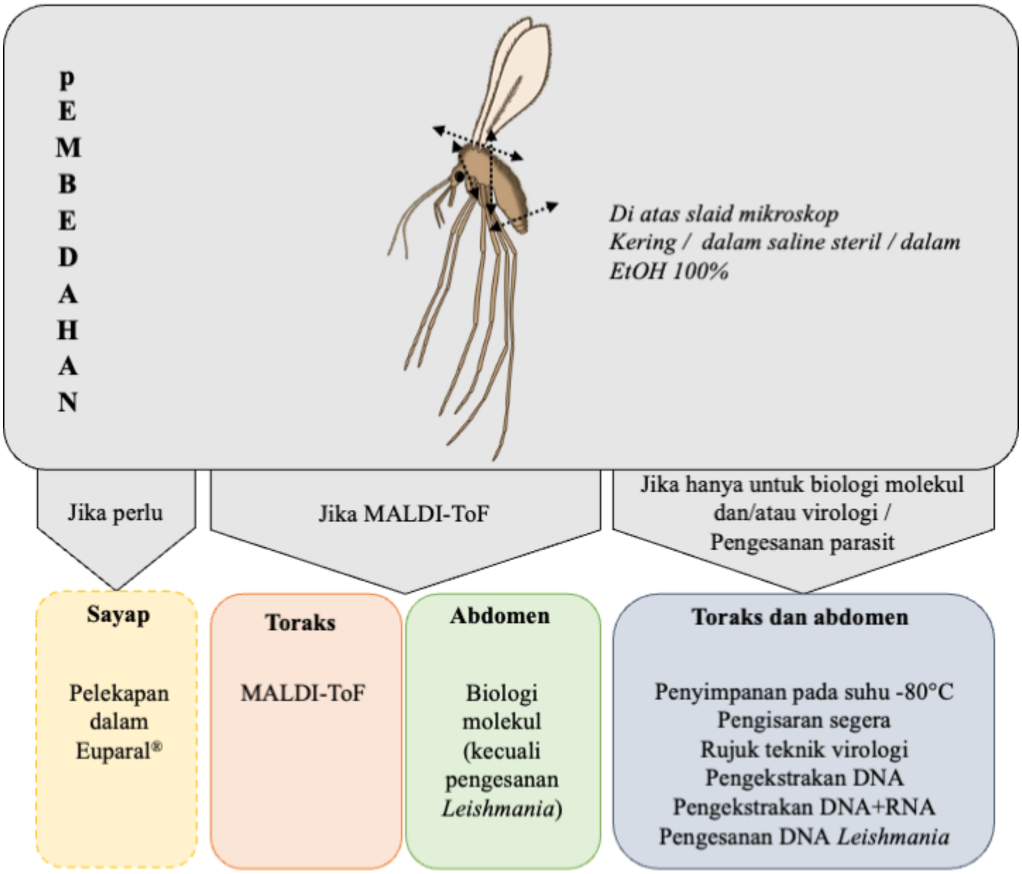

Rajah 6: Pemprosesan lalat pasir untuk aplikasi biologi molekul, proteomik, dan/atau virologi.

5. Pemprosesan spesimen untuk kajian morfologi (Rajah 3, 6, 7 & 8; Lampiran 1, 2, 3 & 4)

Bahagian ini menerangkan prinsip penyediaan spesimen lalat pasir untuk pelekapan yang hanya bertujuan kajian morfologi, diikuti dengan penyesuaian bagi aplikasi di luar morfologi. Walau bagaimanapun, pemahaman terhadap metodologi ini adalah penting kerana prosedur boleh disesuaikan mengikut jenis sampel tertentu apabila perlu.

Rawatan melibatkan langkah-langkah pengosongan dan pengisian secara berturutan menggunakan pipet Pasteur yang dilengkapi dengan mentol getah fleksibel. Bekas kaca berbentuk dasar bulat sangat disyorkan kerana memudahkan operasi ini dengan ketara. Kaca bersifat lengai terhadap semua reagen. Bagi mengelakkan penyejatan reagen, bekas harus dilengkapi dengan penutup dan tidak diisi secara berlebihan untuk mengelakkan tumpahan semasa menutup atau membuka, serta menghalang habuk jatuh ke atas sampel. Bahan kimia yang diperlukan untuk proses penjernihan dan pemprosesan ditunjukkan dalam Jadual 2.

This is an Open Access article distributed under the terms of the Creative Commons Attribution License (https://creativecommons.org/licenses/by/4.0), which permits unrestricted use, distribution, and reproduction in any medium, provided the original work is properly cited.

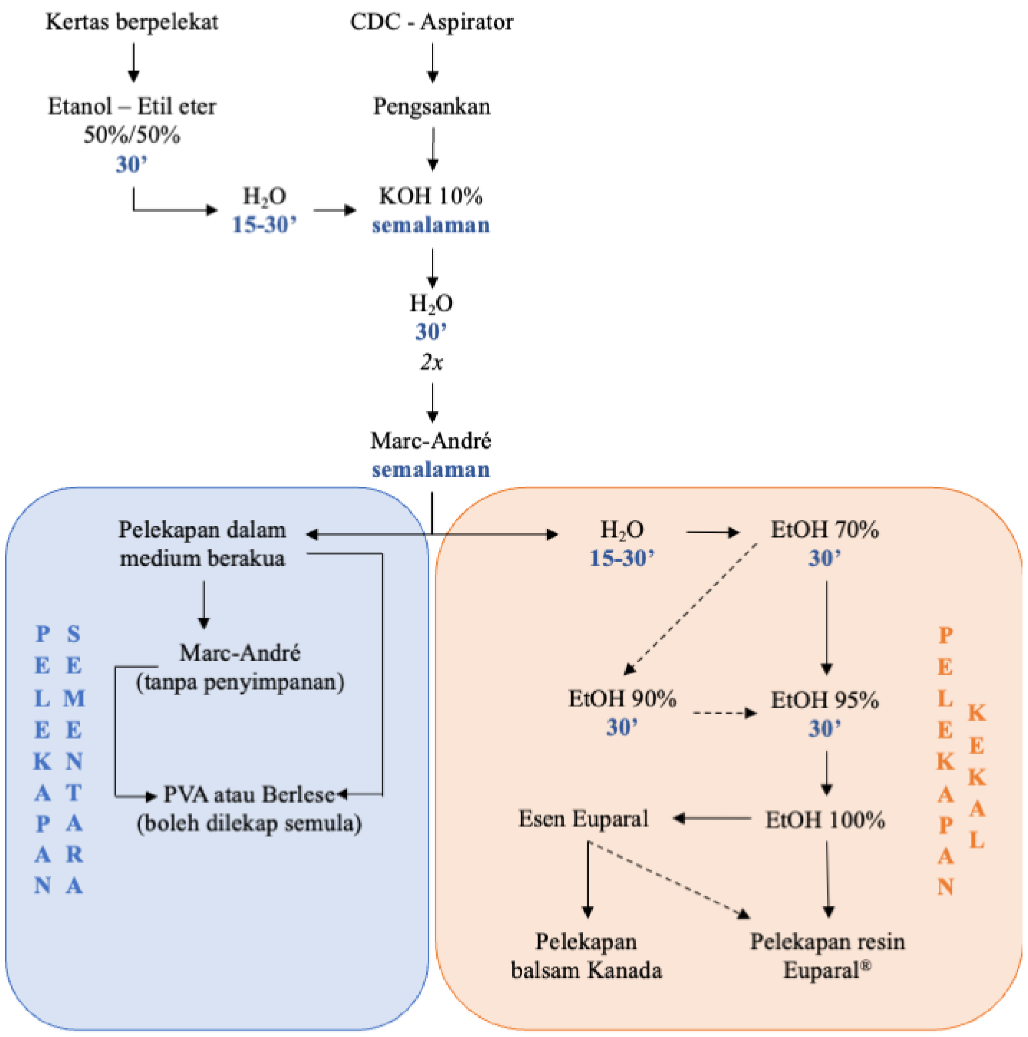

Rajah 7: Kaedah klasik untuk memproses lalat pasir.

Jadual 2: Komposisi reagen yang digunakan.

**Kalium hidroksida 10%**<br>Kalium hidroksida 10 g<br>Air suling cukup untuk menjadikan jumlah 100 mL	**Asid fuchsin 1% dalam air suling**<br>Asid fuchsin (dalam bentuk serbuk) 1 g<br>Air suling 99 mL
**Medium pelekapan gam kloral (Medium Hoyer)**<br>Air suling 50 mL<br>Kloral hidrat 200 g,<br>Gam Arab 50 g<br>Gliserol 20 mL	**Larutan Marc-André berwarna dengan asid fuchsin**<br>Larutan Marc-André 10mL<br>Asid fuchsin 1% 50 µL
**Larutan Marc-André**<br>Kloral hidrat 40 g<br>Asid asetik glasier 30 mL	**Medium Enecê**<br>Kolofoni putih tulen 22 g,<br>Gam damar larut alkohol 12 g

This is an Open Access article distributed under the terms of the Creative Commons Attribution License (https://creativecommons.org/licenses/by/4.0), which permits unrestricted use, distribution, and reproduction in any medium, provided the original work is properly cited.

Air suling 30 mL	Etanol tulen 20 mL
	Kapur barus 10 g
	Esen turpentin 10 mL
	Eukaliptol 26 mL

5.1. Proses penjernihan

Sebelum spesimen lalat pasir dapat disediakan sebagai pelekapan tetap pada slaid mikroskop, spesimen mesti terlebih dahulu dijernihkan melalui pemacatan menggunakan kaedah dan agen penjernihan yang sesuai (contohnya, larutan asid asetik 10% atau larutan Marc-André termasuk kloral hidrat, yang merupakan bahan terhad di banyak negara) bagi menjadikannya telus. Proses penjernihan ini menyingkirkan tisu badan, lemak, rembesan, dan lilin, menjadikan spesimen lutcahaya, yang akan memudahkan pemerhatian struktur eksoskeleton (contohnya, kedudukan sisip seta), ciri permukaan (contohnya, warna), dan ciri dalaman yang kelihatan melalui tegumen (contohnya, spermateka).

Proses penjernihan dua langkah, yang melibatkan penggunaan bes kuat dahulu (seperti kalium hidroksida), diikuti oleh asid lemah (contohnya asid asetik dalam larutan Marc-André), mempunyai tujuan biokimia yang berbeza [74]. Bes memecahkan tisu lembut, seperti protein, lemak, dan otot melalui saponifikasi dan penyahaslian protein, sambil mengekalkan eksoskeleton kitin untuk kejelasan struktur. Asid lemah seterusnya meneutralkan sisa alkali, mencegah pemerosotan lanjut, dan memutihkan kitin untuk meningkatkan ketelusan [74], walaupun membasuh spesimen dua kali dalam air suling selama 15 minit mungkin sudah mencukupi untuk meneutralkan bes. Rawatan bersiri ini menggabungkan penyingkiran tisu yang berkesan dengan pengawetan lembut bagi memastikan keutuhan spesimen optimum untuk pemerhatian mikroskopik.

Dua bilasan selama 20 minit dalam air suling disyorkan sebelum meneruskan ke langkah seterusnya.

5.1.1. Lisis tisu lembut (Rajah 8)

Hidroksida natrium (NaOH) atau hidroksida kalium (KOH) merupakan agen kemasan kimia yang biasa digunakan, dengan kepekatan dan tempoh penggunaan yang berbeza bergantung kepada saiz dan kerapuhan spesimen. Teknik standard dan paling berkesan melibatkan lisis tisu lembut dengan merendam lalat pasir semalaman dalam larutan bes kuat (10% KOH atau NaOH). Kepekatan boleh ditingkatkan untuk mengurangkan tempoh rawatan (contohnya, KOH 20% selama 6 jam) dan boleh dipanaskan pada suhu 37°C.

5.1.2. Proses hasil penjernihan dengan atau tanpa pewarnaan

Langkah ini diikuti dengan rawatan pencerahan, biasanya menggunakan gabungan asid asetik dan kloral hidrat (contohnya, larutan Marc-André). Selepas proses penjernihan, spesimen mesti dibasuh dengan teliti dalam mandian air sekurang-kurangnya dua kali berturut-turut selama 20 minit setiap satu bagi menghilangkan sisa kimia.

Larutan Marc-André adalah agen penjernihan yang lazim digunakan untuk penyediaan spesimen lalat pasir. Keberkesanannya terletak pada kemampuannya memudahkan proses penjernihan sambil meminimumkan kerosakan ketara pada struktur halus seperti sayap dan antena.

Larutan ini harus disediakan secara segar atau disimpan dalam bekas yang kedap untuk mengelakkan penyejatan atau pemerosotan. Penggunaan larutan Marc-André amat sesuai apabila digabungkan dengan teknik pencerahan tambahan atau pewarnaan bagi menonjolkan butiran morfologi tertentu. Butiran komposisi dan penyediaannya disediakan dalam Lampiran 2.

Bagi spesimen lutcahaya yang tinggi, pewarnaan mungkin diperlukan untuk meningkatkan keterlihatan sebelum pelekapan. Terdapat pelbagai jenis pewarna, masing-masing mensasarkan komponen kimia tertentu dalam organisma. Adalah penting untuk memilih pewarna yang serasi dengan kedua-dua spesimen dan medium pelekapan yang digunakan.

Metodologi asas ini boleh disesuaikan mengikut keperluan, contohnya dengan memasukkan 0.1% asid fuchsin ke dalam larutan Marc-André untuk tujuan pewarnaan. Selain itu, spesimen yang disimpan dalam larutan berakua dan bertujuan untuk pelekapan menggunakan resin memerlukan proses penyahhidratan (rujuk Seksyen 5.2 mengenai Penyahhidratan), kerana kebanyakan medium pelekapan resin semula jadi atau sintetik tidak serasi dengan air. New (1974) mencatatkan bahawa sesetengah pewarna boleh mengalami pemerosotan dalam medium pelekapan tertentu [53]. Sebagai contoh, asid fuchsin, yang lazim digunakan bersama balsam Kanada, turut boleh ditetapkan dalam Euparal®. Walau bagaimanapun, spesimen yang diwarnai dengan asid fuchsin berpotensi mengalami pemudaran yang ketara apabila sisa minyak cengkih daripada proses penjernihan akhir masih kekal pada spesimen. Spesimen yang disimpan dalam minyak cengkih cenderung mengalami pemudaran yang ketara dalam beberapa hari.

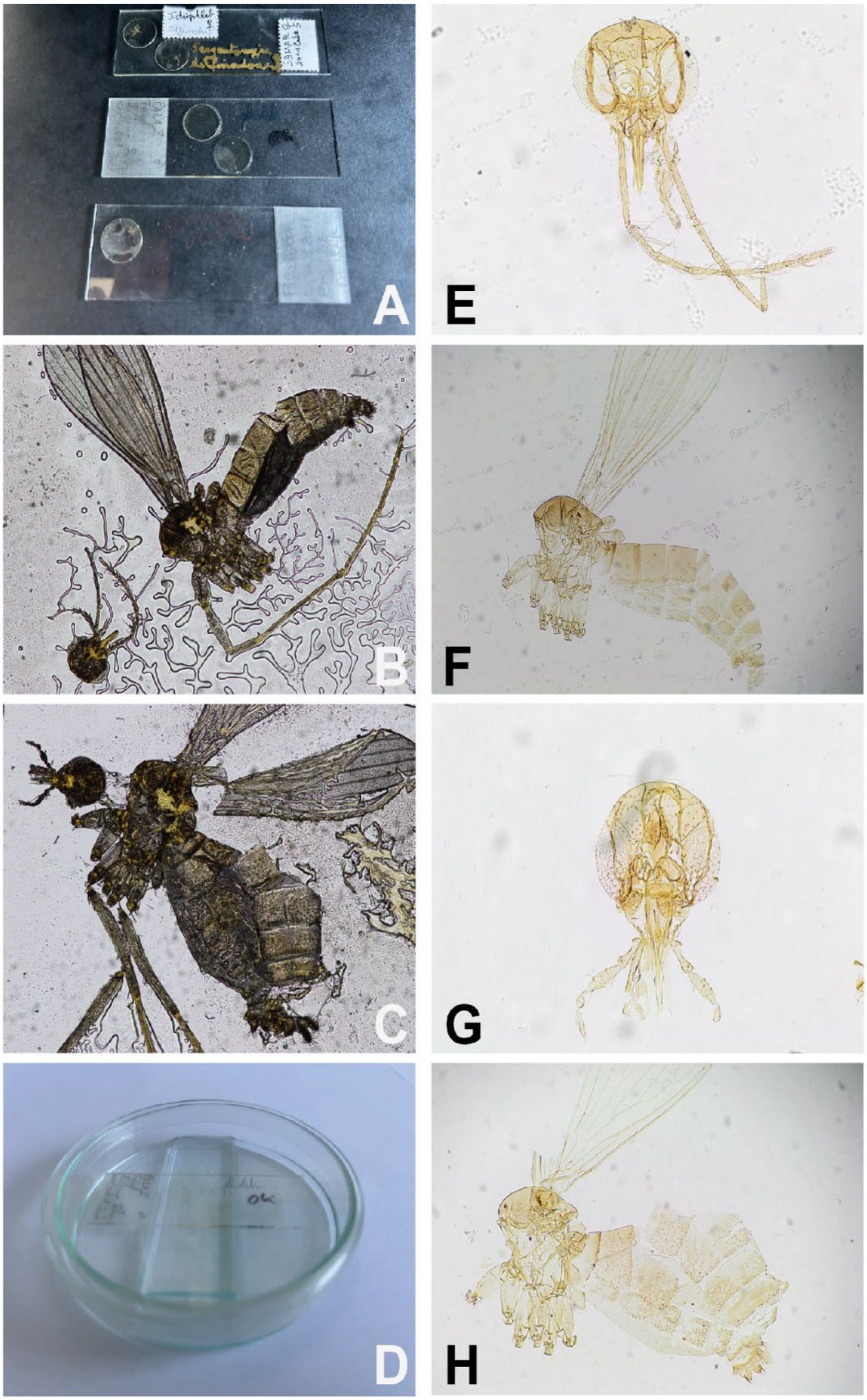

Rajah 8: Perlekapan semula slaid. A: slaid yang rosak dan kering, dilekap dalam Hoyer; B: pandangan mikroskopik lalat pasir yang kering; C: pandangan mikroskopik lalat pasir lain yang rosak; D: kebuk lembap yang mengandungi slaid kering; E: kepala, and F: badan spesimen B selepas pelekapan semula dalam Euparal®; G: kepala, and H: badan spesimen C yang rosak selepas pelekapan semula dalam Euparal®.

5.2. Penyahhidratan

Proses penyahhidratan dijalankan dengan memindahkan spesimen secara berperingkat melalui siri larutan etanol bertingkat: 50%, 70%, 80%, 90% atau 95%, dan akhirnya 100%, dengan setiap rendaman berlangsung sekurang-kurangnya 20 minit. Memandangkan etanol menguap dengan cepat, bekas hendaklah ditutup dengan rapat semasa pemprosesan. Setelah spesimen benar-benar ternyah-hidrat, pemprosesan boleh dihentikan sementara selama beberapa hari dalam esen Euparal®, yang lebih sering dipilih berbanding minyak cengkih. Kreosot bic, yang pernah digunakan secara meluas untuk tujuan ini, kini telah dilarang sepenuhnya kerana sifat toksiknya.

Proses penyahhidratan mesti memastikan cecair dalam spesimen serasi dengan medium pelekapan bagi mengelakkan kelegapan, keruntuhan osmotik, atau herotan yang boleh menjadikan spesimen tidak sesuai untuk kajian taksonomi.

5.3. Medium pelekapan

5.3.1. Pemilihan dan aplikasi bagi penyediaan spesimen

Medium pelekapan sebaiknya mempunyai indeks biasan yang sehampir mungkin dengan kaca, iaitu kira-kira 1.5. Medium tersebut mestilah tidak berwarna, jernih, dan kekal lutsinar sepenuhnya selepas pengeringan serta sepanjang tempoh penyimpanan. Medium juga perlu serasi dengan pewarna yang digunakan dan berupaya menembusi serta meresap ke dalam semua tisu spesimen. Selain itu, medium tidak boleh mengering terlalu cepat atau membentuk kabus semasa proses pelekapan, serta tidak mengalami pengecutan selepas pelekapan. Pemilihan medium pelekapan yang sesuai merupakan aspek asas dalam penyediaan spesimen kerana tiada satu medium yang sesuai untuk semua tujuan. Oleh itu, pemilihan perlu mempertimbangkan beberapa faktor utama berikut:

- **Sifat optik.** Indeks biasan medium pelekapan hendaklah memberikan kontras dan pembiasan yang mencukupi bagi menonjolkan ciri anatomi penting yang digunakan dalam pengecaman taksonomi atau penerangan morfologi, seperti spermateka, askoyd, sensila Newstead, gigi sibarial menegak, dan gigi farinks. Keterlihatan struktur ini bergantung secara langsung kepada sifat optik medium pelekapan.

- **Pengawetan**. Medium pelekapan bagi spesimen rujuk atau bahan untuk koleksi kekal perlu mempunyai kestabilan dan ketahanan jangka panjang. Sebaliknya, bagi kajian inventori atau tinjauan epidemiologi, medium sementara atau separa kekal lazimnya mencukupi kerana pengawetan jangka panjang tidak menjadi keutamaan.

5.3.2. Keperluan bagi medium pelekapan

Pakar sering membangunkan teknik pelekapan khusus dan kompleks yang disesuaikan dengan keperluan penyelidikan tertentu. Walau bagaimanapun, kaedah ini kerap mengabaikan aspek seperti kualiti arkib, keserasian, standardisasi, kemudahan pengendalian, dan pengawetan jangka panjang. Kekurangan standardisasi ini menyukarkan penggabungan koleksi sumbangan serta usaha penjagaan jangka panjang.

Aplikasi saintifik menuntut keperluan yang berbeza bagi medium pelekapan. Ahli taksonomi sering meletakkan keseluruhan spesimen dan lebih memilih medium yang dapat memekatkan organ dalaman secara perlahan untuk meningkatkan keterlihatan struktur kutikel. Indeks biasan cahaya medium harus berbeza secukupnya daripada spesimen dan slaid kaca untuk memaksimumkan kejelasan optik. Medium pelekapan komersial lazimnya diformulasikan dengan indeks biasan yang hampir sama dengan kaca bagi meminimakan pembiasan dan penyebaran cahaya melalui sistem slaid–medium–penutup kaca. Walau bagaimanapun, dalam mikroskopi cahaya terang (brightfield), kontras semula jadi bagi spesimen yang tidak diwarnai boleh diperbaiki dengan memilih medium pelekapan yang mempunyai indeks biasan yang sedikit berbeza daripada spesimen, sekali gus meningkatkan keterlihatan spesimen berbanding latar belakang.

5.3.3. Jenis-jenis medium pelekapan (Jadual 3 & 4)

Mikroskopi memerlukan indeks biasan (*refractive index*, RI) bagi medium pelekapan untuk menentukan bagaimana cahaya diselekohkan melalui slaid, medium, dan spesimen. Apabila RI selaras rapat dengan kaca penutup (≈1.515), cahaya bergerak secara seragam, mengurangkan hamburan dan pengherotan optik, seterusnya meningkatkan resolusi dan keterlihatan struktur halus. Sebaliknya, ketidakpadanan RI boleh menyebabkan kekaburan, halo, atau menyukarkan pemerhatian ciri yang tidak diwarnai. Pemilihan medium pelekapan yang tepat adalah penting untuk mengoptimumkan kontras, kejelasan, dan kualiti imej keseluruhan bagi spesimen tertentu disebabkan perbezaan RI antara pelbagai medium.

Indeks biasan medium pelekapan memberi kesan ketara terhadap keterlihatan struktur halus semasa penyediaan lalat pasir untuk pelekapan pada slaid. Ciri halus dan bersklerotisasi ringan pada lalat pasir, termasuk armatur sibarial, spermateka, segmen antena, dan urat sayap, boleh menjadi sukar untuk diperhatikan dalam medium pelekapan dengan indeks biasan yang tinggi.

Bagi lalat pasir, medium yang biasa digunakan termasuk medium gam-kloral sebagai medium berasaskan air, dan balsam Kanada serta resin Enecê - Nelson Cerqueira (NC) sebagai medium berasaskan pelarut. Rawlins [60] mengkategorikan medium pelekapan kepada dua jenis: (1) medium kekal: mengeras dari masa ke masa dan sesuai untuk pengawetan jangka panjang, dan (2) medium separa kekal: tidak mengeras sepenuhnya dan biasanya digunakan untuk tujuan sementara.

Medium pelekapan boleh berbentuk cecair, berasaskan gam, atau resin, dan larut dalam air, alkohol, atau pelarut lain (contohnya, toluena, xilena) (Jadual 3). Setelah

diaplikasikan, medium perlu dikedap daripada kesan atmosfera dengan menggunakan medium penggelangan yang tidak larut. Bagi membezakan dengan jelas antara jenis medium pelekapan, kategori berikut boleh digunakan:

a. Medium berakua. Medium ini mudah larut dalam air, menjadikannya sesuai untuk pelekapan sementara atau separa kekal. Medium tersebut biasanya mudah dikendalikan, namun mungkin memerlukan proses pengedapan bagi mengelakkan pendedahan kepada kelembapan atmosfera (contohnya, medium berasaskan gam kloral dan alkohol polivinil), terutamanya di kawasan tropika yang lembap.

b. Medium toleransi air terhad. Medium ini kurang terjejas oleh air, namun masih memerlukan perlindungan daripada kelembapan berlebihan. Medium ini turut menawarkan kestabilan jangka panjang yang lebih baik berbanding medium larut air dan sering digunakan untuk pelekapan separa kekal.

c. Medium larut hidrokarbon. Medium ini larut dalam pelarut organik seperti xilena, toluene, atau esen (pelarut Enecê). Medium ini turut direka bentuk untuk pelekapan kekal dengan menawarkan kestabilan jangka panjang yang sangat baik, serta tahan terhadap kelembapan dan pemerosotan, menjadikannya sesuai untuk tujuan arkib (contohnya, balsam Kanada neutral).

Secara ringkas, medium larut air sesuai untuk pelekapan sementara atau apabila spesimen perlu dikeluarkan dengan mudah; medium toleransi air terhad sesuai untuk pelekapan separa kekal dengan ketahanan sederhana; manakala medium larut hidrokarbon lebih menjadi pilihan untuk pelekapan kekal yang bertujuan untuk arkib dan penyimpanan jangka panjang.

Jadual 3: Komposisi medium pelekapan yang terpilih.

Medium pelekapan	Pelarut	Prapolimer atau polimer berpotensi	Catatan
Hoyer = gam kloral	gliserol, air	Sebatian gam arab	Agen pemaseratan: kloral hidrat
CMCP-9 (=selulosa karboksimetil berfenol)	air (CMCP-9: 51−60%)	Alkohol polivinil yang telah dihidrolisis sepenuhnya (CMCP-9: 0−5%)	CMC(P)-9: kelikatan rendah: kelikatan tinggi
DMHF (dimetil hydantoin formaldehid)	air	N,N′- dimetilol dimetil hidrat (di-metilol DMH)<br>Oligomer berjejantas eter/metilena<br>Rangka polimer DMH–formaldehid bersilang	
balsam Kanada	xilena; Komponen separa meruap dalam balsam (Δ^3-karena, asid levopimarik, limonena, mirsena, asid palustrik, β-felandrena, α-pinen, β-pinen)	balsam (abienol, asid abietik, asid isopimarik, asid sandarakopimarik)	peneutralan: Karbonat kalium ; resin daripada *Abies balsamea* (Linné, 1758)
Euparal®	eukaliptol, paraldehid; komponen separa meruap dalam gam sandarak (limonena, α-pinen, β-pinen)	Sebatian gam sandarak (asid komunik, manool, asid polikomunik, asid sandarakopimarik, 12-asetoksi-sandarakopimarik, sugiol, asid torulosik, torulosol, totarol)	agen penjernihan: metil salisilat; warna hijau dalam Euparal®: garam tembaga (tembaga abietinat); resin sandarak daripada *Tetraclinis articulata* (Vahl, 1791)
Enecê	Etanol; dengan kapur barus, eukaliptol dan esen terpentin	Sebatian gam damar dan kolofoni (rosin)	

Jadual 4: Kelebihan dan kekurangan medium pelekapan yang digunakan untuk slaid mikroskop telah dinilai berdasarkan sastera serta pemerhatian tidak diterbitkan oleh pelbagai penyelidik [52].

Nama	Kelebihan	Kekurangan
*Balsam Kanada	Medium ini sangat tahan lama, dengan jangka hayat melebihi 150 tahun.<br>Slaid boleh dilekap menggunakan minyak cengkih atau fenol sebagai agen pelekapan.	Medium ini mengandungi komponen berbahaya dan mesti dikendalikan di bawah tukup wasap.<br>Memerlukan siri penyahhidratan yang lengkap dan memakan masa.<br>Penyahhidratan dengan etanol dan pemindahan menggunakan xilena atau minyak cengkih boleh menjadikan sesetengah taksa rapuh; alternatif lain seperti isopropanol, n-butanol, Cellosolve™, 1,4-dioksana, *Histoclear*, atau terpineol mungkin mengurangkan keretakan.<br>Spesimen boleh menjadi kehitaman jika xilena diganti dengan fenol atau jika terdapat sisa kalium hidroksida.<br>Indeks biasan tinggi boleh menutupi struktur yang tidak diwarnakan, manakala pengeringan sepenuhnya boleh mengambil masa bertahun-tahun tanpa pengeringan menggunakan plat panas.<br>Medium akan menjadi kekuningan dan gelap dari masa ke masa, terutamanya apabila dijernihkan dengan minyak cengkih.<br>Sesetengah pewarna menjadi lemah, dan pewarna kation mungkin pudar jika medium menjadi berasid, keadaan ini boleh berlaku secara spontan dari masa ke masa.
DMHF (dimetil hidantoin formaldehid)	Kelutsinaran tinggi.<br>Indeks biasan yang baik.<br>Membolehkan struktur spesimen dilihat dengan jelas.<br>Menawarkan kestabilan yang agak baik bagi persediaan slaid.<br>Serasi dengan pelbagai teknik pewarnaan.<br>Memberikan perlindungan yang baik kepada sampel.<br>Memastikan lekatan yang baik antara slaid dan kaca penutup slaid.	Berpotensi menjadi kekuningan dari masa ke masa.<br>Boleh mengubah sesetengah pewarna.<br>Tidak sesuai untuk pewarna yang sensitif terhadap formaldehid.<br>Gelembung udara mungkin terbentuk dan proses pengeringan mengambil masa yang agak lama.<br>Medium pelekapan ini sensitif terhadap kelembapan.<br>Proses pelekapan sukar untuk diterbalikkan.<br>Formaldehid merupakan bahan toksik, merengsa, dan bersifat karsinogenik.
*Euparal (lutsinar)	Medium ini tahan lama dengan jangka hayat melebihi 50 tahun.<br>Pelekapan boleh dilakukan secara langsung daripada etanol 80% mengikut cadangan pengeluar.<br>Medium ini tidak menutupi struktur yang tidak diwarnakan serta tidak menguning atau menjadi rapuh dari masa ke masa.<br>Mempunyai indeks biasan yang lebih sesuai berbanding balsam Kanada bagi spesimen	Mengandungi komponen berbahaya dan mesti dikendalikan di bawah tukup wasap.<br>Penyahhidratan menggunakan etanol dan pemindahan melalui esen Euparal boleh menyebabkan sesetengah taksa menjadi rapuh, namun penggunaan isopropanol sebagai alternatif boleh membantu mengurangkan masalah tersebut.

This is an Open Access article distributed under the terms of the Creative Commons Attribution License (https://creativecommons.org/licenses/by/4.0), which permits unrestricted use, distribution, and reproduction in any medium, provided the original work is properly cited.

	Diptera.<br>Berfungsi dengan baik untuk spesimen yang lebih tebal kerana pengecutan yang minimum dan proses pengeringan tanpa pembentukan gelembung.<br>Kekal larut dalam etanol 95%, membolehkan pelekapan semula slaid dilakukan walaupun selepas bertahun-tahun.	
Cecair Hoyer	Spesimen boleh dilekap dalam keadaan hidup atau secara langsung daripada air, etanol, atau formaldehid.<br>Proses pemaseratan menghasilkan kualiti kutikel yang sangat baik. Mempunyai indeks biasan yang sesuai dan boleh dipertingkatkan dengan pewarnaan iodin untuk meningkatkan kontras.<br>Asid asetik dalam formulanya boleh mengembangkan apendaj artropod. Sesetengah spesimen boleh kekal stabil selama 40 hingga 60 tahun. Larut air yang membolehkan pelekapan semula dilakukan dengan mudah.	Spesimen tumbuhan yang halus boleh runtuh melainkan medium ditambahkan secara beransur-ansur, yang menjadikan proses ini memakan masa.<br>Rongga dan hablur boleh terbentuk dalam tempoh kurang daripada 10 tahun.<br>Proses pemaseratan boleh menjadi berlebihan bergantung pada kepekatan kloral hidrat dan tempoh pendedahan.<br>Komponen medium mungkin berpisah, dan penggranulan halus boleh muncul dalam tempoh beberapa bulan atau tahun.<br>Medium menjadi kehitaman juga pernah dilaporkan.
CMCP-9<br><br>(= selulosa karboksimetil berfenol)	Spesimen boleh dilekap secara langsung daripada medium seperti air, etanol, gliserol, atau larutan yang mengandungi formaldehid, dan organ dalaman spesimen boleh dimaserat apabila perlu bagi memudahkan pemeriksaan umum atau penyediaan sampel.	Medium ini boleh membentuk hablur dan menjadi gelap dari masa ke masa, serta kadangkala memaserat spesimen melebihi tahap yang dikehendaki. Jika slaid tidak digelangkan dengan teliti, spesimen yang lebih tebal tidak sesuai dilekap dalam medium ini kerana spesimen boleh mengecut dan menghasilkan ruang di sekeliling tepi kaca penutup slaid. Medium ini juga tidak sesuai untuk spesimen yang diwarnakan atau bahan berkalsium, dan masa pengeringannya lebih lama berbanding CMC.
Eukitt™	Medium ini tahan lama dengan jangka hayat melebihi 30 tahun. Serasi dengan pelbagai pelarut untuk pelekapan seperti aseton, benzena, kloroform, dioksan, eter, isopropanol, metil benzoat, terpineol, toluena dan xilena.<br>Kering dengan cepat, mempunyai pH yang sedikit berasid, dan tidak menunjukkan penggelapan yang ketara apabila meningkat usia.<br>Sesuai digunakan dengan pelbagai pewarna seperti fuksin, hematoksilin, metil hijau, metil ungu dan metilena biru.<br>Spesimen boleh dilekap semula selepas beberapa tahun dengan merendam slaid dalam xilena untuk tempoh yang panjang.	Medium ini mengandungi komponen berbahaya dan mesti dikendalikan di bawah tukup wasap.<br>Memerlukan siri penyahhidratan yang lengkap dan memakan masa.<br>Tidak sesuai untuk spesimen yang lebih tebal kerana pengecutan dan pembentukan gelembung gas.<br>Kaca penutup slaid mungkin terkeluar dari tempatnya dari masa ke masa melainkan slaid kaca dibersihkan dengan baik dan dikedap dengan sempurna. Kemungkinan berlaku polimerisasi tidak sempurna pada kawasan serat kolagen.
Enecê	Medium yang sangat tahan lama, dengan jangka	Memerlukan siri penyahhidratan yang lengkap dan

	hayat sekurang-kurangnya 50 tahun.<br>Tidak bertukar menjadi gelap dari masa ke masa.<br>Bersifat boleh tempa, yang membolehkan pembedahan serangga dalam medium dilakukan serta menyediakan masa yang mencukupi untuk menyusun struktur morfologi dengan tepat.<br>Kos rendah.	memakan masa.<br>Penyahhidratan menggunakan etanol diikuti pemindahan melalui minyak cengkih boleh menyebabkan sesetengah spesimen menjadi rapuh.<br>Proses penjernihan serangga masih berlaku, walaupun dengan kadar yang sangat perlahan; keadaan ini boleh menyukarkan pemerhatian struktur yang sangat kecil, seperti sensila, askoid, dan setae ringkas.

5.3.4. Penerangan mengenai medium pelekapan yang disyorkan (Jadual 3 & 4)

Medium bagi pemerhatian sementara

Gam Kloral = cecair/ medium/larutan Hoyer (RI = 1.48)

Cecair Marc André merupakan medium terbaik untuk pemerhatian jangka pendek (beberapa jam, dan mungkin lebih lama jika slaid disimpan dalam kebuk lembap) terhadap spermateka, termasuk untuk tujuan fotografi (Rajah 4) atau lukisan. Pengawetan spermateka yang telah diperhatikan memerlukan pelekapan semula dalam medium berakua yang membolehkan penyimpanan jangka sederhana. Penyahhidratan bagi pelekapan semula dalam resin tidak digalakkan kerana berisiko menyebabkan kehilangan spesimen. Proses penyahhidratan bagi pemasangan semula dalam medium resin bukanlah mustahil, namun tidak disyorkan kerana berisiko menyebabkan kehilangan spesimen. Gam kloral dan cecair Hoyer dianggap sinonim. Medium ini lazim digunakan untuk pemerhatian organ dalaman kerana kesesuaiannya dengan air, kaedah penggunaannya yang mudah dan cepat, serta indeks biasannya yang memudahkan pemeriksaan struktur halus seperti spermateka. Walau bagaimanapun, gam kloral mempunyai beberapa kekurangan yang ketara sekiranya tidak disediakan dengan sempurna atau tidak disimpan dalam keadaan kelembapan terkawal. Antara masalah yang boleh berlaku ialah penghabluran, perubahan warna, dan kehilangan kelikatan. Penggelangan kaca penutup slaid tidak menyelesaikan masalah ini kerana medium pelekapan boleh mengalami perubahan warna yang ketara (kadangkala hampir hitam) akibat interaksi dengan medium penggelangan tersebut, terutamanya jika Euparal® digunakan. Medium Hoyer dianggap sebagai medium yang paling terbaik dari segi optik untuk lalat pasir Phlebotominae dan secara tradisional telah digunakan bagi tujuan tersebut. Medium ini terdiri daripada beberapa formulasi yang berkait rapat, termasuk gam arab, gliserol, dan kloral hidrat. Pelbagai formulasi telah disalah tafsir dan disalah petik [74].

Walaupun medium Hoyer merupakan medium yang baik untuk pemerhatian spermateka dalam lalat pasir, namun medium tidak sesuai untuk pengawetan jangka panjang. Medium ini sesuai digunakan untuk pemerhatian jangka pendek, termasuk fotografi, lukisan atau imej. Medium berakua sesuai untuk pelekapan sementara, namun tidak dapat menjamin pengawetan jangka panjang. Sebaliknya, pelekapan menggunakan medium resin memberikan ketahanan yang sangat baik dan boleh bertahan sehingga berabad-abad lamanya, tetapi boleh mengaburkan perincian halus spermateka kerana sifat pembiasannya sering hilang.

Medium Hoyer boleh mengalami pemerosotan dari semasa ke semasa akibat penyahhidratan (Rajah 8), yang membawa kepada pembentukan hablur kecil kloral hidrat yang putih dan legap. Walaupun demikian, spesimen masih boleh diperoleh semula daripada slaid yang telah terhablur kerana kutikel kekal utuh secara kimia, walaupun kerosakan fizikal tertentu mungkin berlaku akibat pertumbuhan hablur. Dalam sesetengah keadaan, slaid yang telah terhablur boleh dipulihkan dengan penghidratan semula medium pelekapan dalam persekitaran hangat dan lembap dengan penambahan timol bagi mencegah pertumbuhan kulat. Sebagai alternatif, spesimen boleh dikeluarkan daripada gam kloral dengan merendamnya dalam air, kemudian dinyah-hidratkan dalam asid asetik glasial sebelum dilekap semula dalam balsam Kanada.

DMHF (dimetil hidantoin formaldehid) (RI 1.48)

Medium berasaskan air ini [72] menunjukkan prestasi optik yang sangat baik, serupa dengan Berlese, dan mudah digunakan seperti Berlese. Walau bagaimanapun, berbeza dengan Berlese, medium ini tidak menjadi hitam atau terhablur. Medium ini sesuai digunakan untuk lalat pasir serta serta Psychodidae lain.

CMCP (kamfor-mono-klorofenol) (RI = 1.41)

Medium pelekapan berasaskan gliserin larut air ini digunakan untuk menghasilkan slaid yang lutsinar, kekal bagi spesimen halus, termasuk lalat pasir. Kelebihan medium pelekapan ini ialah spesimen boleh dilekap secara langsung daripada air atau etanol. Medium ini cepat melembutkan dan menjernihkan lalat pasir, melembutkan kutikel bagi membolehkan penempatan spesimen yang tepat, terutamanya berguna untuk mengembangkan sayap atau membedah genitalia. Walaupun dilaporkan dapat membolehkan penyimpanan jangka panjang, tempoh sebenar pengawetannya masih belum diketahui. Had utama medium pelekapan ini terletak pada komponennya yang mengandungi fenol, sebatian toksik dan bahan perengsa yang memerlukan pengendalian yang teliti.

Medium bagi pelekapan kekal

Balsam Kanada (RI = 1.52-1.54)

Balsam Kanada pertama kali diterangkan sebagai medium pelekapan yang sesuai untuk mikroskopi cahaya ditransmisikan oleh Andrew Pritchard pada tahun 1830-an. Medium ini kekal sebagai salah satu medium yang paling banyak digunakan kerana kualiti arkibnya yang terbukti, dengan lebih 150 tahun penggunaan berjaya. Berbeza dengan medium Hoyer, balsam Kanada tidak mengalami penghabluran atau penyerapan kelembapan. Walau bagaimanapun, balsam Kanada mempunyai autofluoresens yang kuat, yang kadangkala boleh menjadi kelemahan bagi sesetengah teknik mikroskopi [60]. Penggunaan pelarut tidak toksik sebagai pengganti xilena dapat mengurangkan risiko keselamatan semasa penyediaan, namun juga mungkin menimbulkan kekangan seperti pengeringan lebih lama dan penggelapan medium lebih awal.

Euparal® (RI = 1.48)

Euparal® merupakan alternatif yang banyak digunakan kepada balsam Kanada untuk pelekapan kekal kerana menawarkan kestabilan jangka panjang yang baik serta indeks biasan yang setanding. Euparal® mempunyai ciri-ciri berikut : (1) Keperluan penyahhidratan: sebelum pemindahan akhir medium pelekapan, spesimen mesti dinyah-hidratkan, biasanya bermula daripada etanol 95% hingga etanol mutlak, dan (2) Masa pemprosesan yang panjang: pelekapan akhir dalam medium resin, sama ada balsam Kanada atau Euparal®, memerlukan proses penyahhidratan, yang memanjangkan keseluruhan tempoh pemprosesan spesimen. Apabila penyahhidratan menggunakan pelarut organik tidak boleh dilaksanakan, sampel yang diekstrak daripada etanol mutlak boleh diletakkan terlebih dahulu dalam larutan perantaraan yang terdiri daripada campuran sama rata Euparal® dan esen Euparal sebelum pelekapan akhir.

Enecê (RI = 1.467)

Enecê ialah medium pelekapan berasaskan resin yang terutamanya digunakan untuk serangga kecil dan amat popular di Brazil. Bes bagi medium ini adalah kolofoni dan gam damar yang dilarutkan dalam alkohol, bersama kapur barus, esen turpentin, dan eukaliptol. Cerqueira [11] menerangkan Enecê sebagai alternatif kepada balsam Kanada bagi pelekapan slaid kekal bagi larva, eksuvia peringkat muda, dan bahkan nyamuk dewasa, dan sejak itu medium ini telah digunakan secara meluas untuk pelekapan lalat pasir. Enecê menawarkan alternatif yang kos efektif untuk pelekapan kekal, dengan kestabilan jangka panjang serta tempoh pengeringan yang mencukupi, membolehkan pembedahan dan penyusunan tepat struktur morfologi.

5.4. Penyediaan dan pengeringan slaid

Pengeringan slaid yang dilekap dengan betul adalah kritikal untuk memastikan kestabilan jangka panjang dan pengawetan spesimen. Slaid perlu dikeringkan sepenuhnya sebelum dipertimbangkan untuk penyimpanan jangka panjang. Bagi mendapatkan keputusan yang optimum, slaid yang dilekap dengan medium pelekapan kekal perlu dikeringkan secara mendatar selama 2–3 minggu, manakala slaid yang disediakan dengan medium separa kekal mungkin hanya memerlukan 1–2 minggu. Penggunaan inkubator pada suhu yang sesuai bagi medium pelekapan yang digunakan, dengan mengelakkan haba berlebihan yang boleh merosakkan spesimen, adalah disyorkan untuk memastikan proses pengeringan yang berkesan. Julat suhu antara 30°C hingga 37°C adalah disarankan. Langkah pengeringan ini amat penting untuk mengelakkan peledingan pada slaid, kemerosotan spesimen, atau ketidakstabilan medium pelekapan semasa penyimpanan.

Medium pelekapan yang digunakan dalam penyediaan slaid hendaklah sentiasa dicatat pada label slaid. Jika boleh, label juga harus menyenaraikan resipi khusus yang digunakan, bersama nama penyedia dan tarikh penyediaan. Slaid yang pada awalnya disediakan sebagai pelekapan sementara dan tidak bertujuan untuk pengawetan jangka panjang. Walau bagaimanapun, jika status spesimen berubah, contohnya ditetapkan sebagai sebahagian daripada siri "rujuk", medium pelekapan yang lebih kekal harus digunakan bagi memastikan pengawetan spesimen untuk kajian taksonomi pada masa hadapan.

5.5. Teknik pelekapan alternatif : pelekapan pada kad.

Pelekapan pada kad merupakan satu teknik yang digunakan bagi beberapa kumpulan serangga, sama ada dipin secara langsung pada kad entomologi atau digamkan pada permukaannya. Walau bagaimanapun, memandangkan saiz lalat pasir yang sangat kecil serta keperluan untuk memerhati organ dalaman bagi tujuan pengecaman spesies melalui proses penjernihan (lihat item 5), kaedah ini tidak sama sekali sesuai digunakan untuk tujuan pelekapan spesimen lalat pasir.

5.6. Pelekapan semula spesimen yang rosak

Bagi spesimen nadir dan bernilai, pendekatan dua langkah disyorkan seperti yang ditunjukkan dalam video : https://zenodo.org/records/18315029. 1) menghidrat semula spesimen tanpa peleraian semula untuk membolehkan pemerhatian preliminari dilakukan. Pemegang untuk beberapa slaid mikroskop perlu diletakkan di dalam piring Petri sebagai sokongan. Slaid yang hendak dihidrat semula kemudian diletakkan di atas pemegang tersebut, dan piring Petri diisi dengan beberapa milimeter pelarut bagi mewujudkan kebuk lembap, dengan memastikan slaid itu sendiri tidak bersentuhan langsung dengan pelarut (Rajah 8D). Tempoh penghidratan semula boleh berbeza, daripada sehari hingga beberapa hari, bergantung kepada keadaan spesimen. Pemantauan harian dan kesabaran adalah penting. Setelah slaid cukup terhidrat semula, slaid tersebut boleh dikeluarkan daripada kebuk lembap dan diletakkan dalam inkubator selama beberapa jam sebelum pemerhatian mikroskopik, fotografi, atau

lukisan. 2) untuk dilekap semula, slaid boleh dikembalikan ke kebuk lembap untuk beberapa jam tambahan atau semalaman. Peleraian semula hendaklah dilakukan di bawah mikroskop binokular. Dengan menggunakan jarum halus, kaca penutup perlu dibuang secara berhati-hati bagi memastikan tiada unsur lalat pasir yang kekal melekat (https://zenodo.org/records/18315029). Seterusnya, unsur lalat pasir yang telah dibedah hendaklah dikutip dan dibilas dengan air dalam telaga kecil, seperti yang digunakan untuk pengekstrakan DNA/RNA memusnah (lihat di bawah), sebelum pendehidratan dan pelekapan semula dalam medium resin. Semasa peleraian semula slaid, adalah penting untuk mengenal pasti medium pelekapan asal bagi memilih pelarut yang bersesuaian. Bagi medium pelekapan berasaskan air, pelarut yang harus digunakan ialah air. Jika medium pelekapan berasaskan resin (contohnya, balsam Kanada atau Euparal®), xilena perlu digunakan, di bawah tukup wasap dan dengan peralatan pelindung diri yang sesuai, termasuk penutup muka.

Pelekapan semula spesimen rujuk atau koleksi hanya boleh dilakukan dengan kebenaran kurator dan/atau institusi yang memiliki spesimen tersebut.

6. Pengecaman spesies

6.1. Morfologi

Pengecaman lalat pasir terutamanya bergantung pada pemerhatian ciri morfologi, termasuk bentuk toraks, sayap, genitalia, sisip seta, dan hubungan morfometrik tertentu antara pelbagai struktur. Para penyelidik menggunakan kunci taksonomi, koleksi rujukan, dan penerangan spesies asal untuk membandingkan spesimen yang dikumpul dengan taksa yang diketahui. Ciri diagnostik utama, seperti urat sayap, morfologi kepala pada kedua-dua jantina, struktur genitalia jantan, dan konfigurasi spermateka betina, amat berguna untuk penentuan spesies. Pengecaman yang tepat biasanya memerlukan pemerhatian mikroskopik terperinci, menggunakan mikroskop majmuk untuk struktur halus seperti genitalia dan spermateka, atau mikroskop stereo bagi ciri morfologi yang lebih meluas. Kemajuan terkini dalam teknologi pengimejan telah memudahkan penggunaan imej digital bagi pengecaman lalat pasir. Gambar resolusi tinggi atau ilustrasi digital ciri utama boleh dibandingkan dengan bahan rujukan atau dianalisis menggunakan sistem pengecaman berasaskan komputer, meningkatkan ketepatan dan kebolehcapaian dalam taksonomi morfologi.

6.2. Geometri sayap

Geometri sayap merupakan ciri penting yang digunakan dalam pengecaman dan pengelasan pelbagai spesies lalat pasir. Sayap lalat pasir mempunyai corak dan struktur yang unik, biasanya panjang dan sempit dengan peruratan yang tersusun dengan baik (Rajah 9 & 10). Susunan urat pada sayap membentuk corak tersendiri yang berbeza antara genus dan spesies, menyediakan ciri diagnostik yang berguna untuk proses pengecaman. Oleh itu, kajian mengenai geometri sayap memberikan maklumat penting bagi tujuan taksonomi.

6.3. Morfometri geometri sayap

Para penyelidik menggunakan pelbagai teknik, termasuk morfometri geometri, untuk menganalisis dan membandingkan bentuk serta saiz sayap di antara spesies atau populasi lalat pasir yang berbeza. Kajian terhadap geometri sayap memberikan maklumat penting mengenai tingkah laku, keutamaan habitat, dan kemampuan untuk terbang.

Dalam pendekatan morfometri geometri, sayap dipembedah dengan teliti, diwarnakan (jika perlu), dan dilekap secara mendatar pada slaid. Slaid yang telah disediakan kemudian difotograf di bawah mikroskop stereo, didigitalkan, dan dianalisis secara morfometrik. Prosedur ini telah diterangkan dengan terperinci dalam sastera [6, 27, 42, 56, 57, 59], berserta cadangan bahawa sayap kanan atau kiri digunakan secara konsisten bagi organ berpasangan untuk mengelakkan kesan alometrik negatif yang berpotensi[62].

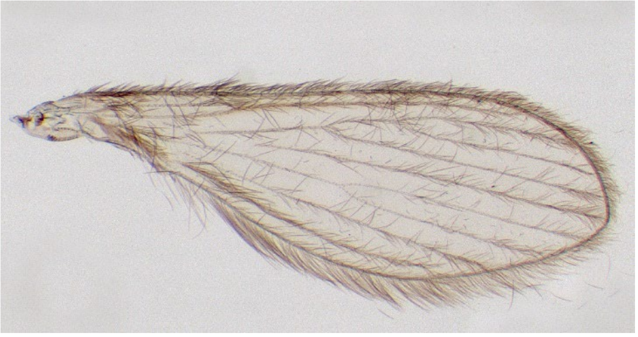

Rajah 9: Sayap *Trichophoromyia ininii* yang belum diproses

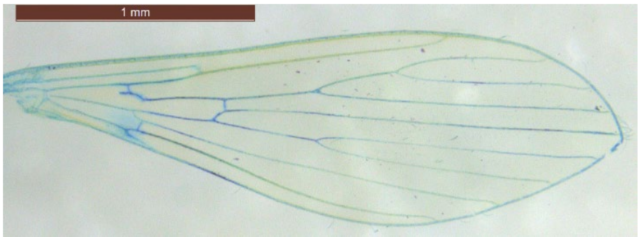

Rajah 10: Sayap *Phlebotomus ariasi* yang telah diwarnakan

Penyediaan sayap untuk analisis morfometri geometri

Bagi visualisasi urat sayap yang optimum, sayap harus dibersihkan daripada sisik dan diwarnakan mengikut kesesuaian. Untuk penyediaan sayap, mulakan dengan mengisi telaga kecil dengan reagen yang diperlukan (metilen biru, etanol, air, dan pengganti xilena). Sayap yang diawet dalam etanol 70% pada suhu bilik diambil dengan

menelangkupkan tiub Eppendorf dan mengosongkannya ke dalam telaga, kemudian angkat sayap secara membujur menggunakan jarum halus melengkung. Lalukan sayap secara singkat dari etanol ke air dan kembali ke etanol untuk menyingkirkan bulu halus. Rendam sayap dalam metilen biru selama 6 minit, dengan memastikan sayap terapung semasa pewarnaan. Angkat sayap dengan berhati-hati dan rendam dalam pengganti xilena selama 2 minit (lebih kurang satu per tiga daripada tempoh perendaman dalam metilen biru). Ketukan lembut jarum pada dinding telaga boleh membantu sayap mendap, manakala xilena bertindak mengekalkan pewarnaan.Akhir sekali, sayap diangkat dan diletakkan pada titisan kecil Euparal® di atas slaid mikroskop. Di bawah kanta pembesar, sayap dibuka dengan perlahan dan kaca penutup slaid diletakkan dengan berhati-hati. Fotografi harus diambil dengan segera sebelum Euparal® mengeras, kerana penyesuaian posisi sayap di bawah kaca penutup mungkin diperlukan untuk penjajaran yang optimum.

6.4. Teknik biologi molekul

Selain daripada kaedah morfologi, teknik molekul semakin penting dalam penyelidikan entomologi, termasuk kajian taksonomi, genetik populasi, dan filogenetik, serta untuk pengesanan DNA/RNA patogen dan penentuan sumber darah hos, yang relevan dalam bidang epidemiologi [70]. Penjujukan DNA boleh digunakan untuk pengesahan spesies atau membezakan spesies yang berkait rapat, menyediakan kaedah pengecaman yang lebih tepat dan boleh dipercayai. Selain itu, teknik molekul canggih seperti PCR, penjujukan DNA, NGS, dan MALDI-ToF MS semakin menonjol untuk pengecaman spesies yang cepat dan tepat, melengkapi kaedah morfologi tradisional [46]. Walaupun terdapat kemajuan ini, pengecaman morfologi kekal sebagai piawai rujukan bagi taksonomi dan asas bagi tafsiran data molekul.

6.4.1. Pengekstrakan asid nukleik memusnah

Pengekstrakan asid nukleik merupakan langkah rutin dalam banyak kajian biologi, dan pelbagai kaedah telah dibangunkan untuk mengasingkan DNA daripada bahan biologi [48]. Terdapat banyak kit pengekstrakan DNA yang tersedia secara komersial direka bentuk untuk memudahkan proses ini [14]. Walau bagaimanapun, kaedah yang lazim digunakan untuk penyediaan spesimen artropod bagi pengecaman morfologi sering mengganggu analisis DNA, kerana teknik ini mungkin merosakkan atau memusnahkan ciri fizikal kritikal spesimen [10]. Kebanyakan protokol pengekstrakan DNA untuk tisu serangga bersifat memusnah secara semula jadi [43], menimbulkan kebimbangan khusus bagi spesimen bersaiz kecil, di mana pengambilan sampel walaupun terhad boleh menjejaskan ciri morfologi penting [72]. Jenis dan keadaan spesimen memainkan peranan utama dalam pemilihan kaedah pengasingan DNA yang sesuai [29].

Keperluan untuk pengecaman lalat pasir yang tepat, pemahaman tentang dinamik populasi, serta pengurangan impak terhadap bukan sasaran telah mendorong pembangunan alat diagnostik molekul [23]. Pendekatan molekul kini kerap digunakan untuk melengkapkan kaedah taksonomi morfologi dalam pengecaman lalat pasir. Sebagai contoh, pendekatan standard untuk pengekodan bar serangga melibatkan pengekstrakan DNA, penjujukan, dan kehilangan spesimen asal. Oleh itu, terdapat keperluan mendesak untuk meneroka kaedah pengekstrakan DNA yang tidak memusnah, yang mengekalkan bahan biologi serta keutuhan morfologi spesimen.

Pelbagai kaedah pengekstrakan asid nukleik telah digunakan pada lalat pasir. Kuantiti atau kualiti asid nukleik yang diperlukan bergantung kepada analisis molekul seterusnya, kerana setiap teknik mempunyai keperluan kepekaan dan ketulenan yang berbeza [9]. Sebagai contoh, mata lalat pasir telah didapati boleh menrencat penggandaan PCR [69]. Selain daripada saringan patogen, DNA lalat pasir secara rutin diekstrak untuk tujuan pengecaman spesies. Pelbagai kaedah pengekstrakan boleh digunakan, walaupun hasil dan kualitinya berbeza mengikut teknik. Sesetengah protokol pengilang telah diubah suai oleh penyelidik untuk lalat pasir [8], bagi meningkatkan hasil dan/atau kualiti asid nukleik yang diekstrak [8, 9, 69], manakala adaptasi lain, yang dibangunkan untuk taksa artropod lain, juga boleh digunakan pada lalat pasir [58, 76]. Pengecaman spesies melalui PCR yang mensasarkan serpihan mitokondria kecil (COI atau CytB) umumnya serasi dengan kaedah pengekstrakan yang melibatkan penyerpihan DNA tinggi. Sebaliknya, teknik NGS jangka panjang lain (Oxford Nanopore dan PacBio) memerlukan penyerpihan minimum dan DNA berkualiti tinggi.

Pengekstrakan menggunakan kolum putar umumnya menghasilkan serpihan DNA genomik sehingga 60 kb, manakala pengekstrakan fenol-kloroform boleh menghasilkan serpihan DNA sehingga 150 kb [77]. Jadual 5 meringkaskan pelbagai teknik pengekstrakan DNA lalat pasir dan menunjukkan sama ada adaptasi metodologi telah dilakukan untuk serangga ini. Hasil (yield) tidak ditunjukkan kerana bergantung kepada saiz spesimen dan kaedah penyediaan. Ruangan modifikasi merujuk kepada adaptasi protokol pengekstrakan untuk lalat pasir atau artropod kecil lain.

Pemilihan kaedah pengekstrakan perlu mengambil kira beberapa kriteria, seperti bilangan sampel, tempoh pengekstrakan, dan teknik yang digunakan pada peringkat seterusnya. Walaupun teknik NGS memerlukan DNA genomik dengan berat molekul yang tinggi, semua kaedah yang dibentangkan di sini boleh digunakan untuk aplikasi berasaskan PCR standard.

Selain itu, beberapa kajian telah meneroka kaedah pengekstrakan DNA yang tidak memusnah untuk artropod terestrial bersaiz kecil, spesimen muzium yang disimpan kering, dan artropod berbadan lembut [19, 26, 28, 55, 63].

Jadual 5: Kos purata, aplikasi, dan penyesuaian protokol bagi pengekstrakan DNA genomik (gDNA) lalat pasir Phlebotomine.

Protokol	Kos	Aplikasi	Penyesuaian protokol bagi artropod bersaiz kecil
Kolum putar	2.5–3.55 US$ [39]	PCR, NGS	[9]
Fenol-klorofom	0.24 US$ [69]	PCR, NGS	[9]
Kaedah HotSHOT	<0.01 US$ [69]	PCR	-
Pemendapan garam	0.12 $ [69]	PCR	-
Kaedah Chelex	0.02 $ [41]	PCR	[41, 76]

6.4.2. Pengekstrakan DNA yang tidak memusnah

Salah satu cabaran utama dalam analisis molekul artropod, terutamanya lalat pasir, ialah pengawetan spesimen bagi tujuan integrasi ke dalam koleksi entomologi. Kebanyakan protokol pengekstrakan DNA memerlukan pemaseratan tisu, seterusnya menjejaskan pengawetan spesimen asal. Kaedah pengekstrakan asid nukleik yang tidak memusnah direka bentuk untuk mengekstrak bahan genetik tanpa merosakkan sampel secara fizikal, menjejaskan kebolehhidupannya, atau mengubah morfologinya. Kaedah ini amat bernilai bagi spesimen yang berharga atau terhad, seperti lalat pasir, kerana mengekalkan keutuhan struktur adalah penting untuk tujuan taksonomi, morfologi, atau diagnostik pada masa hadapan. Salah satu teknik yang kerap digunakan ialah kaedah mandian tidak memusnah, dengan cara lalat pasir dipegunkan dan direndam perlahan-lahan dalam buffer lisis yang mengandungi proteinase K.

Teknik vektolisis ringan telah berjaya digunakan pada lalat pasir, terutamanya spesimen rujuk [24]. Teknik ini menggunakan kit kolum putar konvensional (dalam kes ini, kit Darah Dneasy dan Tisu, QIAGEN, Hilden, Jerman) dengan adaptasi bagi mendapatkan DNA tanpa memusnahkan spesimen. Langkah-langkah lisis yang diubah suai (jumlah buffer lisis dan penambahan langkah penyejubekuaan) [17] membolehkan pembebasan asid nukleik dengan meminimumkan kerosakan morfologi [24].

Bagi lalat pasir, kit pengekstrakan DNA HotSHOT (Bento Bioworks Ltd, London, United Kingdom) juga boleh digunakan [73], kerana cepat dan kos yang rendah, membolehkan pemprosesan sampel secara pantas dan berhemat. Spesimen entomologi yang ditujukan untuk pengecaman morfologi boleh dicuci selepas diproses. Spesimen yang diproses menggunakan kit Darah Dneasy dan Tisu perlu dibersihkan dengan larutan Marc-André, manakala spesimen yang diproses menggunakan kit pengekstrakan DNA HotSHOT cukup jelas untuk dilekap dalam medium berakua, atau lebih baik, dalam resin selepas penyahhidratan, mengikut protokol yang diperincikan dalam artikel ini [73]. Bahan genetik yang telah diekstrak kemudiannya boleh diproses lebih lanjut untuk analisis seterusnya, seperti PCR, bagi menggandakan penanda genetik tertentu. Kaedah pengekstrakan asid nukleik yang tidak memusnah amat penting untuk mengkaji ciri genetik lalat pasir, termasuk pengenalpastian agen penyebab penyakit yang mungkin dibawa. Dengan mengekalkan keutuhan spesimen, penyelidik dapat memperoleh maklumat genetik yang bernilai sambil mengekalkan sampel untuk analisis atau kajian tambahan.

6.5. MALDI-ToF MS

MALDI-ToF MS (Spektrometer jisim masa terbang penyaherapan/pengionan laser dibantu matriks) ialah teknik berasaskan spektrometri jisim yang direka untuk mengesan dan menganalisis profil protein unik (“cap jari”) sampel biologi. Teknik MALDI-ToF semakin diiktiraf sebagai alat penting untuk pengecaman artropod yang mempunyai kepentingan dalam bidang perubatan dan veterinar. Teknik ini telah terbukti berkesan dalam mengenal pasti pelbagai peringkat perkembangan lalat pasir, termasuk peringkat belum matang serta sumber darah hos pada betina yang kenyang darah, dan telah berjaya digunakan untuk membezakan spesies lalat pasir jantan dan betina di bawah pelbagai keadaan penyimpanan dan penghomogenatan [28, 30, 73, 74]. Kaedah ini juga menawarkan keupayaan diskriminasi yang tinggi pada peringkat subgenus, spesies dan populasi. Teknik ini membolehkan penyelidik memperoleh pengecaman spesies secara cepat dan tepat, yang amat diperlukan untuk memahami taburan lalat pasir, tingkah laku lalat pasir serta peranannya dalam penularan penyakit. Dengan membezakan spesies berdasarkan profil protein, MALDI-ToF memainkan peranan penting dalam kajian epidemiologi dan strategi kawalan vektor. Walau bagaimanapun, terdapat dua kekangan utama yang mengehadkan penggunaan rutin teknik ini. Kekangan pertama ialah ketersediaan peralatan spektrometri jisim, yang kosnya sangat tinggi dan sukar diperoleh khusus untuk tujuan pengecaman spesies lalat pasir (atau vektor artropod secara umum). Namun begitu, kekangan ini berpotensi diatasi melalui akses masa penggunaan pada instrumen spektrometer jisim yang kini telah menjadi peralatan penyelidikan standard di fasiliti proteomik dan/atau diagnostik klinikal. Kekangan kedua ialah perwakilan data rujukan lalat pasir yang masih rendah dalam pangkalan data capaian terbuka, sekali gus memerlukan pembinaan pangkalan data dalaman yang mengandungi spektrum rujukan berdasarkan spesimen yang telah dikenal pasti secara jelas, sebaiknya melalui gabungan penilaian morfologi dan penjujukan penanda genetik yang sesuai (COI, cytB atau penanda lain). Kekangan ini diharapkan dapat diatasi dalam masa terdekat melalui kemasukan

Протокол	Цена	Приложение	Адаптация на протокола за дребни членестоноги
Центрофужни колонки	2,5 – 3,55 $ [39]	PCR, NGS	[9]
Фенол-хлороформ	0,12 $ [69]	PCR, NGS	[9]
HotSHOT	<0,01 $ [69]	PCR	-
Изсолване	0,12 $3 [69]	PCR	-
Chelex	0,02 $4 [41] 0,02 $4 [41]	PCR	[41, 76]

secara berperingkat data rujukan lalat pasir yang sebelum ini disimpan secara dalaman ke dalam Platform MSI yang dikendalikan oleh Assistance Publique–Hôpitaux de Paris, Sorbonne University, Perancis, serta koleksi BCCM/IHEM/Sciensano di Brussels, Belgium (https://msi.happy-dev.fr/). Apabila pemprofilan protein MALDI-ToF dirancang untuk digunakan, sampel sebaiknya disimpan dalam keadaan beku kering atau dalam etanol 70% gred molekul, dan tidak terdedah kepada suhu ambien. Memandangkan ketiadaan garis panduan universal bagi penyediaan sampel, pengguna disarankan menggunakan larutan berakua yang mengandungi 60% asetonitril dan 0.3% TFA dengan asid sinapinik (30 mg/mL) sebagai matriks MALDI-ToF, bagi memastikan spektrum protein yang dihasilkan dapat dibandingkan dengan data lalat pasir yang telah diterbitkan sebelum ini.

Penyediaan Sampel untuk MALDI-ToF MS (Rajah 7)

Spesimen serangga yang disimpan di bawah pelbagai keadaan penyimpanan hendaklah terlebih dahulu dikeringkan secara pengeringan udara pada suhu bilik sebelum proses pembedahan dilakukan. Bahagian kepala dan abdomen dikeluarkan bagi mendapatkan bahagian badan yang mengandungi ciri morfologi penting untuk proses pelekapan pada slaid dan analisis morfologi. Bahagian toraks digunakan untuk analisis MALDI-ToF, manakala baki abdomen boleh diawet untuk pengekstrakan DNA. Bagi tujuan pemprofilan protein, toraks dihomogenkan secara manual dalam tuib mikro 1.5 mL menggunakan 10 µL larutan penghomogenatan dengan bantuan penumbuk pakai buang dan pelet. Dua jenis larutan penghomogenatan lazim digunakan, iaitu air suling steril dan asid formik 25%.

7. Kesimpulan

Dalam usaha ini, kajian kami menumpukan kepada penyediaan kaedah pelekapan lalat pasir yang paling berkesan bagi para penyelidik, disesuaikan mengikut objektif penyelidikan tertentu, bagi memudahkan pengecaman spesies secara tepat serta pengesanan patogen. Tiada satu kaedah tunggal yang bersifat universal; sebaliknya, terdapat pelbagai kaedah, masing-masing dengan kelebihan dan keterbatasannya. Dalam lampiran, kami menyediakan protokol terperinci bagi pelbagai teknik pelekapan yang digunakan dalam penyediaan dan pengecaman spesies lalat pasir. Protokol ini, termasuk video panduan yang mengandungi prosedur langkah-demi-langkah yang disesuaikan dengan objektif berbeza bagi memastikan hasil dapatan yang tepat dan diyakini. Dengan menyediakan sumber komprehensif ini, kami berhasrat untuk membantu penyelidik memilih dan melaksanakan teknik pelekapan yang paling sesuai mengikut keperluan penyelidikan mereka.

Penghargaan

Para penulis merakamkan penghargaan kepada Richard Lane dan Zoe Jay Adams dari Natural History Museum of London, UK atas ulasan mereka yang cemerlang, yang meningkatkan kualiti manuskrip ini.

Pembiayaan

Kami mengiktiraf agensi pembangunan Brazil, CNPq (nombor kes: 404395/2024-4) dan Yayasan Araucária (nombor kes: 433/2025 PDI) atas pembiayaan bagi penyelidikan AJA.

Konflik Kepentingan

Jérôme Depaquit merupakan editor bersekutu bagi Parasite; beliau tidak mempunyai pengaruh ke atas proses ulasan dan keputusan berkaitan manuskrip ini. Penulis lain mengisytiharkan bahawa mereka tidak mempunyai sebarang konflik kepentingan.

Pernyataan ketersediaan data

Video-video yang terdapat di Zenodo.

Video 1: https://zenodo.org/records/18198006

Video 2: https://zenodo.org/records/18311158

Video 3: https://zenodo.org/records/18311106

Video 4: https://zenodo.org/records/18311154

Video 5: https://zenodo.org/records/18303014

Video 6: https://zenodo.org/records/18302850

Video 7: https://zenodo.org/records/18315029

Bahan Tambahan

Bahan tambahan untuk artikel ini boleh didapati di: https://www.parasite-journal.org/10.1051/parasite/2026009/olm

Rujukan

1. Alkan C, Allal-Ikhlef AB, Alwassouf S, Baklouti A, Piorkowski G, de Lamballerie X, Izri A, Charrel RN. 2015. Virus isolation, genetic characterization and seroprevalence of Toscana virus in Algeria. Clinical Microbiology and Infection, 21(11), 1040 e1-9.
2. Alten B, Ozbel Y, Ergunay K, Kasap OE, Cull B, Antoniou M, Velo E, Prudhomme J, Molina R, Banuls AL, Schaffner F, Hendrickx G, Van Bortel W, Medlock JM. 2015. Sampling strategies for phlebotomine sand flies (Diptera: Psychodidae) in Europe. Bulletin of Entomological Research 105(6), 664–678.
3. Ayhan N, Baklouti A, Prudhomme J, Walder G, Amaro F, Alten B, Moutailler S, Ergunay K, Charrel RN, Huemer H. 2017. Practical guidelines for studies on sandfly-borne phleboviruses: Part I: Important points to consider *ante* field work. Vector-Borne and Zoonotic Diseases 17(1), 73–80.
4. Bates PA. 1997. Infection of phlebotomine sandflies with *Leishmania*, in The Molecular Biology of Insect Disease Vectors: A Methods Manual. Springer. p. 112–120.5.
5. Baum M, de Castro EA, Pinto MC, Goulart TM, Baura W, Klisiowicz Ddo R, Vieira da Costa-Ribeiro MC. 2015. Molecular detection of the blood meal source of sand flies (Diptera: Psychodidae) in a transmission area of American cutaneous leishmaniasis, Parana State, Brazil. Acta Tropica, 143, 8–12.

6. Belen A, Alten B, Aytekin A. 2004. Altitudinal variation in morphometric and molecular characteristics of *Phlebotomus papatasi* populations. Medical and Veterinary Entomology, 18(4), 343–350.
7. Bhattacharya J, Chandra G, Hati AK. 1991. A simple method for cryopreservation of *Leishmania donovani* promastigotes, Indian Journal of Medical Research, 93, 245–246.
8. Caligiuri LG, Sandoval AE, Miranda JC, Pessoa FA, Santini MS, Salomón OD, Secundino NF, McCarthy CB. 2019. Optimization of DNA extraction from individual sand flies for PCR amplification. Methods and Protocols, 2(2), 36.
9. Casaril AE, de Oliveira LP, Alonso DP, de Oliveira EF, Gomes Barrios SP, de Oliveira Moura Infran J, Fernandes WS, Oshiro ET, Ferreira AMT, Ribolla PEM, de Oliveira AG. 2017. Standardization of DNA extraction from sand flies: Application to genotyping by next generation sequencing. Experimental Parasitology, 177, 66–72.
10. Castalanelli MA, Severtson DL, Brumley CJ, Szito A, Foottit RG, Grimm M, Munyard K, Groth DM. 2010. A rapid non-destructive DNA extraction method for insects and other arthropods. Journal of Asia-Pacific Entomology, 13(3), 243–248.
11. Cerqueira NL. 1943. Um novo meio para montagem de pequenos insetos em lâmina. Memórias do Instituto Oswaldo Cruz, (39), 37–41.
12. Charrel RN, Gallian P, Navarro-Mari JM, Nicoletti L, Papa A, Sanchez-Seco MP, Tenorio A, de Lamballerie X. 2005. Emergence of Toscana virus in Europe. Emerging Infectious Diseases, 11(11), 1657–1663.
13. Chaskopoulou A, Giantsis IA, Demir S, Bon MC. 2016. Species composition, activity patterns and blood meal analysis of sand fly populations (Diptera: Psychodidae) in the metropolitan region of Thessaloniki, an endemic focus of canine leishmaniasis. Acta Tropica, 158, 170–176.
14. Chen H, Rangasamy M, Tan SY, Wang H, Siegfried BD. 2010. Evaluation of five methods for total DNA extraction from western corn rootworm beetles. PLoS One, 5(8), e11963.
15. Depaquit J, Grandadam M, Fouque F, Andry PE, Peyrefitte C. 2010. Arthropod-borne viruses transmitted by Phlebotomine sandflies in Europe: a review. Eurosurveillance, 15(10), 19507.
16. Diamond LS, Herman CM. 1954. Incidence of Trypanosomes in the Canada Goose as revealed by bone marrow culture. Journal of Parasitology, 40(2), 195–202.
17. Ding H, Torno M, Vongphayloth K, Ng G, Tan D, Sng W, Ho K, Randrianambinintsoa FJ, Depaquit J, Tan CH. 2025. Hidden in plain sight: discovery of sand flies in Singapore and description of four species new to science. Parasites & Vectors, 18(1), 402.
18. Es-Sette N, Ajaoud M, Bichaud L, Hamdi S, Mellouki F, Charrel RN, Lemrani M. 2014. *Phlebotomus sergenti* a common vector of *Leishmania tropica* and Toscana virus in Morocco. Journal of Vector Borne Diseases, 51(2), 86–90.
19. Favret C. 2005. A new non-destructive DNA extraction and specimen clearing technique for aphids (Hemiptera). Proceedings of the Entomological Society of Washington, 107(2), 469–470.
20. Galati EAB. 2018. Phlebotominae (Diptera, Psychodidae): Classification, morphology and terminology of adults and identification of American taxa, in Brazilian Sand Flies: Biology, Taxonomy, Medical Importance and Control, Rangel EF, Shaw JJ, Editors. Cham: Springer International Publishing. pp. 9–212.
21. Galati EAB, de Andrade AJ, Perveen F, Loyer M, Vongphayloth K, Randrianambinintsoa FJ, Prudhomme J, Rahola N, Akhoundi M, Shimabukuro PHF, Depaquit J. 2025. Phle-botomine sand flies (Diptera, Psychodidae) of the world. Parasites & Vectors, 18(1), 220.
22. Galati EAB, Galvis-Ovallos F, Lawyer P, Leger N, Depaquit J. 2017. An illustrated guide for characters and terminology used in descriptions of Phlebotominae (Diptera, Psychodidae). Par-asite, 24, 26.
23. Gariepy T, Kuhlmann U, Gillott C, Erlandson M. 2007. Parasitoids, predators and PCR: the use of diagnostic molecular markers in biological control of Arthropods. Journal of Applied Entomology, 131(4), 225–240.
24. Giantsis IA, Chaskopoulou A, Bon MC. 2016. Mild-Vectolysis: A nondestructive DNA extraction method for vouchering sand flies and mosquitoes. Journal of Medical Entomology, 53(3), 692–695.
25. Gidwani K, Picado A, Rijal S, Singh SP, Roy L, Volfova V, Andersen EW, Uranw S, Ostyn B, Sudarshan M, Chakravarty J, Volf P, Sundar S, Boelaert M, Rogers ME. 2011. Serological markers of sand fly exposure to evaluate insecticidal nets against visceral leishmaniasis in India and Nepal: a cluster-randomized trial. PLoS Neglected Tropical Diseases, 5(9), e1296.
26. Gilbert MTP, Moore W, Melchior L, Worobey M. 2007. DNA extraction from dry museum beetles without conferring external morphological damage. PLoS One, 2(3), e272.
27. Giordani BF, Andrade AJ, Galati EAB, Gurgel-Goncalves R. 2017. The role of wing geometric morphometrics in the identification of sandflies within the subgenus *Lutzomyia*. Medical and Veterinary Entomology, 31(4), 373–380.
28. Guzmán-Larralde AJ, Suaste-Dzul AP, Gallou A, Peña-Carrillo KI. 2017. DNA recovery from microhymenoptera using six non-destructive methodologies with considerations for subsequent preparation of museum slides. Genome, 60(1), 85–91.
29. Hajibabaei M, DeWaard JR, Ivanova NV, Ratnasingham S, Dooh RT, Kirk SL, Mackie PM, Hebert PD. 2005. Critical factors for assembling a high volume of DNA barcodes. Philosophical Transactions of the Royal Society B: Biological Sciences, 360(1462), 1959–1967.
30. Haouas N, Pesson B, Boudabous R, Dedet JP, Babba H, Ravel C. 2007. Development of a molecular tool for the identification of *Leishmania* reservoir hosts by blood meal analysis in the insect vectors. American Journal of Tropical Medicine and Hygiene, 77(6), 1054–1059.
31. Hlavackova K, Dvorak V, Chaskopoulou A, Volf P, Halada P. 2019. A novel MALDI-TOF MS-based method for blood meal identification in insect vectors: A proof of concept study on phlebotomine sand flies. PLoS Neglected Tropical Diseases, 13 (9), e0007669.
32. Huemer H, Prudhomme J, Amaro F, Baklouti A, Walder G, Alten B, Moutailler S, Ergunay K, Charrel RN, Ayhan N. 2017. Practical guidelines for studies on sandfly-borne phleboviruses: Part II: Important points to consider for fieldwork and subsequent virological screening. Vector-Borne and Zoonotic Diseases, 17(1), 81–90.
33. Jancarova M, Polanska N, Thiesson A, Arnaud F, Stejskalova M, Rehbergerova M, Kohl A, Viginier B, Volf P, Ratinier M. 2025. Susceptibility of diverse sand fly species to Toscana virus. PLoS Neglected Tropical Diseases, 19(5), e0013031.
34. Kapp JD, Green RE, Shapiro B. 2021. A fast and efficient single-stranded genomic library preparation method optimized for ancient DNA. Journal of Heredity, 112(3), 241–249.
35. Killick-Kendrick R, Maroli M, Killick-Kendrick M. 1991. Bibliography of the colonization of phlebotomine sandflies. Parassitologia, 33(suppl.), 321–333.

36. Lawyer P, Killick-Kendrick M, Rowland T, Rowton E, Volf P. 2017. Laboratory colonization and mass rearing of phlebotomine sand flies (Diptera, Psychodidae). Parasite, 24, 42.
37. Léger N, Pesson B, Madulo-Leblond G. 1986. Les phlébotomes de Grèce : 1ère partie. Bulletin de la Société de Pathologie Exotique, 79, 386–397.
38. Léger N, Pesson B, Madulo-Leblond G. 1986. Les phlébotomes de Grèce : 2ème partie. Bulletin de la Société de Pathologie Exotique, 79, 514–524.
39. Leonel JAF, Vioti G, Alves ML, da Silva DT, Meneghesso PA, Benassi JC, Spada JCP, Galvis-Ovallos F, Soares RM, Oliveira T. 2020. DNA extraction from individual Phlebotomine sand flies (Diptera: Psychodidae: Phlebotominae) specimens: Which is the method with better results? Experimental Parasitology, 218, 107981.
40. Lestinova T, Rohousova I, Sima M, de Oliveira CI, Volf P. 2017. Insights into the sand fly saliva: Blood-feeding and immune interactions between sand flies, hosts, and *Leishmania.* PLoS Neglected Tropical Diseases, 11(7), e0005600.
41. Lienhard A, Schaffer S. 2019. Extracting the invisible: obtaining high quality DNA is a challenging task in small arthropods. PeerJ, 7, e6753.
42. Lozano-Sardaneta YN, Mikery-Pacheco OF, Huerta H, Rojas-Soriano JE, Contreras-Ramos A. 2025. Wing geometric morphometrics is effective to separate sand fly species (Diptera, Psychodidae, Phlebotominae) related with leishmaniasis transmission in Mexico. Acta Tropica, 262, 107523.
43. Mandrioli M. 2008. Insect collections and DNA analyses: how to manage collections? Museum Management and Curatorship, 23(2), 193–199.
44. Maroli M, Feliciangeli MD, Bichaud L, Charrel RN, Gradoni L. 2013. Phlebotomine sandflies and the spreading of leishmaniases and other diseases of public health concern. Medical and Veterinary Entomology, 27(2), 123–147.
45. Marquina D, Buczek M, Ronquist F, Lukasik P. 2021. The effect of ethanol concentration on the morphological and molecular preservation of insects for biodiversity studies. PeerJ, 9, e10799.
46. Mathis A, Depaquit J, Dvorak V, Tuten H, Banuls AL, Halada P, Zapata S, Lehrter V, Hlavackova K, Prudhomme J, Volf P, Sereno D, Kaufmann C, Pfluger V, Schaffner F. 2015. Identification of phlebotomine sand flies using one MALDI-TOF MS reference database and two mass pectrometer systems., Parasites & Vectors, 8, 266.
47. Mekarnia N, Benallal KE, Sadlova J, Vojtkova B, Mauras A, Imbert N, Longhitano M, Harrat Z, Volf P, Loiseau PM, Cojean S. 2024. Effect of *Phlebotomus papatasi* on the fitness, infectivity and antimony-resistance phenotype of antimony-resistant *Leishmania* major Mon-25. International Journal for Parasitology – Drugs and Drug Resistance, 25, 100554.
48. Milligan BG. 1998. Total DNA isolation, in Molecular Genetic Analysis of Population: A Practical Approach, Hoelzel AR, Editor. Oxford: Oxford University Press.
49. Molina R, Jiménez M, Alvar J, González E, Hernández-Taberna S, Ines MM. 2017. Methods in sand fly research. Madrid: Servicio de publicaciones Universidad de Alcalá de Henares, Madrid.
50. Murphy WJ, Eizirik E, O'Brien SJ, Madsen O, Scally M, Douady CJ, Teeling E, Ryder OA, Stanhope MJ, de Jong WW, Springer MS. 2001. Resolution of the early placental mammal radiation using Bayesian phylogenetics. Science, 294(5550), 2348–2351.
51. Nacif-Pimenta R, Pinto LC, Volfova V, Volf P, Pimenta PFP, Secundino NFC. 2020. Conserved and distinct morphological aspects of the salivary glands of sand fly vectors of leishma-niasis: an anatomical and ultrastructural study. Parasites & Vectors, 13(1), 441.
52. Neuhaus B, Schmid T, Riedel J. 2017. Collection management and study of microscope slides: Storage, profiling, deterioration, restoration procedures, and general recommendations. Zootaxa, 4322(1), 1–173.
53. New TR. 1974. Pscoptera. Handbooks for Identification of British Insects (Vol. I). London: Royal Entomological Society of London. 102 pp.
54. Perez-Ruiz M, Collao X, Navarro-Mari JM, Tenorio A. 2007. Reverse transcription, real-time PCR assay for detection of Toscana virus. Journal of Clinical Virology, 39(4), 276–281.
55. Porco D, Rougerie R, Deharveng L, Hebert P. 2010. Coupling non-destructive DNA extraction and voucher retrieval for small soft-bodied Arthropods in a high-throughput context: the example of Collembola. Molecular Ecology Resources, 10(6), 942–945.
56. Prudhomme J, Cassan C, Hide M, Toty C, Rahola N, Vergnes B, Dujardin JP, Alten B, Sereno D, Banuls AL. 2016. Ecology and morphological variations in wings of *Phlebotomus ariasi* (Diptera: Psychodidae) in the region of Roquedur (Gard, France): a geometric morphometrics approach. Parasites & Vectors, 9(1), 578.
57. Prudhomme J, Gunay F, Rahola N, Ouanaimi F, Guernaoui S, Boumezzough A, Banuls AL, Sereno D, Alten B. 2012. Wing size and shape variation of *Phlebotomus papatasi* (Diptera: Psychodidae) populations from the south and north slopes of the Atlas Mountains in Morocco. Journal of Vector Ecology, 37(1), 137–147.
58. Prudhomme J, Toty C, Kasap OE, Rahola N, Vergnes B, Maia C, Campino L, Antoniou M, Jimenez M, Molina R, Cannet A, Alten B, Sereno D, Banuls AL. 2015. New microsatellite markers for multi-scale genetic studies on *Phlebotomus ariasi* Tonnoir, vector of *Leishmania infantum* in the Mediterranean area. Acta Tropica, 142, 79–85.
59. Prudhomme J, Velo E, Bino S, Kadriaj P, Mersini K, Gunay F, Alten B. 2019. Altitudinal variations in wing morphology of *Aedes albopictus* (Diptera, Culicidae) in Albania, the region where it was first recorded in Europe. Parasite, 26, 55.
60. Rawlins DJ. 1992. Light Microscopy: An Introduction to Biotechniques. Oxford: Bios Scientific publishers. 143 pp.
61. Ready PD. 2013. Biology of phlebotomine sand flies as vectors of disease agents. Annual Review of Entomology, 58, 227–250.
62. Rohlf FJ, Slice D. 1990. Extensions of the Procrustes method for the optimal superimposition of landmarks. Systematic Zoology, 39(1), 40–59.
63. Rowley DL, Coddington JA, Gates MW, Norrbom AL, Ochoa RA, Vandenberg NJ, Greenstone MH. 2007. Vouchering DNA-barcoded specimens: Test of a nondestructive extraction proto-col for terrestrial arthropods. Molecular Ecology Notes, 7(6), 915–924.
64. Sábio PB, Andrade AJ, Galati EAB. 2014. Assessment of the taxonomic status of some species included in the *Shannoni* complex, with the description of a new species of *Psathyromyia* (Diptera: Psychodidae: Phlebotominae). Journal of Medical Entomology, 51(2), 331–341.
65. Sadlova J, Yeo M, Seblova V, Lewis MD, Mauricio I, Volf P, Miles MA. 2011. Visualisation of *Leishmania donovani* fluo-rescent hybrids during early stage development in the sand fly vector. PLoS One, 6(5), e19851.

66. Sales K, Miranda DEO, da Silva FJ, Otranto D, Figueredo LA, Dantas-Torres F. 2020. Evaluation of different storage times and preservation methods on phlebotomine sand fly DNA concentration and purity. Parasites & Vectors, 13(1), 399
67. Sales KG, Costa PL, de Morais RC, Otranto D, Brandao-Filho SP, Cavalcanti Mde P, Dantas-Torres F. 2015. Identification of phlebotomine sand fly blood meals by real-time PCR. Parasites& Vectors, 8, 230.
68. Sant'Anna MR, Jones NG, Hindley JA, Mendes-Sousa AF, Dillon RJ, Cavalcante RR, Alexander B, Bates PA. 2008. Blood meal identification and parasite detection in laboratory-fed and field-captured *Lutzomyia longipalpis* by PCR using FTA databasing paper. Acta Tropica, 107(3), 230–237.
69. Senne NA, Santos HA, Araujo TR, Paulino PG, Mendonca LP, Moreira HVS, Camilo TA, da Costa Angelo I. 2022. Robust comparative performance of genomic DNA extraction methods from non-engorged phlebotomine sandflies. Medical and Veterinary Entomology, 36(2), 203–211.
70. Shaw JJ. 2025. A review of Leishmania infections in American Phlebotomine sand flies – Are those that transmit leishmaniasis anthropophilic or anthropportunists? Parasite, 32, 57.
71. Tesh RB, Modi GB. 1983. Growth and transovarial transmission of Chandipura virus (Rhabdoviridae: Vesiculovirus) in *Phlebotomus papatasi.* American Journal of Tropical Medicine and Hygiene, 32(3), 621–623.
72. Thomsen PF, Elias S, Gilbert MTP, Haile J, Munch K, Kuzmina S, Froese DG, Sher A, Holdaway RN, Willerslev E. 2009. Non-destructive sampling of ancient insect DNA. PLoS One, 4(4),e5048
73. Truett GE, Heeger P, Mynatt RL, Truett AA, Walker JA, Warman ML. 2000. Preparation of PCR-quality mouse genomic DNA with hot sodium hydroxide and tris (HotSHOT). Biotechniques, 29(1), 52–54.
74. Upton MS. 1993. Aqueous gum-chloral slide mounting media: an historical review. Bulletin of Entomological Research, 83(2), 267–274.
75. Volf P, Myskova J. 2007. Sand flies and Leishmania: specific versus permissive vectors. Trends in Parasitology, 23(3), 91–92.
76. Wang Q, Wang X. 2012. Comparison of methods for DNA extraction from a single chironomid for PCR analysis. Pakistan Journal of Zoology, 44(2), 421–426.
77. Wang Y, Zhao Y, Bollas A, Wang Y, Au KF. 2021. Nanopore sequencing technology, bioinformatics and applications. Nature Biotechnology, 39(11), 1348–1365.

Cite this article as: Randrianambinintsoa FJ, Augendre L, Prudhomme J, Martinet J-P, Loyer M, Mekarnia N, Kerkoub H, Perveen FK, Huguenin A, Kariya E, Akhoundi M, De Andrade AJ, Berriatua E, Bongiorno G, Boyer S, Christodoulou V, Da Costa-Ribeiro MCV, De Souza LAF, Ding H, Dondji B, Dvořák V, Erisoz Kasap O, Galati EAB, Gállego M, Ballart C, Gouzelou S, Haddad N, Masse RS, Mekuria AH, Ivovic V, Kaczmarek S, Shahar MK, Kirstein OD, Kniha E, Kolářová I, Lincoln T, Lucanas C, Mikov O, Nov K, Özbel Y, Pesson B, Posada Lopez LC, Prasetyo DB, Rahola N, Rebollar-Tellez EA, Rodrigues BL, Roy L, Saini P, Sanjoba C, Shimabukuro PH, Siriyasatien P, Soszynska A, Suleşco T, Sylla M, Torno M, Volf P, Vongphayloth K, Sinh Nam V, Wardhana A, Yessinou E, Zapata S, Gantier J-C & Depaquit J. 2026. Processing and mounting phlebotomine sand flies: a consensus guideline. Parasite xx, xx. https://doi.org/10.1051/parasite/2026009.

PARASiTE

An international open-access, peer-reviewed, online journal publishing high quality papers on all aspects of human and animal parasitology

Reviews, articles and short notes may be submitted. Fields include, but are not limited to: general, medical and veterinary parasitology; morphology, including ultrastructure; parasite systematics, including entomology, acarology, helminthology and protistology, and molecular analyses; molecular biology and biochemistry; immunology of parasitic diseases; host-parasite relationships; ecology and life history of parasites; epidemiology; therapeutics; new diagnostic tools.
All papers in Parasite are published in English. Manuscripts should have a broad interest and must not have been published or submitted elsewhere. No limit is imposed on the length of manuscripts.

Parasite (open-access) continues **Parasite** (print and online editions, 1994-2012) and **Annales de Parasitologie Humaine et Comparée** (1923-1993) and is the official journal of the Société Française de Parasitologie.

Editor-in-Chief:
Jean-Lou Justine, Paris

Submit your manuscript at:
https://www.editorialmanager.com/parasite

Lampiran 1: Asas teori biokimia.

Arthropod yang dibincangkan ialah lalat pasir. Walau bagaimanapun, idea ini boleh diperluaskan kepada arthropod lain yang sering ditemui, yang pengecaman spesiesnya hanya boleh dibuat melalui ciri morfologi dalaman. Secara kebetulan, sesetengah organ dalaman adalah sebahagiannya terkitin dan morfologinya memberikan maklumat yang bernilai. Hal ini menyebabkan pemerhatian terhadap pam makanan, spermateka, dan salurannya menjadi sangat menarik. Sepanjang penggunaan semua reagen yang kami akan kaji, harus diingati bahawa dari fiksasi serangga hingga pemasangan, kami hanya menggunakan tindak balas redoks. Satu-satunya langkah berjaga-jaga atau panduan yang perlu diikuti ialah mengelakkan pencampuran reagen penurunan dengan reagen pengoksida.

Alkohol etil; etanol:

Bahan ini akan digunakan dalam pelbagai cara. Molekul alkohol mempunyai daya tarikan yang kuat terhadap air dan seterusnya menunjukkan kesan penyahhidratan. Walau bagaimanapun, alkohol dengan kepekatan rendah (iaitu terlalu tinggi kandungan air) boleh menyebabkan pemerosotan asid nukleik (air adalah musuh bagi asid nukleik).

Apabila serangga direndam dalam etanol, yang bukan sahaja bertujuan untuk mengawet spesimen, tetapi juga untuk menetapkan tisu. Dalam histologi, biasanya kita membezakan dua konsep penting: kadar penembusan dan kadar fiksasi. Difahami bahawa bahan pengawet yang baik mestilah menembusi tisu dengan cepat sebelum menetapkannya. Bagi etanol 96%, pekali penembusan adalah kira-kira 1.05 (untuk perbandingan, bagi larutan asid pikrat berakua 0.75%, pekali penembusan ialah 0.45, manakala bagi larutan dikromat kalium 3%, pekali penembusan ialah 1.45).

Keinginan untuk mengawet serangga dan arthropod lain secara kekal dalam etanol adalah satu realiti bagi ahli entomologi. Idea ini wajar dipuji, terutamanya untuk menyimpan tangkapan lapangan bagi kajian seterusnya atau untuk penyelidik masa hadapan. Walau bagaimanapun, perkara ini tidak praktikal bagi ahli sitologi atau histologi. Dengan menyimpan sampel terlalu lama dalam bahan penetap, sampel mungkin menjadi hampir mustahil untuk diproses semula. Justeru, sampel yang berusia lebih daripada 10 tahun sukar atau bahkan mustahil untuk digunakan.

Pertimbangan lain ialah nisbah antara jisim arthropod yang hendak ditetapkan dengan isipadu bahan penetap. Dalam amalan zoologi atau perubatan, adalah disyorkan untuk merancang isipadu bahan penetap sekurang-kurangnya 60 kali ganda daripada isipadu spesimen yang hendak ditetapkan. Dalam praktik, bagi mikro-arthropod, untuk satu isipadu spesimen yang hendak ditetapkan, tambahkan sekurang-kurangnya 4–5 kali isipadu etanol. Perlu diingat bahawa kekuatan alkohol akan berkurang apabila alkohol tersebut menyerap semua air yang terdapat dalam tisu arthropod.

Kesimpulannya:

- Alkohol etil merupakan agen kimia penurunan (Justeru tidak sesuai digunakan bersama bahan penetap pengoksida);
- Menyebabkan protein mengendap dengan pantas dan menyahaslikannya;
- Melarutkan beberapa lipid kompleks dan mendakan glikogen;
- Menyebabkan tisu mengecut dengan ketara dan mengeraskannya.

Larutan kalium berbes atau natrium hidroksida:

Penggunaan larutan ini dalam entomologi kebanyakannya tertumpu kepada kalium hidroksida (KOH) tanpa rasional yang jelas. Natrium hidroksida (NaOH) [E524] terdapat dalam larutan pada kepekatan atau normaliti yang berbeza, dan boleh didapati dalam bentuk pelet atau serbuk berkilau. Kelemahan utama natrium hidroksida ialah sangat higroskopik (lebih daripada KOH). Apabila bertindak balas dengan protein, protein akan larut, manakala dengan lipid, lipid ditukar menjadi sabun keras melalui proses saponifikasi (perbezaan utama dengan KOH ialah KOH menghasilkan sabun cecair semasa saponifikasi).

Kalium hidroksida [E525] terdapat dalam bentuk larutan pekat, tetapi kelebihan utamanya ialah tersedia dalam bentuk pelet kira-kira 0.1 g, yang sangat memudahkan penyediaan larutan cair walaupun tanpa neraca yang tepat. Contohnya, 1 pelet 0.1 g dalam 1 mL air suling menghasilkan larutan 10%. Kelebihan kedua penggunaan KOH dalam bentuk pelet ialah ketahanannya yang lebih rendah terhadap pengkarbonatan (larutan KOH mempunyai keafinan tinggi untuk penetapan CO_2, seterusnya menghasilkan garam karbonat).

Bes kuat ini digunakan untuk melarutkan asid lemak dengan menukarkannya menjadi sabun larut air. Perlu diingat bahawa bahan penetap seperti etanol telah melarutkan sebahagian lemak dalam sampel. Walau bagaimanapun, apabila sampel diletakkan semula dalam medium berakua bersama bes kuat, asid lemak (lebih kurang kompleks) akan menjadi mendakan. Oleh itu, bes kuat menjalankan proses saponifikasi sejuk. Dalam beberapa kes, apabila tisu adipos berlebihan, contohnya pada betina, menaikkan suhu kepada 35–40°C dapat mempercepatkan tindak balas, atau jika tidak, masa sentuhan pada suhu bilik perlu dipanjangkan.

Larutan berwarna asid / Larutan Marc-André tanpa warna:

Di sini, kita akan mengulas kelebihan dan kekangan penggunaan larutan Marc-André. Larutan ini terdiri daripada kloral hidrat (triklorasetaldehid monohidrat), asid asetat, dan air. Larutan ini bersifat sangat pengoksida (campuran asid dan aldehid). Larutan ini juga akan meneutralkan lebihan kalium hidroksida yang mungkin masih terdapat dalam sampel, tanpa memendakkan sabun

beralkali yang terbentuk semasa penggunaan kalium hidroksida. Larutan pengoksida ini juga bertindak ke atas fungsi alkohol sekunder glukosamina yang terbentuk dalam kitin dengan mengoksidanya, seterusnya melembutkan kitin. Tindakannya yang lain ialah melarutkan beberapa garam mineral yang terdapat dalam sampel.
Apabila larutan Marc-André diwarnakan terlebih dahulu dengan asid fuksin (dalam keadaan teroksida), larutan ini akan dapat menetap pada fungsi alkohol sekunder struktur tersebut. Selepas tempoh sentuhan larutan Marc-André dan keadaan pewarnaan sampel, bilasan hanya dilakukan menggunakan etanol. Dengan itu, kita memulakan fasa penyahhidratan sampel.

Kelebihan:
- Meneutralkan lebihan larutan beralkali
- Melembutkan kitin
- Mewarnakan kitin untuk memudahkan penilaian struktur dalaman yang berkitin.

Kekangan:
Kloral hidrat bersifat hipnotik dan pernah digunakan dalam perubatan manusia. Penggunaannya mesti di bawah tukup kimia (*chemical hood*), dan rang undang-undang berkaitan risiko bahan kimia mesti dipatuhi.

Larutan Penyahhidratan:
Pengalaman menunjukkan bahawa bagi sampel yang sangat kecil, tidak berkesan untuk mengikuti urutan mandi alkohol dengan kepekatan yang meningkat. Jika sampel besar, proses bermula dengan etanol 80%, kemudian 90%, 95%, dan akhirnya etanol mutlak. Bagi sampel yang sangat kecil, gunakan mandi dengan etanol 90% diikuti dengan perendaman dalam etanol mutlak. Pada tahap ini, perlu sentiasa diingat bahawa etanol mutlak cenderung untuk menetapkan air yang terdapat dalam atmosfera.
Tradisi dalam makmal entomologi ialah menamatkan penyahhidratan sampel dengan mandi kreosot bic. Kini, esen ini, yang dahulunya digunakan secara meluas sebagai racun perosak, antikulat, dan pengawet kayu, sangat tidak digalakkan kerana baunya (hidrokarbon aromatik berpolisi) dan bahan ini turut dianggap sebagai reprotoksik, karsinogenik, pencemar organik berterusan, serta berbahaya kepada organisma akuatik.
Satu penyelesaian yang dicadangkan untuk pelekapan sampel ialah Euparal® dan esen Euparal (dihuraikan dalam perenggan seterusnya). Campuran Euparal® dan esen Euparal diterima dengan baik bagi sampel yang diperoleh selepas mandian etanol 90%.

Lampiran 2: Komposisi reagen yang digunakan.

Kalium hidroksida 10%
Kalium hidroksida 10 g
Air suling cukup untuk menjadikan jumlah 100 mL

Medium pelekapan gam kloral (medium Hoyer)
Air suling 50 mL
Kloral hidrat 200 g
Gam arab 50 g
Gliserol 20 mL

Larutan Marc-André
Kloral hidrat 40 g
Asid asetik glasier 30 mL
Air suling 30 mL

Asid Fuchsin 1% dalam air suling
Serbuk asid fuchsin 1 g
Air suling 99 mL

Larutan Marc-André berwarna dengan asid fuchsin
Larutan Marc-André 10 mL
Asid fuchsin 1% 50 µL

Lampiran 3: Euparal®, balsam Kanada, alkohol polivinil, atau larutan lain untuk pelekapan

Alkohol polivinil: Ini merupakan medium pelekapan yang ideal apabila bahan yang diperlukan untuk proses penyahhidratan yang sempurna tidak tersedia. Alkohol polivinil kemudiannya dicampurkan dengan laktophenol Amman. Walau bagaimanapun, Walau bagaimanapun, kelemahan utama pada penyediaan ini adalah sama ada menjadi kering atau berlaku penghabluran alkohol polivinil akibat penyejatan air, atau menjadi gelap apabila fenol teroksida. Teknik ini masih sesuai digunakan untuk pelekapan jangka pendek.

Balsam Kanada: Penggunaannya untuk pelekapan di antara slaid dan kaca penutup yang memerlukan proses penyahhidratan terhadap spesimen yang hendak dilekapkan. Walau bagaimanapun, penggunaan xilena atau toluena bukanlah tanpa kesulitan.

Medium Enecê: Untuk pelekapan di antara slaid dan kaca penutup, seperti balsam Kanada, medium ini memerlukan penyahhidratan spesimen. Formulasi Enecê: kolofoni putih tulen (22 g); gam damar larut alkohol (12 g); alkohol mutlak (20 mL); kapur barus (10 g); esen turpentin (10 mL); dan eukaliptol (26 mL). Bagi kaedah penyediaannya, satu bekas seperti kelalang Erlenmeyer diperlukan, masukkan alkohol mutlak dan kapur barus. Seterusnya, tambahkan kolofoni dan gam damar. Kelalang kemudian ditutup rapat dengan penyumbat dan digoncang, dan selepas itu dipanaskan dengan teknik bain-marie pada suhu rendah supaya campuran tidak mendidih. Setelah kandungan menjadi cair sepenuhnya, esen turpentin ditambah, kemudian campuran dituras ketika masih panas, dan akhirnya eukaliptol ditambahkan ke dalam turasan. Apabila medium bertukar menjadi kurang likat, cairkan dengan Enecê yang

mempunyai campuran berikut: alkohol mutlak (30 mL); kapur barus (17 g); esen turpentin (15 mL); dan eukaliptol (38 mL) (Cerqueira, 1943).

Euparal®: Ini ialah sejenis resin yang diperoleh daripada pokok sipres Atlas, *Tetraclinis articulata* (Vahl, 1791), dan telah dikaji serta dibangunkan pada tahun 1906 oleh Gilson. Kelebihan utamanya ialah resin ini tidak mempolimer. Spesimen yang dilekapkan di antara slaid dan kaca penutup boleh dipisahkan semula dengan mudah melalui tindak balas alkohol atau, yang lebih berkesan, menggunakan esen Euparal®. Resin ini, yang juga dikenali sebagai damar sandarak, terlarut dengan etanol pada kepekatan 80% dan ke atas.

Penggunaan Triton X100: larutan berakua bukan ion: Triton X-100 ialah dalam bentuk larutan berakua bukan ion (*4-(1,1,3,3-tetrametilbutil*) larutan *fenil-polietilena glikol*, atau *t-oktilfenoksipolietoksietanol*), *etilena glikol tert-oktilfenil eter*), yang banyak digunakan sebagai detergen dalam sel dan biologi molekul. Larutan ini menyebabkan peningkatan terhadap kebolehtelapan sel dan membran nukleus. Spesimen serangga yang diawet dalam alkohol selama bertahun-tahun adalah lazim ditemui. Walau bagaimanapun, pengawetan dalam alkohol bukanlah kaedah yang optimum, dan artropoda yang diawet dengan cara ini menjadi sangat sukar untuk disediakan bagi pemeriksaan mikroskopik. Bekas plastik yang mengandungi spesimen juga sering mengalami pereputan, diikuti oleh penyejatan alkohol. Dalam kedua-dua keadaan, rendaman berpanjangan dalam alkohol atau pengeringan spesimen menimbulkan masalah yang ketara. Pada tahun 2008, Jonque menerbitkan satu catatan mengenai proses penghidratan semula labah-labah menggunakan agen pembasah seperti Agepon yang digunakan dalam pemprosesan filem fotografi [26]. Hal ini mencetuskan idea untuk menggunakan agen pembasah yang bukan merupakan detergen yang kuat.

Berikut merupakan prosedur menggunakan Triton X-100 bukan ion dalam larutan berakua 0.5%:

- Sampel kering diisitepukan dengan alcohol mutlak.
- Tambahkan larutan Triton X-100 berkepekatan 0.5% pada isipadu yang mencukupi supaya keseluruhan sampel terendam sepenuhnya.
- Biarkan sampel berada dalam larutan selama 5 minit hingga beberapa hari, dengan pemantauan berkala. Semua artropod hendaklah terpisah sepenuhnya dalam larutan tersebut.
- Larutan Triton X-100 kemudian disingkirkan dan digantikan dengan larutan kalium hidroksida.

Teknik ini kemudiannya dijalankan seperti yang diterangkan di atas.

Lampiran 4: Langkah demi langkah untuk pelekapan menggunakan medium Euparal® atau balsam Kanada

1. Spesimen mesti dinyahhidratkan (rupa keruh atau berwarna susu menunjukkan penyahhidratan yang tidak mencukupi).
2. Penyahhidratan boleh dicapai melalui peningkatan kepekatan alkohol etil secara berperingkat.
3. Spesimen boleh dipindahkan daripada alkohol 99% atau alkohol mutlak kepada agen penjernih.

Prosedur:

1. Penempatan lalat pasir dewasa dalam etanol 70%.
2. Singkirkan etanol dan gantikan dengan larutan KOH 10%. Tutup lalat pasir dengan slaid kaca.
3. Maserat sehingga serangga menjadi lutsinar.
4. Singkirkan larutan KOH.
5. Rendamkan spesimen dengan air suling dan biarkan selama 30–45 minit.
6. Singkirkan air dan ulangi basuhan dengan air suling setelah 30 minit (Tempoh ini bergantung kepada bilangan spesimen: semakin banyak spesimen diproses serentak, semakin lama masa diperlukan; semakin sedikit, terutamanya jika diproses secara individu, masa boleh dipendekkan).
7. Singkirkan air.
8. Tambah larutan Marc-André (berpotensi untuk diwarnakan dengan asid fuksin) dan biarkan selama 24 jam (1 hari).
9. Singkirkan larutan Marc-André.
10. Rendamkan spesimen dengan air suling dan biarkan selama 30–45 minit.
11. Singkirkan air dan ulangi basuhan dengan air suling selama 30 minit.
12. Singkirkan air.
13. Tambah etanol 70% dan lakukan pembedahan spesimen.
a. Bagi bahagian kepala dan abdomen, tarik perlahan bagi memisahkan kepala atau abdomen daripada toraks.
b. Bagi bahagian toraks, tanggalkan sayap dengan memegang toraks menggunakan sepasang forsep dan menarik pada pangkal apendaj dengan sepasang forsep yang lain. Pembedahan sagital boleh dilakukan dengan membelah toraks kepada bahagian kiri dan kanan, bergantung pada kawasan yang menjadi fokus pemerhatian.
14. Spesimen dinyahhidratkan secara berperingkat melalui siri larutan alcohol etil berakua: bermula pada kepekatan 50% → 80% → 95% sehingga etanol mutlak.
15. Spesimen dinyahhidratkan melalui dua kali pembasuhan, masing-masing selama 10 minit, menggunakan etanol 100%.
16. Singkirkan etanol dan rendam spesimen dengan minyak cengkih selama 15 minit pada suhu bilik.
17. Pindahkan spesimen daripada minyak cengkih ke dalam titisan Euparal® atau balsam Kanada pada slaid kaca yang baru.
18. Susun mengikut keperluan: Kepala, toraks dan abdomen lalat pasir boleh dibedah menggunakan jarum halus atau forsep di bawah mikroskop stereo. Kepala mesti dipisahkan daripada badan untuk dilekap dalam kedudukan ventro-dorsal, iaitu foramen oksipital mesti menghala ke atas supaya sibarium dapat dilihat secara jelas.

Pembedahan dijalankan dalam medium pelekapan lalat pasir.

19. Biarkan spesimen sehingga permukaan menjadi melekit.

20. Basahkan kaca penutup yang bersih dengan alkohol mutlak. Letakkan kaca penutup ke atas balsam Kanada secara menyerong.

21. Simpan slaid dalam kotak kering yang telah dikhaskan untuk penyimpanan
