# Supplementary material for: Processing and mounting phlebotomine sand flies: a consensus guideline
Source: Parasite. 2026 Apr 3;33:18. doi: 10.1051/parasite/2026009 (PMC13047900; doi:10.1051/parasite/2026009)
Supplement: Supplementary file 22 — Nepali translation / नेपाली अनुवाद [file parasite-33-18-s22.pdf]

Parasite **33**, 18 (2026)
© F.J. Randrianambinintsoa et al., published by EDP Sciences, 2026
https://doi.org/10.1051/parasite/2026009

PARASITE
Available online at:
www.parasite-journal.org

REVIEW ARTICLE

OPEN

फ्लेबोटोमाइन भुसुना प्रशोधन तथा माउन्टिङ: एक सहमति आधारित निर्देशिका

Fano José Randrianambinintsoa[1], Laure Augendre[1], Jorian Prudhomme[1], Jean-Philippe Martinet[1], Mathieu Loyer[1], Nalia Mekarnia[1], Hocine Kerkoub[1], Farzana Khan Perveen[1], Antoine Huguenin[1,2], Emilie Kariya[1,2], Mohammad Akhoundi[3], Andrey José de Andrade[4], Eduardo Berriatua[5], Gioia Bongiorno[6], Sébastien Boyer[7,8], Vasiliki Christodoulou[9], Magda Clara Vieira Da Costa-Ribeiro[10], Lucas Alexandre Farias de Souza[10], Huicong Ding[11], Blaise Dondji[12], Vít Dvořák[13], Ozge Erisoz Kasap[14], Eunice Aparecida Bianchi Galati[15], Montserrat Gállego[16], Cristina Ballart[16], Stavroula Gouzelou[17], Nabil Haddad[18], Rezki Sabrina Masse[19], Asrat Hailu Mekuria[20], Vladimir Ivovic[21], Szymon Kaczmarek[22], Mohd Khadri Shahar[19], Oscar D. Kirstein[23], Edwin Kniha[24], Iva Kolářová[13], Lincoln Timinao[25], Cristian Lucanas[26], Ognyan Mikov[27], Kimsear Nov[7], Yusuf Özbel[28], Bernard Pesson[29], Laura Cristina Posada Lopez[30], Didot Budi Prasetyo[1,7], Nil Rahola[31], Eduardo A. Rebollar-Tellez[32], Bruno Leite Rodrigues[15], Lalita Roy[33], Prasanta Saini[34], Chizu Sanjoba[35], Paloma Helena Fernandes Shimabukuro[36], Padet Siriyasatien[37], Agnieszka Soszyńska[22], Tatiana Suleşco[38], Massamba Sylla[39], Majhalia Torno[40], Petr Volf[13], Khamsing Vongphayloth[41], Vu Sinh Nam[42], April Wardhana[43], Eric Yessinou[44], Sonia Zapata[45], Jean-Charles Gantier[1], and Jérôme Depaquit[1,2,*]

[1] Faculté de Pharmacie, Université de Reims Champagne Ardenne, UR ESCAPE-USC ANSES PETARD, 51 rue Cognacq-Jay, 51096 Reims Cedex, France
[2] Pôle de Biologie territoriale, Laboratoire de Parasitologie-Mycologie, Centre Hospitalo-Universitaire, 51092 Reims, France
[3] Parasitology-Mycology Department, Avicenne Hospital, AP-HP, Bobigny, Sorbonne Paris Nord University, France; Unité des Virus Émergents (UVE: Aix-Marseille Univ, Università di Corsica, IRD 190, Inserm 1207, IRBA), 13005 Marseille, France
[4] Parasitology Collection of Basic Pathology, Department of Basic Pathology, Federal University of Paraná, Curitiba 19031, Brazil
[5] Department of Animal Health, University of Murcia, Campus de Espinardo, 30100 Espinardo, Murcia, Spain
[6] Department of Infectious Diseases, Vector-borne Diseases Unit, Istituto Superiore di Sanità, 00166 Rome, Italy
[7] Medical and Veterinary Entomology Unit, Institut Pasteur du Cambodge, Phnom Penh 12201, Cambodia
[8] Ecology & Emergence of Arthropod-borne Pathogens Unit, Department of Global Health, Institut Pasteur, CNRS UMR2000, 75015 Paris, France
[9] Section Veterinary Services (1417), Laboratory for Animal Health Virology, Aglantzia, Nicosia 2109, Cyprus
[10] Insects Vectors and Parasites Laboratory, Department of Basic Pathology and Postgraduate program in Microbiology, Parasitology and Pathology, Federal University of Paraná, 81530-900 Curitiba, Brazil
[11] Department of Biological Sciences, National University of Singapore, 117558, Singapore
[12] Laboratory of the Leishmaniasis Research Project, Mokolo District Hospital, Mokolo, Cameroon; Laboratory of Cellular Immunology and Parasitology, Department of Biological Sciences, Central Washington University, 98926 Ellensburg, WA, USA
[13] Department of Parasitology, Faculty of Science, Charles University, 12800 Prague, Czechia
[14] VERG Laboratories, Department of Biology, Faculty of Science, Hacettepe University, Beytepe, Ankara 06800, Türkiye
[15] Faculdade de Saúde Pública da Universidade de São Paulo (FSP/USP), Pós-graduação em Saúde Pública, 01246-904 São Paulo, Brazil
[16] Secció de Parasitologia, Departament de Biologia, Sanitat i Medi Ambient, Facultat de Farmàcia i Ciències de l'Alimentació, Universitat de Barcelona, & Institut de Salut Global de Barcelona (ISGlobal), Centro de Investigación Biomédica en Red, Enfermedades Infecciosas (CIBERINFEC), 08028 Barcelona, Spain
[17] Laboratory of Infectious Diseases and Public Health, School of Medicine, University of Cyprus, Nicosia, Cyprus & Department of Pediatrics, Archbishop Makarios III Hospital, Nicosia 2115, Cyprus
[18] Faculty of Health Sciences, American University of Beirut, 1107 2020 Beirut, Lebanon
[19] Medical Entomology Unit, Infectious Disease Research Centre, Institute for Medical Research (IMR), National Institutes of Health (NIH), Ministry of Health Malaysia, 40170 Shah Alam, Selangor, Malaysia
[20] School of Medicine, Addis Ababa University, 28017 - 1000 Addis Ababa, Ethiopia
[21] Faculty of Mathematics, Natural Sciences and Information Technologies, University of Primorska, 6000 Koper, Slovenia
[22] University of Lodz, Faculty of Biology and Environmental Protection, Department of Invertebrate Zoology and Hydrobiology, Banacha 12/16, 90-237 Łódź, Poland
[23] Laboratory of Entomology, Ministry of Health, 9134302 Jerusalem, Israel

Edited by Jean-Lou Justine
*corresponding author: jerome.depaquit@univ-reims.fr

[24] Center for Pathophysiology, Infectiology and Immunology, Institute of Specific Prophylaxis and Tropical Medicine, Medical University Vienna, Kinderspitalgasse 15, 1090 Vienna, Austria
[25] Papua New Guinea Institute of Medical Research (PNGIMR) Institute, PO Box 60, Headquarter, Homate Street, 441 Goroka, Eastern Highlands Province, Papua New Guinea
[26] Museum of Natural History, University of the Philippines Los Baños, 4031 Laguna, Philippines
[27] National Centre of Infectious and Parasitic Diseases, 1504 Sofia, Bulgaria
[28] Ege University, Faculty of Medicine, Department of Parasitology, 35040 Bornova/Izmir, Türkiye
[29] Retired, Faculté de Pharmacie, Université de Strasbourg, Strasbourg, 67400 Illkirch-Graffenstaden, France
[30] Program for the Study and Control of Tropical Diseases (PECET), Faculty of Medicine, University of Antioquia, 050010 Medellin, Colombia
[31] MIVEGEC, Univ. Montpellier, CNRS, IRD, 34394 Montpellier, France & Medical Entomology Unit, Institut Pasteur de Madagascar, 101 Antananarivo, Madagascar
[32] Laboratorio de Entomología Médica, Departamento de Zoología de Invertebrados, Facultad de Ciencias Biológicas, Universidad Autónoma de Nuevo León, San Nicolás de los Garza, 66455, NL, México
[33] Tropical and Infectious Disease Centre, BP Koirala Institute of Health Sciences, Dharan 56700, Nepal
[34] ICMR-Vector Control Research Centre, Puducherry 605006, India
[35] Graduate School of Agricultural and Life Sciences, The University of Tokyo, Tokyo 113-8657, Japan
[36] Grupo de estudos em Leishmanioses/Coleção de Flebotomíneos (COLFLEB/Fiocruz-MG), Instituto René Rachou, Fundação Oswaldo Cruz, Belo Horizonte, Minas Gerais, 30190009, Brazil
[37] Center of Excellence in Vector Biology and Vector-Borne Disease, Department of Parasitology, Faculty of Medicine, Chulalongkorn University, Bangkok 10330, Thailand
[38] Department of Arbovirology, Bernhard Nocht Institute for Tropical Medicine, Bernhard Nocht Str. 74, 20359 Hamburg, Germany [39] Laboratory Vectors & Parasites, Department of Livestock Sciences and Techniques, Sine Saloum University El Hadji Ibrahima Niasse (SSUEIN) Kaffrine Campus, C.P. 24600, Senegal.
[40] Environmental Health Institute, National Environment Agency, Singapore 138667, Singapore & Department of Biological Sciences, National University of Singapore, 117558 Singapore
[41] Institut Pasteur du Laos, Laboratory of Vector-Borne Diseases, Samsenhai Road, Ban Kao-Gnot, Sisattanak District, 3560 Vientiane, Lao PDR
[42] National Institute of Hygiene and Epidemiology, 1 Yec-Xanh Street, Hai Ba Trung District, 100000 Hanoi, Vietnam
[43] Indonesian Research Center for Veterinary Science, Indonesian Agency for Agricultural Research and Development, Ministry of Agriculture Republic Indonesia, Bogor 16114, Indonesia & Department of Parasitology, Faculty of Veterinary Medicine, Airlangga University, Surabaya 60115, Indonesia
[44] Laboratory of Research in Applied Biology, Polytechnic School of Abomey-Calavi, University of Abomey-Calavi, 01 P.O. Box 2009, 00000 Cotonou, Benin
[45] Instituto de Microbiología, Colegio de Ciencias Biológicas y Ambientales (COCIBA), Universidad San Francisco de Quito (USFQ), 170901 Quito, Ecuador

Received 1 December 2025, Accepted 29 January 2026, Published online 3 April 2026

सारांश- यस लेखले फ्लेबोटोमाइन भुसुनाका भुसुनाहरूको प्रशोधन तथा माउन्टिङको लागि एक विस्तृत मार्गदर्शन प्रदान गर्दछ, जुन प्रजाति पहिचान र रोगजनक पत्ता लगाउन र पृथकिकरणको लागि महत्वपूर्ण छ। यसले क्षेत्र र प्रयोगशाला सेटिङ दुवैको लागि उपयुक्त प्रविधिहरूको दायराको बारेमा छलफल गर्दछ। यस दिशानिर्देशमा भुसुनाका भुसुनाहरूको सङ्कलन, ह्यान्डलिङ, कभरिङ, र मार्ने (रसायनहरूमा सुख्खा फ्रिजिङ वा CO2 सिफारिस गर्ने) साथै इथेनॉलमा कोल्ड स्टोरेज र संरक्षण जस्ता संरक्षण रणनीतिहरूमा विस्तृत निर्देशनहरू समावेश छन्। निश्चित शारीरिक संरचनाहरू (जननांग अंगहरू, टाउको र पखेटा) को तयारीको गुणस्तर तिनीहरूको उचित सूक्ष्म अवलोकनको लागि आवश्यक छ र यस कार्यमा वर्णन गरिएको छ। लेखले विस्तृत भुसुना प्रशोधन पनि प्रस्तुत गर्दछ, जसमा पोटासियम हाइड्रोक्साइड (Potassium hydroxide) त्यसपछि मार्क-आन्द्रे घोल (Marc-André solution) जस्ता एजेन्टहरू प्रयोग गरेर सफा गर्ने प्रक्रिया समावेश गरिएको छ। माउन्टिङ प्रक्रियाले विभिन्न मिडियाहरूको तुलना गर्दछ, तिनीहरूको अप्टिकल गुणहरू र संरक्षण क्षमतालाई जोड दिन्छ। होयर तरल (Hoyer fluid जसलाई क्लोरल गम-Chloral gum पनि भनिन्छ), यसको पारदर्शीताको कारण, विशेष गरी स्पर्माथिकाको (spermathecae) द्रुत अवलोकनको लागि सिफारिस गरिन्छ, यद्यपि यो दीर्घकालीन भण्डारणको लागि उपयुक्त छैन। छलफल गरिएका अन्य माध्यमहरूमा पोलिभिनाइल अल्कोहल (polyvenyl alcohol), युपरल® (Euparol®) (सीमित पानी सहनशीलताको लागि), र कनाडा बाल्सम (Canada balsam) (Hydrocarbon-घुलनशील माध्यम) समावेश छन्, जसमा पछिल्ला दुईले दीर्घकालीन संरक्षण क्षमताहरू प्रदान गर्दछ। भुसुना प्रशोधनमा विशेष ध्यान आवश्यक पर्ने DNA अनुक्रमण र MALDI-ToF जस्ता नवीन आणविक जीवविज्ञानका दृष्टिकोणहरूलाई पनि सम्बोधन गरिएको छ। यसबाहेक, विभिन्न माउन्टिंग प्रविधिहरू चित्रण गर्ने छोटो भिडियो क्लिपहरू साथै ३३ विभिन्न भाषाहरूमा अनुवादहरू प्रदान गरिएको छ, जसले गर्दा यो निर्देशिका विश्वव्यापी

वैज्ञानिक समुदायको विविध आवश्यकताहरू र अपेक्षाहरू पूरा गर्न मदत गर्दछ।

मुख्य शब्दहरू:

माउन्टिङ, फ्लेबोटोमाइन भुसुना, होयर तरल, मार्क-आन्द्रे घोल, क्लोरल गम, पोलिभिनाइल अल्कोहल, युपरल®, कनाडा बाल्सम, लेशमानिया पृथकिकरण, क्षेत्र अवस्था, संवर्धन, विच्छेदन, आणविक जीवविज्ञान, MALDI-ToF, प्रकार-भुसुनाहरू

Abstract – Processing and mounting phlebotomine sand flies: a consensus guideline. This article provides a comprehensive guide for the processing and mounting of phlebotomine sand fly specimens, which is crucial for species identification and pathogen detection and isolation. It discusses a range of techniques suitable for both field and laboratory settings. The guide includes detailed instructions on sand fly collection, handling, covering, and euthanasia (recommending dry freezing or CO_2 over chemicals) as well as conservation strategies, such as cold storage and preservation in ethanol. The quality of preparation of certain anatomical structures (genital organs, head and wings) is essential for their proper microscopic observation and is described in this work. The article also presents detailed sample processing, including the clearing process with agents such as potassium hydroxide then Marc-André solution. The mounting process compares different media, emphasizing their optical properties and preservation potential. Hoyer fluid (also known as chloral gum) is recommended for quick observation, particularly for spermathecae, due to its clarity, although it is not suitable for long-term storage. Other media discussed include polyvinyl alcohol, Euparal® (for limited water tolerance), and Canada balsam (a hydrocarbon-soluble medium), with the latter two offering long-term preservation capabilities. Innovative molecular biology approaches such as DNA sequencing and MALDI-ToF, which require particular attention to sample processing, are also addressed. Furthermore, short video clips illustrating various mounting techniques as well as translations in many different languages are provided, allowing the guideline to reach the diverse needs and expectations of the global scientific community.

Key words: Mounting, Phlebotomine sand fly, Hoyer fluid, Marc-André solution, Chloral gum, Polyvinyl alcohol, Euparal®, Canada balsam, *Leishmania* isolation, Field conditions, Culture, Dissection, Molecular biology, MALDI-ToF, Type-specimens.

परिचय

फ्लेबोटोमाइन भुसुनाहरू साना डिप्टेरन कीराहरू हुन् जुन साइकोडिडे परिवार, उपपरिवार फ्लेबोटोमिनेसँग सम्बन्धित छन् जसमा कम्तिमा १,०६३ ज्ञात प्रजातिहरू छन् [21]। तिनीहरू रोगजनकहरूका महत्वपूर्ण भेक्टरहरू हुन् (लेशमेनिया, आर्बोभाइरस र बार्टोनेला) क्रमशः लेशमेनियासिस, अर्बोभाइरस संक्रमण र बार्टोनेलोसिस भनिने रोगहरूको लागि जिम्मेवार छन्। तिनीहरूको पहिचान मुख्यतया सावधानीपूर्वक सङ्कलन, उपयुक्त भण्डारण, र सावधानीपूर्वक स्लाइड माउन्टिङद्वारा सहज बनाइएको विस्तृत सूक्ष्म परीक्षणमा आधारित हुन्छ, जसमा धेरै विशिष्ट प्रविधिहरू आवश्यक पर्दछ, प्रत्येकको आफ्नै फाइदा र सीमितताहरू छन्।

वयस्क फ्लेबोटोमाइन भुसुनाको पहिचान बाह्य (जस्तै, एन्टेना, पाल्पी, पुरुष जननेन्द्रिय) र आन्तरिक संरचनाहरू (जस्तै ग्रसनी, सिबेरियम, र स्पर्माथिका) दुवैको अवलोकनमा निर्भर गर्दछ। पछिल्लोको विच्छेदन र पृथकिकरणले तिनीहरूको अवलोकनलाई सहज बनाउँछ र फलस्वरूप, सही पहिचान गर्न मदत गर्दछ। त्यसकारण, लामखुट्टे वा चुसाहा कीराहरू जस्तो नभई, फ्लेबोटोमाइन भुसुनाहरूलाई तिनीहरूको पहिचान गर्नु अघि स्लाइड र कभर स्लिपको बीचमा माउन्ट गर्न आवश्यक पर्दछ।

सन् १९८० को दशकसम्म, फ्लेबोटोमाइन भुसुनाको पहिचानको लागि उपलब्ध एक मात्र विधि सूक्ष्म अवलोकन थियो, र यो आज सबैभन्दा व्यापक रूपमा प्रयोग हुने दृष्टिकोण हो। प्रक्रिया र तयारीको छनोट त्यसैले अपेक्षाकृत सरल थियो र मुख्यतया दुई उद्देश्यका लागि गरिन्थ्यो: एकातिर, भुसुनाको दीर्घकालीन संरक्षणको लागि गरिने निश्चित माउन्टिंग, र अर्कोतिर, दीर्घकालीन संरक्षण सुनिश्चित नगर्ने माध्यममा पहिचानको लागि द्रुत माउन्टिंग। उदाहरणका लागि, अन्तिम चरणमा क्यानाडा बाल्सम जस्ता रेजिनमा माउन्टिंग गर्दा धेरै समय लाग्ने र भुसुनाहरूको पूर्ण निर्जलीकरण गर्न आवश्यक पर्दछ। यसबाहेक, यस माध्यमको अपवर्तक सूचकांक स्पर्माथिकाको सजिलो अवलोकनको लागि सधैं इष्टतम हुँदैन। यसको विपरीत, जलीय माध्यम (जस्तै, होयर तरल) मा माउन्टिंग छिटो हुन्छ र अपवर्तक स्पर्माथिकाको राम्रो दृश्यावलोकन गर्न अनुमति दिन्छ, तर यो जलीय माध्यम भएकोले यसले वायुमण्डलबाट पानी अवशोषित गर्ने गर्छ र माउन्टिंगहरूको दीर्घकालीन संरक्षण गर्न सकिंदैन । एउटा विकल्प भनेको

स्लाइडलाई पूर्ण रूपमा सुकिसकेपछि नेल पालिसले सिल गर्नु हो। यो लाभ-हानिको छनोट आजसम्म पनि कायम छ, जसले तयारीको उद्देश्य अनुरुप माउन्टिंग विधिको छनोटमा प्रभाव पारिरहेको हुन्छ। १९८० को दशकदेखि, भुसुना पहिचान अध्ययनहरूले आकारविज्ञान र जैव रासायनिक दृष्टिकोणहरू संयोजन गरेका छन्। पहिलो क्युटिक्युलर हाइड्रोकार्बन विश्लेषण थियो, जुन चाँडै आणविक जीवविज्ञान प्रविधिहरू (अर्थात्, अनियमित प्रवर्धित बहुरूपी DNA (RAPD), द्वारा प्रतिस्थापन गरिएको थियो।प्रतिबन्ध खण्ड लम्बाइ बहुरूपता (RFLP), DNA प्रवर्धन, र Sanger विधि प्रयोग गरेर अनुक्रमण, साथै अर्को पुस्ता अनुक्रमण (NGS)। आज, आणविक पद्धतिहरू MALDI-ToF जस्ता प्रोटियोमिक विधिहरू द्वाले पूरक बनईरहेका छन्। यसबाहेक, आणविक प्रजाति पहिचानलाई PCR (*Leishmania*, *Trypanosoma*, *Bartonella*, र *Phlebovirus*) द्वारा रोगजनकहरूको पहिचानसँग पनि जोड्न सकिन्छ किनकि यी सबैलाई अन्तिम-बिन्दु र वास्तविक-समय PCR दुवै पद्धतिहरू द्वारा पत्ता लगाउन सकिन्छ, यसका लागि भुसुनाहरूको भुसुना छनोट र भण्डारण प्रक्रियालाई तोकिएको लक्ष्यहरूमा अनुकूलन गर्न आवश्यक पर्दछ [3, 32]। प्रजाति छुट्याउन परम्परागत रूपमा प्रयोग गरिने आकृतिक (रूपात्मक) विशेषताहरू बाहेक, अन्य आकृतिक पद्धतिहरू पनि प्रयोग गर्न सकिन्छ (जस्तै, पखेटाको ज्यामितीय मापन–विङ्ग जियोमर्फोमेट्री))।

लेखकहरूको आफ्नै अनुभव र पहिल्यै प्रकासित तथ्यांकका आधारका, यस अनुसन्धानको उद्देश्य वयस्क फ्लेबोटोमाइन भुसुनाहरूलाई माउन्ट गर्ने तथा प्रशोधन गर्ने प्रक्रियाका लागि मानकीकृत निर्देशिका प्रदान गर्नु थियो, ताकि आकृतिक (रूपात्मक) तथा आणविक विश्लेषणहरूलाई अधिकतम रूपमा अनुकूल बनाउन सकियोस्।

केहि विश्लेषण (जस्तै, आणविक जीवविज्ञान वा MALDI-ToF) गर्नको लागि भुसुनाको त्यो भाग राख्न सकिन्छ जुन आकृतिक पहिचानको लागि आवश्यक छैन, जसले महत्वपूर्ण प्रोटोकल छनोटको आवश्यकता उजागर गर्दछ।
यस लेखमा, हामी जीवित रूपमा समातिएका भुसुनाहरूको बेहोस गर्ने (एनस्थेसिया) र मार्ने (युथनेशिया) विधिहरू, तिनीहरूको संग्रहण, र माउन्ट गर्ने प्रक्रियामा केन्द्रित छौं, ताकि तिनीहरूको छिटो पहिचान गर्न सकियोस् वा दीर्घकालीन संरक्षण सम्भव होस् र पछि गरिने अध्ययनहरूका लागि उपयोग गर्न सकियोस्।

प्रस्तावना: सुरक्षा र नियामक विचारहरूले सम्बन्धित सुरक्षा डेटा पानाहरू (SDS) लाई सन्दर्भ गर्नु आवश्यक छ।

यस निर्देशिकामा प्रस्तुत गरिएका सबै रसायनहरू कडा सुरक्षा सर्तहरू अन्तर्गत ह्यान्डल गरिनुपर्दछ। अनुसन्धान गरिने संस्थाहरूको स्वास्थ्य र सुरक्षा समितिहरूले तपाईंलाई यी रसायनिक पदार्थ जोखिमका बारेमा मात्र नभई तिनीहरूको ह्यान्डलिङ प्रक्रिया र फोहोर व्यवस्थापन सम्बन्धि जानकारी पनि प्रदान गर्न उपलब्ध छन्। यद्यपि, तिनीहरूको प्रयोग र व्यवस्थापन सम्बन्धी सुरक्षा निर्देशनहरू पालना गर्नु अनिवार्य छ। ध्यान दिनुपर्ने कुरा, राम्रो र सुरक्षित प्रयोगशाला अभ्यासहरू र तिनीहरूको देश वा अनुसन्धान संस्थाको लागू हुने कानून र नियमहरूको पालना सुनिश्चित गर्नु सबै प्रयोगकर्ताहरूको जिम्मेवारी हो। यसबाहेक, केही रसायनहरू, वा तिनीहरूका घटकहरू (अर्थात्, क्लोरल हाइड्रेट, Chloral hydrate) केही देशहरूमा नियमन गरिन्छ। यस दिशानिर्देशमा प्रयोग गरिएका संक्षिप्त नामहरूको सूची तालिका १ मा प्रदान गरिएको छ।

तालिका १: संक्षिप्त नामहरूको सूची।

BME	Basal medium Eagle
CDC	Centers for Disease Control and Prevention
CMCP	Camphor-monochlorophenol
CMR	Carcinogenic, mutagenic, reprotoxic substance
COI	Cytochrome c oxidase subunit I gene
CytB	Cytochrome b gene
DNA	Deoxyribonucleic acid
ELISA	Enzyme-linked immunosorbent assay
EtOH	Ethanol
M199	Medium 199
MALDI-ToF MS	Matrix-assisted laser desorption/ionization time-of-flight mass spectrometry
MEM	Minimum essential media
NGS	Next-generation sequencing
NNN	Novy-MacNeal-Nicolle medium
PCR	Polymerase chain reaction
Lao PDR	Lao People’s Democratic Republic
PNOC	Prepronociceptin gene
qPCR	Quantitative PCR (real-time PCR)
RAPD	Random amplified polymorphic DNA
RFLP	Restriction fragment length polymorphism
RI	Refractive index
RNA	Ribonucleic acid
RNases	Ribonucleases
RNASS	RNA stabilization solution
RT-PCR	Reverse transcription PCR
TFA	Trifluoroacetic acid

१. भुसुना समात्ने प्रक्रिया

वयस्क भुसुनाहरूलाई विभिन्न विधिहरू जस्तै CDC लघु प्रकाश पासो, टाँसिने पासो, र श्यानन पासो प्रयोग गरेर, एस्पिरेटरहरू प्रयोग गरेर वा सिधै वातावरणमा आराम गर्ने ठाउँहरू (जस्तै, जनावरहरूको आश्रयस्थल) बाट जीवित वा मृत सङ्कलन गर्न सकिन्छ। यी विधिहरूमा उपयुक्त बासस्थानमा पासो राख्ने, प्रकाश वा अन्य आकर्षित गर्ने माध्यमहरू (CO_2 वा रासायनिक प्रलोभनहरू) द्वारा भुसुनाहरूलाई आकर्षित गर्ने र तिनीहरूलाई थप विश्लेषणको लागि सङ्कलन गर्नु समावेश हुन्छ, जुन विभिन्न प्रकाशनहरूमा वर्णन गरिएको छ [2, 3, 32, 36, 49]।

जीवित भुसुना समात्नाले सबै पछिल्ला चरणहरूमा हुने प्रयोगहरू गर्न सकिन्छ भने मृत अवस्थाको भुसुना सङ्कलन गर्नाले लेशमेनिया वा भाइरस प्रजातिहरू अलग गर्ने काम असम्भव हुन्छ। केही भुसुना समात्ने प्रविधिहरू, जस्तै चिपचिपा टाँसिने कागजहरूमा भुसुनाका अंगहरू (एन्टेना, पाल्प्स, पखेटा, वा खुट्टा) हराउने हुन्छ। साथै, क्यास्टर तेलले लेपिएको चिपचिपा कागजहरूमा भुसुनाहरू टाँसिन्छन् र प्रशोधन सुरुहुनु अघि सामान्यतया बराबर अनुपातमा इथेनॉल र डाइथाइल ईथरको मिश्रणमा १५ मिनेटको भिजाएर यस्तो चिल्लो हटाउनु पर्दछ।

२. भुसुनाहरू मार्ने प्रक्रिया (युथनेशिया)

सङ्कलन पछि, जीवित भुसुनाहरूलाई मार्ने काम गर्नुपर्छ। केही सङ्कलन विधिहरू (जस्तै, टाँसिने कागजहरू, डिटर्जेन्ट वा इथेनॉल भएको जार युक्त CDC प्रकाश जालहरू) प्रयोग गर्दा भुसुनाहरू मृत रूपमा सङ्कलन हुन्छन्। यसरि सङ्कलन गरिएकाहरूलाई सिधै इथेनॉलमा र अन्यमा यदि तिनीहरूलाई सकेसम्म चाँडो इथेनॉलमा भण्डारण गरियो भने उनीहरूको आणविक जीवविज्ञान अध्ययनका लागि सहज हुन्छ।

यद्यपि, यी कुनै पनि मार्ने विधिले MALDI-ToF द्वारा कीरा प्रशोधनको लागि अनुमति दिँदैन। साथै, केही मार्ने विधिहरूले केही निश्चित आकृतिहरू नष्ट गर्न सक्छन्। त्यसैले उचित पहिचान वा दीर्घकालीन भण्डारण सुनिश्चित गर्नका लागि उपयुक्त मानक मार्ने एजेन्ट प्रयोग गर्नु आवश्यक छ। भौचर भुसुनाहरू (अर्थात्, भविष्यको सन्दर्भ वा तुलनाको लागि संरक्षित र भण्डारण गरिएका भुसुनाहरू)। इथाइल एसीटेट (ethyl acetate), इथाइल इथर (ethyl ether), टेट्राक्लोरोइथेन (tetrachloroethane), र क्लोरोफर्म (chloroform) जस्ता रसायनहरू कपासमा भिजाएर भुसुनाहरू राखिएको पात्रमा राखेर भुसुनाहरू मार्न सकिन्छ। यी मार्ने रसायनहरू विषाक्त हुनाले प्रयोग गर्दा निर्माताको सिफारिसहरू पालना गर्नु आवश्यक हुन्छ। तर, हामी क्लोरोफर्म प्रयोग गरेर भुसुनाहरू मार्न सिफारिस गर्दैनौं, किनभने यो हाम्रो अनुभवमा आणविक जीवविज्ञान अध्ययनहरूसँग राम्रोसँग मेल खाँदैन। यी सबै पदार्थहरूको जोखिमपूर्ण प्रकृति र आणविक विश्लेषणका लागि तिनीहरूको उपयुक्तता शंका योग्य भएकाले, यी रासायनिक पदार्थहरूको प्रयोग सामान्यतया निरूत्साहित गरिन्छ।

आकार विज्ञान, डीएनए वा प्रोटिनलाई सुरक्षित राख्ने सबैभन्दा व्यापक रूपमा प्रयोग हुने विधि भनेको भुसुनाहरूलाई सुख्खा फ्रिज गर्नु हो। भुसुनाहरूलाई पूर्ण रूपमा बेहोस बनाउन पर्याप्त लामो समयसम्म फ्रिज गर्नुपर्छ, तर त्यति लामो समयसम्म पनि होइन कि तिनीहरू (i) तिनीहरू सुकेर बिग्रिन्छन्, वा (ii) को व्यवहार्यतामा सम्झौता गर्छन्, यदि उद्देश्य भुसुनाको पाचन मार्गबाट लेशमेनिया अलग गर्ने हो भने तिनीहरूको जीवित क्षमतामा असर पर्छ। **त्यसकारण हामी नियमित रूपमा निगरानी गर्दै -२० डिग्री सेल्सियसमा १५ देखि २० मिनेटको फ्रिजिङ अवधि सिफारिस गर्छौं, ताकि भुसुना बेहोस होस तर लेशमेनिया परजीवीहरू पनि नमरोस।**

यदि फ्रिजर उपलब्ध छैन भने, वैकल्पिक रूपमा CO_2 प्रयोग गरेर कीराहरूलाई बेहोस पार्न सकिन्छ। CO_2 सिलिन्डरहरू प्रयोग गर्न नसकिने क्षेत्रको अवस्थामा, 'सोडा साइफन' (पेय डिस्पेन्सर) मा प्रयोग हुने साना व्यावसायिक CO_2 कन्टेनरहरू प्रयोग गरेर भुसुनाहरू मार्न सकिन्छ, तर हवाई यातायातबाट तिनीहरूको ढुवानी प्रतिबन्धित हुन सक्छन्। अन्तिम उपायको रूपमा, सुर्तीजन्य धुवाँको सम्पर्कमा ल्याएर पनि कीराहरूलाई मार्न सकिन्छ। भुसुनाहरूलाई CDC पासोमा जिउँदै समातिन्छ, एस्पिरेटरको मदतले सङ्कलन गरिन्छ, सिसाको ट्यूबमा राखिन्छ, र सुर्तीजन्य धुवाँको सम्पर्कमा ल्याएपछि तिनीहरूलाई केही सेकेन्डमै मारिन्छन्। यो विधि सबै क्षेत्रका अवस्थाहरूमा लागू हुन्छ, बिशेष गरि कठिन आइसोलेसनमा रहेका क्षेत्रहरूमा पनि। यद्यपि, सिसाको ट्यूब धुवाँले भरिएको हुनाले, यसलाई पूर्ण रूपमा सफा नगरी पछि सङ्कलन गरिने जीवित भुसुनाहरू राख्नका लागि प्रयोग गर्न सकिँदैन। यद्यपि, धुवाँ लागेका एउटै अशुद्ध एस्पिरेटर अन्य पासोहरूबाट भुसुनाहरूलाई संकलन गर्न र बेहोस गर्न प्रयोग गर्न सकिन्छ। एस्पिरेटरबाट सबै भुसुनाहरू हटाइएका छन् कि छैनन् भनेर जाँच गर्न पनि आवश्यक छ। यी विधिहरू भुसुनाको पेट

विच्छेदन मार्फत लेशमेनियाको पृथक्करणका लागि पनि उपयुक्त छन्।

३. भुसुना प्रशोधन गर्नु अघि भण्डारण

प्रशोधन गर्नु अघि फिक्सेसनका पाँच मुख्य तरिकाहरू छन्:

३.१. चिसो

यो विधि -२०°C वा, प्राथमिकतामा, -८०°C मा राम्रोसँग गरिन्छ। यी भण्डारण विधिहरू अब तरल नाइट्रोजन भण्डारण भन्दा बढी व्यापक रूपमा प्रयोग गरिन्छ। सबै अवस्थामा, भुसुनाहरूलाई बेहोस गरेपछि क्रायोप्रिजर्भेसन सकेसम्म चाँडो गर्नुपर्छ। फ्रिजरहरूमा कोल्ड स्टोरेजले भण्डारण अवधिभर पूर्ण रूपमा भुसुनाहरू, साथै आरएनए, डीएनए, र प्रोटीनहरूलाई पूर्ण रूपमा संरक्षण गर्ने गर्दछ। यसको सट्टा, तरल नाइट्रोजनले पखेटा, खुट्टा, पाल्प्स र एन्टेनालाई गम्भीर रूपमा क्षति पुर्‍याउन सक्छ, प्रायः तिनीहरू झर्छन र कहिलेकाहीं प्रमुख आकृतिक संरचना हराउने हुन्छ। सुख्खा फ्रिजर भण्डारण भुसुनाहरूको लागि क्षतिजन्य हुन्छ, तर तिनीहरूको कमजोर अंगहरू संरक्षणको लागि उपयूक्त हुँदैन। महत्वपूर्ण कुरा, पग्लने समयमा, पखेटा, एन्टेना, पाल्प्स, वा खुट्टाहरू शीशीहरूमा टाँसिन सक्छन् र अन्ततः संक्षेपणको कारणले टुटेर झर्न सक्छन्। यद्यपि, क्षेत्रीय अध्ययनहरूमा फ्रिजिङद्वारा संरक्षण सधैं सम्भव हुँदैन किनभने यसलाई फ्रिजर वा तरल नाइट्रोजन कन्टेनरमा पहुँच चाहिन्छ। फ्रिजर भण्डारण संवेदनशीलता गुमाउनु बिना आणविक उपकरणहरू प्रयोग गरेर रोगजनक पत्ता लगाउन पूर्ण रूपमा उपयुक्त छ, यद्यपि आरएनए भाइरस पत्ता लगाउने र अलगाव गर्न -80°C मा वा दीर्घकालीन भण्डारण आवश्यक भएमा तरल नाइट्रोजनमा फ्रिज गर्न आवश्यक पर्दछ। लेशमेनियाको क्रायोप्रिजर्भेसनको अनुकरण गर्दै, यदि भुसुनाहरूलाई पहिले वाष्प चरणमा र त्यसपछि तरल नाइट्रोजनमा (उदाहरणका लागि स्टकिङमा राखिएको शीशीहरूमा) डुबाइएन भने फ्रिज गरिएका भुसुनाहरूको पेट विच्छेदन मार्फत लेशमेनियालाई पृथक्करण गर्न सकिंदैन।

३.२. अल्कोहलमा भण्डारण (इथेनॉल वा आइसोप्रोपाइल अल्कोहल)

भुसुनाहरू भण्डारण गर्न यो सम्भवतः सबैभन्दा व्यापक रूपमा प्रयोग हुने विधि हो। प्रयोगशालाको पहुँच नभएका कठिन परिस्थितिहरूमा पनि यसलाई प्रयोग गर्न सजिलो छ। भण्डारण ट्यूबमा हावाको बुलबुले नभएसम्म कमजोर अंगहरू (पखेटा, खुट्टा, एन्टेना, वा पाल्प्स) अक्षुण्ण रहन्छन्, त्यसैले हामी हावाको बुलबुले हटाउन र कपासको प्लगको माथि लेबल राख्न ट्यूबभित्र सानो कपासको बलले बन्द गर्न सिफारिस गर्छौं (चित्र १)। उपयुक्त अल्कोहल सांद्रतामा सबैको आफ्नै प्राथमिकता हुन सक्छ। सामान्यतया, ७०% भन्दा कम सांद्रता सिफारिस गरिँदैन [४५, ६६]। उच्च सांद्रताले DNA लाई अझ प्रभावकारी रूपमा र लामो समयसम्म सुरक्षित राख्छ तर आकृतिक पहिचानको अध्ययनहरूको लागि भुसुनाहरूलाई अझ कमजोर र भंगुर बनाउँछ। ९६% इथेनॉल (एजियोट्रोप मिश्रण) को प्रयोगले समयसँगै सांद्रताको स्थिरता सुनिश्चित गर्दछ, विशेष गरी उष्णकटिबंधीय देशहरू जस्ता आर्द्र क्षेत्रहरूमा, यद्यपि ९५% इथेनॉल प्राप्त गर्न प्रायः सजिलो हुन्छ। सांद्रताको बावजुद, DNA सामान्यतया इथेनॉलमा राम्रोसँग संरक्षित हुन्छ (यद्यपि फ्रिजिङ विधिहरू भन्दा कम प्रभावकारी रूपमा, विशेष गरी NGS-प्रकारको आणविक विधिहरूको लागि)। प्रोटिनहरू धेरै कम स्थिर हुन्छन्, विशेष गरी प्रोटियोमिक्सको लागि, जस्तै MALDI-ToF अनुप्रयोगहरूमा। केही महिनाको लागि अल्कोहलमा संरक्षित भुसुनाहरू अझै पनि आकृतिक रूपमा पहिचान गर्न सकिन्छ, तर यी भुसुनाहरूबाट सन्दर्भ प्रोटीन स्पेक्ट्रा (reference protein spectra) उत्पन्न गर्नु असम्भव हुन्छ। अल्कोहल वा सुख्खा अवस्थामा भण्डारण गरिएको भुसुनाहरूलाई यदि -२० डिग्री सेल्सियसमा राख्न सकियो भने बिधि अझ परिष्कृत हुन्छ। -२० डिग्री सेल्सियसमा जमेकोले मुख्यतया क्षयीकरण ढिलो गरेर आणविक संरक्षण (जस्तै, न्यूक्लिक एसिड) लाई सुधार गर्दछ र समयसँगै तन्तुको ब्रेकडाउन घटाएर रूपात्मक संरक्षणको लागि माध्यमिक लाभ पनि प्रदान गर्दछ, यद्यपि आकारशास्त्रमा प्रभाव आणविक अखण्डता भन्दा बढी सीमित हुन्छ। केही महिना भन्दा कम समयमा छोटो भण्डारण अवधिको लागि कम्तिमा ७०% को सांद्रतामा इथेनॉल प्रयोग गर्दा DNA र RNA भाइरस पत्ता लगाउनको लागि इथेनॉलमा पनि भण्डारण गर्न सकिन्छ। साथै, आइसोप्रोपाइल अल्कोहल केही देशहरूमा सजिलै उपलब्ध हुन सक्छ र DNA सुरक्षित गर्दछ, तर भुसुनाहरूलाई कडा बनाउँछ। यो इथेनॉल जस्तै ज्वलनशील छैन र त्यसैले सजिलै ढुवानी गर्न सकिन्छ। आवश्यक भएमा, तरल नाइट्रोजन वा सुख्खा-जमेकोमा संरक्षित भुसुनाहरूलाई अल्कोहलमा स्थानान्तरण गर्न सकिन्छ, जसले गर्दा दुवै विधिहरूको कमजोरीहरू घटाउन सकिन्छ।

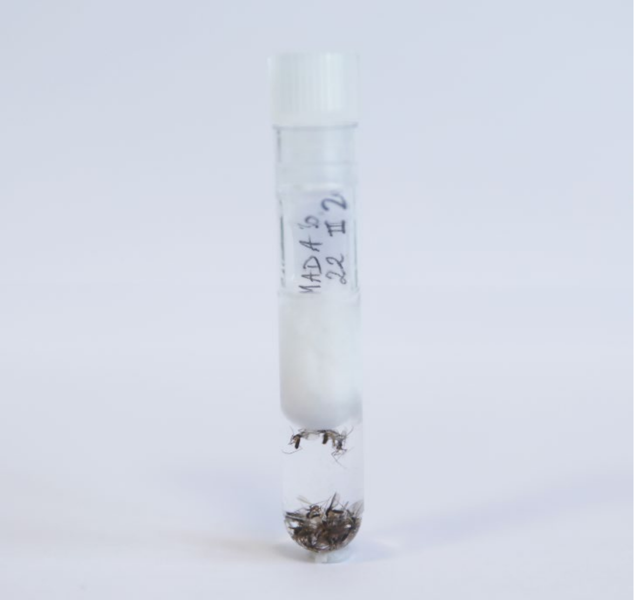

चित्र १: इथेनॉलमा संरक्षित भुसुनाहरू।

३.३. आरएनएएमा भण्डारणस्थिरीकरण समाधान (RNASS)

यो जलीय अभिकर्मक व्यापक रूपमा प्रयोग गरिन्छ, विषाक्त हुदैन, र ताजा तन्तु र कोशिका भुसुनाहरूमा RNA स्थिर र सुरक्षित गर्न डिजाइन गरिएको हुन्छ। यसले भुसुनाहरूमा द्रुत रूपमा प्रवेश गरेर RNases (RNA-degrading enzymes) लाई निष्क्रिय पार्ने कार्य गर्दछ, जसले गर्दा तत्काल फ्रिजिङको आवश्यकता बिना RNA क्षयीकरणलाई रोक्छ। RNASS मा भण्डारण सामान्यतया डाउनस्ट्रीम हिस्टोलोजिकल मूल्याङ्कनको लागि समग्र तन्तु र सेलुलर आकारविज्ञान संरक्षण गर्न प्रभावकारी हुन्छ। RNASS फिक्सेसनको सट्टा RNA स्थिरीकरणको लागि अनुकूलित गरिएको छ, छोटो देखि मध्यम अवधिको भण्डारणले सामान्यतया संरचनात्मक अखण्डता राम्रोसँग कायम राख्छ। RNASS ले भुसुनाहरूलाई कोठाको तापक्रममा ७ दिनसम्म, धेरै हप्ताको लागि ४°C मा, वा दीर्घकालीन संरक्षणको लागि -२०°C/-८०°C मा भण्डारण गर्न अनुमति दिन्छ। यो विधि विशेषगरी क्षेत्रीय कार्य (फिल्डवर्क) वा चिकित्सकीय परिवेशमा, जहाँ कोल्ड चेन पूर्वाधार सीमित हुन्छ, अत्यन्त उपयोगी हुन्छ। RNA छुट्याउनका लागि सामान्यतया भुसुनाहरू अभिकर्मकबाट निकालेर मानक प्रोटोकलअनुसार प्रशोधन गर्नुपर्छ।

३.४. कोठाको तापक्रममा सुख्खा संरक्षण

यो एउटा पुरानो विधि हो जुन, जब इन-टोटो नमुना (माउन्ट गरिएको सम्पूर्ण) मा लागू गरिन्छ, पखेटा, खुट्टा, एन्टेना र पाल्प्स जस्ता कमजोर अंगहरूलाई कमजोर रूपमा संरक्षण गर्ने प्रमुख बेफाइदा हुन्छ। यद्यपि, सिलिका जेल-प्रकारको डेसिकेन्टको साथ फिक्सेसनमा निर्जलीकरण गरिन्छ भने MALDI-ToF प्रयोग गरेर प्रोटोमिक अध्ययनहरू सम्भव रहन्छन्। यसको विपरित, DNA लाई लक्षित गर्ने आणविक विश्लेषणहरू यी भुसुनाहरूमा गर्न गाह्रो हुन्छ, किनभने DNA प्रायः खण्डित र कम मात्रामा हुन्छ, जसको गर्दा विश्लेषणहरू ताजा वा जमेको भुसुनाहरू भन्दा बढी चुनौतीपूर्ण हुन्छ, विशेष गरी आणविक जीनोमहरूको लागि। यद्यपि, यस प्रकारका भुसुनाहरूमा म्युजियोमिक्स जस्ता हालका प्रविधिहरू प्रयोग गर्न सकिन्छ [34]। त्यसकारण, अन्य विकल्प उपलब्ध भएसम्म यो भण्डारण विधि सिफारिस गरिदैन। यसरी सुख्खा संरक्षण गरिएका भुसुनाहरूलाई -२०°C वा -८०°C मा फ्रिजरमा राखेर यसलाई कोल्ड स्टोरेज गर्न सकिन्छ। मुख्य चुनौती भनेको पहिचानको लागि आवश्यक भुसुनाहरू वा शरीरका अंगहरूको उपयुक्त माउन्टिङ प्राप्त गर्नु हो। यो प्राप्त गर्न, पुनर्जलीकरण आवश्यक हुन्छ । हामी Triton X-100 प्रयोग गर्न सिफारिस गर्छौं। नियमित रूपमा सावधानीपूर्वक निगरानी गर्दा, पुनर्जलीकरणको अवधि केही घण्टादेखि धेरै दिनसम्मको हुन सक्छ। पुनर्जलीकरण पछि, भुसुनाहरूलाई लगातार तीन पटक पानीमा पखाल्नु पर्छ।

३.५. फिल्टर पेपरहरूमा संरक्षण

फिल्टर पेपरको मुख्य फाइदा भनेको कोठाको तापक्रममा भण्डारण गरिएका नबनाइएका, सुकेका सम्पूर्ण शरीरका कोषहरू वा रक्त कोषहरू भित्र जीनोमिक डीएनएको दीर्घकालीन स्थिरता हो। फिल्टर पेपर सानो कार्ड साइजमा प्रदान गरिएको छ, जसले गर्दा कोठाको तापक्रममा सानो टेबल ड्राइभरको आकारमा सयौं भुसुनाहरू भण्डारण गर्न सम्भव हुन्छ। फिल्टर पेपर म्याट्रिक्समा संक्रामक एजेन्टहरूलाई विकृत गर्ने एजेन्टहरू हुन्छन्, र यसरी संरक्षण गरिएका भुसुनाहरूलाई अब जैविक जोखिम मानिदैन यसले चयनात्मक जैविक जोखिम सावधानीहरू बिना भुसुनाहरूको भण्डारण र ढुवानी गर्न सकिन्छ [68]।

४. भुसुना विच्छेदन

धेरै अन्य कीराहरू भन्दा फरक, जुन व्यक्तिगत कीराहरूमा देख्न सकिने बाह्य वर्णहरूको आधारमा पहिचान गरिन्छ, भुसुनाहरूलाई प्रजातिको सही पहिचानको लागि शारीरिक विशेषताहरू अध्ययन गर्न विच्छेदन र स्लाइड-माउन्टिंग आवश्यक पर्दछ। छनौट गरिएको तयारी र

माउन्टिंग प्रक्रियाको पर्वाह नगरी, उही विच्छेदन प्रविधि प्रयोग गरिन्छ (चित्र २ र ३) (https://zenodo.org/records/18198006)।

Triton X100 को प्रयोग: गैर-आयनिक जलीय घोल

ध्यान दिनुहोस् कि माउन्टिङले भर्खरै समातिएका वा पर्याप्त रूपमा भण्डारण गरिएका भुसुनाहरूसँग सम्बन्धित छ। धेरै जसो सङ्कलकहरूले कीराका भुसुनाहरू सुक्खा (MALDI-ToF प्रयोगको लागि) वा धेरै वर्षसम्म अल्कोहलमा भण्डारण गर्छन्। दुर्भाग्यवश, अल्कोहलमा संरक्षण वर्षौंको अवधिमा इष्टतम हुँदैन, र यस तरिकाले संरक्षित आर्थ्रोपडहरू सूक्ष्म परीक्षणको लागि तयार गर्न धेरै गाह्रो हुन्छन्। प्रायः हुने एउटा घटना भनेको भुसुनाहरू भएको प्लास्टिकको क्षयीकरण हो, त्यसपछि अल्कोहलको वाष्पीकरण हुन्छ। दुवै अवस्थामा, हामीसँग कुनै विकल्प छैन किनभने भुसुनाहरू अल्कोहलमा धेरै लामो समयसम्म रहन्छन् वा सुक्छन्। त्यसैले भिजाउने एजेन्टहरू प्रयोग गर्ने विचार आयो जुन बलियो डिटर्जेन्ट होइनन्। Triton X100 गैर-आयनिक जलीय घोल (*4-(1,1,3,3-tetramethylbutyl) phenyl-polyethylene glycol solution,* वा *t-octylphenoxypolyethoxyethanol, polyethylene glycol tert-octylphenyl ether*) को रूपमा छ, जुन कोशिका र आणविक जीवविज्ञानमा डिटर्जेन्टको रूपमा व्यापक रूपमा प्रयोग गरिन्छ। यसले कोशिका र आणविक झिल्लीहरूको पारगम्यता सहज बनाउँछ।

तल ०.५% जलीय घोलमा गैर-आयनिक ट्राइटन X१०० प्रयोग गर्ने प्रक्रिया दिइएको छ:

- सुख्खा भुसुना भुसुना लाई पूर्ण अल्कोहलले भिजाउनुहोस्।
- पूर्ण भुसुना भुसुना डुबाउनको लागि आवश्यक मात्रामा ट्राइटन X१०० घोल ०.५% थप्नुहोस्।
- यो प्रक्रियालाई लगभग ५ मिनेटदेखि धेरै दिनसम्म चलाउन दिनुहोस्, नियमित रूपमा निगरानी गर्नुहोस्। सबै आर्थ्रोपोडहरू घोलमा पूर्ण रूपमा अलग हुनुपर्छ।
- Triton X100 घोल हटाउनुहोस् र पोटासियम हाइड्रोक्साइड घोलले बदल्नुहोस्।

४.१. टाउको

स्टेरियोमाइक्रोस्कोप अन्तर्गत मसिना सुई वा कीटविज्ञान पिनहरू प्रयोग गरेर विच्छेदन गर्न सकिन्छ (चित्र २ र ३)। सबैभन्दा बढी प्रयोग हुने सुईहरूमा समावेश छन्: २६G x १/२" (०.४५ × १३ मिमी), ३०G × १/२" (०.३ × १३ मिमी), वा २५G × ५/८" (०.५ × १६ मिमी)। पहिचानको लागि भुसुना तयार गर्न, कम्तिमा, टाउकोलाई शरीरबाट अलग गरिन्छ र सिबेरियम र ग्रसनी देखिन भेन्ट्रल साइड माथि पारेर माउन्ट गरिन्छ, जबकि छाती र पेट विच्छेदन पछि साइड वा तेर्सो पारेर माउन्ट गरिन्छ। टाउकोलाई भेन्ट्रो-डोर्सल स्थितिमा माउन्ट गर्नाले ओसिपिटल फोरेमेन माथितिर हुन्छ, जसले गर्दा सिबेरियम प्रत्यक्ष रूपमा हेर्न सकिन्छ। टाउको पूर्ण रूपमा अलग गरिएको छ भने यी शारीरिक आकृतिहरू हेर्न सजिलो हुन्छ।

४.२. पखेटा र छाती

पखेटाहरू समतल माउन्ट गरिनुपर्छ। प्रत्येक पखेटालाई यसको फेदबाट छुट्याएर स्वतन्त्र रूपमा माउन्ट गर्न सकिन्छ, वा एउटालाई एक्लै र अर्कोलाई छातीमा जोडिएको अवस्थामा माउन्ट गर्न सकिन्छ। यदि ज्यामितीय मोर्फोमेट्री विश्लेषण गर्ने योजना गरिएको छ भने, माउन्ट गर्नु अघि दायाँ र बायाँ पखेटाहरूलाई सही रूपमा पहिचान र लेबल गर्न आवश्यक हुन्छ। छातीलाई धेरै भागहरूमा विभाजित गरिएको हुन्छ, र प्रत्येकमा प्रजाति वर्गीकरण र पहिचानका धेरै महत्त्वपूर्ण जानकारी हुन्छ [20, 64]। सामान्यतया, यो चेटोट्याक्सी (रौं/काँडाहरूको व्यवस्था) र रंग वितरणको जाँच गर्न अनुमति दिन तेर्सो (lateral view) माउन्ट गरिन्छ। छातीको केही क्षेत्रहरूमा ब्रस्टलको दागको उपस्थिति ब्रम्पटोमिया (*Brumptomyia*) जीनसका केही प्रजातिहरू छुट्याउन प्रयोग गर्न सकिन्छ। रंग वितरण जीनस स्तरमा नियोट्रोपिकल भुसुनाहरू (जस्तै, *Bichromomyia*), प्रजाति श्रृंखला (जस्तै, *Pintomyia*), वा एउटै जीनसका प्रजातिहरू (जस्तै, *Micropygomyia*, *Nyssomyia*, *Psathyromyia*, and *Psychodopygus*) [20] छुट्याउन प्रयोग गर्न सकिन्छ। तसर्थ, यदि छाती आणविक विश्लेषणको लागि प्रयोग गरिँदैन भने, यसलाई क्षति नपुगोस् भनेर माउन्ट गर्नुपर्छ। महत्त्वपूर्ण कुरा, यो ध्यान दिनुपर्छ कि रङहरूको तीव्रता महत्त्वपूर्ण छैन, तर छातीभरि तिनीहरूको वितरण महत्त्वपूर्ण हुन्छ। त्यसकारण, स्पष्टीकरण प्रक्रियाले पिग्मेन्टेसन वा यसको ढाँचालाई हटाउने छैन।

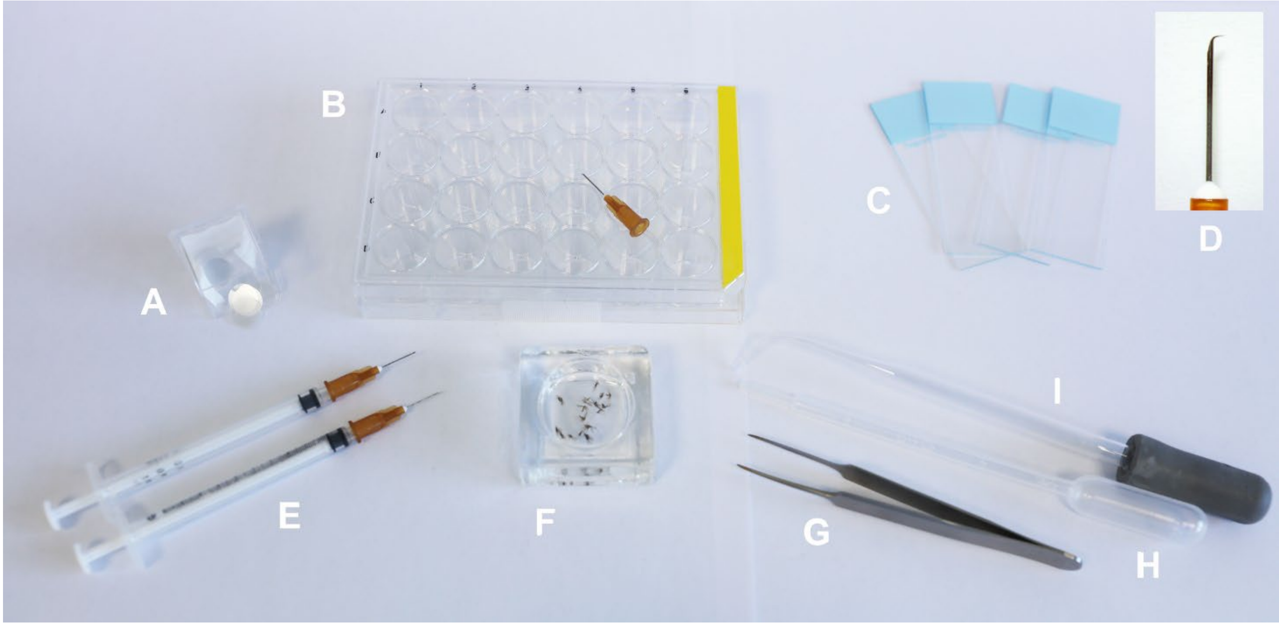

चित्र २: भुसुना जडान गर्न आवश्यक सामग्रीहरू: A: गोलो काँचका कभरस्लिपहरू (१० वा १२ मिमी व्यास); B: २४-वेल प्लेट र हुक गरिएको सुई (यदि तपाईंले भुसुना प्रशोधन गर्न ल्वाङको तेल वा युपरल® एसेन्स प्रयोग गर्नुहुन्छ भने, एक्रिलिक प्लेटहरू प्रयोग नगर्नुहोस् किनभने यसले रासायनिक प्रतिक्रिया गर्दछ र भुसुनाहरू क्षतिग्रस्त हुनेछन्); C: शीशा स्लाइडहरू लेबलिङको लागि उपयुक्त; D: सुईको हुकको विवरण; E: सिरिन्जमा जोडिएका सुईहरू; F: भुसुना राख्ने अवतल काँच प्लेट वा सो सरहको कन्टेनर; G: डुमोन्ट फोर्सेप्स; H: प्लास्टिकको पिपेट; I: कुँडाहरू (वेल्स) भित्र तरल सार्न सजिलो बनाउन तातो दिएर बाङ्गो बनाइएको काँचको पिपेट।

४.३. जननेन्द्रिय

भाले र पोथी दुवैमा जननेन्द्रियहरू मिलाउँदा विशेष सावधानी अपनाउनु पर्छ, किनकि तिनीहरू जेनेरा, सबजेनेरा र प्रजातिहरू पहिचान गर्न महत्त्वपूर्ण हुन्छन्। दुवै लिङ्गहरूमा, जननेन्द्रियहरू जोडीमा हुन्छन्।

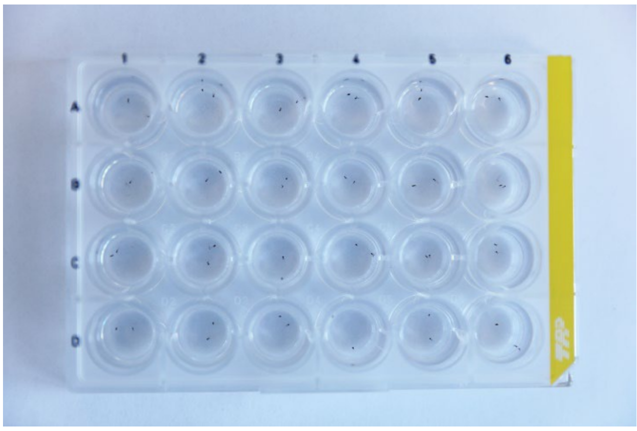

चित्र ३: भुसुनाहरूको टाउको र पेटको टुप्पो भएको २४ वटा कुँडाहरू भएको प्लेट।

४.३.१. भालेहरू

जननेन्द्रियहरू बाह्य हुन्छन् र जोडी फोर्सेप्स मिलेर बनेका हुन्छन्, प्रत्येकमा यसको पृष्ठीय भागमा गोनोकोक्साइट-गोनोस्टाइल आर्टिक्युलेसन र यसको भेन्ट्रल भागमा एप्यान्ड्रियल लोब हुन्छ। गोनोस्टाइलमा काँडा (स्पाइन) हुन्छन् र कहिलेकाहीं सेटे (setae) हुन्छ, जुन गणना गर्न सकिने हुनुपर्छ र जसको सम्मिलन स्थिति स्पष्ट रूपमा देखिनुपर्छ। गोनोकोक्साइटको भित्री सतहलाई ध्यानपूर्वक अवलोकन गर्नु महत्त्वपूर्ण हुन्छ, जसमा सेसाइल सेटेको टुफ्ट वा लोब (= ट्युबरकल) हुन सक्छन् [22]। विच्छेदनमा कम अनुभव भएका सहकर्मीहरूले पेटको छेउबाट जननेन्द्रियलाई अलग नगरी साधारण पार्श्व (lateral) माउन्टिङ गर्न सक्छन् (https://zenodo.org/records/18311158)। यस अवस्थामा, जननेन्द्रियको दुई भागहरूको सुपरपोजिसनले उदाहरणका लागि, गोनोकोक्साइटको आन्तरिक सेटे गणना गर्न गाह्रो हुन सक्छ, तर यसले असफल विच्छेदन मार्फत जननेन्द्रियलाई क्षति पुर्‍याउनबाट जोगाउँछ। अधिक अनुभवी

सहकर्मीहरूले विभाजित गर्न जननेन्द्रियलाई दुई भागमा खोल्ने प्रयास गर्न सक्छन्। यस्तो गर्न, सुईको बेभल्ड साइड (इन्ट्राडर्मल रियाक्सन सुई प्रकार) प्रयोग गरेर, जननांगलाई पूर्ण रूपमा काटेर अलग नगरी गोनोकोक्साइट-गोनोस्टाइल एसेम्बलीहरू (https://zenodo.org/records/18311158) विभाजित गर्न सकिन्छ। यस तरिकाले, तिनीहरूको आन्तरिक अनुहारहरूको अवलोकन सजिलो हुनेछ। यो एसेम्बलीले प्यारामेर र प्यारामेरल शीथहरूको अवलोकनलाई पनि सहज बनाउँछ, जुन अब ओभरल्याप हुँदैनन्। पार्श्व (lateral) माउन्टिङको लागि, जसले अंग सुपरपोजिसनलाई बढावा दिन्छ, भुसुनाहरू पूर्ण रूपमा सफा गर्नुपर्छ।

४.३.२. पोथीहरू

जननेन्द्रिय अंग आन्तरिक हुन्छ, स्पर्माथिका द्वारा बनेको हुन्छ। विच्छेदनको अनुपस्थितिमा, तिनीहरूलाई टेग्युमेन्टहरू मार्फत र पेटलाई भेन्ट्रल स्थितिमा माउन्ट गरेर अवलोकन गर्नुपर्छ। जुन माउन्टिंग माध्यम छनौट गरे पनि, स्पर्माथिका आफैंलाई सामान्यतया सही रूपमा अवलोकन गर्न सकिन्छ, विशेष गरी यदि यो चिल्लो र सफा छैन भने। यद्यपि, कमजोर अपवर्तक मिडियामा चिल्लो, पातलो-तह भएको स्पर्माथिका हेर्न गाह्रो हुन सक्छ। यसबाहेक, पुरानो संसारमा लेशमेनिया इन्फ्यान्टम (*Leishmania infantum*) को मुख्य भेक्टरहरू, सबजेनस लारोसियस (*Larroussius*) [35, 37, 38] जस्ता प्रजाति पहिचानको लागि स्पर्माथिका नलीहरूको आधार अवलोकन गर्नु आवश्यक हुन्छ। यो अवलोकन बिना, भुसुना पहिचान असम्भव हुन्छ। यी संरचनाहरू अवलोकन गर्न कठिन नहोस भने जननेन्द्रिय फर्का-स्पर्माथिका माउन्टिंग पेटबाट हटाउनु पर्छ (https://zenodo.org/records/18311106)। विच्छेदनको समयमा स्पर्माथिका अवलोकन गर्न सामान्यतया गाह्रो हुन्छ, तर जननेन्द्रिय फर्का पत्ता लगाउन अपेक्षाकृत सजिलो हुन्छ। स्पर्माथिका नलिकाहरू जननांग फर्कामा खुल्ने भएकोले, यो फर्काको पृथकीकरणले सामान्यतया स्पर्माथिकालाई पृथक गर्न सहज हुन्छ। यदि यो प्रक्रियाको क्रममा स्पर्माथिका गल्तिले काटिए भने, तिनीहरू हराउँदैनन् र अझै पनि पेटको भित्री भाग भित्र अवलोकन गर्न सकिन्छ (चित्र ४)।

४.४. *लेशमेनिया* पृथक्करणका लागि मिडगट डिसेक्शन

पोथी भुसुनामा लेशमेनिया पत्ता लगाउन र अलग गर्न पाचन पथको विच्छेदन आवश्यक छ। भेक्टोरियल क्षमताको मूल्याङ्कन गर्न यो प्रक्रिया क्षेत्र र प्रयोगशाला दुवै सेटिङहरूमा गर्न सकिन्छ।

भर्खरै बेहोश (युथनाइज) गरिएका पोथीहरूमा काम गर्न सिफारिस गरिन्छ। पोथीहरूलाई पानी वा हल्का डिटर्जेन्ट भएको नुनिलो घोलले धुनुहोस् जसले गर्दा अतिरिक्त रौंहरू हट्छन्। यो चरणले लेशमेनिया आइसोलेसनको लागि एसेप्टिक अवस्था कायम राख्न मद्दत गर्छ, जबकि पहिचानको लागि आवश्यक रूपात्मक विशेषताहरू संरक्षण पनि गर्दछ। लेशमेनिया फेला पार्न र अलग गर्न, मिडगटलाई सावधानीपूर्वक हटाउनुहोस् र यसलाई निर्मलीकृत नुनिलो घोल (०.९% NaCl) को एक थोपामा राख्नुहोस्। प्रकाश माइक्रोस्कोप मुनि गतिशील परजीवीहरू अवलोकन गरेपछि (सिफारिस गरिएको म्याग्निफिकेसन: ~२००×), तिनीहरूलाई संवर्धन माध्यममा स्थानान्तरण गर्न इन्सुलिन सिरिन्ज वा माइक्रोपिपेट प्रयोग गर्नुहोस् (थप विवरणहरूको लागि अध्याय ४.४.३ हेर्नुहोस्)।

टाउको र जननेन्द्रियहरूलाई सफा गर्न सिधै मार्क-एन्ड्रे फ्लुइडमा माउन्ट गर्नुहोस्। महत्त्वपूर्ण: प्रत्यक्ष वा अप्रत्यक्ष रूपमा मार्क-एन्ड्रे फ्लुइडलाई कहिल्यै पनि लेशमेनियाको सम्पर्कमा नल्याउनुहोस - किनकि यो परजीवीहरूको लागि घातक हुन्छ।

पोथी भुसुनाको विच्छेदन एउटा वा दुईटा स्लाइडमा गर्न सकिन्छ; दुवै विकल्पका फाइदा र सीमितताहरू छन् (चित्र ५;https://zenodo.org/records/18311154)।

४.४.१. दुई-स्लाइड विधि

पहिलो विकल्पमा दुई छुट्टाछुट्टै स्लाइडहरूमा काम गर्ने विधि समावेश छ: एउटामा मिडगट निकाल्न निर्मलीकृत सलाइन समावेश गरिएको छ, र अर्कोमा मार्क-एन्ड्रे फ्लुइडमा टाउको र स्पर्माथिका माउन्ट गर्ने। यद्यपि, क्षेत्रको अवस्थामा, दुई वा तीन व्यक्तिहरूले भुसुनाको विच्छेदन गर्छन् र आन्द्रामा लेशमेनिया संक्रमण र भुसुनाको प्रजाति पहिचान र मूल्याङ्कनको लागि सामान्तया जिम्मेवार एकल अनुसन्धानकर्तालाई स्लायडहरू पठाउइन्छ। दुई स्लाइडहरू व्यवस्थापन गर्नुपर्ने हुनाले भुसुना ट्रेसेबिलिटीमा समस्याहरू निम्त्याउन सक्छ र विशेष गरी, यदि लेशमेनिया संक्रमण भएको पेट पत्ता लाग्यो भने कुन भुसुनामा संक्रमण भएको थियो भनेर निश्चित रूपमा निर्धारण गर्न गाह्रो हुन सक्छ (https://zenodo.org/records/18311154)।

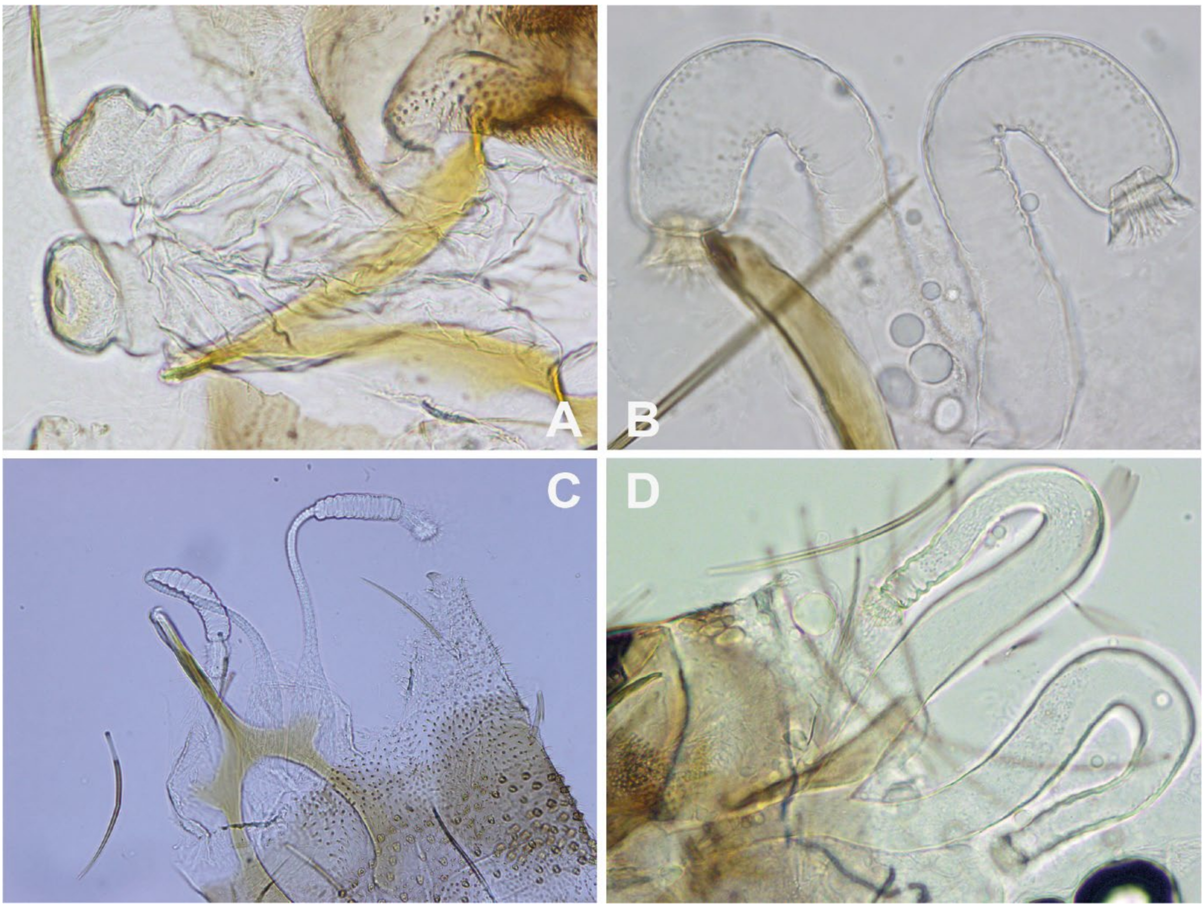

चित्र ४: ताजा भुसुनाहरूबाट स्पर्माथिका विच्छेदन गरी मार्क-आन्द्रे तरल पदार्थमा राखियो। A: इडियोफ्लेबोटोमस लङ्गिफोर्सेप्स (*Idiophlebotomus longiforceps*) (लाओ पीडीआर); B: सर्जेन्टोमिया मिनेटा (*Sergentomyia minuta*) (फ्रान्स); C: फ्लेबोटोमस एरियासी (*Phlebotomus ariasi*) (फ्रान्स); D: सर्जेन्टोमिया एनोडोन्टिस (*Sergentomyia anodontis*) (लाओ पीडीआर)।

४.४.२. एकल-स्लाइड विधि

एउटै स्लाइड प्रयोग गर्नाले नतिजाहरूको ट्रेसेबिलिटी सुनिश्चित हुन्छ। यद्यपि, धेरै सावधानीहरू अपनाउनु पर्छ। यस चरणको समयमा किटाणुरहित वातावरण बनाउन, अपरेटरहरूले नियमित रूपमा हाइड्रोअल्कोहोलिक जेलले आफ्नो हात सफा गर्नुपर्छ। प्रत्येक विच्छेदनको लागि निर्मलीकृत सुईहरू सहित (सुझाव: २५G Ø ०.५ मिमी × १६ मिमी), नन-फ्रोस्टेड स्लाइडहरू र एल्युमिनियम पन्नीमा बेरिएको र सुख्खा तापले निर्मलीकृत पारिएको (पाउपिनेल ओभन प्रयोग गरेर) वर्गाकार कभरस्लिपहरू (२२ x २२ मिमी) प्रयोग गर्नुपर्छ । भुसुनालाई स्लाइडको बीचमा निर्मलीकृत सलाइनको थोपामा राखिएको छ। पहिले टाउको काटिन्छ त्यसपछि पाचन पथ नकाटिकन छैठौं र सातौं पेटको टर्गाइट्स र स्टर्नाइट्स बीच चीरा बनाइँन्छ (यदि धेरै लामो स्पर्माथिका अपेक्षित छ भने उच्च कट बनाउन सकिन्छ)। त्यसपछि, छातीलाई सुईले स्थिर गर्नुपर्छ, र पेट निकाल्नको लागि पेटको पछाडिको खण्डहरू अर्को सुईले बिस्तारै तानिन्छ। यदि यो असफल भयो भने, पेटको छेउलाई सुईले अवरुद्ध गर्ने र पाचन पथको अगाडिको भागबाट तान्नु पर्ने हुन्छ। यदि यो फेरि असफल भयो भने, यसको वरिपरि बाँकी रहेको भागलाई सकेसम्म हटाएर आन्द्रा निकाल्नुपर्छ। आन्द्रा हटाएपछि, पेटका पछाडिका भागहरूलाई पाचन पथबाट काटेर अलग गर्नुपर्छ। त्यसपछि आन्द्रालाई स्लाइडको एक छेउमा राखिएको निर्मलीकृत सलाइनको नयाँ थोपामा राखिन्छ, र त्यसपछि बिस्तारै निर्मलीकृत कभरस्लिपले ढाकिन्छ। टाउको र पेटका अन्तिम भागहरूलाई स्लाइडको अर्को छेउमा राखिएको मार्क-एन्ड्रे तरल पदार्थको सानो थोपामा स्थानान्तरण गरिन्छ, जसले गर्दा लेशमेनियासँग कुनै सम्पर्क हुँदैन। टाउको सही रूपमा उन्मुख हुन्छ (ओसिपिटल फोरेमेन माथितिर), र स्पर्माथिकालाई माथि उल्लेख गरिए अनुसार जननांग फर्कासँग अलग गरिन्छ र सानो गोलो

कभरस्लिप (Ø १२ मिमी, निर्मलीकृत वर्गाकार कभरस्लिपहरूसँग भ्रमित नहुने) ले ढाकिन्छ। बाँकी भुसुनाको शव र पखेटाहरू स्लाइडको बीचमा रहेको लाइनको थोपामा रहन्छन् (https://zenodo.org/records/18311154)। लेसमेनिया देखिएको अवस्थामा, वा वर्गीकरण अन्वेषणको लागि, छाती र पेटलाई आणविक वा प्रोटोमिक अध्ययनको लागि संरक्षित गर्न सकिन्छ, र पखेटाहरूलाई जलीय माध्यममा माउन्ट गर्न सकिन्छ। माउन्टलाई संरक्षित गर्न, मार्क-एन्ड्रे तरल पदार्थको अतिरिक्त मात्रालाई क्लोरल गम (=होयर) वा पोलिभिनाइल अल्कोहल-आधारित माध्यम जस्ता जलीय माउन्टिंग माध्यमले प्रतिस्थापन गर्न सकिन्छ।

यी प्रक्रियाहरू प्रदर्शन गर्ने विस्तृत भिडियोहरू उपलब्ध छन् (भुसुनाको मिडगट डिसेक्शन: https://zenodo.org/records/18303014 र भुसुनाको लार ग्रन्थी विच्छेदन: https://zenodo.org/records/18302850), त्यसैले तिनीहरूलाई यहाँ व्याख्या गरिने छैन।

४.४.३. भुसुनाको आन्द्राबाट *लेशमेनिया* परजीवी पृथक्करण र संवर्धन

संक्रमित पोथी भुसुनाको विच्छेदनबाट परजीवी अलगाव एक नाजुक प्रक्रिया हो जसमा उच्च सीप आवश्यक पर्दछ र सुरुमा परजीवी-मुक्त भुसुनाहरूमा पहिले अभ्यास गर्नुपर्छ। विच्छेदन पछि, आन्द्राहरूलाई निर्मलीकृत नुन पानीको (०.९%) को ताजा थोपा वा धुनको लागि लकको घोलमा (Locke's solution) स्थानान्तरण गरिन्छ [4]। त्यसपछि विच्छेदित आन्द्राहरूलाई दुई तरिकाले प्रशोधन गर्न सकिन्छ: i) स्टोमोडियल भल्भमा विशेष ध्यान दिएर लेशमेनिया प्रोमास्टिगोट्सका विभिन्न चरणहरू र तिनीहरूको स्थान अवलोकन गर्न प्रकाश माइक्रोस्कोपको सहायताले जाँच गर्ने, र ii) प्रोमास्टिगोट्स बाहिर निस्कनको लागि आन्द्रा खोल्ने जसले बृहत रूपमा संबर्धन गर्न सहज बनाउँछ [4]। क्षेत्रीय अवस्थामा संक्रमित भुसुनाको खोजी अपेक्षाकृत दुर्लभ घटना हो तर पनि राम्रो अभ्यास गर्नेहरूले सफलता पूर्वक परजीवीलाई पृथक्करण गर्ने सम्भावना अधिक हुन्छ।

यदि लेशमेनिया परजीवीहरू आन्द्रामा देखिएमा, नयाँ निर्मलीकृत सुईहरू प्रयोग गर्नुपर्छ र तिनीहरूलाई छोड्नको लागि केशिका कार्यद्वारा कभरस्लिप वरिपरि थोरै मात्रामा निर्मलीकृत सलाइन पानी थप्नुपर्छ। परजीवीहरूलाई सलाइनमा छोड्नको लागि आन्द्रालाई सावधानीपूर्वक र छिटो च्यात्नुपर्छ। १०० µL माइक्रोपिपेट वा ट्युबरकुलिन सिरिन्ज प्रयोग गरेर, परजीवीहरूलाई सङ्कलन गर्नुहोस् र तिनीहरूलाई राम्रोसँग लेबल गरिएको कल्चर माध्यममा राख्नुहोस्।

इन भिट्रो लेशमेनिया प्रोमास्टिगोट्सको संवर्धन: पृथक गरिएका परजीवीहरूलाई सुरुमा SNB-9 ब्लड अगर स्लोपहरूमा (blood agar slopes) वा नोभी (Novy), म्याक नील, निकोल (Mc Neal, Nicolle - NNN) ठोस माध्यम [16] मा राखिन्छ जसलाई या त निर्मलीकृत अल्फा-MEM माध्यम [16, 65] वा M199 माध्यमले माथिबाट ढाकिन्छ, जसमध्ये प्रत्येकलाई निम्न तत्वहरू पूरकका रूपमा मिसाईएका हुन्छन १०% ताप-निष्क्रिय निर्मलीकृत भ्रूण बाछोको सीरम [FCS] (परजीवी वृद्धि बढाउन), १% BME भिटामिन, २% निर्मलीकृत मानव पिसाब (सिरिंज फिल्टर Filtropur® प्रयोग गरेर निर्मलीकृत गरिएको S ०.२ µm), २५० µg/mL amikacin (वा ५० µg/mL gentamicin, वा एन्टिबायोटिक र एमिनो एसिडको मिश्रण (L-glutamine २०० mM-penicillin १० ००० U-streptomycin १० mg/mL) [47]। तीन दिन पछि, यदि कुनै संक्रमण छैन भने, कल्चरहरूलाई राम्रोसँग तयार पारिएको फ्रिजिङ माध्यममा निलम्बन गरिन्छ र पछि -८०°C मा १ देखि २ वर्षको लागि वा दीर्घकालीन संरक्षण र भविष्यको प्रयोगात्मक प्रयोगको लागि -१९६°C तरल नाइट्रोजनमा मा भण्डारण गरिन्छ [7]।

४.५. लार ग्रन्थीहरू

भुसुनाको लार ग्रन्थीहरूको विच्छेदन भेक्टर-रोगजनक अन्तरक्रिया अध्ययन गर्ने एक आधारभूत प्रविधि हो, विशेष गरी फ्लेबोभाइरस (जस्तै, टोस्काना भाइरस) जस्ता आर्बोभाइरसहरू पत्ता लगाउनको लागि [44, 75]। भुसुनाको सानो आकारको कारणले गर्दा, यो प्रक्रियालाई स्टेरियोमाइक्रोस्कोपको सहायताले मसिना फोर्सेप्स वा माइक्रोडिसेक्शन सुईहरू प्रयोग गरेर नाजुक लार ग्रन्थीहरूलाई फुट्ने वा प्रदूषण नगरी छुट्याउन सकिन्छ (https://zenodo.org/records/18302850) [51, 61]। भरपर्दो डाउनस्ट्रीम आणविक विश्लेषण सुनिश्चित गर्न पूरै ग्रंथि जोगाउनु महत्वपूर्ण हुन्छ। एक पटक निकालेपछि, ग्रंथिहरूलाई एकरूप (होमोजेनाइज) बनाउन सकिन्छ र RT-PCR, qPCR, वा इम्युनोएसे मार्फत भाइरल RNA वा एन्टिजेनहरू पत्ता लगाउन परीक्षण गर्न सकिन्छ [12]। केवल आन्द्रा वा हेमोसिलमा मात्र नभएर लार ग्रन्थीहरूमा भाइरसहरूको उपस्थितिले परजीवीहरूले यसको बाह्य इन्क्युबेशन अवधि पूरा गरेको छ भन्ने पुष्टि गर्दछ र भुसुनाले रगत चुस्ने क्रममा उक्त रोगजनक स्वस्थ्य

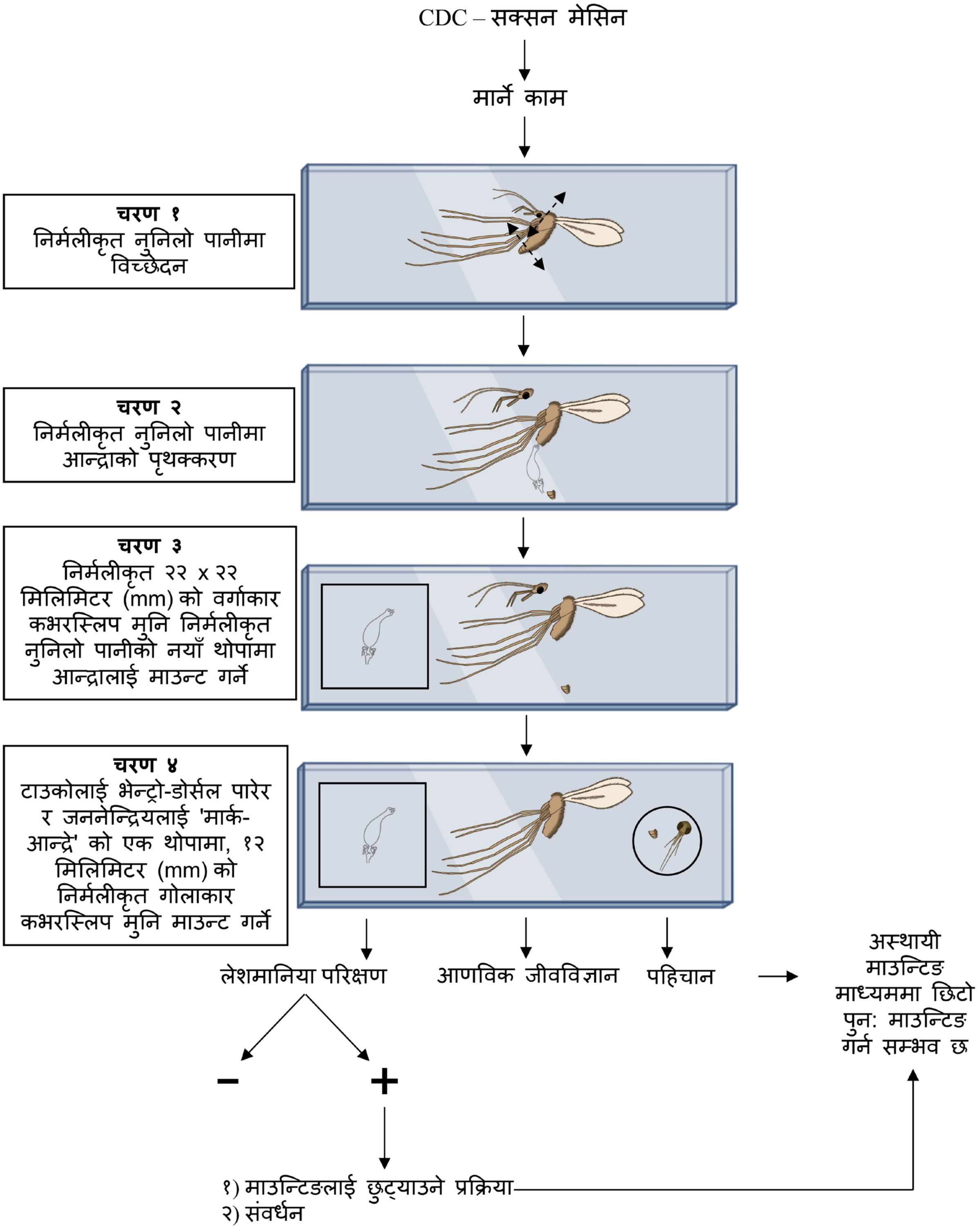

चित्र ५: लेशमेनिया पृथक्करण विधि।

ब्यक्तिमा प्रसारण गर्ने क्षमता राख्दछ [71]।

भुसुनाको लार ग्रन्थीहरूको सानो आकारको कारणले गर्दा विच्छेदन प्रक्रिया प्राविधिक रूपमा कठिन हुन्छ, भुसुनाको नमूना बिग्रनबाट बचाउन महत्त्वपूर्ण विशेषज्ञता चाहिन्छ [1, 51]। अझ, भाइरल भार कम भएकोबेला नेस्टेड PCR वा उच्च-थ्रुपुट अनुक्रमण [54] जस्ता अत्यधिक संवेदनशील पत्ता लगाउने विधिहरू प्रयोग गर्नु आवश्यक हुन्छ। प्रदूषित हुने जस्ता जोखिमहरूले निर्मलीकृत बनाउने प्रविधिहरूको आवश्यकतालाई अझ जोड दिन्छ। प्राविधिक अवरोधहरूभन्दा बाहिर, जैविक कारकहरूले पनि रोगजनक पत्ता लगाउने सफलतालाई प्रभाव पार्छन्; भेक्टर क्षमता भुसुना प्रजातिहरू बीच फरक फरक हुन्छ, र संक्रमण दरहरू पनि पारिस्थितिक र मौसमी अवस्थाहरूले गर्दा थपघट हुन्छन् [33, 61]।

लार ग्रन्थीहरूमा भाइरस पत्ता लगाउनाले प्रसारण जोखिमहरूमा महत्वपूर्ण अन्तर्दृष्टि प्रदान गर्दछ, लक्षित निगरानी र नियन्त्रण उपायहरू छनोट गर्न मद्दत गर्दछ [15]। उदाहरणका लागि, भुसुनामा टोस्काना भाइरस पहिचान गर्नाले स्थानीय क्षेत्रहरूमा निदान प्रोटोकल र सार्वजनिक स्वास्थ्य सल्लाहहरू [18] सूचित गर्न मद्दत गरेको छ। यसबाहेक, भाइरस-लार अन्तरक्रियाको अध्ययनले प्रसारण-अवरुद्ध गर्ने खोपहरू वा उपचारात्मक विकल्पहरूको लागि नयाँ लक्ष्यहरू प्रदान गर्न सक्छ [15, 18]।

भुसुनाको लार ग्रन्थिहरूलाई इम्युनोलोजिकल विधिहरू, विशेष गरी ELISA प्रयोग गरेर भुसुनाको लार विरुद्ध होस्ट एन्टिबडीहरू मापन गर्न एन्टिजेनको स्रोतको रूपमा पनि प्रयोग गर्न सकिन्छ। यो विधिले भुसुनाको टोकाइमा होस्ट एक्सपोजरको मूल्याङ्कनलाई गर्न सक्षम बनाउँछ, यसरी भेक्टर नियन्त्रण विधिहरूको प्रभावकारिताको [25] र लेशमेनिया प्रसारणको जोखिमको पनि मूल्याङ्कन गर्न सहयोग गर्दछ [40]।

४.६. रगतको स्रोतको पहिचान

क्याप्चरबाट अलग गरिएका रगत खाएका पोथीहरूलाई पारस्परिक-दूषित हुनबाट रोक्नको लागि एकल-प्रयोग उपकरण प्रयोग गरेर विच्छेदन गर्नुपर्छ। रगतको खाना पाचनको चरण मूल्याङ्कन गर्न स्टेरियोमाइक्रोस्कोपको सहायताले तिनीहरूको पेटको भाग जाँच गर्नुपर्छ। अण्डा बन्ने कुनै संकेत नदेखाउने, रातो, रातो-खैरो, वा गाढा रातो पेट भएका पोथीहरूलाई मात्र चयन गर्न सिफारिस गरिन्छ। सफा गरेपछि पोथीको स्वरूप वा प्रजाति पहिचान गर्न स्पर्माथिका सहित पेटको टुप्पो हटाउनुहोस्। त्यसपछि पेटको मुख्य भाग (स्पर्माथिका बिना) Eppendorf® ट्यूबहरूमा राख्नुपर्छ र थप विश्लेषण नभएसम्म -20°C मा भण्डारण गर्नुपर्छ। PNOC [5, 30, 50], CytB [67], वा COI [13] जस्ता रगतको खाना पहिचानको लागि सामान्यतया प्रयोग हुने आनुवंशिक मार्करहरू, राम्रोसँग स्थापित छन् र अन्य प्रकाशित लेखहरूमा व्यापक रूपमा वर्णन गरिएका छन्; त्यसैले, तिनीहरूलाई यस पेपरमा थप विस्तृत रूपमा वर्णन गरिने छैन (चित्र 6)। वैकल्पिक रूपमा, रगतको स्रोत पहिचान गर्न, MALDI-ToF पेप्टाइड म्यापिङ गर्न सकिन्छ [31]। यो प्रयोगात्मक रूपमा प्रदर्शन गरिएको छ कि यो प्रविधिले रगत खाएपछि लामो समय भित्र रगतको स्रोत पहिचान गर्न सक्षम बनाउँछ; त्यसकारण, विशेष गरी स्पष्ट रूपमा बढी उन्नत होस्ट रगत पाचन भएका रगत चुसेका पोथी भुसुनाहरूको विश्लेषणको लागि यो छनौटको उपयुक्त विधि हो । भुसुनाहरू आदर्श रूपमा -२०°C वा ४°C मा भण्डारण गरिनुपर्छ, तर छोटो समयको लागि कोठाको तापक्रममा भण्डारण गरिएका भुसुनाहरूबाट पनि राम्रो नतिजा प्राप्त गर्न सकिन्छ। इन्गोर्जड महिलाको पेट विश्लेषणको केही समय अघि शरीरको बाँकी भागबाट विच्छेदन गरिनुपर्छ र आसुत पानीमा एकरूप बनाउनुपर्छ। भुसुनाको शरीरको बाँकी भाग अन्य आणविक र रूपात्मक विश्लेषणहरूको लागि उपलब्ध रहन्छ। MALDI-ToF पेप्टाइड म्यापिङको लागि होमोजनेटबाट एलिकोट लिइसकेपछि, बाँकीलाई होस्ट रगत पहिचान पुष्टि गर्न र/वा लेशमानिया एसपीको उपस्थितिको लागि स्क्रिन गर्न डीएनए अलगावको लागि प्रयोग गर्न सकिन्छ। डीएनए-आधारित आणविक प्रविधिहरूको तुलनामा भुसुना तयारी र विश्लेषणको समग्र समय धेरै छोटो छ।

५. रूपात्मक अध्ययनको लागि भुसुना प्रशोधन (चित्र ३, ६, ७ र ८; परिशिष्ट १, २, ३ र ४)

यस खण्डले केवल आकारविज्ञान अध्ययनको लागि माउन्ट गर्नको लागि भुसुनाको नमूना तयार गर्ने सिद्धान्तहरूको रूपरेखा प्रस्तुत गर्दछ, त्यसपछि आकारविज्ञानभन्दा बाहिरका अनुप्रयोगहरूको लागि अनुकूलन गरिन्छ। यद्यपि, यो पद्धति बुझ्नु महत्त्वपूर्ण छ, किनकि यसले आवश्यक पर्दा विशिष्ट भुसुना प्रकारहरूको लागि प्रक्रियाहरूलाई अनुकूलित गर्न सक्षम बनाउँछ।

उपचारमा लचिलो रबर बल्बहरू युक्त पाश्चर पिपेट्स प्रयोग गरेर क्रमिक खाली गर्ने र भर्ने चरणहरू समावेश छन्। गोलो तल भएका शीशाका कन्टेनरहरूलाई कडाइका साथ सिफारिस गरिन्छ किनभने तिनीहरूले यी कार्यहरूलाई धेरै सहज बनाउँछन्। शीशा सबै अभिकर्मकहरूको लागि निष्क्रिय हुन्छ। अभिकर्मक वाष्पीकरण रोक्नको लागि र भुसुनाहरूलाई धुलो पर्नबाट जोगाउँन, कन्टेनरहरूमा ढक्कनहरू जडान गर्नुपर्छ र कहिल्यै पनि ओभरफिल गर्नु हुँदैन, जसले गर्दा बन्द गर्दा वा खोल्दा ओभरफ्लो होस्। सफा गर्ने र प्रशोधन गर्न आवश्यक रसायनहरू तालिका २ मा देखाइएको छ।

५.१. सफा गर्ने

भुसुनाहरूलाई स्थायी स्लाइड माउन्टको रूपमा तयार गर्नु अघि, तिनीहरूलाई पारदर्शी बनाउनको लागि उपयुक्त विधि र क्लियरिङ्ग एजेन्ट (जस्तै, एसिटिक एसिड १०% घोल वा मार्क-एन्ड्रे घोल सहित क्लोरल हाइड्रेट, जुन धेरै देशहरूमा प्रतिबन्धित रसायन हो) प्रयोग गरेर म्यासेरेसनद्वारा सफा गर्नुपर्छ। क्लियरिङ्ग प्रक्रियाले शरीरका तन्तुहरू, बोसो,

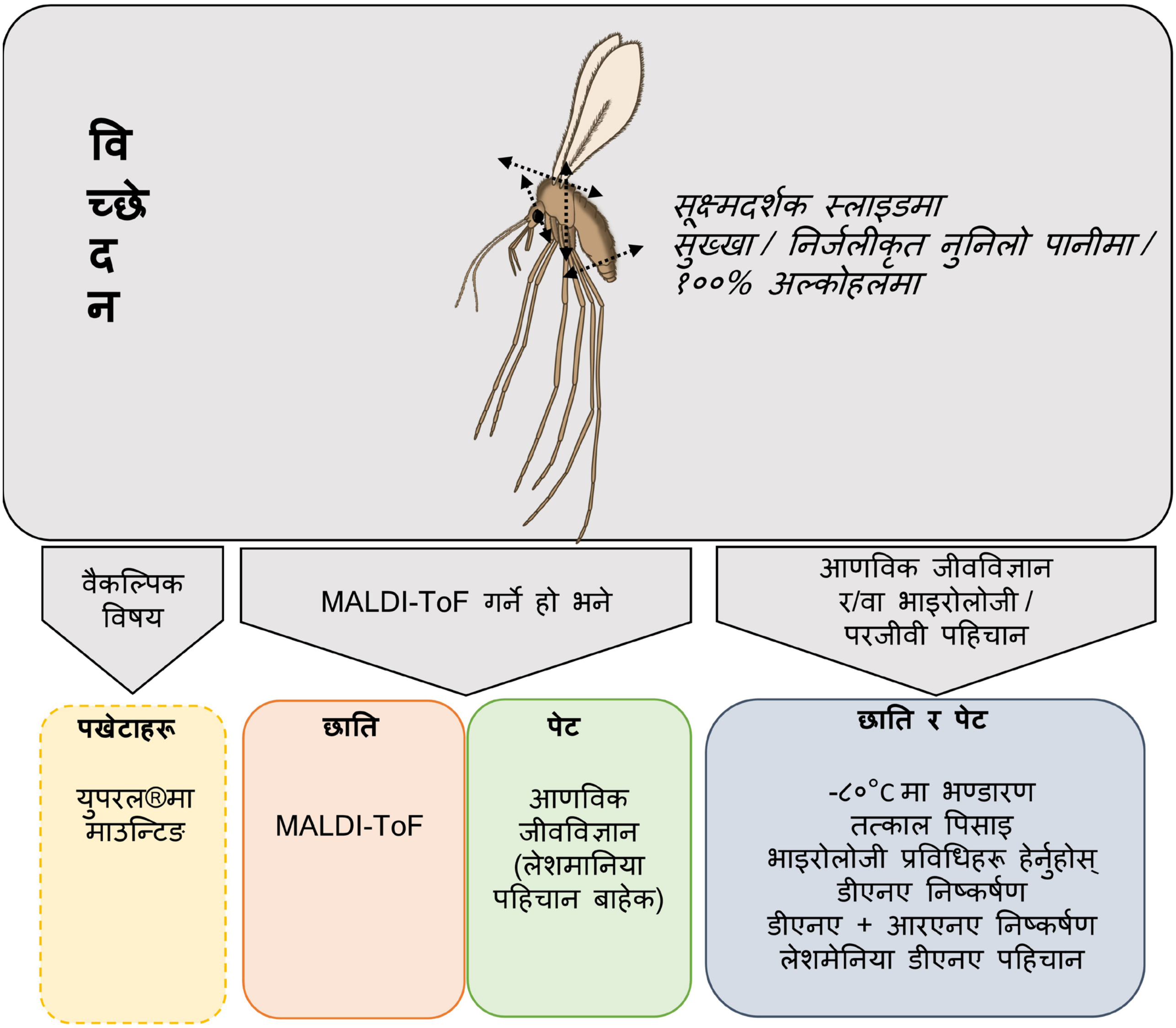

चित्र ६: आणविक जीवविज्ञान, प्रोटियोमिक्स, र/वा भाइरोलोजी अनुप्रयोगहरूको लागि भुसुना प्रशोधन।

स्रावहरू र मोमलाई हटाउँछ, भुसुनालाई पारदर्शी बनाउँछ, जसले गर्दा एक्सोस्केलेटल संरचनाहरू (जस्तै, सेटे सम्मिलन), सतहका विशेषताहरू (जस्तै, रंग) र टेग्युमेन्ट (जस्तै, स्पर्माथिका) मार्फत देखिने आन्तरिक विशेषताहरूको जाँच गर्न सजिलो हुन्छ।

दुई-चरण सफा गर्ने प्रक्रिया, जसमा पहिले बलियो क्षार (जस्तै पोटासियम हाइड्रोक्साइड) प्रयोग गरिन्छ, त्यसपछि कमजोर एसिड (जस्तै मार्क-एन्ड्रे घोलमा एसिटिक एसिड) प्रयोग गरिन्छ, यसले विशिष्ट जैव रासायनिक उद्देश्यहरू पूरा गर्दछ [74]। क्षारले प्रोटीन, बोसो र मांसपेशीहरू जस्ता नरम तन्तुहरूलाई सेपोनिफिकेशन र प्रोटीन डिनेचुरेशन मार्फत तोड्छ, जसले संरचनात्मक स्पष्टताको लागि चिटिन एक्सोस्केलेटनलाई अक्षुण्ण छोड्छ। त्यसपछिको कमजोर एसिडले कुनै पनि बाँकी रहेका क्षारलाई बेअसर गर्छ, थप क्षयलाई रोक्छ, र पारदर्शिता बढाउन चिटिनलाई ब्लीच गर्छ [74], यद्यपि १५ मिनेटको लागि आसुत पानीमा दुई पटक भुसुनाहरूलाई धोएर क्षारलाई बेअसर गर्न पर्याप्त हुन सक्छ। यो क्रमिक उपचारले असरदार रूपमा तन्तु हटाउने र आवश्यक भागहरू संरक्षित गरि सूक्ष्म परीक्षणको लागि उपयुक्त अखण्ड भुसुनाको उपलब्धता सुनिश्चित गर्दछ।

अर्को चरणमा जानु अघि आसुत पानीमा दुई पटक २० मिनेट सम्म पखाल्न सिफारिस गरिन्छ।

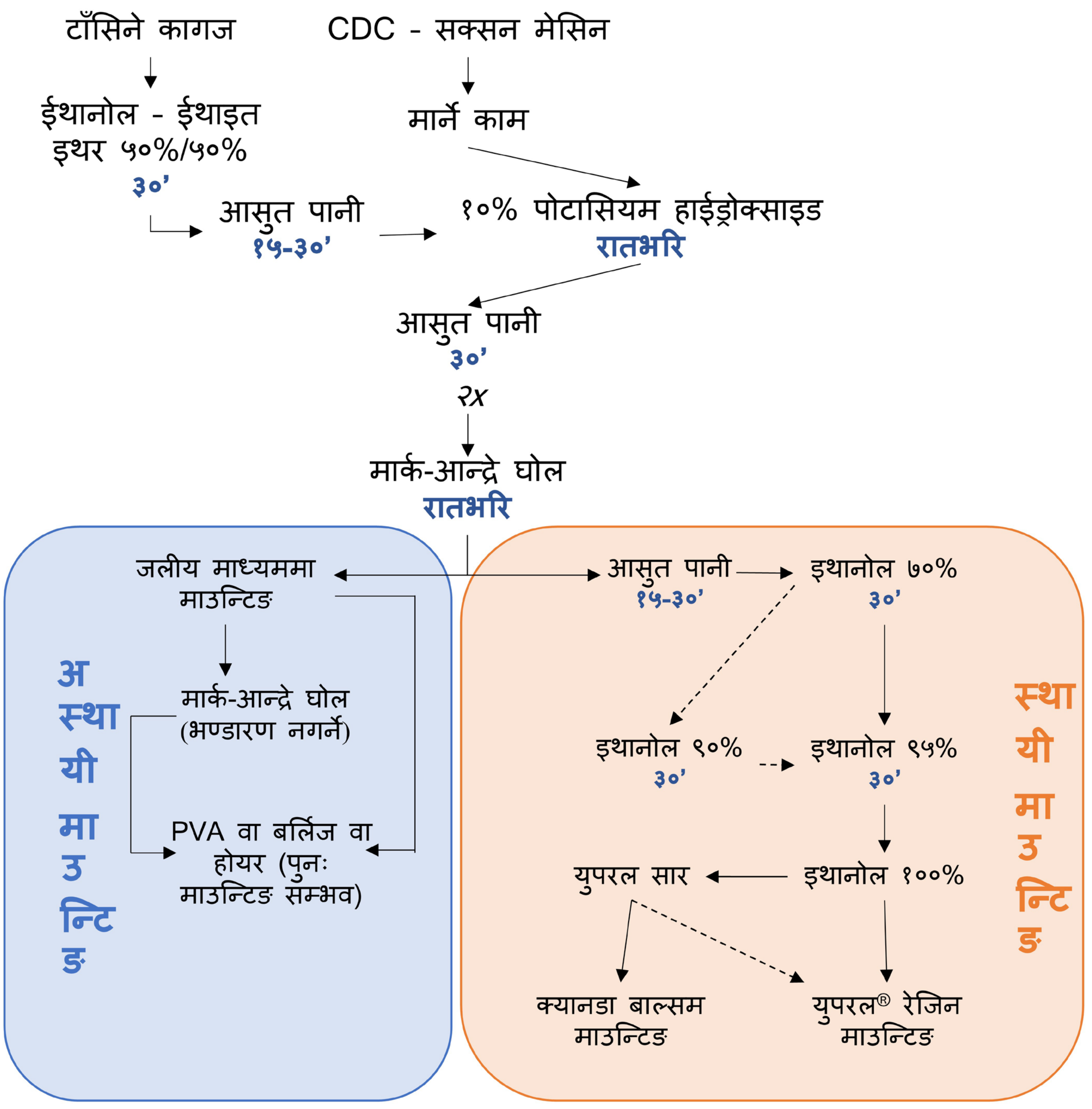

चित्र ७: भुसुना प्रशोधन गर्ने पारम्परिक विधि।

५.१.१. नरम तन्तुको विघटन (चित्र ८)

सोडियम हाइड्रोक्साइड (NaOH) वा पोटासियम हाइड्रोक्साइड (KOH) सामान्यतया प्रयोग हुने रासायनिक म्यासेरेटिङ एजेन्टहरू हुन्, जुन भुसुनाहरूको आकार र नाजुकताको आधारमा फरक सांद्रता र अवधिमा प्रयोग गरिन्छ। मानक र सबैभन्दा प्रभावकारी प्रविधिमा भुसुनालाई बलियो क्षारमा (१०% KOH वा NaOH) रातभर भिजाएर नरम तन्तुहरूलाई भिजाएर राख्नु समावेश गरिएको छ। उपचार अवधि घटाउन (अर्थात्, ६ घण्टाको लागि KOH २०%) साथै ३७°C मा तताउन र सांद्रता बढाउन सकिन्छ।

५.१.२. रंगाएर वा नरंगाई पारदर्शी बनाउने प्रक्रिया

यो चरण पछि लाइटनिङ उपचार गरिन्छ, जसमा सामान्यतया एसिटिक एसिड र क्लोरल हाइड्रेट (जस्तै, मार्क-एन्ड्रे घोल) मिलाइन्छ। सफा गरिसकेपछि, अवशिष्ट रसायनहरू हटाउनको लागि भुसुनाहरूलाई कम्तिमा दुईवटा लगातार २० मिनेट पानीले राम्ररी पखाल्नुपर्छ।

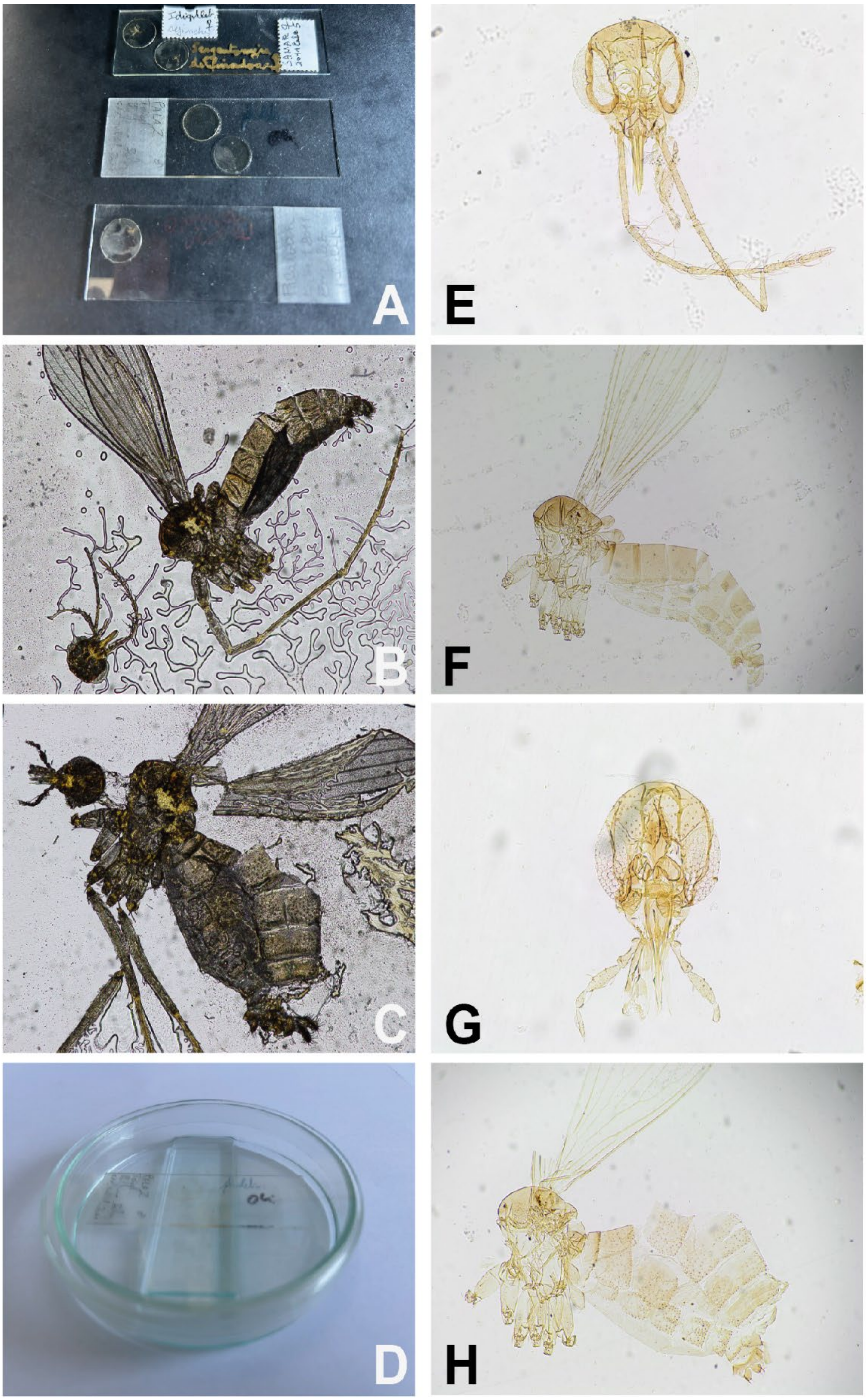

चित्र ८: स्लाइड पुन: माउन्टिंग। A: होयरमा जडान गरिएका क्षतिग्रस्त र सुकेका स्लाइडहरू; B: सुकेको भुसुनाको सूक्ष्म दृश्य; C: अर्को क्षतिग्रस्त भुसुनाको सूक्ष्म दृश्य; D: सुकेको स्लाइड भएको भिजेको कोठा; E: टाउको, र F: युपरल® मा पुन: माउन्ट गरेपछि भुसुना B को शरीर; G: टाउको, र H: युपरल® मा पुन: माउन्ट गरेपछि क्षतिग्रस्त भुसुना C को शरीर।

तालिका २: प्रयोग गरिएका अभिकर्मकहरूको संरचना।

**पोटासियम हाइड्रोक्साइड १०%**	**आसुत पानीमा १% फुचसिन एसिड**
पोटासियम हाइड्रोक्साइड १० ग्राम	एसिड फुचसिन (पाउडरको रूपमा) १ ग्राम
आसुत पानी क्यूएस १०० मिलिलिटर	आसुत पानी ९९ मिलिलिटर
**गम क्लोरल माउन्टिङ मिडिया (होयर मिडिया)**	**मार्क-आन्द्रेको घोलमा एसिड फुचसिनले रंग लगाइएको**
आसुत पानी ५० मिलिलिटर	मार्क-आन्द्रे घोल १० मिलीलीटर
क्लोरल हाइड्रेट २०० ग्राम	फुचसिन १% ५० µL
गम अरबीक ५० ग्राम	
ग्लिसरॉल २० एमएल	
**मार्क-आन्द्रे घोल**	**ईनिस (Enecê)**
क्लोरल हाइड्रेट ४० ग्राम	शुद्ध सेतो कोलोफोनी २२ ग्राम
ग्लेसियल एसिटिक एसिड ३० मिली	अल्कोहलमा घुलनशील कोपल गम १२ ग्राम
आसुत पानी ३० मिलिलिटर	अतिशुद्द इथेनॉल २० एमएल
	कपूर १० ग्राम
	टर्पेन्टाइन एसेन्स १० एमएल
	युकेलिप्टोल २६ एमएल

मार्क-एन्ड्रे घोल, सामान्यतया भुसुनाको नमूना तयार गर्न प्रयोग हुने क्लियरिङ्ग एजेन्ट हो। यसको प्रभावकारिता पखेटा र एन्टेना जस्ता कमजोर संरचनाहरूमा उल्लेखनीय क्षति कम गर्दै क्लियरिङ्ग प्रक्रियालाई सहज बनाउनमा निहित छ।

वाष्पीकरण वा क्षय रोक्नको लागि घोललाई ताजा रूपमा तयार पारिएको वा राम्ररी बन्द गरिएको कन्टेनरमा भण्डारण गर्नुपर्छ। मार्क-एन्ड्रे घोलको प्रयोग विशेष रूपात्मक विवरणहरू बढाउन उज्यालो पार्ने वा रंगाउने प्रविधिहरूसँग मिलाउँदा विशेष रूपमा फाइदाजनक हुन्छ। यसको संरचना र तयारीको विवरण परिशिष्ट २ मा दिइएको छ।

अत्यधिक पारदर्शी भुसुनाहरूको लागि, माउन्ट गर्नु अघि दृश्यता सुधार गर्न रंगाउन आवश्यक हुन सक्छ। धेरै रंगहरू उपलब्ध छन्, र प्रत्येकले जीवको विशिष्ट रासायनिक घटकहरूलाई लक्षित गर्दछ। भुसुना र छनौट गरिएको माउन्टिंग माध्यम दुवैसँग मिल्दो रंगाउने माध्यम चयन गर्नु महत्त्वपूर्ण छ। यो आधारभूत विधिलाई आवश्यकता अनुसार अनुकूलित गर्न सकिन्छ, उदाहरणका लागि, रंगाउनको लागि मार्क-एन्ड्रे घोलमा ०.१% एसिड फुचसिन समावेश गरेर। थप रूपमा, जलीय घोलमा संरक्षित र रेजिनस माउन्टहरूको लागि अभिप्रेरित भुसुनाहरूलाई निर्जलीकरण आवश्यक पर्दछ (डिहाइड्रेसनमा खण्ड ५.२ हेर्नुहोस्), किनकि धेरैजसो प्राकृतिक र सिंथेटिक रेजिन माउन्टिंग मिडिया पानीसँग असंगत हुन्छन्। न्यू (१९७४) ले उल्लेख गरेको छ कि केही रंगाउने पदार्थहरू निश्चित माउन्टिंग मिडियामा बिग्रन सक्छन् [53]। उदाहरणका लागि, क्यानाडा बाल्समसँग सामान्यतया प्रयोग हुने एसिड फुचसिन, युपरल® मा पनि फिक्स गर्न सकिन्छ। यद्यपि, एसिड फुचसिन-रंग गरिएका भुसुनाहरू फिक्का हुने सम्भावना हुन्छ, विशेष गरी जब अन्तिम सफा गर्ने तरल पदार्थको रूपमा प्रयोग हुने ल्वाङको तेलको अवशेषहरू रहन्छन्। ल्वाङको तेलमा भण्डारण गरिएका भुसुनाहरूले केही दिन भित्र फिक्का देखिन सक्छन्।

५.२. डिहाइड्रेसन

निर्जलीकरण क्रमशः भुसुनाहरूलाई ग्रेडेड इथेनॉल घोल शृङ्खला मार्फत स्थानान्तरण गरेर गरिन्छ: ५०%, ७०%, ८०%, ९०%, वा ९५% र अन्तमा १००%, प्रत्येक बाथ कम्तिमा २० मिनेटसम्म रहन्छ। इथेनॉल चाँडै वाष्पीकरण हुने भएकोले, प्रशोधनको क्रममा कन्टेनरलाई राम्ररी बन्द गर्नुपर्छ। एक पटक भुसुना पूर्ण रूपमा निर्जलित भएपछि तपाईंले Euparal® एसेन्समा केही दिनको लागि राख्न सक्नुहुन्छ, जुन ल्वाङको तेल भन्दा राम्रो हुन्छ। यस

उद्देश्यको लागि एक पटक व्यापक रूपमा प्रयोग गरिएको बिच क्रियोसोट, अब यसको विषाक्तताको कारण पूर्ण रूपमा प्रतिबन्धित छ।

निर्जलीकरण प्रक्रियाले भुसुना भित्रको तरल पदार्थ माउन्टिंग माध्यमसँग उपयुक्त छ भनी सुनिश्चित गर्नुपर्छ ताकि अस्पष्टता, ओस्मोटिक पतन, वा विकृति रोक्न सकियोस् जसले भुसुनालाई वर्गीकरण र पहिचानको अध्ययनको लागि अनुपयुक्त बनाउन सक्छ।

५.३. माउन्टिङ मिडिया

५.३.१. भुसुनाको नमुना तयारीको लागि छनोट र प्रयोग

माउन्टिङ माध्यमको अपवर्तन सूचकांक आदर्श रूपमा शीशाको जतिको हुनुपर्छ, जुन लगभग १.५ हुन्छ। यो रंगहीन, स्पष्ट हुनुपर्छ, र सुकेपछि र समयसँगै पूर्ण रूपमा पारदर्शी रहनुपर्छ। यो प्रयोग गरिएका दागहरूसँग मिल्दो हुनुपर्छ र भुसुनाको सबै तन्तुहरूमा प्रवेश गर्न र फैलाउन सक्षम हुनुपर्छ। यो धेरै छिटो सुक्नु हुँदैन वा माउन्टिङ गर्दा धुंधला बन्नु हुँदैन। माउन्टिङ पछि यो संकुचित हुनु हुँदैन। उपयुक्त माउन्टिङ माध्यमको छनोट भुसुना तयारीको आधारभूत पक्ष हो, किनकि कुनै पनि एकल माध्यम सबै उद्देश्यका लागि आदर्श हुँदैन। छनोटले धेरै मुख्य कारकहरूलाई सन्तुलनमा राख्नुपर्छ:

- **अप्टिकल गुणहरू** - माउन्टिंग माध्यमको अपवर्तन सूचकांकले वर्गीकरण पहिचान वा रूपात्मक विवरणको लागि प्रयोग हुने महत्वपूर्ण शारीरिक विशेषताहरूको पर्याप्त कन्ट्रास्ट र अपवर्तन प्रदान गर्नुपर्छ, जस्तै स्पर्माथिका, एस्कोइड्स, न्यूस्टेड सेन्सिला, ठाडो सिबेरियल दाँत, र ग्रसनी दाँत। यी संरचनाहरूको दृश्यता सिधै माउन्टिंग माध्यमको अप्टिकल गुणहरूमा निर्भर गर्दछ।

- **संरक्षण** - स्थायी सङ्कलनको लागि अभिप्रेरित प्रकारका भुसुनाहरू वा सामग्रीहरूको लागि, माध्यमले दीर्घकालीन स्थिरता र स्थायित्व प्रदान गर्नुपर्छ। यसको विपरीत, सूची अध्ययन वा महामारी विज्ञान सर्वेक्षणहरूको लागि, जहाँ दीर्घकालीन संरक्षण कम महत्त्वपूर्ण हुन्छ, अस्थायी वा अर्ध-स्थायी माउन्टिङ मिडिया पर्याप्त हुन सक्छ।

५.३.२. माउन्टिङ मिडियाका लागि आवश्यकताहरू

विशेषज्ञहरूले प्रायः विशिष्ट अनुसन्धान आवश्यकताहरू अनुरूप अनुकूलित र जटिल माउन्टिङ प्रविधिहरू विकास गर्छन्। यद्यपि, यी विधिहरूले प्रायः अभिलेखीय गुणस्तर, अनुकूलता, मानकीकरण, वा ह्यान्डलिङको सहजता र दीर्घकालीन संरक्षण जस्ता पक्षहरूलाई बेवास्ता गर्छन्। मानकीकरणको यो अभावले दान गरिएको संग्रह र दीर्घकालीन क्युरेसन प्रयासहरूको एकीकरणलाई जटिल बनाउँछ।

वैज्ञानिक अनुप्रयोगहरूले माउन्टिङ मिडियाको लागि फरक आवश्यकताहरू लागू गर्छन्। वर्गीकरणविद्हरूले प्रायः सम्पूर्ण भुसुनाहरू माउन्ट गर्छन् र क्युटिक्युलर संरचनाहरूको दृश्यता बढाउन भित्री अंगहरूलाई बिस्तारै म्यासेरेट गर्ने मिडियालाई प्राथमिकता दिन्छन्। अप्टिकल स्पष्टता अधिकतम बनाउन अपवर्तक सूचकांक भुसुना र शीशाको स्लाइड भन्दा पर्याप्त फरक हुनुपर्छ। व्यावसायिक माउन्टिङ मिडियाको अपवर्तक सूचकांक सामान्यतया स्लाइड-माउन्टिङ माध्यम-कभरस्लिप प्रणाली मार्फत प्रकाश अपवर्तन र छरपस्टलाई कम गर्न शीशाको अपवर्तक सूचकांकसंग मिल्दो हुने गरी तयार गरिन्छ। यद्यपि, ब्राइटफिल्ड माइक्रोस्कोपीमा, नरंगाइएको भुसुनाको प्राकृतिक कन्ट्रास्टलाई जानाजानी भुसुनाको भन्दा अलि फरक अपवर्तक सूचकांक भएको माउन्टिंग माध्यम छनौट गरेर हेरफेर गर्न सकिन्छ, जसले गर्दा पृष्ठभूमिमा यसको दृश्यता सुधार हुन्छ।

५.३.३. माउन्टिङ मिडियाका प्रकारहरू (तालिकाहरू ३ र ४)

स्लाइड, माध्यम र भुसुनाको नमुना मार्फत प्रकाश कसरी बांगिन्छ भनेर निर्धारण गर्न माइक्रोस्कोपीलाई माउन्टिङ माध्यमको अपवर्तक सूचकांक (RI) आवश्यक पर्दछ। जब RI कभरस्लिप गिलास (≈≠५१५) सँग नजिकबाट पङ्क्ति बद्ध हुन्छ, प्रकाश समान रूपमा जान्छ, स्क्याटरिङ र अप्टिकल विकृतिहरू घटाउँछ, जसले गर्दा राम्रो संरचनाहरूको रिजोल्युसन र दृश्यतामा सुधार हुन्छ। यसको विपरीत, RI नमिल्दा चित्र धमिलो, वरिपरि घेरा (halo) देखिन सक्छ, वा रंग नलगाईएका संरचनाहरू अस्पष्ट हुन सक्छन्। विभिन्न मिडियाको विशिष्ट RI को कारणले दिइएको भुसुनाको लागि कन्ट्रास्ट, स्पष्टता, र समग्र छवि गुणस्तर अनुकूलन गर्न सही माउन्टिङ माध्यमको चयन महत्त्वपूर्ण हुन्छ।

स्लाइड माउन्टिङको लागि भुसुना तयार गर्दा मसिना संरचनाहरू कति राम्रोसँग देख्न सकिन्छ भन्ने कुरामा

माउन्टिङ माध्यमको अपवर्तन सूचकांकले महत्वपूर्ण प्रभाव पार्छ। भुसुनाहरूको नाजुक र हल्का स्क्लेरोटाइज्ड विशेषताहरू, जसमा सिबेरियल आर्मेचर, स्पर्माथिका, एन्टेनाको खण्डहरू, र पखेटाको भेनेसन समावेश छ, उच्च अपवर्तन सूचकांक माउन्टिङ माध्यममा अवलोकन गर्न गाह्रो हुन सक्छ।

भुसुनाका लागि, सामान्यतया प्रयोग हुने विकल्पहरूमा पानीमा आधारित माउन्टिङ मिडियाको रूपमा गम-क्लोरल मिडिया, र विलायक-आधारित माध्यमको रूपमा क्यानडा बाल्सम र एनेस - नेल्सन सेर्केरा (एनसी) (Enecê - Nelson Cerqueira (NC)) रेजिन समावेश छन्। रॉलिन्स [60] ले माउन्टिङ मिडियालाई दुई प्रकारमा वर्गीकृत गरे: (1) स्थायी मिडिया: यी समयसँगै कडा हुन्छन् र दीर्घकालीन संरक्षणको लागि उपयुक्त हुन्छन्, र (2) अर्ध-स्थायी मिडिया: यी कडा सेट हुँदैनन् र सामान्यतया अस्थायी उद्देश्यका लागि प्रयोग गरिन्छ।

माउन्टिङ मिडिया तरल, गम-आधारित, वा रेजिनस हुन सक्छ, पानी, अल्कोहल वा अन्य विलायकहरूमा घुलनशील (जस्तै, टोल्युइन, जाइलिन) (तालिका ३)। एक पटक प्रयोग गरिसकेपछि, तिनीहरूलाई वायुमण्डलीय प्रभावहरूबाट बचाउन अघुलनशील सिलिंग मिडिया प्रयोग गरेर सिल गर्नुपर्छ। माउन्टिङ मिडियाका प्रकारहरू बीच स्पष्ट रूपमा छुट्याउन, निम्न रूपमा वर्गीकरण गर्न सकिन्छ:

क. जलीय माध्यम - यी मिडियाहरू पानीमा सजिलै घुल्छन्, जसले गर्दा तिनीहरूलाई अस्थायी वा अर्ध-स्थायी माउन्टहरूको लागि उपयुक्त बनाउँछ। तिनीहरू सामान्यतया ह्यान्डल गर्न सजिलो हुन्छन्, तर वायुमण्डलीय आर्द्रता (जस्तै, गम-क्लोरल मिडिया र पोलिभिनाइल अल्कोहल) को सम्पर्कबाट बच्नको लागि सिलिङको आवश्यकता पर्न सक्छ, विशेष गरी उष्णकटिबंधीय आर्द्र मौसममा।

ख. सीमित पानी सहन सक्ने माध्यमहरू - यी माध्यमहरू पानीबाट कम प्रभावित हुन्छन्, तर अझै पनि अत्यधिक आर्द्रताबाट सुरक्षा चाहिन्छ। तिनीहरूले पानीमा घुलनशील विकल्पहरूको तुलनामा लामो समयसम्म स्थिरता प्रदान गर्छन् र प्रायः अर्ध-स्थायी माउन्टहरूमा प्रयोग गरिन्छ।

ग. हाइड्रोकार्बन-घुलनशील माध्यम - यी मिडियाहरू जाइलिन वा टोल्युइन, वा essenecê (enecê विलायक) जस्ता जैविक विलायकहरूमा घुलनशील हुन्छन्। तिनीहरू स्थायी माउन्टिंगको लागि डिजाइन गरिएका छन् र उत्कृष्ट दीर्घकालीन स्थिरता प्रदान गर्छन्, र आर्द्रता र क्षयीकरण रोक्छन्, जसले गर्दा तिनीहरूलाई अभिलेखीय उद्देश्यका लागि उपयुक्त मानिन्छन् (जस्तै, तटस्थ क्यानाडा बाल्सम)।

संक्षेपमा, पानीमा घुलनशील मिडिया अस्थायी माउन्टहरू वा सजिलै भुसुना हटाउन आवश्यक पर्ने केसहरूको लागि उत्तम हुन्छ; सीमित पानी-सहनशील मिडिया मध्यम टिकाउपन चाहिने अर्ध-स्थायी माउन्टहरूको लागि उपयुक्त हुन्छ, र अभिलेखीय र दीर्घकालीन भण्डारणको लागि बनाइएको स्थायी माउन्टहरूको लागि हाइड्रोकार्बन-घुलनशील मिडियालाई प्राथमिकता दिइन्छ।

५.३.४. सिफारिस गरिएको माउन्टिङ मिडियाको विवरण (तालिका ३ र ४)

अस्थायी अवलोकनको लागि मिडिया

क्लोरल गम = होयर तरल पदार्थ/मध्यम/घुलन (आरआई = १.४८)

मार्क एन्ड्रे घोल छोटो अवधिको लागि (केही घण्टा, सायद केही घण्टा यदि स्लाइड आर्द्र कक्षमा भण्डारण गरिएको छ भने) स्पर्माथिका अवलोकनको लागि उत्तम माध्यम हो, जसमा फोटोहरू (चित्र ४) वा रेखाचित्रहरू समावेश छन्। अवलोकन गरिएको स्पर्माथिका संरक्षण गर्न मध्यम अवधिको भण्डारण गर्न सकिने जलीय माध्यममा पुन: माउन्ट गर्न आवश्यक हुन्छ। रेजिनमा पुन: माउन्ट गर्नको लागि तिनीहरूलाई निर्जलीकरण गर्नु असम्भव नभए पनि सिफारिस गरिएको छैन (क्षतिको जोखिम)। क्लोरल गम र होयर फ्लुइडलाई पर्यायवाची मानिन्छ। यो माध्यम सामान्यतया यसको पानीको अनुकूलता, सरलता, द्रुत प्रयोग, र अपवर्तक सूचकांकको कारणले आन्तरिक अंगहरू अवलोकन गर्न प्रयोग गरिन्छ जसले स्पर्माथिका जस्ता नाजुक संरचनाहरूको परीक्षणलाई सहज बनाउँछ। यद्यपि, यदि पूर्ण रूपमा तयार पारिएको छैन वा नियन्त्रित आर्द्रता अवस्थाहरूमा भण्डारण गरिएको छैन भने क्लोरोल गममा महत्त्वपूर्ण कमजोरीहरू छन्। क्रिस्टलाइजेसन, विकृति, र चिपचिपापन यसका प्रमूख समस्याहरू हुन। कभरस्लिपमा घेरा लगाउनाले यी समस्याहरू समाधान गर्दैन, किनकि घेरा लगाउने मिश्रणसंग माउन्ट गर्ने माध्यमको अन्तरक्रियाको कारणले माउन्टिंग मिडिया भारी रूपमा फिक्का (कहिलेकाहीं लगभग कालो) हुन सक्छ, विशेष गरी यदि युपरल® प्रयोग गरिएको छ भने।

तालिका ३: चयन गरिएका माउन्टिङ मिडियाको संरचना।

माउन्टिङ माध्यम	घोलक	सम्भावित पूर्व-पोलिमर(हरू) वा पोलिमर	टिप्पणीहरू
होयर = क्लोरल गम	ग्लिसरॉल, पानी	गम अरबिकका यौगिकहरू	म्यासेरेटिङ एजेन्ट: क्लोरल हाइड्रेट
CMCP-9 (= कार्बोक्सी मिथाइल सेलुलोज फिनोल)	पानी (CMCP-9: ५१–६०%)	पूर्ण रूपमा हाइड्रोलाइज्ड पोलिभिनिल अल्कोहल (CMCP-9: ०–५%)	CMC(P)-9: कम चिपचिपाहट: उच्च चिपचिपाहट
DMHF (डाइमिथाइल हाइडेन्टोइन फॉर्मल्डिहाइड)	पानी	N,N′-डाइमिथाइलोल डाइमिथाइल हाइडान्टोइन (डाइ-मिथाइलोल DMH)<br>इथर-/मिथिलिन-ब्रिज्ड ओलिगोमरहरू<br>क्रसलिङ्क गरिएको DMH-फॉर्मल्डिहाइड पोलिमर नेटवर्क	
क्यानडा बाल्सम	जाइलिन; बाल्समका आंशिक रूपमा वाष्पशील घटकहरू (Δ^3-carene, levopimaric acid, limonene, myrcene, palustric acid, β-phellandrene, α-pinene, β-pinene)	balsam (abienol, abietic एसिड, isopimaric एसिड, Sandaracopimaric एसिड)	तटस्थीकरण: पोटासियम कार्बोनेट; *Abies balsamea* बाट निकालिएको रेजिन (Linné, 1758)
युपरल®	युकेलिप्टोल, प्याराल्डिहाइड; गम स्यान्डाराकका आंशिक रूपमा वाष्पशील घटकहरू (लिमोनेन, α-पाइनेन, β-पाइनेन)	गम स्यान्डाराकका यौगिकहरू (कम्युनिक एसिड, मानूल, पोलिकम्युनिक एसिड, स्यान्डाराकोपिमरिक एसिड, १२-एसिटोक्सी-स्यान्डाराकोपिमरिक एसिड, सुगियोल, टोरुलोसिक एसिड, टोरुलोसोल, टोटारोल)	सफा गर्ने एजेन्ट: मिथाइल सैलिसिलेट; युपरल® हरियो रंगमा: तामाको नुन (तामाको अबिएटिनेट); टेट्राक्लिनिस आर्टिकुलाटा (वाहल, १७९१) बाट स्यान्डाराक रेजिन (Vahl, 1791)
ईनिस (Enecê)	इथाइल अल्कोहल; कपूर, युकेलिप्टोल र टर्पेन्टाइन एसेन्स सहित	कोपल गम र कोलोफोनी (रोसिन) का यौगिकहरू	

होयर माध्यमलाई फ्लेबोटोमाइन भुसुनाको लागि अप्टिकली उत्तम मानिएको छ र परम्परागत रूपमा यी उद्देश्यका लागि प्रयोग गरिएको छ। माध्यममा गम अरेबिक, ग्लिसरॉल र क्लोरल हाइड्रेट सहित सम्बन्धित मिश्रणहरू हुन्छन्। विभिन्न मिश्रणहरूको गलत व्याख्या र गलत उद्धरण गरिएको छ [74]।

भुसुनाहरूमा स्पर्माथिका अवलोकन गर्न होयर राम्रो माध्यम भएतापनि, यो दीर्घकालीन संरक्षणको लागि उपयुक्त छैन। यो फोटोग्राफ, रेखाचित्र, वा छविहरू सहित छोटो अवधिको अवलोकनको लागि उपयुक्त हुन्छ। जलीय माध्यम अस्थायी माउन्टहरूको लागि उपयुक्त छ तर दीर्घकालीन संरक्षण सुनिश्चित गर्न सक्दैन। यसको विपरित, रेजिन माउन्टिंगले उत्कृष्ट स्थायित्व प्रदान गर्दछ जुन प्रायः शताब्दीयौंसम्म रहन्छ, तर तिनीहरूको रिफ्रिन्जेन्स बारम्बार हराउने हुँदा स्पर्माथिकाको सूक्ष्म विवरणहरूलाई प्रष्ट देखाउन सक्दैन।

तालिका ४: विभिन्न व्यक्तिहरूद्वारा माइक्रोस्कोप स्लाइडहरू र अप्रकाशित अवलोकनहरूको बारेमा चयन गरिएका माउन्टिङ मिडियाका फाइदा र बेफाइदाहरू [52]।

नाम	फाइदाहरू	बेफाइदाहरू
* क्यानडा बाल्सम	यो माध्यम अत्यन्तै टिकाउ छ, जसको आयु १५० वर्षभन्दा बढी छ।<br>स्लाइडहरू ल्वाङको तेल वा फिनोललाई माउन्टिङ एजेन्टको रूपमा प्रयोग गरेर माउन्ट गर्न सकिन्छ।	हानिकारक तत्वहरू हुन्छन् र हुड मुनि ह्यान्डल गर्नुपर्छ।<br>पूर्ण, समय खपत गर्ने निर्जलीकरण श्रृंखला आवश्यक छ।<br>इथानोलको निर्जलीकरण र जाइलिन वा ल्वाङको तेल मार्फत स्थानान्तरणले केही ट्याक्सालाई टुक्र्याउन सक्छ; विकल्पहरू (जस्तै, आइसोप्रोपानोल, एन-बुटानोल, सेलोसोल्भ™, १,४-डाइअक्सेन, हिस्टोक्लेयर, टेरपिनोल) ले टुक्रिने समस्या कम गर्न सक्छ।<br>यदि जाइलिनलाई फिनोलले प्रतिस्थापन गरियो वा अवशिष्ट पोटासियम हाइड्रोक्साइड रहयो भने भुसुनाहरू कालो हुन सक्छन्।<br>उच्च अपवर्तन सूचकांकले नरंगाईएको संरचनाहरूलाई अस्पष्ट पार्न सक्छ।<br>हट-प्लेटमा सुकाईएन भने पूर्ण रूपमा सुकाउन वर्षौं लाग्न सक्छ।<br>माध्यमको रंग समयसँगै पहेंलो र गाढा हुँदै जान्छ, विशेष गरी ल्वाङको तेलले सफा गर्दा।<br>केही रंगहरू कमजोर हुन्छन्, र यदि माध्यम अम्लीय भयो भने क्याटनिक रङहरू समयसँगै स्वतःस्फूर्त रूपमा फिक्का हुन सक्छन्।
DMHF (डाइमिथाइल हाइडेन्टोइन फॉर्मल्डिहाइड)	उच्च पारदर्शिता<br>राम्रो अपवर्तन सूचकांक<br>संरचनाहरूको उत्कृष्ट दृश्यता<br>मिश्रणहरूको स्थिरता एकदम राम्रो छ।<br>धेरै रंगाउने प्रविधिहरूसँग उपयुक्त<br>स्लाइड र कभरस्लिप बीच राम्रो आसंजन<br>भुसुनाहरूको राम्रो सुरक्षा	समयसँगै पहेँलो हुन सक्ने सम्भावना<br>केही रंगहरू परिवर्तन हुन सक्छ<br>फर्मल्डिहाइड-संवेदनशील रंगहरूको लागि उपयुक्त छैन<br>हावाका बुलबुले, सुक्ने समय ढिलो<br>आर्द्रताप्रति संवेदनशील माउन्टिङ माध्यम<br>माउन्टिङ उल्टाउन गाह्रो छ<br>फर्मल्डिहाइड विषाक्त, जलन गराउने, क्यान्सरजनक हुन्छ।
* युपरल (पारदर्शी)	५० वर्षभन्दा बढी आयु भएको टिकाउ माध्यम।<br>८०% इथेनॉलबाट सिधै माउन्ट गर्न सम्भव छ (निर्माता सिफारिस)।<br>नरंगाईएका संरचनालाई ओझेल पार्दैन र समयसँगै पहेंलो वा सजिलै टुट्ने हुँदैन।<br>डिप्टेराको लागि क्यानाडा बाल्सम भन्दा उपयुक्त अपवर्तन सूचकांक छ।<br>[illegible]	हानिकारक तत्वहरू हुन्छन् र हुड मुनि ह्यान्डल गर्नुपर्छ।<br>इथानोलको निर्जलीकरण र युपरल एसेन्स मार्फत स्थानान्तरणले केही ट्याक्सालाई टुट्ने बनाउन सक्छ, तर आइसोप्रोपानोल प्रयोग गर्दा यो समस्या कम हुन सक्छ।

तालिका ४: बाँकी

नाम	फाइदाहरू	बेफाइदाहरू
⁎ युपरल (पारदर्शी)	न्यूनतम संकुचन र बबल-रहित सुकाउने भएकोले बाक्लो भुसुनाहरूको लागि राम्रोसँग काम गर्दछ।<br>९५% इथेनॉलमा घुलनशील हुन्छ, जसले गर्दा धेरै वर्ष पछि पनि पुन: माउन्ट गर्न सकिन्छ।	
होयर फ्लुइड	भुसुनाहरू जीवित वा सिधै पानी, इथेनॉल, वा फॉर्मल्डिहाइडबाट माउन्ट गर्न सकिन्छ।<br>मेसेरेसनले उत्कृष्ट क्युटिकल गुणस्तर उत्पादन गर्छ।<br>अनुकूल अपवर्तन सूचकांक छ र उच्च कन्ट्रास्टको लागि आयोडिन स्टेनिङको साथ बढाउन सकिन्छ।<br>मिश्रणमा रहेको एसिटिक एसिडले आर्थ्रोपड एपेन्डेजहरू फैलाउन सक्छ।<br>केही भुसुनाहरू ४०-६० वर्षसम्म स्थिर रहन सक्छन्।<br>पानीमा घुलनशील, सजिलै पुन: माउन्ट गर्न अनुमति दिँन्छ।	बिस्तारै माध्यम हालिएन भने नाजुक भुसुनाहरू बिग्रन सक्छन्, यो तरिका धेरै समय लाग्ने हुन्छ।<br>गुहा र क्रिस्टलहरू १० वर्ष भन्दा कम समयमा बन्न सक्छन्।<br>क्लोरल हाइड्रेटको सांद्रता र एक्सपोजर समयको आधारमा म्यासेरेसन अत्यधिक हुन सक्छ।<br>माध्यमका घटकहरू अलग हुन सक्छन्, र मसिनो दाना महिनौं वा वर्ष भित्र देखा पर्न सक्छ।<br>मिडिया कालो हुन सक्ने पनि रिपोर्ट गरिएको छ।
CMCP-9 (= कार्बोक्सी मिथाइल सेलुलोज फिनोल)	भुसुनाहरू पानी, इथेनॉल, ग्लिसरोल, वा फॉर्मल्डिहाइड युक्त घोल जस्ता माध्यमबाट सिधै माउन्ट गर्न सकिन्छ, र सामान्य परीक्षण वा तयारीलाई सहज बनाउन आवश्यक पर्दा तिनीहरूको आन्तरिक अंगहरूलाई म्यासेरेट गर्न सकिन्छ।	यो माध्यमले क्रिस्टलहरू विकास गर्न सक्छ र समयसँगै गाढा हुन सक्छ, र यसले कहिलेकाहीं भुसुनाहरूलाई उद्देश्यभन्दा बढी म्यासेरेट गर्न सक्छ। स्लाइडलाई सावधानीपूर्वक रिंग नगरेसम्म, बाक्लो भुसुनाहरूलाई यसमा राम्रोसंग सुरक्षित गर्न सकिदैन किनभने तिनीहरू संकुचित हुन सक्छन् र कभरस्लिपको किनारा वरिपरि खाली ठाउँहरू सिर्जना गर्न सक्छन्। यो रंगिएका भुसुनाहरू वा क्याल्सिफाइड सामग्रीहरूको लागि उपयुक्त छैन, र यसको सुक्ने समय CMC भन्दा ढिलो छ।
युकिट™	३० वर्षभन्दा बढी समयसम्म टिक्ने मध्यम।<br>एसीटोन, बेन्जिन, क्लोरोफर्म, डाइअक्सन, ईथर, आइसोप्रोपानोल, मिथाइल बेन्जोएट, टेरपाइनोल, टोल्युइन र जाइलिन सहित माउन्टिङको लागि धेरै विलायकहरूसँग उपयुक्त।<br>[illegible]	हानिकारक तत्वहरू हुन्छन् र हुड मुनि ह्यान्डल गर्नुपर्छ।<br>पूर्ण, समय लाग्ने, निर्जलीकरण श्रृंखला आवश्यक हुन्छ।<br>संकुचन र ग्यास-बबल गठनको कारणले बाक्लो भुसुनाहरूको लागि उपयुक्त होइन।<br>शीशा राम्रोसँग सफा र सिल नगरिए कभरस्लिपहरू समयसँगै छुट्टीन सक्छन्।<br>कोलाजेन फाइबर वरिपरि अपूर्ण पोलिमराइजेशन [illegible]

तालिका ४: बाँकी

नाम	फाइदाहरू	बेफाइदाहरू
युकिट™	चाँडै सुक्छ र थोरै अम्लीय pH हुन्छ।<br>समयसँगै उल्लेखनीय रूपमा कालो हुँदैन।<br>विभिन्न दागहरू (जस्तै, फुचसिन, हेमेटोक्सिलिन, मिथाइल हरियो, मिथाइल बैजनी, मिथाइलीन नीलो) को लागि उपयुक्त।<br>लामो समयसम्म जाइलिनमा भिजाएर भुसुनाहरूलाई वर्षौंपछि पुन: माउन्ट गर्न सकिन्छ।	देखिन सक्छ।
ईनिस (Enecê)	अत्यधिक टिकाउ माध्यम, कम्तिमा ५० वर्ष टिक्ने।<br>समयसँगै अँध्यारो हुँदैन।<br>यो बढी लचिलो छ, जसले माध्यममा कीराहरूको विच्छेदन गर्न अनुमति दिन्छ, साथै रूपात्मक संरचनाहरूको स्थिति निर्धारण गर्न उचित समय प्रदान गर्दछ।<br>कम लागत।	पूर्ण, समय खपत गर्ने निर्जलीकरण श्रृंखला आवश्यक छ।<br>इथानोलको निर्जलीकरण र ल्वाङको तेल मार्फत स्थानान्तरणले केही भुसुनाहरूलाई टुक्र्याउन सक्छ।<br>भुसुनाको नमूना धेरै बिस्तारै भए पनि पारदर्शी हुँदै जान्छ; यसले सेन्सिला, एस्कोइड्स र साधारण सेटे जस्ता धेरै साना संरचनाहरू हेर्न गाह्रो हुन सक्छ।

डिहाइड्रेसनको कारणले गर्दा होयर माध्यम समयसँगै घट्दै जान्छ (चित्र ८), जसले गर्दा साना सेता, अपारदर्शी क्लोरियल हाइड्रेट क्रिस्टलहरू बन्छन्। तैपनि, क्युटिकल रासायनिक रूपमा अक्षुण्ण रहँदा क्रिस्टलाइज्ड स्लाइडहरूबाट भुसुनाहरू पुन: प्राप्त गर्न सकिन्छ, यद्यपि बढ्दो क्रिस्टलहरूबाट केही भौतिक क्षति भने हुन सक्छ। केही अवस्थामा, फंगल वृद्धि रोक्नको लागि थाइमोलको साथ न्यानो, ओसिलो वातावरणमा माउन्टिङ माध्यमलाई पुन: हाइड्रेट गरेर क्रिस्टलाइज्ड स्लाइडहरू पुनर्स्थापित गर्न सकिन्छ। वैकल्पिक रूपमा, भुसुनाहरूलाई गम क्लोरियलबाट पानीमा भिजाउन सकिन्छ, हिमनदी एसिटिक एसिडमा निर्जलित गर्न सकिन्छ, र क्यानडा बाल्सममा पुन: माउन्ट गर्न सकिन्छ।

DMHF (डाइमिथाइल हाइडान्टोइन फॉर्मल्डिहाइड) (आरआई = १.४८)

यो पानीमा आधारित माध्यम [72] ले बर्लीज जस्तै अप्टिकली रूपमा धेरै राम्रो प्रदर्शन गर्दछ, र बर्लीज जस्तै प्रयोग गर्न सजिलो छ। यद्यपि, बर्लीजको विपरीत, यो कालो वा क्रिस्टलाइज हुँदैन। यो भुसुना र अन्य साइकोडायडेको लागि राम्रोसँग काम गर्दछ।

CMCP (क्याम्फर-मोनो-क्लोरोफेनोल) (आरआई = १.४१)

यो ग्लिसरीनमा आधारित, पानीमा घुलनशील माउन्टिंग माध्यम हो जुन नाजुक भुसुनाहरूको पारदर्शी, स्थायी स्लाइडहरू सिर्जना गर्न प्रयोग गरिन्छ। यस माउन्टिंग माध्यमको फाइदा यो हो कि भुसुनाहरू सिधै पानी वा

इथेनॉलबाट माउन्ट गर्न सकिन्छ। यसले भुसुनालाई छिट्टै फैलाउँछ र सफा गर्छ, भुसुनाको उचित स्थिति र स्वरूप देखिन क्युटिकललाई नरम बनाउँछ, जुन विशेष गरी पखेटा फैलाउन वा जननांग विच्छेदन गर्न उपयोगी हुन्छ। यद्यपि यसले दीर्घकालीन भण्डारण गर्न सक्षम हुने रिपोर्ट गरिएको भएता पनि संरक्षणको सही अवधि निश्चित हुदैन। यस माउन्टिंग माध्यममा फिनोल हुन्छ जुन एक विषाक्त र जलनकारी पदार्थ हो र यसलाई सावधानीपूर्वक ह्यान्डलिङ गर्नु पर्ने हुन्छ।

स्थायी माउन्टिङको लागि मिडिया

क्यानडा बाल्सम (आरआई = १.५२-१.५४)

क्यानाडा बाल्समलाई पहिलो पटक १८३० को दशकमा एन्ड्रयू प्रिचर्डले प्रसारित प्रकाश माइक्रोस्कोपीको लागि उपयुक्त माउन्टिंग माध्यमको रूपमा वर्णन गरेका थिए। १५० वर्षभन्दा बढी सफल प्रयोगको साथ, यसको प्रमाणित अभिलेखीय गुणस्तरको कारण यो सबैभन्दा व्यापक रूपमा प्रयोग हुने माध्यमहरू मध्ये एक हो। होयर फ्लुइड मिडियाको विपरीत, क्यानाडा बाल्समले आर्द्रतालाई क्रिस्टलाइज वा अवशोषित गर्दैन। यद्यपि, क्यानाडा बाल्सम दृढ रूपमा अटोफ्लोरोसेन्ट हुन्छ, जुन कहिलेकाहीं निश्चित माइक्रोस्कोपी प्रविधिहरूको लागि उपयुक्त नहुन सक्छ [60]। तयारीको क्रममा जाइलिनको सट्टा गैर-विषाक्त विलायकहरू प्रयोग गर्नाले सुरक्षा जोखिमहरू कम गर्न सकिन्छ, तर यसका केही कमजोरीहरू पनि छन्, जस्तै माध्यम ढिलो सुक्नु र चाँडै गाढा वा कालो देखिन थाल्नु।

युपरल®(आरआई = १.४८)

युपरल® स्थायी माउन्टिङको लागि क्यानाडा बाल्समको व्यापक रूपमा प्रयोग हुने विकल्प हो, जसले उत्कृष्ट दीर्घकालीन स्थिरता र तुलनात्मक अपवर्तक सूचकांक प्रदान गर्दछ। युपरल® मा निम्न विशेषताहरू छन्: (१) निर्जलीकरण आवश्यकता: माउन्टिङ माध्यमको अन्तिम स्थानान्तरण अघि, भुसुना निर्जलित हुनुपर्छ, सामान्यतया ९५% बाट निरपेक्ष इथेनॉलमा हाल्नु पर्ने हुन्छ, र (२) विस्तारित प्रशोधन समय: रेजिनमा अन्तिम संयोजन, चाहे क्यानाडा बाल्सम होस् वा युपरल®, लाई निर्जलीकरण आवश्यक पर्दछ, जसले समग्र भुसुना प्रशोधन समयलाई लामो बनाउँछ। जब जैविक विलायकहरूसँग निर्जलीकरण सम्भव हुँदैन, निरपेक्ष इथेनॉलबाट निकालिएका भुसुनाहरूलाई अन्तिम माउन्टिङ अघि युपरल® र युपरल सारको बराबर मिश्रण भएको मध्यवर्ती घोलमा राख्न सकिन्छ।

ईनिस (आरआई = १.४६७)

ईनिस (Enecê) एक रेजिन-आधारित माउन्टिंग माध्यम हो जुन मुख्यतया साना कीराहरूको लागि प्रयोग गरिन्छ र ब्राजिलमा विशेष गरी लोकप्रिय छ। यसको आधारमा अल्कोहल, कपूर, टर्पेन्टाइनको सार, र युकलिप्टोलमा घुलनशील कोलोफोनी र गम कोपल हुन्छ। Cerqueira [11] ले ईनिस (Enecê) लाई लार्भाको स्थायी स्लाइड, अपरिपक्वहरूको एक्सुभिया, र वयस्क लामखुट्टेहरू पनि माउन्ट गर्न क्यानाडा बाल्समको विकल्पको रूपमा वर्णन गरे, र त्यसपछि यसलाई भुसुना माउन्ट गर्न व्यापक रूपमा प्रयोग गरिएको पाईएको छ। ईनिस (Enecê) स्थायी माउन्टिंगको लागि लागत प्रभावी विकल्पको रूपमा प्रयोग गरिन्छ। यसले दीर्घकालीन स्थिरता र पर्याप्त सुकाउने समय जस्ता गुणहरूले गर्दा विच्छेदन र रूपात्मक संरचनाहरूको सही व्यवस्थापन गर्न सहज गर्दछ।

५.४. स्लाइड तयारी र सुकाउने

दीर्घकालीन स्थिरता र संरक्षण सुनिश्चित गर्न माउन्ट गरिएका स्लाइडहरूलाई उचित रूपमा सुकाउनु महत्त्वपूर्ण छ। दीर्घकालीन भण्डारणको बारेमा विचार गर्नु अघि स्लाइडहरूलाई राम्ररी सुकाउनु पर्छ। इष्टतम परिणामहरूको लागि, स्थायी माउन्टिङ मिडियासँग माउन्ट गरिएका स्लाइडहरूलाई २-३ हप्ताको लागि तेर्सो राखेर सुकाउनु पर्छ, जबकि अर्ध-स्थायी मिडियासँग तयार पारिएका स्लाइडहरूलाई सुक्न केवल १-२ हप्ता मात्र लाग्न सक्छ। प्रभावकारी सुकाउने प्रक्रिया सुनिश्चित गर्न, भुसुनाहरूलाई क्षति पुऱ्याउन सक्ने अत्यधिक तातोबाट बचाउन, प्रयोग गरिएको माउन्टिङ माध्यमको लागि उपयुक्त तापक्रममा सेट गरिएको इन्क्यूबेटर प्रयोग गर्न सिफारिस गरिन्छ। ३०°C र ३७°C को तापमान दायरा सिफारिस गरिन्छ। यो सुकाउने प्रक्रिया अत्यन्त महत्त्वपूर्ण हुन्छ, किनकि यसले भण्डारणको समयमा स्लाइड बाङ्गिनु (warping), भुसुना बिग्रने (specimen deterioration), वा माउन्टिङ माध्यम अस्थिर हुनु जस्ता समस्याहरूबाट जोगाउँछ।

स्लाइड तयारीमा प्रयोग गरिने माउन्टिङ माध्यमलाई सधैं स्लाइड लेबलमा उल्लेख गर्नुपर्छ। यदि सम्भव छ भने, लेबलमा प्रयोग गरिएको विशिष्ट रेसिपी, तयारीकर्ताको नाम र तयारीको मिति पनि समावेश हुनुपर्छ। स्लाइडहरू सुरुमा अस्थायी माउन्टको रूपमा तयार गरिन्छन् र दीर्घकालीन

संरक्षणको लागि होइनन्। यद्यपि, यदि भुसुनाको स्थिति परिवर्तन हुन्छ, जस्तै "प्रकार" श्रृंखलाको भागको रूपमा तोकिएको छ भने, भविष्यमा पहिचान र वर्गीकरण अध्ययनको लागि भुसुनाको संरक्षण सुनिश्चित गर्न थप स्थायी माउन्टिङ माध्यम प्रयोग गर्नुपर्छ।

५.५. वैकल्पिक माउन्टिङ प्रविधिहरू: कार्ड माउन्टिङ

कार्ड माउन्टिङ किराहरूको धेरै समूहहरूको लागि प्रयोग गरिने एक प्रविधि हो जसमा भुसुनाहरूलाई सिधै कीटविज्ञान कार्डहरूमा पिन गर्न सकिन्छ वा सतहमा टाँस्न सकिन्छ। तिनीहरूको सानो आकार र पारदर्शी बनाएर मात्र पहिचानको लागि आन्तरिक अंगहरू अवलोकन गर्नुपर्ने हुँदा (वस्तु ५ हेर्नुहोस्), यो विधि भुसुनाहरू माउन्ट गर्नको लागि बिल्कुल उपयुक्त छैन।

५.६. क्षतिग्रस्त भुसुनाहरू पुन: माउन्ट गर्दै

दुर्लभ वा मूल्यवान भुसुनाहरूको लागि, https://zenodo.org/records/18315029 मा पहुँचयोग्य भिडियो अनुसार दुई-चरण प्रविधि सिफारिस गरिएको छ। १) प्रारम्भिक अवलोकनलाई अनुमति दिनको लागि तिनीहरूलाई विघटन नगरी पुन: हाइड्रेट गर्नुहोस्। धेरै माइक्रोस्कोप स्लाइडहरूको लागि होल्डरलाई पेट्री डिशमा राख्नुपर्छ। त्यसपछि पुन: हाइड्रेट गरिने स्लाइड माथि राखिन्छ, र पेट्री डिशलाई आर्द्र कक्ष सिर्जना गर्न केही मिलिमिटर विलायकले भरिन्छ, जसले स्लाइड आफैं विलायकको सम्पर्कमा नआओस् भनेर सुनिश्चित गर्दछ (चित्र ८ D)। भुसुनाको अवस्थाको आधारमा पुनर्हाइड्रेसनको लागि आवश्यक समय एक देखि धेरै दिनसम्म फरक हुन सक्छ। दैनिक अनुगमन र धैर्यताको खाँचो पर्दछ। स्लाइड पर्याप्त रूपमा पुन: हाइड्रेट भएपछि, यसलाई आर्द्र कक्षबाट हटाउन सकिन्छ र सूक्ष्म परीक्षण, फोटो खिच्न वा चित्र बनाउनु अघि केही घण्टाको लागि इन्क्यूबेटरमा राख्न सकिन्छ। २) पुन: माउन्ट गर्न, स्लाइडलाई केही थप घण्टा वा रातभरको लागि आर्द्र कक्षमा राख्न सकिन्छ। दूरबीन माइक्रोस्कोपको सहायताले विच्छेदन गर्नुपर्छ। मसिना सुईहरू प्रयोग गरेर, कभरस्लिपलाई सावधानीपूर्वक हटाउनु पर्छ, र भुसुनाको कुनै पनि भाग टाँसिएको छैन भनी सुनिश्चित गर्नु पर्दछ (https://zenodo.org/records/18315029)। त्यसपछि, निर्जलीकरण र रेजिन माध्यममा पुन: माउन्ट गर्नु अघि, भुसुना विच्छेद गरिएका भागहरूलाई संकलन गर्नुपर्छ र साना कुँडाकार प्लेटमा पानीले पखाल्नुपर्छ, DNA/RNA नष्ट गरेर निकाल्ने प्रक्रियामा प्रयोग हुने जस्तै (तल हेर्नुहोस्)। स्लाइड छुट्याउँदा, उपयुक्त विलायक चयन गर्न मूल माउन्टिंग माध्यम पहिचान गर्नु महत्वपूर्ण छ। जलीय माउन्टिंग मिडियाको लागि, पानी प्रयोग गर्नुपर्छ। यदि माउन्टिंग माध्यम रेजिन-आधारित छ (जस्तै, क्यानाडा बाल्सम वा युपरल®), भने फ्युम हुड मुनि र मास्क सहित उपयुक्त व्यक्तिगत सुरक्षात्मक उपकरणहरूको साथ जाइलिन प्रयोग गर्नुपर्छ।

प्रकार विशेष वा सङ्कलित भुसुनाहरूको पुन: माउन्टिङ गर्दा क्युरेटर र/वा भुसुनाको स्वामित्व रहेको संस्थाको सहमतिमा मात्र गरिनुपर्छ।

६. भुसुना पहिचान

६.१. आकृति विज्ञान

भुसुनाको पहिचान मुख्यतया तिनीहरूको आकारगत विशेषताहरूको परीक्षणमा निर्भर गर्दछ, जसमा छातीको आकार, पखेटा, जननेन्द्रिय, सेटे, र विभिन्न संरचनाहरू बीचको विशिष्ट आकारगत सम्बन्धहरू समावेश छन्। अनुसन्धानकर्ताहरूले ज्ञात ट्याक्सासँग सङ्कलन गरिएका भुसुनाहरूको तुलना गर्न वर्गीकरण कुञ्जीहरू, सन्दर्भ संग्रहहरू, र मौलिक प्रजाति विवरणहरू प्रयोग गर्छन्। दुवै लिङ्गहरूमा पखेटाको भेनेसन र टाउकोको आकारगत रूप, भाले जननेन्द्रियको संरचना, र पोथी स्पर्माथिकाहरूको कन्फिगरेसन जस्ता प्रमुख निदानात्मक विशेषताहरू प्रजाति निर्धारणको लागि विशेष रूपमा जानकारीमूलक हुन्छन्। सही पहिचानको लागि प्रायः विस्तृत सूक्ष्म परीक्षण आवश्यक पर्दछ, सामान्यतया जननेन्द्रिय र स्पर्माथिका जस्ता सूक्ष्म संरचनाहरू अवलोकन गर्न कम्पाउन्ड माइक्रोस्कोप प्रयोग गरिन्छ, वा फराकिलो आकारगत विशेषताहरूको लागि स्टेरियोमाइक्रोस्कोप प्रयोग गरिन्छ।

इमेजिङ प्रविधिमा हालैका प्रगतिहरूले भुसुना पहिचानको लागि डिजिटल इमेजिङको प्रयोगलाई सहज बनाएको छ। उच्च-रिजोल्युसन तस्बिरहरू वा मुख्य सुविधाहरूको डिजिटल चित्रणहरूलाई सन्दर्भ सामग्रीहरूसँग तुलना गर्न सकिन्छ वा कम्प्युटर-सहायता प्राप्त पहिचान प्रणालीहरू प्रयोग गरेर विश्लेषण गर्न सकिन्छ, जसले गर्दा आकारविज्ञान वर्गीकरणमा शुद्धता र पहुँच दुवै सुधार हुन्छ।

६.२. पखेटा ज्यामिति

पखेटा ज्यामिति विभिन्न भुसुना प्रजातिहरूको पहिचान

र वर्गीकरणमा प्रयोग गरिने एक प्रमुख विशेषता हो। भुसुनाका पखेटाहरूले एक अद्वितीय ढाँचा र संरचना प्रदर्शन गर्छन्, सामान्यतया राम्रोसँग विकसित शिराका साथ लामो र साँघुरो हुन्छन् (चित्र ९ र १०)। नसाहरूको व्यवस्थाले एक विशिष्ट ढाँचा बनाउँछ जुन जेनेरा र प्रजातिहरू बीच फरक हुन सक्छ, र पहिचानको लागि बहुमूल्य निदान गुणहरू प्रदान गर्दछ। फलस्वरूप, वर्गीकरण उद्देश्यका लागि पखेटा ज्यामितिको अध्ययनले बहुमूल्य अन्तर्दृष्टि प्रदान गर्दछ।

६.३. पखेटा ज्यामितीय मोर्फोमेट्रिक्स

अनुसन्धानकर्ताहरूले विभिन्न भुसुनाका प्रजाति वा जनसंख्यामा पखेटाको आकार र आकारको विश्लेषण र तुलना गर्न ज्यामितीय मोर्फोमेट्रिक्स जस्ता विभिन्न प्रविधिहरू प्रयोग गर्छन्। पखेटा ज्यामितिको अध्ययनले व्यवहार, बासस्थान प्राथमिकताहरू, र उडान क्षमताहरूमा बहुमूल्य अन्तर्दृष्टि प्रदान गर्दछ।

ज्यामितीय मोर्फोमेट्रिक प्रविधिमा, पखेटाहरू सावधानीपूर्वक विच्छेदन गरिन्छ, रंगाईन्छ (यदि आवश्यक भएमा), र स्लाइडहरूमा समतल-माउन्ट गरिन्छ। तयार गरिएका स्लाइडहरू त्यसपछि स्टेरियोमाइक्रोस्कोप अन्तर्गत फोटो खिचिन्छन्, डिजिटलाइज गरिन्छन्, र मोर्फोमेट्रिक विश्लेषणको अधीनमा राखिन्छन्। यो प्रक्रियालाई पहिल्यै प्रकासित लेखहरूमा राम्रोसँग वर्णन गरिएको छ [6, 27, 42, 56, 57, 59], जोडी अंगहरूको लागि सम्भावित नकारात्मक एलोमेट्रिक प्रभावहरूबाट बच्न सधैं दायाँ वा बायाँ पखेटा मध्ये एउटै (एकरूप रूपमा) प्रयोग गर्न सिफारिस गरिएको छ [62]।

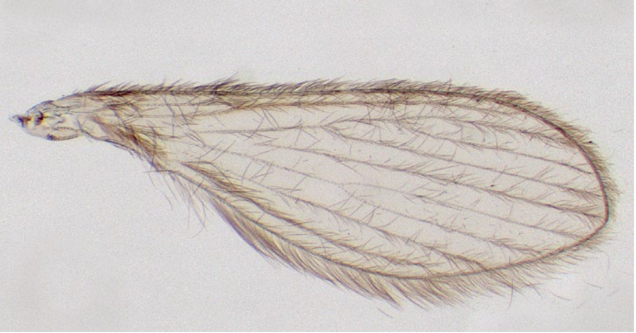

चित्र ९: ट्राइकोफोरोमिया इनिनीको (*Trichophoromyia ininii*) अप्रशोधित पखेटा।

ज्यामितीय मोर्फोमेट्रिक विश्लेषणको लागि पखेटाको तयारी

पखेटाका शिराहरू (veins) स्पष्ट रूपमा देखिनका लागि, पखेटाबाट स्केलहरू हटाई सफा गर्नुपर्छ र उपयुक्त रूपमा रङ्ग (stain) लगाउनुपर्छ। पखेटा तयार गर्न, पहिले आवश्यक अभिकर्मकहरू (मिथिलिन नीलो, इथेनॉल, पानी र जाइलिन विकल्प) ले साना कुँ भर्नुहोस्। एपेनडोर्फ ट्यूबलाई उल्टो पारेर इनारमाथि खाली गरेर कोठाको तापक्रममा ७०% इथेनॉलमा संरक्षित पखेटालाई राम्रो घुमाउरो सुई प्रयोग गरेर पखेटालाई अनुदैर्ध्य रूपमा उठाउनुहोस्। ब्रिस्टलहरू हटाउनका लागि पखेटालाई इथेनॉलबाट पानीमा र पुन: इथेनॉलमा सार्नुहोस्। पखेटालाई ६ मिनेटको लागि मिथिलिन नीलोमा राख्नुहोस्, रंग लगाउदा यो तैरिन्छ भनी सुनिश्चित गर्नुहोस्। पखेटालाई सावधानीपूर्वक पुन: प्राप्त गर्नुहोस् र २ मिनेटको लागि जाइलिनमा डुबाउनुहोस् (मिथिलिन नीलोको लगभग एक तिहाइ समय)। इनारको भित्तामा सुईको कोमल ट्यापले पखेटालाई स्थिर हुन मद्दत गर्न सक्छ; जाइलिनले रंग ठीक गर्न काम गर्छ। अन्तमा, पखेटा उठाउनुहोस् र यसलाई सूक्ष्म स्लाइडमा युपरल® को सानो थोपामा राख्नुहोस्। म्याग्निफाइङ्ग लेन्स मुनि, बिस्तारै पखेटा खोल्नुहोस् र सावधानीपूर्वक कभरस्लिप राख्नुहोस्। युपरल® सेट हुनुभन्दा पहिले तुरुन्तै तस्बिरहरू खिच्नुपर्छ, किनकि उत्तम समरेखन प्राप्त गर्न कभरस्लिप मुनि पखेटाको स्थितिमा थोरै समायोजन आवश्यक पर्न सक्छ।

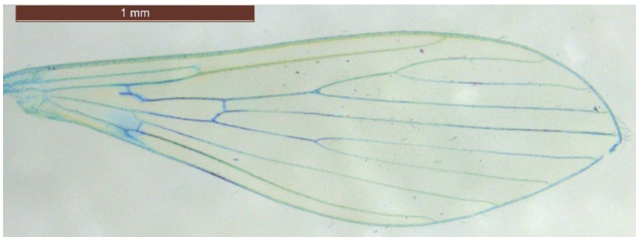

चित्र १०: फ्लेबोटोमस एरियासीको (*Phlebotomus ariasi*) रंगीन पखेटा।

६.४. आणविक जीवविज्ञान प्रविधिहरू

रूपात्मक प्रविधिहरूको अतिरिक्त, कीटशास्त्रीय अनुसन्धानमा आणविक विधिहरू बढ्दो रूपमा आवश्यक छन्, जसमा वर्गीकरण, जनसंख्या आनुवंशिक, र फाइलोजेनेटिक अध्ययनहरू, साथै DNA/RNA द्वारा रोगजनक पत्ता लगाउने, र भुसुनामा रगतको खानाको स्रोत निर्धारण गर्ने, महामारी विज्ञानको क्षेत्रमा भेक्टर व्यवहार महत्त्वपूर्ण हुने [70] समावेश छन्। DNA अनुक्रमण प्रजाति पुष्टिकरणको लागि वा नजिकबाट सम्बन्धित प्रजातिहरूलाई छुट्याउन प्रयोग गर्न सकिन्छ, जसले पहिचानलाई अझ सटीक र भरपर्दो माध्यम प्रदान गर्दछ। यसबाहेक, उन्नत आणविक प्रविधिहरू (जस्तै, PCR, DNA अनुक्रमण, NGS,

आदि) र MALDI-ToF MS ले परम्परागत रूपात्मक विधिहरूको पूरक, सही र द्रुत प्रजाति पहिचानको लागि महत्वपूर्ण [46]। यी प्रगतिहरूको बावजुद, रूपात्मक पहिचान वर्गीकरणको लागि सन्दर्भ मानक हुन्छ र यसकै आधारमा आणविक डेटा व्याख्या गरिएको हुन्छ।

६.४.१. विनाशकारी न्यूक्लिक एसिड निष्कर्षण

धेरै जैविक अध्ययनहरूमा न्यूक्लिक एसिड निष्कर्षण एक नियमित चरण हो, र जैविक सामग्रीहरूबाट डीएनए अलग गर्न विभिन्न विधिहरू विकास गरिएको छ [48]। धेरै व्यावसायिक रूपमा उपलब्ध डीएनए निष्कर्षण किटहरू यस प्रक्रियालाई सहज बनाउन डिजाइन गरिएको छ [14]। यद्यपि, रूपात्मक पहिचानको लागि आर्थ्रोपड भुसुनाहरू तयार गर्न सामान्यतया प्रयोग गरिने विधिहरूले प्रायः डीएनए विश्लेषणमा बाधा पुर्‍याउँछन्, किनकि यी प्रविधिहरूले भुसुनाको महत्वपूर्ण भौतिक विशेषताहरूलाई क्षति पुर्‍याउन वा नष्ट गर्न सक्छन् [10]। कीराको तन्तुहरूको लागि धेरैजसो डीएनए निष्कर्षण प्रोटोकलहरू विनाशकारी प्रकृतिका हुन्छन् [43], जसले थोरै नमूना भुसुनाहरूको लागि विशेष सावधानीको आवश्यकता देखाउँछ, जहाँ सीमित भुसुनाको उपलब्धताले गर्दा महत्त्वपूर्ण रूपात्मक विशेषताहरूमा सम्झौता गर्नु पर्ने हुन सक्छ [72]। उपयुक्त डीएनए अलगाव विधि चयन गर्न भुसुनाको प्रकार र अवस्थाले प्रमुख भूमिका खेल्छ [29]।

भुसुनाहरूको सही पहिचान, जनसंख्या गतिशीलता बुझ्ने र गैर-लक्षित प्रभावहरूलाई कम गर्ने आवश्यकताले आणविक निदान उपकरणहरूको विकासलाई प्रेरित गरेको छ [23]। भुसुनाहरू पहिचान गर्नको लागि रूपात्मक वर्गीकरण विधिहरूलाई पूरक बनाउन आणविक विधिहरू अब बारम्बार प्रयोग गरिन्छ। उदाहरणका लागि, कीरा बारकोडिङको लागि मानक विधिहरूमा DNA निष्कर्षण, अनुक्रमण, र मूल भुसुनाको क्षति समावेश गरिएको हुन्छ। यसरी, जैविक सामग्री र यसको रूपात्मक अखण्डता दुवैलाई जोगाउने गैर-विनाशकारी DNA निष्कर्षण विधिहरू अन्वेषण गर्न तत्काल आवश्यकता छ।

भुसुनाहरूमा धेरै न्यूक्लिक एसिड निष्कर्षण विधिहरू लागू गरिएका छन्। आवश्यक पर्ने न्यूक्लिक एसिडको मात्रा वा गुणस्तर डाउनस्ट्रीम आणविक विश्लेषणमा निर्भर गर्दछ, किनकि विभिन्न प्रविधिहरूमा फरक संवेदनशीलता र शुद्धता आवश्यकताहरू हुन्छन् [9]। उदाहरणका लागि, भुसुनाका आँखाहरूले PCR प्रवर्धनलाई रोक्छ भन्ने पाइएको छ [69]। रोगजनक स्क्रिनिङभन्दा बाहिर, भुसुना DNA नियमित रूपमा प्रजाति पहिचान उद्देश्यका लागि निकालिन्छ। विभिन्न निष्कर्षण विधिहरू प्रयोग गर्न सकिन्छ, यद्यपि प्रविधिहरू बीच उत्पादन र गुणस्तर फरक हुन्छ। केही निर्माताहरूको प्रोटोकलहरू भुसुना [8] को लागि अनुसन्धानकर्ताहरू द्वारा अनुकूलित गरिएको छ, निकालिएको न्यूक्लिक एसिडहरूको उत्पादन र/वा गुणस्तर बढाउँदै [8, 9, 69], जबकि अन्य आर्थ्रोपड ट्याक्साको लागि विकसित अन्य अनुकूलनहरू भुसुनाहरूमा पनि प्रयोग गर्न सकिन्छ [58, 76]। साना माइटोकोन्ड्रियल टुक्राहरू (COI वा CytB) लाई लक्षित गर्ने पहिचान PCR हरू सामान्यतया उच्च DNA खण्डन समावेश गर्ने निष्कर्षण विधिहरूसँग उपयुक्त हुन्छन्। यसको विपरीत, अन्य लामो-पठनीय NGS प्रविधिहरू (अक्सफोर्ड नानोपोर र प्याकबायो) लाई न्यूनतम खण्डन र उच्च-गुणस्तरको DNA चाहिन्छ। स्पिन स्तम्भ निष्कर्षणले सामान्यतया ६० केबी सम्मको जीनोमिक डीएनए टुक्राहरू उत्पादन गर्छ, जबकि फिनोल-क्लोरोफर्म निकासीले १५० केबी सम्मको टुक्राहरू उत्पादन गर्न सक्छ [77]। भुसुना डीएनएको विभिन्न निष्कर्षण प्रविधिहरूको सारांश र यी कीराहरूको लागि विधिगत अनुकूलनहरू गरिएको छ कि छैन भनेर तालिका ५ मा दिईएको छ। डीएनएको उपज मात्रा देखाइएको छैन, किनकि तिनीहरू भुसुना आकार र तयारी विधिमा निर्भर गर्दछन्। परिमार्जन स्तम्भले भुसुना वा अन्य साना आर्थ्रोपोडहरूको लागि निष्कर्षण प्रोटोकलहरूको अनुकूलनलाई जनाउँछ।

निष्कर्षण विधिको छनोट गर्दा धेरै मापदण्डहरू विचार गर्नुपर्छ, जस्तै भुसुनाहरूको संख्या, निष्कर्षण समय, र डाउनस्ट्रीमका लागि प्रयोग गरिएको प्रविधि। NGS प्रविधिहरूलाई उच्च आणविक भार भएको जीनोमिक DNA आवश्यक पर्दछ, यहाँ प्रस्तुत गरिएका सबै विधिहरू मानक PCR-आधारित अनुप्रयोगहरूको लागि प्रयोग गर्न सकिन्छ।

यसबाहेक, धेरै अध्ययनहरूले साना स्थलीय आर्थ्रोपोडहरू, सुख्खा-संरक्षित संग्रहालयका भुसुनाहरू, र नरम-शरीर भएका आर्थ्रोपोडहरूको लागि गैर-विनाशकारी डीएनए निष्कर्षण विधिहरूको अन्वेषण गरेका छन् [19, 26, 28, 55, 63]।

६.४.२. गैर-विनाशकारी न्यूक्लिक एसिड निष्कर्षण

आर्थ्रोपोडहरू, विशेष गरी भुसुनाहरूको आणविक विश्लेषणमा प्रमुख चुनौतीहरू मध्ये एक भनेको कीटशास्त्रीय संग्रहको लागि भुसुनाहरूको संरक्षण गर्नु हो। धेरैजसो डीएनए निष्कर्षण प्रोटोकलहरूमा तन्तुको म्यासेरेसन आवश्यक हुन्छ,

जसले गर्दा मूल भुसुनाको संरक्षणमा सम्झौता हुन्छ। गैर-विनाशकारी न्यूक्लिक एसिड निष्कर्षण विधिहरू, भुसुनालाई भौतिक रूपमा क्षति नगरी, यसको व्यवहार्यतालाई असर नगरी वा यसको आकारविज्ञान परिवर्तन नगरी आनुवंशिक सामग्री निकाल्न डिजाइन गरिएको हो। यी विधिहरू विशेष गरी बहुमूल्य वा सीमित भुसुनाहरू, जो काम गर्दा मूल्यवान हुन्छन्, जहाँ भविष्यको वर्गीकरण, रूपात्मक वा निदान उद्‌देश्यका लागि संरचनात्मक अखण्डता कायम राख्नु आवश्यक हुन्छ। सामान्यतया प्रयोग गरिने गैर-विनाशकारी प्रविधि भिजाउने विधि हो जसमा भुसुनाहरूलाई स्थिर गरिन्छ, र प्रोटीनेज K भएको लिसिस बफरमा बिस्तारै डुबाइन्छ।

भुसुनाहरूमा, विशेष गरी प्रकार विशेष भुसुनाहरूमा हल्का-भेक्टोलिसिस प्रविधि सफलतापूर्वक लागू गरिएको हुन्छ [24]। यो प्रविधिले परम्परागत स्पिन स्तम्भ किट (यस अवस्थामा, DNeasy रगत र टिस्यु किट, QIAGEN, Hilden, जर्मनी) प्रयोग गर्दछ जसमा भुसुनालाई नष्ट नगरी DNA प्राप्त गर्न अनुकूलन हुन्छ। परिमार्जित लाइसिस चरणहरू (लाइसिस बफरको मात्रा र फ्रिजिङ चरणको थप) [17] ले न्यूक्लिक एसिडहरू रिलीज गर्न सहज हुन्छ, र मोर्फोलॉजिकल क्षतिलाई कम गर्दछ [24]। भुसुनाहरूको सन्दर्भमा, हटशट डीएनए निष्कर्षण किट (बेन्टो बायोवर्क्स लिमिटेड, लन्डन, युनाइटेड किंगडम) [73] प्रयोग गर्न पनि सम्भव छ जुन द्रुत र सस्तो हुन्छ, जसले भुसुनाहरूको द्रुत र कम लागतको प्रशोधन गर्न सक्षम हुन्छ। मोर्फोलॉजिकल पहिचानको लागि अभिप्रेरित भुसुनाहरू त्यसपछि धुन सकिन्छ। DNeasy रगत र टिस्यु किटसँग प्रशोधन गरिएकाहरूलाई मार्क-एन्ड्रे घोलले सफा गर्नुपर्छ, जबकि हटशट डीएनए निष्कर्षण किटसँग प्रशोधन गरिएकाहरूलाई जलीय माध्यममा, वा अझ राम्रोसँग, निर्जलीकरण पछि रेजिनमा माउन्ट गर्न पर्याप्त रूपमा पारदर्शी बनाईएको हुन्छ, यस लेखमा विस्तृत प्रोटोकल अनुसार [73]। त्यसपछि निकालिएको आनुवंशिक सामग्रीलाई PCR जस्ता डाउनस्ट्रीम विश्लेषणको लागि थप प्रशोधन गर्न सकिन्छ, जसले गर्दा विशिष्ट आनुवंशिक मार्करहरू बढ्छन्। भुसुनाहरूको आनुवंशिक विशेषताहरूको अध्ययन गर्न गैर-विनाशकारी न्यूक्लिक एसिड निष्कर्षण विधिहरू महत्वपूर्ण छन्, जसमा तिनीहरूले बोक्न सक्ने सम्भावित रोग निम्त्याउने एजेन्टहरू पहिचान गर्ने समावेश छ। भुसुनाको अखण्डता सुरक्षित गरेर, अनुसन्धानकर्ताहरूले थप विश्लेषण वा अध्ययनहरूको लागि भुसुना राख्दै बहुमूल्य आनुवंशिक जानकारी प्राप्त गर्न सकिन्छ।

६.५. MALDI-ToF MS का थप जानकारी

MALDI-ToF MS (म्याट्रिक्स-सहायता प्राप्त लेजर डिसोर्प्शन/आयनाइजेसन टाइम-अफ-फ्लाइट मास स्पेक्ट्रोमेट्री) जैविक भुसुनाहरूको अद्‌वितीय प्रोटीन प्रोफाइल ('औंठाछाप') पत्ता लगाउन र विश्लेषण गर्न डिजाइन गरिएको मास स्पेक्ट्रोमेट्री-आधारित प्रविधि हो। MALDI-ToF लाई चिकित्सा र पशु चिकित्सा महत्त्वका आर्थ्रोपोडहरूको पहिचानको लागि एक महत्वपूर्ण उपकरणको रूपमा बढ्दो रूपमा मान्यता दिइँदैछ। यो प्रविधि भुसुनाका विभिन्न विकास चरणहरू, जसमा अपरिपक्व रूपहरू र पोथीहरूमा रगतको खानाको स्रोत, पहिचान गर्न प्रभावकारी साबित भएको छ, र भण्डारण र एकरूपता अवस्थाहरूमा भुसुनाका प्रजातिहरूमा भाले र पोथी दुवै सफलतापूर्वक छुट्याउन सकिने देखिएको छ [28, 30, 73, 74]। यो विधिले उपजेनेरा, प्रजाति र जनसंख्याको स्तरमा उच्च सफलतापूर्वक छुट्याउन सकिन्छ। यो प्रविधिले अनुसन्धानकर्ताहरूलाई द्रुत

तालिका ५: फ्लेबोटोमाइन भुसुनाको gDNA निष्कर्षणको लागि औसत लागत, प्रयोग, र प्रोटोकल अनुकूलन

प्रोटोकल	लागत	प्रयोग	साना आर्थ्रोपोडहरूको लागि प्रोटोकल अनुकूलन
घुमाउने स्तम्भ	२.५ - ३.५५ अमेरिकी डलर [39]	पीसीआर, एनजीएस	[9]
फेनोल-क्लोरोफर्म	०.२४ अमेरिकी डलर [69]	पीसीआर, एनजीएस	[9]
हटशट	<०.०१ अमेरिकी डलर [69]	पीसीआर	-
नुनद्‌वारा अवक्षेपण विधि	०.१२ अमेरिकी डलर [69]	पीसीआर	-
चेलेक्स	०.०२ अमेरिकी डलर [41]	पीसीआर	[41, 76]

र सही प्रजाति पहिचान प्राप्त गर्न सक्षम बनाउँछ, जुन भुसुनाको वितरण, व्यवहार र रोग प्रसारणमा तिनीहरूको भूमिका बुझ्नको लागि आवश्यक हुन्छ। प्रोटीन प्रोफाइलको आधारमा प्रजातिहरू बीच भिन्नता छुट्याएर, MALDI-ToF ले महामारी विज्ञान अध्ययन र भेक्टर नियन्त्रण रणनीतिहरूमा महत्त्वपूर्ण भूमिका खेल्छ। यस प्रविधिका दुई मुख्य वर्तमान कमजोरीहरू छन् जसले यसको नियमित प्रयोगलाई सीमित गर्दछ। पहिलो भनेको मास स्पेक्ट्रोमेट्री उपकरणको उपलब्धता हो, जुन भुसुनाको प्रजाति पहिचानको उद्देश्यका लागि मात्र प्राप्त गर्न अत्यन्तै महँगो छ (वा सामान्यतया आर्थ्रोपड भेक्टरहरू)। भाग्यवश, प्रोटोमिक सुविधाहरू र/वा क्लिनिकल निदानमा मानक अनुसन्धान उपकरण बनेका मास स्पेक्ट्रोमिटरहरूमा मेसिन समय प्राप्त गरेर पनि यो विधि प्रयोग गर्न सकिन्छ। दोस्रो कुरा, खुला पहुँच डाटाबेसहरूमा उपलब्ध भुसुना सन्दर्भ डेटाको कम प्रतिनिधित्व हुनु, जसले गर्दा आदर्श रूपमा उपयुक्त आनुवंशिक मार्कर (COI, cytB, वा अन्य) को रूपात्मक मूल्याङ्कन र अनुक्रमणको संयोजनद्वारा स्पष्ट रूपमा पहिचान गरिएका भुसुनाहरूमा आधारित सन्दर्भ स्पेक्ट्राको साथ इन-हाउस डाटाबेस निर्माण गर्न आवश्यक देखिन्छ। आशा छ कि सीमितता इन-हाउस भुसुना सन्दर्भ डेटालाई सहायता पब्लिक-होपिटक्स डे पेरिस (Assistance Publique-Hôpitaux de Paris), सोर्बोन विश्वविद्यालय, फ्रान्स (Sorbonne University) र BCCM/IHEM/Sciensano संग्रह, ब्रसेल्स, बेल्जियम द्वारा सञ्चालित MSI प्लेटफर्म (https://msi.happy-dev.fr/) मा क्रमिक रूपमा समावेश गरेर समाधान गरिनेछ। यदि MALDI-ToF प्रोटीन प्रोफाइलिङ विधि प्रयोग गर्ने योजना बनाइएको छ, भुसुनाहरूलाई प्राथमिकताका साथ सुख्खा-फ्रोजन वा आणविक ग्रेडको ७०% इथेनॉलमा भण्डारण गर्नुपर्छ र परिवेशको तापक्रममा राख्न मिल्दैन्। नमुना तयारीको लागि विश्वव्यापी दिशानिर्देशहरूको अभावमा, प्रयोगकर्ताहरूलाई MALDI-ToF म्याट्रिक्स तयारीको लागि जलीय ६०% एसिटोनिट्राइल/०.३% TFA सिनापिनिक एसिड (३० mg/mL) को घोल प्रयोग गर्न सल्लाह दिइन्छ ताकि उनीहरूको प्रोटिन स्पेक्ट्रालाई हालसम्म प्रकाशित भुसुनाको डेटासँग तुलना गर्न सकियोस्।

MALDI-ToF MS को लागि नमुना तयारी (**चित्र ७**)

विभिन्न परिस्थितिहरूमा भण्डारण गरिएका कीराका भुसुनाहरूलाई पहिले कोठाको तापक्रममा हावामा सुकाइन्छ र विच्छेदन गरिन्छ। स्लाइड माउन्टिङ र रूपात्मक विश्लेषणको लागि प्रमुख आकृतिक विशेषताहरू भएका शरीरका भागहरू प्राप्त गर्न टाउको र पेट हटाइन्छ। छातीलाई MALDI-ToF को लागि प्रयोग गर्न सकिन्छ र बाँकी पेटलाई DNA निष्कर्षणको लागि संरक्षित गरिन्छ। प्रोटीन प्रोफाइलिङको लागि, छातीलाई डिस्पोजेबल पेस्टल र पेलेटहरू प्रयोग गरेर १० µL समरूपीकरण घोलको साथ १.५-मिली माइक्रोट्यूबहरूमा म्यानुअल रूपमा समरूपीकरण गरिन्छ। दुई समरूपीकरण समाधानहरू सामान्यतया प्रयोग गरिन्छ: निर्जीवाणुकरण गरिएको आसुत पानी र २५% फर्मिक एसिड।

७. निष्कर्ष

यस निर्देशिकामा हामीले अनुसन्धानकर्ताहरूलाई विशेष अनुसन्धान उद्देश्यहरू अनुरूप, सही पहिचान र रोगजनक पत्ता लगाउन सहज बनाउन भुसुना माउन्ट गर्ने सबैभन्दा प्रभावकारी तरिकाहरू उपलब्ध गर्ने लक्ष्य राखेकाछौं। कुनै एकल, विश्वव्यापी रूपमा सर्वोत्कृष्ट विधि छैन। बरु, धेरै विधिहरू उपलब्ध र प्रयोगमा छन्, र प्रत्येकको आफ्नै फाइदा र सीमितताहरू छन्।

सहायक सामग्रीमा, भुसुनाहरूको तयारी तथा पहिचानका लागि प्रयोग हुने विभिन्न माउन्टिङ प्रविधिहरू सम्बन्धी विस्तृत कार्यविधिहरू प्रस्तुत गरिएको छ। निर्देशनात्मक भिडियोहरू सहित यी कार्यविधिहरूले विभिन्न उद्देश्यहरू अनुरूप चरणबद्ध रूपमा प्रक्रियाहरू बुझ्न मदत गर्दछन्। यी कार्यविधिहरू अनुसार प्रक्रिया पूरा गरेमा सटिक र भरपर्दो परिणामहरू सुनिश्चित हुने गर्दछ। यस समग्र स्रोतको उपलब्धतामार्फत, अनुसन्धानकर्ताहरूलाई आफ्ना विशिष्ट आवश्यकताअनुसार उपयुक्त माउन्टिङ प्रविधिहरू चयन तथा कार्यान्वयन गर्न सहयोग पुर्‍याउने हाम्रो उद्देश्य हो।

कृतज्ञता ज्ञापन

लेखकहरूले बेलायतको लन्डनको प्राकृतिक इतिहास संग्रहालयका रिचर्ड लेन र जो जे एडम्सलाई उनीहरूको उत्कृष्ट समीक्षाको लागि धन्यवाद दिन्छन्, जसले गर्दा यस निर्देशिकाको गुणस्तर बढेको छ।

आर्थिक सहायता

लेखक AJA को अनुसन्धानलाई आर्थिक सहयोग गरेकोमा हामी ब्राजिलियन विकास एजेन्सीहरू CNPq (केस नम्बर: ४०४३९५/२०२४-४) र Araucária Foundation (केस नम्बर: ४३३/२०२५ PDI) लाई धन्यवाद दिन्छौं।

हित द्वन्द वा स्वार्थ संघर्ष

जेरोम डेपाक्विट (Jérôme Depaquit) प्यारासाइटका सह-सम्पादक हुन् यद्यपी यस पाण्डुलिपिको समीक्षा प्रक्रिया र निर्णय लिने प्रक्रियामा उनको कुनै प्रभाव थिएन। अन्य लेखकहरूले पनि उनीहरूको कुनै स्वार्थको संघर्ष नभएको घोषणा गर्दछन्।

डाटा उपलब्धता

जेनोडोमा भिडियोहरू।

भिडियो १: https://zenodo.org/records/18198006

भिडियो २: https://zenodo.org/records/18311158

भिडियो ३: https://zenodo.org/records/18311106

भिडियो ४: https://zenodo.org/records/18311154

भिडियो ५: https://zenodo.org/records/18303014

भिडियो ६: https://zenodo.org/records/18302850

भिडियो ७: https://zenodo.org/records/18315029

पूरक सामग्री

यस लेखको पूरक सामग्री यहाँ उपलब्ध छ।

https://www.parasite-journal.org/10.1051/parasite/2026009/olm

सन्दर्भ सामग्रीहरू

1. Alkan C, Allal-Ikhlef AB, Alwassouf S, Baklouti A, Piorkowski G, de Lamballerie X, Izri A, Charrel RN. 2015. Virus isolation, genetic characterization and seroprevalence of Toscana virus in Algeria. Clinical Microbiology and Infection, 21(11), 1040 e1-9.
2. Alten B, Ozbel Y, Ergunay K, Kasap OE, Cull B, Antoniou M, Velo E, Prudhomme J, Molina R, Banuls AL, Schaffner F, Hendrickx G, Van Bortel W, Medlock JM. 2015. Sampling strategies for phlebotomine sand flies (Diptera: Psychodidae) in Europe. Bulletin of Entomological Research, 105(6), 664-78.
3. Ayhan N, Baklouti A, Prudhomme J, Walder G, Amaro F, Alten B, Moutailler S, Ergunay K, Charrel RN, Huemer H. 2017. Practical guidelines for studies on sandfly-borne phleboviruses: Part I: Important points to consider ante field work. Vector-Borne and Zoonotic Diseases, 17(1), 73-80.
4. Bates PA. 1997. Infection of phlebotomine sandflies with *Leishmania*, in: The Molecular Biology of Insect Disease Vectors: A Methods Manual. Springer. p. 112-120.
5. Baum M, de Castro EA, Pinto MC, Goulart TM, Baura W, Klisiowicz Ddo R, Vieira da Costa-Ribeiro MC. 2015. Molecular detection of the blood meal source of sand flies (Diptera: Psychodidae) in a transmission area of American cutaneous leishmaniasis, Parana State, Brazil. Acta Tropica, 143, 8-12.
6. Belen A, Alten B, Aytekin A. 2004. Altitudinal variation in morphometric and molecular characteristics of *Phlebotomus papatasi* populations. Medical and Veterinary Entomology, 18(4), 343-350.
7. Bhattacharya J, Chandra G, Hati AK. 1991. A simple method for cryopreservation of *Leishmania donovani* promastigotes. Indian Journal of Medical Research, 93, 245-6.
8. Caligiuri LG, Sandoval AE, Miranda JC, Pessoa FA, Santini MS, Salomón OD, Secundino NF, McCarthy CB. 2019. Optimization of DNA extraction from individual sand flies for PCR amplification. Methods and Protocols, 2(2), 36.
9. Casaril AE, de Oliveira LP, Alonso DP, de Oliveira EF, Gomes Barrios SP, de Oliveira Moura Infran J, Fernandes WS, Oshiro ET, Ferreira AMT, Ribolla PEM, de Oliveira AG. 2017. Standardization of DNA extraction from sand flies: Application to genotyping by next generation sequencing. Experimental Parasitology, 177, 66-72.
10. Castalanelli MA, Severtson DL, Brumley CJ, Szito A, Foottit RG, Grimm M, Munyard K, Groth DM. 2010. A rapid non-destructive DNA extraction method for insects and other arthropods. Journal of Asia-Pacific Entomology, 13(3), 243-248.
11. Cerqueira NL. 1943. Um novo meio para montagem de pequenos insetos em lâmina. Memórias do Instituto Oswaldo Cruz, (39), 37-41.
12. Charrel RN, Gallian P, Navarro-Mari JM, Nicoletti L, Papa A, Sanchez-Seco MP, Tenorio A, de Lamballerie X. 2005. Emergence of Toscana virus in Europe. Emerging Infectious Diseases, 11(11), 1657-63.
13. Chaskopoulou A, Giantsis IA, Demir S, Bon MC. 2016. Species composition, activity patterns and blood meal analysis of sand fly populations (Diptera: Psychodidae) in the metropolitan region of Thessaloniki, an endemic focus of canine

leishmaniasis. Acta Tropica, 158, 170-176.
14. Chen H, Rangasamy M, Tan SY, Wang H, Siegfried BD. 2010. Evaluation of five methods for total DNA extraction from western corn rootworm beetles. PLoS One, 5(8), e11963.
15. Depaquit J, Grandadam M, Fouque F, Andry PE, Peyrefitte C. 2010. Arthropod-borne viruses transmitted by Phlebotomine sandflies in Europe: a review. Eurosurveillance, 15(10), 19507.
16. Diamond LS, Herman CM. 1954. Incidence of Trypanosomes in the Canada Goose as revealed by bone marrow culture. Journal of Parasitology, 40(2), 195-202.
17. Ding H, Torno M, Vongphayloth K, Ng G, Tan D, Sng W, Ho K, Randrianambinintsoa FJ, Depaquit J, Tan CH. 2025. Hidden in plain sight: discovery of sand flies in Singapore and description of four species new to science. Parasites & Vectors, 18(1), 402.
18. Es-Sette N, Ajaoud M, Bichaud L, Hamdi S, Mellouki F, Charrel RN, Lemrani M. 2014. *Phlebotomus sergenti* a common vector of *Leishmania tropica* and Toscana virus in Morocco. Journal of Vector Borne Diseases, 51(2), 86-90.
19. Favret C. 2005. A new non-destructive DNA extraction and specimen clearing technique for aphids (Hemiptera). Proceedings of the Entomological Society of Washington, 107(2), 469-470.
20. Galati EAB. 2018. Phlebotominae (Diptera, Psychodidae): Classification, morphology and terminology of adults and identification of American taxa, in: Brazilian Sand Flies: Biology, Taxonomy, Medical Importance and Control, Rangel EF, Shaw JJ, Eds. Springer International Publishing: Cham. p. 9-212.
21. Galati EAB, de Andrade AJ, Perveen F, Loyer M, Vongphayloth K, Randrianambinintsoa FJ, Prudhomme J, Rahola N, Akhoundi M, Shimabukuro PHF, Depaquit J. 2025. Phlebotomine sand flies (Diptera, Psychodidae) of the world. Parasites & Vectors, 18(1), 220.
22. Galati EAB, Galvis-Ovallos F, Lawyer P, Leger N, Depaquit J. 2017. An illustrated guide for characters and terminology used in descriptions of Phlebotominae (Diptera, Psychodidae). Parasite, 24, 26.
23. Gariepy T, Kuhlmann U, Gillott C, Erlandson M. 2007. Parasitoids, predators and PCR: the use of diagnostic molecular markers in biological control of Arthropods. Journal of Applied Entomology, 131(4), 225-240.
24. Giantsis IA, Chaskopoulou A, Bon MC. 2016. Mild-Vectolysis: A nondestructive DNA extraction method for vouchering sand flies and mosquitoes. Journal of Medical Entomology, 53(3), 692-695.
25. Gidwani K, Picado A, Rijal S, Singh SP, Roy L, Volfova V, Andersen EW, Uranw S, Ostyn B, Sudarshan M, Chakravarty J, Volf P, Sundar S, Boelaert M, Rogers ME. 2011. Serological markers of sand fly exposure to evaluate insecticidal nets against visceral leishmaniasis in India and Nepal: a cluster-randomized trial. PLoS Neglected Tropical Diseases, 5(9), e1296.
26. Gilbert MTP, Moore W, Melchior L, Worobey M. 2007. DNA extraction from dry museum beetles without conferring external morphological damage. PLoS One, 2(3), e272.
27. Giordani BF, Andrade AJ, Galati EAB, Gurgel-Goncalves R. 2017. The role of wing geometric morphometrics in the identification of sandflies within the subgenus *Lutzomyia*. Medical and Veterinary Entomology, 31(4), 373-380.
28. Guzmán-Larralde AJ, Suaste-Dzul AP, Gallou A, Peña-Carrillo KI. 2017. DNA recovery from microhymenoptera using six non-destructive methodologies with considerations for subsequent preparation of museum slides. Genome, 60(1), 85-91.
29. Hajibabaei M, DeWaard JR, Ivanova NV, Ratnasingham S, Dooh RT, Kirk SL, Mackie PM, Hebert PD. 2005. Critical factors for assembling a high volume of DNA barcodes. Philosophical Transactions of the Royal Society B: Biological Sciences, 360(1462), 1959-1967.
30. Haouas N, Pesson B, Boudabous R, Dedet JP, Babba H, Ravel C. 2007. Development of a molecular tool for the identification of *Leishmania* reservoir hosts by blood meal analysis in the insect vectors. American Journal of Tropical Medicine and Hygiene, 77(6), 1054-1059.
31. Hlavackova K, Dvorak V, Chaskopoulou A, Volf P, Halada P. 2019. A novel MALDI-TOF MS-based method for blood meal identification in insect vectors: A proof of concept study on phlebotomine sand flies. PLoS Neglected Tropical Diseases,

13(9), e0007669.
32. Huemer H, Prudhomme J, Amaro F, Baklouti A, Walder G, Alten B, Moutailler S, Ergunay K, Charrel RN, Ayhan N. 2017. Practical guidelines for studies on sandfly-borne phleboviruses: Part II: Important points to consider for fieldwork and subsequent virological screening. Vector-Borne and Zoonotic Diseases, 17(1), 81-90.
33. Jancarova M, Polanska N, Thiesson A, Arnaud F, Stejskalova M, Rehbergerova M, Kohl A, Viginier B, Volf P, Ratinier M. 2025. Susceptibility of diverse sand fly species to Toscana virus. PLoS Neglected Tropical Diseases, 19(5), e0013031.
34. Kapp JD, Green RE, Shapiro B. 2021. A fast and efficient single-stranded genomic library preparation method optimized for ancient DNA. Journal of Heredity, 112(3), 241-249.
35. Killick-Kendrick R, Maroli M, Killick-Kendrick M. 1991. Bibliography of the colonization of phlebotomine sandflies. Parassitologia, 33 (suppl.), 321-333.
36. Lawyer P, Killick-Kendrick M, Rowland T, Rowton E, Volf P. 2017. Laboratory colonization and mass rearing of phlebotomine sand flies (Diptera, Psychodidae). Parasite, 24, 42.
37. Léger N, Pesson B, Madulo-Leblond G. 1986. Les phlébotomes de Grèce : 1ère partie. Bulletin de la Société de Pathologie Exotique, 79, 386-397.
38. Léger N, Pesson B, Madulo-Leblond G. 1986. Les phlébotomes de Grèce : 2ème partie. Bulletin de la Société de Pathologie Exotique, 79, 514-524.
39. Leonel JAF, Vioti G, Alves ML, da Silva DT, Meneghesso PA, Benassi JC, Spada JCP, Galvis-Ovallos F, Soares RM, Oliveira T. 2020. DNA extraction from individual Phlebotomine sand flies (Diptera: Psychodidae: Phlebotominae) specimens: Which is the method with better results? Experimental Parasitology, 218, 107981.
40. Lestinova T, Rohousova I, Sima M, de Oliveira CI, Volf P. 2017. Insights into the sand fly saliva: Blood-feeding and immune interactions between sand flies, hosts, and *Leishmania*. PLoS Neglected Tropical Diseases, 11(7), e0005600.
41. Lienhard A, Schaffer S. 2019. Extracting the invisible: obtaining high quality DNA is a challenging task in small arthropods. PeerJ, 7, e6753.
42. Lozano-Sardaneta YN, Mikery-Pacheco OF, Huerta H, Rojas-Soriano JE, Contreras-Ramos A. 2025. Wing geometric morphometrics is effective to separate sand fly species (Diptera, Psychodidae, Phlebotominae) related with leishmaniasis transmission in Mexico. Acta Tropica, 262, 107523.
43. Mandrioli M. 2008. Insect collections and DNA analyses: how to manage collections? Museum Management and Curatorship, 23(2), 193-199.
44. Maroli M, Feliciangeli MD, Bichaud L, Charrel RN, Gradoni L. 2013. Phlebotomine sandflies and the spreading of leishmaniases and other diseases of public health concern. Medical and Veterinary Entomology, 27(2), 123-47.
45. Marquina D, Buczek M, Ronquist F, Lukasik P. 2021. The effect of ethanol concentration on the morphological and molecular preservation of insects for biodiversity studies. PeerJ, 9, e10799.
46. Mathis A, Depaquit J, Dvorak V, Tuten H, Banuls AL, Halada P, Zapata S, Lehrter V, Hlavackova K, Prudhomme J, Volf P, Sereno D, Kaufmann C, Pfluger V, Schaffner F. 2015. Identification of phlebotomine sand flies using one MALDI-TOF MS reference database and two mass spectrometer systems. Parasites & Vectors, 8, 266.
47. Mekarnia N, Benallal KE, Sadlova J, Vojtkova B, Mauras A, Imbert N, Longhitano M, Harrat Z, Volf P, Loiseau PM, Cojean S. 2024. Effect of *Phlebotomus papatasi* on the fitness, infectivity and antimony-resistance phenotype of antimony-resistant *Leishmania* major Mon-25. International Journal for Parasitology - Drugs and Drug Resistance, 25, 100554.
48. Milligan BG. 1998. Total DNA isolation, in: Molecular Genetic Analysis of Population: A Practical Approach, Hoelzel AR, Ed. Oxford University Press: Oxford.
49. Molina R, Jiménez M, Alvar J, González E, Hernández-Taberna S, Ines MM. 2017. Methods in sand fly research. Servicio de publicaciones Universidad de Alcala de Henaresp, Madrid.
50. Murphy WJ, Eizirik E, O'Brien SJ, Madsen O, Scally M, Douady CJ, Teeling E, Ryder OA, Stanhope MJ, de Jong WW, Springer MS. 2001. Resolution of the early placental mammal radiation using Bayesian phylogenetics. Science, 294(5550), 2348-51.
51. Nacif-Pimenta R, Pinto LC, Volfova V, Volf P, Pimenta PFP, Secundino NFC. 2020. Conserved and distinct morphological aspects of the salivary glands of sand fly vectors of leishmaniasis: an

anatomical and ultrastructural study. Parasites & Vectors, 13(1), 441.
52. Neuhaus B, Schmid T, Riedel J. 2017. Collection management and study of microscope slides: Storage, profiling, deterioration, restoration procedures, and general recommendations. Zootaxa, 4322(1), 1-173.
53. New TR. 1974. Pscoptera. Handbooks for Identification of British Insects. Vol. I. London: Royal Entomological Society of London. 102 pp.
54. Perez-Ruiz M, Collao X, Navarro-Mari JM, Tenorio A. 2007. Reverse transcription, real-time PCR assay for detection of Toscana virus. Journal of Clinical Virology, 39(4), 276-81.
55. Porco D, Rougerie R, Deharveng L, Hebert P. 2010. Coupling non-destructive DNA extraction and voucher retrieval for small soft-bodied Arthropods in a high-throughput context: the example of Collembola. Molecular Ecology Resources, 10(6), 942-945.
56. Prudhomme J, Cassan C, Hide M, Toty C, Rahola N, Vergnes B, Dujardin JP, Alten B, Sereno D, Banuls AL. 2016. Ecology and morphological variations in wings of *Phlebotomus ariasi* (Diptera: Psychodidae) in the region of Roquedur (Gard, France): a geometric morphometrics approach. Parasites & Vectors, 9(1), 578.
57. Prudhomme J, Gunay F, Rahola N, Ouanaimi F, Guernaoui S, Boumezzough A, Banuls AL, Sereno D, Alten B. 2012. Wing size and shape variation of *Phlebotomus papatasi* (Diptera: Psychodidae) populations from the south and north slopes of the Atlas Mountains in Morocco. Journal of Vector Ecology, 37(1), 137-47.
58. Prudhomme J, Toty C, Kasap OE, Rahola N, Vergnes B, Maia C, Campino L, Antoniou M, Jimenez M, Molina R, Cannet A, Alten B, Sereno D, Banuls AL. 2015. New microsatellite markers for multi-scale genetic studies on *Phlebotomus ariasi* Tonnoir, vector of *Leishmania infantum* in the Mediterranean area. Acta Tropica, 142, 79-85.
59. Prudhomme J, Velo E, Bino S, Kadriaj P, Mersini K, Gunay F, Alten B. 2019. Altitudinal variations in wing morphology of *Aedes albopictus* (Diptera, Culicidae) in Albania, the region where it was first recorded in Europe. Parasite, 26, 55.
60. Rawlins DJ. 1992. Light Microscopy: An Introduction to Biotechniques., Oxford: Bios Scientific publishers. 143 pp.
61. Ready PD. 2013. Biology of phlebotomine sand flies as vectors of disease agents. Annual Review of Entomology, 58, 227-50.
62. Rohlf FJ, Slice D. 1990. Extensions of the Procrustes method for the optimal superimposition of landmarks. Systematic Zoology, 39(1), 40-59.
63. Rowley DL, Coddington JA, Gates MW, Norrbom AL, Ochoa RA, Vandenberg NJ, Greenstone MH. 2007. Vouchering DNA-barcoded specimens: Test of a nondestructive extraction protocol for terrestrial arthropods. Molecular Ecology Notes, 7(6), 915-924.
64. Sábio PB, Andrade AJ, Galati EAB. 2014. Assessment of the taxonomic status of some species included in the *Shannoni* complex, with the description of a new species of *Psathyromyia* (Diptera: Psychodidae: Phlebotominae). Journal of Medical Entomology, 51(2), 331-341.
65. Sadlova J, Yeo M, Seblova V, Lewis MD, Mauricio I, Volf P, Miles MA. 2011. Visualisation of *Leishmania donovani* fluorescent hybrids during early stage development in the sand fly vector. PLoS One, 6(5), e19851.
66. Sales K, Miranda DEO, da Silva FJ, Otranto D, Figueredo LA, Dantas-Torres F. 2020. Evaluation of different storage times and preservation methods on phlebotomine sand fly DNA concentration and purity. Parasites & Vectors, 13(1), 399.
67. Sales KG, Costa PL, de Morais RC, Otranto D, Brandao-Filho SP, Cavalcanti Mde P, Dantas-Torres F. 2015. Identification of phlebotomine sand fly blood meals by real-time PCR. Parasites & Vectors, 8, 230.
68. Sant'Anna MR, Jones NG, Hindley JA, Mendes-Sousa AF, Dillon RJ, Cavalcante RR, Alexander B, Bates PA. 2008. Blood meal identification and parasite detection in laboratory-fed and field-captured *Lutzomyia longipalpis* by PCR using FTA databasing paper. Acta Tropica, 107(3), 230-7.
69. Senne NA, Santos HA, Araujo TR, Paulino PG, Mendonca LP, Moreira HVS, Camilo TA, da Costa Angelo I. 2022. Robust comparative performance of genomic DNA extraction methods from non-engorged phlebotomine sandflies. Medical and Veterinary Entomology, 36(2), 203-211.
70. Shaw JJ. 2025. A review of *Leishmania* infections in American Phlebotomine sand flies - Are those that transmit leishmaniasis anthropophilic or

anthropportunists ? Parasite, 32, 57.
71. Tesh RB, Modi GB. 1983. Growth and transovarial transmission of Chandipura virus (Rhabdoviridae: Vesiculovirus) in *Phlebotomus papatasi*. American Journal of Tropical Medicine and Hygiene, 32(3), 621-3.
72. Thomsen PF, Elias S, Gilbert MTP, Haile J, Munch K, Kuzmina S, Froese DG, Sher A, Holdaway RN, Willerslev E. 2009. Non-destructive sampling of ancient insect DNA. PLoS One, 4(4), e5048.
73. Truett GE, Heeger P, Mynatt RL, Truett AA, Walker JA, Warman ML. 2000. Preparation of PCR-quality mouse genomic DNA with hot sodium hydroxide and tris (HotSHOT). Biotechniques, 29(1), 52-54.
74. Upton MS. 1993. Aqueous gum-chloral slide mounting media: an historical review. Bulletin of Entomological Research, 83(2), 267-274.
75. Volf P, Myskova J. 2007. Sand flies and *Leishmania*: specific versus permissive vectors. Trends in Parasitology, 23(3), 91-92.
76. Wang Q, Wang X. 2012. Comparison of methods for DNA extraction from a single chironomid for PCR analysis. Pakistan Journal of Zoology, 44(2).
77. Wang Y, Zhao Y, Bollas A, Wang Y, Au KF. 2021. Nanopore sequencing technology, bioinformatics and applications. Nature Biotechnology, 39(11), 1348-1365.

Cite this article as: Randrianambinintsoa FJ, Augendre L, Prudhomme J, Martinet J-P, Loyer M, Mekarnia N, Kerkoub H, Perveen FK, Huguenin A, Kariya E, Akhoundi M, De Andrade AJ, Berriatua E, Bongiorno G, Boyer S, Christodoulou V, Da Costa-Ribeiro MCV, De Souza LAF, Ding H, Dondji B, Dvořák V, Erisoz Kasap O, Galati EAB, Gállego M, Ballart C, Gouzelou S, Haddad N, Masse RS, Mekuria AH, Ivovic V, Kaczmarek S, Shahar MK, Kirstein OD, Kniha E, Kolářová I, Lincoln T, Lucanas C, Mikov O, Nov K, Özbel Y, Pesson B, Posada Lopez LC, Prasetyo DB, Rahola N, Rebollar-Tellez EA, Rodrigues BL, Roy L, Saini P, Sanjoba C, Shimabukuro PH, Siriyasatien P, Soszynska A, Suleşco T, Sylla M, Torno M, Volf P, Vongphayloth K, Sinh Nam V, Wardhana A, Yessinou E, Zapata S, Gantier J-C & Depaquit J. 2026. Processing and mounting phlebotomine sand flies: a consensus guideline. Parasite xx, xx. https://doi.org/10.1051/parasite/2026009.

PARASITE

An international open-access, peer-reviewed, online journal publishing high quality papers on all aspects of human and animal parasitology

Reviews, articles and short notes may be submitted. Fields include, but are not limited to: general, medical and veterinary parasitology; morphology, including ultrastructure; parasite systematics, including entomology, acarology, helminthology and protistology, and molecular analyses; molecular biology and biochemistry; immunology of parasitic diseases; host-parasite relationships; ecology and life history of parasites; epidemiology; therapeutics; new diagnostic tools.
All papers in Parasite are published in English. Manuscripts should have a broad interest and must not have been published or submitted elsewhere. No limit is imposed on the length of manuscripts.

Parasite (open-access) continues **Parasite** (print and online editions, 1994-2012) and **Annales de Parasitologie Humaine et Comparée** (1923-1993) and is the official journal of the Société Française de Parasitologie.

Editor-in-Chief:
Jean-Lou Justine, Paris

Submit your manuscript at:
https://www.editorialmanager.com/parasite

परिशिष्ट १: जैव रासायनिक सैद्धान्तिक आधारहरू।

यस निर्देशिकामा सम्बन्धित आर्थ्रोपोडहरू भुसुनाहरू हुन्। तर, यसलाई अन्य धेरै सामान्य आर्थ्रोपोडहरूमा पनि विस्तार गर्न सकिन्छ जसको पहिचान केवल आन्तरिक लक्षणहरू मार्फत मात्र गर्न सकिन्छ। संयोगवश, केही आन्तरिक अंगहरू आंशिक रूपमा चिटिनाइज्ड हुन्छन् र तिनीहरूको आकृतिले हामीलाई बहुमूल्य जानकारी प्रदान गर्दछ। यसैले खाद्य पम्पहरू, सपर्माथिका र तिनीहरूका नलिकाहरू अवलोकन गर्न धेरै रोचक छ। हामीले प्रयोग गर्ने सबै प्रतिक्रियाशील पदार्थहरू (reagents) सन्दर्भ गर्दा, कीराको स्थिरीकरण (fixation) देखि अन्तिम तयारी (assembly) सम्म, मुख्यतः रेडॉक्स (redox) प्रतिक्रियाहरूको प्रयोग गरिन्छ भन्ने कुरा कहिल्यै भुल्न हुँदैन। एकमात्र सावधानी वा मार्गदर्शनको विचार भनेको रिड्यूसिङ पदार्थ (reducing) र अक्सिडाइजिङ पदार्थ (oxidizing) हरूलाई सँगै मिसिन नदिनु हो।

इथाइल अल्कोहल; इथेनॉल:

अल्कोहल अणुहरू पानीप्रति अत्यन्त आकर्षित हुने भएकाले यसले निर्जलीकरण (dehydrating) प्रभाव देखाउँछ। तर, कम सघनता भएको अल्कोहल (अर्थात् पानी धेरै भएको) न्यूक्लिक एसिडको बिग्रन (degradation) प्रक्रियामा भूमिका खेल्छ, किनकि पानी न्यूक्लिक एसिडको शत्रु हो।

जब कीराहरूलाई इथेनॉलमा राखिन्छ, यो केवल तिनीहरूलाई संरक्षण गर्न मात्र होइन, तर तन्तुहरूलाई ठीक गर्न पनि हो। हिस्टोलोजीमा, हामी सामान्यतया दुई महत्त्वपूर्ण अवधारणाहरू छुट्याउँछौं: प्रवेश दर र फिक्सेशन दर। यो राम्रोसँग बुझिएको छ कि राम्रो संरक्षकले पहिले तन्तुहरूलाई फिक्स गर्नु अघि गहिरो रूपमा प्रवेश गर्नुपर्छ। ९६% मा अल्कोहलको लागि, प्रवेश गुणांक लगभग १.०५ छ (तुलनामा, पिक्रिक एसिडको ०.७५% जलीय घोलको लागि, प्रवेश गुणांक ०.४५ छ, जबकि ३% पोटासियम डाइक्रोमेट घोलको १.४५ छ)।

कीराहरू र अन्य आर्थ्रोपोडहरूलाई इथेनॉलमा अनिश्चित कालसम्म संरक्षण गर्ने चाहना कीटविज्ञानीहरूको लागि वास्तविकता हो। पछिल्ला अध्ययनहरूको लागि वा भविष्यका अनुसन्धानकर्ताहरूको लागि फिल्ड क्याप्चरहरू राख्न चाहनु अझै पनि धेरै सम्मानजनक छ। यद्यपि, यो पद्धति साइटोलोजिस्ट वा हिस्टोलोजिस्टको लागि सम्भव छैन। भुसुनाहरूलाई फिक्सेटिभमा धेरै लामो समयसम्म राख्ने गरेमा, तिनीहरू पुन: काम गर्न व्यावहारिक रूपमा असम्भव हुन सक्छन्। यही कारणले गर्दा १० वर्ष भन्दा पुरानो भुसुनाहरू प्रयोग गर्न गाह्रो वा असम्भव पनि हुन्छन्।

अर्को विचार भनेको फिक्स गर्नुपर्ने आर्थ्रोपडको पिण्ड र फिक्सेटरको आयतन बीचको अनुपात हो। प्राणीशास्त्र वा चिकित्सा अभ्यासमा, निश्चित गर्नुपर्ने टुक्राहरूको आयतन भन्दा ६० गुणा बढी आयतनको योजना बनाउनु उचित हुन्छ। व्यवहारिक रूपमा, सूक्ष्म-आर्थ्रोपडहरूको लागि, निश्चित गर्नुपर्ने भुसुनाहरूको दिइएको मात्राको लागि, कम्तिमा ४-५ मात्रामा अल्कोहल थप्नुहोस्। ध्यान राख्नुहोस् कि अल्कोहलले आफ्नो शक्ति गुमाउनेछ किनकि यसले आर्थ्रोपड तन्तुहरूमा रहेको सबै पानी हटाउँछ।

निष्कर्षमा:

- इथाइल अल्कोहल रिड्यूसिङ रासायनिक पदार्थ हो (त्यसैले अक्सिडेटिभ फिक्सेटिभहरूसँग असंगत);
- यसले प्रोटिनहरूलाई ऊर्जावान रूपमा अवक्षेपण गर्छ र तिनीहरूलाई विकृत बनाउँछ;
- यसले केही जटिल लिपिडहरूलाई पगाल्छ र ग्लाइकोजेन अवक्षेपण गर्छ;
- यसले तन्तुहरूको बलियो संकुचन गराउँछ र तिनीहरूलाई कडा बनाउँछ।

क्षारिय पोटासियम वा सोडियम हाइड्रोक्साइड घोलहरू:

कीट विज्ञानमा, स्पष्ट तर्क बिना नै, यी घोलहरूको प्रयोग मुख्यतया पोटासियम हाइड्रोक्साइडमा केन्द्रित छ।

सोडियम हाइड्रोक्साइड [E524] घोलमा पाइन्छ, या त फरक सांद्रतामा वा फरक सामान्यतामा। यो गोली वा चमकमा आउँछ। यसको प्रमुख बेफाइदा यो हो कि यो धेरै हाइग्रोस्कोपिक छ (KOH भन्दा बढी)। जब यसले प्रोटीनहरूसँग प्रतिक्रिया गर्दछ, यसले तिनीहरूलाई पगाल्छ, र लिपिडहरूसँग यसले सेपोनिफिकेशनको समयमा तिनीहरूलाई कडा साबुनमा रूपान्तरण गर्दछ (यो KOH सँग एक प्रमुख भिन्नता हो, जसले सेपोनिफिकेशनको समयमा तरल साबुन उत्पादन गर्दछ)।

पोटासियम हाइड्रोक्साइड [E525] एक गाढा घोलको रूपमा उपलब्ध हुन्छ, तर सबैभन्दा बढी, यसको फाइदा लगभग ०.१ ग्रामको गोलीहरूमा तयार पारिएको छ, जसले सटीक सन्तुलन निर्दिष्ट नगर्दा पातलो घोलको तयारीलाई धेरै सहज बनाउँछ। उदाहरणका लागि, १ एमएल डिस्टिल्ड पानीमा ०.१ ग्रामको १ गोलीले १०% घोल उत्पादन गर्छ। गोलीहरूको रूपमा पोटासियम हाइड्रोक्साइड को दोस्रो फाइदा भनेको कार्बोनेशनको लागि यसको कम संवेदनशीलता हो (KOH घोलमा CO_2 फिक्स गर्न उच्च आकर्षण हुन्छ, जसले गर्दा कार्बोनेट लवणहरू सिर्जना हुन्छन्)।

यी बलियो क्षारहरू फ्याटी एसिडहरूलाई पानीमा घुलनशील साबुनमा रूपान्तरण गरेर घुलनशील बनाउन प्रयोग गरिन्छ। यो याद राख्नु पर्छ कि फिक्सेटिभ, जस्तै इथेनॉलले, भुसुनामा केही बोसोलाई घुलनशील बनाउँछ।

यद्यपि, जलीय माध्यममा भुसुनालाई बलियो क्षारले प्रतिस्थापन गर्दा, फ्याटी एसिडहरू (कम वा बढी जटिल) अवक्षेपण हुनेछन्। त्यसैले बलियो क्षारले चिसो सेपोनिफिकेशन गर्छन्। केही अवस्थामा, जब एडिपोज तन्तुहरू बढी हुन्छन्, उदाहरणका लागि पोथीहरूमा, प्रतिक्रियालाई सहज बनाउन तापमान 35-40 डिग्री सेल्सियसमा बढाउनु फाइदाजनक हुन्छ, वा अन्यथा कोठाको तापक्रममा बढी समयसम्म राख्नु फाइदाजनक हुन्छ।

रंगीन एसिड घोल/रंगहीन मार्क-एन्ड्रे घोल:

यहाँ, हामी मार्क-एन्ड्रे घोलको प्रयोग गर्नुका फाइदा र कमजोरीहरूको विश्लेषण गर्नेछौं। यो घोल क्लोरल हाइड्रेट (ट्राइक्लोरासेटाल्डिहाइड मोनोहाइड्रेट), एसिटिक एसिड र पानीबाट बनेको हुन्छ। यो घोल धेरै अक्सिडाइजिंग (एसिड र एल्डिहाइडको मिश्रण) हो। यसले पोटासियम हाइड्रोक्साइडको प्रयोगको क्रममा बनेको क्षारीय साबुनहरूलाई अवक्षेपण नगरी भुसुनाहरूमा रहन सक्ने अतिरिक्त पोटासियम हाइड्रोक्साइडलाई बेअसर गर्छन्। यो अक्सिडाइजिंग घोलले चिटिनमा बन्ने ग्लुकोसामाइनहरूको अल्कोहलको अरू कार्यहरूमा पनि प्रभाव पार्छन् जुन तिनीहरूलाई अक्सिडाइज गरेर, र चिटिनलाई नरम पारेर। अर्को कार्य भनेको उपस्थित केही खनिज लवणहरूको विघटन हो।

जब मार्क-एन्ड्रे घोललाई पहिले एसिड फ्युचसिनले रंग लगाइन्छ (यसरी अक्सिडाइज्ड अवस्थामा), यो संरचनाको अल्कोहल प्रकार्यहरूमा फिक्स गर्न सक्षम हुनेछ। मार्क-एन्ड्रे घोलको सम्पर्क समय र भुसुनाहरूको रंगाईने अवस्था पछि, धुने काम केवल इथेनॉलले गरिन्छ। यसरी, हामी भुसुनाहरूको निर्जलीकरण चरण सुरु गर्छौं।

फाइदाहरू:

- अतिरिक्त क्षारिय घोलको तटस्थीकरण
- चिटिनको फैलावट
- चिटिनाइज्ड आन्तरिक संरचनाहरू राम्रोसँग देखिन र मूल्याङ्कन गर्न चिटिनमा रंग लगाउने

कमजोरीहरू:

क्लोरल हाइड्रेट एक निद्राजनक औसधि हो र यसलाई मानव चिकित्सामा प्रयोग गरिएको हुन सक्छ। यसलाई रासायनिक हुड अन्तर्गत प्रयोग गर्नुपर्छ, र रासायनिक जोखिम सम्बन्धी कानूनको पालना गर्नुपर्छ।

निर्जलीकरण तरलहरू:

अनुभवले देखाउँछ कि धेरै साना भुसुनाहरूको लागि, बढ्दो सांद्रता सहित अल्कोहलमा डुबायने अनुक्रम पालना गर्नु उपयोगी हुँदैन। यदि भुसुना ठूलो छ भने, हामी ८०% इथेनॉलबाट सुरू गर्छौं, त्यसपछि ९०%, ९५% र अन्तमा निरपेक्ष इथेनॉलबाट सक्छौ। धेरै साना भुसुनाहरू छन् भने, ९०% इथेनॉलमा डुबाउनुहोस् र त्यसपछि निरपेक्ष इथेनॉलमा डुबाउनुहोस्। यस चरणमा, निरपेक्ष इथेनॉलले वायुमण्डलमा पानीलाई स्थिर गर्न खोज्छ।

कीट विज्ञान प्रयोगशालाहरूमा परम्परागत बिच क्रियोसोट बाथको साथ भुसुनाहरूको निर्जलीकरणलाई गरिन्थ्यो। आज, कीटनाशक, एन्टिफंगल, र काठ संरक्षकको रूपमा व्यापक रूपमा प्रयोग हुने यो सार, यसको गन्ध (पोलिसाइक्लिक एरोमेटिक हाइड्रोकार्बन) को कारणले गर्दा यसको प्रयोग धेरै निरुत्साहित गरिएको छ। यसलाई प्रजननका लागि विषाक्त (reprotoxic), क्यान्सरकारक (carcinogenic), दीर्घकालसम्म वातावरणमा टिकिरहने जैविक प्रदूषक (persistent organic pollutant) तथा जलीय जीवहरूका लागि विषाक्त (ecotoxic) मानिन्छ।

भुसुनाहरू माउन्ट गर्नको लागि हामीले तयार पार्ने एउटा समाधान भनेको युपरल® र युपरल सार हो (तलको अनुच्छेदमा वर्णन गरिएको छ)। ९०% इथेनॉलमा डुबाएर राखिएको वा भिजाएर निकालिएको भुसुनाहरूको माउन्टिङ को लागि युपरल® र युपरल सारको मिश्रण धेरै राम्रो मानिएको छ।

परिशिष्ट २: प्रयोग गरिएका अभिकर्मकहरूको संरचना।

पोटासियम हाइड्रोक्साइड १०%

पोटासियम हाइड्रोक्साइड १० ग्राम
आसुत पानी क्यूएस १०० मिलिलिटर

गम क्लोरल माउन्टिङ माध्यम वा होयर माध्यम

आसुत पानी ५० मिलिलिटर
क्लोरल हाइड्रेट २०० ग्राम
गम अरेबिक ५० ग्राम
ग्लिसरॉल २० एमएल

मार्क-आन्द्रे घोल

क्लोरल हाइड्रेट ४० ग्राम
ग्लेसियल एसिटिक एसिड ३० मिली
आसुत पानी ३० मिलिलिटर

आसुत पानीमा १% फुचसिन एसिड

एसिड फुचसिन पाउडर १ ग्राम
आसुत पानी ९९ मिलिलिटर

फुचसिनले रंगिएको मार्क-आन्द्रे घोल

मार्क-एन्ड्रे घोल १० एमएल
फुचसिन १% ५० µL

परिशिष्ट ३: युपरल®, क्यानाडा बाल्सम, पोलिभिनाइल अल्कोहल वा माउन्टिङको लागि अन्य घोलहरू

पोलिभिनाइल अल्कोहल: उचित निर्जलीकरणको लागि आवश्यक उत्पादनहरू उपलब्ध नभएको बेला यो आदर्श माउन्टिंग माध्यम हो। त्यसपछि पोलिभिनाइल अल्कोहललाई अम्मानको ल्याक्टोफेनोलसँग मिसाइन्छ। यद्यपि, यी संयोजनहरूमा पानीको वाष्पीकरणको कारणले गर्दा पोलिभिनाइल अल्कोहल सुक्ने वा क्रिस्टलाइज हुने वा फिनोल अक्सिडाइज हुँदा कालो हुने प्रमुख बेफाइदाहरू हुन्छन्। यो छोटो अवधिको माउन्टिंगको लागि राम्रो प्रविधि हो।

क्यानडा बाल्सम: स्लाइड र कभरस्लिप बीच माउन्ट गर्न यसको प्रयोगको लागि माउन्ट गरिने भुसुनाहरूको निर्जलीकरण आवश्यक पर्दछ। जाइलिन वा टोल्युइनको प्रयोग असुविधा बिहिन भने छैन।

ईनिस (Enecê): स्लाइड र कभरस्लिप बीच माउन्ट गर्न, जस्तै क्यानाडा बाल्सम, यसलाई भुसुनाको निर्जलीकरण आवश्यक पर्दछ। थप सूत्रीकरण: शुद्ध सेतो कोलोफोनी (२२ ग्राम); अल्कोहल-घुलनशील कोपल गम (१२ ग्राम), निरपेक्ष अल्कोहल (२० एमएल), कपूर (१० ग्राम), टर्पेन्टाइन एसेन्स (१० एमएल), र युकेलिप्टोल (२६ एमएल)। यसको तयारीको लागि, एर्लेनमेयर फ्लास्क जस्ता कन्टेनरमा, निरपेक्ष अल्कोहल र कपूर राख्नुहोस्। त्यसपछि कोलोफोनी र कोपल गम थप्नुहोस्। त्यसपछि फ्लास्कलाई स्टपरले बन्द गरिन्छ र हल्लाइन्छ, र त्यसपछि मात्र यसलाई बेन-मेरीमा (bain-marie) हल्का तापक्रममा तताइन्छ ताकि मिश्रण नउम्लियोस्। सामग्रीहरू पूर्ण रूपमा तरल भएपछि, टर्पेन्टाइन एसेन्स थपिन्छ, त्यसपछि मिश्रण अझै तातो हुँदा फिल्टर गरिन्छ, र अन्तमा, युकेलिप्टोल फिल्ट्रेटमा थपिन्छ। जब माध्यम कम तरल हुन्छ, यसलाई Eenecê ले पातलो गरिन्छ, जसमा निम्न सूत्र हुन्छ: निरपेक्ष अल्कोहल (३० एमएल), कपूर (१७ ग्राम), टर्पेन्टाइन एसेन्स (१५ एमएल), युकेलिप्टोल (३८ एमएल) [11]।

युपरल®: यो एटलस क्षेत्रमा पाईने टेट्राक्लिनिस आर्टिकुलाटा (*Tetraclinis articulata*) (वाहल, १७९१) जातको धूपीबाट आउने रेजिन हो र यसलाई १९०६ मा गिलसनले अध्ययन र विकास गरेका थिए। यसको मुख्य फाइदा यो हो कि यो पोलिमराइज हुँदैन। स्लाइड र कभर-स्लिपहरू बीच राखिएका भुसुनाहरू अल्कोहल वा अझ राम्रोसँग युपरल® सारद्वारा सजिलै पुन: प्राप्त गर्न सकिन्छ। यो रेजिन, जसलाई स्यान्डाराक वा गम जुनिपर पनि भनिन्छ। यो ८०% इथेनॉलमा मज्जाले घुल्छ।

ट्राइटन X१०० को उपयोग: गैर-आयनिक जलीय घोल: Triton X100 एक गैर-आयनिक जलीय घोल (4-(1,1,3,3-tetramethylbutyl) फिनाइल-पोलिथिलीन ग्लाइकोल घोल, वा t-octylphenoxypolyethoxyethanol, polyethylene glycol tert-octylphenyl ईथर) को रूपमा हुन्छ। यो कोषीय र आणविक जीवविज्ञानमा डिटर्जेन्टको रूपमा व्यापक रूपमा प्रयोग गरिन्छ। यसले कोष र केन्द्रका झिल्लीहरूको पारगम्य बनाउन सहयोग गर्दछ।

धेरै वर्षसम्म भुसुनाका नमूनाहरू अल्कोहलमा संरक्षित गरिनु सामान्य मानिन्छन्। दुर्भाग्यवश, अल्कोहलमा संरक्षण अति उपयुक्त भने हुँदैन, र यसरी संरक्षित गरिएका आर्थ्रोपोडहरू सूक्ष्म परीक्षणको लागि तयार गर्न धेरै गाह्रो हुन्छन्। भुसुनाहरू भएको प्लास्टिक प्रायः बिग्रने हुन्छ, त्यसपछि अल्कोहलको वाष्पीकरण सुरू हुन्छ। दुवै अवस्थामा, अल्कोहलसँग लामो समयसम्म सम्पर्कमा रहनु वा भुसुनाहरू सुक्न सक्ने जोखिम हुंदा अल्कोहलले वास्तविक समस्या खडा गर्छ। २००८ मा, जोन्कले फोटोग्राफिक फिल्महरूको लागि प्रयोग गरिने एजेपोन (Agepon) जस्ता भिजाउने एजेन्टको साथ माकुराहरूको पुनर्जलीकरणमा एउटा नोट प्रकाशित गरे [26]। यसले शक्तिशाली डिटर्जेन्ट नभएका

भिजाउने एजेन्टहरू प्रयोग गर्ने विचारलाई निम्त्यायो। तल ०.५% जलीय घोलमा ट्राइटन X१०० प्रयोग गर्ने प्रक्रिया दिइएको छ:

- सुख्खा नमुनालाई पूर्ण अल्कोहलले भिजाउनुहोस्।
- सम्पूर्ण नमुना डुबाउनको लागि आवश्यक मात्रामा ट्राइटन X१०० घोल ०.५% थप्नुहोस्।
- लगभग ५ मिनेट वा सोभन्दा बढी समयसम्म भिज्न दिनुहोस्। सबै आर्थ्रोपोडहरू घोलमा छुट्टीएको हुनुपर्छ।
- ट्राइटन X१०० घोल हटाउने र पोटासियम हाइड्रोक्साइड घोलले प्रतिस्थापन गर्नुहोस्।

त्यसपछि माथि वर्णन गरिए अनुसार प्रविधि अपनाउनुहोस्।

परिशिष्ट ४: युपरल®वा क्यानडा बाल्सम माउन्टिङ मिडिया चरणबद्ध रूपमा

१. भुसुनाहरू निर्जलित हुनुपर्छ (बादल वा दुध जस्तो देखिनुले अपर्याप्त निर्जलीकरणको संकेत गर्दछ)।

२. इथाइल अल्कोहलको सांद्रता बढाएर निर्जलीकरण गर्न सकिन्छ।

३. भुसुनाहरू ९९% अल्कोहल वा पूर्ण अल्कोहलबाट क्लियरिङ्ग एजेन्टमा स्थानान्तरण गर्न सकिन्छ।

प्रक्रिया:

१. वयस्क भुसुनालाई ७०% इथेनॉलमा राख्नुहोस्।

२. इथेनॉल हटाउनुहोस् र १०% KOH ले हाल्नुहोस्। भुसुनालाई सिसाको स्लाइडले छोप्नुहोस्।

३. भुसुनाहरू पारदर्शी नभएसम्म थिच्नुहोस्।

४. KOH हटाउनुहोस्।

५. भुसुनालाई आसुत पानीले भिजाउनुहोस् र ३० देखि ४५ मिनेट पर्खनुहोस्।

६. पानी निकाल्नुहोस् र ३० मिनेटसम्म आसुत पानीले धुनुहोस् (धेरै भुसुनाहरूको समय कार्य: सँगै प्रशोधन गर्ने जति धेरै भुसुनाहरू हुन्छन्, यो समय त्यति नै लामो हुनुपर्छ। जति कम हुन्छन्, विशेष गरी एकल रूपमा उपचार गरिएकाहरूका लागि, यो समय त्यति नै छोटो हुन सक्छ))

७. पानी निकाल्नुहोस्।

८. मार्क-एन्ड्रे घोल (भएसम्म फुचसिन एसिडले रंगिएको) हाल्नुहोस् र २४ घण्टा (एक दिन) पर्खनुहोस्।

९. मार्क-एन्ड्रे घोल निकाल्नुहोस्।

१०. भुसुनालाई आसुत पानीले भिजाउनुहोस् र ३० देखि ४५ मिनेट पर्खनुहोस्।

११. पानी निकाल्नुहोस् र ३० मिनेटसम्म आसुत पानीले फेरि भिजाउनुहोस्।

१२. पानी निकाल्नुहोस्।

१३. ७०% इथेनॉल थप्नुहोस् र भुसुनाको विच्छेदन गर्नुहोस्।

क. टाउको र पेटको लागि, टाउको वा पेटलाई छातीबाट बिस्तारै तान्नुहोस्।

ख. छातीको लागि, एक जोडी फोर्सेप्सले छाती समातेर र अर्को जोडीले एपेन्डेजको आधारमा तानेर पखेटाहरू हटाउनुहोस्। सबैभन्दा चासोको अंगहरूमा विशेष ध्यान दिंदै, छातीलाई बायाँ र दायाँ छेउमा विभाजन गरेर, बाणु विच्छेदन गर्न सम्भव छ।

१४. जलीय इथाइल अल्कोहल घोलको श्रृंखला मार्फत बिस्तारि, ५० - ८० - ९५% सम्म पूर्ण इथेनॉल नपुगुन्जेल, निर्जलीकरण गर्नुहोस्। ।

१५. भुसुनाहरूलाई १००% इथेनॉलले १० मिनेटको अन्तरालमा दुई पटक धोएर निर्जलीकरण गर्नुहोस्।

१६. इथेनॉल निकाल्नुहोस् र कोठाको तापक्रममा १५ मिनेटको लागि ल्वाङको तेलले भुसुनाहरू भिजाउनुहोस्।

१७. ल्वाङको तेलबाट भुसुनाहरूलाई सफा स्लाइडमा Euparal® वा क्यानाडा बाल्समको थोपामा स्थानान्तरण गर्नुहोस्।

१८.इच्छा अनुसार मिलाउनुहोस्: स्यान्ड फ्लाईको टाउको, छाती र पेटलाई विच्छेदन गर्ने माइक्रोस्कोप मुनि मसिना सुई वा फोर्सेप्स प्रयोग गरेर विच्छेदन गर्न सकिन्छ। भेन्ट्रो-डोर्सल स्थितिमा राख्नको लागि टाउकोलाई शरीरबाट विच्छेदन गर्नुपर्छ, अर्थात्, ओसिपिटल फोरेमेन माथितिर उन्मुख हुनुपर्छ ताकि सिबेरियमलाई यसको माध्यमबाट प्रत्यक्ष रूपमा अवलोकन गर्न सकियोस्। विच्छेदन भुसुना माउन्ट गर्ने माध्यममा गरिन्छ।

१९. सतह टाँसिने रहेसम्म भुसुनालाई त्यसै छोड्नुहोस्।

२०. सफा कभरस्लिपलाई पूर्ण अल्कोहलले भिजाउनुहोस्। कभरस्लिपलाई क्यानाडा बाल्सममा मिलाएर राख्नुहोस्।

२१. यस उद्देश्यको लागि बनाइएको सुक्खा बक्समा स्लाइडहरू भण्डार गर्नुहोस्।
