# Supplementary material for: Processing and mounting phlebotomine sand flies: a consensus guideline
Source: Parasite. 2026 Apr 3;33:18. doi: 10.1051/parasite/2026009 (PMC13047900; doi:10.1051/parasite/2026009)
Supplement: Supplementary file 23 — Polish translation / Tłumaczenie polskie [file parasite-33-18-s23.pdf]

Parasite **33**, 18 (2026)
© F.J. Randrianambinintsoa et al., published by EDP Sciences, 2026
https://doi.org/10.1051/parasite/2026009

PARASITE
Available online at:
www.parasite-journal.org

REVIEW ARTICLE OPEN ACCESS

Opracowywanie i preparatyka muchówek z podrodziny Phlebotominae: ujednolicone wytyczne

Fano José Randrianambinintsoa[1], Laure Augendre[1], Jorian Prudhomme[1], Jean-Philippe Martinet[1], Mathieu Loyer[1], Nalia Mekarnia[1], Hocine Kerkoub[1], Farzana Khan Perveen[1], Antoine Huguenin[1,2], Emilie Kariya[1,2], Mohammad Akhoundi[3], Andrey José de Andrade[4], Eduardo Berriatua[5], Gioia Bongiorno[6], Sébastien Boyer[7,8], Vasiliki Christodoulou[9], Magda Clara Vieira Da Costa-Ribeiro[10], Lucas Alexandre Farias de Souza[10], Huicong Ding[11], Blaise Dondji[12], Vít Dvořák[13], Ozge Erisoz Kasap[14], Eunice Aparecida Bianchi Galati[15], Montserrat Gállego[16], Cristina Ballart[16], Stavroula Gouzelou[17], Nabil Haddad[18], Rezki Sabrina Masse[19], Asrat Hailu Mekuria[20], Vladimir Ivovic[21], Szymon Kaczmarek[22], Mohd Khadri Shahar[19], Oscar D. Kirstein[23], Edwin Kniha[24], Iva Kolářová[13], Lincoln Timinao[25], Cristian Lucanas[26], Ognyan Mikov[27], Kimsear Nov[7], Yusuf Özbel[28], Bernard Pesson[29], Laura Cristina Posada Lopez[30], Didot Budi Prasetyo[1,7], Nil Rahola[31], Eduardo A. Rebollar-Tellez[32], Bruno Leite Rodrigues[15], Lalita Roy[33], Prasanta Saini[34], Chizu Sanjoba[35], Paloma Helena Fernandes Shimabukuro[36], Padet Siriyasatien[37], Agnieszka Soszyńska[22], Tatiana Suleşco[38], Massamba Sylla[39], Majhalia Torno[40], Petr Volf[13], Khamsing Vongphayloth[41], Vu Sinh Nam[42], April Wardhana[43], Eric Yessinou[44], Sonia Zapata[45], Jean-Charles Gantier[1], and Jérôme Depaquit[1,2,*]

[1] Faculté de Pharmacie, Université de Reims Champagne Ardenne, UR ESCAPE-USC ANSES PETARD, 51 rue Cognacq-Jay, 51096 Reims Cedex, France
[2] Pôle de Biologie territoriale, Laboratoire de Parasitologie-Mycologie, Centre Hospitalo-Universitaire, 51092 Reims, France
[3] Parasitology-Mycology Department, Avicenne Hospital, AP-HP, Bobigny, Sorbonne Paris Nord University, France; Unité des Virus Émergents (UVE: Aix-Marseille Univ, Università di Corsica, IRD 190, Inserm 1207, IRBA), 13005 Marseille, France
[4] Parasitology Collection of Basic Pathology, Department of Basic Pathology, Federal University of Paraná, Curitiba 19031, Brazil
[5] Department of Animal Health, University of Murcia, Campus de Espinardo, 30100 Espinardo, Murcia, Spain
[6] Department of Infectious Diseases, Vector-borne Diseases Unit, Istituto Superiore di Sanità, 00166 Rome, Italy
[7] Medical and Veterinary Entomology Unit, Institut Pasteur du Cambodge, Phnom Penh 12201, Cambodia
[8] Ecology & Emergence of Arthropod-borne Pathogens Unit, Department of Global Health, Institut Pasteur, CNRS UMR2000, 75015 Paris, France
[9] Section Veterinary Services (1417), Laboratory for Animal Health Virology, Aglantzia, Nicosia 2109, Cyprus
[10] Insects Vectors and Parasites Laboratory, Department of Basic Pathology and Postgraduate program in Microbiology, Parasitology and Pathology, Federal University of Paraná, 81530-900 Curitiba, Brazil
[11] Department of Biological Sciences, National University of Singapore, 117558, Singapore
[12] Laboratory of the Leishmaniasis Research Project, Mokolo District Hospital, Mokolo, Cameroon; Laboratory of Cellular Immunology and Parasitology, Department of Biological Sciences, Central Washington University, 98926 Ellensburg, WA, USA
[13] Department of Parasitology, Faculty of Science, Charles University, 12800 Prague, Czechia
[14] VERG Laboratories, Department of Biology, Faculty of Science, Hacettepe University, Beytepe, Ankara 06800, Türkiye
[15] Faculdade de Saúde Pública da Universidade de São Paulo (FSP/USP), Pós-graduação em Saúde Pública, 01246-904 São Paulo, Brazil
[16] Secció de Parasitologia, Departament de Biologia, Sanitat i Medi Ambient, Facultat de Farmàcia i Ciències de l'Alimentació, Universitat de Barcelona, & Institut de Salut Global de Barcelona (ISGlobal), Centro de Investigación Biomédica en Red, Enfermedades Infecciosas (CIBERINFEC), 08028 Barcelona, Spain
[17] Laboratory of Infectious Diseases and Public Health, School of Medicine, University of Cyprus, Nicosia, Cyprus & Department of Pediatrics, Archbishop Makarios III Hospital, Nicosia 2115, Cyprus
[18] Faculty of Health Sciences, American University of Beirut, 1107 2020 Beirut, Lebanon
[19] Medical Entomology Unit, Infectious Disease Research Centre, Institute for Medical Research (IMR), National Institutes of Health (NIH), Ministry of Health Malaysia, 40170 Shah Alam, Selangor, Malaysia

Edited by Jean-Lou Justine
*Corresponding author: jerome.depaquit@univ-reims.fr

This is an Open Access article distributed under the terms of the Creative Commons Attribution License (https://creativecommons.org/licenses/by/4.0), which permits unrestricted use, distribution, and reproduction in any medium, provided the original work is properly cited.

[20] School of Medicine, Addis Ababa University, 28017 - 1000 Addis Ababa, Ethiopia
[21] Faculty of Mathematics, Natural Sciences and Information Technologies, University of Primorska, 6000 Koper, Slovenia
[22] University of Lodz, Faculty of Biology and Environmental Protection, Department of Invertebrate Zoology and Hydrobiology, Banacha 12/16, 90-237 Łódź, Poland
[23] Laboratory of Entomology, Ministry of Health, 9134302 Jerusalem, Israel
[24] Center for Pathophysiology, Infectiology and Immunology, Institute of Specific Prophylaxis and Tropical Medicine, Medical University Vienna, Kinderspitalgasse 15, 1090 Vienna, Austria
[25] Papua New Guinea Institute of Medical Research (PNGIMR) Institute, PO Box 60, Headquarter, Homate Street, 441 Goroka, Eastern Highlands Province, Papua New Guinea
[26] Museum of Natural History, University of the Philippines Los Baños, 4031 Laguna, Philippines
[27] National Centre of Infectious and Parasitic Diseases, 1504 Sofia, Bulgaria
[28] Ege University, Faculty of Medicine, Department of Parasitology, 35040 Bornova/Izmir, Türkiye
[29] Retired, Faculté de Pharmacie, Université de Strasbourg, Strasbourg, 67400 Illkirch-Graffenstaden, France
[30] Program for the Study and Control of Tropical Diseases (PECET), Faculty of Medicine, University of Antioquia, 050010 Medellin, Colombia
[31] MIVEGEC, Univ. Montpellier, CNRS, IRD, 34394 Montpellier, France & Medical Entomology Unit, Institut Pasteur de Madagascar, 101 Antananarivo, Madagascar
[32] Laboratorio de Entomología Médica, Departamento de Zoología de Invertebrados, Facultad de Ciencias Biológicas, Universidad Autónoma de Nuevo León, San Nicolás de los Garza, 66455, NL, México
[33] Tropical and Infectious Disease Centre, BP Koirala Institute of Health Sciences, Dharan 56700, Nepal
[34] ICMR-Vector Control Research Centre, Puducherry 605006, India
[35] Graduate School of Agricultural and Life Sciences, The University of Tokyo, Tokyo 113-8657, Japan
[36] Grupo de estudos em Leishmanioses/Coleção de Flebotomíneos (COLFLEB/Fiocruz-MG), Instituto René Rachou, Fundação Oswaldo Cruz, Belo Horizonte, Minas Gerais, 30190009, Brazil
[37] Center of Excellence in Vector Biology and Vector-Borne Disease, Department of Parasitology, Faculty of Medicine, Chulalongkorn University, Bangkok 10330, Thailand
[38] Department of Arbovirology, Bernhard Nocht Institute for Tropical Medicine, Bernhard Nocht Str. 74, 20359 Hamburg, Germany [39] Laboratory Vectors & Parasites, Department of Livestock Sciences and Techniques, Sine Saloum University El Hadji Ibrahima Niasse (SSUEIN) Kaffrine Campus, C.P. 24600, Senegal.
[40] Environmental Health Institute, National Environment Agency, Singapore 138667, Singapore & Department of Biological Sciences, National University of Singapore, 117558 Singapore
[41] Institut Pasteur du Laos, Laboratory of Vector-Borne Diseases, Samsenhai Road, Ban Kao-Gnot, Sisattanak District, 3560 Vientiane, Lao PDR
[42] National Institute of Hygiene and Epidemiology, 1 Yec-Xanh Street, Hai Ba Trung District, 100000 Hanoi, Vietnam
[43] Indonesian Research Center for Veterinary Science, Indonesian Agency for Agricultural Research and Development, Ministry of Agriculture Republic Indonesia, Bogor 16114, Indonesia & Department of Parasitology, Faculty of Veterinary Medicine, Airlangga University, Surabaya 60115, Indonesia
[44] Laboratory of Research in Applied Biology, Polytechnic School of Abomey-Calavi, University of Abomey-Calavi, 01 P.O. Box 2009, 00000 Cotonou, Benin
[45] Instituto de Microbiología, Colegio de Ciencias Biológicas y Ambientales (COCIBA), Universidad San Francisco de Quito (USFQ), 170901 Quito, Ecuador

Received 1 December 2025, Accepted 29 January 2026, Published online 3 April 2026

Abstrakt – Niniejszy artykuł stanowi kompleksowy poradnik do preparatyki i tworzenia preparatów mikroskopowych muchówek z podrodziny Phlebotominae, co ma kluczowe znaczenie dla identyfikacji gatunkowej oraz wykrywania i izolacji patogenów. Omówiono w nim szereg technik odpowiednich zarówno do pracy w terenie, jak i w laboratorium. Wytyczne zawierają szczegółowe instrukcje dotyczące odłowu, obchodzenia się z okazami, zabezpieczania oraz uśmiercania owadów (zalecając zamrażanie lub stosowanie CO2 zamiast środków chemicznych), a także strategie konserwacji okazów, takie jak przechowywanie w niskich temperaturach czy w etanolu. W pracy opisano, jak istotna dla poprawnej obserwacji mikroskopowej jest metodologia preparatyki konkretnych struktur anatomicznych (aparatów kopulacyjnych, głowy i skrzydeł). Artykuł przedstawia również szczegółowy proces przygotowania próbek, w tym etap wytrawiania z użyciem odczynników takich jak wodorotlenek potasu (KOH), a następnie roztwór Marc-André. W sekcji dotyczącej preparatyki porównano różne media, kładąc nacisk na ich właściwości optyczne i trwałość. Medium Hoyera (znane również jako guma chloralowa) jest zalecane do szybkich obserwacji, szczególnie w przypadku spermatek, ze względu na jego przejrzystość, choć nie nadaje się on do długoterminowego przechowywania. Omówiono także inne media, w tym alkohol poliwinylowy (PVA), Euparal® (o ograniczonej tolerancji na wodę) oraz balsam kanadyjski (medium rozpuszczalne w węglowodorach), przy czym dwa ostatnie zapewniają możliwość długotrwałego utrwalenia preparatów. W artykule poruszono także nowoczesne podejścia z zakresu biologii molekularnej, takie jak sekwencjonowanie DNA oraz technikę MALDI-ToF, które wymagają szczególnej uwagi

podczas przygotowania próbek. Ponadto dołączono krótkie materiały wideo ilustrujące różne techniki preparatyki oraz tłumaczenia na 33 języki, co pozwala dotrzeć do zróżnicowanych potrzeb i oczekiwań globalnej społeczności naukowej.

Słowa kluczowe: utrwalanie, Phlebotominae, medium Hoyer'a, roztwór Marc-André, guma chloralowa, alkohol poliwinylowy, Euparal®, balsam kanadyjski, izolacja pasożytów *Leishmania*, warunki terenowe, hodowla, sekcja, biologia molekularna, MALDI-ToF, okazy typowe.

Abstract – Processing and mounting phlebotomine sand flies: a consensus guideline. This article provides a comprehensive guide for the processing and mounting of phlebotomine sand fly specimens, which is crucial for species identification and pathogen detection and isolation. It discusses a range of techniques suitable for both field and laboratory settings. The guide includes detailed instructions on sand fly collection, handling, covering, and euthanasia (recommending dry freezing or CO_2 over chemicals) as well as conservation strategies, such as cold storage and preservation in ethanol. The quality of preparation of certain anatomical structures (genital organs, head and wings) is essential for their proper microscopic observation and is described in this work. The article also presents detailed sample processing, including the clearing process with agents such as potassium hydroxide then Marc-André solution. The mounting process compares different media, emphasizing their optical properties and preservation potential. Hoyer fluid (also known as chloral gum) is recommended for quick observation, particularly for spermathecae, due to its clarity, although it is not suitable for long-term storage. Other media discussed include polyvinyl alcohol, Euparal® (for limited water tolerance), and Canada balsam (a hydrocarbon-soluble medium), with the latter two offering long-term preservation capabilities. Innovative molecular biology approaches such as DNA sequencing and MALDI-ToF, which require particular attention to sample processing, are also addressed. Furthermore, short video clips illustrating various mounting techniques as well as translations in many different languages are provided, allowing the guideline to reach the diverse needs and expectations of the global scientific community.

Key words: Mounting, Phlebotomine sand fly, Hoyer fluid, Marc-André solution, Chloral gum, Polyvinyl alcohol, Euparal®, Canada balsam, *Leishmania* isolation, Field conditions, Culture, Dissection, Molecular biology, MALDI-ToF, Type-specimens.

Wstęp

Phlebotominae to podrodzina niewielkich muchówek (Diptera) należąca do rodziny ćmiankowatych (Psychodidae). Grupa ta obejmuje 1063 opisane dotychczas gatunki [21]. Są one istotnymi wektorami patogenów (pierwotniaków z rodzaju *Leishmania*, arbowirusów oraz bakterii *Bartonella*), wywołujące leiszmaniozę, choroby arbowirusowe oraz bartonelozę. Identyfikacja gatunkowa tych owadów opiera się przede wszystkim na szczegółowej analizie morfologicznej spreparowanych okazów pod mikroskopem, uwarunkowanej prawidłowym poborem materiału w terenie, jego właściwym przechowywaniem oraz precyzyjnym wykonaniem trwałych preparatów mikroskopowych. Proces ten wymaga zastosowania specyficznych technik badawczych, z których każda charakteryzuje się odmiennymi zaletami oraz ograniczeniami. Identyfikacja imagines Phlebotominae opiera się na analizie zarówno struktur zewnętrznych (np. czułków, głaszczek, aparatu kopulacyjnego samców), jaki wewnętrznych (np. gardzieli *pharynx*, *cibarium* oraz spermatek). Sekcja i izolacja struktur wewnętrznych ułatwiają ich obserwację, a w konsekwencji – trafną identyfikację gatunkową. Z tego względu, w przeciwieństwie do komarów czy pluskwiaków z podrodziny Triatominae, Phlebotominae wymagają sporządzenia preparatów mikroskopowych (okazów zatopionych w medium, a następnie zamkniętych między szkiełkiem podstawowym a nakrywkowym) jeszcze przed przystąpieniem do identyfikacji. Do lat 80-tych XX wieku analiza mikroskopowa była jedyną dostępną metodą identyfikacji Phlebotominae i do dziś pozostaje najpowszechniej stosowanym podejściem. Wybór procedury przygotowania preparatu był wówczas stosunkowo prosty i sprowadzał się tylko do dwóch możliwości – wykonywano albo preparaty trwałe, umożliwiające zabezpieczenie okazu na długi okres, albo preparaty tymczasowe, sporządzane „na szybko" w medium niezapewniającym takiej trwałości. Wykonanie preparatów trwałych, na przykład w żywicach takich, jak balsam kanadyjski, jest procesem czasochłonnym i wymaga uprzedniej, całkowitej dehydratacji próbek. Ponadto współczynnik załamania światła tego medium nie zawsze jest optymalny dla swobodnej obserwacji spermatek. Zastosowanie uwodnionych mediów (np. medium Hoyera) stanowi szybszą alternatywę i zapewnia lepszą wizualizację silnie załamujących światło struktur spermatek, jednak uniemożliwia długotrwałe przechowywanie ze względu na tendencję do absorpcji wilgoci z otoczenia. Rozwiązaniem tego problemu może być uszczelnienie krawędzi szkiełka nakrywkowego lakierem do paznokci po całkowitym wyschnięciu preparatu. Ten dylemat metodyczny (tzw. coś za coś) pozostaje aktualny do dziś i determinuje wybór techniki utrwalania w zależności od celu badawczego. Od lat 80-tych XX wieku badania nad taksonomią

Phlebotominae łączą analizę morfologiczną z metodami biochemicznymi. Pierwszą z nich była analiza węglowodorów kutykularnych, która została szybko wyparta przez techniki biologii molekularnej, takie jak RAPD (losowa amplifikacja polimorficznego DNA), RFLP (analiza polimorfizmu długości fragmentów restrykcyjnych), amplifikacja DNA połączona z sekwencjonowaniem metodą Sangera oraz sekwencjonowanie nowej generacji (NGS). Obecnie podejścia molekularne są uzupełniane metodami proteomicznymi, m.in. MALDI-ToF. Ponadto molekularna identyfikacja gatunkowa może być łączona z detekcją patogenów z wykorzystaniem PCR (m.in. *Leishmania*, *Trypanosoma*, *Bartonella* oraz *Phlebovirus*). Ponieważ drobnoustroje te mogą być wykrywane zarówno za pomocą klasycznego PCR (end-point), jak i PCR w czasie rzeczywistym (real-time PCR), niezbędne jest dostosowanie protokołów pobierania i przechowywania próbek do specyficznych założeń projektu badawczego [3, 32].

W oparciu głównie na doświadczeniu autorów oraz danych literaturowych celem niniejszego opracowania jest przedstawienie ustandaryzowanych wytycznych dotyczących przygotowania preparatów mikroskopowych oraz obróbki materiału badawczego dorosłych osobników Phlebotominae, mających na celu optymalizację analiz morfologicznych i molekularnych.

Konieczność przeprowadzania określonych analiz, takich jak badania z zakresu biologii molekularnej czy identyfikacja metodą MALDI-ToF, wymaga zachowania części badanego osobnika, która nie jest niezbędna do identyfikacji morfologicznej. Podkreśla to znaczenie prawidłowego doboru odpowiedniego protokołu badawczego. W niniejszym artykule skupiono się na metodach znieczulania i uśmiercania żywo schwytanych Phlebotominae, zasadach ich przechowywania oraz procedurach wykonywania preparatów mikroskopowych, zarówno na potrzeby szybkiej identyfikacji, jak i długoterminowej konserwacji okazów umożliwiającej prowadzenie dalszych badań.

Preambuła: Względy bezpieczeństwa i inne regulacje powinny odnosić się do odpowiednich kart charakterystyki (SDS).

Podczas korzystania z niniejszych wytycznych należy odnosić się do odpowiednich kart charakterystyki substancji chemicznych (SDS). Wszystkie chemikalia wymienione w opracowaniu powinny być stosowane z zachowaniem odpowiednich zasad bezpieczeństwa. Komitety ds. BHP w jednostkach badawczych udzielają informacji nie tylko na temat zagrożeń związanych z tymi substancjami, ale także w zakresie zasad ich użytkowania oraz utylizacji.

Bezwzględnie należy przestrzegać instrukcji dotyczących stosowania i utylizacji chemikaliów. Należy podkreślić, że odpowiedzialność za prawidłowe postępowanie podczas prac laboratoryjnych oraz za przestrzeganie przepisów i regulacji obowiązujących w danym kraju lub instytucji badawczej spoczywa na wszystkich osobach z nich korzystających. Ponadto niektóre odczynniki lub substancje wchodzące w ich skład (np. wodzian chloralu) podlegają w niektórych krajach ograniczeniom prawnym. Wykaz skrótów użytychw tekście zamieszczono w Tabeli 1.

Tabela 1: Wykaz skrótów.

**BME**	Pożywka Eagle'a (Basal medium Eagle)
**CDC**	Centra Kontroli i Prewencji Chorób (Centers for Disease Control and Prevention)
**CMCP**	Kamforowy monochlorofenol
**CMR**	Substancja rakotwórcza, mutagenna lub działająca szkodliwie na płodność i układ rozrodczy
**COI**	Gen podjednostki I oksydazy cytochromu c
**CytB**	Gen cytochromu b
**DNA**	Kwas deoksyrybonukleinowy
**ELISA**	Test immunoenzymatyczny ELISA
**EtOH**	Etanol
**M199**	Medium 199
**MALDI-ToF MS**	Spektrometria mas z jonizacją/desorpcją laserową wspomaganą matrycą z analizą czasu przelotu (Matrix-assisted laser desorption/ionization time-of-flight mass spectrometry)
**MEM**	Podłoże hodowlane/Pożywka MEM (Minimum essential media)
**NGS**	Sekwencjonowanie nowej generacji (Next-generation sequencing)
**NNN**	Podłoże hodowlane/Pożywka Novy-MacNeal-Nicolle
**PCR**	Reakcja łańcuchowa polimerazy
**Lao PDR**	Laotańska Republika Ludowo-Demokratyczna
**PNOC**	Gen prepronocyceptyny
**qPCR**	Ilościowy PCR (PCR w czasie rzeczywistym; Real-time PCR)
**RAPD**	Losowa amplifikacja polimorficznego DNA
**RFLP**	Polimorfizm długości fragmentów restrykcyjnych
**RI**	Współczynnik załamania światła (Refractive index)
**RNA**	Kwas rybonukleinowy
**RNases**	Rybonukleazy
**RNASS**	Roztwór do stabilizacji RNA (RNA stabilization solution)
**RT-PCR**	Reakcja łańcuchowa polimerazy z odwrotną transkrypcją
**TFA**	Kwas trifluorooctowy
**BME**	Pożywka Eagle'a (Basal medium Eagle)
**CDC**	Centra Kontroli i Prewencji Chorób (Centers for Disease Control and Prevention)

1. Odłów Phlebotominae

Dorosłe osobniki Phlebotominae mogą być pozyskiwane żywe lub martwe przy użyciu miniaturowych pułapek świetlnych CDC, pułapek lepowych oraz aspiratorów (stosowanych w połączeniu z pułapkami Shannona lub bezpośrednio w miejscach ich bytowania, np. w pobliżu schronisk dla zwierząt). Metody te polegają na rozmieszczaniu pułapek w odpowiednich siedliskach, wabieniu owadów światłem lub innymi atraktantami (np. CO_2, wabiki chemiczne), a następnie łapaniu do dalszych analiz opisanych w literaturze [2, 3, 32, 36, 49].

Pozyskiwanie żywych osobników umożliwia przeprowadzenie pełnego spektrum analiz badawczych, natomiast materiał martwy nie pozwala na izolację żywych szczepów *Leishmania* ani wirusów. Niektóre techniki odłowu, takie jak lepy, często prowadzą do utraty czułków, głaszczek lub odnóży, bądź do uszkodzenia skrzydeł schwytanych osobników. Dodatkowo olej rycynowy zawarty w lepach zanieczyszcza okazy, przez co wymagane jest jego usunięcia na wstępnym etapie preparatyki, zazwyczaj poprzez 15-minutową kąpiel w mieszaninie etanolu i eteru dietylowego w stosunku 1:1.

2. Uśmiercanie okazów

Po złapaniu, żywe osobniki Phlebotominae muszą zostać uśmiercone. W przypadku niektórych metod poboru (np. stosowania lepów lub pułapek świetlnych typu CDC wyposażonych w pojemnik z detergentem lub etanolem) owady są martwe już w momencie wpadnięcia do pułapki. Analizy z zakresu biologii molekularnej mogą być prowadzone na osobnikach zebranych bezpośrednio do etanolu, a także na okazach złapanych innymi metodami, pod warunkiem, że zostaną one umieszczone w nim możliwie jak najszybciej. Należy jednak pamiętać, że żadna z powyższych metod uśmiercania nie pozwala na późniejsze analizy z wykorzystaniem techniki MALDI-ToF.

Ponadto niektóre sposoby uśmiercania mogą prowadzić do utraty określonych cech morfologicznych. Z tego względu kluczowe jest zastosowanie odpowiedniej, wystandaryzowanej metody, która umożliwi prawidłową identyfikację gatunkową lub przechowywanie osobników jako okazów dowodowych (tj. trwale zakonserwowanych i zarchiwizowanych do przyszłych badań lub porównań). Substancje chemiczne, takie jak octan etylu, eter etylowy, tetrachloroetan czy chloroform, mogą być nanoszone na watę umieszczaną w pojemniku ze schwytanymi muchówkami. Ze względu na toksyczność tych związków należy obchodzić się z nimi z zachowaniem szczególnej ostrożności, ściśle przestrzegając zaleceń producenta.

Z naszego doświadczenia wynika, że uśmiercanie Phlebotominae przy użyciu chloroformu jest niewskazane, ponieważ negatywnie wpływa na wyniki analiz molekularnych. Biorąc pod uwagę szkodliwość wszystkich wymienionych substancji oraz ich ograniczoną przydatność w badaniach molekularnych, stosowanie tych środków chemicznych jest zasadniczo odradzane.

Najczęściej stosowaną metodą, pozwalającą na zachowanie cech morfologicznych, integralności DNA oraz białek, jest zamrażanie okazów „na sucho". Okazy powinny pozostawać zamrożone na tyle długo, aby zostały w stanie anabiozy, lecz nie na tyle, aby: (i) wyschły lub (ii) została naruszona żywotność pasożytów *Leishmania* (gdy celem jest ich izolacja *in vitro* z przewodu pokarmowego muchówki). **Zalecamy zatem mrożenie przez 15–20 minut w temperaturze –20°C, przy regularnym kontrolowaniu stanu okazów. Należy upewnić się, że owady pozostają jedynie w stanie odrętwienia, tak aby nie doprowadzić do uśmiercenia pasożytów *Leishmania*.**

Jeżeli zamrażarka nie jest dostępna, owady mogą być uśmiercane za pomocą CO_2. W warunkach terenowych, gdzie użycie butli z CO_2 jest niemożliwe, można stosować małe naboje z CO_2, wykorzystywane w dystrybutorach napojów gazowanych, jednak ich transport drogą lotniczą może podlegać określonym restrykcjom. W ostateczności owady mogą być uśmiercane poprzez ekspozycję na dym tytoniowy. Phlebotominae są wówczas odławiane przyżyciowo za pomocą pułapek typu CDC, pobierane aspiratorem, umieszczane w szklanej rurce i poddawane działaniu dymu tytoniowego, który zabija je w ciągu kilku sekund. Metoda ta może być stosowana w każdych warunkach terenowych. Należy jednak pamiętać, że szkło szybko nasiąka dymem i bez dokładnego oczyszczenia nie nadaje się do późniejszego zbierania i przechowywania żywych osobników. Niemniej jednak ten sam, nieoczyszczony aspirator może być nadal używany do uśmiercania owadów pozyskiwanych z innych pułapek, o ile planujemy ich utrwalenie. Po zakończeniu pracy ekshaustor należy dokładnie sprawdzić, aby upewnić się, że wszystkie okazy zostały z niego usunięte.

3. Przechowywanie okazów przed ich dalszym opracowywaniem

Przed dalszym opracowaniem materiału stosuje się cztery główne metody jego utrwalania:

3.1. Zamrażanie

Metoda ta jest skuteczna przy przechowywaniu okazów w temperaturze -20°C, a optymalnie -80°C, i obecnie jest stosowana częściej niż przechowywanie w ciekłym azocie. Niezależnie od wybranego podejścia, krioprezerwację należy rozpocząć możliwie jak najszybciej po wstępnym uśpieniu owadów. Przechowywanie w niskiej temperaturze pozwala zachować całe okazy oraz RNA, DNA i białka w praktycznie nienaruszonym stanie. Zastosowanie ciekłego azotu może jednak prowadzić do oderwania lub uszkodzenia skrzydeł, odnóży, głaszczek i czułków, skutkując utratą istotnych cech morfologicznych. Konserwacja „na sucho" w zamrażarkach jest pod tym względem mniej szkodliwa, lecz nadal nie zapewnia pełnej ochrony delikatnych struktur ciała. Dodatkowo podczas

rozmrażania, skrzydła, czułki, głaszczki lub odnóża mogą przyklejać się do ścianek probówek w wyniku kondensacji pary wodnej, co również sprzyja ich odrywaniu. Zamrażanie jest w pełni zgodne z metodami molekularnymi umożliwiającymi bez utraty czułości wykrywanie patogenów. Wykrywanie i izolacja wirusów RNA wymaga jednak przechowywania próbek w temperaturze -80°C lub w ciekłym azocie, jeśli planowana jest ich archiwizacja. Zamrażanie nie pozwala natomiast na izolację pasożytów Leishmania poprzez sekcję przewodu pokarmowego, chyba że Phlebotominae zostaną najpierw umieszczone w fazie gazowej ciekłego azotu, a później w ciekłej (np. w probówkach umieszczonych w pończosze), co symuluje krioprezerwację pasożytów.

3.2. Przechowywanie w alkoholu (etanolu lub alkoholu izopropylowym)

Jest to najczęściej stosowana metoda przechowywania Phlebotominae, łatwa do wdrożenia w warunkach terenowych, nawet bez dostępu do zaplecza laboratoryjnego. Konserwacja w alkoholu jest szczególnie przydatna w analizach morfologicznych, ponieważ delikatne struktury (skrzydła, odnóża, czułki i głaszczki) pozostają nienaruszone, o ile w probówce nie występują pęcherzyki powietrza. W celu ich eliminacji zaleca się uszczelnienie probówki niewielką kulką waty oraz umieszczenie etykiety na jej wierzchu (Rycina 1). Optymalne stężenie alkoholu pozostaje przedmiotem dyskusji, jednak generalnie nie zaleca się stężeń poniżej 70% [45, 66]. Wyższe stężenia skuteczniej i dłużej chronią DNA, ale powodują, że okazy stają się bardziej kruche podczas analiz morfologicznych. Zastosowanie 96% etanolu (mieszaniny azeotropowej) pozwala utrzymać stabilne stężenie alkoholu przez długi czas, szczególnie w wilgotnych warunkach klimatycznych, choć etanol 95% jest często łatwiej dostępny. DNA przechowywane w etanolu zazwyczaj zachowuje dobrą jakość, choć gorszą niż w przypadku zamrażania, zwłaszcza w badaniach molekularnych wykorzystujących NGS. Białka są znacznie mniej trwałe, co ogranicza zastosowanie tej metody w analizach proteomicznych, np. MALDI-ToF. Phlebotominae przechowywane w alkoholu przez kilka miesięcy mogą być nadal identyfikowane morfologicznie, jednak nie nadają się już do uzyskiwania referencyjnych widm białkowych. Procesy degradacji materiału wynikające z przechowywania w alkoholu lub w stanie suchym można spowolnić poprzez dodatkowe zamrożenie próbek w temperaturze -20°C. Mrożenie ogranicza degradację kwasów nukleinowych i rozpad tkanek, choć jego wpływ na zachowanie cech morfologicznych jest ograniczony. Przechowywanie w etanolu o stężeniu co najmniej 70% może być również stosowane do wykrywania wirusów DNA i RNA, o ile materiał był przechowywany krócej niż kilka miesięcy. W niektórych krajach łatwo dostępny jest alkohol izopropylowy, który skutecznie konserwuje DNA, lecz powoduje sztywnienie okazów. Jest on mniej łatwopalny niż etanol, co ułatwia jego transport. W razie potrzeby okazy przechowywane w ciekłym azocie lub zamrożone na sucho mogą zostać przeniesione do alkoholu, jednak takie postępowanie łączy wady obu tych metod.

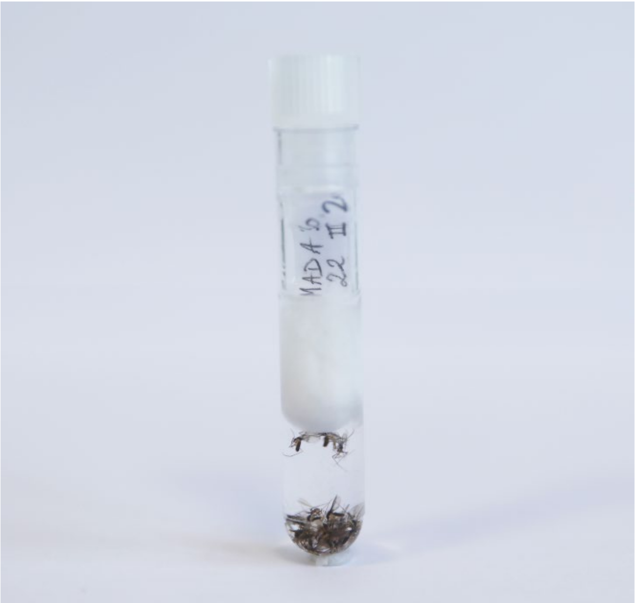

Rycina 1: Phlebotominae zachowane w etanolu.

3.3. Przechowywanie w roztworze stabilizującym RNA (RNASS)

RNASS jest wodnym, nietoksycznym odczynnikiem powszechnie stosowanym do stabilizacji i ochrony RNA w tkankach oraz komórkach. Jego działanie polega na szybkim przenikaniu do wnętrza okazu i inaktywacji RNaz, co zapobiega degradacji RNA bez konieczności natychmiastowego zamrażania próbek.

Przechowywanie w RNASS jest skutecznew zachowaniu ogólnej morfologii tkanek i komórek na potrzeby późniejszych analiz histologicznych. Choć roztwór ten nie jest przeznaczony do utrwalania struktur, krótko i średnioterminowe przechowywanie zazwyczaj pozwala zachować integralność badanego materiału. RNASS umożliwia przechowywanie próbek w temperaturze pokojowej do 7 dni, w temperaturze 4°C przez kilka tygodni oraz w –20°C lub –80°C przez długi czas, co ma swoje zastosowanie w konserwacji okazów. Metoda ta jest szczególnie przydatna podczas prac terenowych lub w warunkach ograniczonego dostępu do urządzeń chłodzących. Ekstrakcja RNA z tak przechowywanych próbek wymaga jedynie wyjęcia próbek z odczynnika i zastosowania standardowych procedur laboratoryjnych.

3.4. Przechowywanie na sucho w temperaturze pokojowej

Jest to starsza metoda, która w przypadku przechowywanie okazów *in toto* (w całości) – najczęściej na szpilkach entomologicznych - ma istotne ograniczenia, ponieważ słabo chroni delikatne struktury, takie jak skrzydła, odnóża, czułki i głaszczki. Mimo to badania proteomiczne z wykorzystaniem MALDI-ToF są nadal możliwe do przeprowadzenia na takich okazach, pod warunkiem, że zostały one odwodnione podczas utrwalania przy użyciu żelu krzemionkowego. Analizy molekularne DNA materiału przechowywanego w ten sposób są znacznie utrudnione, ponieważ DNA bywa silnie pofragmentowane i występuje w niewielkiej ilości. Stanowi to szczególne wyzwanie w badaniach genomów jądrowych. Niemniej jednak w takich przypadkach możliwe jest zastosowanie nowoczesnych metod, takich jak muzeomika [34]. Z tego względu przechowywanie okazów na sucho w temperaturze pokojowej nie jest zalecane, chyba nie ma innej możliwości. Metodę tę można łączyć z przechowywaniem w niskiej temperaturze, umieszczając próbki w zamrażarce (–20°C lub –80°C). Głównym problemem pozostaje uzyskanie odpowiednich preparatów z całych okazów lub ich części niezbędnych do identyfikacji. Wymaga to ponownego nawodnienia (rehydratacji) materiału, do czego zaleca się stosowanie roztworu Triton X-100. Proces ten trwa od kilku godzin do kilku dni i wymaga regularnej kontroli. Po pełnym namoczeniu, okazy należy poddać trzem kąpielom w wodzie.

3.5. Przechowywanie na bibule filtracyjnej

Główną zaletą bibuł filtracyjnych jest możliwość długoterminowego zachowania genomowego DNA w komórkach całego, nieutrwalonego i wysuszonego owada lub w komórkach krwi przechowywanych w temperaturze pokojowej. Arkusze bibuły są niewielkich rozmiarów, co umożliwia przechowywanie setek próbek w objętości odpowiadającej małej kasetce. Matryca bibuły jest nasączona substancjami unieszkodliwiającymi czynniki zakaźne, dzięki czemu próbki przestają stanowić materiał biologicznie niebezpieczny. Pozwala to na ich bezpieczne przechowywanie i transport bez konieczności stosowania rygorystycznych środków ostrożności [68].

4. Preparowanie (sekcja) okazów

W przeciwieństwie do wielu innych owadów, które identyfikuje się na podstawie cech zewnętrznych widocznych na okazach nabitych *in toto* na szpilki entomologiczne, Phlebotominae wymagają wykonania sekcji i sporządzenia preparatów mikroskopowych. Jest to niezbędne do analizy struktur anatomicznych umożliwiających precyzyjną identyfikację gatunkową. Niezależnie od wybranej procedury przygotowania i utrwalania materiału, stosuje się tę samą metodykę sekcji (Ryciny 2 i 3; https://zenodo.org/records/18198006).

Wykorzystanie Triton X-100 (niejonowy roztwór wodny)

Przygotowanie preparatów dotyczy okazów świeżo odłowionych lub prawidłowo przechowywanych. W większości kolekcji entomologicznych Phlebotominae są jednak przechowywane na sucho (np. na potrzeby analiz MALDI-ToF) lub w alkoholu przez wiele lat. Konserwacja alkoholowa nie jest optymalna w dłuższej perspektywie, stawonogi przechowywane w ten sposób stają się trudne do preparowania. Częstym problemem jest degradacja plastikowych pojemników, w których znajdują się okazy, prowadząca do odparowania alkoholu. W obu przypadkach, gdy próbki przez długi czas pozostają w alkoholu lub ulegają wysuszeniu, możliwości dalszych analiz stają mocno ograniczone. Z tego względu zaproponowano zastosowanie środków zwilżających, które nie są silnymi detergentami.

Triton X-100 jest niejonowym roztworem powierzchniowo czynnym *(4-(1,1,3,3-tetrametylobutylo) fenylo-polietylenoglikol; t-oktylofenoksypolietoksyetanol*), powszechnie stosowanym w biologii komórkowej i molekularnej do permeabilizacji błon komórkowych i jądrowych.

Poniżej przedstawiono procedurę wykorzystania 0,5% wodnego roztworu Triton X-100:

Nasącz suchą próbkę alkoholem absolutnym.

Dodaj 0,5% roztwór Triton X-100 w ilości umożliwiającej całkowite zanurzenie okazu.

Pozostaw próbkę na okres od 5 minut do kilku dni, regularnie kontrolując jej stan; wszystkie okazy muszą być całkowicie rozdzielone.

Usuń roztwór Triton X-100 i zastąp go roztworem wodorotlenku potasu.

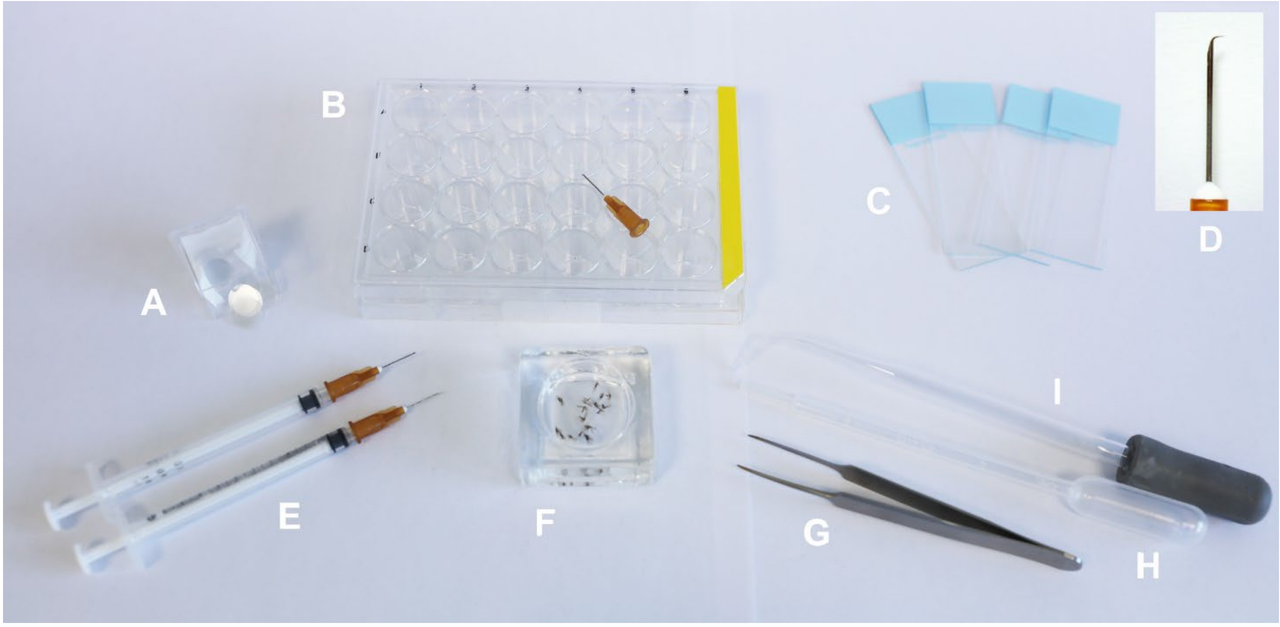

Rycina 2: Materiały niezbędne do wykonywania preparatów mikroskopowych Phlebotominae: A: okrągłe szkiełka nakrywkowe (o średnicy 10 lub 12 mm); B: płytka 24-dołkowa i haczykowato zakończona igła preparacyjna (jeśli jako medium używasz olejku goździkowego lub esencji Euparalu, nie używaj płytek akrylowych, ponieważ wejdą w reakcję chemiczną, doprowadzając do uszkodzenia okazów); C: szkiełka podstawowe z matowym polem do opisu; D: końcówka igły; E: igły osadzone na strzykawkach; F: szkiełko zegarkowe lub inne podobne naczynie na okazy przeznaczone do preparowania; G: pęseta typu Dumont; H: plastikowa pipeta; I: szklana pipeta wygięta przez podgrzanie w celu ułatwienia przenoszenia płynu do dołków.

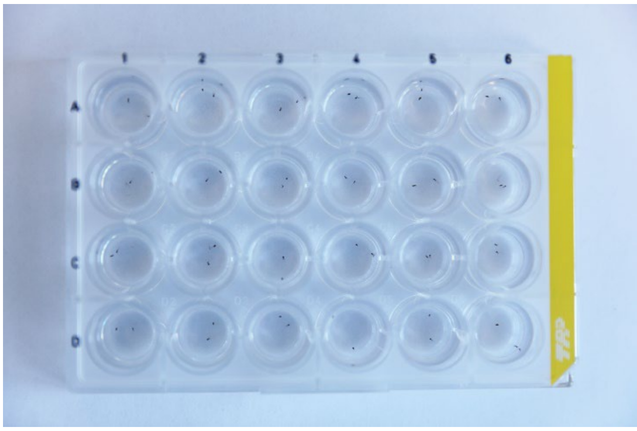

Rycina 3: Płytka z 24 dołkami; w każdym dołku znajduje się głowa oraz ostatnie segmenty odwłoka z genitaliami Phlebotominae.

4.1. Głowa

Głowę oddziela się od reszty ciała za pomocą cienkich igieł preparacyjnych lub szpilek entomologicznych pod mikroskopem stereoskopowym (Ryciny 2 i 3). Najczęściej stosowane są igły: 26G × 1/2″ (0,45 × 13 mm), 30G × 1/2″ (0,30 × 13 mm) lub 25G × 5/8″ (0,50 × 16 mm). Do analiz morfologicznych głowę po oddzieleniu umieszcza się w medium stroną brzuszną (wentralną) ku górze, co umożliwia obserwację *cibarium* oraz gardzieli (*pharynx*). Tułów i odwłok układają się zwykle w pozycji bocznej (lateralnej). Ułożenie głowy w pozycji grzbietowo-brzusznej (wentralno-dorsalnej) powoduje, że otwór potyliczny (*occipital foramen*) skierowany jest ku górze, zapewniając bezpośredni widok na *cibarium*. Dostęp do tych struktur jest znacznie łatwiejszy, gdy głowa zostanie całkowicie oddzielona od tułowia.

4.2. Skrzydła i tułów

Skrzydła należy układać w medium na płasko. Oba skrzydła można odizolować u nasady i preparować osobno lub pozostawić jedno przymocowane do tułowia. W przypadku analiz z wykorzystaniem morfometrii geometrycznej skrzydeł konieczne jest wyizolowanie i oznaczenie prawego oraz lewego skrzydła przed wykonaniem preparatu mikroskopowego. Tułów składa się z kilku segmentów dostarczających istotnych informacji taksonomicznych [20, 64]. Zazwyczaj umieszcza się go w widoku bocznym, aby umożliwić analizę chetotaksji oraz pigmentacji poszczególnych sklerytów. Obecność i ułożenie otworów po szczecinkach w określonych obszarach tułowia może być wykorzystywana do rozróżniania niektórych gatunków rodzaju *Brumptomyia*. Rozmieszczenie barw ma znaczenie diagnostyczne w identyfikacji neotropikalnych Phlebotominae na poziomie rodzaju (np. *Bichromomyia*), serii gatunków (np.

Pintomyia) oraz gatunków w obrębie danego rodzaju (np. *Micropygomyia*, *Nyssomyia*, *Psathyromyia*, *Psychodopygus*) [20]. Jeśli tułów nie jest wykorzystywany do analiz molekularnych, powinien być preparowany w sposób minimalizujący jego uszkodzenie. Istotne jest rozmieszczenie barw, a nie ich intensywność - dlatego wytrawianie okazu nie wpływa na tę cechę.

4.3. Genitalia

Podczas preparowania aparatów kopulacyjnychu samców i samic należy zachować szczególną ostrożność, ponieważ struktury te są kluczowe dla identyfikacji okazów do poziomu rodzaju, podrodzaju i gatunku. U obu płci genitalia wykazują symetrię dwuboczną.

4.3.1. Samce

Aparat kopulacyjny (=genitalia) samców jest zewnętrzny i składa się z parzystych wyrostków obejmujących: od strony grzbietowej gonopodia (połączenie gonokoksytu i gonostylusa) oraz od strony brzusznej płytkę epandrialną. Gonostylus posiada kolce, a czasem również szczecinki, których liczbai rozmieszczenie mają znaczenie diagnostyczne. Szczególną uwagę należy zwrócić na wewnętrzną powierzchnię gonokoksytu, gdzie może występować pęczek sensilli osadzonych na charakterystycznym guzku (*tubercle*) [22]. Osoby mniej doświadczone w preparowaniu mogą przygotować preparat, układając genitalia w widoku bocznym, bez ich oddzielania od końcowych segmentów odwłoka (https://zenodo.org/records/18311158). Metoda ta może jednak powodować nakładanie się pewnych struktur, utrudniając np. zliczenie szczecinek, ale zmniejsza ryzyko uszkodzenia preparatu. Bardziej doświadczeni badacze mogą rozdzielić aparat kopulacyjny na dwie części, używając powierzchni tnącej igły jako skalpela przecinając go tak, aby rozdzielić gonopodia, ale bez całkowitego przecięcia struktur aparatu. Takie podejście ułatwia obserwację powierzchni wewnętrznych gonopodiów oraz paramerów i pochewek parameralnych (parameral sheath).

4.3.2. Samice

Samice mają w dużej mierze genitalia wewnętrzne, a ich kluczową cechą diagnostyczną są spermateki. Jeśli spermateki nie zostały wyizolowane, należy obserwować je przez wytrawione powłoki ciała, układając odwłok na preparacie stroną brzuszną do góry. Niezależnie od zastosowanego medium, spermateki zazwyczaj są wyraźnie widoczne pod mikroskopem, zwłaszcza jeśli nie mają gładkiej powierzchni i nie zostały nadmiernie wytrawione. Obserwacja gładkich spermatek o cienkich ścianach może sprawiać więcej kłopotu w medium o niskim współczynniku załamania światła. Szczególnie istotna jest obserwacja nasady przewodów spermatekalnych, niezbędna do oznaczenia gatunków należących m.in. do podrodzaju *Larroussius* [35, 37, 38], którego przedstawiciele są głównymi wektorami *Leishmania infantum* w regionie Starego Świata. Gdy spermateki nie są widoczne, identyfikacja okazu jest niemożliwa. W przypadku trudności obserwacyjnych, należy usunąć z odwłoka kompleks furki i spermatek (https://zenodo.org/records/18311106). Spermateki są zwykle trudne do zaobserwowania bez ich wcześniejszego wypreparowania, natomiast furka genitalna jest zazwyczaj dobrze widoczna. Ponieważ przewody spermatekalne uchodzą do furki genitalnej, jej wyizolowanie zwykle umożliwia również izolację spermatek. Nawet jeśli spermateki zostaną przypadkowo przecięte podczas tego procesu, można je nadal obserwować przez integumenty odwłoka (Ryc. 4).

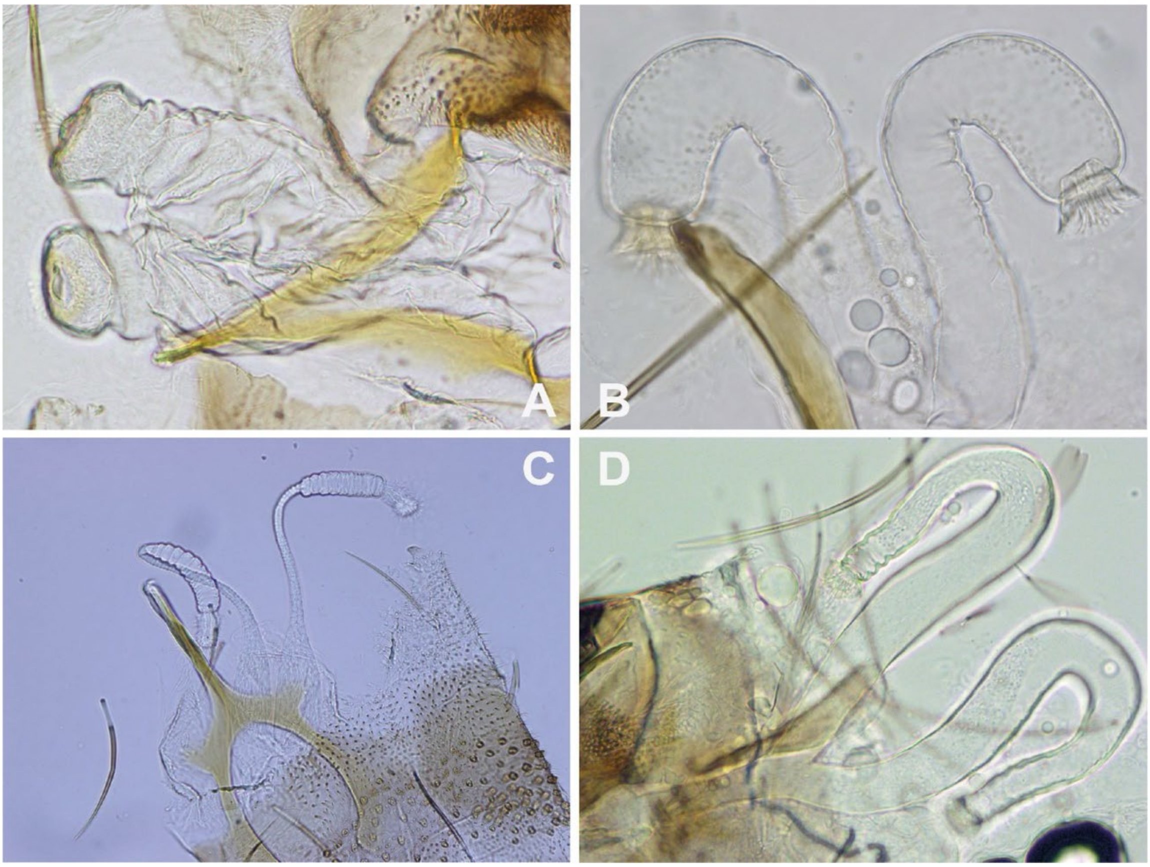

Rycina 4: Wyizolowane spermateki, zachowane w płynie Marc-André ze świeżych okazów. A: *Idiophlebotomus longiforceps* (Lao PDR); B: *Sergentomyia minuta* (Francja); C: *Phlebotomus ariasi* (Francja); D: *Sergentomyia anodontis* (Lao PDR).

4.4. Sekcja jelita środkowego w celu izolacji pasożytów *Leishmania*

Wyizolowanie przewodu pokarmowego jest niezbędne do wykrycia i izolacji *Leishmania* u samic Phlebotominae. Izolacja może być wykonywana zarówno w warunkach terenowych, jak i laboratoryjnych w celu oceny kompetencji wektorowej.

Zaleca się pracować na świeżo uśmierconych samicach. Należy je wypłukać w wodzie lub roztworze soli fizjologicznej zawierającym łagodny detergent, aby usunąć większość szczecinek. Pomaga to zachować sterylność niezbędną do izolacji *Leishmania*, a zarazem chroni cechy morfologiczne potrzebne do identyfikacji badanych muchówek. Aby wykryć i wyizolować *Leishmania*, należy ostrożnie wypreparować jelito środkowe i umieścić je w kropli jałowej soli fizjologicznej (0,9% NaCl). Jeśli pod mikroskopem świetlnym (zalecane powiększenie ok. 200x) widoczne są ruchliwe pasożyty, przenosi się je na podłoże hodowlane za pomocą strzykawki insulinowej lub mikropipety (szczegóły w rozdziale 4.4.3).

Głowę oraz genitalia zabezpiecza się się bezpośrednio w płynie Marc-André w celu ich wytrawienia. Ważne: Płyn Marca-André nie może mieć żadnego kontaktu z pasożytami *Leishmania,* ani bezpośredniego ani pośredniego (np. przez brudne narzędzia), gdyż natychmiast je zabija. Preparowanie samic Phlebotominae można wykonać na jednym lub dwóch szkiełkach; obie metody mają swoje wady i zalety (Ryc. 5; https://zenodo.org/records/18311154).

4.4.1. Metoda dwóch szkiełek

W tej metodzie używa się dwóch osobnych szkiełek: jednego z jałową solą fizjologiczną do izolacji jelita środowego oraz drugiego z płynem Marca-André do zachowania głowy i spermatek. W warunkach terenowych często dzieli się badaczy na dwa zespoły, gdzie kilka osób preparuje okazy, a jedna identyfikuje gatunki i sprawdza obecność *Leishmania*. Obsługa dwóch zestawów szkiełek może jednak wprowadzać chaos w etykietowaniu. Utrudnia to czasem przyporządkowanie danego jelita zawierającego pasożyty do konkretnego osobnika, jeśli pasożyty zostaną wykryte tylko u części badanych okazów (https://zenodo.org/records/18311154).

4.4.2. Metoda jednego szkiełka

Praca na jednym szkiełku ułatwia katalogowanie próbek i kontrolę wyników, wymaga jednak ścisłego przestrzegania zasad ostrożności. Aby zapewnić sterylność, osoby wykonujące izolację muszą regularnie dezynfekować dłonie. Należy korzystać z gładkich szkiełek podstawowych oraz kwadratowych szkiełek nakrywkowych (22 × 22 mm), wcześniej owiniętych w folię i wysterylizowanych gorącym

This is an Open Access article distributed under the terms of the Creative Commons Attribution License (https://creativecommons.org/licenses/by/4.0), which permits unrestricted use, distribution, and reproduction in any medium, provided the original work is properly cited.

powietrzem (np. w sterylizatorze Poupinela). Do każdej sekcji używa się sterylnych igieł (sugerowane: 25G, Ø 0,5 mm × 16 mm). Okaz umieszcza się w kropli sterylnej soli fizjologicznej na środku szkiełka. Głowę oddziela się od tułowia, nacinając jednocześnie odwłok między 6. a 7. segmentem, uważając, by nie przeciąć przewodu pokarmowego (u gatunków z długimi spermatekami tnie się nieco wyżej). Następnie jedną igłą przytrzymuje się tułów, a drugą delikatnie pociąga za koniec odwłoka, by wyizolować jelito. W razie trudności można chwycić końcówkę odwłoka i spróbować wyciągnąć przewód pokarmowy od przodu. Jeśli to nie pomoże, jelito trzeba wypreparować, oczyszczając je z resztek powłok ciała. Po wydobyciu jelita odcina się przewód pokarmowy od końcowych segmentów odwłoka. Jelito przenosi się do nowej kropli soli fizjologicznej z boku szkiełka i przykrywa sterylnym szkiełkiem nakrywkowym. Głowę i końcówkę odwłoka umieszcza się w kropli płynu Marca-André na drugim końcu szkiełka, pilnując, by płyn nie zetknął się z pasożytami. Głowę układa się otworem potylicznym do góry, a spermateki izoluje wraz z widełkami (*furca*) i przykrywa małym, okrągłym szkiełkiem (Ø 12 mm). Reszta tułowia i skrzydła zostają w soli fizjologicznej na środku (https://zenodo.org/records/18311154). Jeśli stwierdzono *Leishmania* lub potrzebne są analizy taksonomiczne, tułów i odwłok można zachować do badań molekularnych/proteomicznych, a ze skrzydeł zrobić preparat w uwodnionym medium (aqueous medium). Aby utrwalić preparat, płyn Marca-André można zastąpić np. gumą chloralową (płyn Hoyera).

Szczegółowe filmy instruktażowe są dostępne online (sekcja jelita: https://zenodo.org/records/18303014, sekcja ślinianek: https://zenodo.org/records/18302850), dlatego pominięto ich szerszy opis.

4.4.3. Izolacja i hodowla pasożytów *Leishmania* z przewodu pokarmowego

Izolacja pasożytów przy sekcji zainfekowanej samicy Phlebotominae wymaga wprawy, dlatego warto ją najpierw przećwiczyć na niezakażonych okazach. Wypreparowane jelita przenosi się do sterylnej kropli roztworu soli fizjologicznej (0,9%) lub płynu Locke'a w celu przemycia [4]. Wypreparowane jelita można badać na dwa sposoby: pod mikroskopem, by ocenić różne stadia rozwojowe i lokalizację promastigotów (zwłaszcza w zastawce wpustowej), albo otworzyć jelito, by uwolnić pasożyty i założyć hodowlę [4]. Ponieważ zainfekowane Phlebotominae w terenie trafiają się rzadko, trening na próbach kontrolnych zwiększa szansę sukcesu izolacji. Jeśli w jelicie widoczne są pasożyty, należy użyć nowych, sterylnych igieł i dodać nieco sterylnej soli fizjologicznej wokół szkiełka nakrywkowego, by ułatwić ich wypłukanie. Jelito rozrywa się ostrożnie, uwalniając *Leishmania* do roztworu. Następnie mikropipetą (100 µL) lub strzykawką tuberkulinową przenosi się pasożyty do pożywki.

Hodowla *in vitro* promastigota *Leishmania*: Pasożyty utrzymuje się na skosach agarowych wzbogaconych krwią (SNB-9) lub podłożu stałym NNN [16], z dodatkiem płynnego medium alpha-MEM [16, 65] lub M199. Każde podłoże musi zawierać 10% inaktywowanej termicznie, sterylnej płodowej surowicy cielęcej [FCS], 1% witamin BME, 2% sterylnej ludzkiej uryny (sterylizowanej przy użyciu filtra strzykawkowego Filtropur® S 0,2 µm) oraz amikacynę w stężeniu 250 µg/ml (zamiennie: gentamycyną 50 µg/ml lub mieszanką antybiotyków i aminokwasów: L-glutamina 200 mg, penicylina 10 000 U, streptomycyna 10 mg/ml) [47].

Jeśli po trzech dniach nie dojdzie do zanieczyszczenia podłoża innymi mikrobami, hodowle mrozi się w odpowiednim medium w -80°C (1–2 lata) lub w ciekłym azocie (-196°C) dla długotrwałej archiwizacji [7].

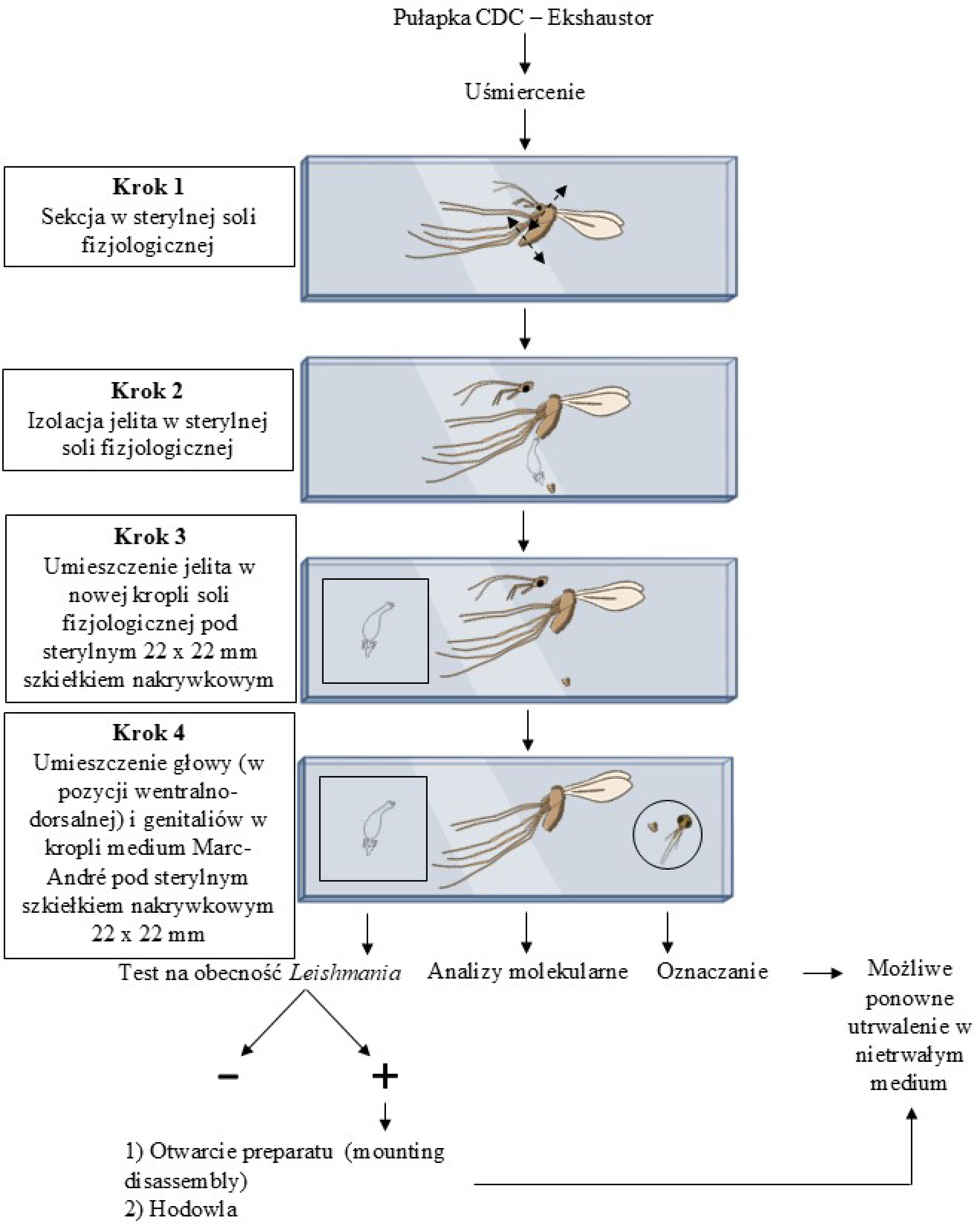

Rycina 5: Metody izolacji pasożytów *Leishmania.*

This is an Open Access article distributed under the terms of the Creative Commons Attribution License (https://creativecommons.org/licenses/by/4.0), which permits unrestricted use, distribution, and reproduction in any medium, provided the original work is properly cited.

4.5. Ślinianki

Izolacja ślinianek Phlebotominae to podstawowa metoda w badaniach interakcji wektor–patogen, zwłaszcza przy wykrywaniu arbowirusów jak *Phlebovirus* (np. wirusa Toscana) [44, 75]. Z uwagi na niewielkie rozmiary badanej grupy muchówek, zabieg ten wymaga precyzji i wprawy w obsługiwaniu narzędzi pod mikroskopem stereoskopowym przy dużym powiększeniu, by nie uszkodzić lub zanieczyścić delikatnych gruczołów (https://zenodo.org/records/18302850) [51, 61]. Ich kompletność i stan zachowania wyznaczają wiarygodność analiz molekularnych. Wyizolowane gruczoły można badać metodami RT-PCR, qPCR, testami immunoenzymatycznymi, aby wykryć przeciwciała czy RNA wirusów [12]. Obecność wirusów w śliniankach (a nie tylko w jelicie) potwierdza, że patogen przeszedł pełny cykl i może zostać przeniesiony na gospodarza przy następnym ugryzieniu przez muchówkę [71].

Proces izolacji ślinianek jest wymagający technicznie, a ryzyko degradacji próbek wysokie, przez co wymagane jest duże doświadczenie by nie zniszczyć materiału [1, 51]. Miano wirusa bywa niskie, dlatego konieczne są czułe metody, jak zagnieżdżone PCR (nested PCR) czy sekwencjonowanie wysokoprzepustowe (high-throughput sequencing) [54]. Należy też bezwzględnie dbać o sterylność środowiska pracy i używanych narzędzi. Na sukces detekcji wirusów wpływają również czynniki biologiczne i ekologiczne: różna kompetencja wektorową poszczególnych gatunków oraz wahania wartości wskaźnika zakażalności (infection rates), które różnią się w zależności od sezonu i miejsca [33, 61].

Wykrywanie wirusów w śliniankach dostarcza danych o ryzyku zakażenia, wspierając prowadzenie odpowiedniego nadzoru i kontroli sanitarnych [15]. Dla przykładu, wykrycie wirusa Toscana w regionach endemicznych wpłynęło na protokoły diagnostyczne i decyzje doradców ds. zdrowia publicznego [18]. Badanie interakcji wirus–ślina może też pomóc w tworzeniu szczepionek blokujących transmisję [15, 18].

Ponadto ślinianki stanowią źródło antygenów w testach ELISA, które służą do pomiaru poziomu przeciwciał przeciwko ślinie muchówek u gospodarza. Pozwala to na ocenę ekspozycji pacjentów na ukąszenia Phlebotominae, co wspiera monitorowanie skuteczności metod zwalczania wektorów [25] oraz szacowanie ryzyka transmisji *Leishmania* [40].

4.6. Identyfikacja żywiciela na podstawie krwi

Opite krwią samice powinny być preparowane przy użyciu jednorazowych narzędzi, aby uniknąć zanieczyszczenia krzyżowego (cross-contamination). Stopień nadtrawienia krwi ocenia się pod mikroskopem stereoskopowym. Do analiz zaleca się wybieranie osobników z czerwonym lub ciemnoczerwonym odwłokiem, bez widocznych oznak rozwoju jaj. Należy odciąć ostatnie segmenty odwłoka ze spermatekami (do identyfikacji gatunkowej po wytrawieniu), a resztę odwłoka umieścić w probówce typu Eppendorf i przechowywać w temperaturze -20°C. Do identyfikacji żywiciela stosuje się sprawdzone markery genetyczne, takie jak PNOC, CytB czy COI, które szczegółowo zostały opisane w literaturze, w związku z czym nie będą szerzej omawiane w niniejszej pracy (Rycina 6). Alternatywą jest mapowanie peptydowe MALDI-ToF [31], które pozwala zidentyfikować krew gospodarza nawet, gdy uległa znacznemu nadtrawieniu. Do tej metody próbki najlepiej przechowywać w temperaturze -20°C lub 4°C, choć dopuszczalne jest krótkotrwałe przechowywanie w temperaturze pokojowej. Odwłok opitej krwią samicy należy oddzielić od reszty ciała i poddać homogenizacji w wodzie destylowanej. Pozostała części muchówki mogą zostać wykorzystane do innych analiz molekularnych i morfologicznych. Po pobraniu fragmentu (alikwoty) z homogenatu na potrzeby mapowania peptydowego MALDI-ToF, pozostałości owada można wykorzystać do izolacji DNA w celu potwierdzenia identyfikacji gospodarza i/lub analiz pod kątem obecności *Leishmania* sp. Całkowity czas przygotowania i analizy próbki jest bardzo krótki w porównaniu z technikami molekularnymi opartymi na DNA.

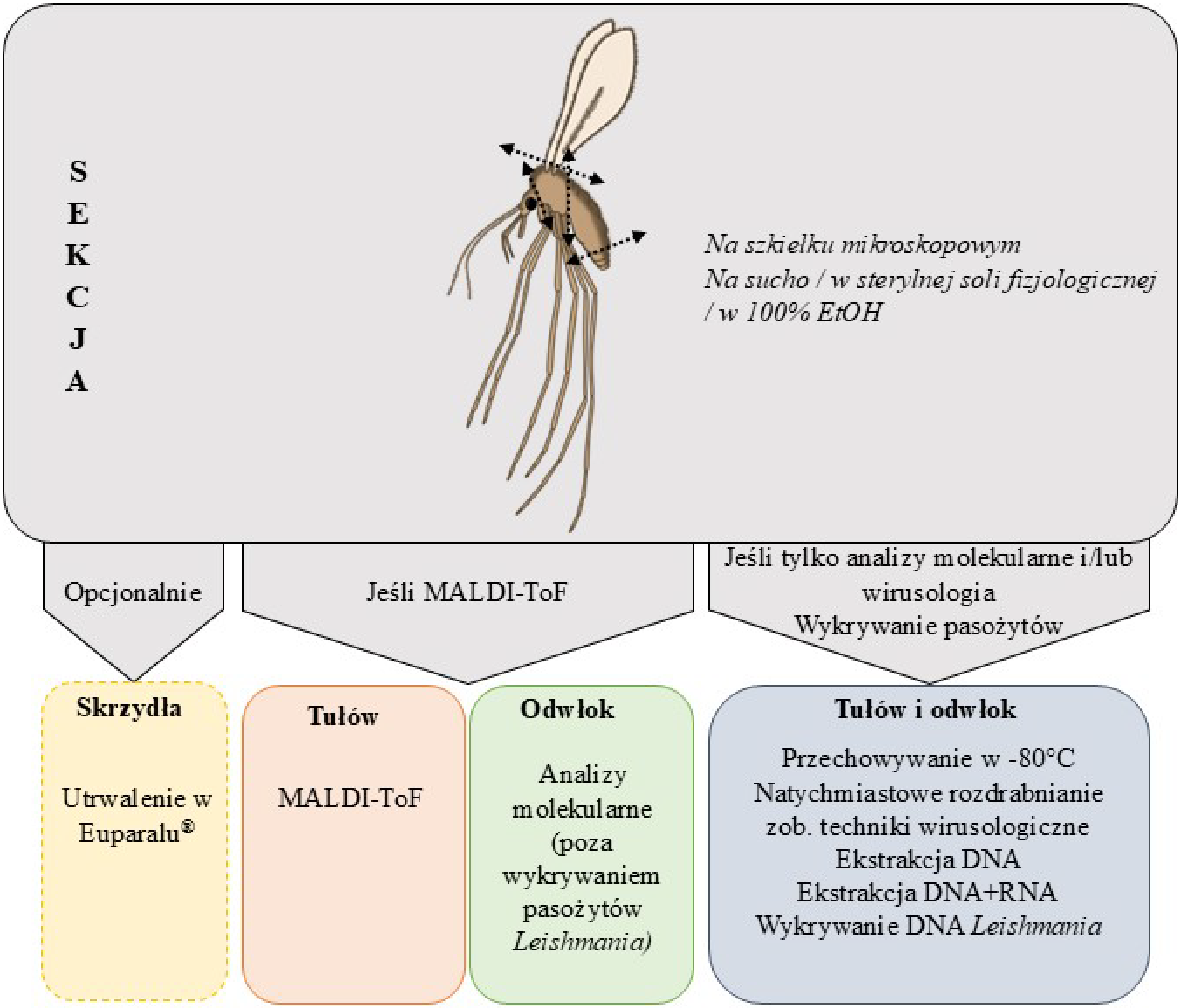

Rycina 6: Procedury służące do przygotowania Phlebotominae do analiz w biologii molekularnej, proteomice i/lub wirusologii.

5. Przygotowanie okazów do badań morfologicznych (Ryciny 3, 6, 7 i 8; Załączniki 1, 2, 3 i 4)

W niniejszym rozdziale omówiono zasady przygotowania preparatów z okazów przeznaczonych do badań morfologicznych oraz sposób ich wykorzystania do innych zastosowań. Zrozumienie tej metodologii jest kluczowe, ponieważ pozwala na sprawne dostosowanie procedur do konkretnych rodzajów próbek. Polega ona na kolejnych etapach opróżniania i napełniania pojemników przy użyciu pipet Pasteura wyposażonych w elastyczne gumowe gruszki. Zdecydowanie zaleca się stosowanie szklanych pojemników o okrągłym dnie, ponieważ znacznie ułatwiają one wykonywanie tych czynności oraz są chemicznie obojętne wobec wszystkich stosowanych odczynników. Aby zapobiec parowaniu odczynników wszystkie pojemniki powinny być szczelnie zamknięte. Nie należy ich również przepełniać, aby uniknąć rozlania zawartości podczas otwierania lub zamykania. Substancje chemiczne wymagane do wytrawienia i dalszej preparacji okazów zestawiono w Tabeli 2.

This is an Open Access article distributed under the terms of the Creative Commons Attribution License (https://creativecommons.org/licenses/by/4.0), which permits unrestricted use, distribution, and reproduction in any medium, provided the original work is properly cited.

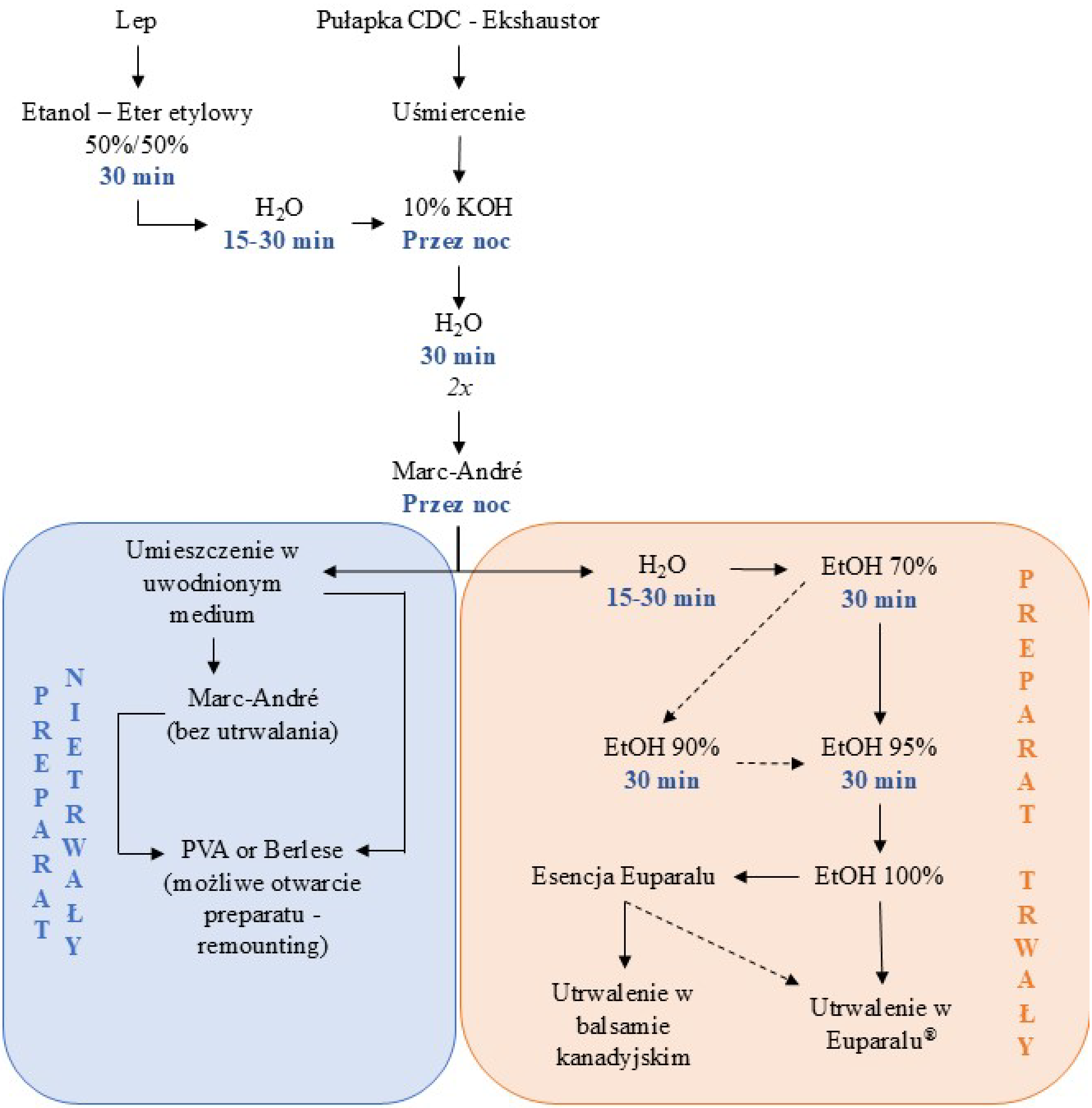

Rycina 7: Typowa metoda preparatyki Phlebotominae.

This is an Open Access article distributed under the terms of the Creative Commons Attribution License (https://creativecommons.org/licenses/by/4.0), which permits unrestricted use, distribution, and reproduction in any medium, provided the original work is properly cited.

Tabela 2: Skład stosowanych odczynników.

**Wodorotlenek potasu 10%**<br>Wodorotlenek potasu: 10g<br>Woda destylowana: *qs* (do objętości) 100 ml	**Fuchsyna kwaśna 1% roztwór w wodzie destylowanej**<br>Fuchsyna kwaśna (proszek): 1 g<br>Woda destylowana: 99 ml
**Guma chloranowa do utrwalania preparatów (medium Hoyera)**<br>Woda destylowana: 50 ml<br>Hydrat chloralu: 200 g<br>Guma arabska: 50 g<br>Glicerol: 20 ml	**Roztwór Marc-André barwiony fuchsyną kwaśną**<br>Roztwór Marc'a-André: 10ml<br>Fuchsyna 1%: 50 µl
**Roztwór Marc-André**<br>Hydrat chloralu: 40 g<br>Schłodzony kwas octowy: 30 ml<br>Woda destylowana: 30 ml	**Medium Enecê**<br>Czysta biała kalafonia: 22 g<br>Rozpuszczalna w alkoholu guma kopalowa: 12 g<br>Etanol absolutny (bezwodny): 20 ml<br>Kamfora: 10 g<br>Olejek terpentynowy (esencja): 10 ml<br>Eukaliptol: 26 ml

5.1. Wytrawianie

Zanim okazy zostaną utrwalone w formie preparatów mikroskopowych, muszą zostać poddane procesowi wytrawiania. Wykorzystuje się do tego celu odpowiednie metody i odczynniki (np. 10% roztwór kwasu octowego lub roztwór Marca-André zawierający hydrat chloralu, który w wielu krajach jest substancją podlegającą ograniczeniom), aby uczynić je przezroczystymi. Proces ten usuwa tkanki miękkie, tłuszcz, wydzieliny oraz woski, dzięki czemu okaz staje się półprzezroczysty. Ułatwia to obserwację struktur egzoszkieletu (np. miejsc osadzenia szczecinek), cech znajdujących się na powierzchni ciała (np. ubarwienia) oraz struktur wewnętrznych widocznych przez powłoki ciała (np. spermatek).

Stosujemy dwuetapowy proces wytrawiania, polegający na użyciu najpierw silnej zasady (takiej jak wodorotlenek potasu), a następnie słabego kwasu (np. kwasu octowego w roztworze Marca-André) [74]. Zasada rozkłada tkanki miękkie, takie jak białka, tłuszcze i mięśnie, poprzez zmydlanie i denaturację białek, pozostawiając nienaruszony chitynowy egzoszkielet, co zapewnia widoczność poszczególnych struktur. Następnie słaby kwas neutralizuje pozostałości alkalii, zapobiegając dalszej degradacji okazu oraz prześwietla chitynę, zwiększając jej przezroczystość [74]. Niekiedy dwukrotne płukanie okazów w wodzie destylowanej przez 15 minut może być wystarczające do neutralizacji zasadowego odczynu okazu po wytrawieniu. Zachowanie takiej sekwencji poszczególnych etapów łączy skuteczne usuwanie tkanek z zachowaniem integralności okazu, przez co nadaje się on do obserwacji mikroskopowych.

Przed przejściem do kolejnego etapu zaleca się dwukrotną kąpiel okazów w wodzie destylowanej po 20 minut.

5.1.1. Liza tkanek miękkich (Rycina 8)

Wodorotlenek sodu (NaOH) lub wodorotlenek potasu (KOH) to powszechnie stosowane środki żrące, używane w różnych stężeniach i czasie ekspozycji na okazy, zależnie od ich wielkości i delikatności. Standardowa i najskuteczniejsza technika polega na lizie tkanek miękkich poprzez pozostawienie Phlebotominae w silnej zasadzie (w rozworze 10% KOH lub NaOH) przez noc. Stężenie zasady można zwiększyć (np. do 20% KOH i zostawić na 6 godzin) lub ogrzać roztwór do temperatury 37°C, aby przyspieszyć ten proces.

5.1.2. Wytrawianie z barwieniem lub bez barwienia

Po tym etapie następuje rozjaśnianie chitynowych struktur, zazwyczaj z wykorzystaniem mieszaniny kwasu octowego i hydratu chloralu (np. roztworu Marca-André). Po wytrawieniu, okazy muszą zostać dokładnie wypłukane

w co najmniej dwukrotnie w kąpielach wodnych (po 20 minut każda), aby usunąć resztki odczynników.

Roztwór Marc'a-André jest powszechnie używanym środkiem wytrawiającym w preparatyce Phlebotominae. Jego skuteczność polega na ułatwieniu procesu trawienia, jednocześnie minimalizując uszkodzenie delikatnych struktur, takich jak skrzydła i czułki. Roztwór ten powinien być świeżo przygotowany lub przechowywany w szczelnie zamkniętym pojemniku, aby zapobiec parowaniu i degradacji. Stosowanie roztworu Marc'a-André jest szczególnie korzystne w połączeniu z technikami wytrawiania lub barwienia, co pozwala uwydatnić specyficzne szczegóły morfologiczne. Szczegóły dotyczące jego składu i przygotowania znajdują się w Załączniku 2.

W przypadku okazów mocno wytrawionych, przed zamknięciem ich w medium może być konieczne barwienie w celu poprawy widoczności struktur. Dostępnych jest wiele barwników, z których każdy celuje w inne komponenty chemiczne organizmu. Ważne jest, aby wybrać odpowiedni barwnik, kompatybilny zarówno z okazem, jak i wybranym medium, w którym zamyka się okaz. Tę metodologię można modyfikować w zależności od potrzeb, na przykład dodając 0,1% fuchsyny kwaśnej do roztworu Marca-André. Dodatkowo okazy przechowywane w roztworach wodnych, a przeznaczone do utrwalania w żywicach syntetycznych wymagają uprzedniej dehydratacji (patrz sekcja 5.2), ponieważ większość naturalnych i syntetycznych żywic nie miesza się z wodą. New (1974) zauważył, że niektóre barwniki mogą blednąć w określonych mediach [53]. Przykładowo, fuchsyna kwaśna, powszechnie stosowana z balsamem kanadyjskim, może być również utrwalana w Euparal®'u. Jednak okazy barwione fuchsyną mają tendencję do blaknięcia, zwłaszcza gdy pozostaną w nich resztki olejku goździkowego używanego do końcowego wytrawiania. Okazy przechowywane w olejku goździkowym mogą wykazywać znaczne wyblaknięcie już po kilku dniach.

Dehydratację przeprowadza się poprzez stopniowe przenoszenie okazów przez szereg roztworów etanolu o rosnącym stężeniu: 50%, 70%, 80%, 90% lub 95%, a na koniec 100%, przy czym każda kąpiel powinna trwać co najmniej 20 minut. Ponieważ etanol szybko paruje, naczynie podczas obróbki powinno być szczelnie zamknięte. Po całkowitym odwodnieniu okazu proces można przerwać na kilka dni, przechowując okazy w Euparal®'u, który jest preferowana względem olejku goździkowego. Kreozot bukowy, niegdyś szeroko stosowany w tym celu, jest obecnie całkowicie zakazany ze względu na swoją toksyczność.

Proces dehydratacji musi gwarantować, że płyn, który dostaje się do wewnątrz okazu jest zgodny z medium do utrwalania preparatów. Pozwala to uniknąć zmętnienia okazów, zapadnięcia się struktur wskutek osmozy lub zniekształceń, które mogłyby sprawić, że nie będzie on nadawał się do badań taksonomicznych.

5.3. Media do preparatyki

5.3.1. Wybór i zastosowanie w preparatyce okazów

Idealne medium powinno mieć współczynnik załamania światła jak najbardziej zbliżony do szkła (ok. 1,5). Musi być bezbarwne, klarowne i pozostawać idealnie przezroczyste po wyschnięciu oraz wraz z upływem czasu, a także kompatybilne ze stosowanymi barwnikami oraz zdolne do penetracji i dyfuzji do wszystkich tkanek okazu. Nie może wysychać zbyt szybko ani tworzyć zamgleń podczas preparowania, ani też kurczyć się po zamknięciu preparatu. Wybór odpowiedniego medium jest kluczowym aspektem preparatyki, ponieważ nie istnieje jedno medium idealne do wszystkich celów. Wybór powinien równoważyć kilka kluczowych czynników:

- **Właściwości optyczne.** Współczynnik załamania światła medium powinien zapewniać wystarczający kontrast i widoczność najważniejszych cech morfologicznych używanych w identyfikacji taksonomicznej (takich jak spermateki, askoidy, sensilla Newsteada, zęby cibarialne i gardzielowe). Widoczność tych struktur zależy bezpośrednio od właściwości optycznych medium.
- **Trwałość.** W przypadku typów opisowych lub materiałów przeznaczonych do stałych kolekcji, medium musi zapewniać długoterminową stabilność i trwałość. Natomiast w badaniach inwentaryzacyjnych lub epidemiologicznych, gdzie długotrwałe przechowywanie spreparowanych okazów jest mniej istotne, wystarczające mogą być media tymczasowe lub półtrwałe.

5.3.2. Wymagania wobec mediów do preparatyki

Specjaliści często opracowują własne, złożone techniki preparacji dostosowane do konkretnych potrzeb badawczych. Metody te jednak często pomijają kwestie takie jak trwałość, standaryzacja procedur czy łatwość opieki i potrzeba długotrwałego zabezpieczenia. Brak standaryzacji procedur utrudnia połączenie intencji przekazywania materiałów do muzeów z koniecznością długotrwałej opieki nad zbiorami.

Różne potrzeby naukowe implikują różne wymagania stawiane mediom utrwalającym preparat. Taksonomowie często przygotowują preparaty z całych okazów i preferują media, które delikatnie wytrawiają narządy wewnętrzne, aby poprawić widoczność struktur kutykularnych. Współczynnik załamania światła takiego medium powinien różnić się na tyle od współczynnika okazu i szkiełka nakrywkowego, aby zmaksymalizować przejrzystość optyczną. Media stosowane komercyjnie zazwyczaj charakteryzują się współczynnikiem załamania światła bliskiemu szkłu (≈1,515), co minimalizuje rozproszenie światła podczas obserwacji przez szkiełko nakrywkowe. Jednak w mikroskopii świetlnej, naturalny kontrast niebarwionego okazu można poprawić, wybierając medium o współczynniku załamania nieco innym niż okazu, co poprawia jego widoczność na tle podłoża.

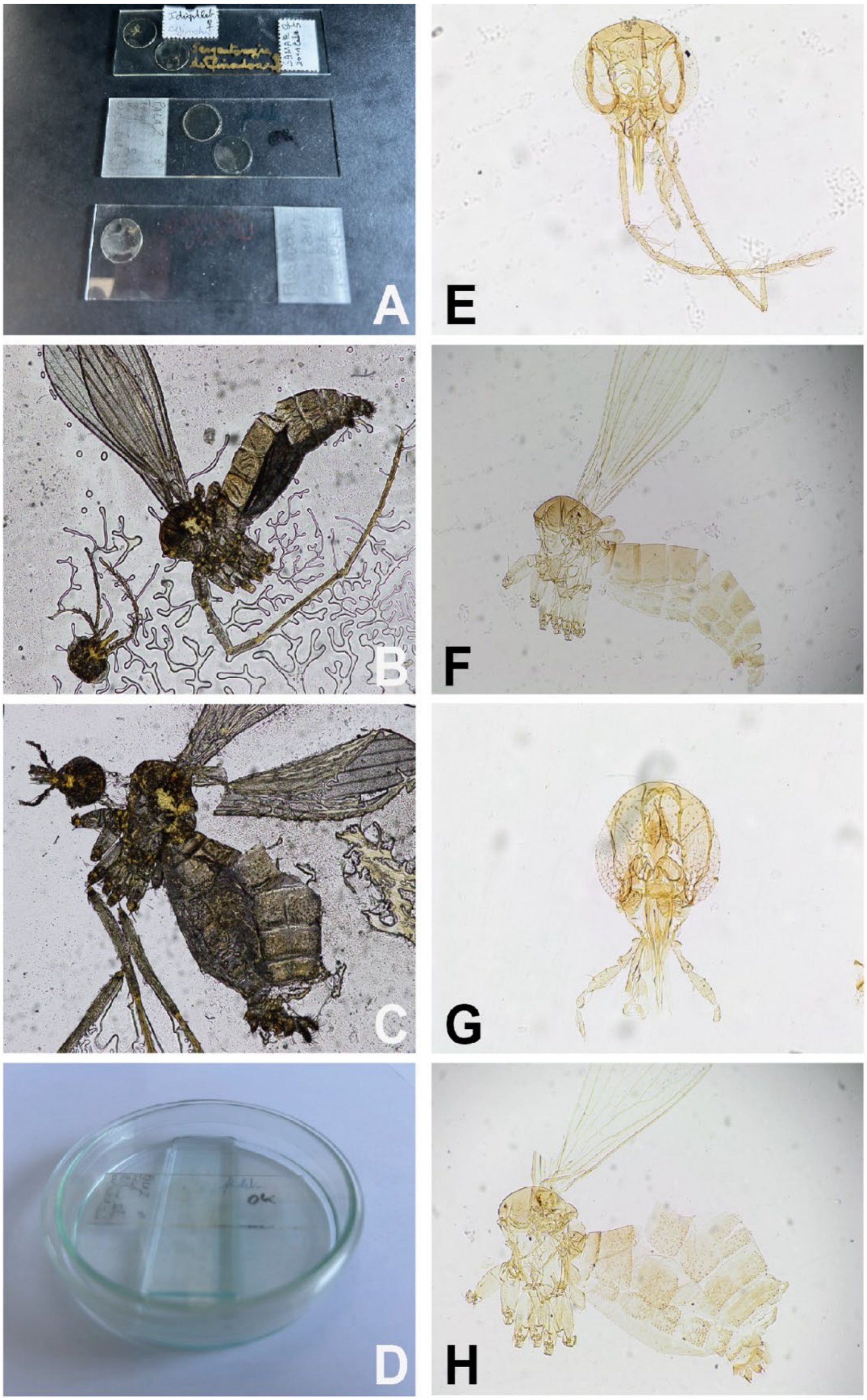

Rycina 8: Ponowne utrwalanie preparatów. A: uszkodzone i wysuszone preparaty po medium Hoyera; B: widok mikroskopowy wysuszonego Phlebotominae; C: widok mikroskopowy innego uszkodzonego Phlebotominae; D: wilgotna komora zawierająca wysuszony preparat; E: głowa i F: okaz B po ponownym utrwaleniu w Euparalu®; G: głowa i H: uszkodzony okazu C po ponownym utrwaleniu w Euparalu®.

5.2. Dehydratacja

5.3.3. Rodzaje mediów używanych w preparatyce (Tabele 3 & 4)

W mikroskopii, współczynnik załamania światła (RI) medium decyduje o tym, jak światło ugięło się przechodząc przez szkiełko nakrywkowe, medium i okaz. Gdy RI jest dopasowany do szkiełka nakrywkowego (≈1,515), światło przechodzi równomiernie, co zmniejsza jego rozproszenie i zniekształcenie optyczne, dając lepszą widoczność detali. I odwrotnie, niedopasowanie RI może powodować rozmycie obrazu, powstawanie efektu "halo" lub słabą widoczność niewybarwionych struktur.

W przypadku Phlebotominae najczęściej stosuje się media chloralowo-gumowe w formie uwodnionej oraz balsam kanadyjski i żywicę Enecê - Nelson Cerqueira (NC) jako środek na bazie rozpuszczalnika. Rawlins [60] podzielił media utrwalające na dwa typy: (1) media trwałe, które twardnieją z czasem i nadają się do długotrwałej konserwacji okazów, oraz (2) media półtrwałe, które nie twardnieją całkowicie i są zazwyczaj używane do celów tymczasowych.

Media do preparatyki mogą występować w formie płynnej, gumy lub żywicy; mogą być rozpuszczalne w wodzie, alkoholu lub innych rozpuszczalnikach (np. toluenie, ksylenie). Po nałożeniu, powinny zostać odizolowane od wpływu czynników atmosferycznych za pomocą nierozpuszczalnych lakierów uszczelniających (ang. *ringing media*). Aby wyraźnie rozróżnić typy mediów, stosuje się następującą kategoryzację:

a. Media uwodnione (aqueous media). Łatwo rozpuszczają się w wodzie, co czyni je odpowiednimi do wykonywania preparatów tymczasowych lub półtrwałych. Są proste w użyciu, ale mogą wymagać uszczelniania brzegów szkiełka, aby zapobiec ich niszczeniu przez wilgoć z powietrza (np. media chloralowo-gumowe i alkohol poliwinylowy), szczególnie gdy są preparaty są wykonywane i przetrzymywane w krajach z wilgotnym klimatem tropikalnym.

b. Media o ograniczonej tolerancji na wodę. W mniejszym stopniu ulegają negatywnemu wpływowi wody, ale nadal wymagają ochrony przed nadmierną wilgotnością. Są bardziej stabilne niż media uwodnione i często używane do preparatów półtrwałych.

c. Media rozpuszczalne w węglowodorach. Są rozpuszczane w rozpuszczalnikach organicznych, takich jak ksylen, toluen czy rozpuszczalnik Enecêa. Przeznaczone są do preparatów trwałych, oferują doskonałą stabilność, odporność na wilgoć i degradację, co czyni je idealnymi do archiwizacji okazów (np. neutralny balsam kanadyjski).

Podsumowując, media uwodnione najlepiej nadają się do tymczasowych preparatów lub przypadków wymagających łatwego otworzenia preparatu; media o ograniczonej tolerancji na wodę nadają się do preparatów półtrwałych wymagających umiarkowanej trwałości, a media rozpuszczalne w węglowodorach są preferowane w przypadku preparatów trwałych przeznaczonych do archiwizacji i długotrwałego przechowywania.

Tabela 3: Skład stosowanych mediów.

Nazwa medium	Rozpuszczalnik	Potencjalne prepolimery lub polimery	Uwagi
Hoyer (guma chloralowa)	glicerol, woda	związki w gumie arabskiej	Substancja wytrawiająca: hydrat chloralu
CMCP-9 (karboksymetyloceluloza-fenol)	woda (CMCP-9: 51−60%)	w pełni zhydrolizowany alkohol poliwinylowy (CMCP-9: 0–5%)	CMC(P)-9: niska lub wysoka lepkość
DMHF (dimetylohydantoinaformaldehyd)	woda	N,N′-dimetylolodimetylohydantoina; oligomery z mostkami eterowymi/metylenowymi; sieć polimerowa DMH-formaldehyd	
Balsam kanadyjski	ksylen; częściowo lotne składniki balsamu (karen, kwas lewopimarowy, limonen, mircen, kwas palustrowy, phellandren, pinen)	balsam (abienol, kwas abietynowy, kwas izopimarowy. kwas sandarakopimarowy)	Neutralizacja: węglan potasu; żywica z jodły balsamicznej Abies balsamea (L., 1758)
Euparal®	eukaliptol, paraldehyd; częściowo lotne substancje z gumy sandarakowa (limonen, α-pineny, β-pineny)	składniki gumy sandarakowej (kwas komunikowy, manool, kwas sandarakopimarowy, kwas 12-acetoksy-sandarakopimarowy, sugiol, kwas torulozowy, torulozol, totarol)	Czynnik wytrawiający: salicylan metylu; odmiana zielona Euparal® zawiera sole miedzi (abietynian miedzi); żywica sandarakowa z Tetraclinis articulata (Vahl, 1791)

This is an Open Access article distributed under the terms of the Creative Commons Attribution License (https://creativecommons.org/licenses/by/4.0), which permits unrestricted use, distribution, and reproduction in any medium, provided the original work is properly cited.

Tabela 4: Zalety i wady wybranych mediów do preparatyki mikroskopowej oraz niepublikowane obserwacje różnych autorów [52].

Nazwa	Zalety	Wady
* Balsam kanadyjski	Medium o bardzo wysokiej trwałości, przekraczającej 150 lat. Preparaty mogą być wykonywane z użyciem olejku goździkowego lub fenolu jako czynników pośrednich.	Zawiera składniki szkodliwe; wymaga pracy pod wyciągiem. Wymaga pełnej, czasochłonnej serii odwodnień okazów przed zatopieniem w balsamie. Odwadnianie etanolem i przenoszenie przez ksylen lub olejek goździkowy może uszkadzać niektóre preparaty; alternatywy (np. izopropanol, n-butanol, Cellosolve™, 1,4-dioksan, Histoclear, terpineol) mogą ograniczyć pękanie okazów. Okazy mogą czernieć, jeśli ksylen zostanie zastąpiony fenolem lub jeśli w preparacie pozostaną resztki wodorotlenku potasu (KOH). Wysoki współczynnik załamania światła może sprawić, że niebarwione struktury będą niewidoczne. Całkowite wyschnięcie może trwać lata, jeżeli nie użyjemy płyty grzewczej. Medium żółknie i ciemnieje z czasem, zwłaszcza, jeśli okazy były wytrawione olejkiem goździkowym. Niektóre barwniki słabną, a barwniki kationowe mogą blaknąć, jeśli medium ulegnie zakwaszeniu (co może nastąpić samoistnie z czasem).
DMHF (dimetylohydantoina formaldehyd)	Wysoka przejrzystość. Odpowiedni współczynnik załamania światła. Doskonała widoczność struktur. Dość dobra stabilność preparatów. Kompatybilny z wieloma technikami barwienia. Dobra ochrona próbek. Gwarantuje odpowiednią adhezję między szkiełkiem podstawowym a nakrywkowym.	Możliwość żółknięcia z upływem czasu. Może zmieniać odcień niektórych barwników. Nieodpowiedni dla barwników wrażliwych na formaldehyd. Przy nakładaniu pojawiają się pęcherzyki powietrza, długi czas schnięcia. Medium wrażliwe na wilgoć. Po wyschnięciu trudno jest go z powrotem rozpuścić. Formaldehyd jest toksyczny, drażniący i rakotwórczy.
* Euparal (przeźroczysty)	Trwałe medium o żywotności powyżej 50 lat. Daje możliwość zatapiania okazów bezpośrednio z 80% etanolu (zalecenie producenta). Nie maskuje niebarwionych struktur, nie żółknie i nie kruszeje z upływem czasu. Posiada współczynnik załamania światła bardziej odpowiedni dla muchówek (Diptera) niż balsam kanadyjski. Dobrze sprawdza się przy grubych okazach dzięki minimalnemu kurczeniu i schnięciu bez wytworzenia pęcherzyków powietrza. Pozostaje rozpuszczalny w 95% etanolu, co pozwala na renowację preparatów nawet po wielu latach.	Zawiera składniki szkodliwe; wymaga pracy pod wyciągiem. Odwadnianie okazów etanolem i przenoszenie bezpośrednio do Euparalu (Euparal Essence) może powodować pękanie (cracking) niektórych okazów, choć użycie izopropanolu może to ograniczyć.
Medium Hoyer'a	Okazy mogą być w nim zatapiane żywe lub przenoszone bezpośrednio z wody, etanolu lub formaldehydu. Wytrawienie przez medium zapewnia doskonałą widoczność oskórka. Ma korzystny współczynnik załamania światła; kontrast można zwiększyć barwieniem jodem. Kwas octowy w składzie może powodować rozprostowywanie (pęcznienie) przydatków stawonogów. Niektóre preparaty zachowują się w stałej formie przez 40–60 lat. Rozpuszczalny w wodzie, co ułatwia renowację preparatów.	Delikatne preparaty roślinne mogą się zapaść, chyba że medium jest dodawane stopniowo (co jest czasochłonne). W ciągu mniej niż 10 lat mogą tworzyć się puste przestrzenie (pustki) i kryształy. Medium może nadmiernie wytrawić okazy, w zależności od stężenia hydratu chloralu i czasu ekspozycji okazu na medium. Składniki mogą się rozwarstwiać, a po miesiącach lub latach może pojawić się drobna granulacja. Odnotowano przypadki czernienia medium.
CMCP-9 (karboksymetyloceluloza-	Okazy mogą być w nim zamykane bezpośrednio z wody, etanolu, glicerolu lub roztworów	Medium może ciemnieć i krystalizować; czasami wytrawia okazy bardziej niż planowano. Jeśli szkiełko

This is an Open Access article distributed under the terms of the Creative Commons Attribution License (https://creativecommons.org/licenses/by/4.0), which permits unrestricted use, distribution, and reproduction in any medium, provided the original work is properly cited.

fenol)	formaldehydu; narządy wewnętrzne mogą ulec wytrawieniu, co ułatwia obserwacje mikroskopowe preparatów.	nie zostanie starannie uszczelnione, grubsze warstwy medium kurczą się, tworząc szczeliny przy krawędziach szkiełka nakrywkowego. Nieodpowiednie dla okazów barwionych i materiałów zwapniałych; schnie wolniej niż CMC.
Eukitt™	Medium trwałe, wytrzymujące ponad 30 lat. Zgodne z wieloma rozpuszczalnikami (aceton, benzen, chloroform, dioksan, eter, izopropanol, benzoesan metylu, terpineol, toluen, ksylen). Szybko schnie, ma lekko kwaśne pH. Nie ciemnieje z wiekiem. Odpowiednie dla różnych barwników (fuchsyna, hematoksylina, zieleń metylowa, fiolet metylowy, błękit metylenowy). Możliwość renowacji preparatów po latach przez moczenie w ksylenie.	Szkodliwy; wymaga pracy pod wyciągiem. Okaz przed umieszczeniem go w medium wymaga pełnej, czasochłonnej serii dehydratacji. Nieodpowiedni do większych okazów ze względu na fakt, że kurczy się i powstają pęcherzyki gazu. Szkiełka nakrywkowe mogą odpadać, jeśli szkło nie było idealnie czyste lub uszczelnione. Może wykazywać niepełną polimeryzację wokół włókien kolagenowych.

5.3.4. Opisy mediów zalecanych do preparatyki okazów (Tabele 3 i 4)

Media do preparatów tymczasowych (nietrwałych)

Guma chloralowa = płyn/medium/roztwór Hoyer'a (RI = 1,48)

Płyn Marca-André jest najlepszym medium do wykonywania krótkotrwałych (kilka godzin, ewentualnie nieco dłużej, jeśli preparat jest przechowywany w wilgotnej komorze) preparatów, zwłaszcza obserwacji spermatek czy wykonywania dokumentacji fotograficznej (Rycina 4) lub rysunkowej okazów. Aby zachować spermateki należy je ponownie zatopić w uwodnionym medium (aqueous medium), co pozwala na ich średnio długie przechowywanie. Odwadnianie w celu przeniesienia do żywicy również jest możliwe, ale odradzane (łatwo o zniszczenie okazu). Guma chloralowa i medium Hoyera to synomimy tego samego medium, które jest powszechnie stosowane do obserwacji narządów wewnętrznych, ponieważ miesza się z wodą, jest proste i szybkie w użyciu oraz posiada współczynnik załamania światła sprzyjający obserwacji delikatnych struktur, takich jak spermateki. Jednakże guma chloralowa ma istotne wady, jeśli nie zostanie prawidłowo przygotowana lub jeśli jest przechowywana w warunkach zmiennej wilgotności. Problemy te obejmują krystalizację, przebarwienia i utratę lepkości. Uszczelnianie krawędzi szkiełka nakrywkowego nie rozwiązuje tych problemów, ponieważ medium może ulec silnemu przebarwieniu (niekiedy prawie do czerni) w wyniku interakcji z substancją uszczelniającą, szczególnie jeśli użyto Euparalu®. Medium Hoyera tradycyjnie uznaje się za optycznie najlepsze dla obserwacji mikroskopowych Phlebotominae i jest najczęściej wykorzystywane do tych celów. Medium Hoyera przygotowuje się z kilku substancji, głównie gumy arabskiej, glicerolu i hydratu chloralu. W literaturze można jednak spotkać wiele błędnie zinterpretowanych lub niedokładnie cytowanych receptur tego medium [74].

Mimo doskonałych właściwości optycznych zwłaszcza do obserwacji spermatek Phlebotominae, medium Hoyera nie nadaje się do długoterminowej konserwacji okazów. Media uwodnione, choć idealne do doraźnych analiz, w tym wykonywania zdjęć, rysunków, nie gwarantują długiej trwałości. W przeciwieństwie do nich, utrwalanie okazów w żywicach zapewnia odporność na ich niszczenie mierzoną w stuleciach, choć kosztem detali, jak spermateki, które w takich mediach mogą stać się słabo widoczne ze względu na utratę zdolności załamywania światła przez ich ściany.

Medium Hoyera degraduje z czasem wskutek odwodnienia (Rycina 8), co prowadzi do powstawania małych, białych, nieprzezroczystych kryształów hydratu chloralu. Niemniej jednak okazy można odzyskać ze skrystalizowanych szkiełek, ponieważ kutykula pozostaje nienaruszona chemicznie, choć narastające kryształy mogą uszkadzać okazy. W niektórych przypadkach skrystalizowane szkiełka można uratować poprzez rehydratację medium w ciepłym, wilgotnym środowisku z dodatkiem tymolu, który zapobiega rozwojowi grzybów. Alternatywnie okazy można odmoczyć z gumy chloralowej w wodzie, odwodnić w lodowatym kwasie octowym i ponownie zatopić w balsamie kanadyjskim.

DMHF (dimetylohydantoina formaldehyd) (RI 1.48)

To medium na bazie wody [72] wykazuje bardzo dobre właściwości optyczne, zbliżone do medium Berlese'a i jest równie łatwe w użyciu. Jednak w przeciwieństwie do Berlese'a nie czernieje i nie krystalizuje. Sprawdza się dobrze w przypadku Phebotominae i innych przedstawicieli Psychodidae.

CMCP (karboksymetyloceluloza-fenol) (RI = 1,41)

Jest to rozpuszczalne w wodzie medium na bazie gliceryny, stosowane do tworzenia przezroczystych, trwałych preparatów z delikatnych owadów, w tym Phlebotominae. Zaletą tego medium jest to, że okazy mogą być umieszczane w nim bezpośrednio z wody lub etanolu. CMCP szybko rozluźnia i wytrawia okaz, zmiękczając kutykulę, co pozwala na właściwe ułożenie – jest to szczególnie przydatne przy preparowaniu skrzydeł lub genitaliów. Chociaż istnieją głosy, że CMCP umożliwia długoterminowe przechowywanie, dokładny czas, przez który pozostaje stabilny jest nieznany. Głównym ograniczeniem tego medium jest to, że zawiera fenol – substancję toksyczną i drażniącą, wymagającą szczególnej ostrożności w stosowaniu.

Media do wykonywania trwałych preparatów

Balsam kanadyjski (RI = 1,52-1,54)

Balsam kanadyjski został po raz pierwszy opisany jako medium używane w mikroskopii przez Andrew Pritcharda w latach 30-tych XIX wieku. Pozostaje on jednym z najczęściej stosowanych mediów, ze względu na swoją trwałość przez bardzo długi czas, z ponad 150-letnią historią wykorzystywania w preparatyce. W przeciwieństwie do medium Hoyera i pokrewnych, balsam kanadyjski nie krystalizuje i nie chłonie wilgoci. Wykazuje on jednak silną autofluorescencję, co niekiedy może być wadą przy niektórych technikach mikroskopowych [60]. Stosowanie nietoksycznych rozpuszczalników zamiast ksylenu może zmniejszyć ryzyko zatrucia się nim przy produkcji balsamu, ale ma wady, takie jak wolniejsze schnięcie i szybsze ciemnienie medium.

Euparal® (RI = 1,48)

Euparal® jest powszechnie stosowaną alternatywą dla balsamu kanadyjskiego do wykonywania preparatów trwałych, oferującą doskonałą trwałość i porównywalny współczynnik załamania światła. Używanie Euparalu® wiąże się z: (1) odwodnieniem okazu: przed ostatecznym przeniesieniem do medium okaz musi zostać odwodniony, zazwyczaj poprzez przejście z etanolu 95% do etanolu 100% oraz (2) dłuższym czasem wykonywania preparatu: umieszczenie okazu w żywicy, niezależnie czy jest to balsam kanadyjski czy Euparal®, wymaga wpierw jego odwodnienia, co wydłuża całkowity czas przygotowania preparatu. Gdy nie ma możliwości odwodnienia rozpuszczalnikami organicznymi, próbki wyjęte z etanolu 100% można umieścić w roztworze pośrednim składającym się z równej mieszaniny Euparalu® i esencji Euparalu przed ostatecznym utrwaleniem.

Enecê (RI = 1,467)

Enecê jest medium na bazie żywicy stosowanym głównie do preparatyki małych owadów, szczególnie popularnym w Brazylii. Jego bazę stanowi kalafonia i niestwardniała żywica (kopal), rozpuszczone w alkoholu, kamforze, esencji terpentynowej i eukaliptolu. Cerqueira [11] opisał Enecê jako alternatywę dla balsamu kanadyjskiego do sporządzania trwałych preparatów larw, wylinek stadiów młodocianych, a nawet dorosłych komarów; od tego czasu medium to zaczęło być również wykorzystywane do preparatyki Phebotominae. Enecê oferuje opłacalną alternatywę dla wykonywania trwałych preparatów, zapewniając dużą trwałość i wystarczająco powolny czas schnięcia, co pozwala na sekcję i precyzyjne rozmieszczenie struktur morfologicznych muchówki.

5.4. Przygotowywanie i suszenie preparatów

Prawidłowe suszenie preparatów mikroskopowych jest kluczowe dla zapewnienia ich długiej trwałości. Preparaty powinny zostać dokładnie osuszone przed przeznaczeniem ich do archiwizacji. Dla uzyskania optymalnych rezultatów, preparaty wykonane z użyciem trwałych mediów mikroskopowych należy suszyć je w pozycji poziomej przez 2–3 tygodnie, natomiast te przygotowane z użyciem mediów półtrwałych wymagają jedynie 1–2 tygodni. Aby zapewnić skuteczny proces suszenia, zaleca się korzystanie z inkubatora ustawionego (w zależności od zastosowanego medium) na odpowiednią temperaturę, unikając nadmiernego nagrzewania preparatu, co mogłoby uszkodzić okazy. Rekomendowany zakres temperatury wynosi 30–37°C. Suszenie ma kluczowe znaczenie, ponieważ zapobiega deformacji szkiełek, degradacji okazów lub destabilizacji medium.

Informacja o zastosowanym medium powinna zawsze znajdować się na etykiecie preparatu. Jeśli to możliwe, etykieta powinna również zawierać informację o konkretnym składzie medium, imię i nazwisko osoby przygotowującej preparat oraz datę jego wykonania. Preparaty są często wykonywane w celach doraźnych, przez co nie są przeznaczone do długoterminowego przechowywania. Jeżeli jednak status okazu ulegnie zmianie, na przykład zostanie on włączony do serii „typowej”, należy wykonać preparat ponownie, stosując trwalsze medium, aby móc zachować okazu do przyszłych badań taksonomicznych.

5.5. Alternatywne techniki preparacyjne: przymocowanie do kartoników

Przymocowywanie okazów do kartoników jest techniką stosowaną w przypadku kilku grup owadów, w które są zbyt delikatne do nabicia bezpośrednio na szpilki entomologiczne. Jednak ze względu na niewielkie rozmiary oraz konieczność obserwacji narządów wewnętrznych w celu identyfikacji po wytrawianiu Phlebotominae (zob. punkt 5), metoda ta nie jest odpowiednia ich do preparowania i archiwizacji.

5.6. Ponowna preparacja uszkodzonych okazów

W przypadku cennych okazów zaleca się podejście dwuetapowe, zgodnie z filmem dostępnym pod linkiem: https://zenodo.org/records/18315029. 1) Okazy należy

This is an Open Access article distributed under the terms of the Creative Commons Attribution License (https://creativecommons.org/licenses/by/4.0), which permits unrestricted use, distribution, and reproduction in any medium, provided the original work is properly cited.

najpierw nawodnić (odmoczyć) bez zdejmowania okazów, aby umożliwić ich wstępną obserwację. Statyw z kilku szkiełek mikroskopowych należy umieścić w szalce Petriego jako podporę. Następnie na nim kładzie się szkiełko przeznaczone do nawodnienia (odmoczenia), a do szalki wlewa się kilka milimetrów rozpuszczalnika w celu wytworzenia wilgotnej komory, dbając o to, aby samo szkiełko nie miało bezpośredniego kontaktu z rozpuszczalnikiem (Rycina 8D). Czas potrzebny do nawodnienia (odmoczenia) może wynosić od jednego do kilku dni, w zależności od stanu okazu. Niezbędne są codzienne kontrole stanu okazów oraz cierpliwość. Po wystarczającym nawodnieniu (odmoczeniu), preparat można wyjąć z wilgotnej komory i umieścić w inkubatorze na kilka godzin przed obserwacją mikroskopową, fotografowaniem lub wykonywaniem rysunków. 2) W celu ponownego utrwalenia, okaz można umieścić ponownie w wilgotnej komorze na kolejne kilka godzin lub na całą noc. Wyciągnięcie okazu z medium należy przeprowadzać pod mikroskopem stereoskopowym. Za pomocą cienkich igieł należy ostrożnie zdjąć szkiełko nakrywkowe, upewniając się, że żadne elementy muchówki nie pozostają do niego przyczepione (https://zenodo.org/records/18315029). Następnie rozczłonkowane części ciała okazu należy zebrać i przepłukać wodą w dołkach np. stosowanych przy ekstrakcji DNA/RNA (zob. poniżej), przed nawodnieniem (odmoczeniem) i ponownym zatopieniem w medium na bazie żywicy. Podczas przenoszenia preparatu kluczowe jest określenie pierwotnie użytego medium w celu dobrania odpowiedniego rozpuszczalnika. W przypadku uwodnionych mediów należy użyć wody. Jeżeli medium jest oparte na żywicy (np. balsam kanadyjski lub Euparal®), należy użyć ksylenu, pracując pod wyciągiem i stosując odpowiednie środki ochrony osobistej, w tym maskę.

Ponowna preparacja okazów typowych lub z muzealnych kolekcji może być wykonywane wyłącznie za zgodą kustosza i/lub instytucji będącej właścicielem okazu.

6. Identyfikacja (oznaczanie) okazów

6.1. Morfologia

Identyfikacja Phlebotominae opiera się przede wszystkim na analizie ich cech morfologicznych, w tym tułowia, skrzydeł, genitaliów, szczecinek oraz specyficznych zależności morfometrycznych między różnymi strukturami. Naukowcy wykorzystują klucze do oznaczania, kolekcje referencyjne oraz oryginalne opisy gatunków, aby porównać zebrane okazy ze znanymi i opisanymi taksonami. Kluczowe cechy diagnostyczne, takie jak użyłkowanie skrzydeł i morfologia głowy u obu płci, budowa męskich narządów rozrodczych oraz układ spermatek u samic, są szczególnie istotne przy oznaczaniu do poziomu gatunku. Dokładna identyfikacja często wymaga szczegółowych analiz mikroskopowych, zazwyczaj przy użyciu mikroskopu świetlnego złożonego do obserwacji drobnych struktur, takich jak genitalia i spermateki, lub mikroskopu stereoskopowego do oceny ogólnych cech morfologicznych.

Rozwój technologii obrazowania w ostatnich latach znacznie ułatwił wykorzystanie obrazowania cyfrowego w identyfikacji Phlebotominae. Fotografie o wysokiej rozdzielczości lub cyfrowe ilustracje kluczowych cech mogą być porównywane z materiałami referencyjnymi lub analizowane za pomocą systemów komputerowego wspomagania identyfikacji, co poprawia zarówno dokładność, jak i dostępność taksonomii morfologicznej.

6.2. Geometria skrzydeł

Geometria skrzydła jest kluczową cechą wykorzystywaną w identyfikacji i taksonomii Phlebotominae. Skrzydła tych owadów wykazują unikalny wzór i budowę; są zazwyczaj długie i wąskie, z dobrze widocznym użyłkowaniem (Ryciny 9 i 10). Układ żyłek tworzy wyraźny wzór, który może różnić się w zależności od rodzaju i gatunku, dostarczając cennych cech diagnostycznych do identyfikacji. W rezultacie badanie geometrii skrzydła dostarcza wiele danych taksonomicznych.

6.3. Geometria skrzydeł - morfometria

Naukowcy stosują różne techniki, takie jak morfometria geometryczna, do analizy i porównywania kształtu oraz wielkości skrzydeł u różnych gatunków lub populacji Phlebotominae. Badanie geometrii skrzydła dostarcza cennych informacji na temat zachowania, preferencji siedliskowych oraz zdolności lotnych tych owadów.

W podejściu morfometryczno-geometrycznym skrzydła są ostrożnie wypreparowywane, wybarwiane (jeśli konieczne) i zatapiane w medium na płasko na szkiełkach podstawowych. Tak przygotowane preparaty są następnie fotografowane pod mikroskopem stereoskopowym, digitalizowane i poddawane analizie morfometrycznej. Procedura ta została dobrze opisana w literaturze [6, 27, 42, 56, 57, 59], z zaleceniem konsekwentnego wykorzystywania prawego lub lewego skrzydła w przypadku narządów parzystych, aby uniknąć potencjalnych negatywnych efektów związanych z allometrią [62].

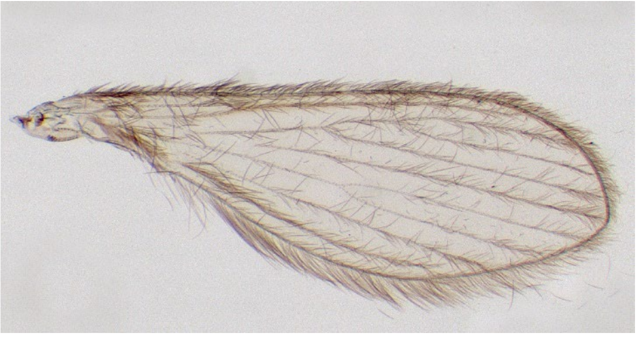

Rycina 9: Niewybarwione skrzydło *Trichophoromyia ininii*.

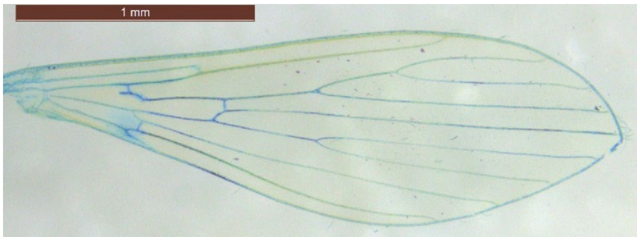

Rycina 10: Skrzydło *Phlebotomus ariasi* po wybarwieniu.

Preparacja skrzydła do analiz morfometrycznych

Aby móc wyraźnie zaobserwować wszystkie żyłki, skrzydła powinny zostać oczyszczone z łusek i odpowiednio wybarwione. Aby przygotować skrzydło, najpierw należy wypełnić małe dołki wymaganymi odczynnikami (błękit metylenowy, etanol, woda i zamiennik ksylenu). Skrzydło przechowywane w 70% etanolu w temperaturze pokojowej należy wyjąć, odwracając probówkę typu Eppendorf i opróżniając nad dołkiem, a następnie podnieść skrzydło za pomocą cienkiej, zakrzywionej na końcu igły. Skrzydło należy na krótko przenieść z etanolu do wody, a następnie z powrotem do etanolu, aby pozbyć się szczecinek. Następnie należy umieścić skrzydło w błękicie metylenowym na 6 minut, upewniając się, że podczas barwienia unosi się ono na powierzchni. Ostrożnie wyjąć skrzydło i zanurzyć je w zamienniku ksylenu na 2 minuty (około jednej trzeciej czasu barwienia błękitem metylenowym). Delikatne stukanie igłą o ścianki studzienki może pomóc skrzydłu opaść na dno; ksylen służy do utrwalenia wybarwienia. Na koniec należy podnieść skrzydło i umieścić je na małej kropli Euparalu® na szkiełku mikroskopowym. Pod binokularem delikatnie rozprostować skrzydło i ostrożnie nałożyć szkiełko nakrywkowe. Zdjęcia należy wykonać niezwłocznie, zanim Euparal® zdąży wyschnąć, ponieważ mogą być konieczne drobne korekty pozycji skrzydła pod szkiełkiem nakrywkowym w celu uzyskania jego optymalnego ułożenia.

6.4. Metody analiz wykorzystujące biologię molekularną

Oprócz analiz morfologicznych, używanie metod molekularnych staje się coraz bardziej istotne w badaniach entomologicznych, w tym w taksonomii, genetyce populacyjnej, filogenetyce, a także w detekcji DNA/RNA patogenów oraz pochodzenia pobranej przez muchówkę krwi, co ma istotne znaczenie w dziedzinie epidemiologii [70]. Sekwencjonowanie DNA może być wykorzystywane do potwierdzenia przynależności do danego gatunku lub różnicowania blisko spokrewnionych gatunków, zapewniając dokładniejszy i bardziej wiarygodny sposób ich identyfikacji. Co więcej, zaawansowane techniki molekularne (tj. PCR, sekwencjonowanie DNA, NGS itp.) oraz MALDI-ToF MS zyskują na znaczeniu w dokładnej i szybkiej identyfikacji gatunków, będąc uzupełnieniem do tradycyjnych analiz morfologicznych [46]. Mimo to, morfologia pozostaje standardem referencyjnym dla taksonomii i podstawą, na której interpretowane są dane molekularne.

6.4.1. Destruktywna izolacja kwasów nukleinowych

Izolacja kwasów nukleinowych jest rutynowym etapem wielu badań biologicznych, opracowano różne metody izolacji DNA z materiału biologicznego [48]. Wiele komercyjnie dostępnych zestawów do izolacji zostało zaprojektowanych w celu ułatwienia tego procesu [14]. Jednakże metody stosowane w preparatyce stawonogów do ich identyfikacji na podstawie morfologii często utrudniają analizę molekularną, ponieważ używane odczynniki mogą uszkodzić lub zniszczyć samo DNA [10]. Większość protokołów izolacji DNA z tkanek owadów ma charakter destruktywny [43], co jest niekorzystne w przypadku małych okazów, u których nawet pobranie niewielkiej ilości próbki może naruszyć ważne cechy morfologiczne i uniemożliwić ich identyfikację [72]. Typ i stan okazu odgrywają kluczową rolę w wyborze odpowiedniej metody izolacji DNA [29].

Potrzeba prawidłowego oznaczenia Phlebotominae, chęć zrozumienia dynamiki populacji i zminimalizowania wpływu na organizmy niebędące przedmiotem zwalczania stała się motorem rozwoju molekularnych narzędzi diagnostycznych [23]. Podejście molekularne jest obecnie często stosowane jako uzupełnienie do analiz morfologicznych w identyfikacji Phlebotominae. Na przykład standardowa procedura barkodingu owadów obejmuje izolację DNA, sekwencjonowanie, a tym samy utratę całości badanego okazu. Istnieje zatem potrzeba opracowania niedestrukcyjnych metod ekstrakcji DNA, które zachowają zarówno materiał biologiczny, jak i jego integralność morfologiczną.

W przypadku Phlebotominae zastosowano liczne metody izolacji kwasów nukleinowych. Wymagana ilość lub jakość kwasów nukleinowych zależy od dalszych analiz, ponieważ różne techniki mają odmienne wymagania dotyczące ich czułości i czystości [9]. Na przykład

stwierdzono, że komórki oczu Phlebotominae hamują amplifikację PCR [69]. Poza poszukiwaniem patogenów, DNA tych muchówek jest rutynowo izolowane w celu identyfikacji gatunkowej. Można stosować różne metody izolacji, choć ich wydajność i jakość różnią się w zależności od wybranej metody. Protokoły niektórych producentów zostały zaadaptowane przez badaczy do pracy z Phlebotominae [8], zwiększając wydajność i/lub jakość ekstrahowanych kwasów nukleinowych [8, 9, 69], choć metody, opracowane dla innych grup owadów, również mogą być stosowane dla Phlebotominae [58, 76]. PCR przeprowadza się celując w małe fragmenty mitochondrialne (COI lub CytB), które są kompatybilne z metodami ekstrakcji silnie pofragmentowanego DNA. Dla kontrastu, inne metody sekwencjonowania długich odczytów (Oxford Nanopore i PacBio) wymagają mało pofragmentowanego i wysokiej jakości DNA. Ekstrakcje kolumienkowe (spin column) zazwyczaj dają fragmenty genomowego DNA o wielkości do 60 kb, a przy zastosowaniu metody fenol-chloroform uzyskiwane są fragmenty do 150 kb [77]. Tabela 5 podsumowuje różne techniki izolacji DNA Phlebotominae i wskazuje, które z nich były wykorzystywane w badaniach tych owadów. Uzyskiwanej wydajności nie podano, ponieważ zależy ona od wielkości okazu i wybranej metody.

Wybór metody ekstrakcji DNA powinien uwzględniać kilka kryteriów, takich jak liczba próbek, czas potrzebny do jej wykonania i techniki stosowane w dalszym etapie. Podczas gdy metody NGS wymagają genomowego DNA o wysokiej masie cząsteczkowej, wszystkie przedstawione tu metody mogą być stosowane w standardowych aplikacjach opartych na PCR. Ponadto, wiele badań przeprowadzono w celu weryfikacji niedestrukcyjnych metod ekstrakcji DNA dla małych stawonogów lądowych, suchych okazów muzealnych oraz stawonogów o miękkim ciele [19, 26, 28, 55, 63].

Tabela 5: Średni koszt, zastosowania i potencjalne wykorzystanie protokołu w badaniach naukowych dla jednej próbki przy ekstrakcji gDNA Phlebotominae

Metoda	Koszt	Zastosowanie	Wykorzystanie protokołu do badań niewielkich stawonogów
Kolumienkowy (Spin column)	2,5 – 3,55 US$ [39]	PCR, NGS	[9]
Fenol-chloroform	0,24 US$ [69]	PCR, NGS	[9]
HotSHOT	<0,01 US$ [69]	PCR	-
Wysalanie (salting out)	0,12 $3 [69]	PCR	-
Chelex	0,02 $4 [41]	PCR	[41, 76]

6.4.2. Niedesktruktywna izolacja kwasów nukleinowych

Jednym z głównych wyzwań w analizie molekularnej stawonogów, zwłaszcza Phlebotominae, jest potrzeba zachowania poszczególnych okazów w celu włączenia ich do kolekcji entomologicznych. Większość protokołów ekstrakcji DNA wymaga maceracji tkanki, co częściowo niszczy zachowany okaz. Metody niedestrukcyjnej ekstrakcji kwasów nukleinowych są jednak zaprojektowane tak, aby izolować materiał genetyczny bez fizycznego uszkadzania okazu, wpływania na jego trwałość czy zmieniania morfologii. Metody te są szczególnie przydatne przy pracy z cennymi lub rzadkimi taksonami, gdzie zachowanie integralności strukturalnej jest niezbędne dla przyszłych analiz taksonomicznych, morfologicznych lub diagnostycznych. Powszechnie stosowaną metodą jest metoda niedestrukcyjnej inkubacji, w której muchówki są unieruchamiane i delikatnie zanurzane w buforze lizującym zawierającym proteinazę K.

Technika łagodnej lizy wektorów (mild-vectolysis) została z powodzeniem zastosowana u Phlebotominae, nawet w przypadku okazów typowych [24]. Technika ta wykorzystuje konwencjonalny zestaw kolumienkowy (w tym przypadku zestaw DNeasy Blood and Tissue, QIAGEN, Hilden, Niemcy) umożliwiający uzyskanie DNA bez uszkadzania okazu. Zmodyfikowane etapy lizy (objętość bufora lizującego i dodanie etapu zamrażania) [17] pozwalają na uwolnienie kwasów nukleinowych, minimalizując uszkodzenia morfologiczne [24]. W przypadku Phlebotominae, możliwe jest również użycie zestawu HotSHOT DNA Extraction kit (Bento Bioworks Ltd, Londyn, Wielka Brytania) [73], który jest szybki i tani, co umożliwia błyskawiczne i niskokosztowe opracowanie próbek. Okazy przeznaczone do identyfikacji morfologicznej można na końcu przepłukać. Okazy opracowane z użyciem zestawu DNeasy Blood and Tissue muszą zostać wytrawione roztworem Marc'a-André, te przy użyciu HotSHOT są wystarczająco wytrawione, aby można je było umieścić w uwodnionym medium lub żywicy po wstępnej dehydratacji, zgodnie z protokołem szczegółowo opisanym w tym artykule [73]. Wyekstrahowany materiał genetyczny można następnie poddać kolejnym procedurom w celu amplifikacji określonych markerów genetycznych. Niedestrukcyjne metody ekstrakcji kwasów nukleinowych są kluczowe dla badania charakterystyki genetycznej Phlebotominae, w tym identyfikacji potencjalnych czynników chorobotwórczych, które mogą przenosić. Dzięki zachowaniu integralności okazu badacze mogą uzyskać cenne informacje genetyczne, jednocześnie zachowując próbkę do dodatkowych analiz lub badań.

6.5. MALDI-ToF MS

MALDI-ToF MS (spektrometria mas z jonizacją laserową wspomaganą matrycą i analizatorem czasu przelotu) to metoda oparta na spektrometrii mas, zaprojektowana do wykrywania i analizy unikalnych profili

białkowych („odcisków palców") próbek biologicznych. MALDI-ToF jest coraz częściej uznawana za ważne narzędzie w identyfikacji owadów o znaczeniu medycznym i weterynaryjnym. Metoda ta okazała się skuteczna w identyfikacji różnych stadiów rozwojowych Phlebotominae, w tym larw, poczwarek oraz krwi pobranej przez samice, i została z powodzeniem zastosowana do odróżniania zarówno samców, jak i samic różnych gatunków [28, 30, 73, 74]. Metoda ta oferuje również wysoką siłę dyskryminacyjną na poziomie podrodzajów, gatunków i populacji. MALDI-ToF umożliwia badaczom szybką i dokładną identyfikację gatunków, co jest niezbędne dla zrozumienia rozprzestrzenienia danych taksonów Phlebotominae, ich zachowania oraz ich roli w przenoszeniu chorób. Poprzez różnicowanie gatunków na podstawie profili białkowych, MALDI-ToF odgrywa kluczową rolę w badaniach epidemiologicznych i strategiach kontroli wektorów.

Obecnie istnieją dwie główne wady tej metody, które ograniczają jej częste wykorzystywanie w badaniach nad Phlebotominae. Po pierwsze, dostępność sprzętu do spektrometrii mas. Jest często za drogi dla placówek badawczych, aby używać go wyłącznie w celach identyfikacji Phlebotominae (lub ogólnie innych stawonogów, które są wektorami chorób). Na szczęście, to ograniczenie można przezwyciężyć, wnioskując o dostęp na spektrometrach mas, które są standardowym narzędziem badawczym w placówkach proteomicznych i/lub diagnostyce klinicznej. Po drugie - niska reprezentacja danych referencyjnych dla Phlebotominae w ogólnodostępnych bazach danych, co skutkuje koniecznością tworzenia własnej biblioteki z widmami referencyjnymi opartymi na zidentyfikowanych okazach, uzyskanej na podstawie analiz morfologicznych i sekwencji odpowiedniego markera genetycznego (COI, CytB lub innego). Mamy nadzieję, że to ograniczenie zostanie wkrótce rozwiązane poprzez stopniowe włączanie dotychczasowych wewnętrznych danych referencyjnych dotyczących Phlebotominae do platformy MSI prowadzonej przez Assistance Publique-Hôpitaux de Paris, Sorbonne University we Francji oraz kolekcję BCCM/IHEM/Sciensano w Brukseli, Belgia (https://msi.happy-dev.fr/).

W przypadku analiz profili białkowych metodą MALDI-ToF, próbki powinny być przechowywane najlepiej zamrażarkach na sucho lub w 70% etanolu do zastosowań molekularnych i nie powinny być wystawiane na działanie temperatury pokojowej. Wobec braku uniwersalnych wytycznych dotyczących przygotowania próbek, zaleca się stosowanie wodnego roztworu kwasu synapinowego (30 mg/mL) w 60% acetonitrylu/0,3% TFA do przygotowania matrycy MALDI-ToF, aby ich widma białkowe były porównywalne z dotychczas opublikowanymi danymi dotyczącymi Phlebotominae.

Przygotowanie próbki do MALDI-ToF MS (Rycina 7)

Okazy są najpierw osuszane w temperaturze pokojowej i odpowiednio preparowane. Głowę i odwłok usuwa się, aby uzyskać części ciała zawierające kluczowe cechy morfologiczne i wykonuje się z nich preparat mikroskopowy do analizy morfologicznej. Tułów można wykorzystać do MALDI-ToF, a pozostałą cześć odwłoka zachować do izolacji DNA. Do analiz profilu białkowego tułów jest ręcznie homogenizowany w mikroprobówkach o pojemności 1,5 ml, zawierających 10 µl roztworu homogenizującego przy użyciu jednorazowych tłuczków. Zazwyczaj stosuje się dwa roztwory homogenizujące: sterylną wodę destylowaną i 25% kwas mrówkowy.

7. Wnioski

W niniejszej pracy dążyliśmy do dostarczenia badaczom najskuteczniejszych metod preparatyki Phlebotominae, dostosowanych do konkretnych celów badawczych, aby ułatwić ich identyfikację i wykrywanie patogenów. Nie istnieje jedna, uniwersalnie optymalna metoda, a raczej kilka metod, z których każda ma swoje zalety i ograniczenia. W danych uzupełniających (Supporting data) zamieściliśmy szczegółowe protokoły różnych technik preparowania stosowanych w oznaczaniu Phlebotominae. Protokoły te, w tym filmy instruktażowe, pokazują krok po kroku procedury dostosowane do różnych celów, zapewniając precyzyjne i wiarygodne wyniki. Oferując te zasoby, chcemy wspierać naukowców w wyborze i stosowaniu odpowiednich technik preparacyjnych do wybranych potrzeb.

Podziękowania

Dziękujemy Richardowi Lane i Zoe Jay Adams z Muzeum Historii Naturalnej w Londynie (Wielka Brytania) za ich doskonałą recenzję, która podniosła jakość niniejszego manuskryptu.

Finansowanie

Dziękujemy brazylijskim agencjom rozwoju CNPq (case number: 404395/2024-4) i Fundacji Araucária (case number: 433/2025 PDI) za sfinansowanie badań AJA.

Konflikt interesów

Jérôme Depaquit jest edytorem pomocniczym czasopisma *Parasite*; nie miał on wpływu na proces recenzji i podejmowanie decyzji dotyczących tego manuskryptu. Pozostali autorzy zaświadczają o braku konfliktu interesów.

Dostępność danych

Materiały wideo na platformie Zenodo.
Video 1: https://zenodo.org/records/18198006
Video 2: https://zenodo.org/records/18311158
Video 3: https://zenodo.org/records/18311106
Video 4: https://zenodo.org/records/18311154
Video 5: https://zenodo.org/records/18303014
Video 6: https://zenodo.org/records/18302850
Video 7: https://zenodo.org/records/18315029

Dodatkowe materiały

Dodatkowe materiały dotyczące tego artykułu są dostępne pod następującym adresem: https://www.parasite-journal.org/10.1051/parasite/2026009/olm

Referencje

1. Alkan C, Allal-Ikhlef AB, Alwassouf S, Baklouti A, Piorkowski G, de Lamballerie X, Izri A, Charrel RN. 2015. Virus isolation, genetic characterization and seroprevalence of Toscana virus in Algeria. Clinical Microbiology and Infection, 21(11), 1040 e1-9.
2. Alten B, Ozbel Y, Ergunay K, Kasap OE, Cull B, Antoniou M, Velo E, Prudhomme J, Molina R, Banuls AL, Schaffner F, Hendrickx G, Van Bortel W, Medlock JM. 2015. Sampling strategies for phlebotomine sand flies (Diptera: Psychodidae) in Europe. Bulletin of Entomological Research 105(6), 664–678.
3. Ayhan N, Baklouti A, Prudhomme J, Walder G, Amaro F, Alten B, Moutailler S, Ergunay K, Charrel RN, Huemer H. 2017. Practical guidelines for studies on sandfly-borne phleboviruses: Part I: Important points to consider *ante* field work. Vector-Borne and Zoonotic Diseases 17(1), 73–80.
4. Bates PA. 1997. Infection of phlebotomine sandflies with *Leishmania*, in The Molecular Biology of Insect Disease Vectors: A Methods Manual. Springer. p. 112–120.5.
5. Baum M, de Castro EA, Pinto MC, Goulart TM, Baura W, Klisiowicz Ddo R, Vieira da Costa-Ribeiro MC. 2015. Molecular detection of the blood meal source of sand flies (Diptera: Psychodidae) in a transmission area of American cutaneous leishmaniasis, Parana State, Brazil. Acta Tropica, 143, 8–12.
6. Belen A, Alten B, Aytekin A. 2004. Altitudinal variation in morphometric and molecular characteristics of *Phlebotomus papatasi* populations. Medical and Veterinary Entomology, 18(4), 343–350.
7. Bhattacharya J, Chandra G, Hati AK. 1991. A simple method for cryopreservation of *Leishmania donovani* promastigotes, Indian Journal of Medical Research, 93, 245–246.
8. Caligiuri LG, Sandoval AE, Miranda JC, Pessoa FA, Santini MS, Salomón OD, Secundino NF, McCarthy CB. 2019. Optimization of DNA extraction from individual sand flies for PCR amplification. Methods and Protocols, 2(2), 36.
9. Casaril AE, de Oliveira LP, Alonso DP, de Oliveira EF, Gomes Barrios SP, de Oliveira Moura Infran J, Fernandes WS, Oshiro ET, Ferreira AMT, Ribolla PEM, de Oliveira AG. 2017. Standardization of DNA extraction from sand flies: Application to genotyping by next generation sequencing. Experimental Parasitology, 177, 66–72.
10. Castalanelli MA, Severtson DL, Brumley CJ, Szito A, Foottit RG, Grimm M, Munyard K, Groth DM. 2010. A rapid non-destructive DNA extraction method for insects and other arthropods. Journal of Asia-Pacific Entomology, 13(3), 243–248.
11. Cerqueira NL. 1943. Um novo meio para montagem de pequenos insetos em lâmina. Memórias do Instituto Oswaldo Cruz, (39), 37–41.
12. Charrel RN, Gallian P, Navarro-Mari JM, Nicoletti L, Papa A, Sanchez-Seco MP, Tenorio A, de Lamballerie X. 2005. Emergence of Toscana virus in Europe. Emerging Infectious Diseases, 11(11), 1657–1663.
13. Chaskopoulou A, Giantsis IA, Demir S, Bon MC. 2016. Species composition, activity patterns and blood meal analysis of sand fly populations (Diptera: Psychodidae) in the metropolitan region of Thessaloniki, an endemic focus of canine leishmaniasis. Acta Tropica, 158, 170–176.
14. Chen H, Rangasamy M, Tan SY, Wang H, Siegfried BD. 2010. Evaluation of five methods for total DNA extraction from western corn rootworm beetles. PLoS One, 5(8), e11963.
15. Depaquit J, Grandadam M, Fouque F, Andry PE, Peyrefitte C. 2010. Arthropod-borne viruses transmitted by Phlebotomine sandflies in Europe: a review. Eurosurveillance, 15(10), 19507.
16. Diamond LS, Herman CM. 1954. Incidence of Trypanosomes in the Canada Goose as revealed by bone marrow culture. Journal of Parasitology, 40(2), 195–202.
17. Ding H, Torno M, Vongphayloth K, Ng G, Tan D, Sng W, Ho K, Randrianambinintsoa FJ, Depaquit J, Tan CH. 2025. Hidden in plain sight: discovery of sand flies in Singapore and description of four species new to science. Parasites & Vectors, 18(1), 402.
18. Es-Sette N, Ajaoud M, Bichaud L, Hamdi S, Mellouki F, Charrel RN, Lemrani M. 2014. *Phlebotomus sergenti* a common vector of *Leishmania tropica* and Toscana virus in Morocco. Journal of Vector Borne Diseases, 51(2), 86–90.
19. Favret C. 2005. A new non-destructive DNA extraction and specimen clearing technique for aphids (Hemiptera). Proceedings of the Entomological Society of Washington, 107(2), 469–470.
20. Galati EAB. 2018. Phlebotominae (Diptera, Psychodidae): Classification, morphology and terminology of adults and identification of American taxa, in Brazilian Sand Flies: Biology, Taxonomy, Medical Importance and Control, Rangel EF, Shaw JJ, Editors. Cham: Springer International Publishing. pp. 9–212.
21. Galati EAB, de Andrade AJ, Perveen F, Loyer M, Vongphayloth K, Randrianambinintsoa FJ, Prudhomme J, Rahola N, Akhoundi M, Shimabukuro PHF, Depaquit J. 2025. Phle-botomine sand flies (Diptera, Psychodidae) of the world. Parasites & Vectors, 18(1), 220.
22. Galati EAB, Galvis-Ovallos F, Lawyer P, Leger N, Depaquit J. 2017. An illustrated guide for characters and terminology used in descriptions of Phlebotominae (Diptera, Psychodidae). Par-asite, 24, 26.
23. Gariepy T, Kuhlmann U, Gillott C, Erlandson M. 2007. Parasitoids, predators and PCR: the use of diagnostic molecular markers in biological control of Arthropods. Journal of Applied Entomology, 131(4), 225–240.
24. Giantsis IA, Chaskopoulou A, Bon MC. 2016. Mild-Vectolysis: A nondestructive DNA extraction method for vouchering sand flies and mosquitoes. Journal of Medical Entomology, 53(3), 692–695.
25. Gidwani K, Picado A, Rijal S, Singh SP, Roy L, Volfova V, Andersen EW, Uranw S, Ostyn B, Sudarshan M, Chakravarty J, Volf P, Sundar S, Boelaert M, Rogers ME. 2011. Serological markers of sand fly exposure to evaluate insecticidal nets against visceral leishmaniasis in India and Nepal: a cluster-randomized trial. PLoS Neglected Tropical Diseases, 5(9), e1296.
26. Gilbert MTP, Moore W, Melchior L, Worobey M. 2007. DNA extraction from dry museum beetles without conferring external morphological damage. PLoS One, 2(3), e272.
27. Giordani BF, Andrade AJ, Galati EAB, Gurgel-Goncalves R. 2017. The role of wing geometric morphometrics in the identification of sandflies within the subgenus *Lutzomyia.* Medical and Veterinary Entomology, 31(4), 373–380.
28. Guzmán-Larralde AJ, Suaste-Dzul AP, Gallou A, Peña-Carrillo KI. 2017. DNA recovery from microhymenoptera using six non-destructive methodologies with considerations for subsequent preparation of museum slides. Genome, 60(1), 85–91.
29. Hajibabaei M, DeWaard JR, Ivanova NV, Ratnasingham S, Dooh RT, Kirk SL, Mackie PM, Hebert PD. 2005. Critical factors for assembling a high volume of DNA barcodes. Philosophical Transactions of the Royal Society B: Biological Sciences, 360(1462), 1959–1967.

This is an Open Access article distributed under the terms of the Creative Commons Attribution License (https://creativecommons.org/licenses/by/4.0), which permits unrestricted use, distribution, and reproduction in any medium, provided the original work is properly cited.

30. Haouas N, Pesson B, Boudabous R, Dedet JP, Babba H, Ravel C. 2007. Development of a molecular tool for the identification of *Leishmania* reservoir hosts by blood meal analysis in the insect vectors. American Journal of Tropical Medicine and Hygiene, 77(6), 1054–1059.
31. Hlavackova K, Dvorak V, Chaskopoulou A, Volf P, Halada P. 2019. A novel MALDI-TOF MS-based method for blood meal identification in insect vectors: A proof of concept study on phlebotomine sand flies. PLoS Neglected Tropical Diseases, 13 (9), e0007669.
32. Huemer H, Prudhomme J, Amaro F, Baklouti A, Walder G, Alten B, Moutailler S, Ergunay K, Charrel RN, Ayhan N. 2017. Practical guidelines for studies on sandfly-borne phleboviruses: Part II: Important points to consider for fieldwork and subsequent virological screening. Vector-Borne and Zoonotic Diseases, 17(1), 81–90.
33. Jancarova M, Polanska N, Thiesson A, Arnaud F, Stejskalova M, Rehbergerova M, Kohl A, Viginier B, Volf P, Ratinier M. 2025. Susceptibility of diverse sand fly species to Toscana virus. PLoS Neglected Tropical Diseases, 19(5), e0013031.
34. Kapp JD, Green RE, Shapiro B. 2021. A fast and efficient single-stranded genomic library preparation method optimized for ancient DNA. Journal of Heredity, 112(3), 241–249.
35. Killick-Kendrick R, Maroli M, Killick-Kendrick M. 1991. Bibliography of the colonization of phlebotomine sandflies. Parassitologia, 33(suppl.), 321–333.
36. Lawyer P, Killick-Kendrick M, Rowland T, Rowton E, Volf P. 2017. Laboratory colonization and mass rearing of phlebotomine sand flies (Diptera, Psychodidae). Parasite, 24, 42.
37. Léger N, Pesson B, Madulo-Leblond G. 1986. Les phlébotomes de Grèce : 1ère partie. Bulletin de la Société de Pathologie Exotique, 79, 386–397.
38. Léger N, Pesson B, Madulo-Leblond G. 1986. Les phlébotomes de Grèce : 2ème partie. Bulletin de la Société de Pathologie Exotique, 79, 514–524.
39. Leonel JAF, Vioti G, Alves ML, da Silva DT, Meneghesso PA, Benassi JC, Spada JCP, Galvis-Ovallos F, Soares RM, Oliveira T. 2020. DNA extraction from individual Phlebotomine sand flies (Diptera: Psychodidae: Phlebotominae) specimens: Which is the method with better results? Experimental Parasitology, 218, 107981.
40. Lestinova T, Rohousova I, Sima M, de Oliveira CI, Volf P. 2017. Insights into the sand fly saliva: Blood-feeding and immune interactions between sand flies, hosts, and *Leishmania.* PLoS Neglected Tropical Diseases, 11(7), e0005600.
41. Lienhard A, Schaffer S. 2019. Extracting the invisible: obtaining high quality DNA is a challenging task in small arthropods. PeerJ, 7, e6753.
42. Lozano-Sardaneta YN, Mikery-Pacheco OF, Huerta H, Rojas-Soriano JE, Contreras-Ramos A. 2025. Wing geometric morphometrics is effective to separate sand fly species (Diptera, Psychodidae, Phlebotominae) related with leishmaniasis transmission in Mexico. Acta Tropica, 262, 107523.
43. Mandrioli M. 2008. Insect collections and DNA analyses: how to manage collections? Museum Management and Curatorship, 23(2), 193–199.
44. Maroli M, Feliciangeli MD, Bichaud L, Charrel RN, Gradoni L. 2013. Phlebotomine sandflies and the spreading of leishmaniases and other diseases of public health concern. Medical and Veterinary Entomology, 27(2), 123–147.
45. Marquina D, Buczek M, Ronquist F, Lukasik P. 2021. The effect of ethanol concentration on the morphological and molecular preservation of insects for biodiversity studies. PeerJ, 9, e10799.
46. Mathis A, Depaquit J, Dvorak V, Tuten H, Banuls AL, Halada P, Zapata S, Lehrter V, Hlavackova K, Prudhomme J, Volf P, Sereno D, Kaufmann C, Pfluger V, Schaffner F. 2015. Identification of phlebotomine sand flies using one MALDI-TOF MS reference database and two mass pectrometer systems., Parasites & Vectors, 8, 266.
47. Mekarnia N, Benallal KE, Sadlova J, Vojtkova B, Mauras A, Imbert N, Longhitano M, Harrat Z, Volf P, Loiseau PM, Cojean S. 2024. Effect of *Phlebotomus papatasi* on the fitness, infectivity and antimony-resistance phenotype of antimony-resistant *Leishmania* major Mon-25. International Journal for Parasitology – Drugs and Drug Resistance, 25, 100554.
48. Milligan BG. 1998. Total DNA isolation, in Molecular Genetic Analysis of Population: A Practical Approach, Hoelzel AR, Editor. Oxford: Oxford University Press.
49. Molina R, Jiménez M, Alvar J, González E, Hernández-Taberna S, Ines MM. 2017. Methods in sand fly research. Madrid: Servicio de publicaciones Universidad de Alcalá de Henares, Madrid.
50. Murphy WJ, Eizirik E, O'Brien SJ, Madsen O, Scally M, Douady CJ, Teeling E, Ryder OA, Stanhope MJ, de Jong WW, Springer MS. 2001. Resolution of the early placental mammal radiation using Bayesian phylogenetics. Science, 294(5550), 2348–2351.
51. Nacif-Pimenta R, Pinto LC, Volfova V, Volf P, Pimenta PFP, Secundino NFC. 2020. Conserved and distinct morphological aspects of the salivary glands of sand fly vectors of leishma-niasis: an anatomical and ultrastructural study. Parasites & Vectors, 13(1), 441.
52. Neuhaus B, Schmid T, Riedel J. 2017. Collection management and study of microscope slides: Storage, profiling, deterioration, restoration procedures, and general recommendations. Zootaxa, 4322(1), 1–173.
53. New TR. 1974. Pscoptera. Handbooks for Identification of British Insects (Vol. I). London: Royal Entomological Society of London. 102 pp.
54. Perez-Ruiz M, Collao X, Navarro-Mari JM, Tenorio A. 2007. Reverse transcription, real-time PCR assay for detection of Toscana virus. Journal of Clinical Virology, 39(4), 276–281.
55. Porco D, Rougerie R, Deharveng L, Hebert P. 2010. Coupling non-destructive DNA extraction and voucher retrieval for small soft-bodied Arthropods in a high-throughput context: the example of Collembola. Molecular Ecology Resources, 10(6), 942–945.
56. Prudhomme J, Cassan C, Hide M, Toty C, Rahola N, Vergnes B, Dujardin JP, Alten B, Sereno D, Banuls AL. 2016. Ecology and morphological variations in wings of *Phlebotomus ariasi* (Diptera: Psychodidae) in the region of Roquedur (Gard, France): a geometric morphometrics approach. Parasites & Vectors, 9(1), 578.
57. Prudhomme J, Gunay F, Rahola N, Ouanaimi F, Guernaoui S, Boumezzough A, Banuls AL, Sereno D, Alten B. 2012. Wing size and shape variation of *Phlebotomus papatasi* (Diptera: Psychodidae) populations from the south and north slopes of the Atlas Mountains in Morocco. Journal of Vector Ecology, 37(1), 137–147.
58. Prudhomme J, Toty C, Kasap OE, Rahola N, Vergnes B, Maia C, Campino L, Antoniou M, Jimenez M, Molina R, Cannet A, Alten B, Sereno D, Banuls AL. 2015. New microsatellite markers for multi-scale genetic studies on *Phlebotomus ariasi* Tonnoir, vector

of *Leishmania infantum* in the Mediterranean area. Acta Tropica, 142, 79–85.
59. Prudhomme J, Velo E, Bino S, Kadriaj P, Mersini K, Gunay F, Alten B. 2019. Altitudinal variations in wing morphology of *Aedes albopictus* (Diptera, Culicidae) in Albania, the region where it was first recorded in Europe. Parasite, 26, 55.
60. Rawlins DJ. 1992. Light Microscopy: An Introduction to Biotechniques. Oxford: Bios Scientific publishers. 143 pp.
61. Ready PD. 2013. Biology of phlebotomine sand flies as vectors of disease agents. Annual Review of Entomology, 58, 227–250.
62. Rohlf FJ, Slice D. 1990. Extensions of the Procrustes method for the optimal superimposition of landmarks. Systematic Zoology, 39(1), 40–59.
63. Rowley DL, Coddington JA, Gates MW, Norrbom AL, Ochoa RA, Vandenberg NJ, Greenstone MH. 2007. Vouchering DNA-barcoded specimens: Test of a nondestructive extraction proto-col for terrestrial arthropods. Molecular Ecology Notes, 7(6), 915–924.
64. Sábio PB, Andrade AJ, Galati EAB. 2014. Assessment of the taxonomic status of some species included in the *Shannoni* complex, with the description of a new species of *Psathyromyia* (Diptera: Psychodidae: Phlebotominae). Journal of Medical Entomology, 51(2), 331–341.
65. Sadlova J, Yeo M, Seblova V, Lewis MD, Mauricio I, Volf P, Miles MA. 2011. Visualisation of *Leishmania donovani* fluo-rescent hybrids during early stage development in the sand fly vector. PLoS One, 6(5), e19851.
66. Sales K, Miranda DEO, da Silva FJ, Otranto D, Figueredo LA, Dantas-Torres F. 2020. Evaluation of different storage times and preservation methods on phlebotomine sand fly DNA concen-tration and purity. Parasites & Vectors, 13(1), 399
67. Sales KG, Costa PL, de Morais RC, Otranto D, Brandao-Filho SP, Cavalcanti Mde P, Dantas-Torres F. 2015. Identification of phlebotomine sand fly blood meals by real-time PCR. Parasites& Vectors, 8, 230.
68. Sant'Anna MR, Jones NG, Hindley JA, Mendes-Sousa AF, Dillon RJ, Cavalcante RR, Alexander B, Bates PA. 2008. Blood meal identification and parasite detection in laboratory-fed and field-captured *Lutzomyia longipalpis* by PCR using FTA databasing paper. Acta Tropica, 107(3), 230–237.
69. Senne NA, Santos HA, Araujo TR, Paulino PG, Mendonca LP, Moreira HVS, Camilo TA, da Costa Angelo I. 2022. Robust comparative performance of genomic DNA extraction methods from non-engorged phlebotomine sandflies. Medical and Veterinary Entomology, 36(2), 203–211.
70. Shaw JJ. 2025. A review of Leishmania infections in American Phlebotomine sand flies – Are those that transmit leishmaniasis anthropophilic or anthropportunists? Parasite, 32, 57.
71. Tesh RB, Modi GB. 1983. Growth and transovarial transmission of Chandipura virus (Rhabdoviridae: Vesiculovirus) in *Phlebotomus papatasi.* American Journal of Tropical Medicine and Hygiene, 32(3), 621–623.
72. Thomsen PF, Elias S, Gilbert MTP, Haile J, Munch K, Kuzmina S, Froese DG, Sher A, Holdaway RN, Willerslev E. 2009. Non-destructive sampling of ancient insect DNA. PLoS One, 4(4),e5048
73. Truett GE, Heeger P, Mynatt RL, Truett AA, Walker JA, Warman ML. 2000. Preparation of PCR-quality mouse genomic DNA with hot sodium hydroxide and tris (HotSHOT). Biotechniques, 29(1), 52–54.
74. Upton MS. 1993. Aqueous gum-chloral slide mounting media: an historical review. Bulletin of Entomological Research, 83(2), 267–274.
75. Volf P, Myskova J. 2007. Sand flies and Leishmania: specific versus permissive vectors. Trends in Parasitology, 23(3), 91–92.
76. Wang Q, Wang X. 2012. Comparison of methods for DNA extraction from a single chironomid for PCR analysis. Pakistan Journal of Zoology, 44(2), 421–426.
77. Wang Y, Zhao Y, Bollas A, Wang Y, Au KF. 2021. Nanopore sequencing technology, bioinformatics and applications. Nature Biotechnology, 39(11), 1348–1365.

Cite this article as: Randrianambinintsoa FJ, Augendre L, Prudhomme J, Martinet J-P, Loyer M, Mekarnia N, Kerkoub H, Perveen FK, Huguenin A, Kariya E, Akhoundi M, De Andrade AJ, Berriatua E, Bongiorno G, Boyer S, Christodoulou V, Da Costa-Ribeiro MCV, De Souza LAF, Ding H, Dondji B, Dvořák V, Erisoz Kasap O, Galati EAB, Gállego M, Ballart C, Gouzelou S, Haddad N, Masse RS, Mekuria AH, Ivovic V, Kaczmarek S, Shahar MK, Kirstein OD, Kniha E, Kolářová I, Lincoln T, Lucanas C, Mikov O, Nov K, Özbel Y, Pesson B, Posada Lopez LC, Prasetyo DB, Rahola N, Rebollar-Tellez EA, Rodrigues BL, Roy L, Saini P, Sanjoba C, Shimabukuro PH, Siriyasatien P, Soszynska A, Suleşco T, Sylla M, Torno M, Volf P, Vongphayloth K, Sinh Nam V, Wardhana A, Yessinou E, Zapata S, Gantier J-C & Depaquit J. 2026. Processing and mounting phlebotomine sand flies: a consensus guideline. Parasite xx, xx. https://doi.org/10.1051/parasite/2026009.

PARASITE

An international open-access, peer-reviewed, online journal publishing high quality papers on all aspects of human and animal parasitology

Reviews, articles and short notes may be submitted. Fields include, but are not limited to: general, medical and veterinary parasitology; morphology, including ultrastructure; parasite systematics, including entomology, acarology, helminthology and protistology, and molecular analyses; molecular biology and biochemistry; immunology of parasitic diseases; host-parasite relationships; ecology and life history of parasites; epidemiology; therapeutics; new diagnostic tools.
All papers in Parasite are published in English. Manuscripts should have a broad interest and must not have been published or submitted elsewhere. No limit is imposed on the length of manuscripts.

Parasite (open-access) continues **Parasite** (print and online editions, 1994-2012) and **Annales de Parasitologie Humaine et Comparée** (1923-1993) and is the official journal of the Société Française de Parasitologie.

Editor-in-Chief:
Jean-Lou Justine, Paris

Submit your manuscript at:
https://www.editorialmanager.com/parasite

Załącznik 1: Biochemia - podstawy teoretyczne.

Omawianymi w tym artykule owadami są Phlebotominae. Jednak ogólny zamysł i treść tego artykułu mogą dotyczyć również innych stawonogów, których identyfikacja jest możliwa jedynie po analizie morfologii struktur wewnętrznych. Niektóre z tych struktur są częściowo chitynowe, a ich morfologia dostarcza nam wiele cennych danych taksonomicznych. Dlatego tak interesująca jest obserwacja aparatów gębowych, spermatek wraz z ich przewodami. Przy wszystkich odczynnikach, które omówimy, nie należy zapominać, że od etapu utrwalania owada do umieszczenia w medium preparatyka opiera się w dużej mierze na reakcjach redoks (oksydacyjno-redukcyjnych). Jedynym środkiem ostrożności i ideą, która powinna nam przyświecać w trakcie preparowania okazów, jest unikanie mieszania odczynników redukujących z utleniającymi.

Alkohol etylowy; etanol: Substancja ta jest wykorzystywana na różne sposoby. Cząsteczki alkoholu wykazują silne powinowactwo do wody, a zatem mają działanie odwadniające. Jednak alkohol o niskim stężeniu (tj. zbyt bogaty w wodę) będzie odgrywał rolę w degradacji kwasów nukleinowych (woda jest wrogiem kwasów nukleinowych). Umieszczanie owadów w etanolu ma na celu nie tylko ich konserwację, ale także utrwalenie tkanek. W histologii zazwyczaj rozróżniamy dwa ważne pojęcia: szybkość penetracji i szybkość utrwalania. Powszechnie wiadomo, że dobry utrwalacz musi najpierw szybko wniknąć w głąb tkanek, zanim je utrwali. Dla alkoholu 96% współczynnik penetracji wynosi około 1,05 (dla porównania, dla 0,75% wodnego roztworu kwasu pikrynowego współczynnik penetracji wynosi 0,45, podczas gdy dla 3% roztworu dwuchromianu potasu wynosi 1,45).

Utrwalanie owadów i innych stawonogów w etanolu jest często normą w pracy entomologa. Idea zachowania próbek zebranych w terenie do późniejszych badań lub dla przyszłych badaczy jest prawidłowa, jednak, z punktu widzenia cytologa czy histologa nie jest to właściwe podejście. Przetrzymywanie próbek w utrwalaczu zbyt długo może sprawić, że staną się one niemożliwe do wykorzystania do dalszych badań. Dlatego próbki starsze niż 10 lat są pod tym względem bardzo problematyczne lub niemal niemożliwe do wykorzystania.

Kolejną kwestią jest stosunek masy utrwalanego stawonoga do objętości utrwalacza. W praktyce zoologicznej lub medycznej zaleca się przechowywanie okazów w objętości 60 razy większej niż ich objętość. W przypadku mikro-stawonogów, na daną objętość okazów używa się co najmniej 4–5x objętości alkoholu. Należy pamiętać, że alkohol straci swoje stężenie, wyciągając całą wodę z tkanek stawonoga.

Podsumowując:

- Alkohol etylowy jest chemicznym czynnikiem redukującym (zatem niekompatybilnym z utrwalaczami utleniającymi);
- Energicznie wytrąca białka i denaturuje je;
- Rozpuszcza niektóre złożone lipidy i wytrąca glikogen;
- Powoduje silne obkurczenie tkanek i ich sztywnienie.

Roztwory wodorotlenku potasu lub sodu

W entomologii do wytrawiania okazów stosowano głównie wodorotlenek potasu, bez jasnego uzasadnienia. Wodorotlenek sodu [E524] występuje w roztworze, w różnych stężeniach lub o różnej normalności. Występuje w postaci granulek lub płatków. Jego główną wadą jest wysoka higroskopijność (większa niż KOH). Reagując z białkami, rozpuszcza je, a lipidy przekształca w mydła stałe (jest to główna różnica w porównaniu z KOH, który podczas zmydlania daje mydła płynne). Wodorotlenek potasu [E525] jest dostępny jako skoncentrowany roztwór, ale przede wszystkim ma tę zaletę, można go również nabyć w postaci granulek o masie około 0,1 g, co znacznie ułatwia przygotowywanie rozcieńczonych roztworów, gdy nie dysponujemy wagą precyzyjną. Na przykład jedna granulka 0,1 g w 1 ml wody destylowanej daje roztwór 10%. Drugą zaletą wodorotlenku potasu w granulkach jest jego mniejsza wrażliwość na karbonatyzację (roztwór KOH ma wysokie powinowactwo do wiązania CO_2, tworząc sole węglanowe).

Te silne zasady będą używane do solubilizacji kwasów tłuszczowych poprzez przekształcenie ich w mydła rozpuszczalne w wodzie. Należy pamiętać, że utrwalacz, taki jak etanol, rozpuścił już część tłuszczów w próbce. Jednakże, poprzez przeniesienie okazu do środowiska wodnego z silną zasadą, kwasy tłuszczowe (mniej lub bardziej złożone) ulegną wytrąceniu. Zatem z użyciem silnej zasady nastąpi zmydlanie na zimno. W niektórych przypadkach, gdy tkanki tłuszczowej jest dużo, na przykład u samic, korzystne będzie podniesienie temperatury do 35–40°C lub wydłużyć czas ekspozycji okazu na zasadę w temperaturze pokojowej.

Zabarwiony roztwór kwaśny/ bezbarwny roztwór Marc-André:

W tym akapicie przeanalizujemy zalety i wady stosowania roztworu Marc-André. Roztwór ten składa się z wodzianu chloralu (monohydrat trichloroacetaldehydu), kwasu octowego i wody. Jest to roztwór silnie utleniający (mieszanina kwasu i aldehydu). Zneutralizuje on nadmiar wodorotlenku potasu, który może pozostać w próbkach, bez wytrącania mydeł alkalicznych powstałych podczas stosowania wodorotlenku potasu. Ten roztwór będzie również działał na drugorzędowe grupy alkoholowe glukozamin tworzące chitynę, utleniając je, a tym samym ją zmiękczając. Ponadto, rozpuszcza on niektóre sole mineralnych obecne w próbce. Gdy roztwór Marc-André jest wcześniej zabarwiony fuksyną kwaśną (zatem w stanie utlenionym), będzie mógł wiązać drugorzędowe alkohole w strukturze. Po kontakcie próbki z roztworem Marc-André i

jej wybarwieniu, płuczemy go wyłącznie w etanolu. W ten sposób rozpoczynamy fazę odwadniania (dehydratacji) próbek.

Zalety:

- Neutralizacja nadmiaru roztworów zasadowych;
- Zmiękczenie chityny;
- Wybarwienie chityny w celu łatwiejszej oceny chitynowych struktur wewnętrznych.

Wady:

Wodzian chloralu ma działanie nasenne, przez co stosowany był w medycynie. Musi być używany pod wyciągiem, przestrzegając wytycznych dotyczących zagrożeń z nim związanych.

Roztwory odwadniające (dehydratacyjne):

Doświadczenie pokazuje, że dla bardzo małych próbek nie ma potrzeby stosowania wielu kąpieli w alkoholu o wzrastających stężeniach. Jeśli jednak próbka jest duża, zaczynamy dehydratację płucząc okazy w 80% etanolu, następnie 90%, 95% i w końcu w czystym etanolu. W przypadku bardzo małych próbek należy płukać w 90% etanolu, a następnie w czystym etanolu. Na tym etapie musimy zawsze pamiętać, że czysty etanol wiąże wodę z powietrza. Tradycją w laboratoriach entomologicznych było zakańczanie odwadniania próbek kąpielą w kreozocie bukowym. Obecnie ta substancja jest stosowana na dużą skalę jako pestycyd, środek przeciwgrzybiczy i konserwant drewna; jest zdecydowanie odradzana w preparatyce ze względu na swój odór (spowodowany wielopierścieniowymi węglowodorami aromatycznymi). Przypuszcza się, że działa szkodliwie na układ rozrodczy, jest rakotwórcza, szkodliwa dla środowiska i ekotoksyczna dla organizmów wodnych. Roztworem, który proponujemy stosować do wykonywania preparatów mikroskopowych z Phlebotominae jest Euparal® i jego esencja (opisane w następnym akapicie). Mieszanina Euparalu® i esencji Euparalu jest często używana; badane okazy można przenosić do tego medium bezpośrednio po kąpieli w 90% etanolu.

Załącznik 2: Skład stosowanych odczynników.

Wodorotlenek potasu 10%
Wodorotlenek potasu 10 g
Woda destylowana dopełnić do 100 ml

Medium na bazie gumy chloralowej (medium Hoyera)
Woda destylowana 50 ml
Wodzian chloralu 200 g
Guma arabska 50 g
Glicerol 20 ml

Roztwór Marc-André
Wodzian chloralu 40 g
Lodowaty kwas octowy 30 ml
Woda destylowana 30 ml

1% roztwór fuksyny kwaśnej w wodzie destylowanej
Fuksyna kwaśna (proszek) 1 g
Woda destylowana 99 ml

Roztwór Marc-André barwiony fuksyną
Roztwór Marc-André 10 ml
Fuksyna 1% 50 µl

Załącznik 3: Euparal®, balsam kanadyjski, alkohol poliwinylowy lub inne roztwory stosowane jako media w preparatyce.

Alkohol poliwinylowy: Jest to idealne medium, gdy substancje niezbędne do odwodnienia próbek nie są dostępne. Alkohol poliwinylowy miesza się wówczas z laktofenolem Ammana. Jednakże takie preparaty wykazują główne wady: szybko wysychają, alkohol poliwinylowy krystalizuje z powodu parowania wody lub czernieją, gdy fenol się utlenia. Alkohol poliwinylowy jest w dalszym ciągu dobrą substancją do wykonywania preparatów tymczasowych.

Balsam kanadyjski: Stosowanie go jako medium wymaga odwodnienia utrwalanych próbek. Użycie ksylenu lub toluenu również nie jest pozbawione niedogodności.

Medium Enecê: Do zamknięcia owada między szkiełkiem podstawowym a nakrywkowym, podobnie jak balsam kanadyjski, wymaga odwodnienia okazu. Do przygotowania tego medium potrzebujemy następujących odczynników: czysta biała kalafonia (22 g); rozpuszczalna w alkoholu żywica kopal (12 g), czysty alkohol (20 ml), kamfora (10 g), esencja terpentynowa (10 ml) i eukaliptol (26 ml). Aby go przygotować, w naczyniu takim jak kolba Erlenmeyera, należy umieścić czysty alkohol i kamforę. Następnie dodać kalafonię i żywicę kopal. Kolbę należy zamknąć korkiem i wstrząsnąć, a dopiero potem ogrzewać w łaźni wodnej w delikatnej temperaturze, aby mieszanina nie wrzała. Gdy zawartość całkowicie się upłynni, dodaje się esencję terpentynową, następnie filtruje, póki mieszanina jest gorąca, a na koniec do przesączu dodaje się eukaliptol. Gdy medium staje się mniej płynne, rozcieńcza się je rozcieńczalnikiem Eenecê o następującym składzie: alkohol absolutny (30 ml), kamfora (17 g), esencja terpentynowa (15 ml), eukaliptol (38 ml) (Cerqueira, 1943).

Euparal®: Jest to żywica pochodząca z cyprzyka czteroklapowego *Tetraclinis articulata* (Vahl, 1791), która została opracowana w 1906 roku przez Gilsona. Jej główną zaletą jest to, że nie polimeryzuje. Próbki umieszczone między szkiełkami można łatwo odzyskać działaniem alkoholu lub esencji Euparalu. Żywica ta, zwana również sandarakiem, nie reaguje drastycznie z etanolem o stężeniu wyższym niż 80%.

Zastosowanie Triton X100: niejonowego roztworu wodnego

Triton X100 to niejonowy roztwór wodny roztwór (*4-(1,1,3,3-tetrametylobutylo) fenylo-polietyleno glikolu lub t-oktylofenoksypolietoksyetanol, eter polietylenoglikolowy tert-oktylofenylu*), szeroko stosowany jako detergent w biologii komórkowej i molekularnej. Pozwala on na permeabilizację błon komórkowych i jądrowych. Próbki owadów często są przechowywane w alkoholu przez wiele lat. Niestety, konserwacja owadów w alkoholu nie jest pozbawiona wad, a przechowywane w nim stawonogi stają się bardzo trudne w przygotowaniu do oglądania pod mikroskopem. Plastikowe fiolki zawierające owady często ulegają degradacji, po czym następuje parowanie alkoholu. W obu przypadkach długotrwały kontakt z alkoholem lub wyschnięcie próbek stanowi poważny problem. W 2008 roku Jonque opublikował notatkę na temat nawodnienia pająków za pomocą środka zwilżającego, takiego jak Agepon, używanego do filmów fotograficznych [26]. Doprowadziło to do pomysłu wykorzystania środków zwilżających, które nie są silnymi detergentami. Poniżej przedstawiono procedurę z użyciem Triton X100 w 0,5% roztworze wodnym stosowaną w preparatyce:

- Zaimpregnować suchą próbkę w czystym alkoholu.
- Dodać odpowiednią ilość roztworu Triton X100 o stężeniu 0,5%, tak aby cała próbka była zanurzona.
- Pozostawić na około 5 minut lub dłużej. Wszystkie okazy muszą swobodnie pływać w roztworze.
- Usunąć roztwór Triton X100 i zastąpić go roztworem wodorotlenku potasu. Następnie postępować zgodnie z techniką opisaną powyżej.

Załącznik 4: Utrwalanie okazów w Euparalu® lub balsamie kanadyjskim – instrukcja „krok po kroku"

1. Okazy muszą być odwodnione (mętny lub mleczny wygląd wskazuje na niewystarczające odwodnienie).
2. Odwodnienie można osiągnąć poprzez wzrastające stężenia alkoholu etylowego.
3. Okazy można przenieść z 99% alkoholu lub czystego alkoholu do środka wytrawiającego.

Procedura:

1. Umieść, Phlebotominae w 70% etanolu.
2. Usuń etanol i zastąp go 10% roztworem KOH. Przykryj szalkę z okazami szkiełkiem.
3. Wytrawiaj do momentu aż owady staną się półprzeźroczyste.
4. Usuń KOH.
5. Zalej owady wodą destylowaną i odczekaj 30 do 45 min.
6. Usuń wodę i powtórz płukanie wodą destylowaną przez 30 min (czas zależny od liczby próbek: im więcej próbek opracowywanych jednocześnie, tym dłuższy powinien być ten etap; im mniej próbek, zwłaszcza opracowywanych indywidualnie, tym krótszy może on być).
7. Usuń wodę.
8. Dodaj roztwór Marc-André (opcjonalnie zabarwiony fuksyną kwaśną) i pozostaw na 24 godziny (1 dobę).
9. Usuń roztwór Marc-André.
10. Zalej preparat wodą destylowaną i odczekaj 30–45 min.
11. Usuń wodę i ponownie płucz wodą destylowaną przez 30 min.
12. Usuń wodę.
13. Dodaj 70% etanol i wykonaj sekcję okazu.

   a. W przypadku głowy i odwłoka delikatnie oddziel głowę lub odwłok od tułowia.

   b. W przypadku tułowia usuń skrzydła, trzymając tułów jedną parą pęset, a drugą pociągając za skrzydło u nasady. Możliwe jest wykonanie preparacji strzałkowej, dzieląc tułów na część lewą i prawą, w zależności od obszarów będących przedmiotem największego zainteresowania.
14. Odwadniaj stopniowo poprzez serię płukania w wodnych roztworach alkoholu etylowego: 50% – 80% – 95%, aż po czysty etanol.
15. Odwodnij okazy, płucząc je dwukrotnie przez 10 min w 100% etanolu.
16. Usuń etanol i zalej okazy olejkiem goździkowym na 15 min w temperaturze pokojowej.
17. Przenieś okazy z olejku goździkowego do kropli Euparalu® lub balsamu kanadyjskiego na czyste szkiełko podstawowe.
18. Ułóż preparat według własnych potrzeb. Głowę, tułów i odwłok muchówki preparuj przy użyciu cienkich igieł lub pęsety pod mikroskopem stereoskopowym. Głowę należy oddzielić od ciała i umieścić w medium w pozycji brzuszno-grzbietowej, tj. otwór potyliczny musi być skierowany ku górze.

   Sekcję wykonuj bezpośrednio w medium preparacyjnym.
19. Pozostaw okaz, do czasu aż jego powierzchnia stanie się lepka.
20. Zwilż szkiełko nakrywkowe czystym alkoholem i nałóż je pod kątem na balsam kanadyjski.
21. Przechowuj preparaty w suchym pudełku wyznaczonym do tego celu.
