# Supplementary material for: Processing and mounting phlebotomine sand flies: a consensus guideline
Source: Parasite. 2026 Apr 3;33:18. doi: 10.1051/parasite/2026009 (PMC13047900; doi:10.1051/parasite/2026009)
Supplement: Supplementary file 24 — Portuguese translation / Tradução para português [file parasite-33-18-s24.pdf]

Parasite **33**, 18 (2026)
© F.J. Randrianambinintsoa et al., published by EDP Sciences, 2026
https://doi.org/10.1051/parasite/2026009

PARASITE
Available online at:
www.parasite-journal.org

REVIEW ARTICLE OPEN ACCESS

Processamento e montagem de flebotomíneos: um guia consensual

Fano José Randrianambinintsoa[1], Laure Augendre[1], Jorian Prudhomme[1], Jean-Philippe Martinet[1], Mathieu Loyer[1], Nalia Mekarnia[1], Hocine Kerkoub[1], Farzana Khan Perveen[1], Antoine Huguenin[1,2], Emilie Kariya[1,2], Mohammad Akhoundi[3], Andrey José de Andrade[4], Eduardo Berriatua[5], Gioia Bongiorno[6], Sébastien Boyer[7,8], Vasiliki Christodoulou[9], Magda Clara Vieira da Costa-Ribeiro[10], Lucas Alexandre Farias de Souza[10], Huicong Ding[11], Blaise Dondji[12], Vít Dvořák[13], Ozge Erisoz Kasap[14], Eunice Aparecida Bianchi Galati[15], Montserrat Gállego[16], Cristina Ballart[16], Stavroula Gouzelou[17], Nabil Haddad[18], Rezki Sabrina Masse[19], Asrat Hailu Mekuria[20], Vladimir Ivovic[21], Szymon Kaczmarek[22], Mohd Khadri Shahar[19], Oscar D. Kirstein[23], Edwin Kniha[24], Iva Kolářová[13], Lincoln Timinao[25], Cristian Lucanas[26], Ognyan Mikov[27], Kimsear Nov[7], Yusuf Özbel[28], Bernard Pesson[29], Laura Cristina Posada Lopez[30], Didot Budi Prasetyo[1,7], Nil Rahola[31], Eduardo A. Rebollar-Tellez[32], Bruno Leite Rodrigues[15], Lalita Roy[33], Prasanta Saini[34], Chizu Sanjoba[35], Paloma Helena Fernandes Shimabukuro[36], Padet Siriyasatien[37], Agnieszka Soszyńska[22], Tatiana Suleşco[38], Massamba Sylla[39], Majhalia Torno[40], Petr Volf[13], Khamsing Vongphayloth[41], Vu Sinh Nam[42], April Wardhana[43], Eric Yessinou[44], Sonia Zapata[45], Jean-Charles Gantier[1], and Jérôme Depaquit[1,2,*]

[1] Faculté de Pharmacie, Université de Reims Champagne Ardenne, UR ESCAPE-USC ANSES PETARD, 51 rue Cognacq-Jay, 51096 Reims Cedex, France
[2] Pôle de Biologie territoriale, Laboratoire de Parasitologie-Mycologie, Centre Hospitalo-Universitaire, 51092 Reims, France
[3] Parasitology-Mycology Department, Avicenne Hospital, AP-HP, Bobigny, Sorbonne Paris Nord University, France; Unité des Virus Émergents (UVE: Aix-Marseille Univ, Università di Corsica, IRD 190, Inserm 1207, IRBA), 13005 Marseille, France
[4] Parasitology Collection of Basic Pathology, Department of Basic Pathology, Federal University of Paraná, Curitiba 19031, Brazil
[5] Department of Animal Health, University of Murcia, Campus de Espinardo, 30100 Espinardo, Murcia, Spain
[6] Department of Infectious Diseases, Vector-borne Diseases Unit, Istituto Superiore di Sanità, 00166 Rome, Italy
[7] Medical and Veterinary Entomology Unit, Institut Pasteur du Cambodge, Phnom Penh 12201, Cambodia
[8] Ecology & Emergence of Arthropod-borne Pathogens Unit, Department of Global Health, Institut Pasteur, CNRS UMR2000, 75015 Paris, France
[9] Section Veterinary Services (1417), Laboratory for Animal Health Virology, Aglantzia, Nicosia 2109, Cyprus
[10] Insects Vectors and Parasites Laboratory, Department of Basic Pathology and Postgraduate program in Microbiology, Parasitology and Pathology, Federal University of Paraná, 81530-900 Curitiba, Brazil
[11] Department of Biological Sciences, National University of Singapore, 117558, Singapore
[12] Laboratory of the Leishmaniasis Research Project, Mokolo District Hospital, Mokolo, Cameroon; Laboratory of Cellular Immunology and Parasitology, Department of Biological Sciences, Central Washington University, 98926 Ellensburg, WA, USA
[13] Department of Parasitology, Faculty of Science, Charles University, 12800 Prague, Czechia
[14] VERG Laboratories, Department of Biology, Faculty of Science, Hacettepe University, Beytepe, Ankara 06800, Türkiye
[15] Faculdade de Saúde Pública da Universidade de São Paulo (FSP/USP), Pós-graduação em Saúde Pública, 01246-904 São Paulo, Brazil
[16] Secció de Parasitologia, Departament de Biologia, Sanitat i Medi Ambient, Facultat de Farmàcia i Ciències de l'Alimentació, Universitat de Barcelona, & Institut de Salut Global de Barcelona (ISGlobal), Centro de Investigación Biomédica en Red, Enfermedades Infecciosas (CIBERINFEC), 08028 Barcelona, Spain
[17] Laboratory of Infectious Diseases and Public Health, School of Medicine, University of Cyprus, Nicosia, Cyprus & Department of Pediatrics, Archbishop Makarios III Hospital, Nicosia 2115, Cyprus
[18] Faculty of Health Sciences, American University of Beirut, 1107 2020 Beirut, Lebanon
[19] Medical Entomology Unit, Infectious Disease Research Centre, Institute for Medical Research (IMR), National Institutes of Health (NIH), Ministry of Health Malaysia, 40170 Shah Alam, Selangor, Malaysia
[20] School of Medicine, Addis Ababa University, 28017 - 1000 Addis Ababa, Ethiopia
[21] Faculty of Mathematics, Natural Sciences and Information Technologies, University of Primorska, 6000 Koper, Slovenia

Edited by Jean-Lou Justine
*Corresponding author: jerome.depaquit@univ-reims.fr

This is an Open Access article distributed under the terms of the Creative Commons Attribution License (https://creativecommons.org/licenses/by/4.0), which permits unrestricted use, distribution, and reproduction in any medium, provided the original work is properly cited.

[22] University of Lodz, Faculty of Biology and Environmental Protection, Department of Invertebrate Zoology and Hydrobiology, Banacha 12/16, 90-237 Łódź, Poland
[23] Laboratory of Entomology, Ministry of Health, 9134302 Jerusalem, Israel
[24] Center for Pathophysiology, Infectiology and Immunology, Institute of Specific Prophylaxis and Tropical Medicine, Medical University Vienna, Kinderspitalgasse 15, 1090 Vienna, Austria
[25] Papua New Guinea Institute of Medical Research (PNGIMR) Institute, PO Box 60, Headquarter, Homate Street, 441 Goroka, Eastern Highlands Province, Papua New Guinea
[26] Museum of Natural History, University of the Philippines Los Baños, 4031 Laguna, Philippines
[27] National Centre of Infectious and Parasitic Diseases, 1504 Sofia, Bulgaria
[28] Ege University, Faculty of Medicine, Department of Parasitology, 35040 Bornova/Izmir, Türkiye
[29] Retired, Faculté de Pharmacie, Université de Strasbourg, Strasbourg, 67400 Illkirch-Graffenstaden, France
[30] Program for the Study and Control of Tropical Diseases (PECET), Faculty of Medicine, University of Antioquia, 050010 Medellin, Colombia
[31] MIVEGEC, Univ. Montpellier, CNRS, IRD, 34394 Montpellier, France & Medical Entomology Unit, Institut Pasteur de Madagascar, 101 Antananarivo, Madagascar
[32] Laboratorio de Entomología Médica, Departamento de Zoología de Invertebrados, Facultad de Ciencias Biológicas, Universidad Autónoma de Nuevo León, San Nicolás de los Garza, 66455, NL, México
[33] Tropical and Infectious Disease Centre, BP Koirala Institute of Health Sciences, Dharan 56700, Nepal
[34] ICMR-Vector Control Research Centre, ICMR-Vector Control Research Centre, Puducherry 605006, India
[35] Graduate School of Agricultural and Life Sciences, The University of Tokyo, Tokyo 113-8657, Japan
[36] Grupo de estudos em Leishmanioses/Coleção de Flebotomíneos (COLFLEB/Fiocruz-MG), Instituto René Rachou, Fundação Oswaldo Cruz, Belo Horizonte, Minas Gerais, 30190009, Brazil
[37] Center of Excellence in Vector Biology and Vector-Borne Disease, Department of Parasitology, Faculty of Medicine, Chulalongkorn University, Bangkok 10330, Thailand
[38] Department of Arbovirology, Bernhard Nocht Institute for Tropical Medicine, Bernhard Nocht Str. 74, 20359 Hamburg, Germany [39] Laboratory Vectors & Parasites, Department of Livestock Sciences and Techniques, Sine Saloum University El Hadji Ibrahima Niasse (SSUEIN) Kaffrine Campus, C.P. 24600, Senegal.
[40] Environmental Health Institute, National Environment Agency, Singapore 138667, Singapore & Department of Biological Sciences, National University of Singapore, 117558 Singapore
[41] Institut Pasteur du Laos, Laboratory of Vector-Borne Diseases, Samsenhai Road, Ban Kao-Gnot, Sisattanak District, 3560 Vientiane, Lao PDR
[42] National Institute of Hygiene and Epidemiology, 1 Yec-Xanh Street, Hai Ba Trung District, 100000 Hanoi, Vietnam
[43] Indonesian Research Center for Veterinary Science, Indonesian Agency for Agricultural Research and Development, Ministry of Agriculture Republic Indonesia, Bogor 16114, Indonesia & Department of Parasitology, Faculty of Veterinary Medicine, Airlangga University, Surabaya 60115, Indonesia
[44] Laboratory of Research in Applied Biology, Polytechnic School of Abomey-Calavi, University of Abomey-Calavi, 01 P.O. Box 2009, 00000 Cotonou, Benin
[45] Instituto de Microbiología, Colegio de Ciencias Biológicas y Ambientales (COCIBA), Universidad San Francisco de Quito (USFQ), 170901 Quito, Ecuador

Received 1 December 2025, Accepted 29 January 2026, Published online 3 April 2026

Resumo – Este artigo fornece um guia abrangente para o processamento e a montagem de espécimes de flebotomíneos, etapa crucial para a identificação de espécies, e detecção e isolamento de patógenos. São discutidas diversas técnicas, adequadas tanto para campo quanto para laboratório. O guia inclui instruções detalhadas sobre coleta de flebotomíneos, manuseio, contenção e eutanásia (recomendando congelamento a seco ou CO_2 em vez de produtos químicos), bem como estratégias de conservação, como armazenamento a frio e preservação em etanol. A qualidade da preparação de determinadas estruturas anatômicas (órgãos genitais, cabeça e asas) é essencial para sua observação microscópica adequada e é descrita neste trabalho. O artigo também apresenta, de forma detalhada, o processamento das amostras, incluindo a clarificação com agentes como hidróxido de potássio, seguido da solução de Marc-André. Quanto ao processo de montagem, comparamos diferentes meios, enfatizando suas propriedades ópticas e capacidade de preservação. O líquido de Hoyer (também conhecido como goma cloral) é recomendado para observação rápida, especialmente das espermatecas, devido à sua nitidez, embora não seja adequado para armazenamento de longo prazo. Outros meios discutidos incluem álcool polivinílico, Euparal® (com tolerância limitada à água) e bálsamo do Canadá (um meio solúvel em hidrocarbonetos), sendo os dois últimos indicados para preservação duradoura. Abordagens inovadoras de biologia molecular, como sequenciamento de DNA e MALDI-ToF, que requerem atenção específica ao processamento das amostras, também são tratadas. Além disso, vídeos curtos ilustrando várias técnicas de montagem, bem como traduções em 33 idiomas, são fornecidos, permitindo que o guia atenda às diversas necessidades da comunidade científica global.

Palavras-chave: Montagem, flebotomíneo, meio de Hoyer, solução de Marc-André, goma cloral, álcool polivinílico, Euparal®, bálsamo do Canadá, isolamento de *Leishmania*, condições de campo, cultivo, dissecação, biologia molecular, MALDI-ToF, espécimes-tipo.

Abstract – Processing and mounting phlebotomine sand flies: a consensus guideline. This article provides a comprehensive guide for the processing and mounting of phlebotomine sand fly specimens, which is crucial for species identification and pathogen detection and isolation. It discusses a range of techniques suitable for both field and laboratory settings. The guide includes detailed instructions on sand fly collection, handling, covering, and euthanasia (recommending dry freezing or CO_2 over chemicals) as well as conservation strategies, such as cold storage and preservation in ethanol. The quality of preparation of certain anatomical structures (genital organs, head and wings) is essential for their proper microscopic observation and is described in this work. The article also presents detailed sample processing, including the clearing process with agents such as potassium hydroxide then Marc-André solution. The mounting process compares different media, emphasizing their optical properties and preservation potential. Hoyer fluid (also known as chloral gum) is recommended for quick observation, particularly for spermathecae, due to its clarity, although it is not suitable for long-term storage. Other media discussed include polyvinyl alcohol, Euparal® (for limited water tolerance), and Canada balsam (a hydrocarbon-soluble medium), with the latter two offering long-term preservation capabilities. Innovative molecular biology approaches such as DNA sequencing and MALDI-ToF, which require particular attention to sample processing, are also addressed. Furthermore, short video clips illustrating various mounting techniques as well as translations in many different languages are provided, allowing the guideline to reach the diverse needs and expectations of the global scientific community.

Key words: Mounting, Phlebotomine sand fly, Hoyer fluid, Marc-André solution, Chloral gum, Polyvinyl alcohol, Euparal®, Canada balsam, *Leishmania* isolation, Field conditions, Culture, Dissection, Molecular biology, MALDI-ToF, Type-specimens.

Introdução

Os flebotomíneos são pequenos insetos dípteros pertencentes à família Psychodidae, subfamília Phlebotominae, com pelo menos 1.063 espécies conhecidas [21]. São vetores importantes de patógenos (*Leishmania*, arbovírus e *Bartonella*), responsáveis por doenças como leishmanioses, arboviroses e bartonelose, respectivamente. Sua identificação baseia-se principalmente no exame microscópico detalhado, facilitado por coleta cuidadosa, armazenamento adequado e montagem apropriada em lâmina, exigindo técnicas específicas, cada qual com vantagens e limitações. A identificação de flebotomíneos adultos depende da observação de estruturas externas (p. ex., antenas, palpos, genitália masculina) e internas (p. ex., faringe, cibário e espermatecas). A dissecção e isolamento destas últimas facilitam sua visualização e, consequentemente, a identificação correta. Portanto, ao contrário de mosquitos ou barbeiros, os flebotomíneos requerem montagem entre lâmina e lamínula para serem identificados. Até a década de 1980, a observação microscópica era o único método disponível para identificação de flebotomíneos, e continua sendo a mais amplamente utilizada. Assim, a escolha da técnica de preparação dos espécimes era relativamente simples, baseada em uma dicotomia: por um lado, montagem definitiva, permitindo preservação de longo prazo; por outro, montagem rápida para identificação, em meio que não garante preservação prolongada.

A montagem final, por exemplo em resina como bálsamo do Canadá, é demorada, exigindo desidratação completa das amostras. Além disso, o índice de refração desse meio nem sempre é ideal para a visualização das espermatecas. Por outro lado, a montagem em meio aquoso (p. ex., líquido de Hoyer) é mais rápida e permite melhor visualização dessas estruturas refrativas, mas não possibilita conservação prolongada, pois tende a absorver água do ambiente. Uma alternativa é selar a lâmina com esmalte de unha após secagem completa.

Esse equilíbrio entre rapidez e durabilidade permanece atual, influenciando a escolha da técnica de montagem conforme o objetivo da preparação. Desde os anos 1980, estudos de identificação de flebotomíneos passaram a combinar morfologia e abordagens bioquímicas. As primeiras incluíam análises de hidrocarbonetos cuticulares, rapidamente substituídas por técnicas de biologia molecular (RAPD, RFLP, amplificação de DNA, sequenciamento Sanger e, mais recentemente, sequenciamento de nova geração — NGS). Hoje, métodos moleculares são complementados por abordagens proteômicas, como MALDI-ToF. Além disso, a identificação molecular pode ser combinada com a detecção de patógenos por PCR (*Leishmania*, *Trypanosoma*, *Bartonella*, *Phlebovirus*), sendo possível por PCR convencional ou em tempo real, o que exige adaptar o processo de coleta e armazenamento conforme o objetivo do estudo [3, 32]. Outras abordagens morfológicas adicionais, como morfometria geométrica de asas, também podem ser aplicadas. Baseado sobretudo na experiência dos autores e na literatura disponível, o objetivo deste trabalho é fornecer diretrizes padronizadas para a montagem e processamento de flebotomíneos adultos, otimizando análises morfológicas e moleculares. A necessidade de realizar determinadas análises (como

biologia molecular ou MALDI-ToF) demanda preservar partes do inseto que não são necessárias para a identificação morfológica, ressaltando a importância da escolha adequada do protocolo. Este artigo aborda métodos de anestesia e eutanásia de flebotomíneos capturados vivos, seu armazenamento e a montagem para identificação rápida ou preservação de longo prazo, permitindo estudos posteriores.

Preâmbulo: Considerações sobre segurança e regulamentos devem fazer referência às Fichas de Dados de Segurança (FDS).

Todos os reagentes apresentados neste guia devem ser manipulados sob estritas condições de segurança. Os comitês de saúde e segurança das instituições de pesquisa podem fornecer informações sobre riscos, procedimentos de manuseio e descarte. É obrigatório seguir as instruções de segurança referentes ao uso e ao descarte de cada substância. Alguns reagentes ou seus componentes (por exemplo, hidrato de cloral) são controlados em determinados países. Uma lista de abreviações utilizadas neste manuscrito é apresentada na Tabela 1.

1. Captura de flebotomíneos

Os flebotomíneos adultos podem ser coletados vivos ou mortos utilizando diversos métodos, como armadilhas luminosas tipo CDC, armadilhas adesivas, capturadores manuais (aspiradores) usados em armadilhas de Shannon ou diretamente em abrigos naturais (por exemplo, abrigos de animais). Esses métodos envolvem a instalação das armadilhas em habitats adequados, atraindo os flebotomíneos com luz ou outros atrativos (CO_2 ou iscas químicas) e coletando-os para posterior análise, conforme descrito em diversas publicações [2, 3, 32, 36, 49].

A captura de flebotomíneos vivos permite todas as aplicações subsequentes, enquanto a coleta de indivíduos mortos impede o isolamento de *Leishmania* ou vírus. Alguns métodos, como papéis adesivos, frequentemente resultam na perda de estruturas importantes dos flebotomíneos (antenas, palpos, asas ou pernas). Além disso, o óleo de rícino utilizado nesses papéis adere aos insetos e deve ser removido no início do processamento, geralmente com um banho de 15 minutos em uma mistura de etanol e éter etílico em partes iguais.

2. Eutanásia dos espécimes

Após a coleta, os flebotomíneos vivos devem ser eutanasiados. Em alguns métodos de coleta (por exemplo, papéis adesivos, armadilhas luminosas CDC equipadas com um frasco contendo detergente ou etanol), os flebotomíneos morrem imediatamente após capturados. Técnicas de biologia molecular podem ser aplicadas aos insetos coletados diretamente em etanol e àqueles que forem rapidamente transferidos para esse meio. No entanto, nenhum desses métodos de eutanásia permite o processamento dos insetos por meio de MALDI-ToF. Além disso, alguns métodos de eutanásia podem causar a perda de certos caracteres morfológicos. Portanto, é essencial utilizar um modo de eutanásia adequado para garantir a identificação correta ou o armazenamento de longo prazo como espécimes testemunho (*voucher specimens*), isto é, espécimes preservados e guardados para referência ou comparação futura. Produtos químicos como acetato de etila, éter etílico, tetracloroetano e clorofórmio podem ser aplicados embebendo-se um pedaço de algodão e colocando-o dentro do recipiente contendo os flebotomíneos a serem eutanasiados. Esses agentes devem ser manipulados com cuidado, seguindo as recomendações do fabricante, devido à sua toxicidade. No entanto, não recomendamos o uso de clorofórmio para eutanasiar flebotomíneos, pois, segundo nossa experiência, ele apresenta baixa compatibilidade com estudos de biologia

Tabela 1. Lista de abreviações.

**BME**	Meio basal de Eagle
**CDC**	Centro de Controle e Prevenção de Doenças
**CMCP**	Canforamonoclorofenol
**CMR**	Carcinogênico, mutagênico e reprotoxicológico
**COI**	Gene da subunidade I do citocromo c oxidase
**CytB**	CytB – Gene do citocromo b
**DNA**	Ácido desoxirribonucleico
**ELISA**	Ensaio imunoenzimático
**EtOH**	Etanol
**M199**	Meio199
**MALDI-ToF MS**	Espectrometria de massa por tempo de voo com ionização/dessorção de laser assistida por matriz
**MEM**	Meio essencial mínimo
**NGS**	Sequenciamento de nova geração
**NNN**	Meio Novy-MacNeal-Nicolle
**PCR**	Reação em cadeia da polimerase
**Lao PDR**	República Democrática Popular do Laos
**PNOC**	Gene prepronoceptina
**qPCR**	PCR quantitativo (PCR em tempo real)
**RAPD**	DNA polimórfico amplificado ao acaso
**RFLP**	Índice de refração
**RI**	Polimorfismo de comprimento de fragmentos de restrição
**RNA**	Ácido ribonucleico
**RNases**	Ribonucleases
**RNASS**	Solução de estabilização de RNA
**RT-PCR**	PCR com transcrição reversa
**TFA**	Ácido trifluoroacético

molecular. Considerando a natureza perigosa desses produtos e sua adequação duvidosa para análises moleculares, o uso desses agentes químicos é geralmente desencorajado.

O método mais amplamente utilizado, que preserva a morfologia, o DNA e as proteínas, é o congelamento a seco dos espécimes. Os insetos devem ser congelados por tempo suficiente para ficarem totalmente anestesiados, mas não por um período tão prolongado que (i) ressequem ou (ii) comprometam a viabilidade da *Leishmania*, caso o objetivo seja isolá-las *in vitro* a partir do trato digestivo. **Recomendamos, portanto, um período de congelamento de 15 a 20 minutos a -20°C, com monitoramento regular para garantir que os insetos estejam apenas anestesiados, sem eliminar os parasitos.**

Se não houver um freezer disponível, os insetos podem, alternativamente, ser eutanasiados usando CO_2. Em condições de campo onde cilindros de CO_2 não possam ser utilizados, os espécimes podem ser mortos utilizando pequenos recipientes comerciais de CO_2 empregados em sifões de refrigerante (dispensadores de bebidas), embora haja possíveis restrições ao transporte aéreo desse tipo de recipiente. Como último recurso, os insetos podem ser mortos pela exposição à fumaça de tabaco. Os flebotomíneos capturados vivos em armadilha CDC, coletados com aspirador, ou mantidos em tubo de vidro, são mortos em segundos, quando expostos à fumaça de tabaco. Esse método é aplicável em todas as condições de campo, inclusive em situações de isolamento difíceis. No entanto, como o vidro fica impregnado pela fumaça, ele não pode ser reutilizado para coleta e manipulação de flebotomíneos vivos sem limpeza completa. Por outro lado, esse aspirador, mesmo sem limpeza, pode ser utilizado para eutanasiar os flebotomíneos coletados em outras armadilhas para fins de fixação. Também é necessário verificar se todos os espécimes foram removidos do aspirador. Esses métodos são compatíveis com o isolamento de *Leishmania* por meio da dissecção do intestino.

3. Armazenamento dos espécimes antes do processamento

Existem cinco métodos principais de preservação antes do processamento:

3.1. Congelamento

Esse método apresenta melhores resultados a -20°C ou, preferencialmente a -80°C. Atualmente, essas formas de armazenamento são mais amplamente utilizadas do que o armazenamento em nitrogênio líquido. Em todos os casos, a criopreservação deve ser realizada o mais rapidamente possível após o atordoamento dos espécimes. O armazenamento sob frio em freezers oferece a vantagem de preservar completamente os insetos, bem como o RNA, o DNA e as proteínas, mantendo sua integridade durante todo o período de conservação. Por outro lado, o nitrogênio líquido pode causar danos severos às asas, pernas, palpos e antenas, frequentemente amputando essas estruturas e, ocasionalmente, removendo caracteres morfológicos importantes. O armazenamento a seco em freezer é menos traumático para os espécimes, mas não é ideal para preservar seus órgãos mais frágeis. É importante destacar que, no momento do descongelamento, asas, antenas, palpos ou pernas podem aderir às paredes dos frascos e eventualmente se romper devido à condensação. Entretanto, a preservação por congelamento nem sempre é viável em estudos de campo, pois exige acesso a um freezer ou a um recipiente de nitrogênio líquido. O armazenamento em freezer é totalmente compatível com a detecção de patógenos por meio de ferramentas moleculares, sem perda de sensibilidade; todavia, quando o armazenamento por longo prazo é necessário, como para a detecção e o isolamento de vírus de RNA, será necessário congelamento a –80°C ou em nitrogênio líquido. No entanto, o congelamento das amostras não permite o isolamento de *Leishmania* por dissecação do intestino, exceto se os flebotomíneos forem primeiro imersos na fase de vapor e, em seguida, no nitrogênio líquido (por exemplo, em frascos colocados dentro de uma meia), simulando a criopreservação de *Leishmania*.

3.2. Armazenamento em álcool (etanol ou álcool isopropílico)

Este é provavelmente o método mais amplamente utilizado para o armazenamento de flebotomíneos. É fácil de implementar em campo, mesmo em condições adversas e sem acesso a laboratório. A preservação em álcool é particularmente adequada para estudos morfológicos, pois os órgãos frágeis (asas, pernas, antenas e palpos) permanecem intactos, desde que não haja bolhas de ar no tubo de armazenamento. Assim, recomendamos vedar o tubo com um pequeno chumaço de algodão para remover quaisquer bolhas de ar e posicionar uma etiqueta sobre o tampão de algodão (Figura 1). A concentração ideal de álcool ainda é tema de debate. De modo geral, concentrações inferiores a 70% não são recomendadas [45, 66]. Concentrações mais altas preservam o DNA de forma mais eficiente e por períodos mais longos, mas tornam os espécimes mais frágeis e quebradiços para estudos morfológicos. O uso de etanol a 96% (mistura azeotrópica) garante estabilidade da concentração ao longo do tempo, especialmente em áreas úmidas, como países tropicais, embora o etanol a 95% seja frequentemente mais fácil de obter. Independentemente da concentração, o DNA geralmente é bem preservado em etanol (embora menos eficazmente do que por meio de métodos de congelamento, especialmente para métodos moleculares do tipo NGS). As proteínas são muito menos estáveis, sobretudo em análises proteômicas, como aplicações de MALDI-ToF. Flebotomíneos preservados em álcool por alguns meses ainda podem ser identificados morfologicamente, mas é impossível gerar espectros proteicos de referência desses espécimes. O armazenamento em álcool ou em condições secas pode ser melhorado se a amostra também for

congelada a –20°C. O congelamento a –20°C melhora principalmente a preservação molecular (por exemplo, ácidos nucleicos) ao desacelerar a degradação. Também fornece um benefício secundário para a preservação morfológica, reduzindo a deterioração tecidual ao longo do tempo, ainda que esse efeito sobre a morfologia seja mais limitado do que sobre a integridade molecular. O armazenamento em etanol também pode ser utilizado para a detecção de vírus de DNA e RNA quando se emprega etanol a uma concentração de pelo menos 70%, por um período curto, inferior a alguns meses. O álcool isopropílico, por sua vez, preserva o DNA, embora torne os espécimes rígidos e pode estar mais facilmente disponível em alguns países. Não é inflamável como o etanol, podendo ser transportado com mais facilidade. Se necessário, flebotomíneos preservados em nitrogênio líquido ou congelados a seco podem ser transferidos para álcool, combinando-se, entretanto, as desvantagens de ambos os métodos.

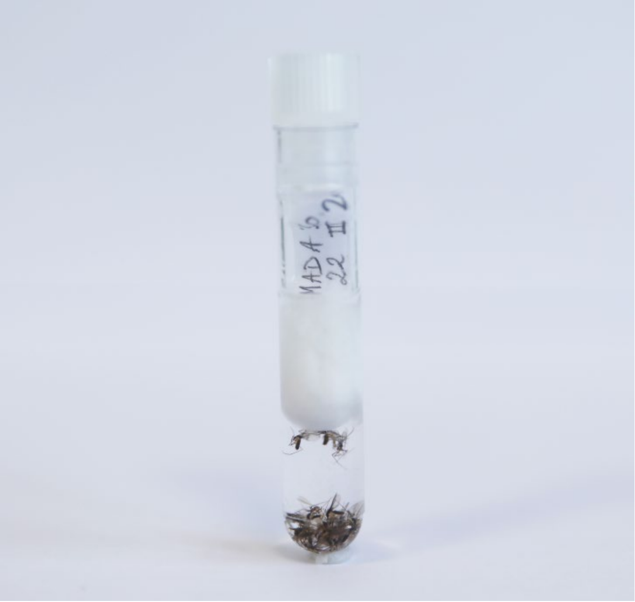

Figura 1: Flebotomíneos preservados em etanol.

3.3. Armazenamento em solução de estabilização de RNA (RNASS)

Este reagente aquoso é amplamente utilizado, não tóxico e desenvolvido para estabilizar e proteger o RNA em amostras frescas de tecidos e células. Atua penetrando rapidamente no espécime e inativando RNases (enzimas que degradam RNA), prevenindo, assim, a degradação do RNA sem a necessidade de congelamento imediato. O armazenamento em RNASS é geralmente eficaz na preservação da morfologia tecidual e celular para análises histológicas subsequentes. Embora a RNASS seja otimizada para estabilização de RNA e não para fixação, o armazenamento de curto a médio prazo normalmente mantém bem a integridade estrutural. A RNASS permite que as amostras sejam armazenadas em temperatura ambiente por até 7 dias, a 4°C por várias semanas, ou a –20°C/–80°C para preservação de longo prazo. Esse método é particularmente valioso em trabalhos de campo ou em ambientes clínicos nos quais a infraestrutura de cadeia fria é limitada. A extração de RNA geralmente requer a remoção das amostras do reagente e seu processamento conforme protocolos padrão.

3.4. Preservação a seco em temperatura ambiente

Este é um método mais antigo que, quando aplicado a um espécime *in toto* (montado por inteiro), apresenta a desvantagem significativa de preservar mal estruturas frágeis, como asas, pernas, antenas e palpos. No entanto, estudos proteômicos utilizando MALDI-ToF continuam viáveis se a desidratação for realizada durante o armazenamento com um dessecante de sílica gel. Em contraste, análises moleculares direcionadas ao DNA permanecem difíceis de realizar nesses tipos de amostras, pois o DNA costuma estar fragmentado e em baixa quantidade, tornando as análises mais desafiadoras do que em amostras frescas ou congeladas, especialmente para genomas nucleares. No entanto, técnicas recentes, como a museômica, podem ser usadas em amostras desse tipo [34]. Portanto, esse método de armazenamento não é recomendado, a menos que não exista alternativa disponível. Ele pode ser combinado com armazenamento a frio, colocando-se os tubos em um freezer a –20°C ou –80°C. O principal desafio é obter a montagem adequada dos espécimes ou das partes do corpo necessárias para a identificação. Para isso, a reidratação é essencial. Recomendamos o uso de uma solução de Triton X-100. A duração da reidratação varia de algumas horas a vários dias, devendo ser monitorada regularmente. Após a reidratação completa, os espécimes devem ser enxaguados em três banhos consecutivos de água.

3.5. Preservação em papéis filtro

A principal vantagem dos papéis filtro é a estabilidade de longo prazo do DNA genômico presente nas células de espécimes inteiros não fixados e desidratados, ou em células sanguíneas armazenadas em temperatura ambiente. O papel filtro é disponibilizado no formato de um pequeno cartão, o que permite armazenar centenas de amostras em temperatura ambiente em um volume equivalente ao de um pequeno porta-documentos. A matriz do papel filtro é impregnada com agentes que desnaturam agentes infecciosos, de modo que as amostras deixem de ser consideradas um risco biológico. Isso possibilita o armazenamento e o transporte das amostras sem a necessidade de precauções específicas de biossegurança [68].

4. Dissecção dos espécimes

Diferentemente de muitos outros insetos, cuja identificação é baseada em caracteres externos observáveis em indivíduos montados inteiros, os flebotomíneos requerem dissecção e montagem em lâmina para o estudo de características anatômicas necessárias à identificação precisa das espécies. Independentemente do método de preparação e montagem escolhido, a mesma técnica de dissecção é empregada (Figuras 2 e 3) (https://zenodo.org/records/18198006).

Uso de Triton X-100: solução aquosa não iônica

Observe que a montagem se refere a espécimes recém-coletados ou adequadamente armazenados. A maioria dos coletores possui amostras de insetos preservadas a seco (para uso em MALDI-ToF) ou armazenadas em álcool por muitos anos. Infelizmente, a preservação em álcool não é ideal ao longo de períodos prolongados, e artrópodes mantidos dessa forma tornam-se muito difíceis de preparar para exame microscópico. Um problema comum é a degradação dos recipientes plásticos que contêm as amostras, seguida da evaporação do álcool. Em ambos os casos, não há alternativas viáveis, pois as amostras permaneceram tempo demais em álcool ou ressecaram completamente.

Assim, surgiu a ideia de utilizar agentes umectantes que não fossem detergentes agressivos. O Triton X-100 é uma solução aquosa não iônica (4-(1,1,3,3-tetrametilbutil) fenil-polietilenoglicol, também conhecido como *t*-octilfenoxipolietoxietanol ou polietilenoglicol *tert*-octilfenil éter), amplamente utilizado como detergente em biologia celular e molecular. Ele permite a permeabilização das membranas celulares e nucleares.

A seguir, apresenta-se um procedimento utilizando Triton X-100 não iônico a 0,5% em solução aquosa:

- Impregnar a amostra seca com álcool absoluto.
- Adicionar o volume necessário da solução de Triton X-100 a 0,5%, de modo que toda a amostra fique imersa.
- Permitir que o processo ocorra de cerca de 5 minutos a vários dias, monitorando regularmente. Todos os artrópodes devem se separar completamente na solução.
- Remover a solução de Triton X-100 e substituí-la por solução de hidróxido de potássio.

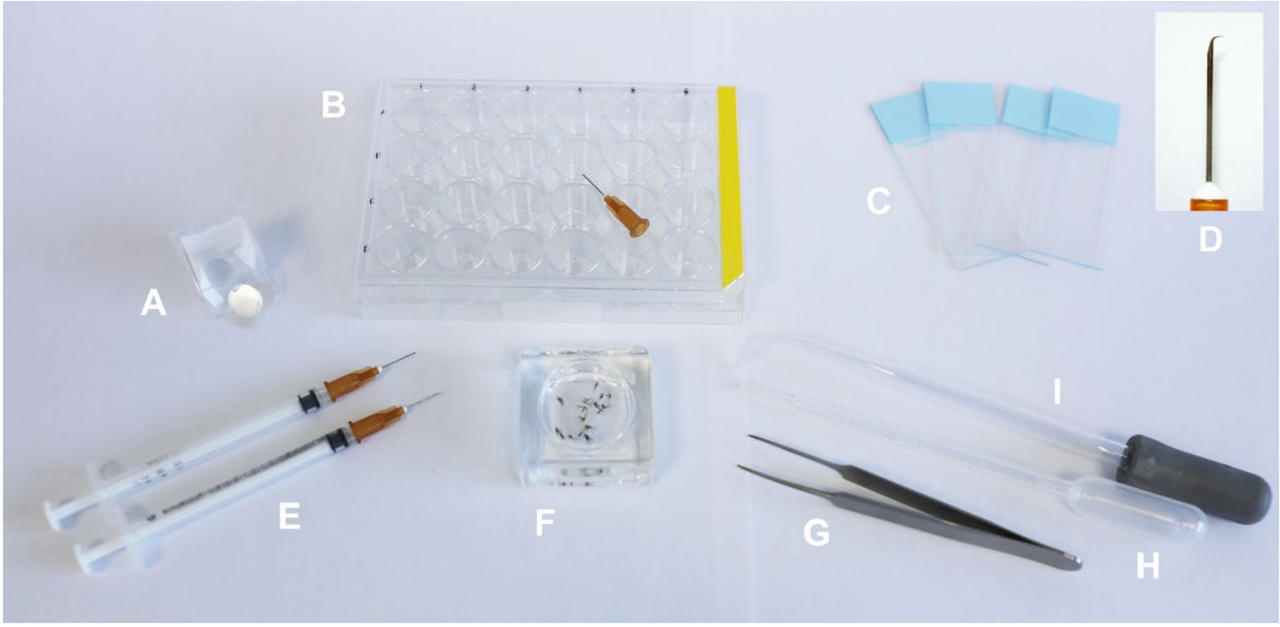

Figura 2: Materiais necessários para a montagem de flebotomíneos: A: lamínulas de vidro circulares (10 ou 12 mm de diâmetro); B: placa de 24 poços e agulha com gancho (se você utilizar óleo de cravo ou essência de Euparal® para processar os flebotomíneos, não use placas de acrílico, pois ocorrerá uma reação química que danificará os espécimes); C: lâminas de vidro adequadas para rotulagem; D: detalhe do gancho da agulha; E: agulhas acopladas a seringas; F: vidro de relógio ou recipiente equivalente contendo os flebotomíneos a serem montados; G: pinça Dumont; H: pipeta plástica; I: pipeta de vidro dobrada por aquecimento para facilitar a transferência de líquido para os poços.

This is an Open Access article distributed under the terms of the Creative Commons Attribution License (https://creativecommons.org/licenses/by/4.0), which permits unrestricted use, distribution, and reproduction in any medium, provided the original work is properly cited.

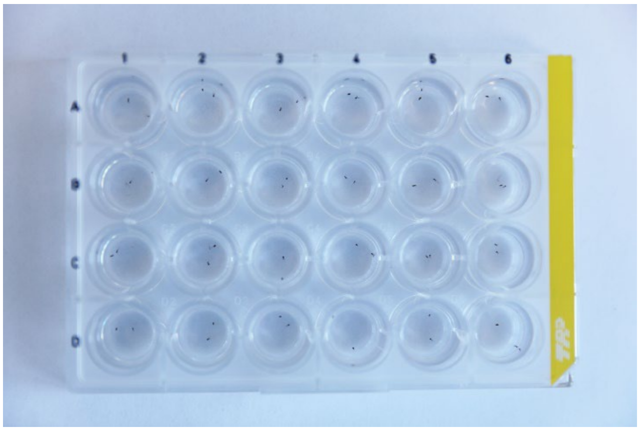

Figura 3: Placa com 24 poços, cada um contendo a cabeça e a extremidade do abdome de flebotomíneos.

4.1. Cabeça

A dissecção pode ser realizada utilizando agulhas finas ou alfinetes entomológicos sob um estereomicroscópio (Figuras 2 e 3). As agulhas mais comumente usadas incluem: 26G x 1/2" (0,45 × 13 mm), 30G × 1/2" (0,3 × 13 mm) ou 25G × 5/8" (0,5 × 16 mm). Para preparar o espécime para identificação, no mínimo, a cabeça deve ser separada do corpo e montada com o lado ventral voltado para cima, de modo a exibir o cibário e a faringe, enquanto o tórax e o abdome são montados lateralmente após a dissecação. Montar a cabeça na posição ventro-dorsal garante que o forame occipital fique orientado para cima, permitindo que o cibário seja observado diretamente. O acesso a essas estruturas anatômicas é facilitado se a cabeça estiver completamente separada.

4.2. Asas e tórax

As asas devem ser montadas planas. Cada asa pode ser destacada na sua base e montada individualmente, ou apenas uma pode ser montada, deixando-se a outra presa ao tórax. Se uma análise de morfometria geométrica for planejada, é essencial identificar e rotular corretamente as asas direita e esquerda antes da montagem. O tórax é dividido em várias partes, e cada uma contém informações taxonômicas muito importantes [20, 64]. Geralmente, ele é montado em vista lateral, permitindo o exame das cerdas e da coloração dos escleritos. A presença de cicatrizes de cerdas em determinadas regiões do tórax pode ser utilizada para distinguir algumas espécies do gênero *Brumptomyia*. A coloração pode ser empregada para separar flebotomíneos neotropicais no nível de gênero (por exemplo, *Bichromomyia*), séries de espécies (por exemplo, *Pintomyia*), ou até mesmo espécies dentro de um mesmo gênero (por exemplo, *Micropygomyia*, *Nyssomyia*, *Psathyromyia* e *Psychodopygus*) [20]. Assim, caso o tórax não seja utilizado para análises moleculares, ele deve ser montado de modo a evitar danos. É importante destacar que não é a intensidade das cores que importa, mas sim a sua distribuição ao longo do tórax. Portanto, o processo de clarificação não elimina a pigmentação nem o seu padrão.

4.3. Genitália

É necessário ter cuidado especial ao montar a genitália tanto de machos quanto de fêmeas, pois essa estrutura é crucial para a identificação de gêneros, subgêneros e espécies. Em ambos os sexos, a genitália é formada por estruturas pareadas.

4.3.1. Machos

A genitália é externa e consiste em estruturas pareadas em forma de pinça; dorsalmente encontram-se articulados o gonocoxito-gonóstilo, e ventralmente, o lobo epandrial. O gonóstilo apresenta espinhos e, às vezes, cerdas, cujos números e posições de inserção precisam estar claramente visíveis. É importante observar cuidadosamente a superfície interna do gonocoxito, que pode apresentar um tufo de cerdas sésseis (implantadas diretamente na superfície) ou cerdas implantadas em lobo (= tubérculo) [22]. Pesquisadores com menos experiência em dissecção podem realizar uma montagem lateral simples, sem destacar a genitália da extremidade do abdome (https://zenodo.org/records/18311158). Nesse caso, a sobreposição das duas partes da genitália pode dificultar a contagem das cerdas internas do gonocoxito, por exemplo — mas esse procedimento evita danos à genitália decorrentes de uma dissecção malsucedida. Pesquisadores mais experientes podem tentar abrir a genitália ao meio, separando as estruturas pares. Para isso, deve-se passar a face chanfrada de uma agulha (do tipo usada para testes intradérmicos), descolando — sem cortar completamente — as estruturas, de modo a dividir os conjuntos gonocoxito–gonóstilo (https://zenodo.org/records/18311158). Dessa forma, a observação das faces internas torna-se facilitada. Essa preparação também facilita a visualização dos parâmeros e das bainhas paramerais, que deixam de estar sobrepostas. Para a montagem lateral, que promove a superposição das estruturas, os espécimes devem estar perfeitamente clareados.

4.3.2. Fêmeas

O aparelho genital é interno, constituído pelas espermatecas. Na ausência de dissecção, elas devem ser observadas através dos tegumentos, montando-se o abdome em posição ventral. Independentemente do meio de montagem escolhido, as espermatecas geralmente podem ser observadas adequadamente, especialmente quando não são lisas e estão clarificadas. Entretanto, a observação de espermatecas lisas e com paredes delgadas pode ser problemática em meios pouco refringentes. Além disso, a observação da base dos dutos das espermatecas é essencial para a identificação de espécies, como no subgênero *Larroussius* [35, 37, 38], principais vetores de *Leishmania infantum* no Velho Mundo. Sem essa observação, a identificação do espécime torna-se impossível. Para superar essas dificuldades, a montagem do conjunto furca genital–espermatecas deve ser removida do abdome (https://zenodo.org/records/18311106). As espermatecas geralmente são difíceis de visualizar durante a dissecção,

This is an Open Access article distributed under the terms of the Creative Commons Attribution License (https://creativecommons.org/licenses/by/4.0), which permits unrestricted use, distribution, and reproduction in any medium, provided the original work is properly cited.

mas a furca genital é relativamente fácil de localizar. Como os ductos das espermatecas desembocam na furca genital, isolar essa estrutura normalmente permite também isolar as espermatecas. Caso as espermatecas sejam acidentalmente cortadas durante o processo, elas não são perdidas e ainda podem ser observadas dentro dos tegumentos abdominais (Figura 4).

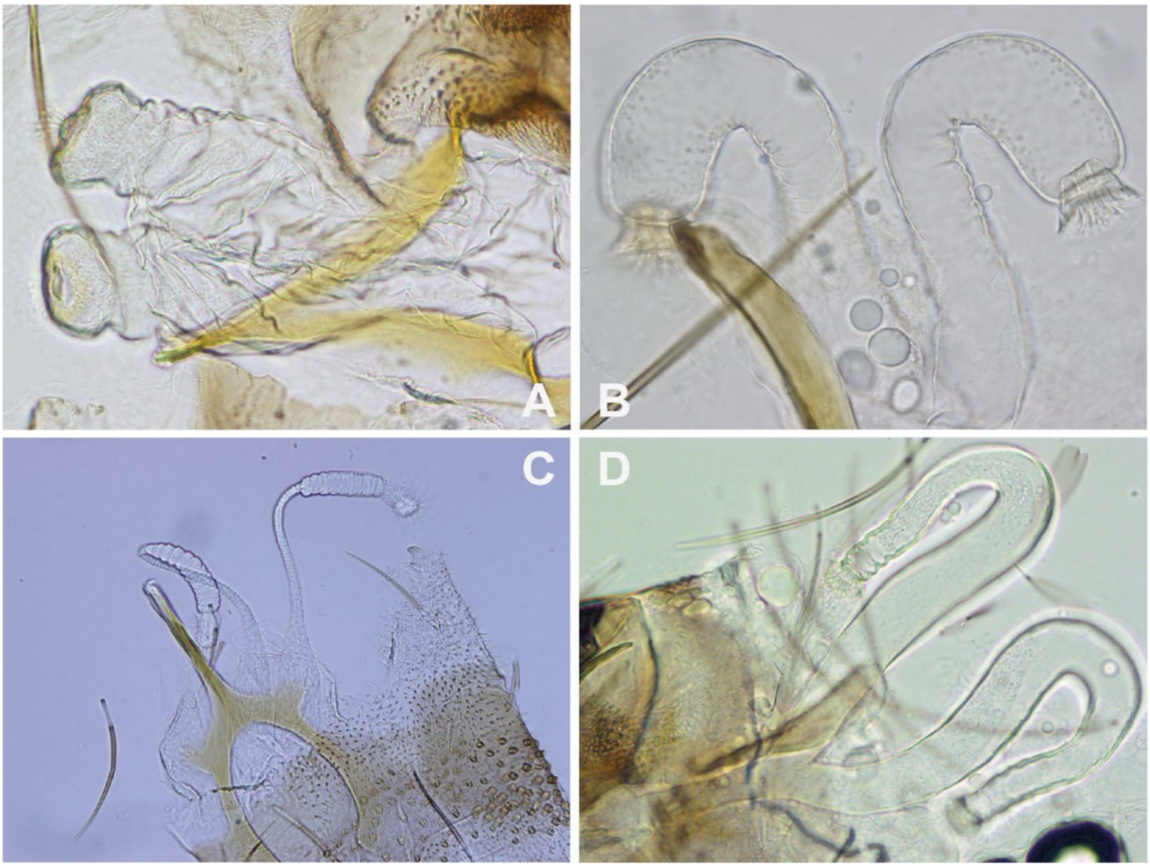

Figura 4: Espermatecas dissecadas e montadas em fluido de Marc-André, provenientes de espécimes frescos. A: *Idiophlebotomus longiforceps* (Lao PDR); B: *Sergentomyia minuta* (França); C: *Phlebotomus ariasi* (França); D: *Sergentomyia anodontis* (Lao PDR).

4.4. Dissecção do intestino médio para isolamento de *Leishmania*

A dissecção do trato digestivo é essencial para a detecção e o isolamento de *Leishmania* em fêmeas de flebotomíneos. O procedimento pode ser realizado tanto em campo quanto em laboratório, a fim de avaliar a competência vetorial.

Recomenda-se trabalhar com fêmeas recém-eutanasiadas. Lave as fêmeas com água ou solução salina contendo um detergente suave para remover o excesso de cerdas. Essa etapa ajuda a manter condições assépticas para o isolamento de *Leishmania*, ao mesmo tempo preservando as características morfológicas necessárias para a identificação. Para encontrar e isolar *Leishmania*, remova cuidadosamente o intestino médio e coloque-o em uma única gota de solução salina estéril (NaCl 0,9%). Após observar parasitos móveis sob microscopia óptica (aumento recomendado: ~200×), utilize uma seringa de insulina ou micropipeta para transferi-los para o meio de cultivo (para mais detalhes, ver Seção 4.4.3).

Monte a cabeça e a genitália diretamente em fluido Marc-André para clarificação. Importante: jamais permita que o fluido Marc-André entre em contato com *Leishmania* — nem direta, nem indiretamente por meio de instrumentos ou agulhas — pois ele é letal para os parasitos. A dissecção de fêmeas de flebotomíneos pode ser realizada em uma ou duas lâminas; ambas as opções apresentam vantagens e limitações (Figura 5; https://zenodo.org/records/18311154).

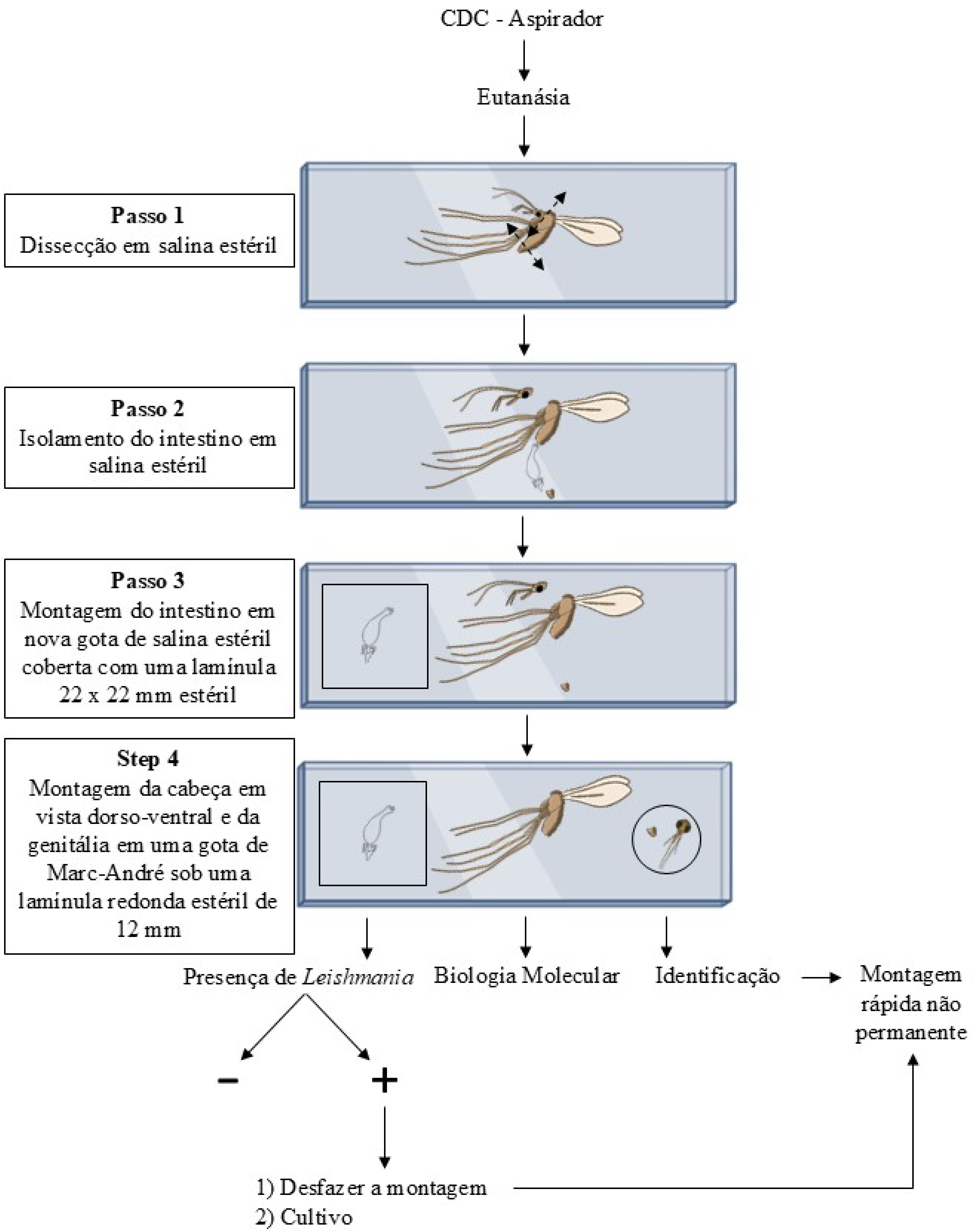

Figura 5: Método para isolamento de *Leishmania*

This is an Open Access article distributed under the terms of the Creative Commons Attribution License (https://creativecommons.org/licenses/by/4.0), which permits unrestricted use, distribution, and reproduction in any medium, provided the original work is properly cited.

4.4.1. Método das duas lâminas

A primeira opção envolve trabalhar com duas lâminas separadas: uma contendo solução salina estéril para a extração do intestino médio, e outra para montar a cabeça e as espermatecas em fluido Marc-André. No entanto, em condições de campo, é comum que duas ou três pessoas dissequem flebotomíneos e encaminhem suas dissecações para um único pesquisador responsável pela identificação das espécies e pela avaliação da infecção por *Leishmania* no intestino. O uso de duas lâminas pode causar problemas de rastreabilidade das amostras e, particularmente, dificultar a determinação precisa de qual flebotomíneo estava infectado quando um intestino positivo é detectado (https://zenodo.org/records/18311154).

4.4.2. Método de uma única lâmina

O uso de uma única lâmina garante a rastreabilidade dos resultados. Entretanto, diversas precauções devem ser tomadas. Para maximizar a esterilidade durante esse procedimento, os operadores devem higienizar regularmente as mãos com gel hidroalcoólico. Devem ser utilizadas lâminas sem fosco e lamínulas quadradas (22 × 22 mm) que devem estar envoltas em papel alumínio e esterilizadas por calor seco (em estufa tipo Poupinel), além de agulhas estéreis para cada dissecção (sugestão: 25G Ø 0,5 mm × 16 mm). O flebotomíneo é colocado em uma gota de solução salina estéril no centro da lâmina. A cabeça é removida, enquanto se realiza uma incisão entre os tergitos e esternitos abdominais 6 e 7, sem cortar o trato digestivo (uma incisão mais anterior pode ser realizada quando se espera encontrar espermatecas muito longas). Em seguida, o tórax deve ser imobilizado com uma agulha, e os últimos segmentos abdominais posteriores puxados suavemente com a outra agulha para extrair o intestino. Caso isso não funcione, é possível bloquear a extremidade do abdome com uma agulha e puxar o trato digestivo pela sua porção anterior. Se ainda assim não funcionar, o intestino deve ser exposto removendo-se o máximo possível do tegumento restante ao seu redor. Após a remoção do intestino, os últimos segmentos abdominais devem ser separados cortando o trato digestivo. O intestino é então colocado em uma nova gota de solução salina estéril posicionada em um dos lados da lâmina, e delicadamente coberto com uma lamínula estéril. A cabeça e os segmentos abdominais finais são transferidos para uma pequena gota de fluido Marc-André colocada na outra extremidade da lâmina, garantindo que não haja qualquer contato com *Leishmania*. A cabeça é orientada corretamente (forame occipital voltado para cima), e as espermatecas são isoladas com a furca genital conforme descrito acima, sendo cobertas com uma lamínula circular pequena (Ø 12 mm, distinta das lamínulas quadradas estéreis). A carcaça restante do flebotomíneo e as asas permanecem na gota de solução salina no centro da lâmina (https://zenodo.org/records/18311154). Em caso de positividade ou para fins de exploração taxonômica, o tórax e o abdome podem ser preservados para estudos moleculares ou de proteômica, e as asas podem ser montadas em meio aquoso. Para preservar a montagem, o excesso de fluido Marc-André pode ser substituído por um meio de montagem aquoso, como goma cloral (= Hoyer) ou um meio à base de álcool polivinílico Vídeos detalhados demonstrando esses procedimentos estão disponíveis (dissecação do intestino médio: https://zenodo.org/records/18303014 e dissecação das glândulas salivares: https://zenodo.org/records/18302850), portanto, eles não serão descritos novamente aqui.

4.4.3. Isolamento e cultivo de *Leishmania* a partir do intestino de flebotomíneos

O isolamento de parasitos a partir da dissecção de fêmeas de flebotomíneos infectadas é um procedimento delicado que requer alta habilidade e deve, inicialmente, ser praticado em espécimes livres de parasitos. Após a dissecção, os intestinos são transferidos para uma gota fresca de solução salina estéril (0,9%) ou solução de Locke para lavagem [4]. Os intestinos dissecados podem então ser processados de duas maneiras: i) examinados ao microscópio óptico para observar os diferentes estágios dos promastigotas de *Leishmania* e sua localização, com atenção especial à válvula estomodeal, ou ii) abrir o intestino para facilitar a saída dos promastigotas, possibilitando seu cultivo em massa [4]. Encontrar flebotomíneos infectados no campo é relativamente raro; portanto, boas sessões de prática aumentarão as chances de isolamento bem-sucedido.

Se for observada *Leishmania* no intestino, devem ser usadas agulhas estéreis novas e adicionada uma pequena quantidade de solução salina estéril ao redor da lamínula por ação capilar para liberar os parasitos. O intestino deve ser cuidadosamente e rapidamente rompido para liberar os parasitos na solução salina. Utilizando uma micropipeta de 100 µL ou seringa tuberculínica, colete os parasitos e inocule-os em meio de cultura devidamente rotulado.

Cultivo *in vitro* de promastigotas de *Leishmania*: os parasitos isolados são inicialmente mantidos em ágar-sangue SNB-9 ou em meio sólido Novy, McNeal, Nicolle (NNN) [16], sobrepostos com meio alfa-MEM estéril [16, 65] ou com meio M199, cada um suplementado com 10% de soro fetal bovino estéril inativado pelo calor (SFB) para promover o crescimento dos parasitos, 1% de vitaminas BME, 2% de urina humana estéril (esterilizada com filtro de seringa Filtropur® S 0,2 µm), 250 µg/mL de amicacina (ou 50 µg/mL de gentamicina) ou uma mistura de antibióticos e aminoácidos (L-glutamina 200 mM, penicilina 10.000 U, estreptomicina 10 mg/mL) [47]. Após três dias, se não houver contaminação, as culturas são suspensas em meio de congelamento preparado adequadamente e armazenadas posteriormente a -80°C por 1 a 2 anos, ou em nitrogênio líquido a -196°C para preservação por longo prazo e uso experimental futuro [7].

4.5. Glândulas salivares

A dissecção das glândulas salivares de flebotomíneos é uma técnica fundamental para o estudo das interações vetor-patógeno, especialmente para a detecção de arbovírus como os *Phlebovirus* (por exemplo, o vírus Toscana) [44, 75]. Devido ao tamanho extremamente reduzido dos flebotomíneos, o procedimento requer grande precisão sob um estereomicroscópio, utilizando pinças finas ou agulhas de microdissecção para isolar as delicadas glândulas salivares sem causar ruptura ou contaminação (https://zenodo.org/records/18302850) [51, 61]. Preservar a integridade das glândulas é essencial para garantir análises moleculares subsequentes confiáveis.

Após a extração, as glândulas podem ser homogeneizadas e testadas por RT-PCR, qPCR ou imunoensaios para detectar RNA viral ou antígenos [12]. A detecção de vírus nas glândulas salivares — e não apenas no intestino ou hemocele — confirma que o patógeno completou seu período de incubação extrínseca e é transmissível durante o repasto sanguíneo [71].

O processo de dissecção é tecnicamente exigente devido ao pequeno tamanho das glândulas salivares, requerendo experiência significativa para evitar a degradação das amostras [1, 51]. Além disso, as cargas virais podem ser baixas, exigindo métodos altamente sensíveis, como PCR (*nested PCR*) ou sequenciamento de alto desempenho [54]. Riscos de contaminação reforçam ainda mais a necessidade de técnicas estéreis. Para além das dificuldades técnicas, fatores biológicos influenciam o sucesso na detecção: a competência vetorial varia entre espécies de flebotomíneos, e as taxas de infecção flutuam de acordo com condições ecológicas e sazonais [33, 61].

A detecção de vírus nas glândulas salivares fornece informações críticas sobre riscos de transmissão, permitindo vigilância e medidas de controle mais específicas [15]. Por exemplo, a identificação do vírus Toscana em flebotomíneos em áreas endêmicas tem orientado protocolos diagnósticos e recomendações de saúde pública [18]. Além disso, o estudo das interações vírus–saliva pode revelar novos alvos para vacinas ou terapias que bloqueiem a transmissão [15, 18].

As glândulas salivares de flebotomíneos também podem ser usadas como fonte de antígenos para medir anticorpos do hospedeiro contra a saliva do vetor por métodos imunológicos, preferencialmente ELISA. Essa abordagem permite avaliar a exposição do hospedeiro às picadas de flebotomíneos, auxiliando na avaliação da eficácia das medidas de controle vetorial [25] e no risco de transmissão de *Leishmania* [40].

4.6. Identificação do repasto sanguíneo

As fêmeas ingurgitadas isoladas das capturas devem ser dissecadas utilizando materiais descartáveis, a fim de evitar contaminação cruzada. O abdome deve ser examinado sob um estereomicroscópio para avaliar o estágio de digestão do sangue. Recomenda-se selecionar apenas fêmeas com abdome vermelho, vermelho-acastanhado ou vermelho-escuro, sem sinais de formação de ovos. A ponta do abdome incluindo as espermatecas, deve ser removida para permitir a identificação morfológica da fêmea após a clarificação. A porção principal do abdome (sem as espermatecas) deve então ser colocada em tubos Eppendorf® e armazenada a –20 °C até análises posteriores. Os marcadores genéticos comumente utilizados para identificação de repasto sanguíneo, como PNOC [5, 30, 50], CytB [67] ou COI [13] são bem estabelecidos e amplamente descritos na literatura; portanto, não serão detalhados neste artigo (Figura 6). Alternativamente, para identificar o sangue do hospedeiro, pode-se utilizar o mapeamento peptídico por MALDI-ToF [31]. Foi demonstrado experimentalmente que essa técnica permite identificar o sangue do hospedeiro por um período mais longo após o repasto sanguíneo; portanto, é um método adequado, especialmente para a análise de fêmeas ingurgitadas com digestão mais avançada do sangue do hospedeiro. As amostras devem idealmente ser armazenadas a -20 °C ou a 4 °C, mas resultados satisfatórios também podem ser obtidos a partir de amostras mantidas em temperatura ambiente por curto período. O abdome da fêmea ingurgitada deve ser separado do restante do corpo pouco antes da análise e homogeneizado em água destilada. O restante do corpo da fêmea permanece disponível para outras análises moleculares e morfológicas. Após retirar uma alíquota da homogeneização para o mapeamento peptídico por MALDI-ToF, o volume restante pode ser utilizado para isolamento de DNA, a fim de confirmar a identificação da fonte sanguínea e/ou verificar a presença de *Leishmania* sp**.** O tempo total de preparação e análise da amostra é mais curto quando comparado ao das técnicas moleculares baseadas em DNA.

This is an Open Access article distributed under the terms of the Creative Commons Attribution License (https://creativecommons.org/licenses/by/4.0), which permits unrestricted use, distribution, and reproduction in any medium, provided the original work is properly cited.

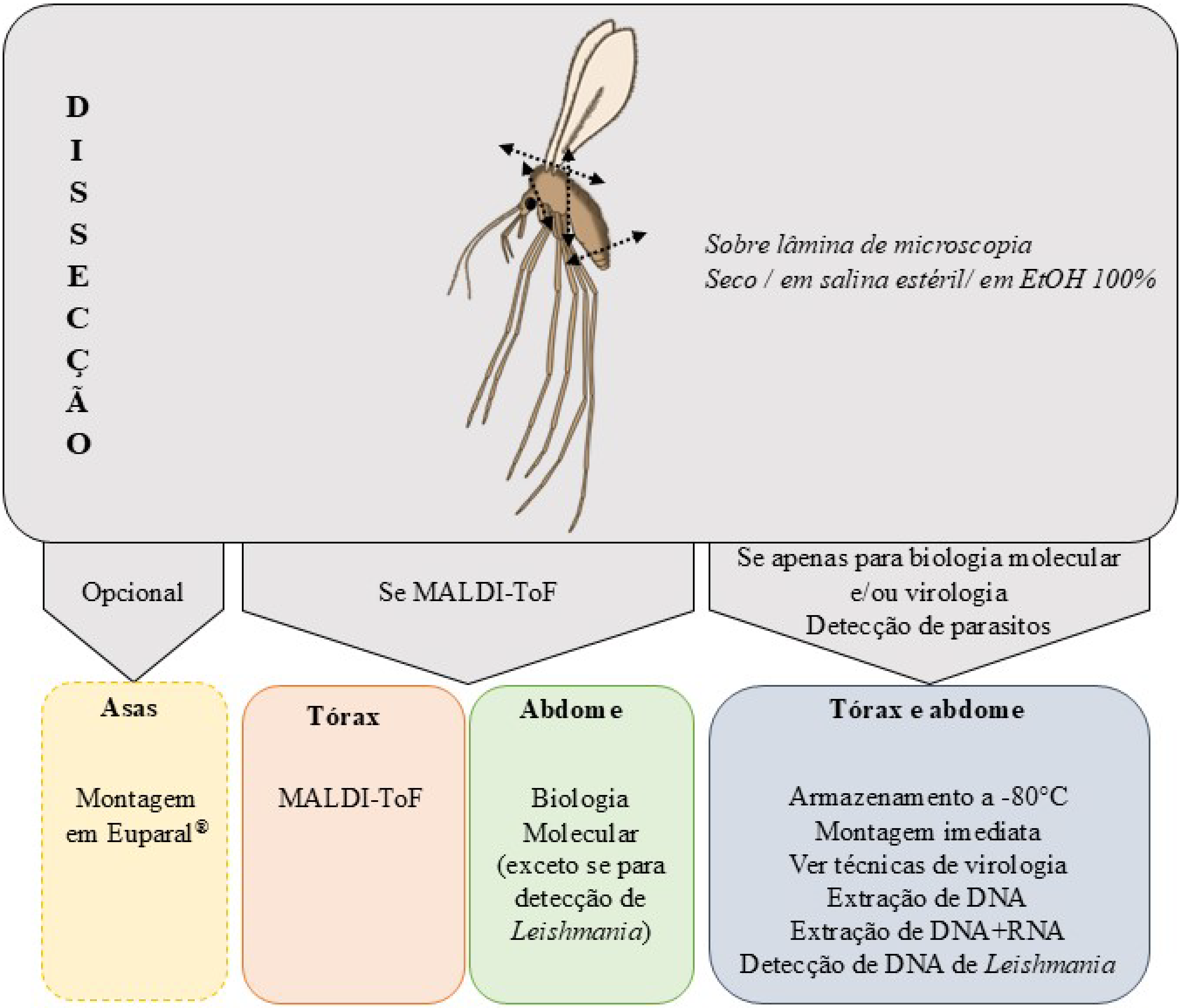

Figura 6: Processamento de flebotomíneos para aplicações em biologia molecular, proteômica e/ou virologia.

5. Processamento de espécimes para estudos morfológicos (Figuras 3, 6, 7 e 8; Apêndices 1, 2, 3 e 4)

Esta seção descreve os princípios para preparar um espécime de flebotomíneo para montagem exclusivamente para estudos morfológicos, seguido da adaptação para aplicações além da morfologia. No entanto, compreender essa metodologia é fundamental, pois permite que os procedimentos sejam adaptados para tipos específicos de amostras quando necessário.

O procedimento envolve etapas sucessivas de esvaziamento e preenchimento utilizando pipetas Pasteur equipadas com bulbos de borracha flexíveis. Recomenda-se fortemente o uso de recipientes de vidro de fundo redondo, pois facilitam consideravelmente essas operações. O vidro é resistente a todos os reagentes. Para prevenir a evaporação dos reagentes, os recipientes devem ser tampados e nunca sobrecarregados, evitando transbordamento ao fechar ou abrir, bem como a deposição de poeira sobre as amostras. Os produtos químicos necessários para a clarificação e o processamento estão apresentados na Tabela 2.

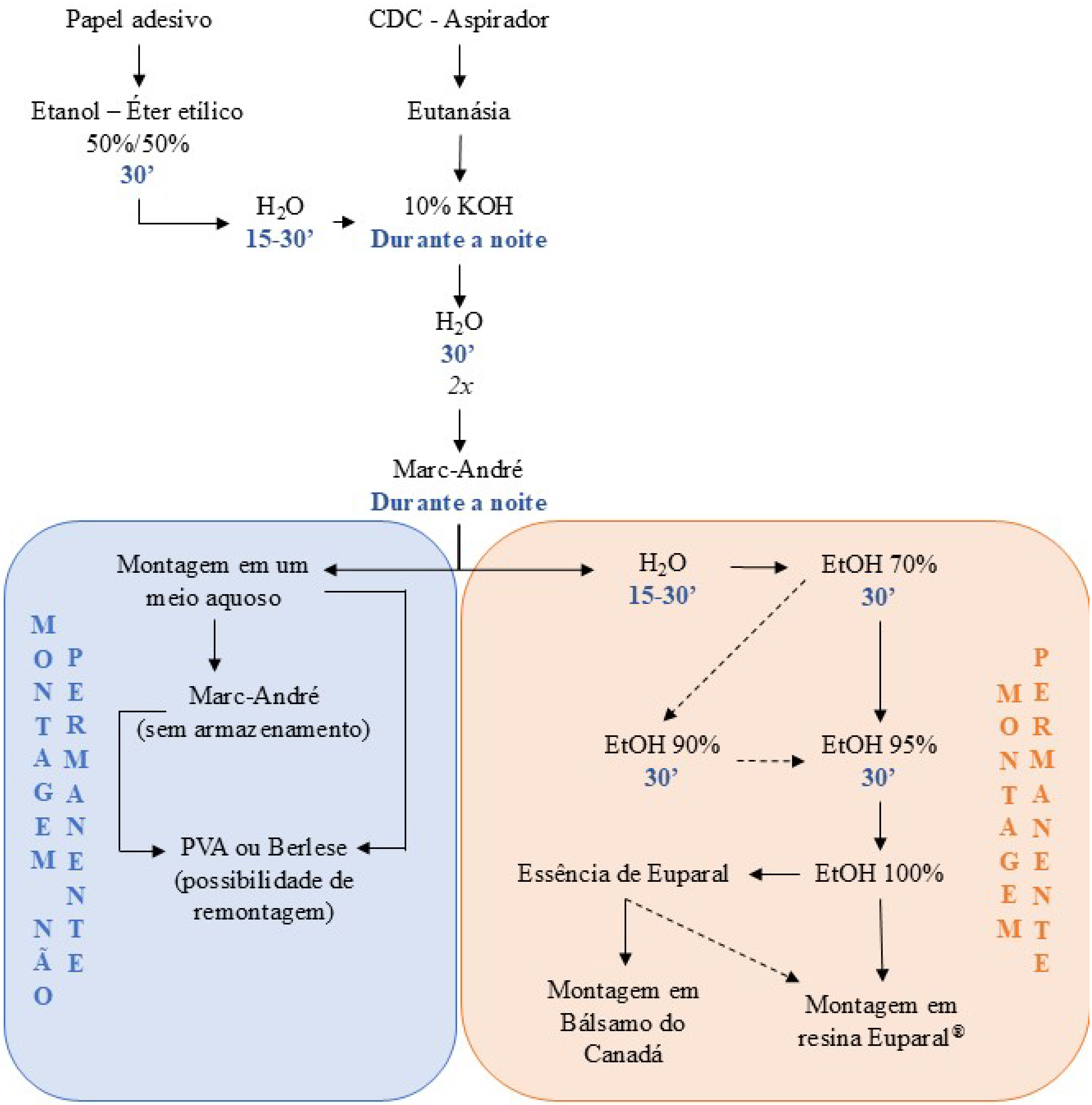

Figura 7: Método clássico de processamento de flebotomíneos.

Tabela 2: Composição dos reagentes utilizados.

**Hidróxido de Potássio 10%**<br>Hidróxido de Potássio 10 g<br>Água destilada qs 100 mL	**Fucsina ácida 1% em água destilada**<br>Fucsina ácida (em pó) 1 g<br>Água destilada 99 mL
**Meio de montagem com goma cloral (meio de Hoyer)**<br>Água destilada 50 mL<br>Hidrato de cloral 200 g<br>Goma Arábica 50 g<br>Glicerol 20 mL	**Solução de Marc-André colorida com fucsina ácida**<br>Solução Marc-André 10 mL<br>Fucsina 1% 50 µL
**Solução Marc-André**<br>Hidrato de cloral 40 g<br>Ácido acético glacial 30 mL<br>Água destilada 30 mL	**Meio Enecê**<br>Colofônia branca pura 22 g<br>Goma de copal solúvel em álcool 12 g<br>Etanol absoluto 20 mL<br>Cânfora 10 g<br>Essência de terebintina 10 mL<br>Eucaliptol 26 mL

5.1. Clarificação

Antes que os espécimes de flebotomíneos possam ser preparados em lâminas permanentes, eles devem primeiro ser clarificados por maceração, utilizando um método e agente clareador apropriados (ou seja, solução de ácido acético 10% ou solução Marc-André contendo hidrato de cloral, que é uma substância controlada em muitos países) para torná-los transparentes. O processo de clarificação remove tecidos corporais, gorduras, secreções e ceras, tornando o espécime translúcido e facilitando o exame de estruturas do exoesqueleto (por exemplo, inserção de cerdas), características da superfície (por exemplo, coloração), e estruturas internas visíveis através do tegumento (por exemplo, espermatecas).

O processo de clarificação em duas etapas, que envolve inicialmente o uso de uma base forte (como hidróxido de potássio), seguido de um ácido fraco (como ácido acético na solução Marc-André), cumpre finalidades bioquímicas distintas [74]. A base degrada os tecidos moles, como proteínas, gorduras e músculos, por saponificação e desnaturação proteica, deixando o exoesqueleto de quitina intacto para garantir clareza estrutural. O ácido fraco subsequente neutraliza qualquer resíduo alcalino, prevenindo degradação adicional, e clareia a quitina para aumentar a transparência [74], embora lavar os espécimes duas vezes em água destilada por 15 minutos também possa ser suficiente para neutralizar a base. Este tratamento sequencial combina a remoção eficaz de tecidos com preservação delicada, garantindo integridade ideal do espécime para exame microscópico. Recomenda-se realizar dois enxágues de 20 minutos em água destilada antes de prosseguir para a etapa seguinte.

5.1.1. Lise de tecidos moles (Figura 8)

Hidróxido de sódio (NaOH) ou hidróxido de potássio (KOH) são agentes químicos comumente usados para maceração, aplicados em diferentes concentrações e tempos de tratamento, dependendo do tamanho e da fragilidade dos espécimes. A técnica padrão e mais eficaz envolve a lise dos tecidos moles, imergindo os flebotomíneos em uma base forte (10% de KOH ou NaOH) durante a noite. A concentração pode ser aumentada para reduzir o tempo de tratamento (por exemplo, KOH 20% por 6 horas), assim como o aquecimento a 37°C.

5.1.2. Clarificação com ou sem coloração

Esta etapa é seguida por um tratamento de clarificação, tipicamente combinando ácido acético e hidrato de cloral (por exemplo, solução Marc-André). Após a clarificação, os espécimes devem ser cuidadosamente enxaguados em pelo menos dois banhos sucessivos de água de 20 minutos cada para remover resíduos químicos.

A solução Marc-André é um agente clareador comumente utilizado para a preparação de espécimes de flebotomíneos. Sua eficácia está em facilitar o processo de clarificação, minimizando danos significativos às estruturas frágeis, como asas e antenas.

A solução deve ser preparada recentemente ou armazenada em recipiente bem fechado para evitar evaporação ou degradação. O uso da solução Marc-André é particularmente vantajoso quando combinado com técnicas de iluminação ou coloração para realçar detalhes morfológicos específicos. Detalhes sobre sua composição e preparo são fornecidos no Apêndice 2.

Para espécimes altamente translúcidos, a coloração pode ser necessária para melhorar a visibilidade antes da montagem. Existem diversos corantes disponíveis, cada um direcionado aos componentes químicos específicos do organismo. É importante selecionar um corante compatível tanto com o espécime quanto com o meio de montagem escolhido. Essa metodologia básica pode ser adaptada conforme necessário, por exemplo, incorporando 0,1% de fucsina ácida à solução Marc-André para coloração.

Além disso, espécimes preservados em soluções aquosas e destinados a montagens em resina requerem desidratação (ver Seção 5.2 sobre Desidratação), pois a maioria dos meios de montagem em resina natural ou sintética é incompatível com água. New (1974) observou que alguns corantes podem se deteriorar em certos meios de montagem [53]. Por exemplo, a fucsina ácida, comumente usada com bálsamo do Canadá, também pode ser usada em Euparal®. No entanto, espécimes corados com fucsina ácida são propensos a desbotamento, particularmente quando resíduos de óleo de cravo, usado como fluido de clarificação final, permanecem. Espécimes armazenados em óleo de cravo podem apresentar desbotamento significativo em poucos dias.

5.2. Desidratação

A desidratação é realizada transferindo gradualmente os espécimes por uma série de soluções etanólicas graduadas: 50%, 70%, 80%, 90% ou 95% e, finalmente, 100%, sendo que cada banho deve durar pelo menos 20 minutos. Como o etanol evapora rapidamente, o recipiente deve ser bem fechado durante o processamento. Uma vez que o espécime esteja completamente desidratado, é possível pausar o processamento por alguns dias em essência de Euparal®, o que é preferível ao óleo de cravo. O creosoto de faia, amplamente usado para esse fim no passado, é atualmente proibido devido à sua toxicidade.

O processo de desidratação deve garantir que o fluido presente no espécime seja compatível com o meio de montagem, prevenindo opacificação, colapso osmótico ou distorção que possam tornar o espécime inadequado para estudos taxonômicos.

5.3. Meios de montagem

5.3.1. Seleção e aplicação para preparação de espécimes

O meio de montagem deve, idealmente, ter um índice de refração o mais próximo possível do vidro, que é aproximadamente 1,5. Deve ser incolor, claro e permanecer perfeitamente transparente após a secagem e com o passar do tempo. Deve ser compatível com os corantes utilizados e capaz de penetrar e se difundir por todos os tecidos do espécime. Não deve secar muito rapidamente nem formar uma névoa durante a montagem, e não deve encolher após a montagem. A escolha de um meio de montagem apropriado é um aspecto fundamental na preparação do espécime, pois nenhum meio é ideal para todos os propósitos. A decisão deve equilibrar vários fatores-chave:

- Propriedades ópticas: O índice de refração do meio de montagem deve fornecer contraste e refração suficientes para destacar características anatômicas críticas utilizadas na identificação taxonômica ou na descrição morfológica, como espermatecas, ascoides, sensilas de Newstead, dentes verticais do cibário e dentes da faringe. A visibilidade dessas estruturas depende diretamente das propriedades ópticas do meio de montagem.

- Preservação: Para espécimes-tipo ou materiais destinados a coleções permanentes, o meio deve oferecer estabilidade e durabilidade por longo prazo. Em contraste, para estudos de inventário ou levantamentos epidemiológicos, em que a preservação prolongada é menos crítica, meios de montagem temporários ou semipermanentes podem ser suficientes.

5.3.2. Requisitos para meios de montagem

Especialistas frequentemente desenvolvem técnicas de montagem personalizadas e complexas, adaptadas a necessidades específicas de pesquisa. No entanto, esses métodos frequentemente negligenciam aspectos como qualidade arquivística, compatibilidade, padronização, facilidade de manuseio e preservação a longo prazo. Essa falta de padronização dificulta a integração de coleções doadas e os esforços de curadoria a longo prazo.

As aplicações científicas impõem requisitos distintos para os meios de montagem. Taxonomistas frequentemente montam espécimes inteiros e preferem meios que macerem suavemente os órgãos internos para melhorar a visibilidade das estruturas cuticulares. O índice de refração deve diferir suficientemente do espécime e da lâmina de vidro para maximizar a clareza óptica.

Meios de montagem comerciais geralmente são formulados com índice de refração próximo ao do vidro, a fim de minimizar a refração e a dispersão da luz através do sistema lâmina–meio de montagem–lamínula. No entanto, em microscopia de campo claro, o contraste natural de um espécime não corado pode ser manipulado ao se escolher deliberadamente um meio de montagem com índice de refração ligeiramente diferente do espécime, melhorando assim sua visibilidade em relação ao fundo.

5.3.3. Tipos de meios de montagem (Tabelas 3 e 4)

A microscopia requer que o índice de refração (IR) do meio de montagem seja considerado para determinar como a luz se propaga através da lâmina, do meio e do espécime. Quando o IR está próximo ao do vidro da lamínula (≈1,515), a luz passa de maneira uniforme, reduzindo a dispersão e distorções ópticas, resultando em melhor resolução e visibilidade de estruturas finas. Por outro lado, uma incompatibilidade de IR pode causar desfoque, halos ou escurecer características não coradas. A escolha do meio de montagem adequado é crucial para otimizar o contraste, a clareza e a qualidade geral da imagem para um dado espécime, devido aos diferentes IRs dos diversos meios.

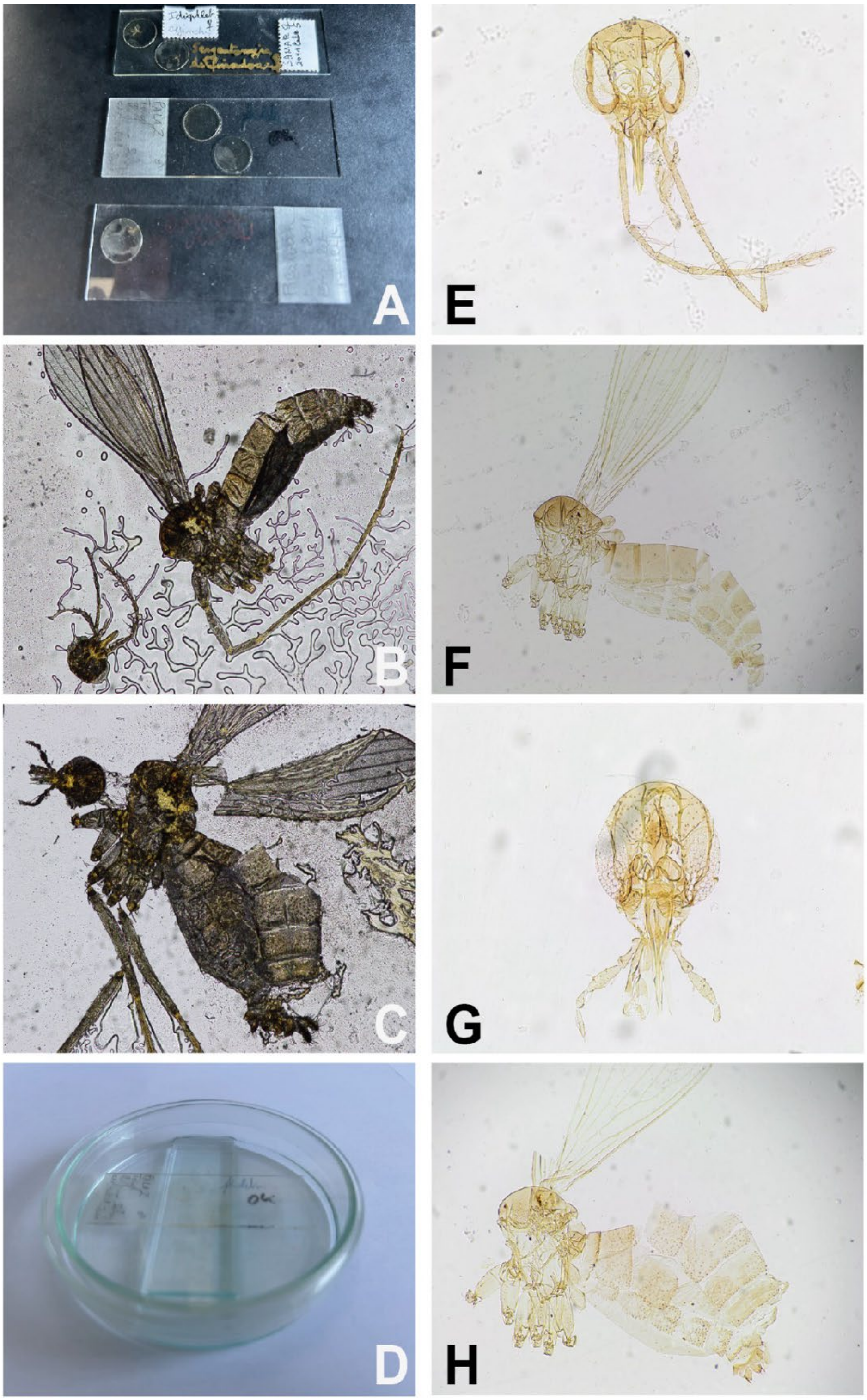

Figura 8: Remontagem de lâminas. A: lâminas danificadas e secas montadas em Hoyer; B: visão microscópica de um flebotomíneo seco; C: visão microscópica de outro flebotomíneo danificado; D: câmara úmida contendo uma lâmina seca; E: cabeça e F: corpo do espécime B após remontagem em Euparal®; G: cabeça e H: corpo do espécime C danificado após remontagem em Euparal®.

O índice de refração do meio de montagem tem impacto significativo na observação das estruturas finas durante a preparação de flebotomíneos para montagem em lâmina. As características delicadas e pouco esclerotizadas dos flebotomíneos, incluindo armadura cibarial, espermatecas, segmentos antenais e nervação das asas, podem ser difíceis de observar em meios de montagem com índice de refração elevado.

Para flebotomíneos, as opções comumente utilizadas incluem meios de goma-cloral como meios de montagem aquosos, bálsamo do Canadá e resina Enecê - Nelson Cerqueira (NC) como meios à base de solvente. Rawlins [60] categorizou os meios de montagem em dois tipos: (1) meio permanente: endurecem com o tempo e são adequados para preservação a longo prazo, e (2) meio semipermanente: não endurece completamente e é tipicamente usado para fins temporários.

Os meios de montagem podem ser líquidos, à base de goma ou resinosos, solúveis em água, álcool ou outros solventes (por exemplo, tolueno, xileno) (Tabela 3). Uma vez aplicados, devem ser selados contra efeitos atmosféricos utilizando meios de vedação não solúveis. Para distinguir claramente entre os tipos de meios de montagem, pode-se usar a seguinte categorização:

a. Meios aquosos: Esses meios se dissolvem facilmente em água, sendo adequados para montagens temporárias ou semipermanentes. Geralmente são fáceis de manusear, mas podem exigir vedação para prevenir a exposição à umidade atmosférica (por exemplo, meios de goma-cloral e álcool polivinílico), especialmente em climas tropicais úmidos.

b. Meios com tolerância limitada à água: Esses meios são menos afetados pela água, mas ainda necessitam de proteção contra umidade excessiva. Oferecem maior estabilidade a longo prazo em comparação com as opções solúveis em água e são frequentemente utilizados em montagens semipermanentes.

c. Meios solúveis em hidrocarbonetos: Esses meios se dissolvem em solventes orgânicos, como xileno, tolueno ou Essenecê (solvente Enecê). São projetados para montagens permanentes, oferecendo excelente estabilidade a longo prazo, resistência à umidade e à degradação, tornando-os ideais para fins arquivísticos (por exemplo, bálsamo do Canadá neutro).

Em resumo, meios solúveis em água são mais indicados para montagens temporárias ou casos que exigem fácil remoção do espécime; meios com tolerância limitada à água são adequados para montagens semipermanentes que requerem durabilidade moderada; e meios solúveis em hidrocarbonetos são preferíveis para montagens permanentes destinadas a armazenamento arquivístico e preservação a longo prazo

Tabela 3: Composição de meios de montagem selecionados.

**Meio de montagem**	**Solvente**	**Pré-polímero(s) ou polímero(s) em potencial**	**Observações**
Hoyer = goma cloral	glicerol, água	compostos de goma arábica	Agente macerante: hidrato de cloral
CMCP-9 (= carboximetil celulose fenol)	água (CMCP-9: 51−60%)	álcool polivinílico totalmente hidrolisado (CMCP-9: 0−5%)	CMC(P)-9: baixa viscosidade: alta viscosidade
DMHF (dimetil hidantoína formaldeído)	água	N,N′-dimetilol dimetil hidantoína (di-metilol DMH)<br>Oligômeros com pontes de éter/metileno<br>Rede polimérica reticulada de DMH-formaldeído	
Bálsamo do Canadá	xileno; componentes parcialmente voláteis do bálsamo (Δ^3-careno, ácido levopimárico, limoneno, mirceno, ácido palústrico, β-felandreno, α-pineno, β-pineno)	bálsamo (abienol, ácido abiético, ácido isopimárico, ácido sandaracopimárico)	neutralização: carbonato de potássio;<br><br>resina de *Abies balsamea* (Linné, 1758)
Euparal®	eucaliptol, paraldeído; componentes parcialmente voláteis da goma sandarac (limoneno, α-pineno, β-pineno)	compostos da goma sandarac (ácido communis, manool, ácido polimunis, ácido sandaracopimárico, ácido 12-acetoxi-sandaracopimárico, sugiol, ácido torulosico, torulosol, totarol)	Agente de clarificação: salicilato de metila; corante verde Euparal®: sal de cobre (abietinato de cobre); resina sandarac de *Tetraclinis articulata* (Vahl, 1791)
Enecê	álcool etílico; com essência de cânfora, eucaliptol e terebintina	compostos de goma de copal e colofônia (resina)	

This is an Open Access article distributed under the terms of the Creative Commons Attribution License (https://creativecommons.org/licenses/by/4.0), which permits unrestricted use, distribution, and reproduction in any medium, provided the original work is properly cited.

Tabela 4: Vantagens e desvantagens de meios de montagem selecionados em lâminas de microscópio e observações não publicadas por diversos autores [52].

Nome	Vantagens	Desvantagens
* Bálsamo do Canadá	O meio é altamente durável, com uma vida útil superior a 150 anos.<br>As lâminas podem ser montadas usando óleo de cravo ou fenol como agentes de montagem.	Contém componentes nocivos e deve ser manuseado sob capela de exaustão.<br>Requer uma série completa e demorada de desidratação.<br>A desidratação com etanol e a transferência via xileno ou óleo de cravo podem tornar alguns táxons quebradiços; alternativas (por exemplo, isopropanol, n-butanol, Cellosolve™, 1,4-dioxano, Histoclear, terpineol) podem reduzir a quebra.<br>Os espécimes podem escurecer se o xileno for substituído por fenol ou se houver resíduos de hidróxido de potássio.<br>Altos índices de refração podem obscurecer estruturas não coradas.<br>A secagem completa pode levar anos sem secagem em placa aquecida.<br>O meio amarela e escurece com o tempo, especialmente quando clareado com óleo de cravo.<br>Algumas manchas enfraquecem e corantes catiônicos podem desbotar se o meio se tornar ácido - o que pode ocorrer espontaneamente com o tempo.
DMHF (dimetil hidantoína formaldeído)	Alta transparência.<br>Bom índice de refração.<br>Excelente visibilidade das estruturas.<br>Boa estabilidade das preparações.<br>Compatível com diversas técnicas de coloração.<br>Boa proteção das amostras.<br>Boa adesão entre a lâmina e a lamínula.	Possível amarelamento com o tempo.<br>Pode alterar algumas colorações.<br>Não adequado para colorações sensíveis ao formaldeído.<br>Bolhas de ar, tempo de secagem lento.<br>Meio de montagem sensível à umidade.<br>A montagem é difícil de reverter.<br>O formaldeído é tóxico, irritante e cancerígeno.
*Euparal (transparente)	Meio durável com vida útil superior a 50 anos.<br>A montagem direta a partir de etanol a 80% é possível (recomendação do fabricante).<br>Não mascara estruturas não coradas e não amarela ou se torna quebradiço com o tempo.<br>Possui um índice de refração mais adequado do que o bálsamo do Canadá para dípteros.<br>Funciona bem para espécimes mais espessos devido à mínima contração e secagem sem bolhas.<br>Permanece solúvel em etanol a 95%, permitindo a remontagem mesmo após muitos anos.	Contém componentes nocivos e deve ser manuseado sob capela de exaustão.<br>A desidratação e transferência com etanol via essência de Euparal podem tornar alguns táxons quebradiços, mas o uso de isopropanol pode reduzir esse problema.
Fluido Hoyer	Os espécimes podem ser montados vivos ou diretamente a partir de água, etanol ou formaldeído.<br>A maceração proporciona excelente qualidade da cutícula.<br>Possui um índice de refração favorável e pode ser realçado com coloração por iodo para maior contraste.<br>O ácido acético na fórmula pode expandir os apêndices dos artrópodes.	Espécimes vegetais delicados podem colapsar, a menos que o meio seja adicionado gradualmente, o que consome muito tempo.<br>Cavidades e cristais podem se formar em menos de 10 anos.<br>A clarificação pode se tornar excessiva, dependendo da concentração de hidrato de cloral e do tempo de exposição.<br>Os componentes do meio podem se separar e uma granulação fina pode aparecer em meses ou anos.

This is an Open Access article distributed under the terms of the Creative Commons Attribution License (https://creativecommons.org/licenses/by/4.0), which permits unrestricted use, distribution, and reproduction in any medium, provided the original work is properly cited.

	Alguns espécimes podem permanecer estáveis por 40 a 60 anos.<br>Solúvel em água, permitindo fácil remontagem.	O escurecimento do meio foi relatado.<br>.
CMCP-9 (= carboximetil celulose fenol)	As amostras podem ser montadas diretamente a partir de meios como água, etanol, glicerol ou soluções contendo formaldeído, e seus órgãos internos podem ser macerados quando necessário para facilitar o exame geral ou a preparação.	Este meio pode desenvolver cristais e escurecer com o tempo, e por vezes pode clarificar as amostras mais do que o pretendido. A menos que a lâmina seja cuidadosamente circundada, amostras mais espessas não se comportarão bem neste meio, pois podem encolher e criar espaços nas bordas da lamínula. Não é adequado para amostras coradas ou materiais calcificados, e seu tempo de secagem é mais lento do que o da carboximetilcelulose (CMC).
Eukitt™	Meio de montagem durável com vida útil superior a 30 anos.<br>Compatível com diversos solventes para montagem, incluindo acetona, benzeno, clorofórmio, dioxano, éter, isopropanol, benzoato de metila, terpineol, tolueno e xileno.<br>Seca rapidamente e possui pH ligeiramente ácido.<br>Não escurece visivelmente com o tempo.<br>Adequado para diversas colorações (por exemplo, fucsina, hematoxilina, verde de metila, violeta de metila, azul de metileno).<br>As amostras podem ser remontadas após anos, mergulhando-as em xileno por um período prolongado.<br>.	Contém componentes nocivos e deve ser manuseado sob capela de exaustão.<br>Requer uma série completa e demorada de desidratação.<br>Não é ideal para espécimes mais espessos devido à retração e formação de bolhas de gás.<br>As lamínulas podem se desprender com o tempo, a menos que o vidro seja bem limpo e selado.<br>Pode apresentar polimerização incompleta ao redor das fibras de colágeno.
Enecê	Meio altamente durável, com vida útil de pelo menos 50 anos.<br>O Enecê não escurece com o tempo.<br>É mais maleável, permitindo a dissecção de insetos no meio, além de proporcionar um tempo razoável para o posicionamento de estruturas morfológicas.<br>Baixo custo.	Requer uma série completa e demorada de desidratação.<br>A desidratação com etanol e a transferência via óleo de cravo podem tornar alguns espécimes quebradiços.<br>O inseto continua a ser clarificado, embora muito lentamente; isso pode dificultar a visualização de estruturas muito pequenas, como sensilas, ascoides e cerdas simples.

5.3.4. Descrição de meios de montagem recomendados (Tabelas 3 e 4)

Meios para observação temporária

Goma-cloral = Fluido/Meio/Solução de Hoyer (IR = 1,48)

O fluido Marc-André é o melhor meio para observação de curto prazo (algumas horas, possivelmente um pouco mais se a lâmina for armazenada em câmara úmida) de espermatecas, incluindo fotografias (Figura 4) ou desenhos. Para preservar as espermatecas observadas, é necessário remontá-las em um meio aquoso que permita armazenamento em médio prazo. Desidratá-las para remontagem em resina não é impossível, mas não é recomendado (risco de perda). A goma-cloral e o fluido de Hoyer são considerados sinônimos.

Este meio é comumente utilizado para observar órgãos internos devido à sua compatibilidade com a água, simplicidade, aplicação rápida e índice de refração que facilita o exame de estruturas delicadas como espermatecas. No entanto, a goma-cloral apresenta desvantagens significativas se não for preparada corretamente ou armazenada sob condições de umidade controlada. Esses problemas incluem cristalização, descoloração e perda de viscosidade. A vedação da lamínula não resolve esses problemas, pois o meio de montagem pode se descolorir intensamente (às vezes quase negro) devido à interação com o meio de vedação, especialmente se o Euparal® for usado.

O meio Hoyer é considerado opticamente o melhor para flebotomíneos e tradicionalmente tem sido usado para esses

fins. O meio consiste em várias formulações relacionadas, incluindo goma arábica, glicerol e hidrato de cloral. Diversas formulações foram mal interpretadas e citadas erroneamente [74].

Embora o Hoyer seja um bom meio para observar espermatecas em flebotomíneos, não é adequado para preservação a longo prazo. É ideal para observações de curto prazo, incluindo fotografias, desenhos ou imagens. Meios aquosos são adequados para montagens temporárias, mas não garantem preservação prolongada. Em contraste, a montagem em resina oferece excelente durabilidade, muitas vezes por séculos, mas pode obscurecer detalhes finos das espermatecas, já que seu índice de refração frequentemente se perde.

O meio Hoyer se degrada com o tempo devido à desidratação (Figura 8), resultando na formação de pequenos cristais brancos opacos de cloral hidrato. No entanto, os espécimes podem ser recuperados de lâminas cristalizadas, pois a cutícula permanece quimicamente intacta, embora algum dano físico possa ocorrer devido ao crescimento dos cristais. Em alguns casos, lâminas cristalizadas podem ser restauradas reidratando o meio de montagem em ambiente quente e úmido com timol para prevenir crescimento fúngico. Alternativamente, os espécimes podem ser retirados da goma-cloral em água, desidratados em ácido acético glacial e remontados em bálsamo do Canadá.

DMHF (dimetil hidantoína formaldeído) (IR = 1,48)

Este meio aquoso [72] apresenta excelente desempenho óptico, semelhante ao Berlese, e é tão fácil de usar quanto este. No entanto, ao contrário do Berlese, não escurece nem cristaliza. Funciona bem para flebotomíneos e outros Psicodídeos.

CMCP (cânfora-mono-clorofenol) (IR = 1,41)

Meio de montagem à base de glicerina e solúvel em água, usado para criar lâminas permanentes transparentes de espécimes delicados, incluindo flebotomíneos. A vantagem deste meio é que os espécimes podem ser montados diretamente da água ou do etanol. Ele relaxa e clarifica rapidamente o flebotomíneo, amolecendo a cutícula para permitir o posicionamento correto do espécime, especialmente útil na abertura das asas ou na dissecação da genitália. Embora se reporte que permita armazenamento a longo prazo, a duração exata da preservação ainda é incerta. A principal limitação deste meio de montagem é sua composição, que contém fenol, uma substância tóxica e irritante que requer manuseio cuidadoso.

Meios para montagem permanente

Bálsamo do Canadá (IR = 1,52–1,54)

O bálsamo do Canadá foi descrito pela primeira vez como um meio de montagem adequado para microscopia de luz transmitida por Andrew Pritchard na década de 1.830. Continua sendo um dos meios mais utilizados devido à sua comprovada estabilidade, com mais de 150 anos de aplicação bem-sucedida. Ao contrário do líquido de Hoyer, o bálsamo do Canadá não cristaliza nem absorve umidade. No entanto, apresenta forte autofluorescência, o que pode ser uma desvantagem para algumas técnicas de microscopia [60]. O uso de solventes não tóxicos em substituição ao xileno pode reduzir riscos à segurança durante a preparação, mas pode introduzir desvantagens como secagem mais lenta e escurecimento precoce do meio.

Euparal® (IR = 1,48)

O Euparal® é uma alternativa amplamente usada ao bálsamo do Canadá para montagem permanente, oferecendo excelente estabilidade a longo prazo e índice de refração comparável. Suas características incluem:

Necessidade de desidratação: antes da transferência final do meio de montagem, o espécime deve ser desidratado, tipicamente transitando de 95% para etanol absoluto.

Tempo de processamento prolongado: a montagem final em resina, seja bálsamo do Canadá ou Euparal®, exige desidratação, o que aumenta o tempo total de processamento do espécime. Quando a desidratação com solventes orgânicos não é viável, amostras retiradas do etanol absoluto podem ser colocadas em solução intermediária consistindo em mistura igual de Euparal® e essência de Euparal®, antes da montagem final.

Enecê (IR = 1,467)

Enecê é um meio de montagem à base de resina, usado principalmente para insetos pequenos e particularmente popular no Brasil. Sua base consiste em colofônia e goma de copal dissolvidas em álcool, cânfora, essência de terebintina e eucaliptol. Cerqueira [11] descreveu o Enecê como uma alternativa ao bálsamo do Canadá para montagem permanente de lâminas de larvas, exúvias de imaturos e até de mosquitos adultos, e desde então tem sido amplamente adotado para flebotomíneos. O Enecê oferece uma alternativa econômica para montagem permanente, proporcionando estabilidade a longo prazo e tempo de secagem suficiente para dissecção e arranjo preciso das estruturas morfológicas.

5.4. Preparação e secagem das lâminas

A secagem adequada das lâminas montadas é fundamental para garantir a estabilidade e preservação a longo prazo. As lâminas devem ser completamente secas antes de serem consideradas para armazenamento prolongado. Para resultados ótimos, lâminas montadas com meios de montagem permanentes devem ser secas na posição horizontal por 2 a 3 semanas, enquanto aquelas preparadas com meios semipermanentes podem requerer apenas 1 a 2 semanas.

Para assegurar um processo de secagem eficaz, recomenda-se o uso de uma incubadora ajustada a uma

temperatura adequada ao meio de montagem utilizado, evitando calor excessivo que possa danificar os espécimes. Recomenda-se uma faixa de temperatura entre 30°C e 37°C. Esta etapa de secagem é crucial para prevenir deformações da lâmina, deterioração do espécime ou instabilidade do meio de montagem durante o armazenamento.

O meio de montagem utilizado na preparação da lâmina deve sempre ser registrado no rótulo da lâmina. Sempre que possível, o rótulo deve incluir também a receita específica utilizada, o nome do preparador e a data de preparação. Inicialmente, as lâminas são preparadas como montagens temporárias e não se destinam à preservação a longo prazo. Contudo, caso o *status* do espécime seja alterado, por exemplo, quando designado como parte de uma série "tipo", deve-se utilizar um meio de montagem mais permanente para garantir a preservação do espécime para futuros estudos taxonômicos.

5.5. Técnicas alternativas de montagem: montagem em cartão

A montagem em cartão é uma técnica utilizada em diversos grupos de insetos, na qual os espécimes podem ser pregados diretamente em cartões entomológicos ou colados à superfície. Devido ao pequeno tamanho dos flebotomíneos e à necessidade de observar órgãos internos para identificação mediante clarificação (ver item 5), este método não é adequado para a sua montagem.

5.6. Remontagem de espécimes danificados

Para espécimes raros ou valiosos, recomenda-se uma abordagem em duas etapas, conforme demonstrado no vídeo acessível em: https://zenodo.org/records/18315029) Reidratação sem desmontar para observação prévia: reidrate os espécimes sem desmontá-los para permitir observação preliminar. Um suporte para várias lâminas de microscópio deve ser colocado dentro de uma placa de Petri para servir de apoio. A lâmina a ser reidratada é então posicionada sobre o suporte, e a placa de Petri é preenchida com alguns milímetros de solvente, criando uma câmara úmida, garantindo que a própria lâmina não entre em contato direto com o solvente (Figura 8 D). O tempo necessário para a reidratação pode variar de um a vários dias, dependendo do estado do espécime. Monitoramento diário e paciência são essenciais. Uma vez que a lâmina esteja suficientemente reidratada, ela pode ser removida da câmara úmida e colocada em incubadora por algumas horas antes da observação microscópica, fotografia ou desenho. 2) Para remontar, a lâmina pode ser retornada à câmara úmida por algumas horas adicionais ou durante a noite. A desmontagem deve ser realizada sob microscópio binocular. Usando agulhas finas, a lamínula deve ser cuidadosamente removida, garantindo que nenhum elemento do flebotomíneo permaneça preso (https://zenodo.org/records/18315029). Em seguida, os elementos dissecados do flebotomíneo devem ser coletados e enxaguados com água em pequenos poços, como os utilizados para extração destrutiva de DNA/RNA (ver abaixo), antes da desidratação e remontagem em meio de resina. Ao desmontar uma lâmina, é crucial identificar o meio de montagem original para selecionar o solvente apropriado. Para meios de montagem aquosos, deve-se usar água. Se o meio de montagem for à base de resina (por exemplo, bálsamo do Canadá ou Euparal®), deve-se usar xileno, sempre sob capela de exaustão e com equipamento de proteção individual adequado, incluindo máscara. A remontagem de espécimes tipo ou de coleção deve ser realizada apenas com o consentimento do curador e/ou da instituição proprietária do espécime.

6. Identificação de espécimes

6.1. Morfologia

A identificação de flebotomíneos baseia-se principalmente na análise de suas características morfológicas, incluindo a forma do tórax, asas, genitália, cerdas e as relações morfométricas específicas entre diversas estruturas. Pesquisadores utilizam chaves taxonômicas, coleções de referência e descrições originais de espécies para comparar os espécimes coletados com táxons já conhecidos. Características diagnósticas-chave, como a nervação das asas e a morfologia da cabeça em ambos os sexos, a estrutura da genitália masculina e a configuração das espermatecas femininas, são particularmente informativas para a determinação da espécie. A identificação precisa frequentemente requer exame microscópico detalhado, tipicamente utilizando um microscópio composto para observar estruturas finas, como genitália e espermatecas, ou um estereomicroscópio para examinar características morfológicas mais amplas.

Avanços recentes em tecnologia de imagem têm facilitado o uso de imagens digitais para a identificação. Fotografias de alta resolução ou ilustrações digitais de características-chave podem ser comparadas com materiais de referência ou analisadas por meio de sistemas informatizados de identificação, melhorando tanto a precisão quanto a acessibilidade na taxonomia morfológica.

6.2. Geometria das asas

A geometria das asas é uma característica-chave utilizada na identificação e classificação de diferentes espécies de flebotomíneos. As asas dos flebotomíneos apresentam um padrão e estrutura únicos, sendo tipicamente longas e estreitas, com nervação bem desenvolvida (Figuras 9 e 10).

O arranjo das veias forma um padrão distinto que pode variar entre gêneros e espécies, fornecendo características diagnósticas valiosas para a identificação. Consequentemente, o estudo da geometria das asas oferece informações relevantes para fins taxonômicos.

6.3. Morfometria geométrica das asas

Pesquisadores utilizam diversas técnicas, como a morfometria geométrica, para analisar e comparar a forma e o tamanho das asas entre diferentes espécies ou populações de flebotomíneos. O estudo da geometria das asas fornece informações valiosas sobre comportamento, preferências de habitat e capacidades de voo.

Na abordagem da morfometria geométrica, as asas são cuidadosamente dissecadas, coradas (quando necessário) e montadas planas em lâminas. As lâminas preparadas são então fotografadas sob estereomicroscópio, digitalizadas e submetidas à análise morfométrica. Este procedimento foi bem descrito na literatura [6, 27, 42, 56, 57, 59], sendo recomendada a utilização consistente da asa direita ou esquerda para órgãos pares, a fim de evitar possíveis efeitos alométricos negativos [62].

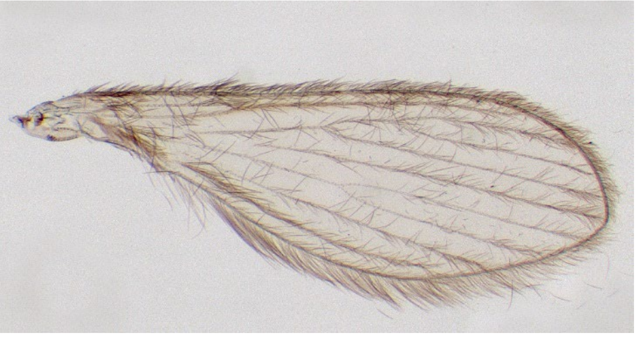

Figura 9: Asa não corada de *Trichophoromyia ininii*.

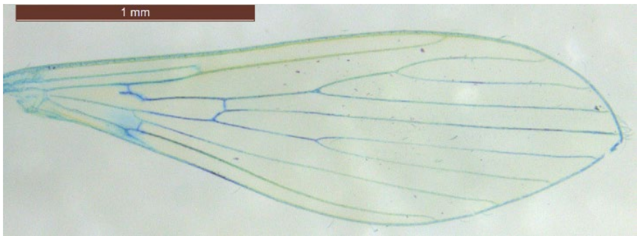

Figura 10: Asa corada de *Phlebotomus ariasi*.

Preparação das asas para análise de morfometria geométrica

Para a visualização ideal das veias das asas, estas devem ser limpas das cerdas e coradas de forma adequada. Para a preparação da asa, preencha primeiro pequenos poços com os reagentes necessários (azul de metileno, etanol, água e substituto de xileno). Retire uma asa preservada em etanol a 70% à temperatura ambiente, invertendo o tubo Eppendorf e esvaziando seu conteúdo sobre o poço, em seguida, levante a asa longitudinalmente utilizando uma agulha fina curva. Passe a asa brevemente do etanol para a água e de volta para o etanol para remover as cerdas. Coloque a asa em azul de metileno por 6 minutos, garantindo que ela flutue durante a coloração. Recupere a asa cuidadosamente e a coloque em imersão em substituto de xileno por 2 minutos (aproximadamente um terço do tempo do azul de metileno). Toques suaves da agulha contra as paredes do poço podem ajudar a asa a se acomodar; o xileno serve para fixar a coloração. Por fim, levante a asa e posicione-a sobre uma pequena gota de Euparal® em uma lâmina de microscópio. Sob uma lente de aumento, desdobre suavemente a asa e posicione cuidadosamente a lamínula. Fotografias devem ser feitas imediatamente antes do endurecimento do Euparal®, pois pequenos ajustes da posição da asa sob a lamínula podem ser necessários para alcançar alinhamento ótimo.

6.4. Técnicas de biologia molecular

Além das técnicas morfológicas, os métodos moleculares tornam-se cada vez mais essenciais na pesquisa entomológica, incluindo estudos taxonômicos, genéticos de populações e filogenéticos, bem como para a detecção de DNA/RNA de patógenos, determinação da origem de repasto sanguíneo e análise do comportamento de vetores, aspectos importantes no campo da epidemiologia [70]. O sequenciamento de DNA pode ser empregado para a confirmação de espécies ou para diferenciar espécies estreitamente relacionadas, fornecendo um método de identificação mais preciso e confiável. Além disso, técnicas moleculares avançadas (como PCR, sequenciamento de DNA, NGS etc.) e MALDI-ToF MS vêm ganhando destaque para a identificação rápida e acurada de espécies, complementando os métodos morfológicos tradicionais [46]. Apesar desses avanços, a identificação morfológica continua sendo o padrão de referência para a taxonomia e a base sobre a qual os dados moleculares são interpretados.

6.4.1. Extração destrutiva de ácidos nucleicos

A extração de ácidos nucleicos é uma etapa rotineira em muitos estudos biológicos, e diversos métodos foram desenvolvidos para isolar DNA a partir de materiais biológicos [48]. Muitos kits comerciais de extração de DNA foram projetados para facilitar esse processo [14]. No entanto, os métodos comumente utilizados para preparar espécimes de artrópodes para identificação morfológica frequentemente dificultam a análise de DNA, pois essas técnicas podem danificar ou destruir características físicas críticas do espécime [10]. A maioria dos protocolos de extração de DNA em tecidos de insetos é destrutiva por natureza [43], levantando preocupações particulares em espécimes pequenos, nos quais até mesmo a coleta limitada pode comprometer características morfológicas importantes [72]. O tipo e o estado do espécime desempenham um papel fundamental na seleção do método adequado de isolamento de DNA [29].

A necessidade de identificação precisa dos flebotomíneos, compreensão da dinâmica populacional e minimização de impactos em organismos não-alvo impulsionou o desenvolvimento de ferramentas moleculares diagnósticas [23]. Atualmente, abordagens

moleculares são frequentemente utilizadas para complementar os métodos taxonômicos morfológicos na identificação de flebotomíneos. Por exemplo, a abordagem padrão para codificação por DNA (DNA *barcoding*) envolve extração de DNA, sequenciamento e perda do espécime original. Assim, há uma necessidade premente de explorar métodos de extração de DNA não destrutivos que preservem tanto o material biológico quanto sua integridade morfológica.

Diversos métodos de extração de ácidos nucleicos foram aplicados a flebotomíneos. A quantidade ou qualidade de ácidos nucleicos necessária depende da análise molecular subsequente, uma vez que diferentes técnicas possuem sensibilidade e requisitos de pureza variados [9]. Por exemplo, foi observado que os olhos de flebotomíneos podem inibir a amplificação por PCR [69]. Além do rastreamento de patógenos, o DNA de flebotomíneos é rotineiramente extraído para fins de identificação de espécies. Diversos métodos de extração podem ser utilizados, embora rendimentos e qualidade variem entre as técnicas. Alguns protocolos comerciais foram adaptados por pesquisadores para flebotomíneos [8], aumentando o rendimento e/ou a qualidade dos ácidos nucleicos extraídos [8, 9, 69], enquanto outras adaptações, desenvolvidas para outros táxons de artrópodes, também podem ser aplicadas a flebotomíneos [58, 76]. Amplificações (PCR) que têm como alvo pequenos fragmentos mitocondriais (COI ou CytB) são geralmente compatíveis com métodos de extração que envolvem alta fragmentação do DNA. Em contraste, técnicas de NGS de leitura longa (Oxford Nanopore e PacBio) exigem DNA de alta qualidade com mínima fragmentação. Extrações com coluna de centrifugação geralmente produzem fragmentos genômicos de até 60 kb, enquanto a extração com fenol-clorofórmio pode gerar fragmentos de até 150 kb [77]. A Tabela 5 resume diferentes técnicas de extração de DNA de flebotomíneos e indica se foram realizadas adaptações metodológicas para esses insetos. Os rendimentos não são apresentados, pois dependem do tamanho do espécime e do método de preparação. A coluna de modificação refere-se a adaptações de protocolos de extração para flebotomíneos ou outros artrópodes pequenos. A escolha do método de extração deve considerar diversos critérios, como número de amostras, tempo de extração e a técnica a ser utilizada posteriormente. Embora técnicas de NGS exijam DNA genômico de alto peso molecular, todos os métodos apresentados aqui podem ser utilizados em aplicações padrão baseadas em PCR.

Além disso, diversos estudos têm explorado métodos de extração de DNA não destrutivos para pequenos artrópodes terrestres, espécimes de museu preservados a seco e artrópodes de corpo mole [19, 26, 28, 55, 63].

Tabela 5: Custo médio, aplicação e adaptação de protocolo para a extração de gDNA de flebotomíneos

Protocolo	Custo	Aplicação	Protocolo adaptado para pequenos artrópodes
Coluna Spin	2.5 – 3.55 US$ [39]	PCR, NGS	[9]
Fenol-clorofórmio	0.24 US$ [69]	PCR, NGS	[9]
HotSHOT	<0.01 US$ [69]	PCR	-
Salting out	0.12 $3 [69]	PCR	-
Chelex	0.02 $4 [41]	PCR	[41, 76]

6.4.2. Extração não destrutiva de ácidos nucleicos

Um dos maiores desafios na análise molecular de artrópodes, especialmente de flebotomíneos, é a preservação dos espécimes para integração em coleções entomológicas. A maioria dos protocolos de extração de DNA requer a maceração do tecido, comprometendo, assim, a preservação do espécime original. Por outro lado, os métodos não destrutivos de extração de ácidos nucleicos foram projetados para extrair material genético sem danificar fisicamente a amostra, afetar sua viabilidade ou alterar sua morfologia. Esses métodos são particularmente valiosos quando se trabalha com espécimes preciosos ou limitados, como flebotomíneos, nos quais a manutenção da integridade estrutural é essencial para futuros estudos taxonômicos, morfológicos ou diagnósticos. Uma técnica comumente empregada é o método de imersão não destrutiva, no qual os flebotomíneos são imobilizados e delicadamente mergulhados em um tampão de lise contendo proteinase K.

A técnica de *mild-vectolysis* foi aplicada com sucesso a flebotomíneos, especialmente a espécimes-tipo [24]. A técnica utiliza um kit convencional de coluna de centrifugação (neste caso, o DNeasy Blood and Tissue kit, QIAGEN, Hilden, Alemanha) com adaptações para obter DNA sem destruir o espécime. As etapas de lise modificadas (volume do tampão de lise e adição de uma etapa de congelamento) [17] permitem a liberação de ácidos nucleicos, minimizando danos morfológicos [24]. Quanto aos flebotomíneos, também é possível utilizar o HotSHOT DNA Extraction kit (Bento Bioworks Ltd, Londres, Reino Unido) [73], que é rápido e econômico, permitindo o processamento ágil e de baixo custo das amostras. Espécimes entomológicos destinados à identificação morfológica podem, então, ser lavados. Aqueles processados com o DNeasy Blood and Tissue kit devem ser clarificados com solução Marc-André, enquanto os processados com o HotSHOT DNA extraction kit já se encontram suficientemente clarificados para serem montados em meio aquoso ou, preferencialmente, em resina

após desidratação, de acordo com o protocolo detalhado neste artigo [73]. O material genético extraído pode então ser processado posteriormente para análises subsequentes, como PCR, para amplificar marcadores genéticos específicos. Os métodos não destrutivos de extração de ácidos nucleicos são cruciais para estudar as características genéticas de flebotomíneos, incluindo a identificação de potenciais agentes causadores de doenças que possam carregar. Ao preservar a integridade do espécime, os pesquisadores podem obter informações genéticas valiosas, mantendo a amostra disponível para análises ou estudos adicionais.

6.5. MALDI-ToF MS

O MALDI-ToF MS (espectrometria de massa por dessorção/ionização a laser assistida por matriz e tempo de voo) é uma técnica baseada em espectrometria de massa projetada para detectar e analisar perfis proteicos únicos ("impressões digitais") de amostras biológicas. O MALDI-ToF é cada vez mais reconhecido como uma ferramenta importante para a identificação de artrópodes de importância médica e veterinária. Essa técnica tem se mostrado eficaz na identificação de diversos estágios de desenvolvimento de flebotomíneos, incluindo formas imaturas e fontes alimentares de fêmeas ingurgitadas, e tem sido aplicada com sucesso para diferenciar espécies de flebotomíneos machos e fêmeas sob uma variedade de condições de armazenamento e homogeneização [28, 30, 73, 74]. O método também oferece alto poder discriminatório nos níveis de subgêneros, espécies e populações. Ele permite aos pesquisadores obter identificação rápida e precisa de espécies, essencial para compreender a distribuição, comportamento dos flebotomíneos e seu papel na transmissão de doenças. Ao diferenciar espécies com base em perfis proteicos, o MALDI-ToF desempenha um papel crucial em estudos epidemiológicos e estratégias de controle vetorial. Atualmente, existem duas limitações principais que restringem a aplicação rotineira desta técnica. A primeira é a disponibilidade de equipamentos de espectrometria de massa, que são proibitivamente caros para aquisição apenas para fins de identificação de espécies de flebotomíneos (ou artrópodes vetores, de modo geral). Felizmente, essa limitação pode ser contornada por meio do acesso a tempo de máquina em espectrômetros de massa, que se tornaram ferramentas padrão em instalações de proteômica e/ou diagnósticos clínicos. A segunda limitação é a baixa representatividade de dados de referência de flebotomíneos disponíveis em bases de dados de acesso aberto, o que gera a necessidade de construir uma base de dados interna com espectros de referência obtidos a partir de espécimes identificados de forma inequívoca, idealmente por uma combinação de avaliação morfológica e sequenciamento de um marcador genético adequado (COI, cytB ou outro). Espera-se que essa limitação seja em breve resolvida com a inclusão gradual de dados de referência de flebotomíneos, até então mantidos internamente, na Plataforma MSI operada pelo *Assistance Publique-Hôpitaux de Paris*, Sorbonne University, França, e na coleção BCCM/IHEM/Sciensano em Bruxelas, Bélgica (https://msi.happy-dev.fr/). Quando a obtenção do perfil proteico por MALDI-ToF for planejada, as amostras devem ser armazenadas preferencialmente congeladas a seco ou em etanol 70%, sem exposição a temperaturas ambientes. Na ausência de diretrizes universais para preparação de amostras, recomenda-se que os usuários utilizem uma solução aquosa de 60% acetonitrila/0,3% TFA com ácido sinapínico (30 mg/mL) para a preparação da matriz MALDI-ToF, de modo a tornar seus espectros proteicos comparáveis com os dados publicados de flebotomíneos até o momento.

Preparação de Amostras para MALDI-ToF MS (Figura 7)

Os espécimes de insetos, armazenados sob diferentes condições, são inicialmente secos sob temperatura ambiente e, em seguida, dissecados. A cabeça e o abdome são removidos para obter partes do corpo contendo características morfológicas-chave, que serão utilizadas para montagem em lâminas e análise morfológica. O tórax pode ser usado para MALDI-ToF, enquanto o abdome restante é preservado para extração de DNA. Para o perfil proteico, o tórax é homogeneizado manualmente em microtubos de 1,5 mL com 10 µL de solução de homogeneização, utilizando pistilos e bastonetes descartáveis. Normalmente, são empregadas duas soluções de homogeneização: água destilada estéril e ácido fórmico a 25%.

7. Conclusão

Neste trabalho, nosso objetivo foi fornecer aos pesquisadores os métodos mais eficazes para a montagem de flebotomíneos, adaptados a objetivos específicos de pesquisa, de modo a facilitar a identificação precisa e a detecção de patógenos. Não existe um método único e universalmente ótimo; ao contrário, diversos métodos estão disponíveis, cada um com suas vantagens e limitações.

Nos dados suplementares, apresentamos protocolos detalhados para várias técnicas de montagem utilizadas na preparação e identificação de flebotomíneos. Esses protocolos, incluindo vídeos instrucionais, oferecem procedimentos passo a passo adaptados a diferentes objetivos, garantindo resultados precisos e confiáveis. Ao disponibilizar esse recurso abrangente, buscamos apoiar os pesquisadores na seleção e aplicação das técnicas de montagem mais adequadas às suas necessidades específicas.

Agradecimentos

Os autores agradecem a Richard Lane e Zoe Jay Adams, do Museu de História Natural de Londres, Reino Unido, pela excelente revisão, que contribuiu significativamente para elevar a qualidade deste manuscrito.

Funding

Agradecemos às agências brasileiras de fomento CNPq (processo nº 404395/2024-4) e Fundação Araucária (processo nº 433/2025 PDI) pelo financiamento das pesquisas de AJA.

Conflitos de interesse

Jérôme Depaquit é editor associado da revista Parasite; ele não teve influência no processo de revisão e na tomada de decisão sobre este manuscrito. Os demais autores declaram não haver conflitos de interesse.

Data availability statement

Vìdeos no Zenodo
Video 1: https://zenodo.org/records/18198006
Video 2: https://zenodo.org/records/18311158
Video 3: https://zenodo.org/records/18311106
Video 4: https://zenodo.org/records/18311154
Video 5: https://zenodo.org/records/18303014
Video 6: https://zenodo.org/records/18302850
Video 7: https://zenodo.org/records/18315029

Materiais adicionais

Mais informações sobre este artigo estão disponíveis no seguinte endereço: https://www.parasite-journal.org/10.1051/parasite/2026009/olm

Referências

1. Alkan C, Allal-Ikhlef AB, Alwassouf S, Baklouti A, Piorkowski G, de Lamballerie X, Izri A, Charrel RN. 2015. Virus isolation, genetic characterization and seroprevalence of Toscana virus in Algeria. Clinical Microbiology and Infection, 21(11), 1040 e1-9.
2. Alten B, Ozbel Y, Ergunay K, Kasap OE, Cull B, Antoniou M, Velo E, Prudhomme J, Molina R, Banuls AL, Schaffner F, Hendrickx G, Van Bortel W, Medlock JM. 2015. Sampling strategies for phlebotomine sand flies (Diptera: Psychodidae) in Europe. Bulletin of Entomological Research 105(6), 664–678.
3. Ayhan N, Baklouti A, Prudhomme J, Walder G, Amaro F, Alten B, Moutailler S, Ergunay K, Charrel RN, Huemer H. 2017. Practical guidelines for studies on sandfly-borne phleboviruses: Part I: Important points to consider *ante* field work. Vector-Borne and Zoonotic Diseases 17(1), 73–80.
4. Bates PA. 1997. Infection of phlebotomine sandflies with *Leishmania*, in The Molecular Biology of Insect Disease Vectors: A Methods Manual. Springer. p. 112–120.5.
5. Baum M, de Castro EA, Pinto MC, Goulart TM, Baura W, Klisiowicz Ddo R, Vieira da Costa-Ribeiro MC. 2015. Molecular detection of the blood meal source of sand flies (Diptera: Psychodidae) in a transmission area of American cutaneous leishmaniasis, Parana State, Brazil. Acta Tropica, 143, 8–12.
6. Belen A, Alten B, Aytekin A. 2004. Altitudinal variation in morphometric and molecular characteristics of *Phlebotomus papatasi* populations. Medical and Veterinary Entomology, 18(4), 343–350.
7. Bhattacharya J, Chandra G, Hati AK. 1991. A simple method for cryopreservation of *Leishmania donovani* promastigotes, Indian Journal of Medical Research, 93, 245–246.
8. Caligiuri LG, Sandoval AE, Miranda JC, Pessoa FA, Santini MS, Salomón OD, Secundino NF, McCarthy CB. 2019. Optimization of DNA extraction from individual sand flies for PCR amplification. Methods and Protocols, 2(2), 36.
9. Casaril AE, de Oliveira LP, Alonso DP, de Oliveira EF, Gomes Barrios SP, de Oliveira Moura Infran J, Fernandes WS, Oshiro ET, Ferreira AMT, Ribolla PEM, de Oliveira AG. 2017. Standardization of DNA extraction from sand flies: Application to genotyping by next generation sequencing. Experimental Parasitology, 177, 66–72.
10. Castalanelli MA, Severtson DL, Brumley CJ, Szito A, Foottit RG, Grimm M, Munyard K, Groth DM. 2010. A rapid non-destructive DNA extraction method for insects and other arthropods. Journal of Asia-Pacific Entomology, 13(3), 243–248.
11. Cerqueira NL. 1943. Um novo meio para montagem de pequenos insetos em lâmina. Memórias do Instituto Oswaldo Cruz, (39), 37–41.
12. Charrel RN, Gallian P, Navarro-Mari JM, Nicoletti L, Papa A, Sanchez-Seco MP, Tenorio A, de Lamballerie X. 2005. Emergence of Toscana virus in Europe. Emerging Infectious Diseases, 11(11), 1657–1663.
13. Chaskopoulou A, Giantsis IA, Demir S, Bon MC. 2016. Species composition, activity patterns and blood meal analysis of sand fly populations (Diptera: Psychodidae) in the metropolitan region of Thessaloniki, an endemic focus of canine leishmaniasis. Acta Tropica, 158, 170–176.
14. Chen H, Rangasamy M, Tan SY, Wang H, Siegfried BD. 2010. Evaluation of five methods for total DNA extraction from western corn rootworm beetles. PLoS One, 5(8), e11963.
15. Depaquit J, Grandadam M, Fouque F, Andry PE, Peyrefitte C. 2010. Arthropod-borne viruses transmitted by Phlebotomine sandflies in Europe: a review. Eurosurveillance, 15(10), 19507.
16. Diamond LS, Herman CM. 1954. Incidence of Trypanosomes in the Canada Goose as revealed by bone marrow culture. Journal of Parasitology, 40(2), 195–202.
17. Ding H, Torno M, Vongphayloth K, Ng G, Tan D, Sng W, Ho K, Randrianambinintsoa FJ, Depaquit J, Tan CH. 2025. Hidden in plain sight: discovery of sand flies in Singapore and description of four species new to science. Parasites & Vectors, 18(1), 402.
18. Es-Sette N, Ajaoud M, Bichaud L, Hamdi S, Mellouki F, Charrel RN, Lemrani M. 2014. *Phlebotomus sergenti* a common vector of *Leishmania tropica* and Toscana virus in Morocco. Journal of Vector Borne Diseases, 51(2), 86–90.
19. Favret C. 2005. A new non-destructive DNA extraction and specimen clearing technique for aphids (Hemiptera). Proceedings of the Entomological Society of Washington, 107(2), 469–470.
20. Galati EAB. 2018. Phlebotominae (Diptera, Psychodidae): Classification, morphology and terminology of adults and identification of American taxa, in Brazilian Sand Flies: Biology, Taxonomy, Medical Importance and Control, Rangel EF, Shaw JJ, Editors. Cham: Springer International Publishing. pp. 9–212.
21. Galati EAB, de Andrade AJ, Perveen F, Loyer M, Vongphayloth K, Randrianambinintsoa FJ, Prudhomme J, Rahola N, Akhoundi M, Shimabukuro PHF, Depaquit J. 2025. Phlebotomine sand flies (Diptera, Psychodidae) of the world. Parasites & Vectors, 18(1), 220.
22. Galati EAB, Galvis-Ovallos F, Lawyer P, Leger N, Depaquit J. 2017. An illustrated guide for characters and terminology used in descriptions of Phlebotominae (Diptera, Psychodidae). Par-asite, 24, 26.
23. Gariepy T, Kuhlmann U, Gillott C, Erlandson M. 2007. Parasitoids, predators and PCR: the use of diagnostic molecular markers in biological control of Arthropods. Journal of Applied Entomology, 131(4), 225–240.
24. Giantsis IA, Chaskopoulou A, Bon MC. 2016. Mild-Vectolysis: A nondestructive DNA extraction method for vouchering sand flies and mosquitoes. Journal of Medical Entomology, 53(3), 692–695.

This is an Open Access article distributed under the terms of the Creative Commons Attribution License (https://creativecommons.org/licenses/by/4.0), which permits unrestricted use, distribution, and reproduction in any medium, provided the original work is properly cited.

25. Gidwani K, Picado A, Rijal S, Singh SP, Roy L, Volfova V, Andersen EW, Uranw S, Ostyn B, Sudarshan M, Chakravarty J, Volf P, Sundar S, Boelaert M, Rogers ME. 2011. Serological markers of sand fly exposure to evaluate insecticidal nets against visceral leishmaniasis in India and Nepal: a cluster-randomized trial. PLoS Neglected Tropical Diseases, 5(9), e1296.
26. Gilbert MTP, Moore W, Melchior L, Worobey M. 2007. DNA extraction from dry museum beetles without conferring external morphological damage. PLoS One, 2(3), e272.
27. Giordani BF, Andrade AJ, Galati EAB, Gurgel-Goncalves R. 2017. The role of wing geometric morphometrics in the identification of sandflies within the subgenus *Lutzomyia.* Medical and Veterinary Entomology, 31(4), 373–380.
28. Guzmán-Larralde AJ, Suaste-Dzul AP, Gallou A, Peña-Carrillo KI. 2017. DNA recovery from microhymenoptera using six non-destructive methodologies with considerations for subsequent preparation of museum slides. Genome, 60(1), 85–91.
29. Hajibabaei M, DeWaard JR, Ivanova NV, Ratnasingham S, Dooh RT, Kirk SL, Mackie PM, Hebert PD. 2005. Critical factors for assembling a high volume of DNA barcodes. Philosophical Transactions of the Royal Society B: Biological Sciences, 360(1462), 1959–1967.
30. Haouas N, Pesson B, Boudabous R, Dedet JP, Babba H, Ravel C. 2007. Development of a molecular tool for the identification of *Leishmania* reservoir hosts by blood meal analysis in the insect vectors. American Journal of Tropical Medicine and Hygiene, 77(6), 1054–1059.
31. Hlavackova K, Dvorak V, Chaskopoulou A, Volf P, Halada P. 2019. A novel MALDI-TOF MS-based method for blood meal identification in insect vectors: A proof of concept study on phlebotomine sand flies. PLoS Neglected Tropical Diseases, 13 (9), e0007669.
32. Huemer H, Prudhomme J, Amaro F, Baklouti A, Walder G, Alten B, Moutailler S, Ergunay K, Charrel RN, Ayhan N. 2017. Practical guidelines for studies on sandfly-borne phleboviruses: Part II: Important points to consider for fieldwork and subsequent virological screening. Vector-Borne and Zoonotic Diseases, 17(1), 81–90.
33. Jancarova M, Polanska N, Thiesson A, Arnaud F, Stejskalova M, Rehbergerova M, Kohl A, Viginier B, Volf P, Ratinier M. 2025. Susceptibility of diverse sand fly species to Toscana virus. PLoS Neglected Tropical Diseases, 19(5), e0013031.
34. Kapp JD, Green RE, Shapiro B. 2021. A fast and efficient single-stranded genomic library preparation method optimized for ancient DNA. Journal of Heredity, 112(3), 241–249.
35. Killick-Kendrick R, Maroli M, Killick-Kendrick M. 1991. Bibliography of the colonization of phlebotomine sandflies. Parassitologia, 33(suppl.), 321–333.
36. Lawyer P, Killick-Kendrick M, Rowland T, Rowton E, Volf P. 2017. Laboratory colonization and mass rearing of phlebotomine sand flies (Diptera, Psychodidae). Parasite, 24, 42.
37. Léger N, Pesson B, Madulo-Leblond G. 1986. Les phlébotomes de Grèce : 1ère partie. Bulletin de la Société de Pathologie Exotique, 79, 386–397.
38. Léger N, Pesson B, Madulo-Leblond G. 1986. Les phlébotomes de Grèce : 2ème partie. Bulletin de la Société de Pathologie Exotique, 79, 514–524.
39. Leonel JAF, Vioti G, Alves ML, da Silva DT, Meneghesso PA, Benassi JC, Spada JCP, Galvis-Ovallos F, Soares RM, Oliveira T. 2020. DNA extraction from individual Phlebotomine sand flies (Diptera: Psychodidae: Phlebotominae) specimens: Which is the method with better results? Experimental Parasitology, 218, 107981.
40. Lestinova T, Rohousova I, Sima M, de Oliveira CI, Volf P. 2017. Insights into the sand fly saliva: Blood-feeding and immune interactions between sand flies, hosts, and *Leishmania.* PLoS Neglected Tropical Diseases, 11(7), e0005600.
41. Lienhard A, Schaffer S. 2019. Extracting the invisible: obtaining high quality DNA is a challenging task in small arthropods. PeerJ, 7, e6753.
42. Lozano-Sardaneta YN, Mikery-Pacheco OF, Huerta H, Rojas-Soriano JE, Contreras-Ramos A. 2025. Wing geometric morphometrics is effective to separate sand fly species (Diptera, Psychodidae, Phlebotominae) related with leishmaniasis transmission in Mexico. Acta Tropica, 262, 107523.
43. Mandrioli M. 2008. Insect collections and DNA analyses: how to manage collections? Museum Management and Curatorship, 23(2), 193–199.
44. Maroli M, Feliciangeli MD, Bichaud L, Charrel RN, Gradoni L. 2013. Phlebotomine sandflies and the spreading of leishmaniases and other diseases of public health concern. Medical and Veterinary Entomology, 27(2), 123–147.
45. Marquina D, Buczek M, Ronquist F, Lukasik P. 2021. The effect of ethanol concentration on the morphological and molecular preservation of insects for biodiversity studies. PeerJ, 9, e10799.
46. Mathis A, Depaquit J, Dvorak V, Tuten H, Banuls AL, Halada P, Zapata S, Lehrter V, Hlavackova K, Prudhomme J, Volf P, Sereno D, Kaufmann C, Pfluger V, Schaffner F. 2015. Identification of phlebotomine sand flies using one MALDI-TOF MS reference database and two mass pectrometer systems., Parasites & Vectors, 8, 266.
47. Mekarnia N, Benallal KE, Sadlova J, Vojtkova B, Mauras A, Imbert N, Longhitano M, Harrat Z, Volf P, Loiseau PM, Cojean S. 2024. Effect of *Phlebotomus papatasi* on the fitness, infectivity and antimony-resistance phenotype of antimony-resistant *Leishmania* major Mon-25. International Journal for Parasitology – Drugs and Drug Resistance, 25, 100554.
48. Milligan BG. 1998. Total DNA isolation, in Molecular Genetic Analysis of Population: A Practical Approach, Hoelzel AR, Editor. Oxford: Oxford University Press.
49. Molina R, Jiménez M, Alvar J, González E, Hernández-Taberna S, Ines MM. 2017. Methods in sand fly research. Madrid: Servicio de publicaciones Universidad de Alcalá de Henares, Madrid.
50. Murphy WJ, Eizirik E, O'Brien SJ, Madsen O, Scally M, Douady CJ, Teeling E, Ryder OA, Stanhope MJ, de Jong WW, Springer MS. 2001. Resolution of the early placental mammal radiation using Bayesian phylogenetics. Science, 294(5550), 2348–2351.
51. Nacif-Pimenta R, Pinto LC, Volfova V, Volf P, Pimenta PFP, Secundino NFC. 2020. Conserved and distinct morphological aspects of the salivary glands of sand fly vectors of leishma-niasis: an anatomical and ultrastructural study. Parasites & Vectors, 13(1), 441.
52. Neuhaus B, Schmid T, Riedel J. 2017. Collection management and study of microscope slides: Storage, profiling, deterioration, restoration procedures, and general recommendations. Zootaxa, 4322(1), 1–173.
53. New TR. 1974. Pscoptera. Handbooks for Identification of British Insects (Vol. I). London: Royal Entomological Society of London. 102 pp.

54. Perez-Ruiz M, Collao X, Navarro-Mari JM, Tenorio A. 2007. Reverse transcription, real-time PCR assay for detection of Toscana virus. Journal of Clinical Virology, 39(4), 276–281.
55. Porco D, Rougerie R, Deharveng L, Hebert P. 2010. Coupling non-destructive DNA extraction and voucher retrieval for small soft-bodied Arthropods in a high-throughput context: the example of Collembola. Molecular Ecology Resources, 10(6), 942–945.
56. Prudhomme J, Cassan C, Hide M, Toty C, Rahola N, Vergnes B, Dujardin JP, Alten B, Sereno D, Banuls AL. 2016. Ecology and morphological variations in wings of *Phlebotomus ariasi* (Diptera: Psychodidae) in the region of Roquedur (Gard, France): a geometric morphometrics approach. Parasites & Vectors, 9(1), 578.
57. Prudhomme J, Gunay F, Rahola N, Ouanaimi F, Guernaoui S, Boumezzough A, Banuls AL, Sereno D, Alten B. 2012. Wing size and shape variation of *Phlebotomus papatasi* (Diptera: Psychodidae) populations from the south and north slopes of the Atlas Mountains in Morocco. Journal of Vector Ecology, 37(1), 137–147.
58. Prudhomme J, Toty C, Kasap OE, Rahola N, Vergnes B, Maia C, Campino L, Antoniou M, Jimenez M, Molina R, Cannet A, Alten B, Sereno D, Banuls AL. 2015. New microsatellite markers for multi-scale genetic studies on *Phlebotomus ariasi* Tonnoir, vector of *Leishmania infantum* in the Mediterranean area. Acta Tropica, 142, 79–85.
59. Prudhomme J, Velo E, Bino S, Kadriaj P, Mersini K, Gunay F, Alten B. 2019. Altitudinal variations in wing morphology of *Aedes albopictus* (Diptera, Culicidae) in Albania, the region where it was first recorded in Europe. Parasite, 26, 55.
60. Rawlins DJ. 1992. Light Microscopy: An Introduction to Biotechniques. Oxford: Bios Scientific publishers. 143 pp.
61. Ready PD. 2013. Biology of phlebotomine sand flies as vectors of disease agents. Annual Review of Entomology, 58, 227–250.
62. Rohlf FJ, Slice D. 1990. Extensions of the Procrustes method for the optimal superimposition of landmarks. Systematic Zoology, 39(1), 40–59.
63. Rowley DL, Coddington JA, Gates MW, Norrbom AL, Ochoa RA, Vandenberg NJ, Greenstone MH. 2007. Vouchering DNA-barcoded specimens: Test of a nondestructive extraction proto-col for terrestrial arthropods. Molecular Ecology Notes, 7(6), 915–924.
64. Sábio PB, Andrade AJ, Galati EAB. 2014. Assessment of the taxonomic status of some species included in the *Shannoni* complex, with the description of a new species of *Psathyromyia* (Diptera: Psychodidae: Phlebotominae). Journal of Medical Entomology, 51(2), 331–341.
65. Sadlova J, Yeo M, Seblova V, Lewis MD, Mauricio I, Volf P, Miles MA. 2011. Visualisation of *Leishmania donovani* fluorescent hybrids during early stage development in the sand fly vector. PLoS One, 6(5), e19851.
66. Sales K, Miranda DEO, da Silva FJ, Otranto D, Figueredo LA, Dantas-Torres F. 2020. Evaluation of different storage times and preservation methods on phlebotomine sand fly DNA concentration and purity. Parasites & Vectors, 13(1), 399
67. Sales KG, Costa PL, de Morais RC, Otranto D, Brandao-Filho SP, Cavalcanti Mde P, Dantas-Torres F. 2015. Identification of phlebotomine sand fly blood meals by real-time PCR. Parasites & Vectors, 8, 230.
68. Sant'Anna MR, Jones NG, Hindley JA, Mendes-Sousa AF, Dillon RJ, Cavalcante RR, Alexander B, Bates PA. 2008. Blood meal identification and parasite detection in laboratory-fed and field-captured *Lutzomyia longipalpis* by PCR using FTA databasing paper. Acta Tropica, 107(3), 230–237.
69. Senne NA, Santos HA, Araujo TR, Paulino PG, Mendonca LP, Moreira HVS, Camilo TA, da Costa Angelo I. 2022. Robust comparative performance of genomic DNA extraction methods from non-engorged phlebotomine sandflies. Medical and Veterinary Entomology, 36(2), 203–211.
70. Shaw JJ. 2025. A review of *Leishmania* infections in American Phlebotomine sand flies – Are those that transmit leishmaniasis anthropophilic or anthropportunists? Parasite, 32, 57.
71. Tesh RB, Modi GB. 1983. Growth and transovarial transmission of Chandipura virus (Rhabdoviridae: Vesiculovirus) in *Phlebotomus papatasi.* American Journal of Tropical Medicine and Hygiene, 32(3), 621–623.
72. Thomsen PF, Elias S, Gilbert MTP, Haile J, Munch K, Kuzmina S, Froese DG, Sher A, Holdaway RN, Willerslev E. 2009. Non-destructive sampling of ancient insect DNA. PLoS One, 4(4),e5048
73. Truett GE, Heeger P, Mynatt RL, Truett AA, Walker JA, Warman ML. 2000. Preparation of PCR-quality mouse genomic DNA with hot sodium hydroxide and tris (HotSHOT). Biotechniques, 29(1), 52–54.
74. Upton MS. 1993. Aqueous gum-chloral slide mounting media: an historical review. Bulletin of Entomological Research, 83(2), 267–274.
75. Volf P, Myskova J. 2007. Sand flies and *Leishmania*: specific versus permissive vectors. Trends in Parasitology, 23(3), 91–92.
76. Wang Q, Wang X. 2012. Comparison of methods for DNA extraction from a single chironomid for PCR analysis. Pakistan Journal of Zoology, 44(2), 421–426.
77. Wang Y, Zhao Y, Bollas A, Wang Y, Au KF. 2021. Nanopore sequencing technology, bioinformatics and applications. Nature Biotechnology, 39(11), 1348–1365.

Cite this article as: Randrianambinintsoa FJ, Augendre L, Prudhomme J, Martinet J-P, Loyer M, Mekarnia N, Kerkoub H, Perveen FK, Huguenin A, Kariya E, Akhoundi M, De Andrade AJ, Berriatua E, Bongiorno G, Boyer S, Christodoulou V, Da Costa-Ribeiro MCV, De Souza LAF, Ding H, Dondji B, Dvořák V, Erisoz Kasap O, Galati EAB, Gállego M, Ballart C, Gouzelou S, Haddad N, Masse RS, Mekuria AH, Ivovic V, Kaczmarek S, Shahar MK, Kirstein OD, Kniha E, Kolářová I, Lincoln T, Lucanas C, Mikov O, Nov K, Özbel Y, Pesson B, Posada Lopez LC, Prasetyo DB, Rahola N, Rebollar-Tellez EA, Rodrigues BL, Roy L, Saini P, Sanjoba C, Shimabukuro PH, Siriyasatien P, Soszynska A, Suleşco T, Sylla M, Torno M, Volf P, Vongphayloth K, Sinh Nam V, Wardhana A, Yessinou E, Zapata S, Gantier J-C & Depaquit J. 2026. Processing and mounting phlebotomine sand flies: a consensus guideline. Parasite xx, xx. https://doi.org/10.1051/parasite/2026009.

PARASITE

An international open-access, peer-reviewed, online journal publishing high quality papers on all aspects of human and animal parasitology

Reviews, articles and short notes may be submitted. Fields include, but are not limited to: general, medical and veterinary parasitology; morphology, including ultrastructure; parasite systematics, including entomology, acarology, helminthology and protistology, and molecular analyses; molecular biology and biochemistry; immunology of parasitic diseases; host-parasite relationships; ecology and life history of parasites; epidemiology; therapeutics; new diagnostic tools.
All papers in Parasite are published in English. Manuscripts should have a broad interest and must not have been published or submitted elsewhere. No limit is imposed on the length of manuscripts.

Parasite (open-access) continues **Parasite** (print and online editions, 1994-2012) and **Annales de Parasitologie Humaine et Comparée** (1923-1993) and is the official journal of the Société Française de Parasitologie.

Editor-in-Chief:
Jean-Lou Justine, Paris

Submit your manuscript at:
https://www.editorialmanager.com/parasite

Apêndice 1: Fundamentos teóricos bioquímicos.

Os artrópodes em questão são os flebotomíneos. No entanto, a ideia geral pode ser estendida a outros artrópodes muito comuns, para os quais a identificação só pode ser realizada com base em caracteres morfológicos internos. Por acaso, alguns órgãos internos são parcialmente quitinizados, e suas morfologias nos fornecem informações valiosas. É por isso que é muito interessante observar as bombas alimentares, as espermatecas e seus dutos. Com todos os reagentes que revisaremos, nunca se deve esquecer que, desde a etapa de fixação do inseto até a montagem, estaremos simplesmente aplicando reações redox. A única precaução ou princípio a ser seguido é evitar a mistura de reagentes redutores com reagentes oxidantes.

Álcool etílico; etanol:

Esta substância será utilizada de diferentes maneiras. As moléculas de álcool apresentam forte afinidade com a água e, portanto, exercem um efeito desidratante. No entanto, um álcool de baixa concentração (isto é, com excesso de água) desempenhará um papel na degradação de ácidos nucleicos (a água é inimiga dos ácidos nucleicos).

Quando os insetos são colocados em etanol, o objetivo não é apenas preservá-los, mas também fixar os tecidos. Em histologia, geralmente diferenciamos dois conceitos importantes: a taxa de penetração e a taxa de fixação. É bem compreendido que um bom preservativo deve primeiro penetrar rapidamente nos tecidos antes de fixá-los. Para álcool a 96%, o coeficiente de penetração é aproximadamente 1,05 (em comparação, para uma solução aquosa de ácido pícrico a 0,75%, o coeficiente de penetração é 0,45, enquanto para uma solução de dicromato de potássio a 3% é 1,45).

O desejo de preservar insetos e outros artrópodes indefinidamente em etanol é uma realidade para os entomologistas. A intenção de manter as capturas de campo para estudos subsequentes ou para futuros pesquisadores ainda é muito louvável. No entanto, essa prática não é viável para um citologista ou histologista. Ao manter as amostras no fixador por tempo excessivo, elas podem tornar-se praticamente impossíveis de serem reprocessadas. Por isso, amostras com mais de 10 anos são difíceis ou até impossíveis de serem utilizadas.

Outra consideração é a proporção entre a massa do artrópode a ser fixado e o volume do fixador. Na prática zoológica ou médica, recomenda-se prever um volume 60 vezes maior do que o volume das peças a serem fixadas. Na prática, para microartrópodes, para um determinado volume de espécimes a serem fixados, deve-se adicionar pelo menos 4–5 volumes de álcool. É importante ter em mente que o álcool perde sua força à medida que remove toda a água presente nos tecidos do artrópode.

Em conclusão:
- O álcool etílico é um agente químico redutor (portanto, incompatível com fixadores oxidativos);
- Precipita energicamente as proteínas e as desnatura;
- Dissolve certos lipídios complexos e precipita glicogênio;
- Provoca forte contração dos tecidos e os endurece.

Soluções básicas de hidróxido de potássio ou sódio:

O uso dessas soluções em entomologia tem se concentrado principalmente no hidróxido de potássio (KOH), sem uma justificativa clara.

O hidróxido de sódio (NAOH) [E524], também conhecido como soda cáustica, está disponível em solução, podendo apresentar diferentes concentrações ou normalidades. É encontrado em forma de escamas ou pequenos grânulos brilhantes. Sua maior desvantagem é ser altamente higroscópico (mais do que o KOH). Ao reagir com proteínas, dissolve-as, e com lipídios, transforma-os em sabões sólidos durante a saponificação (esta é uma diferença importante em relação ao KOH, que produz sabões líquidos durante a saponificação).

O hidróxido de potássio [E525] está disponível como solução concentrada, mas, acima de tudo, possui a vantagem de ser formulado em pastilhas de aproximadamente 0,1 g, o que facilita bastante a preparação de soluções diluídas quando não se dispõe de uma balança de precisão. Por exemplo, 1 pastilha de 0,1 g em 1 mL de água destilada gera uma solução a 10%. A segunda vantagem do hidróxido de potássio em pastilha é sua menor sensibilidade à carbonatação (uma solução de KOH possui alta afinidade para fixar CO_2, formando assim sais carbonatados).

Essas bases fortes serão utilizadas para solubilizar ácidos graxos, transformando-os em sabões solúveis em água. Deve-se lembrar que o fixador, como o etanol, já solubilizou parte das gorduras presentes na amostra. Entretanto, ao colocar a amostra em um meio aquoso contendo uma base forte, os ácidos graxos (mais ou menos complexos) precipitarão. A base forte realizará, portanto, uma saponificação a frio. Em alguns casos, quando os tecidos adiposos estiverem em excesso — por exemplo, em fêmeas — será vantajoso elevar a temperatura para 35–40°C a fim de facilitar a reação, ou então prolongar o tempo de contato em temperatura ambiente.

Solução ácida colorida / solução de Marc-André incolor:

Aqui, exploraremos as vantagens e inconvenientes do uso da solução de Marc-André. Esta solução é composta por hidrato de cloral (tricloroacetaldeído monohidratado), ácido acético e água. Trata-se de uma solução altamente oxidante (mistura de ácido e aldeído). Ela neutraliza o excesso de hidróxido de potássio que possa permanecer nas amostras, sem precipitar os sabões alcalinos formados durante o uso do hidróxido de potássio.

Esta solução oxidante também atua sobre as funções de álcool secundário das glucosaminas que compõem a quitina, oxidando-as e, assim, amolecendo a quitina. Outra ação importante é a dissolução de certos sais minerais presentes.

Quando a solução de Marc-André é previamente colorida com fucsina ácida (portanto em estado oxidado), ela consegue fixar-se nas funções de álcool secundário da

estrutura. Após o tempo de contato da solução de Marc-André e o estado de coloração das amostras, a lavagem será realizada apenas com etanol. Assim, inicia-se a fase de desidratação das amostras.

Benefícios:
- Neutralização do excesso de soluções básicas
- Relaxamento da quitina
- Coloração da quitina para melhor avaliação das estruturas internas quitinizadas

Desvantagens:
O cloral hidrato é hipnótico e já foi utilizado em medicina humana. Deve ser manipulado em capela química, respeitando-se a legislação vigente sobre riscos químicos.

Soluções de desidratação:
A experiência mostra que, para amostras muito pequenas, não é útil seguir a sequência de banhos de álcool com concentrações crescentes. Se a amostra for grande, inicia-se com etanol a 80%, seguido por 90%, 95% e, finalmente, etanol absoluto. Para amostras muito pequenas, utiliza-se um banho de etanol a 90%, seguido da imersão em etanol absoluto. Nesta etapa, deve-se sempre ter em mente que o etanol absoluto tende a fixar a água presente na atmosfera.

A tradição em laboratórios de entomologia era finalizar a desidratação das amostras com um banho de creosoto de faia. Atualmente, esta essência, amplamente utilizada como pesticida, antifúngico e conservante de madeira, é fortemente desaconselhada devido ao seu odor (hidrocarbonetos aromáticos policíclicos) e presume-se que seja nefrotóxica, carcinogênica, um poluente orgânico persistente e ecotóxica para organismos aquáticos.

Uma solução que propomos para preparar as amostras para montagem é uma mistura de Euparal® e essência de Euparal (descrita no parágrafo seguinte). Uma combinação de Euparal® com sua essência é muito bem aceita, especialmente para amostras obtidas após um banho de etanol a 90%.

Apêndice 2: Composição dos reagentes utilizados.

Hidróxido de potássio 10%
Hidróxido de potássio 10 g
Água destilada q.s.p. 100 mL

Meio de montagem goma-cloral (Hoyer)
Água destilada 50 mL
Cloral hidrato 200 g
Goma arábica 50 g
Glicerol 20 mL

Solução Marc-André
Hidrato de cloral 40 g
Ácido acético glacial 30 mL
Água destilada 30 mL

Ácido fucsina 1% em água destilada
Fucsina ácida em pó 1 g
Água destilada 99 mL

Solução Marc-André colorida com fucsina
Solução Marc-André 10 mL
Fucsina 1% 50 µ

Apêndice 3: Euparal®, Bálsamo do Canadá, álcool polivinílico ou outras soluções para montagem.

Álcool polivinílico: Este é o meio de montagem ideal quando os produtos necessários para a desidratação adequada não estão disponíveis. O álcool polivinílico é então misturado com solução de lactofenol de Amman. No entanto, essas preparações apresentam as principais desvantagens de secarem rapidamente ou de o álcool polivinílico cristalizar devido à evaporação da água, ou ainda escurecer quando o fenol oxida. Apesar disso, permanece uma boa técnica para montagens de curto prazo.

Bálsamo do Canadá: Seu uso para montagem entre lâmina e lamínula requer a desidratação prévia dos espécimes a serem montados. O uso de xileno ou tolueno não está isento de inconvenientes.

Meio Enecê: Para montagem entre lâmina e lamínula, semelhante ao Bálsamo do Canadá, é necessário desidratar o espécime. Formulação do Enecê: colofônia branca pura (22 g); goma de copal solúvel em álcool (12 g); etanol absoluto (20 mL); cânfora (10 g); essência de terebintina (10 mL); e eucaliptol (26 mL).

Para sua preparação, em um recipiente, como um frasco Erlenmeyer, coloca-se primeiro o álcool absoluto e a cânfora. Em seguida, adicionam-se a colofônia e a goma de copal. O frasco é então tampado e agitado, sendo aquecido em banho-maria a temperatura branda, evitando a ebulição da mistura. Uma vez que os componentes estejam completamente liquefeitos, adiciona-se a essência de terebintina, filtra-se a mistura ainda quente e, por fim, adiciona-se o eucaliptol ao filtrado. Quando o meio se torna menos fluido, ele é diluído com Enecê, utilizando a seguinte fórmula: etanol absoluto (30 mL), cânfora (17 g), essência de terebintina (15 mL), e eucaliptol (38 mL) (Cerqueira, 1943).

Euparal®: Trata-se de uma resina proveniente do Cipreste-do-Atlas *Tetraclinis articulata* (Vahl, 1791), estudada e desenvolvida em 1906 por Gilson. Sua principal vantagem é que não se polimeriza. As amostras montadas entre lâminas e lamínulas podem ser facilmente recuperadas pela ação do álcool ou, ainda melhor, da essência de **Euparal®**. Esta resina, também chamada sandaric ou sandáraca, é compatível com etanol a partir de 80%.

Utilização de Triton X-100: solução aquosa não-iônica

O Triton X-100 encontra-se na forma de solução aquosa não-iônica (solução de 4-(1,1,3,3-tetrametilbutil) fenil-polietilenoglicol, ou t-octilfenoxipolietoxietanol, éter de polietilenoglicol-tert-octilfenil), amplamente utilizado como detergente em biologia celular e molecular. Ele permite a permeabilização das membranas celulares e nucleares.

A preservação de insetos em álcool por muitos anos é comum. Infelizmente, a conservação em álcool não é ideal, e os artrópodes preservados dessa forma tornam-se muito difíceis de preparar para exame microscópico. Plásticos contendo amostras frequentemente se degradam, seguidos da evaporação do álcool. Em ambos os casos, o contato prolongado com álcool ou a secagem das amostras representa um problema real. Em 2008, Jonque publicou uma nota sobre a reidratação de aranhas utilizando um agente umectante, como o Agepon usado para filmes fotográficos [26]. Isso levou à ideia de utilizar agentes umectantes que não sejam detergentes potentes.

A seguir, um procedimento utilizando Triton X-100 em solução aquosa a 0,5%:

- Impregnar a amostra seca com álcool absoluto.
- Adicionar o volume necessário da solução de Triton X-100 a 0,5%, de modo que toda a amostra fique imersa.
- Deixar em repouso por cerca de 5 minutos ou mais. Todos os artrópodes devem tornar-se independentes na solução.
- Remover a solução de Triton X-100 e substituí-la pela solução de hidróxido de potássio.

Em seguida, a técnica é seguida conforme descrito anteriormente.

Apêndice 4: Meios de montagem Euparal® ou Bálsamo do Canadá – passo a passo

1. Os espécimes devem ser desidratados (a aparência turva ou leitosa indica desidratação inadequada).
2. A desidratação pode ser realizada por meio de concentrações crescentes de álcool etílico.
3. Os espécimes podem ser transferidos do álcool a 99% ou absoluto para um agente de clarificação.

Procedimento:

1. Coloque flebotomíneos adultos em etanol 70%.
2. Remover o etanol e substituir por KOH a 10%. Cobrir os flebotomíneos com uma lâmina de vidro.
3. Macerar até que os insetos se tornem transparentes.
4. Remover o KOH.
5. Cobrir o espécime com água destilada e aguardar 30 a 45 minutos.
6. Remover a água e repetir a lavagem com água destilada por 30 minutos (o tempo depende do número de amostras: quanto mais amostras processadas juntas, maior deve ser o tempo; para amostras individuais, o tempo pode ser menor).
7. Remover a água.
8. Adicionar solução Marc-André (eventualmente corada com ácido fucsina) e aguardar 24 horas (um dia).
9. Remover a solução Marc-André.
10. Cobrir o espécime com água destilada e aguardar 30 a 45 minutos.
11. Remover a água e repetir a lavagem com água destilada por 30 minutos.
12. Remover a água.
13. Adicionar etanol 70% e dissecar o espécime.
    a. Para a cabeça e o abdome, puxar delicadamente a cabeça ou o abdome do tórax.
    b. Para o tórax, remover as asas segurando o tórax com um par de pinças e puxando a base dos apêndices com outro par. É possível realizar uma dissecação sagital, dividindo o tórax em lados esquerdo e direito, dependendo das regiões de maior interesse.
14. Desidratar gradualmente através de uma série de soluções aquosas de álcool etílico: 50 – 80 – 95% até atingir etanol absoluto.
15. Desidratar os espécimes lavando-os duas vezes, 10 minutos cada, com etanol 100%.
16. Remover o etanol e cobrir os espécimes com óleo de cravo por 15 minutos à temperatura ambiente.
17. Transferir os espécimes do óleo de cravo para uma gota de Euparal® ou Bálsamo do Canadá em uma lâmina limpa.
18. Arranjar conforme desejado: a cabeça, o tórax e o abdome do flebotomíneo podem ser dissecados usando agulhas finas ou pinças sob um microscópio de dissecação. A cabeça deve ser dissecada do corpo para ser montada em posição ventro-dorsal, ou seja, o forame occipital deve estar voltado para cima para que o cibário possa ser observado diretamente através dele. A dissecação é realizada no meio de montagem do flebotomíneo.
19. Deixar o espécime até que a superfície fique pegajosa.
20. Umedecer uma lamínula limpa com álcool absoluto. Depositar a lamínula sobre o Bálsamo do Canadá em um ângulo.
21. Armazenar as lâminas em uma caixa seca destinada a este fim.
