# Supplementary material for: Processing and mounting phlebotomine sand flies: a consensus guideline
Source: Parasite. 2026 Apr 3;33:18. doi: 10.1051/parasite/2026009 (PMC13047900; doi:10.1051/parasite/2026009)
Supplement: Supplementary file 25 — Russian translation / Перевод на русский [file parasite-33-18-s25.pdf]

Parasite **33**, 18 (2026)
© F.J. Randrianambinintsoa et al., published by EDP Sciences, 2026
https://doi.org/10.1051/parasite/2026009

PARASITE
Available online at:
www.parasite-journal.org

REVIEW ARTICLE OPEN ACCESS

# Обработка и монтирование Phlebotominae москитов: согласованное руководство

Fano José Randrianambinintsoa[1], Laure Augendre[1], Jorian Prudhomme[1], Jean-Philippe Martinet[1], Mathieu Loyer[1], Nalia Mekarnia[1], Hocine Kerkoub[1], Farzana Khan Perveen[1], Antoine Huguenin[1,2], Emilie Kariya[1,2], Mohammad Akhoundi[3], Andrey José de Andrade[4], Eduardo Berriatua[5], Gioia Bongiorno[6], Sébastien Boyer[7,8], Vasiliki Christodoulou[9], Magda Clara Vieira Da Costa-Ribeiro[10], Lucas Alexandre Farias de Souza[10], Huicong Ding[11], Blaise Dondji[12], Vít Dvořák[13], Ozge Erisoz Kasap[14], Eunice Aparecida Bianchi Galati[15], Montserrat Gállego[16], Cristina Ballart[16], Stavroula Gouzelou[17], Nabil Haddad[18], Rezki Sabrina Masse[19], Asrat Hailu Mekuria[20], Vladimir Ivovic[21], Szymon Kaczmarek[22], Mohd Khadri Shahar[19], Oscar D. Kirstein[23], Edwin Kniha[24], Iva Kolářová[13], Lincoln Timinao[25], Cristian Lucanas[26], Ognyan Mikov[27], Kimsear Nov[7], Yusuf Özbel[28], Bernard Pesson[29], Laura Cristina Posada Lopez[30], Didot Budi Prasetyo[1,7], Nil Rahola[31], Eduardo A. Rebollar-Tellez[32], Bruno Leite Rodrigues[15], Lalita Roy[33], Prasanta Saini[34], Chizu Sanjoba[35], Paloma Helena Fernandes Shimabukuro[36], Padet Siriyasatien[37], Agnieszka Soszyńska[22], Tatiana Suleşco[38], Massamba Sylla[39], Majhalia Torno[40], Petr Volf[13], Khamsing Vongphayloth[41], Vu Sinh Nam[42], April Wardhana[43], Eric Yessinou[44], Sonia Zapata[45], Jean-Charles Gantier[1], and Jérôme Depaquit[1,2,*]

1 Faculté de Pharmacie, Université de Reims Champagne Ardenne, UR ESCAPE-USC ANSES PETARD, 51 rue Cognacq-Jay, 51096 Reims Cedex, France
2 Pôle de Biologie territoriale, Laboratoire de Parasitologie-Mycologie, Centre Hospitalo-Universitaire, 51092 Reims, France
3 Parasitology-Mycology Department, Avicenne Hospital, AP-HP, Bobigny, Sorbonne Paris Nord University, France; Unité des Virus Émergents (UVE: Aix-Marseille Univ, Università di Corsica, IRD 190, Inserm 1207, IRBA), 13005 Marseille, France
4 Parasitology Collection of Basic Pathology, Department of Basic Pathology, Federal University of Paraná, Curitiba 19031, Brazil
5 Department of Animal Health, University of Murcia, Campus de Espinardo, 30100 Espinardo, Murcia, Spain
6 Department of Infectious Diseases, Vector-borne Diseases Unit, Istituto Superiore di Sanità, 00166 Rome, Italy
7 Medical and Veterinary Entomology Unit, Institut Pasteur du Cambodge, Phnom Penh 12201, Cambodia
8 Ecology & Emergence of Arthropod-borne Pathogens Unit, Department of Global Health, Institut Pasteur, CNRS UMR2000, 75015 Paris, France
9 Section Veterinary Services (1417), Laboratory for Animal Health Virology, Aglantzia, Nicosia 2109, Cyprus
10 Insects Vectors and Parasites Laboratory, Department of Basic Pathology and Postgraduate program in Microbiology, Parasitology and Pathology, Federal University of Paraná, 81530-900 Curitiba, Brazil
11 Department of Biological Sciences, National University of Singapore, 117558, Singapore
12 Laboratory of the Leishmaniasis Research Project, Mokolo District Hospital, Mokolo, Cameroon; Laboratory of Cellular Immunology and Parasitology, Department of Biological Sciences, Central Washington University, 98926 Ellensburg, WA, USA
13 Department of Parasitology, Faculty of Science, Charles University, 12800 Prague, Czechia
14 VERG Laboratories, Department of Biology, Faculty of Science, Hacettepe University, Beytepe, Ankara 06800, Türkiye
15 Faculdade de Saúde Pública da Universidade de São Paulo (FSP/USP), Pós-graduação em Saúde Pública, 01246-904 São Paulo, Brazil
16 Secció de Parasitologia, Departament de Biologia, Sanitat i Medi Ambient, Facultat de Farmàcia i Ciències de l'Alimentació, Universitat de Barcelona, & Institut de Salut Global de Barcelona (ISGlobal), Centro de Investigación Biomédica en Red, Enfermedades Infecciosas (CIBERINFEC), 08028 Barcelona, Spain
17 Laboratory of Infectious Diseases and Public Health, School of Medicine, University of Cyprus, Nicosia, Cyprus & Department of Pediatrics, Archbishop Makarios III Hospital, Nicosia 2115, Cyprus
18 Faculty of Health Sciences, American University of Beirut, 1107 2020 Beirut, Lebanon
19 Medical Entomology Unit, Infectious Disease Research Centre, Institute for Medical Research (IMR), National Institutes of Health (NIH), Ministry of Health Malaysia, 40170 Shah Alam, Selangor, Malaysia

Edited by Jean-Lou Justine
*Corresponding author: jerome.depaquit@univ-reims.fr

This is an Open Access article distributed under the terms of the Creative Commons Attribution License (https://creativecommons.org/licenses/by/4.0), which permits unrestricted use, distribution, and reproduction in any medium, provided the original work is properly cited.

20 School of Medicine, Addis Ababa University, 28017 - 1000 Addis Ababa, Ethiopia
21 Faculty of Mathematics, Natural Sciences and Information Technologies, University of Primorska, 6000 Koper, Slovenia
22 University of Lodz, Faculty of Biology and Environmental Protection, Department of Invertebrate Zoology and Hydrobiology, Banacha 12/16, 90-237 Łódź, Poland
23 Laboratory of Entomology, Ministry of Health, 9134302 Jerusalem, Israel
[24] Center for Pathophysiology, Infectiology and Immunology, Institute of Specific Prophylaxis and Tropical Medicine, Medical University Vienna, Kinderspitalgasse 15, 1090 Vienna, Austria
[25] Papua New Guinea Institute of Medical Research (PNGIMR) Institute, PO Box 60, Headquarter, Homate Street, 441 Goroka, Eastern Highlands Province, Papua New Guinea
[26] Museum of Natural History, University of the Philippines Los Baños, 4031 Laguna, Philippines
[27] National Centre of Infectious and Parasitic Diseases, 1504 Sofia, Bulgaria
[28] Ege University, Faculty of Medicine, Department of Parasitology, 35040 Bornova/Izmir, Türkiye
[29] Retired, Faculté de Pharmacie, Université de Strasbourg, Strasbourg, 67400 Illkirch-Graffenstaden, France
[30] Program for the Study and Control of Tropical Diseases (PECET), Faculty of Medicine, University of Antioquia, 050010 Medellin, Colombia
[31] MIVEGEC, Univ. Montpellier, CNRS, IRD, 34394 Montpellier, France & Medical Entomology Unit, Institut Pasteur de Madagascar, 101 Antananarivo, Madagascar
[32] Laboratorio de Entomología Médica, Departamento de Zoología de Invertebrados, Facultad de Ciencias Biológicas, Universidad Autónoma de Nuevo León, San Nicolás de los Garza, 66455, NL, México
[33] Tropical and Infectious Disease Centre, BP Koirala Institute of Health Sciences, Dharan 56700, Nepal
[34] ICMR-Vector Control Research Centre, Puducherry 605006, India
[35] Graduate School of Agricultural and Life Sciences, The University of Tokyo, Tokyo 113-8657, Japan
[36] Grupo de estudos em Leishmanioses/Coleção de Flebotomíneos (COLFLEB/Fiocruz-MG), Instituto René Rachou, Fundação Oswaldo Cruz, Belo Horizonte, Minas Gerais, 30190009, Brazil
[37] Center of Excellence in Vector Biology and Vector-Borne Disease, Department of Parasitology, Faculty of Medicine, Chulalongkorn University, Bangkok 10330, Thailand
[38] Department of Arbovirology, Bernhard Nocht Institute for Tropical Medicine, Bernhard Nocht Str. 74, 20359 Hamburg, Germany
[39] Laboratory Vectors & Parasites, Department of Livestock Sciences and Techniques, Sine Saloum University El Hadji Ibrahima Niasse (SSUEIN) Kaffrine Campus, C.P. 24600, Senegal.
[40] Environmental Health Institute, National Environment Agency, Singapore 138667, Singapore & Department of Biological Sciences, National University of Singapore, 117558 Singapore
[41] Institut Pasteur du Laos, Laboratory of Vector-Borne Diseases, Samsenhai Road, Ban Kao-Gnot, Sisattanak District, 3560 Vientiane, Lao PDR
[42] National Institute of Hygiene and Epidemiology, 1 Yec-Xanh Street, Hai Ba Trung District, 100000 Hanoi, Vietnam
[43] Indonesian Research Center for Veterinary Science, Indonesian Agency for Agricultural Research and Development, Ministry of Agriculture Republic Indonesia, Bogor 16114, Indonesia & Department of Parasitology, Faculty of Veterinary Medicine, Airlangga University, Surabaya 60115, Indonesia
[44] Laboratory of Research in Applied Biology, Polytechnic School of Abomey-Calavi, University of Abomey-Calavi, 01 P.O. Box 2009, 00000 Cotonou, Benin
[45] Instituto de Microbiología, Colegio de Ciencias Biológicas y Ambientales (COCIBA), Universidad San Francisco de Quito (USFQ), 170901 Quito, Ecuador

Received 1 December 2025, Accepted 29 January 2026, Published online 3 April 2026

**Абстракт** – В данной статье представлены исчерпывающие рекомендации по обработке и подготовке микроскопических препаратов (монтирование) москитов, что имеет решающее значение для их видовой идентификации, обнаружения и выделения патогенов. В ней рассматривается ряд методов, подходящих как для полевых, так и для лабораторных условий. Руководство включает подробные инструкции по сбору, обращению и эвтаназии москитов (рекомендуется сухая заморозка или $CO_2$ вместо химикатов), а также стратегии консервации, такие как холодное хранение и консервирование в этаноле. Качество подготовки определённых анатомических структур (половых органов, головы и крыльев) имеет важное значение для их правильного микроскопического наблюдения и описано в данной работе. В статье также представлена детальная обработка образцов, включая процесс просветления с помощью таких агентов, как гидроксид калия, а затем раствора Марка-Андре. В процессе монтирования сравниваются различные среды, подчёркиваются их оптические свойства и потенциал консервации. Жидкость Хойера рекомендуется для быстрого наблюдения, особенно сперматек, благодаря своей прозрачности, хотя она не подходит для длительного хранения. Другие рассмотренные среды включают поливиниловый спирт, Euparal® (с ограниченной водостойкостью) и канадский бальзам (растворим в углеводородах), причём последние две обеспечивают возможность длительного сохранения. Также рассматриваются инновационные подходы молекулярной биологии, такие как секвенирование ДНК и MALDI-ToF, требующие особого внимания к обработке образцов. Кроме того,

прилагаются короткие видеоролики, иллюстрирующие различные методы монтирования образцов, а также переводы на 33 языка, что позволяет данному руководству учитывать разнообразные потребности и ожидания мирового научного сообщества.

**Ключевые слова:** Монтирование, Phlebotominae москит, жидкость Хойера, раствор Марка-Андре, хлораловая камедь, поливиниловый спирт, Euparal®, канадский бальзам, выделение *Leishmania*, полевые условия, культура, препарирование, молекулярная биология, MALDI-ToF, типовые образцы.

**Abstract – Processing and mounting phlebotomine sand flies: a consensus guideline.** This article provides a comprehensive guide for the processing and mounting of phlebotomine sand fly specimens, which is crucial for species identification and pathogen detection and isolation. It discusses a range of techniques suitable for both field and laboratory settings. The guide includes detailed instructions on sand fly collection, handling, covering, and euthanasia (recommending dry freezing or $CO_2$ over chemicals) as well as conservation strategies, such as cold storage and preservation in ethanol. The quality of preparation of certain anatomical structures (genital organs, head and wings) is essential for their proper microscopic observation and is described in this work. The article also presents detailed sample processing, including the clearing process with agents such as potassium hydroxide then Marc-André solution. The mounting process compares different media, emphasizing their optical properties and preservation potential. Hoyer fluid (also known as chloral gum) is recommended for quick observation, particularly for spermathecae, due to its clarity, although it is not suitable for long-term storage. Other media discussed include polyvinyl alcohol, Euparal® (for limited water tolerance), and Canada balsam (a hydrocarbon-soluble medium), with the latter two offering long-term preservation capabilities. Innovative molecular biology approaches such as DNA sequencing and MALDI-ToF, which require particular attention to sample processing, are also addressed. Furthermore, short video clips illustrating various mounting techniques as well as translations in many different languages are provided, allowing the guideline to reach the diverse needs and expectations of the global scientific community.

**Key words:** Mounting, Phlebotomine sand fly, Hoyer fluid, Marc-André solution, Chloral gum, Polyvinyl alcohol, Euparal®, Canada balsam, *Leishmania* isolation, Field conditions, Culture, Dissection, Molecular biology, MALDI-ToF, Type-specimens.

## Введение

Москиты — это мелкие двукрылые насекомые, принадлежащие к семейству Psychodidae, подсемейству Phlebotominae, насчитывающему как минимум 1063 известных вида [21]. Они являются важными переносчиками патогенов (*Leishmania*, арбовирусов и *Bartonella*), вызывающих заболевания, называемые соответственно лейшманиозом, арбовирусными инфекциями и бартонеллёзом. Их идентификация, главным образом, основана на детальном микроскопическом исследовании, облегчённая тщательным сбором, надлежащим хранением и аккуратным монтированием препаратов на предметных стёклах, требующие применения нескольких специфических методов, каждый из которых имеет свои преимущества и ограничения.

Идентификация взрослых москитов основана на наблюдении как внешних (напр. антенны, пальпы, гениталии самцов), так и внутренних структур (напр. глотка, цибариум и сперматека). Препарирование и выделение последних облегчает их наблюдение и, следовательно, точную идентификацию. Поэтому, в отличие от комаров или триатомовых клопов, перед идентификацией, москитов необходимо монтировать между предметным и покровным стёклами.

До 1980-х годов микроскопическое наблюдение было единственным доступным методом идентификации москитов, и оно остаётся наиболее широко используемым подходом и сегодня. Поэтому, выбор процесса и подготовки был относительно простым и, главным образом, основывался на дихотомии: с одной стороны, окончательное монтирование, позволяющее долговременно сохранять образец и, с другой стороны, быстрое монтирование для идентификации в среде, которая не обеспечивает долговременного сохранения. Окончательное монтирование, например, в смоле, такой как канадский бальзам, занимает много времени и требует полного обезвоживания образцов. Более того, показатель преломления этой среды не всегда оптимален для лёгкой визуализации сперматек. Монтирование в водорастворимой среде (напр. в жидкости Хойера), напротив, быстрее и позволяет лучше рассмотреть преломляющие свет сперматеки, но не позволяет долговременно сохранять препараты, поскольку она имеет тенденцию поглощать воду из атмосферы. Один из вариантов — запечатать предметное стекло лаком для ногтей после полного высыхания препарата. Этот компромиссный выбор сохраняется и по сей день, влияя на выбор метода монтирования, в зависимости от предполагаемого назначения препарата. Начиная с 1980-х годов, исследования по идентификации москитов сочетают морфологические и биохимические подходы. Первым был анализ кутикулярных

углеводородов, который быстро был заменён методами молекулярной биологии (напр. случайная амплификация полиморфной ДНК (RAPD), полиморфизм длины рестрикционных фрагментов (RFLP), амплификация ДНК и секвенирование по методу Sanger, а также секвенирование следующего поколения (NGS)). Сегодня молекулярные подходы дополняются протеомными методами, такими как MALDI-ToF. Более того, молекулярная идентификация видов может сочетаться с обнаружением патогенов

методом ПЦР (*Leishmania*, *Trypanosoma*, *Bartonella*, и *Phlebovirus*), поскольку все они могут быть обнаружены как с помощью ПЦР с детекцией по конечной точке, так и с помощью ПЦР в реальном времени, что требует адаптации процесса сбора и хранения к поставленным целям [3, 32]. В дополнение к морфологическим признакам, традиционно используемым для определения видов, могут применяться и другие морфологические подходы (напр. геоморфометрия крыльев).

Целью данного исследования, основанного главным образом на собственном опыте авторов и данных литературы, было разработать стандартизированные рекомендации по монтированию и обработке имаго москитов для оптимизации морфологического и молекулярного анализа.

Необходимость проведения определённого анализа (напр. молекулярная биология или MALDI-ToF) требует сохранения части москита, не нужного для морфологического определения, что подчёркивает необходимость критического выбора протокола.

В данной статье мы сосредоточимся на методах анестезии и эвтаназии москитов, пойманных живыми, их хранении и процессе монтирования для быстрого определения или долгосрочного хранения, позволяющего проводить последующие исследования.

## Преамбула: Вопросы безопасности и соблюдения нормативных требований должны ссылаться на соответствующие паспорта безопасности (SDS).

Все химические вещества, представленные в данном руководстве, должны использоваться в строгом соответствии с правилами техники безопасности. Комитеты по охране труда и технике безопасности исследовательских учреждений готовы предоставить вам информацию не только об опасностях этих химических веществ, но и о процедурах обращения с ними, и утилизации отходов. Однако, обязательно соблюдение инструкций по технике безопасности при их использовании и утилизации. Следует отметить, что все пользователи несут ответственность за соблюдение надлежащей и безопасной лабораторной практики, а также применимого законодательства и нормативных актов своей страны или исследовательского учреждения. Кроме того, некоторые химические вещества или их компоненты (напр. хлоралгидрат) регулируются законодательством некоторых стран. Список сокращений, используемых в данном документе, приведен в Таблице 1.

**Таблица 1:** Список сокращений.

| | |
|---|---|
| **BME** | Basal medium Eagle (Базальная среда Eagle) |
| **CDC** | Центры по контролю и профилактике заболеваний |
| **CMCP** | Camphor-monochlorophenol (Камфора-монохлорфенол) |
| **CMR** | Канцерогенное, мутагенное, репродуктивно-токсичное вещество |
| **COI** | Ген субъединицы I цитохром c-оксидазы |
| **CytB** | Ген цитохрома b |
| **ELISA** | Иммуноферментный анализ (ИФА) |
| **EtOH** | Этанол |
| **M199** | Среда M199 |
| **MALDI-ToF MS** | Масс-спектрометрия с времяпролетным анализом и лазерной десорбцией/ионизацией с использованием матрицы |
| **MEM** | Minimum essential media (Минимальная основная среда) |
| **NGS** | Next-generation sequencing (Секвенирование нового поколения) |
| **NNN** | Среда Novy-MacNeal-Nicolle |
| **PNOC** | Prepronociceptin gene (Ген препроноцицептина) |
| **RAPD** | Произвольно амплифицированная полиморфная ДНК (ПАПД) |
| **RFLP** | Полиморфизм длины рестрикционных фрагментов (ПДРФ) |
| **RI** | Refractive index (Показатель преломления) |
| **RNASS** | RNA stabilization solution (Раствор для стабилизации РНК) |
| **ТФУК** | Трифторуксусная кислота |
| **ДНК** | Дезоксирибонуклеиновая кислота |
| **ЛНДР** | Лаосская Народно-Демократическая Республика |
| **кПЦР** | Количественная ПЦР (ПЦР в реальном времени) |
| **ОТ-ПЦР** | ПЦР с обратной транскрипцией |
| **ПЦР** | Полимеразная цепная реакция |
| **РНК** | Рибонуклеиновая кислота |
| **РНКазы** | Рибонуклеазы |
| **BME** | Basal medium Eagle (Базальная среда Eagle) |
| **CDC** | Центры по контролю и профилактике заболеваний |

## 1. Отлов москитов

Имаго москитов можно собирать живыми или мёртвыми различными методами, такими как миниатюрные световловушки типа CDC, клейкие ловушки и аспираторы, ловушки Шеннона, или непосредственно в местах их отдыха в окружающей среде (напр. в приютах для животных). Эти методы включают размещение ловушек в подходящих местах обитания, привлечение москитов светом или другими аттрактантами ($CO_2$ или химические приманки) и их сбор для дальнейшего анализа, как описано в ряде публикаций [2, 3, 32, 36, 49].

Отлов живых москитов позволяет использовать их во всех последующих процессах, тогда как сбор мёртвых особей предотвращает выделение *Leishmania* или штаммов вирусов. Некоторые методы отлова, такие как клейкая бумага, регулярно приводят к потере органов у москитов (антенны, пальпы, крылья или ноги). Кроме того, касторовое масло, покрывающее клейкую бумагу, прилипает к москитам и должно быть удалено в начале обработки, обычно с помощью 15-минутной экспозиции в смеси этанола и диэтилового эфира в равных частях.

## 2. Эвтаназия образцов

После сбора, живых москитов необходимо умертвить. При некоторых методах сбора (напр. липкая бумага, световоловушки CDC, оснащённые банкой с моющим средством или этанолом) москиты погибают сразу после сбора. Молекулярная биология может быть применена к тем, которые были собраны непосредственно в этанол и к другим, если они, как можно быстрее, были помещены в этанол для хранения. Однако, ни один из этих методов умерщвления не позволяет проводить обработку насекомых методом MALDI-ToF. В дополнение, некоторые методы умерщвления могут привести к потере определённых морфологических признаков. Поэтому, крайне важно использовать соответствующее стандартное средство для умерщвления, чтобы обеспечить правильную идентификацию или длительное хранение в качестве ваучер образцов (т.е. образцы, законсервированные и хранящиеся для дальнейшего использования или сравнения). Такие химические вещества, как этилацетат, этиловый эфир, тетрахлорэтан и хлороформ, можно пропитать ватой и поместить в ёмкость с москитами для эвтаназии. С этими средствами умерщвления следует обращаться осторожно, следуя рекомендациям производителя, из-за их токсичности. Однако, мы не рекомендуем умерщвлять москитов хлороформом, поскольку, по нашему опыту, он плохо подходит для исследований в области молекулярной биологии. Учитывая опасный характер всех этих продуктов и их сомнительную пригодность для молекулярных анализов, использование этих химикатов, как правило, не рекомендуется.

Наиболее распространённый метод, позволяющий сохранить морфологию, ДНК или белки, — это сухая заморозка образцов. Образцы должны быть заморожены достаточно долго, чтобы полностью анестезировать их, но не настолько долго, чтобы они (i) высохли или (ii) жизнеспособность лейшмании была под угрозой, если цель состоит в том, чтобы выделить их *in vitro* из пищеварительного тракта москита. **Поэтому мы рекомендуем замораживание в течение 15–20 минут при -20°C, регулярно контролируя образцы, чтобы убедиться, что они лишь оцепенели, а паразиты *Leishmania* не погибли.**

Если морозильник недоступен, насекомых можно умертвить с помощью $CO_2$. В полевых условиях, где нельзя использовать баллоны с $CO_2$, образцы можно умертвить с помощью маленьких коммерческих баллонов с $CO_2$, используемых в «сифонах для газировки» (диспенсеры для напитков), но могут существовать ограничения на их транспортировку по воздуху. В крайнем случае, насекомых можно умертвить путём воздействия табачного дыма. Москитов отлавливают живыми в ловушку типа CDC, собирают с помощью аспиратора, удерживают в стеклянной трубке и подвергают воздействию табачного дыма, который убивает их в течение нескольких секунд. Этот метод применим во всех полевых условиях, даже в сложных условиях изоляции. Однако, поскольку стеклянная трубка пропитывается дымом, её нельзя использовать для последующего сбора и обработки живых москитов без тщательной очистки. Тем не менее, тот же неочищенный аспиратор можно использовать для эвтаназии москитов из других ловушек с целью фиксации. Также необходимо проверить, были ли все особи удалены из аспиратора. Эти методы совместимы с выделением лейшмании путём вскрытия кишечника.

## 3. Хранение образцов перед обработкой

Существует четыре основных метода фиксации перед манипуляцией:

### 3.1. Заморозка

Этот метод лучше всего проводить при температуре -20°C или, предпочтительно, -80°C. В настоящее время эти методы хранения используются шире, чем хранение в жидком азоте. Во всех случаях криоконсервацию необходимо проводить как можно быстрее после оцепенения образцов. Холодное хранение в морозильниках имеет преимущество в том, что позволяет полностью сохранить самих насекомых, а также РНК, ДНК и белки в целостности на протяжении всего периода хранения. Напротив, жидкий азот может серьёзно повредить крылья, ноги, пальпы и антенны,

часто ампутируя их и, иногда, удаляя ключевые морфологические признаки. Сухое морозильное хранение менее травматично для образцов, но не идеально для сохранения их хрупких органов. Важно отметить, что во время размораживания крылья, антенны, пальпы или ноги могут прилипнуть к пробиркам и в конечном итоге оторваться из-за конденсации. Однако, консервация путём замораживания не всегда осуществима в полевых исследованиях, поскольку требует доступа к морозильнику или контейнеру с жидким азотом. Замораживание полностью совместимо с обнаружением патогенов с помощью молекулярных методов без потери чувствительности, хотя для обнаружения и выделения РНК-вирусов требуется замораживание при -80°C или в жидком азоте, если необходимо длительное хранение. Однако, заморозка образцов не позволяет выделить лейшмании путём препарирования кишечника, за исключением случаев, когда москитов сначала погружают в паровую фазу, а затем в жидкий азот (напр. в пробирки, помещённые в чулок), имитируя криоконсервацию лейшмании.

## 3.2. Хранение в спирте (этанол или изопропиловый спирт)

Это, вероятно, наиболее распространённый метод хранения москитов. Он легко реализуем в полевых условиях, даже в сложных условиях без доступа к лаборатории. Консервация в спирте особенно подходит для морфологических исследований, поскольку хрупкие органы (крылья, ноги, антенны или пальпы) остаются неповреждёнными, если в пробирке нет пузырьков воздуха. Поэтому мы рекомендуем запечатать пробирку небольшим ватным шариком, чтобы удалить пузырьки воздуха, и поместить этикетку поверх ватного тампона (рис. 1). Оптимальная концентрация спирта остаётся предметом дискуссий. В целом, концентрации ниже 70% не рекомендуются [45, 66]. Более высокие концентрации эффективнее и на более длительный срок сохраняют ДНК, но делают образцы более хрупкими и ломкими для морфологических исследований. Использование 96% этанола (азеотропная смесь) обеспечивает стабильность концентрации с течением времени, особенно во влажных районах, таких как тропические страны, хотя 95% этанол часто легче получить. Независимо от концентрации, ДНК, в целом, хорошо сохраняется в этаноле (хотя и менее эффективно, чем при методах замораживания, особенно для молекулярных методов типа NGS). Белки гораздо менее стабильны, особенно для протеомики, например, при применении MALDI-ToF. Москиты, зафиксированные в спирте в течение нескольких месяцев, всё ещё могут быть идентифицированы морфологически, но получить эталонные спектры белков из этих образцов невозможно. Хранение в спирте или в сухих условиях можно улучшить, если образец также заморозить при -20 °C. Заморозка при -20 °C, в основном, улучшает молекулярную сохранность (напр. нуклеиновые кислоты) за счёт замедления деградации, а также обеспечивает дополнительное преимущество для сохранения морфологии за счёт уменьшения разрушения тканей с течением времени, хотя влияние на морфологию более ограничено, чем на молекулярную целостность. Хранение в этаноле также может применяться для обнаружения ДНК и РНК-вирусов при использовании этанола в концентрации не менее 70% в течение короткого периода хранения, менее нескольких месяцев. Также, изопропиловый спирт может быть легко доступен в некоторых странах и сохраняет ДНК, но делает образцы жёсткими. Он не воспламеняется, как этанол, и поэтому его легко транспортировать. При необходимости москитов, сохранённых в жидком азоте или подвергнутых сухой заморозке, можно перенести в спирт, тем самым объединив недостатки обоих методов.

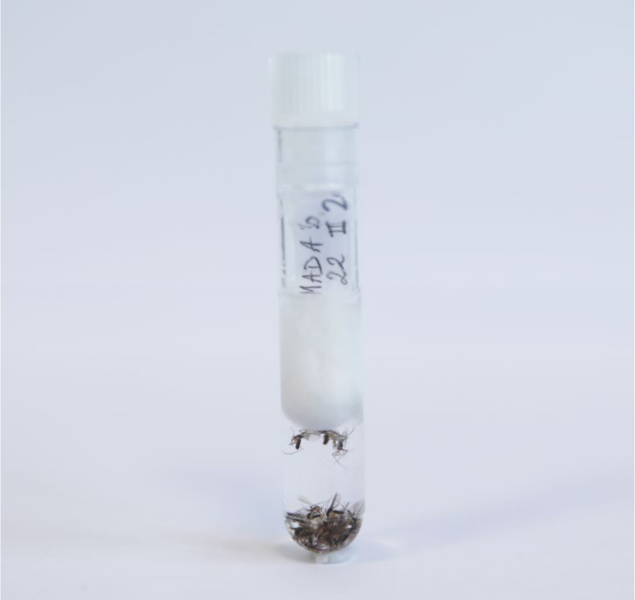

**Рисунок 1:** Москиты, сохранённые в этаноле.

## 3.3. Хранение в растворе для стабилизации РНК (RNASS)

Этот водный реагент широко используется, нетоксичен и предназначен для стабилизации и защиты РНК в свежих образцах тканей и клеток. Он действует быстро, проникая в образец и инактивируя РНКазы (ферменты, расщепляющие РНК), тем самым предотвращая деградацию РНК без необходимости немедленного замораживания. Хранение в RNASS, в целом, эффективно для сохранения общей морфологии тканей и клеток для последующей гистологической оценки. Хотя, RNASS оптимизирован для стабилизации

РНК, а не для фиксации, краткосрочное и среднесрочное хранение обычно хорошо сохраняет структурную целостность. RNASS позволяет хранить образцы при комнатной температуре до 7 дней, при 4°C в течение нескольких недель или при -20°C/-80°C для длительного хранения. Этот метод особенно ценен в полевых или клинических условиях, где инфраструктура холодовой цепи ограничена. Экстракция РНК обычно требует извлечения образцов из реагента и их обработку в соответствии со стандартными протоколами.

### 3.4. Сухое хранение при комнатной температуре

Это более старый метод, который при применении к цельному образцу (смонтированному целиком) имеет существенный недостаток, плохо сохраняя хрупкие органы, такие как крылья, ноги, антенны и пальпы. Однако, протеомные исследования, с использованием MALDI-ToF, остаются осуществимыми, если обезвоживание проводится после фиксации с помощью осушителя типа силикагеля. В отличие от этого, проведение молекулярных анализов ДНК с такими образцами остаётся сложным, поскольку ДНК часто фрагментирована и в малом количестве, что означает, что анализы остаются более сложными, чем со свежими или замороженными образцами, особенно для ядерных геномов. Однако, на образцах такого типа можно использовать современные методы, такие как музеомика [34]. Поэтому, этот метод хранения не рекомендуется, если нет альтернативы. Его можно комбинировать с холодным хранением, поместив пробирки в морозильник при -20°C или -80°C. Главная проблема заключается в обеспечении надлежащего монтирования образцов или частей тела, необходимых для идентификации. Чтобы этого добиться, необходима регидратация. Мы рекомендуем использовать раствор Triton X-100. Продолжительность регидратации варьируется от нескольких часов до нескольких дней при условии регулярного тщательного контроля. После полной регидратации, образцы следует промыть в трёх последовательных водяных банях.

### 3.5. Хранение на фильтровальной бумаге

Главное преимущество фильтровальной бумаги заключается в долговременной стабильности геномной ДНК внутри клеток нефиксированного, высушенного целого тела или клеток крови, хранящихся при комнатной температуре. Фильтровальная бумага поставляется в виде небольших карточек, что позволяет хранить несколько сотен образцов при комнатной температуре в объёме, эквивалентном объёму небольшого офисного ящика. Матрица фильтровальной бумаги пропитана веществами, денатурирующими инфекционные агенты, поэтому, образцы больше не считаются биологически опасными. Это позволяет хранить и транспортировать образцы без избирательных мер предосторожности против биологической опасности [68].

## 4. Препарирование образца

В отличие от многих других насекомых, идентификация которых основана на внешних признаках, видимых у отдельных насекомых, приколотых целиком, для точной идентификации вида москитов необходимо препарирование и монтирование на предметные стекла для изучения анатомических структур. Независимо от выбранной процедуры подготовки и монтирования, используется одна и та же техника препарирования (Рисунки 2 и 3) (https://zenodo.org/records/18198006).

**Применение Triton X-100 : неионогенный водный раствор**

Следует отметить, что речь идёт о монтировании свежеотловленных или надлежащим образом хранившихся образцов. Большинство коллекционеров хранят образцы насекомых в сухом виде (для использования в MALDI-ToF) или в спирте в течение многих лет. К сожалению, консервация в спирте на протяжении нескольких лет, не является оптимальной и членистоногих, зафиксированных таким образом, становится очень трудно подготовить к микроскопическому исследованию. Часто происходит деградация пластика, содержащего образцы, с последующим испарением спирта. В результате, образцы либо слишком долго остаются в спирте или высыхают. Поэтому, возникла идея использовать смачивающие вещества, которые не являются сильными детергентами. Triton X-100 представляет собой неионогенный водный раствор (*раствор 4-(1,1,3,3-тетраметилбутил) фенилполиэтиленгликоля* или *т-октилфеноксиполиэтоксиэтанол*, *трет-октилфениловый эфир полиэтиленгликоля*), широко используемый в качестве детергента в клеточной и молекулярной биологии. Он позволяет повысить проницаемость клеточных и ядерных мембран.

Ниже описана процедура с использованием неионогенного раствора Triton X-100 в 0,5% водном растворе:

Пропитайте сухой образец абсолютным спиртом.

Добавьте необходимый объем 0,5% раствора Triton X-100 так, чтобы весь образец был погружен в него.

Оставьте действовать от 5 минут до нескольких дней, регулярно контролируя процесс. Все членистоногие должны быть полностью отделены от субстрата в растворе.

Удалите раствор Triton X-100 и замените его раствором гидроксида калия.

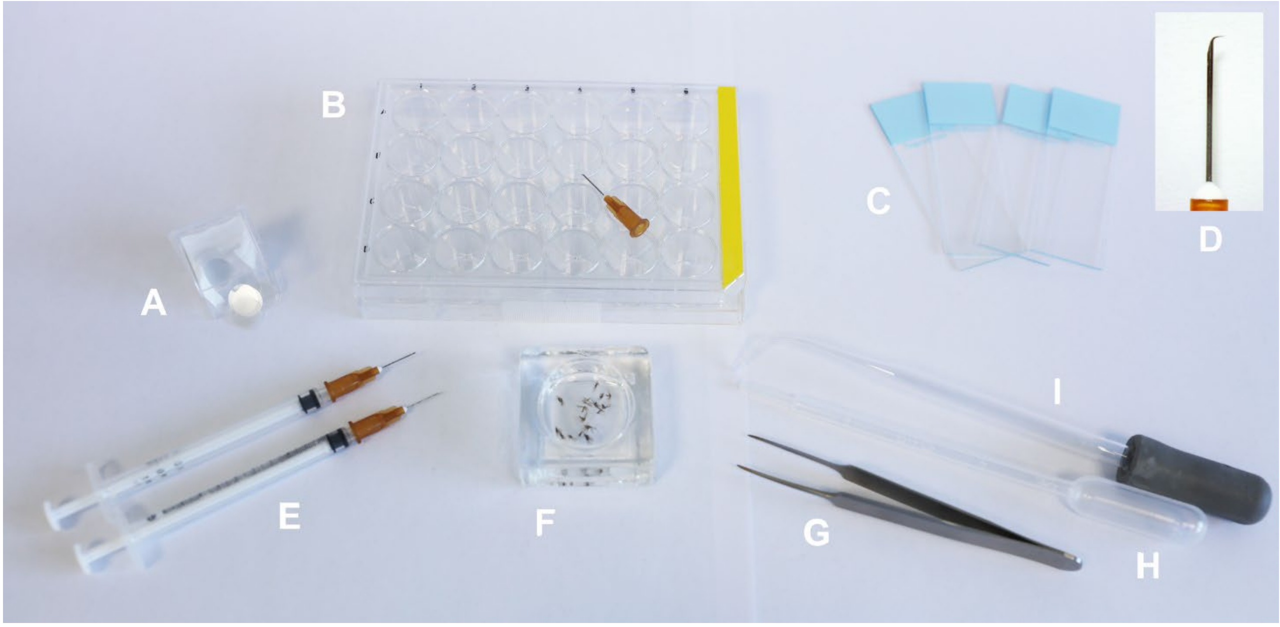


**Рисунок 2**: Материалы, необходимые для монтирования москитов: A: круглые стеклянные покровные стекла (диаметром 10 или 12 мм); B: 24-луночный планшет и игла с крючком (если вы используете гвоздичное масло или эссенцию Euparal® для обработки москитов, не используйте акриловые планшеты, так как произойдёт химическая реакция, и образцы будут повреждены); C: стеклянные предметные стекла, подходящие для этикетирования; D: деталь крючка иглы; E: иглы, прикреплённые к шприцам; F: часовое стекло или аналогичный контейнер с москитами для монтирования; G: пинцет Dumont; H: пластиковая пипетка; I: стеклянная пипетка, согнутая путём нагревания для облегчения переноса жидкости в лунки.

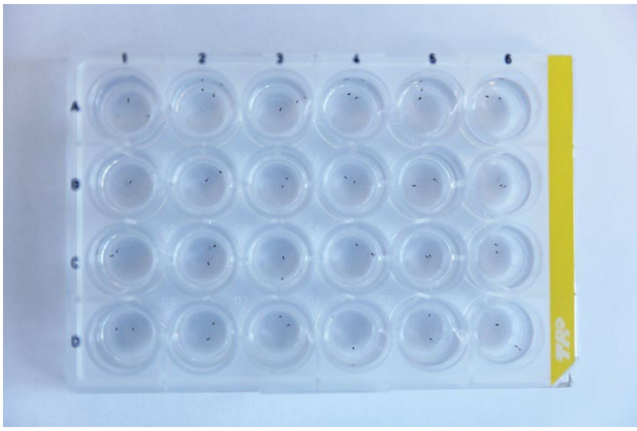

**Рисунок 3**: Планшет с 24 лунками, в каждой из которых находятся голова и кончик брюшка москитов.

## 4.1. Голова

Препарирование можно проводить с помощью тонких игл или энтомологических булавок под стереомикроскопом (Рисунки 2 и 3). Наиболее часто используемые иглы: 26G × 1/2" (0,45 × 13 мм), 30G × 1/2" (0,3 × 13 мм) или 25G × 5/8" (0,5 × 16 мм). При подготовки образца для определения, как минимум, голову отделяют от тела и монтируют вентральной стороной вверх, чтобы показать цибариум и глотку, в то время как грудь и брюшко монтируют латерально после препарирования. Монтирование головы в вентро-дорсальном положении обеспечивает ориентацию затылочного отверстия вверх, что позволяет лучше визуализировать цибариум. Доступ к этим анатомическим структурам облегчается, если голова полностью отделена.

## 4.2. Крылья и грудь

Крылья должны быть смонтированы в плоском положении. Каждое крыло может быть отделено у его основания и смонтировано независимо, или же может быть смонтировано только одно крыло, а другое оставлено прикреплённым к груди. Если планируется анализ геометрической морфометрии, необходимо правильно идентифицировать и обозначить правое и левое крылья перед монтированием. Грудь состоит из несколько частей и каждая содержит очень важную таксономическую информацию [20, 64]. Обычно её закрепляют в латеральной проекции, чтобы позволить изучить хетотаксию и распределение окраски. Наличие мест прикрепления щетинок в определённых областях груди может быть использовано для различения некоторых видов рода *Brumptomyia*. Распределение окраски может быть использовано для разделения

неотропических москитов на уровне рода (напр. *Bichromomyia*), видовой серии (напр. *Pintomyia*) или даже видов одного рода (напр. *Micropygomyia*, *Nyssomyia*, *Psathyromyia* и *Psychodopygus*) [20]. Таким образом, если грудь не используется для молекулярного анализа, её следует закрепить таким образом, чтобы не повредить. Важно отметить, что имеет значение не интенсивность окраски, а их распределение на груди. Поэтому, процесс осветления не устраняет пигментацию или её рисунок.

## 4.3. Гениталии

Особое внимание следует уделять монтированию гениталий как у самцов, так и у самок, поскольку они имеют решающее значение для идентификации родов, подродов и видов. У обоих полов гениталии парные.

### 4.3.1. Самцы

Гениталии наружные и состоят из парных щипцов, каждый из которых включает в себя сочленение гонококсита и гоностиля в дорсальной части, а в вентральной части располагается эпандриальная лопасть. Гоностиль несёт шипы и иногда щетинки, которые должны поддаваться подсчёту, а места их прикрепления должны быть чётко видны. Важно внимательно осмотреть внутреннюю поверхность гонококсита, на которой может находиться пучок сидячих щетинок или щетинки, расположенные на лопасти (= бугорке) [22]. Коллеги с меньшим опытом в препарировании могут выполнить простое боковое препарирование, не отделяя гениталии от конца брюшка (https://zenodo.org/records/18311158). В этом случае наложение двух частей гениталий может, например, затруднить подсчёт внутренних щетинок гонококсита, но это позволяет избежать повреждения гениталий из-за неудачного препарирования. Более опытные коллеги могут попытаться разрезать гениталии на две части, чтобы отделить их. Для этого скошенная сторона иглы (игла для внутрикожных тестов) должна пройти сквозь, отделив, но не полностью разрезав гениталий, чтобы разделить гонококсито-гоностильную связку (https://zenodo.org/records/18311158). Таким образом, улучшится наблюдение за их внутренними поверхностями. Эта техника также позволяет лучше визуализировать парамеры и парамеральные оболочки, которые больше не перекрываются. Для латеральной фиксации, которая часто приводит к наложению органов, образцы должны быть идеально просветлены.

### 4.3.2. Самки

Половой аппарат внутренний и состоит из сперматек. При отсутствии препарирования их необходимо наблюдать через тегументы, монтируя брюшко в вентральном положении. Независимо от выбранной среды для монтирования, сами сперматеки, как правило, можно наблюдать правильно, особенно если они не гладкие и прозрачные. Однако, визуализация гладких, тонкостенных сперматек может быть проблематична в средах с низкой преломляющей способностью. Кроме того, визуализация основания протоков сперматек имеет важное значение для идентификации видов, например, у подвида *Larroussius* [35, 37, 38], основных переносчиков *Leishmania infantum* в Старом Свете. Без этого наблюдения определение образца остаётся невозможным. Для преодоления этих трудностей, необходимо извлечь комплекс «генитальная фурка-сперматеки» из брюшной полости (https://zenodo.org/records/18311106). Сперматеки, как правило, трудно визуализировать во время препарирования, но генитальную фурку относительно легко обнаружить. Поскольку семяпроводы открываются в половую фурку, отделение этой фурки обычно позволяет изолировать сперматеки. Если сперматеки случайно повреждаются в процессе, они не теряются и их все ещё можно визуализировать в брюшном покрове (рис. 4).

## 4.4. Препарирование средней кишки для выделения паразита *Leishmania*

Препарирование пищеварительного тракта необходимо для обнаружения и выделения лейшмании у самок москитов. Процедура может проводиться как в полевых, так и в лабораторных условиях для оценки векторной компетентности.

Рекомендуется работать со свежеумершвлёнными самками. Промойте самок водой или физиологическим раствором, содержащим мягкое моющее средство, чтобы удалить излишки волосков. Этот шаг помогает поддержать асептические условия для выделения лейшмании, сохраняя при этом морфологические признаки, необходимые для идентификации. Для обнаружения и выделения лейшманий, аккуратно удалите среднюю кишку и поместите её в одну каплю стерильного физиологического раствора (0,9% NaCl). После визуализации подвижных паразитов под световым микроскопом (рекомендуемое увеличение: ~200×) используйте инсулиновый шприц или микропипетку для переноса их в среду для культивирования (более подробно в главе 4.4.3).

Поместите голову и гениталии в раствор Марка-Андре для их просветления. Важно: ни в коем случае не допускайте контакта раствора Марка-Андре с лейшманиями – ни напрямую, ни косвенно через инструменты или иглы – так как это смертельно для паразитов.

Препарирование самок москитов можно проводить как на одном, так и на двух предметных стёклах; оба варианта имеют свои преимущества и ограничения (рис. 5; https://zenodo.org/records/18311154).

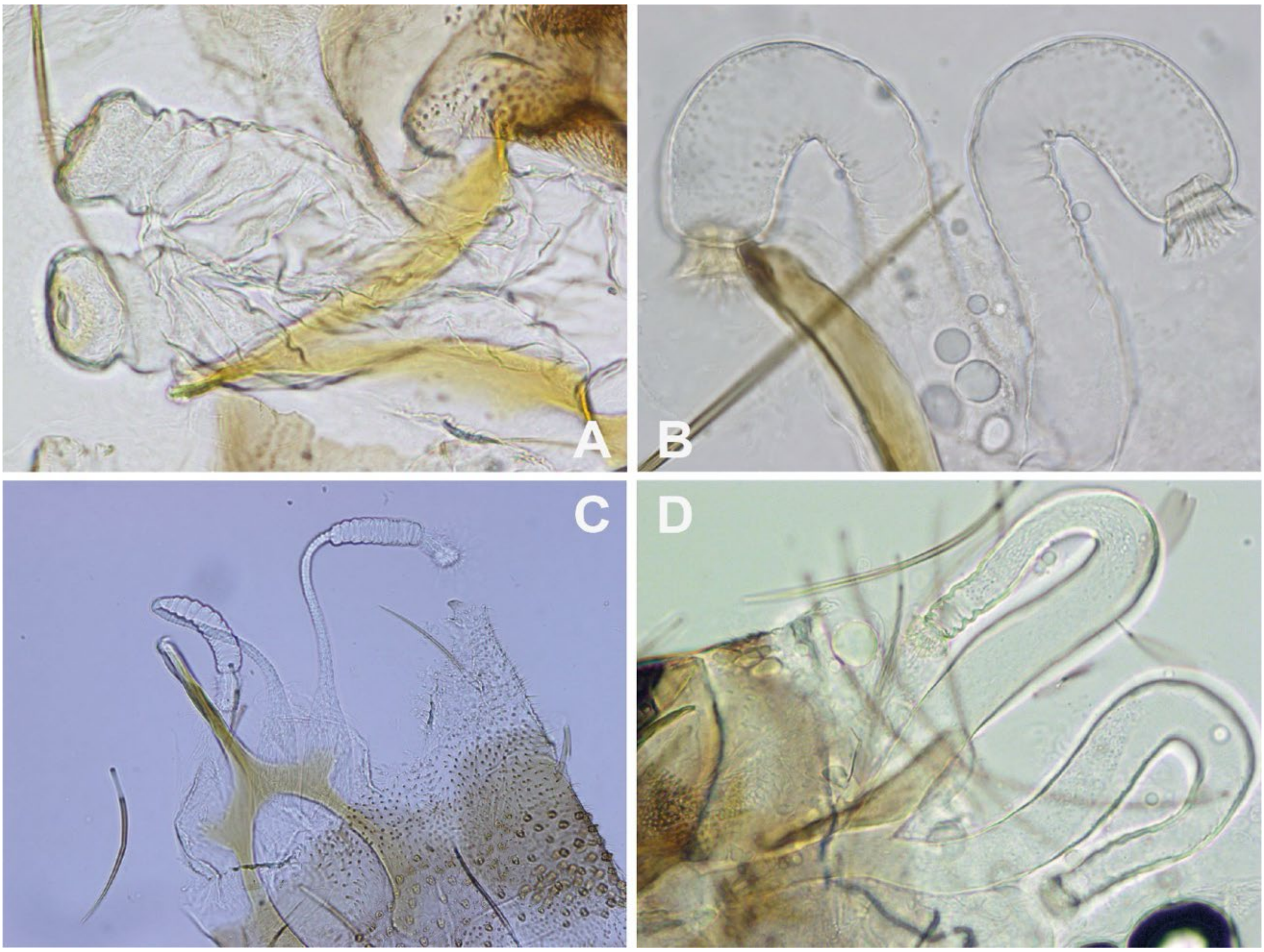


**Рисунок 4**: Сперматеки, препарированные из свежих образцов и помещённые в раствор Марка-Андре. A: *Idiophlebotomus longiforceps* (ЛНДР); B: *Sergentomyia minuta* (Франция); C: *Phlebotomus ariasi* (Франция); D: *Sergentomyia anodontis* (ЛНДР).

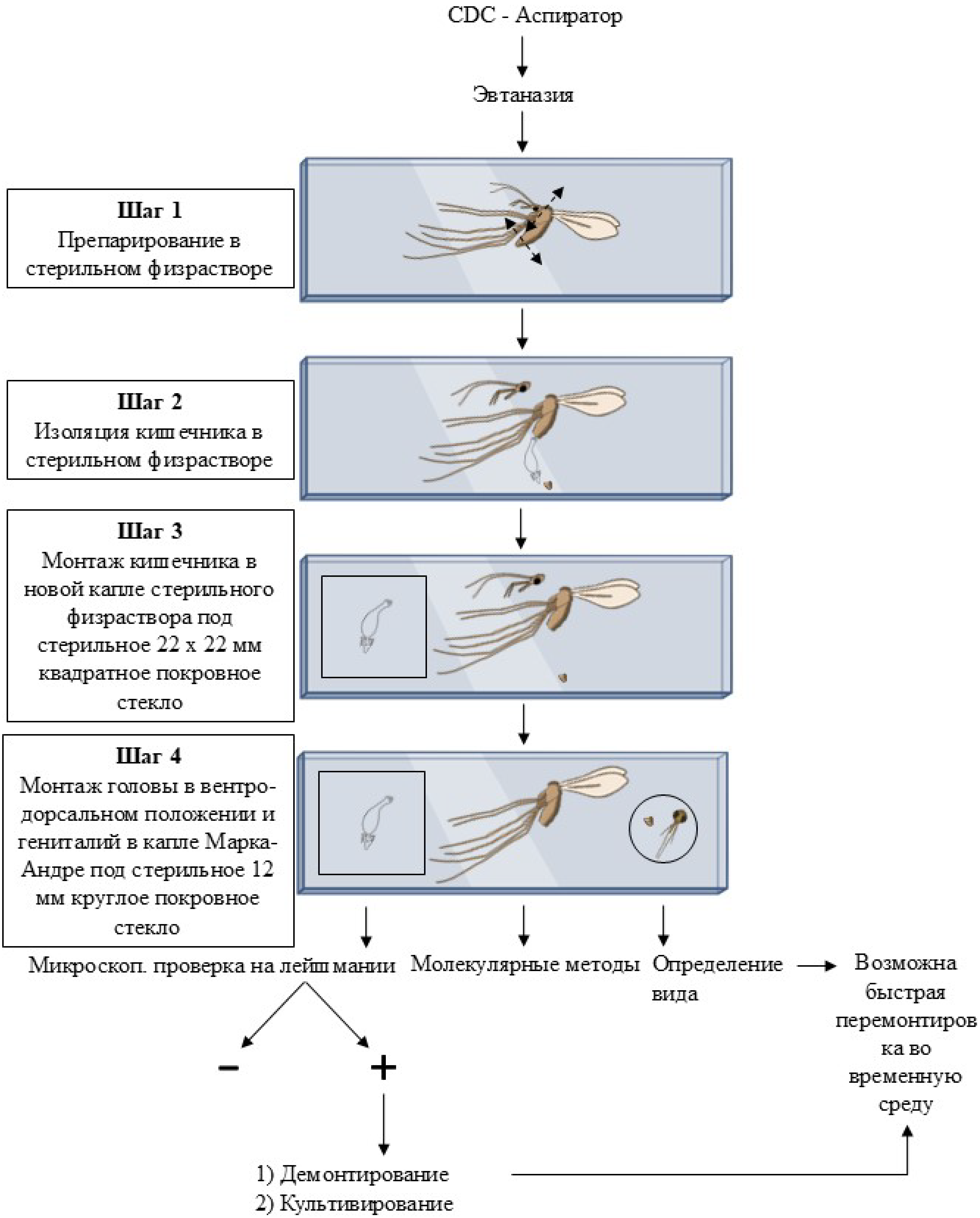


**Рисунок 5:** Метод выделения паразита *Leishmania*

This is an Open Access article distributed under the terms of the Creative Commons Attribution License (https://creativecommons.org/licenses/by/4.0), which permits unrestricted use, distribution, and reproduction in any medium, provided the original work is properly cited.

#### 4.4.1. Метод двух стёкол

Первый вариант предполагает работу с двумя отдельными предметными стёклами: одно содержит стерильный физиологический раствор для извлечения средней кишки, а другое — для фиксации головы и сперматек в растворе Марка-Андре. Однако, в полевых условиях, обычно два или три человека препарируют москитов и передают результаты своих препарирований одному исследователю, ответственному за идентификацию вида и оценку инфицирования кишечника паразитом *Leishmania*. Работа с двумя предметными стёклами может создавать проблемы с отслеживаемостью образцов и, в частности, затруднять точное определение того, какой именно москит был инфицирован, если обнаружен положительный результат на инфекцию кишечника (https://zenodo.org/records/18311154
).

#### 4.4.2. Метод одного стекла

Использование одного предметного стекла обеспечивает прослеживаемость результатов. Однако, следует соблюдать ряд мер предосторожности. Для обеспечения максимальной стерильности на этом этапе, работники должны регулярно мыть руки гидроспиртовым гелем. Необходимо использовать предметные стекла без матового покрытия и квадратные покровные стекла (22 x 22 мм), завёрнутые в алюминиевую фольгу и стерилизованные сухим жаром (с использованием печи Пупинеля), а также стерильные иглы для каждого препарирования (рекомендуется: 25G Ø 0,5 мм × 16 мм). Москит помещается в каплю стерильного физиологического раствора в центре предметного стекла. Голова отсекается, после этого делается разрез между 6-м и 7-м брюшными тергитами и стернитами, не перерезая пищеварительный тракт (можно сделать разрез выше, если ожидаются очень длинные сперматеки). Затем, грудь необходимо обездвижить иглой, а последние задние брюшные сегменты осторожно оттягиваются другой иглой для извлечения кишечника. Если это не удаётся, можно перекрыть конец брюшка иглой и извлечь пищеварительный тракт из его передней части. Если и это не удаётся, необходимо извлечь кишечник, удалив как можно больше оставшейся оболочки вокруг него. После извлечения кишечника, необходимо отделить последние сегменты брюшка, разрезав пищеварительный тракт. Затем кишечник помещают в новую каплю стерильного физиологического раствора, расположенную с одной стороны предметного стекла, и аккуратно накрывают стерильным покровным стеклом. Голову и последние сегменты брюшка переносят в небольшую каплю раствора Марка-Андре, расположенную на другом конце предметного стекла, избегая контакта с лейшманиями. Голову правильно ориентируют (затылочное отверстие вверх), а сперматеки изолируют вместе с генитальной фуркой, как указано выше, и накрывают небольшим круглым покровным стеклом (Ø 12 мм, не путать со стерильными квадратными покровными стёклами). Оставшееся тело москита и крылья остаются в капле физиологического раствора в центре предметного стекла (https://zenodo.org/records/18311154). В случае положительного результата или для таксономического исследования грудь и брюшко можно сохранить для молекулярных или протеомных исследований, а крылья можно смонтировать в среду на водной основе. Для обеспечения сохранности препарата излишки раствора Марка-Андре можно заменить средой на водной основе для монтирования, такой как хлоралова камедь (= жидкость Хойера) или средой на основе поливинилового спирта (ПВС).

Подробные видеоролики, демонстрирующие эти процедуры, доступны (препарирование средней кишки москита: https://zenodo.org/records/18303014 и препарирование слюнных желез москита: https://zenodo.org/records/18302850), поэтому здесь они не будут объясняться.

#### 4.4.3. Выделение и культивирование паразита *Leishmania* из кишечника москитов

Выделение паразита путём препарирования инфицированных самок москитов является деликатной процедурой, требующей высокого мастерства, и её следует сначала отработать на образцах, свободных от паразитов. После препарирования, кишечник переносится в свежую каплю стерильного физиологического раствора (0,9 %) или раствора Локка (Lock solution) для промывания [4]. Затем, препарированный кишечник можно обрабатывать двумя способами: i) исследовать под световым микроскопом для визуализации различных стадий промастигот лейшмании и их локализацию, уделяя особое внимание стомодеальному клапану, и ii) вскрыть кишечник для облегчения выхода промастигот, что облегчает их массовое культивирование [4]. Обнаружение инфицированных москитов в полевых условиях является относительно редким явлением, поэтому хорошая практика позволит максимально увеличить шансы на успешное выделение.

Если в кишечнике обнаружены лейшмании, следует использовать новые стерильные иглы и добавить небольшое количество стерильного физиологического раствора вокруг покровного стекла для их высвобождения за счёт капиллярного действия. Чтобы выпустить паразитов в солевой раствор, необходимо осторожно и быстро вскрыть пищеварительный тракт. Затем, с помощью микропипетки объёмом 100 мкл или туберкулинового шприца, паразитов собирают и инокулируют в, соответствующим образом, обозначенную среду для культивирования.

*In vitro* культивирование промастигот *Leishmania*: выделенные паразиты первоначально поддерживаются на агаре SNB-9 с прослойкой крови или на твёрдой среде Novy, McNeal, Nicolle (NNN) [16], покрытой либо стерильной средой alpha-MEM [16, 65], либо средой M199, каждая из которых дополнена 10% стерильной, инактивированной нагреванием фетальной телячьей сывороткой (FCS), 1% витамином BME, 2% стерильной человеческой мочой (стерилизованной фильтрацией через шприц Filtropur® S с порами 0,2 мкм) и 250 мкг/мл амикацина (или 50 мкг/мл гентамицина, или смесь антибиотиков и аминокислот (200 мМ L-глутамина, 10 000 ЕД пенициллина, 10 мг/мл стрептомицина)) [47]. Через три дня, при отсутствии загрязнения, культуры суспендируют в подходящей среде для замораживания, а затем хранят при -80°C в течение 1–2 лет или в жидком азоте при -196°C для длительного хранения и последующего экспериментального использования [7]

## 4.5. Слюнные железы

Препарирование слюнных желез москитов является фундаментальным методом изучения взаимодействия переносчика и патогена, особенно для обнаружения арбовирусов рода *Phlebovirus* (напр. вирус Тоскана) [44, 75]. Из-за крошечных размеров москитов эта процедура требует большой точности под стереомикроскопом, с использованием тонких щипцов или препаровальных игл для выделения нежных слюнных желез, не повреждая и не загрязняя их (https://zenodo.org/records/18302850) [51, 61]. Сохранение целостности желез имеет решающее значение для обеспечения надёжного последующего молекулярного анализа. После извлечения, железы можно гомогенизировать и протестировать с помощью ОТ-ПЦР, кПЦР или иммуноанализа для обнаружения вирусной РНК или антигенов [12]. Присутствие вирусов в слюнных железах, а не только в кишечнике или гемоцеле, подтверждает, что патоген завершил своё развитие в переносчике и может передаваться во время кровососания [71].

Процесс препарирования технически сложен из-за небольшого размера слюнных желез москитов, что требует значительной квалификации для предотвращения деградации образцов [1, 51]. Кроме того, вирусная нагрузка в слюнных железах может быть низкой, что требует применения высокочувствительных методов обнаружения, таких как вложенная ПЦР или высокопроизводительное секвенирование [54]. Риск заражения ещё раз подчёркивает необходимость использования стерильных методов. Помимо технических трудностей, на успех обнаружения влияют биологические факторы; векторная компетентность различается у разных видов москитов, а показатели инфицирования колеблются в зависимости от экологических и сезонных условий [33, 61].

Обнаружение вирусов в слюнных железах даёт критически важную информацию о рисках передачи, позволяя проводить целенаправленный мониторинг и меры контроля [15]. Например, идентификация вируса Тоскана в москитах в эндемичных регионах повлияла на разработку диагностических протоколов и рекомендаций по охране общественного здоровья [18]. Более того, изучение взаимодействия вируса со слюной москитов может выявить новые мишени для разработки вакцин или терапевтических стратегий, направленных на блокирование передачи [15, 18].

Слюнные железы москитов также могут быть использованы в качестве источника антигенов для изучения антител хозяина к слюне москитов с помощью иммунологических методов, предпочтительно ELISA. Этот метод позволяет оценить степень подверженности хозяина укусам москитов, тем самым способствуя оценке эффективности методов борьбы с переносчиками [25] и риска передачи лейшмании [40].

## 4.6. Идентификация источников питания кровью

Напитавшихся кровью самок, отделённых от пойманных особей, следует препарировать с помощью одноразового оборудования, чтобы предотвратить перекрёстное загрязнение. Их брюшко следует осмотреть под стереомикроскопом для оценки стадии переваривания крови. Рекомендуется отбирать только самок с красным, красновато-коричневым или тёмно-красным брюшком, не проявляющих признаков образования яиц. Удалите кончик брюшка, включая сперматеки, чтобы после просветления можно было провести морфологическую идентификацию самки. Основная часть брюшка (без сперматек) затем должна быть помещена в пробирки Eppendorf® и храниться при -20°C до дальнейшего анализа. Генетические маркеры, обычно используемые для идентификации источника крови, такие как PNOC [5, 30, 50], CytB [67] или COI [13], хорошо известны и подробно описаны в литературе; поэтому они не будут подробно рассматриваться в данной статье (рис. 6). В качестве альтернативы, для идентификации крови хозяина может быть использовано пептидное картирование MALDI-ToF [31]. Экспериментально доказано, что этот метод позволяет идентифицировать кровь хозяина в течение более длительного периода времени после приёма крови; поэтому он является подходящим методом, особенно для анализа насытившихся кровью самок с заметно более выраженным перевариванием крови хозяина. Образцы в идеале следует хранить при -20°C или 4°C, но хорошие результаты могут быть получены и из образцов, хранившихся при комнатной температуре в течение короткого времени. Брюшко насытившейся кровью самки следует отделить от

остальной части тела незадолго до анализа и гомогенизировать в дистиллированной воде. Остальная часть тела москита остаётся доступной для других молекулярных и морфологических анализов. После отбора аликвоты из гомогената для пептидного картирования методом MALDI-ToF, остальная часть может быть использована для выделения ДНК с целью подтверждения идентификации крови хозяина и/или скрининга на наличие *Leishmania* sp. Общее время подготовки и анализа образцов очень короткое по сравнению с молекулярными методами, основанными на ДНК анализе.

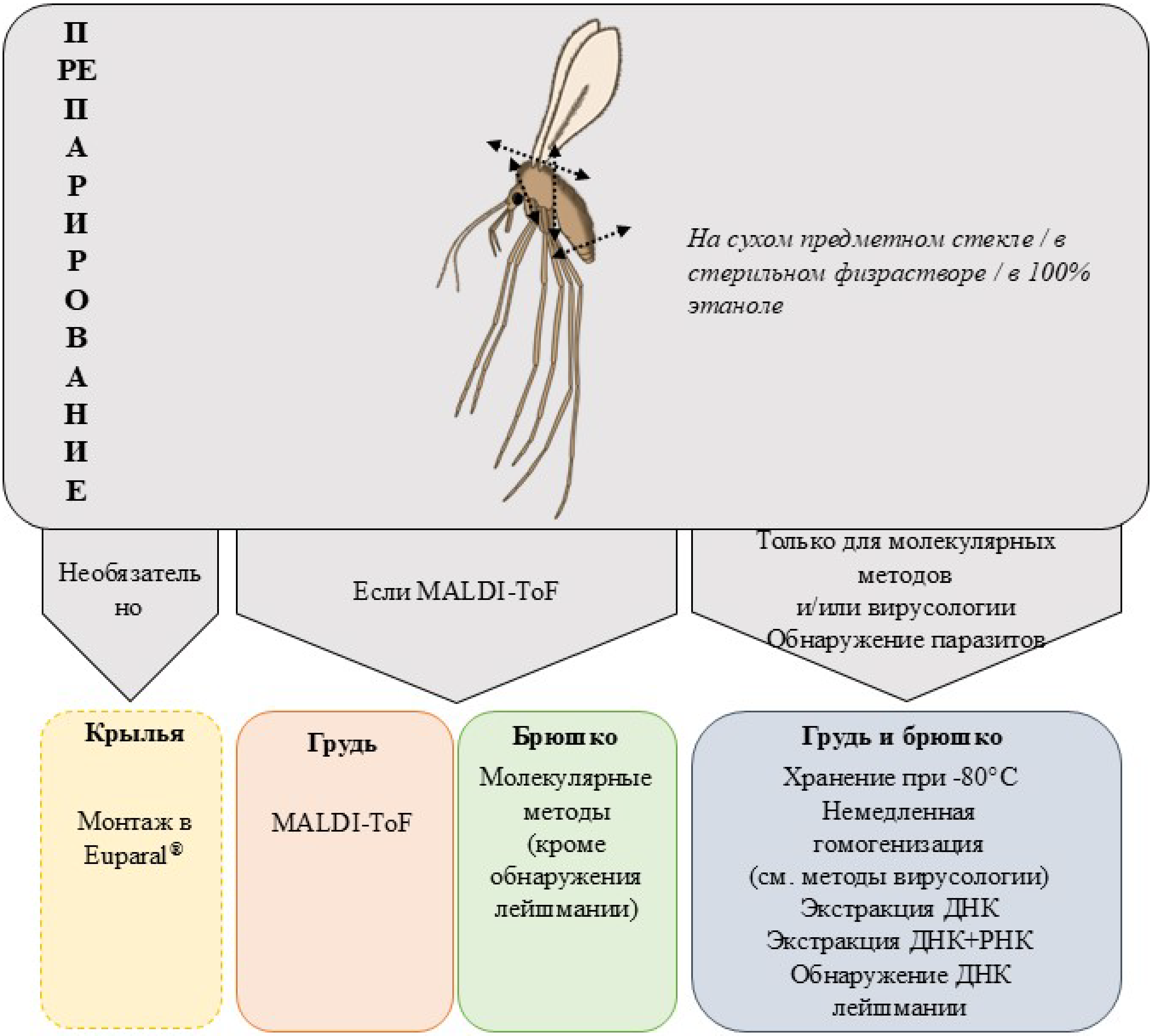


**Рисунок 6**: Обработка москитов для применения в молекулярной биологии, протеомике и/или вирусологии.

## 5. Обработка образцов для морфологических исследований (Рисунки 3, 6, 7 и 8; Приложения 1, 2, 3 и 4)

В этом разделе изложены принципы подготовки образцов москитов для монтирования исключительно в целях морфологических исследований, а также возможные модификации для применения за пределами морфологических исследований. Однако, понимание этой методологии имеет важное значение, поскольку,

при необходимости, позволяет адаптировать процедуры к конкретным типам образцов.

Обработка включает последовательные этапы опорожнения и наполнения с использованием пипеток Пастера, оснащённых гибкими резиновыми грушами. Настоятельно рекомендуется использовать стеклянные контейнеры с круглым дном, поскольку они значительно облегчают эти операции. Стекло инертно ко всем реагентам. Чтобы предотвратить испарение реагентов, контейнеры должны быть снабжены крышками и никогда не переполняться, что приводит к переливу при закрытии или открытии, а также чтобы предотвратить попадание пыли на образцы. Химические вещества, необходимые для просветления и обработки, приведены в Таблице 2.

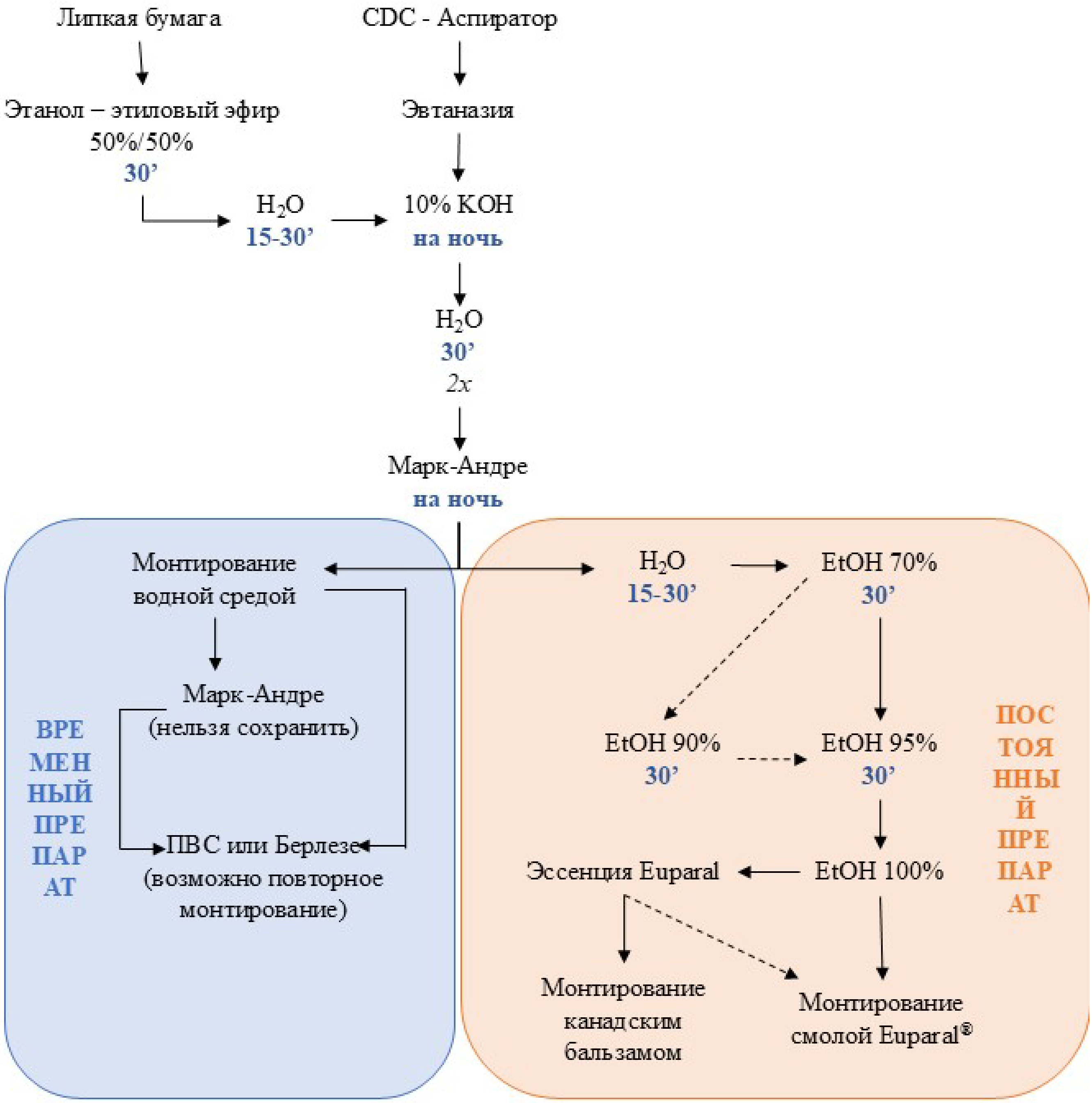


**Рисунок 7**: Классический метод обработки москитов.

**Таблица 2:** Состав используемых реагентов.

| **10% Гидроксид калия** | **1% Фуксиновая кислота в дистиллированной воде** |
|---|---|
| Гидроксид калия 10 г | Фуксиновая кислота (в виде порошка) 1 г |
| Дистиллированная вода до 100 мл | Дистиллированная вода 99 мл |
| **Хлораловая камедь (Жидкость Хойера)** | **Раствор Марка-Андре, окрашенный кислым фуксином** |
| Дистиллированная вода 50 мл | Раствор Марка-Андре 10 мл |
| Хлоралгидрат 200 г | 1% Фуксин 50 мкл |
| Гуммиарабик 50 г | |
| Глицерин 20 мл | |
| **Раствор Марка-Андре** | **Среда Епесê** |
| Хлоралгидрат 40 г | Чистая белая канифоль 22 г |
| Ледяная уксусная кислота 30 мл | Спирторастворимая копаловая камедь 12 г |
| Дистиллированная вода 30 мл | Абсолютный этанол 20 мл |
| | Камфора 10 г |
| | Скипидарная эссенция 10 мл |
| | Эвкалиптол 26 мл |

## 5.1. Очистка

Перед заключением москитов в постоянные препараты на предметных стёклах, их необходимо сначала просветлить с использованием соответствующего метода и осветляющего агента (напр. 10%-ный раствор уксусной кислоты или раствор Марка-Андре, содержащего хлоралгидрат, вещество, регулируемое во многих странах), чтобы сделать их прозрачными. Процесс осветления удаляет ткани тела, жиры, секреты и воск, делая образец полупрозрачным и облегчая исследование экзоскелетных структур (напр. мест прикрепления щетинок), особенностей поверхности (напр. окраска) и внутренних структур, видимых через тегумент (напр. сперматеки).

Двухэтапный процесс осветления, который включает в себя сначала использование сильной щёлочи (напр. гидроксида калия), а затем слабой кислоты (напр. уксусная кислота в растворе Марка-Андре), служит различным биохимическим целям [74]. Щёлочь вызывает деградацию мягких тканей, включая белки, липиды и мышцы, посредством омыления и денатурации белков, при этом хитиновый экзоскелет остаётся неповреждённым, что обеспечивает структурную ясность. Последующая обработка слабой кислотой нейтрализует оставшуюся щёлочь, предотвращая дальнейшую деградацию, и отбеливает хитин, повышая его прозрачность [74], хотя двукратное промывание образцов в дистиллированной воде в течение 15 минут также может быть достаточным для нейтрализации щёлочи. Эта последовательная обработка сочетает эффективное удаление ткани с бережным сохранением, обеспечивая оптимальную целостность образца для микроскопического исследования. Перед переходом к следующему шагу рекомендуется два двадцатиминутных полоскания образца в дистиллированной воде.

### 5.1.1. Лизис мягких тканей (рисунок 8)

Гидроксид натрия (NaOH) или гидроксид калия (KOH) являются широко используемыми химическими мацерирующими агентами, применяемыми в различных концентрациях и в течение разного времени в зависимости от размера и хрупкости образцов. Стандартная и наиболее эффективная методика включает лизис мягких тканей путём замачивания москитов в сильном щелочном растворе (10% KOH или NaOH) на ночь. Концентрацию можно увеличить для сокращения продолжительности обработки (напр. 20% KOH в течение 6 часов), а также нагревание до 37°C.

### 5.1.2. Просветление с окрашиванием или без него

За этим этапом следует просветляющая обработка, обычно с использованием уксусной кислоты и хлоралгидрата (напр. раствор Марка-Андре). После просветления образцы необходимо тщательно промыть, как минимум, в двух последовательных экспозициях в воде по 20 минут каждая, чтобы удалить остатки химических веществ.

Раствор Марка-Андре — широко используемый просветляющий агент для подготовки образцов москитов. Его эффективность заключается в облегчении процесса просветления при минимизации значительного повреждения хрупких структур, таких как крылья и антенны.

Раствор следует готовить непосредственно перед использованием или хранить в плотно закрытой ёмкости, чтобы предотвратить испарение или деградацию. Использование раствора Марка-Андре особенно удачно в сочетании с методами просветления или окрашивания для выделения специфических морфологических деталей. Подробности о его составе и приготовлении приведены в Приложении 2.

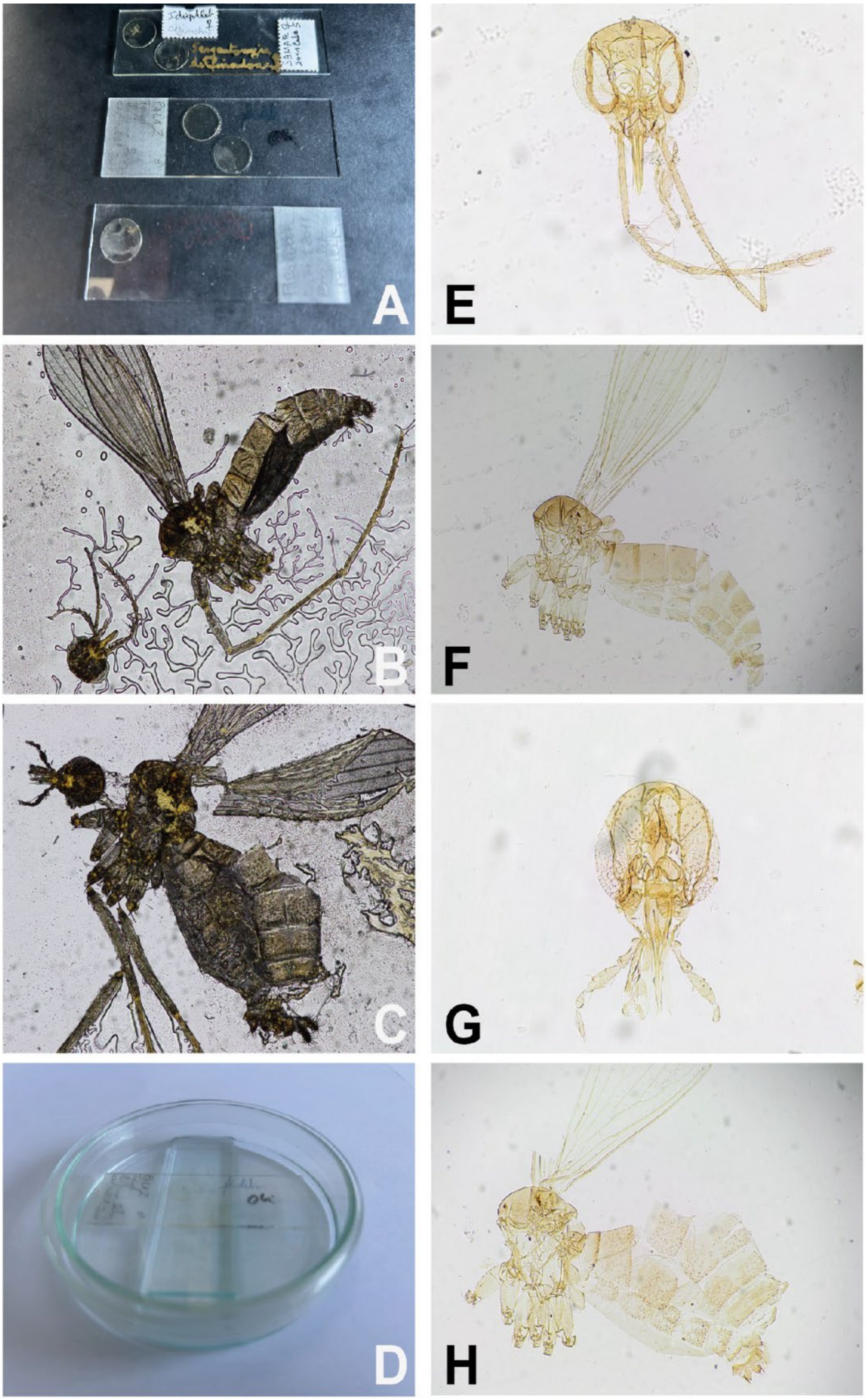


**Рисунок 8**: Перемонтировка препарата. A: поврежденные и высушенные предметные стекла в жидкости Хойера; B: микроскопическое изображение высушенного москита; C: микроскопическое изображение другого повреждённого москита; D: влажная камера, содержащая высушенное предметное стекло; E: голова и F: тело образца B после перемонтировки в среде Euparal®.

Для улучшения видимости очень прозрачных образцов может потребоваться их окрашивание перед монтированием. Существует множество красителей, каждый из которых связывается со специфическими химическими компонентами организма. Важно выбрать краситель, совместимый как с образцом, так и с выбранной монтирующей средой. Эту базовую методику можно модифицировать для цели окрашивания, например, добавив 0,1% кислого фуксина в раствор Марка-Андре. Образцы, хранящиеся в водных растворах и предназначенные для заключения в смольные среды, требуют обезвоживания (см. раздел 5.2 Дегидратация), поскольку большинство природных и синтетических смолистых сред для монтирования несовместимы с водой. New (1974) отметил, что некоторые красители могут разрушаться в определённых монтирующих средах [53]. Например, кислый фуксин, обычно используемый с канадским бальзамом, также может быть зафиксирован в препарате Euparal®. Однако образцы, окрашенные кислым фуксином, подвержены выцветанию, особенно если остаются остатки гвоздичного масла, используемого в качестве окончательной просветляющей жидкости. Образцы, хранившиеся в гвоздичном масле, могут значительно выцвести в течение нескольких дней.

## 5.2. Дегидратация

Дегидратация достигается путём последовательного пропускания образцов через этанол возрастающей концентрации: 50%, 70%, 80%, 90% или 95%, и, наконец, 100%, при этом каждая ванна длится не менее 20 минут. Поскольку этанол быстро испаряется, контейнеры должны быть плотно закрыты во время процесса. После полного обезвоживания образца, процесс можно приостановить на несколько дней, оставив его в эссенции Euparal®, которая предпочтительнее гвоздичного масла. Древесный креозот, некогда широко использовавшийся для этой цели, теперь полностью запрещён из-за его токсичности.

Процесс дегидратации должен гарантировать совместимость жидкости внутри образца с монтирующей средой. Это позволит предотвратить помутнение, осмотический коллапс или деформацию, которые могут сделать образец непригодным для таксономического исследования.

## 5.3. Монтирующие среды

### 5.3.1. Выбор и применение для подготовки образцов

В идеале, монтирующая среда должна иметь показатель преломления, максимально близкий к показателю преломления стекла, который составляет приблизительно 1,5. Она должна быть бесцветной, прозрачной и оставаться идеально прозрачной после высыхания и в течение длительного времени. Она должна быть совместима с используемыми красителями и способна проникать и диффундировать во все ткани образца. Она не должна слишком быстро высыхать или мутнеть во время монтировки. Она не должна сжиматься после монтировки. Выбор подходящей монтирующей среды является фундаментальным аспектом при подготовке образца, поскольку ни одна среда не является идеальной для всех целей. При выборе следует учитывать несколько ключевых факторов:

- **Оптические свойства.** Показатель преломления монтирующей среды должен обеспечивать достаточный контраст и преломление важных анатомических признаков, используемых для таксономической идентификации или морфологического описания, таких как сперматеки, аскоиды, сенсиллы Ньюстеда, вертикальные цибариальные зубцы и фарингиальные (глоточные) зубцы. Видимость этих структур напрямую зависит от оптических свойств монтирующей среды.
- **Сохранение.** Для типовых образцов или материалов, предназначенных для постоянных коллекций, среда должна обеспечивать длительную стабильность и прочность. В отличие от этого, для инвентаризационных исследований или эпидемиологических обследований, где долгосрочное сохранение менее критично, могут быть достаточны временные или полупостоянные монтирующие среды.

### 5.3.2. Требования к монтирующим средам

Специалисты часто разрабатывают индивидуальные и сложные методы монтирования, адаптированные к конкретным исследовательским потребностям. Однако, эти методы часто упускают из виду такие аспекты, как архивное качество, совместимость, стандартизация, удобство обращения и долговременное хранение. Отсутствие стандартизации затрудняет интеграцию пожертвованных коллекций и долгосрочное кураторское обслуживание.

Научные приложения предъявляют особые требования к средам для монтирования. Таксономисты часто монтируют целые образцы и предпочитают среды, которые мягко мацерируют внутренние органы для улучшения видимости кутикулярных структур. Показатель преломления должен достаточно отличаться от показателя преломления образца и предметного стекла, чтобы максимизировать оптическую чёткость. Коммерческие монтирующие среды обычно имеют показатель преломления, близкий к показателю преломления стекла, чтобы минимизировать преломление и рассеяние света в системе «предметное стекло-монтирующая среда-покровное стекло». Однако, в светлопольной микроскопии, естественный контраст неокрашенного образца можно регулировать, намеренно выбирая

монтирующую среду с показателем преломления, немного отличающимся от показателя преломления образца, тем самым улучшая его видимость на фоне.

### 5.3.3. Типы монтирующих сред (Таблицы 3 и 4)

Для микроскопии необходимо знать показатель преломления (RI) монтирующей среды, чтобы определить, как свет преломляется через предметное стекло, среду и образец. Когда RI близко совпадает с показателем преломления покровного стекла (≈1.515), свет проходит равномерно, уменьшая рассеяние и оптические искажения, что приводит к улучшению разрешения и видимости тонких структур. И наоборот, несоответствие RI может вызвать размытие, ореолы или скрыть неокрашенные детали. Из-за различных показателей преломления разных сред, выбор правильной монтирующей среды имеет решающее значение для оптимизации контраста, чёткости и общего качества изображения для данного образца.

Показатель преломления монтирующей среды оказывает существенное влияние на то, насколько хорошо можно увидеть тонкие структуры при подготовке москитов к заключению под покровное стекло. Тонкие и слабо склеротизированные структуры москитов, включая цибариальную арматуру, сперматеки, сегменты усиков и жилкование крыльев, могут быть трудноразличимы в среде с высоким показателем преломления.

Для москитов обычно используются среды на водной основе с гуммиарабиком и хлоралгидратом, а также канадский бальзам или смола Enecê - Nelson Cerqueira (NC), растворимые в неполярных растворителях. Rawlins [60] разделил монтирующие среды на два типа: (1) постоянные среды: они затвердевают со временем и подходят для длительного хранения, и (2) полупостоянные среды: они не затвердевают полностью и обычно используются для временного хранения. Монтирующие среды могут быть растворимы в воде, спирте или других растворителях (напр. толуоле, ксилоле) (Таблица 3). После нанесения, их следует герметизировать от воздействия атмосферных условий с помощью нерастворимых герметизирующих лаков. Для чёткого различения типов монтирующих сред, можно использовать следующую классификацию:

**a. Водорастворимые среды (далее водные среды)**. Эти среды легко растворяются в воде, что делает их пригодными для временных или полупостоянных препаратов. Обычно с ними легко работать, но для предотвращения воздействия атмосферной влаги (напр. среды на основе хлоралгидрата и поливинилового спирта) может потребоваться их герметичное ограждение, особенно в тропическом влажном климате.

**b. Среды с ограниченной водостойкостью**. Эти среды менее подвержены воздействию воды, но всё же требуют защиты от чрезмерной влажности. Они обеспечивают большую долговременную стабильность по сравнению с водорастворимыми вариантами и часто используются в полупостоянных препаратах.

**c. Углеводородорастворимые среды**. Эти среды растворяются в органических растворителях, таких как ксилол или толуол. Они предназначены для постоянных препаратов и обладают превосходной долговременной стабильностью, а также устойчивы к влаге и деградации, что делает их идеальными для архивного хранения (напр. нейтральный канадский бальзам).

В целом, водные среды лучше всего подходят для временных препаратов или случаев, требующих лёгкого извлечения образца; среды с ограниченной водостойкостью подходят для препаратов, требующих средней долговечности, а углеводородорастворимые среды предпочтительны для постоянных препаратов, предназначенных для архивного и долгосрочного хранения.

**Таблица 3**: Состав выбранных материалов для монтирования.

| Монтирующая среда | Растворитель | Полимер | Примечания |
|---|---|---|---|
| Жидкость Хойера | глицерин, вода | соединения гуммиарабика | просветляющий компонент: хлоралгидрат |
| CMCP-9 (= фенол карбоксиметил целлюлозы) | вода (CMCP-9: 51–60%) | полностью гидролизованный поливиниловый спирт (CMCP-9: 0–5%) | CMC(P)-9: низкая вязкость; CMC(P)-10: высокая вязкость |
| DMHF (диметилгидантоинформальдегид) | вода | N'N′-диметилол-диметилгидантоин (диметилол DMH) Олигомеры с эфирными/метиленовыми мостиками Сшитая полимерная сетка DMH-формальдегид. | |
| Канадский бальзам | ксилол; частично летучие компоненты бальзама (Δ³-карен, левопимаровая и палюстровая кислоты, лимонен, мирцен, β-фелландрен, α- и β-пинен) | бальзам (абиенол, абиетиновая изопимаровая и сандаракопимаровая кислоты) | нейтрализация карбонатом калия; смола пихты *Abies balsamea* (Linné, 1758) |
| Euparal® (эупарал) | эвкалиптол, паральдегид; частично летучие компоненты сандараковой камеди (лимонен, α- и β-пинен) | соединения сандараковой камеди (коммуниковая кислота, маноол, поликоммуниковая кислота, сандаракопимаровая кислота, 12-ацетокси-сандаракопимаровая кислота, сугиол, торулозиновая кислота, торулозол, тотарол) | Просветляющий компонент: метилсалицилат; краситель в Euparal® зеленый: соль меди (абиетинат меди); смола сандарака из *Tetraclinis articulata* (Vahl, 1791) |
| Enecê | этиловый спирт, камфора, эвкалиптом и скипидарная эссенция | Соединения копаловой смолы и канифоли | |

**Таблица 4:** Преимущества и недостатки некоторых сред для монтирования микроскопических препаратов и неопубликованные наблюдения различных авторов [52].

| Название | Преимущества | Недостатки |
|---|---|---|
| Канадский бальзам | Чрезвычайно прочная среда, срок службы которой превышает 150 лет.<br>Препараты можно монтировать с использованием гвоздичного масла или фенола/ксилена в качестве монтирующей среды. | Содержит вредные компоненты и требует работы под вытяжным шкафом.<br>Трудоёмкая серия дегидратаций занимает много времени.<br>Дегидратация этанолом и перенос через ксилол или гвоздичное масло могут вызвать хрупкость; альтернативные варианты (напр. изопропанол, н-бутанол, Cellosolve™, 1,4-диоксан, Histoclear, терпинеол) могут уменьшить ломкость.<br>Образцы могут почернеть, если ксилол заменить фенолом или если останется остаточный гидроксид калия.<br>Высокие показатели преломления могут затруднить наблюдение за неокрашенными структурами.<br>Полное высыхание может занять годы без сушки на нагревательной плите.<br>Со временем среда желтеет и темнеет, особенно при просветлении гвоздичным маслом.<br>Некоторые красители ослабевают, а катионные красители могут выцветать, если среда становится кислой, что может происходить спонтанно со временем. |
| DMHF (диметилгидантоинформальдегид) | Высокая прозрачность.<br>Хороший показатель преломления.<br>Отличная видимость структур.<br>Достаточно хорошая стабильность препаратов.<br>Совместимость со многими методами окрашивания.<br>Хорошая защита образцов.<br>Хорошее сцепление между предметным и покровным стёклами. | Возможно пожелтение со временем.<br>Может изменить некоторые виды окрашивания.<br>Не подходит для техник окрашивания, чувствительных к формальдегиду.<br>Образование пузырьков воздуха, медленное высыхание.<br>Монтирующая среда чувствительна к влаге.<br>Сложность повторного монтажа.<br>Формальдегид токсичен, вызывает раздражение и является канцерогенным. |
| Euparal (прозрачный) | Прочный материал со сроком службы более 50 лет.<br>Возможно монтирование непосредственно из 80% этанола (рекомендация производителя).<br>Не маскирует неокрашенные структуры, не желтеет и не становится хрупким со временем.<br>Имеет показатель преломления, более подходящий для двукрылых насекомых, чем канадский бальзам.<br>Хорошо подходит для более толстых образцов благодаря минимальной усадке и высыханию без пузырьков.<br>Сохраняет растворимость в 95% этаноле, что позволяет повторно монтировать образцы даже спустя много лет. | Содержит вредные компоненты и требует работы под вытяжным шкафом.<br>Обезвоживание этанолом и его перенос через эссенцию Euparal могут сделать некоторые таксоны хрупкими, но использование изопропанола может уменьшить эту проблему. |

| | | |
|---|---|---|
| Жидкость Хойера | Образцы можно монтировать живыми или непосредственно из воды, этанола или формальдегида.<br>Мацерация обеспечивает отличное качество кутикулы.<br>Обладает благоприятным показателем преломления, который может быть улучшен окрашиванием йодом для повышения контрастности.<br>Уксусная кислота в составе может способствовать расширению конечностей членистоногих.<br>Некоторые образцы могут оставаться стабильными в течение 40–60 лет.<br>Водорастворимая, что позволяет легко повторно монтировать образцы. | Нежные образцы растений могут разрушиться, если монтирующая среда не будет добавляться постепенно, что занимает много времени.<br>Полости и кристаллы могут образоваться менее чем через 10 лет.<br>Мацерация может стать чрезмерной в зависимости от концентрации хлоралгидрата и времени воздействия.<br>Компоненты среды могут разделиться и мелкая грануляция может появиться в течение нескольких месяцев или лет.<br>Сообщалось о почернении среды. |
| CMCP-9 (= фенол карбоксиметил целлюлозы) | Образцы можно монтировать непосредственно из таких сред, как вода, этанол, глицерин или растворы, содержащие формальдегид, а их внутренние органы при необходимости могут быть мацерированы для облегчения визуального наблюдения или подготовки. | Со временем может кристаллизоваться и темнеть.<br>Иногда происходит чрезмерная мацерация образцов<br>Толстые образцы сжимаются и образуют пустоты, если край предметного стекла не будет тщательно окаймлён.<br>Не подходит для окрашенных или кальцинированных образцов.<br>Более длительное время сушки, чем у сред на основе CMC |
| Eukitt™ | Высокопрочная материал, сохраняющий свойства не менее 30 лет.<br>Совместим со многими растворителями (ацетон, бензол, хлороформ, диоксан, эфир, изопропанол, метилбензоат, терпинеол, толуол, ксилол).<br>Быстросохнущий, со слабокислым pH.<br>Незначительно темнеет со временем.<br>Совместим со многими красителями (напр. фуксином, гематоксилином, метилзелёным, метилфиолетовым, метиленовым синим). Спустя годы образцы можно повторно смонтировать, вымачивая их в ксилоле в течение длительного времени. | Содержит токсичные компоненты, требующие работы под вытяжным шкафом.<br>Требует тщательной и длительной дегидратации.<br>Не подходит для толстых образцов (усадка, пузырьки газа).<br>Стёкла могут отслаиваться со временем, если они неправильно очищены или не герметично закрыты. Полимеризация может быть неполной вокруг волокон, богатых коллагеном. |
| Enecê | Высокопрочный материал, сохраняющий свои свойства не менее 50 лет.<br>Enecê не темнеет со временем.<br>Он более пластичен, что позволяет проводить препарирование насекомых в этой среде, а также обеспечивает достаточное время для позиционирования морфологических структур.<br>Низкая стоимость. | Требуется тщательная и длительная дегидратация.<br>Дегидратация этанолом и перенос через гвоздичное масло могут сделать некоторые экземпляры хрупкими.<br>Просветление происходит очень медленно, что может затруднить наблюдение за очень тонкими структурами (сенсиллами, аскоидами, простыми щетинками). |

#### 5.3.4. Описание рекомендуемых монтирующих сред (Таблицы 3 и 4)

*Среды для временных препаратов*

**Хлораловая камедь = жидкость/среда/раствор Хойера** (RI = 1.48)

Раствор Марка-Андре является лучшей средой для очень кратковременного наблюдения (несколько часов или немного дольше, если предметное стекло хранится во влажной камере) сперматек, особенно для фотографирования (рис. 4) или создания рисунков. Для более длительного хранения наблюдаемых сперматек требуется монтирование в водную среду, подходящую для среднесрочного хранения. Дегидратация с последующим монтированием с использованием смолы технически возможна, но не рекомендуется из-за высокого риска потери материала. Жидкость Хойера обычно используется для наблюдения за внутренними органами из-за её совместимости с водой, простоты приготовления, быстрого нанесения и показателя преломления, который благоприятен для исследования таких деликатных структур, как сперматеки. Однако, жидкость Хойера имеет существенные недостатки, если она не идеально приготовлена или не хранится в условиях контролируемой влажности, включая кристаллизацию, изменение цвета и потерю вязкости. Окантовка покровного стекла лаком не устраняет эти дефекты, поскольку монтирующая среда может значительно изменить цвет (иногда почти почернеть) из-за взаимодействия с лаком, особенно при использовании препарата Euparal®. Жидкость Хойера долгое время считалась обладающей наилучшими оптическими свойствами для изучения москитов и широко использовалась для этой цели. Хлораловая камедь и жидкость Хойера считаются синонимами. Они содержат гуммиарабик, глицерин и хлоралгидрат, и являются вариантом так называемой жидкости Берлезе. В прошлом, различные составы были неверно истолкованы или неправильно процитированы в литературе [74].

Хотя жидкость Хойера хорошо подходит для наблюдения за сперматеками москитов, она не подходит для длительного хранения. Она идеально подходит для кратковременных наблюдений, включая фотографирование, зарисовку или получение изображений. В целом, водные среды подходят для временных препаратов, но не гарантируют долговременной стабильности. Напротив, смолистые среды обладают превосходной долговечностью, часто сохраняясь в течение столетий, но могут скрывать мелкие детали сперматек из-за частой потери показателя преломления.

Со временем жидкость Хойера деградирует из-за обезвоживания (рис. 8), в результате чего образуются мелкие, непрозрачные, белые кристаллы хлоралгидрата. Тем не менее, образцы можно извлечь из закристаллизованных препаратов, при этом кутикула остаётся химически неповреждённой, хотя из-за роста кристаллов могут возникнуть физические повреждения. В некоторых случаях закристаллизованные препараты можно восстановить путём регидратации монтирующей среды в тёплой, влажной среде с добавлением тимола для предотвращения роста грибков. В качестве альтернативы, образцы можно извлечь из жидкости Хойера путём погружения в воду, обезвоживания в ледяной уксусной кислоте, а затем повторно монтировать в канадский бальзам.

**DMHF (диметилгидантоинформальдегид)** (RI 1.48)

Эта водная среда [72] демонстрирует превосходные оптические характеристики, сравнимые с характеристиками жидкости Хойера, и при этом так же проста в использовании. В отличие от последнего, DMHF не темнеет и не кристаллизуется со временем. Она хорошо подходит для монтирования москитов и других представителей семейства Psychodidae.

**Рисунок 8**: Перемонтировка препарата. A: повреждённые и высушенные предметные стекла в жидкости Хойера; B: микроскопическое изображение высушенного москита; C: микроскопическое изображение другого повреждённого москита; D: влажная камера, содержащая высушенное предметное стекло; E: голова и F: тело образца B после перемонтировки в среде Euparal®; G: голова и H: тело повреждённого образца C после перемонтировки в среде Euparal®.

**CMCP (камфор-монохлорфенол)** (RI = 1.41)

Это водорастворимая среда на основе глицерина, используемая для создания прозрачных и постоянных препаратов деликатных образцов, особенно москитов. Одним из её главных преимуществ является возможность монтирования образцов непосредственно из воды или этанола. Она быстро просветляет москита, размягчая кутикулу и облегчая правильное расположение анатомических частей, особенно расправление крыльев или препарирование гениталий. Хотя сообщается, что она обеспечивает длительное хранение, фактическая продолжительность сохранения остаётся неопределённой. Её основным ограничением является наличие фенола, токсичного и раздражающего вещества, требующего строгих мер предосторожности при обращении с образцами.

*Среды для постоянных препаратов*

**Канадский бальзам** (RI = 1.52-1.54)

Канадский бальзам был впервые описан как подходящая среда для микроскопии в проходящем

свете ещё в 1830-х годах Эндрю Притчардом (Andrew Pritchard). Он остаётся одной из наиболее широко используемых сред благодаря своим доказанным архивным качествам, успешно используемым более 150 лет. В отличие от сред на основе Хойера и Берлезе, он не кристаллизуется и не поглощает атмосферную влагу. Однако он проявляет выраженную автофлуоресценцию, что может быть недостатком для некоторых методов микроскопии [60]. Использование нетоксичных растворителей вместо ксилола снижает риски для здоровья, но может привести к более медленному высыханию и более раннему потемнению среды.

**Euparal®** (RI = 1.48)

Euparal® — широко используемая альтернатива канадскому бальзаму для постоянных препаратов, обладающая превосходной долговременной стабильностью и сопоставимым показателем преломления. Его использование имеет два основных ограничения: (1) необходимость дегидратации: перед окончательным заключением образцов в монтирующую среду, образец необходимо обезвоживать, обычно переходя от 95%-ного к абсолютному этанолу, и (2) увеличенное время обработки: окончательная монтировка в смолу, будь то канадский бальзам или Euparal®, требует обезвоживания, что увеличивает общее время обработки образца. Если обезвоживание с помощью органических растворителей невозможно, образцы, хранящиеся в абсолютном этаноле, перед окончательным монтированием, можно поместить в промежуточный раствор, состоящий из смеси препарата Euparal® и эссенции Euparal® в соотношении 1:1.

**Enecê** (RI = 1.467)

Enecê — это смолистая монтирующая среда, используемая в основном для мелких насекомых и особенно популярная в Бразилии. Она состоит из канифоли и копаловой камеди, растворённых в спирте, камфоре, скипидаре и эвкалиптоле. Cerqueira [11] описал среду Enecê как альтернативу канадскому бальзаму для монтирования постоянных препаратов личинок, экзувий неполовозрелых стадий развития и даже взрослых комаров. С тех пор она широко используется для монтирования москитов. Enecê предлагает экономически выгодную альтернативу для изготовления постоянных препаратов, обеспечивая долговременную стабильность и достаточное время высыхания, что позволяет проводить препарирование и точное расположение морфологических структур

## 5.4. Подготовка и сушка препаратов

Правильная сушка смонтированных препаратов имеет решающее значение для обеспечения их долговременной стабильности и сохранности. Перед длительным хранением предметные стекла следует тщательно высушить. Препараты, приготовленные с использованием постоянных монтирующих сред, следует сушить в горизонтальном положении в течение 2–3 недель, тогда как для препаратов, приготовленных с использованием временных сред, может понадобиться 1–2 недели. Для обеспечения эффективного процесса сушки рекомендуется использовать инкубатор, установленный на соответствующую температуру для используемой монтирующей среды, избегая чрезмерного нагрева, который может повредить образцы. Рекомендуемый температурный диапазон составляет от 30°C до 37°C. Этот этап сушки крайне важен для предотвращения деформации предметных стёкол, ухудшения состояния образцов или нестабильности монтирующей среды во время хранения.

На этикетке предметного стекла всегда должна быть указана используемая монтирующая среда. По возможности, на этикетке также следует указать точный состав среды, имя изготовителя и дату приготовления. Препараты часто изначально готовятся как временные. Однако, если статус образца меняется (напр. он становится частью типовой серии), рекомендуется повторное монтирование в постоянную монтирующую среду, для обеспечения сохранности образца для будущих таксономических исследований.

## 5.5. Альтернативные способы монтирования: монтирование на плашку

Метод монтирования на картонные плашки используется для некоторых групп насекомых, при котором экземпляры могут быть либо наколоты булавками на энтомологические плашки, либо приклеены к их поверхности. Из-за небольшого размера москитов и необходимости осмотра внутренних органов для идентификации (см. пункт 5) этот метод совершенно не подходит для их монтирования.

## 5.6. Восстановление повреждённых образцов

Для редких или ценных образцов рекомендуется двухэтапный подход, как показано в видеоролике, доступном по адресу: https://zenodo.org/records/18315029. 1) Регидратация без демонтирования для предварительного наблюдения. Опору, составленную из нескольких предметных стёкол следует поместить в чашку Петри. Затем, препарат, подлежащий регидратации, помещают сверху, и чашку Петри заполняют несколькими миллиметрами растворителя для создания влажной камеры. При этом следует избегать контакта препарата с растворителем (рис. 8 D). Время, необходимое для регидратации, может варьировать от одного до

нескольких дней в зависимости от состояния образца. Ежедневный мониторинг и терпение крайне важны. После достаточной регидратации предметное стекло можно извлечь из влажной камеры и поместить в инкубатор на несколько часов перед микроскопическим исследованием, фотографированием или зарисовкой. 2) Для повторного монтирования предметное стекло можно вернуть во влажную камеру ещё на несколько часов или на ночь. Демонтирование следует проводить под стереомикроскопом. С помощью тонких игл аккуратно следует снять покровное стекло, убедившись, что к нему не прикреплены части москита (https://zenodo.org/records/18315029). Затем, препарированные части москита следует собрать и промыть водой в небольших лунках, подобных тем, которые используются для деструктивной экстракции ДНК/РНК (см. ниже), перед дегидратацией и повторным монтированием в смоляную среду. При демонтировании препарата крайне важно определить исходную монтирующую среду, чтобы выбрать подходящий растворитель. Для водных монтирующих сред следует использовать воду. Если монтирующая среда основана на смоле (напр. канадский бальзам или Euparal®), следует использовать ксилол, под вытяжным шкафом с использованием соответствующих средств индивидуальной защиты, включая маску.

Повторное монтирование типовых или коллекционных образцов должно производиться только с согласия куратора и/или учреждения, владеющего образцом.

# 6. Идентификация образца

## 6.1. Морфология

Идентификация москитов прежде всего основана на изучении их морфологических характеристик, включая форму груди, крыльев, гениталий, щетинок и определённые морфометрические соотношения между различными структурами. Исследователи используют таксономические ключи, эталонные коллекции и оригинальные описания видов для сравнения собранных образцов с известными таксонами. Ключевые диагностические характеристики, такие как жилкование крыльев и морфология головы у обоих полов, структура гениталий самцов и конфигурация сперматек у самок, особенно информативны для идентификации вида. Надёжная идентификация часто требует детального микроскопического исследования, как правило, с использованием светового микроскопа для наблюдения за тонкими структурами (гениталии, сперматеки) или стереомикроскопа для более общих морфологических характеристик. Недавние достижения в области визуализации облегчили использование цифровой визуализации для определения москитов. Фотографии высокого разрешения или цифровые иллюстрации ключевых признаков могут быть сравнены с эталонными материалами или проанализированы с помощью систем компьютерной идентификации, что повышает как точность, так и доступность морфологической таксономии.

## 6.2. Геометрия крыла

Геометрия крыла является ключевой характеристикой для идентификации и классификации различных видов москитов. Крылья москитов имеют характерную морфологию, как правило, длинные и узкие, с хорошо развитым жилкованием (Рисунки 9 и 10).

Расположение жилок образует характерный рисунок, который может варьировать в зависимости от рода и вида, что обеспечивает ценные диагностические признаки для идентификации. Таким образом, изучение геометрии крыла предоставляет важную информацию для таксономических целей.

## 6.3. Геометрическая морфометрия крыла

Исследователи используют различные методы, такие как геометрическая морфометрия, для анализа и сравнения формы и размера крыльев у разных видов или популяций москитов. Изучение геометрии крыльев даёт ценные сведения о поведении, предпочтениях в среде обитания и лётных способностях.

В методе геометрической морфометрии крылья тщательно препарируют, окрашивают (при необходимости) и монтируют на предметные стекла. Затем, подготовленные предметные стекла фотографируются под стереомикроскопом, оцифровываются и подвергаются морфометрическому анализу. Эта процедура хорошо описана в литературе [6, 27, 42, 56, 57, 59], с рекомендацией использовать последовательно правое или левое крыло, чтобы избежать потенциальных негативных аллометрических эффектов для парных органов [62].

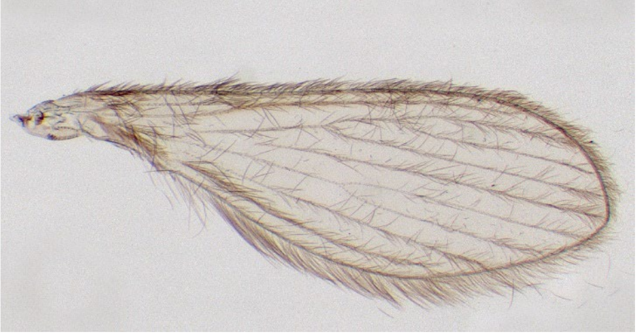

**Рисунок 9:** Свежее крыло *Trichophoromyia ininii.*

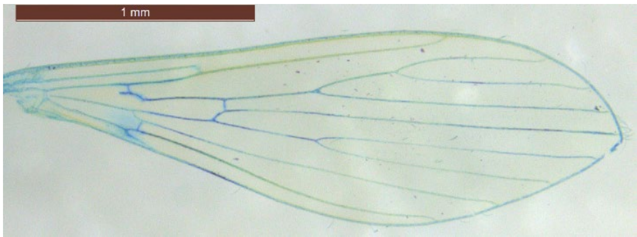


**Рисунок 10:** Окрашенное крыло *Phlebotomus ariasi.*

*Подготовка крыла для анализа с использованием геометрической морфометрии.*

Для оптимальной визуализации жилок крыла, крылья следует очистить от чешуек и, соответствующим образом, окрасить. Для подготовки крыла сначала заполните небольшие лунки необходимыми реагентами (метиленовый синий, этанол, вода и заменитель ксилола). Достаньте крыло, законсервированное в 70% этаноле при комнатной температуре, перевернув пробирку Эппендорфа и вылив содержимое в лунку, затем поднимите крыло продольно с помощью тонкой изогнутой иглы. Кратковременно перенесите крыло из этанола в воду и обратно в этанол, чтобы удалить щетинки. Поместите крыло в метиленовый синий на 6 минут, следя за тем, чтобы оно оставалось на плаву во время окрашивания. Осторожно извлеките крыло и погрузите его в заменитель ксилола на 2 минуты (примерно одну треть времени окрашивания метиленовым синим). Лёгкое постукивание иглой по стенкам лунки может помочь крылу опуститься; ксилол служит для закрепления красителя. Наконец, поднимите крыло и поместите его на небольшую каплю среды Euparal® на предметном стекле микроскопа. Под увеличительным стеклом осторожно разверните крыло и аккуратно накройте покровным стеклом. Фотографии следует сделать незамедлительно, до полного застывания Euparal®, так как для достижения оптимального выравнивания может потребоваться небольшая корректировка положения крыла под покровным стеклом.

## 6.4. Методы молекулярной биологии

Помимо морфологических подходов, молекулярные методы стали неотъемлемой частью энтомологии, особенно для таксономических, популяционно-генетических и филогенетических исследований, а также для обнаружения ДНК/РНК патогенов и определения происхождения крови, поскольку поведение переносчиков является ключевым элементом в эпидемиологии [70]. Секвенирование ДНК может быть использовано для подтверждения видовой идентификации или дифференциации близкородственных видов, предлагая таким образом более точный и надёжный способ определения. Кроме того, передовые молекулярные методы (ПЦР, секвенирование ДНК, NGS и др.) и масс-спектрометрия MALDI-ToF приобретают все большее значение для быстрой и точной идентификации видов, дополняя традиционные морфологические методы [46]. Несмотря на эти достижения, морфологическая идентификация остаётся золотым стандартом в таксономии и составляет основу, на которой интерпретируются молекулярные данные.

### 6.4.1. Деструктивная экстракция нуклеиновых кислот

Выделение нуклеиновых кислот является рутинным этапом во многих биологических исследованиях. Были разработаны различные методы выделения ДНК из биологических материалов [48]. Многие коммерчески доступные наборы для выделения ДНК предназначены для облегчения этого процесса [14]. Однако методы, классически используемые для подготовки экземпляров членистоногих для морфологической идентификации, часто затрудняют анализ ДНК, поскольку эти методы могут повредить или разрушить важные физические характеристики образца [10]. Большинство протоколов выделения ДНК из тканей насекомых носят деструктивный характер [43], что создаёт особые проблемы для мелких образцов, где даже ограниченный отбор проб может поставить под угрозу важные морфологические особенности [72]. Тип и состояние сохранности образца играют ключевую роль в выборе подходящего метода выделения ДНК [29].

Необходимость точной идентификации москитов, понимания динамики популяций и минимизации нецелевого воздействия стимулировала разработку молекулярно-диагностических инструментов [23]. Молекулярные подходы в настоящее время часто используются для дополнения морфологических таксономических методов идентификации москитов. Например, стандартный метод штрихкодирования насекомых включает выделение и секвенирование ДНК, что приводит к потере исходного образца. Поэтому, существует острая необходимость в разработке и изучении неразрушающих методов выделения ДНК, которые сохраняют как биологический материал, так и морфологическую целостность образца.

Применяется множество методов для выделения нуклеиновых кислот из москитов. Количество и качество необходимых нуклеиновых кислот зависят от последующего молекулярного анализа, поскольку разные методы имеют разные требования к чувствительности и чистоте [9]. Например, было установлено, что глаза москитов потенциально могут подавлять амплификацию ПЦР [69]. Помимо скрининга патогенов, ДНК москитов обычно выделяют для видовой идентификации. Могут использоваться различные методы выделения, с различной

эффективностью и качеством в зависимости от метода. Некоторые протоколы производителей были адаптированы исследователями для москитов [8], что позволило увеличить выход и/или качество выделенных нуклеиновых кислот [8, 9, 69], в то время как другие адаптации, первоначально разработанные для других групп членистоногих, также могут быть применены для москитов [58, 76]. Идентификационные ПЦР, нацеленные на небольшие митохондриальные фрагменты (COI или CytB), как правило, совместимы с методами выделения, которые приводят к высокой фрагментации ДНК. В отличие от этого, некоторые NGS технологии секвенирования длинных цепей (Oxford Nanopore и PacBio) требуют минимальной фрагментации и высококачественной ДНК. Экстракция с помощью центрифужных колонок обычно даёт фрагменты геномной ДНК размером до 60 кб, тогда как экстракция фенол-хлороформом может давать фрагменты размером до 150 кб [77]. В таблице 5 приведены различные методы экстракции ДНК москитов и указано, были ли внесены методологические изменения для этих насекомых. Выход продукта не указан, поскольку он зависит от размера образца и метода подготовки. В столбце с модификациями указаны изменения в протоколах экстракции для москитов или других мелких членистоногих.

При выборе метода экстракции следует учитывать несколько критериев, таких как количество образцов, время экстракции и используемая последующая методика. Хотя методы NGS требуют высокомолекулярной геномной ДНК, все представленные здесь методы могут быть использованы для стандартных приложений на основе ПЦР.

Кроме того, в ряде исследований были изучены неразрушающие методы экстракции ДНК для мелких наземных членистоногих, сухих музейных образцов и мягкотелых членистоногих [19, 26, 28, 55, 63].

**Таблица 5**: Средняя стоимость, область применения и модификация протокола для выделения геномной ДНК москитов.

| Протокол | Цена | Применение | Модификация протокола для мелких членистоногих |
|---|---|---|---|
| Центрифужная колонка | 2.5 – 3.55 US$ [39] | ПЦР, NGS | [9] |
| Фенол-хлороформ | 0.24 US$ [69] | ПЦР, NGS | [9] |
| HotSHOT | <0.01 US$ [69] | ПЦР | - |
| Солевой метод | 0.12 $3 [69] | ПЦР | - |
| Chelex | 0.02 $4 [41] | ПЦР | [41, 76] |

### 6.4.2. Неразрушающая экстракция нуклеиновых кислот

Одной из главных проблем молекулярного анализа членистоногих, особенно москитов, является сохранение образцов для включения в энтомологические коллекции. Большинство протоколов экстракции ДНК требуют мацерации ткани, что ставит под угрозу сохранность исходного образца. Однако, неразрушающие методы экстракции нуклеиновых кислот разработаны для извлечения генетического материала без физического повреждения образца, влияния на его жизнеспособность или морфологию. Эти методы особенно ценны при работе с ценными или ограниченными образцами, такими как москиты, где сохранение структурной целостности имеет важное значение для будущих таксономических, морфологических или диагностических целей. Распространенным способом является неразрушающий метод, при котором москиты иммобилизуются и осторожно погружаются в лизисный буфер, содержащий протеиназу K.

Метод мягкого векторного лизиса успешно применялся к москитам, особенно к типовым образцам [24]. Этот метод использует обычный набор центрифужных колонок (спин-колонок) (в данном случае, набор DNeasy Blood and Tissue, QIAGEN, Хильден, Германия), модифицированный для получения ДНК без разрушения образца. Модифицированные этапы лизиса (объем лизисного буфера и добавление этапа замораживания) [17]

позволяют высвободить нуклеиновые кислоты, минимизируя морфологические повреждения [24]. Для москитов также можно использовать набор для экстракции ДНК HotSHOT (Bento Bioworks Ltd, Лондон, Великобритания) [73], который позволяет быстро и недорого обрабатывать образцы. Энтомологические образцы, предназначенные для морфологической идентификации, затем можно промыть. Образцы, обработанные с помощью набора DNeasy Blood and Tissue, должны быть осветлены раствором Марка-Андре, тогда как образцы, обработанные с помощью набора для экстракции ДНК HotSHOT, достаточно просветлены, чтобы их можно было поместить в водную среду или, предпочтительно, в смолу после дегидратации, согласно протоколу, подробно описанному в этой статье [73]. Извлечённый генетический материал затем может быть дополнительно обработан для последующего анализа, например, ПЦР для амплификации специфических генетических маркеров. Неразрушающие методы экстракции нуклеиновых кислот имеют решающее значение для изучения генетических характеристик москитов, включая выявление потенциальных возбудителей заболеваний, которых они могут переносить. Сохраняя целостность образца, исследователи могут получить ценную генетическую информацию, сохраняя при этом образец для дополнительных анализов или исследований.

## 6.5. MALDI-ToF MS

MALDI-ToF MS (масс-спектрометрия с матрично-ассистированной лазерной десорбцией/ионизацией и времяпролетным анализом) — это метод масс-спектрометрии, предназначенный для обнаружения и анализа уникальных белковых профилей («отпечатков пальцев») биологических образцов. MALDI-ToF всё чаще признаётся важным инструментом для идентификации членистоногих, имеющих медицинское и ветеринарное значение. Было показано, что этот метод эффективен для идентификации взрослых особей и личинок москитов, а также крови хозяина у сытых самок, и успешно использовался для определения видовой принадлежности самцов и самок москитов в различных условиях хранения и гомогенизации [28, 30, 73, 74]. Этот метод также обладает высокой разрешающей способностью на уровне подродов, видов и популяций. Этот метод позволяет исследователям быстро и точно идентифицировать виды, что имеет важное значение для понимания распространения москитов, их поведения и роли в передаче заболеваний. Благодаря возможности дифференцировать виды на основе белковых профилей, MALDI-ToF играет решающую роль в эпидемиологических исследованиях и стратегиях борьбы с переносчиками заболеваний. В настоящее время существуют два основных недостатка этого метода, ограничивающих его рутинное применение. Во-первых, это доступность оборудования для масс-спектрометрии, которое является непомерно дорогим и труднодоступным исключительно для идентификации видов москитов (или членистоногих переносчиков в целом). К счастью, это ограничение можно преодолеть, получив дополнительное время работы на масс-спектрометрах, которые стали стандартным исследовательским инструментом в протеомных лабораториях и/или клинической диагностике. Во-вторых, это ограниченная доступность эталонных спектров москитах в общедоступных базах данных, что приводит к необходимости создания собственной базы данных с эталонными спектрами на основе уникально идентифицированных образцов, в идеале — путём сочетания морфологической оценки и секвенирования подходящего генетического маркера (COI, cytB и т. д.). Надеемся, что это ограничение вскоре будет устранено путём постепенного включения имеющихся на данный момент эталонных данных по москитам в платформу MSI, которая управляется Assistance Publique-Hôpitaux de Paris, Сорбоннским университетом (Франция) и коллекцией BCCM/IHEM/Sciensano в Брюсселе (Бельгия) (https://msi.happy-dev.fr/). При планировании проведения профилирования белков методом MALDI-ToF, образцы следует хранить, предпочтительно, в сухом замороженном состоянии или в 70% этаноле молекулярной чистоты и не подвергать воздействию колебаний температур. В связи с отсутствием универсальных стандартов по подготовке образцов, рекомендуется использовать водный раствор, состоящий из 60% ацетонитрила и 0,3% ТФК, содержащий синапиновую кислоту (30 мг/мл), в качестве матрицы для MALDI-ToF, чтобы обеспечить сопоставимость белковых спектров с существующими опубликованными данными по москитам.

*Подготовка образцов для MALDI-ToF MS (рисунок 7)*

Образцы насекомых, хранившиеся в различных условиях, сначала высушивают на воздухе при комнатной температуре и препарируют. Голову и брюшко удаляют, чтобы сохранить части, содержащие основные морфологические признаки, для монтирования препаратов и морфологического анализа. Грудь можно использовать для MALDI-ToF, а оставшуюся часть брюшка сохраняют для выделения ДНК. Для профилирования белков грудь гомогенизируют вручную в микропробирках объемом 1,5 мл с 10 мкл гомогенизирующего раствора, используя одноразовые пестики и гранулы. Обычно используются два гомогенизирующих раствора: стерильная дистиллированная воду и 25% муравьиная кислота.

# 7. Вывод

В данной работе мы стремились предоставить исследователям наиболее эффективные методы подготовки москитов, адаптированные к конкретным исследовательским задачам, для обеспечения точной идентификации и обнаружения патогенов. Единого, универсально оптимального метода не существует; доступно несколько методов, каждый со своими преимуществами и ограничениями.

В дополнительных материалах мы предоставили подробные протоколы для различных методов подготовки и идентификации москитов. Эти протоколы, включая обучающие видеоролики, предлагают пошаговые процедуры, адаптированные к различным целям, обеспечивая точные и надёжные результаты. Предлагая этот всеобъемлющий ресурс, мы стремимся помочь исследователям в выборе и применении наиболее подходящих методов подготовки для их конкретных нужд.

## Благодарности

Авторы выражают благодарность Ричарду Лейну (Richard Lane) и Зои Джей Адамс (Zoe Jay Adams) из Музея естественной истории в Лондоне (Великобритания) за их превосходное рецензирование, которое помогло повысить качество данной рукописи.

## Финансирование

Мы благодарим бразильские агентства по развитию CNPq (номер дела: 404395/2024-4) и Фонд Араукарии (Araucária) (номер дела: 433/2025 PDI) за финансирование исследовательской работы AJA.

## Конфликты интересов

Жером Депаки (Jérôme Depaquit) является заместителем редактора журнала *Parasite*; он не оказывал влияния на процесс рецензирования и принятия решения по данной рукописи. Остальные авторы заявляют об отсутствии конфликтов интересов.

## Заявление о доступности данных

Видеоролики на Zenodo
Видео 1: https://zenodo.org/records/18198006
Видео 2: https://zenodo.org/records/18311158
Видео 3: https://zenodo.org/records/18311106
Видео 4: https://zenodo.org/records/18311154
Видео 5: https://zenodo.org/records/18303014
Видео 6: https://zenodo.org/records/18302850
Видео 7: https://zenodo.org/records/18315029

## Дополнительные материалы

Более подробная информация об этой статье доступна по следующему адресу: https://www.parasite-journal.org/10.1051/parasite/2026009/olm

## Список литературы

1. Alkan C, Allal-Ikhlef AB, Alwassouf S, Baklouti A, Piorkowski G, de Lamballerie X, Izri A, Charrel RN. 2015. Virus isolation, genetic characterization and seroprevalence of Toscana virus in Algeria. Clinical Microbiology and Infection, 21(11), 1040 e1-9.
2. Alten B, Ozbel Y, Ergunay K, Kasap OE, Cull B, Antoniou M, Velo E, Prudhomme J, Molina R, Banuls AL, Schaffner F, Hendrickx G, Van Bortel W, Medlock JM. 2015. Sampling strategies for phlebotomine sand flies (Diptera: Psychodidae) in Europe. Bulletin of Entomological Research 105(6), 664–678.
3. Ayhan N, Baklouti A, Prudhomme J, Walder G, Amaro F, Alten B, Moutailler S, Ergunay K, Charrel RN, Huemer H. 2017. Practical guidelines for studies on sandfly-borne phleboviruses: Part I: Important points to consider *ante* field work. Vector-Borne and Zoonotic Diseases 17(1), 73–80.
4. Bates PA. 1997. Infection of phlebotomine sandflies with *Leishmania*, in The Molecular Biology of Insect Disease Vectors: A Methods Manual. Springer. p. 112–120.5.
5. Baum M, de Castro EA, Pinto MC, Goulart TM, Baura W, Klisiowicz Ddo R, Vieira da Costa-Ribeiro MC. 2015. Molecular detection of the blood meal source of sand flies (Diptera: Psychodidae) in a transmission area of American cutaneous leishmaniasis, Parana State, Brazil. Acta Tropica, 143, 8–12.
6. Belen A, Alten B, Aytekin A. 2004. Altitudinal variation in morphometric and molecular characteristics of *Phlebotomus papatasi* populations. Medical and Veterinary Entomology, 18(4), 343–350.
7. Bhattacharya J, Chandra G, Hati AK. 1991. A simple method for cryopreservation of *Leishmania donovani* promastigotes, Indian Journal of Medical Research, 93, 245–246.
8. Caligiuri LG, Sandoval AE, Miranda JC, Pessoa FA, Santini MS, Salomón OD, Secundino NF, McCarthy CB. 2019. Optimization of DNA extraction from individual sand flies for PCR amplification. Methods and Protocols, 2(2), 36.
9. Casaril AE, de Oliveira LP, Alonso DP, de Oliveira EF, Gomes Barrios SP, de Oliveira Moura Infran J, Fernandes WS, Oshiro ET, Ferreira AMT, Ribolla PEM, de Oliveira AG. 2017. Standardization of DNA extraction from sand flies: Application to genotyping by next generation sequencing. Experimental Parasitology, 177, 66–72.
10. Castalanelli MA, Severtson DL, Brumley CJ, Szito A, Foottit RG, Grimm M, Munyard K, Groth DM. 2010. A rapid non-destructive DNA extraction method for insects and other arthropods. Journal of Asia-Pacific Entomology, 13(3), 243–248.
11. Cerqueira NL. 1943. Um novo meio para montagem de pequenos insetos em lâmina. Memórias do Instituto Oswaldo Cruz, (39), 37–41.
12. Charrel RN, Gallian P, Navarro-Mari JM, Nicoletti L, Papa A, Sanchez-Seco MP, Tenorio A, de Lamballerie X. 2005. Emergence of Toscana virus in Europe. Emerging Infectious Diseases, 11(11), 1657–1663.
13. Chaskopoulou A, Giantsis IA, Demir S, Bon MC. 2016. Species composition, activity patterns and blood meal analysis of sand fly

populations (Diptera: Psychodidae) in the metropolitan region of Thessaloniki, an endemic focus of canine leishmaniasis. Acta Tropica, 158, 170–176.

14. Chen H, Rangasamy M, Tan SY, Wang H, Siegfried BD. 2010. Evaluation of five methods for total DNA extraction from western corn rootworm beetles. PLoS One, 5(8), e11963.
15. Depaquit J, Grandadam M, Fouque F, Andry PE, Peyrefitte C. 2010. Arthropod-borne viruses transmitted by Phlebotomine sandflies in Europe: a review. Eurosurveillance, 15(10), 19507.
16. Diamond LS, Herman CM. 1954. Incidence of Trypanosomes in the Canada Goose as revealed by bone marrow culture. Journal of Parasitology, 40(2), 195–202.
17. Ding H, Torno M, Vongphayloth K, Ng G, Tan D, Sng W, Ho K, Randrianambinintsoa FJ, Depaquit J, Tan CH. 2025. Hidden in plain sight: discovery of sand flies in Singapore and description of four species new to science. Parasites & Vectors, 18(1), 402.
18. Es-Sette N, Ajaoud M, Bichaud L, Hamdi S, Mellouki F, Charrel RN, Lemrani M. 2014. *Phlebotomus sergenti* a common vector of *Leishmania tropica* and Toscana virus in Morocco. Journal of Vector Borne Diseases, 51(2), 86–90.
19. Favret C. 2005. A new non-destructive DNA extraction and specimen clearing technique for aphids (Hemiptera). Proceedings of the Entomological Society of Washington, 107(2), 469–470.
20. Galati EAB. 2018. Phlebotominae (Diptera, Psychodidae): Classification, morphology and terminology of adults and identification of American taxa, in Brazilian Sand Flies: Biology, Taxonomy, Medical Importance and Control, Rangel EF, Shaw JJ, Editors. Cham: Springer International Publishing. pp. 9–212.
21. Galati EAB, de Andrade AJ, Perveen F, Loyer M, Vongphayloth K, Randrianambinintsoa FJ, Prudhomme J, Rahola N, Akhoundi M, Shimabukuro PHF, Depaquit J. 2025. Phle-botomine sand flies (Diptera, Psychodidae) of the world. Parasites & Vectors, 18(1), 220.
22. Galati EAB, Galvis-Ovallos F, Lawyer P, Leger N, Depaquit J. 2017. An illustrated guide for characters and terminology used in descriptions of Phlebotominae (Diptera, Psychodidae). Par-asite, 24, 26.
23. Gariepy T, Kuhlmann U, Gillott C, Erlandson M. 2007. Parasitoids, predators and PCR: the use of diagnostic molecular markers in biological control of Arthropods. Journal of Applied Entomology, 131(4), 225–240.
24. Giantsis IA, Chaskopoulou A, Bon MC. 2016. Mild-Vectolysis: A nondestructive DNA extraction method for vouchering sand flies and mosquitoes. Journal of Medical Entomology, 53(3), 692–695.
25. Gidwani K, Picado A, Rijal S, Singh SP, Roy L, Volfova V, Andersen EW, Uranw S, Ostyn B, Sudarshan M, Chakravarty J, Volf P, Sundar S, Boelaert M, Rogers ME. 2011. Serological markers of sand fly exposure to evaluate insecticidal nets against visceral leishmaniasis in India and Nepal: a cluster-randomized trial. PLoS Neglected Tropical Diseases, 5(9), e1296.
26. Gilbert MTP, Moore W, Melchior L, Worobey M. 2007. DNA extraction from dry museum beetles without conferring external morphological damage. PLoS One, 2(3), e272.
27. Giordani BF, Andrade AJ, Galati EAB, Gurgel-Goncalves R. 2017. The role of wing geometric morphometrics in the identification of sandflies within the subgenus *Lutzomyia.* Medical and Veterinary Entomology, 31(4), 373–380.
28. Guzmán-Larralde AJ, Suaste-Dzul AP, Gallou A, Peña-Carrillo KI. 2017. DNA recovery from microhymenoptera using six non-destructive methodologies with considerations for subsequent preparation of museum slides. Genome, 60(1), 85–91.
29. Hajibabaei M, DeWaard JR, Ivanova NV, Ratnasingham S, Dooh RT, Kirk SL, Mackie PM, Hebert PD. 2005. Critical factors for assembling a high volume of DNA barcodes. Philosophical Transactions of the Royal Society B: Biological Sciences, 360(1462), 1959–1967.
30. Haouas N, Pesson B, Boudabous R, Dedet JP, Babba H, Ravel C. 2007. Development of a molecular tool for the identification of *Leishmania* reservoir hosts by blood meal analysis in the insect vectors. American Journal of Tropical Medicine and Hygiene, 77(6), 1054–1059.
31. Hlavackova K, Dvorak V, Chaskopoulou A, Volf P, Halada P. 2019. A novel MALDI-TOF MS-based method for blood meal identification in insect vectors: A proof of concept study on phlebotomine sand flies. PLoS Neglected Tropical Diseases, 13 (9), e0007669.
32. Huemer H, Prudhomme J, Amaro F, Baklouti A, Walder G, Alten B, Moutailler S, Ergunay K, Charrel RN, Ayhan N. 2017. Practical guidelines for studies on sandfly-borne phleboviruses: Part II: Important points to consider for fieldwork and subsequent virological screening. Vector-Borne and Zoonotic Diseases, 17(1), 81–90.
33. Jancarova M, Polanska N, Thiesson A, Arnaud F, Stejskalova M, Rehbergerova M, Kohl A, Viginier B, Volf P, Ratinier M. 2025. Susceptibility of diverse sand fly species to Toscana virus. PLoS Neglected Tropical Diseases, 19(5), e0013031.
34. Kapp JD, Green RE, Shapiro B. 2021. A fast and efficient single-stranded genomic library preparation method optimized for ancient DNA. Journal of Heredity, 112(3), 241–249.
35. Killick-Kendrick R, Maroli M, Killick-Kendrick M. 1991. Bibliography of the colonization of phlebotomine sandflies. Parassitologia, 33(suppl.), 321–333.
36. Lawyer P, Killick-Kendrick M, Rowland T, Rowton E, Volf P. 2017. Laboratory colonization and mass rearing of phlebotomine sand flies (Diptera, Psychodidae). Parasite, 24, 42.
37. Léger N, Pesson B, Madulo-Leblond G. 1986. Les phlébotomes de Grèce : 1ère partie. Bulletin de la Société de Pathologie Exotique, 79, 386–397.
38. Léger N, Pesson B, Madulo-Leblond G. 1986. Les phlébotomes de Grèce : 2ème partie. Bulletin de la Société de Pathologie Exotique, 79, 514–524.
39. Leonel JAF, Vioti G, Alves ML, da Silva DT, Meneghesso PA, Benassi JC, Spada JCP, Galvis-Ovallos F, Soares RM, Oliveira T. 2020. DNA extraction from individual Phlebotomine sand flies (Diptera: Psychodidae: Phlebotominae) specimens: Which is the method with better results? Experimental Parasitology, 218, 107981.
40. Lestinova T, Rohousova I, Sima M, de Oliveira CI, Volf P. 2017. Insights into the sand fly saliva: Blood-feeding and immune interactions between sand flies, hosts, and *Leishmania.* PLoS Neglected Tropical Diseases, 11(7), e0005600.
41. Lienhard A, Schaffer S. 2019. Extracting the invisible: obtaining high quality DNA is a challenging task in small arthropods. PeerJ, 7, e6753.
42. Lozano-Sardaneta YN, Mikery-Pacheco OF, Huerta H, Rojas-Soriano JE, Contreras-Ramos A. 2025. Wing geometric morphometrics is effective to separate sand fly species (Diptera, Psychodidae, Phlebotominae) related with leishmaniasis transmission in Mexico. Acta Tropica, 262, 107523.
43. Mandrioli M. 2008. Insect collections and DNA analyses: how to manage collections? Museum Management and Curatorship, 23(2), 193–199.

44. Maroli M, Feliciangeli MD, Bichaud L, Charrel RN, Gradoni L. 2013. Phlebotomine sandflies and the spreading of leishmaniases and other diseases of public health concern. Medical and Veterinary Entomology, 27(2), 123–147.
45. Marquina D, Buczek M, Ronquist F, Lukasik P. 2021. The effect of ethanol concentration on the morphological and molecular preservation of insects for biodiversity studies. PeerJ, 9, e10799.
46. Mathis A, Depaquit J, Dvorak V, Tuten H, Banuls AL, Halada P, Zapata S, Lehrter V, Hlavackova K, Prudhomme J, Volf P, Sereno D, Kaufmann C, Pfluger V, Schaffner F. 2015. Identification of phlebotomine sand flies using one MALDI-TOF MS reference database and two mass pectrometer systems., Parasites & Vectors, 8, 266.
47. Mekarnia N, Benallal KE, Sadlova J, Vojtkova B, Mauras A, Imbert N, Longhitano M, Harrat Z, Volf P, Loiseau PM, Cojean S. 2024. Effect of *Phlebotomus papatasi* on the fitness, infectivity and antimony-resistance phenotype of antimony-resistant *Leishmania* major Mon-25. International Journal for Parasitology – Drugs and Drug Resistance, 25, 100554.
48. Milligan BG. 1998. Total DNA isolation, in Molecular Genetic Analysis of Population: A Practical Approach, Hoelzel AR, Editor. Oxford: Oxford University Press.
49. Molina R, Jiménez M, Alvar J, González E, Hernández-Taberna S, Ines MM. 2017. Methods in sand fly research. Madrid: Servicio de publicaciones Universidad de Alcalá de Henares, Madrid.
50. Murphy WJ, Eizirik E, O'Brien SJ, Madsen O, Scally M, Douady CJ, Teeling E, Ryder OA, Stanhope MJ, de Jong WW, Springer MS. 2001. Resolution of the early placental mammal radiation using Bayesian phylogenetics. Science, 294(5550), 2348–2351.
51. Nacif-Pimenta R, Pinto LC, Volfova V, Volf P, Pimenta PFP, Secundino NFC. 2020. Conserved and distinct morphological aspects of the salivary glands of sand fly vectors of leishma-niasis: an anatomical and ultrastructural study. Parasites & Vectors, 13(1), 441.
52. Neuhaus B, Schmid T, Riedel J. 2017. Collection management and study of microscope slides: Storage, profiling, deterioration, restoration procedures, and general recommendations. Zootaxa, 4322(1), 1–173.
53. New TR. 1974. Pscoptera. Handbooks for Identification of British Insects (Vol. I). London: Royal Entomological Society of London. 102 pp.
54. Perez-Ruiz M, Collao X, Navarro-Mari JM, Tenorio A. 2007. Reverse transcription, real-time PCR assay for detection of Toscana virus. Journal of Clinical Virology, 39(4), 276–281.
55. Porco D, Rougerie R, Deharveng L, Hebert P. 2010. Coupling non-destructive DNA extraction and voucher retrieval for small soft-bodied Arthropods in a high-throughput context: the example of Collembola. Molecular Ecology Resources, 10(6), 942–945.
56. Prudhomme J, Cassan C, Hide M, Toty C, Rahola N, Vergnes B, Dujardin JP, Alten B, Sereno D, Banuls AL. 2016. Ecology and morphological variations in wings of *Phlebotomus ariasi* (Diptera: Psychodidae) in the region of Roquedur (Gard, France): a geometric morphometrics approach. Parasites & Vectors, 9(1), 578.
57. Prudhomme J, Gunay F, Rahola N, Ouanaimi F, Guernaoui S, Boumezzough A, Banuls AL, Sereno D, Alten B. 2012. Wing size and shape variation of *Phlebotomus papatasi* (Diptera: Psychodidae) populations from the south and north slopes of the Atlas Mountains in Morocco. Journal of Vector Ecology, 37(1), 137–147.
58. Prudhomme J, Toty C, Kasap OE, Rahola N, Vergnes B, Maia C, Campino L, Antoniou M, Jimenez M, Molina R, Cannet A, Alten B, Sereno D, Banuls AL. 2015. New microsatellite markers for multi-scale genetic studies on *Phlebotomus ariasi* Tonnoir, vector of *Leishmania infantum* in the Mediterranean area. Acta Tropica, 142, 79–85.
59. Prudhomme J, Velo E, Bino S, Kadriaj P, Mersini K, Gunay F, Alten B. 2019. Altitudinal variations in wing morphology of *Aedes albopictus* (Diptera, Culicidae) in Albania, the region where it was first recorded in Europe. Parasite, 26, 55.
60. Rawlins DJ. 1992. Light Microscopy: An Introduction to Biotechniques. Oxford: Bios Scientific publishers. 143 pp.
61. Ready PD. 2013. Biology of phlebotomine sand flies as vectors of disease agents. Annual Review of Entomology, 58, 227–250.
62. Rohlf FJ, Slice D. 1990. Extensions of the Procrustes method for the optimal superimposition of landmarks. Systematic Zoology, 39(1), 40–59.
63. Rowley DL, Coddington JA, Gates MW, Norrbom AL, Ochoa RA, Vandenberg NJ, Greenstone MH. 2007. Vouchering DNA-barcoded specimens: Test of a nondestructive extraction proto-col for terrestrial arthropods. Molecular Ecology Notes, 7(6), 915–924.
64. Sábio PB, Andrade AJ, Galati EAB. 2014. Assessment of the taxonomic status of some species included in the *Shannoni* complex, with the description of a new species of *Psathyromyia* (Diptera: Psychodidae: Phlebotominae). Journal of Medical Entomology, 51(2), 331–341.
65. Sadlova J, Yeo M, Seblova V, Lewis MD, Mauricio I, Volf P, Miles MA. 2011. Visualisation of *Leishmania donovani* fluo-rescent hybrids during early stage development in the sand fly vector. PLoS One, 6(5), e19851.
66. Sales K, Miranda DEO, da Silva FJ, Otranto D, Figueredo LA, Dantas-Torres F. 2020. Evaluation of different storage times and preservation methods on phlebotomine sand fly DNA concen-tration and purity. Parasites & Vectors, 13(1), 399
67. Sales KG, Costa PL, de Morais RC, Otranto D, Brandao-Filho SP, Cavalcanti Mde P, Dantas-Torres F. 2015. Identification of phlebotomine sand fly blood meals by real-time PCR. Parasites& Vectors, 8, 230.
68. Sant'Anna MR, Jones NG, Hindley JA, Mendes-Sousa AF, Dillon RJ, Cavalcante RR, Alexander B, Bates PA. 2008. Blood meal identification and parasite detection in laboratory-fed and field-captured *Lutzomyia longipalpis* by PCR using FTA databasing paper. Acta Tropica, 107(3), 230–237.
69. Senne NA, Santos HA, Araujo TR, Paulino PG, Mendonca LP, Moreira HVS, Camilo TA, da Costa Angelo I. 2022. Robust comparative performance of genomic DNA extraction methods from non-engorged phlebotomine sandflies. Medical and Veterinary Entomology, 36(2), 203–211.
70. Shaw JJ. 2025. A review of Leishmania infections in American Phlebotomine sand flies – Are those that transmit leishmaniasis anthropophilic or anthropportunists? Parasite, 32, 57.
71. Tesh RB, Modi GB. 1983. Growth and transovarial transmission of Chandipura virus (Rhabdoviridae: Vesiculovirus) in *Phlebotomus papatasi.* American Journal of Tropical Medicine and Hygiene, 32(3), 621–623.
72. Thomsen PF, Elias S, Gilbert MTP, Haile J, Munch K, Kuzmina S, Froese DG, Sher A, Holdaway RN, Willerslev E. 2009. Non-destructive sampling of ancient insect DNA. PLoS One, 4(4),e5048
73. Truett GE, Heeger P, Mynatt RL, Truett AA, Walker JA, Warman ML. 2000. Preparation of PCR-quality mouse genomic

DNA with hot sodium hydroxide and tris (HotSHOT). Biotechniques, 29(1), 52–54.
74. Upton MS. 1993. Aqueous gum-chloral slide mounting media: an historical review. Bulletin of Entomological Research, 83(2), 267–274.
75. Volf P, Myskova J. 2007. Sand flies and Leishmania: specific versus permissive vectors. Trends in Parasitology, 23(3), 91–92.
76. Wang Q, Wang X. 2012. Comparison of methods for DNA extraction from a single chironomid for PCR analysis. Pakistan Journal of Zoology, 44(2), 421–426.
77. Wang Y, Zhao Y, Bollas A, Wang Y, Au KF. 2021. Nanopore sequencing technology, bioinformatics and applications. Nature Biotechnology, 39(11), 1348–1365.

**Cite this article as:** Randrianambinintsoa FJ, Augendre L, Prudhomme J, Martinet J-P, Loyer M, Mekarnia N, Kerkoub H, Perveen FK, Huguenin A, Kariya E, Akhoundi M, De Andrade AJ, Berriatua E, Bongiorno G, Boyer S, Christodoulou V, Da Costa-Ribeiro MCV, De Souza LAF, Ding H, Dondji B, Dvořák V, Erisoz Kasap O, Galati EAB, Gállego M, Ballart C, Gouzelou S, Haddad N, Masse RS, Mekuria AH, Ivovic V, Kaczmarek S, Shahar MK, Kirstein OD, Kniha E, Kolářová I, Lincoln T, Lucanas C, Mikov O, Nov K, Özbel Y, Pesson B, Posada Lopez LC, Prasetyo DB, Rahola N, Rebollar-Tellez EA, Rodrigues BL, Roy L, Saini P, Sanjoba C, Shimabukuro PH, Siriyasatien P, Soszynska A, Suleşco T, Sylla M, Torno M, Volf P, Vongphayloth K, Sinh Nam V, Wardhana A, Yessinou E, Zapata S, Gantier J-C & Depaquit J. 2026. Processing and mounting phlebotomine sand flies: a consensus guideline. Parasite xx, xx. https://doi.org/10.1051/parasite/2026009

PARASITE

An international open-access, peer-reviewed, online journal publishing high quality papers on all aspects of human and animal parasitology

Reviews, articles and short notes may be submitted. Fields include, but are not limited to: general, medical and veterinary parasitology; morphology, including ultrastructure; parasite systematics, including entomology, acarology, helminthology and protistology, and molecular analyses; molecular biology and biochemistry; immunology of parasitic diseases; host-parasite relationships; ecology and life history of parasites; epidemiology; therapeutics; new diagnostic tools.
All papers in Parasite are published in English. Manuscripts should have a broad interest and must not have been published or submitted elsewhere. No limit is imposed on the length of manuscripts.

**Parasite** (open-access) continues **Parasite** (print and online editions, 1994-2012) and **Annales de Parasitologie Humaine et Comparée** (1923-1993) and is the official journal of the Société Française de Parasitologie.

Editor-in-Chief:
Jean-Lou Justine, Paris

Submit your manuscript at:
https://www.editorialmanager.com/parasite

## Приложение 1: Биохимические теоретические основы.

В данной работе речь идёт о членистоногих, в частности о москитах. Однако, общие принципы могут быть распространены и на других очень распространённых членистоногих, идентификация которых возможна только по внутренним морфологическим признакам. К счастью, некоторые внутренние органы частично хитинизированы, и их морфология предоставляет нам ценную информацию. Именно поэтому очень интересно наблюдать за пищевыми насосами, сперматеками и их протоками. При работе со всеми здесь рассматриваемыми реагентами, не следует забывать, что от стадии фиксации насекомого до монтирования мы, по сути, применяем окислительно-восстановительные реакции. Единственная мера предосторожности или рекомендация — избегать смешивания восстанавливающих реагентов с окисляющими.

**Этиловый спирт; этанол**:

Это вещество используется различными способами. Молекулы спирта обладают сильным сродством к воде и, следовательно, проявляют обезвоживающий эффект. Однако спирт в низкой концентрации (т.е., слишком богатый водой) будет способствовать деградации нуклеиновых кислот (вода является врагом нуклеиновых кислот).

Когда насекомых помещают в этанол, это делается не только для их сохранения, но и для фиксации тканей. В гистологии обычно различают два важных понятия: скорость проникновения и скорость фиксации. Хорошо известно, что хороший консервант должен сначала быстро проникнуть глубоко в ткани, прежде чем зафиксировать их. Для 96% спирта коэффициент проникновения составляет приблизительно 1,05 (для сравнения, для 0,75% водного раствора пикриновой кислоты коэффициент проникновения составляет 0,45, а для 3% раствора дихромата калия — 1,45).

Желание сохранять насекомых и других членистоногих в этаноле неограниченно долго является реальностью для энтомологов. Идея сохранения полевых образцов для последующих исследований или для будущих учёных по-прежнему очень актуальна. Однако такой подход несовместим с требованиями цитолога или гистолога. Попытка слишком долгого хранения образцов в фиксаторе может сделать их практически непригодными для дальнейшей работы. Именно поэтому образцы старше 10 лет трудно или даже невозможно использовать.

Ещё один фактор, который следует учитывать, — это соотношение между массой фиксируемых членистоногих и объёмом фиксатора. В зоологической или медицинской практике рекомендуется использовать объем фиксатора в 60 раз больше объёма фиксируемых образцов. На практике, для микрочленистоногих, на заданный объем фиксируемых образцов следует добавлять не менее 4–5 объёмов спирта. Следует помнить, что спирт теряет свою концентрацию, поглощая всю воду, присутствующую в тканях членистоногих.

В заключение:

- Этиловый спирт является восстанавливающим химическим агентом (следовательно, несовместим с окислительными фиксаторами);
- Он вызывает интенсивное осаждение и денатурацию белков;
- Он растворяет некоторые сложные липиды и осаждает гликоген;
- Он вызывает сильное сокращение тканей и приводит к их затвердению.

**Основные растворы гидроксида калия или натрия:**

Использование этих растворов в энтомологии в основном сосредоточено на гидроксиде калия без чёткого обоснования.

Гидроксид натрия [E524] доступен в растворе различной концентрации или нормальности, а также в виде гранул или хлопьев. Его главным недостатком является высокая гигроскопичность (выше, чем у KOH). При взаимодействии с белками он растворяет их, а с липидами, превращает их в твёрдые мыла в процессе омыления (существенное отличие от KOH, который в этой реакции образует жидкие мыла).

Гидроксид калия [E525] доступен в виде концентрированного раствора, но особенно в виде гранул массой приблизительно 0,1 г, что значительно облегчает приготовление разбавленных растворов, когда нет возможности использовать прецизионные весы. Например, 1 гранула весом 0,1 г в 1 мл дистиллированной воды даёт 10%-ный раствор. Ещё одним преимуществом гидроксида калия в гранулах заключается в его меньшей чувствительности к карбонизации (раствор KOH обладает высокой способностью связывать $CO_2$, образуя таким образом карбонатные соли).

Эти сильные щёлочи используются для растворения жирных кислот, превращая их в водорастворимые мыла. Следует помнить, что фиксатор, такой как этанол, растворяет некоторые жиры в образце. Однако, когда образец помещают в водную среду, содержащую сильную щёлочь, жирные кислоты (различной сложности) выпадают в осадок. Таким образом, сильная щёлочь осуществляет холодное омыление. В некоторых случаях, когда жировой ткани избыток, например, у самок, будет целесообразно повысить температуру до 35–40 °C для облегчения реакции или увеличить время контакта при комнатной температуре.

**Раствор Марка-Андре, окрашенный фуксином или неокрашенный:**

Здесь мы рассмотрим преимущества и недостатки использования раствора Марка-Андре. Этот раствор состоит из хлоралгидрата (моногидрата трихлорацетальдегида), уксусной кислоты и воды. Этот раствор обладает высокой окислительной способностью (смесь кислоты и альдегида). Он нейтрализует избыток гидроксида калия, который может оставаться в образцах, не вызывая осаждения щелочных мыл, образующихся при использовании гидроксида калия. Этот окислительный раствор также воздействует на вторичные спиртовые группы глюкозаминов, входящих в состав хитина, окисляя их и тем самым размягчая хитин. Он также растворяет некоторые присутствующие минеральные соли.

Если раствор Марка-Андре предварительно окрашен кислым фуксином (следовательно, находится в окисленном состоянии), он способен связываться со вторичными спиртовыми группами хитиновых структур. По истечении времени контакта с раствором Марка-Андре и окрашивания образцов, проводится промывка только этанолом. Это запускает фазу дегидратации образцов.

**Преимущества:**
- Нейтрализация избытка щелочных растворов
- Размягчение хитина
- Окрашивание хитина позволяет лучше рассмотреть внутренние хитинизированные структуры.

**Недостатки:**

Хлоралгидрат обладает снотворным действием, исторически используемым в медицине. Его применение должно осуществляться под вытяжным шкафом и в строгом соответствии с правилами, касающимися химических рисков.

**Растворы для обезвоживания:**

Опыт показывает, что для очень мелких образцов нет необходимости следовать последовательной обработке спиртовыми растворами возрастающей концентрации. Если образец большой, следует начать с 80% этанола, затем перейти к 90%, 95% и, наконец, к абсолютному этанолу. Для очень малых образцов достаточно 90% этанола с последующим погружением в абсолютный этанол. На этом этапе всегда важно помнить, что абсолютный этанол имеет тенденцию поглощать воду из атмосферы.

Традиционно, в энтомологических лабораториях, окончательная дегидратация образцов проводилась с использованием креозота бука. Сегодня эта эссенция, когда-то широко используемая в качестве пестицида, фунгицида и консерванта древесины, крайне не рекомендуется из-за своего запаха (полициклические ароматические углеводороды), и предполагаемой токсичности: репродуктивно токсична, канцерогенна, является стойким органическим загрязнителем и экотоксична для водных организмов.

Предлагаемый нами способ монтирования образцов — использование Euparal® и эссенции Euparal (описано в следующем абзаце). Смесь Euparal® и эссенции Euparal хорошо переносится образцами, которые предварительно прошли обработку в 90% этаноле.

## Приложение 2: Состав используемых реагентов.

**10% Гидроксид калия**
Гидроксид калия 10 г
Дистиллированная вода до 100 мл

**Монтирующая среда хлораловая камедь (жидкость Хойера)**
Дистиллированная вода 50 мл
Хлоралгидрат 200 г
Гуммиарабик 50 г
Глицерин 20 мл

**Раствор Марка-Андре**
Хлоралгидрат 40 г
Ледяная уксусная кислота 30 мл
Дистиллированная вода 30 мл

**1% Фуксиновая кислота в дистиллированной воде**
Порошок кислого фуксина 1 г
Дистиллированная вода 99 мл

**Раствор Марка-Андре, окрашенный фуксином.**
Раствор Марка-Андре 10 мл
1% Фуксин 50 мкл

## Приложение 3: Euparal®, канадский бальзам, поливиниловый спирт или другие монтирующие растворы.

**Поливиниловый спирт:** Он является идеальной монтирующей средой для образцов, когда отсутствуют необходимые для надлежащей дегидратации продукты. В этом случае поливиниловый спирт смешивают с лактофенолом Аманна. Однако, у этой смеси есть существенные недостатки: либо среда высыхает, либо поливиниловый спирт кристаллизуется из-за испарения воды, либо препарат темнеет при окислении фенола. Тем не менее, этот метод остаётся подходящим для кратковременной фиксации.

**Канадский бальзам:** Использование канадского бальзама для заключения образца между предметным и покровным стёкол требует предварительной дегидратации образца. Использование ксилола или толуола на этом этапе имеет свои недостатки.

**Среда Enecê** Как и канадский бальзам, среда Enecê требует предварительной дегидратации образцов перед монтированием на предметное стекло. Состав среды Enecê: чистая белая канифоль (22 г); растворимая в спирте копаловая камедь (12 г); абсолютный спирт (20 мл); камфора (10 г); скипидарная эссенция (10 мл); эвкалиптол (26 мл). Приготовление: В подходящую ёмкость (напр. колбу Эрленмейера) помещают абсолютный спирт и камфору, затем добавляют канифоль и копаловую камедь. Затем колбу закрывают пробкой и встряхивают, и только после этого нагревают на водяной бане при умеренной температуре, чтобы избежать кипения. После полного разжижения содержимого добавляют скипидарную эссенцию. Затем смесь фильтруют в горячем состоянии, и к фильтрату добавляют эвкалиптол. Когда среда становится менее текучей, её разбавляют раствором Enecê, приготовленный по следующей формуле: абсолютный спирт (30 мл), камфора (17 г), скипидарная эссенция (15 мл) и эвкалиптол (38 мл) (Cerqueira, 1943).

**Euparal®:** Это смола, получаемая из атласского кипариса, *Tetraclinis articulata* (Vahl, 1791), изученная и разработанная Гилсоном в 1906 году. Её главное преимущество заключается в том, что она не полимеризуется. Образцы, заключённые между предметным и покровным стеклом, легко извлекаются с помощью спирта или, что ещё лучше, эссенции Euparal®. Эта смола, также известная как сандарак, выдерживает концентрацию этанола 80% и выше.

**Применение Triton X-100: неионогенный водный раствор**

Triton X-100 представляет собой неионогенный водный раствор (*раствор 4-(1,1,3,3-тетраметилбутил) фенилполиэтиленгликоля* или *т-октилфеноксиполиэтоксиэтанол*, *трет-октилфениловый эфир полиэтиленгликоля*), широко используемый в качестве детергента в клеточной и молекулярной биологии. Он позволяет повысить проницаемость клеточных и ядерных мембран.

Образцы насекомых, хранящиеся в спирте в течение многих лет, являются обычным явлением. К сожалению, консервация в спирте не является оптимальной, и членистоногие, сохранённые таким образом, становятся очень сложными для подготовки к микроскопическому исследованию. Пластик, содержащий образцы, часто разрушается, что приводит к испарению спирта. В обоих случаях длительный контакт со спиртом или высыхание образцов представляют собой серьёзную проблему. В 2008 году Жонк (Jonque) опубликовал заметку о регидратации пауков с помощью смачивающего агента, такого как Агепон, используемый для фотопленок [26]. Это привело к идее использования смачивающих агентов, которые не являются сильными детергентами.

Ниже описана процедура с использованием 0,5% водного раствора Triton X-100:

- Пропитайте сухой образец абсолютным спиртом.
- Добавьте необходимый объем 0,5% раствора Triton X-100 так, чтобы весь образец был погружен в него.
- Оставьте на 5 минут или дольше. Все членистоногие должны отделиться друг от друга в растворе.
- Раствор Triton X-100 удаляется и заменяется раствором гидроксида калия.

Затем применяется описанная выше методика.

## Приложение 4: Пошаговое руководство по применению монтирующих сред Euparal® или канадского бальзама

1. Образцы должны быть обезвожены (мутный или молочный вид указывает на недостаточную дегидратацию).
2. Обезвоживание может быть достигнуто путём повышения концентрации этилового спирта.
3. Образцы можно переносить из 99% спирта или абсолютного спирта в просветляющее средство.

**Процедура:**

1. Поместите взрослых москитов в 70% этаноле.
2. Удалите этанол и замените его 10% раствором KOH. Накройте москитов предметным стеклом.
3. Оставьте настаиваться до тех пор, пока насекомые не станут полупрозрачными.
4. Удалите KOH.
5. Залейте образец дистиллированной водой и подождите от 30 до 45 минут.
6. Удалите воду и повторите промывку дистиллированной водой в течение 30 минут (время зависит от количества одновременно обрабатываемых образцов: чем больше образцов, тем дольше необходимо соблюдать это время; наоборот, для небольшого количества образцов, особенно обрабатываемых индивидуально, это время можно сократить).
7. Удалите воду.
8. Добавьте раствор Марка-Андре (возможно, окрашенный фуксином) и подождите 24 часа (один день).
9. Удалите раствор Марка-Андре.
10. Залейте образец дистиллированной водой и подождите от 30 до 45 минут.
11. Слейте воду и повторите промывку дистиллированной водой в течение 30 минут.
12. Удалите воду.
13. Добавьте 70% этанол и проведите препарирование образца.

    a. Чтобы отделить голову и брюшко от груди, осторожно потяните их.

    b. Для препарирования груди, удалите крылья, удерживая грудь одной парой пинцетов и оттягивая основание придатков другой парой. Также возможно

выполнить сагиттальный разрез, разделив грудную клетку на левую и правую части.

14. Постепенно обезвоживайте, последовательно пропуская через ряд водных растворов этилового спирта: 50 – 80 – 95%, пока не достигните абсолютного этанола.

15. Завершите обезвоживание образцов, дважды выдерживая их в 100% этаноле по 10 минут каждый раз.

16. Удалите этанол и поместите образцы в гвоздичное масло на 15 минут при комнатной температуре.

17. Перенесите образцы из гвоздичного масла в каплю Euparal® или канадского бальзама на чистом предметном стекле.

18. Расположите образцы в соответствии с желаемой ориентацией: голову, грудь и брюшко москита можно препарировать с помощью тонких игл или пинцета под стереомикроскопом. Голову необходимо отделить от тела и закрепить в вентро-дорсальном положении, то есть затылочным отверстием вверх, чтобы через него можно было увидеть цибариум.

Препарирование проводится в монтирующей среде для москитов.

19. Оставьте образец до тех пор, пока его поверхность не станет липкой.

20. Смочите чистое покровное стекло чистым спиртом. Поместите покровное стекло на канадский бальзам или Euparal® под углом.

21. Храните предметные стекла в специально предназначенном для этого сухом боксе.
