# Supplementary material for: Processing and mounting phlebotomine sand flies: a consensus guideline
Source: Parasite. 2026 Apr 3;33:18. doi: 10.1051/parasite/2026009 (PMC13047900; doi:10.1051/parasite/2026009)
Supplement: Supplementary file 26 — Serbian translation / Превод на српски [file parasite-33-18-s26.pdf]

Parasite **33**, 18 (2026)
© F.J. Randrianambinintsoa et al., published by EDP Sciences, 2026
https://doi.org/10.1051/parasite/2026009

PARASITE
Available online at:
www.parasite-journal.org

REVIEW ARTICLE OPEN ACCESS

Priprema i prepariranje flebotomina: smernice zasnovane na konsenzusu

Fano José Randrianambinintsoa[1], Laure Augendre[1], Jorian Prudhomme[1], Jean-Philippe Martinet[1], Mathieu Loyer[1], Nalia Mekarnia[1], Hocine Kerkoub[1], Farzana Khan Perveen[1], Antoine Huguenin[1,2], Emilie Kariya[1,2], Mohammad Akhoundi[3], Andrey José de Andrade[4], Eduardo Berriatua[5], Gioia Bongiorno[6], Sébastien Boyer[7,8], Vasiliki Christodoulou[9], Magda Clara Vieira Da Costa-Ribeiro[10], Lucas Alexandre Farias de Souza[10], Huicong Ding[11], Blaise Dondji[12], Vít Dvořák[13], Ozge Erisoz Kasap[14], Eunice Aparecida Bianchi Galati[15], Montserrat Gállego[16], Cristina Ballart[16], Stavroula Gouzelou[17], Nabil Haddad[18], Rezki Sabrina Masse[19], Asrat Hailu Mekuria[20], Vladimir Ivovic[21], Szymon Kaczmarek[22], Mohd Khadri Shahar[19], Oscar D. Kirstein[23], Edwin Kniha[24], Iva Kolářová[13], Lincoln Timinao[25], Cristian Lucanas[26], Ognyan Mikov[27], Kimsear Nov[7], Yusuf Özbel[28], Bernard Pesson[29], Laura Cristina Posada Lopez[30], Didot Budi Prasetyo[1,7], Nil Rahola[31], Eduardo A. Rebollar-Tellez[32], Bruno Leite Rodrigues[15], Lalita Roy[33], Prasanta Saini[34], Chizu Sanjoba[35], Paloma Helena Fernandes Shimabukuro[36], Padet Siriyasatien[37], Agnieszka Soszyńska[22], Tatiana Suleşco[38], Massamba Sylla[39], Majhalia Torno[40], Petr Volf[13], Khamsing Vongphayloth[41], Vu Sinh Nam[42], April Wardhana[43], Eric Yessinou[44], Sonia Zapata[45], Jean-Charles Gantier[1], and Jérôme Depaquit[1,2,*]

[1] Faculté de Pharmacie, Université de Reims Champagne Ardenne, UR ESCAPE-USC ANSES PETARD, 51 rue Cognacq-Jay, 51096 Reims Cedex, France
[2] Pôle de Biologie territoriale, Laboratoire de Parasitologie-Mycologie, Centre Hospitalo-Universitaire, 51092 Reims, France
[3] Parasitology-Mycology Department, Avicenne Hospital, AP-HP, Bobigny, Sorbonne Paris Nord University, France; Unité des Virus Émergents (UVE: Aix-Marseille Univ, Università di Corsica, IRD 190, Inserm 1207, IRBA), 13005 Marseille, France
[4] Parasitology Collection of Basic Pathology, Department of Basic Pathology, Federal University of Paraná, Curitiba 19031, Brazil
[5] Department of Animal Health, University of Murcia, Campus de Espinardo, 30100 Espinardo, Murcia, Spain
[6] Department of Infectious Diseases, Vector-borne Diseases Unit, Istituto Superiore di Sanità, 00166 Rome, Italy
[7] Medical and Veterinary Entomology Unit, Institut Pasteur du Cambodge, Phnom Penh 12201, Cambodia
[8] Ecology & Emergence of Arthropod-borne Pathogens Unit, Department of Global Health, Institut Pasteur, CNRS UMR2000, 75015 Paris, France
[9] Section Veterinary Services (1417), Laboratory for Animal Health Virology, Aglantzia, Nicosia 2109, Cyprus
[10] Insects Vectors and Parasites Laboratory, Department of Basic Pathology and Postgraduate program in Microbiology, Parasitology and Pathology, Federal University of Paraná, 81530-900 Curitiba, Brazil
[11] Department of Biological Sciences, National University of Singapore, 117558, Singapore
[12] Laboratory of the Leishmaniasis Research Project, Mokolo District Hospital, Mokolo, Cameroon; Laboratory of Cellular Immunology and Parasitology, Department of Biological Sciences, Central Washington University, 98926 Ellensburg, WA, USA
[13] Department of Parasitology, Faculty of Science, Charles University, 12800 Prague, Czechia
[14] VERG Laboratories, Department of Biology, Faculty of Science, Hacettepe University, Beytepe, Ankara 06800, Türkiye
[15] Faculdade de Saúde Pública da Universidade de São Paulo (FSP/USP), Pós-graduação em Saúde Pública, 01246-904 São Paulo, Brazil
[16] Secció de Parasitologia, Departament de Biologia, Sanitat i Medi Ambient, Facultat de Farmàcia i Ciències de l'Alimentació, Universitat de Barcelona, & Institut de Salut Global de Barcelona (ISGlobal), Centro de Investigación Biomédica en Red, Enfermedades Infecciosas (CIBERINFEC), 08028 Barcelona, Spain
[17] Laboratory of Infectious Diseases and Public Health, School of Medicine, University of Cyprus, Nicosia, Cyprus & Department of Pediatrics, Archbishop Makarios III Hospital, Nicosia 2115, Cyprus
[18] Faculty of Health Sciences, American University of Beirut, 1107 2020 Beirut, Lebanon
[19] Medical Entomology Unit, Infectious Disease Research Centre, Institute for Medical Research (IMR), National Institutes of Health (NIH), Ministry of Health Malaysia, 40170 Shah Alam, Selangor, Malaysia

Edited by Jean-Lou Justine
*Corresponding author: jerome.depaquit@univ-reims.fr

This is an Open Access article distributed under the terms of the Creative Commons Attribution License (https://creativecommons.org/licenses/by/4.0), which permits unrestricted use, distribution, and reproduction in any medium, provided the original work is properly cited.

[20] School of Medicine, Addis Ababa University, 28017 - 1000 Addis Ababa, Ethiopia
[21] Faculty of Mathematics, Natural Sciences and Information Technologies, University of Primorska, 6000 Koper, Slovenia
[22] University of Lodz, Faculty of Biology and Environmental Protection, Department of Invertebrate Zoology and Hydrobiology, Banacha 12/16, 90-237 Łódź, Poland
[23] Laboratory of Entomology, Ministry of Health, 9134302 Jerusalem, Israel
[24] Center for Pathophysiology, Infectiology and Immunology, Institute of Specific Prophylaxis and Tropical Medicine, Medical University Vienna, Kinderspitalgasse 15, 1090 Vienna, Austria
[25] Papua New Guinea Institute of Medical Research (PNGIMR) Institute, PO Box 60, Headquarter, Homate Street, 441 Goroka, Eastern Highlands Province, Papua New Guinea
[26] Museum of Natural History, University of the Philippines Los Baños, 4031 Laguna, Philippines
[27] National Centre of Infectious and Parasitic Diseases, 1504 Sofia, Bulgaria
[28] Ege University, Faculty of Medicine, Department of Parasitology, 35040 Bornova/Izmir, Türkiye
[29] Retired, Faculté de Pharmacie, Université de Strasbourg, Strasbourg, 67400 Illkirch-Graffenstaden, France
[30] Program for the Study and Control of Tropical Diseases (PECET), Faculty of Medicine, University of Antioquia, 050010 Medellin, Colombia
[31] MIVEGEC, Univ. Montpellier, CNRS, IRD, 34394 Montpellier, France & Medical Entomology Unit, Institut Pasteur de Madagascar, 101 Antananarivo, Madagascar
[32] Laboratorio de Entomología Médica, Departamento de Zoología de Invertebrados, Facultad de Ciencias Biológicas, Universidad Autónoma de Nuevo León, San Nicolás de los Garza, 66455, NL, México
[33] Tropical and Infectious Disease Centre, BP Koirala Institute of Health Sciences, Dharan 56700, Nepal
[34] ICMR-Vector Control Research Centre, Puducherry 605006, India
[35] Graduate School of Agricultural and Life Sciences, The University of Tokyo, Tokyo 113-8657, Japan
[36] Grupo de estudos em Leishmanioses/Coleção de Flebotomíneos (COLFLEB/Fiocruz-MG), Instituto René Rachou, Fundação Oswaldo Cruz, Belo Horizonte, Minas Gerais, 30190009, Brazil
[37] Center of Excellence in Vector Biology and Vector-Borne Disease, Department of Parasitology, Faculty of Medicine, Chulalongkorn University, Bangkok 10330, Thailand
[38] Department of Arbovirology, Bernhard Nocht Institute for Tropical Medicine, Bernhard Nocht Str. 74, 20359 Hamburg, Germany [39] Laboratory Vectors & Parasites, Department of Livestock Sciences and Techniques, Sine Saloum University El Hadji Ibrahima Niasse (SSUEIN) Kaffrine Campus, C.P. 24600, Senegal.
[40] Environmental Health Institute, National Environment Agency, Singapore 138667, Singapore & Department of Biological Sciences, National University of Singapore, 117558 Singapore
[41] Institut Pasteur du Laos, Laboratory of Vector-Borne Diseases, Samsenhai Road, Ban Kao-Gnot, Sisattanak District, 3560 Vientiane, Lao PDR
[42] National Institute of Hygiene and Epidemiology, 1 Yec-Xanh Street, Hai Ba Trung District, 100000 Hanoi, Vietnam
[43] Indonesian Research Center for Veterinary Science, Indonesian Agency for Agricultural Research and Development, Ministry of Agriculture Republic Indonesia, Bogor 16114, Indonesia & Department of Parasitology, Faculty of Veterinary Medicine, Airlangga University, Surabaya 60115, Indonesia
[44] Laboratory of Research in Applied Biology, Polytechnic School of Abomey-Calavi, University of Abomey-Calavi, 01 P.O. Box 2009, 00000 Cotonou, Benin
[45] Instituto de Microbiología, Colegio de Ciencias Biológicas y Ambientales (COCIBA), Universidad San Francisco de Quito (USFQ), 170901 Quito, Ecuador

Received 1 December 2025, Accepted 29 January 2026, Published online 3 April 2026

Sažetak – Ovaj rad pruža sveobuhvatni vodič za obradu i prepariranje primeraka flebotomina, što je od ključnog značaja za identifikaciju vrsta, kao i za detekciju i izolaciju patogena. Opisane su različite tehnike pogodne kako za terenske, tako i za laboratorijske uslove. Smernice uključuju detaljna uputstva za sakupljanje flebotomina, rukovanje, anesteziju i eutanaziju (uz preporuku suvog zamrzavanja ili CO_2 umesto hemijskih sredstava), kao i strategije čuvanja uzoraka, poput skladištenja na niskim temperaturama i konzervacije u etanolu. Kvalitet pripreme pojedinih anatomskih struktura (genitalnih organa, glave i krila) od presudnog je značaja za njihovo pravilno mikroskopsko posmatranje i detaljno je opisan u ovom radu. Takođe su predstavljeni detaljni postupci obrade uzoraka, uključujući proces prosvetljavanja pomoću sredstava kao što su kalijum-hidroksid i Marc-André rastvor. U delu koji se odnosi na prepariranje upoređeni su različiti medijumi, uz poseban naglasak na njihova optička svojstva i potencijal za dugotrajno očuvanje preparata. Hoyerov medijum (poznat i kao hloralna guma) preporučuje se za brza posmatranja, naročito spermateka, zbog svoje prozirnosti, iako nije pogodan za dugotrajno čuvanje. Ostali razmatrani medijumi uključuju polivinil-alkohol, Euparal® (sa ograničenom tolerancijom na vodu) i kanada balzam (medijum rastvorljiv u ugljovodonicima), pri čemu poslednja dva omogućavaju dugotrajnu konzervaciju preparata. Takođe su obrađeni savremeni pristupi molekularne biologije, kao što su DNK sekvenciranje i MALDI-ToF, koji zahtevaju posebnu pažnju u obradi uzoraka. Pored toga, dostupni su kratki video-snimci koji ilustruju različite tehnike prepariranja, kao i prevodi na 33 jezika, čime se ove smernice prilagođavaju raznovrsnim potrebama i očekivanjima globalne naučne zajednice.

Ključne reči: Prepariranje, flebotomine, Hoyerov medijum, Marc-André rastvor, hloralna guma, polivinil-alkohol, Euparal®, kanada balzam, izolacija Leishmania, terenski uslovi, kultura, disekcija, molekularna biologija, MALDI-ToF, tipski primerci.

Abstract – Processing and mounting phlebotomine sand flies: a consensus guideline. This article provides a comprehensive guide for the processing and mounting of phlebotomine sand fly specimens, which is crucial for species identification and pathogen detection and isolation. It discusses a range of techniques suitable for both field and laboratory settings. The guide includes detailed instructions on sand fly collection, handling, covering, and euthanasia (recommending dry freezing or CO_2 over chemicals) as well as conservation strategies, such as cold storage and preservation in ethanol. The quality of preparation of certain anatomical structures (genital organs, head and wings) is essential for their proper microscopic observation and is described in this work. The article also presents detailed sample processing, including the clearing process with agents such as potassium hydroxide then Marc-André solution. The mounting process compares different media, emphasizing their optical properties and preservation potential. Hoyer fluid (also known as chloral gum) is recommended for quick observation, particularly for spermathecae, due to its clarity, although it is not suitable for long-term storage. Other media discussed include polyvinyl alcohol, Euparal® (for limited water tolerance), and Canada balsam (a hydrocarbon-soluble medium), with the latter two offering long-term preservation capabilities. Innovative molecular biology approaches such as DNA sequencing and MALDI-ToF, which require particular attention to sample processing, are also addressed. Furthermore, short video clips illustrating various mounting techniques as well as translations in many different languages are provided, allowing the guideline to reach the diverse needs and expectations of the global scientific community.

Key words: Mounting, Phlebotomine sand fly, Hoyer fluid, Marc-André solution, Chloral gum, Polyvinyl alcohol, Euparal®, Canada balsam, *Leishmania* isolation, Field conditions, Culture, Dissection, Molecular biology, MALDI-ToF, Type-specimens.

Uvod

Flebotomine su mali dvokrilni insekti koji pripadaju familiji Psychodidae, podfamiliji Phlebotominae, sa najmanje 1.063 do sada opisanih vrsta. Predstavljaju važne prenosioce patogena (rod *Leishmania*, arbovirusi i *Bartonella*) odgovornih za bolesti poznate kao lišmanijaze, arbovirusne infekcije i bartoneloze. Njihova identifikacija se prvenstveno zasniva na detaljnom mikroskopskom pregledu, koji zahteva pažljivo sakupljanje, odgovarajuće čuvanje i pravilno prepariranje uzoraka, pri čemu se primenjuje više specifičnih tehnika, od kojih svaka ima svoje prednosti i ograničenja.

Identifikacija adultnih flebotomina zasniva se na posmatranju spoljašnjih (npr. antene, palpi, genitalije mužjaka) i unutrašnjih struktura (npr. ždrelo, cibarium i spermateke). Disekcija i izolacija unutrašnjih struktura olakšavaju njihovo posmatranje i, samim tim, preciznu identifikaciju. Za razliku od komaraca ili stenica, flebotomine se pre identifikacije moraju preparirati između predmetnog i pokrovnog stakalca.

Do osamdesetih godina prošlog veka mikroskopsko posmatranje bilo je jedina dostupna metoda za identifikaciju flebotomina i ona i danas ostaje najčešće korišćen pristup. Izbor postupka obrade i prepariranja bio je stoga relativno jednostavan i uglavnom se svodio na dihotomiju: s jedne strane, trajno prepariranje koje omogućava dugotrajno čuvanje primerka, a s druge strane brzo prepariranje radi identifikacije u medijumu koji ne obezbeđuje dugotrajnu stabilnost preparata. Konačno prepariranje, na primer u smoli poput kanadskog balzama, vremenski je zahtevno i podrazumeva potpunu dehidrataciju uzoraka. Pored toga, indeks prelamanja ovog medijuma nije uvek optimalan za posmatranje spermateka. Suprotno tome, prepariranje u vodenim medijumima (npr. Hoyerov rastvor) je brže i omogućava bolje uočavanje spermateka, ali ne obezbeđuje dugotrajno očuvanje preparata, zbog toga što ovi medijumi imaju tendenciju da apsorbuju vlagu iz atmosfere. Jedna od mogućnosti jeste zaptivanje preparata bezbojnim lakom za nokte nakon potpunog sušenja. Ova kompromisna odluka i danas utiče na izbor metode prepariranja u zavisnosti od namene preparata. Od osamdesetih godina prošlog veka, studije identifikacije flebotomina kombinuju morfološke i biohemijske pristupe. Prvi takav pristup obuhvatao je analizu kutikularnih ugljovodonika, koju su ubrzo zamenile tehnike molekularne biologije, uključujući RAPD, RFLP, amplifikaciju DNK i sekvenciranje Sangerovom metodom, kao i metode sekvenciranja nove generacije (NGS). Danas su molekularni pristupi dopunjeni proteomskim metodama, poput MALDI-ToF. Pored toga, molekularna identifikacija vrsta može se kombinovati sa detekcijom patogena PCR metodama (rod *Leishmania*, *Trypanosoma*, *Bartonella* i flebovirusi), pri čemu se svi oni mogu detektovati klasičnom ili real-time PCR metodom, što zahteva prilagođavanje načina uzorkovanja i čuvanja materijala u skladu sa planiranim analizama. Pored morfoloških karakteristika koje se tradicionalno koriste za razlikovanje vrsta, mogu se primeniti i drugi morfološki pristupi, poput geometrijske morfometrije krila. Na osnovu iskustva autora i dostupnih podataka iz literature, cilj ovog rada je da se predlože standardizovane smernice za obradu i prepariranje adultnih primeraka flebotomina, u cilju optimizacije morfoloških i molekularnih analiza.

Potreba za sprovođenjem određenih analiza (npr. molekularnih metoda ili MALDI-ToF) zahteva očuvanje dela flebotomine koji nije neophodan za morfološku identifikaciju, što ukazuje na značaj pažljivog izbora protokola.

U ovom radu fokus je stavljen na metode anestezije i eutanazije živih flebotomina, njihovo čuvanje i postupke prepariranja, bilo radi brze identifikacije, bilo radi dugotrajnog očuvanja preparata koji omogućava naknadne analize.

Uvodna napomena: Bezbednosni i regulatorni aspekti rada sa hemikalijama treba da budu usklađeni sa odgovarajućim bezbednosno-tehničkim protokolima (Safety Data Sheets – SDS).

Sve hemikalije navedene u ovim smernicama moraju se koristiti uz strogo poštovanje bezbednosnih mera. Odbori za zaštitu zdravlja i bezbednost na radu u istraživačkim ustanovama dostupni su radi pružanja informacija ne samo o opasnostima koje ove hemikalije predstavljaju, već i o pravilnim postupcima rukovanja i odlaganja otpada. Ipak, obavezno je pridržavanje svih bezbednosnih uputstava koja se odnose na njihovu upotrebu i zbrinjavanje. Važno je naglasiti da je odgovornost svih korisnika da obezbede usklađenost sa dobrom i bezbednom laboratorijskom praksom, kao i sa važećim zakonodavstvom i propisima svoje zemlje ili istraživačke institucije. Pored toga, neke od navedenih hemikalija, ili njihovi sastojci (npr. hloral-hidrat), u pojedinim državama podležu regulatornim ograničenjima. Spisak skraćenica korišćenih u ovom radu dat je u Tabeli 1.

1. Sakupljanje flebotomina

Odrasli primerci flebotomina se mogu sakupljati živi ili mrtvi, primenom različitih metoda, kao što su CDC minijaturne svetlosne klopke, lepljive klopke i aspiratori koji se koriste u okviru Šenonovih klopki, ili direktnim sakupljanjem sa mesta mirovanja u prirodnoj sredini (npr. objekti za smeštaj životinja). Ove metode podrazumevaju postavljanje klopki u odgovarajuća staništa, privlačenje flebotomina svetlom ili drugim atraktantima (CO_2 ili hemijski mamci) i njihovo prikupljanje radi dalje analize, kako je opisano u više publikacija. Sakupljanje živih flebotomina omogućava primenu svih daljih analiza, dok prikupljanje uginulih jedinki onemogućava izolaciju sojeva Leishmania ili virusa. Neke metode sakupljanja, poput lepljivih papira, često dovode do gubitka delova tela flebotomina (antena, palpi, krila ili noge). Pored toga, ricinusovo ulje kojim su lepljivi papiri premazani prijanja uz telo flebotomina i mora se ukloniti na početku obrade uzoraka, najčešće potapanjem u trajanju od približno 15 minuta u mešavinu etanola i dietil-etra u jednakim zapreminskim odnosima.

2. Usmrćivanje primeraka

Tabela 1. Spisak skraćenica.

**BME**	Bazalni medijum Eagle
**CDC**	Centri za kontrolu i prevenciju bolesti
**CMCP**	Kamfor–monohlorofenol
**CMR**	Karcinogena, mutagena i reprotoksična supstanca
**COI**	Gen za podjedinicu I citohrom-c oksidaze
**CytB**	Gen za citohrom b
**DNA**	Dezoksiribonukleinska kiselina
**ELISA**	Imunoenzimski test (enzimski imunosorbentni test)
**EtOH**	Etanol
**M199**	Medijum 199
**MALDI-ToF MS**	Masena spektrometrija sa laserskom desorpcijom/jonizacijom uz vremenski prelet
**MEM**	Minimalni esencijalni medijum
**NGS**	Sekvenciranje sledeće generacije
**NNN**	Novy-MacNeal-Nicolle medijum
**PCR**	Lančana reakcija polimeraze
**Lao PDR**	Narodna Demokratska Republika Laos
**PNOC**	Gen za prepronociceptin
**qPCR**	Kvantitativni PCR (real-time PCR)
**RAPD**	Nasumično amplifikovana polimorfna DNK
**RFLP**	Polimorfizam dužine restrikcionih fragmenata
**RI**	Indeks prelamanja
**RNA**	Ribonukleinska kiselina
**RNases**	Ribonukleaze
**RNASS**	Rastvor za stabilizaciju RNK
**RT-PCR**	PCR sa reverznom transkripcijom
**TFA**	Trifluorosirćetna kiselina

Nakon sakupljanja, žive flebotomine moraju se eutanazirati. Kod pojedinih metoda sakupljanja (npr. lepljivi papiri, CDC svetlosne klopke opremljene posudom sa deterdžentom ili etanolom), flebotomine su već uginule u trenutku prikupljanja. Na takvim uzorcima moguće je primeniti metode molekularne biologije ukoliko su prikupljeni direktno u etanol ili ako se u etanol smeste u najkraćem mogućem roku. Međutim, nijedan od ovih načina usmrćivanja ne omogućava dalju obradu insekata za MALDI-ToF analizu. Pored toga, pojedini postupci eutanazije mogu dovesti do gubitka određenih morfoloških karaktera. Zbog toga je neophodno koristiti odgovarajuće,

standardizovane metode usmrćivanja kako bi se obezbedila pravilna identifikacija ili dugotrajno čuvanje primeraka kao referentnog materijala (voucher specimens).

Hemijska sredstva poput etil-acetata, dietil-etra, tetrakloroetana i hloroforma mogu se naneti na vatu koja se potom stavlja u posudu sa flebotominama radi njihove eutanazije. Ova sredstva moraju se koristiti sa velikim oprezom i u skladu sa preporukama proizvođača, zbog njihove toksičnosti. Ipak, ne preporučuje se usmrćivanje flebotomina hloroformom, jer je, prema iskustvu autora, slabo kompatibilan sa metodama molekularne biologije. Imajući u vidu opasna svojstva ovih supstanci, kao i njihovu ograničenu primenljivost u molekularnim analizama, upotreba hemijskih sredstava za eutanaziju se generalno ne preporučuje.

Najčešće korišćena metoda, koja u najvećoj meri čuva morfologiju, DNK i proteine, jeste suvo zamrzavanje primeraka. Flebotomine treba zamrzavati dovoljno dugo da budu potpuno anestezirane, ali ne predugo, kako bi se izbeglo (i) isušivanje primeraka ili (ii) narušavanje vitalnosti parazita *Leishmania*, ukoliko je cilj njihova izolacija *in vitro* iz digestivnog trakta flebotomina. **Zbog toga se preporučuje zamrzavanje u trajanju od 15 do 20 minuta na temperaturi od –20 °C, uz redovno praćenje stanja primeraka kako bi se osiguralo da su samo omamljeni, a da *Leishmania* paraziti ostanu živi**.

Ukoliko zamrzivač nije dostupan, insekti se alternativno mogu eutanazirati pomoću CO_2. U terenskim uslovima, gde upotreba boca sa CO_2 nije moguća, primerci se mogu usmrćivati pomoću malih komercijalnih CO_2 patrona koje se koriste u sifonima za gazirana pića, uz napomenu da njihov transport avionom može biti ograničen. Kao krajnje rešenje, insekti se mogu usmrćivati izlaganjem duvanskom dimu. U tom slučaju, flebotomine se žive sakupljaju u CDC klopkama, prenose aspiratorom, zadržavaju u staklenoj cevčici i izlažu duvanskom dimu, što dovodi do njihove smrti u roku od nekoliko sekundi. Ova metoda je primenljiva u svim terenskim uslovima, čak i u veoma izolovanim područjima. Međutim, staklena cevčica se impregnira dimom i ne može se ponovo koristiti za sakupljanje i rukovanje živim flebotominama bez temeljnog čišćenja. Ipak, isti neoprani aspirator može se koristiti za eutanaziju flebotomina iz drugih klopki u svrhu njihove fiksacije. Takođe je neophodno proveriti da li su svi primerci uklonjeni iz aspiratora. Navedene metode kompatibilne su sa izolacijom parazita iz roda *Leishmania* putem disekcije digestivnog trakta.

3. Čuvanje primeraka pre obrade

Postoje pet osnovna načina fiksacije i čuvanja flebotomina pre njihove dalje obrade:

3.1. Zamrzavanje

Ovaj način čuvanja je najbolji na temperaturama od –20 °C ili, po mogućstvu, na –80 °C. Ova metoda skladištenja danas se koristi češće od čuvanja u tečnom azotu. U svim slučajevima, krioprezervacija mora biti sprovedena što je moguće brže nakon omamljivanja primeraka. Čuvanje na niskim temperaturama u zamrzivačima ima prednost potpunog očuvanja samih insekata, kao i RNK, DNK i proteina, uz očuvanje njihovog punog integriteta tokom perioda skladištenja. Nasuprot tome, tečni azot može ozbiljno oštetiti krila, noge, palpe i antene, često dovodeći do njihovog otpadanja i povremenog gubitka ključnih morfoloških karaktera. Suvo zamrzavanje u zamrzivaču manje je traumatično za primerke, ali ipak nije idealno za očuvanje njihovih krhkih delova tela. Važno je istaći da se tokom odmrzavanja krila, antene, palpi ili noge mogu zalepiti za zidove epruveta usled kondenzacije i potom otkinuti. Ipak, čuvanje zamrzavanjem nije uvek izvodljivo u terenskim istraživanjima, jer zahteva pristup zamrzivaču ili posudi sa tečnim azotom. Ovaj način čuvanja u potpunosti je kompatibilan sa detekcijom patogena molekularnim metodama, bez gubitka osetljivosti, iako detekcija i izolacija RNK virusa zahteva čuvanje na –80 °C ili u tečnom azotu ukoliko je potrebno dugotrajno skladištenje. Međutim, zamrzavanje uzoraka ne omogućava izolaciju parazita *Leishmania* disekcijom digestivnog trakta, osim ukoliko se flebotomine najpre izlože gasovitoj fazi, a zatim potapaju u tečni azot (na primer u epruvetama smeštenim u čarapu), čime se simulira krioprezervacija parazita *Leishmania*.

3.2. Čuvanje u alkoholu (etanol ili izopropil-alkohol)

Ovo je verovatno najčešće korišćen način čuvanja flebotomina. Lako se primenjuje u terenskim uslovima, čak i u situacijama kada ne postoji pristup laboratoriji. Konzervacija u alkoholu naročito je pogodna za morfološka ispitivanja, jer krhki delovi tela (krila, noge, antene ili palpi) ostaju očuvani, pod uslovom da u epruveti nema mehurića vazduha. Zbog toga se preporučuje zatvaranje epruvete malim pamučnim čepom kako bi se uklonio vazduh, kao i postavljanje etikete iznad pamučnog čepa (Slika 1). Odgovarajuća koncentracija alkohola i dalje je predmet rasprave. Uopšteno, koncentracije ispod 70 % se ne preporučuju. Veće koncentracije omogućavaju bolje i dugotrajnije očuvanje DNK, ali istovremeno čine primerke krhkijim i lomljivijim za morfološka ispitivanja. Upotreba 96 % etanola (azeotropna smeša) obezbeđuje stabilnost koncentracije tokom vremena, naročito u vlažnim sredinama, kao što su tropske oblasti, iako je 95 % etanol često lakše dostupan. Bez obzira na koncentraciju, DNK se u etanolu uglavnom dobro čuva (iako manje efikasno nego pri zamrzavanju, naročito za molekularne metode zasnovane na NGS tehnologiji). Proteini su znatno manje stabilni, posebno u kontekstu proteomskih analiza, kao što je MALDI-ToF. Flebotomine čuvane u alkoholu tokom nekoliko meseci i dalje se mogu morfološki identifikovati, ali iz takvih primeraka nije moguće dobiti referentne proteinske spektre. Čuvanje u alkoholu ili u suvim uslovima može se unaprediti dodatnim zamrzavanjem uzoraka na –20 °C. Zamrzavanje na –20 °C pre svega poboljšava očuvanje

molekularnog materijala (npr. nukleinskih kiselina), a u manjoj meri doprinosi i očuvanju morfologije smanjenjem razgradnje tkiva tokom vremena. Čuvanje u etanolu može se primeniti i za detekciju DNK i RNK virusa, ukoliko se koristi etanol u koncentraciji od najmanje 70 % i ako je period skladištenja kraći od nekoliko meseci. Izopropil-alkohol je u nekim zemljama lako dostupan i omogućava očuvanje DNK, ali dovodi do rigidnosti tkiva primeraka. Za razliku od etanola, nije zapaljiv i zbog toga se lakše transportuje. Ukoliko je potrebno, flebotomine koje su prethodno čuvane u tečnom azotu ili suvo zamrzavane mogu se naknadno prebaciti u alkohol, čime se, nažalost, kombinuju nedostaci oba načina čuvanja.

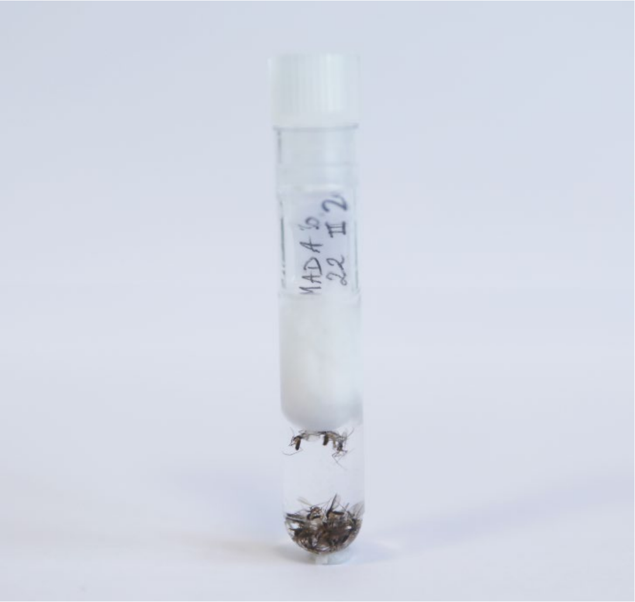

Slika 1: Flebotomine u etanolu.

3.3. Čuvanje u rastvoru za stabilizaciju RNK (RNASS)

Ovaj vodeni reagens je široko rasprostranjen, netoksičan i namenjen stabilizaciji i zaštiti RNK u svežim uzorcima tkiva i ćelija. Deluje tako što brzo prodire u uzorak i inaktivira ribonukleaze (enzime koji razgrađuju RNK), čime sprečava degradaciju RNK bez potrebe za trenutnim zamrzavanjem. Čuvanje u RNASS-u je generalno efikasno u očuvanju ukupne morfologije tkiva i ćelija za potrebe daljih histoloških ispitivanja. Iako je RNASS prvenstveno optimizovan za stabilizaciju RNK, kratkoročno i srednjoročno čuvanje obično omogućava dobro očuvanje strukturnog integriteta. RNASS omogućava skladištenje uzoraka na sobnoj temperaturi do 7 dana, na 4 °C tokom nekoliko nedelja, ili na –20 °C / –80 °C za dugotrajno čuvanje. Ovaj način čuvanja naročito je značajan u terenskim istraživanjima ili kliničkim uslovima u kojima je kontinuirano održavanje niske temperature (cold chain) nedostupno. Ekstrakcija RNK obično zahteva uklanjanje uzoraka iz rastvora i njihovu dalju obradu u skladu sa standardnim protokolima.

3.4. Suvo čuvanje na sobnoj temperaturi

Ovo je stariji način čuvanja koji, kada se primenjuje na cele primerke (preparirane in toto), ima veliki nedostatak u slabom očuvanju krhkih delova tela, kao što su krila, noge, antene i palpi. Ipak, proteomska ispitivanja primenom MALDI-ToF metode i dalje su izvodljiva ukoliko se dehidratacija sprovodi u trenutku fiksacije uz upotrebu sredstva za sušenje, poput silikagela. Nasuprot tome, molekularne analize usmerene na DNK teško se sprovode na ovako čuvanim uzorcima, jer je DNK često fragmentisana i prisutna u malim količinama, zbog čega su analize znatno zahtevnije nego kod svežih ili zamrznutih uzoraka, naročito kada je reč o genetskom materijalu jedra. Ipak, savremene tehnike, poput muzeomike (museomics), mogu se primeniti i na ovakve uzorke. Zbog navedenih ograničenja, ovaj način čuvanja se ne preporučuje, osim u slučajevima kada ne postoji druga alternativa. Suvo čuvanje može se kombinovati sa čuvanjem na niskim temperaturama, postavljanjem epruveta u zamrzivač na –20°C ili –80°C. Glavni izazov predstavlja adekvatno prepariranje primeraka ili delova tela neophodnih za identifikaciju. U tu svrhu neophodna je rehidratacija uzoraka i u tu svrhu se preporučuje upotreba rastvora Triton X-100. Trajanje rehidratacije varira od nekoliko sati do nekoliko dana i zahteva redovno praćenje. Nakon potpune rehidratacije, primerke treba isprati tri puta u destilovanoj vodi.

3.5. Čuvanje na filter-papirima

Glavna prednost filter-papira jeste dugoročna stabilnost genomske DNK unutar ćelija nefiksiranih, osušenih celih primeraka ili krvnih ćelija koje se čuvaju na sobnoj temperaturi. Filter-papir se isporučuje u obliku kartica malog formata, što omogućava skladištenje više stotina uzoraka na sobnoj temperaturi u zapremini veličine manje kutije. Matrica filter-papira impregnisana je supstancama koje denaturišu infektivne agense, te se uzorci više ne smatraju biološki rizičnim. Ovo omogućava čuvanje i transport uzoraka bez potrebe za posebnim merama biološke bezbednosti [68].

4. Disekcija primeraka

Za razliku od mnogih drugih insekata, koji se identifikuju na osnovu spoljašnjih karaktera vidljivih na celim, in toto prepariranim jedinkama pričvršćenim iglom, flebotomine zahtevaju disekciju i prepariranje preparata na mikroskopske pločice kako bi se proučile anatomske strukture neophodne za tačnu identifikaciju vrste. Bez obzira na izabrani postupak pripreme i prepariranja, primenjuje se ista tehnika disekcije (Slike 2 i 3) (https://zenodo.org/records/18198006).

Upotreba Triton X-100: nejonizujući vodeni rastvor

Važno je napomenuti da se prepariranje odnosi na sveže sakupljene ili adekvatno čuvane primerke. Većina sakupljača poseduje insekte koji su dugo vremena čuvani suvi (za potrebe MALDI-ToF analiza) ili u alkoholu. Nažalost, dugotrajno čuvanje u alkoholu nije optimalno i insekti čuvani na ovaj način postaju veoma teški za pripremu za mikroskopsko posmatranje. Čest problem je degradacija plastičnih posuda u kojima se uzorci čuvaju, nakon čega dolazi do isparavanja alkohola. U oba slučaja, primerci ili ostaju predugo u alkoholu ili se potpuno isuše. Zbog toga se javila ideja o upotrebi sredstava za kvašenje koja nisu jaki deterdženti. Triton X-100 je nejonizujući vodeni rastvor (4-(1,1,3,3-tetrametilbutil) fenil-polietilen-glikol rastvor, odnosno t-octylphenoxypolyethoxyethanol, polietilen-glikol tert-octylphenyl ether), koji se široko koristi kao deterdžent u ćelijskoj i molekularnoj biologiji. Omogućava permeabilizaciju ćelijskih i jedarnih membrana.

U nastavku je prikazan postupak korišćenja nejoničnog Triton X-100 u vodenom rastvoru koncentracije 0,5 %:
- Suvi uzorak se impregniše apsolutnim alkoholom.
- Doda se potrebna zapremina 0,5 % rastvora Triton X-100 tako da je ceo uzorak potopljen.
- Postupak se ostavlja da traje od približno 5 minuta do nekoliko dana, uz redovno praćenje; svi insekti moraju se u potpunosti razdvojiti u rastvoru.
- Rastvor Triton X-100 se uklanja i zamenjuje rastvorom kalijum-hidroksida.

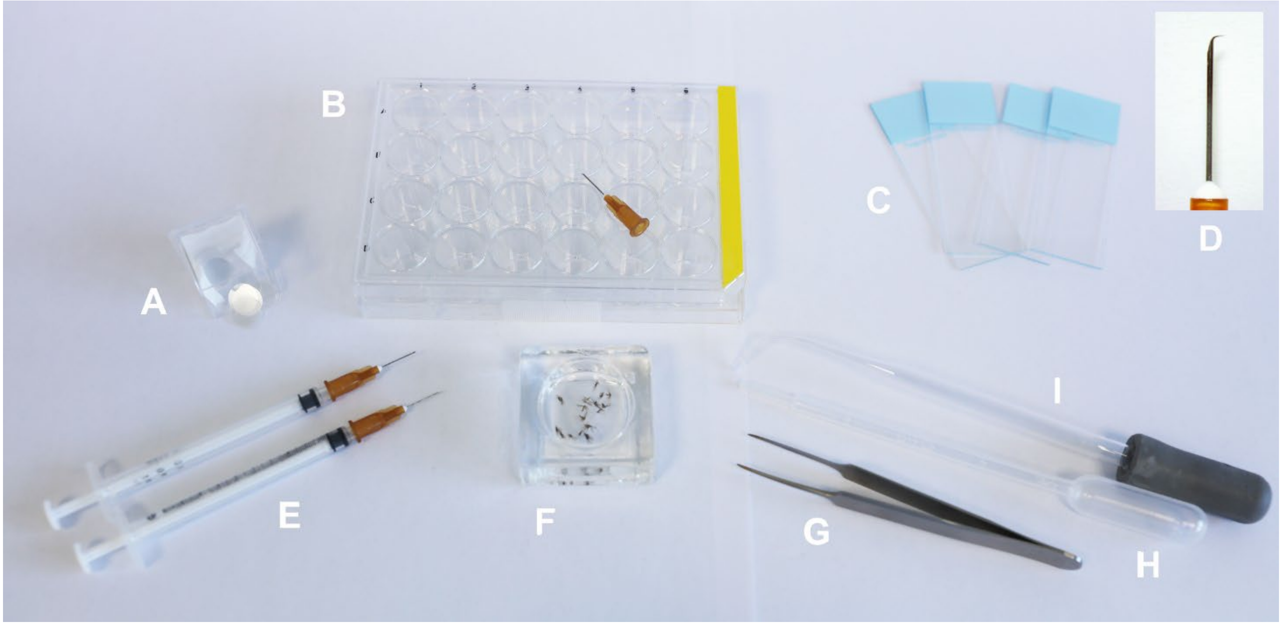

Slika 2: Materijal potreban za prepariranje flebotomina: A: okrugla staklena pokrovna stakalca (prečnika 10 ili 12 mm); B: ploča sa 24 udubljenja i igla sa kukom (ukoliko se za obradu flebotomina koriste karanfilićevo ulje ili esencija Euparal®, ne treba koristiti akrilne ploče jer će doći do hemijske reakcije i primerci će biti oštećeni); C: staklena predmetna stakalca pogodna za obeležavanje; D: detalj kuke na igli; E: igle pričvršćene na špriceve; F: satno stakalce ili odgovarajuća posuda sa flebotominama namenjenim za prepariranje; G: Dumont pinceta; H: plastična pipeta; I: staklena pipeta savijena zagrevanjem radi lakšeg prenosa tečnosti u udubljenja.

4.1. Glava

Disekcija se može obavljati pomoću finih igala ili entomoloških iglica pod stereomikroskopom (Slike 2 i 3). Najčešće korišćene igle uključuju: 26G × 1/2" (0,45 × 13 mm), 30G × 1/2" (0,3 × 13 mm) ili 25G × 5/8" (0,5 × 16 mm). Za pripremu primerka radi identifikacije, najmanje što je potrebno jeste odvajanje glave od tela i njeno prepariranje u ventralnom položaju kako bi se omogućio prikaz cibariuma i ždrela, dok se grudni koš i abdomen prepariraju lateralno nakon disekcije. Prepariranje glave u ventro-dorzalnom položaju obezbeđuje da je okcipitalni otvor orijentisan nagore, čime se omogućava direktno posmatranje cibariuma. Pristup ovim anatomskim strukturama dodatno je olakšan ukoliko je glava u potpunosti odvojena od tela

.

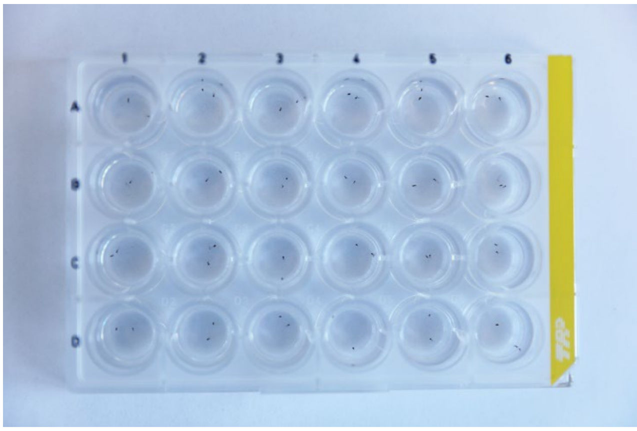

Slika 3: Ploča sa 24 udubljenja, od kojih svako sadrži glavu i vrh abdomena flebotomina.

4.2. Krila i toraks (grudi)

Krila moraju biti preparirana ravno. Svako krilo može se odvojiti u osnovi i preparirati zasebno, ili se jedno krilo može preparirati samostalno, dok drugo ostaje pričvršćeno za toraks. Ukoliko je planirana analiza geometrijske morfometrije, neophodno je pre prepariranja pravilno odrediti i obeležiti desno i levo krilo. Toraks je podeljen na više delova, od kojih svaki sadrži veoma važne taksonomske informacije. Uobičajeno se pozicionira u lateralnom položaju, kako bi se omogućilo ispitivanje hetotaksije i rasporeda boja. Prisustvo ožiljaka od čekinja u određenim regionima toraksa može se koristiti za razlikovanje pojedinih vrsta iz roda *Brumptomyia*. Raspored boja može se koristiti za razdvajanje neotropskih flebotomina na nivou roda (npr. *Bichromomyia*), serija vrsta (npr. *Pintomyia*), pa čak i vrsta unutar istog roda (npr. *Micropygomyia*, *Nyssomyia*, *Psathyromyia* i *Psychodopygus*). Stoga, ukoliko se toraks ne koristi za molekularne analize, treba ga preparirati tako da se ne ošteti. Važno je napomenuti da nije bitan intenzitet boja, već njihov raspored na toraksu. Zbog toga proces prosvetljavanja neće ukloniti pigmentaciju niti njen obrazac.

4.3. Genitalije

Posebnu pažnju treba posvetiti prepariranju genitalija kod mužjaka i ženki, jer su one od ključnog značaja za identifikaciju rodova, podrodova i vrsta. Kod oba pola, genitalije se sastoje od parnih struktura.

4.3.1. Mužjaci

Genitalije su spoljašnje i sastoje se od parnih klešta, od kojih se svako sastoji od spoja gonokoksita i gonostila u dorzalnom delu i epandrijalnog režnja u ventralnom delu. Gonostil nosi bodlje i ponekad sete, koje moraju biti prebrojive i čija mesta insercije moraju biti jasno vidljiva. Važno je pažljivo posmatrati unutrašnju površinu gonokoksita, koja može nositi snopić sedećih seta ili seta koje se nalaze na režnju (= tuberkulu) [22]. Kolege sa manje iskustva u disekciji mogu primeniti jednostavno lateralno prepariranje, bez odvajanja genitalija od završnog dela abdomena (https://zenodo.org/records/18311158). U tom slučaju, preklapanje dva dela genitalija može otežati, na primer, brojanje unutrašnjih seta gonokoksita, ali se time izbegava oštećenje genitalija usled neuspele disekcije. Iskusnije kolege mogu pokušati da genitalije razdvoje na dva dela. Da bi se to postiglo, zakošena strana igle (igla za intradermalne inekcije) mora se provući kroz genitalije, pri čemu se one razdvajaju bez potpunog presecanja, kako bi se razdvojili sklopovi gonokoksit–gonostil (https://zenodo.org/records/18311158). Na ovaj način, posmatranje njihovih unutrašnjih površina postaje jednostavno. Ovakav način prepariranja takođe olakšava posmatranje paramera i parameralnih ovojnica, koje se više ne preklapaju. Kod lateralnog pozicioniranja na pločici, kada dolazi do preklapanje organa, primerci moraju biti savršeno prosvetljeni.

4.3.2. Ženke

Genitalni apparat ženki je unutrašnji i čine ga spermateke. U odsustvu disekcije, one se moraju posmatrati kroz tegumente, pri čemu se abdomen preparira u ventralnom položaju. Bez obzira na izabrani medijum za prepariranje, same spermateke se uglavnom mogu pravilno posmatrati, naročito ukoliko nisu glatke i ukoliko su prosvetljene. Međutim, posmatranje glatkih spermateka sa tankim zidovima može biti problematično u slabo refraktilnim medijumima. Pored toga, posmatranje osnove kanala spermateka (genitalnog atrijuma) od suštinskog je značaja za identifikaciju vrsta, kao što je to slučaj kod podroda *Larroussius* [35, 37, 38], glavnih vektora *Leishmania infantum* u Starom svetu. Bez ovog posmatranja, identifikacija primeraka ostaje nemoguća. Kako bi se prevazišle ove poteškoće u posmatranju, kompleks genitalna furka–spermateke treba izdvojiti iz abdomena (https://zenodo.org/records/18311106). Spermateke je tokom disekcije uglavnom teško uočiti, ali je genitalna furka relativno lako prepoznatljiva. Pošto se spermatekalni kanali otvaraju u bazi genitalne furke, izolacija ove furke obično omogućava i izolaciju spermateka. Ukoliko se spermateke slučajno preseku tokom postupka, one se ne gube i dalje se mogu posmatrati unutar abdominalnih segmenata (Slika 4).

This is an Open Access article distributed under the terms of the Creative Commons Attribution License (https://creativecommons.org/licenses/by/4.0), which permits unrestricted use, distribution, and reproduction in any medium, provided the original work is properly cited.

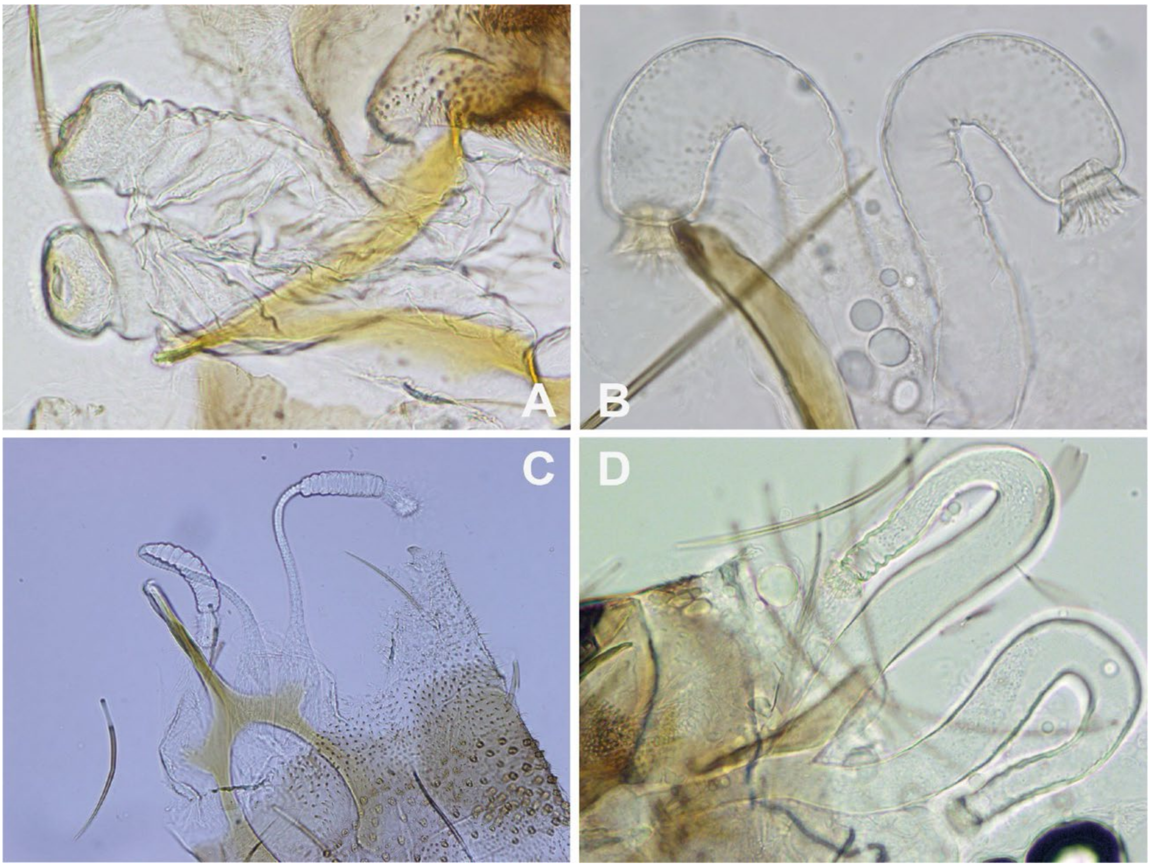

Slika 4: Spermateke disekovane i preparirane u Marc-André rastvoru iz svežih primeraka. A: *Idiophlebotomus longiforceps* (NR Laos); B: *Sergentomyia minuta* (Francuska); C: *Phlebotomus ariasi* (Francuska); D: *Sergentomyia anodontis* (NR Laos).

4.4. Disekcija srednjeg creva radi izolacije *Leishmania*

Disekcija digestivnog trakta je neophodna za detekciju i izolaciju parazita iz roda *Leishmania* kod ženki flebotomina. Postupak se može izvoditi i u terenskim i u laboratorijskim uslovima, radi procene vektorske kompetencije. Preporučuje se rad sa sveže eutanaziranim ženkama. Ženke treba oprati vodom ili fiziološkim rastvorom koji sadrži blagi deterdžent, kako bi se uklonile suvišne dlačice. Ovaj korak pomaže u održavanju aseptičnih uslova za izolaciju *Leishmania*, uz istovremeno očuvanje morfoloških karakteristika neophodnih za identifikaciju. Da bi se pronašli i izoloval paraziti iz roda *Leishmania*, srednje crevo treba pažljivo izdvojiti i staviti u jednu kap sterilnog fiziološkog rastvora (0,9% NaCl). Nakon posmatranja pokretnih parazita pod svetlosnim mikroskopom (preporučeno uvećanje: ~200×), oni se insulinskim špricem ili mikropipetom prenose u hranljivi medijum za kultivaciju (za više detalja videti Poglavlje 4.4.3).

Glava i genitalije se prepariraju direktno u Marc-André rastvor radi prosvetljavanja. **Važno**: Marc-André rastvor nikada ne sme doći u kontakt sa parazitima *Leishmania* – ni direktno, ni indirektno preko alata ili igala – jer je smrtonosan za parazite. Disekcija ženki flebotomina može se izvoditi na jednom ili na dva predmetna stakalca; obe opcije imaju svoje prednosti i ograničenja (Slika 5; https://zenodo.org/records/18311154)

.

This is an Open Access article distributed under the terms of the Creative Commons Attribution License (https://creativecommons.org/licenses/by/4.0), which permits unrestricted use, distribution, and reproduction in any medium, provided the original work is properly cited.

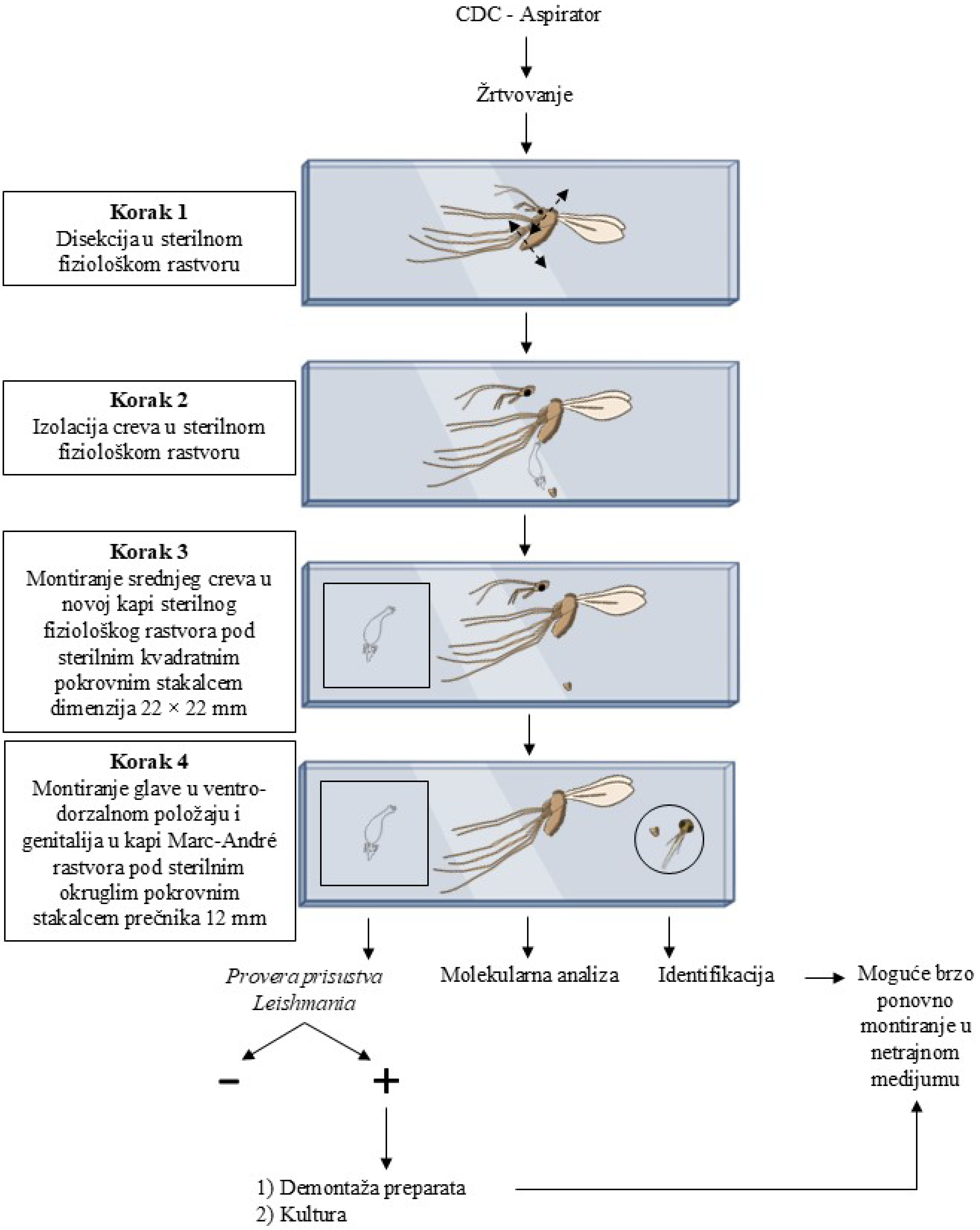

Slika 5: Metoda za izolaciju parazita *Leishmania*

This is an Open Access article distributed under the terms of the Creative Commons Attribution License (https://creativecommons.org/licenses/by/4.0), which permits unrestricted use, distribution, and reproduction in any medium, provided the original work is properly cited.

4.4.1. Metoda sa dva stakalca

Prva opcija podrazumeva rad na dva odvojena predmetna stakalca: na jednom se nalazi sterilni fiziološki rastvor za izdvajanje srednjeg creva, dok se na drugom prepariraju glava i spermateke u Marc-André rastvoru. Međutim, u terenskim uslovima je uobičajeno da dve ili tri osobe disekuju flebotomine i prosleđuju disekovane primerke jednom istraživaču zaduženom za identifikaciju vrste i procenu prisustva parazita iz roda *Leishmania* u crevu. Upravljanje sa dva stakalca može dovesti do problema sa sledljivošću uzoraka i, naročito, otežati pouzdano utvrđivanje koja je jedinka bila zaražena u slučaju detekcije pozitivnog creva (https://zenodo.org/records/18311154).

4.4.2. Metoda sa jednim stakalcem

Upotreba jednog predmetnog stakalca obezbeđuje sledljivost rezultata. Međutim, potrebno je preduzeti nekoliko mera opreza. Kako bi se postigla maksimalna sterilnost tokom ovog koraka, operateri moraju redovno dezinfikovati ruke hidroalkoholnim gelom. Moraju se koristiti nebrušena predmetna stakalca i kvadratna pokrovna stakalca (22 × 22 mm), umotana u aluminijumsku foliju i sterilizovana suvim zagrevanjem (u Poupinel peći), kao i sterilne igle za svaku disekciju (preporuka: 25G Ø 0,5 mm × 16 mm). Flebotomina se postavlja u kap sterilnog fiziološkog rastvora u sredini stakalca. Glava se odvaja, dok se pravi rez između 6 i 7 abdominalnog tergita i sternita, bez presecanja digestivnog trakta (rez može biti viši ukoliko se očekuju veoma duge spermateke). Zatim se toraks imobilizuje jednom iglom, a završni zadnji segmenti abdomena se drugom iglom pažljivo povlače kako bi se izvuklo crevo. Ukoliko to ne uspe, moguće je blokirati kraj abdomena iglom i povući digestivni trakt sa njegove prednje strane. Ako ni to ne uspe, crevo se mora izdvojiti uklanjanjem što većeg dela preostalog tegumenta oko njega. Nakon izdvajanja creva, završni abdominalni segmenti se odvajaju presecanjem digestivnog trakta. Crevo se zatim stavlja u novu kap sterilnog fiziološkog rastvora, postavljenu na jednoj strani stakalca, i pažljivo se prekriva sterilnim pokrovnim stakalcem. Glava i završni abdominalni segmenti se prenose u malu kap Marc-André rastvora postavljenu na drugom kraju stakalca, uz obavezno izbegavanje kontakta sa parazitima *Leishmania*. Glava se pravilno orijentiše (okcipitalni otvor okrenut nagore), a spermateke se izdvajaju zajedno sa genitalnom furkom, kao što je ranije opisano, i prekrivaju se malim okruglim pokrovnim stakalcem (Ø 12 mm, ne treba ga mešati sa sterilnim kvadratnim pokrovnim stakalcima). Preostali deo tela flebotomine i krila ostaju u kapi fiziološkog rastvora u sredini stakalca (https://zenodo.org/records/18311154). U slučaju pozitivnog nalaza, ili u svrhu taksonomskih istraživanja, toraks i abdomen mogu se sačuvati za molekularna ili proteomska ispitivanja, a krila se mogu preparirati u vodenom medijumu. Radi očuvanja preparata, višak Marc-André rastvora može se zameniti vodenim medijumom za prepariranje, kao što je hloralna guma (= Hoyer) ili medijum na bazi polivinil-alkohola.

Detaljni video-zapisi koji prikazuju ove postupke dostupni su preko interneta (disekcija srednjeg creva flebotomina: https://zenodo.org/records/18303014 i disekcija pljuvačnih žlezda flebotomina: https://zenodo.org/records/18302850), te ovde neće biti dodatno objašnjavani.

4.4.3. Izolacija i kultivacija parazita *Leishmania* iz creva flebotomina

Izolacija parazita disekcijom inficiranih ženki flebotomina predstavlja delikatan postupak koji zahteva visok nivo veštine i koji bi u početku trebalo uvežbavati na primercima bez parazita. Nakon disekcije, creva se prenose u svežu kap sterilnog fiziološkog rastvora (0,9 %) ili Locke-ovog rastvora radi ispiranja [4]. Disekovana creva se zatim mogu obraditi na dva načina: i) posmatranjem pod svetlosnim mikroskopom radi uočavanja različitih stadijuma promastigota *Leishmania* i njihove lokalizacije, uz posebnu pažnju posvećenu stomodealnom zalisku, i ii) otvaranjem creva kako bi se olakšao izlazak promastigota, čime se omogućava njihova masovna kultivacija [4]. Pronalaženje infektivnih flebotomina u prirodi relativno je retka pojava, te dobra praksa i vežbanje značajno povećavaju šanse za uspešnu izolaciju. Ukoliko se u crevu uoče paraziti *Leishmania*, potrebno je koristiti nove sterilne igle i dodati malu količinu sterilnog fiziološkog rastvora oko pokrovnog stakalca, kapilarnim delovanjem, kako bi se paraziti oslobodili. Crevo treba pažljivo i brzo pocepati kako bi se paraziti oslobodili u fiziološki rastvor. Pomoću mikropipete zapremine 100 µL ili tuberkulinskog šprica, paraziti se sakupljaju i inokulišu u odgovarajući, pravilno obeležen hranljivi medijum.

In vitro kultivacija promastigota *Leishmania*: izolovani paraziti se u početku održavaju na kosinama SNB-9 krvnog agara ili u čvrstom medijumu Novy–McNeal–Nicolle (NNN) [16], preko kojih se dodaje ili sterilni alfa-MEM medijum [16, 65] ili M199 medijum, pri čemu su oba obogaćena sa 10 % termički inaktivisanim sterilnim fetalnim telećim serumom [FCS] (radi podsticanja rasta parazita), 1 % BME vitamina, 2 % sterilnog humanog urina (sterilisanog pomoću špric-filtra Filtropur® S 0,2 µm), 250 µg/mL amikacina (ili 50 µg/mL gentamicina, ili mešavinom antibiotika i amino-kiselina (L-glutamin 200 mM – penicilin 10 000 U – streptomicin 10 mg/mL) [47]. Nakon tri dana, ukoliko nema kontaminacije, kulture se rastvaraju u odgovarajuće pripremljenom medijumu za zamrzavanje i potom čuvaju na –80 °C u trajanju od 1 do 2 godine ili u tečnom azotu na –196 °C radi dugotrajnog čuvanja i buduće eksperimentalne upotrebe [7].

4.5. Pljuvačne žlezde

Disekcija pljuvačnih žlezda flebotomina predstavlja osnovnu tehniku za proučavanje interakcija vektora i patogena, naročito za detekciju arbovirusa kao što su flebovirusi (npr. Toscana virus) [44, 75]. Zbog izuzetno male veličine flebotomina, postupak zahteva veliku preciznost pod stereomikroskopom, uz upotrebu finih pinceta ili igala za mikrodisekciju, kako bi se osetljive pljuvačne žlezde izdvojile bez njihovog pucanja ili kontaminacije (https://zenodo.org/records/18302850) [51, 61]. Očuvanje integriteta žlezda je od ključnog značaja za pouzdanu dalju molekularnu analizu. Nakon izdvajanja, žlezde se mogu homogenizovati i analizirati primenom RT-PCR, qPCR ili imunoeseja radi detekcije virusne RNK ili antigena [12]. Prisustvo virusa u pljuvačnim žlezdama, a ne samo u crevu ili hemocelu, potvrđuje da je patogen završio ekstrinzični inkubacioni period i da je prenosiv tokom krvnog obroka [71].

Postupak disekcije je tehnički zahtevan zbog male veličine pljuvačnih žlezda flebotomina i zahteva značajno iskustvo kako bi se izbegla degradacija uzorka [1, 51]. Dodatno, virusno opterećenje može biti nisko, što zahteva primenu visoko osetljivih metoda detekcije, kao što su ugnježdeni PCR ili sekvenciranje visokog protoka [54]. Rizik od kontaminacije dodatno naglašava potrebu za sterilnim tehnikama rada. Pored tehničkih izazova, na uspešnost detekcije utiču i biološki faktori; vektorska kompetencija varira među vrstama flebotomina, a stope infekcije se menjaju u zavisnosti od ekoloških i sezonskih uslova [33, 61].

Detekcija virusa u pljuvačnim žlezdama pruža ključne informacije o riziku prenosa, omogućavajući ciljani nadzor i mere kontrole [15]. Na primer, identifikacija Toscana virusa u flebotominama u endemskim područjima doprinela je unapređenju dijagnostičkih protokola i preporuka za javno zdravlje [18]. Pored toga, proučavanje interakcija virusa i pljuvačke može otkriti nove ciljeve za vakcine ili terapije koje blokiraju prenos [15, 18].

Pljuvačne žlezde flebotomina mogu se koristiti i kao izvor antigena za merenje antitela domaćina na pljuvačku flebotomina primenom imunoloških metoda, po mogućstvu ELISA. Ovaj pristup omogućava procenu izloženosti domaćina ujeda flebotomina, čime se podržava evaluacija efikasnosti mera kontrole vektora [25] i procena rizika prenosa Leishmania [40].

4.6. Identifikacija krvnog obroka

Nasisane ženke izdvojene tokom sakupljanja treba disekovati uz upotrebu jednokratne opreme, kako bi se sprečila unakrsna kontaminacija. Njihov abdomen treba pregledati pod stereomikroskopom radi procene stepena digestije krvnog obroka. Preporučuje se izbor isključivo ženki sa crvenim, crvenkasto-braon ili tamnocrvenim abdomenom, bez znakova formiranja jaja. Vrh abdomena, uključujući spermateke, treba ukloniti radi morfološke identifikacije ženke nakon prosvetljavanja. Glavni deo abdomena (bez spermateka) zatim se stavlja u mikroepruvete (1.5 ili 2 ml) i čuva na –20 °C do dalje analize. Genetički markeri koji se najčešće koriste za identifikaciju krvnog obroka, kao što su PNOC [5, 30, 50], CytB [67] ili COI [13], dobro su uspostavljeni i detaljno opisani u literaturi; stoga se u ovom radu neće dodatno razmatrati (Slika 6). Alternativno, za identifikaciju krvi domaćina može se primeniti MALDI-ToF mapiranje peptida [31]. Eksperimentalno je pokazano da ova tehnika omogućava identifikaciju krvi domaćina i u dužem vremenskom periodu nakon uzimanja krvnog obroka; stoga predstavlja odgovarajući metod izbora, naročito za analizu nasisanih ženki kod kojih je digestija krvi domaćina vidljivo uznapredovala. Uzorci bi idealno trebalo da se čuvaju na –20 °C ili 4 °C, ali se dobri rezultati mogu dobiti i iz uzoraka koji su kratko vreme čuvani na sobnoj temperaturi. Abdomen nasisane ženke treba disekovati od ostatka tela neposredno pre analize i homogenizovati u destilovanoj vodi. Naovaj način ostatak tela flebotomine ostaje dostupan za druge molekularne i morfološke analize. Nakon uzimanja alikvota iz homogenata za MALDI-ToF mapiranje peptida, preostali deo se može koristiti za izolaciju DNK radi potvrde identifikacije krvi domaćina i/ili analizu prisustva *Leishmania* sp. Ukupno vreme pripreme uzoraka i analize znatno je kraće u poređenju sa molekularnim tehnikama zasnovanim na DNK.

This is an Open Access article distributed under the terms of the Creative Commons Attribution License (https://creativecommons.org/licenses/by/4.0), which permits unrestricted use, distribution, and reproduction in any medium, provided the original work is properly cited.

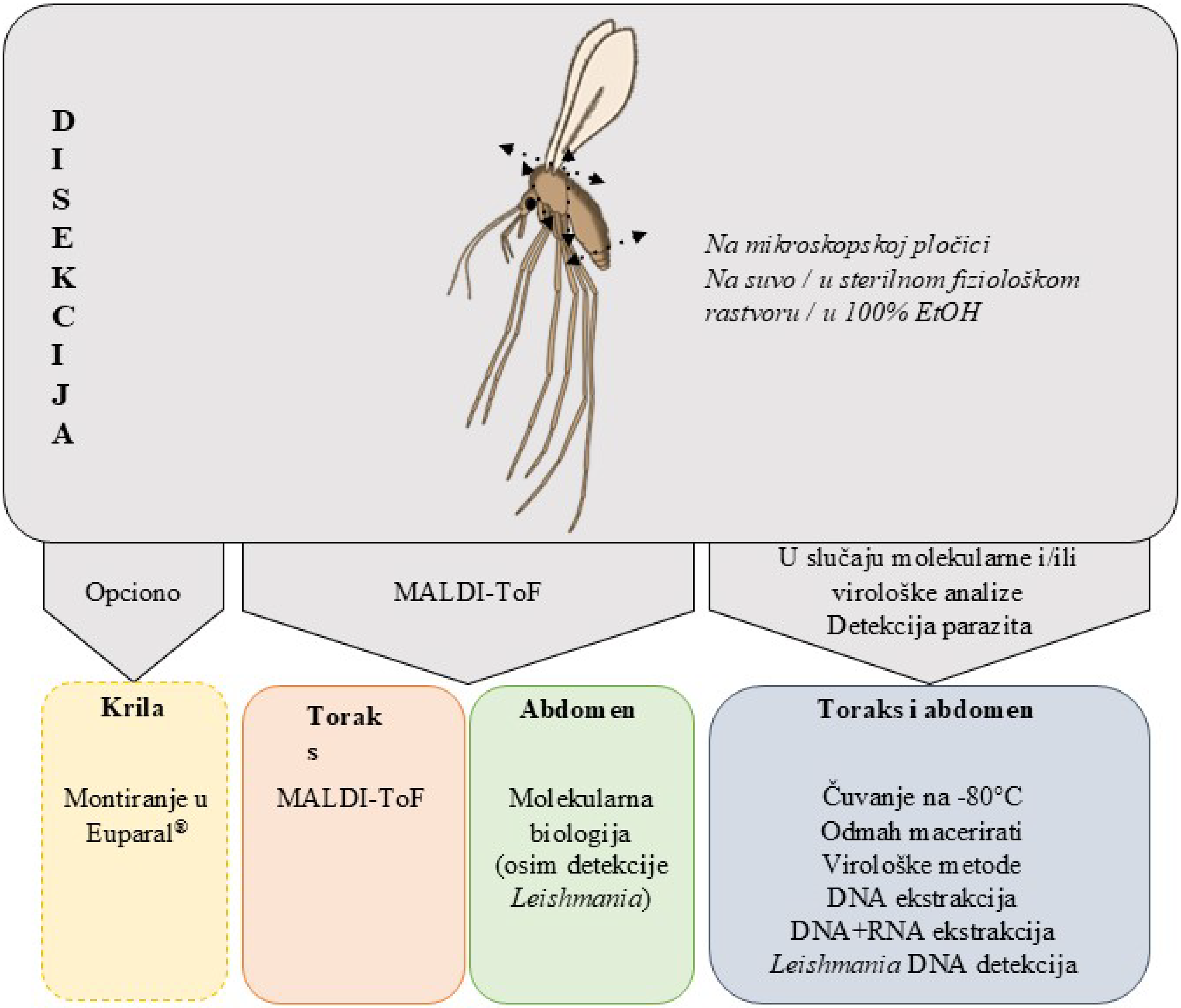

Slika 6: Obrada flebotomina za primenu u molekularnoj biologiji, proteomici i/ili virologiji.

5. Obrada primeraka za morfološka ispitivanja (Slike 3, 6, 7 i 8; Dodaci 1, 2, 3 i 4)

Ovo poglavlje opisuje principe pripreme primerka flebotomine za prepariranje isključivo u svrhu morfoloških ispitivanja, nakon čega sledi prilagođavanje postupka za dodatne analize. Međutim, razumevanje ove metodologije je od ključnog značaja, jer omogućava prilagođavanje postupaka specifičnim tipovima uzoraka kada je to potrebno. Postupak obuhvata uzastopne korake pražnjenja i punjenja, uz upotrebu Pasterovih pipeta opremljenih fleksibilnim gumenim balonima. Preporučuju se staklene posude sa zaobljenim, jer značajno olakšavaju ove operacije, a pre svega zbog toga što je staklo inertno prema svim reagensima. Kako bi se sprečilo isparavanje reagenasa, posude treba da budu opremljene poklopcima i nikada ne smeju biti prepunjene, da ne bi došlo do prelivanja prilikom zatvaranja ili otvaranja, kao i da bi se sprečilo taloženje prašine na uzorcima. Hemikalije potrebne za prosvetljavanje i obradu prikazane su u Tabeli 2.

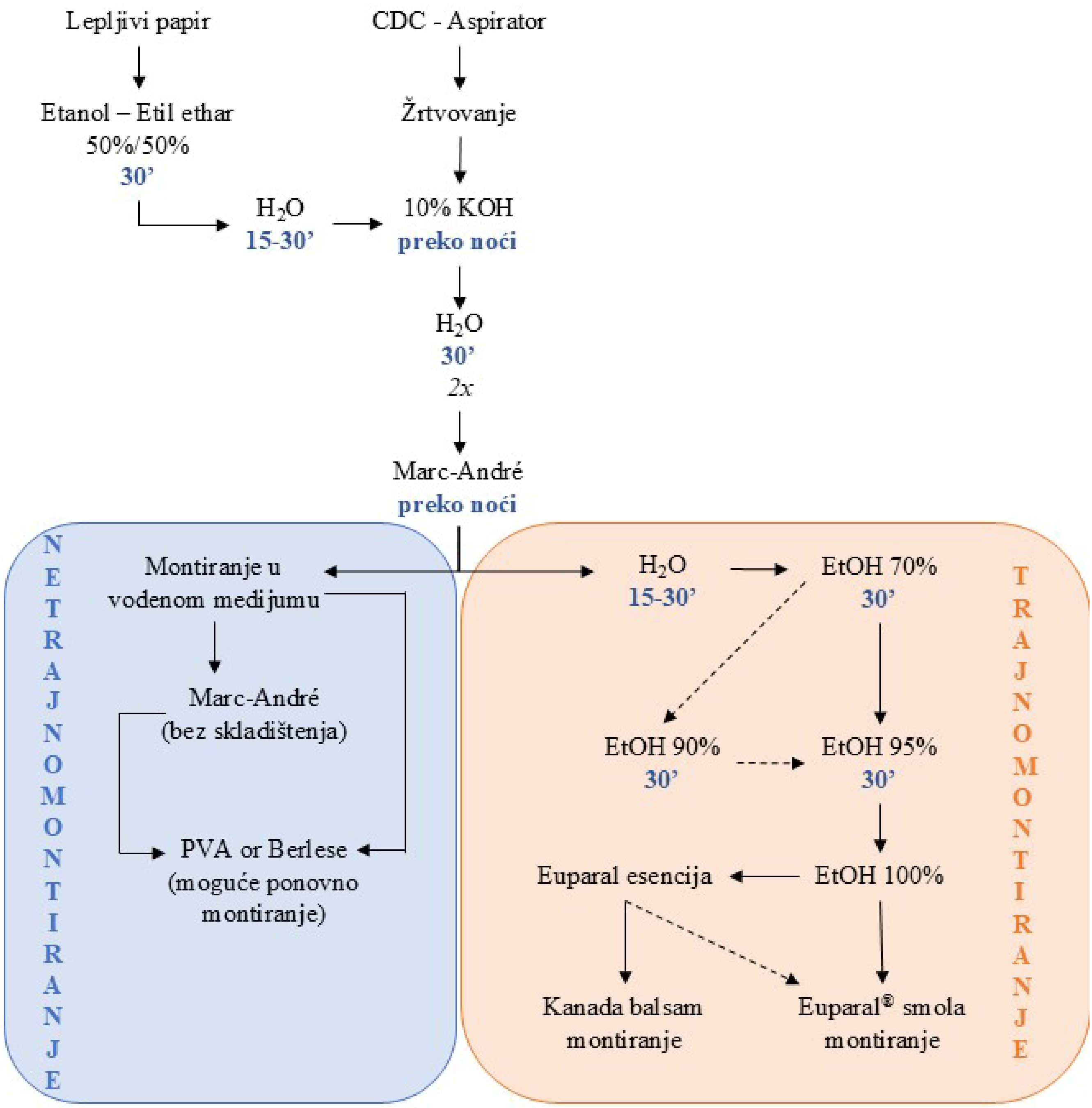

Slika 7: Klasična metoda obrade flebotomina.

5.1. Prosvetljavanje

Pre nego što se primerci flebotomina mogu pripremiti kao trajni mikroskopski preparati, moraju se najpre prosvetliti maceracijom primenom odgovarajuće metode i sredstva za prosvetljavanje (npr. 10% rastvor sirćetne kiseline ili Marc-André rastvor koji sadrži hloral-hidrat, hemikaliju čija je upotreba u mnogim zemljama ograničena), kako bi postali providni. Proces prosvetljavanja uklanja telesna tkiva, masti, sekrete i voskove, čineći primerak prozirnim i olakšavajući ispitivanje struktura egzoskeleta (npr. mesta insercije seta), površinskih karakteristika (npr. obojenost) i unutrašnjih struktura vidljivih kroz tegument (npr. spermateke).

Tabela 2: Sastav korišćenih reagenasa.

**Kalijum-hidroksid 10%**<br>Kalijum-hidroksid 10 g<br>Destilovana voda do 100 mL	**Kiseli fuksin 1% u destilovanoj vodi**<br>Kiseli fuksin (u prahu) 1 g<br>Destilovana voda 99 mL
**Hloralna guma za prepariranje (Hoyerov medijum)**<br>Destilovana voda 50 mL<br>Hloral-hidrat 200 g<br>Gumiarabika 50 g<br>Glicerol 20 mL	**Marc-André rastvor obojen kiselim fuksinom**<br>Marc-André rastvor 10 mL<br><br>Fuksin 1% 50 µL
**Solution de Marc-André**<br>Hloral-hidrat 40 g<br>Glacijalna sirćetna kiselina 30 mL<br>Destilovana voda 30 mL	**Enecé medijum**<br>Čista bela kopalna smola 22 g<br>Alkohol-rastvorljiva kopalna smola 12 g<br>Apsolutni etanol 20 mL<br>Kamfor 10 g<br>Terpentinsko ulje 10 mL<br>Eucaliptol 26 mL

Postupak prosvetljavanja u dva koraka, koji podrazumeva početnu primenu jake baze (kao što je kalijum-hidroksid), praćenu slabom kiselinom (kao što je sirćetna kiselina u Marc-André rastvoru), ima jasno razdvojene biohemijske svrhe [74]. Baza razgrađuje meka tkiva, kao što su proteini, masti i mišići, putem saponifikacije i denaturacije proteina, ostavljajući hitinski egzoskelet očuvanim radi jasnog uvida u strukturu. Naknadna primena slabe kiseline neutrališe preostalu bazu, sprečavajući dalju degradaciju, i izbeljuje hitin čime se povećava transparentnost [74], iako ispiranje primeraka dva puta u destilovanoj vodi u trajanju od 15 minuta takođe može biti dovoljno za neutralizaciju baze. Ovaj sekvencijalni tretman kombinuje efikasno uklanjanje tkiva sa blagim očuvanjem struktura, obezbeđujući optimalni integritet primeraka za mikroskopsko ispitivanje.

Pre prelaska na sledeći korak preporučuju se dva ispiranja u destilovanoj vodi u trajanju od po 20 minuta.

5.1.1. Liza mekih tkiva (Slika 8)

Natrijum-hidroksid (NaOH) ili kalijum-hidroksid (KOH) su hemijska sredstva za maceraciju koja se najčešće koriste, primenjujući se u različitim koncentracijama i tokom različitog vremena, u zavisnosti od veličine i krhkosti primeraka. Standardna i najefikasnija tehnika podrazumeva liziranje mekih tkiva potapanjem flebotomina u jaku bazu (10% KOH ili NaOH) tokom noći. Koncentracija se može povećati radi skraćenja trajanja tretmana (npr. 20% KOH tokom 6 sati), kao i primenom zagrevanja na 37 °C.

5.1.2. Prosvetljavanje sa ili bez bojenja

Zatim sledi postupak posvetljivanja, koji obično kombinuje sirćetnu kiselinu i hloral-hidrat (npr. Marc-André rastvor). Nakon prosvetljavanja, primerci se moraju temeljno isprati u najmanje dve vode, u trajanju od po 20 minuta, kako bi se uklonili ostaci hemikalija.

Marc-André rastvor je često korišćeno sredstvo za prosvetljavanje u pripremi preparata flebotomina. Njegova efikasnost ogleda se u olakšavanju procesa prosvetljavanja uz istovremeno minimiziranje značajnog oštećenja krhkih struktura, kao što su krila i antene.

Rastvor treba pripremiti neposredno pre upotrebe ili čuvati u dobro zatvorenoj posudi kako bi se sprečilo isparavanje ili degradacija. Upotreba Marc-André rastvora je naročito pogodna kada se kombinuje sa tehnikama posvetljivanja ili bojenja radi naglašavanja pojedinih morfoloških detalja. Detalji o njegovom sastavu i pripremi dati su u Dodatku 2.

Kod izrazito prozirnih primeraka, bojenje može biti neophodno radi poboljšanja vidljivosti pre prepariranja. Dostupan je veliki broj boja, od kojih svaka cilja specifične hemijske komponente organizma. Važno je odabrati boju koja je kompatibilna i sa preparatom i sa izabranim medijumom za prepariranje. Ova osnovna metodologija može se po potrebi prilagoditi, na primer dodavanjem 0,1% kiselog fuksina u Marc-André rastvor radi bojenja. Pored toga, primerci čuvani u vodenim rastvorima i namenjeni prepariranju u smolaste medijume zahtevaju dehidrataciju (videti Odeljak 5.2 o dehidrataciji), jer većina prirodnih i sintetičkih smolastih medijuma za prepariranje nije kompatibilna sa vodom. New (1974) je naveo da se neke boje mogu degradirati u određenim medijumima za prepariranje [53]. Na primer, kiseli fuksin, koji se često koristi sa kanadam balzamom, može se fiksirati i u Euparal®-u. Međutim, preparati obojeni kiselim fuksinom skloni su izbleđivanju, naročito kada ostaci karanfilićevog ulja, koje se koristi kao završno sredstvo za prosvetljavanje, ostanu prisutni. Primerci čuvani u karanfilićevom ulju mogu pokazati značajno izbleđivanje već nakon nekoliko dana.

This is an Open Access article distributed under the terms of the Creative Commons Attribution License (https://creativecommons.org/licenses/by/4.0), which permits unrestricted use, distribution, and reproduction in any medium, provided the original work is properly cited.

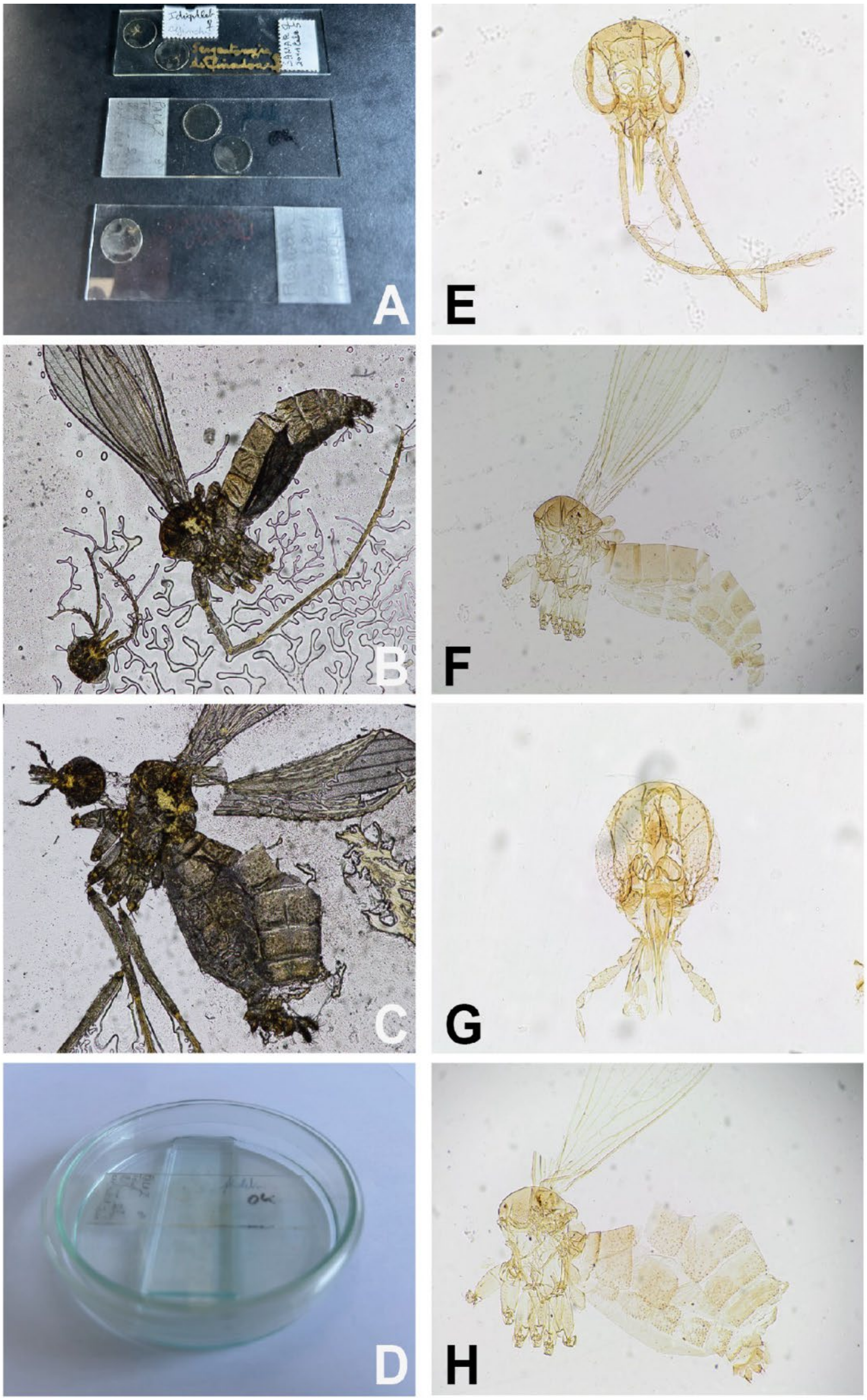

Slika 8: Ponovno prepariranje preparata. A: oštećeni i osušeni preparati u Hoyerovom medijumu; B: mikroskopski prikaz osušenog primerka flebotomine; C: mikroskopski prikaz drugog oštećenog primerka flebotomine; D: vlažna komora koja sadrži osušeni preparat; E: glava i F: telo primerka B nakon ponovnog prepariranja u Euparal®; G: glava i H: telo oštećenog primerka C nakon ponovnog prepariranja u Euparal®..

5.2. Dehidratacija

Dehidratacija se sprovodi postepenim premeštanjem uzoraka kroz seriju etanolnih rastvora rastuće koncentracije: 50%, 70%, 80%, 90% ili 95% i konačno 100%, pri čemu svako ispiranje traje najmanje 20 minuta. S obzirom na to da etanol brzo isparava, posuda mora biti čvrsto zatvorena tokom postupka. Nakon što je primerak potpuno dehidriran, postupak se može privremeno obustaviti na nekoliko dana u Euparal®, koji je pogodniji od karanfilićevog ulja. Bukov kreozot, koji se ranije široko koristio u ovu svrhu, danas je u potpunosti zabranjen zbog svoje toksičnosti.

Proces dehidratacije mora obezbediti da tečnost unutar primerka bude kompatibilna sa medijumom za prepariranje, kako bi se sprečila pojava zamućenja, osmotskog kolapsa ili deformacija koje bi mogle učiniti primerak neupotrebljivim za taksonomska istraživanja.

5.3. Medijumi za prepariranje

5.3.1. Izbor i primena medijuma u pripremi preparata

Medijum za prepariranje bi idealno trebalo da ima indeks prelamanja što bliži indeksu stakla, koji iznosi približno 1,5. Mora biti bezbojan, bistar i da ostane potpuno providan nakon sušenja i tokom vremena. Mora biti kompatibilan sa korišćenim bojama i sposoban da prodre i ravnomerno se rasporedi u sva tkiva primerka. Ne sme se sušiti prebrzo niti stvarati zamućenje tokom prepariranja. Takođe ne sme se skupljati nakon prepariranja. Izbor odgovarajućeg medijuma za prepariranje predstavlja osnovni aspekt pripreme preparata, jer nijedan pojedinačni medijum nije idealan za sve namene. Izbor treba da uspostavi ravnotežu između nekoliko ključnih faktora:

- Optička svojstva. Indeks prelamanja medijuma za prepariranje treba da obezbedi dovoljan kontrast i prelamanje svetlosti ključnih anatomskih struktura koje se koriste za taksonomsku identifikaciju ili morfološki opis, kao što su spermateke, askoidi, Njustedove senzile, vertikalni cibarialni zubi i faringealni zubi. Vidljivost ovih struktura direktno zavisi od optičkih svojstava medijuma za prepariranje.

- Očuvanje. Za tipske primerke ili materijal namenjen trajnim kolekcijama, medijum mora obezbediti dugoročnu stabilnost i trajnost. Nasuprot tome, za inventarizacione studije ili epidemiološka istraživanja, gde dugotrajno očuvanje nije od presudnog značaja, privremeni ili polutrajni medijumi za prepariranje mogu biti dovoljni.

5.3.2. Zahtevi za medijume za prepariranje

Stručnjaci često razvijaju prilagođene i složene tehnike prepariranja, usmerene ka specifičnim istraživačkim potrebama. Međutim, ove metode često zanemaruju aspekte kao što su arhivski kvalitet, kompatibilnost, standardizacija ili lakoća rukovanja i dugoročno očuvanje. Ovaj nedostatak standardizacije otežava integraciju doniranih kolekcija i napore dugoročnog kustodiranja.

Naučne primene nameću različite zahteve za medijume za prepariranje. Taksonomi često prepariraju cele primerke i preferiraju medijume koji blago razgrađuju unutrašnje organe kako bi se poboljšala vidljivost kutikularnih struktura. Indeks prelamanja treba da se dovoljno razlikuje od indeksa primerka i predmetnog stakalca kako bi se postigla maksimalna optička jasnoća. Komercijalni medijumi za prepariranje obično su formulisani sa indeksom prelamanja bliskim indeksu stakla, kako bi se smanjilo prelamanje i rasipanje svetlosti kroz sistem predmetno stakalce–medijum–pokrovno stakalce. Međutim, u svetlosnoj mikroskopiji u propuštenom svetlu (brightfield), prirodni kontrast neobojenog preparata može se modifikovati namernim izborom medijuma za prepariranje sa indeksom prelamanja koji se blago razlikuje od indeksa primerka, čime se poboljšava njegova vidljivost u odnosu na pozadinu.

5.3.3. Tipovi medijuma za prepariranje (Tabele 3 i 4)

U mikroskopiji, indeks prelamanja (IP) medijuma za prepariranje određuje način na koji se svetlost lomi prilikom prolaska kroz predmetno stakalce, medijum i preparat. Kada je IP blizak indeksu pokrovnog stakla (≈1,515), svetlost prolazi ravnomerno, smanjujući rasipanje i optičke distorzije, što rezultuje boljom rezolucijom i vidljivošću finih struktura. Suprotno tome, nepodudaranje indeksa prelamanja može dovesti do zamućenja, pojave halo-efekta ili prikrivanja neobojenih struktura. Zbog različitih vrednosti IP kod različitih medijuma, izbor odgovarajućeg medijuma za prepariranje od presudnog je značaja za optimizaciju kontrasta, jasnoće i ukupnog kvaliteta slike za dati preparat.

Indeks prelamanja medijuma za prepariranje ima značajan uticaj na vidljivost finih struktura prilikom pripreme flebotomina za mikroskopske preparate. Delikatne i slabo sklerotizovane strukture flebotomina, uključujući cibarium, spermateke, antenalne segmente i nervaturu krila, mogu biti teško uočljive u medijumima za prepariranje sa visokim indeksom prelamanja.

Kod flebotomina se najčešće koriste hloralno-gumeni medijumi kao vodeni medijumi za prepariranje, kao i kanada balzam i smola Enecê – Nelson Cerqueira (NC) kao medijumi rastvorljivi u organskim rastvaračima. Rawlins [60] je podelio medijume za prepariranje u dve kategorije: (1) trajni medijumi, koji se tokom vremena stvrdnjavaju i pogodni su za dugotrajno čuvanje, i (2) polutrajni medijumi, koji se ne stvrdnjavaju u potpunosti i obično se koriste u privremene svrhe.

Medijumi za prepariranje mogu biti tečni, na bazi gume ili smolasti, rastvorljivi u vodi, alkoholu ili drugim rastvaračima (npr. toluen, ksilen) (Tabela 3). Nakon

nanošenja, preparati treba da budu zaštićeni od uticaja atmosfere zaptivanjem pomoću nerastvorljivih sredstava za obrubljivanje. Radi jasnog razlikovanja tipova medijuma za prepariranje, može se koristiti sledeća podela:

a. Vodeni medijumi. Ovi medijumi se lako rastvaraju u vodi, što ih čini pogodnim za privremene ili polutrajne preparate. Obično su laki za rukovanje, ali mogu zahtevati zaptivanje kako bi se sprečilo izlaganje atmosferskoj vlazi (npr. hloralno-gumeni medijumi i polivinil-alkohol), naročito u tropskim, vlažnim klimatskim uslovima.

b. Medijumi ograničene tolerancije na vodu. Ovi medijumi su manje osetljivi na vodu, ali i dalje zahtevaju zaštitu od prekomerne vlažnosti. Obezbeđuju veću dugoročnu stabilnost u poređenju sa vodorastvorljivim opcijama i često se koriste za polutrajne preparate.

c. Medijumi rastvorljivi u ugljovodonicima. Ovi medijumi se rastvaraju u organskim rastvaračima kao što su ksilen ili toluen, ili esenecê (rastvarač enecê). Namenjeni su trajnom prepariranju i pružaju odličnu dugoročnu stabilnost, uz otpornost na vlagu i degradaciju, što ih čini idealnim za arhivske svrhe (npr. neutralni kanada balzam).

Ukratko, medijumi rastvorljivi u vodi su najpogodniji za privremene preparate ili slučajeve u kojima je potrebno lako uklanjanje primerka; medijumi ograničene tolerancije na vodu pogodni su za polutrajne preparate koji zahtevaju umerenu trajnost, dok su medijumi rastvorljivi u ugljovodonicima poželjni za trajne preparate namenjene arhiviranju i dugoročnom čuvanju.

Tabela 3: Sastav pojedinih medijuma za prepariranje.

Medijum za prepariranje	Rastvarač	Potencijalni pre-polimer(i) ili polimer	Napomene
Hoyer = hloralna guma	glicerol, voda	jedinjenja arapske gume	Sredstvo za maceraciju: hloral-hidrat
CMCP-9 (= karboksimetil-celuloza fenol)	voda (CMCP-9: 51–60%)	potpuno hidrolizovani polivinil-alkohol (CMCP-9: 0–5%)	CMCP-9: niska viskoznost; visoka viskoznost
DMHF (dimetil-hidantoin formaldehid)	voda	N,N′-dimetilol dimetil-hidantoin (di-metilol DMH); oligomeri povezani etarskim i metilenskim mostovima; umrežena polimerna mreža DMH–formaldehid	
Kanada balzam	ksilen; delimično isparljive komponente balzama (Δ^3-karen, levopimarska kiselina, limonen, mircen, palustrinska kiselina, β-felandren, α-pinen, β-pinen)	balzam (abienol, abietinska kiselina, izopimarska kiselina, sandarakopimarska kiselina)	neutralizacija: kalijum-karbonat; smola iz Abies balsamea (Linné, 1758)
Euparal®	eukaliptol, paraldehid; delimično isparljive komponente sandarak-gume (limonen, α-pinen, β-pinen)	jedinjenja sandarak-gume (komunska kiselina, manool, polikomunska kiselina, sandarakopimarska kiselina, 12-acetoksi-sandarakopimarska kiselina, sugiol, torulozna kiselina, torulosol, totarol)	sredstvo za prosvetljavanje: metil-salicilat; boja u Euparal® green: bakarna so (bakrov abietinat); sandarak-smola iz Tetraclinis articulata (Vahl, 1791)
Enecê	etil-alkohol; sa kamforom, eukaliptolom i terpentinovom esencijom	jedinjenja kopalne smole i kolofonijuma (rosin)	

Tabela 4: Prednosti i nedostaci odabranih medijuma za prepariranje na mikroskopskim preparatima i neobjavljena zapažanja različitih autora [52].

Naziv	Prednosti	Nedostatci
* Kanada balzam	Medijum je veoma trajan, sa vekom trajanja koji premašuje 150 godina.<br>Preparati se mogu preparirati korišćenjem karanfilićevog ulja ili fenola kao sredstava za prepariranje.	Sadrži štetne komponente i mora se koristiti pod digestorom.<br>Zahteva kompletnu, vremenski zahtevnu seriju dehidratacije.<br>Dehidratacija etanolom i prelazak preko ksilena ili karanfilićevog ulja mogu učiniti neke taksone krhkim; alternative (npr. izopropanol, n-butanol, Cellosolve™, 1,4-dioksan, Histoclear, terpeneol) mogu smanjiti lomljenje.<br>Primerci mogu potamniti ukoliko se ksilen zameni fenolom ili ako zaostane kalijum-hidroksid.<br>Visok indeks prelamanja može prikriti neobojene strukture.<br>Potpuno sušenje može trajati godinama ukoliko se ne koristi grejna ploča.<br>Medijum vremenom žuti i tamni, naročito ako je prosvetljavanje vršeno karanfilićevim uljem.<br>Neke boje slabe, a kationske boje mogu izbledeti ako medijum postane kiseo — što se može spontano desiti tokom vremena.
DMHF (dimetil-hidantoin formaldehid)	Visoka providnost<br>Dobar indeks prelamanja<br>Odlična vidljivost struktura<br>Relativno dobra stabilnost preparata<br>Kompatibilnost sa mnogim tehnikama bojenja<br>Dobra zaštita uzoraka<br>Dobro prianjanje između predmetnog i pokrovnog stakalca	Moguće žućenje tokom vremena<br>Može promeniti neke boje<br>Nije pogodno za boje osetljive na formaldehid<br>Pojava mehurića vazduha, sporo sušenje<br>Medijum za prepariranje osetljiv na vlagu<br>Prepariranje je teško reverzibilno<br>Formaldehid je toksičan, iritantan i kancerogen
* Euparal (proziran)	Trajan medijum sa vekom trajanja dužim od 50 godina.<br>Moguće je prepariranje direktno iz 80% etanola (preporuka proizvođača).<br>Ne prikriva neobojene strukture i ne žuti niti postaje krt tokom vremena.<br>Ima indeks prelamanja pogodniji od kanadskog balzama za Diptera.<br>Dobro funkcioniše za deblje primerke zbog minimalnog skupljanja i sušenja bez mehurića.<br>Ostaje rastvorljiv u 95% etanolu, što omogućava ponovno prepariranje čak i nakon mnogo godina.	Sadrži štetne komponente i mora se koristiti pod digestorom.<br>Dehidratacija etanolom i prelazak preko Euparal esencije mogu učiniti neke taksone krhkim, ali upotreba izopropanola može ublažiti ovaj problem.
Hoyer tečnost	Primerci se mogu preparirati živi ili direktno iz vode, etanola ili formaldehida.<br>Maceracija daje izuzetan kvalitet kutikule.<br>Ima povoljan indeks prelamanja i može se unaprediti bojenjem jodom radi većeg kontrasta.<br>Sirćetna kiselina u sastavu može proširiti telesne nastavke insekata.<br>Neki preparati mogu ostati stabilni 40–60 godina.<br>Rastvorljiv u vodi, što omogućava lako ponovno prepariranje.	Osetljivi biljni primerci mogu kolabirati ukoliko se medijum dodaje postepeno, što je vremenski zahtevno.<br>Šupljine i kristali mogu se formirati za manje od 10 godina.<br>Maceracija može postati preterana u zavisnosti od koncentracije hloral-hidrata i vremena izlaganja.<br>Komponente medijuma se mogu razdvajati, a fina granulacija se može pojaviti tokom meseci ili godina.<br>Zabeleženo je potamnjivanje medijuma.

This is an Open Access article distributed under the terms of the Creative Commons Attribution License (https://creativecommons.org/licenses/by/4.0), which permits unrestricted use, distribution, and reproduction in any medium, provided the original work is properly cited.

CMCP-9 (= karboksimetil-celuloza fenol)	Primerci se mogu preparirati direktno iz medijuma kao što su voda, etanol, glicerol ili rastvori koji sadrže formaldehid, a njihovi unutrašnji organi se po potrebi mogu macerirati kako bi se olakšao opšti pregled ili priprema.	Ovaj medijum može tokom vremena formirati kristale i potamniti, a ponekad može macerirati primerke više nego što je planirano. Ukoliko preparat nije pažljivo obrubljen, deblji uzorci u njemu neće biti stabilni jer se mogu skupljati i stvarati praznine oko ivica pokrovnog stakalca. Nije pogodan za obojene primerke niti za kalcifikovane materijale, a vreme sušenja je sporije nego kod CMC.
Eukitt™	Trajan medijum sa vekom trajanja dužim od 30 godina.<br>Kompatibilan sa mnogim rastvaračima za prepariranje, uključujući aceton, benzen, hloroform, dioksan, etar, izopropanol, metil-benzoat, terpeneol, toluen i ksilen.<br>Brzo se suši i ima blago kiseo pH.<br>Ne pokazuje primetno tamnjenje tokom starenja.<br>Pogodan za različite boje (npr. fuksin, hematoksilin, metil-zeleno, metil-ljubičasto, metilen-plavo).<br>Primerci se mogu ponovo preparirati i nakon više godina potapanjem u ksilen tokom dužeg vremenskog perioda.	Sadrži štetne komponente i mora se koristiti pod digestorom.<br>Zahteva kompletnu, vremenski zahtevnu seriju dehidratacije.<br>Nije idealan za deblje primerke zbog skupljanja i stvaranja gasnih mehurića.<br>Pokrovna stakalca se mogu vremenom odvojiti ukoliko staklo nije dobro očišćeno i zaptiveno.<br>Može doći do nepotpune polimerizacije oko kolagenih vlakana.
Enecê	Veoma trajan medijum, sa vekom trajanja od najmanje 50 godina.<br>Enecê ne tamni tokom vremena.<br>Podatan je, omogućava disekciju insekata unutar medijuma, kao i razumno vreme za pozicioniranje morfoloških struktura.<br>Niska cena.	Zahteva kompletnu, vremenski zahtevnu seriju dehidratacije.<br>Dehidratacija etanolom i prelazak preko karanfilićevog ulja mogu učiniti neke primerke krhkim.<br>Insekt nastavlja da se prosvetljava, iako veoma sporo; to može otežati uočavanje veoma sitnih struktura, kao što su senzile, askoidi i jednostavne sete.

5.3.4. Opis preporučenih medijuma za prepariranje (Tabele 3 i 4)

Medijumi za privremeno posmatranje

Chloral gum = Hoyer fluid/medium/solution (RI = 1.48)

Marc-André rastvor je najbolji medijum za kratkotrajno (nekoliko sati, eventualno nešto duže ukoliko se preparat čuva u vlažnoj komori) posmatranje spermateka, uključujući fotografisanje (Slika 4) ili izradu crteža. Očuvanje posmatranih spermateka zahteva njihovo ponovno prepariranje u vodeni medijum koji omogućava srednjoročno čuvanje. Dehidratacija radi ponovnog prepariranja u smolasti medijum nije nemoguća, ali se ne preporučuje (rizik od gubitka). Hloralna guma i Hoyerov rastvor smatraju se sinonimima. Ovaj medijum se često koristi za posmatranje unutrašnjih organa zbog svoje kompatibilnosti sa vodom, jednostavne primene, brze upotrebe i indeksa prelamanja koji olakšava ispitivanje delikatnih struktura kao što su spermateke. Međutim, hloralna guma ima značajne nedostatke ukoliko nije savršeno pripremljena ili ukoliko se ne čuva u kontrolisanim uslovima vlažnosti. Ovi problemi uključuju kristalizaciju, promenu boje i gubitak viskoznosti. Obrubljivanje pokrovnog stakalca ne rešava ove probleme, jer medijum za prepariranje može postati snažno obojen (ponekad gotovo crn) usled interakcije sa sredstvom za obrubljivanje, naročito ako se koristi Euparal®.

Hoyerov medijum se smatrao optički najboljim za flebotomine i tradicionalno se koristio u te svrhe. Medijum se sastoji od više međusobno sličnih formulacija koje uključuju gumiarabiku, glicerol i hloral-hidrat. Različite formulacije su pogrešno tumačene i pogrešno citirane [74].

Iako je Hoyerov medijum dobar za posmatranje spermateka kod flebotomina, nije pogodan za dugotrajno čuvanje. Idealan je za kratkotrajna posmatranja, uključujući fotografije, crteže ili snimke. Vodeni medijumi su pogodni za privremene preparate, ali ne mogu obezbediti dugoročno očuvanje. Nasuprot tome, prepariranje u smolaste medijume obezbeđuje izuzetnu trajnost, često u trajanju od više vekova, ali može prikriti fine detalje spermateka, jer se njihova refringencija često gubi.

Hoyerov medijum vremenom degradira usled dehidratacije (Slika 8), što dovodi do formiranja malih belih, neprovidnih kristala hloral-hidrata. Ipak, primerci se mogu povratiti iz kristalizovanih preparata, jer kutikula ostaje hemijski očuvana, iako može doći do određenog fizičkog oštećenja usled rasta kristala. U pojedinim slučajevima, kristalizovani preparati mogu se obnoviti rehidratacijom medijuma za prepariranje u toploj, vlažnoj sredini uz dodatak timola radi sprečavanja rasta gljivica. Alternativno, primerci se mogu izvaditi iz hloralne gume potapanjem u vodu, dehidratovati

This is an Open Access article distributed under the terms of the Creative Commons Attribution License (https://creativecommons.org/licenses/by/4.0), which permits unrestricted use, distribution, and reproduction in any medium, provided the original work is properly cited.

u ledenoj sirćetnoj kiselini i ponovo preparirati u kanada balzam.

DMHF (dimetil-hidantoin formaldehid) (IP = 1,48)

Ovaj vodeni medijum [72] pokazuje veoma dobre optičke osobine, slično kao Berleseov medijum, i jednako je jednostavan za upotrebu. Međutim, za razliku od Berleseovog medijuma, ne potamni niti kristališe. Dobro funkcioniše za flebotomine i druge Psychodidae.

CMCP (kamfor–mono-hlorofenol) (IP = 1,41)

Ovo je medijum za prepariranje na bazi glicerola, rastvorljiv u vodi, koji se koristi za izradu providnih, trajnih mikroskopskih preparata osetljivih primeraka, uključujući flebotomine. Prednost ovog medijuma za prepariranje je u tome što se primerci mogu preparirati direktno iz vode ili etanola. Brzo relaksira i prosvetljava flebotomine, omekšavajući kutikulu i omogućavajući pravilno pozicioniranje primerka, što je naročito korisno pri raširivanju krila ili disekciji genitalija. Iako se navodi da omogućava dugotrajno čuvanje, tačno trajanje očuvanja ostaje neizvesno. Glavno ograničenje ovog medijuma za prepariranje leži u njegovom sastavu koji sadrži fenol, toksičnu i iritantnu supstancu koja zahteva pažljivo rukovanje.

Medijumi za trajno prepariranje

Kanada balzam (IP = 1,52–1,54)

Kanada balzam je prvi put opisan kao pogodan medijum za prepariranje u mikroskopiji u propuštenom svetlu od strane Andrewa Pritcharda tokom 1830-ih godina. Ostaje jedan od najčešće korišćenih medijuma zahvaljujući dokazanim arhivskim kvalitetima, sa više od 150 godina uspešne primene. Za razliku od Hoyerovih medijuma, kanada balzam ne kristališe niti apsorbuje vlagu. Međutim, kanada balzam je snažno autofluorescentan, što u nekim slučajevima može predstavljati nedostatak za određene mikroskopske tehnike [60]. Upotreba netoksičnih rastvarača umesto ksilena može smanjiti bezbednosne rizike tokom pripreme, ali istovremeno može dovesti do određenih nedostataka, kao što su sporije sušenje i ranije tamnjenje medijuma.

Euparal® (IP = 1,48)

Euparal® je široko korišćena alternativa kanadskom balzamu za trajno prepariranje, koja nudi odličnu dugoročnu stabilnost i uporediv indeks prelamanja. Euparal® ima sledeće karakteristike: (1) zahtev za dehidratacijom: pre konačnog prenosa u medijum za prepariranje, primerak mora biti dehidratovan, obično prelaskom iz 95% u apsolutni etanol, i (2) produženo vreme obrade: konačno prepariranje u smolasti medijum, bilo da je u pitanju kanada balzam ili Euparal®, zahteva dehidrataciju, što produžava ukupno vreme obrade uzoraka. Kada dehidratacija organskim rastvaračima nije izvodljiva, uzorci izdvojeni iz apsolutnog etanola mogu se postaviti u intermedijarni rastvor koji se sastoji od jednake smeše Euparal®-a i Euparal esencije, pre konačnog prepariranja.

Enecê (IP = 1,467)

Enecê je smolasti medijum za prepariranje koji se prvenstveno koristi za male insekte i naročito je popularan u Brazilu. Njegova osnova sastoji se od kolofonijuma i kopalne smole rastvorenih u alkoholu, kamforu, terpentinovoj esenciji i eukaliptolu. Cerqueira [11] je opisao Enecê kao alternativu kanadskom balzamu za prepariranje trajnih preparata larvi, egzuvija juvenilnih stadijuma, pa čak i odraslih komaraca, a od tada je široko prihvaćen i za prepariranje flebotomina. Enecê predstavlja isplativu alternativu za trajno prepariranje, obezbeđujući dugoročnu stabilnost i dovoljno vremena sušenja, što omogućava disekciju i precizno raspoređivanje morfoloških struktura.

5.4. Priprema mikroskopskih preparata i sušenje

Pravilno sušenje preparata od ključnog je značaja za obezbeđivanje dugoročne stabilnosti i očuvanja. Preparati treba da budu temeljno osušeni pre dugoročnog skladištenja. Radi optimalnih rezultata, preparati koji su pripremljeni u trajnim medijumima treba da se suše u horizontalnom položaju tokom 2–3 nedelje, dok preparati pripremljeni polutrajnim medijumima mogu zahtevati samo 1–2 nedelje. Kako bi se obezbedio efikasan proces sušenja, preporučuje se upotreba inkubatora podešenog na odgovarajuću temperaturu za konkretan medijum za prepariranje, uz izbegavanje prekomerne toplote koja bi mogla oštetiti primerke. Preporučuje se temperaturni opseg od 30 °C do 37 °C. Ovaj korak sušenja je od presudnog značaja kako bi se sprečilo krivljenje preparata, propadanje primeraka ili nestabilnost medijuma za prepariranje tokom skladištenja.

Medijum za prepariranje korišćen u pripremi preparata uvek treba navesti na etiketi preparata. Ukoliko je moguće, etiketa treba da sadrži i tačan recept koji je korišćen, kao i ime osobe koja je preparat pripremila i datum pripreme. Preparati se u početku pripremaju kao privremeni i nisu namenjeni dugoročnom čuvanju. Međutim, ukoliko se status primerka promeni, na primer ako bude određen kao deo „tipske“ serije, potrebno je koristiti trajniji medijum za prepariranje kako bi se obezbedilo očuvanje primerka za buduća taksonomska istraživanja.

5.5. Alternativne tehnike prepariranja: prepariranje na kartice

Prepariranje na kartice je tehnika koja se koristi kod više grupa insekata, pri čemu se primerci mogu direktno pribosti entomološkim iglama na kartice ili zalepiti za njihovu površinu. S obzirom na malu veličinu flebotomina i potrebu za posmatranjem unutrašnjih organa radi identifikacije

nakon prosvetljavanja (videti tačku 5), ova metoda uopšte nije pogodna za prepariranje flebotomina.

5.6. Ponovno prepariranje oštećenih primeraka

Za retke ili vredne primerke preporučuje se dvostepeni pristup, u skladu sa video-zapisom dostupnim na: https://zenodo.org/records/18315029.

1) Rehidratacija bez rastavljanja, kako bi se omogućilo preliminarno posmatranje. Držač za više mikroskopskih preparata treba postaviti u Petrijevu posudu kako bi služio kao nosač. Preparat koji se rehidrira zatim se postavlja odozgo, a Petrijeva posuda se puni sa nekoliko milimetara rastvarača kako bi se formirala vlažna komora, pri čemu se mora obezbediti da sam preparat ne dođe u kontakt sa rastvaračem (Slika 8 D). Vreme potrebno za rehidrataciju može varirati od jednog do nekoliko dana, u zavisnosti od stanja primerka. Svakodnevno praćenje i strpljenje su neophodni. Kada je preparat dovoljno rehidratovan, može se izvaditi iz vlažne komore i postaviti u inkubator na nekoliko sati pre mikroskopskog posmatranja, fotografisanja ili crtanja.

2) Za ponovno prepariranje, preparat se može vratiti u vlažnu komoru na još nekoliko sati ili preko noći. Rastavljanje se mora obaviti pod binokularnim mikroskopom. Upotrebom finih igala, pokrovno stakalce se mora pažljivo ukloniti, vodeći računa da nijedan deo flebotomine ne ostane zalepljen (https://zenodo.org/records/18315029). Zatim se disekovani delovi flebotomine sakupljaju i ispiraju vodom u malim udubljenjima, poput onih koja se koriste za destruktivnu ekstrakciju DNK/RNK (videti niže), pre dehidratacije i ponovnog prepariranja u smolasti medijum. Prilikom rastavljanja preparata, od ključnog je značaja identifikovati originalni medijum za prepariranje kako bi se izabrao odgovarajući rastvarač. Za vodene medijume za prepariranje treba koristiti vodu. Ukoliko je medijum za prepariranje smolast (npr. kanada balzam ili Euparal®), treba koristiti ksilen, uz rad pod digestorom i uz odgovarajuću ličnu zaštitnu opremu, uključujući masku.

Ponovno prepariranje tipskih ili kolekcijskih primeraka sme se obavljati isključivo uz saglasnost kustosa i/ili institucije koja poseduje primerak.

6. Identifikacija flebotomina

6.1. Morfologija

Identifikacija flebotomina se prvenstveno zasniva na ispitivanju njihovih morfoloških karakteristika, uključujući oblik toraksa, krila, genitalija, seta i specifične morfometrijske odnose između različitih struktura. Istraživači koriste taksonomske ključeve, referentne zbirke i originalne opise vrsta kako bi uporedili sakupljene primerke sa poznatim taksonima. Ključne dijagnostičke osobine, kao što su nervatura krila i morfologija glave kod oba pola, struktura muških genitalija i konfiguracija ženskih spermateka, naročito su informativne za određivanje vrste. Precizna identifikacija često zahteva detaljno mikroskopsko ispitivanje, najčešće uz upotrebu svetlosnog mikroskopa za posmatranje finih struktura poput genitalija i spermateka, ili stereomikroskopa za šire morfološke osobine.

Nedavni napreci u tehnologiji snimanja omogućili su upotrebu digitalne vizualizacije u identifikaciji flebotomina. Fotografije visoke rezolucije ili digitalne ilustracije ključnih karakteristika mogu se upoređivati sa referentnim materijalima ili analizirati pomoću računarski potpomognutih sistema za identifikaciju, čime se unapređuju i tačnost i dostupnost u morfološkoj taksonomiji.

6.2. Geometrija krila

Geometrija krila predstavlja jednu od ključnih karakteristika koje se koriste u identifikaciji i klasifikaciji različitih vrsta flebotomina. Krila flebotomina imaju jedinstven obrazac i strukturu, obično su duga i uska, sa dobro razvijenom nervaturom (Slike 9 i 10). Raspored nervi formira karakterističan obrazac koji može varirati između rodova i vrsta, pružajući vredne dijagnostičke osobine za identifikaciju. Stoga, proučavanje geometrije krila daje značajne uvide u taksonomske analize.

6.3. Geometrijska morfometrija krila

Istraživači koriste različite tehnike, kao što je geometrijska morfometrija, za analizu i poređenje oblika i veličine krila između različitih vrsta ili populacija flebotomina. Proučavanje geometrije krila pruža dragocene uvide u ponašanje, preferencije staništa i sposobnosti letenja.

U pristupu geometrijske morfometrije, krila se pažljivo disekuju, po potrebi boje i prepariraju ravno na mikroskopska stakalca. Pripremljeni preparati se zatim fotografišu pod stereomikroskopom, digitalizuju i podvrgavaju morfometrijskoj analizi. Ovaj postupak je detaljno opisan u literaturi [6, 27, 42, 56, 57, 59], uz preporuku da se kod uparenih organa dosledno koristi desno ili levo krilo, kako bi se izbegli mogući negativni alometrijski efekti [62].

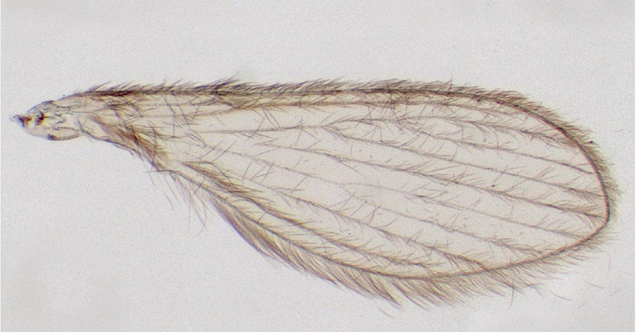

Slika 9: Neobrađeno krilo vrste *Trichophoromyia ininii*.

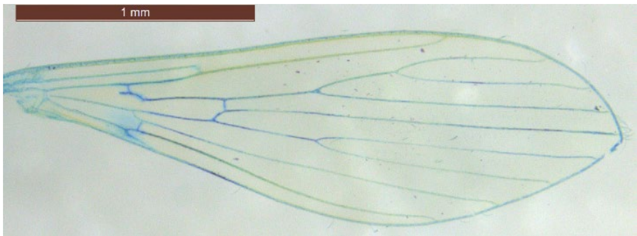

Slika 10: Obojeno krilo vrste *Phlebotomus ariasi.*

Priprema krila za geometrijsku morfometrijsku analizu

Radi optimalne vizualizacije nervature krila, krila treba očistiti od ljuski i odgovarajuće obojiti. Za pripremu krila, najpre se mala udubljenja napune potrebnim reagensima (metilen-plavo, etanol, voda i zamena za ksilen). Krilo sačuvano u 70% etanolu na sobnoj temperaturi uzima se okretanjem Eppendorf® epruvete i njenim pražnjenjem iznad udubljenja, a zatim se krilo uzdužno podiže finom zakrivljenom iglom. Krilo se kratko prenosi iz etanola u vodu i nazad u etanol radi uklanjanja čekinja. Zatim se krilo stavlja u metilen-plavo na 6 minuta, vodeći računa da tokom bojenja pluta. Krilo se pažljivo preuzima i potapa u zamenu za ksilen na 2 minuta (otprilike jednu trećinu vremena bojenja u metilen-plavom). Blagi dodiri iglom o zidove udubljenja mogu pomoći da se krilo pravilno smesti; ksilen služi za fiksiranje boje. Na kraju, krilo se podiže i postavlja na malu kap Euparal®-a na predmetnom stakalcu. Pod lupom se krilo pažljivo razvuče i pažljivo se postavlja pokrovno stakalce. Fotografije treba snimiti odmah, pre nego što se Euparal® stegne, jer mogu biti potrebna manja podešavanja položaja krila ispod pokrovnog stakalca kako bi se postiglo optimalno poravnanje.

6.4. Molekularno biološke tehnike

Pored morfoloških tehnika, molekularne metode postaju sve značajnije u entomološkim istraživanjima, uključujući taksonomske, populaciono-genetičke i filogenetske studije, kao i detekciju DNK/RNK patogena i određivanje porekla krvnog obroka, pri čemu je ponašanje vektora od posebnog značaja u oblasti epidemiologije [70]. Sekvenciranje DNK može se koristiti za potvrdu vrste ili za razdvajanje blisko srodnih vrsta, pružajući preciznije i pouzdanije sredstvo identifikacije. Pored toga, napredne molekularne tehnike (npr. PCR, sekvenciranje DNK, NGS itd.) i MALDI-ToF MS sve više dobijaju na značaju za preciznu i brzu identifikaciju vrsta, dopunjujući tradicionalne morfološke metode [46]. Uprkos ovim naprecima, morfološka identifikacija ostaje referentni standard u taksonomiji i osnova na kojoj se tumače molekularni podaci.

6.4.1. Destruktivna ekstrakcija nukleinskih kiselina

Ekstrakcija nukleinskih kiselina predstavlja rutinski korak u mnogim biološkim istraživanjima, a razvijeni su brojni postupci za izolaciju DNK iz bioloških materijala [48]. Mnogi komercijalno dostupni kompleti za ekstrakciju DNK osmišljeni su da olakšaju ovaj proces [14]. Međutim, metode koje se uobičajeno koriste za pripremu insekata radi morfološke identifikacije često otežavaju analizu DNK, jer mogu oštetiti ili uništiti ključne fizičke karakteristike primerka [10]. Većina protokola za ekstrakciju DNK iz tkiva insekata po svojoj prirodi je destruktivna [43], što predstavlja poseban problem kod sitnih primeraka, gde čak i ograničeno uzorkovanje može ugroziti važne morfološke osobine [72]. Tip i stanje primerka imaju ključnu ulogu pri izboru odgovarajuće metode izolacije DNK [29].

Potreba za tačnom identifikacijom flebotomina, razumevanjem populacione dinamike i minimizovanjem uticaja na neciljne organizme podstakla je razvoj molekularnih dijagnostičkih alata [23]. Molekularni pristupi se danas često koriste kao dopuna morfološkim taksonomskim metodama za identifikaciju flebotomina. Na primer, standardni pristup DNK barkodiranju insekata podrazumeva ekstrakciju DNK, sekvenciranje i gubitak originalnog primerka. Zbog toga postoji izražena potreba za razvojem nedestruktivnih metoda ekstrakcije DNK koje bi očuvale i biološki materijal i njegovu morfološku celovitost.

Brojne metode ekstrakcije nukleinskih kiselina primenjivane su kod flebotomina. Količina i kvalitet nukleinskih kiselina koji su potrebni zavise od daljih molekularnih analiza, jer različite tehnike imaju različite zahteve u pogledu osetljivosti i čistoće [9]. Na primer, utvrđeno je da oči flebotomina mogu inhibirati PCR amplifikaciju [69]. Pored skrininga patogena, DNK flebotomina se rutinski izdvaja i u svrhu identifikacije vrsta. Mogu se koristiti različite metode ekstrakcije, pri čemu se prinos i kvalitet razlikuju u zavisnosti od tehnike. Neki protokoli proizvođača prilagođeni su od strane istraživača za flebotomine [8], čime je povećan prinos i/ili kvalitet izdvojenih nukleinskih kiselina [8, 9, 69], dok se druge prilagođene metode, razvijene za druge taksone insekata, takođe mogu primeniti na flebotominama [58, 76]. Identifikacioni PCR protokoli usmereni na kratke mitohondrijalne fragmente (COI ili CytB) uglavnom su kompatibilni sa metodama ekstrakcije koje dovode do visokog stepena fragmentacije DNK. Nasuprot tome, NGS tehnike dugog čitanja (Oxford Nanopore i PacBio) zahtevaju minimalnu fragmentaciju i DNK visokog kvaliteta. Ekstrakcije pomoću spin-kolona obično daju fragmente genomske DNK do 60 kb, dok fenol-hloroformska ekstrakcija može proizvesti fragmente do 150 kb [77]. U Tabeli 5 prikazane su različite tehnike ekstrakcije DNK iz flebotomina, uz naznaku da li su metodološke prilagodbe rađene posebno za ove insekte. Prinosi nisu prikazani, jer zavise od veličine primerka i načina pripreme. Kolona „modifikacija“ odnosi se na prilagođavanja protokola ekstrakcije za flebotomine ili druge male insekte.

Pri izboru metode ekstrakcije treba uzeti u obzir više kriterijuma, kao što su broj uzoraka, vreme potrebno za ekstrakciju i tehnika koja će se primenjivati u daljoj analizi. Dok NGS tehnike zahtevaju genomsku DNK velike molekulske mase, sve ovde predstavljene metode mogu se koristiti za standardne PCR-bazirane primene.

Pored toga, više studija se bavilo razvojem nedestruktivnih metoda ekstrakcije DNK za sitne kopnene zglavkare, suvo čuvane muzejske primerke i insekte sa mekim telom [19, 26, 28, 55, 63].

Tabela 5: Prosečni trošak, primena i prilagođavanje protokola za ekstrakciju gDNK flebotominskih peščanih mušica

Protokol	Cena	Aplikacija	Adaptacija protokola za male insekte
Spin kolona	2.5 – 3.55 US$ [39]	PCR, NGS	[9]
Fenol - hloroform	0.24 US$ [69]	PCR, NGS	[9]
HotSHOT	<0.01 US$ [69]	PCR	-
Salting out	0.12 $3 [69]	PCR	-
Chelex	0.02 $4 [41]	PCR	[41, 76]

6.4.2. Nedestruktivna ekstrakcija nukleinskih kiselina

Jedan od glavnih izazova u molekularnoj analizi insekata, naročito flebotomina, jeste očuvanje primeraka radi njihovog uključivanja u entomološke zbirke. Većina protokola za ekstrakciju DNK zahteva maceraciju tkiva, čime se narušava očuvanost originalnog primerka. Nedestruktivne metode ekstrakcije nukleinskih kiselina, međutim, osmišljene su tako da omoguće izdvajanje genetskog materijala bez fizičkog oštećenja uzorka, bez uticaja na njegovu upotrebljivost ili promene morfologije. Ove metode su naročito dragocene pri radu sa vrednim ili ograničenim primercima, kao što su flebotomine, gde je očuvanje strukturnog integriteta od suštinskog značaja za buduća taksonomska, morfološka ili dijagnostička istraživanja. Jedna od često korišćenih tehnika jeste nedestruktivna metoda potapanja, pri kojoj se flebotomine imobilizuju i nežno potapaju u litički pufer koji sadrži proteinazu K.

Tehnika blage vektolize uspešno je primenjena kod flebotomina, naročito kod tipskih primeraka [24]. Ova tehnika koristi konvencionalni spin-kolona komplet (u ovom slučaju DNeasy Blood and Tissue kit, QIAGEN, Hilden, Nemačka), uz prilagođavanje koje omogućava dobijanje DNK bez uništavanja primerka. Modifikovani koraci lize (zapremina litičkog pufera i uvođenje koraka zamrzavanja) [17] omogućavaju oslobađanje nukleinskih kiselina uz minimalno morfološko oštećenje [24]. Kada su u pitanju flebotomine, moguće je koristiti i HotSHOT komplet za ekstrakciju DNK (Bento Bioworks Ltd, London, Ujedinjeno Kraljevstvo) [73], koji je brz i jeftin i omogućava brzo i niskobudžetno procesiranje uzoraka. Entomološki primerci namenjeni morfološkoj identifikaciji mogu se potom isprati. Primerci obrađeni pomoću DNeasy Blood and Tissue kompleta moraju se prosvetliti Marc-André rastvorom, dok su primerci obrađeni HotSHOT kompletom za ekstrakciju DNK dovoljno prosvetljeni da se mogu preparirati u vodeni medijum ili, još bolje, u smolasti medijum nakon dehidratacije, u skladu sa protokolom detaljno opisanim u ovom radu [73]. Izdvojeni genetski materijal zatim se može dalje koristiti za naknadne analize, kao što je PCR, radi amplifikacije specifičnih genetskih markera. Nedestruktivne metode ekstrakcije nukleinskih kiselina od ključnog su značaja za proučavanje genetskih karakteristika flebotomina, uključujući identifikaciju potencijalnih uzročnika bolesti koje mogu prenositi. Očuvanjem integriteta primerka, istraživači mogu dobiti dragocene genetske informacije, uz zadržavanje uzorka za dodatne analize ili istraživanja.

6.5. MALDI-ToF MS

MALDI-ToF MS (matriksom potpomognuta laserska desorpcija/jonizacija uz merenje vremena preleta – masena spektrometrija) predstavlja tehniku zasnovanu na masenoj spektrometriji, namenjenu detekciji i analizi jedinstvenih proteinskih profila („otisaka prstiju“) bioloških uzoraka. MALDI-ToF se sve više prepoznaje kao važan alat za identifikaciju insekata od medicinskog i veterinarskog značaja. Ova tehnika se pokazala efikasnom u identifikaciji različitih razvojnih stadijuma flebotomina, uključujući nezrele oblike i krvne obroke nahranjenih ženki, i uspešno je primenjena za razlikovanje i muških i ženskih jedinki flebotomina u različitim uslovima skladištenja i homogenizacije [28, 30, 73, 74]. Ova metoda takođe pruža visoku diskriminatornu moć na nivou podrodova, vrsta i populacija. Tehnika omogućava istraživačima brzo i precizno određivanje vrsta, što je ključno za razumevanje rasprostranjenosti flebotomina, njihovog ponašanja i uloge u prenošenju bolesti. Razlikovanjem vrsta na osnovu proteinskih profila, MALDI-ToF ima značajnu ulogu u epidemiološkim istraživanjima i strategijama kontrole vektora.

Postoje dva glavna nedostatka ove tehnike koja trenutno ograničavaju njenu rutinsku primenu. Prvi se odnosi na dostupnost masenih spektrometara, koji su izuzetno skupi i teško se nabavljaju isključivo u svrhu identifikacije vrsta flebotomina (ili artropodnih vektora uopšte). Srećom, ovo ograničenje se može prevazići korišćenjem vremena na masenim spektrometrima koji su postali standardni istraživački alati u proteomskim laboratorijama i/ili kliničkoj dijagnostici. Drugi nedostatak je slaba zastupljenost referentnih podataka za flebotomine u bazama podataka sa otvorenim pristupom, što nameće potrebu za formiranjem sopstvene (in-house) baze podataka sa referentnim spektrima, zasnovane na nedvosmisleno identifikovanim primercima, idealno kombinacijom morfološke analize i sekvenciranja odgovarajućeg genetskog markera (COI, cytB ili drugog). Ovo ograničenje će se, nadamo se, uskoro prevazići postepenim uključivanjem do sada internih referentnih podataka o flebotominama u MSI Platformu koju vode Assistance

Publique–Hôpitaux de Paris, Sorbonne University, Francuska, kao i kolekciju BCCM/IHEM/Sciensano u Briselu, Belgija (https://msi.happy-dev.fr/).

Kada se planira primena MALDI-ToF proteinskog profilisanja, uzorke je poželjno čuvati suvo-zamrznute ili u 70% etanolu molekularnog kvaliteta i ne izlagati ih sobnim temperaturama. U nedostatku univerzalnih smernica za pripremu uzoraka, korisnicima se preporučuje upotreba vodene otopine od 60% acetonitrila/0,3% TFA sa sinapinskom kiselinom (30 mg/mL) za pripremu MALDI-ToF matriksa, kako bi dobijeni proteinski spektri bili uporedivi sa do sada objavljenim podacima za flebotomine.

Priprema uzoraka za MALDI-ToF MS (Slika 7)

Primerci insekata, čuvani pod različitim uslovima, najpre se suše na vazduhu na sobnoj temperaturi i zatim disekuju. Glava i abdomen se uklanjaju kako bi se dobili delovi tela koji sadrže ključne morfološke karakteristike za prepariranje na preparate i morfološku analizu. Toraks se može koristiti za MALDI-ToF, dok se preostali deo abdomena može sačuvati za ekstrakciju DNK. Za proteinsko profilisanje, toraks se ručno homogenizuje u mikrocevkama zapremine 1,5 mL sa 10 µL homogenizacionog rastvora, korišćenjem jednokratnih tučkova i peleta. Najčešće se koriste dva homogenizaciona rastvora: sterilna destilovana voda i 25% mravlja kiselina.

7. Zaključak

U ovom radu imali smo za cilj da istraživačima ponudimo najefikasnije metode za prepariranje flebotomina, prilagođene specifičnim istraživačkim ciljevima, kako bi se olakšala tačna identifikacija i detekcija patogena. Ne postoji jedna univerzalno optimalna metoda; umesto toga, dostupno je više metoda, od kojih svaka ima svoje prednosti i ograničenja.

U dodatnim podacima pružili smo detaljne protokole za različite tehnike prepariranja koje se koriste u pripremi i identifikaciji flebotomina. Ovi protokoli, uključujući i edukativne video-zapise, nude postupke korak po korak, prilagođene različitim ciljevima, obezbeđujući precizne i pouzdane rezultate. Nudeći ovaj sveobuhvatan resurs, nastojimo da podržimo istraživače u izboru i primeni najodgovarajućih tehnika prepariranja u skladu sa njihovim specifičnim potrebama.

Zahvalnice

Autori zahvaljuju Richardu Laneu i Zoe Jay Adams sa Prirodnjačkog muzeja u Londonu, Ujedinjeno Kraljevstvo, na izvrsnoj recenziji koja je doprinela unapređenju kvaliteta ovog rukopisa.

Finansiranje

Zahvaljujemo brazilskim razvojnim agencijama CNPq (broj projekta: 404395/2024-4) i Fondaciji Araucária (broj projekta: 433/2025 PDI) na finansijskoj podršci istraživanju AJA.

Sukob interesa

Jérôme Depaquit je pridruženi urednik časopisa Parasite; nije imao nikakav uticaj na recenzentski postupak niti na donošenje odluka u vezi sa ovim rukopisom. Ostali autori izjavljuju da nemaju sukob interesa.

Data availability statement

Videi na Zenodo
Video 1: https://zenodo.org/records/18198006
Video 2: https://zenodo.org/records/18311158
Video 3: https://zenodo.org/records/18311106
Video 4: https://zenodo.org/records/18311154
Video 5: https://zenodo.org/records/18303014
Video 6: https://zenodo.org/records/18302850
Video 7: https://zenodo.org/records/18315029

Materijali dodatka

Više informacija o ovom članku dostupno je na sljedećoj adresi: https://www.parasite-journal.org/10.1051/parasite/2026009/olm

Referências

1. Alkan C, Allal-Ikhlef AB, Alwassouf S, Baklouti A, Piorkowski G, de Lamballerie X, Izri A, Charrel RN. 2015. Virus isolation, genetic characterization and seroprevalence of Toscana virus in Algeria. Clinical Microbiology and Infection, 21(11), 1040 e1-9.
2. Alten B, Ozbel Y, Ergunay K, Kasap OE, Cull B, Antoniou M, Velo E, Prudhomme J, Molina R, Banuls AL, Schaffner F, Hendrickx G, Van Bortel W, Medlock JM. 2015. Sampling strategies for phlebotomine sand flies (Diptera: Psychodidae) in Europe. Bulletin of Entomological Research 105(6), 664–678.
3. Ayhan N, Baklouti A, Prudhomme J, Walder G, Amaro F, Alten B, Moutailler S, Ergunay K, Charrel RN, Huemer H. 2017. Practical guidelines for studies on sandfly-borne phleboviruses: Part I: Important points to consider *ante* field work. Vector-Borne and Zoonotic Diseases 17(1), 73–80.
4. Bates PA. 1997. Infection of phlebotomine sandflies with *Leishmania*, in The Molecular Biology of Insect Disease Vectors: A Methods Manual. Springer. p. 112–120.5.
5. Baum M, de Castro EA, Pinto MC, Goulart TM, Baura W, Klisiowicz Ddo R, Vieira da Costa-Ribeiro MC. 2015. Molecular detection of the blood meal source of sand flies (Diptera: Psychodidae) in a transmission area of American cutaneous leishmaniasis, Parana State, Brazil. Acta Tropica, 143, 8–12.
6. Belen A, Alten B, Aytekin A. 2004. Altitudinal variation in morphometric and molecular characteristics of *Phlebotomus papatasi* populations. Medical and Veterinary Entomology, 18(4), 343–350.
7. Bhattacharya J, Chandra G, Hati AK. 1991. A simple method for cryopreservation of *Leishmania donovani* promastigotes, Indian Journal of Medical Research, 93, 245–246.
8. Caligiuri LG, Sandoval AE, Miranda JC, Pessoa FA, Santini MS, Salomón OD, Secundino NF, McCarthy CB. 2019.

Optimization of DNA extraction from individual sand flies for PCR amplification. Methods and Protocols, 2(2), 36.
9. Casaril AE, de Oliveira LP, Alonso DP, de Oliveira EF, Gomes Barrios SP, de Oliveira Moura Infran J, Fernandes WS, Oshiro ET, Ferreira AMT, Ribolla PEM, de Oliveira AG. 2017. Standardization of DNA extraction from sand flies: Application to genotyping by next generation sequencing. Experimental Parasitology, 177, 66–72.
10. Castalanelli MA, Severtson DL, Brumley CJ, Szito A, Foottit RG, Grimm M, Munyard K, Groth DM. 2010. A rapid non-destructive DNA extraction method for insects and other arthropods. Journal of Asia-Pacific Entomology, 13(3), 243–248.
11. Cerqueira NL. 1943. Um novo meio para montagem de pequenos insetos em lâmina. Memórias do Instituto Oswaldo Cruz, (39), 37–41.
12. Charrel RN, Gallian P, Navarro-Mari JM, Nicoletti L, Papa A, Sanchez-Seco MP, Tenorio A, de Lamballerie X. 2005. Emergence of Toscana virus in Europe. Emerging Infectious Diseases, 11(11), 1657–1663.
13. Chaskopoulou A, Giantsis IA, Demir S, Bon MC. 2016. Species composition, activity patterns and blood meal analysis of sand fly populations (Diptera: Psychodidae) in the metropolitan region of Thessaloniki, an endemic focus of canine leishmaniasis. Acta Tropica, 158, 170–176.
14. Chen H, Rangasamy M, Tan SY, Wang H, Siegfried BD. 2010. Evaluation of five methods for total DNA extraction from western corn rootworm beetles. PLoS One, 5(8), e11963.
15. Depaquit J, Grandadam M, Fouque F, Andry PE, Peyrefitte C. 2010. Arthropod-borne viruses transmitted by Phlebotomine sandflies in Europe: a review. Eurosurveillance, 15(10), 19507.
16. Diamond LS, Herman CM. 1954. Incidence of Trypanosomes in the Canada Goose as revealed by bone marrow culture. Journal of Parasitology, 40(2), 195–202.
17. Ding H, Torno M, Vongphayloth K, Ng G, Tan D, Sng W, Ho K, Randrianambinintsoa FJ, Depaquit J, Tan CH. 2025. Hidden in plain sight: discovery of sand flies in Singapore and description of four species new to science. Parasites & Vectors, 18(1), 402.
18. Es-Sette N, Ajaoud M, Bichaud L, Hamdi S, Mellouki F, Charrel RN, Lemrani M. 2014. *Phlebotomus sergenti* a common vector of *Leishmania tropica* and Toscana virus in Morocco. Journal of Vector Borne Diseases, 51(2), 86–90.
19. Favret C. 2005. A new non-destructive DNA extraction and specimen clearing technique for aphids (Hemiptera). Proceedings of the Entomological Society of Washington, 107(2), 469–470.
20. Galati EAB. 2018. Phlebotominae (Diptera, Psychodidae): Classification, morphology and terminology of adults and identification of American taxa, in Brazilian Sand Flies: Biology, Taxonomy, Medical Importance and Control, Rangel EF, Shaw JJ, Editors. Cham: Springer International Publishing. pp. 9–212.
21. Galati EAB, de Andrade AJ, Perveen F, Loyer M, Vongphayloth K, Randrianambinintsoa FJ, Prudhomme J, Rahola N, Akhoundi M, Shimabukuro PHF, Depaquit J. 2025. Phle-botomine sand flies (Diptera, Psychodidae) of the world. Parasites & Vectors, 18(1), 220.
22. Galati EAB, Galvis-Ovallos F, Lawyer P, Leger N, Depaquit J. 2017. An illustrated guide for characters and terminology used in descriptions of Phlebotominae (Diptera, Psychodidae). Par-asite, 24, 26.
23. Gariepy T, Kuhlmann U, Gillott C, Erlandson M. 2007. Parasitoids, predators and PCR: the use of diagnostic molecular markers in biological control of Arthropods. Journal of Applied Entomology, 131(4), 225–240.
24. Giantsis IA, Chaskopoulou A, Bon MC. 2016. Mild-Vectolysis: A nondestructive DNA extraction method for vouchering sand flies and mosquitoes. Journal of Medical Entomology, 53(3), 692–695.
25. Gidwani K, Picado A, Rijal S, Singh SP, Roy L, Volfova V, Andersen EW, Uranw S, Ostyn B, Sudarshan M, Chakravarty J, Volf P, Sundar S, Boelaert M, Rogers ME. 2011. Serological markers of sand fly exposure to evaluate insecticidal nets against visceral leishmaniasis in India and Nepal: a cluster-randomized trial. PLoS Neglected Tropical Diseases, 5(9), e1296.
26. Gilbert MTP, Moore W, Melchior L, Worobey M. 2007. DNA extraction from dry museum beetles without conferring external morphological damage. PLoS One, 2(3), e272.
27. Giordani BF, Andrade AJ, Galati EAB, Gurgel-Goncalves R. 2017. The role of wing geometric morphometrics in the identification of sandflies within the subgenus *Lutzomyia.* Medical and Veterinary Entomology, 31(4), 373–380.
28. Guzmán-Larralde AJ, Suaste-Dzul AP, Gallou A, Peña-Carrillo KI. 2017. DNA recovery from microhymenoptera using six non-destructive methodologies with considerations for subsequent preparation of museum slides. Genome, 60(1), 85–91.
29. Hajibabaei M, DeWaard JR, Ivanova NV, Ratnasingham S, Dooh RT, Kirk SL, Mackie PM, Hebert PD. 2005. Critical factors for assembling a high volume of DNA barcodes. Philosophical Transactions of the Royal Society B: Biological Sciences, 360(1462), 1959–1967.
30. Haouas N, Pesson B, Boudabous R, Dedet JP, Babba H, Ravel C. 2007. Development of a molecular tool for the identification of *Leishmania* reservoir hosts by blood meal analysis in the insect vectors. American Journal of Tropical Medicine and Hygiene, 77(6), 1054–1059.
31. Hlavackova K, Dvorak V, Chaskopoulou A, Volf P, Halada P. 2019. A novel MALDI-TOF MS-based method for blood meal identification in insect vectors: A proof of concept study on phlebotomine sand flies. PLoS Neglected Tropical Diseases, 13 (9), e0007669.
32. Huemer H, Prudhomme J, Amaro F, Baklouti A, Walder G, Alten B, Moutailler S, Ergunay K, Charrel RN, Ayhan N. 2017. Practical guidelines for studies on sandfly-borne phleboviruses: Part II: Important points to consider for fieldwork and subsequent virological screening. Vector-Borne and Zoonotic Diseases, 17(1), 81–90.
33. Jancarova M, Polanska N, Thiesson A, Arnaud F, Stejskalova M, Rehbergerova M, Kohl A, Viginier B, Volf P, Ratinier M. 2025. Susceptibility of diverse sand fly species to Toscana virus. PLoS Neglected Tropical Diseases, 19(5), e0013031.
34. Kapp JD, Green RE, Shapiro B. 2021. A fast and efficient single-stranded genomic library preparation method optimized for ancient DNA. Journal of Heredity, 112(3), 241–249.
35. Killick-Kendrick R, Maroli M, Killick-Kendrick M. 1991. Bibliography of the colonization of phlebotomine sandflies. Parassitologia, 33(suppl.), 321–333.
36. Lawyer P, Killick-Kendrick M, Rowland T, Rowton E, Volf P. 2017. Laboratory colonization and mass rearing of phlebotomine sand flies (Diptera, Psychodidae). Parasite, 24, 42.
37. Léger N, Pesson B, Madulo-Leblond G. 1986. Les phlébotomes de Grèce : 1ère partie. Bulletin de la Société de Pathologie Exotique, 79, 386–397.
38. Léger N, Pesson B, Madulo-Leblond G. 1986. Les phlébotomes de Grèce : 2ème partie. Bulletin de la Société de Pathologie Exotique, 79, 514–524.

39. Leonel JAF, Vioti G, Alves ML, da Silva DT, Meneghesso PA, Benassi JC, Spada JCP, Galvis-Ovallos F, Soares RM, Oliveira T. 2020. DNA extraction from individual Phlebotomine sand flies (Diptera: Psychodidae: Phlebotominae) specimens: Which is the method with better results? Experimental Parasitology, 218, 107981.
40. Lestinova T, Rohousova I, Sima M, de Oliveira CI, Volf P. 2017. Insights into the sand fly saliva: Blood-feeding and immune interactions between sand flies, hosts, and *Leishmania.* PLoS Neglected Tropical Diseases, 11(7), e0005600.
41. Lienhard A, Schaffer S. 2019. Extracting the invisible: obtaining high quality DNA is a challenging task in small arthropods. PeerJ, 7, e6753.
42. Lozano-Sardaneta YN, Mikery-Pacheco OF, Huerta H, Rojas-Soriano JE, Contreras-Ramos A. 2025. Wing geometric morphometrics is effective to separate sand fly species (Diptera, Psychodidae, Phlebotominae) related with leishmaniasis transmission in Mexico. Acta Tropica, 262, 107523.
43. Mandrioli M. 2008. Insect collections and DNA analyses: how to manage collections? Museum Management and Curatorship, 23(2), 193–199.
44. Maroli M, Feliciangeli MD, Bichaud L, Charrel RN, Gradoni L. 2013. Phlebotomine sandflies and the spreading of leishmaniases and other diseases of public health concern. Medical and Veterinary Entomology, 27(2), 123–147.
45. Marquina D, Buczek M, Ronquist F, Lukasik P. 2021. The effect of ethanol concentration on the morphological and molecular preservation of insects for biodiversity studies. PeerJ, 9, e10799.
46. Mathis A, Depaquit J, Dvorak V, Tuten H, Banuls AL, Halada P, Zapata S, Lehrter V, Hlavackova K, Prudhomme J, Volf P, Sereno D, Kaufmann C, Pfluger V, Schaffner F. 2015. Identification of phlebotomine sand flies using one MALDI-TOF MS reference database and two mass pectrometer systems., Parasites & Vectors, 8, 266.
47. Mekarnia N, Benallal KE, Sadlova J, Vojtkova B, Mauras A, Imbert N, Longhitano M, Harrat Z, Volf P, Loiseau PM, Cojean S. 2024. Effect of *Phlebotomus papatasi* on the fitness, infectivity and antimony-resistance phenotype of antimony-resistant *Leishmania* major Mon-25. International Journal for Parasitology – Drugs and Drug Resistance, 25, 100554.
48. Milligan BG. 1998. Total DNA isolation, in Molecular Genetic Analysis of Population: A Practical Approach, Hoelzel AR, Editor. Oxford: Oxford University Press.
49. Molina R, Jiménez M, Alvar J, González E, Hernández-Taberna S, Ines MM. 2017. Methods in sand fly research. Madrid: Servicio de publicaciones Universidad de Alcalá de Henares, Madrid.
50. Murphy WJ, Eizirik E, O'Brien SJ, Madsen O, Scally M, Douady CJ, Teeling E, Ryder OA, Stanhope MJ, de Jong WW, Springer MS. 2001. Resolution of the early placental mammal radiation using Bayesian phylogenetics. Science, 294(5550), 2348–2351.
51. Nacif-Pimenta R, Pinto LC, Volfova V, Volf P, Pimenta PFP, Secundino NFC. 2020. Conserved and distinct morphological aspects of the salivary glands of sand fly vectors of leishma-niasis: an anatomical and ultrastructural study. Parasites & Vectors, 13(1), 441.
52. Neuhaus B, Schmid T, Riedel J. 2017. Collection management and study of microscope slides: Storage, profiling, deterioration, restoration procedures, and general recommendations. Zootaxa, 4322(1), 1–173.
53. New TR. 1974. Pscoptera. Handbooks for Identification of British Insects (Vol. I). London: Royal Entomological Society of London. 102 pp.
54. Perez-Ruiz M, Collao X, Navarro-Mari JM, Tenorio A. 2007. Reverse transcription, real-time PCR assay for detection of Toscana virus. Journal of Clinical Virology, 39(4), 276–281.
55. Porco D, Rougerie R, Deharveng L, Hebert P. 2010. Coupling non-destructive DNA extraction and voucher retrieval for small soft-bodied Arthropods in a high-throughput context: the example of Collembola. Molecular Ecology Resources, 10(6), 942–945.
56. Prudhomme J, Cassan C, Hide M, Toty C, Rahola N, Vergnes B, Dujardin JP, Alten B, Sereno D, Banuls AL. 2016. Ecology and morphological variations in wings of *Phlebotomus ariasi* (Diptera: Psychodidae) in the region of Roquedur (Gard, France): a geometric morphometrics approach. Parasites & Vectors, 9(1), 578.
57. Prudhomme J, Gunay F, Rahola N, Ouanaimi F, Guernaoui S, Boumezzough A, Banuls AL, Sereno D, Alten B. 2012. Wing size and shape variation of *Phlebotomus papatasi* (Diptera: Psychodidae) populations from the south and north slopes of the Atlas Mountains in Morocco. Journal of Vector Ecology, 37(1), 137–147.
58. Prudhomme J, Toty C, Kasap OE, Rahola N, Vergnes B, Maia C, Campino L, Antoniou M, Jimenez M, Molina R, Cannet A, Alten B, Sereno D, Banuls AL. 2015. New microsatellite markers for multi-scale genetic studies on *Phlebotomus ariasi* Tonnoir, vector of *Leishmania infantum* in the Mediterranean area. Acta Tropica, 142, 79–85.
59. Prudhomme J, Velo E, Bino S, Kadriaj P, Mersini K, Gunay F, Alten B. 2019. Altitudinal variations in wing morphology of *Aedes albopictus* (Diptera, Culicidae) in Albania, the region where it was first recorded in Europe. Parasite, 26, 55.
60. Rawlins DJ. 1992. Light Microscopy: An Introduction to Biotechniques. Oxford: Bios Scientific publishers. 143 pp.
61. Ready PD. 2013. Biology of phlebotomine sand flies as vectors of disease agents. Annual Review of Entomology, 58, 227–250.
62. Rohlf FJ, Slice D. 1990. Extensions of the Procrustes method for the optimal superimposition of landmarks. Systematic Zoology, 39(1), 40–59.
63. Rowley DL, Coddington JA, Gates MW, Norrbom AL, Ochoa RA, Vandenberg NJ, Greenstone MH. 2007. Vouchering DNA-barcoded specimens: Test of a nondestructive extraction proto-col for terrestrial arthropods. Molecular Ecology Notes, 7(6), 915–924.
64. Sábio PB, Andrade AJ, Galati EAB. 2014. Assessment of the taxonomic status of some species included in the *Shannoni* complex, with the description of a new species of *Psathyromyia* (Diptera: Psychodidae: Phlebotominae). Journal of Medical Entomology, 51(2), 331–341.
65. Sadlova J, Yeo M, Seblova V, Lewis MD, Mauricio I, Volf P, Miles MA. 2011. Visualisation of *Leishmania donovani* fluo-rescent hybrids during early stage development in the sand fly vector. PLoS One, 6(5), e19851.
66. Sales K, Miranda DEO, da Silva FJ, Otranto D, Figueredo LA, Dantas-Torres F. 2020. Evaluation of different storage times and preservation methods on phlebotomine sand fly DNA concen-tration and purity. Parasites & Vectors, 13(1), 399
67. Sales KG, Costa PL, de Morais RC, Otranto D, Brandao-Filho SP, Cavalcanti Mde P, Dantas-Torres F. 2015. Identification of phlebotomine sand fly blood meals by real-time PCR. Parasites& Vectors, 8, 230.

68. Sant'Anna MR, Jones NG, Hindley JA, Mendes-Sousa AF, Dillon RJ, Cavalcante RR, Alexander B, Bates PA. 2008. Blood meal identification and parasite detection in laboratory-fed and field-captured *Lutzomyia longipalpis* by PCR using FTA databasing paper. Acta Tropica, 107(3), 230–237.
69. Senne NA, Santos HA, Araujo TR, Paulino PG, Mendonca LP, Moreira HVS, Camilo TA, da Costa Angelo I. 2022. Robust comparative performance of genomic DNA extraction methods from non-engorged phlebotomine sandflies. Medical and Veterinary Entomology, 36(2), 203–211.
70. Shaw JJ. 2025. A review of Leishmania infections in American Phlebotomine sand flies – Are those that transmit leishmaniasis anthropophilic or anthropportunists? Parasite, 32, 57.
71. Tesh RB, Modi GB. 1983. Growth and transovarial transmission of Chandipura virus (Rhabdoviridae: Vesiculovirus) in *Phlebotomus papatasi*. American Journal of Tropical Medicine and Hygiene, 32(3), 621–623.
72. Thomsen PF, Elias S, Gilbert MTP, Haile J, Munch K, Kuzmina S, Froese DG, Sher A, Holdaway RN, Willerslev E. 2009. Non-destructive sampling of ancient insect DNA. PLoS One, 4(4),e5048
73. Truett GE, Heeger P, Mynatt RL, Truett AA, Walker JA, Warman ML. 2000. Preparation of PCR-quality mouse genomic DNA with hot sodium hydroxide and tris (HotSHOT). Biotechniques, 29(1), 52–54.
74. Upton MS. 1993. Aqueous gum-chloral slide mounting media: an historical review. Bulletin of Entomological Research, 83(2), 267–274.
75. Volf P, Myskova J. 2007. Sand flies and Leishmania: specific versus permissive vectors. Trends in Parasitology, 23(3), 91–92.
76. Wang Q, Wang X. 2012. Comparison of methods for DNA extraction from a single chironomid for PCR analysis. Pakistan Journal of Zoology, 44(2), 421–426.
77. Wang Y, Zhao Y, Bollas A, Wang Y, Au KF. 2021. Nanopore sequencing technology, bioinformatics and applications. Nature Biotechnology, 39(11), 1348–1365.

Cite this article as: Randrianambinintsoa FJ, Augendre L, Prudhomme J, Martinet J-P, Loyer M, Mekarnia N, Kerkoub H, Perveen FK, Huguenin A, Kariya E, Akhoundi M, De Andrade AJ, Berriatua E, Bongiorno G, Boyer S, Christodoulou V, Da Costa-Ribeiro MCV, De Souza LAF, Ding H, Dondji B, Dvořák V, Erisoz Kasap O, Galati EAB, Gállego M, Ballart C, Gouzelou S, Haddad N, Masse RS, Mekuria AH, Ivovic V, Kaczmarek S, Shahar MK, Kirstein OD, Kniha E, Kolářová I, Lincoln T, Lucanas C, Mikov O, Nov K, Özbel Y, Pesson B, Posada Lopez LC, Prasetyo DB, Rahola N, Rebollar-Tellez EA, Rodrigues BL, Roy L, Saini P, Sanjoba C, Shimabukuro PH, Siriyasatien P, Soszynska A, Suleşco T, Sylla M, Torno M, Volf P, Vongphayloth K, Sinh Nam V, Wardhana A, Yessinou E, Zapata S, Gantier J-C & Depaquit J. 2026. Processing and mounting phlebotomine sand flies: a consensus guideline. Parasite xx, xx. https://doi.org/10.1051/parasite/2026009.

PARASITE

An international open-access, peer-reviewed, online journal publishing high quality papers on all aspects of human and animal parasitology

Reviews, articles and short notes may be submitted. Fields include, but are not limited to: general, medical and veterinary parasitology; morphology, including ultrastructure; parasite systematics, including entomology, acarology, helminthology and protistology, and molecular analyses; molecular biology and biochemistry; immunology of parasitic diseases; host-parasite relationships; ecology and life history of parasites; epidemiology; therapeutics; new diagnostic tools.
All papers in Parasite are published in English. Manuscripts should have a broad interest and must not have been published or submitted elsewhere. No limit is imposed on the length of manuscripts.

Parasite (open-access) continues **Parasite** (print and online editions, 1994-2012) and **Annales de Parasitologie Humaine et Comparée** (1923-1993) and is the official journal of the Société Française de Parasitologie.

Editor-in-Chief:
Jean-Lou Justine, Paris

Submit your manuscript at:
https://www.editorialmanager.com/parasite

Dodatak 1: Biohemijske teorijske osnove.

Metode pripreme i prepariranja u ovoj studiji se odnose flebotomine. Međutim, mogu se proširiti i na druge vrlo česte insekte kod kojih se identifikacija može obaviti isključivo na osnovu unutrašnjih morfoloških struktura. Na primer, neki unutrašnji organi su delimično hitinizovani i njihova morfologija nam pruža dragocene informacije. Zbog toga je od posebnog značaja posmatranje digestivnog trakta, spermateka i njihovih izvodnih kanala. Sa svim reagensima koje ćemo razmotriti, nikada ne treba zaboraviti da od faze fiksacije insekta do prepariranja preparata u suštini primenjujemo redoks reakcije. Glavna mera opreza je izbegavanje mešanja redukujućih reagenasa sa oksidujućim reagensima.

Etil alkohol; etanol:

Ova supstanca će se koristiti na različite načine. Molekuli alkohola imaju snažan afinitet prema vodi i zbog toga imaju dehidrataciono dejstvo. Međutim, alkohol niske koncentracije (tj. previše bogat vodom) ima ulogu u degradaciji nukleinskih kiselina (voda je neprijatelj nukleinskih kiselina).

Kada se insekti stavljaju u etanol, to nije samo radi njihovog očuvanja, već i radi fiksacije tkiva. U histologiji se obično razlikuju dva važna pojma: brzina prodiranja i brzina fiksacije. Dobro je poznato da dobar fiksativ mora najpre brzo prodreti duboko u tkiva pre nego što dođe do njihove fiksacije. Za alkohol od 96%, koeficijent prodiranja iznosi približno 1,05 (poređenja radi, za 0,75% vodeni rastvor pikrinske kiseline koeficijent prodiranja je 0,45, dok je za 3% rastvor kalijum-dihromata 1,45).

Želja da se insekti i drugi zglavkari neograničeno dugo čuvaju u etanolu realnost je u entomologiji. Ideja da se primerci ulovljeni u prirodi sačuvaju za naknadna istraživanja u osnovi je vrlo časna. Ipak, za citologa ili histologa to nije prihvatljivo. Ukoliko se uzorci predugo drže u fiksativu, oni mogu postati praktično neupotrebljivi za dalju obradu. Zbog toga su uzorci stariji od 10 godina često teško upotrebljivi ili čak potpuno neupotrebljivi.

Problem takođe može biti odnos između mase insekata koji se fiksira i zapremine fiksativa. U zoološkoj ili medicinskoj praksi preporučuje se da zapremina fiksativa bude oko 60 puta veća od zapremine uzoraka koji se fiksiraju. U praksi, kod malih vrsta insekata, za datu zapreminu primeraka koje treba fiksirati, treba dodati najmanje 4–5 zapremina alkohola. Treba imati u vidu da će alkohol gubiti svoju jačinu jer iz tkiva insekata izvlači svu prisutnu vodu.

Zaključak:
- Etil-alkohol je redukujući hemijski agens (samim tim nije kompatibilan sa oksidujućim fiksativima);
- Snažno taloži proteine i dovodi do njihove denaturacije;
- Rastvara određene složene lipide i taloži glikogen;
- Izaziva snažnu kontrakciju tkiva i dovodi do njihovog očvršćavanja.

Bazni rastvori kalijum- ili natrijum-hidroksida:

Upotreba ovih rastvora u entomologiji uglavnom je bila usmerena na kalijum-hidroksid, bez jasnog obrazloženja.

Natrijum-hidroksid [E524] se javlja u obliku rastvora, bilo u različitim koncentracijama ili sa različitom normalnošću. Dostupan je u obliku peleta ili pahuljica. Njegov glavni nedostatak je izrazita higroskopnost (veća nego kod KOH). Kada reaguje sa proteinima, rastvara ih, dok sa lipidima tokom procesa saponifikacije stvara čvrste sapune (što predstavlja značajnu razliku u odnosu na KOH, koji tokom saponifikacije daje tečne sapune).

Kalijum-hidroksid [E525] dostupan je kao koncentrovani rastvor, ali pre svega ima prednost što se proizvodi u obliku peleta mase oko 0,1 g, što znatno olakšava pripremu razblaženih rastvora kada nije dostupna precizna vaga. Na primer, jedan pelet od 0,1 g rastvoren u 1 mL destilovane vode daje 10% rastvor. Druga prednost kalijum-hidroksida u obliku peleta jeste njegova manja osetljivost na karbonatizaciju (rastvor KOH ima visok afinitet za vezivanje CO_2, pri čemu nastaju karbonatne soli).

Ove jake baze koriste se za rastvaranje masnih kiselina njihovim pretvaranjem u vodorastvorljive sapune. Treba imati u vidu da je fiksativ, kao što je etanol, već rastvorio deo masti prisutnih u uzorku. Međutim, kada se uzorak prebaci u vodeni medijum sa jakom bazom, masne kiseline (više ili manje složene) dolazi do taloženja. Jaka baza će, dakle, dovesti do hladne saponifikacije. U nekim slučajevima, kada su masna tkiva prisutna u višku, na primer kod ženki, može biti korisno povisiti temperaturu na 35–40 °C kako bi se olakšala reakcija, ili produžiti vreme kontakta na sobnoj temperaturi.

Obojeni kiseli rastvor / bezbojni Marc-André rastvor:

Ovde ćemo razmotriti prednosti i nedostatke upotrebe Marc-André rastvora. Ovaj rastvor se sastoji od hloral-hidrata (trihloroacetaldehid monohidrat), sirćetne kiseline i vode. Reč je o veoma oksidujućem rastvoru (mešavina kiseline i aldehida). On će neutralisati višak kalijum-hidroksida koji može zaostati u uzorcima, a da pritom ne taloži alkalne sapune nastale tokom upotrebe kalijum-hidroksida. Ovaj oksidujući rastvor takođe deluje na sekundarne alkoholne funkcije glukozamina koji ulaze u sastav hitina, oksidujući ih i na taj način omekšavajući hitin. Dodatno dejstvo ogleda se u rastvaranju određenih mineralnih soli prisutnih u uzorku.

Kada je Marc-André rastvor prethodno obojen kiselim fuksinom (dakle u oksidovanom stanju), on je u stanju da se veže za sekundarne alkoholne funkcije strukture. Nakon vremena kontakta Marc-André rastvora i stepena obojenosti uzoraka, ispiranje se vrši isključivo etanolom. Time započinje faza dehidratacije uzoraka.

Prednosti:
- neutralizacija viška baznih rastvora
- relaksacija hitina

- bojenje hitina radi boljeg uočavanja hitinizovanih unutrašnjih struktura

Nedostaci:

Hloral-hidrat je hipnotik i korišćen je u humanoj medicini. Mora se koristiti pod hemijskim digestorom i uz poštovanje važeće zakonske regulative u vezi sa hemijskim rizicima.

Rastvori za dehidrataciju:

Iskustvo pokazuje da kod veoma malih uzoraka nije korisno slediti niz alkoholnih kupki sa postepeno rastućim koncentracijama. Ukoliko je uzorak veći, započinje se sa 80% etanolom, zatim 90%, 95% i na kraju apsolutnim etanolom. Kod veoma malih uzoraka koristi se kupka sa 90% etanolom, nakon čega sledi potapanje u apsolutni etanol. U ovoj fazi uvek treba imati na umu da apsolutni etanol nastoji da veže vodu iz atmosfere.

Tradicionalno, u entomološkim laboratorijama dehidratacija uzoraka završavala se kupkom u bukovom kreozotu. Danas se ova esencija, koja je bila široko korišćena kao pesticid, antifungalno sredstvo i konzervans za drvo, snažno ne preporučuje zbog svog mirisa (policiklični aromatični ugljovodonici), kao i zbog pretpostavke da je reprotoksična, kancerogena, postojani organski zagađivač i ekotoksična za vodene organizme.

Rešenje koje predlažemo za pripremu uzoraka za prepariranje jeste upotreba Euparal®-a i Euparal esencije (opisane u narednom pasusu). Mešavina Euparal®-a i Euparal esencije veoma je pogodna; uzorci dobijeni nakon kupke u 90% etanolu dobro se prihvataju u ovom medijumu

Dodatak 2: Sastav korišćenih reagenasa.

Kalijum-hidroksid 10%
Kalijum-hidroksid 10 g
Destilovana voda q.s. do 100 mL

Hloralna guma – medijum za prepariranje (Hoyerov medijum)
Destilovana voda 50 mL
Hloral-hidrat 200 g
Gumiarabika 50 g
Glicerol 20 mL

Marc-André rastvor
Hloral-hidrat 40 g
Ledena sirćetna kiselina 30 mL
Destilovana voda 30 mL

Kiseli fuksin 1% u destilovanoj vodi
Kiseli fuksin (prah) 1 g
Destilovana voda 99 mL

Marc-André rastvor obojen fuksinom
Marc-André rastvor 10 mL
Fuksin 1% 50 µL

Dodatak 3: Euparal®, kanada balzam, polivinil-alkohol i drugi rastvori za prepariranje.

Polivinil-alkohol: Ovo je idealan medijum za prepariranje kada ne postoje dostupni neophodni proizvodi za pravilnu dehidrataciju. Polivinil-alkohol se tada meša sa Ammanovim laktfenolom. Međutim, ovakvi preparati imaju velike nedostatke: ili dolazi do njihovog isušivanja, ili polivinil-alkohol kristališe usled isparavanja vode, ili dolazi do potamnjivanja kada fenol oksiduje. Ipak, ovo ostaje dobra tehnika za kratkotrajno prepariranje.

Kanada balzam: Njegova upotreba za prepariranje između predmetnog i pokrovnog stakalca zahteva dehidrataciju uzoraka koji se prepariraju. Upotreba ksilena ili toluena nije bez određenih nedostataka.

Medijum Enecê: Za prepariranje između predmetnog i pokrovnog stakalca, kao i kod kanadskog balzama, zahteva dehidrataciju primerka. Formulacija Enecê medijuma: čista bela kolofonija (22 g); alkoholno rastvorljiva kopalna smola (12 g); apsolutni alkohol (20 mL); kamfor (10 g); terpentinova esencija (10 mL) i eukaliptol (26 mL). Za pripremu, u posudu, kao što je Erlenmajer, dodaju se apsolutni alkohol i kamfor. Zatim se dodaju kolofonija i kopalna smola. Tikvica se zatvara čepom i mućka, a potom se zagreva u vodenom kupatilu na blagoj temperaturi, tako da smeša ne proključa. Kada se sadržaj potpuno rastvori, dodaje se terpentinova esencija, zatim se smeša filtrira dok je još topla, i na kraju se filtratu dodaje eukaliptol. Kada medijum postane manje tečan, razblažuje se Enecê-om, sledećeg sastava: apsolutni alkohol (30 mL), kamfor (17 g), terpentinova esencija (15 mL), eukaliptol (38 mL) (Cerqueira, 1943).

Euparal®: Ovo je smola poreklom od atlaskog čempresa *Tetraclinis articulata* (Vahl, 1791), koju je proučavao i razvio Gilson 1906. godine. Njena glavna prednost je što ne polimerizuje. Uzorci preparirani između predmetnog i pokrovnog stakalca mogu se lako ponovo izdvojiti dejstvom alkohola ili, još bolje, Euparal® esencije. Ova smola, poznata i kao sandarak, prihvata etanol od 80%.

Upotreba Triton X100: nejonizujući vodeni rastvor: Triton X100 je u obliku nejonizujućeg vodenog rastvora (4-(1,1,3,3-tetrametilbutil) fenil-polietilen-glikol rastvor, odnosno t-oktilfenoksipolietoksietanol, polietilen-glikol tert-oktilfenil etar) i široko se koristi kao deterdžent u ćelijskoj i molekularnoj biologiji. Omogućava permeabilizaciju ćelijskih i jedarnih membrana.

Uzorci insekata čuvani u alkoholu tokom više godina česta su pojava. Nažalost, čuvanje u alkoholu nije optimalno i tako očuvani insekti postaju veoma teški za pripremu za mikroskopsko ispitivanje. Plastične posude koje sadrže uzorke često degradiraju, nakon čega dolazi do isparavanja alkohola. U oba slučaja, dugotrajan kontakt sa alkoholom ili isušivanje uzoraka predstavlja ozbiljan problem. Jonque je

2008. godine objavio belešku o rehidrataciji paukova pomoću sredstva za vlaženje kao što je Agepon, koji se koristi za fotografske filmove [26]. To je dovelo do ideje o upotrebi sredstava za vlaženje koja nisu snažni deterdženti.

U nastavku je opisan postupak korišćenjem Triton X100 u 0,5% vodenom rastvoru:
- suvi uzorak se impregnira apsolutnim alkoholom;
- dodaje se potrebna zapremina 0,5% rastvora Triton X100 tako da ceo uzorak bude potopljen;
- ostavi se da stoji oko 5 minuta ili duže; svi insekti moraju biti odvojeni u rastvoru;
- rastvor Triton X100 se uklanja i zamenjuje rastvorom kalijum-hidroksida.

Dalji postupak se zatim nastavlja kako je gore opisano.

Dodatak 4: Prepariranje u Euparal® ili kanada balzamu, korak po korak.

1. Primerci moraju biti dehidratovani (zamućen ili mlečan izgled ukazuje na neadekvatnu dehidrataciju).
2. Dehidratacija se može postići primenom etil-alkohola u rastućim koncentracijama.
3. Primerci se mogu preneti iz 99% alkohola ili apsolutnog alkohola u sredstvo za prosvetljavanje.

Postupak:

1. Stavite adultne flebotomine u 70% etanolu.
2. Ukloniti etanol i zameniti ga 10% KOH. Prekriti flebotomine predmetnim stakalcem.
3. Macerirati dok insekti ne postanu providni.
4. Ukloniti KOH.
5. Prekriti primerak destilovanom vodom i sačekati 30–45 min.
6. Ukloniti vodu i ponoviti ispiranje destilovanom vodom u trajanju od 30 min (vreme zavisi od broja uzoraka: što je više uzoraka koji se obrađuju zajedno, to se ovo vreme mora duže poštovati; što ih je manje, naročito kod pojedinačne obrade, to vreme može biti kraće).
7. Ukloniti vodu.
8. Dodati Marc-André rastvor (po potrebi obojen kiselim fuksinom) i sačekati 24 sata (jedan dan).
9. Ukloniti Marc-André rastvor.
10. Prekriti primerak destilovanom vodom i sačekati 30–45 min.
11. Ukloniti vodu i ponoviti ispiranje destilovanom vodom u trajanju od 30 min.
12. Ukloniti vodu.
13. Dodati 70% etanol i disekovati primerak.
    a. Za glavu i abdomen: nežno povući glavu ili abdomen sa toraksa.
    b. Za toraks: ukloniti krila tako što se toraks drži jednim parom pinceta, a u osnovi nastavaka povlači drugim parom. Moguća je i sagitalna disekcija, pri čemu se toraks deli na levu i desnu stranu, u zavisnosti od regiona od najvećeg interesa.
14. Postepeno dehidratovati kroz niz vodenih rastvora etil-alkohola: 50% – 80% – 95%, sve do apsolutnog etanola.
15. Dehidratovati primerke ispiranjem dva puta, po 10 min svaki put, u 100% etanolu.
16. Ukloniti etanol i prekriti primerke karanfilićevim uljem u trajanju od 15 min na sobnoj temperaturi.
17. Preneti primerke iz karanfilićevog ulja u kap Euparal®-a ili kanada balzama na čisto predmetno stakalce.
18. Rasporediti po želji: glava, toraks i abdomen flebotomine mogu se disek ovati finim iglama ili pincetama pod disekcionim mikroskopom. Glava se mora odvojiti od tela kako bi se pozicionirala I preparirala u ventro-dorzalnom položaju, tj. okcipitalni foramen mora biti orijentisan nagore, kako bi se cibarium mogao direktno posmatrati kroz njega.
    Disekcija se izvodi u medijumu za prepariranje flebotomina.
19. Ostaviti primerak dok površina ne postane lepljiva.
20. Navlažiti čisto pokrovno stakalce apsolutnim alkoholom. Spustiti pokrovno stakalce na kanada balzam pod uglom.
21. Čuvati preparate u suvoj kutiji namenjenoj za tu svrhu.
