# Supplementary material for: Processing and mounting phlebotomine sand flies: a consensus guideline
Source: Parasite. 2026 Apr 3;33:18. doi: 10.1051/parasite/2026009 (PMC13047900; doi:10.1051/parasite/2026009)
Supplement: Supplementary file 27 — Slovenian translation / Prevod v slovenščino [file parasite-33-18-s27.pdf]

Parasite **33**, 18 (2026)
© F.J. Randrianambinintsoa et al., published by EDP Sciences, 2026
https://doi.org/10.1051/parasite/2026009

PARASITE
Available online at:
www.parasite-journal.org

REVIEW ARTICLE OPEN ACCESS

# Smernice za obdelavo in pripravo mikroskopskih preparatov peščenih muh

Fano José Randrianambinintsoa[1], Laure Augendre[1], Jorian Prudhomme[1], Jean-Philippe Martinet[1], Mathieu Loyer[1], Nalia Mekarnia[1], Hocine Kerkoub[1], Farzana Khan Perveen[1], Antoine Huguenin[1,2], Emilie Kariya[1,2], Mohammad Akhoundi[3], Andrey José de Andrade[4], Eduardo Berriatua[5], Gioia Bongiorno[6], Sébastien Boyer[7,8], Vasiliki Christodoulou[9], Magda Clara Vieira Da Costa-Ribeiro[10], Lucas Alexandre Farias de Souza[10], Huicong Ding[11], Blaise Dondji[12], Vít Dvořák[13], Ozge Erisoz Kasap[14], Eunice Aparecida Bianchi Galati[15], Montserrat Gállego[16], Cristina Ballart[16], Stavroula Gouzelou[17], Nabil Haddad[18], Rezki Sabrina Masse[19], Asrat Hailu Mekuria[20], Vladimir Ivovic[21], Szymon Kaczmarek[22], Mohd Khadri Shahar[19], Oscar D. Kirstein[23], Edwin Kniha[24], Iva Kolářová[13], Lincoln Timinao[25], Cristian Lucanas[26], Ognyan Mikov[27], Kimsear Nov[7], Yusuf Özbel[28], Bernard Pesson[29], Laura Cristina Posada Lopez[30], Didot Budi Prasetyo[1,7], Nil Rahola[31], Eduardo A. Rebollar-Tellez[32], Bruno Leite Rodrigues[15], Lalita Roy[33], Prasanta Saini[34], Chizu Sanjoba[35], Paloma Helena Fernandes Shimabukuro[36], Padet Siriyasatien[37], Agnieszka Soszyńska[22], Tatiana Suleşco[38], Massamba Sylla[39], Majhalia Torno[40], Petr Volf[13], Khamsing Vongphayloth[41], Vu Sinh Nam[42], April Wardhana[43], Eric Yessinou[44], Sonia Zapata[45], Jean-Charles Gantier[1], and Jérôme Depaquit[1,2,*]

[1] Faculté de Pharmacie, Université de Reims Champagne Ardenne, UR ESCAPE-USC ANSES PETARD, 51 rue Cognacq-Jay, 51096 Reims Cedex, France
[2] Pôle de Biologie territoriale, Laboratoire de Parasitologie-Mycologie, Centre Hospitalo-Universitaire, 51092 Reims, France
[3] Parasitology-Mycology Department, Avicenne Hospital, AP-HP, Bobigny, Sorbonne Paris Nord University, France; Unité des Virus Émergents (UVE: Aix-Marseille Univ, Università di Corsica, IRD 190, Inserm 1207, IRBA), 13005 Marseille, France
[4] Parasitology Collection of Basic Pathology, Department of Basic Pathology, Federal University of Paraná, Curitiba 19031, Brazil
[5] Department of Animal Health, University of Murcia, Campus de Espinardo, 30100 Espinardo, Murcia, Spain
[6] Department of Infectious Diseases, Vector-borne Diseases Unit, Istituto Superiore di Sanità, 00166 Rome, Italy
[7] Medical and Veterinary Entomology Unit, Institut Pasteur du Cambodge, Phnom Penh 12201, Cambodia
[8] Ecology & Emergence of Arthropod-borne Pathogens Unit, Department of Global Health, Institut Pasteur, CNRS UMR2000, 75015 Paris, France
[9] Section Veterinary Services (1417), Laboratory for Animal Health Virology, Aglantzia, Nicosia 2109, Cyprus
[10] Insects Vectors and Parasites Laboratory, Department of Basic Pathology and Postgraduate program in Microbiology, Parasitology and Pathology, Federal University of Paraná, 81530-900 Curitiba, Brazil
[11] Department of Biological Sciences, National University of Singapore, 117558, Singapore
[12] Laboratory of the Leishmaniasis Research Project, Mokolo District Hospital, Mokolo, Cameroon; Laboratory of Cellular Immunology and Parasitology, Department of Biological Sciences, Central Washington University, 98926 Ellensburg, WA, USA
[13] Department of Parasitology, Faculty of Science, Charles University, 12800 Prague, Czechia
[14] VERG Laboratories, Department of Biology, Faculty of Science, Hacettepe University, Beytepe, Ankara 06800, Türkiye
[15] Faculdade de Saúde Pública da Universidade de São Paulo (FSP/USP), Pós-graduação em Saúde Pública, 01246-904 São Paulo, Brazil
[16] Secció de Parasitologia, Departament de Biologia, Sanitat i Medi Ambient, Facultat de Farmàcia i Ciències de l'Alimentació, Universitat de Barcelona, & Institut de Salut Global de Barcelona (ISGlobal), Centro de Investigación Biomédica en Red, Enfermedades Infecciosas (CIBERINFEC), 08028 Barcelona, Spain
[17] Laboratory of Infectious Diseases and Public Health, School of Medicine, University of Cyprus, Nicosia, Cyprus & Department of Pediatrics, Archbishop Makarios III Hospital, Nicosia 2115, Cyprus
[18] Faculty of Health Sciences, American University of Beirut, 1107 2020 Beirut, Lebanon
[19] Medical Entomology Unit, Infectious Disease Research Centre, Institute for Medical Research (IMR), National Institutes of Health (NIH), Ministry of Health Malaysia, 40170 Shah Alam, Selangor, Malaysia
[20] School of Medicine, Addis Ababa University, 28017 - 1000 Addis Ababa, Ethiopia
[21] Faculty of Mathematics, Natural Sciences and Information Technologies, University of Primorska, 6000 Koper, Slovenia

Edited by Jean-Lou Justine
* odgovorni avtor: jerome.depaquit@univ-reims.fr

This is an Open Access article distributed under the terms of the Creative Commons Attribution License (https://creativecommons.org/licenses/by/4.0), which permits unrestricted use, distribution, and reproduction in any medium, provided the original work is properly cited.

[22] University of Lodz, Faculty of Biology and Environmental Protection, Department of Invertebrate Zoology and Hydrobiology, Banacha 12/16, 90-237 Łódź, Poland
[23] Laboratory of Entomology, Ministry of Health, 9134302 Jerusalem, Israel
[24] Center for Pathophysiology, Infectiology and Immunology, Institute of Specific Prophylaxis and Tropical Medicine, Medical University Vienna, Kinderspitalgasse 15, 1090 Vienna, Austria
[25] Papua New Guinea Institute of Medical Research (PNGIMR) Institute, PO Box 60, Headquarter, Homate Street, 441 Goroka, Eastern Highlands Province, Papua New Guinea
[26] Museum of Natural History, University of the Philippines Los Baños, 4031 Laguna, Philippines
[27] National Centre of Infectious and Parasitic Diseases, 1504 Sofia, Bulgaria
[28] Ege University, Faculty of Medicine, Department of Parasitology, 35040 Bornova/Izmir, Türkiye
[29] Retired, Faculté de Pharmacie, Université de Strasbourg, Strasbourg, 67400 Illkirch-Graffenstaden, France
[30] Program for the Study and Control of Tropical Diseases (PECET), Faculty of Medicine, University of Antioquia, 050010 Medellin, Colombia
[31] MIVEGEC, Univ. Montpellier, CNRS, IRD, 34394 Montpellier, France & Medical Entomology Unit, Institut Pasteur de Madagascar, 101 Antananarivo, Madagascar
[32] Laboratorio de Entomología Médica, Departamento de Zoología de Invertebrados, Facultad de Ciencias Biológicas, Universidad Autónoma de Nuevo León, San Nicolás de los Garza, 66455, NL, México
[33] Tropical and Infectious Disease Centre, BP Koirala Institute of Health Sciences, Dharan 56700, Nepal
[34] ICMR-Vector Control Research Centre, Puducherry 605006, India
[35] Graduate School of Agricultural and Life Sciences, The University of Tokyo, Tokyo 113-8657, Japan
[36] Grupo de estudos em Leishmanioses/Coleção de Flebotomíneos (COLFLEB/Fiocruz-MG), Instituto René Rachou, Fundação Oswaldo Cruz, Belo Horizonte, Minas Gerais, 30190009, Brazil
[37] Center of Excellence in Vector Biology and Vector-Borne Disease, Department of Parasitology, Faculty of Medicine, Chulalongkorn University, Bangkok 10330, Thailand
[38] Department of Arbovirology, Bernhard Nocht Institute for Tropical Medicine, Bernhard Nocht Str. 74, 20359 Hamburg, Germany [39] Laboratory Vectors & Parasites, Department of Livestock Sciences and Techniques, Sine Saloum University El Hadji Ibrahima Niasse (SSUEIN) Kaffrine Campus, C.P. 24600, Senegal.
[40] Environmental Health Institute, National Environment Agency, Singapore 138667, Singapore & Department of Biological Sciences, National University of Singapore, 117558 Singapore
[41] Institut Pasteur du Laos, Laboratory of Vector-Borne Diseases, Samsenhai Road, Ban Kao-Gnot, Sisattanak District, 3560 Vientiane, Lao PDR
[42] National Institute of Hygiene and Epidemiology, 1 Yec-Xanh Street, Hai Ba Trung District, 100000 Hanoi, Vietnam
[43] Indonesian Research Center for Veterinary Science, Indonesian Agency for Agricultural Research and Development, Ministry of Agriculture Republic Indonesia, Bogor 16114, Indonesia & Department of Parasitology, Faculty of Veterinary Medicine, Airlangga University, Surabaya 60115, Indonesia
[44] Laboratory of Research in Applied Biology, Polytechnic School of Abomey-Calavi, University of Abomey-Calavi, 01 P.O. Box 2009, 00000 Cotonou, Benin
[45] Instituto de Microbiología, Colegio de Ciencias Biológicas y Ambientales (COCIBA), Universidad San Francisco de Quito (USFQ), 170901 Quito, Ecuador

Received 1 December 2025, Accepted 29 January 2026, Published online 3 April 2026

**Povzetek** – Ta članek predstavlja celovit vodnik za obdelavo in prepariranje peščenih muh, kar je ključno za identifikacijo vrst ter odkrivanje in izolacijo patogenov. Obravnava vrsto tehnik, primernih za terenske in laboratorijske razmere. Predstavljena so podrobna navodila za zbiranje, rokovanje, prepariranje ter usmrtitev peščenih muh (s priporočilom suhega zamrzovanja ali $CO_2$ namesto kemikalij) ter strategiji hladnega shranjevanja in konzerviranja v etanolu. Kakovost priprave določenih anatomskih struktur (spolni organi, glava in krila) je bistvena za ustrezno mikroskopsko opazovanje peščenih muh, ki je tudi podrobno predstavljeno v tem prispevku. Članek predstavlja tudi podrobno obdelavo vzorcev, vključno z uporabo reagentov, kot sta kalijev hidroksid in Marc-Andréjeva raztopina za doseganje prosojnosti preparatov. V prispevku smo primerjali različna preparirna sredstva, njihove optične lastnosti in potencial za ohranjanje preparatov. Hoyerjevo raztopino (znana tudi kot kloralna smola) zaradi učinka prosojnosti priporočamo za hitro opazovanje, zlasti spermatek, vendar ni primerna za dolgoročno shranjevanje. Med drugimi obravnavanimi sredstvi so polivinilni alkohol, Euparal® (z omejeno toleranco do vode) in kanadski balzam (v ogljikovodikih topno sredstvo), pri čemer zadnja dva sredstva omogočata dolgoročno ohranjanje. Obravnavani so tudi sodobni pristopi molekularne biologije, kot sta sekveniranje DNA in MALDI-ToF, ki zahtevajo posebno pozornost pri obdelavi vzorcev. Poleg tega so na voljo kratki videoposnetki, ki prikazujejo različne tehnike prepariranja, ter prevodi v 33 različnih jezikov, ki omogočajo, da smernice dosežejo raznolike potrebe in pričakovanja globalne znanstvene skupnosti.

**Ključne besede:** Prepariranje, peščene muhe, Hoyerjeva raztopina, Marc-Andréjeva raztopina, kloralna smola, polivinilni alkohol, Euparal®, kanadski balzam, izolacija Leishmania, terenske razmere, gojenje, disekcija, molekularna biologija, MALDI-ToF, tipski primerki

**Abstract – Processing and mounting phlebotomine sand flies: a consensus guideline.** This article provides a comprehensive guide for the processing and mounting of phlebotomine sand fly specimens, which is crucial for species identification and pathogen detection and isolation. It discusses a range of techniques suitable for both field and laboratory settings. The guide includes detailed instructions on sand fly collection, handling, covering, and euthanasia (recommending dry freezing or $CO_2$ over chemicals) as well as conservation strategies, such as cold storage and preservation in ethanol. The quality of preparation of certain anatomical structures (genital organs, head and wings) is essential for their proper microscopic observation and is described in this work. The article also presents detailed sample processing, including the clearing process with agents such as potassium hydroxide then Marc-André solution. The mounting process compares different media, emphasizing their optical properties and preservation potential. Hoyer fluid (also known as chloral gum) is recommended for quick observation, particularly for spermathecae, due to its clarity, although it is not suitable for long-term storage. Other media discussed include polyvinyl alcohol, Euparal® (for limited water tolerance), and Canada balsam (a hydrocarbon-soluble medium), with the latter two offering long-term preservation capabilities. Innovative molecular biology approaches such as DNA sequencing and MALDI-ToF, which require particular attention to sample processing, are also addressed. Furthermore, short video clips illustrating various mounting techniques as well as translations in many different languages are provided, allowing the guideline to reach the diverse needs and expectations of the global scientific community.

**Key words:** Mounting, Phlebotomine sand fly, Hoyer fluid, Marc-André solution, Chloral gum, Polyvinyl alcohol, Euparal®, Canada balsam, *Leishmania* isolation, Field conditions, Culture, Dissection, Molecular biology, MALDI-ToF, Type-specimens.

## Uvod

Peščene muhe so majhni dvokrilci (Diptera), ki pripadajo družini Psychodidae, poddružini Phlebotominae, z najmanj 1063 znanimi vrstami [21]. So pomembni prenašalci patogenov (Leishmania, arbovirusi in Bartonella), ki povzročajo bolezni, imenovane leishmanioza, arbovirusne okužbe in bartoneloza. Identifikacija peščenih muh temelji predvsem na podrobnem mikroskopskem pregledu, ki ga omogočajo skrbno zbiranje, ustrezno shranjevanje in skrbno prepariranje na predmetnem stekelcu, kar zahteva več specifičnih tehnik, od katerih ima vsaka svoje prednosti in omejitve.

Določanje odraslih peščenih muh temelji na opazovanju tako zunanjih (npr. tipalnice, palpi, moški spolni organi) kot notranjih struktur (npr. žrelo, cibarium in spermateke). Sekcija in izolacija teh struktur olajšata njihovo opazovanje in posledično natančno določanje. Zato peščene muhe, za razliko od komarjev ali stenic, pred določanjem zahtevajo namestitev med predmetno in krovno stekelce. Do osemdesetih let prejšnjega stoletja je bilo mikroskopsko opazovanje edina razpoložljiva metoda za določanje peščenih muh in ostaja še danes najpogosteje uporabljen pristop. Izbira postopka in priprave je bila zato razmeroma preprosta in je temeljila predvsem na dihotomiji: na eni strani trajna preparacija, ki omogoča dolgoročno ohranjanje osebka, na drugi strani hitro prepariranje za določanje v mediju, ki ne zagotavlja dolgoročnega shranjevanja. Trajno prepariranje, na primer v smoli, kot je kanadski balzam, je časovno zahtevno, saj zahteva popolno dehidracijo vzorcev. Poleg tega lomni količnik tega medija ni vedno optimalen za enostavno opazovanje spermatek. Prepariranje v vodnem mediju (npr. Hoyerjeva raztopina) je hitrejše in omogoča lažje opazovanje spermatek, vendar ne omogoča dolgoročnega ohranjanja preparatov, ker ta medij absorbira vlago iz zraka. Ena od možnosti je zatesnitev stekelca z lakom za nohte, ko je preparat popolnoma suh. Ta kompromisna izbira še danes vpliva na izbiro metode prepariranja glede na predvideni namen priprave. Od osemdesetih let dalje so študije določanja peščenih muh združevale morfološke in biokemične pristope. Ti pristopi so vključevali analize kutikularnih ogljikovodikov, ki so jih hitro nadomestile tehnike molekularne biologije (npr. naključno pomnožena polimorfna DNA (RAPD), polimorfizem dolžin restrikcijskih fragmentov (RFLP), pomnoževanje DNA in sekveniranje po Sangerjevi metodi ter sekveniranje naslednje generacije (NGS)). Danes molekularne pristope dopolnjujejo proteomske metode, kot je MALDI-ToF. Poleg tega je molekularno določanje vrst

mogoče združiti z odkrivanjem patogenov s PCR (*Leishmania*, *Trypanosoma*, *Bartonella* in *Phlebovirus*), saj je vse mogoče zaznati tako z konvencionalnim PCR kot s PCR v realnem času, kar zahteva prilagoditev postopka vzorčenja in shranjevanja zastavljenim ciljem [3, 32]. Poleg morfoloških značilnosti, ki se tradicionalno uporabljajo za razlikovanje vrst, se lahko uporabljajo tudi drugi morfološki pristopi (npr. geometrijska morfometrija kril). Na podlagi lastnih izkušenj avtorjev in podatkov iz literature je bil cilj te raziskave pripraviti standardizirane smernice za prepariranje in obdelavo odraslih peščenih muh z namenom optimizacije morfoloških in molekularnih analiz. Potreba po izvedbi določenih analiz (npr. molekularna biologija ali MALDI-ToF) zahteva ohranitev dela peščene muhe, ki ni nujen za morfološko določanje, kar poudarja potrebo po premišljeni izbiri ustreznega protokola. V tem članku se osredotočamo na metode anestezije in evtanazije živih peščenih muh, njihovo shranjevanje ter postopek prepariranja za hitro določanje ali za dolgoročno ohranjanje, ki omogoča nadaljnje raziskave.

## Predgovor: Varnostni in regulativni vidiki naj se sklicujejo na ustrezne varnostne liste (SDS)

Z vsemi kemikalijami, predstavljenimi v teh smernicah, je treba ravnati ob strogem upoštevanju varnostnih pogojev. Komisije za varnost in zdravje pri delu v raziskovalnih ustanovah lahko zagotovijo informacije ne le o nevarnostih teh kemikalij, temveč tudi o postopkih ravnanja z njimi in postopkih odstranjevanja odpadkov. Obvezno je upoštevati varnostna navodila glede njihove uporabe in odstranjevanja. Opozarjamo, da je odgovornost vseh uporabnikov zagotoviti skladnost z dobrimi in varnimi laboratorijskimi praksami ter z veljavno zakonodajo in predpisi svoje države ali raziskovalne ustanove. Poleg tega so nekatere kemikalije ali njihove sestavine (npr. kloral hidrat) v nekaterih državah regulirane. Seznam okrajšav, uporabljenih v tem rokopisu, je naveden v Preglednici 1.

**Preglednica 1:** Seznam okrajšav.

| | |
|---|---|
| BME | Osnovni gojiščni medij Eagle |
| CDC | Centri za nadzor in preprečevanje bolezni |
| CMCP | Kamfor-monoklorofenol |
| CMR | Rakotvorna, mutagena, reproduktivno toksična snov |
| COI | Gen za podenoto I citokrom c oksidaze |
| CytB | Gen za citokrom b |
| DNA | Deoksiribonukleinska kislina |
| ELISA | Encimsko vezani imunski test |
| EtOH | Etanol |
| M199 | Medij 199 |
| MALDI-ToF MS | Masna spektrometrija z matrično podprto lasersko desorpcijo/ionizacijo s časom preleta |
| MEM | Minimalno esencialno gojišče |
| NGS | Sekvenciranje naslednje generacije |
| NNN | Gojišče Novy-MacNeal-Nicolle |
| PCR | Verižna reakcija s polimerazo |
| Lao PDR | Ljudska demokratična republika Laos |
| PNOC | Gen za prepronociceptin |
| qPCR | Kvantitativna verižna reakcija s polimerazo (PCR v realnem času) |
| RAPD | Naključno pomnožena polimorfna DNA |
| RFLP | Polimorfizem dolžin restrikcijskih fragmentov |
| RI | Lomni količnik |
| RNA | Ribonukleinska kislina |
| RNases | Ribonukleaze |
| RNASS | Raztopina za stabilizacijo RNA |
| RT-PCR | Verižna reakcija s polimerazo po reverzni transkripciji |
| TFA | Trifluoroocetna kislina |

## 1. Vzorčenje peščenih muh

Odrasle peščene muhe je mogoče zbirati žive ali mrtve z različnimi metodami, kot so miniaturne svetlobne pasti CDC, lepljive pasti in aspiratorji v kombinaciji s Shannonovimi pastmi, ali neposredno z mest počivanja v okolju (npr. v zavetiščih za živali). Te metode vključujejo nameščanje pasti v primerne habitate, privabljanje peščenih muh s svetlobo ali drugimi vabami ($CO_2$ ali kemične vabe) ter njihovo zbiranje za nadaljnje analize, kot je opisano v

več publikacijah [2, 3, 32, 36, 49].
Ulov živih peščenih muh omogoča vse nadaljnje postopke, medtem ko zbiranje mrtvih muh preprečuje izolacijo virusov in sevov rodu *Leishmania*. Nekatere tehnike lova, kot so lepljivi papirji, povzročajo izgubo telesnih delov peščenih muh (tipalnice, palpi, krila ali noge). Poleg tega se ricinusovo olje, s katerim so premazani lepljivi papirji, oprime peščenih muh in ga je treba odstraniti na začetku obdelave, običajno s 15-minutnim namakanjem v mešanici etanola in dietiletra v razmerju 1 : 1.

## 2. Usmrtitev peščenih muh

Po ulovu je treba žive peščene muhe usmrtiti. Pri nekaterih metodah zbiranja (npr. lepljivi papirji, svetlobne pasti CDC, opremljene s posodo z detergentom ali etanolom) so peščene muhe ob zbiranju že mrtve. Molekularnobiološke analize se lahko izvajajo na osebkih, zbranih neposredno v etanolu, ter tudi na drugih, če so čim hitreje prenešeni v etanol. Vendar nobena od teh metod usmrtitve ne omogoča obdelave žuželk z metodo MALDI-ToF. Poleg tega lahko nekatere metode usmrtitve povzročijo izgubo določenih morfoloških znakov. Zato je nujno uporabiti ustrezno standardno sredstvo za usmrtitev, da se zagotovi pravilno določanje ali dolgoročno shranjevanje za referenčne osebke (to so osebki, ohranjeni in shranjeni za prihodnjo primerjavo ali referenco). Kemikalije, kot so etilacetat, dietileter, tetrakloroetan in kloroform, se lahko vpijejo v vato in namestijo v posodo z živimi peščenimi muhami z namenom evtanazije. S temi sredstvi je treba ravnati previdno in v skladu s priporočili proizvajalca zaradi njihove toksičnosti. Vendar usmrtitev peščenih muh s kloroformom ni priporočljiva, saj je po naših izkušnjah slabo združljiva z molekularnobiološkimi raziskavami. Glede na nevarno naravo teh snovi in njihovo vprašljivo primernost za molekularne analize se uporaba teh kemikalij na splošno odsvetuje. Najpogosteje uporabljena metoda, ki ohranja morfologijo, DNA ali beljakovine, je zamrzovanje osebkov v suhem stanju. Osebke je treba zamrzovati dovolj dolgo, da so popolnoma anestezirani, vendar ne tako dolgo, da se (i) izsušijo ali (ii) ogrozi vitalnost *Leishmania*, če je cilj njihova *in vitro* izolacija iz prebavnega trakta peščene muhe. **Zato priporočamo zamrzovanje 15 do 20 minut pri –20 °C, ob rednem preverjanju, da so peščene muhe le omamljene in da paraziti rodu Leishmania niso uničeni.**

Če zamrzovalnik ni na voljo, je mogoče žuželke evtanazirati z uporabo $CO_2$. V terenskih razmerah, kjer jeklenk s $CO_2$ ni mogoče uporabiti, je mogoče živali usmrtiti z majhnimi komercialnimi posodami s $CO_2$, ki se uporabljajo v napravah za gaziranje pijač, vendar lahko obstajajo omejitve pri njihovem letalskem prevozu. Kot skrajno možnost je mogoče žuželke usmrtiti z izpostavitvijo tobačnemu dimu. Peščene muhe se ujamejo žive v pasti CDC, zberejo z aspiratorjem, zadržijo v stekleni cevki in izpostavijo tobačnemu dimu, ki jih usmrti v nekaj sekundah. Ta metoda je uporabna v vseh terenskih razmerah, tudi v pogojih otežene izolacije. Ker se steklo prepoji z dimom, ga brez temeljitega čiščenja ni mogoče uporabljati za nadaljnje zbiranje in ravnanje z živimi peščenimi muhami. Kljub temu se isti neočiščeni aspirator lahko še vedno uporablja za evtanazijo peščenih muh iz drugih pasti za namene fiksacije. Prav tako je treba preveriti, ali so bili iz aspiratorja odstranjeni vsi osebki. Te metode so združljive z izolacijo *Leishmania* s sekcijo prebavnega trakta peščenih muh.

## 3. Shranjevanje peščenih muh pred obdelavo

Pred obdelavo obstajajo pet glavne metode fiksacije:

### 3.1. Zamrzovanje

Ta metoda se najbolje izvaja pri –20 °C ali, po možnosti, pri –80 °C. Ti načini shranjevanja se danes uporabljajo pogosteje kot shranjevanje v tekočem dušiku. V vseh primerih je treba kriokonzervacijo izvesti čim prej po omamljanju osebkov. Hladno shranjevanje v zamrzovalnikih omogoča popolno ohranitev žuželk samih ter RNA, DNA in beljakovin z ohranjeno integriteto skozi celotno obdobje shranjevanja. Tekoči dušik lahko močno poškoduje krila, noge, palpe in tipalnice ter jih pogosto amputira in občasno odstrani ključne morfološke znake. Shranjevanje v suhem zamrzovalniku je za osebke lahko primerno, vendar ni idealno za ohranjanje njihovih krhkih organov. Pomembno je, da se ob odmrzovanju krila, tipalnice, palpi ali noge lahko prilepijo na epruvete in se zaradi kondenzacije sčasoma odtrgajo. Ohranjanje z zamrzovanjem pa v terenskih raziskavah ni vedno izvedljivo, saj zahteva dostop do zamrzovalnika ali posode s tekočim dušikom. Shranjevanje v zamrzovalniku je združljivo z odkrivanjem patogenov z uporabo molekularnih orodij brez izgube občutljivosti, čeprav odkrivanje in izolacija RNA virusov pri dolgoročnem shranjevanju zahtevata zamrzovanje pri –80 °C ali v tekočem dušiku. Zamrzovanje vzorcev ne omogoča izolacije *Leishmania* s sekcijo črevesa, razen če so peščene muhe najprej potopljene v plinsko fazo in nato v tekoči

dušik (na primer v vialah, nameščenih v nogavico), s čimer se posnema kriokonzervacija rodu *Leishmania*.

## 3.2. Shranjevanje v alkoholu (etanol ali izopropilni alkohol)

To je verjetno najpogosteje uporabljena metoda za shranjevanje peščenih muh. Je enostavno izvedljiva na terenu, tudi v zahtevnih razmerah brez dostopa do laboratorija. Ohranjanje v alkoholu je posebej primerno za morfološke raziskave, saj krhki organi (krila, noge, tipalnice ali palpi) ostanejo nepoškodovani, če v epruveti ni zračnih mehurčkov. Zato priporočamo, da epruveto zaprete z majhno kroglico vate, da odstranite zračne mehurčke, in na vrh namestite etiketo (Slika 1). Ustrezna koncentracija alkohola ostaja predmet razprave. Na splošno se koncentracije pod 70 % ne priporočajo [45, 66]. Višje koncentracije učinkoviteje in dalj časa ohranjajo DNA, vendar živali naredijo bolj krhke in lomljive za morfološke raziskave. Uporaba 96 % etanola (azeotropna zmes) zagotavlja stabilnost koncentracije skozi čas, zlasti v vlažnih območjih, kot so tropske države, čeprav je 95 % etanol pogosto lažje dostopen. Ne glede na koncentracijo je DNA v etanolu na splošno dobro ohranjena (čeprav manj učinkovito kot pri zamrzovanju, zlasti za molekularne metode tipa NGS). Beljakovine so bistveno manj stabilne, zlasti za proteomske analize, kot je MALDI-ToF. Peščene muhe, shranjene v alkoholu nekaj mesecev, je še vedno mogoče morfološko določiti, vendar iz teh osebkov ni mogoče pridobiti referenčnih beljakovinskih spektrov. Shranjevanje v alkoholu ali v suhih pogojih se lahko izboljša, če se vzorec dodatno zamrzne pri –20 °C. Zamrzovanje pri –20 °C predvsem izboljša molekularno ohranitev (npr. nukleinskih kislin) z upočasnitvijo razgradnje in ima tudi sekundarni učinek na morfološko ohranitev z zmanjšanjem razpada tkiv, čeprav je vpliv na morfologijo manj obsežen kot na molekularno integriteto. Shranjevanje v etanolu se lahko uporablja tudi za odkrivanje DNA in RNA virusov, kadar se uporablja etanol v koncentraciji najmanj 70 % za kratkotrajno shranjevanje, krajše od nekaj mesecev. Izopropilni alkohol je v nekaterih državah lahko lažje dostopen in ohranja DNA, vendar osebke otrdi. Ni vnetljiv kot etanol, zato ga je mogoče lažje prevažati. Po potrebi se lahko peščene muhe, shranjene v tekočem dušiku ali suho zamrznjene, prenesejo v alkohol, s čimer se združijo pomanjkljivosti obeh metod.

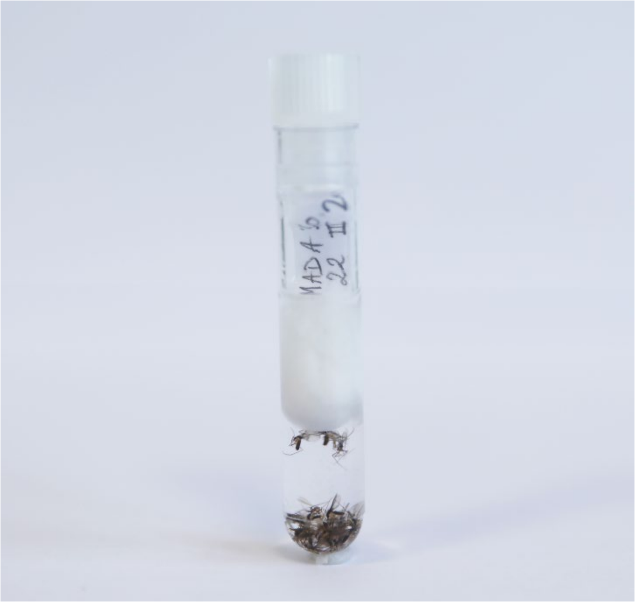

**Slika 1:** Peščene muhe, shranjene v etanolu.

## 3.3. Shranjevanje v raztopini za stabilizacijo RNA (RNASS)

Ta vodni reagent je široko uporaben, netoksičen in namenjen stabilizaciji ter zaščiti RNA v svežih tkivnih in celičnih vzorcih. Deluje tako, da hitro prodre v osebek in inaktivira RNAze (encime, ki razgrajujejo RNA), s čimer prepreči razgradnjo RNA brez potrebe po takojšnjem zamrzovanju. Shranjevanje v RNASS je na splošno učinkovito pri ohranjanju celotne tkivne in celične morfologije za nadaljnjo histološko analizo. Čeprav je RNASS optimiziran za stabilizacijo RNA in ne za fiksacijo, kratkoročno do srednjeročno shranjevanje običajno dobro ohranja strukturno integriteto. RNASS omogoča shranjevanje vzorcev pri sobni temperaturi do 7 dni, pri 4 °C več tednov ali pri –20 °C/–80 °C za dolgoročno ohranjanje. Ta metoda je posebej uporabna pri terenskem delu ali v kliničnih okoljih, kjer je hladna veriga omejena. Izolacija RNA običajno zahteva odstranitev vzorcev iz reagenta in njihovo obdelavo v skladu s standardnimi protokoli.

## 3.4. Suho ohranjanje pri sobni temperaturi

Gre za starejšo metodo, ki ima pri uporabi na celi živali (preparirani v celoti) glavno pomanjkljivost v slabem ohranjanju krhkih organov, kot so krila, noge, tipalnice in palpi. Vendar so proteomske raziskave z uporabo MALDI-ToF še vedno izvedljive, če se ob fiksaciji izvede

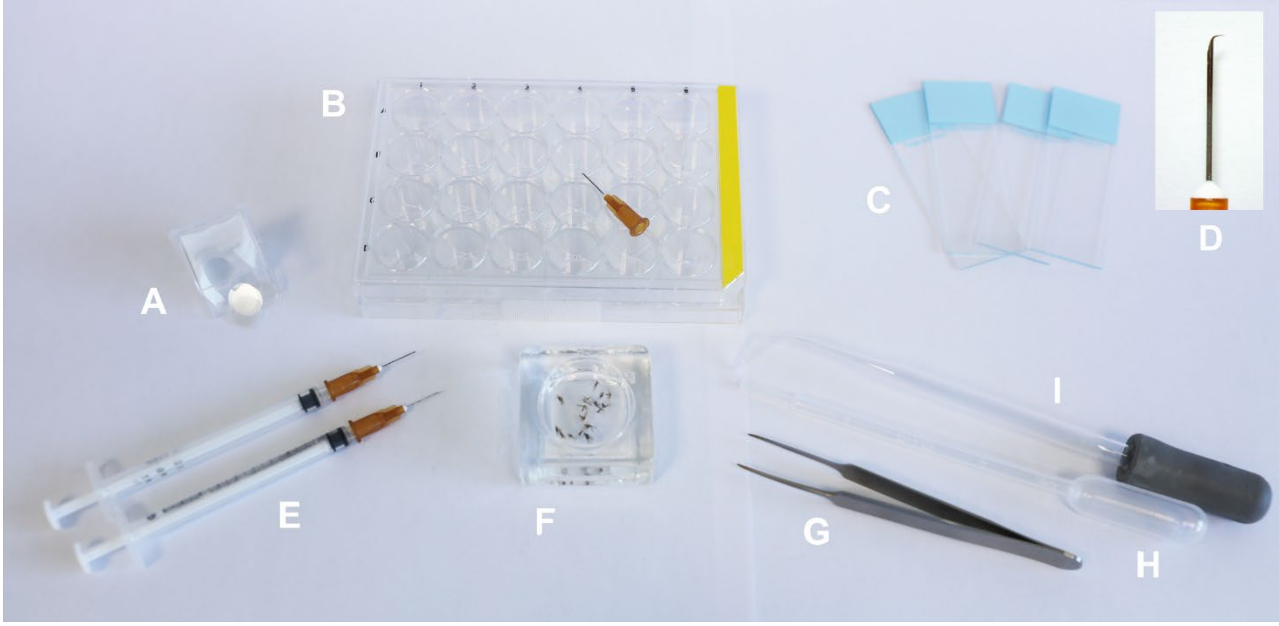


**Slika 2:** Material za prepariranje peščenih muh: A: okrogla steklena krovna stekelca (premera 10 ali 12 mm); B: plošča s 24 vdolbinami in igla s kavljem (če za obdelavo peščenih muh uporabljate olje nageljnovih žbic ali Euparal® esenco, ne uporabljajte akrilnih plošč, ker pride do kemične reakcije in poškodbe osebkov); C: predmetna stekelca, primerna za označevanje; D: detajl kavlja na igli; E: igle, pritrjene na brizge; F: urne stekelce ali enakovredna posoda z osebki, namenjenimi prepariranju; G: Dumontova pinceta; H: plastična pipeta; I: steklena pipeta, upognjena s segrevanjem za lažji prenos tekočine v vdolbine.

dehidracija z uporabo sušilnega sredstva na osnovi silikagela. Nasprotno pa so molekularne analize, usmerjene na DNA, na takih vzorcih težavne, saj je DNA pogosto fragmentirana in v nizki količini, kar pomeni, da so analize zahtevnejše kot pri svežih ali zamrznjenih vzorcih, zlasti za jedrne genome. Vendar se lahko na takih vzorcih uporabijo novejše tehnike, kot je muzeomika [34]. Zato se ta metoda shranjevanja ne priporoča, razen če ni druge možnosti. Lahko se kombinira s hladnim shranjevanjem tako, da se epruvete namestijo v zamrzovalnik pri –20 °C ali –80 °C. Glavni izziv je zagotoviti ustrezno preparacijo osebkov ali delov telesa, ki jih potrebujemo za določanje. Za to je nujna rehidracija. Priporočljiva je uporaba raztopine Triton X-100. Trajanje rehidracije se giblje od nekaj ur do več dni ob rednem nadzoru. Po popolni rehidraciji je treba osebke sprati v treh zaporednih vodnih kopelih.

## 3.5. Ohranjanje na filtrirnem papirju

Glavna prednost filtrirnih papirjev je dolgoročna stabilnost genomske DNA v celicah nefiksiranega, posušenega celotnega telesa ali krvnih celic, shranjenih pri sobni temperaturi. Filtrirni papir je na voljo v obliki majhnega kvadratka, kar omogoča shranjevanje več sto vzorcev pri sobni temperaturi v prostornini velikosti namiznega predala. Matrika filtrirnega papirja je impregnirana s sredstvi, ki denaturirajo povzročitelje okužb, zato se vzorci ne obravnavajo več kot biološko nevarni. To omogoča shranjevanje in transport vzorcev brez posebnih biološkovarnostnih ukrepov [68].

## 4. Sekcija peščenih muh

Za razliko od mnogih drugih žuželk, ki jih je mogoče določati do vrst na podlagi zunanjih znakov, peščene muhe za preučevanje anatomskih značilnosti in natančno določanje vrst zahtevajo sekcijo in prepariranje na predmetnem stekelcu. Ne glede na izbrani postopek priprave in prepariranja se uporablja enaka tehnika sekcije (Sliki 2 in 3) (https://zenodo.org/records/18198006).

**Uporaba Triton X100: neionska vodna raztopina**

Prepariranje se nanaša na sveže ulovljene ali ustrezno shranjene osebke. Večina zbirateljev ima vzorce žuželk, ohranjene v suhih zbirkah (za uporabo z MALDI-ToF) ali shranjene v alkoholu več let. Žal ohranjanje v alkoholu skozi daljše obdobje ni optimalno in tako ohranjeni členonožci postanejo zelo težavni za pripravo za mikroskopski pregled. Pogost pojav je razgradnja plastike,

v kateri so shranjeni vzorci, čemur sledi izhlapevanje alkohola. Oba primera shranjevanja sta lahko problematična, ker vzorci ostanejo predolgo v alkoholu ali se izsušijo. Zato se je pojavila ideja o uporabi vlažilnih sredstev, ki niso močni detergenti. Triton X100 je v obliki neionske vodne raztopine (*4-(1,1,3,3-tetrametilbutil) fenil-polietilen glikol (t-oktilfenoksipolietoksietanol; polietilen glikol tert-oktilfenil eter*)) in se široko uporablja kot detergent v celični in molekularni biologiji. Omogoča permeabilizacijo celičnih in jedrnih membran.

Podan je postopek z uporabo 0,5 % vodne raztopine neionskega Triton X100:

- Suh vzorec impregnirajte z absolutnim alkoholom.
- Dodajte ustrezno količino 0,5 % raztopine Triton X100, tako da je celoten vzorec potopljen.
- Postopek naj traja približno 5 minut do več dni ob rednem nadzoru. Vsi osebki morajo biti v raztopini popolnoma ločeni.
- Odstranite raztopino Triton X100 in jo nadomestite z raztopino kalijevega hidroksida.

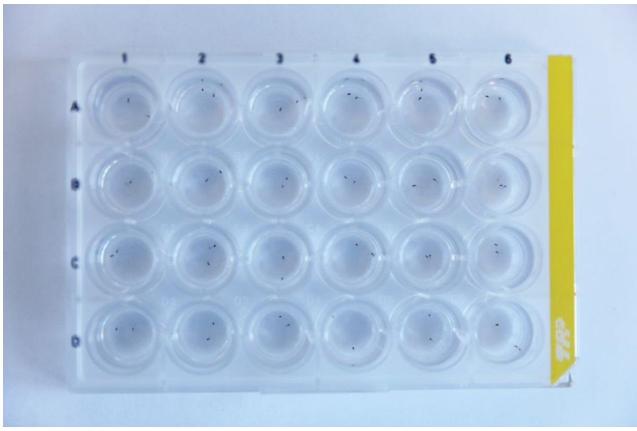

**Slika 3:** Plošča s 24 vdolbinami, od katerih vsaka vsebuje glavo in zadnji del zadka peščenih muh.

## 4.1. Glava

Sekcija se lahko izvaja z uporabo finih igel ali entomoloških bucik pod stereomikroskopom (Sliki 2 in 3). Najpogosteje uporabljene igle vključujejo: 26G × 1/2" (0,45 × 13 mm), 30G × 1/2" (0,3 × 13 mm) ali 25G × 5/8" (0,5 × 16 mm). Za pripravo osebka za določanje se loči glava od telesa in preparira z ventralno stranjo navzgor, da sta vidna cibarium in žrelo, medtem ko se oprsje in zadek po sekciji preparirata v lateralnem položaju. Prepariranje glave v ventralno-dorzalnem položaju zagotavlja, da je zatilna odprtina usmerjena navzgor, kar omogoča neposredno opazovanje cibariuma. Dostop do teh anatomskih struktur je lažji, če je glava popolnoma ločena.

## 4.2. Krila in oprsje

Krila morajo biti preparirana ravno. Vsako krilo je mogoče ločiti pri bazi in preparirati samostojno ali pa se eno preparira ločeno, drugo pa ostane pritrjeno na oprsje. Če je načrtovana analiza z geometrijsko morfometrijo, je bistveno, da se pred prepariranjem pravilno identificirata in označita desno in levo krilo. Oprsje je razdeljeno na več delov, od katerih vsak vsebuje zelo pomembne taksonomske informacije [20, 64]. Običajno se preparira v lateralnem položaju, da je mogoče pregledati hetotaksijo (razporeditev ščetin) in porazdelitev barv. Prisotnost narastišč ščetin v določenih predelih oprsja se lahko uporablja za razlikovanje nekaterih vrst v rodu *Brumptomyia*. Porazdelitev barv se lahko uporablja za ločevanje neotropskih peščenih muh na ravni rodu (npr. *Bichromomyia*), serij vrst (npr. *Pintomyia*) ali celo vrst znotraj istega rodu (npr. *Micropygomyia*, *Nyssomyia*, *Psathyromyia* in *Psychodopygus*) [20]. Če se oprsje ne uporablja za molekularno analizo, ga je treba preparirati tako, da se ne poškoduje. Pomembno je poudariti, da ni pomembna intenzivnost barv, temveč njihova porazdelitev po oprsju. Postopek vzpostavitve prosojnosti zato ne odstrani pigmentacije ali njenega vzorca.

## 4.3. Spolni organi

Pri prepariranju spolnih organov samcev in samic je potrebna posebna skrb, saj so ključni za določanje rodov, podrodov in vrst. Pri obeh spolih so spolni organi parni.

### 4.3.1. Samci

Spolni organi so zunanji in so sestavljeni iz parnih klešč, pri čemer je vsak sestavljen iz sklepa gonokoksit–gonostil v dorzalnem delu in epandrialnega režnja v ventralnem delu. Gonostil nosi trne, včasih tudi ščetine, ki morajo biti preštete in katerih mesta pritrditve morajo biti jasno vidna. Pomembno je skrbno opazovati notranjo površino gonokoksita, ki lahko nosi šop nepecljatih ščetin ali ščetine, ki so nameščene na režnju (= tuberkul) [22]. Tisti z manj izkušnjami pri sekcijah lahko izvedejo preprosto lateralno prepariranje, ne da bi spolne organe ločili od konca zadka (https://zenodo.org/records/18311158). V tem primeru

na

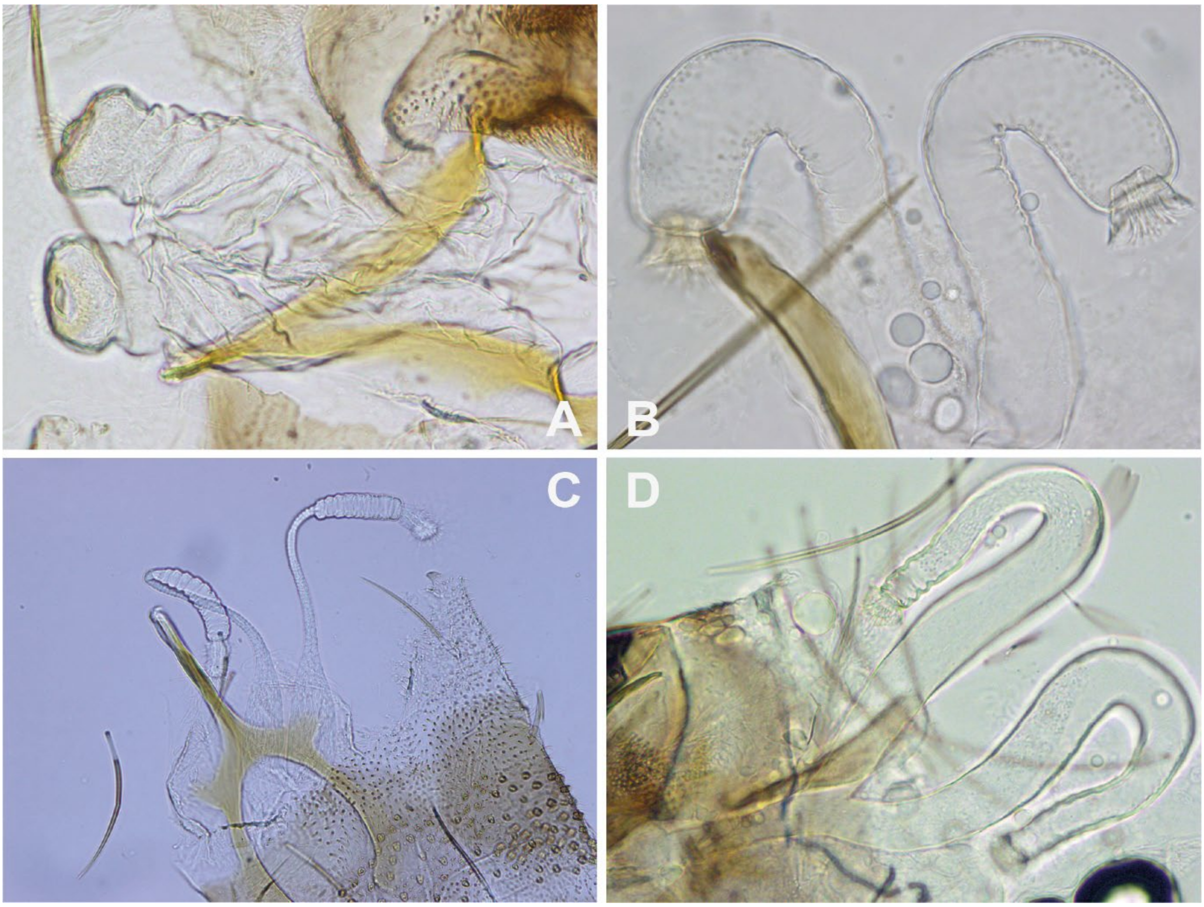


**Slika 4:** Spermateke, secirane in preparirane v Marc-Andréjevi raztopini iz svežih osebkov. A: *Idiophlebotomus longiforceps* (Laos PDR); B: *Sergentomyia minuta* (Francija); C: *Phlebotomus ariasi* (Francija); D: *Sergentomyia anodontis* (Laos PDR).

lahko prekrivanje obeh delov spolnih organov oteži štetje notranjih ščetin gonokoksita, vendar se s tem prepreči poškodba spolnih organov zaradi neuspele sekcije. Bolj izkušeni lahko poskusijo spolne organe razpreti oziroma razdeliti na dva dela. V ta namen je treba skozi spolne organe potisniti poševni rob igle (tip igle za intradermalno aplikacijo), pri čemer se spolni organi ločijo, ne da bi bili popolnoma prerezani, s čimer se razdelijo sklopi gonokoksit–gonostil (https://zenodo.org/records/18311158). Na ta način je opazovanje njihovih notranjih površin lažje. Ta razporeditev prav tako olajša opazovanje paramer in paramernih ovojnic, ki se ne prekrivajo več. Pri lateralnem prepariranju, kjer pride do prekrivanja organov, morajo biti osebki popolnoma prosojni.

#### 4.3.2. Samice

Spolni aparat je notranji in ga tvorijo spermateke. Če sekcija ni izvedena, jih je treba opazovati skozi tegumente, pri čemer je zadek prepariran v ventralnem položaju. Ne glede na izbrano preparirno sredstvo je spermateke na splošno mogoče pravilno opazovati, zlasti če niso gladke in so prosojne. Vendar je opazovanje gladkih, tankostenskih spermatek lahko problematično v slabo refraktilnih sredstvih. Poleg tega je opazovanje baze spermatekalnih vodov bistveno za določanje vrste, na primer pri podrodu *Larroussius* [35, 37, 38], ki predstavlja glavne prenašalce *Leishmania infantum* v Starem svetu. Brez tega opazovanja določanje osebka ni mogoče. Za premagovanje teh težav pri opazovanju je treba sitem genitalna furka-spermateki odstraniti iz zadka (https://zenodo.org/records/18311106).

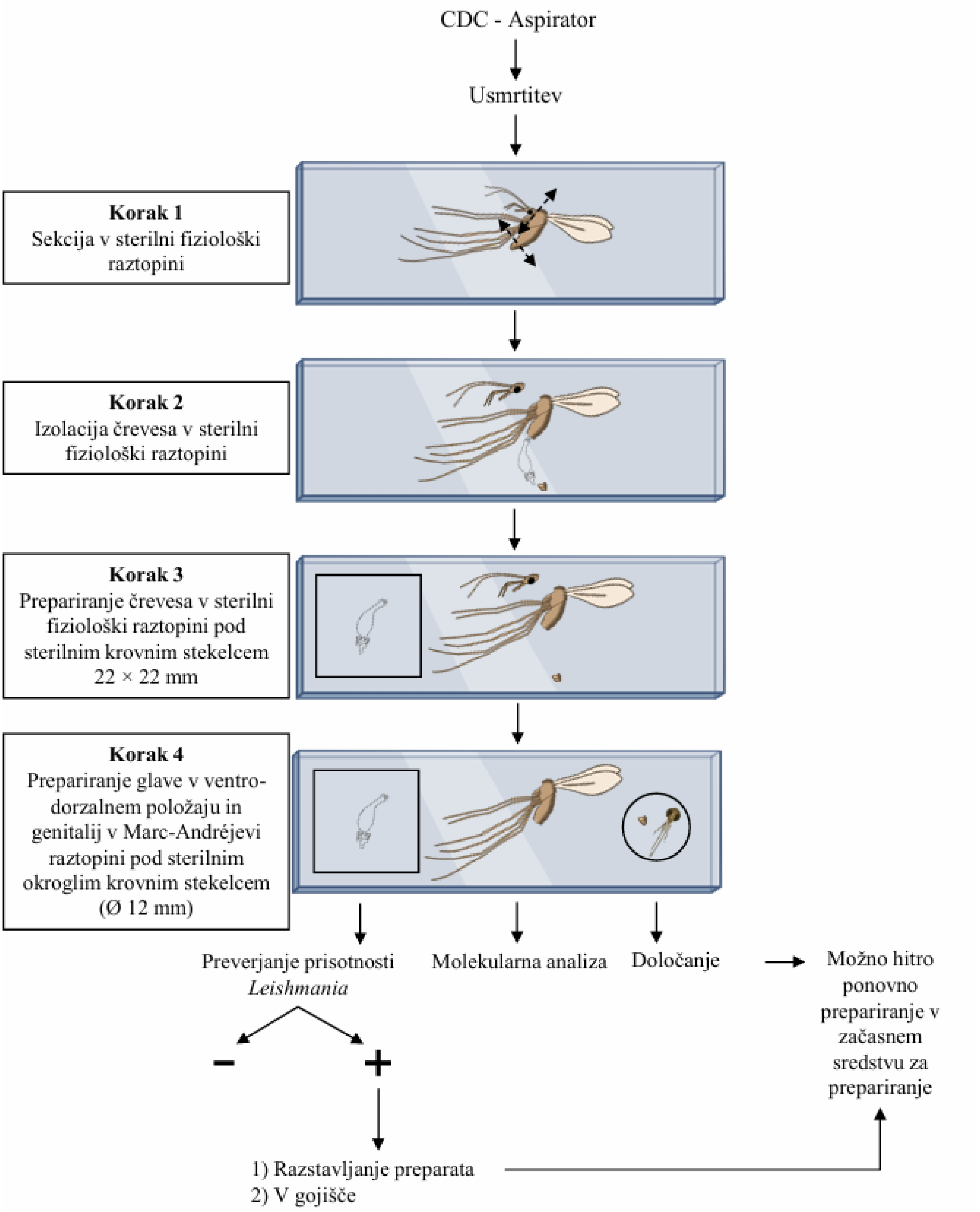


**Slika 5**: Metoda za izolacijo *Leishmania*.

Spermateke je med sekcijo praviloma težko opazovati, medtem ko je genitalno furko razmeroma lahko najti. Ker se spermatekalni vodi odpirajo v genitalno furko, izolacija te furke običajno omogoča izolacijo spermatek. Če so spermateke med postopkom po nesreči prerezane, niso izgubljene in jih je še vedno mogoče opazovati znotraj abdominalnih tegumentov (Slika 4).

## 4.4. Sekcija srednjega črevesa za izolacijo *Leishmania*

Sekcija prebavnega trakta je bistvena za odkrivanje in izolacijo *Leishmania* pri samicah peščenih muh. Postopek

se lahko izvaja tako v terenskih kot laboratorijskih razmerah za oceno vektorske kompetentnosti.

Priporočljivo je delati s sveže evtanaziranimi samicami. Samice sperite z vodo ali fiziološko raztopino, ki vsebuje blag detergent, da odstranite odvečne dlačice. Ta korak pomaga ohranjati aseptične pogoje za izolacijo *Leishmania*, hkrati pa ohranja morfološke značilnosti, potrebne za določanje. Za odkrivanje in izolacijo *Leishmania* previdno odstranite srednje črevo in ga položite v eno kapljico sterilne fiziološke raztopine (0,9 % NaCl). Po opazovanju gibljivih parazitov pod svetlobnim mikroskopom (priporočena povečava: ~200×) jih z inzulinsko brizgo ali mikropipeto prenesite v gojišče (za podrobnosti glejte poglavje 4.4.3).

Glavo in spolne organe neposredno preparirajte v Marc-Andréjevi raztopini za vzpotavitev prosojnosti. Pomembno: Marc-Andréjeva raztopina nikoli ne sme priti v stik z *Leishmania* – ne neposredno ne posredno prek orodij ali igel – saj je za parazite smrtna.

Sekcija samic peščenih muh se lahko izvaja na enem ali dveh predmetnih stekelcih; obe možnosti imata prednosti in omejitve (Slika 5; https://zenodo.org/records/18311154).

### 4.4.1. Metoda z dvema stekelcema

Prva možnost vključuje delo na dveh ločenih stekelcih: eno vsebuje sterilno fiziološko raztopino za izolacijo srednjega črevesa, drugo pa za prepariranje glave in spermatek v Marc-Andréjevi raztopini. V terenskih razmerah pa je pogosto, da dve ali tri osebe izvajajo sekcije peščenih muh in svoje preparate posredujejo enemu raziskovalcu, odgovornemu za določanje vrste in analizo okužbe s *Leishmania* v črevesu. Upravljanje dveh stekelc lahko povzroči težave pri sledljivosti vzorcev in zlasti oteži zanesljivo določitev, kateri osebek je bil okužen, če se odkrije pozitivno črevo (https://zenodo.org/records/18311154).

### 4.4.2. Metoda z enim stekelcem

Uporaba enega stekelca zagotavlja sledljivost rezultatov. Vendar je treba upoštevati več previdnostnih ukrepov. Za čim večjo sterilnost med tem korakom morajo izvajalci redno razkuževati roke z hidroalkoholnim gelom. Uporabiti je treba nebrušena predmetna stekelca in kvadratna krovna stekelca (22 × 22 mm), zavita v aluminijasto folijo in sterilizirana s suho toploto (v sušilni peči tipa Poupinel), ter sterilne igle za vsako sekcijo (priporočilo: 25G Ø 0,5 mm × 16 mm). Peščena muha se položi v kapljico sterilne fiziološke raztopine na sredini stekelca. Glava se odreže, medtem ko se med 6. in 7. abdominalnim tergitom in sternitom naredi rez, ne da bi se prerezal prebavni trakt (če pričakujemo zelo dolge spermateke, se lahko rez naredi višje). Nato je treba oprsje imobilizirati z eno iglo, zadnje posteriorne abdominalne segmente pa z drugo iglo nežno potegniti, da se izloči črevo. Če to ne uspe, je mogoče konec zadka blokirati z iglo in prebavni trakt potegniti iz njegovega anteriornega dela. Če tudi to ne uspe, je treba črevo izločiti z odstranitvijo čim več preostalega tegumenta okoli njega. Ko je črevo odstranjeno, je treba zadnje abdominalne segmente ločiti s prerezom prebavnega trakta. Črevo se nato prenese v novo kapljico sterilne fiziološke raztopine, nameščeno na eni strani stekelca, in nežno prekrije s sterilnim krovnim stekelcem. Glava in zadnji abdominalni segmenti se prenesejo v majhno kapljico Marc-Andréjeve raztopine na drugem koncu stekelca, pri čemer je treba zagotoviti, da ni stika z *Leishmania*. Glava se pravilno orientira (zatilna odprtina navzgor), spermateke pa se izolirajo skupaj z genitalno furko, kot je opisano zgoraj, in prekrijejo z majhnim okroglim krovnim stekelcem (Ø 12 mm; ne zamenjati s sterilnimi kvadratnimi krovnimi stekelci). Preostanek trupa peščene muhe in krila ostanejo v kapljici fiziološke raztopine na sredini stekelca (https://zenodo.org/records/18311154). V primeru pozitivnega rezultata ali za taksonomske raziskave se lahko oprsje in zadek ohranita za molekularne ali proteomske študije, krila pa se lahko preparirajo v vodnem preparirnem sredstvu. Za ohranitev preparata se lahko presežna količina Marc-Andréjeve raztopine nadomesti z vodnim sredstvom za prepariranje, kot sta kloralna smola (= Hoyer) ali sredstvo na osnovi polivinilnega alkohola. Podrobni videoposnetki, ki prikazujejo te postopke (sekcija srednjega črevesa peščene muhe: https://zenodo.org/records/18303014 in sekcija slinavk peščene muhe: https://zenodo.org/records/18302850), so na voljo, zato tukaj niso dodatno pojasnjeni.

### 4.4.3. Izolacija in gojenje parazitov Leishmania iz čreves peščenih muh

Izolacija parazitov iz okuženih samic peščenih muh je občutljiv postopek, ki zahteva visoko usposobljenost in ga je priporočljivo najprej vaditi na neokuženih osebkih. Po sekciji se črevesa prenesejo v svežo kapljico sterilne fiziološke raztopine (0,9 %) ali Lockejeve raztopine za izpiranje [4]. Sekcionirana črevesa se lahko nato obdelajo na dva načina: i) pregledajo se pod svetlobnim mikroskopom za opazovanje različnih razvojnih stopenj promastigot *Leishmania* in njihove lokalizacije, s posebnim

poudarkom na žrelni zaklopki (stomodeum), in ii) odpre se črevo, da se omogoči izhod promastigotov in s tem njihovo množično gojenje [4]. Najdba infektivnih peščenih muh na terenu je razmeroma redka, zato dobra praksa poveča možnosti uspešne izolacije.

Če se v črevesu opazi parazite *Leishmania*, je treba uporabiti nove sterilne igle in ob rob krovnega stekelca s kapilarnim učinkom dodati majhno količino sterilne fiziološke raztopine, da se sprostijo. Črevo je treba previdno in hitro raztrgati, da se paraziti sprostijo v fiziološko raztopino. Z mikropipeto (100 µL) ali tuberkulinsko brizgo zberite parazite in jih inokulirajte v ustrezno označeno gojišče.

*In vitro* gojenje promastigot *Leishmania*: izolirani paraziti se sprva gojijo na krvnih agarnih poševnih gojiščih SNB-9 ali v trdnem gojišču Novy-MacNeal-Nicolle (NNN) [16], prekritem bodisi s sterilnim gojiščem alfa-MEM [16, 65] bodisi z gojiščem M199, obogatena z 10 % toplotno inaktiviranim sterilnim fetalnim telečjim serumom (FCS) (za pospešitev rasti parazitov), 1 % vitaminov BME, 2 % sterilnega človeškega urina (steriliziranega s filtrirno brizgo Filtropur® S 0,2 µm), 250 µg/mL amikacina (ali 50 µg/mL gentamicina ali zmesjo antibiotikov in aminokislin (L-glutamin 200 mM–penicilin 10 000 U–streptomicin 10 mg/mL) [47]). Po treh dneh, če ni kontaminacije, se kulture suspendirajo v ustrezno pripravljenem zamrzovalnem gojišču in se nato shranijo pri –80 °C za 1 do 2 leti ali v tekočem dušiku pri –196 °C za dolgoročno ohranitev in prihodnjo eksperimentalno uporabo [7].

## 4.5. Slinavke

Sekcija slinavk peščenih muh je temeljna tehnika za preučevanje interakcij med vektorjem in patogenom, zlasti za odkrivanje arbovirusov, kot so flebovirusi (npr. virus Toscana) [44, 75]. Zaradi majhne velikosti peščenih muh postopek zahteva natančnost pod stereomikroskopom z uporabo finih pincet ali mikropreparirnih igel za izolacijo občutljivih slinavk brez raztrganja ali kontaminacije (https://zenodo.org/records/18302850) [51, 61]. Ohranitev integritete žlez je ključna za zanesljive nadaljnje molekularne analize. Po izolaciji se lahko žleze homogenizirajo in analizirajo z RT-PCR, qPCR ali imunološkimi testi za odkrivanje virusne RNA ali antigenov [12]. Prisotnost virusov v slinavkah, in ne zgolj v črevesu ali hemocelu, potrjuje, da je patogen zaključil svojo ekstrinzično inkubacijsko dobo in je prenosljiv med krvnim obrokom [71].

Postopek sekcije je tehnično zahteven zaradi majhne velikosti slinavk peščenih muh in zahteva znatno strokovno znanje, da se prepreči degradacija vzorca [1, 51]. Poleg tega je lahko virusna obremenitev nizka, zato so potrebne zelo občutljive metode odkrivanja, kot sta ugnezdeni PCR ali sekveniranje z visokim pretokom [54]. Zaradi tveganja za kontaminacijo so nujne sterilne tehnike. Poleg tehničnih izzivov na uspešnost odkrivanja vplivajo tudi biološki dejavniki; vektorska kompetentnost se med vrstami peščenih muh razlikuje, stopnje okužbe pa nihajo glede na ekološke in sezonske razmere [33, 61].

Odkrivanje virusov v slinavkah omogoča ključne vpoglede v tveganje prenosa ter podpira ciljno usmerjeno spremljanje in nadzorne ukrepe [15]. Na primer, identifikacija virusa Toscana v peščenih muhah na endemičnih območjih je prispevala k oblikovanju diagnostičnih protokolov in javnozdravstvenih priporočil [18]. Poleg tega lahko preučevanje interakcij med virusom in slino razkrije nove tarče za cepiva ali terapevtske pristope, usmerjene v preprečevanje prenosa [15, 18].

Slinavke peščenih muh se lahko uporabljajo tudi kot vir antigenov za merjenje protiteles gostitelja proti slini peščenih muh z imunološkimi metodami, predvsem z metodo ELISA. Ta pristop omogoča oceno izpostavljenosti gostitelja pikom peščenih muh ter podpira vrednotenje učinkovitosti ukrepov za nadzor vektorjev [25] in tveganja prenosa *Leishmania* [40].

## 4.6. Identifikacija krvnega obroka

Napitih samic, izoliranih pri vzorčenju, je treba preparirati z opremo za enkratno uporabo, da se prepreči navzkrižna kontaminacija. Njihov zadek je treba pregledati pod stereomikroskopom za oceno stopnje prebavljenosti krvnega obroka. Priporočljivo je izbrati le samice z rdečim, rdečerjavim ali temno rdečim zadkom, brez znakov razvoja jajčec. Odstranite vrh zadka, vključno s spermatekami, da omogočite morfološko določanje samice po prosojanju. Glavni del zadka (brez spermatek) nato prenesite v epruvete Eppendorf® in shranite pri –20 °C do nadaljnje analize. Genetski označevalci, ki se običajno uporabljajo za identifikacijo krvnega obroka, kot so PNOC [5, 30, 50], CytB [67] ali COI [13], so dobro uveljavljeni in podrobno opisani v literaturi, zato v tem članku niso dodatno predstavljeni (Slika 6). Alternativno se lahko za identifikacijo gostiteljeve krvi uporabi preslikava peptidov z MALDI-ToF [31]. Eksperimentalno je bilo dokazano, da ta tehnika omogoča identifikacijo gostiteljske krvi tudi

daljši čas po zaužitju krvnega obroka; zato je primerna metoda izbire, zlasti za analizo napitih samic z vidno napredovano prebavo krvi. Vzorce je priporočljivo shranjevati pri –20 °C ali 4 °C, vendar je mogoče dobre rezultate doseči tudi z vzorci, ki so bili kratek čas shranjeni pri sobni temperaturi. Zadek napite samice je treba tik pred analizo ločiti od preostalega telesa in homogenizirati v destilirani vodi. Preostali del telesa peščene muhe ostane na voljo za druge molekularne in morfološke analize. Po odvzemu alikvota iz homogenata za določanje peptidnega profila z metodo MALDI-TOF MS lahko preostanek uporabimo za izolacijo DNK, s katero potrdimo identifikacijo gostiteljske krvi in/ali preverimo prisotnost vrst rodu *Leishmania*. Skupni čas priprave in analize vzorca je zelo kratek v primerjavi z molekularnimi tehnikami, ki temeljijo na DNA.

## 5. Obdelava peščenih muh za morfološke raziskave (Slike 3, 6, 7 in 8; Priloge 1, 2, 3 in 4)

To poglavje opisuje načela priprave osebka peščene muhe za prepariranje izključno za morfološke raziskave, nato pa prilagoditve za uporabo, ki presega morfologijo. Razumevanje te metodologije je ključno, saj omogoča prilagoditev postopkov posebnim tipom vzorcev, kadar je to potrebno.

Postopek vključuje zaporedne korake praznjenja in polnjenja posodic z muhami z uporabo Pasteurjevih pipet, opremljenih s prožnimi gumijastimi nastavki. Zelo priporočljiva je uporaba steklenih posod z okroglim dnom, saj bistveno olajšajo te postopke. Steklo je inertno glede na vse reagente. Da se prepreči izhlapevanje reagentov, morajo biti posode opremljene s pokrovi in nikoli prepolne, saj lahko pri zapiranju ali odpiranju pride do prelivanja; hkrati je treba preprečiti padanje prahu na vzorce.

Kemikalije, potrebne za prosojanje in obdelavo, so prikazane v Preglednici 2

### 5.1. Prosojenje (clearing)

Preden je mogoče osebke peščenih muh preparirati kot trajne mikroskopske preparate, jih je treba najprej prosojiti z maceracijo z uporabo ustrezne metode in sredstva za prosojenje (npr. 10 % raztopina ocetne kisline ali Marc-Andréjeva raztopina, ki vsebuje kloral hidrat, kemikalijo, nadzorovano z zakonom v številnih državah), da postanejo prosojni. Postopek prosojenja odstrani telesna tkiva, maščobe, izločke in voske, zaradi česar postane osebek prosojen, kar omogoča pregled eksoskeletnih struktur (npr. mesta za pritrditev ščetin), površinskih značilnosti (npr. obarvanost) ter notranjih struktur, vidnih skozi tegument (npr. spermateke). Dvostopenjski postopek prosojenja, ki vključuje najprej uporabo močne baze (npr. kalijev hidroksid), nato pa šibke kisline (npr. ocetne kisline v Marc-Andréjevi raztopini), ima različne biokemične namene [74]. Baza razgradi mehka tkiva, kot so beljakovine, maščobe in mišice, s saponifikacijo in denaturacijo beljakovin, pri čemer ostane hitinski eksoskelet ohranjen za jasno strukturno opazovanje. Nadaljnja uporaba šibke kisline nevtralizira preostalo bazo, prepreči nadaljnjo razgradnjo in pobeli hitin ter tako poveča prosojnost [74], čeprav je lahko za nevtralizacijo baze zadostno tudi dvakratno izpiranje osebkov v destilirani vodi po 15 minut. To zaporedno obdelovanje združuje učinkovito odstranjevanje tkiv z nežnim ohranjanjem struktur ter zagotavlja optimalno ohranitev osebkov za mikroskopski pregled.

Pred naslednjim korakom sta priporočeni dve 20-minutni izpiranji v destilirani vodi.

#### 5.1.1. Razgradnja mehkih tkiv (Slika 8)

Natrijev hidroksid (NaOH) ali kalijev hidroksid (KOH) sta pogosto uporabljeni kemični maceracijski sredstvi, ki se uporabljata v različnih koncentracijah in trajanju glede na velikost in krhkost osebkov. Standardna in najučinkovitejša tehnika vključuje razgradnjo mehkih tkiv z namakanjem peščenih muh v močni bazi (10 % KOH ali NaOH) čez noč. Koncentracijo je mogoče povečati za skrajšanje trajanja obdelave (npr. 20 % KOH za 6 ur), prav tako pa je mogoče uporabiti segrevanje pri 37 °C.

#### 5.1.2. Prosojenje z barvanjem ali brez njega

Temu sledi postopek prosojenja, običajno s kombinacijo ocetne kisline in kloral hidrata (npr. Marc-Andréjeva raztopina). Po prosojenju je treba osebke temeljito sprati v najmanj dveh zaporednih vodnih kopelih po 20 minut, da se odstranijo ostanki kemikalij.

Marc-Andréjeva raztopina je pogosto uporabljeno sredstvo za prosojenje pri pripravi osebkov peščenih muh. Njena učinkovitost temelji na povečanju prosojnosti ob hkratnem zmanjšanju poškodb krhkih struktur, kot so krila in tipalnice.

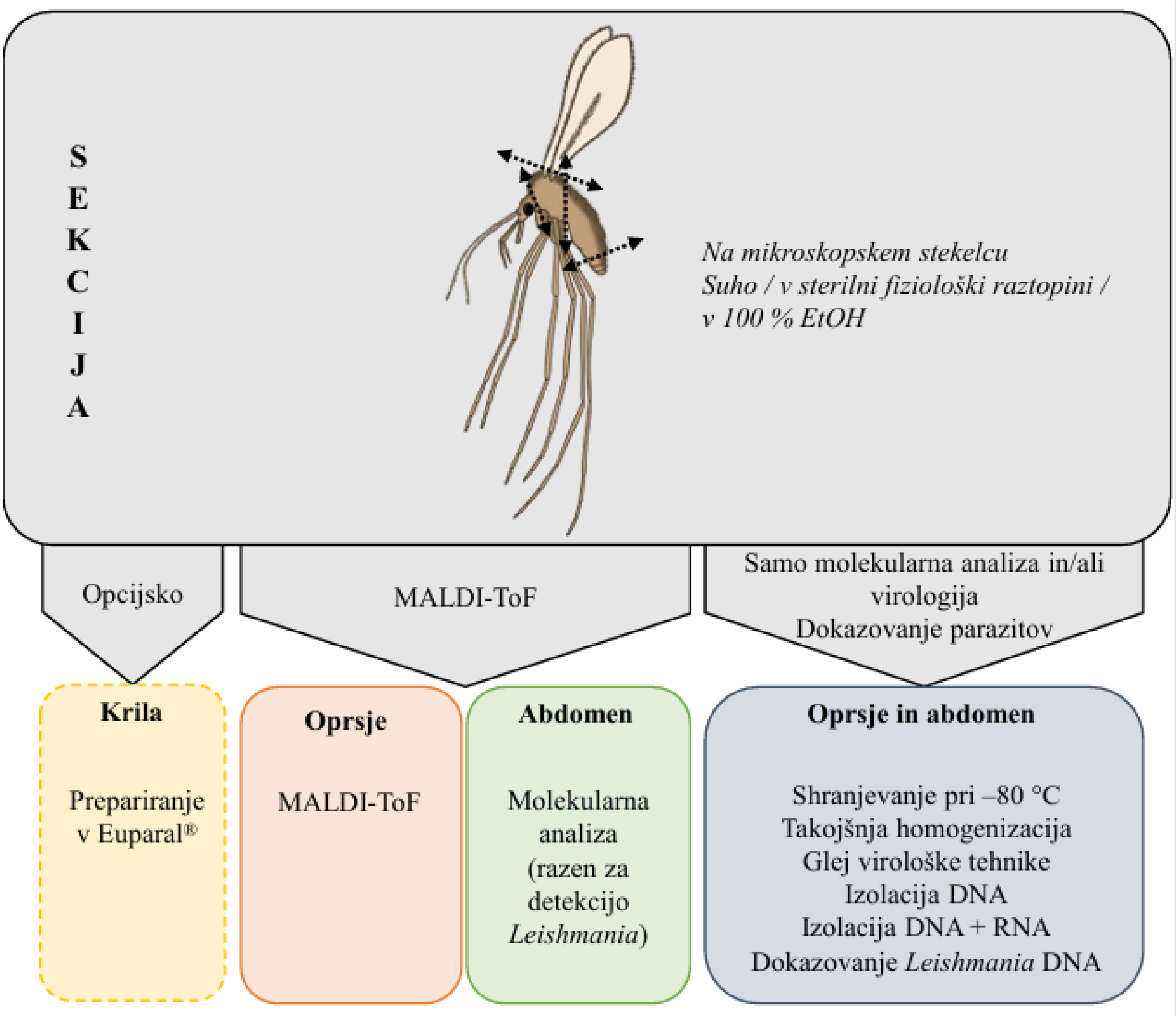


**Slika 6:** Obdelava peščenih muh za uporabo v molekularni biologiji, proteomiki in/ali virologiji.

.

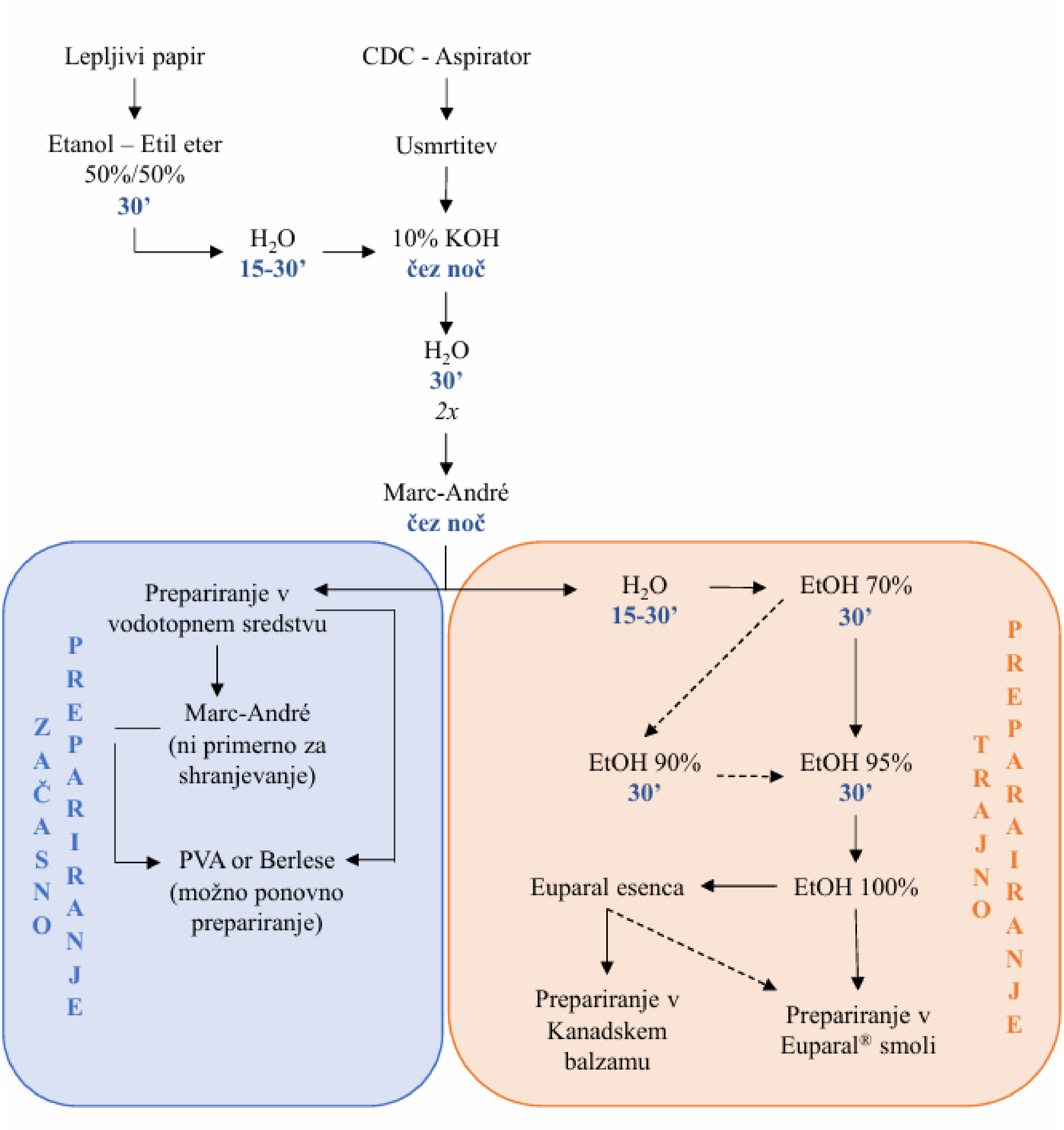


**Slika 7:** Klasična metoda obdelave peščenih muh

**Preglednica 2:** Sestava reagentov.

| | |
|---|---|
| **Kalijev hidroksid 10 %**<br>Kalijev hidroksid 10 %<br>Destilirana voda do 100 mL | **Kisli fuksin 1 % v destilirani vodi**<br>Kisli fuksin (v prahu) 1 g<br>Destilirana voda 99 mL |
| **Sredstvo za prepariranje kloralna smola (Hoyerjevo sredstvo)**<br>Destilirana voda 50 mL<br>Kloral hidrat 200 g<br>Gumiarabika 50 g<br>Glicerol 20 mL | **Marc-Andréjeva raztopina, obarvana s kislim fuksinom**<br>Marc-Andréjeva raztopina 10 mL<br>Fuksin 1 % 50 µL |
| **Marc-Andréjeva raztopina**<br>Kloral hidrat 40 g<br>Ledena ocetna kislina 30 mL<br>Destilirana voda 30 mL | **Enecejevo sredstvo**<br>Čista bela kolofonija 22 g<br>V alkoholu topna kopalova smola 12 g<br>Absolutni etanol 20 mL<br>Kamfor 10 g<br>Terpentinsko olje 10 mL<br>Evkaliptol 26 mL |

Raztopino je treba pripraviti svežo ali jo hraniti v tesno zaprti posodi, da se prepreči izhlapevanje ali razgradnja. Uporaba Marc-Andréjeve raztopine je posebej primerna v kombinaciji s postopki prosojenja ali barvanja za poudarjanje določenih morfoloških podrobnosti. Podrobnosti o sestavi in pripravi so navedene v Prilogi 2.

Pri zelo prosojnih osebkih je lahko pred prepariranjem potrebno barvanje za izboljšanje vidljivosti. Na voljo je veliko barvil, od katerih vsako cilja na določene kemične komponente organizma. Pomembno je izbrati barvilo, ki je združljivo tako z osebkom kot z izbranim sredstvom za prepariranje. Osnovno metodologijo je mogoče po potrebi prilagoditi, na primer z dodajanjem 0,1-odstotnega kislega fuksina v Marc-Andréjevo raztopino za barvanje. Poleg tega osebki, shranjeni v vodnih raztopinah in namenjeni prepariranju v smolnatih sredstvih, zahtevajo dehidracijo (glejte poglavje 5.2 o dehidraciji), saj večina naravnih in sintetičnih smolnatih sredstev za prepariranje ni združljiva z vodo. New (1974) poroča, da se lahko nekatera barvila v določenih sredstvih za prepariranje razgradijo [53]. Na primer, kisli fuksin, ki se pogosto uporablja s kanadskim balzamom, je mogoče fiksirati tudi v Euparalu®. Vendar so osebki, obarvani s kislim fuksinom, nagnjeni k bledenju, zlasti če ostanejo ostanki olja nageljnovih žbic, uporabljenega kot končna tekočina za prosojenje. Osebki, shranjeni v olju nageljnovih žbic, lahko že v nekaj dneh znatno zbledijo.

## 5.2. Dehidracija

Dehidracija se izvaja s postopnim prenašanjem vzorcev skozi zaporedje etanolnih raztopin z naraščajočo koncentracijo: 50 %, 70 %, 80 %, 90 % ali 95 % in nazadnje 100 %, pri čemer vsaka kopel traja najmanj 20 minut. Ker etanol hitro izhlapeva, mora biti posoda med postopkom tesno zaprta. Ko je osebek popolno dehidriran, je mogoče postopek za nekaj dni prekiniti v Euparal® esenci, ki je primernejša od olja nageljnovih žbic. Bukov kreozot, ki se je nekoč pogosto uporabljal v ta namen, je danes zaradi toksičnosti popolno prepovedan.

Postopek dehidracije mora zagotoviti, da je tekočina v notranjosti osebka združljiva z izbranim sredstvom za prepariranje, da se preprečijo motnost, osmotski kolaps ali deformacije, ki bi lahko naredile osebek neprimeren za taksonomske raziskave.

## 5.3. Sredstva za prepariranje

### 5.3.1. Izbor in uporaba pri prepariranju

Sredstvo za prepariranje naj ima idealno lomni količnik čim bližje lomnemu količniku stekla, ki znaša približno 1,5. Biti mora brezbarvno, jasno in po sušenju ter skozi čas popolnoma prozorno. Združljivo mora biti z uporabljenimi barvili ter sposobno prodirati in se razpršiti v vsa tkiva osebka. Ne sme se sušiti prehitro ali med prepariranjem tvoriti motnosti. Po prepariranju se ne sme krčiti. Izbor ustreznega sredstva za prepariranje je temeljni vidik

priprave osebkov, saj nobeno sredstvo ni idealno za vse namene. Izbira mora uravnotežiti več ključnih dejavnikov:

- **Optične lastnosti**. Lomni količnik sredstva za prepariranje mora zagotavljati zadosten kontrast in lom svetlobe ključnih anatomskih struktur, ki se uporabljajo za taksonomsko določanje ali morfološki opis, kot so spermateke, askodi, Newsteadovi čutilni organi, vertikalni cibarialni zobci in faringealni zobci. Vidljivost teh struktur je neposredno odvisna od optičnih lastnosti sredstva za prepariranje.

- **Ohranjanje**. Pri tipskih primerkih ali materialu, namenjenem trajnim zbirkam, mora sredstvo za prepariranje zagotavljati dolgotrajno stabilnost in obstojnost. Nasprotno pa so pri inventarnih raziskavah ali epidemioloških pregledih, kjer dolgoročno ohranjanje ni ključno, lahko zadostna začasna ali poltrajna sredstva za prepariranje.

### 5.3.2. Zahteve za sredstva za prepariranje

Strokovnjaki pogosto razvijajo prilagojene in kompleksne tehnike prepariranja, prilagojene specifičnim raziskovalnim potrebam. Vendar te metode pogosto zanemarjajo vidike, kot so arhivska kakovost, združljivost, standardizacija ali enostavnost uporabe ter dolgoročno ohranjanje. Pomanjkanje standardizacije otežuje vključevanje podarjenih zbirk in dolgoročno kuriranje.

Znanstvene aplikacije postavljajo različne zahteve za sredstva za prepariranje. Taksonomi pogosto preparirajo cele osebke in dajejo prednost sredstvom, ki nežno macerirajo notranje organe ter tako izboljšajo vidljivost kutikularnih struktur. Lomni količnik naj se dovolj razlikuje od lomnega količnika osebka in predmetnega stekelca, da se poveča optična jasnost. Komercialna sredstva za prepariranje so običajno formulirana z lomnim količnikom, ki je blizu lomnemu količniku stekla, da se zmanjša lom in sipanje svetlobe skozi sistem predmetno stekelce–sredstvo za prepariranje–krovno stekelce. V svetlobni mikroskopiji v svetlem polju pa je mogoče naravni kontrast neobarvanega osebka prilagoditi z namensko izbiro sredstva za prepariranje z nekoliko drugačnim lomnim količnikom od lomnega količnika osebka, s čimer se izboljša njegova vidljivost glede na ozadje.

### 5.3.3. Vrste sredstev za prepariranje (Preglednici 3 in 4)

Mikroskopija zahteva poznavanje lomnega količnika (RI) sredstva za prepariranje, da se določi, kako se svetloba lomi skozi predmetno stekelce, sredstvo in osebek. Kadar je lomni količnik usklajen z lomnim količnikom stekla krovnega stekelca (≈1,515), svetloba prehaja enakomerno, kar zmanjšuje sipanje in optične popačitve ter izboljša ločljivost in vidljivost finih struktur. Neskladje lomnih količnikov lahko povzroči zamegljenost, svetlobne obroče ali prikrije neobarvane strukture. Izbira ustreznega sredstva za prepariranje je zato ključna za optimizacijo kontrasta, jasnosti in splošne kakovosti slike pri določenem osebku, saj imajo različna sredstva različne lomne količnike.

Lomni količnik sredstva za prepariranje pomembno vpliva na vidljivost finih struktur pri prepariranju peščenih muh na predmetnem stekelcu. Občutljive in rahlo sklerotizirane strukture peščenih muh, vključno s cibarialnim aparatom, spermatekami, antenalnimi členi in žilami na krilih, je lahko težko opazovati v sredstvu za prepariranje z visokim lomnim količnikom.

Pri peščenih muhah se pogosto uporabljajo vodna sredstva za prepariranje na osnovi kloralne smole ter kanadski balzam in smola Enecê – Nelson Cerqueira (NC) kot topilna sredstva za prepariranje. Rawlins [60] je sredstva za prepariranje razdelil v dve skupini: (1) trajna sredstva – ta se sčasoma strdijo in so primerna za dolgoročno ohranjanje; (2) poltrajna sredstva – ta se ne strdijo popolnoma in se običajno uporabljajo za začasne namene.

Sredstva za prepariranje so lahko tekoča, na osnovi gum ali smolnata, topna v vodi, alkoholu ali drugih topilih (npr. toluen, ksilen) (Preglednica 3). Po nanosu jih je treba zaščititi pred vplivi ozračja z uporabo netopnih obročnih tesnilnih sredstev.

Za jasno razlikovanje med vrstami sredstev za prepariranje se lahko uporabi naslednja razvrstitev:

**a. Vodna sredstva**. Ta sredstva se zlahka raztapljajo v vodi, zato so primerna za začasne ali poltrajne preparate. Običajno so enostavna za uporabo, vendar je zaradi izpostavljenosti zračni vlagi pogosto nujno tesnjenje (npr. kloralna smola in polivinilni alkohol), zlasti v tropskih vlažnih podnebjih.

**b. Sredstva z omejeno toleranco na vodo**. Ta sredstva so manj občutljiva na vodo, vendar še vedno zahtevajo zaščito pred prekomerno vlago. Zagotavljajo večjo dolgoročno stabilnost v primerjavi z vodotopnimi možnostmi in se pogosto uporabljajo za poltrajne preparate.

**c. Sredstva, topna v ogljikovodikih**. Ta sredstva se raztapljajo v organskih topilih, kot sta ksilen ali toluen, ali

**Preglednica 3:** Sestava izbranih sredstev za prepariranje.

| Sredstvo za prepariranje | Topilo | Potencialni predpolimer(i) ali polimer | Opombe |
|---|---|---|---|
| Hoyer = kloralna smola | glicerol, voda | Spojine gumiarabike | maceracijsko sredstvo: kloral hidrat |
| CMCP-9 (= karboksimetilcelulo za fenol) | voda (CMCP-9: 51–60 %) | Popolnoma hidroliziran polivinilni alkohol (CMCP-9: 0–5 %) | CMC(P)-9: nizka viskoznost; visoka viskoznost |
| DMHF (dimetilhidantoin formaldehid | eau | N,N′-dimetilol dimetilhidantoin (di-metilol DMH); etrsko-/metilensko-povezani oligomeri; zamrežena polimerna mreža DMH–formaldehid | |
| Kanadski balzam | ksilen; delno hlapne sestavine balzama ($\Delta^3$-karen, levopimarska kislina, limonen, mircen, palustrinska kislina, β-felandren, α-pinen, β-pinen) | balzam (abienol, abietinska kislina, izopimarska kislina, sandarakopimarska kislina) | nevtralizacija: kalijev karbonat; smola iz *Abies balsamea* (Linné, 1758) |
| Euparal® | evkaliptol, paraldehid; delno hlapne sestavine smole sandarak (limonen, α-pinen, β-pinen) | Spojine smole sandarak (komunska kislina, manool, polikomunska kislina, sandarakopimarska kislina, 12-acetoksi-sandarakopimarska kislina, sugiol, torulozna kislina, torulozol, totarol) | Sredstvo za prosojenje: metil salicilat; barva Euparal® green: bakrova sol (bakrov abietinat); smola sandarak iz *Tetraclinis articulata* (Vahl, 1791) |
| Enecê | etilni alkohol; s kamforom, evkaliptolom in terpentinskim oljem | Spojine kopalne smole in kolofonije (rosin) | |

v topilu enecê. Namenjena so trajnemu prepariranju ter zagotavljajo odlično dolgoročno stabilnost in odpornost proti vlagi ter razgradnji, zato so primerna za arhivsko hrambo (npr. nevtralni kanadski balzam).

Vodotopna sredstva za prepariranje so torej najprimernejša za začasne preparate ali v primerih, ko je potrebna enostavna odstranitev osebka; sredstva z omejeno toleranco na vodo so primerna za poltrajne preparate z zmerno zahtevano obstojnostjo, medtem ko so sredstva, topna v ogljikovodikih, najprimernejša za trajne preparate, namenjene arhiviranju in dolgoročnemu shranjevanju.

### 5.3.4. Opis priporočenih sredstev za prepariranje (Preglednici 3 in 4)

<u>*Sredstva za začasno opazovanje*</u>

**Kloralna smola = Hoyerjeva tekočina/sredstvo/raztopina** (RI = 1,48)

Marc-Andréjeva raztopina je najboljše sredstvo za kratkotrajno (nekaj ur, morda nekoliko dlje, če je preparat shranjen v vlažni komori) opazovanje spermatek, vključno s fotografiranjem (Slika 4) ali risanjem. Za ohranitev spermatek je potrebno njihovo ponovno prepariranje v vodnem sredstvu, ki omogoča srednjeročno shranjevanje. Dehidracija za ponovno prepariranje v smoli ni nemogoča, vendar ni priporočljiva (tveganje izgube). Kloralna smola in Hoyerjeva tekočina se štejeta za sinonima. To sredstvo se pogosto uporablja za opazovanje notranjih organov zaradi združljivosti z vodo, enostavne uporabe, hitrega nanašanja in lomnega količnika, ki omogoča pregled občutljivih struktur, kot so spermateke. Vendar ima kloralna smola pomembne pomanjkljivosti, če ni popolnoma pravilno pripravljena ali shranjena v nadzorovanih pogojih vlage. Te vključujejo kristalizacijo, razbarvanje in izgubo viskoznosti. Tesnjenje krovnega stekelca teh težav ne odpravi, saj lahko sredstvo za prepariranje močno potemni (včasih skoraj počrni) zaradi interakcije s tesnilnim sredstvom, zlasti če se uporablja Euparal®. Hoyerjevo sredstvo velja za optično najprimernejše za peščene muhe in se tradicionalno uporablja v ta namen. Sestavljeno je iz več sorodnih formulacij, ki vključujejo gumiarabiko, glicerol in kloral hidrat. Nekatere formulacije so bile napačno interpretirane in napačno citirane [74].

Čeprav je Hoyer dobro sredstvo za opazovanje spermatek pri peščenih muhah, ni primerno za dolgoročno ohranjanje. Idealno je za kratkotrajna opazovanja, vključno s fotografijami, risbami ali drugimi slikovnimi zapisi. Vodna sredstva za prepariranje so primerna za začasne preparate, vendar ne zagotavljajo dolgotrajnega ohranjanja. Nasprotno pa preparati v smolah zagotavljajo odlično obstojnost, pogosto več stoletij, vendar lahko prikrijejo fine podrobnosti spermatek, saj se njihov lom svetlobe pogosto izgubi.

Hoyerjevo sredstvo se sčasoma razgradi zaradi dehidracije (Slika 8), kar povzroči nastanek majhnih belih, neprozornih kristalov kloral hidrata. Kljub temu je mogoče osebke rešiti iz kristaliziranih preparatov, saj kutikula ostane kemično ohranjena, čeprav lahko pride do mehanskih poškodb zaradi rastočih kristalov. V nekaterih primerih je mogoče kristalizirane preparate obnoviti z rehidracijo sredstva za prepariranje v toplem, vlažnem okolju z dodatkom timola za preprečevanje rasti gliv. Alternativno je mogoče osebke odstraniti iz kloralne smole z namakanjem v vodi, jih dehidrirati v ledeni ocetni kislini in ponovno preparirati v kanadskem balzamu.

**DMHF (dimetilhidantoin formaldehid)** (RI = 1,48)

To vodotopno sredstvo [72] ima zelo dobre optične lastnosti, podobno kot Berlesejevo sredstvo, in je enako enostavno za uporabo. V nasprotju z Berlesejevim sredstvom ne počrni in ne kristalizira. Dobro se obnese pri peščenih muhah in drugih predstavnikih družine Psychodidae.

**CMCP (kamfor-monoklorofenol)** (RI = 1,41)

Gre za glicerinsko, vodotopno sredstvo za prepariranje, ki se uporablja za izdelavo prozornih, trajnih preparatov občutljivih osebkov, vključno s peščenimi muhami. Prednost tega sredstva je, da je osebke mogoče preparirati neposredno iz vode ali etanola. Hitro zmehča in prosoji peščeno muho ter zmehča kutikulo, kar omogoča pravilno namestitev osebka, zlasti pri razpiranju kril ali sekciji spolnih organov. Čeprav naj bi omogočalo dolgoročno shranjevanje, natančno trajanje ohranitve ni znano. Glavna omejitev tega sredstva je vsebnost fenola, ki je toksična in dražilna snov ter zahteva previdno ravnanje.

<u>*Sredstva za trajno prepariranje*</u>

**Kanadski balzam** (RI = 1,52–1,54)

Kanadski balzam je kot primerno sredstvo za prepariranje v presevni svetlobni mikroskopiji prvi opisal Andrew Pritchard v 30. letih 19. stoletja. Še vedno ostaja eno najpogosteje uporabljenih sredstev zaradi dokazane arhivske kakovosti in več kot 150-letne uspešne uporabe. V nasprotju s Hoyerjevimi sredstvi kanadski balzam ne kristalizira in ne absorbira vlage. Vendar je močno avtofluorescenten, kar je lahko v nekaterih mikroskopskih tehnikah pomanjkljivost [60]. Uporaba netoksičnih topil namesto ksilena lahko zmanjša varnostna tveganja pri pripravi, vendar lahko povzroči pomanjkljivosti, kot sta počasnejše sušenje in zgodnejša potemnitev sredstva.

**Preglednica 4:** Prednosti in pomanjkljivosti izbranih sredstev za prepariranje pri pripravi mikroskopskih preparatov ter neobjavljena opažanja različnih avtorjev [52].

| Naziv | Prednosti | Pomanjkljivosti |
|---|---|---|
| * Kanadski balzam | Sredstvo je zelo obstojno, z življenjsko dobo, daljšo od 150 let. Preparati se lahko izdelajo z uporabo olja nageljnovih žbic ali fenola kot vmesnega sredstva. | Vsebuje škodljive sestavine in z njim je treba ravnati v digestoriju. Zahteva popolno in časovno zahtevno dehidracijsko serijo. Dehidracija v etanolu in prenos prek ksilena ali olja nageljnovih žbic lahko nekatere taksone naredi krhke; alternative (npr. izopropanol, n-butanol, Cellosolve™, 1,4-dioksan, Histoclear, terpineol) lahko zmanjšajo lomljivost. Osebki lahko počrnijo, če se ksilen nadomesti s fenolom ali če ostane preostanek kalijevega hidroksida. Visok lomni količnik lahko prikrije neobarvane strukture. Popolno sušenje brez sušenja na grelni plošči lahko traja več let. Sredstvo sčasoma porumeni in potemni, zlasti če je bilo uporabljeno olje nageljnovih žbic. Nekatera barvila oslabijo, kationska barvila pa lahko zbledijo, če sredstvo postane kislo, kar se lahko sčasoma spontano zgodi. |
| DMHF (dimetilhidantoin formaldehid) | Visoka prosojnost. Dober lomni količnik. Odlična vidljivost struktur. Razmeroma dobra stabilnost preparatov. Združljivost s številnimi tehnikami barvanja. Dobra zaščita vzorcev. Dobra adhezija med predmetnim in krovnim stekelcem. | Možno rumenenje sčasoma. Lahko spremeni nekatera barvila. Ni primerno za barvila, občutljiva na formaldehid. Nastanek zračnih mehurčkov, počasno sušenje. Občutljivo na vlago. Prepariranje je težko reverzibilno. Formaldehid je toksičen, dražilen in kancerogen. |
| * Euparal (prozoren) | Obstojno sredstvo z življenjsko dobo nad 50 let. Prepariranje neposredno iz 80 % etanola je možno (po priporočilu proizvajalca). Ne zakriva neobarvanih struktur in sčasoma ne porumeni ali postane krhko. Ima primernejši lomni količnik kot kanadski balzam za Diptera. Primerno za debelejše osebke zaradi minimalnega krčenja in sušenja brez mehurčkov. Ostaja topno v 95 % etanolu, kar omogoča ponovno prepariranje tudi po več letih. | Vsebuje škodljive sestavine in zahteva delo v digestoriju. Dehidracija v etanolu in prenos prek Euparal® esence lahko nekatere taksone naredi krhke, vendar lahko uporaba izopropanola ta učinek zmanjša. |
| Hoyerjeva tekočina | Osebke je mogoče preparirati žive ali neposredno iz vode, etanola ali formaldehida. Maceracija omogoča odlično kakovost kutikule. Ugoden lomni količnik, ki ga je mogoče izboljšati z jodnim barvanjem za večji kontrast. Ocetna kislina v sestavi lahko razširi okončine členonožcev. Nekateri preparati lahko ostanejo stabilni 40–60 let. Vodotopno sredstvo, ki omogoča enostavno ponovno prepariranje. | Občutljivi rastlinski osebki se lahko sesedejo, če se sredstvo ne dodaja postopoma, kar je časovno zahtevno. V manj kot 10 letih se lahko tvorijo votline in kristali. Maceracija je lahko prekomerna glede na koncentracijo kloral hidrata in čas izpostavljenosti. Sestavine sredstva se lahko ločijo, fina granulacija pa se lahko pojavi po nekaj mesecih ali letih. Poročano je bilo o potemnitvi sredstva. |
| CMCP-9 (= karboksimetilceluloza fenol) | Osebke je mogoče preparirati neposredno iz medijev, kot so voda, etanol, glicerol ali raztopine s formaldehidom; po potrebi se lahko notranji organi macerirajo za lažji splošni pregled ali pripravo. | Sredstvo lahko sčasoma razvije kristale in potemni ter lahko osebke macerira bolj, kot je predvideno. Če stekelce ni skrbno zatesnjeno, debelejši vzorci niso primerni, saj se lahko skrčijo in ustvarijo vrzeli ob robovih krovnega stekelca. Ni primerno za obarvane osebke ali kalcificirane materiale. Čas sušenja je daljši kot pri CMC. |

| Naziv | Prednosti | Pomanjkljivosti |
|---|---|---|
| Eukitt™ | Obstojno sredstvo z življenjsko dobo nad 30 let. Združljivo s številnimi topili za prepariranje, vključno z acetonom, benzenom, kloroformom, dioksanom, etrom, izopropanolom, metil benzoatom, terpineolom, toluenom in ksilenom. Hitro se suši in ima rahlo kisel pH. S staranjem ne potemni opazno. Primerno za različna barvila (npr. fuksin, hematoksilin, metilno zeleno, metil vijolično, metilensko modrilo). Osebke je mogoče ponovno preparirati po letih z dolgotrajnim namakanjem v ksilenu. | Vsebuje škodljive sestavine in zahteva delo pod digestorijem. Zahteva popolno in časovno zahtevno dehidracijsko serijo. Ni idealno za debelejše osebke zaradi krčenja in nastajanja plinskih mehurčkov. Krovna stekelca se lahko sčasoma odlepijo, če steklo ni dobro očiščeno in zatesnjeno. Lahko pride do nepopolne polimerizacije okoli kolagenskih vlaken. |
| Enecê | Zelo obstojno sredstvo, ki traja vsaj 50 let. Sčasoma ne potemni. Je bolj plastično, kar omogoča sekcijo žuželk neposredno v sredstvu in daje dovolj časa za pravilno namestitev morfoloških struktur. Nizka cena. | Zahteva popolno in časovno zahtevno dehidracijsko serijo. Dehidracija v etanolu in prenos prek olja nageljnovih žbic lahko nekatere osebke naredi krhke. Osebek se še naprej zelo počasi prosoji, kar lahko oteži opazovanje zelo majhnih struktur, kot so senzile, askoidi in enostavne ščetine. |

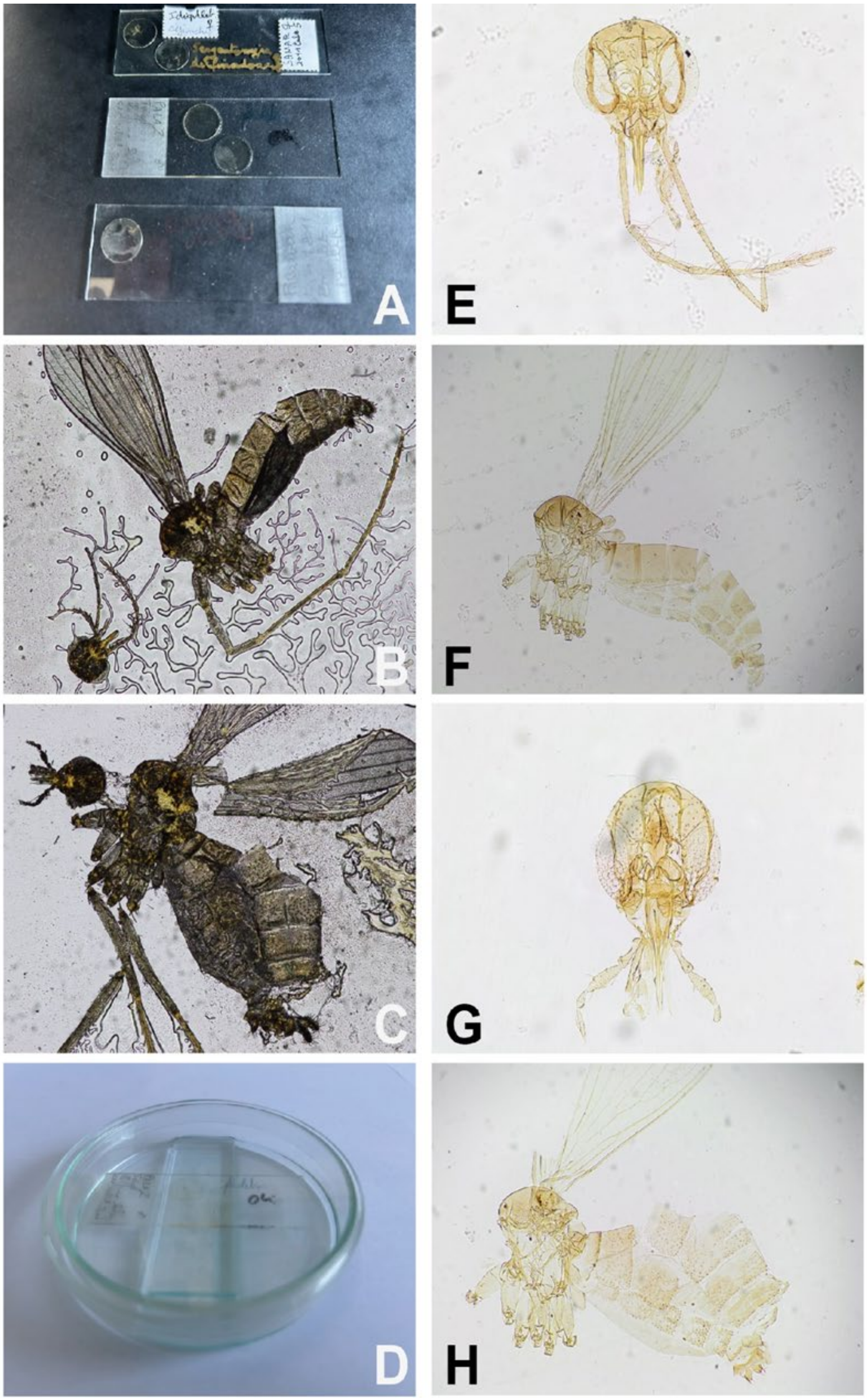


**Slika 8:** Ponovno prepariranje preparata. A: poškodovani in posušeni preparati, preparirani v Hoyerjevem sredstvu; B: mikroskopski prikaz posušene peščene muhe; C: mikroskopski prikaz poškodovane peščene muhe; D: vlažna komora s posušenim preparatom; E: glava in F: telo osebka B po ponovnem prepariranju v Euparalu®; G: glava in H: telo poškodovanega osebka C po ponovnem prepariranju v Euparalu®.

**Euparal®** (RI = 1.48)

Euparal® je pogosto uporabljena alternativa kanadskemu balzamu za trajno prepariranje, saj zagotavlja odlično dolgoročno stabilnost in primerljiv lomni količnik. Zanj sta značilni: (1) zahteva po dehidraciji: pred končnim prenosom v sredstvo za prepariranje je treba osebek dehidrirati, običajno s prehodom iz 95 % v absolutni etanol, in (2) podaljšan čas obdelave: končna priprava v smoli, bodisi kanadskem balzamu bodisi Euparalu®, zahteva dehidracijo, kar podaljša celoten postopek obdelave vzorca. Kadar dehidracija z organskimi topili ni izvedljiva, je mogoče vzorce, prenesene iz absolutnega etanola, najprej postaviti v vmesno raztopino, sestavljeno iz enake mešanice Euparala® in Euparal® esence, nato pa jih končno preparirati.

**Enecê** (RI = 1.467)

Enecê je smolnato sredstvo za prepariranje, ki se uporablja predvsem za majhne žuželke in je posebej priljubljeno v Braziliji. Njegova osnova je kolofonija in kopalna smola, raztopljeni v alkoholu, kamforju, terpentinskem olju in evkaliptolu. Cerqueira [11] je Enecê opisal kot alternativo kanadskemu balzamu za trajne preparate ličink, eksuvijev nezrelih stadijev in celo odraslih komarjev; od takrat se pogosto uporablja tudi za prepariranje peščenih muh. Enecê predstavlja cenovno ugodno alternativo za trajno prepariranje, saj zagotavlja dolgoročno stabilnost in dovolj dolg čas sušenja, kar omogoča sekcijo in natančno razporeditev morfoloških struktur.

## 5.4. Priprava preparatov in sušenje

Pravilno sušenje preparatov je ključno za zagotavljanje dolgoročne stabilnosti in ohranitve. Preparati morajo biti temeljito posušeni, preden se pripravijo za dolgoročno shranjevanje. Za optimalne rezultate je treba preparate, izdelane s trajnim sredstvom za prepariranje, sušiti v vodoravnem položaju 2–3 tedne, medtem ko preparati, pripravljeni s poltrajnim sredstvom, običajno zahtevajo 1–2 tedna sušenja. Za učinkovito sušenje je priporočljiva uporaba inkubatorja, nastavljenega na ustrezno temperaturo glede na uporabljeno sredstvo za prepariranje, pri čemer se je treba izogibati previsokim temperaturam, ki bi lahko poškodovale osebke. Priporočeno temperaturno območje je med 30 °C in 37 °C. Ta faza sušenja je bistvena za preprečevanje ukrivljanja preparatov, propadanja osebkov ali nestabilnosti sredstva za prepariranje med shranjevanjem.

Sredstvo za prepariranje, uporabljeno pri pripravi preparata, mora biti vedno navedeno na etiketi. Če je mogoče, naj etiketa vključuje tudi natančen recept, ime osebe, ki je preparat pripravila, ter datum priprave. Preparati so pogosto sprva pripravljeni kot začasni in niso namenjeni dolgoročnemu shranjevanju. Če pa se status osebka spremeni, na primer če postane del tipske serije, je treba uporabiti trajnejše sredstvo za prepariranje, da se zagotovi ohranitev osebka za prihodnje taksonomske raziskave.

## 5.5. Alternativne tehnike prepariranja: prepariranje na kartončkih

Prepariranje na kartončkih je tehnika, ki se uporablja pri več skupinah žuželk, pri kateri se osebki bodisi neposredno pribodejo na entomološke kartončke bodisi se nanje prilepijo. Glede na njihovo majhnost in potrebo po opazovanju notranjih organov za določanje po postopku prosojenja (glejte poglavje 5) ta metoda nikakor ni primerna za prepariranje peščenih muh.

## 5.6. Ponovno prepariranje poškodovanih osebkov

Pri redkih ali dragocenih osebkih je priporočljiv dvostopenjski pristop v skladu z videoposnetkom, dostopnim na: https://zenodo.org/records/18315029.

1) Rehidracija, ne da bi ločili sestavne dele, da se omogoči predhodno opazovanje. Nosilec za več mikroskopskih preparatov se namesti v petrijevko kot opora. Preparat, ki ga je treba rehidrirati, se položi na vrh nosilca, nato pa se v petrijevko doda nekaj milimetrov topila, da se ustvari vlažna komora, pri čemer preparat ne sme priti v neposreden stik s topilom (Slika 8 D). Čas rehidracije se lahko razlikuje od enega do več dni, odvisno od stanja osebka. Potrebna sta vsakodnevno spremljanje in potrpežljivost. Ko je preparat dovolj rehidriran, se odstrani iz vlažne komore in za nekaj ur postavi v inkubator pred mikroskopskim pregledom, fotografiranjem ali risanjem.

2) Ponovno prepariranje. Preparat se lahko za nekaj dodatnih ur ali čez noč ponovno vrne v vlažno komoro. Razstavljanje je treba izvesti pod stereomikroskopom. Z

uporabo finih igel je treba krovno stekelce previdno odstraniti, pri čemer je treba zagotoviti, da noben del peščene muhe ne ostane pritrjen nanj (https://zenodo.org/records/18315029). Nato je treba secirane dele peščene muhe prenesti in sprati z vodo v majhnih vdolbinicah, ki se uporabljajo tudi pri destruktivni izolaciji DNA/RNA (glejte spodaj), pred dehidracijo in ponovnim prepariranjem v smoli. Pri razstavljanju preparata je ključno prepoznati prvotno uporabljeno sredstvo za prepariranje, da se izbere ustrezno topilo. Pri vodnih sredstvih za prepariranje je treba uporabiti vodo. Če je sredstvo za prepariranje smolnato (npr. kanadski balzam ali Euparal®), je treba uporabiti ksilen, pri čemer je delo treba izvajati v digestoriju in z ustrezno osebno varovalno opremo, vključno z masko.

Ponovno prepariranje tipskih ali zbirkovnih osebkov je dovoljeno le s soglasjem kustosa in/ali ustanove, ki je lastnik osebka.

## 6. Določanje vrst peščenih muh

### 6.1. Morfologija

Določanje peščenih muh temelji predvsem na pregledu njihovih morfoloških značilnosti, vključno z obliko oprsja, kril, spolnih organov, ščetin ter specifičnih morfometričnih razmerij med posameznimi strukturami. Raziskovalci uporabljajo taksonomske ključe, referenčne zbirke in izvirne opise vrst za primerjavo zbranih osebkov z že opisanimi taksoni. Ključne diagnostične značilnosti, kot so oživljenost kril in morfologija glave pri obeh spolih, zgradba moških spolnih organov ter razporeditev in oblika spermatek pri samicah, so posebej informativne za določanje vrste. Natančno določanje pogosto zahteva podroben mikroskopski pregled, običajno z uporabo svetlobnega mikroskopa za opazovanje finih struktur, kot so spolni organi in spermateke, ali stereomikroskopa za pregled širših morfoloških značilnosti.

Nedavni napredek v slikovni tehnologiji je omogočil uporabo digitalnega slikanja pri določanju peščenih muh. Visokoločljive fotografije ali digitalne ilustracije ključnih struktur je mogoče primerjati z referenčnim gradivom ali analizirati z računalniško podprtimi sistemi za določanje, kar izboljšuje tako natančnost kot dostopnost morfološke taksonomije.

### 6.2. Geometrija kril

Geometrija kril je pomembna značilnost pri določanju in razvrščanju različnih vrst peščenih muh. Krila peščenih muh imajo značilen vzorec in zgradbo, običajno so dolga in ozka z dobro razvitimi žilami (Sliki 9 in 10). Razporeditev žil tvori poseben vzorec, ki se lahko razlikuje med rodovi in vrstami ter predstavlja dragocene diagnostične znake za določanje. Zato preučevanje geometrije kril nudi pomembne informacije za taksonomske namene.

### 6.3. Geometrijska morfometrija kril

Raziskovalci uporabljajo različne tehnike, kot je geometrijska morfometrija, za analizo in primerjavo oblike ter velikosti kril med različnimi vrstami ali populacijami peščenih muh. Preučevanje geometrije kril lahko nudi tudi vpogled v vedenje, habitatne preference in letalne sposobnosti.

Pri pristopu geometrijske morfometrije se krila skrbno secirajo, po potrebi obarvajo in plosko preparirajo na predmetnih stekelcih. Pripravljeni preparati se nato fotografirajo pod stereomikroskopom, digitalizirajo in analizirajo z morfometričnimi metodami. Postopek je podrobno opisan v literaturi [6, 27, 42, 56, 57, 59], pri čemer je priporočljivo dosledno uporabljati desno ali levo krilo pri parnih organih, da se preprečijo morebitni negativni alometrični učinki [62].

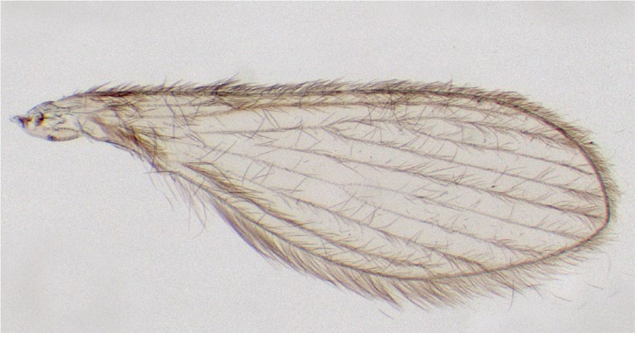

**Slika 9:** Neobdelano krilo vrste *Trichophoromyia ininii*.

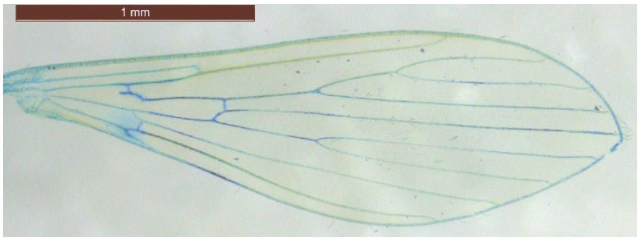


**Slika 10:** Obarvano krilo vrste *Phlebotomus ariasi*.

*Priprava kril za geometrijsko morfometrično analizo*

Za optimalno vidljivost krilnih žil je treba s kril odstraniti luske in jih ustrezno obarvati. Za pripravo kril najprej napolnite majhne vdolbinice s potrebnimi reagenti (metilensko modrilo, etanol, voda in nadomestek za ksilen). Krilo, shranjeno v 70 % etanolu pri sobni temperaturi, prenesite tako, da obrnete epruveto Eppendorf in vsebino izpraznite nad vdolbinico, nato pa krilo vzdolžno dvignite s fino ukrivljeno iglo. Krilo na kratko prenesite iz etanola v vodo in nazaj v etanol, da odstranite ščetine. Nato ga za 6 minut položite v metilensko modrilo, pri čemer zagotovite, da med barvanjem plava. Krilo previdno ponovno poberite in ga za 2 minuti potopite v nadomestek za ksilen (približno tretjino časa barvanja z metilenskim modrilom). Nežno tapkanje z iglo ob stene vdolbinice lahko pomaga, da se krilo umiri; ksilen služi za fiksacijo obarvanosti. Na koncu krilo dvignite in ga položite na majhno kapljico Euparala® na predmetnem stekelcu. Pod povečevalnim steklom krilo nežno razprite in previdno namestite krovno stekelce. Fotografije je treba posneti takoj, preden se Euparal® strdi, saj bo morda potrebno rahlo prilagoditi položaj krila pod krovnim stekelcem za dosego optimalne poravnave.

## 6.4. Molekularno biološke metode

Poleg morfoloških metod postajajo molekularne metode vse pomembnejše v entomoloških raziskavah, vključno s taksonomskimi, populacijsko-genetskimi in filogenetskimi študijami, pa tudi za odkrivanje patogenov na ravni DNA/RNA ter določanje izvora krvnega obroka in vedenja vektorjev, kar je pomembno na področju epidemiologije [70]. Sekveniranje DNA se lahko uporablja za potrditev vrste ali za razlikovanje med tesno sorodnimi vrstami ter omogoča natančnejše in zanesljivejše določanje. Poleg tega napredne molekularne tehnike (npr. PCR, sekveniranje DNA, NGS itd.) in MALDI-ToF MS vse bolj pridobivajo pomen pri hitrem in natančnem določanju vrst ter dopolnjujejo tradicionalne morfološke metode [46]. Kljub tem napredkom ostaja morfološko določanje referenčni standard v taksonomiji in osnova za interpretacijo molekularnih podatkov.

### 6.4.1. Destruktivna izolacija nukleinskih kislin

Izolacija nukleinskih kislin je rutinski korak v številnih bioloških raziskavah in razvite so bile različne metode za izolacijo DNA iz biološkega materiala [48]. Številni komercialno dostopni kompleti za izolacijo DNA so namenjeni poenostavitvi tega postopka [14]. Vendar metode, ki se običajno uporabljajo za pripravo členonožcev za morfološko določanje, pogosto ovirajo analizo DNA, saj lahko poškodujejo ali uničijo ključne morfološke značilnosti osebka [10]. Večina protokolov za izolacijo DNA iz tkiv žuželk je destruktivne narave [43], kar predstavlja posebno težavo pri majhnih osebkih, kjer lahko že omejeno vzorčenje ogrozi pomembne morfološke strukture [72]. Vrsta in stanje osebka imata ključno vlogo pri izbiri ustrezne metode izolacije DNA [29].

Potreba po natančnem določanju peščenih muh, razumevanju populacijske dinamike in zmanjšanju vplivov na neciljne organizme je spodbudila razvoj molekularnih diagnostičnih orodij [23]. Molekularni pristopi se danes pogosto uporabljajo kot dopolnilo morfološkim taksonomskim metodam. Na primer, standardni pristop pri DNA-barkodiranju žuželk vključuje izolacijo DNA, sekveniranje in izgubo osebka. Zato je nujno raziskovati nedestruktivne metode izolacije DNA, ki ohranjajo tako biološki material kot morfološko celovitost.

Številne metode izolacije nukleinskih kislin so bile uporabljene pri peščenih muhah. Količina in kakovost nukleinskih kislin sta odvisni od nadaljnje molekularne analize, saj imajo različne tehnike različne zahteve glede občutljivosti in čistosti [9]. Ugotovljeno je bilo na primer, da oči peščenih muh lahko zavirajo amplifikacijo PCR [69]. Poleg presejanja patogenov se DNA peščenih muh rutinsko ekstrahira tudi za namene določanja vrst. Uporabiti je mogoče različne metode izolacije, vendar se izplen in kakovost med tehnikami razlikujeta. Nekateri proizvajalčevi protokoli so bili prilagojeni za peščene muhe [8], kar je povečalo izplen in/ali kakovost pridobljenih nukleinskih kislin [8, 9, 69], medtem ko se lahko uporabljajo tudi prilagoditve, razvite za druge taksone členonožcev [58, 76]. Identifikacijski PCR, usmerjeni na

kratke mitohondrijske fragmente (COI ali CytB), so praviloma združljivi z metodami izolacije, ki povzročajo visoko fragmentacijo DNA. Nasprotno pa dolgodosežne NGS-tehnike (Oxford Nanopore in PacBio) zahtevajo minimalno fragmentacijo in visoko kakovost DNA. Izolacije s kolonskimi sistemi (spin column) običajno dajejo genomske fragmente do 60 kb, medtem ko lahko fenol-kloroformska izolacija omogoči fragmente do 150 kb [77]. Preglednica 5 povzema različne tehnike izolacije DNA peščenih muh in navaja, ali so bile metode prilagojene za te žuželke. Izpleni niso prikazani, saj so odvisni od velikosti osebka in načina priprave. Stolpec »prilagoditev« se nanaša na spremembe protokolov izolacije za peščene muhe ali druge majhne členonožce.

Izbira metode izolacije mora upoštevati več meril, kot so število vzorcev, čas izolacije in nadaljnja uporabljena tehnika. Medtem ko NGS-tehnike zahtevajo genomsko DNA visoke molekulske mase, so vse tukaj predstavljene metode primerne za standardne aplikacije na osnovi PCR.

Poleg tega je več raziskav obravnavalo nedestruktivne metode izolacije DNA pri majhnih kopenskih členonožcih, suho ohranjenih muzejskih osebkih in členonožcih s tanko kutikulo [19, 26, 28, 55, 63].

#### 6.4.2. Nedestruktivna izolacija nukleinskih kislin

Eden glavnih izzivov molekularne analize členonožcev, zlasti peščenih muh, je ohranitev osebkov za vključitev v entomološke zbirke. Večina protokolov za izolacijo DNA zahteva maceracijo tkiva, kar ogrozi ohranitev osebka. Nedestruktivne metode izolacije nukleinskih kislin so zasnovane tako, da omogočajo pridobivanje genetskega materiala brez fizične poškodbe vzorca, vpliva na njegovo uporabnost ali spremembe njegove morfologije. Te metode so posebej dragocene pri delu z redkimi ali omejenimi vzorci, kot so peščene muhe, kjer je ohranjanje strukturne celovitosti bistveno za prihodnje taksonomske, morfološke ali diagnostične namene. Pogosto uporabljena tehnika je nedestruktivna metoda kopeli, pri kateri se peščene muhe imobilizirajo in nežno potopijo v lizni pufer, ki vsebuje proteinazo K.

Tehnika blage vektolize je bila uspešno uporabljena pri peščenih muhah, zlasti pri tipskih osebkih [24]. Tehnika uporablja standardni komplet s kolonskim sistemom (v tem primeru DNeasy Blood and Tissue kit, QIAGEN, Hilden, Nemčija) s prilagoditvami za pridobitev DNA brez uničenja osebka. Spremenjeni koraki razgradnje (količina liznega pufra in dodatek zamrzovalnega koraka) [17] omogočajo sproščanje nukleinskih kislin ob minimalnih morfoloških poškodbah [24]. Pri peščenih muhah je mogoče uporabiti tudi komplet za izolacijo DNA HotSHOT (Bento Bioworks Ltd, London, Združeno kraljestvo) [73], ki je hiter in cenovno ugoden ter omogoča hitro in ekonomično obdelavo vzorcev. Osebke, namenjene morfološkemu določanju, je nato mogoče sprati. Osebke, obdelane s kompletom DNeasy Blood and Tissue, je treba prosojiti z Marc-Andréjevo raztopino, medtem ko so tisti, obdelani s kompletom HotSHOT, dovolj prosojeni za prepariranje v vodnem sredstvu ali, še bolje, po dehidraciji v smolnatem sredstvu, v skladu s protokolom, opisanim v tem članku [73]. Izolirani genetski material je nato mogoče uporabiti za nadaljnje analize, kot je PCR za pomnoževanje specifičnih genetskih označevalcev. Nedestruktivne metode izolacije nukleinskih kislin so ključne za preučevanje genetskih značilnosti peščenih muh, vključno z identifikacijo potencialnih povzročiteljev bolezni, ki jih lahko prenašajo. Z ohranjanjem celovitosti osebka raziskovalci pridobijo dragocene genetske podatke, hkrati pa ohranijo vzorec za dodatne analize ali študije.

## 6.5. MALDI-ToF MS

MALDI-ToF MS (masna spektrometrija z matrično podprto lasersko desorpcijo/ionizacijo s časom preleta) je tehnika, ki temelji na masni spektrometriji in je namenjena zaznavanju ter analizi značilnih beljakovinskih profilov bioloških vzorcev. MALDI-ToF je vse bolj prepoznan kot pomembno orodje za določanje členonožcev medicinskega in veterinarskega pomena. Tehnika se je izkazala za učinkovito pri določanju različnih razvojnih stopenj peščenih muh, vključno z nezrelimi oblikami ter krvnim obrokom napitih samic, uspešno pa je bila uporabljena tudi za razlikovanje med samci in samicami različnih vrst peščenih muh pri različnih pogojih shranjevanja in homogenizacije [28, 30, 73, 74]. Metoda omogoča visoko ločljivost na ravni podrodov, vrst in populacij. Raziskovalcem omogoča hitro in natančno določanje vrst, kar je bistveno za razumevanje razširjenosti peščenih muh, njihovega vedenja in njihove vloge pri prenosu bolezni. Z razlikovanjem vrst na podlagi beljakovinskih profilov ima MALDI-ToF ključno vlogo v epidemioloških raziskavah in strategijah nadzora vektorjev. Trenutno obstajata dve glavni pomanjkljivosti, ki omejujeta rutinsko uporabo te tehnike. Prva je dostopnost opreme za masno spektrometrijo, ki je finančno zahtevna in je običajno ni mogoče pridobiti izključno za namene določanja peščenih muh (ali drugih členonožcev). To omejitev je mogoče preseči z dostopom do merilnega časa na napravah, ki so danes standardno raziskovalno orodje v proteomskih laboratorijih in/ali klinični diagnostiki. Druga pomanjkljivost je omejena zastopanost referenčnih podatkov o peščenih muhah v bazah podatkov z odprtim dostopom, zaradi česar je pogosto treba vzpostaviti lastno referenčno zbirko spektralnih podatkov, ki temelji na nedvoumno določenih osebkih, idealno s kombinacijo morfološke presoje in sekveniranja ustreznega genetskega označevalca (COI, cytB ali drugega). To omejitev naj bi postopoma odpravili z vključevanjem obstoječih referenčnih podatkov o peščenih muhah v platformo MSI (Assistance Publique–Hôpitaux de Paris, Sorbonne University, Francija) ter zbirko BCCM/IHEM/Sciensano v Bruslju, Belgija (https://msi.happy-dev.fr/). Če je načrtovana uporaba MALDI-ToF profiliranja beljakovin, je treba vzorce shranjevati prednostno suho zamrznjene ali v 70 % etanolu molekularne kakovosti in jih ne izpostavljati sobnim temperaturam. Ker univerzalne smernice za pripravo vzorcev še ne obstajajo, se priporoča uporaba vodne raztopine 60 % acetonitrila/0,3 % TFA s sinapinsko kislino (30 mg/mL) za pripravo matriksa MALDI-ToF, da bodo pridobljeni spektri primerljivi z doslej objavljenimi podatki o peščenih muhah.

*Priprava vzorcev za MALDI-ToF MS* (Slika 7)

Vzorce peščenih muh, shranjene pod različnimi pogoji, najprej posušimo na zraku pri sobni temperaturi in nato prepariramo. Glava in zadek se odstranita, da se pridobijo deli telesa, ki vsebujejo ključne morfološke značilnosti za prepariranje na predmetnih stekelcih in morfološko analizo. Oprsje se lahko uporabi za MALDI-ToF, zadek pa se ohrani za izolacijo DNA. Za profiliranje beljakovin se oprsje ročno homogenizira v 1,5-mL mikrocevkah z 10 µL

**Preglednica 5:** Povprečni stroški, uporaba in prilagoditve protokolov za izolacijo genomske DNA peščenih muh.

| Protokol | Strošek | Uporaba | Prilagoditev protokola za majhne členon |
|---|---|---|---|
| Spin kolona | 2,5 – 3,55 US$ [39] | PCR, NGS | [9] |
| Fenol-kloroform | 0,24 US$ [69] | PCR, NGS | [9] |
| HotSHOT | < 0,01 US$ [69] | PCR | - |
| Salting out | 0,12 US$ [69] | PCR | - |
| Chelex | 0,02 US$ [41] | PCR | [41, 76] |

This is an Open Access article distributed under the terms of the Creative Commons Attribution License (https://creativecommons.org/licenses/by/4.0), which permits unrestricted use, distribution, and reproduction in any medium, provided the original work is properly cited.

homogenizacijske raztopine z uporabo enkratnih pestilov. Običajno se uporabljata dve homogenizacijski raztopini: sterilna destilirana voda in 25 % mravljična kislina.

## 7. Zaključek

V tem prispevku smo si prizadevali raziskovalcem predstaviti najučinkovitejše metode za prepariranje peščenih muh, prilagojene specifičnim raziskovalnim ciljem, z namenom natančne določitve vrst in odkrivanja patogenov. Ne obstaja ena sama, univerzalno optimalna metoda; na voljo je več pristopov, vsak s svojimi prednostmi in pomanjkljivostmi. V prilogah smo podali podrobne protokole za različne tehnike prepariranja, uporabljene pri pripravi in določanju peščenih muh. Ti protokoli, vključno z izobraževalnimi videoposnetki, vsebujejo postopke po korakih, prilagojene različnim ciljem, ter omogočajo natančne in zanesljive rezultate. S tem celovitim virom želimo raziskovalcem pomagati pri izbiri in uporabi najprimernejših tehnik prepariranja glede na njihove specifične potrebe.

### Zahvala

Avtorji se zahvaljujejo Richardu Lanu in Zoe Jay Adams iz Prirodoslovnega muzeja v Londonu, Združeno kraljestvo, za njun temeljit pregled, ki je prispeval k izboljšanju kakovosti tega rokopisa

### Financiranje

Zahvaljujemo se brazilskima razvojno-raziskovalnima agencijama CNPq (št. zadeve: 404395/2024-4) in Fundaciji Araucária (št. zadeve: 433/2025 PDI) za financiranje raziskave AJA.

### Nasprotje interesov

Jérôme Depaquit je pridruženi urednik revije Parasite; ni imel nobenega vpliva na recenzijski postopek ali odločanje o tem rokopisu. Ostali avtorji izjavljajo, da nimajo nasprotja interesov.

### Izjava o dostopnosti podatkov

Videi na Zenodo:

Video 1: https://zenodo.org/records/18198006
Video 2: https://zenodo.org/records/18311158
Video 3: https://zenodo.org/records/18311106
Video 4: https://zenodo.org/records/18311154
Video 5: https://zenodo.org/records/18303014
Video 6: https://zenodo.org/records/18302850
Video 7: https://zenodo.org/records/18315029

### Dodatno gradivo

Dodatno gradivo k temu članku je dostopno na https://www.parasite-journal.org/10.1051/parasite/2026009/olm

### Literatura

1. Alkan C, Allal-Ikhlef AB, Alwassouf S, Baklouti A, Piorkowski G, de Lamballerie X, Izri A, Charrel RN. 2015. Virus isolation, genetic characterization and seroprevalence of Toscana virus in Algeria. Clinical Microbiology and Infection, 21(11), 1040 e1-9.
2. Alten B, Ozbel Y, Ergunay K, Kasap OE, Cull B, Antoniou M, Velo E, Prudhomme J, Molina R, Banuls AL, Schaffner F, Hendrickx G, Van Bortel W, Medlock JM. 2015. Sampling strategies for phlebotomine sand flies (Diptera: Psychodidae) in Europe. Bulletin of Entomological Research 105(6), 664–678.
3. Ayhan N, Baklouti A, Prudhomme J, Walder G, Amaro F, Alten B, Moutailler S, Ergunay K, Charrel RN, Huemer H. 2017. Practical guidelines for studies on sandfly-borne phleboviruses: Part I: Important points to consider *ante* field work. Vector-Borne and Zoonotic Diseases 17(1), 73–80.
4. Bates PA. 1997. Infection of phlebotomine sandflies with *Leishmania*, in The Molecular Biology of Insect Disease Vectors: A Methods Manual. Springer. p. 112–120.5.
5. Baum M, de Castro EA, Pinto MC, Goulart TM, Baura W, Klisiowicz Ddo R, Vieira da Costa-Ribeiro MC. 2015. Molecular detection of the blood meal source of sand flies (Diptera: Psychodidae) in a transmission area of American cutaneous leishmaniasis, Parana State, Brazil. Acta Tropica, 143, 8–12.
6. Belen A, Alten B, Aytekin A. 2004. Altitudinal variation in morphometric and molecular characteristics of *Phlebotomus papatasi* populations. Medical and Veterinary Entomology, 18(4), 343–350.
7. Bhattacharya J, Chandra G, Hati AK. 1991. A simple method for cryopreservation of *Leishmania donovani* promastigotes, Indian Journal of Medical Research, 93, 245–246.
8. Caligiuri LG, Sandoval AE, Miranda JC, Pessoa FA, Santini MS, Salomón OD, Secundino NF, McCarthy CB. 2019. Optimization of DNA extraction from individual sand flies for PCR amplification. Methods and Protocols, 2(2), 36.

9. Casaril AE, de Oliveira LP, Alonso DP, de Oliveira EF, Gomes Barrios SP, de Oliveira Moura Infran J, Fernandes WS, Oshiro ET, Ferreira AMT, Ribolla PEM, de Oliveira AG. 2017. Standardization of DNA extraction from sand flies: Application to genotyping by next generation sequencing. Experimental Parasitology, 177, 66–72.
10. Castalanelli MA, Severtson DL, Brumley CJ, Szito A, Foottit RG, Grimm M, Munyard K, Groth DM. 2010. A rapid non-destructive DNA extraction method for insects and other arthropods. Journal of Asia-Pacific Entomology, 13(3), 243–248.
11. Cerqueira NL. 1943. Um novo meio para montagem de pequenos insetos em lâmina. Memórias do Instituto Oswaldo Cruz, (39), 37–41.
12. Charrel RN, Gallian P, Navarro-Mari JM, Nicoletti L, Papa A, Sanchez-Seco MP, Tenorio A, de Lamballerie X. 2005. Emergence of Toscana virus in Europe. Emerging Infectious Diseases, 11(11), 1657–1663.
13. Chaskopoulou A, Giantsis IA, Demir S, Bon MC. 2016. Species composition, activity patterns and blood meal analysis of sand fly populations (Diptera: Psychodidae) in the metropolitan region of Thessaloniki, an endemic focus of canine leishmaniasis. Acta Tropica, 158, 170–176.
14. Chen H, Rangasamy M, Tan SY, Wang H, Siegfried BD. 2010. Evaluation of five methods for total DNA extraction from western corn rootworm beetles. PLoS One, 5(8), e11963.
15. Depaquit J, Grandadam M, Fouque F, Andry PE, Peyrefitte C. 2010. Arthropod-borne viruses transmitted by Phlebotomine sandflies in Europe: a review. Eurosurveillance, 15(10), 19507.
16. Diamond LS, Herman CM. 1954. Incidence of Trypanosomes in the Canada Goose as revealed by bone marrow culture. Journal of Parasitology, 40(2), 195–202.
17. Ding H, Torno M, Vongphayloth K, Ng G, Tan D, Sng W, Ho K, Randrianambinintsoa FJ, Depaquit J, Tan CH. 2025. Hidden in plain sight: discovery of sand flies in Singapore and description of four species new to science. Parasites & Vectors, 18(1), 402.
18. Es-Sette N, Ajaoud M, Bichaud L, Hamdi S, Mellouki F, Charrel RN, Lemrani M. 2014. *Phlebotomus sergenti* a common vector of *Leishmania tropica* and Toscana virus in Morocco. Journal of Vector Borne Diseases, 51(2), 86–90.
19. Favret C. 2005. A new non-destructive DNA extraction and specimen clearing technique for aphids (Hemiptera). Proceedings of the Entomological Society of Washington, 107(2), 469–470.
20. Galati EAB. 2018. Phlebotominae (Diptera, Psychodidae): Classification, morphology and terminology of adults and identification of American taxa, in Brazilian Sand Flies: Biology, Taxonomy, Medical Importance and Control, Rangel EF, Shaw JJ, Editors. Cham: Springer International Publishing. pp. 9–212.
21. Galati EAB, de Andrade AJ, Perveen F, Loyer M, Vongphayloth K, Randrianambinintsoa FJ, Prudhomme J, Rahola N, Akhoundi M, Shimabukuro PHF, Depaquit J. 2025. Phle-botomine sand flies (Diptera, Psychodidae) of the world. Parasites & Vectors, 18(1), 220.
22. Galati EAB, Galvis-Ovallos F, Lawyer P, Leger N, Depaquit J. 2017. An illustrated guide for characters and terminology used in descriptions of Phlebotominae (Diptera, Psychodidae). Par-asite, 24, 26.
23. Gariepy T, Kuhlmann U, Gillott C, Erlandson M. 2007. Parasitoids, predators and PCR: the use of diagnostic molecular markers in biological control of Arthropods. Journal of Applied Entomology, 131(4), 225–240.
24. Giantsis IA, Chaskopoulou A, Bon MC. 2016. Mild-Vectolysis: A nondestructive DNA extraction method for vouchering sand flies and mosquitoes. Journal of Medical Entomology, 53(3), 692–695.
25. Gidwani K, Picado A, Rijal S, Singh SP, Roy L, Volfova V, Andersen EW, Uranw S, Ostyn B, Sudarshan M, Chakravarty J, Volf P, Sundar S, Boelaert M, Rogers ME. 2011. Serological markers of sand fly exposure to evaluate insecticidal nets against visceral leishmaniasis in India and Nepal: a cluster-randomized trial. PLoS Neglected Tropical Diseases, 5(9), e1296.
26. Gilbert MTP, Moore W, Melchior L, Worobey M. 2007. DNA extraction from dry museum beetles without conferring external morphological damage. PLoS One, 2(3), e272.
27. Giordani BF, Andrade AJ, Galati EAB, Gurgel-Goncalves R. 2017. The role of wing geometric morphometrics in the identification of sandflies within the subgenus *Lutzomyia.* Medical and Veterinary Entomology, 31(4), 373–380.
28. Guzmán-Larralde AJ, Suaste-Dzul AP, Gallou A, Peña-Carrillo KI. 2017. DNA recovery from microhymenoptera using six non-destructive methodologies with considerations for subsequent preparation of museum slides. Genome, 60(1), 85–91.
29. Hajibabaei M, DeWaard JR, Ivanova NV, Ratnasingham S, Dooh RT, Kirk SL, Mackie PM, Hebert PD. 2005. Critical factors for assembling a high volume of DNA barcodes. Philosophical Transactions of the Royal Society B: Biological Sciences, 360(1462), 1959–1967.
30. Haouas N, Pesson B, Boudabous R, Dedet JP, Babba H, Ravel C. 2007. Development of a molecular tool for the identification of *Leishmania* reservoir hosts by blood meal analysis in the insect vectors. American Journal of Tropical Medicine and Hygiene, 77(6), 1054–1059.
31. Hlavackova K, Dvorak V, Chaskopoulou A, Volf P, Halada P. 2019. A novel MALDI-TOF MS-based method for blood meal identification in insect vectors: A proof of concept study on phlebotomine sand flies. PLoS Neglected Tropical Diseases, 13 (9), e0007669.
32. Huemer H, Prudhomme J, Amaro F, Baklouti A, Walder G, Alten B, Moutailler S, Ergunay K, Charrel RN, Ayhan N. 2017. Practical guidelines for studies on sandfly-borne phleboviruses: Part II: Important points to consider for fieldwork and subsequent virological screening. Vector-Borne and Zoonotic Diseases, 17(1), 81–90.
33. Jancarova M, Polanska N, Thiesson A, Arnaud F, Stejskalova M, Rehbergerova M, Kohl A, Viginier B, Volf P, Ratinier M. 2025. Susceptibility of diverse sand fly species to Toscana virus. PLoS Neglected Tropical Diseases, 19(5), e0013031.
34. Kapp JD, Green RE, Shapiro B. 2021. A fast and efficient single-stranded genomic library preparation method optimized for ancient DNA. Journal of Heredity, 112(3), 241–249.
35. Killick-Kendrick R, Maroli M, Killick-Kendrick M. 1991. Bibliography of the colonization of phlebotomine sandflies. Parassitologia, 33(suppl.), 321–333.
36. Lawyer P, Killick-Kendrick M, Rowland T, Rowton E, Volf P. 2017. Laboratory colonization and mass rearing of phlebotomine sand flies (Diptera, Psychodidae). Parasite, 24, 42.
37. Léger N, Pesson B, Madulo-Leblond G. 1986. Les phlébotomes de Grèce : 1ère partie. Bulletin de la Société de Pathologie Exotique, 79, 386–397.
38. Léger N, Pesson B, Madulo-Leblond G. 1986. Les phlébotomes de Grèce : 2ème partie. Bulletin de la Société de Pathologie Exotique, 79, 514–524.
39. Leonel JAF, Vioti G, Alves ML, da Silva DT, Meneghesso PA, Benassi JC, Spada JCP, Galvis-Ovallos F, Soares RM, Oliveira T.

2020. DNA extraction from individual Phlebotomine sand flies (Diptera: Psychodidae: Phlebotominae) specimens: Which is the method with better results? Experimental Parasitology, 218, 107981.
40. Lestinova T, Rohousova I, Sima M, de Oliveira CI, Volf P. 2017. Insights into the sand fly saliva: Blood-feeding and immune interactions between sand flies, hosts, and *Leishmania*. PLoS Neglected Tropical Diseases, 11(7), e0005600.
41. Lienhard A, Schaffer S. 2019. Extracting the invisible: obtaining high quality DNA is a challenging task in small arthropods. PeerJ, 7, e6753.
42. Lozano-Sardaneta YN, Mikery-Pacheco OF, Huerta H, Rojas-Soriano JE, Contreras-Ramos A. 2025. Wing geometric morphometrics is effective to separate sand fly species (Diptera, Psychodidae, Phlebotominae) related with leishmaniasis transmission in Mexico. Acta Tropica, 262, 107523.
43. Mandrioli M. 2008. Insect collections and DNA analyses: how to manage collections? Museum Management and Curatorship, 23(2), 193–199.
44. Maroli M, Feliciangeli MD, Bichaud L, Charrel RN, Gradoni L. 2013. Phlebotomine sandflies and the spreading of leishmaniases and other diseases of public health concern. Medical and Veterinary Entomology, 27(2), 123–147.
45. Marquina D, Buczek M, Ronquist F, Lukasik P. 2021. The effect of ethanol concentration on the morphological and molecular preservation of insects for biodiversity studies. PeerJ, 9, e10799.
46. Mathis A, Depaquit J, Dvorak V, Tuten H, Banuls AL, Halada P, Zapata S, Lehrter V, Hlavackova K, Prudhomme J, Volf P, Sereno D, Kaufmann C, Pfluger V, Schaffner F. 2015. Identification of phlebotomine sand flies using one MALDI-TOF MS reference database and two mass pectrometer systems., Parasites & Vectors, 8, 266.
47. Mekarnia N, Benallal KE, Sadlova J, Vojtkova B, Mauras A, Imbert N, Longhitano M, Harrat Z, Volf P, Loiseau PM, Cojean S. 2024. Effect of *Phlebotomus papatasi* on the fitness, infectivity and antimony-resistance phenotype of antimony-resistant *Leishmania* major Mon-25. International Journal for Parasitology – Drugs and Drug Resistance, 25, 100554.
48. Milligan BG. 1998. Total DNA isolation, in Molecular Genetic Analysis of Population: A Practical Approach, Hoelzel AR, Editor. Oxford: Oxford University Press.
49. Molina R, Jiménez M, Alvar J, González E, Hernández-Taberna S, Ines MM. 2017. Methods in sand fly research. Madrid: Servicio de publicaciones Universidad de Alcalá de Henares, Madrid.
50. Murphy WJ, Eizirik E, O'Brien SJ, Madsen O, Scally M, Douady CJ, Teeling E, Ryder OA, Stanhope MJ, de Jong WW, Springer MS. 2001. Resolution of the early placental mammal radiation using Bayesian phylogenetics. Science, 294(5550), 2348–2351.
51. Nacif-Pimenta R, Pinto LC, Volfova V, Volf P, Pimenta PFP, Secundino NFC. 2020. Conserved and distinct morphological aspects of the salivary glands of sand fly vectors of leishma-niasis: an anatomical and ultrastructural study. Parasites & Vectors, 13(1), 441.
52. Neuhaus B, Schmid T, Riedel J. 2017. Collection management and study of microscope slides: Storage, profiling, deterioration, restoration procedures, and general recommendations. Zootaxa, 4322(1), 1–173.
53. New TR. 1974. Pscoptera. Handbooks for Identification of British Insects (Vol. I). London: Royal Entomological Society of London. 102 pp.
54. Perez-Ruiz M, Collao X, Navarro-Mari JM, Tenorio A. 2007. Reverse transcription, real-time PCR assay for detection of Toscana virus. Journal of Clinical Virology, 39(4), 276–281.
55. Porco D, Rougerie R, Deharveng L, Hebert P. 2010. Coupling non-destructive DNA extraction and voucher retrieval for small soft-bodied Arthropods in a high-throughput context: the example of Collembola. Molecular Ecology Resources, 10(6), 942–945.
56. Prudhomme J, Cassan C, Hide M, Toty C, Rahola N, Vergnes B, Dujardin JP, Alten B, Sereno D, Banuls AL. 2016. Ecology and morphological variations in wings of *Phlebotomus ariasi* (Diptera: Psychodidae) in the region of Roquedur (Gard, France): a geometric morphometrics approach. Parasites & Vectors, 9(1), 578.
57. Prudhomme J, Gunay F, Rahola N, Ouanaimi F, Guernaoui S, Boumezzough A, Banuls AL, Sereno D, Alten B. 2012. Wing size and shape variation of *Phlebotomus papatasi* (Diptera: Psychodidae) populations from the south and north slopes of the Atlas Mountains in Morocco. Journal of Vector Ecology, 37(1), 137–147.
58. Prudhomme J, Toty C, Kasap OE, Rahola N, Vergnes B, Maia C, Campino L, Antoniou M, Jimenez M, Molina R, Cannet A, Alten B, Sereno D, Banuls AL. 2015. New microsatellite markers for multi-scale genetic studies on *Phlebotomus ariasi* Tonnoir, vector of *Leishmania infantum* in the Mediterranean area. Acta Tropica, 142, 79–85.
59. Prudhomme J, Velo E, Bino S, Kadriaj P, Mersini K, Gunay F, Alten B. 2019. Altitudinal variations in wing morphology of *Aedes albopictus* (Diptera, Culicidae) in Albania, the region where it was first recorded in Europe. Parasite, 26, 55.
60. Rawlins DJ. 1992. Light Microscopy: An Introduction to Biotechniques. Oxford: Bios Scientific publishers. 143 pp.
61. Ready PD. 2013. Biology of phlebotomine sand flies as vectors of disease agents. Annual Review of Entomology, 58, 227–250.
62. Rohlf FJ, Slice D. 1990. Extensions of the Procrustes method for the optimal superimposition of landmarks. Systematic Zoology, 39(1), 40–59.
63. Rowley DL, Coddington JA, Gates MW, Norrbom AL, Ochoa RA, Vandenberg NJ, Greenstone MH. 2007. Vouchering DNA-barcoded specimens: Test of a nondestructive extraction proto-col for terrestrial arthropods. Molecular Ecology Notes, 7(6), 915–924.
64. Sábio PB, Andrade AJ, Galati EAB. 2014. Assessment of the taxonomic status of some species included in the *Shannoni* complex, with the description of a new species of *Psathyromyia* (Diptera: Psychodidae: Phlebotominae). Journal of Medical Entomology, 51(2), 331–341.
65. Sadlova J, Yeo M, Seblova V, Lewis MD, Mauricio I, Volf P, Miles MA. 2011. Visualisation of *Leishmania donovani* fluo-rescent hybrids during early stage development in the sand fly vector. PLoS One, 6(5), e19851.
66. Sales K, Miranda DEO, da Silva FJ, Otranto D, Figueredo LA, Dantas-Torres F. 2020. Evaluation of different storage times and preservation methods on phlebotomine sand fly DNA concen-tration and purity. Parasites & Vectors, 13(1), 399
67. Sales KG, Costa PL, de Morais RC, Otranto D, Brandao-Filho SP, Cavalcanti Mde P, Dantas-Torres F. 2015. Identification of phlebotomine sand fly blood meals by real-time PCR. Parasites& Vectors, 8, 230.
68. Sant'Anna MR, Jones NG, Hindley JA, Mendes-Sousa AF, Dillon RJ, Cavalcante RR, Alexander B, Bates PA. 2008. Blood meal identification and parasite detection in laboratory-fed and

field-captured *Lutzomyia longipalpis* by PCR using FTA databasing paper. Acta Tropica, 107(3), 230–237.
69. Senne NA, Santos HA, Araujo TR, Paulino PG, Mendonca LP, Moreira HVS, Camilo TA, da Costa Angelo I. 2022. Robust comparative performance of genomic DNA extraction methods from non-engorged phlebotomine sandflies. Medical and Veterinary Entomology, 36(2), 203–211.
70. Shaw JJ. 2025. A review of Leishmania infections in American Phlebotomine sand flies – Are those that transmit leishmaniasis anthropophilic or anthropportunists? Parasite, 32, 57.
71. Tesh RB, Modi GB. 1983. Growth and transovarial transmission of Chandipura virus (Rhabdoviridae: Vesiculovirus) in *Phlebotomus papatasi.* American Journal of Tropical Medicine and Hygiene, 32(3), 621–623.
72. Thomsen PF, Elias S, Gilbert MTP, Haile J, Munch K, Kuzmina S, Froese DG, Sher A, Holdaway RN, Willerslev E. 2009. Non-destructive sampling of ancient insect DNA. PLoS One, 4(4),e5048
73. Truett GE, Heeger P, Mynatt RL, Truett AA, Walker JA, Warman ML. 2000. Preparation of PCR-quality mouse genomic DNA with hot sodium hydroxide and tris (HotSHOT). Biotechniques, 29(1), 52–54.
74. Upton MS. 1993. Aqueous gum-chloral slide mounting media: an historical review. Bulletin of Entomological Research, 83(2), 267–274.
75. Volf P, Myskova J. 2007. Sand flies and Leishmania: specific versus permissive vectors. Trends in Parasitology, 23(3), 91–92.
76. Wang Q, Wang X. 2012. Comparison of methods for DNA extraction from a single chironomid for PCR analysis. Pakistan Journal of Zoology, 44(2), 421–426.
77. Wang Y, Zhao Y, Bollas A, Wang Y, Au KF. 2021. Nanopore sequencing technology, bioinformatics and applications. Nature Biotechnology, 39(11), 1348–1365.

**Cite this article as:** Randrianambinintsoa FJ, Augendre L, Prudhomme J, Martinet J-P, Loyer M, Mekarnia N, Kerkoub H, Perveen FK, Huguenin A, Kariya E, Akhoundi M, De Andrade AJ, Berriatua E, Bongiorno G, Boyer S, Christodoulou V, Da Costa-Ribeiro MCV, De Souza LAF, Ding H, Dondji B, Dvořák V, Erisoz Kasap O, Galati EAB, Gállego M, Ballart C, Gouzelou S, Haddad N, Masse RS, Mekuria AH, Ivovic V, Kaczmarek S, Shahar MK, Kirstein OD, Kniha E, Kolářová I, Lincoln T, Lucanas C, Mikov O, Nov K, Özbel Y, Pesson B, Posada Lopez LC, Prasetyo DB, Rahola N, Rebollar-Tellez EA, Rodrigues BL, Roy L, Saini P, Sanjoba C, Shimabukuro PH, Siriyasatien P, Soszynska A, Suleşco T, Sylla M, Torno M, Volf P, Vongphayloth K, Sinh Nam V, Wardhana A, Yessinou E, Zapata S, Gantier J-C & Depaquit J. 2026. Processing and mounting phlebotomine sand flies: a consensus guideline. Parasite xx, xx. https://doi.org/10.1051/parasite/2026009.

PARASITE

An international open-access, peer-reviewed, online journal publishing high quality papers on all aspects of human and animal parasitology

Reviews, articles and short notes may be submitted. Fields include, but are not limited to: general, medical and veterinary parasitology; morphology, including ultrastructure; parasite systematics, including entomology, acarology, helminthology and protistology, and molecular analyses; molecular biology and biochemistry; immunology of parasitic diseases; host-parasite relationships; ecology and life history of parasites; epidemiology; therapeutics; new diagnostic tools.
All papers in Parasite are published in English. Manuscripts should have a broad interest and must not have been published or submitted elsewhere. No limit is imposed on the length of manuscripts.

**Parasite** (open-access) continues **Parasite** (print and online editions, 1994-2012) and **Annales de Parasitologie Humaine et Comparée** (1923-1993) and is the official journal of the Société Française de Parasitologie.

Editor-in-Chief:
Jean-Lou Justine, Paris

Submit your manuscript at:
[illegible]

## Priloga 1: Teoretične biokemijske osnove.

Obravnavani členonožci so peščene muhe. Vendar je splošno načelo mogoče razširiti tudi na druge, zelo pogoste členonožce, pri katerih je določanje mogoče le na podlagi notranjih morfoloških znakov. Nekateri notranji organi so delno hitinizirani, njihova morfologija pa zagotavlja pomembne diagnostične informacije. Zato je posebej pomembno opazovanje sesalnih črpalk, spermatek in njihovih vodov. Pri vseh reagentih, ki jih obravnavamo, je treba upoštevati, da od faze fiksacije žuželke do končne sestave preparata potekajo predvsem oksidacijsko-redukcijske reakcije. Osnovno načelo, ki ga je treba upoštevati, je izogibanje mešanju redukcijskih reagentov z oksidacijskimi reagenti.

**Etilni alkohol; etanol:**

Etanol se uporablja na različne načine. Molekule alkohola imajo veliko afiniteto do vode, zato delujejo dehidracijsko. Alkohol z nizko koncentracijo (tj. z visoko vsebnostjo vode) pa lahko prispeva k razgradnji nukleinskih kislin, saj je voda dejavnik njihove degradacije. Ko žuželke potopimo v etanol, to ni namenjeno zgolj njihovemu shranjevanju, temveč tudi fiksaciji tkiv. V histologiji ločimo dva pomembna pojma: hitrost penetracije in hitrost fiksacije. Učinkovit fiksativ mora najprej hitro prodreti globoko v tkiva, nato pa jih fiksirati. Pri 96 % alkoholu je koeficient penetracije približno 1,05 (za primerjavo: pri 0,75 % vodni raztopini pikrinske kisline je 0,45, pri 3 % raztopini kalijevega dikromata pa 1,45). Pri entomologih je dolgotrajno shranjevanje žuželk in drugih členonožcev v etanolu običajna praksa. Namen je ohraniti terenske vzorce za nadaljnje raziskave ali prihodnje generacije raziskovalcev. Vendar s citološkega ali histološkega vidika tak pristop ni ustrezen. Predolgo hranjenje vzorcev v fiksativu lahko povzroči, da jih je pozneje skoraj nemogoče ponovno obdelati. Zato so vzorci, starejši od 10 let, pogosto težko ali celo neuporabni za nadaljnje analize. Pomembno je tudi razmerje med maso členonožca in volumnom fiksativa. V zoološki ali medicinski praksi se priporoča uporaba volumna fiksativa, ki je približno 60-krat večji od volumna fiksiranega materiala. Pri mikročlenonožcih je v praksi priporočljivo, da se za določen volumen osebkov doda vsaj 4–5-krat večji volumen alkohola. Upoštevati je treba, da alkohol postopoma izgublja koncentracijo, saj absorbira vodo iz tkiv členonožcev.

Zaključek:

- Etilni alkohol je redukcijsko delujoča kemijska snov (zato ni združljiv z oksidacijskimi fiksativi);
- Povzroča hitro precipitacijo in denaturacijo beljakovin;
- Raztaplja nekatere kompleksne lipide in precipitira glikogen;
- Povzroča izrazito kontrakcijo in utrjevanje tkiv.

**Bazične raztopine kalijevega ali natrijevega hidroksida:**

V entomologiji se je uporaba teh raztopin tradicionalno osredotočala predvsem na kalijev hidroksid, brez jasno utemeljenega razloga.

Natrijev hidroksid [E524] se uporablja v raztopini, pri različnih koncentracijah ali normalnostih. Na voljo je v obliki peletov ali kosmičev. Njegova glavna pomanjkljivost je izrazita higroskopnost (večja kot pri KOH). Ob reakciji z beljakovinami jih raztaplja, z lipidi pa med procesom saponifikacije tvori trda mila (kar predstavlja pomembno razliko v primerjavi s KOH, ki pri saponifikaciji tvori tekoča mila).

Kalijev hidroksid [E525] je na voljo kot koncentrirana raztopina, predvsem pa ima prednost, da se dobavlja v obliki peletov mase približno 0,1 g, kar močno olajša pripravo razredčenih raztopin tudi brez analitske tehtnice. Na primer, en pelet mase 0,1 g, raztopljen v 1 mL destilirane vode, daje 10 % raztopino. Druga prednost kalijevega hidroksida v obliki peletov je manjša občutljivost na karbonatizacijo (raztopina KOH ima veliko afiniteto do vezave $CO_2$ in s tem tvori karbonatne soli).

Ti močni bazi se uporabljata za raztapljanje maščobnih kislin s pretvorbo v vodotopna mila. Upoštevati je treba, da je fiksativ, kot je etanol, že raztopil del maščob v vzorcu. Ko pa vzorec prenesemo v vodno okolje z močno bazo, se maščobne kisline (bolj ali manj kompleksne) lahko precipitira-jo. Močna baza tako omogoča hladno saponifikacijo. V primerih, ko je maščobno tkivo prisotno v večji količini, na primer pri samicah, je lahko koristno zvišati temperaturo na 35–40 °C za pospešitev reakcije ali podaljšati čas delovanja pri sobni temperaturi.

**Obarvana kisla raztopina / brezbarvna Marc-Andréjeva raztopina:**

V tem prispevku obravnavamo prednosti in pomanjkljivosti uporabe Marc-Andréjeve raztopine. Raztopina je

This is an Open Access article distributed under the terms of the Creative Commons Attribution License (https://creativecommons.org/licenses/by/4.0), which permits unrestricted use, distribution, and reproduction in any medium, provided the original work is properly cited.

sestavljena iz kloral hidrata (trikloroacetaldehid monohidrat), ocetne kisline in vode. Gre za izrazito oksidacijsko raztopino (mešanica kisline in aldehida). Nevtralizira presežek kalijevega hidroksida, ki lahko ostane v vzorcih, ne da bi pri tem precipitirala alkalna mila, nastala med uporabo kalijevega hidroksida. Oksidacijska raztopina deluje tudi na sekundarne alkoholne skupine glukozaminov, ki sestavljajo hitin, saj jih oksidira in s tem zmehča hitin. Dodatno lahko raztaplja nekatere prisotne mineralne soli. Če je Marc-Andréjeva raztopina predhodno obarvana z kislim fuksinom (v oksidiranem stanju), se lahko veže na sekundarne alkoholne skupine struktur. Po določenem času delovanja Marc-Andréjeve raztopine in doseženi stopnji obarvanosti vzorcev se izpiranje izvede izključno z etanolom. S tem se začne faza dehidracije vzorcev.

**Prednosti:**

- Nevtralizacija presežka bazičnih raztopin;
- Mehčanje hitina;
- Obarvanje hitina za boljšo vidljivost hitiniziranih notranjih struktur.
-

**Pomanjkljivosti:**

Kloral hidrat ima hipnotični učinek in se je uporabljal v humani medicini. Uporabljati ga je treba pod digestorijem ter v skladu z veljavno zakonodajo o kemijskih tveganjih.

**Raztopine za dehidracijo**:

Izkušnje kažejo, da pri zelo majhnih vzorcih ni nujno slediti zaporedju alkoholnih kopeli z naraščajočo koncentracijo. Pri večjih vzorcih se začne z 80 % etanolom, nato nadaljuje z 90 %, 95 % in na koncu z absolutnim etanolom. Pri zelo majhnih vzorcih zadostuje kopel v 90 % etanolu, ki ji sledi potopitev v absolutni etanol. Na tej stopnji je treba upoštevati, da absolutni etanol veže vlago iz zraka.

V entomoloških laboratorijih je bila tradicionalna praksa, da se dehidracija zaključi s kopeljo v bukovem kreozotu. Danes je ta snov, ki se je uporabljala kot pesticid, fungicid in sredstvo za zaščito lesa, zaradi vonja (policiklični aromatski ogljikovodiki) in domnevnih reprotoksičnih ter kancerogenih učinkov močno odsvetovana. Poleg tega velja za obstojno organsko onesnaževalo in je ekotoksična za vodne organizme.

Kot primerno raztopino za nadaljnje prepariranje vzorcev predlagamo Euparal® in Euparal® esenco (opisano v naslednjem odstavku). Mešanica Euparala® in Euparal® esence je dobro združljiva z vzorci, pridobljenimi po kopeli v 90 % etanolu.

## Priloga 2: Sestava uporabljenih reagentov

**Kalijev hidroksid 10 %**
Kalijev hidroksid 10 g
Destilirana voda do 100 mL (q.s. ad 100 mL)

**Kloralno-gumijasto sredstvo za prepariranje (Hoyerjevo sredstvo)**
Destilirana voda 50 mL
Kloral hidrat 200 g
Gumiarabika 50 g
Glicerol 20 mL

**Marc-Andréjeva raztopina**
Kloral hidrat 40 g
Ledena ocetna kislina 30 mL
Destilirana voda 30 mL

**Kisli fuksin 1 % v destilirani vodi**
Kisli fuksin (prah) 1 g
Destilirana voda 99 mL

**Marc-Andréjeva raztopina, obarvana s fuksinom**
Marc-Andréjeva raztopina 10 mL
Fuksin 1 % 50 µL

## Priloga 3: Euparal®, kanadski balzam, polivinil alkohol in druge raztopine za prepariranje

**Polivinil alkohol**: To je primerno sredstvo za prepariranje, kadar niso na voljo reagenti za ustrezno dehidracijo. Polivinil alkohol se meša z Ammanovim laktofenolom. Tako pripravljeni preparati imajo pomembne pomanjkljivosti: lahko se izsušijo ali pa polivinil alkohol kristalizira zaradi izhlapevanja vode oziroma potemni zaradi oksidacije fenola. Metoda je primerna predvsem za kratkotrajno prepariranje.

**Kanadski balsam**: Uporaba za prepariranje med predmetnim in krovnim stekelcem zahteva predhodno dehidracijo vzorcev. Uporaba ksilena ali toluena ni brez pomanjkljivosti.

**Sredstvo Enecê**: Tako kot pri kanadskem balzamu je za prepariranje med predmetnim in krovnim stekelcem potrebna dehidracija osebka. Sestava Enecê: čista bela kolofonija (22 g), v alkoholu topna kopalna smola (12 g), absolutni alkohol (20 mL), kamfor (10 g), terpentinska esenca (10 mL) in evkaliptol (26 mL). Priprava: V posodo, na primer erlenmajerico, dodamo absolutni alkohol in kamfor, nato kolofonijo in kopalno smolo. Posodo zapremo, pretresemo in nato segrevamo v vodni kopeli pri zmerni temperaturi, tako da mešanica ne zavre. Ko se vsebina popolnoma utekočini, dodamo terpentinsko esenco, mešanico še vročo filtriramo in filtratu dodamo evkaliptol. Ko sredstvo postane manj tekoče, ga razredčimo z raztopino Enecê naslednje sestave: absolutni alkohol (30 mL), kamfor (17 g), terpentinska esenca (15 mL) in evkaliptol (38 mL) (Cerqueira, 1943).

**Euparal®**: Gre za smolo, pridobljeno iz atlaskega cipresa Tetraclinis articulata (Vahl, 1791), ki jo je leta 1906 proučeval in razvil Gilson. Njena glavna prednost je, da ne polimerizira. Osebke, preparirane med predmetnim in krovnim stekelcem, je mogoče razmeroma enostavno ponovno pridobiti z delovanjem alkohola ali še bolje z Euparal® esenco. Ta smola, imenovana tudi sandarak, je združljiva z etanolom koncentracije od 80 % dalje.

**Uporaba Triton X100: neionska vodna raztopina**. Triton X100 je neionska vodna raztopina (4-(1,1,3,3-tetrametilbutil) fenil-polietilen glikol oziroma t-oktilfenoksipolietoksietanol, polietilen glikol tert-oktilfenil eter), ki se pogosto uporablja kot detergent v celični in molekularni biologiji. Omogoča permeabilizacijo celičnih in jedrnih membran. Vzorci žuželk, shranjeni v alkoholu več let, so pogosti. Dolgotrajno shranjevanje v alkoholu ni optimalno, zato tako ohranjeni členonožci postanejo težko pripravljivi za mikroskopski pregled. Plastika, ki vsebuje vzorce, se lahko razgradi, čemur sledi izhlapevanje alkohola. V obeh primerih dolgotrajen stik z alkoholom ali izsušitev vzorcev predstavljata resen problem. Leta 2008 je Jonque objavil opombo o rehidraciji pajkov z uporabo omočevalnega sredstva, kot je Agepon, ki se uporablja pri fotografskih filmih [26]. To je spodbudilo uporabo vlažilnih sredstev, ki niso močni detergenti.

Postopek z uporabo 0,5 % vodne raztopine Triton X100:

- Suh vzorec impregniramo z absolutnim alkoholom.
- Dodati ustrezen volumen 0,5 % raztopine Triton X100, tako da je celoten vzorec potopljen.
- Pustiti, da deluje približno 5 minut ali dlje, pri čemer morajo biti vsi členonožci v raztopini popolnoma ločeni.
- Raztopino Triton X100 odstraniti in jo nadomestiti z raztopino kalijevega hidroksida.

Nadaljnji postopek poteka, kot je opisano zgoraj.

## Priloga 4: Sredstvi za prepariranje Euparal® ali kanadski balzam – postopek po korakih

1. Osebki morajo biti dehidrirani (motna ali mlečna pojavnost kaže na neustrezno dehidracijo).
2. Dehidracija se izvede s postopnim povečevanjem koncentracije etilnega alkohola.
3. Osebke je mogoče prenesti iz 99 % ali absolutnega alkohola v sredstvo za prosojenje.

Postopek:

1. Odrasle peščene muhe prepariramo v 70 % etanolu.
2. Odstranimo etanol in ga nadomestimo z 10 % KOH. Peščene muhe prekrijemo s predmetnim stekelcem.
3. Maceriramo, dokler žuželke ne postanejo prosojne.
4. Odstranimo KOH.
5. Osebek prekrijemo z destilirano vodo in počakamo 30 do 45 minut.
6. Odstranimo vodo in ponovimo izpiranje z destilirano vodo 30 minut (čas je odvisen od števila vzorcev: več kot jih obdelujemo hkrati, daljši mora biti ta čas; pri manjšem številu, zlasti pri individualni obdelavi, je lahko krajši). 7. Odstranimo vodo.
8. Dodamo Marc-Andréjevo raztopino (po potrebi obarvano z acidnim fuksinom) in počakamo 24 ur (en dan).
9. Odstranimo Marc-Andréjevo raztopino.
10. Osebek prekrijemo z destilirano vodo in počakamo 30 do 45 minut.
11. Odstranimo vodo in ponovimo izpiranje z destilirano vodo 30 minut.
12. Odstranimo vodo.
13. Dodamo 70 % etanol in sekcioniramo osebek.
a. Pri glavi in zadku nežno ločimo glavo ali zadek od oprsja.
b. Pri oprsju odstranimo krila tako, da oprsje primemo z eno pinceto, z drugo pa povlečemo na bazi okončin. Možno je izvesti sagitalno sekcijo in oprsje razdeliti na levo in desno polovico, glede na področja največjega interesa.
14. Osebke postopno dehidriramo skozi serijo vodnih raztopin etilnega alkohola 50–80–95 % do absolutnega etanola.
15. Osebke dehidriramo z dvema izpiranjem v 100 % etanolu, po 10 minut vsako.

16. Odstranimo etanol in osebke prekrijemo s klinčkovim oljem za 15 minut pri sobni temperaturi.
17. Osebke prenesemo iz klinčkovega olja v kapljico Euparala® ali kanadskega balzama na čistem predmetnem stekelcu.
18. Razporedimo po potrebi: glavo, oprsje in zadek peščene muhe lahko prepariramo z drobnimi iglami ali pincetami pod stereomikroskopom. Glavo je treba ločiti od telesa, da jo lahko prepariramo v ventro-dorzalnem položaju, to pomeni, da mora biti zatilna odprtina usmerjena navzgor, tako da je cibarium mogoče neposredno opazovati skozi njo. Sekcija poteka v sredstvu za prepariranje peščenih muh.
19. Osebek pustimo, dokler površina ne postane lepljiva.
20. Čisto krovno stekelce navlažimo z absolutnim alkoholom. Krovno stekelce pod kotom spustimo na kanadski balzam.
21. Preparat shranimo v suho škatlo, namenjeno za ta namen.
