# Supplementary material for: Processing and mounting phlebotomine sand flies: a consensus guideline
Source: Parasite. 2026 Apr 3;33:18. doi: 10.1051/parasite/2026009 (PMC13047900; doi:10.1051/parasite/2026009)
Supplement: Supplementary file 28 — Spanish translation / Traducción al español [file parasite-33-18-s28.pdf]

Parasite **33**, 18 (2026)
© F.J. Randrianambinintsoa et al., published by EDP Sciences, 2026
https://doi.org/10.1051/parasite/2026009

PARASITE
Available online at:
www.parasite-journal.org

REVIEW ARTICLE OPEN ACCESS

Procesamiento y montaje de flebotomíneos: una guía de consenso

Fano José Randrianambinintsoa[1], Laure Augendre[1], Jorian Prudhomme[1], Jean-Philippe Martinet[1], Mathieu Loyer[1], Nalia Mekarnia[1], Hocine Kerkoub[1], Farzana Khan Perveen[1], Antoine Huguenin[1,2], Emilie Kariya[1,2], Mohammad Akhoundi[3], Andrey José de Andrade[4], Eduardo Berriatua[5], Gioia Bongiorno[6], Sébastien Boyer[7,8], Vasiliki Christodoulou[9], Magda Clara Vieira Da Costa-Ribeiro[10], Lucas Alexandre Farias de Souza[10], Huicong Ding[11], Blaise Dondji[12], Vít Dvořák[13], Ozge Erisoz Kasap[14], Eunice Aparecida Bianchi Galati[15], Montserrat Gállego[16], Cristina Ballart[16], Stavroula Gouzelou[17], Nabil Haddad[18], Rezki Sabrina Masse[19], Asrat Hailu Mekuria[20], Vladimir Ivovic[21], Szymon Kaczmarek[22], Mohd Khadri Shahar[19], Oscar D. Kirstein[23], Edwin Kniha[24], Iva Kolářová[13], Lincoln Timinao[25], Cristian Lucanas[26], Ognyan Mikov[27], Kimsear Nov[7], Yusuf Özbel[28], Bernard Pesson[29], Laura Cristina Posada Lopez[30], Didot Budi Prasetyo[1,7], Nil Rahola[31], Eduardo A. Rebollar-Tellez[32], Bruno Leite Rodrigues[15], Lalita Roy[33], Prasanta Saini[34], Chizu Sanjoba[35], Paloma Helena Fernandes Shimabukuro[36], Padet Siriyasatien[37], Agnieszka Soszyńska[22], Tatiana Suleşco[38], Massamba Sylla[39], Majhalia Torno[40], Petr Volf[13], Khamsing Vongphayloth[41], Vu Sinh Nam[42], April Wardhana[43], Eric Yessinou[44], Sonia Zapata[45], Jean-Charles Gantier[1], and Jérôme Depaquit[1,2,*]

[1] Faculté de Pharmacie, Université de Reims Champagne Ardenne, UR ESCAPE-USC ANSES PETARD, 51 rue Cognacq-Jay, 51096 Reims Cedex, France
[2] Pôle de Biologie territoriale, Laboratoire de Parasitologie-Mycologie, Centre Hospitalo-Universitaire, 51092 Reims, France
[3] Parasitology-Mycology Department, Avicenne Hospital, AP-HP, Bobigny, Sorbonne Paris Nord University, France; Unité des Virus Émergents (UVE: Aix-Marseille Univ, Università di Corsica, IRD 190, Inserm 1207, IRBA), 13005 Marseille, France
[4] Parasitology Collection of Basic Pathology, Department of Basic Pathology, Federal University of Paraná, Curitiba 19031, Brazil
[5] Department of Animal Health, University of Murcia, Campus de Espinardo, 30100 Espinardo, Murcia, Spain
[6] Department of Infectious Diseases, Vector-borne Diseases Unit, Istituto Superiore di Sanità, 00166 Rome, Italy
[7] Medical and Veterinary Entomology Unit, Institut Pasteur du Cambodge, Phnom Penh 12201, Cambodia
[8] Ecology & Emergence of Arthropod-borne Pathogens Unit, Department of Global Health, Institut Pasteur, CNRS UMR2000, 75015 Paris, France
[9] Section Veterinary Services (1417), Laboratory for Animal Health Virology, Aglantzia, Nicosia 2109, Cyprus
[10] Insects Vectors and Parasites Laboratory, Department of Basic Pathology and Postgraduate program in Microbiology, Parasitology and Pathology, Federal University of Paraná, 81530-900 Curitiba, Brazil
[11] Department of Biological Sciences, National University of Singapore, 117558, Singapore
[12] Laboratory of the Leishmaniasis Research Project, Mokolo District Hospital, Mokolo, Cameroon; Laboratory of Cellular Immunology and Parasitology, Department of Biological Sciences, Central Washington University, 98926 Ellensburg, WA, USA
[13] Department of Parasitology, Faculty of Science, Charles University, 12800 Prague, Czechia
[14] VERG Laboratories, Department of Biology, Faculty of Science, Hacettepe University, Beytepe, Ankara 06800, Türkiye
[15] Faculdade de Saúde Pública da Universidade de São Paulo (FSP/USP), Pós-graduação em Saúde Pública, 01246-904 São Paulo, Brazil
[16] Secció de Parasitologia, Departament de Biologia, Sanitat i Medi Ambient, Facultat de Farmàcia i Ciències de l'Alimentació, Universitat de Barcelona, & Institut de Salut Global de Barcelona (ISGlobal), Centro de Investigación Biomédica en Red, Enfermedades Infecciosas (CIBERINFEC), 08028 Barcelona, Spain
[17] Laboratory of Infectious Diseases and Public Health, School of Medicine, University of Cyprus, Nicosia, Cyprus & Department of Pediatrics, Archbishop Makarios III Hospital, Nicosia 2115, Cyprus
[18] Faculty of Health Sciences, American University of Beirut, 1107 2020 Beirut, Lebanon
[19] Medical Entomology Unit, Infectious Disease Research Centre, Institute for Medical Research (IMR), National Institutes of Health (NIH), Ministry of Health Malaysia, 40170 Shah Alam, Selangor, Malaysia
[20] School of Medicine, Addis Ababa University, 28017 - 1000 Addis Ababa, Ethiopia
[21] Faculty of Mathematics, Natural Sciences and Information Technologies, University of Primorska, 6000 Koper, Slovenia
[22] University of Lodz, Faculty of Biology and Environmental Protection, Department of Invertebrate Zoology and Hydrobiology, Banacha 12/16, 90-237 Łódź, Poland

Edited by Jean-Lou Justine
*Corresponding author: jerome.depaquit@univ-reims.fr

This is an Open Access article distributed under the terms of the Creative Commons Attribution License (https://creativecommons.org/licenses/by/4.0), which permits unrestricted use, distribution, and reproduction in any medium, provided the original work is properly cited.

[23] Laboratory of Entomology, Ministry of Health, 9134302 Jerusalem, Israel
[24] Center for Pathophysiology, Infectiology and Immunology, Institute of Specific Prophylaxis and Tropical Medicine, Medical University Vienna, Kinderspitalgasse 15, 1090 Vienna, Austria
[25] Papua New Guinea Institute of Medical Research (PNGIMR) Institute, PO Box 60, Headquarter, Homate Street, 441 Goroka, Eastern Highlands Province, Papua New Guinea
[26] Museum of Natural History, University of the Philippines Los Baños, 4031 Laguna, Philippines
[27] National Centre of Infectious and Parasitic Diseases, 1504 Sofia, Bulgaria
[28] Ege University, Faculty of Medicine, Department of Parasitology, 35040 Bornova/Izmir, Türkiye
[29] Retired, Faculté de Pharmacie, Université de Strasbourg, Strasbourg, 67400 Illkirch-Graffenstaden, France
[30] Program for the Study and Control of Tropical Diseases (PECET), Faculty of Medicine, University of Antioquia, 050010 Medellin, Colombia
[31] MIVEGEC, Univ. Montpellier, CNRS, IRD, 34394 Montpellier, France & Medical Entomology Unit, Institut Pasteur de Madagascar, 101 Antananarivo, Madagascar
[32] Laboratorio de Entomología Médica, Departamento de Zoología de Invertebrados, Facultad de Ciencias Biológicas, Universidad Autónoma de Nuevo León, San Nicolás de los Garza, 66455, NL, México
[33] Tropical and Infectious Disease Centre, BP Koirala Institute of Health Sciences, Dharan 56700, Nepal
[34] ICMR-Vector Control Research Centre, Puducherry 605006, India
[35] Graduate School of Agricultural and Life Sciences, The University of Tokyo, Tokyo 113-8657, Japan
[36] Grupo de estudos em Leishmanioses/Coleção de Flebotomíneos (COLFLEB/Fiocruz-MG), Instituto René Rachou, Fundação Oswaldo Cruz, Belo Horizonte, Minas Gerais, 30190009, Brazil
[37] Center of Excellence in Vector Biology and Vector-Borne Disease, Department of Parasitology, Faculty of Medicine, Chulalongkorn University, Bangkok 10330, Thailand
[38] Department of Arbovirology, Bernhard Nocht Institute for Tropical Medicine, Bernhard Nocht Str. 74, 20359 Hamburg, Germany [39] Laboratory Vectors & Parasites, Department of Livestock Sciences and Techniques, Sine Saloum University El Hadji Ibrahima Niasse (SSUEIN) Kaffrine Campus, C.P. 24600, Senegal.
[40] Environmental Health Institute, National Environment Agency, Singapore 138667, Singapore & Department of Biological Sciences, National University of Singapore, 117558 Singapore
[41] Institut Pasteur du Laos, Laboratory of Vector-Borne Diseases, Samsenhai Road, Ban Kao-Gnot, Sisattanak District, 3560 Vientiane, Lao PDR
[42] National Institute of Hygiene and Epidemiology, 1 Yec-Xanh Street, Hai Ba Trung District, 100000 Hanoi, Vietnam
[43] Indonesian Research Center for Veterinary Science, Indonesian Agency for Agricultural Research and Development, Ministry of Agriculture Republic Indonesia, Bogor 16114, Indonesia & Department of Parasitology, Faculty of Veterinary Medicine, Airlangga University, Surabaya 60115, Indonesia
[44] Laboratory of Research in Applied Biology, Polytechnic School of Abomey-Calavi, University of Abomey-Calavi, 01 P.O. Box 2009, 00000 Cotonou, Benin
[45] Instituto de Microbiología, Colegio de Ciencias Biológicas y Ambientales (COCIBA), Universidad San Francisco de Quito (USFQ), 170901 Quito, Ecuador

Recibido el 1 de diciembre 2025, Aceptado el 29 de enero 2026, Publicado online 3 de abril de 2026

Resumen – Este artículo proporciona una guía completa para el procesamiento y montaje de especímenes de flebotominos, crucial para la identificación de especies y la detección y aislamiento de patógenos. Describe diversas técnicas adecuadas tanto para el campo como para el laboratorio. La guía incluye instrucciones detalladas sobre la recolección, manipulación, cubertura y eutanasia de flebotominos (recomendando la congelación en seco o el CO_2 en lugar de productos químicos), así como estrategias de conservación, como el almacenamiento en frío y la conservación en etanol. La calidad de la preparación de ciertas estructuras anatómicas (órganos genitales, cabeza y alas) es esencial para su correcta observación microscópica y se describe en este trabajo. El artículo también presenta el procesamiento detallado de las muestras, incluyendo el proceso de clarificación con agentes como el hidróxido de potasio y la solución de Marc-André. El proceso de montaje compara diferentes medios, destacando sus propiedades ópticas y su potencial de conservación. El medio de Hoyer (también conocido como goma cloral) se recomienda para la observación rápida, especialmente de espermatecas, debido a su claridad, aunque no es adecuado para el almacenamiento a largo plazo. Otros medios analizados incluyen el alcohol polivinílico, Euparal® (para tolerancia limitada al agua) y el bálsamo de Canadá (un medio soluble en hidrocarburos); estos dos últimos ofrecen capacidades de conservación a largo plazo. También se abordan enfoques innovadores de biología molecular, como la secuenciación de ADN y MALDI-ToF, que requieren especial atención al procesamiento de las muestras. Además, se incluyen breves vídeos que ilustran diversas técnicas de montaje, así como traducciones a 33 idiomas diferentes, lo que permite que la guía satisfaga las diversas necesidades y expectativas de la comunidad científica mundial.

Palabras clave: Montaje, flebotomino, medio de Hoyer, solución de Marc-André, goma cloral, alcohol polivinílico, Euparal®, bálsamo de Canadá, aislamiento de Leishmania, condiciones de campo, cultivo, disección, biología molecular, MALDI-ToF, especímenes tipo.

Abstract – Processing and mounting phlebotomine sand flies: a consensus guideline. This article provides a comprehensive guide for the processing and mounting of phlebotomine sand fly specimens, which is crucial for species identification and pathogen detection and isolation. It discusses a range of techniques suitable for both field and laboratory settings. The guide includes detailed instructions on sand fly collection, handling, covering, and euthanasia (recommending dry freezing or CO_2 over chemicals) as well as conservation strategies, such as cold storage and preservation in ethanol. The quality of preparation of certain anatomical structures (genital organs, head and wings) is essential for their proper microscopic observation and is described in this work. The article also presents detailed sample processing, including the clearing process with agents such as potassium hydroxide then Marc-André solution. The mounting process compares different media, emphasizing their optical properties and preservation potential. Hoyer fluid (also known as chloral gum) is recommended for quick observation, particularly for spermathecae, due to its clarity, although it is not suitable for long-term storage. Other media discussed include polyvinyl alcohol, Euparal® (for limited water tolerance), and Canada balsam (a hydrocarbon-soluble medium), with the latter two offering long-term preservation capabilities. Innovative molecular biology approaches such as DNA sequencing and MALDI-ToF, which require particular attention to sample processing, are also addressed. Furthermore, short video clips illustrating various mounting techniques as well as translations in many different languages are provided, allowing the guideline to reach the diverse needs and expectations of the global scientific community.

Key words: Mounting, Phlebotomine sand fly, Hoyer fluid, Marc-André solution, Chloral gum, Polyvinyl alcohol, Euparal®, Canada balsam, *Leishmania* isolation, Field conditions, Culture, Dissection, Molecular biology, MALDI-ToF, Type-specimens.

Introducción

Los flebotominos son pequeños insectos dípteros pertenecientes a la familia Psychodidae, subfamilia Phlebotominae, con al menos 1063 especies conocidas [21]. Son importantes vectores de patógenos (*Leishmania,* arbovirus y *Bartonella*), responsables de enfermedades como la leishmaniasis, las arbovirosis y la bartonelosis, respectivamente. Su identificación se basa principalmente en un examen microscópico detallado, facilitado por una recolección cuidadosa, un almacenamiento adecuado y un montaje cuidadoso en portaobjetos. Esto requiere diversas técnicas específicas, cada una con sus propias ventajas y limitaciones.

La identificación de los flebotominos adultos se basa en la observación de las estructuras externas (p. ej., antenas, palpos, genitales masculinos) e internas (p. ej., faringe, cibario y espermatecas). La disección y el aislamiento de estas últimas facilitan su observación y, en consecuencia, una identificación precisa. Por lo tanto, a diferencia de los mosquitos o las vinchucas, los flebotominos requieren un montaje entre un portaobjetos y un cubreobjetos antes de su identificación.

Hasta la década de 1980, la observación microscópica era el único método disponible para la identificación de los flebotominos, y sigue siendo el más utilizado en la actualidad. Por lo tanto, la elección del proceso y la preparación fue relativamente sencilla y se basó principalmente en una dicotomía: por un lado, el montaje definitivo, que permite la conservación a largo plazo de la muestra, y por otro, un montaje rápido para la identificación en un medio que no garantiza la conservación a largo plazo. El montaje final, por ejemplo, en una resina como el bálsamo de Canadá, requiere mucho tiempo y la deshidratación completa de las muestras. Además, el índice de refracción de este medio no siempre es óptimo para facilitar la observación de las espermatecas. El montaje en un medio acuoso (p. ej., medio de Hoyer), en cambio, es más rápido y permite una mejor visualización de las espermatecas refractivas, pero no permite la conservación a largo plazo de los montajes porque tiende a absorber agua de la atmósfera. Una opción es sellar el portaobjetos con esmalte de uñas una vez que esté completamente seco. Esta disyuntiva persiste en la actualidad y condiciona la elección del método de montaje en función del objetivo de la preparación. Desde la década de 1980, los estudios de identificación de flebotominos han combinado la morfología y los enfoques bioquímicos. El primero de ellos fueron los análisis de hidrocarburos cuticulares, que fueron rápidamente reemplazados por técnicas de biología molecular (es decir, ADN polimórfico amplificado aleatoriamente [RAPD], polimorfismo de longitud de fragmentos de restricción [RFLP], amplificación de ADN y

secuenciación mediante el método de Sanger, así como la secuenciación de nueva generación [NGS]). Hoy en día, los enfoques moleculares se complementan con métodos proteómicos como MALDI-ToF. Además, la identificación molecular de especies se puede combinar con la detección de patógenos por PCR (*Leishmania, Trypanosoma, Bartonella* y Phlebovirus), ya que todos pueden detectarse tanto por PCR de punto final como por PCR en tiempo real, lo que requiere la adaptación del proceso de muestreo y almacenamiento a los objetivos asignados [3, 32]. Además de las características morfológicas tradicionalmente utilizadas para la discriminación de especies, se pueden aplicar otros enfoques morfológicos (por ejemplo, la geomorfometría del ala).

Basándose principalmente en la propia experiencia de los autores y en datos bibliográficos, el objetivo de esta investigación fue proporcionar directrices estandarizadas para el montaje y procesamiento de flebotominos adultos con el fin de optimizar los análisis morfológicos y moleculares.

La necesidad de realizar ciertos análisis (p. ej., biología molecular o MALDI-ToF) requiere conservar parte del flebotomino que no es necesaria para la identificación morfológica, lo que pone de relieve la importancia de una elección crítica del protocolo.

En este artículo, nos centramos en los métodos de anestesia y eutanasia de flebotominos capturados vivos, su almacenamiento y su proceso de montaje, para una rápida identificación o para la conservación a largo plazo que permita estudios posteriores.

Preámbulo: Las consideraciones de seguridad y normativas deben consultar las Fichas de Datos de Seguridad (FDS) pertinentes

Todos los productos químicos presentados en esta guía deben manipularse bajo estrictas condiciones de seguridad. Los comités de salud y seguridad de los centros de investigación están a su disposición para proporcionarle información no solo sobre los peligros de estos productos químicos, sino también sobre sus procedimientos de manipulación y eliminación de residuos. Sin embargo, es obligatorio seguir las instrucciones de seguridad relativas a su uso y eliminación. Cabe destacar que es responsabilidad de todos los usuarios garantizar el cumplimiento de las buenas prácticas de laboratorio y de la legislación y normativas aplicables de su país o institución de investigación. Además, algunas sustancias químicas o sus componentes (por ejemplo, el hidrato de cloral) están reguladas en algunos países. En la Tabla 1 se incluye una lista de las abreviaturas utilizadas en este manuscrito.

Tabla 1: Lista de abreviaturas.

**BME**	Águila mediana basal
**CDC**	Centros para el Control y la Prevención de Enfermedades
**CMCP**	Alcanfor-monoclorofenol
**CMR**	Sustancia cancerígena, mutagénica y tóxica para la reproducción
**COI**	Gen de la subunidad I de citocromo oxidasa
**CytB**	Gen del citocromo b
**DNA**	Ácido desoxirribonucleico
**ELISA**	Ensayo inmunoabsorbente ligado a enzimas
**EtOH**	Etanol
**M199**	Medio 199
**MALDI-ToF MS**	Espectrometría de masas de tiempo de vuelo por desorción/ionización láser asistida por matriz
**MEM**	Medios esenciales mínimos
**NGS**	Secuenciación de próxima generación
**NNN**	Medio Novy-MacNeal-Nicolle
**PCR**	Reacción en cadena de la polimerasa
**Lao PDR**	República Democrática Popular Lao
**PNOC**	Gen de la prepronociceptina
**qPCR**	PCR cuantitativa (PCR en tiempo real)
**RAPD**	ADN polimórfico amplificado aleatoriamente
**RFLP**	Polimorfismo de longitud de fragmentos de restricción
**RI**	Índice de refracción
**RNA**	Ácido ribonucleico
**RNases**	Ribonucleasas
**RNASS**	Solución de estabilización de ARN
**RT-PCR**	PCR de transcripción inversa
**TFA**	Ácido trifluoroacético

1 Captura de flebotominos

Los flebotominos adultos pueden recolectarse vivos o muertos mediante diversos métodos, como trampas de luz en miniatura CDC, trampas adhesivas y aspiradores con trampas Shannon, o directamente de lugares de descanso en el entorno (por ejemplo, refugios para animales). Estos métodos implican colocar las trampas en hábitats adecuados, atraer a los flebotominos con luz u otros atrayentes (CO_2 o cebos químicos) y recolectarlos para su posterior análisis, como se describe en varias publicaciones [2, 3, 32, 36, 49].

La captura de flebotominos vivos permite todas las aplicaciones posteriores, mientras que la recolección de individuos muertos impide el aislamiento de cepas de *Leishmania* o virus. Algunas técnicas de captura, como los papeles adhesivos, suelen provocar la pérdida de órganos del flebotomino (antenas, palpos, alas o patas). Además, el

aceite de ricino que recubre los papeles adhesivos se adhiere a los flebotominos y debe retirarse al inicio del procesamiento, generalmente mediante un baño de 15 minutos en una mezcla de etanol y éter dietílico a partes iguales.

2 Eutanasia de especímenes

Tras la recolección, los flebotominos vivos deben ser sacrificados. Con algunos métodos de recolección (p. ej., papeles adhesivos, trampas de luz CDC equipadas con un frasco con detergente o etanol), los flebotominos mueren al momento de la recolección. La biología molecular puede aplicarse a los flebotominos recolectados directamente en etanol y a los demás si se almacenan en etanol lo más rápido posible. Sin embargo, ninguno de estos métodos de sacrificio permite el procesamiento de insectos mediante MALDI-ToF. Además, algunos métodos pueden causar la pérdida de ciertas características morfológicas. Por lo tanto, es esencial utilizar sustancias letales estándar adecuadas para garantizar una correcta identificación o el almacenamiento a largo plazo como especímenes de referencia (es decir, especímenes preservados y almacenados para futuras referencias o comparaciones). Productos químicos como acetato de etilo, éter etílico, tetracloroetano y cloroformo pueden impregnarse en algodón y colocarse en un recipiente que contenga los flebotominos para la eutanasia. Estos agentes exterminadores deben manipularse con cuidado, siguiendo las recomendaciones del fabricante debido a su toxicidad. Sin embargo, no recomendamos matar flebotominos con cloroformo, ya que, según nuestra experiencia, es poco compatible con los estudios de biología molecular. Dada la peligrosidad de todos estos productos y su dudosa idoneidad para los análisis moleculares, generalmente se desaconseja su uso.

El método más utilizado, que preserva la morfología, el ADN o las proteínas, es la congelación en seco de las muestras. Los especímenes deben congelarse el tiempo suficiente para quedar completamente anestesiados, pero no tanto como para que (i) se deshidraten o (ii) se comprometa la viabilidad de *Leishmania*, si el objetivo es aislarlos in vitro a partir del tracto digestivo del flebotomino. Por lo tanto, recomendamos una congelación de 15 a 20 minutos a -20 °C, monitoreándolas regularmente para garantizar que solo se aturdan sin matar los parásitos de *Leishmania*.

Si no se dispone de un congelador, los insectos pueden ser eutanasiados con CO_2. En condiciones de campo donde no se pueden usar cilindros de CO2, los especímenes pueden ser sacrificados usando pequeños contenedores comerciales de CO2 usados en "sifones de soda" (dispensadores de bebidas), pero puede haber restricciones en su transporte aéreo. Como último recurso, los insectos pueden ser sacrificados por exposición al humo de tabaco. Los flebotominos que se capturan vivos en trampas CDC, se recolectan con un aspirador, se retienen en el tubo de vidrio y se exponen al humo de tabaco, que los mata en segundos. Este método es aplicable en todas las condiciones de campo, incluso en condiciones de aislamiento difíciles. Sin embargo, debido a que el vidrio se impregna de humo, no puede usarse para la posterior recolección y manipulación de flebotominos vivos sin una limpieza a fondo. No obstante, el mismo aspirador sin limpiar puede usarse para sacrificar flebotominos de otras trampas para fines de fijación. También es necesario verificar que se hayan retirado todos los especímenes del aspirador. Estos métodos son compatibles con el aislamiento de *Leishmania* mediante disección intestinal.

3 Almacenamiento de muestras antes del procesamiento

Existen cinco métodos principales de fijación antes del procesamiento:

3.1 Congelación

Este método se realiza mejor a -20 °C o, preferiblemente, a -80 °C. Estos métodos de almacenamiento se utilizan actualmente más que el almacenamiento con nitrógeno líquido. En todos los casos, la criopreservación debe implementarse lo antes posible después del aturdimiento de las muestras. El almacenamiento en frío en congeladores ofrece la ventaja de preservar completamente los insectos, así como el ARN, el ADN y las proteínas con total integridad durante todo el período de almacenamiento. En cambio, el nitrógeno líquido puede dañar gravemente las alas, patas, palpos y antenas, a menudo amputándolos y, en ocasiones, eliminando caracteres morfológicos clave. El almacenamiento en congelador seco es menos traumático para las muestras, pero no es ideal para preservar sus frágiles órganos. Cabe destacar que, al descongelar, las alas, antenas, palpos o patas pueden adherirse a los viales y eventualmente desprenderse debido a la condensación. Sin embargo, la conservación mediante congelación no siempre es viable en estudios de campo, ya que requiere acceso a un congelador o a un contenedor de nitrógeno líquido. El almacenamiento en congelador es totalmente compatible con la detección de patógenos mediante herramientas moleculares sin pérdida de sensibilidad, aunque la detección y el aislamiento del virus ARN requieren congelación a -80° C o en nitrógeno líquido si se requiere un almacenamiento prolongado. Sin embargo, la congelación de las muestras no permite el aislamiento de *Leishmania* mediante disección intestinal, salvo cuando se emplea nitrógeno líquido. En este caso, es necesario exponer primero los flebotominos a la fase de vapor del nitrógeno líquido antes de introducirlos en

el líquido propiamente dicho (por ejemplo, en viales colocados dentro de una media), simulando así la criopreservación de *Leishmania*.

3.2 Almacenamiento en alcohol (etanol o alcohol isopropílico)

Este es probablemente el método más utilizado para almacenar flebotominos. Es fácil de implementar en campo, incluso en condiciones difíciles sin acceso a un laboratorio. La conservación en alcohol es especialmente adecuada para estudios morfológicos, ya que los órganos frágiles (alas, patas, antenas o palpos) permanecen intactos si no hay burbujas de aire en el tubo de almacenamiento. Por lo tanto, recomendamos sellar el tubo con una bolita de algodón para eliminar las burbujas de aire y colocar una etiqueta sobre el tapón de algodón (Figura 1). La concentración adecuada de alcohol sigue siendo objeto de debate. Generalmente, no se recomiendan concentraciones inferiores al 70 % [45, 66]. Concentraciones más altas conservan el ADN de forma más eficaz y durante más tiempo, pero hacen que las muestras sean más frágiles y quebradizas para los estudios morfológicos. El uso de etanol al 96 % (la mezcla azeotrópica) garantiza la estabilidad de la concentración a lo largo del tiempo, especialmente en zonas húmedas como los países tropicales, aunque el etanol al 95 % suele ser más fácil de obtener. Independientemente de la concentración, el ADN generalmente se conserva bien en etanol (aunque con menor eficacia que con métodos de congelación, en particular para métodos moleculares de tipo NGS). Las proteínas son mucho menos estables, especialmente para la proteómica, como las aplicaciones MALDI-ToF. Los flebotominos conservados en alcohol durante unos meses aún pueden identificarse morfológicamente, pero es imposible generar espectros de proteínas de referencia a partir de estas muestras. El almacenamiento en alcohol o en condiciones secas puede mejorarse si la muestra también se congela a -20 °C. La congelación a -20 °C mejora principalmente la conservación molecular (p. ej., ácidos nucleicos) al ralentizar la degradación y también proporciona un beneficio secundario para la conservación morfológica al reducir la degradación tisular con el tiempo, aunque el efecto sobre la morfología es más limitado que sobre la integridad molecular. El almacenamiento en etanol también puede aplicarse para la detección de virus de ADN y ARN cuando se utiliza etanol a una concentración de al menos el 70 % durante un período de almacenamiento corto, inferior a unos pocos meses. Además, el alcohol isopropílico puede estar fácilmente disponible en algunos países y conserva el ADN, pero endurece las muestras. No es inflamable como el etanol y, por lo tanto, se transporta fácilmente. Si es necesario, los flebotominos conservados en nitrógeno líquido o congelados en seco pueden transferirse al alcohol, combinando así las desventajas de ambos métodos.

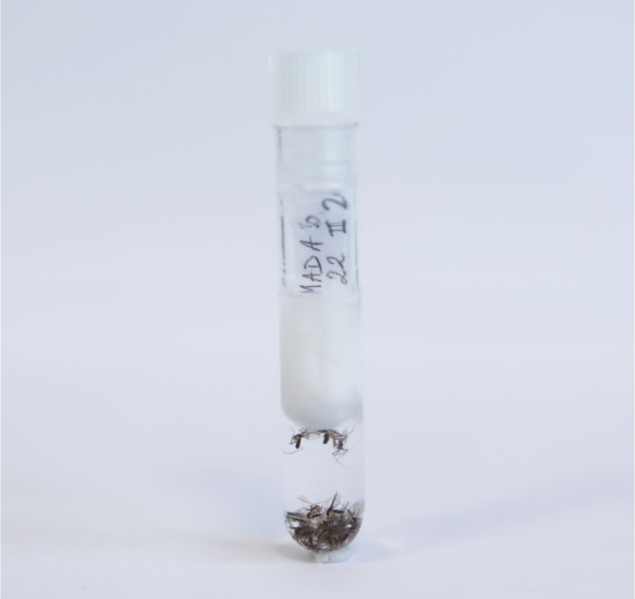

Figura 1: Flebotominos conservados en etanol.

3.3 Almacenamiento en solución de estabilización de ARN (RNASS)

Este tipo de reactivos acuosos son ampliamente utilizados, no tóxicos y está diseñados para estabilizar y proteger el ARN en muestras frescas de tejido y células. Actúa penetrando rápidamente en la muestra e inactivando las ARNasas (enzimas que degradan el ARN), previniendo así la degradación del ARN sin necesidad de congelación inmediata. El almacenamiento en RNASS suele ser eficaz para preservar la morfología tisular y celular general para la evaluación histológica posterior. Si bien el RNASS está optimizado para la estabilización del ARN en lugar de la fijación, el almacenamiento a corto y mediano plazo suele mantener una buena integridad estructural. El RNASS permite almacenar las muestras a temperatura ambiente hasta 7 días, a 4 °C durante varias semanas o a -20 °C/-80 °C para la conservación a largo plazo. Este método es especialmente valioso en el trabajo de campo o en entornos clínicos donde la infraestructura de la cadena de frío es limitada. La extracción de ARN generalmente requiere retirar las muestras del reactivo y procesarlas según protocolos estándar.

3.4 Conservación en seco a temperatura ambiente

Este método, más antiguo, al aplicarse a una muestra in toto (montada entera), presenta la importante desventaja de no conservar adecuadamente órganos frágiles como alas, patas, antenas y palpos. Sin embargo, los estudios proteómicos mediante MALDI-ToF siguen siendo viables si la deshidratación se realiza tras la fijación con un desecante de gel de sílice. Por el contrario, los análisis moleculares dirigidos al ADN siguen siendo difíciles de realizar en estas muestras, ya que el ADN suele estar fragmentado y en baja cantidad, lo que implica que los análisis son más complejos que con muestras frescas o congeladas, especialmente en el caso de genomas nucleares. No obstante, técnicas recientes como la museomómica podrían utilizarse en muestras de este tipo [34]. Por lo tanto, este método de almacenamiento no se recomienda, a menos que no haya otra alternativa disponible. Puede combinarse con el almacenamiento en frío colocando los tubos en un congelador a -20 °C o -80 °C. El principal reto reside en lograr un montaje adecuado de las muestras o las partes del cuerpo necesarias para la identificación. Para ello, la rehidratación es esencial. Recomendamos utilizar una solución de Triton X-100. La duración de la rehidratación varía de unas pocas horas a varios días, bajo estricta supervisión. Tras la rehidratación completa, las muestras deben enjuagarse en tres baños de agua consecutivos.

3.5 Conservación en papel de filtro

La principal ventaja de los papeles de filtro es la estabilidad a largo plazo del ADN genómico en las células de cuerpos completos desecados y no fijados, o en células sanguíneas almacenadas a temperatura ambiente. El papel de filtro se presenta en un formato de tarjeta pequeño, lo que permite almacenar cientos de muestras a temperatura ambiente en un volumen equivalente al de un pequeño destornillador de mesa. La matriz del papel de filtro está impregnada con agentes que desnaturalizan los agentes infecciosos, por lo que las muestras ya no se consideran un riesgo biológico. Esto permite el almacenamiento y transporte de muestras sin precauciones selectivas de riesgo biológico [68].

4 Disección de la muestra

A diferencia de otros insectos, que se identifican basándose en caracteres externos observables en insectos individuales fijados en su totalidad, los flebotominos requieren disección y montaje en portaobjetos para estudiar las características anatómicas e identificar con precisión la especie. Independientemente del procedimiento de preparación y montaje elegido, se emplea la misma técnica de disección (Figuras 2 y 3) (https://zenodo.org/records/18198006).

Uso de Triton X100: solución acuosa no iónica

Tenga en cuenta que el montaje se refiere a especímenes recién capturados o adecuadamente almacenados. La mayoría de los coleccionistas conservan muestras de insectos en seco (para su uso en MALDI-ToF) o en alcohol durante muchos años. Desafortunadamente, la conservación en alcohol no es óptima durante años, y los artrópodos conservados de esta manera se vuelven muy difíciles de preparar para el examen microscópico. Un incidente frecuente es la degradación de los plásticos que contienen las muestras, seguida de la evaporación del alcohol. En ambos casos, no tenemos opciones porque las muestras permanecen demasiado tiempo en alcohol o se secan. Por lo tanto, surgió la idea de utilizar agentes humectantes que no sean detergentes fuertes. Triton X100 se presenta en forma de una solución acuosa no iónica (solución de 4-(1,1,3,3-tetrametilbutil) fenil-polietilenglicol, o t-octilfenoxipolietoxietanol, éter terc-octilfenílico de polietilenglicol), ampliamente utilizada como detergente en biología celular y molecular. Permite la permeabilización de las membranas celulares y nucleares.

A continuación, se describe un procedimiento utilizando Triton X100 no iónico en solución acuosa al 0,5 %:

- Impregnar la muestra seca con alcohol absoluto.
- Añadir el volumen necesario de la solución de Triton X100 al 0,5 % de modo que toda la muestra quede sumergida.
- Dejar que el proceso transcurra durante aproximadamente 5 minutos hasta varios días, monitoreando regularmente. Todos los artrópodos deben quedar completamente separados en la solución.
- Retirar la solución de Triton X100 y reemplazarla por una solución de hidróxido de potasio.

4.1 Cabeza

La disección puede realizarse utilizando agujas finas o alfileres entomológicos bajo un estereomicroscopio (Figuras 2 y 3). Las agujas más comúnmente utilizadas incluyen: 26G × 1/2" (0,45 × 13 mm), 30G × 1/2" (0,3 × 13 mm) o 25G × 5/8" (0,5 × 16 mm). Para preparar el espécimen para la identificación, como mínimo, la cabeza se separa del cuerpo y se monta con la cara ventral hacia arriba para mostrar el cibario y la faringe, mientras que el tórax y el abdomen se montan lateralmente tras la disección. Montar la cabeza en posición ventro-dorsal asegura que el foramen occipital quede orientado hacia arriba, de modo que el cibario pueda observarse directamente. El acceso a estas características anatómicas se facilita si la cabeza se separa completamente.

This is an Open Access article distributed under the terms of the Creative Commons Attribution License (https://creativecommons.org/licenses/by/4.0), which permits unrestricted use, distribution, and reproduction in any medium, provided the original work is properly cited.

4.2 Alas y tórax

Las alas deben montarse planas. Cada ala puede separarse en su base y montarse de forma independiente, o bien montarse solo una, dejando la otra unida al tórax. Si se prevé realizar análisis de morfometría geométrica, es esencial identificar y etiquetar correctamente las alas derecha e izquierda antes del montaje. El tórax se divide en varias partes, y cada una contiene información taxonómica muy importante [20, 64]. Generalmente se monta en vista lateral, para permitir el examen de la quetotaxia y la distribución del color. La presencia de cicatrices de cerdas en determinadas regiones del tórax puede utilizarse para distinguir algunas especies del género *Brumptomyia*. La distribución del color puede emplearse para separar flebotominos neotropicales a nivel de género (p. ej., *Bichromomyia*), de series de especies (p. ej., *Pintomyia*), o incluso de especies dentro del mismo género (p. ej., *Micropygomyia*, *Nyssomyia*, *Psathyromyia* y *Psychodopygus*) [20]. Por lo tanto, si el tórax no se utiliza para análisis moleculares, debe montarse de manera que no se dañe. Es importante señalar que no es la intensidad de los colores lo que importa, sino su distribución en el tórax. Por consiguiente, el proceso de aclaramiento no eliminará la pigmentación ni su patrón.

4.3 Genitalia

Debe tenerse especial cuidado al montar la genitalia tanto en machos como en hembras, ya que es crucial para la identificación de géneros, subgéneros y especies. En ambos sexos, la genitalia es pareada.

4.3.1 Machos

La genitalia es externa y consiste en pinzas pareadas, cada una formada por la articulación gonocoxito–gonoestilo en su parte dorsal y el lóbulo epandrial en su parte ventral. El gonostilo porta espinas y, a veces, setas, que deben poder contarse y cuyas posiciones de inserción deben ser claramente visibles. Es importante observar cuidadosamente la superficie interna del gonocoxito, que

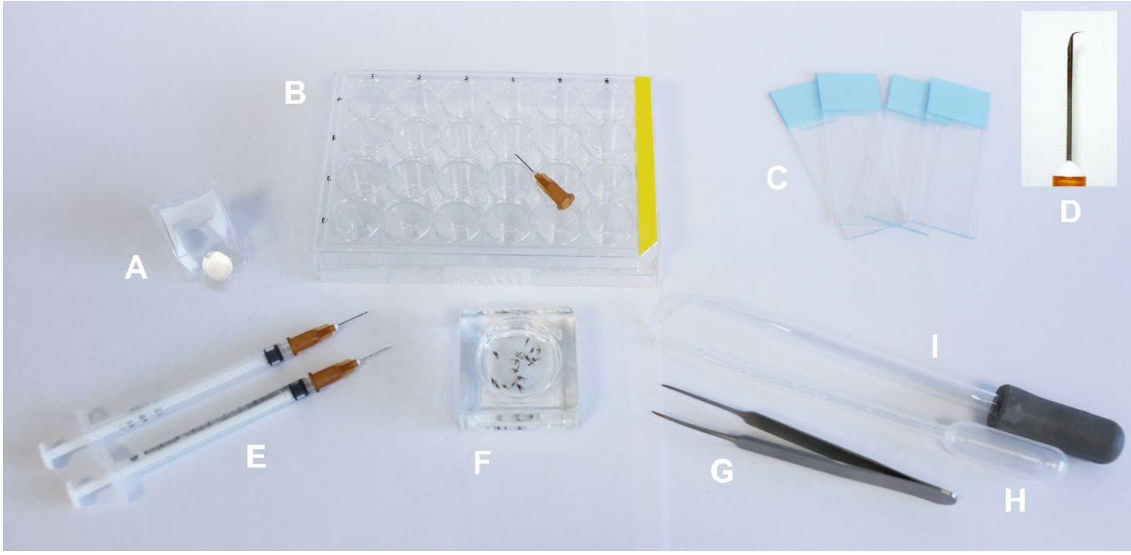

Figura 2: Materiales necesarios para el montaje de flebotominos: A: cubreobjetos redondos de vidrio (10 o 12 mm de diámetro); B: placa de 24 pocillos y aguja con gancho (si se utiliza aceite de clavo o esencia de Euparal® para procesar los flebotominos, no se deben usar placas acrílicas, ya que se producirá una reacción química y los especímenes se dañarán); C: portaobjetos de vidrio adecuados para el etiquetado; D: detalle del gancho de la aguja; E: agujas acopladas a jeringas; F: vidrio de reloj o recipiente equivalente que contiene los flebotominos a montar; G: pinzas Dumont; H: pipeta de plástico; I: pipeta de vidrio doblada mediante calentamiento para facilitar la transferencia de líquido a los pocillos.

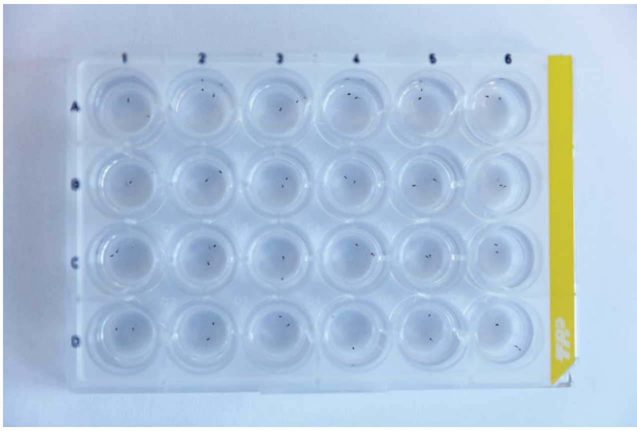

Figura 3: Placa con 24 pocillos, cada uno que contiene la cabeza y la punta del abdomen de flebotominos.

puede presentar un penacho de setas sésiles o setas portadas por un lóbulo (= tubérculo) [22]. Los colegas con menor experiencia en disecciones pueden realizar un montaje lateral simple, sin separar la genitalia del extremo del abdomen (https://zenodo.org/records/18311158). En este caso, la superposición de las dos partes de la genitalia puede dificultar, por ejemplo, el recuento de las setas internas del gonocoxito, pero se evita dañar la genitalia por una disección fallida. Los colegas más experimentados pueden intentar abrir la genitalia en dos, separándola. Para ello, el lado biselado de una aguja (tipo aguja para reacción intradérmica) debe pasarse a través, desprendiendo sin cortar completamente la genitalia para separar los conjuntos gonocoxito–gonóstilo (https://zenodo.org/records/18311158). De este modo, la observación de sus caras internas será sencilla. Este montaje también facilita la observación de los parámeros y las vainas paramerales, que ya no se superponen. Para el montaje lateral, que favorece la superposición de órganos, los especímenes deben estar perfectamente aclarados.

4.3.2 Hembras

El aparato genital es interno y está constituido por las espermatecas. En ausencia de disección, deben observarse a través de los tegumentos montando el abdomen en posición ventral. Independientemente del medio de montaje elegido, las espermatecas en sí, generalmente pueden observarse correctamente, si no son lisas y están aclaradas. Sin embargo, la observación de espermatecas lisas y de pared delgada puede ser problemática en medios con baja refracción. Además, la observación de la base de los conductos espermatecales es esencial para la identificación de especies, como en el subgénero *Larroussius* [35, 37, 38], los principales vectores de *Leishmania infantum* en el Viejo

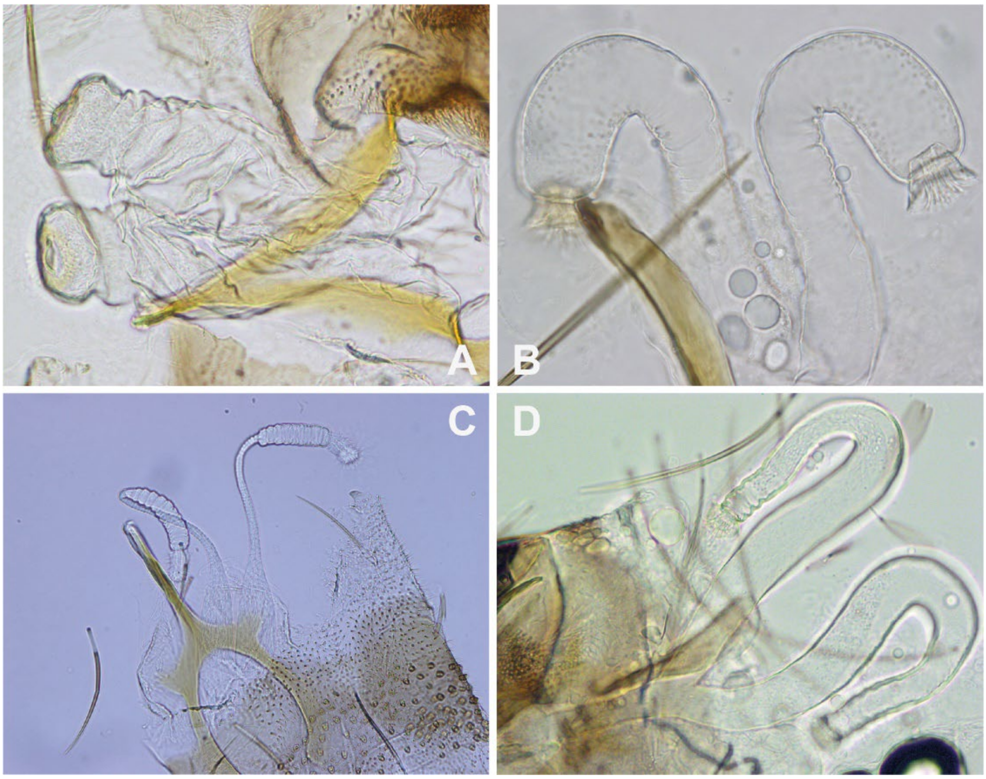

Figura 4: Espermatecas disecadas y montadas en la solución de Marc-André a partir de especímenes frescos. A: *Idiophlebotomus longiforceps* (RDP Lao); B: *Sergentomyia minuta* (Francia); C: *Phlebotomus ariasi* (Francia); D: *Sergentomyia anodontis* (RDP Lao).

This is an Open Access article distributed under the terms of the Creative Commons Attribution License (https://creativecommons.org/licenses/by/4.0), which permits unrestricted use, distribution, and reproduction in any medium, provided the original work is properly cited.

Mundo. Sin esta observación, la identificación del espécimen resulta imposible. Para superar estas dificultades, el conjunto furca genital–espermatecas debe separarse del abdomen (https://zenodo.org/records/18311106). Las espermatecas suelen ser difíciles de observar durante la disección, pero la furca genital es relativamente fácil de localizar. Dado que los conductos espermatecales desembocan en la furca genital, aislar esta furca normalmente permite aislar las espermatecas. Si las espermatecas se cortan accidentalmente durante el proceso, no se pierden y aún pueden observarse dentro de los tegumentos abdominales (Figura 4).

4.4 Disección del intestino medio para el aislamiento de *Leishmania*

La disección del tracto digestivo es esencial para detectar y aislar *Leishmania* en los flebotominos hembra. El procedimiento puede realizarse tanto en campo como en laboratorio, para evaluar la competencia vectorial.

Se recomienda trabajar con hembras recién sacrificadas. Lave las hembras con agua o solución salina que contenga un detergente suave para eliminar el exceso de pelos. Este paso ayuda a mantener condiciones asépticas para el aislamiento de *Leishmania*, al tiempo que preserva las características morfológicas necesarias para la identificación. Para localizar y aislar *Leishmania*, retire cuidadosamente el intestino medio y colóquelo en una sola gota de solución salina estéril (NaCl al 0,9 %). Tras observar parásitos móviles bajo un microscopio óptico (aumento recomendado: ~200×), utilice una jeringa de insulina o una micropipeta para transferirlos al medio de cultivo (para más detalles, véase el Capítulo 4.4.3).

Monte la cabeza y la genitalia directamente en solución de Marc-André para aclararlas. Importante: nunca permita que la solución de Marc-André entre en contacto con *Leishmania*, ni directa ni indirectamente a través de herramientas o agujas, ya que es letal para los parásitos.

La disección de flebotominos hembra puede realizarse en uno o dos portaobjetos de vidrio; ambas opciones tienen ventajas y limitaciones (Figura 5; https://zenodo.org/records/18311154).

4.4.1 Método de dos portaobjetos

La primera opción consiste en trabajar con dos portaobjetos separados: un que contiene solución salina estéril para extraer el intestino medio, y el otro para montar la cabeza y las espermatecas en solución de Marc-André. Sin embargo, en condiciones de campo es habitual que dos o tres personas diseccionen flebotominos y vayan pasando sus disecciones a un único investigador responsable de la

This is an Open Access article distributed under the terms of the Creative Commons Attribution License (https://creativecommons.org/licenses/by/4.0), which permits unrestricted use, distribution, and reproduction in any medium, provided the original work is properly cited.

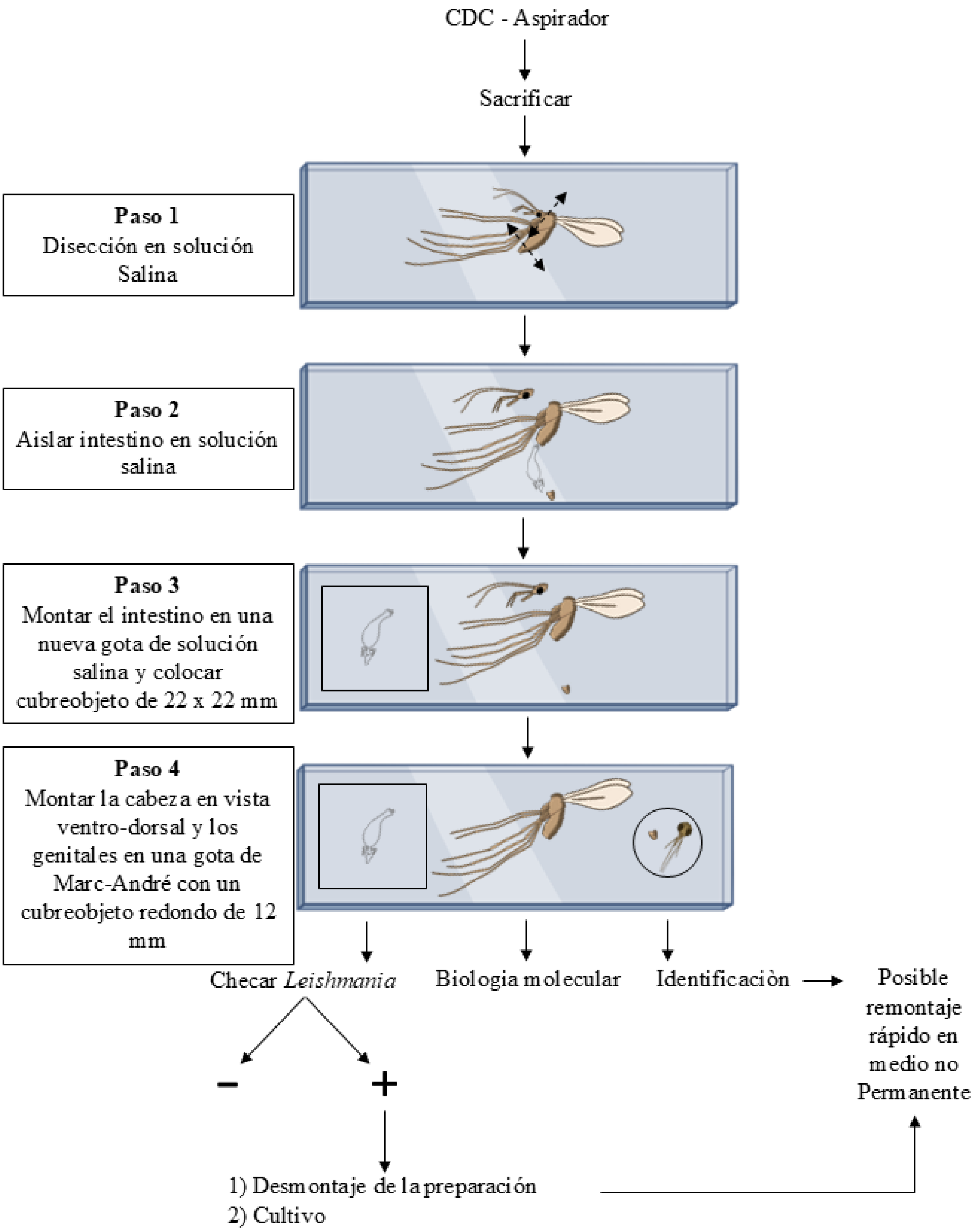

Figura 5: Método para el aislamiento de *Leishmania*.

identificación de especies y de la evaluación de la infección por *Leishmania* en el intestino. El método de dos portaobjetos puede generar problemas de trazabilidad de las muestras y, en particular, dificultar la determinación con certeza de qué flebotomino individual estaba infectado si se detecta un intestino positivo (https://zenodo.org/records/18311154).

4.4.2 Método de un solo portaobjetos

El uso de un solo portaobjetos de vidrio garantiza la trazabilidad de los resultados. No obstante, deben tomarse varias precauciones. Para maximizar la esterilidad durante este paso, los operadores deben limpiarse regularmente las manos con gel hidroalcohólico. Deben utilizarse portaobjetos no esmerilados y cubreobjetos cuadrados (22 × 22 mm) envueltos en papel de aluminio y esterilizados por

This is an Open Access article distributed under the terms of the Creative Commons Attribution License (https://creativecommons.org/licenses/by/4.0), which permits unrestricted use, distribution, and reproduction in any medium, provided the original work is properly cited.

calor seco (utilizando un horno Poupinel), junto con agujas estériles para cada disección (sugerencia: 25G Ø 0,5 mm × 16 mm). El flebotomino se coloca en una gota de solución salina estéril en el centro de la lámina. Se secciona la cabeza mientras se realiza una incisión entre los tergitos y esternitos abdominales 6º y 7º sin cortar el tracto digestivo (puede realizarse un corte más alto si se esperan espermatecas muy largas). A continuación, el tórax debe inmovilizarse con una aguja y los últimos segmentos abdominales posteriores se tiran suavemente con la otra aguja para extraer el intestino. Si esto falla, existe la posibilidad de bloquear el extremo del abdomen con una aguja y tirar del tracto digestivo desde su parte anterior. Si esto vuelve a fallar, el intestino debe extraerse retirando la mayor parte posible del tegumento restante a su alrededor. Cuando el intestino se extrae, los últimos segmentos abdominales deben separarse cortando el tracto digestivo. El intestino se coloca entonces en una nueva gota de solución salina estéril, situada a un lado del portaobjetos y se cubre suavemente con un cubreobjetos estéril. La cabeza y los últimos segmentos abdominales se transfieren a una pequeña gota de la solución de Marc-André colocada en el otro extremo del portaobjetos, asegurando que no haya contacto con *Leishmania*. La cabeza se orienta correctamente (foramen occipital hacia arriba), y las espermatecas se aíslan con la furca genital como se indicó anteriormente y se cubren con un pequeño cubreobjetos redondo (Ø 12 mm, no confundir con los cubreobjetos cuadrados estériles). El resto del cuerpo del flebotomino y las alas permanecen en la gota de solución salina en el centro de la lámina (https://zenodo.org/records/18311154). En caso de positividad, o para exploración taxonómica, el tórax y el abdomen pueden conservarse para estudios moleculares o proteómicos, y las alas pueden montarse en un medio acuoso. Para preservar el montaje, el volumen extra de líquido de Marc-André puede reemplazarse por un medio de montaje acuoso como goma cloral (= Hoyer) o un medio a base de alcohol polivinílico.

Existen vídeos detallados que demuestran estos procedimientos (disección del intestino medio del flebotomino: https://zenodo.org/records/18303014 y disección de glándulas salivales del flebotomino: https://zenodo.org/records/18302850), por lo que no se explicarán aquí.

4.4.3 Aislamiento y cultivo de *Leishmania* a partir de intestinos de flebotominos

El aislamiento de parásitos a partir de la disección de flebotominos hembra infectados es un procedimiento delicado que requiere gran habilidad y debe practicarse inicialmente con especímenes libres de parásitos. Tras la disección, los intestinos se transfieren a una gota fresca de solución salina estéril (0,9 %) o solución de Locke para el lavado [4]. Los intestinos disecados pueden procesarse de dos maneras: i) examinarse bajo un microscopio óptico para observar los diferentes estadios de promastigotes de Leishmania y su localización, con especial atención a la válvula estomodeal, y ii) abrir el intestino para facilitar la salida de los promastigotes y permitir su cultivo masivo [4]. Encontrar flebotominos infectivos en el campo es relativamente poco frecuente y, por lo tanto, buenas sesiones de práctica maximizarán las probabilidades de un aislamiento exitoso.

Si se observan parásitos de *Leishmania* en el intestino, deben utilizarse agujas estériles nuevas y añadirse una pequeña cantidad de solución salina estéril alrededor del cubreobjetos por acción capilar para liberarlos. El intestino debe desgarrarse cuidadosa y rápidamente para liberar los parásitos en la solución salina. Utilizando una micropipeta de 100 µL o una jeringa de insulina, recoja los parásitos e inocúlelos en un medio de cultivo correctamente etiquetado.

Cultivo in vitro de promastigotes de *Leishmania*: los parásitos aislados se mantienen inicialmente en tubos de agar sangre inclinado SNB-9 o en medio sólido Novy, McNeal, Nicolle (NNN) [16], cubiertos con medio alfa-MEM estéril [16, 65] o con medio M199, cada uno suplementado con 10 % de suero fetal bovino estéril inactivado por calor [SFB] (para favorecer el crecimiento parasitario), 1 % de vitaminas BME, 2 % de orina humana estéril (esterilizada mediante un filtro de jeringa Filtropur® S 0,2 µm), 250 µg/mL de amikacina (o 50 µg/mL de gentamicina, o una mezcla de antibióticos y aminoácidos (L-glutamina 200 mM–penicilina 10 000 U–estreptomicina 10 mg/mL) [47]). Tras tres días, si no hay contaminación, los cultivos se suspenden en un medio de congelación adecuadamente preparado y posteriormente se almacenan a −80 °C durante 1 a 2 años o en nitrógeno líquido a −196 °C para su conservación a largo plazo y uso experimental futuro [7].

4.5 Glándulas salivales

La disección de las glándulas salivales de los flebotominos es una técnica fundamental para estudiar las interacciones vector-patógeno, particularmente para la detección de arbovirus como los flebovirus (p. ej., virus Toscana) [44, 75]. Debido al tamaño diminuto de los flebotominos, el procedimiento requiere precisión bajo un estereomicroscopio, utilizando pinzas finas o agujas de microdisección para aislar las delicadas glándulas salivales sin causar ruptura ni contaminación (https://zenodo.org/records/18302850) [51, 61]. La preservación de la integridad glandular es crucial para garantizar análisis moleculares posteriores fiables. Una vez extraídas, las glándulas pueden homogenizarse y analizarse mediante RT-PCR, qPCR o inmunoensayos para detectar

ARN viral o antígenos [12]. La presencia de virus en las glándulas salivales, y no solo en el intestino o el hemocele, confirma que el patógeno ha completado su período de incubación extrínseco y es transmisible durante la alimentación sanguínea [71].

El proceso de disección es técnicamente exigente debido al pequeño tamaño de las glándulas salivales de los flebotominos, lo que requiere una experiencia considerable para evitar la degradación de las muestras [1, 51]. Además, las cargas virales pueden ser bajas, lo que hace necesarios métodos de detección altamente sensibles, como PCR anidada o secuenciación de alto rendimiento [54]. Los riesgos de contaminación refuerzan aún más la necesidad de técnicas estériles. Más allá de los desafíos técnicos, los factores biológicos influyen en el éxito de la detección; la competencia vectorial varía entre especies de flebotominos, y las tasas de infección fluctúan según las condiciones ecológicas y la estación [33, 61].

La detección de virus en las glándulas salivales proporciona información crítica sobre los riesgos de transmisión, lo que permite una vigilancia y medidas de control dirigidas [15] ha permitido optimizar los protocolos diagnósticos y reforzar las estrategias de vigilancia y comunicación en salud pública. [18]. Además, el estudio de las interacciones virus–saliva podría revelar nuevos objetivos para vacunas o terapias que bloqueen la transmisión [15, 18].

Las glándulas salivales de los flebotominos también pueden utilizarse como fuente de antígenos para medir anticuerpos del hospedador contra la saliva de flebotominos mediante métodos inmunológicos, preferentemente ELISA. Este método permite evaluar la exposición del hospedador a la picadura de flebotominos, apoyando así la evaluación de la eficacia de los métodos de control vectorial [25] y del riesgo de transmisión de Leishmania [40].

4.6 Identificación de la ingesta de sangre

En las hembras ingurgitadas se puede extraer el ADN de la sangre para identificar la especie/s de hospedador de la que se alimentó el flebotomino. Para su disección, se debe emplear material de un solo uso para evitar la contaminación cruzada. Su abdomen debe examinarse bajo un estereomicroscopio para evaluar el estadio de digestión de la sangre en el abdomen. Se recomienda seleccionar únicamente hembras con abdomen rojo, marrón rojizo u oscuro, que no muestren signos de formación de huevos. Retire la punta del abdomen, incluidas las espermatecas, para identificar morfológicamente a la hembra tras el aclaramiento. La parte principal del abdomen (sin las espermatecas) debe colocarse en tubos Eppendorf® y almacenarse a −20 °C hasta su análisis posterior. Los marcadores genéticos comúnmente utilizados para la identificación de la fuente sanguínea, como PNOC [5, 30, 50], CytB [67] o COI [13], están bien establecidos y ampliamente descritos en la literatura; por lo tanto, no se detallarán más en este trabajo (Figura 6). Alternativamente, para identificar la sangre del hospedador, puede emplearse el mapeo peptídico MALDI-TOF [31]. Se ha demostrado experimentalmente que esta técnica permite identificar la sangre del hospedador en un intervalo de tiempo más largo tras la ingestión de la comida sanguínea; por lo tanto, es un método adecuado, especialmente para el análisis de hembras ingurgitadas con digestión de sangre del hospedador visiblemente más avanzada. Idealmente, las muestras deben almacenarse a −20 °C o 4 °C, pero también pueden obtenerse buenos resultados a partir de muestras almacenadas a temperatura ambiente durante un corto período. El abdomen de una hembra ingurgitada debe diseccionarse del resto del cuerpo poco antes del análisis y homogenizarse en agua destilada. El resto del cuerpo del flebotomino queda disponible para otros análisis moleculares y morfológicos. Tras tomar la alícuota del homogenizado para el mapeo peptídico MALDI-TOF, el resto puede utilizarse para la extracción de ADN con el fin de confirmar la identificación de la sangre del hospedador y/o detectar la presencia de *Leishmania* spp. El tiempo total de preparación y análisis de las muestras para la técnica MALDI-TOF es muy corto en comparación con las técnicas moleculares basadas en ADN.

5 Procesamiento de especímenes para estudios morfológicos (Figuras 3, 6, 7 y 8; Apéndices 1, 2, 3 y 4)

Esta sección describe los principios para preparar un espécimen (flebotomino) para su montaje, destinado exclusivamente a estudios morfológicos, seguidos de su adaptación para aplicaciones más allá de la morfología. Sin embargo, comprender esta metodología es crucial, ya que permite adaptar los procedimientos a tipos de muestras específicos cuando sea necesario.

El tratamiento implica pasos sucesivos de vaciado y llenado utilizando pipetas Pasteur equipadas con peras de goma flexibles. Se recomiendan encarecidamente los recipientes de vidrio con fondo redondeado, ya que facilitan enormemente estas operaciones. El vidrio es inerte frente a todos los reactivos. Para evitar la evaporación de los reactivos, los recipientes deben estar provistos de tapas y nunca sobrellenarse, lo que podría provocar derrames al cerrar o abrir, ni permitir que caiga polvo sobre las muestras. Los productos químicos necesarios para el aclaramiento y procesamiento se muestran en la Tabla 2.

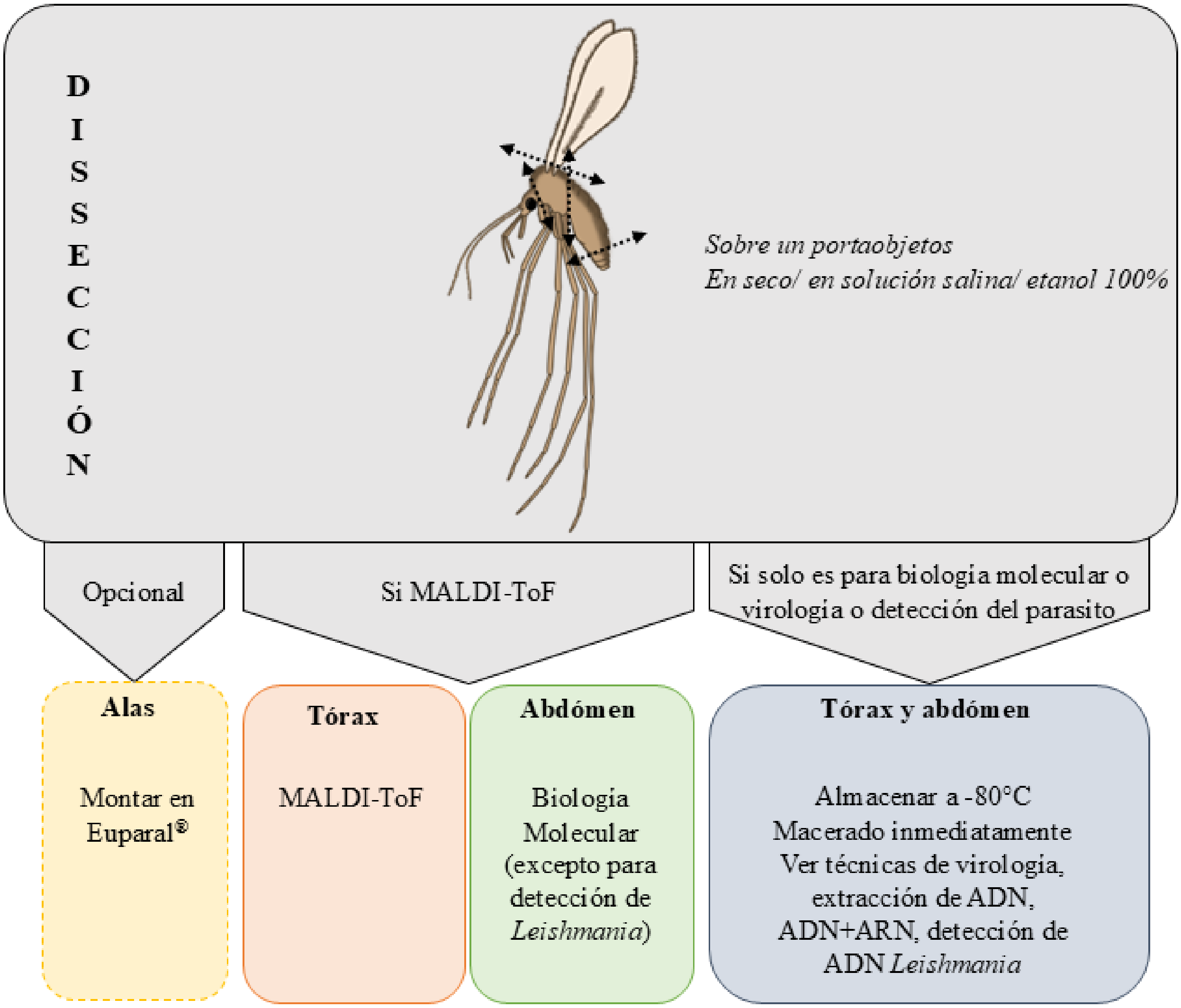

Figura 6: Procesamiento de flebotominos para aplicaciones de biología molecular, proteómica y/o virología.

5.1 Aclaramiento

Antes de que los especímenes de flebotominos puedan prepararse como montajes permanentes en portaobjetos, primero deben aclararse mediante maceración utilizando un método y un agente de aclaramiento adecuados (es decir, solución de ácido acético al 10 % o solución de Marc-André que incluye hidrato de cloral, un compuesto químico restringido en muchos países) para hacerlos transparentes. El proceso de aclaramiento elimina los tejidos corporales, grasas, secreciones y ceras, volviendo el espécimen translúcido y facilitando el examen de las estructuras del exoesqueleto (p. ej., inserción de setas), las características superficiales (p. ej., coloración) y las estructuras internas visibles a través del tegumento (p. ej., espermatecas).

El proceso de aclaramiento en dos pasos, que implica primero el uso de una base fuerte (como el hidróxido de potasio), seguido de un ácido débil (como el ácido acético en la solución de Marc-André), cumple propósitos bioquímicos distintos [74]. La base descompone los tejidos blandos, como proteínas, grasas y músculos, mediante saponificación y desnaturalización proteica, dejando intacto el exoesqueleto de quitina para una mayor claridad estructural. El ácido débil posterior neutraliza cualquier resto de álcali, evitando una degradación adicional, y blanquea la quitina para mejorar la transparencia [74], aunque el lavado de los especímenes dos veces en agua destilada durante 15 minutos también puede ser suficiente para neutralizar la base. Este tratamiento secuencial combina una eliminación eficaz de tejidos con una conservación que mantiene la integridad del espécimen, para el examen microscópico.

Se recomiendan dos enjuagues de 20 minutos en agua destilada antes de proceder al siguiente paso.

5.1.1 Lisis de tejidos blandos (Figura 8)

El hidróxido de sodio (NaOH) o el hidróxido de potasio (KOH) son agentes químicos de maceración comúnmente

This is an Open Access article distributed under the terms of the Creative Commons Attribution License (https://creativecommons.org/licenses/by/4.0), which permits unrestricted use, distribution, and reproduction in any medium, provided the original work is properly cited.

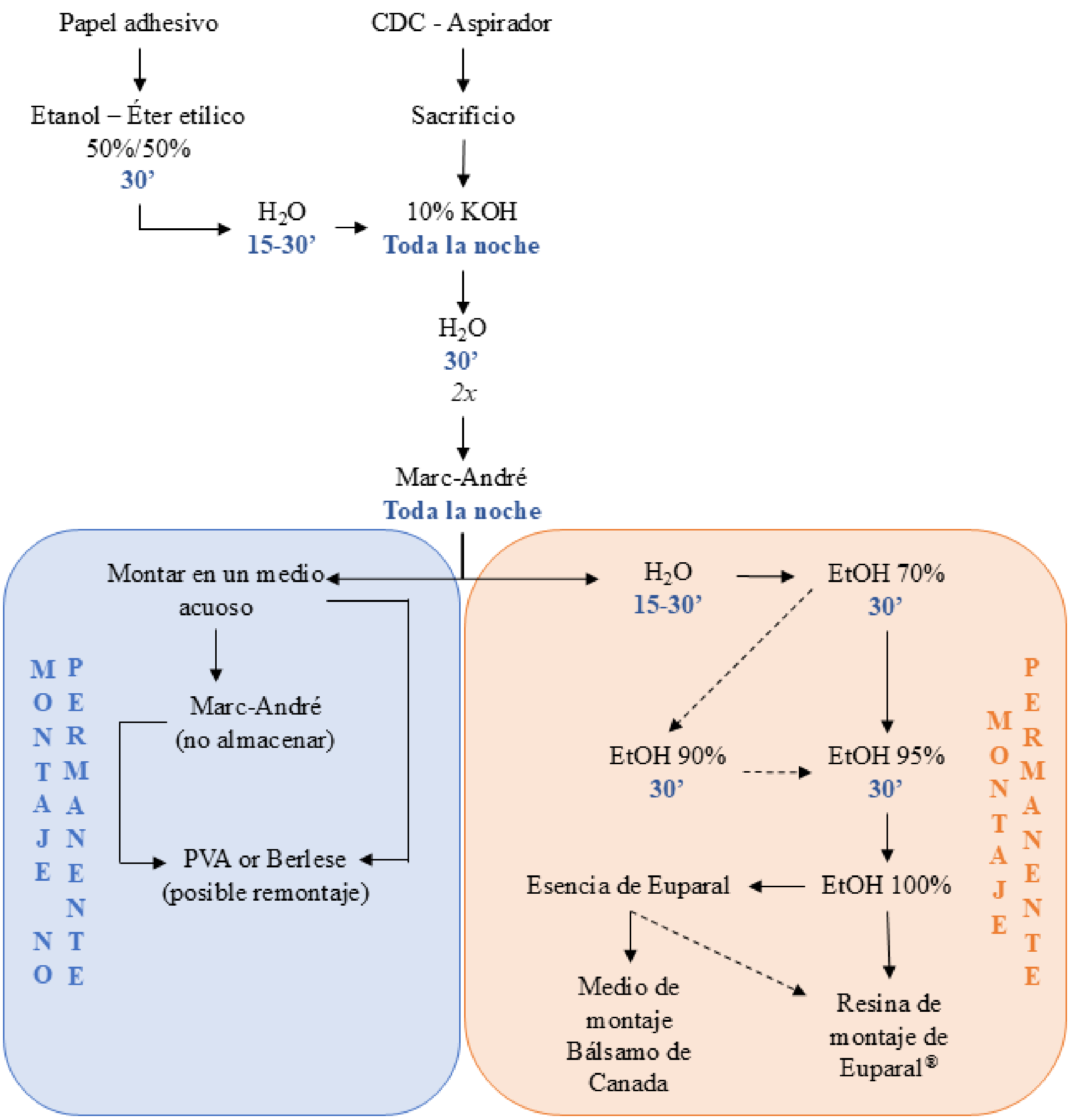

Figura 7: Método clásico para procesar flebotominos.

utilizados, aplicados a diferentes concentraciones y durante distintos períodos según el tamaño y la fragilidad de los especímenes. La técnica estándar y más eficaz consiste en lisar los tejidos blandos mediante la inmersión de los flebotominos en una base fuerte (KOH o NaOH al 10 %) durante toda la noche. La concentración puede incrementarse para reducir la duración del tratamiento (p. ej., KOH al 20 % durante 6 horas), así como mediante calentamiento a 37 °C.

5.1.2 Aclaramiento con o sin tinción

Este paso va seguido de un tratamiento de aclaramiento o blanqueamiento, que normalmente combina ácido acético e hidrato de cloral (por ejemplo, solución de Marc-André). Tras el aclaramiento, los especímenes deben enjuagarse cuidadosamente en al menos dos baños sucesivos de agua, de 20 minutos cada uno, para eliminar los residuos químicos.

La solución de Marc-André es un agente de aclaramiento ampliamente utilizado para la preparación de especímenes de flebotominos. Su eficacia radica en facilitar el proceso de aclaramiento mientras minimiza daños significativos en estructuras frágiles, como alas y antenas.

La solución debe prepararse recientemente o almacenarse en un recipiente herméticamente cerrado para evitar su evaporación o degradación. El uso de la solución de Marc-André es particularmente ventajoso cuando se combina con técnicas de aclaramiento o tinción para resaltar detalles morfológicos específicos. Los detalles sobre su composición y preparación se presentan en el Apéndice 2.

En especímenes altamente translúcidos, puede ser necesario aplicar una tinción para mejorar la visibilidad antes del montaje. Existen numerosos colorantes, cada uno dirigido a componentes químicos específicos del organismo

This is an Open Access article distributed under the terms of the Creative Commons Attribution License (https://creativecommons.org/licenses/by/4.0), which permits unrestricted use, distribution, and reproduction in any medium, provided the original work is properly cited.

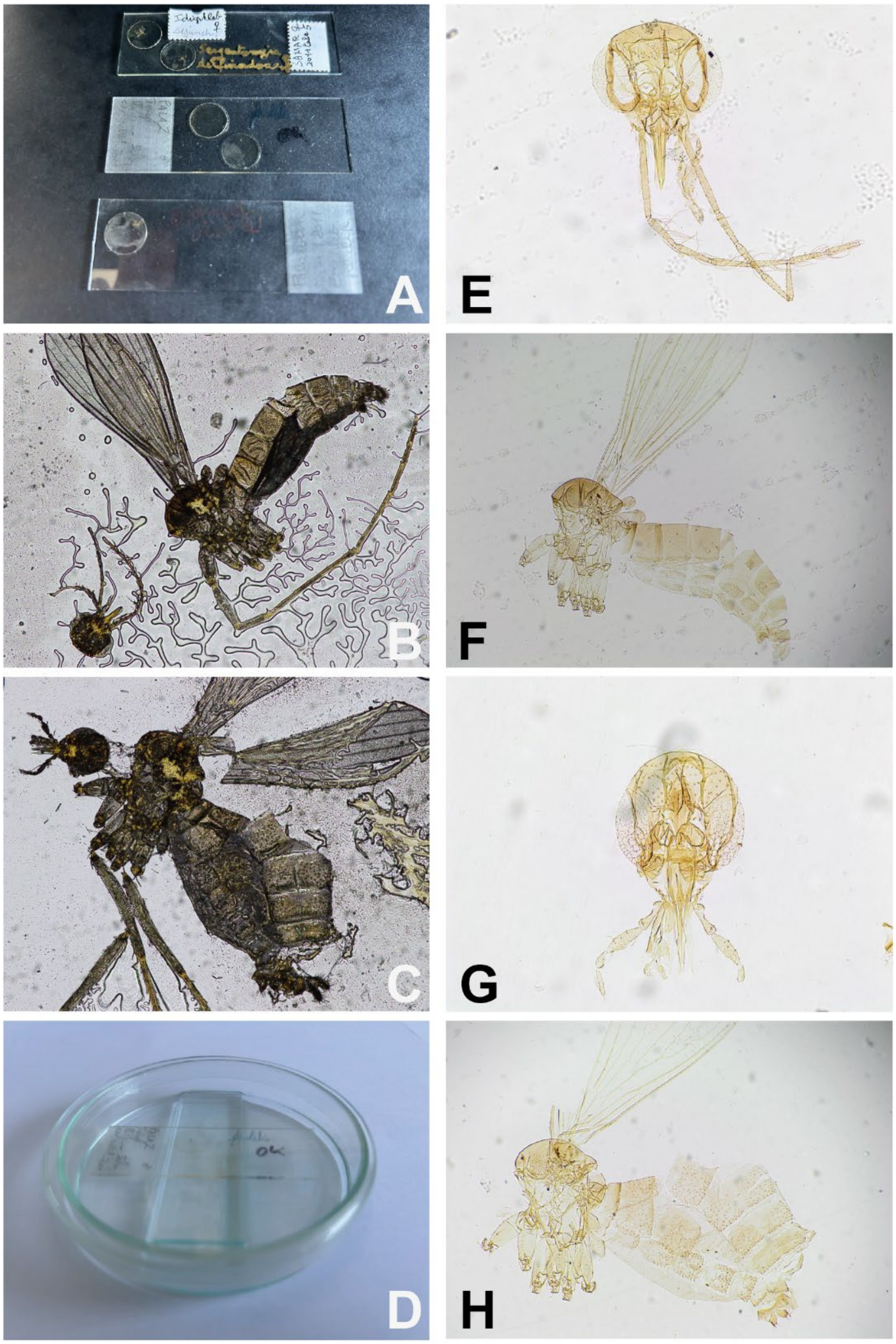

Figura 8: Remontaje de portaobjetos. A: portaobjetos dañados y secos montados en Hoyer; B: vista microscópica de un flebotino seco; C: vista microscópica de otro flebotomino dañado; D: cámara húmeda con un portaobjetos seco; E: cabeza y F: cuerpo del espécimen B tras su montaje en Euparal®; G: cabeza y H: cuerpo del espécimen C dañado tras su montaje en Euparal®.

Es importante seleccionar un colorante compatible tanto con el espécimen como con el medio de montaje elegido. Esta metodología básica puede adaptarse según sea necesario, por ejemplo, incorporando fucsina ácida al 0.1 % en la solución de Marc-André para la tinción. Además, los especímenes conservados en soluciones acuosas y destinados a montajes en medios resinosos requieren deshidratación (véase la Sección 5.2 sobre deshidratación), ya que la mayoría de los medios de montaje resinosos, naturales o sintéticos, son incompatibles con el agua. New (1974) señaló que algunos colorantes pueden deteriorarse en determinados medios de montaje [53]. Por ejemplo, la fucsina ácida, comúnmente utilizada con bálsamo de Canadá, también puede fijarse en Euparal®. Sin embargo,

This is an Open Access article distributed under the terms of the Creative Commons Attribution License (https://creativecommons.org/licenses/by/4.0), which permits unrestricted use, distribution, and reproduction in any medium, provided the original work is properly cited.

Tabla 2: Composición de los reactivos utilizados.

Hidróxido de potasio 10%	Fucsina ácida 1% en agua destilada
Hidróxido de potasio 10 g	Fucsina ácida (en polvo) 1 g
Agua destilada 100 mL	Agua destilada 99 mL
Medio de montaje con goma cloral (medio de Hoyer)	Solución de Marc-André coloreada con fucsina ácida
Agua destilada 50 mL	Solución de Marc-André 10 mL
Hidrato de cloral 200 g	Fucsina 1 % 50 µL
Goma arábiga 50 g	
Glicerol 20 mL	
Solución de Marc-André	Medio Enecê
Hidrato de cloral 40 g	Colofonia blanca pura 22 g
Ácido acético glacial 30 mL	Goma copal (Copal Manila) soluble en alcohol 12 g
Agua destilada 30 mL	Etanol absoluto 20 Ml
	Alcanfor 10 g
	Esencia de trementina 10 mL
	Eucaliptol 26 mL

los especímenes teñidos con fucsina ácida son propensos a la decoloración, especialmente cuando permanecen restos de aceite de clavo, utilizado como fluido final de aclaramiento. Los especímenes almacenados en aceite de clavo pueden mostrar una decoloración significativa en pocos días.

5.2 Deshidratación

La deshidratación se lleva a cabo transfiriendo gradualmente las muestras a través de una serie de soluciones de etanol de concentración creciente: 50%, 70%, 80%, 90% o 95% y, finalmente, 100%, manteniendo cada baño durante al menos 20 minutos. Dado que el etanol se evapora rápidamente, el recipiente debe mantenerse herméticamente cerrado durante el procesamiento. Una vez que el espécimen está completamente deshidratado, el procesamiento puede interrumpirse durante algunos días en esencia de Euparal®, la cual es preferible al aceite de clavo. El creosoto de haya, ampliamente utilizado en el pasado para este fin, se encuentra actualmente totalmente prohibido debido a su toxicidad.

El proceso de deshidratación debe garantizar que el fluido presente en el interior del espécimen sea compatible con el medio de montaje, con el fin de evitar opacidad, colapso osmótico o distorsiones que podrían hacer que el espécimen resulte inadecuado para estudios taxonómicos.

5.3 Medios de montaje

5.3.1 Selección y aplicación para la preparación de especímenes

Idealmente, el medio de montaje debe tener un índice de refracción lo más cercano posible al del vidrio, que es aproximadamente 1,5. Debe ser incoloro, claro y permanecer perfectamente transparente tras el secado y con el paso del tiempo. Debe ser compatible con los colorantes utilizados y capaz de penetrar y difundirse en todos los tejidos del espécimen. No debe secarse demasiado rápido ni formar velos u opacidades durante el montaje, ni contraerse después de la preparación.

La selección de un medio de montaje adecuado constituye un aspecto fundamental de la preparación de especímenes, ya que no existe un único medio ideal para todos los propósitos. La elección debe equilibrar varios factores clave:

- Propiedades ópticas. El índice de refracción del medio de montaje debe proporcionar suficiente contraste y refracción de las estructuras anatómicas críticas utilizadas para la identificación taxonómica o la descripción morfológica, tales como las espermatecas, los ascoides, las sensilas de Newstead, los dientes verticales del cibario y los dientes de la faringe. La visibilidad de estas estructuras depende directamente de las propiedades ópticas del medio de montaje.
- Preservación. En el caso de especímenes tipo o materiales destinados a colecciones permanentes, el medio debe ofrecer estabilidad y durabilidad a largo plazo. En contraste, para estudios de inventario o epidemiológicos, donde la preservación a largo plazo es menos crítica, pueden ser suficientes los medios de montaje temporales o semipermanentes.

5.3.2 Requisitos de los medios de montaje

Los especialistas suelen desarrollar técnicas de montaje personalizadas y complejas, adaptadas a necesidades de investigación específicas. Sin embargo, estos métodos con frecuencia pasan por alto aspectos como la calidad archivística, la compatibilidad, la estandarización o la facilidad de manejo y preservación a largo plazo. Esta falta

This is an Open Access article distributed under the terms of the Creative Commons Attribution License (https://creativecommons.org/licenses/by/4.0), which permits unrestricted use, distribution, and reproduction in any medium, provided the original work is properly cited.

Tabla 3. Composición de algunos medios de montaje seleccionados.

Medio de montaje	Solvente	Prepolímero(s) o polímero(s) potencial(es)	Observaciones
Hoyer = goma de cloral	Glicerol, agua	Compuestos de goma arábiga	Agente macerante: hidrato de cloral
CMCP-9 (=carboximetil celulose fenol)	Agua (CMCP-9: 51−60%)	Alcohol polivinílico totalmente hidrolizado (CMCP-9: 0−5%)	CMC(P)-9: baja viscosidad / alta viscosidad
DMHF (dimetil hidantoína formaldehído)	Agua	N,N′-dimetilol dimetil hidantoína (di-metilol DMH) oligómeros unidos por puentes<br>Eter/metileno; red polimérica de DMH-formaldehído reticulada	
Bálsamo de Canadá	Xileno; componentes parcialmente volátiles del bálsamo (Δ³-careno, ácido levopimárico, limoneno, mirceno, ácido palústrico, β-felandreno, α-pineno, β-pineno)	bálsamo (abienol, ácido abiético, ácido isopimárico, ácido sandaracopimárico)	Neutralización: carbonato de potasio; resina de *Abies balsamea* (Linné, 1758)
Euparal®	Eucaliptol, paraldehído; componentes parcialmente volátiles de la goma sandáraca (limoneno, α-pineno, β-pineno)	Compuestos de la goma sandáraca (ácido comunínico, manool, ácido policomunínico, ácido sandaracopimárico, ácido 12-acetoxi-sandaracopimárico, sugiol, ácido torulósico, torulosol, totarol)	Agente aclarante: salicilato de metilo; color del Euparal® verde: sal de cobre (abietinato de cobre); resina sandáraca de *Tetraclinis articulata* (Vahl, 1791)
Enecê	Alcohol etílico; con alcanfor, eucaliptol y esencia de trementina	Compuestos de goma copal y colofonia (rosina)	

de estandarización dificulta la integración de colecciones donadas y los esfuerzos de curaduría a largo plazo.

Las aplicaciones científicas imponen requisitos distintos para los medios de montaje. Los taxónomos suelen montar especímenes completos y prefieren medios que maceren suavemente los órganos internos para mejorar la visibilidad de las estructuras cuticulares. El índice de refracción del medio de montaje debe diferir lo suficiente del índice del espécimen y del portaobjetos de vidrio, con el fin de maximizar la claridad óptica. Los medios de montaje comerciales suelen formularse con un índice de refracción cercano al del vidrio, con el fin de minimizar la refracción y dispersión de la luz a través del sistema portaobjetos–medio de montaje–cubreobjetos. No obstante, en microscopía de campo claro, el contraste natural de un espécimen sin teñir puede manipularse deliberadamente mediante la elección de un medio de montaje con un índice de refracción ligeramente diferente al del espécimen, mejorando así su visibilidad frente al fondo.

5.3.3 Tipos de medios de montaje (Tablas 3 y 4)

La microscopía requiere considerar el índice de refracción (IR) del medio de montaje para determinar cómo se desvía la luz a través del portaobjetos, el medio y el espécimen. Cuando el IR está estrechamente alineado con el del vidrio del cubreobjetos (≈1,515), la luz se transmite de manera uniforme, disminuyendo la dispersión y las distorsiones ópticas, lo que resulta en una mejor resolución y visibilidad de las estructuras finas. Por el contrario, una discrepancia en el IR puede provocar desenfoque, halos u ocultar características no teñidas. Debido a los distintos índices de refracción de los diferentes medios, la selección del medio de montaje adecuado es crucial para optimizar el contraste, la claridad y la calidad general de la imagen para un espécimen determinado.

El índice de refracción del medio de montaje tiene un impacto significativo en la observación de estructuras finas durante la preparación de flebotominos en montajes permanentes. Las estructuras delicadas y débilmente esclerotizadas de los flebotominos, como la armadura cibarial, las espermatecas, los segmentos antenales y la venación alar, pueden resultar difíciles de observar en medios de montaje con un índice de refracción elevado.

En flebotominos, las opciones más utilizadas incluyen medios a base de goma cloral como medios de montaje acuosos, y el bálsamo de Canadá y la resina Enecê – Nelson

Cerqueira (NC) como medios de montaje a base de solventes. Rawlins [60] clasificó los medios de montaje en dos tipos: (1) medios permanentes: que se endurecen con el tiempo y son adecuados para la conservación a largo plazo, y (2) medios semipermanentes, que no endurecen completamente y se utilizan generalmente con fines temporales.

Los medios de montaje pueden ser líquidos, a base de gomas o resinosos, y ser solubles en agua, alcohol u otros solventes (p. ej., tolueno, xileno) (Tabla 3). Una vez aplicados, deben sellarse frente a los efectos atmosféricos mediante medios de sellado no solubles. Para distinguir claramente entre los distintos tipos de medios de montaje, puede utilizarse la siguiente categorización:

(a) *Medios acuosos.* Estos medios se disuelven fácilmente en agua, lo que los hace adecuados para montajes temporales o semipermanentes. Generalmente son fáciles de manipular, pero pueden requerir sellado para evitar la exposición a la humedad atmosférica (es decir, medios goma–cloral y alcohol polivinílico), especialmente en climas tropicales húmedos.

(b) *Medios con tolerancia limitada al agua*. Estos medios son menos afectados por el agua, aunque aún requieren protección frente a una humedad excesiva. Proporcionan una mayor estabilidad a largo plazo en comparación con las opciones solubles en agua y se utilizan con frecuencia en montajes semipermanentes.

(c) *Medios solubles en hidrocarburos*. Estos medios se disuelven en solventes orgánicos como xileno o tolueno, o en essencê (solvente enecê). Están diseñados para montajes permanentes y ofrecen una excelente estabilidad a largo plazo; además, resisten la humedad y la degradación, lo que los hace ideales para fines de archivo (p. ej., bálsamo de Canadá neutral).

En resumen, los medios solubles en agua son los más adecuados para montajes temporales o para casos en los que se requiera una fácil remoción del espécimen; los medios con tolerancia limitada al agua son apropiados para montajes semipermanentes que requieren una durabilidad moderada; y los medios solubles en hidrocarburos son preferidos para montajes permanentes destinados a conservación archivística y almacenamiento a largo plazo.

5.3.4 Descripción de los medios de montaje recomendados (Tablas 3 y 4)

Medios para observación temporal

Goma de cloral = fluido/medio/solución Hoyer (IR = 1,48). El medio de Marc André es el mejor medio para la observación a corto plazo (unas horas, quizá algunas más si el portaobjetos se almacena en una cámara húmeda) de espermatecas, incluyendo fotografías (Figura 4) o dibujos. La conservación de las espermatecas observadas requiere su remontaje en un medio acuoso que permita su almacenamiento a medio plazo. Deshidratarlas para remontaje en resina no es imposible, pero no se recomienda (riesgo de pérdida). La goma de cloral y el medio de Hoyer se consideran sinónimos. Este medio se utiliza comúnmente para la observación de órganos internos debido a su compatibilidad con el agua, simplicidad, rápida aplicación e índice de refracción que facilita el examen de estructuras delicadas como las espermatecas. Sin embargo, la goma de cloral presenta inconvenientes significativos si no se prepara correctamente o se almacena en condiciones de humedad controlada. Estos problemas incluyen cristalización, decoloración y pérdida de viscosidad. El anillado del cubreobjetos no resuelve estos problemas, ya que el medio de montaje puede decolorarse considerablemente (a veces casi negro) debido a la interacción con el medio de anillado, especialmente si se utiliza Euparal®.

El medio Hoyer se ha considerado ópticamente el mejor para los flebotominos y se ha utilizado tradicionalmente para estos fines. El medio consta de varias formulaciones estrechamente relacionadas, como la goma arábiga, el glicerol y el hidrato de cloral. Varias formulaciones han sido malinterpretadas y citadas erróneamente [74].

Aunque el medio Hoyer es un buen medio para observar espermatecas en los flebotominos, no es adecuado para la conservación a largo plazo. Es ideal para observaciones a corto plazo, como fotografías, dibujos o imágenes. Los medios acuosos son adecuados para montajes temporales, pero no garantizan una conservación a largo plazo. Por el contrario, el montaje con resina ofrece una excelente durabilidad, que a menudo dura siglos, pero puede ocultar detalles finos de las espermatecas, ya que su refringencia se pierde con frecuencia. El medio Hoyer se degrada con el tiempo debido a la deshidratación (Figura 8), lo que resulta en la formación de pequeños cristales blancos y opacos de hidrato de cloral. Sin embargo, se pueden recuperar muestras de portaobjetos cristalizados, ya que la cutícula permanece químicamente intacta, aunque el crecimiento de los cristales puede causar algún daño físico. En algunos casos, los portaobjetos cristalizados se pueden restaurar rehidratando el medio de montaje en un ambiente cálido y húmedo con timol para prevenir el crecimiento de hongos. Como alternativa, las muestras se pueden remojar en agua para eliminar la goma cloral, deshidratarlas en ácido acético glacial y volver a montarlas en bálsamo de Canadá.

DMHF (dimetilhidantoína formaldehído) (RI 1,48). Este medio a base de agua [72] presenta un excelente

Tabla 4. Ventajas y desventajas de algunos medios de montaje para preparaciones microscópicas, basadas en observaciones no publicadas de diversas personas [52].

Nombre	Ventajas	Desventajas
* Bálsamo de Canadá	Medio altamente duradero, con una vida útil superior a 150 años.<br>Las preparaciones pueden montarse utilizando aceite de clavo o fenol como agentes de montaje.	Contiene componentes nocivos y debe manipularse bajo campana de extracción.<br>Requiere una serie completa y prolongada de deshidratación.<br>La deshidratación en etanol y la transferencia mediante xileno o aceite de clavo pueden volver frágiles algunos taxones; alternativas (p. ej., isopropanol, *n*-butanol, Cellosolve™, 1,4-dioxano, Histoclear, terpineol) pueden reducir la rotura.<br>Los especímenes pueden ennegrecerse si el xileno se sustituye por fenol o si permanecen residuos de hidróxido de potasio.<br>Los altos índices de refracción pueden ocultar estructuras no teñidas.<br>El secado completo puede tardar años si no se utiliza placa calefactora.<br>El medio amarillea y se oscurece con el tiempo, especialmente cuando se aclara con aceite de clavo.<br>Algunas tinciones se debilitan y los colorantes catiónicos pueden desvanecerse si el medio se vuelve ácido, lo cual puede ocurrir espontáneamente con el tiempo.
DMHF (dimetilhidantoína formaldehído)	Alta transparencia<br>Buen índice de refracción<br>Excelente visibilidad de las estructuras<br>Estabilidad bastante buena de las preparaciones<br>Compatible con diversas técnicas de tinción<br>Buena protección de las muestras<br>Buena adhesión entre el portaobjetos y el cubreobjetos	Posible amarilleamiento con el tiempo<br>Puede alterar algunas manchas<br>No apto para manchas sensibles al formaldehído<br>Burbujas de aire, secado lento<br>Medio de montaje sensible a la humedad<br>El montaje es difícil de revertir<br>El formaldehído es tóxico, irritante y cancerígeno
*Euparal (transparente)	Medio duradero, con una vida útil superior a 50 años.<br>Permite el montaje directo desde etanol al 80 % (recomendación del fabricante).<br>No enmascara estructuras no teñidas y no amarillea ni se vuelve frágil con el tiempo.<br>Posee un índice de refracción más adecuado que el bálsamo de Canadá para Dípteros.<br>Funciona bien con especímenes más gruesos debido a la mínima contracción y al secado sin burbujas.<br>Permanece soluble en etanol al 95 %, lo que permite el remontaje incluso tras muchos años.	Contiene componentes nocivos y debe manipularse bajo campana de extracción.<br>La deshidratación en etanol y la transferencia mediante esencia de Euparal pueden volver frágiles algunos taxones, aunque el uso de isopropanol puede reducir este problema.

(Continua en la página siguiente)

Tabla 4. (Continuación)

Nombre	Ventajas	Desventajas
Fluido de Hoyer	Los especímenes pueden montarse vivos o directamente desde agua, etanol o formaldehído.<br>La maceración produce una excelente calidad de la cutícula.<br>Posee un índice de refracción favorable y puede mejorarse con tinción con yodo para mayor contraste.<br>El ácido acético de la fórmula puede expandir los apéndices de los artrópodos.<br>Algunos especímenes pueden permanecer estables durante 40–60 años.<br>Soluble en agua, lo que permite un remontaje sencillo.	Los especímenes vegetales delicados pueden colapsar si el medio no se añade gradualmente, lo cual es un proceso lento.<br>Pueden formarse cavidades y cristales en menos de 10 años.<br>La maceración puede ser excesiva según la concentración de hidrato de cloral y el tiempo de exposición.<br>Los componentes del medio pueden separarse y aparecer una granulación fina en meses o años.<br>Se ha reportado ennegrecimiento del medio.
CMCP-9 (=carboximetil celulosa fenol)	Los especímenes pueden montarse directamente desde medios como agua, etanol, glicerol o soluciones con formaldehído, y los órganos internos pueden macerarse cuando sea necesario para facilitar el examen general o la preparación.	El medio puede formar cristales y oscurecerse con el tiempo, y en ocasiones puede macerar los especímenes más de lo deseado.<br>Si la preparación no se sella cuidadosamente, las muestras gruesas se contraen y generan espacios alrededor del cubreobjetos.<br>No es adecuado para especímenes teñidos ni para materiales calcificados.<br>El tiempo de secado es más lento que el de la CMC.
Eukitt™	Medio duradero, con una vida útil superior a 30 años.<br>Compatible con numerosos solventes de montaje, incluyendo acetona, benceno, cloroformo, dioxano, éter, isopropanol, benzoato de metilo, terpineol, tolueno y xileno.<br>Seca rápidamente y presenta un pH ligeramente ácido.<br>No se oscurece de forma apreciable con el envejecimiento.<br>Adecuado para diversas tinciones (p. ej., fucsina, hematoxilina, verde de metilo, violeta de metilo, azul de metileno).<br>Los especímenes pueden remontarse tras años de inmersión prolongada en xileno.	Contiene componentes nocivos y debe manipularse bajo campana de extracción.<br>Requiere una serie completa y prolongada de deshidratación.<br>No es ideal para especímenes gruesos debido a la contracción y la formación de burbujas de gas.<br>Los cubreobjetos pueden desprenderse con el tiempo si el vidrio no se limpia bien y no se sella adecuadamente.<br>Puede presentar polimerización incompleta alrededor de fibras de colágeno.
Enecê	Medio altamente duradero, con una vida útil de al menos 50 años.<br>No se oscurece con el tiempo.<br>Es más maleable, lo que permite la disección de insectos dentro del medio y proporciona un tiempo razonable para posicionar las estructuras morfológicas.<br>Bajo costo.	Requiere una serie completa y prolongada de deshidratación.<br>La deshidratación en etanol y la transferencia mediante aceite de clavo pueden volver frágiles algunos especímenes.<br>El insecto continúa aclarándose, aunque muy lentamente; esto puede dificultar la observación de estructuras muy pequeñas, como sensilas, ascoides y setas simples.

This is an Open Access article distributed under the terms of the Creative Commons Attribution License (https://creativecommons.org/licenses/by/4.0), which permits unrestricted use, distribution, and reproduction in any medium, provided the original work is properly cited.

rendimiento óptico, similar al del Berlese, y es tan fácil de usar como este. Sin embargo, a diferencia del Berlese, no se ennegrece ni cristaliza. Funciona bien para flebotominos y otros Psychodidae.

CMCP (alcanfor-monoclorofenol) (IR = 1,41). Este medio de montaje, soluble en agua y a base de glicerina, se utiliza para crear portaobjetos transparentes y permanentes de especímenes delicados, incluyendo flebotominos. La ventaja de este medio de montaje es que los especímenes se pueden montar directamente en agua o etanol. También relaja y limpia rápidamente el flebotomino, ablandando la cutícula para permitir una correcta colocación del espécimen, lo cual es especialmente útil para extender las alas o diseccionar los genitales. Aunque se ha informado que permite el almacenamiento a largo plazo, la duración exacta de la conservación sigue siendo incierta. La principal limitación de este medio de montaje reside en su composición, que contiene fenol, una sustancia tóxica e irritante que requiere una manipulación cuidadosa.

Medios para montaje permanente

Bálsamo de Canadá (IR = 1,52-1,54). El bálsamo de Canadá fue descrito por primera vez como un medio de montaje adecuado para microscopía de luz transmitida por Andrew Pritchard en la década de 1830. Sigue siendo uno de los medios más utilizados debido a su probada calidad de archivo, con más de 150 años de aplicación exitosa. A diferencia de los medios fluidos Hoyer, el bálsamo de Canadá no cristaliza ni absorbe humedad. Sin embargo, el bálsamo de Canadá es fuertemente autofluorescente, lo que a veces puede ser una desventaja para ciertas técnicas de microscopía [60]. El uso de disolventes no tóxicos en lugar de xileno puede reducir los riesgos de seguridad durante la preparación, pero también puede presentar inconvenientes como un secado más lento y un oscurecimiento más temprano del medio.

Euparal® (IR = 1,48). Euparal® es una alternativa ampliamente utilizada al bálsamo de Canadá para montaje permanente, que ofrece una excelente estabilidad a largo plazo y un índice de refracción comparable. Euparal® presenta las siguientes características: (1) Requisito de deshidratación: antes de la transferencia final del medio de montaje, la muestra debe deshidratarse, generalmente pasando de etanol al 95 % a etanol absoluto. Además, (2) Tiempo de procesamiento prolongado: el ensamblaje final en una resina, ya sea bálsamo de Canadá o Euparal®, requiere deshidratación, lo que prolonga el tiempo total de procesamiento de la muestra. Cuando la deshidratación con disolventes orgánicos no es factible, las muestras extraídas del etanol absoluto pueden colocarse en una solución intermedia compuesta por una mezcla a partes iguales de Euparal® y esencia Euparal® antes del montaje final.

Enecê (IR = 1,467). El enecê es un medio de montaje a base de resina, utilizado principalmente para insectos pequeños, y es particularmente popular en Brasil. Su base consiste en colofonia y goma copal disueltas en alcohol, alcanfor, esencia de trementina y eucaliptol. Cerqueira [11] describió el enecê como una alternativa al bálsamo de Canadá para el montaje de preparaciones permanentes de larvas, exuvias de inmaduros e incluso mosquitos adultos, y desde entonces se ha adoptado ampliamente para el montaje de flebotominos. El enecê ofrece una alternativa rentable para el montaje permanente, proporcionando estabilidad a largo plazo y un tiempo de secado suficiente, lo que permite la disección y la disposición precisa de las estructuras morfológicas.

5.4 Preparación y secado de las preparaciones

El secado adecuado de las preparaciones montadas es fundamental para garantizar su estabilidad y conservación a largo plazo. Las preparaciones deben secarse completamente antes de considerar su almacenamiento a largo plazo. Para obtener resultados óptimos, los portaobjetos montados con medios de montaje permanentes deben secarse horizontalmente durante 2 a 3 semanas, mientras que los preparados con medios semipermanentes pueden requerir solo 1 a 2 semanas. Para garantizar un secado eficaz, se recomienda utilizar una incubadora a una temperatura adecuada para el medio de montaje utilizado, evitando el calor excesivo que podría dañar las muestras. Se recomienda un rango de temperatura de 30°C a 37°C. Este paso de secado es crucial para evitar la deformación de los portaobjetos, el deterioro de las muestras o la inestabilidad del medio de montaje durante el almacenamiento.

El medio de montaje utilizado en la preparación de los portaobjetos siempre debe indicarse en la etiqueta. Si es posible, la etiqueta también debe incluir la receta específica utilizada, junto con el nombre de la persona que realiza la preparación y la fecha de preparación. Los portaobjetos se preparan inicialmente como montajes temporales y no están destinados a la conservación a largo plazo. Sin embargo, si el estado del espécimen cambia, por ejemplo, si se designa como parte de una serie "tipo", se debe utilizar un medio de montaje más permanente para garantizar su conservación para futuros estudios taxonómicos.

5.5 Técnicas alternativas de montaje: montaje en tarjetas

El montaje en tarjetas es una técnica utilizada para varios grupos de insectos, en la que los especímenes pueden fijarse directamente sobre tarjetas entomológicas o pegarse a la superficie. Dado su pequeño tamaño y la necesidad de observar los órganos internos para su identificación mediante aclaración (véase el punto 5), este método no es adecuado para el montaje de flebotominos.

This is an Open Access article distributed under the terms of the Creative Commons Attribution License (https://creativecommons.org/licenses/by/4.0), which permits unrestricted use, distribution, and reproduction in any medium, provided the original work is properly cited.

5.6 Remontaje de muestras dañadas

Para muestras raras o valiosas, se recomienda un enfoque de dos pasos, según el video disponible en: https://zenodo.org/records/18315029. 1) Rehidrátelas sin desmontarlas para permitir una observación preliminar. Se debe colocar un soporte para varios portaobjetos en una placa de Petri. El portaobjetos que se va a rehidratar se coloca encima y la placa de Petri se llena con unos pocos milímetros de disolvente para crear una cámara húmeda, asegurando que el propio portaobjetos no entre en contacto con el disolvente (Figura 8 D). El tiempo necesario para la rehidratación puede variar de uno a varios días, dependiendo del estado de la muestra. La monitorización diaria y la paciencia son esenciales. Una vez que el portaobjetos esté suficientemente rehidratado, se puede retirar de la cámara húmeda y colocarlo en una incubadora durante unas horas antes de examinarlo al microscopio, fotografiarlo o dibujarlo. 2) Para volver a montarlo, el portaobjetos puede devolverse a la cámara húmeda durante unas horas más o durante la noche. El desmontaje debe realizarse bajo un microscopio binocular. Con agujas finas, se debe retirar cuidadosamente el cubreobjetos, asegurándose de que no queden adheridos elementos del flebotomino (https://zenodo.org/records/18315029). A continuación, los elementos disecados del flebotomino se deben recolectar y enjuagar con agua en pocillos pequeños, como los utilizados para la extracción destructiva de ADN/ARN (véase más adelante), antes de deshidratarlos y volver a montarlos en un medio de resina. Al desmontar un portaobjetos, es fundamental identificar el medio de montaje original para seleccionar el disolvente adecuado. Para medios de montaje acuosos, se debe utilizar agua. Si el medio de montaje es a base de resina (p. ej., bálsamo de Canadá o Euparal®), se debe utilizar xileno, bajo una campana extractora y con el equipo de protección personal adecuado, incluida una mascarilla.

El montaje de especímenes tipo o de colección solo debe realizarse con el consentimiento del conservador o de la institución propietaria del espécimen.

6 Identificación del espécimen

6.1 Morfología

La identificación de los flebotominos se basa principalmente en el examen de sus características morfológicas, incluyendo la forma del tórax, las alas, los genitales, las setas y las relaciones morfométricas específicas entre diversas estructuras. Los investigadores utilizan claves taxonómicas, colecciones de referencia y descripciones originales de especies para comparar los especímenes recolectados con taxones conocidos. Características diagnósticas clave, como la venación del ala y la morfología de la cabeza en ambos sexos, la estructura de los genitales masculinos y la configuración de las espermatecas femeninas, son especialmente informativas para la determinación de la especie. Una identificación precisa a menudo requiere un examen microscópico detallado, generalmente utilizando un microscopio compuesto para observar estructuras finas como los genitales y las espermatecas, o un estereomicroscopio para características morfológicas más amplias.

Los avances recientes en la tecnología de imagen han facilitado el uso de imágenes digitales para la identificación de flebotominos. Fotografías de alta resolución o ilustraciones digitales de características clave pueden compararse con materiales de referencia o analizarse mediante sistemas de identificación asistidos por computadora, lo que mejora la precisión y la accesibilidad en la taxonomía morfológica.

6.2 Geometría del ala

La geometría del ala es una característica clave para la identificación y clasificación de las diferentes especies de flebotominos. Las alas de los flebotominos presentan un patrón y una estructura únicos, típicamente largos y estrechos, con una venación bien desarrollada (Figuras 9 y 10). La disposición de las venas forma un patrón distintivo que puede variar entre géneros y especies, lo que proporciona valiosas características diagnósticas para la identificación. Por consiguiente, el estudio de la geometría del ala proporciona información valiosa para fines taxonómicos.

6.3 Morfometría geométrica del ala

Los investigadores utilizan diversas técnicas, como la morfometría geométrica, para analizar y comparar la forma y el tamaño de las alas en diferentes especies o poblaciones de flebotominos. El estudio de la geometría de las alas proporciona información valiosa sobre el comportamiento, las preferencias de hábitat y las habilidades de vuelo.

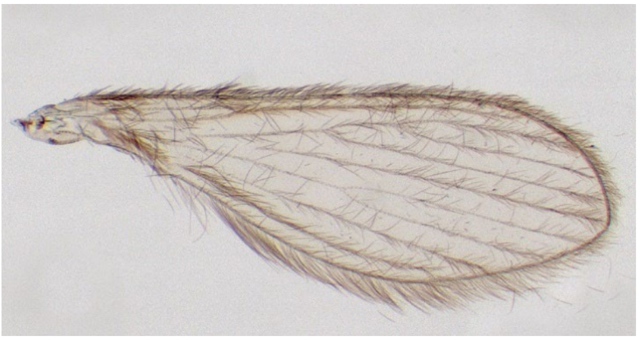

Figura 9: Ala sin procesar de *Trichophoromyia ininii.*

En el método morfométrico geométrico, las alas se disecan cuidadosamente, se tiñen (si es necesario) y se montan en portaobjetos. Los portaobjetos preparados se fotografían con un estereomicroscopio, se digitalizan y se someten a análisis morfométrico. Este procedimiento ha sido ampliamente descrito en la literatura [6, 27, 42, 56, 57, 59], y se recomienda utilizar el ala derecha o izquierda de forma uniforme para los órganos pareados a fin de evitar posibles efectos alométricos negativos [62].

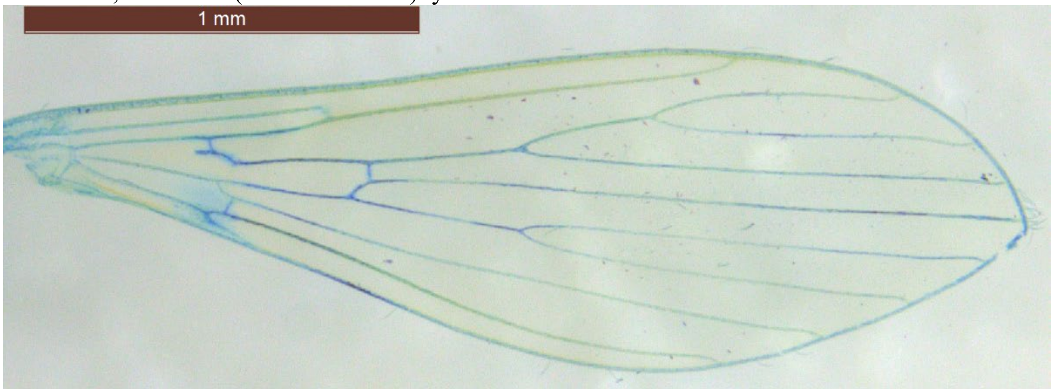

Figura 10: Ala coloreada de *Phlebotomus ariasi.*

Tabla 5: Costo promedio, aplicación y adaptación de protocolos para la extracción de ADNg de flebotominos

Protocolo	Costo	Aplicación	Adaptación del protocolo para artrópodos pequeños
Spin column	2.5–3.55 US$ [39]	PCR, NGS	[9]
Fenol-cloroformo	0.24 US$ [69]	PCR, NGS	[9]
HotSHOT	<0.01 US$ [69]	PCR	–
Salting out	0.12 US$ [69]	PCR	–
Chelex	0.02 – 0.04 US$ [41]	PCR	[41, 76]

Preparación de alas para análisis morfométrico geométrico

Para una visualización óptima de las venas alares, las alas deben limpiarse de escamas y teñirse adecuadamente. Para la preparación del ala, primero llene pequeños pocillos con los reactivos necesarios (azul de metileno, etanol, agua y sustituto de xileno). Recupere un ala conservada en etanol al 70% a temperatura ambiente invirtiendo el tubo Eppendorf y vaciándolo sobre el pocillo; luego, levante el ala longitudinalmente utilizando una aguja curva fina. Pase brevemente el ala de etanol a agua y nuevamente a etanol para eliminar las quetas. Coloque el ala en azul de metileno durante 6 minutos, asegurándose de que flote durante la tinción. Recupere el ala con cuidado y sumérjala en el sustituto de xileno durante 2 minutos (aproximadamente un tercio del tiempo del azul de metileno). Golpecitos suaves de la aguja contra las paredes del pocillo pueden ayudar a que el ala se asiente; el xileno sirve para fijar la coloración. Finalmente, levante el ala y colóquela sobre una pequeña gota de Euparal® en un portaobjetos de microscopio. Bajo una lupa, desdoble suavemente el ala y coloque cuidadosamente un cubreobjetos. Las fotografías deben tomarse rápidamente antes de que el Euparal® se fije, ya que pueden ser necesarios pequeños ajustes de la posición del ala bajo el cubreobjetos para lograr una alineación óptima.

6.4 Técnicas de biología molecular

Además de las técnicas morfológicas, los métodos moleculares son cada vez más esenciales en la investigación entomológica, incluyendo estudios taxonómicos, de genética de poblaciones y filogenéticos, así como para la detección de patógenos de ADN/ARN y para determinar el origen de las ingestas de sangre, siendo el comportamiento de los vectores importante en el campo de la epidemiología [70]. La secuenciación de ADN puede emplearse para la confirmación de especies o para diferenciar especies estrechamente relacionadas, proporcionando un medio de identificación más preciso y fiable. Además, técnicas moleculares avanzadas (como PCR, secuenciación de ADN, NGS, etc.) y la MALDI-ToF MS están ganando protagonismo para una identificación precisa y rápida de especies, complementando los métodos morfológicos tradicionales [46]. A pesar de estos avances, la

identificación morfológica sigue siendo el estándar de referencia para la taxonomía y la base sobre la que se interpretan los datos moleculares.

6.4.1 Extracción destructiva de ácidos nucleicos

La extracción de ácidos nucleicos es un paso rutinario en muchos estudios biológicos, y se han desarrollado diversos métodos para aislar ADN de material biológico [48]. Muchos kits de extracción de ADN disponibles comercialmente están diseñados para facilitar este proceso [14]. Sin embargo, los métodos comúnmente utilizados para preparar especímenes de artrópodos para identificación morfológica a menudo dificultan el análisis de ADN, ya que estas técnicas pueden dañar o destruir características físicas críticas del espécimen [10]. La mayoría de los protocolos de extracción de ADN de tejidos de insectos son de naturaleza destructiva [43], lo que plantea preocupaciones particulares para especímenes pequeños, donde incluso un muestreo limitado puede comprometer características morfológicas importantes [72]. El tipo y la condición del espécimen juegan un papel clave en la selección de un método de aislamiento de ADN apropiado [29].

La necesidad de identificar flebotominos con precisión, comprender la dinámica de sus poblaciones y minimizar los impactos no deseados ha impulsado el desarrollo de herramientas de diagnóstico molecular [23]. Los enfoques moleculares ahora se utilizan con frecuencia para complementar los métodos taxonómicos morfológicos en la identificación de flebotominos. Por ejemplo, el enfoque estándar para el código de barras de insectos implica la extracción y secuenciación de ADN, y la pérdida del espécimen original. Por lo tanto, existe una necesidad apremiante de explorar métodos de extracción de ADN no destructivos que preserven tanto el material biológico como su integridad morfológica.

Se han aplicado numerosos métodos de extracción de ácidos nucleicos a flebotominos. La cantidad o calidad de los ácidos nucleicos requeridos depende del análisis molecular posterior, ya que diferentes técnicas tienen requisitos de sensibilidad y pureza variables [9]. Por ejemplo, se ha observado que los ojos de los flebotominos inhiben la amplificación por PCR [69]. Más allá del cribado de patógenos, el ADN de flebotominos se extrae de forma rutinaria para fines de identificación de especies. Se pueden utilizar diversos métodos de extracción, aunque el rendimiento y la calidad difieren entre técnicas. Algunos protocolos de fabricantes han sido adaptados por investigadores para flebotominos [8], aumentando el rendimiento y/o la calidad de los ácidos nucleicos extraídos [8, 9, 69], mientras que otras adaptaciones, desarrolladas para otros taxones de artrópodos, también pueden usarse en flebotominos [58, 76].

Las PCR de identificación dirigidas a pequeños fragmentos mitocondriales (COI o CytB) son generalmente compatibles con métodos de extracción que implican una alta fragmentación del ADN. Por el contrario, otras técnicas de secuenciación de nueva generación (NGS) de lectura larga (Oxford Nanopore y PacBio) requieren una fragmentación mínima y ADN de alta calidad. Las extracciones por columnas de filtración generalmente producen fragmentos de ADN genómico de hasta 60 kb, mientras que la extracción con fenol-cloroformo puede producir fragmentos de hasta 150 kb [77]. La Tabla 5 resume diferentes técnicas de extracción de ADN de flebotominos e indica si se han realizado adaptaciones metodológicas para estos insectos. No se muestran los rendimientos, ya que dependen del tamaño del espécimen y del método de preparación. La columna de modificación se refiere a las adaptaciones de los protocolos de extracción para flebotominos u otros artrópodos pequeños.

La elección del método de extracción debe considerar varios criterios, como el número de muestras, el tiempo de extracción y la técnica a utilizar posteriormente. Si bien las técnicas de NGS requieren ADN genómico de alto peso molecular, todos los métodos aquí presentados pueden utilizarse para aplicaciones estándar basadas en PCR.

Además, varios estudios han explorado métodos de extracción de ADN no destructivos para artrópodos terrestres pequeños, especímenes de museo preservados en seco y artrópodos de cuerpo blando [19, 26, 28, 55, 63].

6.4.2 Extracción no destructiva de ácidos nucleicos

Uno de los principales desafíos en el análisis molecular de artrópodos, especialmente flebotominos, es la preservación de especímenes para su integración en colecciones entomológicas. La mayoría de los protocolos de extracción de ADN requieren la maceración del tejido, lo que compromete la conservación del espécimen original. Sin embargo, los métodos de extracción de ácidos nucleicos no destructivos están diseñados para obtener material genético sin dañar físicamente la muestra, afectar su viabilidad o alterar su morfología. Estos métodos son especialmente valiosos cuando se trabaja con especímenes preciosos o escasos, como los flebotominos, donde mantener la integridad estructural es esencial para futuros fines taxonómicos, morfológicos o de diagnóstico. Una técnica comúnmente empleada es el método de baño no destructivo, en el que los flebotominos son inmovilizados e inmersos suavemente en un tampón de lisis que contiene proteinasa K.

La técnica de vectólisis suave se ha aplicado con éxito a flebotominos, particularmente a especímenes tipo [24]. Esta técnica utiliza un kit convencional de columnas de filtración (en este caso, un kit DNeasy Blood and Tissue, QIAGEN, Hilden, Alemania) adaptado para obtener ADN

sin destruir el espécimen. Los pasos de lisis modificados (volumen del tampón de lisis y adición de una etapa de congelación) [17] permiten la liberación de ácidos nucleicos, minimizando el daño morfológico [24]. En cuanto a los flebotominos, también es posible utilizar un kit de extracción de ADN HotSHOT (Bento Bioworks Ltd, Londres, Reino Unido) [73], que es rápido y económico, permitiendo procesar muestras de manera ágil y a bajo costo.

Posteriormente, los especímenes entomológicos destinados a identificación morfológica pueden enjuagarse. Aquellos procesados con un kit DNeasy Blood and Tissue deben aclararse con solución de Marc-André, mientras que los procesados con el kit de extracción de ADN HotSHOT quedan suficientemente clarificados para montarse en un medio acuoso o, preferiblemente, en resina después de la deshidratación, de acuerdo con el protocolo detallado en este artículo [73].

El material genético extraído puede luego procesarse para análisis posteriores, como PCR, para amplificar marcadores genéticos específicos. Los métodos de extracción de ácidos nucleicos no destructivos son cruciales para estudiar las características genéticas de los flebotominos, incluyendo la identificación de posibles agentes patógenos que puedan portar. Al preservar la integridad del espécimen, los investigadores pueden obtener valiosa información genética, manteniendo la muestra disponible para análisis o estudios adicionales.

6.5 MALDI-ToF MS

MALDI-ToF MS (espectrometría de masas por desorción/ionización láser asistida por matriz con analizador de tiempo de vuelo) es una técnica basada en espectrometría de masas diseñada para detectar y analizar los perfiles proteicos únicos ("huellas digitales") de muestras biológicas. MALDI-ToF es cada vez más reconocida como una herramienta importante para la identificación de artrópodos de importancia médica y veterinaria. Esta técnica ha demostrado ser eficaz para identificar diferentes estadios de desarrollo de los flebotominos, incluidas las formas inmaduras y las fuentes sanguíneas de hembras ingurgitadas, y se ha aplicado con éxito para diferenciar especies de flebotominos tanto machos como hembras bajo una variedad de condiciones de almacenamiento y homogenización [28, 30, 73, 74]. Además, este método ofrece un alto poder de discriminación a nivel de subgéneros, especies y poblaciones.

Esta técnica permite a los investigadores lograr una identificación rápida y precisa de las especies, lo cual es esencial para comprender la distribución y el comportamiento de los flebotominos, así como su papel en la transmisión de enfermedades. Al diferenciar especies en función de sus perfiles proteicos, MALDI-ToF desempeña un papel crucial en los estudios epidemiológicos y en las estrategias de control de vectores.

Existen actualmente dos principales limitaciones de esta técnica que restringen su aplicación rutinaria. La primera es la disponibilidad de equipos de espectrometría de masas, que son prohibitivamente costosos para ser adquiridos únicamente con fines de identificación de especies de flebotominos (o de vectores artrópodos en general). Afortunadamente, esta limitación puede superarse mediante el acceso a tiempo de uso en espectrómetros de masas que se han convertido en una herramienta estándar en instalaciones de proteómica y/o diagnóstico clínico. La segunda limitación es la baja representación de datos de referencia de flebotominos disponibles en bases de datos de acceso abierto, lo que genera la necesidad de construir una base de datos propia con espectros de referencia basados en especímenes identificados de manera inequívoca, idealmente mediante una combinación de evaluación morfológica y secuenciación de un marcador genético adecuado (COI, cytB u otro).

Se espera que esta limitación se resuelva próximamente mediante la inclusión gradual de datos de referencia de flebotominos, hasta ahora de uso interno, en la plataforma MSI gestionada por Assistance Publique–Hôpitaux de Paris, Universidad de la Sorbona, Francia, y en la colección BCCM/IHEM/Sciensano en Bruselas, Bélgica (https://msi.happy-dev.fr/).

Cuando se planifique el uso del perfilado proteico por MALDI-ToF, las muestras deben almacenarse preferentemente congeladas en seco o en etanol al 70% de grado molecular y no exponerse a temperaturas ambientales. En ausencia de directrices universales para la preparación de muestras, se recomienda a los usuarios emplear una solución acuosa de acetonitrilo al 60%/0,3% de TFA de ácido sinapínico (30 mg/mL) para la preparación de la matriz MALDI-ToF, con el fin de hacer comparables sus espectros proteicos con los datos de flebotominos publicados hasta la fecha.

Preparación de muestras para MALDI-ToF MS (Fig. 7)

Los especímenes de insectos, almacenados bajo diversas condiciones, son primero secados al aire a temperatura ambiente y disecados. La cabeza y el abdomen son retirados para obtener partes del cuerpo que contienen características morfológicas clave para el montaje en portaobjetos y el análisis morfológico. El tórax puede ser utilizado para MALDI-ToF y el abdomen restante es preservado para la extracción de ADN. Para el perfil de proteínas, el tórax es homogenizado manualmente en microtubos de 1,5 mL con 10 µL de solución de homogeneización utilizando morteros y gránulos desechables. Por lo general se utilizan dos soluciones de homogeneización: agua destilada estéril y ácido fórmico al 25%.

7 Conclusión

En este trabajo, nuestro objetivo fue proporcionar a los investigadores los métodos más eficaces para el montaje de flebotominos, adaptados a objetivos de investigación específicos, con el fin de facilitar una identificación precisa y la detección de patógenos. No existe un único método universalmente óptimo; más bien, existen varios métodos, cada uno con sus propias ventajas y limitaciones.

En los Datos Suplementarios, proporcionamos protocolos detallados para diversas técnicas de montaje utilizadas en la preparación e identificación de flebotominos. Estos protocolos, que incluyen videos instructivos, ofrecen procedimientos paso a paso adaptados a distintos objetivos, garantizando resultados precisos y confiables. Al ofrecer este recurso integral, buscamos apoyar a los investigadores en la selección y aplicación de las técnicas de montaje más adecuadas para sus necesidades específicas.

Agradecimientos

Los autores agradecen a Richard Lane y a Zoe Jay Adams, del Museo de Historia Natural de Londres, Reino Unido, por su excelente revisión, que contribuyó a mejorar la calidad de este manuscrito.

Financiamiento

Reconocemos a las agencias brasileñas de desarrollo CNPq (número de proceso: 404395/2024-4) y a la Fundación Araucária (número de proceso: 433/2025 PDI) por la financiación de la investigación de AJA.

Conflicto de intereses

Jérôme Depaquit es editor asociado de Parasite; no tuvo ninguna influencia en el proceso de revisión ni en la toma de decisiones sobre este manuscrito. Los demás autores declaran que no tienen conflictos de interés.

Declaración de disponibilidad de datos

Videos Zenodo
Video 1: https://zenodo.org/records/18198006
Video 2: https://zenodo.org/records/18311158
Video 3: https://zenodo.org/records/18311106
Video 4: https://zenodo.org/records/18311154
Video 5: https://zenodo.org/records/18303014
Video 6: https://zenodo.org/records/18302850
Video 7: https://zenodo.org/records/18315029

Material suplementario

El material suplementario de este articulo está disponible en http://www.parasite-journal.org/10.1051/parasite/2026009/olm.

References

1. Alkan C, Allal-Ikhlef AB, Alwassouf S, Baklouti A, Piorkowski G, de Lamballerie X, Izri A, Charrel RN. 2015. Virus isolation, genetic characterization and seroprevalence of Toscana virus in Algeria. Clinical Microbiology and Infection, 21(11), 1040 e1-9.
2. Alten B, Ozbel Y, Ergunay K, Kasap OE, Cull B, Antoniou M, Velo E, Prudhomme J, Molina R, Banuls AL, Schaffner F, Hendrickx G, Van Bortel W, Medlock JM. 2015. Sampling strategies for phlebotomine sand flies (Diptera: Psychodidae) in Europe. Bulletin of Entomological Research 105(6), 664–678.
3. Ayhan N, Baklouti A, Prudhomme J, Walder G, Amaro F, Alten B, Moutailler S, Ergunay K, Charrel RN, Huemer H. 2017. Practical guidelines for studies on sandfly-borne phleboviruses: Part I: Important points to consider *ante* field work. Vector-Borne and Zoonotic Diseases 17(1), 73–80.
4. Bates PA. 1997. Infection of phlebotomine sandflies with *Leishmania*, in The Molecular Biology of Insect Disease Vectors: A Methods Manual. Springer. p. 112–120.5.
5. Baum M, de Castro EA, Pinto MC, Goulart TM, Baura W, Klisiowicz Ddo R, Vieira da Costa-Ribeiro MC. 2015. Molecular detection of the blood meal source of sand flies (Diptera: Psychodidae) in a transmission area of American cutaneous leishmaniasis, Parana State, Brazil. Acta Tropica, 143, 8–12.
6. Belen A, Alten B, Aytekin A. 2004. Altitudinal variation in morphometric and molecular characteristics of *Phlebotomus papatasi* populations. Medical and Veterinary Entomology, 18(4), 343–350.
7. Bhattacharya J, Chandra G, Hati AK. 1991. A simple method for cryopreservation of *Leishmania donovani* promastigotes, Indian Journal of Medical Research, 93, 245–246.
8. Caligiuri LG, Sandoval AE, Miranda JC, Pessoa FA, Santini MS, Salomón OD, Secundino NF, McCarthy CB. 2019. Optimization of DNA extraction from individual sand flies for PCR amplification. Methods and Protocols, 2(2), 36.
9. Casaril AE, de Oliveira LP, Alonso DP, de Oliveira EF, Gomes Barrios SP, de Oliveira Moura Infran J, Fernandes WS, Oshiro ET, Ferreira AMT, Ribolla PEM, de Oliveira AG. 2017. Standardization of DNA extraction from sand flies: Application to genotyping by next generation sequencing. Experimental Parasitology, 177, 66–72.
10. Castalanelli MA, Severtson DL, Brumley CJ, Szito A, Foottit RG, Grimm M, Munyard K, Groth DM. 2010. A rapid non-destructive DNA extraction method for insects and other arthropods. Journal of Asia-Pacific Entomology, 13(3), 243–248.
11. Cerqueira NL. 1943. Um novo meio para montagem de pequenos insetos em lâmina. Memórias do Instituto Oswaldo Cruz, (39), 37–41.
12. Charrel RN, Gallian P, Navarro-Mari JM, Nicoletti L, Papa A, Sanchez-Seco MP, Tenorio A, de Lamballerie X. 2005. Emergence of Toscana virus in Europe. Emerging Infectious Diseases, 11(11), 1657–1663.

13. Chaskopoulou A, Giantsis IA, Demir S, Bon MC. 2016. Species composition, activity patterns and blood meal analysis of sand fly populations (Diptera: Psychodidae) in the metropolitan region of Thessaloniki, an endemic focus of canine leishmaniasis. Acta Tropica, 158, 170–176.
14. Chen H, Rangasamy M, Tan SY, Wang H, Siegfried BD. 2010. Evaluation of five methods for total DNA extraction from western corn rootworm beetles. PLoS One, 5(8), e11963.
15. Depaquit J, Grandadam M, Fouque F, Andry PE, Peyrefitte C. 2010. Arthropod-borne viruses transmitted by Phlebotomine sandflies in Europe: a review. Eurosurveillance, 15(10), 19507.
16. Diamond LS, Herman CM. 1954. Incidence of Trypanosomes in the Canada Goose as revealed by bone marrow culture. Journal of Parasitology, 40(2), 195–202.
17. Ding H, Torno M, Vongphayloth K, Ng G, Tan D, Sng W, Ho K, Randrianambinintsoa FJ, Depaquit J, Tan CH. 2025. Hidden in plain sight: discovery of sand flies in Singapore and description of four species new to science. Parasites & Vectors, 18(1), 402.
18. Es-Sette N, Ajaoud M, Bichaud L, Hamdi S, Mellouki F, Charrel RN, Lemrani M. 2014. *Phlebotomus sergenti* a common vector of *Leishmania tropica* and Toscana virus in Morocco. Journal of Vector Borne Diseases, 51(2), 86–90.
19. Favret C. 2005. A new non-destructive DNA extraction and specimen clearing technique for aphids (Hemiptera). Proceedings of the Entomological Society of Washington, 107(2), 469–470.
20. Galati EAB. 2018. Phlebotominae (Diptera, Psychodidae): Classification, morphology and terminology of adults and identification of American taxa, in Brazilian Sand Flies: Biology, Taxonomy, Medical Importance and Control, Rangel EF, Shaw JJ, Editors. Cham: Springer International Publishing. pp. 9–212.
21. Galati EAB, de Andrade AJ, Perveen F, Loyer M, Vongphayloth K, Randrianambinintsoa FJ, Prudhomme J, Rahola N, Akhoundi M, Shimabukuro PHF, Depaquit J. 2025. Phle-botomine sand flies (Diptera, Psychodidae) of the world. Parasites & Vectors, 18(1), 220.
22. Galati EAB, Galvis-Ovallos F, Lawyer P, Leger N, Depaquit J. 2017. An illustrated guide for characters and terminology used in descriptions of Phlebotominae (Diptera, Psychodidae). Par-asite, 24, 26.
23. Gariepy T, Kuhlmann U, Gillott C, Erlandson M. 2007. Parasitoids, predators and PCR: the use of diagnostic molecular markers in biological control of Arthropods. Journal of Applied Entomology, 131(4), 225–240.
24. Giantsis IA, Chaskopoulou A, Bon MC. 2016. Mild-Vectolysis: A nondestructive DNA extraction method for vouchering sand flies and mosquitoes. Journal of Medical Entomology, 53(3), 692–695.
25. Gidwani K, Picado A, Rijal S, Singh SP, Roy L, Volfova V, Andersen EW, Uranw S, Ostyn B, Sudarshan M, Chakravarty J, Volf P, Sundar S, Boelaert M, Rogers ME. 2011. Serological markers of sand fly exposure to evaluate insecticidal nets against visceral leishmaniasis in India and Nepal: a cluster-randomized trial. PLoS Neglected Tropical Diseases, 5(9), e1296.
26. Gilbert MTP, Moore W, Melchior L, Worobey M. 2007. DNA extraction from dry museum beetles without conferring external morphological damage. PLoS One, 2(3), e272.
27. Giordani BF, Andrade AJ, Galati EAB, Gurgel-Goncalves R. 2017. The role of wing geometric morphometrics in the identification of sandflies within the subgenus *Lutzomyia.* Medical and Veterinary Entomology, 31(4), 373–380.
28. Guzmán-Larralde AJ, Suaste-Dzul AP, Gallou A, Peña-Carrillo KI. 2017. DNA recovery from microhymenoptera using six non-destructive methodologies with considerations for subsequent preparation of museum slides. Genome, 60(1), 85–91.
29. Hajibabaei M, DeWaard JR, Ivanova NV, Ratnasingham S, Dooh RT, Kirk SL, Mackie PM, Hebert PD. 2005. Critical factors for assembling a high volume of DNA barcodes. Philosophical Transactions of the Royal Society B: Biological Sciences, 360(1462), 1959–1967.
30. Haouas N, Pesson B, Boudabous R, Dedet JP, Babba H, Ravel C. 2007. Development of a molecular tool for the identification of *Leishmania* reservoir hosts by blood meal analysis in the insect vectors. American Journal of Tropical Medicine and Hygiene, 77(6), 1054–1059.
31. Hlavackova K, Dvorak V, Chaskopoulou A, Volf P, Halada P. 2019. A novel MALDI-TOF MS-based method for blood meal identification in insect vectors: A proof of concept study on phlebotomine sand flies. PLoS Neglected Tropical Diseases, 13 (9), e0007669.
32. Huemer H, Prudhomme J, Amaro F, Baklouti A, Walder G, Alten B, Moutailler S, Ergunay K, Charrel RN, Ayhan N. 2017. Practical guidelines for studies on sandfly-borne phleboviruses: Part II: Important points to consider for fieldwork and subsequent virological screening. Vector-Borne and Zoonotic Diseases, 17(1), 81–90.
33. Jancarova M, Polanska N, Thiesson A, Arnaud F, Stejskalova M, Rehbergerova M, Kohl A, Viginier B, Volf P, Ratinier M. 2025. Susceptibility of diverse sand fly species to Toscana virus. PLoS Neglected Tropical Diseases, 19(5), e0013031.
34. Kapp JD, Green RE, Shapiro B. 2021. A fast and efficient single-stranded genomic library preparation method optimized for ancient DNA. Journal of Heredity, 112(3), 241–249.
35. Killick-Kendrick R, Maroli M, Killick-Kendrick M. 1991. Bibliography of the colonization of phlebotomine sandflies. Parassitologia, 33(suppl.), 321–333.
36. Lawyer P, Killick-Kendrick M, Rowland T, Rowton E, Volf P. 2017. Laboratory colonization and mass rearing of phlebotomine sand flies (Diptera, Psychodidae). Parasite, 24, 42.
37. Léger N, Pesson B, Madulo-Leblond G. 1986. Les phlébotomes de Grèce : 1ère partie. Bulletin de la Société de Pathologie Exotique, 79, 386–397.
38. Léger N, Pesson B, Madulo-Leblond G. 1986. Les phlébotomes de Grèce : 2ème partie. Bulletin de la Société de Pathologie Exotique, 79, 514–524.
39. Leonel JAF, Vioti G, Alves ML, da Silva DT, Meneghesso PA, Benassi JC, Spada JCP, Galvis-Ovallos F, Soares RM, Oliveira T. 2020. DNA extraction from individual Phlebotomine sand flies (Diptera: Psychodidae: Phlebotominae) specimens: Which is the method with better results? Experimental Parasitology, 218, 107981.
40. Lestinova T, Rohousova I, Sima M, de Oliveira CI, Volf P. 2017. Insights into the sand fly saliva: Blood-feeding and immune interactions between sand flies, hosts, and *Leishmania.* PLoS Neglected Tropical Diseases, 11(7), e0005600.
41. Lienhard A, Schaffer S. 2019. Extracting the invisible: obtaining high quality DNA is a challenging task in small arthropods. PeerJ, 7, e6753.
42. Lozano-Sardaneta YN, Mikery-Pacheco OF, Huerta H, Rojas-Soriano JE, Contreras-Ramos A. 2025. Wing geometric morphometrics is effective to separate sand fly species (Diptera, Psychodidae, Phlebotominae) related with leishmaniasis transmission in Mexico. Acta Tropica, 262, 107523.

43. Mandrioli M. 2008. Insect collections and DNA analyses: how to manage collections? Museum Management and Curatorship, 23(2), 193–199.
44. Maroli M, Feliciangeli MD, Bichaud L, Charrel RN, Gradoni L. 2013. Phlebotomine sandflies and the spreading of leishmaniases and other diseases of public health concern. Medical and Veterinary Entomology, 27(2), 123–147.
45. Marquina D, Buczek M, Ronquist F, Lukasik P. 2021. The effect of ethanol concentration on the morphological and molecular preservation of insects for biodiversity studies. PeerJ, 9, e10799.
46. Mathis A, Depaquit J, Dvorak V, Tuten H, Banuls AL, Halada P, Zapata S, Lehrter V, Hlavackova K, Prudhomme J, Volf P, Sereno D, Kaufmann C, Pfluger V, Schaffner F. 2015. Identification of phlebotomine sand flies using one MALDI-TOF MS reference database and two mass pectrometer systems., Parasites & Vectors, 8, 266.
47. Mekarnia N, Benallal KE, Sadlova J, Vojtkova B, Mauras A, Imbert N, Longhitano M, Harrat Z, Volf P, Loiseau PM, Cojean S. 2024. Effect of *Phlebotomus papatasi* on the fitness, infectivity and antimony-resistance phenotype of antimony-resistant *Leishmania* major Mon-25. International Journal for Parasitology – Drugs and Drug Resistance, 25, 100554.
48. Milligan BG. 1998. Total DNA isolation, in Molecular Genetic Analysis of Population: A Practical Approach, Hoelzel AR, Editor. Oxford: Oxford University Press.
49. Molina R, Jiménez M, Alvar J, González E, Hernández-Taberna S, Ines MM. 2017. Methods in sand fly research. Madrid: Servicio de publicaciones Universidad de Alcalá de Henares, Madrid.
50. Murphy WJ, Eizirik E, O'Brien SJ, Madsen O, Scally M, Douady CJ, Teeling E, Ryder OA, Stanhope MJ, de Jong WW, Springer MS. 2001. Resolution of the early placental mammal radiation using Bayesian phylogenetics. Science, 294(5550), 2348–2351.
51. Nacif-Pimenta R, Pinto LC, Volfova V, Volf P, Pimenta PFP, Secundino NFC. 2020. Conserved and distinct morphological aspects of the salivary glands of sand fly vectors of leishma-niasis: an anatomical and ultrastructural study. Parasites & Vectors, 13(1), 441.
52. Neuhaus B, Schmid T, Riedel J. 2017. Collection management and study of microscope slides: Storage, profiling, deterioration, restoration procedures, and general recommendations. Zootaxa, 4322(1), 1–173.
53. New TR. 1974. Pscoptera. Handbooks for Identification of British Insects (Vol. I). London: Royal Entomological Society of London. 102 pp.
54. Perez-Ruiz M, Collao X, Navarro-Mari JM, Tenorio A. 2007. Reverse transcription, real-time PCR assay for detection of Toscana virus. Journal of Clinical Virology, 39(4), 276–281.
55. Porco D, Rougerie R, Deharveng L, Hebert P. 2010. Coupling non-destructive DNA extraction and voucher retrieval for small soft-bodied Arthropods in a high-throughput context: the example of Collembola. Molecular Ecology Resources, 10(6), 942–945.
56. Prudhomme J, Cassan C, Hide M, Toty C, Rahola N, Vergnes B, Dujardin JP, Alten B, Sereno D, Banuls AL. 2016. Ecology and morphological variations in wings of *Phlebotomus ariasi* (Diptera: Psychodidae) in the region of Roquedur (Gard, France): a geometric morphometrics approach. Parasites & Vectors, 9(1), 578.
57. Prudhomme J, Gunay F, Rahola N, Ouanaimi F, Guernaoui S, Boumezzough A, Banuls AL, Sereno D, Alten B. 2012. Wing size and shape variation of *Phlebotomus papatasi* (Diptera: Psychodidae) populations from the south and north slopes of the Atlas Mountains in Morocco. Journal of Vector Ecology, 37(1), 137–147.
58. Prudhomme J, Toty C, Kasap OE, Rahola N, Vergnes B, Maia C, Campino L, Antoniou M, Jimenez M, Molina R, Cannet A, Alten B, Sereno D, Banuls AL. 2015. New microsatellite markers for multi-scale genetic studies on *Phlebotomus ariasi* Tonnoir, vector of *Leishmania infantum* in the Mediterranean area. Acta Tropica, 142, 79–85.
59. Prudhomme J, Velo E, Bino S, Kadriaj P, Mersini K, Gunay F, Alten B. 2019. Altitudinal variations in wing morphology of *Aedes albopictus* (Diptera, Culicidae) in Albania, the region where it was first recorded in Europe. Parasite, 26, 55.
60. Rawlins DJ. 1992. Light Microscopy: An Introduction to Biotechniques. Oxford: Bios Scientific publishers. 143 pp.
61. Ready PD. 2013. Biology of phlebotomine sand flies as vectors of disease agents. Annual Review of Entomology, 58, 227–250.
62. Rohlf FJ, Slice D. 1990. Extensions of the Procrustes method for the optimal superimposition of landmarks. Systematic Zoology, 39(1), 40–59.
63. Rowley DL, Coddington JA, Gates MW, Norrbom AL, Ochoa RA, Vandenberg NJ, Greenstone MH. 2007. Vouchering DNA-barcoded specimens: Test of a nondestructive extraction proto-col for terrestrial arthropods. Molecular Ecology Notes, 7(6), 915–924.
64. Sábio PB, Andrade AJ, Galati EAB. 2014. Assessment of the taxonomic status of some species included in the *Shannoni* complex, with the description of a new species of *Psathyromyia* (Diptera: Psychodidae: Phlebotominae). Journal of Medical Entomology, 51(2), 331–341.
65. Sadlova J, Yeo M, Seblova V, Lewis MD, Mauricio I, Volf P, Miles MA. 2011. Visualisation of *Leishmania donovani* fluo-rescent hybrids during early stage development in the sand fly vector. PLoS One, 6(5), e19851.
66. Sales K, Miranda DEO, da Silva FJ, Otranto D, Figueredo LA, Dantas-Torres F. 2020. Evaluation of different storage times and preservation methods on phlebotomine sand fly DNA concen-tration and purity. Parasites & Vectors, 13(1), 399
67. Sales KG, Costa PL, de Morais RC, Otranto D, Brandao-Filho SP, Cavalcanti Mde P, Dantas-Torres F. 2015. Identification of phlebotomine sand fly blood meals by real-time PCR. Parasites& Vectors, 8, 230.
68. Sant'Anna MR, Jones NG, Hindley JA, Mendes-Sousa AF, Dillon RJ, Cavalcante RR, Alexander B, Bates PA. 2008. Blood meal identification and parasite detection in laboratory-fed and field-captured *Lutzomyia longipalpis* by PCR using FTA databasing paper. Acta Tropica, 107(3), 230–237.
69. Senne NA, Santos HA, Araujo TR, Paulino PG, Mendonca LP, Moreira HVS, Camilo TA, da Costa Angelo I. 2022. Robust comparative performance of genomic DNA extraction methods from non-engorged phlebotomine sandflies. Medical and Veterinary Entomology, 36(2), 203–211.
70. Shaw JJ. 2025. A review of Leishmania infections in American Phlebotomine sand flies – Are those that transmit leishmaniasis anthropophilic or anthropportunists? Parasite, 32, 57.
71. Tesh RB, Modi GB. 1983. Growth and transovarial transmission of Chandipura virus (Rhabdoviridae: Vesiculovirus) in *Phlebotomus papatasi.* American Journal of Tropical Medicine and Hygiene, 32(3), 621–623.
72. Thomsen PF, Elias S, Gilbert MTP, Haile J, Munch K, Kuzmina S, Froese DG, Sher A, Holdaway RN, Willerslev E. 2009. Non-

destructive sampling of ancient insect DNA. PLoS One, 4(4),e5048
73. Truett GE, Heeger P, Mynatt RL, Truett AA, Walker JA, Warman ML. 2000. Preparation of PCR-quality mouse genomic DNA with hot sodium hydroxide and tris (HotSHOT). Biotechniques, 29(1), 52–54.
74. Upton MS. 1993. Aqueous gum-chloral slide mounting media: an historical review. Bulletin of Entomological Research, 83(2), 267–274.
75. Volf P, Myskova J. 2007. Sand flies and Leishmania: specific versus permissive vectors. Trends in Parasitology, 23(3), 91–92.
76. Wang Q, Wang X. 2012. Comparison of methods for DNA extraction from a single chironomid for PCR analysis. Pakistan Journal of Zoology, 44(2), 421–426.
77. Wang Y, Zhao Y, Bollas A, Wang Y, Au KF. 2021. Nanopore sequencing technology, bioinformatics and applications. Nature Biotechnology, 39(11), 1348–1365.

Cite este articulo como: Randrianambinintsoa FJ, Augendre L, Prudhomme J, Martinet J-P, Loyer M, Mekarnia N, Kerkoub H, Perveen FK, Huguenin A, Kariya E, Akhoundi M, De Andrade AJ, Berriatua E, Bongiorno G, Boyer S, Christodoulou V, Da Costa-Ribeiro MCV, De Souza LAF, Ding H, Dondji B, Dvořák V, Erisoz Kasap O, Galati EAB, Gállego M, Ballart C, Gouzelou S, Haddad N, Masse RS, Mekuria AH, Ivovic V, Kaczmarek S, Shahar MK, Kirstein OD, Kniha E, Kolářová I, Lincoln T, Lucanas C, Mikov O, Nov K, Özbel Y, Pesson B, Posada Lopez LC, Prasetyo DB, Rahola N, Rebollar-Tellez EA, Rodrigues BL, Roy L, Saini P, Sanjoba C, Shimabukuro PH, Siriyasatien P, Soszynska A, Suleşco T, Sylla M, Torno M, Volf P, Vongphayloth K, Sinh Nam V, Wardhana A, Yessinou E, Zapata S, Gantier J-C & Depaquit J. 2026. Processing and mounting phlebotomine sand flies: a consensus guideline. Parasite xx, xx. https://doi.org/10.1051/parasite/2026009.

PARASiTE

An international open-access, peer-reviewed, online journal publishing high quality papers on all aspects of human and animal parasitology

Reviews, articles and short notes may be submitted. Fields include, but are not limited to: general, medical and veterinary parasitology; morphology, including ultrastructure; parasite systematics, including entomology, acarology, helminthology and protistology, and molecular analyses; molecular biology and biochemistry; immunology of parasitic diseases; host-parasite relationships; ecology and life history of parasites; epidemiology; therapeutics; new diagnostic tools.
All papers in Parasite are published in English. Manuscripts should have a broad interest and must not have been published or submitted elsewhere. No limit is imposed on the length of manuscripts.

Parasite (open-access) continues **Parasite** (print and online editions, 1994-2012) and **Annales de Parasitologie Humaine et Comparée** (1923-1993) and is the official journal of the Société Française de Parasitologie.

Editor-in-Chief:
Jean-Lou Justine, Paris

Submit your manuscript at:
https://www.editorialmanager.com/parasite

Apéndice 1: Fundamentos teóricos bioquímicos

Los artrópodos en cuestión son los flebotomíneos (sand flies). Sin embargo, la idea general puede extenderse a otros artrópodos muy comunes cuya identificación solo puede realizarse mediante caracteres morfológicos internos. Por fortuna, algunos órganos internos están parcialmente quitinizados y su morfología nos proporcionan información valiosa. Por esta razón es muy interesante observar las bombas alimentarias, las espermatecas y sus conductos. Con todos los reactivos que revisaremos, nunca debe olvidarse que, desde la etapa de fijación del insecto hasta el montaje, simplemente aplicaremos reacciones redox. La única precaución o principio que debemos seguir es evitar mezclar reactivos reductores con reactivos oxidantes.

Alcohol etílico; etanol:

Esta sustancia se utilizará de diferentes maneras. Las moléculas de alcohol tienen una fuerte afinidad por el agua y por lo tanto presentan un efecto deshidratante. Sin embargo, un alcohol de baja concentración (es decir, demasiado rico en agua) jugará un papel en la degradación de los ácidos nucleicos (el agua es enemiga de los ácidos nucleicos).

Cuando los insectos se colocan en etanol, no es solamente para preservarlos, sino también para fijar los tejidos. En histología se suelen diferenciar dos conceptos importantes: velocidad de penetración y velocidad de fijación. Se entiende bien que un buen preservante debe primero penetrar rápidamente en profundidad en los tejidos antes de fijarlos. Para alcohol al 96%, el coeficiente de penetración es aproximadamente 1,05 (para comparación: una solución acuosa de ácido pícrico al 0,75% tiene un coeficiente de penetración de 0,45, mientras que una solución de dicromato de potasio al 3% tiene 1,45).

Para los entomólogos es una práctica común intentar preservar insectos y otros artrópodos indefinidamente en etanol. La idea es muy honorable, ya que permite conservar las capturas de campo para estudios posteriores o para futuros investigadores. Sin embargo, esta idea no es posible para un citólogo o histólogo. Si se intenta mantener las muestras demasiado tiempo en el fijador, pueden volverse prácticamente imposibles de reprocesar. Por esta razón, muestras con más de 10 años son difíciles o incluso imposibles de utilizar.

Otro aspecto a considerar es la relación entre la masa del artrópodo a fijar y el volumen del fijador. En práctica zoológica o médica se recomienda utilizar un volumen 60 veces mayor que el volumen del material a fijar. En la práctica, para microartrópodos, para un volumen dado de especímenes, añadir al menos 4–5 volúmenes de alcohol. Debe tenerse en cuenta que el alcohol pierde su eficacia al absorber toda el agua presente en los tejidos del artrópodo.

En conclusión:

- El alcohol etílico es un agente químico reductor (por lo tanto, incompatible con fijadores oxidantes):
- Precipita enérgicamente las proteínas y las desnaturaliza;
- Disuelve ciertos lípidos complejos y precipita el glucógeno;
- Produce una fuerte contracción de los tejidos y su endurecimiento

Soluciones básicas de hidróxido de potasio o sodio:

El uso de estas soluciones en entomología se ha centrado principalmente en el hidróxido de potasio, sin una justificación clara. El Hidróxido de sodio (NaOH) [E524] se encuentra en solución con diferentes concentraciones o normalidades. También se presenta en pellets o escamas. Su principal desventaja es que es muy higroscópico (más que el KOH). Cuando reacciona con las proteínas las disuelve, con los lípidos los transforma en jabones duros durante la saponificación (esto es una diferencia importante con el KOH, que produce jabones líquidos durante la saponificación).
El Hidróxido de potasio (KOH) [E525] está disponible como solución concentrada, pero sobre todo tiene la ventaja de presentarse en pellets de aproximadamente 0,1 g, lo cual facilita mucho la preparación de soluciones diluidas cuando no se dispone de una balanza de precisión. Por ejemplo, 1 pellet de 0,1 g en 1 mL de agua destilada produce una solución al 10%. Otra ventaja es su menor sensibilidad a la carbonatación (las soluciones de KOH tienen una gran afinidad por fijar CO_2, formando sales carbonatadas).
Estas bases fuertes se utilizan para solubilizar ácidos grasos transformándolos en jabones solubles en agua. Debe recordarse que el fijador (como el etanol) ya ha solubilizado parte de las grasas del espécimen. Sin embargo, al colocar la muestra en un medio acuoso con una base fuerte, los ácidos grasos (más o menos complejos) precipitan. La base fuerte realiza entonces una saponificación en frío. En algunos casos, cuando los tejidos adiposos están en exceso (por ejemplo en hembras), será ventajoso aumentar la temperatura a 35–40 °C o prolongar el tiempo de contacto a temperatura ambiente.

Solución ácida coloreada / solución Marc-André incolora:
Aquí exploraremos las ventajas y desventajas del uso de la solución Marc-André. Esta solución está compuesta por hidrato de cloral (tricloracetaldehído monohidratado), ácido acético y agua. Es una solución muy oxidante (mezcla de ácido y aldehído). Neutralizará el exceso de hidróxido de potasio que pueda quedar en las muestras, sin precipitar los jabones alcalinos formados durante el uso del hidróxido de potasio. Esta solución oxidante también tendrá una acción sobre las funciones alcohol secundario de las glucosaminas que forman parte de la quitina, oxidándolas y por lo tanto ablandando la quitina. Otra acción es la disolución de ciertas sales minerales presentes.

This is an Open Access article distributed under the terms of the Creative Commons Attribution License (https://creativecommons.org/licenses/by/4.0), which permits unrestricted use, distribution, and reproduction in any medium, provided the original work is properly cited.

Cuando la solución Marc-André se colorea previamente con fucsina ácida (por lo tanto en estado oxidado), podrá fijarse sobre las funciones alcohol secundario de la estructura. Después del tiempo de contacto de la solución Marc-André y del estado de tinción de las muestras, el enjuague se realizará únicamente con etanol. De este modo se inicia la fase de deshidratación de las muestras.

Beneficios:

- neutralización del exceso de soluciones básicas;
- relajación de la quitina;
- tinción de la quitina para evaluar mejor las estructuras internas quitinizadas.

Inconvenientes:
El hidrato de cloral es hipnótico y ha sido utilizado en medicina humana. Debe utilizarse bajo campana química y debe cumplirse la legislación sobre riesgos químicos.

Soluciones de deshidratación:
La experiencia demuestra que para muestras muy pequeñas no es útil seguir la secuencia de baños de alcohol con concentraciones crecientes. Si la muestra es grande, comenzaremos con etanol al 80 %, luego 90 %, 95 % y finalmente etanol absoluto. Para muestras muy pequeñas, utilizar un baño con etanol al 90 % seguido de una inmersión en etanol absoluto. En esta etapa siempre recordaremos que el etanol absoluto busca fijar el agua presente en la atmósfera. La tradición en los laboratorios de entomología era finalizar la deshidratación de las muestras con un baño de creosota de haya. Hoy en día, esta esencia, ampliamente utilizada como pesticida, antifúngico y preservante de madera, está muy fuertemente desaconsejada debido a su olor (hidrocarburos aromáticos policíclicos) y se supone que es reprotóxica, carcinogénica, un contaminante orgánico persistente y ecotóxica para los organismos acuáticos.
Una solución que proponemos preparar para el montaje de las muestras es Euparal® y esencia de Euparal (descrita en el siguiente párrafo). Una mezcla de Euparal® y esencia de Euparal es muy bien aceptada para las muestras obtenidas después de un baño de etanol al 90 %.

Apéndice 2: Composición de los reactivos utilizados.

Hidróxido de potasio al 10 %
Hidróxido de potasio 10 g
Agua destilada c.s.p. 100 mL

Medio de montaje de goma cloral, medio de Hoyer
Agua destilada 50 mL
Hidrato de cloral 200 g
Goma arábiga 50 g
Glicerol 20 mL

Solución Marc-André
Hidrato de cloral 40 g
Ácido acético glacial 30 mL
Agua destilada 30 mL

Fucsina ácida al 1 % en agua destilada
Polvo de fucsina ácida 1 g
Agua destilada 99 mL

Solución Marc-André coloreada con fucsina
Solución Marc-André 10 mL
Fucsina al 1 % 50 µL

Apéndice 3: Euparal®, bálsamo de Canadá, alcohol polivinílico u otras soluciones de montaje

Alcohol polivinílico: este es el medio de montaje ideal cuando no se dispone de los productos necesarios para una deshidratación adecuada. El alcohol polivinílico se mezcla entonces con lactofenol de Amman. Sin embargo, estos montajes presentan importantes inconvenientes: pueden secarse o el alcohol polivinílico puede cristalizar debido a la evaporación del agua o ennegrecerse cuando el fenol se oxida. Sigue siendo una buena técnica para montajes de corta duración.

Bálsamo de Canadá: su uso para el montaje entre portaobjetos y cubreobjetos requiere la deshidratación de las muestras que se van a montar. El uso de xileno o tolueno no está exento de inconvenientes.

Medio Enecê: para el montaje entre portaobjetos y cubreobjetos, como el bálsamo de Canadá, requiere la deshidratación del espécimen. Formulación del Enecê: colofonia blanca pura (22 g); goma copal soluble en alcohol (12 g), alcohol absoluto (20 mL), alcanfor (10 g), esencia de trementina (10 mL) y eucaliptol (26 mL). Para su preparación, en un recipiente, como un matraz Erlenmeyer, colocar el alcohol absoluto y el alcanfor. Luego añadir la colofonia y la goma copal. El matraz se cierra con un tapón y se agita, y solo entonces se calienta al baño maría a temperatura suave para que la mezcla no hierva. Una vez que el contenido está completamente licuado, se añade la esencia de trementina, luego se filtra mientras la mezcla aún está caliente y finalmente se añade el eucaliptol al filtrado. Cuando el medio se vuelve menos fluido, se diluye con Enecê, que tiene la siguiente fórmula: alcohol absoluto (30 mL), alcanfor (17 g), esencia de trementina (15 mL), eucaliptol (38 mL) (Cerqueira, 1943).

Euparal®: es una resina que proviene del ciprés del Atlas *Tetraclinis articulata* (Vahl, 1791) y fue estudiada y desarrollada en 1906 por Gilson. Su principal ventaja es que no polimeriza. Las muestras montadas entre portaobjetos y cubreobjetos pueden recuperarse fácilmente por la acción del alcohol o, mejor aún, de la esencia de Euparal®. Esta resina, también llamada sandáraca, acepta etanol desde el 80 %.

Uso de Triton X100: solución acuosa no iónica: El Triton X100 se presenta en forma de solución acuosa no iónica (solución de 4-(1,1,3,3-tetrametilbutil) fenil-polietilenglicol, o t-octilfenoxipolietoxietanol, éter de polietilenglicol tert-octilfenilo), ampliamente utilizada como detergente en biología celular y molecular. Permite la permeabilización de las membranas celulares y nucleares.
Las muestras de insectos preservadas en alcohol durante muchos años son algo común. Desafortunadamente, la conservación en alcohol no es óptima y los artrópodos así preservados se vuelven muy difíciles de preparar para el examen microscópico. Los plásticos que contienen las muestras a menudo se degradan, seguido de la evaporación del alcohol. En ambos casos, el contacto prolongado con el alcohol o el secado de las muestras plantea un problema real. En 2008, Jonque publicó una nota sobre la rehidratación de arañas con un agente humectante como Agepon utilizado para películas fotográficas [26]. Esto llevó a la idea de utilizar agentes humectantes que no sean detergentes potentes.
A continuación, se describe un procedimiento utilizando Triton X100 en solución acuosa al 0,5 %:

- impregnar la muestra seca con alcohol absoluto;
- añadir el volumen necesario de solución de Triton X100 al 0,5 % para que toda la muestra quede sumergida;
- dejar reposar durante aproximadamente 5 minutos o más; todos los artrópodos deben quedar independientes en la solución;
- la solución de Triton X100 se retira y se reemplaza por la solución de hidróxido de potasio.

La técnica se continúa entonces como se describió anteriormente.

Apéndice 4: Medios de montaje con Euparal® o bálsamo de Canadá paso a paso

1. Los especímenes deben estar deshidratados (una apariencia turbia o lechosa indica una deshidratación inadecuada).
2. La deshidratación puede lograrse mediante concentraciones crecientes de alcohol etílico.
3. Los especímenes pueden transferirse desde alcohol al 99% o alcohol absoluto a un agente de aclaramiento.

Procedimiento:

1. Diseccionar flebotomíneos adultos en etanol al 70 %.
2. Retirar el etanol y reemplazarlo con KOH al 10 %. Cubrir los flebotomíneos con un portaobjetos.
3. Macerar hasta que los insectos se vuelvan transparentes.
4. Retirar el KOH.
5. Cubrir el espécimen con agua destilada y esperar de 30 a 45 min.
6. Retirar el agua y repetir el lavado con agua destilada durante 30 min (el tiempo depende del número de muestras: cuanto más numerosas sean las muestras procesadas juntas, más debe respetarse este tiempo; cuanto menos numerosas sean, especialmente para las tratadas individualmente, más corto puede ser este tiempo).
7. Retirar el agua.
8. Añadir la solución Marc-André (potencialmente coloreada con fucsina ácida) y esperar 24 horas (un día).
9. Retirar la solución Marc-André.
10. Cubrir el espécimen con agua destilada y esperar de 30 a 45 min.
11. Retirar el agua y repetir el lavado con agua destilada durante 30 min.
12. Retirar el agua.
13. Añadir etanol al 70 % y diseccionar el espécimen.

a. Para la cabeza y el abdomen, tirar suavemente de la cabeza o del abdomen para separarlos del tórax.

b. Para el tórax, retirar las alas sosteniendo el tórax con un par de pinzas y tirando en la base de los apéndices con otro par. Es posible realizar una disección sagital, dividiendo el tórax en lados izquierdo y derecho, según las regiones de mayor interés.

14. Deshidratar gradualmente mediante una serie de soluciones acuosas de alcohol etílico: 50 – 80 – 95% hasta llegar al etanol absoluto.

15. Deshidratar los especímenes lavándolos dos veces, 10 min cada vez, con etanol al 100%.

16. Retirar el etanol y cubrir los especímenes con aceite de clavo durante 15 min a temperatura ambiente.

17. Transferir los especímenes del aceite de clavo a una gota de Euparal® o bálsamo de Canadá sobre un portaobjetos limpio.

18. Disponer según se desee: la cabeza, el tórax y el abdomen del flebotomíneo pueden disecarse utilizando agujas finas o pinzas bajo un microscopio estereoscópico. La cabeza debe separarse del cuerpo para montarse en posición ventro-dorsal, es decir, el foramen occipital debe orientarse hacia arriba para que el cibario pueda observarse directamente a través de él. La disección se realiza en el medio de montaje del flebotomíneo.

19. Dejar el espécimen hasta que la superficie se vuelva pegajosa.

20. Humedecer un cubreobjetos limpio con alcohol absoluto. Colocar el cubreobjetos sobre el bálsamo de Canadá en ángulo.

21. Guardar las láminas en una caja seca destinada para este propósito.
