# Supplementary material for: Processing and mounting phlebotomine sand flies: a consensus guideline
Source: Parasite. 2026 Apr 3;33:18. doi: 10.1051/parasite/2026009 (PMC13047900; doi:10.1051/parasite/2026009)
Supplement: Supplementary file 29 — Thai translation / คำแปลภาษาไทย [file parasite-33-18-s29.pdf]

Parasite **33**, 18 (2026)
© F.J. Randrianambinintsoa et al., published by EDP Sciences, 2026
https://doi.org/10.1051/parasite/2026009

PARASITE
Available online at:
www.parasite-journal.org

REVIEW ARTICLE OPEN ACCESS

การเตรียมตัวอย่างและการทำสไลด์ริ้นฝอยทราย: แนวทางปฏิบัติร่วม

Fano José Randrianambinintsoa[1], Laure Augendre[1], Jorian Prudhomme[1], Jean-Philippe Martinet[1], Mathieu Loyer[1], Nalia Mekarnia[1], Hocine Kerkoub[1], Farzana Khan Perveen[1], Antoine Huguenin[1,2], Emilie Kariya[1,2], Mohammad Akhoundi[3], Andrey José de Andrade[4], Eduardo Berriatua[5], Gioia Bongiorno[6], Sébastien Boyer[7,8], Vasiliki Christodoulou[9], Magda Clara Vieira Da Costa-Ribeiro[10], Lucas Alexandre Farias de Souza[10], Huicong Ding[11], Blaise Dondji[12], Vít Dvořák[13], Ozge Erisoz Kasap[14], Eunice Aparecida Bianchi Galati[15], Montserrat Gállego[16], Cristina Ballart[16], Stavroula Gouzelou[17], Nabil Haddad[18], Rezki Sabrina Masse[19], Asrat Hailu Mekuria[20], Vladimir Ivovic[21], Szymon Kaczmarek[22], Mohd Khadri Shahar[19], Oscar D. Kirstein[23], Edwin Kniha[24], Iva Kolářová[13], Lincoln Timinao[25], Cristian Lucanas[26], Ognyan Mikov[27], Kimsear Nov[7], Yusuf Özbel[28], Bernard Pesson[29], Laura Cristina Posada Lopez[30], Didot Budi Prasetyo[1,7], Nil Rahola[31], Eduardo A. Rebollar-Tellez[32], Bruno Leite Rodrigues[15], Lalita Roy[33], Prasanta Saini[34], Chizu Sanjoba[35], Paloma Helena Fernandes Shimabukuro[36], Padet Siriyasatien[37], Agnieszka Soszyńska[22], Tatiana Suleşco[38], Massamba Sylla[39], Majhalia Torno[40], Petr Volf[13], Khamsing Vongphayloth[41], Vu Sinh Nam[42], April Wardhana[43], Eric Yessinou[44], Sonia Zapata[45], Jean-Charles Gantier[1], and Jérôme Depaquit[1,2,*]

[1] Faculté de Pharmacie, Université de Reims Champagne Ardenne, UR ESCAPE-USC ANSES PETARD, 51 rue Cognacq-Jay, 51096 Reims Cedex, France
[2] Pôle de Biologie territoriale, Laboratoire de Parasitologie-Mycologie, Centre Hospitalo-Universitaire, 51092 Reims, France
[3] Parasitology-Mycology Department, Avicenne Hospital, AP-HP, Bobigny, Sorbonne Paris Nord University, France; Unité des Virus Émergents (UVE: Aix-Marseille Univ, Università di Corsica, IRD 190, Inserm 1207, IRBA), 13005 Marseille, France
[4] Parasitology Collection of Basic Pathology, Department of Basic Pathology, Federal University of Paraná, Curitiba 19031, Brazil
[5] Department of Animal Health, University of Murcia, Campus de Espinardo, 30100 Espinardo, Murcia, Spain
[6] Department of Infectious Diseases, Vector-borne Diseases Unit, Istituto Superiore di Sanità, 00166 Rome, Italy
[7] Medical and Veterinary Entomology Unit, Institut Pasteur du Cambodge, Phnom Penh 12201, Cambodia
[8] Ecology & Emergence of Arthropod-borne Pathogens Unit, Department of Global Health, Institut Pasteur, CNRS UMR2000, 75015 Paris, France
[9] Section Veterinary Services (1417), Laboratory for Animal Health Virology, Aglantzia, Nicosia 2109, Cyprus
[10] Insects Vectors and Parasites Laboratory, Department of Basic Pathology and Postgraduate program in Microbiology, Parasitology and Pathology, Federal University of Paraná, 81530-900 Curitiba, Brazil
[11] Department of Biological Sciences, National University of Singapore, 117558, Singapore
[12] Laboratory of the Leishmaniasis Research Project, Mokolo District Hospital, Mokolo, Cameroon; Laboratory of Cellular Immunology and Parasitology, Department of Biological Sciences, Central Washington University, 98926 Ellensburg, WA, USA
[13] Department of Parasitology, Faculty of Science, Charles University, 12800 Prague, Czechia
[14] VERG Laboratories, Department of Biology, Faculty of Science, Hacettepe University, Beytepe, Ankara 06800, Türkiye
[15] Faculdade de Saúde Pública da Universidade de São Paulo (FSP/USP), Pós-graduação em Saúde Pública, 01246-904 São Paulo, Brazil
[16] Secció de Parasitologia, Departament de Biologia, Sanitat i Medi Ambient, Facultat de Farmàcia i Ciències de l'Alimentació, Universitat de Barcelona, & Institut de Salut Global de Barcelona (ISGlobal), Centro de Investigación Biomédica en Red, Enfermedades Infecciosas (CIBERINFEC), 08028 Barcelona, Spain
[17] Laboratory of Infectious Diseases and Public Health, School of Medicine, University of Cyprus, Nicosia, Cyprus & Department of Pediatrics, Archbishop Makarios III Hospital, Nicosia 2115, Cyprus
[18] Faculty of Health Sciences, American University of Beirut, 1107 2020 Beirut, Lebanon
[19] Medical Entomology Unit, Infectious Disease Research Centre, Institute for Medical Research (IMR), National Institutes of Health (NIH), Ministry of Health Malaysia, 40170 Shah Alam, Selangor, Malaysia
[20] School of Medicine, Addis Ababa University, 28017 - 1000 Addis Ababa, Ethiopia
[21] Faculty of Mathematics, Natural Sciences and Information Technologies, University of Primorska, 6000 Koper, Slovenia

Edited by Jean-Lou Justine
*Corresponding author: jerome.depaquit@univ-reims.fr

This is an Open Access article distributed under the terms of the Creative Commons Attribution License (https://creativecommons.org/licenses/by/4.0), which permits unrestricted use, distribution, and reproduction in any medium, provided the original work is properly cited.

[22] University of Lodz, Faculty of Biology and Environmental Protection, Department of Invertebrate Zoology and Hydrobiology, Banacha 12/16, 90-237 Łódź, Poland
[23] Laboratory of Entomology, Ministry of Health, 9134302 Jerusalem, Israel
[24] Center for Pathophysiology, Infectiology and Immunology, Institute of Specific Prophylaxis and Tropical Medicine, Medical University Vienna, Kinderspitalgasse 15, 1090 Vienna, Austria
[25] Papua New Guinea Institute of Medical Research (PNGIMR) Institute, PO Box 60, Headquarter, Homate Street, 441 Goroka, Eastern Highlands Province, Papua New Guinea
[26] Museum of Natural History, University of the Philippines Los Baños, 4031 Laguna, Philippines
[27] National Centre of Infectious and Parasitic Diseases, 1504 Sofia, Bulgaria
[28] Ege University, Faculty of Medicine, Department of Parasitology, 35040 Bornova/Izmir, Türkiye
[29] Retired, Faculté de Pharmacie, Université de Strasbourg, Strasbourg, 67400 Illkirch-Graffenstaden, France
[30] Program for the Study and Control of Tropical Diseases (PECET), Faculty of Medicine, University of Antioquia, 050010 Medellin, Colombia
[31] MIVEGEC, Univ. Montpellier, CNRS, IRD, 34394 Montpellier, France & Medical Entomology Unit, Institut Pasteur de Madagascar, 101 Antananarivo, Madagascar
[32] Laboratorio de Entomología Médica, Departamento de Zoología de Invertebrados, Facultad de Ciencias Biológicas, Universidad Autónoma de Nuevo León, San Nicolás de los Garza, 66455, NL, México
[33] Tropical and Infectious Disease Centre, BP Koirala Institute of Health Sciences, Dharan 56700, Nepal
[34] ICMR-Vector Control Research Centre, Puducherry 605006, India
[35] Graduate School of Agricultural and Life Sciences, The University of Tokyo, Tokyo 113-8657, Japan
[36] Grupo de estudos em Leishmanioses/Coleção de Flebotomíneos (COLFLEB/Fiocruz-MG), Instituto René Rachou, Fundação Oswaldo Cruz, Belo Horizonte, Minas Gerais, 30190009, Brazil
[37] Center of Excellence in Vector Biology and Vector-Borne Disease, Department of Parasitology, Faculty of Medicine, Chulalongkorn University, Bangkok 10330, Thailand
[38] Department of Arbovirology, Bernhard Nocht Institute for Tropical Medicine, Bernhard Nocht Str. 74, 20359 Hamburg, Germany [39] Laboratory Vectors & Parasites, Department of Livestock Sciences and Techniques, Sine Saloum University El Hadji Ibrahima Niasse (SSUEIN) Kaffrine Campus, C.P. 24600, Senegal.
[40] Environmental Health Institute, National Environment Agency, Singapore 138667, Singapore & Department of Biological Sciences, National University of Singapore, 117558 Singapore
[41] Institut Pasteur du Laos, Laboratory of Vector-Borne Diseases, Samsenhai Road, Ban Kao-Gnot, Sisattanak District, 3560 Vientiane, Lao PDR
[42] National Institute of Hygiene and Epidemiology, 1 Yec-Xanh Street, Hai Ba Trung District, 100000 Hanoi, Vietnam
[43] Indonesian Research Center for Veterinary Science, Indonesian Agency for Agricultural Research and Development, Ministry of Agriculture Republic Indonesia, Bogor 16114, Indonesia & Department of Parasitology, Faculty of Veterinary Medicine, Airlangga University, Surabaya 60115, Indonesia
[44] Laboratory of Research in Applied Biology, Polytechnic School of Abomey-Calavi, University of Abomey-Calavi, 01 P.O. Box 2009, 00000 Cotonou, Benin
[45] Instituto de Microbiología, Colegio de Ciencias Biológicas y Ambientales (COCIBA), Universidad San Francisco de Quito (USFQ), 170901 Quito, Ecuador

Received 1 December 2025, Accepted 29 January 2026, Published online 3 April 2026

บทคัดย่อ – บทความฉบับนี้จัดทำขึ้นเพื่อเป็นแนวทางสำหรับการเตรียมตัวอย่าง และการทำสไลด์ริ้นฝอยทรายซึ่งมีความสำคัญอย่างยิ่งต่อการจำแนกสายพันธุ์ของริ้นฝอยทราย ตลอดจนการตรวจ และการแยกเชื้อก่อโรคที่มีริ้นฝอยทรายเป็นพาหะ บทความนี้ครอบคลุมเทคนิคและขั้นตอนที่เหมาะสมสำหรับการปฏิบัติงานทั้งในภาคสนามและในห้องปฏิบัติการ เพื่อให้ได้ตัวอย่างที่มีคุณภาพและเหมาะสมต่อการวิเคราะห์ในระดับต่าง ๆ ซึ่งเนื้อหาประกอบด้วยคำแนะนำโดยละเอียดเกี่ยวกับการเก็บตัวอย่าง การจัดการ การดูแล และการทำให้ริ้นฝอยทรายสลบและตาย โดยแนะนำวิธีการทำให้แห้งด้วยการแช่เยือกแข็ง หรือการใช้ก๊าซคาร์บอนไดออกไซด์เป็นทางเลือกแทนการใช้สารเคมี รวมถึงแนวทางการเก็บรักษาตัวอย่าง เช่น การแช่เย็นและการเก็บในเอทานอล ทั้งนี้คุณภาพของการจัดวางโครงสร้างทางกายวิภาคที่สำคัญ เช่น อวัยวะสืบพันธุ์ ส่วนหัว และปีก มีผลโดยตรงต่อความชัดเจนในการจำแนกสายพันธุ์ของริ้นฝอยทรายด้วยกล้องจุลทรรศน์ ซึ่งได้มีการอธิบายรายละเอียดและข้อควรระวังในแต่ละขั้นตอนไว้อย่างเป็นระบบ นอกจากนี้บทความยังมีการนำเสนอขั้นตอนการเตรียมตัวอย่างอย่างละเอียด โดยครอบคลุมกระบวนการทำให้ตัวอย่างใสด้วยสารละลายต่าง ๆ เช่น โพแทสเซียมไฮดรอกไซด์ และการปรับสภาพตัวอย่างด้วยสารละลายมาร์ก-อ็องเดร (Marc-André) ในส่วนของการจัดทำสไลด์ถาวร ได้มีการเปรียบเทียบคุณสมบัติของสารตัวกลางสำหรับปิดแผ่นสไลด์ (mounting media) หลายประเภท โดยพิจารณาจากสมบัติทางแสงและประสิทธิภาพในการเก็บรักษา พบว่าสารละลายฮอยเออร์ (Hoyer) หรือ คลอรอล กัม (chloral gum) มีคุณสมบัติที่เหมาะสมสำหรับการตรวจวิเคราะห์ตัวอย่างแบบเร่งด่วนที่ต้องการความรวดเร็ว โดยเฉพาะการดูลักษณะทางสัณฐานวิทยาของถุงเก็บน้ำเชื้อ (spermathecae) เนื่องจากมีความใสสูง อย่างไรก็ตาม สารดังกล่าวไม่เหมาะสมต่อการเก็บในระยะยาว บทความนี้ยังได้กล่าวถึงสารตัวกลางสำหรับปิดแผ่นสไลด์ชนิดอื่น ๆ

ได้แก่ โพลีไวนิลแอลกอฮอล์ (polyvinyl alcohol) ยูพารอล (Euparal®) ซึ่งมีความสามารถในการทนต่อน้ำในระดับจำกัด และ แคนาดาบาลซัม (Canada balsam) ซึ่งละลายในตัวทำละลายไฮโดรคาร์บอน โดยสาร Euparal® และ Canada balsam มีคุณสมบัติที่เหมาะสมต่อการเก็บรักษาสไลด์ในระยะยาว นอกจากนี้เอกสารนี้ได้เพิ่มแนวทางและเทคนิคสมัยใหม่ทางอณูชีววิทยา เช่น การวิเคราะห์ลำดับนิวคลีโอไทด์ และเทคนิคมาลดิ-ทอฟ (MALDI-ToF) ซึ่งต้องอาศัยความพิถีพิถันเป็นพิเศษในกระบวนการเตรียมตัวอย่างเพื่อรักษาคุณภาพของสารพันธุกรรมและข้อมูลทางชีวโมเลกุล อีกทั้งยังได้จัดทำวิดีโอสาธิตขั้นตอนการจัดทำสไลด์ในรูปแบบต่าง ๆ เพื่อเสริมสร้างความเข้าใจในการปฏิบัติงานจริง พร้อมทั้งจัดทำคำบรรยายและคำแปลใน 33 ภาษา เพื่อรองรับการใช้งานของนักวิจัยและผู้ปฏิบัติงานจากหลากหลายประเทศ อันเป็นการตอบสนองต่อความต้องการและความคาดหวังของเครือข่ายนักวิทยาศาสตร์ทั่วโลกในระดับสากลอย่างแท้จริง

คำสำคัญ: ทำสไลด์, ริ้นฝอยทราย, สารละลาย Hoyer, สารละลาย Marc-André, สาร chloral gum, สาร polyvinyl alcohol, สาร Euparal®, สาร Canada balsam, การแยกเชื้อลิชมาเนีย, ออกภาคสนาม, การเพาะเชื้อ, การผ่าตัวอย่างแมลง, อณูชีววิทยา, เทคนิค MALDI-ToF, ตัวอย่างต้นแบบ

Abstract – Processing and mounting phlebotomine sand flies: a consensus guideline. This article provides a comprehensive guide for the processing and mounting of phlebotomine sand fly specimens, which is crucial for species identification and pathogen detection and isolation. It discusses a range of techniques suitable for both field and laboratory settings. The guide includes detailed instructions on sand fly collec-tion, handling, covering, and euthanasia (recommending dry freezing or CO_2 over chemicals) as well as conservation strategies, such as cold storage and preservation in ethanol. The quality of preparation of certain anatomical structures (genital organs, head and wings) is essential for their proper microscopic observation and is described in this work. The article also presents detailed sample processing, including the clearing process with agents such as potassium hydrox-ide then Marc-André solution. The mounting process compares different media, emphasizing their optical properties and preservation potential. Hoyer fluid (also known as chloral gum) is recommended for quick observation, particularly for spermathecae, due to its clarity, although it is not suitable for long-term storage. Other media discussed include polyvinyl alcohol, Euparal® (for limited water tolerance), and Canada balsam (a hydrocarbon-soluble medium), with the latter two offering long-term preservation capabilities. Innovative molecular biology approaches such as DNA sequencing and MALDI-ToF, which require particular attention to sample processing, are also addressed. Furthermore, short video clips illustrating various mounting techniques as well as translations in many different languages are provided, allowing the guideline to reach the diverse needs and expectations of the global scientific community.

Key words: Mounting, Phlebotomine sand fly, Hoyer fluid, Marc-André solution, Chloral gum, Polyvinyl alcohol, Euparal®, Canada balsam, *Leishmania* isolation, Field conditions, Culture, Dissection, Molecular biology, MALDI-ToF, Type-specimens.

บทนำ

ริ้นฝอยทราย (Phlebotomine sand flies) เป็นแมลงขนาดเล็กในอันดับ Diptera วงศ์ Psychodidae วงศ์ย่อย Phlebotominae ซึ่งในปัจจุบันมีการรายงานการพบแล้วอย่างน้อย 1,063 สายพันธุ์ทั่วโลก [21] แมลงกลุ่มนี้มีบทบาทสำคัญทางสาธารณสุข เนื่องจากเป็นพาหะของเชื้อก่อโรคหลายชนิด ได้แก่ เชื้อลิชมาเนีย (*Leishmania* spp.) เชื้อไวรัสในกลุ่มอาร์โบไวรัส (arboviruses) และเชื้อแบคทีเรียสกุล *Bartonella* ซึ่งก่อให้เกิดโรคลิชมาเนีย (leishmaniasis) การติดเชื้ออาร์โบไวรัส และโรคบาร์โทเนลโลซิส (bartonellosis) ตามลำดับ การจำแนกสายพันธุ์ของริ้นฝอยทรายต้องอาศัยการตรวจอย่างละเอียดภายใต้กล้องจุลทรรศน์ ซึ่งจำเป็นต้องผ่านกระบวนการเก็บรวบรวมที่ประณีต การเก็บรักษาที่เหมาะสม และการจัดทำสไลด์ตัวอย่างที่มีคุณภาพ โดยใช้เทคนิคเฉพาะทางหลายประการซึ่งแต่ละวิธีมีข้อดีและข้อจำกัดแตกต่างกัน

การจำแนกสายพันธุ์ของริ้นฝอยทรายตัวเต็มวัยพิจารณาจากลักษณะทางสัณฐานวิทยาทั้งโครงสร้างภายนอก เช่น หนวด ระยางค์ปาก และอวัยวะสืบพันธุ์เพศผู้ รวมถึงโครงสร้างภายใน เช่น คอหอย (pharynx) กระเปาะอาหาร (cibarium) และถุงเก็บน้ำเชื้อ (spermathecae) การผ่าและแยกโครงสร้างภายในเหล่านี้ช่วยให้สามารถเห็นได้อย่างชัดเจนยิ่งขึ้นและนำไปสู่การจำแนกสายพันธุ์ของแมลงที่มีความแม่นยำสูง ด้วยลักษณะเฉพาะดังกล่าว การจำแนกสายพันธุ์ของริ้นฝอยทรายจึงแตกต่างจากแมลงดูดกินเลือดกลุ่มอื่น ๆ เช่น ยุง หรือมวนเพชฌฆาต โดยจำเป็นต้องมีการผนึกตัวอย่างระหว่างแผ่นสไลด์และกระจกปิดสไลด์ก่อนการดูผ่านกล้องเพื่อจำแนกสายพันธุ์ของริ้นฝอยทราย

ช่วงทศวรรษที่ 1980 การศึกษาลักษณะทางสัณฐานวิทยาภายใต้กล้องจุลทรรศน์ถือเป็นวิธีการเพียงวิธีเดียวในการจำแนกสายพันธุ์ของริ้นฝอยทราย และยังคงเป็นวิธีที่ใช้กันอย่างแพร่หลายที่สุดในปัจจุบัน ในอดีตการเลือกกระบวนการเตรียมตัวอย่างจึงค่อนข้างตรงไปตรงมา โดยสามารถแบ่งออกเป็นสองแนวทางหลัก ได้แก่ การทำสไลด์ถาวรเพื่อการเก็บตัวอย่างในระยะยาว และการทำสไลด์แบบรวดเร็วเพื่อการจำแนกแต่ไม่สามารถเก็บในระยะยาวได้ ยกตัวอย่างเช่น การใช้เรซินจำพวก Canada balsam เป็นขั้นตอนที่ต้องใช้เวลามากเนื่องจากต้องไล่น้ำออกจากตัวอย่างให้หมด นอกจากนี้ค่าดัชนีหักเหแสงของสารชนิดนี้อาจไม่เอื้อต่อการดูถุงเก็บน้ำเชื้อ (spermathecae) ได้โดยง่าย ในทางกลับกันการทำสไลด์ในสารที่มีน้ำเป็นตัวทำละลาย เช่น สารละลาย Hoyer จะรวดเร็วกว่าและช่วยให้เห็นถุงเก็บน้ำเชื้อ (spermathecae) ได้ชัดเจนกว่า แต่ไม่สามารถเก็บได้ในระยะยาวเนื่องจากสารมีแนวโน้มที่จะดูดความชื้นจากอากาศ อย่างไรก็ตามสามารถแก้ไขได้โดยการปิดผนึกขอบกระจกสไลด์ด้วยน้ำยาทาเล็บเมื่อตัวอย่างแห้งสนิท ข้อจำกัดและการเลือกใช้วิธีการเหล่านี้ยังคงมีผลต่อการตัดสินใจเลือกวิธี

ทำสไลด์ถาวรของตัวอย่างในปัจจุบัน โดยขึ้นอยู่กับวัตถุประสงค์ของการเตรียมตัวอย่างนั้น ๆ ตั้งแต่ทศวรรษที่ 1980 เป็นต้นมา การศึกษาจำแนกสายพันธุ์ของริ้นฝอยทรายได้เริ่มนำแนวทางทางสัณฐานวิทยามาผสมผสานกับแนวทางทางชีวเคมี วิธีแรกที่นำมาใช้คือการวิเคราะห์สารประกอบไฮโดรคาร์บอนที่ผนังลำตัว (cuticular hydrocarbon analysis) ซึ่งต่อมาถูกแทนที่ด้วยเทคนิคทางอณูชีววิทยาอย่างรวดเร็ว เช่น เทคนิคใช้ไพรเมอร์สุ่ม (random amplified polymorphic DNA: RAPD) เทคนิคใช้เอนไซม์ตัดจำเพาะ (restriction fragment length polymorphism: RFLP) การเพิ่มปริมาณสารพันธุกรรม และการหาลำดับนิวคลีโอไทด์ด้วยวิธีแซงเกอร์ (sanger sequencing) รวมถึงการหาลำดับนิวคลีโอไทด์ในยุคใหม่ หรือที่เรียกว่า next-generation sequencing; NGS ซึ่งในปัจจุบันแนวทางทางอณูชีววิทยายังถูกเสริมด้วยวิธีการวิเคราะห์โปรตีน เช่น MALDI-ToF นอกจากนี้ การจำแนกสายพันธุ์ด้วยวิธีทางโมเลกุลยังสามารถทำควบคู่ไปกับการตรวจหาเชื้อก่อโรคด้วยเทคนิคพีซีอาร์ (Polymerase Chain Reaction; PCR) เช่น *Leishmania*, *Trypanosoma*, *Bartonella* และ *Phlebovirus* เนื่องจากสามารถตรวจพบได้ทั้งวิธีพีซีอาร์แบบดั้งเดิม (End-point PCR) และ พีซีอาร์แบบเรียลไทม์ (Real-time PCR) ซึ่งต้องมีการปรับกระบวนการเก็บตัวอย่างและวิธีการเก็บให้สอดคล้องกับเป้าหมายการวิจัย [3, 32] นอกเหนือจากลักษณะทางสัณฐานวิทยาแบบดั้งเดิมแล้ว ยังมีการประยุกต์ใช้แนวทางทางสัณฐานวิทยาอื่น ๆ เพิ่มเติม เช่น การวัดสัณฐานแบบเรขาคณิตบนปีก (wing geomorphometry)

บทความนี้มีวัตถุประสงค์เพื่อเสนอแนวทางมาตรฐานในการทำสไลด์และการเตรียมตัวอย่างริ้นฝอยทรายตัวเต็มวัย เพื่อเพิ่มประสิทธิภาพในการวิเคราะห์ทางสัณฐานวิทยาและอณูชีววิทยา โดยอาศัยประสบการณ์ของผู้นิพนธ์และข้อมูลจากการทบทวนวรรณกรรม

ความจำเป็นในการวิเคราะห์บางประเภท เช่น อณูชีววิทยา หรือ MALDI-ToF ทำให้ต้องมีการเก็บชิ้นส่วนของตัวอย่างริ้นฝอยทรายที่ไม่ได้ใช้ในการจำแนกทางสัณฐานวิทยา ซึ่งย้ำให้เห็นถึงความสำคัญในการเลือกวิธีการอย่างถี่ถ้วน

บทความฉบับนี้มุ่งเน้นที่วิธีการทำให้แมลงสลบและการทำให้แมลงตายในตัวอย่างริ้นฝอยทรายที่เก็บได้ในขณะมีชีวิต การเก็บรักษาตัวอย่าง และการเตรียมสไลด์ เพื่อการจำแนกสายพันธุ์อย่างรวดเร็วหรือเพื่อการเก็บในระยะยาวสำหรับการศึกษาในอนาคต

บทนำเบื้องต้น: การพิจารณาด้านความปลอดภัยและข้อกำหนดทางกฎหมายควรอ้างอิงตามเอกสารข้อมูลความปลอดภัยของสารเคมี (Safety Data Sheets; SDS) ที่เกี่ยวข้อง

สารเคมีทั้งหมดที่นำเสนอในคู่มือนี้ต้องได้รับการจัดการภายใต้เงื่อนไขความปลอดภัยที่เข้มงวด คณะกรรมการด้านอาชีวอนามัยและความปลอดภัยของสถาบันวิจัยพร้อมให้ข้อมูลเกี่ยวกับอันตรายของสารเคมี ขั้นตอนการจัดการ และการกำจัดของเสีย อย่างไรก็ตาม ผู้ใช้งานจำเป็นต้องปฏิบัติตามคำแนะนำด้านความปลอดภัยในการใช้งานและการกำจัดอย่างเคร่งครัด ทั้งนี้ เป็นความรับผิดชอบของผู้ใช้ทุกคนที่จะต้องมั่นใจว่าการดำเนินงานเป็นไปตามแนวทางปฏิบัติที่ดีในห้องปฏิบัติการ (Good Laboratory Practices; GLP) รวมถึงกฎหมายและข้อบังคับที่บังคับใช้ในประเทศหรือสถาบันวิจัยนั้น ๆ นอกจากนี้ สารเคมีบางชนิดหรือส่วนประกอบบางอย่าง เช่น คลอรอลไฮเดรต (chloral hydrate) อาจมีการควบคุมทางกฎหมายในบางประเทศ โดยรายการคำย่อที่ใช้ในต้นฉบับนี้ได้แสดงไว้ในตารางที่ 1

ตารางที่ 1 รายการคำย่อ

BME	Basal medium Eagle
CDC	Centers for Disease Control and Prevention
CMCP	Camphor-monochlorophenol
CMR	Carcinogenic, mutagenic, reprotoxic substance
COI	Cytochrome c oxidase subunit I gene
CytB	Cytochrome b gene
DNA	Deoxyribonucleic acid
ELISA	Enzyme-linked immunosorbent assay
EtOH	Ethanol
M199	Medium 199
MALDI-ToF MS	Matrix-assisted laser desorption/ionization time-of-flight mass spectrometry
MEM	Minimum essential media
NGS	Next-generation sequencing
NNN	Novy-MacNeal-Nicolle medium
PCR	Polymerase chain reaction
Lao PDR	Lao People’s Democratic Republic
PNOC	Prepronociceptin gene
qPCR	Quantitative PCR (real-time PCR)
RAPD	Random amplified polymorphic DNA
RFLP	Restriction fragment length polymorphism
RI	Refractive index
RNA	Ribonucleic acid
RNases	Ribonucleases
RNASS	RNA stabilization solution
RT-PCR	Reverse transcription PCR
TFA	Trifluoroacetic acid

1. การเก็บตัวอย่างริ้นฝอยทราย

ริ้นฝอยทรายตัวเต็มวัยสามารถเก็บได้ทั้งในสภาพที่ยังมีชีวิตและในสภาพที่ตายแล้ว โดยใช้วิธีการหลากหลายรูปแบบ อาทิ กับดักแสงไฟขนาดเล็กแบบ CDC (CDC miniature light traps) กับดักกาว (sticky traps) และเครื่องดูดจับแมลง (aspirators) ซึ่งอาจใช้ร่วมกับกับดักแชนนอน (Shannon traps) หรือการเก็บโดยตรงจากแหล่งพักนอนตามธรรมชาติหรือสิ่งแวดล้อมที่เกี่ยวข้อง เช่น คอกสัตว์ วิธีการเหล่านี้เกี่ยวข้องกับการเลือกตำแหน่งวางกับดักในแหล่งที่อยู่อาศัยที่เหมาะสม การดึงดูดริ้นฝอยทรายด้วยแสงหรือสารล่ออื่น ๆ เช่น ก๊าซคาร์บอนไดออกไซด์ (CO_2) หรือสารเคมีล่อแมลง และการเก็บรวบรวมตัวอย่างเพื่อนำไปวิเคราะห์ในขั้นตอนต่อไป ตามที่ได้มีการอธิบายรายละเอียดไว้ในเอกสารสิ่งพิมพ์หลายฉบับ [2, 3, 32, 36, 49] การเก็บตัวอย่างริ้นฝอยทรายในสภาพที่ยังมีชีวิตมีข้อได้เปรียบ เนื่องจากสามารถนำตัวอย่างไปประยุกต์ใช้ในการศึกษาต่อเนื่องได้ทุกรูปแบบ รวมถึงการแยกเชื้อและการวิเคราะห์ทางอณูชีววิทยา ในขณะที่การเก็บรวบรวมตัวอย่างที่ตายแล้วไม่เหมาะสมต่อการแยกเชื้อ ลิชมาเนีย หรือไวรัส อย่างไรก็ตามเทคนิคการเก็บบางวิธี เช่น การใช้กระดาษกาว มักก่อให้เกิดความเสียหายต่อโครงสร้างทางกายวิภาคของริ้นฝอยทราย ได้แก่ หนวด ระยางค์ปาก ปีก หรือขา ซึ่งอาจส่งผลต่อความแม่นยำในการจำแนกสายพันธุ์ นอกจากนี้ น้ำมันละหุ่ง (castor oil) ที่ใช้เคลือบกระดาษกาวมีลักษณะเหนียวและติดแน่นกับลำตัวของแมลง จำเป็นต้องกำจัดออกตั้งแต่ระยะเริ่มต้นของกระบวนการเตรียมตัวอย่าง โดยทั่วไปนิยมใช้

วิธีการแช่ตัวอย่างในสารผสมของเอทานอลและไดเอทิลอีเทอร์ในอัตราส่วนเท่ากันเป็นระยะเวลาประมาณ 15 นาที เพื่อขจัดคราบน้ำมันดังกล่าวก่อนทำในขั้นตอนถัดไป

2. การทำให้ตัวอย่างริ้นฝอยทรายสลบและตาย

หลังจากเก็บตัวอย่างริ้นฝอยทรายแล้ว ริ้นฝอยทรายที่มีชีวิตจำเป็นต้องได้รับการทำให้สลบและตายด้วยวิธีที่เหมาะสมและสอดคล้องกับวัตถุประสงค์ของการศึกษา ทั้งนี้วิธีการเก็บตัวอย่างบางรูปแบบ เช่น การใช้กับดักกาว หรือกับดักแสงไฟแบบ CDC ที่บรรจุสารซักฟอกหรือเอทานอล จะทำให้ริ้นฝอยทรายตายทันทีในระหว่างการเก็บตัวอย่าง ตัวอย่างที่เก็บในเอทานอลสามารถนำไปใช้ในการวิเคราะห์ทางอณูชีววิทยาได้โดยตรง หรือควรนำไปแช่ในเอทานอลโดยเร็วที่สุดหลังการเก็บ อย่างไรก็ตาม วิธีการดังกล่าวไม่เหมาะสมสำหรับการวิเคราะห์ด้วยเทคนิค MALDI-ToF วิธีการทำให้ตัวอย่างตายบางวิธีอาจส่งผลให้ลักษณะทางสัณฐานวิทยาบางอย่างเสียหายไป จึงมีความจำเป็นอย่างยิ่งที่จะต้องเลือกใช้วิธีมาตรฐานที่สามารถรักษาลักษณะสำคัญของตัวอย่างไว้ โดยเฉพาะในกรณีที่ต้องการจำแนกสายพันธุ์อย่างถูกต้อง หรือเก็บเป็นตัวอย่างอ้างอิง (voucher specimens) ในระยะยาว สารเคมีที่นิยมใช้ในการทำให้แมลงสลบและตาย ได้แก่ เอทิลอะซิเตต (ethyl acetate) เอทิลอีเทอร์ (ethyl ether) เททระคลอโรอีเทน (tetrachloroethane) และคลอโรฟอร์ม (chloroform) โดยมักหยดสารลงบนสำลีและวางไว้ภายในภาชนะบรรจุตัวอย่าง อย่างไรก็ตาม สารเคมีดังกล่าวมีความเป็นพิษสูง จึงต้องใช้งานด้วยความระมัดระวังและปฏิบัติตามคำแนะนำด้านความปลอดภัยอย่างเคร่งครัด จากประสบการณ์ของผู้นิพนธ์ ไม่แนะนำให้ใช้คลอโรฟอร์ม เนื่องจากพบว่าส่งผลเสียต่อคุณภาพของการศึกษาทางอณูชีววิทยา อีกทั้งเมื่อพิจารณาถึงอันตรายต่อผู้ปฏิบัติงานและความไม่แน่นอนของผลการวิเคราะห์ในระดับโมเลกุล จึงควรหลีกเลี่ยงการใช้สารเคมีดังกล่าวโดยทั่วไป

วิธีการที่ได้รับความนิยมและสามารถรักษาสภาพของลักษณะทางสัณฐานวิทยา สารพันธุกรรมดีเอ็นเอ และโปรตีนได้ดีที่สุด คือ ทำให้แห้งด้วยการแช่เยือกแข็ง (dry freezing) โดยควรแช่ตัวอย่างให้นานพอจนแมลงสลบอย่างสมบูรณ์ แต่ไม่ควรนานเกินไปจนทำให้ (1) ตัวอย่างแห้งกรอบ หรือ (2) ส่งผลกระทบต่อความมีชีวิตของเชื้อลิชมาเนีย ในกรณีที่มีวัตถุประสงค์เพื่อแยกเชื้อลิชมาเนียจากทางเดินอาหารของแมลง ผู้นิพนธ์แนะนำให้แช่แข็งที่อุณหภูมิ -20 องศาเซลเซียส เป็นระยะเวลา 15-20 นาที และควรตรวจสอบตัวอย่างเป็นระยะเพื่อให้แน่ใจว่าแมลงเพียงอยู่ในสภาวะสลบ ขณะที่เชื้อปรสิตยังคงมีชีวิต

ในกรณีที่ไม่สามารถใช้ตู้แช่แข็งได้ สามารถใช้ก๊าซคาร์บอนไดออกไซด์เป็นทางเลือก โดยในภาคสนามอาจใช้หลอดก๊าซคาร์บอนไดออกไซด์ขนาดเล็กที่ใช้กับเครื่องทำน้ำโซดาแทน (soda siphons) ทั้งนี้ควรคำนึงถึงข้อจำกัดด้านการขนส่งทางอากาศ หากไม่มีทางเลือกอื่น วิธีรมควันบุหรี่สามารถนำมาใช้ได้ โดยกักแมลงไว้ในหลอดแก้วและพ่นควันเข้าไป ซึ่งจะทำให้แมลงตายภายในไม่กี่วินาที วิธีนี้มีประสิทธิภาพในพื้นที่ห่างไกล อย่างไรก็ตาม ควันบุหรี่จะเคลือบผิวหลอดแก้ว จึงไม่ควรนำหลอดเดิมกลับมาใช้เก็บแมลงมีชีวิตอีก หากยังไม่ได้ทำความสะอาดอย่างละเอียด วิธีการทั้งหมดที่กล่าวมาข้างต้นสามารถใช้ร่วมกับการผ่าทางเดินอาหารเพื่อแยกเชื้อลิชมาเนียในริ้นฝอยทรายได้

3. การเก็บรักษาตัวอย่างก่อนนำไปศึกษา

การเก็บรักษาตัวอย่างก่อนนำไปทำต่อสามารถแบ่งออกเป็น 5 วิธีหลัก ดังนี้

3.1. การแช่แข็ง (freezing)

ควรเก็บที่อุณหภูมิ -20 องศาเซลเซียส หรือหากเป็นไปได้ควรเก็บที่ -80 องศาเซลเซียส ซึ่งปัจจุบันเป็นที่นิยมมากกว่าการเก็บในไนโตรเจนเหลว การแช่แข็งควรทำทันทีหลังจากทำให้แมลงสลบ ข้อดีของการเก็บในเครื่องแช่แข็งคือสามารถรักษาสภาพตัวอย่าง รวมถึงความสมบูรณ์ของสารพันธุกรรมอาร์เอ็นเอ (RNA) สารพันธุกรรมดีเอ็นเอ (DNA) และโปรตีนได้ดีตลอดระยะเวลาการเก็บรักษา ในขณะที่ไนโตรเจนเหลวอาจทำให้ปีก ขา ระยางค์ปาก และหนวดเกิดความเสียหายรุนแรงจนหลุดขาด ส่งผลให้สูญเสียลักษณะทางสัณฐานที่สำคัญ การทำให้แห้งด้วยการแช่เยือกแข็งก่อให้เกิดความเสียหายน้อยกว่า แต่ในขั้นตอนการละลายตัวอย่าง อวัยวะที่เปราะบางอาจติดกับผนังหลอดเนื่องจากการควบแน่นของน้ำ จึงทำให้ฉีกขาดได้ นอกจากนี้ การแช่แข็งอาจไม่สะดวกในงานภาคสนามเนื่องจากข้อจำกัดด้านอุปกรณ์ วิธีนี้เหมาะสมอย่างยิ่งสำหรับการตรวจหาเชื้อก่อโรคด้วยเทคนิคทางโมเลกุล อย่างไรก็ตาม หากต้องการตรวจหาและแยกเชื้อไวรัสชนิดอาร์เอ็นเอ (RNA viruses) ในระยะยาว จำเป็นต้องเก็บที่ -80 องศาเซลเซียส หรือในไนโตรเจนเหลว ทั้งนี้ การแช่แข็งตัวอย่างมักไม่เหมาะสมสำหรับการผ่าตัวแมลงเพื่อแยกเชื้อลิชมาเนียจากทางเดินอาหารของริ้นฝอยทราย

3.2. การเก็บตัวอย่างในแอลกอฮอล์ เช่น เอทานอล (ethanol alcohol) หรือ ไอโซโพรพิลแอลกอฮอล์ (isopropyl alcohol)

วิธีการเก็บริ้นฝอยทรายในแอลกอฮอล์ถือเป็นวิธีที่ถูกใช้อย่างแพร่หลายมากที่สุด เนื่องจากสามารถนำไปใช้ได้ง่ายในภาคสนาม แม้ในสภาพแวดล้อมที่ยากลำบากหรือไม่มีห้องปฏิบัติการรองรับ การเก็บในแอลกอฮอล์มีความเหมาะสมเป็นพิเศษสำหรับการศึกษาทางสัณฐานวิทยา เนื่องจากอวัยวะที่เปราะบาง เช่น ปีก ขา หนวด หรือปาล์ป (palps) จะยังคงสภาพสมบูรณ์ หากไม่มีฟองอากาศอยู่ภายในหลอดเก็บตัวอย่าง ดังนั้นจึงแนะนำให้ปิดผนึกหลอดด้วยสำลีก้อนเล็ก ๆ เพื่อกำจัดฟองอากาศ และติดฉลากไว้ด้านบนของจุกสำลี (รูปที่ 1) ความเข้มข้นของแอลกอฮอล์ที่เหมาะสมสำหรับการเก็บรักษาตัวอย่างยังคงเป็นประเด็นที่มีการถกเถียงกันอยู่ โดยทั่วไปไม่แนะนำให้ใช้แอลกอฮอล์ที่มีความเข้มข้นต่ำกว่า 70% [45, 66] แอลกอฮอล์ที่มีความเข้มข้นสูงสามารถรักษาสภาพของสารพันธุกรรมดีเอ็นเอได้อย่างมีประสิทธิภาพมากกว่าและเป็นระยะเวลานานกว่า อย่างไรก็ตาม ความเข้มข้นที่สูงขึ้นอาจทำให้ตัวอย่างมีความเปราะและแตกหักง่าย ส่งผลให้ไม่เหมาะสมต่อการศึกษาทางสัณฐานวิทยา การใช้เอทานอลความเข้มข้น 96% ซึ่งเป็นสารผสมแบบอะซีโอโทรป (azeotrope) สามารถช่วยให้ความเข้มข้นของแอลกอฮอล์คงที่ในระยะยาว โดยเฉพาะในพื้นที่ที่มีความชื้นสูง เช่น ประเทศในเขตร้อน แม้ว่าเอทานอลความเข้มข้น 95% จะหาได้ง่ายกว่าและถูกนำมาใช้อย่างแพร่หลายมากกว่า ไม่ว่าความเข้มข้นของแอลกอฮอล์จะเป็นเท่าใด โดยทั่วไปสารพันธุกรรมดีเอ็นเอสามารถคงสภาพได้ดีเมื่อเก็บในเอทานอล แม้ว่าประสิทธิภาพจะต่ำกว่าวิธีการเก็บด้วยการแช่แข็ง โดยเฉพาะอย่างยิ่งสำหรับการวิเคราะห์ทางชีวโมเลกุลขั้นสูง เช่น เทคนิคการหาลำดับนิวคลีโอไทด์แบบ next-generation sequencing (NGS) ในทางตรงกันข้าม โปรตีนมีความเสถียรต่ำกว่ามาก โดยเฉพาะการศึกษาด้านโปรตีโอมิกส์ (proteomics) เช่น การประยุกต์ใช้เทคนิค MALDI-ToF ริ้นฝอยทรายที่เก็บในแอลกอฮอล์เป็นระยะเวลาหลายเดือนยังคงสามารถนำมาจำแนกสายพันธุ์ทางสัณฐานวิทยาได้ แต่ไม่เหมาะสมสำหรับการสร้างฐานข้อมูลสเปกตรัมโปรตีนอ้างอิงได้ การเก็บตัวอย่างในแอลกอฮอล์หรือในสภาพแห้งสามารถปรับปรุงคุณภาพได้ หากมีการเก็บตัวอย่างร่วมกับการแช่แข็งที่อุณหภูมิ –20 องศาเซลเซียส การแช่แข็งที่อุณหภูมิ –20

องศาเซลเซียส ช่วยเพิ่มประสิทธิภาพในการรักษาสภาพทางชีวโมเลกุล โดยเฉพาะกรดนิวคลีอิกผ่านการชะลอการเสื่อมสลายของโมเลกุล อีกทั้งยังให้ประโยชน์รองลงมาต่อการคงสภาพทางสัณฐานวิทยา โดยช่วยลดการเสื่อมสภาพของเนื้อเยื่อในระยะยาว แม้ว่าผลต่อการคงสภาพทางสัณฐานวิทยาจะมีความจำกัดมากกว่าผลความสมบูรณ์ของข้อมูลระดับโมเลกุล การเก็บในเอทานอลยังสามารถประยุกต์ใช้สำหรับการตรวจหาดีเอ็นเอไวรัส (DNA virus) และ อาร์เอ็นเอไวรัส (RNA virus) ได้ โดยใช้เอทานอลที่มีความเข้มข้นอย่างน้อย 70% และเหมาะสำหรับการเก็บในระยะเวลาสั้น ไม่เกินไม่กี่เดือน นอกจากนี้ ในบางประเทศ ไอโซโพรพิลแอลกอฮอล์อาจหาได้ง่ายและสามารถรักษาสภาพของสารพันธุกรรมดีเอ็นเอได้ อย่างไรก็ตามสารชนิดนี้ทำให้ตัวอย่างมีความแข็งตัวมากขึ้น ไอโซโพรพิลแอลกอฮอล์ไม่มีคุณสมบัติติดไฟเช่นเดียวกับเอทานอล จึงสามารถขนส่งได้สะดวก หากมีความจำเป็น ริ้นฝอยทรายที่เก็บในไนโตรเจนเหลวหรือด้วยการแช่แข็งแบบแห้งสามารถนำมาเปลี่ยนไปเก็บในแอลกอฮอล์ได้ในภายหลัง อย่างไรก็ตาม วิธีการดังกล่าวจะเป็นการรวมข้อจำกัดของทั้งสองวิธีเข้าด้วยกัน

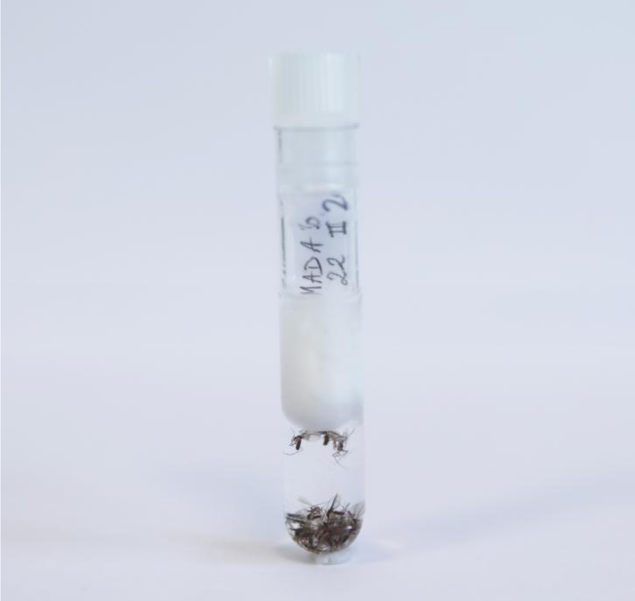

รูปที่ 1. การเก็บตัวอย่างริ้นฝอยทรายในเอทานอล

3.3. การเก็บตัวอย่างในสารละลายรักษาสภาพสารพันธุกรรมอาร์เอ็นเอ (RNA Stabilization Solution; RNASS)

RNASS เป็นสารละลายชนิดมีน้ำเป็นตัวทำละลาย ไม่เป็นพิษ และออกแบบมาเพื่อรักษาสภาพสารพันธุกรรมอาร์เอ็นเอในเนื้อเยื่อสด โดยสามารถซึมเข้าสู่ตัวอย่างได้อย่างรวดเร็ว และยับยั้งเอนไซม์ RNases ส่งผลให้สามารถรักษาสารพันธุกรรมอาร์เอ็นเอ ได้โดยไม่จำเป็นต้องแช่แข็งทันที นอกจากนี้ RNASS ยังช่วยคงสภาพโครงสร้างเนื้อเยื่อและสัณฐานวิทยาของเซลล์ ตัวอย่างที่เก็บใน RNASS สามารถเก็บไว้ที่อุณหภูมิห้องได้นานถึง 7 วัน ที่ 4 องศาเซลเซียส ได้นานหลายสัปดาห์ หรือที่ -20 องศาเซลเซียส หรือ -80 องศาเซลเซียส สำหรับการเก็บระยะยาว ซึ่งมีประโยชน์อย่างยิ่งในงานภาคสนามที่ขาดระบบความเย็น ซึ่งโดยปกติแล้วในการสกัดสารพันธุกรรมอาร์เอ็นเอจำเป็นต้องนำตัวอย่างออกจากสารละลายรักษาสภาพดังกล่าวเสียก่อน แล้วจึงเข้าสู่กระบวนการสกัดตามเกณฑ์วิธีมาตรฐานต่อไป

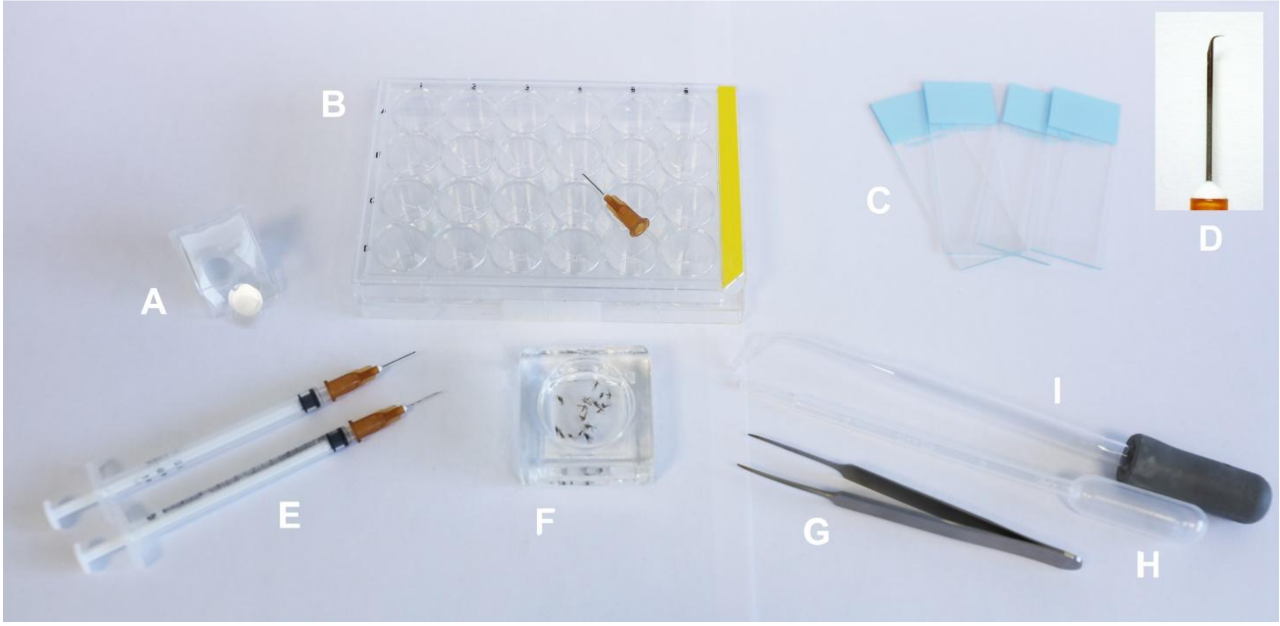

รูปที่ 2. อุปกรณ์ที่จำเป็นสำหรับการทำสไลด์ของริ้นฝอยทราย A: กระจกปิดสไลด์แก้วแบบกลม (เส้นผ่านศูนย์กลาง 10 หรือ 12 มิลลิเมตร); B: ถาดหลุม 24 หลุม และเข็มปลายงอ หมายเหตุ: หากใช้น้ำมันกานพลูหรือ Euparal® essence ในการเตรียมตัวอย่าง ไม่ควรใช้ถาดอะคริลิก เนื่องจากอาจเกิดปฏิกิริยาทางเคมีและทำให้ตัวอย่างเสียหาย; C: แผ่นสไลด์แก้วที่สามารถเขียนฉลากได้; D: ลักษณะของเข็มปลายงอ; E: เข็มที่ต่อกับกระบอกฉีดยา; F: กระจกนาฬิกาหรือภาชนะที่เหมาะสมสำหรับบรรจุตัวอย่างที่รอการทำสไลด์ถาวร; G: ปากคีบ Dumont; H: ปิเปตพลาสติก; I: ปิเปตแก้วที่ดัดโค้งด้วยความร้อน เพื่อความสะดวกในการหยดสารละลายลงในหลุม

3.4. การเก็บตัวอย่างแบบแห้งที่อุณหภูมิห้อง

การเก็บตัวอย่างแบบแห้งเป็นวิธีดั้งเดิมที่ใช้กับตัวอย่างทั้งตัว (*in toto*) โดยมีข้อจำกัดสำคัญคืออาจทำให้อวัยวะที่เปราะบางเสียหายได้ง่าย เช่น ปีก ขา หนวด และปาล์ป อย่างไรก็ตาม การศึกษาด้านโปรตีโอมิกส์ด้วยเทคนิค MALDI-ToF ยังคงสามารถทำได้ หากทำให้ตัวอย่างแห้งสนิทโดยใช้สารดูดความชื้น เช่น ซิลิกาเจล ในทางตรงกันข้ามการวิเคราะห์สารพันธุกรรมดีเอ็นเอ จากตัวอย่างแบบแห้งทำได้ยาก เนื่องจากสารพันธุกรรมดีเอ็นเอมักจะแตกหักและมีปริมาณต่ำกว่าตัวอย่างสดหรือแช่แข็ง อย่างไรก็ตามเทคโนโลยีสมัยใหม่ เช่น มิวเซโอมิกส์ (museomics) [34] คือ ศาสตร์ที่ผสมผสานเทคนิคทางวิทยาศาสตร์โอมิกส์ เช่น จีโนมิกส์และโปรตีโอมิกส์เข้ากับการศึกษาวัตถุจัดแสดงทางประวัติศาสตร์และตัวอย่างในพิพิธภัณฑ์ เพื่อวิเคราะห์ข้อมูลชีวโมเลกุล ซึ่งอาจนำมาใช้กับตัวอย่างกลุ่มนี้ได้ แต่ผู้นิพนธ์ไม่แนะนำให้ใช้วิธีนี้ เว้นแต่จะไม่มีทางเลือกอื่น วิธีการดังกล่าวสามารถนำมาใช้ร่วมกับการเก็บตัวอย่างในสภาพเย็นได้ โดยการนำหลอดตัวอย่างไปเก็บในตู้แช่แข็งที่อุณหภูมิ –20 องศาเซลเซียส หรือ –80 องศาเซลเซียส ความท้าทายของวิธีนี้คือการทำสไลด์ชิ้นส่วนให้ครบถ้วน และจัดวางในตำแหน่งที่เหมาะสม เพื่อให้การระบุสายพันธุ์เป็นไปอย่างแม่นยำและถูกต้อง วิธีนี้จำเป็นต้องมีขั้นตอนการคืนตัว (rehydration) โดยแนะนำให้ใช้สารละลาย Triton X-100 ระยะเวลาของการคืนตัวอาจแตกต่างกันตั้งแต่ไม่กี่ชั่วโมงจนถึงหลายวัน ทั้งนี้ต้องมีการเฝ้าติดตามอย่างใกล้ชิดและสม่ำเสมอ หลังจากการคืนสภาพความชุ่มชื้นเสร็จสมบูรณ์แล้ว ควรล้างตัวอย่างด้วยน้ำสะอาดในอ่างน้ำติดต่อกันจำนวนสามครั้ง

3.5. การเก็บตัวอย่างบนกระดาษกรอง

ข้อดีหลักของการใช้กระดาษกรองในการเก็บตัวอย่างคือความเสถียรในระยะยาวของจีโนมิกดีเอ็นเอ (genomic DNA) ภายในเซลล์ของตัวอย่างที่อยู่ในสภาพแห้งและไม่ได้ผ่านการตรึงสภาพ รวมถึงเซลล์เม็ดเลือดที่สามารถเก็บไว้ที่อุณหภูมิห้องได้ กระดาษกรองถูกผลิตในรูปแบบการ์ดขนาดเล็ก ทำให้สามารถเก็บตัวอย่างได้หลายร้อยตัวอย่างที่อุณหภูมิห้อง โดยใช้พื้นที่เพียงเทียบเท่าลิ้นชักโต๊ะขนาดเล็ก นอกจากนี้เนื้อกระดาษกรองยังถูกเคลือบด้วยสารที่มีฤทธิ์ทำให้เชื้อก่อโรคเสียสภาพ ส่งผลให้ตัวอย่างที่เก็บไว้ไม่ถูกจัดว่าเป็นขยะติดเชื้อ (biohazard) อีกต่อไป คุณสมบัติดังกล่าวเอื้อให้สามารถจัดเก็บและขนส่งตัวอย่างได้โดยไม่จำเป็นต้องใช้มาตรการควบคุมหรือการป้องกันขยะติดเชื้อเป็นพิเศษ [68]

4. การผ่าตัวอย่างริ้นฝอยทราย

ริ้นฝอยทรายมีลักษณะแตกต่างจากแมลงชนิดอื่นที่สามารถจำแนกสายพันธุ์ได้จากลักษณะภายนอกโดยการปักเข็มบนตัวแมลง แต่สำหรับริ้นฝอยทราย การจำแนกสายพันธุ์อย่างถูกต้องและแม่นยำจำเป็นต้องอาศัยการผ่าและการจัดทำสไลด์เพื่อศึกษาลักษณะทางกายวิภาคภายใต้กล้องจุลทรรศน์ ไม่ว่าจะเลือกใช้เทคนิคการเตรียมตัวอย่างหรือสารตัวกลางสำหรับปิดแผ่นสไลด์ชนิดใด หลักการพื้นฐานของการผ่าตัวริ้นฝอยทรายยังคงเป็นมาตรฐานเดียวกัน (รูปที่ 2 และ รูปที่ 3) (https://zenodo.org/records/18198006)

การใช้ Triton X-100 (สารละลายชนิดไม่มีประจุ)

โดยทั่วไปการทำสไลด์มักทำกับตัวอย่างที่เพิ่งเก็บมาใหม่หรือเก็บไว้ภายใต้สภาวะที่เหมาะสม อย่างไรก็ตาม ในทางปฏิบัตินักวิจัยมักมีตัวอย่างริ้นฝอยทรายที่เก็บรักษาแบบแห้ง เช่น ตัวอย่างที่ใช้สำหรับการวิเคราะห์ด้วย MALDI-ToF หรือเก็บในแอลกอฮอล์เป็นระยะเวลานานหลายปี การเก็บในแอลกอฮอล์เป็นเวลานานเกินไปมักส่งผลให้เนื้อเยื่อแข็งตัว ทำให้การเตรียมตัวอย่างเพื่อการตรวจสอบภายใต้กล้องจุลทรรศน์ทำได้ยากมาก ปัญหาที่พบได้บ่อย ได้แก่ การเสื่อมสภาพของภาชนะพลาสติกซึ่งนำไปสู่การระเหยของแอลกอฮอล์ รวมถึงการเกิดตัวอย่างแห้งกรอบหรือแข็ง ไม่ว่าจะเป็นกรณีของการแช่แอลกอฮอล์เป็นเวลานานหรือตัวอย่างที่เก็บแบบแห้ง ตัวอย่างเหล่านี้ล้วนจัดการได้ยากและเสี่ยงต่อการสูญเสียโครงสร้างทางสัณฐานวิทยาที่สำคัญ ด้วยเหตุนี้ จึงมีการนำแนวคิดการใช้ "สารช่วยเปียก หรือ สารลดแรงตึงผิว" (wetting agents) ที่ไม่ก่อให้เกิดการทำลายเนื้อเยื่ออย่างรุนแรงมาใช้ โดยเลือกใช้สารลลະลาย Triton X-100 ซึ่งเป็นสารลดแรงตึงผิวชนิดไม่มีประจุ (non-ionic detergent) มีชื่อทางเคมีว่า 4-(1,1,3,3-tetramethylbutyl) phenyl-polyethylene glycol หรือ t-octylphenoxypolyethoxyethanol สารชนิดนี้นิยมใช้ในงานชีววิทยาของเซลล์และอณูชีววิทยา เนื่องจากสามารถเพิ่มการซึมผ่านของเยื่อหุ้มเซลล์และเยื่อหุ้มนิวเคลียสได้อย่างมีประสิทธิภาพ โดยไม่ทำลายโครงสร้างพื้นฐานของเนื้อเยื่อ

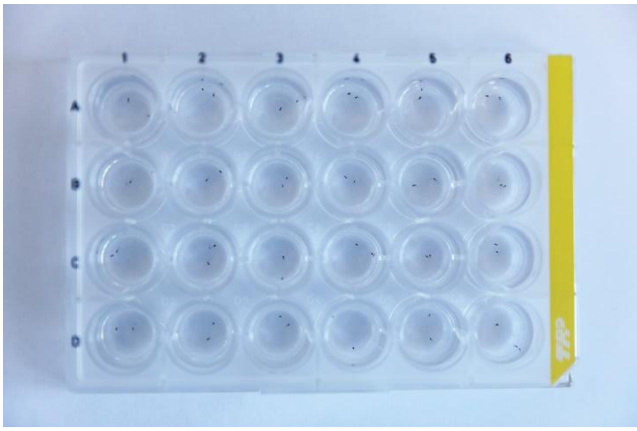

รูปที่ 3. ถาดหลุม 24 หลุม โดยแต่ละหลุมบรรจุส่วนหัวและส่วนปลายของส่วนท้องของริ้นฝอยทราย

4.1. ส่วนหัว (head)

การผ่าตัวริ้นฝอยทรายสามารถทำได้โดยใช้เข็มขนาดเล็กหรือเข็มหมุดที่ใช้กับงานด้านกีฏวิทยา โดยทำภายใต้กล้องจุลทรรศน์สเตอริโอ (รูปที่ 2 และ รูปที่ 3) เข็มที่นิยมใช้มากที่สุด ได้แก่ เข็มขนาด 26G × 1/2 นิ้ว (0.45 × 13 มิลลิเมตร) 30G × 1/2 นิ้ว (0.3 × 13 มิลลิเมตร) หรือ 25G × 5/8 นิ้ว (0.5 × 16 มิลลิเมตร) ซึ่งมีความเหมาะสมต่อการควบคุมการเคลื่อนไหวและลดความเสียหายต่อโครงสร้างขนาดเล็กในการเตรียมตัวอย่างเพื่อการจำแนกสายพันธุ์ อย่างน้อยที่สุดจำเป็นต้องแยกส่วนหัวออกจากลำตัว และทำสไลด์โดยจัดวางในตำแหน่งหงายด้านท้อง (ventral side) เพื่อให้สามารถดูโครงสร้างสำคัญ ได้แก่ กระเปาะอาหาร (cibarium) และคอหอย (pharynx) ได้อย่างชัดเจน ในขณะที่ส่วนอกและส่วนท้องมักวางลงสไลด์ในมุมด้านข้าง (lateral view) หลังจากผ่าแมลงแยกส่วนแล้ว การทำสไลด์จะวางส่วนหัวในตำแหน่งหงายตามแนว ventro-dorsal จะทำให้ช่อง occipital foramen อยู่ในตำแหน่งหงาย ส่งผลให้สามารถตรวจสอบไซบาเรียมได้โดยตรง การแยกส่วนหัวออกจากลำตัวอย่างสมบูรณ์จะช่วยให้เข้าถึงลักษณะทางกายวิภาคเหล่านี้ได้สะดวก และลดการบดบังของโครงสร้างอื่นได้

ขั้นตอนการใช้สารละลาย Triton X-100 ความเข้มข้น 0.5%

- นำตัวอย่างริ้นฝอยทรายที่แห้งมาแช่ในแอลกอฮอล์บริสุทธิ์ (absolute alcohol) จนตัวอย่างชุ่มอย่างทั่วถึง
- เติมสารละลาย Triton X-100 ความเข้มข้น 0.5% ในน้ำในปริมาณที่เพียงพอเพื่อให้ตัวอย่างจมอยู่ในสารละลายทั้งหมด
- ปล่อยให้ตัวอย่างอยู่ในสารละลายเป็นระยะเวลาประมาณ 5 นาที จนถึงหลายวัน โดยต้องตรวจสอบเป็นระยะ ทั้งนี้ระยะเวลาที่เหมาะสมขึ้นอยู่กับสภาพของตัวอย่าง
- เมื่อสามารถแยกส่วนต่าง ๆ ของแมลงออกจากกันได้อย่างสมบูรณ์ในสารละลายแล้ว ให้ดูดสารละลาย Triton X-100 ออก เติมสารละลายโพแทสเซียมไฮดรอกไซด์ (KOH) เพื่อดำเนินกระบวนการในขั้นตอนถัดไปของการเตรียมตัวอย่าง

4.2. ส่วนปีกและส่วนอก (wings and thorax)

ปีกของริ้นฝอยทรายต้องถูกวางบนสไลด์ให้อยู่ในลักษณะแบนราบ โดยสามารถแยกปีกแต่ละข้างออกจากส่วนฐานเพื่อวางบนสไลด์แยกกันอย่างอิสระ หรืออาจเลือกทำสไลด์โดยปีกเพียงข้างเดียวและคงอีกข้างหนึ่งไว้ให้ติดกับส่วนอกก็ได้ ทั้งนี้ หากมีแผนการวิเคราะห์ด้วยวิธีวัดสัณฐานแบบเรขาคณิต (geometric morphometry) จำเป็นต้องระบุและทำเครื่องหมายแยกปีกซ้ายและขวาอย่างชัดเจนก่อนการทำสไลด์ ส่วนอกประกอบด้วยโครงสร้างย่อยหลายส่วน ซึ่งมีข้อมูลทางอนุกรมวิธานที่สำคัญอย่างยิ่ง [20, 64] โดยทั่วไปจะวางในมุมมองด้านข้าง เพื่อให้สามารถดูตำแหน่งและรูปแบบของขน (chaetotaxy) รวมถึงการกระจายตัวของสีได้อย่างครบถ้วน การตรวจพบรอยแผลของขนแข็ง (scars of bristles) ในบางตำแหน่งของส่วนอกสามารถใช้เป็นลักษณะวินิจฉัยสำหรับการจำแนกสายพันธุ์ในสกุล *Brumptomyia* ได้ นอกจากนี้ รูปแบบการกระจายตัวของสีบนส่วนอกยังมีบทบาทสำคัญในการจำแนกริ้นฝอยทรายในเขตครอบคลุมพื้นที่ในทวีปอเมริกาใต้ อเมริกากลาง หมู่เกาะแคริบเบียน และทางตอนใต้ของทวีปอเมริกาเหนือ ในประเทศเม็กซิโก หรือเรียกว่า เขตนีโอทรอปิคอล (neotropical) สามารถจำแนกได้ตั้งแต่ระดับสกุล เช่น *Brichromomyia* ระดับสายพันธุ์ เช่น *Pintomyia* ไปจนถึงระดับสายพันธุ์ภายในสกุลเดียวกัน เช่น *Micropygomyia*, *Nyssomyia*, *Psathyromyia* และ *Psychodopygus* [20] ดังนั้น หากไม่มีความจำเป็นต้องนำส่วนอกไปใช้ในการวิเคราะห์ทางอณูชีววิทยา ควรทำสไลด์ส่วนอกด้วยความระมัดระวังเพื่อหลีกเลี่ยงการทำลายโครงสร้างสำคัญ ข้อควรตระหนักคือ ลักษณะทางอนุกรมวิธานที่สำคัญไม่ได้ขึ้นอยู่กับความเข้มของสี แต่ขึ้นอยู่กับรูปแบบการกระจายตัวของสีบนส่วนอก ดังนั้น กระบวนการทำให้ตัวอย่างใส จะไม่ส่งผลกระทบต่อความถูกต้องในการจำแนกสายพันธุ์ในส่วนนี้

4.3. อวัยวะสืบพันธุ์ (genitalia)

อวัยวะสืบพันธุ์เป็นโครงสร้างที่มีความสำคัญอย่างยิ่งต่อการจำแนกในระดับสกุล สกุลย่อย และสายพันธุ์ จึงจำเป็นต้องใช้ความระมัดระวังเป็นพิเศษในการผ่าและการทำสไลด์ทั้งในเพศผู้และเพศเมีย โดยอวัยวะสืบพันธุ์ของริ้นฝอยทรายทั้งสองเพศมีลักษณะเป็นโครงสร้างคู่

4.3.1. เพศผู้ (males)

อวัยวะสืบพันธุ์เพศผู้เป็นโครงสร้างภายนอก ประกอบด้วยรยางค์คู่ (paired forceps) ซึ่งแต่ละข้างประกอบด้วย gonocoxite และ gonostyle ในส่วนด้านบน (dorsal part) และ epandrial lobe ในส่วนด้านล่าง (ventral part) บริเวณ gonostyle จะมีหนาม (spines) และในบางชนิดอาจมีขนแข็ง (setae) ซึ่งต้องสามารถนับจำนวนและตรวจสอบตำแหน่งการแทรกตัวได้อย่างชัดเจน ลักษณะสำคัญอีกประการหนึ่งคือพื้นผิวด้านในของ gonocoxite ซึ่งอาจมีกลุ่มขนแข็งแบบไม่มีก้าน (sessile setae) หรือขนแข็งที่อยู่บนติ่งเนื้อ (lobe หรือ tubercle) [22] สำหรับผู้ที่ยังมีประสบการณ์ในการผ่าตัวแมลงไม่มาก อาจเลือกใช้วิธีการวางบนสไลด์ด้านข้างโดยไม่แยกอวัยวะสืบพันธุ์ออกจากส่วนปลายของส่วนท้อง (https://zenodo.org/records/18311158) แม้วิธีนี้อาจทำให้เกิดการวางซ้อนกันของอวัยวะสืบพันธุ์ทั้งสองด้าน ส่งผลให้การนับขนแข็งภายใน gonocoxite ทำได้ยากขึ้น แต่จะช่วยลดความเสี่ยงของความเสียหายที่เกิดจากการผ่าตัวแมลงผิดพลาด สำหรับผู้ที่มีความชำนาญ สามารถเปิดอวัยวะสืบพันธุ์ออกเป็นสองส่วนโดยใช้ด้านที่ลาดเอียงของเข็ม ใช้เข็มสอดผ่านเพื่อแยกอวัยวะสืบพันธุ์ออกจากกันโดยไม่ตัดให้ขาดจากกันโดยสมบูรณ์ ทั้งนี้เพื่อแยกชุดของโกโนคอกไซต์-โกโนสไตล์ (gonocoxite-gonostyle assemblies) ออกจากกัน (https://zenodo.org/records/18311158) วิธีการนี้ช่วยให้สามารถเห็นพื้นผิวด้านในของโครงสร้างดังกล่าวได้ง่ายขึ้นทั้งนี้ นอกจากนี้ การจัดวางอวัยวะในลักษณะดังกล่าวยังเอื้อต่อการดูพาราเมียร์ (parameres) และปลอกพาราเมียร์ (parameral sheaths) เนื่องจากโครงสร้างเหล่านี้จะไม่ซ้อนทับกันอีกต่อไป สำหรับการทำสไลด์แบบด้านข้าง ซึ่งมีแนวโน้มก่อให้เกิดการซ้อนทับของอวัยวะ ตัวอย่างจำเป็นต้องผ่านกระบวนการทำให้ใส (clearing) อย่างสมบูรณ์ก่อนการทำสไลด์

4.3.2. เพศเมีย (females)

ระบบสืบพันธุ์เพศเมีย เป็นโครงสร้างภายใน ประกอบด้วยถุงเก็บน้ำเชื้อ (spermathecae) ซึ่งในกรณีที่ไม่มีการผ่าตัวแมลง สามารถดูได้โดยการดูผ่านผนังลำตัว (teguments) โดยวางส่วนท้องในตำแหน่งหงาย (ventral position) โดยทั่วไปถุงเก็บน้ำเชื้อ (spermathecae) สามารถดูได้ค่อนข้างชัดเจน โดยเฉพาะในตัวอย่างที่ผ่านกระบวนการทำให้ใสแล้ว และมีพื้นผิวไม่เรียบ อย่างไรก็ตาม ถุงเก็บน้ำเชื้อ (spermathecae) ที่มีผนังบางและพื้นผิวเรียบอาจดูได้ยาก โดยเฉพาะเมื่อใช้สารตัวกลางสำหรับปิดแผ่นสไลด์ที่มีค่าดัชนีหักเหแสงต่ำ นอกจากนี้ การตรวจสอบส่วนฐานของท่อส่ง (base of the ducts) ซึ่งมีความจำเป็นต่อการจำแนกสายพันธุ์บางกลุ่ม เช่น สกุลย่อย *Larroussius* [35, 37, 38] ซึ่งเป็นพาหะหลักของเชื้อ *Leishmania infantum* ในกลุ่มโลกเก่า มักทำได้ยากหรือไม่สามารถทำได้เลยหากไม่มีการผ่าตัวแมลง เพื่อแก้ไขข้อจำกัดดังกล่าว ควรทำการแยกส่วน genital furca และ ถุงเก็บน้ำเชื้อ (spermathecae) ออกจากส่วนท้อง (https://zenodo.org/records/18311106) โดยทั่วไป genital furca สามารถระบุตำแหน่งได้ง่ายกว่าถุงเก็บน้ำเชื้อ (spermathecae) เนื่องจากท่อส่งของถุงเก็บน้ำเชื้อ (spermathecae) จะเปิดเข้าสู่โครงสร้างนี้ การแยก furca ออกมาจึงมักทำให้ได้ถุงเก็บน้ำเชื้อ (spermathecae) ติดออกมาด้วย หากถุงเก็บน้ำเชื้อ (spermathecae) ถูกตัดขาดโดยไม่ตั้งใจในระหว่างการผ่าแมลง ตัวอย่างยังไม่ถือว่าสูญเสียไปทั้งหมด เนื่องจากยังสามารถดูโครงสร้างดังกล่าวได้ภายในผนังลำตัวของส่วนท้อง (**รูปที่ 4**)

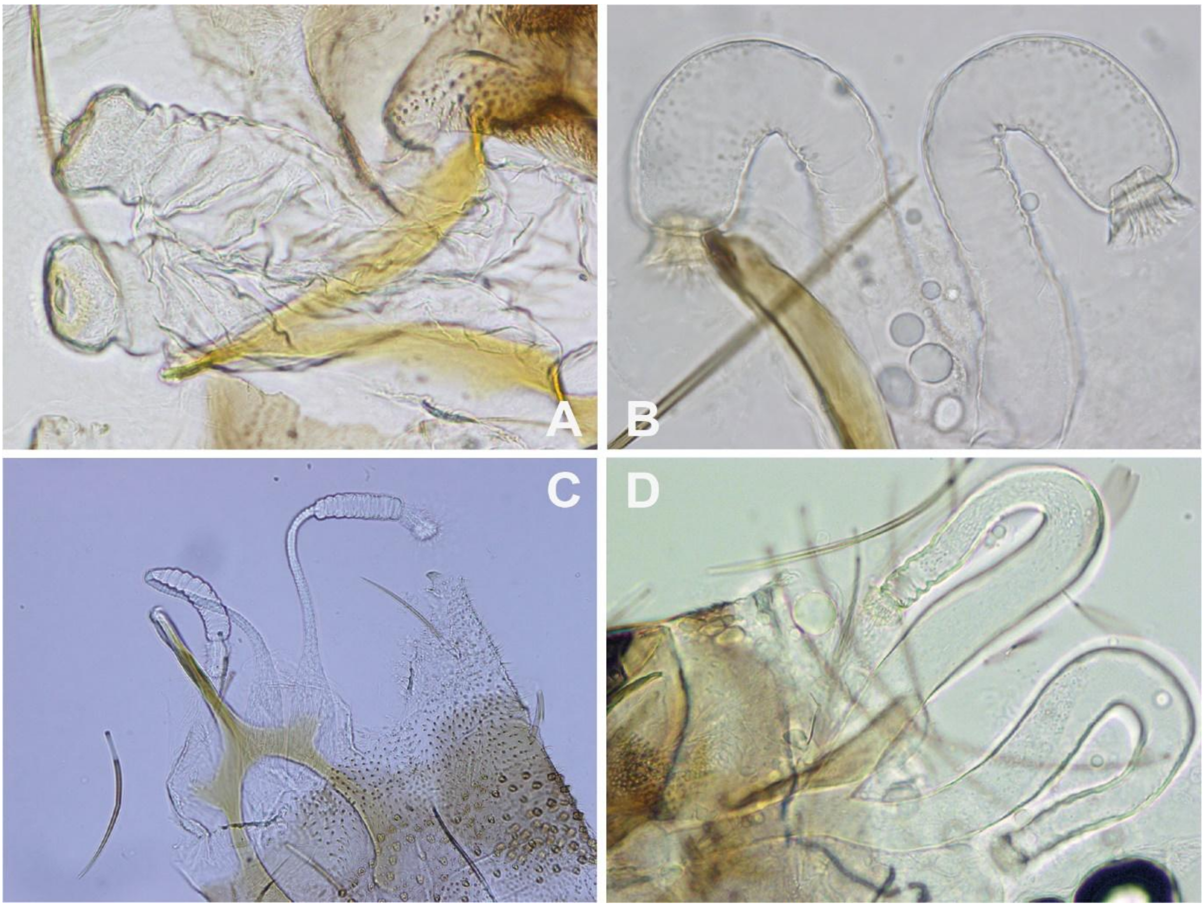

รูปที่ 4. ถุงเก็บน้ำเชื้อ (spermathecae) ที่ผ่านผ่าและทำสไลด์โดยใช้สารละลาย Marc-André จากตัวอย่างสด A: *Idiophlebotomus longiforceps* (Lao PDR) จากสาธารณรัฐประชาธิปไตยประชาชนลาว; B: *Sergentomyia minuta* (France) จากประเทศฝรั่งเศส; C: *Phlebotomus ariasi* (France) จากประเทศฝรั่งเศส; D: *Sergentomyia anodontis* (Lao PDR) จากสาธารณรัฐประชาธิปไตยประชาชนลาว

4.4. การผ่าทางเดินอาหารส่วนกลาง (midgut) ของริ้นฝอยทรายเพื่อการแยกเชื้อลิชมาเนีย

การผ่าทางเดินอาหารส่วนกลางของริ้นฝอยทรายมีความสำคัญอย่างยิ่งต่อการตรวจหาและแยกเชื้อลิชมาเนียในริ้นฝอยทรายเพศเมีย โดยสามารถทำได้ทั้งในภาคสนามและในห้องปฏิบัติการ ทั้งนี้เพื่อใช้ในการประเมินความสามารถในการเป็นพาหะ (vectorial competence) ของริ้นฝอยทรายแต่ละสายพันธุ์ ข้อแนะนำในการปฏิบัติควรเลือกใช้ริ้นฝอยทรายเพศเมียที่เพิ่งถูกทำให้ตายใหม่ ๆ จากนั้นล้างตัวแมลงด้วยน้ำสะอาดหรือน้ำเกลือผสมสารซักฟอกฤทธิ์อ่อน เพื่อกำจัดขนและสิ่งปนเปื้อนภายนอก ขั้นตอนดังกล่าวช่วยคงสภาพการทำงานภายใต้สภาวะปลอดเชื้อสำหรับการแยกเชื้อลิชมาเนีย และในขณะเดียวกันยังช่วยรักษาลักษณะทางสัณฐานวิทยาที่จำเป็นต่อการจำแนกสายพันธุ์ ในการตรวจและแยกเชื้อลิชมาเนีย ให้ดึงทางเดินอาหารส่วนกลางออกมาอย่างระมัดระวัง แล้ววางลงในหยดน้ำเกลือปลอดเชื้อ (0.9% NaCl) ปริมาตรหนึ่งหยด เมื่อตรวจพบเชื้อที่ยังคงมีการเคลื่อนไหวภายใต้กล้องจุลทรรศน์ชนิดใช้แสงกำลังขยายที่แนะนำประมาณ 200 เท่า ให้ใช้เข็มฉีดยาสำหรับอินซูลินหรือไมโครปิเปตในการย้ายเชื้อเข้าสู่อาหารเลี้ยงเชื้อ (รายละเอียดเพิ่มเติมดูในบทที่ 4.4.3) สำหรับการจำแนกสายพันธุ์ ให้นำส่วนหัวและอวัยวะสืบพันธุ์ไปวางในสารละลาย Marc-André โดยตรงเพื่อทำให้ตัวอย่างโปร่งใส ข้อควรระวังสำคัญ ห้ามมิให้สารละลาย Marc-André สัมผัสกับเชื้อลิชมาเนียโดยเด็ดขาด ไม่ว่าจะเป็นการสัมผัสโดยตรงหรือโดยอ้อมผ่านอุปกรณ์หรือเข็ม เนื่องจากสารดังกล่าวมีฤทธิ์ทำลายเชื้อ การผ่าริ้นฝอยทรายเพศเมียสามารถทำได้โดยใช้สไลด์แก้วเพียงแผ่นเดียวหรือสองแผ่น ซึ่งแต่ละวิธีมีข้อดีและข้อจำกัดแตกต่างกัน (**รูปที่ 5**; https://zenodo.org/records/18311154)

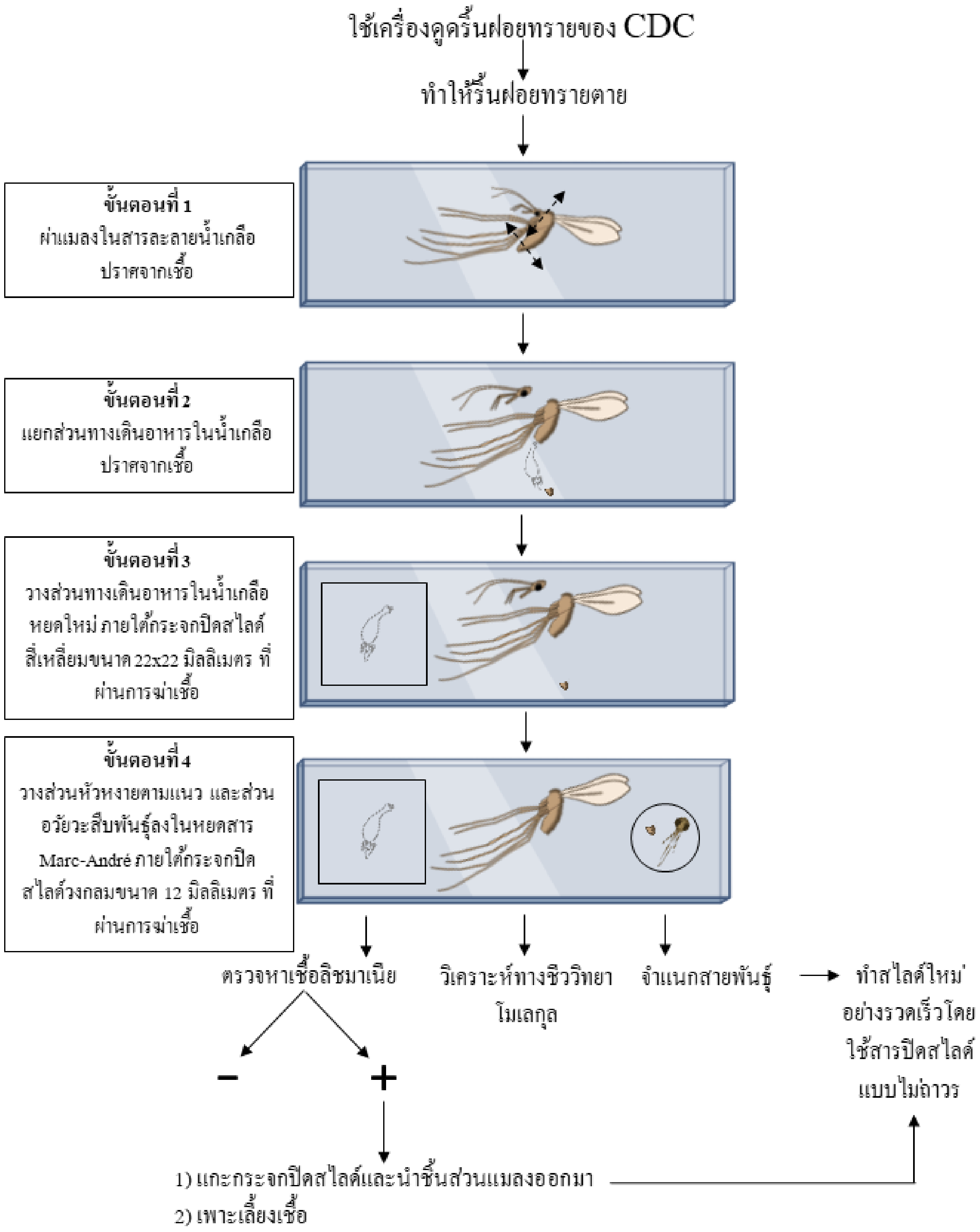

รูปที่ 5. วิธีการแยกเชื้อลิชมาเนียจากทางเดินอาหารส่วนกลางของริ้นฝอยทราย

4.4.1. วิธีใช้สไลด์สองแผ่น (two-slides method)

วิธีนี้เป็นการปฏิบัติงานโดยใช้สไลด์แก้วสองแผ่นแยกจากกัน โดยแผ่นแรกบรรจุน้ำเกลือปลอดเชื้อสำหรับการแยกทางเดินอาหารส่วนกลาง ส่วนแผ่นที่สองใช้สำหรับทำสไลด์ส่วนหัวและถุงเก็บน้ำเชื้อ (spermathecae) ในสารละลาย Marc-André อย่างไรก็ตาม ในการทำงานภาคสนามซึ่งมักมีผู้ปฏิบัติงานหลายคนร่วมกันผ่าตัวอย่าง และส่งต่อชิ้นส่วนให้แก่ผู้รับผิดชอบหลักเพียงคนเดียวเพื่อทำการจำแนกสายพันธุ์และประเมินการติดเชื้อ การใช้สไลด์สองแผ่นอาจก่อให้เกิดปัญหาในการติดตามแหล่งที่มาของตัวอย่าง (traceability) และทำให้ยากต่อการระบุอย่างชัดเจนว่าทางเดินอาหารที่ตรวจพบการติดเชื้อนั้นเป็นของริ้นฝอยทรายตัวใด

(https://zenodo.org/records/18311154)

This is an Open Access article distributed under the terms of the Creative Commons Attribution License (https://creativecommons.org/licenses/by/4.0), which permits unrestricted use, distribution, and reproduction in any medium, provided the original work is properly cited.

4.4.2. วิธีใช้สไลด์แผ่นเดียว (single-slide method)

การใช้สไลด์แก้วเพียงแผ่นเดียวช่วยเพิ่มความแม่นยำในการติดตามผลการวิเคราะห์ของตัวอย่างแต่ละตัว อย่างไรก็ตาม วิธีนี้จำเป็นต้องอาศัยความระมัดระวังสูงเพื่อคงสภาพการทำงานภายใต้สภาวะปลอดเชื้อ ผู้ปฏิบัติงานควรทำความสะอาดมือด้วยเจลแอลกอฮอล์อย่างสม่ำเสมอ ใช้สไลด์แก้วชนิดไม่มีแถบฝ้า และกระจกปิดสไลด์รูปสี่เหลี่ยมขนาด 22 × 22 มิลลิเมตร ซึ่งผ่านการห่อด้วยฟอยล์อะลูมิเนียมและฆ่าเชื้อด้วยความร้อนแห้งโดยใช้ตู้อบลมร้อน (poupinel) รวมทั้งใช้เข็มใหม่ที่ผ่านการฆ่าเชื้อสำหรับการผ่าแมลงแต่ละครั้ง (แนะนำเข็มขนาด 25G เส้นผ่านศูนย์กลาง 0.5 มิลลิเมตร × 16 มิลลิเมตร) ขั้นตอนการดำเนินงาน ให้วางริ้นฝอยทรายลงในหยดน้ำเกลือปลอดเชื้อบริเวณกึ่งกลางสไลด์ ตัดส่วนหัวออก จากนั้นกรีดบริเวณระหว่างปล้องท้อง tergites และ sternites ที่ 6 และ 7 โดยระมัดระวังไม่ให้ท่อทางเดินอาหารเสียหาย โดยสามารถกรีดในตำแหน่งที่สูงขึ้นได้ในกรณีที่คาดว่าถุงเก็บน้ำเชื้อ (spermathecae) มีความยาวมาก ใช้เข็มหนึ่งเล่มตรึงส่วนนอกให้คงที่ และใช้เข็มอีกเล่มดึงส่วนปลายท้องออกมาอย่างเบามือเพื่อดึงทางเดินอาหารออกมา หากไม่สามารถดึงออกมาได้ ให้ตรึงส่วนปลายท้องไว้และดึงทางเดินอาหารจากด้านหน้าแทน หากยังไม่สำเร็จ ให้เอาผนังลำตัวที่เหลืออยู่รอบ ๆ ออกให้มากที่สุดจนสามารถแยกทางเดินอาหารออกมาได้ เมื่อแยกทางเดินอาหารได้แล้ว ให้ตัดแยกส่วนปล้องท้องสุดท้ายออก และย้ายทางเดินอาหารไปวางในหยดน้ำเกลือปลอดเชื้อหยดใหม่ที่ตำแหน่งด้านหนึ่งของสไลด์ จากนั้นปิดด้วยกระจกปิดสไลด์ปลอดเชื้ออย่างระมัดระวัง ส่วนหัวและปล้องท้องสุดท้ายให้นำไปวางในหยดสารละลาย Marc-André ขนาดเล็กที่ปลายอีกด้านของสไลด์ โดยต้องหลีกเลี่ยงการปนเปื้อนกับเชื้อลิชมาเนีย จัดวางส่วนหัวให้ถูกทิศทางโดยหงายช่อง occipital foramen ขึ้น และแยกถุงเก็บน้ำเชื้อ (spermathecae) พร้อมกับ genital furca จากนั้นปิดด้วยกระจกปิดสไลด์แบบกลมขนาดเส้นผ่านศูนย์กลาง 12 มิลลิเมตร เพื่อป้องกันความสับสนกับกระจกปิดสไลด์รูปสี่เหลี่ยมปลอดเชื้อ ส่วนซากที่เหลือและปีกให้คงไว้ในหยดน้ำเกลือบริเวณกึ่งกลางสไลด์ (https://zenodo.org/records/18311154)

ในกรณีที่ตรวจพบผลบวก หรือเพื่อการศึกษาทางอนุกรมวิธานเพิ่มเติม สามารถเก็บส่วนอกและส่วนท้องไว้สำหรับการศึกษาทางอณูชีววิทยาหรือโปรตีโอมิกส์ได้ นอกจากนี้สามารถทำสไลด์ปิกด้วยสารตัวกลางสำหรับปิดแผ่นสไลด์ชนิดน้ำ (aqueous medium) เพื่อการเก็บรักษาระยะยาว โดยอาจใช้สารตัวกลางสำหรับปิดแผ่นสไลด์ชนิดน้ำ เช่น chloral gum หรือ Hoyer หรือสารตัวกลางสำหรับปิดแผ่นสไลด์ที่มีส่วนผสมของ polyvinyl alcohol แทนสารละลาย Marc-André สามารถศึกษาขั้นตอนการปฏิบัติโดยละเอียดเพิ่มเติมได้จากวิดีโอสาธิต ได้แก่ การผ่าทางเดินอาหารส่วนกลาง: https://zenodo.org/records/18303014 และการผ่าต่อมน้ำลาย: https://zenodo.org/records/18302850

จึงไม่อธิบายรายละเอียดเพิ่มเติมในที่นี้

4.4.3. การแยกเชื้อและการเพาะเลี้ยงเชื้อลิชมาเนียจากทางเดินอาหารของริ้นฝอยทราย

การแยกเชื้อลิชมาเนียจากริ้นฝอยทรายเพศเมียเป็นกระบวนการที่ต้องอาศัยความละเอียดรอบคอบและทักษะเชิงเทคนิคขั้นสูง ผู้ปฏิบัติงานควรได้รับการฝึกฝนกับตัวอย่างที่ไม่ติดเชื้อในระยะแรก เพื่อเพิ่มความชำนาญและลดความเสียหายของตัวอย่าง ก่อนทำกับตัวอย่างจริง ภายหลังการผ่าแมลงให้ย้ายส่วนทางเดินอาหารที่แยกได้ไปวางบนหยดสารละลายน้ำเกลือปราศจากเชื้อ (0.9% NaCl) หรือสารละลายล็อค (Locke's solution) เพื่อทำการล้างตัวอย่าง [4] ทางเดินอาหารที่ผ่านการผ่าสามารถนำไปทำต่อได้ 2 แนวทาง ดังนี้ 1) การตรวจวิเคราะห์ภายใต้กล้องจุลทรรศน์แบบใช้แสง เพื่อดูระยะต่าง ๆ ของเชื้อลิชมาเนียในรูปแบบโปรมาสติโกต (promastigotes) และระบุตำแหน่งการอาศัยของเชื้อภายในทางเดินอาหาร โดยควรให้ความสำคัญเป็นพิเศษกับบริเวณลิ้นเปิด-ปิดส่วนต้นของทางเดินอาหาร (stomodeal valve) ซึ่งเป็นตำแหน่งสำคัญต่อความสามารถในการแพร่เชื้อของพาหะ 2) การเปิดทางเดินอาหารเพื่อปลดปล่อยเชื้อ เป็นวิธีที่ช่วยให้โปรมาสติโกตเคลื่อนที่ออกจากทางเดินอาหาร ทำให้สามารถเก็บเชื้อไปใช้ในการเพาะเลี้ยงเชื้อปริมาณมากได้สะดวกยิ่งขึ้น [4] เนื่องจากการพบริ้นฝอยทรายที่ติดเชื้อลิชมาเนียตามธรรมชาติมีโอกาสเกิดขึ้นค่อนข้างต่ำ การฝึกฝนจนเกิดความชำนาญจึงมีความสำคัญอย่างยิ่งต่อการเพิ่มอัตราความสำเร็จในการแยกเชื้อ

เมื่อยืนยันการพบเชื้อในทางเดินอาหารแล้ว ให้ใช้เข็มฉีดยาปราศจากเชื้ออันใหม่ และหยดสารละลายน้ำเกลือปราศจากเชื้อปริมาณเล็กน้อยบริเวณขอบกระจกปิดสไลด์ เพื่ออาศัยแรงแคพิลลารี (capillary action) ช่วยในการปลดปล่อยเชื้อ จากนั้นให้ฉีกทางเดินอาหารอย่างระมัดระวังและรวดเร็ว เพื่อให้เชื้อปรสิตออกมาอยู่ในสารละลาย แล้วใช้ไมโครปิเปตขนาด 100 ไมโครลิตร หรือกระบอกฉีดยาชนิดทูเบอร์คูลิน (tuberculin syringe) ดูดเก็บเชื้อ เพื่อนำไปถ่ายเชื้อ (inoculation) ลงในอาหารเลี้ยงเชื้อที่มีการระบุรายละเอียดอย่างถูกต้อง

การเพาะเลี้ยงเชื้อลิชมาเนียโปรมาสติโกตในห้องปฏิบัติการ (*In vitro* culture) เชื้อที่แยกได้ในระยะแรกควรเลี้ยงในอาหารเลี้ยงเชื้อชนิดแข็ง SNB-9 หรือ NNN [16] และเติมอาหารเลี้ยงเชื้อชนิดเหลวทับหน้าด้วย alpha-MEM [16, 65] หรือ M199 โดยอาหารเลี้ยงเชื้อเหลวต้องเสริมองค์ประกอบดังต่อไปนี้ เซรั่มลูกวัว (Fetal Calf Serum; FCS) ที่ผ่านการยับยั้งฤทธิ์ด้วยความร้อน 10% วิตามิน BME 1% ปัสสาวะมนุษย์ปราศจากเชื้อ 2% (กรองผ่าน syringe filter ขนาด 0.2 ไมโครเมตร) ยาปฏิชีวนะ Amikacin 250 ไมโครกรัม/มิลลิลิตร หรือ Gentamycin 50 ไมโครกรัม/มิลลิลิตร (หรือชุดยาปฏิชีวนะ เช่น L-glutamine 200 มิลลิโมลาร์ Penicillin 10,000 ยูนิต และ Streptomycin 10 มิลลิกรัม/มิลลิลิตร) [47] หากไม่พบการปนเปื้อนภายหลังการเพาะเลี้ยงเป็นเวลา 3 วัน ให้เตรียมสารแขวนลอยในอาหารสำหรับแช่แข็งและเก็บที่อุณหภูมิ -80 องศาเซลเซียส ซึ่งสามารถเก็บได้นาน 1-2 ปี หรือเก็บในไนโตรเจนเหลวที่อุณหภูมิ -196 องศาเซลเซียส เพื่อเก็บเชื้อในระยะยาวและใช้ประโยชน์ในการศึกษาวิจัยในอนาคต [7]

4.5. ต่อมน้ำลายของริ้นฝอยทราย (salivary glands)

การผ่าต่อมน้ำลายของริ้นฝอยทรายเป็นเทคนิคพื้นฐานที่มีความสำคัญอย่างยิ่งในการศึกษาปฏิสัมพันธ์ระหว่างพาหะและเชื้อก่อโรค โดยเฉพาะการตรวจหาเชื้อไวรัสที่มีแมลงเป็นพาหะ เช่น ฟลิโบไวรัส (Phlebovirus) อาทิ ไวรัสทอสคานา (Toscana virus) [44, 75] เนื่องจากริ้นฝอยทรายมีขนาดเล็กมาก การผ่าแมลงจึงต้องทำภายใต้กล้องจุลทรรศน์สเตอริโอ โดยใช้ฟอร์เซปปลายละเอียด (fine forceps) หรือเข็มขนาดเล็กพิเศษสำหรับผ่า (microdissection needles) เพื่อแยกต่อมน้ำลายที่บอบบางโดยไม่ให้เกิดการแตกหรือปนเปื้อน (https://zenodo.org/records/18302850) [51, 61] การรักษาความสมบูรณ์ของต่อมน้ำลายมีความสำคัญอย่างยิ่งต่อความน่าเชื่อถือของการวิเคราะห์ในระดับโมเลกุล หลังจากแยกต่อมน้ำลายแล้ว สามารถนำตัวอย่างไปทำให้เป็นเนื้อเดียวกัน (homogenization) และตรวจวิเคราะห์ด้วยวิธี RT-PCR วิธี qPCR หรือการทดสอบทางภูมิคุ้มกัน เพื่อยืนยันการมีอยู่ของอาร์เอ็นเอไวรัส (RNA virus) หรือแอนติเจน [12] การตรวจพบไวรัสในต่อมน้ำลาย แทนการพบเพียงในทางเดินอาหาร (gut) หรือช่องลำตัว (hemocoel) ถือเป็นหลักฐานยืนยันว่าเชื้อได้ผ่านระยะฟักตัวภายนอก (extrinsic incubation period) อย่างสมบูรณ์ และมีศักยภาพในการแพร่เชื้อระหว่างการดูดเลือด [71]

กระบวนการผ่าต่อมน้ำลายมีความซับซ้อนทางเทคนิค เนื่องจากขนาดของอวัยวะมีขนาดเล็กมากและเสื่อมสภาพได้ง่าย จึงจำเป็นต้องอาศัยความเชี่ยวชาญสูง [1, 51] อีกทั้งปริมาณไวรัสในตัวอย่างอาจอยู่ในระดับต่ำ ทำให้ต้องใช้เทคนิคการตรวจที่มีความไวสูง

เช่น เทคนิค nested-PCR หรือการหาลำดับพันธุกรรมสมรรถนะสูง (high-throughput sequencing) [54] นอกจากนี้ ความสามารถในการเป็นพาหะและอัตราการติดเชื้อยังขึ้นอยู่กับสายพันธุ์ของริ้นฝอยทราย รวมถึงปัจจัยด้านนิเวศวิทยาและฤดูกาล [33, 61]
การตรวจพบไวรัสในต่อมน้ำลายช่วยเพิ่มความเข้าใจเกี่ยวกับความเสี่ยงในการแพร่เชื้อและสนับสนุนการพัฒนามาตรการเฝ้าระวังและควบคุมโรคอย่างมีประสิทธิภาพ [15] ตัวอย่างเช่น การตรวจพบไวรัสทอสคานาในริ้นฝอยทรายในพื้นที่เสี่ยง สามารถนำไปใช้กำหนดแนวทางการวินิจฉัยและมาตรการด้านสาธารณสุข [18] ยิ่งไปกว่านั้น การศึกษาปฏิสัมพันธ์ระหว่างไวรัสและองค์ประกอบของน้ำลายอาจนำไปสู่การระบุเป้าหมายใหม่สำหรับการพัฒนาวัคซีนหรือแนวทางการรักษา เพื่อยับยั้งการถ่ายทอดเชื้อ [15, 18] ต่อมน้ำลายของริ้นฝอยทรายยังสามารถใช้เป็นแหล่งแอนติเจนสำหรับการตรวจวัดระดับแอนติบอดีของโฮสต์ต่อการถูกริ้นฝอยทรายกัด ด้วยเทคนิคทางภูมิคุ้มกันวิทยา เช่น ELISA (enzyme-linked immunosorbent assay) ซึ่งเป็นประโยชน์ในการประเมินระดับการสัมผัสของโฮสต์ต่อพาหะ ประสิทธิภาพของมาตรการควบคุมแมลง [25] และความเสี่ยงต่อการแพร่เชื้อลิชมาเนีย [40]

4.6. การระบุชนิดของเลือดในริ้นฝอยทราย

ริ้นฝอยทรายเพศเมียที่มีเลือดเต็มท้อง ซึ่งคัดแยกได้จากการดักจับภาคสนาม นำมาผ่าโดยใช้อุปกรณ์แบบใช้ครั้งเดียวทิ้ง เพื่อป้องกันการปนเปื้อน ก่อนการผ่าแมลงให้ตรวจสอบลำตัวส่วนท้องภายใต้กล้องจุลทรรศน์สเตอริโอ เพื่อประเมินระยะการย่อยเลือด โดยแนะนำให้เลือกเฉพาะตัวเมียที่มีส่วนท้องสีแดง สีน้ำตาลแดง หรือสีแดงเข้ม และต้องไม่ปรากฏลักษณะของการสร้างไข่ (oogenesis) ให้ทำการตัดส่วนปลายของท้องรวมถึงถุงเก็บน้ำเชื้อ (spermathecae) ออก เพื่อนำไปใช้ในการระบุสายพันธุ์ของริ้นฝอยทรายด้วยลักษณะทางสัณฐานวิทยาภายหลังการทำให้ใส ส่วนท้องหลักที่ไม่มีถุงเก็บน้ำเชื้อ (spermathecae) ควรเก็บลงในหลอดทดลอง และเก็บที่อุณหภูมิ -20 องศาเซลเซียส จนกว่าจะวิเคราะห์ต่อไป การระบุชนิดของเลือดโฮสต์สามารถทำได้โดยใช้เครื่องหมายทางพันธุกรรมที่ใช้กันอย่างแพร่หลาย เช่น ยีน *PNOC* [5, 30, 50] ยีน *CytB* [67] หรือ ยีน *COI* [13] ซึ่งมีมาตรฐานและระเบียบวิธีที่ได้รับการอธิบายไว้อย่างชัดเจนในเอกสารทางวิชาการแล้ว ดังนั้นจึงไม่ได้นำเสนอรายละเอียดเพิ่มเติมในคู่มือนี้ (รูปที่ 6) อีกทางเลือกหนึ่งในการระบุชนิดเลือดของโฮสต์ คือการใช้เทคนิค MALDI-ToF peptide mapping [31] ซึ่งมีหลักฐานจากการทดลองสนับสนุนว่าสามารถระบุชนิดเลือดของโฮสต์ได้ในช่วงเวลาที่ยาวนานกว่าหลังการกินเลือด เมื่อเทียบกับวิธีทางโมเลกุลที่อาศัยสารพันธุกรรมดีเอ็นเอเป็นหลัก เทคนิคนี้จึงเหมาะสมอย่างยิ่งสำหรับตัวอย่างริ้นฝอยทรายเพศเมียที่มีการย่อยเลือดของโฮสต์ไปแล้วในระดับสูงมาก ตัวอย่างสามารถเก็บไว้ที่อุณหภูมิ -20 องศาเซลเซียส หรือ 4 องศาเซลเซียสได้ อย่างไรก็ตาม สามารถได้ผลลัพธ์ที่ดีจากตัวอย่างที่เก็บไว้ที่อุณหภูมิห้องในช่วงเวลาสั้นๆ ได้เช่นกัน ส่วนท้องของริ้นฝอยทรายที่มีเลือดควรถูกผ่าออกจากส่วนที่เหลือของลำตัวไม่นานก่อนนำมาวิเคราะห์ และนำมาบดในน้ำกลั่นปลอดเชื้อ ส่วนลำตัวที่เหลือของริ้นฝอยทรายยังสามารถนำไปใช้ในการวิเคราะห์ทางโมเลกุลหรือการศึกษาสัณฐานวิทยาอื่น ๆ ได้ และภายหลังการแบ่งสารละลายจากตัวอย่างที่ทำให้เป็นเนื้อเดียวกันไปใช้ในการวิเคราะห์ด้วย MALDI-ToF แล้ว ส่วนที่เหลือสามารถนำไปสกัดสารพันธุกรรมดีเอ็นเอ เพื่อยืนยันชนิดเลือดโฮสต์ และ/หรือ ตรวจหาเชื้อลิชมาเนียได้ ทั้งนี้ระยะเวลาโดยรวมในการเตรียมตัวอย่างและการวิเคราะห์ด้วยวิธีดังกล่าวถือว่าสั้นกว่าวิธีทางโมเลกุลที่ใช้สารพันธุกรรมดีเอ็นเอเป็นหลักอย่างมีนัยสำคัญ

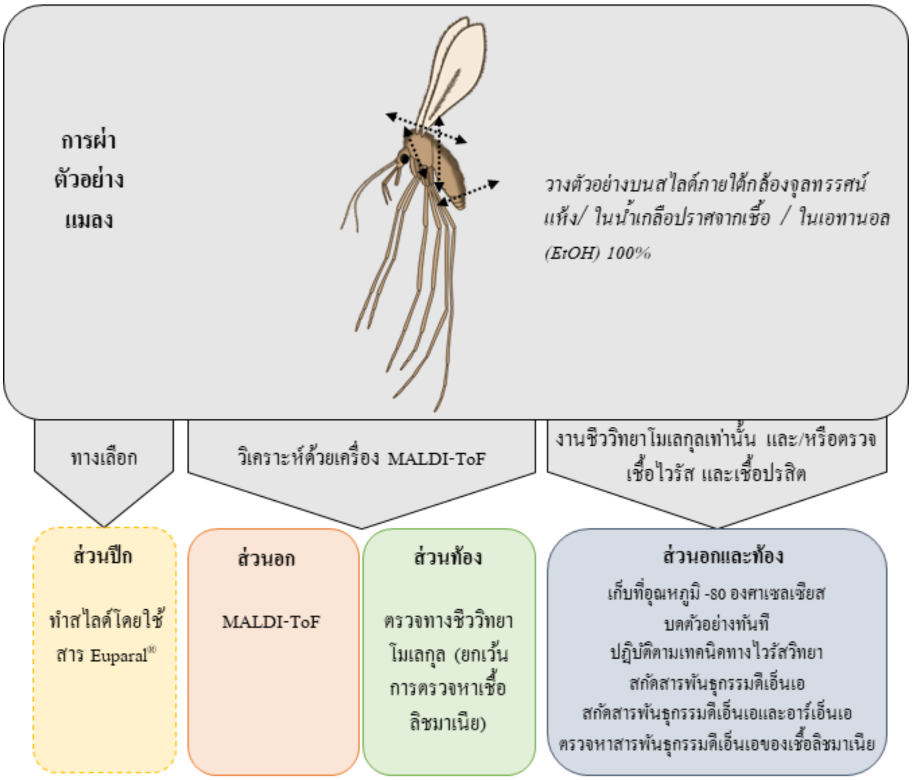

รูปที่ 6. กระบวนการจัดเตรียมตัวอย่างริ้นฝอยทรายสำหรับการประยุกต์ใช้ในด้านชีววิทยาโมเลกุล โปรตีโอมิกส์ และ/หรือ ไวรัสวิทยา

5. การเตรียมตัวอย่างสำหรับการศึกษาทางสัณฐานวิทยา (รูปที่ 3, 6, 7 และ 8; เอกสารเพิ่มเติม 1, 2, 3 และ 4)

หัวข้อนี้ได้อธิบายหลักการและขั้นตอนพื้นฐานในการเตรียมตัวอย่างริ้นฝอยทรายสำหรับการทำสไลด์ถาวร เพื่อการศึกษาทางสัณฐานวิทยาเป็นหลัก รวมถึงแนวทางการประยุกต์ใช้กับการศึกษาด้านอื่น ๆ นอกเหนือจากสัณฐานวิทยา ความเข้าใจในระเบียบวิธีดังกล่าวมีความสำคัญอย่างยิ่ง เนื่องจากช่วยให้สามารถปรับเปลี่ยนขั้นตอนให้เหมาะสมกับชนิดของตัวอย่างและวัตถุประสงค์ของการศึกษาเฉพาะด้านได้ กระบวนการเตรียมตัวอย่างประกอบด้วยการล้างและการเปลี่ยนสารละลายหลายขั้นตอน โดยใช้พาสเจอร์ปิเปตแบบใช้จุกยาง แนะนำให้ใช้ภาชนะแก้วก้นกลมสำหรับการปฏิบัติงาน เนื่องจากมีความทนทานต่อสารเคมีและช่วยอำนวยความสะดวกในการจัดการตัวอย่าง ควรปิดฝาภาชนะทุกครั้งเพื่อป้องกันการระเหยของสารเคมี และไม่ควรเติมสารละลายจนเต็มภาชนะ เพื่อหลีกเลี่ยงการล้นเมื่อเปิดหรือปิดฝา รวมถึงลดความเสี่ยงจากการปนเปื้อนของฝุ่นละออง สารเคมีที่ใช้สำหรับการทำให้ใส และขั้นตอนการเตรียมสไลด์ต่าง ๆ แสดงรายละเอียดไว้ในตารางที่ 2

ตารางที่ 2 ส่วนประกอบของสารเคมีที่ใช้ในการเตรียมตัวอย่าง

**Potassium hydroxide 10%**	**Fuchsin acid 1% in distilled water**
Potassium hydroxide 10 กรัม	Acid fuchsin (as a powder) 1 กรัม
Distilled water *qs* 100 มิลลิลิตร	Distilled water 99 มิลลิลิตร
**Gum chloral mounting media (Hoyer medium)**	**Marc-André solution colored with acid fuchsin**
Distilled water 50 มิลลิลิตร	Marc-André solution 10 มิลลิลิตร
Chloral hydrate 200 กรัม	Fuchsin 1% 50 ไมโครลิตร
Gum Arabic 50 กรัม	
Glycerol 20 มิลลิลิตร	
**Marc-André solution**	**Enecê medium**
Chloral hydrate 40 กรัม	Pure white colophony 22 กรัม
Glacial acetic acid 30 มิลลิลิตร	Alcohol-soluble copal gum 12 กรัม
Distilled water 30 มิลลิลิตร	Absolute ethanol 20 มิลลิลิตร
	Camphor 10 กรัม
	Turpentine essence 10 มิลลิลิตร
	Eucalyptol 26 มิลลิลิตร

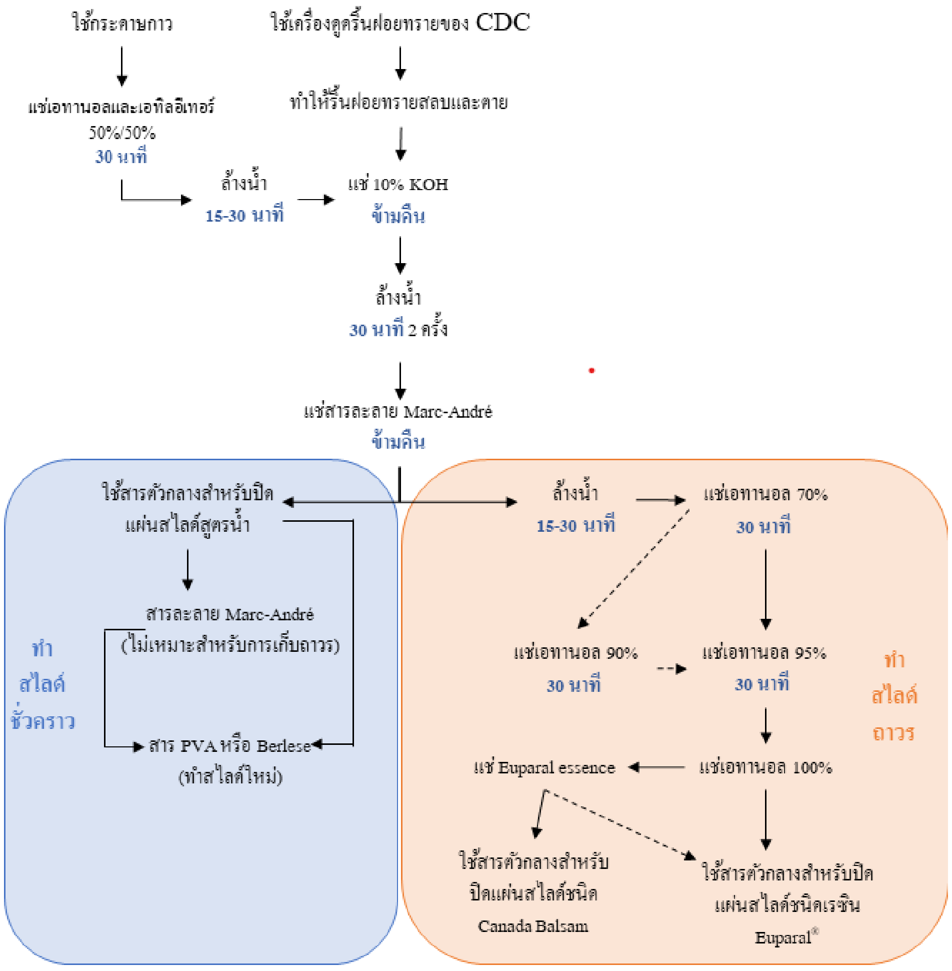

รูปที่ 7. วิธีมาตรฐานแบบดั้งเดิมในการเตรียมตัวอย่างริ้นฝอยทราย (classical method)

This is an Open Access article distributed under the terms of the Creative Commons Attribution License (https://creativecommons.org/licenses/by/4.0), which permits unrestricted use, distribution, and reproduction in any medium, provided the original work is properly cited.

5.1. การทำให้ใส (clearing)

ก่อนการเตรียมตัวอย่างริ้นฝอยทรายเป็นแผ่นสไลด์ ตัวอย่างจะต้องผ่านกระบวนการทำให้ใส โดยการแช่ให้ตัวอย่างเปื่อย (maceration) ด้วยสารเคมีและระเบียบวิธีที่เหมาะสม เช่น สารละลายกรดอะซิติก 10% หรือสารละลาย Marc-André ซึ่งมีส่วนประกอบหลักคือคลอรัลไฮเดรต ทั้งนี้ คลอรัลไฮเดรตจัดเป็นสารเคมีควบคุมในหลายประเทศ ผู้ปฏิบัติงานจึงต้องปฏิบัติตามข้อกำหนดด้านความปลอดภัยและกฎหมายที่เกี่ยวข้องอย่างเคร่งครัด กระบวนการทำให้ใสมีวัตถุประสงค์เพื่อกำจัดเนื้อเยื่ออ่อน ไขมัน สิ่งคัดหลั่ง และไข ออกจากตัวแมลง ทำให้ตัวอย่างมีความโปร่งแสง ซึ่งช่วยเพิ่มความชัดเจนในการตรวจสอบลักษณะทางสัณฐานวิทยาที่สำคัญ เช่น โครงสร้างเปลือกนอกและตำแหน่งการยึดเกาะของขนแข็ง (setae insertion) ลักษณะพื้นผิวและการกระจายของสี และโครงสร้างภายในที่มองเห็นผ่านผนังลำตัว เช่น ถุงเก็บน้ำเชื้อ (spermathecae)

การทำให้ใสแบบสองขั้นตอน ประกอบด้วยการใช้ด่างแก่ เช่น โพแทสเซียมไฮดรอกไซด์ (KOH) ตามด้วยกรดอ่อน เช่น กรดอะซิติกในสารละลาย Marc-André ซึ่งมีวัตถุประสงค์ทางชีวเคมีที่แตกต่างกัน [74] ด่างแก่จะทำลายเนื้อเยื่ออ่อน เช่น โปรตีน ไขมัน และกล้ามเนื้อ ผ่านกระบวนการเกิดสบู่ หรือเรียกว่า ซาพอนิฟิเคชัน (saponification) และการเสียสภาพของโปรตีน (protein denaturation) ส่งผลให้เหลือโครงสร้างโครงร่างภายนอกที่ประกอบด้วยไคตินคงสภาพอยู่ ซึ่งเอื้อต่อความชัดเจนในการดูโครงสร้างทางกายวิภาค จากนั้นกรดอ่อนจะช่วยสะเทินฤทธิ์ด่างที่ตกค้าง ลดความเสี่ยงต่อการเสื่อมสภาพของตัวอย่าง และช่วยฟอกสีไคตินให้โปร่งใสมากขึ้น [74] ในบางกรณีการล้างตัวอย่างด้วยน้ำกลั่น 2 ครั้ง ครั้งละ 15 นาที อาจเพียงพอสำหรับการสะเทินฤทธิ์ด่างได้เช่นกัน ลำดับดังกล่าวเป็นการผสมผสานระหว่างประสิทธิภาพในการกำจัดเนื้อเยื่อกับการรักษาโครงสร้างของตัวอย่าง เพื่อให้ได้ตัวอย่างที่มีความสมบูรณ์สูงสุดสำหรับการตรวจด้วยกล้องจุลทรรศน์

ข้อแนะนำ: ควรล้างตัวอย่างด้วยน้ำกลั่นอย่างน้อย 2 ครั้ง ครั้งละประมาณ 20 นาที ก่อนทำในขั้นตอนถัดไป

5.1.1. การย่อยสลายเนื้อเยื่ออ่อน (soft tissue lysis) (รูปที่ 8)

โซเดียมไฮดรอกไซด์ (NaOH) หรือโพแทสเซียมไฮดรอกไซด์ (KOH) เป็นสารด่างแก่ที่นิยมใช้ในการแช่ให้ตัวอย่างเปื่อย ความเข้มข้นและระยะเวลาการแช่ควรปรับให้เหมาะสมกับขนาดและความบอบบางของตัวอย่าง แนวทางมาตรฐานที่ใช้กันอย่างแพร่หลายคือ การแช่ตัวอย่างริ้นฝอยทรายในสารละลายด่างแก่ความเข้มข้น 10% (KOH หรือ NaOH) เป็นเวลาข้ามคืน ทั้งนี้ สามารถเพิ่มความเข้มข้นของด่างเพื่อลดระยะเวลาการแช่ได้ เช่น การใช้ 20% KOH เป็นเวลา 6 ชั่วโมง หรือใช้การให้ความร้อนร่วมที่อุณหภูมิประมาณ 37 องศาเซลเซียส เพื่อเร่งกระบวนการย่อยสลาย ทั้งนี้ต้องควบคุมอย่างระมัดระวังเพื่อหลีกเลี่ยงการทำลายโครงสร้างไคตินที่สำคัญ

5.1.2. การทำให้ใสและการย้อมสี

ภายหลังการย่อยสลายเนื้อเยื่ออ่อน ตัวอย่างจะเข้าสู่ขั้นตอนการทำให้ใสและเพิ่มความสว่าง โดยทั่วไปใช้สารละลายที่ประกอบด้วยกรดอะซิติกและคลอรัลไฮเดรต เช่น สารละลาย Marc-André หลังจากการแช่ใส ควรล้างตัวอย่างในน้ำอย่างน้อย 2 ครั้ง ครั้งละประมาณ 20 นาที เพื่อขจัดสารเคมีตกค้าง สารละลาย Marc-André เป็นสารแช่ใสที่นิยมใช้ในการเตรียมตัวอย่างริ้นฝอยทราย เนื่องจากมีประสิทธิภาพสูงในการทำให้ตัวอย่างโปร่งใส ขณะเดียวกันก็ลดความเสียหายต่อโครงสร้างที่บอบบาง เช่น ปีกและหนวด ควรเตรียมสารละลายดังกล่าวใหม่ก่อนการใช้งาน หรือเก็บไว้ในภาชนะที่ปิดผนึกอย่างแน่นหนา เพื่อป้องกันการระเหยหรือการเสื่อมสภาพของสาร การใช้สารละลาย Marc-André มีข้อได้เปรียบเป็นพิเศษเมื่อใช้ร่วมกับเทคนิคการเพิ่มความสว่างหรือการย้อมสี เพื่อเพิ่มความชัดเจนของรายละเอียดทางสัณฐานวิทยาเฉพาะส่วน โดยรายละเอียดเกี่ยวกับองค์ประกอบและวิธีการเตรียมสารละลายดังกล่าวได้ระบุไว้ในส่วนเพิ่มเติมที่ 2

สำหรับตัวอย่างที่มีความโปร่งแสงสูง การย้อมสีอาจจำเป็นเพื่อเพิ่มความเปรียบต่างและความชัดเจนของโครงสร้าง สีย้อมแต่ละชนิดจะจับกับองค์ประกอบทางเคมีที่แตกต่างกัน ผู้ปฏิบัติงานจึงควรเลือกสีย้อมที่เหมาะสมและเข้ากันได้กับตัวกลางสำหรับการทำสไลด์ ตัวอย่างหนึ่งของการประยุกต์ใช้คือการเติม acid fuchsin ความเข้มข้น 0.1% ลงในสารละลาย Marc-André เพื่อช่วยย้อมสีโครงสร้างที่ต้องการ ในกรณีที่ตัวอย่างถูกเก็บรักษาในสารละลายสูตรน้ำและมีแผนจะทำสไลด์ด้วยตัวกลางประเภทเรซิน จำเป็นต้องผ่านกระบวนการดึงน้ำออกก่อน (ดูหัวข้อ 5.2 การดึงน้ำออก) เนื่องจากตัวกลางเรซินทั้งชนิดธรรมชาติและสังเคราะห์ส่วนใหญ่ไม่สามารถเข้ากันน้ำได้ New (1974) รายงานว่าสีย้อมบางชนิดอาจเกิดการเสื่อมสภาพได้เมื่อใช้ร่วมกับสารตัวกลางสำหรับการทำสไลด์บางประเภท [53] ตัวอย่างเช่น แอซิดฟุคซิน (acid fuchsin) ซึ่งนิยมใช้ร่วมกับแคนาดาบัลซัม (Canada balsam) และสามารถใช้กับ Euparal® ได้เช่นกัน อย่างไรก็ตามตัวอย่างที่ย้อมด้วย acid fuchsin มีแนวโน้มเกิดการซีดจาง โดยเฉพาะเมื่อมีน้ำมันกานพลู (clove oil) ตกค้างจากการใช้เป็นสารแช่ใสขั้นตอนสุดท้าย ซึ่งอาจทำให้สีซีดจางอย่างเห็นได้ชัดภายในระยะเวลาเพียงไม่กี่วัน

5.2. การดึงน้ำออก (dehydration)

การดึงน้ำออกจากตัวอย่างทำโดยการถ่ายโอนตัวอย่างผ่านลำดับความเข้มข้นของสารละลายเอทานอลแบบเพิ่มระดับเริ่มตั้งแต่ความเข้มข้น 50%, 70%, 80%, 90% หรือ 95% และสิ้นสุดที่เอทานอล 100% โดยแช่ตัวอย่างในแต่ละความเข้มข้นเป็นเวลาไม่น้อยกว่า 20 นาที ทั้งนี้ เนื่องจากเอทานอลมีคุณสมบัติระเหยได้รวดเร็ว จึงควรปิดฝาภาชนะให้สนิทตลอดระยะเวลาดำเนินการ ภายหลังการดึงน้ำออกอย่างสมบูรณ์ สามารถพักตัวอย่างไว้ได้เป็นระยะเวลาสองถึงสามวันใน Euparal® ซึ่งมีความเหมาะสมมากกว่าน้ำมันกานพลู สำหรับขั้นตอนเตรียมสไลด์ถาวร ทั้งนี้ บีชครีโอโซต (beech creosote) ซึ่งเคยนิยมใช้ในอดีต ปัจจุบันถูกสั่งห้ามใช้อย่างเด็ดขาด เนื่องจากมีความเป็นพิษสูง กระบวนการดึงน้ำออกต้องทำอย่างระมัดระวัง เพื่อให้ของเหลวภายในตัวอย่างสามารถเข้ากันได้กับตัวกลางที่ใช้ในการสไลด์ หากการดึงน้ำออกไม่สมบูรณ์ อาจก่อให้เกิดความขุ่นของตัวอย่าง การยุบตัวอันเนื่องมาจากแรงดันออสโมติกหรือการบิดเบี้ยวของโครงสร้าง ซึ่งจะส่งผลให้ตัวอย่างไม่เหมาะสมต่อการศึกษาทางอนุกรมวิธาน

5.3. สารตัวกลางสำหรับปิดแผ่นสไลด์

5.3.1. หลักการเลือกและการประยุกต์ใช้สารตัวกลางสำหรับปิดแผ่นสไลด์ในการเตรียมตัวอย่าง

โดยหลักการสารตัวกลางสำหรับปิดแผ่นสไลด์ควรมีดัชนีหักเหของแสง (refractive index; RI) ใกล้เคียงกับแก้ว ซึ่งมีค่าประมาณ 1.5 สารตัวกลางสำหรับปิดแผ่นสไลด์ต้องมีลักษณะใส ไม่มีสี และสามารถคงความโปร่งใสได้อย่างถาวรภายหลังการแห้ง นอกจากนี้ สารตัวกลางสำหรับปิดแผ่นสไลด์ควรเข้ากันได้กับสีย้อมที่ใช้ สามารถแทรกซึมเข้าสู่เนื้อเยื่อของตัวอย่างได้อย่างทั่วถึง ไม่แห้งเร็วเกินไป ไม่เกิดฝ้าหรือความขุ่นในระหว่างการสไลด์ และไม่เกิดการหดตัวเมื่อแข็งตัวสมบูรณ์ เนื่องจากไม่มีสารตัวกลางสำหรับปิดแผ่นสไลด์ชนิดใดที่เหมาะสมที่สุดสำหรับทุกวัตถุประสงค์ การเลือกใช้จึงต้องพิจารณาจากปัจจัยหลักดังต่อไปนี้:

- **คุณสมบัติทางแสง (optical properties)** ดัชนีหักเหของตัวกลางควรเอื้อต่อการสร้างความต่างระดับสี และการหักเหของแสงที่เพียงพอ เพื่อให้สามารถดูโครงสร้างทางกายวิภาคที่สำคัญต่อการจำแนกสายพันธุ์และการศึกษาทางสัณฐานวิทยาได้อย่างชัดเจน เช่น ถุงเก็บน้ำเชื้อ (spermathecae) แอสคอยด์ (ascoids) นิวสเตดเซนซิลลา (newstead sensilla) ฟันบริเวณกระเปาะอาหาร (cibarial teeth) และฟันบริเวณคอหอย (pharyngeal teeth) ความชัดเจนของโครงสร้างเหล่านี้ขึ้นอยู่โดยตรงกับคุณสมบัติทางแสงของตัวกลางสำหรับปิดแผ่นสไลด์

This is an Open Access article distributed under the terms of the Creative Commons Attribution License (https://creativecommons.org/licenses/by/4.0), which permits unrestricted use, distribution, and reproduction in any medium, provided the original work is properly cited.

- การเก็บรักษา (preservation) ตัวอย่างสำหรับตัวอย่างต้นแบบ (type specimens) หรือตัวอย่างที่จัดเก็บแบบถาวร สารตัวกลางสำหรับปิดแผ่นสไลด์ต้องมีความเสถียรและคงสภาพได้ดีในระยะยาว ในขณะที่งานสำรวจทางระบาดวิทยาหรือการจัดทำบัญชีรายชื่อ ซึ่งมิได้เน้นการเก็บรักษาในระยะยาว อาจเลือกใช้ตัวกลางแบบชั่วคราวหรือกึ่งถาวรได้

5.3.2. ข้อกำหนดทางวิชาการสำหรับสารตัวกลางสำหรับปิดแผ่นสไลด์

แม้ว่านักวิจัยหรือผู้เชี่ยวชาญบางรายจะพัฒนาเทคนิคการทำสไลด์ที่มีความเฉพาะเจาะจงตามความต้องการของงานวิจัย แต่เทคนิคดังกล่าวมักละเลยประเด็นด้านความเข้ากันได้มาตรฐานสากล ความสะดวกในการจัดการ และคุณภาพของการเก็บในระยะยาว การขาดมาตรฐานเหล่านี้อาจก่อให้เกิดปัญหาในการรวบรวมจากแหล่งต่าง ๆ และการดูแลรักษาในระยะยาว

ในงานอนุกรมวิธาน นักอนุกรมวิธานมักทำสไลด์ตัวอย่างของแมลงทั้งตัว และนิยมใช้ตัวกลางสำหรับปิดแผ่นสไลด์ที่สามารถแช่ให้อวัยวะภายในของแมลงเปื่อยอย่างอ่อนโยนเพื่อเพิ่มความชัดเจนของโครงสร้างเปลือกนอก ตัวกลางสำหรับปิดแผ่นสไลด์ควรมีดัชนีหักเหที่แตกต่างจากตัวอย่างและแผ่นสไลด์แก้วในระดับที่เหมาะสม เพื่อให้ได้คุณภาพทางแสงสูงสุด ตัวกลางสไลด์ที่มีขายอยู่ส่วนใหญ่มักถูกออกแบบให้มีค่าดัชนีหักเหของแสงใกล้เคียงกับแก้ว เพื่อช่วยลดการหักเหและการกระเจิงของแสงผ่านระบบสไลด์-ตัวกลาง-กระจกปิดสไลด์ อย่างไรก็ตาม ในการใช้กล้องจุลทรรศน์แบบ brightfield ความต่างระดับสีตามธรรมชาติของตัวอย่างที่ไม่ได้ย้อมสีสามารถเพิ่มขึ้นได้จากการเลือกตัวกลางที่มีค่าดัชนีหักเหของแสงแตกต่างจากตัวอย่างเล็กน้อย ซึ่งช่วยเพิ่มความชัดเจนของโครงสร้างเมื่อเทียบกับพื้นหลัง

5.3.3. ประเภทของตัวกลางสำหรับปิดแผ่นสไลด์ (ตารางที่ 3 และ 4)

การตรวจด้วยกล้องจุลทรรศน์จำเป็นต้องอาศัยค่าดัชนีหักเหของแสง (refractive index; RI) ของสารตัวกลางสำหรับปิดแผ่นสไลด์ เพื่อกำหนดลักษณะการหักเหของแสงขณะผ่านแผ่นสไลด์ สารตัวกลางสำหรับปิดแผ่นสไลด์และตัวอย่าง เมื่อค่าดัชนีหักเหของแสงของสารทำสไลด์มีความใกล้เคียงกับค่าดัชนีหักเหของแสงของกระจกแผ่นปิดสไลด์ (≈1.515) แสงจะสามารถผ่านได้อย่างสม่ำเสมอ ส่งผลให้การกระเจิงของแสงและความบิดเบือนทางแสงลดลง และช่วยเพิ่มความละเอียดและความชัดเจนของโครงสร้างขนาดเล็ก ในทางตรงกันข้าม ความไม่สอดคล้องของค่าดัชนีหักเหของแสง อาจทำให้เกิดภาพเบลอ การเกิดวงแสง (halos) หรือบดบังโครงสร้างที่ไม่ได้ผ่านการย้อมสี ดังนั้น การเลือกใช้สารทำสไลด์ที่เหมาะสมจึงมีความสำคัญอย่างยิ่งต่อการเพิ่มประสิทธิภาพของคอนทราสต์ ความชัดเจน และคุณภาพของภาพโดยรวมของตัวอย่างแต่ละประเภท เนื่องจากสารทำสไลด์แต่ละชนิดมีค่าดัชนีหักเหของแสง ที่แตกต่างกัน

ค่าดัชนีหักเหของแสงของสารทำสไลด์มีผลอย่างมากต่อความสามารถในการเห็นโครงสร้างขนาดเล็กเมื่อเตรียมริ้นฝอยทรายสำหรับการทำสไลด์ เนื่องจากลักษณะโครงสร้างที่บอบบางและมีการแข็งตัวของผนังเซลล์ต่ำ (lightly sclerotized) ของริ้นฝอยทราย เช่น โครงสร้างฟันของกระเปาะอาหาร (cibarial armature) ถุงเก็บน้ำเชื้อ (spermathecae) ปล้องหนวด และเส้นปีก อาจดูได้ยากเมื่อใช้สารทำสไลด์ที่มีค่าดัชนีหักเหของแสงสูง

สำหรับริ้นฝอยทราย สารทำสไลด์ที่นิยมใช้ ได้แก่ สารสารตัวกลางสำหรับปิดแผ่นสไลด์สูตรน้ำ (water-based mountants) เช่น กลุ่มกัม–คลอรัล (gum–chloral media) และสารตัวกลางสำหรับปิดแผ่นสไลด์สูตรตัวทำละลาย (solvent-based mountants) เช่น แคนาดาบัลซัม (Canada balsam) และเรซิน Enecê-Nelson Cerqueira (NC) โดย Rawlins [60] ได้จำแนกตัวกลางสำหรับปิดแผ่นสไลด์ออกเป็น 2 ประเภท ได้แก่ (1) ตัวกลางสำหรับปิดแผ่นสไลด์สำหรับปิดแผ่นสไลด์แบบถาวร (permanent mountants) ซึ่งจะแข็งตัวเมื่อเวลาผ่านไปและเหมาะสำหรับการเก็บระยะยาว และ (2) ตัวกลางสำหรับปิดแผ่นสไลด์สำหรับปิดแผ่นสไลด์แบบกึ่งถาวร (semi-permanent mountants) ซึ่งไม่แข็งตัวสมบูรณ์ และมักใช้สำหรับวัตถุประสงค์ชั่วคราว

ตารางที่ 3: ส่วนประกอบของสารตัวกลาง

สารตัวกลางสำหรับปิดแผ่นสไลด์	ตัวทำละลาย (solvent)	พอลิเมอร์/สารก่อตัว (polymer)	หมายเหตุ
Hoyer = chloral gum	Glycerol, water	Compounds of gum arabic	Macerating agent: chloral hydrate
CMCP-9 (= carboxy methyl cellulose phenol)	Water (CMCP-9: 51−60%)	Fully hydrolyzed polyvinyl alcohol (CMCP-9: 0−5%)	CMC(P)-9: low viscosity: high viscosity
DMHF (dimethyl hydantoin formaldehyde)	Water	N,N′-dimethylol dimethyl hydantoin (di-methylol DMH)<br>Ether-/methylene-bridged oligomers<br>Crosslinked DMH–formaldehyde polymer network	
Canada balsam	Xylene; partly volatile components of balsam (Δ^3-carene, levopimaric acid, limonene, myrcene, palustric acid, β-phellandrene, α-pinene, β-pinene)	Balsam (abienol, abietic acid, isopimaric acid, sandaracopimaric acid)	Neutralization: potassium carbonate;<br>resin from Abies balsamea (Linné, 1758)

Euparal®	Eucalyptol, paraldehyde; partly volatile components of gum sandarac (limonene, α-pinene, β-pinene)	Compounds of gum sandarac (communic acid, manool, polycommunic acid, sandaracopimaric acid, 12-acetoxy-sandaracopimaric acid, sugiol, torulosic acid, torulosol, totarol)	Clearing agent: methyl salicylate; color in Euparal® green: copper salt (copper abietinate); sandarac resin from Tetraclinis articulata (Vahl, 1791)
Enecê	Ethyl alcohol; with camphor, eucalyptol and turpentine essence	Compounds of copal gum and colophony (rosin)	

สารตัวกลางสำหรับปิดแผ่นสไลด์อาจอยู่ในรูปของเหลว กัม (gum-based) หรือเรซิน (resinous) โดยละลายได้ในน้ำ แอลกอฮอล์ หรือตัวทำละลายอินทรีย์ เช่น โทลูอีนหรือไซลีน (ตารางที่ 3) ภายหลังการสไลด์ ควรปิดผนึกขอบสไลด์ (sealing) ด้วยสารที่ไม่ละลายน้ำ เพื่อป้องกันผลกระทบจากความชื้นและสภาวะบรรยากาศ โดยสามารถจำแนกตัวกลางได้ดังนี้

a. สารตัวกลางสำหรับปิดแผ่นสไลด์สูตรน้ำ (aqueous media) ละลายในน้ำ ใช้สะดวก เหมาะกับสไลด์ชั่วคราวหรือกึ่งถาวร แต่จำเป็นต้องปิดผนึกขอบสไลด์ โดยเฉพาะในเขตร้อนชื้น เช่น gum-chloral และ polyvinyl alcohol (PVA)

b. สารตัวกลางสำหรับปิดแผ่นสไลด์ทนน้ำได้จำกัด (limited water-tolerant media) สารกลุ่มนี้ได้รับผลกระทบจากน้ำในระดับที่น้อยกว่า อย่างไรก็ตาม ยังคงจำเป็นต้องมีการป้องกันความชื้นที่มากเกินไป สารดังกล่าวมีความเสถียรในระยะยาวสูงกว่าสารตัวกลางสำหรับปิดแผ่นสไลด์ที่ละลายน้ำได้ และมักถูกนำมาใช้ในการทำสไลด์แบบกึ่งถาวร

c. สารตัวกลางสำหรับปิดแผ่นสไลด์ที่ละลายในไฮโดรคาร์บอน (hydrocarbon-soluble media) สารกลุ่มนี้ละลายในตัวทำละลายอินทรีย์ เช่น ไซลีน (xylene) โทลูอีน (toluene) หรือเอสเซเนเซ (essenecê; ตัวทำละลาย enecê) โดยได้รับการออกแบบมาเพื่อการทำสไลด์ถาวร มีความเสถียรในระยะยาวสูง และทนต่อความชื้นและการเสื่อมสภาพ จึงเหมาะสมอย่างยิ่งสำหรับการเก็บแบบถาวรเพื่อเป็นหลักฐาน (archival purposes) เช่น แคนาดาบัลซัมชนิดเป็นกลาง (neutral Canada balsam) และ

โดยสรุป สารทำสไลด์ชนิดละลายน้ำเหมาะสำหรับการทำสไลด์ชั่วคราว หรือกรณีที่จำเป็นต้องเอาตัวอย่างออกได้ง่าย สารทำสไลด์ที่ทนต่อน้ำในระดับจำกัดเหมาะสำหรับการทำสไลด์แบบกึ่งถาวรซึ่งต้องการความคงทนในระดับปานกลาง ขณะที่สารตัวกลางสำหรับปิดแผ่นสไลด์ชนิดละลายในไฮโดรคาร์บอนเหมาะสำหรับการทำสไลด์ถาวรที่มุ่งเน้นการเก็บในระยะยาวหรือเพื่อวัตถุประสงค์เก็บแบบถาวรเพื่อเป็นหลักฐาน

ตารางที่ 4 ข้อดีและข้อจำกัดของสารตัวกลางสำหรับปิดแผ่นสไลด์ รวมทั้งข้อมูลจากการประสบการณ์ที่ยังไม่ได้ตีพิมพ์ของนักวิจัยหลายท่าน [52]

ชื่อ	ข้อดี	ข้อจำกัด
***Canada balsam**	มีความคงทนสูงมาก อายุการใช้งานมากกว่า 150 ปี<br>สามารถทำสไลด์โดยใช้ clove oil หรือ phenol เป็นตัวกลางสำหรับปิดแผ่นสไลด์ได้	มีส่วนประกอบที่เป็นอันตราย จำเป็นต้องปฏิบัติงานภายใต้ตู้ดูดควันต้องผ่านกระบวนการดึงน้ำออก (dehydration) อย่างสมบูรณ์ ซึ่งใช้เวลานานการดึงน้ำด้วยเอทานอลและการส่งผ่านไซลีนหรือ clove oil อาจทำให้ตัวอย่างบางชนิดมีความเปราะ สารทางเลือกอื่น ๆ เช่น isopropanol, n-butanol, Cellosolve™, 1 ,4 - dioxane, Histoclear, terpineol) may reduce breakage. อาจช่วยลดการแตกหักของตัวอย่างได้ตัวอย่างอาจมีสีคล้ำหากใช้ phenol แทนไซลีน หรือหากมี KOH หลงเหลืออยู่<br>ค่าดัชนีหักเหแสงสูงอาจบดบังโครงสร้างที่ไม่ได้ย้อมสี<br>การแห้งสนิทอาจใช้เวลาหลายปีหากไม่ใช้ความร้อนช่วย<br>ตัวกลางอาจเกิดการเหลืองและคล้ำลงตามกาลเวลา และสีย้อมบางชนิดอาจซีดจางเมื่อสารมีความเป็นกรด
**DMHF** (dimethyl hydantoin formaldehyde)	มีความโปร่งใสสูง<br>มีดัชนีการหักเหของแสงที่เหมาะสม<br>มองเห็นโครงสร้างต่าง ๆ ได้อย่างชัดเจน<br>การเตรียมสไลด์มีความคงสภาพค่อนข้างดี<br>สามารถใช้ร่วมกับเทคนิคการย้อมสีได้หลายชนิด	อาจเปลี่ยนเป็นสีเหลืองเมื่อเวลาผ่านไป<br>อาจทำให้สีของสารย้อมบางชนิดเปลี่ยนแปลง<br>ไม่เหมาะสำหรับสารย้อมที่ไวต่อฟอร์มาลดีไฮด์อาจเกิดฟองอากาศ และใช้เวลาแห้งช้า<br>เป็นสารตัวกลางที่ไวต่อความชื้น

***Euparal** (ชนิดใส)	ให้การปกป้องตัวอย่างได้ดี<br>มีการยึดติดระหว่างแผ่นสไลด์และแผ่นปิดสไลด์ได้ดี<br>มีความคงทนสูง อายุการใช้งานมากกว่า 50 ปี<br>สามารถทำสไลด์ได้โดยตรงจากเอทานอล 80% (ตามคำแนะนำของผู้ผลิต)ไม่บดบังโครงสร้างที่ไม่ได้ย้อมสี และไม่เหลืองหรือเปราะตามกาลเวลามีค่าดัชนีหักเหแสงที่เหมาะสมกับแมลงอันดับ Diptera มากกว่า Canada balsamเหมาะกับตัวอย่างที่มีความหนา เนื่องจากการหดตัวต่ำและไม่เกิดฟองอากาศสามารถละลายได้ในเอทานอล 95% ทำให้สามารถนำมาทำสไลด์ใหม่ได้แม้ผ่านไปหลายปี	เป็นสารตัวกลางที่จะแก้ไขหรือนำออกได้ยาก<br>ฟอร์มาลดีไฮด์เป็นสารพิษ ระคายเคือง และก่อมะเร็ง<br>มีส่วนประกอบที่เป็นอันตราย จำเป็นต้องจัดการภายใต้ตู้ดูดควัน การดึงน้ำด้วยเอทานอลและการส่งผ่าน Euparal essence อาจทำให้ตัวอย่างบางชนิดมีความเปราะ (การใช้ไอโซโพรพานอลอาจช่วยลดผลกระทบดังกล่าว)
**Hoyer fluid**	สามารถทำสไลด์ได้จากตัวอย่างมีชีวิต หรือโดยตรงจากน้ำ เอทานอล หรือฟอร์มาลดีไฮด์<br>การแช่เปื่อยยุ่ย (maceration) ให้คุณภาพของคิวทิเคิลที่ดีมาก<br>มีค่าดัชนีหักเหแสงเหมาะสม และสามารถเพิ่มความแตกต่างของภาพด้วยการย้อมไอโอดีน<br>กรดอะซิติกในสูตรช่วยให้รยางค์ของอาร์โทรพอดกางออก<br>ตัวอย่างบางส่วนอาจยังคงสภาพคงตัวได้นานถึง 40-60 ปี<br>ละลายน้ำได้ ทำให้สามารถนำมาทำสไลด์ใหม่ได้ง่าย	ตัวอย่างที่บอบบางอาจเกิดการฟีบยุบ หากไม่ได้เติมสารละลายอย่างค่อยเป็นค่อยไป อาจเกิดช่องว่างและการตกผลึกภายในระยะเวลาน้อยกว่า 10 ปี<br>ระดับการแช่เปื่อยยุ่ยขึ้นกับความเข้มข้นของ chloral hydrate และระยะเวลาในการแช่<br>ส่วนประกอบอาจแยกตัวหรือเกิดตะกอนเม็ดละเอียดภายในไม่กี่เดือนถึงไม่กี่ปี<br>มีรายงานการเปลี่ยนสีเป็นสีดำของตัวกลาง
**CMCP-9** (carboxymethyl cellulose phenol)	สามารถทำสไลด์ได้โดยตรงจากน้ำ เอทานอล กลีเซอรอล หรือฟอร์มาลดีไฮด์<br>สามารถแช่ยุ่ยอวัยวะภายในเมื่อจำเป็น เพื่ออำนวยความสะดวกในการตรวจและเตรียมตัวอย่าง	อาจเกิดการตกผลึกและการคล้ำลงตามกาลเวลา และอาจแช่ยุ่ยตัวอย่างมากเกินไป หากไม่ปิดผนึกขอบสไลด์อย่างเหมาะสม ตัวอย่างที่มีความหนาอาจเกิดการหดตัวและมีช่องว่างบริเวณขอบกระจกปิดสไลด์ ไม่เหมาะสำหรับตัวอย่างที่ย้อมสีหรือมีแคลเซียม และแห้งช้ากว่า CMC
**Eukitt™**	มีความคงทนสูง อายุการใช้งานมากกว่า 30 ปีเข้ากันได้กับตัวทำละลายหลายชนิด เช่น อะซิโตน ไซลีน และไอโซโพรพานอล แห้งเร็ว มีค่า pH เป็นกรดอ่อน และไม่คล้ำลงอย่างชัดเจนเมื่อเวลาผ่านไปเหมาะสมกับสีย้อมหลายชนิด เช่น fuchsin, hematoxylin, methyl green, methyl violet, methylene blue และสามารถนำมาทำสไลด์ใหม่ได้โดยการแช่ในไซลีนเป็นเวลานาน	มีส่วนประกอบที่เป็นอันตราย จำเป็นต้องจัดการภายใต้ตู้ดูดควันต้องผ่านขั้นตอนการดึงน้ำออกซึ่งใช้เวลานานไม่เหมาะสำหรับตัวอย่างที่มีความหนา เนื่องจากการหดตัวและการเกิดฟองก๊าซกระจกปิดสไลด์อาจหลุดออกได้ หากการทำความสะอาดพื้นผิวไม่เพียงพอ
**Enecê**	มีความคงทนสูงมาก อายุการใช้งานอย่างน้อย 50 ปี<br>ไม่คล้ำลงตามกาลเวลา<br>มีความอ่อนตัว (malleable) ทำให้สามารถผ่าแยกแมลงภายในตัวกลางได้โดยตรง และมีเวลามากพอสำหรับการจัดวางตำแหน่งโครงสร้างมีต้นทุนต่ำ	ต้องผ่านกระบวนการดึงน้ำออกซึ่งใช้เวลานาน การดึงน้ำด้วยเอทานอลและการส่งผ่าน clove oil อาจทำให้ตัวอย่างมีความเปราะตัวอย่างจะค่อย ๆ ถูกทำให้ใสเพิ่มขึ้นตามเวลา ซึ่งอาจทำให้การดูโครงสร้างขนาดเล็ก เช่น sensilla และ ascoids ทำได้ยากขึ้น

5.3.4. รายละเอียดของสารตัวกลางสำหรับปิดแผ่นสไลด์ที่นิยมใช้ (ตารางที่ 3 และ ตารางที่ 4)

<u>สารตัวกลางสำหรับปิดแผ่นสไลด์สำหรับการดูชั่วคราว</u>

Chloral gum = Hoyer fluid/medium/solution (ดัชนีหักเหแสง RI = 1.48)

สารละลาย Marc André ถือเป็นตัวกลางที่เหมาะสมที่สุดสำหรับการดูถุงเก็บน้ำเชื้อ (spermathecae) ในระยะสั้น ไม่กี่ชั่วโมง และอาจยืดออกได้อีกเล็กน้อยหากเก็บสไลด์ไว้ในห้องควบคุมความชื้น รวมถึงการถ่ายภาพ (รูปที่ 4) หรือการวาดภาพประกอบ การเก็บถุงเก็บน้ำเชื้อ (spermathecae) ที่ได้จากการดูดังกล่าวจำเป็นต้องนำมาทำสไลด์ใหม่ในตัวกลางชนิดใช้น้ำ ซึ่งเอื้อให้สามารถเก็บได้ในระยะปานกลาง แม้ว่าการดึงน้ำออกเพื่อทำสไลด์ใหม่ในเรซินจะไม่ใช่สิ่งที่เป็นไปไม่ได้ แต่ไม่แนะนำให้ทำ เนื่องจากมีความเสี่ยงสูงต่อการสูญหายของตัวอย่าง สารตัวกลางสำหรับปิดแผ่นสไลด์ชนิด chloral gum และ Hoyer ถือเป็นสารชนิดเดียวกัน นิยมใช้อย่างแพร่หลายในการดูอวัยวะภายในของแมลง เนื่องจากคุณสมบัติที่เข้ากับน้ำได้ดี ใช้งานง่าย รวดเร็ว และมีค่าดัชนีหักเหที่ช่วยให้ตรวจวิเคราะห์โครงสร้างที่บอบบางได้ง่าย เช่น ถุงเก็บน้ำเชื้อ (spermathecae) อย่างไรก็ตาม chloral gum มีข้อเสียสำคัญหากเตรียมได้ไม่สมบูรณ์หรือเก็บในสภาวะความชื้นที่ไม่เหมาะสม ปัญหาที่พบ ได้แก่ การเกิดผลึก การเปลี่ยนสี และการสูญเสียความหนืด ซึ่งการใช้สารปิดขอบสไลด์ ไม่สามารถ

แก้ปัญหาเหล่านี้ได้ เนื่องจากตัวกลางอาจเกิดการเปลี่ยนสีอย่างรุนแรง บางครั้งสามารถเปลี่ยนเป็นสีดำจากปฏิกิริยากับสารปิดขอบ โดยเฉพาะอย่างยิ่งหากใช้ Euparal® ถึงแม้ว่า Hoyer ได้รับการยอมรับว่าดีที่สุดสำหรับการศึกษาเกี่ยวกับริ้นฝอยทราย และมีการใช้งานในลักษณะนี้ตามธรรมเนียมปฏิบัติมาอย่างยาวนาน ตัวกลางนี้ประกอบด้วยสูตรผสมหลายสูตรที่มีความใกล้เคียงกัน ซึ่งรวมถึงส่วนผสมของ สาร gum arabic กลีเซอรอล และคลอรัลไฮเดรต ทั้งนี้สูตรผสมต่าง ๆ มักมีการตีความและอ้างอิงที่คลาดเคลื่อนอยู่บ่อยครั้ง [74] แม้ว่า Hoyer จะเป็นตัวกลางที่ดีที่สุดสำหรับการดูถุงเก็บน้ำเชื้อ (spermathecae) ของริ้นฝอยทราย แต่ไม่เหมาะสำหรับการเก็บตัวอย่างในระยะยาว จึงเหมาะสำหรับการใช้ดูในระยะสั้น เช่น การถ่ายภาพ หรือการวาดภาพประกอบ ตัวกลางสูตรน้ำเหมาะสำหรับการทำสไลด์ชั่วคราวเท่านั้นและไม่สามารถรับประกันการเก็บในระยะยาวได้ ในทางตรงกันข้าม การใช้เรซินจะให้ความทนทานที่ดีเยี่ยมยาวนานนับศตวรรษ แต่อาจบดบังรายละเอียดที่ละเอียดอ่อนของถุงเก็บน้ำเชื้อ (spermathecae) เนื่องจากการหักเหของแสงมักสูญเสียไป Hoyer จะเสื่อมสภาพตามกาลเวลาเนื่องจากการสูญเสียน้ำ (รูปที่ 8) ส่งผลให้เกิดผลึกคลอรัลไฮเดรตสีขาวขุ่น อย่างไรก็ตาม ยังคงสามารถกู้คืนตัวอย่างจากสไลด์ที่เกิดผลึกได้ เนื่องจากผนังลำตัวของแมลงยังคงสภาพสมบูรณ์ทางเคมี แต่อาจเกิดความเสียหายทางกายภาพจากการขยายตัวของผลึก ในบางกรณีสามารถซ่อมแซมสไลด์ได้โดยการคืนความชื้น (rehydration) ในสภาพแวดล้อมที่อุ่นและชื้น พร้อมเติมสารไทมอล (thymol) เพื่อป้องกันการเจริญเติบโตของเชื้อรา หรืออาจนำตัวอย่างแช่น้ำเพื่อล้างตัวกลางเดิมออก แล้วจึงดึงน้ำออกด้วยกรดกลาเชียลอะซิติกก่อนนำไปทำสไลด์ใหม่ด้วย Canada balsam

DMHF (Dimethyl Hydantoin Formaldehyde) (ดัชนีหักเหแสง RI = 1.48)

เป็นตัวกลางสูตรน้ำ [72] ที่ให้ประสิทธิภาพดูกล้องดีเยี่ยมใกล้เคียงกับ Berlese และใช้งานง่าย แต่ข้อดีที่เหนือกว่าคือตัวกลางชนิดนี้จะไม่เปลี่ยนเป็นสีดำหรือเกิดการตกผลึก ใช้งานได้ดีกับริ้นฝอยทรายและแมลงในวงศ์ Psychodidae อื่นๆ

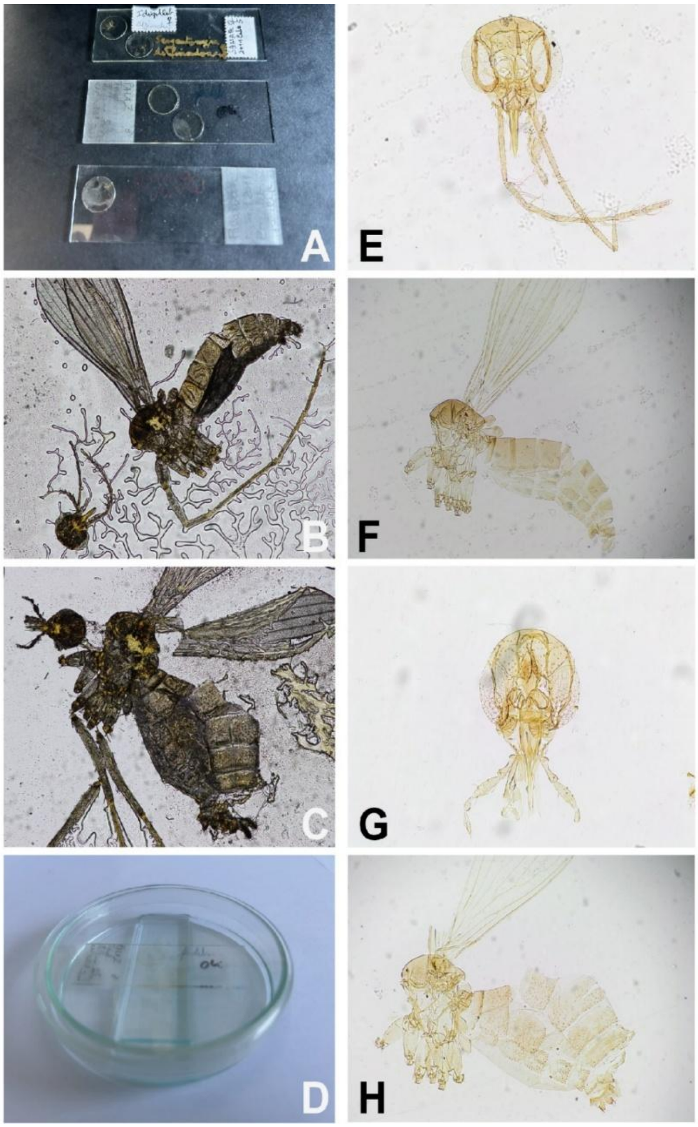

รูปที่ 8 การทำสไลด์ใหม่ (slides remounting) A: สไลด์ที่เสียหายและแห้งซึ่งใช้ตัวกลาง Hoyer; B: มุมมองผ่านกล้องจุลทรรศน์ของริ้นฝอยทรายที่แห้ง; C: มุมมองผ่านกล้องจุลทรรศน์ของตัวอย่างที่เสียหายอีกตัวหนึ่ง; D: ถาดควบคุมความชื้น (wet chamber) ที่บรรจุสไลด์ที่แห้ง; E: ส่วนหัว และ F: ส่วนลำตัวของตัวอย่าง B ภายหลังการทำสไลด์ใหม่ใน Euparal®; G: ส่วนหัว และ H: ส่วนลำตัวของตัวอย่าง C ภายหลังการทำสไลด์ใหม่ใน Euparal®

CMCP (Camphor-mono-chlorophenol) (ดัชนีหักเหแสง RI = 1.41)

CMCP เป็นสารตัวกลางสำหรับปิดแผ่นสไลด์สูตรกลีเซอรอลที่ละลายน้ำได้ เหมาะสำหรับการเตรียมแผ่นสไลด์ถาวรชนิดโปร่งแสงของตัวอย่างที่มีความบอบบาง รวมถึงริ้นฝอยทราย จุดเด่นของตัวกลางชนิดนี้คือสามารถนำตัวอย่างลงสไลด์ได้โดยตรงจากน้ำหรือเอทานอล โดยไม่จำเป็นต้องผ่านกระบวนการดึงน้ำออกอย่างซับซ้อน CMCP ช่วยให้ตัวอย่างคลายตัวและมีความโปร่งใสได้อย่างรวดเร็ว พร้อมทั้งทำให้ผนังลำตัวอ่อนนุ่มลง ซึ่งเอื้อต่อการจัดตำแหน่งตัวอย่างได้อย่างเหมาะสม โดยเฉพาะในขั้นตอนการกางปีก การจัดแนวหนวด หรือการผ่าแยกอวัยวะสืบพันธุ์เพื่อการศึกษาทางอนุกรมวิธาน แม้จะมีรายงานว่าสามารถใช้เก็บตัวอย่างได้ในระยะยาว แต่ช่วงเวลาการเก็บที่แน่นอนยังไม่สามารถระบุได้อย่างชัดเจน ข้อจำกัดสำคัญของ CMCP คือมีส่วนประกอบของฟีนอล (phenol) ซึ่งเป็นสารพิษและก่อให้เกิดการระคายเคืองต่อผิวหนังและระบบทางเดินหายใจ ดังนั้น การใช้งานควรทำภายใต้ตู้ดูดควัน และสวมอุปกรณ์ป้องกันส่วนบุคคลอย่างเหมาะสม

<u>สารตัวกลางสำหรับปิดแผ่นสไลด์ถาวร</u>

Canada balsam (ดัชนีหักเหแสง RI = 1.52-1.54)

Canada balsam ได้รับการอธิบายครั้งแรกว่าเป็นตัวกลางที่เหมาะสมสำหรับการส่องกล้องจุลทรรศน์แบบแสงส่องผ่าน รายงานโดย Andrew Pritchard ในช่วงทศวรรษที่ 1830 และยังคงเป็นหนึ่งในตัวกลางที่ใช้กันอย่างแพร่หลายที่สุดจนถึงปัจจุบัน เนื่องจากมีคุณสมบัติด้านการเก็บตัวอย่างที่ยอดเยี่ยม โดยมีสไลด์ที่ยังคงสภาพสมบูรณ์ได้นานกว่า 150 ปี เมื่อเปรียบเทียบกับตัวกลางสูตรน้ำ เช่น Hoyer และ Canada balsam ไม่มีแนวโน้มเกิดการตกผลึกหรือดูดซับความชื้นจากบรรยากาศ อย่างไรก็ตาม ตัวกลางชนิดนี้มีคุณสมบัติการเรืองแสงเอง (autofluorescence) ค่อนข้างสูง ซึ่งอาจเป็นข้อจำกัดในการใช้งานร่วมกับเทคนิคกล้องจุลทรรศน์บางประเภท โดยเฉพาะการถ่ายภาพเรืองแสง [60] แม้ว่าการใช้ตัวทำละลายที่มีความเป็นพิษต่ำกว่าไซลีน (xylene) จะช่วยลดความเสี่ยงด้านความปลอดภัยในห้องปฏิบัติการได้ แต่ก็อาจก่อให้เกิดข้อเสีย เช่น ระยะเวลาการแห้งของสไลด์ที่ยาวนานขึ้น และแนวโน้มที่ตัวกลางจะมีสีคล้ำเร็วขึ้นเมื่อเก็บในระยะยาว

Euparal® (ดัชนีหักเหแสง RI = 1.48)

Euparal® เป็นตัวกลางสำหรับการทำสไลด์ถาวรที่นิยมใช้เป็นทางเลือกแทน Canada balsam โดยมีความเสถียรในระยะยาวสูงและมีดัชนีหักเหแสงใกล้เคียงกัน เหมาะอย่างยิ่งสำหรับการเตรียมสไลด์แมลงอันดับ Diptera รวมถึงริ้นฝอยทราย ข้อพิจารณาสำคัญในการใช้ Euparal® ได้แก่ 1) ข้อกำหนดด้านการดึงน้ำออก ตัวอย่างต้องผ่านกระบวนการดึงน้ำออกก่อนการสไลด์ขั้นสุดท้าย โดยทั่วไปจะเปลี่ยนผ่านจากเอทานอล 95% ไปสู่เอทานอลบริสุทธิ์ (absolute ethanol) 2) ระยะเวลาการเตรียมที่ยาวขึ้น เช่นเดียวกับ Canada balsam การทำสไลด์ด้วย Euparal® จำเป็นต้องผ่านขั้นตอนการดึงน้ำออก ซึ่งทำให้ระยะเวลาโดยรวมของกระบวนการเตรียมตัวอย่างเพิ่มขึ้น ในกรณีที่ไม่สามารถถ่ายโอนตัวอย่างจาก absolute ethanol ลงสู่ Euparal® ได้โดยตรง สามารถพักตัวอย่างในสารละลายขั้นกลางที่ประกอบด้วย Euparal® และ Euparal essence ในอัตราส่วน 1:1 ก่อนการสไลด์จริง เพื่อช่วยลดความเสียหายต่อโครงสร้างที่บอบบาง

Enecê (ดัชนีหักเหแสง RI = 1.467)

Enecê เป็นตัวกลางสำหรับปิดแผ่นสไลด์ประเภทเรซินที่พัฒนาขึ้นสำหรับการเตรียมสไลด์แมลงขนาดเล็ก และได้รับความนิยมอย่างแพร่หลายในประเทศบราซิล ส่วนประกอบหลักประกอบด้วย colophony และ gum copal ละลายในเอทานอล ร่วมกับการบูร (camphor) น้ำมันสน (turpentine) และยูคาลิปตอล (eucalyptol) Cerqueira [11] ได้เสนอให้ใช้ Enecê เป็นทางเลือกแทน Canada balsam สำหรับการเตรียมสไลด์ถาวรของตัวอ่อน คราบลอก (exuviae) และแมลงตัวเต็มวัย ซึ่งต่อมาได้รับการยอมรับอย่างกว้างขวางในการเตรียมสไลด์ของริ้นฝอยทราย Enecê มีต้นทุนต่ำ ให้ความเสถียรในระยะยาว และมีอัตราการแห้งที่เหมาะสม ทำให้สามารถผ่าแยกและจัดวางโครงสร้างทางสัณฐานวิทยาได้อย่างแม่นยำก่อนที่ตัวกลางจะแข็งตัว

5.4. การเตรียมสไลด์และการทำให้แห้ง (slide preparation and drying)

การทำให้สไลด์แห้งอย่างเหมาะสมเป็นขั้นตอนสำคัญยิ่ง เพื่อให้มั่นใจถึงเสถียรภาพของสารตัวกลางสำหรับปิดแผ่นสไลด์และเก็บตัวอย่างในระยะยาว สไลด์ทุกแผ่นต้องถูกทำให้แห้งสนิทก่อนนำไปจัดเก็บในคลังตัวอย่าง โดยทั่วไปสไลด์ที่เตรียมด้วย ตัวกลางสำหรับปิดแผ่นสไลด์ชนิดถาวร (permanent mountants) ควรวางในแนวราบและปล่อยให้แห้งเป็นระยะเวลาอย่างน้อย 2-3 สัปดาห์ ในขณะที่สไลด์ที่เตรียมด้วยตัวกลางสำหรับปิดแผ่นสไลด์ชนิดกึ่งถาวร (semi-permanent mountants) อาจใช้ระยะเวลาเพียง 1-2 สัปดาห์ ทั้งนี้ ระยะเวลาการแห้งขึ้นอยู่กับชนิดของตัวกลาง ความหนาของชั้นสไลด์ และสภาพแวดล้อม เพื่อเพิ่มประสิทธิภาพและความสม่ำเสมอของการแห้ง แนะนำให้ใช้ ตู้อบ (incubator) โดยตั้งอุณหภูมิให้เหมาะสมกับตัวกลางที่ใช้ และหลีกเลี่ยงความร้อนที่สูงเกินไปซึ่งอาจทำให้ตัวอย่างบิดเบี้ยวหรือเกิดความเสียหาย อุณหภูมิที่เหมาะสมโดยทั่วไปอยู่ในช่วง 30 และ 37 องศาเซลเซียส ขั้นตอนนี้มีความสำคัญอย่างยิ่งในการป้องกันการหดตัวของตัวกลาง การเคลื่อนตำแหน่งของตัวอย่าง และความไม่เสถียรของสไลด์ในระยะยาว สไลด์ทุกแผ่นควรมีฉลากระบุ ชนิดของตัวกลางสำหรับปิดแผ่นสไลด์ที่ใช้อย่างชัดเจน และหากเป็นไปได้ควรระบุ สูตรเฉพาะ ชื่อผู้เตรียม และวันที่จัดทำ เพื่อความถูกต้องและสามารถตรวจสอบย้อนหลังได้ สไลด์บางส่วนอาจถูกเตรียมขึ้นในระยะแรกเพื่อการตรวจสอบเบื้องต้นเท่านั้น อย่างไรก็ตาม หากตัวอย่างดังกล่าวถูกกำหนดให้เป็นส่วนหนึ่งของชุด "ตัวอย่างต้นแบบหรือตัวอย่างอ้างอิง" ควรเปลี่ยนไปใช้ตัวกลางสำหรับปิดแผ่นสไลด์ชนิดถาวรเพื่อให้มั่นใจในการเก็บตัวอย่างสำหรับการศึกษาทางอนุกรมวิธานในอนาคต

5.5. เทคนิคทางเลือกในการเตรียมตัวอย่างแบบการติดบนกระดาษ หรือ card mounting

การติดบนกระดาษเป็นเทคนิคที่ใช้กันทั่วไปในแมลงหลายกลุ่ม โดยอาจเป็นการปักเข็มผ่านแผ่นกระดาษ หรือการติดตัวอย่างลงบนแผ่นรองด้วยกาว อย่างไรก็ตามเนื่องจากริ้นฝอยทรายมีขนาดเล็กมาก และการระบุสายพันธุ์จำเป็นต้องอาศัยการตรวจสอบโครงสร้างภายในหลังผ่านกระบวนการทำให้ใส (ดูข้อ 5) วิธีการติดบนกระดาษจึงไม่เหมาะสมอย่างยิ่ง สำหรับการเตรียมตัวอย่างริ้นฝอยทราย

5.6. การทำสไลด์ใหม่สำหรับตัวอย่างที่เสียหาย (remounting damaged specimens)

สำหรับตัวอย่างที่หายาก มีคุณค่าทางวิชาการ หรือเป็นตัวอย่างอ้างอิง แนะนำให้ใช้กระบวนการทำสไลด์ใหม่แบบสองขั้นตอน โดยอ้างอิงแนวทางสาธิตในวิดีโอ (https://zenodo.org/records/18315029. 1) ดังนี้ 1) **การคืนความชื้น (rehydration)**

ในขั้นตอนแรกให้คืนความชื้นแก่สไลด์ โดยยังไม่แยกชิ้นส่วน เพื่อประเมินสภาพของตัวอย่างเบื้องต้น วิธีการคือ วางแท่นรองสไลด์ไว้ในจาน (petri dish) จากนั้นวางสไลด์ที่ต้องการคืนความชื้นไว้ด้านบน เติมตัวทำละลายลงในจานเพียงเล็กน้อยเพื่อสร้างสภาวะห้องชื้น (humid chamber) โดยต้องระวังไม่ให้ตัวสไลด์สัมผัสกับของเหลวโดยตรง (รูปที่ 8D) ระยะเวลาในการคืนความชื้นอาจใช้ตั้งแต่ 1 วันจนถึงหลายวัน ขึ้นอยู่กับสภาพของสไลด์และชนิดของสารตัวกลางสำหรับปิดแผ่นสไลด์เดิม จำเป็นต้องตรวจสอบสไลด์ทุกวันอย่างสม่ำเสมอ เมื่อคืนความชื้นได้เพียงพอแล้ว ให้นำสไลด์ออกจากห้องชื้นและวางในตู้อบเป็นเวลาหลายชั่วโมง ก่อนนำไปตรวจด้วยกล้องจุลทรรศน์

This is an Open Access article distributed under the terms of the Creative Commons Attribution License (https://creativecommons.org/licenses/by/4.0), which permits unrestricted use, distribution, and reproduction in any medium, provided the original work is properly cited.

การถ่ายภาพ หรือการวาดภาพประกอบ 2) การทำสไลด์ใหม่ (remounting) หากจำเป็นต้องทำสไลด์ใหม่ ให้คืนความชื้นสไลด์อีกครั้งเป็นเวลาหลายชั่วโมงหรือข้ามคืน ขั้นตอนการแยกกระจกปิดสไลด์ควรทำภายใต้กล้องจุลทรรศน์ โดยใช้เข็มปลายละเอียดแซะออกอย่างระมัดระวัง เพื่อหลีกเลี่ยงการสูญเสียชิ้นส่วนของตัวอย่าง จากนั้นนำชิ้นส่วนที่แยกออกมาล้างในน้ำสะอาดในหลุมขนาดเล็ก เช่น หลุมที่ใช้ในการสกัดสารพันธุกรรมดีเอ็นเอ/อาร์เอ็นเอ ก่อนดึงน้ำออกและทำสไลด์ใหม่ด้วยตัวกลางประเภทเรซิน การเลือกตัวทำละลายสำหรับการแยกสไลด์เดิมต้องขึ้นอยู่กับชนิดของสารตัวกลางสำหรับปิดแผ่นสไลด์เดิม ดังนี้ สารตัวกลางสำหรับปิดแผ่นสไลด์สูตรน้ำจะใช้น้ำเป็นตัวทำละลาย และตัวกลางประเภทเรซิน เช่น Canada balsam หรือ Euparal® ให้ใช้ไซลีน โดยต้องปฏิบัติงานภายใต้ตู้ดูดควัน และสวมอุปกรณ์ป้องกันอันตรายส่วนบุคคลที่เหมาะสม

ข้อควรระวัง: การทำสไลด์ใหม่สำหรับตัวอย่างต้นแบบหรือตัวอย่างอ้างอิงจากพิพิธภัณฑ์ ต้องได้รับความเห็นชอบจากภัณฑารักษ์และ/หรือสถาบันเจ้าของตัวอย่างก่อนดำเนินการทุกครั้ง

6. การจำแนกสายพันธุ์ของริ้นฝอยทราย (specimen identification)

6.1. การระบุสายพันธุ์โดยอาศัยลักษณะทางสัณฐานวิทยา

การระบุสายพันธุ์ของริ้นฝอยทรายทำโดยอาศัยการวิเคราะห์ลักษณะทางสัณฐานวิทยาเป็นหลัก ซึ่งครอบคลุมลักษณะของส่วนอก ปีก อวัยวะสืบพันธุ์ ขน รวมถึงความสัมพันธ์เชิงสัณฐานมิติระหว่างโครงสร้างต่าง ๆ ของตัวอย่าง การวิเคราะห์ดังกล่าวต้องอาศัยการเปรียบเทียบกับคีย์อนุกรมวิธาน (taxonomic keys) ตัวอย่างอ้างอิงและเอกสารคำบรรยายลักษณะดั้งเดิมของสายพันธุ์ เพื่อจัดจำแนกตัวอย่างที่เก็บรวบรวมได้เข้าสู่หน่วยอนุกรมวิธาน ที่ทราบสายพันธุ์แล้วอย่างถูกต้อง ลักษณะสำคัญที่ใช้ในการจำแนกสายพันธุ์ ได้แก่ รูปแบบเส้นปีก (wing venation) ลักษณะทางสัณฐานของส่วนหัวในทั้งสองเพศ โครงสร้างอวัยวะสืบพันธุ์ของเพศผู้ (male genitalia) และรูปร่างลักษณะของถุงเก็บน้ำเชื้อในเพศเมีย (female spermathecae) ซึ่งถือเป็นลักษณะวินิจฉัยที่มีความจำเพาะสูงและมีความสำคัญอย่างยิ่งต่อการจำแนกสายพันธุ์ การระบุสายพันธุ์อย่างแม่นยำจำเป็นต้องตรวจสอบภายใต้กล้องจุลทรรศน์ โดยทั่วไปใช้กล้องจุลทรรศน์แบบใช้แสง สำหรับการดูโครงสร้างที่มีความละเอียดสูง เช่น อวัยวะสืบพันธุ์และถุงเก็บน้ำเชื้อ (spermathecae) ขณะที่กล้องจุลทรรศน์สเตอริโอเหมาะสำหรับการตรวจลักษณะทางสัณฐานวิทยาในภาพรวมของตัวอย่าง ปัจจุบันความก้าวหน้าทางเทคโนโลยีด้านการสร้างภาพได้สนับสนุนการใช้ภาพถ่ายดิจิทัลความละเอียดสูงและภาพวาดดิจิทัลในการระบุสายพันธุ์ โดยสามารถนำมาใช้เปรียบเทียบกับตัวอย่างอ้างอิงหรือประยุกต์ร่วมกับระบบการระบุสายพันธุ์ด้วยคอมพิวเตอร์ ซึ่งช่วยเพิ่มความแม่นยำ ความรวดเร็ว และการเข้าถึงข้อมูลอนุกรมวิธานเชิงสัณฐานวิทยา

6.2. การวัดสัณฐานแบบเรขาคณิตบนปีก (wing geometry)

การวัดสัณฐานแบบเรขาคณิตบนปีกเป็นลักษณะสำคัญที่ใช้ในการระบุและจำแนกสายพันธุ์ของริ้นฝอยทราย ปีกของริ้นฝอยทรายโดยทั่วไปมีลักษณะยาวและแคบ พร้อมด้วยเส้นปีกที่พัฒนาอย่างสมบูรณ์ (รูปที่ 9 และ รูปที่ 10) การเรียงตัวและความสัมพันธ์ของเส้นปีกก่อให้เกิดรูปแบบเฉพาะที่แตกต่างกันไปตามสกุล (genus) และสายพันธุ์ (species) รูปแบบเรขาคณิตของปีกจึงถือเป็นเครื่องมือที่มีประโยชน์ต่อการจำแนกสายพันธุ์แมลงที่มีคุณค่าสูงในเชิงอนุกรมวิธาน และสามารถนำมาใช้สนับสนุนการระบุสายพันธุ์โดยอาศัยลักษณะทางสัณฐานวิทยาอื่น ๆ ได้อย่างมีประสิทธิภาพ

6.3. การวิเคราะห์สัณฐานมิติเชิงเรขาคณิตของปีก (wing geometric morphometrics)

นักวิจัยใช้เทคนิคหลากหลายประการ เช่น การวิเคราะห์สัณฐานมิติเชิงเรขาคณิต (geometric morphometrics) เพื่อวิเคราะห์สัณฐานมิติเชิงเรขาคณิตเป็นเทคนิคเชิงปริมาณที่ใช้ในการศึกษาและเปรียบเทียบรูปร่างและขนาดของปีกในริ้นฝอยทรายระหว่างสายพันธุ์ต่าง ๆ หรือระหว่างประชากรในพื้นที่ที่แตกต่างกัน วิธีการดังกล่าวช่วยให้เข้าใจความแปรผันทางสัณฐานที่อาจเกี่ยวข้องกับพฤติกรรมการบิน แหล่งที่อยู่อาศัย หรือศักยภาพในการเป็นพาหะของเชื้อโรค ในกระบวนการวิเคราะห์ ปีกจะถูกแยกออกจากลำตัวอย่างระมัดระวัง อาจผ่านการย้อมสีหากจำเป็น จากนั้นจัดวางบนแผ่นสไลด์ให้อยู่ในตำแหน่งราบและเหมาะสมต่อการถ่ายภาพ ภาพปีกจะถูกบันทึกด้วยกล้องจุลทรรศน์สเตอริโอและแปลงเป็นข้อมูลดิจิทัลเพื่อนำไปวิเคราะห์ทางสัณฐานมิติต่อไป ขั้นตอนและหลักการวิเคราะห์ดังกล่าวมีการอธิบายไว้อย่างละเอียดในเอกสารอ้างอิง [6, 27, 42, 56, 57, 59] ทั้งนี้ เพื่อความสม่ำเสมอของข้อมูล แนะนำให้เลือกใช้ปีกด้านเดียวกัน (ด้านซ้ายหรือด้านขวา) ตลอดการศึกษาในอวัยวะที่เป็นคู่ เพื่อลดอิทธิพลจากความแปรผันที่เกิดจากการเจริญเติบโตแบบต่างส่วน [62]

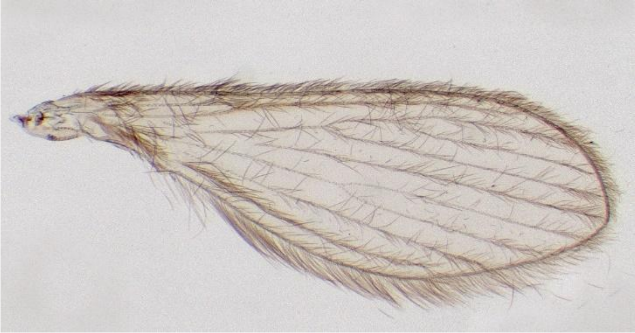

รูปที่ 9. ลักษณะปีกสดของริ้นฝอยทรายสายพันธุ์ *Trichophoromyia ininii*

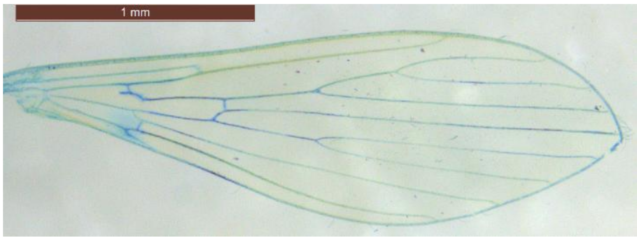

รูปที่ 10. ลักษณะปีกที่ผ่านการย้อมสีของริ้นฝอยทรายสายพันธุ์ *Phlebotomus ariasi*

<u>*การเตรียมส่วนปีกของแมลงสำหรับการวิเคราะห์สัณฐานมิติเชิงเรขาคณิต*</u>

เพื่อให้สามารถดูและวิเคราะห์เส้นปีกได้อย่างชัดเจน จำเป็นต้องกำจัดเกล็ด (scales) และขนแข็ง (bristles) ออกจากปีก รวมถึงย้อมสีด้วยวิธีที่เหมาะสมก่อนการถ่ายภาพและการวิเคราะห์สัณฐานมิติเชิงเรขาคณิต เริ่มต้นด้วยการเตรียมหลุมทดสอบขนาดเล็ก โดยเติมสารเคมีที่เกี่ยวข้อง ได้แก่ เมทิลีนบลู เอทานอล น้ำกลั่น และสารทดแทนไซลีน ลงในหลุมแยกตามลำดับขั้นตอน นำปีกที่เก็บไว้ในเอทานอลความเข้มข้น 70% ณ อุณหภูมิห้อง ออกมาโดยการคว่ำหลอดลงบนหลุมทดสอบ จากนั้นใช้เข็มปลายโค้งขนาดเล็กช้อนปีกขึ้นมาอย่างระมัดระวัง โดยจัดแนวตามความยาวของปีกเพื่อลดการ

บิดงอหรือเสียรูป ปีกจะถูกนำผ่านสารเอทานอลและน้ำสลับกันในระยะเวลาสั้น ๆ ก่อนนำกลับเข้าสู่เอทานอลอีกครั้ง เพื่อช่วยกำจัดขนแข็งที่ติดอยู่บนพื้นผิวปีก หลังจากนั้นนำปีกแช่ในสารละลายเมทิลีนบลูเป็นเวลา 6 นาที โดยต้องตรวจสอบให้แน่ใจว่าปีกลอยตัวอยู่ตลอดระยะเวลาการย้อมสี เพื่อให้การย้อมสีเป็นไปอย่างสม่ำเสมอ เมื่อครบกำหนดให้ช้อนปีกขึ้นอย่างระมัดระวังและนำไปแช่ในสารทดแทนไซลีนเป็นเวลา 2 นาที หรือประมาณหนึ่งในสามของระยะเวลาที่ใช้ในการย้อมเมทิลีนบลู การเคาะเข็มเบา ๆ กับผนังหลุมทดสอบสามารถช่วยให้ปีกจมลงในสารละลายได้ โดยสารทดแทนไซลีนจะทำหน้าที่ช่วยคงสภาพสีของปีกในขั้นตอนสุดท้าย ให้ยกปีกขึ้นและวางลงบนหยดสาร Euparal® บนสไลด์แก้ว ภายใต้กล้องขยาย ให้คลี่ปีกออกอย่างนุ่มนวลจนได้ตำแหน่งที่เหมาะสม จากนั้นวางกระจกปิดสไลด์อย่างระมัดระวัง ควรถ่ายภาพทันที ก่อนที่สาร Euparal® จะแข็งตัว เนื่องจากอาจจำเป็นต้องปรับตำแหน่งของปีกเล็กน้อยเพื่อให้ได้การจัดวางที่เหมาะสมที่สุดสำหรับการวิเคราะห์

6.4. เทคนิคทางชีวโมเลกุล (molecular biology techniques)

นอกเหนือจากการวิเคราะห์ทางสัณฐานวิทยาแล้ว เทคนิคทางชีวโมเลกุลมีบทบาทสำคัญเพิ่มมากขึ้นในงานวิจัยทางกีฏวิทยา โดยครอบคลุมการศึกษาในด้านอนุกรมวิธาน พันธุศาสตร์ประชากร ความสัมพันธ์เชิงวิวัฒนาการ การตรวจหาเชื้อก่อโรคด้วยสารพันธุกรรมดีเอ็นเอหรือสารพันธุกรรมอาร์เอ็นเอ ตลอดจนการวิเคราะห์แหล่งที่มาของเลือดที่แมลงดูดกิน ซึ่งข้อมูลเหล่านี้มีความสำคัญอย่างยิ่งต่อการศึกษาทางระบาดวิทยา [70] การวิเคราะห์ลำดับนิวคลีโอไทด์ สามารถนำมาใช้ยืนยันสายพันธุ์หรือแยกสายพันธุ์ที่มีลักษณะทางสัณฐานใกล้เคียงกันมาก ช่วยเพิ่มความถูกต้องและความน่าเชื่อถือของการระบุสายพันธุ์ นอกจากนี้เทคนิคทางโมเลกุลขั้นสูง เช่น polymerase chain reaction (PCR), DNA sequencing, next-generation sequencing (NGS) รวมถึงการวิเคราะห์ด้วย MALDI-ToF กำลังได้รับความนิยมมากขึ้น เนื่องจากสามารถให้ผลการระบุสายพันธุ์ที่รวดเร็วและแม่นยำ และช่วยเสริมประสิทธิภาพของการตรวจวิเคราะห์ทางสัณฐานวิทยาแบบดั้งเดิม [46] อย่างไรก็ตามแม้เทคโนโลยีทางชีวโมเลกุลจะมีความก้าวหน้าอย่างต่อเนื่อง การระบุสายพันธุ์โดยอาศัยลักษณะทางสัณฐานวิทยายังคงถือเป็นมาตรฐานอ้างอิง (reference standard) สำหรับงานด้านอนุกรมวิธาน และเป็นพื้นฐานสำคัญในการตีความและตรวจสอบความถูกต้องของข้อมูลทางโมเลกุล

6.4.1. การสกัดกรดนิวคลิอิกแบบทำลายตัวอย่าง (destructive nucleic acid extraction)

การสกัดกรดนิวคลิอิกเป็นขั้นตอนพื้นฐานที่ใช้กันอย่างแพร่หลายในงานวิจัยทางชีววิทยา โดยมีการพัฒนาเทคนิคและวิธีการหลากหลายรูปแบบเพื่อแยกสารพันธุกรรมดีเอ็นเอออกจากตัวอย่างชีวภาพ [48] ปัจจุบันมีชุดสกัดสารพันธุกรรมดีเอ็นเอสำเร็จรูปจำนวนมากที่ได้รับการออกแบบมาเพื่อเพิ่มความสะดวกและลดความซับซ้อนของกระบวนการสกัด [14] อย่างไรก็ตามวิธีการมาตรฐานที่ใช้ในการเตรียมตัวอย่างสัตว์ขาปล้องสำหรับการระบุสายพันธุ์ทางสัณฐานวิทยามักก่อให้เกิดข้อจำกัดต่อการวิเคราะห์ทางโมเลกุล เนื่องจากขั้นตอนดังกล่าวอาจทำให้โครงสร้างทางกายภาพที่มีความสำคัญเชิงอนุกรมวิธานเสียหายหรือถูกทำลาย [10] วิธีการสกัดสารพันธุกรรมดีเอ็นเอจากเนื้อเยื่อแมลงส่วนใหญ่จัดอยู่ในกลุ่มวิธีการสกัดแบบทำลายตัวอย่าง [43] ซึ่งเป็นประเด็นที่น่ากังวลโดยเฉพาะในกรณีของตัวอย่างขนาดเล็ก เช่น ริ้นฝอยทราย เนื่องจากการสุ่มตัวอย่างเนื้อเยื่อแม้เพียงเล็กน้อยอาจส่งผลกระทบต่อคุณลักษณะทางสัณฐานวิทยาที่ใช้ในการระบุสายพันธุ์ [72] ด้วยเหตุนี้ ประเภท ขนาด และสภาพการเก็บรักษาของตัวอย่างจึงเป็นปัจจัยสำคัญในการพิจารณาเลือกวิธีการสกัดสารพันธุกรรมดีเอ็นเอที่เหมาะสม [29]

ความจำเป็นในการระบุสายพันธุ์ของริ้นฝอยทรายอย่างถูกต้อง การทำความเข้าใจพลวัตของประชากร และการลดผลกระทบต่อสิ่งมีชีวิตที่ไม่ใช่เป้าหมาย จึงเป็นแรงผลักดันสำคัญให้เกิดการพัฒนาและประยุกต์ใช้เครื่องมือวินิจฉัยทางโมเลกุล [23] ในทางปฏิบัติมักมีการใช้เทคนิคทางโมเลกุลร่วมกับการจำแนกสายพันธุ์โดยอาศัยลักษณะทางสัณฐานวิทยา ตัวอย่างเช่น แนวทางมาตรฐานของการทำรหัสดีเอ็นเอ (DNA barcoding) ในแมลง ประกอบด้วยขั้นตอนการสกัดสารพันธุกรรมดีเอ็นเอ การหาลำดับนิวคลีโอไทด์ และส่งผลให้ตัวอย่างต้นฉบับสูญเสียความสมบูรณ์ทางสัณฐานไปโดยสิ้นเชิง ด้วยเหตุนี้จึงเกิดความสนใจอย่างต่อเนื่องในการพัฒนาวิธีการสกัดสารพันธุกรรมดีเอ็นเอแบบไม่ทำลายตัวอย่าง (non-destructive DNA extraction) เพื่อให้สามารถเก็บข้อมูลชีวภาพและข้อมูลทางสัณฐานวิทยาไว้ควบคู่กัน มีการนำวิธีการสกัดกรดนิวคลิอิกหลายรูปแบบมาประยุกต์ใช้กับริ้นฝอยทราย ทั้งนี้ปริมาณและคุณภาพของกรดนิวคลิอิกที่ต้องการขึ้นอยู่กับประเภทของการวิเคราะห์ในขั้นตอนถัดไป เนื่องจากเทคนิคทางโมเลกุลแต่ละชนิดมีระดับความไว (sensitivity) และความต้องการความบริสุทธิ์ของสารพันธุกรรมดีเอ็นเอแตกต่างกัน [9] ตัวอย่างเช่น มีรายงานว่าตาของริ้นฝอยทรายอาจมีสารยับยั้งปฏิกิริยาพีซีอาร์ (PCR inhibitors) ซึ่งส่งผลต่อประสิทธิภาพการเพิ่มปริมาณสารพันธุกรรมดีเอ็นเอ [69] นอกเหนือจากการตรวจคัดกรองเชื้อก่อโรคแล้ว การสกัดสารพันธุกรรมดีเอ็นเอจากริ้นฝอยทรายยังคงทำกันเป็นกิจวัตรเพื่อวัตถุประสงค์ด้านการระบุสายพันธุ์ โดยสามารถเลือกใช้วิธีการสกัดได้หลากหลาย แม้ว่าปริมาณผลผลิตและคุณภาพของสารพันธุกรรมดีเอ็นเอจะมีความแปรผันตามเทคนิคที่ใช้ นักวิจัยหลายรายได้ปรับปรุงวิธีการจากผู้ผลิตให้เหมาะสมกับลักษณะเฉพาะของริ้นฝอยทราย [8] เพื่อเพิ่มทั้งปริมาณและคุณภาพของกรดนิวคลิอิกที่สกัดได้ [8, 9, 69] ขณะเดียวกัน วิธีการสกัดที่พัฒนาขึ้นสำหรับสัตว์ขาปล้องกลุ่มอื่นก็สามารถนำมาประยุกต์ใช้กับริ้นฝอยทรายได้เช่นกัน [58, 76] โดยทั่วไปการทำพีซีอาร์เพื่อการระบุสายพันธุ์ที่มุ่งเป้าไปยังยีนไมโทคอนเดรียขนาดสั้น เช่น ยีน cytochrome c oxidase subunit I (*COI*) หรือ ยีน cytochrome b (*CytB*) สามารถใช้ร่วมกับวิธีการสกัดที่ก่อให้เกิดการแตกหักของสารพันธุกรรมดีเอ็นเอในระดับสูงได้ อย่างไรก็ตาม เทคนิคการหาลำดับนิวคลีโอไทด์ยุคใหม่แบบอ่านสายยาว (long-read next-generation sequencing) เช่น Oxford Nanopore© และ PacBio© มีความต้องการสารพันธุกรรมดีเอ็นเอที่มีการแตกหักน้อยที่สุดและมีคุณภาพสูง วิธีการสกัดแบบใช้คอลัมน์หมุนเหวี่ยง (spin column extraction) มักให้ชิ้นส่วนสารพันธุกรรมดีเอ็นเอที่มีขนาดสูงสุดประมาณ 60 กิโลเบส (kb) ในขณะที่การสกัดด้วยวิธีฟีนอล-คลอโรฟอร์ม (phenol-chloroform extraction) สามารถให้ชิ้นส่วนสารพันธุกรรมดีเอ็นเอที่มีขนาดใหญ่ได้ถึงประมาณ 150 กิโลเบส [77]. ตารางที่ 5 แสดงภาพรวมของเทคนิคการสกัดสารพันธุกรรมดีเอ็นเอที่ใช้กับริ้นฝอยทรายในรูปแบบต่าง ๆ พร้อมระบุว่ามีการปรับปรุงวิธีการให้เหมาะสมกับแมลงกลุ่มนี้หรือไม่ ทั้งนี้ไม่ได้แสดงค่าปริมาณผลผลิต เนื่องจากขึ้นอยู่กับขนาดตัวอย่างและวิธีการเตรียมตัวอย่างเป็นสำคัญ โดยคอลัมน์ "การปรับปรุง" หมายถึงการปรับเปลี่ยนวิธีการเพื่อให้เหมาะสมกับริ้นฝอยทรายหรือสัตว์ขาปล้องขนาดเล็กอื่น ๆ การเลือกวิธีการสกัดกรดนิวคลิอิกควรพิจารณาจากหลายปัจจัย ได้แก่ จำนวนตัวอย่าง ระยะเวลาในการทำ และเทคนิคที่จะใช้ในการวิเคราะห์ในขั้นตอนถัดไป แม้ว่าเทคนิค NGS จะต้องการสารพันธุกรรมดีเอ็นเอที่มีน้ำหนักโมเลกุลสูง แต่วิธีการสกัดทั้งหมดที่กล่าวถึงในส่วนนี้สามารถนำไปใช้กับการประยุกต์พื้นฐานที่อาศัยการทำพีซีอาร์ได้ นอกจากนี้ยังมีงานวิจัยจำนวนมากที่มุ่งศึกษาวิธีการสกัดสารพันธุกรรมดีเอ็นเอแบบไม่ทำลายตัวอย่างในสัตว์ขาปล้องขนาดเล็ก ตัวอย่างพิพิธภัณฑ์ที่เก็บรักษาแบบแห้ง และสัตว์ขาปล้องที่มีลำตัวอ่อนนิ่ม [19, 26, 28, 55, 63].

6.4.2. การสกัดกรดนิวคลิอิกแบบไม่ทำลายตัวอย่าง (non-destructive nucleic acid extraction)

หนึ่งในความท้าทายสำคัญของการวิเคราะห์ทางโมเลกุลในสัตว์ขาปล้อง โดยเฉพาะอย่างยิ่งในริ้นฝอยทราย คือการรักษาสภาพตัวอย่างให้สามารถนำไปใช้เป็นตัวอย่างอ้างอิงในทางกีฏวิทยาได้ วิธีการสกัดสารพันธุกรรมดีเอ็นเอส่วนใหญ่มักอาศัยการบดเนื้อเยื่อ ซึ่งส่งผลให้โครงสร้างทางกายภาพของตัวอย่างถูกทำลายและไม่สามารถนำไปใช้ในการศึกษาทางสัณฐานวิทยาในภายหลังได้ วิธีการสกัดกรดนิวคลิอิกแบบไม่ทำลายตัวอย่างได้รับการพัฒนาขึ้นเพื่อตอบโจทย์ข้อจำกัดดังกล่าว โดยมีวัตถุประสงค์เพื่อแยกสารพันธุกรรมออกจากตัวอย่างโดยไม่ก่อให้เกิดความเสียหายทางกายภาพ ไม่กระทบต่อความมีชีวิต (ในกรณีที่ใช้กับตัวอย่างสด) และไม่เปลี่ยนแปลงลักษณะทางสัณฐานวิทยาที่มีความสำคัญเชิงอนุกรมวิธาน วิธีการเหล่านี้มีคุณค่าอย่างยิ่งเมื่อต้องทำงานกับตัวอย่างที่หาได้ยาก มีจำนวนจำกัด หรือเป็นตัวอย่างต้นแบบ เช่น ริ้นฝอยทราย ซึ่งจำเป็นต้องรักษาความสมบูรณ์ของโครงสร้างเพื่อการศึกษาและการอ้างอิงในอนาคต หนึ่งในแนวทางที่นิยมใช้คือ วิธีการทดสอบแบบไม่ทำลายตัวอย่าง (non-destructive bathing method) โดยจะทำการตรึงตัวอย่างให้อยู่กับที่ และแช่ในสารละลายสำหรับการย่อยสลาย (lysis buffer) ที่มีเอนไซม์โปรติเนส เค (proteinase K) ภายใต้สภาวะที่ควบคุมอย่างเหมาะสม เพื่อให้กรดนิวคลิอิกถูกปลดปล่อยออกมาโดยไม่ทำลายโครงสร้างภายนอกของตัวอย่าง

เทคนิคการย่อยสลายพาหะอย่างอ่อน ได้รับการนำมาประยุกต์ใช้กับริ้นฝอยทรายได้อย่างดี โดยเฉพาะในกรณีของตัวอย่างต้นแบบ [24] เทคนิคนี้อาศัยชุดสกัดสารพันธุกรรมดีเอ็นเอแบบคอลัมน์หมุนเหวี่ยงมาตรฐาน เช่น DNeasy Blood and Tissue (Qiagen, ประเทศเยอรมนี) ร่วมกับการปรับปรุงขั้นตอนการย่อยสลาย ได้แก่ การปรับปริมาณบัฟเฟอร์และการเพิ่มขั้นตอนการแช่เยือกแข็ง [17] ซึ่งช่วยให้สามารถสกัดกรดนิวคลิอิกได้ในขณะที่ลดความเสียหายต่อสัณฐานวิทยาของตัวอย่างให้น้อยที่สุด [24] นอกจากนี้ ยังมีการรายงานการใช้ชุดสกัดสารพันธุกรรมดีเอ็นเอแบบ HotSHOT (Bento Lab) กับริ้นฝอยทราย [73] ซึ่งเป็นวิธีที่รวดเร็ว มีต้นทุนต่ำ และเหมาะสำหรับการเตรียมตัวอย่างจำนวนมาก แม้ว่าวิธีดังกล่าวจะให้สารพันธุกรรมดีเอ็นเอที่เหมาะสมสำหรับการวิเคราะห์ด้วยพีซีอาร์เป็นหลัก แต่ก็มีข้อได้เปรียบด้านความสะดวกและความประหยัด ตัวอย่างทางกีฏวิทยาที่ผ่านกระบวนการสกัดสารพันธุกรรมดีเอ็นเอแบบไม่ทำลายตัวอย่าง ยังสามารถนำไปเตรียมเพื่อการระบุสายพันธุ์ทางสัณฐานวิทยาได้ต่อไป โดยตัวอย่างที่ผ่านการสกัดด้วยชุด DNeasy Blood and Tissue จำเป็นต้องผ่านขั้นตอนการทำให้ใส ด้วยวิธี Marc-André ขณะที่ตัวอย่างที่ผ่านกระบวนการด้วยชุด Hotshot จะมีความใสเพียงพอสำหรับการนำไปทำสไลด์ถาวรในตัวกลางที่มีน้ำเป็นองค์ประกอบหรืออาจเลือกใช้การแช่ในเรซินหลังการกำจัดน้ำตามวิธีการที่มีการรายงานไว้ [73] กรดนิวคลิอิกที่สกัดได้จากวิธีการดังกล่าวสามารถนำไปใช้ในการวิเคราะห์ในขั้นตอนถัดไป เช่น การทำพีซีอาร์เพื่อเพิ่มปริมาณเครื่องหมายทางพันธุกรรมที่จำเพาะ วิธีการสกัดแบบไม่ทำลายตัวอย่างจึงมีบทบาทสำคัญอย่างยิ่งในการศึกษาลักษณะทางพันธุกรรมของริ้นฝอยทราย การตรวจหาเชื้อก่อโรคที่อาจถูกถ่ายทอดโดยพาหะนำโรค รวมถึงการรักษาสภาพตัวอย่างสำหรับการศึกษาและการวินิจฉัยเพิ่มเติมในอนาคต

ตารางที่ 5 ค่าใช้จ่ายเฉลี่ย การประยุกต์ใช้ และการปรับปรุงวิธีการสำหรับการสกัดสารพันธุกรรมดีเอ็นเอจากตัวอย่างริ้นฝอยทราย

วิธีการ	ค่าใช้จ่าย (ดอลลาร์สหรัฐ)	การประยุกต์ใช้	การปรับปรุงวิธีการสำหรับสัตว์ขาปล้องขนาดเล็ก
Spin column	2.5 – 3.55 US$ [39]	PCR, NGS	[9]
Phenol- chloroform	0.12 US$ [69]	PCR, NGS	[9]
HotSHOT	<0.01 US$ [69]	PCR	-
Salting out	0.12 $3 [69]	PCR	-
Chelex	0.02 $4 [41]	PCR	[41, 76]

6.5. เทคนิค MALDI-ToF MS

MALDI-ToFMS (Matrix-Assisted Laser Desorption/ Ionization Time-of-Flight Mass Spectrometry) เป็นเทคนิคการวิเคราะห์มวลสารที่ใช้ในการตรวจวัดและวิเคราะห์รูปแบบโปรไฟล์ของโปรตีน (protein profiles) ซึ่งมีลักษณะจำเพาะต่อชนิดของสิ่งมีชีวิต โดยโปรไฟล์ดังกล่าวสามารถใช้เป็น “ลายนิ้วมือทางชีวภาพ” (biological fingerprints) สำหรับการระบุสายพันธุ์ของตัวอย่างทางชีวภาพได้อย่างมีประสิทธิภาพ ในช่วงทศวรรษที่ผ่านมาเทคนิค MALDI-ToF ได้รับการยอมรับอย่างกว้างขวางในฐานะเครื่องมือสำคัญสำหรับการจำแนกสายพันธุ์ของสัตว์ขาปล้องที่มีความสำคัญทางการแพทย์และสัตวแพทย์ มีรายงานจำนวนมากแสดงให้เห็นว่า MALDI-ToF สามารถใช้ระบุสายพันธุ์ของริ้นฝอยทรายได้อย่างแม่นยำในระยะการเจริญเติบโตที่หลากหลาย รวมถึงระยะตัวอ่อน อีกทั้งยังสามารถใช้จำแนกแหล่งที่มาของเลือดในริ้นฝอยทรายเพศเมียที่ดูดเลือดแล้วได้อย่างมีประสิทธิภาพ นอกจากนี้เทคนิคดังกล่าวยังประสบความสำเร็จในการจำแนกสายพันธุ์ของริ้นฝอยทรายทั้งเพศผู้และเพศเมียภายใต้เงื่อนไขการเก็บรักษาและวิธีการทำให้ตัวอย่างเป็นเนื้อเดียวกันที่แตกต่างกัน [28, 30, 73, 74] MALDI-ToF มีความสามารถในการจำแนกสายพันธุ์ของแมลงสูงครอบคลุมตั้งแต่ระดับสกุลย่อย (subgenera) ระดับสายพันธุ์ (species) ไปจนถึงระดับกลุ่มประชากรส่งผลให้สามารถระบุสายพันธุ์ได้อย่างรวดเร็วและแม่นยำ คุณสมบัติดังกล่าวมีความสำคัญอย่างยิ่งต่อการศึกษาด้านอนุกรมวิธานเชิงประยุกต์ การทำความเข้าใจการกระจายตัว พฤติกรรม และบทบาทของริ้นฝอยทรายในฐานะแมลงพาหะของเชื้อก่อโรค ดังนั้นการจำแนกสายพันธุ์ด้วยโปรไฟล์โปรตีนจาก MALDI-ToF จึงมีบทบาทสำคัญในงานระบาดวิทยาและการกำหนดกลยุทธ์ในการควบคุมแมลงพาหะ การประยุกต์ใช้ MALDI-ToF ในงานประจำ (routine use) ยังคงมีข้อจำกัดที่สำคัญ 2 ประการ ได้แก่. 1) ความพร้อมของเครื่องมือวิเคราะห์มวลสาร โดยเครื่อง MALDI-ToF มีต้นทุนการจัดซื้อและบำรุงรักษาสูง จึงอาจไม่คุ้มค่าในการจัดหาเพื่อใช้สำหรับการระบุสายพันธุ์ของริ้นฝอยทรายหรือแมลงพาหะเพียงอย่างเดียว ข้อจำกัดนี้สามารถลดลงได้โดยการใช้ทรัพยากรร่วมกับหน่วยงานด้านโปรตีโอมิกส์หรือห้องปฏิบัติการวินิจฉัยทางคลินิก ซึ่งในปัจจุบันเครื่องมือดังกล่าวถือเป็นอุปกรณ์มาตรฐานที่มีการใช้งานอย่างแพร่หลายแล้ว 2) ความจำกัดของข้อมูลอ้างอิงในฐานข้อมูลสาธารณะ โปรไฟล์โปรตีนอ้างอิงของริ้นฝอยทรายในฐานข้อมูลสาธารณะยังมีจำนวนจำกัด ส่งผลให้ผู้ใช้งานจำเป็นต้องจัดทำฐานข้อมูลภายใน (in-house database) โดยใช้สเปกตรัมอ้างอิงจากตัวอย่างที่ได้รับการระบุสายพันธุ์อย่างถูกต้อง ซึ่งตามหลักควรอาศัยการยืนยันผลแบบบูรณาการระหว่างการตรวจสอบ

ทางสัณฐานวิทยาและการวิเคราะห์ลำดับนิวคลีโอไทด์ของเครื่องหมายทางพันธุกรรมที่เหมาะสม เช่น *COI*, *cytB* หรือเครื่องหมายอื่น ๆ

ทั้งนี้ คาดว่าข้อจำกัดดังกล่าวจะลดลงในอนาคตจากการรวบรวมข้อมูลอ้างอิงเข้าสู่แพลตฟอร์ม MSI ซึ่งดำเนินงานโดยเครือข่ายความร่วมมือในกรุงปารีสและกรุงบรัสเซลส์ (Platform run by Assistance Publique-Hôpitaux de Paris, Sorbonne University, France and the BCCM/IHEM/Sciensano collection in Brussels, Belgium (https://msi.happy-dev.fr/) สำหรับการเตรียมตัวอย่างเพื่อการสร้างโปรไฟล์โปรตีนด้วย MALDI-ToF แนะนำให้เก็บตัวอย่างในสภาวะแช่เยือกแข็งแบบแห้ง (dry-frozen) หรือเก็บในเอทานอลเกรดชีวโมเลกุลความเข้มข้น 70% และควรหลีกเลี่ยงการเก็บที่อุณหภูมิห้อง เนื่องจากอาจส่งผลต่อความคงตัวของโปรตีน ในกรณีที่ยังไม่มีแนวปฏิบัติมาตรฐานสากลสำหรับการเตรียมตัวอย่าง แนะนำให้ใช้สารละลาย sinapinic acid ความเข้มข้น 30 มิลลิกรัม/มิลลิลิตร ในตัวทำละลาย 60% acetonitrile และ 0.3% trifluoroacetic acid (TFA) เป็นสารเมทริกซ์ (MALDI matrix) เพื่อให้สเปกตรัมโปรตีนที่ได้สามารถนำไปเปรียบเทียบกับข้อมูลอ้างอิงที่มีการเผยแพร่แล้วได้อย่างเหมาะสม

การเตรียมตัวอย่างแมลงสำหรับการวิเคราะห์ด้วยเทคนิค MALDI-ToF MS (รูปที่ 7)

ตัวอย่างแมลงที่เก็บภายใต้สภาวะต่าง ๆ จะต้องนำมาผึ่งให้แห้งที่อุณหภูมิห้องก่อนการเตรียมตัวอย่างทุกครั้งเพื่อขจัดความชื้นหรือสารละลายตกค้าง จากนั้นจึงผ่าตัวอย่างโดยแยกส่วนหัวและส่วนท้องออกจากกันเพื่อคงสภาพโครงสร้างที่มีลักษณะสำคัญทางสัณฐานวิทยาไว้สำหรับการทำสไลด์ถาวรและการวิเคราะห์ทางอนุกรมวิธาน ส่วนอกของตัวอย่างจะถูกนำไปใช้สำหรับการวิเคราะห์ด้วยเทคนิค MALDI-ToF เนื่องจากเป็นบริเวณที่มีปริมาณโปรตีนเหมาะสมต่อการสร้างโปรไฟล์โปรตีน ในขณะที่ส่วนท้องที่เหลือสามารถเก็บไว้เพื่อนำไปใช้ในการสกัดสารพันธุกรรมดีเอ็นเอและการวิเคราะห์ทางอณูชีววิทยาในขั้นตอนถัดไป สำหรับการเตรียมตัวอย่างเพื่อวิเคราะห์โปรไฟล์โปรตีน ส่วนอกจะถูกนำมาทำให้เป็นเนื้อเดียวกันด้วยมือ (manual homogenization) ภายในหลอดไมโครเซนตริฟิวจ์ขนาด 1.5 มิลลิลิตร โดยเติมสารละลายสำหรับการทำให้เป็นเนื้อเดียวกันปริมาตร 10 ไมโครลิตร และใช้แท่งบดตัวอย่างแบบใช้แล้วทิ้ง (disposable pestles) เป็นอุปกรณ์ในการบดตัวอย่าง สารละลายที่ใช้ในการทำให้เป็นเนื้อเดียวกันโดยทั่วไปแบ่งออกเป็น 2 ประเภท ได้แก่ น้ำกลั่นปราศจากเชื้อ (sterile distilled water) และ กรดฟอร์มิก (formic acid) ความเข้มข้น 25% การเลือกใช้สารละลายขึ้นอยู่กับวัตถุประสงค์ของการวิเคราะห์และวิธีการที่ใช้ในแต่ละห้องปฏิบัติการ ทั้งนี้การเตรียมตัวอย่างต้องทำอย่างระมัดระวังเพื่อหลีกเลี่ยงการปนเปื้อน ซึ่งอาจส่งผลต่อคุณภาพและความสามารถในการเปรียบเทียบสเปกตรัมโปรตีนที่ได้จากการวิเคราะห์ด้วย MALDI-ToF

7. บทสรุป

การศึกษานี้มีวัตถุประสงค์เพื่อนำเสนอแนวทางและวิธีการเตรียมตัวอย่างริ้นฝอยทรายสำหรับการทำสไลด์ถาวรที่มีประสิทธิภาพ เพื่อสนับสนุนการระบุสายพันธุ์และการตรวจหาเชื้อก่อโรคให้มีความถูกต้องและเชื่อถือได้ ทั้งนี้ วิธีการเตรียมตัวอย่างแต่ละรูปแบบถูกออกแบบให้สอดคล้องกับวัตถุประสงค์เฉพาะของงานวิจัยที่แตกต่างกัน โดยไม่มีวิธีการใดวิธีการหนึ่งที่ถือเป็นมาตรฐานสากลที่เหมาะสมที่สุดสำหรับทุกบริบทการศึกษา ในส่วนของข้อมูลสนับสนุน ผู้นิพนธ์ได้จัดทำรายละเอียดของวิธีการทำสไลด์ในรูปแบบต่าง ๆ ที่ใช้ในการเตรียมและระบุสายพันธุ์ของริ้นฝอยทราย โดยนำเสนอขั้นตอนการปฏิบัติงานอย่างเป็นลำดับ พร้อมทั้งสื่อประกอบในรูปแบบวีดีโอสาธิต เพื่อให้ได้ผลลัพธ์ที่มีความเที่ยงตรงและสามารถทำซ้ำได้ การจัดเตรียมทรัพยากรดังกล่าวมีเป้าหมายเพื่อสนับสนุนให้นักวิจัยสามารถเลือกและประยุกต์ใช้เทคนิคการทำสไลด์ที่เหมาะสมกับความต้องการเฉพาะของงานวิจัยของตนเองได้อย่างมีประสิทธิภาพ

Acknowledgments

ผู้เขียนขอขอบคุณ Richard Lane และ Zoe Jay Adams จากพิพิธภัณฑ์ประวัติศาสตร์ธรรมชาติแห่งกรุงลอนดอน สหราชอาณาจักร (Natural History Museum of London, UK) สำหรับการทบทวนต้นฉบับอย่างละเอียด ซึ่งมีส่วนช่วยยกระดับคุณภาพของต้นฉบับฉบับนี้อย่างมีนัยสำคัญ

Funding

งานวิจัยนี้ได้รับการสนับสนุนจาก The Brazilian development agencies CNPq (case number: 404395/2024-4) และ the Araucária Foundation (case 1007 number: 433/2025 PDI) for funding AJA' research

Conflicts of interest

Jérôme Depaquit ดำรงตำแหน่งบรรณาธิการสมทบ (associate editor) ของวารสาร Parasite อย่างไรก็ตาม เขาไม่มีส่วนเกี่ยวข้องใด ๆ ต่อกระบวนการพิจารณาทบทวนต้นฉบับและการตัดสินใจเกี่ยวกับบทความฉบับนี้ ผู้เขียนท่านอื่นขอแสดงว่าไม่มีผลประโยชน์ทับซ้อนใด ๆ

Data availability statement

Video 1: https://zenodo.org/records/18198006
Video 2: https://zenodo.org/records/18311158
Video 3: https://zenodo.org/records/18311106
Video 4: https://zenodo.org/records/18311154
Video 5: https://zenodo.org/records/18303014
Video 6: https://zenodo.org/records/18302850
Video 7: https://zenodo.org/records/18315029

Supplementary material

The supplementary material of this article is available at https://www.parasite-journal.org/10.1051/parasite/2026009/olm

References

1. Alkan C, Allal-Ikhlef AB, Alwassouf S, Baklouti A, Piorkowski G, de Lamballerie X, Izri A, Charrel RN. 2015. Virus isolation, genetic characterization and seroprevalence of Toscana virus in Algeria. Clinical Microbiology and Infection, 21(11), 1040 e1-9.
2. Alten B, Ozbel Y, Ergunay K, Kasap OE, Cull B, Antoniou M, Velo E, Prudhomme J, Molina R, Banuls AL, Schaffner F, Hendrickx G, Van Bortel W, Medlock JM. 2015. Sampling strategies for phlebotomine sand flies (Diptera: Psychodidae) in Europe. Bulletin of Entomological Research 105(6), 664–678.
3. Ayhan N, Baklouti A, Prudhomme J, Walder G, Amaro F, Alten B, Moutailler S, Ergunay K, Charrel RN, Huemer H. 2017. Practical guidelines for studies on sandfly-borne phleboviruses: Part I: Important points to consider *ante* field work. Vector-Borne and Zoonotic Diseases 17(1), 73–80.
4. Bates PA. 1997. Infection of phlebotomine sandflies with *Leishmania*, in The Molecular Biology of Insect Disease Vectors: A Methods Manual. Springer. p. 112–120.5.
5. Baum M, de Castro EA, Pinto MC, Goulart TM, Baura W, Klisiowicz Ddo R, Vieira da Costa-Ribeiro MC. 2015. Molecular detection of the blood meal source of sand flies (Diptera: Psychodidae) in a transmission area of American cutaneous leishmaniasis, Parana State, Brazil. Acta Tropica, 143, 8–12.
6. Belen A, Alten B, Aytekin A. 2004. Altitudinal variation in morphometric and molecular characteristics of *Phlebotomus papatasi* populations. Medical and Veterinary Entomology, 18(4), 343–350.
7. Bhattacharya J, Chandra G, Hati AK. 1991. A simple method for cryopreservation of *Leishmania donovani* promastigotes, Indian Journal of Medical Research, 93, 245–246.
8. Caligiuri LG, Sandoval AE, Miranda JC, Pessoa FA, Santini MS, Salomón OD, Secundino NF, McCarthy CB. 2019. Optimization of DNA extraction from individual sand flies for PCR amplification. Methods and Protocols, 2(2), 36.
9. Casaril AE, de Oliveira LP, Alonso DP, de Oliveira EF, Gomes Barrios SP, de Oliveira Moura Infran J, Fernandes WS, Oshiro ET, Ferreira AMT, Ribolla PEM, de Oliveira AG. 2017. Standardization of DNA extraction from sand flies: Application to genotyping by next generation sequencing. Experimental Parasitology, 177, 66–72.
10. Castalanelli MA, Severtson DL, Brumley CJ, Szito A, Foottit RG, Grimm M, Munyard K, Groth DM. 2010. A rapid non-destructive DNA extraction method for insects and other arthropods. Journal of Asia-Pacific Entomology, 13(3), 243–248.
11. Cerqueira NL. 1943. Um novo meio para montagem de pequenos insetos em lâmina. Memórias do Instituto Oswaldo Cruz, (39), 37–41.
12. Charrel RN, Gallian P, Navarro-Mari JM, Nicoletti L, Papa A, Sanchez-Seco MP, Tenorio A, de Lamballerie X. 2005. Emergence of Toscana virus in Europe. Emerging Infectious Diseases, 11(11), 1657–1663.
13. Chaskopoulou A, Giantsis IA, Demir S, Bon MC. 2016. Species composition, activity patterns and blood meal analysis of sand fly populations (Diptera: Psychodidae) in the metropolitan region of Thessaloniki, an endemic focus of canine leishmaniasis. Acta Tropica, 158, 170–176.
14. Chen H, Rangasamy M, Tan SY, Wang H, Siegfried BD. 2010. Evaluation of five methods for total DNA extraction from western corn rootworm beetles. PLoS One, 5(8), e11963.
15. Depaquit J, Grandadam M, Fouque F, Andry PE, Peyrefitte C. 2010. Arthropod-borne viruses transmitted by Phlebotomine sandflies in Europe: a review. Eurosurveillance, 15(10), 19507.
16. Diamond LS, Herman CM. 1954. Incidence of Trypanosomes in the Canada Goose as revealed by bone marrow culture. Journal of Parasitology, 40(2), 195–202.
17. Ding H, Torno M, Vongphayloth K, Ng G, Tan D, Sng W, Ho K, Randrianambinintsoa FJ, Depaquit J, Tan CH. 2025. Hidden in plain sight: discovery of sand flies in Singapore and description of four species new to science. Parasites & Vectors, 18(1), 402.
18. Es-Sette N, Ajaoud M, Bichaud L, Hamdi S, Mellouki F, Charrel RN, Lemrani M. 2014. *Phlebotomus sergenti* a common vector of *Leishmania tropica* and Toscana virus in Morocco. Journal of Vector Borne Diseases, 51(2), 86–90.
19. Favret C. 2005. A new non-destructive DNA extraction and specimen clearing technique for aphids (Hemiptera). Proceedings of the Entomological Society of Washington, 107(2), 469–470.
20. Galati EAB. 2018. Phlebotominae (Diptera, Psychodidae): Classification, morphology and terminology of adults and identification of American taxa, in Brazilian Sand Flies: Biology, Taxonomy, Medical Importance and Control, Rangel EF, Shaw JJ, Editors. Cham: Springer International Publishing. pp. 9–212.
21. Galati EAB, de Andrade AJ, Perveen F, Loyer M, Vongphayloth K, Randrianambinintsoa FJ, Prudhomme J, Rahola N, Akhoundi M, Shimabukuro PHF, Depaquit J. 2025. Phle-botomine sand flies (Diptera, Psychodidae) of the world. Parasites & Vectors, 18(1), 220.
22. Galati EAB, Galvis-Ovallos F, Lawyer P, Leger N, Depaquit J. 2017. An illustrated guide for characters and terminology used in descriptions of Phlebotominae (Diptera, Psychodidae). Par-asite, 24, 26.
23. Gariepy T, Kuhlmann U, Gillott C, Erlandson M. 2007. Parasitoids, predators and PCR: the use of diagnostic molecular markers in biological control of Arthropods. Journal of Applied Entomology, 131(4), 225–240.
24. Giantsis IA, Chaskopoulou A, Bon MC. 2016. Mild-Vectolysis: A nondestructive DNA extraction method for vouchering sand flies and mosquitoes. Journal of Medical Entomology, 53(3), 692–695.
25. Gidwani K, Picado A, Rijal S, Singh SP, Roy L, Volfova V, Andersen EW, Uranw S, Ostyn B, Sudarshan M, Chakravarty J, Volf P, Sundar S, Boelaert M, Rogers ME. 2011. Serological markers of sand fly exposure to evaluate insecticidal nets against visceral leishmaniasis in India and Nepal: a cluster-randomized trial. PLoS Neglected Tropical Diseases, 5(9), e1296.
26. Gilbert MTP, Moore W, Melchior L, Worobey M. 2007. DNA extraction from dry museum beetles without conferring external morphological damage. PLoS One, 2(3), e272.
27. Giordani BF, Andrade AJ, Galati EAB, Gurgel-Goncalves R. 2017. The role of wing geometric morphometrics in the identification of sandflies within the subgenus *Lutzomyia*. Medical and Veterinary Entomology, 31(4), 373–380.
28. Guzmán-Larralde AJ, Suaste-Dzul AP, Gallou A, Peña-Carrillo KI. 2017. DNA recovery from microhymenoptera using six non-destructive methodologies with considerations for subsequent preparation of museum slides. Genome, 60(1), 85–91.
29. Hajibabaei M, DeWaard JR, Ivanova NV, Ratnasingham S, Dooh RT, Kirk SL, Mackie PM, Hebert PD. 2005. Critical factors for assembling a high volume of DNA barcodes. Philosophical Transactions of the Royal Society B: Biological Sciences, 360(1462), 1959–1967.

30. Haouas N, Pesson B, Boudabous R, Dedet JP, Babba H, Ravel C. 2007. Development of a molecular tool for the identification of *Leishmania* reservoir hosts by blood meal analysis in the insect vectors. American Journal of Tropical Medicine and Hygiene, 77(6), 1054–1059.
31. Hlavackova K, Dvorak V, Chaskopoulou A, Volf P, Halada P. 2019. A novel MALDI-TOF MS-based method for blood meal identification in insect vectors: A proof of concept study on phlebotomine sand flies. PLoS Neglected Tropical Diseases, 13 (9), e0007669.
32. Huemer H, Prudhomme J, Amaro F, Baklouti A, Walder G, Alten B, Moutailler S, Ergunay K, Charrel RN, Ayhan N. 2017. Practical guidelines for studies on sandfly-borne phleboviruses: Part II: Important points to consider for fieldwork and subsequent virological screening. Vector-Borne and Zoonotic Diseases, 17(1), 81–90.
33. Jancarova M, Polanska N, Thiesson A, Arnaud F, Stejskalova M, Rehbergerova M, Kohl A, Viginier B, Volf P, Ratinier M. 2025. Susceptibility of diverse sand fly species to Toscana virus. PLoS Neglected Tropical Diseases, 19(5), e0013031.
34. Kapp JD, Green RE, Shapiro B. 2021. A fast and efficient single-stranded genomic library preparation method optimized for ancient DNA. Journal of Heredity, 112(3), 241–249.
35. Killick-Kendrick R, Maroli M, Killick-Kendrick M. 1991. Bibliography of the colonization of phlebotomine sandflies. Parassitologia, 33(suppl.), 321–333.
36. Lawyer P, Killick-Kendrick M, Rowland T, Rowton E, Volf P. 2017. Laboratory colonization and mass rearing of phlebotomine sand flies (Diptera, Psychodidae). Parasite, 24, 42.
37. Léger N, Pesson B, Madulo-Leblond G. 1986. Les phlébotomes de Grèce : 1ère partie. Bulletin de la Société de Pathologie Exotique, 79, 386–397.
38. Léger N, Pesson B, Madulo-Leblond G. 1986. Les phlébotomes de Grèce : 2ème partie. Bulletin de la Société de Pathologie Exotique, 79, 514–524.
39. Leonel JAF, Vioti G, Alves ML, da Silva DT, Meneghesso PA, Benassi JC, Spada JCP, Galvis-Ovallos F, Soares RM, Oliveira T. 2020. DNA extraction from individual Phlebotomine sand flies (Diptera: Psychodidae: Phlebotominae) specimens: Which is the method with better results? Experimental Parasitology, 218, 107981.
40. Lestinova T, Rohousova I, Sima M, de Oliveira CI, Volf P. 2017. Insights into the sand fly saliva: Blood-feeding and immune interactions between sand flies, hosts, and *Leishmania*. PLoS Neglected Tropical Diseases, 11(7), e0005600.
41. Lienhard A, Schaffer S. 2019. Extracting the invisible: obtaining high quality DNA is a challenging task in small arthropods. PeerJ, 7, e6753.
42. Lozano-Sardaneta YN, Mikery-Pacheco OF, Huerta H, Rojas-Soriano JE, Contreras-Ramos A. 2025. Wing geometric morphometrics is effective to separate sand fly species (Diptera, Psychodidae, Phlebotominae) related with leishmaniasis transmission in Mexico. Acta Tropica, 262, 107523.
43. Mandrioli M. 2008. Insect collections and DNA analyses: how to manage collections? Museum Management and Curatorship, 23(2), 193–199.
44. Maroli M, Feliciangeli MD, Bichaud L, Charrel RN, Gradoni L. 2013. Phlebotomine sandflies and the spreading of leishmaniases and other diseases of public health concern. Medical and Veterinary Entomology, 27(2), 123–147.
45. Marquina D, Buczek M, Ronquist F, Lukasik P. 2021. The effect of ethanol concentration on the morphological and molecular preservation of insects for biodiversity studies. PeerJ, 9, e10799.
46. Mathis A, Depaquit J, Dvorak V, Tuten H, Banuls AL, Halada P, Zapata S, Lehrter V, Hlavackova K, Prudhomme J, Volf P, Sereno D, Kaufmann C, Pfluger V, Schaffner F. 2015. Identification of phlebotomine sand flies using one MALDI-TOF MS reference database and two mass pectrometer systems., Parasites & Vectors, 8, 266.
47. Mekarnia N, Benallal KE, Sadlova J, Vojtkova B, Mauras A, Imbert N, Longhitano M, Harrat Z, Volf P, Loiseau PM, Cojean S. 2024. Effect of *Phlebotomus papatasi* on the fitness, infectivity and antimony-resistance phenotype of antimony-resistant *Leishmania* major Mon-25. International Journal for Parasitology – Drugs and Drug Resistance, 25, 100554.
48. Milligan BG. 1998. Total DNA isolation, in Molecular Genetic Analysis of Population: A Practical Approach, Hoelzel AR, Editor. Oxford: Oxford University Press.
49. Molina R, Jiménez M, Alvar J, González E, Hernández-Taberna S, Ines MM. 2017. Methods in sand fly research. Madrid: Servicio de publicaciones Universidad de Alcalá de Henares, Madrid.
50. Murphy WJ, Eizirik E, O'Brien SJ, Madsen O, Scally M, Douady CJ, Teeling E, Ryder OA, Stanhope MJ, de Jong WW, Springer MS. 2001. Resolution of the early placental mammal radiation using Bayesian phylogenetics. Science, 294(5550), 2348–2351.
51. Nacif-Pimenta R, Pinto LC, Volfova V, Volf P, Pimenta PFP, Secundino NFC. 2020. Conserved and distinct morphological aspects of the salivary glands of sand fly vectors of leishma-niasis: an anatomical and ultrastructural study. Parasites & Vectors, 13(1), 441.
52. Neuhaus B, Schmid T, Riedel J. 2017. Collection management and study of microscope slides: Storage, profiling, deterioration, restoration procedures, and general recommendations. Zootaxa, 4322(1), 1–173.
53. New TR. 1974. Pscoptera. Handbooks for Identification of British Insects (Vol. I). London: Royal Entomological Society of London. 102 pp.
54. Perez-Ruiz M, Collao X, Navarro-Mari JM, Tenorio A. 2007. Reverse transcription, real-time PCR assay for detection of Toscana virus. Journal of Clinical Virology, 39(4), 276–281.
55. Porco D, Rougerie R, Deharveng L, Hebert P. 2010. Coupling non-destructive DNA extraction and voucher retrieval for small soft-bodied Arthropods in a high-throughput context: the example of Collembola. Molecular Ecology Resources, 10(6), 942–945.
56. Prudhomme J, Cassan C, Hide M, Toty C, Rahola N, Vergnes B, Dujardin JP, Alten B, Sereno D, Banuls AL. 2016. Ecology and morphological variations in wings of *Phlebotomus ariasi* (Diptera: Psychodidae) in the region of Roquedur (Gard, France): a geometric morphometrics approach. Parasites & Vectors, 9(1), 578.
57. Prudhomme J, Gunay F, Rahola N, Ouanaimi F, Guernaoui S, Boumezzough A, Banuls AL, Sereno D, Alten B. 2012. Wing size and shape variation of *Phlebotomus papatasi* (Diptera: Psychodidae) populations from the south and north slopes of the Atlas Mountains in Morocco. Journal of Vector Ecology, 37(1), 137–147.
58. Prudhomme J, Toty C, Kasap OE, Rahola N, Vergnes B, Maia C, Campino L, Antoniou M, Jimenez M, Molina R, Cannet A, Alten B, Sereno D, Banuls AL. 2015. New microsatellite markers for multi-scale genetic studies on *Phlebotomus ariasi* Tonnoir, vector

of *Leishmania infantum* in the Mediterranean area. Acta Tropica, 142, 79–85.
59. Prudhomme J, Velo E, Bino S, Kadriaj P, Mersini K, Gunay F, Alten B. 2019. Altitudinal variations in wing morphology of *Aedes albopictus* (Diptera, Culicidae) in Albania, the region where it was first recorded in Europe. Parasite, 26, 55.
60. Rawlins DJ. 1992. Light Microscopy: An Introduction to Biotechniques. Oxford: Bios Scientific publishers. 143 pp.
61. Ready PD. 2013. Biology of phlebotomine sand flies as vectors of disease agents. Annual Review of Entomology, 58, 227–250.
62. Rohlf FJ, Slice D. 1990. Extensions of the Procrustes method for the optimal superimposition of landmarks. Systematic Zoology, 39(1), 40–59.
63. Rowley DL, Coddington JA, Gates MW, Norrbom AL, Ochoa RA, Vandenberg NJ, Greenstone MH. 2007. Vouchering DNA-barcoded specimens: Test of a nondestructive extraction proto-col for terrestrial arthropods. Molecular Ecology Notes, 7(6), 915–924.
64. Sábio PB, Andrade AJ, Galati EAB. 2014. Assessment of the taxonomic status of some species included in the *Shannoni* complex, with the description of a new species of *Psathyromyia* (Diptera: Psychodidae: Phlebotominae). Journal of Medical Entomology, 51(2), 331–341.
65. Sadlova J, Yeo M, Seblova V, Lewis MD, Mauricio I, Volf P, Miles MA. 2011. Visualisation of *Leishmania donovani* fluo-rescent hybrids during early stage development in the sand fly vector. PLoS One, 6(5), e19851.
66. Sales K, Miranda DEO, da Silva FJ, Otranto D, Figueredo LA, Dantas-Torres F. 2020. Evaluation of different storage times and preservation methods on phlebotomine sand fly DNA concen-tration and purity. Parasites & Vectors, 13(1), 399
67. Sales KG, Costa PL, de Morais RC, Otranto D, Brandao-Filho SP, Cavalcanti Mde P, Dantas-Torres F. 2015. Identification of phlebotomine sand fly blood meals by real-time PCR. Parasites& Vectors, 8, 230.
68. Sant'Anna MR, Jones NG, Hindley JA, Mendes-Sousa AF, Dillon RJ, Cavalcante RR, Alexander B, Bates PA. 2008. Blood meal identification and parasite detection in laboratory-fed and field-captured *Lutzomyia longipalpis* by PCR using FTA databasing paper. Acta Tropica, 107(3), 230–237.
69. Senne NA, Santos HA, Araujo TR, Paulino PG, Mendonca LP, Moreira HVS, Camilo TA, da Costa Angelo I. 2022. Robust comparative performance of genomic DNA extraction methods from non-engorged phlebotomine sandflies. Medical and Veterinary Entomology, 36(2), 203–211.
70. Shaw JJ. 2025. A review of Leishmania infections in American Phlebotomine sand flies – Are those that transmit leishmaniasis anthropophilic or anthropportunists? Parasite, 32, 57.
71. Tesh RB, Modi GB. 1983. Growth and transovarial transmission of Chandipura virus (Rhabdoviridae: Vesiculovirus) in *Phlebotomus papatasi.* American Journal of Tropical Medicine and Hygiene, 32(3), 621–623.
72. Thomsen PF, Elias S, Gilbert MTP, Haile J, Munch K, Kuzmina S, Froese DG, Sher A, Holdaway RN, Willerslev E. 2009. Non-destructive sampling of ancient insect DNA. PLoS One, 4(4),e5048
73. Truett GE, Heeger P, Mynatt RL, Truett AA, Walker JA, Warman ML. 2000. Preparation of PCR-quality mouse genomic DNA with hot sodium hydroxide and tris (HotSHOT). Biotechniques, 29(1), 52–54.
74. Upton MS. 1993. Aqueous gum-chloral slide mounting media: an historical review. Bulletin of Entomological Research, 83(2), 267–274.
75. Volf P, Myskova J. 2007. Sand flies and Leishmania: specific versus permissive vectors. Trends in Parasitology, 23(3), 91–92.
76. Wang Q, Wang X. 2012. Comparison of methods for DNA extraction from a single chironomid for PCR analysis. Pakistan Journal of Zoology, 44(2), 421–426.
77. Wang Y, Zhao Y, Bollas A, Wang Y, Au KF. 2021. Nanopore sequencing technology, bioinformatics and applications. Nature Biotechnology, 39(11), 1348–1365.

Cite this article as: Randrianambinintsoa FJ, Augendre L, Prudhomme J, Martinet J-P, Loyer M, Mekarnia N, Kerkoub H, Perveen FK, Huguenin A, Kariya E, Akhoundi M, De Andrade AJ, Berriatua E, Bongiorno G, Boyer S, Christodoulou V, Da Costa-Ribeiro MCV, De Souza LAF, Ding H, Dondji B, Dvořák V, Erisoz Kasap O, Galati EAB, Gállego M, Ballart C, Gouzelou S, Haddad N, Masse RS, Mekuria AH, Ivovic V, Kaczmarek S, Shahar MK, Kirstein OD, Kniha E, Kolářová I, Lincoln T, Lucanas C, Mikov O, Nov K, Özbel Y, Pesson B, Posada Lopez LC, Prasetyo DB, Rahola N, Rebollar-Tellez EA, Rodrigues BL, Roy L, Saini P, Sanjoba C, Shimabukuro PH, Siriyasatien P, Soszynska A, Suleşco T, Sylla M, Torno M, Volf P, Vongphayloth K, Sinh Nam V, Wardhana A, Yessinou E, Zapata S, Gantier J-C & Depaquit J. 2026. Processing and mounting phlebotomine sand flies: a consensus guideline. Parasite xx, xx. https://doi.org/10.1051/parasite/2026009.

PARASITE

An international open-access, peer-reviewed, online journal publishing high quality papers on all aspects of human and animal parasitology

Reviews, articles and short notes may be submitted. Fields include, but are not limited to: general, medical and veterinary parasitology; morphology, including ultrastructure; parasite systematics, including entomology, acarology, helminthology and protistology, and molecular analyses; molecular biology and biochemistry; immunology of parasitic diseases; host-parasite relationships; ecology and life history of parasites; epidemiology; therapeutics; new diagnostic tools.
All papers in Parasite are published in English. Manuscripts should have a broad interest and must not have been published or submitted elsewhere. No limit is imposed on the length of manuscripts.

Parasite (open-access) continues **Parasite** (print and online editions, 1994-2012) and **Annales de Parasitologie Humaine et Comparée** (1923-1993) and is the official journal of the Société Française de Parasitologie.

Editor-in-Chief:
Jean-Lou Justine, Paris

Submit your manuscript at:
https://www.editorialmanager.com/parasite

Appendix

เอกสารเพิ่มเติม 1: พื้นฐานทางทฤษฎีชีวเคมี

สัตว์ขาปล้อง (arthropods) ที่กล่าวถึงในที่นี้คือริ้นฝอยทราย อย่างไรก็ตาม แนวคิดทั่วไปสามารถประยุกต์ใช้กับสัตว์ขาปล้องชนิดอื่น ๆ ที่พบได้ทั่วไป ซึ่งการจำแนกสายพันธุ์อาศัยลักษณะทางสัณฐานวิทยาภายในเป็นหลัก ซึ่งอวัยวะภายในบางส่วนมีไคติน (chitin) เป็นองค์ประกอบ และสัณฐานวิทยาของโครงสร้างเหล่านี้ให้ข้อมูลที่มีคุณค่าอย่างยิ่ง ด้วยเหตุนี้การศึกษาส่วนที่เก็บอาหาร (food pumps) ถุงเก็บน้ำเชื้อ (spermathecae) และท่อของมันจึงมีความสำคัญเป็นพิเศษ สำหรับสารเคมีทั้งหมดที่จะกล่าวถึงในเอกสารนี้ สิ่งสำคัญที่ไม่ควรมองข้ามคือ ตั้งแต่ขั้นตอนการตรึงสภาพของแมลง (fixation) ไปจนถึงการประกอบตัวอย่างบนสไลด์ (assembly) ล้วนเป็นการประยุกต์ใช้ปฏิกิริยารีดอกซ์ (redox reactions) ทั้งสิ้น หลักการสำคัญที่ใช้เป็นแนวทางคือ การหลีกเลี่ยงการใช้สารรีดิวซ์ (reducing reagents) ร่วมกับสารออกซิไดซ์ (oxidizing reagents) เนื่องจากอาจก่อให้เกิดปฏิกิริยาที่ไม่พึงประสงค์และส่งผลเสียต่อโครงสร้างของตัวอย่าง

เอทิลแอลกอฮอล์หรือเอทานอล (Ethyl alcohol; ethanol)

เอทานอลถูกนำมาใช้ในหลายบริบทของกระบวนการเตรียมตัวอย่าง โมเลกุลของแอลกอฮอล์มีความชอบน้ำสูงจึงมีคุณสมบัติในการดึงน้ำออกจากเนื้อเยื่อ อย่างไรก็ตามเอทานอลที่มีความเข้มข้นต่ำ หรือมีน้ำเป็นองค์ประกอบสูง อาจส่งผลให้กรดนิวคลิอิกเสื่อมสภาพได้ เนื่องจากน้ำเป็นปัจจัยสำคัญที่ทำลายเสถียรภาพของกรดนิวคลิอิก การแช่แมลงในเอทานอลไม่ได้มีจุดประสงค์เพียงเพื่อการเก็บรักษาเท่านั้น แต่ยังทำหน้าที่เป็นสารตรึงสภาพ (fixative) ของเนื้อเยื่อด้วย ในทางพยาธิวิทยามีการแยกแนวคิดสำคัญออกเป็นสองประการ ได้แก่ อัตราการแทรกซึมของสาร (penetration rate) และอัตราการตรึงสภาพ (fixation rate) เป็นที่ยอมรับกันว่าสารตรึงสภาพที่มีประสิทธิภาพต้องสามารถแทรกซึมเข้าสู่เนื้อเยื่อได้อย่างรวดเร็วก่อนที่จะเกิดการตรึงสภาพอย่างสมบูรณ์ สำหรับเอทานอลความเข้มข้น 96% มีค่าสัมประสิทธิ์การแทรกซึมประมาณ 1.05 เมื่อเปรียบเทียบกับกรดพิกริก 0.75% ซึ่งมีค่า 0.45 และโพแทสเซียมไดโครเมต 3% ซึ่งมีค่า 1.45 การเก็บรักษาแมลงและสัตว์ขาปล้องอื่น ๆ ในเอทานอลเป็นระยะเวลานานเป็นแนวปฏิบัติที่พบได้ทั่วไปในทางกีฏวิทยา โดยเฉพาะอย่างยิ่งสำหรับตัวอย่างที่เก็บจากภาคสนามเพื่อใช้ในการศึกษาภายหลังหรือเพื่อการอ้างอิงในอนาคต อย่างไรก็ตาม แนวคิดดังกล่าวไม่เหมาะสมสำหรับงานด้านเซลล์วิทยาหรือพยาธิวิทยา เนื่องจากการเก็บตัวอย่างไว้ในสารตรึงสภาพเป็นเวลานานเกินไปอาจทำให้เนื้อเยื่อแข็งตัวอย่างถาวรและไม่สามารถนำมาปรับสภาพหรือเตรียมใหม่ได้ ตัวอย่างที่มีอายุเกินกว่า 10 ปีจึงมักยากหรือไม่สามารถนำมาใช้งานได้อีก อีกปัจจัยหนึ่งที่มีความสำคัญคือ อัตราส่วนระหว่างมวลของสัตว์ขาปล้องต่อปริมาตรของสารตรึงสภาพ ในทางสัตววิทยาและเวชปฏิบัติ แนะนำให้ใช้ปริมาตรของสารตรึงสภาพมากกว่าปริมาตรของชิ้นเนื้ออย่างน้อย 60 เท่า อย่างไรก็ตาม สำหรับสัตว์ขาปล้องขนาดเล็ก ในทางปฏิบัติควรเติมเอทานอลอย่างน้อย 4-5 เท่าของปริมาตรตัวอย่าง ทั้งนี้ควรคำนึงว่าเอทานอลจะสูญเสียความเข้มข้นลงเมื่อทำการดึงน้ำออกจากเนื้อเยื่อของสัตว์ขาปล้อง

สรุปคุณสมบัติของเอทิลแอลกอฮอล์

- เอทิลแอลกอฮอล์เป็นสารเคมีในกลุ่ม รีดิวซ์ และไม่ควรใช้ร่วมกับสารตรึงสภาพประเภทออกซิไดซ์
- ทำให้โปรตีนตกตะกอนและเสียสภาพ (protein denaturation) อย่างรุนแรง
- สามารถละลายลิปิดเชิงซ้อนบางชนิด และทำให้ไกลโคเจนตกตะกอน
- ทำให้เนื้อเยื่อเกิดการหดตัวอย่างชัดเจนและมีลักษณะแข็งตัว

สารละลายเบสโพแทสเซียมไฮดรอกไซด์ หรือโซเดียมไฮดรอกไซด์

การใช้สารละลายเหล่านี้ในงานกีฏวิทยาส่วนใหญ่มุ่งเน้นไปที่การใช้โพแทสเซียมไฮดรอกไซด์ (KOH) โดยไม่มีเหตุผลที่ชัดเจนรองรับ

โซเดียมไฮดรอกไซด์ [E524] โซเดียมไฮดรอกไซด์มีอยู่ในรูปของสารละลาย ทั้งในความเข้มข้นหรือค่าความปกติที่แตกต่างกัน รวมถึงในรูปเม็ด (pellets) หรือเกล็ด (glitter) ข้อเสียสำคัญคือมีคุณสมบัติดูดความชื้นสูงมาก (มากกว่า KOH) เมื่อทำปฏิกิริยากับโปรตีน จะทำให้โปรตีนละลาย และเมื่อทำปฏิกิริยากับลิปิด จะเปลี่ยนลิปิดเหล่านั้นให้กลายเป็นสบู่แข็งในระหว่างกระบวนการเปลี่ยนเป็นสบู่ (saponification) ซึ่งแตกต่างจาก KOH อย่างชัดเจน เนื่องจาก KOH ให้ผลิตภัณฑ์เป็นสบู่เหลวในระหว่างกระบวนการนี้

โพแทสเซียมไฮดรอกไซด์ [E525] โพแทสเซียมไฮดรอกไซด์มีจำหน่ายในรูปสารละลายเข้มข้น แต่เหนือสิ่งอื่นใด มีข้อได้เปรียบคือถูกผลิตในรูปเม็ดที่มีน้ำหนักประมาณ 0.1 กรัม ซึ่งช่วยอำนวยความสะดวกอย่างมากในการเตรียมสารละลายเจือจางในกรณีที่ไม่มีเครื่องชั่งที่มีความแม่นยำ ตัวอย่างเช่น เม็ด KOH น้ำหนัก 0.1 กรัม จำนวน 1 เม็ด ละลายในน้ำกลั่น 1 มิลลิลิตร จะได้สารละลายความเข้มข้น 10% ข้อได้เปรียบประการที่สองของ KOH ในรูปเม็ดคือมีความไวต่อการเกิดคาร์บอเนตต่ำกว่า (สารละลาย KOH มีความสามารถสูงในการจับกับคาร์บอนไดออกไซด์ ทำให้เกิดเกลือคาร์บอเนต)

เบสแก่เหล่านี้จะถูกใช้เพื่อทำให้กรดไขมันละลาย โดยเปลี่ยนกรดไขมันให้เป็นสบู่ที่ละลายน้ำได้ ควรระลึกไว้ว่าสารตรึงสภาพ เช่น เอทานอล ได้ละลายไขมันบางส่วนในตัวอย่างไปแล้ว อย่างไรก็ตาม เมื่อแทนที่ตัวอย่างในตัวกลางที่เป็นน้ำด้วยเบสแก่ กรดไขมัน (ซึ่งอาจมีความซับซ้อนมากหรือน้อย) จะตกตะกอน ดังนั้น เบสแก่จึงทำหน้าที่ในการเปลี่ยนเป็นสบู่แบบเย็น (cold saponification) ในบางกรณี เมื่อเนื้อเยื่อไขมันมีปริมาณมากเกินไป เช่น ในตัวอย่างเพศเมีย การเพิ่มอุณหภูมิเป็น 35–40°C จะช่วยอำนวยให้ปฏิกิริยาเกิดได้ง่ายขึ้น หรืออีกทางหนึ่งคือการยืดระยะเวลาการสัมผัสสารที่อุณหภูมิห้อง

สารละลายกรดแบบมีสี/ สารละลายมาร์ก-อองเดร (Marc-André solution) แบบไม่มีสี

ในส่วนนี้ จะกล่าวถึงข้อดีและข้อจำกัดของการใช้สารละลายมาร์ก-อองเดร (Marc-André solution) สารละลายนี้ประกอบด้วยคลอรัลไฮเดรต (trichloracetaldehyde monohydrate) กรดอะซิติก และน้ำ สารละลายดังกล่าวมีฤทธิ์ออกซิไดซ์สูงมาก (เป็นส่วนผสมระหว่างกรดและอัลดีไฮด์) สารละลายนี้จะทำหน้าที่สะเทินโพแทสเซียมไฮดรอกไซด์ส่วนเกินที่อาจหลงเหลืออยู่ในตัวอย่าง โดยไม่ทำให้สบู่ที่เป็นด่างซึ่งเกิดขึ้นระหว่างการใช้โพแทสเซียมไฮดรอกไซด์ตกตะกอน นอกจากนี้ สารละลายออกซิไดซ์นี้ยังออกฤทธิ์ต่อหมู่ฟังก์ชันแอลกอฮอล์ลำดับที่สองของกลูโคซามีนที่เป็นองค์ประกอบของไคติน ผ่านกระบวนการออกซิเดชัน ส่งผลให้ไคตินอ่อนนุ่มลง อีกทั้งยังช่วยละลายเกลือแร่บางชนิดที่มีอยู่

เมื่อสารละลาย Marc-André solution เติมสีล่วงหน้าด้วยกรดฟุคซิน (fuchsine acid) ซึ่งอยู่ในสภาวะออกซิไดซ์ สารละลายจะสามารถเข้าจับกับหมู่ฟังก์ชันแอลกอฮอล์ลำดับที่สองของโครงสร้างได้ หลังจากสิ้นสุดระยะเวลาการสัมผัสสารละลายมาร์ก-อองเดรและกระบวนการย้อมสีตัวอย่างแล้ว การล้างจะกระทำโดยใช้เอทานอลเท่านั้น ซึ่งถือเป็นจุดเริ่มต้นของขั้นตอนการดึงน้ำออก (dehydration) ของตัวอย่าง

ข้อดี (benefits)

- การสะเทินสารละลายเบสส่วนเกิน
- การทำให้โครงสร้างไคตินคลายตัว
- การย้อมสีไคตินเพื่อช่วยให้สามารถประเมินโครงสร้างภายในที่มีไคตินได้ชัดเจนยิ่งขึ้น

ข้อจำกัด (drawbacks)
คลอรัลไฮเดรตเป็นสารที่มีฤทธิ์เป็นยานอนหลับและเคยถูกใช้ในทางการแพทย์กับมนุษย์ การใช้งานจำเป็นต้องกระทำภายใต้ตู้ดูดควันสารเคมี และต้องปฏิบัติตามกฎหมายและข้อกำหนดด้านความเสี่ยงทางสารเคมีอย่างเคร่งครัด

สารละลายสำหรับการดึงน้ำออก (dehydration solutions)
จากประสบการณ์พบว่าสำหรับตัวอย่างที่มีขนาดเล็กมาก ไม่จำเป็นต้องใช้ลำดับของแอลกอฮอล์ที่มีความเข้มข้นเพิ่มขึ้นตามขั้นตอน หากตัวอย่างมีขนาดใหญ่ จะเริ่มต้นด้วยเอทานอล 80% จากนั้น 90%, 95% และสุดท้ายคือเอทานอลบริสุทธิ์ (absolute ethanol) สำหรับตัวอย่างที่มีขนาดเล็กมาก ให้ใช้เอทานอล 90% ตามด้วยการแช่ในเอทานอลบริสุทธิ์
ในขั้นตอนนี้ ควรระลึกไว้เสมอว่าเอทานอลบริสุทธิ์มีแนวโน้มในการดูดจับน้ำจากบรรยากาศ
ตามธรรมเนียมในห้องปฏิบัติการกีฏวิทยา การดึงน้ำออกจากตัวอย่างมักจะสิ้นสุดด้วยการแช่ใน beech creosote อย่างไรก็ตาม ปัจจุบันสารชนิดนี้ซึ่งเคยถูกใช้อย่างแพร่หลายเป็นสารกำจัดศัตรูพืช สารต้านเชื้อรา และสารกันเน่าไม้ ถูกไม่แนะนำอย่างยิ่ง เนื่องจากมีกลิ่นรุนแรง ซึ่งประกอบด้วยสารไฮโดรคาร์บอนอะโรมาติกหลายวง และถูกสันนิษฐานว่ามีความเป็นพิษต่อระบบสืบพันธุ์ ก่อมะเร็ง เป็นสารมลพิษอินทรีย์ที่คงทน และเป็นพิษต่อสิ่งมีชีวิตน้ำ
สารละลายที่เสนอให้ใช้ในการเตรียมตัวอย่างสำหรับการปิดสไลด์คือ Euparal® และ Euparal essence (อธิบายในย่อหน้าถัดไป) ส่วนผสมของ Euparal® และ Euparal essence สามารถใช้ได้อย่างเหมาะสม โดยเฉพาะสำหรับตัวอย่างที่ผ่านการแช่ในเอทานอล 90% มาแล้ว

เอกสารเพิ่มเติม 2: องค์ประกอบของสารละลายที่ใช้

Potassium hydroxide 10%
Potassium hydroxide 10 กรัม
Distilled water q.s. 100 มิลลิลิตร

Gum chloral mounting medium Hoyer medium
Distilled water 50 มิลลิลิตร
Chloral hydrate 200 กรัม
Gum arabic 50 กรัม
Glycerol 20 มิลลิลิตร

Marc-André solution
Chloral hydrate 40 กรัม
Glacial acetic acid 30 มิลลิลิตร
Distilled water 30 มิลลิลิตร

Fuchsin acid 1% in distilled water
Acid fuchsin powder 1 กรัม
Distilled water 99 มิลลิลิตร

Marc-André solution colored with fuchsin
Marc-André solution 10 มิลลิลิตร
Fuchsin 1% 50 ไมโครลิตร

เอกสารเพิ่มเติม 3: Euparal®, Canada balsam, polyvinyl alcohol หรือสารละลายอื่น ๆ สำหรับการปิดแผ่นสไลด์

โพลีไวนิลแอลกอฮอล์ (polyvinyl alcohol) โพลีไวนิลแอลกอฮอล์เป็นตัวกลางสำหรับปิดสไลด์ที่เหมาะสมในกรณีที่ไม่สามารถจัดหาสารเคมีที่จำเป็นสำหรับการดึงน้ำออกอย่างเหมาะสมได้ โดยจะนำโพลีไวนิลแอลกอฮอล์มาผสมกับ Amman’s lactophenol อย่างไรก็ตามการเตรียมสไลด์ด้วยวิธีนี้มีข้อจำกัดสำคัญคือ ตัวอย่างอาจแห้ง หรือโพลีไวนิลแอลกอฮอล์อาจตกผลึกเนื่องจากการระเหยของน้ำ หรือเกิดการคล้ำดำเมื่อฟีนอลเกิดการออกซิไดซ์ วิธีนี้จึงยังคงเหมาะสมสำหรับการปิดสไลด์ในระยะสั้นเท่านั้น
แคนาดาบัลซัม (Canada balsam) การใช้แคนาดาบัลซัมสำหรับการปิดตัวอย่างระหว่างแผ่นสไลด์และกระจกปิดสไลด์ จำเป็นต้องผ่านกระบวนการดึงน้ำออกจากตัวอย่างก่อน การใช้ไซลีนหรือโทลูอีนในขั้นตอนดังกล่าวมีข้อจำกัดและความไม่สะดวกบางประการ
Enecê medium เช่นเดียวกับแคนาดาบัลซัม การใช้ตัวกลาง Enecê สำหรับการปิดตัวอย่างระหว่างแผ่นสไลด์และกระจกปิดสไลด์ จำเป็นต้องผ่านกระบวนการดึงน้ำออกจากตัวอย่าง องค์ประกอบของ Enecê ประกอบด้วย โคโลโฟนีสีขาวบริสุทธิ์ (colophony) จำนวน 22 กรัม กัมโคพอลที่ละลายในแอลกอฮอล์ (copal gum) จำนวน 12 กรัม เอทานอลบริสุทธิ์ จำนวน 20 มิลลิลิตร การบูร จำนวน 10 กรัม น้ำมันสน จำนวน 10 มิลลิลิตร และยูคาลิปทอล จำนวน 26 มิลลิลิตร
วิธีเตรียม: ใส่เอทานอลบริสุทธิ์และการบูรลงในภาชนะ เช่น ขวดเออร์เลนเมเยอร์ จากนั้นเติมโคโลโฟนีและกัมโคพอล ปิดภาชนะด้วยจุกและเขย่า แล้วนำไปให้ความร้อนในอ่างน้ำ (bain-marie) ที่อุณหภูมิไม่สูงจนส่วนผสมเดือด เมื่อส่วนผสมละลายเป็นเนื้อเดียวกันแล้ว ให้เติมน้ำมันสน จากนั้นกรองในขณะที่ส่วนผสมยังร้อนอยู่ และสุดท้ายเติมยูคาลิปทอลลงในสารกรอง เมื่อเนื้อสารมีความหนืดมากขึ้น ให้เจือจางด้วยสาร Eenecê ซึ่งมีองค์ประกอบดังนี้: เอทานอลบริสุทธิ์ จำนวน 30 มิลลิลิตร การบูร จำนวน 17 กรัม น้ำมันสน จำนวน 15 มิลลิลิตร และยูคาลิปทอล จำนวน 38 มิลลิลิตร (Cerqueira, 1943)
ยูพาราล® (Euparal®) ยูพาราลเป็นเรซินที่ได้จากต้นไซเปรสแห่งเทือกเขาแอตลาส *Tetraclinis articulata* (Vahl, 1791) และได้รับการศึกษาและพัฒนาในปี ค.ศ. 1906 โดย Gilson ข้อได้เปรียบหลักของเรซินชนิดนี้คือไม่เกิดการพอลิเมอไรเซชัน ตัวอย่างที่ปิดระหว่างแผ่นสไลด์และกระจกปิดสไลด์สามารถนำกลับออกมาได้ง่ายโดยการใช้แอลกอฮอล์ หรือดียิ่งขึ้นโดยใช้ Euparal® essence เรซิน

ชนิดนี้ซึ่งเรียกอีกชื่อหนึ่งว่า sandarac สามารถใช้กับเอทานอลที่มีความเข้มข้นตั้งแต่ 80% ขึ้นไป

การใช้ Triton X-100 (สารละลายน้ำชนิดไม่มีประจุ)

Triton X-100 อยู่ในรูปของสารละลายน้ำชนิดไม่มีประจุ (4-(1,1,3,3-tetramethylbutyl) phenyl-polyethylene glycol solution หรือ t-octylphenoxypolyethoxyethanol, polyethylene glycol tert-octylphenyl ether) ซึ่งถูกใช้อย่างแพร่หลายในฐานะสารทำความสะอาดในงานชีววิทยาระดับเซลล์และโมเลกุล โดยมีคุณสมบัติช่วยเพิ่มการซึมผ่านของเยื่อหุ้มเซลล์และเยื่อหุ้มนิวเคลียส ตัวอย่างแมลงที่ถูกเก็บรักษาไว้ในแอลกอฮอล์เป็นเวลาหลายปีพบได้ทั่วไป อย่างไรก็ตาม การเก็บรักษาในแอลกอฮอล์ไม่ใช่วิธีที่เหมาะสมที่สุด และสัตว์ขาปล้องที่ถูกเก็บรักษาในลักษณะนี้มักจะเตรียมตัวอย่างเพื่อการตรวจด้วยกล้องจุลทรรศน์ได้ยาก ภาชนะพลาสติกที่บรรจุตัวอย่างมักเสื่อมสภาพตามเวลา ส่งผลให้แอลกอฮอล์ระเหยออกไป ทั้งการสัมผัสแอลกอฮอล์เป็นเวลานานและการแห้งของตัวอย่างล้วนเป็นปัญหาสำคัญ ในปี ค.ศ. 2008 Jonque ได้รายงานวิธีการคืนสภาพความชุ่มชื้นให้กับแมงมุมโดยใช้สารช่วยลดแรงตึงผิว เช่น Agepon ซึ่งใช้ในอุตสาหกรรมฟิล์มถ่ายภาพ [26] แนวคิดนี้นำไปสู่การประยุกต์ใช้สารช่วยลดแรงตึงผิวที่ไม่ใช่สารทำความสะอาดที่มีฤทธิ์รุนแรง

ขั้นตอนการใช้ Triton X-100 ในสารละลายน้ำความเข้มข้น 0.5%

- ชุบตัวอย่างแห้งด้วยเอทานอลบริสุทธิ์
- เติมสารละลาย Triton X-100 ความเข้มข้น 0.5% ในปริมาตรที่เพียงพอให้ตัวอย่างจมอยู่ทั้งหมด
- ปล่อยทิ้งไว้ประมาณ 5 นาทีหรือมากกว่า จนกระทั่งสัตว์ขาปล้องทั้งหมดแยกตัวออกจากกันในสารละลาย
- เทสารละลาย Triton X-100 ออก และแทนที่ด้วยสารละลายโพแทสเซียมไฮดรอกไซด์ (KOH)

จากนั้นดำเนินการตามขั้นตอนที่ได้อธิบายไว้ก่อนหน้านี้

เอกสารเพิ่มเติม 4: ขั้นตอนการใช้ Euparal® หรือ Canada balsam เป็นตัวกลางสำหรับการปิดสไลด์ทีละขั้นตอน

1. ตัวอย่างต้องผ่านกระบวนการดึงน้ำออกอย่างสมบูรณ์ (ลักษณะขุ่นหรือคล้ายน้ำนมบ่งชี้ว่าการดึงน้ำออกไม่เพียงพอ)
2. การดึงน้ำออกสามารถทำได้ใช้เอทิลแอลกอฮอล์ที่มีความเข้มข้นเพิ่มขึ้นตามลำดับ
3. ตัวอย่างสามารถย้ายจากเอทานอล 99% หรือเอทานอลบริสุทธิ์ไปยังสารทำให้ใส

ขั้นตอนปฏิบัติ (Procedure)

1. การตกตะกอนของแมลงวันทรายตัวเต็มวัยในเอทานอล 70%
2. เอาเอทานอลออกและแทนที่ด้วยสารละลายโพแทสเซียมไฮดรอกไซด์ (KOH) ความเข้มข้น 10% จากนั้นปิดตัวอย่างริ้นฝอยทรายด้วยแผ่นสไลด์แก้ว
3. แช่ให้เกิดการเปื่อยยุ่ย จนกระทั่งตัวแมลงมีลักษณะโปร่งใส
4. เอาสารละลาย KOH ออก
5. ปิดคลุมตัวอย่างด้วยน้ำกลั่นและรอเป็นเวลา 30-45 นาที
6. เอาน้ำออก และล้างซ้ำด้วยน้ำกลั่นเป็นเวลา 30 นาที (ระยะเวลาขึ้นอยู่กับจำนวนตัวอย่างที่ทำพร้อมกัน ยิ่งมีตัวอย่างจำนวนมาก ระยะเวลานี้ต้องยืดออก หากมีตัวอย่างน้อย โดยเฉพาะกรณีทำทีละตัว ระยะเวลานี้สามารถสั้นลงได้)
7. เอาน้ำออก
8. เติมสารละลายมาร์ก-อองเดร (Marc-André solution) โดยอาจเติมกรดฟุคซิน (fuchsin acid) เพื่อให้มีสี และทิ้งไว้เป็นเวลา 24 ชั่วโมง หรือ 1 วัน
9. เอาสารละลายมาร์ก-อองเดร (Marc-André solution) ออก
10. ปิดคลุมตัวอย่างด้วยน้ำกลั่นและรอเป็นเวลา 30-45 นาที
11. เอาน้ำออก และล้างซ้ำด้วยน้ำกลั่นเป็นเวลา 30 นาที
12. เอาน้ำออก
13. เติมเอทานอล 70% และผ่าแยกตัวอย่าง
    a. สำหรับส่วนหัวและท้อง ให้ดึงส่วนหัวหรือท้องออกจากอกอย่างระมัดระวัง
    b. สำหรับส่วนอก ให้ดึงปีกออกโดยใช้ปากคีบคู่หนึ่งจับส่วนอกไว้ และใช้อีกคู่หนึ่งดึงที่โคนของอวัยวะยื่นออกมา นอกจากนี้ สามารถผ่าในแนวซาจิททัล (sagittal dissection) โดยแบ่งส่วนอกออกเป็นด้านซ้ายและขวาได้ ขึ้นอยู่กับบริเวณที่ต้องการศึกษามากที่สุด
14. ดึงน้ำออกจากตัวอย่างอย่างค่อยเป็นค่อยไป โดยผ่านชุดสารละลายเอทิลแอลกอฮอล์ที่เป็นน้ำในความเข้มข้น 50 - 80 - 95% จนถึงเอทานอลบริสุทธิ์
15. ดึงน้ำออกจากตัวอย่างโดยล้างด้วยเอทานอล 100% จำนวน 2 ครั้ง ครั้งละ 10 นาที
16. เอาเอทานอลออกและปิดตัวอย่างด้วยน้ำมันกานพลู (clove oil) เป็นเวลา 15 นาที ที่อุณหภูมิห้อง
17. ย้ายตัวอย่างจากน้ำมันกานพลูไปยังหยดของ Euparal® หรือ Canada balsam บนแผ่นสไลด์ที่สะอาด
18. จัดวางตัวอย่างตามต้องการ ส่วนหัว อก และท้องของริ้นฝอยทรายสามารถผ่าแยกได้โดยใช้เข็มขนาดเล็กหรือปากคีบ ภายใต้กล้องสเตอริโอไมโครสโคป ส่วนหัวต้องถูกแยกออกจากลำตัวเพื่อจัดวางในแนวด้านท้อง-ด้านหลัง (ventro-dorsal position) กล่าวคือ ช่องเปิดท้ายทอย (occipital foramen) ต้องหันขึ้นด้านบน เพื่อให้สามารถสังเกตไซบาเรียม (cibarium) ได้โดยตรงผ่านช่องเปิดนี้

การผ่าแยกดำเนินการภายในตัวกลางสำหรับปิดสไลด์ของริ้นฝอยทรา

19. ปล่อยตัวอย่างไว้จนกระทั่งพื้นผิวของตัวกลางเริ่มมีลักษณะเหนียว
20. ชุบกระจกปิดสไลด์ที่สะอาดด้วยเอทานอลบริสุทธิ์ และวางกระจกปิดสไลด์ลงบน Canada balsam โดยเอียงเล็กน้อย
21. เก็บสไลด์ไว้ในกล่องแห้งที่จัดเตรียมไว้สำหรับวัตถุประสงค์นี้
