# Supplementary material for: Processing and mounting phlebotomine sand flies: a consensus guideline
Source: Parasite. 2026 Apr 3;33:18. doi: 10.1051/parasite/2026009 (PMC13047900; doi:10.1051/parasite/2026009)
Supplement: Supplementary file 30 — Tok Pisin translation of the title, summary, and an appendix / Het tok, samari na wanpela appendix long Tok Pisin [file parasite-33-18-s30.pdf]

Parasite **33**, 18 (2026)
© F.J. Randrianambinintsoa et al., published by EDP Sciences, 2026
https://doi.org/10.1051/parasite/2026009

PARASITE
Available online at:
www.parasite-journal.org

REVIEW ARTICLE OPEN ACCESS

Pepa soim ol wei long processim na hau long putim sand fly antap long wanpela glas long behian taim long mekim wok panimaut

Fano José Randrianambinintsoa[1], Laure Augendre[1], Jorian Prudhomme[1], Jean-Philippe Martinet[1], Mathieu Loyer[1], Nalia Mekarnia[1], Hocine Kerkoub[1], Farzana Khan Perveen[1], Antoine Huguenin[1,2], Emilie Kariya[1,2], Mohammad Akhoundi[3], Andrey José de Andrade[4], Eduardo Berriatua[5], Gioia Bongiorno[6], Sébastien Boyer[7,8], Vasiliki Christodoulou[9], Magda Clara Vieira Da Costa-Ribeiro[10], Lucas Alexandre Farias de Souza[10], Huicong Ding[11], Blaise Dondji[12], Vít Dvořák[13], Ozge Erisoz Kasap[14], Eunice Aparecida Bianchi Galati[15], Montserrat Gállego[16], Cristina Ballart[16], Stavroula Gouzelou[17], Nabil Haddad[18], Rezki Sabrina Masse[19], Asrat Hailu Mekuria[20], Vladimir Ivovic[21], Szymon Kaczmarek[22], Mohd Khadri Shahar[19], Oscar D. Kirstein[23], Edwin Kniha[24], Iva Kolářová[13], Lincoln Timinao[25], Cristian Lucanas[26], Ognyan Mikov[27], Kimsear Nov[7], Yusuf Özbel[28], Bernard Pesson[29], Laura Cristina Posada Lopez[30], Didot Budi Prasetyo[1,7], Nil Rahola[31], Eduardo A. Rebollar-Tellez[32], Bruno Leite Rodrigues[15], Lalita Roy[33], Prasanta Saini[34], Chizu Sanjoba[35], Paloma Helena Fernandes Shimabukuro[36], Padet Siriyasatien[37], Agnieszka Soszyńska[22], Tatiana Suleşco[38], Massamba Sylla[39], Majhalia Torno[40], Petr Volf[13], Khamsing Vongphayloth[41], Vu Sinh Nam[42], April Wardhana[43], Eric Yessinou[44], Sonia Zapata[45], Jean-Charles Gantier[1], and Jérôme Depaquit[1,2,*]

[1] Faculté de Pharmacie, Université de Reims Champagne Ardenne, UR ESCAPE-USC ANSES PETARD, 51 rue Cognacq-Jay, 51096 Reims Cedex, France
[2] Pôle de Biologie territoriale, Laboratoire de Parasitologie-Mycologie, Centre Hospitalo-Universitaire, 51092 Reims, France
[3] Parasitology-Mycology Department, Avicenne Hospital, AP-HP, Bobigny, Sorbonne Paris Nord University, France; Unité des Virus Émergents (UVE: Aix-Marseille Univ, Università di Corsica, IRD 190, Inserm 1207, IRBA), 13005 Marseille, France
[4] Parasitology Collection of Basic Pathology, Department of Basic Pathology, Federal University of Paraná, Curitiba 19031, Brazil
[5] Department of Animal Health, University of Murcia, Campus de Espinardo, 30100 Espinardo, Murcia, Spain
[6] Department of Infectious Diseases, Vector-borne Diseases Unit, Istituto Superiore di Sanità, 00166 Rome, Italy
[7] Medical and Veterinary Entomology Unit, Institut Pasteur du Cambodge, Phnom Penh 12201, Cambodia
[8] Ecology & Emergence of Arthropod-borne Pathogens Unit, Department of Global Health, Institut Pasteur, CNRS UMR2000, 75015 Paris, France
[9] Section Veterinary Services (1417), Laboratory for Animal Health Virology, Aglantzia, Nicosia 2109, Cyprus
[10] Insects Vectors and Parasites Laboratory, Department of Basic Pathology and Postgraduate program in Microbiology, Parasitology and Pathology, Federal University of Paraná, 81530-900 Curitiba, Brazil
[11] Department of Biological Sciences, National University of Singapore, 117558, Singapore
[12] Laboratory of the Leishmaniasis Research Project, Mokolo District Hospital, Mokolo, Cameroon; Laboratory of Cellular Immunology and Parasitology, Department of Biological Sciences, Central Washington University, 98926 Ellensburg, WA, USA
[13] Department of Parasitology, Faculty of Science, Charles University, 12800 Prague, Czechia
[14] VERG Laboratories, Department of Biology, Faculty of Science, Hacettepe University, Beytepe, Ankara 06800, Türkiye
[15] Faculdade de Saúde Pública da Universidade de São Paulo (FSP/USP), Pós-graduação em Saúde Pública, 01246-904 São Paulo, Brazil
[16] Secció de Parasitologia, Departament de Biologia, Sanitat i Medi Ambient, Facultat de Farmàcia i Ciències de l'Alimentació, Universitat de Barcelona, & Institut de Salut Global de Barcelona (ISGlobal), Centro de Investigación Biomédica en Red, Enfermedades Infecciosas (CIBERINFEC), 08028 Barcelona, Spain
[17] Laboratory of Infectious Diseases and Public Health, School of Medicine, University of Cyprus, Nicosia, Cyprus & Department of Pediatrics, Archbishop Makarios III Hospital, Nicosia 2115, Cyprus
[18] Faculty of Health Sciences, American University of Beirut, 1107 2020 Beirut, Lebanon
[19] Medical Entomology Unit, Infectious Disease Research Centre, Institute for Medical Research (IMR), National Institutes of Health (NIH), Ministry of Health Malaysia, 40170 Shah Alam, Selangor, Malaysia

Edited by Jean-Lou Justine
*Corresponding author: jerome.depaquit@univ-reims.fr

This is an Open Access article distributed under the terms of the Creative Commons Attribution License (https://creativecommons.org/licenses/by/4.0), which permits unrestricted use, distribution, and reproduction in any medium, provided the original work is properly cited.

[20] School of Medicine, Addis Ababa University, 28017 - 1000 Addis Ababa, Ethiopia
[21] Faculty of Mathematics, Natural Sciences and Information Technologies, University of Primorska, 6000 Koper, Slovenia
[22] University of Lodz, Faculty of Biology and Environmental Protection, Department of Invertebrate Zoology and Hydrobiology, Banacha 12/16, 90-237 Łódź, Poland
[23] Laboratory of Entomology, Ministry of Health, 9134302 Jerusalem, Israel
[24] Center for Pathophysiology, Infectiology and Immunology, Institute of Specific Prophylaxis and Tropical Medicine, Medical University Vienna, Kinderspitalgasse 15, 1090 Vienna, Austria
[25] Papua New Guinea Institute of Medical Research (PNGIMR) Institute, PO Box 60, Headquarter, Homate Street, 441 Goroka, Eastern Highlands Province, Papua New Guinea
[26] Museum of Natural History, University of the Philippines Los Baños, 4031 Laguna, Philippines
[27] National Centre of Infectious and Parasitic Diseases, 1504 Sofia, Bulgaria
[28] Ege University, Faculty of Medicine, Department of Parasitology, 35040 Bornova/Izmir, Türkiye
[29] Retired, Faculté de Pharmacie, Université de Strasbourg, Strasbourg, 67400 Illkirch-Graffenstaden, France
[30] Program for the Study and Control of Tropical Diseases (PECET), Faculty of Medicine, University of Antioquia, 050010 Medellin, Colombia
[31] MIVEGEC, Univ. Montpellier, CNRS, IRD, 34394 Montpellier, France & Medical Entomology Unit, Institut Pasteur de Madagascar, 101 Antananarivo, Madagascar
[32] Laboratorio de Entomología Médica, Departamento de Zoología de Invertebrados, Facultad de Ciencias Biológicas, Universidad Autónoma de Nuevo León, San Nicolás de los Garza, 66455, NL, México
[33] Tropical and Infectious Disease Centre, BP Koirala Institute of Health Sciences, Dharan 56700, Nepal
[34] ICMR-Vector Control Research Centre, Puducherry 605006, India
[35] Graduate School of Agricultural and Life Sciences, The University of Tokyo, Tokyo 113-8657, Japan
[36] Grupo de estudos em Leishmanioses/Coleção de Flebotomíneos (COLFLEB/Fiocruz-MG), Instituto René Rachou, Fundação Oswaldo Cruz, Belo Horizonte, Minas Gerais, 30190009, Brazil
[37] Center of Excellence in Vector Biology and Vector-Borne Disease, Department of Parasitology, Faculty of Medicine, Chulalongkorn University, Bangkok 10330, Thailand
[38] Department of Arbovirology, Bernhard Nocht Institute for Tropical Medicine, Bernhard Nocht Str. 74, 20359 Hamburg, Germany [39] Laboratory Vectors & Parasites, Department of Livestock Sciences and Techniques, Sine Saloum University El Hadji Ibrahima Niasse (SSUEIN) Kaffrine Campus, C.P. 24600, Senegal.
[40] Environmental Health Institute, National Environment Agency, Singapore 138667, Singapore & Department of Biological Sciences, National University of Singapore, 117558 Singapore
[41] Institut Pasteur du Laos, Laboratory of Vector-Borne Diseases, Samsenhai Road, Ban Kao-Gnot, Sisattanak District, 3560 Vientiane, Lao PDR
[42] National Institute of Hygiene and Epidemiology, 1 Yec-Xanh Street, Hai Ba Trung District, 100000 Hanoi, Vietnam
[43] Indonesian Research Center for Veterinary Science, Indonesian Agency for Agricultural Research and Development, Ministry of Agriculture Republic Indonesia, Bogor 16114, Indonesia & Department of Parasitology, Faculty of Veterinary Medicine, Airlangga University, Surabaya 60115, Indonesia
[44] Laboratory of Research in Applied Biology, Polytechnic School of Abomey-Calavi, University of Abomey-Calavi, 01 P.O. Box 2009, 00000 Cotonou, Benin
[45] Instituto de Microbiología, Colegio de Ciencias Biológicas y Ambientales (COCIBA), Universidad San Francisco de Quito (USFQ), 170901 Quito, Ecuador

Received 1 December 2025, Accepted 29 January 2026, Published online 3 April 2026

Samari – Dispela pepa i givim somepela tintin long hau mipela processim ol sand fly long luksave long wanem kain sand fly i stap long area bilong yu na to long luksave long ol binatang nogut wei i stap insait long ol sand fly wei ol i ken kamapim birowa long ol man-meri. Dispela pepa bai tok aut long ol way wer yu can wok instait long wanpla lab or sapos yu la ketchim ol sand fly outsait. Dispela pepa bai givim sampela tintin long hau bai yu ken ketchim ol sand fly, hau bai yu putim ol i stap, hau bai yu karamapi na hau yu bai kilim ol wantaim ol kemikol. Na to hausat bai yu putim ol insait long ol ice bokis or wanpela kemikol ol i kolim ethanol. Mipela laikim yu long ketchim na putim dispela ol sand fly gut so dat ol bodi pats bilong dispela sand fly ino buruk nambout na bai yu ken luksave long em taim yu lukluk long wanpela mikroskop. Dispela pepa em i tokaut long hausat ol sand fly wei yu i ketchim em yu processim wantaim ol keikol ol i kolim potassium hydroxide na Marc-Andre solution. Bai mipela i luksave long hausat ol sand fly i kamap gut taim mipela i mixim wantaim ol kainkain kemikol. Wanpela kemikol ol i kolim Hoyer fluid em ol lain sa usim long luksave long wanpla bodi pat bilong sand fly ol i kolim spermathecae tasol em ino gutpla tumas long holim gut dispela ol sand fly long longpela taim. Tupela narapela kemikol ol i kolim polyvinyl alcohol o Euparal na Canada balsam em i orait moa long holim gut ol sand fly long longpela taim. Dispela pepa em i toktok long hausat bai mipela wok wantaim DNA bilong ol sand flies long kamapim ol tintin long hau bai ol sand fly wei yu i kolectim bai

yu stretim. Na tu wanpela sotpla piksa wei i soim hau long putim ol sand fly insait long ol kemikol bai mipela i givim wantaim olgeta infomesen wei mipela i tanim tok i go long 33 pela tokples so dat planti lain ken luksave long dispela wokpanim aut..

Ol ki wod: Putim ol sand fly antap long glas, sand fly, Hoyer fluid, Marc-André solution, chloral gum, polyvinyl alcohol, Euparal®, Canada balsam, Leishmania isolation, wok painimaut long komunititi, lukautim binatang, katim ol sand fly, lukluk long ol DNA bilong ol sand fly, MALDI-ToF, Type-specimens.

Abstract – Processing and mounting phlebotomine sand flies: a consensus guideline. This article provides a comprehensive guide for the processing and mounting of phlebotomine sand fly specimens, which is crucial for species identification and pathogen detection and isolation. It discusses a range of techniques suitable for both field and laboratory settings. The guide includes detailed instructions on sand fly collection, handling, covering, and euthanasia (recommending dry freezing or CO_2 over chemicals) as well as conservation strategies, such as cold storage and preservation in ethanol. The quality of preparation of certain anatomical structures (genital organs, head and wings) is essential for their proper microscopic observation and is described in this work. The article also presents detailed sample processing, including the clearing process with agents such as potassium hydroxide then Marc-André solution. The mounting process compares different media, emphasizing their optical properties and preservation potential. Hoyer fluid (also known as chloral gum) is recommended for quick observation, particularly for spermathecae, due to its clarity, although it is not suitable for long-term storage. Other media discussed include polyvinyl alcohol, Euparal® (for limited water tolerance), and Canada balsam (a hydrocarbon-soluble medium), with the latter two offering long-term preservation capabilities. Innovative molecular biology approaches such as DNA sequencing and MALDI-ToF, which require particular attention to sample processing, are also addressed. Furthermore, short video clips illustrating various mounting techniques as well as translations in many different languages are provided, allowing the guideline to reach the diverse needs and expectations of the global scientific community.

Key words: Mounting, Phlebotomine sand fly, Hoyer fluid, Marc-André solution, Chloral gum, Polyvinyl alcohol, Euparal®, Canada balsam, *Leishmania* isolation, Field conditions, Culture, Dissection, Molecular biology, MALDI-ToF, Type-specimens.

Appendix 4: Euparal® or Canada Balsam mounting media step by step

1. Spesimen mesti dinyahhidratkan (rupa keruh atau berwarna susu menunjukkan penyahhidratan yang tidak mencukupi).
2. Penyahhidratan boleh dicapai melalui peningkatan kepekatan alkohol etil secara berperingkat.
3. Spesimen boleh dipindahkan daripada alkohol 99% atau alkohol mutlak kepada agen penjernih.

Prosedur:

1. Penempatan lalat pasir dewasa dalam etanol 70%.
2. Singkirkan etanol dan gantikan dengan larutan KOH 10%. Tutup lalat pasir dengan slaid kaca.
3. Maserat sehingga serangga menjadi lutsinar.
4. Singkirkan larutan KOH.
5. Rendamkan spesimen dengan air suling dan biarkan selama 30–45 minit.
6. Singkirkan air dan ulangi basuhan dengan air suling setelah 30 minit (Tempoh ini bergantung kepada bilangan spesimen: semakin banyak spesimen diproses serentak, semakin lama masa diperlukan; semakin sedikit, terutamanya jika diproses secara individu, masa boleh dipendekkan).
7. Singkirkan air.
8. Tambah larutan Marc-André (berpotensi untuk diwarnakan dengan asid fuksin) dan biarkan selama 24 jam (1 hari).
9. Singkirkan larutan Marc-André.
10. Rendamkan spesimen dengan air suling dan biarkan selama 30–45 minit.
11. Singkirkan air dan ulangi basuhan dengan air suling selama 30 minit.
12. Singkirkan air.
13. Tambah etanol 70% dan lakukan pembedahan spesimen.
    a. Bagi bahagian kepala dan abdomen, tarik perlahan bagi memisahkan kepala atau abdomen daripada toraks.
    b. Bagi bahagian toraks, tanggalkan sayap dengan memegang toraks menggunakan sepasang forsep dan menarik pada pangkal apendaj dengan sepasang forsep yang lain. Pembedahan sagital boleh dilakukan dengan membelah toraks kepada bahagian kiri dan kanan, bergantung pada kawasan yang menjadi fokus pemerhatian.
14. Spesimen dinyahhidratkan secara berperingkat melalui siri larutan alcohol etil berakua: bermula pada kepekatan 50% → 80% → 95% sehingga etanol mutlak.
15. Spesimen dinyahhidratkan melalui dua kali pembasuhan, masing-masing selama 10 minit, menggunakan etanol 100%.
16. Singkirkan etanol dan rendam spesimen dengan minyak

cengkih selama 15 minit pada suhu bilik.
17. Pindahkan spesimen daripada minyak cengkih ke dalam titisan Euparal® atau balsam Kanada pada slaid kaca yang baru.
18. Susun mengikut keperluan: Kepala, toraks dan abdomen lalat pasir boleh dibedah menggunakan jarum halus atau forsep di bawah mikroskop stereo. Kepala mesti dipisahkan daripada badan untuk dilekap dalam kedudukan ventro-dorsal, iaitu foramen oksipital mesti menghala ke atas supaya sibarium dapat dilihat secara jelas.
Pembedahan dijalankan dalam medium pelekapan lalat pasir.
19. Biarkan spesimen sehingga permukaan menjadi melekit.
20. Basahkan kaca penutup yang bersih dengan alkohol mutlak. Letakkan kaca penutup ke atas balsam Kanada secara menyerong.
21. Simpan slaid dalam kotak kering yang telah dikhaskan untuk penyimpanan
