# Supplementary material for: Processing and mounting phlebotomine sand flies: a consensus guideline
Source: Parasite. 2026 Apr 3;33:18. doi: 10.1051/parasite/2026009 (PMC13047900; doi:10.1051/parasite/2026009)
Supplement: Supplementary file 31 — Turkish translation / Türkçe çeviri [file parasite-33-18-s31.pdf]

Parasite **33**, 18 (2026)
© F.J. Randrianambinintsoa et al., published by EDP Sciences, 2026
https://doi.org/10.1051/parasite/2026009

PARASITE
Available online at:
www.parasite-journal.org

REVIEW ARTICLE OPEN ACCESS

Phlebotomine kum sineklerinin işlenmesi ve preparasyonu: Konsensüs kılavuzu

Fano José Randrianambinintsoa[1], Laure Augendre[1], Jorian Prudhomme[1], Jean-Philippe Martinet[1], Mathieu Loyer[1], Nalia Mekarnia[1], Hocine Kerkoub[1], Farzana Khan Perveen[1], Antoine Huguenin[1,2], Emilie Kariya[1,2], Mohammad Akhoundi[3], Andrey José de Andrade[4], Eduardo Berriatua[5], Gioia Bongiorno[6], Sébastien Boyer[7,8], Vasiliki Christodoulou[9], Magda Clara Vieira Da Costa-Ribeiro[10], Lucas Alexandre Farias de Souza[10], Huicong Ding[11], Blaise Dondji[12], Vít Dvořák[13], Ozge Erisoz Kasap[14], Eunice Aparecida Bianchi Galati[15], Montserrat Gállego[16], Cristina Ballart[16], Stavroula Gouzelou[17], Nabil Haddad[18], Rezki Sabrina Masse[19], Asrat Hailu Mekuria[20], Vladimir Ivovic[21], Szymon Kaczmarek[22], Mohd Khadri Shahar[19], Oscar D. Kirstein[23], Edwin Kniha[24], Iva Kolářová[13], Lincoln Timinao[25], Cristian Lucanas[26], Ognyan Mikov[27], Kimsear Nov[7], Yusuf Özbel[28], Bernard Pesson[29], Laura Cristina Posada Lopez[30], Didot Budi Prasetyo[1,7], Nil Rahola[31], Eduardo A. Rebollar-Tellez[32], Bruno Leite Rodrigues[15], Lalita Roy[33], Prasanta Saini[34], Chizu Sanjoba[35], Paloma Helena Fernandes Shimabukuro[36], Padet Siriyasatien[37], Agnieszka Soszyńska[22], Tatiana Suleşco[38], Massamba Sylla[39], Majhalia Torno[40], Petr Volf[13], Khamsing Vongphayloth[41], Vu Sinh Nam[42], April Wardhana[43], Eric Yessinou[44], Sonia Zapata[45], Jean-Charles Gantier[1], and Jérôme Depaquit[1,2,*]

[1] Faculté de Pharmacie, Université de Reims Champagne Ardenne, UR ESCAPE-USC ANSES PETARD, 51 rue Cognacq-Jay, 51096 Reims Cedex, France
[2] Pôle de Biologie territoriale, Laboratoire de Parasitologie-Mycologie, Centre Hospitalo-Universitaire, 51092 Reims, France
[3] Parasitology-Mycology Department, Avicenne Hospital, AP-HP, Bobigny, Sorbonne Paris Nord University, France; Unité des Virus Émergents (UVE: Aix-Marseille Univ, Università di Corsica, IRD 190, Inserm 1207, IRBA), 13005 Marseille, France
[4] Parasitology Collection of Basic Pathology, Department of Basic Pathology, Federal University of Paraná, Curitiba 19031, Brazil
[5] Department of Animal Health, University of Murcia, Campus de Espinardo, 30100 Espinardo, Murcia, Spain
[6] Department of Infectious Diseases, Vector-borne Diseases Unit, Istituto Superiore di Sanità, 00166 Rome, Italy
[7] Medical and Veterinary Entomology Unit, Institut Pasteur du Cambodge, Phnom Penh 12201, Cambodia
[8] Ecology & Emergence of Arthropod-borne Pathogens Unit, Department of Global Health, Institut Pasteur, CNRS UMR2000, 75015 Paris, France
[9] Section Veterinary Services (1417), Laboratory for Animal Health Virology, Aglantzia, Nicosia 2109, Cyprus
[10] Insects Vectors and Parasites Laboratory, Department of Basic Pathology and Postgraduate program in Microbiology, Parasitology and Pathology, Federal University of Paraná, 81530-900 Curitiba, Brazil
[11] Department of Biological Sciences, National University of Singapore, 117558, Singapore
[12] Laboratory of the Leishmaniasis Research Project, Mokolo District Hospital, Mokolo, Cameroon; Laboratory of Cellular Immunology and Parasitology, Department of Biological Sciences, Central Washington University, 98926 Ellensburg, WA, USA
[13] Department of Parasitology, Faculty of Science, Charles University, 12800 Prague, Czechia
[14] VERG Laboratories, Department of Biology, Faculty of Science, Hacettepe University, Beytepe, Ankara 06800, Türkiye
[15] Faculdade de Saúde Pública da Universidade de São Paulo (FSP/USP), Pós-graduação em Saúde Pública, 01246-904 São Paulo, Brazil
[16] Secció de Parasitologia, Departament de Biologia, Sanitat i Medi Ambient, Facultat de Farmàcia i Ciències de l'Alimentació, Universitat de Barcelona, & Institut de Salut Global de Barcelona (ISGlobal), Centro de Investigación Biomédica en Red, Enfermedades Infecciosas (CIBERINFEC), 08028 Barcelona, Spain
[17] Laboratory of Infectious Diseases and Public Health, School of Medicine, University of Cyprus, Nicosia, Cyprus & Department of Pediatrics, Archbishop Makarios III Hospital, Nicosia 2115, Cyprus
[18] Faculty of Health Sciences, American University of Beirut, 1107 2020 Beirut, Lebanon
[19] Medical Entomology Unit, Infectious Disease Research Centre, Institute for Medical Research (IMR), National Institutes of Health (NIH), Ministry of Health Malaysia, 40170 Shah Alam, Selangor, Malaysia
[20] School of Medicine, Addis Ababa University, 28017 - 1000 Addis Ababa, Ethiopia
[21] Faculty of Mathematics, Natural Sciences and Information Technologies, University of Primorska, 6000 Koper, Slovenia

Edited by Jean-Lou Justine
* sorumlu yazar: jerome.depaquit@univ-reims.fr

This is an Open Access article distributed under the terms of the Creative Commons Attribution License (https://creativecommons.org/licenses/by/4.0), which permits unrestricted use, distribution, and reproduction in any medium, provided the original work is properly cited.

[22] University of Lodz, Faculty of Biology and Environmental Protection, Department of Invertebrate Zoology and Hydrobiology, Banacha 12/16, 90-237 Łódź, Poland
[23] Laboratory of Entomology, Ministry of Health, 9134302 Jerusalem, Israel
[24] Center for Pathophysiology, Infectiology and Immunology, Institute of Specific Prophylaxis and Tropical Medicine, Medical University Vienna, Kinderspitalgasse 15, 1090 Vienna, Austria
[25] Papua New Guinea Institute of Medical Research (PNGIMR) Institute, PO Box 60, Headquarter, Homate Street, 441 Goroka, Eastern Highlands Province, Papua New Guinea
[26] Museum of Natural History, University of the Philippines Los Baños, 4031 Laguna, Philippines
[27] National Centre of Infectious and Parasitic Diseases, 1504 Sofia, Bulgaria
[28] Ege University, Faculty of Medicine, Department of Parasitology, 35040 Bornova/Izmir, Türkiye
[29] Retired, Faculté de Pharmacie, Université de Strasbourg, Strasbourg, 67400 Illkirch-Graffenstaden, France
[30] Program for the Study and Control of Tropical Diseases (PECET), Faculty of Medicine, University of Antioquia, 050010 Medellin, Colombia
[31] MIVEGEC, Univ. Montpellier, CNRS, IRD, 34394 Montpellier, France & Medical Entomology Unit, Institut Pasteur de Madagascar, 101 Antananarivo, Madagascar
[32] Laboratorio de Entomología Médica, Departamento de Zoología de Invertebrados, Facultad de Ciencias Biológicas, Universidad Autónoma de Nuevo León, San Nicolás de los Garza, 66455, NL, México
[33] Tropical and Infectious Disease Centre, BP Koirala Institute of Health Sciences, Dharan 56700, Nepal
[34] ICMR-Vector Control Research Centre, Puducherry 605006, India
[35] Graduate School of Agricultural and Life Sciences, The University of Tokyo, Tokyo 113-8657, Japan
[36] Grupo de estudos em Leishmanioses/Coleção de Flebotomíneos (COLFLEB/Fiocruz-MG), Instituto René Rachou, Fundação Oswaldo Cruz, Belo Horizonte, Minas Gerais, 30190009, Brazil
[37] Center of Excellence in Vector Biology and Vector-Borne Disease, Department of Parasitology, Faculty of Medicine, Chulalongkorn University, Bangkok 10330, Thailand
[38] Department of Arbovirology, Bernhard Nocht Institute for Tropical Medicine, Bernhard Nocht Str. 74, 20359 Hamburg, Germany [39] Laboratory Vectors & Parasites, Department of Livestock Sciences and Techniques, Sine Saloum University El Hadji Ibrahima Niasse (SSUEIN) Kaffrine Campus, C.P. 24600, Senegal.
[40] Environmental Health Institute, National Environment Agency, Singapore 138667, Singapore & Department of Biological Sciences, National University of Singapore, 117558 Singapore
[41] Institut Pasteur du Laos, Laboratory of Vector-Borne Diseases, Samsenhai Road, Ban Kao-Gnot, Sisattanak District, 3560 Vientiane, Lao PDR
[42] National Institute of Hygiene and Epidemiology, 1 Yec-Xanh Street, Hai Ba Trung District, 100000 Hanoi, Vietnam
[43] Indonesian Research Center for Veterinary Science, Indonesian Agency for Agricultural Research and Development, Ministry of Agriculture Republic Indonesia, Bogor 16114, Indonesia & Department of Parasitology, Faculty of Veterinary Medicine, Airlangga University, Surabaya 60115, Indonesia
[44] Laboratory of Research in Applied Biology, Polytechnic School of Abomey-Calavi, University of Abomey-Calavi, 01 P.O. Box 2009, 00000 Cotonou, Benin
[45] Instituto de Microbiología, Colegio de Ciencias Biológicas y Ambientales (COCIBA), Universidad San Francisco de Quito (USFQ), 170901 Quito, Ecuador

Received 1 December 2025, Accepted 29 January 2026, Published online 3 April 2026

Özet: Bu makale, phlebotomine kum sineği örneklerinin işlenmesi ve preparat haline getrilimesi ile ilgili kapsamlı bir rehber sunmakta olup, bu süreçler kum sineği örneklerinin tür teşhisi, kum sineklerinde patojen tespiti ve izolasyonu için kritik bir öneme sahiptir. Arazi ve laboratuvar koşullarında uygulanabilecek çeşitli tekniklerin ele alındığı bu makale, kum sineklerinin örneklenmesi, taşınması ve immobilizasyonu (kimyasallar yerine kuru olarak dondurma ya da CO_2 kullanımının önerildiği) ile soğukta ya da ethanolde saklama gibi muhafaza stratejileri için detaylı talimatlar sunmaktadır. Belirli anatomik yapıların (genital organlar, baş ve kanatlar) preparasyon kalitesi, bunların mikroskobik olarak doğru şekilde incelenebilmesi için kritik öneme sahiptir ve bu çalışmada ayrıntılı olarak açıklanmaktadır. Makalede ayrıca, potasyum hidroksit gibi kimyasallarla yapılan saydamlaştırma işlemi ve bunu takiben uygulanan Marc-André solüsyonu dâhil olmak üzere, örneklerin ayrıntılı işlenme basamakları sunulmaktadır. Kapatma aşaması için farklı maddeler karşılaştırılmakta; bu maddelerin optik özellikleri ve uzun süreli koruma potansiyelleri vurgulanmaktadır. Hoyer sıvısı (kloral zamkı olarak da bilinir), özellikle spermatekaların hızlı gözlemi için yüksek şeffaflığı nedeniyle önerilmekle birlikte, uzun süreli muhafaza için uygun değildir. Tartışılan diğer maddeler arasında polivinil alkol, sınırlı su toleransına sahip Euparal® ve hidrokarbonlarda çözünebilen bir madde olan Kanada balsamı yer almakta; son iki madde uzun dönemli koruma açısından avantaj sunmaktadır. Ayrıca, örnek işleme süreçlerinde

özel dikkat gerektiren DNA dizileme ve MALDI-ToF gibi yenilikçi moleküler biyoloji yaklaşımlarına da değinilmektedir. Bunun yanı sıra, çeşitli kapama tekniklerini gösteren kısa video klipler ile 33 farklı dile yapılan çeviriler sunulmakta; böylece kılavuzun küresel bilim camiasının farklı ihtiyaç ve beklentilerine ulaşması sağlanmaktadır.

Anahtar kelimeler:

Preparat, phlebotomine kum sinekleri, Hoyer sıvısı, Marc-André solüsyonu, kloral gum, polivinil alkol, Euparal®, Kanada balsamı, *Leishmania* izolasyonu, arazi koşulları, kültür, diseksiyon, moleküler biyoloji, MALDI-ToF, tip örnekleri

Abstract – Processing and mounting phlebotomine sand flies: a consensus guideline. This article provides a comprehensive guide for the processing and mounting of phlebotomine sand fly specimens, which is crucial for species identification and pathogen detection and isolation. It discusses a range of techniques suitable for both field and laboratory settings. The guide includes detailed instructions on sand fly collection, handling, covering, and euthanasia (recommending dry freezing or CO_2 over chemicals) as well as conservation strategies, such as cold storage and preservation in ethanol. The quality of preparation of certain anatomical structures (genital organs, head and wings) is essential for their proper microscopic observation and is described in this work. The article also presents detailed sample processing, including the clearing process with agents such as potassium hydroxide then Marc-André solution. The mounting process compares different media, emphasizing their optical properties and preservation potential. Hoyer fluid (also known as chloral gum) is recommended for quick observation, particularly for spermathecae, due to its clarity, although it is not suitable for long-term storage. Other media discussed include polyvinyl alcohol, Euparal® (for limited water tolerance), and Canada balsam (a hydrocarbon-soluble medium), with the latter two offering long-term preservation capabilities. Innovative molecular biology approaches such as DNA sequencing and MALDI-ToF, which require particular attention to sample processing, are also addressed. Furthermore, short video clips illustrating various mounting techniques as well as translations in many different languages are provided, allowing the guideline to reach the diverse needs and expectations of the global scientific community.

Key words: Mounting, Phlebotomine sand fly, Hoyer fluid, Marc-André solution, Chloral gum, Polyvinyl alcohol, Euparal®, Canada balsam, *Leishmania* isolation, Field conditions, Culture, Dissection, Molecular biology, MALDI-ToF, Type-specimens.

Giriş

Phlebotominae alt familyasına ait, Psychodidae familyasında yer alan phlemotomine kum sinekleri, en az 1.063 bilinen türü bulunan, Diptera takımına ait küçük böceklerdir [21]. *Leishmania*, arbovirüsler ve *Bartonella* gibi patojenlerin önemli vektörleridir ve sırasıyla leishmaniasis, arbovirüs enfeksiyonları ve bartonellozis hastalıklarına neden olurlar. Teşhisleri esas olarak dikkatli örnek toplama, uygun saklama ve özenli preparat hazırlama ile desteklenen ayrıntılı mikroskobik incelemeye dayanır. Bu süreç, her birinin kendine özgü avantaj ve sınırlılıkları olan çeşitli özel teknikler gerektirir.

Ergin phlemotomine kum sineklerinin teşhis edilmesi hem dış (örn. antenler, palpler, erkek genitalyası) hem de iç yapıların (örn. farinks, cibarium ve spermatekalar) gözlemlenmesine dayanır. İç yapıların diseksiyonu ve izolasyonu, bunların gözlemlenmesini ve dolayısıyla doğru teşhisini kolaylaştırır. Bu nedenle sivrisinekler ya da triatomin böceklerden farklı olarak phlebotomine kum sinekleri, teşhis öncesinde lam ile lamel arasına alınarak preparat haline getirilmelidir.

Mikroskobik gözlem, 1980'li yıllara kadar kum sineklerinin teşhisinde mevcut tek yöntemdi ve günümüzde de en yaygın kullanılan yaklaşım olmaya devam etmektedir. Bu nedenle işlem ve preparasyon seçimi nispeten basitti ve temel olarak ikiye ayrılıyordu: uzun süreli muhafazaya olanak sağlayan kalıcı preparat hazırlanması ve uzun süreli saklama sağlamayan kapama kimyasalı içinde hızlı preparat hazırlanması. Örneğin Kanada balsamı gibi bir reçine ile yapılan kalıcı preparat zaman alıcıdır ve örneklerin tamamen dehidrate edilmesini gerektirir. Ayrıca bu kapama kimyasalının kırılma indisi, spermatekaların kolay gözlemlenmesi için her zaman optimal değildir. Buna karşılık sulu bir kapama maddesinde (örn. Hoyer sıvısı) kapama daha hızlıdır ve refraktif spermatekaların daha iyi görüntülenmesini sağlar; ancak atmosferden su absorbe etme eğiliminde olduğu için uzun süreli saklama sağlamaz. Bir seçenek, lam tamamen kuruduktan sonra oje ile kenarlarını kapatmaktır. Bu tercih ikilemi günümüzde de devam etmekte olup, preparat hazırlama yönteminin seçimi,

preparatın amacına bağlıdır. 1980'lerden itibaren kum sineği teşhis çalışmaları morfolojik ve biyokimyasal yaklaşımları birleştirmiştir. İlk olarak kutiküler hidrokarbon analizleri uygulanmış, ancak bu yöntem kısa sürede moleküler biyoloji teknikleri (RAPD, RFLP, DNA amplifikasyonu, Sanger dizileme ve yeni nesil dizileme – NGS) ile yer değiştirmiştir. Günümüzde moleküler yaklaşımlar MALDI-ToF gibi proteomik yöntemlerle tamamlanmaktadır. Ayrıca moleküler tür teşhisi, PCR ile patojen tespiti (*Leishmania*, *Trypanosoma*, *Bartonella* ve Phlebovirus) ile birleştirilebilir; tümü hem konvansiyonel PCR hem de gerçek zamanlı PCR ile saptanabilir. Bu durum, örnekleme ve saklama sürecinin hedeflenen amaçlara göre uyarlanmasını gerektirir [3, 32]. Tür ayrımında geleneksel olarak kullanılan morfolojik karakterlere ek olarak kanat geometrik morfometrisi gibi diğer morfolojik yaklaşımlar da uygulanabilir.

Bu çalışma, büyük ölçüde yazarların deneyimlerine ve literatür verilerine dayanarak, ergin phlebotomine kum sineklerinin morfolojik ve moleküler analizlerini optimize etmek amacıyla preparat hazırlama ve ileri işlemler için standartlaştırılmış kılavuzlar sunmayı amaçlamaktadır.

Bazı analizlerin (örn. moleküler biyoloji ya da MALDI-ToF) yapılabilmesi, morfolojik tanımlama için gerekli olmayan vücut kısımlarının korunmasını gerektirir; bu da uygun protokol seçiminin önemini ortaya koymaktadır.

Bu makalede canlı yakalanan kum sineklerinin anestezi ve ötanazi yöntemlerine, saklanmasına ve hızlı tanımlama ya da uzun süreli muhafaza için preparat hazırlama süreçlerine odaklanılmaktadır.

Önsöz: Güvenlik ve mevzuata ilişkin hususlarda ilgili Güvenlik Bilgi Formlarına (GBF) atıfta bulunulmalıdır.

Bu kılavuzda sunulan tüm kimyasallar sıkı güvenlik koşulları altında kullanılmalıdır. Araştırma kurumlarının iş sağlığı ve güvenliği birimleri, bu kimyasalların tehlikeleri, kullanım prosedürleri ve atık bertarafı hakkında bilgi sağlamaktadır. Ancak kullanım ve bertarafla ilgili güvenlik talimatlarına uyulması zorunludur. İyi ve güvenli laboratuvar uygulamalarına ve ilgili ülke ya da kurum mevzuatına uyum sağlamak tüm kullanıcıların sorumluluğundadır. Ayrıca bazı kimyasallar ya da bileşenleri (örn. kloral hidrat) bazı ülkelerde düzenlemeye tabidir. Bu makalede kullanılan kısaltmalar Tablo 1'de verilmiştir.

Tablo 1: Kısaltmalar listesi

**BME**	Basal medium Eagle
**CDC**	Hastalık Kontrol ve Öneme Merkezi
**CMCP**	Camphor-monochlorophenol
**CMR**	Kanserojen, mutajen, reprodüktif toksik madde
**COI**	Sitokrom c oksidaz alt ünite I geni
**CytB**	Sitokrom b gen,
**DNA**	Deoksiribonükleik asit
**ELISA**	Enzim bağlantılı immünosorbent analizi
**EtOH**	Etanol
**M199**	Medium 199
**MALDI-ToF MS**	Matris destekli lazer desorpsiyon/iyonizasyon uçuş zamanı kütle spektrometrisi
**MEM**	Minimum essential media
**NGS**	Yeni Nesil Dizileme
**NNN**	Novy-MacNeal-Nicolle medium
**PCR**	Polimeraz Zincir Reaksiyonu
**Lao PDR**	Laos
**PNOC**	Prepronociceptin geni
**qPCR**	Kantitatif PCR (Gerçek zamanlı PCR)
**RAPD**	Rastgele Çoğaltılmış Polimorfik DNA
**RFLP**	Restriksiyon Fragment Uzunluk Polimorfizmi
**RI**	Kırılma İndisi
**RNA**	Ribonükleik asit
**RNaz**	Ribonükleaz
**RNASS**	RNA stabilizasyon solüsyonu
**RT-PCR**	Ters transkripsiyon PCR
**TFA**	Trifloroasetik asit

1. Kum sineklerinin örneklenmesi

Ergin kum sinekleri canlı ya da ölü olarak; CDC minyatür ışık tuzakları, yapışkan tuzaklar (yağlı kağıt) ve aspiratör yardımıyla Shannon tuzaklarını kullanarak ya da yine aspiratör ile çevredeki dinlenme alanlarından (örn. hayvan barınakları) doğrudan toplama gibi çeşitli yöntemlerle toplanabilirler [2, 3, 32, 36, 49]. Bu yöntemler uygun habitatlara tuzak yerleştirilmesini, kum sineklerinin ışık ya da diğer çekicilerle (CO_2 ya da diğer kimyasal çekiciler) çekilmesini ve analiz için toplanmasını içerir.

Canlı örnekleme tüm ileri uygulamalara olanak sağlarken, ölü bireylerin toplanması *Leishmania* ya da virüs suşlarının izolasyonunu engeller. Yağlı kağıtlar gibi bazı yöntemler anten, palpus, kanat ya da bacak kaybına yol açabilir. Ayrıca hint yağı kaplı yapışkan kağıtlar kum sineklerine

yapışır ve işleme başlamadan önce genellikle eşit oranlarda etanol ve dietil eter karışımında 15 dakikalık bir banyoyla temizleme işlemini gerektirir.

2. Örneklerin immobilizasyonu

Örneklenen canlı kum sinekleri öldürülmelidir. Bazı yöntemlerde (örn. yağlı kağıtlar, deterjan ya da etanol içeren kapları bulunan CDC ışık tuzakları) bireyler toplandığında zaten ölüdür. Doğrudan etanol içine toplanan örnekler için moleküler biyolojik yöntemler uygulanabilir; diğer yöntemlerle toplanan örnekler ise mümkün olan en kısa sürede etanole içine alınıp, saklanmalıdır. Ancak bu öldürme yöntemleri MALDI-ToF uygulamalarına uygun değildir ve bazı morfolojik karakterlerin kaybına yol açabilir. Bu nedenle doğru teşhis ya da uzun süreli referans örneği saklama için uygun bir standart öldürme yöntemi kullanılması gereklidir. Etil asetat, etil eter, tetrakloroetan ve kloroform pamuk üzerine emdirilerek kullanılabilir; ancak toksisiteleri nedeniyle dikkatli kullanılmalıdır. Deneyimlerimize göre kloroform moleküler çalışmalarla uyumsuz olduğundan önerilmez. Bu kimyasalların tehlikeli doğası ve moleküler analizlere uygunluklarının sınırlı olması nedeniyle kullanımları genel olarak önerilmez.

Morfoloji, DNA ve proteinleri koruyan en yaygın yöntem kuru dondurmadır. Örnekler tamamen immobilize olacak kadar, ancak (i) kuruyacak ya da (ii) amaç, kum sineğinin sindirim kanalından *in vitro* izolasyon yapmak ise, *Leishmania* canlılığını olumsuz etkileyecek kadar uzun süre dondurulmamalıdır. **Bu nedenle -20 °C'de 15–20 dakika dondurma ve düzenli kontrol önerilir.**

Dondurucu bulunmadığı durumlarda, böcekler alternatif olarak CO_2 kullanılarak öldürülebilir. Arazi koşullarında CO_2 tüplerinin kullanılamadığı durumlarda, 'Soda sifonlarında' (içecek dağıtıcıları) kullanılan küçük ticari CO_2 kartuşları ile örnekler öldürülebilir; ancak bunların hava yoluyla taşınmasında kısıtlamalar olabilir. Son çare olarak, böcekler tütün dumanına maruz bırakılarak öldürülebilir: Kum sinekleri CDC tuzağında canlı olarak yakalanır, bir aspiratör ile toplanır, cam tüp içerisinde tutulur ve birkaç saniye içinde ölmelerini sağlayan tütün dumanına maruz bırakılır. Bu yöntem, zorlu izolasyon koşulları da dahil olmak üzere tüm arazi şartlarında uygulanabilir. Ancak cam yüzey dumanla kaplandığından, iyice temizlenmeden canlı kum sineklerinin sonraki toplama ve manipülasyon işlemlerinde kullanılamaz. Bununla birlikte, aynı temizlenmemiş aspiratör, diğer tuzaklardan elde edilen kum sineklerinin fiksasyon amacıyla öldürülmesinde kullanılabilir. Ayrıca, aspiratör içerisindeki tüm örneklerin çıkarıldığından emin olunması gerekir. Bu yöntemler, bağırsak diseksiyonu yoluyla *Leishmania* izolasyonu ile uyumludur.

3. İşlem öncesi örnek saklama

İşlem öncesinde uygulanan beş temel fiksasyon yöntemi bulunmaktadır:

3.1. Dondurma

Bu yöntem en iyi -20 °C'de ya da tercihen -80 °C'de uygulanır. Bu saklama yöntemleri günümüzde sıvı azotla saklamaya kıyasla daha yaygın olarak kullanılmaktadır. Tüm durumlarda, örnekler sersemletildikten sonra kriyoprezervasyon mümkün olan en kısa sürede uygulanmalıdır. Dondurucuda soğuk saklama, böceklerin yanı sıra RNA, DNA ve proteinlerin de saklama süresi boyunca tam olarak korunmasını sağlar. Buna karşılık, sıvı azot kanatlara, bacaklara, palp ve antenlere ciddi zarar verebilir; sıklıkla bu yapıların kopmasına ve zaman zaman önemli morfolojik karakterlerin kaybına yol açabilir. Dondurucuda kuru olarak saklama örnekler için daha az travmatiktir, ancak hassas organların korunması açısından ideal değildir. Özellikle çözündürme sırasında, kanatlar, antenler, palpler veya bacaklar yoğuşma nedeniyle tüplere yapışabilir ve sonuçta kopabilir. Bununla birlikte, arazi çalışmalarında dondurarak muhafaza her zaman uygulanabilir değildir; çünkü bir dondurucuya ya da sıvı azot tankına erişim gerektirir. Dondurucuda saklama, moleküler yöntemlerle patojen tespitiyle duyarlılık kaybı olmaksızın tamamen uyumludur. Ancak RNA virüslerinin tespiti ve izolasyonu, uzun süreli saklama gerekiyorsa, -80 °C'de ya da sıvı azotta muhafazayı gerektirir. Öte yandan, örneklerin dondurulması, kum sinekleri önce buhar fazına ve ardından sıvı azota (örneğin bir çorap içine yerleştirilmiş flakonlar içinde) daldırılarak *Leishmania* kriyoprezervasyonunu simüle etmedikçe, bağırsak diseksiyonu yoluyla *Leishmania* izolasyonuna olanak tanımaz.

3.2. Alkolde saklama (etanol ya da izopropanol)

Bu yöntem muhtemelen kum sineklerinin saklanmasında en yaygın kullanılan yöntemdir. Laboratuvara erişimin olmadığı zorlu koşullar da dahil olmak üzere, arazi çalışmalarında uygulanması kolaydır. Alkolde muhafaza, özellikle morfolojik çalışmalar için uygundur; çünkü saklama tüpünde hava kabarcığı bulunmadığı sürece hassas yapılar (kanatlar, bacaklar, antenler ya da palpler) bütünlüğünü korur. Bu nedenle, hava kabarcıklarını gidermek amacıyla tüpün küçük bir pamuk tıkaç ile kapatılması ve etiketin pamuk tıkaç üzerine yerleştirilmesi önerilir (Şekil 1).

Uygun alkol konsantrasyonu hâlen tartışmalıdır. Genel olarak %70'in altındaki konsantrasyonlar önerilmez [45, 66]. Daha yüksek konsantrasyonlar DNA'yı daha etkili ve daha uzun süre korur; ancak örnekleri morfolojik çalışmalar açısından daha kırılgan ve gevrek hâle getirir. %96 etanolün (azeotrop karışım) kullanımı, özellikle tropikal ülkeler gibi

nemli bölgelerde zaman içinde konsantrasyon stabilitesini sağlar; ancak %95 etanol çoğu zaman daha kolay temin edilebilir. Konsantrasyondan bağımsız olarak, DNA genellikle etanol içinde iyi korunur (ancak özellikle NGS tipi moleküler yöntemler için, dondurma yöntemlerine kıyasla daha az etkilidir). Proteinler ise çok daha az stabildir; özellikle MALDI-ToF gibi proteomik uygulamalarda. Alkolde birkaç ay saklanan kum sinekleri morfolojik olarak hâlâ teşhis edilebilir; ancak bu örneklerden referans protein spektrumları elde etmek mümkün değildir. Alkolde veya kuru koşullarda saklama, örneklerin ayrıca -20°C'de dondurulmasıyla iyileştirilebilir. -20°C'de dondurma, esas olarak moleküler korunumu (örneğin nükleik asitler) artırır; bozunmayı yavaşlatır ve zamanla doku yıkımını azaltarak morfolojik korunuma da ikincil bir katkı sağlar; ancak morfoloji üzerindeki etkisi, moleküler bütünlük üzerindeki etkisine kıyasla daha sınırlıdır. Etanolde saklama, en az %70 konsantrasyonda ve birkaç aydan kısa süreli depolamalarda DNA ve RNA virüslerinin tespiti için de uygulanabilir. Ayrıca, izopropil alkol bazı ülkelerde kolaylıkla temin edilebilir ve DNA'yı korur; ancak örnekleri sertleştirir. Etanol gibi yanıcı değildir ve bu nedenle taşınması daha kolaydır. Gerekli durumlarda, sıvı azotta ya da kuru dondurulmuş olarak saklanan kum sinekleri alkole aktarılabilir; ancak bu durumda her iki yöntemin dezavantajları bir arada ortaya çıkabilir.

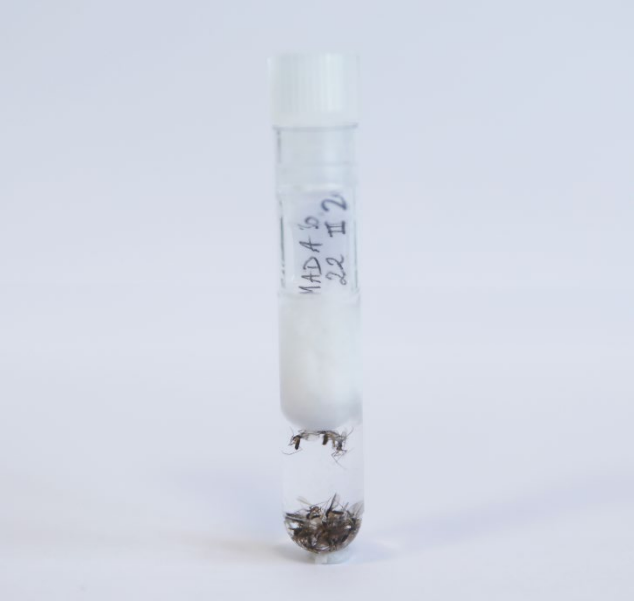

Şekil 1: Etanolde muhafaza edilmiş kum sinekleri.

3.3. RNA stabilizasyon solüsyonunda (RNASS) saklama

Yaygın olarak kullanılan bu sulu reaktif, toksik değildir ve taze doku ile hücre örneklerinde RNA'yı stabilize etmek ve korumak üzere tasarlanmıştır. Örneğe hızla penetre olarak RNazları (RNA'yı parçalayan enzimler) inaktive eder ve böylece RNA'nın, hemen dondurma gerektirmeksizin, bozulmasını önler. RNASS içinde saklama, sonraki histolojik değerlendirmeler için genel doku ve hücresel morfolojinin korunmasında genellikle etkilidir. RNASS, fiksasyondan ziyade RNA stabilizasyonu için optimize edilmiş olsa da, kısa ve orta vadeli saklamalarda yapısal bütünlük çoğunlukla iyi düzeyde korunur. RNASS, örneklerin oda sıcaklığında 7 güne kadar, 4°C'de birkaç hafta boyunca ya da -20°C/-80°C'de uzun süreli olarak saklanmasına olanak tanır. Bu yöntem, soğuk zincir altyapısının sınırlı olduğu arazi çalışmaları ya da klinik ortamlarda özellikle değerlidir. RNA ekstraksiyonu genellikle örneklerin reaktiften çıkarılmasını ve standart protokollere göre işlenmesini gerektirir.

3.4. Oda sıcaklığında kuru saklama

Daha eski bir yöntem olan oda sıcaklığında kuru saklama, örneğin bütün hâlde (in toto, tüm vücut olarak) preparat haline getirilmesi durumunda, kanatlar, bacaklar, antenler ve palpler gibi hassas yapıların zayıf korunması gibi önemli bir dezavantaja sahiptir. Bununla birlikte, fiksasyon sırasında silika jel tipi bir desikant ile dehidrasyon uygulanırsa, MALDI-ToF kullanılarak proteomik çalışmalar yapmak yine de mümkündür. Buna karşılık, DNA'yı hedefleyen moleküler analizlerin bu örneklerde gerçekleştirilmesi zordur; çünkü DNA çoğu zaman parçalanmıştır ve miktarı azalmıştır. Bu durum, özellikle nükleer genom çalışmalarında, taze ya da dondurulmuş örneklere kıyasla analizleri daha güç hâle getirir. Ancak müseomik (museomics) gibi yeni teknikler bu tür örneklerde uygulanabilir [34]. Bu nedenle, başka bir seçenek bulunmadıkça bu saklama yöntemi önerilmez. Tüplerin -20 °C veya -80 °C'de dondurucuya yerleştirilmesiyle soğuk saklama ile kombine edilebilir. Başlıca zorluk, teşhis için gerekli olan örneklerin ya da vücut parçalarının uygun şekilde preparat haline getirilmesidir. Bunun için rehidrasyon zorunludur. Triton X-100 çözeltisinin kullanılması önerilir. Rehidrasyon süresi, düzenli ve dikkatli izleme altında birkaç saatten birkaç güne kadar değişebilir. Tam rehidrasyon sağlandıktan sonra örnekler ardışık üç su banyosunda durulanmalıdır.

3.5. Filtre kağıdında saklama

Filtre kâğıtlarının başlıca avantajı, fikse edilmemiş, kurutulmuş tüm vücut ya da kan hücreleri içindeki genomik DNA'nın oda sıcaklığında uzun süre stabil kalabilmesidir. Filtre kâğıtlarının küçük kart boyutlarında olması küçük bir masa çekmecesi hacminde yüzlerce örneğin oda

sıcaklığında saklanmasına olanak tanır. Filtre kâğıdı matriksine, enfeksiyöz etkenleri denatüre eden maddeler emdirilmiştir; bu nedenle örnekler artık biyotehlike olarak değerlendirilmez. Bu durum, örneklerin özel biyogüvenlik önlemleri olmaksızın saklanmasına ve taşınmasına imkân sağlar [68].

4. Örnek disseksiyonu

Birçok böcek türü, bütün hâlde (in toto) iğnelenmiş bireyler üzerinde gözlemlenebilen dış morfolojik karakterlere göre teşhis edilirken, kum sineklerinde türün doğru şekilde tanımlanabilmesi için anatomik yapıların incelenmesi amacıyla diseksiyon ve lam üzerine kapama gereklidir. Seçilen preparasyon ve kapama yöntemi ne olursa olsun, aynı diseksiyon tekniği uygulanır (Şekil 2 ve 3) (https://zenodo.org/records/18198006).

Triton X100 kullanımı: non-iyonik sulu çözelti

Kapama işleminin taze yakalanmış ya da uygun şekilde muhafaza edilmiş örnekler için geçerli olduğu unutulmamalıdır. Çoğu araştırmacı örnekleri kuru olarak (MALDI-ToF kullanımı için) ya da uzun yıllar alkole alınmış şekilde saklar. Ne yazık ki, alkolde yıllarca muhafaza ideal değildir ve bu şekilde saklanan arthropodların mikroskobik incelemeye hazırlanması oldukça güçleşir. Sık karşılaşılan bir durum, örnekleri içeren plastik materyalin zamanla bozulması ve ardından alkolün buharlaşmasıdır. Her iki durumda da örnekler ya çok uzun süre alkole maruz kalmış ya da tamamen kurumuş olduğundan seçeneklerimiz sınırlıdır. Bu nedenle, güçlü deterjan özelliği olmayan ıslatıcı maddelerin kullanımı gündeme gelmiştir. Triton X100, non-iyonik bir sulu çözelti formundadır (*4-(1,1,3,3-tetrametilbutil) fenil-polietilen glikol çözeltisi ya da t-oktilfenoksipolietoksietanol, polietilen glikol tert-oktilfenil eter*) ve sitolojik ve moleküler çalışmalarda sıklıkla deterjan olarak kullanılmaktadır. Hücre ve çekirdek zarlarının geçirgen olmasını sağlar.

Aşağıda %0,5'lik sulu non-iyonik Triton X100 çözeltisi kullanılarak uygulanan bir prosedür verilmiştir:

- Kuru örneği mutlaka alkol ile muamele edin.
- Örneğin tamamı çözelti içinde kalacak şekilde gerekli hacimde %0,5 Triton X100 çözeltisi ekleyin.
- İşlemi düzenli kontrol altında yaklaşık 5 dakikadan birkaç güne kadar sürdürün. Tüm örnekler çözeltide tamamen ayrışmış hâle gelmelidir.
- Triton X100 çözeltisini uzaklaştırın ve yerine potasyum hidroksit çözeltisi ekleyin.

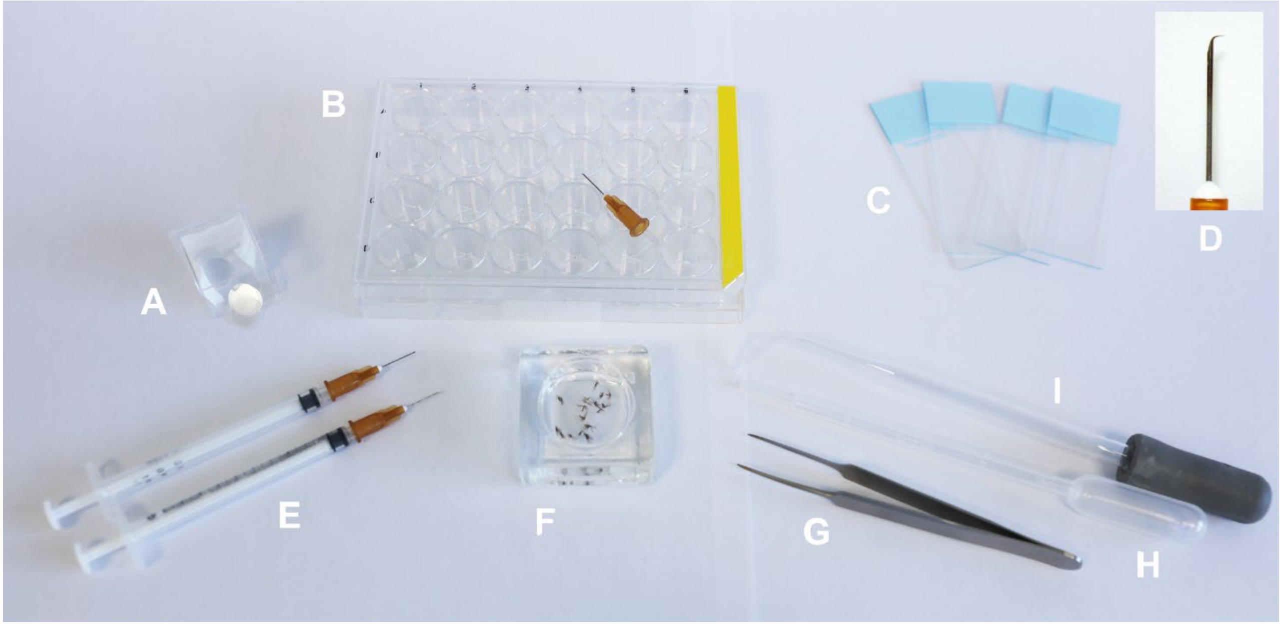

Şekil 2: Kum sineği preparatlarının hazırlanması için gerekli materyaller: A: Yuvarlak cam lameller (10 veya 12 mm çapında); B: 24 kuyucuklu plak ve kancalı iğne (kum sineklerinin işlenmesinde karanfil yağı veya Euparal® esansı kullanılıyorsa akrilik plak kullanılmamalıdır; kimyasal reaksiyon meydana gelebilir ve örnekler zarar görebilir); C: Etiketlemeye uygun cam lamlar; D: İğne kancasının detayı; E: Enjektöre takılı iğneler; F: Preparat haline getirilecek kum sineklerini içeren saat camı veya eşdeğer bir kap; G: Dumont penset; H: Plastik pipet; I: Sıvının kuyucuklara aktarılmasını kolaylaştırmak amacıyla ısıtılarak bükülmüş cam pipet.

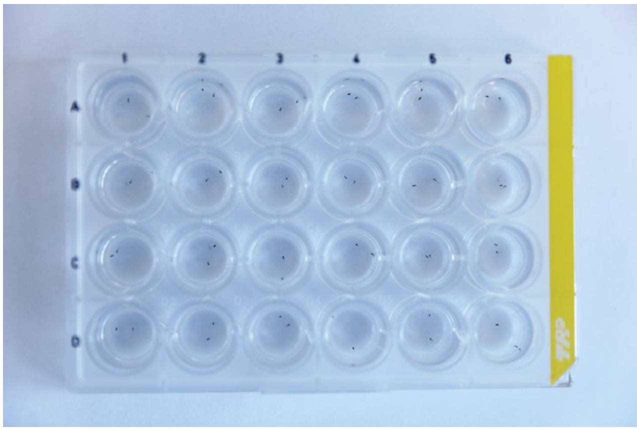

Şekil 3: Her bir kuyucukta kum sineklerinin başı ve abdomen ucunun bulunduğu 24 kuyucuklu bir plak.

4.1. Baş

Diseksiyon, bir stereomikroskop altında ince iğneler veya entomolojik iğneler kullanılarak gerçekleştirilebilir (Şekil 2 ve 3). En yaygın kullanılan iğneler şunlardır: 26G × 1/2" (0,45 × 13 mm), 30G × 1/2" (0,3 × 13 mm) veya 25G × 5/8" (0,5 × 16 mm). Örneğin teşhise hazırlanabilmesi için en azından baş kısmı gövdeden ayrılmalı ve cibarium ile farinksin görülebilmesi amacıyla ventral yüzü üste gelecek şekilde kapatılmalıdır. Toraks ve abdomen ise diseksiyonun ardından lateral pozisyonda kapatılır. Başın ventro-dorsal pozisyonda yerleştirilmesi, foramen occipitale'nin yukarı bakacak şekilde konumlanmasını sağlar; böylece cibarium doğrudan gözlemlenebilir. Bu anatomik yapılara erişim, başın tamamen ayrılmasıyla daha da kolaylaşır.

4.2. Kanatlar ve toraks

Kanatlar düz bir şekilde kapatılmalıdır. Her bir kanat tabanından ayrılarak ayrı ayrı kapatılabilir ya da biri tek başına kapatılıp diğeri toraksa bağlı bırakılabilir. Eğer geometrik morfometri analizi planlanıyorsa, kapamadan önce sağ ve sol kanatların doğru şekilde tanımlanması ve etiketlenmesi zorunludur. Toraks birkaç bölümden oluşur ve her biri son derece önemli taksonomik bilgiler içerir [20, 64]. Genellikle, ketotaksi (kıl dizilimi) ve renk dağılımının incelenebilmesi için lateral pozisyonda kapatılır. Toraksın belirli bölgelerindeki kıl izlerinin varlığı, *Brumptomyia* cinsine ait bazı türlerin ayırt edilmesinde kullanılabilir. Renk dağılımı ise Neotropikal kum sineklerinin cins düzeyinde (örneğin *Bichromomyia*), tür serisi düzeyinde (örneğin *Pintomyia*) ya da aynı cins içindeki türlerin ayrımında (örneğin *Micropygomyia*, *Nyssomyia*, *Psathyromyia* ve *Psychodopygus*) kullanılabilir [20]. Bu nedenle, toraks moleküler analiz için kullanılmayacaksa, zarar görmeyecek şekilde kapatılmalıdır. Önemle belirtilmelidir ki burada önemli olan renklerin yoğunluğu değil, toraks üzerindeki dağılımıdır. Dolayısıyla, şeffaflaştırma (klarifikasyon) işlemi pigmentasyonu ya da pigment desenini ortadan kaldırmaz.

4.3. Genitalya

Erkek ve dişilerde genital yapıların kapatılmasında özellikle dikkatli olunmalıdır; çünkü bu yapılar cins, alt cins ve tür düzeyinde teşhis için kritik öneme sahiptir. Her iki eşeyde de genital yapılar çifttir.

4.3.1. Erkekler

Genital yapılar eksternaldir ve dorsal kısmında gonokoksit–gonostil eklemini, ventral kısmında ise epandrial lobu içeren çift forsepslerden oluşur. Gonostil üzerinde dikenler ve bazen setalar bulunur; bunların sayılabilir olması ve yerleşim noktalarının açıkça görülebilmesi gerekir. Gonokoksitin iç yüzeyi dikkatle incelenmelidir; çünkü burada sapsız bir seta demeti ya da bir lob (= tüberkül) üzerinde taşınan setalar bulunabilir [22]. Diseksiyon konusunda daha az deneyimli araştırmacılar, genital yapıları abdomen ucundan ayırmadan basit bir lateral kapama yapabilirler (https://zenodo.org/records/18311158). Bu durumda genital yapıların iki parçasının üst üste gelmesi, örneğin gonokoksitin iç setalarının sayımını zorlaştırabilir; ancak başarısız bir diseksiyon sonucu genital yapıların zarar görmesini önler. Daha deneyimli araştırmacılar genital yapıları ikiye açarak ayırmayı deneyebilirler. Bunun için iğnenin eğik (bevel) yüzü — intradermal reaksiyon iğnesi tipinde — genital yapılar tamamen kesilmeden içinden geçirilerek gonokoksit–gonostil komplekslerinin birbirinden ayrılması sağlanır (https://zenodo.org/records/18311158). Bu şekilde iç yüzeylerin gözlemlenmesi kolaylaşır. Ayrıca bu düzenleme, artık üst üste binmeyen parametrelerin ve parametre kılıflarının incelenmesini de kolaylaştırır. Organların üst üste gelmesine yol açan lateral kapama yapılacaksa, örneklerin mükemmel şekilde şeffaflaştırılmış olması gerekir.

4.3.2. Dişiler

Genital yapılar internaldir ve bir çift spermatekadan oluşur. Diseksiyon yapılmadığı durumlarda, bu yapılar tegumentler (vücut örtüleri) üzerinden ve abdomenin ventral pozisyonda kapatılmasıyla gözlemlenmelidir. Seçilen kapama maddesinden bağımsız olarak, özellikle yüzeyi düzgün olmayan ve yeterince şeffaflaştırılmış spermatekalar genellikle doğru şekilde gözlemlenebilir. Ancak yüzeyi düzgün, ince duvarlı spermatekaların düşük kırılma indisine sahip (zayıf refraktif) maddelerde gözlemlenmesi sorunlu

This is an Open Access article distributed under the terms of the Creative Commons Attribution License (https://creativecommons.org/licenses/by/4.0), which permits unrestricted use, distribution, and reproduction in any medium, provided the original work is properly cited.

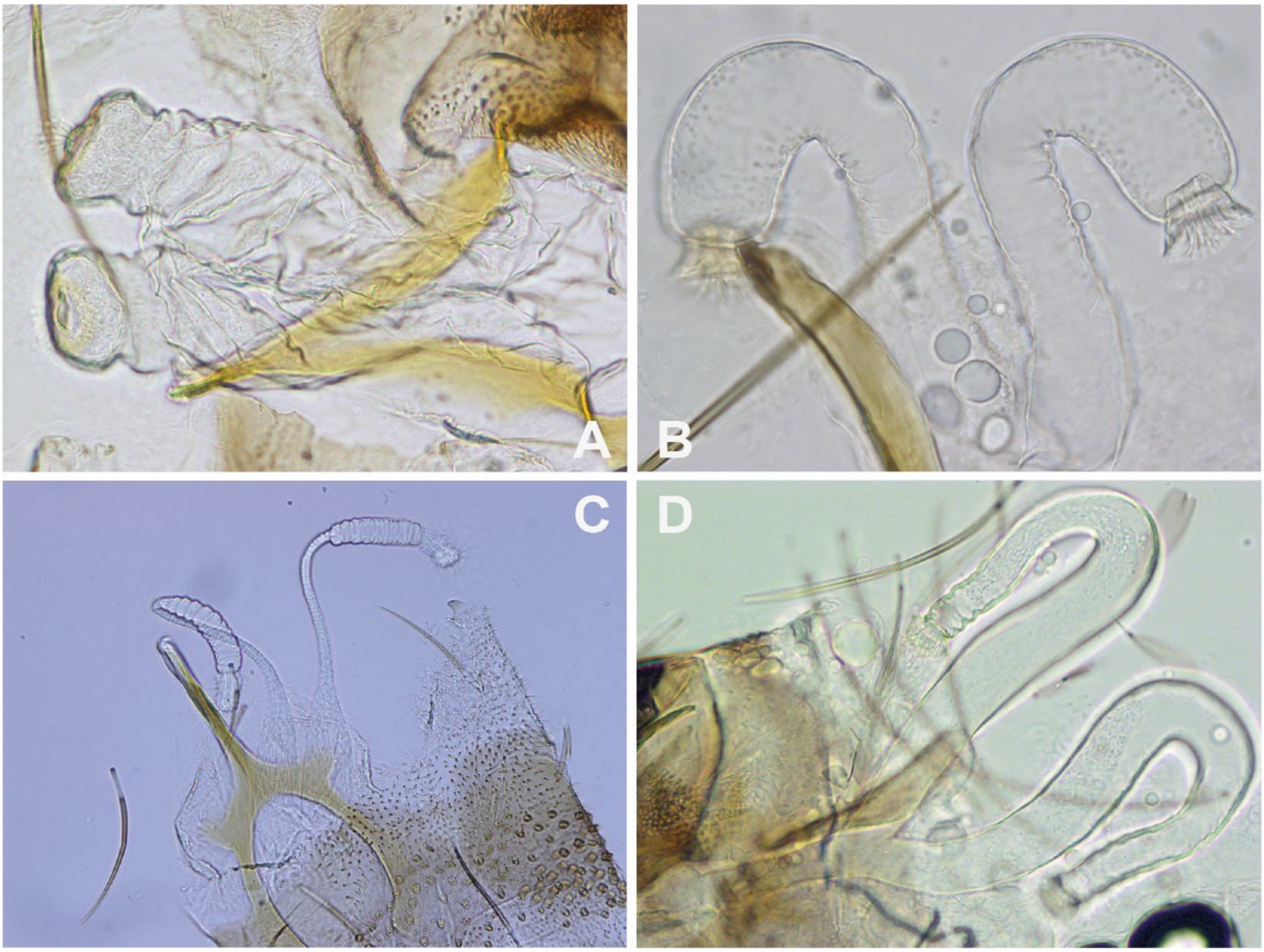

Şekil 4: Taze örneklerden diseke edilerek Marc-André sıvısında kapatılmış spermatekalar. A: *Idiophlebotomus longiforceps* (Lao PDR); B: *Sergentomyia minuta* (Fransa); C: *Phlebotomus ariasi* (Fransa); D: *Sergentomyia anodontis* (Lao PDR).

olabilir. Ayrıca, spermateka kanallarının tabanlarının gözlemlenmesi tür teşhisi açısından zorunludur; örneğin, Eski Dünya'da *Leishmania infantum*'un başlıca vektörleri olan *Larroussius* altcinsinde bu özellik kritik öneme sahiptir [35, 37, 38]. Bu yapılar incelenmeden örneklerin doğru teşhisi mümkün değildir. Bu gözlem güçlüklerini aşmak için genital furka–spermateka kompleksinin abdomenden ayrılarak kapatılması önerilir (https://zenodo.org/records/18311106). Diseksiyon sırasında spermatekaların bulunması genellikle zordur; ancak genital furka nispeten kolay tespit edilir. Spermatekal kanallar genital furkaya açıldığından, bu yapının izole edilmesi çoğunlukla spermatekaların da izole edilmesini sağlar. İşlem sırasında spermatekalar kazara kesilse bile kaybolmaz; abdomen integumentleri içinde yine de gözlemlenebilirler (Şekil 4).

4.4. *Leishmania* izolasyonu için orta bağırsak diseksiyonu

Dişi kum sineklerinde *Leishmania*'nın saptanması ve izolasyonu için sindirim kanalının diseksiyonu zorunludur. Bu işlem hem arazi hem de laboratuvar koşullarında vektörel kapasitenin değerlendirilmesi amacıyla gerçekleştirilebilir. Taze olarak öldürülmüş dişilerle çalışılması önerilir. Fazla kılları uzaklaştırmak için örnekler su veya hafif deterjan içeren fizyolojik tuzlu su ile yıkanmalıdır. Bu adım, tür teşhisi için gerekli morfolojik özellikleri korurken *Leishmania* izolasyonu için aseptik koşulların sağlanmasına yardımcı olur. *Leishmania*'yı bulmak ve izole etmek için orta bağırsak dikkatlice çıkarılmalı ve steril fizyolojik tuzlu su (%0,9 NaCl) içeren tek bir damla içine yerleştirilmelidir. Hareketli parazitler ışık mikroskobunda (önerilen büyütme: yaklaşık 200×) gözlemlendikten sonra, bir insülin enjektörü ya da mikropipet yardımıyla kültür besiyerine aktarılmalıdır

(ayrıntılar için bkz. Bölüm 4.4.3). Baş ve genital yapılar, şeffaflaştırma amacıyla doğrudan Marc-André sıvısında kapatılmalıdır. Önemli: Marc-André sıvısının *Leishmania* ile — doğrudan ya da dolaylı olarak (aletler veya iğneler aracılığıyla) — temas etmesine asla izin verilmemelidir; çünkü bu sıvı parazitler için öldürücüdür. Dişi kum sineklerinin diseksiyonu tek lam üzerinde ya da iki ayrı lamda gerçekleştirilebilir; her iki yöntemin de kendine özgü avantajları ve sınırlılıkları vardır (Şekil 5; https://zenodo.org/records/18311154).

4.4.1. İki lam yöntemi

İlk seçenek, iki ayrı lam üzerinde çalışmayı içerir: biri orta bağırsağın çıkarılması için steril fizyolojik tuzlu su içeren lam, diğeri ise baş ve spermatekaların Marc-André sıvısında kapatılması için kullanılır. Ancak arazi koşullarında genellikle iki ya da üç kişi kum sineklerini diseke eder ve hazırladıkları örnekleri, tür teşhisi ile bağırsakta *Leishmania* enfeksiyonunun değerlendirilmesinden sorumlu tek bir araştırmacıya iletir. Bu durumda iki ayrı lamın yönetimi, örneklerin izlenebilirliği açısından sorunlara yol açabilir ve özellikle pozitif bir bağırsak tespit edildiğinde, enfekte olan bireyin kesin olarak hangi kum sineği olduğunu belirlemeyi güçleştirebilir (https://zenodo.org/records/18311154).

4.4.2. Tek lam yöntemi

Tek bir lamın kullanılması, sonuçların izlenebilirliğini güvence altına alır. Ancak bu yöntemde bazı önlemler alınmalıdır. Bu aşamada steriliteyi en üst düzeye çıkarmak için araştırmacılar ellerini düzenli olarak hidroalkolik jel ile temizlemelidir. Buzlu (frosted) olmayan lamlar ve 22 × 22 mm boyutunda kare lameller kullanılmalı; bunlar alüminyum folyo ile sarılarak kuru hava sterilizatöründe (Poupinel etüvü) sterilize edilmelidir. Ayrıca her diseksiyon için steril iğneler kullanılmalıdır (öneri: 25G Ø 0,5 mm × 16 mm). Kum sineği, lamın ortasında bulunan steril fizyolojik tuzlu su damlası içine yerleştirilir. Baş kesilirken, sindirim kanalı kesilmeden 6. ve 7. abdominal tergitler ve sternitler arasından bir insizyon yapılır (spermatekaların çok uzun olması bekleniyorsa daha yukarıdan kesim yapılabilir). Daha sonra toraks bir iğne ile sabitlenmeli ve diğer iğne ile abdomenin en arka segmentleri nazikçe çekilerek bağırsak çıkarılmalıdır. Bu işlem başarısız olursa, abdomen ucunu bir iğne ile sabitleyip sindirim kanalını anterior kısmından çekmek mümkündür. Bu da başarısız olursa, bağırsak çevresindeki tegument mümkün olduğunca uzaklaştırılarak çıkarılmalıdır. Bağırsak çıkarıldıktan sonra, son abdominal segmentler sindirim kanalı kesilerek ayrılır. Bağırsak, lamın bir kenarına yerleştirilmiş yeni bir steril fizyolojik tuzlu su damlasına aktarılır ve nazikçe steril kare lamel ile kapatılır. Baş ve son abdominal segmentler, lamın diğer ucunda bulunan küçük bir Marc-André sıvısı damlasına aktarılır; bu sırada *Leishmania* ile herhangi bir temas olmamasına dikkat edilmelidir. Baş doğru şekilde konumlandırılır (foramen occipitale yukarı bakacak şekilde) ve spermatekalar, yukarıda belirtildiği gibi genital furka ile birlikte izole edilerek küçük yuvarlak bir lamel (Ø 12 mm; steril kare lamellerle karıştırılmamalıdır) ile kapatılır. Kum sineğinin geri kalan dokuları ve kanatları lamın ortasındaki fizyolojik tuzlu su damlasında kalır (https://zenodo.org/records/18311154). Pozitiflik durumunda veya taksonomik inceleme amacıyla toraks ve abdomen moleküler ya da proteomik çalışmalar için saklanabilir; kanatlar ise sulu bir ortamda kapatılabilir. Preparatı kalıcı hâle getirmek için, fazla Marc-André sıvısı uzaklaştırılarak yerine kloral gam (= Hoyer) ya da polivinil alkol bazlı bir kapama maddesi konulabilir.

Bu prosedürleri ayrıntılı olarak gösteren videolar mevcuttur (kum sineği orta bağırsak diseksiyonu: https://zenodo.org/records/18303014 ve kum sineği tükürük bezlerinin diseksiyonu: https://zenodo.org/records/18302850), bu nedenle burada açıklanmayacaktır.

4.4.3. Kum sineği bağırsaklarından *Leishmania* parazitinin izolasyonu ve kültürü

Enfekte dişi kum sineklerinin diseksiyonu yoluyla parazit izolasyonu, yüksek beceri gerektiren hassas bir işlemdir ve başlangıçta parazitsiz örnekler üzerinde pratik yapılması önerilir. Diseksiyon sonrası bağırsaklar, yıkama amacıyla taze bir damla steril fizyolojik tuzlu su (%0,9) ya da Locke çözeltisine aktarılır [4]. Diseke edilen bağırsaklar iki şekilde işlenebilir: i) Işık mikroskobunda incelenerek *Leishmania* promastigotlarının farklı evreleri ve lokalizasyonları gözlemlenir (özellikle stomodeal valv dikkatle değerlendirilmelidir);

ii) Promastigotların dışarı çıkışını kolaylaştırmak ve kitlesel kültür elde etmek amacıyla bağırsak açılır [4]. Doğada enfektif kum sineklerine nadiren rastlanır; bu nedenle iyi bir uygulama, başarılı izolasyon olasılığını artırır.

Bağırsakta *Leishmania* parazitleri gözlemlenirse, yeni steril iğneler kullanılmalı ve lamel kenarından kapiler etkiyle az miktarda steril fizyolojik tuzlu su eklenerek parazitlerin serbest kalması sağlanmalıdır. Bağırsak dikkatli ve hızlı bir şekilde yırtılarak parazitlerin salin içine geçmesi sağlanır. 100 µL'lik bir mikropipet ya da tüberkülin enjektörü kullanılarak parazitler toplanır ve uygun şekilde etiketlenmiş kültür besiyerine inoküle edilir.

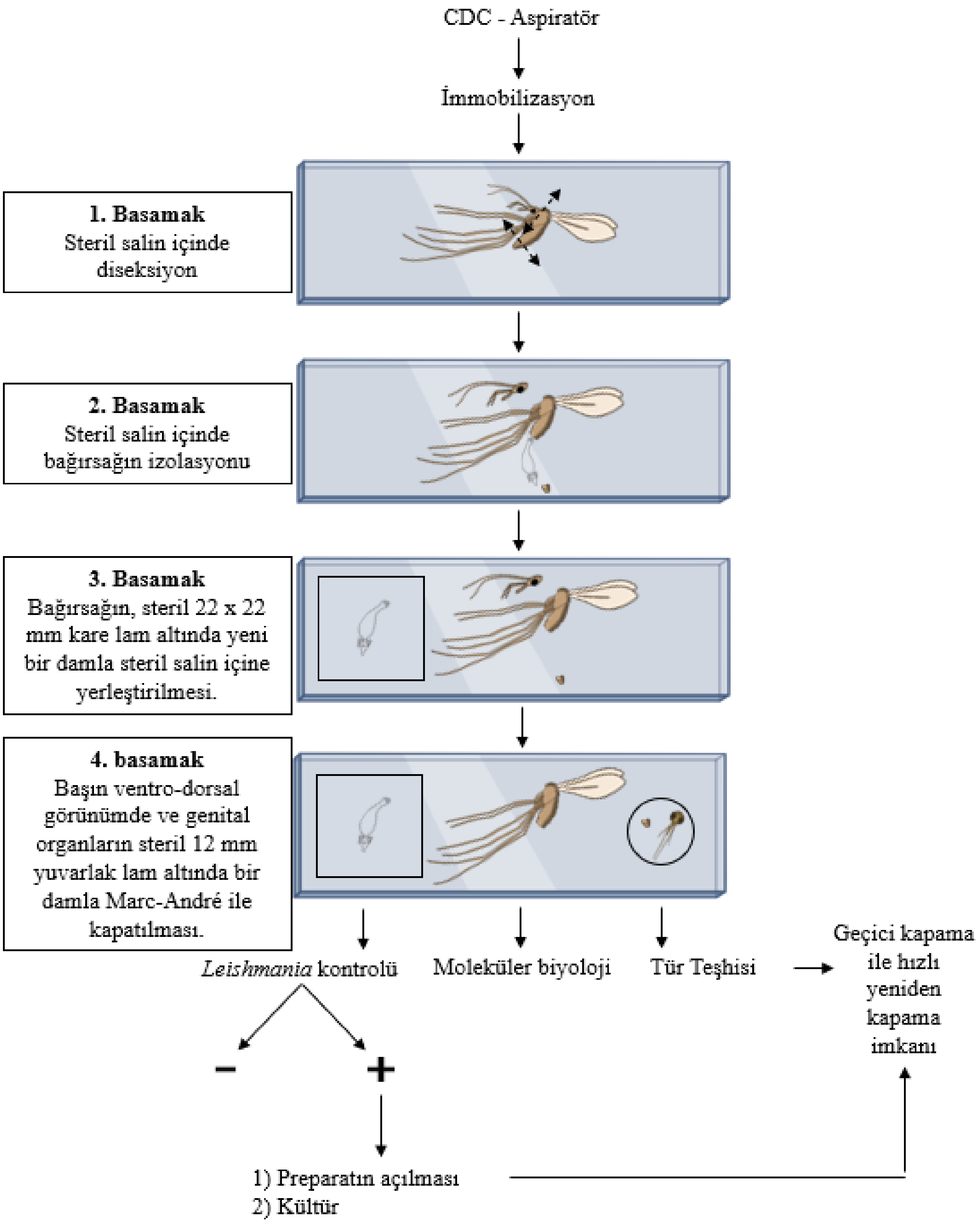

Şekil 5: *Leishmania* izolasyon yöntemi.

Leishmania promastigotlarının *in vitro* kültürü: İzole edilen parazitler başlangıçta SNB-9 kanlı agar eğik tüp kültürü ya da Novy McNeal Nicolle medium (NNN) katı besiyerinde [16] tutulur; bu maddelerin üzeri steril alfa-MEM [16, 65] ya da M199 besiyeri ile örtülür. Her iki sıvı madde de %10 ısı ile inaktive edilmiş steril fetal buzağı serumu (FCS), %1 BME vitaminleri, %2 steril insan idrarı (Filtropur® S 0,2 µm enjektör filtresi ile sterilize edilmiş), 250 µg/mL amikasin (veya 50 µg/mL gentamisin ya da L-glutamin 200 mM–penisilin 10.000 U–streptomisin 10 mg/mL içeren antibiyotik ve amino asit karışımı) [47] ile desteklenir. Üç gün sonra kontaminasyon gözlenmezse, kültürler uygun şekilde hazırlanmış dondurma ortamına aktarılır ve -80°C'de 1–2 yıl süreyle ya da uzun süreli saklama ve ileri deneysel kullanım için -196°C'de sıvı azotta muhafaza edilir [7].

4.5. Tükürük bezleri

Kum sineklerinin tükürük bezlerinin diseksiyonu, özellikle *Phlebovirus* (örneğin Toscana virüsü) gibi arbovirüslerin tespitinde vektör-parazit etkileşimlerini incelemek için temel bir tekniktir [44, 75]. Kum sineklerinin çok küçük boyutları nedeniyle, stereomikroskop altında hassasiyet gerektiren bu işlem ile ince forseps veya mikrodiseksiyon iğneleri kullanılarak tükürük bezleri zarar görmeden ve kontamine olmadan izole edilmelidir (https://zenodo.org/records/18302850) [51, 61]. Bez bütünlüğünün korunması, güvenilir moleküler analizler için kritik öneme sahiptir. İzole edildikten sonra tükürük bezleri homojenize edilip RT-PCR, qPCR veya immünoassay yöntemleri ile viral RNA veya antijenlerin varlığı test edilebilir [12]. Virüslerin yalnızca bağırsakta veya hemosölde değil, tükürük bezlerinde de bulunması, patojenin ekstrinsik inkübasyon dönemini tamamladığını ve kan emme sırasında bulaştırılabilir olduğunu doğrular [71]. Diseksiyon süreci teknik olarak zordur; tükürük bezlerinin küçük boyutu nedeniyle örneklerin bozulmasını önlemek yüksek uzmanlık gerektirir [1, 51]. Ayrıca viral yük düşük olabileceğinden, nested PCR veya yüksek verimli dizileme (high-throughput sequencing) gibi yüksek hassasiyette tespit yöntemlerin uygulanmasını gerektirebilir [54]. Kontaminasyon riskleri de steril çalışmanın önemini vurgular. Teknik zorlukların ötesinde, vektör kapasitesinin kum sineği türleri arasında değşiklik göstermesi ve enfeksiyon oranlarının ekolojik ve mevsimsel koşullara bağlı olarak dalgalanması gibi biyolojik faktörler de tespit başarısını etkiler [33, 61].

Tükürük bezlerinde virüslerin tespiti, bulaş riskine dair kritik bilgiler sağlar, sürveyans ve kontrol yöntemlerinin geliştirilmesine olanak tanır [15]. Örneğin, endemik bölgelerde Toscana virüsünün kum sineklerinde tespit edilmesi, tanı protokollerinin ve halk sağlığı uyarılarının hazırlanmasına katkı sağlamıştır [18]. Ayrıca, virüs-tükürük etkileşimlerinin incelenmesi, bulaşı engelleyici aşı veya terapötik hedeflerin belirlenmesine olanak tanıyabilir [15, 18].

Kum sineği tükürük bezleri ayrıca, konakların kum sineği ısırığına maruziyetini değerlendirmek amacıyla ELISA başta olmak üzere immünolojik yöntemler için antijen kaynağı olarak kullanılabilir. Bu yöntem, vektör kontrol yöntemlerinin etkinliğinin değerlendirilmesini [25] ve *Leishmania* bulaş riskinin izlenmesini destekler [40].

4.6. Kan emmiş dişilerde konak tayini

Örneklenen kum sinekleri arasından seçilen kan emmiş dişi kum sinekleri, çapraz kontaminasyonu önlemek için tek kullanımlık ekipman ile diseke edilmelidir. Abdomenleri, kan sindiriminin hangi evrede olduğunun belirlenmesi amacıyla bir stereomikroskop altında incelenmelidir. Sadece kırmızı, kırmızımsı-kahverengi ya da koyu kırmızı abdomenli ve yumurta oluşumu belirtisi göstermeyen dişilerin seçilmesi önerilir. Dişiyi morfolojik olarak tanımlamak için spermatekaları da içerecek şekilde abdomenin ucu diseke edilmelidir. Abdomenin ana kısmı (spermatekalar hariç) Eppendorf® tüplerine aktarılmalı ve daha sonraki analizler için -20°C'de saklanmalıdır. Konağın belirlenmesinde yaygın olarak kullanılan genetik belirteçler, örneğin PNOC [5, 30, 50], CytB [67] ya da COI [13], literatürde kapsamlı bir şekilde tanımlanmıştır; bu nedenle bu makalede ayrıntılarına girilmeyecektir (Şekil 6). Alternatif olarak, konağı belirlemek için MALDI-ToF peptid haritalama yöntemi de uygulanabilir [31]. Deneysel olarak bu tekniğin, kan emme sonrası daha uzun bir süre geçtikten sonra bile konak kanının tespitine olanak sağladığı gösterilmiştir; bu nedenle, özellikle kanın sindiriminin daha ileri seviyede olduğu dişi örneklerin analizinde uygun bir yöntemdir. Örnekler ideal olarak -20°C ya da 4°C'de saklanmalıdır; ancak kısa süre için oda sıcaklığında saklanan örnekler için de iyi sonuçlar elde edilebilir. Kan emmiş bir dişinin abdomeni, analizden hemen önce vücuttan ayrılmalı ve distile su içinde homojenize edilmelidir. Kum sineğinin kalan vücudu, diğer moleküler ve morfolojik analizler için kullanılmaya devam edebilir. Homojenattan MALDI-ToF peptid haritalama için alikotun alınmasını takiben, kalan kısım DNA izolasyonu için kullanılabilir; bu sayede hem kanın hangi konağa ait olduğu doğrulanabilir hem de *Leishmania* varlığı taranabilir. Örnek hazırlama ve analiz süresi, DNA tabanlı moleküler tekniklerle kıyaslandığında oldukça kısadır.

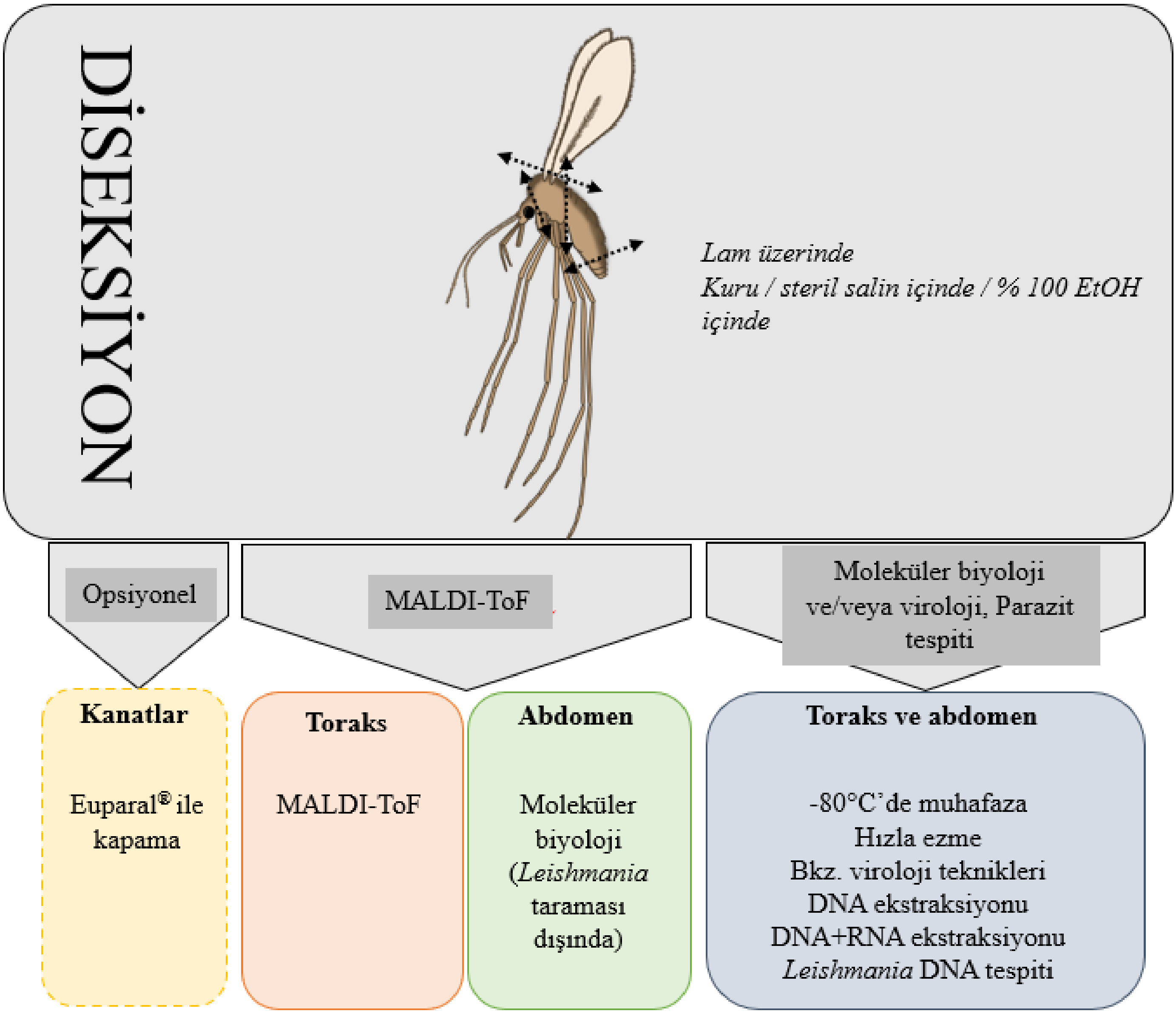

Şekil 6: Moleküler biyoloji, proteomik ve/veya viroloji uygulamaları için kum sineği işleme süreci.

5. Morfolojik Çalışmalar İçin Örnek Hazırlama (Şekiller 3, 6, 7 & 8; Ekler 1, 2, 3 & 4)

Bu bölüm, kum sineği örneklerinin yalnızca morfolojik inceleme amacıyla preparat haline getirilmesi için temel prensipleri ve ardından morfoloji dışı uygulamalara uyarlanmasını açıklar. Bu metodolojiyi anlamak önemlidir; çünkü gerektiğinde prosedürler farklı örnek tiplerine uyarlanabilir.

Örnek işleme, esnek lastik balonlu Pasteur pipetleri kullanılarak ardışık boşaltma ve dolum adımlarını içerir. Yuvarlak tabanlı cam kaplar şiddetle önerilir; çünkü bu kaplar işlemleri önemli ölçüde kolaylaştırır. Ayrıca cam, tüm reaktiflere karşı inerttir. Reaktiflerin buharlaşmasını önlemek için kaplar kapaklı olmalı ve kapak kapatılırken veya açılırken taşmayı önlemek amacıyla aşırı doldurulmamalıdır. Örneklere toz düşmesini engellemek için de kapaklı kullanım önemlidir. Kum sineklerini temizleme ve işleme sırasında gereken kimyasallar Tablo 2'de gösterilmiştir.

This is an Open Access article distributed under the terms of the Creative Commons Attribution License (https://creativecommons.org/licenses/by/4.0), which permits unrestricted use, distribution, and reproduction in any medium, provided the original work is properly cited.

Tablo 2: Kullanılan Reaktiflerin Bileşimi

**Potasyum hidroksit %10**<br>Potasyum hidroksit 10gr<br>Distile Su (qs 100 mL)	**Fuchsin Asidi %1 (Distile Suda)**<br>Fuchsin Asidi (Toz) 1 g<br>Distile Su 99 mL
**Kloral gum kapama solüsyonu (Hoyer solüsyonu)**<br>Distile su 50 mL<br>Kloral hidrat 200 g<br>Gum Arabic 50 g<br>Gliserol 20 mL	**Marc-André solüsyonu (Asit Fuchsin ile Renklendirilmiş)**<br>Marc-André solüsyonu 10mL<br>Fuchsin %1 50 µL
**Marc-André solüsyonu**<br>Kloral hidrat 40 g<br>Glasiyel asetik asit 30 mL<br>Distile su 30 mL	**Enecê solüsyonu**<br>Saf Beyaz Kolofoni 22 g<br>Alkolde Çözünebilen Kopal Reçinesi 12 g<br>Saf Etanol 20 mL<br>Kafur 10 g<br>Terebentin Esansı<br>Okaliptol 26 mL

5.1.2. Boyalı ya da boyasız şeffaflaştırma

Bu aşamayı, genellikle asetik asit ve kloral hidratı (örn. Marc-André solüsyonu) birleştiren bir şeffaflaştırma işlemi takip eder. Temizleme işleminden sonra, kalan kimyasalları gidermek için örnekler en az iki kez ardışık olarak uygulanan 20 dakikalık su banyosunda iyice durulanmalıdır.

Marc-André solüsyonu, kum sineği örneklerinin hazırlanmasında yaygın olarak kullanılan bir şeffaflaştırma maddesidir. Kanatlar ve antenler gibi hassas yapılara önemli ölçüde zarar vermeden şeffaflaştırma sürecini kolaylaştırdığı için etkinliği yüksektir.

Solüsyon, buharlaşmayı ya da bozulmayı önlemek için taze hazırlanmalı ya da sıkıca kapatılmış bir kapta saklanmalıdır. Marc-André solüsyonunun kullanımı, özellikle belirli morfolojik detayları belirginleştirmek için şeffaflaştırma ya da boyama teknikleriyle birleştirildiğinde avantajlıdır. Bileşimi ve hazırlanışına ilişkin ayrıntılar Ek 2'de verilmiştir.

Çok saydam örnekler için, kapamadan önce görünürlüğü artırmak amacıyla boyama gerekebilir. Her biri organizmanın belirli kimyasal bileşenlerini hedefleyen birçok boya mevcuttur. Hem örnekle hem de seçilen kapama maddesiyle uyumlu bir boya seçmek önemlidir. Bu temel yöntem, örneğin boyama için Marc-André solüsyonuna %0,1 asit Fuchsin eklenerek gerektiği gibi uyarlanabilir. Ek olarak, sulu çözeltilerde muhafaza edilen ve reçineli preparatlar için tasarlanan örneklerin dehidrasyonu gereklidir (Dehidrasyon hakkındaki Bölüm 5.2'ye bakınız), çünkü çoğu doğal ve sentetik reçine kapama maddesi su ile uyumlu değildir. New (1974), bazı lekelerin belirli kapama maddelerinde bozulabileceğini belirtmiştir [53]. Örneğin, Kanada balsamı ile yaygın olarak kullanılan asit Fuchsin, Euparal®'da da sabitlenebilir. Bununla birlikte, asit Fuchsin ile boyanmış örnekler, özellikle son temizleme sıvısı olarak kullanılan karanfil yağının kalıntıları kaldığında solmaya eğilimlidir. Karanfil yağında saklanan örnekler birkaç gün içinde önemli ölçüde solma gösterebilir.

5.2. Dehidrasyon

Dehidratasyon, örneklerin kademeli olarak derecelendirilmiş bir etanol çözeltisi serisinden geçirilmesiyle gerçekleştirilir: %50, %70, %80, %90 ya da %95 ve son olarak %100; her banyo en az 20 dakika sürer. Etanol hızla buharlaştığı için, işlem sırasında kap sıkıca kapatılmalıdır. Örnek tamamen dehidrate edildikten sonra, karanfil yağı yerine Euparal® özü kullanılarak işleme birkaç gün ara verilebilir. Bir zamanlar bu amaçla yaygın olarak kullanılan kayın kreozotu, toksikliği nedeniyle artık tamamen yasaklanmıştır.

Dehidrasyon işlemi, örneğin içindeki sıvının, opaklığı, ozmotik çökmeyi ya da örneği taksonomik çalışma için uygunsuz hale getirebilecek bozulmayı önlemek için kapama maddesiyle uyumlu olmasını sağlamalıdır.

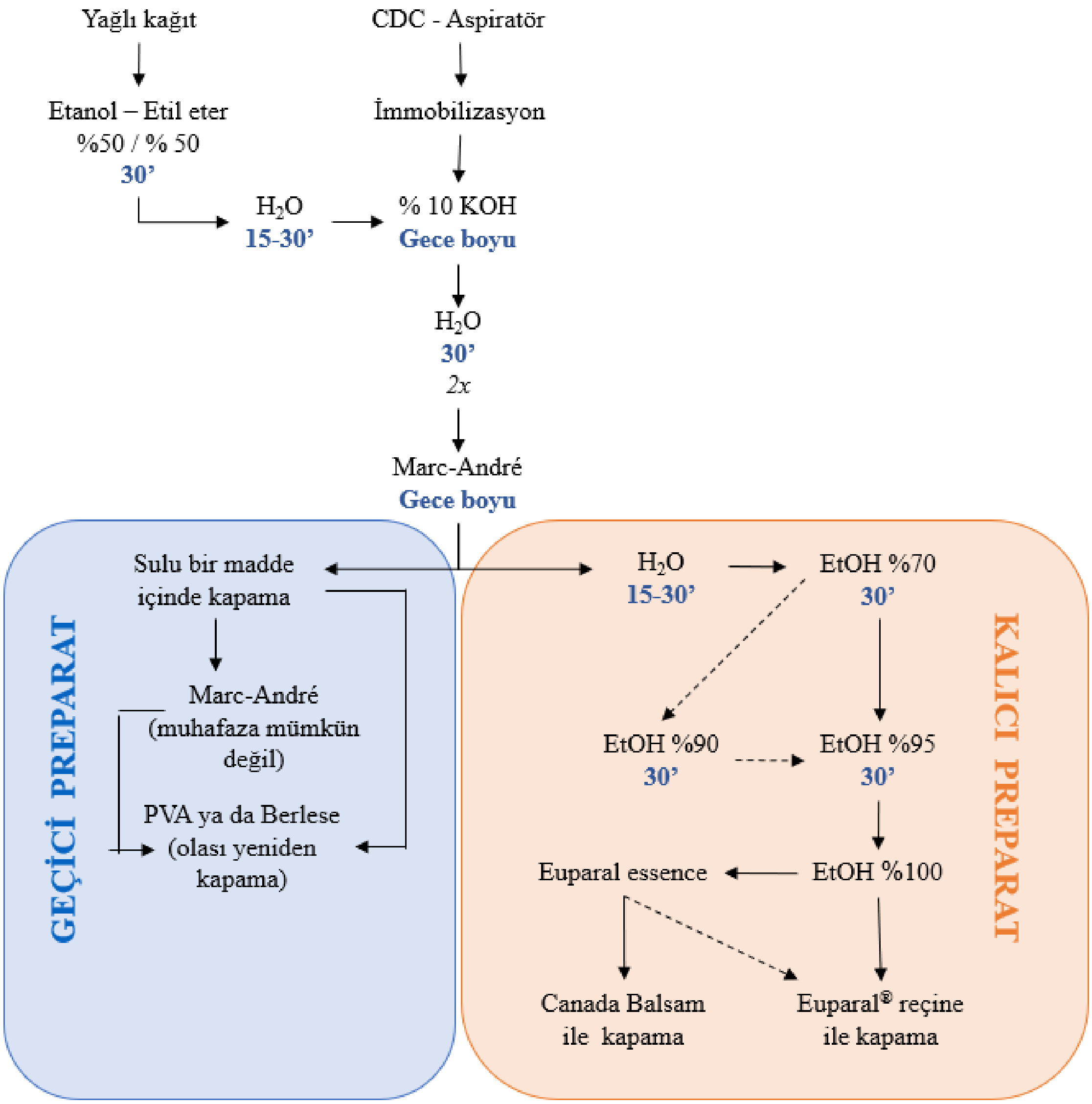

Şekil 7: Kum sineklerini işlemek için izlenen klasik yöntem.

5.3. Kapama maddeleri

5.3.1. Örneğin hazırlanması için seçim ve uygulama

İdeal olarak, kapama maddesinin kırılma indisi, yaklaşık 1,5 olan camınkine mümkün olduğunca yakın olmalıdır. Renksiz, berrak olmalı ve kuruduktan sonra ve zaman içinde tamamen şeffaf kalmalıdır. Kullanılan boyalarla uyumlu olmalı ve örneğin tüm dokularına nüfuz edip yayılabilmelidir. Çok hızlı kurumaması ya da kapama sırasında bulanıklık oluşturmaması gerekir. Kapamadan sonra büzülmemelidir. Uygun bir kapama maddesinin seçimi, örnek hazırlığının temel bir yönüdür, çünkü tek bir madde tüm amaçlar için ideal değildir. Seçim, birkaç önemli faktörü dengeleme:

- **Optik özellikler**. Kapama maddesinin kırılma indisi, spermateka, askoidler, Newstead duyu organları, dikey ağız dişleri ve faringeal dişler gibi taksonomik tanımlama ya da morfolojik tanımlama için kullanılan kritik anatomik özelliklerin yeterli derecede kontrastını ve kırılmasını sağlamalıdır. Bu yapıların görünürlüğü doğrudan kapama maddesinin optik özelliklerine bağlıdır.
- **Koruma**. Tip örnekleri ya da kalıcı koleksiyonlar için tasarlanan materyaller için, kapama maddesi uzun süreli stabilite ve dayanıklılık sunmalıdır. Buna karşılık, uzun süreli korumanın daha az kritik olduğu envanter çalışmaları ya da epidemiyolojik araştırmalar için geçici ya da yarı kalıcı kapama maddeleri yeterli olabilir.

5.3.2. Kapama maddeleri için gereksinimler

Uzmanlar genellikle belirli araştırma ihtiyaçlarına göre uyarlanmış, özelleştirilmiş ve karmaşık kapama teknikleri geliştirirler. Bununla birlikte, bu yöntemler sıklıkla arşiv kalitesi, uyumluluk, standardizasyon ya da kullanım kolaylığı ve uzun vadeli koruma gibi hususları göz ardı eder. Bu standardizasyon eksikliği, bağışlanan koleksiyonların entegrasyonunu ve uzun vadeli küratörlük çalışmalarını zorlaştırmaktadır.

Bilimsel uygulamalar, örnek hazırlama maddeleri için farklı gereksinimler ortaya koymaktadır. Taksonomistler genellikle örneklerin tamamını hazırlarlar ve kütiküler yapıların görünürlüğünü artırmak için iç organları hafifçe parçalayan maddeleri tercih ederler. Optik netliği en üst düzeye çıkarmak için kırılma indisi, örneğin ve lam ile lamelin kırılma indisinden yeterince farklı olmalıdır. Ticari kapama maddeleri genellikle, preparat-kapama maddesi-lamel sisteminde ışık kırılmasını ve saçılmasını en aza indirmek için camınkine yakın bir kırılma indisiyle formüle edilir. Bununla birlikte, parlak alan mikroskobunda, boyanmamış bir örneğin doğal kontrastı, örneğin kırılma indisinden biraz farklı bir kırılma indisine sahip bir kapama maddesi kasıtlı olarak seçilerek manipüle edilebilir ve böylece arka plana karşı görünürlüğü artırılabilir.

5.3.3. Kapama maddeleri (Tablo 3, 4)

Mikroskopi, ışığın slayt, kapama maddesi ve örnek içinden nasıl kırıldığını belirlemek için bir kapama maddesinin kırılma indeksine (RI) ihtiyaç duyar. RI, lamın kırılma indeksiyle (≈1,515) aynı olduğunda, ışık düzgün bir şekilde geçer, saçılma ve optik bozulmalar azalır, bu da çözünürlüğün ve ince yapıların görünürlüğünün iyileşmesine yol açar. Öte yandan, kırılma indisi uyumsuzluğu bulanıklığa, halelere ya da boyanmamış özelliklerin gizlenmesine neden olabilir. Farklı maddelerin farklı kırılma indisleri nedeniyle, doğru kapama maddesinin seçimi, belirli bir örnek için kontrastı, netliği ve genel görüntü kalitesini optimize etmek açısından çok önemlidir.

Kapama maddesinin kırılma indisi, kum sineklerini lam üzerine yerleştirmek için hazırlarken ince yapıların ne kadar iyi görülebileceği üzerinde önemli bir etkiye sahiptir. Cibarial armatür, spermateka, anten segmentleri ve kanat damarlanması da dahil olmak üzere kum sineklerinin hassas ve hafif sertleşmiş özellikleri, yüksek kırılma indisine sahip bir kapama maddesinde gözlemlenmesi zor olabilir.

Kum sinekleri için yaygın olarak kullanılan seçenekler arasında su bazlı kapama maddesi olarak gum-kloral ve çözücü bazlı madde olarak Kanada balsamı ve Enecê - Nelson Cerqueira (NC) reçinesi yer almaktadır. Rawlins [60] kapama maddelerini iki türe ayırmıştır: (1) kalıcı maddeler: bunlar zamanla sertleşir ve uzun süreli koruma için uygundur ve (2) yarı kalıcı maddeler: bunlar sertleşmez ve tipik olarak geçici amaçlar için kullanılır.

Tablo 3: Seçili kapama solüsyonlarının bileşimleri.

Kapama solüsyonu	Çözücü	Potansiyel ön polimer(ler) ya da polimerler	Açıklamalar
Hoyer = kloral gum	Gliserol, su	Gum Arabik bileşenleri	Maserasyon yapan madde: kloral hidrat
CMCP-9 (= karboksi metil selüloz fenol)	Su (CMCP-9: 51−60%)	Tamamen hidrolize polivinil alkol (CMCP-9: 0−5%)	CMC(P)-9: düşük viskozite: yüksek viskozite
DMHF (dimetil hidantoin formaldehit)	Su	N,N′-dimethylol dimethyl hydantoin (di-methylol DMH)<br>Eter/metilen köprülü oligomerler<br>Çapraz bağlı DMH–formaldehit polimer ağı	
Kanada balsamı	Xylene; balsamın kısmen uçucu bileşenleri (Δ^3-karen, levopimarik asit, limonen, mirsen, palustrik asit, β-phellandren, α-pinen, β-pinen)	Balsam (abienol, abietik asit, isopimarik asit, sandarakopimarik asit)	Nötralizasyon: potasyum karbonat; *Abies balsamea'*dan resin (Linné, 1758)
Euparal®	Okaliptol, paraldehit; Gum sandarakın kısmen uçucu bileşenleri (limonene, α-pinene, β-pinene)	Gum sandarakının bileşikleri (communik asit, manool, polycommunik asit, sandaracopimarik asit, 12-acetoxy-sandaracopimarik asit, sugiol, torulosik asit, torulosol, totarol)	Şeffaflaştırıcı madde: metil salisilat; renkleri Euparal® yeşili: bakır tuzu (bakır abietinat); sandarak, *Tetraclinis articulata'dan* (Vahl, 1791) alınmıştır.
Enecê	Etil alkol; kafur, okaliptol ve terpentin özü ile	Kopal sakız ve kolofoni (reçine) bileşikleri	

This is an Open Access article distributed under the terms of the Creative Commons Attribution License (https://creativecommons.org/licenses/by/4.0), which permits unrestricted use, distribution, and reproduction in any medium, provided the original work is properly cited.

Kapama maddesi sıvı, sakız bazlı ya da reçineli olabilir; su, alkol ya da diğer çözücülerde (örneğin, toluen, ksilen) çözünebilir (Tablo 3). Uygulandıktan sonra, çözünmeyen halkalama ortamı kullanılarak atmosfer etkilerinden korunmalıdır. Kapama maddesi türlerini net bir şekilde ayırt etmek için aşağıdaki kategarizasyon kullanılabilir:

a. Sulu maddeler. Bu maddeler suda kolayca çözünür, bu da geçici ya da yarı kalıcı preparatlar için uygundur. Genellikle kullanımı kolaydır, ancak özellikle tropikal nemli iklimlerde atmosfer nemine (örn. gum-kloral ve polivinil alkol) maruz kalmayı önlemek için sızdırmazlık gerektirebilir.

b. Sınırlı suya dayanıklı madde. Bu maddeler sudan daha az etkilenir, ancak aşırı neme karşı korunmaya gereksinimi vardır. Suya çözünür seçeneklere kıyasla uzun vadeli daha yüksek dayanıklılık sağlarlar ve yarı kalıcı praparatlarda sıkça kullanılırlar.

Tablo 4: Mikroskop slaytları ve çeşitli kişiler tarafından yayımlanmamış gözlemler hakkında seçili kapama malzemelerinin avantajları ve dezavantajları [52].

**İsim**	**Avantajlar**	**Dezavantajlar**
Kanada balsamı	Bu madde son derece dayanıklıdır ve ömrü 150 yılı aşmaktadır.<br>Preparatlar, kapama maddesi olarak karanfil yağı ya da fenol kullanılarak hazırlanabilir.	Zararlı bileşenler içerir ve çeker ocak altında işlem görmelidir.<br>Tam ve zaman alıcı bir dehidrasyon serisi gerektirir.<br>Etanol dehidrasyonu ve ksilen ya da karanfil yağı yoluyla transfer, bazı taksonları kırılgan hale getirebilir; alternatifler (örneğin, izopropanol, n-butanol, Cellosolve™, 1,4-dioksan, Histoclear, terpineol) kırılmayı azaltabilir.<br>Ksilen yerine fenol kullanılırsa ya da artık potasyum hidroksit kalırsa örnekler kararabilir.<br>Yüksek kırılma indisleri, boyanmamış yapıları gizleyebilir.<br>Isıtmalı kurutma yapılmadığı takdirde tam kuruma yıllar sürebilir.<br>Özellikle karanfil yağı ile temizlendiğinde, kapama maddesi zamanla sararır ve koyulaşır.<br>Bazı lekeler zayıflar ve ortam asidik hale gelirse (ki bu zamanla kendiliğinden olabilir) katyonik boyalar solabilir.
DMHF (dimetil hidantoin formaldehit)	Yüksek şeffaflık<br>İyi kırılma indisi<br>Yapıların mükemmel görünürlüğü<br>Preparatların stabilitesinin oldukça iyi olması<br>Birçok boyama tekniğiyle uyumlu<br>Numunelerin iyi korunması<br>Lam ve lamel arasında iyi yapışma	Zamanla sararma olasılığı.<br>Bazı lekelerin görünümünü değiştirebilir.<br>Formaldehit hassasiyeti olan lekeler için uygun değildir.<br>Hava kabarcıkları, yavaş kuruma süresi.<br>Kapama malzemesi neme karşı hassastır.<br>Preparatın geri alınması zordur.<br>Formaldehit toksik, tahriş edici ve kanserojendir.

This is an Open Access article distributed under the terms of the Creative Commons Attribution License (https://creativecommons.org/licenses/by/4.0), which permits unrestricted use, distribution, and reproduction in any medium, provided the original work is properly cited.

Euparal (şeffaf)	50 yıldan fazla ömürlü dayanıklı kapama maddesi.<br>Doğrudan %80 etanolden kapama yapılabilir (üretici önerisi).<br>Lekelenmemiş yapıları maskelemez ve zamanla sararmaz ya da kırılgan olmaz.<br>Diptera için Kanada balsamından daha uygun bir kırılma indisine sahiptir.<br>Minimum büzülme ve kabarcıksız kurutma sayesinde kalın örnekler için iyi çalışır.<br>%95'lik etanolde çözünebilirliğini koruduğu için, yıllar sonra bile yeniden kullanılabilir.	Zararlı bileşenler içerdiği için çeker ocak altında kullanılmalıdır.<br>Etanol ile dehidrasyon ve Euparal Essence yoluyla transfer, bazı taksonları kırılgan hale getirebilir, ancak izopropanol kullanımı bu sorunu azaltabilir.
Hoyer solüsyonu	Örnekler canlı olarak ya da doğrudan su, etanol ya da formaldehitten alınıp preparat haline getirilebilir.<br><br>Maserasyon mükemmel kütikül kalitesi sağlar.<br>Uygun bir kırılma indeksine sahiptir ve daha yüksek kontrast için iyot boyamasıyla iyileştirilebilir.<br><br>Formüldeki asetik asit, arthropod uzuvlarını genişletebilir.<br><br>Bazı örnekler 40-60 yıl boyunca stabil kalabilir.<br><br>Suda çözünür, bu da yeniden kapamayı kolaylaştırır.	Hassas bitki örnekleri, kapama maddesi kademeli olarak eklenmediği takdirde çökebilir; bu da zaman alıcıdır.<br>10 yıldan kısa sürede boşluklar ve kristaller oluşabilir.<br>Kloral hidrat konsantrasyonuna ve maruz kalma süresine bağlı olarak maserasyon aşırı olabilir.<br>Maddenin bileşenleri ayrılabilir ve aylar ya da yıllar içinde ince granülasyon oluşabilir.<br>Maddenin karardığı da bildirilmiştir.
CMCP-9<br>(= karboksi metil selüloz fenol)	Örnekler, su, etanol, gliserol ya da formaldehit içeren çözeltilerden doğrudan hazırlanabilir ve genel inceleme ya da hazırlığı kolaylaştırmak için gerektiğinde iç organlar parçalanabilir.	Bu madde zamanla kristaller oluşturabilir ve koyulaşabilir, ayrıca bazen örnekleri amaçlanandan daha fazla parçalayabilir. Lam dikkatlice halkalanmadığı takdirde, daha kalın numuneler bu kapama maddesinde iyi sonuç vermez çünkü büzülerek lamın kenarlarında boşluklar oluşturabilirler. Boyalı örnekler ya da kalsifiye malzemeler için uygun değildir ve kuruma süresi CMC'den daha yavaştır.

Eukitt™	30 yıldan fazla dayanıklı, uzun ömürlü bir kapama maddesi.<br><br>Aseton, benzen, kloroform, dioksan, eter, izopropanol, metil benzoat, terpineol, toluen ve ksilen dahil olmak üzere birçok kapama çözücüsüyle uyumludur.<br><br>Hızlı kurur ve hafif asidik bir pH'a sahiptir.<br>Yaşlandıkça belirgin şekilde koyulaşmaz.<br>Çeşitli boyalar için uygundur (örneğin, fuksin, hematoksilin, metil yeşil, metil mor, metilen mavisi).<br>Örnekler, uzun süre ksilende bekletilerek yıllar sonra tekrar preparat haline getirilebilir.	Zararlı bileşenler içerdiği için çeker ocak altında kullanılmalıdır.<br>Tam ve zaman alıcı bir dehidrasyon serisi gerektirir.<br>Büzülme ve gaz kabarcığı oluşumu nedeniyle daha kalın örnekler için ideal değildir.<br>Cam iyi temizlenip kapatılmadığı takdirde, lameller zamanla ayrılabilir.<br>Kollajen liflerinin etrafında eksik polimerizasyon görülebilir.
Enecê	Son derece dayanıklı bir kapama maddesi olup en az 50 yıl dayanır.<br>Enecê zamanla kararmaz.<br>Daha esnektir, bu da böceklerin kapama maddesi içinde diseksiyonuna olanak tanır ve morfolojik yapıların konumlandırılması için yeterli süre sağlar.<br>Düşük maliyetlidir.	Tam ve zaman alıcı bir dehidrasyon serisi gerektirir.<br>Etanol dehidrasyonu ve karanfil yağı yoluyla aktarım, bazı örnekleri kırılgan hale getirebilir.<br>Böcek çok yavaş da olsa şeffaflaştırmaya devam eder; bu, duyu organları, askoidler ve basit setalar (kıllar) gibi çok küçük yapıları görmeyi zorlaştırabilir.

c. Hidrokarbonda çözünür maddeler. Bu maddeler, ksilen ya da toluen gibi organik çözücülerde ya da essenecê (enecê çözücü) içinde çözünür. Kalıcı preparat için tasarlanmıştır ve uzun vadede mükemmel bir stabilite sunar, nem ve bozulmaya dirençlidirler, bu da onları arşiv amaçlı kullanım için ideal kılar (örn. nötr Kanada balsamı).
Özetle, suda çözünen maddeler, geçici preparatlar ya da örneğin kolayca çıkarılmasını gerektiren durumlar için en iyisidir; sınırlı suya dayanıklı maddeler, orta dayanıklılık gerektiren yarı kalıcı preparatlar için uygundur ve hidrokarbonda çözünür maddeler ise arşiv ve uzun süreli muhafaza için tasarlanmış kalıcı preparatlar için tercih edilir.

5.3.4. Tavsiye edilen kapama maddelerinin tanımı (Tablo 3, 4)

<u>*Geçici gözlem için kullanılan maddeler*</u>

Kloral gum = Hoyer sıvısı/ortamı/solüsyonu (RI = 1.48)
Marc André solüsyonu, spermatekaların kısa süreli (birkaç saat, slayt nemli bir odada saklanırsa belki birkaç saat daha) gözlemlenmesi için en iyi maddedir; ayrıca fotoğraf (Şekil 4) ya da çizimler için de uygundur. Görülen spermatekaların görünür kalması, orta vadeli saklamaya olanak sağlayan sulu bir ortamda yeniden kapatılmalarını gerektirir. Spermatekalar reçinede yeniden kapatılmak için kurutulabilir, ancak kayıp riski nedeniyle önerilmez. Kloral gum ve Hoyer sıvısı eşanlamlı kabul edilir. Bu madde, suyla uyumluluğu, basitliği, hızlı uygulanabilirliği ve spermateka gibi hassas yapıların incelenmesini kolaylaştıran kırılma indisi nedeniyle iç organların gözlemlenmesinde yaygın olarak kullanılır. Ancak kloral gum, mükemmel şekilde hazırlanmadığı ya da kontrollü nem koşulları altında saklanmadığı takdirde, kristalleşme, renk değişimi ve viskozite kaybı gibi önemli dezavantajlara sahiptir. Lamelin halkalanması bu sorunları çözmez, çünkü kapama maddesi, özellikle Euparal® kullanılıyorsa, halkalama ortamıyla etkileşimi nedeniyle ciddi şekilde renk değiştirebilir (bazen neredeyse siyahlaşabilir).
Hoyer solüsyonu, Phlebotomine kum sinekleri için optik olarak en iyi solüsyon olarak kabul edilmiş ve geleneksel olarak bu amaçlarla kullanılmıştır. Bu solüsyon, gum

arabik, gliserol ve kloral hidrat dahil olmak üzere birbirine çok benzeyen çeşitli formülasyonlardan oluşmaktadır. Çeşitli formülasyonlar yanlış yorumlanmış ve yanlış alıntılanmıştır [74]. Hoyer solüsyonu, kum sineklerindeki spermatekaları gözlemlemek için iyi bir madde olsa da, uzun süreli saklama için uygun değildir. Fotoğraflar, çizimler ya da resimler de dahil olmak üzere kısa süreli gözlemler için idealdir.
Sulu solüsyonlar geçici preparatlar için uygundur, ancak uzun süreli koruma sağlayamazlar. Buna karşılık, reçine ile preparat hazırlama, genellikle yüzyıllarca süren mükemmel bir dayanıklılık sağlar, ancak kırılma özellikleri sıklıkla kaybolduğu için spermatekaların ince ayrıntılarını gizleyebilir.
Hoyer solüsyonu dehidrasyon nedeniyle zamanla bozulur (Şekil 8); bunun sonucunda küçük, beyaz, opak kloral hidrat kristalleri oluşur. Bununla birlikte, kristalleşmiş preparatlardan örnekler elde edilebilir çünkü kütikül kimyasal olarak bozulmadan kalır, ancak büyüyen kristallerden dolayı bazı fiziksel hasarlar meydana gelebilir. Bazı durumlarda, kristalleşmiş preparatlar, mantar oluşumunu önlemek için sıcak ve nemli bir ortamda timol ile yeniden nemlendirilerek eski hâline getirilebilir. Alternatif olarak, örnekler gum kloraldan suda arındırılabilir, buzlu asetik asitte dehidrate edilebilir ve Kanada balsamı ile yeniden kapatılabilir.

DMHF (dimetil hydantoin formaldehit) (RI 1.48)
Bu su bazlı madde [72] optik olarak Berlese'ye çok benzer şekilde çok iyi performans gösterir ve Berlese kadar kullanımı kolaydır. Ancak Berlese'nin aksine siyahlaşmaz ya da kristalleşmez. Kum sinekleri ve diğer Psychodidae türleri için iyi sonuç verir.

Şekil 8: Preparatların yeniden kapatılması. A: Hoyer'le kapatılmış hasarlı ve kurumuş slaytlar; B: Kurumuş bir kum sineğinin mikroskopik görüntüsü; C: Hasarlı başka bir kum sineğinin mikroskopik görüntüsü; D: Kurumuş bir preparat içeren ıslak oda; E: Euparal® ile yeniden kapatıldıktan sonra B örneğinin başı ve F: gövdesi; G: Euparal® ile yeniden kapatıldıktan sonra hasarlı C örneğinin başı ve H: gövdesi.

CMCP (kampor-mono-klorofenol) (RI = 1.41)
Bu, kum sinekleri de dahil olmak üzere hassas örneklerin şeffaf, kalıcı preparatlarını oluşturmak için kullanılan gliserin bazlı, suda çözünebilen bir maddedir. Bu maddenin avantajı, örneklerin doğrudan su ya da etanolden preparat haline getirilebilmesidir. Kum sineğini hızla gevşetir ve şeffaflaştırır, kütikülü yumuşatarak örneğin doğru konumlandırılmasına olanak tanır; bu, özellikle kanatların açılması ya da genital organların diseksiyonu için kullanışlıdır. Uzun süreli saklamayı mümkün kıldığı bildirilse de koruma süresinin tam olarak ne kadar olduğu belirsizdir. CMCP'nin en büyük sınırlaması, dikkatli kullanım gerektiren toksik ve tahriş edici bir madde olan fenol içermesidir.

<u>*Kalıcı preparat için kullanılan maddeler*</u>
Kanada balsamı (RI = 1.52-1.54)
Kanada balsamı, ilk olarak 1830'larda Andrew Pritchard tarafından ışık mikroskobu için uygun bir kapama maddesi olarak tanımlanmıştır. 150 yılı aşkın süredir başarılı bir şekilde uygulanan, kanıtlanmış arşiv kalitesi nedeniyle en yaygın kullanılan maddelerden biri olmaya devam etmektedir. Hoyer sıvı solüsyonunun aksine, Kanada balsamı kristalleşmez ya da nemi emmez. Bununla birlikte, Kanada balsamı güçlü bir şekilde otofloresan özelliktedir, bu da bazı mikroskopi teknikleri için bazen dezavantaj olabilir [60]. Ksilen yerine toksik olmayan çözücüler kullanmak, hazırlık sırasında güvenlik risklerini azaltabilir, ancak aynı zamanda kapama maddesinin daha yavaş kuruması ve daha erken kararması gibi dezavantajlar da getirebilir.

Euparal® (RI = 1.48)
Euparal®, kalıcı preparatlar için Kanada balsamına yaygın olarak kullanılan bir alternatiftir ve mükemmel uzun vadeli stabilite ve karşılaştırılabilir bir kırılma indisi sunar. Euparal®'ın özellikleri şöyle sıralanabilir: (1) Dehidrasyon gereksinimi: Kapama maddesinin son transferinden önce, numunenin dehidrate edilmesi gerekir, tipik olarak %95'ten mutlak etanole geçiş yapılır ve (2) Uzun işlem süresi: İster Kanada balsamı ister Euparal® olsun, reçine içinde son kapama dehidrasyon gerektirir, bu da örneğin genel işlem süresini uzatır. Organik çözücülerle dehidrasyon mümkün olmadığında, mutlak etanolden ekstrakte edilen örnekler, son kapamadan önce Euparal® ve Euparal özütünün eşit karışımından oluşan bir ara çözeltiye yerleştirilebilir.

Enecê (RI = 1.467)
Enecê, esas olarak küçük böcekler için kullanılan ve özellikle Brezilya'da popüler olan reçine bazlı bir kapama maddesidir. Temeli, alkol, kafur, terebentin özü ve ökaliptolde çözülmüş kolofon ve kopal sakızından oluşur. Cerqueira [11], Enecê'yi larvaların, ergin öncesi döneme ait bireylerin dış kabuklarının ve hatta ergin sivrisineklerin kalıcı preparatlarının hazırlanması için Kanada balsamına alternatif olarak tanımlamış ve o zamandan beri kum sineği preparatları için yaygın olarak benimsenmiştir. Enecê, kalıcı preparat için uygun maliyetli bir alternatif sunar, uzun süreli stabilite ve yeterli kuruma süresi sağlar, morfolojik yapıların diseksiyonuna ve doğru düzenlenmesine olanak tanır.

5.4. Preparasyonun hazırlanması ve kurutulması

Hazırlanan preparatların uzun süreli stabilitesi ve korunması için uygun şekilde kurutulması çok önemlidir. Uzun süreli saklama düşünülmeden önce preparatlar iyice kurutulmalıdır. En iyi sonuçlar için, kalıcı kapama maddesi ile hazırlanan preparatlar 2-3 hafta, yarı kalıcı madde ile hazırlananlar ise sadece 1-2 hafta boyunca yatay olarak kurutulmalıdır. Etkili bir kurutma işlemi sağlamak için, kullanılan kapama maddesine uygun bir sıcaklığa ayarlanmış bir inkübatör kullanılması ve örneklere zarar verebilecek aşırı ısıdan kaçınılması önerilir. 30°C ile 37°C arasında bir sıcaklık aralığı önerilir. Bu kurutma adımı, saklama sırasında preparatın bükülmesini, örneğin bozulmasını ya da kapama maddesinin kararsızlığını önlemek için çok önemlidir.

Preparatın hazırlığında kullanılan kapama maddesi her zaman preparat etiketinde belirtilmelidir. Mümkünse, etiket ayrıca kullanılan özel tarifi, hazırlayan kişinin adını ve hazırlık tarihini de içermelidir. Preparatlar başlangıçta genelde geçici kapama ile hazırlanır, uzun süreli saklama için tasarlanmaz. Bununla birlikte, örneğin bir "tip" serisinin parçası olarak belirlenmesi gibi, örneğin durumu değişirse, gelecekteki taksonomik çalışmalar için örneğin korunmasını sağlamak amacıyla daha kalıcı bir kapama maddesi kullanılması gereklidir.

5.5. Alternatif kapama teknikleri: kart üzerine yerleştirme

Kart üzerine yerleştirme, örneklerin doğrudan entomolojik kartlara iğnelenebildiği ya da yüzeye yapıştırılabildiği, çeşitli böcek grupları için kullanılan bir tekniktir. Küçük boyutları ve tanımlama için iç organların incelenmesi gerekliliği (bkz. madde 5) nedeniyle, bu yöntem kum sinekleri için hiç uygun değildir.

5.6. Zarar görmüş örneklerin yeniden kapatılması

Nadir ya da değerli örnekler için, https://zenodo.org/records/18315029 adresinden erişilebilen videoya göre iki aşamalı bir yaklaşım önerilir: 1) Ön gözlem yapılmasına olanak sağlamak için örneklerin sökülmeden yeniden nemlendirilmesi. Preparat için destek görevi görmesi amacıyla bir Petri kabına lamın üzerine konulacağı tutucular yerleştirilip nemlendirilecek lam bunların üzerine konulması ve nemli bir ortam oluşturmak için Petri içine birkaç milimetre çözücü konulmasıdır; bu sırada preparatın çözücü ile temas etmemesi sağlanmalıdır (Şekil 8 D). Nemlendirme için gereken süre, örneğin durumuna bağlı olarak bir günden birkaç güne kadar değişebilir. Günlük izleme ve sabır şarttır. Preparat yeterince nemlendirildikten sonra, nemli ortamdan çıkarılabilir ve mikroskobik inceleme, fotoğraf çekimi ya da çizimden önce birkaç saatliğine inkübatöre yerleştirilebilir. 2) Yeniden kapatmak için, slayt birkaç saat daha ya da gece boyunca nemli ortama geri konulabilir. Sökme işlemi binoküler mikroskop altında yapılmalıdır. İnce iğneler kullanılarak, kum sineği parçalarının yapışık kalmadığından emin olunarak, lamel dikkatlice çıkarılmalıdır (https://zenodo.org/records/18315029). Daha sonra, kum sineğinden alınan parçalar toplanmalı ve dehidrasyon ve reçineye yeniden yerleştirme işleminden önce, yıkıcı DNA/RNA ekstraksiyonunda kullanılanlara benzer küçük kuyucuklarda suyla durulanmalıdır (aşağıya bakınız). Bir preparatı sökerken, uygun bir çözücü seçmek için orijinal kapama maddesini belirlemek çok önemlidir. Sulu kapama maddeleri için su kullanılmalıdır. Kapama maddesi reçine bazlı ise (örneğin, Kanada balsamı ya da Euparal®), bir çeker ocak altında ve maske de dahil olmak üzere uygun kişisel koruyucu ekipman kullanılarak ksilen kullanılmalıdır.

Tip ya da koleksiyon örneklerinin yeniden sergilenmesi, yalnızca küratörün ve/veya örneğin sahibi olan kurumun izniyle yapılmalıdır.

6. Örneğin identifikasyonu (tür teşhisi)

6.1. Morfoloji

Kum sineklerinin tanımlanması öncelikle göğüs, kanatlar, üreme organları, kıllar ve çeşitli yapılar arasındaki spesifik morfometrik ilişkiler de dahil olmak üzere morfolojik özelliklerinin incelenmesine dayanır. Araştırmacılar, toplanan örnekleri bilinen taksonlarla karşılaştırmak için taksonomik anahtarlar, referans koleksiyonları ve orijinal tür tanımlarını kullanırlar. Her iki cinsiyette de kanat damarlanması ve baş morfolojisi, erkek üreme organlarının yapısı ve dişi spermatekalarının konfigürasyonu gibi temel teşhis özellikleri, tür tayini yapılması için kullanılmaktadır. Doğru tanımlama genellikle ayrıntılı mikroskobik inceleme gerektirir; bu incelemede genellikle üreme organları ve spermatekalar gibi ince yapıları gözlemlemek için normal mikroskop ya da daha geniş morfolojik özellikler için stereo mikroskop kullanılır.

Görüntüleme teknolojisindeki son gelişmeler, kum sineği tanımlamasında dijital görüntülemenin kullanımını kolaylaştırmıştır. Temel özelliklerin yüksek çözünürlüklü fotoğrafları ya da dijital çizimleri, referans materyallerle karşılaştırılabilir ya da bilgisayar destekli tanımlama sistemleri kullanılarak analiz edilebilir; bu da morfolojik taksonomide hem doğruluğu hem de erişilebilirliği artırır.

6.2. Kanat geometrisi

Kanat geometrisi, farklı kum sineği türlerinin tanımlanmasında ve sınıflandırılmasında kullanılan önemli bir özelliktir. Kum sineklerinin kanatları, tipik olarak uzun ve dar olup iyi gelişmiş damarlanmaya sahip benzersiz bir desen ve yapı sergiler (Şekil 9 ve 10). Damarların dizilimi, cinsler ve türler arasında değişebilen belirgin bir desen oluşturarak tanımlama için değerli teşhis özellikleri sağlar. Sonuç olarak, kanat geometrisinin incelenmesi taksonomik amaçlar için değerli bilgiler sağlar.

6.3. Kanat geometrik morfometrisi

Araştırmacılar, farklı kum sineği türleri ya da popülasyonları arasında kanat şekli ve boyutunu analiz etmek ve karşılaştırmak için geometrik morfometri gibi çeşitli teknikler kullanmaktadır. Kanat geometrisini incelemek, davranış, habitat tercihleri ve uçuş yetenekleri hakkında değerli bilgiler sağlar.

Geometrik morfometri yaklaşımında, kanatlar dikkatlice kesilir, boyanır (gerekirse) ve düz bir şekilde preparat yapılır. Hazırlanan preparatlar daha sonra stereomikroskop altında fotoğraflanır, dijitalleştirilir ve morfometrik analize tabi tutulur. Bu prosedür literatürde iyi bir şekilde açıklanmıştır [6, 27, 42, 56, 57, 59] ve potansiyel negatif allometrik etkilerden kaçınmak için eşleştirilmiş organlar için tutarlı bir şekilde sağ ya da sol kanat kullanılması önerilmektedir [62].

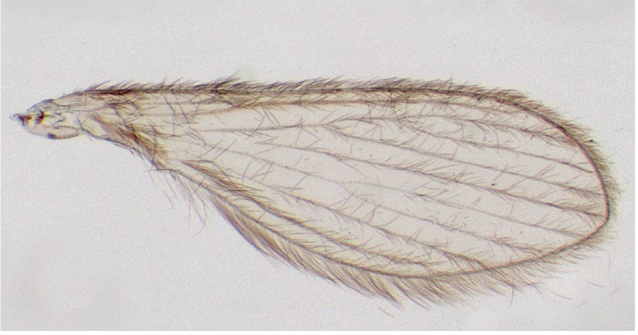

Şekil 9: *Trichophoromyia ininii*'nın işlem görmemiş kanadı

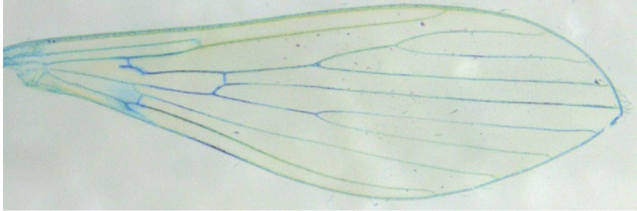

Şekil 10: *Phlebotomus ariasi*'nin boyanmış kanadı

<u>*Geometrik morfometrik analiz için kanadın hazırlanması*</u>

Kanat damarlarının en iyi şekilde görüntülenmesi için, kanatlar kıllardan temizlenmeli ve uygun şekilde boyanmalıdır. Kanat hazırlığı için, önce küçük kuyucukları gerekli reaktiflerle (metilen mavisi, etanol, su ve ksilen ikamesi) doldurun. Eppendorf tüpünü ters çevirip kuyucuğun üzerine boşaltarak oda sıcaklığında %70 etanolde muhafaza edilmiş bir kanadı alın, ardından ince kavisli bir iğne kullanarak kanadı uzunlamasına kaldırın. Kılları temizlemek için kanadı kısa süreliğine etanolden suya ve tekrar etanole geçirin. Kanadı 6 dakika boyunca metilen mavisine koyun ve boyama sırasında yüzdüğünden emin olun. Kanadı dikkatlice çıkarın ve 2 dakika boyunca (metilen mavisinin süresinin yaklaşık üçte biri kadar) ksilen ikamesine batırın. İğnenin kuyucuk duvarlarına hafifçe vurulması kanadın yerleşmesine yardımcı olabilir; ksilen, renklendirmeyi sabitlemeye yarar. Son olarak, kanadı kaldırın ve mikroskop lamı üzerindeki küçük bir damla Euparal® üzerine yerleştirin. Büyüteç altında, kanadı nazikçe açın ve dikkatlice bir lamel yerleştirin. Euparal® sertleşmeden hemen önce fotoğraflar çekilmelidir, çünkü optimum hizalamayı sağlamak için lamelin altında bulunan kanadın pozisyonunda küçük ayarlamalar gerekebilir.

6.4. Moleküler biyolojik teknikler

Morfolojik tekniklere ek olarak, taksonomik, popülasyon genetiği ve filogenetik çalışmaların yanı sıra DNA/RNA patojen tespiti ve kan besin analizi de dahil olmak üzere entomolojik araştırmalarda moleküler yöntemler giderek daha önemli hale gelmektedir; vektör davranışı epidemiyolojide oldukça önemlidir [70]. DNA dizileme, tür doğrulaması ya da yakından ilişkili türleri ayırt etmek için kullanılabilir ve daha doğru ve güvenilir bir tanımlama yöntemi sağlar. Dahası, gelişmiş moleküler teknikler (yani PCR, DNA dizileme, NGS, vb.) ve MALDI-ToF MS, geleneksel morfolojik yöntemleri tamamlayarak doğru ve hızlı tür tanımlaması için önem kazanmaktadır [46]. Bu gelişmelere rağmen, morfolojik tanımlama taksonomi için referans standart ve moleküler verilerin yorumlanmasının temeli olmaya devam etmektedir.

6.4.1. Yıkıcı nükleik asit ekstrasyonu

Nükleik asit ekstraksiyonu birçok biyolojik çalışmada rutin bir adımdır ve biyolojik materyallerden DNA'yı izole etmek için çeşitli yöntemler geliştirilmiştir [48]. Piyasada bulunan birçok DNA ekstraksiyon kiti bu süreci kolaylaştırmak için tasarlanmıştır [14]. Bununla birlikte, arthropod örneklerinin morfolojik tanımlama için hazırlanmasında yaygın olarak kullanılan yöntemler örneğin kritik fiziksel özelliklerine zarar verebileceği ya da bunları yok edebileceği için DNA analizini genelde engellemektedir [10]. Böcek dokuları için DNA ekstraksiyon protokollerinin çoğu doğası gereği tahrip edicidir [43], bu da özellikle küçük örnekler için endişe yaratmaktadır; burada sınırlı örnekleme bile önemli morfolojik özellikleri tehlikeye atabilir [72]. Örneğin türü

ve durumu, uygun bir DNA ekstraksiyon yönteminin seçilmesinde önemli bir rol oynamaktadır [29].

Kum sineklerinin doğru tanımlanması, popülasyon dinamiklerinin anlaşılması ve hedef dışı etkilerin en aza indirilmesi ihtiyacı, moleküler tanı araçlarının geliştirilmesini tetiklemiştir [23]. Moleküler yaklaşımlar artık kum sineklerinin tanımlanması için morfolojik taksonomik yöntemleri tamamlamak amacıyla sıklıkla kullanılmaktadır. Örneğin, böcek barkodlama için standart yaklaşım, DNA ekstraksiyonu, dizileme ve orijinal örneğin kaybını içerir. Bu nedenle, hem biyolojik materyali hem de morfolojik bütünlüğünü koruyan tahrip edici olmayan DNA ekstraksiyon yöntemlerini araştırmak için acil bir ihtiyaç vardır.

Kum sineklerine çok sayıda nükleik asit ekstraksiyon yöntemi uygulanmıştır. Gerekli nükleik asitlerin miktarı ya da kalitesi, farklı tekniklerin değişen hassasiyet ve saflık gereksinimlerine bağlı olarak, sonraki moleküler analize bağlıdır [9]. Örneğin, kum sineği gözlerinin PCR amplifikasyonunu inhibe ettiği bulunmuştur [69]. Patojen taramasının ötesinde, kum sineği DNA'sı tür tanımlama amacıyla rutin olarak çıkarılmaktadır. Çeşitli ekstraksiyon yöntemleri kullanılabilir, ancak verim ve kalite teknikler arasında farklılık gösterir. Bazı üreticilerin protokolleri araştırmacılar tarafından kum sinekleri için uyarlanmıştır [8], bu da çıkarılan nükleik asitlerin verimini ve/ya da kalitesini artırır [8, 9, 69], diğer arthropod taksonları için geliştirilen diğer uyarlamalar da kum sineklerinde kullanılabilir [58, 76]. Küçük mitokondriyal parçaları (COI ya da CytB) hedefleyen PCR analizleri genellikle yüksek DNA parçalanması içeren ekstraksiyon yöntemleriyle uyumludur. Buna karşılık, diğer uzun okuma NGS teknikleri (Oxford Nanopore ve PacBio) minimum parçalanma ve yüksek kaliteli DNA gerektirir. Spin kolon ekstraksiyonları genellikle 60 kb'ye kadar genomik DNA parçaları üretirken, fenol-kloroform ekstraksiyonu 150 kb'ye kadar parçalar üretebilir [77]. Tablo 5, kum sineği DNA'sının farklı ekstraksiyon tekniklerini özetlemekte ve bu böcekler için metodolojik uyarlamaların yapılıp yapılmadığını göstermektedir. Verim, örnek boyutuna ve hazırlama yöntemine bağlı olduğundan gösterilmemiştir. Modifikasyon sütunu, kum sinekleri ya da diğer küçük arthropodlar için ekstraksiyon protokollerine ait uyarlamaları ifade etmektedir. Ekstraksiyon yöntemi, örnek sayısı, ekstraksiyon süresi ve sonrasında kullanılan teknik gibi çeşitli kriterleri dikkate alarak seçilmelidir. NGS teknikleri yüksek moleküler ağırlıklı genomik DNA gerektirirken, burada sunulan tüm yöntemler standart PCR tabanlı uygulamalar için kullanılabilir.

Ayrıca, küçük karasal arthropodlar, kuru olarak korunmuş müze örnekleri ve yumuşak gövdeli arthropodlar için tahrip edici olmayan DNA ekstraksiyon yöntemlerini araştıran çeşitli çalışmalar da bulunmaktadır [19, 26, 28, 55, 63].

6.4.2. Yıkıcı olmayan nükleik asit ekstraksiyonu

Arthropodların, özellikle kum sineklerinin moleküler analizindeki en büyük zorluklardan biri, örneklerin entomolojik koleksiyonlara entegre edilmesi için korunmasıdır. Çoğu DNA ekstraksiyon protokolü, dokunun parçalanmasını gerektirir ve bu da orijinal örneğin korunmasını tehlikeye atar. Ancak, tahrip edici olmayan nükleik asit ekstraksiyon yöntemleri, örneğe fiziksel olarak zarar vermeden, canlılığını etkilemeden ya da morfolojisini değiştirmeden genetik materyali çıkarmak için tasarlanmıştır. Bu yöntemler, özellikle kum sinekleri gibi değerli ya da sınırlı örneklerle çalışırken, yapısal bütünlüğün korunmasının gelecekteki taksonomi, morfolojik ya da tanısal amaçlar için hayati önem taşıdığı durumlarda son derece değerlidir. Sıklıkla kullanılan bir teknik, kum sineklerinin hareketsiz hale getirildiği ve proteinaz K içeren bir lizis tamponuna yavaşça daldırıldığı, tahrip edici olmayan banyo yöntemidir. Hafif vektoliz tekniği, özellikle tip örnekleri olmak üzere kum sineklerine başarıyla uygulanmıştır [24]. Bu teknik, numuneyi tahrip etmeden DNA elde etmek için uyarlanmış geleneksel bir spin kolon kiti (bu durumda, bir DNeasy Kan ve Doku kiti, QIAGEN, Hilden, Almanya) kullanır. Değiştirilmiş lizis adımları (lizis tamponunun hacmi ve dondurma adımının eklenmesi) [17], nükleik asitlerin salınmasına olanak tanıyarak morfolojik hasarı en aza indirir [24]. Kum sinekleri ile ilgili olarak, hızlı ve ucuz olan ve örneklerin hızlı ve düşük maliyetli işlenmesini sağlayan bir HotSHOT DNA Ekstraksiyon kiti (Bento Bioworks Ltd, Londra, Birleşik Krallık) [73] kullanmak da mümkündür. Morfolojik tanımlama için tasarlanmış entomolojik örnekler daha sonra durulanabilir. DNeasy Kan ve Doku kiti ile işlenenler Marc-André solüsyonu ile temizlenmelidir, oysa HotSHOT DNA ekstraksiyon kiti ile işlenenler, bu makalede ayrıntılı olarak açıklanan protokole göre [73], sulu bir ortamda ya da daha iyisi, dehidrasyondan sonra bir reçinede kapatılacak kadar yeterince temizlenir. Çıkarılan genetik materyal daha sonra, belirli genetik belirteçleri çoğaltmak için PCR gibi ileri analizler için daha fazla işlenebilir. Yıkıcı olmayan nükleik asit ekstraksiyon yöntemleri, kum sineklerinin genetik özelliklerini incelemek, taşıyabilecekleri potansiyel hastalık ajanlarını belirlemek de dahil olmak üzere çok önemlidir. Örnek bütünlüğünü koruyarak, araştırmacılar değerli genetik bilgiler elde edebilirken, örneği ek analizler ya da çalışmalar için saklayabilirler.

Tablo 5: Phlebotomine kum sineklerinin gDNA'sının ekstraksiyonu için ortalama maliyet, uygulama ve protokol uyarlaması.

Protokol	Maliyet	Uygulama	Küçük arthropodlar için uyarlanmış protokol
Spin kolon	2.5 – 3.55 US$ [39]	PCR, NGS	[9]
Fenol-kloroform	0.24 US$ [69]	PCR, NGS	[9]
HotSHOT	<0.01 US$ [69]	PCR	-
Salting out (Tuzlama)	0.12 $3 [69]	PCR	-
Chelex	0.02 $4 [41]	PCR	[41, 76]

6.5. MALDI-ToF MS (MALDI-TOF Kütle Spektrofotometresi)

MALDI-ToF MS (matris destekli lazer desorpsiyon/iyonizasyon uçuş süresi kütle spektrometrisi), biyolojik örneklerin benzersiz protein profillerini ('parmak izlerini') tespit etmek ve analiz etmek için tasarlanmış, kütle spektrometrisi tabanlı bir tekniktir. MALDI-ToF, tıbbi ve veterinerlik açısından önem taşıyan arthropodların tanımlanmasında giderek daha önemli bir araç olarak kabul edilmektedir. Bu tekniğin ergin öncesi evrelere ait bireylerin tür seviyesinde teşhisi ve kan emmiş dişilerde konak tayinini de içeren çeşitli analizlerde başarılı sonuç verdiği kanıtlanmış ve çeşitli saklama ve homojenizasyon koşulları altında hem erkek hem de dişi kum sineği türlerini ayırt etmek için başarıyla uygulandığı gösterilmiştir [28, 30, 73, 74]. Bu yöntem, alt cins, tür ve popülasyon düzeylerinde de yüksek düzeyde ayırt edicidir. Bu teknik, araştırmacıların kum sineği dağılımını, davranışını ve hastalık bulaştırmadaki rolünü anlamaları için gerekli olan hızlı ve doğru tür teşhisine olanak tanır. MALDI-ToF, protein profillerine dayanarak türler arasında ayrım yapabilme özelliği sayesinde epidemiyolojik çalışmalarda ve vektör kontrol stratejilerinde kritik bir rol oynamaktadır. Bu tekniğin rutin uygulamasını sınırlayan iki ana dezavantajı vardır. Birincisi, kütle spektrometresi ekipmanının bulunabilirliğidir; bu ekipman, yalnızca kum sineklerinin (ya da genel olarak arthropod vektörlerin) tür tanımlaması amacıyla kolayca temin edilemeyecek kadar pahalıdır. Neyse ki, bu sınırlama, proteomik laboratuvarlarda ve/veya klinik tanı merkezlerinde standart araştırma aracı hâline gelmiş kütle spektrometrelerinin hizmet alımı yoluyla kullanılması sayesinde aşılabilir. İkinci olarak, açık erişimli veri tabanlarında kum sineği referans verileri az miktardadır; bu da ideal olarak morfolojik değerlendirme ve uygun bir genetik belirtecin (COI, cytB ya da diğer) dizilenmesinin bir kombinasyonuyla, kesin olarak tanımlanmış örneklere dayalı referans spektrumları içeren kurum içi bir veri tabanı oluşturma ihtiyacına yol açmaktadır. Bu sınırlamanın, Fransa'daki Assistance Publique-Hôpitaux de Paris, Sorbonne Üniversitesi ve Belçika'daki BCCM/IHEM/Sciensano koleksiyonu tarafından yürütülen MSI Platformu'na (https://msi.happy-dev.fr/) bugüne kadar kurum içi olarak elde edilen kum sineği referans verilerinin kademeli olarak dahil edilmesiyle yakında çözüleceği umulmaktadır. MALDI-ToF protein profillemesi planlandığında, numuneler tercihen kuru olarak dondurulmalı ya da %70 etanolde saklanmalı ve çevre sıcaklığına maruz bırakılmamalıdır. Evrensel örnek hazırlama kılavuzlarının bulunmaması nedeniyle, kullanıcıların protein spektrumlarını şimdiye kadar yayınlanmış kum sineği verileriyle karşılaştırılabilir hale getirmek ve MALDI-ToF matris hazırlığı için %60 asetonitril/%0,3 TFA'dan oluşan sulu sinapinik asit çözeltisi (30 mg/mL) kullanmaları önerilir.

MALDI-ToF Kütle Spektrofotometrisi için örnek hazırlanması (Şekil 7)
Çeşitli koşullar altında saklanan arthropod örnekleri önce oda sıcaklığında havayla kurutulur ve diseksiyon işlemine tabi tutulur. Baş ve abdomen, preparat yapmak ve morfolojik analiz için önemli morfolojik özellikler içeren vücut parçalarını elde etmek üzere çıkarılır. Toraks, MALDI-ToF için kullanılabilir ve geri kalan abdominal segmentler DNA ekstraksiyonu için saklanır. Protein profillemesi için, toraks, tek kullanımlık ezici kullanılarak 1,5 mL'lik mikrotüplerde 10 µL homojenizasyon çözeltisi ile elle homojenize edilir. Tipik olarak iki homojenizasyon çözeltisi kullanılır: steril distile su ve %25 formik asit.

7. Sonuç

Bu çalışmada, araştırmacılara, belirli araştırma hedeflerine uygun olarak, kum sineklerinin doğru tanımlanmasına ve patojen tespitine olanak tanıyan en etkili yöntemleri sunmayı amaçladık. Evrensel olarak tek bir en iyi yöntem

yoktur; aksine, her birinin kendi avantajları ve sınırlamaları olan çeşitli yöntemler mevcuttur. Ek dosyalar kısmında, kum sineklerinin hazırlanması ve tanımlanmasında kullanılan çeşitli preparasyon teknikleri için ayrıntılı protokoller sunduk. Bu protokoller, öğretici videoları da içererek, farklı amaçlara göre uyarlanmış adım adım prosedürler sunarak hassas ve güvenilir sonuçlar sağlamaktadır. Bu kapsamlı kaynağı sunarak, araştırmacıların kendi özel ihtiyaçlarına en uygun preparasyon tekniklerini seçmelerine ve uygulamalarına destek olmayı amaçlamadık.

Teşekkürler

Yazarlar, bu makalenin kalitesini artıran değerlendirmeleri için İngiltere, Londra Doğal Tarih Müzesi'nden Richard Lane ve Zoe Jay Adams'a teşekkür ederler.

Finansman

AJA'nın araştırmasına sağladıkları finansman için Brezilya kalkınma ajansları CNPq'ye (vaka numarası: 404395/2024-4) ve Araucária Vakfı'na (vaka numarası: 433/2025 PDI) teşekkür ederiz.

Çıkar çatışmaları

Jérôme Depaquit, Parasite dergisinin yardımcı editörüdür; bu makalenin değerlendirme sürecinde ve karar alma aşamasında hiçbir etkisi olmamıştır. Diğer yazarlar çıkar çatışması olmadığını beyan ederler.

Veri kullanılabilirliği bildirimi

Zenodo'daki videolar.

Video 1: https://zenodo.org/records/18198006
Video 2: https://zenodo.org/records/18311158
Video 3: https://zenodo.org/records/18311106
Video 4: https://zenodo.org/records/18311154
Video 5: https://zenodo.org/records/18303014
Video 6: https://zenodo.org/records/18302850
Video 7: https://zenodo.org/records/18315029

Ek dosyalar

https://www.parasite-journal.org/10.1051/parasite/2026009/olm

Bibliyografik referanslar

1. Alkan C, Allal-Ikhlef AB, Alwassouf S, Baklouti A, Piorkowski G, de Lamballerie X, Izri A, Charrel RN. 2015. Virus isolation, genetic characterization and seroprevalence of Toscana virus in Algeria. Clinical Microbiology and Infection, 21(11), 1040 e1-9.
2. Alten B, Ozbel Y, Ergunay K, Kasap OE, Cull B, Antoniou M, Velo E, Prudhomme J, Molina R, Banuls AL, Schaffner F, Hendrickx G, Van Bortel W, Medlock JM. 2015. Sampling strategies for phlebotomine sand flies (Diptera: Psychodidae) in Europe. Bulletin of Entomological Research 105(6), 664–678.
3. Ayhan N, Baklouti A, Prudhomme J, Walder G, Amaro F, Alten B, Moutailler S, Ergunay K, Charrel RN, Huemer H. 2017. Practical guidelines for studies on sandfly-borne phleboviruses: Part I: Important points to consider *ante* field work. Vector-Borne and Zoonotic Diseases 17(1), 73–80.
4. Bates PA. 1997. Infection of phlebotomine sandflies with *Leishmania*, in The Molecular Biology of Insect Disease Vectors: A Methods Manual. Springer. p. 112–120.5.
5. Baum M, de Castro EA, Pinto MC, Goulart TM, Baura W, Klisiowicz Ddo R, Vieira da Costa-Ribeiro MC. 2015. Molecular detection of the blood meal source of sand flies (Diptera: Psychodidae) in a transmission area of American cutaneous leishmaniasis, Parana State, Brazil. Acta Tropica, 143, 8–12.
6. Belen A, Alten B, Aytekin A. 2004. Altitudinal variation in morphometric and molecular characteristics of *Phlebotomus papatasi* populations. Medical and Veterinary Entomology, 18(4), 343–350.
7. Bhattacharya J, Chandra G, Hati AK. 1991. A simple method for cryopreservation of *Leishmania donovani* promastigotes, Indian Journal of Medical Research, 93, 245–246.
8. Caligiuri LG, Sandoval AE, Miranda JC, Pessoa FA, Santini MS, Salomón OD, Secundino NF, McCarthy CB. 2019. Optimization of DNA extraction from individual sand flies for PCR amplification. Methods and Protocols, 2(2), 36.
9. Casaril AE, de Oliveira LP, Alonso DP, de Oliveira EF, Gomes Barrios SP, de Oliveira Moura Infran J, Fernandes WS, Oshiro ET, Ferreira AMT, Ribolla PEM, de Oliveira AG. 2017. Standardization of DNA extraction from sand flies: Application to genotyping by next generation sequencing. Experimental Parasitology, 177, 66–72.
10. Castalanelli MA, Severtson DL, Brumley CJ, Szito A, Foottit RG, Grimm M, Munyard K, Groth DM. 2010. A rapid non-destructive DNA extraction method for insects and other arthropods. Journal of Asia-Pacific Entomology, 13(3), 243–248.
11. Cerqueira NL. 1943. Um novo meio para montagem de pequenos insetos em lâmina. Memórias do Instituto Oswaldo Cruz, (39), 37–41.
12. Charrel RN, Gallian P, Navarro-Mari JM, Nicoletti L, Papa A, Sanchez-Seco MP, Tenorio A, de Lamballerie X. 2005. Emergence of Toscana virus in Europe. Emerging Infectious Diseases, 11(11), 1657–1663.
13. Chaskopoulou A, Giantsis IA, Demir S, Bon MC. 2016. Species composition, activity patterns and blood meal analysis of sand fly populations (Diptera: Psychodidae) in the metropolitan region of Thessaloniki, an endemic focus of canine leishmaniasis. Acta Tropica, 158, 170–176.
14. Chen H, Rangasamy M, Tan SY, Wang H, Siegfried BD. 2010. Evaluation of five methods for total DNA extraction from western corn rootworm beetles. PLoS One, 5(8), e11963.
15. Depaquit J, Grandadam M, Fouque F, Andry PE, Peyrefitte C. 2010. Arthropod-borne viruses transmitted by Phlebotomine sandflies in Europe: a review. Eurosurveillance, 15(10), 19507.
16. Diamond LS, Herman CM. 1954. Incidence of Trypanosomes in the Canada Goose as revealed by bone marrow culture. Journal of Parasitology, 40(2), 195–202.
17. Ding H, Torno M, Vongphayloth K, Ng G, Tan D, Sng W, Ho K, Randrianambinintsoa FJ, Depaquit J, Tan CH. 2025. Hidden in plain sight: discovery of sand flies in Singapore and description of four species new to science. Parasites & Vectors, 18(1), 402.

18. Es-Sette N, Ajaoud M, Bichaud L, Hamdi S, Mellouki F, Charrel RN, Lemrani M. 2014. *Phlebotomus sergenti* a common vector of *Leishmania tropica* and Toscana virus in Morocco. Journal of Vector Borne Diseases, 51(2), 86–90.
19. Favret C. 2005. A new non-destructive DNA extraction and specimen clearing technique for aphids (Hemiptera). Proceedings of the Entomological Society of Washington, 107(2), 469–470.
20. Galati EAB. 2018. Phlebotominae (Diptera, Psychodidae): Classification, morphology and terminology of adults and identification of American taxa, in Brazilian Sand Flies: Biology, Taxonomy, Medical Importance and Control, Rangel EF, Shaw JJ, Editors. Cham: Springer International Publishing. pp. 9–212.
21. Galati EAB, de Andrade AJ, Perveen F, Loyer M, Vongphayloth K, Randrianambinintsoa FJ, Prudhomme J, Rahola N, Akhoundi M, Shimabukuro PHF, Depaquit J. 2025. Phle-botomine sand flies (Diptera, Psychodidae) of the world. Parasites & Vectors, 18(1), 220.
22. Galati EAB, Galvis-Ovallos F, Lawyer P, Leger N, Depaquit J. 2017. An illustrated guide for characters and terminology used in descriptions of Phlebotominae (Diptera, Psychodidae). Par-asite, 24, 26.
23. Gariepy T, Kuhlmann U, Gillott C, Erlandson M. 2007. Parasitoids, predators and PCR: the use of diagnostic molecular markers in biological control of Arthropods. Journal of Applied Entomology, 131(4), 225–240.
24. Giantsis IA, Chaskopoulou A, Bon MC. 2016. Mild-Vectolysis: A nondestructive DNA extraction method for vouchering sand flies and mosquitoes. Journal of Medical Entomology, 53(3), 692–695.
25. Gidwani K, Picado A, Rijal S, Singh SP, Roy L, Volfova V, Andersen EW, Uranw S, Ostyn B, Sudarshan M, Chakravarty J, Volf P, Sundar S, Boelaert M, Rogers ME. 2011. Serological markers of sand fly exposure to evaluate insecticidal nets against visceral leishmaniasis in India and Nepal: a cluster-randomized trial. PLoS Neglected Tropical Diseases, 5(9), e1296.
26. Gilbert MTP, Moore W, Melchior L, Worobey M. 2007. DNA extraction from dry museum beetles without conferring external morphological damage. PLoS One, 2(3), e272.
27. Giordani BF, Andrade AJ, Galati EAB, Gurgel-Goncalves R. 2017. The role of wing geometric morphometrics in the identification of sandflies within the subgenus *Lutzomyia.* Medical and Veterinary Entomology, 31(4), 373–380.
28. Guzmán-Larralde AJ, Suaste-Dzul AP, Gallou A, Peña-Carrillo KI. 2017. DNA recovery from microhymenoptera using six non-destructive methodologies with considerations for subsequent preparation of museum slides. Genome, 60(1), 85–91.
29. Hajibabaei M, DeWaard JR, Ivanova NV, Ratnasingham S, Dooh RT, Kirk SL, Mackie PM, Hebert PD. 2005. Critical factors for assembling a high volume of DNA barcodes. Philosophical Transactions of the Royal Society B: Biological Sciences, 360(1462), 1959–1967.
30. Haouas N, Pesson B, Boudabous R, Dedet JP, Babba H, Ravel C. 2007. Development of a molecular tool for the identification of *Leishmania* reservoir hosts by blood meal analysis in the insect vectors. American Journal of Tropical Medicine and Hygiene, 77(6), 1054–1059.
31. Hlavackova K, Dvorak V, Chaskopoulou A, Volf P, Halada P. 2019. A novel MALDI-TOF MS-based method for blood meal identification in insect vectors: A proof of concept study on phlebotomine sand flies. PLoS Neglected Tropical Diseases, 13 (9), e0007669.
32. Huemer H, Prudhomme J, Amaro F, Baklouti A, Walder G, Alten B, Moutailler S, Ergunay K, Charrel RN, Ayhan N. 2017. Practical guidelines for studies on sandfly-borne phleboviruses: Part II: Important points to consider for fieldwork and subsequent virological screening. Vector-Borne and Zoonotic Diseases, 17(1), 81–90.
33. Jancarova M, Polanska N, Thiesson A, Arnaud F, Stejskalova M, Rehbergerova M, Kohl A, Viginier B, Volf P, Ratinier M. 2025. Susceptibility of diverse sand fly species to Toscana virus. PLoS Neglected Tropical Diseases, 19(5), e0013031.
34. Kapp JD, Green RE, Shapiro B. 2021. A fast and efficient single-stranded genomic library preparation method optimized for ancient DNA. Journal of Heredity, 112(3), 241–249.
35. Killick-Kendrick R, Maroli M, Killick-Kendrick M. 1991. Bibliography of the colonization of phlebotomine sandflies. Parassitologia, 33(suppl.), 321–333.
36. Lawyer P, Killick-Kendrick M, Rowland T, Rowton E, Volf P. 2017. Laboratory colonization and mass rearing of phlebotomine sand flies (Diptera, Psychodidae). Parasite, 24, 42.
37. Léger N, Pesson B, Madulo-Leblond G. 1986. Les phlébotomes de Grèce : 1ère partie. Bulletin de la Société de Pathologie Exotique, 79, 386–397.
38. Léger N, Pesson B, Madulo-Leblond G. 1986. Les phlébotomes de Grèce : 2ème partie. Bulletin de la Société de Pathologie Exotique, 79, 514–524.
39. Leonel JAF, Vioti G, Alves ML, da Silva DT, Meneghesso PA, Benassi JC, Spada JCP, Galvis-Ovallos F, Soares RM, Oliveira T. 2020. DNA extraction from individual Phlebotomine sand flies (Diptera: Psychodidae: Phlebotominae) specimens: Which is the method with better results? Experimental Parasitology, 218, 107981.
40. Lestinova T, Rohousova I, Sima M, de Oliveira CI, Volf P. 2017. Insights into the sand fly saliva: Blood-feeding and immune interactions between sand flies, hosts, and *Leishmania.* PLoS Neglected Tropical Diseases, 11(7), e0005600.
41. Lienhard A, Schaffer S. 2019. Extracting the invisible: obtaining high quality DNA is a challenging task in small arthropods. PeerJ, 7, e6753.
42. Lozano-Sardaneta YN, Mikery-Pacheco OF, Huerta H, Rojas-Soriano JE, Contreras-Ramos A. 2025. Wing geometric morphometrics is effective to separate sand fly species (Diptera, Psychodidae, Phlebotominae) related with leishmaniasis transmission in Mexico. Acta Tropica, 262, 107523.
43. Mandrioli M. 2008. Insect collections and DNA analyses: how to manage collections? Museum Management and Curatorship, 23(2), 193–199.
44. Maroli M, Feliciangeli MD, Bichaud L, Charrel RN, Gradoni L. 2013. Phlebotomine sandflies and the spreading of leishmaniases and other diseases of public health concern. Medical and Veterinary Entomology, 27(2), 123–147.
45. Marquina D, Buczek M, Ronquist F, Lukasik P. 2021. The effect of ethanol concentration on the morphological and molecular preservation of insects for biodiversity studies. PeerJ, 9, e10799.
46. Mathis A, Depaquit J, Dvorak V, Tuten H, Banuls AL, Halada P, Zapata S, Lehrter V, Hlavackova K, Prudhomme J, Volf P, Sereno D, Kaufmann C, Pfluger V, Schaffner F. 2015. Identification of phlebotomine sand flies using one MALDI-TOF MS reference database and two mass pectrometer systems., Parasites & Vectors, 8, 266.
47. Mekarnia N, Benallal KE, Sadlova J, Vojtkova B, Mauras A, Imbert N, Longhitano M, Harrat Z, Volf P, Loiseau PM, Cojean S. 2024. Effect of *Phlebotomus papatasi* on the fitness,

infectivity and antimony-resistance phenotype of antimony-resistant *Leishmania* major Mon-25. International Journal for Parasitology – Drugs and Drug Resistance, 25, 100554.

48. Milligan BG. 1998. Total DNA isolation, in Molecular Genetic Analysis of Population: A Practical Approach, Hoelzel AR, Editor. Oxford: Oxford University Press.
49. Molina R, Jiménez M, Alvar J, González E, Hernández-Taberna S, Ines MM. 2017. Methods in sand fly research. Madrid: Servicio de publicaciones Universidad de Alcalá de Henares, Madrid.
50. Murphy WJ, Eizirik E, O'Brien SJ, Madsen O, Scally M, Douady CJ, Teeling E, Ryder OA, Stanhope MJ, de Jong WW, Springer MS. 2001. Resolution of the early placental mammal radiation using Bayesian phylogenetics. Science, 294(5550), 2348–2351.
51. Nacif-Pimenta R, Pinto LC, Volfova V, Volf P, Pimenta PFP, Secundino NFC. 2020. Conserved and distinct morphological aspects of the salivary glands of sand fly vectors of leishma-niasis: an anatomical and ultrastructural study. Parasites & Vectors, 13(1), 441.
52. Neuhaus B, Schmid T, Riedel J. 2017. Collection management and study of microscope slides: Storage, profiling, deterioration, restoration procedures, and general recommendations. Zootaxa, 4322(1), 1–173.
53. New TR. 1974. Pscoptera. Handbooks for Identification of British Insects (Vol. I). London: Royal Entomological Society of London. 102 pp.
54. Perez-Ruiz M, Collao X, Navarro-Mari JM, Tenorio A. 2007. Reverse transcription, real-time PCR assay for detection of Toscana virus. Journal of Clinical Virology, 39(4), 276–281.
55. Porco D, Rougerie R, Deharveng L, Hebert P. 2010. Coupling non-destructive DNA extraction and voucher retrieval for small soft-bodied Arthropods in a high-throughput context: the example of Collembola. Molecular Ecology Resources, 10(6), 942–945.
56. Prudhomme J, Cassan C, Hide M, Toty C, Rahola N, Vergnes B, Dujardin JP, Alten B, Sereno D, Banuls AL. 2016. Ecology and morphological variations in wings of *Phlebotomus ariasi* (Diptera: Psychodidae) in the region of Roquedur (Gard, France): a geometric morphometrics approach. Parasites & Vectors, 9(1), 578.
57. Prudhomme J, Gunay F, Rahola N, Ouanaimi F, Guernaoui S, Boumezzough A, Banuls AL, Sereno D, Alten B. 2012. Wing size and shape variation of *Phlebotomus papatasi* (Diptera: Psychodidae) populations from the south and north slopes of the Atlas Mountains in Morocco. Journal of Vector Ecology, 37(1), 137–147.
58. Prudhomme J, Toty C, Kasap OE, Rahola N, Vergnes B, Maia C, Campino L, Antoniou M, Jimenez M, Molina R, Cannet A, Alten B, Sereno D, Banuls AL. 2015. New microsatellite markers for multi-scale genetic studies on *Phlebotomus ariasi* Tonnoir, vector of *Leishmania infantum* in the Mediterranean area. Acta Tropica, 142, 79–85.
59. Prudhomme J, Velo E, Bino S, Kadriaj P, Mersini K, Gunay F, Alten B. 2019. Altitudinal variations in wing morphology of *Aedes albopictus* (Diptera, Culicidae) in Albania, the region where it was first recorded in Europe. Parasite, 26, 55.
60. Rawlins DJ. 1992. Light Microscopy: An Introduction to Biotechniques. Oxford: Bios Scientific publishers. 143 pp.
61. Ready PD. 2013. Biology of phlebotomine sand flies as vectors of disease agents. Annual Review of Entomology, 58, 227–250.
62. Rohlf FJ, Slice D. 1990. Extensions of the Procrustes method for the optimal superimposition of landmarks. Systematic Zoology, 39(1), 40–59.
63. Rowley DL, Coddington JA, Gates MW, Norrbom AL, Ochoa RA, Vandenberg NJ, Greenstone MH. 2007. Vouchering DNA-barcoded specimens: Test of a nondestructive extraction proto-col for terrestrial arthropods. Molecular Ecology Notes, 7(6), 915–924.
64. Sábio PB, Andrade AJ, Galati EAB. 2014. Assessment of the taxonomic status of some species included in the *Shannoni* complex, with the description of a new species of *Psathyromyia* (Diptera: Psychodidae: Phlebotominae). Journal of Medical Entomology, 51(2), 331–341.
65. Sadlova J, Yeo M, Seblova V, Lewis MD, Mauricio I, Volf P, Miles MA. 2011. Visualisation of *Leishmania donovani* fluo-rescent hybrids during early stage development in the sand fly vector. PLoS One, 6(5), e19851.
66. Sales K, Miranda DEO, da Silva FJ, Otranto D, Figueredo LA, Dantas-Torres F. 2020. Evaluation of different storage times and preservation methods on phlebotomine sand fly DNA concen-tration and purity. Parasites & Vectors, 13(1), 399
67. Sales KG, Costa PL, de Morais RC, Otranto D, Brandao-Filho SP, Cavalcanti Mde P, Dantas-Torres F. 2015. Identification of phlebotomine sand fly blood meals by real-time PCR. Parasites& Vectors, 8, 230.
68. Sant'Anna MR, Jones NG, Hindley JA, Mendes-Sousa AF, Dillon RJ, Cavalcante RR, Alexander B, Bates PA. 2008. Blood meal identification and parasite detection in laboratory-fed and field-captured *Lutzomyia longipalpis* by PCR using FTA databasing paper. Acta Tropica, 107(3), 230–237.
69. Senne NA, Santos HA, Araujo TR, Paulino PG, Mendonca LP, Moreira HVS, Camilo TA, da Costa Angelo I. 2022. Robust comparative performance of genomic DNA extraction methods from non-engorged phlebotomine sandflies. Medical and Veterinary Entomology, 36(2), 203–211.
70. Shaw JJ. 2025. A review of Leishmania infections in American Phlebotomine sand flies – Are those that transmit leishmaniasis anthropophilic or anthropportunists? Parasite, 32, 57.
71. Tesh RB, Modi GB. 1983. Growth and transovarial transmission of Chandipura virus (Rhabdoviridae: Vesiculovirus) in *Phlebotomus papatasi.* American Journal of Tropical Medicine and Hygiene, 32(3), 621–623.
72. Thomsen PF, Elias S, Gilbert MTP, Haile J, Munch K, Kuzmina S, Froese DG, Sher A, Holdaway RN, Willerslev E. 2009. Non-destructive sampling of ancient insect DNA. PLoS One, 4(4),e5048
73. Truett GE, Heeger P, Mynatt RL, Truett AA, Walker JA, Warman ML. 2000. Preparation of PCR-quality mouse genomic DNA with hot sodium hydroxide and tris (HotSHOT). Biotechniques, 29(1), 52–54.
74. Upton MS. 1993. Aqueous gum-chloral slide mounting media: an historical review. Bulletin of Entomological Research, 83(2), 267–274.
75. Volf P, Myskova J. 2007. Sand flies and Leishmania: specific versus permissive vectors. Trends in Parasitology, 23(3), 91–92.
76. Wang Q, Wang X. 2012. Comparison of methods for DNA extraction from a single chironomid for PCR analysis. Pakistan Journal of Zoology, 44(2), 421–426.
77. Wang Y, Zhao Y, Bollas A, Wang Y, Au KF. 2021. Nanopore sequencing technology, bioinformatics and applications. Nature Biotechnology, 39(11), 1348–1365.

Cite this article as: Randrianambinintsoa FJ, Augendre L, Prudhomme J, Martinet J-P, Loyer M, Mekarnia N, Kerkoub H, Perveen FK, Huguenin A, Kariya E, Akhoundi M, De Andrade AJ, Berriatua E, Bongiorno G, Boyer S, Christodoulou V, Da Costa-Ribeiro MCV, De Souza LAF, Ding H, Dondji B, Dvořák V, Erisoz Kasap O, Galati EAB, Gállego M, Ballart C, Gouzelou S, Haddad N, Masse RS, Mekuria AH, Ivovic V, Kaczmarek S, Shahar MK, Kirstein OD, Kniha E, Kolářová I, Lincoln T, Lucanas C, Mikov O, Nov K, Özbel Y, Pesson B, Posada Lopez LC, Prasetyo DB, Rahola N, Rebollar-Tellez EA, Rodrigues BL, Roy L, Saini P, Sanjoba C, Shimabukuro PH, Siriyasatien P, Soszynska A, Suleşco T, Sylla M, Torno M, Volf P, Vongphayloth K, Sinh Nam V, Wardhana A, Yessinou E, Zapata S, Gantier J-C & Depaquit J. 2026. Processing and mounting phlebotomine sand flies: a consensus guideline. Parasite xx, xx. https://doi.org/10.1051/parasite/2026009.

PARASiTE

An international open-access, peer-reviewed, online journal publishing high quality papers on all aspects of human and animal parasitology

Reviews, articles and short notes may be submitted. Fields include, but are not limited to: general, medical and veterinary parasitology; morphology, including ultrastructure; parasite systematics, including entomology, acarology, helminthology and protistology, and molecular analyses; molecular biology and biochemistry; immunology of parasitic diseases; host-parasite relationships; ecology and life history of parasites; epidemiology; therapeutics; new diagnostic tools.
All papers in Parasite are published in English. Manuscripts should have a broad interest and must not have been published or submitted elsewhere. No limit is imposed on the length of manuscripts.

Parasite (open-access) continues **Parasite** (print and online editions, 1994-2012) and **Annales de Parasitologie Humaine et Comparée** (1923-1993) and is the official journal of the Société Française de Parasitologie.

Editor-in-Chief:
Jean-Lou Justine, Paris

Submit your manuscript at:
https://www.editorialmanager.com/parasite

This is an Open Access article distributed under the terms of the Creative Commons Attribution License (https://creativecommons.org/licenses/by/4.0), which permits unrestricted use, distribution, and reproduction in any medium, provided the original work is properly cited.

Appendix

Ek 1: Biyokimyasal teorik temeller.

Burada ele alınan arthropodlar kum sinekleridir. Bununla birlikte, genel fikir, tanımlaması yalnızca iç morfolojik özelliklere dayanarak yapılabilen diğer çok yaygın arthropodlara da genişletilebilir. Tesadüfen, bazı iç organlar kısmen kitinleşmiştir ve morfolojileri bize değerli bilgiler sağlar. Bu nedenle besin pompalarını, spermateka ve kanallarını gözlemlemek tür tayini için gereklidir. İnceleyeceğimiz tüm reaktiflerle, böcek sabitleme aşamasından preparasyona kadar sadece redoks reaksiyonları uygulayacağımızı asla unutmamalıyız. Bizi yönlendirecek tek önlem ya da fikir, indirgeyici reaktifleri oksitleyici reaktiflerle karıştırmaktan kaçınmaktır.

Etil alkol; etanol:
Bu madde farklı şekillerde kullanılacaktır. Alkol molekülleri suya karşı güçlü bir çekime sahiptir ve bu nedenle dehidrasyon etkisi gösterirler. Bununla birlikte, düşük konsantrasyonlu (yani çok fazla su içeren) bir alkol, nükleik asitlerin bozulmasında rol oynayacaktır (su, nükleik asitlerin düşmanıdır).
Böceklerin etanolde saklanması, sadece onların muhafaza edilmesini değil, aynı zamanda dokularının fiksasyonunu da sağlar. Histolojide genellikle iki önemli kavramı birbirinden ayırırız: penetrasyon hızı ve fiksasyon hızı. İyi bir koruyucunun, dokuları fikse etmeden önce hızla derinlere nüfuz etmesi gerektiği iyi bilinmektedir. %96'lık alkol için penetrasyon katsayısı yaklaşık 1,05'tir (karşılaştırma olarak, %0,75'lik sulu pikrik asit çözeltisi için penetrasyon katsayısı 0,45 iken, %3'lük potasyum dikromat çözeltisi için bu değer 1,45'tir).
Entomologlar, böcekleri ve diğer arthropodları etanolde süresiz olarak muhafaza etmek isterler. Araziden toplanan örnekleri daha sonraki çalışmalar ya da gelecekteki araştırmacılar için saklama fikri hâlâ çok saygıdeğerdir. Bununla birlikte, bu fikir bir sitolog veya histolog için mümkün değildir. Örnekleri fiksatif içinde çok uzun süre tutmayı hedeflemek, yeniden işlenmelerini pratik olarak olanaksız hale getirebilir. Bu nedenle 10 yıldan eski örneklerin kullanımı zor, hatta olanaksızdır.
Bir diğer husus ise fikse edilecek arthropod kütlesi ile fiksatif hacmi arasındaki orandır. Zoolojik ya da tıbbi uygulamada, fikse edilecek parçaların hacminden 60 kat daha büyük bir hacim planlamak tavsiye edilir. Uygulamada, mikro arthropodlar için, fikse edilecek örneklerin belirli bir hacmi için en az 4-5 hacim alkol ekleyin. Alkolün arthropod dokularında bulunan tüm suyu uzaklaştırdıkça gücünü kaybedeceğini unutmayın.
Sonuç olarak:

- Etil alkol indirgeyici bir kimyasal maddedir (bu nedenle oksidatif fiksatiflerle uyumlu değildir);
- Proteinleri enerjik olarak çökeltir ve denatüre eder;
- Bazı karmaşık lipitleri çözer ve glikojeni çökeltir.;
- Dokuların güçlü bir şekilde kasılmasına ve sertleşmesine neden olur.

Temel potasyum ve sodium hidroksit solüsyonları:
Bu solüsyonların entomolojide kullanımı, net bir gerekçe olmadığı halde esas olarak potasyum hidroksit üzerine odaklanmıştır. Sodyum hidroksit [E524], farklı konsantrasyonlarda ya da farklı normalitede solüsyon halinde bulunur. Pelet ya da sim halinde gelir. En büyük dezavantajı, çok higroskopik olmasıdır (KOH'dan daha fazla). Proteinlerle reaksiyona girdiğinde onları çözer ve lipitlerle reaksiyona girdiğinde sabunlaşma sırasında onları sert sabunlara dönüştürür (bu, sabunlaşma sırasında sıvı sabunlar veren KOH'tan önemli bir farktır).
Potasyum hidroksit [E525], konsantre bir çözelti olarak mevcuttur, ancak her şeyden önemlisi, yaklaşık 0,1 g'lık peletler halinde formüle edilmiş olması avantajına sahiptir; bu da hassas bir terazi olmadığı durumlarda seyreltilmiş çözeltilerin hazırlanmasını büyük ölçüde kolaylaştırır. Örneğin, 1 mL damıtılmış suda 0,1 g'lık 1 pelet, %10'luk bir çözelti verir. Pelet halindeki potasyum hidroksitin ikinci avantajı, karbonatlaşmaya karşı daha düşük hassasiyetidir (KOH çözeltisi CO_2'yi bağlama konusunda yüksek bir eğilime sahiptir ve bu nedenle karbonat tuzları oluşturur).
Bu güçlü bazlar, yağ asitlerini suda çözünebilir sabunlara dönüştürerek çözünmelerini sağlamak için kullanılacaktır. Unutulmamalıdır ki, etanol gibi sabitleyici madde, örnekteki yağların bir kısmını çözmüştür. Bununla birlikte, sulu ortamdaki örneği güçlü bir bazla değiştirerek, yağ asitleri (az ya da çok kompleks) çöker ve bu nedenle güçlü baz, soğuk sabunlaştırma işlemi gerçekleştirir. Bazı durumlarda, örneğin dişilerde olduğu gibi, yağ dokuları fazla olduğunda, reaksiyonu kolaylaştırmak için sıcaklığı 35-40°C'ye yükseltmek ya da oda sıcaklığında temas süresini uzatmak avantajlı olacaktır.

Renkli asit solüsyonu/renksiz Marc-André solüsyonu:
Bu kısımda Marc-André solüsyonunun kullanımının avantajlarını ve dezavantajlarını inceleyeceğiz. Bu solüsyon kloral hidrat (trikloretaldehit monohidrat), asetik asit ve sudan oluşmaktadır. Bu solüsyon oldukça oksitleyicidir (asit ve aldehit karışımı). Potasyum hidroksit kullanımı sırasında oluşan alkali sabunları çökeltmeden, numunelerde kalabilecek fazla potasyum hidroksiti nötralize edecektir. Bu oksitleyici solüsyon, kitinde oluşan glukozaminlerin ikincil alkol fonksiyonları üzerinde de etki göstererek onları oksitleyip kitini yumuşatır. Bir diğer etkisi ise mevcut bazı mineral tuzlarının çözünmesidir. Marc-André solüsyonu önceden Asit Fuchsin ile renklendirildiğinde (yani oksitlenmiş haldeyken), yapının

This is an Open Access article distributed under the terms of the Creative Commons Attribution License (https://creativecommons.org/licenses/by/4.0), which permits unrestricted use, distribution, and reproduction in any medium, provided the original work is properly cited.

ikincil alkol fonksiyonlarına tutunabilir. Marc-André solüsyonunun temas süresi ve örneklerin boyanma durumundan sonra, durulama sadece etanol ile yapılır. Böylece numunelerin dehidrasyon aşamasına başlanır.

Faydaları:

- Fazla bazik çözeltilerin nötrleştirilmesi
- Kitinin gevşemesi
- Kitinleşmiş iç yapıların daha iyi değerlendirilmesi için kitinin boyanması

Dezavantajları:

Kloral hidrat hipnotik etkiye sahiptir ve insan tıbbında kullanılmaktadır. Kimyasal bir çeker ocak altında kullanılmalı ve kimyasal risklere ilişkin mevzuata uyulmalıdır.

Dehidrasyon solüsyonları:

Deneyimler gösteriyor ki, az sayıdaki örnekler için artan konsantrasyonlarda alkol banyoları sırasını takip etmek faydalı değildir. Örnek sayısı fazlaysa, %80 etanol ile başlayıp, ardından %90, %95 ve son olarak mutlak etanol ile devam edilir. Çok az sayıdaki örnekler için, %90 etanol ile bir banyo yapıp ardından mutlak etanole daldırma işlemi uygulanır. Bu aşamada, mutlak etanolün atmosferdeki suyu bağlamaya çalıştığını unutmamak gerekir.

Entomoloji laboratuvarlarında geleneksel yöntem, örneklerin dehidrasyonunu kayın kreozot banyosu (beech creosote bath) ile tamamlamaktı. Günümüzde, insektisit, anti-fungal ve ahşap koruyucu olarak yaygın olarak kullanılan bu özüt, kokusu (polisiklik aromatik hidrokarbonlar) nedeniyle pek tavsiye edilmemekte olup, üreme sistemine zararlı, kanserojen, kalıcı organik kirletici ve sucul organizmalar için ekotoksik bir madde olduğu varsayılmaktadır.

Örneklerin kapatılması için hazırlamayı önerdiğimiz çözelti Euparal® ve Euparal özüdür (aşağıdaki paragrafta açıklanmıştır). Euparal® ve Euparal özü karışımı çok iyi sonuç vermektedir; %90 etanol banyosundan sonra elde edilen örnekler kullanılır.

Ek 2: Kullanılan reaktiflerin bileşimi.

Potasyum hidroksit %10
Potasyum hidroksit 10 g
Distile su. 100 mL

Gum kloral kapama çözeltisi Hoyer çözeltisi
Distile su 50 mL
Kloral hidrat 200 g
Gum arabik 50 g
Gliserol 20 mL

Marc-André solüsyonu
Kloral hidrat 40 g
Glasiyal asetik asit 30 mL
Distile su 30 mL

%1 distile suda Asit Fuchsin
Asit Fuchsin tozu 1 g
Distile su 99 mL

Asit Fuchsin ile renklendirilmiş Marc-André solüsyonu
Marc-André solüsyonu 10 mL
Asit Fuchsin 1% 50 µL

Ek 3: Euparal®, Kanada balsamı, polivinil alkol ya da kapamada kullanılan diğer solüsyonlar

Polivinil alkol: Bu, uygun dehidrasyon için gerekli ürünler bulunmadığında ideal bir kapama maddesidir. Polivinil alkol daha sonra Amman laktofenolü ile karıştırılır. Bununla birlikte, bu karışımlar ya kuruma ya da suyun buharlaşması nedeniyle polivinil alkolün kristalleşmesi ya da fenol oksitlendiğinde kararma gibi önemli dezavantajlar sergiler. Geçici preparat hazırlamak için iyi bir tekniktir.

Kanada Balsamı: Bunun kullanılması için örneklerin dehidrate edilmesi gereklidir. Ksilen ya da toluen kullanımı ise bazı sakıncaları beraberinde getirir.

Enecê solüsyonu: Kanada Balsamı gibi bunun kullanılması için de örneklerin dehidrate edilmesi gereklidir. Enecê formülasyonu: saf beyaz reçine (22 g); alkolde çözünen kopal zamkı (gum copal) (12 g), mutlak alkol (20 mL), kafur (10 g), terebentin esansı (10 mL) ve ökaliptol (26 mL). Hazırlanması için, Erlenmeyer şişesi gibi bir kaba mutlak alkol ve kafur konulur. Ardından reçine ve kopal zamkı eklenir. Şişe tıpa ile kapatılır ve çalkalanır; ancak bundan sonra karışım kaynamayacak şekilde kısık ateşte benmari usulü ısıtılır. İçerik tamamen sıvılaştıktan sonra terebentin özü eklenir, karışım hâlâ sıcakken süzülür ve son olarak süzüntüye ökaliptol eklenir. Solüsyon daha az akışkan hale geldiğinde, aşağıdaki formüle sahip Eenecê ile seyreltilir: mutlak alkol (30 mL), kafur (17 g), terebentin özü (15 mL), ökaliptol (38 mL) (Cerqueira, 1943).

Euparal®: Bu, Atlas Dağları'ndaki *Tetraclinis articulata* (Vahl, 1791) selvi ağacından elde edilen ve 1906 yılında Gilson tarafından incelenip geliştirilen bir reçinedir. Başlıca avantajı polimerleşmemesidir. Lam ve lamel arasına yerleştirilen örnekler, alkol ya da daha iyisi Euparal® özütü ile kolayca geri kazanılabilir. Sandarak

olarak da adlandırılan bu reçine, %80'den fazla etanolü emer.

Triton X100'ün Kullanımı: iyonik olmayan sulu çözelti: Triton X100, hücre ve moleküler biyolojide deterjan olarak yaygın olarak kullanılan, iyonik olmayan sulu bir çözelti (4-(1,1,3,3-tetrametilbutil) fenil-polietilen glikol çözeltisi ya da t-oktilfenoksipolietoksietanol, polietilen glikol tert-oktilfenil eter) formundadır. Hücre ve çekirdek zarlarının geçirgenliğini artırır. Böcek örneklerinin uzun yıllar boyunca çeşitli nedenlerle alkolde saklamak yaygın bir uygulamadır. Ancak, alkolde saklamak pek uygun olmadığı için bu şekilde saklanan arthropodların mikroskobik inceleme için hazırlanması çok zorlaşır. Örnekleri içeren plastikler genellikle bozulur ve ardından alkol buharlaşır. Her iki durumda da alkolle uzun süreli temas ya da örneklerin kuruması gerçek bir sorun teşkil eder. 2008 yılında Jonque, fotoğraf filmlerinde kullanılan Agepon gibi bir ıslatıcı madde ile örümceklerin yeniden nemlendirilmesi üzerine bir not yayınlamış [26], bu da güçlü deterjan olmayan ıslatıcı maddelerin kullanılması fikrinin doğmasını sağlamıştır.
Aşağıda %0,5'lik sulu çözelti içinde Triton X100 kullanılarak yapılan işlem verilmiştir:

- Kuru örnek mutlak alkolle emdirilir.
- Örneğin tamamını suya batırmaya yetecek kadar miktarda %0,5'lik Triton X100 solüsyonu eklenir.
- Yaklaşık 5 dakika ya da daha fazla bekletin. Tüm arthropodların solüsyon içinde bağımsız hale gelmesi gerekir.
- Triton X100 solüsyonu uzaklaştırılır ve yerine potasyum hidroksit solüsyonu konulur.

Ardından yukarıda açıklandığı gibi teknik uygulanır.

Ek 4: Euparal® ya da Kanada Balsamı ile kapama işleminin basamakları

1. Örnekler kurutulmalıdır (bulanık ya da sütlü görünüm yetersiz kurutmayı gösterir).
2. Dehidrasyon, etil alkol konsantrasyonunun artırılmasıyla sağlanabilir.
3. Örnekler %99 alkolden ya da mutlak alkolden bir şeffaflaştırıcı maddeye aktarılabilir.

İşlem:
1. Ergin kum sineklerini %70'lik etanolün içine koyun
2. Etanolü uzaklaştırıp yerine %10 KOH ekleyin. Örneği lamel ile kapatın.
3. Örnek şeffaf hale gelene kadar masere edin.
4. KOH'u uzaklaştırın.
5. Örneği distile su ile kaplayın ve 30-45 dk. bekleyin.
6. Suyu uzaklaştırın ve distile suyla yıkama işlemini 30 dakika boyunca tekrarlayın (süre, örnek sayısına bağlıdır: birlikte işlenecek örnek sayısı ne kadar fazlaysa, bu süre o kadar uzun olmalıdır. Örnek sayısı ne kadar azsa, özellikle tek tek işlenenler için, bu süre o kadar kısa olabilir).
7. Suyu uzaklaştırın.
8. Marc-André solüsyonunu (muhtemelen asit Fuchsin ile renklendirilmiş) ekleyin ve 24 saat (bir gün) bekleyin.
9. Marc-André solüsyonunu uzaklaştırın.
10. Örneği distile su ile kaplayın ve 30-45 dk. bekleyin.
11. Suyu uzaklaştırın ve distile su ile yıkama işlemini 30 dakika boyunca tekrarlayın.
12. Suyu uzaklaştırın
13. %70 etanol ekleyin ve örneği diseke edin.
a. Baş ve abdomen bölgesi için, başı ya da abdomeni torakstan nazikçe çekerek ayırın.
b. Toraks için, toraksı bir pense ile tutup, kanatlarının tabanından diğer bir pense ile kanatları çekerek çıkarın. İlgi duyulan bölgelere bağlı olarak, toraksı sagital olarak diseke ederek sol ve sağ taraflara ayırmak da mümkündür.
14. Sulu etil alkol çözeltileri serisiyle kademeli olarak dehidratasyon işlemi yapılır. Mutlak etanole ulaşana kadar %50 – %80 – %95 oranında işlem uygulanır.
15. Örnekleri %100 etanol ile iki kez, her seferinde 10 dakika süreyle yıkayarak dehidrate edin.
16. Etanolü uzaklaştırın ve örnekleri oda sıcaklığında 15 dakika boyunca karanfil yağı ile kaplayın.
17. Örnekleri karanfil yağından temiz bir lam üzerine damlatılan Euparal® ya da Kanada Balsamı'na aktarın.
18. İstenildiği gibi düzenleyin: Kum sineğinin başı, toraksı ve abdomeni, diseksiyon mikroskobu altında ince iğneler ya da forsepsler kullanılarak diseke edilebilir. Baş, ventro-dorsal olarak kapatılabilmesi için gövdeden ayrılır; yani, oksipital foramen yukarı doğru yönlendirilerek cibaryumun doğrudan gözlenmesi sağlanır. Diseksiyon kum sineği kapama maddesi kullanılarak tamamlanır.
19. Örneği yüzeyi yapışkan hale gelene kadar bekletin.
20. Temiz bir lameli saf alkolle ıslatın. Lameli Kanada balsamının üzerine açılı bir şekilde bırakın.
21. Preparatları bu amaç için tasarlanmış kuru bir kutuda saklayın.
