# Supplementary material for: Processing and mounting phlebotomine sand flies: a consensus guideline
Source: Parasite. 2026 Apr 3;33:18. doi: 10.1051/parasite/2026009 (PMC13047900; doi:10.1051/parasite/2026009)
Supplement: Supplementary file 32 — Urdu translation / اردو ترجمہ [file parasite-33-18-s32.pdf]

Parasite **33**, 18 (2026)
© F.J. Randrianambinintsoa et al., published by EDP Sciences, 2026
https://doi.org/10.1051/parasite/2026009

PARASITE
Available online at:
www.parasite-journal.org

REVIEW ARTICLE — OPEN ACCESS

فلیبوٹومین ریتیلی مکھیوں کی پراسیسنگ اور ماؤنٹنگ: ایک اتفاق رائے سے ہدایت نامہ

Fano José Randrianambinintsoa[1], Laure Augendre[1], Jorian Prudhomme[1], Jean-Philippe Martinet[1], Mathieu Loyer[1], Nalia Mekarnia[1], Hocine Kerkoub[1], Farzana Khan Perveen[1], Antoine Huguenin[1,2], Emilie Kariya[1,2], Mohammad Akhoundi[3], Andrey José de Andrade[4], Eduardo Berriatua[5], Gioia Bongiorno[6], Sébastien Boyer[7,8], Vasiliki Christodoulou[9], Magda Clara Vieira Da Costa-Ribeiro[10], Lucas Alexandre Farias de Souza[10], Huicong Ding[11], Blaise Dondji[12], Vít Dvořák[13], Ozge Erisoz Kasap[14], Eunice Aparecida Bianchi Galati[15], Montserrat Gállego[16], Cristina Ballart[16], Stavroula Gouzelou[17], Nabil Haddad[18], Rezki Sabrina Masse[19], Asrat Hailu Mekuria[20], Vladimir Ivovic[21], Szymon Kaczmarek[22], Mohd Khadri Shahar[19], Oscar D. Kirstein[23], Edwin Kniha[24], Iva Kolářová[13], Lincoln Timinao[25], Cristian Lucanas[26], Ognyan Mikov[27], Kimsear Nov[7], Yusuf Özbel[28], Bernard Pesson[29], Laura Cristina Posada Lopez[30], Didot Budi Prasetyo[1,7], Nil Rahola[31], Eduardo A. Rebollar-Tellez[32], Bruno Leite Rodrigues[15], Lalita Roy[33], Prasanta Saini[34], Chizu Sanjoba[35], Paloma Helena Fernandes Shimabukuro[36], Padet Siriyasatien[37], Agnieszka Soszyńska[22], Tatiana Suleşco[38], Massamba Sylla[39], Majhalia Torno[40], Petr Volf[13], Khamsing Vongphayloth[41], Vu Sinh Nam[42], April Wardhana[43], Eric Yessinou[44], Sonia Zapata[45], Jean-Charles Gantier[1], and Jérôme Depaquit[1,2,*]

[1] Faculté de Pharmacie, Université de Reims Champagne Ardenne, UR ESCAPE-USC ANSES PETARD, 51 rue Cognacq-Jay, 51096 Reims Cedex, France
[2] Pôle de Biologie territoriale, Laboratoire de Parasitologie-Mycologie, Centre Hospitalo-Universitaire, 51092 Reims, France
[3] Parasitology-Mycology Department, Avicenne Hospital, AP-HP, Bobigny, Sorbonne Paris Nord University, France; Unité des Virus Émergents (UVE: Aix-Marseille Univ, Università di Corsica, IRD 190, Inserm 1207, IRBA), 13005 Marseille, France
[4] Parasitology Collection of Basic Pathology, Department of Basic Pathology, Federal University of Paraná, Curitiba 19031, Brazil
[5] Department of Animal Health, University of Murcia, Campus de Espinardo, 30100 Espinardo, Murcia, Spain
[6] Department of Infectious Diseases, Vector-borne Diseases Unit, Istituto Superiore di Sanità, 00166 Rome, Italy
[7] Medical and Veterinary Entomology Unit, Institut Pasteur du Cambodge, Phnom Penh 12201, Cambodia
[8] Ecology & Emergence of Arthropod-borne Pathogens Unit, Department of Global Health, Institut Pasteur, CNRS UMR2000, 75015 Paris, France
[9] Section Veterinary Services (1417), Laboratory for Animal Health Virology, Aglantzia, Nicosia 2109, Cyprus
[10] Insects Vectors and Parasites Laboratory, Department of Basic Pathology and Postgraduate program in Microbiology, Parasitology and Pathology, Federal University of Paraná, 81530-900 Curitiba, Brazil
[11] Department of Biological Sciences, National University of Singapore, 117558, Singapore
[12] Laboratory of the Leishmaniasis Research Project, Mokolo District Hospital, Mokolo, Cameroon; Laboratory of Cellular Immunology and Parasitology, Department of Biological Sciences, Central Washington University, 98926 Ellensburg, WA, USA
[13] Department of Parasitology, Faculty of Science, Charles University, 12800 Prague, Czechia
[14] VERG Laboratories, Department of Biology, Faculty of Science, Hacettepe University, Beytepe, Ankara 06800, Türkiye
[15] Faculdade de Saúde Pública da Universidade de São Paulo (FSP/USP), Pós-graduação em Saúde Pública, 01246-904 São Paulo, Brazil
[16] Secció de Parasitologia, Departament de Biologia, Sanitat i Medi Ambient, Facultat de Farmàcia i Ciències de l'Alimentació, Universitat de Barcelona, & Institut de Salut Global de Barcelona (ISGlobal), Centro de Investigación Biomédica en Red, Enfermedades Infecciosas (CIBERINFEC), 08028 Barcelona, Spain
[17] Laboratory of Infectious Diseases and Public Health, School of Medicine, University of Cyprus, Nicosia, Cyprus & Department of Pediatrics, Archbishop Makarios III Hospital, Nicosia 2115, Cyprus
[18] Faculty of Health Sciences, American University of Beirut, 1107 2020 Beirut, Lebanon
[19] Medical Entomology Unit, Infectious Disease Research Centre, Institute for Medical Research (IMR), National Institutes of Health (NIH), Ministry of Health Malaysia, 40170 Shah Alam, Selangor, Malaysia
[20] School of Medicine, Addis Ababa University, 28017 - 1000 Addis Ababa, Ethiopia
[21] Faculty of Mathematics, Natural Sciences and Information Technologies, University of Primorska, 6000 Koper, Slovenia
[22] University of Lodz, Faculty of Biology and Environmental Protection, Department of Invertebrate Zoology and Hydrobiology, Banacha 12/16, 90-237 Łódź, Poland

Edited by Jean-Lou Justine
*Corresponding author: jerome.depaquit@univ-reims.fr

This is an Open Access article distributed under the terms of the Creative Commons Attribution License (https://creativecommons.org/licenses/by/4.0), which permits unrestricted use, distribution, and reproduction in any medium, provided the original work is properly cited.

[23] Laboratory of Entomology, Ministry of Health, 9134302 Jerusalem, Israel
[24] Center for Pathophysiology, Infectiology and Immunology, Institute of Specific Prophylaxis and Tropical Medicine, Medical University Vienna, Kinderspitalgasse 15, 1090 Vienna, Austria
[25] Papua New Guinea Institute of Medical Research (PNGIMR) Institute, PO Box 60, Headquarter, Homate Street, 441 Goroka, Eastern Highlands Province, Papua New Guinea
[26] Museum of Natural History, University of the Philippines Los Baños, 4031 Laguna, Philippines
[27] National Centre of Infectious and Parasitic Diseases, 1504 Sofia, Bulgaria
[28] Ege University, Faculty of Medicine, Department of Parasitology, 35040 Bornova/Izmir, Türkiye
[29] Retired, Faculté de Pharmacie, Université de Strasbourg, Strasbourg, 67400 Illkirch-Graffenstaden, France
[30] Program for the Study and Control of Tropical Diseases (PECET), Faculty of Medicine, University of Antioquia, 050010 Medellin, Colombia
[31] MIVEGEC, Univ. Montpellier, CNRS, IRD, 34394 Montpellier, France & Medical Entomology Unit, Institut Pasteur de Madagascar, 101 Antananarivo, Madagascar
[32] Laboratorio de Entomología Médica, Departamento de Zoología de Invertebrados, Facultad de Ciencias Biológicas, Universidad Autónoma de Nuevo León, San Nicolás de los Garza, 66455, NL, México
[33] Tropical and Infectious Disease Centre, BP Koirala Institute of Health Sciences, Dharan 56700, Nepal
[34] ICMR-Vector Control Research Centre, Puducherry 605006, India
[35] Graduate School of Agricultural and Life Sciences, The University of Tokyo, Tokyo 113-8657, Japan
[36] Grupo de estudos em Leishmanioses/Coleção de Flebotomíneos (COLFLEB/Fiocruz-MG), Instituto René Rachou, Fundação Oswaldo Cruz, Belo Horizonte, Minas Gerais, 30190009, Brazil
[37] Center of Excellence in Vector Biology and Vector-Borne Disease, Department of Parasitology, Faculty of Medicine, Chulalongkorn University, Bangkok 10330, Thailand
[38] Department of Arbovirology, Bernhard Nocht Institute for Tropical Medicine, Bernhard Nocht Str. 74, 20359 Hamburg, Germany [39] Laboratory Vectors & Parasites, Department of Livestock Sciences and Techniques, Sine Saloum University El Hadji Ibrahima Niasse (SSUEIN) Kaffrine Campus, C.P. 24600, Senegal.
[40] Environmental Health Institute, National Environment Agency, Singapore 138667, Singapore & Department of Biological Sciences, National University of Singapore, 117558 Singapore
[41] Institut Pasteur du Laos, Laboratory of Vector-Borne Diseases, Samsenhai Road, Ban Kao-Gnot, Sisattanak District, 3560 Vientiane, Lao PDR
[42] National Institute of Hygiene and Epidemiology, 1 Yec-Xanh Street, Hai Ba Trung District, 100000 Hanoi, Vietnam
[43] Indonesian Research Center for Veterinary Science, Indonesian Agency for Agricultural Research and Development, Ministry of Agriculture Republic Indonesia, Bogor 16114, Indonesia & Department of Parasitology, Faculty of Veterinary Medicine, Airlangga University, Surabaya 60115, Indonesia
[44] Laboratory of Research in Applied Biology, Polytechnic School of Abomey-Calavi, University of Abomey-Calavi, 01 P.O. Box 2009, 00000 Cotonou, Benin
[45] Instituto de Microbiología, Colegio de Ciencias Biológicas y Ambientales (COCIBA), Universidad San Francisco de Quito (USFQ), 170901 Quito, Ecuador

Received 1 December 2025, Accepted 29 January 2026, Published online 3 April 2026

خلاصه

یہ مقالہ فلیبوٹومین ریتیلی مکھیوں کے نمونوں کی پروسیسنگ اور ماؤنٹنگ کے لیے ایک جامع رہنمائی فراہم کرتا ہے، جو انواع کی شناخت اور پیتھوجن کی تشخیص و علیحدگی کے لیے انتہائی اہم ہے۔ اس میں میدانی اور تجربہ گاہی دونوں حالات کے لیے موزوں تکنیکوں پر بحث کی گئی ہے۔ رہنما خطوط میں ریتیلی مکھیوں کے جمع کرنے، ہینڈلنگ، ڈھانپنے اور یوتھینیشیا (کیمیائی مواد کے بجائے خشک انجماد یا کاربن ڈائی آکسائیڈ کی سفارش) کے ساتھ ساتھ تحفظ کی حکمت عملیوں، جیسے که سرد ذخیرہ کاری اور ایتھنول میں محفوظ کرنے کے تفصیلی ہدایات شامل ہیں۔ بعض تشریحی ڈھانچوں (جنسی اعضاء، سر اور پر) کی تیاری کا معیار ان کے صحیح خردبینی مشاہدے کے لیے ضروری ہے اور اس مقالے میں اس کی وضاحت کی گئی ہے۔ اس میں تفصیلی نمونو ں کی پروسیسنگ بھی پیش کی گئی ہے، جس میں صاف کرنے کا عمل جیسے پوٹاشیم ہائیڈرو آکسائیڈ اور پھر مارک اینڈرے محلول جیسے ایجنٹس شامل ہیں ۔ ماؤنٹنگ کے عمل میں مختلف میڈیا کا موازنہ کیا گیا ہے، جس میں ان کی بصری خصوصیات اور تحفظ کی صلاحیت پر زور دیا گیا ہے۔ ہوئر فلوئڈ (جسے کلورل گم بھی کہا جاتا ہے) کی فوری مشاہدے کے لیے سفارش کی جاتی ہے، خاص طور پر ا سپرماتھیکی کے لیے، اپنی شفافیت کی وجہ سے، حالانکه کلورل گم طویل مدتی ذخیرہ کاری کے لیے موزوں نہیں ہے۔ دیگر میڈیا میں پولی وینائل الکحل، یوپارل® (محدود پانی برداشت کے لیے) اور کینیڈا بالسم (ایک ہائیڈرو کاربن حل پذیر میڈیم) شامل ہیں، جن میں سے آخری دو طویل مدتی تحفظ کی صلاحیت پیش کرتے ہیں۔ جدید سالماتی حیاتیاتی طریقوں جیسے ڈی این اے سیکوئنسنگ اور میلڈی-ٹوف کو بھی زیرِ بحث لایا گیا ہے، جن کے لیے نمونہ کی پروسیسنگ میں خاص توجه درکار ہوتی ہے۔

مزید برآں، مختلف ماؤنٹنگ تکنیکوں کی عکاسی کرنے والی مختصر ویڈیو کلپس کے ساتھ ساتھ 33 مختلف زبانوں میں تراجم بھی فراہم کیے گئے ہیں، جو اس رہنما خطوط کو عالمی سائنسی برادری کی متنوع ضروریات و توقعات تک پہنچنے میں مدد دیتے ہیں۔

کلیدی الفاظ

ماؤنٹنگ (چسپاں/لگائو) ، فلیبوٹومین ریتیلی مکھی، ہوئر فلوئڈ (ہوئر مائع) ، مارک اینڈری محلول (مارک اینڈرے سلوشن) ، کلورل گم ، پولی وینائل الکحل، یوپارل®، کینیڈا بالسم ، لیشمینیا کی علیحدگی، میدانی حالات (فیلڈ کنڈیشنز) ، کلچر (پرورش) ، تشریح (چیرپھاڑ/تشریح) ، مالیکیولر بائیولوجی (سالماتی حیاتیات) ، میلڈی-ٹوف (مالدی ٹی او ایف) ، نمونہ نوع (ٹائپ . نمونے)

Abstract – Processing and mounting phlebotomine sand flies: a consensus guideline. This article provides a comprehensive guide for the processing and mounting of phlebotomine sand fly specimens, which is crucial for species identification and pathogen detection and isolation. It discusses a range of techniques suitable for both field and laboratory settings. The guide includes detailed instructions on sand fly collection, handling, covering, and euthanasia (recommending dry freezing or CO_2 over chemicals) as well as conservation strategies, such as cold storage and preservation in ethanol. The quality of preparation of certain anatomical structures (genital organs, head and wings) is essential for their proper microscopic observation and is described in this work. The article also presents detailed sample processing, including the clearing process with agents such as potassium hydroxide then Marc-André solution. The mounting process compares different media, emphasizing their optical properties and preservation potential. Hoyer fluid (also known as chloral gum) is recommended for quick observation, particularly for spermathecae, due to its clarity, although it is not suitable for long-term storage. Other media discussed include polyvinyl alcohol, Euparal® (for limited water tolerance), and Canada balsam (a hydrocarbon-soluble medium), with the latter two offering long-term preservation capabilities. Innovative molecular biology approaches such as DNA sequencing and MALDI-ToF, which require particular attention to sample processing, are also addressed. Furthermore, short video clips illustrating various mounting techniques as well as translations in many different languages are provided, allowing the guideline to reach the diverse needs and expectations of the global scientific community.

Key words: Mounting, Phlebotomine sand fly, Hoyer fluid, Marc-André solution, Chloral gum, Polyvinyl alcohol, Euparal®, Canada balsam, *Leishmania* isolation, Field conditions, Culture, Dissection, Molecular biology, MALDI-ToF, Type-specimens.

تعارف

فلیبوٹومین ریتیلی مکھیاں سائیکوڈیڈی خاندان سے تعلق رکھنے والے چھوٹے ڈپٹیرن کیڑے ہیں، ذیلی خاندان فلیبوٹومینی ہے ، اور جن میں سے کم از کم 1,063 معلوم انواع ہیں [21] ۔ وہ پیتھوجینز کے اہم ویکٹر ہیں ان میں لیشمینیا، آربو وائرسز، اور بارٹونیلا ہیں۔ جو بالترتیب لیشمینیا س، آربو وائرس انفیکشن اور بارٹونیلوسس کی بیماریوں کے ذمہ دار ہیں۔ ان کی شناخت بنیادی طور پر تفصیلی خوردبینی امتحان پر مبنی ہے۔ یہ احتیاط سے جمع کرنے، مناسب ذخیرہ کرنے، اور سلائیڈ پر احتیاط سے نصب کرنے پر مبنی ہے، اور اس کے لیے متعدد مخصوص تکنیکوں کی ضرورت ہے، جن میں سے ہر ایک کے اپنے فوائد اور حدود ہیں۔

بالغ فلیبوٹومین ریتیلی مکھیوں کی شناخت بیرونی (مثلاً اینٹینا، پالپی، مردانہ اعضاء) اور اندرونی (مثلاً فارینکس،ا سیبریم، اور سپرماتھیکا) کے ڈھانچے کے مشاہدے پر مبنی ہے۔ ان کا ڈسیکشن (تقسیم) اور علیحدگی ان کے مشاہدے کو آسان بناتی ہے اور اس کے نتیجے میں درست شناخت کی اجازت دیتی ہے۔ لہذا، مچھروں یا بوسہ لینے والے کیڑوں کے برعکس، فلیبوٹومین ریتیلی مکھیوں کی شناخت پہلے اس بات پر ہوتی ہے کہ انہیں سلائیڈ اور کور سلپ کے درمیان نصب کرنے کی ضرورت ہے۔

مزید برآں، اس مواد کی عکاسی فلیبوٹومین ریتیلی مکھیوں کے آسان مشاہدے کے لیے ہمیشہ موزوں نہیں ہوتی۔ اس کے برعکس، ایک آبی میڈیم (جیسے ہوئر مائع) میں چڑھنا تیز تر ہوتا ہے اور اضطراری ا سپر میتھیکا کو بہتر انداز میں دیکھنے کی اجازت دیتا ہے، لیکن یہ ماونٹنگز کو طویل عرصے تک محفوظ نہیں رکھتا کیونکہ یہ درمیانے درجے سے پانی جذب کرتا ہے۔ ایک آپشن یہ ہے کہ سلائیڈ کو مکمل طور پر خشک ہونے کے بعد نیل پالش سے بند کر دیا جائے۔ یہ طریقہ آج بھی عام ہے۔

1980کی دہائی تک، مائکروسکوپک مشاہدہ فلیبوٹومین ریتیلی مکھیوں کی شناخت کے لیے واحد دستیاب طریقہ تھا، اور یہ آج بھی سب سے زیادہ استعمال ہونے والا طریقہ ہے۔ لہذا، عمل اور تیاری کا انتخاب نسبتاً آسان تھا اور بنیادی طور پر دو قسموں پر مشتمل تھا: ایک طرف، مستقل طور پر نصب کرنا جو نمونہ کو طویل عرصے تک محفوظ رکھنے کی اجازت دیتا ہے، اور دوسری طرف، فوری طور پر نصب کیا جاتا ہے جو کسی ایسے مواد میں شناخت کے لیے کیا جاتا ہے جو طویل مدتی تحفظ کو یقینی نہیں بناتا ہے۔ مثال کے طور پر، فائنل ماؤنٹنگ، جیسے کینیڈا بلسم جیسے رال میں، یہ طریقہ وقت طلب ہے اور اس میں نمونوں کی مکمل ہائیڈریشن کی ضرورت ہوتی ہے۔

1980 کی دہائی سے، فلیبوٹومین ریتیلی مکھیوں کی شناخت کے مطالعہ نے مورفولوجیکل اور بائیو کیمیکل طریقوں کو یکجا کیا ہے۔ سب سے پہلے کوٹیکولر ہائیڈرو کاربن کے تجزیے تھے، جنہیں جلد ہی مالیکیولر بائیولوجی کی تکنیکوں (یعنی بے ترتیب ایمپلیفائیڈ پولیمورفک ڈی این اے (آر اے پی ڈی)، پابندی کے ٹکڑے کی لمبائی پولیمورفزم (آر ایف ایل پی) ، ڈی این اے ایمپلیفیکیشن، اور سینگر کا طریقہ استعمال کرتے ہوئے سیکوئنسنگ، نیز نیکسٹ جنریشن سیکوئنسنگ (این جی ایس) نے لے لی۔ آج، مالیکیولر طریقوں کو پروٹومک طریقوں جیسے کہ میلڈی ٹوف (مالدی ٹی او ایف) سے مکمل کیا جاتا ہے مزید براں، سالماتی پرجاتیوں کی شناخت پی سی آر کے ساتھ مل کر پیتھوجینز (لیشمینیا، ٹریپینوسوما، بارٹونیلا، اور فلیبو وائرس) کا استعمال کیا جا سکتا ہے کیونکہ ان سب کو آخری پوائنٹ اور ریئل ٹائم پی سی آر کی ضرورت ہوتی ہے [32، 3] ۔ انواع کے امتیاز کے لیے روایتی طور پر استعمال ہونے والی مورفولوجیکل خصوصیات کے علاوہ، دیگر مورفولوجیکل طریقوں کو بھی اپنایا جا سکتا ہے(مثال کے طور پر، پنکھوں کی جیومورفومیٹری)۔

زیادہ تر مصنفین کے اپنے تجربات اور ادبی اعداد و شمار کی بنیاد پر، اس تحقیق کا مقصد بالغ فلیبوٹومین ریتیلی مکھیوں کو ماؤنٹ کرنے اور پراسیس کرنے کے لیے معیاری رہنما اصول فراہم کرنا تھا تاکہ مورفولوجیکل اور مالیکیولر تجزیوں کو بہتر بنایا جا سکے۔

مخصوص تجزیات (مثلاً مالیکیولر بائیولوجی یا میلڈی ٹوف: مالدی ٹی او ایف) کے لیے ریتیلی مکھیوں کے اس حصے کو محفوظ کرنا ضروری ہوتا ہے جو مورفولوجیکل شناخت کے لیے ضروری نہیں ہوتا، جس سے مناسب پروٹوکول کے انتخاب کی اہمیت کو اجاگرکرتی ہے۔ اس مضمون میں ہم زندہ پکڑے گئے فلیبوٹومائن ریتیلی مکھیوں کو بے ہوش کرنے اور ہمدردانہ ہلاکت کے طریقوں، ان کے ذخیرہ کرنے اور ماؤنٹنگ کے عمل پر توجہ مرکوز کرتے ہیں، تاکہ تیز شناخت یا طویل مدتی تحفظ کے ذریعے بعد کی تحقیقات ممکن ہوں۔

تمہید: حفاظتی اور ضابطہ جاتی امور کے لیے متعلقہ سیفٹی ڈیٹا شیٹس (ایس ڈی ایس) کا حوالہ دیا جانا چاہیے۔

اس رہنما اصول میں پیش کیے گئے تمام کیمیکلز کو سخت حفاظتی حالات میں سنبھالا جانا چاہیے۔ تحقیقی سہولیات کی صحت اور حفاظتی کمیٹیاں نہ صرف ان کیمیکلز کے خطرات کے بارے میں، بلکہ ان کے استعمال کے طریقہ کار اور فضلہ کے ٹھکانے کے بارے میں بھی معلومات فراہم کرنے کے لیے دستیاب ہیں۔ تاہم، ان کے استعمال اور تلف کے حوالے سے حفاظتی ہدایات پر عمل کرنا لازمی ہے۔ قابل ذکر ہے کہ یہ تمام صارفین کی ذمہ داری ہے کہ وہ اپنے ملک یا تحقیقی ادارے کے قابل اطلاق قوانین اور ضوابط کے ساتھ ساتھ اچھی اور محفوظ لیبارٹری کی پریکٹسز کی تعمیل کو یقینی بنائیں۔ ۔ مزید براں، بعض کیمیکلز یا ان کے اجزاء (مثلاً، کلورل ہائیڈریٹ) کو بعض ممالک میں ریگولیٹ کیے جاتے ہیں۔ اس مسودے میں بیان کردہ استعمال ہونے والی اختصارات کی فہرست **جدول 1** میں دی گئی ہے۔

جدول 1): اختصارات کی فہرست۔

سیریل نمبر	اختصارات	تفصیل
1):	**بی ایم ای**	ایگل کا بنیادی میڈیم
2):	**سی ڈی سی**	امریکی ادارہ برائے بیماریوں کے کنٹرول اور روک تھام
3):	**سی ایم سی پی**	کیمفرمونوکلوروپینول
4):	**سی ایم آر**	سرطان پیدا کرنے والا، میوٹاجینک، تولیدی زہریلا مادہ
5):	**سی او آئی**	سائٹوکروم سی کا جین I آکسیڈیز سب یونٹ
6):	**سائٹ بی**	سائٹوکروم بی جین
7):	**ڈی این اے**	ڈی آکسی رائبونیوکلیک ایسڈ
8):	**ایلیسا**	انزائم سے منسلک امیونوسوربینٹ اسائی
9):	**ای ٹی او ایچ**	ایتھانول
10):	**ایم 199**	میڈیم 199
11):	**مالدی ٹی او ایف**	آئنائزیشن ٹائم آف فلائیٹ
12):	**مالدی ٹوف ایم ایس**	آئنائزیشن ٹائم آف فلائیٹ ماس /میٹرکس سے معاون لیزر ڈیسرپشن اسپیکٹرومیٹری
13):	**ایم ای ایم**	کم از کم ضروری میڈیا
14):	**این جی ایس**	نیکسٹ جنریشن سیکوئنسنگ
15):	**این این این**	نووی-میک نیل- نکول میڈیم
16):	**پی سی آر**	پولیمریز چین ری ایکشن
17):	**لاؤ پی ڈی آر**	لاؤ عوامی جمہوریہ
18):	**پی این او سی**	پری پرو نوسیپٹن جین
19):	**کیو پی سی آر**	کوانٹیٹیٹو پی سی آر(ریئل ٹائم پی سی آر)
20):	**آر اے پی ڈی**	ترتیب بڑھایا گیا پولیمورفک ڈی این اے
21):	**آر ایف ایل پی**	ریسٹرکِشن فریگمنٹ لینتھ پولیمورفزم
22):	**آر آئی**	انعکاسی نما
23):	**آر این اے**	رائبونوکلیک ایسڈ
24):	**ار نےسس**	رائبونیوکلیز
25):	**آر این اے ایس ایس**	آر این اے استحکام محلول
26):	**آر ٹی پی سی آر**	ریورس ٹرانسکرپشن پی سی آر
27):	**ایس ڈی ایس**	سیفٹی ڈیٹا شیٹس
28):	**ٹی ایف اے**	ٹری فلوررو ایسٹک ایسڈ

مواد اور طریقہ کار

1): ریتیلی مکھیاں پکڑنا

بالغ ریتیلی مکھیاں زندہ یا مردہ مختلف طریقوں سے جمع کی جا سکتی ہیں، جیسے سی ڈی سی ، منی ایچر لائٹ ٹریپس، چپچپے ٹریپس، شیننون ٹریپس کے ساتھ ایسپیریٹرز، یا ماحول میں آرام کی جگہوں (مثلاً جانوروں کے پناہ گاہوں) سے براہِ راست۔ ان طریقوں میں مناسب رہائش گاہوں میں پھندے لگانا، روشنی یا دیگر کشش (کاربن ڈائی آکسائیڈ یا کیمیائی لؤرز) کے ذریعے ریتیلی مکھیاں اپنی طرف متوجہ کرنا، اور مزید تجزیے کے لیے انہیں جمع کرنا شامل ہے، جیسا کہ متعدد اشاعتوں [2، 3، 32، 36، 49] میں بیان کیا گیا ہے۔

زندہ ریتیلی مکھیاں کو پکڑنے سے تمام بعد کے اطلاقات ممکن ہوتے ہیں، جبکہ مردہ ریتیلی مکھیوں کو جمع کرنے سے لیشمینیا یا وائرس کے اسٹرینز کو الگ کرنا ممکن نہیں ہوتا۔ ریتیلی مکھیاں پکڑنے کی تکنیکیں، جیسے چپچپے کاغذ، جو باقاعدگی سے ریتیلی مکھیاں کے اعضاء (آنٹینا، پالپس، پروں یا ٹانگوں) کو نقصان کا باعث بنتی ہیں۔ اس کے علاوہ، کاسٹر آئل کی کوٹنگ والے چپچپے کاغذ ریتیلی مکھیوں سے چپک جاتے ہیں اور عملدرآمد کے آغاز میں انہیں ہٹانا ضروری ہوتا ہے، عام طور پر برابر کے حصوں میں ایتھانول اور ڈائی ایتھل ایتھر کے 15 منٹ کے غسل کے ذریعے۔

2):نمونے کی ہلاکت

جمع کرنے کے بعد زندہ ریتیلی مکھیوں کو ہمدردانہ طور پر مارنا ضروری ہے۔ کچھ طریقوں (مثلاً چپچپے کاغذ، ڈیٹرجنٹ یا ایتھانول سے بھرے جار کے ساتھ لیس سی ڈی سی لائٹ ٹریپس) سے ریتیلی مکھیاں جمع ہوتے ہی مر جاتی ہیں۔ مالیکیولر بائیولوجی ان نمونوں پر لاگو کی جا سکتی ہے جو براہِ راست ایتھانول میں جمع کیے گئے ہوں یا اگر دوسرے نمونے جلد از جلد ایتھانول میں محفوظ کیے جائیں۔ تاہم، ان میں سے کوئی بھی مارنے کے طریقے کیڑوں کو میلڈی-ٹوف کے ذریعے پراسیس کرنے کی اجازت نہیں دیتے۔ مزید برآں، کچھ مارنے کے طریقے مخصوص مورفولوجیکل خصوصیات کے ضائع ہونے کا سبب بن سکتے ہیں۔ لہٰذا مناسب شناخت یا طویل مدتی ذخیرہ کے لیے معیاری ہلاکت کے ایجنٹ کا استعمال ضروری ہے (یعنی وہ نمونے جو مستقبل کے حوالے یا موازنہ کے لیے محفوظ اور ذخیرہ کیے جاتے ہیں)۔ ایسے کیمیکلز جیسے ایتھائل ایسٹیٹ، ایتھائل ایتھر ، ٹرائی کلوروایتھین اور کلورو فارم روئی میں جذب کیے جا سکتے ہیں اور ریتیلی مکھیوں کے حامل برتن میں رکھ کر انہیں ہلاک کیا جا سکتا ہے۔ ان ہلا ک کر دینے والے مادوں کو ان کی زہریلی خصوصیات کے پیش نظر، بنانے والے کی ہدایات کے مطابق احتیاط سے استعمال کرنا چاہیے۔ تاہم، ہم کلورو فارم سے ریتیلی مکھیوں کو مارنے کی سفارش نہیں کرتے، کیونکہ ہمارے تجربے میں یہ مولیکیولر بائیولوجی کے مطالعات کے لیے موزوں نہیں ہے۔ ان تمام مصنوعات کی خطرناک نوعیت اور مولیکیولر تجزیوں کے لیے ان کی مشکوک موزونیت کو مدنظر رکھتے ہوئے، ان کیمیکلز کے استعمال سے عمومی طور پر منع کیا جاتا ہے۔
سب سے زیادہ استعمال ہونے والا طریقہ، جو مورفولوجی، ڈی این اے یا پروٹینز کو محفوظ رکھتا ہے، ریتیلی مکھیوں کے نمونوں کو خشک منجمد کرنا ہے۔ نمونوں کو اتنی دیر تک منجمد کرنا چاہیے کہ وہ مکمل طور پر بے حس ہو جائیں، لیکن اتنی دیر تک نہیں کہ (i) وہ خشک ہو جائیں، یا (ii) لیشمینیا کی بقا متاثر ہو، اگر مقصد ریتیلی مکھیوں کے نظام ہضم سے وٹرو میں (*in vitro*) الگ کرنا ہو۔ لہٰذا ہم -20°C پر 15 سے 20 منٹ تک منجمد رکھنے کی سفارش کرتے ہیں، اور باقاعدگی

سے نگرانی کرتے رہیں تاکہ یہ یقینی بنایا جا سکے کہ وہ لیشمینیا کے پرجیویوں / طفیلی (پیراسائٹس) صرف بے ہوش ہوں، مارے نہ جائیں۔ اگر فریزر دستیاب نہ ہو تو کیڑوں کو متبادل طور پر کاربن ڈائی آکسائیڈ کے ذریعے بے درد ہلاک کیا جا سکتا ہے۔ فیلڈ کے حالات میں جہاں کاربن ڈائی آکسائیڈ سلینڈراستعمال نہیں کیے جا سکتے، نمونے 'سوڈا سائفونز' (مشروبات کے ڈسپنسر) میں استعمال ہونے والے چھوٹے تجارتی کاربن ڈائی آکسائیڈ کنٹینرز کے ذریعے مارے جا سکتے ہیں، لیکن ان کی ہوائی نقل و حمل پر پابندیاں ہو سکتی ہیں۔ آخری چارہ کے طور پر، ریتیلی مکھیوں کو تمباکو کے دھوئیں کے سامنے رکھ کر ہلاک کیا جا سکتا ہے۔ ریتیلی مکھیاں سی ڈی سی ٹریپ میں زندہ پکڑی جاتی ہیں، ایک ایسپیریٹر کے ذریعے جمع کی جاتی ہیں، شیشے کی نلی میں محفوظ کی جاتی ہیں، اور تمباکو کے دھوئیں کے سامنے لائی جاتی ہیں جو چند سیکنڈز میں انہیں ہلاک کر دیتا ہے۔ یہ طریقہ کار تمام میدانی حالات میں، حتیٰ کہ مشکل علیحدگی کے حالات میں بھی قابلِ عمل ہے۔ تاہم چونکہ ایسپیریٹرکا شیشہ دھوئیں سے آلودہ ہو جاتا ہے، بغیر مکمل صفائی کے اسے بعد میں زندہ ریتیلی مکھیاں جمع کرنے اور سنبھالنے کے لیے استعمال نہیں کیا جا سکتا۔ تاہم، اسی بغیر صاف کیے گئے ایسپیریٹر کو دیگر جالوں سے ریتیلی مکھیوں کو فکسیشن کے مقاصد کے لیے اور ہلاک کرنے کے لیے استعمال کیا جا سکتا ہے۔ یہ بھی ضروری ہے کہ چیک کیا جائے کہ آیا تمام نمونے ایسپیریٹر سے نکال دیے گئے ہیں۔ یہ طریقے لیشمینیا کے آئسولیشن کے لیے آنتوں کی تشریح کے ساتھ مطابقت رکھتے ہیں۔

نگرانی کرتے رہیں تاکہ یہ یقینی بنایا جا سکے کہ وہ لیشمینیا کے پرجیویوں /طفیلی (پیراسائٹس) صرف بے ہوش ہوں، مارے نہ جائیں۔
اگر فریزر دستیاب نہ ہو تو کیڑوں کو متبادل طور پر کاربن ڈائی آکسائیڈ کے ذریعے بے درد ہلاک کیا جا سکتا ہے۔ فیلڈ کے حالات میں جہاں کاربن ڈائی آکسائیڈ سلینڈراستعمال نہیں کیے جا سکتے، نمونے 'سوڈا سائفونز' (مشروبات کے ڈسپنسر) میں استعمال ہونے والے چھوٹے تجارتی کاربن ڈائی آکسائیڈ کنٹینرز کے ذریعے مارے جا سکتے ہیں، لیکن ان کی ہوائی نقل و حمل پر پابندیاں ہو سکتی ہیں۔ آخری چارہ کے طور پر، ریتیلی مکھیوں کو تمباکو کے دھوئیں کے سامنے رکھ کر ہلاک کیا جا سکتا ہے۔ ریتیلی مکھیاں سی ڈی سی ٹریپ میں زندہ پکڑی جاتی ہیں، ایک ایسپیریٹر کے ذریعے جمع کی جاتی ہیں، شیشے کی نلی میں محفوظ کی جاتی ہیں، اور تمباکو کے دھوئیں کے سامنے لائی جاتی ہیں جو چند سیکنڈز میں انہیں ہلاک کر دیتا ہے۔ یہ طریقہ کار تمام میدانی حالات میں، حتیٰ کہ مشکل علیحدگی کے حالات میں بھی قابلِ عمل ہے۔ تاہم چونکہ ایسپیریٹرکا شیشہ دھوئیں سے آلودہ ہو جاتا ہے، بغیر مکمل صفائی کے اسے بعد میں زندہ ریتیلی مکھیاں جمع کرنے اور سنبھالنے کے لیے استعمال نہیں کیا جا سکتا۔ تاہم، اسی بغیر صاف کیے گئے ایسپیریٹر کو دیگر جالوں سے ریتیلی مکھیوں کو فکسیشن کے مقاصد کے لیے اور ہلاک کرنے کے لیے استعمال کیا جا سکتا ہے۔ یہ بھی ضروری ہے کہ چیک کیا جائے کہ آیا تمام نمونے ایسپیریٹر سے نکال دیے گئے ہیں۔ یہ طریقے لیشمینیا کے آئسولیشن کے لیے آنتوں کی تشریح کے ساتھ مطابقت رکھتے ہیں۔

3): عملدرآمد سے قبل نمونوں کا ذخیرہ

عملدرآمد سے پہلے ٹھہراؤ کے پانچ (5) بنیادی طریقے ہیں:

3.1): جمنا

یہ طریقہ -20°C پر یا ترجیحاً -80°C پر بہترین طریقے سے کیا جاتا ہے۔ یہ ذخیرہ کرنے کے طریقے اب مائع نائٹروجن اسٹوریج کے مقابلے میں زیادہ

یہ طریقہ -20°C پر یا ترجیحاً -80°C پر بہترین طریقے سے کیا جاتا ہے۔ یہ

ہے۔ ایتھانول استعمال کرکے ڈی این اے اور آر این اے وائرس کی تشخیص

ذخیرہ کرنے کے طریقے اب مائع نائٹروجن اسٹوریج کے مقابلے میں زیادہ وسیع پیمانے پر استعمال ہوتے ہیں۔ تمام صورتوں میں، نمونوں کو بے ہوش کرنے کے بعد جتنا جلدی ممکن ہو کرایو پریزرویشن نافذ کرنا ضروری ہے۔ فریزر میں سرد ذخیرہ ریتیلی مکھیوں کو خود، نیز آر این اے، ڈی این اے اور پروٹینز کو ذخیرہ کے پورے عرصے کے دوران مکمل سالمیت کے سا تھ محفوظ رکھنے کا فائدہ فراہم کرتا ہے۔ اس کے بجائے، مائع نائٹروجن پروں، ٹانگوں، پالپس اور اینٹینا کو شدید نقصان پہنچا سکتا ہے، اکثر انہیں کاٹ دیتا ہے اور کبھی کبھار اہم مورفولوجیکل خصوصیات کو ہٹا دیتا ہے۔ خشک فریزر میں ذخیرہ کرنا نمونوں کے لیے کم تکلیف دہ ہے، لیکن ان کے نازک اعضاء کو محفوظ کرنے کے لیے مثالی نہیں ہے۔ اہم بات یہ ہے کہ پگھلنے کے وقت، پروں، اینٹینا، پالپس یا ٹانگیں بوتلوں سے چپک سکتی ہیں اور آخر کار نمی کی تشکیل کی وجہ سے پھٹ سکتی ہیں۔ تاہم، فیلڈ کے مطالعات میں نمونوں کو منجمد کر کے محفوظ کرنا ہمیشہ ممکن نہیں ہوتا کیونکہ اس کے لیے فریزر یا مائع نائٹروجن کے کنٹینر تک رسائی درکار ہوتی ہے۔ فریزر میں ذخیرہ کرنا حساسیت کے نقصان کے بغیر سالماتی اوزاروں کے ذریعے پیتھوجن کی تشخیص کے لیے مکمل طور پر موزوں ہے، اگرچہ آر این اے وائرس کی تشخیص اور علیحدگی کے لیے اگر طویل مدتی ذخیرہ ضروری ہو تو -80°C پر یا مائع نائٹروجن میں منجمد کرنے کی ضرورت ہوتی ہے۔ تاہم، نمونوں کو منجمد کرنے سے لیشمینیا کو آنتوں کی تشریح کے ذریعے الگ نہیں کیا جا سکتا، سوائے اس کے کہ ریتیلی مکھیاں پہلے بخارات کے مرحلے میں اور پھر مائع نائٹروجن میں ڈبوئی جائیں (مثال کے طور پر جراب میں رکھے گئے وائلز میں)، تاکہ لیشمینیا کے کرایو پریزرویشن کی نقل کی جا سکے۔

3.2): الکحل (ایتھانول یا آئیسوپروپائل الکحل) میں ذخیرہ

یہ شاید ریتیلی مکھیاں محفوظ کرنے کا سب سے زیادہ استعمال ہونے والا طریقہ ہے۔ اسے میدان میں نافذ کرنا آسان ہے، یہاں تک کہ مشکل حالات میں بھی جب لیبارٹری تک رسائی نہ ہو۔ الکحل میں تحفظ مورفولوجیکل مطالعات کے لیے خاص طور پر موزوں ہے، کیونکہ نازک اعضاء (پر، ٹانگیں، اینٹینے یا پالپس) سالم رہتے ہیں، بشرطیکہ ذخیرہ کرنے والی ٹیوب میں ہوا کے بلبلے نہ ہوں۔ لہٰذا، ہم تجویز کرتے ہیں کہ ٹیوب کو ایک چھوٹے روئی کے گولے سے سیل کر دیا جائے تاکہ ہوا کے بلبلے خارج ہو جائیں اور روئی کے پلگ کے اوپر لیبل لگا دیا جائے **(شکل 1)**۔ الکحل کی مناسب ارتکاز ایک زیرِ بحث معاملہ ہے۔ عام طور پر 70% سے کم ارتکاز کی سفارش نہیں کی جاتی [45, 66]- زیادہ ارتکاز ڈی این اے کو زیادہ مؤثر طریقے سے اور طویل عرصے تک محفوظ رکھتا ہے لیکن نمونوں کو مورفولوجیکل مطالعات کے لیے زیادہ نازک اورٹوٹنے والا بنا دیتا ہے۔ 96% ایتھانول (ایزیوٹروپ مکسچر) کے استعمال کے وقت اس کے سا تھ ارتکاز میں استحکام برقرار رہتا ہے، خاص طور پر نمی والے علاقوں جیسے استوائی ممالک میں، حالانکہ 95% ایتھانول عموماً حاصل کرنا آسان ہوتا ہے۔ ارتکاز سے قطع نظر، ڈی این اے عام طور پر ایتھانول میں اچھی طرح محفوظ رہتا ہے (اگرچہ منجمد کرنے کے طریقوں کے مقابلے میں کم مؤثر طریقے سے، خاص طور پر -این جی ایس- طرز کے مالیکیولر طریقوں کے لیے)۔پروٹینز بہت کم مستحکم ہوتے ہیں، خاص طور پر پروٹومیکس، جیسے مالدی ٹوف اطلاقات کے لیے۔ چند ماہ کے لیے الکحل میں محفوظ کیے گئے ریتیلی مکھیاں اب بھی مورفولوجیکل طور پر شناخت کی جا سکتی ہیں، لیکن ان نمونوں سے حوالہ جاتی پروٹین سپیکٹرا تیار کرنا ناممکن ہے۔ الکحل یا خشک حالات میں ذخیرہ کو بہتر بنایا جا سکتا ہے اگر نمونہ کو -20°C پر منجمد بھی کیا جائے۔ -20°C پر منجمد کرنا بنیادی طور پر مالیکیولر تحفظ (مثلاً نیوکلیک ایسڈز) کو بہتر بناتا ہے کیونکہ یہ ان کی خرابی کو سست کر دیتا ہے اور وقت کے سا تھ ٹشو ز کے ٹوٹ پھوٹ کو کم کرکے مورفولوجیکل تحفظ کے لیے ثانوی فائدہ بھی فراہم کرتا ہے، اگرچہ مورفولوجی پر اس کا اثر مالیکیولر سالمیت کے مقابلے میں زیادہ محدود ہوتا ہے۔ مختصر مدت (چند ماہ سے کم) کے لیے کم از کم 70% ارتکاز پر لیے بھی ذخیرہ کیا جا سکتا ہے۔ مزید برآں، آئسوپروپائل الکحل بعض ممالک میں آسانی سے دستیاب ہو سکتا ہے اور ڈی این اے کو محفوظ رکھتا ہے، لیکن نمونوں کو سخت کر دیتا ہے۔ یہ ایتھانول کی طرح قابلِ اشتعال نہیں ہوتا اور اس لیے آسانی سے منتقل کیا جا سکتا ہے۔ اگر ضرورت ہو تو مائع نائٹروجن یا خشک منجمد حالت میں محفوظ شدہ ریتیلی مکھیاں الکحل میں منتقل کی جا سکتی ہیں، جس سے دونوں طریقوں کے نقصانات یکجا ہو جاتے ہیں۔

3.3):آر این اے استحکام بخش محلول (RNASS) میں ذخیرہ

یہ آبی محلول وسیع پیمانے پر استعمال ہوتا ہے، غیر زہریلا ہے، اور تازہ بافتوں اور خلیاتی نمونوں میں آر این اے کو مستحکم اور محفوظ کرنے کے لیے ڈیزائن کیا گیا ہے۔ یہ نمونے میں تیزی سے سرایت کر کے آر این اےز (آر این اے کو تحلیل کرنے والے انزائمز) کو غیر فعال کر دیتا ہے، اس طرح فوری منجمد کرنے کی ضرورت کے بغیر آر این اے کی تحلیل سے بچاتا ہے۔ آر این اےز میں ذخیرہ کرنے سے عام طور پر ٹشوز اور خلیاتی ساخت کو بعد ازاں ہسٹولوجیکل جائزے کے لیے ہے۔ اگرچہ آر این اےز کو آر این اے کے استحکام کے لیے بہتر بنایا گیا ہے نہ کہ فکسیشن کے لیے، مختصر تا درمیانی مدت کے ذخیرے میں ساختی سالمیت عموماً برقرار رہتی ہے۔ آر این اےز نمونوں کو کمرے کے درجہ حرارت پر 7 دن تک، 4°C پر کئی ہفتوں تک، یا طویل مدتی ذخیرہ کے لیے -80°C /-20°C پر محفوظ کرنے کی اجازت دیتا ہے۔ یہ طریقہ خاص طور پر فیلڈ ورک یا کلینیکل ماحول میں قیمتی ہے، جہاں کولڈ چین کے انفراسٹرکچر کی کمی ہوتی ہے۔ آر این اے کی نکاسی کے لیے عام طور پر نمونوں کو ریجنٹ سے نکال کر معیاری پروٹوکول کے مطابق پراسیس کرنے کی ضرورت ہوتی ہے۔

3.4): کمرے کے درجہ حرارت پر خشک تحفظ

یہ ایک پرانا طریقہ ہے جو جب پورے نمونے (*in toto*) پر لاگو کیا جاتا ہے تو اس کا سب سے بڑا نقصان یہ ہے کہ یہ نازک اعضاء جیسے پروں، ٹانگوں، اینٹینا اور پالپس کو مناسب طریقے سے محفوظ نہیں رکھتا۔ تاہم، اگر فکسیشن کے دوران سلیکا جیل نما خشک کن (ڈیسیکینٹ) کے ساتھ نمونے کو خشک کیا جائے تو میلڈی ٹوف (مالدی ٹی او ایف) کے ذریعے پروٹومیک مطالعے ممکن ہیں۔ اس کے برعکس، ڈی این اے کو ہدف بنانے والی مالیکیولر تجزیات ان نمونوں پر کرنا مشکل ہے، کیونکہ ڈی این اے اکثر ٹکڑوں میں تقسیم شدہ اور کم مقدار میں ہوتا ہے، جس کا مطلب ہے کہ تجزیات تازہ یا منجمد نمونوں کے مقابلے میں زیادہ مشکل ہیں، خاص طور پر نیوکلیئر جینومز کے لیے۔ تاہم، حالیہ تکنیکیں جیسے میوزومیکس اس قسم کے نمونوں پر استعمال کی جا سکتی ہیں [34]- لہٰذا، اس طریقہ ذخیرہ کی سفارش نہیں کی جاتی، جب تک کوئی متبادل دستیاب نہ ہو۔ اسے ٹیوبوں کو -20°C یا -80°C پر فریزر میں رکھ کر سرد ذخیرہ کے ساتھ بھی ملایا جا سکتا ہے۔ سب سے بڑا چیلنج شے یا شناخت کے لیے ضروری جسمانی حصوں کی مناسب ماؤنٹنگ کرنا ہے۔ اس مقصد کے لیے، ری ہائیڈریشن ضروری ہے۔ ہم ٹرائٹون X-100 کے محلول کے استعمال کی سفارش کرتے ہیں۔ ری ہائیڈریشن کا دورانیہ چند گھنٹوں سے لے کر کئی دنوں تک ہوتا ہے، جس کے دوران باقاعدہ اور قریبی نگرانی ضروری ہے۔ مکمل ری ہائیڈریشن کے بعد، نمونوں کو تین متواتر پانی کے غسلوں میں دھویا جانا چاہیے۔

3.5):فلٹر پیپرز پر محفوظ کرنا

فلٹر پیپرز کا بنیادی فائدہ غیر فکسڈ، سوکھے ہوئے پورے جسم، یا کمرے کے درجہ حرارت پر محفوظ خون کے خلیات کے اندر جینومک ڈی این اے کا طویل مدتی استحکام ہے۔ فلٹر پیپر چھوٹے کارڈ سائز میں فراہم کیا جاتا ہے، جس سے کمرے کے درجہ حرارت پر ایک چھوٹے ٹیبل ڈرائیور کے حجم کے سائز میں کئی سو نمونوں کو ذخیرہ کرنا ممکن ہوتا ہے۔ فلٹر پیپر میٹرکس کو ایسے ایجنٹوں سے رنگین کیا جاتا ہے جو متعدی ایجنٹوں کی

نشاندہی کرتے ہیں، اور اس طرح نمونوں کو اب حیاتیاتی خطرہ نہیں سمجھا جاتا ہے۔ یہ انتخابی بائیو ہارڈ احتیاطی تدابیر کے بغیر نمونوں کو ذخیرہ کرنے اور نقل و حمل کی اجازت دیتا ہے [68]۔

4):نمونہ کا اخراج (ڈ سیکشن)

بہت سے دوسرے حشرات کے برعکس، جن کی شناخت بیرونی حروف کی بنیاد پر کی جاتی ہے جو مکمل طور پر انفرادی کیڑوں پر مشاہدہ کیے جاتے ہیں، ریتیلی مکھیوں کو انواع کی درست شناخت کے لیے جسمانی خصوصیات کا مطالعہ کرنے کے لیے ڈسکشن اور سلائیڈ ماؤنٹنگ کی ضرورت ہوتی ہے۔ منتخب کردہ تیاری اور بڑھتے ہوئے طریقہ کار سے قطع نظر، اسی ڈسیکشن تکنیک کا استعمال کیا جاتا ہے (**شکل 2 اور 3**) اور(**ویڈیو کلپ 1**): (https://zenodo.org/records/18198006)۔

ٹرائٹن X100 کا استعمال: غیر آئنک آبی محلول

نوٹ کریں کہ بڑھتے ہوئے تازہ پکڑے گئے یا مناسب طریقے سے ذخیرہ شدہ نمونوں سے متعلق خدشات ہیں۔ زیادہ تر جمع کرنے والوں کے پاس ریتیلی مکھیوں کے نمونے خشک (میلڈی ٹوف استعمال کے لیے) یا کئی سالوں تک الکحل میں محفوظ ہوتے ہیں۔ بدقسمتی سے، الکحل میں سال کے عرصے کے دوران زیادہ سے زیادہ محفوظ نہیں ہے، اور اس طرح سے محفوظ کیے گئے آرتھروپوڈز کو خوردبینی امتحان کے لیے تیار کرنا بہت مشکل ہو جاتا ہے۔ ایک واقعہ جو اکثر پیش آتا ہے وہ ہے نمونوں پر مشتمل پلاسٹک کا انحطاط، اس کے بعد الکحل کا بخارات بننا۔ دونوں صورتوں میں، ہمارے پاس کوئی اختیار نہیں ہے کیونکہ نمونے الکحل میں بہت لمبے عرصے کے لیے رہتے ہیں اور خشک ہوجاتے ہیں۔ لہذا یہ خیال ہے کہ گیلے کرنے والے ایجنٹوں کو استعمال کیا جائے جو مضبوط ڈٹرجنٹ نہیں ہیں۔ ٹرائٹن X100 ایک غیر آئنک آبی محلول (polyethylene glycol 4 (1,1,3,3-tetramethylbutyl) phenyl-polyethylene glycol محلول، یا t-octylphenoxypolyethoxyethanol، (polyethylene glycol tert-octylphenyl ether کی شکل میں ہے، بڑے پیمانے پر ڈی سیلولر اور ڈی سیلولرولوجی میں استعمال کیا جاتا ہے۔ یہ سیل اور جوہری جھلیوں کے پارمیبلائزیشن کو قابل بناتا ہے۔

ذیل میں 0.5% آبی محلول میں غیر آئنک ٹرائٹن X100 کا استعمال کرنے والا طریقہ کار ہے۔

1) : خشک نمونے کو مطلق الکحل کے ساتھ امپریگنیٹ کریں۔
2) : ٹرائٹن X100 محلول کا ضروری حجم 0.5% پر شامل کریں تاکہ پورا نمونہ ڈوب جائے۔
3) :عمل کو تقریباً 5 منٹ سے کئی دنوں تک چلنے دیں، باقاعدگی سے نگرانی کریں۔ محلول میں تمام آرتھروپوڈس کو مکمل طور پر الگ ہونا چاہیے۔
4) : ٹرائٹن X100 محلول کو ہٹا دیں اور پوٹاشیم ہائیڈرو آکسائیڈ محلول سے تبدیل کریں۔

4.1): سر

تشریح باریک سوئیوں یا حشرہ شناسی کے پنوں کے ذریعے سٹیریو مائیکروسکوپ (اشکال 2 اور 3) کے نیچے کی جا سکتی ہے۔ سب سے زیادہ استعمال ہونے والی سوئیاں درج ذیل ہیں: 13 × 0.45 ("1/2 × 26G ملی میٹر)، 13 × 0.3 ("1/2 × 30G ملی میٹر)، یا 0.5 ("5/8 × 25G × 16 ملی میٹر)۔ نمونہ کی شناخت کے لیے تیار کرنے کے لیے، کم از کم سر کو جسم سے الگ کر کے پیٹ کی جانب اوپر کی طرف رکھا جاتا ہے تاکہ سیبریئم اور فیرنکس دکھائی دیں، جبکہ سینہ اور پیٹ کو تشریح کے بعد پہلو کی جانب نصب کیا جاتا ہے۔

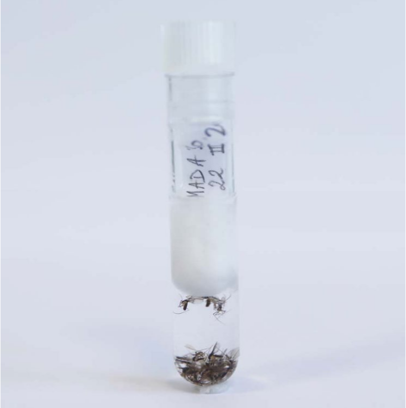

شکل1): ایتھانول میں محفوظ شدہ ریتیلی مکھیاں۔

سر کو پیٹ اور پیٹھ کی جانب نصب کرنے سے اوسیپیٹل سوراخ اوپر کی جانب رہتا ہے، تاکہ سیبریئم براہِ راست دیکھا جا سکے۔ اگر سر کو مکمل طور پر الگ کیا جائے تو ان اناٹومیکل خصوصیات تک رسائی آسان ہو جاتی ہے۔

4.1): پرَ اور سینہ

فلیبوٹومین ریتیلی مکھیوں کے پنکھوں کو فلیٹ نصب کیا جانا چاہئے. ہر بازو کو اس کی بنیاد سے الگ کیا جا سکتا ہے اور آزادانہ طور پر نصب کیا جا سکتا ہے، یا ایک کو اکیلے نصب کیا جا سکتا ہے، جبکہ دوسرے کو چھاتی سے منسلک چھوڑ کر۔ اگر جیومیٹرک مورفومیٹری تجزیہ کی منصوبہ بندی کی گئی ہے تو، نصب کرنے سے پہلے دائیں اور بائیں بازو کی درست شناخت اور لیبل لگانا ضروری ہے۔ فلیبوٹومین ریتیلی مکھیوں کی چھاتی کو کئی حصوں میں تقسیم کیا گیا ہے، اور ہر ایک میں بہت اہم ٹیکونومک معلومات ہیں [20، 64]۔ عام طور پر، اسے لیٹرل ویو میں نصب کیا جاتا ہے، تاکہ چیٹو ٹیکسی اور رنگ کی تقسیم کی جانچ کی جا سکے۔ چھاتی کے بعض علاقوں میں برسلز کے نشانات کی موجودگی کو برمپٹومیا جینس کی کچھ انواع میں فرق کرنے کے لیے استعمال کیا جا سکتا ہے۔ رنگوں کی تقسیم کو نسل کی سطح پر نیوٹروپک فلیبوٹومین ریتیلی مکھیوں کو الگ کرنے کے لیے ، پرجاتیوں کی (مثال کے طور پر، بائیکرومومیا استعمال کیا جا سکتا ہے ، یا یہاں تک کہ ایک ہی نسل کی نسلوں(مثال کے طور پر، پنٹومیا) سیریز ۔ اس [20] (سائیکوپیگس ,ساتھیرومیا، اور ,نیسومیا)جیسے، مائکروپیگومیا،) طرح، اگر چھاتی کو سالماتی تجزیہ کے لیے استعمال نہیں کیا جاتا ہے، تو اسے نصب کیا جانا چاہیے تاکہ اسے نقصان نہ پہنچے۔ اہم بات یہ ہے کہ یہ بات ذہن نشین کر لینی چاہیے کہ رنگوں کی شدت اہمیت نہیں رکھتی بلکہ سینے میں ان کی تقسیم ہوتی ہے۔ لہٰذا، وضاحتی عمل رنگت یا اس کے پیٹرن کو ختم نہیں کرے گا

4.3): جنسی اعضاء

نر اور مادہ دونوں میں جنسی اعضاء کو نصب کرتے وقت خاص احتیاط برتی جانی چاہیے، کیونکہ یہ جنسوں، ذیلی جنسوں اور انواع کی شناخت کے لیے انتہائی اہم ہیں۔ دونوں جنسوں میں جنسی اعضاء جوڑوں کی صورت میں ہوتے ہیں۔

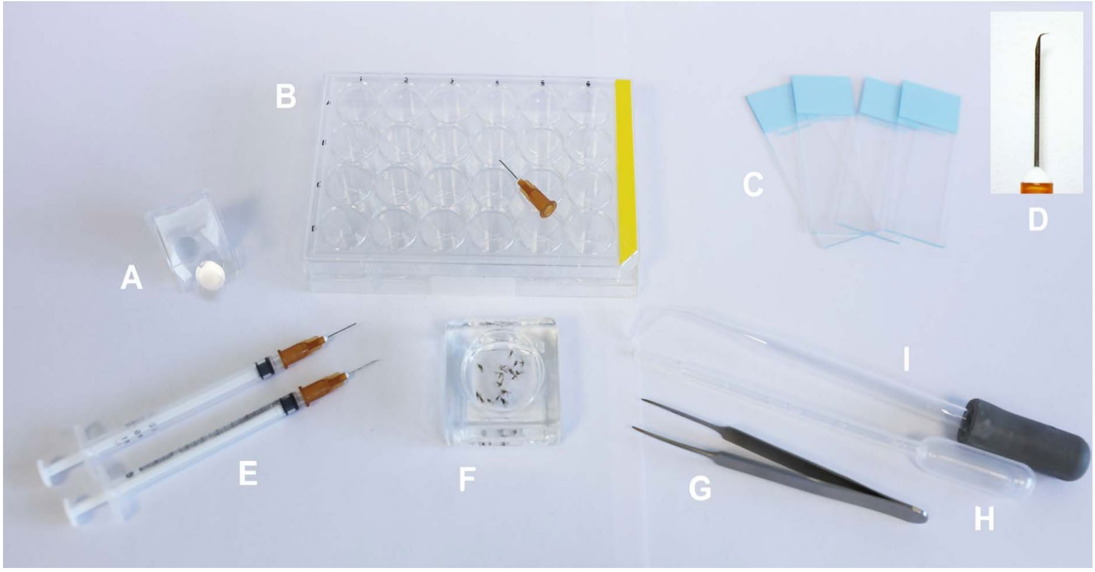

شکل 2): ریت کی مکھیوں کو چڑھانے کے لیے درکار مواد: A): گول شیشے کی کور سلپس (10 یا 12 ملی میٹر قطر); B): 24 کنویں کی پلیٹ اور جھکی ہوئی سوئی (اگر آپ ریتیلی مکھیوں کو پروسیس کرنے کے لیے لونگ کا تیل یا یوپرل ایسنس استعمال کرتے ہیں، تو ایکریلک پلیٹیں استعمال نہ کریں کیونکہ کیمیائی رد عمل ہو گا اور نمونوں کو نقصان پہنچے گا)؛ C): لیبلنگ کے لیے موزوں شیشے کی سلائیڈز۔ ;D): سوئی ہک کی تفصیل؛ E): سرنجوں سے منسلک سوئیاں؛F): گھڑی کا گلاس (واچ گلاس) یا مساوی کنٹینر جس میں ریتیلی مکھیوں کو نصب کیا جائے؛ G): ڈومونٹ فورسپس; H): پلاسٹک پائپیٹ; I): کنویں میں مائع کی منتقلی کو آسان بنانے کے لیے گرم کرکے شیشے کی پیپٹ کو جھکا ہوا ہے۔

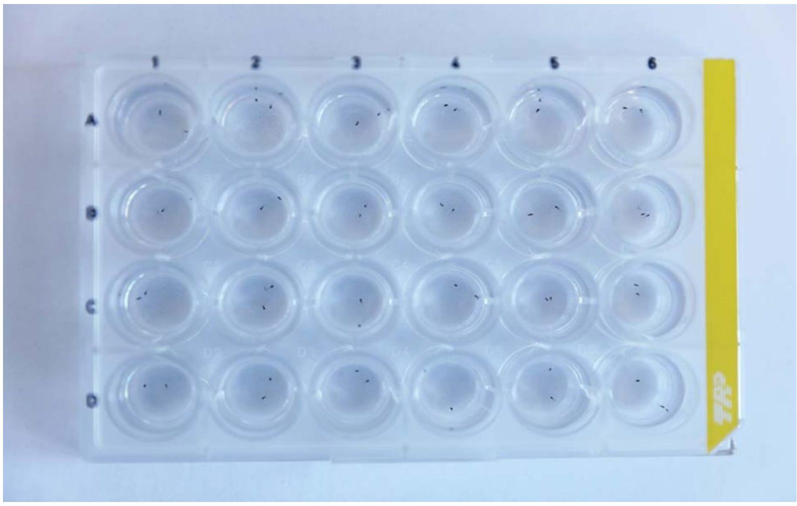

شکل 3): ایک پلیٹ جس میں 24 خانے ہیں، ہر خانے میں ریتیلی مکھیوں کے سر اور پیٹ کی نوک موجود ہے۔

4.3.1: نر

نر میں جنسی اعضاء بیرونی ہیں اور جوڑے دار فورسیپس پر مشتمل ہوتے ہیں، ہر ایک کے ڈورسل حصے میں گونوکوکسائٹ-گونوسٹائل جوڑ اور وینٹرل حصے میں ایپانڈریائی لوب ہوتا ہے۔ گونوسٹائل پر کانٹے اور بعض اوقات سیٹے ہوتے ہیں، جنہیں گننا ضروری ہے اور جن کی جڑنے کی جگہیں واضح طور پر دکھائی دینی چاہئیں۔ یہ ضروری ہے کہ گونوکوکسائٹ کی اندرونی سطح کو احتیاط سے دیکھا جائے، جس پر ایک جھرمٹ ہے ٹانگ سیٹے یا وہ سیٹے جو کسی لوب (یعنی ٹوبرکل) کے ذریعے اٹھائے گئے ہوں، موجود ہوں دیکھے جا سکتے ہیں [22]. انسانی تجربے کے کم رکھنے والے ساتھی ایک سادہ اطراف پر ماؤنٹنگ کر سکتے ہیں، بغیر جنسی اعضاء کو پیٹ کے آخر سے الگ کیے - **(ویڈیو کلپ-2):** (https://zenodo.org/records/18311158)اس صورت میں، جنسی اعضاء کے دو حصوں کا اوپر ایک دوسرے پر آنا مثال کے طور پر گونوکوکسائٹ کے اندرونی سیٹا کی گنتی کو مشکل بنا سکتا ہے، لیکن اس سے ناکام تشریح کے ذریعے جنسی اعضاء کو نقصان پہنچنے سے بچا جاتا ہے۔ زیادہ تجربہ کار ساتھی دو حصوں میں کھول کر انہیں الگ کرنے کی کوشش کر سکتے ہیں۔ ایسا کرنے کے لیے، ایک سوئی (انٹراڈرمل ری ایکشن نیڈل کی قسم) کے بیول والے کنارے کو گونوکوکسائٹ-گونوسٹائل اسمبلیوں کو مکمل طور پر کاٹے بغیر الگ کرنے کے لیے گزارا جاتا ہے۔ **(ویڈیو کلپ 2)** (https://zenodo.org/records/18311158) - اس طرح ان کے اندرونی حصوں کا مشاہدہ آسان ہو جائے گا۔ یہ ترتیب پیرامیرز اور پیرامیئل غلافوں کے مشاہدے کو بھی آسان بناتی ہے، جو اب ایک دوسرے پر نہیں چڑھتے۔ جانبی ماؤنٹنگ کے لیے، جو اعضاء کی تہہ بندی کو فروغ دیتی ہے، نمونوں کو مکمل طور پر صاف کرنا ضروری ہے۔

4.3.2:مادہ

مادہ میں جنسی نظام اندرونی ہے اور سپرماتھیکا پر مشتمل ہوتا ہے۔ بغیر تشریح کے انہیں ٹیگو مینٹس کے ذریعے اور پیٹ کو شکمی حالت میں ماؤنٹ کر کے دیکھا جاتا ہے۔ چاہے کوئی بھی ماؤنٹنگ میڈیم استعمال کیا جائے، سپرماتھیکا خود عموماً درست طور پر دیکھی جا سکتی ہے، خاص طور پر اگر وہ ہموار اور صاف نہ ہو۔ تاہم ہموار، پتلی دیوار والی سپرماتھیکا کو کم ریفریکٹیو میڈیا میں دیکھنا مشکل ہو سکتا ہے۔ مزید برآں، سپرماتھیکل نالیوں کی بنیاد کا مشاہدہ نوع کی شناخت کے لیے ضروری ہے، جیسا کہ سبینس لاروسیس [35, 37, 38] میں، جو پرانی دنیا میں لیشمینیا انفانتم کے اہم حامل ہیں۔ اس مشاہدے کے بغیر، نمونے کی شناخت ناممکن رہتی ہے۔ ان مشاہداتی دشواریوں پر قابو پانے کے لیے، جنسی فورکا-سپرماتھیکا ماؤنٹنگ کو پیٹ سے نکالا جانا چاہیے **(ویڈیو کلپ 3):** - (https://zenodo.org/records/18311106)سپرماتھیکا عام طور پر دیکھنے میں مشکل ہوتی ہیں۔
تشریح/ ڈ سیکشن کے دوران، لیکن جنسی فورکا تلاش کرنا نسبتاً آسان ہوتا ہے۔ چونکہ سپرماتھیکل نالیاں جنسی فورکا میں کھلتی ہیں، اس لیے اس فورکا کو الگ کرنے سے عام طور پر سپرماتھیکا کو بھی الگ کیا جا سکتا ہے۔ اگر اس عمل کے دوران سپرماتھیکا غلطی سے کٹ بھی جائیں، تو وہ ضائع نہیں ہوتیں اور انہیں اب بھی پیٹ کے پردوں کے اندر دیکھا جا سکتا ہے **(شکل 4)**۔

4.4: لیشمینیا کے علیحدہ کرنے کے لیے درمیانی آنت کی تشریح (ڈسیکشن)

مادہ فلیبوٹومین ریتیلی مکھیوں میں لیشمینیا کا پتہ لگانے اور الگ کرنے کے لیے نظامِ ہضم کی تشریح ضروری ہے۔ یہ طریقہ کار فیلڈ اور لیبارٹری دونوں میں انجام دیا جا سکتا ہے تاکہ ویکٹر کی اہلیت کا اندازہ لگایا جا سکے۔ تازہ بلاک شدہ مادہ پر کام کرنے کی سفارش کی جاتی ہے۔ مادہ کو پانی یا ہلکے ڈٹرجنٹ والے نمکین محلول (سا لا ین) سے دھوئیں تاکہ اضافی بال ہٹ جائیں۔ یہ مرحلہ لیشمینیا کے الگ کرنے کے لیے بے جراثیم حالات برقرار رکھنے میں مدد دیتا ہے، جبکہ شناخت کے لیے ضروری ساختی خصوصیات کو محفوظ رکھتا ہے۔ لیشمینیا کو تلاش کرنے اور الگ کرنے کے لیے، احتیاط سے درمیانی آنت نکالیں اور اسے ایک قطرہ جراثیم کش نمکین محلول (0.9 NaCl%) میں رکھیں۔ روشنی کے خوردبین کے تحت متحرک پرجیویوں(پیراسائٹس) کا مشاہدہ کرنے کے بعد (تجویز کردہ بڑھائی: تقریباً 200×)، انسولین سرنج یا مائیکروپیپیٹ استعمال کرتے ہوئے انہیں کاشت کے میڈیم میں منتقل کریں (مزید تفصیلات کے لیے باب 4.4.3 دیکھیں)۔ سر اور جنسی اعضاء کو صاف کرنے کے لیے براہِ راست مارک-اینڈرے سیال میں نصب کریں۔ **اہم:** مارک-اینڈرے سیال کو لیشمینیا کے رابطے میں آنے نہ دیں— نہ براہِ راست اور نہ ہی اوزار یا سوئیوں کے ذریعے—کیونکہ یہ پیراسائٹس کے لیے جان لیوا ہے۔ مادہ ریتیلی مکھیوں کی تشریح ایک یا دو سلائیڈز پر کی جا سکتی ہے؛ دونوں اختیارات کے اپنے فوائد اور حدود ہیں **(شکل 5)** -

4.4.1):دو سلائیڈز کا طریقہ

پہلا آپشن دو الگ الگ سلائیڈز پر کام کرنے پر مشتمل ہے: ایک میں مڈگٹ نکالنے کے لیے اسٹرائل سا لا ین ہوتا ہے، اور دوسری میں سر اور سپرماتھیکا کو مارک-اینڈرے سیال میں ماؤنٹ کیا جاتا ہے۔ تاہم، فیلڈ کے حالات میں عام طور پر دو یا تین افراد ریتیلی مکھیوں کی تشریح کرتے ہیں اور اپنی تشریحات ایک ہی محقق کو بھیجتے ہیں جو انواع کی شناخت اور آنت میں لیشمینیا کے انفیکشن کے جائزے کا ذمہ دار ہوتا ہے۔ دوسلائیڈز کا انتظام نمونے کی ٹریس ایبلٹی کے مسائل پیدا کر سکتا ہے اور خاص طور پر اس بات کا تعین مشکل بنا دیتا ہے کہ اگر آنت میں مثبت انفیکشن پایا جائے تو کون سی انفرادی ریتیلی مکھیاں متاثر تھی- **(ویڈیو کلپ 4):** (https://zenodo.org/records/18311154).

4.4.2): ایک سلائیڈ کا طریقہ

ایک ہی سلائیڈ کے استعمال سے نتائج کی ٹریس ایبلٹی یقینی ہوتی ہے۔ تاہم، متعدد احتیاطی تدابیر اختیار کرنا ضروری ہے۔ اس مرحلے میں جراثیم سے پاک رہنے کے لیے آپریٹرز کو باقاعدگی سے اپنے ہا تھ ہائیڈرو الکوحلک جیل سے صاف کرنے چاہئیں۔ غیر فرسٹڈ سلائیڈز اور مربع کور سلپس جو ایلومینیم فوائل میں لپٹی ہوں اور خشک حرارت (ملی میٹر22 × 22) سے جراثیم کش کی گئی ہوں، استعمال) پوپینیل اوون استعمال کرتے ہوئے تجویز(کی جانی چاہئیں، نیز ہر تشریح کے لیے جراثیم سے پاک سوئیاں استعمال کی جائیں۔ ریتیلی مکھیّ) ملی میٹر16 × ملی میٹر 25G Ø 0.5 کے ایک)٪(0.9کو سلائیڈ کے وسط میں جراثیم سے اسٹرائل سا لا ین جاتا ہے۔ سر کو اس وقت کاٹا جاتا ہے جب چھٹے اور - قطرے میں رکھا (ساتویں سیگمنٹ کے درمیان ایک چیر لگائی جاتی ہے۔
کلپ ویڈیو 4): (https://zenodo.org/records/18311154)

معدے کے ٹرگائٹس اور سٹرنائٹس کو ہاضمے کی نالی کاٹے بغیر نکالا جاتا ہے (اگر بہت لمبی سپرماتھیکا متوقع ہوں تو زیادہ اوپر کٹ کیا جا سکتا ہے)۔ پھر تھوراکس کو ایک سوئی سے بے حرکت کیا جاتا ہے اور دوسری سوئی سے پیٹ کے آخری پچھلے حصے کو نرمی سے کھینچ کر آنت نکالی جاتی ہے۔ اگر یہ ناکام ہو جائے تو پیٹ کے آخر کو سوئی سے بلاک کر کے ہاضمے کی نالی کو اس کے اگلے حصے سے کھینچا جا سکتا ہے۔

اگر یہ دوبارہ ناکام ہو جائے تو آنت کو نکالنے کے لیے اس کے گرد باقی ماندہ ٹیگو مینٹ کو زیادہ سے زیادہ ہٹانا ہوگا۔ جب آنت نکال لی جائے تو آنت کے آخری حصے کو کاٹ کر ہاضمے کی نالی سے الگ کرنا ہوگا۔ پھر آنت کو سلائڈ کے ایک طرف رکھے گئے اسٹرائل سا لا ین کے نئے قطرے میں رکھا جاتا ہے اور پھر نرمی سے اسٹرائل کورسلپ سے ڈھانپ دیا جاتا ہے۔ سر اور پیٹ کے آخری حصے سلائیڈ کے دوسرے سرے پر رکھی گئی مارک-اینڈرے مائع کے ایک چھوٹے سے قطرے میں منتقل کیے جاتے ہیں، اس بات کو یقینی بناتے ہوئے کہ لیشمینیا کے ساتھ کوئی رابطہ نہ ہو۔ سر کو صحیح سمت میں رکھا جاتا ہے (پس منظر کا سوراخ اوپر کی طرف)، اور سپرماتھیکا کو جینیٹل فرکا کے ساتھ جیسا کہ اوپر بتایا گیا ہے الگ کیا جاتا ہے اور ایک چھوٹی گول کورسلپ (12 Ø ملی میٹر، جسے اسٹرائل چوکور کورسلپس کے ساتھ الجھایا نہ جائے) سے ڈھانپ دیا جاتا ہے۔ باقی ماندہ ریتیلی مکھی کی لاش اور پروں کو سلائیڈ کے بیچ میں نمکین پانی/سا لا ین کے قطرے میں رکھا جاتا ہے۔ **(ویڈیو کلپ 4):** (https://zenodo.org/records/18311154)۔ اگر مثبت نتیجہ آئے، یا ٹیکسونومک تحقیق کے لیے، سینہ اور پیٹ کو مالیکولر یا پروٹومیک مطالعات کے لیے محفوظ کیا جا سکتا ہے، اور پروں کو آبی ماحول میں نصب کیا جا سکتا ہے۔ ماؤنٹ کو محفوظ کرنے کے لیے، مارک-اینڈرے مائع کے اضافی حجم کو کلورل گم (=ہویئر) یا پولی وینائل الکحل پر مبنی ماؤنٹنگ میڈیم جیسے آبی ماؤنٹنگ میڈیم سے تبدیل کیا جا سکتا ہے۔

ان طریقہ کارکو دکھانے والی تفصیلی ویڈیوزدستیاب ہیں۔ ریتیلی مکھی کی وسطی آنت کی تشریح **(ویڈیو کلپ 5):**
اس کے علاوہ لعابی غدود کی اور تشریح **(ویڈیو کلپ 6):**
(https://zenodo.org/records/18303014)
(https://zenodo.org/records/18302850) میں کی گئی ہیں۔ لٰہذا یہاں ان کی وضاحت نہیں کی جائے گی۔

4.4.3: ریتیلی مکھی کے آنتوں سے لیشمینیا کے پرجیوی کا علیحدہ کرنا اور افزائش

متاثرہ مادہ ریتیلی مکھی کی تشریح سے پیراسائٹس کا علیحدہ کرنا ایک نازک عمل ہے جس میں اعلیٰ مہارت درکار ہوتی ہے اور ابتدا میں اسے پیراسائٹس سے پاک نمونوں پر آزمانا چاہیے۔ تشریح کے بعد آنتوں کو تازہ قطرے میں اسٹرائل سا لا ین (0.9٪) یا لوک کے محلول میں دھونے کے لیے منتقل کیا جاتا ہے [4]۔ تراشے ہوئے آنتوں کو پھر دو طریقوں سے پراسیس کیا جا سکتا ہے : i) روشنی کے خوردبین کے نیچے دیکھ کر لیشمینیا پروماسٹیگوٹس کے مختلف مراحل اور ان کی جگہ کا مشاہدہ کرنا، خاص طور پر اسٹومیڈیئل والو پر توجہ کے ساتھ ، اور ii) آنت کو کھول کر پروماسٹیگوٹس کے اخراج کو آسان بنانا تاکہ ان کی بڑے پیمانے پر کاشت ممکن ہو سکے [4]۔ فیلڈ میں متعدی مادہ ریتیلی مکھی کا ملنا نسبتاً نایاب واقعہ ہے، اس لیے اچھی عملی مشقیں کامیاب علیحدگی کے امکانات کو زیادہ سے زیادہ کر دیتی ہیں۔

اگر آنت میں لیشمینیا کے پیراسائٹس دیکھے جائیں تو نئی جراثیم کش سوئیاں استعمال کریں اور کیپلیری ایکشن کے ذریعے کور سلپ کے گرد تھوڑی سی مقدار میں جراثیم کش نمکین محلول شامل کریں تاکہ وہ آزاد ہو جائیں۔ آنت کو احتیاط سے اور تیزی سے پھاڑ کر پیراسائٹس کو سا لا ین میں خارج کریں۔ 100 μL مائیکروپیپیٹ یا ٹوبرکلین سرنج استعمال کرتے ہوئے پیراسائٹس کو جمع کریں اور مناسب لیبل شدہ SAM-9 بلڈ آگر میڈیم میں لگائیں۔

لیشمانیا پروماسٹیگوٹس کی ان ویٹرو کاشت

الگ کیے گئے پیراسائٹس ابتدائی طور پر SNB-9 بلڈ ایگر کے ڈھلوانوں یا Novy, Mc Neal, Nicolle (NNN) ٹھوس میڈیم [16] پر برقرار رکھے جاتے ہیں، جس کے اوپر یا تو جراثیم کش الفا-MEM میڈیم [16, 65] یا M199 میڈیم ڈالا جاتا ہے، اور ہر ایک میں 10% حرارتی طور پر غیر فعال شدہ جراثیم کش فیٹل کیل سرم [FCS] شامل کیا جاتا ہے۔ (پیراسائٹ کی نشوونما کو بڑھانے کے لیے)، 1% BME وٹامنز، 2% اسٹرائل انسانی پیشاب (سنجری فلٹر 0.2 μm Filtropur® S کے ذریعے اسٹرائل کیا گیا) 250 μg/mL ایمپکاسین (یا 50 μg/mL جینٹامائسن، یا اینٹی بائیوٹکس اور امینو ایسڈز کا مرکب (ایل-گلوٹامین 200 mM-پینسلِن 10 000 U-سٹریپٹومائسِن (10 mg/mL [47])– تین دن بعد، اگر آلودگی نہ ہو تو کلچرز کو صحیح طریقے سے تیار کردہ فریزنگ میڈیم میں معلق کر کے بعد ازاں 1 سے 2 سال کے لیے -80°C پر یا طویل مدتی تحفظ اور مستقبل کے تجرباتی استعمال کے لیے -196°C پر مائع نائٹروجن میں محفوظ کیا جاتا ہے [7]۔

4.5: لار کے (لعابی) غدود

ریتیلی مکھی کے لعابی غدود کی تشریح ویکٹر-پیتھوجن تعاملات کے مطالعے کے لیے ایک بنیادی تکنیک ہے، خاص طور پر فلیبو وائرس (مثلاً ٹوسکانا وائرس) جیسے آربو وائرس کا پتہ لگانے کے لیے [44, 75]. ریتیلی مکھوں کے انتہائی چھوٹے سائز کی وجہ سے، اس عمل کے لیے سٹیریو مائیکروسکوپ کے نیچے باریک فورسپس یا مائیکروڈائیسیکشن سوئیوں کا استعمال کرتے ہوئے نازک لعابی غدود کو پھاڑ یا آلودگی کے بغیر الگ کرنے میں باریکی درکار ہوتی ہے [51, 61] ۔

ویڈیو کلپ 6): [https://zenodo.org/records/18302850], غدود کی سالمیت کو برقرار رکھنا بعد میں ہونے والے سالماتی تجزیے کی درستگی کے لیے نہایت ضروری ہے۔ ایک بار نکالے جانے کے بعد، ان غدود کو ہم آمیز (homogenized) کر کے qPCR، RT-PCR، یا امیونو تیزاب کے ذریعے وائرل RNA یا اینٹی جینز کا پتہ لگانے کے لیے جانچا جا سکتا ہے [12]۔ صرف آنت یا ہیموکیل کے بجائے لعابی غدود میں وائرس کی موجودگی اس بات کی تصدیق کرتی ہے کہ پیتھوجن نے اپنی بیرونی انکیوبیشن مدت مکمل کر لی ہے اور خون چوسنے کے دوران منتقل ہونے کے قابل ہے [71]۔

ریتیلی مکھی کے لعابی غدود کے چھوٹے سائز کی وجہ سے تشریح کا عمل تکنیکی طور پر مشکل ہے، جس کے لیے نمونے کی خرابی سے بچنے کے لیے خاطر خواہ مہارت درکار ہوتی ہے [1, 51]. مزید برآں، وائرل لوڈ کم ہو سکتا ہے، جس کے لیے انتہائی حساس تشخیصی طریقے جیسے نیسٹڈ پی سی آر یا بائی تھروپٹ سیکوینسنگ ضروری ہیں [54]۔ آلودگی کے خطرات اس بات کو مزید اجاگر کرتے ہیں کہ جراثیم سے پاک طریقے اپنانا ضروری ہے۔ تکنیکی رکاوٹوں کے علاوہ، حیاتیاتی عوامل بھی شناخت کی کامیابی پر اثرانداز ہوتے ہیں؛ مختلف ریتیلی مکھیاں انواع میں ویکٹر صلاحیت مختلف ہوتی ہے، اور انفیکشن کی شرحیں ماحولیاتی اور موسمی حالات کے مطابق بدلتے رہتی ہیں [33, 61].
لاروں کے غدود میں وائرس کا پتہ لگانا منتقلی کے خطرات کے بارے میں اہم بصیرت فراہم کرتا ہے، جس سے ہدف شدہ نگرانی اور کنٹرول کے اقدامات ممکن ہوتے ہیں [15]۔ مثال کے طور پر، وبائی علاقوں میں ریتیلی مکھیوں میں ٹاسکانا وائرس کی شناخت نے تشخیصی پروٹوکولز اور عوامی صحت کے مشوروں کو آگاہ کیا ہے [18]۔ مزید برآں، وائرس-تھوک کے تعاملات کا مطالعہ منتقلی روکنے والی ویکسینز یا علاجی ادویات کے لیے نئے اہداف کا انکشاف کر سکتا ہے [15, 18]۔ ریتیلی مکھیاں کے لعابی غدود کو اینٹی جینز کے ماخذ کے طور پر بھی استعمال کیا جا سکتا ہے

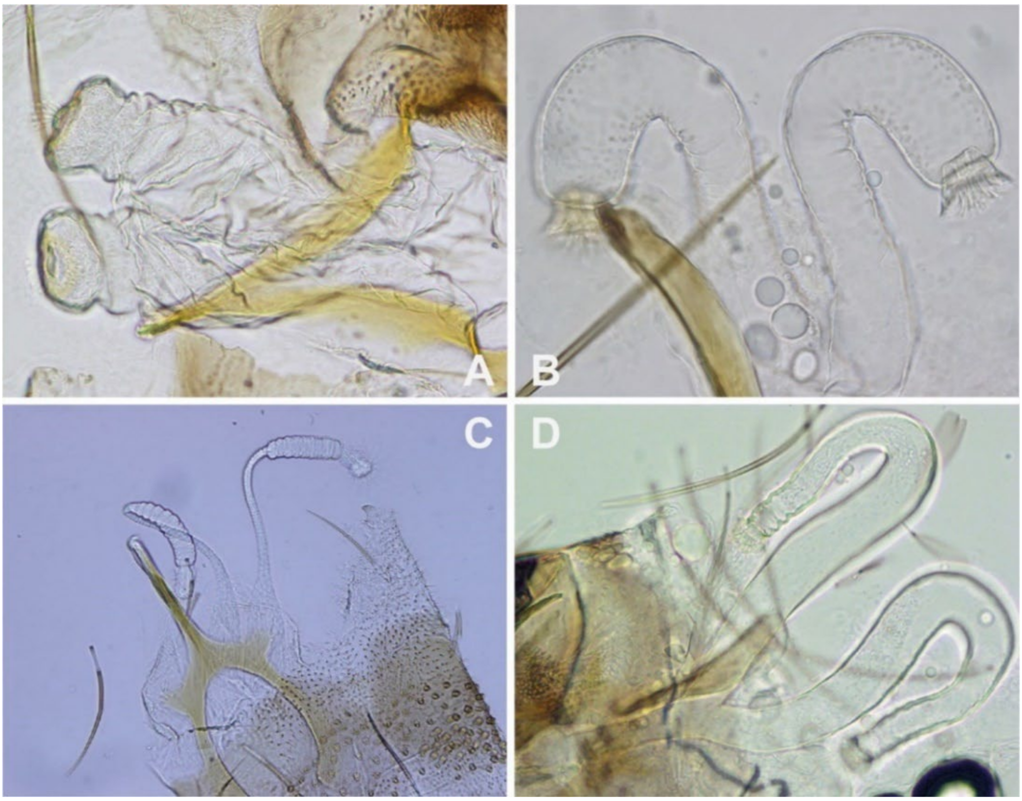

شکل 4): تازہ نمونوں سے نکالی گئی اور مارک-اینڈری سیال میں محفوظ کی گئی سپرماتھیکا۔
A): *Idiophlebotomus longiforceps* (لاوپی ڈی آر); B): *Sergentomyia minuta* (فرانس);
C): *Phlebotomus ariasi* (فرانس); D): *Sergentomyia anodontis* (لاوپی ڈی آر).

تاکہ امیونولوجیکل طریقوں، ترجیحاً ای ایل آئی ایس اے کے ذریعے، میزبان کے ریتیلی مکھیاں کے لعاب کے خلاف اینٹی باڈیز کی مقدار ناپی جا سکے۔ یہ طریقہ میزبان کے ریتیلی مکھیاں کے کاٹنے کے سامنے آنے کا اندازہ لگانے کے قابل بناتا ہے، یوں یہ ویکٹر کنٹرول کے طریقوں کی مؤثریت [25] اور لیشمینیا کے انتقال کے خطرے [40] کے جائزے میں معاون ثابت ہوتا ہے۔

4.6): خونی غذا کی شناخت

گرفتاری سے حاصل کردہ سوجی ہوئی مادوّں کو کراس کنٹیمینیشن سے بچنے کے لیے یک بار استعمال ہونے والے آلات کے ساتھ تشریح کیا جانا چاہیے۔ ان کے پیٹ کا معائنہ سٹیریو مائیکروسکوپ کے تحت کیا جائے تاکہ خون کے کھانے کے ہضم کے مرحلے کا اندازہ لگایا جا سکے۔ صرف ان مادوّں کا انتخاب کریں جن کا پیٹ سرخ، سرخ مائل بھورا یا گہرا سرخ ہو اور جن میں انڈوں کی تشکیل کے کوئی آثار نہ ہوں۔ صفائی کے بعد مادےّ کی مورفولوجیکل شناخت کے لیے سپرماتھیکا سمیت پیٹ کی نوک کو ہٹا دیں۔ پیٹے کے مرکزی حصے (سپرماتھیکا کے بغیر) کو پھر ایپنڈورف® ٹیوبز میں رکھ کر مزید تجزیے تک -20°C پر محفوظ کیا جانا چاہیے۔ خونی غذا کی شناخت کے لیے عام طور پر استعمال ہونے والے جینیاتی مارکر، جیسے PNOC [5, 30, 50]، CytB [67], یا COI (13)، اچھی طرح قائم شدہ ہیں اور ادبیات میں تفصیل سے بیان کیے گئے ہیں؛ لہٰذا، اس مقالے میں ان کی مزید تفصیل نہیں دی جائے گی (**شکل 6**). متبادل طور پر، میزبان کے خون کی شناخت کے لیے میلڈی-ٹوف پیپٹائڈ میپنگ استعمال کی جا سکتی ہے [31]. تجرباتی طور پر یہ ثابت ہو چکا ہے کہ یہ تکنیک خون کے کھانے کے بعد طویل عرصے تک میزبان کے خون کی شناخت ممکن بناتی ہے؛ لہٰذا، یہ ایک موزوں طریقہ انتخاب ہے۔ خاص طور پر ایسے بھرے ہوئے مادہ ریتیلی مکھی کے تجزیے کے لیے جن میں میزبان خون کا ہضم واضح طور پر زیادہ ترقی یافتہ ہو۔ نمونوں کو مثالی طور پر -20°C یا 4°C پر محفوظ کیا جانا چاہیے، لیکن مختصر مدت کے لیے کمرے کے درجہ حرارت پر رکھنے سے بھی اچھے نتائج حاصل کیے جا سکتے ہیں۔ بھرے ہوئے مادہ کے پیٹ کو تجزیے سے کچھ دیر قبل باقی جسم سے الگ کر کے مقطر پانی میں ہم آہنگ کیا جانا چاہیے۔ باقی ماندہ ریتیلی مکھی کی جسمانی ساخت دیگر مالیکیولر اور مورفولوجیکل تجزیوں کے لیے دستیاب رہتی ہے۔ ہوموجینیٹ سے میلڈی-ٹوف پیپٹائڈ میپنگ کے لیے علیحدہ نمونہ لینے کے بعد، باقی حصہ میزبان خون کی شناخت کی تصدیق اور/یا لیشمینیا کی موجودگی کے لیے ڈی این اے علیحدگی کے لیے استعمال کیا جا سکتا ہے۔ ڈی این اے پر مبنی مالیکیولر تکنیکوں کے مقابلے میں نمونہ کی تیاری اور تجزیے کا مجموعی وقت بہت کم ہوتا ہے

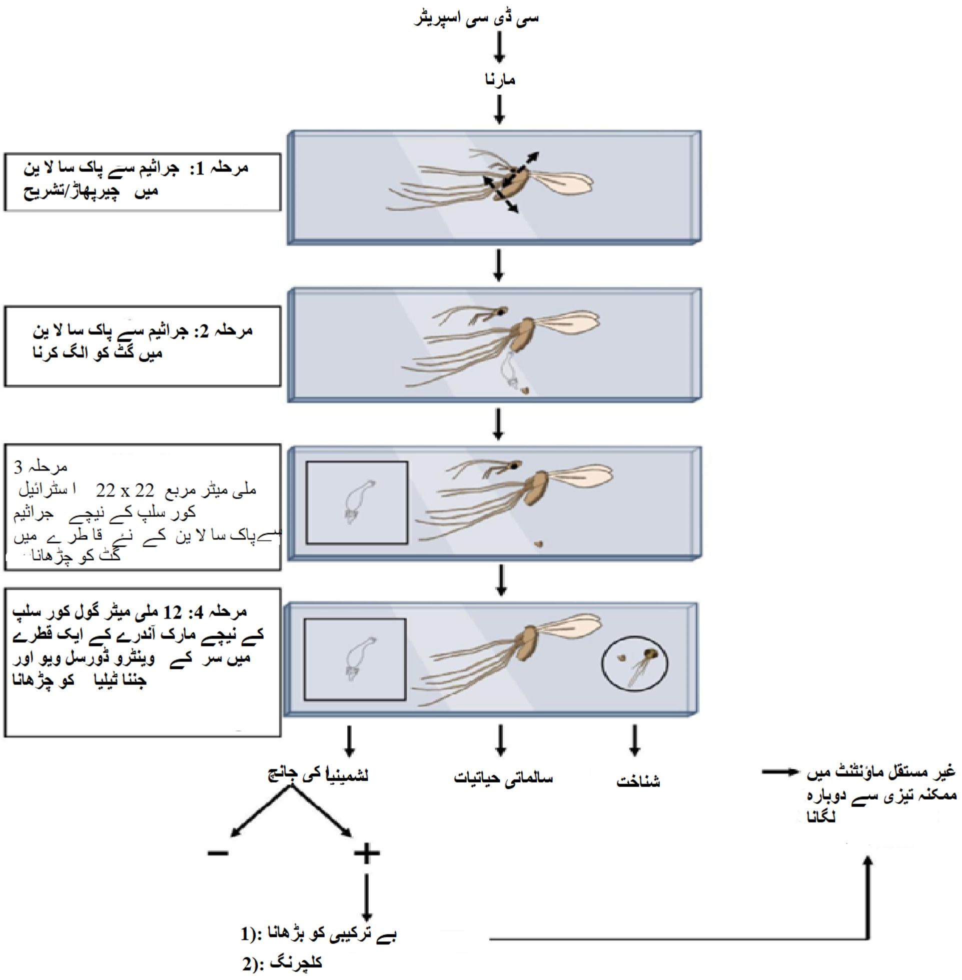

شکل 5): لیشمینیا کے علیحدہ کرنے کا طریقہ۔

5): شکلاتی مطالعات کے لیے نمونہ کی تیاری (شکل 3، 6، 7 اور 8؛ ضمیمہ جات A، B، C اور D)

یہ سیکشن صرف مورفولوجیکل مطالعات کے لیے ریتیلی مکھی کے نمونے کی تیاری کے اصول بیان کرتا ہے، اور اس کے بعد مورفولوجی سے آگے کے اطلاقات کے لیے اس کی موافقت بھی پیش کی گئی ہے۔ تاہم، اس طریقہ کار کو سمجھنا نہایت ضروری ہے، کیونکہ اس سے ضرورت پڑنے پر مخصوص نمونوں کے لیے طریقہ کار کو ڈھالنا ممکن ہوتا ہے۔ اس عمل میں لچکدار ربڑ کے بلبلوں سے لیس پاسٹر پائپٹس کے ذریعے بار بار خالی کرنے اور بھرنے کے مراحل شامل ہیں۔ گول تہہ والے شیشے کے برتن سختی سے تجویز کیے جاتے ہیں کیونکہ یہ ان عملیات کو بہت آسان بناتے ہیں۔ شیشہ تمام ریجنٹس کے لیے غیر فعال ہوتا ہے۔ ریجنٹس کے بخارات کو روکنے کے لیے برتنوں پر ڈھکن لگا کر رکھنا چاہیے اور انہیں کبھی زیادہ بھرنا نہیں چاہیے تاکہ بند یا کھولتے وقت اوور فلو نہ ہو اور نمونوں پر گرد نہ گرے۔ صفائی اور پراسیسنگ کے لیے درکار کیمیکلز **جدول 2** میں دکھائے گئے ہیں۔

5.1): صفائی

ریتیلی مکھیوں کے نمونے مستقل سلائیڈ ماؤنٹس کی تیاری سے پہلے مناسب طریقہ اور کلیئرنگ ایجنٹ (مثلاً 10% ایسٹک ایسڈ کا محلول یا مارک-اینڈرے محلول جس میں کلورل ہائیڈریٹ شامل ہے، جو کئی ممالک میں محدود کیمیکل ہے) کے ذریعے میسریشن کے ذریعے شفاف کیے جاتے ہیں۔ صفائی کے عمل سے جسم کے بافتیں، چربی، اخراجات اور موم ہٹ جاتے ہیں، جس سے نمونہ نیم شفاف ہو جاتا ہے اور بیرونی ڈھانچے (مثلاً سیٹیوں کا داخلہ)، سطحی خصوصیات (مثلاً رنگت) اور ٹیگو مینٹ کے ذریعے دکھائی دینے والی اندرونی خصوصیات (مثلاً سپرماتھیکا) کا معائنہ آسان ہو جاتا ہے۔ دو مرحلوں پر مشتمل صفائی کا عمل، جس میں پہلے ایک مضبوط الکلی (مثلاً پوٹاشیم ہائیڈروکسائیڈ) استعمال کیا جاتا ہے اور پھر ایک کمزور تیزاب (مثلاً مارک-آندرے محلول میں ایسٹک ایسڈ)، مختلف حیاتیاتی مقاصد کے لیے کام کرتا ہے [74]۔ الکلی نرم بافتوں، جیسے پروٹین، چربی اور پٹھوں کو صابن بنانے (saponification) اور پروٹین کی ساختی تبدیلی (denaturation) کے ذریعے تحلیل کر دیتا ہے، جس سے ساختی وضاحت کے لیے چیتھن کا بیرونی ڈھانچہ سالم رہتا ہے۔ بعد ازاں کمزور تیزاب باقی ماندہ الکلی کو نیوٹرلائز کر کے مزید تحلیل کو روکتا ہے

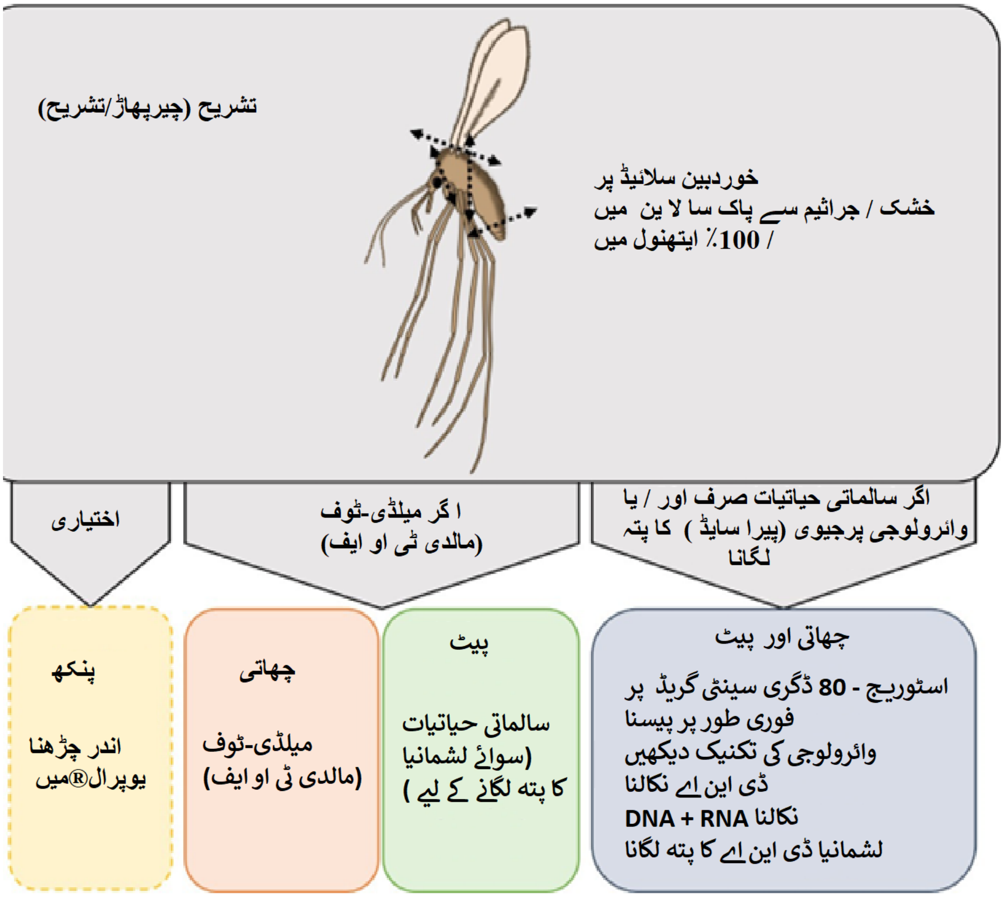

شکل 6): ریتیلی مکھی کی پراسیسنگ مالیکیولر بائیولوجی، پروٹومیکس، اور/یا وائرولوجی کے اطلاقات کےلیے۔

اور شفافیت بڑھانے کے لیے کائٹن کو سفید کرتا ہے [74]، اگرچہ نمونوں کو دو مرتبہ 15 منٹ کے لیے مقطر پانی میں دھونا بھی الکلی کو نیوٹرلائز کرنے کے لیے کافی ہو سکتا ہے۔ یہ تسلسل وار عمل مؤثر بافتی ہٹانے اور نرمی سے تحفظ کو یکجا کرتا ہے، جس سے خوردبینی معائنے کے لیے نمونوں کی سالمیت بہترین رہتی ہے۔ اگلے مرحلے پر جانے سے پہلے مقطر پانی میں دو مرتبہ 20 منٹ کے لیے دھونے کی سفارش کی جاتی ہے۔

5.1.1): نرم بافتوں کا لائسس (شکل 8)

سوڈیم ہائیڈروکسائیڈ (NaOH) یا پوٹاشیم ہائیڈروکسائیڈ (KOH) عام طور پر استعمال ہونے والے کیمیائی میسریٹنگ ایجنٹس ہیں، جو نمونوں کے سائز اور نازک پن کے مطابق مختلف ارتکاز اور دورانیے کے لیے استعمال کیے جاتے ہیں۔ معیاری اور سب سے مؤثر تکنیک میں نرم بافتوں کو تحلیل کرنے کے لیے ریتیلی مکھیوں کو رات بھر ایک مضبوط الکلی (10% KOH یا NaOH) میں بھگونا شامل ہے۔ علاج کے دورانیے کو کم کرنے کے لیے ارتکاز بڑھایا جا سکتا ہے (مثلاً 6 گھنٹے کے لیے 20٪ KOH) اور اسے 37°C پر گرم بھی کیا جا سکتا ہے۔

5.1.2): وضاحت رنگ آمیزی کے ساتھ یا اس کے بغیر

اس مرحلے کے بعد روشنی کا عمل کیا جاتا ہے، عام طور پر ایسٹک ایسڈ اور کلورل ہائیڈریٹ (مثلاً مارک-اینڈرے محلول) کے امتزاج سے۔ صفائی کے بعد نمونوں کو باقی ماندہ کیمیائی مادوں کے خاتمے کے لیے کم از کم دو متواتر پانی کے غسلوں میں ہر ایک میں بیس منٹ کے لیے اچھی طرح دھویا جانا چاہیے۔ مارک-اینڈرے محلول ریتیلی مکھیاں کے نمونے تیار کرنے کے لیے ایک عام طور پر استعمال ہونے والا صفائی کرنے والا ایجنٹ ہے۔ اس کی مؤثریت صفائی کے عمل کو آسان بنانے میں ہے، جبکہ نازک ڈھانچوں، جیسے پروں اور اینٹینا کو خاطر خواہ نقصان سے بچاتا ہے۔ اس محلول کو بخارات بننے یا خراب ہونے سے بچانے کے لیے تازہ تیار کیا جانا چاہیے یا کسی مضبوطی سے بند ڈبے یا بوتل میں محفوظ کیا جانا چاہیے۔ مارک-اینڈرے محلول کا استعمال خاص طور پر اس وقت فائدہ مند ہوتا ہے جب اسے مخصوص مورفولوجیکل تفصیلات کو بہتر بنانے کے لیے روشن کرنے یا رنگنے کی تکنیکوں کے ساتھ ملایا جائے۔ اس کے اجزاء اور تیاری کی تفصیلات **ضمیمہ B** میں دی گئی ہیں۔

بہت زیادہ شفاف نمونوں کے لیے، ماؤنٹ کرنے سے پہلے دیکھنے کی صلاحیت بہتر بنانے کے لیے رنگائی ضروری ہو سکتی ہے۔ کئی قسم کے رنگ دستیاب

ہیں،

جدول 2): استعمال شدہ محلولوں کی ترکیب۔

سیریل نمبر	محلول کا نام اور اس کی ترکیب	سیریل نمبر	محلول کا نام اور اس کی ترکیب
1):	**پوٹاشیم ہائیڈرو آکسائیڈ 10%**<br>پوٹاشیم ہائیڈروکسائیڈ 10 گرام<br>آبی مقطر حسبِ ضرورت 100 ملی لیٹر	4):	**فوکسن ایسڈ 1% مقطر پانی میں**<br>ایسڈ فوشین (پاؤڈر کی صورت میں) 1 گرام<br>آبی مقطر 99 ملی لیٹر
2):	**گم کلورل ماؤنٹنگ میڈیا (ہوئیر میڈیم)**<br>پانیِ مقطر 50 ملی لیٹر<br>کلورل ہائیڈریٹ 200 گرام<br>گم عربیک 50 گرام<br>گلیسرول 20 ملی لیٹر	5):	**ایسڈ فوشین سے رنگین مارک-آندرے محلول**<br>مارک-اینڈرے محلول 10 ملی لیٹر<br>فوکسین 1% 50 مائکرو لیٹر
3):	**مارک-اینڈرے محلول**<br>کلورل ہائیڈریٹ 40 گرام<br>گلیشیل ایسٹک ایسڈ 30 ملی لیٹر<br>مقطر شدہ پانی 30 ملی لیٹر	6):	**اینیسے میڈیم**<br>خالص سفید کولوفونی 22 گرام<br>الکحل میں حل پذیر کوپال گم 12 گرام<br>مطلق ایتھانول 20 ملی لیٹر<br>کافور 10 گرام<br>ٹارپینٹائن جوہر کا عرق 10 ملی لیٹر<br>یوکالیپٹول 26 ملی لیٹر

ہر ایک جاندار کے مخصوص کیمیائی اجزاء کو نشانہ بناتا ہے۔ یہ ضروری ہے کہ ایسا رنگ منتخب کیا جائے جو نمونے اور منتخب شدہ ماؤنٹنگ میڈیم دونوں کے ساتھ مطابقت رکھتا ہو۔ یہ بنیادی طریقہ کار ضرورت کے مطابق ڈھالا جا سکتا ہے، مثال کے طور پر رنگنے کے لیے مارک-اینڈرے محلول میں 0.1% ایسڈ فوشین شامل کر کے۔ مزید برآں، آبی محلولوں میں محفوظ کیے گئے اور ریزینی ماؤنٹس کے لیے مخصوص نمونوں کو ڈی ہائیڈریشن کی ضرورت ہوتی ہے (دیکھیں سیکشن 5.2: ڈی ہائیڈریشن)، کیونکہ زیادہ تر قدرتی اور مصنوعی ریزن ماؤنٹنگ میڈیم پانی کے ساتھ مطابقت نہیں رکھتے۔ نیو (1974) نے نوٹ کیا کہ بعض اسٹین مخصوص ماؤنٹنگ میڈیا میں خراب ہو سکتے ہیں [53]. مثال کے طور پر، ایسڈ فوشین، جو عام طور پر کینیڈا بالسم کے ساتھ استعمال ہوتا ہے، کو یوپارل® میں بھی فکس کیا جا سکتا ہے۔ تاہم، ایسڈ فوشین سے رنگے گئے نمونے مدھم پڑنے کے شکار ہوتے ہیں، خاص طور پر جب لونگ کے تیل کے باقیات، جو آخری صفائی کے سیال کے طور پر استعمال ہوتا ہے، موجود رہیں۔ لونگ کے تیل میں محفوظ کیے گئے نمونے چند دنوں کے اندر نمایاں طور پر مدھم ہو سکتے ہیں۔

5.2): پانی کو خشک کرنا (ڈی ہائیڈریشن)

نمونے کو بتدریج درجہ وار ایتھانول محلول کی سیریز (50٪ ، 70٪، 80٪، 90٪ یا 95٪ اور آخر میں 100٪) سے گزار کر خشک کیا جاتا ہے، اور ہر محلول میں کم از کم 20 منٹ کے لیے رکھا جاتا ہے۔ چونکہ ایتھانول تیزی سے بخارات بن جاتا ہے، اس لیے عمل کے دوران برتن کو مضبوطی سے بند رکھنا چاہیے۔ ایک بار نمونہ مکمل طور پر خشک ہو جائے تو آپ عمل کو چند دنوں کے لیے یوپارل ®ایسنس میں معطل کر سکتے ہیں، جو کہ لونگ کے تیل کے مقابلے میں بہتر ہے۔ بیچ کریوزوٹ، جو کبھی اس مقصد کے لیے وسیع پیمانے پر استعمال ہوتا تھا، اب اپنی زہریلے پن کی وجہ سے مکمل طور پر ممنوع ہے۔ ڈی ہائیڈریشن کے عمل کو اس بات کو یقینی بنانا چاہیے کہ نمونے کے اندر موجود سیال ماؤنٹنگ میڈیم کے ساتھ مطابقت رکھتا ہو تاکہ غیر شفافیت، اسموٹک انہدام، یا تحریف سے بچا جا سکے جو نمونہ کو ٹیکسونومک مطالعے کے لیے نامناسب بنا سکتی ہے۔

5.3): ماؤنٹنگ میڈیا

5.3.1): نمونہ کی تیاری کے لیے انتخاب اور استعمال

ماؤنٹنگ میڈیم کا مثالی طور پر شیشے کے تقریباً 1.5 کے قریب انعکاسی اشاریہ ہونا چاہیے۔ یہ بے رنگ، شفاف اور خشک ہونے کے بعد اور وقت کے ساتھ مکمل طور پر شفاف رہنا چاہیے۔ یہ استعمال ہونے والے رنگوں کے ساتھ مطابقت رکھتا ہو اور نمونے کے تمام بافتوں میں سرایت اور پھیلاؤ کی صلاحیت رکھتا ہو۔ ماؤنٹنگ کے دوران یہ بہت تیزی سے خشک نہیں ہونا چاہیے اور نہ ہی دھندلا پن پیدا کرنا چاہیے۔ ماؤنٹنگ کے بعد یہ سکڑنا نہیں چاہیے۔ مناسب ماؤنٹنگ میڈیم کا انتخاب نمونہ کی تیاری کا ایک بنیادی پہلو ہے، کیونکہ کوئی ایک میڈیم تمام مقاصد کے لیے مثالی نہیں ہوتا۔ انتخاب میں کئی اہم عوامل کا توازن ہونا چاہیے:

1)- **بصری خصوصیات**. ماؤنٹنگ میڈیم کا انعکاسی اشاریہ (refractive index) ٹیکسونومک شناخت یا مورفولوجیکل وضاحت کے لیے استعمال ہونے والی اہم تشریحی خصوصیات، جیسے سپرماتھیکا (spermathecae)، ایسکوئڈز (ascoids)، نیوسٹیڈ سینسیلا کافی لیے کے (pharyngeal teeth) دانت فیرنجیل اور ، (vertical cibarial teeth)دانت سیبریل عمودی ، (Newstead sensilla)انعکاس فراہم کرنا چاہیے۔ ان ڈھانچوں کی نمائش براہ راست ماؤنٹنگ میڈیم کی بصری خصوصیات پر منحصر ہوتیہے۔

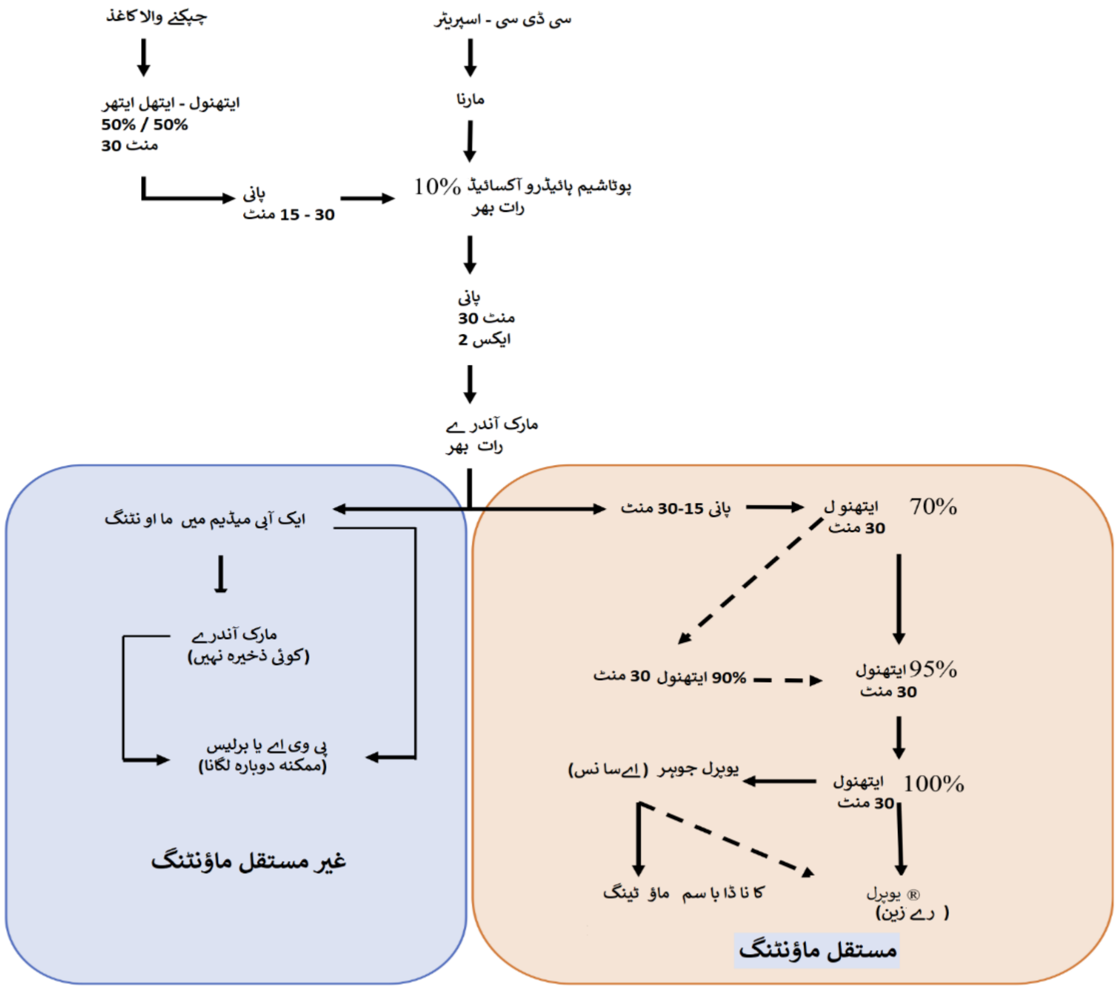

شکل 7): ریتیلی مکھیوں کا پراسیس کرنے کا کلاسیکی طریقہ۔

2)- **محفوظیت**۔ ٹائپ نمونوں یا مستقل مجموعوں کے لیے مخصوص مواد کے لیے، میڈیم کو طویل مدتی استحکام اور پائیداری فراہم کرنی چاہیے۔ اس کے برعکس، انوینٹری مطالعات یا وبائیاتی سروے کے لیے، جہاں طویل مدتی محفوظی کم اہم ہو، عارضی یا نیم مستقل ماؤنٹنگ میڈیا کافی ہو سکتے ہیں۔

5.3.2): ماؤنٹنگ میڈیا کے لیے ضروریات

ماہرین اکثر مخصوص تحقیقی ضروریات کے مطابق حسب ضرورت اور پیچیدہ ماؤنٹنگ تکنیکیں تیار کرتے ہیں۔ تاہم، یہ طریقے اکثر آرکائیول معیار، مطابقت، معیاری کاری یا استعمال میں آسانی جیسے پہلوؤں کو نظر انداز کر دیتے ہیں۔ اور طویل مدتی تحفظ اس معیاری فقدان نے عطیہ شدہ مجموعات کے انضمام اور طویل مدتی نگہداشت کی کوششوں کو پیچیدہ بنا دیا ہے۔ سائنسی اطلاقات ماؤنٹنگ میڈیا کے لیے مخصوص تقاضے عائد کرتی ہیں۔ ٹیکسونومسٹ اکثر پورے نمونے ماؤنٹ کرتے ہیں اور ایسے میڈیا کو ترجیح دیتے ہیں جو اندرونی اعضاء کو نرمی سے نرم کر کے کٹیکولر ڈھانچوں کی وضاحت بڑھا دے۔ بصری وضاحت کو زیادہ سے زیادہ کرنے کے لیے انڈیکس انعکاس نور کا نمونے اور شیشے کی سلائیڈ سے کافی حد تک مختلف ہونا چاہیے۔ تجارتی ماؤنٹنگ میڈیا عموماً شیشے کے قریب شکنی نما اشارے کے سا تھ تیار کیا جاتا ہے تاکہ سلائیڈ–ماؤنٹنگ میڈیم–کوور سلپ کے نظام میں روشنی کی شکنی اور پھیلاؤ کم سے کم ہو۔ تاہم، برائٹ فیلڈ مائیکروسکوپی میں، بغیر رنگے نمونے کے قدرتی تضاد کو جان بوجھ کر ایسے ماؤنٹنگ میڈیم کے انتخاب سے تبدیل کیا جا سکتا ہے جس کا شکنی نما اشارہ نمونے سے قدرے مختلف ہو، جس سے پس منظر کے مقابلے میں اس کی نمائش بہتر ہوتی ہے۔

5.3.3): ماؤنٹنگ میڈیا کی اقسام (جدول 3 اور 4)

مائیکروسکوپی میں مائونٹنگ میڈیم کے انعکاسی نما (RI) کی ضرورت ہوتی ہے تاکہ یہ معلوم کیا جا سکے کہ روشنی سلائیڈ، میڈیم اور نمونے کے ذریعے کیسے مڑتی ہے۔ جب RI کا قدر تقریباً کور سلپ شیشے (≈1.515) کے برابر ہو، تو روشنی یکساں طور پر گزرتی ہے، جس سے بکھراؤ اور

جدول 3): منتخب ماؤنٹنگ میڈیا کی ترکیب۔

سیریل نمبر	ماؤنٹنگ میڈیم	سالوینٹس	ممکنہ پری پولیمر(ز) یا پولیمر	تشریحات
**1):**	ہویئر = کلورل گم	گلیسرول، پانی	گم عربیک کے مرکبات	نرلائی کرنے والا ایجنٹ (پھانسی دینے والا ایجنٹ): کلورائل ہائیڈریٹ
**2):**	سی ایم سی پی-9 (=کاربوکسی میتھائل سیلولوز فینول)	پانی (60%−51 :9- سی ایم سی پی)	مکمل طور پر ہائیڈرولائزڈ پولی وینائل الکحل :9- سی ایم سی پی%) 0−5)	سی ایم سی (پی) 9- کم چپچپاہٹ: زیادہ لغزش
**3):**	ڈی ایم ایچ ایف (ڈائی میتھائل ہائیڈانٹوئن فارمالڈیہائیڈ)	پانی	N,N ′-ڈائی میتھول ڈائی میتھائل ہائیڈانٹوئن (ڈائی-میتھول ڈی ایم ایچ) ایتھر/میتھیلین سے منسلک اولیگومرز کراس لنکڈ– ڈی ایم ایچ فارمالڈہائیڈ پولیمر نیٹ ورک	
**4):**	کینیڈا بالسم	xylene بالسم کے جزوی طور پر اتار چڑھاؤ والے اجزاء (Δ^3- کیرین، لیووپیمیرک ایسڈ، لیمونین، مائرسین، پیلسٹرک ایسڈ، β- فیلنڈرین، α-پینین، β-پینین)	بالسم (ابیینول، ابیٹک ایسڈ، آئسو پیمیرک ایسڈ، سینڈاراکوپیمیرک ایسڈ)	پوٹاشیم کاربونیٹ کا تخفیف (ابیس بلسمیا رال) (1758 ,لینیئس)
**5):**	یوپرال®	یوکلپٹول ، پیرا-ایل ڈیہا ایڈ ؛ گم سینڈراک کے جزوی طور پر اتار چڑھاؤ والے اجزاء (لیمونین، α- پینین، β-پینین)	گم سینڈارک کے مرکبات (کمیونک ایسڈ، مانول، پولی کمیونک ایسڈ، سینڈاراکوپیمیرک ایسڈ، 12- ایسیٹوکسائی- سینڈاراکوپیمیرک ایسڈ، سوگیول، ٹورولوسک ایسڈ، ٹورولوسول، ٹوٹارول) کوپال گم اور کولوفونی (روسن) کے مرکبات	صفائی کرنے والا ایجنٹ: میتھل سالیسیلیٹ؛ یوپرال® میں سبز رنگ: تانبے کا نمک (تانبے ایبیینیٹ)؛ ٹیٹرا کلینس ارتیکلاتا سے صندل کی لکڑی کی رال (Vahl, 1791)
**6):**	اینیکے/ اینیسے	ایتھیل الکحل؛ کافور، یوکلپٹس اور تارپین نمکیات	کوپل گم اور کالوفونی کے مرکبات (روزن)	

بصری تحریفات کم ہوتی ہیں، اور باریک ساختوں کی قرارداد اور وضاحت بہتر ہوتی ہے۔ اس کے برعکس، RI میں عدم مطابقت دھندلا پن، بالوز، یا بغیر رنگے ہوئے اجزاء کو پوشیدہ کر سکتی ہے۔ مختلف میڈیا کے مختلف RI کی وجہ سے کسی مخصوص نمونے کے لیے درست ماؤنٹنگ میڈیم کا انتخاب کنٹراسٹ، وضاحت، اور مجموعی تصویر کے معیار کو بہتر بنانے کے لیے انتہائی ضروری ہے۔ ماؤنٹنگ میڈیم کا انعکاسی اشاریہ سلیڈ ماؤنٹنگ کے لیے ریتیلی مکھیوں کی تیاری کے دوران باریک ڈھانچوں کی وضاحت پر نمایاں اثر ڈالتا ہے۔ ریتیلی مکھیوں کی نازک اور ہلکی سی سکلروٹائزڈ خصوصیات، جن میں سیبریل آرمچر، سپرماتھیکا، اینٹینل سیگمنٹس اور پروں کی رگوں کا جال شامل ہیں، زیادہ انعکاسی اشاریہ والے ماؤنٹنگ میڈیم میں دیکھنا مشکل ہو سکتا ہے۔ ریتیلی مکھیوں کے لیے عام طور پر استعمال ہونے والے اختیارات میں پانی پر مبنی

ماؤنٹنگ میڈیا کے طور پر گمَ-کلورل میڈیا، اور سالوینٹ پر مبنی ماؤنٹنگ میڈیا کے طور پر کینیڈا بالسم اور اینیکے-نیلسن سیرکیرا (NC) ریزن شامل ہیں۔ رولنز [60] نے ماؤنٹنگ میڈیا کو دو اقسام میں تقسیم کیا ہے: (1) مستقل میڈیا: یہ وقت کے ساتھ سخت ہو جاتے ہیں اور طویل مدتی تحفظ کے لیے موزوں ہیں، اور (2) نیم مستقل میڈیا: یہ سخت نہیں ہوتے اور عام طور پر عارضی مقاصد کے لیے استعمال ہوتے ہیں۔

ماؤنٹنگ میڈیا مائع، گوند پر مبنی یا ریزینی ہو سکتا ہے، جو پانی، الکحل یا دیگر محلولوں (مثلاً ٹولوین، زائلین) میں حل پذیر ہوتا ہے (**جدول 3**) -ایک بار لگائے جانے کے بعد انہیں غیر ناقابل حل رنگنگ میڈیا کے ذریعے فضائی اثرات سے محفوظ رکھا جانا چاہیے۔ ماؤنٹنگ میڈیا کی اقسام میں واضح فرق کرنے کے لیے درج ذیل درجہ بندی استعمال کی جا سکتی ہے:

a): آبی میڈیا: یہ میڈیا پانی میں آسانی سے حل ہو جاتے ہیں، جس کی وجہ سے یہ عارضی یا نیم مستقل ماؤنٹس کے لیے موزوں ہیں۔ یہ عام طور پر استعمال میں آسان ہوتے ہیں، لیکن فضائی نمی سے بچاؤ کے لیے سیل کرنے کی ضرورت پڑ سکتی ہے (مثلاً گمَ-کلورل میڈیا اور پولی وینائل الکحل)، خاص طور پر استوائی مرطوب آب و ہوا میں۔

b): محدود پانی برداشت کرنے والے میڈیا: یہ میڈیا پانی سے کم متاثر ہوتے ہیں، لیکن پھر بھی زیادہ نمی سے تحفظ کی ضرورت ہوتی ہے۔ یہ پانی میں حل ہونے والے اختیارات کے مقابلے میں طویل مدتی استحکام فراہم کرتے ہیں اور اکثر جزوی طور پر مستقل ماؤنٹنگ میں استعمال ہوتے ہیں۔

c): ہائیڈروکاربن میں حل ہونے والے میڈیا: یہ میڈیا نامیاتی محلولوں جیسے زائلین یا ٹولین، یا ایسینیسے (اینیسے سالوینٹ) میں تحلیل کیے جاتے ہیں۔ یہ مستقل ماؤنٹنگ کے لیے ڈیزائن کیے گئے ہیں اور طویل مدتی استحکام فراہم کرتے ہیں، نیز نمی اور زوال سے مزاحمت کرتے ہیں، جس سے یہ آرکائیول (آثار قدیمہ) مقاصد کے لیے مثالی ہیں (مثلاً نیوٹرل کینیڈا بالسم، ڈی پی ایکس)۔
خلاصہ یہ کہ پانی میں حل ہونے والے میڈیا عارضی ماؤنٹس یا ایسے کیسز کے لیے بہترین ہیں جن میں نمونہ آسانی سے نکالا جا سکتا ہو؛ محدود پانی برداشت کرنے والے میڈیا نیم مستقل ماؤنٹس کے لیے موزوں ہیں جنہیں معتدل پائیداری درکار ہوتی ہے، اور ہائیڈروکاربن میں حل ہونے والے میڈیا مستقل ماؤنٹس کے لیے ترجیح دیے جاتے ہیں جو آرکائیول اور طویل مدتی ذخیرہ کے لیے ہوں۔

5.3.4): تجویز کردہ ماؤنٹنگ میڈیا کی تفصیل (جدول 3 اور 4)

A): عارضی مشاہدے کے لیے میڈیا

a): کلورل گم = ہوئیر فلوئڈ/میڈیم/سولیوشن (RI = 1.48)

مارک اینڈرے سیال سپرماتھیکا کے قلیل مدتی (چند گھنٹے، شاید چند مزید اگر سلائیڈ کو مرطوب چیمبر میں محفوظ کیا جائے) مشاہدے کے لیے بہترین ذریعہ ہے، بشمول تصاویر (**شکل 4**) یا خاکے۔ مشاہدہ شدہ سپرماتھیکا کو محفوظ کرنے کے لیے انہیں آبی مادے میں دوبارہ ماؤنٹ کرنا ضروری ہے جو درمیانی مدت کے ذخیرے کی اجازت دیتا ہے۔ انہیں ریزن میں دوبارہ ماؤنٹ کرنے کے لیے خشک کرنا ناممکن نہیں لیکن سفارش نہیں کی جاتی (گم ہونے کا خطرہ)۔ کلورل گم اور ہوئیر فلوئڈ کو مترادف سمجھا جاتا ہے۔ یہ مادہّ اپنی پانی میں مطابقت، سادگی، تیز استعمال، اور انعکاسی اشاریے (refractive index) کی وجہ سے اندرونی اعضاء کے مشاہدے کے لیے عام طور پر استعمال ہوتا

مارک اینڈرے سیال سپرماتھیکا کے قلیل مدتی (چند گھنٹے، شاید چند مزید اگر سلائیڈ کو مرطوب چیمبر میں محفوظ کیا جائے) مشاہدے کے لیے بہترین ذریعہ ہے، بشمول تصاویر (**شکل 4**) یا خاکے۔ مشاہدہ شدہ سپرماتھیکا کو محفوظ کرنے کے لیے انہیں آبی مادے میں دوبارہ ماؤنٹ کرنا ضروری ہے جو درمیانی مدت کے ذخیرے کی اجازت دیتا ہے۔ انہیں ریزن میں دوبارہ ماؤنٹ کرنے کے لیے خشک کرنا ناممکن نہیں لیکن سفارش نہیں کی جاتی (گم ہونے کا خطرہ)۔ کلورل گم اور ہوئیر فلوئڈ کو مترادف سمجھا جاتا ہے۔ یہ مادہّ اپنی پانی میں مطابقت، سادگی، تیز استعمال، اور انعکاسی اشاریے (refractive index) کی وجہ سے اندرونی اعضاء کے مشاہدے کے لیے عام طور پر استعمال ہوتا ہے، جو نازک ساختوں جیسے سپرماتھیکا (spermathecae) کے معائنے میں آسانی فراہم کرتا ہے۔ تاہم، اگر کلورل گم کو بالکل درست طریقے سے تیار نہ کیا جائے یا کنٹرول شدہ نمی کی صورتِ حال میں محفوظ نہ کیا جائے تو اس کے نمایاں نقصانات ہیں۔ ان مسائل میں کرسٹال بننا، رنگت میں تبدیلی، اور گاڑھاپن کا ختم ہونا شامل ہیں۔ کور سلپ کے گرد رنگ لگانے سے یہ مسائل حل نہیں ہوتے، کیونکہ ماؤنٹنگ میڈیا رنگ لگانے والے مادے کے ساتھ تعامل کی وجہ سے شدید طور پر بد رنگ ہو سکتا ہے (کبھی کبھی تقریباً سیاہ)، خاص طور پر اگر یوپارل ®استعمال کیا گیا ہو۔ ہوئیر میڈیم کو فلیبوٹومین ریتیلی مکھیوں کے لیے بصری اعتبار سے بہترین سمجھا گیا ہے اور روایتی طور پر ان مقاصد کے لیے استعمال ہوتا رہا ہے۔ یہ میڈیم چند باہم قریبی منسلک فارمولیشنز پر مشتمل ہے، جن میں گمَ عربک، گلیسرول، اور کلورائل ہائیڈریٹ شامل ہیں۔ مختلف فارمولیشنز کی غلط تشریح اور غلط حوالہ دیا گیا ہے [74]۔ اگرچہ ہوئیر ریتیلی مکھیوں میں سپرماتھیکا کے مشاہدے کے لیے ایک اچھا میڈیم ہے، یہ طویل مدتی تحفظ کے لیے موزوں نہیں ہے۔ یہ مختصر مدتی مشاہدات کے لیے مثالی ہے، جن میں تصاویر، خاکے یا ڈرائنگ شامل ہیں۔ آبی میڈیا عارضی ماؤنٹس کے لیے موزوں ہیں لیکن طویل مدتی تحفظ کو یقینی نہیں بنا سکتے۔ اس کے برعکس، ریزن ماؤنٹنگ بہترین پائیداری فراہم کرتی ہے جو اکثر صدیوں تک رہتی ہے، لیکن یہ سپرماتھیکا کی باریک تفصیلات کو دھندلا سکتی ہے، کیونکہ ان کی انعکاس پذیری اکثر کھو جاتی ہے۔ ہوئیر میڈیم وقت کے ساتھ پانی کی کمی (ڈی ہائیڈریشن) کی وجہ سے خراب ہو جاتا ہے (**شکل 8**)، جس کے نتیجے میں چھوٹے سفید، غیر شفاف کلورل ہائیڈریٹ کرسٹل بن جاتے ہیں۔ تاہم، چونکہ کٹیکل کیمیائی طور پر سالم رہتی ہے، نمونے کرسٹلائزڈ سلائیڈز سے بازیاب کیے جا سکتے ہیں، اگرچہ بڑھتے ہوئے کرسٹلوں کی وجہ سے کچھ جسمانی نقصان ہو سکتا ہے۔ بعض صورتوں میں، فنگل نمو کو روکنے کے لیے تھائیمول کے ساتھ گرم، مرطوب ماحول میں ماؤنٹنگ میڈیم کو دوبارہ ہائیڈریٹ کر کے کرسٹلائزڈ سلائیڈز کو بحال کیا جا سکتا ہے۔ متبادل طور پر، نمونوں کو گمَ کلورل سے پانی میں نکالا جا سکتا ہے، گلیشیل ایسٹک ایسڈ میں خشک کیا جا سکتا ہے، اور کینیڈا بالسم میں دوبارہ ماؤنٹ کیا جا سکتا ہے۔

b): ڈی ایم ایچ ایف (ڈائی میتھائل ہائیڈانٹوئن فارمل ڈی ہائیڈ) (RI = 1.48)

یہ پانی پر مبنی میڈیم [72] بصری اعتبار سے بہت اچھا کام کرتا ہے، بالکل برلیز کی طرح، اور استعمال میں بھی اتنا ہی آسان ہے۔ تاہم، برلیزکے برعکس، یہ سیاہ نہیں ہوتا اور نہ ہی کرسٹالائز ہوتا ہے۔ یہ ریتیلی مکھیاں اور دیگر سائیکوڈیڈی کے لیے اچھی طرح کام کرتا ہے۔

b): ڈی ایم ایچ ایف (ڈائی میتھائل ہائیڈانٹوئن فارمل ڈی ہائیڈ) (RI = 1.48)

یہ پانی پر مبنی میڈیم [72] بصری اعتبار سے بہت اچھا کام کرتا ہے، بالکل برلیز کی طرح، اور استعمال میں بھی اتنا ہی آسان ہے۔ تاہم، برلیزکے برعکس، یہ سیاہ نہیں ہوتا اور نہ ہی کرسٹالائز ہوتا ہے۔ یہ ریتیلی مکھیاں

جدول 4): مائیکروسکوپ سلائیڈز کے لیے منتخب ماؤنٹنگ میڈیا کے فوائد اور نقصانات اور مختلف افراد کے غیر شائع شدہ مشاہدات **[52]**-

سیریل نمبر	نام	فوائد	نقصانات
1):	* کینیڈا بالسم	یہ میڈیم انتہائی پائیدار ہے، جس کی عمر 150 سال سے زیادہ ہے۔ لونگ کے تیل یا فینول کو بڑھتے ہوئے ماؤنٹنگ ایجنٹ کے طور پر استعمال کرتے ہوئے سلائیڈیں لگائی جا سکتی ہیں۔	اس میں ایسے اجزاء ہوتے ہیں جو نقصان دہ ہیں اور ایک ہڈ کے نیچے سنبھالا جانا چاہئے۔ اس کے لیے ایک وقت طلب ڈی ہائیڈریشن کا ایک مکمل سلسلہ درکار ہے۔ اور ایتھنول کے ساتھ ڈی ہائیڈریشن اور زائلین یا لونگ کے تیل کے ذریعے منتقلی ٹیکساس کو کسی حد تک نازک بنا سکتی ہے۔ متبادل سالوینٹس (مثلاً آئسوپروپانول ، -این - بیو ٹا نول ، ہسٹوکلور ، سائی کلو ہیکس زین ، 1,4 - ڈائی آکسین , ٹیرپینول) ساخت کے ٹو ٹینے کو کم کر سکتے ہیں۔ اگر زائلین کی بجائے فینول استعمال کیا جائے یا اگر باقی پوٹاشیم ہائیڈرو آکسائیڈ موجود ہو تو نمونے سیاہ ہو سکتے ہیں۔ انتہائی عکاس اشارے رنگین ڈھانچے کو چھپا سکتے ہیں۔ اسے گرم پلیٹ میں خشک کیے بغیر مکمل خشک ہونے میں سال لگ سکتے ہیں۔ درمیانہ وقت کے ساتھ اس کا رنگ پیلا اور سیاہ ہو جائے گا، خاص طور پر جب لونگ کے تیل سے صاف کیا جائے۔ کچھ داغ دھندلا ہو جائیں گے، اور تیزاب کے سامنے آنے پر روغن ختم ہو سکتا ہے - جو وقت کے ساتھ ساتھ خود ہی ہو جائے گا۔
2):	ڈی ایم ایچ ایف: ڈائمتھائل ہائیڈ نٹائن فارملڈی ہائڈ	- اعلی شفافیت۔<br>- اچھا اضطراری انڈیکس۔<br>- ڈھانچے کی عمدہ نمائش -<br>- تیاریوں کا کافی اچھا استحکام۔<br>داغ لگانے کی بہت سی تکنیکوں کے ساتھ ہم آہنگ۔<br>- نمونے کی اچھی حفاظت۔<br>سلائیڈ اور کور سلپ کے درمیان اچھی آسنجن۔	وقت کے ساتھ زرد پڑنا ممکن ہے۔<br>کچھ داغ بدل سکتے ہیں۔<br>فارملڈی ہائڈ حساس داغوں کے لیے موزوں نہیں ہے۔<br>ہوا کے بلبلے، آہستہ خشک ہونے کا وقت بڑھتے ہوئے درمیانے درجے کی نمی کے لیے حساس ماؤنٹنگ کو ریورس کرنا مشکل ہے۔<br>فارملڈی ہائڈ زہریلا، چڑچڑاپن، سرطان پیدا کرنے والا ہے۔
3):	* یوپارل® (شفاف)	50 سال سے زیادہ کی عمر کے ساتھ پائیدار میڈیم۔<br>ایتھنول (مینوفیکچرر کی سفارش) کے 80% ساتھ نصب کیا جا سکتا ہے۔<br>غیر داغدار سطحوں کا احاطہ نہیں کرتا ہے اور وقت کے ساتھ پیلے یا ٹوٹنے والا نہیں ہے۔<br>سلائیڈز کے لیے کینیڈا بالسم سے زیادہ مناسب ریفلیکشن انڈیکس ہے۔<br>موٹی نمونوں کے لئے اچھی کارکردگی فراہم کرتا ہے<br>95 ٪ ایتھانول میں حل رہتا ہے،<br>جس سے کئی سالوں بعد بھی دوبارہ ماؤنٹ کرنا ممکن ہوتا ہے۔	نقصان دہ اجزاء پر مشتمل ہے اور اسے ہڈ کے نیچے ہینڈل کیا جانا چاہئے۔<br>یوپارل ا یسانس کے ذریعے ایتھنول کی ڈی ہائیڈریشن اور منتقلی کچھ ٹیکساس کو ٹوٹ پھوٹ کا باعث بن سکتی ہے، لیکن آئسوپروپانول کا استعمال اس مسئلے کو کم کر سکتا ہے۔
4):	ہوئیر فلوئڈ	نمونے زندہ حالت میں یا براہِ راست پانی، ایتھانول یا فارملڈیہائیڈ سے ماؤنٹ کیے	نازک پودوں کے نمونے گر سکتے ہیں جب تک کہ میڈیم آہستہ آہستہ شامل نہ کیا

		جا سکتے ہیں۔<br>میسریشن سے کٹیکل کا معیار بہترین ہوتا ہے۔<br>اس کا انعکاسی انڈیکس موافق ہے اور زیادہ تضاد کے لیے اسے آیوڈین اسٹیننگ سے بہتر بنایا جا سکتا ہے۔<br>فارمولے میں موجود ایسٹک ایسڈ آرتھوپوڈ کے اعضاء کو پھیلاتا ہے۔<br>کچھ نمونے 40–60 سال تک مستحکم رہ سکتے ہیں۔<br>پانی میں حل ہونے والا، دوبارہ ماؤنٹ کرنے میں آسانی فراہم کرتا ہے۔	جائے، جو وقت طلب ہے۔<br>10 سال سے کم عرصے میں خلا اور کرسٹل بن سکتے ہیں۔<br>کلورل ہائیڈریٹ کی مقدار اور نمائش کے وقت کے مطابق میسریشن حد سے زیادہ ہو سکتی ہے۔<br>میڈیم کے اجزاء الگ ہو سکتے ہیں، اور چند ماہ یا سالوں میں باریک دانے ظاہر ہو سکتے ہیں۔<br>میڈیا کے سیاہ پڑنے کی اطلاع بھی ملی ہے۔
5):	CMCP-9<br>(= کاربوکسی میتھل سیلولوز فینول)	نمونے پانی، ایتھانول، گلیسرول یا فارملڈیہائیڈ پر مشتمل محلولوں جیسے میڈیا سے براہِ راست ماؤنٹ کیے جا سکتے ہیں، اور ان کے اندرونی اعضاء کو عمومی معائنہ یا تیاری کو آسان بنانے کے لیے ضروری ہونے پر میسریٹ کیا جا سکتا ہے۔	یہ میڈیم وقت کے ساتھ کرسٹل بنا سکتا ہے اور سیاہ ہو سکتا ہے، اور بعض اوقات نمونوں کو مطلوبہ حد سے زیادہ نرم کر دیتا ہے۔ جب تک سلائیڈ کو احتیاط سے رنگ نہ کیا جائے، موٹے نمونے اس میں ٹھیک نہیں رہیں گے کیونکہ وہ سکڑ کر کور سلپ کے کناروں کے گرد خلا پیدا کر سکتے ہیں۔<br>یہ رنگے ہوئے نمونوں یا کیلسیفائیڈ مادوں کے لیے موزوں نہیں ہے، اور اس کے خشک ہونے کا وقت سی ایم سی کے مقابلے میں زیادہ سست ہے۔
6):	یوکٹِ™	یہ ایک پائیدار میڈیم ہے جو 30سال سے زیادہ عرصہ برقرار رہتا ہے۔<br>ماؤنٹنگ کے لیے بہت سے سالوینٹس کے ساتھ مطابقت رکھتا ہے، جن میں ایسٹون، بینزین، کلورو فارم، ڈائی آکسین، ایتھر ، آئسوپروپانول، میتھل بینزوئیٹ، ٹرپینول، ٹولین، اور زائلین شامل ہیں۔ یہ تیزی سے خشک ہوتا ہے اور اس کا pH قدرے تیزابی ہوتا ہے۔<br>عمر کے ساتھ نمایاں طور پر گہرا نہیں ہوتا۔<br>مختلف اسٹینز (مثلاً فوکسن، ہیماٹوکسیلین، میتھائل گرین، میتھائل وائلٹ، میتھائلین بلیو) کے لیے موزوں۔<br>نمونوں کو برسوں بعد بھی زیلیین میں طویل عرصے تک بھگونے کے بعد دوبارہ ماؤنٹ کیا جا سکتا ہے۔	اس میں نقصان دہ اجزاء شامل ہیں اور اسے ہڈ کے نیچے ہی استعمال کرنا ضروری ہے۔<br>مکمل، وقت طلب ڈی ہائیڈریشن سیریز درکار ہے۔<br>سکڑاؤ اور گیس کے بلبلوں کے بننے کی وجہ سے موٹے نمونوں کے لیے موزوں نہیں۔<br>کور سلپس وقت کے ساتھ الگ ہو سکتے ہیں جب تک شیشے کو اچھی طرح صاف اور سیل نہ کیا جائے۔<br>کولیجن ریشوں کے گرد نامکمل پولیمرائزیشن ظاہر ہو سکتی ہے۔
7):	اینیکے	انتہائی پائیدار میڈیم، جو کم از کم 50 سال تک رہتا ہے۔<br>یہ وقت کے ساتھ سیاہ نہیں ہوتا ہے۔<br>یہ بہت لچکدار ہے، جو مواد میں کیڑوں کی چیر پھاڑ/تشریح کی اجازت دیتا ہے۔<br>اس کے ساتھ ساتھ اس کو شکلاتی ساختیں پوزیشننگ کے لئے مناسب مقدار میں وقت کی ضرورت ہوتی ہے۔<br>یہ کم قیمت بھی ہو تا ہے ۔	اس کے لیے ایک مکمل، وقت گزارنے والی ہائیڈریشن سیریز کی ضرورت ہے۔ ایتھنول کے ساتھ ہائیڈریشن اور لونگ کے تیل سے ٹرانسفیکشن کچھ نمونوں کو نازک بنا سکتا ہے۔ کیڑا نظر آتا ہے، اگرچہ بہت آہستہ؛ یہ اس کے چھوٹے سائز کی وجہ سے دیکھنا مشکل بنا سکتا ہے، جیسے کہ سنسیلا، اسکویڈ، اور سادہ سیٹی۔

اور دیگر سائیکوڈیڈی کے لیے اچھی طرح کام کرتا ہے۔

c): CMCP (کیمفور-مونو-کلوروپینول) (RI = 1.41)

یہ گلیسرین پر مبنی، پانی میں حل ہونے والا ماؤنٹنگ میڈیم نازک نمونوں، بشمول ریتیلی مکھیوں ، کی شفاف اور مستقل سلائیڈیں بنانے کے لیے استعمال ہوتا ہے۔ اس ماؤنٹنگ میڈیم کا فائدہ یہ ہے کہ نمونوں کو براہِ راست پانی یا ایتھانول سے ماؤنٹ کیا جا سکتا ہے۔ یہ ریتیلی مکھیوں کو تیزی سے نرم کر کے شفاف کر دیتا ہے، جس سے کٹیکل نرم ہو کر نمونے کو صحیح پوزیشن میں رکھنے میں مدد ملتی ہے، جو خاص طور پر پروں کو پھیلانے یا جنسی اعضاء کی تشریح میں بہت مفید ہے۔ اگرچہ کہا جاتا ہے کہ یہ طویل مدتی ذخیرہ ممکن بناتا ہے، تاہم تحفظ کی درست مدت نامعلوم ہے۔ اس ماؤنٹنگ میڈیم کی سب سے بڑی محدودیت اس کے اجزاء میں موجود فینول ہے، جو ایک زہریلا اور جلن پیدا کرنے والا مادہ ہے اور جس کے استعمال میں احتیاط برتنی پڑتی ہے۔

B): مستقل ماؤنٹنگ کے لیے میڈیا

a): کینیڈا بالسم (RI = 1.52-1.54)

کینیڈا بالسلم کو پہلی بار 1830 کی دہائی میں اینڈریو پرچرڈ نے منتقل شدہ روشنی خوردبینی معائنے کے لیے ایک موزوں ماؤنٹنگ میڈیم کے طور پر بیان کیا تھا۔ یہ اپنی ثابت شدہ آرکائیول کوالٹی کی وجہ سے 150 سال سے زائد کامیاب استعمال کے ساتھ اب بھی سب سے زیادہ استعمال ہونے والے میڈیا میں سے ایک ہے۔ ہوئیر فلوئڈ میڈیا کے برعکس، کینیڈا بالسلم نہ تو کرسٹالائز ہوتا ہے اور نہ ہی نمی جذب کرتا ہے۔ تاہم، کینیڈا بالسلم شدید آٹوفلوورسینٹ ہے، جو بعض اوقات مخصوص مائیکروسکوپی تکنیکوں کے لیے نقصان دہ ثابت ہو سکتا ہے [60]۔ زائلین کے بجائے غیر زہریلے سالوینٹس کے استعمال سے تیاری کے دوران حفاظتی خطرات کم ہو سکتے ہیں، لیکن اس کے نتیجے میں میڈیم کے سست خشک ہونے اور جلد سیاہ ہونے جیسے نقصانات بھی سامنے آ سکتے ہیں۔

b): یوپارل® (RI = 1.48)

یوپارال® مستقل ماؤنٹنگ کے لیے کینیڈا بالسم کا ایک وسیع پیمانے پر استعمال ہونے والا متبادل ہے، جو بہترین طویل مدتی استحکام اور موازنہ جاتی انعکاسِ ضریب فراہم کرتا ہے۔ یوپارال®کی درج ذیل خصوصیات ہیں:
(1): ڈی ہائیڈریشن کی ضرورت: ماؤنٹنگ میڈیم کی حتمی منتقلی سے قبل نمونہ کو ڈی ہائیڈریٹ کرنا ضروری ہے، عام طور پر 95% الکحل سے مطلق الکحل تک منتقل کیا جاتا ہے، اور
(2): طویل پراسیسنگ وقت :چاہے کینیڈا بالسم ہو یا یوپارال®، ریزن میں حتمی اسمبلی کے لیے ڈی ہائیڈریشن درکار ہوتی ہے، جو مجموعی نمونہ پراسیسنگ کے وقت کو بڑھا دیتی ہے۔ جب نامیاتی محلولوں کے ساتھ ڈی ہائیڈریشن ممکن نہ ہو، تو مطلق ایتھانول سے نکالے گئے نمونوں کو حتمی ماؤنٹنگ سے قبل یوپارال® اور یوپارال ایسنس (essence) کے برابر ملاپ پر مشتمل ایک عبوری محلول میں رکھا جا سکتا ہے۔

c): اینیکے (RI = 1.467)

اینیکے ایک رال پر مبنی ماؤنٹنگ میڈیم ہے جو بنیادی طور پر چھوٹے کیڑوں کے لیے استعمال ہوتا ہے اور خاص طور پر برازیل میں مقبول ہے۔ اس کی بنیاد کولوفونی اور گم کوپال پر مشتمل ہوتی ہے جو الکحل، کیمفور، ٹرپنٹائن کے عرق اور یوکالیپٹول میں حل کیے جاتے ہیں۔ سرکیرا (Cerqueira) [11] نے اینیسی کو لاروؤں، نابالغوں کے خول اور بالغ مچھروں کی مستقل سلائیڈز بنانے کے لیے کینیڈا بالسم کا متبادل قرار دیا، اور تب سے اسے ریتیلی مکھیوں کی سلائیڈ سازی کے لیے وسیع پیمانے پر اپنایا گیا ہے۔ اینیسی مستقل ماؤنٹنگ کے لیے ایک کم لاگت متبادل فراہم کرتا ہے، جو طویل مدتی استحکام اور مناسب خشک ہونے کا وقت فراہم کرتا ہے، جس سے تشریح اور مورفولوجیکل ڈھانچوں کی درست ترتیب ممکن ہوتی ہے۔

5.4): سلائیڈ کی تیاری اور خشک کرنا

منسلک شدہ سلائیڈز کو مناسب طریقے سے خشک کرنا طویل مدتی استحکام اور تحفظ کو یقینی بنانے کے لیے انتہائی ضروری ہے۔ سلائیڈز کو خشک کرنا چاہیے-طویل المدتی ذخیرہ کرنے پر غور کرنے سے پہلے اچھی طرح خشک کریں۔ بہترین نتائج کے لیے، مستقل ماؤنٹنگ میڈیا سے تیار کردہ سلائیڈز کو 2–3 ہفتے تک افقی طور پر خشک کرنا چاہیے، جبکہ نیم مستقل میڈیا سے تیار کردہ سلائیڈز کو صرف 1–2 ہفتے درکار ہو سکتے ہیں۔ مؤثر خشک کرنے کے عمل کو یقینی بنانے کے لیے، استعمال ہونے والے ماؤنٹنگ میڈیا کے لیے مناسب درجہ حرارت پر سیٹ شدہ انکیوبیٹر استعمال کرنے کی سفارش کی جاتی ہے، تاکہ ضرورت سے زیادہ حرارت سے نمونوں کو نقصان نہ پہنچے۔ 30°C سے 37°C کے درجہ حرارت کی حد تجویز کی جاتی ہے۔ یہ خشک کرنے کا مرحلہ ذخیرہ کے دوران سلائیڈ کے مڑنے، نمونوں کی بگاڑ، یا ماؤنٹنگ میڈیم کی غیر مستحکمیت سے بچنے کے لیے انتہائی اہم ہے۔ سلائیڈ کی تیاری میں استعمال ہونے والا ماونٹنگ میڈیم ہمیشہ سلائیڈ لیبل پر درج ہونا چاہیے۔ اگر ممکن ہو تو لیبل میں استعمال شدہ مخصوص ترکیب کے ساتھ ساتھ تیار کرنے والے شخص (پریپریٹیٹر) کا نام اور تیاری کی تاریخ بھی شامل ہونی چاہیے۔ سلائیڈز ابتدائی طور پر عارضی ماؤنٹس کے طور پر تیار کی جاتی ہیں اور طویل مدتی تحفظ کے لیے نہیں ہوتیں۔ تاہم، اگر نمونے کی حیثیت تبدیل ہو جائے، جیسے اسے "ٹائپ" سیریز کا حصہ قرار دیا جائے، تو مستقبل میں ٹیکسونومک مطالعے کے لیے نمونے کے تحفظ کو یقینی بنانے کے لیے زیادہ مستقل ماؤنٹنگ میڈیم استعمال کرنا چاہیے۔

5.5): متبادل ماؤنٹنگ تکنیکیں: کارڈ ماؤنٹنگ

کارڈ ماؤنٹنگ ایک ایسی تکنیک ہے جو ریتیلی مکھیوں کے کئی گروہوں کے لیے استعمال ہوتی ہے، جس میں نمونوں کو یا تو براہِ راست حشرہ شناسی کے کارڈز پر پن کیا جاتا ہے یا سطح پر چپکایا جاتا ہے۔ ان کے چھوٹے سائز اور شناخت کے لیے اندرونی اعضاء کے مشاہدے کی ضرورت (وضاحت کے لیے دیکھیں شق 5) کے پیش نظر، یہ طریقہ ریتیلی مکھیوں کو ماؤنٹ کرنے کے لیے بالکل بھی مناسب نہیں ہے۔

5.6): خراب شدہ نمونوں کی دوبارہ ترتیب

نایاب یا قیمتی نمونوں کے لیے **(ویڈیو کلپ 6):** (https://zenodo.org/records/18315029)کے مطابق دو مرحلوں پر مشتمل طریقہ کار تجویز کیا جاتا ہے۔ (1) : ابتدائی مشاہدے کے لیے انہیں جدا کیے بغیر دوبارہ ہائیڈریٹ کریں۔ سپورٹ کے طور پر متعدد مائیکروسکوپ سلائیڈز کے لیے ایک ہولڈر پیٹری ڈش میں رکھا جانا چاہیے۔ پھر جس سلائیڈ کو دوبارہ ہائیڈریٹ کرنا ہے اسے اوپر رکھا جاتا ہے، اور پیٹری ڈش کو چند ملی میٹر محلول سے بھر دیا جاتا ہے تاکہ ایک مرطوب خانہ بن جائے، اس بات کو یقینی بناتے ہوئے کہ سلائیڈ خود محلول کے رابطے میں نہ آئے **(شکل 8 D**۔ دوبارہ ہائیڈریشن کے لیے درکار وقت نمونے کی حالت کے مطابق ایک سے چند دنوں تک مختلف ہو سکتا ہے۔ روزانہ نگرانی اور صبر ضروری ہیں۔ ایک بار جب سلائیڈ مناسب طور پر ری ہائیڈریٹ ہو جائے، تو اسے نمی والے چیمبر سے نکالا جا سکتا ہے اور خوردبینی معائنہ، فوٹوگرافی یا خاکہ بنانے سے پہلے چند گھنٹوں کے لیے انکیوبیٹر میں رکھا جا سکتا ہے۔ (2) :دوبارہ ماؤنٹ کرنے کے لیے، سلائیڈ کو چند اضافی گھنٹوں یا رات بھر کے لیے نمی والے چیمبر میں واپس رکھا جا سکتا ہے۔ اجزاء کی علیحدگی بائنوکولر خوردبین کے نیچے کی جانی چاہیے۔ باریک سوئیوں کی مدد سے کور سلپ کو احتیاط سے ہٹایا جانا چاہیے، اس بات کو یقینی بناتے ہوئے کہ کوئی بھی ریتیلی مکھی کے اجزاء منسلک نہ

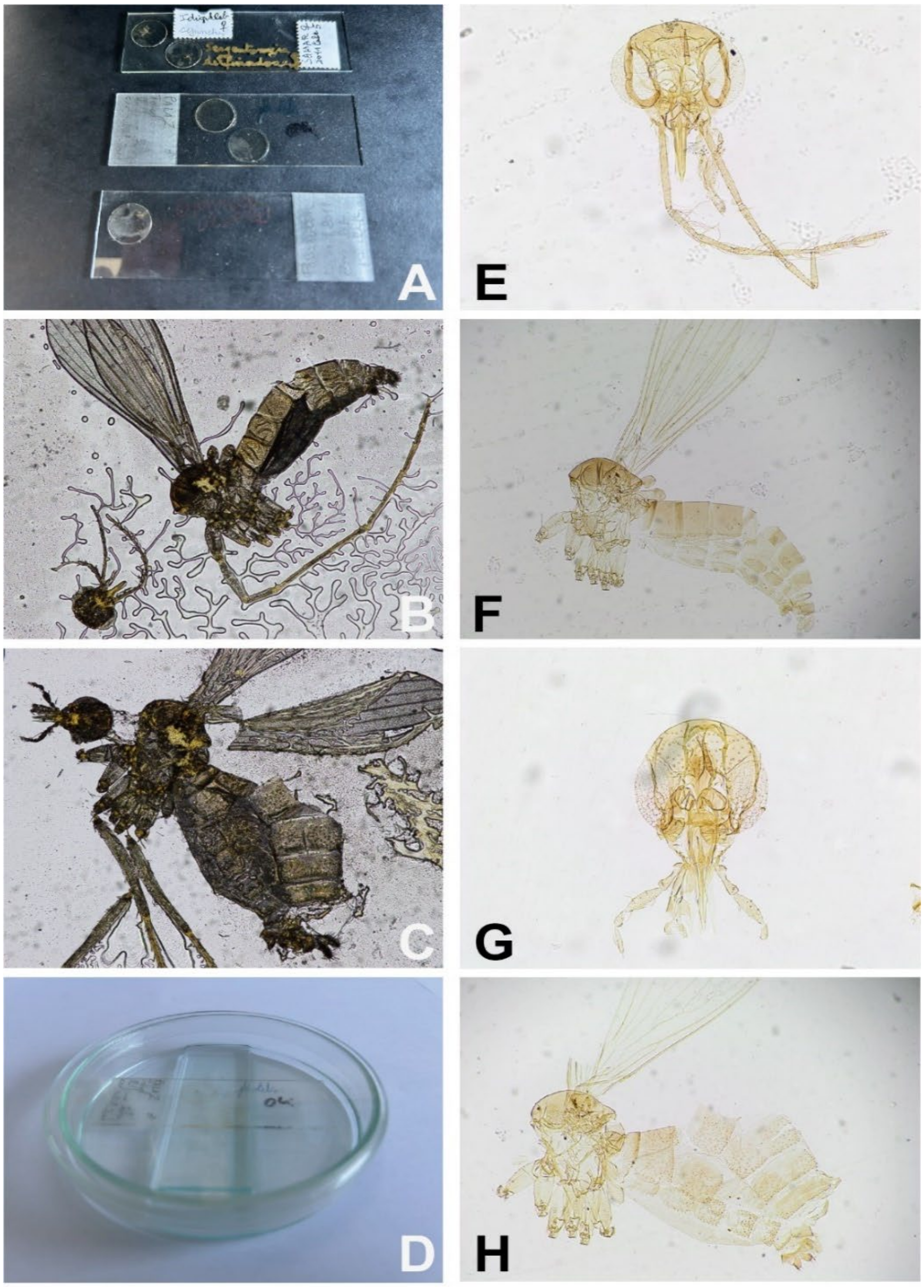

شکل 8): سلائیڈ کی دوبارہ ماؤنٹنگ۔ A): ہویئر میں ماؤنٹ کی گئی خراب اور خشک سلائیڈیں؛ B): خشک ریتیلی مکھی کی خوردبینی تصویر؛ C): ایک اور خراب شدہ ریتیلی مکھی کی خوردبینی تصویر؛ D): ایک خشک سلائیڈ پر مشتمل گیلی چیمبر؛ E): سر، اور F): نمونہ B کا جسم یوپارل®میں دوبارہ ماؤنٹ کرنے کے بعد ؛ G): سر، اور H: خراب شدہ نمونہ C کا جسم یوپارل® میں دوبارہ ماؤنٹ کرنے کے بعد۔

رہیں- (**ویڈیو کلپ 7**): (https://zenodo.org/records/18315029)- اس کے بعد، ریتیلی مکھی کے ڈائیسیکٹڈ اجزاء کو جمع کر کے چھوٹے ویلز میں پانی سے دھویا جانا چاہیے، جیسا کہ تباہ کن ڈی این اے/آر این اے ایکسٹریکشن کے لیے استعمال ہونے والے ویلز (نیچے دیکھیں)، قبل اس کے کہ انہیں ڈی ہائیڈریشن کے بعد ریزن میڈیم میں دوبارہ ماؤنٹ کیا جائے۔ سلائڈ کو الگ کرتے وقت، ایک مناسب محلول چننے کے لیے اصل ماؤنٹنگ میڈیم کی شناخت کرنا انتہائی ضروری ہے۔ آبی ماؤنٹنگ میڈیا کے لیے پانی استعمال کرنا چاہیے۔ اگر ماؤنٹنگ میڈیم ریزن پر مبنی ہو (مثلاً کینیڈا بالسم یا یوپارل®)، تو ایک فیوم ہڈ کے نیچے اور مناسب ذاتی حفاظتی سازوسامان، بشمول ماسک، کے ساتھ زائلین استعمال کرنا چاہیے۔ ٹائپ یا مجموعے کے نمونوں کی دوبارہ ماؤنٹنگ صرف کیوریٹر اور/یا نمونے کے مالک ادارے کی اجازت سے ہی کی جانی چاہیے۔

6): نمونے کی شناخت

6.1): شکل و صورت

ریتیلی مکھیوں کی شناخت بنیادی طور پر ان کی مورفولوجیکل خصوصیات کے معائنے پر منحصر ہوتی ہے، جن میں تھوراکس (سینے) کی شکل، پروں، تولیدی اعضاء، سیٹا (setae)، اور مختلف ساختوں کے درمیان مخصوص مورفومیٹرک تعلقات شامل ہیں۔ محققین جمع شدہ نمونوں کا موازنہ معروف ٹیکسا سے کرنے کے لیے ٹیکسونومک کیز، حوالہ جاتی مجموعوں، اور اصل نوع کی وضاحتوں کا استعمال کرتے ہیں۔ اہم تشخیصی خصوصیات، جیسے دونوں جنسوں میں پروں کی رگوں کا جال اور سر کی ساخت، نر کے جنسی اعضاء کی ساخت، اور مادہ کے سپرماتھیکا (spermathecae) کی ترتیب، نوع کی شناخت کے لیے خاص طور پر معاون ہوتی ہیں۔ درست شناخت کے لیے اکثر تفصیلی خوردبینی معائنہ ضروری ہوتا ہے، عام طور پر مرکب خوردبین (compound microscope) کا استعمال کیا جاتا ہے تاکہ باریک ساختیں جیسے جنسی اعضاء اور سپرماتھیکا دیکھے جا سکیں، یا وسیع تر مورفولوجیکل خصوصیات کے لیے سٹیریو خوردبین (stereomicroscope) استعمال کیا جاتا ہے۔ تصویری ٹیکنالوجی میں حالیہ پیش رفت نے ریتیلی مکھیاں کی شناخت کے لیے ڈیجیٹل امیجنگ کے استعمال کو آسان بنا دیا ہے۔ اعلیٰ قرارداد والی تصاویر یا کلیدی خصوصیات کی ڈیجیٹل خاکوں کا موازنہ حوالہ جاتی مواد سے کیا جا سکتا ہے یا کمپیوٹر سے معاونت یافتہ شناخت کے نظام کے ذریعے تجزیہ کیا جا سکتا ہے، جس سے مورفولوجیکل ٹیکسونومی میں درستگی اور رسائی دونوں میں بہتری آتی ہے۔

6.2): پر کی شکل و صورت

پر کی شکل مختلف ریتیلی مکھیاں کی انواع کی شناخت اور درجہ بندی میں استعمال ہونے والی ایک اہم خصوصیت ہے۔ ریتیلی مکھیاں کے پروں میں ایک منفرد نمونہ اور ساخت ہوتی ہے، جو عموماً لمبے اور تنگ ہوتے ہیں اور جن میں رگوں کا نظام اچھی طرح ترقی یافتہ ہوتا ہے (**شکل 9 اور 10**)۔ رگوں کی ترتیب ایک مخصوص نمونہ بناتی ہے جو جینرا اور انواع کے درمیان مختلف ہو سکتی ہے، اور شناخت کے لیے قیمتی تشخیصی خصوصیات فراہم کرتی ہے۔ نتیجتاً، پر کی شکل کا مطالعہ ٹیکسونومک مقاصد کے لیے قیمتی بصیرت فراہم کرتا ہے۔

6.3): پر کی جیومیٹرک مورفومیٹرکس

محققین مختلف تکنیکوں، جیسے جیومیٹرک مورفومیٹرکس، کا استعمال مختلف ریتیلی مکھیاں کی انواع یا آبادیوں میں پروں کی شکل اور سائز کا تجزیہ اور موازنہ کرنے کے لیے کرتے ہیں۔ پروں کی جیومیٹری کا مطالعہ رویے، رہائش کی ترجیحات، اور پرواز کی صلاحیتوں کے بارے میں قیمتی بصیرت فراہم کرتا ہے۔ جہتی مورفومیٹرک طریقہ کار میں پروں کو احتیاط سے کاٹ کر الگ کیا جاتا ہے، رنگا جاتا ہے (اگر ضروری ہو)، اور سلائیڈز پر چپٹا کر نصب کیا جاتا ہے۔ تیار کردہ سلائیڈز کو پھر سٹیریو مائیکروسکوپ کے نیچے فوٹوگراف کیا جاتا ہے، ڈیجیٹائز کیا جاتا ہے، اور مورفومیٹرک تجزیے کے لیے پیش کیا جاتا ہے۔ اس طریقہ کار کو ادبیات [6, 27, 42, 56, 57, 59] میں بخوبی بیان کیا گیا ہے، اور جوڑے والے اعضاء کے لیے ممکنہ منفی الومیٹرک اثرات سے بچنے کے لیے دائیں یا بائیں پروں کے مستقل استعمال کی سفارش کی گئی ہے [62]۔

6.3.1): ہندسی مورفومیٹرک تجزیے کے لیے پروں کی تیاری

ریتیلی مکھیوں پرَ کی رگوں کی بہترین نمائش کے لیے، پرَ کو پپُوٹوں سے صاف کرنا چاہیے اور مناسب طریقے سے رنگنا چاہیے۔ پرَ کے لیے تیاری کے لیے سب سے پہلے چھوٹے کنووں کو مطلوبہ مادوں (میتھیلائن بلیو، ایتھانول، پانی اور زائلین کے متبادل) سے بھر دیں۔ کمرے کے درجۂ حرارت پر 70% ایتھانول میں محفوظ شدہ پروں کو اپینڈورف ٹیوب کو الٹا کر کے کنوے میں خالی کریں، پھر باریک خمیدہ سوئی کی مدد سے پروں کو لمبائی میں اٹھا لیں۔ پر کو برشز (ریشے نما اجزاء) ہٹانے کے لیے مختصراً ایتھانول سے پانی میں اور پھر واپس ایتھانول میں ڈالیں۔ پر کو 6 منٹ کے لیے میتھیلین بلیو میں رکھیں، اس بات کو یقینی بناتے ہوئے کہ رنگنے کے دوران یہ تیرتا رہے۔ پر کو احتیاط سے نکال کر 2 منٹ کے لیے زائلین سبسٹیٹیوٹ میں ڈبو دیں (تقریباً میتھیلین بلیو کے وقت کا ایک تہائی)۔ سوئی کو ویل کی دیواروں سے ہلکے ہلکے ٹپکا کر پر کو بٹھانے میں مدد مل سکتی ہے؛ زائلین رنگ کو مستحکم کرتا ہے۔ آخر میں، پروں کو اٹھا کر مائیکروسکوپ سلائیڈ پر® یوپارال کے ایک چھوٹے سے قطرے پر رکھیں۔ بڑے عدسے کے نیچے، نرمی سے پروں کو کھولیں اور احتیاط سے کور سلپ رکھیں۔ ®یوپارال کے جمنے سے پہلے فوری طور پر تصاویر لینی چاہئیں، کیونکہ بہترین ترتیب کے لیے کور سلپ کے نیچے پروں کی پوزیشن میں معمولی ایڈجسٹمنٹ کی ضرورت پڑ سکتی ہے۔

6.4): مالیکیولر بائیولوجی کی تکنیکیں

شکلاتی تکنیکوں کے علاوہ، سالماتی طریقے حشرہ شناسی کی تحقیق میں تیزی سے ضروری ہوتے جا رہے ہیں، جن میں درجہ بندی، آبادیاتی جینیات، اور نسبتی مطالعے کے ساتھ ساتھ ڈی این اے/آر این اے پیتھوجین کی شناخت اور خون کے ماخذ کا تعین شامل ہے، جبکہ وبائیات (وبائی بیماریاں)کے شعبے میں ویکٹر کے رویے کو سمجھنا بھی اہم ہے [70]. ڈی این اے سیکوینسنگ کو انواع کی تصدیق یا قریبی متعلقہ انواع میں امتیاز کے لیے استعمال کیا جا سکتا ہے، جو شناخت کا زیادہ درست اور قابلِ اعتماد ذریعہ فراہم کرتی ہے۔ مزید برآں، جدید مالیکیولر تکنیکیں (مثلاً پی سی آر، ڈی این اے سیکوئنسنگ، این جی ایس وغیرہ) اور مالدی ٹوف ایم ایس درست اور تیز نوع کی شناخت کے لیے اہمیت اختیار کر رہی ہیں، جو روایتی مورفولوجیکل طریقوں کا تکمیلی کردار ادا کرتی ہیں [46]۔ ان پیش رفتوں کے باوجود، مورفولوجیکل شناخت ٹیکسونومی کے لیے حوالہ جاتی معیار اور وہ بنیاد ہے جس کی روشنی میں مالیکیولر ڈیٹا کی تشریح کی جاتی ہے۔

6.4.1): تباہ کن نیوکلیک ایسڈ کی نکاسی

نیوکلیک ایسڈ کی استخراج بہت سے حیاتیاتی مطالعات میں ایک معمول کا مرحلہ ہے، اور حیاتیاتی مادوں سے ڈی این اے الگ کرنے کے لیے مختلف طریقے تیار کیے گئے ہیں [48]۔ کمرشل طور پر دستیاب ڈی این اے استخراج کے کٹس اس عمل کو آسان بنانے کے لیے ڈیزائن کیے گئے ہیں [14]۔ تاہم، آر تھر ویوڈ نمونوں کو مورفولوجیکل شناخت کے لیے تیار کرنے میں عام طور پر استعمال ہونے والے طریقے اکثر ڈی این اے کے

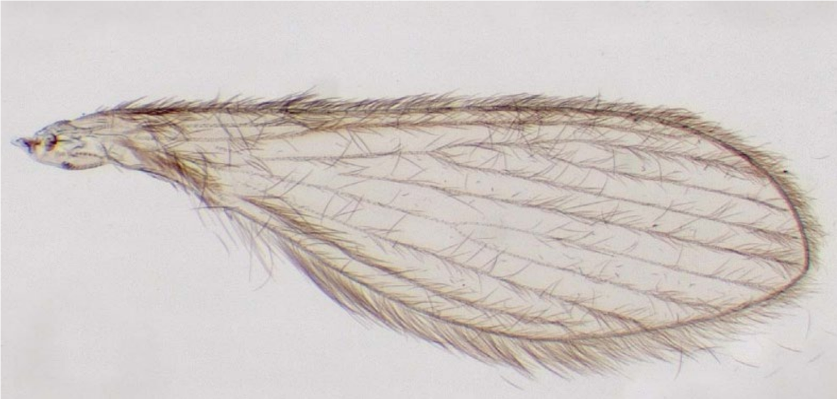

شکل9): (ٹریکوفورومیا اینینیؔ (*Trichophoromyia ininii*) کے خام پر۔

تجزیے میں رکاوٹ بنتے ہیں، کیونکہ یہ تکنیکیں نمونے کی اہم جسمانی خصوصیات کو نقصان پہنچا سکتی ہیں یا انہیں تباہ کر سکتی ہیں [10]۔ ریتیلی مکھیوں کے ٹشوز کے لیے زیادہ تر ڈی این اے استخراج کے پروٹوکول فطرتاً تباہ کن ہوتے ہیں [43]، جو چھوٹے نمونوں کے لیے خاص تشویش کا باعث ہیں، جہاں محدود نمونہ بھی اہم مورفولوجیکل خصوصیات کو متاثر کر سکتا ہے [72]۔ نمونے کی قسم اور حالت مناسب ڈی این اے علیحدگی کے طریقہ کار کے انتخاب میں کلیدی کردار ادا کرتی ہیں [29]۔ ریتیلی مکھیوں کی درست شناخت، آبادی کے ارتقاء کو سمجھنے، اور غیر ہدف اثرات کو کم کرنے کی ضرورت نے مالیکیولر تشخیصی آلات کی ترقی کو فروغ دیا ہے [23]. اب مولیکیولر طریقے اکثر ریتیلی مکھیاں شناخت کرنے کے لیے مورفولوجیکل ٹیکسونومک طریقوں کے ساتھ ملاپ کے طور پر استعمال کیے جاتے ہیں۔ مثال کے طور پر، کیڑوں کی بارکوڈنگ کے لیے معیاری طریقہ کار میں ڈی این اے نکالنا، سیکوینسنگ، اور اصل نمونے کا ضائع ہونا شامل ہے۔ لہٰذا، اس بات کی اشد ضرورت ہے کہ ایسے غیر تباہ کن ڈی این اے نکالنے کے طریقے تلاش کیے جائیں جو حیاتیاتی مادے اور اس کی مورفولوجیکل سالمیت دونوں کو برقرار رکھیں۔

کئی نیوکلیک ایسڈ نکالنے کے طریقے ریتیلی مکھیوں پر استعمال کیے گئے ہیں۔ مطلوبہ نیوکلیک ایسڈ کی مقدار یا معیار بعد میں کیے جانے والے مالیکیولر تجزیے پر منحصر ہوتا ہے، کیونکہ مختلف تکنیکوں کی حساسیت اور خالصت کے تقاضے مختلف ہوتے ہیں [9]۔ مثال کے طور پر، ریتیلی مکھیوں کی آنکھوں میں پی سی آر ایمپلیفیکیشن کو روکنے والی صلاحیت پائی گئی ہے [69]۔ پیتھوجین سے آگے اسکریننگ کے لیے، ریتیلی مکھیوں کا ڈی این اے معمول کے مطابق نوع کی شناخت کے مقاصد کے لیے نکالا جاتا ہے۔ مختلف استخراجی طریقے استعمال کیے جا سکتے ہیں، اگرچہ تکنیکوں کے درمیان پیداوار اور معیار مختلف ہوتا ہے۔ کچھ صنعت کاروں کے پروٹوکول کو محققین نے ریتیلی مکھیوں کے لیے اپنایا ہے [8]، جس سے نکالی گئی نیوکلیک ایسڈز کی پیداوار اور/یا معیار میں اضافہ ہوا ہے [8, 9, 69]، جبکہ دیگر ترمیمات، جو دیگر آرتھروپوڈ ٹیکسا کے لیے تیار کی گئی ہیں، ریتیلی مکھیوں پر بھی استعمال کی جا سکتی ہیں [58, 76]۔ چھوٹے ماٹوکونڈریل ٹکڑوں (COI یا CytB) کو ہدف بنانے والی شناخت پی سی آر عموماً ایسے استخراجی طریقوں کے ساتھ مطابقت رکھتی ہیں جن میں ڈی این اے کی زیادہ تقسیم شامل ہوتی ہے۔ اس کے برعکس، دیگر طویل ریڈ این جی ایس تکنیکیں (آکسفورڈ نینوپور اور پیکیوبیو) کم سے کم تقسیم اور اعلیٰ معیار کے ڈی این اے کی متقاضی ہوتی ہیں۔ اسپن کالم استخراج عموماً 60 کلو بیس تک کے جینومک ڈی این اے کے ٹکڑے فراہم کرتا ہے، جبکہ فینول-کلورو فارم استخراج 150 کلو بیس تک کے ٹکڑے پیدا کر سکتا ہے [77]. **جدول 5** میں ریتیلی مکھیاں کے ڈی این اے نکالنے کی مختلف تکنیکوں کا خلاصہ پیش کیا گیا ہے اور بتایا گیا ہے کہ آیا ان کیڑوں کے لیے طریقہ کار میں کوئی تبدیلیاں کی گئی ہیں۔ پیداوار دکھائی نہیں گئی کیونکہ یہ نمونے کے سائز اور تیاری کے طریقہ کار پر منحصر ہوتی ہے۔ ترمیمی کالم سے مراد ریتیلی مکھیاں یا دیگر چھوٹے آرتھروپوڈز کے لیے نکالنے کے پروٹوکول میں کی جانے والی تبدیلیاں ہیں۔ استخراج کے طریقہ کار کے انتخاب میں کئی معیارات کو مدنظر رکھنا چاہیے، جیسے نمونوں کی تعداد، استخراج کا وقت، اور بعد میں استعمال ہونے والی تکنیک. اگرچہ NGS تکنیکوں کے لیے زیادہ سالماتی وزن والا جینومک ڈی این اے درکار ہوتا ہے، یہاں پیش کردہ تمام طریقے معیاری PCR پر مبنی اطلاقات کے لیے استعمال کیے جا سکتے ہیں۔ مزید برآں، متعدد مطالعات نے چھوٹے زمینی آرتھروپوڈز، خشک محفوظ شدہ میوزیم نمونوں، اور نرم جسم والے آرتھروپوڈز کے لیے غیر تباہ کن ڈی این اے استخراج کے طریقوں کا جائزہ لیا ہے [19, 26, 28, 55, 63] -

6.4.2 غیر تباہ کن نیوکلیک ایسڈ کا استخراج

جوڑ پیر والے حشرات، خاص طور پر ریتیلی مکھیوں کے مالیکیولر تجزیے میں ایک بڑا چیلنج نمونوں کو حشرہ شناسی کے مجموعوں میں

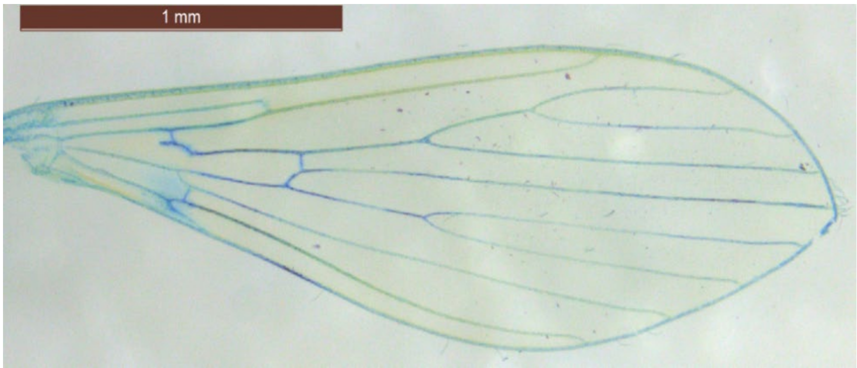

شکل 10): ریتیلی مکھیوں آریاسی (*Phlebotomus ariasi*) کے رنگین پر۔

شامل کرنے کے لیے محفوظ رکھنا ہے۔ زیادہ تر ڈی این اے استخراج کے پروٹوکول ٹشو کو نرم کرنے (میکیریشن) کا تقاضا کرتے ہیں، جس سے اصل نمونے کی حفاظت متاثر ہوتی ہے۔ تاہم، غیر تباہ کن نیوکلیک ایسڈ استخراج کے طریقے ایسے ہیں جو نمونے کو جسمانی طور پر نقصان پہنچائے بغیر، اس کی بقا یا ساخت میں تبدیلی کیے بغیر جینیاتی مواد نکالنے کے لیے ڈیزائن کیے گئے ہیں۔ یہ طریقے خاص طور پر قیمتی یا محدود نمونوں، جیسے کہ ریتیلی مکھیوں، کے ساتھ کام کرتے وقت انتہائی قیمتی ہیں، جہاں ساختی سالمیت کو برقرار رکھنا مستقبل کی درجہ بندی، مورفولوجیکل یا تشخیصی مقاصد کے لیے ضروری ہے۔ ایک عام طور پر استعمال ہونے والا طریقہ غیر تباہ کن غسل کا طریقہ ہے جس میں ریتیلی مکھیاں غیر متحرک کی جاتی ہیں اور انہیں احتیاط سے پروٹینیز کے ساتھ لائسس بفر میں ڈبو دیا جاتا ہے۔

مِلِڈ ویکٹولائسس تکنیک کو ریتیلی مکھیوں ، خاص طور پر ٹائپ نمونوں پر کامیابی کے ساتھ لاگو کیا گیا ہے [24]۔ یہ تکنیک ایک روایتی اسپن کالم کٹ (اس صورت میں ڈی نیسی ٹشو اور خون کی کٹ، QIAGEN، ہِلڈَن، جرمنی) استعمال کرتی ہے جسے نمونہ کو نقصان پہنچائے بغیر ڈی این اے حاصل کرنے کے لیے ڈھالا گیا ہے۔ تبدیل شدہ لائسس کے مراحل (لائسس بفر کے حجم میں اضافہ اور منجمد کرنے کے مرحلے کا اضافہ) [17] نیوکلیک ایسڈز کی رہائی ممکن بناتے ہیں اور مورفولوجیکل نقصان کو کم سے کم کرتے ہیں [24]۔ ریتیلی مکھیوں کے ، حوالے سے [HotSHOT DNA Extraction kit) Bento Bioworks Ltd, (173) London, United Kingdom کا استعمال بھی ممکن ہے۔

جو تیز اور سستی کٹ ہے اور نمونوں کی فوری اور کم لاگت پراسیسنگ کو ممکن بناتی ہے۔ شکل شناسی کی شناخت کے لیے ریتیلی مکھیوں کے نمونے بعد میں دھوئے جا سکتے ہیں۔ جو نمونے ڈی نیسی ٹشو اور خون کی کٹ کے ساتھ پراسیس کیے گئے ہیں، انہیں مارک-اینڈرے محلول سے صاف کرنا ضروری ہے، جبکہ HotSHOT DNA ایکسٹریکشن کٹ کے ساتھ پراسیس کیے گئے نمونے اس مقالے [73] میں بیان کردہ پروٹوکول کے مطابق ڈی ہائیڈریشن کے بعد ریزن میں ماؤنٹ کرنے کے لیے کافی حد تک شفاف ہو جاتے ہیں۔ نکالا گیا جینیاتی مادہ بعد ازاں مزید عمل کے لیے استعمال کیا جا سکتا ہے، جیسے PCR کے ذریعے مخصوص جینیاتی مارکر کو بڑھانا۔ غیر تباہ کن نیوکلیک ایسڈ خارج کرنے کے طریقے ریتیلی مکھیوں کی جینیاتی خصوصیات کا مطالعہ کرنے کے لیے انتہائی اہم ہیں، جن میں وہ ممکنہ بیماری پیدا کرنے والے عوامل کی شناخت بھی شامل ہے جو وہ اپنے ساتھ لے جا سکتی ہیں۔ نمونے کی سالمیت کو برقرار رکھ کر، محققین قیمتی جینیاتی معلومات حاصل کر سکتے ہیں، جبکہ اضافی تجزیوں یا مطالعات کے لیے نمونہ کو محفوظ بھی رکھ سکتے ہیں۔

جدول 5): فلیبٹومین ریت مکوڑوں کے gDNA کی استخراج کے لیے اوسط لاگت، اطلاق، اور پروٹوکول کی موافقت۔

سیریل نمبر	پروٹوکول	لاگت	درخواست	چھوٹے آرتھروپوڈز کے لیے پروٹوکول کی موافقت
1):	گھماؤ کالم	2.5 – 3.55 US$ [39]	این , پی سی آر جی ایس	[9]
2):	فینول کلوروفارم	0.24 US$ [69]	این , پی سی آر جی ایس	[9]
3):	ہاٹ شاٹ	<0.01 US$ [69]	پی سی آر	-
4):	نمکین نکالنا	0.12 $3 [69]	پی سی آر	-
5):	چیلیکس	0.02 $ [41]	پی سی آر	[41, 76]

6.5): میلڈی ٹوف (مالدی ٹی او ایف) ایم ایس

میلڈی ٹوف (مالدی ٹی او ایف) ایم ایس (میٹرکس اسسٹڈ لیزر ڈیسورپشن/آئنائزیشن ٹائم آف فلائیٹ ماس اسپیکٹرو میٹری) ایک ماس اسپیکٹرو میٹری پر مبنی تکنیک ہے جو حیاتیاتی نمونوں کے منفرد پروٹین پروفائلز (‘فنگر پرنٹس’) کا پتہ لگانے اور تجزیہ کرنے کے لیے ڈیزائن کی گئی ہے۔ میلڈی ٹوف کو طبی اور ویٹرنری اہمیت کے حامل آرتھروپوڈز کی شناخت کے لیے ایک اہم آلہ کے طور پر تیزی سے تسلیم کیا جا رہا ہے۔ یہ تکنیک ریتیلی مکھیوں کے مختلف ترقیاتی مراحل، بشمول ناتواں شکلوں ، خون سے بھرے مادہ اور مکھیوں کے خون کے کھانے کی شناخت میں مؤثر ثابت ہوئی ہے، اور اسے ذخیرہ اور ہم آہنگی کے مختلف حالات میں نر اور مادہ دونوں ریتیلی مکھیوں کی انواع میں فرق کرنے کے لیے کامیابی کے ساتھ استعمال کیا گیا ہے [28, 30, 73, 74]. یہ طریقہ ذیلی جنس، انواع اور آبادیوں کی سطح پر بھی اعلیٰ امتیازی صلاحیت فراہم کرتا ہے۔ یہ تکنیک محققین کو انواع کی تیز اور درست شناخت ممکن بناتی ہے، جو کہ ریتیلی مکھیوں کی تقسیم، رویے اور بیماریوں کے انتقال میں ان کے کردار کو سمجھنے کے لیے ضروری ہے۔ پروٹین پروفائلز کی بنیاد پر انواع کے درمیان فرق کرکے، میلڈی ٹوف وبائیاتی مطالعات اور ویکٹر کنٹرول حکمت عملیوں میں ایک اہم کردار ادا کرتا ہے۔ اس تکنیک کے دو اہم موجودہ نقصانات ہیں جو اس کے معمول کے استعمال کو محدود کرتے ہیں۔ پہلا مسئلہ ماس اسپیکٹرومیٹری کے آلات کی دستیابی ہے، جو صرف ریتیلی مکھیوں (یا عام طور پر آرتھروپوڈ ویکٹرز) کی انواع کی شناخت کے مقصد کے لیے حاصل کرنا انتہائی مہنگا ہے۔ خوش قسمتی سے، اس حد کو ان ماس اسپیکٹرو میٹرز پر مشین ٹائم حاصل کرکے ختم کیا جا سکتا ہے جو پروٹومیک سہولیات اور/یا کلینیکل تشخیص میں ایک معیاری تحقیقی آلہ بن چکے ہیں۔ دوسری کمی کھلے رسائی والے ڈیٹا بیس میں ریتیلی مکھیوں کے حوالہ جاتی ڈیٹا کی کم دستیابی ہے، جس کے نتیجے میں ایک اندرون خانہ ڈیٹا بیس بنانے کی ضرورت پیش آتی ہے، جس میں واضح طور پر شناخت شدہ نمونوں پر مبنی حوالہ جاتی سپیکٹرا ہوں، مثالی طور پر مورفولوجیکل تشخیص اور کسی مناسب جینیاتی مارکر (COI، cytB) یا دیگر) کی سیکوئنسنگ کے امتزاج کے ذریعے۔ امید ہے کہ اس محدودیت کو جلد ہی دور کر دیا جائے گا، اس کے لیے اب تک کے اندرونِ خانہ ریفرنس ڈیٹا کو بتدریج MSI پلیٹ فارم میں شامل کیا جائے گا، جو de بوپیٹو-پبلک اسسٹنس – پیرس سوربون

یونیورسٹی ,فرانس اور بیلجیم ,برسلز، برائے کلیکشن
BCCM/IHEM/Sciensano (/https://msi.happy-dev.fr) کے زیر انتظام ہے۔

جب میلڈی ٹوف پروٹین پروفائلنگ کو نافذ کرنے کا منصوبہ ہو تو نمونوں کو ترجیحاً خشک منجمد حالت میں یا مالیکولر گریڈ کے 70% ایتھانول میں محفوظ کیا جانا چاہیے۔ اور ماحولیاتی درجہ حرارت سے متاثر نہیں ہوتا ہے۔ نمونہ کی تیاری کے لیے عالمی رہنما اصولوں کی عدم موجودگی میں، صارفین کو مشورہ دیا جاتا ہے کہ وہ میلڈی ٹوف میٹرکس کی تیاری کے لیے سیناپینک ایسڈ (30 ملی گرام/ملی لیٹر) کا 60% ایکینوٹرائل/0.3% TFA آبی محلول استعمال کریں تاکہ ان کے پروٹین سپیکٹرا اب تک شائع شدہ ریتیلی مکھیاں کے ڈیٹا کے ساتھ موازنہ پذیر ہوں۔

6.5.1): MALDI-ToF MS کے لیے نمونہ کی تیاری (شکل 7)

ریتیلی مکھیوں کے نمونے، جو مختلف حالات میں محفوظ کیے گئے ہوں، سب سے پہلے کمرے کے درجہ حرارت پر ہوا میں خشک کیے جاتے ہیں اور ان کی تشریح کی جاتی ہے۔ سر اور پیٹ کو سلائیڈ پر چڑھانے اور مورفولوجیکل تجزیے کے لیے اہم مورفولوجیکل خصوصیات پر مشتمل جسمانی حصے حاصل کرنے کے لیے نکالا جاتا ہے۔ تھوراکس کو میلڈی ٹوف کے لیے استعمال کیا جا سکتا ہے اور باقی ماندہ پیٹ کو ڈی این اے نکالنے کے لیے محفوظ کیا جا سکتا ہے۔ پروٹین پروفائلنگ کے لیے، تھوراکس کو دستی طور پر 1.5 ملی لیٹر مائیکرو ٹیوبز میں 10 µL ہوموجنائزیشن محلول کے ساتھ ڈسپوزیبل پیسٹلز اور پیلیٹس استعمال کرتے ہوئے ہوموجنائز کیا جاتا ہے۔ عام طور پر دو ہوموجنائزیشن محلول استعمال کیے جاتے ہیں: جراثیم سے پاک مقطر پانی اور 25% فارمک ایسڈ۔

7): نتیجہ

اس کام میں، ہمارا مقصد محققین کو ریتیلی مکھیوں کو ماؤنٹ کرنے کے لیے سب سے مؤثر طریقے فراہم کرنا تھا، جو مخصوص تحقیقی مقاصد کے مطابق ڈیزائن کیے گئے ہیں، تاکہ درست شناخت اور پیتھوجین کی تشخیص میں آسانی ہو۔ کوئی ایک، عالمگیر طور پر مثالی طریقہ نہیں ہے؛ بلکہ کئی طریقے موجود ہیں، جن میں سے ہر ایک کے اپنے فوائد اور خامیاں ہیں۔ مددگار ڈیٹا میں، ہم نے ریتیلی مکھیاں تیار کرنے اور ان کی شناخت کے لیے استعمال ہونے والی مختلف ماؤنٹنگ تکنیکوں کے مفصل پروٹوکول فراہم کیے ہیں۔ ان پروٹوکولز میں ہدایتی ویڈیوز بھی شامل ہیں جو مختلف مقاصد کے لیے مرحلہ وار طریقہ کار پیش کرتی ہیں اور درست و قابلِ اعتماد نتائج کو یقینی بناتی ہیں۔ اس جامع وسیلے کی فراہمی کے ذریعے، ہمارا مقصد محققین کو ان کی مخصوص ضروریات کے مطابق سب سے مناسب ماؤنٹنگ تکنیکوں کے انتخاب اور اطلاق میں معاونت فراہم کرنا ہے۔

تقدیمات

مصنفین برطانیہ کے نیچرل ہسٹری میوزیم کے رچرڈ لین اور زوئی جے ایڈمز کے تہہ دل سے شکر گزار ہیں جنہوں نے اپنی بہترین نظرثانی سے اس مسودے کے معیار کو بہتر بنایا۔

فنڈنگ

ہم برازیلین ترقیاتی ایجنسیوں CNPq (کیس نمبر: 4-2024/404395) اور Araucária فاؤنڈیشن (کیس نمبر: PDI 2025/433) کے شکرگزار ہیں جنہوں نے AJA کی تحقیق کو فنڈ فراہم کیا۔

مفادات کے تصادم

جیروم ڈیپاکوئٹ جو جرنل پیراسائٹس کے ایسوسی ایٹ ایڈیٹر ہیں؛ اس مسودے کے جائزہ کے عمل اور فیصلہ سازی پر ان کا کوئی اثر نہیں تھا۔ دیگر مصنفین اعلان کرتے ہیں کہ ان کے مفادات کا کوئی تصادم نہیں ہے۔

ڈیٹا کی دستیابی کا بیان

ویڈیو کلپس Zenodo پر۔
ویڈیو 1): https://zenodo.org/records/18198006
ویڈیو 2): https://zenodo.org/records/18311158
ویڈیو 3): https://zenodo.org/records/18311106
ویڈیو 4): https://zenodo.org/records/18311154
ویڈیو 5): https://zenodo.org/records/18303014
ویڈیو 6): https://zenodo.org/records/18302850
ویڈیو7): https://zenodo.org/records/18315029

اضافی مواد

https://www.parasite-journal.org/10.1051/parasite/2026009/olm
اس مضمون کے لیے اضافی مواد درج ذیل لنک پر آن لائن دستیاب ہیں۔

حوالہ جات

1): Alkan C, Allal-Ikhlef AB, Alwassouf S, Baklouti A, Piorkowski G, de Lamballerie X, Izri A, Charrel RN. 2015. Virus isolation, genetic characterization and seroprevalence of Toscana virus in Algeria. Clinical Microbiology and Infection, 21(11), 1040 e1-9.

2): Alten B, Ozbel Y, Ergunay K, Kasap OE, Cull B, Antoniou M, Velo E, Prudhomme J, Molina R, Banuls AL, Schaffner F, Hendrickx G, Van Bortel W, Medlock JM. 2015. Sampling strategies for phlebotomine sand flies (Diptera: Psychodidae) in Europe. Bulletin of Entomological Research 105(6), 664–678.

3): Ayhan N, Baklouti A, Prudhomme J, Walder G, Amaro F, Alten B, Moutailler S, Ergunay K, Charrel RN, Huemer H. 2017. Practical guidelines for studies on sandfly-borne phleboviruses: Part I: Important points to consider *ante* field work. Vector-Borne and Zoonotic Diseases 17(1), 73–80.

4): Bates PA. 1997. Infection of phlebotomine sandflies with *Leishmania*, in The Molecular Biology of Insect Disease Vectors: A Methods Manual. Springer. p. 112–120.5.

5): Baum M, de Castro EA, Pinto MC, Goulart TM, Baura W, Klisiowicz Ddo R, Vieira da Costa-Ribeiro MC. 2015. Molecular detection of the blood meal source of sand flies (Diptera: Psychodidae) in a transmission area of American cutaneous leishmaniasis, Parana State, Brazil. Acta Tropica, 143, 8–12.

6): Belen A, Alten B, Aytekin A. 2004. Altitudinal variation in morphometric and molecular characteristics of *Phlebotomus papatasi* populations. Medical and Veterinary Entomology, 18(4), 343–350.

7): Bhattacharya J, Chandra G, Hati AK. 1991. A simple method for cryopreservation of *Leishmania donovani* promastigotes, Indian Journal of Medical Research, 93, 245–246.

8): Caligiuri LG, Sandoval AE, Miranda JC, Pessoa FA, Santini MS, Salomón OD, Secundino NF, McCarthy CB. 2019. Optimization of DNA extraction from individual sand flies for PCR amplification. Methods and Protocols, 2(2), 36.

9): Casaril AE, de Oliveira LP, Alonso DP, de Oliveira EF, Gomes Barrios SP, de Oliveira Moura Infran J, Fernandes WS, Oshiro ET, Ferreira AMT, Ribolla PEM, de Oliveira AG. 2017. Standardization of DNA extraction from sand flies: Application to genotyping by next generation sequencing. Experimental Parasitology, 177, 66–72.

10): Castalanelli MA, Severtson DL, Brumley CJ, Szito A, Foottit RG, Grimm M, Munyard K, Groth DM. 2010. A rapid non-destructive DNA extraction method for insects and other arthropods. Journal of Asia-Pacific Entomology, 13(3), 243–248.

11): Cerqueira NL. 1943. Um novo meio para montagem de pequenos insetos em lâmina. Memórias do Instituto Oswaldo Cruz, (39), 37–41.

12): Charrel RN, Gallian P, Navarro-Mari JM, Nicoletti L, Papa A, Sanchez-Seco MP, Tenorio A, de Lamballerie X. 2005. Emergence of Toscana virus in Europe. Emerging Infectious Diseases, 11(11), 1657–1663.

13): Chaskopoulou A, Giantsis IA, Demir S, Bon MC. 2016. Species composition, activity patterns and blood meal analysis of sand fly populations (Diptera: Psychodidae) in the metropolitan region of Thessaloniki, an endemic focus of canine leishmaniasis. Acta Tropica, 158, 170–176.

14): Chen H, Rangasamy M, Tan SY, Wang H, Siegfried BD. 2010. Evaluation of five methods for total DNA extraction from western corn rootworm beetles. PLoS One, 5(8), e11963.

15): Depaquit J, Grandadam M, Fouque F, Andry PE, Peyrefitte C. 2010. Arthropod-borne viruses transmitted by Phlebotomine sandflies in Europe: a review. Eurosurveillance, 15(10), 19507.

16): Diamond LS, Herman CM. 1954. Incidence of Trypanosomes in the Canada Goose as revealed by bone marrow culture. Journal of Parasitology, 40(2), 195–202.

17): Ding H, Torno M, Vongphayloth K, Ng G, Tan D, Sng W, Ho K, Randrianambinintsoa FJ, Depaquit J, Tan CH. 2025. Hidden in plain sight: discovery of sand flies in Singapore and description of four species new to science. Parasites & Vectors, 18(1), 402.

18): Es-Sette N, Ajaoud M, Bichaud L, Hamdi S, Mellouki F, Charrel RN, Lemrani M. 2014. *Phlebotomus sergenti* a common vector of *Leishmania tropica* and Toscana virus in Morocco. Journal of Vector Borne Diseases, 51(2), 86–90.

19): Favret C. 2005. A new non-destructive DNA extraction and specimen clearing technique for aphids (Hemiptera). Proceedings of the Entomological Society of Washington, 107(2), 469–470.

20): Galati EAB. 2018. Phlebotominae (Diptera, Psychodidae): Classification, morphology and terminology of adults and identification of American taxa, in Brazilian Sand Flies: Biology, Taxonomy, Medical Importance and Control, Rangel EF, Shaw JJ, Editors. Cham: Springer International Publishing. pp. 9–212.

21): Galati EAB, de Andrade AJ, Perveen F, Loyer M, Vongphayloth K, Randrianambinintsoa FJ, Prudhomme J, Rahola N, Akhoundi M, Shimabukuro PHF, Depaquit J. 2025. Phle-botomine sand flies (Diptera, Psychodidae) of the world. Parasites & Vectors, 18(1), 220.

22): Galati EAB, Galvis-Ovallos F, Lawyer P, Leger N, Depaquit J. 2017. An illustrated guide for characters and terminology used in descriptions of Phlebotominae (Diptera, Psychodidae). Par-asite, 24, 26.

23): Gariepy T, Kuhlmann U, Gillott C, Erlandson M. 2007. Parasitoids, predators and PCR: the use of diagnostic molecular markers in biological control of Arthropods. Journal of Applied Entomology, 131(4), 225–240.

24): Giantsis IA, Chaskopoulou A, Bon MC. 2016. Mild-Vectolysis: A nondestructive DNA extraction method for vouchering sand flies and mosquitoes. Journal of Medical Entomology, 53(3), 692–695.

25): Gidwani K, Picado A, Rijal S, Singh SP, Roy L, Volfova V, Andersen EW, Uranw S, Ostyn B, Sudarshan M, Chakravarty J, Volf P, Sundar S, Boelaert M, Rogers ME. 2011. Serological markers of sand fly exposure to evaluate insecticidal nets against visceral leishmaniasis in India and Nepal: a cluster-randomized trial. PLoS Neglected Tropical Diseases, 5(9), e1296.

26): Gilbert MTP, Moore W, Melchior L, Worobey M. 2007. DNA extraction from dry museum beetles without conferring external morphological damage. PLoS One, 2(3), e272.

27): Giordani BF, Andrade AJ, Galati EAB, Gurgel-Goncalves R. 2017. The role of wing geometric morphometrics in the identification of sandflies within the subgenus *Lutzomyia.*

This is an Open Access article distributed under the terms of the Creative Commons Attribution License (https://creativecommons.org/licenses/by/4.0), which permits unrestricted use, distribution, and reproduction in any medium, provided the original work is properly cited.

to manage collections? Museum Management and Curatorship, 23(2), 193–199.
44): Maroli M, Feliciangeli MD, Bichaud L, Charrel RN, Gradoni L. 2013. Phlebotomine sandflies and the spreading of leishmaniases and other diseases of public health concern. Medical and Veterinary Entomology, 27(2), 123–147.
45): Marquina D, Buczek M, Ronquist F, Lukasik P. 2021. The effect of ethanol concentration on the morphological and molecular preservation of insects for biodiversity studies. PeerJ, 9, e10799.
46): Mathis A, Depaquit J, Dvorak V, Tuten H, Banuls AL, Halada P, Zapata S, Lehrter V, Hlavackova K, Prudhomme J, Volf P, Sereno D, Kaufmann C, Pfluger V, Schaffner F. 2015. Identification of phlebotomine sand flies using one MALDI-TOF MS reference database and two mass pectrometer systems., Parasites & Vectors, 8, 266.
47): Mekarnia N, Benallal KE, Sadlova J, Vojtkova B, Mauras A, Imbert N, Longhitano M, Harrat Z, Volf P, Loiseau PM, Cojean S. 2024. Effect of *Phlebotomus papatasi* on the fitness, infectivity and antimony-resistance phenotype of antimony-resistant *Leishmania* major Mon-25. International Journal for Parasitology – Drugs and Drug Resistance, 25, 100554.
48): Milligan BG. 1998. Total DNA isolation, in Molecular Genetic Analysis of Population: A Practical Approach, Hoelzel AR, Editor. Oxford: Oxford University Press.
49): Molina R, Jiménez M, Alvar J, González E, Hernández-Taberna S, Ines MM. 2017. Methods in sand fly research. Madrid: Servicio de publicaciones Universidad de Alcalá de Henares, Madrid.
50): Murphy WJ, Eizirik E, O'Brien SJ, Madsen O, Scally M, Douady CJ, Teeling E, Ryder OA, Stanhope MJ, de Jong WW, Springer MS. 2001. Resolution of the early placental mammal radiation using Bayesian phylogenetics. Science, 294(5550), 2348–2351.
51): Nacif-Pimenta R, Pinto LC, Volfova V, Volf P, Pimenta PFP, Secundino NFC. 2020. Conserved and distinct morphological aspects of the salivary glands of sand fly vectors of leishmaniasis: an anatomical and ultrastructural study. Parasites & Vectors, 13(1), 441.
52): Neuhaus B, Schmid T, Riedel J. 2017. Collection management and study of microscope slides: Storage, profiling, deterioration, restoration procedures, and general recommendations. Zootaxa, 4322(1), 1–173.
53): New TR. 1974. Pscoptera. Handbooks for Identification of British Insects (Vol. I). London: Royal Entomological Society of London. 102 pp.
54): Perez-Ruiz M, Collao X, Navarro-Mari JM, Tenorio A. 2007. Reverse transcription, real-time PCR assay for detection of Toscana virus. Journal of Clinical Virology, 39(4), 276–281.
55): Porco D, Rougerie R, Deharveng L, Hebert P. 2010. Coupling non-destructive DNA extraction and voucher retrieval for small soft-bodied Arthropods in a high-throughput context: the example of Collembola. Molecular Ecology Resources, 10(6), 942–945.
56): Prudhomme J, Cassan C, Hide M, Toty C, Rahola N, Vergnes B, Dujardin JP, Alten B, Sereno D, Banuls AL. 2016. Ecology and morphological variations in wings of *Phlebotomus ariasi* (Diptera: Psychodidae) in the region of Roquedur (Gard, France): a geometric morphometrics approach. Parasites & Vectors, 9(1), 578
Medical and Veterinary Entomology, 31(4), 373–380.
28): Guzmán-Larralde AJ, Suaste-Dzul AP, Gallou A, Peña-Carrillo KI. 2017. DNA recovery from microhymenoptera using six non-destructive methodologies with considerations for subsequent preparation of museum slides. Genome, 60(1), 85–91.
29): Hajibabaei M, DeWaard JR, Ivanova NV, Ratnasingham S, Dooh RT, Kirk SL, Mackie PM, Hebert PD. 2005. Critical factors for assembling a high volume of DNA barcodes. Philosophical Transactions of the Royal Society B: Biological Sciences, 360(1462), 1959–1967.
30): Haouas N, Pesson B, Boudabous R, Dedet JP, Babba H, Ravel C. 2007. Development of a molecular tool for the identification of *Leishmania* reservoir hosts by blood meal analysis in the insect vectors. American Journal of Tropical Medicine and Hygiene, 77(6), 1054–1059.
31): Hlavackova K, Dvorak V, Chaskopoulou A, Volf P, Halada P. 2019. A novel MALDI-TOF MS-based method for blood meal identification in insect vectors: A proof of concept study on phlebotomine sand flies. PLoS Neglected Tropical Diseases, 13 (9), e0007669.
32): Huemer H, Prudhomme J, Amaro F, Baklouti A, Walder G, Alten B, Moutailler S, Ergunay K, Charrel RN, Ayhan N. 2017. Practical guidelines for studies on sandfly-borne phleboviruses: Part II: Important points to consider for fieldwork and subsequent virological screening. Vector-Borne and Zoonotic Diseases, 17(1), 81–90.
33): Jancarova M, Polanska N, Thiesson A, Arnaud F, Stejskalova M, Rehbergerova M, Kohl A, Viginier B, Volf P, Ratinier M. 2025. Susceptibility of diverse sand fly species to Toscana virus. PLoS Neglected Tropical Diseases, 19(5), e0013031.
34): Kapp JD, Green RE, Shapiro B. 2021. A fast and efficient single-stranded genomic library preparation method optimized for ancient DNA. Journal of Heredity, 112(3), 241–249.
35): Killick-Kendrick R, Maroli M, Killick-Kendrick M. 1991. Bibliography of the colonization of phlebotomine sandflies. Parassitologia, 33(suppl.), 321–333.
36): Lawyer P, Killick-Kendrick M, Rowland T, Rowton E, Volf P. 2017. Laboratory colonization and mass rearing of phlebotomine sand flies (Diptera, Psychodidae). Parasite, 24, 42.
37): Léger N, Pesson B, Madulo-Leblond G. 1986. Les phlébotomes de Grèce : 1ère partie. Bulletin de la Société de Pathologie Exotique, 79, 386–397.
38): Léger N, Pesson B, Madulo-Leblond G. 1986. Les phlébotomes de Grèce : 2ème partie. Bulletin de la Société de Pathologie Exotique, 79, 514–524.
39): Leonel JAF, Vioti G, Alves ML, da Silva DT, Meneghesso PA, Benassi JC, Spada JCP, Galvis-Ovallos F, Soares RM, Oliveira T. 2020. DNA extraction from individual Phlebotomine sand flies (Diptera: Psychodidae: Phlebotominae) specimens: Which is the method with better results? Experimental Parasitology, 218, 107981.
40): Lestinova T, Rohousova I, Sima M, de Oliveira CI, Volf P. 2017. Insights into the sand fly saliva: Blood-feeding and immune interactions between sand flies, hosts, and *Leishmania.* PLoS Neglected Tropical Diseases, 11(7), e0005600.
41): Lienhard A, Schaffer S. 2019. Extracting the invisible: obtaining high quality DNA is a challenging task in small arthropods. PeerJ, 7, e6753.
42): Lozano-Sardaneta YN, Mikery-Pacheco OF, Huerta H, Rojas-Soriano JE, Contreras-Ramos A. 2025. Wing
43): Mandrioli M. 2008. Insect collections and DNA analyses: how

68): Sant'Anna MR, Jones NG, Hindley JA, Mendes-Sousa AF, Dillon RJ, Cavalcante RR, Alexander B, Bates PA. 2008. Blood meal identification and parasite detection in laboratory-fed and field-captured *Lutzomyia longipalpis* by PCR using FTA databasing paper. Acta Tropica, 107(3), 230–237.

69): Senne NA, Santos HA, Araujo TR, Paulino PG, Mendonca LP, Moreira HVS, Camilo TA, da Costa Angelo I. 2022. Robust comparative performance of genomic DNA extraction methods from non-engorged phlebotomine sandflies. Medical and Veterinary Entomology, 36(2), 203–211.

70): Shaw JJ. 2025. A review of Leishmania infections in American Phlebotomine sand flies – Are those that transmit leishmaniasis anthropophilic or anthropportunists? Parasite, 32, 57.

71): Tesh RB, Modi GB. 1983. Growth and transovarial transmission of Chandipura virus (Rhabdoviridae: Vesiculovirus) in *Phlebotomus papatasi.* American Journal of Tropical Medicine and Hygiene, 32(3), 621–623.

72): Thomsen PF, Elias S, Gilbert MTP, Haile J, Munch K, Kuzmina S, Froese DG, Sher A, Holdaway RN, Willerslev E. 2009. Non-destructive sampling of ancient insect DNA. PLoS One, 4(4),e5048

73): Truett GE, Heeger P, Mynatt RL, Truett AA, Walker JA, Warman ML. 2000. Preparation of PCR-quality mouse genomic DNA with hot sodium hydroxide and tris (HotSHOT). Biotechniques, 29(1), 52–54.

74): Upton MS. 1993. Aqueous gum-chloral slide mounting media: an historical review. Bulletin of Entomological Research, 83(2), 267–274.

75): Volf P, Myskova J. 2007. Sand flies and Leishmania: specific versus permissive vectors. Trends in Parasitology, 23(3), 91–92.

76): Wang Q, Wang X. 2012. Comparison of methods for DNA extraction from a single chironomid for PCR analysis. Pakistan Journal of Zoology, 44(2), 421–426.

77): Wang Y, Zhao Y, Bollas A, Wang Y, Au KF. 2021. Nanopore sequencing technology, bioinformatics and applications. Nature Biotechnology, 39(11), 1348–1365.

57): Prudhomme J, Gunay F, Rahola N, Ouanaimi F, Guernaoui S, Boumezzough A, Banuls AL, Sereno D, Alten B. 2012. Wing size and shape variation of *Phlebotomus papatasi* (Diptera: Psychodidae) populations from the south and north slopes of the Atlas Mountains in Morocco. Journal of Vector Ecology, 37(1), 137–147.

58): Prudhomme J, Toty C, Kasap OE, Rahola N, Vergnes B, Maia C, Campino L, Antoniou M, Jimenez M, Molina R, Cannet A, Alten B, Sereno D, Banuls AL. 2015. New microsatellite markers for multi-scale genetic studies on *Phlebotomus ariasi* Tonnoir, vector of *Leishmania infantum* in the Mediterranean area. Acta Tropica, 142, 79–85.

59): Prudhomme J, Velo E, Bino S, Kadriaj P, Mersini K, Gunay F, Alten B. 2019. Altitudinal variations in wing morphology of *Aedes albopictus* (Diptera, Culicidae) in Albania, the region where it was first recorded in Europe. Parasite, 26, 55.

60): Rawlins DJ. 1992. Light Microscopy: An Introduction to Biotechniques. Oxford: Bios Scientific publishers. 143 pp.

61): Ready PD. 2013. Biology of phlebotomine sand flies as vectors of disease agents. Annual Review of Entomology, 58, 227–250.

62): Rohlf FJ, Slice D. 1990. Extensions of the Procrustes method for the optimal superimposition of landmarks. Systematic Zoology, 39(1), 40–59.

63): Rowley DL, Coddington JA, Gates MW, Norrbom AL, Ochoa RA, Vandenberg NJ, Greenstone MH. 2007. Vouchering DNA-barcoded specimens: Test of a nondestructive extraction proto-col for terrestrial arthropods. Molecular Ecology Notes, 7(6), 915–924.

64): Sábio PB, Andrade AJ, Galati EAB. 2014. Assessment of the taxonomic status of some species included in the *Shannoni* complex, with the description of a new species of *Psathyromyia* (Diptera: Psychodidae: Phlebotominae). Journal of Medical Entomology, 51(2), 331–341.

65): Sadlova J, Yeo M, Seblova V, Lewis MD, Mauricio I, Volf P, Miles MA. 2011. Visualisation of *Leishmania donovani* fluorescent hybrids during early stage development in the sand fly vector. PLoS One, 6(5), e19851.

66): Sales K, Miranda DEO, da Silva FJ, Otranto D, Figueredo LA, Dantas-Torres F. 2020. Evaluation of different storage times and preservation methods on phlebotomine sand fly DNA concen-tration and purity. Parasites & Vectors, 13(1), 399

67): Sales KG, Costa PL, de Morais RC, Otranto D, Brandao-Filho SP, Cavalcanti Mde P, Dantas-Torres F. 2015. Identification of phlebotomine sand fly blood meals by real-time PCR. Parasites& Vectors, 8, 230.

Cite this article as: Randrianambinintsoa FJ, Augendre L, Prudhomme J, Martinet J-P, Loyer M, Mekarnia N, Kerkoub H, Perveen FK, Huguenin A, Kariya E, Akhoundi M, De Andrade AJ, Berriatua E, Bongiorno G, Boyer S, Christodoulou V, Da Costa-Ribeiro MCV, De Souza LAF, Ding H, Dondji B, Dvořák V, Erisoz Kasap O, Galati EAB, Gállego M, Ballart C, Gouzelou S, Haddad N, Masse RS, Mekuria AH, Ivovic V, Kaczmarek S, Shahar MK, Kirstein OD, Kniha E, Kolářová I, Lincoln T, Lucanas C, Mikov O, Nov K, Özbel Y, Pesson B, Posada Lopez LC, Prasetyo DB, Rahola N, Rebollar-Tellez EA, Rodrigues BL, Roy L, Saini P, Sanjoba C, Shimabukuro PH, Siriyasatien P, Soszynska A, Suleşco T, Sylla M, Torno M, Volf P, Vongphayloth K, Sinh Nam V, Wardhana A, Yessinou E, Zapata S, Gantier J-C & Depaquit J. 2026. Processing and mounting phlebotomine sand flies: a consensus guideline. Parasite xx, xx. https://doi.org/10.1051/parasite/2026009.

PARASITE

An international open-access, peer-reviewed, online journal publishing high quality papers on all aspects of human and animal parasitology

Reviews, articles and short notes may be submitted. Fields include, but are not limited to: general, medical and veterinary parasitology; morphology, including ultrastructure; parasite systematics, including entomology, acarology, helminthology and protistology, and molecular analyses; molecular biology and biochemistry; immunology of parasitic diseases; host-parasite relationships; ecology and life history of parasites; epidemiology; therapeutics; new diagnostic tools.
All papers in Parasite are published in English. Manuscripts should have a broad interest and must not have been published or submitted elsewhere. No limit is imposed on the length of manuscripts.

Parasite (open-access) continues **Parasite** (print and online editions, 1994-2012) and **Annales de Parasitologie Humaine et Comparée** (1923-1993) and is the official journal of the Société Française de Parasitologie.

Editor-in-Chief:
Jean-Lou Justine, Paris

Submit your manuscript at:
https://www.editorialmanager.com/parasite

ضمیمہ A): حیاتی کیمیائی نظریاتی بنیادیں

متعلقہ آرتھروپوڈ ریتیلی مکھیاں ہیں۔ تاہم، یہ عمومی تصور دوسرے بہت عام آرتھروپوڈز پر بھی لاگو کیا جا سکتا ہے جن کی شناخت صرف اندرونی مورفولوجیکل خصوصیات کی بنیاد پر ہی ممکن ہے۔ اتفاقاً کچھ اندرونی اعضاء جزوی طور پر کائٹنائزڈ ہوتے ہیں اور ان کی ساخت ہمیں قیمتی معلومات فراہم کرتی ہے۔ اسی لیے خوراک کے پمپس، سپرماتھیکا اور ان کے نالیوں کا مشاہدہ کرنا بہت دلچسپ ہے۔ تمام ریجنٹس جن کا ہم جائزہ لیں گے، انہیں کبھی فراموش نہیں کرنا چاہیے کہ ریتیلی مکھیاں کے فکسیشن کے مرحلے سے لے کر اسمبلی تک ہم صرف ریڈوکس ردعمل ہی استعمال کریں گے۔ واحد احتیاط یا رہنما اصول یہ ہے کہ کم کرنے والے ریجنٹس کو آکسائیڈ کرنے والے ریجنٹس کے ساتھ نہ ملایا جائے۔

ایتھائل الکحل (ایتھانول)

یہ مادہ مختلف طریقوں سے استعمال ہوگا۔ الکحل کے مالیکیولز پانی کے ساتھ مضبوط کشش رکھتے ہیں اور اس لیے پانی نکالنے والا اثر دکھاتے ہیں۔ تاہم، کم ارتکاز والا الکحل (یعنی پانی میں بہت زیادہ حل شدہ) نیوکلیک ایسڈز کی تحلیل میں کردار ادا کرے گا (پانی نیوکلیک ایسڈز کا دشمن ہے)۔
جب ریتیلی مکھیاں ایتھانول میں رکھے جاتے ہیں تو یہ نہ صرف انہیں محفوظ کرنے کے لیے ہوتا ہے بلکہ بافتوں کو مستحکم کرنے کے لیے بھی ہوتا ہے۔ ہسٹولوجی میں ہم عموماً دو اہم تصورات میں فرق کرتے ہیں: نفوذ کی شرح اور استحکام کی شرح۔ یہ بخوبی سمجھا جاتا ہے کہ ایک اچھا محفوظ کنندہ پہلے تیزی سے گہرائی تک بافتوں میں داخل ہونا چاہیے اور پھر انہیں مستحکم کرنا چاہیے۔ 96 فیصد الکحل کے لیے نفوذ ضریب تقریباً 1.05 ہے (موازنہ کے طور پر، 0.75 فیصد آبی پکِرک ایسڈ کے محلول کے لیے نفوذ ضریب 0.45 ہے، جبکہ 3 فیصد پوٹاشیم ڈائی کرومیٹ کے محلول کے لیے یہ 1.45 ہے)۔
ریتیلی مکھیاں اور دیگر جوڑپایوں کو ایتھانول میں لامتناہی طور پر محفوظ کرنا ریتیلی مکھیاں کے ماہرین کے لیے ایک حقیقت ہے۔ یہ خیال اب بھی بہت قابلِ احترام ہے کہ میدان سے حاصل کردہ نمونے بعد کی تحقیقات یا مستقبل کے محققین کے لیے محفوظ رکھے جائیں۔ تاہم، سائٹولوجسٹ یا ہسٹولوجسٹ کے لیے یہ ممکن نہیں ہوتا۔ اگر نمونوں کو طویل عرصے تک فکسٹیو میں رکھا جائے تو وہ عملی طور پر دوبارہ استعمال کے قابل نہیں رہتے۔ اسی لیے دس سال سے پرانے نمونے استعمال کرنا مشکل یا ناممکن ہو جاتا ہے۔
ایک اور پہلو جوڑ پاؤں والے جاندار کے وزن اور فکسٹر کے حجم کا تناسب ہے۔ حیوانیات یا طبی عمل میں مشورہ دیا جاتا ہے کہ فکس کیے جانے والے ٹکڑوں کے حجم سے 60 گنا زیادہ حجم کا منصوبہ بنایا جائے۔ عملی طور پر مائیکرو جوڑ پاؤں والے جانداروں کے لیے، دیے گئے حجم کے نمونوں کے ساتھ کم از کم 4–5 گنا الکحل شامل کریں۔ یاد رکھیں کہ الکحل اپنی قوت کھو دے گا کیونکہ یہ آرتھروپوڈ کے بافتوں میں موجود تمام پانی کو ہٹا دیتا ہے۔

خلاصہ

- ایتھل الکحل ایک ریڈوکشن کرنے والا کیمیائی ایجنٹ ہے (اسی لیے آکسیڈیٹو فکسٹیو کے ساتھ مطابقت نہیں رکھتا)؛
- یہ تیزی سے پروٹینز کو تہہ نشین کر دیتا ہے اور ان کی ساخت کو بگاڑ دیتا ہے؛
- یہ بعض پیچیدہ لپڈز کو حل کرتا ہے اور گلائکوجن کو تہہ نشین کر دیتا ہے؛
- یہ بافتوں میں شدید سکڑاؤ پیدا کرتا ہے اور انہیں سخت کر دیتا ہے۔

بنیادی پوٹاشیم یا سوڈیم ہائیڈروکسائیڈ محلول

حشرہ شناسی میں ان محلولوں کے استعمال کا زیادہ تر توجہ پوٹاشیم ہائیڈروکسائیڈ پر مرکوز رہا ہے، بغیر کسی واضح جواز کے۔ سوڈیم ہائیڈروکسائیڈ [E524] محلول کی صورت میں پایا جاتا ہے، یا تو مختلف ارتکازات میں یا مختلف نارملیٹی کے ساتھ یہ آتا ہے- یہ گولیوں یا چمکدار ذرات کی شکل میں دستیاب ہے۔ اس کا سب سے بڑا نقصان یہ ہے کہ یہ بہت زیادہ نمی جذب کرنے والا ہے (KOH سے زیادہ)۔ جب یہ پروٹینز کے ساتھ تعامل کرتا ہے تو انہیں گھول دیتا ہے، اور لپڈز کے ساتھ صابن بننے کے عمل کے دوران انہیں سخت صابنوں میں تبدیل کر دیتا ہے (یہ KOH کے مقابلے میں ایک بڑا فرق ہے، جو صابن بننے کے عمل کے دوران مائع صابن پیدا کرتا ہے)۔
پوٹاشیم ہائیڈروکسائیڈ [E525] ایک مرتکز محلول کی صورت میں دستیاب ہے، لیکن سب سے بڑا فائدہ یہ ہے کہ اسے تقریباً 0.1 گرام کے دانوں کی شکل میں تیار کیا گیا ہے، جو بغیر درست ترازو کے پتلے محلول بنانے میں بہت آسانی فراہم کرتا ہے۔ مثال کے طور پر، 0.1 گرام کے ایک پیلیٹ کو 1 ملی لیٹر مقطر پانی میں گھولنے سے 10 فیصد محلول تیار ہوتا ہے۔ پیلیٹس کی صورت میں پوٹاشیم ہائیڈروکسائیڈ کا دوسرا فائدہ کاربونیٹ ہونے کے تئیں کم حساسیت ہے (KOH کا محلول CO_2 کو جذب کرنے کی زیادہ صلاحیت رکھتا ہے، جس سے کاربونیٹ نمک بن جاتے ہیں)۔
یہ مضبوط اڈے چکنائیوں کو پانی میں حل ہونے والے صابنوں میں تبدیل کرکے تحلیل کرنے کے لیے استعمال کیے جائیں گے۔ یہ بات یاد رکھنی چاہیے کہ فکسٹیو، جیسے ایتھانول، نے نمونے میں موجود کچھ چکنائیوں کو حل کر دیا تھا۔ تاہم، نمونے کو آبی ماحول میں مضبوط اڈے سے بدلنے پر، چکنائی کے تیزاب (زیادہ یا کم پیچیدہ) تہہ نشین ہو جائیں گے۔ لہٰذا یہ مضبوط الکلی ٹھنڈی صابن سازی (cold saponification) کا عمل انجام دے گا۔ بعض صورتوں میں، جب چربی کے بافت (adipose tissues) زیادہ ہوں، مثلاً مادہ میں، تو ردِ عمل کو آسان بنانے کے لیے درجۂ حرارت کو 35–40°C تک بڑھانا یا کمرے کے درجۂ حرارت پر رابطے کے وقت میں اضافہ کرنا فائدہ مند ہوگا۔

رنگی تیزاب محلول/ بے رنگ مارک-اینڈرے محلول

یہاں ہم مارک-آندرے محلول کے استعمال کے فوائد اور نقصانات کا جائزہ لیں گے۔ یہ محلول کلورل ہائیڈریٹ (ٹرائیکلوروایسیٹالڈیہائیڈ مونوہائیڈریٹ)، ایسٹک ایسڈ اور پانی پر مشتمل ہے۔ یہ محلول بہت آکسائڈائزنگ ہے (تیزاب اور الڈیہائیڈ کا مرکب)۔ یہ نمونوں میں موجود اضافی پوٹاشیم ہائیڈروکسائیڈ کو نیوٹرلائز کرے گا، بغیر ان الکلائن صابنوں کے جو پوٹاشیم ہائیڈروکسائیڈ کے استعمال کے دوران بنتے ہیں، کو تہہ نشین کیے بغیر۔ یہ آکسائڈائزنگ محلول کائٹن (chitin) میں بننے والے گلوکوزامین کے ثانوی الکحل فنکشنز پر بھی آکسیڈائز کر کے اثر انداز ہوگا، یوں کائٹن کو نرم کرے گا۔ ایک اور عمل موجود بعض معدنی نمکوں کا تحلیل ہونا ہے۔

جب مارک-آندرے محلول کو پہلے ایسڈ فوشین سے رنگا جائے (یعنی آکسائڈائزنگ شدہ حالت میں)، تو یہ ساخت میں ثانوی الکحل کے افعال پر ٹھہرنے کے قابل ہوگا۔ مارک-آندرے محلول کے رابطے کے وقت اور نمونوں کی رنگائی کی حالت کے بعد، صرف ایتھانول سے دھویا جائے گا۔ اس طرح ہم نمونوں کے ڈی ہائیڈریشن کے مرحلے کا آغاز کرتے ہیں۔

فوائد

- اضافی بنیادی محلولوں کا غیر جانبدار کرنا۔
- کائٹن کا نرم ہونا۔
- کائٹن کا رنگ کرنا تاکہ اندرونی کیٹین شدہ ڈھانچوں کا بہتر انداز میں جائزہ لیا جا سکے۔

نقصانات

کلورل ہائیڈریٹ ایک نیند لانے والی دوا ہے اور انسانی طب میں استعمال ہوتی رہی ہے۔ اسے کیمیائی ہڈ کے نیچے استعمال کرنا ضروری ہے اور کیمیائی خطرات سے متعلق قوانین کی پابندی کرنا لازمی ہے۔

ڈی ہائیڈریشن محلول

تجربہ سے معلوم ہوتا ہے کہ بہت چھوٹے نمونوں کے لیے بڑھتی ہوئی ارتکاز والے الکحل غسلوں کے تسلسل پر عمل کرنا مفید نہیں ہوتا۔ اگر نمونہ بڑا ہو تو ہم 80% ایتھانول سے شروع کریں گے، پھر 90%، 95% اور آخر میں مطلق ایتھانول۔ کے لیے بہت چھوٹے نمونوں کے لیے 90% ایتھانول کے غسل کا استعمال کریں اور پھر مطلق ایتھانول میں ڈبوئیں۔ اس مرحلے پر ہم ہمیشہ یاد رکھیں گے کہ مطلق ایتھانول ماحول میں موجود پانی کو جمانے کی کوشش کرتا ہے۔

حشره شناسی کی لیبارٹریوں میں روایتی طور پر نمونوں کی ڈی ہائیڈریشن کو بیچ کریوزوٹ کے غسل سے مکمل کیا جاتا تھا۔ آج یہ تیل، جو کیڑوں مار دوا، فنگس روکنے والی دوا اور لکڑی کے تحفظ کے لیے وسیع پیمانے پر استعمال ہوتا ہے، اپنی بو (پولی سائیکلک ارومیٹک ہائیڈروکاربنز) کی وجہ سے سختی سے منع کیا جاتا ہے اور اسے تولیدی زہریلا، کینسر پیدا کرنے والا، ایک مستقل نامیاتی آلودگی پھیلانے والا اور آبی جانداروں کے لیے ماحولیاتی طور پر زہریلا تصور کیا جاتا ہے۔

ایک محلول جو ہم نمونوں کو ماؤنٹ کرنے کے لیے تیار کرنے کی تجویز پیش کرتے ہیں وہ ® یوپارل اور یوپارل ایسنس ہے (جس کا ذکر اگلے پیراگراف میں کیا گیا ہے)۔ ® یوپارل اور یوپارل ایسنس کا مرکب بہت مقبول ہے؛ 90% ایتھانول کے غسل کے بعد حاصل کردہ نمونے بہت اچھے ہوتے ہیں۔

ضمیمہ B): استعمال شدہ ریجنٹس کے اجزاء

پوٹاشیم ہائیڈروکسائیڈ 10%
پوٹاشیم ہائیڈروکسائیڈ 10 گرام،
مقطر پانی q.s. 100 ملی لیٹر-

گم کلورل ماؤنٹنگ میڈیم
ہویئر میڈیم آبی مقطر 50 ملی لیٹر،
کلورل ہائیڈریٹ 200 گرام،
گمَ عربی 50 گرام،
گلیسرول 20 ملی لیٹر-

مارک-اینڈرے محلول
کلورل ہائیڈریٹ 40 گرام،
گلیشیل ایسٹک ایسڈ 30 ملی لیٹر،
مقطر پانی 30 ملی لیٹر-

فوکسن ایسڈ 1% مقطر پانی میں
ایسڈ فوکسن پاؤڈر 1 گرام،
مقطر پانی 99 ملی لیٹر-

فوکسن سے رنگین مارک
آندرے محلول مارک-آندرے محلول 10 ملی لیٹر،
فوشین 1% 50 مائیکرو لیٹر-

ضمیمہ C): یوپارال®، کینیڈا بالسم، پولی وینائل الکحل یا ماؤنٹنگ کے لیے دیگر محلول

پولی وینائل الکحل: جب مناسب ڈی ہائیڈریشن کے لیے ضروری مصنوعات دستیاب نہ ہوں تو یہ ماؤنٹنگ کے لیے مثالی مادہ ہے۔ پولی وینائل الکحل کو پھر ایمان کے لییکٹوفینول کے ساتھ ملایا جاتا ہے۔ تاہم، ان اسمبلیوں میں بڑے نقصانات یہ ہیں کہ یا تو یہ خشک ہو جاتی ہیں یا پانی کے بخارات بننے کی وجہ سے پولی وینائل الکحل کرسٹالائز ہو جاتا ہے یا فینول کے آکسیڈائز ہونے پر یہ سیاہ ہو جاتا ہے۔ یہ مختصر مدتی ماؤنٹنگ کے لیے ایک اچھی تکنیک ہے۔

کینیڈا بالسم: سلائیڈ اور کور سلپ کے درمیان ماؤنٹنگ کے لیے اس کے استعمال کے لیے نمونوں کو ڈی ہائیڈریٹ کرنا ضروری ہوتا ہے۔ زائلین یا ٹولوین کے استعمال میں بھی مشکلات ہیں۔

اینیکے میڈیم: سلائیڈ اور کور سلپ کے درمیان لگانے کے لیے، جیسے کینیڈا بالسم ، نمونے کی ڈی ہائیڈریشن درکار ہوتی ہے۔

اینیکے ترکیب: خالص سفید کولوفونی (22 گرام)؛ الکحل میں حل پذیر کوپال گم (12 گرام)، مطلق الکحل (20 ملی لیٹر)، کافور (10 گرام)، ٹرپنٹائن ایسنس (10 ملی لیٹر)، اور یوکالیپٹول (26 ملی لیٹر)۔ تیاری کے لیے، ایک برتن مثلاً ارلن مائر فلاسک میں مطلق الکحل اور کیمفر ڈالیں۔ پھر کولوفونی اور کوپال گم شامل کریں۔ اس کے بعد فلاسک کو اسٹاپر سے بند کر کے ہلایا جاتا ہے، اور پھر اسے بین- ماری میں ہلکی آنچ پر گرم کیا جاتا ہے تاکہ مکسچر ابُال نہ کھائے۔ جب اجزاء مکمل طور پر پگھل جائیں، تو ٹرپنٹائن ایسنس شامل کیا جاتا ہے، پھر مکسچر کے گرم ہونے کی حالت میں اسے چھانا جاتا ہے، اور آخر میں، چھانے ہوئے محلول میں یوکالیپٹول شامل کیا جاتا ہے۔ جب میڈیم کم سیال ہو جائے تو اسے Eenecê کے ساتھ پتلا کیا جاتا ہے، جس کا فارمولا درج ذیل ہے: مطلق الکحل (30 ملی لیٹر)، کافور (17 گرام)، ٹرپنٹائن کا عرق (15 ملی لیٹر)، یوکالیپٹول (38 ملی لیٹر) (Cerqueira, 1943)۔

یوپارال®: یہ ایک رال ہے جو اٹلس کے صنوبر (*Tetraspinia articulata*) (Vahl, 1791) سے حاصل ہوتی ہے اور 1906 میں گلسن نے اس کا مطالعہ اور ترقی کی۔ اس کا بنیادی فائدہ یہ ہے کہ یہ پولیمرائز نہیں ہوتی۔ سلائڈز اور کور سلپس کے درمیان رکھے گئے نمونے آسانی سے الکحل یا بہتر طور پر یوپارال®ایسنس کے اثر سے بازیاب کیے جا سکتے ہیں۔ یہ ریزن، جسے سینڈریک بھی کہا جاتا ہے، 80% ایتھانول قبول کرتا ہے۔

ٹریٹون X100 کا استعمال: غیر آئنک آبی محلول

ٹریٹن X100 ایک غیر آئنک آبی محلول (4-(1,1,3,3-ٹائٹرا میتھل بٹائل) فینائل-پولی ایتھیلین گلائکول محلول، یا t-آکٹیلفینوکسیپولیتھوکسی ایتھانول، پولی ایتھیلین گلائکول ٹرٹ-آکٹیلفینائل ایتھر) کی شکل میں ہے، جو خلیاتی اور مالیکیولر حیاتیات میں ڈیٹرجنٹ کے طور پر وسیع پیمانے پر استعمال ہوتا ہے۔ یہ خلیاتی اور نیوکلیئر جھلیوں کو نفوذپذیر بناتا ہے۔ کئی سالوں سے الکحل میں محفوظ کیے گئے حشری نمونے عام ہیں۔ بدقسمتی سے، الکحل میں تحفظ مثالی نہیں ہے، اور اس طرح محفوظ کیے گئے جوڑ پیر والے جانور خوردبینی معائنے کے لیے تیار کرنا بہت مشکل ہو جاتے ہیں۔ نمونوں پر مشتمل پلاسٹک اکثر خراب ہو جاتا ہے، جس کے بعد الکحل بھی بخارات بن کر اڑ جاتا ہے۔ دونوں صورتوں میں، الکحل کے ساتھ طویل رابطے یا نمونوں کے خشک ہونے سے ایک حقیقی مسئلہ پیدا ہوتا ہے۔ 2008 میں جونک نے Agepon جیسے فوٹوگرافک فلموں کے لیے استعمال ہونے والے گیلا کرنے والے ایجنٹ کے ذریعے ریتیلی مکھیوں کی دوبارہ ہائیڈریشن پر ایک نوٹ شائع کیا [26]۔ اس سے ایسے گیلا کرنے والے ایجنٹس کے استعمال کا خیال پیدا ہوا جو طاقتور ڈٹرجنٹس نہ ہوں۔

ذیل میں 0.5% آبی محلول میں ٹرائیٹون X100 کے استعمال کا طریقہ کار دیا گیا ہے:

- خشک نمونے کو مطلق الکحل سے سیراب کریں۔
- پورے نمونے کے ڈوب جانے کے لیے 0.5% ٹرائیٹون X100 محلول کی ضروری مقدار شامل کریں۔
- تقریباً 5 منٹ یا اس سے زیادہ کے لیے رکھیں۔
- تمام ریتیلی مکھیوں کو محلول میں آزاد ہونا چاہیے۔
- ٹریٹون X100 کے محلول کو نکال کر اس کی جگہ پوٹاشیم ہائیڈروکسائیڈ کا محلول ڈال دیا جاتا ہے۔ پھر اوپر بیان کردہ طریقہ کار کے مطابق عمل کیا جاتا ہے۔

ضمیمہ D): ® یوپارل یا کینیڈا بالسلم ماؤنٹنگ میڈیا مرحلہ وار

1. نمونوں کو ڈی ہائیڈریٹ کرنا ضروری ہے (دھندلا یا دودھیا دکھائی دینا ناکافی ڈی ہائیڈریشن کی نشاندہی کرتا ہے)۔
2. پانی نکالنے کا عمل ایتھائل الکحل کی بڑھتی ہوئی ارتکاز کے ذریعے کیا جا سکتا ہے۔
3. نمونوں کو 99% الکحل یا مطلق الکحل سے صفائی کے محلول میں منتقل کیا جا سکتا ہے

۔ طریقہ کار:

1. بالغ ریتیلی مکھیوں کو 70% ایتھانول میں کاٹ کر الگ کریں۔
2. ایتھانول نکالیں اور اس کی جگہ 10% KOH ڈالیں۔ ریتیلی مکھیاں شیشے کی سلائیڈ سے ڈھانپیں۔
3. کیڑوں کے شفاف ہونے تک بھگونے دیں۔
4. کے او ایچ ہٹا دیں۔
5. نمونہ کو مقطر پانی سے ڈھانپیں اور 30 سے 45 منٹ انتظار کریں۔
6. پانی نکالیں اور 30 منٹ کے لیے مقطر پانی سے دھونے کا عمل دہرائیں (یہ وقت متعدد نمونوں کی تعداد پر منحصر ہے: جتنے زیادہ نمونے ایک ساتھ پراسیس کیے جائیں گے، اتنا ہی زیادہ وقت درکار ہوگا۔ جتنے کم ہوں گے، خاص طور پر جو انفرادی طورپر ٹریٹ کیے جاتے ہیں، اتنا ہی کم وقت درکار ہوگا۔
7. پانی نکال دیں
8. مارک-آندرے محلول (ممکنہ طور پر فوکسن ایسڈ سے رنگین) شامل کریں اور 24 گھنٹے (ایک دن) انتظار کریں۔
9. مارک-اینڈرے محلول ہٹا دیں۔
10. نمونہ کو مقطر پانی سے ڈھانپیں اور 30 سے 45 منٹ انتظار کریں۔
11. پانی نکالیں اور 30 منٹ کے لیے دوبارہ مقطر پانی سے دھوئیں۔
12. پانی نکالیں
13. 70% ایتھانول شامل کریں اور نمونہ کاٹ کر کھولیں۔
a. سر اور پیٹ کے لیے، نرمی سے سر یا پیٹ کو تھوراکس سے الگ کریں۔
b. سینے کے لیے، ایک فورسپس سے سینے کو پکڑیں اور دوسری فورسپس سے اعضاء کی جڑ پر کھینچ کر پروں کو ہٹا دیں۔ اگر زیادہ دلچسپی کے علاقوں کے مطابق ہو تو سینے کو بائیں اور دائیں حصوں میں تقسیم کر کے سیگیٹل تشریح بھی کی جا سکتی ہے۔
14. آبی ایتھائل الکحل کے محلولوں کی ایک سیریز کے ذریعے بتدریج خشک کریں۔ 50 – 80 – 95 فیصد تک جب تک مطلق ایتھانول نہ پہنچ جائے۔
15. نمونوں کو 100% ایتھانول سے دو مرتبہ، ہر بار 10 منٹ کے لیے دھو کر خشک کریں۔
16. ایتھانول ہٹا دیں اور نمونوں کو کمرے کے درجہ حرارت پر 15 منٹ کے لیے لونگ کے تیل سے ڈھانپیں۔
17. نمونوں کو لونگ کے تیل سے نکال کر ایک صاف سلائیڈ پر یوپارال®یا کینیڈا بالسلم کے ایک قطرے میں منتقل کریں۔
18. من چاہی ترتیب دیں: ریتیلی مکھیوں کی سر، سینہ اور پیٹ کو باریک سوئیوں یا فورسپس کے ذریعے ڈسیکشن خوردبین کے نیچے الگ کیا جا سکتا ہے۔ سر کو جسم سے الگ کرنا ضروری ہے تاکہ اسے وینٹرو-ڈورسل پوزیشن میں ماؤنٹ کیا جا سکے، یعنی اوسیپیٹل فورامین کو اوپر کی جانب رکھا جائے تاکہ سیبریئم براہِ راست اس کے ذریعے دیکھا جا سکے۔ یہ عمل ریتیلی مکھیوں کے ماؤنٹنگ میڈیم میں کیا جاتا ہے۔
19. نمونہ کو اتنا رکھیں کہ سطح چپچپی ہو جائے۔
20. ایک صاف کور سلپ کو مطلق الکحل سے گیلا کریں۔ کور سلپ کو زاویے پر کینیڈا بالسم پر گرا دیں۔
21. سلائڈز کو اس مقصد کے لیے مخصوص خشک ڈبے میں محفوظ کریں۔
