# Supplementary material for: Processing and mounting phlebotomine sand flies: a consensus guideline
Source: Parasite. 2026 Apr 3;33:18. doi: 10.1051/parasite/2026009 (PMC13047900; doi:10.1051/parasite/2026009)
Supplement: Supplementary file 33 — Vietnamese translation / Bản dịch tiếng Việt [file parasite-33-18-s33.pdf]

Parasite **33**, 18 (2026)
© F.J. Randrianambinintsoa et al., published by EDP Sciences, 2026
https://doi.org/10.1051/parasite/2026009

PARASITE
Available online at:
www.parasite-journal.org

REVIEW ARTICLE OPEN ACCESS

Quy trình xử lý và gắn tiêu bản muỗi cát *Phlebotominae*: hướng dẫn dựa trên đồng thuận chuyên gia

Fano José Randrianambinintsoa[1], Laure Augendre[1], Jorian Prudhomme[1], Jean-Philippe Martinet[1], Mathieu Loyer[1], Nalia Mekarnia[1], Hocine Kerkoub[1], Farzana Khan Perveen[1], Antoine Huguenin[1,2], Emilie Kariya[1,2], Mohammad Akhoundi[3], Andrey José de Andrade[4], Eduardo Berriatua[5], Gioia Bongiorno[6], Sébastien Boyer[7,8], Vasiliki Christodoulou[9], Magda Clara Vieira Da Costa-Ribeiro[10], Lucas Alexandre Farias de Souza[10], Huicong Ding[11], Blaise Dondji[12], Vít Dvořák[13], Ozge Erisoz Kasap[14], Eunice Aparecida Bianchi Galati[15], Montserrat Gállego[16], Cristina Ballart[16], Stavroula Gouzelou[17], Nabil Haddad[18], Rezki Sabrina Masse[19], Asrat Hailu Mekuria[20], Vladimir Ivovic[21], Szymon Kaczmarek[22], Mohd Khadri Shahar[19], Oscar D. Kirstein[23], Edwin Kniha[24], Iva Kolářová[13], Lincoln Timinao[25], Cristian Lucanas[26], Ognyan Mikov[27], Kimsear Nov[7], Yusuf Özbel[28], Bernard Pesson[29], Laura Cristina Posada Lopez[30], Didot Budi Prasetyo[1,7], Nil Rahola[31], Eduardo A. Rebollar-Tellez[32], Bruno Leite Rodrigues[15], Lalita Roy[33], Prasanta Saini[34], Chizu Sanjoba[35], Paloma Helena Fernandes Shimabukuro[36], Padet Siriyasatien[37], Agnieszka Soszyńska[22], Tatiana Suleşco[38], Massamba Sylla[39], Majhalia Torno[40], Petr Volf[13], Khamsing Vongphayloth[41], Vu Sinh Nam[42], April Wardhana[43], Eric Yessinou[44], Sonia Zapata[45], Jean-Charles Gantier[1], and Jérôme Depaquit[1,2,*]

[1] Faculté de Pharmacie, Université de Reims Champagne Ardenne, UR ESCAPE-USC ANSES PETARD, 51 rue Cognacq-Jay, 51096 Reims Cedex, France
[2] Pôle de Biologie territoriale, Laboratoire de Parasitologie-Mycologie, Centre Hospitalo-Universitaire, 51092 Reims, France
[3] Parasitology-Mycology Department, Avicenne Hospital, AP-HP, Bobigny, Sorbonne Paris Nord University, France; Unité des Virus Émergents (UVE: Aix-Marseille Univ, Università di Corsica, IRD 190, Inserm 1207, IRBA), 13005 Marseille, France
[4] Parasitology Collection of Basic Pathology, Department of Basic Pathology, Federal University of Paraná, Curitiba 19031, Brazil
[5] Department of Animal Health, University of Murcia, Campus de Espinardo, 30100 Espinardo, Murcia, Spain
[6] Department of Infectious Diseases, Vector-borne Diseases Unit, Istituto Superiore di Sanità, 00166 Rome, Italy
[7] Medical and Veterinary Entomology Unit, Institut Pasteur du Cambodge, Phnom Penh 12201, Cambodia
[8] Ecology & Emergence of Arthropod-borne Pathogens Unit, Department of Global Health, Institut Pasteur, CNRS UMR2000, 75015 Paris, France
[9] Section Veterinary Services (1417), Laboratory for Animal Health Virology, Aglantzia, Nicosia 2109, Cyprus
[10] Insects Vectors and Parasites Laboratory, Department of Basic Pathology and Postgraduate program in Microbiology, Parasitology and Pathology, Federal University of Paraná, 81530-900 Curitiba, Brazil
[11] Department of Biological Sciences, National University of Singapore, 117558, Singapore
[12] Laboratory of the Leishmaniasis Research Project, Mokolo District Hospital, Mokolo, Cameroon; Laboratory of Cellular Immunology and Parasitology, Department of Biological Sciences, Central Washington University, 98926 Ellensburg, WA, USA
[13] Department of Parasitology, Faculty of Science, Charles University, 12800 Prague, Czechia
[14] VERG Laboratories, Department of Biology, Faculty of Science, Hacettepe University, Beytepe, Ankara 06800, Türkiye
[15] Faculdade de Saúde Pública da Universidade de São Paulo (FSP/USP), Pós-graduação em Saúde Pública, 01246-904 São Paulo, Brazil
[16] Secció de Parasitologia, Departament de Biologia, Sanitat i Medi Ambient, Facultat de Farmàcia i Ciències de l'Alimentació, Universitat de Barcelona, & Institut de Salut Global de Barcelona (ISGlobal), Centro de Investigación Biomédica en Red, Enfermedades Infecciosas (CIBERINFEC), 08028 Barcelona, Spain
[17] Laboratory of Infectious Diseases and Public Health, School of Medicine, University of Cyprus, Nicosia, Cyprus & Department of Pediatrics, Archbishop Makarios III Hospital, Nicosia 2115, Cyprus
[18] Faculty of Health Sciences, American University of Beirut, 1107 2020 Beirut, Lebanon

Edited by Jean-Lou Justine
*Corresponding author: `jerome.depaquit@univ-reims.fr`

This is an Open Access article distributed under the terms of the Creative Commons Attribution License (https://creativecommons.org/licenses/by/4.0), which permits unrestricted use, distribution, and reproduction in any medium, provided the original work is properly cited.

[19] Medical Entomology Unit, Infectious Disease Research Centre, Institute for Medical Research (IMR), National Institutes of Health (NIH), Ministry of Health Malaysia, 40170 Shah Alam, Selangor, Malaysia
[20] School of Medicine, Addis Ababa University, 28017 - 1000 Addis Ababa, Ethiopia
[21] Faculty of Mathematics, Natural Sciences and Information Technologies, University of Primorska, 6000 Koper, Slovenia
[22] University of Lodz, Faculty of Biology and Environmental Protection, Department of Invertebrate Zoology and Hydrobiology, Banacha 12/16, 90-237 Łódź, Poland
[23] Laboratory of Entomology, Ministry of Health, 9134302 Jerusalem, Israel
[24] Center for Pathophysiology, Infectiology and Immunology, Institute of Specific Prophylaxis and Tropical Medicine, Medical University Vienna, Kinderspitalgasse 15, 1090 Vienna, Austria
[25] Papua New Guinea Institute of Medical Research (PNGIMR) Institute, PO Box 60, Headquarter, Homate Street, 441 Goroka, Eastern Highlands Province, Papua New Guinea
[26] Museum of Natural History, University of the Philippines Los Baños, 4031 Laguna, Philippines
[27] National Centre of Infectious and Parasitic Diseases, 1504 Sofia, Bulgaria
[28] Ege University, Faculty of Medicine, Department of Parasitology, 35040 Bornova/Izmir, Türkiye
[29] Retired, Faculté de Pharmacie, Université de Strasbourg, Strasbourg, 67400 Illkirch-Graffenstaden, France
[30] Program for the Study and Control of Tropical Diseases (PECET), Faculty of Medicine, University of Antioquia, 050010 Medellin, Colombia
[31] MIVEGEC, Univ. Montpellier, CNRS, IRD, 34394 Montpellier, France & Medical Entomology Unit, Institut Pasteur de Madagascar, 101 Antananarivo, Madagascar
[32] Laboratorio de Entomología Médica, Departamento de Zoología de Invertebrados, Facultad de Ciencias Biológicas, Universidad Autónoma de Nuevo León, San Nicolás de los Garza, 66455, NL, México
[33] Tropical and Infectious Disease Centre, BP Koirala Institute of Health Sciences, Dharan 56700, Nepal
[34] ICMR-Vector Control Research Centre, Puducherry 605006, India
[35] Graduate School of Agricultural and Life Sciences, The University of Tokyo, Tokyo 113-8657, Japan
[36] Grupo de estudos em Leishmanioses/Coleção de Flebotomíneos (COLFLEB/Fiocruz-MG), Instituto René Rachou, Fundação Oswaldo Cruz, Belo Horizonte, Minas Gerais, 30190009, Brazil
[37] Center of Excellence in Vector Biology and Vector-Borne Disease, Department of Parasitology, Faculty of Medicine, Chulalongkorn University, Bangkok 10330, Thailand
[38] Department of Arbovirology, Bernhard Nocht Institute for Tropical Medicine, Bernhard Nocht Str. 74, 20359 Hamburg, Germany [39] Laboratory Vectors & Parasites, Department of Livestock Sciences and Techniques, Sine Saloum University El Hadji Ibrahima Niasse (SSUEIN) Kaffrine Campus, C.P. 24600, Senegal.
[40] Environmental Health Institute, National Environment Agency, Singapore 138667, Singapore & Department of Biological Sciences, National University of Singapore, 117558 Singapore
[41] Institut Pasteur du Laos, Laboratory of Vector-Borne Diseases, Samsenhai Road, Ban Kao-Gnot, Sisattanak District, 3560 Vientiane, Lao PDR
[42] National Institute of Hygiene and Epidemiology, 1 Yec-Xanh Street, Hai Ba Trung District, 100000 Hanoi, Vietnam
[43] Indonesian Research Center for Veterinary Science, Indonesian Agency for Agricultural Research and Development, Ministry of Agriculture Republic Indonesia, Bogor 16114, Indonesia & Department of Parasitology, Faculty of Veterinary Medicine, Airlangga University, Surabaya 60115, Indonesia
[44] Laboratory of Research in Applied Biology, Polytechnic School of Abomey-Calavi, University of Abomey-Calavi, 01 P.O. Box 2009, 00000 Cotonou, Benin
[45] Instituto de Microbiología, Colegio de Ciencias Biológicas y Ambientales (COCIBA), Universidad San Francisco de Quito (USFQ), 170901 Quito, Ecuador

Received 1 December 2025, Accepted 29 January 2026, Published online 3 April 2026

Tóm tắt – Bài báo này cung cấp hướng dẫn toàn diện về quy trình xử lý và gắn tiêu bản muỗi cát thuộc phân họ *Phlebotominae*, điều có ý nghĩa then chốt đối với định danh loài cũng như phát hiện và phân lập tác nhân gây bệnh. Tài liệu trình bày nhiều kỹ thuật khác nhau, phù hợp cho cả điều kiện thực địa và phòng thí nghiệm.
Hướng dẫn bao gồm các chỉ dẫn chi tiết về thu thập, thao tác, đậy nắp và gây chết muỗi cát (khuyến nghị đông khô hoặc sử dụng CO_2 thay vì hóa chất), cũng như các chiến lược bảo quản như lưu trữ lạnh và bảo quản trong ethanol. Chất lượng chuẩn bị một số cấu trúc giải phẫu (cơ quan sinh dục, đầu và cánh) là yếu tố thiết yếu để quan sát hiển vi chính xác, và được mô tả cụ thể trong công trình này.
Bài báo cũng trình bày chi tiết quy trình xử lý mẫu, bao gồm bước làm trong bằng các chất như kali hydroxide, sau đó là dung dịch Marc-André. Phần gắn tiêu bản so sánh các môi trường gắn khác nhau, nhấn mạnh đặc tính quang học và khả năng bảo quản của từng loại. Dung dịch Hoyer (còn gọi là chloral gum) được khuyến nghị cho quan sát nhanh, đặc biệt đối với túi chứa tinh (spermathecae), nhờ độ trong suốt cao, mặc dù không phù hợp cho lưu trữ dài hạn.

Các môi trường khác được đề cập bao gồm polyvinyl alcohol, Euparal® (có khả năng dung nạp nước hạn chế), và Canada balsam (môi trường tan trong hydrocarbon); trong đó hai loại sau thích hợp cho bảo quản lâu dài.
Ngoài ra, các phương pháp sinh học phân tử hiện đại như giải trình tự DNA và MALDI-ToF — vốn đòi hỏi quy trình xử lý mẫu đặc thù — cũng được đề cập. Bài báo còn cung cấp các video minh họa ngắn về các kỹ thuật gắn tiêu bản khác nhau, cùng bản dịch sang 33 ngôn ngữ, giúp hướng dẫn này đáp ứng nhu cầu và kỳ vọng đa dạng của cộng đồng khoa học toàn cầu.

Từ khóa: Làm tiêu bản, muỗi cát *phlebotomine*, dung dịch Hoyer, dung dịch Marc-André, keo chloral, cồn olyvinyl, Euparal®, nhựa thơm Canada, phân lập *Leishmania*, điều kiện thực địa, nuôi cấy, mổ, sinh học phân tử, MALDI-ToF, dạng tiêu bản

Abstract – Processing and mounting phlebotomine sand flies: a consensus guideline. This article provides a comprehensive guide for the processing and mounting of phlebotomine sand fly specimens, which is crucial for species identification and pathogen detection and isolation. It discusses a range of techniques suitable for both field and laboratory settings. The guide includes detailed instructions on sand fly collection, handling, covering, and euthanasia (recommending dry freezing or CO_2 over chemicals) as well as conservation strategies, such as cold storage and preservation in ethanol. The quality of preparation of certain anatomical structures (genital organs, head and wings) is essential for their proper microscopic observation and is described in this work. The article also presents detailed sample processing, including the clearing process with agents such as potassium hydroxide then Marc-André solution. The mounting process compares different media, emphasizing their optical properties and preservation potential. Hoyer fluid (also known as chloral gum) is recommended for quick observation, particularly for spermathecae, due to its clarity, although it is not suitable for long-term storage. Other media discussed include polyvinyl alcohol, Euparal® (for limited water tolerance), and Canada balsam (a hydrocarbon-soluble medium), with the latter two offering long-term preservation capabilities. Innovative molecular biology approaches such as DNA sequencing and MALDI-ToF, which require particular attention to sample processing, are also addressed. Furthermore, short video clips illustrating various mounting techniques as well as translations in many different languages are provided, allowing the guideline to reach the diverse needs and expectations of the global scientific community.

Key words: Mounting, Phlebotomine sand fly, Hoyer fluid, Marc-André solution, Chloral gum, Polyvinyl alcohol, Euparal®, Canada balsam, *Leishmania* isolation, Field conditions, Culture, Dissection, Molecular biology, MALDI-ToF, Type-specimens.

Giới thiệu

Muỗi cát *Phlebotominae* là các côn trùng hai cánh nhỏ thuộc họ *Psychodidae*, phân họ *Phlebotomina*e với ít nhất 1.063 loài đã được ghi nhận [21]. Chúng là các véc tơ quan trọng của nhiều tác nhân gây bệnh (*Leishmania*, arbovirus và Bartonella) gây ra các bệnh tương ứng là *leishmaniasis*, nhiễm arbovirus và bartonellosis. Việc định danh loài chủ yếu dựa trên quan sát hiển vi chi tiết, được hỗ trợ bởi việc thu thập cẩn thận, bảo quản thích hợp và gắn lam kính chính xác, đòi hỏi một số kỹ thuật chuyên biệt, mỗi kỹ thuật đều có những ưu điểm và hạn chế riêng.

Việc định danh muỗi cát *Phlebotominae* trưởng thành dựa trên quan sát cả các cấu trúc bên ngoài (ví dụ: râu, xúc biện, cơ quan sinh dục đực) và các cấu trúc bên trong (ví dụ: hầu, khoang miệng (cibarium) và túi chứa tinh). Việc giải phẫu và tách riêng các cấu trúc bên trong này giúp quan sát rõ hơn và do đó nâng cao độ chính xác trong định danh. Vì vậy, không giống như muỗi hay bọ xít hút máu, muỗi cát *Phlebotomina*e cần được gắn giữa lam kính và lamen trước khi tiến hành định danh.

Cho đến những năm 1980, quan sát hiển vi là phương pháp duy nhất để định danh muỗi cát và đến nay vẫn là cách tiếp cận được sử dụng rộng rãi nhất. Do đó, việc lựa chọn quy trình và phương pháp chuẩn bị mẫu tương đối đơn giản và chủ yếu dựa trên hai cách: một bên là gắn tiêu bản vĩnh viễn cho phép bảo quản mẫu lâu dài, và bên kia là gắn nhanh để định danh trong môi trường không đảm bảo bảo quản lâu dài. Ví dụ, gắn tiêu bản cuối cùng trong nhựa như Canada balsam tốn nhiều thời gian, đòi hỏi khử nước hoàn toàn mẫu vật. Hơn nữa, chỉ số khúc xạ (RI) của môi trường này không phải lúc nào cũng tối ưu để quan sát dễ dàng túi chứa tinh. Ngược lại, gắn trong môi trường nền nước (ví dụ: dung dịch Hoyer) nhanh hơn và cho phép quan sát tốt hơn các túi chứa tinh có tính khúc xạ cao, nhưng không cho phép bảo quản tiêu bản lâu dài vì môi trường này có xu hướng hấp thụ nước từ khí quyển. Một lựa chọn là bịt kín lam kính bằng sơn móng tay khi đã khô hoàn toàn. Cách làm này vẫn tồn tại đến nay, ảnh hưởng đến việc lựa chọn phương pháp gắn tiêu

bản tùy theo mục đích sử dụng của mẫu chuẩn bị. Từ những năm 1980, các nghiên cứu định danh muỗi cát đã kết hợp giữa hình thái học và các cách tiếp cận sinh hóa. Phương pháp đầu tiên là phân tích hydrocarbon biểu bì, sau đó nhanh chóng được thay thế bằng các kỹ thuật sinh học phân tử (tức là RAPD, RFLP, khuếch đại DNA và giải trình tự theo phương pháp Sanger, cũng như giải trình tự thế hệ mới (NGS)). Hiện nay, các cách tiếp cận phân tử được bổ sung bằng các phương pháp protein học như phổ khối MALDI-ToF. Hơn nữa, định danh loài bằng phương pháp phân tử có thể được kết hợp với phát hiện tác nhân gây bệnh bằng PCR (*Leishmania, Trypanosoma, Bartonella* và *Phlebovirus*), vì tất cả đều có thể được phát hiện bằng PCR điểm cuối và qPCR, đòi hỏi phải điều chỉnh quy trình lấy mẫu và bảo quản phù hợp với mục tiêu đề ra [3, 32]. Ngoài các đặc điểm hình thái truyền thống được sử dụng để phân biệt loài, các cách tiếp cận hình thái khác cũng có thể được áp dụng (tức là hình thái hình học cánh).

Phần lớn dựa trên kinh nghiệm của chính các tác giả và dữ liệu từ tài liệu tham khảo, mục tiêu của nghiên cứu này là cung cấp các hướng dẫn chuẩn hóa về gắn tiêu bản và xử lý muỗi cát *Phlebotominae* trưởng thành nhằm tối ưu hóa phân tích hình thái và phân tích sinh học phân tử.

Nhu cầu thực hiện một số phân tích nhất định (ví dụ: sinh học phân tử hoặc MALDI-ToF) đòi hỏi phải giữ lại một phần của muỗi cát không cần thiết cho định danh hình thái, điều này nhấn mạnh sự cần thiết phải lựa chọn quy trình một cách cẩn trọng.

Trong bài báo này, chúng tôi tập trung vào các phương pháp gây mê và gây chết muỗi cát bắt sống, cách bảo quản chúng và quy trình gắn tiêu bản, phục vụ cho định danh nhanh hoặc cho bảo quản lâu dài nhằm cho phép các nghiên cứu tiếp theo.

Lời mở đầu: Các vấn đề liên quan đến an toàn và tuân thủ quy định cần được tham chiếu theo các Bảng dữ liệu an toàn hóa chất (Safety Data Sheets – SDS) tương ứng đối với từng hóa chất và thuốc thử được sử dụng.

Tất cả các hóa chất được trình bày trong hướng dẫn này phải được xử lý trong điều kiện an toàn nghiêm ngặt. Các ủy ban an toàn và sức khỏe của cơ sở nghiên cứu có thể cung cấp thông tin không chỉ về các nguy cơ của các hóa chất này mà còn về quy trình xử lý và thải bỏ chúng. Tuy nhiên, bắt buộc phải tuân thủ các hướng dẫn an toàn liên quan đến việc sử dụng và thải bỏ chúng. Cần lưu ý rằng trách nhiệm của tất cả người sử dụng là đảm bảo tuân thủ thực hành phòng thí nghiệm tốt và an toàn cũng như các quy định pháp luật hiện hành của quốc gia hoặc cơ sở nghiên cứu của mình. Ngoài ra, một số hóa chất hoặc thành phần của chúng (ví dụ: chloral hydrate) bị quản lý tại một số quốc gia. Danh sách các chữ viết tắt được sử dụng trong bản thảo này được trình bày trong Bảng 1.

Bảng 1. Thuật ngữ viết tắt

**BME**	Basal medium Eagle
**CDC**	Centers for Disease Control and Prevention
**CMCP**	Camphor-monochlorophenol
**CMR**	Carcinogenic, mutagenic, reprotoxic substance
**COI**	Cytochrome c oxidase subunit I gene
**CytB**	Cytochrome b gene
**DNA**	Deoxyribonucleic acid
**ELISA**	Enzyme-linked immunosorbent assay
**EtOH**	Ethanol
**M199**	Medium 199
**MALDI-ToF MS**	Matrix-assisted laser desorption/ionization time-of-flight mass spectrometry
**MEM**	Minimum essential media
**NGS**	Next-generation sequencing
**NNN**	Novy-MacNeal-Nicolle medium
**PCR**	Polymerase chain reaction
**Lao PDR**	Lao People's Democratic Republic
**PNOC**	Prepronociceptin gene
**qPCR**	Quantitative PCR (real-time PCR)
**RAPD**	Random amplified polymorphic DNA
**RFLP**	Restriction fragment length polymorphism
**RI**	Refractive index
**RNA**	Ribonucleic acid
**RNases**	Ribonucleases
**RNASS**	RNA stabilization solution
**RT-PCR**	Reverse transcription PCR
**TFA**	Trifluoroacetic acid

1. Thu thập muỗi cát

Muỗi cát trưởng thành có thể được thu thập ở trạng thái sống hoặc chết bằng nhiều phương pháp khác nhau như bẫy đèn CDC cỡ nhỏ, bẫy dính, và máy hút côn trùng sử dụng bẫy Shannon, hoặc thu trực tiếp từ nơi cư trú trong môi trường (ví dụ: chuồng trại động vật). Các phương pháp này bao gồm việc đặt bẫy tại các sinh cảnh phù hợp, thu hút ruồi cát bằng ánh sáng hoặc các chất dẫn dụ khác (CO_2 hoặc chất dẫn dụ hóa học), và thu mẫu để phân tích tiếp theo, như đã mô tả trong nhiều công bố [2, 3, 32, 36, 49].

Việc bắt muỗi cát còn sống cho phép thực hiện tất cả các ứng dụng phân tích tiếp theo, trong khi thu thập cá thể đã chết sẽ không thể phân lập chủng *Leishmania* hoặc vi rút. Một số kỹ thuật bắt mẫu, như giấy dính, thường xuyên dẫn đến mất một số bộ phận của muỗi cát (râu, xúc biện, cánh hoặc chân). Ngoài ra, lớp dầu thầu dầu phủ trên giấy dính bám vào cơ thể ruồi cát và phải được loại bỏ ở giai đoạn đầu

xử lý, thường bằng cách ngâm 15 phút trong hỗn hợp ethanol và diethyl ether theo tỷ lệ bằng nhau.

2. Gây chết mẫu vật

Sau khi thu thập, muỗi cát còn sống phải được gây chết. Với một số phương pháp thu mẫu (ví dụ: giấy dính, bẫy đèn CDC có gắn lọ chứa chất tẩy rửa hoặc ethanol), muỗi cát đã chết ngay khi được thu. Các mẫu thu trực tiếp vào ethanol có thể được sử dụng cho sinh học phân tử, và các mẫu khác cũng có thể áp dụng nếu được bảo quản trong ethanol càng sớm càng tốt. Tuy nhiên, không phương pháp gây chết nào trong số này cho phép xử lý côn trùng bằng MALDI-ToF. Ngoài ra, một số phương pháp gây chết có thể làm mất một số đặc điểm hình thái. Do đó, việc sử dụng cách gây chết tiêu chuẩn phù hợp là điều thiết yếu nhằm đảm bảo định danh chính xác hoặc bảo quản lâu dài dưới dạng mẫu lưu trữ đối chứng (tức là các mẫu được bảo quản và lưu giữ để tham chiếu hoặc so sánh trong tương lai). Các hóa chất như ethyl acetate, ethyl ether, tetrachloroethane và chloroform có thể được thấm vào bông gòn và đặt trong vật chứa có muỗi cát để gây chết. Các chất gây chết này phải được xử lý cẩn thận theo khuyến cáo của nhà sản xuất do tính độc của chúng. Tuy nhiên, chúng tôi không khuyến nghị sử dụng chloroform để gây chết muỗi cát vì theo kinh nghiệm của chúng tôi, chất này kém tương thích với các nghiên cứu sinh học phân tử. Do tính chất nguy hiểm của tất cả các sản phẩm này và sự phù hợp còn nghi ngờ đối với các phân tích phân tử, việc sử dụng các hóa chất này nhìn chung không được khuyến khích.

Phương pháp được sử dụng rộng rãi nhất, bảo tồn được hình thái, DNA hoặc protein, là đông lạnh khô mẫu vật. Mẫu phải được làm lạnh đủ lâu để gây mê hoàn toàn, nhưng không quá lâu đến mức (i) bị khô, hoặc (ii) ảnh hưởng đến khả năng sống của *Leishmania* nếu mục tiêu là phân lập chúng in vitro từ ống tiêu hóa của muỗi cát. Do đó, chúng tôi khuyến nghị thời gian đông lạnh từ 15 đến 20 phút ở -20°C, theo dõi thường xuyên để đảm bảo chỉ làm bất động mà không tiêu diệt ký sinh trùng *Leishmania*.

Nếu không có tủ đông, côn trùng có thể được gây chết bằng CO_2. Trong điều kiện thực địa không thể sử dụng bình CO_2, mẫu có thể được gây chết bằng các bình CO_2 thương mại nhỏ dùng cho “Soda siphons” (dụng cụ pha đồ uống), tuy nhiên có thể bị hạn chế khi vận chuyển bằng đường hàng không. Trong trường hợp bất khả kháng, côn trùng có thể được gây chết bằng cách tiếp xúc với khói thuốc lá. Muỗi cát được bắt sống trong bẫy CDC, thu bằng máy hút côn trùng, giữ trong ống thủy tinh và cho tiếp xúc với khói thuốc lá, khiến chúng chết trong vài giây. Phương pháp này có thể áp dụng trong mọi điều kiện thực địa, kể cả trong điều kiện cách ly khó khăn. Tuy nhiên, do ống thủy tinh bị ám khói, không thể sử dụng lại để thu và xử lý muỗi cát sống nếu không được làm sạch kỹ lưỡng. Dù vậy, cùng một ống hút chưa được làm sạch vẫn có thể được sử dụng để gây chết ruồi cát từ các bẫy khác nhằm mục đích cố định mẫu. Cũng cần kiểm tra để đảm bảo tất cả mẫu đã được lấy ra khỏi ống hút. Các phương pháp này tương thích với việc phân lập *Leishmania* thông qua giải phẫu ruột.

3. Bảo quản mẫu vật trước khi xử lý

Có 5 phương pháp cố định chính trước khi xử lý mẫu:

3.1. Làm lạnh

Phương pháp này được thực hiện tốt nhất ở -20°C hoặc, ưu tiên hơn, ở -80°C. Hiện nay, các phương pháp bảo quản bằng tủ đông được sử dụng rộng rãi hơn so với bảo quản bằng nitơ lỏng. Trong mọi trường hợp, bảo quản lạnh sâu phải được tiến hành càng sớm càng tốt sau khi làm bất động mẫu. Bảo quản lạnh trong tủ đông có ưu điểm là bảo tồn hoàn toàn côn trùng cũng như RNA, DNA và protein với tính toàn vẹn đầy đủ trong suốt thời gian lưu trữ. Ngược lại, nitơ lỏng có thể gây tổn thương nghiêm trọng cho cánh, chân, xúc biện và râu, thường làm đứt rời các bộ phận này và đôi khi làm mất các đặc điểm hình thái quan trọng. Bảo quản trong tủ đông khô ít gây chấn thương hơn cho mẫu, nhưng không lý tưởng cho việc bảo tồn các cơ quan mỏng manh. Đáng lưu ý, tại thời điểm rã đông, cánh, râu, xúc biện hoặc chân có thể dính vào thành ống và cuối cùng bị rách do ngưng tụ. Tuy nhiên, bảo quản bằng đông lạnh không phải lúc nào cũng khả thi trong các nghiên cứu thực địa vì cần có tủ đông hoặc bình chứa nitơ lỏng. Bảo quản trong tủ đông hoàn toàn tương thích với phát hiện tác nhân gây bệnh bằng các công cụ phân tử mà không làm giảm độ nhạy, mặc dù việc phát hiện và phân lập vi rút RNA đòi hỏi phải đông lạnh ở -80°C hoặc trong nitơ lỏng nếu cần bảo quản lâu dài. Tuy nhiên, đông lạnh mẫu không cho phép phân lập *Leishmania* thông qua giải phẫu ruột, trừ khi muỗi cát trước tiên được đặt trong pha hơi và sau đó trong nitơ lỏng (ví dụ trong các ống đặt trong tất lưới), mô phỏng bảo quản lạnh sâu *Leishmania*.

3.2. Bảo quản trong cồn

Đây có lẽ là phương pháp được sử dụng rộng rãi nhất để bảo quản muỗi cát. Phương pháp này dễ thực hiện trong điều kiện thực địa, ngay cả khi không có phòng thí nghiệm. Bảo quản trong cồn đặc biệt phù hợp cho các nghiên cứu hình thái, vì các cơ quan mỏng manh (cánh, chân, râu hoặc xúc biện) vẫn được giữ nguyên vẹn nếu không có bọt khí trong ống bảo quản. Do đó, chúng tôi khuyến nghị bịt kín ống bằng một nút bông nhỏ để loại bỏ bọt khí và đặt nhãn lên trên nút bông (Hình 1). Nồng độ cồn thích hợp vẫn còn là vấn đề được tranh luận. Nói chung, không khuyến nghị sử dụng nồng độ dưới 70% [45, 66]. Nồng độ cao hơn bảo tồn DNA hiệu quả hơn và trong thời gian dài hơn nhưng làm

mẫu vật trở nên giòn và dễ vỡ hơn đối với nghiên cứu hình thái. Việc sử dụng cồn 96% (hỗn hợp azeotrope) đảm bảo độ ổn định nồng độ theo thời gian, đặc biệt ở các khu vực ẩm như các quốc gia nhiệt đới, mặc dù cồn 95% thường dễ kiếm hơn. Bất kể nồng độ nào, DNA nhìn chung được bảo tồn tốt trong cồn (mặc dù kém hiệu quả hơn so với phương pháp đông lạnh, đặc biệt đối với các phương pháp phân tử kiểu NGS). Protein kém ổn định hơn nhiều, đặc biệt đối với protein học như các ứng dụng MALDI-ToF. Muỗi cát được bảo quản trong cồn trong vài tháng vẫn có thể được định danh hình thái, nhưng không thể tạo phổ protein tham chiếu từ các mẫu này. Bảo quản trong cồn hoặc điều kiện khô có thể được cải thiện nếu mẫu đồng thời được đông lạnh ở -20°C. Đông lạnh ở -20°C chủ yếu cải thiện bảo tồn phân tử (ví dụ: acid nucleic) bằng cách làm chậm quá trình phân hủy và đồng thời mang lại lợi ích thứ cấp cho bảo tồn hình thái bằng cách giảm phân rã mô theo thời gian, mặc dù tác động lên hình thái hạn chế hơn so với tính toàn vẹn phân tử. Bảo quản trong cồn cũng có thể áp dụng cho phát hiện DNA và vi rút RNA khi sử dụng cồn ethanol với nồng độ ít nhất 70% trong thời gian bảo quản ngắn, dưới vài tháng. Ngoài ra, cồn isopropyl có thể sẵn có ở một số quốc gia và bảo tồn DNA, nhưng làm mẫu trở nên cứng. Chất này không dễ cháy như ethanol và do đó có thể dễ dàng vận chuyển. Nếu cần thiết, muỗi cát được bảo quản trong nitơ lỏng hoặc đông khô có thể được chuyển sang cồn, qua đó kết hợp các nhược điểm của cả hai phương pháp.

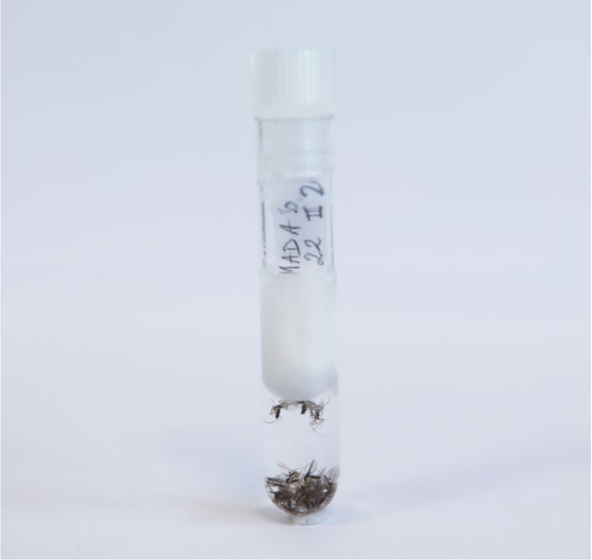

Hình 1. Muỗi cát bảo quản trong cồn

3.3. Bảo quản trong dung dịch bảo vệ RNA

Dung dịch này được sử dụng rộng rãi, không độc và được thiết kế để ổn định và bảo vệ RNA trong mô tươi và mẫu tế bào. Dung dịch hoạt động bằng cách nhanh chóng thấm vào mẫu và bất hoạt RNase (enzyme phân hủy RNA), qua đó ngăn chặn sự thoái hóa RNA mà không cần đông lạnh ngay lập tức. Bảo quản trong RNASS nhìn chung có hiệu quả trong việc duy trì hình thái tổng thể của mô và tế bào cho các đánh giá mô học tiếp theo. Mặc dù RNASS được tối ưu hóa cho ổn định RNA hơn là cố định mô, bảo quản ngắn đến trung hạn thường vẫn duy trì tốt tính toàn vẹn cấu trúc. RNASS cho phép bảo quản mẫu ở nhiệt độ phòng đến 7 ngày, ở 4°C trong vài tuần, hoặc ở -20°C/-80°C để bảo quản lâu dài. Phương pháp này đặc biệt có giá trị trong nghiên cứu thực địa hoặc môi trường lâm sàng nơi cơ sở hạ tầng chuỗi lạnh bị hạn chế. Tách chiết RNA thường yêu cầu lấy mẫu ra khỏi dung dịch và xử lý theo các quy trình chuẩn.

3.4. Bảo quản khô ở nhiệt độ phòng

Đây là một phương pháp cũ, khi áp dụng cho mẫu nguyên vẹn (gắn toàn bộ), có nhược điểm lớn là bảo tồn kém các cơ quan mỏng manh như cánh, chân, râu và xúc biện. Tuy nhiên, các nghiên cứu protein học sử dụng MALDI-ToF vẫn khả thi nếu quá trình khử nước được thực hiện khi cố định bằng chất hút ẩm loại silica gel. Ngược lại, các phân tích phân tử nhắm vào DNA vẫn khó thực hiện trên các mẫu này vì DNA thường bị phân mảnh và có hàm lượng thấp, khiến việc phân tích khó khăn hơn so với mẫu tươi hoặc đông lạnh, đặc biệt đối với bộ gen nhân. Tuy nhiên, các kỹ thuật gần đây như museomics có thể được áp dụng cho loại mẫu này [34]. Do đó, phương pháp bảo quản này không được khuyến nghị, trừ khi không có lựa chọn thay thế. Có thể kết hợp với bảo quản lạnh bằng cách đặt các ống trong tủ đông ở -20°C hoặc -80°C. Thách thức chính là đạt được việc gắn tiêu bản thích hợp của mẫu hoặc các phần cơ thể cần thiết cho định danh. Để thực hiện điều này, tái ngậm nước là bước thiết yếu. Chúng tôi khuyến nghị sử dụng dung dịch Triton X-100. Thời gian tái ngậm nước thay đổi từ vài giờ đến vài ngày, cần theo dõi sát thường xuyên. Sau khi tái ngậm nước hoàn toàn, mẫu nên được rửa qua ba bể nước liên tiếp.

3.5. Bản quản trong giấy lọc

Ưu điểm chính của giấy lọc là tính ổn định lâu dài của DNA hệ gen trong các tế bào của cơ thể nguyên vẹn chưa cố định, được làm khô và bảo quản ở nhiệt độ phòng. Giấy lọc được cung cấp ở dạng thẻ nhỏ, cho phép lưu trữ vài trăm mẫu ở nhiệt độ phòng trong một thể tích tương đương một hộp nhỏ. Ma trận giấy lọc được tẩm các chất làm biến tính các tác nhân gây nhiễm, do đó mẫu không còn được xem là nguy cơ sinh học. Điều này cho phép lưu trữ và vận chuyển mẫu mà không cần các biện pháp phòng ngừa nguy cơ sinh học đặc biệt [68].

4. Giải phẫu tiêu bản

Không giống nhiều côn trùng khác, được định danh dựa trên các đặc điểm bên ngoài có thể quan sát trên cá thể ghim nguyên vẹn, muỗi cát yêu cầu giải phẫu và gắn lam kính để nghiên cứu các đặc điểm giải phẫu nhằm định danh loài chính xác. Bất kể quy trình chuẩn bị và gắn tiêu bản được lựa chọn, cùng một kỹ thuật giải phẫu được áp dụng (Hình 2 & 3) (https://zenodo.org/records/18198006).

Sử dụng Triton X100: dung dịch nước không ion

Lưu ý rằng việc gắn tiêu bản áp dụng cho các mẫu mới bắt hoặc đã được bảo quản thích hợp. Phần lớn người thu mẫu có côn trùng được bảo quản khô (để sử dụng MALDI-ToF) hoặc lưu trữ trong cồn nhiều năm. Thật không may, bảo quản trong cồn không tối ưu trong thời gian nhiều năm, và các động vật chân đốt được bảo quản theo cách này trở nên rất khó chuẩn bị cho quan sát hiển vi. Một sự cố thường xảy ra là sự phân hủy của ống nhựa chứa mẫu, sau đó là cồn bay hơi. Trong cả hai trường hợp, chúng tôi không có lựa chọn nào vì mẫu đã ở trong cồn quá lâu hoặc bị khô. Do đó, ý tưởng sử dụng các chất làm ướt không phải chất tẩy rửa mạnh đã được đề xuất. Triton X100 tồn tại dưới dạng dung dịch nước không ion (4-(1,1,3,3-tetramethylbutyl) phenyl-polyethylene glycol solution, hay t-octylphenoxypolyethoxyethanol, polyethylene glycol tert-octylphenyl ether), được sử dụng rộng rãi như một chất tẩy rửa trong sinh học tế bào và sinh học phân tử. Chất này cho phép làm tăng tính thấm của màng tế bào và màng nhân.

Dưới đây là quy trình sử dụng Triton X100 không ion trong dung dịch nước 0,5%:

Tẩm mẫu khô bằng cồn tuyệt đối.

Thêm thể tích dung dịch Triton X100 0,5% cần thiết sao cho toàn bộ mẫu được ngập hoàn toàn.

Để quá trình diễn ra trong khoảng 5 phút đến vài ngày, theo dõi thường xuyên. Tất cả động vật chân đốt phải được tách rời hoàn toàn trong dung dịch.

Loại bỏ dung dịch Triton X100 và thay bằng dung dịch kali hydroxit (KOH).

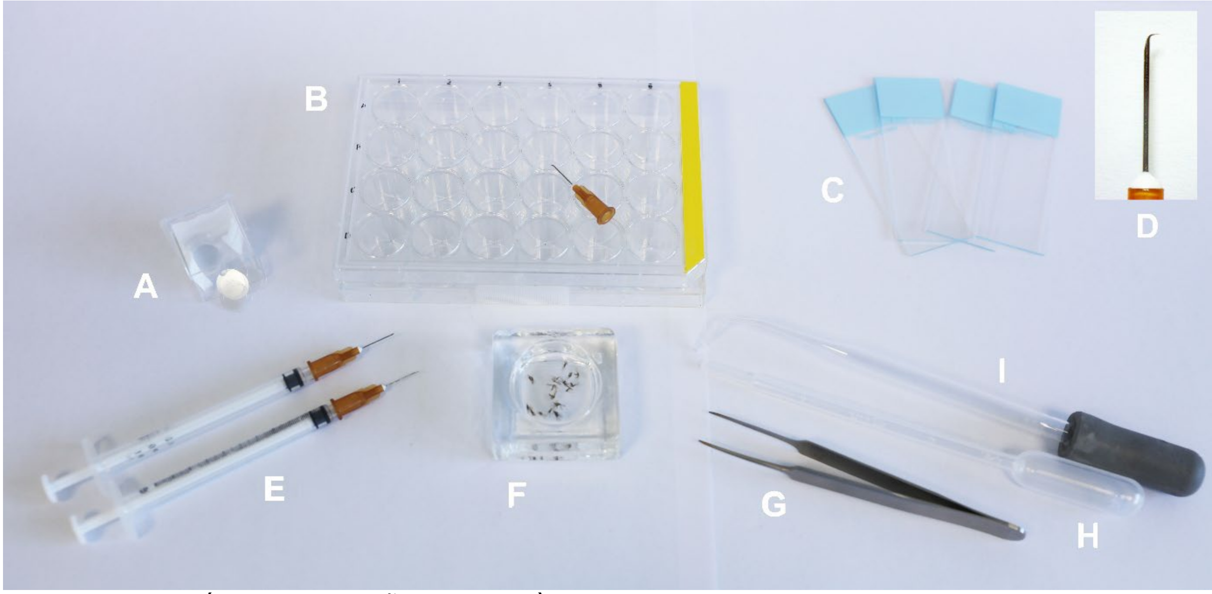

Hình 2. Vật liệu cần thiết để gắn tiêu bản muỗi cát

A: Lamen kính tròn (đường kính 10 hoặc 12 mm).

B: Đĩa 24 giếng và kim móc (nếu sử dụng tinh dầu đinh hương hoặc dung dịch Euparal® để xử lý ruồi cát, không sử dụng đĩa acrylic vì sẽ xảy ra phản ứng hóa học làm hỏng mẫu).

C: Lam kính phù hợp để ghi nhãn.

D: Chi tiết đầu kim móc.

E: Kim gắn vào bơm tiêm.

F: Đĩa đồng hồ hoặc dụng cụ tương đương chứa ruồi cát cần gắn.

G: Nhíp Dumont.

H: Pipette nhựa.

I: Pipette thủy tinh được uốn cong bằng nhiệt để thuận tiện cho việc chuyển dung dịch vào các giếng.

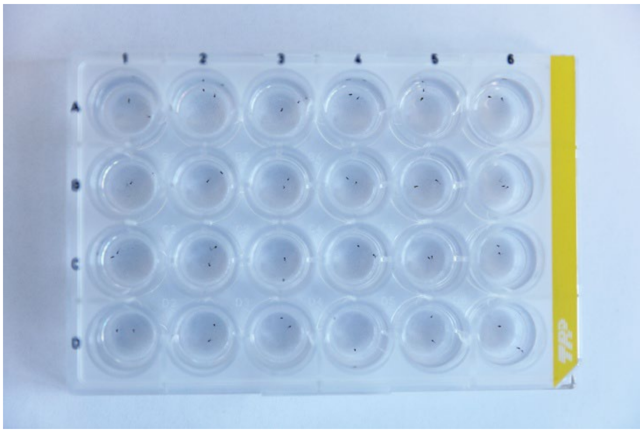

Hình 3: Đĩa 24 giếng, mỗi giếng chứa đầu và phần chót bụng của muỗi cát.

4.1. Đầu

Giải phẫu có thể được thực hiện bằng kim mảnh hoặc kim côn trùng học dưới kính hiển vi soi nổi (Hình 2 & 3). Các loại kim thường được sử dụng bao gồm: 26G x 1/2" (0,45 × 13 mm), 30G × 1/2" (0,3 × 13 mm), hoặc 25G × 5/8" (0,5 × 16 mm). Để chuẩn bị mẫu cho định danh, tối thiểu cần tách đầu khỏi thân và gắn ở tư thế mặt bụng hướng lên để hiển thị khoang miệng (cibarium) và hầu, trong khi ngực và bụng được gắn ở tư thế bên sau khi giải phẫu. Gắn đầu ở tư thế bụng–lưng đảm bảo lỗ chẩm hướng lên trên, nhờ đó khoang miệng có thể được quan sát trực tiếp. Việc tiếp cận các đặc điểm giải phẫu này trở nên dễ dàng hơn nếu đầu được tách hoàn toàn.

4.2. Cánh và ngực

Cánh phải được gắn phẳng. Mỗi cánh có thể được tách tại gốc và gắn độc lập, hoặc chỉ gắn một cánh trong khi để cánh còn lại gắn với ngực. Nếu dự kiến thực hiện phân tích hình thái hình học cánh, cần xác định và ghi nhãn chính xác cánh phải và cánh trái trước khi gắn. Ngực được chia thành nhiều phần, mỗi phần chứa thông tin phân loại học rất quan trọng [20, 64]. Thông thường, ngực được gắn ở tư thế bên để cho phép quan sát sự phân bố lông và phân bố màu sắc. Sự hiện diện của sẹo lông ở một số vùng của ngực có thể được sử dụng để phân biệt một số loài thuộc giống *Brumptomyia*. Phân bố màu sắc có thể được sử dụng để phân tách muỗi cát vùng Tân nhiệt đới ở mức giống (ví dụ: *Bichromomyia*), chuỗi loài (ví dụ: *Pintomyia*), hoặc thậm chí các loài trong cùng một giống (ví dụ: *Micropygomyia, Nyssomyia, Psathyromyia* và *Psychodopygus*) [20]. Do đó, nếu ngực không được sử dụng cho phân tích phân tử, cần gắn tiêu bản sao cho không làm hỏng cấu trúc này. Quan trọng là cần lưu ý rằng điều quan trọng không phải là cường độ màu sắc, mà là sự phân bố của chúng trên ngực. Vì vậy, quá trình làm trong sẽ không loại bỏ sắc tố hoặc kiểu phân bố của chúng.

4.3. Cơ quan sinh dục

Cần đặc biệt cẩn trọng khi gắn cơ quan sinh dục ở cả con đực và con cái, vì đây là các cấu trúc then chốt để định danh giống, phân giống và loài. Ở cả hai giới, cơ quan sinh dục là cấu trúc đôi.

4.3.1. Cơ quan sinh dục đực

Cơ quan sinh dục là cấu trúc ngoài và gồm các kìm đôi, mỗi kìm bao gồm khớp gonocoxite–gonostyle ở phần lưng và thùy epandrium ở phần bụng. Gonostyle mang các gai và đôi khi có lông, cần phải đếm được và vị trí bám của chúng phải quan sát rõ ràng. Cần quan sát kỹ bề mặt trong của gonocoxite, có thể mang chùm lông không cuống hoặc lông gắn trên một thùy (= củ) [22]. Các đồng nghiệp ít kinh nghiệm trong giải phẫu có thể thực hiện gắn bên đơn giản mà không tách cơ quan sinh dục khỏi cuối bụng (https://zenodo.org/records/18311158). Trong trường hợp này, sự chồng lấp của hai phần cơ quan sinh dục có thể gây khó khăn khi đếm các lông bên trong của gonocoxite, nhưng tránh được nguy cơ làm hỏng cơ quan sinh dục do giải phẫu không thành công. Các đồng nghiệp có kinh nghiệm hơn có thể tách đôi cơ quan sinh dục. Để thực hiện, cần đưa cạnh vát của kim (loại kim phản ứng trong da) xuyên qua, tách mà không cắt hoàn toàn cơ quan sinh dục để phân tách các phức hợp gonocoxite–gonostyle (https://zenodo.org/records/18311158). Bằng cách này, việc quan sát các mặt trong sẽ trở nên dễ dàng. Cách bố trí này cũng tạo điều kiện quan sát paramer và bao paramer, vốn không còn chồng lấp. Đối với gắn bên, vốn làm tăng sự chồng lấp cơ quan, mẫu phải được làm trong hoàn toàn.

4.3.2. Cơ quan sinh dục cái

Bộ máy sinh dục là cấu trúc trong, bao gồm các túi chứa tinh. Khi không giải phẫu, chúng phải được quan sát xuyên qua lớp biểu bì ngoài và bụng được gắn ở tư thế mặt bụng hướng lên. Bất kể môi trường gắn được lựa chọn, bản thân túi chứa tinh nhìn chung có thể được quan sát chính xác, đặc biệt nếu chúng không nhẵn và đã được làm trong. Tuy nhiên, việc quan sát các túi chứa tinh nhẵn, thành mỏng có thể gặp khó khăn trong các môi trường có tính khúc xạ kém. Hơn nữa, việc quan sát phần gốc của túi chứa tinh là quan trọng để định danh loài, chẳng hạn trong phân giống *Larroussius*

This is an Open Access article distributed under the terms of the Creative Commons Attribution License (https://creativecommons.org/licenses/by/4.0), which permits unrestricted use, distribution, and reproduction in any medium, provided the original work is properly cited.

[35, 37, 38], là các véc tơ chính của *Leishmania infan*tum tại Cựu Thế giới. Nếu không quan sát được cấu trúc này, việc định danh mẫu là không thể. Để khắc phục các khó khăn này, nên tách phức hợp càng sinh dục–túi chứa tinh khỏi bụng để gắn riêng (https://zenodo.org/records/18311106). Túi chứa tinh thường khó quan sát trong quá trình giải phẫu, nhưng càng sinh dục tương đối dễ xác định. Vì các ống túi chứa tinh đổ vào càng sinh dục, việc tách càng này thường cho phép tách luôn túi chứa tinh. Nếu túi chứa tinh vô tình bị cắt trong quá trình thao tác, chúng không bị mất và vẫn có thể quan sát trong lớp biểu bì bụng (Hình 4).

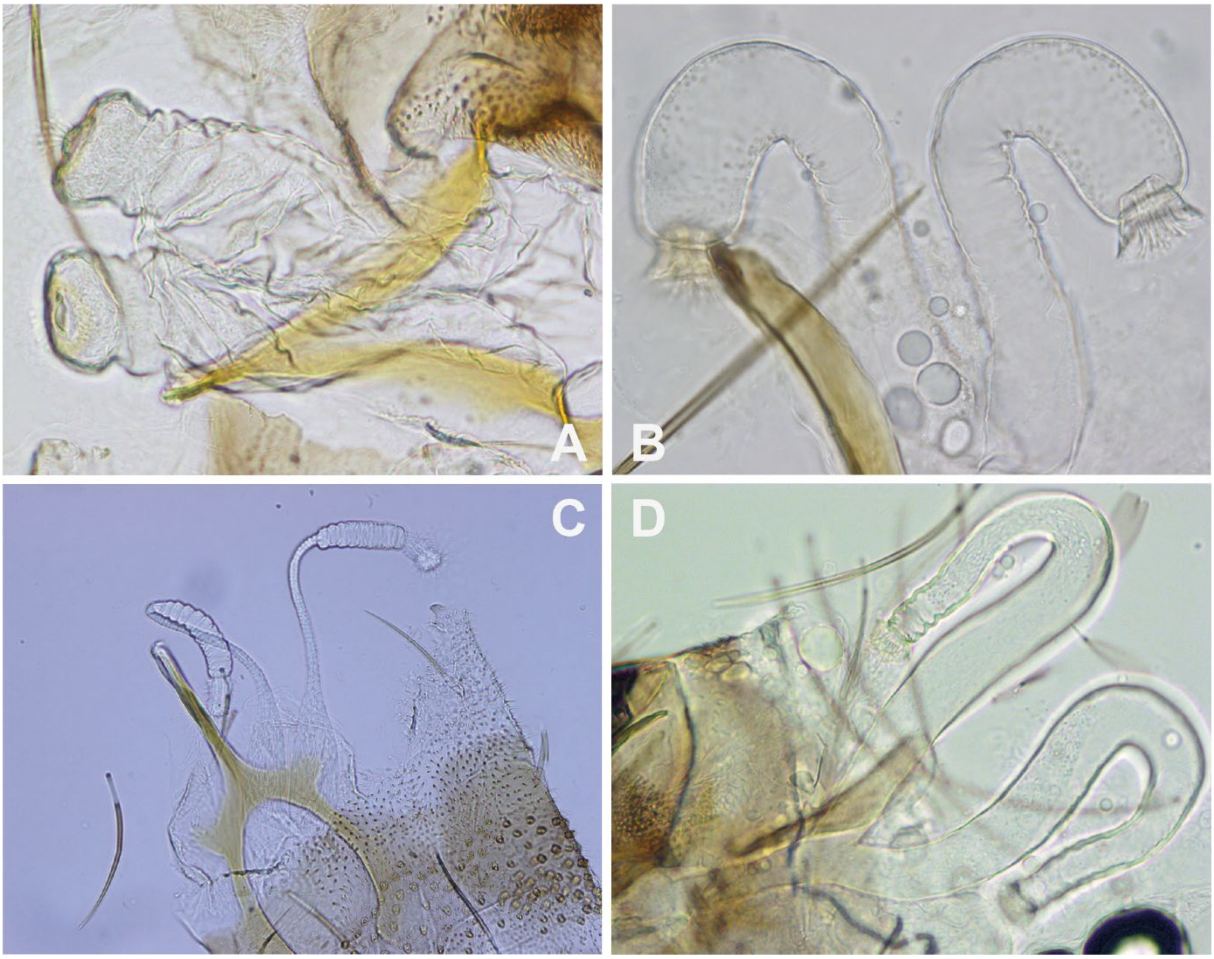

Hình 4. Túi chứa tinh được giải phẫu và gắn trong dịch Marc-André từ mẫu tươi A
A: *Idiophlebotomus longiforceps* (Lào).
B: *Sergentomyia minuta* (Pháp).
C: *Phlebotomus ariasi* (Pháp).
D: *Sergentomyia anodontis* (Lào).

4.4. Giải phẫu ruột giữa để phân lập *Leishmania*

Giải phẫu ống tiêu hóa là bước thiết yếu để phát hiện và phân lập *Leishmania* ở muỗi cát cái. Quy trình này có thể được thực hiện cả tại thực địa và trong phòng thí nghiệm nhằm đánh giá năng lực truyền bệnh (vectorial competence).

Khuyến nghị làm việc trên các cá thể cái vừa được gây chết. Rửa cá thể cái bằng nước hoặc dung dịch muối sinh lý có chứa chất tẩy rửa nhẹ để loại bỏ lông dư thừa. Bước này giúp duy trì điều kiện vô trùng cho việc phân lập *Leishmania*, đồng thời bảo tồn các đặc điểm hình thái cần thiết cho định danh. Để tìm và phân lập *Leishmani*a, cẩn thận lấy ruột giữa và đặt vào một giọt dung dịch muối sinh lý vô trùng (NaCl 0,9%). Sau khi quan sát ký sinh trùng di động dưới kính hiển vi quang học (độ phóng đại khuyến nghị: ~200×), sử dụng bơm kim tiêm insulin hoặc

micropipette để chuyển chúng vào môi trường nuôi cấy (xem thêm chi tiết tại Chương 4.4.3)

Gắn đầu và cơ quan sinh dục trực tiếp trong dịch Marc-André để làm trong. Quan trọng: tuyệt đối không để dịch Marc-André tiếp xúc với *Leishmania* – dù trực tiếp hay gián tiếp qua dụng cụ hoặc kim – vì dịch này gây chết ký sinh trùng.

Giải phẫu muỗi cát cái có thể được thực hiện trên một lam kính hoặc hai lam kính; cả hai phương án đều có ưu điểm và hạn chế (Hình 5; https://zenodo.org/records/18311154).

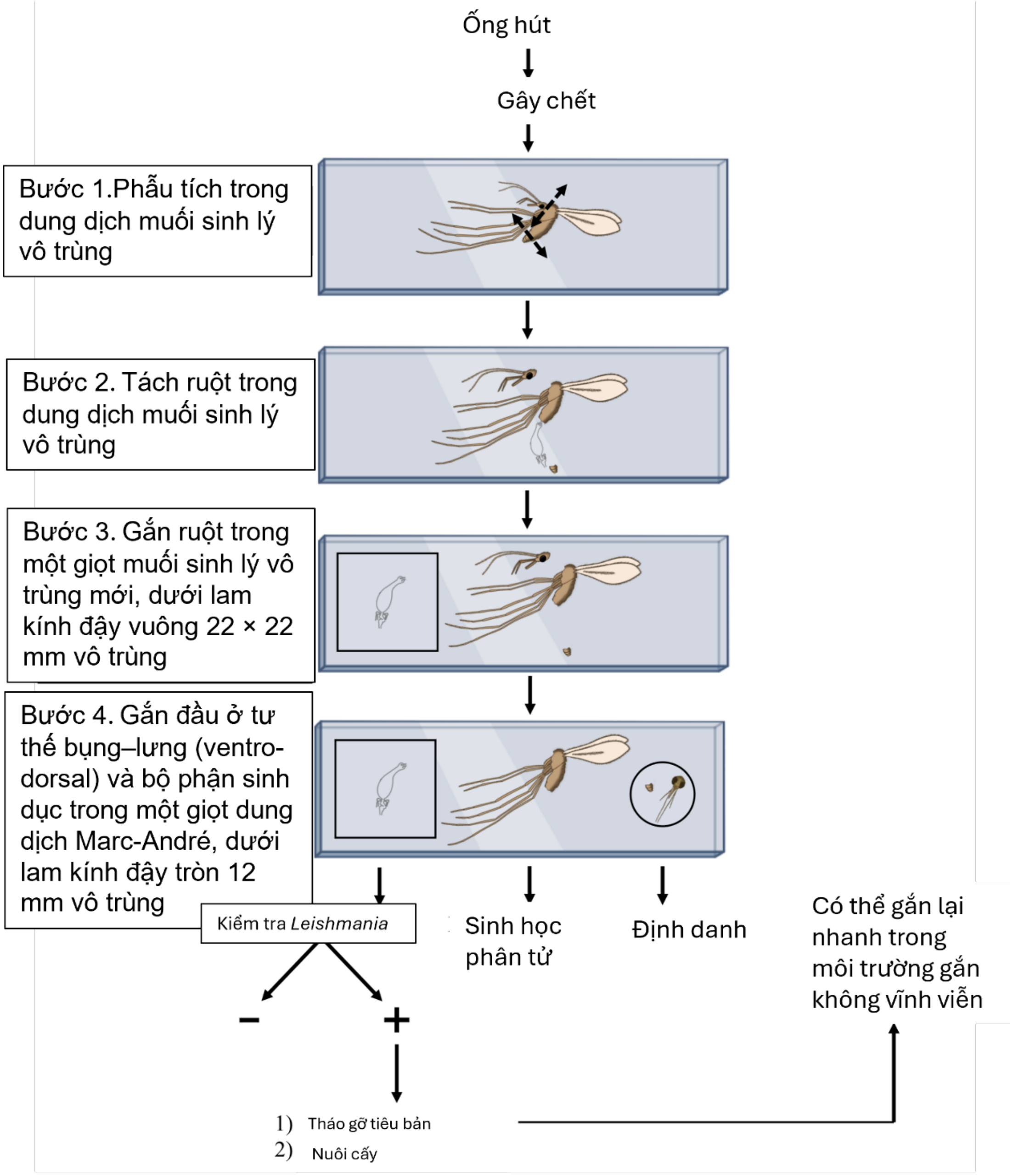

Hình 5: Phương pháp phân lập *Leishmania*.

4.4.1. Phương pháp hai lam kính

Phương án thứ nhất là làm việc trên hai lam kính riêng biệt: một lam chứa dung dịch muối sinh lý vô trùng để lấy ruột giữa, và lam còn lại để gắn đầu và túi chứa tinh trong dịch Marc-André. Tuy nhiên, trong điều kiện thực địa, thường có hai hoặc ba người giải phẫu ruồi cát và chuyển các mẫu giải phẫu của họ cho một nhà nghiên cứu duy nhất chịu trách nhiệm định danh loài và đánh giá nhiễm *Leishmania* trong ruột. Việc quản lý hai lam kính có thể gây ra vấn đề về truy xuất mẫu và đặc biệt gây khó khăn trong việc xác định chính xác cá thể ruồi cát nào bị nhiễm khi phát hiện một ruột dương tính (https://zenodo.org/records/18311154).

4.4.2. Phương pháp một lam kính

Sử dụng một lam kính duy nhất đảm bảo khả năng truy xuất kết quả. Tuy nhiên, cần thực hiện một số biện pháp phòng ngừa. Để tối đa hóa tính vô trùng trong bước này, người thao tác phải thường xuyên làm sạch tay bằng gel hydroalcoholic. Cần sử dụng lam kính không mài mờ và lamen kính vuông (22 x 22 mm) được bọc trong giấy nhôm và tiệt trùng bằng nhiệt khô (sử dụng tủ Poupinel), cùng với kim vô trùng cho mỗi lần giải phẫu (gợi ý: 25G Ø 0,5mm × 16mm). Muỗi cát được đặt trong một giọt dung dịch muối sinh lý vô trùng ở giữa lam kính. Đầu được cắt rời đồng thời rạch một đường giữa đốt bụng thứ 6 và thứ 7 (tergite và sternite) mà không cắt ống tiêu hóa (có thể cắt cao hơn nếu dự kiến có túi chứa tinh rất dài). Sau đó, cần cố định ngực bằng một kim, và nhẹ nhàng kéo các đốt bụng sau cùng bằng kim còn lại để lấy ruột. Nếu không thành công, có thể cố định phần cuối bụng bằng một kim và kéo ống tiêu hóa từ phía trước. Nếu vẫn không thành công, cần lấy ruột bằng cách loại bỏ càng nhiều lớp biểu bì còn lại xung quanh càng tốt. Khi ruột được lấy ra, các đốt bụng cuối phải được tách bằng cách cắt ống tiêu hóa. Ruột sau đó được đặt vào một giọt dung dịch muối sinh lý vô trùng mới ở một bên lam kính, rồi nhẹ nhàng đậy bằng lamen kính vô trùng. Đầu và các đốt bụng cuối được chuyển sang một giọt nhỏ dịch Marc-André đặt ở đầu còn lại của lam kính, đảm bảo không tiếp xúc với *Leishmania*. Đầu được định hướng đúng (lỗ chẩm hướng lên trên), và túi chứa tinh được tách cùng với càng sinh dục như đã nêu ở trên và được đậy bằng một lamen kính tròn nhỏ (Ø 12 mm, không nhầm với lá kính vuông vô trùng). Phần xác còn lại của muỗi cát và cánh nằm trong giọt dung dịch muối sinh lý ở giữa lam kính (https://zenodo.org/records/18311154). Trong trường hợp dương tính, hoặc phục vụ nghiên cứu phân loại học, ngực và bụng có thể được bảo quản cho các nghiên cứu phân tử hoặc proteomics, và cánh có thể được gắn trong môi trường nước. Để bảo quản tiêu bản, thể tích dư của dịch Marc-André có thể được thay thế bằng môi trường gắn nước như keo chloral (=Hoyer) hoặc môi trường dựa trên polyvinyl alcohol.

Các video chi tiết minh họa các quy trình này có sẵn (giải phẫu ruột giữa ruồi cát: https://zenodo.org/records/18303014 và giải phẫu tuyến nước bọt ruồi cát: https://zenodo.org/records/18302850), vì vậy sẽ không được trình bày tại đây.

4.4.3. Phân lập và nuôi cấy ký sinh trùng *Leishmania* từ ruột muỗi cát

Phân lập ký sinh trùng từ giải phẫu muỗi cát cái nhiễm là một quy trình tinh vi, đòi hỏi kỹ năng cao và ban đầu nên được thực hành trên các mẫu không nhiễm ký sinh trùng. Sau khi giải phẫu, ruột được chuyển sang một giọt dung dịch muối sinh lý vô trùng mới (0,9%) hoặc dung dịch Locke để rửa [4]. Ruột đã giải phẫu sau đó có thể được xử lý theo hai cách: i) quan sát dưới kính hiển vi quang học để nhận diện các giai đoạn khác nhau của promastigote *Leishmania* và vị trí của chúng, đặc biệt chú ý đến van stomodeal, và ii) mở ruột để tạo điều kiện cho promastigote thoát ra nhằm nuôi cấy hàng loạt [4]. Việc tìm thấy muỗi cát nhiễm có khả năng lây truyền trong thực địa là tương đối hiếm, do đó các buổi thực hành tốt sẽ tối đa hóa cơ hội phân lập thành công.

Nếu quan sát thấy ký sinh trùng *Leishmania* trong ruột, cần sử dụng kim vô trùng mới và thêm một lượng nhỏ dung dịch muối sinh lý vô trùng xung quanh lamen kính bằng hiện tượng mao dẫn để giải phóng chúng. Ruột cần được xé cẩn thận và nhanh chóng để giải phóng ký sinh trùng vào dung dịch muối sinh lý. Sử dụng micropipette 100 µL hoặc bơm tiêm tuberculin, thu ký sinh trùng và cấy vào môi trường nuôi cấy được ghi nhãn đúng cách.

Nuôi cấy in vitro promastigote *Leishmania*: các ký sinh trùng phân lập ban đầu được duy trì trên thạch máu SNB-9 hoặc môi trường rắn Novy, Mc Neal, Nicolle (NNN) [16] được phủ bằng môi trường alpha-MEM vô trùng [16, 65] hoặc môi trường M199, mỗi loại được bổ sung 10% huyết thanh bê thai vô trùng đã bất hoạt nhiệt [FCS] (nhằm tăng sinh trưởng ký sinh trùng), 1% vitamin BME, 2% nước tiểu người vô trùng (được tiệt trùng bằng màng lọc bơm tiêm Filtropur® S 0,2 µm), 250 µg/mL amikacin (hoặc 50 µg/mL gentamicin, hoặc hỗn hợp kháng sinh và axit amin (L-glutamine 200 mM–penicillin 10 000 U–streptomycin 10 mg/mL) [47]. Sau ba ngày, nếu không có nhiễm tạp, các dòng nuôi cấy được treo trong môi trường đông lạnh được chuẩn bị thích hợp và sau đó bảo quản ở -80°C trong 1 đến 2 năm hoặc trong nitơ lỏng ở -196°C để bảo tồn lâu dài và sử dụng cho các thí nghiệm sau này [7].

4.5. Tuyến nước bọt

Giải phẫu tuyến nước bọt của muỗi cát là một kỹ thuật cơ bản để nghiên cứu tương tác véc tơ–tác nhân gây bệnh, đặc biệt để phát hiện các arbovirus như *Phlebovirus* (ví dụ: vi rút Toscana) [44, 75]. Do kích thước rất nhỏ của muỗi cát, quy trình này đòi hỏi độ chính xác cao dưới kính hiển vi soi nổi, sử dụng kẹp mảnh hoặc kim vi phẫu để tách tuyến

This is an Open Access article distributed under the terms of the Creative Commons Attribution License (https://creativecommons.org/licenses/by/4.0), which permits unrestricted use, distribution, and reproduction in any medium, provided the original work is properly cited.

nước bọt mỏng manh mà không gây vỡ hoặc nhiễm bẩn (https://zenodo.org/records/18302850) [51, 61]. Bảo tồn tính toàn vẹn của tuyến là yếu tố then chốt để đảm bảo các phân tích phân tử tiếp theo đáng tin cậy. Sau khi tách, các tuyến có thể được nghiền và kiểm tra bằng RT-PCR, qPCR hoặc các xét nghiệm miễn dịch để phát hiện RNA vi rút hoặc kháng nguyên [12]. Sự hiện diện của vi rút trong tuyến nước bọt, thay vì chỉ trong ruột hoặc khoang cơ thể (hemocoel), xác nhận rằng tác nhân gây bệnh đã hoàn thành thời kỳ ủ ngoại sinh và có khả năng truyền trong quá trình hút máu [71].

Quá trình giải phẫu đòi hỏi kỹ thuật cao do kích thước nhỏ của tuyến nước bọt muỗi cát, yêu cầu chuyên môn đáng kể để tránh suy giảm chất lượng mẫu [1, 51]. Ngoài ra, tải lượng vi rút có thể thấp, đòi hỏi các phương pháp phát hiện có độ nhạy cao như PCR lồng hoặc giải trình tự thông lượng cao [54]. Nguy cơ nhiễm chéo càng nhấn mạnh nhu cầu về kỹ thuật vô trùng nghiêm ngặt. Bên cạnh các thách thức kỹ thuật, các yếu tố sinh học cũng ảnh hưởng đến thành công phát hiện; năng lực truyền bệnh khác nhau giữa các loài muỗi cát, và tỷ lệ nhiễm biến động theo điều kiện sinh thái và mùa vụ [33, 61].

Phát hiện vi rút trong tuyến nước bọt cung cấp những hiểu biết quan trọng về nguy cơ truyền bệnh, cho phép triển khai các biện pháp giám sát và kiểm soát có mục tiêu [15]. Ví dụ, việc xác định vi rút Toscana ở muỗi cát tại các vùng lưu hành đã góp phần xây dựng quy trình chẩn đoán và khuyến cáo y tế công cộng [18]. Hơn nữa, nghiên cứu tương tác vi rút–nước bọt có thể mở ra các mục tiêu mới cho vắc xin hoặc liệu pháp ngăn chặn lây truyền [15, 18].

Tuyến nước bọt muỗi cát cũng có thể được sử dụng làm nguồn kháng nguyên để đo kháng thể của vật chủ chống lại nước bọt muỗi cát bằng các phương pháp miễn dịch học, ưu tiên ELISA. Phương pháp này cho phép đánh giá mức độ phơi nhiễm của vật chủ với vết đốt muỗi cát, qua đó hỗ trợ đánh giá hiệu quả của các biện pháp kiểm soát véc tơ [25] và nguy cơ truyền *Leishmania* [40].

4.6. Xác định nguồn máu

Các cá thể cái đã hút máu được tách ra từ các bẫy thu phải được giải phẫu bằng dụng cụ dùng một lần để ngăn ngừa nhiễm chéo. Bụng của chúng cần được quan sát dưới kính hiển vi soi nổi để đánh giá giai đoạn tiêu hóa máu. Khuyến nghị chỉ chọn những cá thể cái có bụng màu đỏ, đỏ nâu hoặc đỏ sẫm, không có dấu hiệu hình thành trứng. Cắt bỏ phần cuối bụng bao gồm cả túi chứa tinh để định danh hình thái học cá thể cái sau khi làm trong. Phần chính của bụng (không bao gồm túi chứa tinh) sau đó được đặt vào ống Eppendorf® và bảo quản ở -20°C cho đến khi phân tích tiếp theo. Các chỉ thị di truyền thường được sử dụng để xác định nguồn máu, như PNOC [5, 30, 50], CytB [67] hoặc COI [13], đã được thiết lập vững chắc và mô tả rộng rãi trong tài liệu; do đó, sẽ không được trình bày chi tiết thêm trong bài báo này (Hình 6). Ngoài ra, để xác định máu vật chủ, có thể triển khai lập bản đồ peptide MALDI-ToF [31]. Đã được chứng minh thực nghiệm rằng kỹ thuật này cho phép xác định máu vật chủ trong khoảng thời gian dài hơn sau khi hút máu; do đó, đây là phương pháp phù hợp, đặc biệt đối với phân tích các cá thể cái đã hút máu có mức độ tiêu hóa máu rõ rệt hơn. Mẫu lý tưởng nên được bảo quản ở -20°C hoặc 4°C, nhưng kết quả tốt cũng có thể đạt được từ các mẫu được lưu trữ ở nhiệt độ phòng trong thời gian ngắn. Bụng của cá thể cái đã hút máu nên được tách khỏi phần còn lại của cơ thể ngay trước khi phân tích và được nghiền trong nước cất. Phần còn lại của cơ thể muỗi cát vẫn sẵn có cho các phân tích phân tử và hình thái học khác. Sau khi lấy phần mẫu từ dịch nghiền cho phân tích bản đồ peptide MALDI-ToF, phần còn lại có thể được sử dụng để tách chiết DNA nhằm xác định nguồn máu vật chủ và/hoặc sàng lọc sự hiện diện của *Leishmania* sp. Tổng thời gian chuẩn bị và phân tích mẫu rất ngắn so với các kỹ thuật phân tử dựa trên DNA.

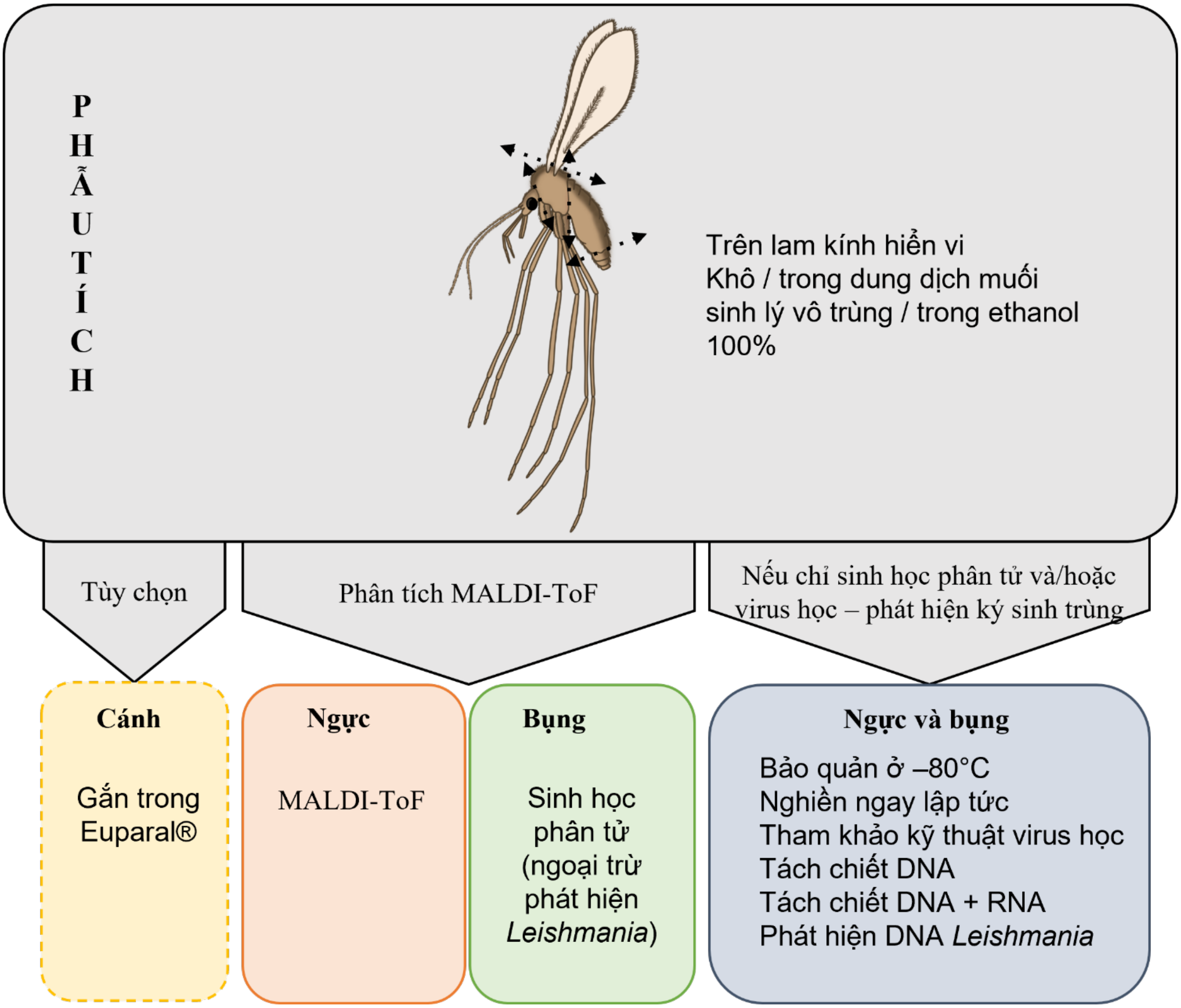

Hình 6: Xử lý muỗi cát cho các ứng dụng sinh học phân tử, proteomics và/hoặc vi rút học.

5. Xử lý mẫu vật cho nghiên cứu hình thái **(Hình 3, 6, 7 & 8; Phụ lục 1, 2, 3 & 4)**

Phần này trình bày các nguyên tắc chuẩn bị mẫu muỗi cát để gắn tiêu bản chỉ phục vụ nghiên cứu hình thái, sau đó là các điều chỉnh cho những ứng dụng ngoài phạm vi hình thái học. Tuy nhiên, việc hiểu rõ phương pháp này là rất quan trọng vì cho phép điều chỉnh quy trình phù hợp với từng loại mẫu cụ thể khi cần thiết.

Quy trình xử lý bao gồm các bước rút bỏ và bổ sung dung dịch liên tiếp bằng pipette Pasteur có gắn bóng cao su mềm. Khuyến nghị mạnh mẽ sử dụng lọ thủy tinh đáy tròn vì chúng tạo điều kiện thuận lợi đáng kể cho các thao tác này. Thủy tinh trơ với tất cả các thuốc thử. Để ngăn bay hơi thuốc thử, các lọ cần được đậy kín và không được đổ quá đầy nhằm tránh tràn khi đóng hoặc mở, đồng thời ngăn bụi rơi vào mẫu. Các hóa chất cần thiết cho quá trình làm trong và xử lý được trình bày trong Bảng 2.

This is an Open Access article distributed under the terms of the Creative Commons Attribution License (https://creativecommons.org/licenses/by/4.0), which permits unrestricted use, distribution, and reproduction in any medium, provided the original work is properly cited.

Bảng 2: Thành phần hóa chất sử dụng

**Potassium hydroxide 10%**<br>Potassium hydroxide 10 g<br>Nước cất *qs* 100 mL	**Fuchsin acid 1% trong nước cất**<br>Acid fuchsin (as a powder) 1 g<br>Nước cất 99 mL
**Môi trường keo gắn tiêu bản chloral (Hoyer medium)**<br>Nước cất 50 mL<br>Chloral hydrate 200 g<br>Gum Arabic 50 g<br>Glycerol 20 mL	**Marc-André solution colored with acid fuchsin**<br>Dung dịch Marc-André 10mL<br>Fuchsin 1% 50 µL
**Dung dịch Marc-André**<br>Chloral hydrate 40 g<br>Glacial acetic acid 30 mL<br>Nước cất 30 mL	**Enecê medium**<br>Pure white colophony 22 g<br>Alcohol-soluble copal gum 12 g<br>Absolute ethanol 20 mL<br>Camphor 10 g<br>Turpentine essence 10 mL<br>Eucalyptol 26 mL

5.1. Làm trong

Trước khi mẫu muỗi cát có thể được chuẩn bị thành tiêu bản vĩnh viễn trên lam kính, trước tiên chúng phải được làm trong bằng phương pháp tiêu hủy mô mềm sử dụng các chất thích hợp (ví dụ: dung dịch axit acetic 10% hoặc dung dịch Marc-André có chứa chloral hydrate – một hóa chất bị hạn chế tại nhiều quốc gia) nhằm làm mẫu trở nên trong suốt. Quá trình làm trong loại bỏ mô cơ thể, mỡ, dịch tiết và lớp sáp, làm mẫu bán trong, tạo điều kiện quan sát các cấu trúc ngoại xương (ví dụ: vị trí gắn lông), đặc điểm bề mặt (ví dụ: màu sắc) và các cấu trúc bên trong nhìn thấy qua biểu bì (ví dụ: túi chứa tinh).

Quy trình làm trong hai bước, bao gồm trước tiên sử dụng bazơ mạnh (như potassium hydroxide), sau đó là axit yếu (như axit acetic trong dung dịch Marc-André), phục vụ các mục đích sinh hóa khác nhau [74]. Bazơ phân giải mô mềm như protein, mỡ và cơ thông qua xà phòng hóa và biến tính protein, trong khi vẫn giữ nguyên bộ xương kitin để đảm bảo độ rõ cấu trúc. Axit yếu tiếp theo trung hòa kiềm còn sót lại, ngăn phân hủy tiếp diễn và tẩy nhạt kitin nhằm tăng độ trong suốt [74], mặc dù việc rửa mẫu hai lần trong nước cất mỗi lần 15 phút cũng có thể đủ để trung hòa bazơ. Xử lý tuần tự này kết hợp hiệu quả loại bỏ mô với bảo tồn nhẹ nhàng, đảm bảo tính toàn vẹn tối ưu cho quan sát hiển vi.

Khuyến nghị rửa hai lần, mỗi lần 20 phút trong nước cất trước khi chuyển sang bước tiếp theo.

5.1.1. Tiêu hủy mô mềm (Hình 8)

Sodium hydroxide (NaOH) hoặc potassium hydroxide (KOH) là các chất tiêu hủy mô hóa học thường được sử dụng, với nồng độ và thời gian xử lý thay đổi tùy theo kích thước và độ mỏng manh của mẫu. Kỹ thuật tiêu chuẩn và hiệu quả nhất là tiêu hủy mô mềm bằng cách ngâm muỗi cát trong bazơ mạnh (KOH hoặc NaOH 10%) qua đêm. Có thể tăng nồng độ để rút ngắn thời gian xử lý (ví dụ: KOH 20% trong 6 giờ), cũng như kết hợp gia nhiệt ở 37°C.

5.1.2. Làm sáng với nhuộm hoặc không nhuộm

Sau đó là bước làm sáng, thường kết hợp axit acetic và chloral hydrate (ví dụ: dung dịch Marc-André). Sau khi làm trong, mẫu phải được rửa kỹ trong ít nhất hai bể nước liên tiếp, mỗi bể 20 phút để loại bỏ hóa chất dư.

Dung dịch Marc-André là chất làm trong thường được sử dụng để chuẩn bị mẫu muỗi cát. Hiệu quả của nó nằm ở khả năng hỗ trợ quá trình làm trong đồng thời hạn chế gây tổn hại đáng kể đến các cấu trúc mỏng manh như cánh và râu.

Dung dịch cần được pha mới hoặc bảo quản trong lọ đậy kín để tránh bay hơi hoặc suy giảm chất lượng. Việc sử dụng dung dịch Marc-André đặc biệt có lợi khi kết hợp với kỹ thuật làm sáng hoặc nhuộm nhằm tăng cường chi tiết hình thái cụ thể. Thành phần và cách pha được trình bày chi tiết trong Phụ lục 2.

Đối với các mẫu có độ trong cao, có thể cần nhuộm trước khi gắn tiêu bản để tăng khả năng quan sát. Có nhiều loại thuốc nhuộm, mỗi loại tác động lên các thành phần hóa học khác nhau của cơ thể. Cần lựa chọn thuốc nhuộm tương thích với cả mẫu và môi trường gắn. Phương pháp cơ bản

này có thể điều chỉnh khi cần, ví dụ bằng cách bổ sung acid fuchsin 0,1% vào dung dịch Marc-André để nhuộm. Ngoài ra, các mẫu bảo quản trong dung dịch nước và dự định gắn trong môi trường nhựa cần được khử nước (xem mục 5.2), vì hầu hết môi trường gắn nhựa tự nhiên hoặc tổng hợp không tương thích với nước. New (1974) lưu ý rằng một số thuốc nhuộm có thể suy giảm trong một số môi trường gắn [53]. Ví dụ, acid fuchsin thường dùng với Canada balsam cũng có thể cố định trong Euparal®. Tuy nhiên, các mẫu nhuộm acid fuchsin dễ bị phai màu, đặc biệt khi còn sót lại dầu đinh hương – dung dịch làm trong cuối cùng. Các mẫu lưu trữ trong dầu đinh hương có thể phai đáng kể chỉ sau vài ngày.

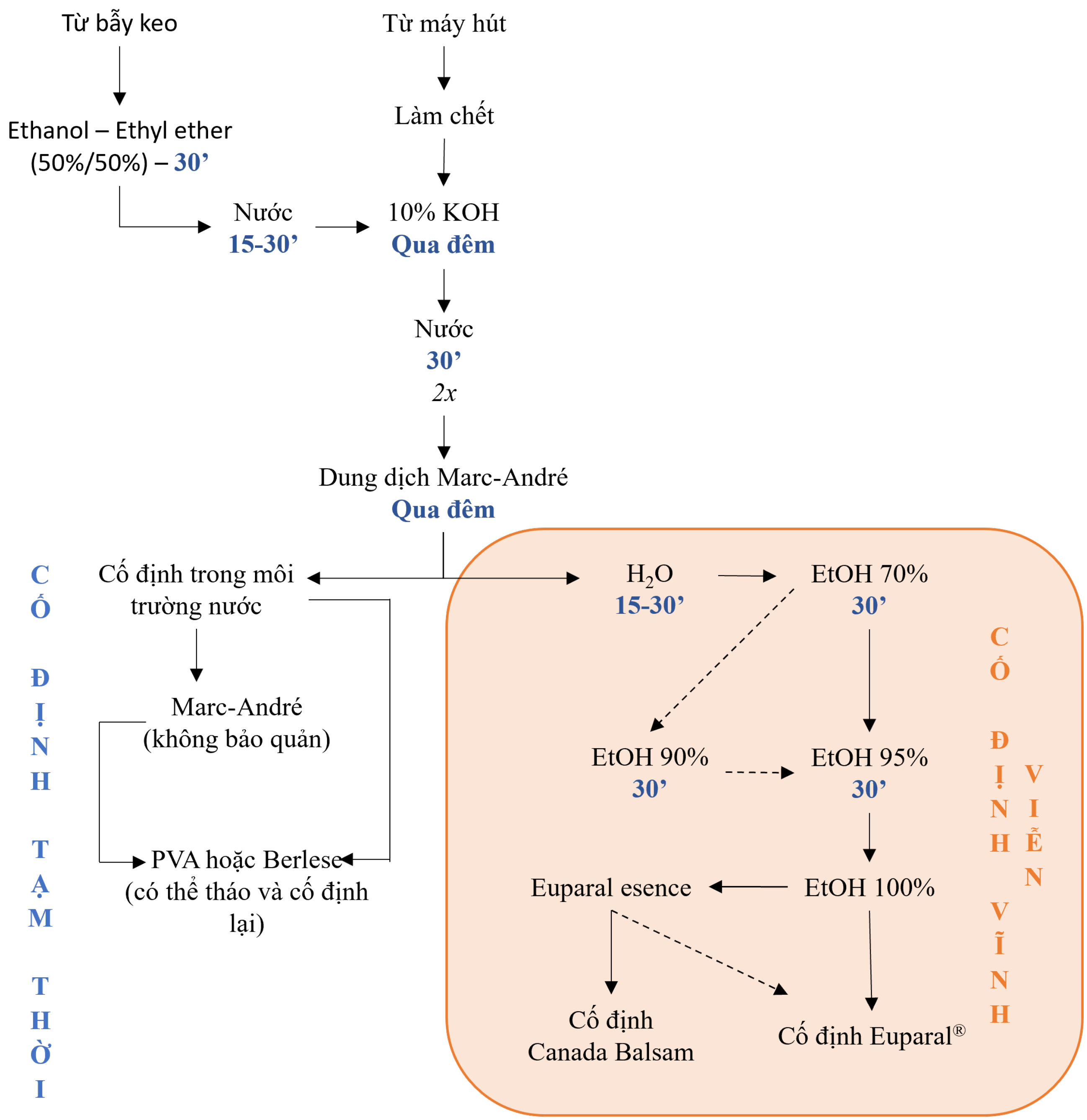

Hình 7: Phương pháp cổ điển xử lý muỗi cát.

5.2. Khử nước

Khử nước được thực hiện bằng cách chuyển mẫu qua dãy ethanol tăng dần: 50%, 70%, 80%, 90% hoặc 95% và cuối cùng 100%, mỗi bể tối thiểu 20 phút. Do ethanol bay hơi nhanh, lọ chứa phải được đậy kín trong quá trình xử lý. Khi mẫu đã khử nước hoàn toàn, có thể tạm dừng xử lý vài ngày trong Euparal® essence, được ưu tiên hơn dầu đinh hương. Beech creosote, trước đây được sử dụng rộng rãi cho mục đích này, hiện đã bị cấm hoàn toàn do độc tính.

Quá trình khử nước phải đảm bảo dịch trong mẫu tương thích với môi trường gắn nhằm tránh hiện tượng đục, xẹp do thẩm thấu hoặc biến dạng khiến mẫu không phù hợp cho nghiên cứu phân loại học.

5.3. Môi trường gắn

5.3.1. Lựa chọn và ứng dụng trong chuẩn bị mẫu

Môi trường gắn lý tưởng nên có chiết suất càng gần với thủy tinh càng tốt (khoảng 1,5). Phải không màu, trong suốt và duy trì độ trong hoàn toàn sau khi khô và theo thời gian. Phải tương thích với thuốc nhuộm sử dụng và có khả năng thấm và khuếch tán vào mọi mô của mẫu. Không được khô quá nhanh hoặc tạo mờ trong quá trình gắn. Không được co rút sau khi gắn. Việc lựa chọn môi trường gắn thích hợp là yếu tố cơ bản trong chuẩn bị mẫu, vì không có môi trường nào phù hợp cho mọi mục đích. Sự lựa chọn cần cân bằng các yếu tố sau:

- Tính chất quang học. Chiết suất của môi trường gắn phải tạo đủ độ tương phản và khúc xạ cho các đặc điểm giải phẫu quan trọng dùng trong định danh hoặc mô tả hình thái, như túi chứa tinh, ascoid, Newstead sensilla, răng cibarial dọc và răng hầu. Khả năng quan sát các cấu trúc này phụ thuộc trực tiếp vào tính chất quang học của môi trường gắn.
- Bảo tồn. Đối với mẫu chuẩn hoặc mẫu dự kiến lưu trữ lâu dài, môi trường phải đảm bảo tính ổn định và độ bền dài hạn. Ngược lại, trong nghiên cứu kiểm kê hoặc điều tra dịch tễ học, nơi bảo tồn lâu dài ít quan trọng hơn, môi trường tạm thời hoặc bán vĩnh viễn có thể đủ.

5.3.2. Yêu cầu đối với môi trường gắn

Các chuyên gia thường phát triển các kỹ thuật gắn tùy chỉnh và phức tạp theo nhu cầu nghiên cứu cụ thể. Tuy nhiên, các phương pháp này thường bỏ qua các khía cạnh như chất lượng lưu trữ, tính tương thích, tiêu chuẩn hóa hoặc khả năng thao tác và bảo quản dài hạn. Sự thiếu tiêu chuẩn hóa này gây khó khăn cho việc tích hợp bộ sưu tập cho tặng và quản lý lâu dài.

Ứng dụng khoa học đặt ra yêu cầu khác nhau cho môi trường gắn. Các nhà phân loại học thường gắn toàn bộ mẫu và ưa chuộng môi trường có khả năng tiêu hủy nhẹ cơ quan nội tạng nhằm tăng khả năng quan sát cấu trúc biểu bì. Chỉ số khúc xạ phải khác biệt đủ lớn so với chỉ số khúc xạ của mẫu vật và lam kính để tối đa hóa độ rõ quang học. Môi trường gắn thương mại thường được điều chỉnh chiết suất gần với thủy tinh nhằm giảm khúc xạ và tán xạ ánh sáng trong hệ lam–gắn mẫu môi trường–lamen. Tuy nhiên, trong hiển vi trường sáng, độ tương phản tự nhiên của mẫu không nhuộm có thể được điều chỉnh bằng cách chọn môi trường có chiết suất hơi khác so với mẫu, từ đó cải thiện khả năng quan sát trên nền.

5.3.3. Các loại môi trường gắn (Bảng 3 & 4)

Kính hiển vi đòi hỏi chỉ số khúc xạ (RI) của môi trường gắn để xác định cách ánh sáng bị bẻ cong qua lam kính, môi trường và mẫu. Khi RI gần với thủy tinh lamen kính (≈1,515), ánh sáng truyền đều, giảm tán xạ và biến dạng quang học, từ đó cải thiện độ phân giải và khả năng quan sát cấu trúc tinh vi. Ngược lại, sai khác RI có thể gây mờ, quầng sáng hoặc che khuất cấu trúc không nhuộm. Việc lựa chọn môi trường phù hợp là yếu tố then chốt để tối ưu hóa độ tương phản và chất lượng hình ảnh.

Đối với muỗi cát, các lựa chọn phổ biến bao gồm môi trường gum-chloral gốc nước, và Canada balsam hoặc nhựa Enecê – Nelson Cerqueira (NC) gốc dung môi. Rawlins [60] phân loại môi trường gắn thành: (1) môi trường vĩnh viễn – đông cứng theo thời gian và phù hợp bảo tồn dài hạn; và (2) môi trường bán vĩnh viễn – không đông cứng hoàn toàn, thường dùng tạm thời.

Các môi trường gắn tiêu bản có thể ở dạng lỏng, dạng keo hoặc dạng nhựa, hòa tan trong nước, alcohol hoặc các dung môi khác (ví dụ: toluene, xylene) (Bảng 3). Sau khi áp dụng, chúng cần được niêm kín khỏi tác động của môi trường khí quyển bằng các môi trường viền kín không tan. Để phân biệt rõ ràng giữa các loại môi trường gắn tiêu bản, có thể sử dụng cách phân loại sau:

a. Môi trường nước (Aqueous media). Các môi trường này hòa tan dễ dàng trong nước, phù hợp cho tiêu bản tạm thời hoặc bán vĩnh viễn. Chúng thường dễ thao tác, nhưng có thể cần hàn kín để tránh tiếp xúc với độ ẩm khí quyển (ví dụ: môi trường gum-chloral và polyvinyl alcohol), đặc biệt trong khí hậu nhiệt đới ẩm.

b. Môi trường chịu nước hạn chế (Limited water-tolerant media). Các môi trường này ít bị ảnh hưởng bởi nước hơn, nhưng vẫn cần được bảo vệ khỏi độ ẩm quá mức. Chúng mang lại độ ổn định lâu dài cao hơn so với các lựa chọn hòa tan trong nước và thường được sử dụng cho tiêu bản bán vĩnh viễn.

c. Môi trường hòa tan trong hydrocarbon (Hydrocarbon-soluble media). Các môi trường này được hòa tan trong dung môi hữu cơ như xylene, toluene hoặc essenecê (dung môi enecê). Chúng được thiết kế cho gắn tiêu bản vĩnh viễn và mang lại độ ổn định lâu dài rất cao, chống ẩm và chống suy thoái, do đó lý tưởng cho mục đích lưu trữ lâu dài (ví dụ: Canada balsam trung tính).

This is an Open Access article distributed under the terms of the Creative Commons Attribution License (https://creativecommons.org/licenses/by/4.0), which permits unrestricted use, distribution, and reproduction in any medium, provided the original work is properly cited.

Bảng 3. Thành phần môi trường gắn tiêu bản được lựa chọn

Môi trường gắn	Dung môi	Tiền polyme tiềm năng hoặc polyme	Ghi chú
Hoyer = chloral gum	glycerol, nước	Các hợp chất gum arabic	Chất ngâm tiêu bản: chloral hydrate
CMCP-9 (= carboxy methyl cellulose phenol)	nước (CMCP-9: 51−60%)	Thủy phân hoàn toàn polyvinyl alcohol (CMCP-9: 0−5%)	CMC(P)-9: độ nhớt thấp, độ nhớt cao
DMHF (dimethyl hydantoin formaldehyde)	nước	N,N′-dimethylol dimethyl hydantoin (di-methylol DMH)<br>Ether-/methylene-bridged oligomers<br>Crosslinked DMH–formaldehyde polymer network	
Canada balsam	xylene; thành phần dễ bay hơi của một số balsam (Δ^3-carene, levopimaric acid, limonene, myrcene, palustric acid, β-phellandrene, α- pinene, β-pinene)	balsam (abienol, abietic acid, isopimaric acid, sandaracopimaric acid)	Trung hòa: potassium carbonate;<br>resin từ *Abies balsamea* (Linné, 1758)
Euparal®	eucalyptol, paraldehyde; các thành phần dễ bay hơi của gum sandarac (limonene, α-pinene, β-pinene)	Các hợp chất của gum sandarac (communic acid, manool, polycommunic acid, sandaracopimaric acid, 12-acetoxy-sandaracopimaric acid, sugiol, torulosic acid, torulosol, totarol)	Chất làm trong: methyl salicylate; color in Euparal® green: copper salt (copper abietinate); sandarac resin from *Tetraclinis articulata* (Vahl, 1791)
Enecê	ethyl alcohol; với camphor, eucalyptol and turpentine essence	Các hợp chất của copal và colophony (rosin)	

Tóm lại, các môi trường hòa tan trong nước phù hợp nhất cho tiêu bản tạm thời hoặc các trường hợp cần dễ dàng tháo gỡ mẫu; môi trường chịu nước hạn chế phù hợp cho tiêu bản bán vĩnh viễn yêu cầu độ bền trung bình; và môi trường hòa tan trong hydrocarbon được ưu tiên cho tiêu bản vĩnh viễn phục vụ lưu trữ và bảo quản lâu dài.

Bảng 4. Ưu điểm và nhược điểm của một số môi trường gắn tiêu bản được lựa chọn đối với lam kính hiển vi và các quan sát chưa công bố của nhiều tác giả [52]

Tên	Ưu điểm	Nhược điểm
* Canada balsam	Môi trường có độ bền rất cao, với tuổi thọ vượt quá 150 năm. Có thể gắn tiêu bản bằng dầu đinh hương hoặc phenol làm chất trung gian.	Chứa các thành phần độc hại và phải được xử lý dưới tủ hút. Yêu cầu quy trình khử nước đầy đủ, tốn nhiều thời gian. Khử nước bằng ethanol và chuyển qua xylene hoặc dầu đinh hương có thể làm một số taxon trở nên giòn; các lựa chọn thay thế (ví dụ: isopropanol, n-butanol, Cellosolve™, 1,4-dioxane, Histoclear, terpineol) có thể làm giảm hiện tượng gãy vỡ. Mẫu vật có thể bị sẫm đen nếu thay xylene bằng phenol hoặc nếu còn dư potassium hydroxide. Chỉ số khúc xạ cao có thể che khuất các cấu trúc không nhuộm. Quá trình khô hoàn toàn có thể mất nhiều năm nếu không sử dụng bàn gia nhiệt. Môi trường có thể ngả vàng và sẫm màu theo thời gian, đặc biệt khi làm trong bằng dầu đinh hương. Một số thuốc nhuộm bị suy yếu, và các thuốc nhuộm cation có thể phai màu nếu môi trường trở nên có tính acid — điều này có thể xảy ra tự phát theo thời gian.
DMHF (dimethyl hydantoin formaldehyde)	Cung cấp khả năng chống nhăn và chống nếp gấp xuất sắc với độ bền duy trì sau giặt. Có tính phản ứng cao, đóng rắn nhanh ở nhiệt độ trung bình. Duy trì độ bền vải tốt hơn so với các nhựa formaldehyde thế hệ cũ. Tan trong nước và dễ phối chế trong các hệ thống hoàn tất vải tiêu chuẩn. Hiệu quả về chi phí với hiệu suất công nghiệp ổn định. Linh hoạt cho vải cotton, viscose, linen và các loại vải pha. Có sẵn các cấp hàm lượng formaldehyde tự do thấp để đáp ứng nhiều quy định.	Giải phóng formaldehyde → gây lo ngại về sức khỏe, an toàn và quy định pháp lý. Chịu hạn chế theo các quy định và nhãn sinh thái. Cần sử dụng chất xúc tác acid, có nguy cơ gây hư hại vải và ăn mòn. Có thể gây ố vàng trên vải sáng màu. Có thể làm giảm độ mềm của vải. Yêu cầu kiểm soát quy trình nghiêm ngặt. Không phù hợp trong trường hợp yêu cầu không chứa formaldehyde (zero-formaldehyde).

This is an Open Access article distributed under the terms of the Creative Commons Attribution License (https://creativecommons.org/licenses/by/4.0), which permits unrestricted use, distribution, and reproduction in any medium, provided the original work is properly cited.

Bảng 4. (tiếp theo)

Tên	Ưu điểm	Nhược điểm
* Euparal (trong suốt)	Môi trường bền, với tuổi thọ trên 50 năm.<br>Có thể gắn trực tiếp từ ethanol 80% (theo khuyến cáo của nhà sản xuất).<br>Không che khuất các cấu trúc không nhuộm và không ngả vàng hoặc trở nên giòn theo thời gian.<br>Có chỉ số khúc xạ phù hợp hơn Canada balsam đối với Diptera.<br>Phù hợp với mẫu dày do co rút tối thiểu và khô không tạo bọt khí.<br>Vẫn hòa tan trong ethanol 95%, cho phép gắn lại tiêu bản ngay cả sau nhiều năm.	Chứa các thành phần độc hại và phải được xử lý dưới tủ hút. Khử nước bằng ethanol và chuyển qua Euparal Essence có thể làm một số taxon trở nên giòn, nhưng sử dụng isopropanol có thể giảm vấn đề này.
Dung dịch Hoyer	Có thể gắn tiêu bản khi còn sống hoặc trực tiếp từ nước, ethanol hoặc formaldehyde.<br>Quá trình tiêu bản hóa (maceration) cho chất lượng biểu bì rất tốt.<br>Có chỉ số khúc xạ thuận lợi và có thể tăng cường bằng nhuộm iodine để tăng độ tương phản.<br>A xít Acetic trong công thức có thể làm giãn các phần phụ của động vật chân khớp.<br>Một số tiêu bản có thể ổn định trong 40–60 năm.<br>Tan trong nước, cho phép gắn lại tiêu bản dễ dàng.	Mẫu thực vật mỏng manh có thể bị xẹp trừ khi môi trường được thêm dần từng bước, điều này tốn thời gian.<br>Có thể hình thành hốc rỗng và tinh thể trong vòng chưa đầy 10 năm.<br>Quá trình tiêu bản hóa có thể trở nên quá mức tùy thuộc vào nồng độ chloral hydrate và thời gian tiếp xúc.<br>Các thành phần của môi trường có thể tách lớp và xuất hiện hạt mịn trong vài tháng hoặc vài năm.<br>Đã có báo cáo về hiện tượng môi trường bị sẫm đen.
CMCP-9 (= carboxy methyl cellulose phenol)	Có thể gắn trực tiếp từ các môi trường như nước, ethanol, glycerol hoặc các dung dịch chứa formaldehyde, và các cơ quan bên trong có thể được tiêu bản hóa khi cần thiết để thuận tiện cho việc quan sát tổng quát hoặc chuẩn bị mẫu..	Môi trường này có thể hình thành tinh thể và sẫm màu theo thời gian, và đôi khi có thể làm tiêu bản hóa mẫu quá mức so với mong muốn. Nếu lam không được viền kín cẩn thận, các mẫu dày sẽ không phù hợp vì chúng có thể co rút và tạo khe hở quanh mép lamen. Không phù hợp với mẫu đã nhuộm hoặc vật liệu có vôi hóa, và thời gian khô chậm hơn so với CMC.

Bảng 4. (tiếp theo)

Tên	Ưu điểm	Nhược điểm
Eukitt™	Môi trường bền, kéo dài trên 30 năm. Tương thích với nhiều dung môi dùng để gắn, bao gồm acetone, benzene, chloroform, dioxan, ether, isopropanol, methyl benzoate, terpineol, toluene và xylene. Khô nhanh và có pH hơi acid. Không sẫm màu đáng kể theo thời gian. Phù hợp với nhiều thuốc nhuộm khác nhau (ví dụ: fuchsin, hematoxylin, methyl green, methyl violet, methylene blue). Có thể gắn lại tiêu bản sau nhiều năm bằng cách ngâm trong xylene trong thời gian dài.	Chứa các thành phần độc hại và phải được xử lý dưới tủ hút. Yêu cầu quy trình khử nước đầy đủ, tốn nhiều thời gian. Không lý tưởng cho mẫu dày do co rút và hình thành bọt khí. Lamen có thể bong theo thời gian nếu kính không được làm sạch kỹ và niêm kín. Có thể xuất hiện hiện tượng polymer hóa không hoàn toàn quanh các sợi collagen.
Enecê	Môi trường rất bền, kéo dài ít nhất 50 năm. Enecê không sẫm màu theo thời gian. Có tính dẻo hơn, cho phép giải phẫu côn trùng trong môi trường, đồng thời cung cấp khoảng thời gian hợp lý để định vị các cấu trúc hình thái. Chi phí thấp.	Yêu cầu quy trình khử nước đầy đủ, tốn nhiều thời gian. Khử nước bằng ethanol và chuyển qua dầu đinh hương có thể làm một số mẫu trở nên giòn. Côn trùng tiếp tục được làm trong mặc dù rất chậm; điều này có thể gây khó khăn khi quan sát các cấu trúc rất nhỏ như sensilla, ascoids và lông riêng rẽ.

5.3.4. Mô tả các môi trường gắn được khuyến nghị (Bảng 3 & 4)

Môi trường cho quan sát tạm thời

Gôm Chloral = Hoyer fluid/medium/solution (RI = 1,48)

Dung dịch Marc André là môi trường tốt nhất cho quan sát ngắn hạn (vài giờ, có thể lâu hơn nếu bảo quản trong buồng ẩm) túi chứa tinh, bao gồm chụp ảnh (Figure 4) hoặc vẽ. Để bảo tồn lâu hơn, cần gắn lại trong môi trường nước cho lưu trữ trung hạn. Khử nước để gắn nhựa không được khuyến nghị (nguy cơ mất mẫu). Gôm Chloral và dịch Hoyer được coi là đồng nghĩa. Môi trường này thường được sử dụng để quan sát các cơ quan nội tạng do khả năng tương thích với nước, tính đơn giản, ứng dụng nhanh chóng và chỉ số khúc xạ tạo điều kiện thuận lợi cho việc kiểm tra các cấu trúc mỏng manh như túi tinh. Tuy nhiên, gôm chloral có những nhược điểm đáng kể nếu không được chuẩn bị hoàn hảo hoặc bảo quản trong điều kiện độ ẩm được kiểm soát. Những vấn đề này bao gồm kết tinh, đổi màu và mất độ nhớt. Việc làm sạch lam kính không giải quyết được những vấn đề này, vì môi trường gắn kết có thể bị đổi màu nặng (đôi khi gần như đen) do tương tác với môi trường làm sạch, đặc biệt nếu sử dụng Euparal®. Môi trường Hoyer được coi là tốt nhất về mặt quang học cho muỗi cát *phlebotomine* và theo truyền thống đã được sử dụng cho các mục đích này. Môi trường này bao gồm một số công thức có liên quan chặt chẽ, bao gồm gôm arabic, glycerol và chloral hydrate. Nhiều công thức đã bị hiểu sai và trích dẫn sai [74].

Mặc dù Hoyer là môi trường tốt để quan sát túi chứa tinh của muỗi cát, nhưng không phù hợp cho bảo tồn dài hạn. Nó lý tưởng cho việc quan sát ngắn hạn, bao gồm chụp ảnh, vẽ hoặc hình ảnh. Môi trường nước chỉ thích hợp cho tiêu bản tạm thời, nhưng không thể đảm bảo bảo quản lâu dài. Ngược lại, phương pháp gắn mẫu bằng nhựa mang lại độ bền tuyệt vời, thường kéo dài hàng thế kỷ, nhưng có thể làm mờ các chi tiết nhỏ của túi tinh, vì khả năng khúc xạ ánh sáng của chúng thường bị mất đi.

Theo thời gian, Hoyer bị suy giảm do mất nước (Figure 8), hình thành các tinh thể chloral hydrate trắng đục. Tuy nhiên, có thể phục hồi mẫu bằng cách tái ngậm ẩm trong môi trường ẩm có bổ sung thymol để ngăn nấm, hoặc lấy mẫu khỏi gôm chloral, khử nước bằng axit acetic băng và gắn lại trong Canada balsam.

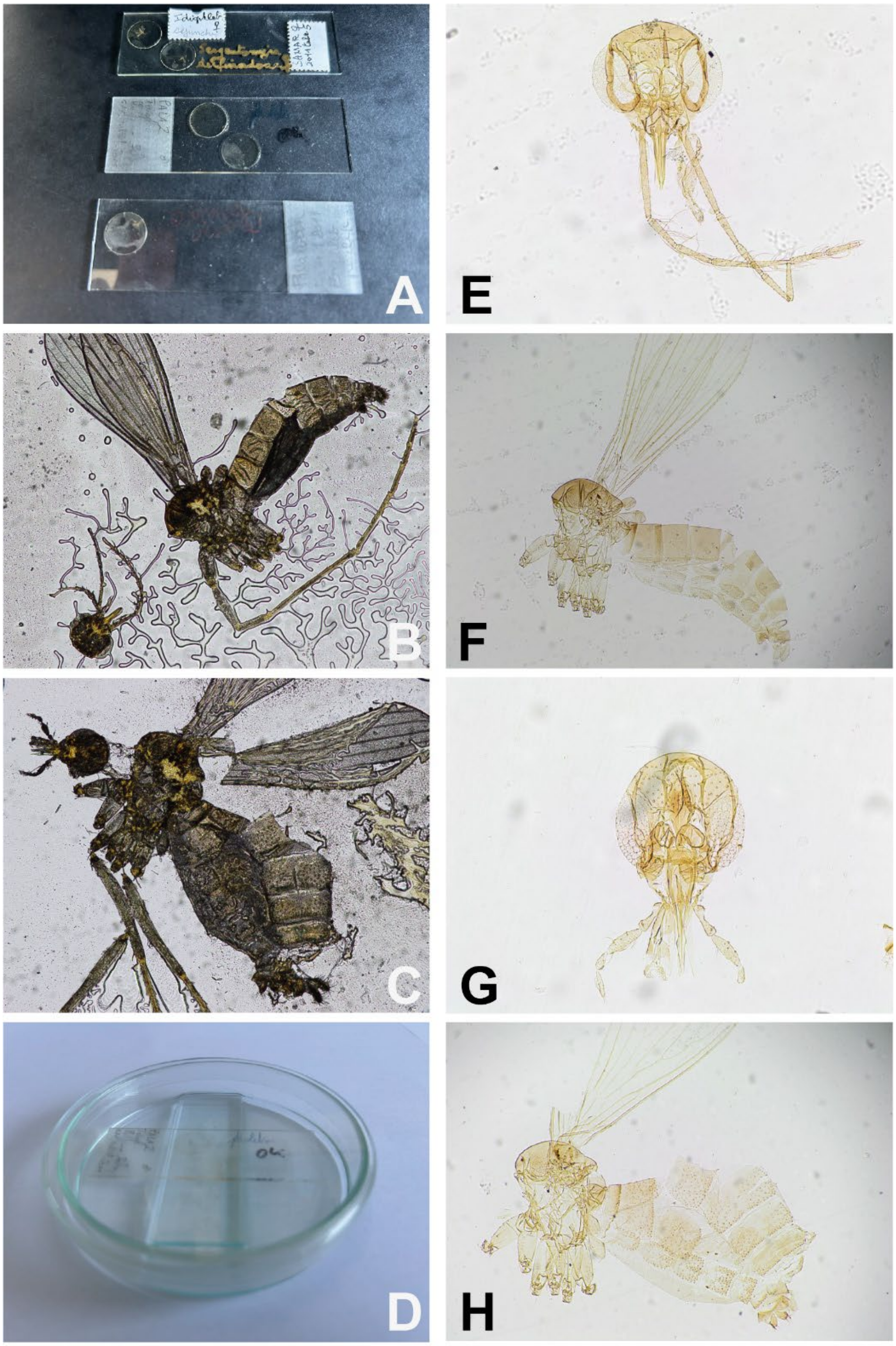

Hình 8: Gắn tiêu bản trên lam kính. A: lam kính bị hư hỏng và khô được gắn trong Hoyer; B: hình ảnh hiển vi của một muỗi cát khô; C: hình ảnh hiển vi của một muỗi cát bị hư hỏng khác; D: buồng ẩm chứa một lam kính đã khô; E: đầu, và F: thân của mẫu B sau khi được gắn lại trong Euparal®; G: đầu, và H: thân của mẫu C bị hư hỏng sau khi được gắn lại trong Euparal®.

This is an Open Access article distributed under the terms of the Creative Commons Attribution License (https://creativecommons.org/licenses/by/4.0), which permits unrestricted use, distribution, and reproduction in any medium, provided the original work is properly cited.

DMHF (dimethyl hydantoin formaldehyde) (RI 1,48)

Môi trường gốc nước này [72] có chất lượng quang học rất tốt, tương tự Berlese nhưng không chuyển đen hoặc kết tinh. Phù hợp cho muỗi cát và các loài Psychodidae khác.

CMCP (RI = 1,41)

Đây là môi trường gắn gốc glycerin, hòa tan trong nước, dùng để tạo tiêu bản trong suốt của các mẫu mỏng manh, bao gồm muỗi cát. Ưu điểm là có thể gắn trực tiếp từ nước hoặc ethanol. Tuy nhiên, môi trường này có chứa phenol – một chất độc và gây kích ứng – cần thao tác cẩn trọng.

Môi trường gắn vĩnh viễn

Canada balsam (RI = 1,52–1,54)

Canada balsam lần đầu tiên được mô tả là một môi trường gắn tiêu bản phù hợp cho kính hiển vi ánh sáng truyền qua bởi Andrew Pritchard vào những năm 1830. Cho đến nay, đây vẫn là một trong những môi trường được sử dụng rộng rãi nhất nhờ chất lượng lưu trữ đã được chứng minh, với hơn 150 năm ứng dụng thành công. Không giống như môi trường Hoyer, Canada balsam không kết tinh hoặc hấp thụ độ ẩm. Tuy nhiên, Canada balsam có hiện tượng tự huỳnh quang mạnh, đôi khi có thể là bất lợi đối với một số kỹ thuật hiển vi nhất định [60]. Việc sử dụng các dung môi không độc thay cho xylene có thể giảm rủi ro an toàn trong quá trình chuẩn bị, nhưng cũng có thể gây ra các hạn chế như thời gian khô chậm hơn và môi trường sẫm màu sớm hơn.

Euparal® (RI = 1,48)

Euparal® là một lựa chọn thay thế được sử dụng rộng rãi cho Canada balsam trong gắn tiêu bản vĩnh viễn, mang lại độ ổn định lâu dài xuất sắc và chỉ số khúc xạ tương đương. Euparal® có các đặc điểm sau: (1) yêu cầu khử nước: trước khi chuyển cuối cùng sang môi trường gắn tiêu bản, mẫu phải được khử nước, thường chuyển từ ethanol 95% sang ethanol tuyệt đối; và (2) thời gian xử lý kéo dài: quá trình lắp ráp cuối cùng trong nhựa, dù là Canada balsam hay Euparal®, đều yêu cầu khử nước, làm tăng tổng thời gian xử lý mẫu. Khi không thể thực hiện khử nước bằng dung môi hữu cơ, các mẫu lấy từ ethanol tuyệt đối có thể được đặt vào dung dịch trung gian gồm hỗn hợp bằng nhau của Euparal® và Euparal essence trước khi gắn tiêu bản cuối cùng.

Enecê (RI = 1,467)

Enecê là một môi trường gắn tiêu bản dạng nhựa, chủ yếu được sử dụng cho các côn trùng nhỏ và đặc biệt phổ biến tại Brazil. Thành phần cơ bản của nó gồm nhựa thông và nhựa copal hòa tan trong alcohol, camphor, tinh dầu nhựa thông và bạch đàn. Cerqueira [11] đã mô tả Enecê như một giải pháp thay thế cho Canada balsam trong gắn tiêu bản vĩnh viễn của ấu trùng, vỏ lột xác của các giai đoạn trước trưởng thành và thậm chí cả muỗi trưởng thành, và từ đó đã được áp dụng rộng rãi để gắn tiêu bản muỗi cát. Enecê mang lại một lựa chọn kinh tế cho gắn tiêu bản vĩnh viễn, cung cấp độ ổn định lâu dài và thời gian khô đủ dài, cho phép giải phẫu và sắp xếp chính xác các cấu trúc hình thái.

5.4. Chuẩn bị lam kính và làm khô

Việc làm khô đúng cách các lam kính đã gắn tiêu bản có vai trò then chốt nhằm đảm bảo độ ổn định và bảo quản lâu dài. Lam kính cần được làm khô hoàn toàn trước khi đưa vào lưu trữ dài hạn. Để đạt kết quả tối ưu, các lam gắn bằng môi trường vĩnh viễn nên được làm khô ở tư thế nằm ngang trong 2–3 tuần, trong khi các lam chuẩn bị bằng môi trường bán vĩnh viễn có thể chỉ cần 1–2 tuần. Để đảm bảo quá trình làm khô hiệu quả, khuyến nghị sử dụng tủ ấm được cài đặt ở nhiệt độ phù hợp với môi trường gắn đang sử dụng, tránh nhiệt độ quá cao có thể làm hỏng mẫu. Khoảng nhiệt độ từ 30°C đến 37°C được khuyến nghị. Bước làm khô này là thiết yếu nhằm ngăn ngừa cong vênh lam, hỏng mẫu hoặc mất ổn định môi trường gắn trong quá trình lưu trữ.

Môi trường gắn tiêu bản sử dụng trong quá trình chuẩn bị lam phải luôn được ghi rõ trên nhãn lam. Nếu có thể, nhãn cũng nên bao gồm công thức cụ thể đã sử dụng, cùng với tên người chuẩn bị và ngày chuẩn bị. Ban đầu, các lam thường được chuẩn bị như tiêu bản tạm thời và không nhằm mục đích bảo quản lâu dài. Tuy nhiên, nếu tình trạng của mẫu thay đổi, ví dụ được chỉ định là một phần của bộ mẫu "type", thì nên sử dụng môi trường gắn vĩnh viễn hơn để đảm bảo bảo tồn mẫu cho các nghiên cứu phân loại trong tương lai.

5.5. Kỹ thuật gắn tiêu bản thay thế: gắn trên thẻ (card mounting)

Gắn trên thẻ là một kỹ thuật được sử dụng cho một số nhóm côn trùng, trong đó mẫu có thể được ghim trực tiếp lên thẻ côn trùng học hoặc dán lên bề mặt. Do kích thước rất nhỏ của muỗi cát và nhu cầu quan sát các cơ quan bên trong để định danh thông qua quá trình làm trong (xem mục 5), phương pháp này hoàn toàn không phù hợp để gắn tiêu bản muỗi cát.

5.6. Gắn lại các mẫu bị hư hỏng

Đối với các mẫu hiếm hoặc có giá trị, khuyến nghị áp dụng phương pháp hai bước theo video có thể truy cập tại: https://zenodo.org/records/18315029.

1. Tái ngậm nước mà không tháo rời để cho phép quan sát sơ bộ. Một giá đỡ nhiều lam kính hiển vi nên được đặt trong đĩa Petri để làm giá đỡ. Lam cần tái ngậm nước sau đó được đặt phía trên, và đĩa Petri được thêm vài milimet dung môi để tạo buồng ẩm, bảo đảm lam không tiếp xúc trực tiếp với dung môi (Hình 8 D). Thời gian cần thiết để tái ngậm nước có thể dao động từ một đến vài ngày, tùy

thuộc vào tình trạng mẫu. Việc theo dõi hàng ngày và sự kiên nhẫn là điều thiết yếu. Khi lam đã được tái ngậm nước đầy đủ, có thể lấy ra khỏi buồng ẩm và đặt vào tủ ấm trong vài giờ trước khi quan sát hiển vi, chụp ảnh hoặc vẽ.

2. Để gắn lại, lam có thể được đưa trở lại buồng ẩm trong vài giờ hoặc qua đêm. Việc tháo rời cần được thực hiện dưới kính hiển vi soi nổi. Sử dụng kim mảnh, lamen phải được tháo cẩn thận, bảo đảm không còn bất kỳ phần muỗi cát nào bám lại (https://zenodo.org/records/18315029). Tiếp theo, các phần đã giải phẫu của muỗi cát cần được thu gom và rửa bằng nước trong các giếng nhỏ, tương tự như loại dùng cho tách chiết DNA/RNA phá hủy (xem bên dưới), trước khi khử nước và gắn lại trong môi trường nhựa. Khi tháo rời lam, điều quan trọng là phải xác định môi trường gắn ban đầu để lựa chọn dung môi phù hợp. Đối với môi trường gắn hòa tan trong nước, sử dụng nước. Nếu môi trường gắn là dạng nhựa (ví dụ: Canada balsam hoặc Euparal®), cần sử dụng xylene, dưới tủ hút và với trang bị bảo hộ cá nhân phù hợp, bao gồm khẩu trang.

Việc gắn lại các mẫu thuộc bộ mẫu type hoặc mẫu của bộ sưu tập chỉ được thực hiện khi có sự đồng ý của người quản lý bộ sưu tập và/hoặc cơ quan sở hữu mẫu.

6. Định danh mẫu

6.1. Hình thái học

Việc định danh muỗi cát chủ yếu dựa trên việc quan sát các đặc điểm hình thái của chúng, bao gồm hình dạng ngực, cánh, cơ quan sinh dục, lông (setae) và các mối quan hệ hình thái đo đạc cụ thể giữa các cấu trúc khác nhau. Các nhà nghiên cứu sử dụng khóa định loại, bộ sưu tập tham chiếu và mô tả loài gốc để so sánh các mẫu thu thập với các taxon đã biết. Các đặc điểm chẩn đoán quan trọng, như hệ gân cánh và hình thái đầu ở cả hai giới, cấu trúc cơ quan sinh dục đực và cấu hình của túi chứa tinh ở con cái, đặc biệt hữu ích cho việc xác định loài. Định danh chính xác thường yêu cầu quan sát chi tiết dưới kính hiển vi, thường sử dụng kính hiển vi quang học để quan sát các cấu trúc tinh vi như cơ quan sinh dục và túi chứa tinh, hoặc kính hiển vi soi nổi để quan sát các đặc điểm hình thái tổng quát hơn.

Những tiến bộ gần đây trong công nghệ hình ảnh đã tạo điều kiện cho việc sử dụng hình ảnh số trong định danh muỗi cát. Các ảnh chụp độ phân giải cao hoặc minh họa số của các đặc điểm quan trọng có thể được so sánh với tài liệu tham chiếu hoặc phân tích bằng các hệ thống định danh có hỗ trợ máy tính, góp phần nâng cao cả độ chính xác và khả năng tiếp cận trong phân loại hình thái.

6.2. Hình học cánh

Hình học cánh là một đặc điểm quan trọng được sử dụng trong định danh và phân loại các loài muỗi cát khác nhau. Cánh của muỗi cát có kiểu hình và cấu trúc đặc trưng, thường dài và hẹp với hệ gân phát triển rõ (Hình 9 & 10). Sự sắp xếp của các gân tạo thành một mô hình riêng biệt có thể khác nhau giữa các giống và loài, cung cấp các đặc điểm chẩn đoán có giá trị cho định danh. Do đó, nghiên cứu hình học cánh mang lại những hiểu biết quan trọng cho mục đích phân loại học.

6.3. Hình thái đo hình học cánh (Wing geometric morphometrics)

Các nhà nghiên cứu sử dụng nhiều kỹ thuật khác nhau, chẳng hạn như hình thái đo hình học, để phân tích và so sánh hình dạng và kích thước cánh giữa các loài hoặc quần thể muỗi cát khác nhau. Việc nghiên cứu hình học cánh cung cấp những hiểu biết giá trị về hành vi, sở thích sinh cảnh và khả năng bay.

Trong cách tiếp cận hình thái đo hình học, cánh được giải phẫu cẩn thận, nhuộm (nếu cần) và gắn phẳng trên lam kính. Các lam đã chuẩn bị sau đó được chụp ảnh dưới kính hiển vi soi nổi, số hóa và tiến hành phân tích hình thái đo. Quy trình này đã được mô tả đầy đủ trong tài liệu [6, 27, 42, 56, 57, 59], với khuyến nghị sử dụng nhất quán cánh phải hoặc cánh trái đối với các cơ quan đôi nhằm tránh các hiệu ứng đo tỷ lệ âm tiềm tàng [62].

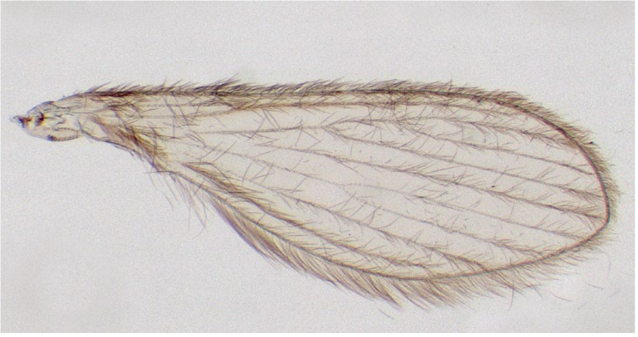

Hình 9: Cánh nguyên bản của *Trichophoromyia ininii*.

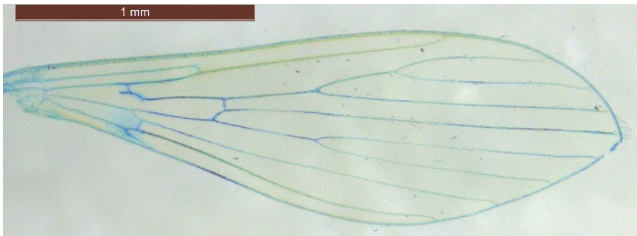

Hình 10: Cánh nhuộm màu của *Phlebotomus ariasi*.

Chuẩn bị cánh cho phân tích hình thái đo hình học

Để quan sát tối ưu hệ gân cánh, cánh cần được làm sạch vảy và nhuộm thích hợp. Đối với chuẩn bị cánh, trước tiên chuẩn bị các giếng nhỏ chứa các thuốc thử cần thiết (methylene blue, ethanol, nước và chất thay thế xylene). Lấy một cánh được bảo quản trong ethanol 70% ở nhiệt độ phòng bằng cách lật ngược ống Eppendorf và đổ vào giếng, sau đó dùng kim cong mảnh nhấc cánh theo chiều dọc. Chuyển cánh nhanh từ ethanol sang nước và trở lại ethanol để loại bỏ lông nhỏ. Đặt cánh vào methylene blue trong 6 phút, bảo đảm cánh nổi trong quá trình nhuộm. Thu hồi cánh cẩn thận và ngâm trong chất thay thế xylene trong 2 phút (khoảng một phần ba thời gian của methylene blue). Gõ nhẹ kim vào thành giếng có thể giúp cánh lắng xuống; xylene có tác dụng cố định màu nhuộm. Cuối cùng, nhấc cánh và đặt lên một giọt nhỏ Euparal® trên lam kính hiển vi. Dưới kính lúp, nhẹ nhàng trải phẳng cánh và cẩn thận đặt lamen. Cần chụp ảnh ngay trước khi Euparal® đông lại, vì có thể cần điều chỉnh nhẹ vị trí cánh dưới lamen để đạt sự căn chỉnh tối ưu.

6.4. Các kỹ thuật sinh học phân tử

Bên cạnh các kỹ thuật hình thái, các phương pháp phân tử ngày càng đóng vai trò thiết yếu trong nghiên cứu côn trùng học, bao gồm các nghiên cứu phân loại học, di truyền quần thể và phát sinh chủng loài, cũng như phát hiện tác nhân gây bệnh DNA/RNA và xác định nguồn máu hút, trong đó hành vi véc tơ có ý nghĩa quan trọng trong lĩnh vực dịch tễ học [70]. Giải trình tự DNA có thể được sử dụng để xác nhận loài hoặc phân biệt các loài có quan hệ gần gũi, cung cấp phương tiện định danh chính xác và đáng tin cậy hơn. Ngoài ra, các kỹ thuật phân tử tiên tiến (ví dụ: PCR, giải trình tự DNA, NGS, v.v.) và MALDI-ToF MS ngày càng được sử dụng rộng rãi cho định danh loài nhanh chóng và chính xác, bổ sung cho các phương pháp hình thái truyền thống [46]. Mặc dù có những tiến bộ này, định danh hình thái vẫn là tiêu chuẩn tham chiếu trong phân loại học và là cơ sở để diễn giải dữ liệu phân tử.

6.4.1. Tách chiết acid nucleic theo phương pháp phá hủy

Tách chiết acid nucleic là một bước thường quy trong nhiều nghiên cứu sinh học, và nhiều phương pháp đã được phát triển để phân lập DNA từ vật liệu sinh học [48]. Nhiều bộ kit tách chiết DNA thương mại được thiết kế để tạo thuận lợi cho quá trình này [14]. Tuy nhiên, các phương pháp thường dùng để chuẩn bị mẫu chân đốt cho định danh hình thái thường cản trở phân tích DNA, do các kỹ thuật này có thể làm hỏng hoặc phá hủy các đặc điểm hình thái quan trọng của mẫu [10]. Phần lớn các quy trình tách chiết DNA từ mô côn trùng có tính phá hủy [43], gây lo ngại đặc biệt đối với các mẫu nhỏ, nơi ngay cả việc lấy mẫu hạn chế cũng có thể làm tổn hại các đặc điểm hình thái quan trọng [72]. Loại và tình trạng của mẫu đóng vai trò quan trọng trong việc lựa chọn phương pháp phân lập DNA phù hợp [29].

Nhu cầu định danh chính xác muỗi cát, hiểu động thái quần thể và giảm thiểu tác động không chủ đích đã thúc đẩy sự phát triển của các công cụ chẩn đoán phân tử [23]. Các cách tiếp cận phân tử hiện nay thường được sử dụng để bổ sung cho các phương pháp phân loại hình thái trong định danh muỗi cát. Ví dụ, phương pháp tiêu chuẩn cho DNA barcoding côn trùng bao gồm tách chiết DNA, giải trình tự và mất đi mẫu vật gốc. Do đó, có nhu cầu cấp thiết trong việc phát triển các phương pháp tách chiết DNA không phá hủy nhằm bảo tồn cả vật liệu sinh học và tính toàn vẹn hình thái của mẫu.

Nhiều phương pháp tách chiết acid nucleic đã được áp dụng cho muỗi cát. Số lượng hoặc chất lượng acid nucleic cần thiết phụ thuộc vào phân tích phân tử tiếp theo, do các kỹ thuật khác nhau có yêu cầu về độ nhạy và độ tinh sạch khác nhau [9]. Ví dụ, mắt muỗi cát đã được ghi nhận là có thể ức chế khuếch đại PCR [69]. Ngoài sàng lọc tác nhân gây bệnh, DNA muỗi cát thường được tách chiết cho mục đích định danh loài. Có thể sử dụng nhiều phương pháp tách chiết khác nhau, mặc dù sản lượng và chất lượng khác nhau giữa các kỹ thuật. Một số quy trình của nhà sản xuất đã được điều chỉnh cho muỗi cát [8], giúp tăng sản lượng và/hoặc chất lượng acid nucleic thu được [8, 9, 69], trong khi các điều chỉnh khác được phát triển cho các taxon chân đốt khác cũng có thể áp dụng cho muỗi cát [58, 76]. Các PCR định danh nhắm vào các đoạn ty thể nhỏ (COI hoặc CytB) thường tương thích với các phương pháp tách chiết gây phân mảnh DNA cao. Ngược lại, các kỹ thuật NGS đọc dài khác (Oxford Nanopore và PacBio) yêu cầu DNA chất lượng cao và mức độ phân mảnh tối thiểu. Phương pháp tách chiết bằng cột quay (spin column) thường tạo ra các đoạn DNA hệ gen lên đến 60 kb, trong khi tách chiết phenol-chloroform có thể tạo ra các đoạn lên đến 150 kb [77]. Bảng 5 tóm tắt các kỹ thuật tách chiết DNA muỗi cát khác nhau và cho biết liệu có điều chỉnh phương pháp cho nhóm côn trùng này hay không. Sản lượng không được trình bày vì phụ thuộc vào kích thước mẫu và phương pháp chuẩn bị. Cột "modification" đề cập đến các điều chỉnh quy trình tách chiết cho muỗi cát hoặc các chân đốt nhỏ khác.

Việc lựa chọn phương pháp tách chiết cần cân nhắc một số tiêu chí như số lượng mẫu, thời gian tách chiết và kỹ thuật phân tích tiếp theo. Trong khi các kỹ thuật NGS yêu cầu DNA hệ gen có khối lượng phân tử cao, tất cả các phương pháp được trình bày ở đây đều có thể sử dụng cho các ứng dụng PCR tiêu chuẩn.

Ngoài ra, nhiều nghiên cứu đã khảo sát các phương pháp tách chiết DNA không phá hủy cho các chân đốt trên cạn kích thước nhỏ, mẫu bảo quản khô trong bảo tàng và các chân đốt thân mềm [19, 26, 28, 55, 63].

6.4.2. Tách chiết acid nucleic không phá hủy

Một trong những thách thức lớn trong phân tích phân tử các chân đốt, đặc biệt là muỗi cát, là bảo tồn mẫu để tích hợp vào các bộ sưu tập côn trùng học. Phần lớn các quy trình tách chiết DNA yêu cầu tiêu bản hóa mô, do đó làm ảnh hưởng đến việc bảo quản mẫu gốc. Ngược lại, các phương pháp tách chiết acid nucleic không phá hủy được thiết kế để thu nhận vật liệu di truyền mà không gây hư hại vật lý cho mẫu, không ảnh hưởng đến tính khả thi hoặc làm thay đổi hình thái. Các phương pháp này đặc biệt có giá trị khi làm việc với các mẫu quý hiếm hoặc số lượng hạn chế, như muỗi cát, nơi việc duy trì tính toàn vẹn cấu trúc là thiết yếu cho các mục đích phân loại, hình thái hoặc chẩn đoán trong tương lai. Một kỹ thuật thường được sử dụng là phương pháp ngâm không phá hủy, trong đó muỗi cát được cố định bất động và nhẹ nhàng ngâm trong dung dịch ly giải chứa proteinase K.

Kỹ thuật mild-vectolysis đã được áp dụng thành công cho muỗi cát, đặc biệt là các mẫu type [24]. Kỹ thuật này sử dụng bộ kit spin column thông thường (trong trường hợp này là DNeasy Blood and Tissue kit, QIAGEN, Hilden, Đức) với các điều chỉnh để thu DNA mà không phá hủy mẫu. Các bước ly giải được sửa đổi (thể tích dung dịch ly giải và bổ sung bước đông lạnh) [17] cho phép giải phóng acid nucleic đồng thời giảm thiểu tổn thương hình thái [24]. Đối với muỗi cát, cũng có thể sử dụng bộ kit HotSHOT DNA Extraction (Bento Bioworks Ltd, London, Vương quốc Anh) [73], nhanh và chi phí thấp, cho phép xử lý mẫu nhanh chóng và tiết kiệm. Các mẫu côn trùng dự định định danh hình thái sau đó có thể được rửa sạch. Các mẫu xử lý bằng DNeasy Blood and Tissue kit cần được làm trong bằng dung dịch Marc-André, trong khi các mẫu xử lý bằng HotSHOT DNA extraction kit được làm trong đủ để có thể gắn trong môi trường nước, hoặc tốt hơn là trong môi trường nhựa sau khi khử nước, theo quy trình chi tiết trong bài viết này [73]. Vật liệu di truyền thu được sau đó có thể được xử lý tiếp cho các phân tích tiếp theo, chẳng hạn như PCR để khuếch đại các marker di truyền cụ thể. Các phương pháp tách chiết acid nucleic không phá hủy có vai trò then chốt trong nghiên cứu các đặc điểm di truyền của muỗi cát, bao gồm xác định các tác nhân gây bệnh tiềm tàng mà chúng có thể mang theo. Bằng cách bảo tồn tính toàn vẹn của mẫu, các nhà nghiên cứu có thể thu được thông tin di truyền giá trị đồng thời giữ lại mẫu cho các phân tích hoặc nghiên cứu bổ sung.

Bảng 5: Chi phí trung bình, ứng dụng và điều chỉnh quy trình cho tách chiết gDNA của muỗi cát *Phlebotominae*

Quy trình	Giá	Áp dụng	Quy trình tương thích cho chân đốt nhỏ
Cột quay	2.5 – 3.55 US$ [39]	PCR, NGS	[9]
Phenol-chloroform	0.24 US$ [69]	PCR, NGS	[9]
HotSHOT	<0.01 US$ [69]	PCR	-
Salting out	0.12 $3 [69]	PCR	-
Chelex	0.02 $4 [41]	PCR	[41, 76]

6.5. MALDI-ToF MS

MALDI-ToF MS (matrix-assisted laser desorption/ionization time-of-flight mass spectrometry) là một kỹ thuật dựa trên khối phổ được thiết kế để phát hiện và phân tích các cấu hình protein đặc trưng (“dấu vân tay”) của các mẫu sinh học. MALDI-ToF ngày càng được công nhận là một công cụ quan trọng trong định danh các chân đốt có ý nghĩa y học và thú y. Kỹ thuật này đã chứng minh hiệu quả trong việc định danh các giai đoạn phát triển khác nhau của muỗi cát, bao gồm cả các giai đoạn trước trưởng thành và nguồn máu của con cái hút máu, và đã được áp dụng thành công để phân biệt muỗi cát đực và cái trong nhiều điều kiện bảo quản và đồng nhất hóa khác nhau [28, 30, 73, 74]. Phương pháp này cũng mang lại khả năng phân biệt cao ở cấp phân giống, loài và quần thể. Kỹ thuật này cho phép các nhà nghiên cứu đạt được định danh loài nhanh chóng và chính xác, điều thiết yếu để hiểu phân bố, hành vi của muỗi cát và vai trò của chúng trong truyền bệnh. Bằng cách phân biệt các loài dựa trên cấu hình protein, MALDI-ToF đóng vai trò then chốt trong các nghiên cứu dịch tễ học và chiến lược kiểm soát véc tơ. Hiện nay có hai hạn chế chính làm giảm khả năng áp dụng thường quy của kỹ thuật này. Thứ nhất là tính sẵn có của thiết bị khối phổ, vốn có chi phí rất cao nếu chỉ sử dụng cho mục đích định danh loài muỗi cát (hoặc véc tơ chân đốt nói chung). May mắn thay, hạn chế này có thể được khắc phục bằng cách tiếp cận thời gian sử dụng thiết bị tại các phòng thí nghiệm proteomics và/hoặc chẩn đoán lâm sàng, nơi khối phổ đã trở thành công cụ nghiên cứu tiêu chuẩn. Thứ hai là sự đại diện thấp của dữ liệu tham chiếu muỗi cát trong các cơ sở dữ liệu truy cập mở, dẫn đến nhu cầu xây dựng cơ sở dữ liệu nội bộ với các phổ tham chiếu dựa trên các mẫu được định danh rõ ràng, lý tưởng là bằng cách kết hợp đánh giá hình thái và giải trình tự marker di truyền phù hợp (COI, cytB hoặc khác). Hạn chế này hy vọng sẽ sớm được giải quyết thông qua việc dần dần tích hợp các dữ liệu tham chiếu muỗi cát hiện còn lưu trữ nội bộ vào Nền tảng MSI do Assistance Publique–Hôpitaux de Paris, Sorbonne University, Pháp và bộ sưu tập BCCM/IHEM/Sciensano tại Brussels, Bỉ vận hành (https://msi.happy-dev.fr/). Khi dự kiến áp dụng định dạng

protein MALDI-ToF, mẫu nên được bảo quản ưu tiên bằng đông khô hoặc trong ethanol 70% cấp phân tử và không để tiếp xúc với nhiệt độ môi trường. Trong bối cảnh chưa có hướng dẫn chung về chuẩn bị mẫu, người sử dụng được khuyến nghị dùng dung dịch a xít sinapinic (30 mg/mL) trong 60% acetonitrile/0.3% TFA để chuẩn bị matrix MALDI-ToF nhằm đảm bảo phổ protein thu được có thể so sánh với các dữ liệu muỗi cát đã công bố.

Chuẩn bị mẫu cho MALDI-ToF MS (Hình 7)

Các mẫu côn trùng, được bảo quản trong điều kiện khác nhau, trước hết được làm khô trong không khí ở nhiệt độ phòng và giải phẫu. Đầu và bụng được tách ra để thu các phần cơ thể chứa các đặc điểm hình thái quan trọng cho việc gắn lam và phân tích hình thái. Ngực có thể được sử dụng cho MALDI-ToF và phần bụng còn lại được bảo quản để tách chiết DNA. Đối với phân tích cấu hình protein, ngực được đồng nhất hóa thủ công trong ống microtube 1,5 mL với 10 µL dung dịch đồng nhất hóa bằng chày nghiền dùng một lần và viên nghiền. Hai dung dịch đồng nhất hóa thường được sử dụng: nước cất vô trùng và a xít formic 25%.

7. Kết luận

Trong nghiên cứu này, chúng tôi hướng đến việc cung cấp cho các nhà nghiên cứu những phương pháp gắn tiêu bản muỗi cát hiệu quả nhất, phù hợp với từng mục tiêu nghiên cứu cụ thể, nhằm tạo điều kiện cho định danh chính xác và phát hiện tác nhân gây bệnh. Không có một phương pháp tối ưu duy nhất mang tính phổ quát; thay vào đó, tồn tại nhiều phương pháp, mỗi phương pháp đều có những ưu điểm và hạn chế riêng.

Trong phần Dữ liệu bổ sung, chúng tôi đã cung cấp các quy trình chi tiết cho nhiều kỹ thuật gắn tiêu bản khác nhau được sử dụng trong chuẩn bị và định danh muỗi cát. Các quy trình này, bao gồm cả video hướng dẫn, trình bày các bước thực hiện cụ thể phù hợp với từng mục tiêu khác nhau, nhằm đảm bảo kết quả chính xác và đáng tin cậy. Thông qua việc cung cấp nguồn tài liệu toàn diện này, chúng tôi mong muốn hỗ trợ các nhà nghiên cứu trong việc lựa chọn và áp dụng các kỹ thuật gắn tiêu bản phù hợp nhất với nhu cầu cụ thể của mình.

Acknowledgments

Các tác giả xin cảm ơn Richard Lane và Zoe Jay Adams từ Natural History Museum of London, Vương quốc Anh, vì đã phản biện xuất sắc, góp phần nâng cao chất lượng của bản thảo này.

Funding

Chúng tôi ghi nhận sự tài trợ cho nghiên cứu của AJA từ các cơ quan phát triển Brazil gồm CNPq (mã hồ sơ: 404395/2024-4) và Araucária Foundation (mã hồ sơ: 433/2025 PDI).

Conflicts of interest

Jérôme Depaquit là phó tổng biên tập của Parasite; ông không có bất kỳ ảnh hưởng nào đối với quá trình phản biện và ra quyết định đối với bản thảo này. Các tác giả còn lại tuyên bố không có xung đột lợi ích.

Data availability statement

Video trên Zenodo
Video 1: https://zenodo.org/records/18198006
Video 2: https://zenodo.org/records/18311158
Video 3: https://zenodo.org/records/18311106
Video 4: https://zenodo.org/records/18311154
Video 5: https://zenodo.org/records/18303014
Video 6: https://zenodo.org/records/18302850
Video 7: https://zenodo.org/records/18315029

Supplementary material

https://www.parasite-journal.org/10.1051/parasite/2026009/olm

References

1. Alkan C, Allal-Ikhlef AB, Alwassouf S, Baklouti A, Piorkowski G, de Lamballerie X, Izri A, Charrel RN. 2015. Virus isolation, genetic characterization and seroprevalence of Toscana virus in Algeria. Clinical Microbiology and Infection, 21(11), 1040 e1-9.
2. Alten B, Ozbel Y, Ergunay K, Kasap OE, Cull B, Antoniou M, Velo E, Prudhomme J, Molina R, Banuls AL, Schaffner F, Hendrickx G, Van Bortel W, Medlock JM. 2015. Sampling strategies for phlebotomine sand flies (Diptera: Psychodidae) in Europe. Bulletin of Entomological Research 105(6), 664–678.
3. Ayhan N, Baklouti A, Prudhomme J, Walder G, Amaro F, Alten B, Moutailler S, Ergunay K, Charrel RN, Huemer H. 2017. Practical guidelines for studies on sandfly-borne phleboviruses: Part I: Important points to consider *ante* field work. Vector-Borne and Zoonotic Diseases 17(1), 73–80.
4. Bates PA. 1997. Infection of phlebotomine sandflies with *Leishmania*, in The Molecular Biology of Insect Disease Vectors: A Methods Manual. Springer. p. 112–120.5.
5. Baum M, de Castro EA, Pinto MC, Goulart TM, Baura W, Klisiowicz Ddo R, Vieira da Costa-Ribeiro MC. 2015. Molecular detection of the blood meal source of sand flies (Diptera:

Psychodidae) in a transmission area of American cutaneous leishmaniasis, Parana State, Brazil. Acta Tropica, 143, 8–12.

6. Belen A, Alten B, Aytekin A. 2004. Altitudinal variation in morphometric and molecular characteristics of *Phlebotomus papatasi* populations. Medical and Veterinary Entomology, 18(4), 343–350.
7. Bhattacharya J, Chandra G, Hati AK. 1991. A simple method for cryopreservation of *Leishmania donovani* promastigotes, Indian Journal of Medical Research, 93, 245–246.
8. Caligiuri LG, Sandoval AE, Miranda JC, Pessoa FA, Santini MS, Salomón OD, Secundino NF, McCarthy CB. 2019. Optimization of DNA extraction from individual sand flies for PCR amplification. Methods and Protocols, 2(2), 36.
9. Casaril AE, de Oliveira LP, Alonso DP, de Oliveira EF, Gomes Barrios SP, de Oliveira Moura Infran J, Fernandes WS, Oshiro ET, Ferreira AMT, Ribolla PEM, de Oliveira AG. 2017. Standardization of DNA extraction from sand flies: Application to genotyping by next generation sequencing. Experimental Parasitology, 177, 66–72.
10. Castalanelli MA, Severtson DL, Brumley CJ, Szito A, Foottit RG, Grimm M, Munyard K, Groth DM. 2010. A rapid non-destructive DNA extraction method for insects and other arthropods. Journal of Asia-Pacific Entomology, 13(3), 243–248.
11. Cerqueira NL. 1943. Um novo meio para montagem de pequenos insetos em lâmina. Memórias do Instituto Oswaldo Cruz, (39), 37–41.
12. Charrel RN, Gallian P, Navarro-Mari JM, Nicoletti L, Papa A, Sanchez-Seco MP, Tenorio A, de Lamballerie X. 2005. Emergence of Toscana virus in Europe. Emerging Infectious Diseases, 11(11), 1657–1663.
13. Chaskopoulou A, Giantsis IA, Demir S, Bon MC. 2016. Species composition, activity patterns and blood meal analysis of sand fly populations (Diptera: Psychodidae) in the metropolitan region of Thessaloniki, an endemic focus of canine leishmaniasis. Acta Tropica, 158, 170–176.
14. Chen H, Rangasamy M, Tan SY, Wang H, Siegfried BD. 2010. Evaluation of five methods for total DNA extraction from western corn rootworm beetles. PLoS One, 5(8), e11963.
15. Depaquit J, Grandadam M, Fouque F, Andry PE, Peyrefitte C. 2010. Arthropod-borne viruses transmitted by Phlebotomine sandflies in Europe: a review. Eurosurveillance, 15(10), 19507.
16. Diamond LS, Herman CM. 1954. Incidence of Trypanosomes in the Canada Goose as revealed by bone marrow culture. Journal of Parasitology, 40(2), 195–202.
17. Ding H, Torno M, Vongphayloth K, Ng G, Tan D, Sng W, Ho K, Randrianambinintsoa FJ, Depaquit J, Tan CH. 2025. Hidden in plain sight: discovery of sand flies in Singapore and description of four species new to science. Parasites & Vectors, 18(1), 402.
18. Es-Sette N, Ajaoud M, Bichaud L, Hamdi S, Mellouki F, Charrel RN, Lemrani M. 2014. *Phlebotomus sergenti* a common vector of *Leishmania tropica* and Toscana virus in Morocco. Journal of Vector Borne Diseases, 51(2), 86–90.
19. Favret C. 2005. A new non-destructive DNA extraction and specimen clearing technique for aphids (Hemiptera). Proceedings of the Entomological Society of Washington, 107(2), 469–470.
20. Galati EAB. 2018. Phlebotominae (Diptera, Psychodidae): Classification, morphology and terminology of adults and identification of American taxa, in Brazilian Sand Flies: Biology, Taxonomy, Medical Importance and Control, Rangel EF, Shaw JJ, Editors. Cham: Springer International Publishing. pp. 9–212.
21. Galati EAB, de Andrade AJ, Perveen F, Loyer M, Vongphayloth K, Randrianambinintsoa FJ, Prudhomme J, Rahola N, Akhoundi M, Shimabukuro PHF, Depaquit J. 2025. Phle-botomine sand flies (Diptera, Psychodidae) of the world. Parasites & Vectors, 18(1), 220.
22. Galati EAB, Galvis-Ovallos F, Lawyer P, Leger N, Depaquit J. 2017. An illustrated guide for characters and terminology used in descriptions of Phlebotominae (Diptera, Psychodidae). Par-asite, 24, 26.
23. Gariepy T, Kuhlmann U, Gillott C, Erlandson M. 2007. Parasitoids, predators and PCR: the use of diagnostic molecular markers in biological control of Arthropods. Journal of Applied Entomology, 131(4), 225–240.
24. Giantsis IA, Chaskopoulou A, Bon MC. 2016. Mild-Vectolysis: A nondestructive DNA extraction method for vouchering sand flies and mosquitoes. Journal of Medical Entomology, 53(3), 692–695.
25. Gidwani K, Picado A, Rijal S, Singh SP, Roy L, Volfova V, Andersen EW, Uranw S, Ostyn B, Sudarshan M, Chakravarty J, Volf P, Sundar S, Boelaert M, Rogers ME. 2011. Serological markers of sand fly exposure to evaluate insecticidal nets against visceral leishmaniasis in India and Nepal: a cluster-randomized trial. PLoS Neglected Tropical Diseases, 5(9), e1296.
26. Gilbert MTP, Moore W, Melchior L, Worobey M. 2007. DNA extraction from dry museum beetles without conferring external morphological damage. PLoS One, 2(3), e272.
27. Giordani BF, Andrade AJ, Galati EAB, Gurgel-Goncalves R. 2017. The role of wing geometric morphometrics in the identification of sandflies within the subgenus *Lutzomyia*. Medical and Veterinary Entomology, 31(4), 373–380.
28. Guzmán-Larralde AJ, Suaste-Dzul AP, Gallou A, Peña-Carrillo KI. 2017. DNA recovery from microhymenoptera using six non-destructive methodologies with considerations for subsequent preparation of museum slides. Genome, 60(1), 85–91.
29. Hajibabaei M, DeWaard JR, Ivanova NV, Ratnasingham S, Dooh RT, Kirk SL, Mackie PM, Hebert PD. 2005. Critical factors for assembling a high volume of DNA barcodes. Philosophical Transactions of the Royal Society B: Biological Sciences, 360(1462), 1959–1967.
30. Haouas N, Pesson B, Boudabous R, Dedet JP, Babba H, Ravel C. 2007. Development of a molecular tool for the identification of *Leishmania* reservoir hosts by blood meal analysis in the insect vectors. American Journal of Tropical Medicine and Hygiene, 77(6), 1054–1059.
31. Hlavackova K, Dvorak V, Chaskopoulou A, Volf P, Halada P. 2019. A novel MALDI-TOF MS-based method for blood meal identification in insect vectors: A proof of concept study on phlebotomine sand flies. PLoS Neglected Tropical Diseases, 13 (9), e0007669.
32. Huemer H, Prudhomme J, Amaro F, Baklouti A, Walder G, Alten B, Moutailler S, Ergunay K, Charrel RN, Ayhan N. 2017. Practical guidelines for studies on sandfly-borne phleboviruses: Part II: Important points to consider for fieldwork and subsequent virological screening. Vector-Borne and Zoonotic Diseases, 17(1), 81–90.
33. Jancarova M, Polanska N, Thiesson A, Arnaud F, Stejskalova M, Rehbergerova M, Kohl A, Viginier B, Volf P, Ratinier M. 2025. Susceptibility of diverse sand fly species to Toscana virus. PLoS Neglected Tropical Diseases, 19(5), e0013031.
34. Kapp JD, Green RE, Shapiro B. 2021. A fast and efficient single-stranded genomic library preparation method optimized for ancient DNA. Journal of Heredity, 112(3), 241–249.

35. Killick-Kendrick R, Maroli M, Killick-Kendrick M. 1991. Bibliography of the colonization of phlebotomine sandflies. Parassitologia, 33(suppl.), 321–333.
36. Lawyer P, Killick-Kendrick M, Rowland T, Rowton E, Volf P. 2017. Laboratory colonization and mass rearing of phlebotomine sand flies (Diptera, Psychodidae). Parasite, 24, 42.
37. Léger N, Pesson B, Madulo-Leblond G. 1986. Les phlébotomes de Grèce : 1ère partie. Bulletin de la Société de Pathologie Exotique, 79, 386–397.
38. Léger N, Pesson B, Madulo-Leblond G. 1986. Les phlébotomes de Grèce : 2ème partie. Bulletin de la Société de Pathologie Exotique, 79, 514–524.
39. Leonel JAF, Vioti G, Alves ML, da Silva DT, Meneghesso PA, Benassi JC, Spada JCP, Galvis-Ovallos F, Soares RM, Oliveira T. 2020. DNA extraction from individual Phlebotomine sand flies (Diptera: Psychodidae: Phlebotominae) specimens: Which is the method with better results? Experimental Parasitology, 218, 107981.
40. Lestinova T, Rohousova I, Sima M, de Oliveira CI, Volf P. 2017. Insights into the sand fly saliva: Blood-feeding and immune interactions between sand flies, hosts, and *Leishmania.* PLoS Neglected Tropical Diseases, 11(7), e0005600.
41. Lienhard A, Schaffer S. 2019. Extracting the invisible: obtaining high quality DNA is a challenging task in small arthropods. PeerJ, 7, e6753.
42. Lozano-Sardaneta YN, Mikery-Pacheco OF, Huerta H, Rojas-Soriano JE, Contreras-Ramos A. 2025. Wing geometric morphometrics is effective to separate sand fly species (Diptera, Psychodidae, Phlebotominae) related with leishmaniasis transmission in Mexico. Acta Tropica, 262, 107523.
43. Mandrioli M. 2008. Insect collections and DNA analyses: how to manage collections? Museum Management and Curatorship, 23(2), 193–199.
44. Maroli M, Feliciangeli MD, Bichaud L, Charrel RN, Gradoni L. 2013. Phlebotomine sandflies and the spreading of leishmaniases and other diseases of public health concern. Medical and Veterinary Entomology, 27(2), 123–147.
45. Marquina D, Buczek M, Ronquist F, Lukasik P. 2021. The effect of ethanol concentration on the morphological and molecular preservation of insects for biodiversity studies. PeerJ, 9, e10799.
46. Mathis A, Depaquit J, Dvorak V, Tuten H, Banuls AL, Halada P, Zapata S, Lehrter V, Hlavackova K, Prudhomme J, Volf P, Sereno D, Kaufmann C, Pfluger V, Schaffner F. 2015. Identification of phlebotomine sand flies using one MALDI-TOF MS reference database and two mass pectrometer systems., Parasites & Vectors, 8, 266.
47. Mekarnia N, Benallal KE, Sadlova J, Vojtkova B, Mauras A, Imbert N, Longhitano M, Harrat Z, Volf P, Loiseau PM, Cojean S. 2024. Effect of *Phlebotomus papatasi* on the fitness, infectivity and antimony-resistance phenotype of antimony-resistant *Leishmania* major Mon-25. International Journal for Parasitology – Drugs and Drug Resistance, 25, 100554.
48. Milligan BG. 1998. Total DNA isolation, in Molecular Genetic Analysis of Population: A Practical Approach, Hoelzel AR, Editor. Oxford: Oxford University Press.
49. Molina R, Jiménez M, Alvar J, González E, Hernández-Taberna S, Ines MM. 2017. Methods in sand fly research. Madrid: Servicio de publicaciones Universidad de Alcalá de Henares, Madrid.
50. Murphy WJ, Eizirik E, O'Brien SJ, Madsen O, Scally M, Douady CJ, Teeling E, Ryder OA, Stanhope MJ, de Jong WW, Springer MS. 2001. Resolution of the early placental mammal radiation using Bayesian phylogenetics. Science, 294(5550), 2348–2351.
51. Nacif-Pimenta R, Pinto LC, Volfova V, Volf P, Pimenta PFP, Secundino NFC. 2020. Conserved and distinct morphological aspects of the salivary glands of sand fly vectors of leishma-niasis: an anatomical and ultrastructural study. Parasites & Vectors, 13(1), 441.
52. Neuhaus B, Schmid T, Riedel J. 2017. Collection management and study of microscope slides: Storage, profiling, deterioration, restoration procedures, and general recommendations. Zootaxa, 4322(1), 1–173.
53. New TR. 1974. Pscoptera. Handbooks for Identification of British Insects (Vol. I). London: Royal Entomological Society of London. 102 pp.
54. Perez-Ruiz M, Collao X, Navarro-Mari JM, Tenorio A. 2007. Reverse transcription, real-time PCR assay for detection of Toscana virus. Journal of Clinical Virology, 39(4), 276–281.
55. Porco D, Rougerie R, Deharveng L, Hebert P. 2010. Coupling non-destructive DNA extraction and voucher retrieval for small soft-bodied Arthropods in a high-throughput context: the example of Collembola. Molecular Ecology Resources, 10(6), 942–945.
56. Prudhomme J, Cassan C, Hide M, Toty C, Rahola N, Vergnes B, Dujardin JP, Alten B, Sereno D, Banuls AL. 2016. Ecology and morphological variations in wings of *Phlebotomus ariasi* (Diptera: Psychodidae) in the region of Roquedur (Gard, France): a geometric morphometrics approach. Parasites & Vectors, 9(1), 578.
57. Prudhomme J, Gunay F, Rahola N, Ouanaimi F, Guernaoui S, Boumezzough A, Banuls AL, Sereno D, Alten B. 2012. Wing size and shape variation of *Phlebotomus papatasi* (Diptera: Psychodidae) populations from the south and north slopes of the Atlas Mountains in Morocco. Journal of Vector Ecology, 37(1), 137–147.
58. Prudhomme J, Toty C, Kasap OE, Rahola N, Vergnes B, Maia C, Campino L, Antoniou M, Jimenez M, Molina R, Cannet A, Alten B, Sereno D, Banuls AL. 2015. New microsatellite markers for multi-scale genetic studies on *Phlebotomus ariasi* Tonnoir, vector of *Leishmania infantum* in the Mediterranean area. Acta Tropica, 142, 79–85.
59. Prudhomme J, Velo E, Bino S, Kadriaj P, Mersini K, Gunay F, Alten B. 2019. Altitudinal variations in wing morphology of *Aedes albopictus* (Diptera, Culicidae) in Albania, the region where it was first recorded in Europe. Parasite, 26, 55.
60. Rawlins DJ. 1992. Light Microscopy: An Introduction to Biotechniques. Oxford: Bios Scientific publishers. 143 pp.
61. Ready PD. 2013. Biology of phlebotomine sand flies as vectors of disease agents. Annual Review of Entomology, 58, 227–250.
62. Rohlf FJ, Slice D. 1990. Extensions of the Procrustes method for the optimal superimposition of landmarks. Systematic Zoology, 39(1), 40–59.
63. Rowley DL, Coddington JA, Gates MW, Norrbom AL, Ochoa RA, Vandenberg NJ, Greenstone MH. 2007. Vouchering DNA-barcoded specimens: Test of a nondestructive extraction proto-col for terrestrial arthropods. Molecular Ecology Notes, 7(6), 915–924.
64. Sábio PB, Andrade AJ, Galati EAB. 2014. Assessment of the taxonomic status of some species included in the *Shannoni* complex, with the description of a new species of *Psathyromyia*

(Diptera: Psychodidae: Phlebotominae). Journal of Medical Entomology, 51(2), 331–341.
65. Sadlova J, Yeo M, Seblova V, Lewis MD, Mauricio I, Volf P, Miles MA. 2011. Visualisation of *Leishmania donovani* fluorescent hybrids during early stage development in the sand fly vector. PLoS One, 6(5), e19851.
66. Sales K, Miranda DEO, da Silva FJ, Otranto D, Figueredo LA, Dantas-Torres F. 2020. Evaluation of different storage times and preservation methods on phlebotomine sand fly DNA concentration and purity. Parasites & Vectors, 13(1), 399
67. Sales KG, Costa PL, de Morais RC, Otranto D, Brandao-Filho SP, Cavalcanti Mde P, Dantas-Torres F. 2015. Identification of phlebotomine sand fly blood meals by real-time PCR. Parasites& Vectors, 8, 230.
68. Sant'Anna MR, Jones NG, Hindley JA, Mendes-Sousa AF, Dillon RJ, Cavalcante RR, Alexander B, Bates PA. 2008. Blood meal identification and parasite detection in laboratory-fed and field-captured *Lutzomyia longipalpis* by PCR using FTA databasing paper. Acta Tropica, 107(3), 230–237.
69. Senne NA, Santos HA, Araujo TR, Paulino PG, Mendonca LP, Moreira HVS, Camilo TA, da Costa Angelo I. 2022. Robust comparative performance of genomic DNA extraction methods from non-engorged phlebotomine sandflies. Medical and Veterinary Entomology, 36(2), 203–211.
70. Shaw JJ. 2025. A review of Leishmania infections in American Phlebotomine sand flies – Are those that transmit leishmaniasis anthropophilic or anthropportunists? Parasite, 32, 57.
71. Tesh RB, Modi GB. 1983. Growth and transovarial transmission of Chandipura virus (Rhabdoviridae: Vesiculovirus) in *Phlebotomus papatasi*. American Journal of Tropical Medicine and Hygiene, 32(3), 621–623.
72. Thomsen PF, Elias S, Gilbert MTP, Haile J, Munch K, Kuzmina S, Froese DG, Sher A, Holdaway RN, Willerslev E. 2009. Non-destructive sampling of ancient insect DNA. PLoS One, 4(4),e5048
73. Truett GE, Heeger P, Mynatt RL, Truett AA, Walker JA, Warman ML. 2000. Preparation of PCR-quality mouse genomic DNA with hot sodium hydroxide and tris (HotSHOT). Biotechniques, 29(1), 52–54.
74. Upton MS. 1993. Aqueous gum-chloral slide mounting media: an historical review. Bulletin of Entomological Research, 83(2), 267–274.
75. Volf P, Myskova J. 2007. Sand flies and Leishmania: specific versus permissive vectors. Trends in Parasitology, 23(3), 91–92.
76. Wang Q, Wang X. 2012. Comparison of methods for DNA extraction from a single chironomid for PCR analysis. Pakistan Journal of Zoology, 44(2), 421–426.
77. Wang Y, Zhao Y, Bollas A, Wang Y, Au KF. 2021. Nanopore sequencing technology, bioinformatics and applications. Nature Biotechnology, 39(11), 1348–1365.

Cite this article as: Randrianambinintsoa FJ, Augendre L, Prudhomme J, Martinet J-P, Loyer M, Mekarnia N, Kerkoub H, Perveen FK, Huguenin A, Kariya E, Akhoundi M, De Andrade AJ, Berriatua E, Bongiorno G, Boyer S, Christodoulou V, Da Costa-Ribeiro MCV, De Souza LAF, Ding H, Dondji B, Dvořák V, Erisoz Kasap O, Galati EAB, Gállego M, Ballart C, Gouzelou S, Haddad N, Masse RS, Mekuria AH, Ivovic V, Kaczmarek S, Shahar MK, Kirstein OD, Kniha E, Kolářová I, Lincoln T, Lucanas C, Mikov O, Nov K, Özbel Y, Pesson B, Posada Lopez LC, Prasetyo DB, Rahola N, Rebollar-Tellez EA, Rodrigues BL, Roy L, Saini P, Sanjoba C, Shimabukuro PH, Siriyasatien P, Soszynska A, Suleşco T, Sylla M, Torno M, Volf P, Vongphayloth K, Sinh Nam V, Wardhana A, Yessinou E, Zapata S, Gantier J-C & Depaquit J. 2026. Processing and mounting phlebotomine sand flies: a consensus guideline. Parasite xx, xx. https://doi.org/10.1051/parasite/2026009.

PARASiTE

An international open-access, peer-reviewed, online journal publishing high quality papers on all aspects of human and animal parasitology

Reviews, articles and short notes may be submitted. Fields include, but are not limited to: general, medical and veterinary parasitology; morphology, including ultrastructure; parasite systematics, including entomology, acarology, helminthology and protistology, and molecular analyses; molecular biology and biochemistry; immunology of parasitic diseases; host-parasite relationships; ecology and life history of parasites; epidemiology; therapeutics; new diagnostic tools.
All papers in Parasite are published in English. Manuscripts should have a broad interest and must not have been published or submitted elsewhere. No limit is imposed on the length of manuscripts.

Parasite (open-access) continues **Parasite** (print and online editions, 1994-2012) and **Annales de Parasitologie Humaine et Comparée** (1923-1993) and is the official journal of the Société Française de Parasitologie.

Editor-in-Chief:
Jean-Lou Justine, Paris

Submit your manuscript at:
https://www.editorialmanager.com/parasite

Phu luc

Phụ lục 1: Cơ sở lý thuyết sinh hóa

Các động vật chân đốt được đề cập ở đây là muỗi cát. Tuy nhiên, nguyên lý chung có thể mở rộng cho các nhóm chân đốt phổ biến khác mà việc định danh chỉ có thể thực hiện dựa trên các đặc điểm hình thái bên trong. Ngẫu nhiên, một số cơ quan nội tạng được kitin hóa một phần và hình thái của chúng cung cấp nhiều thông tin giá trị. Vì vậy, việc quan sát bơm thức ăn, túi chứa tinh (spermathecae) và các ống dẫn của chúng là rất quan trọng.
Với tất cả các thuốc thử sẽ được trình bày dưới đây, cần luôn ghi nhớ rằng từ giai đoạn cố định côn trùng đến khi gắn tiêu bản, về bản chất chúng ta đang áp dụng các phản ứng oxy hóa – khử (redox). Nguyên tắc quan trọng nhất là tránh trộn lẫn các thuốc thử khử với các thuốc thử oxy hóa.

Ethyl alcohol; ethanol
Chất này được sử dụng theo nhiều cách khác nhau. Phân tử rượu có ái lực mạnh với nước nên có tác dụng khử nước. Tuy nhiên, rượu nồng độ thấp (tức là chứa nhiều nước) lại góp phần làm thoái hóa a xít nucleic (nước là "kẻ thù" của a xít nucleic).
Khi côn trùng được đặt trong ethanol, mục đích không chỉ là bảo quản mà còn để cố định mô. Trong mô học, thường phân biệt hai khái niệm quan trọng: tốc độ thấm và tốc độ cố định. Một chất bảo quản tốt phải nhanh chóng thấm sâu vào mô trước khi cố định chúng. Với ethanol 96%, hệ số thấm xấp xỉ 1,05 (so sánh: dung dịch a xít picric 0,75% có hệ số 0,45; dung dịch kali dicromat 3% có hệ số 1,45).
Việc bảo quản côn trùng và các chân đốt khác vô thời hạn trong ethanol là thực tế phổ biến trong côn trùng học. Mục tiêu lưu giữ mẫu thu thập ngoài thực địa cho các nghiên cứu sau này là hoàn toàn chính đáng. Tuy nhiên, đối với nhà tế bào học hoặc mô học, điều này không khả thi. Nếu mẫu được giữ quá lâu trong dung dịch cố định, chúng có thể trở nên gần như không thể xử lý lại. Vì vậy, các mẫu trên 10 năm tuổi thường khó hoặc không thể sử dụng.
Một yếu tố khác là tỷ lệ giữa khối lượng mẫu và thể tích dung dịch cố định. Trong thực hành động vật học hoặc y học, nên dùng thể tích dung dịch gấp 60 lần thể tích mẫu. Trong thực tế với vi chân đốt, nên thêm ít nhất 4–5 thể tích ethanol so với thể tích mẫu. Lưu ý rằng ethanol sẽ giảm nồng độ khi hấp thu nước từ mô.

Kết luận:

- Ethanol là tác nhân khử (không tương thích với chất cố định oxy hóa);
- Làm kết tủa mạnh protein và gây biến tính;
- Hòa tan một số lipid phức và làm kết tủa glycogen;
- Gây co rút mạnh và làm cứng mô.

Dung dịch kiềm mạnh: kali hoặc natri hydroxide
Trong côn trùng học, việc sử dụng các dung dịch kiềm mạnh chủ yếu tập trung vào kali hydroxide (KOH), dù không phải lúc nào cũng có giải thích rõ ràng cho sự lựa chọn này.
Natri hydroxide [E524] tồn tại ở dạng dung dịch với nhiều nồng độ hoặc độ chuẩn khác nhau; cũng có ở dạng viên hoặc dạng hạt. Nhược điểm chính là tính hút ẩm rất mạnh (mạnh hơn KOH). Khi phản ứng với protein, NaOH hòa tan chúng; với lipid, nó chuyển lipid thành xà phòng rắn trong quá trình xà phòng hóa (đây là điểm khác biệt quan trọng so với KOH, vốn tạo ra xà phòng lỏng trong quá trình này).
Kali hydroxide [E525] có sẵn dưới dạng dung dịch đậm đặc, nhưng đặc biệt thuận lợi khi được bào chế ở dạng viên khoảng 0,1 g, giúp dễ dàng pha dung dịch loãng khi không có cân chính xác. Ví dụ: 1 viên 0,1 g hòa tan trong 1 mL nước cất sẽ tạo dung dịch 10%. Ưu điểm thứ hai của KOH dạng viên là ít nhạy với quá trình carbonat hóa hơn (dung dịch KOH có ái lực cao với CO_2, tạo thành muối carbonat).
Các bazơ mạnh này được sử dụng để hòa tan a xít béo bằng cách chuyển chúng thành xà phòng tan trong nước. Cần nhớ rằng chất cố định như ethanol đã hòa tan một phần lipid trong mẫu. Tuy nhiên, khi chuyển mẫu sang môi trường nước có chứa bazơ mạnh, các a xít béo (ít nhiều phức tạp) có thể kết tủa. Bazơ mạnh sẽ thực hiện quá trình xà phòng hóa nguội. Trong một số trường hợp, khi mô mỡ quá nhiều (ví dụ ở con cái), nên tăng nhiệt độ lên 35–40°C để thúc đẩy phản ứng, hoặc kéo dài thời gian tiếp xúc ở nhiệt độ phòng.

Dung dịch a xít có màu / dung dịch Marc-André không màu
Ở đây, chúng ta xem xét những ưu điểm và hạn chế của dung dịch Marc-André. Dung dịch này gồm chloral hydrate (trichloroacetaldehyde monohydrate), a xít acetic và nước. Đây là dung dịch có tính oxy hóa mạnh (hỗn hợp a xít và aldehyde).
Dung dịch này sẽ:

- Trung hòa lượng kali hydroxide dư còn lại trong mẫu, mà không làm kết tủa xà phòng kiềm đã hình thành;
- Oxy hóa các nhóm alcohol bậc hai của glucosamine trong cấu trúc kitin, từ đó làm mềm kitin;
- Hòa tan một số muối khoáng hiện diện trong mẫu.

Khi dung dịch Marc-André được nhuộm trước bằng a xít fuchsin (ở trạng thái oxy hóa), nó có thể gắn vào các nhóm alcohol bậc hai trong cấu trúc. Sau thời gian tiếp xúc thích hợp và đạt mức độ nhuộm mong muốn, mẫu chỉ được rửa

This is an Open Access article distributed under the terms of the Creative Commons Attribution License (https://creativecommons.org/licenses/by/4.0), which permits unrestricted use, distribution, and reproduction in any medium, provided the original work is properly cited.

bằng ethanol. Khi đó, chúng ta bắt đầu giai đoạn khử nước của mẫu.

Lợi ích:

- Trung hòa dung dịch kiềm dư;
- Làm mềm (giãn) kitin;
- Nhuộm kitin giúp đánh giá rõ hơn các cấu trúc bên trong đã kitin hóa.

Hạn chế:

- Chloral hydrate có tác dụng gây ngủ và từng được sử dụng trong y học; phải thao tác trong tủ hút hóa chất và tuân thủ đầy đủ quy định về an toàn hóa chất.

Dung dịch khử nước

Kinh nghiệm cho thấy đối với mẫu rất nhỏ, không cần thiết phải thực hiện đầy đủ chuỗi bể ethanol tăng dần nồng độ. Nếu mẫu lớn, nên bắt đầu với ethanol 80%, sau đó 90%, 95% và cuối cùng là ethanol tuyệt đối. Với mẫu rất nhỏ, chỉ cần bể ethanol 90% rồi chuyển sang ethanol tuyệt đối. Ở giai đoạn này, cần luôn ghi nhớ rằng ethanol tuyệt đối có xu hướng hấp thu nước từ không khí.
Theo truyền thống trong các phòng thí nghiệm côn trùng học, quá trình khử nước mẫu vật thường được hoàn tất bằng cách ngâm mẫu trong dung dịch creosote gỗ sồi. Hiện nay, việc sử dụng chất này bị khuyến cáo mạnh mẽ do mùi khó chịu (chứa hydrocarbon thơm đa vòng) và do được cho là có khả năng gây độc sinh sản, gây ung thư, là chất ô nhiễm hữu cơ bền và độc hại với sinh vật thủy sinh.
Giải pháp được đề xuất để chuẩn bị mẫu gắn là sử dụng Euparal® và tinh chất Euparal® (được mô tả ở phần sau). Hỗn hợp Euparal® và tinh chất Euparal® đặc biệt phù hợp cho các mẫu sau khi đã qua bể ethanol 90%.

Phụ lục 2: Thành phần thuốc thử

Kali hydroxide 10%
Kali hydroxide 10 g
Nước cất vừa đủ 100 mL
Môi trường gắn Hoyer (Gôm chloral)
Nước cất 50 mL
Chloral hydrate 200 g
Gôm arabic 50 g
Glycerol 20 mL
Dung dịch Marc-André
Chloral hydrate 40 g
A xít acetic băng 30 mL
Nước cất 30 mL
Fuchsin acid 1%
Bột a xít fuchsin 1 g
Nước cất 99 mL
Dung dịch Marc-André nhuộm fuchsin
Dung dịch Marc-André 10 mL
Fuchsin 1% 50 µL

Phụ lục 3: Euparal®, Canada Balsam, polyvinyl alcohol và các dung dịch khác

Polyvinyl alcohol (PVA)
Đây là môi trường gắn lý tưởng khi không có đầy đủ hóa chất cần thiết để thực hiện khử nước đúng quy trình. Polyvinyl alcohol được trộn với lactophenol của Amman. Tuy nhiên, các tiêu bản này có nhược điểm lớn là dễ bị khô, hoặc PVA kết tinh do nước bay hơi, hoặc bị sẫm màu khi phenol bị oxy hóa. Dù vậy, đây vẫn là kỹ thuật phù hợp cho gắn tiêu bản ngắn hạn.

Canada Balsam
Việc sử dụng để gắn giữa lam kính và lamen đòi hỏi mẫu phải được khử nước hoàn toàn. Việc sử dụng xylene hoặc toluene không phải không có bất tiện, đặc biệt liên quan đến an toàn và độc tính.

Môi trường Enecê
Tương tự Canada Balsam, khi gắn giữa lam kính và lamen, cần phải khử nước mẫu trước.
Công thức Enecê:
- Colophony trắng tinh khiết: 22 g
- Nhựa copal tan trong cồn: 12 g
- Cồn tuyệt đối: 20 mL
- Long não: 10 g
- Tinh dầu thông (turpentine essence): 10 mL
- Eucalyptol: 26 mL

Cách pha chế:
Trong một bình (ví dụ bình tam giác Erlenmeyer), cho cồn tuyệt đối và long não trước. Sau đó thêm colophony và nhựa copal. Đậy kín bình, lắc đều rồi đun cách thủy ở nhiệt độ nhẹ, tránh để sôi. Khi hỗn hợp hóa lỏng hoàn toàn, thêm tinh dầu thông, lọc khi còn nóng, và cuối cùng thêm eucalyptol vào dịch lọc.
Khi môi trường trở nên kém lỏng, có thể pha loãng bằng dung dịch Enecê có công thức:
- Cồn tuyệt đối: 30 mL
- Long não: 17 g
- Tinh dầu thông: 15 mL
- Eucalyptol: 38 mL

(Cerqueira, 1943).

Euparal®
Đây là một loại nhựa thu được từ cây bách Atlas *Tetraclinis articulata* (Vahl, 1791), được Gilson nghiên cứu và phát triển năm 1906. Ưu điểm chính là không polyme hóa. Các mẫu gắn giữa lam kính và lamen có thể dễ dàng thu hồi lại bằng ethanol hoặc tốt hơn là bằng tinh chất Euparal®. Loại nhựa này, còn gọi là sandarac, chấp nhận mẫu từ ethanol 80%.

Sử dụng Triton X100 (dung dịch nước không ion)
Triton X100 là dung dịch nước không ion (4-(1,1,3,3-tetramethylbutyl) phenyl-polyethylene glycol, còn gọi là t-octylphenoxypolyethoxyethanol hoặc polyethylene glycol tert-octylphenyl ether), được sử dụng rộng rãi như một chất tẩy rửa trong sinh học tế bào và phân tử. Nó giúp làm tăng tính thấm của màng tế bào và màng nhân.
Mẫu côn trùng được bảo quản trong ethanol nhiều năm là điều rất phổ biến. Tuy nhiên, bảo quản lâu trong ethanol không tối ưu, khiến các chân đốt trở nên khó xử lý khi quan sát hiển vi. Hộp nhựa chứa mẫu có thể xuống cấp, dẫn đến bay hơi ethanol. Trong cả hai trường hợp (tiếp xúc lâu với ethanol hoặc mẫu bị khô), việc chuẩn bị tiêu bản gặp nhiều khó khăn.
Năm 2008, Jonque công bố một ghi chú về tái hydrat hóa nhện bằng chất làm ướt Agepon (dùng cho phim ảnh). Điều này gợi ý sử dụng các chất làm ướt không phải là chất tẩy rửa mạnh.

Quy trình sử dụng Triton X100 0,5%:
- Tẩm mẫu khô bằng ethanol tuyệt đối.
- Thêm dung dịch Triton X100 0,5% đủ để ngập hoàn toàn mẫu.
- Để yên khoảng 5 phút hoặc hơn, đến khi các chân đốt tách rời hoàn toàn trong dung dịch.
- Loại bỏ dung dịch Triton X100 và thay bằng dung dịch KOH.
- Tiếp tục quy trình như đã mô tả ở trên.

Phụ lục 4: Quy trình gắn Euparal® hoặc Canada Balsam từng bước

1. Mẫu phải được khử nước hoàn toàn (xuất hiện đục hoặc trắng sữa là dấu hiệu khử nước chưa đủ).
2. Khử nước bằng dãy nồng độ ethanol tăng dần.
3. Có thể chuyển mẫu từ ethanol 99% hoặc tuyệt đối sang chất làm trong.

Quy trình

1. Cho ruồi cát trưởng thành trong ethanol 70%.
2. Loại bỏ ethanol, thay bằng KOH 10%. Đậy lam kính lên mẫu.
3. Làm nhũn (macerate) đến khi côn trùng trở nên trong suốt.
4. Loại bỏ KOH.
5. Phủ mẫu bằng nước cất, chờ 30–45 phút.
6. Loại bỏ nước và rửa lại bằng nước cất trong 30 phút (thời gian phụ thuộc số lượng mẫu: càng nhiều mẫu xử lý cùng lúc, thời gian cần càng dài; xử lý riêng lẻ có thể rút ngắn).
7. Loại bỏ nước.
8. Thêm dung dịch Marc-André (có thể nhuộm a xít fuchsin) và chờ 24 giờ.
9. Loại bỏ dung dịch Marc-André.
10. Phủ nước cất, chờ 30–45 phút.
11. Loại bỏ nước và rửa lại bằng nước cất trong 30 phút.
12. Loại bỏ nước.
13. Thêm ethanol 70% và tiến hành phẫu tích:
    a. Với đầu và bụng, nhẹ nhàng tách khỏi ngực.
    b. Với ngực, tách cánh bằng cách giữ ngực bằng một kim mổ và kéo phần gốc phụ bộ bằng kim khác. Có thể phẫu tích dọc (sagittal), chia ngực thành hai nửa trái – phải tùy mục đích quan sát.
14. Khử nước dần qua dãy ethanol 50% – 80% – 95% – đến tuyệt đối.
15. Rửa hai lần bằng ethanol 100%, mỗi lần 10 phút.
16. Loại bỏ ethanol và ngâm trong dầu đinh hương 15 phút ở nhiệt độ phòng.
17. Chuyển mẫu từ dầu đinh hương sang một giọt Euparal® hoặc Canada Balsam trên lam kính sạch.
18. Sắp xếp theo yêu cầu: đầu, ngực và bụng có thể phẫu tích dưới kính soi nổi bằng kim mảnh hoặc nhíp. Đầu phải được gắn ở tư thế bụng–lưng (ventro-dorsal), tức là lỗ chẩm (occipital foramen) hướng lên trên để có thể quan sát trực tiếp cibarium qua đó.

Việc phẫu tích được thực hiện ngay trong môi trường gắn.

19. Để mẫu đến khi bề mặt trở nên dính.
20. Làm ướt lamen sạch bằng ethanol tuyệt đối. Đặt lamen nghiêng lên giọt Canada Balsam.
21. Bảo quản lam kính trong hộp khô chuyên dụng.
