# Supplementary material for: Processing and mounting phlebotomine sand flies: a consensus guideline
Source: Parasite. 2026 Apr 3;33:18. doi: 10.1051/parasite/2026009 (PMC13047900; doi:10.1051/parasite/2026009)
Supplement: Supplementary file 34 — Wolof translation / Tekki ci lakku wolof [file parasite-33-18-s34.pdf]

Parasite **33**, 18 (2026)
© F.J. Randrianambinintsoa et al., published by EDP Sciences, 2026
https://doi.org/10.1051/parasite/2026009

PARASITE
Available online at:
www.parasite-journal.org

REVIEW ARTICLE OPEN ACCESS

Naka lañuy liggéeyee ak samp yuul yi (yooy yi): ab gindikaay bu ñépp dëppoo

Fano José Randrianambinintsoa[1], Laure Augendre[1], Jorian Prudhomme[1], Jean-Philippe Martinet[1], Mathieu Loyer[1], Nalia Mekarnia[1], Hocine Kerkoub[1], Farzana Khan Perveen[1], Antoine Huguenin[1,2], Emilie Kariya[1,2], Mohammad Akhoundi[3], Andrey José de Andrade[4], Eduardo Berriatua[5], Gioia Bongiorno[6], Sébastien Boyer[7,8], Vasiliki Christodoulou[9], Magda Clara Vieira Da Costa-Ribeiro[10], Lucas Alexandre Farias de Souza[10], Huicong Ding[11], Blaise Dondji[12], Vít Dvořák[13], Ozge Erisoz Kasap[14], Eunice Aparecida Bianchi Galati[15], Montserrat Gállego[16], Cristina Ballart[16], Stavroula Gouzelou[17], Nabil Haddad[18], Rezki Sabrina Masse[19], Asrat Hailu Mekuria[20], Vladimir Ivovic[21], Szymon Kaczmarek[22], Mohd Khadri Shahar[19], Oscar D. Kirstein[23], Edwin Kniha[24], Iva Kolářová[13], Lincoln Timinao[25], Cristian Lucanas[26], Ognyan Mikov[27], Kimsear Nov[7], Yusuf Özbel[28], Bernard Pesson[29], Laura Cristina Posada Lopez[30], Didot Budi Prasetyo[1,7], Nil Rahola[31], Eduardo A. Rebollar-Tellez[32], Bruno Leite Rodrigues[15], Lalita Roy[33], Prasanta Saini[34], Chizu Sanjoba[35], Paloma Helena Fernandes Shimabukuro[36], Padet Siriyasatien[37], Agnieszka Soszyńska[22], Tatiana Suleşco[38], Massamba Sylla[39], Majhalia Torno[40], Petr Volf[13], Khamsing Vongphayloth[41], Vu Sinh Nam[42], April Wardhana[43], Eric Yessinou[44], Sonia Zapata[45], Jean-Charles Gantier[1], and Jérôme Depaquit[1,2,*]

[1] Faculté de Pharmacie, Université de Reims Champagne Ardenne, UR ESCAPE-USC ANSES PETARD, 51 rue Cognacq-Jay, 51096 Reims Cedex, France
[2] Pôle de Biologie territoriale, Laboratoire de Parasitologie-Mycologie, Centre Hospitalo-Universitaire, 51092 Reims, France
[3] Parasitology-Mycology Department, Avicenne Hospital, AP-HP, Bobigny, Sorbonne Paris Nord University, France; Unité des Virus Émergents (UVE: Aix-Marseille Univ, Università di Corsica, IRD 190, Inserm 1207, IRBA), 13005 Marseille, France
[4] Parasitology Collection of Basic Pathology, Department of Basic Pathology, Federal University of Paraná, Curitiba 19031, Brazil
[5] Department of Animal Health, University of Murcia, Campus de Espinardo, 30100 Espinardo, Murcia, Spain
[6] Department of Infectious Diseases, Vector-borne Diseases Unit, Istituto Superiore di Sanità, 00166 Rome, Italy
[7] Medical and Veterinary Entomology Unit, Institut Pasteur du Cambodge, Phnom Penh 12201, Cambodia
[8] Ecology & Emergence of Arthropod-borne Pathogens Unit, Department of Global Health, Institut Pasteur, CNRS UMR2000, 75015 Paris, France
[9] Section Veterinary Services (1417), Laboratory for Animal Health Virology, Aglantzia, Nicosia 2109, Cyprus
[10] Insects Vectors and Parasites Laboratory, Department of Basic Pathology and Postgraduate program in Microbiology, Parasitology and Pathology, Federal University of Paraná, 81530-900 Curitiba, Brazil
[11] Department of Biological Sciences, National University of Singapore, 117558, Singapore
[12] Laboratory of the Leishmaniasis Research Project, Mokolo District Hospital, Mokolo, Cameroon; Laboratory of Cellular Immunology and Parasitology, Department of Biological Sciences, Central Washington University, 98926 Ellensburg, WA, USA
[13] Department of Parasitology, Faculty of Science, Charles University, 12800 Prague, Czechia
[14] VERG Laboratories, Department of Biology, Faculty of Science, Hacettepe University, Beytepe, Ankara 06800, Türkiye
[15] Faculdade de Saúde Pública da Universidade de São Paulo (FSP/USP), Pós-graduação em Saúde Pública, 01246-904 São Paulo, Brazil
[16] Secció de Parasitologia, Departament de Biologia, Sanitat i Medi Ambient, Facultat de Farmàcia i Ciències de l'Alimentació, Universitat de Barcelona, & Institut de Salut Global de Barcelona (ISGlobal), Centro de Investigación Biomédica en Red, Enfermedades Infecciosas (CIBERINFEC), 08028 Barcelona, Spain
[17] Laboratory of Infectious Diseases and Public Health, School of Medicine, University of Cyprus, Nicosia, Cyprus & Department of Pediatrics, Archbishop Makarios III Hospital, Nicosia 2115, Cyprus
[18] Faculty of Health Sciences, American University of Beirut, 1107 2020 Beirut, Lebanon

Edited by Jean-Lou Justine
* Kii ngéen di jokkol ci bépu faana bu aji ci teere bi: jerome.depaquit@univ-reims.fr

This is an Open Access article distributed under the terms of the Creative Commons Attribution License (https://creativecommons.org/licenses/by/4.0), which permits unrestricted use, distribution, and reproduction in any medium, provided the original work is properly cited.

[19] Medical Entomology Unit, Infectious Disease Research Centre, Institute for Medical Research (IMR), National Institutes of Health (NIH), Ministry of Health Malaysia, 40170 Shah Alam, Selangor, Malaysia
[20] School of Medicine, Addis Ababa University, 28017 - 1000 Addis Ababa, Ethiopia
[21] Faculty of Mathematics, Natural Sciences and Information Technologies, University of Primorska, 6000 Koper, Slovenia
[22] University of Lodz, Faculty of Biology and Environmental Protection, Department of Invertebrate Zoology and Hydrobiology, Banacha 12/16, 90-237 Łódź, Poland
[23] Laboratory of Entomology, Ministry of Health, 9134302 Jerusalem, Israel
[24] Center for Pathophysiology, Infectiology and Immunology, Institute of Specific Prophylaxis and Tropical Medicine, Medical University Vienna, Kinderspitalgasse 15, 1090 Vienna, Austria
[25] Papua New Guinea Institute of Medical Research (PNGIMR) Institute, PO Box 60, Headquarter, Homate Street, 441 Goroka, Eastern Highlands Province, Papua New Guinea
[26] Museum of Natural History, University of the Philippines Los Baños, 4031 Laguna, Philippines
[27] National Centre of Infectious and Parasitic Diseases, 1504 Sofia, Bulgaria
[28] Ege University, Faculty of Medicine, Department of Parasitology, 35040 Bornova/Izmir, Türkiye
[29] Retired, Faculté de Pharmacie, Université de Strasbourg, Strasbourg, 67400 Illkirch-Graffenstaden, France
[30] Program for the Study and Control of Tropical Diseases (PECET), Faculty of Medicine, University of Antioquia, 050010 Medellin, Colombia
[31] MIVEGEC, Univ. Montpellier, CNRS, IRD, 34394 Montpellier, France & Medical Entomology Unit, Institut Pasteur de Madagascar, 101 Antananarivo, Madagascar
[32] Laboratorio de Entomología Médica, Departamento de Zoología de Invertebrados, Facultad de Ciencias Biológicas, Universidad Autónoma de Nuevo León, San Nicolás de los Garza, 66455, NL, México
[33] Tropical and Infectious Disease Centre, BP Koirala Institute of Health Sciences, Dharan 56700, Nepal
[34] ICMR-Vector Control Research Centre, Puducherry 605006, India
[35] Graduate School of Agricultural and Life Sciences, The University of Tokyo, Tokyo 113-8657, Japan
[36] Grupo de estudos em Leishmanioses/Coleção de Flebotomíneos (COLFLEB/Fiocruz-MG), Instituto René Rachou, Fundação Oswaldo Cruz, Belo Horizonte, Minas Gerais, 30190009, Brazil
[37] Center of Excellence in Vector Biology and Vector-Borne Disease, Department of Parasitology, Faculty of Medicine, Chulalongkorn University, Bangkok 10330, Thailand
[38] Department of Arbovirology, Bernhard Nocht Institute for Tropical Medicine, Bernhard Nocht Str. 74, 20359 Hamburg, Germany [39] Laboratory Vectors & Parasites, Department of Livestock Sciences and Techniques, Sine Saloum University El Hadji Ibrahima Niasse (USSEIN) Kaffrine Campus, C.P. 24600, Senegal.
[40] Environmental Health Institute, National Environment Agency, Singapore 138667, Singapore & Department of Biological Sciences, National University of Singapore, 117558 Singapore
[41] Institut Pasteur du Laos, Laboratory of Vector-Borne Diseases, Samsenhai Road, Ban Kao-Gnot, Sisattanak District, 3560 Vientiane, Lao PDR
[42] National Institute of Hygiene and Epidemiology, 1 Yec-Xanh Street, Hai Ba Trung District, 100000 Hanoi, Vietnam
[43] Indonesian Research Center for Veterinary Science, Indonesian Agency for Agricultural Research and Development, Ministry of Agriculture Republic Indonesia, Bogor 16114, Indonesia & Department of Parasitology, Faculty of Veterinary Medicine, Airlangga University, Surabaya 60115, Indonesia
[44] Laboratory of Research in Applied Biology, Polytechnic School of Abomey-Calavi, University of Abomey-Calavi, 01 P.O. Box 2009, 00000 Cotonou, Benin
[45] Instituto de Microbiología, Colegio de Ciencias Biológicas y Ambientales (COCIBA), Universidad San Francisco de Quito (USFQ), 170901 Quito, Ecuador

Received 1 December 2025, Accepted 29 January 2026, Published online 3 April 2026

Tënk – Jukki bii dafay joxe ab gindikaay bu mat sëkk ci naka lañuy liggéeyee yuul yi, di ay jéego yu am solo lool ngir xàmmee xeeti yuul yi ak ngir gis walla tàggale doomi jangoro yu bon yi. Dafa lëmbe ay xarala yu wuute yu baax ngir jëfandikoo ci aal bi ak ci laboratuwaar bi. Jukki bi am na ay tektal yu leer ci ni ñuy dajale, jàppe, aar ak raye yuul yi (bolle ci digle ñu jëfandikoo seddal gu wow walla CO_2 te bañ a jëfandikoo ay jëniy simi, ak itam ay fànna yu ñu leen mana dence yu melni seddal ak denc ci biir éthanol. Waajal ay cér yu rañeku yu wóor (melni jëmm, bopp ak laaf) ngir seet ko ci mikroskoop, ñu ngi ko fësal bu baax ci feemi pess yi. Këyit gi itam dafa joxe ay leeral yu mat ci ni ñuy waajalee dieumi gunoor bi, bokk na ci leeral ak ay saafara yu melni potash ak ndoxum Marc-André. Ci doxalinu montaas bi, dañuy méngale ay ndox yu wuute, ci fësal seeni melokaan ci wàllu leeral ak ni ñu mënee yàgge cib denc.

Liwidu walla diwlini Hoyer (ñu xame ko itam ci turu chloral gum) lañuy digle ngir saytu gu gaaw, rawatina suddee spermathek yi, ndaxte leer na, wante baaxul ngir nanguwul denc ko lu yàgg. Ñu ngi waxtaane yeneen xeeti ndox, bokk na ci polyvinyl alcohol, Euparal® (ngir mu mën a dékku ndox lu néew) ak Canada balsam (ab media buy diig ci hydrocarbures yi), te ñaari media yu mujj yi dañuy maye ñu denc ko lu yàgg. Feem yu bees ci wàllu biyoloji molekilëer, niki sékanssaj ADN ak espektrometri de mass MALDI-ToF, yuy laaj ñu jox leen cër bu wér bu ñuy jëfandikoo yuul yi, ñoom itam ñu ngi leen fiy wonee. Ci li ci topp, boole na ñu ci ay wideo yu gàtt yuy wone xeeti montaas mbaa samp yu wuute, and ak ay tekki ci 33 làkk walla kalama you bokul, loolu di tax ñu mën a fàjj soxla ak yaakaar yu wuute yu tukke ci mbootaayu borom xam-xam yi ci àdduna bi.

Baati màndarga yi:

Dajale, yuull yi, Liwidu Hoyer, Ndoxum Marc-André, gomu kloral, alkol polivinil, Euparal®,

Abstract – Processing and mounting phlebotomine sand flies: a consensus guideline. This article provides a comprehensive guide for the processing and mounting of phlebotomine sand fly specimens, which is crucial for species identification and pathogen detection and isolation. It discusses a range of techniques suitable for both field and laboratory settings. The guide includes detailed instructions on sand fly collection, handling, covering, and euthanasia (recommending dry freezing or CO_2 over chemicals) as well as conservation strategies, such as cold storage and preservation in ethanol. The quality of preparation of certain anatomical structures (genital organs, head and wings) is essential for their proper microscopic observation and is described in this work. The article also presents detailed sample processing, including the clearing process with agents such as potassium hydroxide then Marc-André solution. The mounting process compares different media, emphasizing their optical properties and preservation potential. Hoyer fluid (also known as chloral gum) is recommended for quick observation, particularly for spermathecae, due to its clarity, although it is not suitable for long-term storage. Other media discussed include polyvinyl alcohol, Euparal® (for limited water tolerance), and Canada balsam (a hydrocarbon-soluble medium), with the latter two offering long-term preservation capabilities. Innovative molecular biology approaches such as DNA sequencing and MALDI-ToF, which require particular attention to sample processing, are also addressed. Furthermore, short video clips illustrating various mounting techniques as well as translations in many different languages are provided, allowing the guideline to reach the diverse needs and expectations of the global scientific community.

Key words: Mounting, Phlebotomine sand fly, Hoyer fluid, Marc-André solution, Chloral gum, Polyvinyl alcohol, Euparal®, Canada balsam, *Leishmania* isolation, Field conditions, Culture, Dissection, Molecular biology, MALDI-ToF, Type-specimens.

Ubbité

Yuul yi ak xeeti gunoor la ñu yu banxasoo ci njabootu Psychodidae, eumb-xeet wu suuffe di su-njabootu Phlebotominae, di lim lu mat 1063 xeet yu ñu xaamee [21]. Ñu di ay nass-kat yu am solo ci ay doomi jangoro yu bon (*Leishmania*, *arbovirus* yi ak *Bartonella*), waralit ay jàngoro yu melni *leishmaniasis*, arbovirus ak bartonellosis. Raññee leen dafa sukkandiku lu ëpp ci seet bu xóot bu ñuy def ak mikroskoop, loolu di lu man a nekk ginaw senoub dajale gu mat sëkk, denc bu mucc ayib, gu yeyoo ak samp gu ñu leen di samp ci ay laam, te loolu dafa laaj ay xarala yu wuute yu bare, bu nekk ak ay njariñam ak ay àtteem. Xamme mbindéefi yuul yu matt yi, dañu koy sukkandi ci seetlu seen melokaanu biti (lu melni seeni antëŋ ak seeni palps (mudi ay ñox), ak seeni awra you góor yi). Dañuy seet itam seen melokaanu biir (lu melni seen farinks, seen sibarium ak seen spermathek). Senoum peess ak texale seeni ceer yu biir dafay yombal seen seetlu, te loolu di tax ñu mën leen a xamme ci anam yu wóor. Kon, ci senouk wuute ak yoo yi walla triatome yi, fòw da ñu leen di tass ci diggante benn laamu mikroskoop, këp ca ass laam balaa ñu leen di mën a xàmmee. Ba ci atum 1980 yi, seetlu ak mikroskoop rekk moo doonoon péexe bi amoon ngir xàmmee yuulyi, te ba tey mooy péexe bi ñu gën a jëfandikoo. Tànneefu doxalin yi ak waajal yi kon yombon na te lallu won ci ñaari fana you tàggatiko : ci genn wet, samp gu sax ngir aar misaal yi lu yàgg cib denc ; ci geneen wet, samp bu gaaw ci anam yuy waral nga xàmmee ko, waaye denc ba du yàgg. Samp bu sax, misaal ci biir resiin bu melni Canada balsam, aw doxalin la wuy jël ay waxtu te dafa laaj ñu wowal yuul yi ba ndox mi ci seen yaram jeex takk. Rax ci dolli, indice refractioŋ bu medium bii du saa yu nekk lay baax ngir yombal seetluk spermathek yi. Waaye, montass ci biir ndox (lu melni, Hoyer) moo gëna gaaw te dafay tax ñu gëna mëna gis spermathek yi, waaye garantiwul ni waajal bi dina ñu yàgg, ndax medium bi dafay xëcci guus ci diaaw dji. Benn ci matuwaay yi mooy nga tëj laam bi ak vernis bamu wow bu baax. Deggo boobu mingi wéy di am solo te dafay indi jafe-jafe ci tànneefi xeetu montaass bi, lépp di aju ci mébetu waajal bi. Lu ko dalee ci ati 1980, njàngum ràññee yuul yi, dañu boole njàngum melokann ak biosimi.

Benn ci xeeti xam-xam yooyu dafa sukkandikoo ci jàngat hydrocarbure yi ci yaram wi, bi ñu gaawe wecce ak pexem biyologi molekilëer (maanaam, yokk ADN polymorphique [RAPD], polymorphism guddaayi fragment [RFLP], yokk ADN Sanger ak toppalante [NGS]). Tay, xeetu molecule yooyu dañu leen a mottali ak pexey proteomik yu melni Matrix-Assisted Laser Desorption-Time of Flight (MALDI-ToF). Rax ci dolli, ràññeekaay molecule bu xeetu mbindéef yi mën nañu ko boole ak gis doomi jangoro yi ak PCR (*Leishmania, Trypanosoma, Bartonella*, ak *Phlebovirus*), te yepp mën nañu leen a gis ci PCR buñ miin ci saa si, te loolu dafay laaj ñu méngale seeni misaal ak pexe yi ñu leen di diappe, yu adio ci liñou yaakara djiss. Lu weesu màndarga yaram yi ñuy faral di jëfandikoo ngir raññe xeet yi, mën nañu jëfandikoo yeneen xeeti xamekaay you adjiou ci yaram vi ba legui (maanaam, xayma nattub laaf yi).

Buñu sukkandiko ci jaar-jaaru bindkat yi ak ci li bawoo ci téere yi, jubluwaay bi ci jàngat bii mooy joxe tegtal yuñ yamale ngir waajaluk liggéeyou yuul yu matt yi ngir gëna mëna jàngat sen mbindin ak senuk saytu ci wallu molekilëer.

Dafay am solo ñu def yenn jàngat (lu melni, biyologi molekilëer wala MALDI-ToF),te lollu dafay laaj ñu denc genn wàll ci yuul wi, di wall vu amul benn njariñ ci ràññeekoo ci melokaannam, loolu dafay wone njariñu tànneef ci protokol yu leer.

Ci xët mii, dañuy wax ci anam yi ñuy nelawale ak rayy yuul yi ñu jàpp ñuy dundu, ni ñu leen di dence, ak ni ñu leen di tasse ci diggante laam bi ak laam si, ngir gaaw leena ràññee wala ngir denc leen lu yàgg suko defee ñu mën cie deffat ab jàngat euleuk.

Njitlaay : Kaaraange ak sàrt yiñu tëral dañu leen a wara tunk ci kayit

Bépp jëniy simi buñu tuddu ci téere bii dañu ko wara jëfandikoo ci anam bu wóor. Mbollo yiy saytu wérgi-yaram ak kaaraange ci barabi gëstu yi mën naño joxe ay leeral ci loraange yiy tukke ci jëniy simi yooyu, waaye itam ci ni ñu leen di jëfandikoo ak nu ñuy watto mbalit mi ci tukke bo loraange du ci top ken. Waaye fàww nga topp tegtali kaaraange yiñu tëral ngir jëfandikoo leen ak sakal leen pexe. Dafay am solo lool ñu xamni wareefu jëfandikukat bu nekk mooy topp xeeti jëf yu baax yiñu tëral ci laborotouwar yi, sàrti kaaraange yi, ak yoon yi, ak sàrt yiñu tëral ci seen réew wala seen barabu gëstu kaay. Rax ci dolli, yenn jëniy simi yi wala yenn ci seeni mbir (lu melni, chloral hydrate) dañu leen di yamale ci yenn réew yi.

1.Njàppum yuul

Yuul yu matt yi mën nañu leen a jàpp ñuy dundu wala ñu dee ci anam yu bari, lu ci melni fiir yu ndaw yu melni CDC, fiir yuy kole nga xam ni yuul wi da ci naan ñapp, mucu kaay yu ñuy jëfandikoo di fiiru Shannon, wala ñu jàpp leen ci seen barab yu ñuy nekk (lu melni, gettu baayima yi). Pexe yooyu mingi adiou ci dugal ay fiir ci seeni dëkkuwaay, xëcc yuul yi ak leeraay wala yeneen mbir yuy xëcc (CO_2 wala xëcc simik), ba noppi dajale leen ngir jàngat leen ci benen yoon, ni ñu ko leeralee ci téere yu bari [2, 3, 32, 36, 49]. Jàpp yuul yuy dundu dafay tax ñu mëna jëfandikoo pexe yi ñuy wone ci kanam yépp, waaye dajale yuul yu dee dafay tere ñu xàjjale *Leishmania* wala ay xeeti virus. Yenn pexey jàpp, lu ci melni këyit yuy kole, dañuy faral di indi cëri yuul yi di dam (ñox yi, laaf, wala tànk). Rax ci dolli, diwu ricin bi ñuy jëfandikoo diko diw thi këyit yi, yuul yi dañu ciy kole, mo tax ñu war ko raxass numu gana gawe, di faral di leen xooj 15 simili ci njaxasu etanol ak diethyl ether yu tolloo.

2. Rayinu yuul yi

Ginaaw ñu dajalee yuul yi ba noppi, dañu leen wara ray. Ak yenn pexe yu ñu len di jàppe (lu melni, këyit yu kole wala fiir CDC yu leer yu am butéel bu def xetu saabu wala etanol), yuul yi dañuy dee ba pare bi ñu len di jël. Biyologi molekilëer mën nañu ko jëfandikoo ci xeetu mbindéef yiñu jëlee ci etanol, ak itam ci yeneen sudee dañu leen a denc ci etanol ci nimu gëna gaawe. Waaye benn ci xeeti doxalin yooyu nangu wul nga tek ci di jafandikoo MALDI-ToF. Rax ci dolli, yenn xeeti rayin yi mën naa tax ñu ñàkk yenn màndarga ci yarami gunoor yi. Kon dafay am solo lool ñu jëfandikoo tooke ju mana ray ci anam you mucci euyib, ngir mëna ràññee bu baax sen melokaan wala ñu denc len lu yàgg ci misaali royuwaay (maanaam, denc ko ngir royuwaay wala méngale ëlëg).

Jëniy simi yu melni asetat etil, eter etil, tetrachloroetan, ak chloroform mën nañu cee xooj wëtteen ba noppi dugal ko ci potte bi deff yuul yi ngir ray leen. Dañu wara moytu jëfandikoo jëniy simi yooyu, topp tegtali defarkat bi, ndax daño am tookke. Dunu digale ñu jëfandikoo chloroform ngir ray yuul, ndax ci sunu jaar-jaar, day galangkoor gëstub biyologi molekilëer. Boo jittale loraange yi mana tukke ci jëniy simi yooyu yépp ak senuk japndal ci gëstu wallu molekilëer, dañuy daadi daax jëfandikoo leen.

Pexe miñu gëna jëfandikoo ngir sam mandargayi, ADN ak protein mooy seeral leen guuy, bañu wow kong. Daño wara seeral yuul yi lu yàgg ngir ñu mën leena faat, waaye nak moytu ñu baña (i) wow (ii) wala yàq dundu *Leishmania*, sudee liñu bëgg mooy ñu tàqale leen ci wàllu mbax gui walla buttit yi. Moo tax dañu leen di digal ngeen bàyyi ko ci -20°C ci dirub 15 ba 20 simili, ngeen diko saytu saa yu nekk ba gunor yi bili mbaane tee *Leishmania* bañ ce dee.

Sudee congelatër amul, mën naño ray gunóor yi ci CO_2. Ci anam yu bari, fuñu mënatule jëfandikoo mbandum CO_2, mën nañu ray xeetu mbindéef yi ci jëfandikoo ay kartoussi CO_2 yu ndaw yu melni yi ñuy jëfandikoo ci sifon soda, waaye nak seenub dem ak dikk ci jawwu ji aki roplane man naa indi ay tere. Li mujjee mooy mën naño ray yuul yi suñu nekkee ci saxaarus poon. Dañuy jàpp yuul yi ñuy dundu ci fiir bu CDC, ñu jëfandikoo muccu kaay ngir dindi leen, tëye leen ci biir pote bu weer, ba noppi ñu dugal leen ci saxaarus poon, ñu dee ci diir bu gàtt. Bokk na ci anam yi ñuy jëfandikoo ci aal bi, ci weetay bu metti. Waaye ginaaw saxaar si dafay fees dell ci pote bu weer bi, dotu ñu ko mëna jëfandikoo ngir jàpp ak jëfandikoo yuul yuy dundu sudul nee dañu ko raxas bu baax, bamu set wecc. Teerewul, mën naño jëfandikoo benn muccu kaay buñu raxasul ngir ray yuul yi ci yeneen fiir ngir fikse leen. Dafay am solo itam nga xool ndax dindi nañu xeetu mbindéef yépp ci muccu kaay bi (aspiratër bi). Pexe yooyu daño méngoo ak ni ñuy xàjjale *Leishmania* yi ci bax wala buttitu yuul yi.

3. Sàmm yuul yi laata ñu leen di liggéey

Am na 5 yoon yu fëss yu ñuy jëfandikoo ngir dëgëral laata ñu leen di liggéey:

3.1. Seral

Yoon wii, li gën mooy ñu def ko ci -20°C walla, li gën a baax, ci -80°C. Yoon yii ñu sàmm, léegi ñoo gën a siiw ci jëfandikoo, rawatina denc ci nitrojen bu saw. Ci lépp, denc gi ci sedd gu metti war naño ko def ci saw, gannaaw bi ñu daaneelee rab yi. Denc gi ci sedd bu metti ci biir konjelatëer am na njariñ lool, ndaxte dafay denc bu baax rab yi ci seen bopp, ak seen RNA, DNA ak seen poroteyin yi, te du yaax dara ci sen yaram dirub jamono ja ñu leen fa denc. Ci geneen wàll, nitrojèn bu ndoxe mën na yàq bu baax laaf yi, tànk yi, niox yi, loolu di faral di tax ñu dog leen, te yenn saa yi, ñàkk melokaan yu am solo ci yaram wi.

Denc bu sedd guy ci firigidëer dafa leen di lor tuuti, waaye baaxul ngir sàmm seeni cér yu doyodi. Am na solo ñu xam ne bu ñu leen di sawale, laaf yi, niox yi (antëni yi, palp yi) walla tànk yi mën nañoo tax ci mbarum tiyo yi te dagg ndax ndox mu sedd mi fay dajaloo. Waaye, denc ci sedd du lu man a nekk saa su nekk ci biir gëstu yi ñuy def ci biti, ndaxte dafa laaj am firigidëer walla mband bu am nitrojen bu ndoxe. Denc ci konjelatëër mën na ànd ak fëggu jàngoroy yi, ak jumtukaay yu molekilëer yi te du wàññi dooley fëgg gi, waaye fëgg gi ak tàggale wiris RNA yi dafa laaj ñu konjele ko ci -80°C walla ci nitrojèn likid su fekkee denc gu yàgg lañu ko bëgg denc. Waaye, seddal ci konjelatëër du tax ñu mën a xàjjale *Leishmania* yi ci biir bi, lu dul bu ñu njëkkee xooj yul yi ci coolak nitrojen, teg ci nitrojen bu ndoxe (ci misaal, ci ay butel yu ñu def ci biir kawas), loolu di niru ni ñuy seddale Leishmania yi ngir denc ko.

3.2. Denc ci alkol (etanol walla alkol isopropil)

Xëy na, lii mooy fànna bi ñu gëna jëfandikoo ngir denc yull yi. Yomb na lool a def ci aal bi, ba sax ci ay anam yu jafe te amul laboratuwar. Denc gi ci alkol baax na lool ngir ay jàngat yu jëm ci melokaanu yaram wi, ndaxte cér yu doyodi yi (laaf yi, tànk yi, ak niox yi) duñu yàqu su amul ngelaw ci biir tiyo bi. Looloo tax ñu digle ngeen tëj tiyo bi ak wëtten bu tuuti ngir génne ngelaw li ci biir te tek benn etiket mbaa këyit wuy raaññee ci kaw wëtten wi (Nataal 1). Tolluwaayu alkol bi gën a baax, loolu ba léegi ñu ngi koy waxtaane. Ci lu ëpp, diglewuñu tolluwaay bu yeess 70% [45, 66]. Tolluwaay yu gën a kawe ñoo gën a sàmm DNA bi ci diir bu yàgg, waaye dañuy tax yuul yi gën a gaawa damm te lollu day galangkor njàngatum melokaan wi. Jëfandikoo etanol bu 96% (boole ci azeotroopik) dafay tax tolluwaay bi di sax ci diir bu yàgg, rawatina ci gox yu tooy yi mel ni réewi tàngoor yi, donte etanol bu 95% moo gëna yomb a am. Ak tolluwaay bumu mana doon, ADN bi dafay faral di sàmmu bu baax ci etanol (waaye du ni sàmmo bu ñu ko seddalee, rawatina ci fànnay molekil yi niki NGS). Ci geneen wàll, proteyin yi ñoom duñu sax lu tollu noonu, rawatina ci jëfandikoo yu proteyomik yu melni MALDI-ToF. Mbindaafan yi ñu denc ci alkol ay weer mën nañu leen a xàmmee ci seen melokaan, waaye mënul nekk ñu jële ci ñoom ay spektru proteyin yu royuwaay.

Denc gi ci alkol lu tollo ak ay weer du tee ñu man a raaññe yuul yi ci seeni melokan, waaye do tuñu mana am ay spektru proteyin yu royuwaay ci yuul yoo yi. Denc bu wow mën na gën a baax su fekkee ne yuul yi dañu leen a seddal itam ci -20°C. Gël ci -20°C ; li mu njëkk a gëne mooy sàmmug molekil yi (li melni asid nukleik yi) ndaxte dafay wàññi yàqu-yàqu bi, te itam am na beneen njariñ bu topp ci sàmm melokaanu mbindéef mi, ndaxte dafay wàññi yàqug suux yi lumu yaag yaag, wante njeexit li mu am ci melokaanu mbindéef mi dafa gën a tuuti bu ñu ko méngalee ak sàmmug molekil yi. Mën naño jëfandikoo itam denc gi ci etanol ngir gis wiris yu DNA ak RNA, su fekkee etanol bi am na lu tollu ci 70% ci biir te denc gi weesuwul ay weer yu néew. Ci beneen faana, alkol isopropil mën naa diaap ndi ci yenn réew yi te mën na denc DNA, waaye dafay tax yuul yi di gaawa damm. Gaawu la taak ni etanol, looloo tax ñu mën koo yóbbu ci anam yu gën a yomb. Suko aajo wo, mën nañu jël yuul yi denc leen ci nitrojèn bu sedd lool walla yu ñu wowal, soob leen ci alkol, loolu di boole jafe-jafey ñaari fànna yépp.

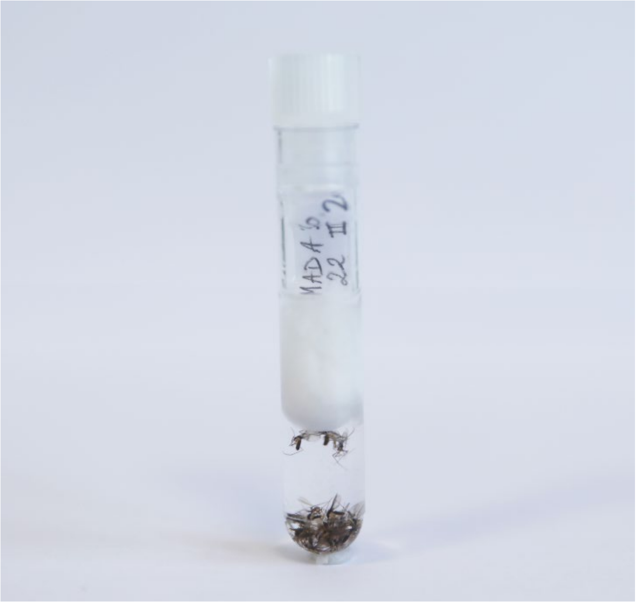

Nataal 1: Yuul yu ñu denc ci etanol.

3.3. Denc ci saafara buy dëgëral RNA (RNASS)

Jumtukaay bu ñu bare di jëfandikoo lool te amul tooke bii, dañu ko defar ngir mu dëgëral te aar RNA bi ci suux yi ak selil yu bees yi. Dafa doxee ci ni muy gaaw a dugg ci biir yuul yi te day luggu RNases yi (di ay enzyme yuy yàq RNA), loolu tax mu tere RNA bi yàqu te soxlawul ñu gaaw koo seddal. Denc ci biir RNASS lu ci ëpp baax na lool ngir sàmm melokaanu suux yi ak selil yi, te gannaaw loolu dana ñu ci mana def ci seen seetlu histologi. Dontee ndox momu dañu ko defar ngir mu taxawal RNA te du ko fiksé, denc ko ci diir bu gàtt walla bu digg-dóomu dafay doy ngir mu sàmm melokaanu yuul yi. RNASS dafay maye ñu denc ay mbindéef ci tàngaayu (nuggayu) neeg lu mat 7 fan, ci 4°C mba lu mat ayu-bés, walla ci -20°C/-80°C ngir denc gu yàgg. Yoon wii am na solo lool ci liggéeyu biti walla ci barabi fajukaay yi, fa nga xam ne jumtukaay yu taxawu sedd gi bariwul. Jële RNA bi, li mu laaj mooy ñu jële ay yuul ci saafara si, te defar leen ci anam yu topp yoon yu ñu baaxoo.

3.4. Denc gu wow ci tàngaayu(nuggayu) néeg bi

Lii aw yoon la wu yàgg, te buñu ko jëfee ci ay yuul yuñu denc lëm (yuñu montaass ñu mat), li mu ëpp ayib mooy du denc bu baax cér yu doyodi yi niki laaf yi, tànk yi, ak niox yi (antén yi ak palps yi). Waaye, jàngum proteomik yiy jëfandikoo MALDI-ToF mën nañu koo def ba tey, su fekkee ni dañuy wowal ci saa si ginnaaw bi ñu taxawee, te jëfandikoo lu melni gel silice ngir wowal. Ci beneen wet gi, saytu yu jëm ci molekil yi tey seet DNA dafa jafe ci mbirum nattukaay bu mel nii, ndaxte DNA bi dafay faral di dam-dame te nekk ci lim bu tuuti, loolu tax saytu yi gën a jafe ci nattukaay yu tooy walla yu ñu seddal, rawatina ci génome bu noyoo bi. Waaye, xarala yu bees yu melni museomics mën nañu ko jëfandikoo ci mbirum nattukaay bu mel nii [34]. Looloo tax, xeetu sàmm bii, digee wuñu ko, lu dul bu meneen péexe amul. Mën nañu koo boole ak denc ci sedd gu metti, ci ñu dugal tiyo yi ci ab konjelatëer ci -20°C walla -80°C. Jafe-jafe bi gën a réy mooy am montaas bu baax ci wàllu yaram walla mbir yu ñu soxla ngir mën leen a xàmmee. Ngir def loolu, gajfalaat leen am na solo lool. Dañuy digle ñu jëfandikoo Triton X-100. Waxtuyi gajfalaat bi di jël mën naa tollu ci ay waxtu yu néew ba ci ay fan yu bare te dafa laaj ñu koy topp bu baax. Ginnaaw bu gajfalaat bi jeexee, war nañoo xooj yuul yi, leng xalli lenn ci ñetti ndox yu toftaloo.

3.5. Denc ci kayit uk seggukaay

Njariñ li gën a mag ci kayit uk seggukaay mooy ni ADN génomik bi day yàgg te du yàqu ci biir selil yi, ci yaram wu matt yu ñu fiksewul te wowalu ñu ko, walla selili dereet yu ñu denc ci tàngaayu néeg. Këyitu seggukaay bi, dañu ko defar ci melokaanu kaart yu ndaw, loolu tax ñu mën ko denc ci tàngaayu néeg bi ay téeméeri sampiŋ ci barab bu tollu ni bu benn tirooru biro bu ndaw. Këyituk segukaay gi dañu koo xooj ci ak ay jëniy simi yuy yàq doomi jangoro yi, loolu moo tax sampal yi dootu ñu leen jàppe ne ñu lambo tooke. Lii dafay tax ñu mën a denc ak a yóbbu sampal yi te du laaj ay matuwaay yu wóor yu jëm ci fitnay andak tooke [68].

4. Peessum yuul yi

Ci lu wutte lool ak yennen xeeti gunoor yu bare, yu ni ñuum tei ñu maan leen a xamee ci xool seen melokaanu biti, yuul yi ñom dañuy laaj ñu feess leen te samp leen ci ay laam ngir jàng seen melokaanu biir yi am solo ngir man a xam seen xeet bu weer. Ak pexem waajal ak samp mumu mana doon, man a doon, doxalin wuñu tànn ngir waajal ak samp, benn xarala buñuy jëfandikoo ci wallu feess lañuy séddalee (Nataal 2 ak 3) (https://zenodo.org/records/18198006).

Jëfandikoo Triton X-100: saafara bu amul ion ci ndox

War naño xam ne samp gi, dañu ko defa ak ay mbidéeff yu ñu mujjee jàpp walla yu ñu sàmmon len bu baax. Waaye, dajaleekatu gunoor yu bare am nañu ay royukaayu yu ñu sàmm ci wow (ngir jëfandikoo leen ci MALDI-ToF) walla yu ñu denc ko ci alkol ay at yu bare. Waaye, denc bu yàgg lool ci alkol baaxul, te mbidéeff yu ñu sàmm noonu dafay jafe lool ñu waajal leen ngir seet leen ci mikroskob. Benn jafe-jafe bu ñu faral di gis mooy plastig yi ñuy dence yuul yi dañuy yàqu, ginnaaw loolu alkol bi di wow. Ci ñaari nekkin yépp, deff ay pexe ngir mussal leen jafe na, ndax li

ñu leen dencoon ci alkol lu yàgg lool, bañu wow kong. Ci kaw loolu la xalaatu jëfandikoo ay ndox yuy tooyal, yu dul ay saabu yuy yaax judo. Triton X-100 mi ngi ñëwe ci melokaanu safara bu amul ion ci ndox (safara bu 4-(1,1,3,3-tetramethylbutyl)phenyl-polyethylene glycol, walla t-octylphenoxypolyethoxyethanol, polyethylene glycol tert-octylphenyl ether), ñu koy jëfandikoo bu baax niki setalukaay ci bioloji selil ak molekil. Dafay tax deeri selil yi ak yu nukleyeer yi di gën a ubbeeku.

Jukki bii ci topp dafay leeral naka lañuy jëfandikoo Triton X-100 ci biir ndox mu tollu ci 0.5%:

- Tóoyal mbidéeff bu wow bi ak alkol bu ken raxul, bu set wecc.
- Yokkal ci tolluwayu ndoxum saafara Triton X-100 bu 0.5% bi, ba mbidéeff bi muuru lépp.
- Bàyyil mu liggéey diirub 5 simili ba ci ay fan, di ko saytu saa su nekk. Xeeti gunoor yépp war nañoo tàqalikoo bu baax ci biir saafara si.
- Jëleel saafara Triton X100 bi te wuutu ko ak saafara potassium hydroxide.

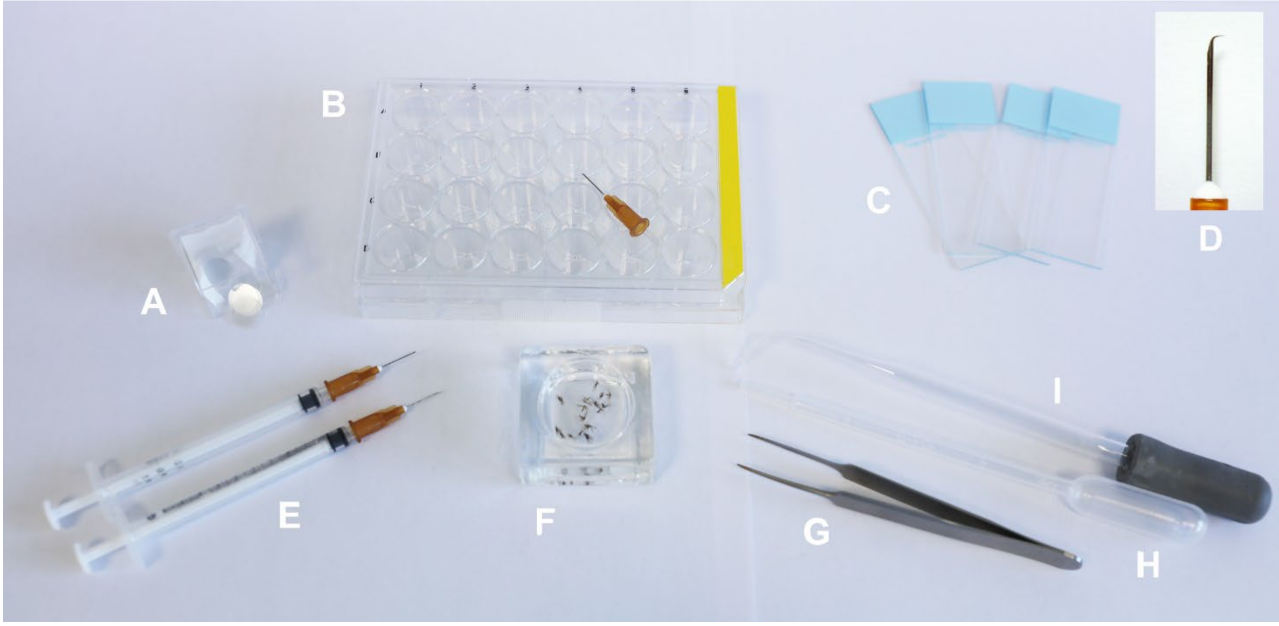

Nataal 2: Jumtukaay yi ñu sooxla ngir samp yuul yi: A: laam ci yu wërngëlu yu ñuy tek ci kaw (diameetar 10 walla 12 mm); B: Plak bu am 24 pax ak pikk bu am kàcc (su fekkee diwlinu girofe walla Euparal® lañuy jëfandikoo ngir samp yuul yi, buleen jëfandikoo plak akrilik, ndaxte ab reaksiyoŋ simik mën na yàq mbideef yi); C: Ay lam yu weer yu nangu bind ci kawam; D: Detay bu ñeel kùru pikk mi; E: Ay pikk yu ñu nass ci ay sereng; F: Weeru montor walla lu ko nirool luy ñuy deff mbideff yi ñu war a samp; G: Kepp Dumont; H: Muccukaay bu plastik; I: Muccukaay bu weer bu ñu lakk ba mu dëng ngir yombal yóbbu ndox mi ci pax yi.

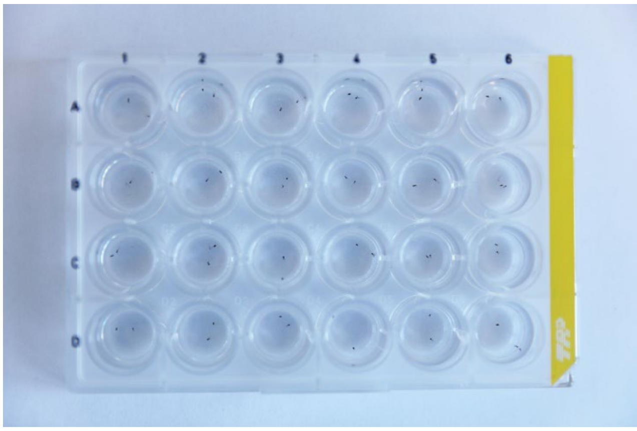

Nataal 3: Plak bu am 24 pax, bu nekk am bopp ak biirub mbindaan.

4.1. Bopp

Peess mi mën nañu ko defee ak ay pikk yu sew walla ay pikk yu ñu jagleel gunoor yi, ci suufu stereomikroskop (Nataal 2 ak 3). Pikëer yi ñu gëna jëfandikoo ñooy: 26G × 1/2" (0.45 × 13 mm), 30G × 1/2" (0.3 × 13 mm) walla 25G × 5/8" (0.5 × 16 mm). Ngir waajal ab royukaay ngir ñu mën koo xàmmee, lu néew-néewa, dañuy dagg bopp bi ci yaram bi te samp ko wetu biir bi di diëm kaw ngir feessal cibarium bi ak gorob bi, fekk nañuy samp dënn bi ak biir bi ci wet gi ginnaaw bi ñu leen teng xee. Teguk bopp bi ci jubluwaay bu jëm kaw dafay tax xuru bopp bi di jëm fess, loolu di tax ñu

mën a gis sibariyom bi ci lu jub. Yomb na lool ñu jot ci cér yu yaram yii su ñu taxalee bopp bi bu baax ak desitu yaram wi.

4.2. Laaf yi ak dënn bi

Laaf yi dañu leen a wara samp ba ñu talli. Laaf bu nekk mën nañu koo dindi ci reenam, samp ko moom kenn, walla benn laaf rekk lañu mën a samp, te beneen bi des takh ci dënn bi. Su fekkee dañu bëgg def analisu morformometriy bu jevomeetrik, am na solo lool ñu xàmmee bu baax te texale laafu ndeyjoor ak bu càmmooñ laata ñu leen di samp. Dënn bi, dañu ko séddale ci ay xaaj yu bare, te bu nekk am na ay xibaar yu am solo lool ci wàllu ràññee xeet yi [20, 64]. Ci lu ëpp, dañu koy samp ci wet gi ngir ñu mën a seetlu chetotaxy (manam mellow karaw yi) ak ni wirgo yi séddalikoo. Mën nañu jëfandikoo barab yi karaw yi sampoo ci dënn bi ngir ràññee yenn xeet yi ci biir xeet wu mag wii di *Brumptomyia.* Man nañu jëfandikoo ni melo yi séddalikoo ngir séddale yuuli Neotropical yi ci tolluwaayu xeet (misal, *Bichromomyia*), ci ay mbooloom xeet (misal, *Pintomyia*), ak sax ci biir xeet bi (misal, *Micropygomyia, Nyssomyia, Psathyromyia,* ak *Psychodopygus*) [20]. Looloo tax, bu ñu bëggul def analizi molekilëer ci dënn bi, war nañu koo montaass ci anam gu tax du yàqu. War naño xam ne du doole wirgo yi moo am solo, waaye ni ñu tasaaroo ci dënn bi mooy joxe ay tektal. Looloo tax, prosesus bu leeral bi warula dindi wirgo wi mbaa ni mu tasaaroo.

4.3. Awra yi

Fàww ñu bàyyi xel bu baax ci ni ñu teege awra yi ci góor ñi ak ci jigéen ñi, ndaxte loolu am na solo lool ngir xàmmee xeet yi, xeet yu ndaw yi ak sàq yi. Muy ci góor gi di jigéen gi, ñaari séyu kaay yi, ñaar-ñaar lañu.

4.3.1. Góor yi

Cër yi ñeel wallu séy bi ci biti lañu nekk te ñaari forceps yu ñu boole lañu. Benn bu nekk, ci wetu ginnaawam, am na boolekaayu gonocoxite-gonostyle, te ci wetu kanamam, am na xottu epandrial. Gonostyle bi am na ay degg ak yenn saa yi ay karaw yu gatt, yu war a limu te barab yi ñu soxe ko war a feeñ bu baax. Am na solo ñu seet bu baax gonocoxite bi ci suufam, ndaxte mën na fa am sëxub karaw yu gatt yu ñu samp ci ab giir (= tubercle) [22]. Nit ñi sen xam-xam mattewul lool bu baax ci peess, mën nañoo def montaas bu yomb ci wet gi te duñu dindi awra yi ci catu biir bi (https://zenodo.org/records/18311158). Ci noonu, bu ñaari xaaji awra wi tegallo, mën na jafeel waññi, misaal, karawi biir yu gonokosisit bi, waaye yoon wii dafay tax ba duñu yàq céri awra yi su fekkee ne peess mi baaxul. Ñi gën a xarañ mën nañoo jéem a ubbi awra wi ci ñaari xaaj ngir tàqale leen. Ngir def loolu, dañuy dugal ndànk-ndànk wet gu ñu waññi ci pikk (ni pikk bu ñuy defaree ping ci biir der bi), loolu di tax ñu mën a xotti awra yi te duñu leen dagg ba mu dog, ngir mën a séddale mbooloom gonokoksit-gonostil yi (https://zenodo.org/records/18311158). Jubluwaay bii dafay yombal seetlu wàlli biir yi te itam di tax ñu gën a gis paramérs yi ak seeni mbubb, yu dootul tegallo. Ngir samp gu wet gi, gi faral di tax cér yi tegallo, war naños leerale royukaay yi bu baax.

4.3.2. Jigéen ñi

Jëmmukaayu sëy bi ci biir la nekk te ay spermathek lañu. Ci luddul am peess, war nañu leen seetlu ci biir der bi, ci yékkati biir bi ba mu xëccu ci suuf. Fu mu man a tollu ak jumtukaay bu ñu mana jëfandikoo ngir samp ko, spermathek yi ci seen bopp manees na leen a gis bu baax ci lu ëpp, rawatina su fekkee ne yënguwuñu te leerul lool. Waaye, gis spermathek yu yëngu te am ay mbar yu sew man na doon jafe-jafe ci ay bërëb yu seen leer néew. Tamit, seetlu cosaanu yooni spermathkeca yi am na solo lool ngir xam xeet wi bu baax, rawatina ci xeeti subgenus *Larroussius* [35, 37, 38], ñoo di nass-kati *Leishmania infantum* yi gën a fës ci Àdduna bu Yàgg ba. Su seetlu googu amul, xaame mbindéef yi mënul nekk. Ngir jàll jafe-jafe yooyu, fàww ñu génne ci biir bi kompleksi furka genital-spermathek bi (https://zenodo.org/records/18311106). Ci lu ëpp, jafe na nga seet spermathek yi ci biir peess mi, waaye furka awra bi moom yomb na a gis. Ndaxte yooni spermathek yi ci furca awra bi lañuy ubbeekoo, bu ñu berree furca bi, loolu dafay tax ñu mën a ber itam spermathek yi. Su fekkee ci njuumte daño dagg spermathek yi ci biir liggéey bi, duñu leen ñàkk te mën nañu leen a gis ba tey ci biir deru biir bi (Nataal 4).

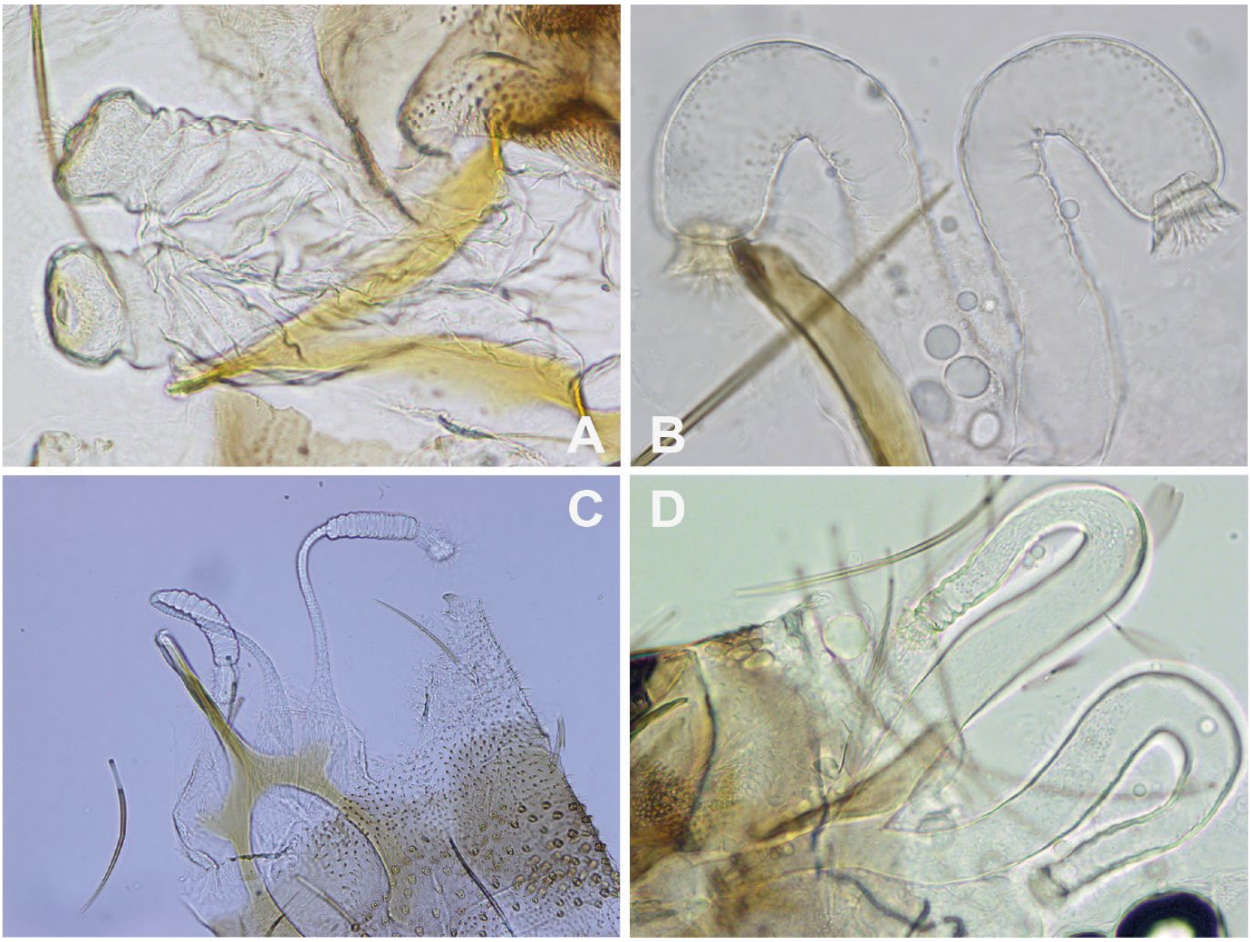

Nataal 4: Spermathek yi ñu dagg te samp leen ci ndoxum Marc-André ñu loogge ci ay mbindéef yuy doora dee**.** A: *Idiophlebotomus longiforceps* (Laos); B: *Sergentomyia minuta* (France); C: *Phlebotomus ariasi* (France); D: *Sergentomyia anodontis* (Laos).

4.4. Peessum baax gi ngir wut *Leishmania*

Peessum baax gi am na solo lool ngir gis ak tàggale *Leishmania* yi ci yull yu jigéeni yi. Doxalin wowu mën nañu ko def ci béréb bi ak ci laboratuwaar bi ngir saytu kàttanu yull yi ci wallu nassee jàngoro ji.

Digle nañu ñu deff liggéey bobu ci jigéen ñi ñu rey léegi. War nañu raxas jigéen ñi ak ndox walla ci ndoxum xorom bu am saabu bu yam ngir dindi karaw yu bare yi. Jéego bii dafay tax ba bërëb bi set wecc te du ame ay doomi jangoro, ngir man a génne *Leishmania*, boolle ci di sàmm itam melokaan yi ñu soxla ngir xàmmee ko. Ngir gis te ber *Leishmania*, fàww ñu génne biir bu diggu gi ci anam yu ñaw te def ko ci benn ndox mu sell (0.9% NaCl). Ginnaaw bi ñu seetaanee saan yi (doomi jangoro yi) ñu yangu yi ci biir mikroskob optik (magnifisikasiyoŋ bu ñu digle: ~200×), dañu leen di jël yóbbu ci benn bërëb, suxat leen, jëfandikoo pistolet insulin walla mikropipet (ngir gën a xam, seetal xaaj wala dog 4.4.3).

Bopp bi ak awra yi war nañu leen samp ci ndoxum Marc-André mi ngir ñu leer. Lu am solo : Ndoxum Marc-André bi warul laal *Leishmania* yi mukk, ci ben anam, jaare ko ci jumtukaay mbaa pikkür, ndaxte dafay rey saan yi.

Pessum jigéen ñi, mën nañu ko def ci benn laam walla ci ñaari laam yu wuute; ñaari tànneef yépp am nañu seeni njariñ ak seeni àtte (Nataal 5; https://zenodo.org/records/18311154).

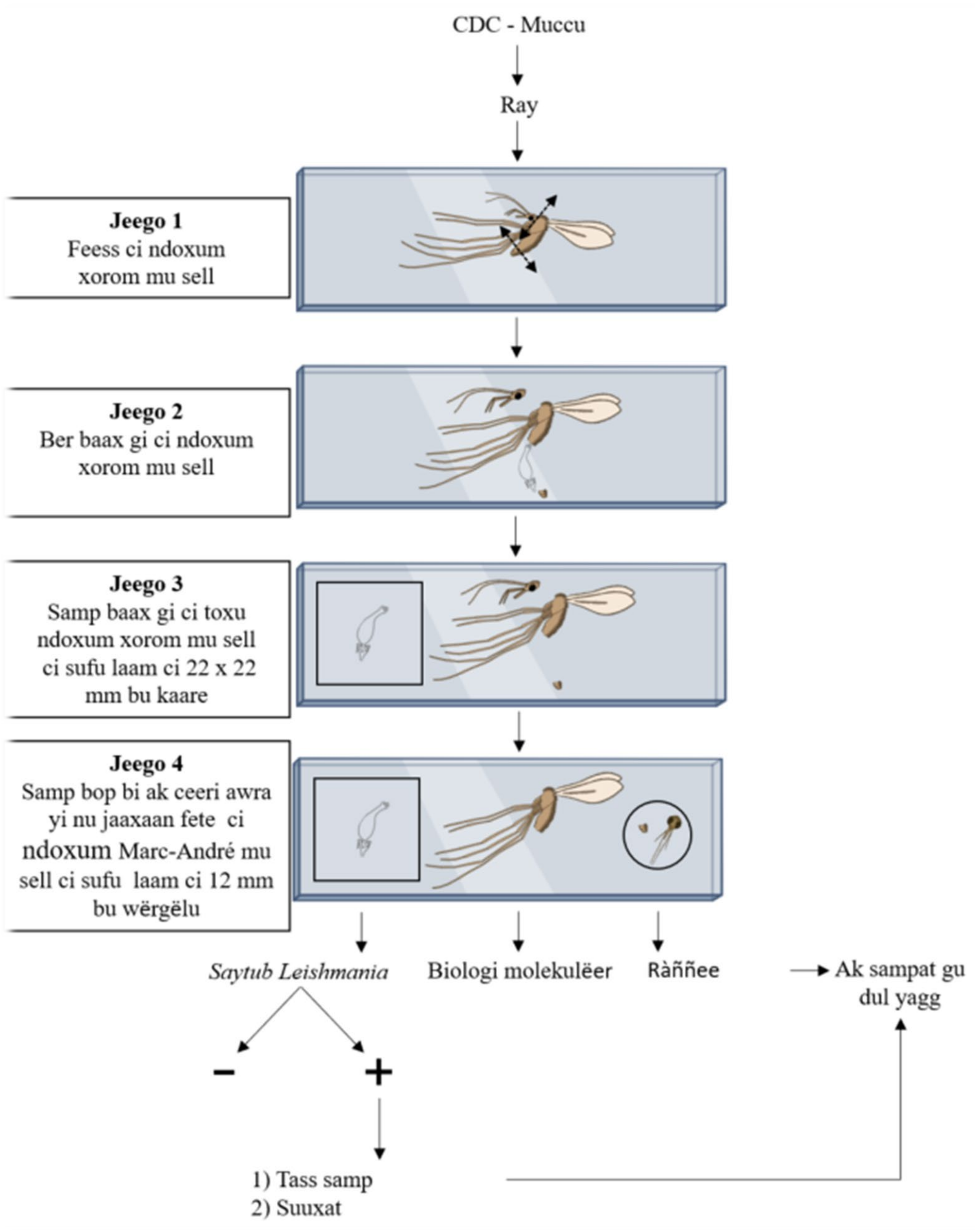

Nataal 5: Pexe ngir ber *Leishmania* yi.

4.4.1. Jaffandikok ñaari laam

Tànneef bu njëkk bi mooy liggéey ci ñaari laam yu wuute: benn bi am ndoxum xorom bu set ngir génne baax gi, beneen bi ngir samp bopp bi ak spermathek yi ci ndoxum Marc-André. Waaye, ci barabi liggéeyukaay yi, lu yàgg la ñu gis ñaar walla ñetti nit ñuy feess ba noppi yónnee seeni waajtaay benn boroom xam-xam rekk, bu sasso ranee xet wi bu wóor, ak bolle saytu doomi jangoroy *Leishmania* yi ci biir baxx gi. Yora ndo ñaari laam yooyu mën na indi ay jafe-jafe ci topptoo nattukaay yi, rawatina, mën na tax mu jafe lool ñu mën a wëral ne kenn ku nekk moo am doomi jangoro ji, su ñu gisee ne am na *Leishmania* ci baxx gi (https://zenodo.org/records/18311154).

This is an Open Access article distributed under the terms of the Creative Commons Attribution License (https://creativecommons.org/licenses/by/4.0), which permits unrestricted use, distribution, and reproduction in any medium, provided the original work is properly cited.

4.4.2. Jaffandiko ben lam

Jëfandikoo benn laam dafay tax ñu gën a mën a topp li ci génn, waaye dafa laaj ay matuwaay yu bare. Ngir gën a setal ci jéego bii, ñiy liggéey war nañoo setal seeni yoxo saa su nekk ak ndoxum alkoll. Laam yu ñu namul ak laam ci you am ñenti kogn (yu kaare) (22 × 22 mm), yu ñu laxass ci alimiñom te sellal ak tàngoor wu wow (ci furno bu melni bu Poupinel), lañu war a jëfandikoo, ak itam pikiir yu set ngir peess mu nekk (xalaat: 25G Ø 0.5 mm × 16 mm). Ñu teg yuul wi ci biir tuuti ndoxum xorom bu set ci diggu laam bi. Dañuy dagg bopp bi, dagg itam ci diggante juróom-bennéel ak juróom-ñaareelu tergite ak sternite yu biir bi, waaye duñu dagg mbax mi (man naño dagg fu gën a jege kanam su fekkee dañuy xaar ay spermathek yu gudd lool). Gannaaw loolu, dañuy sampale deun bi ak ab pikkür, te di xëcc ak ndank tenxo yu mujj yi ci biir bi ak beneen pikkür ngir génne bax gi ak buttit yi. Su loolu jàllul, mën nañu sampale buntub biir bi ak pikkür te xëcc biir bi mu juge ci kanam.

Su jéem gii tamit jàllul, fàww ñu génne mbax mi ak buttit yi, ci dindi lu bare ci der bu ko wër bi des, li ñu mën. Bu ñu génnee mbax mi ak buttit yi ba noppi, fàww ñu dagg ko ngir xàjjale xaaj yu mujj yu biir bi. Gannaaw loolu, dañuy teg mbax mi ak buttit yi ci beneen bóllu ndoxum xorom bu set ci benn wetu laam bi, te muur ko ak muurukaay bu tuuti bu weex (Ø 12 mm) te bañ koo jaawale. Bopp bi ak pàcc yu mujj yi ci biir bi, dañu leen di yóbbu ci bóllu ndoxum Marc-André bu tuuti bu ñu teg ci beneen wetu laam bi, di moytu bu baax ba duñu laal *Leishmania* bi. Bopp bi dañu koy jubal bu baax (xuru ginnaaw gi jëm kaw), te ñu ber spermathek yi ak furca awra bi ni ñu ko waxe ci kaw, soog ko muur ak benn muurukaay bu ndaw bu wert (Ø 12 mm, buñu ko jaawaleek muurukaay yu kaare yu set yi). Desitu néewu yuul wi ak laaf yi dañuy des ci biir tuuti ndoxum xorom ci diggu laam bi (https://zenodo.org/records/18311154). Su fekkee am na njariñ, walla ngir gëstub xajale xeet, mën nañu sàmm dënn bi ak biir bi ngir ay gëstu molekilëer walla proteomik, te laaf yi mën nañu leen samp ci biir ndox. Ngir wóoral ne li ñu samp du yàqu, mën nañu wuutu ndoxum Marc-André bi ëpp ak beneen jumtukaay bu ndox bu ñuy sampee, lu melni chloral gum (= jumtukaayu Hoyer) walla beneen jumtukaay bu ñu defaree ak polyvinyl alcohol.

Am na ay video yuy leerale bu baax doxalin yooyu (peessum baxug yull yi: https://zenodo.org/records/18303014 ak peessum poftanum teflitt yi: https://zenodo.org/records/18302850), moo tax duñu ko fi leerale bu baax.

4.4.3. Tàggale ak suxatt *Leishmania* yi ci mbax mi ak buttit yi

Tàggale saan yi ci jigéen yu ñu feess yi te ñu yanu jangoro ji, liggéey bu jaffe la bu laaj xam-xam bu wér, te war nañu ko jëkk a jéem ci ay mbindéef yu amul saan. Ginnaaw bi ñu ko ubbee, dañuy jël mbax mi ak buttit yi teg ko ci benn pott bu bees bu am ndoxum xorom bu set (0.9%) walla ndoxum Locke ngir raxas ko [4]. Gannaaw loolu, manees na saytu butit yu ñu ubbi yi ci ñaari anam: i) seetlu leen ci biir mikroskob ngir gis tolluwaay yu wuute yu promastigot *Leishmania* yi ak fan lañu nekk, te bàyyi xel bu baax ci supap stomodeaal bi; ii) ubbi leen ngir yombal génnug promastigot yi ak seenug suuxat ñoom ñiepp [4]. Ndaxte gis yull yu am jàngoro boo nekke ci aal bi lu jafe la, njàngale ak naffar bu dëgër mooy yokk bu baax ñaw gi ngir man leen a ber.

Soo gisee *Leishmania* ci biir mbax mi ak buttit yi, war naño jëfandikoo pikk yu bees yu set te yokk ci tuuti ndoxum xorom, ci wetu weer bi ngir mu xóot te génne leen. War nañu xotti mbax mi ak buttit yi ci anam yu sell te gaaw ngir génne saan yi ci biir saabu saline bi. Ci jëfandikoo benn mikropipet (as muccukaay) bu 100 µL walla pingub tuberkulin, dañuy dajale parasit yi, teg leen ci benn barab, buñu bind turam bu leer, suxat leen.

Suuxat promastigot yu *Leishmania* in vitro : parasit yi ñu tànn, ci njëlbéen gi dañu leen di sàmm ci derett SNB-agar walla ci melokann bu dëgër bu Novy, McNeal, Nicolle (NNN) [16], ñu muur ko ak media alpha-MEM bu set [16, 65] walla media M199, bu nekk ñu yokk ci 10% serum fetal bu set te ñu rey tàngooram (FCS), 1% witamin BME, 2% sawum nit mu set (buñu setal ci segukaay Filtropur® S 0.2 µm), ak 250 µg/mL amikacin (walla 50 µg/mL gentamisin, walla njaxasug antibiyotik ak asid amine (L-glutamine 200 mM, penisilin 10,000 U, streptomisin 10 mg/mL)) [47]. Ginnaaw ñetti fan, su amul benn suub, dañuy jaxase kiltir yi ci benn suuxat bu baax ngir seddal te denc ko ci -80°C diirub benn ba ñaari at, walla ci nitrojen bu -196°C ngir denc ko lu yàgg te jëfandikoo ko ci ay nattukaay yu ñëw [7].

4.5. Poftani tiflitt yi

Pessum poftani tiflitt yi ab xarala la bu am solo ngir jàng ni jàngoro ak li koy yóbbu di jokkoo, rawatina ngir gis ay arbovirus yu melni *Phlebovirus* yi (misal, virus Toscana) [44, 75]. Yuul yi senub dayoo dafa tuutii lool, ba waral senub liggéey laaj ñu def ko ak yëg-yëg, akug xarala ci ron mikroskoop stereoskopik, di jëfandikoo pik yu ñaw ak pusso yu sew, ngir man a ber poftani tifflit yi, te ban leena tocc wala ñu leen di taxal leneen (https://zenodo.org/records/18302850) [51, 61]. Sàmm poftani tifflit yi am na solo lool ngir wóoral wóorug saytu molekil yu ci topp yi. Buñu leen génnee ba noppi, mën nañu leen boole (diaxassee len) te saytu leen ak RT-PCR, qPCR walla saytu immunoloji ngir gis ARN bu wiris bi, walla ay antijèn [12]. Fesug wiris bi ci poftani tiflitt yi, te du rekk ci mbax mi ak buttit yi, dafay firndeel ne domu jangoroji mattal na ak maggam, ba nope man na loor mbidaafan guinaw bu yuul wi maatte mbindeff wala nitt ngir naan dereet [71].

Doxalinu peess mi bi dafa jafe ci wàllu xarala ndaxte yuul yi daño tuuti, te dafa laaj xam-xam bu takku ngir bañ mu yàqu [1, 51]. Ci topp, limu wiris bi mën naa neew, loolu tax ñu war a jëfandikoo ay fànna yu gën a ñaw ngir gis ko, niki nested PCR walla high-throughput sequencing [54]. Xeeti loru ci walle yi tamit ñoo gën a dëgëral soxla bi am ci jëfandikoo ay xarala yu set wecc. Lu weesu jafe-jafey xarala yi, am na ay mbir yu aju ci wàllu dundin yu am solo ci ndamul gis gi: kàttanu yebbi-jangoro ji dafay wuutte ci xeet yi, te tolluwaayu eumb jangoro ji day soppiku ci ni àll bi mel ak jamono ji [33, 61].

Gis wiris yi ci poftani tifflit yi dafay joxe ay xibaar yu am solo ci xeeti loru yi ci wàllante bi te di tax ñu mën a taxawal ay matuwaay yu jublu ci saytu ak aar [15]. Ci misaal, bi ñu xamee wirisu Toscana ci yuul yi ci gox yi muy faral di am, loolu jàppale na ci sosug ay day (protokol) yu ñuy seetee ak ay digle ci wàllu wergu-yaramu askan wi [18]. Bole ci loolu, jàng jëflante bi am ci diggante wiris yi ak poftani tiflittyi di mën na feeñal ay jubluwaay yu bees ngir defar ay saafara walla ay feemi faj yu tax ñu mën a gantal wallante gi [15, 18].

Poftani tifflitt yi mën nañu leen jëfandikoo itam niki ab cëslaayu antigen ngir natt, jaare ko ci ay fànnay immunoloji, rawatina ELISA, antikor yu mbindéef mi di am te jëm ci ndoxum gémmiñu yull yi. Jubluwaay bii dafay tax ñu mën a xam ndax mbindéef yi mucc na ci jangoroy yull yi, te di jàppale ci natt ni xeeti xeex yi ñuy jëfandikoo ngir aaru ci mat matub gunoor yi [25], ak itam xeet looru bi am ci wallug *Leishmania* [40].

4.6. Xamme jan deret la yuul wi naan

Jigéen ñi ñu jàpp, war nañu leen ubbi ak jumtukaay yu ñu jëfandikoo benn yoon rekk, ngir bañ am jaxasoo ci doomi jangoro yi. Seen biir war nañu ko seet ci biir mikroskoop bu am ñaari gët ngir xam ndax deret ji ñu naan mingi reess ak fumu tollu. Li ñu digle mooy ñu tànn rekk jigéen ñi am biir bu xonq, xonq-kaani walla xonq bu dëgër, te amul benn firnde bu wone ne ñu ngi def ay nen. Cattu biir bi, ak spermathek yi, war nañu ko dindi ngir man a xàmmee jigéen ji ci melokaanam ginnaaw bu ñu ko setalee. Li ëpp ci biir bi (bu ñu ci boolewul spermathek yi) dañu koy def ci ay tiyo Eppendorf® te denc ko ci -20°C ba keroog ñu koy gëstuwaat.

Mandargay ndono yi (Markër jéneetik yi) ñu faral di jëfandikoo ngir xàmmee lekkug deret, niki PNOC [5, 30, 50], CytB [67] walla COI [13], xam nañu leen bu baax te leeral nañu leen bu yaatu ci téere yi; moo tax, duñu ci gën a yaatala wax ci jukki bii (Nataal 6). Walla, mën naño xàmmee dereeti rab wimu matt, jaare ko ci mapping peptide bu MALDI-ToF [31]. Ci genen fana, wone nañu ne xarala bii dafay tax ñu mën a xàmmee dereeti rab wimu matt ci diir bu gën a yàgg ginnaaw bi mu naanee dereet; looloo tax mu nekk xarala bu ñu gën a taamu, rawatina ngir saytu jigéen ñi fees dell ak dereet te seen reessalum dereet gën a fës. Li gën mooy ñu denc ay yuul ci -20°C walla -4°C, waaye mën nañu am itam ay njëriñ yu baax ci ay yuul yu ñu denc ci tàngaayu néeg bi ab diir bu gàtt. Biirub jigéen ju feess lool ji, war nañu ko taaxale ak yeneen cér yi ci lu jittu saytu bi, te buñu ko defee, ñu jaxase ko ci ndox mu ñu setal. Yeneen cér yi ci yaramu mbindafan mi mën nañu leen jëfandikoo ngir yeneen saytu yu jëm ci melokaanam ak ci wàllu molekilër. Gannaaw bu ñu jëlee benn xaaj ci li ñu boole woon ngir seetlu peptid yi ak MALDI-ToF, li ci des ci sampal bi mën nañu koo jëfandikoo ngir génne ADN bi, ngir wóoral xam-xamu dereeti rab wimu matt walla ngir seet ndax am na *Leishmania*. Waxtu yi muy jël ngir waajal ak seetlu sampal bi gàtt na lool suñu ko méngalee ak xarala yu molekër yu sukkandiku ci ADN.

5. Waajal yuul yi ngir saytu seen melokaan **(Nataal 3, 6, 7 ak 8; Téerey yokkute 1, 2, 3 ak 4)**

Xët wii dafay wone ponk yu mag yi ngir waajal yuul yi ngir montaas, rawatina ngir gëstu yu jëm ci melokaan, ak itam ni ñu ko mënee jëfandikoo ci yeneen anam yu weesu gëstu yooyu. Waaye, xam doxalin wii am na solo lool, ndaxte dafay tax ñu mën a dëppale doxalin yi ak xeeti nattukaay yi su soxlawoon.

Jëflante bi dafa sukkandiku ci ay jéego yu bare yu ñuy tuure ak di feesal, te loolu ñu koy defee ak ay muccukaay (pipet) Pasteur yu am poñu kawssu yu nooy. Dañoo digle bu baax jëfandikoo ay bool yu ñu defaree weer te am suuf gu weurngeul, ndaxte loolu dafay yombal lool jëflante yooyu. Weer bi du laal benn cafka bi ñuy jëfandikoo te looloo tax mu wóoral ne dina ànd bu baax ak jëniy simi yépp. Ngir wàññi naawuk jëniy simi yi, war nañu muur ay boyet te bañ leen a feesal ba lool, loolu dina tax du sottiku suñu leen ubbee walla suñu leen tëjee, te itam dina tax pënd du dal ci santiyong yi.

Jëniy simi yi ñu soxla ngir jéego yu leerale ak yu waajjal, ñoo ngi leen lim ci Allouwa 2.

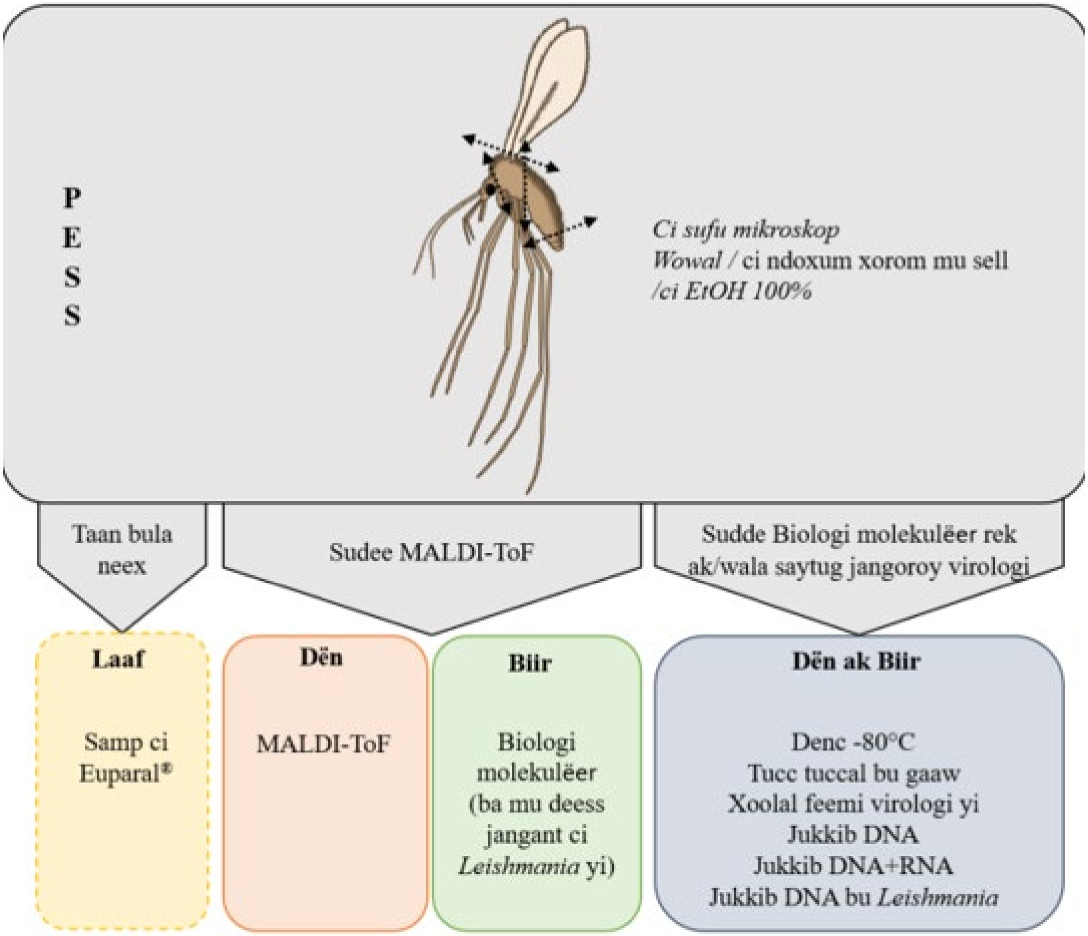

Nataal 6: Waajal yuul yi ngir jëfandikoo len ci bioloji molekilëer, proteomik ak/walla wiroloji.

Allouwa 2: Mbir yi dajje ci jëniy simi

Hydroxyd potassium 10 %
Hydroxyd potassium 10g
Ndox mu ñu segg *qsp* 100 mL

Wër-Wëntu bi ñuy sampe ak gomme chloral (Wër-Wëntu bu Hoyer)
Ndox mu ñu segg 50 mL
Hydrat chloral 200 g
Daakande 50 g
Glycérol 20 mL

Ndoxum Marc-André
Hydrat chloral 40 g
Acid acétik bu sedd lool 30 mL
Ndox mu ñu segg 30 mL

Fuchsin acide 1% ci ndox mu ñu segg
Fuchsin acide (sunguf) 1 g
Ndox mu ñu segg 99 mL

Ndoxum Marc-André munu suub ak fuchsin bu acid
Ndoxum Marc-André 10mL
Fuchsin 1 % 50 µL

Milieu Enecê
Colophan bu weex te raxul 22 g
Gomm copal buy diigg ci alkol 12 g
Éthanol bu ken raxul 20 mL
Camphr 10 g
Essenss térébenthin 10 mL
Eucalyptol 26 mL

This is an Open Access article distributed under the terms of the Creative Commons Attribution License (https://creativecommons.org/licenses/by/4.0), which permits unrestricted use, distribution, and reproduction in any medium, provided the original work is properly cited.

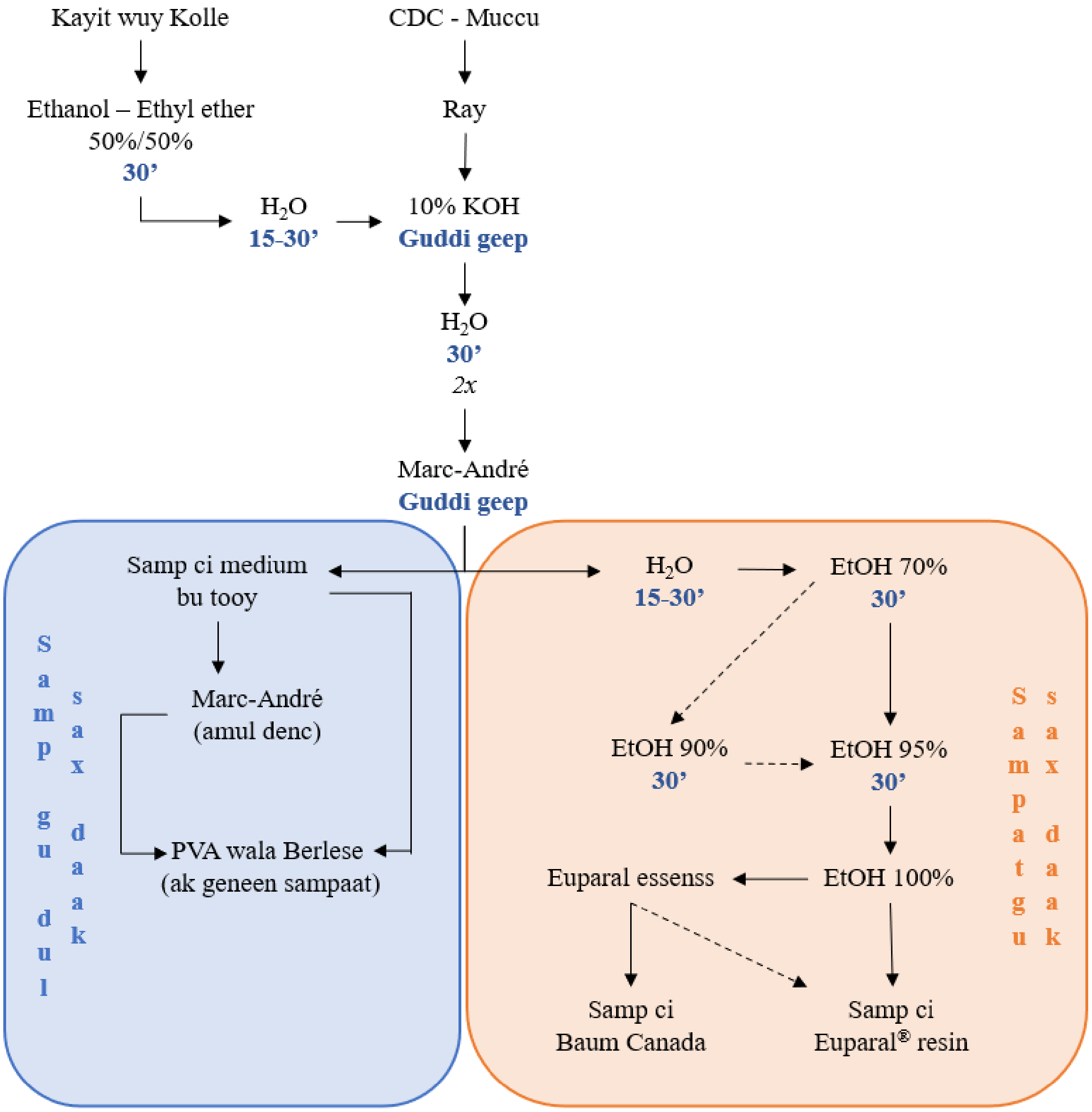

Nataal 7: Pexe bu ñu baaxoo jëfandikoo ngir waajal yuul yi.

5.1. Leeral

Bala ñoo man a waajal yuul yi ci ay laam ak laam ci yu sax, fàww ñu jëkk leen a leeral ci soop lenn ci ndoxum weexal bu baax (misal, diggante 10% acetic acid solution walla diggante Marc-André bi am chloral hydrate, di benn mbir bu ñu saytu ci réew yu bare), ngir leeralaat. Mbir mi leen setal dafay dindi suuxi yaram wi, diwgi, ay niaxi (sékresiyoŋ) sir yi, di tax yuul wi leer te day yombal seetlu mbir yi nekk ci biti yaram bi (misal, fa karaw yi soxe ko), melokaanu kaw gi (wirgo yi, melo) ak melokaanu biir gi ñu gis ci der bi (ni melni, spermathek).

Yoonu leeral bi ci ñaari jéego, bi nga xam ne dañuy jëkk jëfandikoo lu wex lu am doole (ni melni potassium hydroxide), teg ci gannaawam lu wex lu doyadi (ni melni acetic acid bi nekk ci saafara Marc-André), am na ay njariñ yu wuute ci wàllu biosimi [74]. Bàas bi mooy tax suux yu nooy yi di yàqu, bokk na ci poroteyin yi, diwlin yi ak sidit yi, loolu di jaar ci saponifikasioŋ ak dénaturasioŋ poroteyin, te di bàyyi deeru biti bi, lolu moo tax melokaanu mbideeff mi leer bu baax and ak defaruk kitin bi. Asid bu doyadi bi mooy luggu desiit yu alkalin yi, di tere yeneen yàqu-yàqu, te di jàppale ci weexalu kitin bi ngir mu gën a leer [74]. War nañu xam ne ñaari raxas yu toppante yu 15 simili ci ndox

mu ñu segg (distile) mën na doy itam ngir luggu baas bi. Toppato gii di toppante dafay boole dindi suux yi ci anam gu jàppandi ak sàmm melokaan yi ci anam gu dal, loolu di tax nattukaay bi des di wér ngir ñu mën koo seetlu bu baax ci mikroskoop.

Ñu ngi digle ñu raxas ko ñaari yoon, bu nekk 20 simili, ci ndox mu ñu seeg laata ñuy dem ci jéego bi ci topp.

5.1.1. Yàqug suux yu nooy yi (Nataal 8)

Sodium hydroxide (NaOH) walla potassium hydroxide (KOH) lañu faral di jëfandikoo niki ay jëniy simi yuñuy boolee, ci ay tolluwaay ak diir yu wuute, te loolu day aju ci réyaay ak nooyug yuul yi. Jubluwaay bi gën a siiw te gën a jàppandi mooy ñoruk suux yu nooy yi ci diggal mbindeff yi ci biir luur bu dëgër (10% KOH walla NaOH) guddi gi yépp. Mën nañu yokk dooley bi ngir wàññi diirub waajal bi (misal: 20% KOH ci diirub 6 waxtu), te mën nañu ko boole ak tàngal ba mu àgg 37°C.

5.1.2. Weexal ak suub walla ñaka suub

Jéego bii, dañu ciy toftal weexal, li ci ëpp dañ koy defaree boole asid asetig ak kloral idraat (misal: solisiyoŋ Marc-André). Ginnaaw weexal gi, war nañu raxas yull yi bu baax ci ñaari cangàay yu toftalloo ndox mu ñu segg yu toftaloo, bu nekk 20 simili, ngir dindi lépp lu des ci ay jëniy simi. Saafara Marc-André, ab jumtukaay la bu ñu bare di jëfandikoo ngir leeral yull yi. Li tax mu am njariñ mooy mën na leeral ay royukaay te du yàq lu bari ay melokaan yu doyodi, melni laaf yi ak niox yi.

Fàww ñu waajal saafara si léegi walla ñu denc ko ci lu tëju bu baax ngir mu bañ a wow walla mu yàqu. Jëfandikoo saabu Marc-André bi am na ay njariñ yu réy rawatina suñu ko boolee ak yeneen xarala yu leerale walla yu rëdd ngir gën a feeñal yenn melokaanu yaram yi. Leral yu mat ci limu dajjale ci jëniy simi ak ni ñu koy waajalee, ñu ngi ko fësal ci Téereb yokkute 2.

Ngir ay mbir yu leer lool, man na laaj ñu suub leen ngir gën a feeñ laata ñu leen di samp. Am na ay tàkkukaay yu bare yu am, te bu nekk jëm ci ay cér yu ñu xam ci wàllu simi ci biir mbindeef mi. Am na solo lool ñu tànn tàkkukaay bu dëppoo ak yuul wi ak itam ak li ñu ko bëgg taqal. Man nañoo soppi doxalin wu jëkk wii, misaal, ci boole ci saafara Marc-André bi 0.1% ci fuchsine bu aseedi ngir am tàkkukaay. Ginnaaw loolu, ay mbideeff yu ñu denc ci ay dencukaay yu ndox te bëgg leen a montaass ci ay dencukaay yu resin, dafa laaj ñu wowal leen bu njëkk (seetal xaaj 5.2), ndaxte li ëpp ci dencukaay yu resin yu naturel walla yu synthétik duñu ànd ak ndox. New (1974) xamle na ne am na ay tàkkukaay yuy man a yàqu ci yenn dencukaay yu resin yi [53].

Ci misaal, fuchsin bu asid, bi ñu faral di jëfandikoo ak Canada balsam, mën nañu koo fiksé itam ci Euparal®. Waaye, mbideeff yi ñu suub ak fuchsin bu asid, dañuy faral di soppiku ci seen melo ndànk-ndànk, rawatina su desit yu diwlinu girofle, bi ñu jëfandikoo ngir leeral ko ci mujj gi, desee. Misaal yi ñu denc ci diwlinu giwlet mën nañoo soppiku wirgo bu baax ci diirub ay fan yu néew.

5.2. Genne ndox mi

Wàññi ndox mi, dañu koy defee ci diggal mbindéef yi ci ay safara yu etanol yu seen konsantrasyoŋ di gën a yokku: 50%, 70%, 80%, 90% walla 95%, ba noppi 100%, te safara bu nekk war na yàgg lu tollu ci 20 simili. Ndaxte etanol dafa gaaw a naaw, war nañu tëj bu baax pot yu ñu koy defaree diirub waajal gi. Bu mbindéef mi wowee ba noppi, mën nañu taxawal liggéey bi ay fan, bàyyi ko ci biir essence Euparal®, loolu moo gën diwlinu girofle. Creosote bu Hetre, bi ñu daan jëfandikoo bu baax ngir lii, léegi tere nañu ko tere bu wér ndaxte dafa am tooke.

Jëfu wowal gi war na wóoral ne ndox mi nekk ci biir mbindéef bi mën na ànd ak jumtukaayu samp bi, ngir moytu mu lëndëm, mu yàqu ci biir, walla mu soppiku, loolu lépp di lu mëna tax mbindéef mi du baax ngir jàngale taxonomi di rañee ci biiru xeet.

5.3. Jumtukaay yu ñuy sampee

5.3.1. Tànn ak jëfandikoo ngir waajal mbindéef

Li ñuy sampe war na am, ci li gën, ab indisu refracsiyoŋ bu jege lool bu weer, maanaam lu tollu ci 1.5. War na nekk lu amul wirgo, lu leer te des di leer nàññ bu wowee ak lumu yaag yaag. War na mën a ànd ak suub yi ñuy jëfandikoo te mën a dugg te tasaaroo ci biir suuxu mbindéef bi. Warula gaawa wow lool walla mu defar ab lëund ci biir montaas bi, te warul waññeeku ginnaaw bi ñu ko teggee. Tànn ab jumtukaay bu baax ngir montaas am na solo lool ci waajal mbindéef bi, ndaxte amul benn jumtukaay bu mat sëkk ngir jëfandikoo yépp. Tànneef bi dafa war a doon ab déggoo ci diggante ay mbir yu am solo yu bare:

- Jikko yu aju ci gis-gis. Indis de réfracsyoŋ bu medium bu ñu samp bi war na joxe kontras ak réfracsyoŋ yu doy ngir man a seetlu ay melokaanu yaram yu am solo yu ñuy jëfandikoo ngir xàmmee xeetu rab wi walla ngir melokaanam, niki spermathek, ascoids, ak deggi Newstead, ay bëñi cibarium yu taxaw ak ay bëñi pharyngsa. Gisug melokaan yooyu dafa aju ci ni medium bu ñu samp bi di leen wonee.
- Denc. Ngir ay royukaay yu ñu ràññee walla ay jumtukaay yu ñu bëgg denc ba fàww, li ñu leen di dencale war na tax ñu sax te bañ a yàqu ci diir bu yàgg. Waaye, ngir ay jàngat yu ñuy def ci lim walla ay seetlu ci wallu jangoro, foofu denc bu yàgg amul solo lool, ay jumtukaay yu ñuy takk yu diir bu gàtt walla yu diir bu gudd tuuti mën na doy.

5.3.2. Warrefi jumtukaay yu ñuy sampee

Boroom xam-xam yi faral nañoo sos seeni xarala yu ñu leen jagleel, te yenn saa yi jafe, ngir mu dëppoo ak seeni jubluwaayi gëstu. Waaye, xarala yooyu lu ci ëpp duñu bàyyi

xel ci ay mbir yu melni baax gi mu war a am ngir man koo denc lu yàgg, ni xarala yi di àndante, ni ñu ko war a defe ci anam gu ñépp bokk, ni mu yomb a jëfandikoo, walla denc gi ci diir bu gudd. Ñàkk gi am ci anam gu ñépp bokk gi dafay jafeel boole ay mbooloom jumtukaay ak jéego yi ñuy def ngir denc ko lu yàgg.

Jëfandikoo yu xam-xam yi dañuy laaj ay jumtukaay yu wuute ngir samp. Raññee kat yi, lu ci ëpp, dañuy samp ay royukaay yu mat sëkk te di tànn jumtukaay yu mën a yàq tuuti ay cér yu biir ngir gën a feeñal melokaanu der bu ñu ëci bi. Dooley réfracsyoŋ bi dafa wara wuute bu baax ak bu mbindéef mi ak bu laam bi ngir gën a leer nàññ. Li ñuy jaay ciy jumtukaay ngir taakal, li ci ëpp dañu leen defaree ak dooley réfracsyoŋ bu jege bu weer ngir wàññi réfracsyoŋ bi ak tasaaroog leer gi ci biir laam bi-ak ndoxu montass bi. Waaye, ci mikroskoop bu leer (naañ ba yaatu), wuute gu cosaanu gu ab mbindéef bu ñu subul mën nañu koo soppi su ñu tànnee ci anam gu ñu xalaat ab jumtukaayu takk bu am ab indisu refraasiyoŋ bu wuute tuuti ak bu mbindéef bi, loolu di gën a yokk ni mu feeñee ci gannaaw.

5.3.3. Xeeti jumtukaay yu ñuy sampe (Allouwa 3 ak 4)

Ci mikroskoop, indisu reysiyoŋ (RI) bu jumtukaay bu ñuy taqale bi mooy dogal naka la leer giy jaar ci biir laam, jumtukaay bi ak mbindéef bi. Su RI bi jegee bu baax bu weeru kaw bi (≈1.515), leer gi day jaar ci boolee seen ci anam bu yomb, loolu day wàññi tasaaroo gi ak yàqu-yàqu yu leer gi, tey gën a baaxal resolusiyoŋ bi ak feeñug melokaan yu sew yi. Ci geneen wàll, su indisu reflesiyoŋ yi dëppowul, mën na jur ak lëndëm, ay leer-leer ci wet, walla mu nëbb melokaan yi ñu subul. Tànn jumtukaayu samp bu baax bi am na solo lool ngir gën a baaxal wuute gi, leer gi ak baaxug nataal bi ci jëmm ji ñu jël, ndaxte indisu refraŋsiyoŋ yu wuute yi ci jumtukaay yu wuute yi.

Indisu refraŋsiyoŋ bu jumtukaayu samp bi am na solo lool ci ni ñu man a gisee melokaan yu ñaw yi su ñuy waajal yuul yi ngir samp leen ci laam. Jëmm yu ñaw te dëgërul bu baax yu yuul yi, niki teginu cibarium bi, spermathecae, xaaj-xaaji niox yi ak sidit yu laaf yi, mën na jafe lool ñu gis leen ci jumtukaay yu ñu sampale yu am indisu refracsiyoŋ bu kawe.

Alluwa 3: Ay tànnef ci liy and ci jumtukaay yu ñuy sampe

Mediumi sampyi	Liy diiggal	Pré-polymère(s) ou polymère(s) potentiel(s)	Taxaw seetlu
Hoyer = gomm chloral	glycérol, ndox	Ndaakande lañu ko defare	Day diiggal : hydrat chloral
CMCP-9 (= carboxyméthyl-cellulose phénol)	Ndox (CMCP-9: 51−60%)	alkol polyvinyl buñu tooyal leep (CMCP-9: 0−5%)	CMCP-9: farul lool: Far lool
DMHF (diméthyl-hydantoïne-formaldéhyde)	Ndox	N,N′-diméthylol-diméthyl-hydantoïne (di-méthylol DMH) Oligomères pontés par éther/méthylène Rabbu DMH–formaldéhyde bu fassante	
Baume Canada	xylène; wër-wëntu yu gaawa naaw cig wet (Δ^3-carène, acide lévopimarique, limonène, myrcène, acide palustrique, β-phéllandrène, α-pinène, β-pinène)	baum (abiénol, acide abiétique, acide isopimarique, acide sandaracopimarique)	neutralisation : carbonate potassium ; résine bu bayiko ci *Abies balsamea* (Linné, 1758)
Euparal®	eucalyptol, paraldéhyde ; wër-wëntu yu gaawa naaw cig wet gommu sandarak (limonène, α-pinène, β-pinène)	Boole (acide communik, manool, acide polycommunique, acide sandaracopimarik, acide 12-acétoxy-sandaracopimarik, sugiol, acide torulosik, torulosol, totarol)	Day leeral : salicylate de méthyle ; wirgo bu Euparal® wert : xoromass cuivre (abiétinate de cuivre) ; résine de sandarak bu bayiko ci *Tetraclinis articulata* (Vahl, 1791)
Enecê	alkol éthyl, ak camphr, essenssou xotu boutel ak térébenthine	Boole gomme copal ak colophane (résine)	

Bo dieule yuul yi, jumtukaay yi ñu gën a jëfandikoo ñooy: gomu kloral ni jumtukaay bu am ndox, ak itam baal sam bu Kanada ak reesin bu Enecê-Nelson Cerqueira (NC) ni jumtukaay yu am ndox. Rawlins [60] séddale na jumtukaay yu ñuy sampe ci ñaari xeet: (1) yu sax yi, yi di gën a dëgër ak jamono te baax ngir denc gu yàgg, ak (2) yu wékk-sax yi, yi dëgërul ba pare te ñu leen di gën a jëfandikoo ngir lu jàppandi.

Li ñuy sampe mën naa nekk lu ndoxe, lu ñu defaree gom walla reesin, te mu man a saw ci ndox, alkol walla yeneen sawukaay (misal: toluen walla xilen) (Alluwa 3). Ginnaaw buñu leen jëfandikoo, fàww ñu aar leen ci luy jaar ci jawwu ji, jëfandikoo werniis yu ñuy tëje yu dul saw. Ngir man a ràññee bu baax xeeti jumtukaay yu ñuy sampee yi, manees na jëfandikoo raññale gii ci gi toppante:

a. Jumtukaay yu am ndox. Jumtukaay yooyu dañuy saw ci ndox ci lu yomb, looloo tax ñu baax ci samp yu diir bu gàtt walla yu diir bu yem. Ci lu ëpp, yomb naño jëfandikoo waaye man na laaj ñu muur leen ngir bañ ñu jàkkaarlook tooy-tooyu jawwu ji (maanaam, jumtukaay yu ñu defaree gum chloral ak polyvinyl alcohol), rawatina ci gox yu tàng te tooy.

b. Ay jumtukaay yu nangu ndox tuuti. Jumtukaay yii, ndox du leen yàq bu baax, waaye fàww ñu aar leen ci tooy-tooy bu ëpp. Dañoo gën a sax ci lu yàgg bo len tëkëlee ak yu mën a saw ci ndox te ñu ngi leen di faral di jëfandikoo ngir montaass yu dul yàgg lool.

c. Wër-Wëntu yu man a saw ci idrokarbiir. Wër-Wëntu yooyu, ci ay sawukaay organik lañu leen di sawale, lu melni xylene, toluene walla enecê. Defar nañu leen ngir takk (samp) gu sax te am nañu taxawaay bu baax ci diir bu yàgg. Dañoo mën a dékku tooy ak yàqu, looloo tax ñu baax lool ngir denc (maanaam, Canada balsam bu digg-dóomu).

Ngir tënk, Wër-Wëntu yu ndox mën a saw lañu gën a jëfandikoo ngir takk yu diir bu gàtt walla ci anam yu laaj ñu mën a jële mbindéef mi ci lu yomb; Materiyo yi nga xam ne duñu mëna muñ ndox lu bare, baax nañu ngir montaas yu sax tuuti te soxla ag dëgër gu digg-dóomu; mujjantal, materiyo yi mëna saw ci idrokarbiir ñoo gën a baax ngir montaas yu sax dàkk te ñu leen bëgg a denc ci Kaggu yi dirub lu yàgg.

Alluwa 4: Njariñ yi ak ñakk-njariñ yi ci yenn jumtukaay yu ñuy taqale ngir waajal mikroskoop, ci gëstuy ay boroom xam-xam yu bare yu ñu siiwalul [52].

Tur	Njariñ	Jafe-jafe
* Baume bu Canada	Wër-wëntu buy yàag, ag yaggayam man na romb 150 at.<br>Laam yi man nañu leeena samp ginaaw ak leeral ci essenssu Xorom poole wala phenol.	Ëmb na ay jëniy simi yu lambo tooke, mën nañu leen jëfandikoo ci suufu hotte.<br>Dafay soxla ñàkk am ndox mu mat sëkk, diir bu yàgg.<br>Dindim ndox mi ak etanol, tek ci toxal gi ci xylene wala diwu xorom polle mën na tax yenn mbindéef yi woyof lool bag awa yàqu, yeneen suumb (isopropanol, n-butanol, cellosolveTM, 1,4-dioxane, histoclear, terpineol) mën naño wàññi yàqu-yàqu bi.<br>Yen mbindéef yi mën naño ñuul sudee ñu daño wecci xylene ak phenol wala sudee KOH dessandi na ci.<br>Index refractif bu yéeg bi mën na lëndëmloo mandargo bu amul suub.<br>Mën nañu ko weer bamu woow ci dirub ay at yu bari, te du ñu am plaat bu tàng. Dafay mboq ndànk ba noppi ñuul, rawatina ginaaw buñu ko raxasee ak diwu xorom polle.<br>Yenn suub yi dañuy yàqu; suubi cation yi mën naño furi sudee wër-wëntu dafay asid ci kanam.

This is an Open Access article distributed under the terms of the Creative Commons Attribution License (https://creativecommons.org/licenses/by/4.0), which permits unrestricted use, distribution, and reproduction in any medium, provided the original work is properly cited.

DMHF (diméthyl-hydantoïne-formaldéhyde)	Leer gu rëy.<br>Index de refractioŋ bu baax.<br>Gis-gis bu baax ci melokaan yi.<br>waajal gu tanne.<br>Dafay méngoo ak pexey suub yu bari.<br>Aar mbindéef bu baax.<br>Ñaap gu baax ci digganté laam ak laam ci.	Mën na mboq ci dirub jamono.<br>Mën na soppi yenn cuub yi.<br>Du méngoo ak tinte yiy gaawa jël formaldehyde. Ay mbuussi ngelaw yu bari di forme, di wow ndànk.<br>Medium bu samp bu gawa jël toy toy.<br>Samp bu jafee delloosi la.<br>Formaldehyde daa am tooke, dafay indi xasan xasan ci nit, te mën na indi cancer.
* Euparal (leer)	Medium buy yàgg te mëna dundu lu ëpp 50 at. Mën nañu ko samp ci etanol 80% (li defarkat bi digale). Du lëndëm jëmmal bu amul melo te du mboq wala mu dagg ci lu yàgg. Index de refractioŋ moo gëna baax bu balsam Canada ci Jëmi gunoor yi am ñiari laaf.<br>Jeggo bu baax ak mbindéef yu tal ti (du fat leer, mbussi ngelaw yu néew).<br>Dafay diig ci 95% ci etanol, suko defee ñu mëna ko sampt ginaaw ay at yu bari.	Dafa am ay mbir yu am tooke kon dañuko wara jëfandikoo ci suufu hotte.<br>Dindim ndox mi ak etanol , tek ci toxal ci essence Euparal® mën na néewal doole yenn mbindéef; isopropanol mën na wàññi jafe-jafe yii.
Ndoxum Hoyer	Mbindéef yi manes na leen a samp ci lu gaaw wala nu jële len ci ndox, etanol wala formaldehyde<br>Diggal buy lissal dafay defar deer bu baax.<br>Index refractif bu baax, buñu mëna yokk ci cuusug iod.<br>Asid asetik mën na yokk yaatuwaayu ceeri gunoor yi.<br>Yenn xeetu mbindéef yi mën nañu leen a jëfandikoo ci 40 ba 60 at.<br>Dafay diig ci ndox, yombal samp ak sampaat.	Gañcax gu woyof gi mën nañu daanu sudee duñu si fekke ne yokk bi demul ndànk-ndànk.<br>Lu melni ay pax aki peer mën nañ feeñ ci diir bu yess 10 at. Diggal buy lissal bu ëpp mën na am mingi aju ci bariwaayu hydrate chloral bi ak diir bi ñu koy tëye. Tàqaloog jëniy simi yi ak feeñug doom yu woyof ci diirub jamono. Amna ay yoon yu medium bi dafay lëndëm.
CMCP-9<br>(= carboxyméthyl-cellulose phénol)	Mbindéef yi manes na leen a samp ci lu gaaw, jële len ci ndox, etanol, gliserol wala formaldehyde<br>Ceeri biir yi mën nañu leen a diggal ngir mu yombal seetlu bi wala waajal bi.	Mën na peer tek ci lëndëm ci dirub jamono. Yenn saa diggal bu ëpp dafay am.<br>Mbindéef yi dañuy fassu ba noppi wacci ay bët sudee laam bi amul kadre bu baax.<br>Du méngoo ak mbindéef yu am melo wala yuñu kalsifiye.<br>Diiru wowal bi gëna gudd bu media yiñu defare CMC.
Eukitt™	Medium buy yàgg (≥ 30 at). te ànd ak solvant yu bari (aseton, benzèn, kloroform, dioksan, eter, isopropanol, benzoat metil, terpineol, toluène, xylène).<br>Dafay gaaw wow, pH asid tuuti.	Dafa am ay mbir yu am tookke yu waral ñu koy jëfandikoo ci suufu hotte.<br>Dafay laaj ñàkka am ndox mu bari te yàgg.<br>Du méngoo bu baax ak xeetu mbindéef yu dëll (day rassu, di am ay mbussi gaz).<br>Laam yi mën naño tàqaloo ci dirub jamono su fekke ne laam du nu ko fomp, wala tëju

This is an Open Access article distributed under the terms of the Creative Commons Attribution License (https://creativecommons.org/licenses/by/4.0), which permits unrestricted use, distribution, and reproduction in any medium, provided the original work is properly cited.

	Du lëndëm bu baax ci dirub jamono. Dafay ànd ak ay xeeti cuub yu bari (lu melni, fuchsin, hematoxylin, wert metil, violet metil, baxa metil). Mën nañu ko defaraat ginaaw at yu bari suñu ko diggale ci xylene lu yàgg.	Ñu ko bu baax. Polimerisasioŋ mën na baña mat ci fibre yu bari kolagen.
Enecê	Medium buy yàgg lool (≥ 50 at). Du lëndëm lu yàgg. Dafa gëna yomba jëfandikoo, day yombal pees mi, te ceer yi dañuy neexa tek ci diir bu gaaw. Njëgg lu diaap ndi.	Dafay laaj ñàkka am ndox ci lu yàgg. Dindim ndox mi ak etanol ba noppi jara leko ci esssenssu xorom polle day tax yeen ceeri mbindéef yi gaawa dam. Leral gi dafay yeex, loolu tax na seetlu yeen ceeri mbindéef yi (sensilla, ascoid, karaw yu seew yi) yombul lool.

5.3.4. Melokaanu jumtukaay yuñu digle ngir samp (Alluwas 3 ak 4)

Jumtukaay ngir saytu bu gàtt

Chloral gum = ndoxee/jumtukaay/solusiyoŋu Hoyer (IR = 1.48)

Ndoxum Marc-André mooy ndox bi gën ngir seetlu gu gaawa-gaaw (ay waxtu rekk, walla lu ko ëpp tuuti suñu sampee laam bi ci bérab bu tooy) spermathek yi, rawatina ngir jël ay nataal (Nataal 4) walla rëdd ko. Ngir denc spermathek yi ñu seetlu ci diir bu gën a yàgg, dafa laaj ñu sampaat leen ci diggante bu am ndox, bu maye ñu mën koo denc ci diir bu digg-dóomu. Mën nañu koo wowal ba pare samp ko ci reesin, waaye laaj na xarala bu kawe te digale wuñu ko ndaxte am na xeeti loru bu réy ci ñakk li ñuy seet. Gom Kloral ak ndoxum Hoyer dañu leen a jàppe ni ñaar yu bokk benn maanaa. Ndox momu, dañu koy faral di jëfandikoo ngir seetlu cér yu biir yi, ndaxte dafa ànd ak ndox, yomb a waajal, gaaw a jëfe, ak itam ndax ni muy wonee melokaanu leer bi, loolu di baax ngir seetlu ay melokaan yu nooy ni melni spermathek yi. Waaye, gom koloral am na ay ñakk yu mag suñu ko waajalul ni mu ware walla suñu ko dencul ci ay anam yu saytu tooyoor bi, bokk na ci mu sedd ba defar ay peer, mu soppiku kulëër, ak mu ñàkk a fat.

Wërngël laam ci du jubbanti ayib woovu, ndaxte li ñu ko tegale mën na soppiku bu baax ba ñuul lool (yenn saa yi mu mel ni lu ñuul kukk) ndax jëflante gi mu am ak li ñu ko tegale, rawatina su ñu jëfandikoo Euparal®. Li ñu naan Medium Hoyer, yàgg nañu ko jàppee ne mooy li gën a baax ci wàllu gis-gis ngir jàngat ci yull yi te jëfandikoo nañu ko bu baax ci loolu. Am na ay formilasyoŋ yu bare yu jegeente te ñu defaree leen ci gomu arabiik, gliserol ak kloral idraat, te am na ci yenn yi ñu jàngate ak njuumte walla ñu tudde leen ci téere yi ci ludul yoon [74].

Donte milieu bu Hoyer baax na lool ngir seetlu spermathecae yi ci yull yi, baaxul ngir denc gu yàgg. Bax na lool ngir seetlu yu gàtt, bokk na ci nataal, walla rëdd. Ci lu ëpp, jumtukaay yu ndox yi bax nañu ngir samp yu diir bu gàtt waaye wóorul ne dinañu sax diir bu yàgg. Ci geneen wàll, jumtukaay yu resin yi dañuy sax lool, lu ci ëpp ay xarnu, waaye mën nañu nëbb ay melokaan yu ñaw yu spermathek yi ndaxte dañuy ñàkk seen kàttanu leeral.

Ci ab diir) , li ñuy wax medium Hoyer day yàqu ndax wow (Nataal 8), loolu di jur ay peer yu weex, yu ndaw te leerul yu chloral hydrate. Waaye, manees na jëlaat ay mbindéef ci ay laam yu kristalise, ndaxte deer bi du yàqu ci wàllu simi, donte man na am ay yàqu-yàqu yu am ci wàllu melokaan ndax peer yi di yokku. Ci yenn saa yi, mën nañu defaraat laam yu peer yi ci tooyalaat li ñu leen taqale woon ci bérab bu tàng te tooy, te di ci yokk tuuti timol ngir bañ ay yamar xaalla yu sew di fa sax. Ci wenen waal, mën nañu jële ay royukaay ci gomu kloral bi, ci kaw ñu suuxal leen ci ndox, tooyalaat leen ci asid asetig glasiaal, ba noppi taqalewaat leen ci balsaam Kanada.

DMHF (dimethylhydantoin formaldehyde) (IR = 1.48)

Milieu bu ndoxe bi [72] am na gis-gis bu rafet lool, bu tollu ni bu dëggërinu Berlese, te itam yomb na jëfandikoo ni moom. Lu wuute ak dëggërinu Berlese, DMHF du ñuul walla mu sopiku ay peer ak jamono. Bax na lool ngir samp yull yi ak yeneen Psychodidae itam.

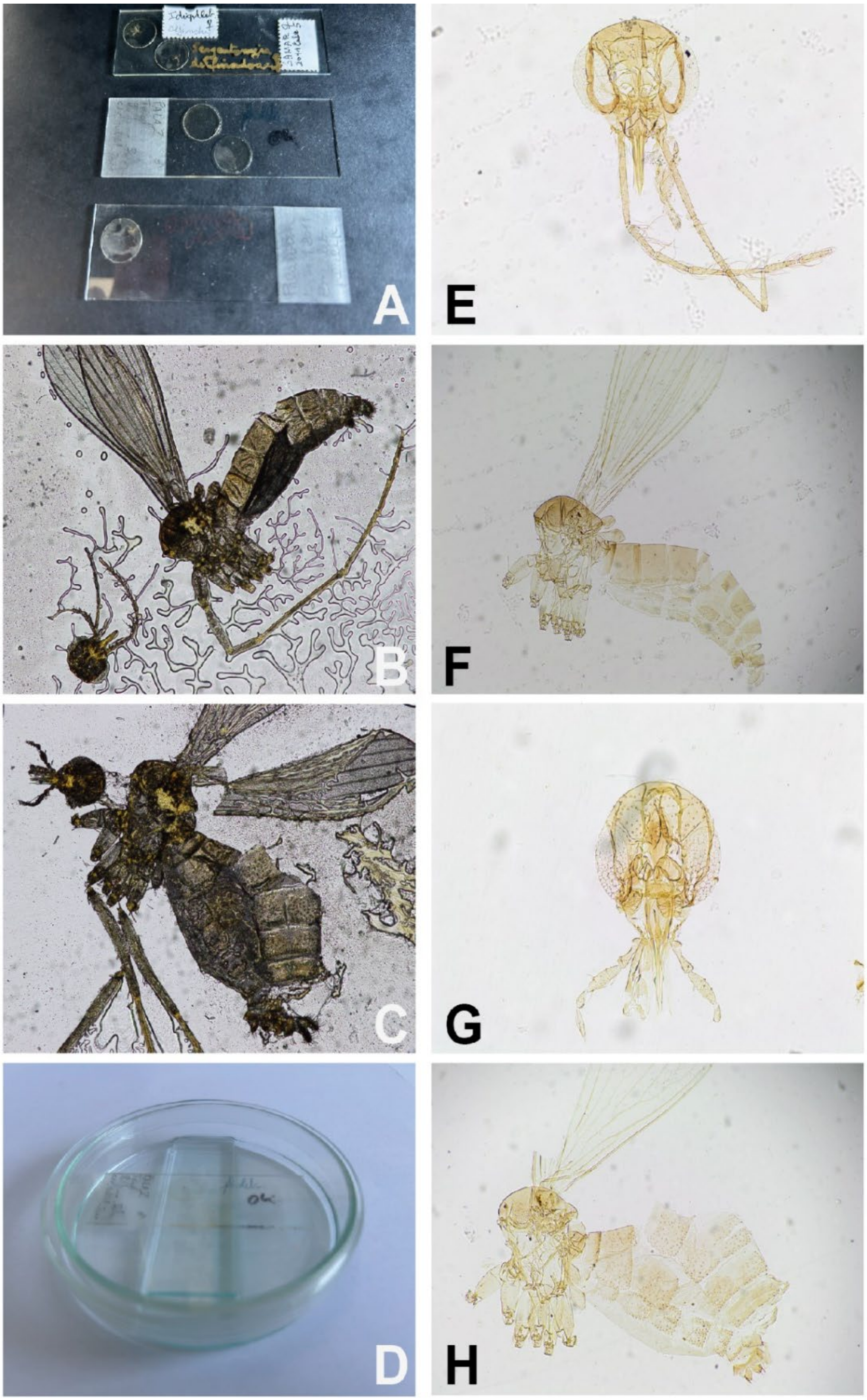

Nataal 8: Dajaleeti ay laam. A: Lam yu yàqu te wow yu ñu samp ak saafara Hoyer; B: Gis-gisu yuul wu wov ci mikroskoop; C: Gis-gisu yuul wu yàqu ci mikroskoop; D: néeg bu tooy bu denc laam bu wow; E: bopp ak F: yaramu mbindéef B ginnaaw bi ñu ko sampate ci Euparal®; G: bopp ak H: yaramu mbindéef C ginnaaw bi ñu ko sampate ci Euparal®.

This is an Open Access article distributed under the terms of the Creative Commons Attribution License (https://creativecommons.org/licenses/by/4.0), which permits unrestricted use, distribution, and reproduction in any medium, provided the original work is properly cited.

CMCP (camphor-mono-chlorophenol) (IR = 1.41)

Lii ab jumtukaay la buñu defaree glycerol tey saw ci ndox, ñu koy jëfandikoo ngir defar ay nataal yu leer tey sax yu ay mbideff yu ñaw, rawatina ay yuul. Benn ci ay njëriñam yu mag mooy mën nañu samp ay royukaay ci anam gu jub jóge ci ndox walla etanol. Dafay dalal te leeralaat gunoor yi ci lu gaaw, di nooyal der bi tey yombal taxawal gu jub gu cér yi, rawatina tasaare laaf yi walla bér awra yi. Donte wax nañu ne jumtukaay bii mën na tax ñu denc lu yàgg, waaye yàgg-yàggu denc googu xamu ñu ci lu bari. Li ko gën a galankor mooy am gi mu am phenol, di lu bon tey indib xasan, tey laaj ñu jël ay matuwaay yu dëgër buñu koy jëfandikoo.

Médium ngir samp bu sax

Canada balsam (IR = 1.52-1.54)

Andrew Pritchard, ci ati 1830 yi, moo njëkk a wax ne Canada balsam ab medium bu baax la ngir samp ci mikroskoop bu leer buy jaar ci biir. Ba léegi, bokk na ci yi ñu gëna jëfandikoo ndaxte wone na boppam ne du yàqu ci diir bu yàgg, te jëfandikoo nañu ko lu ëpp téeméer ak juróom-fukki at ci anam gu jàll. Du melni Wër-Wëntu Hoyer yi, du peer te du muccu ndoxum jawwu ji. Waaye, dafa amug leeral ci bopam gu fés, loolu man naa doon ab gàllankoor ci yenn xaralay mikroskop yi [60]. Jëfandikoo ay sawukaay yu amul tooke ngir wuutu xylene dafay wàññi xeeti loru yi ci wérgu-yaram, waaye mën na tax mu wow bu yéex te gën a gaaw a ñuul, li muy tekki mooy dafay gën a yàqu.

Euparal® (IR = 1.48)

Euparal® mooy wuutu gu ñu bare di jëfandikoo mu wuutu Canada balsam ngir samp sax yi, di joxe ag sax gu yàgg ak ab indisu refracsiyoŋ bu niroo. Jëfandikoo gi am na ñaari sart yu mag: (1) faww ñu wowal mbideff yi laata ñu leen di samp bu mujj bi, li ci ëpp mooy ñu jaarale leen ci etanol 95% tek ci etanol absolu; (2) jéego bii di wañãi ndox dafay guddal waxtu wi yépp di jël ngir liggéey bi. Su fekkee ne mënul nekk ñu wañãi ndox ak ay saafara yu organik, mën nañu jël ay mbideff yu ñu denc ci etanol bu sett wecc, teg leen ci beneen saafara bu digg-dóomu bu ñu defaree Euparal® ak Euparal essence ci anam yu yam, laata ñuy def samp bu mujj bi.

Enecê (IR = 1.467)

Enecê ab medium la buñuy gëna jëfandikoo ngir fikse ay gunóor yu ndaw te ak jëfandikoom yaatu na lool ci Bresil. Dafa am kolofaŋ ak gom copal buñ raxas ci alkol, kanfor, terpentin ak eucalyptol. Cerqueira [11] dafa wax ni Enecê mooy wuutu balsam bu Canada ngir sampuk walax njaan yi, ak ruge kug walax njaan yoo yu, ba ci yoo yu matt yi. Booba ba leegi ñu ngi koy jëfandikoo bu baax ngir ray yuul yi. Enecê nekk na tànneef bu am njariñ ci wàllu koom-koom ngir samp yu yàgg, di joxe taxawaay bu baax ci diir bu yàgg ak diir bu doy ngir mëna dagg ak teg ci barab bu jaar yoon ceer yiy mandargaal.

5.4. Waajal ak weer laam yi

Wowal bu jaar yoon ci laam yiñu samp lu am solo la ngir ak dëgër ak mëna yàggug xeetu mbideeff yi. Laam yi daño wara wow bu baax balaa ñu leen di denc. Waajal yi ñu defaree ay ndoxi samp yu sax dañu leen a wara weer ba ñu taali ñareet ci diirub 2 ba 3 ayu-bis, waaye ñiy jëfandikoo ndox yi nga xam ni senug yaag daa dig domu, dañuy laaj 1 ba 2 ayu-bis. Li gëna wóor mooy nga weer ko ci fuur bu am tàngoor wu méngoo ak ndox moomii ñuy jëfandikoo, nga moytu tàngoor wu ëpp lu mëna yàq xeetu mbideeff yi. Tàngoor wu dox diggante 30°C ba 37°C moo baax. Jéego boobu dafay am solo ngir moytu laam bi moy, xeetu yàqu-yàqu, wala ndoxu samp mu dallul ci diiru dencukaay bi.

Daño wara bind ndoxu samp mi ñuy jëfandikoo ngir fikse ko ci kayit wi dikkale ak laam bi. Su ko mënee, daño wara bind day bi gëna jubal ci medium bi, turu ki koy defar, ak bis bi ñu ko defaree. Laam yi dañu leen di njëkka waajal ñu nekk samp yudul yàgg; waaye, sudee tolluwaayu benn xeetu mbindéef soppeeku na (ci misaal, ñu teg ko muy bokk ci xeetu seeri tiggi), dañoo digle ñu defaraat ko ci medium bu nara yàgg ngir mëna ko denc ngir njàngum raññatle xeet yi ci kanam.

5.5. Xeetu pexey samp yu ñu mana wuutale: Samp ci kàrt

Samp ci kàrt pexe la bu ñuy jëfandikoo ci mbolem gunóor yu bari, maanaam dañuy fikse xeetu gunóor yi ci kàrt entomologik wala ñu kol leen ci seen kaw. Ci kaw rëyaayu yuul yu tuuti yi lool, dañoo soxla xool bu baax ci seen biir ngir mëna xàmmee (xoolal pàcc 5), jëfandikoo anam wii du lu gën ci yuul yi.

5.6. Dellu sampat yuul yu yàqu yi

Ngir xeetu mbindéef yu bariwul wala yu am solo, dañu leen di digal ngeen jëfandikoo ñaari jéego, lépp di méngoo ak protokol biñ wane ci wideo bi diaap ndi ci: https://zenodo.org/records/18315029.

1) Gacc faalaat te duñu ko dindi. Laam yi dañu leen di njëkka tooyal ngir mëna am ab seetlu bu njëkk. Dañuy dugal benn tegu kayu laam yu bari ci biir plaatu Petri, ba noppi ñu def laam biñ wara toppatoo ci kawam. Dañuy yokk ay milimeet yu néew ci solvant ngir defar néeg bu xawa tooy, nga moytu lam bi laal ndox mi (Figure 8 D). Diirub nandalaat bi mingi tàmbalee ci benn fan wala fan yu bari, lépp di aju ci ni xeetu mbindéef bi di doxee. Diko saytu bis bu nekk ak nanggo xaar lu am solo la ci. Su laam bi tooyee lu doy, mën nañu ko dugal ci incubatër waxtu yu néew balaa ñu koy xool ci mikroskoop, foto wala ñu koy nataal ci rëd.

This is an Open Access article distributed under the terms of the Creative Commons Attribution License (https://creativecommons.org/licenses/by/4.0), which permits unrestricted use, distribution, and reproduction in any medium, provided the original work is properly cited.

2) Sooy sampaat, mën nga delloosi laam bi ci néeg bu xawa tooy bi ay waxtu yu néew wala mu fanaan ca. Booy dindi samp bi dañu ko wara def ci biir mikroskop bu am yaari gët. Jëfandikoo puso yu sew, ñu dindi laam ci ndànk, ngir moytu benn cër ci yuul wi deess, tax ci (https://zenodo.org/records/18315029). Ginaaw loolu ñu dajale cër yiñu dagg, raxas leen ci ndox ak ay puussoo yu ndaw, lu ci melni yi ñuy jëfandikoo ngir dindi ADN/ARN buy yàq (xoolal ci suuf), balaa ñuy ñàkk ndox ba noppi ñu defaraat leen ci biir medium bu am resin. Sooy dindi laam, fàww nga xam medium bi nga njëkka jëfandikoo ngir mëna tànn solvant bi war. Ndox lañuy jëfandikoo ci medium yu ndoxe, waaye xylene lañu soxla ci medium yu am resin (lu melni, balsam Canada wala Euparal®). Xylene dañu ko wara jëfandikoo ci suufu hot bi ak jumtukaayi kaaraange bopp yu war, lu ci melni kaaraangey noyyi. Samp xeetu mbideeff yi, dañu ko wara def ci ak deggo ak ki ko wara saam wala kuréel gi yor maakaan mi ko moom.

6. Xamme mbideeff yi

6.1. Melokaan

Li gëna am solo ngir xàmmee yuul yi mooy xool seeni màndargay yaram, lu ci melni jëmmu dënn bi, laaf yi, cër yi, karaw yi, ak yenn lëkkaloo ci wallu diggante, ci xeet yu wuute yi. Gëstukat yi dañuy jëfandikoo caabi ràññee yi, dajale royuwaay yi, ak tegtali xeetu mbindéef yi ngir méngale xeetu mbindéef yiñ dajale ak yiñu jota xam. Màndarga yi gëna am solo ngir saytu leen, lu ci melni rëdi laaf yi ak melokanu bopp bi ci góor ñi ak jigéen ñi, cëri awra yu góor yi, ak yu jigéen ñi, dañu am solo lool ngir ràññee xeetu mbindéef yi. Ngir ràññee ko bu baax fàww ñu saytu ko bu baax ci mikroskoop, ñu koy faral di jëfandikoo mikroskoop optik ngir xool mbir yu rafet yi (awra yi, spermathek), wala loupe bu am yaari gët ngir gëna xam màndarga yaram yi. Ay jéego yu am solo yuñ amal ci wàllu nataal yombal nañu jëfandikoo nataal you portale ngir xàmmee yuul yi. Nataal yu am resolusioŋ bu kawe wala tegtali portale yu màndarga yi gëna am solo mën nañu leen méngale ak ay jumtukaayi royuwaay wala ñu jàngat leen ci sistemu ràññeekaay bu ordinatër di jàppale, loolu mooy gëna yombal njubte ak yombal taxonomi mandarga.

6.2. Mbidum laaf yi

Mbidum laaf mooy màndarga bi gëna am solo ngir xàmmee ak xaajale xeeti yuul yu bari yi. Laafi yuul yi dañu am jëmm ju ñuy ràññee, dañuy gudd ba noppi sew, am sidit yu màgg (Nataal 9 ak 10). Rabbinou rëd yi ci laaf yi day wone ay mandarga yu wutte ci biru xeet, ak ci xàmmee leen. Kon jàngat rëdinu laaf yi dafay joxe leeral yu am solo ci wàllu ràññee.

6.3. Tolluwayu rëd yi ak seenub seddelikoo ci laaf yi

Barina pexe yu ñuy jëfandikoo, lu ci melni tolluwayu rëd yi ak seenub seddelikoo, ngir jàngat ak méngale jëmmu laaf yi ak dayo yi ci diggante xeeti yuul yi, wala seenuw askan. Jàngat tolluwayu rëd yi dafay jàngale itam ni ñuy doxalee, seeni tànneef ci dëkkuwaay, ak ni ñuy mënee naawe.

Ci anamu tolluwayu rëd yi ak seenub seddelikoo, dañuy teng xi laaf yi ndank, suub leen ba noppi ñu tass leen, ba ñu talli ñiareet ci digante laam bi ak laam ci. Ginaaw loolu ñu jël laam yiñu waajal, foto leen ci mikroskoop bu am yaari gët, runk leen ci ordinatër yi, ba noppi ñu jàngat leen ci tolluwayu rëdi laaf yi ak seenub seddelikoo. Xeetu xam-xam boobu dañu ko fësal bu baax ci biir téere yi [6, 27, 42, 56, 57, 59], ak digale ñu jëfandikoo ci anam wu xajale, laaf bu ndeyjoor wala bu cammo ñ ngir cër yuñ boole ngir moytu jafe-jafe nattin yu baaxul [62].

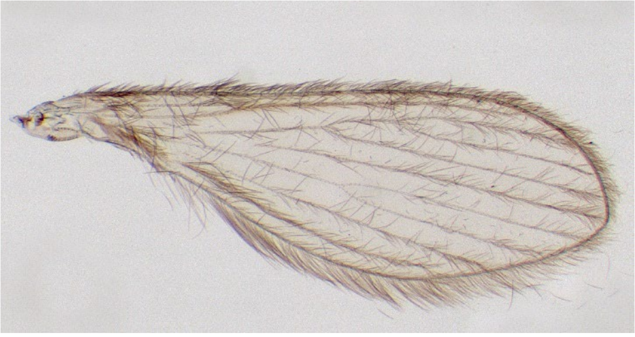

Nataalu 9: Laafu *Trichophoromyia iniinii* bu ni ñum

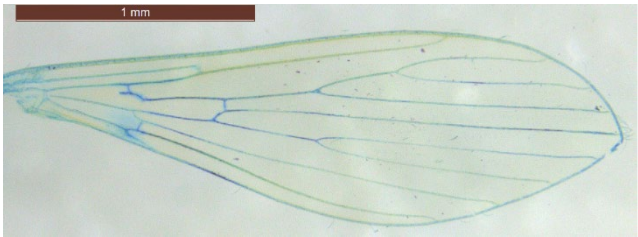

Nataalu 10: Laafu *Phlebotomus ariasi* bu am meloy wirgo.

Waajal laaf ngir jàngat tolluwayu rëd yi ak seenub seddelikoo

Ngir mëna gis sidit mbaa rëd yi ci laaf yi bu baax, fàww da nuy xuuf karaw yeep ba noppi suub leen. Soo leen di waajal, dangay njëkka feesal pott yu ndaw yi ak reactif yi nga soxla (bleu méthylene, éthanol, ndox, ak luy wecci xylene). Wutal laaf buñ denc ci 70% etanol ci tàngooru

néeg, nga wëlbati tube Eppendorf bi nga sotti ko ci kaw pott bi, ba noppi nga yëkkati laaf bi ci guddaay bi nga jëfandikoo puso bu seww te xawa sëgg. Wutal laaf ci etanol nga dugal ko ci ndox, ba noppi nga delloo ko ci etanol ngir ruussal karaw yi. Bi ci topp, nga dugal laaf wi ci methylene wu melo ni baxa ci diiru 6 simili, nga fexe ba mu des ci ndox mi balaa ngay def melo bi. Dindil laaf wi ndànk nga dugal ko ci luy wecci xylene ci diiru 2 simili (lu tollu ci benn ci ñatti pàcc yi ci diiru methylene wu melo ni baxa). Fëggal puso bi tuuti ci wetu finga dell laaf wi, mu daldi suux; xylene bi fi nekk dafay dëgërël wirgo yi. Soo noppee nga yëkkati laaf wi, tek ko ci kaw tuuti Euparal® , tek ko ci laam bi. Tekk ci saytu koo ci mikroskoop bi. Ci suufu benn loupe bu am yaari gët, ubbil laaf wi ndànk, tekk ci kawam laam ci. Dañuy daadi wara jël nataal yi ci saa si, balaa Euparal® bi di gëna jëm ca kanam, ndax fàww nga yengëll laaf wi tuuti bamu taalli ñarreet.

6.4. Pexem Bioloji Molekulëer

Lu weesu gis-gisu melookan, anami molekilëer nekk na lu am solo ci njàngatum gunoor, rawatina ci xeet, ndonoy askan, ak njàngatum mbook, ak itam ngir gis ADN / ARN bu doomi jangoro yi ak xam ci ban xeettu mbdideff lañu naane deret, ak seenuk aay ci joxe ay jangoro [70]. Mën naño jëfandikoo toppalante ADN ngir firndeel ràññeekaay xeetu mbindéef yi wala ngir wuutale xeetu mbindéef yi gëna bokkoo, loolu mooy tax ñu mëna ràññee mbindéef yi ci anam wu gëna jub te wóor. Rax ci dolli, pexey molekilëer yu xarañ yi (PCR, toppalante ADN, NGS, ak ñoom seen) ak spectrometri masse MALDI-ToF ñu ngi gëna am solo ngir ràññee xeetu mbindéef yi ci anam wu gaaw te jaar yoon, di mottali pexey melookan yu yàgg yi [46]. Doonte amna yokkute yooyu, ràññeekaay melookan mooy gëna am solo ci taxalee leen, te mooy nekk royuwaay bi ñuy jëfandikoo ngir tekki ak fiirndell li ñu giis ci wallu molekilëer.

6.4.1. Jukki buy yàq asid nucleik

Jukki asid nucleik jéego la bu ñuy faral di def ci njàngum biologi yu bari, te barina pexe yuñ defar ngir tàqale ADN ci mbirum biologi [48]. Barina kit yu ñuy jaay ngir dindi ADN ngir yombal liggéey bii [14]. Waaye, anam yi ñuy jëfandikoo bu yàgg ngir waajal xeeti gunoor yi ngir ràññee melookan dañuy faral di tere jàngat molekilëer yi ndax mën naño yàq màndarga yu am solo yi ci xeetu mbindéef bi [10]. Protocolu extraction ADN yu bari yi ñuy jëfandikoo ci suxxi gunóor yi dañuy yàq [43], te loolu dafay indi jafe-jafe yu gëna mag ci xeetu gunóor yu ndaw yi, ndax doonte lu néew nga dindi ci yaram wi, mën na yàq màndargay melookan yu am solo [72]. Xeet ak meelow dencukaay bi dañu am solo lool ci tànneef njubluwaay bu méngoo ak ADN bi [29]. Soxla ràññeekaay yuul yi bu wóor, xam anam yi askan wi di doxee, ak wàññi jafe-jafe yi ñu bëggul woon, moo waral ñu defar jumtukaayi diagnostic molekilëer [23]. Leegi dañuy faral di jëfandikoo xeeti molecule ngir mottali xeetu taggale ci mandargay ràññee yuul yi. Ci misaal, anam wi ñuy jëfandikoo ngir kod barre ci gunóor mooy jël ADN bi ba noppi toppalante ko, te loolu mooy tax ñu ñàkk xeetu gunóor bi yepp. Kon dafay am solo lool ñu taxawal am pexe mu dul yàq ADN boole di same yaram and melookanu mbindéef yi.

Barina pexe yuñ jëfandikoo ngir segg asid nucleik ci yuul yi. Bariwaayu ak mucc ayibuk ADN biñu soxla te aju ci saytu molekilëer, ak pexe yu bari yu am day laaj yu wuute ci wàllu dëppo ak setal [9]. Ci misaal, bëti yuul gis nañu ni mën nañu tere PCR jakk [69]. Ginaaw ràññeek doomi jangoro yi, dañuy faral di jël ADN yuul ngir ràññee xeet yi tamit. Barina pexe yuñ mëna jëfandikoo dindi ADN, ci anam yu baax te seel. Yenn tektal yi defarkat yi tànn, gëstukat yi dañu leen di jëfandikoo ci yuul yi [8], yokk njuréef ak / seeluk asid nucleik yi ñuy génne [8, 9, 69], ci yeneen jëfandikoo, ñu njëkka defar ngir yeneen mboolooy gunoor, mën nañu ko jëfandikoo itam ci yuul yi [58, 7]. PCR yiy xàmmee daggiti mitochondri yu ndaw yi (COI wala CytB) dañuy méngoo bu baax ak pexe yi ñuy jëlee ci ADN bi, te loolu dafay jur daggato bu bari ci ADN bi. Waaye yenn xaralay toppalante buum yu gudd (NGS), lu ci melni Oxford Nanopore wala PacBio, dañu soxla ADN bu baax te du xaajaloo bu baax. Li ñuy jëlee ci ay jëni silice dafay defar ay pàccu ADN genomik yu yegg ba 60 kb, waaye li ñuy jëlee ci phenol-chloroform mën na defar ay pàcc yu yegg ba 150 kb [77]. Alluwa 5 dafay tënk pexe yu bari yi ñuy jëfandikoo ngir dindi ADN ci yuul yi, ba noppi wone ndax am nañu ay coppite ci pexe yiñ jëfandikoo. Li ci genne wone wuñu ko ndax dañu aju ci dayo xeetu mbindéef mi ak ci anam wi ñu koy waajalee. Carub "soppi" dafay wax ci méngale protokolu jël gi, ci yuul yi wala yeneen xetti gunor yu ndaw.

Sooy tànn xeetu génne gi, fàww nga bàyyi xel ci mbir yu bari, lu ci melni limu mbideff yi, diirub génne bi, ak pexe yi ci topp. Doonte pexem NGS bi dañuy laaj ADN genomik bu barib natt ci molekilëer, pexe yi ñuy wane fii yépp méngoo nañu ak jëfandikoo PCR yiñu gëna xam.

Rax ci dolli, jàngat yu bari dañu jàngat pexe yi amul yàqu-yàqu ngir dindi ADN ci xeeti gunor yu ndaw yiy dundu ci kaw suuf, ci misaali yuñ denc ci lu wow ci ay kaggu, ak ci xeeti gunor yu am yaram wu woyof [19, 26, 28, 55, 63].

6.4.2. Jukkig asid nucleik budul yàq

Benn ci jafe-jafe yi gëna gàlankoor jàngat molekilëer yi ci xeeti gunor yi, rawatina ci yuul yi, mooy ñu wara denc xeetu mbindéef yi ngir boole leen ci ndajalem xeet yeep. Yoonu jukkig ADN yu bari dañuy laaj tucc tuccal ceer yi, suko defee loolu day yàq meelokan wu jëkk wu mbideeff mi. Pexem dindi asid nucleik budul yàq, dañu ko tëral ngir dindi te bana yàq mbideff mbàa mokal ay ceeram. Pexe

yooyu dañu am solo lool ci xeetu mbindéef yu bariwul wala yu néew, lu ci melni yuul, ndax foofu mën naño baña yàq njubte gi ci wàllu ràññee melokann, wala ngir saytu jangoro yi. Benn ci pexem yi ñuy gëna jëfandikoo mooy xeetu tooyal budul yàq, muy tëj yuul yi ba noppi dugal leen ci ndox muy togg sen yaram bu am proteinase K. Pexem Vectorlyse bu woyof lañu jëfandikoo ak ndam ci yuul yi, rawatina ci xeet yu ràññee ku yi [24]. Bokk na ci anam yi ñuy jëfandikoo kit kolon silisium buñ miin (ci misaal, kit DNeasy Blood and Tissue, QIAGEN, Hilden, Allemagne), ak ay coppite yuy tax ñu mëna dindi ADN te du yàq mbindéef mi. Jéego yi ñu deff ci soppali (dayyob tampon bi ak yokk benn jéego ci seeral bi [17] dañuy may ñu bàyyi asid nucleik yi ci noonu lañuy wàññee yàqu-yàqu yi ci melokaan [24]. Ci yuul yi, mën naño jëfandikoo kit biy dindi ADN HotSHOT (Bento Bioworks Ltd, Londres, UK); Dafa gaaw te diapndi, suko defee ñu mëna defar misaal ci anam wu gaaw te yomb. Mbindéef yi jem ci walu raaññe ci xeet, man nañu leen a raxass.

Yi ñi ligeye ak kit DNeasy Blood ak Tissue dañu leena wara leeral ci jëfandikoo solution Marc-André, waaye ñi nu ligëggeyee ak kit HotSHOT dañu leen a wara leeral bu baax ngir ñu mën leen a samp ci medium bu tooy, wala ci lu ñu gëna taamu ci resin ginaaw bi ñu waaññe sen ndox, buñu sukkandikoo ci feem wi ñu leeral ci xët wii [73]. Suko defee ñu mëna jëfandikoo macceer genetik biñu jëlee ci jàngat ya ca waalla, lu ci melni PCR, ngir gëna am màrker genetik yuñu tànn. Pexem dindi asid nucleik budul yàq lu am solo la ngir jàngat màndarga genetik yi ci yuul yi, rawatina ngir ràññee doomi jangoro yi ñu mëna yóbbaale. Ci kaw saam ñongug mbindéef yi, anam yooyu dañuy may ñu am xibaar bu am solo ci wàllu genetik, boole ci samaale xeetu mbindéef mi ngir gën ce mana toftal yeneen jàngatat.

Allouwa 5: Njëg lu digg domu, jëfandikoo gi, ak méngale feem yi ngir jël ADN genomik (gDNA) ci yuul yi

Feem	Njëg	Doxalin	Tëggat pexxe yi bamu mengo ak xeeti gunoor yu tuut yi
Ay **Jëni** silice	2,5 – 3,55 US$ [39]	PCR, NGS	[9]
Phénol–chloroform	0,24 US$ [69]	PCR, NGS	[9]
HotSHOT	< 0,01 US$ [69]	PCR	-
Salage	0,12 US$ [69]	PCR	-
Chelex	0,02 US$ [41]	PCR	[41, 76]

6.5. Spektrometri masse MALDI-ToF

Spektrometri masse (MALDI-ToF) pexem jàngat la bu sukkandiko ci Spektrometri masse, ñu defar ko ngir jàngat ay missal ci protein yi ("mandarga baraami protein") ci ay mbindéef ci biologi. MALDI-ToF ñu ngi koy gëna xàmmee ni jumtukaay la bu am solo ngir xàmmee xeeti gunoor yu am solo ci wàllu pajum nitt ak baayima. Mii pexe dafa wone ni am na njariñ ci ràññee màndarga yu bari ci màgg gi ci yuul yi, lu ci melni walax njaan yi ak deret ji nga xamni yuul yu jigéen yu matt yi moom lanu naan, te jëfandikoo nañu ko itam bu baax ngir wuutale xeetu góor ak jigéen ci anami dencukaay yu bari [28, 30, 73, 74]. Dafay maye itam kàttan gu dëgër ci ràññatlee su-genre yi (xeet yu suuffe yi), xeetu mbindéef yi ak ci askan wi. Kon MALDI-ToF dafay tax ñu mëna xàmmee xeetu mbindéef yi ci anam wu gaaw te jaar yoon, te loolu dafay am solo ngir xam yuul yi tasaaroo ci dend bi, seeni doxalin ak seen wareef ci joxe fjangoro yi. Suñu ràññee xeetu yi ci seeni melokaani protein, anam wii dafa am solo lool ci njàngum epidemiologi ak ci pexe yi ñuy jëfandikoo ngir saytu ñi koy joxe. Waaye, ba leegi ñaari mbir yu am solo ñoo galangkoor seenuk jëfandikoo. Bi njëkk mooy diapndik jumtukaayi Spektrometri masse, njëg gi yomb lool ngir ñu mën ko jënd ngir ràññee yuul yi (wala yenen xeti gunoor yi ci lu ëpp). Teerewul mën nañu moytu jafe-jafe yii suñu jëfandikoo spektrometri masse yu jota am ci dendub proteomik wala ci labo yi ñuy gindikoo, fi jumtuwaay yo yu nekkee luñu miin. Ñaareelu jafe-jafe bi mooy ñaka diapndik tekktal yi ñu jota xam ci yuul yi te ñu tunk leen ci ay kaggu wala way ndere yu ubbeeku yi. Loolu dafay laaj ñu defar ay kaggu yu biir, yu sukkandiko ci tàani leer yuñ jëlee ci xeetu mbindéef yiñ xàmmee bu baax, gëna wóor ci boole ràññeekaay melokaan ak toppalante màrker genetik bu méngoo (COI, cytB, wala leneen). Dañu wara dindi jafe-jafe yii ndànk-ndànk jaaraleko ci boole kagguy xam xam yiñuy jëfandikoo ci biir dendub MSI, bi Assistance Publique-Hôpitaux de Paris ak Daara ju kawe ju Sorbonne (France) yor, ak itam ci dajalu BCCM/IHEM/Sciensano ci Bruxelles (Belgique). Sudee dañuy xalaat mandargall protein ci MALDI-ToF, li gëna wóor mooy ñu denc mbindéef yi ci anam yu wow, ci Kongelatëer wala ci 70% ci etanol bu am grade molekilëer, te waru ñu ko bàyyi mu nekk ci tàngoor wi ko wër. Bi ñu demee ba amul feem yu yaatu yuñu mëna jëfandikoo ngir waajal yuul yi, dañoo digale ñu jëfandikoo ndox mu am 60% acetonitrile ak 0.3% TFA bu am asid sinapinik (30 mg/mL) muy matrix MALDI-ToF, ngir mëna méngale tàani leeri protein yi ak xam xam yu diapndi yiñu siiwal ci yuul yi.

Misaalu waajal spektrométri masse MALDI-ToF (Nataal 7)

Xeetu gunóor yiñuu denc ci anam yu bari, dañu leen di njëkka weer ci ngelaw ci tàngooru néeg, daal di leen feess. Bopp bi ak biir bi dañu leen di dindi ngir baña yàq wàll yi am màndarga yaram yu am solo ngir samp laam ak jàngat melokaan. Dën bi lañuy jëfandikoo ngir jàngat MALDI-ToF, waaye dessitu biir bi mën nañu ko tëye ngir jël ADN bi. Ngir missalu protein, dañuy saaji dën bi ci biir ay pote (microtube) yu 1,5 mL ak 10 µL yu koy diggal, ñu jëfandikoo ay kuur yu ndaw benn yoon kese, yuñuy sani buñi noppe. Ñaari pexe yu ñuy faral di jëfandikoo mooy: ndox muñu segg mu sell, te amul ben sikk ak 25% asid formik.

7. Mujjental ak tunk

Ci liggéey bii, linu bëggoon mooy jox gëstukat yi pexe yi gëna am njariñ ngir samp yuul yi, ñu méngale ko ak mébetu gëstu bi, ngir yombal ràññeekaayi yuul yi ak gis doomi jangoro yi. Amul benn xeetu pexe bu ñéep mengo and, ci àdduna bi yépp; barina xeeti feem yu jàppandi, bu nekk ak lu ci baax ak lu ci baaxul. Ci wàllu xamm xam yi ñu ci toftal (Supporting data), dañuy fessal ay pexe yu leer yu bari yu ñuy jëfandikoo ngir waajal ak ràññee yuul yi. Feem yooyu, boole na wideo yiy jàngale, di joxe ay jéego, ñu méngale ko ak mébet yu wuute, lépp ngir am njariñ yu jaar yoon, te mën nañu ko sotti. Sunu joxee jumtukaay bu mat sëkk bii, noo ngi am yaakaar jàppale gëstukat yi ñu tànn ba noppi jëfandikoo pexem samp yi gëna mengo ak ci seeni soxla.

8. Jërëjëf mbaa thiant ak ngërëm

Gni solloo terebi bi gnigui gërëm Richard Lane ak Zoe Jay Adams ñu faroo ci Musée d'Histoire Naturelle, London (UK), ci seen jàngat bu baax bi ñu def, loolu jàppale na mbind mi mu gëna ñong.

9. Xaalis walla kopar yi yombal liggey bi

Noo ngi sant kuréel yiy liggéey ci yokkute ci Bresil, CNPq (nimero dosiye: 404395/2024-4) ak Fondation Araucária (nimero dosiye: 433/2025 PDI) ndax jàppale AJA ci gëstu bi.

10. Xeex mbaa xëtio, walla ngayoo bu mana tukke ci sollos liggéey bi

Jérôme Depaquit bokk na ci ñiy saytu surnaal bi tuddu Parasite; amul benn kàttan waalaa sañ sañ ci ni ñuy xoolaate mbind mi, saytu ko, wala jël ci ab dogal. Ñeneen ñi sex mbind mi itam wax nañu ni amul benn coow, mbaa luy nirou ab xecco mba ngaayo ci seen biir.

Diapndik li gene ci liggëy bi

Wideo yuy wax ci Zenodo.

Wideo 1: https://zenodo.org/records/18198006
Wideo 2: https://zenodo.org/records/18311158
Wideo 3: https://zenodo.org/records/18311106
Wideo 4: https://zenodo.org/records/18311154
Wideo 5: https://zenodo.org/records/18303014
Wideo 6: https://zenodo.org/records/18302850
Wideo 7: https://zenodo.org/records/18315029

Li ci dollee ku

https://www.parasite-journal.org/10.1051/parasite/2026009/olm

References

1. Alkan C, Allal-Ikhlef AB, Alwassouf S, Baklouti A, Piorkowski G, de Lamballerie X, Izri A, Charrel RN. 2015. Virus isolation, genetic characterization and seroprevalence of Toscana virus in Algeria. Clinical Microbiology and Infection, 21(11), 1040 e1-9.
2. Alten B, Ozbel Y, Ergunay K, Kasap OE, Cull B, Antoniou M, Velo E, Prudhomme J, Molina R, Banuls AL, Schaffner F, Hendrickx G, Van Bortel W, Medlock JM. 2015. Sampling strategies for phlebotomine sand flies (Diptera: Psychodidae) in Europe. Bulletin of Entomological Research 105(6), 664–678.
3. Ayhan N, Baklouti A, Prudhomme J, Walder G, Amaro F, Alten B, Moutailler S, Ergunay K, Charrel RN, Huemer H. 2017. Practical guidelines for studies on sandfly-borne phleboviruses: Part I: Important points to consider *ante* field work. Vector-Borne and Zoonotic Diseases 17(1), 73–80.
4. Bates PA. 1997. Infection of phlebotomine sandflies with *Leishmania*, in The Molecular Biology of Insect Disease Vectors: A Methods Manual. Springer. p. 112–120.5.
5. Baum M, de Castro EA, Pinto MC, Goulart TM, Baura W, Klisiowicz Ddo R, Vieira da Costa-Ribeiro MC. 2015. Molecular detection of the blood meal source of sand flies (Diptera: Psychodidae) in a transmission area of American cutaneous leishmaniasis, Parana State, Brazil. Acta Tropica, 143, 8–12.
6. Belen A, Alten B, Aytekin A. 2004. Altitudinal variation in morphometric and molecular characteristics of *Phlebotomus*

papatasi populations. Medical and Veterinary Entomology, 18(4), 343–350.
7. Bhattacharya J, Chandra G, Hati AK. 1991. A simple method for cryopreservation of *Leishmania donovani* promastigotes, Indian Journal of Medical Research, 93, 245–246.
8. Caligiuri LG, Sandoval AE, Miranda JC, Pessoa FA, Santini MS, Salomón OD, Secundino NF, McCarthy CB. 2019. Optimization of DNA extraction from individual sand flies for PCR amplification. Methods and Protocols, 2(2), 36.
9. Casaril AE, de Oliveira LP, Alonso DP, de Oliveira EF, Gomes Barrios SP, de Oliveira Moura Infran J, Fernandes WS, Oshiro ET, Ferreira AMT, Ribolla PEM, de Oliveira AG. 2017. Standardization of DNA extraction from sand flies: Application to genotyping by next generation sequencing. Experimental Parasitology, 177, 66–72.
10. Castalanelli MA, Severtson DL, Brumley CJ, Szito A, Foottit RG, Grimm M, Munyard K, Groth DM. 2010. A rapid non-destructive DNA extraction method for insects and other arthropods. Journal of Asia-Pacific Entomology, 13(3), 243–248.
11. Cerqueira NL. 1943. Um novo meio para montagem de pequenos insetos em lâmina. Memórias do Instituto Oswaldo Cruz, (39), 37–41.
12. Charrel RN, Gallian P, Navarro-Mari JM, Nicoletti L, Papa A, Sanchez-Seco MP, Tenorio A, de Lamballerie X. 2005. Emergence of Toscana virus in Europe. Emerging Infectious Diseases, 11(11), 1657–1663.
13. Chaskopoulou A, Giantsis IA, Demir S, Bon MC. 2016. Species composition, activity patterns and blood meal analysis of sand fly populations (Diptera: Psychodidae) in the metropolitan region of Thessaloniki, an endemic focus of canine leishmaniasis. Acta Tropica, 158, 170–176.
14. Chen H, Rangasamy M, Tan SY, Wang H, Siegfried BD. 2010. Evaluation of five methods for total DNA extraction from western corn rootworm beetles. PLoS One, 5(8), e11963.
15. Depaquit J, Grandadam M, Fouque F, Andry PE, Peyrefitte C. 2010. Arthropod-borne viruses transmitted by Phlebotomine sandflies in Europe: a review. Eurosurveillance, 15(10), 19507.
16. Diamond LS, Herman CM. 1954. Incidence of Trypanosomes in the Canada Goose as revealed by bone marrow culture. Journal of Parasitology, 40(2), 195–202.
17. Ding H, Torno M, Vongphayloth K, Ng G, Tan D, Sng W, Ho K, Randrianambinintsoa FJ, Depaquit J, Tan CH. 2025. Hidden in plain sight: discovery of sand flies in Singapore and description of four species new to science. Parasites & Vectors, 18(1), 402.
18. Es-Sette N, Ajaoud M, Bichaud L, Hamdi S, Mellouki F, Charrel RN, Lemrani M. 2014. *Phlebotomus sergenti* a common vector of *Leishmania tropica* and Toscana virus in Morocco. Journal of Vector Borne Diseases, 51(2), 86–90.
19. Favret C. 2005. A new non-destructive DNA extraction and specimen clearing technique for aphids (Hemiptera). Proceedings of the Entomological Society of Washington, 107(2), 469–470.
20. Galati EAB. 2018. Phlebotominae (Diptera, Psychodidae): Classification, morphology and terminology of adults and identification of American taxa, in Brazilian Sand Flies: Biology, Taxonomy, Medical Importance and Control, Rangel EF, Shaw JJ, Editors. Cham: Springer International Publishing. pp. 9–212.
21. Galati EAB, de Andrade AJ, Perveen F, Loyer M, Vongphayloth K, Randrianambinintsoa FJ, Prudhomme J, Rahola N, Akhoundi M, Shimabukuro PHF, Depaquit J. 2025. Phle-botomine sand flies (Diptera, Psychodidae) of the world. Parasites & Vectors, 18(1), 220.
22. Galati EAB, Galvis-Ovallos F, Lawyer P, Leger N, Depaquit J. 2017. An illustrated guide for characters and terminology used in descriptions of Phlebotominae (Diptera, Psychodidae). Par-asite, 24, 26.
23. Gariepy T, Kuhlmann U, Gillott C, Erlandson M. 2007. Parasitoids, predators and PCR: the use of diagnostic molecular markers in biological control of Arthropods. Journal of Applied Entomology, 131(4), 225–240.
24. Giantsis IA, Chaskopoulou A, Bon MC. 2016. Mild-Vectolysis: A nondestructive DNA extraction method for vouchering sand flies and mosquitoes. Journal of Medical Entomology, 53(3), 692–695.
25. Gidwani K, Picado A, Rijal S, Singh SP, Roy L, Volfova V, Andersen EW, Uranw S, Ostyn B, Sudarshan M, Chakravarty J, Volf P, Sundar S, Boelaert M, Rogers ME. 2011. Serological markers of sand fly exposure to evaluate insecticidal nets against visceral leishmaniasis in India and Nepal: a cluster-randomized trial. PLoS Neglected Tropical Diseases, 5(9), e1296.
26. Gilbert MTP, Moore W, Melchior L, Worobey M. 2007. DNA extraction from dry museum beetles without conferring external morphological damage. PLoS One, 2(3), e272.
27. Giordani BF, Andrade AJ, Galati EAB, Gurgel-Goncalves R. 2017. The role of wing geometric morphometrics in the identification of sandflies within the subgenus *Lutzomyia*. Medical and Veterinary Entomology, 31(4), 373–380.
28. Guzmán-Larralde AJ, Suaste-Dzul AP, Gallou A, Peña-Carrillo KI. 2017. DNA recovery from microhymenoptera using six non-destructive methodologies with considerations for subsequent preparation of museum slides. Genome, 60(1), 85–91.
29. Hajibabaei M, DeWaard JR, Ivanova NV, Ratnasingham S, Dooh RT, Kirk SL, Mackie PM, Hebert PD. 2005. Critical factors for assembling a high volume of DNA barcodes. Philosophical Transactions of the Royal Society B: Biological Sciences, 360(1462), 1959–1967.
30. Haouas N, Pesson B, Boudabous R, Dedet JP, Babba H, Ravel C. 2007. Development of a molecular tool for the identification of *Leishmania* reservoir hosts by blood meal analysis in the insect vectors. American Journal of Tropical Medicine and Hygiene, 77(6), 1054–1059.
31. Hlavackova K, Dvorak V, Chaskopoulou A, Volf P, Halada P. 2019. A novel MALDI-TOF MS-based method for blood meal identification in insect vectors: A proof of concept study on phlebotomine sand flies. PLoS Neglected Tropical Diseases, 13 (9), e0007669.
32. Huemer H, Prudhomme J, Amaro F, Baklouti A, Walder G, Alten B, Moutailler S, Ergunay K, Charrel RN, Ayhan N. 2017. Practical guidelines for studies on sandfly-borne phleboviruses: Part II: Important points to consider for fieldwork and subsequent virological screening. Vector-Borne and Zoonotic Diseases, 17(1), 81–90.
33. Jancarova M, Polanska N, Thiesson A, Arnaud F, Stejskalova M, Rehbergerova M, Kohl A, Viginier B, Volf P, Ratinier M. 2025. Susceptibility of diverse sand fly species to Toscana virus. PLoS Neglected Tropical Diseases, 19(5), e0013031.
34. Kapp JD, Green RE, Shapiro B. 2021. A fast and efficient single-stranded genomic library preparation method optimized for ancient DNA. Journal of Heredity, 112(3), 241–249.
35. Killick-Kendrick R, Maroli M, Killick-Kendrick M. 1991. Bibliography of the colonization of phlebotomine sandflies. Parassitologia, 33(suppl.), 321–333.
36. Lawyer P, Killick-Kendrick M, Rowland T, Rowton E, Volf P. 2017. Laboratory colonization and mass rearing of phlebotomine sand flies (Diptera, Psychodidae). Parasite, 24, 42.

37. Léger N, Pesson B, Madulo-Leblond G. 1986. Les phlébotomes de Grèce : 1ère partie. Bulletin de la Société de Pathologie Exotique, 79, 386–397.
38. Léger N, Pesson B, Madulo-Leblond G. 1986. Les phlébotomes de Grèce : 2ème partie. Bulletin de la Société de Pathologie Exotique, 79, 514–524.
39. Leonel JAF, Vioti G, Alves ML, da Silva DT, Meneghesso PA, Benassi JC, Spada JCP, Galvis-Ovallos F, Soares RM, Oliveira T. 2020. DNA extraction from individual Phlebotomine sand flies (Diptera: Psychodidae: Phlebotominae) specimens: Which is the method with better results? Experimental Parasitology, 218, 107981.
40. Lestinova T, Rohousova I, Sima M, de Oliveira CI, Volf P. 2017. Insights into the sand fly saliva: Blood-feeding and immune interactions between sand flies, hosts, and *Leishmania.* PLoS Neglected Tropical Diseases, 11(7), e0005600.
41. Lienhard A, Schaffer S. 2019. Extracting the invisible: obtaining high quality DNA is a challenging task in small arthropods. PeerJ, 7, e6753.
42. Lozano-Sardaneta YN, Mikery-Pacheco OF, Huerta H, Rojas-Soriano JE, Contreras-Ramos A. 2025. Wing geometric morphometrics is effective to separate sand fly species (Diptera, Psychodidae, Phlebotominae) related with leishmaniasis transmission in Mexico. Acta Tropica, 262, 107523.
43. Mandrioli M. 2008. Insect collections and DNA analyses: how to manage collections? Museum Management and Curatorship, 23(2), 193–199.
44. Maroli M, Feliciangeli MD, Bichaud L, Charrel RN, Gradoni L. 2013. Phlebotomine sandflies and the spreading of leishmaniases and other diseases of public health concern. Medical and Veterinary Entomology, 27(2), 123–147.
45. Marquina D, Buczek M, Ronquist F, Lukasik P. 2021. The effect of ethanol concentration on the morphological and molecular preservation of insects for biodiversity studies. PeerJ, 9, e10799.
46. Mathis A, Depaquit J, Dvorak V, Tuten H, Banuls AL, Halada P, Zapata S, Lehrter V, Hlavackova K, Prudhomme J, Volf P, Sereno D, Kaufmann C, Pfluger V, Schaffner F. 2015. Identification of phlebotomine sand flies using one MALDI-TOF MS reference database and two mass pectrometer systems., Parasites & Vectors, 8, 266.
47. Mekarnia N, Benallal KE, Sadlova J, Vojtkova B, Mauras A, Imbert N, Longhitano M, Harrat Z, Volf P, Loiseau PM, Cojean S. 2024. Effect of *Phlebotomus papatasi* on the fitness, infectivity and antimony-resistance phenotype of antimony-resistant *Leishmania* major Mon-25. International Journal for Parasitology – Drugs and Drug Resistance, 25, 100554.
48. Milligan BG. 1998. Total DNA isolation, in Molecular Genetic Analysis of Population: A Practical Approach, Hoelzel AR, Editor. Oxford: Oxford University Press.
49. Molina R, Jiménez M, Alvar J, González E, Hernández-Taberna S, Ines MM. 2017. Methods in sand fly research. Madrid: Servicio de publicaciones Universidad de Alcalá de Henares, Madrid.
50. Murphy WJ, Eizirik E, O'Brien SJ, Madsen O, Scally M, Douady CJ, Teeling E, Ryder OA, Stanhope MJ, de Jong WW, Springer MS. 2001. Resolution of the early placental mammal radiation using Bayesian phylogenetics. Science, 294(5550), 2348–2351.
51. Nacif-Pimenta R, Pinto LC, Volfova V, Volf P, Pimenta PFP, Secundino NFC. 2020. Conserved and distinct morphological aspects of the salivary glands of sand fly vectors of leishma-niasis: an anatomical and ultrastructural study. Parasites & Vectors, 13(1), 441.
52. Neuhaus B, Schmid T, Riedel J. 2017. Collection management and study of microscope slides: Storage, profiling, deterioration, restoration procedures, and general recommendations. Zootaxa, 4322(1), 1–173.
53. New TR. 1974. Pscoptera. Handbooks for Identification of British Insects (Vol. I). London: Royal Entomological Society of London. 102 pp.
54. Perez-Ruiz M, Collao X, Navarro-Mari JM, Tenorio A. 2007. Reverse transcription, real-time PCR assay for detection of Toscana virus. Journal of Clinical Virology, 39(4), 276–281.
55. Porco D, Rougerie R, Deharveng L, Hebert P. 2010. Coupling non-destructive DNA extraction and voucher retrieval for small soft-bodied Arthropods in a high-throughput context: the example of Collembola. Molecular Ecology Resources, 10(6), 942–945.
56. Prudhomme J, Cassan C, Hide M, Toty C, Rahola N, Vergnes B, Dujardin JP, Alten B, Sereno D, Banuls AL. 2016. Ecology and morphological variations in wings of *Phlebotomus ariasi* (Diptera: Psychodidae) in the region of Roquedur (Gard, France): a geometric morphometrics approach. Parasites & Vectors, 9(1), 578.
57. Prudhomme J, Gunay F, Rahola N, Ouanaimi F, Guernaoui S, Boumezzough A, Banuls AL, Sereno D, Alten B. 2012. Wing size and shape variation of *Phlebotomus papatasi* (Diptera: Psychodidae) populations from the south and north slopes of the Atlas Mountains in Morocco. Journal of Vector Ecology, 37(1), 137–147.
58. Prudhomme J, Toty C, Kasap OE, Rahola N, Vergnes B, Maia C, Campino L, Antoniou M, Jimenez M, Molina R, Cannet A, Alten B, Sereno D, Banuls AL. 2015. New microsatellite markers for multi-scale genetic studies on *Phlebotomus ariasi* Tonnoir, vector of *Leishmania infantum* in the Mediterranean area. Acta Tropica, 142, 79–85.
59. Prudhomme J, Velo E, Bino S, Kadriaj P, Mersini K, Gunay F, Alten B. 2019. Altitudinal variations in wing morphology of *Aedes albopictus* (Diptera, Culicidae) in Albania, the region where it was first recorded in Europe. Parasite, 26, 55.
60. Rawlins DJ. 1992. Light Microscopy: An Introduction to Biotechniques. Oxford: Bios Scientific publishers. 143 pp.
61. Ready PD. 2013. Biology of phlebotomine sand flies as vectors of disease agents. Annual Review of Entomology, 58, 227–250.
62. Rohlf FJ, Slice D. 1990. Extensions of the Procrustes method for the optimal superimposition of landmarks. Systematic Zoology, 39(1), 40–59.
63. Rowley DL, Coddington JA, Gates MW, Norrbom AL, Ochoa RA, Vandenberg NJ, Greenstone MH. 2007. Vouchering DNA-barcoded specimens: Test of a nondestructive extraction proto-col for terrestrial arthropods. Molecular Ecology Notes, 7(6), 915–924.
64. Sábio PB, Andrade AJ, Galati EAB. 2014. Assessment of the taxonomic status of some species included in the *Shannoni* complex, with the description of a new species of *Psathyromyia* (Diptera: Psychodidae: Phlebotominae). Journal of Medical Entomology, 51(2), 331–341.
65. Sadlova J, Yeo M, Seblova V, Lewis MD, Mauricio I, Volf P, Miles MA. 2011. Visualisation of *Leishmania donovani* fluo-rescent hybrids during early stage development in the sand fly vector. PLoS One, 6(5), e19851.
66. Sales K, Miranda DEO, da Silva FJ, Otranto D, Figueredo LA, Dantas-Torres F. 2020. Evaluation of different storage times and

preservation methods on phlebotomine sand fly DNA concentration and purity. Parasites & Vectors, 13(1), 399
67. Sales KG, Costa PL, de Morais RC, Otranto D, Brandao-Filho SP, Cavalcanti Mde P, Dantas-Torres F. 2015. Identification of phlebotomine sand fly blood meals by real-time PCR. Parasites& Vectors, 8, 230.
68. Sant'Anna MR, Jones NG, Hindley JA, Mendes-Sousa AF, Dillon RJ, Cavalcante RR, Alexander B, Bates PA. 2008. Blood meal identification and parasite detection in laboratory-fed and field-captured *Lutzomyia longipalpis* by PCR using FTA databasing paper. Acta Tropica, 107(3), 230–237.
69. Senne NA, Santos HA, Araujo TR, Paulino PG, Mendonca LP, Moreira HVS, Camilo TA, da Costa Angelo I. 2022. Robust comparative performance of genomic DNA extraction methods from non-engorged phlebotomine sandflies. Medical and Veterinary Entomology, 36(2), 203–211.
70. Shaw JJ. 2025. A review of Leishmania infections in American Phlebotomine sand flies – Are those that transmit leishmaniasis anthropophilic or anthropportunists? Parasite, 32, 57.
71. Tesh RB, Modi GB. 1983. Growth and transovarial transmission of Chandipura virus (Rhabdoviridae: Vesiculovirus) in *Phlebotomus papatasi.* American Journal of Tropical Medicine and Hygiene, 32(3), 621–623.
72. Thomsen PF, Elias S, Gilbert MTP, Haile J, Munch K, Kuzmina S, Froese DG, Sher A, Holdaway RN, Willerslev E. 2009. Non-destructive sampling of ancient insect DNA. PLoS One, 4(4),e5048
73. Truett GE, Heeger P, Mynatt RL, Truett AA, Walker JA, Warman ML. 2000. Preparation of PCR-quality mouse genomic DNA with hot sodium hydroxide and tris (HotSHOT). Biotechniques, 29(1), 52–54.
74. Upton MS. 1993. Aqueous gum-chloral slide mounting media: an historical review. Bulletin of Entomological Research, 83(2), 267–274.
75. Volf P, Myskova J. 2007. Sand flies and Leishmania: specific versus permissive vectors. Trends in Parasitology, 23(3), 91–92.
76. Wang Q, Wang X. 2012. Comparison of methods for DNA extraction from a single chironomid for PCR analysis. Pakistan Journal of Zoology, 44(2), 421–426.
77. Wang Y, Zhao Y, Bollas A, Wang Y, Au KF. 2021. Nanopore sequencing technology, bioinformatics and applications. Nature Biotechnology, 39(11), 1348–1365.

Cite this article as: Randrianambinintsoa FJ, Augendre L, Prudhomme J, Martinet J-P, Loyer M, Mekarnia N, Kerkoub H, Perveen FK, Huguenin A, Kariya E, Akhoundi M, De Andrade AJ, Berriatua E, Bongiorno G, Boyer S, Christodoulou V, Da Costa-Ribeiro MCV, De Souza LAF, Ding H, Dondji B, Dvořák V, Erisoz Kasap O, Galati EAB, Gállego M, Ballart C, Gouzelou S, Haddad N, Masse RS, Mekuria AH, Ivovic V, Kaczmarek S, Shahar MK, Kirstein OD, Kniha E, Kolářová I, Lincoln T, Lucanas C, Mikov O, Nov K, Özbel Y, Pesson B, Posada Lopez LC, Prasetyo DB, Rahola N, Rebollar-Tellez EA, Rodrigues BL, Roy L, Saini P, Sanjoba C, Shimabukuro PH, Siriyasatien P, Soszynska A, Suleșco T, Sylla M, Torno M, Volf P, Vongphayloth K, Sinh Nam V, Wardhana A, Yessinou E, Zapata S, Gantier J-C & Depaquit J. 2026. Processing and mounting phlebotomine sand flies: a consensus guideline. Parasite xx, xx. https://doi.org/10.1051/parasite/2026009.

PARASITE

An international open-access, peer-reviewed, online journal publishing high quality papers on all aspects of human and animal parasitology

Reviews, articles and short notes may be submitted. Fields include, but are not limited to: general, medical and veterinary parasitology; morphology, including ultrastructure; parasite systematics, including entomology, acarology, helminthology and protistology, and molecular analyses; molecular biology and biochemistry; immunology of parasitic diseases; host-parasite relationships; ecology and life history of parasites; epidemiology; therapeutics; new diagnostic tools.
All papers in Parasite are published in English. Manuscripts should have a broad interest and must not have been published or submitted elsewhere. No limit is imposed on the length of manuscripts.

Parasite (open-access) continues **Parasite** (print and online editions, 1994-2012) and **Annales de Parasitologie Humaine et Comparée** (1923-1993) and is the official journal of the Société Française de Parasitologie.

Editor-in-Chief:
Jean-Lou Justine, Paris

Submit your manuscript at:
https://www.editorialmanager.com/parasite

Annexes

Annexe 1: Ceslaay yu nu biiral ci Biochimik.

Xeeti gunoor yi ñu soxal fii ñoy yuul yi. Waaye, mën naño jëfandikoo njàngale mi ci yeneen xeeti gunoor yu bari yu senub ràññeekaay aju ci màndarga yaram yi ci biir. Amna ñu diagleg, ràññee ay gunoor yu, senii cëri biir dañuy am chitin, te seen melookaan dafay joxe leeral yu am solo. Moo tax xool pompu ñam yi, spermathek yi ak seeni yoon lu am solo la. Ak reactif yi ñuy xoolaat yépp, fàww ñu fàttali ni, daale ko ci fana fiksation guunor bi ba ci samp bi, dañuy jëfandikoo reaction oxidasioŋ. Benn matuwaay bi gëna am solo mooy nga moytu jaxase reactif buy wañña ak jëniy simi yuy oxidee.

Alkol etilik; etanol:

Barina anam yu ñuy jëfandikoo mbir moomu. Molecule alcool yi daño bëgg ndox lool, moo tax dañuy mang xa ndox ci yaram wi. Waaye, alkol bu bariwul (maanaam bari ndox lool) dina am solo ci yàq asid nucleik yi (ndox mooy noonu asid nucleik yi).

Suñu defee ay gunóor ci etanol, du ngir denc rek, waaye ngir fikse suux yi itam. Ci histologi, ñaari mbir yu am solo lañuy ràññee: gaawaayu dugg gi ak gaawaayu fiksee bi. Daño xam bu baax ni fiksee bu baax dafa wara dugg bu baax ci suux yi ci lu gaaw balaa muy deff liggey bi. Bu dee alkol 96%, gaawayu dugg bi mingi tollu ci 1,05 (ngir méngale, dig gi ci ndox mu 0,75% asid pikrik amna koeffisient duggu bu 0,45, waaye dig gi ci dichromate potassium bu 3% am koeffisient 1,45).

Ñi seen xam xam macci ci gunoor, daño bëgg ñu denc yoo yi ak yeneen xeetu gunoor yi ci etanol ba fàww. Xalaat bi ñu am ci denc li ñu diappe ci tool yi (aal bi) ngir ñu jàngat ko ëlëg wala ngir deff ci ay gëstu ci kanam, lu jara gërëm la. Waaye, anam wii ñu koy defee mënul ànd ak li cytologi wala histologi bëgg. Sooy jéema tëye mbindéef yi ci biir fikse kat bi lu yàgg, mën naño yàqu ba doo mëna liggéeyaat ak ñoom. Loolu moo waral mbindéef yu weesu 10 ci ay at dañuy naxaree liggeye, wala sax ken doo tu ko mëna jëfandikoo.

Leneen luñu wara bàyyi xel mooy ceer bi am ci diggante diissayu gunoor yi ñu wara fiksee ak tolluwaayu ndox miy fikse. Ci wàllu zoologi wala ci wàllu paj, daño diggele ñu jëfandikoo tolluwaayu ndox miy fikse bamu ëpp 60 ca li nga wara fikse. Ci jëf, ngir gunnor you tuut yi lool, dangay dieul lumu gatt gatt nient ca, wala diourom ca ci lu tollok gunoor yi nga wara fikse.

Nañu baayi xel ci nee alkol bi dafay xëcci ndox mi ci suxu gunoor, te loolu day bëp dangaram.
Ci gàttal:

- Alcool ethylik dafay wàññi (te mënul ànd ak fikse oxidant yi);
- Dafay diggal yen protein yi di wàññi sen doole, ba nopi soppi len;
- Dafay diggal yen diw yi, ak glucogen bi;
- Dafay dajale suux yi ba noppi gën lena dëgëral.

Solution yu yomb yu hydroxyd potassium wala sodium:

Jëfandikoo pexe yooyu ci xam xamu guunor dafa njëkka lalu ci hydroxide potassium, te amul benn sabab bu leer. Hydroxyd bu sodium [E524] mën nañu ko am mu ndoxee, ci konsentrasoŋ yu bari wala ci anam wu jaar yoon, mën nañu ko am ci mellow tangal mba ay taparka yu ndaw. Gàllankoor bi mu am mooy hygroscopicity bu rëy bi (gëna rëy bu KOH). Su amee jaxasoo ak protein yi, daf a leen di dindi, sudde lipid yi, daf a leen di soppi def saabu yu dëgër ci diiru saponification (muy wuute bu mag ak KOH, biy defar saabu yu ndoxe ci diiru jaxasoo bi).

Hydroxyde potassium [E525] mën nañu ko am ci xeetu solution bu far, waaye li ci njëkk mooy ci xeetu tangal yuy tollu ci 0.1 g, loolu dafay yombal bu baax waajal diggal bi sudee amul balans bu jaar yoon. Ci misaal, 0.1 g tangal buñu raxas ci 1 ml ndox mu ñu segg settan tal ko dafay am 10%. Meneen mbir mu am solo ci KOH ci xeetu tangal mooy gëna néew luñu koy gaañ ci karbonaasioŋ (solution KOH amna lëko bu dëgër ak CO_2, moo waral ñu am carbonate).

Base yu am doole yooyu dañu leen di jëfandikoo ngir dindi asid graa yi, ñu soppi leen ñu nekk saabu yuy diig ci ndox. Dafay baax ñu xamni fikse kaay bi, lu melni etanol, dafay diigal yenn lipid yi ci mbideff yi. Waaye, suñu jaarale mbideeff bi ci biir ndox mu am base bu dëgër, asid graa yi (yu am jafe-jafe yu wuute) dañuy seey. Bàas bu dëgër bi dafay def saponificatioŋ bu sedd. Amna numuy deme, sudee sux yu nooy yi barina ñu lool, lu ci melni ci jigéen ñi, mën na nekk lu baax ñu yokk tàngoor wi ba 35–40 °C ngir yombal reaction bi, wala ñu yokk diir bi ñuy jëflante ci nugayoub néeg.

Marc-André, buñu soop ci fuchsin wala deet :

Fii dañuy waxtaane njariñ ak loraange yi ci jëfandikoo pexem Marc-André. Ndox momu dafa am hidrat chloral (monhidrat trikloracetaldehyde), asid asetik ak ndox. Ndox la mu am oxide bu baax (njaxasu asid ak aldehyde). Dafay dindi hydroxyde potassium bu bari bi mëna des ci mbindéef yi te du diggal saabu alkaline yiy tukke ci jëfandikoo KOH. Ndoxum oxidant bi dafay liggéey itam ci ñaareelu alkol yi di glucosamines yi defar chitin, daal di leen oxide ba noppi nooyal sen chitin bi. Dafay may itam yenn xorom yu am mineroo ñu seey.
Suñu njëkkee soob Marc-André ci fuchsin asid (maanaam, ci melokaan wu oxidee), mën na lëkkaloo ak ñaareelu mbooloy alcool yi taxawal chitin bi. Ginaaw sen diirub

jaxasso, ak melow mbindéef yi, ñuy jëfandikoo etanol kese ngir raxas leen. Ginaaw loolu la faanay dehydration ci mbindéef yi tàmbali.

Njariñ yi :

- Nassaxal ndoxum alkalin mu ëpp
- Nooyal chitin
- Cuubug chitin bi, ngir ñu gëna gis mello yi ci biir

Loraange yi :

Chloral hydrate jëff la juy mandal te ñu yàgga jëfandikoo ci pajum nit ñi. Jëfandikoo gi dañu ko wara amal ci suufu hotte simi, ba noppi ñu topp sàrt yiñ tëral ci wàllu xeeti loru ci jëniy simi yi.

Yoon yi ngir dindi ndox :

Jaar-jaar yi dañu wone ni, ci misaal yu tuuti lool, jarul ñu topp ay xeetu suub ci alkol yu dangar ji di gënë kawe. Sudee mbideff mi dafa yaatu, tàmbalil ci 80% etanol, ginaaw ga 90%, 95%, nga mujjee ci etanol bu ken raxul. Lu aju ci mbideff yu ndaw yi lool, jo len ci alkol 90% ba noppi nga dugal lenn ci etanol bu ken raxul, lolu doyna seuk . Ci tolluwaay bobu, fàww ñu bàyyi xel ci ni etanol absolu dafay xëcci vapër ndox mi ci jawwu ji.

Bu njëkkoon, ci labo yi ñuy saytoo gunoor yi, dañu daan jëfandikoo kreosote bu hêtre ngir dindi ndox ci mbindéef yi. Tay, looli nga xam ni daan nañu ko jëfandikoo lu bari ngir faggagal guunor yi ak yamar xaalla yu tuut yi, di ci aar itam denk yi ñuy denc, dañu ko baaye jëfandikoo bu baax ndax xetam (muy jëm ci hydrocarbure aromatik polycyclic) ak toksisitem biñu koy jiiñ : reprotoxik, di joxe cancer, te itam bayima yiy dund ci xetti ndox yiiy taa ci dend bi.

Li ñuy xalaat ngir samp ay mbideff mooy jëfandikoo Euparal® ak diwu Euparal (ñu leeral ko ci xët mi ci topp). Njaxasu Euparal® ak diwu Euparal du yaax dara ci mbideff yi ñu njëkka def ci 90% ci etanol.

Annexe 2 : Li ñu boole ci xeeti ndox yi ñu jëfandiko

Hydroxyd potassium 10 %
Hydroxyd potassium : 10 g
Ndox mu ñu seeg: q.s.p. 100 mL

Medium samp bu gom chloral (Medium Hoyer)
Ndox mu ñu seeg: 50 mL
Hydrate chloral : 200 g
Daakande : 50 g
Glycérol : 20 mL

Ndoxum Marc-André
Hydrat chloral : 40 g
Acid acétik bu sedd guuy : 30 mL
Ndox mu ñu seeg: 30 mL

Fuchsin acide 1 % ci biir Ndox mu ñu seeg
Fuchsin acid (sunguf) : 1 g
Ndox mu ñu seeg : 99 mL

Ndoxum Marc-André mu ñu soob ak fuchsin
Ndoxum Marc-André: 10 mL
Fuchsin acid 1% 50 µL

Annexe 3 : Euparal®, baume du Canada, alcool polyvinylik et ak yennen xeeti ndoxi montaass®

Alcool polyvinyl **:** Alcool polyvinyl mooy medium bi gëna baax ngir samp sudee produit yi war ngir waañi ndoxmi bu baax amul. Su demee nii, dañuy jaxase alkol polyvinyl ak lactophenol bu Amann. Waaye, xeetu samp boobu amna lu ci baaxul: mën na nekk medium bi dafay wow, wala alcool polyvinyl bi dafay peer ndax naawug ndox mi, wala samp bi dafay lëndëm su phenol bi oxide. Waaye pexe bii baaxna lool ci samp yu gàtt.
Balsam Canada: Jëfandikoo balsam Canada ngir fikse laam ak laam ci fàww nga njëkka dindi ndox mi ci xeetu mbindéef bi. Jëfandikoo xylene wala toluene ci jéego bii amul benn njariñ.

Enecê Medium **:** Ci lu melni balsam bu Canada, Enecê Medium dafay laaj ñu njëkka fiirru ndox mi ci xeetu mbideff yi balaa ñu koy samp ci diggënte laam ak lam ci. Enecê boole bu yam: kolofon bu weex bu sell (22 g); gomme copal buy seey ci alkol (12 g); alkol bu ken raxul (20 ml); kamfor (10 g); terpentin (10 mL); eukalipt: 26 mL. Ni ñu koy waajalee: Def ci bool bu méngoo (lu melni, pottub Erlenmeyer), nga sotti ci alkol bi ak camphor bi, ba noppi nga sotti ci kolofon bi ak gomme copal bi. Ginaaw loolu nga saañ bool bi, yëngal ko, ba noppi nga dugal ko ci ndox mu nugg, te baxul. Su li ci biir tàmbalee ndoxe, ñu sotti ci diwu terpentine. Ginaaw loolu nga segg njaxas moomu, ba noppi nga sotti eucalyptol ci seggitt wi. Sudee ndox mi wàññeeku na, dañu ciy yook diwu Enecê, bi ñu defaree ci formul bii: alkol bu raxul (30 ml), kamfor (17 g), diwu terpentin (15 ml), eukalipt (38 ml) (Cerqueira, 1943).

Euparal® **:** Lii ab resin la bu bawoo ci garab gu mag gi di atlas, *Tetraclinis articulata* (1791), Gilson moo ko jàngat ba noppi defar ko ci 1906. Njariñam li gëna mag mooy du polimerise. Mbindéef yi ñu tass ci diggänté laam ak laam ci mën nañu leen jëlaat ci alkol bi, wala lu gëna baax, essence Euparal®. Resin boobu ñuy woowe itam sandarac, dina nangu etanol ci 80% ci konsentrasoŋ bi.

Jëfandikoo Triton X100: ndoxe te amul iyon :

Triton X100 am ndox la mu amul iyon *(4-(1,1,3,3-tetrametilbutil)fenil-polietilen glikol, wala tert-oktilfenoksipolietoksietanol, tert-oktilfenil polietilen glikol eter)*, ñu koy jëfandikoo bu bari ci selul yi ak ci moleculeer bi. Dafay yombal dugg gi ci biir selul yi ak deer wi eubm nuwaayoo bi.

Misaalu gunóor yuñ denc ay at yu bari ci alkol barina luñu koy gis. Li leer mooy anam wi ñu koy dence nekkul lu baax, te xetu gunoor yi ñuy denc ci anam woowu dañuy jafe waajal ngir ñu saytu leen ci mikroskoop. Dencukaay yu plastik yi mën nañu yàqu, moo waral alkol bi di naaw. Ci ñari fana yooyu yépp, nekk ci alkol bi lu yàgg wala wowuk mbindéef yi, day indi jafe-jafe bu mag. Ci atum 2008, Jonque siiwal na bataaxal buy wax ni ñuy jëfandikoo luy tooyal jargoñ yi ci lu melni Agepon, ñu koy jëfandikoo ngirr jël ay nataal [26]. Seetlu boobu moo jur xalaatu jëfandikoo luy tooyal lu amul detergent bu am doole.

Doxalin wiy jëfandikoo 0.5% ci Triton X100 :

- Dugalal mbideff yu wow yi ci alkol bu raxul.
- Def ci 0.5% Triton X100 ba mu woor lane mbindéef yi suux nañu bu baax ci ndox mi.
- Nga bàyyi ko diir bu tollu ci 5 simili wala lu gëna yàgg. Xeetu gunoor yépp war naño taaxalikoo ci biir ndox mi.
- Dindil Triton X100 bi nga wecci ko ak hydroxyd potassium.

Ginaaw loolu ñu wéyal doxalin wi ci anam wi ñu ko bindee ci kaw.

Annexe 4: Montass ci ndank ak ndank ak Euparal® wala balsam Canada

1. Daño wara mang xa ndox mi ci xeetu mbindéef yi (su amee niir wala meew, loolu dafay wone ni ndox mi dañul).
2. Mën naño wàññi ndox ci yaram suñu yokkee alkol ethyl ci biir yaram wi.
3. Mën nañu jële mbindéef yi ci alkol 99% wala alkol bu raxul, joo len ci luy leeral.

Tëralin:
1. Dugalal yuul yu matt yi ci etanol bu 70%.
2. Dindil etanol bi nga wecci ko ak 10% KOH. Tëjal yuul yi al laamu weer.
3. Nga bàyyi ko mu seey ba gunóor yi leer.
4. Dindil KOH bi.
5. Sottilen ndox muñu segg bamu sen yaram wepp muru, xaaral 30 ba 45 simili.
6. Dindil ndox mi, nga baamtu raxas bi ci ndox mu ñu segg mi dirub 30 simili (diir bi mingi aju ci limu mbindéef yi ñuy raxas benn yoon: lu mbindéef yi gëna bari, diir bi gëna gudd; ci geneen wàll, lu limu mbideff yi gana néew, diir bi gëna gatt, rawatina suñu leen di raxas benn-benn, diir bi mën na wàññeeku lool).
7. Dindil ndox mi.
8. Def ci ndoxu Marc-André (soo bëggee fuchsin asid) nga bàyyi ko mu taxaw 24 waxtu (benn bis).
9. Dindil ndoxum Marc-André.
10.Xoojal mbindéef yi ci ndox muñu segg, nga xaar 30 ba 45 simili.
11. Nga dindi ndox mi, raxasat ko ci ndox mu seeg ci diiru 30 simili.
12. Dindil ndox mi.
13. Def ci etanol 70%, boo noppe nak nga feess mbindéef yi.
Sooy jëfandikoo bopp bi ak dën bi, dindil ndànk bopp bi wala biir bi, xëc leen ndànk bañu taaxali ko.
b. Ci dën bi, dindil laaf yi ndànk, nga jàpp dën bi ak benn peeru mungass nga xëcc ci suufu laaf yi ak beneen mungass. Wala mën nañu koo fees ci dig dën bi, xaaj ko ñaari pàcc, cammoñ ak ndijoor, lépp di aju ci barab biñ bëgga xool.
14. Nga dindi ndox mi ndànk-ndànk ci alkol ethyl bu ànd ak ndox: 50 – 80 – 95%, ba ci alkol bu ken raxul.
15. Yeggalel dindi ndox mi ci yaram wi, ci soob leen ñaari yoon yu toppalante ci dirub 10 simili ci etanol 100%.
16. Dindil etanol bi nga teg ci diwu xorom polle 15 simili ci tàngooru néeg.
17. Jëlee leen ci diwu xorom polle, joo leen ci toxuk Euparal® bo tek ci kaw laam bu sett.
18. Teggal tenxo yi ci ni nga ko bëgge, bop, biir ak dënu yuul wi man nga leena fess ak xeeti pik yu seew ci loup bu am yaari gët.
Bopp bi dafa wara tàqaloo ak yaram wi, ñu jaaxaanal ko, maanaam foramen bi ci jë bi fette kaw, suko defee ñu mëna xool cibarium bi ci biir. Dañu koy def ci biir medium bi ñuy defee sampuk yuul yi.
19. Boole ci ab neggandiku ab diir ba kaw samp bi nekk luy kole tuuti.
20. Tooyal laam bu set ak alkol bu ken raxul. Tegal laam ci ci kaw balsam bu Canada wala Euparal® wengal ko tuutti.
21. Dencal laam yi ci booyet yu wow te set yu ñu deffar ngir loolu.
